# Supplementary material for: Expanding the landscape of BREX diversity: uncovering multi-layered functional frameworks and identification of novel BREX-related defense systems
Source: Nucleic Acids Res. 2026 Jan 27;54(3):gkag035. doi: 10.1093/nar/gkag035 (PMC12839542; doi:10.1093/nar/gkag035)
Supplement: gkag035_Supplemental_Files [file gkag035_supplemental_files.zip › Supplementary_Data_S1.pdf]

Supplementary Data S1

This PDF contains the conserved gene neighbourhoods with corresponding NCBI node information parsed for all ten systems examined in this study. Following these, the original treefiles used for the main figures, generated with IQ-TREE bootstrap analyses, are provided in Newick format.

1. Type-1 BREX systems……………………………………………………2-59
2. Type-5 BREX systems……………………………………………………59-61
3. Type-6 BREX systems……………………………………………………61-66
4. Type-2 BREX/Pgl systems……………………………………………67-80
5. Type-3 BREX systems…………………………………………………..81-92
6. Type-4 BREX systems…………………………………………………..93-98
7. Type-1 DUF499 centered BREX-related systems…………………99-140
8. Type-2 DUF499 centered BREX-related systems…………………141-155
9. Type-3 DUF499 centered BREX-related systems…………………156-163
10. BREX related capture systems………………………………………164
11. Newick tree (BrxC-ATPase)…………………………………………..165
12. Newick tree (PglZ)……………………………………………………166
13. Newick tree (DUF499-ATPase)………………………………………167

Note: Titles are internally hyperlinked. Click on the titles to access the material

<table>
<tr><th>Protein-ID</th><th>System</th><th>Gene-Neighbourhoods</th><th>Length</th><th>Taxonomy</th><th>Assembly-ID</th><th>Strain</th><th>Locus-Tag</th><th>Genomic-coordinates</th><th>TaxID</th><th>Fasta-defline</th></tr>
<tr><td>--</td><td></td><td></td><td></td><td></td><td></td><td></td><td></td><td></td><td></td><td></td></tr>
<tr><td>--Type-1-BREX</td><td></td><td></td><td></td><td></td><td></td><td></td><td></td><td></td><td></td><td></td></tr>
<tr><td>--Canonical-BREX</td><td></td><td></td><td></td><td></td><td></td><td></td><td></td><td></td><td></td><td></td></tr>
<tr><td>--</td><td></td><td></td><td></td><td></td><td></td><td></td><td></td><td></td><td></td><td></td></tr>
<tr><td>MCC7571311.1</td><td>Type1.BREX</td><td>nSTAND1+HNH->HNH->TrwI->?->?->VirB4-FtsK->Lysozyme+NLPC->BrxA-DNA-binding->BrxB-BrxF-iSTAND->DUF6079*->N6-METHYLASE->PglZ->SIGMA+OB-Fold+AAA-ATPase+Lon-Protease-BrxL->?->?->wHTH+WYL+ [illegible]</td><td>1210</td><td>Archaea>DPANN_group>Candidatus_Micrarchaeota>unclassified_Candidatus_Micrarchaeota</td><td>GCA_020854835.1</td><td>Candidatus_Micrarchaeota_archaeon</td><td>KO465_08330</td><td>10159..13791</td><td>2250274</td><td>BREX-system-P-loop-protein-BrxC-[Candidatus-Micrarchaeota-archaeon]</td></tr>
<tr><td>MBT4111448.1</td><td>Type1.BREX</td><td><-HIT||CopG/RHH->ZnR->?->DnaB->?->BrxA-DNA-binding->BrxB-BrxF-iSTAND->DUF6079-p*-></td><td>882</td><td>Archaea>DPANN_group>Candidatus_Woesearchaeota</td><td>GCA_018650945.1</td><td>Candidatus_Woesearchaeota_archaeon</td><td>HOE37_06330</td><td>27255..29901</td><td>2026803</td><td>BREX-system-P-loop-protein-BrxC-[Candidatus-Woesearchaeota-archaeon]</td></tr>
<tr><td>MBT4336377.1</td><td>Type1.BREX</td><td><-HIT||CopG/RHH->ZnR->?->DnaB->?->BrxA-DNA-binding->BrxB-BrxF-iSTAND->DUF6079*->N6-METHYLASE->PglZ->SIGMA+OB-Fold+AAA-ATPase+Lon-Protease-BrxL->SF1-SF2-Helicase+REase->?-><-?<-?<- [illegible]</td><td>1173</td><td>Archaea>DPANN_group>Candidatus_Woesearchaeota</td><td>GCA_018652365.1</td><td>Candidatus_Woesearchaeota_archaeon</td><td>HOD66_03370</td><td>27503..31024</td><td>2026803</td><td>BREX-system-P-loop-protein-BrxC-[Candidatus-Woesearchaeota-archaeon]</td></tr>
<tr><td>MBT3814300.1</td><td>Type1.BREX</td><td><-DUF6079-p*<-BrxB-BrxF-iSTAND<-BrxA-DNA-binding<-?<-DnaB<-?<-ZnR<-CopG/RHH||HIT-></td><td>353</td><td>Archaea>DPANN_group>Candidatus_Woesearchaeota</td><td>GCA_018654495.1</td><td>Candidatus_Woesearchaeota_archaeon</td><td>HOG07_02565</td><td>1..1059</td><td>2026803</td><td>BREX-system-P-loop-protein-BrxC-[Candidatus-Woesearchaeota-archaeon]</td></tr>
<tr><td>MBT4469968.1</td><td>Type1.BREX</td><td>Phage_integrase->?->?-><-?<-SF1-SF2-Helicase+REase<-SIGMA+OB-Fold+AAA-ATPase+Lon-Protease-BrxL<-PglZ<-N6-METHYLASE<-DUF6079*<-BrxB-BrxF-iSTAND<-BrxA-DNA-binding<-?<-DnaB<-?<-ZnR<- [illegible]</td><td>1173</td><td>Archaea>DPANN_group>Candidatus_Woesearchaeota</td><td>GCA_018656325.1</td><td>Candidatus_Woesearchaeota_archaeon</td><td>HOC62_06290</td><td>58853..62374</td><td>2026803</td><td>BREX-system-P-loop-protein-BrxC-[Candidatus-Woesearchaeota-archaeon]</td></tr>
<tr><td>MBT6744308.1</td><td>Type1.BREX</td><td><-HIT||CopG/RHH->ZnR->?->DnaB->?->BrxA-DNA-binding->BrxB-BrxF-iSTAND->DUF6079*->N6-METHYLASE->PglZ->SIGMA+OB-Fold+AAA-ATPase+Lon-Protease-BrxL->SF1-SF2-Helicase+REase->?-><-?<-?<- [illegible]</td><td>1173</td><td>Archaea>DPANN_group>Candidatus_Woesearchaeota</td><td>GCA_018696595.1</td><td>Candidatus_Woesearchaeota_archaeon</td><td>HOA70_03205</td><td>27503..31024</td><td>2026803</td><td>BREX-system-P-loop-protein-BrxC-[Candidatus-Woesearchaeota-archaeon]</td></tr>
<tr><td>MCL2156391.1</td><td>Type1.BREX</td><td>BrxA-DNA-binding->BrxB-BrxF-iSTAND->DUF6079*->Phage_integrase+Phage_integrase-><-N6-METHYLASE||N6-METHYLASE->?->PIN->PglZ->SIGMA+OB-Fold+AAA-ATPase+Lon-Protease-BrxL-></td><td>1194</td><td>Archaea>Euryarchaeota>Methanomada_group>Methanobacteria>Methanobacteriales>Methanobacteriaceae</td><td>GCA_009776495.1</td><td>Methanobrevibacter_sp.</td><td>FWH54_00015</td><td>1416..5000</td><td>66852</td><td>BREX-system-P-loop-protein-BrxC-[Methanobrevibacter-sp.]</td></tr>
<tr><td>MBE6500276.1</td><td>Type1.BREX</td><td>N6-METHYLASE->BrxA-DNA-binding->BrxB-BrxF-iSTAND->DUF6079*->?->?->PglZ->SIGMA+OB-Fold+AAA-ATPase+Lon-Protease-BrxL-></td><td>1195</td><td>Archaea>Euryarchaeota>Methanomada_group>Methanobacteria>Methanobacteriales>Methanobacteriaceae</td><td>GCA_015062925.1</td><td>Methanobrevibacter_thaueri</td><td>E7Z80_07025</td><td>4963..8550</td><td>190975</td><td>BREX-system-P-loop-protein-BrxC-[Methanobrevibacter-thaueri]</td></tr>
<tr><td>MBR3112546.1</td><td>Type1.BREX</td><td><-TPR+TPR+TPR+TPR<-?<-OLD-ABC<-SF1-SF2-Helicase+REase<-?<-?<-SIGMA+OB-Fold+AAA-ATPase+Lon-Protease-BrxL<-PglZ<-?<-DUF6079*<-BrxB-BrxF-iSTAND<-BrxA-DNA-binding<-N6-METHYLASE|| [illegible]</td><td>1187</td><td>Archaea>Euryarchaeota>Methanomada_group>Methanobacteria>Methanobacteriales>Methanobacteriaceae</td><td>GCA_017545145.1</td><td>Methanobrevibacter_sp.</td><td>IKH29_02390</td><td>52350..55913</td><td>66852</td><td>BREX-system-P-loop-protein-BrxC-[Methanobrevibacter-sp.]</td></tr>
<tr><td>MBR5504577.1</td><td>Type1.BREX</td><td><-SbcC<-?||?->McrC-NTD+REase-><- [illegible]</td><td>1186</td><td>Archaea>Euryarchaeota>Methanomada_group>Methanobacteria>Methanobacteriales>Methanobacteriaceae</td><td>GCA_017558445.1</td><td>Methanobrevibacter_sp.</td><td>IKV87_09095</td><td>7776..11336</td><td>66852</td><td>BREX-system-P-loop-protein-BrxC-[Methanobrevibacter-sp.]</td></tr>
<tr><td>MBS6828080.1</td><td>Type1.BREX</td><td><-N6-METHYLASE<-DUF6079-p*<- [illegible]</td><td>905</td><td>Archaea>Euryarchaeota>Methanomada_group>Methanobacteria>Methanobacteriales>Methanobacteriaceae</td><td>GCA_018379875.1</td><td>Methanobrevibacter_smithii</td><td>KH189_08115</td><td>2976..5693</td><td>2173</td><td>BREX-system-P-loop-protein-BrxC-[Methanobrevibacter-smithii]</td></tr>
<tr><td>WP_011954749.1</td><td>Type1.BREX</td><td>Phage_integrase+Phage_integrase-><- [illegible]</td><td>1194</td><td>Archaea>Euryarchaeota>Methanomada_group>Methanobacteria>Methanobacteriales>Methanobacteriaceae</td><td>GCF_000016525.1</td><td>Methanobrevibacter</td><td>MSM_RS08755</td><td>1809971..1813555</td><td>2172</td><td>MULTISPECIES:-BREX-system-P-loop-protein-BrxC-[Methanobrevibacter]</td></tr>
<tr><td>WP_143744747.1</td><td>Type1.BREX</td><td><-SIGMA+OB-Fold+AAA-ATPase+ [illegible]</td><td>1174</td><td>Archaea>Euryarchaeota>Methanomada_group>Methanobacteria>Methanobacteriales>Methanobacteriaceae</td><td>GCF_002208625.1</td><td>Methanobrevibacter_sp._87.7</td><td>BGI41_RS00855</td><td>14881..18405</td><td>387957</td><td>BREX-system-P-loop-protein-BrxC-[Methanobrevibacter-sp.-87.7]</td></tr>
<tr><td>WP_100907789.1</td><td>Type1.BREX</td><td>EVE->BrxD-AAA->SF1-SF2-Helicase+wHTH+ [illegible]</td><td>1197</td><td>Archaea>Euryarchaeota>Methanomada_group>Methanobacteria>Methanobacteriales>Methanobacteriaceae</td><td>GCF_002813675.1</td><td>Methanobacterium_sp._MZ-A1</td><td>BK008_RS11195</td><td>2292330..2295923</td><td>1911685</td><td>BREX-system-P-loop-protein-BrxC-[Methanobacterium-sp.-MZ-A1]</td></tr>
<tr><td>WP_100909051.1</td><td>Type1.BREX</td><td>BrxD-AAA->SF1-SF2-Helicase+wHTH+ [illegible]</td><td>1197</td><td>Archaea>Euryarchaeota>Methanomada_group>Methanobacteria>Methanobacteriales>Methanobacteriaceae</td><td>GCF_002813695.1</td><td>Methanobacterium_subterraneum</td><td>BK009_RS03450</td><td>727019..730612</td><td>59277</td><td>BREX-system-P-loop-protein-BrxC-[Methanobacterium-subterraneum]</td></tr>
<tr><td>WP_191216096.1</td><td>Type1.BREX</td><td><-DTXR-HTH||?->?-><-?||?->?-><- [illegible]</td><td>1183</td><td>Archaea>Euryarchaeota>Methanomada_group>Methanobacteria>Methanobacteriales>Methanobacteriaceae</td><td>GCF_009914355.1</td><td>Methanothermobacter_sp._THM-1</td><td>FZP57_06355</td><td>1234960..1238511</td><td>2606911</td><td>BREX-system-P-loop-protein-BrxC-[Methanothermobacter-sp.-THM-1]</td></tr>
<tr><td>WP_071906589.1</td><td>Type1.BREX</td><td><-McrC-NTD+REase<-McrB-NTD+McrB-AAA|| [illegible]</td><td>1198</td><td>Archaea>Euryarchaeota>Methanomada_group>Methanobacteria>Methanobacteriales>Methanobacteriaceae</td><td>GCF_900095295.1</td><td>Methanobacterium_congolense</td><td>MCBB_RS04215</td><td>888415..892011</td><td>118062</td><td>BREX-system-P-loop-protein-BrxC-[Methanobacterium-congolense]</td></tr>
<tr><td>WP_042694044.1</td><td>Type1.BREX</td><td>TRD-><-SIGMA+OB-Fold+AAA-ATPase+ [illegible]</td><td>1193</td><td>Archaea>Euryarchaeota>Methanomada_group>Methanobacteria>Methanobacteriales>Methanobacteriaceae</td><td>GCF_900289035.1</td><td>Methanobrevibacter_oralis</td><td>C9989_RS00250</td><td>18115..21696</td><td>66851</td><td>BREX-system-P-loop-protein-BrxC-[Methanobrevibacter-oralis]</td></tr>
<tr><td>WP_052331840.1</td><td>Type1.BREX</td><td>TRD-><-SIGMA+OB-Fold+AAA-ATPase+ [illegible]</td><td>1188</td><td>Archaea>Euryarchaeota>Methanomada_group>Methanobacteria>Methanobacteriales>Methanobacteriaceae</td><td>GCF_902384065.1</td><td>Methanobrevibacter_oralis</td><td>TY05_RS07165</td><td>191000..194566</td><td>66851</td><td>BREX-system-P-loop-protein-BrxC-[Methanobrevibacter-oralis]</td></tr>
<tr><td>ERJ05676.1</td><td>Type1.BREX</td><td><-N6-METHYLASE-p||viral-protein-><-?<- [illegible]</td><td>1231</td><td>Archaea>Euryarchaeota>Stenosarchaea_group>Halobacteria>Halobacteriales>Haloarculaceae</td><td>GCA_000215915.3</td><td>Halorhabdus_tiamatea_SARL4B</td><td>HLRTI_002298</td><td>10094..13789</td><td>1033806</td><td>hypothetical-protein-HLRTI_002298-[Halorhabdus-tiamatea-SARL4B]</td></tr>
<tr><td>EMA17933.1</td><td>Type1.BREX</td><td><-Exonuc_V_gamma+REase<-TUDOR+ [illegible]</td><td>1280</td><td>Archaea>Euryarchaeota>Stenosarchaea_group>Halobacteria>Halobacteriales>Haloarculaceae</td><td>GCA_000336895.1</td><td>Haloarcula_argentinensis_DSM_12282</td><td>C443_20652</td><td>152002..155844</td><td>1230451</td><td>hypothetical-protein-C443_20652-[Haloarcula-argentinensis-DSM-12282]</td></tr>
<tr><td>EMA17935.1</td><td>Type1.BREX</td><td><-PglZ<-N6-METHYLASE<-REase<-wHTH<- [illegible]</td><td>1227</td><td>Archaea>Euryarchaeota>Stenosarchaea_group>Halobacteria>Halobacteriales>Haloarculaceae</td><td>GCA_000336895.1</td><td>Haloarcula_argentinensis_DSM_12282</td><td>C443_20662</td><td>156419..160102</td><td>1230451</td><td>hypothetical-protein-C443_20662-[Haloarcula-argentinensis-DSM-12282]</td></tr>
<tr><td>ELZ19699.1</td><td>Type1.BREX</td><td><-N6-METHYLASE-p||?->?->?-><-PIN||?-> [illegible]</td><td>1198</td><td>Archaea>Euryarchaeota>Stenosarchaea_group>Halobacteria>Halobacteriales>Haloarculaceae</td><td>GCA_000337455.1</td><td>Halosimplex_carlsbadense_2-9-1</td><td>C475_22179</td><td>59263..62859</td><td>797114</td><td>hypothetical-protein-C475_22179-[Halosimplex-carlsbadense-2-9-1]</td></tr>
<tr><td>ELZ19701.1</td><td>Type1.BREX</td><td><-PIN||?-><-REase<-?||?-><-DUF6079<- [illegible]</td><td>1248</td><td>Archaea>Euryarchaeota>Stenosarchaea_group>Halobacteria>Halobacteriales>Haloarculaceae</td><td>GCA_000337455.1</td><td>Halosimplex_carlsbadense_2-9-1</td><td>C475_22189</td><td>63665..67411</td><td>797114</td><td>hypothetical-protein-C475_22189-[Halosimplex-carlsbadense-2-9-1]</td></tr>
<tr><td>WP_015789649.1</td><td>Type1.BREX</td><td><-REase<-?<-?||DOC+zhth-><-BirA-HTH<- [illegible]</td><td>1236</td><td>Archaea>Euryarchaeota>Stenosarchaea_group>Halobacteria>Halobacteriales>Haloarculaceae</td><td>GCF_000023945.1</td><td>Halorhabdus_utahensis</td><td>HUTA_RS09330</td><td>1919731..1923441</td><td>146826</td><td>hypothetical-protein-[Halorhabdus-utahensis]</td></tr>
<tr><td>WP_015789651.1</td><td>Type1.BREX</td><td>DOC+zhth-><-BirA-HTH<-?||?-> [illegible]</td><td>1280</td><td>Archaea>Euryarchaeota>Stenosarchaea_group>Halobacteria>Halobacteriales>Haloarculaceae</td><td>GCF_000023945.1</td><td>Halorhabdus_utahensis</td><td>HUTA_RS09340</td><td>1924016..1927858</td><td>146826</td><td>hypothetical-protein-[Halorhabdus-utahensis]</td></tr>
<tr><td>WP_008308279.1</td><td>Type1.BREX</td><td>ParA-Soj-PloopNTPase-> [illegible]</td><td>1227</td><td>Archaea>Euryarchaeota>Stenosarchaea_group>Halobacteria>Halobacteriales>Haloarculaceae</td><td>GCF_000336615.1</td><td>Haloarcula_amylolytica</td><td>C442_RS04815</td><td>261082..264765</td><td>396317</td><td>hypothetical-protein-[Haloarcula-amylolytica]</td></tr>
<tr><td>WP_008308281.1</td><td>Type1.BREX</td><td>ParA-Soj-PloopNTPase-> [illegible]</td><td>959</td><td>Archaea>Euryarchaeota>Stenosarchaea_group>Halobacteria>Halobacteriales>Haloarculaceae</td><td>GCF_000336615.1</td><td>Haloarcula_amylolytica</td><td>C442_RS04825</td><td>265340..268215</td><td>396317</td><td>hypothetical-protein,-partial-[Haloarcula-amylolytica]</td></tr>
<tr><td>WP_049931199.1</td><td>Type1.BREX</td><td><-PglZ<-N6-METHYLASE<-PIN<-?||?-><- [illegible]</td><td>1272</td><td>Archaea>Euryarchaeota>Stenosarchaea_group>Halobacteria>Halobacteriales>Haloarculaceae</td><td>GCF_000337455.1</td><td>Halosimplex_carlsbadense</td><td>C475_RS21845</td><td>59263..63081</td><td>171164</td><td>hypothetical-protein-[Halosimplex-carlsbadense]</td></tr>
<tr><td>WP_049931237.1</td><td>Type1.BREX</td><td><-PIN<-?||?-><-Peptidase_M1<-REase<- [illegible]</td><td>1239</td><td>Archaea>Euryarchaeota>Stenosarchaea_group>Halobacteria>Halobacteriales>Haloarculaceae</td><td>GCF_000337455.1</td><td>Halosimplex_carlsbadense</td><td>C475_RS21855</td><td>63665..67384</td><td>171164</td><td>hypothetical-protein-[Halosimplex-carlsbadense]</td></tr>
<tr><td>WP_245545399.1</td><td>Type1.BREX</td><td><-SLATT<-SMODS<-SMODS||CopG/RHH-><-?|| [illegible]</td><td>991</td><td>Archaea>Euryarchaeota>Stenosarchaea_group>Halobacteria>Halobacteriales>Haloarculaceae</td><td>GCF_000379085.1</td><td>Halomicrobium_katesii</td><td>HALKA_RS0114555</td><td>2756089..2759064</td><td>437163</td><td>hypothetical-protein-[Halomicrobium-katesii]</td></tr>
<tr><td>WP_018258688.1</td><td>Type1.BREX</td><td><-SMODS||CopG/RHH-><-?||MarR-HTH-><- [illegible]</td><td>1248</td><td>Archaea>Euryarchaeota>Stenosarchaea_group>Halobacteria>Halobacteriales>Haloarculaceae</td><td>GCF_000379085.1</td><td>Halomicrobium_katesii</td><td>HALKA_RS0114565</td><td>2760536..2764282</td><td>437163</td><td>hypothetical-protein-[Halomicrobium-katesii]</td></tr>
<tr><td>WP_008528537.1</td><td>Type1.BREX</td><td><-SbcC<-?<-N6-METHYLASE<- [illegible]</td><td>1279</td><td>Archaea>Euryarchaeota>Stenosarchaea_group>Halobacteria>Halobacteriales>Haloarculaceae</td><td>GCF_000470655.1</td><td>Halorhabdus_tiamatea</td><td>HTIA_RS15355</td><td>321396..325235</td><td>430914</td><td>hypothetical-protein-[Halorhabdus-tiamatea]</td></tr>
<tr><td>WP_008528535.1</td><td>Type1.BREX</td><td><-N6-METHYLASE<-zf-IS66+DDE_Tnp_IS66|| [illegible]</td><td>1226</td><td>Archaea>Euryarchaeota>Stenosarchaea_group>Halobacteria>Halobacteriales>Haloarculaceae</td><td>GCF_000470655.1</td><td>Halorhabdus_tiamatea</td><td>HTIA_RS15365</td><td>325810..329490</td><td>430914</td><td>hypothetical-protein-[Halorhabdus-tiamatea]</td></tr>
<tr><td>WP_058991492.1</td><td>Type1.BREX</td><td><-TUDOR+SF1-SF2-Helicase+iREase+CTD<- [illegible]</td><td>1280</td><td>Archaea>Euryarchaeota>Stenosarchaea_group>Halobacteria>Halobacteriales>Haloarculaceae</td><td>GCF_001485575.1</td><td>Haloarcula_sp._CBA1127</td><td>AV059_RS01020</td><td>247527..251369</td><td>1765055</td><td>hypothetical-protein-[Haloarcula-sp.-CBA1127]</td></tr>
<tr><td>WP_058991494.1</td><td>Type1.BREX</td><td><-PglZ<-?<-N6-METHYLASE<-REase<- [illegible]</td><td>1227</td><td>Archaea>Euryarchaeota>Stenosarchaea_group>Halobacteria>Halobacteriales>Haloarculaceae</td><td>GCF_001485575.1</td><td>Haloarcula_sp._CBA1127</td><td>AV059_RS01030</td><td>251944..255627</td><td>1765055</td><td>hypothetical-protein-[Haloarcula-sp.-CBA1127]</td></tr>
<tr><td>WP_108383896.1</td><td>Type1.BREX</td><td><-SF1-SF2-Helicase-p||?-><-?<-?<-?<- [illegible]</td><td>1236</td><td>Archaea>Euryarchaeota>Stenosarchaea_group>Halobacteria>Halobacteriales>Haloarculaceae</td><td>GCF_003058365.1</td><td>Halococcoides_cellulosivorans</td><td>HARCEL1_RS12430</td><td>2549904..2553614</td><td>1679096</td><td>hypothetical-protein-[Halococcoides-cellulosivorans]</td></tr>
<tr><td>WP_108383898.1</td><td>Type1.BREX</td><td><-SF1-SF2-Helicase-p||?-><-?<-?<-?<- [illegible]</td><td>1280</td><td>Archaea>Euryarchaeota>Stenosarchaea_group>Halobacteria>Halobacteriales>Haloarculaceae</td><td>GCF_003058365.1</td><td>Halococcoides_cellulosivorans</td><td>HARCEL1_RS12440</td><td>2554189..2558031</td><td>1679096</td><td>hypothetical-protein-[Halococcoides-cellulosivorans]</td></tr>
<tr><td>WP_129754743.1</td><td>Type1.BREX</td><td>ParA-Soj-PloopNTPase-> [illegible]</td><td>1227</td><td>Archaea>Euryarchaeota>Stenosarchaea_group>Halobacteria>Halobacteriales>Haloarculaceae</td><td>GCF_004150385.1</td><td>Haloarcula_hispanica</td><td>ELS20_RS00155</td><td>36123..39806</td><td>51589</td><td>hypothetical-protein-[Haloarcula-hispanica]</td></tr>
<tr><td>WP_136689168.1</td><td>Type1.BREX</td><td><-35exo<-35exo<-MNS+REase<-?<-ARG||?-> [illegible]</td><td>1249</td><td>Archaea>Euryarchaeota>Stenosarchaea_group>Halobacteria>Halobacteriales>Haloarculaceae</td><td>GCF_004944975.1</td><td>Halorhabdus_amylolytica</td><td>E3374_RS11205</td><td>84563..88312</td><td>2559573</td><td>hypothetical-protein-[Halorhabdus-amylolytica]</td></tr>
<tr><td>WP_136689170.1</td><td>Type1.BREX</td><td><-MNS+REase<-?<-ARG||?->?-> [illegible]</td><td>1279</td><td>Archaea>Euryarchaeota>Stenosarchaea_group>Halobacteria>Halobacteriales>Haloarculaceae</td><td>GCF_004944975.1</td><td>Halorhabdus_amylolytica</td><td>E3374_RS11215</td><td>88926..92765</td><td>2559573</td><td>hypothetical-protein-[Halorhabdus-amylolytica]</td></tr>
<tr><td>WP_152120782.1</td><td>Type1.BREX</td><td>BrxB-BrxF-iSTAND->DUF6079*-> [illegible]</td><td>1215</td><td>Archaea>Euryarchaeota>Stenosarchaea_group>Halobacteria>Halobacteriales>Haloarculaceae</td><td>GCF_009184545.1</td><td>Halosegnis_rubeus</td><td>DMP03_RS11345</td><td>583..4230</td><td>2212850</td><td>BREX-system-P-loop-protein-BrxC-[Halosegnis-rubeus]</td></tr>
<tr><td>WP_188853474.1</td><td>Type1.BREX</td><td><-ABC-ATPase+SbcC+VirB11+ABC-ATPase+ [illegible]</td><td>1227</td><td>Archaea>Euryarchaeota>Stenosarchaea_group>Halobacteria>Halobacteriales>Haloarculaceae</td><td>GCF_014647095.1</td><td>Haloarcula_salaria</td><td>IEY10_RS16005</td><td>45657..49340</td><td>575195</td><td>hypothetical-protein-[Haloarcula-salaria]</td></tr>
<tr><td>WP_188853476.1</td><td>Type1.BREX</td><td><-Calcineurin<-?<-?||?->?->?-> [illegible]</td><td>1280</td><td>Archaea>Euryarchaeota>Stenosarchaea_group>Halobacteria>Halobacteriales>Haloarculaceae</td><td>GCF_014647095.1</td><td>Haloarcula_salaria</td><td>IEY10_RS16015</td><td>49915..53757</td><td>575195</td><td>hypothetical-protein-[Haloarcula-salaria]</td></tr>
<tr><td>WP_188997387.1</td><td>Type1.BREX</td><td>MarR-HTH-><-SF1-SF2-Helicase+REase<- [illegible]</td><td>1251</td><td>Archaea>Euryarchaeota>Stenosarchaea_group>Halobacteria>Halobacteriales>Haloarculaceae</td><td>GCF_014647235.1</td><td>Halomicroarcula_pellucida</td><td>IEY28_RS11100</td><td>280416..284171</td><td>1427151</td><td>hypothetical-protein-[Halomicroarcula-pellucida]</td></tr>
<tr><td>WP_188997391.1</td><td>Type1.BREX</td><td>MarR-HTH-><-SF1-SF2-Helicase+REase<- [illegible]</td><td>1288</td><td>Archaea>Euryarchaeota>Stenosarchaea_group>Halobacteria>Halobacteriales>Haloarculaceae</td><td>GCF_014647235.1</td><td>Halomicroarcula_pellucida</td><td>IEY28_RS11110</td><td>284755..288621</td><td>1427151</td><td>hypothetical-protein-[Halomicroarcula-pellucida]</td></tr>
<tr><td>WP_229112049.1</td><td>Type1.BREX</td><td><-TUDOR+SF1-SF2-Helicase+iREase+CTD<- [illegible]</td><td>1278</td><td>Archaea>Euryarchaeota>Stenosarchaea_group>Halobacteria>Halobacteriales>Haloarculaceae</td><td>GCF_017094525.1</td><td>Halapricum_desulfuricans</td><td>HSR122_RS06875</td><td>1337225..1341061</td><td>2841257</td><td>hypothetical-protein-[Halapricum-desulfuricans]</td></tr>
<tr><td>WP_229112051.1</td><td>Type1.BREX</td><td><-N6-METHYLASE-p<-?<-DDE_Tnp_1<- [illegible]</td><td>1248</td><td>Archaea>Euryarchaeota>Stenosarchaea_group>Halobacteria>Halobacteriales>Haloarculaceae</td><td>GCF_017094525.1</td><td>Halapricum_desulfuricans</td><td>HSR122_RS06885</td><td>1341681..1345427</td><td>2841257</td><td>hypothetical-protein-[Halapricum-desulfuricans]</td></tr>
<tr><td>WP_129754745.1</td><td>Type1.BREX</td><td>ParA-Soj-PloopNTPase-> [illegible]</td><td>1280</td><td>Archaea>Euryarchaeota>Stenosarchaea_group>Halobacteria>Halobacteriales>Haloarculaceae</td><td>GCF_022807515.1</td><td>Haloarcula_hispanica</td><td>ELS20_RS00165</td><td>40381..44223</td><td>51589</td><td>hypothetical-protein-[Haloarcula-hispanica]</td></tr>
<tr><td>WP_274325353.1</td><td>Type1.BREX</td><td><-PIN<-?||?-><-Peptidase_M1<-REase<- [illegible]</td><td>1126</td><td>Archaea>Euryarchaeota>Stenosarchaea_group>Halobacteria>Halobacteriales>Haloarculaceae</td><td>GCF_028747785.1</td><td>Halosimplex_sp._XZYJT29</td><td>PSC72_RS07975</td><td>1604962..1608342</td><td>3026162</td><td>hypothetical-protein-[Halosimplex-sp.-XZYJT29]</td></tr>
<tr><td>WP_135666499.1</td><td>Type1.BREX</td><td><-PglZ<-RelE<-?<-N6-METHYLASE<- [illegible]</td><td>1277</td><td>Archaea>Euryarchaeota>Stenosarchaea_group>Halobacteria>Halobacteriales>Haloarculaceae</td><td>GCF_900880625.1</td><td>Halorhabdus_rudnickae</td><td>BN2694_RS13550</td><td>37861..41694</td><td>1775544</td><td>hypothetical-protein-[Halorhabdus-rudnickae]</td></tr>
<tr><td>WP_135666504.1</td><td>Type1.BREX</td><td><-N6-METHYLASE<-TBP+TBP||TBP+TBP->?-> [illegible]</td><td>1248</td><td>Archaea>Euryarchaeota>Stenosarchaea_group>Halobacteria>Halobacteriales>Haloarculaceae</td><td>GCF_900880625.1</td><td>Halorhabdus_rudnickae</td><td>BN2694_RS13560</td><td>42308..46054</td><td>1775544</td><td>hypothetical-protein-[Halorhabdus-rudnickae]</td></tr>
<tr><td>WP_049892750.1</td><td>Type1.BREX</td><td><-N6-METHYLASE||OLD-ABC+TOPRIM-><- [illegible]</td><td>1280</td><td>Archaea>Euryarchaeota>Stenosarchaea_group>Halobacteria>Halobacteriales>Halobacteriaceae</td><td>GCF_000069025.1</td><td>Halobacterium_salinarum</td><td>OE_RS13050</td><td>230465..234307</td><td>2242</td><td>hypothetical-protein-[Halobacterium-salinarum]</td></tr>
<tr><td>WP_049892751.1</td><td>Type1.BREX</td><td><-DDE_Tnp_1||?-><-?<- [illegible]</td><td>1227</td><td>Archaea>Euryarchaeota>Stenosarchaea_group>Halobacteria>Halobacteriales>Halobacteriaceae</td><td>GCF_000069025.1</td><td>Halobacterium_salinarum</td><td>OE_RS13060</td><td>234882..238565</td><td>2242</td><td>hypothetical-protein-[Halobacterium-salinarum]</td></tr>
<tr><td>WP_123076504.1</td><td>Type1.BREX</td><td><-TUDOR+SF1-SF2-Helicase+iREase+CTD<- [illegible]</td><td>1277</td><td>Archaea>Euryarchaeota>Stenosarchaea_group>Halobacteria>Halobacteriales>Halobacteriaceae</td><td>GCF_003721175.2</td><td>Halarchaeum_sp._CBA1220</td><td>EFA46_RS09075</td><td>1680640..1684473</td><td>1853682</td><td>hypothetical-protein-[Halarchaeum-sp.-CBA1220]</td></tr>
<tr><td>WP_123076500.1</td><td>Type1.BREX</td><td><-PglZ<-N6-METHYLASE||?->?-><-?||?-> [illegible]</td><td>1248</td><td>Archaea>Euryarchaeota>Stenosarchaea_group>Halobacteria>Halobacteriales>Halobacteriaceae</td><td>GCF_003721175.2</td><td>Halarchaeum_sp._CBA1220</td><td>EFA46_RS09085</td><td>1685087..1688833</td><td>1853682</td><td>hypothetical-protein-[Halarchaeum-sp.-CBA1220]</td></tr>
<tr><td>WP_135823790.1</td><td>Type1.BREX</td><td>N6-METHYLASE->?-><-DUF6079*<- [illegible]</td><td>1264</td><td>Archaea>Euryarchaeota>Stenosarchaea_group>Halobacteria>Halobacteriales>Halobacteriaceae</td><td>GCF_004765785.1</td><td>Halorussus_ruber</td><td>EPL01_RS03100</td><td>202199..205993</td><td>1126238</td><td>hypothetical-protein-[Halorussus-ruber]</td></tr>
<tr><td>WP_135823792.1</td><td>Type1.BREX</td><td>N6-METHYLASE->?-><-DUF6079<- [illegible]</td><td>1304</td><td>Archaea>Euryarchaeota>Stenosarchaea_group>Halobacteria>Halobacteriales>Halobacteriaceae</td><td>GCF_004765785.1</td><td>Halorussus_ruber</td><td>EPL01_RS03110</td><td>206652..210566</td><td>1126238</td><td>hypothetical-protein-[Halorussus-ruber]</td></tr>
<tr><td>WP_136361239.1</td><td>Type1.BREX</td><td><-HTH+BirA-HTH<-?<-DDE_Tnp_1|| [illegible]</td><td>1233</td><td>Archaea>Euryarchaeota>Stenosarchaea_group>Halobacteria>Halobacteriales>Halobacteriaceae</td><td>GCF_004799605.1</td><td>Halobacterium_salinarum</td><td>HBSAL_RS04975</td><td>932815..936516</td><td>2242</td><td>hypothetical-protein-[Halobacterium-salinarum]</td></tr>
<tr><td>WP_136361241.1</td><td>Type1.BREX</td><td><-DDE_Tnp_1||Classical-AAA->?-><- [illegible]</td><td>1231</td><td>Archaea>Euryarchaeota>Stenosarchaea_group>Halobacteria>Halobacteriales>Halobacteriaceae</td><td>GCF_004799605.1</td><td>Halobacterium_salinarum</td><td>HBSAL_RS04985</td><td>937132..940827</td><td>2242</td><td>hypothetical-protein-[Halobacterium-salinarum]</td></tr>
<tr><td>WP_233265034.1</td><td>Type1.BREX</td><td><-NTP_transf||?->?-><-PIWI<- [illegible]</td><td>962</td><td>Archaea>Euryarchaeota>Stenosarchaea_group>Halobacteria>Halobacteriales>Halobacteriaceae</td><td>GCF_009791395.1</td><td>Halomarina_oriensis</td><td>GQS65_RS04705</td><td>84531..87418</td><td>671145</td><td>hypothetical-protein,-partial-[Halomarina-oriensis]</td></tr>
<tr><td>WP_205252856.1</td><td>Type1.BREX</td><td><-Exonuc_V_gamma+REase<-TUDOR+ [illegible]</td><td>1280</td><td>Archaea>Euryarchaeota>Stenosarchaea_group>Halobacteria>Halobacteriales>Halobacteriaceae</td><td>GCF_016917835.2</td><td>Halobacterium_sp._BOL4-2</td><td>JRZ79_RS24035</td><td>52137..55979</td><td>2810537</td><td>hypothetical-protein-[Halobacterium-sp.-BOL4-2]</td></tr>
<tr><td>WP_205252782.1</td><td>Type1.BREX</td><td><-HEPN<-PglZ<-?<-N6-METHYLASE||?->?-> [illegible]</td><td>1227</td><td>Archaea>Euryarchaeota>Stenosarchaea_group>Halobacteria>Halobacteriales>Halobacteriaceae</td><td>GCF_016917835.2</td><td>Halobacterium_sp._BOL4-2</td><td>JRZ79_RS24045</td><td>56554..60237</td><td>2810537</td><td>hypothetical-protein-[Halobacterium-sp.-BOL4-2]</td></tr>
<tr><td>WP_227231659.1</td><td>Type1.BREX</td><td>SF1-SF2-Helicase+REase-> [illegible]</td><td>1231</td><td>Archaea>Euryarchaeota>Stenosarchaea_group>Halobacteria>Halobacteriales>Halobacteriaceae</td><td>GCF_020567425.1</td><td>Salinirubrum_litoreum</td><td>LI337_RS19795</td><td>90015..93710</td><td>1126234</td><td>hypothetical-protein-[Salinirubrum-litoreum]</td></tr>
<tr><td>WP_227231661.1</td><td>Type1.BREX</td><td>SF1-SF2-Helicase+REase-> [illegible]</td><td>1054</td><td>Archaea>Euryarchaeota>Stenosarchaea_group>Halobacteria>Halobacteriales>Halobacteriaceae</td><td>GCF_020567425.1</td><td>Salinirubrum_litoreum</td><td>LI337_RS19805</td><td>94326..97490</td><td>1126234</td><td>hypothetical-protein,-partial-[Salinirubrum-litoreum]</td></tr>
<tr><td>WP_231187339.1</td><td>Type1.BREX</td><td>BrxA-DNA-binding->DUF6079*-> [illegible]</td><td>1228</td><td>Archaea>Euryarchaeota>Stenosarchaea_group>Halobacteria>Halobacteriales>Halobacteriaceae</td><td>GCF_021083305.1</td><td>Haladaptatus_sp._DYF46</td><td>LJF34_RS20420</td><td>52158..55844</td><td>2886041</td><td>hypothetical-protein-[Haladaptatus-sp.-DYF46]</td></tr>
<tr><td>WP_231187343.1</td><td>Type1.BREX</td><td>BrxA-DNA-binding->DUF6079-> [illegible]</td><td>1283</td><td>Archaea>Euryarchaeota>Stenosarchaea_group>Halobacteria>Halobacteriales>Halobacteriaceae</td><td>GCF_021083305.1</td><td>Haladaptatus_sp._DYF46</td><td>LJF34_RS20430</td><td>56419..60270</td><td>2886041</td><td>hypothetical-protein-[Haladaptatus-sp.-DYF46]</td></tr>
<tr><td>WP_235167905.1</td><td>Type1.BREX</td><td><-Phage_int_SAM_5<-?<- [illegible]</td><td>1248</td><td>Archaea>Euryarchaeota>Stenosarchaea_group>Halobacteria>Halobacteriales>Halobacteriaceae</td><td>GCF_021503975.1</td><td>Halobacterium_salinarum</td><td>KI372_RS02110</td><td>10921..14667</td><td>2242</td><td>hypothetical-protein-[Halobacterium-salinarum]</td></tr>
<tr><td>WP_235262714.1</td><td>Type1.BREX</td><td><-TUDOR+SF1-SF2-Helicase+iREase+CTD<- [illegible]</td><td>1279</td><td>Archaea>Euryarchaeota>Stenosarchaea_group>Halobacteria>Halobacteriales>Halobacteriaceae</td><td>GCF_021503975.1</td><td>Halobacterium_salinarum</td><td>KVP02_RS02090</td><td>31387..35226</td><td>2242</td><td>hypothetical-protein-[Halobacterium-salinarum]</td></tr>
<tr><td>WP_267638768.1</td><td>Type1.BREX</td><td><-HEPN<-Peptidase_M1||?->?-> [illegible]</td><td>1251</td><td>Archaea>Euryarchaeota>Stenosarchaea_group>Halobacteria>Halobacteriales>Halobacteriaceae</td><td>GCF_026586665.1</td><td>Halovenus_rubra</td><td>N5098_RS15400</td><td>10573..14328</td><td>869890</td><td>hypothetical-protein-[Halovenus-rubra]</td></tr>
<tr><td>WP_121821034.1</td><td>Type1.BREX</td><td><-Calcineurin<-?||BrxA-DNA-binding-> [illegible]</td><td>1237</td><td>Archaea>Euryarchaeota>Stenosarchaea_group>Halobacteria>Halobacteriales>Natronoarchaeaceae</td><td>GCF_003675855.1</td><td>Halostella_salina</td><td>D8896_RS05250</td><td>383144..386857</td><td>1547897</td><td>hypothetical-protein-[Halostella-salina]</td></tr>
<tr><td>WP_121821036.1</td><td>Type1.BREX</td><td><-Calcineurin<-?||BrxA-DNA-binding-> [illegible]</td><td>1280</td><td>Archaea>Euryarchaeota>Stenosarchaea_group>Halobacteria>Halobacteriales>Natronoarchaeaceae</td><td>GCF_003675855.1</td><td>Halostella_salina</td><td>D8896_RS05260</td><td>387432..391274</td><td>1547897</td><td>hypothetical-protein-[Halostella-salina]</td></tr>
<tr><td>WP_008387699.1</td><td>Type1.BREX</td><td><-N6-METHYLASE<-REase<-?<- [illegible]</td><td>1270</td><td>Archaea>Euryarchaeota>Stenosarchaea_group>Halobacteria>Haloferacales>Haloferacaceae</td><td>GCF_000337095.1</td><td>Halogeometricum_pallidum</td><td>C474_RS13405</td><td>6094..9906</td><td>411361</td><td>hypothetical-protein-[Halogeometricum-pallidum]</td></tr>
<tr><td>WP_008387702.1</td><td>Type1.BREX</td><td><-N6-METHYLASE<-REase<-?<- [illegible]</td><td>1243</td><td>Archaea>Euryarchaeota>Stenosarchaea_group>Halobacteria>Haloferacales>Haloferacaceae</td><td>GCF_000337095.1</td><td>Halogeometricum_pallidum</td><td>C474_RS13415</td><td>10487..14218</td><td>411361</td><td>hypothetical-protein-[Halogeometricum-pallidum]</td></tr>
<tr><td>WP_049935523.1</td><td>Type1.BREX</td><td>ABC-ATPase+SbcC->?->?->?->DTXR-HTH-> [illegible]</td><td>1271</td><td>Archaea>Euryarchaeota>Stenosarchaea_group>Halobacteria>Haloferacales>Haloferacaceae</td><td>GCF_000427685.1</td><td>Haloplanus_natans</td><td>HALNA_RS06110</td><td>731546..735361</td><td>376171</td><td>hypothetical-protein-[Haloplanus-natans]</td></tr>
<tr><td>WP_049935526.1</td><td>Type1.BREX</td><td>DTXR-HTH->tRNA-><-?<-PglZ<- [illegible]</td><td>1253</td><td>Archaea>Euryarchaeota>Stenosarchaea_group>Halobacteria>Haloferacales>Haloferacaceae</td><td>GCF_000427685.1</td><td>Haloplanus_natans</td><td>HALNA_RS06120</td><td>735945..739706</td><td>376171</td><td>hypothetical-protein-[Haloplanus-natans]</td></tr>
<tr><td>WP_122106903.1</td><td>Type1.BREX</td><td><-DDE_Tnp_1<-?<-?||MarR-HTH-><-?<- [illegible]</td><td>1248</td><td>Archaea>Euryarchaeota>Stenosarchaea_group>Halobacteria>Haloferacales>Haloferacaceae</td><td>GCF_003697845.1</td><td>Halobellus_captivus</td><td>EAO78_RS16655</td><td>16343..20089</td><td>2592614</td><td>hypothetical-protein-[Halobellus-captivus]</td></tr>
<tr><td>WP_122106905.1</td><td>Type1.BREX</td><td>MarR-HTH-><-?<-ParA-Soj-PloopNTPase<- [illegible]</td><td>1279</td><td>Archaea>Euryarchaeota>Stenosarchaea_group>Halobacteria>Haloferacales>Haloferacaceae</td><td>GCF_003697845.1</td><td>Halobellus_captivus</td><td>EAO78_RS16665</td><td>20703..24542</td><td>2592614</td><td>hypothetical-protein-[Halobellus-captivus]</td></tr>
<tr><td>WP_248897121.1</td><td>Type1.BREX</td><td>BirA-HTH->?-><-?<-?||?-> [illegible]</td><td>1247</td><td>Archaea>Euryarchaeota>Stenosarchaea_group>Halobacteria>Haloferacales>Haloferacaceae</td><td>GCF_023276355.1</td><td>Haloplanus_sp._XH21</td><td>MXB53_RS09475</td><td>1803325..1807068</td><td>2934938</td><td>hypothetical-protein-[Haloplanus-sp.-XH21]</td></tr>
<tr><td>WP_248897123.1</td><td>Type1.BREX</td><td>BirA-HTH->?-><-?<-?||?-> [illegible]</td><td>1277</td><td>Archaea>Euryarchaeota>Stenosarchaea_group>Halobacteria>Haloferacales>Haloferacaceae</td><td>GCF_023276355.1</td><td>Haloplanus_sp._XH21</td><td>MXB53_RS09485</td><td>1807682..1811515</td><td>2934938</td><td>hypothetical-protein-[Haloplanus-sp.-XH21]</td></tr>
<tr><td>WP_256422112.1</td><td>Type1.BREX</td><td><-N6-METHYLASE||?-><-?<-?<- [illegible]</td><td>1248</td><td>Archaea>Euryarchaeota>Stenosarchaea_group>Halobacteria>Haloferacales>Haloferacaceae</td><td>GCF_024494595.1</td><td>Halobellus_rarus</td><td>NO876_RS13095</td><td>193250..196996</td><td>1126237</td><td>hypothetical-protein-[Halobellus-rarus]</td></tr>
<tr><td>ELZ39021.1</td><td>Type1.BREX</td><td>OrfB_IS605+OrfB_Zn_ribbon->DDE_Tnp_1-> [illegible]</td><td>1280</td><td>Archaea>Euryarchaeota>Stenosarchaea_group>Halobacteria>Haloferacales>Halorubraceae</td><td>GCA_000336875.1</td><td>Halorubrum_californiense_DSM_19288</td><td>C463_17695</td><td>23296..27138</td><td>1227465</td><td>hypothetical-protein-C463_17695-[Halorubrum-californiense-DSM-19288]</td></tr>
<tr><td>PHQ38249.1</td><td>Type1.BREX</td><td>SF1-SF2-Helicase->SF1-SF2-Helicase+ [illegible]</td><td>1227</td><td>Archaea>Euryarchaeota>Stenosarchaea_group>Halobacteria>Haloferacales>Halorubraceae</td><td>GCA_002727125.1</td><td>Halorubrum_persicum</td><td>DJ69_12930</td><td>85034..88717</td><td>1383844</td><td>hypothetical-protein-DJ69_12930-[Halorubrum-persicum]</td></tr>
<tr><td>SFP79187.1</td><td>Type1.BREX</td><td><-PglZ||ZnR+REase->OLD-ABC+TOPRIM-><- [illegible]</td><td>1232</td><td>Archaea>Euryarchaeota>Stenosarchaea_group>Halobacteria>Haloferacales>Halorubraceae</td><td>GCA_900115675.1</td><td>Halolamina_pelagica</td><td>SAMN05216277_10870</td><td>87547..91245</td><td>699431</td><td>hypothetical-protein-SAMN05216277_10870-[Halolamina-pelagica]</td></tr>
<tr><td>WP_015911586.1</td><td>Type1.BREX</td><td><-N6-METHYLASE-p||MazE_antitoxin-> [illegible]</td><td>1270</td><td>Archaea>Euryarchaeota>Stenosarchaea_group>Halobacteria>Haloferacales>Halorubraceae</td><td>GCF_000022205.1</td><td>Halorubrum_lacusprofundi</td><td>CF318_RS14700</td><td>1993..5805</td><td>2247</td><td>hypothetical-protein-[Halorubrum-lacusprofundi]</td></tr>
<tr><td>WP_015911588.1</td><td>Type1.BREX</td><td><-TUDOR+SF1-SF2-Helicase+iREase+CTD<- [illegible]</td><td>1239</td><td>Archaea>Euryarchaeota>Stenosarchaea_group>Halobacteria>Haloferacales>Halorubraceae</td><td>GCF_000022205.1</td><td>Halorubrum_lacusprofundi</td><td>CF318_RS14710</td><td>6389..10108</td><td>2247</td><td>hypothetical-protein-[Halorubrum-lacusprofundi]</td></tr>
<tr><td>WP_008446367.1</td><td>Type1.BREX</td><td>OrfB_IS605+OrfB_Zn_ribbon->|| [illegible]</td><td>1227</td><td>Archaea>Euryarchaeota>Stenosarchaea_group>Halobacteria>Haloferacales>Halorubraceae</td><td>GCF_000336875.1</td><td>Halorubrum_californiense</td><td>C463_RS17190</td><td>19038..22721</td><td>416585</td><td>hypothetical-protein-[Halorubrum-californiense]</td></tr>
<tr><td>WP_239638934.1</td><td>Type1.BREX</td><td>OrfB_IS605+OrfB_Zn_ribbon->|| [illegible]</td><td>1235</td><td>Archaea>Euryarchaeota>Stenosarchaea_group>Halobacteria>Haloferacales>Halorubraceae</td><td>GCF_000336875.1</td><td>Halorubrum_californiense</td><td>C463_RS17200</td><td>23431..27138</td><td>416585</td><td>hypothetical-protein-[Halorubrum-californiense]</td></tr>
<tr><td>WP_235271349.1</td><td>Type1.BREX</td><td><-Phage_integrase<- [illegible]</td><td>1111</td><td>Archaea>Euryarchaeota>Stenosarchaea_group>Halobacteria>Haloferacales>Halorubraceae</td><td>GCF_000700025.1</td><td>Halorubrum_saccharovorum</td><td>FK85_RS03110</td><td>45328..48663</td><td>2248</td><td>hypothetical-protein-[Halorubrum-saccharovorum]</td></tr>
<tr><td>WP_235271352.1</td><td>Type1.BREX</td><td><-PglZ<-PglZ<-N6-METHYLASE||?->?-><- [illegible]</td><td>692</td><td>Archaea>Euryarchaeota>Stenosarchaea_group>Halobacteria>Haloferacales>Halorubraceae</td><td>GCF_000700025.1</td><td>Halorubrum_saccharovorum</td><td>FK85_RS18475</td><td>49771..51849</td><td>2248</td><td>hypothetical-protein-[Halorubrum-saccharovorum]</td></tr>
<tr><td>WP_094582515.1</td><td>Type1.BREX</td><td><-N6-METHYLASE<-DUF6079*<- [illegible]</td><td>1277</td><td>Archaea>Euryarchaeota>Stenosarchaea_group>Halobacteria>Haloferacales>Halorubraceae</td><td>GCF_002252835.1</td><td>Halorubrum_ezzemoulense</td><td>DJ78_RS02240</td><td>3110..6943</td><td>337243</td><td>hypothetical-protein-[Halorubrum-ezzemoulense]</td></tr>
<tr><td>WP_094582519.1</td><td>Type1.BREX</td><td><-N6-METHYLASE<-DUF6079<- [illegible]</td><td>1226</td><td>Archaea>Euryarchaeota>Stenosarchaea_group>Halobacteria>Haloferacales>Halorubraceae</td><td>GCF_002252835.1</td><td>Halorubrum_ezzemoulense</td><td>DJ78_RS02250</td><td>7518..11198</td><td>337243</td><td>hypothetical-protein-[Halorubrum-ezzemoulense]</td></tr>
<tr><td>WP_096396418.1</td><td>Type1.BREX</td><td><-ParA-Soj-PloopNTPase<-PADR-HTH<-?|| [illegible]</td><td>1227</td><td>Archaea>Euryarchaeota>Stenosarchaea_group>Halobacteria>Haloferacales>Halorubraceae</td><td>GCF_002355655.1</td><td>Halorubrum_trapanicum</td><td>CPZ01_RS14385</td><td>137266..140949</td><td>29284</td><td>hypothetical-protein-[Halorubrum-trapanicum]</td></tr>
<tr><td>WP_099288831.1</td><td>Type1.BREX</td><td><-GDResolvase||?->?->?->?-> [illegible]</td><td>1248</td><td>Archaea>Euryarchaeota>Stenosarchaea_group>Halobacteria>Haloferacales>Halorubraceae</td><td>GCF_002727095.1</td><td>Halorubrum_sp._C191</td><td>Z052_RS03555</td><td>9147..12893</td><td>1383842</td><td>hypothetical-protein-[Halorubrum-sp.-C191]</td></tr>
<tr><td>WP_099288832.1</td><td>Type1.BREX</td><td><-GDResolvase||?->?->?->?-> [illegible]</td><td>1280</td><td>Archaea>Euryarchaeota>Stenosarchaea_group>Halobacteria>Haloferacales>Halorubraceae</td><td>GCF_002727095.1</td><td>Halorubrum_sp._C191</td><td>Z052_RS03565</td><td>13507..17349</td><td>1383842</td><td>hypothetical-protein-[Halorubrum-sp.-C191]</td></tr>
<tr><td>WP_245851676.1</td><td>Type1.BREX</td><td>SF1-SF2-Helicase->SF1-SF2-Helicase+ [illegible]</td><td>1139</td><td>Archaea>Euryarchaeota>Stenosarchaea_group>Halobacteria>Haloferacales>Halorubraceae</td><td>GCF_002727125.1</td><td>Halorubrum_persicum</td><td>DJ69_RS13035</td><td>85298..88717</td><td>1383844</td><td>hypothetical-protein-[Halorubrum-persicum]</td></tr>
<tr><td>WP_099255996.1</td><td>Type1.BREX</td><td>HTH_28->NTP_transf_5-><-?||?-><-?||<- [illegible]</td><td>1278</td><td>Archaea>Euryarchaeota>Stenosarchaea_group>Halobacteria>Haloferacales>Halorubraceae</td><td>GCF_002727125.1</td><td>Halorubrum_persicum</td><td>DJ69_RS13045</td><td>89292..93128</td><td>1383844</td><td>hypothetical-protein-[Halorubrum-persicum]</td></tr>
<tr><td>WP_112078641.1</td><td>Type1.BREX</td><td>ABC-ATPase+SbcC->?->BPD_transp_2->?-> [illegible]</td><td>1251</td><td>Archaea>Euryarchaeota>Stenosarchaea_group>Halobacteria>Haloferacales>Halorubraceae</td><td>GCF_003287355.1</td><td>Halorubrum_sp._48-1-W</td><td>DQW50_RS07315</td><td>54126..57881</td><td>2249761</td><td>hypothetical-protein-[Halorubrum-sp.-48-1-W]</td></tr>
<tr><td>WP_112078643.1</td><td>Type1.BREX</td><td>ABC-ATPase+SbcC->?->BPD_transp_2->?-> [illegible]</td><td>1277</td><td>Archaea>Euryarchaeota>Stenosarchaea_group>Halobacteria>Haloferacales>Halorubraceae</td><td>GCF_003287355.1</td><td>Halorubrum_sp._48-1-W</td><td>DQW50_RS07325</td><td>58459..62292</td><td>2249761</td><td>hypothetical-protein-[Halorubrum-sp.-48-1-W]</td></tr>
<tr><td>WP_137716102.1</td><td>Type1.BREX</td><td>Phage_integrase-><-Phage_integrase|| [illegible]</td><td>1248</td><td>Archaea>Euryarchaeota>Stenosarchaea_group>Halobacteria>Haloferacales>Halorubraceae</td><td>GCF_005435055.1</td><td>Halorubrum_sp._SS7</td><td>EXE44_RS03240</td><td>153853..157599</td><td>2518119</td><td>hypothetical-protein-[Halorubrum-sp.-SS7]</td></tr>
<tr><td>WP_137715939.1</td><td>Type1.BREX</td><td><-Phage_integrase||?->HD->?->?-><-?|| [illegible]</td><td>1277</td><td>Archaea>Euryarchaeota>Stenosarchaea_group>Halobacteria>Haloferacales>Halorubraceae</td><td>GCF_005435055.1</td><td>Halorubrum_sp._SS7</td><td>EXE44_RS03250</td><td>158213..162046</td><td>2518119</td><td>hypothetical-protein-[Halorubrum-sp.-SS7]</td></tr>
<tr><td>WP_137705565.1</td><td>Type1.BREX</td><td><-ParA-Soj-PloopNTPase||?-><-PADR-HTH<- [illegible]</td><td>1227</td><td>Archaea>Euryarchaeota>Stenosarchaea_group>Halobacteria>Haloferacales>Halorubraceae</td><td>GCF_005435115.1</td><td>Halorubrum_sp._ARQ200</td><td>EXE50_RS16210</td><td>7684..11367</td><td>1855872</td><td>hypothetical-protein-[Halorubrum-sp.-ARQ200]</td></tr>
<tr><td>WP_137705567.1</td><td>Type1.BREX</td><td><-PADR-HTH<-?<-?<-?<-?|| [illegible]</td><td>1280</td><td>Archaea>Euryarchaeota>Stenosarchaea_group>Halobacteria>Haloferacales>Halorubraceae</td><td>GCF_005435115.1</td><td>Halorubrum_sp._ARQ200</td><td>EXE50_RS16220</td><td>11942..15784</td><td>1855872</td><td>hypothetical-protein-[Halorubrum-sp.-ARQ200]</td></tr>
<tr><td>WP_210114021.1</td><td>Type1.BREX</td><td><-N6-METHYLASE-p||BrxA-DNA-binding-> [illegible]</td><td>1233</td><td>Archaea>Euryarchaeota>Stenosarchaea_group>Halobacteria>Haloferacales>Halorubraceae</td><td>GCF_017873555.1</td><td>Halorubrum_trapanicum</td><td>J2744_RS15020</td><td>2668..6369</td><td>29284</td><td>hypothetical-protein-[Halorubrum-trapanicum]</td></tr>
<tr><td>WP_210114022.1</td><td>Type1.BREX</td><td><-N6-METHYLASE-p||BrxA-DNA-binding-> [illegible]</td><td>1230</td><td>Archaea>Euryarchaeota>Stenosarchaea_group>Halobacteria>Haloferacales>Halorubraceae</td><td>GCF_017873555.1</td><td>Halorubrum_trapanicum</td><td>J2744_RS15030</td><td>6985..10677</td><td>29284</td><td>hypothetical-protein-[Halorubrum-trapanicum]</td></tr>
<tr><td>WP_271958997.1</td><td>Type1.BREX</td><td>TOPRIM->?-><-SF1-SF2-Helicase+REase<- [illegible]</td><td>1226</td><td>Archaea>Euryarchaeota>Stenosarchaea_group>Halobacteria>Haloferacales>Halorubraceae</td><td>GCF_028211185.1</td><td>Halorubrum_ezzemoulense</td><td>PM020_RS15300</td><td>39362..43042</td><td>337243</td><td>hypothetical-protein-[Halorubrum-ezzemoulense]</td></tr>
<tr><td>WP_271952852.1</td><td>Type1.BREX</td><td><-DUF6079<-BrxB-BrxF-iSTAND<- [illegible]</td><td>1248</td><td>Archaea>Euryarchaeota>Stenosarchaea_group>Halobacteria>Haloferacales>Halorubraceae</td><td>GCF_028211255.1</td><td>Halorubrum_ezzemoulense</td><td>PM030_RS17190</td><td>6805..10551</td><td>337243</td><td>hypothetical-protein-[Halorubrum-ezzemoulense]</td></tr>
<tr><td>WP_271959521.1</td><td>Type1.BREX</td><td><-N6-METHYLASE||BrxA-DNA-binding-> [illegible]</td><td>1233</td><td>Archaea>Euryarchaeota>Stenosarchaea_group>Halobacteria>Haloferacales>Halorubraceae</td><td>GCF_028211295.1</td><td>Halorubrum_ezzemoulense</td><td>PM026_RS14195</td><td>3267..6968</td><td>337243</td><td>hypothetical-protein-[Halorubrum-ezzemoulense]</td></tr>
<tr><td>WP_271959526.1</td><td>Type1.BREX</td><td><-N6-METHYLASE||BrxA-DNA-binding-> [illegible]</td><td>1230</td><td>Archaea>Euryarchaeota>Stenosarchaea_group>Halobacteria>Haloferacales>Halorubraceae</td><td>GCF_028211295.1</td><td>Halorubrum_ezzemoulense</td><td>PM026_RS14205</td><td>7584..11276</td><td>337243</td><td>hypothetical-protein-[Halorubrum-ezzemoulense]</td></tr>
<tr><td>WP_074878712.1</td><td>Type1.BREX</td><td><-TUDOR+SF1-SF2-Helicase+iREase+CTD<- [illegible]</td><td>1279</td><td>Archaea>Euryarchaeota>Stenosarchaea_group>Halobacteria>Haloferacales>Halorubraceae</td><td>GCF_900115675.1</td><td>Halolamina</td><td>BMZ83_RS11295</td><td>83133..86972</td><td>1075397</td><td>MULTISPECIES:-hypothetical-protein-[Halolamina]</td></tr>
<tr><td>WP_074878715.1</td><td>Type1.BREX</td><td><-PglZ||ZnR+REase->OLD-ABC+TOPRIM-><- [illegible]</td><td>1227</td><td>Archaea>Euryarchaeota>Stenosarchaea_group>Halobacteria>Haloferacales>Halorubraceae</td><td>GCF_900115675.1</td><td>Halolamina</td><td>BMZ83_RS11305</td><td>87547..91230</td><td>1075397</td><td>MULTISPECIES:-hypothetical-protein-[Halolamina]</td></tr>
<tr><td>WP_013878506.1</td><td>Type1.BREX</td><td><-ParB+N6_N4_METHYLASE|| [illegible]</td><td>1280</td><td>Archaea>Euryarchaeota>Stenosarchaea_group>Halobacteria>Natrialbales>Natrialbaceae</td><td>GCF_000217715.1</td><td>Halopiger_xanaduensis</td><td>HALXA_RS01375</td><td>280540..284382</td><td>387343</td><td>hypothetical-protein-[Halopiger-xanaduensis]</td></tr>
<tr><td>WP_006184162.1</td><td>Type1.BREX</td><td><-BrxB-BrxF-iSTAND<-DUF6079*<- [illegible]</td><td>1243</td><td>Archaea>Euryarchaeota>Stenosarchaea_group>Halobacteria>Natrialbales>Natrialbaceae</td><td>GCF_000337615.1</td><td>Natrinema_pallidum</td><td>C487_RS02820</td><td>457..4188</td><td>69527</td><td>hypothetical-protein-[Natrinema-pallidum]</td></tr>
<tr><td>WP_138651678.1</td><td>Type1.BREX</td><td><-PglZ<-N6-METHYLASE||?->?-><-REase|| [illegible]</td><td>1280</td><td>Archaea>Euryarchaeota>Stenosarchaea_group>Halobacteria>Natrialbales>Natrialbaceae</td><td>GCF_005890195.1</td><td>Natrinema_pallidum</td><td>FGF80_RS00910</td><td>194921..198763</td><td>69527</td><td>hypothetical-protein-[Natrinema-pallidum]</td></tr>
<tr><td>WP_226042478.1</td><td>Type1.BREX</td><td>RelE-><-TUDOR+SF1-SF2-Helicase+iREase+ [illegible]</td><td>1280</td><td>Archaea>Euryarchaeota>Stenosarchaea_group>Halobacteria>Natrialbales>Natrialbaceae</td><td>GCF_020405225.1</td><td>Natrinema_sp._DC36</td><td>LDH74_RS10650</td><td>2032265..2036107</td><td>2878680</td><td>hypothetical-protein-[Natrinema-sp.-DC36]</td></tr>
<tr><td>WP_226042480.1</td><td>Type1.BREX</td><td><-REase+METHYLASE||?-><-PglZ<- [illegible]</td><td>1227</td><td>Archaea>Euryarchaeota>Stenosarchaea_group>Halobacteria>Natrialbales>Natrialbaceae</td><td>GCF_020405225.1</td><td>Natrinema_sp._DC36</td><td>LDH74_RS10660</td><td>2036682..2040365</td><td>2878680</td><td>hypothetical-protein-[Natrinema-sp.-DC36]</td></tr>
<tr><td>WP_252697945.1</td><td>Type1.BREX</td><td><-REase<-HTH+REase<-?<-SbcC||?->?->?-> [illegible]</td><td>1226</td><td>Archaea>Euryarchaeota>Stenosarchaea_group>Halobacteria>Natrialbales>Natrialbaceae</td><td>GCF_023973145.1</td><td>Natribaculum_sp._CGA3</td><td>NGM68_RS09840</td><td>2053676..2057356</td><td>2953881</td><td>hypothetical-protein-[Natribaculum-sp.-CGA3]</td></tr>
<tr><td>WP_252697948.1</td><td>Type1.BREX</td><td><-SbcC||?->?->?->?->BrxA-DNA-binding-> [illegible]</td><td>1284</td><td>Archaea>Euryarchaeota>Stenosarchaea_group>Halobacteria>Natrialbales>Natrialbaceae</td><td>GCF_023973145.1</td><td>Natribaculum_sp._CGA3</td><td>NGM68_RS09850</td><td>2057949..2061803</td><td>2953881</td><td>hypothetical-protein-[Natribaculum-sp.-CGA3]</td></tr>
<tr><td>WP_254767378.1</td><td>Type1.BREX</td><td><-TUDOR+SF1-SF2-Helicase+iREase+CTD<- [illegible]</td><td>1278</td><td>Archaea>Euryarchaeota>Stenosarchaea_group>Halobacteria>Natrialbales>Natrialbaceae</td><td>GCF_024296665.1</td><td>Natribaculum_sp._AD-4</td><td>NKG98_RS17310</td><td>3290404..3294240</td><td>2961596</td><td>hypothetical-protein-[Natribaculum-sp.-AD-4]</td></tr>
<tr><td>WP_254767380.1</td><td>Type1.BREX</td><td><-REase<-PglZ<-N6-METHYLASE<-SLATT<- [illegible]</td><td>1265</td><td>Archaea>Euryarchaeota>Stenosarchaea_group>Halobacteria>Natrialbales>Natrialbaceae</td><td>GCF_024296665.1</td><td>Natribaculum_sp._AD-4</td><td>NKG98_RS17320</td><td>3294854..3298651</td><td>2961596</td><td>hypothetical-protein-[Natribaculum-sp.-AD-4]</td></tr>
<tr><td>WP_255191907.1</td><td>Type1.BREX</td><td><-REC+CheB-Methylesterase<-REC+ [illegible]</td><td>1261</td><td>Archaea>Euryarchaeota>Stenosarchaea_group>Halobacteria>Natrialbales>Natrialbaceae</td><td>GCF_024362485.1</td><td>Halovivax_sp._WLHSJ27</td><td>NMQ09_RS17725</td><td>3493167..3496952</td><td>2963433</td><td>hypothetical-protein-[Halovivax-sp.-WLHSJ27]</td></tr>
<tr><td>WP_255191909.1</td><td>Type1.BREX</td><td>HTH_23+HTH_32+HTH_33-><-McrC-NTD+ [illegible]</td><td>1273</td><td>Archaea>Euryarchaeota>Stenosarchaea_group>Halobacteria>Natrialbales>Natrialbaceae</td><td>GCF_024362485.1</td><td>Halovivax_sp._WLHSJ27</td><td>NMQ09_RS17735</td><td>3497545..3501366</td><td>2963433</td><td>hypothetical-protein-[Halovivax-sp.-WLHSJ27]</td></tr>
<tr><td>WP_090620839.1</td><td>Type1.BREX</td><td>GDResolvase->?->?->?->?->?-><-?<- [illegible]</td><td>1227</td><td>Archaea>Euryarchaeota>Stenosarchaea_group>Halobacteria>Natrialbales>Natrialbaceae</td><td>GCF_900110865.1</td><td>Natrinema_salaciae</td><td>BMX07_RS18655</td><td>46177..49860</td><td>1186196</td><td>hypothetical-protein-[Natrinema-salaciae]</td></tr>
<tr><td>WP_090620841.1</td><td>Type1.BREX</td><td><-ParB+N6_N4_METHYLASE|| [illegible]</td><td>1280</td><td>Archaea>Euryarchaeota>Stenosarchaea_group>Halobacteria>Natrialbales>Natrialbaceae</td><td>GCF_900110865.1</td><td>Natrinema_salaciae</td><td>BMX07_RS18665</td><td>50435..54277</td><td>1186196</td><td>hypothetical-protein-[Natrinema-salaciae]</td></tr>
<tr><td>KUK70180.1</td><td>Type1.BREX</td><td><-SIGMA+OB-Fold+AAA-ATPase+ [illegible]</td><td>1157</td><td>Archaea>Euryarchaeota>Stenosarchaea_group>Methanomicrobia>Methanomicrobiales>Methanocalculaceae</td><td>GCA_001508455.1</td><td>Methanocalculus_sp._52_23</td><td>XD88_0819</td><td>8030..11503</td><td>1641382</td><td>MAG:-Uncharacterized-protein-XD88_0819-[Methanocalculus-sp.-52_23]</td></tr>
<tr><td>MBR1368475.1</td><td>Type1.BREX</td><td><-HTH_Tnp_1||OLD-ABC+TOPRIM-> [illegible]</td><td>1155</td><td>Archaea>Euryarchaeota>Stenosarchaea_group>Methanomicrobia>Methanomicrobiales>Methanocalculaceae</td><td>GCA_018132105.1</td><td>Methanocalculus_chunghsingensis</td><td>RJ53_02730</td><td>5881..9348</td><td>156457</td><td>hypothetical-protein-[Methanocalculus-chunghsingensis]</td></tr>
<tr><td>WP_246495968.1</td><td>Type1.BREX</td><td>OLD-ABC+TOPRIM->SF1-SF2-Helicase-> [illegible]</td><td>1148</td><td>Archaea>Euryarchaeota>Stenosarchaea_group>Methanomicrobia>Methanomicrobiales>Methanocalculaceae</td><td>GCF_018132105.1</td><td>Methanocalculus_chunghsingensis</td><td>RJ53_RS02795</td><td>5902..9348</td><td>156457</td><td>BREX-system-P-loop-protein-BrxC-[Methanocalculus-chunghsingensis]</td></tr>
<tr><td>WP_253460960.1</td><td>Type1.BREX</td><td><-TPR<-TBP+TBP<-ASCH|| [illegible]</td><td>1180</td><td>Archaea>Euryarchaeota>Stenosarchaea_group>Methanomicrobia>Methanomicrobiales>Methanocalculaceae</td><td>GCF_024170265.1</td><td>Methanocalculus_sp._AMF5</td><td>J2T61_RS09750</td><td>4070..7612</td><td>1198257</td><td>BREX-system-P-loop-protein-BrxC-[Methanocalculus-sp.-AMF5]</td></tr>
<tr><td>WP_253488285.1</td><td>Type1.BREX</td><td><-SIGMA+OB-Fold+AAA-ATPase+ [illegible]</td><td>1155</td><td>Archaea>Euryarchaeota>Stenosarchaea_group>Methanomicrobia>Methanomicrobiales>Methanocalculaceae</td><td>GCF_024170505.1</td><td>Methanocalculus_alkaliphilus</td><td>J2T58_RS06410</td><td>9528..12995</td><td>768730</td><td>BREX-system-P-loop-protein-BrxC-[Methanocalculus-alkaliphilus]</td></tr>
<tr><td>WP_245241072.1</td><td>Type1.BREX</td><td>HTH+REase->?->?-><-SIGMA+OB-Fold+ [illegible]</td><td>1180</td><td>Archaea>Euryarchaeota>Stenosarchaea_group>Methanomicrobia>Methanomicrobiales>Methanocorpusculaceae</td><td>GCF_012062385.1</td><td>Methanocorpusculum_sp._GPch4</td><td>EYA77_RS00170</td><td>16814..20356</td><td>2527877</td><td>BREX-system-P-loop-protein-BrxC-[Methanocorpusculum-sp.-GPch4]</td></tr>
<tr><td>HIH85712.1</td><td>Type1.BREX</td><td><-SIGMA+OB-Fold+AAA-ATPase+ [illegible]</td><td>1155</td><td>Archaea>Euryarchaeota>Stenosarchaea_group>Methanomicrobia>Methanomicrobiales>Methanomicrobiaceae</td><td>GCA_013329555.1</td><td>Methanoculleus_sp.</td><td>HA342_02595</td><td>8043..11510</td><td>90427</td><td>BREX-system-P-loop-protein-BrxC-[Methanoculleus-sp.]</td></tr>
<tr><td>QYZ80392.1</td><td>Type1.BREX</td><td>ArdCN+MPTase->?-><-?<-Glyco_hydro|| [illegible]</td><td>1159</td><td>Archaea>Euryarchaeota>Stenosarchaea_group>Methanomicrobia>Methanomicrobiales>Methanomicrobiaceae</td><td>GCA_019633745.1</td><td>Methanofollis_formosanus</td><td>E2N92_03980</td><td>901654..905133</td><td>299308</td><td>BREX-system-P-loop-protein-BrxC-[Methanofollis-formosanus]</td></tr>
<tr><td>MCK4269451.1</td><td>Type1.BREX</td><td>BrxA-DNA-binding->BrxB-BrxF-iSTAND-> [illegible]</td><td>1207</td><td>Archaea>Euryarchaeota>Stenosarchaea_group>Methanomicrobia>Methanomicrobiales>Methanomicrobiaceae</td><td>GCA_023128355.1</td><td>Methanogenium_sp.</td><td>KAW93_03130</td><td>3124..6747</td><td>2196</td><td>BREX-system-P-loop-protein-BrxC-[Methanogenium-sp.]</td></tr>
<tr><td>WP_004077401.1</td><td>Type1.BREX</td><td>wHTH+WYL-><-SIGMA+OB-Fold+AAA-ATPase+ [illegible]</td><td>1180</td><td>Archaea>Euryarchaeota>Stenosarchaea_group>Methanomicrobia>Methanomicrobiales>Methanomicrobiaceae</td><td>GCF_000243255.1</td><td>Methanoplanus_limicola</td><td>METLIM_RS07750</td><td>1605385..1608927</td><td>2315</td><td>BREX-system-P-loop-protein-BrxC-[Methanoplanus-limicola]</td></tr>
<tr><td>WP_004078487.1</td><td>Type1.BREX</td><td><-CR-ATPase5+REase||MORC+REase-p-><- [illegible]</td><td>1155</td><td>Archaea>Euryarchaeota>Stenosarchaea_group>Methanomicrobia>Methanomicrobiales>Methanomicrobiaceae</td><td>GCF_000243255.1</td><td>Methanoplanus_limicola</td><td>METLIM_RS10810</td><td>2304713..2308180</td><td>2315</td><td>BREX-system-P-loop-protein-BrxC-[Methanoplanus-limicola]</td></tr>
<tr><td>WP_004038977.1</td><td>Type1.BREX</td><td><-ASCH<-REase+DUF5655<-EVE<- [illegible]</td><td>1222</td><td>Archaea>Euryarchaeota>Stenosarchaea_group>Methanomicrobia>Methanomicrobiales>Methanomicrobiaceae</td><td>GCF_000275865.1</td><td>Methanofollis_liminatans</td><td>METLI_RS06530</td><td>1309221..1312889</td><td>2201</td><td>BREX-system-P-loop-protein-BrxC-[Methanofollis-liminatans]</td></tr>
<tr><td>WP_014866844.1</td><td>Type1.BREX</td><td><-HEPN<-EVE<-REase+DUF5655<-ASCH<- [illegible]</td><td>1155</td><td>Archaea>Euryarchaeota>Stenosarchaea_group>Methanomicrobia>Methanomicrobiales>Methanomicrobiaceae</td><td>GCF_000304355.2</td><td>Methanoculleus_bourgensis</td><td>BN140_RS04760</td><td>992975..996442</td><td>83986</td><td>BREX-system-P-loop-protein-BrxC-[Methanoculleus-bourgensis]</td></tr>
<tr><td>WP_048152685.1</td><td>Type1.BREX</td><td>Schlafen+HATPase+wHTH-><-?||?-><- [illegible]</td><td>1179</td><td>Archaea>Euryarchaeota>Stenosarchaea_group>Methanomicrobia>Methanomicrobiales>Methanomicrobiaceae</td><td>GCF_000784355.1</td><td>Methanolacinia_paynteri</td><td>METPAY_RS11475</td><td>99003..102542</td><td>230356</td><td>BREX-system-P-loop-protein-BrxC-[Methanolacinia-paynteri]</td></tr>
<tr><td>WP_250317376.1</td><td>Type1.BREX</td><td><-PolB||?-><-SbcC<-Calcineurin||?-> [illegible]</td><td>1034</td><td>Archaea>Euryarchaeota>Stenosarchaea_group>Methanomicrobia>Methanomicrobiales>Methanomicrobiaceae</td><td>GCF_018495055.1</td><td>Methanoculleus_bourgensis</td><td>KH990_RS12090</td><td>14004..17108</td><td>83986</td><td>BREX-system-P-loop-protein-BrxC-[Methanoculleus-bourgensis]</td></tr>
</table>

Protein-ID	System	Gene-Neighbourhoods	Length	Taxonomy	Assembly-ID	Strain	Locus-Tag	Genomic-coordinates	TaxID	Fasta-define
WP_250317375.1	Type1.BREX	<-PolB<-PolB||?-><-SbcC<-Calcineurin||?->REase->BrxA-DNA-binding->BrxB-BrxF-iSTAND->DUF6079-p*->DUF6079->N6-METHYLASE->PglZ->SIGMA+OB-Fold+AAA-ATPase+Lon-Protease-BrxL->EVE->ZDNA-HTH-[illegible]	149	Archaea>Euryarchaeota>Stenosarchaea_group>Methanomicrobia>Methanomicrobiales>Methanomicrobiaceae	GCF_018495055.1	Methanoculleus_bourgensis	KH990_RS13175	13640..14089	83986	hypothetical-protein-[Methanoculleus-bourgensis]
WP_246589305.1	Type1.BREX	ArdCN+MPTase->?-><-?<-Glyco_hydro||?->ORC-CDC6+HTH->?->BrxA-DNA-binding->BrxB-BrxF-iSTAND->DUF6079*->N6-METHYLASE->?->PglZ->SIGMA+OB-Fold+AAA-ATPase+Lon-Protease-BrxL->	1160	Archaea>Euryarchaeota>Stenosarchaea_group>Methanomicrobia>Methanomicrobiales>Methanomicrobiaceae	GCF_019633745.1	Methanofollis_formosanus	E2N92_RS04005	901651..905133	299308	BREX-system-P-loop-protein-BrxC-[Methanofollis-formosanus]
WP_257742634.1	Type1.BREX	<-REase||PIN-><-?||Peptidase_M42->BrxA-DNA-binding->BrxB-BrxF-iSTAND->DUF6079*->N6-METHYLASE->HTH+REase->DDE_Tnp_1->?->PglZ->SIGMA+OB-Fold+AAA-ATPase+Lon-Protease-BrxL->?-><-DDE_Tnp	1155	Archaea>Euryarchaeota>Stenosarchaea_group>Methanomicrobia>Methanomicrobiales>Methanomicrobiaceae	GCF_024662215.1	Methanoplanus_endosymbiosus	L6E24_RS14385	3075748..3079215	33865	BREX-system-P-loop-protein-BrxC-[Methanoplanus-endosymbiosus]
WP_074369579.1	Type1.BREX	35exo->?->HTH+REase-><-TBP+TBP<-ASCH<-EVE||BrxA-DNA-binding->BrxB-BrxF-iSTAND->DUF6079*->N6-METHYLASE->?->PglZ->SIGMA+OB-Fold+AAA-ATPase+Lon-Protease-BrxL->REase-><-DOC+HTH	1160	Archaea>Euryarchaeota>Stenosarchaea_group>Methanomicrobia>Methanomicrobiales>Methanomicrobiaceae	GCF_900095385.1	Methanoculleus_chikugoensis	L21_RS05950	37665..41147	118126	BREX-system-P-loop-protein-BrxC-[Methanoculleus-chikugoensis]
ABD40832.1	Type1.BREX	<-N6-METHYLASE<-?<-SIGMA+OB-Fold+AAA-ATPase+Lon-Protease-BrxL<-PglZ<-TOPRIM<-OLD-ABC<-N6-METHYLASE<-RHH<-DUF6079*<-BrxB-BrxF-iSTAND<-BrxA-DNA-binding<-?<-?<-Glyco_hydro	1166	Archaea>Euryarchaeota>Stenosarchaea_group>Methanomicrobia>Methanomicrobiales>Methanospirillaceae	GCA_000013445.1	Methanospirillum_hungatei_JF-1	Mhun_1083	1223800..1227300	323259	hypothetical-protein-Mhun_1083-[Methanospirillum-hungatei-JF-1]
MBN1165665.1	Type1.BREX	TPR+TPR+TPR->BrxA-DNA-binding->BrxB-BrxF-iSTAND->DUF6079*->N6-METHYLASE-p->N6-METHYLASE->?->?->ParA-Soj-PloopNTPase->?->HTH+REase->PglZ->SIGMA+OB-Fold+AAA-ATPase+Lon-Protease-BrxL->	1218	Archaea>Euryarchaeota>Stenosarchaea_group>Methanomicrobia>Methanomicrobiales>Methanospirillaceae	GCA_016926505.1	Methanospirillaceae_archaeon	JXA44_00885	48354..52010	2813030	BREX-system-P-loop-protein-BrxC-[Methanospirillaceae-archaeon]
MCA1916608.1	Type1.BREX	<-N6-METHYLASE-p<-Schlafen+HATPase+wHTH<-N6-METHYLASE<-DUF6079*<-BrxB-BrxF-iSTAND||OLD-ABC->TPR->	1178	Archaea>Euryarchaeota>Stenosarchaea_group>Methanomicrobia>Methanomicrobiales>Methanospirillaceae	GCA_020216685.1	Methanospirillum_hungatei	LDL35_09440	4587..8123	2203	BREX-system-P-loop-protein-BrxC-[Methanospirillum-hungatei]
WP_052288823.1	Type1.BREX	<-SIGMA+OB-Fold+AAA-ATPase+Lon-Protease-BrxL<-PglZ<-TOPRIM<-OLD-ABC<-N6-METHYLASE<-?<-RHH<-DUF6079*<-BrxB-BrxF-iSTAND<-BrxA-DNA-binding<-?<-?<-?<-Glyco_hydro	1159	Archaea>Euryarchaeota>Stenosarchaea_group>Methanomicrobia>Methanomicrobiales>Methanospirillaceae	GCF_000013445.1	Methanospirillum_hungatei	MHUN_RS05665	1223800..1227279	2203	BREX-system-P-loop-protein-BrxC-[Methanospirillum-hungatei]
WP_011448873.1	Type1.BREX	CR-ATPase8+REase-><-TPR+TPR<-TPR<-TOPRIM<-OLD-ABC<-?||BrxB-BrxF-iSTAND->DUF6079*->N6-METHYLASE->?->?->?->HATPase+wHTH->HTH+REase->N6-METHYLASE-p-><-DDE_Tnp||N6-METHYLASE-p->	1178	Archaea>Euryarchaeota>Stenosarchaea_group>Methanomicrobia>Methanomicrobiales>Methanospirillaceae	GCF_000013445.1	Methanospirillum_hungatei	MHUN_RS09850	2137740..2141276	2203	BREX-system-P-loop-protein-BrxC-[Methanospirillum-hungatei]
WP_214418671.1	Type1.BREX	<-SIGMA+OB-Fold+AAA-ATPase+Lon-Protease-BrxL<-PglZ<-N6-METHYLASE-p<-HTH+REase<-Schlafen+HATPase+wHTH<-N6-METHYLASE<-DUF6079*<-BrxB-BrxF-iSTAND<-zf-IS66<-N6-METHYLASE<-?<-RNase<-?<-REase||EVE->	1171	Archaea>Euryarchaeota>Stenosarchaea_group>Methanomicrobia>Methanomicrobiales>Methanospirillaceae	GCF_018502485.1	Methanospirillum_sp._J.3.6.1-F.2.7.3	KHC33_RS10925	2269426..2272941	2834276	BREX-system-P-loop-protein-BrxC-[Methanospirillum-sp.-J.3.6.1-F.2.7.3]
WP_218608642.1	Type1.BREX	<-TBP+TBP<-?<-HD<-Peptidase_S8<-Classical-AAA<-N6-METHYLASE||?->?-><-?<-DUF6079*<-BrxB-BrxF-iSTAND<-BrxA-DNA-binding<-?||?-><-HicB_lk_antitox<-HicA_toxin||?->?->Schlafen+HATPase+wHTH->	1214	Archaea>Euryarchaeota>Stenosarchaea_group>Methanomicrobia>Methanomicrobiales>Methanospirillaceae	GCF_019263745.1	Methanospirillum_hungatei	KSK55_RS06665	1421419..1425063	2203	BREX-system-P-loop-protein-BrxC-[Methanospirillum-hungatei]
MCE8425368.1	Type1.BREX	<-PglZ<-Schlafen+HATPase+wHTH<-N6-METHYLASE<-DUF6079*<-BrxB-BrxF-iSTAND<-BrxA-DNA-binding||SF1-SF2-Helicase+REase-><-ADH_N+ADH_zinc_N_2	1196	Archaea>Euryarchaeota>Stenosarchaea_group>Methanomicrobia>Methanosarcinales>Candidatus_Methanoperedenaceae	GCA_021409295.1	Candidatus_Methanoperedens_sp.	J5U17_06270	6093..9683	2032688	BREX-system-P-loop-protein-BrxC-[Candidatus-Methanoperedens-sp.]
AAM05760.1	Type1.BREX	<-Calcineurin<-SIGMA+OB-Fold+AAA-ATPase+Lon-Protease-BrxL<-PglZ<-MNS+REase||PHD_like+HicB_lk_antitox-><-Schlafen+HATPase+wHTH<-Schlafen+HATPase+wHTH<-HDRnase<-N6-METHYLASE<-DUF6079*<-BrxB-BrxF-iSTAND||?->PolB->?-><-?||?-><-GT4+DSBH<-Glyco_hydro_130	1201	Archaea>Euryarchaeota>Stenosarchaea_group>Methanomicrobia>Methanosarcinales>Methanosarcinaceae	GCA_000007345.1	Methanosarcina_acetivorans_C2A	MA_2373	2935402..2939007	188937	predicted-protein-[Methanosarcina-acetivorans-C2A]
AFV22360.1	Type1.BREX	MNS->?->McrB-AAA->McrC-NTD+REase-><-DNA-glycosylase||?->CarD+HTH->CarD+HTH->BrxB-BrxF-iSTAND->DUF6079*->N6-METHYLASE->Schlafen+HATPase+wHTH->N6-METHYLASE-p->ASCH->HD->PglZ->SIGMA+OB-Fold+AAA-ATPase+Lon-Protease-BrxL-><-REase<-SF1-SF2-Helicase	1185	Archaea>Euryarchaeota>Stenosarchaea_group>Methanomicrobia>Methanosarcinales>Methanosarcinaceae	GCA_000306725.1	Methanolobus_psychrophilus_R15	Mpsy_0147	147504..151061	1094980	hypothetical-protein-Mpsy_0147-[Methanolobus-psychrophilus-R15]
AKB55571.1	Type1.BREX	<-SIGMA+OB-Fold+AAA-ATPase+Lon-Protease-BrxL<-PglZ<-Schlafen-p<-TIR<-N6-METHYLASE<-DUF6079-p*<-DUF6079-p<-BrxB-BrxF-iSTAND||?-><-tRNA<-tRNA<-?<-?<-?<-?<-?<-ABC-ATPase+SbcC	797	Archaea>Euryarchaeota>Stenosarchaea_group>Methanomicrobia>Methanosarcinales>Methanosarcinaceae	GCA_000970025.1	Methanosarcina_barkeri_MS	MSBRM_2573	3399926..3402319	1434108	hypothetical-protein-MSBRM_2573-[Methanosarcina-barkeri-MS]
AKB55572.1	Type1.BREX	<-SIGMA+OB-Fold+AAA-ATPase+Lon-Protease-BrxL<-PglZ<-Schlafen-p<-TIR<-N6-METHYLASE<-DUF6079-p<-DUF6079-p*<-BrxB-BrxF-iSTAND||?-><-tRNA<-tRNA<-?<-?<-?<-?<-?<-ABC-ATPase+SbcC	353	Archaea>Euryarchaeota>Stenosarchaea_group>Methanomicrobia>Methanosarcinales>Methanosarcinaceae	GCA_000970025.1	Methanosarcina_barkeri_MS	MSBRM_2574	3402467..3403528	1434108	ATPase-like-protein-[Methanosarcina-barkeri-MS]
KXS44097.1	Type1.BREX	<-SIGMA+OB-Fold+AAA-ATPase+Lon-Protease-BrxL<-PglZ<-TOPRIM<-OLD-ABC<-?<-N6-METHYLASE-p<-Schlafen+HATPase+wHTH<-N6-METHYLASE<-DUF6079*<-BrxB-BrxF-iSTAND<-McrC-NTD+REase<-EVE+McrB-AAA<-?||McrC-NTD+REase->MNS+REase->RADICAL-SAM->	1185	Archaea>Euryarchaeota>Stenosarchaea_group>Methanomicrobia>Methanosarcinales>Methanosarcinaceae	GCA_001577565.1	Methanolobus_sp._T82-4	AWU59_730	16022..19579	1794908	MAG:-hypothetical-protein-AWU59_730-[Methanolobus-sp.-T82-4]
OBZ35401.1	Type1.BREX	BrxB-BrxF-iSTAND->DUF6079*->Schlafen+HATPase+wHTH->N6-METHYLASE->?->PglZ->SIGMA+OB-Fold+AAA-ATPase+Lon-Protease-BrxL->RNaseH-><-?<-REase<-McrB-AAA	1187	Archaea>Euryarchaeota>Stenosarchaea_group>Methanomicrobia>Methanosarcinales>Methanosarcinaceae	GCA_001683555.1	Methanohalophilus_sp._DAL1	A9957_07285	3150..6713	1864608	MAG:-hypothetical-protein-A9957_07285-[Methanohalophilus-sp.-DAL1]
NPE28183.1	Type1.BREX	<-SIGMA+OB-Fold+AAA-ATPase+Lon-Protease-BrxL<-PglZ<-TOPRIM<-OLD-ABC<-TIR<-N6-METHYLASE-p<-Schlafen+HATPase+wHTH<-N6-METHYLASE<-DUF6079*<-BrxB-BrxF-iSTAND<-URI-DNase<-?<-SF1-SF2-Helicase+REase||<-?||?->HNH->	1185	Archaea>Euryarchaeota>Stenosarchaea_group>Methanomicrobia>Methanosarcinales>Methanosarcinaceae	GCA_013166765.1	Methanococcoides_sp._SA1	HNV12_09470	12693..16250	2735871	BREX-system-P-loop-protein-BrxC-[Methanococcoides-sp.-SA1]
HII01702.1	Type1.BREX	<-SF1-SF2-Helicase<-?<-OLD-ABC+TOPRIM<-?<-N6-METHYLASE<-DUF6079*<-BrxB-BrxF-iSTAND||?->SAM-METHYLASE->?->MNS-><-?||HAD+HAD-><-TPR+TPR+TPR+TPR<-REC+OmpR-HTH	1196	Archaea>Euryarchaeota>Stenosarchaea_group>Methanomicrobia>Methanosarcinales>Methanosarcinaceae	GCA_013329415.1	Methanosarcinaceae_archaeon	HA351_08670	9336..12926	1945595	BREX-system-P-loop-protein-BrxC-[Methanosarcinaceae-archaeon]
HIH93569.1	Type1.BREX	<-PglZ<-?<-MNS+REase||PHD_like+HicB_lk_antitox->HicA_toxin-><-Schlafen+HATPase+wHTH<-Schlafen-p<-HDRnase<-?<-DUF6079*<-BrxB-BrxF-iSTAND	1124	Archaea>Euryarchaeota>Stenosarchaea_group>Methanomicrobia>Methanosarcinales>Methanosarcinaceae	GCA_013329655.1	Methanosarcina_acetivorans	HA338_05875	28858..32230	2214	BREX-system-P-loop-protein-BrxC-[Methanosarcina-acetivorans]
MBN2489135.1	Type1.BREX	<-ArdCN+MPTase||?->URI-DNase->DNA-glycosylase-><-SF1-SF2-Helicase+REase||BrxB-BrxF-iSTAND->DUF6079-p*->	551	Archaea>Euryarchaeota>Stenosarchaea_group>Methanomicrobia>Methanosarcinales>Methanosarcinaceae	GCA_016935475.1	Methanosarcinaceae_archaeon	JXA98_08910	15184..16838	1945595	BREX-system-P-loop-protein-BrxC-[Methanosarcinaceae-archaeon]
MCD4820926.1	Type1.BREX	<-ASCH||Phage_int_SAM_4+Phage_integrase->?-><-?<-SIGMA+OB-Fold+AAA-ATPase+Lon-Protease-BrxL<-PglZ<-?<-N6-METHYLASE-p<-Schlafen+HATPase+wHTH<-N6-METHYLASE<-DUF6079*<-BrxB-BrxF-iSTAND<-URI-DNase<-SF1-SF2-Helicase+REase||HEPN+PAS+DUF4011+SF1-SF2-Helicase+REase->[illegible]	1185	Archaea>Euryarchaeota>Stenosarchaea_group>Methanomicrobia>Methanosarcinales>Methanosarcinaceae	GCA_021108275.1	Methanococcoides_sp.	K8R11_02360	106094..109651	1966350	BREX-system-P-loop-protein-BrxC-[Methanococcoides-sp.]
WP_011032108.1	Type1.BREX	<-D5N+viral-AAA+CDC6-HTH<-?<-?<-Phage_int_SAM_4+Phage_integrase||BrxB-BrxF-iSTAND->DUF6079*-><-TPR+TPR+TPR+TPR+TPR+TPR+TPR||N6-METHYLASE->Schlafen-p->Schlafen+HATPase+wHTH-p->HATPase+wHTH->PadR-HTH-><-?||Mrr_N+ACET-><-HicA_toxin	1212	Archaea>Euryarchaeota>Stenosarchaea_group>Methanomicrobia>Methanosarcinales>Methanosarcinaceae	GCF_000007065.1	Methanosarcina_mazei	MM_RS00835	194588..198226	2209	BREX-system-P-loop-protein-BrxC-[Methanosarcina-mazei]
WP_048065383.1	Type1.BREX	PHD_like+HicB_lk_antitox->HicA_toxin-><-?<-HATPase_c<-Schlafen+HATPase+wHTH<-Schlafen-p<-Schlafen+HATPase+wHTH<-HDRnase<-N6-METHYLASE<-DUF6079*<-BrxB-BrxF-iSTAND<-tRNA||?->PolB-><-?||HEPN->?-><-GT4+DSBH<-Glyco_hydro_130	1198	Archaea>Euryarchaeota>Stenosarchaea_group>Methanomicrobia>Methanosarcinales>Methanosarcinaceae	GCF_000007345.1	Methanosarcina_acetivorans	MA_RS12305	2935402..2938998	2214	BREX-system-P-loop-protein-BrxC-[Methanosarcina-acetivorans]
WP_013898237.1	Type1.BREX	<-SIGMA+OB-Fold+AAA-ATPase+Lon-Protease-BrxL<-PglZ<-HD<-N6-METHYLASE<-Schlafen+HATPase+wHTH<-DUF6079*<-BrxB-BrxF-iSTAND-p<-BrxB-BrxF-iSTAND<-HKD-DNase+MED12||?->?->?->?->HAD+HAD->	1188	Archaea>Euryarchaeota>Stenosarchaea_group>Methanomicrobia>Methanosarcinales>Methanosarcinaceae	GCF_000217995.1	Methanosalsum_zhilinae	MZHIL_RS04755	1026278..1029844	39669	BREX-system-P-loop-protein-BrxC-[Methanosalsum-zhilinae]
WP_015324772.1	Type1.BREX	<-TOPRIM<-OLD-ABC<-?<-?||?-><-N6-METHYLASE-p<-?<-?<-N6-METHYLASE<-DUF6079*<-BrxB-BrxF-iSTAND||?->?->TPR+TPR-><-?||DSBH->?-><-?||35exo->	1187	Archaea>Euryarchaeota>Stenosarchaea_group>Methanomicrobia>Methanosarcinales>Methanosarcinaceae	GCF_000328665.1	Methanomethylovorans_hollandica	METHO_RS06665	1392190..1395753	101192	BREX-system-P-loop-protein-BrxC-[Methanomethylovorans-hollandica]
WP_015410898.1	Type1.BREX	PRTase-><-?||?->?->tRNA->BrxB-BrxF-iSTAND->DUF6079*-><-TPR+TPR+TPR+TPR+TPR+TPR+TPR||N6-METHYLASE->N6-METHYLASE-p->Schlafen-p->Schlafen+HATPase+wHTH-p->HATPase+wHTH->PadR-HTH-><-?||Mrr_N+ACET->	1212	Archaea>Euryarchaeota>Stenosarchaea_group>Methanomicrobia>Methanosarcinales>Methanosarcinaceae	GCF_000341715.1	Methanosarcina_mazei	MMTUC01_RS00750	161332..164970	2209	BREX-system-P-loop-protein-BrxC-[Methanosarcina-mazei]
WP_048118458.1	Type1.BREX	BrxA-DNA-binding->BrxB-BrxF-iSTAND->DUF6079*->N6-METHYLASE->PglZ->SIGMA+OB-Fold+AAA-ATPase+Lon-Protease-BrxL-><-DUF488<-Glycos_transf_1+Glyco_trans_1_2<-?<-DSBH	1143	Archaea>Euryarchaeota>Stenosarchaea_group>Methanomicrobia>Methanosarcinales>Methanosarcinaceae	GCF_000969905.1	Methanosarcina_vacuolata	MSVAZ_RS04205	977468..980899	2215	BREX-system-P-loop-protein-BrxC-[Methanosarcina-vacuolata]
WP_048158954.1	Type1.BREX	DDE_Tnp_1->DDE_Tnp_1-><-Schlafen+HATPase+wHTH<-Schlafen-p<-Schlafen+HATPase+wHTH<-?<-?<-TIR<-N6-METHYLASE<-DUF6079*<-BrxB-BrxF-iSTAND||?->RRXRR+HNH->PglZ->SIGMA+OB-Fold+AAA-ATPase+Lon-Protease-BrxL->Calcineurin->SbcC-><-SF1-SF2-Helicase+REase	1223	Archaea>Euryarchaeota>Stenosarchaea_group>Methanomicrobia>Methanosarcinales>Methanosarcinaceae	GCF_000969965.1	Methanosarcina_sp._WWM596	MSWHS_RS09215	2091991..2095662	1434103	BREX-system-P-loop-protein-BrxC-[Methanosarcina-sp.-WWM596]
WP_048171288.1	Type1.BREX	PHD_like+HicB_lk_antitox->HicA_toxin-><-HATPase+wHTH<-Schlafen+HATPase+wHTH<-Schlafen-p<-Schlafen+HATPase+wHTH<-SMODS<-SLATT<-N6-METHYLASE<-DUF6079*<-BrxB-BrxF-iSTAND<-tRNA<-?||?->?->?->DnaB-><-REC+OmpR-HTH<-DnaB||tRNA->ACET->	1220	Archaea>Euryarchaeota>Stenosarchaea_group>Methanomicrobia>Methanosarcinales>Methanosarcinaceae	GCF_000970085.1	Methanosarcina_siciliae	MSSIT_RS07430	1736961..1740623	38027	BREX-system-P-loop-protein-BrxC-[Methanosarcina-siciliae]
WP_148705147.1	Type1.BREX	<-HATPase+wHTH<-Schlafen+HATPase+wHTH<-Schlafen-p<-Schlafen+HATPase+wHTH<-?<-?<-TIR<-N6-METHYLASE||TPR+TPR+TPR+TPR+TPR+TPR+TPR-><-DUF6079*<-BrxB-BrxF-iSTAND<-tRNA<-?||?-><-?||?->?->DnaB-><-REC+OmpR-HTH<-DnaB	1222	Archaea>Euryarchaeota>Stenosarchaea_group>Methanomicrobia>Methanosarcinales>Methanosarcinaceae	GCF_000970125.1	Methanosarcina_siciliae	MSSIH_RS07150	1687969..1691637	38027	BREX-system-P-loop-protein-BrxC-[Methanosarcina-siciliae]
WP_048181889.1	Type1.BREX	SF1-SF2-Helicase-><-SbcC<-Calcineurin<-SIGMA+OB-Fold+AAA-ATPase+Lon-Protease-BrxL<-PglZ<-SF1-SF2-Helicase<-SbcC<-N6-METHYLASE<-DUF6079*<-BrxB-BrxF-iSTAND<-tRNA<-?||?-><-?||RVT->RVT->ABC-ATPase+SbcC->Peptidase->	1212	Archaea>Euryarchaeota>Stenosarchaea_group>Methanomicrobia>Methanosarcinales>Methanosarcinaceae	GCF_000970145.1	Methanosarcina_siciliae	MSSAC_RS09150	2102967..2106605	38027	BREX-system-P-loop-protein-BrxC-[Methanosarcina-siciliae]
WP_048052927.1	Type1.BREX	<-Mrr_N+ACET||?-><-PadR-HTH<-?<-HATPase+wHTH<-Schlafen+HATPase+wHTH-p<-Schlafen-p<-N6-METHYLASE||TPR+TPR+TPR+TPR+TPR+TPR+TPR-><-DUF6079*<-BrxB-BrxF-iSTAND<-tRNA<-?<-?||?-><-PRTase	1212	Archaea>Euryarchaeota>Stenosarchaea_group>Methanomicrobia>Methanosarcinales>Methanosarcinaceae	GCF_000970165.1	Methanosarcina_mazei	MSMAW_RS06205	1427636..1431274	2209	BREX-system-P-loop-protein-BrxC-[Methanosarcina-mazei]
WP_048046369.1	Type1.BREX	HicA_toxin-><-Mrr_N+ACET||?-><-PadR-HTH<-HATPase+wHTH<-Schlafen+HATPase+wHTH-p<-Schlafen-p<-N6-METHYLASE||TPR+TPR+TPR+TPR+TPR+TPR+TPR-><-DUF6079*<-BrxB-BrxF-iSTAND<-DDE_Tnp_1<-?<-OLD-ABC<-Cas_Cas1<-?||?->?->HNH->	1212	Archaea>Euryarchaeota>Stenosarchaea_group>Methanomicrobia>Methanosarcinales>Methanosarcinaceae	GCF_000970205.1	Methanosarcina_mazei	MSMAS_RS06220	1426586..1430224	2209	BREX-system-P-loop-protein-BrxC-[Methanosarcina-mazei]
WP_048040167.1	Type1.BREX	<-MNS+REase<-?<-ACET<-?<-HATPase+wHTH<-Schlafen-p<-TIR<-N6-METHYLASE||TPR+TPR+TPR+TPR+TPR+TPR+TPR-><-DUF6079*<-BrxB-BrxF-iSTAND<-tRNA<-?<-?||?-><-PRTase	1212	Archaea>Euryarchaeota>Stenosarchaea_group>Methanomicrobia>Methanosarcinales>Methanosarcinaceae	GCF_000970225.1	Methanosarcina_mazei	MSMAL_RS06195	1422644..1426282	2209	BREX-system-P-loop-protein-BrxC-[Methanosarcina-mazei]
WP_048038520.1	Type1.BREX	HicA_toxin-><-Mrr_N+ACET||?-><-PadR-HTH<-HATPase+wHTH<-Schlafen+HATPase+wHTH-p<-Schlafen-p<-N6-METHYLASE||TPR+TPR+TPR+TPR+TPR+TPR+TPR-><-DUF6079*<-BrxB-BrxF-iSTAND<-tRNA<-?<-?||?-><-PRTase	1212	Archaea>Euryarchaeota>Stenosarchaea_group>Methanomicrobia>Methanosarcinales>Methanosarcinaceae	GCF_000970245.1	Methanosarcina_mazei	MSMAC_RS13360	3106154..3109792	2209	BREX-system-P-loop-protein-BrxC-[Methanosarcina-mazei]
WP_048125616.1	Type1.BREX	RRXRR+HNH->RNase->?->PolB->HEPN-><-DDE_Tnp_1||?-><-?||BrxB-BrxF-iSTAND->DUF6079*->N6-METHYLASE->PglZ->SIGMA+OB-Fold+AAA-ATPase+Lon-Protease-BrxL->SF1-SF2-Helicase->?->ParB+HNH-><-BirA-HTH	1252	Archaea>Euryarchaeota>Stenosarchaea_group>Methanomicrobia>Methanosarcinales>Methanosarcinaceae	GCF_000970265.1	Methanosarcina_lacustris	MSLAZ_RS07215	1686822..1690580	170861	BREX-system-P-loop-protein-BrxC-[Methanosarcina-lacustris]
WP_048126494.1	Type1.BREX	<-DDE_Tnp_1<-DDE_Tnp_1||<-SIGMA+OB-Fold+AAA-ATPase+Lon-Protease-BrxL<-PglZ<-SF1-SF2-Helicase<-OLD-ABC+TOPRIM<-?<-N6-METHYLASE||TPR+TPR+TPR+TPR+TPR+TPR+TPR-><-DUF6079*<-BrxB-BrxF-iSTAND<-?<-?||?-><-TPR+TPR+TPR+TPR	1211	Archaea>Euryarchaeota>Stenosarchaea_group>Methanomicrobia>Methanosarcinales>Methanosarcinaceae	GCF_000970265.1	Methanosarcina_lacustris	MSLAZ_RS10160	2470398..2474033	170861	BREX-system-P-loop-protein-BrxC-[Methanosarcina-lacustris]
WP_048048522.1	Type1.BREX	HicA_toxin-><-Mrr_N+ACET||?-><-PadR-HTH<-HATPase+wHTH<-Schlafen+HATPase+wHTH-p<-Schlafen-p<-N6-METHYLASE||TPR+TPR+TPR+TPR+TPR+TPR+TPR-><-DUF6079*<-BrxB-BrxF-iSTAND<-tRNA<-?<-?||?-><-PRTase	1212	Archaea>Euryarchaeota>Stenosarchaea_group>Methanomicrobia>Methanosarcinales>Methanosarcinaceae	GCF_000979015.1	Methanosarcina_mazei	DU63_RS00595	17697..21335	2209	BREX-system-P-loop-protein-BrxC-[Methanosarcina-mazei]
WP_048048860.1	Type1.BREX	<-ACET||?->?->?->?->?->Glyco_hydro_130->GT4+DSBH->||BrxB-BrxF-iSTAND->DUF6079*->N6-METHYLASE->Schlafen-p->Schlafen+HATPase+wHTH-p->HATPase+wHTH->?->PadR-HTH-><-?||Mrr_N+ACET-><-HicA_toxin	1212	Archaea>Euryarchaeota>Stenosarchaea_group>Methanomicrobia>Methanosarcinales>Methanosarcinaceae	GCF_000979185.1	Methanosarcina_mazei	DU71_RS16355	152756..156394	2209	BREX-system-P-loop-protein-BrxC-[Methanosarcina-mazei]
WP_048133224.1	Type1.BREX	<-SIGMA+OB-Fold+AAA-ATPase+Lon-Protease-BrxL<-PglZ<-HTH_Tnp_1<-?<-?<-?<-?<-?<-TIR<-N6-METHYLASE<-DUF6079*<-BrxB-BrxF-iSTAND	1243	Archaea>Euryarchaeota>Stenosarchaea_group>Methanomicrobia>Methanosarcinales>Methanosarcinaceae	GCF_000979295.1	Methanosarcina_sp._1.H.T.1A.1	EO95_RS06145	15439..19170	1483602	BREX-system-P-loop-protein-BrxC-[Methanosarcina-sp.-1.H.T.1A.1]
WP_048041771.1	Type1.BREX	PIN-><-?<-?||?-><-Phage_int_SAM_4+Phage_integrase||BrxB-BrxF-iSTAND->DUF6079*-><-TPR+TPR+TPR+TPR+TPR+TPR+TPR||N6-METHYLASE->Schlafen-p->Schlafen+HATPase+wHTH-p->HATPase+wHTH->PadR-HTH-><-?||Mrr_N+ACET-><-HicA_toxin	1212	Archaea>Euryarchaeota>Stenosarchaea_group>Methanomicrobia>Methanosarcinales>Methanosarcinaceae	GCF_000979335.1	Methanosarcina_mazei	DU82_RS15310	140764..144402	2209	BREX-system-P-loop-protein-BrxC-[Methanosarcina-mazei]
WP_048037387.1	Type1.BREX	HicA_toxin-><-Mrr_N+ACET||?-><-PadR-HTH<-HATPase+wHTH<-Schlafen+HATPase+wHTH-p<-Schlafen-p<-N6-METHYLASE||TPR+TPR+TPR+TPR+TPR+TPR+TPR-><-DUF6079*<-BrxB-BrxF-iSTAND||<-GT4+DSBH<-Glyco_hydro_130<-?<-?<-?||Y1_Tnp->HTH_OrfB_IS605+OrfB_IS605+OrfB_Zn_ribbon->	1212	Archaea>Euryarchaeota>Stenosarchaea_group>Methanomicrobia>Methanosarcinales>Methanosarcinaceae	GCF_000979345.1	Methanosarcina	DU57_RS09710	64540..68178	2207	MULTISPECIES:-BREX-system-P-loop-protein-BrxC-[Methanosarcina]
WP_048045678.1	Type1.BREX	PRTase-><-?||?->?->tRNA->BrxB-BrxF-iSTAND->DUF6079*-><-TPR+TPR+TPR+TPR+TPR+TPR+TPR||N6-METHYLASE->Schlafen-p->HATPase+wHTH->PadR-HTH->Mrr_N+ACET-><-PHD_like+HicB_lk_antitox||MNS+REase->	1212	Archaea>Euryarchaeota>Stenosarchaea_group>Methanomicrobia>Methanosarcinales>Methanosarcinaceae	GCF_000979375.1	Methanosarcina_mazei	DU40_09105	234397..238035	2209	BREX-system-P-loop-protein-BrxC-[Methanosarcina-mazei]
WP_048049967.1	Type1.BREX	PRTase-><-?||?->?->tRNA->BrxB-BrxF-iSTAND->DUF6079*-><-TPR+TPR+TPR+TPR+TPR+TPR+TPR||N6-METHYLASE->TIR->Schlafen-p->Schlafen+HATPase+wHTH-p->HATPase+wHTH->PadR-HTH-><-?||Mrr_N+ACET->	1212	Archaea>Euryarchaeota>Stenosarchaea_group>Methanomicrobia>Methanosarcinales>Methanosarcinaceae	GCF_000979685.1	Methanosarcina_mazei	DU55_RS15175	41781..45419	2209	BREX-system-P-loop-protein-BrxC-[Methanosarcina-mazei]
WP_048042209.1	Type1.BREX	HicA_toxin-><-Mrr_N+ACET||?-><-PadR-HTH<-HATPase+wHTH<-Schlafen+HATPase+wHTH-p<-Schlafen-p<-N6-METHYLASE||TPR+TPR+TPR+TPR+TPR+TPR+TPR-><-DUF6079*<-BrxB-BrxF-iSTAND<-tRNA<-?<-NACHT<-?||<-HKD-DNase<-tRNA<-?<-?||?-><-PRTase	1212	Archaea>Euryarchaeota>Stenosarchaea_group>Methanomicrobia>Methanosarcinales>Methanosarcinaceae	GCF_000979755.1	Methanosarcina_mazei	DU69_RS16895	67838..71476	2209	BREX-system-P-loop-protein-BrxC-[Methanosarcina-mazei]
WP_048162601.1	Type1.BREX	Glyco_hydro_130->GT4+DSBH-><-?<-?||tRNA-><-NUDIX+DUF429||?->BrxB-BrxF-iSTAND->DUF6079*->N6-METHYLASE->HTH+REase->N6-METHYLASE-p->?->Schlafen+HATPase+wHTH->?->Mrr_N+ACET-><-TPR+TPR+TPR+TPR	1223	Archaea>Euryarchaeota>Stenosarchaea_group>Methanomicrobia>Methanosarcinales>Methanosarcinaceae	GCF_000979975.1	Methanosarcina_sp._1.H.A.2.2	EO93_RS11285	63696..67367	1483601	BREX-system-P-loop-protein-BrxC-[Methanosarcina-sp.-1.H.A.2.2]
WP_096712430.1	Type1.BREX	RADICAL-SAM->tRNA->tRNA-><-?<-?<-?||?-><-SF1-SF2-Helicase||BrxB-BrxF-iSTAND->DUF6079*->N6-METHYLASE->?->SbcC->PglZ->SIGMA+OB-Fold+AAA-ATPase+Lon-Protease-BrxL->RNaseH-><-REase<-McrB-AAA<-McrC-NTD+REase	1188	Archaea>Euryarchaeota>Stenosarchaea_group>Methanomicrobia>Methanosarcinales>Methanosarcinaceae	GCF_001726015.1	Methanohalophilus_euhalobius	CP947_RS06580	29477..33043	51203	BREX-system-P-loop-protein-BrxC-[Methanohalophilus-euhalobius]
WP_094227763.1	Type1.BREX	TPR+TPR+TPR+TPR->?-><-DDE_Tnp<-?||?->?->SF1-SF2-Helicase->SF1-SF2-Helicase->BrxB-BrxF-iSTAND->DUF6079*->N6-METHYLASE->?->SbcC->SF1-SF2-Helicase-><-DDE_Tnp_1||HD->OLD-ABC->	1185	Archaea>Euryarchaeota>Stenosarchaea_group>Methanomicrobia>Methanosarcinales>Methanosarcinaceae	GCF_002243045.1	Methanolobus_psychrotolerans	BB781_RS06865	19266..22823	1874706	BREX-system-P-loop-protein-BrxC-[Methanolobus-psychrotolerans]
WP_123137046.1	Type1.BREX	VirB11->?->?->RADICAL-SAM->tRNA->tRNA->?->?-><-?<-SF1-SF2-Helicase||BrxB-BrxF-iSTAND->DUF6079*->Schlafen+HATPase+wHTH->N6-METHYLASE->?->PglZ->SIGMA+OB-Fold+AAA-ATPase+Lon-Protease-BrxL-><-?<-REase<-SF1-SF2-Helicase+REase<-RNase+DSRBD	1187	Archaea>Euryarchaeota>Stenosarchaea_group>Methanomicrobia>Methanosarcinales>Methanosarcinaceae	GCF_003722075.1	Methanohalophilus_sp._RSK	EFE42_RS09705	37539..41102	2485783	BREX-system-P-loop-protein-BrxC-[Methanohalophilus-sp.-RSK]
WP_128754626.1	Type1.BREX	RADICAL-SAM->tRNA->tRNA->?-><-?<-?<-?||?-><-SF1-SF2-Helicase||BrxB-BrxF-iSTAND->DUF6079*->N6-METHYLASE->HD->PglZ->SIGMA+OB-Fold+AAA-ATPase+Lon-Protease-BrxL->RNaseH-><-SF1-SF2-Helicase+REase||CRISPR_Cas6->?->RAMPs->	1188	Archaea>Euryarchaeota>Stenosarchaea_group>Methanomicrobia>Methanosarcinales>Methanosarcinaceae	GCF_004104135.1	Methanohalophilus_sp._WG1-DM	CI957_RS08065	29040..32606	2491675	BREX-system-P-loop-protein-BrxC-[Methanohalophilus-sp.-WG1-DM]
WP_163645423.1	Type1.BREX	HicA_toxin-><-Mrr_N+ACET||?-><-PadR-HTH<-?<-HATPase+wHTH<-Schlafen+HATPase+wHTH-p<-Schlafen-p<-N6-METHYLASE<-DUF6079*<-BrxB-BrxF-iSTAND||<-GT4+DSBH<-Glyco_hydro_130<-?<-?<-?||?->Phage_int_SAM_4+Phage_integrase->	1212	Archaea>Euryarchaeota>Stenosarchaea_group>Methanomicrobia>Methanosarcinales>Methanosarcinaceae	GCF_010706455.1	Methanosarcina_mazei	FQU78_RS06260	1447297..1450935	2209	BREX-system-P-loop-protein-BrxC-[Methanosarcina-mazei]
WP_229393447.1	Type1.BREX	DDE_Tnp_IS66->?->?->BrxB-BrxF-iSTAND->DUF6079*->N6-METHYLASE-><-Schlafen+HATPase+wHTH||Mrr_N+ACET->PglZ->SIGMA+OB-Fold+AAA-ATPase+Lon-Protease-BrxL->MNS+REase-><-?||Calcineurin->SbcC->	1202	Archaea>Euryarchaeota>Stenosarchaea_group>Methanomicrobia>Methanosarcinales>Methanosarcinaceae	GCF_020804205.1	Methanosarcina_sp._DH1	FXW07_RS01525	42111..45719	2605695	BREX-system-P-loop-protein-BrxC-[Methanosarcina-sp.-DH1]
WP_229395640.1	Type1.BREX	BrxA-DNA-binding->BrxB-BrxF-iSTAND->DUF6079*->N6-METHYLASE->ParB+HNH->PglZ->SIGMA+OB-Fold+AAA-ATPase+Lon-Protease-BrxL-><-DUF488<-Glycos_transf_1+Glyco_trans_1_2<-?<-DSBH	1143	Archaea>Euryarchaeota>Stenosarchaea_group>Methanomicrobia>Methanosarcinales>Methanosarcinaceae	GCF_020804205.1	Methanosarcina_sp._DH1	FXW07_RS12025	174817..178248	2605695	BREX-system-P-loop-protein-BrxC-[Methanosarcina-sp.-DH1]
WP_229391422.1	Type1.BREX	BrxD-AAA->SF1-SF2-Helicase+wHTH+Small-3-barrel+DUF1998-><-SIGMA+OB-Fold+AAA-ATPase+Lon-Protease-BrxL<-PglZ||TPR+TPR+TPR+TPR-><-PadR-HTH<-Schlafen+HATPase+wHTH<-TIR<-N6-METHYLASE<-DUF6079*<-BrxB-BrxF-iSTAND<-?||||?->?->?->?-><-?<-N6-METHYLASE-p	1201	Archaea>Euryarchaeota>Stenosarchaea_group>Methanomicrobia>Methanosarcinales>Methanosarcinaceae	GCF_020804225.1	Methanosarcina_sp._DH2	FXV91_RS14640	168630..172235	2605639	BREX-system-P-loop-protein-BrxC-[Methanosarcina-sp.-DH2]
WP_250868420.1	Type1.BREX	SF1-SF2-Helicase->?-><-URI-DNase<-SF1-SF2-Helicase+REase||SF1-SF2-Helicase->BrxB-BrxF-iSTAND->DUF6079-p*->DUF6079-p->?->?->PglZ->SIGMA+OB-Fold+AAA-ATPase+Lon-Protease-BrxL-><-tRNA<-tRNA<-RADICAL-SAM	749	Archaea>Euryarchaeota>Stenosarchaea_group>Methanomicrobia>Methanosarcinales>Methanosarcinaceae	GCF_023684405.1	Methanococcoides_seepicolus	KDK67_RS08780	13773..16022	2828780	BREX-system-P-loop-protein-BrxC-[Methanococcoides-seepicolus]
WP_256623626.1	Type1.BREX	EVE+McrB-AAA->McrC-NTD+REase->?->URI-DNase->BrxB-BrxF-iSTAND->DUF6079*->N6-METHYLASE->Schlafen+HATPase+wHTH->N6-METHYLASE-p->SbcC-><-?||?->?->OLD-ABC->TOPRIM->	1185	Archaea>Euryarchaeota>Stenosarchaea_group>Methanomicrobia>Methanosarcinales>Methanosarcinaceae	GCF_024500045.1	Methanolobus_chelungpuianus	PV02_RS11875	317319..320876	502115	BREX-system-P-loop-protein-BrxC-[Methanolobus-chelungpuianus]
WP_270096529.1	Type1.BREX	SF1-SF2-Helicase+REase->HNH-><-SIGMA+OB-Fold+AAA-ATPase+Lon-Protease-BrxL<-PglZ<-TOPRIM<-OLD-ABC<-HD<-N6-METHYLASE-p<-N6-METHYLASE-p<-DUF6079*<-BrxB-BrxF-iSTAND<-REase<-?<-?<-RNase||Classical-AAA->SF1-SF2-Helicase->	1190	Archaea>Euryarchaeota>Stenosarchaea_group>Methanomicrobia>Methanosarcinales>Methanosarcinaceae	GCF_027594145.1	Methanococcoides_alaskense	PBV53_RS07490	737341..740913	325778	BREX-system-P-loop-protein-BrxC-[Methanococcoides-alaskense]
WP_091932407.1	Type1.BREX	SF1-SF2-Helicase+REase->?-><-SIGMA+OB-Fold+AAA-ATPase+Lon-Protease-BrxL<-PglZ<-TOPRIM<-OLD-ABC<-N6-METHYLASE-p<-Schlafen+HATPase+wHTH<-N6-METHYLASE<-DUF6079*<-BrxB-BrxF-iSTAND||McrC-NTD+REase->McrB-AAA->SF1-SF2-Helicase-><-DDE_Tnp_1+DDE_Tnp_1_2	1184	Archaea>Euryarchaeota>Stenosarchaea_group>Methanomicrobia>Methanosarcinales>Methanosarcinaceae	GCF_900114835.1	Methanolobus_profundi	BM008_RS01885	368008..371562	487685	BREX-system-P-loop-protein-BrxC-[Methanolobus-profundi]
WP_244603415.1	Type1.BREX	<-SIR2<-SIGMA+OB-Fold+AAA-ATPase+Lon-Protease-BrxL<-PglZ<-SF1-SF2-Helicase<-OLD-ABC+TOPRIM<-N6-METHYLASE-p<-N6-METHYLASE-p<-DUF6079*<-BrxB-BrxF-iSTAND<-URI-DNase<-?<-?||?->TPR->TPR-><-ZnR+HTH_Tnp_ISL3+DDE_Tnp_ISL3	1181	Archaea>Euryarchaeota>Stenosarchaea_group>Methanomicrobia>Methanosarcinales>Methanosarcinaceae	GCF_900774285.1	Methanococcoides_sp._NM1	E7X56_RS05470	155089..158634	1201013	BREX-system-P-loop-protein-BrxC-[Methanococcoides-sp.-NM1]
WP_172363658.1	Type1.BREX	<-SIGMA+OB-Fold+AAA-ATPase+Lon-Protease-BrxL<-PglZ<-TOPRIM<-OLD-ABC<-TIR<-N6-METHYLASE-p<-Schlafen+HATPase+wHTH<-N6-METHYLASE<-DUF6079*<-BrxB-BrxF-iSTAND<-URI-DNase<-?<-SF1-SF2-Helicase+REase||<-?||?->HNH->	1185	Archaea>Euryarchaeota>Stenosarchaea_group>Methanomicrobia>Methanosarcinales>Methanosarcinaceae	-	Methanococcoides_sp._SA1	HNV12_09470	12693..16250	2735871	BREX-system-P-loop-protein-BrxC-[Methanococcoides-sp.-SA1]
WP_048171216.1	Type1.BREX	Glyco_hydro_130->GT4+DSBH-><-?<-?||tRNA-><-NUDIX+DUF429||?->BrxB-BrxF-iSTAND->DUF6079*->N6-METHYLASE->?->OLD-ABC+TOPRIM->SF1-SF2-Helicase->Schlafen-p->	1204	Archaea>Euryarchaeota>Stenosarchaea_group>Methanomicrobia>Methanosarcinales>Methanosarcinaceae	-	Methanosarcina_sp._2.H.A.1B.4	EO92_RS09675	12629..16243	1483600	BREX-system-P-loop-protein-BrxC-[Methanosarcina-sp.-2.H.A.1B.4]
OFV66969.1	Type1.BREX	<-PglZ<-N6-METHYLASE-p<-?<-?<-SbcC<-?<-N6-METHYLASE<-DUF6079*<-BrxB-BrxF-iSTAND<-BrxA-DNA-binding<-SIGMA+OB-Fold+AAA-ATPase+Lon-Protease-BrxL||?-><-?<-?<-nSTAND	1184	Archaea>Euryarchaeota>Stenosarchaea_group>Methanomicrobia>Methanosarcinales>Methanosarcinales_incertae_sedis	GCA_001766825.1	Candidatus_Syntrophoarchaeum_butanivorans	SBU_000262	234688..238242	1839936	ATPase-[Candidatus-Syntrophoarchaeum-butanivorans]
MCD4767073.1	Type1.BREX	<-N6-METHYLASE-p<-BirA-HTH<-DrHyd<-N6-METHYLASE<-DUF6079*<-BrxB-BrxF-iSTAND	1179	Archaea>Euryarchaeota>Stenosarchaea_group>Methanomicrobia>Methanosarcinales>unclassified_Methanosarcinales	GCA_021107935.1	Methanosarcinales_archaeon	K8R34_11245	4319..7858	2250255	BREX-system-P-loop-protein-BrxC-[Methanosarcinales-archaeon]
MCD4843299.1	Type1.BREX	<-AIPR<-HD<-HTH+REase<-Schlafen-p<-TIR<-N6-METHYLASE-p<-?<-DrHyd+HTH<-N6-METHYLASE<-DUF6079*<-BrxB-BrxF-iSTAND<-?<-HicA_toxin<-HicB_lk_antitox<-?<-SAM-METHYLASE	1180	Archaea>Euryarchaeota>Stenosarchaea_group>Methanomicrobia>Methanosarcinales>unclassified_Methanosarcinales	GCA_021108405.1	Methanosarcinales_archaeon	K8R25_02310	18778..22320	2250255	BREX-system-P-loop-protein-BrxC-[Methanosarcinales-archaeon]
QNO42144.1	Type1.BREX	<-SIGMA+OB-Fold+AAA-ATPase+Lon-Protease-BrxL<-PglZ<-Schlafen+HATPase+wHTH<-SF1-SF2-Helicase<-OLD-ABC+TOPRIM<-N6-METHYLASE<-DUF6079-p<-DUF6079-p*<-BrxB-BrxF-iSTAND||DDE_Tnp_1->	488	Archaea>Euryarchaeota>Stenosarchaea_group>Methanomicrobia>Methanosarcinales>unclassified_Methanosarcinales	-	Methanosarcinales_archaeon_ANME-2c_ERB4	CAOPPJJI_00010	16805..18271	2759911	hypothetical-protein-CAOPPJJI_00010-[Methanosarcinales-archaeon-ANME-2c-ERB4]
OPY50193.1	Type1.BREX	DUF772+DDE_Tnp_1+DDE_Tnp_1_6->REase->?-><-?<-SIGMA+OB-Fold+AAA-ATPase+Lon-Protease-BrxL<-PglZ<-N6-METHYLASE<-DUF6079*<-BrxB-BrxF-iSTAND<-BrxA-DNA-binding<-?<-?<-Phage_int_SAM_4+Phage_integrase	1204	Archaea>Euryarchaeota>Stenosarchaea_group>Methanomicrobia>Methanotrichales>Methanotrichaceae	GCA_002067365.1	Methanosaeta_sp._PtaU1.Bin060	A4E48_01974	19816..23430	1811688	MAG:-hypothetical-protein-A4E48_01974-[Methanosaeta-sp.-PtaU1.Bin060]
MBN1236634.1	Type1.BREX	<-SIGMA+OB-Fold+AAA-ATPase+Lon-Protease-BrxL<-PglZ<-?<-N6-METHYLASE||SbcC->?-><-DUF6079*<-BrxB-BrxF-iSTAND<-BrxA-DNA-binding	1217	Archaea>Euryarchaeota>Stenosarchaea_group>Methanomicrobia>Methanotrichales>Methanotrichaceae	GCA_016927055.1	Methanotrichaceae_archaeon	JW999_11415	12015..15668	2595067	BREX-system-P-loop-protein-BrxC-[Methanotrichaceae-archaeon]
MBP7068907.1	Type1.BREX	<-SIGMA+OB-Fold+AAA-ATPase+Lon-Protease-BrxL<-PglZ<-?<-N6-METHYLASE<-DUF6079*<-BrxB-BrxF-iSTAND<-BrxA-DNA-binding<-REase	1215	Archaea>Euryarchaeota>Stenosarchaea_group>Methanomicrobia>Methanotrichales>Methanotrichaceae	GCA_017994095.1	Methanothrix_sp.	KBB04_11615	10819..14466	90426	BREX-system-P-loop-protein-BrxC-[Methanothrix-sp.]
MBU4220824.1	Type1.BREX	PAIREDC-HTH->?->?->?->?-><-?||BrxB-BrxF-iSTAND->DUF6079*->N6-METHYLASE->TIR->Schlafen-p->HEPN->Schlafen+HATPase+wHTH->?->?->PglZ-p->	1180	Archaea>Euryarchaeota>unclassified_Euryarchaeota	GCA_018898145.1	Euryarchaeota_archaeon	KKA10_04250	6252..9794	2026739	BREX-system-P-loop-protein-BrxC-[Euryarchaeota-archaeon]
EMR73247.1	Type1.BREX	BrxA-DNA-binding->BrxB-BrxF-iSTAND->DUF6079*->N6-METHYLASE->PglZ->?->SIGMA+OB-Fold+AAA-ATPase+Lon-Protease-BrxL-p->	1207	Archaea>TACK_group>Nitrososphaerota>unclassified_Nitrososphaerota	GCA_000349625.1	Thaumarchaeota_archaeon_SCGC_AB-539-E09	MCGE09_00285	3053..6676	1198115	hypothetical-protein-MCGE09_00285-[Thaumarchaeota-archaeon-SCGC-AB-539-E09]
MBA3712418.1	Type1.BREX	<-GDResolvase+Recombinase<-GDResolvase||<-?<-?||GspA-ATPase->?->TOPRIM+Topoisom_bac->BrxA-DNA-binding->BrxB-BrxF-iSTAND->DUF6079-p*->	967	Bacteria>Acidobacteriota>Blastocatellia>Blastocatellales>Pyrinomonadaceae>unclassified_Pyrinomonadaceae	GCA_013816505.1	Pyrinomonadaceae_bacterium	HOW76_08205	12993..15893	2283092	BREX-system-P-loop-protein-BrxC-[Pyrinomonadaceae-bacterium]
MBD0371455.1	Type1.BREX	VirB11->PSBP->?-><-SIGMA+OB-Fold+AAA-ATPase+Lon-Protease-BrxL<-PglZ<-ParB+HNH<-NERD<-N6-METHYLASE<-HEPN<-DUF6079*<-BrxB-BrxF-iSTAND<-BrxA-DNA-binding	1218	Bacteria>Acidobacteriota>Blastocatellia>Blastocatellales>Pyrinomonadaceae>unclassified_Pyrinomonadaceae	GCA_014534425.1	Pyrinomonadaceae_bacterium	ICV60_11505	29495..33151	2283092	BREX-system-P-loop-protein-BrxC-[Pyrinomonadaceae-bacterium]
MBK9154354.1	Type1.BREX	Phage_integrase->DUF488-><-?<-?<-?||HTH_3->Peptidase_M78->BrxA-DNA-binding->BrxB-BrxF-iSTAND->DUF6079-p*->?->DUF6079-p->AIPR->?->PglZ->PglZ->Exonuc_V_gamma+REase->SF1-SF2-Helicase->SF1-SF2-Helicase->	481	Bacteria>Acidobacteriota>Blastocatellia>Chloracidobacterium>unclassified_Chloracidobacterium	GCA_016716365.1	Chloracidobacterium_sp.	IPM25_09080	169835..171280	2099375	BREX-system-P-loop-protein-BrxC-[Chloracidobacterium-sp.]
MCI0490100.1	Type1.BREX	BrxA-DNA-binding->BrxB-BrxF-iSTAND->DUF6079*->HEPN->Four-helical-protein-><-?<-?||N6-METHYLASE->Schlafen+HATPase+wHTH-p->	1218	Bacteria>Acidobacteriota>Blastocatellia>unclassified_Blastocatellia	GCA_022615215.1	Blastocatellia_bacterium	L0229_26200	2085..5741	2052146	BREX-system-P-loop-protein-BrxC-[Blastocatellia-bacterium]
WP_005031444.1	Type1.BREX	HEPN+PAS+DUF4011+SF1-SF2-Helicase+REase-><-SIGMA+OB-Fold+AAA-ATPase+Lon-Protease-BrxL<-PglZ<-Schlafen+HATPase+wHTH<-N6-METHYLASE<-Trypsin<-DUF6079*<-BrxB-BrxF-iSTAND<-BrxA-DNA-binding||?->ZetaToxin->wHTH+SAD+HNH->?-><-DOC+HTH	1205	Bacteria>Acidobacteriota>Holophagae>Holophagales>Holophagaceae>Holophaga	GCF_000242615.2	Holophaga_foetida	HOLFODRAFT_RS0203665	863160..866777	35839	BREX-system-P-loop-protein-BrxC-[Holophaga-foetida]
MXX75209.1	Type1.BREX	Cysteine-Desulfurase-><-?||BrxA-DNA-binding->BrxB-BrxF-iSTAND->DUF6079*->HTH+MPTase->?->?->?->N6-METHYLASE->PglZ->SIGMA+OB-Fold+AAA-ATPase+Lon-Protease-BrxL->?-><-ABhydrolase	1173	Bacteria>Acidobacteriota>Holophagae>Holophagales>unclassified_Holophagales	GCA_009837885.1	Holophagales_bacterium	F4Y77_07375	64446..67967	2478486	BREX-system-P-loop-protein-BrxC-[Holophagales-bacterium]
MBK9374878.1	Type1.BREX	<-NtrC-AAA+FIS-HTH||?->?-><-?||?->?->BrxA-DNA-binding->DUF6079*->N6-METHYLASE-p->?->PglZ->Tox-RelE->HTH_37->	1147	Bacteria>Acidobacteriota>Holophagae>Holophagales>unclassified_Holophagales	GCA_016719485.1	Holophagales_bacterium	IPN03_14410	526057..529500	2478486	BREX-system-P-loop-protein-BrxC-[Holophagales-bacterium]
MBK9968089.1	Type1.BREX	<-HAD+HAD<-?<-?<-?<-?||XIS-HTH->BrxA-DNA-binding->BrxB-BrxF-iSTAND->DUF6079*->N6-METHYLASE->DOC+HTH->PglZ->SIGMA+OB-Fold+AAA-ATPase+Lon-Protease-BrxL->	1245	Bacteria>Acidobacteriota>Holophagae>Holophagales>unclassified_Holophagales	GCA_016722225.1	Holophagales_bacterium	IPP07_25730	57868..61605	2478486	BREX-system-P-loop-protein-BrxC-[Holophagales-bacterium]
MBZ2183842.1	Type1.BREX	<-UbiA<-?||?->ABhydrolase-><-?||?-><-?||BrxA-DNA-binding->BrxB-BrxF-iSTAND->DUF6079*->N6-METHYLASE->ArdCN+MPTase->Schlafen+HATPase+wHTH->ParA-Soj-PloopNTPase->PglZ->SIGMA+OB-Fold+AAA-ATPase+Lon-Protease-BrxL->?->?->ROK-HTH->	1166	Bacteria>Acidobacteriota>Terriglobia>Bryobacterales>Bryobacteraceae>Bryobacter	GCA_019931185.1	Bryobacter_sp.	K7J46_03960	13925..17425	1962911	BREX-system-P-loop-protein-BrxC-[Bryobacter-sp.]
WP_238326045.1	Type1.BREX	<-SIGMA+OB-Fold+AAA-ATPase+Lon-Protease-BrxL<-PglZ<-PHP+SbcC<-N6-METHYLASE<-DUF6079*<-BrxB-BrxF-iSTAND<-BrxA-DNA-binding||?->AbiEii->	1159	Bacteria>Acidobacteriota>Terriglobia>Bryobacterales>Bryobacteraceae>Bryobacter	GCF_000702445.1	Bryobacter_aggregatus	M017_RS0125570	1351195..1354674	360054	BREX-system-P-loop-protein-BrxC-[Bryobacter-aggregatus]
GIU77527.1	Type1.BREX	Phage_int_SAM_4+Phage_integrase->HTH_21+rve-><-?<-?||?->HTH_21+rve->?-><-PHP+SbcC<-SIGMA+OB-Fold+AAA-ATPase+Lon-Protease-BrxL<-PglZ<-N6-METHYLASE<-DUF6079*<-BrxB-BrxF-iSTAND<-BrxA-DNA-binding<-?||DUF772+DDE_Tnp_1+DDE_Tnp_1_6-><-XIS-HTH||MULE-TRANSPOSASE->HIN-HTH+rve+rve_3->?->DUF772+DDE_Tnp_1+DDE_Tnp_1_6->	1157	Bacteria>Acidobacteriota>Terriglobia>Bryobacterales>Bryobacteraceae>unclassified_Bryobacteraceae	GCA_026002875.1	Bryobacteraceae_bacterium	KatS3mg005_0765	905101..908574	2212468	MAG:-hypothetical-protein-KatS3mg005_0765-[Bryobacteraceae-bacterium]
MBK9165938.1	Type1.BREX	XIS-HTH-><-?<-SIGMA+OB-Fold+AAA-ATPase+Lon-Protease-BrxL<-PglZ<-PglZ<-PglZ<-?<-N6-METHYLASE<-HEPN<-DUF6079*<-BrxB-BrxF-iSTAND<-BrxA-DNA-binding<-CopG/RHH||?-><-ParD_antitoxin<-HTH_37||?->?->Tox-RelE->	1234	Bacteria>Acidobacteriota>Terriglobia>Bryobacterales>unclassified_Bryobacterales	GCA_016716385.1	Bryobacterales_bacterium	IPM24_00570	108049..111753	2026791	BREX-system-P-loop-protein-BrxC-[Bryobacterales-bacterium]
MCC6390067.1	Type1.BREX	ParB->ParB->TPR->?->MACRODOMAIN<-SIGMA+OB-Fold+AAA-ATPase+Lon-Protease-BrxL<-PglZ<-N6-METHYLASE-p<-DOC+HTH<-N6-METHYLASE<-DUF6079*<-BrxB-BrxF-iSTAND<-BrxA-DNA-binding<-?||?-><-?<-ART-DarT<-SF1-SF2-Helicase<-HTH+MPTase	1241	Bacteria>Acidobacteriota>Terriglobia>Bryobacterales>unclassified_Bryobacterales	GCA_020847895.1	Bryobacterales_bacterium	IT167_05665	14981..18706	2026791	BREX-system-P-loop-protein-BrxC-[Bryobacterales-bacterium]
WP_089839623.1	Type1.BREX	ParB->TIR->Peptidase_C14-><-?<-REase<-SIGMA+OB-Fold+AAA-ATPase+Lon-Protease-BrxL<-PglZ<-N6-METHYLASE<-DUF6079*<-BrxB-BrxF-iSTAND<-BrxA-DNA-binding<-?||?-><-?<-MODE-HTH+PBPII||XIS-HTH->Phage_int_SAM_3+Phage_integrase->	1149	Bacteria>Acidobacteriota>Terriglobia>Terriglobales>Acidobacteriaceae>Granulicella	GCF_900114625.1	Granulicella_pectinivorans	BM400_RS13360	3371671..3375120	474950	BREX-system-P-loop-protein-BrxC-[Granulicella-pectinivorans]
MBV9502366.1	Type1.BREX	CopG/RHH->BrxA-DNA-binding->BrxB-BrxF-iSTAND->DUF6079-p*->SIGMA+OB-Fold+AAA-ATPase+Lon-Protease-BrxL->SIGMA+OB-Fold+AAA-ATPase+Lon-Protease-BrxL->ArdCN+MPTase->	537	Bacteria>Acidobacteriota>Terriglobia>Terriglobales>Acidobacteriaceae>unclassified_Acidobacteriaceae	GCA_019244055.1	Acidobacteriaceae_bacterium	J0138_23605	3237..4850	2052142	hypothetical-protein-[Acidobacteriaceae-bacterium]
MBZ5499154.1	Type1.BREX	Phage_int_SAM_1+Phage_integrase-><-?<-SIGMA+OB-Fold+AAA-ATPase+Lon-Protease-BrxL<-PglZ<-Schlafen+HATPase+wHTH<-SbcC<-N6-METHYLASE<-?<-TIR<-DUF6079*<-BrxB-BrxF-iSTAND<-BrxA-DNA-binding||XIS-HTH->	1147	Bacteria>Acidobacteriota>Terriglobia>unclassified_Terriglobia	GCA_020072565.1	Acidobacteria_bacterium	LAP85_22370	28879..32322	2081523	BREX-system-P-loop-protein-BrxC-[Acidobacteria-bacterium]
MBP7799662.1	Type1.BREX	<-CR-ATPase5+REase<-REase+SF1-SF2-Helicase<-?||DDE_Tnp-><-SIGMA+OB-Fold+AAA-ATPase+Lon-Protease-BrxL<-PglZ<-Schlafen+HATPase+wHTH<-N6-METHYLASE<-?<-DUF6079*<-BrxB-BrxF-iSTAND<-BrxA-DNA-binding	1146	Bacteria>Acidobacteriota>Thermoanaerobaculia>Thermoanaerobaculales>Thermoanaerobaculaceae>unclassified_Thermoanaerobaculaceae	GCA_018000955.1	Thermoanaerobaculaceae_bacterium	KA072_13665	19624..23064	2699758	BREX-system-P-loop-protein-BrxC-[Thermoanaerobaculaceae-bacterium]
OFW00070.1	Type1.BREX	HTH_21+rve+rve_3-><-?<-?<-?<-?||DDE_Tnp_1_4-><-SIGMA+OB-Fold+AAA-ATPase+Lon-Protease-BrxL<-PglZ<-N6-METHYLASE<-DUF6079*<-BrxB-BrxF-iSTAND<-BrxA-DNA-binding<-REase<-?<-?||HNH->ParB+N6_N4_METHYLASE->	1145	Bacteria>Acidobacteriota>unclassified_Acidobacteriota	GCA_001767075.1	Acidobacteria_bacterium_RIFCSPLOWO2_12_FULL_54_10	A3F68_05125	19518..22955	1797186	hypothetical-protein-A3F68_05125-[Acidobacteria-bacterium-RIFCSPLOWO2_12_FULL_54_10]
OFW17305.1	Type1.BREX	<-SF1-SF2-Helicase<-SIGMA+OB-Fold+AAA-ATPase+Lon-Protease-BrxL<-PglZ<-HATPase+wHTH<-HATPase_c_4<-Schlafen-p<-N6-METHYLASE<-HEPN<-PolB<-DUF6079*<-BrxB-BrxF-iSTAND<-BrxA-DNA-binding||wHTH+AbiEi_2->AbiEii-><-?<-?<-PIN	1156	Bacteria>Acidobacteriota>unclassified_Acidobacteriota	GCA_001767195.1	Acidobacteria_bacterium_RIFCSPLOWO2_02_FULL_67_21	A3H29_10520	11544..15014	1797183	hypothetical-protein-A3H29_10520-[Acidobacteria-bacterium-RIFCSPLOWO2_02_FULL_67_21]
OFW39743.1	Type1.BREX	PIN->?->?-><-AbiEii<-wHTH+AbiEi_2||BrxA-DNA-binding->BrxB-BrxF-iSTAND->DUF6079*->PolB->HEPN->N6-METHYLASE->Schlafen+HATPase+wHTH->HATPase+wHTH->PglZ->SIGMA+OB-Fold+AAA-ATPase+Lon-Protease-BrxL->SF1-SF2-Helicase->DDE_Tnp_1_4->	1156	Bacteria>Acidobacteriota>unclassified_Acidobacteriota	GCA_001767515.1	Acidobacteria_bacterium_RIFCSPLOWO2_12_FULL_67_14	A3F70_01760	7438..10908	1797192	hypothetical-protein-A3F70_01760-[Acidobacteria-bacterium-RIFCSPLOWO2_12_FULL_67_14]
PYQ26570.1	Type1.BREX	BrxA-DNA-binding->BrxB-BrxF-iSTAND->DUF6079*->N6-METHYLASE->DOC+HTH->PglZ->SIGMA+OB-Fold+AAA-ATPase+Lon-Protease-BrxL->?-><-?||ACET+ACET->SIGMA-HTH->	1155	Bacteria>Acidobacteriota>unclassified_Acidobacteriota	GCA_003223645.1	Acidobacteriota_bacterium	DMF56_23440	3756..7223	1978231	BREX-system-P-loop-protein-BrxC-[Acidobacteriota-bacterium]
MYN66269.1	Type1.BREX	BrxA-DNA-binding->BrxB-BrxF-iSTAND->DUF6079*->OLD-ABC->TOPRIM->Schlafen+HATPase+wHTH->N6-METHYLASE->?->?->SbcC->PHP+SbcC->Schlafen+HATPase+wHTH->	1283	Bacteria>Acidobacteriota>unclassified_Acidobacteriota	GCA_009861545.1	Acidobacteriota_bacterium	F4X11_14760	2943..6794	1978231	BREX-system-P-loop-protein-BrxC-[Acidobacteriota-bacterium]
NLV30311.1	Type1.BREX	DNA-glycosylase-><-?<-SH3||wHTH+WYL+WCX->SF1-SF2-Helicase->BrxA-DNA-binding->BrxB-BrxF-iSTAND->DUF6079*->Schlafen+HATPase+wHTH+HTH->N6-METHYLASE->PglZ->SIGMA+OB-Fold+AAA-ATPase+HKD-DNase-BrxL->HigB_toxin->HTH+MPTase->SF1-SF2-Helicase->?-><-DSBA+Thioredoxin	1169	Bacteria>Acidobacteriota>unclassified_Acidobacteriota	GCA_012729555.1	Acidobacteriota_bacterium	GXY47_04070	27594..31103	1978231	BREX-system-P-loop-protein-BrxC-[Acidobacteriota-bacterium]
MBI2837087.1	Type1.BREX	<-SIGMA+OB-Fold+AAA-ATPase+Lon-Protease-BrxL<-PglZ<-Schlafen+HATPase+wHTH<-PHP+SbcC<-Schlafen+HATPase+wHTH<-PHP+SbcC<-N6-METHYLASE<-DUF6079*<-BrxB-BrxF-iSTAND<-BrxA-DNA-binding<-XIS-HTH	1224	Bacteria>Acidobacteriota>unclassified_Acidobacteriota	GCA_016191015.1	Acidobacteriota_bacterium	HYX75_02140	17742..21416	1978231	BREX-system-P-loop-protein-BrxC-[Acidobacteriota-bacterium]
MBI4904787.1	Type1.BREX	Sulfatase+DUF4994-><-?<-?<-?||XIS-HTH->BrxA-DNA-binding->BrxB-BrxF-iSTAND->DUF6079*->N6-METHYLASE->REase+AIPR->PglZ->SIGMA+OB-Fold+AAA-ATPase+Lon-Protease-BrxL->?->?->?-><-?<-HTH_3	1144	Bacteria>Acidobacteriota>unclassified_Acidobacteriota	GCA_016213775.1	Acidobacteriota_bacterium	HY820_14195	24664..28098	1978231	BREX-system-P-loop-protein-BrxC-[Acidobacteriota-bacterium]
MBS1828808.1	Type1.BREX	D5N+D5ATPase-><-?||ParB-><-?<-Phage_integrase+Phage_integrase<-?||CopG/RHH->BrxA-DNA-binding->BrxB-BrxF-iSTAND->DUF6079*->HEPN->N6-METHYLASE->PglZ->SIGMA+OB-Fold+AAA-ATPase+Lon-Protease-BrxL->REase->HEPN->?->Phage_integrase->	1226	Bacteria>Acidobacteriota>unclassified_Acidobacteriota	GCA_018268935.1	Acidobacteriota_bacterium	JST93_26130	24024..27704	1978231	BREX-system-P-loop-protein-BrxC-[Acidobacteriota-bacterium]
MBV8891344.1	Type1.BREX	HTH_Tnp_1+HTH_21+rve+rve_3->?->CopG/RHH->BrxA-DNA-binding->BrxB-BrxF-iSTAND->DUF6079-p*->	334	Bacteria>Acidobacteriota>unclassified_Acidobacteriota	GCA_019243065.1	Acidobacteriota_bacterium	J0266_05105	5695..6697	1978231	BREX-system-P-loop-protein-BrxC-[Acidobacteriota-bacterium]
MCI0392588.1	Type1.BREX	NtrC-AAA+FIS-HTH->BrxA-DNA-binding->BrxB-BrxF-iSTAND->DUF6079*->HKD-DNase+DUF3150->N6-METHYLASE->	1160	Bacteria>Acidobacteriota>unclassified_Acidobacteriota	GCA_022614935.1	Acidobacteriota_bacterium	MOB07_27970	3246..6728	1978231	BREX-system-P-loop-protein-BrxC-[Acidobacteriota-bacterium]
MCI0393103.1	Type1.BREX	BrxA-DNA-binding->BrxB-BrxF-iSTAND->DUF6079*-><-?||HEPN->N6-METHYLASE->Schlafen+HATPase+wHTH+HTH->PglZ->SIGMA+OB-Fold+AAA-ATPase+Lon-Protease-BrxL-><-MPTase<-HTH_3	1220	Bacteria>Acidobacteriota>unclassified_Acidobacteriota	GCA_022614935.1	Acidobacteriota_bacterium	MOB07_30595	1910..5572	1978231	BREX-system-P-loop-protein-BrxC-[Acidobacteriota-bacterium]
MCI0407271.1	Type1.BREX	HNH->URI-DNase->?->REase->TPR+TPR->?->CHTH->BrxA-DNA-binding->BrxB-BrxF-iSTAND->DUF6079*->N6-METHYLASE->PglZ->SIGMA+OB-Fold+AAA-ATPase+Lon-Protease-BrxL->METHYLASE->Radical-SAM->?->?->XIS-HTH->Thermonuclease->	1222	Bacteria>Acidobacteriota>unclassified_Acidobacteriota	GCA_022615035.1	Acidobacteriota_bacterium	L0191_01695	12711..16379	1978231	BREX-system-P-loop-protein-BrxC-[Acidobacteriota-bacterium]
MCI0624094.1	Type1.BREX	<-HHH_5+RecR+TOPRIM<-?<-RFC-AAA+RuvB-AAA<-ncRNA<-PglZ<-PIN<-PhdYeFM_antitox<-REase<-DUF6079*<-BrxB-BrxF-iSTAND<-BrxA-DNA-binding||?->?->?-><-AA_kinase+ACT+ACT_7<-NAD_binding_3+Homoserine_dh<-REC+NtrC-AAA+FIS-HTH<-GAF+PAS+HISKIN	1197	Bacteria>Acidobacteriota>unclassified_Acidobacteriota	GCA_022615935.1	Acidobacteriota_bacterium	L0387_20995	11986..15579	1978231	BREX-system-P-loop-protein-BrxC-[Acidobacteriota-bacterium]
MCI0628306.1	Type1.BREX	TraJ-RHH->VirD4-TraG+VirD4-FtsK-><-Schlafen+PIWI<-SIGMA+OB-Fold+AAA-ATPase+Lon-Protease-BrxL<-PglZ<-N6-METHYLASE-p<-DOC+HTH<-N6-METHYLASE<-DUF6079*	1127	Bacteria>Acidobacteriota>unclassified_Acidobacteriota	GCA_022615935.1	Acidobacteriota_bacterium	L0387_42755	64096..67479	1978231	BREX-system-P-loop-protein-BrxC-[Acidobacteriota-bacterium]
MCI0724954.1	Type1.BREX	DUF6079-p*->N6-METHYLASE->DOC+HTH->N6-METHYLASE-p->PglZ->SIGMA+OB-Fold+AAA-ATPase+Lon-Protease-BrxL-p->	736	Bacteria>Acidobacteriota>unclassified_Acidobacteriota	GCA_022616615.1	Acidobacteriota_bacterium	L0338_39220	1..2211	1978231	hypothetical-protein-[Acidobacteriota-bacterium]
PKP62156.1	Type1.BREX	SbcC+SbcC->BrxA-DNA-binding->BrxB-BrxF-iSTAND->DUF6079*->N6-METHYLASE->PglZ->SIGMA+OB-Fold+AAA-ATPase+Lon-Protease-BrxL->HNH->STYKIN->TPR+TPR->TPR->	1193	Bacteria>Atribacterota>unclassified_Atribacterota	GCA_002841055.1	Candidatus_Atribacteria_bacterium_HGW-Atribacteria-1	CVT91_00360	3425..7006	2013676	BREX-system-P-loop-protein-BrxC-[Candidatus-Atribacteria-bacterium-HGW-Atribacteria-1]
PKP59611.1	Type1.BREX	BrxB-BrxF-iSTAND-p->DUF6079-p->DUF6079*->N6-METHYLASE->PglZ->	1026	Bacteria>Atribacterota>unclassified_Atribacterota	GCA_002841055.1	Candidatus_Atribacteria_bacterium_HGW-Atribacteria-1	CVT91_06440	727..3807	2013676	BREX-system-P-loop-protein-BrxC-[Candidatus-Atribacteria-bacterium-HGW-Atribacteria-1]
PKP59550.1	Type1.BREX	<-PglZ<-N6-METHYLASE<-DUF6079*<-BrxB-BrxF-iSTAND-p	1170	Bacteria>Atribacterota>unclassified_Atribacterota	GCA_002841055.1	Candidatus_Atribacteria_bacterium_HGW-Atribacteria-1	CVT91_06535	4911..8423	2013676	BREX-system-P-loop-protein-BrxC-[Candidatus-Atribacteria-bacterium-HGW-Atribacteria-1]
PKP57580.1	Type1.BREX	<-DUF6079*<-BrxB-BrxF-iSTAND<-DUF4007<-Mrr_N<-N6-METHYLASE<-?<-?||rve->	1366	Bacteria>Atribacterota>unclassified_Atribacterota	GCA_002841055.1	Candidatus_Atribacteria_bacterium_HGW-Atribacteria-1	CVT91_10260	255..4355	2013676	hypothetical-protein-CVT91_10260-[Candidatus-Atribacteria-bacterium-HGW-Atribacteria-1]
MBN2413375.1	Type1.BREX	BrxA-DNA-binding->BrxB-BrxF-iSTAND->DUF6079*->PglZ->N6-METHYLASE->SIR2->AbiEi_4->AbiEii->SIGMA+OB-Fold+AAA-ATPase+Lon-Protease-BrxL->?->HTH_3->HIN-HTH->	1212	Bacteria>Bacteria_incertae_sedis>Bacteria_candidate_phyla>candidate_division_KSB1>unclassified_candidate_division_KSB1	GCA_016934825.1	candidate_division_KSB1_bacterium	JXQ31_16970	1525..5163	2172550	BREX-system-P-loop-protein-BrxC-[candidate-division-KSB1-bacterium]
MCI0698477.1	Type1.BREX	<-Schlafen+HATPase+wHTH<-HEPN<-DUF6079*<-BrxB-BrxF-iSTAND<-BrxA-DNA-binding<-AbiEii+ZnR<-AbiEi_4+AbiEi_1	1215	Bacteria>Bacteria_incertae_sedis>Bacteria_candidate_phyla>candidate_division_KSB1>unclassified_candidate_division_KSB1	GCA_022616415.1	candidate_division_KSB1_bacterium	L0337_41575	2047..5694	2172550	BREX-system-P-loop-protein-BrxC-[candidate-division-KSB1-bacterium]
KUK86067.1	Type1.BREX	<-SIGMA+OB-Fold+AAA-ATPase+Lon-Protease-BrxL<-PglZ<-N6-METHYLASE<-DUF6079*<-BrxB-BrxF-iSTAND<-BrxA-DNA-binding	1197	Bacteria>Bacteria_incertae_sedis>Bacteria_candidate_phyla>candidate_division_TA06	GCA_001508575.1	candidate_division_TA06_bacterium_34_109	XE03_1737	9245..12838	1635277	hypothetical-protein-XE03_1737-[candidate-division-TA06-bacterium-34_109]
NIM12892.1	Type1.BREX	BrxA-DNA-binding->BrxB-BrxF-iSTAND->DUF6079*->N6-METHYLASE->PglZ->SIGMA+OB-Fold+AAA-ATPase+Lon-Protease-BrxL->?-><-?<-?<-TPR+TPR<-?<-TPR	1215	Bacteria>Bacteria_incertae_sedis>Bacteria_candidate_phyla>Candidatus_Aminicenantes>unclassified_Aminicenantes	GCA_011771225.1	Candidatus_Aminicenantes_bacterium	GT081_12995	3544..7191	2052149	BREX-system-P-loop-protein-BrxC-[Candidatus-Aminicenantes-bacterium]
NIM13883.1	Type1.BREX	<-SIGMA+OB-Fold+AAA-ATPase+Lon-Protease-BrxL<-PglZ<-N6-METHYLASE<-DUF6079*<-BrxB-BrxF-iSTAND<-BrxA-DNA-binding||?->?-><-?<-?||HTH_3->?->HTH_3->	1218	Bacteria>Bacteria_incertae_sedis>Bacteria_candidate_phyla>Candidatus_Aminicenantes>unclassified_Aminicenantes	GCA_011771225.1	Candidatus_Aminicenantes_bacterium	GT081_18040	9506..13162	2052149	BREX-system-P-loop-protein-BrxC-[Candidatus-Aminicenantes-bacterium]
HDN19844.1	Type1.BREX	BrxA-DNA-binding->BrxB-BrxF-iSTAND->DUF6079*->N6-METHYLASE->?->?->N6-METHYLASE->PglZ->SIGMA+OB-Fold+AAA-ATPase+Lon-Protease-BrxL->?->HTH_36->	1231	Bacteria>Bacteria_incertae_sedis>Bacteria_candidate_phyla>Candidatus_Bipolaricaulota>unclassified_Candidatus_Bipolaricaulota	GCA_011042375.1	Candidatus_Acetothermia_bacterium	ENF46_01950	2856..6551	2053493	BREX-system-P-loop-protein-BrxC-[Candidatus-Acetothermia-bacterium]
MCD5408800.1	Type1.BREX	BrxB-BrxF-iSTAND->DUF6079-p*->DUF6079-p->N6-METHYLASE->PglZ->SIGMA+OB-Fold+AAA-ATPase+Lon-Protease-BrxL->?->?-><-?||FURR-HTH->	174	Bacteria>Bacteria_incertae_sedis>Bacteria_candidate_phyla>Candidatus_Bipolaricaulota>unclassified_Candidatus_Bipolaricaulota	GCA_021129895.1	Candidatus_Bipolaricaulota_bacterium	LR090_06240	1440..1964	2306012	hypothetical-protein-[Candidatus-Bipolaricaulota-bacterium]
MCD5408801.1	Type1.BREX	BrxB-BrxF-iSTAND->DUF6079-p->DUF6079-p*->N6-METHYLASE->PglZ->SIGMA+OB-Fold+AAA-ATPase+Lon-Protease-BrxL->?->?-><-?||FURR-HTH->?->ABC-ATPase+SbcC->	995	Bacteria>Bacteria_incertae_sedis>Bacteria_candidate_phyla>Candidatus_Bipolaricaulota>unclassified_Candidatus_Bipolaricaulota	GCA_021129895.1	Candidatus_Bipolaricaulota_bacterium	LR090_06245	1904..4891	2306012	BREX-system-P-loop-protein-BrxC-[Candidatus-Bipolaricaulota-bacterium]
MCC6649984.1	Type1.BREX	<-BrxD-AAA<-PglZ<-Schlafen+HATPase+wHTH<-RNase+PHP||?->?-><-N6-METHYLASE<-?<-?<-DUF6079-p*	705	Bacteria>Bacteria_incertae_sedis>Bacteria_candidate_phyla>Candidatus_Eisenbacteria	GCA_020849095.1	Candidatus_Eisenbacteria_bacterium	IT348_02410	16201..18318	2212470	hypothetical-protein-[Candidatus-Eisenbacteria-bacterium]
MCK4303758.1	Type1.BREX	BrxA-DNA-binding->BrxB-BrxF-iSTAND->DUF6079*->N6-METHYLASE->Schlafen+HATPase+wHTH-><-FGS<-?||?-><-DDE_Tnp	1190	Bacteria>Bacteria_incertae_sedis>Bacteria_candidate_phyla>Candidatus_Eisenbacteria>unclassified_Candidatus_Eisenbacteria	GCA_023128935.1	Candidatus_Eisenbacteria_sp.	KAY24_05935	1288..4860	2814261	BREX-system-P-loop-protein-BrxC-[Candidatus-Eisenbacteria-sp.]
MCK4412365.1	Type1.BREX	<-ACET||?->Radical_SAM->?-><-SIGMA+OB-Fold+AAA-ATPase+Lon-Protease-BrxL<-PglZ<-N6-METHYLASE<-HATPase+wHTH<-?<-DUF6079*<-BrxB-BrxF-iSTAND<-BrxA-DNA-binding<-?||?->?-><-?<-DSBH	1148	Bacteria>Bacteria_incertae_sedis>Bacteria_candidate_phyla>Candidatus_Eisenbacteria>unclassified_Candidatus_Eisenbacteria	GCA_023129715.1	Candidatus_Eisenbacteria_sp.	KAY32_02355	86405..89851	2814261	BREX-system-P-loop-protein-BrxC-[Candidatus-Eisenbacteria-sp.]
OGG45687.1	Type1.BREX	<-DOC+HTH<-PIN<-wHTH<-HTH_3<-N6-METHYLASE<-DUF6079*<-BrxB-BrxF-iSTAND<-BrxA-DNA-binding	1145	Bacteria>Bacteria_incertae_sedis>Bacteria_candidate_phyla>Candidatus_Handelsmanbacteria	GCA_001781055.1	Candidatus_Handelsmanbacteria_bacterium_RIFCSPLOWO2_12_FULL_64_10	A3F84_14490	8296..11733	1817868	hypothetical-protein-A3F84_14490-[Candidatus-Handelsmanbacteria-bacterium-RIFCSPLOWO2_12_FULL_64_10]
MCK9474444.1	Type1.BREX	BrxA-DNA-binding->BrxB-BrxF-iSTAND->DUF6079*->OLD-ABC->NERD->N6-METHYLASE->PglZ->SIGMA+OB-Fold+AAA-ATPase+HKD-DNase-BrxL->?->ZnR->?->PadR-HTH->	1170	Bacteria>Bacteria_incertae_sedis>Bacteria_candidate_phyla>Candidatus_Muirbacteria>Candidatus_Muirbacterium	GCA_023228995.1	Candidatus_Muirbacterium_halophilum	MOR46_00860	28955..32467	2053465	BREX-system-P-loop-protein-BrxC-[Candidatus-Muirbacterium-halophilum]
MBD3180773.1	Type1.BREX	BrxA-DNA-binding->BrxB-BrxF-iSTAND->DUF6079-p*->DUF6079-p->N6-METHYLASE->	557	Bacteria>Bacteria_incertae_sedis>Bacteria_candidate_phyla>Candidatus_Poribacteria>unclassified_Candidatus_Poribacteria	GCA_014730235.1	Candidatus_Poribacteria_bacterium	GF312_00675	1397..3070	2026781	BREX-system-P-loop-protein-BrxC-[Candidatus-Poribacteria-bacterium]
MCD6506891.1	Type1.BREX	<-N6-METHYLASE<-?<-Schlafen+HATPase+wHTH<-N6-METHYLASE<-DUF6079*<-BrxB-BrxF-iSTAND<-BrxA-DNA-binding<-SIGMA+OB-Fold+AAA-ATPase+Lon-Protease-BrxL	1172	Bacteria>Bacteria_incertae_sedis>Bacteria_candidate_phyla>Candidatus_Poribacteria>unclassified_Candidatus_Poribacteria	GCA_021162805.1	Candidatus_Poribacteria_bacterium	J7M22_09745	5721..9239	2026781	BREX-system-P-loop-protein-BrxC-[Candidatus-Poribacteria-bacterium]
PKL50472.1	Type1.BREX	<-MPTase<-HTH_19<-?||BrxA-DNA-binding->BrxB-BrxF-iSTAND->DUF6079*->Schlafen+HATPase+wHTH+HTH->OLD-ABC->REase->N6-METHYLASE->PglZ->SIGMA+OB-Fold+AAA-ATPase+HKD-DNase-BrxL->	1185	Bacteria>Bacteria_incertae_sedis>Bacteria_candidate_phyla>Candidatus_Riflebacteria>unclassified_Candidatus_Riflebacteria	GCA_002839435.1	Candidatus_Riflebacteria_bacterium_HGW-Riflebacteria-2	CVV42_02960	62937..66494	2013830	MAG:-BREX-system-P-loop-protein-BrxC-[Candidatus-Riflebacteria-bacterium-HGW-Riflebacteria-2]
MBF0500206.1	Type1.BREX	IG+IG-><-ANK<-?<-ANK+ANK+ANK<-?||EVE->BrxA-DNA-binding->BrxB-BrxF-iSTAND->DUF6079*->N6-METHYLASE->OLD-ABC->TOPRIM->N6-METHYLASE->PglZ->SIGMA+OB-Fold+AAA-ATPase+Lon-Protease-BrxL->DDE_Tnp_IS66-><-SbcC	1142	Bacteria>Bacteria_incertae_sedis>Bacteria_candidate_phyla>Candidatus_Riflebacteria>unclassified_Candidatus_Riflebacteria	GCA_015233155.1	Candidatus_Riflebacteria_bacterium	HQM09_08735	64284..67712	2053586	BREX-system-P-loop-protein-BrxC-[Candidatus-Riflebacteria-bacterium]
OGL14724.1	Type1.BREX	REase-><-?<-ArdCN+MPTase<-SIGMA+OB-Fold+AAA-ATPase+Lon-Protease-BrxL<-PglZ<-Schlafen+HATPase+wHTH<-RNase+PHP<-N6-METHYLASE<-DUF6079*<-BrxB-BrxF-iSTAND<-BrxA-DNA-binding	1145	Bacteria>Bacteria_incertae_sedis>Bacteria_candidate_phyla>Candidatus_Rokubacteria>unclassified_Candidatus_Rokubacteria	GCA_001790335.1	Candidatus_Rokubacteria_bacterium_RIFCSPLOWO2_12_FULL_71_22	A3F92_03770	25373..28810	1802110	MAG:-hypothetical-protein-A3F92_03770-[Candidatus-Rokubacteria-bacterium-RIFCSPLOWO2_12_FULL_71_22]
OIP86292.1	Type1.BREX	SLATT-><-HerA/FtsK||ZnR+HARE-HTH->ABhydrolase-><-?||BrxA-DNA-binding->BrxB-BrxF-iSTAND->DUF6079*->N6-METHYLASE->PglZ->SIGMA+OB-Fold+AAA-ATPase+Lon-Protease-BrxL->?->?-><-HerA/FtsK	1102	Bacteria>Bacteria_incertae_sedis>Bacteria_candidate_phyla>Candidatus_Saccharibacteria>unclassified_Candidatus_Saccharibacteria	GCA_001873625.1	Candidatus_Saccharibacteria_bacterium_CG2_30_41_52	AUK57_00775	137723..141031	1805375	hypothetical-protein-AUK57_00775-[Candidatus-Saccharibacteria-bacterium-CG2_30_41_52]
NCC19592.1	Type1.BREX	<-SIGMA+OB-Fold+AAA-ATPase+Lon-Protease-BrxL<-PglZ<-?<-N6-METHYLASE<-DUF6079*<-BrxB-BrxF-iSTAND<-BrxA-DNA-binding<-?<-ZnR+HARE-HTH<-HerA/FtsK	1102	Bacteria>Bacteria_incertae_sedis>Bacteria_candidate_phyla>Candidatus_Saccharibacteria>unclassified_Candidatus_Saccharibacteria	GCA_009929655.1	Candidatus_Saccharibacteria_bacterium	EOM33_00865	8194..11502	2026720	BREX-system-P-loop-protein-BrxC-[Candidatus-Saccharibacteria-bacterium]
MBQ3440720.1	Type1.BREX	<-Glycos_transf_1+Glyco_trans_1_2<-?<-?||?-><-?<-?<-?||BrxA-DNA-binding->BrxB-BrxF-iSTAND->DUF6079*->N6-METHYLASE->PglZ->SIGMA+OB-Fold+AAA-ATPase+Lon-Protease-BrxL->?->ZnR->	1108	Bacteria>Bacteria_incertae_sedis>Bacteria_candidate_phyla>Candidatus_Saccharibacteria>unclassified_Candidatus_Saccharibacteria	GCA_017406285.1	Candidatus_Saccharibacteria_bacterium	IJG27_00165	27802..31128	2026720	BREX-system-P-loop-protein-BrxC-[Candidatus-Saccharibacteria-bacterium]
MBO7131877.1	Type1.BREX	ZnR->?-><-?||DOC+HTH-><-SIGMA+OB-Fold+AAA-ATPase+Lon-Protease-BrxL<-PglZ<-N6-METHYLASE<-DUF6079*<-BrxB-BrxF-iSTAND<-BrxA-DNA-binding<-ParB+HNH<-?||HD->	1109	Bacteria>Bacteria_incertae_sedis>Bacteria_candidate_phyla>Candidatus_Saccharibacteria>unclassified_Candidatus_Saccharibacteria	GCA_017645955.1	Candidatus_Saccharibacteria_bacterium	J6V85_01240	41463..44792	2026720	BREX-system-P-loop-protein-BrxC-[Candidatus-Saccharibacteria-bacterium]
MCA9347639.1	Type1.BREX	<-ACET<-DUF6079*<-BrxB-BrxF-iSTAND<-BrxA-DNA-binding<-?<-ZnR+HARE-HTH<-ABhydrolase||ABC-ATPase+SbcC+ABC-ATPase+SbcC->AAA-ATPase->	1102	Bacteria>Bacteria_incertae_sedis>Bacteria_candidate_phyla>Candidatus_Saccharibacteria>unclassified_Candidatus_Saccharibacteria	GCA_020428535.1	Candidatus_Saccharibacteria_bacterium	KC930_03630	351..3659	2026720	BREX-system-P-loop-protein-BrxC-[Candidatus-Saccharibacteria-bacterium]
MBI2566519.1	Type1.BREX	NtrC-AAA+FIS-HTH->?->?-><-?<-?||BrxA-DNA-binding->BrxB-BrxF-iSTAND->DUF6079*->?->N6-METHYLASE->HEPN->ParA-Soj-PloopNTPase->?->PglZ->HNH-><-?<-NtrC-AAA+FIS-HTH	1109	Bacteria>Bacteria_incertae_sedis>Bacteria_candidate_phyla>Candidatus_Schekmanbacteria	GCA_016188575.1	Candidatus_Schekmanbacteria_bacterium	HYV63_05760	102186..105515	2419984	BREX-system-P-loop-protein-BrxC-[Candidatus-Schekmanbacteria-bacterium]
MBI2568435.1	Type1.BREX	RADICAL-SAM->METHYLASE->RADICAL-SAM->RADICAL-SAM->Glycos_transf_1+Glyco_trans_1_2-><-REase||?->BrxA-DNA-binding->BrxB-BrxF-iSTAND->DUF6079*->REase->N6-METHYLASE->DOC+HTH->N6-METHYLASE-p->?->?->PglZ->SIGMA+OB-Fold+AAA-ATPase+Lon-Protease-BrxL->	1243	Bacteria>Bacteria_incertae_sedis>Bacteria_candidate_phyla>Candidatus_Schekmanbacteria	GCA_016188575.1	Candidatus_Schekmanbacteria_bacterium	HYV63_15550	53896..57627	2419984	BREX-system-P-loop-protein-BrxC-[Candidatus-Schekmanbacteria-bacterium]
PKK90022.1	Type1.BREX	<-Amidohydro_1<-?<-?||BrxA-DNA-binding->BrxB-BrxF-iSTAND->DUF6079*->HEPN->N6-METHYLASE-p->?->HEPN->	1176	Bacteria>Bacteria_incertae_sedis>Bacteria_candidate_phyla>Candidatus_Wallbacteria	GCA_002838975.1	Candidatus_Wallbacteria_bacterium_HGW-Wallbacteria-1	CVV64_11925	128140..131670	2013854	BREX-system-P-loop-protein-BrxC-[Candidatus-Wallbacteria-bacterium-HGW-Wallbacteria-1]
MCB9789984.1	Type1.BREX	MarR-HTH->?-><-?||ArdCN+MPTase->?->?->HTH_3->BrxA-DNA-binding->BrxB-BrxF-iSTAND->DUF6079-p*->?->?->N6-METHYLASE-p->N6-METHYLASE->N6-METHYLASE-p->?->PglZ->SIGMA+OB-Fold+AAA-ATPase+Lon-Protease-BrxL->REase->	654	Bacteria>Bacteria_incertae_sedis>Bacteria_candidate_phyla>Patescibacteria_group>Parcubacteria_group>Candidatus_Nomurabacteria	GCA_020632435.1	Candidatus_Nomurabacteria_bacterium	H6762_03295	728705..730669	2052152	BREX-system-P-loop-protein-BrxC-[Candidatus-Nomurabacteria-bacterium]
USN96045.1	Type1.BREX	HNH-><-?||?-><-?<-?||BrxA-DNA-binding->BrxB-BrxF-iSTAND->DUF6079*->N6-METHYLASE->PglZ->SIGMA+OB-Fold+AAA-ATPase+Lon-Protease-BrxL->SbcC->YTCD-HTH-><-?||?->VirB6->	1102	Bacteria>Bacteria_incertae_sedis>Bacteria_candidate_phyla>Patescibacteria_group>Parcubacteria_group>Candidatus_Nomurabacteria	GCA_023898645.1	Candidatus_Nomurabacteria_bacterium	H6797_03115	608928..612236	2052152	MAG:-BREX-system-P-loop-protein-BrxC-[Candidatus-Nomurabacteria-bacterium]
MCB9093205.1	Type1.BREX	<-NtrC-AAA+FIS-HTH||?->S1COLD->N6-METHYLASE->DUF4007->LON-AAA->DUF6079*->PglZ->SIGMA+OB-Fold+AAA-ATPase-BrxL-><-REase<-ParB+HNH<-ParB+HNH	1375	Bacteria>Bdellovibrionota>Bacteriovoracia>Bacteriovoracales>Halobacteriovoraceae>unclassified_Halobacteriovoraceae	GCA_020635155.1	Halobacteriovoraceae_bacterium	H6620_11660	12013..16140	2026745	chitobiase/beta-hexosaminidase-C-terminal-domain-containing-protein-[Halobacteriovoraceae-bacterium]
NUM59087.1	Type1.BREX	Phage_integrase->Phage_integrase->Phage_int_SAM_1+Phage_integrase->?-><-?<-SbcC+SbcC<-SIGMA+OB-Fold+AAA-ATPase+HKD-DNase-BrxL<-PglZ<-N6-METHYLASE<-DUF6079*<-BrxB-BrxF-iSTAND<-BrxA-DNA-binding<-XIS-HTH<-?<-?<-ABhydrolase<-REC+NtrC-AAA+FIS-HTH<-HISKIN+MORC	1157	Bacteria>Bdellovibrionota>Bdellovibrionia>Bdellovibrionales>Pseudobdellovibrionaceae>unclassified_Pseudobdellovibrionaceae	GCA_013359135.1	Pseudobdellovibrionaceae_bacterium	HUU56_10670	15517..18990	2026715	BREX-system-P-loop-protein-BrxC-[Pseudobdellovibrionaceae-bacterium]
PIT99446.1	Type1.BREX	<-ABhydrolase<-REC+NtrC-AAA+FIS-HTH<-HISKIN+MORC<-?<-?||XIS-HTH->BrxA-DNA-binding->BrxB-BrxF-iSTAND->DUF6079*->N6-METHYLASE->PglZ->SIGMA+OB-Fold+AAA-ATPase+HKD-DNase-BrxL->SIR2-><-MuB-ATPase<-HTH_28+HTH_32+rve+rve_3	1164	Bacteria>Bdellovibrionota>Bdellovibrionia>Bdellovibrionales>unclassified_Bdellovibrionales	GCA_002778785.1	Bdellovibrionales_bacterium_CG10_big_fil_rev_8_21_14_0_10_45_34	COT74_10625	394529..398023	1973927	BREX-system-P-loop-protein-BrxC-[Bdellovibrionales-bacterium-CG10_big_fil_rev_8_21_14_0_10_45_34]
NLI42049.1	Type1.BREX	<-YTCD-HTH<-?<-?<-SIGMA+OB-Fold+AAA-ATPase+Lon-Protease-BrxL<-PglZ<-N6-METHYLASE<-DUF6079*<-BrxB-BrxF-iSTAND<-BrxA-DNA-binding	1102	Bacteria>Caldisericota/Cryosericota_group>Caldisericota>Caldisericia>Caldisericales>unclassified_Caldisericales	GCA_012517765.1	Caldisericales_bacterium	GX421_12905	11000..14308	2614251	BREX-system-P-loop-protein-BrxC-[Caldisericales-bacterium]
MCK6622791.1	Type1.BREX	<-ABhydrolase<-?<-?<-SIGMA+OB-Fold+AAA-ATPase+Lon-Protease-BrxL<-PglZ<-DOC+HTH<-N6-METHYLASE<-Y1_Tnp<-DUF6079*<-BrxB-BrxF-iSTAND<-BrxA-DNA-binding	1161	Bacteria>Calditrichota>Calditrichia>unclassified_Calditrichia	GCA_023151135.1	Calditrichia_bacterium	L6Q94_20070	39359..42844	2268178	BREX-system-P-loop-protein-BrxC-[Calditrichia-bacterium]
KAA3613611.1	Type1.BREX	<-PIN||?->?->BrxA-DNA-binding->BrxB-BrxF-iSTAND->DUF6079*->N6-METHYLASE->?->PglZ->SIGMA+OB-Fold+AAA-ATPase+HKD-DNase-BrxL->REase->CARF+NtrC-AAA+FIS-HTH->REase->	1159	Bacteria>Calditrichota>unclassified_Calditrichota	GCA_008501795.1	Calditrichota_bacterium	DWQ03_12295	106728..110207	2212469	BREX-system-P-loop-protein-BrxC-[Calditrichota-bacterium]

Protein-ID	System	Gene-Neighbourhoods	Length	Taxonomy	Assembly-ID	Strain	Locus-Tag	Genomic-coordinates	TaxID	Fasta-define
WP_046997142.1	Type1.BREX	[illegible]	1202	Bacteria>Campylobacterota>Epsilonproteobacteria>Campylobacterales>Arcobacteraceae>Aliarcobacter	GCF_001010585.1	Aliarcobacter_butzleri	AA20_RS09495	10373..13981	28197	BREX-system-P-loop-protein-BrxC-[Aliarcobacter-butzleri]
WP_141050577.1	Type1.BREX	[illegible]	1162	Bacteria>Campylobacterota>Epsilonproteobacteria>Campylobacterales>Arcobacteraceae>Aliarcobacter	GCF_006503615.1	Aliarcobacter_cryaerophilus	AV936_RS08795	11744..15232	28198	BREX-system-P-loop-protein-BrxC-[Aliarcobacter-cryaerophilus]
WP_187475233.1	Type1.BREX	[illegible]	1166	Bacteria>Campylobacterota>Epsilonproteobacteria>Campylobacterales>Arcobacteraceae>Aliarcobacter	GCA_014352915.1	Aliarcobacter_cryaerophilus	HOO34_RS05640	1060903..1064403	28198	BREX-system-P-loop-protein-BrxC-[Aliarcobacter-cryaerophilus]
WP_260949077.1	Type1.BREX	[illegible]	1162	Bacteria>Campylobacterota>Epsilonproteobacteria>Campylobacterales>Arcobacteraceae>Aliarcobacter	GCF_025334565.1	Aliarcobacter_cryaerophilus	N5T87_RS09120	17819..21307	28198	BREX-system-P-loop-protein-BrxC-[Aliarcobacter-cryaerophilus]
WP_260942902.1	Type1.BREX	[illegible]	1162	Bacteria>Campylobacterota>Epsilonproteobacteria>Campylobacterales>Arcobacteraceae>Aliarcobacter	GCF_025334745.1	Aliarcobacter_cryaerophilus	N5T78_RS08220	26893..30381	28198	BREX-system-P-loop-protein-BrxC-[Aliarcobacter-cryaerophilus]
WP_260896239.1	Type1.BREX	[illegible]	1166	Bacteria>Campylobacterota>Epsilonproteobacteria>Campylobacterales>Arcobacteraceae>Aliarcobacter	GCF_025335025.1	Aliarcobacter_cryaerophilus	N5T57_RS09580	13633..17133	28198	BREX-system-P-loop-protein-BrxC-[Aliarcobacter-cryaerophilus]
WP_260916976.1	Type1.BREX	[illegible]	1162	Bacteria>Campylobacterota>Epsilonproteobacteria>Campylobacterales>Arcobacteraceae>Aliarcobacter	GCF_025335525.1	Aliarcobacter_butzleri	N5U17_RS09665	19417..22905	28197	BREX-system-P-loop-protein-BrxC-[Aliarcobacter-butzleri]
WP_260968564.1	Type1.BREX	[illegible]	1162	Bacteria>Campylobacterota>Epsilonproteobacteria>Campylobacterales>Arcobacteraceae>Aliarcobacter	GCF_025335945.1	Aliarcobacter_cryaerophilus	N5S77_RS10385	37284..40772	28198	BREX-system-P-loop-protein-BrxC-[Aliarcobacter-cryaerophilus]
WP_260934490.1	Type1.BREX	[illegible]	1162	Bacteria>Campylobacterota>Epsilonproteobacteria>Campylobacterales>Arcobacteraceae>Aliarcobacter	GCF_025336285.1	Aliarcobacter_cryaerophilus	N5T58_RS09420	6714..10202	28198	BREX-system-P-loop-protein-BrxC-[Aliarcobacter-cryaerophilus]
WP_152054592.1	Type1.BREX	[illegible]	1162	Bacteria>Campylobacterota>Epsilonproteobacteria>Campylobacterales>Arcobacteraceae>Aliarcobacter	GCF_902500815.1	Aliarcobacter_butzleri	GDI74_RS00980	25690..29178	28197	BREX-system-P-loop-protein-BrxC-[Aliarcobacter-butzleri]
RXJ95785.1	Type1.BREX	[illegible]	1162	Bacteria>Campylobacterota>Epsilonproteobacteria>Campylobacterales>Arcobacteraceae>Arcobacter	GCA_004116575.1	Arcobacter_sp._CECT_9299	CRU94_03995	49858..53346	2044506	hypothetical-protein-CRU94_03995-[Arcobacter-sp.-CECT-9299]
WP_129105531.1	Type1.BREX	[illegible]	1162	Bacteria>Campylobacterota>Epsilonproteobacteria>Campylobacterales>Arcobacteraceae>Arcobacter	GCF_004116575.1	Arcobacter_sp._CECT_9299	CRU94_RS03995	49858..53346	2044506	BREX-system-P-loop-protein-BrxC-[Arcobacter-sp.-CECT-9299]
WP_164968523.1	Type1.BREX	[illegible]	1160	Bacteria>Campylobacterota>Epsilonproteobacteria>Campylobacterales>Arcobacteraceae>Arcobacter	GCF_013201725.1	Arcobacter_defluvii	ADFLV_RS07175	1422397..1425879	873191	BREX-system-P-loop-protein-BrxC-[Arcobacter-defluvii]
MCD8478249.1	Type1.BREX	[illegible]	544	Bacteria>Campylobacterota>Epsilonproteobacteria>Campylobacterales>Sulfurospirillaceae>Sulfurospirillum	GCA_021206415.1	Sulfurospirillum_sp.	LRY68_10520	4304..5938	2053622	BREX-system-P-loop-protein-BrxC-[Sulfurospirillum-sp.]
OZA03558.1	Type1.BREX	[illegible]	1158	Bacteria>Campylobacterota>Epsilonproteobacteria>Campylobacterales>Thiovulaceae>Sulfuricurvum	GCA_002282015.1	Sulfuricurvum_sp._17-40-25	B7X89_02520	35980..39456	1970439	MAG:-hypothetical-protein-B7X89_02520-[Sulfuricurvum-sp.-17-40-25]
WP_013460546.1	Type1.BREX	[illegible]	1163	Bacteria>Campylobacterota>Epsilonproteobacteria>Campylobacterales>Thiovulaceae>Sulfuricurvum	GCF_000183725.1	Sulfuricurvum_kujiense	SULKU_RS08475	1692937..1696428	148813	BREX-system-P-loop-protein-BrxC-[Sulfuricurvum-kujiense]
PHS59105.1	Type1.BREX	[illegible]	1158	Bacteria>Campylobacterota>Epsilonproteobacteria>Campylobacterales>Thiovulaceae>Sulfurimonas	GCA_002733155.1	Sulfurimonas_sp.	COB17_00805	100943..104419	2022749	MAG:-hypothetical-protein-COB17_00805-[Sulfurimonas-sp.]
QFR43750.1	Type1.BREX	[illegible]	1163	Bacteria>Campylobacterota>Epsilonproteobacteria>Campylobacterales>Thiovulaceae>Sulfurimonas	GCA_009258045.1	Sulfurimonas_xiamenensis	FJR47_07440	1472966..1476457	2590021	BREX-system-P-loop-protein-BrxC-[Sulfurimonas-xiamenensis]
MBT5934549.1	Type1.BREX	[illegible]	988	Bacteria>Campylobacterota>Epsilonproteobacteria>Campylobacterales>Thiovulaceae>Sulfurimonas	GCA_018698455.1	Sulfurimonas_sp.	HOG88_04280	23331..26296	2022749	BREX-system-P-loop-protein-BrxC-[Sulfurimonas-sp.]
WP_188093712.1	Type1.BREX	[illegible]	1160	Bacteria>Campylobacterota>Epsilonproteobacteria>Campylobacterales>Thiovulaceae>Sulfurimonas	GCF_009258045.1	Sulfurimonas_xiamenensis	FJR47_RS07440	1472975..1476457	2590021	BREX-system-P-loop-protein-BrxC-[Sulfurimonas-xiamenensis]
WP_193150356.1	Type1.BREX	[illegible]	1160	Bacteria>Campylobacterota>Epsilonproteobacteria>Campylobacterales>Thiovulaceae>Sulfurimonas	GCF_014905115.1	Sulfurimonas_sediminis	FJR45_RS09735	1812721..1816203	2590020	BREX-system-P-loop-protein-BrxC-[Sulfurimonas-sediminis]
WP_226069221.1	Type1.BREX	[illegible]	1201	Bacteria>Campylobacterota>Epsilonproteobacteria>Campylobacterales>Thiovulaceae>Sulfurimonas	GCF_020410885.1	Sulfurimonas_sp._SWIR-19	LCX93_RS05490	1055837..1059442	2878390	BREX-system-P-loop-protein-BrxC-[Sulfurimonas-sp.-SWIR-19]
OYZ34277.1	Type1.BREX	[illegible]	1013	Bacteria>Campylobacterota>Epsilonproteobacteria>Campylobacterales>unclassified_Campylobacterales	GCA_002281685.1	Campylobacterales_bacterium_16-40-21	B7Y30_07055	21520..24561	1970501	hypothetical-protein-B7Y30_07055-[Campylobacterales-bacterium-16-40-21]
MBD3789167.1	Type1.BREX	[illegible]	671	Bacteria>Campylobacterota>Epsilonproteobacteria>Campylobacterales>unclassified_Campylobacterales	GCA_014763895.1	Campylobacterales_bacterium	IE885_02155	92297..94311	2268180	BREX-system-P-loop-protein-BrxC-[Campylobacterales-bacterium]
OQX77395.1	Type1.BREX	[illegible]	1159	Bacteria>Campylobacterota>unclassified_Campylobacterota	GCA_002085305.1	Epsilonproteobacteria_bacterium_4484_65	B6D54_02040	4837..8316	1970777	hypothetical-protein-B6D54_02040-[Epsilonproteobacteria-bacterium-4484_65]
WP_013452128.1	Type1.BREX	[illegible]	1161	Bacteria>Deferribacterota>Deferribacteres>Deferribacterales>Calditerrivibrionaceae>Calditerrivibrio	GCF_000183405.1	Calditerrivibrio_nitroreducens	CALNI_RS10260	2104217..2107702	477976	BREX-system-P-loop-protein-BrxC-[Calditerrivibrio-nitroreducens]
MBZ4644075.1	Type1.BREX	[illegible]	1168	Bacteria>Deferribacterota>Deferribacteres>Deferribacterales>Deferribacteraceae>unclassified_Deferribacteraceae	GCA_020054865.1	Deferribacteraceae_bacterium	JG767_1684	7812..11318	2053543	hypothetical-protein-[Deferribacteraceae-bacterium]
WP_242820841.1	Type1.BREX	[illegible]	1168	Bacteria>Deferribacterota>Deferribacteres>Deferribacterales>Deferrivibrionaceae>Deferrivibrio	GCF_020480685.1	Deferrivibrio_essentukiensis	LF845_RS09815	29861..33367	2880922	BREX-system-P-loop-protein-BrxC-[Deferrivibrio-essentukiensis]
MBF0239771.1	Type1.BREX	[illegible]	1178	Bacteria>delta/epsilon_subdivisions>Deltaproteobacteria>Deltaproteobacteria_incertae_sedis>SAR324_cluster	GCA_015231535.1	SAR324_cluster_bacterium	HQM12_18890	8068..11604	2024889	BREX-system-P-loop-protein-BrxC-[SAR324-cluster-bacterium]
EFK06105.1	Type1.BREX	[illegible]	1215	Bacteria>delta/epsilon_subdivisions>Deltaproteobacteria>unclassified_Deltaproteobacteria	GCA_000179315.1	delta_proteobacterium_NaphS2	NPH_2628	46790..50437	88274	conserved-hypothetical-protein-[delta-proteobacterium-NaphS2]
OGR04765.1	Type1.BREX	[illegible]	983	Bacteria>delta/epsilon_subdivisions>Deltaproteobacteria>unclassified_Deltaproteobacteria	GCA_001799225.1	Deltaproteobacteria_bacterium_RIFOXYD12_FULL_50_9	A2511_13070	2145..5095	1797902	ATPase,-partial-[Deltaproteobacteria-bacterium-RIFOXYD12_FULL_50_9]
MAE16011.1	Type1.BREX	[illegible]	1175	Bacteria>delta/epsilon_subdivisions>Deltaproteobacteria>unclassified_Deltaproteobacteria	GCA_002685875.1	Deltaproteobacteria_bacterium	CL911_01040	1115..4642	2026735	hypothetical-protein-[Deltaproteobacteria-bacterium]
PIV21173.1	Type1.BREX	[illegible]	1215	Bacteria>delta/epsilon_subdivisions>Deltaproteobacteria>unclassified_Deltaproteobacteria	GCA_002780715.1	Deltaproteobacteria_bacterium_CG03_land_8_20_14_0_80_45_14	COS40_08745	11135..14782	1973969	BREX-system-P-loop-protein-BrxC-[Deltaproteobacteria-bacterium-CG03_land_8_20_14_0_80_45_14]
PKN56508.1	Type1.BREX	[illegible]	1206	Bacteria>delta/epsilon_subdivisions>Deltaproteobacteria>unclassified_Deltaproteobacteria	GCA_002841945.1	Deltaproteobacteria_bacterium_HGW-Deltaproteobacteria-14	CVU56_15695	1564..5184	2013741	BREX-system-P-loop-protein-BrxC-[Deltaproteobacteria-bacterium-HGW-Deltaproteobacteria-14]
PXF57197.1	Type1.BREX	[illegible]	1215	Bacteria>delta/epsilon_subdivisions>Deltaproteobacteria>unclassified_Deltaproteobacteria	GCA_003194495.1	Deltaproteobacteria_bacterium	C4B58_10725	19842..23489	2026735	BREX-system-P-loop-protein-BrxC-[Deltaproteobacteria-bacterium]
HBL23376.1	Type1.BREX	[illegible]	888	Bacteria>delta/epsilon_subdivisions>Deltaproteobacteria>unclassified_Deltaproteobacteria	GCA_003506795.1	Deltaproteobacteria_bacterium	DDZ40_04590	1..2666	2026735	BREX-system-P-loop-protein-BrxC-[Deltaproteobacteria-bacterium]
RLC18759.1	Type1.BREX	[illegible]	1189	Bacteria>delta/epsilon_subdivisions>Deltaproteobacteria>unclassified_Deltaproteobacteria	GCA_003647425.1	Deltaproteobacteria_bacterium	DRI57_08000	870..4439	2026735	BREX-system-P-loop-protein-BrxC-[Deltaproteobacteria-bacterium]
TDB40153.1	Type1.BREX	[illegible]	1180	Bacteria>delta/epsilon_subdivisions>Deltaproteobacteria>unclassified_Deltaproteobacteria	GCA_004348915.1	Deltaproteobacteria_bacterium	D9V46_00535	112112..115654	2026735	BREX-system-P-loop-protein-BrxC-[Deltaproteobacteria-bacterium]
MBC8466717.1	Type1.BREX	[illegible]	1172	Bacteria>delta/epsilon_subdivisions>Deltaproteobacteria>unclassified_Deltaproteobacteria	GCA_014384065.1	Deltaproteobacteria_bacterium	H8D55_02650	10326..13844	2026735	BREX-system-P-loop-protein-BrxC-[Deltaproteobacteria-bacterium]
MBF0105032.1	Type1.BREX	[illegible]	1166	Bacteria>delta/epsilon_subdivisions>Deltaproteobacteria>unclassified_Deltaproteobacteria	GCA_015228875.1	Deltaproteobacteria_bacterium	HQM16_06860	10380..13880	2026735	BREX-system-P-loop-protein-BrxC-[Deltaproteobacteria-bacterium]
MBF0105437.1	Type1.BREX	[illegible]	1155	Bacteria>delta/epsilon_subdivisions>Deltaproteobacteria>unclassified_Deltaproteobacteria	GCA_015228875.1	Deltaproteobacteria_bacterium	HQM16_08940	21511..24978	2026735	BREX-system-P-loop-protein-BrxC-[Deltaproteobacteria-bacterium]
MBI2354427.1	Type1.BREX	[illegible]	1182	Bacteria>delta/epsilon_subdivisions>Deltaproteobacteria>unclassified_Deltaproteobacteria	GCA_016182975.1	Deltaproteobacteria_bacterium	HYV06_05270	743..4291	2026735	BREX-system-P-loop-protein-BrxC-[Deltaproteobacteria-bacterium]
MBI4517718.1	Type1.BREX	[illegible]	1149	Bacteria>delta/epsilon_subdivisions>Deltaproteobacteria>unclassified_Deltaproteobacteria	GCA_016210005.1	Deltaproteobacteria_bacterium	HY699_18080	1797..5246	2026735	BREX-system-P-loop-protein-BrxC-[Deltaproteobacteria-bacterium]
MBI5514929.1	Type1.BREX	[illegible]	1224	Bacteria>delta/epsilon_subdivisions>Deltaproteobacteria>unclassified_Deltaproteobacteria	GCA_016218725.1	Deltaproteobacteria_bacterium	HY909_14230	59420..63094	2026735	BREX-system-P-loop-protein-BrxC-[Deltaproteobacteria-bacterium]
MBI5606167.1	Type1.BREX	[illegible]	1147	Bacteria>delta/epsilon_subdivisions>Deltaproteobacteria>unclassified_Deltaproteobacteria	GCA_016219225.1	Deltaproteobacteria_bacterium	HY879_22775	11994..15437	2026735	BREX-system-P-loop-protein-BrxC-[Deltaproteobacteria-bacterium]
MBK7759463.1	Type1.BREX	[illegible]	766	Bacteria>delta/epsilon_subdivisions>Deltaproteobacteria>unclassified_Deltaproteobacteria	GCA_016706815.1	Deltaproteobacteria_bacterium	IPI35_24325	2903..5203	2026735	BREX-system-P-loop-protein-BrxC-[Deltaproteobacteria-bacterium]
MBN1905588.1	Type1.BREX	[illegible]	1190	Bacteria>delta/epsilon_subdivisions>Deltaproteobacteria>unclassified_Deltaproteobacteria	GCA_016931115.1	Deltaproteobacteria_bacterium	JW927_10865	38502..42074	2026735	BREX-system-P-loop-protein-BrxC-[Deltaproteobacteria-bacterium]
MBP8650772.1	Type1.BREX	[illegible]	1145	Bacteria>delta/epsilon_subdivisions>Deltaproteobacteria>unclassified_Deltaproteobacteria	GCA_018053065.1	Deltaproteobacteria_bacterium	KBI48_08700	29985..33422	2026735	BREX-system-P-loop-protein-BrxC-[Deltaproteobacteria-bacterium]
MBT3255199.1	Type1.BREX	[illegible]	1216	Bacteria>delta/epsilon_subdivisions>Deltaproteobacteria>unclassified_Deltaproteobacteria	GCA_018644785.1	Deltaproteobacteria_bacterium	HN366_01900	5574..9224	2026735	BREX-system-P-loop-protein-BrxC-[Deltaproteobacteria-bacterium]
MBT4264397.1	Type1.BREX	[illegible]	409	Bacteria>delta/epsilon_subdivisions>Deltaproteobacteria>unclassified_Deltaproteobacteria	GCA_018651965.1	Deltaproteobacteria_bacterium	HOD85_09330	22894..24123	2026735	BREX-system-P-loop-protein-BrxC-[Deltaproteobacteria-bacterium]
MBW1741678.1	Type1.BREX	[illegible]	1106	Bacteria>delta/epsilon_subdivisions>Deltaproteobacteria>unclassified_Deltaproteobacteria	GCA_019306505.1	Deltaproteobacteria_bacterium	JRJ42_11205	451..3771	2026735	BREX-system-P-loop-protein-BrxC-[Deltaproteobacteria-bacterium]
MBW1799340.1	Type1.BREX	[illegible]	188	Bacteria>delta/epsilon_subdivisions>Deltaproteobacteria>unclassified_Deltaproteobacteria	GCA_019306865.1	Deltaproteobacteria_bacterium	JRJ85_01290	1..565	2026735	BREX-system-P-loop-protein-BrxC-[Deltaproteobacteria-bacterium]
MBW1938726.1	Type1.BREX	[illegible]	1178	Bacteria>delta/epsilon_subdivisions>Deltaproteobacteria>unclassified_Deltaproteobacteria	GCA_019307905.1	Deltaproteobacteria_bacterium	JRI67_08190	4087..7623	2026735	BREX-system-P-loop-protein-BrxC-[Deltaproteobacteria-bacterium]
MBW1967272.1	Type1.BREX	[illegible]	1154	Bacteria>delta/epsilon_subdivisions>Deltaproteobacteria>unclassified_Deltaproteobacteria	GCA_019308155.1	Deltaproteobacteria_bacterium	JRI48_07780	2430..5894	2026735	BREX-system-P-loop-protein-BrxC-[Deltaproteobacteria-bacterium]
MBW2031933.1	Type1.BREX	[illegible]	162	Bacteria>delta/epsilon_subdivisions>Deltaproteobacteria>unclassified_Deltaproteobacteria	GCA_019308745.1	Deltaproteobacteria_bacterium	JRJ31_23030	2222..2706	2026735	hypothetical-protein-[Deltaproteobacteria-bacterium]
MBW2308571.1	Type1.BREX	[illegible]	1220	Bacteria>delta/epsilon_subdivisions>Deltaproteobacteria>unclassified_Deltaproteobacteria	GCA_019310535.1	Deltaproteobacteria_bacterium	JRG73_16720	7753..11415	2026735	BREX-system-P-loop-protein-BrxC-[Deltaproteobacteria-bacterium]
MCC6503799.1	Type1.BREX	[illegible]	1181	Bacteria>delta/epsilon_subdivisions>Deltaproteobacteria>unclassified_Deltaproteobacteria	GCA_020846435.1	Deltaproteobacteria_bacterium	IT362_11790	16699..20244	2026735	BREX-system-P-loop-protein-BrxC-[Deltaproteobacteria-bacterium]
MCD6185271.1	Type1.BREX	[illegible]	852	Bacteria>delta/epsilon_subdivisions>Deltaproteobacteria>unclassified_Deltaproteobacteria	GCA_021157555.1	Deltaproteobacteria_bacterium	J7K84_05705	1..2556	2026735	BREX-system-P-loop-protein-BrxC-[Deltaproteobacteria-bacterium]
MCD6306396.1	Type1.BREX	[illegible]	1148	Bacteria>delta/epsilon_subdivisions>Deltaproteobacteria>unclassified_Deltaproteobacteria	GCA_021159525.1	Deltaproteobacteria_bacterium	J7M32_08925	1514..4960	2026735	BREX-system-P-loop-protein-BrxC-[Deltaproteobacteria-bacterium]
MCD6532985.1	Type1.BREX	[illegible]	691	Bacteria>delta/epsilon_subdivisions>Deltaproteobacteria>unclassified_Deltaproteobacteria	GCA_021163445.1	Deltaproteobacteria_bacterium	J7L25_02755	20972..23047	2026735	BREX-system-P-loop-protein-BrxC-[Deltaproteobacteria-bacterium]
MCK9263307.1	Type1.BREX	[illegible]	1179	Bacteria>delta/epsilon_subdivisions>Deltaproteobacteria>unclassified_Deltaproteobacteria	GCA_023230665.1	Deltaproteobacteria_bacterium	MOR18_05785	10308..13847	2026735	BREX-system-P-loop-protein-BrxC-[Deltaproteobacteria-bacterium]
MCP4158880.1	Type1.BREX	[illegible]	1006	Bacteria>delta/epsilon_subdivisions>Deltaproteobacteria>unclassified_Deltaproteobacteria	GCA_024224625.1	Deltaproteobacteria_bacterium	GY760_02295	25697..28718	2026735	BREX-system-P-loop-protein-BrxC-[Deltaproteobacteria-bacterium]
MCP4577326.1	Type1.BREX	[illegible]	1196	Bacteria>delta/epsilon_subdivisions>Deltaproteobacteria>unclassified_Deltaproteobacteria	GCA_024231575.1	Deltaproteobacteria_bacterium	GY846_13700	13452..17042	2026735	BREX-system-P-loop-protein-BrxC-[Deltaproteobacteria-bacterium]
OGR40953.1	Type1.BREX	[illegible]	1147	Bacteria>Elusimicrobiota>unclassified_Elusimicrobiota	GCA_001799575.1	Elusimicrobia_bacterium_GWA2_56_46	A2X28_06390	23644..27087	1797922	nucleoside-triphosphate-hydrolase-[Elusimicrobia-bacterium-GWA2_56_46]
MBK7688407.1	Type1.BREX	[illegible]	415	Bacteria>Elusimicrobiota>unclassified_Elusimicrobiota	GCA_016709555.1	Elusimicrobiota_bacterium	IPJ35_05275	9900..11147	2030800	hypothetical-protein-[Elusimicrobiota-bacterium]
MBK7688408.1	Type1.BREX	[illegible]	466	Bacteria>Elusimicrobiota>unclassified_Elusimicrobiota	GCA_016709555.1	Elusimicrobiota_bacterium	IPJ35_05280	11117..12517	2030800	hypothetical-protein-[Elusimicrobiota-bacterium]
MXY08911.1	Type1.BREX	[illegible]	1183	Bacteria>FCB_group>Bacteroidetes/Chlorobi_group>Bacteroidota>Bacteroidetes_incertae_sedis>Rhodothermaceae	GCA_009838245.1	Rhodothermaceae_bacterium	F4Y61_09720	3347..6898	2026787	hypothetical-protein-[Rhodothermaceae-bacterium]
MCS4056789.1	Type1.BREX	[illegible]	1165	Bacteria>FCB_group>Bacteroidetes/Chlorobi_group>Bacteroidota>Bacteroidetes_incertae_sedis>Rhodothermaceae	GCA_024809315.1	Salinibacter_ruber	GGP75_002131	31231..34728	146919	hypothetical-protein-[Salinibacter-ruber]
WP_119843039.1	Type1.BREX	[illegible]	1201	Bacteria>FCB_group>Bacteroidetes/Chlorobi_group>Bacteroidota>Bacteroidetes_incertae_sedis>Rhodothermaceae	GCF_002290345.1	Salinibacter_ruber	B7W84_RS15115	76596..80201	146919	BREX-system-P-loop-protein-BrxC-[Salinibacter-ruber]
WP_103028550.1	Type1.BREX	[illegible]	1168	Bacteria>FCB_group>Bacteroidetes/Chlorobi_group>Bacteroidota>Bacteroidetes_incertae_sedis>Rhodothermaceae	GCF_002894625.1	Salinibacter_altiplanensis	B7R94_RS01140	2369..5875	1803181	BREX-system-P-loop-protein-BrxC-[Salinibacter-altiplanensis]
WP_259084851.1	Type1.BREX	[illegible]	1143	Bacteria>FCB_group>Bacteroidetes/Chlorobi_group>Bacteroidota>Bacteroidetes_incertae_sedis>Rhodothermaceae	GCF_024808015.1	Salinibacter_ruber	GGP69_RS05925	149309..152740	146919	BREX-system-P-loop-protein-BrxC-[Salinibacter-ruber]
WP_259111349.1	Type1.BREX	[illegible]	1145	Bacteria>FCB_group>Bacteroidetes/Chlorobi_group>Bacteroidota>Bacteroidetes_incertae_sedis>Rhodothermaceae	GCF_024808065.1	Salinibacter_ruber	GGP79_RS14420	5761..9198	146919	BREX-system-P-loop-protein-BrxC-[Salinibacter-ruber]
WP_259049434.1	Type1.BREX	[illegible]	1145	Bacteria>FCB_group>Bacteroidetes/Chlorobi_group>Bacteroidota>Bacteroidetes_incertae_sedis>Rhodothermaceae	GCF_024808355.1	Salinibacter_ruber	GGP97_RS08705	20679..24116	146919	BREX-system-P-loop-protein-BrxC-[Salinibacter-ruber]
WP_259099618.1	Type1.BREX	[illegible]	1143	Bacteria>FCB_group>Bacteroidetes/Chlorobi_group>Bacteroidota>Bacteroidetes_incertae_sedis>Rhodothermaceae	GCF_024808405.1	Salinibacter_ruber	GGP77_002965	30959..34390	146919	BREX-system-P-loop-protein-BrxC-[Salinibacter-ruber]
WP_259094322.1	Type1.BREX	[illegible]	1143	Bacteria>FCB_group>Bacteroidetes/Chlorobi_group>Bacteroidota>Bacteroidetes_incertae_sedis>Rhodothermaceae	GCF_024809025.1	Salinibacter_ruber	GGP87_RS12315	32840..36271	146919	BREX-system-P-loop-protein-BrxC-[Salinibacter-ruber]
WP_259115052.1	Type1.BREX	[illegible]	1167	Bacteria>FCB_group>Bacteroidetes/Chlorobi_group>Bacteroidota>Bacteroidetes_incertae_sedis>Rhodothermaceae	GCF_024809315.1	Salinibacter_ruber	GGP75_RS10390	31231..34734	146919	BREX-system-P-loop-protein-BrxC-[Salinibacter-ruber]
WP_259076695.1	Type1.BREX	[illegible]	1167	Bacteria>FCB_group>Bacteroidetes/Chlorobi_group>Bacteroidota>Bacteroidetes_incertae_sedis>Rhodothermaceae	GCF_024809595.1	Salinibacter_ruber	GGP73_RS15155	19341..22844	146919	BREX-system-P-loop-protein-BrxC-[Salinibacter-ruber]
WP_259210815.1	Type1.BREX	[illegible]	1145	Bacteria>FCB_group>Bacteroidetes/Chlorobi_group>Bacteroidota>Bacteroidetes_incertae_sedis>Rhodothermaceae	GCF_024809615.1	Salinibacter_ruber	GGP43_RS13275	33115..36552	146919	BREX-system-P-loop-protein-BrxC-[Salinibacter-ruber]
WP_259195582.1	Type1.BREX	[illegible]	1144	Bacteria>FCB_group>Bacteroidetes/Chlorobi_group>Bacteroidota>Bacteroidetes_incertae_sedis>Rhodothermaceae	GCF_024809635.1	Salinibacter_ruber	GGP50_RS04130	10424..13858	146919	BREX-system-P-loop-protein-BrxC-[Salinibacter-ruber]
WP_259087839.1	Type1.BREX	[illegible]	1168	Bacteria>FCB_group>Bacteroidetes/Chlorobi_group>Bacteroidota>Bacteroidetes_incertae_sedis>Rhodothermaceae	GCF_024809725.1	Salinibacter_ruber	GGQ07_RS07865	19343..22849	146919	BREX-system-P-loop-protein-BrxC-[Salinibacter-ruber]
WP_251951689.1	Type1.BREX	[illegible]	1143	Bacteria>FCB_group>Bacteroidetes/Chlorobi_group>Bacteroidota>Bacteroidetes_incertae_sedis>Rhodothermaceae	GCF_943908255.1	Salinibacter_ruber	NGL47_RS12295	24332..27763	146919	BREX-system-P-loop-protein-BrxC-[Salinibacter-ruber]
WP_263820582.1	Type1.BREX	[illegible]	1167	Bacteria>FCB_group>Bacteroidetes/Chlorobi_group>Bacteroidota>Bacteroidetes_incertae_sedis>Rhodothermaceae	GCF_947077735.1	Salinibacter_sp.	OJA43_RS08805	45420..48923	2065818	BREX-system-P-loop-protein-BrxC-[Salinibacter-sp.]
WP_259248797.1	Type1.BREX	[illegible]	1167	Bacteria>FCB_group>Bacteroidetes/Chlorobi_group>Bacteroidota>Bacteroidetes_incertae_sedis>Rhodothermaceae	-	Salinibacter_ruber	GGP55_RS14300	49306..52809	146919	BREX-system-P-loop-protein-BrxC-[Salinibacter-ruber]
HHU27149.1	Type1.BREX	[illegible]	343	Bacteria>FCB_group>Bacteroidetes/Chlorobi_group>Bacteroidota>Bacteroidia>Bacteroidales	-	Bacteroidales_bacterium	GXZ56_11115	1..1030	2030927	BREX-system-P-loop-protein-BrxC-[Bacteroidales-bacterium]
WP_204433169.1	Type1.BREX	[illegible]	1195	Bacteria>FCB_group>Bacteroidetes/Chlorobi_group>Bacteroidota>Bacteroidia>Bacteroidales	-	Bacteroides_mediterraneensis	H6B28_RS04775	126749..130336	1841856	BREX-system-P-loop-protein-BrxC-[Bacteroides-mediterraneensis]
MCS3257591.1	Type1.BREX	[illegible]	733	Bacteria>FCB_group>Bacteroidetes/Chlorobi_group>Bacteroidota>Bacteroidia>Bacteroidales	-	Bacteroides_thetaiotaomicron	NXV10_10775	1863438..1865639	818	BREX-system-P-loop-protein-BrxC-[Bacteroides-thetaiotaomicron]
WP_118696414.1	Type1.BREX	[illegible]	938	Bacteria>FCB_group>Bacteroidetes/Chlorobi_group>Bacteroidota>Bacteroidia>Bacteroidales	-	Bacteroides_uniformis	KSX92_08935	3061..5877	820	BREX-system-P-loop-protein-BrxC-[Bacteroides-uniformis]
EEZ19147.1	Type1.BREX	[illegible]	1213	Bacteria>FCB_group>Bacteroidetes/Chlorobi_group>Bacteroidota>Bacteroidia>Bacteroidales	GCA_000162195.1	Bacteroides_sp._3_1_33FAA	HMPREF0105_4547	25602..29243	457391	hypothetical-protein-HMPREF0105_4547-[Bacteroides-sp.-3_1_33FAA]
CDD81792.1	Type1.BREX	[illegible]	1217	Bacteria>FCB_group>Bacteroidetes/Chlorobi_group>Bacteroidota>Bacteroidia>Bacteroidales	GCA_000435095.1	Bacteroides_sp._CAG:462	BN666_01223	46564..50217	1262740	putative-uncharacterized-protein-[Bacteroides-sp.-CAG:462]
CDD50103.1	Type1.BREX	[illegible]	1195	Bacteria>FCB_group>Bacteroidetes/Chlorobi_group>Bacteroidota>Bacteroidia>Bacteroidales	GCA_000435415.1	Bacteroides_sp._CAG:875	BN800_01988	17775..21362	1262752	putative-uncharacterized-protein-[Bacteroides-sp.-CAG:875]
CDD33290.1	Type1.BREX	[illegible]	1195	Bacteria>FCB_group>Bacteroidetes/Chlorobi_group>Bacteroidota>Bacteroidia>Bacteroidales	GCA_000436055.1	Bacteroides_sp._CAG:714	BN762_00981	3639..7226	1262749	putative-uncharacterized-protein-[Bacteroides-sp.-CAG:714]
CDE09669.1	Type1.BREX	[illegible]	1221	Bacteria>FCB_group>Bacteroidetes/Chlorobi_group>Bacteroidota>Bacteroidia>Bacteroidales	GCA_000437495.1	Prevotella_sp._CAG:485	BN677_00784	6933..10598	1262927	putative-uncharacterized-protein-[Prevotella-sp.-CAG:485]
OCW94638.1	Type1.BREX	[illegible]	1202	Bacteria>FCB_group>Bacteroidetes/Chlorobi_group>Bacteroidota>Bacteroidia>Bacteroidales	GCA_001704385.2	Macellibacteroides_sp._HH-ZS	A9168_06455	108865..112473	1857568	hypothetical-protein-A9168_06455-[Macellibacteroides-sp.-HH-ZS]
OKZ04598.1	Type1.BREX	[illegible]	1202	Bacteria>FCB_group>Bacteroidetes/Chlorobi_group>Bacteroidota>Bacteroidia>Bacteroidales	GCA_001915435.1	Bacteroides_sp._41_26	BHV71_10470	85707..89315	1896973	MAG:-hypothetical-protein-BHV71_10470-[Bacteroides-sp.-41_26]
OKZ20119.1	Type1.BREX	[illegible]	1185	Bacteria>FCB_group>Bacteroidetes/Chlorobi_group>Bacteroidota>Bacteroidia>Bacteroidales	GCA_001915545.1	Bacteroides_sp._43_108	BHV77_10980	38384..41941	1896974	MAG:-hypothetical-protein-BHV77_10980-[Bacteroides-sp.-43_108]
OKZ19958.1	Type1.BREX	[illegible]	1213	Bacteria>FCB_group>Bacteroidetes/Chlorobi_group>Bacteroidota>Bacteroidia>Bacteroidales	GCA_001915545.1	Bacteroides_sp._43_108	BHV77_11420	12434..16075	1896974	MAG:-hypothetical-protein-BHV77_11420-[Bacteroides-sp.-43_108]
HAH90337.1	Type1.BREX	[illegible]	1222	Bacteria>FCB_group>Bacteroidetes/Chlorobi_group>Bacteroidota>Bacteroidia>Bacteroidales	GCA_003450425.1	Prevotella_sp.	DCL96_01170	7620..11288	59823	BREX-system-P-loop-protein-BrxC-[Prevotella-sp.]
HAH91106.1	Type1.BREX	[illegible]	1202	Bacteria>FCB_group>Bacteroidetes/Chlorobi_group>Bacteroidota>Bacteroidia>Bacteroidales	GCA_003450425.1	Prevotella_sp.	DCL96_05230	7787..11395	59823	BREX-system-P-loop-protein-BrxC-[Prevotella-sp.]
RHC75801.1	Type1.BREX	[illegible]	691	Bacteria>FCB_group>Bacteroidetes/Chlorobi_group>Bacteroidota>Bacteroidia>Bacteroidales	GCA_003468465.1	Bacteroides_uniformis	DW831_02855	19890..21965	820	BREX-system-P-loop-protein-BrxC-[Bacteroides-uniformis]
RHK76870.1	Type1.BREX	[illegible]	1208	Bacteria>FCB_group>Bacteroidetes/Chlorobi_group>Bacteroidota>Bacteroidia>Bacteroidales	GCA_003474155.1	Phocaeicola_vulgatus	DW047_18670	2653..6279	821	BREX-system-P-loop-protein-BrxC-[Phocaeicola-vulgatus]
RHR54760.1	Type1.BREX	[illegible]	1216	Bacteria>FCB_group>Bacteroidetes/Chlorobi_group>Bacteroidota>Bacteroidia>Bacteroidales	GCA_003479205.1	Parabacteroides_sp._AF17-28	DWW90_15085	22918..26568	2292241	BREX-system-P-loop-protein-BrxC-[Parabacteroides-sp.-AF17-28]
HBT86696.1	Type1.BREX	[illegible]	1062	Bacteria>FCB_group>Bacteroidetes/Chlorobi_group>Bacteroidota>Bacteroidia>Bacteroidales	GCA_003510605.1	Porphyromonadaceae_bacterium	DEB12_12440	1..3185	2049046	BREX-system-P-loop-protein-BrxC-[Porphyromonadaceae-bacterium]
HBQ57053.1	Type1.BREX	[illegible]	1176	Bacteria>FCB_group>Bacteroidetes/Chlorobi_group>Bacteroidota>Bacteroidia>Bacteroidales	GCA_003511405.1	Porphyromonadaceae_bacterium	DD786_07890	28635..32165	2049046	BREX-system-P-loop-protein-BrxC-[Porphyromonadaceae-bacterium]
HCI54649.1	Type1.BREX	[illegible]	227	Bacteria>FCB_group>Bacteroidetes/Chlorobi_group>Bacteroidota>Bacteroidia>Bacteroidales	GCA_003521565.1	Bacteroidales_bacterium	DFI01_01845	1..682	2030927	BREX-system-P-loop-protein-BrxC-[Bacteroidales-bacterium]
HCY74134.1	Type1.BREX	[illegible]	1205	Bacteria>FCB_group>Bacteroidetes/Chlorobi_group>Bacteroidota>Bacteroidia>Bacteroidales	GCA_003537735.1	Rikenellaceae_bacterium	DHU75_08775	2145..5762	2049048	BREX-system-P-loop-protein-BrxC-[Rikenellaceae-bacterium]
HCV16199.1	Type1.BREX	[illegible]	581	Bacteria>FCB_group>Bacteroidetes/Chlorobi_group>Bacteroidota>Bacteroidia>Bacteroidales	GCA_003542255.1	Rikenellaceae_bacterium	DF637_07690	3098..4843	2049048	hypothetical-protein-[Rikenellaceae-bacterium]
ROS89081.1	Type1.BREX	[illegible]	1215	Bacteria>FCB_group>Bacteroidetes/Chlorobi_group>Bacteroidota>Bacteroidia>Bacteroidales	GCA_003762635.1	Muribaculaceae_bacterium_Isolate-043_(Harlan)	EEL36_13385	16553..20200	2486463	BREX-system-P-loop-protein-BrxC-[Muribaculaceae-bacterium-Isolate-043-(Harlan)]
ROT16603.1	Type1.BREX	[illegible]	1195	Bacteria>FCB_group>Bacteroidetes/Chlorobi_group>Bacteroidota>Bacteroidia>Bacteroidales	GCA_003762745.1	Muribaculaceae_bacterium_Isolate-105_(HZI)	EEL50_01930	18926..22513	2486472	BREX-system-P-loop-protein-BrxC-[Muribaculaceae-bacterium-Isolate-105-(HZI)]
ROT18783.1	Type1.BREX	[illegible]	1223	Bacteria>FCB_group>Bacteroidetes/Chlorobi_group>Bacteroidota>Bacteroidia>Bacteroidales	GCA_003833095.1	Muribaculaceae_bacterium_Isolate-114_(HZI)	EEL53_11930	25441..29112	2486475	BREX-system-P-loop-protein-BrxC-[Muribaculaceae-bacterium-Isolate-114-(HZI)]
KAA3824840.1	Type1.BREX	[illegible]	1065	Bacteria>FCB_group>Bacteroidetes/Chlorobi_group>Bacteroidota>Bacteroidia>Bacteroidales	GCA_008565655.1	Bacteroides_ovatus	F3F58_10380	1..3200	28116	BREX-system-P-loop-protein-BrxC,-partial-[Bacteroides-ovatus]
KAA3823632.1	Type1.BREX	[illegible]	131	Bacteria>FCB_group>Bacteroidetes/Chlorobi_group>Bacteroidota>Bacteroidia>Bacteroidales	GCA_008565655.1	Bacteroides_ovatus	F3F58_17080	102919..103310	28116	BREX-system-P-loop-protein-BrxC,-partial-[Bacteroides-ovatus]
KAA3934629.1	Type1.BREX	[illegible]	197	Bacteria>FCB_group>Bacteroidetes/Chlorobi_group>Bacteroidota>Bacteroidia>Bacteroidales	GCA_008565965.1	Bacteroides_ovatus	F3F50_10370	48351..48941	28116	BREX-system-P-loop-protein-BrxC,-partial-[Bacteroides-ovatus]
KAA3965513.1	Type1.BREX	[illegible]	437	Bacteria>FCB_group>Bacteroidetes/Chlorobi_group>Bacteroidota>Bacteroidia>Bacteroidales	GCA_008566245.1	Bacteroides_ovatus	F3F43_12105	5385..6696	28116	BREX-system-P-loop-protein-BrxC,-partial-[Bacteroides-ovatus]
KAA4088264.1	Type1.BREX	[illegible]	1069	Bacteria>FCB_group>Bacteroidetes/Chlorobi_group>Bacteroidota>Bacteroidia>Bacteroidales	GCA_008566385.1	Bacteroides_ovatus	F3F49_00290	70210..73420	28116	BREX-system-P-loop-protein-BrxC,-partial-[Bacteroides-ovatus]
KAA4085421.1	Type1.BREX	[illegible]	134	Bacteria>FCB_group>Bacteroidetes/Chlorobi_group>Bacteroidota>Bacteroidia>Bacteroidales	GCA_008566385.1	Bacteroides_ovatus	F3F49_09110	1..402	28116	BREX-system-P-loop-protein-BrxC,-partial-[Bacteroides-ovatus]
KAA4007861.1	Type1.BREX	[illegible]	1208	Bacteria>FCB_group>Bacteroidetes/Chlorobi_group>Bacteroidota>Bacteroidia>Bacteroidales	GCA_008566415.1	Bacteroides_ovatus	F3F37_14290	84100..87726	28116	BREX-system-P-loop-protein-BrxC-[Bacteroides-ovatus]
KAA4743085.1	Type1.BREX	[illegible]	1056	Bacteria>FCB_group>Bacteroidetes/Chlorobi_group>Bacteroidota>Bacteroidia>Bacteroidales	GCA_008569225.1	Bacteroides_fragilis	F3B36_09960	278156..281326	817	BREX-system-P-loop-protein-BrxC-[Bacteroides-fragilis]
KAB5317657.1	Type1.BREX	[illegible]	1196	Bacteria>FCB_group>Bacteroidetes/Chlorobi_group>Bacteroidota>Bacteroidia>Bacteroidales	GCA_009025145.1	Bacteroides_stercoris	F9949_11590	61674..65264	46506	BREX-system-P-loop-protein-BrxC-[Bacteroides-stercoris]
NBJ07681.1	Type1.BREX	[illegible]	1223	Bacteria>FCB_group>Bacteroidetes/Chlorobi_group>Bacteroidota>Bacteroidia>Bacteroidales	GCA_009911575.1	Alistipes_sp._Z76	D1647_16085	15947..19618	2304565	BREX-system-P-loop-protein-BrxC-[Alistipes-sp.-Z76]
NLA16135.1	Type1.BREX	[illegible]	1174	Bacteria>FCB_group>Bacteroidetes/Chlorobi_group>Bacteroidota>Bacteroidia>Bacteroidales	GCA_012510445.1	Bacteroidales_bacterium	GX877_06335	9723..13247	2030927	BREX-system-P-loop-protein-BrxC-[Bacteroidales-bacterium]
NLN95595.1	Type1.BREX	[illegible]	1176	Bacteria>FCB_group>Bacteroidetes/Chlorobi_group>Bacteroidota>Bacteroidia>Bacteroidales	GCA_012522795.1	Bacteroidales_bacterium	GX128_05430	111716..115246	2030927	BREX-system-P-loop-protein-BrxC-[Bacteroidales-bacterium]
HHU96164.1	Type1.BREX	[illegible]	1202	Bacteria>FCB_group>Bacteroidetes/Chlorobi_group>Bacteroidota>Bacteroidia>Bacteroidales	GCA_012839475.1	Petrimonas_sp.	GXX67_02725	36773..40381	2023866	BREX-system-P-loop-protein-BrxC-[Petrimonas-sp.]
QKG81082.1	Type1.BREX	[illegible]	1206	Bacteria>FCB_group>Bacteroidetes/Chlorobi_group>Bacteroidota>Bacteroidia>Bacteroidales	GCA_013265555.1	Tenuifilum_thalassicum	FHG85_12670	3070622..3074242	2590900	BREX-system-P-loop-protein-BrxC-[Tenuifilum-thalassicum]
MBD5181931.1	Type1.BREX	[illegible]	1193	Bacteria>FCB_group>Bacteroidetes/Chlorobi_group>Bacteroidota>Bacteroidia>Bacteroidales	GCA_014800235.1	Bacteroidales_bacterium	HDS98_05210	20716..24297	2030927	BREX-system-P-loop-protein-BrxC-[Bacteroidales-bacterium]
MBD5263514.1	Type1.BREX	[illegible]	1221	Bacteria>FCB_group>Bacteroidetes/Chlorobi_group>Bacteroidota>Bacteroidia>Bacteroidales	GCA_014801365.1	Bacteroides_sp.	HDS39_05520	20086..23751	29523	BREX-system-P-loop-protein-BrxC-[Bacteroides-sp.]
MBD5304899.1	Type1.BREX	[illegible]	1182	Bacteria>FCB_group>Bacteroidetes/Chlorobi_group>Bacteroidota>Bacteroidia>Bacteroidales	GCA_014801805.1	Bacteroides_sp.	HDS12_01205	15900..19448	29523	BREX-system-P-loop-protein-BrxC-[Bacteroides-sp.]
MBD5349669.1	Type1.BREX	[illegible]	1219	Bacteria>FCB_group>Bacteroidetes/Chlorobi_group>Bacteroidota>Bacteroidia>Bacteroidales	GCA_014802205.1	Bacteroides_sp.	HDR89_02130	36441..40100	29523	BREX-system-P-loop-protein-BrxC-[Bacteroides-sp.]
MBD5357714.1	Type1.BREX	[illegible]	1203	Bacteria>FCB_group>Bacteroidetes/Chlorobi_group>Bacteroidota>Bacteroidia>Bacteroidales	GCA_014802265.1	Bacteroides_sp.	HDR88_12035	22916..26527	29523	BREX-system-P-loop-protein-BrxC-[Bacteroides-sp.]
MBD5419378.1	Type1.BREX	[illegible]	1201	Bacteria>FCB_group>Bacteroidetes/Chlorobi_group>Bacteroidota>Bacteroidia>Bacteroidales	GCA_014803765.1	Bacteroides_sp.	HDR48_05020	1391..4996	29523	BREX-system-P-loop-protein-BrxC-[Bacteroides-sp.]
MBE6254618.1	Type1.BREX	[illegible]	1211	Bacteria>FCB_group>Bacteroidetes/Chlorobi_group>Bacteroidota>Bacteroidia>Bacteroidales	GCA_015060555.1	Prevotella_sp.	E7105_03785	25107..28742	59823	BREX-system-P-loop-protein-BrxC-[Prevotella-sp.]
MBN2915288.1	Type1.BREX	[illegible]	971	Bacteria>FCB_group>Bacteroidetes/Chlorobi_group>Bacteroidota>Bacteroidia>Bacteroidales	GCA_016939035.1	Prevotella_sp.	JTJ26_04215	25012..27927	59823	BREX-system-P-loop-protein-BrxC-[Prevotella-sp.]
MBQ2195595.1	Type1.BREX	[illegible]	1009	Bacteria>FCB_group>Bacteroidetes/Chlorobi_group>Bacteroidota>Bacteroidia>Bacteroidales	GCA_017389665.1	Prevotella_sp.	II407_01515	3565..6594	59823	BREX-system-P-loop-protein-BrxC-[Prevotella-sp.]
MBQ2852763.1	Type1.BREX	[illegible]	1215	Bacteria>FCB_group>Bacteroidetes/Chlorobi_group>Bacteroidota>Bacteroidia>Bacteroidales	GCA_017409275.1	Bacteroidales_bacterium	IJE76_05160	110084..113731	2030927	hypothetical-protein-[Bacteroidales-bacterium]
MBQ6178640.1	Type1.BREX	[illegible]	1202	Bacteria>FCB_group>Bacteroidetes/Chlorobi_group>Bacteroidota>Bacteroidia>Bacteroidales	GCA_017429185.1	Bacteroidales_bacterium	IJK32_03880	18867..22475	2030927	BREX-system-P-loop-protein-BrxC-[Bacteroidales-bacterium]
MBR0492955.1	Type1.BREX	[illegible]	1215	Bacteria>FCB_group>Bacteroidetes/Chlorobi_group>Bacteroidota>Bacteroidia>Bacteroidales	GCA_017430125.1	Muribaculaceae_bacterium	IJJ83_04260	18614..22261	2498093	BREX-system-P-loop-protein-BrxC-[Muribaculaceae-bacterium]
MBQ6872144.1	Type1.BREX	[illegible]	1190	Bacteria>FCB_group>Bacteroidetes/Chlorobi_group>Bacteroidota>Bacteroidia>Bacteroidales	GCA_017435685.1	Bacteroidales_bacterium	IJQ23_07295	3964..7536	2030927	BREX-system-P-loop-protein-BrxC-[Bacteroidales-bacterium]
MBQ6577354.1	Type1.BREX	[illegible]	744	Bacteria>FCB_group>Bacteroidetes/Chlorobi_group>Bacteroidota>Bacteroidia>Bacteroidales	GCA_017438445.1	Bacteroidales_bacterium	IJL91_06365	8459..10695	2030927	hypothetical-protein-[Bacteroidales-bacterium]
MBR0262391.1	Type1.BREX	[illegible]	992	Bacteria>FCB_group>Bacteroidetes/Chlorobi_group>Bacteroidota>Bacteroidia>Bacteroidales	GCA_017443345.1	Prevotella_sp.	IJQ60_00735	1..2978	59823	BREX-system-P-loop-protein-BrxC-[Prevotella-sp.]
MBQ9588226.1	Type1.BREX	[illegible]	1183	Bacteria>FCB_group>Bacteroidetes/Chlorobi_group>Bacteroidota>Bacteroidia>Bacteroidales	GCA_017446745.1	Bacteroidales_bacterium	IJR26_10275	19779..23330	2030927	BREX-system-P-loop-protein-BrxC-[Bacteroidales-bacterium]
MBR0254975.1	Type1.BREX	[illegible]	1217	Bacteria>FCB_group>Bacteroidetes/Chlorobi_group>Bacteroidota>Bacteroidia>Bacteroidales	GCA_017447265.1	Bacteroidales_bacterium	IJQ69_03550	66425..70078	2030927	BREX-system-P-loop-protein-BrxC-[Bacteroidales-bacterium]
MBQ7672328.1	Type1.BREX	[illegible]	1193	Bacteria>FCB_group>Bacteroidetes/Chlorobi_group>Bacteroidota>Bacteroidia>Bacteroidales	GCA_017450385.1	Paludibacteraceae_bacterium	IJS49_04535	76772..80353	2748031	BREX-system-P-loop-protein-BrxC-[Paludibacteraceae-bacterium]
MBQ8673311.1	Type1.BREX	[illegible]	1180	Bacteria>FCB_group>Bacteroidetes/Chlorobi_group>Bacteroidota>Bacteroidia>Bacteroidales	GCA_017471925.1	Bacteroides_sp.	IJ511_04565	26677..30219	29523	BREX-system-P-loop-protein-BrxC-[Bacteroides-sp.]
MBR2237166.1	Type1.BREX	[illegible]	1217	Bacteria>FCB_group>Bacteroidetes/Chlorobi_group>Bacteroidota>Bacteroidia>Bacteroidales	GCA_017475265.1	Prevotella_sp.	IJ887_04725	43951..47604	59823	BREX-system-P-loop-protein-BrxC-[Prevotella-sp.]

Protein-ID	System	Gene-Neighbourhoods	Length	Taxonomy	Assembly-ID	Strain	Locus-Tag	Genomic-coordinates	TaxID	Fasta-defline
MBR3491242.1	Type1.BREX	<-CopG/RHH<-SIGMA-HTH+MA<-KAP-NTPase||BrxA-DNA-binding->BrxB-BrxF-iSTAND->DUF6079*->N6-METHYLASE-p->	1195	Bacteria>FCB_group>Bacteroidetes/Chlorobi_group>Bacteroidota>Bacteroidia>Bacteroidales	GCA_017508895.1	Bacteroidales_bacterium	IKH44_02940	7264..10851	2030927	BREX-system-P-loop-protein-BrxC-[Bacteroidales-bacterium]
MBR6936560.1	Type1.BREX	<-SIGMA+OB-Fold+AAA-ATPase+Lon-Protease-BrxL<-PglZ<-Schlafen+HATPase+wHTH<-N6-METHYLASE<-?<-OLD-ABC<-ParB<-DUF6079-p*<-DUF6079-p	827	Bacteria>FCB_group>Bacteroidetes/Chlorobi_group>Bacteroidota>Bacteroidia>Bacteroidales	GCA_017519645.1	Prevotella_sp.	IKH63_03150	127462..129945	59823	hypothetical-protein-[Prevotella-sp.]
MBR6937288.1	Type1.BREX	HTH_3->Couple_hipA->HipA_C->Phage_integrase->?->HTH_3->BrxA-DNA-binding->BrxB-BrxF-iSTAND->DUF6079-p*->	288	Bacteria>FCB_group>Bacteroidetes/Chlorobi_group>Bacteroidota>Bacteroidia>Bacteroidales	GCA_017519645.1	Prevotella_sp.	IKH63_06950	44867..45729	59823	hypothetical-protein-[Prevotella-sp.]
MBR4647494.1	Type1.BREX	MNS+CR-ATPase5-><-?||?->?->Calcineurin->SbcC+CCDC158->BrxA-DNA-binding->BrxB-BrxF-iSTAND->DUF6079*->OLD-ABC->PIN->N6-METHYLASE->PglZ->SIGMA+OB-Fold+AAA-ATPase+Lon-Protease-BrxL->?->?- [illegible]	1174	Bacteria>FCB_group>Bacteroidetes/Chlorobi_group>Bacteroidota>Bacteroidia>Bacteroidales	GCA_017525245.1	Bacteroidales_bacterium	IKO75_10320	20247..23771	2030927	BREX-system-P-loop-protein-BrxC-[Bacteroidales-bacterium]
MBR3727503.1	Type1.BREX	<-Response_reg<-SIGMA+OB-Fold+AAA-ATPase+Lon-Protease-BrxL<-PglZ<-DOC+HTH<-N6-METHYLASE<-SbcC<-SbcC<-DUF6079*<-BrxB-BrxF-iSTAND<-BrxA-DNA-binding<-HTH_3||LRR_5+LRR_5+LRR_5+LRR_5->IG+I [illegible]	1215	Bacteria>FCB_group>Bacteroidetes/Chlorobi_group>Bacteroidota>Bacteroidia>Bacteroidales	GCA_017530125.1	Muribaculaceae_bacterium	IKN10_00790	15216..18863	2498093	BREX-system-P-loop-protein-BrxC-[Muribaculaceae-bacterium]
MBR3711479.1	Type1.BREX	<-CR-ATPase5+REase||BrxA-DNA-binding->BrxB-BrxF-iSTAND->DUF6079*->N6-METHYLASE-p->	1195	Bacteria>FCB_group>Bacteroidetes/Chlorobi_group>Bacteroidota>Bacteroidia>Bacteroidales	GCA_017530365.1	Bacteroidales_bacterium	IKM99_11065	8978..12565	2030927	BREX-system-P-loop-protein-BrxC-[Bacteroidales-bacterium]
MBR6863890.1	Type1.BREX	<-ParB+HNH<-wHTH+WYL+WCX<-?||?->Couple_hipA+HIRAN->EVE->HD->BrxA-DNA-binding->BrxB-BrxF-iSTAND->DUF6079*->N6-METHYLASE->PglZ->SIGMA+OB-Fold+AAA-ATPase+HKD-DNase-BrxL->?->SF1-SF2-Helic [illegible]	1202	Bacteria>FCB_group>Bacteroidetes/Chlorobi_group>Bacteroidota>Bacteroidia>Bacteroidales	GCA_017531205.1	Bacteroidales_bacterium	IKM75_03420	106682..110290	2030927	BREX-system-P-loop-protein-BrxC-[Bacteroidales-bacterium]
MBR4380326.1	Type1.BREX	<-HTH+REase<-Phage_int_SAM_5+Phage_integrase<-NACHT+nSTAND4<-?<-SIGMA+OB-Fold+AAA-ATPase+Lon-Protease-BrxL<-PglZ<-Schlafen+HATPase+wHTH<-N6-METHYLASE<-DUF6079*<-BrxB-BrxF-iSTAND<-BrxA [illegible]	1216	Bacteria>FCB_group>Bacteroidetes/Chlorobi_group>Bacteroidota>Bacteroidia>Bacteroidales	GCA_017534535.1	Bacteroidaceae_bacterium	IKP48_03630	27978..31628	2212467	BREX-system-P-loop-protein-BrxC-[Bacteroidaceae-bacterium]
MBR5893775.1	Type1.BREX	<-SIGMA+OB-Fold+AAA-ATPase+HKD-DNase-BrxL<-PglZ<-DOC+HTH<-N6-METHYLASE<-?<-DUF6079*<-BrxB-BrxF-iSTAND<-BrxA-DNA-binding<-?<-HAD+HAD	1185	Bacteria>FCB_group>Bacteroidetes/Chlorobi_group>Bacteroidota>Bacteroidia>Bacteroidales	GCA_017554605.1	Bacteroidaceae_bacterium	IKZ37_09095	11422..14979	2212467	BREX-system-P-loop-protein-BrxC-[Bacteroidaceae-bacterium]
MBR5630362.1	Type1.BREX	<-CopG/RHH||BrxA-DNA-binding->BrxB-BrxF-iSTAND->DUF6079*->N6-METHYLASE->PglZ->SIGMA+OB-Fold+AAA-ATPase+HKD-DNase-BrxL->	1171	Bacteria>FCB_group>Bacteroidetes/Chlorobi_group>Bacteroidota>Bacteroidia>Bacteroidales	GCA_017557685.1	Bacteroidales_bacterium	IKW82_01820	70807..74322	2030927	BREX-system-P-loop-protein-BrxC-[Bacteroidales-bacterium]
MBR5576788.1	Type1.BREX	<-LRR_5+LRR_5+LRR_5+LRR_5<-?<-?<-?<-?<-?<-?||BrxA-DNA-binding->BrxB-BrxF-iSTAND->DUF6079*->N6-METHYLASE->SbcC+SbcC->?->ACET+TPR->?->SF1-SF2-Helicase->PglZ->SIGMA+OB-Fold+AAA-ATPase+HK [illegible]	1209	Bacteria>FCB_group>Bacteroidetes/Chlorobi_group>Bacteroidota>Bacteroidia>Bacteroidales	GCA_017558255.1	Bacteroidaceae_bacterium	IKW22_05890	14217..17846	2212467	BREX-system-P-loop-protein-BrxC-[Bacteroidaceae-bacterium]
MBO7470104.1	Type1.BREX	BrxA-DNA-binding->BrxB-BrxF-iSTAND->DUF6079*->N6-METHYLASE->?->?->PglZ->SIGMA+OB-Fold+AAA-ATPase+HKD-DNase-BrxL->	1169	Bacteria>FCB_group>Bacteroidetes/Chlorobi_group>Bacteroidota>Bacteroidia>Bacteroidales	GCA_017649945.1	Bacteroidales_bacterium	J6T81_08275	1593..5102	2030927	BREX-system-P-loop-protein-BrxC-[Bacteroidales-bacterium]
MBO7636706.1	Type1.BREX	<-Peptidase_C39_2<-N6-METHYLASE<-SIR2<-N6-METHYLASE<-DUF6079*<-BrxB-BrxF-iSTAND<-BrxA-DNA-binding	1214	Bacteria>FCB_group>Bacteroidetes/Chlorobi_group>Bacteroidota>Bacteroidia>Bacteroidales	GCA_017651825.1	Paludibacteraceae_bacterium	J6S89_09025	9410..13054	2748031	BREX-system-P-loop-protein-BrxC-[Paludibacteraceae-bacterium]
MBP6870975.1	Type1.BREX	<-SF1-SF2-Helicase<-STYKinase-like<-TRPR-HTH||HTH_3->Couple_hipA->HipA_C->?->BrxA-DNA-binding->BrxB-BrxF-iSTAND->DUF6079*->?->?->?->CR-Kinase+Classical-AAA->?->?->?->N6-METHYLASE->	1166	Bacteria>FCB_group>Bacteroidetes/Chlorobi_group>Bacteroidota>Bacteroidia>Bacteroidales	GCA_017991835.1	Bacteroidales_bacterium	KBC43_03120	368781..372281	2030927	BREX-system-P-loop-protein-BrxC-[Bacteroidales-bacterium]
MBP9983647.1	Type1.BREX	<-N6-METHYLASE<-DUF6079*<-BrxB-BrxF-iSTAND<-BrxA-DNA-binding<-HTH_3<-SMS-KaiC<-XIS-HTH<-?<-Phage_int_SAM_5+Phage_integrase<-XIS-HTH+XIS-HTH+XIS-HTH+XIS-HTH<-sGTP	1184	Bacteria>FCB_group>Bacteroidetes/Chlorobi_group>Bacteroidota>Bacteroidia>Bacteroidales	GCA_018064125.1	Prevotella_sp.	KBF13_00815	5388..8942	59823	BREX-system-P-loop-protein-BrxC-[Prevotella-sp.]
MBS6238336.1	Type1.BREX	<-PAS+HISKIN||?-><-AraC-HTH+AraC-HTH<-SIGMA+OB-Fold+AAA-ATPase+HKD-DNase-BrxL<-PglZ<-Schlafen+HATPase+wHTH<-ParB+HNH<-ParB+HNH<-N6-METHYLASE<-DUF6079*<-BrxB-BrxF-iSTAND<-BrxA-DNA-bind [illegible]	1205	Bacteria>FCB_group>Bacteroidetes/Chlorobi_group>Bacteroidota>Bacteroidia>Bacteroidales	GCA_018374845.1	Bacteroides_sp.	KH897_08200	204452..208069	29523	BREX-system-P-loop-protein-BrxC-[Bacteroides-sp.]
MBV1818013.1	Type1.BREX	HNH->?-><-D5N+D5ATPase<-?<-SF1-SF2-Helicase+RdgC+REase<-SIGMA+OB-Fold+AAA-ATPase+Lon-Protease-BrxL-p<-PglZ<-N6-METHYLASE<-DUF6079*<-BrxB-BrxF-iSTAND<-BrxA-DNA-binding<-?<-REase<-SF1-S [illegible]	1195	Bacteria>FCB_group>Bacteroidetes/Chlorobi_group>Bacteroidota>Bacteroidia>Bacteroidales	GCA_019084065.1	Bacteroidales_bacterium_MSK.15.36	KUA25_08040	15312..18899	2850325	BREX-system-P-loop-protein-BrxC-[Bacteroidales-bacterium-MSK.15.36]
MBV1819082.1	Type1.BREX	<-RNase<-PglZ<-N6-METHYLASE<-DUF6079*<-BrxB-BrxF-iSTAND<-BrxA-DNA-binding	1185	Bacteria>FCB_group>Bacteroidetes/Chlorobi_group>Bacteroidota>Bacteroidia>Bacteroidales	GCA_019084065.1	Bacteroidales_bacterium_MSK.15.36	KUA25_13485	7035..10592	2850325	BREX-system-P-loop-protein-BrxC-[Bacteroidales-bacterium-MSK.15.36]
MBW6491485.1	Type1.BREX	<-HTH_3||XIS-HTH->?->?->?->BrxA-DNA-binding->BrxB-BrxF-iSTAND->DUF6079*->HTH+REase->N6-METHYLASE->AbiEii->PglZ->SIGMA+OB-Fold+AAA-ATPase+Lon-Protease-BrxL->REase->HEPN->ClpABN-AAA+Clp [illegible]	1144	Bacteria>FCB_group>Bacteroidetes/Chlorobi_group>Bacteroidota>Bacteroidia>Bacteroidales	GCA_019429275.1	Lentimicrobium_sp.	KOB15_09875	47933..51367	2034841	BREX-system-P-loop-protein-BrxC-[Lentimicrobium-sp.]
MCB9017748.1	Type1.BREX	<-ACET||?-><-SIGMA+OB-Fold+AAA-ATPase+HKD-DNase-BrxL<-PglZ<-N6-METHYLASE-p<-HTH+REase<-?<-Schlafen+HATPase+wHTH<-DUF6079*<-BrxB-BrxF-iSTAND<-BrxA-DNA-binding<-?<-PALP	1207	Bacteria>FCB_group>Bacteroidetes/Chlorobi_group>Bacteroidota>Bacteroidia>Bacteroidales	GCA_020636675.1	Prevotellaceae_bacterium	H6544_03995	62697..66320	2049047	BREX-system-P-loop-protein-BrxC-[Prevotellaceae-bacterium]
HJF82219.1	Type1.BREX	<-SF1-SF2-Helicase-p<-HTH_3<-?<-?||BrxA-DNA-binding->BrxB-BrxF-iSTAND->DUF6079*->OLD-ABC->TOPRIM->N6-METHYLASE->SIR2+SIR2->PglZ->SIGMA+OB-Fold+AAA-ATPase+HKD-DNase-BrxL->	1195	Bacteria>FCB_group>Bacteroidetes/Chlorobi_group>Bacteroidota>Bacteroidia>Bacteroidales	GCA_020742805.1	Phocaeicola_plebeius	K8V40_11345	4107..7694	310297	BREX-system-P-loop-protein-BrxC-[Phocaeicola-plebeius]
HJF93068.1	Type1.BREX	<-SIR2+SIR2<-N6-METHYLASE<-DUF6079*<-BrxB-BrxF-iSTAND<-BrxA-DNA-binding<-HTH_3	1217	Bacteria>FCB_group>Bacteroidetes/Chlorobi_group>Bacteroidota>Bacteroidia>Bacteroidales	GCA_020742905.1	Mediterranea_massiliensis	K8W02_11910	6041..9694	1841865	BREX-system-P-loop-protein-BrxC-[Mediterranea-massiliensis]
MCC8037087.1	Type1.BREX	<-HAD+PRTase+PRTase+REa [illegible]	1219	Bacteria>FCB_group>Bacteroidetes/Chlorobi_group>Bacteroidota>Bacteroidia>Bacteroidales	GCA_020859755.1	Bacteroidales_bacterium	LIP02_02980	18317..21976	2030927	BREX-system-P-loop-protein-BrxC-[Bacteroidales-bacterium]
MCC8072421.1	Type1.BREX	<-TOPRIM+Topoisom_bac<- [illegible]	1191	Bacteria>FCB_group>Bacteroidetes/Chlorobi_group>Bacteroidota>Bacteroidia>Bacteroidales	GCA_020859845.1	Bacteroidales_bacterium	LIO90_11575	54243..57818	2030927	BREX-system-P-loop-protein-BrxC-[Bacteroidales-bacterium]
MCC8115076.1	Type1.BREX	PglZ->SIGMA+OB-Fold+AAA [illegible]	159	Bacteria>FCB_group>Bacteroidetes/Chlorobi_group>Bacteroidota>Bacteroidia>Bacteroidales	GCA_020860445.1	Bacteroidales_bacterium	LIP03_14010	6329..6808	2030927	hypothetical-protein-[Bacteroidales-bacterium]
MCC8115077.1	Type1.BREX	PglZ->SIGMA+OB-Fold+AAA [illegible]	1040	Bacteria>FCB_group>Bacteroidetes/Chlorobi_group>Bacteroidota>Bacteroidia>Bacteroidales	GCA_020860445.1	Bacteroidales_bacterium	LIP03_14015	6789..9911	2030927	BREX-system-P-loop-protein-BrxC-[Bacteroidales-bacterium]
MCC8119638.1	Type1.BREX	<-PglZ<-Schlafen+HATPas [illegible]	1189	Bacteria>FCB_group>Bacteroidetes/Chlorobi_group>Bacteroidota>Bacteroidia>Bacteroidales	GCA_020860515.1	Bacteroidales_bacterium	LIP09_12950	29133..32702	2030927	BREX-system-P-loop-protein-BrxC-[Bacteroidales-bacterium]
MCD8313154.1	Type1.BREX	<-N6-METHYLASE-p<-DUF60 [illegible]	762	Bacteria>FCB_group>Bacteroidetes/Chlorobi_group>Bacteroidota>Bacteroidia>Bacteroidales	GCA_021204895.1	Bacteroidales_bacterium	LUC24_03250	1675..3962	2030927	BREX-system-P-loop-protein-BrxC-[Bacteroidales-bacterium]
MCD8387916.1	Type1.BREX	<-AbiEii<-?<-?<-?<-SIGM [illegible]	1201	Bacteria>FCB_group>Bacteroidetes/Chlorobi_group>Bacteroidota>Bacteroidia>Bacteroidales	GCA_021205605.1	Bacteroidales_bacterium	LUD17_13690	15132..18737	2030927	BREX-system-P-loop-protein-BrxC-[Bacteroidales-bacterium]
MCE5179211.1	Type1.BREX	<-SIGMA+OB-Fold+AAA-ATP [illegible]	1197	Bacteria>FCB_group>Bacteroidetes/Chlorobi_group>Bacteroidota>Bacteroidia>Bacteroidales	GCA_021372935.1	Porphyromonadaceae_bacterium	LLF81_08720	99386..102979	2049046	BREX-system-P-loop-protein-BrxC-[Porphyromonadaceae-bacterium]
MCF0219894.1	Type1.BREX	CR-Kinase+Classical-AAA [illegible]	1215	Bacteria>FCB_group>Bacteroidetes/Chlorobi_group>Bacteroidota>Bacteroidia>Bacteroidales	GCA_021443165.1	Muribaculaceae_bacterium	HUK14_08940	85831..89478	2498093	BREX-system-P-loop-protein-BrxC-[Muribaculaceae-bacterium]
MCF2581772.1	Type1.BREX	<-SIGMA+OB-Fold+AAA-ATP [illegible]	1182	Bacteria>FCB_group>Bacteroidetes/Chlorobi_group>Bacteroidota>Bacteroidia>Bacteroidales	GCA_021530995.1	Bacteroides_caecigallinarum	I6E24_07160	15567..19115	1411144	BREX-system-P-loop-protein-BrxC-[Bacteroides-caecigallinarum]
MCF8307437.1	Type1.BREX	<-HTH_3<-?<-?<-N6-METHY [illegible]	1165	Bacteria>FCB_group>Bacteroidetes/Chlorobi_group>Bacteroidota>Bacteroidia>Bacteroidales	GCA_021739995.1	Bacteroidales_bacterium	K9I68_00355	30814..34311	2030927	BREX-system-P-loop-protein-BrxC-[Bacteroidales-bacterium]
MCH4018978.1	Type1.BREX	XIS-HTH->?->AAA+-><-?|| [illegible]	1198	Bacteria>FCB_group>Bacteroidetes/Chlorobi_group>Bacteroidota>Bacteroidia>Bacteroidales	GCA_022483425.1	Prevotella_sp.	LKE74_11130	2793807..2797403	59823	BREX-system-P-loop-protein-BrxC-[Prevotella-sp.]
MCI1742488.1	Type1.BREX	<-HTH_31||tRNA-><-?<-SI [illegible]	1206	Bacteria>FCB_group>Bacteroidetes/Chlorobi_group>Bacteroidota>Bacteroidia>Bacteroidales	GCA_022647785.1	Prevotella_sp.	LKI18_09155	39395..43015	59823	BREX-system-P-loop-protein-BrxC-[Prevotella-sp.]
MCI2087046.1	Type1.BREX	<-SIGMA+OB-Fold+AAA-ATP [illegible]	1198	Bacteria>FCB_group>Bacteroidetes/Chlorobi_group>Bacteroidota>Bacteroidia>Bacteroidales	GCA_022653375.1	Prevotella_sp.	LKK10_02020	11419..15015	59823	BREX-system-P-loop-protein-BrxC-[Prevotella-sp.]
MCI6111298.1	Type1.BREX	ABC-ATPase+SbcC-><-?<-P [illegible]	1219	Bacteria>FCB_group>Bacteroidetes/Chlorobi_group>Bacteroidota>Bacteroidia>Bacteroidales	GCA_022771115.1	Prevotella_sp.	MR706_06730	27310..30969	59823	BREX-system-P-loop-protein-BrxC-[Prevotella-sp.]
MCI6081496.1	Type1.BREX	<-METHYLASE<-?<-?<-?<-? [illegible]	1215	Bacteria>FCB_group>Bacteroidetes/Chlorobi_group>Bacteroidota>Bacteroidia>Bacteroidales	GCA_022771625.1	Prevotella_sp.	MR724_08080	18194..21841	59823	BREX-system-P-loop-protein-BrxC-[Prevotella-sp.]
MCI6195664.1	Type1.BREX	<-PglZ<-SIR2+SIR2<-Pept [illegible]	1190	Bacteria>FCB_group>Bacteroidetes/Chlorobi_group>Bacteroidota>Bacteroidia>Bacteroidales	GCA_022773545.1	Prevotella_sp.	MR670_00305	49581..53153	59823	BREX-system-P-loop-protein-BrxC-[Prevotella-sp.]
MCI7017346.1	Type1.BREX	<-SIGMA+OB-Fold+AAA-ATP [illegible]	1189	Bacteria>FCB_group>Bacteroidetes/Chlorobi_group>Bacteroidota>Bacteroidia>Bacteroidales	GCA_022775925.1	Prevotella_sp.	MR923_09655	21723..25292	59823	BREX-system-P-loop-protein-BrxC-[Prevotella-sp.]
MCI6403340.1	Type1.BREX	AAA-ATPase-p-><-?||BrxA [illegible]	1217	Bacteria>FCB_group>Bacteroidetes/Chlorobi_group>Bacteroidota>Bacteroidia>Bacteroidales	GCA_022777225.1	Prevotella_sp.	MR820_04565	20272..23925	59823	BREX-system-P-loop-protein-BrxC-[Prevotella-sp.]
MCI6372654.1	Type1.BREX	<-N6-METHYLASE-p<-DUF60 [illegible]	1205	Bacteria>FCB_group>Bacteroidetes/Chlorobi_group>Bacteroidota>Bacteroidia>Bacteroidales	GCA_022777445.1	Paraprevotella_sp.	MR792_05245	1364..4981	2049036	BREX-system-P-loop-protein-BrxC-[Paraprevotella-sp.]
MCI6336437.1	Type1.BREX	<-DUF6079-p*<-BrxB-BrxF [illegible]	337	Bacteria>FCB_group>Bacteroidetes/Chlorobi_group>Bacteroidota>Bacteroidia>Bacteroidales	GCA_022777825.1	Prevotella_sp.	MR802_00605	1..1013	59823	BREX-system-P-loop-protein-BrxC-[Prevotella-sp.]
MCI6336457.1	Type1.BREX	PIWI->?->BrxA-DNA-bindi [illegible]	1215	Bacteria>FCB_group>Bacteroidetes/Chlorobi_group>Bacteroidota>Bacteroidia>Bacteroidales	GCA_022777825.1	Prevotella_sp.	MR802_00705	5343..8990	59823	BREX-system-P-loop-protein-BrxC-[Prevotella-sp.]
MCI6336758.1	Type1.BREX	DUF6079-p*->OLD-ABC->TO [illegible]	873	Bacteria>FCB_group>Bacteroidetes/Chlorobi_group>Bacteroidota>Bacteroidia>Bacteroidales	GCA_022777825.1	Prevotella_sp.	MR802_02260	1..2624	59823	BREX-system-P-loop-protein-BrxC-[Prevotella-sp.]
MCI7256454.1	Type1.BREX	<-SIGMA+OB-Fold+AAA-ATP [illegible]	1195	Bacteria>FCB_group>Bacteroidetes/Chlorobi_group>Bacteroidota>Bacteroidia>Bacteroidales	GCA_022780235.1	Prevotella_sp.	MR559_03870	8872..12459	59823	BREX-system-P-loop-protein-BrxC-[Prevotella-sp.]
MCI7250520.1	Type1.BREX	ABC-ATPase+SbcC-><-?<-P [illegible]	275	Bacteria>FCB_group>Bacteroidetes/Chlorobi_group>Bacteroidota>Bacteroidia>Bacteroidales	GCA_022781125.1	Prevotella_sp.	MR548_05545	54684..55511	59823	hypothetical-protein-[Prevotella-sp.]
MCI7548156.1	Type1.BREX	BrxA-DNA-binding->BrxB- [illegible]	1222	Bacteria>FCB_group>Bacteroidetes/Chlorobi_group>Bacteroidota>Bacteroidia>Bacteroidales	GCA_022785425.1	Bacteroides_sp.	MSA48_06355	2201..5869	29523	BREX-system-P-loop-protein-BrxC-[Bacteroides-sp.]
MCI7614186.1	Type1.BREX	<-SF1-SF2-Helicase<-?|| [illegible]	1215	Bacteria>FCB_group>Bacteroidetes/Chlorobi_group>Bacteroidota>Bacteroidia>Bacteroidales	GCA_022786925.1	Bacteroidales_bacterium	MSS61_02370	10942..14589	2030927	BREX-system-P-loop-protein-BrxC-[Bacteroidales-bacterium]
MCK4345284.1	Type1.BREX	<-Phage_integrase||?->H [illegible]	1141	Bacteria>FCB_group>Bacteroidetes/Chlorobi_group>Bacteroidota>Bacteroidia>Bacteroidales	GCA_023128755.1	Bacteroidales_bacterium	KAX05_08355	4669..8094	2030927	BREX-system-P-loop-protein-BrxC-[Bacteroidales-bacterium]
MCK9398992.1	Type1.BREX	PIN-><-?||DDE_Tnp_1-><- [illegible]	1163	Bacteria>FCB_group>Bacteroidetes/Chlorobi_group>Bacteroidota>Bacteroidia>Bacteroidales	GCA_023229505.1	Bacteroidales_bacterium	MOQ51_03210	70739..74230	2030927	BREX-system-P-loop-protein-BrxC-[Bacteroidales-bacterium]
MCK9310968.1	Type1.BREX	ABC-ATPase+SbcC->?->?-> [illegible]	1143	Bacteria>FCB_group>Bacteroidetes/Chlorobi_group>Bacteroidota>Bacteroidia>Bacteroidales	GCA_023230125.1	Bacteroidales_bacterium	MOP26_01690	7742..11173	2030927	BREX-system-P-loop-protein-BrxC-[Bacteroidales-bacterium]
MCM1267126.1	Type1.BREX	<-SbcC<-?<-HNH<-SIGMA+O [illegible]	1173	Bacteria>FCB_group>Bacteroidetes/Chlorobi_group>Bacteroidota>Bacteroidia>Bacteroidales	GCA_023665065.1	Bacteroidales_bacterium	NC302_04400	49162..52683	2030927	BREX-system-P-loop-protein-BrxC-[Bacteroidales-bacterium]
MCQ2071598.1	Type1.BREX	<-TOPRIM<-?<-?||Peptida [illegible]	1190	Bacteria>FCB_group>Bacteroidetes/Chlorobi_group>Bacteroidota>Bacteroidia>Bacteroidales	GCA_024398655.1	Bacteroidaceae_bacterium	MJY96_00535	107887..111459	2212467	BREX-system-P-loop-protein-BrxC-[Bacteroidaceae-bacterium]
MCS2813929.1	Type1.BREX	<-Peptidase_C39_2<-ACET [illegible]	246	Bacteria>FCB_group>Bacteroidetes/Chlorobi_group>Bacteroidota>Bacteroidia>Bacteroidales	GCA_024791005.1	Bacteroides_ovatus	NXX52_14680	3516528..3517268	28116	hypothetical-protein-[Bacteroides-ovatus]
MCS2813930.1	Type1.BREX	<-ACET+TPR<-N6-METHYLAS [illegible]	131	Bacteria>FCB_group>Bacteroidetes/Chlorobi_group>Bacteroidota>Bacteroidia>Bacteroidales	GCA_024791005.1	Bacteroides_ovatus	NXX52_14685	3517247..3517642	28116	DUF6079-family-protein-[Bacteroides-ovatus]
WP_156315612.1	Type1.BREX	XIS-HTH-><-?<-?||?->?-> [illegible]	925	Bacteria>FCB_group>Bacteroidetes/Chlorobi_group>Bacteroidota>Bacteroidia>Bacteroidales	GCA_030820635.1	Phocaeicola_coprophilus	JCM13816_RS17810	17247..20024	387090	BREX-system-P-loop-protein-BrxC-[Phocaeicola-coprophilus]
WP_118354616.1	Type1.BREX	<-N6-METHYLASE-p<-DOC+H [illegible]	1194	Bacteria>FCB_group>Bacteroidetes/Chlorobi_group>Bacteroidota>Bacteroidia>Bacteroidales	GCA_030820635.1	Prevotella_stercorea	FXX22_RS02010	13543..17127	363265	BREX-system-P-loop-protein-BrxC-[Prevotella-stercorea]
SCH71957.1	Type1.BREX	<-SIGMA+OB-Fold+AAA-ATP [illegible]	1179	Bacteria>FCB_group>Bacteroidetes/Chlorobi_group>Bacteroidota>Bacteroidia>Bacteroidales	GCA_900066445.1	uncultured_Bacteroides_sp.	SAMEA3545309_01215	56504..60043	162156	Uncharacterised-protein-[uncultured-Bacteroides-sp.]
SCI35540.1	Type1.BREX	<-N6-METHYLASE<-TOPRIM< [illegible]	1195	Bacteria>FCB_group>Bacteroidetes/Chlorobi_group>Bacteroidota>Bacteroidia>Bacteroidales	GCA_900066455.1	uncultured_Bacteroides_sp.	OCV81_RS14585	3983..7570	162156	Uncharacterised-protein-[uncultured-Bacteroides-sp.]
SHF69941.1	Type1.BREX	<-RADICAL-SAM<-?||tRNA- [illegible]	1182	Bacteria>FCB_group>Bacteroidetes/Chlorobi_group>Bacteroidota>Bacteroidia>Bacteroidales	GCA_900128905.1	Bacteroides_luti	SAMN05444405_11263	81108..84656	1297750	hypothetical-protein-SAMN05444405_11263-[Bacteroides-luti]
SHK68061.1	Type1.BREX	<-Classical-AAA<-?||tRN [illegible]	1206	Bacteria>FCB_group>Bacteroidetes/Chlorobi_group>Bacteroidota>Bacteroidia>Bacteroidales	GCA_900142525.1	Prevotella_ruminicola	SAMN05216463_10942	46265..49885	839	hypothetical-protein-SAMN05216463_10942-[Prevotella-ruminicola]
WP_008760280.1	Type1.BREX	D5ATPase+DUF3874-><-?|| [illegible]	1193	Bacteria>FCB_group>Bacteroidetes/Chlorobi_group>Bacteroidota>Bacteroidia>Bacteroidales	GCF_000162515.1	Bacteroides_thetaiotaomicron	HMPREF9007_RS20215	181866..185447	818	MULTISPECIES:-BREX-system-P-loop-protein-BrxC-[Bacteroides]
WP_004312187.1	Type1.BREX	<-SIGMA+OB-Fold+AAA-ATP [illegible]	1208	Bacteria>FCB_group>Bacteroidetes/Chlorobi_group>Bacteroidota>Bacteroidia>Bacteroidales	GCF_000178275.1	Bacteroidaceae	K0G46_RS13725	52737..56363	815	MULTISPECIES:-BREX-system-P-loop-protein-BrxC-[Bacteroidaceae]
WP_006745116.1	Type1.BREX	<-MODE-HTH+PBPII||?-><- [illegible]	1192	Bacteria>FCB_group>Bacteroidetes/Chlorobi_group>Bacteroidota>Bacteroidia>Bacteroidales	GCF_000212915.1	Bacteroides_coprosuis	BCOP_RS09060	2132867..2136445	151276	BREX-system-P-loop-protein-BrxC-[Bacteroides-coprosuis]
WP_018109295.1	Type1.BREX	<-FURR-HTH<-?<-Peptidas [illegible]	1202	Bacteria>FCB_group>Bacteroidetes/Chlorobi_group>Bacteroidota>Bacteroidia>Bacteroidales	GCF_000375405.1	Bacteroides_propionicifaciens	B091_RS0106980	23999..27607	392838	BREX-system-P-loop-protein-BrxC-[Bacteroides-propionicifaciens]
WP_002561973.1	Type1.BREX	35exo+SF1-SF2-Helicase- [illegible]	1202	Bacteria>FCB_group>Bacteroidetes/Chlorobi_group>Bacteroidota>Bacteroidia>Bacteroidales	GCF_000382465.1	Bacteroides_sp._HPS0048	HMPREF1214_RS19625	483033..486641	1078089	BREX-system-P-loop-protein-BrxC-[Bacteroides-sp.-HPS0048]
WP_024993540.1	Type1.BREX	<-wHTH+WYL+WCX<-wHTH+WY [illegible]	1210	Bacteria>FCB_group>Bacteroidetes/Chlorobi_group>Bacteroidota>Bacteroidia>Bacteroidales	GCF_000613805.1	Phocaeicola_paurosaccharolyticus	JCM15092_RS04015	53285..56917	732242	BREX-system-P-loop-protein-BrxC-[Phocaeicola-paurosaccharolyticus]
WP_042520323.1	Type1.BREX	DDE_Tnp_ISL3-><-PARG+MA [illegible]	1204	Bacteria>FCB_group>Bacteroidetes/Chlorobi_group>Bacteroidota>Bacteroidia>Bacteroidales	GCF_000833975.1	Prevotella_pectinovora	ST44_RS13175	10747..14361	1602169	BREX-system-P-loop-protein-BrxC-[Prevotella-pectinovora]
WP_044073670.1	Type1.BREX	HTH_3->BrxA-DNA-binding [illegible]	1206	Bacteria>FCB_group>Bacteroidetes/Chlorobi_group>Bacteroidota>Bacteroidia>Bacteroidales	GCF_000834015.1	Prevotella_pectinovora	ST42_RS12125	1627..5247	1602169	BREX-system-P-loop-protein-BrxC-[Prevotella-pectinovora]
WP_094389547.1	Type1.BREX	LRR_5->?->?->?-><-?||?- [illegible]	1228	Bacteria>FCB_group>Bacteroidetes/Chlorobi_group>Bacteroidota>Bacteroidia>Bacteroidales	GCF_002251345.1	Prevotella_sp._P4-119	CIK89_RS00860	20388..24074	2024218	BREX-system-P-loop-protein-BrxC-[Prevotella-sp.-P4-119]
WP_094382632.1	Type1.BREX	<-SIGMA+OB-Fold+AAA-ATP [illegible]	1215	Bacteria>FCB_group>Bacteroidetes/Chlorobi_group>Bacteroidota>Bacteroidia>Bacteroidales	GCF_002251435.1	Prevotella_sp._P2-180	CIK98_RS02120	248745..252392	2024224	BREX-system-P-loop-protein-BrxC-[Prevotella-sp.-P2-180]
WP_094881847.1	Type1.BREX	<-TOPRIM<-OLD-ABC<-DUF6 [illegible]	1206	Bacteria>FCB_group>Bacteroidetes/Chlorobi_group>Bacteroidota>Bacteroidia>Bacteroidales	GCF_002265625.1	Prevotella_sp._885	CHL74_RS14190	4100..7720	2022527	BREX-system-P-loop-protein-BrxC-[Prevotella-sp.-885]
WP_233544533.1	Type1.BREX	ABC-ATPase+SbcC->?-><-? [illegible]	1083	Bacteria>FCB_group>Bacteroidetes/Chlorobi_group>Bacteroidota>Bacteroidia>Bacteroidales	GCF_003468525.1	Prevotella_sp._AM34-19LB	DW830_RS13215	15981..19232	2292364	BREX-system-P-loop-protein-BrxC-[Prevotella-sp.-AM34-19LB]
WP_233544532.1	Type1.BREX	ABC-ATPase+SbcC->?-><-? [illegible]	126	Bacteria>FCB_group>Bacteroidetes/Chlorobi_group>Bacteroidota>Bacteroidia>Bacteroidales	GCF_003468525.1	Prevotella_sp._AM34-19LB	DW830_RS16695	15625..16005	2292364	hypothetical-protein-[Prevotella-sp.-AM34-19LB]
WP_118255350.1	Type1.BREX	PRTase-><-?<-?<-?<-?||H [illegible]	1206	Bacteria>FCB_group>Bacteroidetes/Chlorobi_group>Bacteroidota>Bacteroidia>Bacteroidales	GCF_003471795.1	Prevotella_copri	DW192_RS11885	19147..22767	165179	BREX-system-P-loop-protein-BrxC-[Prevotella-copri]
WP_118441918.1	Type1.BREX	HTH_Tnp_1+HTH_21+rve+rv [illegible]	1195	Bacteria>FCB_group>Bacteroidetes/Chlorobi_group>Bacteroidota>Bacteroidia>Bacteroidales	GCF_003474535.1	Phocaeicola_plebeius	DW041_RS13375	4564..8151	310297	BREX-system-P-loop-protein-BrxC-[Phocaeicola-plebeius]
WP_122342696.1	Type1.BREX	Arm-DNA-bind_5+Phage_in [illegible]	1211	Bacteria>FCB_group>Bacteroidetes/Chlorobi_group>Bacteroidota>Bacteroidia>Bacteroidales	GCF_003475265.1	Parabacteroides_merdae	DWZ81_RS06115	100594..104229	46503	BREX-system-P-loop-protein-BrxC-[Parabacteroides-merdae]
WP_135993587.1	Type1.BREX	<-SF1-SF2-Helicase<-SIG [illegible]	1221	Bacteria>FCB_group>Bacteroidetes/Chlorobi_group>Bacteroidota>Bacteroidia>Bacteroidales	GCF_004793655.1	Muribaculum_intestinale	E5330_RS10970	18051..21716	1796646	BREX-system-P-loop-protein-BrxC-[Muribaculum-intestinale]
WP_172774764.1	Type1.BREX	OLD-ABC->TOPRIM->XIS-HT [illegible]	1217	Bacteria>FCB_group>Bacteroidetes/Chlorobi_group>Bacteroidota>Bacteroidia>Bacteroidales	GCF_012976275.1	Phocaeicola_vulgatus	HKQ53_RS16515	79396..83049	821	BREX-system-P-loop-protein-BrxC-[Phocaeicola-vulgatus]
WP_173076490.1	Type1.BREX	ABhydrolase-><-PDZ+Pept [illegible]	1195	Bacteria>FCB_group>Bacteroidetes/Chlorobi_group>Bacteroidota>Bacteroidia>Bacteroidales	GCF_013265555.1	Tenuifilum_thalassicum	FHG85_RS12675	3070655..3074242	2590900	BREX-system-P-loop-protein-BrxC-[Tenuifilum-thalassicum]
WP_175324259.1	Type1.BREX	<-SIGMA+OB-Fold+AAA-ATP [illegible]	1208	Bacteria>FCB_group>Bacteroidetes/Chlorobi_group>Bacteroidota>Bacteroidia>Bacteroidales	GCF_013361175.1	Phocaeicola_vulgatus	HUV02_RS04070	75655..79281	821	BREX-system-P-loop-protein-BrxC-[Phocaeicola-vulgatus]
WP_179400143.1	Type1.BREX	<-Phage_holin||?->tRNA- [illegible]	1192	Bacteria>FCB_group>Bacteroidetes/Chlorobi_group>Bacteroidota>Bacteroidia>Bacteroidales	GCF_013409575.1	Macellibacteroides_fermentans	F5613_RS13465	149892..153470	879969	BREX-system-P-loop-protein-BrxC-[Macellibacteroides-fermentans]
WP_181213825.1	Type1.BREX	TOPRIM->?->TOPRIM->?->< [illegible]	1212	Bacteria>FCB_group>Bacteroidetes/Chlorobi_group>Bacteroidota>Bacteroidia>Bacteroidales	GCF_013618865.1	Phocaeicola_faecicola	H3001_RS00990	112732..116370	2739389	BREX-system-P-loop-protein-BrxC-[Phocaeicola-faecicola]
WP_115498725.1	Type1.BREX	<-SIGMA+OB-Fold+AAA-ATP [illegible]	1211	Bacteria>FCB_group>Bacteroidetes/Chlorobi_group>Bacteroidota>Bacteroidia>Bacteroidales	GCF_014385205.1	Parabacteroides_acidifaciens	H8784_RS05825	88295..91930	2290935	BREX-system-P-loop-protein-BrxC-[Parabacteroides-acidifaciens]
WP_004319896.1	Type1.BREX	BrxA-DNA-binding->BrxB- [illegible]	1202	Bacteria>FCB_group>Bacteroidetes/Chlorobi_group>Bacteroidota>Bacteroidia>Bacteroidales	GCF_015548155.1	Bacteroides	PO193_RS10880	44218..47826	816	MULTISPECIES:-BREX-system-P-loop-protein-BrxC-[Bacteroides]
WP_195290998.1	Type1.BREX	Calcineurin-><-?<-SIGMA [illegible]	1188	Bacteria>FCB_group>Bacteroidetes/Chlorobi_group>Bacteroidota>Bacteroidia>Bacteroidales	GCF_015548275.1	Alistipes_finegoldii	I2C47_RS09460	177258..180824	214856	BREX-system-P-loop-protein-BrxC-[Alistipes-finegoldii]
WP_118403962.1	Type1.BREX	<-Phage_int_SAM_5+Phage [illegible]	1211	Bacteria>FCB_group>Bacteroidetes/Chlorobi_group>Bacteroidota>Bacteroidia>Bacteroidales	GCF_015553065.1	Bacteroides_cellulosilyticus	I2F01_RS18130	73980..77615	246787	BREX-system-P-loop-protein-BrxC-[Bacteroides-cellulosilyticus]
WP_195571580.1	Type1.BREX	BrxA-DNA-binding->BrxB- [illegible]	1202	Bacteria>FCB_group>Bacteroidetes/Chlorobi_group>Bacteroidota>Bacteroidia>Bacteroidales	GCF_015557365.1	Bacteroides_nordii	I2G99_RS18035	12742..16350	291645	BREX-system-P-loop-protein-BrxC-[Bacteroides-nordii]
WP_195628684.1	Type1.BREX	<-MORC+REase<-?<-REase+ [illegible]	769	Bacteria>FCB_group>Bacteroidetes/Chlorobi_group>Bacteroidota>Bacteroidia>Bacteroidales	GCF_015558155.1	Bacteroides_finegoldii	I2H47_RS02655	98961..101270	338188	hypothetical-protein-[Bacteroides-finegoldii]
WP_195663259.1	Type1.BREX	<-SIGMA+OB-Fold+AAA-ATP [illegible]	1206	Bacteria>FCB_group>Bacteroidetes/Chlorobi_group>Bacteroidota>Bacteroidia>Bacteroidales	GCF_015559525.1	Bacteroides_congonensis	I2I22_RS15360	96982..100602	1871006	BREX-system-P-loop-protein-BrxC-[Bacteroides-congonensis]
WP_203041477.1	Type1.BREX	Phage_lysozyme->PRTase- [illegible]	1207	Bacteria>FCB_group>Bacteroidetes/Chlorobi_group>Bacteroidota>Bacteroidia>Bacteroidales	GCF_016803185.1	Prevotella_copri	F8Q96_RS13415	22671..26294	165179	BREX-system-P-loop-protein-BrxC-[Prevotella-copri]
WP_204430319.1	Type1.BREX	<-NADAR<-SIGMA+OB-Fold+ [illegible]	1217	Bacteria>FCB_group>Bacteroidetes/Chlorobi_group>Bacteroidota>Bacteroidia>Bacteroidales	GCF_016899805.1	Bacteroides_gallinaceum	H6B13_RS12495	13542..17195	1462571	BREX-system-P-loop-protein-BrxC-[Bacteroides-gallinaceum]
WP_205111283.1	Type1.BREX	OLD-ABC->XIS-HTH-><-?<- [illegible]	1217	Bacteria>FCB_group>Bacteroidetes/Chlorobi_group>Bacteroidota>Bacteroidia>Bacteroidales	GCF_016899955.1	Marseilla_massiliensis	H6B30_RS12995	19775..23428	1841864	BREX-system-P-loop-protein-BrxC-[Marseilla-massiliensis]
WP_204392825.1	Type1.BREX	<-SbcC<-HKD-DNase<-SIGM [illegible]	1185	Bacteria>FCB_group>Bacteroidetes/Chlorobi_group>Bacteroidota>Bacteroidia>Bacteroidales	GCF_016901995.1	Bacteroides_caecigallinarum	H6A61_RS08410	10706..14263	1411144	BREX-system-P-loop-protein-BrxC-[Bacteroides-caecigallinarum]
WP_204506195.1	Type1.BREX	HTH_3->BrxA-DNA-binding [illegible]	1187	Bacteria>FCB_group>Bacteroidetes/Chlorobi_group>Bacteroidota>Bacteroidia>Bacteroidales	GCF_016902095.1	Phocaeicola_plebeius	H6A37_RS09755	27157..30720	310297	BREX-system-P-loop-protein-BrxC-[Phocaeicola-plebeius]
WP_204401091.1	Type1.BREX	Calcineurin+Calcineurin [illegible]	1179	Bacteria>FCB_group>Bacteroidetes/Chlorobi_group>Bacteroidota>Bacteroidia>Bacteroidales	GCF_016902515.1	Bacteroides_caecigallinarum	H6A30_RS00985	221918..225457	1411144	BREX-system-P-loop-protein-BrxC-[Bacteroides-caecigallinarum]
WP_211451926.1	Type1.BREX	Peptidase_M23-><-?<-?<- [illegible]	1208	Bacteria>FCB_group>Bacteroidetes/Chlorobi_group>Bacteroidota>Bacteroidia>Bacteroidales	GCF_018289355.1	Phocaeicola_vulgatus	INE85_RS07560	1903040..1906666	821	BREX-system-P-loop-protein-BrxC-[Phocaeicola-vulgatus]
WP_215652320.1	Type1.BREX	Phage_lysozyme->PRTase- [illegible]	1206	Bacteria>FCB_group>Bacteroidetes/Chlorobi_group>Bacteroidota>Bacteroidia>Bacteroidales	GCF_018784085.1	Prevotella_copri	GPK65_RS08775	33741..37361	165179	BREX-system-P-loop-protein-BrxC-[Prevotella-copri]
WP_215708904.1	Type1.BREX	Arm-DNA-bind_5-><-UDG<- [illegible]	1215	Bacteria>FCB_group>Bacteroidetes/Chlorobi_group>Bacteroidota>Bacteroidia>Bacteroidales	GCF_018785865.1	Bacteroides_uniformis	GRD73_RS11230	15032..18679	820	BREX-system-P-loop-protein-BrxC-[Bacteroides-uniformis]
WP_216951216.1	Type1.BREX	<-HTH_Tnp<-?<-?||HTH_3- [illegible]	1208	Bacteria>FCB_group>Bacteroidetes/Chlorobi_group>Bacteroidota>Bacteroidia>Bacteroidales	GCF_019042015.1	Phocaeicola_vulgatus	KTG13_RS11225	6191..9817	821	BREX-system-P-loop-protein-BrxC-[Phocaeicola-vulgatus]
WP_217745553.1	Type1.BREX	<-HIRAN<-RADICAL-SAM<-A [illegible]	1206	Bacteria>FCB_group>Bacteroidetes/Chlorobi_group>Bacteroidota>Bacteroidia>Bacteroidales	GCF_019127365.1	Prevotella_copri	KSW85_RS06175	41108..44728	165179	BREX-system-P-loop-protein-BrxC-[Prevotella-copri]
WP_217718390.1	Type1.BREX	TOPRIM->XIS-HTH-><-?<-? [illegible]	1217	Bacteria>FCB_group>Bacteroidetes/Chlorobi_group>Bacteroidota>Bacteroidia>Bacteroidales	GCF_019127825.1	Bacteroides_uniformis	KSX11_RS12350	118043..121696	820	BREX-system-P-loop-protein-BrxC-[Bacteroides-uniformis]
WP_217725669.1	Type1.BREX	<-BACON+Peptidase_M64|| [illegible]	1202	Bacteria>FCB_group>Bacteroidetes/Chlorobi_group>Bacteroidota>Bacteroidia>Bacteroidales	GCF_019128915.1	unclassified_Bacteroides	KSX87_RS10005	229853..233461	2646097	MULTISPECIES:-BREX-system-P-loop-protein-BrxC-[unclassified-Bacteroides]
WP_217719995.1	Type1.BREX	DUF4007->BrxB-BrxF-iSTA [illegible]	265	Bacteria>FCB_group>Bacteroidetes/Chlorobi_group>Bacteroidota>Bacteroidia>Bacteroidales	GCF_019412375.1	Bacteroides_uniformis	KSX92_RS08930	2230..3027	820	DUF6079-family-protein-[Bacteroides-uniformis]
WP_115473019.1	Type1.BREX	<-OLD-ABC||?-><-Phage_i [illegible]	1211	Bacteria>FCB_group>Bacteroidetes/Chlorobi_group>Bacteroidota>Bacteroidia>Bacteroidales	GCF_019733435.1	Bacteroides_fragilis	G7060_RS07670	242068..245703	817	BREX-system-P-loop-protein-BrxC-[Bacteroides-fragilis]
WP_120489887.1	Type1.BREX	BrxA-DNA-binding->BrxB- [illegible]	1202	Bacteria>FCB_group>Bacteroidetes/Chlorobi_group>Bacteroidota>Bacteroidia>Bacteroidales	GCF_019733535.1	Bacteroides_ovatus	G7086_RS03160	85646..89254	28116	BREX-system-P-loop-protein-BrxC-[Bacteroides-ovatus]
WP_130041244.1	Type1.BREX	Polbeta+HEPN->D5ATPase+ [illegible]	1175	Bacteria>FCB_group>Bacteroidetes/Chlorobi_group>Bacteroidota>Bacteroidia>Bacteroidales	GCF_019733625.1	Bacteroides_thetaiotaomicron	EAJ10_RS04945	124743..128270	818	BREX-system-P-loop-protein-BrxC-[Bacteroides-thetaiotaomicron]
WP_221729077.1	Type1.BREX	<-DUF772+DDE_Tnp_1+DDE_ [illegible]	1206	Bacteria>FCB_group>Bacteroidetes/Chlorobi_group>Bacteroidota>Bacteroidia>Bacteroidales	GCF_019733655.1	Parabacteroides_merdae	HCH02_RS20805	4560..8180	46503	BREX-system-P-loop-protein-BrxC-[Parabacteroides-merdae]
WP_060386337.1	Type1.BREX	<-SF1-SF2-Helicase<-?|| [illegible]	1213	Bacteria>FCB_group>Bacteroidetes/Chlorobi_group>Bacteroidota>Bacteroidia>Bacteroidales	GCF_020091485.1	Bacteroides_stercoris	K6V31_RS06895	1552414..1556055	46506	BREX-system-P-loop-protein-BrxC-[Bacteroides-stercoris]
WP_225659484.1	Type1.BREX	Polbeta+HEPN->D5ATPase+ [illegible]	1175	Bacteria>FCB_group>Bacteroidetes/Chlorobi_group>Bacteroidota>Bacteroidia>Bacteroidales	GCF_020313445.1	Bacteroides_thetaiotaomicron	KFX61_RS09580	34477..38004	818	BREX-system-P-loop-protein-BrxC-[Bacteroides-thetaiotaomicron]
WP_227088012.1	Type1.BREX	ArdCN+MPTase->TOPRIM->? [illegible]	1176	Bacteria>FCB_group>Bacteroidetes/Chlorobi_group>Bacteroidota>Bacteroidia>Bacteroidales	GCF_020560105.1	Bacteroides_cellulosilyticus	LI190_RS20855	41329..44859	246787	BREX-system-P-loop-protein-BrxC-[Bacteroides-cellulosilyticus]
WP_217720628.1	Type1.BREX	<-NUDIX+NUDIX<-?<-?<-?| [illegible]	1208	Bacteria>FCB_group>Bacteroidetes/Chlorobi_group>Bacteroidota>Bacteroidia>Bacteroidales	GCF_020827675.1	Bacteroidaceae	HZY58_RS04175	119839..123465	815	MULTISPECIES:-BREX-system-P-loop-protein-BrxC-[Bacteroidaceae]
WP_234117060.1	Type1.BREX	<-BACON+Peptidase_M64|| [illegible]	1202	Bacteria>FCB_group>Bacteroidetes/Chlorobi_group>Bacteroidota>Bacteroidia>Bacteroidales	GCF_021410745.1	Bacteroides_ovatus	K0G75_RS22190	19952..23560	28116	BREX-system-P-loop-protein-BrxC-[Bacteroides-ovatus]
WP_235327427.1	Type1.BREX	<-SIGMA+OB-Fold+AAA-ATP [illegible]	1179	Bacteria>FCB_group>Bacteroidetes/Chlorobi_group>Bacteroidota>Bacteroidia>Bacteroidales	GCF_021530995.1	Bacteroides_caecigallinarum	I6E24_RS07160	15567..19106	1411144	BREX-system-P-loop-protein-BrxC-[Bacteroides-caecigallinarum]
WP_235417293.1	Type1.BREX	BrxA-DNA-binding->BrxB- [illegible]	1176	Bacteria>FCB_group>Bacteroidetes/Chlorobi_group>Bacteroidota>Bacteroidia>Bacteroidales	GCF_021531515.1	Prevotella_stercorea	I6E41_RS10105	1347..4877	363265	BREX-system-P-loop-protein-BrxC-[Prevotella-stercorea]
WP_243323291.1	Type1.BREX	XIS-HTH-><-?||TraJ-RHH- [illegible]	1142	Bacteria>FCB_group>Bacteroidetes/Chlorobi_group>Bacteroidota>Bacteroidia>Bacteroidales	GCF_022809355.1	Parabacteroides_sp._AGMB00274	MUN53_RS02605	134848..138276	2924040	hypothetical-protein-[Parabacteroides-sp.-AGMB00274]
WP_243326763.1	Type1.BREX	<-DUF6079-p*<-BrxB-BrxF [illegible]	90	Bacteria>FCB_group>Bacteroidetes/Chlorobi_group>Bacteroidota>Bacteroidia>Bacteroidales	GCF_022809355.1	Parabacteroides_sp._AGMB00274	MUN53_RS17900	89..361	2924040	hypothetical-protein-[Parabacteroides-sp.-AGMB00274]
WP_249093422.1	Type1.BREX	TOPRIM->XIS-HTH-><-?<-? [illegible]	1217	Bacteria>FCB_group>Bacteroidetes/Chlorobi_group>Bacteroidota>Bacteroidia>Bacteroidales	GCF_023337025.1	Mediterranea_sp._ET5	M3087_RS02205	173446..177099	2939419	BREX-system-P-loop-protein-BrxC-[Mediterranea-sp.-ET5]
WP_250803006.1	Type1.BREX	<-SIGMA+OB-Fold+AAA-ATP [illegible]	1208	Bacteria>FCB_group>Bacteroidetes/Chlorobi_group>Bacteroidota>Bacteroidia>Bacteroidales	GCF_023668475.1	Phocaeicola_massiliensis	NCY86_RS00900	193015..196641	204516	BREX-system-P-loop-protein-BrxC-[Phocaeicola-massiliensis]
WP_250702187.1	Type1.BREX	<-GDResolvase+HIN-HTH<- [illegible]	1215	Bacteria>FCB_group>Bacteroidetes/Chlorobi_group>Bacteroidota>Bacteroidia>Bacteroidales	GCF_023668595.1	Bacteroides_ovatus	NCZ26_RS08955	97357..101004	28116	BREX-system-P-loop-protein-BrxC-[Bacteroides-ovatus]
WP_252760249.1	Type1.BREX	<-TIR<-?<-SIGMA+OB-Fold [illegible]	1205	Bacteria>FCB_group>Bacteroidetes/Chlorobi_group>Bacteroidota>Bacteroidia>Bacteroidales	GCF_024054555.1	Prevotella_cerevisiae	NG821_RS03330	12038..15655	2053716	BREX-system-P-loop-protein-BrxC-[Prevotella-cerevisiae]
WP_252760927.1	Type1.BREX	DDE_Tnp_ISL3->XIS-HTH-> [illegible]	1206	Bacteria>FCB_group>Bacteroidetes/Chlorobi_group>Bacteroidota>Bacteroidia>Bacteroidales	GCF_024054555.1	Prevotella_cerevisiae	NG821_RS06905	6531..10151	2053716	BREX-system-P-loop-protein-BrxC-[Prevotella-cerevisiae]
WP_258327305.1	Type1.BREX	<-HHH_5+SF1-SF2-Helicas [illegible]	1217	Bacteria>FCB_group>Bacteroidetes/Chlorobi_group>Bacteroidota>Bacteroidia>Bacteroidales	GCF_024733785.1	Barnesiella_sp._ET7	NW211_RS06370	166743..170396	2972460	BREX-system-P-loop-protein-BrxC-[Barnesiella-sp.-ET7]
WP_258898999.1	Type1.BREX	<-SIGMA+OB-Fold+AAA-ATP [illegible]	1208	Bacteria>FCB_group>Bacteroidetes/Chlorobi_group>Bacteroidota>Bacteroidia>Bacteroidales	GCF_024759365.1	Bacteroides_ovatus	NXX44_RS13110	3467956..3471582	28116	BREX-system-P-loop-protein-BrxC-[Bacteroides-ovatus]
WP_258900416.1	Type1.BREX	<-PglZ<-SF1-SF2-Helicas [illegible]	1208	Bacteria>FCB_group>Bacteroidetes/Chlorobi_group>Bacteroidota>Bacteroidia>Bacteroidales	GCF_024760065.1	Bacteroides_thetaiotaomicron	NXX51_RS12095	3253003..3256629	818	BREX-system-P-loop-protein-BrxC-[Bacteroides-thetaiotaomicron]
WP_234146092.1	Type1.BREX	Arm-DNA-bind_5+Phage_in [illegible]	1180	Bacteria>FCB_group>Bacteroidetes/Chlorobi_group>Bacteroidota>Bacteroidia>Bacteroidales	GCF_024760085.1	Bacteroides_uniformis	J8872_RS07820	80453..83995	820	BREX-system-P-loop-protein-BrxC-[Bacteroides-uniformis]
WP_258991265.1	Type1.BREX	<-SIGMA+OB-Fold+AAA-ATP [illegible]	1208	Bacteria>FCB_group>Bacteroidetes/Chlorobi_group>Bacteroidota>Bacteroidia>Bacteroidales	GCF_024790585.1	Phocaeicola_vulgatus	NXX11_RS15345	3784105..3787731	821	BREX-system-P-loop-protein-BrxC-[Phocaeicola-vulgatus]
WP_259011403.1	Type1.BREX	BrxA-DNA-binding->BrxB- [illegible]	1202	Bacteria>FCB_group>Bacteroidetes/Chlorobi_group>Bacteroidota>Bacteroidia>Bacteroidales	GCF_024791585.1	Bacteroides_sp._BFG-638	NXX54_RS14145	3701947..3705555	2972765	BREX-system-P-loop-protein-BrxC-[Bacteroides-sp.-BFG-638]
WP_258973412.1	Type1.BREX	Polbeta+HEPN->D5ATPase+ [illegible]	1133	Bacteria>FCB_group>Bacteroidetes/Chlorobi_group>Bacteroidota>Bacteroidia>Bacteroidales	GCF_024793205.1	Bacteroides_thetaiotaomicron	NXX32_RS11560	3197619..3201020	818	BREX-system-P-loop-protein-BrxC-[Bacteroides-thetaiotaomicron]
WP_258979859.1	Type1.BREX	BrxA-DNA-binding->BrxB- [illegible]	780	Bacteria>FCB_group>Bacteroidetes/Chlorobi_group>Bacteroidota>Bacteroidia>Bacteroidales	GCF_024793605.1	Bacteroides_sp._BFG-637	NXX38_RS14295	3701972..3704314	2972764	BREX-system-P-loop-protein-BrxC-[Bacteroides-sp.-BFG-637]
WP_009000708.1	Type1.BREX	BrxA-DNA-binding->BrxB- [illegible]	1202	Bacteria>FCB_group>Bacteroidetes/Chlorobi_group>Bacteroidota>Bacteroidia>Bacteroidales	GCF_025147085.1	Bacteroides	NQ505_RS12870	3648371..3651979	816	MULTISPECIES:-BREX-system-P-loop-protein-BrxC-[Bacteroides]
WP_262301578.1	Type1.BREX	<-SIGMA+OB-Fold+AAA-ATP [illegible]	1218	Bacteria>FCB_group>Bacteroidetes/Chlorobi_group>Bacteroidota>Bacteroidia>Bacteroidales	GCF_025460925.1	Prevotella_hominis	OCW41_RS08900	100342..103998	2518605	BREX-system-P-loop-protein-BrxC-[Prevotella-hominis]
WP_148330685.1	Type1.BREX	<-N6-METHYLASE<-TOPRIM< [illegible]	1195	Bacteria>FCB_group>Bacteroidetes/Chlorobi_group>Bacteroidota>Bacteroidia>Bacteroidales	GCF_025567125.1	Phocaeicola_fibrisolvens	OCV81_RS14585	3983..7570	2981793	BREX-system-P-loop-protein-BrxC-[Phocaeicola-fibrisolvens]
WP_195661285.1	Type1.BREX	BrxA-DNA-binding->BrxB- [illegible]	1202	Bacteria>FCB_group>Bacteroidetes/Chlorobi_group>Bacteroidota>Bacteroidia>Bacteroidales	GCF_025914175.1	Bacteroides_nordii	KQP55_RS20540	5436855..5440463	291645	BREX-system-P-loop-protein-BrxC-[Bacteroides-nordii]
WP_264912090.1	Type1.BREX	<-HIRAN<-RADICAL-SAM<-A [illegible]	1206	Bacteria>FCB_group>Bacteroidetes/Chlorobi_group>Bacteroidota>Bacteroidia>Bacteroidales	GCF_026015575.1	Prevotella_copri	ONS98_RS12175	17740..21360	165179	BREX-system-P-loop-protein-BrxC-[Prevotella-copri]
WP_264965401.1	Type1.BREX	ABC-ATPase+ABC-ATPase+A [illegible]	1194	Bacteria>FCB_group>Bacteroidetes/Chlorobi_group>Bacteroidota>Bacteroidia>Bacteroidales	GCF_026015685.1	Prevotella_copri	ONT16_RS03155	729578..733162	165179	BREX-system-P-loop-protein-BrxC-[Prevotella-copri]
WP_270558549.1	Type1.BREX	<-HISKIN<-?<-Polbeta<-M [illegible]	1216	Bacteria>FCB_group>Bacteroidetes/Chlorobi_group>Bacteroidota>Bacteroidia>Bacteroidales	GCF_027686315.1	Bacteroides_uniformis	PG022_RS08930	563554..567204	820	BREX-system-P-loop-protein-BrxC-[Bacteroides-uniformis]
WP_271687043.1	Type1.BREX	<-SIGMA+OB-Fold+AAA-ATP [illegible]	1208	Bacteria>FCB_group>Bacteroidetes/Chlorobi_group>Bacteroidota>Bacteroidia>Bacteroidales	GCF_028199265.1	Bacteroides_xylanisolvens	PL435_RS07875	93897..97523	371601	BREX-system-P-loop-protein-BrxC-[Bacteroides-xylanisolvens]
WP_271704578.1	Type1.BREX	HTH_3->MORC+DUF3684+REa [illegible]	1209	Bacteria>FCB_group>Bacteroidetes/Chlorobi_group>Bacteroidota>Bacteroidia>Bacteroidales	GCF_028200085.1	Bacteroides_xylanisolvens	PL493_RS08910	20513..24142	371601	BREX-system-P-loop-protein-BrxC-[Bacteroides-xylanisolvens]
WP_272709339.1	Type1.BREX	<-DUF4365+REase<-NUDIX+ [illegible]	1208	Bacteria>FCB_group>Bacteroidetes/Chlorobi_group>Bacteroidota>Bacteroidia>Bacteroidales	GCF_028528025.1	Bacteroides	PQG94_RS11850	55098..58724	816	MULTISPECIES:-BREX-system-P-loop-protein-BrxC-[Bacteroides]
WP_274224871.1	Type1.BREX	Arm-DNA-bind_5+Phage_in [illegible]	1213	Bacteria>FCB_group>Bacteroidetes/Chlorobi_group>Bacteroidota>Bacteroidia>Bacteroidales	GCF_028743275.1	Bacteroides_fragilis	PT255_RS11125	2684967..2688608	817	BREX-system-P-loop-protein-BrxC-[Bacteroides-fragilis]
WP_032560932.1	Type1.BREX	SF1-SF2-Helicase->?-><- [illegible]	1211	Bacteria>FCB_group>Bacteroidetes/Chlorobi_group>Bacteroidota>Bacteroidia>Bacteroidales	GCF_029872615.1	Bacteroides_fragilis	M068_RS14675	124538..128173	817	BREX-system-P-loop-protein-BrxC-[Bacteroides-fragilis]

Protein-ID	System	Gene-Neighbourhoods	Length	Taxonomy	Assembly-ID	Strain	Locus-Tag	Genomic-coordinates	TaxID	Fasta-define
WP_154043608.1	Type1.BREX	<-SbcC<-SIGMA+OB-Fold+AAA-ATPase+Lon-Protease-BrxL<-PglZ<-Schlafen+HATPase+wHTH<-N6-METHYLASE<-SLOG+BirA-HTH<-SF1-SF2-Helicase+PRTase<-HNH<-OLD-ABC<-DUF6079*<-BrxB-BrxF-iSTAND<-BrxA-DNA-binding<-?<-?<-?<-?<-?<-Calcineurin+Calcineurin-conf	1179	Bacteria>FCB_group>Bacteroidetes/Chlorobi_group>Bacteroidota>Bacteroidia>Bacteroidales	GCF_900066445.1	Bacteroides_cellulolyticus	OCV82_RS05980	56504..60043	2981780	BREX-system-P-loop-protein-BrxC-[Bacteroides-cellulolyticus]
WP_074557116.1	Type1.BREX	<-HAD+PRTase+PRTase+REase+SF1-SF2-Helicase<-?<-TPR+TPR<-?<-Schlafen+HATPase+wHTH<-TrwF||BrxA-DNA-binding->BrxB-BrxF-iSTAND->DUF6079*->N6-METHYLASE->SIR2->Schlafen+HATPase+wHTH->PglZ->SIGMA+OB-Fold+AAA-ATPase+HKD-DNase-BrxL-><-35exo+SF1-SF2-Helicase||BACON+Peptidase_M64->	1202	Bacteria>FCB_group>Bacteroidetes/Chlorobi_group>Bacteroidota>Bacteroidia>Bacteroidales	GCF_900102645.1	Bacteroides_ovatus	BLR14_RS05840	106864..110472	28116	BREX-system-P-loop-protein-BrxC-[Bacteroides-ovatus]
WP_072542576.1	Type1.BREX	<-AAA-ATPase<-HTH+rve<-?<-SIGMA+OB-Fold+AAA-ATPase+HKD-DNase-BrxL<-PglZ<-SIR2+SIR2<-N6-METHYLASE<-TOPRIM<-OLD-ABC<-DUF6079*<-BrxB-BrxF-iSTAND<-BrxA-DNA-binding||?->?->?->?-><-?<-?<-OLD-ABC	1195	Bacteria>FCB_group>Bacteroidetes/Chlorobi_group>Bacteroidota>Bacteroidia>Bacteroidales	GCF_900128455.1	Bacteroides_mediterraneensis	BQ7480_RS08760	2129051..2132638	1841856	BREX-system-P-loop-protein-BrxC-[Bacteroides-mediterraneensis]
WP_073402639.1	Type1.BREX	<-RADICAL-SAM<-?||tRNA-><-SIGMA+OB-Fold+AAA-ATPase+Lon-Protease-BrxL<-PglZ<-Schlafen+HATPase+wHTH<-ParB+HNH<-N6-METHYLASE<-?<-DUF6079*<-BrxB-BrxF-iSTAND<-BrxA-DNA-binding	1188	Bacteria>FCB_group>Bacteroidetes/Chlorobi_group>Bacteroidota>Bacteroidia>Bacteroidales	GCF_900128905.1	Bacteroides_luti	BUC21_RS11790	81108..84674	1297750	BREX-system-P-loop-protein-BrxC-[Bacteroides-luti]
WP_254795118.1	Type1.BREX	<-Classical-AAA<-?||tRNA-><-?<-?<-HTH+DOC||?->HTH_3->BrxA-DNA-binding->BrxB-BrxF-iSTAND->DUF6079*->N6-METHYLASE->SbcC+SbcC->?->SIR2+SIR2->PglZ->SIGMA+OB-Fold+AAA-ATPase+HKD-DNase-BrxL->?->?->ParB+HNH->	1209	Bacteria>FCB_group>Bacteroidetes/Chlorobi_group>Bacteroidota>Bacteroidia>Bacteroidales	GCF_900142525.1	Prevotella_ruminicola	BUB86_RS07050	46256..49885	839	BREX-system-P-loop-protein-BrxC-[Prevotella-ruminicola]
WP_077197246.1	Type1.BREX	DDE_Tnp_IS66+DDE_Tnp_IS66_C-><-?<-?<-?<-?<-?||HTH_3->BrxA-DNA-binding->BrxB-BrxF-iSTAND->DUF6079*->N6-METHYLASE->Schlafen+HATPase+wHTH->PglZ->SIGMA+OB-Fold+AAA-ATPase+Lon-Protease-BrxL->?->||HTH_38+rve-><-?||DDE_Tnp_1->HTH_3->	1176	Bacteria>FCB_group>Bacteroidetes/Chlorobi_group>Bacteroidota>Bacteroidia>Bacteroidales	GCF_900155655.1	Prevotella_ihumii	CZ367_RS13300	3061778..3065308	1917878	BREX-system-P-loop-protein-BrxC-[Prevotella-ihumii]
WP_176134075.1	Type1.BREX	<-Phage_holin||?->tRNA-><-?||?->?->ABC-ATPase+SbcC-><-?||BrxA-DNA-binding->BrxB-BrxF-iSTAND->DUF6079*->N6-METHYLASE->OLD-ABC+TOPRIM->SF1-SF2-Helicase->?->N6-METHYLASE-p->ParB+HNH->ParB+HNH->PglZ->SIGMA+OB-Fold+AAA-ATPase+Lon-Protease-BrxL->	1192	Bacteria>FCB_group>Bacteroidetes/Chlorobi_group>Bacteroidota>Bacteroidia>Bacteroidales	GCF_900168155.1	Parabacteroides_chartae	B5X57_RS12575	48804..52382	1037355	BREX-system-P-loop-protein-BrxC-[Parabacteroides-chartae]
WP_130829424.1	Type1.BREX	<-SIGMA+OB-Fold+AAA-ATPase+Lon-Protease-BrxL<-PglZ<-Schlafen+HATPase+wHTH<-Schlafen<-N6-METHYLASE<-DUF6079*<-BrxB-BrxF-iSTAND<-BrxA-DNA-binding<-HTH_3<-?||?-><-PTSEIIA1||?->PRTase+MutS->	1153	Bacteria>FCB_group>Bacteroidetes/Chlorobi_group>Bacteroidota>Bacteroidia>Bacteroidales	GCF_900607215.1	Prevotella	ETF27_RS09605	9675..13136	838	MULTISPECIES:-BREX-system-P-loop-protein-BrxC-[Prevotella]
WP_125105113.1	Type1.BREX	<-HHH_5+SF1-SF2-Helicase<-HIRAN<-RADICAL-SAM<-NADAR<-SIGMA+OB-Fold+AAA-ATPase+HKD-DNase-BrxL<-PglZ<-Schlafen+HATPase+wHTH<-SIR2<-N6-METHYLASE<-DUF6079*<-BrxB-BrxF-iSTAND<-BrxA-DNA-binding<-HTH_3||?->?->?->?-><-XIS-HTH<-TOPRIM	1217	Bacteria>FCB_group>Bacteroidetes/Chlorobi_group>Bacteroidota>Bacteroidia>Bacteroidales	GCF_900625065.1	Prevotella_marseillensis	EJL04_RS12230	3058927..3062580	2479840	BREX-system-P-loop-protein-BrxC-[Prevotella-marseillensis]
WP_144153273.1	Type1.BREX	<-HIRAN<-RADICAL-SAM<-ARG<-?<-?||HTH_3->wHTH+WYL+WCX->BrxA-DNA-binding->BrxB-BrxF-iSTAND->DUF6079*->N6-METHYLASE->SIR2+SIR2->PglZ->SIGMA+OB-Fold+AAA-ATPase+HKD-DNase-BrxL->McrB-AAA->McrC-NTD+REase->	1194	Bacteria>FCB_group>Bacteroidetes/Chlorobi_group>Bacteroidota>Bacteroidia>Bacteroidales	GCF_902167755.1	Prevotella_copri	BILOI45876EF_RS09275	107988..111572	165179	BREX-system-P-loop-protein-BrxC-[Prevotella-copri]
WP_032543214.1	Type1.BREX	<-METHYLASE<-ACET<-DOC+HTH<-URI-DNase+RAMA<-?<-SIGMA+OB-Fold+AAA-ATPase+HKD-DNase-BrxL<-PglZ<-Schlafen+HATPase+wHTH<-N6-METHYLASE<-DUF6079*<-BrxB-BrxF-iSTAND<-BrxA-DNA-binding<-HTH_3<-?<-?<-TraJ-RHH<-ZnR+TOPRIM<-CR-ATPase4<-XIS-HTH	1211	Bacteria>FCB_group>Bacteroidetes/Chlorobi_group>Bacteroidota>Bacteroidia>Bacteroidales	GCF_902364655.1	Bacteroides_fragilis	PDN68_RS18445	100181..103816	817	BREX-system-P-loop-protein-BrxC-[Bacteroides-fragilis]
WP_060407611.1	Type1.BREX	<-SIGMA+OB-Fold+AAA-ATPase+HKD-DNase-BrxL<-PglZ<-Schlafen+HATPase+wHTH<-SIR2<-N6-METHYLASE-p<-N6-METHYLASE<-DUF6079*<-BrxB-BrxF-iSTAND<-BrxA-DNA-binding<-HTH_3<-?<-?<-?||SIGMA-HTH->	1205	Bacteria>FCB_group>Bacteroidetes/Chlorobi_group>Bacteroidota>Bacteroidia>Bacteroidales	GCF_902387305.1	Bacteroides_cellulosilyticus	FYC30_RS03960	75039..78656	246787	BREX-system-P-loop-protein-BrxC-[Bacteroides-cellulosilyticus]
WP_118419043.1	Type1.BREX	BrxA-DNA-binding->BrxB-BrxF-iSTAND->DUF6079*->N6-METHYLASE->SIR2+SIR2->Schlafen+HATPase+wHTH->PglZ->SIGMA+OB-Fold+AAA-ATPase+HKD-DNase-BrxL-><-35exo+SF1-SF2-Helicase||BACON+Peptidase_M64->	1202	Bacteria>FCB_group>Bacteroidetes/Chlorobi_group>Bacteroidota>Bacteroidia>Bacteroidales	GCF_918279245.1	Bacteroides_ovatus	PO203_RS09380	101158..104766	28116	BREX-system-P-loop-protein-BrxC-[Bacteroides-ovatus]
WP_117985926.1	Type1.BREX	XIS-HTH->SMS-KaiC-><-SF1-SF2-Helicase<-SIGMA+OB-Fold+AAA-ATPase+HKD-DNase-BrxL<-PglZ<-Schlafen+HATPase+wHTH<-SF1-SF2-Helicase<-OLD-ABC+TOPRIM<-N6-METHYLASE<-DUF6079*<-BrxB-BrxF-iSTAND<-BrxA-DNA-binding<-HTH_3<-AbiEi_4+AbiEi_1<-STYKinase-like<-TRPR-HTH||SbcC->DEDD_Tnp_IS110+Transposase_20->	1210	Bacteria>FCB_group>Bacteroidetes/Chlorobi_group>Bacteroidota>Bacteroidia>Bacteroidales	GCF_934726555.1	Bacteroides	O1446_RS12920	68574..72206	816	MULTISPECIES:-BREX-system-P-loop-protein-BrxC-[Bacteroides]
WP_158582848.1	Type1.BREX	ACET-><-?<-Cysteine-Desulfurase||tRNA-><-?<-?<-?<-?||BrxA-DNA-binding->BrxB-BrxF-iSTAND->DUF6079*->N6-METHYLASE->Schlafen+HATPase+wHTH->PglZ->SIGMA+OB-Fold+AAA-ATPase+HKD-DNase-BrxL-><-?||?->PolB->HEPN->	1210	Bacteria>FCB_group>Bacteroidetes/Chlorobi_group>Bacteroidota>Bacteroidia>Bacteroidales	GCF_938033175.1	Parabacteroides_sp._AF17-28	DWW90_RS15090	22936..26568	2292241	BREX-system-P-loop-protein-BrxC-[Parabacteroides-sp.-AF17-28]
WP_118139864.1	Type1.BREX	<-HIRAN<-RADICAL-SAM<-ARG<-?<-?||HTH_3->wHTH+WYL+WCX->BrxA-DNA-binding->BrxB-BrxF-iSTAND->DUF6079*->OLD-ABC->TOPRIM->N6-METHYLASE->N6-METHYLASE-p->?->SF1-SF2-Helicase->PglZ->SIGMA+OB-Fold+AAA-ATPase+HKD-DNase-BrxL->McrB-AAA->	1202	Bacteria>FCB_group>Bacteroidetes/Chlorobi_group>Bacteroidota>Bacteroidia>Bacteroidales	-	Prevotella_copri	DWW60_RS07145	31878..35486	165179	BREX-system-P-loop-protein-BrxC-[Prevotella-copri]
WP_153112500.1	Type1.BREX	HAD+HAD->?->?->?->BrxA-DNA-binding->BrxB-BrxF-iSTAND->DUF6079*->N6-METHYLASE->SIGMA+OB-Fold+AAA-ATPase+Lon-Protease-BrxL->PglZ-><-?<-?<-Calcineurin+Calcineurin-conf||?-><-?<-YTCD-HTH	1188	Bacteria>FCB_group>Bacteroidetes/Chlorobi_group>Bacteroidota>Bacteroidia>Bacteroidales	-	Prevotella_copri	F7D34_RS01710	38543..42109	165179	BREX-system-P-loop-protein-BrxC-[Prevotella-copri]
WP_264909062.1	Type1.BREX	<-HAD+HAD<-SIGMA+OB-Fold+AAA-ATPase+Lon-Protease-BrxL<-PglZ<-N6-METHYLASE<-?<-?<-?<-PIN<-HTH+MPTase<-DUF6079*<-BrxB-BrxF-iSTAND<-BrxA-DNA-binding||?->D5ATPase+DUF3874-><-?<-CRISPR_Cas2<-Cas_Cas1<-Cas9+HNH+RuvC_III<-HTH+MPTase	1222	Bacteria>FCB_group>Bacteroidetes/Chlorobi_group>Bacteroidota>Bacteroidia>Bacteroidales	-	Prevotella_copri	ONT11_RS09740	2183354..2187022	165179	BREX-system-P-loop-protein-BrxC-[Prevotella-copri]
WP_264908465.1	Type1.BREX	<-McrC-NTD+REase<-McrB-AAA<-SIGMA+OB-Fold+AAA-ATPase+HKD-DNase-BrxL<-PglZ<-SIR2+SIR2<-N6-METHYLASE<-N6-METHYLASE<-DUF6079*<-BrxB-BrxF-iSTAND<-BrxA-DNA-binding<-wHTH+WYL+WCX<-HTH_3||?->?->ARG->RADICAL-SAM->HIRAN->	1206	Bacteria>FCB_group>Bacteroidetes/Chlorobi_group>Bacteroidota>Bacteroidia>Bacteroidales	-	Prevotella_copri	ONT12_RS10110	2444120..2447740	165179	BREX-system-P-loop-protein-BrxC-[Prevotella-copri]
WP_264951499.1	Type1.BREX	SLOG+BirA-HTH-><-?||AT-HTH-HYD2->AT-HTH-HYD2-><-HNH<-OLD-ABC<-?||BrxA-DNA-binding->BrxB-BrxF-iSTAND->DUF6079*->HTH+MPTase->PIN->?->?->?->N6-METHYLASE->PglZ->SIGMA+OB-Fold+AAA-ATPase+Lon-Protease-BrxL->HAD+HAD->	1222	Bacteria>FCB_group>Bacteroidetes/Chlorobi_group>Bacteroidota>Bacteroidia>Bacteroidales	-	Prevotella_copri	ONT17_RS13510	3302536..3306204	165179	BREX-system-P-loop-protein-BrxC-[Prevotella-copri]
WP_264904325.1	Type1.BREX	<-HIRAN<-RADICAL-SAM<-ARG<-?<-?||HTH_3->wHTH+WYL+WCX->BrxA-DNA-binding->BrxB-BrxF-iSTAND->DUF6079*->N6-METHYLASE-p-><-DEDD_Tnp_IS110+Transposase_20<-DEDD_Tnp_IS110+Transposase_20||N6-METHYLASE->SIR2+SIR2->PglZ->SIGMA+OB-Fold+AAA-ATPase+HKD-DNase-BrxL->McrB-AAA->McrC-NTD+REase->	1206	Bacteria>FCB_group>Bacteroidetes/Chlorobi_group>Bacteroidota>Bacteroidia>Bacteroidales	-	Prevotella_copri	ONT18_RS04485	1079147..1082767	165179	BREX-system-P-loop-protein-BrxC-[Prevotella-copri]
WP_073207711.1	Type1.BREX	<-Classical-AAA<-?||tRNA-><-?<-?<-HTH+DOC||?->HTH_3->BrxA-DNA-binding->BrxB-BrxF-iSTAND->DUF6079*->N6-METHYLASE->SbcC+SbcC->?->SIR2+SIR2->PglZ->SIGMA+OB-Fold+AAA-ATPase+HKD-DNase-BrxL->?->ParB+HNH->ParB+HNH->	1206	Bacteria>FCB_group>Bacteroidetes/Chlorobi_group>Bacteroidota>Bacteroidia>Bacteroidales	-	Prevotella_ruminicola	SAMN05216463_10942	46265..49885	839	BREX-system-P-loop-protein-BrxC-[Prevotella-ruminicola]
WP_181980669.1	Type1.BREX	TOPRIM->OLD-ABC+TOPRIM->SF1-SF2-Helicase->MORC+DUF3684+REase-><-SIGMA+OB-Fold+AAA-ATPase+HKD-DNase-BrxL<-PglZ<-N6-METHYLASE<-REase<-OLD-ABC<-DUF6079*<-BrxB-BrxF-iSTAND<-BrxA-DNA-binding<-HTH_3<-SMS-KaiC<-XIS-HTH<-?<-Phage_int_SAM_5+Phage_integrase<-XIS-HTH+XIS-HTH+XIS-HTH+XIS-HTH<-sGTP	1192	Bacteria>FCB_group>Bacteroidetes/Chlorobi_group>Bacteroidota>Bacteroidia	GCF_015558535.1	Bacteroidales	QH473_RS20265	26845..30423	171549	MULTISPECIES:-BREX-system-P-loop-protein-BrxC-[Bacteroidales]
WP_057098610.1	Type1.BREX	<-KAP-NTPase<-SIGMA+OB-Fold+AAA-ATPase+Lon-Protease-BrxL<-PglZ<-Schlafen+HATPase+wHTH<-N6-METHYLASE<-DUF6079*<-BrxB-BrxF-iSTAND<-BrxA-DNA-binding<-HTH_3||XIS-HTH->XIS-HTH-><-?<-?<-ABhydrolase	1212	Bacteria>FCB_group>Bacteroidetes/Chlorobi_group>Bacteroidota>Bacteroidia	GCF_017309675.2	Bacteroidales	DWX60_RS07020	13179..16817	171549	MULTISPECIES:-BREX-system-P-loop-protein-BrxC-[Bacteroidales]
WP_046151576.1	Type1.BREX	<-wHTH+WYL+WCX<-ParB+HNH<-SIR2<-SIGMA+OB-Fold+AAA-ATPase+Lon-Protease-BrxL<-PglZ<-?<-Schlafen+HATPase+wHTH<-REase<-N6-METHYLASE<-DUF6079*<-BrxB-BrxF-iSTAND<-BrxA-DNA-binding<-HTH_3||SIR2->HerA/FtsK-><-Phage_lysozyme<-VirD4-TraG+VirD4-FtsK	1184	Bacteria>FCB_group>Bacteroidetes/Chlorobi_group>Bacteroidota>Bacteroidia	GCF_900102645.1	Bacteroidales	BLR14_RS08960	69320..72874	171549	MULTISPECIES:-BREX-system-P-loop-protein-BrxC-[Bacteroidales]
WP_009275691.1	Type1.BREX	<-NUDIX+NUDIX<-?<-?<-?||HTH_3->wHTH+WYL+WCX->BrxA-DNA-binding->BrxB-BrxF-iSTAND->DUF6079*->N6-METHYLASE-p->	1208	Bacteria>FCB_group>Bacteroidetes/Chlorobi_group>Bacteroidota>Bacteroidia	GCF_949084405.1	Bacteroidales	KSZ59_RS23265	10034..13660	171549	MULTISPECIES:-BREX-system-P-loop-protein-BrxC-[Bacteroidales]
BBE18573.1	Type1.BREX	TPR+TPR->BrxA-DNA-binding->BrxB-BrxF-iSTAND->DUF6079*->N6-METHYLASE->?->DOC+HTH->Pkinase->REase->PglZ->SIGMA+OB-Fold+AAA-ATPase+Lon-Protease-BrxL->	1211	Bacteria>FCB_group>Bacteroidetes/Chlorobi_group>Bacteroidota>Bacteroidia>Marinilabiliales	-	Aquipluma_nitroreducens	AQPE_2736	3049074..3052709	2010828	hypothetical-protein-AQPE_2736-[Aquipluma-nitroreducens]
BBE20443.1	Type1.BREX	TraJ-RHH->TraI-VirD2-RCRE-><-wHTH+WYL+WCX||?->SMODS->?->HTH_3->BrxA-DNA-binding->BrxB-BrxF-iSTAND->DUF6079*->N6-METHYLASE->PglZ->HKD-DNase->AbiEi_4->AbiEii->SIGMA+OB-Fold+AAA-ATPase+HKD-DNase-BrxL->	1173	Bacteria>FCB_group>Bacteroidetes/Chlorobi_group>Bacteroidota>Bacteroidia>Marinilabiliales	-	Aquipluma_nitroreducens	AQPE_4635	5186015..5189536	2010828	hypothetical-protein-AQPE_4635-[Aquipluma-nitroreducens]
MCF8362389.1	Type1.BREX	<-SIGMA+OB-Fold+AAA-ATPase+HKD-DNase-BrxL<-PglZ<-PglZ<-Schlafen+HATPase+wHTH<-N6-METHYLASE<-N6-METHYLASE-p<-DUF6079*<-BrxB-BrxF-iSTAND<-BrxA-DNA-binding<-HTH_3<-Schlafen+HATPase+wHTH-p	1180	Bacteria>FCB_group>Bacteroidetes/Chlorobi_group>Bacteroidota>Bacteroidia>Marinilabiliales	GCA_021740345.1	Prolixibacteraceae_bacterium	K9G70_07170	11489..15031	2053594	BREX-system-P-loop-protein-BrxC-[Prolixibacteraceae-bacterium]
MCK9411439.1	Type1.BREX	Peptidase_A8->Peptidase_A8-><-wHTH+WYL+WCX||?->SMODS->?->HTH_3->BrxA-DNA-binding->BrxB-BrxF-iSTAND->DUF6079*->N6-METHYLASE->Schlafen+HATPase+wHTH->?->PglZ->AbiEi_4->SIGMA+OB-Fold+AAA-ATPase+HKD-DNase-BrxL->?->REC+LYTTR->	1173	Bacteria>FCB_group>Bacteroidetes/Chlorobi_group>Bacteroidota>Bacteroidia>Marinilabiliales	GCA_023229575.1	Prolixibacteraceae_bacterium	MOQ53_04000	25065..28586	2053594	BREX-system-P-loop-protein-BrxC-[Prolixibacteraceae-bacterium]
MCU4173933.1	Type1.BREX	<-REase+RNase||?->?->?->?->?->HTH_3->BrxA-DNA-binding->BrxB-BrxF-iSTAND->DUF6079*->N6-METHYLASE->PglZ->SIGMA+OB-Fold+AAA-ATPase+HKD-DNase-BrxL->AbiEii->?-><-?||VWA+REase->	1167	Bacteria>FCB_group>Bacteroidetes/Chlorobi_group>Bacteroidota>Bacteroidia>Marinilabiliales	GCA_025499625.1	Marinilabiliaceae_bacterium_N1Y90	J1N11_02510	10462..13965	2816964	BREX-system-P-loop-protein-BrxC-[Marinilabiliaceae-bacterium-N1Y90]
WP_027470973.1	Type1.BREX	wHTH+WYL+WCX->HTH_3->BrxA-DNA-binding->BrxB-BrxF-iSTAND->DUF6079*->?->SbcC->?->?->SbcC->?->N6-METHYLASE-p->	1172	Bacteria>FCB_group>Bacteroidetes/Chlorobi_group>Bacteroidota>Bacteroidia>Marinilabiliales	GCF_000583675.1	Saccharicrinis_fermentans	JCM21142_RS14985	5014..8532	982	BREX-system-P-loop-protein-BrxC-[Saccharicrinis-fermentans]
WP_062122677.1	Type1.BREX	ABhydrolase->tRNA-><-SIGMA+OB-Fold+AAA-ATPase+HKD-DNase-BrxL<-PglZ<-Schlafen+HATPase+wHTH<-N6-METHYLASE<-DUF6079-p<-DUF499<-DUF6079-p*<-BrxB-BrxF-iSTAND<-BrxA-DNA-binding<-HTH_3||?-><-?<-?<-?<-SIGMA-HTH	329	Bacteria>FCB_group>Bacteroidetes/Chlorobi_group>Bacteroidota>Bacteroidia>Marinilabiliales	GCF_000974365.1	Geofilum_rubicundum	JCM15548_RS04470	156242..157231	472113	hypothetical-protein-[Geofilum-rubicundum]
WP_075603947.1	Type1.BREX	TPR+TPR+TPR+TPR->?->XIS-HTH->?->CHC2+TOPRIM-DNAG->?->BrxA-DNA-binding->BrxB-BrxF-iSTAND->DUF6079*->N6-METHYLASE->?->?->ParB+HNH->PglZ->SIGMA+OB-Fold+AAA-ATPase+Lon-Protease-BrxL->ZDNA-HTH->	1204	Bacteria>FCB_group>Bacteroidetes/Chlorobi_group>Bacteroidota>Bacteroidia>Marinilabiliales	GCF_001660705.2	Saccharicrinis_aurantiacus	A8B35_RS21005	65274..68888	1849719	BREX-system-P-loop-protein-BrxC-[Saccharicrinis-aurantiacus]
WP_107823954.1	Type1.BREX	<-STYKinase-like<-Couple_hipA<-HTH_3<-SIGMA+OB-Fold+AAA-ATPase+HKD-DNase-BrxL<-PglZ<-N6-METHYLASE<-Schlafen+HATPase+wHTH<-DUF6079*<-BrxB-BrxF-iSTAND<-BrxA-DNA-binding<-HTH_3	1168	Bacteria>FCB_group>Bacteroidetes/Chlorobi_group>Bacteroidota>Bacteroidia>Marinilabiliales	GCF_003046625.1	Mangrovibacterium_marinum	C8N47_RS17935	11546..15052	1639118	BREX-system-P-loop-protein-BrxC-[Mangrovibacterium-marinum]
WP_163710191.1	Type1.BREX	HTH_3->BrxA-DNA-binding->BrxB-BrxF-iSTAND->DUF6079*->Schlafen+HATPase+wHTH->SbcC->?->N6-METHYLASE->SF1-SF2-Helicase->PglZ->SIGMA+OB-Fold+AAA-ATPase+HKD-DNase-BrxL->	1168	Bacteria>FCB_group>Bacteroidetes/Chlorobi_group>Bacteroidota>Bacteroidia>Marinilabiliales	GCF_010882175.1	Mangrovibacterium_lignilyticum	GJV22_RS06145	1519093..1522599	2668052	BREX-system-P-loop-protein-BrxC-[Mangrovibacterium-lignilyticum]
WP_250725046.1	Type1.BREX	DDE_Tnp_ISL3->DUF4372+DDE_Tnp_1-><-SIGMA+OB-Fold+AAA-ATPase+HKD-DNase-BrxL<-PglZ<-Couple_hipA+HipA_C<-HTH_3<-N6-METHYLASE-p||DDE_Tnp_1_4-><-N6-METHYLASE<-DUF6079*<-BrxB-BrxF-iSTAND<-BrxA-DNA-binding<-HTH_3||ZnR->DDE_Tnp_ISL3-><-sGTP||?->SIR2->	1173	Bacteria>FCB_group>Bacteroidetes/Chlorobi_group>Bacteroidota>Bacteroidia>Marinilabiliales	GCF_023674465.1	Alkaliflexus_sp._Ai-910	M9189_RS04830	1155477..1158998	2949635	BREX-system-P-loop-protein-BrxC-[Alkaliflexus-sp.-Ai-910]
OJV92258.1	Type1.BREX	RNase->?->TatD_DNase->?->tRNA-><-SIGMA+OB-Fold+AAA-ATPase+HKD-DNase-BrxL<-PglZ<-?<-N6-METHYLASE<-DUF6079*<-BrxB-BrxF-iSTAND<-BrxA-DNA-binding<-HTH_3	1200	Bacteria>FCB_group>Bacteroidetes/Chlorobi_group>Bacteroidota>Bacteroidia>unclassified_Bacteroidia	GCA_001897595.1	Bacteroidia_bacterium_44-10	BGO34_14870	43076..46678	1895721	hypothetical-protein-BGO34_14870-[Bacteroidia-bacterium-44-10]
OSZ81159.1	Type1.BREX	BrxA-DNA-binding->BrxB-BrxF-iSTAND->DUF6079*->N6-METHYLASE->McrB-AAA->McrC-NTD+REase->PglZ->SIGMA+OB-Fold+AAA-ATPase+Lon-Protease-BrxL-><-Arm-DNA-bind_5+Phage_int_SAM_5+Phage_integrase<-?<-REC+OmpR-HTH<-REC+CITB-HTH	1144	Bacteria>FCB_group>Bacteroidetes/Chlorobi_group>Bacteroidota>Chitinophagia>Chitinophagales	GCA_002127295.1	Chitinophagaceae_bacterium_IBVUCB2	CAP36_07965	1792100..1795534	1985174	hypothetical-protein-CAP36_07965-[Chitinophagaceae-bacterium-IBVUCB2]
PJE46487.1	Type1.BREX	<-DOC+HTH<-?<-?<-HerA/FtsK||wHTH+WYL+WCX->?->REase+RNase->BrxA-DNA-binding->BrxB-BrxF-iSTAND->DUF6079*->N6-METHYLASE->PglZ->SIGMA+OB-Fold+AAA-ATPase+HKD-DNase-BrxL->?->?->STYKIN->SF1-SF2-Helicase->HTH+HEPN->McrB-AAA->	1169	Bacteria>FCB_group>Bacteroidetes/Chlorobi_group>Bacteroidota>Chitinophagia>Chitinophagales	GCA_002786355.1	Sediminibacterium_sp._FEMGT703S	CUR34_07570	66784..70293	2080766	MAG:-hypothetical-protein-CUR34_07570-[Sediminibacterium-sp.]
QEC55281.1	Type1.BREX	<-AbiEi_4<-MORC+SF1-SF2-Helicase<-TatD_DNase<-?<-KAP-glycosylase<-KAP-NTPase<-SIGMA+OB-Fold+AAA-ATPase+HKD-DNase-BrxL<-PglZ<-N6-METHYLASE<-DUF6079*<-BrxB-BrxF-iSTAND<-BrxA-DNA-binding<-?<-wHTH+WYL+WCX	1177	Bacteria>FCB_group>Bacteroidetes/Chlorobi_group>Bacteroidota>Chitinophagia>Chitinophagales	GCA_007970805.1	Flavisolibacter_ginsenosidimutans	FSB75_04955	1196460..1199993	661481	BREX-system-P-loop-protein-BrxC-[Flavisolibacter-ginsenosidimutans]
NTS43012.1	Type1.BREX	<-SIGMA+OB-Fold+AAA-ATPase+HKD-DNase-BrxL<-PglZ<-N6-METHYLASE<-DUF6079*<-BrxB-BrxF-iSTAND<-BrxA-DNA-binding<-CASPASE<-?<-?<-?<-wHTH+WYL+WCX	1173	Bacteria>FCB_group>Bacteroidetes/Chlorobi_group>Bacteroidota>Chitinophagia>Chitinophagales	GCA_013327885.1	Flavisolibacter_longurius	HRG84_19150	28734..32255	2740583	BREX-system-P-loop-protein-BrxC-[Flavisolibacter-longurius]
MBL7764603.1	Type1.BREX	HTH->BrxA-DNA-binding->BrxB-BrxF-iSTAND->DUF6079*->N6-METHYLASE->McrB-AAA->McrC-NTD+REase->PglZ->SIGMA+OB-Fold+AAA-ATPase+Lon-Protease-BrxL->	1146	Bacteria>FCB_group>Bacteroidetes/Chlorobi_group>Bacteroidota>Chitinophagia>Chitinophagales	GCA_016786845.1	Chitinophagaceae_bacterium	JNJ58_00790	47471..50911	1869212	BREX-system-P-loop-protein-BrxC-[Chitinophagaceae-bacterium]
MBP8114974.1	Type1.BREX	<-SIGMA+OB-Fold+AAA-ATPase+Lon-Protease-BrxL-p<-PglZ<-?<-N6-METHYLASE<-DUF6079*	1136	Bacteria>FCB_group>Bacteroidetes/Chlorobi_group>Bacteroidota>Chitinophagia>Chitinophagales	GCA_018005575.1	Chitinophagaceae_bacterium	KAY50_06430	6842..10254	1869212	BREX-system-P-loop-protein-BrxC-[Chitinophagaceae-bacterium]
MCB9043396.1	Type1.BREX	sGTP->?->HTH->BrxA-DNA-binding->BrxB-BrxF-iSTAND->DUF6079*->Four-helical-protein->N6-METHYLASE->AbiEi_4->AbiEii->PglZ->SIGMA+OB-Fold+AAA-ATPase+Lon-Protease-BrxL->?->?->DDE_Tnp->	1197	Bacteria>FCB_group>Bacteroidetes/Chlorobi_group>Bacteroidota>Chitinophagia>Chitinophagales	GCA_020636475.1	Chitinophagales_bacterium	H6554_06480	28579..32172	2448779	BREX-system-P-loop-protein-BrxC-[Chitinophagales-bacterium]
MCK9404665.1	Type1.BREX	<-HARE-HTH+REase<-SIGMA+OB-Fold+AAA-ATPase+Lon-Protease-BrxL<-PglZ<-?<-N6-METHYLASE<-DUF6079*<-BrxB-BrxF-iSTAND<-BrxA-DNA-binding<-REase	1144	Bacteria>FCB_group>Bacteroidetes/Chlorobi_group>Bacteroidota>Chitinophagia>Chitinophagales	GCA_023229465.1	Chitinophagaceae_bacterium	MOQ26_14830	11931..15365	1869212	BREX-system-P-loop-protein-BrxC-[Chitinophagaceae-bacterium]
WP_172623061.1	Type1.BREX	<-AbiEi_4<-MORC+SF1-SF2-Helicase<-TatD_DNase<-?<-KAP-glycosylase<-KAP-NTPase<-SIGMA+OB-Fold+AAA-ATPase+HKD-DNase-BrxL<-PglZ<-N6-METHYLASE<-DUF6079*<-BrxB-BrxF-iSTAND<-BrxA-DNA-binding<-?<-wHTH+WYL+WCX	1174	Bacteria>FCB_group>Bacteroidetes/Chlorobi_group>Bacteroidota>Chitinophagia>Chitinophagales	GCF_007970805.1	Flavisolibacter_ginsenosidimutans	FSB75_RS04955	1196460..1199984	661481	BREX-system-P-loop-protein-BrxC-[Flavisolibacter-ginsenosidimutans]
WP_072366579.1	Type1.BREX	<-HTH_Tnp_1||HTH_3->BrxA-DNA-binding->BrxB-BrxF-iSTAND->DUF6079*->?->N6-METHYLASE->McrB-AAA->McrC-NTD+REase->PglZ->SIGMA+OB-Fold+AAA-ATPase+Lon-Protease-BrxL->	1142	Bacteria>FCB_group>Bacteroidetes/Chlorobi_group>Bacteroidota>Chitinophagia>Chitinophagales	GCF_900119105.1	Chitinophaga_sancti	BT118_RS32550	2428..5856	1004	BREX-system-P-loop-protein-BrxC-[Chitinophaga-sancti]
ADB40822.1	Type1.BREX	GDResolvase+HIN-HTH-><-?||?-><-?||?-><-?||URI-DNase->BrxA-DNA-binding->BrxB-BrxF-iSTAND->DUF6079*->MarR-HTH->AbiEii->N6-METHYLASE->PglZ->SIGMA+OB-Fold+AAA-ATPase+HKD-DNase-BrxL-><-DUF4096+DDE_Tnp_1+DDE_Tnp_1_2||HTH+rve->AAA-ATPase->	1186	Bacteria>FCB_group>Bacteroidetes/Chlorobi_group>Bacteroidota>Cytophagia>Cytophagales	GCA_000024525.1	Spirosoma_linguale_DSM_74	Slin_4844	5894993..5898553	504472	hypothetical-protein-Slin_4844-[Spirosoma-linguale-DSM-74]
UOQ83254.1	Type1.BREX	<-pPIWI+pPIWI||HEPN-><-REase<-?||Phage_integrase+Phage_integrase->NUMOD4+HNH->HTH_3->BrxA-DNA-binding->BrxB-BrxF-iSTAND->DUF6079*->N6-METHYLASE-p->N6-METHYLASE->N6-METHYLASE-p->PglZ->SIGMA+OB-Fold+AAA-ATPase+HKD-DNase-BrxL-><-SF1-SF2-Helicase+REase||NtrC-AAA+FIS-HTH->LexA-protease->	1148	Bacteria>FCB_group>Bacteroidetes/Chlorobi_group>Bacteroidota>Cytophagia>Cytophagales	GCA_022919455.1	Hymenobacter_sp._5414T-23	MUN83_21225	87571..91017	2932252	BREX-system-P-loop-protein-BrxC-[Hymenobacter-sp.-5414T-23]
SKB69504.1	Type1.BREX	D5ATPase->?->?->?->?->CopG/RHH->HTH->BrxA-DNA-binding->BrxB-BrxF-iSTAND->DUF6079*->N6-METHYLASE->PglZ->SIGMA+OB-Fold+AAA-ATPase+Lon-Protease-BrxL->MORC+DUF3684+REase->?->SF1-SF2-Helicase-><-REase<-?||DOC+HTH->	1201	Bacteria>FCB_group>Bacteroidetes/Chlorobi_group>Bacteroidota>Cytophagia>Cytophagales	GCA_900167945.1	Dyadobacter_psychrophilus	SAMN05660293_01532	106911..110516	651661	hypothetical-protein-SAMN05660293_01532-[Dyadobacter-psychrophilus]
WP_013929909.1	Type1.BREX	Peptidase_S51->?-><-SIGMA+OB-Fold+AAA-ATPase+Lon-Protease-BrxL<-PglZ<-Schlafen+HATPase+wHTH<-HipA_C<-Couple_hipA<-HTH_3<-N6-METHYLASE<-DUF6079*<-BrxB-BrxF-iSTAND<-BrxA-DNA-binding<-DDE_Tnp<-?<-DDE_Tnp_1_assoc<-MULE-TRANSPOSASE<-SF1-SF2-Helicase<-?<-HEPN+DUF499	1173	Bacteria>FCB_group>Bacteroidetes/Chlorobi_group>Bacteroidota>Cytophagia>Cytophagales	GCF_000218895.1	Runella_slithyformis	RUNSL_RS20935	4933430..4936951	106	BREX-system-P-loop-protein-BrxC-[Runella-slithyformis]
WP_008625904.1	Type1.BREX	Calcineurin->?->SbcC->?-><-SF1-SF2-Helicase||?->?->BrxA-DNA-binding->BrxB-BrxF-iSTAND->DUF6079*->N6-METHYLASE->McrB-AAA->McrC-NTD+REase->PglZ->SIGMA+OB-Fold+AAA-ATPase+Lon-Protease-BrxL-><-URI-DNase<-?<-?<-SF1-SF2-Helicase+RNase+RNase	1146	Bacteria>FCB_group>Bacteroidetes/Chlorobi_group>Bacteroidota>Cytophagia>Cytophagales	GCF_000330725.2	Mariniradius_saccharolyticus	C943_RS07085	84904..88344	1245591	BREX-system-P-loop-protein-BrxC-[Mariniradius-saccharolyticus]
WP_018619849.1	Type1.BREX	<-SAM-METHYLASE||?->HTH_Tnp_1+HTH_21+rve+rve_3-><-SIGMA+OB-Fold+AAA-ATPase+Lon-Protease-BrxL<-PglZ<-?<-?||?-><-N6-METHYLASE<-DUF6079*<-BrxB-BrxF-iSTAND<-BrxA-DNA-binding||HTH+REase->REase->?->wHTH+WYL->	1142	Bacteria>FCB_group>Bacteroidetes/Chlorobi_group>Bacteroidota>Cytophagia>Cytophagales	GCF_000374065.1	Spirosoma_luteum	B156_RS0111470	131208..134636	431553	BREX-system-P-loop-protein-BrxC-[Spirosoma-luteum]
WP_028665035.1	Type1.BREX	DSBH+AraC-HTH->?-><-?<-CNMP+CRP-HTH<-?||BrxA-DNA-binding->BrxB-BrxF-iSTAND->DUF6079*->AbiEi_4+AbiEi_1->AbiEii->N6-METHYLASE->PglZ->SIGMA+OB-Fold+AAA-ATPase+HKD-DNase-BrxL->TPR+TPR+TPR-><-?<-SIGMA-HTH	1171	Bacteria>FCB_group>Bacteroidetes/Chlorobi_group>Bacteroidota>Cytophagia>Cytophagales	GCF_000423565.1	Runella_zeae	G563_RS0108985	25803..29318	94255	BREX-system-P-loop-protein-BrxC-[Runella-zeae]
WP_053405044.1	Type1.BREX	<-SIGMA+OB-Fold+AAA-ATPase+HKD-DNase-BrxL<-PglZ||ParB+HNH-><-N6-METHYLASE<-DUF6079*<-BrxB-BrxF-iSTAND<-BrxA-DNA-binding<-AAA-ToxA||?->?-><-ParB<-ParA-Soj-PloopNTPase<-?<-AAA-ATPase	1179	Bacteria>FCB_group>Bacteroidetes/Chlorobi_group>Bacteroidota>Cytophagia>Cytophagales	GCF_001274635.1	Persicobacter_sp._CCB-QB2	NG22_RS06825	1502189..1505728	1561025	BREX-system-P-loop-protein-BrxC-[Persicobacter-sp.-CCB-QB2]
WP_073850520.1	Type1.BREX	<-HNH+SAVED<-?<-?<-?<-?<-SIGMA+OB-Fold+AAA-ATPase+HKD-DNase-BrxL<-PglZ<-N6-METHYLASE<-DUF6079*<-BrxB-BrxF-iSTAND<-BrxA-DNA-binding<-HTH_3||?->ZnR+TPR->TPR-><-HTH_3<-HTH_36<-XIS-HTH	1179	Bacteria>FCB_group>Bacteroidetes/Chlorobi_group>Bacteroidota>Cytophagia>Cytophagales	GCF_001907195.1	Pontibacter_flavimaris	A3841_RS01550	388287..391826	1797110	BREX-system-P-loop-protein-BrxC-[Pontibacter-flavimaris]
WP_084816172.1	Type1.BREX	GDResolvase->REase->?-><-MIT_C<-SIGMA+OB-Fold+AAA-ATPase+Lon-Protease-BrxL<-PglZ-p<-PglZ<-N6-METHYLASE<-DUF6079*<-BrxB-BrxF-iSTAND<-BrxA-DNA-binding||GDResolvase->	1165	Bacteria>FCB_group>Bacteroidetes/Chlorobi_group>Bacteroidota>Cytophagia>Cytophagales	GCF_002261125.1	Siphonobacter_sp._BAB-5385	BWI93_RS24020	10112..13609	1864822	BREX-system-P-loop-protein-BrxC-[Siphonobacter-sp.-BAB-5385]
WP_273212250.1	Type1.BREX	<-AbiEii<-wHTH+AbiEi_2<-SIGMA+OB-Fold+AAA-ATPase+Lon-Protease-BrxL<-PglZ<-Schlafen+HATPase+wHTH<-N6-METHYLASE<-HNH<-OLD-ABC<-DUF6079*<-BrxB-BrxF-iSTAND<-BrxA-DNA-binding<-?<-?<-D5ATPase<-XIS-HTH<-?<-Arm-DNA-bind_5+Phage_int_SAM_5+Phage_integrase	1173	Bacteria>FCB_group>Bacteroidetes/Chlorobi_group>Bacteroidota>Cytophagia>Cytophagales	GCF_002483185.1	Runella_zeae	B4E63_RS12460	307458..310979	94255	BREX-system-P-loop-protein-BrxC-[Runella-zeae]
WP_100629834.1	Type1.BREX	<-SbcC||BrxA-DNA-binding->BrxB-BrxF-iSTAND->DUF6079-p*->DUF6079-p->N6-METHYLASE->REase->?->?->?->?->?->REase->	553	Bacteria>FCB_group>Bacteroidetes/Chlorobi_group>Bacteroidota>Cytophagia>Cytophagales	GCF_002807035.1	Algoriphagus_formosus	CDH51_RS17530	460328..461989	2007308	BREX-system-P-loop-protein-BrxC-[Algoriphagus-formosus]
WP_119666207.1	Type1.BREX	<-IG+IG<-?<-NTP_transf_5<-MarR-HTH<-?||METHYLASE->ChlI+Lon_C+AAA->BrxA-DNA-binding->BrxB-BrxF-iSTAND->DUF6079*-><-AbiEii<-AbiEi_4+AbiEi_1||REase->N6-METHYLASE-><-?||?->?->?->PglZ->	1143	Bacteria>FCB_group>Bacteroidetes/Chlorobi_group>Bacteroidota>Cytophagia>Cytophagales	GCF_003583365.1	Fibrisoma_montanum	DYU11_RS03320	792110..795541	2305895	BREX-system-P-loop-protein-BrxC-[Fibrisoma-montanum]
WP_122934661.1	Type1.BREX	<-ParB<-ParA-Soj-PloopNTPase||AbiEi_4->AbiEii->BrxA-DNA-binding->BrxB-BrxF-iSTAND->DUF6079*->N6-METHYLASE->McrC-NTD+REase->PglZ->SIGMA+OB-Fold+AAA-ATPase+Lon-Protease-BrxL->?->?->Peptidase_S8->	1142	Bacteria>FCB_group>Bacteroidetes/Chlorobi_group>Bacteroidota>Cytophagia>Cytophagales	GCF_003711225.1	Runella_sp._SP2	DTQ70_RS30140	43566..46994	2268026	BREX-system-P-loop-protein-BrxC-[Runella-sp.-SP2]
WP_133557368.1	Type1.BREX	<-SF1-SF2-Helicase<-HKD-DNase<-?||?->HAD+PRTase+PRTase+REase+SF1-SF2-Helicase->?->?->BrxA-DNA-binding->BrxB-BrxF-iSTAND->DUF6079*->SIGMA-HTH->N6-METHYLASE->?->wHTH+WYL+WCX-p->SF1-SF2-Helicase->?->?->?->PSBP->	1167	Bacteria>FCB_group>Bacteroidetes/Chlorobi_group>Bacteroidota>Cytophagia>Cytophagales	GCF_004362805.1	Algoriphagus_boseongensis	DFQ04_RS15130	300982..304485	1442587	BREX-system-P-loop-protein-BrxC-[Algoriphagus-boseongensis]
WP_138994213.1	Type1.BREX	TraN->?->?->?->GDResolvase+HIN-HTH->BrxA-DNA-binding->BrxB-BrxF-iSTAND->DUF6079*->N6-METHYLASE->Schlafen+HATPase+wHTH->PglZ->SIGMA+OB-Fold+AAA-ATPase+Lon-Protease-BrxL->MORC+DUF3684+REase->HEPN+PAS+DUF4011+SF1-SF2-Helicase+REase-><-SF1-SF2-Helicase+wHTH+Small-B-barrel+DUF1998<-BrxD-AAA	1214	Bacteria>FCB_group>Bacteroidetes/Chlorobi_group>Bacteroidota>Cytophagia>Cytophagales	GCF_006149005.1	Larkinella_sp._C7	FHG71_RS25315	15156..18800	2576607	BREX-system-P-loop-protein-BrxC-[Larkinella-sp.-C7]
WP_146895591.1	Type1.BREX	<-wHTH+WYL+WCX||SF1-SF2-Helicase->RVT->?-><-SIGMA+OB-Fold+AAA-ATPase+Lon-Protease-BrxL<-PglZ<-N6-METHYLASE<-DUF6079*<-BrxB-BrxF-iSTAND<-BrxA-DNA-binding||McrB-AAA->McrC-NTD+REase-><-?<-?<-?<-?<-HTH_3	1143	Bacteria>FCB_group>Bacteroidetes/Chlorobi_group>Bacteroidota>Cytophagia>Cytophagales	GCF_007991635.1	Adhaeribacter_aerolatus	AAEO2nite_RS05160	90394..93825	670289	BREX-system-P-loop-protein-BrxC-[Adhaeribacter-aerolatus]
WP_146899771.1	Type1.BREX	<-SIGMA+OB-Fold+AAA-ATPase+HKD-DNase-BrxL<-PglZ<-N6-METHYLASE<-DUF6079*<-BrxB-BrxF-iSTAND<-BrxA-DNA-binding<-?<-?||?->DNA-glycosylase->?-><-TPR	1182	Bacteria>FCB_group>Bacteroidetes/Chlorobi_group>Bacteroidota>Cytophagia>Cytophagales	GCF_007991635.1	Adhaeribacter_aerolatus	AAEO2nite_RS16235	111241..114789	670289	BREX-system-P-loop-protein-BrxC-[Adhaeribacter-aerolatus]
WP_159471214.1	Type1.BREX	<-XIS-HTH<-AbiEii<-AbiEi_4+AbiEi_1<-REase<-SIGMA+OB-Fold+AAA-ATPase+Lon-Protease-BrxL<-PglZ<-N6-METHYLASE<-HNH<-OLD-ABC<-DUF6079*<-BrxB-BrxF-iSTAND<-BrxA-DNA-binding||CHTH->?->?->?-><-CopG/RHH	1179	Bacteria>FCB_group>Bacteroidetes/Chlorobi_group>Bacteroidota>Cytophagia>Cytophagales	GCF_009801115.1	Dyadobacter_sp._3J3	FZF08_RS17845	89009..92548	2606600	BREX-system-P-loop-protein-BrxC-[Dyadobacter-sp.-3J3]
WP_168675169.1	Type1.BREX	<-CASPASE<-DDE_Tnp_4+DDE_Tnp_1_2||Phage_integrase+Phage_integrase-><-McrC-NTD+REase<-McrB-AAA<-?<-SIGMA+OB-Fold+AAA-ATPase+HKD-DNase-BrxL<-PglZ<-N6-METHYLASE<-DUF6079*<-BrxB-BrxF-iSTAND<-BrxA-DNA-binding||NtrC-AAA+FIS-HTH->SMODS->?->Classical-AAA->?->LexA-protease->	1172	Bacteria>FCB_group>Bacteroidetes/Chlorobi_group>Bacteroidota>Cytophagia>Cytophagales	GCF_012275535.1	Hymenobacter_artigasi	HBN54_RS20840	55199..58717	2719616	BREX-system-P-loop-protein-BrxC-[Hymenobacter-artigasi]
WP_181308258.1	Type1.BREX	HEPN->RNaseH-><-?<-HU-HIG+REase<-REase+N-glycosylase||BrxA-DNA-binding->BrxB-BrxF-iSTAND->DUF6079*->N6-METHYLASE->PglZ->SIGMA+OB-Fold+AAA-ATPase+Lon-Protease-BrxL-><-?||GDResolvase->RVT->	1144	Bacteria>FCB_group>Bacteroidetes/Chlorobi_group>Bacteroidota>Cytophagia>Cytophagales	GCF_013623775.1	Rufibacter_sp._XAAS-G3-1	HHD50_RS18475	14255..17689	2729134	BREX-system-P-loop-protein-BrxC-[Rufibacter-sp.-XAAS-G3-1]
WP_183972373.1	Type1.BREX	D5ATPase->?->?->HKD-DNase-><-?<-?||BrxA-DNA-binding->BrxB-BrxF-iSTAND->DUF6079*->REase->N6-METHYLASE->HKD-DNase->?->?->STYKIN->?->?->Schlafen+HATPase+wHTH->	1202	Bacteria>FCB_group>Bacteroidetes/Chlorobi_group>Bacteroidota>Cytophagia>Cytophagales	GCF_014195535.1	Runella_defluvii	FHS57_RS08095	111516..115124	370973	BREX-system-P-loop-protein-BrxC-[Runella-defluvii]
WP_185195977.1	Type1.BREX	<-TIR<-?<-HTH+MPTase<-?||AbiEi_4->AbiEii-><-SIGMA+OB-Fold+AAA-ATPase+HKD-DNase-BrxL<-PglZ<-N6-METHYLASE<-DUF6079*<-BrxB-BrxF-iSTAND<-BrxA-DNA-binding<-HTH_3||?->ZnR+TPR->ParB+HNH-><-GDResolvase+Recombinase	1179	Bacteria>FCB_group>Bacteroidetes/Chlorobi_group>Bacteroidota>Cytophagia>Cytophagales	GCF_014212495.1	Pontibacter_sp._Tf4	H7F15_RS05560	22760..26299	2761620	BREX-system-P-loop-protein-BrxC-[Pontibacter-sp.-Tf4]
WP_187455529.1	Type1.BREX	<-N6-METHYLASE<-?||XIS-HTH-><-SIGMA+OB-Fold+AAA-ATPase+HKD-DNase-BrxL<-PglZ<-N6-METHYLASE<-DUF6079*<-BrxB-BrxF-iSTAND<-BrxA-DNA-binding<-?<-?<-HEPN<-?<-?<-HNH	1178	Bacteria>FCB_group>Bacteroidetes/Chlorobi_group>Bacteroidota>Cytophagia>Cytophagales	GCF_014349165.1	Hymenobacter_sp._BT491	H9I52_RS21275	49708..53244	2766779	BREX-system-P-loop-protein-BrxC-[Hymenobacter-sp.-BT491]
WP_188169955.1	Type1.BREX	<-OLD-ABC<-Arm-DNA-bind_5+Phage_int_SAM_5+Phage_integrase<-MACRODOMAIN<-ART-DarT<-SF1-SF2-Helicase<-SIGMA+OB-Fold+AAA-ATPase+Lon-Protease-BrxL<-PglZ<-OLD-ABC+TOPRIM<-N6-METHYLASE<-DUF6079*<-BrxB-BrxF-iSTAND<-BrxA-DNA-binding<-HTH_3<-?<-?<-Pkinase||Calcineurin->	1216	Bacteria>FCB_group>Bacteroidetes/Chlorobi_group>Bacteroidota>Cytophagia>Cytophagales	GCF_014534745.1	Flammeovirga_sp._EKP202	IC221_RS16125	83755..87405	2770592	BREX-system-P-loop-protein-BrxC-[Flammeovirga-sp.-EKP202]
WP_188933335.1	Type1.BREX	<-wHTH+WYL+WCX||?->TIR-><-HTH_28+HTH_32+rve+rve_3<-SF1-SF2-Helicase<-SIGMA+OB-Fold+AAA-ATPase+HKD-DNase-BrxL<-PglZ<-N6-METHYLASE<-DUF6079*<-BrxB-BrxF-iSTAND<-BrxA-DNA-binding<-HTH_3||Preader17-><-ABhydrolase<-Arm-DNA-bind_5+Phage_int_SAM_5+Phage_integrase	1165	Bacteria>FCB_group>Bacteroidetes/Chlorobi_group>Bacteroidota>Cytophagia>Cytophagales	GCF_014641595.1	Dyadobacter_endophyticus	IEY24_RS14735	618400..621897	1749036	BREX-system-P-loop-protein-BrxC-[Dyadobacter-endophyticus]
WP_189584875.1	Type1.BREX	AbiGi+seqA+RAMA->BrxA-DNA-binding->BrxB-BrxF-iSTAND->DUF6079*->N6-METHYLASE->McrC-NTD+REase->PglZ->SIGMA+OB-Fold+AAA-ATPase+Lon-Protease-BrxL-><-?<-?<-URI-DNase<-TPR+TPR	1146	Bacteria>FCB_group>Bacteroidetes/Chlorobi_group>Bacteroidota>Cytophagia>Cytophagales	GCF_014652135.1	Mongoliitalea_lutea	IE080_RS15850	2275..5715	849756	BREX-system-P-loop-protein-BrxC-[Mongoliitalea-lutea]
WP_191042738.1	Type1.BREX	TraJ-RHH->TraI-VirD2-RCRE-><-NtrC-AAA+FIS-HTH||35exo+wHTH+SF1-SF2-Helicase+HTH->N6-METHYLASE->?->BrxA-DNA-binding->BrxB-BrxF-iSTAND->DUF6079*->N6-METHYLASE->HigB->HTH_3->?->?->?->PglZ->SIGMA+OB-Fold+AAA-ATPase+HKD-DNase-BrxL->	1206	Bacteria>FCB_group>Bacteroidetes/Chlorobi_group>Bacteroidota>Cytophagia>Cytophagales	GCF_014699035.1	Spirosoma_validum	IC230_RS29845	51060..54680	2771355	BREX-system-P-loop-protein-BrxC-[Spirosoma-validum]
WP_201982535.1	Type1.BREX	HEPN->CHTH->TOPRIM->Sp-><-ParB<-OLD-ABC+TOPRIM<-SIGMA+OB-Fold+AAA-ATPase+Lon-Protease-BrxL<-PglZ<-N6-METHYLASE<-DUF6079*<-BrxB-BrxF-iSTAND<-BrxA-DNA-binding<-?||REase+PNTD1+PNTD2->PIWI-><-?<-Phage_integrase+Phage_integrase<-ACET||35exo->	1143	Bacteria>FCB_group>Bacteroidetes/Chlorobi_group>Bacteroidota>Cytophagia>Cytophagales	GCF_016734815.1	Hymenobacter_rubidus	JKL48_RS11490	20898..24329	1441626	BREX-system-P-loop-protein-BrxC-[Hymenobacter-rubidus]
WP_205503392.1	Type1.BREX	<-wHTH+WYL+WCX||SF1-SF2-Helicase->RVT->?-><-SIGMA+OB-Fold+AAA-ATPase+Lon-Protease-BrxL<-PglZ<-N6-METHYLASE<-DUF6079*<-BrxB-BrxF-iSTAND<-BrxA-DNA-binding<-McrC-NTD+REase<-McrB-AAA||?->DDE_Tnp_4+DDE_Tnp_1_2-><-tRNA||HAD+HAD->Peptidase_M50->	1180	Bacteria>FCB_group>Bacteroidetes/Chlorobi_group>Bacteroidota>Cytophagia>Cytophagales	GCF_016924645.1	Rufibacter_sp._SYSU_D00308	JT718_RS14320	179098..182640	2812556	BREX-system-P-loop-protein-BrxC-[Rufibacter-sp.-SYSU-D00308]
WP_234861365.1	Type1.BREX	STYKIN->?<-?||?->GDResolvase-><-?||TPR+TPR->BrxA-DNA-binding->BrxB-BrxF-iSTAND->DUF6079*->N6-METHYLASE->OLD-ABC+TOPRIM->PglZ->SIGMA+OB-Fold+AAA-ATPase+HKD-DNase-BrxL->TIR->?->?->?->ParB+N6_N4_METHYLASE->	1174	Bacteria>FCB_group>Bacteroidetes/Chlorobi_group>Bacteroidota>Cytophagia>Cytophagales	GCF_021491425.1	Mariniradius_sp._RY-2	LOU89_RS09865	251856..255380	2909237	BREX-system-P-loop-protein-BrxC-[Mariniradius-sp.-RY-2]
WP_241368547.1	Type1.BREX	<-ParB+N6_N4_METHYLASE<-?<-?<-?<-SIGMA+OB-Fold+AAA-ATPase+HKD-DNase-BrxL<-PglZ<-OLD-ABC+TOPRIM<-N6-METHYLASE<-DUF6079*<-BrxB-BrxF-iSTAND<-BrxA-DNA-binding<-TPR+TPR<-?<-?<-GDResolvase	1174	Bacteria>FCB_group>Bacteroidetes/Chlorobi_group>Bacteroidota>Cytophagia>Cytophagales	GCF_022549775.1	Belliella_sp._DSM_111904	MM239_RS12315	24692..28216	2923435	BREX-system-P-loop-protein-BrxC-[Belliella-sp.-DSM-111904]
WP_244713422.1	Type1.BREX	<-XIS-HTH||HAD+PRTase+PRTase+REase+SF1-SF2-Helicase->?-><-?<-SIGMA+OB-Fold+AAA-ATPase+Lon-Protease-BrxL<-Preader17<-PglZ<-N6-METHYLASE<-DUF6079*<-BrxB-BrxF-iSTAND<-BrxA-DNA-binding||?->?-><-McrC-NTD+REase<-McrB-AAA||HKD-DNase->HNH->Phage_integrase+Phage_integrase->	1145	Bacteria>FCB_group>Bacteroidetes/Chlorobi_group>Bacteroidota>Cytophagia>Cytophagales	GCF_022919135.1	Hymenobacter_sp._5116S-27	MUN80_RS13030	3065695..3069132	2932249	BREX-system-P-loop-protein-BrxC-[Hymenobacter-sp.-5116S-27]
WP_262922299.1	Type1.BREX	<-pPIWI+pPIWI||HEPN-><-REase<-?||Phage_integrase+Phage_integrase->NUMOD4+HNH->HTH_3->BrxA-DNA-binding->BrxB-BrxF-iSTAND->DUF6079*->N6-METHYLASE-p->N6-METHYLASE->N6-METHYLASE-p->PglZ->SIGMA+OB-Fold+AAA-ATPase+HKD-DNase-BrxL-><-SF1-SF2-Helicase+REase||NtrC-AAA+FIS-HTH->LexA-protease->	1164	Bacteria>FCB_group>Bacteroidetes/Chlorobi_group>Bacteroidota>Cytophagia>Cytophagales	GCF_022919455.1	Hymenobacter_sp._5414T-23	MUN83_RS21225	87523..91017	2932252	BREX-system-P-loop-protein-BrxC-[Hymenobacter-sp.-5414T-23]
WP_255036052.1	Type1.BREX	XIS-HTH->D5ATPase->?->?->?->?->BrxA-DNA-binding->BrxB-BrxF-iSTAND->DUF6079*->Schlafen+HATPase+wHTH->N6-METHYLASE->?->PglZ->SIGMA+OB-Fold+AAA-ATPase+Lon-Protease-BrxL->REase+DUF5655->?->?->SF1-SF2-Helicase->	1199	Bacteria>FCB_group>Bacteroidetes/Chlorobi_group>Bacteroidota>Cytophagia>Cytophagales	GCF_024343775.1	Lacihabitans_soyangensis	EGI31_RS04905	50707..54306	869394	BREX-system-P-loop-protein-BrxC-[Lacihabitans-soyangensis]
WP_269011923.1	Type1.BREX	HTH_3->Couple_hipA+HipA_C-><-?<-HNH+Polbeta<-SIGMA+OB-Fold+AAA-ATPase+HKD-DNase-BrxL<-PglZ<-N6-METHYLASE<-DUF6079*<-BrxB-BrxF-iSTAND<-BrxA-DNA-binding<-?<-?<-?<-?<-?<-wHTH+WYL+WCX||TPR+TPR+TPR->	1166	Bacteria>FCB_group>Bacteroidetes/Chlorobi_group>Bacteroidota>Cytophagia>Cytophagales	GCF_026938185.1	Aquirufa_antheringensis	G9H55_RS04755	131184..134684	2516559	BREX-system-P-loop-protein-BrxC-[Aquirufa-antheringensis]
WP_082214111.1	Type1.BREX	XIS-HTH->D5ATPase->?->?->?->CopG/RHH->HTH->BrxA-DNA-binding->BrxB-BrxF-iSTAND->DUF6079*->N6-METHYLASE->PglZ->SIGMA+OB-Fold+AAA-ATPase+Lon-Protease-BrxL->MORC+DUF3684+REase->?->SF1-SF2-Helicase-><-REase||DOC+HTH->Arm-DNA-bind_5+Phage_int_SAM_5+Phage_integrase->	1201	Bacteria>FCB_group>Bacteroidetes/Chlorobi_group>Bacteroidota>Cytophagia>Cytophagales	GCF_900167945.1	Dyadobacter_psychrophilus	B5X68_RS07585	106911..110516	651661	BREX-system-P-loop-protein-BrxC-[Dyadobacter-psychrophilus]
WP_084121639.1	Type1.BREX	<-TraJ-RHH||?->TPR+TPR->MazG-><-?<-SIGMA+OB-Fold+AAA-ATPase+Lon-Protease-BrxL<-PglZ<-McrC-NTD+REase<-N6-METHYLASE<-DUF6079*<-BrxB-BrxF-iSTAND<-BrxA-DNA-binding<-MPTase<-?<-?||SF1-SF2-Helicase->	1146	Bacteria>FCB_group>Bacteroidetes/Chlorobi_group>Bacteroidota>Cytophagia>Cytophagales	GCF_900176595.1	Aquiflexum_balticum	B9A52_RS17175	4064780..4068220	280473	BREX-system-P-loop-protein-BrxC-[Aquiflexum-balticum]
WP_244679416.1	Type1.BREX	<-pPIWI+pPIWI||HEPN-><-REase<-?||Phage_integrase+Phage_integrase->NUMOD4+HNH->HTH_3->BrxA-DNA-binding->BrxB-BrxF-iSTAND->DUF6079*->N6-METHYLASE-p->N6-METHYLASE->N6-METHYLASE-p->PglZ->SIGMA+OB-Fold+AAA-ATPase+HKD-DNase-BrxL-><-SF1-SF2-Helicase+REase||NtrC-AAA+FIS-HTH->LexA-protease->	1148	Bacteria>FCB_group>Bacteroidetes/Chlorobi_group>Bacteroidota>Cytophagia>Cytophagales	-	Hymenobacter_sp._5414T-23	MUN83_21225	87571..91017	2932252	BREX-system-P-loop-protein-BrxC-[Hymenobacter-sp.-5414T-23]
MBT5528618.1	Type1.BREX	HEPN-><-Phage_integrase||BrxA-DNA-binding->BrxB-BrxF-iSTAND->DUF6079-p*->	473	Bacteria>FCB_group>Bacteroidetes/Chlorobi_group>Bacteroidota>Cytophagia>unclassified_Cytophagia	GCA_018665725.1	Cytophagia_bacterium	HOK35_05630	3390..4810	2026730	BREX-system-P-loop-protein-BrxC-[Cytophagia-bacterium]
OIQ21186.1	Type1.BREX	CHTH+LexA-protease-><-GDResolvase+Recombinase+ZnR<-?||?-><-?||BrxA-DNA-binding->BrxB-BrxF-iSTAND->DUF6079*->N6-METHYLASE->SF1-SF2-Helicase->?->PglZ->SIGMA+OB-Fold+AAA-ATPase+HKD-DNase-BrxL->ParB+HNH->URI-DNase->?->GDResolvase->	1165	Bacteria>FCB_group>Bacteroidetes/Chlorobi_group>Bacteroidota>Flavobacteriia>Flavobacteriales	GCA_001874145.1	Lacinutrix_sp._MedPE-SW	BM549_09430	19615..23112	1860087	MAG:-hypothetical-protein-BM549_09430-[Lacinutrix-sp.-MedPE-SW]
GAW88409.1	Type1.BREX	XIS-HTH->HTH_36->Classical-AAA->HTH_3->BrxA-DNA-binding->BrxB-BrxF-iSTAND->BrxB-BrxF-iSTAND-p->DUF6079-p*->DUF6079-p->N6-METHYLASE->?->ParB->?->ParB+HNH->HEPN->	385	Bacteria>FCB_group>Bacteroidetes/Chlorobi_group>Bacteroidota>Flavobacteriia>Flavobacteriales	GCA_002207815.1	Flavobacterium_psychrophilum	FPS14_contig00004-0074	61867..63024	96345	hypothetical-protein-FPS14_contig00004-0074-[Flavobacterium-psychrophilum]
GAW88410.1	Type1.BREX	XIS-HTH->HTH_36->Classical-AAA->HTH_3->BrxA-DNA-binding->BrxB-BrxF-iSTAND->BrxB-BrxF-iSTAND-p->DUF6079-p->DUF6079-p*->N6-METHYLASE->?->ParB->?->ParB+HNH->HEPN->	768	Bacteria>FCB_group>Bacteroidetes/Chlorobi_group>Bacteroidota>Flavobacteriia>Flavobacteriales	GCA_002207815.1	Flavobacterium_psychrophilum	FPS14_contig00004-0075	63069..65375	96345	hypothetical-protein-FPS14_contig00004-0075-[Flavobacterium-psychrophilum]
OYX86687.1	Type1.BREX	<-RADICAL-SAM+TRAM||?->Arm-DNA-bind_5+Phage_int_SAM_5+Phage_integrase->?->XIS-HTH->D5ATPase+DUF3874->BrxA-DNA-binding->BrxB-BrxF-iSTAND->DUF6079*->?->SF1-SF2-Helicase->?->N6-METHYLASE->?->AbiEi_4->AbiEii->	1199	Bacteria>FCB_group>Bacteroidetes/Chlorobi_group>Bacteroidota>Flavobacteriia>Flavobacteriales	GCA_002280815.1	Flavobacteriales_bacterium_32-34-25	B7Y83_00570	11855..15454	1970508	hypothetical-protein-B7Y83_00570-[Flavobacteriales-bacterium-32-34-25]
MAM30277.1	Type1.BREX	<-ParB+N6_N4_METHYLASE||XIS-HTH-><-SIGMA+OB-Fold+AAA-ATPase+HKD-DNase-BrxL<-PglZ<-TPR+S1<-NERD+SF1-SF2-Helicase<-N6-METHYLASE<-ParB+HNH<-DUF6079*<-BrxB-BrxF-iSTAND<-BrxA-DNA-binding<-HTH_3||GDResolvase->?->?->ABC-ATPase+SbcC->N6-METHYLASE->	1165	Bacteria>FCB_group>Bacteroidetes/Chlorobi_group>Bacteroidota>Flavobacteriia>Flavobacteriales	GCA_002693005.1	Flavobacteriaceae_bacterium	CMC13_14765	19860..23357	1871037	hypothetical-protein-[Flavobacteriaceae-bacterium]
MAL23063.1	Type1.BREX	<-METHYLASE<-?<-SF1-SF2-Helicase<-?<-?<-REase+DUF5655<-N6-METHYLASE<-?<-Schlafen+HATPase+wHTH<-DUF6079*<-BrxB-BrxF-iSTAND<-BrxA-DNA-binding<-HTH_3<-?<-HipA_C<-Couple_hipA<-HTH_3	1157	Bacteria>FCB_group>Bacteroidetes/Chlorobi_group>Bacteroidota>Flavobacteriia>Flavobacteriales	GCA_002694765.1	Xanthomarina_sp.	CMP04_07455	107327..110800	1931211	hypothetical-protein-[Xanthomarina-sp.]
HBR11728.1	Type1.BREX	<-GDResolvase+Recombinase+ZnR<-?||REase+HTH+35exo+Small-B-barrel+SF1-SF2-Helicase-><-DOC+HTH<-SIGMA+OB-Fold+AAA-ATPase+Lon-Protease-BrxL<-PglZ<-N6-METHYLASE<-DUF6079*<-BrxB-BrxF-iSTAND<-BrxA-DNA-binding	1210	Bacteria>FCB_group>Bacteroidetes/Chlorobi_group>Bacteroidota>Flavobacteriia>Flavobacteriales	GCA_003511565.1	Chryseobacterium_sp.	DD740_05880	144786..148418	1871047	BREX-system-P-loop-protein-BrxC-[Chryseobacterium-sp.]
HCC95656.1	Type1.BREX	Arm-DNA-bind_5+Phage_int_SAM_5+Phage_integrase-><-?<-PglZ<-PglZ<-Couple_hipA+HipA_C<-HTH_3<-N6-METHYLASE<-N6-METHYLASE-p<-DUF6079-p*<-DUF6079-p<-BrxB-BrxF-iSTAND-p	980	Bacteria>FCB_group>Bacteroidetes/Chlorobi_group>Bacteroidota>Flavobacteriia>Flavobacteriales	GCA_003517615.1	Flavobacteriaceae_bacterium	DEQ26_15305	12785..15728	1871037	BREX-system-P-loop-protein-BrxC-[Flavobacteriaceae-bacterium]
ROI04325.1	Type1.BREX	<-HTH_3<-?||?->?-><-?<-?<-SIGMA+OB-Fold+AAA-ATPase+HKD-DNase-BrxL<-PglZ<-N6-METHYLASE<-DUF6079*<-BrxB-BrxF-iSTAND<-BrxA-DNA-binding<-Schlafen+HATPase+wHTH||?->ChlI+Lon_C+AAA-><-?||?->SLOG->	1174	Bacteria>FCB_group>Bacteroidetes/Chlorobi_group>Bacteroidota>Flavobacteriia>Flavobacteriales	GCA_003729975.1	Kaistella_haifensis	EGH90_10140	55406..58930	421525	BREX-system-P-loop-protein-BrxC-[Kaistella-haifensis]
AZB21330.1	Type1.BREX	<-SLOG<-?||?-><-ChlI+Lon_C+AAA||BrxA-DNA-binding->BrxB-BrxF-iSTAND->DUF6079-p->DUF6079-p*->N6-METHYLASE->PglZ->SIGMA+OB-Fold+AAA-ATPase+HKD-DNase-BrxL->?->?->ZnR->DDE_Tnp_ISL3->	808	Bacteria>FCB_group>Bacteroidetes/Chlorobi_group>Bacteroidota>Flavobacteriia>Flavobacteriales	GCA_003816015.1	Kaistella_haifensis	EG338_03940	799177..801603	421525	BREX-system-P-loop-protein-BrxC-[Kaistella-haifensis]
RZJ34503.1	Type1.BREX	<-GDResolvase||HTH_3->BrxA-DNA-binding->BrxB-BrxF-iSTAND->DUF6079*->SF1-SF2-Helicase->?->PglZ->SIGMA+OB-Fold+AAA-ATPase+HKD-DNase-BrxL-><-URI-DNase||tRNA-><-?<-REC<-AraC-HTH	1165	Bacteria>FCB_group>Bacteroidetes/Chlorobi_group>Bacteroidota>Flavobacteriia>Flavobacteriales	GCA_004210845.1	Flavobacterium_sp.	EOD51_09215	4934..8431	239	MAG:-BREX-system-P-loop-protein-BrxC-[Flavobacterium-sp.]
GEM55955.1	Type1.BREX	<-SIGMA+OB-Fold+AAA-ATPase+Lon-Protease-BrxL<-PglZ<-AbiEii<-AbiEi_4<-ParB+HNH<-ParB+HNH<-N6-METHYLASE<-DUF6079*<-BrxB-BrxF-iSTAND<-BrxA-DNA-binding<-tRNA||Arm-DNA-bind_5+Phage_int_SAM_5+Phage_integrase->?->XIS-HTH->?->DUF488->GDResolvase->	1216	Bacteria>FCB_group>Bacteroidetes/Chlorobi_group>Bacteroidota>Flavobacteriia>Flavobacteriales	GCA_007990815.1	Flavobacterium_branchiophilum_NBRC_15030_=_ATCC_35035	FB1_21760	14247..17897	1218110	hypothetical-protein-FB1_21760-[Flavobacterium-branchiophilum-NBRC-15030-=-ATCC-35035]
NJY61426.1	Type1.BREX	Arm-DNA-bind_5+Phage_int_SAM_5+Phage_integrase-><-?<-SIGMA+OB-Fold+AAA-ATPase+Lon-Protease-BrxL<-PglZ<-Schlafen+HATPase+wHTH<-N6-METHYLASE<-DUF6079*<-BrxB-BrxF-iSTAND<-BrxA-DNA-binding<-ANK+ANK<-?<-TraI-VirD2-RCRE<-TraJ-RHH<-ZnR+TOPRIM<-D5ATPase<-XIS-HTH	1202	Bacteria>FCB_group>Bacteroidetes/Chlorobi_group>Bacteroidota>Flavobacteriia>Flavobacteriales	GCA_012038135.1	Salinimicrobium_nanhaiense	HC174_01475	311288..314896	2722701	BREX-system-P-loop-protein-BrxC-[Salinimicrobium-nanhaiense]
NOQ74050.1	Type1.BREX	<-REC+LuxR-HTH<-?||sGTP-><-35exo+SF1-SF2-Helicase||HTH_3->BrxA-DNA-binding->BrxB-BrxF-iSTAND->DUF6079*->N6-METHYLASE->	1203	Bacteria>FCB_group>Bacteroidetes/Chlorobi_group>Bacteroidota>Flavobacteriia>Flavobacteriales	GCA_013138735.1	Crocinitomix_sp.	GQ574_18735	89954..93565	2039232	BREX-system-P-loop-protein-BrxC-[Crocinitomix-sp.]
MBQ0768324.1	Type1.BREX	<-N6-METHYLASE<-Schlafen+HATPase+wHTH<-DUF6079*<-BrxB-BrxF-iSTAND<-BrxA-DNA-binding	1160	Bacteria>FCB_group>Bacteroidetes/Chlorobi_group>Bacteroidota>Flavobacteriia>Flavobacteriales	GCA_018067115.1	Bizionia_sp.	KBT58_03470	3917..7399	1954480	BREX-system-P-loop-protein-BrxC-[Bizionia-sp.]
MBW7869163.1	Type1.BREX	HTH_3->BrxA-DNA-binding->BrxB-BrxF-iSTAND->DUF6079*->N6-METHYLASE->?->Schlafen+HATPase+wHTH->KAP-NTPase->PglZ->SIGMA+OB-Fold+AAA-ATPase+HKD-DNase-BrxL->	1166	Bacteria>FCB_group>Bacteroidetes/Chlorobi_group>Bacteroidota>Flavobacteriia>Flavobacteriales	GCA_019455205.1	Brumimicrobium_sp.	H3C31_12675	1666..5166	2029867	BREX-system-P-loop-protein-BrxC-[Brumimicrobium-sp.]
MBZ0328278.1	Type1.BREX	<-SIGMA+OB-Fold+AAA-ATPase+HKD-DNase-BrxL<-PglZ<-?<-SF1-SF2-Helicase<-?<-HKD-DNase<-N6-METHYLASE<-DUF6079*<-BrxB-BrxF-iSTAND<-BrxA-DNA-binding<-HTH_3||?-><-?<-?||?->GDResolvase->	1164	Bacteria>FCB_group>Bacteroidetes/Chlorobi_group>Bacteroidota>Flavobacteriia>Flavobacteriales	GCA_019913785.1	Altibacter_sp.	K8F54_11775	128036..131530	2024823	BREX-system-P-loop-protein-BrxC-[Altibacter-sp.]
WCC43686.1	Type1.BREX	<-DUF6079-p<-DUF6079-p*<-BrxB-BrxF-iSTAND<-BrxA-DNA-binding<-ZnR+TOPRIM<-VirD4-TraG<-?<-?<-Mob_Pre	780	Bacteria>FCB_group>Bacteroidetes/Chlorobi_group>Bacteroidota>Flavobacteriia>Flavobacteriales	GCA_028010105.1	Tenacibaculum_finnmarkense	PJW08_00020	1487..3829	2781243	BREX-system-P-loop-protein-BrxC-[Tenacibaculum-finnmarkense]
WP_014083579.1	Type1.BREX	<-SIGMA+OB-Fold+AAA-ATPase+Lon-Protease-BrxL<-PglZ<-AbiEii<-AbiEi_4<-TIR<-?<-TIR<-N6-METHYLASE<-DUF6079*<-BrxB-BrxF-iSTAND<-BrxA-DNA-binding	1199	Bacteria>FCB_group>Bacteroidetes/Chlorobi_group>Bacteroidota>Flavobacteriia>Flavobacteriales	GCF_000253275.1	Flavobacterium_branchiophilum	FBFL15_RS05125	1197053..1200652	55197	BREX-system-P-loop-protein-BrxC-[Flavobacterium-branchiophilum]
WP_051224566.1	Type1.BREX	<-TPR+TPR<-?<-SIGMA+OB-Fold+AAA-ATPase+HKD-DNase-BrxL<-PglZ<-?<-N6-METHYLASE<-?<-DUF6079*<-BrxB-BrxF-iSTAND<-BrxA-DNA-binding<-HTH_3||?->GDResolvase+Recombinase+ZnR->?-><-?<-Peptidase_M49	1168	Bacteria>FCB_group>Bacteroidetes/Chlorobi_group>Bacteroidota>Flavobacteriia>Flavobacteriales	GCF_000425485.1	Flavobacterium_tegetincola	H602_RS18955	62387..65893	150172	BREX-system-P-loop-protein-BrxC-[Flavobacterium-tegetincola]
WP_028123425.1	Type1.BREX	<-XIS-HTH||?-><-?<-?||HTH_3->BrxA-DNA-binding->BrxB-BrxF-iSTAND->DUF6079*->N6-METHYLASE->PglZ->SIGMA+OB-Fold+AAA-ATPase+HKD-DNase-BrxL->	1188	Bacteria>FCB_group>Bacteroidetes/Chlorobi_group>Bacteroidota>Flavobacteriia>Flavobacteriales	GCF_000428485.1	Epilithonimonas_tenax	F564_RS0115480	6701..10267	191577	BREX-system-P-loop-protein-BrxC-[Epilithonimonas-tenax]
WP_026451333.1	Type1.BREX	VirB5->TrwI->TraK->?->TraN->?->HTH_3->BrxA-DNA-binding->BrxB-BrxF-iSTAND->DUF6079*->Schlafen+HATPase+wHTH->?->?->SF1-SF2-Helicase->?->METHYLASE->PglZ->	1160	Bacteria>FCB_group>Bacteroidetes/Chlorobi_group>Bacteroidota>Flavobacteriia>Flavobacteriales	GCF_000429125.1	Aequorivita_capsosiphonis	G440_RS0110860	108295..111777	487317	BREX-system-P-loop-protein-BrxC-[Aequorivita-capsosiphonis]
WP_034657463.1	Type1.BREX	REase->?->HEPN->REase->?-><-?||HTH_3->BrxA-DNA-binding->BrxB-BrxF-iSTAND->DUF6079*->N6-METHYLASE->?->TPR+REase->?->SF1-SF2-Helicase->?->METHYLASE->PglZ->SIGMA+OB-Fold+AAA-ATPase+HKD-DNase-BrxL->	1160	Bacteria>FCB_group>Bacteroidetes/Chlorobi_group>Bacteroidota>Flavobacteriia>Flavobacteriales	GCF_000468595.1	Cellulophaga_baltica	M665_RS0118760	90424..93906	76594	BREX-system-P-loop-protein-BrxC-[Cellulophaga-baltica]
WP_025613698.1	Type1.BREX	Relaxase->VirD4-TraG+VirD4-FtsK->MORC+DUF3684+REase->Classical-AAA-><-Peptidase_S8<-SIGMA+OB-Fold+AAA-ATPase+Lon-Protease-BrxL<-PglZ<-N6-METHYLASE<-DUF6079*<-BrxB-BrxF-iSTAND<-BrxA-DNA-binding<-HTH<-?<-TraN<-?<-TraK<-TrwI<-VirB5	1199	Bacteria>FCB_group>Bacteroidetes/Chlorobi_group>Bacteroidota>Flavobacteriia>Flavobacteriales	GCF_000477035.2	Cellulophaga_baltica	M667_RS02035	472716..476315	76594	BREX-system-P-loop-protein-BrxC-[Cellulophaga-baltica]
WP_034059321.1	Type1.BREX	<-SAM-METHYLASE<-?<-PglZ<-METHYLASE<-?<-SF1-SF2-Helicase<-?<-N6-METHYLASE<-Schlafen+HATPase+wHTH<-DUF6079*<-BrxB-BrxF-iSTAND<-BrxA-DNA-binding<-HTH_3<-?<-TPR+TPR+TPR||ABhydrolase->SIGMA-HTH->	1160	Bacteria>FCB_group>Bacteroidetes/Chlorobi_group>Bacteroidota>Flavobacteriia>Flavobacteriales	GCF_000797445.1	Lacinutrix_jangbogonensis	RH82_RS07100	67712..71194	1469557	BREX-system-P-loop-protein-BrxC-[Lacinutrix-jangbogonensis]
WP_059014619.1	Type1.BREX	<-HEPN<-ParB+HNH<-N6-METHYLASE<-DUF6079*<-BrxB-BrxF-iSTAND<-BrxA-DNA-binding<-HTH_3<-Classical-AAA<-HTH_36<-HTH_36<-XIS-HTH<-Arm-DNA-bind_5+Phage_int_SAM_5+Phage_integrase	1169	Bacteria>FCB_group>Bacteroidetes/Chlorobi_group>Bacteroidota>Flavobacteriia>Flavobacteriales	GCF_001485515.1	Flavobacterium_psychrophilum	FPK15_RS07995	6803..10312	96345	BREX-system-P-loop-protein-BrxC-[Flavobacterium-psychrophilum]
WP_076662677.1	Type1.BREX	DOC+HTH-><-?<-?<-?<-SIGMA+OB-Fold+AAA-ATPase+Lon-Protease-BrxL<-PglZ<-ParB+HNH<-?<-N6-METHYLASE<-DUF6079*<-BrxB-BrxF-iSTAND<-BrxA-DNA-binding<-HNH<-sGTP<-GTPase-AIG<-?<-?||wHTH+WYL+WCX->	1201	Bacteria>FCB_group>Bacteroidetes/Chlorobi_group>Bacteroidota>Flavobacteriia>Flavobacteriales	GCF_001975575.1	Mangrovimonas_sp._DI_80	BKM32_RS04295	828004..831609	1779330	BREX-system-P-loop-protein-BrxC-[Mangrovimonas-sp.-DI-80]
WP_088323652.1	Type1.BREX	<-XIS-HTH<-?<-ParB+N6_N4_Mtase<-?<-GDResolvase<-?<-SIGMA+OB-Fold+AAA-ATPase+HKD-DNase-BrxL<-PglZ<-N6-METHYLASE<-DUF6079*<-BrxB-BrxF-iSTAND<-BrxA-DNA-binding<-HTH_3||?->REase+DUF4268->?->?->?-><-Phage_int_SAM_5+Phage_integrase	1158	Bacteria>FCB_group>Bacteroidetes/Chlorobi_group>Bacteroidota>Flavobacteriia>Flavobacteriales	GCF_002201235.1	Polaribacter_tangerinus	CCB00_RS05195	350003..353479	1920034	BREX-system-P-loop-protein-BrxC-[Polaribacter-tangerinus]
WP_094415998.1	Type1.BREX	<-PIN<-?<-SIGMA+OB-Fold+AAA-ATPase+Lon-Protease-BrxL<-PglZ<-N6-METHYLASE<-Schlafen+HATPase+wHTH<-DUF6079*<-BrxB-BrxF-iSTAND<-BrxA-DNA-binding<-HTH<-?<-XIS-HTH	1197	Bacteria>FCB_group>Bacteroidetes/Chlorobi_group>Bacteroidota>Flavobacteriia>Flavobacteriales	GCF_002251835.1	Flavobacterium_cyanobacteriorum	CHU92_RS12285	18327..21920	2022802	BREX-system-P-loop-protein-BrxC-[Flavobacterium-cyanobacteriorum]
WP_107011557.1	Type1.BREX	<-PglZ<-?<-HAD+PRTase+PRTase+REase+SF1-SF2-Helicase<-?<-?<-REase+DUF5655<-REase+DUF5655<-N6-METHYLASE<-DUF6079*<-BrxB-BrxF-iSTAND<-BrxA-DNA-binding||?->?->SF1-SF2-Helicase->?->?->?->XIS-HTH->	1166	Bacteria>FCB_group>Bacteroidetes/Chlorobi_group>Bacteroidota>Flavobacteriia>Flavobacteriales	GCF_003024155.1	Gramella_fulva	C7S20_RS05585	1214513..1218013	2126553	BREX-system-P-loop-protein-BrxC-[Christiangramia-fulva]
WP_108879107.1	Type1.BREX	MoxR-AAA->?->wHTH+WYL+WCX->ZnR+CC+REase->BrxA-DNA-binding->BrxB-BrxF-iSTAND->DUF6079*->N6-METHYLASE->PglZ->SIGMA+OB-Fold+AAA-ATPase+HKD-DNase-BrxL->?->MORC+DUF3684+REase->SF1-SF2-Helicase+PIN->?-><-AAA-ToxA||wHTH+WYL+WCX->	1166	Bacteria>FCB_group>Bacteroidetes/Chlorobi_group>Bacteroidota>Flavobacteriia>Flavobacteriales	GCF_003095655.1	Dokdonia_sp._Dokd-P16	DCS32_RS15465	3477371..3480871	2173169	BREX-system-P-loop-protein-BrxC-[Dokdonia-sp.-Dokd-P16]
WP_129016554.1	Type1.BREX	<-SIGMA+OB-Fold+AAA-ATPase+Lon-Protease-BrxL<-PglZ<-Schlafen+HATPase+wHTH<-N6-METHYLASE<-DUF6079*<-BrxB-BrxF-iSTAND<-BrxA-DNA-binding||?-><-ACET<-SIGMA-HTH	1202	Bacteria>FCB_group>Bacteroidetes/Chlorobi_group>Bacteroidota>Flavobacteriia>Flavobacteriales	GCF_004115975.1	Gelidibacter_gilvus	ESZ48_RS06605	20961..24569	59602	BREX-system-P-loop-protein-BrxC-[Gelidibacter-gilvus]
WP_129366655.1	Type1.BREX	<-HNH<-SbcC<-SIGMA+OB-Fold+AAA-ATPase+HKD-DNase-BrxL<-PglZ<-N6-METHYLASE<-DUF6079*<-BrxB-BrxF-iSTAND<-BrxA-DNA-binding<-Classical-AAA	1168	Bacteria>FCB_group>Bacteroidetes/Chlorobi_group>Bacteroidota>Flavobacteriia>Flavobacteriales	GCF_004121075.1	Lutibacter_sp._HS1-25	EC396_RS10485	12877..16383	2485000	BREX-system-P-loop-protein-BrxC-[Lutibacter-sp.-HS1-25]
WP_132215847.1	Type1.BREX	<-CARF+REase<-CARF<-HKD-DNase+HKD-DNase<-?<-S1+SF1-SF2-Helicase+S1<-SIGMA+OB-Fold+AAA-ATPase+HKD-DNase-BrxL<-PglZ<-ParB+HNH<-N6-METHYLASE<-DUF6079*<-BrxB-BrxF-iSTAND<-BrxA-DNA-binding<-ZnR+CC+REase<-wHTH+WYL+WCX<-Arm-DNA-bind_5+Phage_int_SAM_5+Phage_integrase<-ZnR||Phage_integrase+Phage_integrase-><-Zn_Tnp_IS1595+DDE_Tnp_IS1595<-REC+LuxR-HTH	1169	Bacteria>FCB_group>Bacteroidetes/Chlorobi_group>Bacteroidota>Flavobacteriia>Flavobacteriales	GCF_004341235.1	Mariniflexile_fucanivorans	EV196_RS05490	443037..446546	264023	BREX-system-P-loop-protein-BrxC-[Mariniflexile-fucanivorans]
WP_131908313.1	Type1.BREX	<-XIS-HTH<-?||?-><-SIGMA+OB-Fold+AAA-ATPase+Lon-Protease-BrxL<-PglZ<-AbiEii<-AbiEi_4<-ParB+HNH<-N6-METHYLASE<-DUF6079*<-BrxB-BrxF-iSTAND<-BrxA-DNA-binding||?-><-?||HTH_18+HTH_AraC->	1203	Bacteria>FCB_group>Bacteroidetes/Chlorobi_group>Bacteroidota>Flavobacteriia>Flavobacteriales	GCF_004349085.1	Flavobacterium_caseinilyticum	EOF89_RS02690	598586..602197	2541732	BREX-system-P-loop-protein-BrxC-[Flavobacterium-caseinilyticum]
WP_133546801.1	Type1.BREX	<-XIS-HTH<-?<-?<-?<-AraC-HTH<-Arm-DNA-bind_5+Phage_int_SAM_5+Phage_integrase||?->BrxA-DNA-binding->BrxB-BrxF-iSTAND->DUF6079*->REase+DUF5655->NERD+SF1-SF2-Helicase->TPR+S1->PglZ->SIGMA+OB-Fold+AAA-ATPase+HKD-DNase-BrxL->HEPN->?-><-HTH_36	1167	Bacteria>FCB_group>Bacteroidetes/Chlorobi_group>Bacteroidota>Flavobacteriia>Flavobacteriales	GCF_004362395.1	Salegentibacter_sp._24	DET49_RS03950	90042..93545	2183986	BREX-system-P-loop-protein-BrxC-[Salegentibacter-sp.-24]
WP_136564624.1	Type1.BREX	XIS-HTH->?->AbiEi_1->AbiEii+ZnR->wHTH+WYL+WCX->ZnR+CC+REase->HTH_3->BrxA-DNA-binding->BrxB-BrxF-iSTAND->DUF6079*->N6-METHYLASE->HKD-DNase->NERD+SF1-SF2-Helicase->TPR+S1->PglZ->SIGMA+OB-Fold+AAA-ATPase+HKD-DNase-BrxL->?->SMODS->	1164	Bacteria>FCB_group>Bacteroidetes/Chlorobi_group>Bacteroidota>Flavobacteriia>Flavobacteriales	GCF_004916895.1	Muricauda_alvinocaridis	EZV76_RS00375	77026..80520	2530200	BREX-system-P-loop-protein-BrxC-[Muricauda-alvinocaridis]
WP_141421442.1	Type1.BREX	<-HAD+PRTase+PRTase+REase+SF1-SF2-Helicase<-?<-SIGMA+OB-Fold+AAA-ATPase+HKD-DNase-BrxL<-PglZ<-TPR+S1<-NERD+SF1-SF2-Helicase<-N6-METHYLASE<-ParB+HNH<-DUF6079*<-BrxB-BrxF-iSTAND<-BrxA-DNA-binding<-TPR+TPR<-?<-?<-?<-Phage_int+DNA-glycosylase<-HNH<-OLD-ABC	1165	Bacteria>FCB_group>Bacteroidetes/Chlorobi_group>Bacteroidota>Flavobacteriia>Flavobacteriales	GCF_006546625.1	Haloflavibacter_putidus	FKR84_RS06225	21354..24851	2576776	BREX-system-P-loop-protein-BrxC-[Haloflavibacter-putidus]
WP_019974718.1	Type1.BREX	<-SIGMA+OB-Fold+AAA-ATPase+HKD-DNase-BrxL<-PglZ<-N6-METHYLASE<-DUF6079*<-BrxB-BrxF-iSTAND<-BrxA-DNA-binding<-HTH_3	1191	Bacteria>FCB_group>Bacteroidetes/Chlorobi_group>Bacteroidota>Flavobacteriia>Flavobacteriales	GCF_007990795.1	Empedobacter_brevis	EB1_RS03060	16539..20114	247	BREX-system-P-loop-protein-BrxC-[Empedobacter-brevis]
WP_089081509.1	Type1.BREX	<-SIGMA+OB-Fold+AAA-ATPase+Lon-Protease-BrxL<-PglZ<-AbiEii<-AbiEi_4<-ParB+HNH<-ParB+HNH<-N6-METHYLASE<-DUF6079*<-BrxB-BrxF-iSTAND<-BrxA-DNA-binding<-tRNA||Arm-DNA-bind_5+Phage_int_SAM_5+Phage_integrase->?->XIS-HTH->?->DUF488->GDResolvase->	1199	Bacteria>FCB_group>Bacteroidetes/Chlorobi_group>Bacteroidota>Flavobacteriia>Flavobacteriales	GCF_007990815.1	Flavobacterium_branchiophilum	FB1_RS11100	14247..17846	55197	BREX-system-P-loop-protein-BrxC-[Flavobacterium-branchiophilum]
WP_153266434.1	Type1.BREX	HTH_36->Classical-AAA->TRPR-HTH->STYKinase-like->HTH_3->?->HTH_3->BrxA-DNA-binding->BrxB-BrxF-iSTAND->DUF6079*->N6-METHYLASE->PglZ->SIGMA+OB-Fold+AAA-ATPase+HKD-DNase-BrxL->HigB->HTH_3->?-><-XIS-HTH	1157	Bacteria>FCB_group>Bacteroidetes/Chlorobi_group>Bacteroidota>Flavobacteriia>Flavobacteriales	GCF_009498295.1	Oceanihabitans_sp._IOP_32	FEZ18_RS00165	31374..34847	2529032	BREX-system-P-loop-protein-BrxC-[Oceanihabitans-sp.-IOP_32]
WP_158973850.1	Type1.BREX	<-ANK+ANK||wHTH+WYL+WCX->BrxA-DNA-binding->BrxB-BrxF-iSTAND->DUF6079*->N6-METHYLASE->PglZ->SIGMA+OB-Fold+AAA-ATPase+HKD-DNase-BrxL->?->?->SF1-SF2-Helicase->?-><-D5ATPase	1170	Bacteria>FCB_group>Bacteroidetes/Chlorobi_group>Bacteroidota>Flavobacteriia>Flavobacteriales	GCF_009797025.1	Cellulophaga_sp._L1A9	GQR94_RS02145	525105..528617	2686362	BREX-system-P-loop-protein-BrxC-[Cellulophaga-sp.-L1A9]
WP_165150638.1	Type1.BREX	<-ABhydrolase<-REase+RNase<-?<-wHTH+WYL+WCX||?->?->BrxA-DNA-binding->BrxB-BrxF-iSTAND->DUF6079*->N6-METHYLASE->ParB+HNH->PglZ->SIGMA+OB-Fold+AAA-ATPase+Lon-Protease-BrxL->HEPN->?->?-><-FGase<-tRNA||SKI->	1198	Bacteria>FCB_group>Bacteroidetes/Chlorobi_group>Bacteroidota>Flavobacteriia>Flavobacteriales	GCF_011065475.1	Aequorivita_sp._KMM_9714	G3A46_RS13570	21223..24819	2707173	BREX-system-P-loop-protein-BrxC-[Aequorivita-sp.-KMM-9714]
WP_177760874.1	Type1.BREX	HTH_3-><-McrC-NTD+REase||HTH+rve->AAA-ATPase-><-TPR+TPR<-?<-SIGMA+OB-Fold+AAA-ATPase+Lon-Protease-BrxL<-PglZ<-N6-METHYLASE<-DUF6079*<-BrxB-BrxF-iSTAND<-BrxA-DNA-binding<-HTH_3<-HigB_toxin<-?<-AbiEii	1217	Bacteria>FCB_group>Bacteroidetes/Chlorobi_group>Bacteroidota>Flavobacteriia>Flavobacteriales	GCF_013389595.1	Flavobacterium_sp._I3-2	HW119_RS00890	194599..198252	2748319	BREX-system-P-loop-protein-BrxC-[Flavobacterium-sp.-I3-2]
WP_163432971.1	Type1.BREX	<-LexA-protease<-?||?->XIS-HTH->PIN->GDResolvase+HIN-HTH->HTH_3->BrxA-DNA-binding->BrxB-BrxF-iSTAND->DUF6079*->N6-METHYLASE->PglZ->HEPN->?-><-?||?->Relaxase->VirD4-TraG+VirD4-FtsK->	1189	Bacteria>FCB_group>Bacteroidetes/Chlorobi_group>Bacteroidota>Flavobacteriia>Flavobacteriales	GCF_014050215.1	unclassified_Flavobacterium	G7BO0_RS14255	32052..35621	196869	MULTISPECIES:-BREX-system-P-loop-protein-BrxC-[unclassified-Flavobacterium]
WP_073117772.1	Type1.BREX	SLATT_1-><-METHYLASE-p<-?<-SMODS<-?<-?<-SIGMA+OB-Fold+AAA-ATPase+HKD-DNase-BrxL<-PglZ<-N6-METHYLASE<-DUF6079*<-BrxB-BrxF-iSTAND<-BrxA-DNA-binding<-ZnR+CC+REase<-wHTH+WYL+WCX<-HipA_C<-Couple_hipA<-HTH_3<-?<-Classical-AAA	1170	Bacteria>FCB_group>Bacteroidetes/Chlorobi_group>Bacteroidota>Flavobacteriia>Flavobacteriales	GCF_014636515.1	Wenyingzhuangia_marina	IEV03_RS11465	186450..189962	1195760	BREX-system-P-loop-protein-BrxC-[Wenyingzhuangia-marina]
WP_093139951.1	Type1.BREX	<-SF1-SF2-Helicase<-SIGMA+OB-Fold+AAA-ATPase+Lon-Protease-BrxL<-PglZ<-Schlafen+HATPase+wHTH<-?<-N6-METHYLASE<-DUF6079*<-BrxB-BrxF-iSTAND<-BrxA-DNA-binding||?-><-?<-?<-?||?->?->HisKin->	1202	Bacteria>FCB_group>Bacteroidetes/Chlorobi_group>Bacteroidota>Flavobacteriia>Flavobacteriales	GCF_014651275.1	Ulvibacter_litoralis	IE050_RS03690	820645..824253	227084	BREX-system-P-loop-protein-BrxC-[Ulvibacter-litoralis]
WP_194850146.1	Type1.BREX	<-SF1-SF2-Helicase<-OLD-ABC+TOPRIM<-SIGMA+OB-Fold+AAA-ATPase+HKD-DNase-BrxL<-PglZ<-METHYLASE<-?<-SF1-SF2-Helicase<-?<-N6-METHYLASE<-DUF6079*<-BrxB-BrxF-iSTAND<-BrxA-DNA-binding<-?<-TraN<-?<-TraK<-TrwI<-VirB5<-HerA/FtsK	1160	Bacteria>FCB_group>Bacteroidetes/Chlorobi_group>Bacteroidota>Flavobacteriia>Flavobacteriales	GCF_015356755.1	Nonlabens_antarcticus	IQ282_RS01355	76947..80429	392714	BREX-system-P-loop-protein-BrxC-[Nonlabens-antarcticus]
WP_198639224.1	Type1.BREX	REase->SF1-SF2-Helicase+REase->NERD+SF1-SF2-Helicase->HNH-><-?<-SIGMA+OB-Fold+AAA-ATPase+Lon-Protease-BrxL<-PglZ<-ParB+HNH<-N6-METHYLASE<-DUF6079*<-BrxB-BrxF-iSTAND<-BrxA-DNA-binding<-?<-?<-?<-?<-?<-wHTH+WYL+WCX	1201	Bacteria>FCB_group>Bacteroidetes/Chlorobi_group>Bacteroidota>Flavobacteriia>Flavobacteriales	GCF_016236975.1	Salegentibacter_maritimus	I6U50_RS13470	35086..38691	2794347	BREX-system-P-loop-protein-BrxC-[Salegentibacter-maritimus]
WP_199600307.1	Type1.BREX	Relaxase->VirD4-TraG+VirD4-FtsK->Classical-AAA-><-Peptidase_S8<-SIGMA+OB-Fold+AAA-ATPase+HKD-DNase-BrxL<-PglZ<-N6-METHYLASE<-DEOR-HTH<-Schlafen+HATPase+wHTH<-DUF6079*<-BrxB-BrxF-iSTAND<-BrxA-DNA-binding<-HTH_3||?-><-?<-TraN<-?<-TraK<-TrwI	1160	Bacteria>FCB_group>Bacteroidetes/Chlorobi_group>Bacteroidota>Flavobacteriia>Flavobacteriales	GCF_016469035.1	Gelidibacter_salicanalis	JEM65_RS13235	170248..173730	291193	BREX-system-P-loop-protein-BrxC-[Gelidibacter-salicanalis]
WP_203057453.1	Type1.BREX	<-DUF4011+SF1-SF2-Helicase+REase<-SIGMA+OB-Fold+AAA-ATPase+HKD-DNase-BrxL<-?<-HAD+PRTase+PRTase+REase+SF1-SF2-Helicase<-?<-SIGMA-HTH<-N6-METHYLASE<-DUF6079*<-BrxB-BrxF-iSTAND<-BrxA-DNA-binding<-?||ZnR+CC+REase->?-><-?<-?<-MACRODOMAIN<-ART-DarT	1178	Bacteria>FCB_group>Bacteroidetes/Chlorobi_group>Bacteroidota>Flavobacteriia>Flavobacteriales	GCF_016786255.1	Robertkochia_sediminum	IS446_RS01110	192082..195618	2785326	BREX-system-P-loop-protein-BrxC-[Robertkochia-sediminum]
WP_203251454.1	Type1.BREX	XIS-HTH-><-SIGMA+OB-Fold+AAA-ATPase+HKD-DNase-BrxL<-PglZ<-TPR+S1<-NERD+SF1-SF2-Helicase<-N6-METHYLASE<-N6-METHYLASE<-DUF6079*<-BrxB-BrxF-iSTAND<-BrxA-DNA-binding<-HTH_3<-REase<-?<-SF1-SF2-Helicase+DUF1998<-?<-wHTH+WYL+WCX<-tRNA<-Glyoxalase_4	1160	Bacteria>FCB_group>Bacteroidetes/Chlorobi_group>Bacteroidota>Flavobacteriia>Flavobacteriales	GCF_016811105.1	Aurantibacter_crassamenti	JQC67_RS03370	810968..814450	1837375	BREX-system-P-loop-protein-BrxC-[Aurantibacter-crassamenti]
WP_179314673.1	Type1.BREX	<-ParB+HNH<-ParB+HNH<-PglZ<-METHYLASE<-?<-SF1-SF2-Helicase<-?<-N6-METHYLASE<-DUF6079*<-BrxB-BrxF-iSTAND<-BrxA-DNA-binding<-HTH_3<-Classical-AAA<-HTH_36<-XIS-HTH	1160	Bacteria>FCB_group>Bacteroidetes/Chlorobi_group>Bacteroidota>Flavobacteriia>Flavobacteriales	GCF_019278425.1	Winogradskyella	HOI32_RS04080	939044..942526	286104	MULTISPECIES:-BREX-system-P-loop-protein-BrxC-[Winogradskyella]
WP_224137560.1	Type1.BREX	<-REC<-N6-METHYLASE<-ASCH<-HIRAN<-SIGMA+OB-Fold+AAA-ATPase+HKD-DNase-BrxL<-PglZ<-N6-METHYLASE<-DUF6079*<-BrxB-BrxF-iSTAND<-BrxA-DNA-binding||wHTH+WYL+WCX->?->?->?->?->REase-><-Classical-AAA	1174	Bacteria>FCB_group>Bacteroidetes/Chlorobi_group>Bacteroidota>Flavobacteriia>Flavobacteriales	GCF_020084865.1	Candidatus_Kaistella_beijingensis	J4771_RS06115	1314125..1317649	2820270	BREX-system-P-loop-protein-BrxC-[Candidatus-Kaistella-beijingensis]
WP_224474755.1	Type1.BREX	<-SIGMA+OB-Fold+AAA-ATPase+HKD-DNase-BrxL<-PglZ<-METHYLASE<-?<-SF1-SF2-Helicase<-?<-REase+DUF5655<-N6-METHYLASE<-DUF6079*<-BrxB-BrxF-iSTAND<-BrxA-DNA-binding||?->?->?->?->?->HNH	1167	Bacteria>FCB_group>Bacteroidetes/Chlorobi_group>Bacteroidota>Flavobacteriia>Flavobacteriales	GCF_020164485.1	Salegentibacter_tibetensis	LB467_RS14420	15605..19108	2873600	BREX-system-P-loop-protein-BrxC-[Salegentibacter-tibetensis]

Protein-ID	System	Gene-Neighbourhoods	Length	Taxonomy	Assembly-ID	Strain	Locus-Tag	Genomic-coordinates	TaxID	Fasta-defline
WP_226063322.1	Type1.BREX	SF1-SF2-Helicase->GDResolvase+HIN-HTH->URI-DNase-><-?<-?<-?<-SIGMA+OB-Fold+AAA-ATPase+HKD-DNase-BrxL<-PglZ<-N6-METHYLASE<-DUF6079*<-BrxB-BrxF-iSTAND<-BrxA-DNA-binding||?->?->REase+35exo->	1175	Bacteria>FCB_group>Bacteroidetes/Chlorobi_group>Bacteroidota>Flavobacteriia>Flavobacteriales	GCF_020410745.1	Kaistella_sp._GW4-15	LCB14_RS07510	1609850..1613377	2878534	BREX-system-P-loop-protein-BrxC-[Kaistella-sp.-GW4-15]
WP_232121385.1	Type1.BREX	<-ArdCN+MPTase<-?<-?||<-TPR+TPR<-SIGMA+OB-Fold+AAA-ATPase+HKD-DNase-BrxL<-PglZ<-N6-METHYLASE<-DUF6079*<-BrxB-BrxF-iSTAND<-BrxA-DNA-binding<-ZnR+TOPRIM<-?<-ZnR+TOPRIM<-VirD4-TraG<-?<-?<-TraI-VirD2-RCRE	1176	Bacteria>FCB_group>Bacteroidetes/Chlorobi_group>Bacteroidota>Flavobacteriia>Flavobacteriales	GCF_021205915.1	Tenacibaculum_dicentrarchi	LNI89_RS11375	20484..24014	669041	BREX-system-P-loop-protein-BrxC-[Tenacibaculum-dicentrarchi]
WP_232125551.1	Type1.BREX	<-ArdCN+MPTase<-?<-?||?-><-TPR+TPR<-SIGMA+OB-Fold+AAA-ATPase+HKD-DNase-BrxL<-PglZ<-N6-METHYLASE<-DUF6079*<-BrxB-BrxF-iSTAND<-BrxA-DNA-binding<-ZnR+TOPRIM<-?<-ZnR+TOPRIM<-VirD4-TraG<-?<-?<-TraI-VirD2-RCRE	1176	Bacteria>FCB_group>Bacteroidetes/Chlorobi_group>Bacteroidota>Flavobacteriia>Flavobacteriales	GCF_021206215.1	Tenacibaculum_finnmarkense	LNJ08_RS11655	18832..22362	2781243	BREX-system-P-loop-protein-BrxC-[Tenacibaculum-finnmarkense]
WP_233899778.1	Type1.BREX	Arm-DNA-bind_5+Phage_int_SAM_5+Phage_integrase->D5ATPase->XIS-HTH->?->D5ATPase->BrxA-DNA-binding->BrxB-BrxF-iSTAND->DUF6079*->N6-METHYLASE->PglZ->SIGMA+OB-Fold+AAA-ATPase+HKD-DNase-BrxL-><-HipA_C<-Couple_hipA<-HTH_3	1176	Bacteria>FCB_group>Bacteroidetes/Chlorobi_group>Bacteroidota>Flavobacteriia>Flavobacteriales	GCF_021390795.1	Tenacibaculum_piscium	LOU78_RS02170	464400..467930	1458515	BREX-system-P-loop-protein-BrxC-[Tenacibaculum-piscium]
WP_232123632.1	Type1.BREX	ZnR+TOPRIM->BrxA-DNA-binding->BrxB-BrxF-iSTAND->DUF6079*->N6-METHYLASE->PglZ->SIGMA+OB-Fold+AAA-ATPase+HKD-DNase-BrxL->TPR+TPR->	1176	Bacteria>FCB_group>Bacteroidetes/Chlorobi_group>Bacteroidota>Flavobacteriia>Flavobacteriales	GCF_022381915.1	Tenacibaculum_finnmarkense	G1K59_RS11550	3814..7344	2781243	BREX-system-P-loop-protein-BrxC-[Tenacibaculum-finnmarkense]
WP_193709240.1	Type1.BREX	TraI-VirD2-RCRE->?->?->VirD4-TraG->ZnR+TOPRIM->?->ZnR+TOPRIM->BrxA-DNA-binding->BrxB-BrxF-iSTAND->DUF6079*->N6-METHYLASE->PglZ->SIGMA+OB-Fold+AAA-ATPase+HKD-DNase-BrxL->TPR+TPR-><-?||?->?->ArdCN+MPTase->	1176	Bacteria>FCB_group>Bacteroidetes/Chlorobi_group>Bacteroidota>Flavobacteriia>Flavobacteriales	GCF_022382115.1	Tenacibaculum_finnmarkense	PJJ26_RS00320	69854..73384	2781243	BREX-system-P-loop-protein-BrxC-[Tenacibaculum-finnmarkense]
WP_244340081.1	Type1.BREX	<-PglZ<-Couple_hipA+HipA_C<-HTH_3<-?<-URI-DNase<-ParB+HNH<-N6-METHYLASE<-?<-N6-METHYLASE<-DUF6079*<-BrxB-BrxF-iSTAND<-BrxA-DNA-binding<-HTH_3||TraJ-RHH->?->?->?->Peptidase_S9+Hydrolase_4->	1169	Bacteria>FCB_group>Bacteroidetes/Chlorobi_group>Bacteroidota>Flavobacteriia>Flavobacteriales	GCF_022836875.1	Flavobacterium	LZF87_RS14585	3214137..3217646	237	MULTISPECIES:-BREX-system-P-loop-protein-BrxC-[Flavobacterium]
WP_249986967.1	Type1.BREX	<-DOC+HTH<-ANK+ANK||HTH_3-><-REase<-HNH<-OLD-ABC<-SIGMA+OB-Fold+AAA-ATPase+HKD-DNase-BrxL<-PglZ<-N6-METHYLASE<-DUF6079*<-BrxB-BrxF-iSTAND<-BrxA-DNA-binding<-?<-?<-?<-?<-?<-?<-ParA-Soj-PloopNTPase	1176	Bacteria>FCB_group>Bacteroidetes/Chlorobi_group>Bacteroidota>Flavobacteriia>Flavobacteriales	GCF_023555435.1	Polaribacter_sp._Z014	MPF19_RS04515	308529..312059	2927126	BREX-system-P-loop-protein-BrxC-[Polaribacter-sp.-Z014]
WP_264521759.1	Type1.BREX	GDResolvase+Recombinase+Zn_ribbon_recom-><-MarR-HTH||?->HTH_3->BrxA-DNA-binding->BrxB-BrxF-iSTAND->DUF6079*->N6-METHYLASE->?->PglZ->SIGMA+OB-Fold+AAA-ATPase+HKD-DNase-BrxL->?-><-?<-HTH_3||?->GDResolvase->	1166	Bacteria>FCB_group>Bacteroidetes/Chlorobi_group>Bacteroidota>Flavobacteriia>Flavobacteriales	GCF_025947145.1	Flavobacterium_sp._N1994	OLM53_RS03950	842577..846077	2986827	BREX-system-P-loop-protein-BrxC-[Flavobacterium-sp.-N1994]
WP_267609146.1	Type1.BREX	<-Phage_int_SAM_1+Phage_integrase<-?<-SIGMA+OB-Fold+AAA-ATPase+HKD-DNase-BrxL<-PglZ<-N6-METHYLASE<-DUF6079*<-BrxB-BrxF-iSTAND<-BrxA-DNA-binding||Phage_integrase-><-XIS-HTH<-?<-ParB<-?<-GDResolvase+HIN-HTH	1173	Bacteria>FCB_group>Bacteroidetes/Chlorobi_group>Bacteroidota>Flavobacteriia>Flavobacteriales	GCF_026626535.1	Chryseobacterium_sp._CY353	OUU28_RS14320	10693..14214	2997334	BREX-system-P-loop-protein-BrxC-[Chryseobacterium-sp.-CY353]
WP_225581981.1	Type1.BREX	Arm-DNA-bind_5+Phage_int_SAM_5+Phage_integrase->XIS-HTH->HTH_3->BrxA-DNA-binding->BrxB-BrxF-iSTAND->DUF6079*->N6-METHYLASE->PglZ->McrB-AAA->McrC-NTD+REase->?-><-SF1-SF2-Helicase<-?||DEDD_Tnp_IS110+Transposase_20->	1189	Bacteria>FCB_group>Bacteroidetes/Chlorobi_group>Bacteroidota>Flavobacteriia>Flavobacteriales	GCF_030324595.1	Myroides_odoratimimus	IF087_RS16265	4194..7763	76832	BREX-system-P-loop-protein-BrxC-[Myroides-odoratimimus]
WP_089756260.1	Type1.BREX	<-RHS+RHS<-?<-?||NUMOD4+HNH->NUMOD4+HNH-><-SIGMA+OB-Fold+AAA-ATPase+HKD-DNase-BrxL<-PglZ<-ParB+HNH<-N6-METHYLASE<-DUF6079*<-BrxB-BrxF-iSTAND<-BrxA-DNA-binding<-HTH_3<-HTH_19||SIGMA-HTH-><-REase<-ParB+HNH<-?<-TraI-VirD2-RCRE	1176	Bacteria>FCB_group>Bacteroidetes/Chlorobi_group>Bacteroidota>Flavobacteriia>Flavobacteriales	GCF_900100115.1	Chryseobacterium_soldanellicola	BLT04_RS13255	831929..835459	311333	BREX-system-P-loop-protein-BrxC-[Chryseobacterium-soldanellicola]
WP_089663492.1	Type1.BREX	BrxA-DNA-binding->BrxB-BrxF-iSTAND->DUF6079*->N6-METHYLASE->?->?->HAD+PRTase+PRTase+REase+SF1-SF2-Helicase->?->PglZ->SIGMA+OB-Fold+AAA-ATPase+HKD-DNase-BrxL->	1167	Bacteria>FCB_group>Bacteroidetes/Chlorobi_group>Bacteroidota>Flavobacteriia>Flavobacteriales	GCF_900105035.1	Gramella_echinicola	BLT93_RS14455	3168257..3171760	279359	BREX-system-P-loop-protein-BrxC-[Christiangramia-echinicola]
WP_103999283.1	Type1.BREX	<-GDResolvase+Recombinase+ZnR||?-><-?||?-><-?<-?||HTH_3->BrxA-DNA-binding->BrxB-BrxF-iSTAND->DUF6079*->N6-METHYLASE->ParB+HNH->URI-DNase->RADICAL-SAM->METHYLASE+RNA-Helicase->HTH_3->PglZ->SIGMA+OB-Fold+AAA-ATPase+HKD-DNase-BrxL-><-Arm-DNA-bind_5+Phage_int_SAM_5+Phage_integrase	1170	Bacteria>FCB_group>Bacteroidetes/Chlorobi_group>Bacteroidota>Flavobacteriia>Flavobacteriales	GCF_900108015.1	Flavobacterium_urumqiense	C4S35_RS05860	195084..198596	935224	BREX-system-P-loop-protein-BrxC-[Flavobacterium-urumqiense]
WP_074938106.1	Type1.BREX	<-PglZ<-METHYLASE<-?<-SF1-SF2-Helicase<-?<-?<-TPR+REase<-?<-N6-METHYLASE<-DUF6079*<-BrxB-BrxF-iSTAND<-BrxA-DNA-binding<-HTH_3	1160	Bacteria>FCB_group>Bacteroidetes/Chlorobi_group>Bacteroidota>Flavobacteriia>Flavobacteriales	GCF_900112395.1	Algibacter_lectus	BM246_RS11755	97299..100781	221126	BREX-system-P-loop-protein-BrxC-[Algibacter-lectus]
WP_091208763.1	Type1.BREX	<-SIGMA+OB-Fold+AAA-ATPase+HKD-DNase-BrxL<-PglZ<-TPR+S1<-NERD+SF1-SF2-Helicase<-HKD-DNase<-?<-?<-N6-METHYLASE<-?<-DUF6079*<-BrxB-BrxF-iSTAND<-BrxA-DNA-binding<-HTH_3||?->HIN-HTH->	1172	Bacteria>FCB_group>Bacteroidetes/Chlorobi_group>Bacteroidota>Flavobacteriia>Flavobacteriales	GCF_900112975.1	Flavobacterium_xueshanense	BMZ76_RS14825	46469..49987	935223	BREX-system-P-loop-protein-BrxC-[Flavobacterium-xueshanense]
WP_090838887.1	Type1.BREX	<-SAM-METHYLASE<-?<-ParB+HNH<-ParB+HNH<-PglZ<-TPR+S1<-NERD+SF1-SF2-Helicase<-N6-METHYLASE<-DUF6079*<-BrxB-BrxF-iSTAND<-BrxA-DNA-binding<-HTH_3	1160	Bacteria>FCB_group>Bacteroidetes/Chlorobi_group>Bacteroidota>Flavobacteriia>Flavobacteriales	GCF_900114005.1	Olleya_namhaensis	BMZ09_RS07050	330310..333792	1144750	BREX-system-P-loop-protein-BrxC-[Olleya-namhaensis]
WP_089819303.1	Type1.BREX	<-ARG<-?<-HEPN<-?<-?||BrxA-DNA-binding->BrxB-BrxF-iSTAND->DUF6079*->N6-METHYLASE->PglZ->SIGMA+OB-Fold+AAA-ATPase+HKD-DNase-BrxL->?->HTH_Tnp_1->HTH_Tnp_1+HTH_21+rve+rve_3->	1174	Bacteria>FCB_group>Bacteroidetes/Chlorobi_group>Bacteroidota>Flavobacteriia>Flavobacteriales	GCF_900114045.1	Kaistella_treverensis	BMZ55_RS05400	1141726..1145250	631455	BREX-system-P-loop-protein-BrxC-[Kaistella-treverensis]
WP_159447525.1	Type1.BREX	<-HISKIN+REC<-?<-?||wHTH+WYL+WCX->ZnR+CC+REase->BrxA-DNA-binding->BrxB-BrxF-iSTAND->DUF6079-p*->DUF6079-p->N6-METHYLASE-p->N6-METHYLASE->ParB+HNH->HTH_3->Couple_hipA+HipA_C->PglZ->SIGMA+OB-Fold+AAA-ATPase+HKD-DNase-BrxL->	719	Bacteria>FCB_group>Bacteroidetes/Chlorobi_group>Bacteroidota>Flavobacteriia>Flavobacteriales	GCF_900167935.1	Maribacter_arcticus	B5X55_RS04985	472839..474998	561365	BREX-system-P-loop-protein-BrxC-[Maribacter-arcticus]
WP_094162522.1	Type1.BREX	<-CR-Kinase<-?<-SIGMA+OB-Fold+AAA-ATPase+Lon-Protease-BrxL<-PglZ<-AbiEii<-AbiEi_4<-N6-METHYLASE<-DUF6079*<-BrxB-BrxF-iSTAND<-BrxA-DNA-binding<-D5ATPase+DUF3874	1198	Bacteria>FCB_group>Bacteroidetes/Chlorobi_group>Bacteroidota>Flavobacteriia>Flavobacteriales	GCF_900186645.1	Flavobacterium_psychrophilum	CI037_RS06110	11651..15247	96345	BREX-system-P-loop-protein-BrxC-[Flavobacterium-psychrophilum]
WP_095070899.1	Type1.BREX	<-AbiEii<-AbiEi_4||XIS-HTH->HTH_36->Classical-AAA-><-wHTH+WYL+WCX||BrxA-DNA-binding->BrxB-BrxF-iSTAND->DUF6079*->N6-METHYLASE->PglZ->SIGMA+OB-Fold+AAA-ATPase+HKD-DNase-BrxL->ASCH->HIRAN->ASCH->ZnR+CC+REase->ARG->	1178	Bacteria>FCB_group>Bacteroidetes/Chlorobi_group>Bacteroidota>Flavobacteriia>Flavobacteriales	GCF_900187185.1	Chryseobacterium_taklimakanense	CKV81_RS04675	977692..981228	536441	BREX-system-P-loop-protein-BrxC-[Chryseobacterium-taklimakanense]
WP_089379946.1	Type1.BREX	RHH-><-SIGMA+OB-Fold+AAA-ATPase+HKD-DNase-BrxL<-PglZ<-?<-SF1-SF2-Helicase<-?<-wHTH+WYL+WCX-p<-N6-METHYLASE<-REase+DUF5655<-DUF6079*<-BrxB-BrxF-iSTAND<-BrxA-DNA-binding<-HTH_3<-Phage_integrase+Phage_integrase||?->Radical-SAM->	1168	Bacteria>FCB_group>Bacteroidetes/Chlorobi_group>Bacteroidota>Flavobacteriia>Flavobacteriales	GCF_900188235.1	Lutibacter_agarilyticus	SAMN06265371_101285	359460..362966	1109740	BREX-system-P-loop-protein-BrxC-[Lutibacter-agarilyticus]
WP_089376855.1	Type1.BREX	<-GDResolvase+Recombinase+ZnR||?-><-?<-?<-Radical-SAM<-?||Phage_integrase+Phage_integrase->BrxA-DNA-binding->BrxB-BrxF-iSTAND->DUF6079*->REase+DUF5655->N6-METHYLASE->?->?->?->?->SF1-SF2-Helicase->?->METHYLASE->	1165	Bacteria>FCB_group>Bacteroidetes/Chlorobi_group>Bacteroidota>Flavobacteriia>Flavobacteriales	GCF_900188355.1	Lutibacter_flavus	CHB63_RS02565	583517..587014	691689	BREX-system-P-loop-protein-BrxC-[Lutibacter-flavus]
WP_213195478.1	Type1.BREX	TraI-VirD2-RCRE->?->TRPR-HTH->STYKinase-like->wHTH+WYL+WCX->ZnR+CC+REase->HIRAN->BrxA-DNA-binding->BrxB-BrxF-iSTAND->DUF6079*->N6-METHYLASE->HTH_3->Couple_hipA+HipA_C->PglZ->SIGMA+OB-Fold+AAA-ATPase+HKD-DNase-BrxL->?->?->?->wHTH+WYL+WCX->	1176	Bacteria>FCB_group>Bacteroidetes/Chlorobi_group>Bacteroidota>Flavobacteriia>Flavobacteriales	GCF_907163105.1	Cloacibacterium_caeni	KKQ76_RS01235	263131..266661	2004710	BREX-system-P-loop-protein-BrxC-[Cloacibacterium-caeni]
WP_271393637.1	Type1.BREX	<-ParB+HNH<-PIWI<-SIGMA+OB-Fold+AAA-ATPase+HKD-DNase-BrxL<-PglZ<-N6-METHYLASE<-DUF6079*<-BrxB-BrxF-iSTAND<-BrxA-DNA-binding<-HTH<-?<-?<-XIS-HTH<-XIS-HTH<-?<-Arm-DNA-bind_5+Phage_int_SAM_5+Phage_integrase	1168	Bacteria>FCB_group>Bacteroidetes/Chlorobi_group>Bacteroidota>Flavobacteriia>Flavobacteriales	GCF_947492035.1	Aequorivita_sinensis	PQR58_RS10295	126765..130271	1382458	BREX-system-P-loop-protein-BrxC-[Aequorivita-sinensis]
WP_271393758.1	Type1.BREX	<-SIGMA+OB-Fold+AAA-ATPase+HKD-DNase-BrxL<-PglZ<-?<-SF1-SF2-Helicase<-?<-N6-METHYLASE<-ParB+HNH<-DUF6079*<-BrxB-BrxF-iSTAND<-BrxA-DNA-binding<-?<-STYKinase-like<-TRPR-HTH<-?<-Classical-AAA<-HTH_36<-XIS-HTH	1165	Bacteria>FCB_group>Bacteroidetes/Chlorobi_group>Bacteroidota>Flavobacteriia>Flavobacteriales	GCF_947492035.1	Aequorivita_sinensis	PQR58_RS11110	23754..27251	1382458	BREX-system-P-loop-protein-BrxC-[Aequorivita-sinensis]
WP_272023608.1	Type1.BREX	<-ParB+HNH<-PglZ<-METHYLASE<-?<-SF1-SF2-Helicase<-?<-TPR+REase<-?<-N6-METHYLASE<-DUF6079*<-BrxB-BrxF-iSTAND<-BrxA-DNA-binding<-HTH_3<-?<-?<-?<-?<-ParA-Soj-PloopNTPase<-Relaxase	1160	Bacteria>FCB_group>Bacteroidetes/Chlorobi_group>Bacteroidota>Flavobacteriia>Flavobacteriales	GCF_947496445.1	Olleya_namhaensis	PQG05_RS12495	26845..30327	1144750	BREX-system-P-loop-protein-BrxC-[Olleya-namhaensis]
MBK6861507.1	Type1.BREX	<-METHYLASE-p||?-><-MACRODOMAIN<-ART-DarT<-SF1-SF2-Helicase<-PIWI<-SIGMA+OB-Fold+AAA-ATPase+Lon-Protease-BrxL<-PglZ<-DUF6079*<-BrxB-BrxF-iSTAND<-BrxA-DNA-binding<-HTH<-?<-?<-Arm-DNA-bind_5+Phage_int_SAM_5+Phage_integrase<-XIS-HTH||?-><-sGTP	1144	Bacteria>FCB_group>Bacteroidetes/Chlorobi_group>Bacteroidota>Saprospiria>Saprospirales	GCA_016702565.1	Saprospiraceae_bacterium	IPG95_14640	1781325..1784759	2202734	BREX-system-P-loop-protein-BrxC-[Saprospiraceae-bacterium]
MBK8736252.1	Type1.BREX	<-N6-METHYLASE-p<-N6-METHYLASE-p<-DUF6079-p*<-?<-?<-BrxB-BrxF-iSTAND<-BrxA-DNA-binding<-?<-XIS-HTH<-?<-Arm-DNA-bind_5+Phage_int_SAM_5+Phage_integrase	919	Bacteria>FCB_group>Bacteroidetes/Chlorobi_group>Bacteroidota>Saprospiria>Saprospirales	GCA_016710585.1	Saprospiraceae_bacterium	IPL98_10255	1583..4342	2202734	BREX-system-P-loop-protein-BrxC-[Saprospiraceae-bacterium]
MBK8109385.1	Type1.BREX	<-PglZ<-PglZ<-AbiEii<-AbiEi_4||?-><-DUF6079-p<-DUF6079-p<-DUF6079-p*<-BrxB-BrxF-iSTAND<-BrxA-DNA-binding<-S1COLD<-S1COLD<-?<-Glyco_hydro_38<-DOC+HTH<-?||SSB->	128	Bacteria>FCB_group>Bacteroidetes/Chlorobi_group>Bacteroidota>Saprospiria>Saprospirales	GCA_016712925.1	Saprospiraceae_bacterium	IPK46_03100	78067..78453	2202734	hypothetical-protein-[Saprospiraceae-bacterium]
MBK8623789.1	Type1.BREX	<-AraC-HTH<-?<-?<-PIWI<-SIGMA+OB-Fold+AAA-ATPase+Lon-Protease-BrxL<-PglZ<-AbiEii<-AbiEi_4<-N6-METHYLASE<-DUF6079*<-BrxB-BrxF-iSTAND<-BrxA-DNA-binding<-HTH<-?<-XIS-HTH<-?<-Arm-DNA-bind_5+Phage_int_SAM_5+Phage_integrase<-XIS-HTH<-sGTP	1197	Bacteria>FCB_group>Bacteroidetes/Chlorobi_group>Bacteroidota>Saprospiria>Saprospirales	GCA_016714895.1	Saprospiraceae_bacterium	IPN79_19055	313073..316666	2202734	BREX-system-P-loop-protein-BrxC-[Saprospiraceae-bacterium]
MBK8854955.1	Type1.BREX	<-Arm-DNA-bind_5+Phage_int_SAM_5+Phage_integrase||Phage_int_SAM_5+Phage_integrase->?->?->IG+IG->?->HTH->BrxA-DNA-binding->BrxB-BrxF-iSTAND->DUF6079-p*->DUF6079-p->DUF6079-p->?->N6-METHYLASE->AbiEi_4->AbiEii->PglZ->SH3->	155	Bacteria>FCB_group>Bacteroidetes/Chlorobi_group>Bacteroidota>Saprospiria>Saprospirales	GCA_016715785.1	Saprospiraceae_bacterium	IPN10_12845	55975..56442	2202734	hypothetical-protein-[Saprospiraceae-bacterium]
MBK8854956.1	Type1.BREX	Phage_int_SAM_5+Phage_integrase->?->?->IG+IG->?->HTH->BrxA-DNA-binding->BrxB-BrxF-iSTAND->DUF6079-p->DUF6079-p*->DUF6079-p->?->N6-METHYLASE->AbiEi_4->AbiEii->PglZ->SH3-><-?<-XIS-HTH	772	Bacteria>FCB_group>Bacteroidetes/Chlorobi_group>Bacteroidota>Saprospiria>Saprospirales	GCA_016715785.1	Saprospiraceae_bacterium	IPN10_12850	56456..58774	2202734	hypothetical-protein-[Saprospiraceae-bacterium]
MBK9103294.1	Type1.BREX	<-CR-Kinase+Classical-AAA<-?<-?<-?<-?||SF1-SF2-Helicase->BrxA-DNA-binding->BrxB-BrxF-iSTAND->DUF6079-p*->DUF6079-p->N6-METHYLASE-><-?<-?||HTH_3->Couple_hipA->HipA_C->PglZ->SIGMA+OB-Fold+AAA-ATPase+Lon-Protease-BrxL->	591	Bacteria>FCB_group>Bacteroidetes/Chlorobi_group>Bacteroidota>Saprospiria>Saprospirales	GCA_016716665.1	Saprospiraceae_bacterium	IPL92_01655	365771..367546	2202734	BREX-system-P-loop-protein-BrxC-[Saprospiraceae-bacterium]
MBK9689678.1	Type1.BREX	<-DUF6079-p<-?<-DUF6079-p<-DUF6079-p*<-BrxB-BrxF-iSTAND<-BrxA-DNA-binding<-BrxA-DNA-binding-p<-S1COLD<-S1COLD<-?<-Glyco_hydro_38	236	Bacteria>FCB_group>Bacteroidetes/Chlorobi_group>Bacteroidota>Saprospiria>Saprospirales	GCA_016717655.1	Saprospiraceae_bacterium	IP065_18840	1629..2339	2202734	hypothetical-protein-[Saprospiraceae-bacterium]
MBL0081467.1	Type1.BREX	<-PglZ<-PglZ<-?<-?<-AbiEii<-?<-?<-DUF6079-p<-DUF6079-p*<-BrxB-BrxF-iSTAND<-BrxA-DNA-binding-p<-BrxA-DNA-binding-p<-S1COLD<-?<-Glyco_hydro_38<-?<-Glyco_hydro_38	143	Bacteria>FCB_group>Bacteroidetes/Chlorobi_group>Bacteroidota>Saprospiria>Saprospirales	GCA_016721645.1	Saprospiraceae_bacterium	IPP37_03060	15607..16038	2202734	hypothetical-protein-[Saprospiraceae-bacterium]
MBK9994339.1	Type1.BREX	<-N6-METHYLASE-p<-DUF6079*<-BrxB-BrxF-iSTAND<-BrxA-DNA-binding<-?<-XIS-HTH<-?<-Arm-DNA-bind_5+Phage_int_SAM_5+Phage_integrase<-XIS-HTH<-sGTP	1197	Bacteria>FCB_group>Bacteroidetes/Chlorobi_group>Bacteroidota>Saprospiria>Saprospirales	GCA_016722295.1	Saprospiraceae_bacterium	IPP01_10235	1480..5073	2202734	BREX-system-P-loop-protein-BrxC-[Saprospiraceae-bacterium]
MBP6234856.1	Type1.BREX	HNH->wHTH+WYL+WCX->NERD+ZnR-><-ArdCN+MPTase<-wHTH+WYL+WCX||REase+RNase->BrxA-DNA-binding->BrxB-BrxF-iSTAND->DUF6079*->N6-METHYLASE->?->?->?->?->?->?-><-ACET	1152	Bacteria>FCB_group>Bacteroidetes/Chlorobi_group>Bacteroidota>Saprospiria>Saprospirales	GCA_017986015.1	Saprospiraceae_bacterium	KA536_01890	136824..140282	2202734	BREX-system-P-loop-protein-BrxC-[Saprospiraceae-bacterium]
MCB0517804.1	Type1.BREX	<-SIGMA+OB-Fold+AAA-ATPase+HKD-DNase-BrxL<-PglZ<-N6-METHYLASE<-DUF6079*<-BrxB-BrxF-iSTAND<-BrxA-DNA-binding<-?<-HNH||REC<-?<-?<-SF1-SF2-Helicase+RNase<-?<-RVT	1174	Bacteria>FCB_group>Bacteroidetes/Chlorobi_group>Bacteroidota>Saprospiria>Saprospirales	GCA_020436325.1	Saprospiraceae_bacterium	KDD27_02635	9612..13136	2202734	BREX-system-P-loop-protein-BrxC-[Saprospiraceae-bacterium]
MCB0520339.1	Type1.BREX	URI-DNase-><-SIGMA+OB-Fold+AAA-ATPase+Lon-Protease-BrxL<-PglZ<-McrC-NTD+REase<-McrB-AAA<-N6-METHYLASE||CR-ATPase5+REase-><-DUF6079*<-BrxB-BrxF-iSTAND<-BrxA-DNA-binding<-tRNA||?-><-?<-DTXR-HTH<-?<-Peptidase_M29	1145	Bacteria>FCB_group>Bacteroidetes/Chlorobi_group>Bacteroidota>Saprospiria>Saprospirales	GCA_020436325.1	Saprospiraceae_bacterium	KDD27_15480	21267..24704	2202734	BREX-system-P-loop-protein-BrxC-[Saprospiraceae-bacterium]
MCB0524505.1	Type1.BREX	SF1-SF2-Helicase+RNase+RNase->?->REase+35exo->?->?->BrxA-DNA-binding->BrxB-BrxF-iSTAND->DUF6079-p->DUF6079*->N6-METHYLASE->?->McrB-NTD+HNH->Polbeta->?->CR-ATPase5+REase->PglZ->SIGMA+OB-Fold+AAA-ATPase+HKD-DNase-BrxL->	1094	Bacteria>FCB_group>Bacteroidetes/Chlorobi_group>Bacteroidota>Saprospiria>Saprospirales	GCA_020436385.1	Saprospiraceae_bacterium	KDC86_08270	14975..18259	2202734	BREX-system-P-loop-protein-BrxC-[Saprospiraceae-bacterium]
MCB0625399.1	Type1.BREX	ACET->?->XIS-HTH->BrxA-DNA-binding->BrxB-BrxF-iSTAND->DUF6079*->N6-METHYLASE->McrB-AAA->REase->DUF4007->DnaA->CR-ATPase9->PglZ->SIGMA+OB-Fold+AAA-ATPase+Lon-Protease-BrxL->	1151	Bacteria>FCB_group>Bacteroidetes/Chlorobi_group>Bacteroidota>Saprospiria>Saprospirales	GCA_020436745.1	Saprospiraceae_bacterium	KDC43_16150	4651..8106	2202734	BREX-system-P-loop-protein-BrxC-[Saprospiraceae-bacterium]
MCB9352089.1	Type1.BREX	<-PglZ<-?<-?<-OLD-ABC<-S1COLD<-?<-Polbeta<-?<-?<-DUF6079*<-BrxB-BrxF-iSTAND<-BrxA-DNA-binding<-SF1-SF2-Helicase<-RNase<-?<-?<-SF1-SF2-Helicase+REase||NtrC-AAA+FIS-HTH->	1176	Bacteria>FCB_group>Bacteroidetes/Chlorobi_group>Bacteroidota>Saprospiria>Saprospirales	GCA_020636095.1	Lewinellaceae_bacterium	H6573_32040	43547..47077	2760807	BREX-system-P-loop-protein-BrxC-[Lewinellaceae-bacterium]
MCB9352382.1	Type1.BREX	HMA+HAD+HAD->?-><-?||?-><-?<-NtrC-AAA+FIS-HTH||?->BrxA-DNA-binding->BrxB-BrxF-iSTAND->DUF6079*->N6-METHYLASE->PglZ->SIGMA+OB-Fold+AAA-ATPase+Lon-Protease-BrxL->RVT->?->?-><-?<-Glyco_hydro_130	1214	Bacteria>FCB_group>Bacteroidetes/Chlorobi_group>Bacteroidota>Saprospiria>Saprospirales	GCA_020636095.1	Lewinellaceae_bacterium	H6573_33575	34149..37793	2760807	BREX-system-P-loop-protein-BrxC-[Lewinellaceae-bacterium]
MCB9053313.1	Type1.BREX	<-PglZ<-TOPRIM<-OLD-ABC<-?<-NACHT<-?<-REase<-TPR+S1<-NERD+SF1-SF2-Helicase<-DUF6079*<-BrxB-BrxF-iSTAND<-BrxA-DNA-binding<-?<-RNase<-SF1-SF2-Helicase+REase||NtrC-AAA+FIS-HTH->OLD-ABC->TOPRIM->	1180	Bacteria>FCB_group>Bacteroidetes/Chlorobi_group>Bacteroidota>Saprospiria>Saprospirales	GCA_020636405.1	Lewinellaceae_bacterium	H6556_28080	325530..329072	2760807	BREX-system-P-loop-protein-BrxC-[Lewinellaceae-bacterium]
WP_013769021.1	Type1.BREX	CR-Kinase+Classical-AAA->?-><-SIGMA+OB-Fold+AAA-ATPase+Lon-Protease-BrxL<-PglZ<-McrC-NTD+REase<-McrB-AAA<-N6-METHYLASE<-DUF6079*<-BrxB-BrxF-iSTAND<-BrxA-DNA-binding<-?<-McrB-NTD<-MACRODOMAIN<-DarT+Peptidase	1144	Bacteria>FCB_group>Bacteroidetes/Chlorobi_group>Bacteroidota>Saprospiria>Saprospirales	GCF_000212735.1	Haliscomenobacter_hydrossis	HALHY_RS33450	27488..30922	2350	BREX-system-P-loop-protein-BrxC-[Haliscomenobacter-hydrossis]
WP_020534907.1	Type1.BREX	N6-METHYLASE->?-><-?<-?||?->?->HTH_3->BrxA-DNA-binding->BrxB-BrxF-iSTAND->DUF6079*->N6-METHYLASE->OLD-ABC->TOPRIM->?->HAD+PRTase+PRTase+REase+SF1-SF2-Helicase->?->PglZ->SIGMA+OB-Fold+AAA-ATPase+HKD-DNase-BrxL->Peptidase->	1159	Bacteria>FCB_group>Bacteroidetes/Chlorobi_group>Bacteroidota>Saprospiria>Saprospirales	GCF_000379805.1	Lewinella_cohaerens	A3EU_RS29375	116498..119977	70995	BREX-system-P-loop-protein-BrxC-[Lewinella-cohaerens]
WP_044220103.1	Type1.BREX	<-HTH_3||?->?->?->NtrC-AAA+FIS-HTH||SF1-SF2-Helicase+REase->RNase->BrxA-DNA-binding->BrxB-BrxF-iSTAND->DUF6079*->N6-METHYLASE->?->REase->HTH+rve->AAA-ATPase->HTH_3->DDE_Tnp_1_4-><-?||SbcC->	1186	Bacteria>FCB_group>Bacteroidetes/Chlorobi_group>Bacteroidota>Saprospiria>Saprospirales	GCF_000759025.1	Phaeodactylibacter_xiamenensis	IX84_RS10880	11358..14918	1524460	BREX-system-P-loop-protein-BrxC-[Phaeodactylibacter-xiamenensis]
WP_147165921.1	Type1.BREX	<-HTH_3<-REase||?->PIN-><-?<-HTH_3<-HigB<-REase<-N6-METHYLASE<-DUF6079*<-BrxB-BrxF-iSTAND<-BrxA-DNA-binding<-REase<-SF1-SF2-Helicase+REase||NtrC-AAA+FIS-HTH-><-?<-?<-?||HTH_3->	1186	Bacteria>FCB_group>Bacteroidetes/Chlorobi_group>Bacteroidota>Saprospiria>Saprospirales	GCF_007993045.1	Phaeodactylibacter_luteus	FRY97_RS02895	87702..91262	1564516	BREX-system-P-loop-protein-BrxC-[Phaeodactylibacter-luteus]
WP_147932457.1	Type1.BREX	Calcineurin->RADICAL-SAM->METHYLASE+RNA-Helicase->?->?->DnaJ->BrxA-DNA-binding->BrxB-BrxF-iSTAND->DUF6079*->?->N6-METHYLASE->?->HNH->?->URI-DNase->wHTH+AbiEi_2->AbiEii->PglZ->	1198	Bacteria>FCB_group>Bacteroidetes/Chlorobi_group>Bacteroidota>Saprospiria>Saprospirales	GCF_008014675.1	Neolewinella_aurantiaca	FUA23_RS19525	18155..21751	2602767	BREX-system-P-loop-protein-BrxC-[Neolewinella-aurantiaca]
WP_168040578.1	Type1.BREX	<-REC+LYTTR<-HisKin<-Peptidase_M3||?-><-?||BrxA-DNA-binding->BrxB-BrxF-iSTAND->DUF6079*->N6-METHYLASE->HTH+DOC->PglZ->SIGMA+OB-Fold+AAA-ATPase+HKD-DNase-BrxL->	1211	Bacteria>FCB_group>Bacteroidetes/Chlorobi_group>Bacteroidota>Saprospiria>Saprospirales	GCF_011927835.1	Neolewinella_antarctica	GGR27_RS19565	12164..15799	442734	BREX-system-P-loop-protein-BrxC-[Neolewinella-antarctica]
WP_236977640.1	Type1.BREX	RNase+DSRBD->?-><-?<-?||tRNA->BrxA-DNA-binding->BrxB-BrxF-iSTAND->DUF6079*->?->N6-METHYLASE->MORC+DUF3684+HNH->PglZ->SIGMA+OB-Fold+AAA-ATPase+HKD-DNase-BrxL-><-?||?->?->PIN->	1159	Bacteria>FCB_group>Bacteroidetes/Chlorobi_group>Bacteroidota>Saprospiria>Saprospirales	GCF_021729145.1	Membranihabitans_sp._C281	LVD47_RS14080	21527..25006	2904244	BREX-system-P-loop-protein-BrxC-[Membranihabitans-maritimus]
OWY22675.1	Type1.BREX	<-N6-METHYLASE<-?<-DUF6079*<-BrxB-BrxF-iSTAND<-BrxA-DNA-binding<-?<-?<-HAD<-?<-?<-?||DnaJ->	1168	Bacteria>FCB_group>Bacteroidetes/Chlorobi_group>Bacteroidota>Sphingobacteriia>Sphingobacteriales	GCA_002212725.1	Sphingobacteriales_bacterium_TSM_CSM	BVG80_10190	6474..9980	1932005	hypothetical-protein-BVG80_10190-[Sphingobacteriales-bacterium-TSM_CSM]
OYD42847.1	Type1.BREX	wHTH+WYL+WCX-><-?<-?||?-><-?||sGTP->HTH_3->BrxA-DNA-binding->BrxB-BrxF-iSTAND->DUF6079*->N6-METHYLASE->ParB+HNH->PglZ->SIGMA+OB-Fold+AAA-ATPase+HKD-DNase-BrxL->	1175	Bacteria>FCB_group>Bacteroidetes/Chlorobi_group>Bacteroidota>Sphingobacteriia>Sphingobacteriales	GCA_002245855.1	Sphingobacterium_cellulitidis	CHT99_08530	557163..560690	1768011	hypothetical-protein-CHT99_08530-[Sphingobacterium-cellulitidis]
QQS30389.1	Type1.BREX	<-SIGMA+OB-Fold+AAA-ATPase+Lon-Protease-BrxL<-?<-PglZ<-AbiEii<-AbiEi_4<-?<-N6-METHYLASE<-Four-helical-protein<-DUF6079*<-BrxB-BrxF-iSTAND<-BrxA-DNA-binding<-HTH||ABhydrolase-><-MerR-HTH	1202	Bacteria>FCB_group>Bacteroidetes/Chlorobi_group>Bacteroidota>Sphingobacteriia>Sphingobacteriales	GCA_016700115.1	Sphingobacteriales_bacterium	IPM47_05415	1549850..1553458	2044944	BREX-system-P-loop-protein-BrxC-[Sphingobacteriales-bacterium]
WP_157278947.1	Type1.BREX	Arm-DNA-bind_5+Phage_int_SAM_5+Phage_integrase->?->XIS-HTH->TraJ-RHH->?->?->HTH_3->BrxA-DNA-binding->BrxB-BrxF-iSTAND->DUF6079*->N6-METHYLASE->Schlafen+HATPase+wHTH->PglZ->SIGMA+OB-Fold+AAA-ATPase+HKD-DNase-BrxL->?-><-HTH_21+rve+rve_3	1169	Bacteria>FCB_group>Bacteroidetes/Chlorobi_group>Bacteroidota>Sphingobacteriia>Sphingobacteriales	GCF_000427965.1	Olivibacter_sitiensis	A37S_RS0120635	7227..10736	376470	BREX-system-P-loop-protein-BrxC-[Olivibacter-sitiensis]
WP_094282244.1	Type1.BREX	<-SIGMA+OB-Fold+AAA-ATPase+HKD-DNase-BrxL<-PglZ<-ParB+HNH<-N6-METHYLASE<-N6-METHYLASE-p<-DUF6079*<-BrxB-BrxF-iSTAND<-BrxA-DNA-binding<-HTH_3<-sGTP||?-><-?||SIGMA-HTH->	1175	Bacteria>FCB_group>Bacteroidetes/Chlorobi_group>Bacteroidota>Sphingobacteriia>Sphingobacteriales	GCF_002245765.1	Sphingobacterium_cellulitidis	CHU00_RS15775	18381..21908	1768011	BREX-system-P-loop-protein-BrxC-[Sphingobacterium-cellulitidis]
WP_070561019.1	Type1.BREX	TPR+TPR+TPR+TPR+TPR->?->ANK->ANK+ANK+ANK-><-?<-SIGMA+OB-Fold+AAA-ATPase+HKD-DNase-BrxL<-PglZ<-HTH+REase<-N6-METHYLASE<-DUF6079*<-BrxB-BrxF-iSTAND<-BrxA-DNA-binding<-HTH_3<-?||?->XIS-HTH->HTH_36->?->Relaxase->	1187	Bacteria>FCB_group>Bacteroidetes/Chlorobi_group>Bacteroidota>Sphingobacteriia>Sphingobacteriales	GCF_002339225.1	Sphingobacterium_sp._HMSC13C05	HMPREF3127_RS01230	95716..99279	1581095	BREX-system-P-loop-protein-BrxC-[Sphingobacterium-sp.-HMSC13C05]
WP_165305702.1	Type1.BREX	SLATT_1->?->HEPN+REase->?->?->HTH_3->BrxA-DNA-binding->BrxB-BrxF-iSTAND->DUF6079*->N6-METHYLASE->HTH+REase->PglZ->SIGMA+OB-Fold+AAA-ATPase+HKD-DNase-BrxL-><-?<-HTH_3	1187	Bacteria>FCB_group>Bacteroidetes/Chlorobi_group>Bacteroidota>Sphingobacteriia>Sphingobacteriales	GCF_011064245.1	Sphingobacterium_sp._DR205	G6053_RS05210	1244063..1247626	2713573	BREX-system-P-loop-protein-BrxC-[Sphingobacterium-sp.-DR205]
WP_200066585.1	Type1.BREX	TrwC+SF1-SF2-Helicase->?->SF1-SF2-Helicase+REase->ParB->HTH->BrxA-DNA-binding->BrxB-BrxF-iSTAND->DUF6079*->N6-METHYLASE->PglZ->SIGMA+OB-Fold+AAA-ATPase+Lon-Protease-BrxL->?-><-?||?-><-?||SIGMA-HTH->	1199	Bacteria>FCB_group>Bacteroidetes/Chlorobi_group>Bacteroidota>Sphingobacteriia>Sphingobacteriales	GCF_016522075.1	Mucilaginibacter_segetis	I5M19_RS12090	547285..550884	2793071	BREX-system-P-loop-protein-BrxC-[Mucilaginibacter-segetis]
WP_090698254.1	Type1.BREX	<-HTH_19||?->?-><-?||ANK+ANK->BrxA-DNA-binding->BrxB-BrxF-iSTAND->DUF6079*->N6-METHYLASE->PglZ->SIGMA+OB-Fold+AAA-ATPase+HKD-DNase-BrxL->?-><-PHP+SbcC	1169	Bacteria>FCB_group>Bacteroidetes/Chlorobi_group>Bacteroidota>Sphingobacteriia>Sphingobacteriales	GCF_900103545.1	Daejeonella_rubra	BLR27_RS01455	316504..320013	990371	BREX-system-P-loop-protein-BrxC-[Daejeonella-rubra]
MCE1168906.1	Type1.BREX	<-N6-METHYLASE<-Four-helical-protein<-DUF6079*<-BrxB-BrxF-iSTAND<-BrxA-DNA-binding<-HTH||<-?<-SIGMA+OB-Fold+AAA-ATPase+Lon-Protease-BrxL	1202	Bacteria>FCB_group>Bacteroidetes/Chlorobi_group>Bacteroidota>Sphingobacteriia>unclassified_Sphingobacteriia	GCA_021286705.1	Sphingobacteriia_bacterium	LWX70_12500	2695..6303	2035813	BREX-system-P-loop-protein-BrxC-[Sphingobacteriia-bacterium]
KPP94735.1	Type1.BREX	SAM-METHYLASE->tRNA-><-SIGMA+OB-Fold+AAA-ATPase+HKD-DNase-BrxL<-PglZ<-Schlafen+HATPase+wHTH<-N6-METHYLASE<-?<-DUF6079*<-BrxB-BrxF-iSTAND<-BrxA-DNA-binding<-?<-SF1-SF2-Helicase<-?<-?<-DnaB+SMS-KaiC<-HTH_36<-XIS-HTH	1203	Bacteria>FCB_group>Bacteroidetes/Chlorobi_group>Bacteroidota>unclassified_Bacteroidota	GCA_001314545.1	Bacteroidetes_bacterium_HLUCCA01	HLUCCA01_08750	188157..191768	1666909	Brex-phage-defense-system-ATPase-BrxC-[Bacteroidetes-bacterium-HLUCCA01]
OPZ99879.1	Type1.BREX	<-DUF6079*<-BrxB-BrxF-iSTAND<-BrxA-DNA-binding<-tRNA||?->?->?->?->Aminotran_1_2-><-?||Glyco_hydro_15->	1147	Bacteria>FCB_group>Bacteroidetes/Chlorobi_group>Bacteroidota>unclassified_Bacteroidota	GCA_002069345.1	Bacteroidetes_bacterium_ADurb.Bin416	BWY72_00109	505..3948	1852814	hypothetical-protein-BWY72_00109-[Bacteroidetes-bacterium-ADurb.Bin416]
PKP36801.1	Type1.BREX	<-HDRnase||BrxA-DNA-binding->BrxB-BrxF-iSTAND->DUF6079*->N6-METHYLASE-p->	1199	Bacteria>FCB_group>Bacteroidetes/Chlorobi_group>Bacteroidota>unclassified_Bacteroidota	GCA_002842655.1	Bacteroidetes_bacterium_HGW-Bacteroidetes-14	CVT97_08015	1837..5436	2013682	BREX-system-P-loop-protein-BrxC-[Bacteroidetes-bacterium-HGW-Bacteroidetes-14]
PSR13219.1	Type1.BREX	<-DUF6079*<-BrxB-BrxF-iSTAND<-BrxA-DNA-binding<-?||?->REase->?-><-DOC+HTH<-?||ABhydrolase->	1045	Bacteria>FCB_group>Bacteroidetes/Chlorobi_group>Bacteroidota>unclassified_Bacteroidota	GCA_003023605.1	Bacteroidota_bacterium	C7N36_08840	1..3135	1898104	BREX-system-P-loop-protein-BrxC,-partial-[Bacteroidota-bacterium]
PTM08477.1	Type1.BREX	DOC+HTH-><-?<-REase<-?||?->BrxA-DNA-binding->BrxB-BrxF-iSTAND->DUF6079*->N6-METHYLASE-p->	1159	Bacteria>FCB_group>Bacteroidetes/Chlorobi_group>Bacteroidota>unclassified_Bacteroidota	GCA_003045895.1	Bacteroidota_bacterium	DA408_20510	6731..10210	1898104	BREX-system-P-loop-protein-BrxC-[Bacteroidota-bacterium]
KAF0128492.1	Type1.BREX	<-SIGMA+OB-Fold+AAA-ATPase+Lon-Protease-BrxL<-PglZ<-McrC-NTD+REase<-McrB-AAA<-Schlafen+HATPase+wHTH<-N6-METHYLASE<-DUF6079*<-BrxB-BrxF-iSTAND<-BrxA-DNA-binding	1144	Bacteria>FCB_group>Bacteroidetes/Chlorobi_group>Bacteroidota>unclassified_Bacteroidota	GCA_009773165.1	Bacteroidota_bacterium	FD155_3234	38823..42257	1898104	hypothetical-protein-FD155_3234-[Bacteroidota-bacterium]
NQT60364.1	Type1.BREX	DUF4007->LON-AAA->N6-METHYLASE->?->ACET->?->PIN->HATPase+wHTH->HTH+REase->DUF6079*->PglZ->SIGMA+OB-Fold+AAA-ATPase-BrxL->?->Y1_Tnp->	1348	Bacteria>FCB_group>Bacteroidetes/Chlorobi_group>Bacteroidota>unclassified_Bacteroidota	GCA_013202335.1	Bacteroidota_bacterium	HQ557_15395	13554..17600	1898104	hypothetical-protein-[Bacteroidota-bacterium]
NUQ82570.1	Type1.BREX	<-GspA-ATPase<-TrkH<-?<-?||?->BrxA-DNA-binding->BrxB-BrxF-iSTAND->DUF6079*->N6-METHYLASE->Schlafen+HATPase+wHTH->PglZ->SIGMA+OB-Fold+AAA-ATPase+Lon-Protease-BrxL->DSBH+AraC-HTH+AraC-HTH->?->?->?->?-><-ABhydrolase	1197	Bacteria>FCB_group>Bacteroidetes/Chlorobi_group>Bacteroidota>unclassified_Bacteroidota	GCA_013360915.1	Bacteroidota_bacterium	HUU10_13230	47380..50973	1898104	BREX-system-P-loop-protein-BrxC-[Bacteroidota-bacterium]
MBK7107567.1	Type1.BREX	<-REase<-SIGMA+OB-Fold+AAA-ATPase+Lon-Protease-BrxL<-PglZ<-DUF6079*<-BrxB-BrxF-iSTAND<-BrxA-DNA-binding<-HTH<-?<-XIS-HTH<-?<-?<-Phage_integrase<-Arm-DNA-bind_5+Phage_int_SAM_5	1197	Bacteria>FCB_group>Bacteroidetes/Chlorobi_group>Bacteroidota>unclassified_Bacteroidota	GCA_016708205.1	Bacteroidota_bacterium	IPH61_00035	12776..16369	1898104	BREX-system-P-loop-protein-BrxC-[Bacteroidota-bacterium]
MBK7967322.1	Type1.BREX	<-N6-METHYLASE-p<-N6-METHYLASE-p<-N6-METHYLASE-p<-?<-DUF6079-p<-DUF6079-p<-?<-DUF6079-p<-DUF6079-p*<-BrxB-BrxF-iSTAND	197	Bacteria>FCB_group>Bacteroidetes/Chlorobi_group>Bacteroidota>unclassified_Bacteroidota	GCA_016713445.1	Bacteroidota_bacterium	IPK10_19950	46949..47542	1898104	hypothetical-protein-[Bacteroidota-bacterium]
MBL0279470.1	Type1.BREX	sGTP->XIS-HTH->Arm-DNA-bind_5+Phage_int_SAM_5+Phage_integrase->?->XIS-HTH->?->HTH->BrxA-DNA-binding->BrxB-BrxF-iSTAND->DUF6079*->PglZ->SIGMA+OB-Fold+AAA-ATPase+Lon-Protease-BrxL->REase+DUF5655->PIWI->	1197	Bacteria>FCB_group>Bacteroidetes/Chlorobi_group>Bacteroidota>unclassified_Bacteroidota	GCA_016720495.1	Bacteroidota_bacterium	IPQ11_03885	937205..940798	1898104	BREX-system-P-loop-protein-BrxC-[Bacteroidota-bacterium]
MBM3454071.1	Type1.BREX	N6-METHYLASE->BrxA-DNA-binding->BrxB-BrxF-iSTAND->DUF6079*->N6-METHYLASE->PglZ->SIGMA+OB-Fold+AAA-ATPase+Lon-Protease-BrxL-><-HTH_21+rve+rve_3||HIN-HTH->	1144	Bacteria>FCB_group>Bacteroidetes/Chlorobi_group>Bacteroidota>unclassified_Bacteroidota	GCA_016868905.1	Bacteroidota_bacterium	FJX80_02865	4277..7711	1898104	BREX-system-P-loop-protein-BrxC-[Bacteroidota-bacterium]
MBN8706499.1	Type1.BREX	Phage_int_SAM_4+Phage_integrase->35exo+URI-DNase->tRNA->HTH->BrxA-DNA-binding->BrxB-BrxF-iSTAND->DUF6079*->OLD-ABC->HNH->N6-METHYLASE->?->Schlafen+HATPase+wHTH->PglZ->SIGMA+OB-Fold+AAA-ATPase+Lon-Protease-BrxL->SbcC->	1173	Bacteria>FCB_group>Bacteroidetes/Chlorobi_group>Bacteroidota>unclassified_Bacteroidota	GCA_017303995.1	Bacteroidota_bacterium	JOL62_11545	8364..11885	1898104	BREX-system-P-loop-protein-BrxC-[Bacteroidota-bacterium]
MBS1911241.1	Type1.BREX	<-SIGMA+OB-Fold+AAA-ATPase+Lon-Protease-BrxL<-PglZ<-HNH<-OLD-ABC<-ParB+HNH<-N6-METHYLASE<-DUF6079*<-BrxB-BrxF-iSTAND<-BrxA-DNA-binding<-tRNA<-?||?-><-?||SIGMA-HTH->	1145	Bacteria>FCB_group>Bacteroidetes/Chlorobi_group>Bacteroidota>unclassified_Bacteroidota	GCA_018266795.1	Bacteroidota_bacterium	JST22_04585	55920..59357	1898104	BREX-system-P-loop-protein-BrxC-[Bacteroidota-bacterium]
MBT4727235.1	Type1.BREX	REase->?->?->?->HEPN-><-Phage_integrase||BrxA-DNA-binding->BrxB-BrxF-iSTAND->DUF6079-p*->	481	Bacteria>FCB_group>Bacteroidetes/Chlorobi_group>Bacteroidota>unclassified_Bacteroidota	GCA_018658345.1	Bacteroidota_bacterium	HOB88_02235	7916..9358	1898104	BREX-system-P-loop-protein-BrxC-[Bacteroidota-bacterium]
MBT7826368.1	Type1.BREX	<-Phage_integrase||BrxA-DNA-binding->BrxB-BrxF-iSTAND->DUF6079*->N6-METHYLASE-p->	1165	Bacteria>FCB_group>Bacteroidetes/Chlorobi_group>Bacteroidota>unclassified_Bacteroidota	GCA_018675135.1	Bacteroidota_bacterium	HN600_07220	2065..5562	1898104	BREX-system-P-loop-protein-BrxC-[Bacteroidota-bacterium]
MBU2649683.1	Type1.BREX	<-DUF6079*<-BrxB-BrxF-iSTAND<-BrxA-DNA-binding<-Arm-DNA-bind_5+Phage_int_SAM_5+Phage_integrase<-?||?-><-RFC-AAA+RuvB-AAA	1189	Bacteria>FCB_group>Bacteroidetes/Chlorobi_group>Bacteroidota>unclassified_Bacteroidota	GCA_018831055.1	Bacteroidota_bacterium	KKA81_02005	1..3566	1898104	BREX-system-P-loop-protein-BrxC-[Bacteroidota-bacterium]
MCB0536651.1	Type1.BREX	BrxA-DNA-binding->BrxB-BrxF-iSTAND->DUF6079*->N6-METHYLASE->PglZ->SIGMA+OB-Fold+AAA-ATPase+Lon-Protease-BrxL->REase+DUF5655->	1197	Bacteria>FCB_group>Bacteroidetes/Chlorobi_group>Bacteroidota>unclassified_Bacteroidota	GCA_020436205.1	Bacteroidota_bacterium	KDE33_03895	1012..4605	1898104	BREX-system-P-loop-protein-BrxC-[Bacteroidota-bacterium]
MCH7965547.1	Type1.BREX	<-Aminotran_3||?-><-?||?-><-SIGMA+OB-Fold+AAA-ATPase+HKD-DNase-BrxL<-PglZ<-HTH+REase<-N6-METHYLASE<-DUF6079*<-BrxB-BrxF-iSTAND<-BrxA-DNA-binding	1161	Bacteria>FCB_group>Bacteroidetes/Chlorobi_group>Bacteroidota>unclassified_Bacteroidota	GCA_022564295.1	Bacteroidota_bacterium	IH852_16610	28479..31964	1898104	BREX-system-P-loop-protein-BrxC-[Bacteroidota-bacterium]
MCR9155224.1	Type1.BREX	DUF4007->?->REase->SF1-SF2-Helicase+REase->BrxA-DNA-binding->BrxB-BrxF-iSTAND->DUF6079*->N6-METHYLASE->McrB-AAA->McrC-NTD+REase->PglZ->SIGMA+OB-Fold+AAA-ATPase+Lon-Protease-BrxL-><-RNase<-?<-SF1-SF2-Helicase+Classical-AAA<-wHTH+WYL+WCX	1141	Bacteria>FCB_group>Bacteroidetes/Chlorobi_group>Bacteroidota>unclassified_Bacteroidota	GCA_024742495.1	Bacteroidota_bacterium	NXI09_14035	83336..86761	1898104	BREX-system-P-loop-protein-BrxC-[Bacteroidota-bacterium]
MAO64836.1	Type1.BREX	Phage_int_SAM_4+Phage_integrase-><-?<-SIGMA+OB-Fold+AAA-ATPase+HKD-DNase-BrxL<-PglZ<-MORC+DUF3684+HNH<-REase+DUF4268<-Trypsin<-N6-METHYLASE<-SIGMA-HTH<-DUF6079*<-BrxB-BrxF-iSTAND<-REase<-SF1-SF2-Helicase+Classical-AAA<-?<-HNH	1168	Bacteria>FCB_group>Bacteroidetes/Chlorobi_group>Balneolota>Balneolia>Balneolales	GCA_002696035.1	Balneola_sp.	CL666_07525	664531..668037	2024824	hypothetical-protein-[Balneola-sp.]
TVQ65167.1	Type1.BREX	<-N6-METHYLASE-p<-AbiEi<-DUF6079*<-BrxB-BrxF-iSTAND<-BrxA-DNA-binding||HTH_3->Couple_hipA->HipA_C-><-?<-FGS	1164	Bacteria>FCB_group>Bacteroidetes/Chlorobi_group>Balneolota>Balneolia>Balneolales	GCA_007693745.1	Balneolaceae_bacterium	EA360_10455	1062..4556	2053516	MAG:-BREX-system-P-loop-protein-BrxC-[Balneolaceae-bacterium]
MCC5907043.1	Type1.BREX	<-REase<-?<-?<-?||TPR+TPR->BrxA-DNA-binding->BrxB-BrxF-iSTAND->DUF6079*->AbiEi->AbiEii->N6-METHYLASE->Schlafen+HATPase+wHTH->PglZ->SIGMA+OB-Fold+AAA-ATPase+HKD-DNase-BrxL->REase-><-?||Thymidylate_kin->	1167	Bacteria>FCB_group>Bacteroidetes/Chlorobi_group>Balneolota>Balneolia>Balneolales	GCA_020832285.1	Balneolaceae_bacterium	JJU13_12585	29362..32865	2053516	BREX-system-P-loop-protein-BrxC-[Balneolaceae-bacterium]
MCH2449130.1	Type1.BREX	Phage_int_SAM_4+Phage_integrase->TRPR-HTH->?->HTH_3->BrxA-DNA-binding->BrxB-BrxF-iSTAND->DUF6079*->N6-METHYLASE->PglZ->SIGMA+OB-Fold+AAA-ATPase+HKD-DNase-BrxL-><-?<-HD||REase->MULE-TRANSPOSASE->	1151	Bacteria>FCB_group>Bacteroidetes/Chlorobi_group>Balneolota>Balneolia>Balneolales	GCA_022450045.1	Gracilimonas_sp.	MK198_03190	295077..298532	1974203	BREX-system-P-loop-protein-BrxC-[Gracilimonas-sp.]
WP_095606906.1	Type1.BREX	<-REase||?->?-><-SIGMA+OB-Fold+AAA-ATPase+Lon-Protease-BrxL<-PglZ<-N6-METHYLASE<-DUF6079*<-BrxB-BrxF-iSTAND<-BrxA-DNA-binding<-HEPN||?-><-XIS-HTH<-?<-?<-?<-Phage_int_SAM_5+Phage_integrase	1213	Bacteria>FCB_group>Bacteroidetes/Chlorobi_group>Balneolota>Balneolia>Balneolales	GCF_002287075.1	Aliifodinibius_sp._WN023	CK503_RS11215	230173..233814	2032627	BREX-system-P-loop-protein-BrxC-[Aliifodinibius-sp.-WN023]
WP_210512160.1	Type1.BREX	Phage_int_SAM_5+Phage_integrase->?->?->REase->?->REase->BrxA-DNA-binding->BrxB-BrxF-iSTAND->DUF6079*->N6-METHYLASE-><-AbiEii<-AbiEi_4||XIS-HTH->PIN->PglZ->SIGMA+OB-Fold+AAA-ATPase+Lon-Protease-BrxL->	1141	Bacteria>FCB_group>Bacteroidetes/Chlorobi_group>Balneolota>Balneolia>Balneolales	GCF_017921895.1	Natronogracilivirga_saccharolytica	NATSA_RS09880	86175..89600	2812953	BREX-system-P-loop-protein-BrxC-[Natronogracilivirga-saccharolytica]
WP_237852616.1	Type1.BREX	<-SIGMA+OB-Fold+AAA-ATPase+HKD-DNase-BrxL<-PglZ||SF1-SF2-Helicase-><-SF1-SF2-Helicase<-?<-N6-METHYLASE<-AbiEii<-AbiEi<-DUF6079*<-BrxB-BrxF-iSTAND<-BrxA-DNA-binding<-REase||?->?->?->?-><-HEPN	1164	Bacteria>FCB_group>Bacteroidetes/Chlorobi_group>Balneolota>Balneolia>Balneolales	GCF_022012475.1	Rhodohalobacter_sp._WB101	L6773_RS04280	217944..221438	2911366	BREX-system-P-loop-protein-BrxC-[Rhodohalobacter-sp.-WB101]
WP_265763895.1	Type1.BREX	<-HEPN<-SIGMA+OB-Fold+AAA-ATPase+HKD-DNase-BrxL<-PglZ<-HTH+REase<-REase<-?<-N6-METHYLASE<-DUF6079*<-BrxB-BrxF-iSTAND<-BrxA-DNA-binding<-tRNA||?->?->?-><-?<-?<-ABC-ATPase+SbcC	1165	Bacteria>FCB_group>Bacteroidetes/Chlorobi_group>Balneolota>Balneolia>Balneolales	GCF_026229185.1	Aliifodinibius_sp._1BSP15-2V2	J6I44_RS00130	34405..37902	2820877	BREX-system-P-loop-protein-BrxC-[Aliifodinibius-sp.-1BSP15-2V2]
ANT65175.1	Type1.BREX	<-HTH_21+rve+rve_3<-HTH_Tnp_1||DUF772+DDE_Tnp_1_2-><-SIGMA+OB-Fold+AAA-ATPase+Lon-Protease-BrxL<-PglZ<-Schlafen+HATPase+wHTH<-SbcC<-N6-METHYLASE<-Schlafen+HATPase+wHTH<-DUF6079*<-BrxB-BrxF-iSTAND<-BrxA-DNA-binding<-XIS-HTH<-RVT<-RVT||AAA-ATPase-p-><-?||?-><-Thioredoxin	1144	Bacteria>FCB_group>Bacteroidetes/Chlorobi_group>Chlorobiota>Chlorobiia>Chlorobiales	GCA_001687065.1	Prosthecochloris_sp._CIB_2401	Ptc2401_01411	1430885..1434319	1868325	hypothetical-protein-Ptc2401_01411-[Prosthecochloris-sp.-CIB-2401]
TNJ34090.1	Type1.BREX	HIN-HTH->HTH_Tnp_1+HTH_21+rve+rve_3-><-SIGMA+OB-Fold+AAA-ATPase+Lon-Protease-BrxL<-PglZ<-TOPRIM<-OLD-ABC<-N6-METHYLASE<-Schlafen+HATPase+wHTH<-DUF6079*<-BrxB-BrxF-iSTAND<-BrxA-DNA-binding<-XIS-HTH<-RVT<-RVT	1144	Bacteria>FCB_group>Bacteroidetes/Chlorobi_group>Chlorobiota>Chlorobiia>Chlorobiales	GCA_006265245.1	Prosthecochloris_vibrioformis	FGF68_10440	14740..18174	1098	BREX-system-P-loop-protein-BrxC-[Prosthecochloris-vibrioformis]
KAA6232806.1	Type1.BREX	<-McrC-NTD+REase<-McrB-AAA<-HARE-HTH+REase<-SIGMA+OB-Fold+AAA-ATPase+HKD-DNase-BrxL<-PglZ||DDE_Tnp_1_4-><-N6-METHYLASE<-REase<-ABC-ATPase<-DUF6079*<-BrxB-BrxF-iSTAND<-BrxA-DNA-binding<-XIS-HTH<-?<-?<-?||Nudix_N_2+NUDIX-><-?<-HAMP+PAS+HISKIN	1204	Bacteria>FCB_group>Bacteroidetes/Chlorobi_group>Chlorobiota>Chlorobiia>Chlorobiales	GCA_008634005.1	Chlorobium_phaeovibrioides	FP507_06840	1409593..1413207	1094	BREX-system-P-loop-protein-BrxC-[Chlorobium-phaeovibrioides]
NMW20007.1	Type1.BREX	DUF6079*->Schlafen+HATPase+wHTH+HTH->N6-METHYLASE-p->N6-METHYLASE->Schlafen+HATPase+wHTH->	1160	Bacteria>FCB_group>Bacteroidetes/Chlorobi_group>Chlorobiota>Chlorobiia>Chlorobiales	GCA_012972825.1	Chlorobiaceae_bacterium	HKK66_13560	1..3483	2067991	BREX-system-P-loop-protein-BrxC-[Chlorobiaceae-bacterium]
MBV5326244.1	Type1.BREX	BrxA-DNA-binding->BrxB-BrxF-iSTAND->DUF6079*->Schlafen+HATPase+wHTH->OLD-ABC->REase->	1179	Bacteria>FCB_group>Bacteroidetes/Chlorobi_group>Chlorobiota>Chlorobiia>Chlorobiales	GCA_019162945.1	Chlorobium_sp.	JZU65_01205	2432..5971	1095	BREX-system-P-loop-protein-BrxC-[Chlorobium-sp.]
MBV5326252.1	Type1.BREX	<-ParB+HNH<-N6-METHYLASE-p<-RVT<-Schlafen+HATPase+wHTH+HTH<-DUF6079*<-BrxB-BrxF-iSTAND<-BrxA-DNA-binding<-XIS-HTH<-?<-tRNA<-HslUClpX-AAA<-?<-?<-?<-His_Phos_1	1179	Bacteria>FCB_group>Bacteroidetes/Chlorobi_group>Chlorobiota>Chlorobiia>Chlorobiales	GCA_019162945.1	Chlorobium_sp.	JZU65_01250	9464..13003	1095	BREX-system-P-loop-protein-BrxC-[Chlorobium-sp.]
MBV5328349.1	Type1.BREX	<-SIGMA+OB-Fold+AAA-ATPase+Lon-Protease-BrxL<-PglZ<-TOPRIM<-OLD-ABC<-N6-METHYLASE<-DUF6079*<-BrxB-BrxF-iSTAND<-BrxA-DNA-binding<-XIS-HTH	1208	Bacteria>FCB_group>Bacteroidetes/Chlorobi_group>Chlorobiota>Chlorobiia>Chlorobiales	GCA_019162945.1	Chlorobium_sp.	JZU65_12040	10298..13924	1095	BREX-system-P-loop-protein-BrxC-[Chlorobium-sp.]
MBV5329669.1	Type1.BREX	<-N6-METHYLASE-p<-Schlafen+HATPase+wHTH<-DUF6079*<-BrxB-BrxF-iSTAND<-BrxA-DNA-binding<-XIS-HTH<-HTH_3||XIS-HTH->XIS-HTH->SMS-KaiC->	1150	Bacteria>FCB_group>Bacteroidetes/Chlorobi_group>Chlorobiota>Chlorobiia>Chlorobiales	GCA_019162945.1	Chlorobium_sp.	JZU65_18935	2439..5891	1095	BREX-system-P-loop-protein-BrxC-[Chlorobium-sp.]
MBV5304386.1	Type1.BREX	BrxA-DNA-binding->BrxB-BrxF-iSTAND->DUF6079*->N6-METHYLASE->PglZ->SIGMA+OB-Fold+AAA-ATPase+Lon-Protease-BrxL->	1138	Bacteria>FCB_group>Bacteroidetes/Chlorobi_group>Chlorobiota>Chlorobiia>Chlorobiales	GCA_019163115.1	Chlorobium_sp.	JZU70_09345	1849..5265	1095	BREX-system-P-loop-protein-BrxC-[Chlorobium-sp.]
MCF8215288.1	Type1.BREX	HslUClpX-AAA->tRNA->MNS+MarR-HTH->?-><-?<-METHYLASE||?->XIS-HTH->BrxA-DNA-binding->BrxB-BrxF-iSTAND->DUF6079*->Schlafen+HATPase+wHTH->N6-METHYLASE->SbcC->Schlafen+HATPase+wHTH->PglZ->SIGMA+OB-Fold+AAA-ATPase+Lon-Protease-BrxL->RVT-><-Calcineurin||RADICAL-SAM->	1145	Bacteria>FCB_group>Bacteroidetes/Chlorobi_group>Chlorobiota>Chlorobiia>Chlorobiales	GCA_021738525.1	Chlorobium_sp.	K9I96_00250	58948..62385	1095	BREX-system-P-loop-protein-BrxC-[Chlorobium-sp.]
WP_012508724.1	Type1.BREX	<-DDE_Tnp_1+DDE_Tnp_1||Tox-RelE->HTH_3->BrxL->?->?->BrxA-DNA-binding->BrxB-BrxF-iSTAND->DUF6079*->N6-METHYLASE->HTH+REase->PglZ->SIGMA+OB-Fold+AAA-ATPase+Lon-Protease-BrxL->RelB-><-HipA_C<-HTH_3	1211	Bacteria>FCB_group>Bacteroidetes/Chlorobi_group>Chlorobiota>Chlorobiia>Chlorobiales	GCF_000020645.1	Pelodictyon_phaeoclathratiforme	PPHA_RS10105	2123667..2127302	34090	BREX-system-P-loop-protein-BrxC-[Pelodictyon-phaeoclathratiforme]
WP_204208384.1	Type1.BREX	ABhydrolase->?->Aminotran_1_2-><-?||DUF772+DDE_Tnp_1_2->BrxA-DNA-binding->BrxB-BrxF-iSTAND->DUF6079-p*->?->Schlafen+HATPase+wHTH+HTH->N6-METHYLASE->DUF4372+DDE_Tnp_1->N6-METHYLASE-p->ParB+HNH->RVT->PglZ->SIGMA+OB-Fold+AAA-ATPase+HKD-DNase-BrxL->	997	Bacteria>FCB_group>Bacteroidetes/Chlorobi_group>Chlorobiota>Chlorobiia>Chlorobiales	GCF_001687065.1	Prosthecochloris_sp._CIB_2401	Ptc2401_RS05035	1057250..1060243	1868325	BREX-system-P-loop-protein-BrxC-[Prosthecochloris-sp.-CIB-2401]
WP_204208337.1	Type1.BREX	<-HTH_21+rve+rve_3<-HTH_Tnp_1||DUF772+DDE_Tnp_1_2-><-SIGMA+OB-Fold+AAA-ATPase+Lon-Protease-BrxL<-PglZ<-Schlafen+HATPase+wHTH<-SbcC<-N6-METHYLASE<-Schlafen+HATPase+wHTH<-DUF6079*<-BrxB-BrxF-iSTAND<-BrxA-DNA-binding<-XIS-HTH||AAA-ATPase-p->||?-><-Thioredoxin	1145	Bacteria>FCB_group>Bacteroidetes/Chlorobi_group>Chlorobiota>Chlorobiia>Chlorobiales	GCF_001687065.1	Prosthecochloris_sp._CIB_2401	Ptc2401_RS06780	1430885..1434322	1868325	BREX-system-P-loop-protein-BrxC-[Prosthecochloris-sp.-CIB-2401]
WP_076791024.1	Type1.BREX	<-SIGMA+OB-Fold+AAA-ATPase+HKD-DNase-BrxL<-PglZ<-DOC+HTH<-?<-?<-Schlafen+HATPase+wHTH+HTH<-DUF6079*<-BrxB-BrxF-iSTAND<-BrxA-DNA-binding||?->RelE-ParE-><-?<-Peptidase	1179	Bacteria>FCB_group>Bacteroidetes/Chlorobi_group>Chlorobiota>Chlorobiia>Chlorobiales	GCF_001975465.1	Chlorobium_sp._KB01	BOD20_RS04865	12313..15852	1917528	BREX-system-P-loop-protein-BrxC-[Chlorobium-sp.-KB01]
WP_085659853.1	Type1.BREX	<-REase||HIN-HTH->HTH_Tnp_1+HTH_21+rve+rve_3-><-HARE-HTH+REase<-SIGMA+OB-Fold+AAA-ATPase+Lon-Protease-BrxL<-PglZ<-MACRODOMAIN<-DarT+Peptidase<-N6-METHYLASE<-DUF6079*<-BrxB-BrxF-iSTAND<-BrxA-DNA-binding<-XIS-HTH<-?<-?<-?||Nudix_N_2+NUDIX->	1209	Bacteria>FCB_group>Bacteroidetes/Chlorobi_group>Chlorobiota>Chlorobiia>Chlorobiales	GCF_002113825.1	Prosthecochloris_sp._HL-130-GSB	B9H02_RS07545	1603101..1606730	1974213	BREX-system-P-loop-protein-BrxC-[Prosthecochloris-sp.-HL-130-GSB]
WP_139626761.1	Type1.BREX	<-PglZ<-ParB+HNH<-N6-METHYLASE<-N6-METHYLASE-p<-Schlafen+HATPase+wHTH+HTH<-DUF6079*<-BrxB-BrxF-iSTAND<-BrxA-DNA-binding||<-Aminotran_1_2<-?<-ABhydrolase	1204	Bacteria>FCB_group>Bacteroidetes/Chlorobi_group>Chlorobiota>Chlorobiia>Chlorobiales	GCF_006265245.1	Prosthecochloris_vibrioformis	FGF68_RS08505	9816..13430	1098	BREX-system-P-loop-protein-BrxC-[Prosthecochloris-vibrioformis]
WP_211340437.1	Type1.BREX	HIN-HTH->HTH_Tnp_1+HTH_21+rve+rve_3-><-GDResolvase<-SIGMA+OB-Fold+AAA-ATPase+Lon-Protease-BrxL<-PglZ<-TOPRIM<-ParB+SbcC<-N6-METHYLASE<-Schlafen+HATPase+wHTH<-DUF6079*<-BrxB-BrxF-iSTAND<-BrxA-DNA-binding<-XIS-HTH	1145	Bacteria>FCB_group>Bacteroidetes/Chlorobi_group>Chlorobiota>Chlorobiia>Chlorobiales	GCF_006265245.1	Prosthecochloris_vibrioformis	FGF68_RS10435	14740..18177	1098	BREX-system-P-loop-protein-BrxC-[Prosthecochloris-vibrioformis]
WP_192496514.1	Type1.BREX	<-McrB-AAA<-HARE-HTH+REase<-SIGMA+OB-Fold+AAA-ATPase+HKD-DNase-BrxL<-PglZ<-N6-METHYLASE-p||DDE_Tnp_1_4-><-N6-METHYLASE<-REase<-ABC-ATPase<-DUF6079*<-BrxB-BrxF-iSTAND<-BrxA-DNA-binding<-XIS-HTH<-?<-?<-?<-?||Nudix_N_2+NUDIX->	1203	Bacteria>FCB_group>Bacteroidetes/Chlorobi_group>Chlorobiota>Chlorobiia>Chlorobiales	GCF_008634005.1	Chlorobium_phaeovibrioides	FP507_RS07315	1409593..1413204	1094	BREX-system-P-loop-protein-BrxC-[Chlorobium-phaeovibrioides]
WP_151419733.1	Type1.BREX	BrxA-DNA-binding->BrxB-BrxF-iSTAND->DUF6079*->REase+iREase->?->N6-METHYLASE->HTH+REase->REase->PglZ->SIGMA+OB-Fold+AAA-ATPase+HKD-DNase-BrxL->HTH_Tnp_1->rve->	1183	Bacteria>FCB_group>Bacteroidetes/Chlorobi_group>Chlorobiota>Chlorobiia>Chlorobiales	GCF_008634005.1	Chlorobium_phaeovibrioides	FP507_RS10470	13780..17331	1094	BREX-system-P-loop-protein-BrxC-[Chlorobium-phaeovibrioides]
MBM3423463.1	Type1.BREX	HTH+REase->BrxA-DNA-binding->BrxB-BrxF-iSTAND->DUF6079*->Schlafen+HATPase+wHTH+HTH->	1182	Bacteria>FCB_group>Bacteroidetes/Chlorobi_group>Chlorobiota>unclassified_Chlorobiota	GCA_016868665.1	Chlorobiota_bacterium	FJY09_09520	5435..8983	2268192	BREX-system-P-loop-protein-BrxC-[Chlorobiota-bacterium]
QOJ30044.1	Type1.BREX	SF1-SF2-Helicase+PRTase-><-?<-?<-PGK<-?||BrxA-DNA-binding->BrxB-BrxF-iSTAND->DUF6079*->Schlafen+HATPase+wHTH->N6-METHYLASE->PglZ->SIGMA+OB-Fold+AAA-ATPase+Lon-Protease-BrxL-><-?<-?<-ABC-ATPase+SbcC	1198	Bacteria>FCB_group>Bacteroidetes/Chlorobi_group>Ignavibacteriota>Ignavibacteria>Ignavibacteriales	GCA_015709675.1	Ignavibacteriales_bacterium	HRU80_14675	3719207..3722803	2049428	BREX-system-P-loop-protein-BrxC-[Ignavibacteriales-bacterium]
MBV6512081.1	Type1.BREX	SF1-SF2-Helicase+PRTase-><-?<-?<-PGK<-?||BrxA-DNA-binding->BrxB-BrxF-iSTAND->DUF6079*->Schlafen+HATPase+wHTH->N6-METHYLASE->PglZ->SIGMA+OB-Fold+AAA-ATPase+Lon-Protease-BrxL-><-?<-?<-ABC-ATPase+SbcC	1198	Bacteria>FCB_group>Bacteroidetes/Chlorobi_group>Ignavibacteriota>Ignavibacteria>Ignavibacteriales	GCA_019187025.1	Ignavibacteriaceae_bacterium	FMNOHCHN_01571	145578..149174	2651163	hypothetical-protein-[Ignavibacteriaceae-bacterium]
MBV6422081.1	Type1.BREX	URI-DNase->tRNA->Phage_int_SAM_5+Phage_integrase-><-TIR<-?<-SIGMA+OB-Fold+AAA-ATPase+Lon-Protease-BrxL<-PglZ<-DUF6079*<-N6-METHYLASE<-BrxB-BrxF-iSTAND	1214	Bacteria>FCB_group>Bacteroidetes/Chlorobi_group>Ignavibacteriota>Ignavibacteria>Ignavibacteriales	GCA_019187565.1	Ignavibacteriaceae_bacterium	DAHOPDDO_03370	33171..36815	2651163	hypothetical-protein-[Ignavibacteriaceae-bacterium]
MCB9211065.1	Type1.BREX	<-AAA-ATPase<-?<-SIGMA+OB-Fold+AAA-ATPase-BrxL<-PglZ<-DUF6079*<-BrxB-BrxF-iSTAND<-DUF4007<-N6-METHYLASE<-SbcC+SbcC<-Calcineurin<-OLD-ABC+TOPRIM<-REase+RNase	1377	Bacteria>FCB_group>Bacteroidetes/Chlorobi_group>Ignavibacteriota>Ignavibacteria>Ignavibacteriales	GCA_020635365.1	Ignavibacteriales_bacterium	H6609_16995	10432..14565	2049428	chitobiase/beta-hexosaminidase-C-terminal-domain-containing-protein-[Ignavibacteriales-bacterium]
MCK6605711.1	Type1.BREX	S1+GTPase-AIG-><-ADH_N+ADH_zinc_N||Calcineurin->HAD+HAD->?->BrxA-DNA-binding->BrxB-BrxF-iSTAND->DUF6079*->N6-METHYLASE->TPR+REase->PglZ->SIGMA+OB-Fold+AAA-ATPase+Lon-Protease-BrxL->?->URI-DNase-><-SAM-METHYLASE	1179	Bacteria>FCB_group>Bacteroidetes/Chlorobi_group>Ignavibacteriota>Ignavibacteria>Ignavibacteriales	GCA_023151225.1	Ignavibacteriaceae_bacterium	L6Q59_12515	25884..29423	2651163	BREX-system-P-loop-protein-BrxC-[Ignavibacteriaceae-bacterium]
MBL0321410.1	Type1.BREX	Phage_int_SAM_5->Phage_integrase->AbiGi-><-ANK+ANK||?->TPR+TPR->TPR+TPR->BrxA-DNA-binding->BrxB-BrxF-iSTAND->DUF6079-p*->DUF6079-p->N6-METHYLASE->PglZ->HTH+rve->AAA-ATPase->?->?->SF1-SF2-Helicase->SF1-SF2-Helicase->	737	Bacteria>FCB_group>Bacteroidetes/Chlorobi_group>Ignavibacteriota>Ignavibacteria>unclassified_Ignavibacteria	GCA_016722455.1	Ignavibacteria_bacterium	IPP80_03355	309754..311967	2053306	BREX-system-P-loop-protein-BrxC-[Ignavibacteria-bacterium]
MBK7413718.1	Type1.BREX	<-PhdYeFM_antitox||tRNA->Phage_int_SAM_5-><-ANK<-?||?->TPR+TPR+TPR->TPR+TPR->BrxA-DNA-binding->BrxB-BrxF-iSTAND->DUF6079*->N6-METHYLASE-p->N6-METHYLASE-p->PglZ->PglZ->?->HTH+rve->AAA-ATPase->	1183	Bacteria>FCB_group>Bacteroidetes/Chlorobi_group>Ignavibacteriota>Ignavibacteria>unclassified_Ignavibacteria	-	Ignavibacteria_bacterium	IPI29_14305	1380288..1383839	2053306	BREX-system-P-loop-protein-BrxC-[Ignavibacteria-bacterium]
WP_157621530.1	Type1.BREX	<-N6-METHYLASE-p<-DUF6079*<-BrxB-BrxF-iSTAND<-DUF6079*<-BrxA-DNA-binding<-HTH_32+DDE_3	1117	Bacteria>FCB_group>Bacteroidetes/Chlorobi_group>Rhodothermota>Rhodothermia>Rhodothermales	GCF_000419585.1	Salisaeta_longa	SALLO_RS0113970	2150..5503	503170	hypothetical-protein-[Salisaeta-longa]
WP_040606398.1	Type1.BREX	<-N6-METHYLASE-p<-DUF6079<-BrxB-BrxF-iSTAND<-DUF6079*<-BrxA-DNA-binding<-HTH_32+DDE_3<-HTH_32+DDE_3<-?||?-><-?||?->NTP_transf_5->	1247	Bacteria>FCB_group>Bacteroidetes/Chlorobi_group>Rhodothermota>Rhodothermia>Rhodothermales	GCF_000419585.1	Salisaeta_longa	SALLO_RS0113980	6164..9907	503170	hypothetical-protein-[Salisaeta-longa]
WP_098060820.1	Type1.BREX	Peptidase->?->?->?->BrxA-DNA-binding->BrxB-BrxF-iSTAND->DUF6079*->N6-METHYLASE->PglZ->SIGMA+OB-Fold+AAA-ATPase+HKD-DNase-BrxL->CHTH->Tox-RES->Thioredoxin->	1206	Bacteria>FCB_group>Bacteroidetes/Chlorobi_group>Rhodothermota>Rhodothermia>Rhodothermales	GCF_002554705.1	Longimonas_halophila	CRI93_RS01465	364524..368144	1469170	BREX-system-P-loop-protein-BrxC-[Longimonas-halophila]
MBP9037128.1	Type1.BREX	<-N6-METHYLASE<-?<-HTH+REase<-BrxB-BrxF-iSTAND<-BrxA-DNA-binding<-XIS-HTH<-?<-HNH<-SIS_2+SIS	1152	Bacteria>FCB_group>Candidatus_Cloacimonetes>Candidatus_Cloacimonetes>unclassified_Candidatus_Cloacimonas	GCA_018057045.1	Candidatus_Cloacimonas_sp.	KBG38_04995	2613..6071	2053534	BREX-system-P-loop-protein-BrxC-[Candidatus-Cloacimonas-sp.]
OQC05381.1	Type1.BREX	BrxA-DNA-binding->BrxB-BrxF-iSTAND->DUF6079*->OLD-ABC->REase->N6-METHYLASE->PglZ->SIGMA+OB-Fold+AAA-ATPase+HKD-DNase-BrxL->HEPN->	1162	Bacteria>FCB_group>Candidatus_Cloacimonetes>unclassified_Candidatus_Cloacimonetes	GCA_002070875.1	Candidatus_Cloacimonetes_bacterium_ADurb.Bin089	BWX76_00750	1812..5300	1852836	MAG:-hypothetical-protein-BWX76_00750-[Candidatus-Cloacimonetes-bacterium-ADurb.Bin089]
OQC10188.1	Type1.BREX	N6-METHYLASE->?->PVC-AAA->NERD->?->?->?->BrxA-DNA-binding->BrxB-BrxF-iSTAND->DUF6079*->N6-METHYLASE->SbcC->?->ParB->ParB->PglZ->SIGMA+OB-Fold+AAA-ATPase+Lon-Protease-BrxL->ARG->	1149	Bacteria>FCB_group>Candidatus_Cloacimonetes>unclassified_Candidatus_Cloacimonetes	GCA_002070895.1	Candidatus_Cloacimonetes_bacterium_ADurb.Bin088	BWX75_00589	19726..23175	1852835	MAG:-hypothetical-protein-BWX75_00589-[Candidatus-Cloacimonetes-bacterium-ADurb.Bin088]
MCB5286841.1	Type1.BREX	<-IG+IG||?->?-><-tRNA||?->?->XIS-HTH->BrxA-DNA-binding->BrxB-BrxF-iSTAND->DUF6079*->OLD-ABC->PIN->N6-METHYLASE->BirA-HTH->PglZ->SIGMA+OB-Fold+AAA-ATPase+Lon-Protease-BrxL->	1145	Bacteria>FCB_group>Candidatus_Cloacimonetes>unclassified_Candidatus_Cloacimonetes	GCA_020532145.1	Candidatus_Cloacimonetes_bacterium	LHW64_03450	9843..13280	2030808	BREX-system-P-loop-protein-BrxC-[Candidatus-Cloacimonadota-bacterium]
MCB5271576.1	Type1.BREX	KAP-NTPase->KAP-glycosylase->?->TatD_DNase-><-SIGMA+OB-Fold+AAA-ATPase+HKD-DNase-BrxL<-PglZ<-N6-METHYLASE<-REase<-OLD-ABC<-DUF6079*<-BrxB-BrxF-iSTAND<-BrxA-DNA-binding<-NERD+ZnR	1171	Bacteria>FCB_group>Candidatus_Cloacimonetes>unclassified_Candidatus_Cloacimonetes	GCA_020532545.1	Candidatus_Cloacimonetes_bacterium	LHW56_07005	18816..22331	2030808	BREX-system-P-loop-protein-BrxC-[Candidatus-Cloacimonadota-bacterium]
KZD20046.1	Type1.BREX	AbiEi_4->nSTAND1+HNH->?-><-?||?->BrxA-DNA-binding->BrxB-BrxF-iSTAND->N6-METHYLASE->DUF6079*->N6-METHYLASE->MarR-HTH->Polbeta->PglZ->SIGMA+OB-Fold+AAA-ATPase+Lon-Protease-BrxL-><-?<-AAA-ATPase<-HTH_7+rve+rve_3||Exonuc_V_gamma+REase->	1146	Bacteria>FCB_group>Candidatus_Fermentibacteria>Candidatus_Fermentibacteria_(class)>Candidatus_Fermentibacterales>Candidatus_Fermentibacteraceae	GCA_001618625.1	Candidatus_Fermentibacter_daniensis	AO396_01425	11808..15248	1729713	hypothetical-protein-AO396_01425-[Candidatus-Fermentibacter-daniensis]
MCD6589067.1	Type1.BREX	<-DUF6079*<-BrxB-BrxF-iSTAND<-BrxA-DNA-binding<-?<-?<-CHTH<-RelE<-?<-ANK+ANK+ANK	1169	Bacteria>FCB_group>Candidatus_Fermentibacteria>Candidatus_Fermentibacteria_(class)>Candidatus_Fermentibacterales>Candidatus_Fermentibacteraceae	GCA_021162285.1	Candidatus_Fermentibacteraceae_bacterium	J7K88_11010	26..3535	2774599	BREX-system-P-loop-protein-BrxC-[Candidatus-Fermentibacteraceae-bacterium]
NLV46851.1	Type1.BREX	Mrr_N->Mrr_N->Mrr_N->DUF4007->LON-AAA->N6-METHYLASE->DUF6079*->PglZ->SIGMA+OB-Fold+AAA-ATPase-BrxL->HEPN->?->Radical-SAM->MPTase->?->?->REase->	1339	Bacteria>FCB_group>Candidatus_Hydrogenedentes>unclassified_Candidatus_Hydrogenedentes	GCA_012730105.1	Candidatus_Hydrogenedentes_bacterium	GXY07_20415	9749..13768	2030809	hypothetical-protein-[Candidatus-Hydrogenedentes-bacterium]
NUM53512.1	Type1.BREX	<-SF1-SF2-Helicase<-OLD-ABC+TOPRIM<-?<-SIGMA+OB-Fold+AAA-ATPase+Lon-Protease-BrxL<-PglZ<-ArdCN+MPTase<-?<-N6-METHYLASE<-HEPN<-DUF6079*<-BrxB-BrxF-iSTAND<-BrxA-DNA-binding||?->HTH_3->MPTase->	1149	Bacteria>FCB_group>Candidatus_Hydrogenedentes>unclassified_Candidatus_Hydrogenedentes	GCA_013359265.1	Candidatus_Hydrogenedentes_bacterium	HUU46_07705	154693..158142	2030809	BREX-system-P-loop-protein-BrxC-[Candidatus-Hydrogenedentes-bacterium]
MCC6145266.1	Type1.BREX	<-GrpE<-SIGMA+OB-Fold+AAA-ATPase+Lon-Protease-BrxL<-PglZ<-?<-SbcC<-N6-METHYLASE<-Couple_hipA+HipA_C<-HTH_3<-DUF6079*<-BrxB-BrxF-iSTAND<-BrxA-DNA-binding<-?||REase+RNase-><-TPR<-?<-?<-Ntox49<-ARG	1156	Bacteria>FCB_group>Candidatus_Hydrogenedentes>unclassified_Candidatus_Hydrogenedentes	GCA_020845535.1	Candidatus_Hydrogenedentes_bacterium	IT368_15785	16372..19842	2030809	BREX-system-P-loop-protein-BrxC-[Candidatus-Hydrogenedentes-bacterium]
MCC6156024.1	Type1.BREX	<-SIGMA+OB-Fold+AAA-ATPase+Lon-Protease-BrxL<-PglZ<-ArdCN+MPTase<-N6-METHYLASE<-HEPN<-DUF6079*	1142	Bacteria>FCB_group>Candidatus_Hydrogenedentes>unclassified_Candidatus_Hydrogenedentes	GCA_020846215.1	Candidatus_Hydrogenedentes_bacterium	IT367_19820	10018..13447	2030809	BREX-system-P-loop-protein-BrxC-[Candidatus-Hydrogenedentes-bacterium]
RKY59330.1	Type1.BREX	SIGMA+OB-Fold+AAA-ATPase+Lon-Protease-BrxL->BrxA-DNA-binding->BrxB-BrxF-iSTAND->DUF6079*->N6-METHYLASE->Schlafen+HATPase+wHTH->N6-METHYLASE->	1192	Bacteria>FCB_group>Candidatus_Latescibacteria>unclassified_Candidatus_Latescibacteria	GCA_003645885.1	Candidatus_Latescibacteria_bacterium	DRP94_03915	4031..7609	2053570	BREX-system-P-loop-protein-BrxC-[Candidatus-Latescibacteria-bacterium]
MCC7260964.1	Type1.BREX	<-Cas_NE0113<-CRISPR_Cas6<-?||TPR->BrxA-DNA-binding->BrxB-BrxF-iSTAND->DUF6079*->N6-METHYLASE->	1146	Bacteria>FCB_group>Candidatus_Latescibacteria>unclassified_Candidatus_Latescibacteria	GCA_020852055.1	Candidatus_Latescibacteria_bacterium	IT369_00445	7937..11377	2053570	BREX-system-P-loop-protein-BrxC-[Candidatus-Latescibacteria-bacterium]
MBT4282223.1	Type1.BREX	<-N6-METHYLASE-p<-DUF6079*<-BrxB-BrxF-iSTAND<-BrxA-DNA-binding<-?<-?<-?||ABC-ATPase+SbcC+ABC-ATPase+SbcC->	1156	Bacteria>FCB_group>Candidatus_Marinimicrobia	GCA_018651785.1	Candidatus_Marinimicrobia_bacterium	HOD98_02880	1407..4877	2026760	BREX-system-P-loop-protein-BrxC-[Candidatus-Marinimicrobia-bacterium]
MBT4361892.1	Type1.BREX	DUF6079*->N6-METHYLASE->PglZ->SIGMA+OB-Fold+AAA-ATPase+HKD-DNase-BrxL->REase+DUF5655->	1148	Bacteria>FCB_group>Candidatus_Marinimicrobia	GCA_018653465.1	Candidatus_Marinimicrobia_bacterium	HOD11_13855	1..3448	2026760	BREX-system-P-loop-protein-BrxC-[Candidatus-Marinimicrobia-bacterium]
MBT5758478.1	Type1.BREX	<-N6-METHYLASE-p<-DUF6079*<-BrxB-BrxF-iSTAND<-BrxA-DNA-binding<-?<-?<-?||ABC-ATPase+SbcC+ABC-ATPase+SbcC->	1156	Bacteria>FCB_group>Candidatus_Marinimicrobia	GCA_018667415.1	Candidatus_Marinimicrobia_bacterium	HOI55_02355	1576..5046	2026760	BREX-system-P-loop-protein-BrxC-[Candidatus-Marinimicrobia-bacterium]
OWV23454.1	Type1.BREX	DNA_pol3_beta+DNA_pol3_beta_2+DNA_pol3_beta_3->?-><-?<-?<-?<-SIGMA+OB-Fold+AAA-ATPase+Lon-Protease-BrxL<-PglZ<-Schlafen+HATPase+wHTH<-N6-METHYLASE<-DUF6079*<-BrxB-BrxF-iSTAND<-BrxA-DNA-binding<-?<-MODE-HTH+PBPII||?->?->Phos_pyr_kin->RADICAL-SAM->	1174	Bacteria>FCB_group>Fibrobacterota>Fibrobacteria>Fibrobacterales>Fibrobacteraceae	GCA_002210425.1	Fibrobacter_sp._UWB2	B7982_07645	365376..368900	1964358	ATPase-[Fibrobacter-sp.-UWB2]
NLW30564.1	Type1.BREX	HNH->?->BrxA-DNA-binding->BrxB-BrxF-iSTAND->DUF6079*->?->ParB+HNH->PglZ->SIGMA+OB-Fold+AAA-ATPase+HKD-DNase-BrxL->	1185	Bacteria>FCB_group>Fibrobacterota>Fibrobacteria>Fibrobacterales>Fibrobacteraceae	GCA_012728995.1	Fibrobacter_sp.	GXY77_03805	4326..7883	35828	BREX-system-P-loop-protein-BrxC-[Fibrobacter-sp.]
NLW30641.1	Type1.BREX	BrxA-DNA-binding->BrxB-BrxF-iSTAND->DUF6079*->Schlafen+HATPase+wHTH+HTH->N6-METHYLASE->PglZ->SIGMA+OB-Fold+AAA-ATPase+Lon-Protease-BrxL->ArdCN+MPTase->	1213	Bacteria>FCB_group>Fibrobacterota>Fibrobacteria>Fibrobacterales>Fibrobacteraceae	GCA_012728995.1	Fibrobacter_sp.	GXY77_04195	2375..6016	35828	BREX-system-P-loop-protein-BrxC-[Fibrobacter-sp.]
SHK42502.1	Type1.BREX	<-VirE<-?<-XIS-HTH||?->?->?->BrxA-DNA-binding->BrxB-BrxF-iSTAND->DUF6079*->ParB+HNH->N6-METHYLASE->Schlafen+HATPase+wHTH->PglZ->SIGMA+OB-Fold+AAA-ATPase+Lon-Protease-BrxL->TIR->?->REase->Phage_int_SAM_1+Phage_integrase->	1186	Bacteria>FCB_group>Fibrobacterota>Fibrobacteria>Fibrobacterales>Fibrobacteraceae	GCA_900142505.1	Fibrobacter_sp._UWH5	SAMN05720764_101395	444346..447906	1896211	hypothetical-protein-SAMN05720764_101395-[Fibrobacter-sp.-UWH5]
WP_212668349.1	Type1.BREX	<-VirE<-?<-XIS-HTH||?->?->?->BrxA-DNA-binding->BrxB-BrxF-iSTAND->DUF6079*->ParB+HNH->N6-METHYLASE->Schlafen+HATPase+wHTH->PglZ->SIGMA+OB-Fold+AAA-ATPase+Lon-Protease-BrxL->REase->REase->Phage_int_SAM_1+Phage_integrase->	1186	Bacteria>FCB_group>Fibrobacterota>Fibrobacteria>Fibrobacterales>Fibrobacteraceae	GCF_002210355.1	Fibrobacter_sp._UWH3	B7993_RS04325	153736..157296	1964353	BREX-system-P-loop-protein-BrxC-[Fibrobacter-sp.-UWH3]
WP_198953245.1	Type1.BREX	DNA_pol3_beta+DNA_pol3_beta_2+DNA_pol3_beta_3->?-><-?<-?<-?<-SIGMA+OB-Fold+AAA-ATPase+Lon-Protease-BrxL<-PglZ<-Schlafen+HATPase+wHTH<-N6-METHYLASE<-DUF6079*<-BrxB-BrxF-iSTAND<-BrxA-DNA-binding<-?<-MODE-HTH+PBPII||?->?->Phos_pyr_kin->RADICAL-SAM->	1176	Bacteria>FCB_group>Fibrobacterota>Fibrobacteria>Fibrobacterales>Fibrobacteraceae	GCF_002210425.1	Fibrobacter_sp._UWB2	B7982_RS07630	365376..368906	1964358	BREX-system-P-loop-protein-BrxC-[Fibrobacter-sp.-UWB2]
WP_073283173.1	Type1.BREX	<-VirE<-?<-XIS-HTH||?->?->?->BrxA-DNA-binding->BrxB-BrxF-iSTAND->DUF6079*->ParB+HNH->N6-METHYLASE->Schlafen+HATPase+wHTH->PglZ->SIGMA+OB-Fold+AAA-ATPase+Lon-Protease-BrxL->REase->REase->Phage_int_SAM_1+Phage_integrase->	1184	Bacteria>FCB_group>Fibrobacterota>Fibrobacteria>Fibrobacterales>Fibrobacteraceae	GCF_900142465.1	Fibrobacter_sp._UWH6	BUB73_RS01535	351031..354585	1896212	BREX-system-P-loop-protein-BrxC-[Fibrobacter-sp.-UWH6]

Protein-ID	System	Gene-Neighbourhoods	Length	Taxonomy	Assembly-ID	Strain	Locus-Tag	Genomic-coordinates	TaxID	Fasta-defline
WP_217650997.1	Type1.BREX	[illegible]	1176	Bacteria>FCB_group>Fibrobacterota>Fibrobacteria>Fibrobacterales>Fibrobacteraceae	GCF_900142475.1	Fibrobacter_sp._UWH4	BUA93_RS10220	85922..89452	1896210	BREX-system-P-loop-protein-BrxC-[Fibrobacter-sp.-UWH4]
WP_073156756.1	Type1.BREX	[illegible]	1184	Bacteria>FCB_group>Fibrobacterota>Fibrobacteria>Fibrobacterales>Fibrobacteraceae	GCF_900142505.1	Fibrobacter_sp._UWH5	BUB38_RS01950	444352..447906	1896211	BREX-system-P-loop-protein-BrxC-[Fibrobacter-sp.-UWH5]
WP_073424902.1	Type1.BREX	ABhydrolase->?-><-SIGMA+OB-Fold+AAA-ATPase+HKD-DNase-BrxL<-PglZ<-N6-METHYLASE<-DUF6079*<-BrxB-BrxF-iSTAND<-BrxA-DNA-binding<-LRR_5<-PIN<-RelB	1212	Bacteria>FCB_group>Fibrobacterota>Fibrobacteria>Fibrobacterales>Fibrobacteraceae	GCF_900142945.1	Fibrobacter_sp._UWB7	BUC30_RS10995	309469..313107	1896206	BREX-system-P-loop-protein-BrxC-[Fibrobacter-sp.-UWB7]
MBK8058263.1	Type1.BREX	[illegible]	1035	Bacteria>FCB_group>Gemmatimonadota>unclassified_Gemmatimonadota	GCA_016713015.1	Gemmatimonadota_bacterium	IPK33_10390	16210..19317	2026742	BREX-system-P-loop-protein-BrxC-[Gemmatimonadota-bacterium]
MBK8060292.1	Type1.BREX	[illegible]	1154	Bacteria>FCB_group>Gemmatimonadota>unclassified_Gemmatimonadota	GCA_016713015.1	Gemmatimonadota_bacterium	IPK33_21145	255603..259067	2026742	BREX-system-P-loop-protein-BrxC-[Gemmatimonadota-bacterium]
MBK9977127.1	Type1.BREX	[illegible]	79	Bacteria>FCB_group>Gemmatimonadota>unclassified_Gemmatimonadota	GCA_016721935.1	Gemmatimonadota_bacterium	IPP20_04220	1050046..1050285	2026742	hypothetical-protein-[Gemmatimonadota-bacterium]
WP_185874362.1	Type1.BREX	[illegible]	1243	Bacteria>Fusobacteriota>Fusobacteriia>Fusobacteriales>Fusobacteriaceae>Cetobacterium	GCF_014250675.1	Cetobacterium_sp._8H	H5J22_RS00820	148071..151802	2759681	BREX-system-P-loop-protein-BrxC-[Cetobacterium-sp.-8H]
CDA09060.1	Type1.BREX	[illegible]	1250	Bacteria>Fusobacteriota>Fusobacteriia>Fusobacteriales>Fusobacteriaceae>Fusobacterium	GCA_000433695.1	Fusobacterium_sp._CAG:649	BN748_00884	23068..26820	1262900	putative-uncharacterized-protein-[Fusobacterium-sp.-CAG:649]
ALF25292.1	Type1.BREX	[illegible]	1250	Bacteria>Fusobacteriota>Fusobacteriia>Fusobacteriales>Fusobacteriaceae>Fusobacterium	GCA_001296185.1	Fusobacterium_nucleatum_subsp._nucleatum	RN95_02150	455192..458944	76856	cobalamin-adenosyltransferase-[Fusobacterium-nucleatum-subsp.-nucleatum]
KAB0576672.1	Type1.BREX	[illegible]	1217	Bacteria>Fusobacteriota>Fusobacteriia>Fusobacteriales>Fusobacteriaceae>Fusobacterium	GCA_008801825.1	Fusobacterium_naviforme	F7P78_08540	57971..61624	77917	BREX-system-P-loop-protein-BrxC-[Fusobacterium-naviforme]
MCF0162780.1	Type1.BREX	PrmA+METHYLASE->?->?->METHYLASE->?->?->?->DUF6079*->N6-METHYLASE->?-><-ABC-ATPase||AlcRN+AraC-HTH+AraC-HTH->	1240	Bacteria>Fusobacteriota>Fusobacteriia>Fusobacteriales>Fusobacteriaceae>Fusobacterium	GCA_021443685.1	Fusobacterium_necrophorum	HUJ88_RS09480	48208..51930	859	BREX-system-P-loop-protein-BrxC-[Fusobacterium-necrophorum]
MCF2639759.1	Type1.BREX	DUF6079*->N6-METHYLASE->PglZ->BrxA-DNA-binding->BrxB-BrxF-iSTAND->CR-ATPase5+REase->DOC->Calcineurin->HIN-HTH->	1244	Bacteria>Fusobacteriota>Fusobacteriia>Fusobacteriales>Fusobacteriaceae>Fusobacterium	GCA_021531615.1	Fusobacterium_varium	I6E31_07220	5895..9629	856	BREX-system-P-loop-protein-BrxC-[Fusobacterium-varium]
WP_008797395.1	Type1.BREX	[illegible]	1249	Bacteria>Fusobacteriota>Fusobacteriia>Fusobacteriales>Fusobacteriaceae>Fusobacterium	GCF_000163915.2	Fusobacterium_nucleatum	HMPREF0405_RS05640	1192926..1196675	851	BREX-system-P-loop-protein-BrxC-[Fusobacterium-vincentii]
WP_005903411.1	Type1.BREX	[illegible]	1250	Bacteria>Fusobacteriota>Fusobacteriia>Fusobacteriales>Fusobacteriaceae>Fusobacterium	GCF_000178895.1	Fusobacterium_nucleatum	C4N14_RS08075	1667383..1671135	851	BREX-system-P-loop-protein-BrxC-[Fusobacterium-nucleatum]
WP_008692035.1	Type1.BREX	[illegible]	1250	Bacteria>Fusobacteriota>Fusobacteriia>Fusobacteriales>Fusobacteriaceae>Fusobacterium	GCF_000218655.1	Fusobacterium_nucleatum	HMPREF0401_RS10120	557323..561075	851	BREX-system-P-loop-protein-BrxC-[Fusobacterium-animalis]
WP_008700610.1	Type1.BREX	[illegible]	1250	Bacteria>Fusobacteriota>Fusobacteriia>Fusobacteriales>Fusobacteriaceae>Fusobacterium	GCF_000234075.2	Fusobacterium_nucleatum	FSDG_RS04805	1011636..1015388	851	MULTISPECIES:-BREX-system-P-loop-protein-BrxC-[Fusobacterium]
WP_009423276.1	Type1.BREX	Peptidase_M20->Peptidase_M3_N+Peptidase_M3->?->?-><-?<-?||DUF6079*->	1219	Bacteria>Fusobacteriota>Fusobacteriia>Fusobacteriales>Fusobacteriaceae>Fusobacterium	GCF_000235465.1	Fusobacterium_sp._oral_taxon_370	HMPREF9093_RS02800	9924..13583	712288	BREX-system-P-loop-protein-BrxC-[Fusobacterium-sp.-oral-taxon-370]
WP_005959274.1	Type1.BREX	PrmA+METHYLASE->?->?->METHYLASE->?->?->?->DUF6079*->N6-METHYLASE->?->PglZ->BrxA-DNA-binding->BrxB-BrxF-iSTAND->	1240	Bacteria>Fusobacteriota>Fusobacteriia>Fusobacteriales>Fusobacteriaceae>Fusobacterium	GCF_000262225.1	Fusobacterium_necrophorum	HMPREF1049_0421	64292..68014	859	BREX-system-P-loop-protein-BrxC-[Fusobacterium-necrophorum]
WP_029493951.1	Type1.BREX	[illegible]	1250	Bacteria>Fusobacteriota>Fusobacteriia>Fusobacteriales>Fusobacteriaceae>Fusobacterium	GCF_000455885.1	Fusobacterium_hwasookii	J143_RS0110850	68335..72087	1583098	BREX-system-P-loop-protein-BrxC-[Fusobacterium-hwasookii]
WP_029598126.1	Type1.BREX	SWIM+DUF3670+SF1-SF2-Helicase->?-><-?<-?<-?<-?||DUF6079*->OLD-ABC->TOPRIM->N6-METHYLASE->KAP-NTPase->HNH->SbcC->SIR2->HerA/FtsK->	1250	Bacteria>Fusobacteriota>Fusobacteriia>Fusobacteriales>Fusobacteriaceae>Fusobacterium	GCF_000455985.1	Fusobacterium_nucleatum	J141_RS0104310	22455..26207	851	BREX-system-P-loop-protein-BrxC-[Fusobacterium-animalis]
WP_023036666.1	Type1.BREX	[illegible]	1250	Bacteria>Fusobacteriota>Fusobacteriia>Fusobacteriales>Fusobacteriaceae>Fusobacterium	GCF_000479185.1	Fusobacterium_nucleatum	HMPREF1767_RS09525	33414..37166	851	BREX-system-P-loop-protein-BrxC-[Fusobacterium-nucleatum]
WP_032838665.1	Type1.BREX	[illegible]	1250	Bacteria>Fusobacteriota>Fusobacteriia>Fusobacteriales>Fusobacteriaceae>Fusobacterium	GCF_000524215.1	Fusobacterium_sp._CM1	HMPREF1498_RS02575	43304..47056	936561	BREX-system-P-loop-protein-BrxC-[Fusobacterium-sp.-CM1]
WP_032836039.1	Type1.BREX	[illegible]	1250	Bacteria>Fusobacteriota>Fusobacteriia>Fusobacteriales>Fusobacteriaceae>Fusobacterium	GCF_000524235.1	Fusobacterium_sp._OBRC1	HMPREF1501_RS01100	63475..67227	1032505	BREX-system-P-loop-protein-BrxC-[Fusobacterium-sp.-OBRC1]
WP_032846252.1	Type1.BREX	[illegible]	1250	Bacteria>Fusobacteriota>Fusobacteriia>Fusobacteriales>Fusobacteriaceae>Fusobacterium	GCF_000524395.1	Fusobacterium_sp._CM22	HMPREF1500_RS09190	124439..128191	936563	BREX-system-P-loop-protein-BrxC-[Fusobacterium-sp.-CM22]
WP_035935618.1	Type1.BREX	[illegible]	1240	Bacteria>Fusobacteriota>Fusobacteriia>Fusobacteriales>Fusobacteriaceae>Fusobacterium	GCF_000691685.1	Fusobacterium_necrophorum	FUS05_RS04310	3773..7495	859	BREX-system-P-loop-protein-BrxC-[Fusobacterium-necrophorum]
WP_035906561.1	Type1.BREX	[illegible]	1240	Bacteria>Fusobacteriota>Fusobacteriia>Fusobacteriales>Fusobacteriaceae>Fusobacterium	GCF_000691745.1	Fusobacterium_necrophorum	FUS08_RS05985	5507..9229	859	BREX-system-P-loop-protein-BrxC-[Fusobacterium-necrophorum]
WP_060676307.1	Type1.BREX	[illegible]	1250	Bacteria>Fusobacteriota>Fusobacteriia>Fusobacteriales>Fusobacteriaceae>Fusobacterium	GCF_001296085.1	Fusobacterium_nucleatum	RN98_RS07270	1461443..1465195	851	BREX-system-P-loop-protein-BrxC-[Fusobacterium-animalis]
WP_062627844.1	Type1.BREX	[illegible]	1240	Bacteria>Fusobacteriota>Fusobacteriia>Fusobacteriales>Fusobacteriaceae>Fusobacterium	GCF_001596495.1	Fusobacterium_necrophorum	A2J05_RS04010	64534..68256	859	BREX-system-P-loop-protein-BrxC-[Fusobacterium-necrophorum]
WP_070766192.1	Type1.BREX	[illegible]	1250	Bacteria>Fusobacteriota>Fusobacteriia>Fusobacteriales>Fusobacteriaceae>Fusobacterium	GCF_001815715.1	Fusobacterium_sp._HMSC064B11	HMPREF3051_RS04300	251838..255590	1739543	BREX-system-P-loop-protein-BrxC-[Fusobacterium-sp.-HMSC064B11]
WP_088387741.1	Type1.BREX	[illegible]	1250	Bacteria>Fusobacteriota>Fusobacteriia>Fusobacteriales>Fusobacteriaceae>Fusobacterium	GCF_002204435.1	Fusobacterium_nucleatum	CA839_RS02150	467099..470851	851	BREX-system-P-loop-protein-BrxC-[Fusobacterium-polymorphum]
WP_158411765.1	Type1.BREX	[illegible]	1250	Bacteria>Fusobacteriota>Fusobacteriia>Fusobacteriales>Fusobacteriaceae>Fusobacterium	GCF_002573475.1	Fusobacterium_nucleatum	RN90_RS06260	1289254..1293006	851	BREX-system-P-loop-protein-BrxC-[Fusobacterium-animalis]
WP_098702328.1	Type1.BREX	[illegible]	1250	Bacteria>Fusobacteriota>Fusobacteriia>Fusobacteriales>Fusobacteriaceae>Fusobacterium	GCF_002573625.1	Fusobacterium_nucleatum	RN96_RS03470	669523..673275	851	BREX-system-P-loop-protein-BrxC-[Fusobacterium-polymorphum]
WP_098997105.1	Type1.BREX	[illegible]	1250	Bacteria>Fusobacteriota>Fusobacteriia>Fusobacteriales>Fusobacteriaceae>Fusobacterium	GCF_002591465.1	Fusobacterium_nucleatum	CBG56_RS03320	143954..147706	851	MULTISPECIES:-BREX-system-P-loop-protein-BrxC-[Fusobacterium]
WP_098973854.1	Type1.BREX	[illegible]	1250	Bacteria>Fusobacteriota>Fusobacteriia>Fusobacteriales>Fusobacteriaceae>Fusobacterium	GCF_002591515.1	Fusobacterium_nucleatum	CBG58_RS00340	77641..81393	851	BREX-system-P-loop-protein-BrxC-[Fusobacterium-polymorphum]
WP_099972121.1	Type1.BREX	[illegible]	1250	Bacteria>Fusobacteriota>Fusobacteriia>Fusobacteriales>Fusobacteriaceae>Fusobacterium	GCF_002761935.1	Fusobacterium_pseudoperiodonticum	CTM69_RS07785	481250..485002	2663009	BREX-system-P-loop-protein-BrxC-[Fusobacterium-pseudoperiodonticum]
WP_099959237.1	Type1.BREX	[illegible]	1250	Bacteria>Fusobacteriota>Fusobacteriia>Fusobacteriales>Fusobacteriaceae>Fusobacterium	GCF_002761955.1	Fusobacterium_pseudoperiodonticum	CTM71_RS09955	1967192..1970944	2663009	BREX-system-P-loop-protein-BrxC-[Fusobacterium-pseudoperiodonticum]
WP_158410489.1	Type1.BREX	[illegible]	1250	Bacteria>Fusobacteriota>Fusobacteriia>Fusobacteriales>Fusobacteriaceae>Fusobacterium	GCF_002762015.1	Fusobacterium_nucleatum	CI111_RS06870	6549..10301	851	BREX-system-P-loop-protein-BrxC-[Fusobacterium-animalis]
WP_100024172.1	Type1.BREX	[illegible]	1250	Bacteria>Fusobacteriota>Fusobacteriia>Fusobacteriales>Fusobacteriaceae>Fusobacterium	GCF_002763625.1	Fusobacterium_pseudoperiodonticum	CTM72_RS01085	199877..203629	2663009	BREX-system-P-loop-protein-BrxC-[Fusobacterium-pseudoperiodonticum]
WP_099987862.1	Type1.BREX	[illegible]	1250	Bacteria>Fusobacteriota>Fusobacteriia>Fusobacteriales>Fusobacteriaceae>Fusobacterium	GCF_002763695.1	Fusobacterium_pseudoperiodonticum	CTM74_RS09005	1721055..1724807	2663009	BREX-system-P-loop-protein-BrxC-[Fusobacterium-pseudoperiodonticum]
WP_099989146.1	Type1.BREX	[illegible]	1250	Bacteria>Fusobacteriota>Fusobacteriia>Fusobacteriales>Fusobacteriaceae>Fusobacterium	GCF_002763735.1	Fusobacterium_pseudoperiodonticum	CTM78_RS04805	952520..956272	2663009	BREX-system-P-loop-protein-BrxC-[Fusobacterium-pseudoperiodonticum]
WP_106894939.1	Type1.BREX	[illegible]	1240	Bacteria>Fusobacteriota>Fusobacteriia>Fusobacteriales>Fusobacteriaceae>Fusobacterium	GCF_003019715.1	Fusobacterium_necrophorum	C4N15_RS06225	1221345..1225067	859	BREX-system-P-loop-protein-BrxC-[Fusobacterium-necrophorum]
WP_118234412.1	Type1.BREX	[illegible]	1247	Bacteria>Fusobacteriota>Fusobacteriia>Fusobacteriales>Fusobacteriaceae>Fusobacterium	GCF_003470805.1	Fusobacterium_mortiferum	DW663_RS07335	52452..56195	850	BREX-system-P-loop-protein-BrxC-[Fusobacterium-mortiferum]
WP_062624739.1	Type1.BREX	[illegible]	1240	Bacteria>Fusobacteriota>Fusobacteriia>Fusobacteriales>Fusobacteriaceae>Fusobacterium	GCF_003732505.1	Fusobacterium_necrophorum	QDG35_RS01290	242452..246174	859	BREX-system-P-loop-protein-BrxC-[Fusobacterium-necrophorum]
WP_124794840.1	Type1.BREX	[illegible]	1250	Bacteria>Fusobacteriota>Fusobacteriia>Fusobacteriales>Fusobacteriaceae>Fusobacterium	GCF_003859915.1	Fusobacterium_canifelinum	EII27_RS00780	173024..176776	285729	BREX-system-P-loop-protein-BrxC-[Fusobacterium-canifelinum]
WP_129491631.1	Type1.BREX	[illegible]	1240	Bacteria>Fusobacteriota>Fusobacteriia>Fusobacteriales>Fusobacteriaceae>Fusobacterium	GCF_004134945.1	Fusobacterium_necrophorum	EPT53_RS09505	5167..8889	859	BREX-system-P-loop-protein-BrxC-[Fusobacterium-necrophorum]
WP_198480265.1	Type1.BREX	[illegible]	1250	Bacteria>Fusobacteriota>Fusobacteriia>Fusobacteriales>Fusobacteriaceae>Fusobacterium	GCF_016127595.1	Fusobacterium_canifelinum	I6H56_RS08610	1734646..1738398	285729	BREX-system-P-loop-protein-BrxC-[Fusobacterium-canifelinum]
WP_211495174.1	Type1.BREX	[illegible]	1240	Bacteria>Fusobacteriota>Fusobacteriia>Fusobacteriales>Fusobacteriaceae>Fusobacterium	GCF_018205295.1	Fusobacterium_necrophorum	IX293_RS07190	26353..30075	859	BREX-system-P-loop-protein-BrxC-[Fusobacterium-necrophorum]
WP_211494658.1	Type1.BREX	[illegible]	1240	Bacteria>Fusobacteriota>Fusobacteriia>Fusobacteriales>Fusobacteriaceae>Fusobacterium	GCF_018206635.1	Fusobacterium_necrophorum	IX329_RS07975	11683..15405	859	BREX-system-P-loop-protein-BrxC-[Fusobacterium-necrophorum]
WP_220302801.1	Type1.BREX	[illegible]	1250	Bacteria>Fusobacteriota>Fusobacteriia>Fusobacteriales>Fusobacteriaceae>Fusobacterium	GCF_019552045.1	Fusobacterium_nucleatum	JY397_RS08300	1731485..1735237	851	BREX-system-P-loop-protein-BrxC-[Fusobacterium-polymorphum]
WP_220282655.1	Type1.BREX	[illegible]	1250	Bacteria>Fusobacteriota>Fusobacteriia>Fusobacteriales>Fusobacteriaceae>Fusobacterium	GCF_019552065.1	Fusobacterium_nucleatum	JY402_RS06730	1356729..1360481	851	BREX-system-P-loop-protein-BrxC-[Fusobacterium-polymorphum]
WP_220297607.1	Type1.BREX	[illegible]	1250	Bacteria>Fusobacteriota>Fusobacteriia>Fusobacteriales>Fusobacteriaceae>Fusobacterium	GCF_019552085.1	Fusobacterium_nucleatum	JY398_RS09460	1985091..1988843	851	BREX-system-P-loop-protein-BrxC-[Fusobacterium-animalis]
WP_220308657.1	Type1.BREX	[illegible]	1250	Bacteria>Fusobacteriota>Fusobacteriia>Fusobacteriales>Fusobacteriaceae>Fusobacterium	GCF_019552125.1	Fusobacterium_nucleatum	JY401_RS04550	954810..958562	851	BREX-system-P-loop-protein-BrxC-[Fusobacterium-animalis]
WP_238959505.1	Type1.BREX	[illegible]	1250	Bacteria>Fusobacteriota>Fusobacteriia>Fusobacteriales>Fusobacteriaceae>Fusobacterium	GCF_022340045.1	Fusobacterium_nucleatum	K5I20_00280	65514..69266	851	BREX-system-P-loop-protein-BrxC-[Fusobacterium-nucleatum]
WP_238972693.1	Type1.BREX	[illegible]	1250	Bacteria>Fusobacteriota>Fusobacteriia>Fusobacteriales>Fusobacteriaceae>Fusobacterium	GCF_022340165.1	Fusobacterium_nucleatum	K5I19_RS02505	127932..131684	851	BREX-system-P-loop-protein-BrxC-[Fusobacterium-nucleatum]
WP_060798221.1	Type1.BREX	[illegible]	1250	Bacteria>Fusobacteriota>Fusobacteriia>Fusobacteriales>Fusobacteriaceae>Fusobacterium	GCF_023383525.1	Fusobacterium_nucleatum	YWH7054_RS02310	21716..25468	851	BREX-system-P-loop-protein-BrxC-[Fusobacterium-nucleatum]
WP_022069638.1	Type1.BREX	[illegible]	1250	Bacteria>Fusobacteriota>Fusobacteriia>Fusobacteriales>Fusobacteriaceae>Fusobacterium	GCF_023383545.1	Fusobacterium_nucleatum	YWH7053_RS01985	23867..27619	851	BREX-system-P-loop-protein-BrxC-[Fusobacterium-nucleatum]
WP_249524183.1	Type1.BREX	[illegible]	1250	Bacteria>Fusobacteriota>Fusobacteriia>Fusobacteriales>Fusobacteriaceae>Fusobacterium	GCF_023383605.1	Fusobacterium_nucleatum	YWH7056_RS07045	28639..32391	851	BREX-system-P-loop-protein-BrxC-[Fusobacterium-nucleatum]
WP_249526950.1	Type1.BREX	[illegible]	1250	Bacteria>Fusobacteriota>Fusobacteriia>Fusobacteriales>Fusobacteriaceae>Fusobacterium	GCF_023383645.1	Fusobacterium_nucleatum	YWH7199_RS06535	22791..26543	851	BREX-system-P-loop-protein-BrxC-[Fusobacterium-nucleatum]
WP_023036012.1	Type1.BREX	[illegible]	1249	Bacteria>Fusobacteriota>Fusobacteriia>Fusobacteriales>Fusobacteriaceae>Fusobacterium	GCF_024140205.1	Fusobacterium_nucleatum	BKN39_RS05495	10931..14680	851	MULTISPECIES:-BREX-system-P-loop-protein-BrxC-[Fusobacterium]
WP_265152268.1	Type1.BREX	[illegible]	1250	Bacteria>Fusobacteriota>Fusobacteriia>Fusobacteriales>Fusobacteriaceae>Fusobacterium	GCF_026089295.1	Fusobacterium_simiae	OCK72_RS06710	29932..33684	855	BREX-system-P-loop-protein-BrxC-[Fusobacterium-simiae]
WP_270426686.1	Type1.BREX	[illegible]	1243	Bacteria>Fusobacteriota>Fusobacteriia>Fusobacteriales>Fusobacteriaceae>Fusobacterium	GCF_027666755.1	Fusobacterium_mortiferum	PGR85_RS01105	267990..271721	850	BREX-system-P-loop-protein-BrxC-[Fusobacterium-mortiferum]
WP_271333596.1	Type1.BREX	[illegible]	1250	Bacteria>Fusobacteriota>Fusobacteriia>Fusobacteriales>Fusobacteriaceae>Fusobacterium	GCF_027947475.1	Fusobacterium_nucleatum	PGW91_RS03610	747072..750824	851	BREX-system-P-loop-protein-BrxC-[Fusobacterium-nucleatum]
WP_274179672.1	Type1.BREX	[illegible]	1250	Bacteria>Fusobacteriota>Fusobacteriia>Fusobacteriales>Fusobacteriaceae>Fusobacterium	GCF_028735995.1	Fusobacterium_nucleatum	PSC67_RS07260	1410490..1414242	851	BREX-system-P-loop-protein-BrxC-[Fusobacterium-nucleatum]
WP_005980883.1	Type1.BREX	[illegible]	1242	Bacteria>Fusobacteriota>Fusobacteriia>Fusobacteriales>Fusobacteriaceae>Fusobacterium	GCF_902373815.1	Fusobacterium_ulcerans	FXX38_RS11815	320598..324326	861	BREX-system-P-loop-protein-BrxC-[Fusobacterium-ulcerans]
WP_008800858.1	Type1.BREX	[illegible]	1240	Bacteria>Fusobacteriota>Fusobacteriia>Fusobacteriales>Fusobacteriaceae>Fusobacterium	GCF_902373895.1	Fusobacterium_gonidiaformans	FY288_RS06315	242664..246386	849	BREX-system-P-loop-protein-BrxC-[Fusobacterium-gonidiaformans]
WP_008793539.1	Type1.BREX	[illegible]	1250	Bacteria>Fusobacteriota>Fusobacteriia>Fusobacteriales>Fusobacteriaceae>Fusobacterium	GCF_902373915.1	Fusobacterium_periodonticum	FY302_RS02975	615184..618936	860	BREX-system-P-loop-protein-BrxC-[Fusobacterium-periodonticum]
WP_023041227.1	Type1.BREX	[illegible]	884	Bacteria>Fusobacteriota>Fusobacteriia>Fusobacteriales>Fusobacteriaceae>Fusobacterium	GCF_902375195.1	Fusobacterium_nucleatum	FY391_RS05415	47998..50649	851	BREX-system-P-loop-protein-BrxC,-partial-[Fusobacterium-nucleatum]
VTX48853.1	Type1.BREX	[illegible]	1192	Bacteria>Fusobacteriota>Fusobacteriia>Fusobacteriales>Leptotrichiaceae>Leptotrichia	GCA_901873485.1	uncultured_Leptotrichia_sp.	FPIDKHFM_01499	24244..27822	159271	Uncharacterised-protein-[uncultured-Leptotrichia-sp.]
WP_021768239.1	Type1.BREX	[illegible]	1193	Bacteria>Fusobacteriota>Fusobacteriia>Fusobacteriales>Leptotrichiaceae>Leptotrichia	GCF_000469525.1	Leptotrichia_sp._oral_taxon_225	HMPREF9108_RS00960	38226..41807	671213	BREX-system-P-loop-protein-BrxC-[Leptotrichia-sp.-oral-taxon-225]
WP_146995744.1	Type1.BREX	[illegible]	1206	Bacteria>Fusobacteriota>Fusobacteriia>Fusobacteriales>Leptotrichiaceae>Leptotrichia	GCF_007990425.1	Leptotrichia_trevisanii	FVE79_RS00395	92064..95684	109328	BREX-system-P-loop-protein-BrxC-[Leptotrichia-trevisanii]
WP_026745399.1	Type1.BREX	[illegible]	1193	Bacteria>Fusobacteriota>Fusobacteriia>Fusobacteriales>Leptotrichiaceae>Leptotrichia	GCF_007990525.1	Leptotrichia_hofstadii	FVE77_RS00755	171012..174593	157688	BREX-system-P-loop-protein-BrxC-[Leptotrichia-hofstadii]
WP_018499190.1	Type1.BREX	[illegible]	1192	Bacteria>Fusobacteriota>Fusobacteriia>Fusobacteriales>Leptotrichiaceae>Leptotrichia	GCF_007990545.2	Leptotrichia_wadei	FVE73_RS10040	2180563..2184141	157687	BREX-system-P-loop-protein-BrxC-[Leptotrichia-wadei]
WP_147006310.1	Type1.BREX	[illegible]	1192	Bacteria>Fusobacteriota>Fusobacteriia>Fusobacteriales>Leptotrichiaceae>Leptotrichia	GCF_007990635.1	Leptotrichia_hongkongensis	FVE75_RS10120	2124318..2127896	554406	BREX-system-P-loop-protein-BrxC-[Leptotrichia-hongkongensis]
WP_178935823.1	Type1.BREX	[illegible]	1193	Bacteria>Fusobacteriota>Fusobacteriia>Fusobacteriales>Leptotrichiaceae>Leptotrichia	GCF_013394795.1	Leptotrichia_sp._oral_taxon_223	HW275_RS06835	1386069..1389650	712363	BREX-system-P-loop-protein-BrxC-[Leptotrichia-sp.-oral-taxon-223]
WP_011422284.1	Type1.BREX	[illegible]	1153	Bacteria>Myxococcota>Myxococcia>Myxococcales>Cystobacterineae>Anaeromyxobacteraceae	GCF_000013385.1	Anaeromyxobacter_dehalogenans	ADEH_RS16690	3696197..3699658	161493	BREX-system-P-loop-protein-BrxC-[Anaeromyxobacter-dehalogenans]
EPX63269.1	Type1.BREX	[illegible]	1148	Bacteria>Myxococcota>Myxococcia>Myxococcales>Cystobacterineae>Archangiaceae	GCA_000335475.2	Cystobacter_fuscus_DSM_2262	D187_005675	86120..89566	1242864	hypothetical-protein-D187_005675-[Cystobacter-fuscus-DSM-2262]
WP_051256264.1	Type1.BREX	[illegible]	1149	Bacteria>Myxococcota>Myxococcia>Myxococcales>Cystobacterineae>Archangiaceae	GCF_000335475.2	Cystobacter_fuscus	D187_RS07930	86120..89569	43	BREX-system-P-loop-protein-BrxC-[Cystobacter-fuscus]
WP_075010321.1	Type1.BREX	[illegible]	1230	Bacteria>Myxococcota>Myxococcia>Myxococcales>Cystobacterineae>Archangiaceae	GCF_900109545.1	Stigmatella_aurantiaca	BMZ62_RS31345	37883..41575	41	BREX-system-P-loop-protein-BrxC-[Stigmatella-aurantiaca]
WP_126934601.1	Type1.BREX	[illegible]	1152	Bacteria>Myxococcota>Myxococcia>Myxococcales>Cystobacterineae>Myxococcaceae	-	Corallococcus_sp._AB018	D7Y11_21050	14759..18217	2316715	BREX-system-P-loop-protein-BrxC-[Corallococcus-sp.-AB018]
MCA3011281.1	Type1.BREX	[illegible]	1152	Bacteria>Myxococcota>Myxococcia>Myxococcales>Cystobacterineae>Myxococcaceae	GCA_020247085.1	Myxococcaceae_bacterium	INH41_02665	329..3787	2484252	BREX-system-P-loop-protein-BrxC-[Myxococcaceae-bacterium]
WP_121754709.1	Type1.BREX	[illegible]	1152	Bacteria>Myxococcota>Myxococcia>Myxococcales>Cystobacterineae>Myxococcaceae	GCA_003668965.1	Corallococcus_sp._AB030	D7Y15_RS30065	6215..9673	2316716	BREX-system-P-loop-protein-BrxC-[Corallococcus-sp.-AB030]
WP_223643830.1	Type1.BREX	[illegible]	1152	Bacteria>Myxococcota>Myxococcia>Myxococcales>Cystobacterineae>Myxococcaceae	GCF_019968905.1	Corallococcus_sp._EGB	KYK13_RS09920	2348903..2352361	1521117	BREX-system-P-loop-protein-BrxC-[Corallococcus-sp.-EGB]
HCF60732.1	Type1.BREX	[illegible]	871	Bacteria>Myxococcota>Myxococcia>Myxococcales>unclassified_Myxococcales	GCA_003534605.1	Myxococcales_bacterium	DFS52_22370	11512..14124	2026763	BREX-system-P-loop-protein-BrxC-[Myxococcales-bacterium]
MCA9633383.1	Type1.BREX	[illegible]	1193	Bacteria>Myxococcota>Myxococcia>Myxococcales>unclassified_Myxococcales	GCA_020430865.1	Myxococcales_bacterium	KC766_37295	7322..10903	2026763	BREX-system-P-loop-protein-BrxC-[Myxococcales-bacterium]
MBE7449254.1	Type1.BREX	[illegible]	1153	Bacteria>Myxococcota>Polyangia>Haliangiales>Kofleriaceae>unclassified_Kofleriaceae	GCA_015075455.1	Kofleriaceae_bacterium	HS111_10245	215767..219228	2212474	BREX-system-P-loop-protein-BrxC-[Kofleriaceae-bacterium]
MBP9169874.1	Type1.BREX	[illegible]	1258	Bacteria>Myxococcota>Polyangia>Haliangiales>Kofleriaceae>unclassified_Kofleriaceae	GCA_018057825.1	Kofleriaceae_bacterium	KBG48_20895	5357..9133	2212474	BREX-system-P-loop-protein-BrxC-[Kofleriaceae-bacterium]
MBA3547589.1	Type1.BREX	[illegible]	1231	Bacteria>Myxococcota>Polyangia>Nannocystales>Nannocystaceae>Nannocystis	GCA_013812955.1	Nannocystis_sp.	HOT76_13980	6923..10618	1962667	BREX-system-P-loop-protein-BrxC-[Nannocystis-sp.]
MBA3548104.1	Type1.BREX	[illegible]	1231	Bacteria>Myxococcota>Polyangia>Nannocystales>Nannocystaceae>Nannocystis	GCA_013812955.1	Nannocystis_sp.	HOT76_16605	30110..33805	1962667	BREX-system-P-loop-protein-BrxC-[Nannocystis-sp.]
MBK8265326.1	Type1.BREX	[illegible]	1160	Bacteria>Myxococcota>Polyangia>Nannocystales>Nannocystaceae>Nannocystis	GCA_016712315.1	Nannocystis_sp.	IPK80_28855	46380..49862	1962667	BREX-system-P-loop-protein-BrxC-[Nannocystis-sp.]
WP_267777334.1	Type1.BREX	[illegible]	502	Bacteria>Myxococcota>Polyangia>Nannocystales>Nannocystaceae>Nannocystis	GCF_026626665.1	Nannocystis_pusilla	OV079_RS49390	11161170..11162678	889268	hypothetical-protein-[Nannocystis-pusilla]
MBN2191668.1	Type1.BREX	[illegible]	869	Bacteria>Myxococcota>Polyangia>Polyangiales>Polyangiaceae>unclassified_Polyangiaceae	GCA_016933275.1	Polyangiaceae_bacterium	JW751_02535	24054..26663	2268199	BREX-system-P-loop-protein-BrxC-[Polyangiaceae-bacterium]
MCK6536698.1	Type1.BREX	[illegible]	1149	Bacteria>Myxococcota>Polyangia>Polyangiales>Polyangiaceae>unclassified_Polyangiaceae	GCA_023150915.1	Polyangiaceae_bacterium	L6Q84_27330	8687..12136	2268199	BREX-system-P-loop-protein-BrxC-[Polyangiaceae-bacterium]
MCK6587209.1	Type1.BREX	[illegible]	1258	Bacteria>Myxococcota>Polyangia>Polyangiales>Polyangiaceae>unclassified_Polyangiaceae	GCA_023150955.1	Polyangiaceae_bacterium	L6Q76_06450	1533..5309	2268199	BREX-system-P-loop-protein-BrxC-[Polyangiaceae-bacterium]
MCB9617769.1	Type1.BREX	[illegible]	1156	Bacteria>Myxococcota>Polyangia>Polyangiales>Sandaracinaceae>Sandaracinus	GCA_020633155.1	Sandaracinus_sp.	H6722_35485	14055..17525	2024858	BREX-system-P-loop-protein-BrxC-[Sandaracinus-sp.]
MCK6544286.1	Type1.BREX	[illegible]	1246	Bacteria>Myxococcota>unclassified_Myxococcota	GCA_023150785.1	Myxococcota_bacterium	L6R52_00300	47328..51068	2818507	BREX-system-P-loop-protein-BrxC-[Myxococcota-bacterium]
MCK6512397.1	Type1.BREX	[illegible]	1145	Bacteria>Myxococcota>unclassified_Myxococcota	GCA_023150835.1	Myxococcota_bacterium	L6R29_20905	15899..19336	2818507	BREX-system-P-loop-protein-BrxC-[Myxococcota-bacterium]
WP_053765323.1	Type1.BREX	[illegible]	1214	Bacteria>Nitrospirota>Nitrospiria>Nitrospirales>Nitrospiraceae>Leptospirillum	GCF_001280545.1	Leptospirillum_ferriphilum	AOA54_RS10310	13451..17095	178606	BREX-system-P-loop-protein-BrxC-[Leptospirillum-ferriphilum]
NOS82785.1	Type1.BREX	[illegible]	1196	Bacteria>Nitrospirota>Nitrospiria>Nitrospirales>Nitrospiraceae>Nitrospira	GCA_013140535.1	Nitrospira_sp.	HOP32_14505	14147..17737	70125	BREX-system-P-loop-protein-BrxC-[Nitrospira-sp.]
MBX3319023.1	Type1.BREX	[illegible]	1193	Bacteria>Nitrospirota>Nitrospiria>Nitrospirales>Nitrospiraceae>Nitrospira	GCA_019636895.1	Nitrospira_sp.	KF890_04020	66850..70431	70125	BREX-system-P-loop-protein-BrxC-[Nitrospira-sp.]
MCG3775230.1	Type1.BREX	[illegible]	1096	Bacteria>Nitrospirota>Nitrospiria>Nitrospirales>Nitrospiraceae>Nitrospira	GCA_022117495.1	Nitrospira_sp.	JW395_2061	2579..5869	70125	hypothetical-protein-[Nitrospira-sp.]
WP_121988995.1	Type1.BREX	[illegible]	1193	Bacteria>Nitrospirota>Nitrospiria>Nitrospirales>Nitrospiraceae>Nitrospira	GCF_900403705.1	Nitrospira_lenta	NITLEN_RS07580	263981..267562	1436998	BREX-system-P-loop-protein-BrxC-[Nitrospira-lenta]
MBX9657254.1	Type1.BREX	[illegible]	1193	Bacteria>Nitrospirota>Nitrospiria>Nitrospirales>Nitrospiraceae>unclassified_Nitrospiraceae	GCA_019748255.1	Nitrospiraceae_bacterium	K2X00_01715	152268..155849	2026770	BREX-system-P-loop-protein-BrxC-[Nitrospiraceae-bacterium]
HDH04529.1	Type1.BREX	[illegible]	1182	Bacteria>Nitrospirota>unclassified_Nitrospirota	GCA_011040095.1	Nitrospirota_bacterium	ENH01_02275	6887..10435	2026887	BREX-system-P-loop-protein-BrxC-[Nitrospirota-bacterium]
MBI5192441.1	Type1.BREX	[illegible]	1181	Bacteria>Nitrospirota>unclassified_Nitrospirota	GCA_016212215.1	Nitrospirota_bacterium	HZA08_03235	28914..32459	2026887	BREX-system-P-loop-protein-BrxC-[Nitrospirota-bacterium]
MCC6543788.1	Type1.BREX	[illegible]	1181	Bacteria>Nitrospirota>unclassified_Nitrospirota	GCA_020846775.1	Nitrospirota_bacterium	IT392_04715	12437..15982	2026887	BREX-system-P-loop-protein-BrxC-[Nitrospirota-bacterium]
MCC6543975.1	Type1.BREX	[illegible]	1143	Bacteria>Nitrospirota>unclassified_Nitrospirota	GCA_020846775.1	Nitrospirota_bacterium	IT392_05655	165915..169346	2026887	BREX-system-P-loop-protein-BrxC-[Nitrospirota-bacterium]
WP_225487034.1	Type1.BREX	[illegible]	1146	Bacteria>Pseudomonadota>Acidithiobacillia>Acidithiobacillales>Acidithiobacillaceae>Acidithiobacillus	-	Acidithiobacillus_ferrooxidans	LDB30_08025	1563873..1567313	920	BREX-system-P-loop-protein-BrxC-[Acidithiobacillus-ferrooxidans]
WP_206385353.1	Type1.BREX	[illegible]	1179	Bacteria>Pseudomonadota>Acidithiobacillia>Acidithiobacillales>Acidithiobacillaceae>Acidithiobacillus	-	Acidithiobacillus_sp._MC6.1	JKG47_07195	11507..15046	2801580	BREX-system-P-loop-protein-BrxC-[Acidithiobacillus-sp.-MC6.1]
WP_172437661.1	Type1.BREX	[illegible]	1203	Bacteria>Pseudomonadota>Acidithiobacillia>Acidithiobacillales>Acidithiobacillaceae>Acidithiobacillus	-	Acidithiobacillus_sp._SH	HF670_09010	8231..11842	187490	MULTISPECIES:-BREX-system-P-loop-protein-BrxC-[Acidithiobacillus]
MBU2839699.1	Type1.BREX	[illegible]	1203	Bacteria>Pseudomonadota>Acidithiobacillia>Acidithiobacillales>Acidithiobacillaceae>Acidithiobacillus	GCA_018854655.1	Acidithiobacillus_thiooxidans	HF670_09010	8231..11842	930	BREX-system-P-loop-protein-BrxC-[Acidithiobacillus-thiooxidans]
WP_014029717.1	Type1.BREX	[illegible]	1179	Bacteria>Pseudomonadota>Acidithiobacillia>Acidithiobacillales>Acidithiobacillaceae>Acidithiobacillus	GCF_000214095.2	Acidithiobacillus_ferrivorans	ACIFE_RS11315	2362677..2366216	160808	BREX-system-P-loop-protein-BrxC-[Acidithiobacillus-ferrivorans]
WP_064218004.1	Type1.BREX	[illegible]	1187	Bacteria>Pseudomonadota>Acidithiobacillia>Acidithiobacillales>Acidithiobacillaceae>Acidithiobacillus	GCF_001652185.1	Acidithiobacillus_ferrooxidans	A4H96_RS01790	31399..34962	920	BREX-system-P-loop-protein-BrxC-[Acidithiobacillus-ferrooxidans]
WP_163056479.1	Type1.BREX	[illegible]	1203	Bacteria>Pseudomonadota>Acidithiobacillia>Acidithiobacillales>Acidithiobacillaceae>Acidithiobacillus	GCF_010577825.1	Acidithiobacillus_ferrooxidans	X857_RS08730	38026..41637	920	BREX-system-P-loop-protein-BrxC-[Acidithiobacillus-ferrooxidans]
WP_054609107.1	Type1.BREX	[illegible]	1180	Bacteria>Pseudomonadota>Acidithiobacillia>Acidithiobacillales>Acidithiobacillaceae>Acidithiobacillus	GCF_020887015.1	Acidithiobacillus_ferrooxidans	EC577_RS04340	17685..21227	920	BREX-system-P-loop-protein-BrxC-[Acidithiobacillus-ferrooxidans]
WP_271780036.1	Type1.BREX	[illegible]	1179	Bacteria>Pseudomonadota>Acidithiobacillia>Acidithiobacillales>Acidithiobacillaceae>Acidithiobacillus	GCF_028215975.1	Acidithiobacillus_ferriphilus	PJU76_RS00595	119550..123089	1689834	BREX-system-P-loop-protein-BrxC-[Acidithiobacillus-ferriphilus]
WP_101538034.1	Type1.BREX	[illegible]	1204	Bacteria>Pseudomonadota>Acidithiobacillia>Acidithiobacillales>Acidithiobacillaceae	GCF_018854655.1	Acidithiobacillus	HF670_RS09000	8228..11842	119977	MULTISPECIES:-BREX-system-P-loop-protein-BrxC-[Acidithiobacillus]
WP_023449856.1	Type1.BREX	[illegible]	1177	Bacteria>Pseudomonadota>Alphaproteobacteria>Caulobacterales>Caulobacteraceae>Asticcacaulis	GCF_000495835.1	Asticcacaulis_sp._AC402	ABAC402_RS31845	123615..127148	1282361	BREX-system-P-loop-protein-BrxC-[Asticcacaulis-sp.-AC402]
WP_114817812.1	Type1.BREX	[illegible]	1179	Bacteria>Pseudomonadota>Alphaproteobacteria>Caulobacterales>Caulobacteraceae>Brevundimonas	GCF_003350205.1	Brevundimonas_bullata	DXK21_RS10945	61535..65074	13160	BREX-system-P-loop-protein-BrxC-[Brevundimonas-bullata]
MBA4013265.1	Type1.BREX	[illegible]	1180	Bacteria>Pseudomonadota>Alphaproteobacteria>Caulobacterales>Caulobacteraceae>Phenylobacterium	GCA_013822795.1	Phenylobacterium_sp.	C0481_15475	50366..53908	1871053	BREX-system-P-loop-protein-BrxC-[Phenylobacterium-sp.]
MAS04597.1	Type1.BREX	[illegible]	1153	Bacteria>Pseudomonadota>Alphaproteobacteria>Hyphomicrobiales>Ahrensiaceae>Ahrensia	GCA_002698425.1	Ahrensia_sp.	CL534_07875	15621..19082	1872419	ATP-binding-protein-[Ahrensia-sp.]
WP_131311644.1	Type1.BREX	[illegible]	1198	Bacteria>Pseudomonadota>Alphaproteobacteria>Hyphomicrobiales>Ancalomicrobiaceae>Siculibacillus	GCF_004328075.1	Siculibacillus_lacustris	EYW49_RS21275	17320..20916	1549641	BREX-system-P-loop-protein-BrxC-[Siculibacillus-lacustris]
EAS50782.1	Type1.BREX	[illegible]	1206	Bacteria>Pseudomonadota>Alphaproteobacteria>Hyphomicrobiales>Aurantimonadaceae>Aurantimonas	GCA_000153465.1	Aurantimonas_manganoxydans_SI85-9A1	SI859A1_00907	1076027..1079647	287752	putative-ATPase-like-protein-[Aurantimonas-manganoxydans-SI85-9A1]
WP_040325304.1	Type1.BREX	[illegible]	1203	Bacteria>Pseudomonadota>Alphaproteobacteria>Hyphomicrobiales>Aurantimonadaceae>Aurantimonas	GCF_001463865.1	Aurantimonas_manganoxydans	AUL25_RS15575	29742..33353	651183	BREX-system-P-loop-protein-BrxC-[Aurantimonas-manganoxydans]
WP_061974428.1	Type1.BREX	[illegible]	1181	Bacteria>Pseudomonadota>Alphaproteobacteria>Hyphomicrobiales>Aurantimonadaceae>Aureimonas	GCF_001463725.1	Aureimonas_sp._AU20	AUL37_RS03985	274964..278509	1349819	BREX-system-P-loop-protein-BrxC-[Aureimonas-sp.-AU20]
WP_007065363.1	Type1.BREX	[illegible]	1204	Bacteria>Pseudomonadota>Alphaproteobacteria>Hyphomicrobiales>Aurantimonadaceae>Fulvimarina	GCF_001463845.1	Fulvimarina_pelagi	FP2506_RS00480	107157..110771	217511	BREX-system-P-loop-protein-BrxC-[Fulvimarina-pelagi]
WP_268883456.1	Type1.BREX	[illegible]	617	Bacteria>Pseudomonadota>Alphaproteobacteria>Hyphomicrobiales>Aurantimonadaceae>Jiella	GCF_026898155.1	Jiella_sp._HL-NP1	OH818_RS13650	2639917..2641770	2986949	BREX-system-P-loop-protein-BrxC-[Jiella-sp.-HL-NP1]
WP_230722685.1	Type1.BREX	[illegible]	1177	Bacteria>Pseudomonadota>Alphaproteobacteria>Hyphomicrobiales>Aurantimonadaceae>Martelella	GCF_021021415.1	Martelella_mediterranea	K7H91_RS23870	16033..19566	293089	BREX-system-P-loop-protein-BrxC-[Martelella-mediterranea]
HBQ31644.1	Type1.BREX	[illegible]	1180	Bacteria>Pseudomonadota>Alphaproteobacteria>Hyphomicrobiales>Brucellaceae>Brucella/Ochrobactrum_group	GCA_003528245.1	Brucella_anthropi	DD739_02835	9201..12743	529	BREX-system-P-loop-protein-BrxC-[Brucella-anthropi]
MBA8845725.1	Type1.BREX	[illegible]	181	Bacteria>Pseudomonadota>Alphaproteobacteria>Hyphomicrobiales>Brucellaceae>Brucella/Ochrobactrum_group	GCA_014138125.1	Ochrobactrum_sp._RH1CCR137	FHW01_003637	23274..23819	2587043	hypothetical-protein-[Ochrobactrum-sp.-RH1CCR137]
WP_094540671.1	Type1.BREX	[illegible]	1179	Bacteria>Pseudomonadota>Alphaproteobacteria>Hyphomicrobiales>Brucellaceae>Brucella/Ochrobactrum_group	GCF_002252505.1	Brucella_grignonensis	CEV33_RS11715	211751..215290	94627	BREX-system-P-loop-protein-BrxC-[Brucella-grignonensis]
WP_113731560.1	Type1.BREX	[illegible]	1179	Bacteria>Pseudomonadota>Alphaproteobacteria>Hyphomicrobiales>Brucellaceae>Brucella/Ochrobactrum_group	GCF_003261015.1	Brucella_anthropi	F9L04_RS18285	14553..18092	529	BREX-system-P-loop-protein-BrxC-[Brucella-anthropi]
WP_151554984.1	Type1.BREX	[illegible]	1205	Bacteria>Pseudomonadota>Alphaproteobacteria>Hyphomicrobiales>Brucellaceae>Brucella/Ochrobactrum_group	GCF_008932275.1	Brucella_tritici	F9K85_RS05045	164979..168596	94626	BREX-system-P-loop-protein-BrxC-[Brucella-tritici]
WP_151664132.1	Type1.BREX	[illegible]	1179	Bacteria>Pseudomonadota>Alphaproteobacteria>Hyphomicrobiales>Brucellaceae>Brucella/Ochrobactrum_group	GCF_008932705.1	Brucella_anthropi	F9L04_RS20265	51555..55094	529	BREX-system-P-loop-protein-BrxC-[Brucella-anthropi]
WP_151665645.1	Type1.BREX	[illegible]	1180	Bacteria>Pseudomonadota>Alphaproteobacteria>Hyphomicrobiales>Brucellaceae>Brucella/Ochrobactrum_group	GCF_008932825.1	Brucella_anthropi	F9K96_RS24475	19243..22785	529	BREX-system-P-loop-protein-BrxC-[Brucella-anthropi]
WP_109366634.1	Type1.BREX	[illegible]	1179	Bacteria>Pseudomonadota>Alphaproteobacteria>Hyphomicrobiales>Brucellaceae>Brucella/Ochrobactrum_group	GCF_900243435.1	[Ochrobactrum]_soli	OHAE_RS05910	16979..20518	2448455	BREX-system-P-loop-protein-BrxC-[[Ochrobactrum]
WP_184220677.1	Type1.BREX	[illegible]	1179	Bacteria>Pseudomonadota>Alphaproteobacteria>Hyphomicrobiales>Brucellaceae>Paenochrobactrum	GCF_014205685.1	Paenochrobactrum_gallinarii	FHS77_RS04490	13414..16953	643673	BREX-system-P-loop-protein-BrxC-[Paenochrobactrum-gallinarii]
WP_075657534.1	Type1.BREX	[illegible]	1179	Bacteria>Pseudomonadota>Alphaproteobacteria>Hyphomicrobiales>Brucellaceae>Pseudochrobactrum	GCF_001939785.1	Pseudochrobactrum_sp._B5	BKD14_RS17970	55973..59512	1289478	BREX-system-P-loop-protein-BrxC-[Pseudochrobactrum-sp.-B5]
WP_113944570.1	Type1.BREX	[illegible]	1179	Bacteria>Pseudomonadota>Alphaproteobacteria>Hyphomicrobiales>Brucellaceae>Pseudochrobactrum	GCF_003314995.1	Pseudochrobactrum_asaccharolyticum	DFR47_RS11470	41745..45284	354351	BREX-system-P-loop-protein-BrxC-[Pseudochrobactrum-asaccharolyticum]
MBX3547065.1	Type1.BREX	[illegible]	777	Bacteria>Pseudomonadota>Alphaproteobacteria>Hyphomicrobiales>Chelatococcaceae>Chelatococcus	GCA_019635905.1	Chelatococcus_sp.	KF818_30290	46824..49157	1953771	BREX-system-P-loop-protein-BrxC-[Chelatococcus-sp.]
MBL8596415.1	Type1.BREX	[illegible]	1166	Bacteria>Pseudomonadota>Alphaproteobacteria>Hyphomicrobiales>Devosiaceae>Devosia	GCA_016793625.1	Devosia_sp.	JNL14_01620	65385..68885	1871048	BREX-system-P-loop-protein-BrxC-[Devosia-sp.]
WP_240230050.1	Type1.BREX	[illegible]	1166	Bacteria>Pseudomonadota>Alphaproteobacteria>Hyphomicrobiales>Devosiaceae>Devosia	GCF_022429625.1	Devosia_sp._JXJ_CY_41	MF606_RS14450	2929921..2933421	2917991	BREX-system-P-loop-protein-BrxC-[Devosia-sp.-JXJ-CY-41]
WP_203065852.1	Type1.BREX	[illegible]	1179	Bacteria>Pseudomonadota>Alphaproteobacteria>Hyphomicrobiales>Devosiaceae>Paradevosia	GCF_016801025.1	Paradevosia_shaoguanensis	JNE37_RS07680	1630850..1634389	1335043	BREX-system-P-loop-protein-BrxC-[Paradevosia-shaoguanensis]
VXB71403.1	Type1.BREX	[illegible]	1238	Bacteria>Pseudomonadota>Alphaproteobacteria>Hyphomicrobiales>Hyphomicrobiales_incertae_sedis>Enhydrobacter	GCA_902506225.1	Enhydrobacter_sp._8BJ	ENHYD8BJ_80433	457912..461628	2653137	conserved-hypothetical-protein-[Enhydrobacter-sp.-8BJ]

Protein-ID	System	Gene-Neighbourhoods	Length	Taxonomy	Assembly-ID	Strain	Locus-Tag	Genomic-coordinates	TaxID	Fasta-define
WP_266332120.1	Type1.BREX	<-SF1-SF2-Helicase<-HKD-DNase+HKD-DNase<-wHTH<-SF1-SF2-Helicase+REase<-SF1-SF2-Helicase+REase<-SIGMA+OB-Fold+AAA-ATPase+HKD-DNase-BrxL<-PglZ<-N6-METHYLASE<-DUF6079*<-BrxB-BrxF-iSTAND<-BrxA-DNA-binding<-wHTH+WYL+WCX<-MODE-HTH+PBPII||TetR-HTH-><-RHH<-VirB10<-VirB9<-VirB8	1179	Bacteria>Pseudomonadota>Alphaproteobacteria>Hyphomicrobiales>Kaistiaceae>Kaistia	GCF_026343955.1	Kaistia_defluvii	OSH10_RS12655	370045..373584	410841	BREX-system-P-loop-protein-BrxC-[Kaistia-defluvii]
WP_056266971.1	Type1.BREX	<-SIGMA+OB-Fold+AAA-ATPase+Lon-Protease-BrxL<-PglZ<-N6-METHYLASE<-DUF6079*<-BrxB-BrxF-iSTAND<-BrxA-DNA-binding	1150	Bacteria>Pseudomonadota>Alphaproteobacteria>Hyphomicrobiales>Methylobacteriaceae>Methylobacterium	GCF_001422775.1	Methylobacterium_sp._Leaf87	ASF22_RS18370	367037..370489	1736243	BREX-system-P-loop-protein-BrxC-[Methylobacterium-sp.-Leaf87]
WP_147828804.1	Type1.BREX	Arm-DNA-bind_3+Phage_integrase->?->Phage_integrase-><-SIGMA+OB-Fold+AAA-ATPase+Lon-Protease-BrxL<-PglZ<-N6-METHYLASE<-DUF6079*<-BrxB-BrxF-iSTAND<-BrxA-DNA-binding<-?<-SF1-SF2-Helicase<-AAA-ATPase<-REase||rve+rve_3->ParB+HNH->ParB+HNH->	1149	Bacteria>Pseudomonadota>Alphaproteobacteria>Hyphomicrobiales>Methylobacteriaceae>Methylobacterium	GCF_008040045.1	Methylobacterium_sp._WL8	FV234_RS09050	16496..19945	2603899	BREX-system-P-loop-protein-BrxC-[Methylobacterium-sp.-WL8]
WP_009764060.1	Type1.BREX	<-Phage_integrase||PRTase-><-?||?->?->||BrxA-DNA-binding->BrxB-BrxF-iSTAND->DUF6079*->N6-METHYLASE->PglZ->SIGMA+OB-Fold+AAA-ATPase+Lon-Protease-BrxL->REase->	1149	Bacteria>Pseudomonadota>Alphaproteobacteria>Hyphomicrobiales>Methylobacteriaceae>Microvirga	GCF_000262405.1	Microvirga_lotononidis	MICLODRAFT_RS22145	1808535..1811984	864069	BREX-system-P-loop-protein-BrxC-[Microvirga-lotononidis]
WP_202057660.1	Type1.BREX	Phage_integrase+Phage_integrase->REase->DnaK->?->?->?->BrxA-DNA-binding->BrxB-BrxF-iSTAND->DUF6079*->N6-METHYLASE->PglZ->SIGMA+OB-Fold+AAA-ATPase+HKD-DNase-BrxL->?->?->?-><-?<-tRNA<-?<-CDP-OH_P_transf	1176	Bacteria>Pseudomonadota>Alphaproteobacteria>Hyphomicrobiales>Methylobacteriaceae>Microvirga	GCF_016743775.1	Microvirga_aerilata	JKG68_RS07685	69600..73130	670292	BREX-system-P-loop-protein-BrxC-[Microvirga-aerilata]
WP_036280608.1	Type1.BREX	HKD-DNase->SF1-SF2-Helicase-><-Small-B-barrel+SF1-SF2-Helicase+RdgC+REase<-SF1-SF2-Helicase+REase<-35Exo+REase||wHTH+WYL+WCX->BrxA-DNA-binding->BrxB-BrxF-iSTAND->DUF6079*->OLD-ABC->REase->N6-METHYLASE->PglZ->SIGMA+OB-Fold+AAA-ATPase+HKD-DNase-BrxL->HIN-HTH->?->LZ_Tnp_IS66+zf-IS66+DDE_Tnp_IS66+DDE_Tnp_IS66_C->	1178	Bacteria>Pseudomonadota>Alphaproteobacteria>Hyphomicrobiales>Methylocystaceae>Methylocystis	GCF_000188155.2	Methylocystis_sp._ATCC_49242	MET49242_RS03150	256459..259995	622637	BREX-system-P-loop-protein-BrxC-[Methylocystis-sp.-ATCC-49242]
WP_024514760.1	Type1.BREX	HerA/FtsK->?-><-?<-?||?-><-SIGMA+OB-Fold+AAA-ATPase+Lon-Protease-BrxL<-PglZ<-N6-METHYLASE<-DUF6079*<-BrxB-BrxF-iSTAND<-BrxA-DNA-binding||McrB-AAA->OLD-ABC+TOPRIM->SF1-SF2-Helicase->	1155	Bacteria>Pseudomonadota>Alphaproteobacteria>Hyphomicrobiales>Nitrobacteraceae>Bradyrhizobium	GCF_000472425.1	Bradyrhizobium_sp._Tv2a-2	A3AI_RS0106300	29834..33301	113395	BREX-system-P-loop-protein-BrxC-[Bradyrhizobium-sp.-Tv2a-2]
WP_027571504.1	Type1.BREX	<-Thermonuclease<-?||ACET-><-AAA-ATPase<-REase+HTH+35exo+Small-B-barrel+SF1-SF2-Helicase||BrxA-DNA-binding->BrxB-BrxF-iSTAND->DUF6079*->N6-METHYLASE->PglZ->SIGMA+OB-Fold+AAA-ATPase+Lon-Protease-BrxL-><-?<-HTH+HKD-DNase<-Pkinase+SF1-SF2-Helicase<-SF1-SF2-Helicase	1149	Bacteria>Pseudomonadota>Alphaproteobacteria>Hyphomicrobiales>Nitrobacteraceae>Bradyrhizobium	GCF_000473065.1	Bradyrhizobium_sp._WSM1743	YU9_RS0103440	276500..279949	318996	BREX-system-P-loop-protein-BrxC-[Bradyrhizobium-sp.-WSM1743]
WP_027565284.1	Type1.BREX	<-MarR-HTH<-?<-CR-ATPase<-GDResolvase+HIN-HTH<-?<-SIGMA+OB-Fold+AAA-ATPase+Lon-Protease-BrxL<-PglZ<-N6-METHYLASE<-DUF6079*<-BrxB-BrxF-iSTAND<-BrxA-DNA-binding<-?<-Phage_integrase<-tRNA<-?||S4+PSYN->SIGMA-HTH+SIGMA-HTH->	1144	Bacteria>Pseudomonadota>Alphaproteobacteria>Hyphomicrobiales>Nitrobacteraceae>Bradyrhizobium	GCF_000518345.1	Bradyrhizobium_sp._URHA0013	N551_RS0100500	109615..113049	1380352	BREX-system-P-loop-protein-BrxC-[Bradyrhizobium-sp.-URHA0013]
WP_044590041.1	Type1.BREX	<-Peptidase+SF1-SF2-Helicase+REase<-?<-SIGMA-HTH<-SbcC+CCDC158<-Calcineurin-SbcD||BrxA-DNA-binding->BrxB-BrxF-iSTAND->DUF6079*->N6-METHYLASE->PglZ->SIGMA+OB-Fold+AAA-ATPase+Lon-Protease-BrxL-><-?<-?<-Thermonuclease<-SF1-SF2-Helicase	1149	Bacteria>Pseudomonadota>Alphaproteobacteria>Hyphomicrobiales>Nitrobacteraceae>Bradyrhizobium	GCF_000938235.1	Bradyrhizobium_sp._LTSPM299	UP10_RS26195	631176..634625	1619233	BREX-system-P-loop-protein-BrxC-[Bradyrhizobium-sp.-LTSPM299]
WP_085384424.1	Type1.BREX	DUF772+DDE_Tnp_1+DDE_Tnp_1_6->?->?->?->?->BrxA-DNA-binding->BrxB-BrxF-iSTAND->DUF6079*->N6-METHYLASE->PglZ->SIGMA+OB-Fold+AAA-ATPase+Lon-Protease-BrxL->	1199	Bacteria>Pseudomonadota>Alphaproteobacteria>Hyphomicrobiales>Nitrobacteraceae>Bradyrhizobium	GCF_002108905.1	Bradyrhizobium_canariense	BSR47_RS02850	9232..12831	255045	BREX-system-P-loop-protein-BrxC-[Bradyrhizobium-canariense]
WP_130224749.1	Type1.BREX	<-ArdCN+MPTase<-HEPN<-Small-B-barrel+SF1-SF2-Helicase+RdgC+REase<-35Exo+REase<-?||wHTH+WYL+WCX->BrxA-DNA-binding->BrxB-BrxF-iSTAND->DUF6079*->N6-METHYLASE->PglZ->SIGMA+OB-Fold+AAA-ATPase+HKD-DNase-BrxL->AbiEi_4->AbiEii->OLD-ABC+TOPRIM->SF1-SF2-Helicase->REase-><-ABhydrolase	1181	Bacteria>Pseudomonadota>Alphaproteobacteria>Hyphomicrobiales>Nitrobacteraceae>Bradyrhizobium	GCF_004212675.1	Bradyrhizobium_sp._Leo121	CWO90_RS07090	48469..52014	1571195	BREX-system-P-loop-protein-BrxC-[Bradyrhizobium-sp.-Leo121]
WP_152104118.1	Type1.BREX	BrxA-DNA-binding->BrxB-BrxF-iSTAND->DUF6079*->N6-METHYLASE->PglZ->SIGMA+OB-Fold+AAA-ATPase+Lon-Protease-BrxL->?->?->dCache_1+HisKA+HATPase_c->REC+NtrC-AAA+FIS-HTH->	1199	Bacteria>Pseudomonadota>Alphaproteobacteria>Hyphomicrobiales>Nitrobacteraceae>Bradyrhizobium	GCF_009176665.1	Bradyrhizobium_ottawaense	GDN72_RS19495	4098964..4102563	931866	BREX-system-P-loop-protein-BrxC-[Bradyrhizobium-ottawaense]
WP_157343672.1	Type1.BREX	ABC-ATPase+SbcC->?->?-><-tRNA<-Small-B-barrel+SF1-SF2-Helicase+RdgC+REase<-SF1-SF2-Helicase+REase<-35Exo+REase||wHTH+WYL+WCX->BrxA-DNA-binding->BrxB-BrxF-iSTAND->DUF6079*->N6-METHYLASE->PglZ->SIGMA+OB-Fold+AAA-ATPase+HKD-DNase-BrxL-><-?<-HTH+REase<-Arm-DNA-bind_3+Phage_integrase<-?||?-><-Peptidase_M24	1181	Bacteria>Pseudomonadota>Alphaproteobacteria>Hyphomicrobiales>Nitrobacteraceae>Bradyrhizobium	GCF_009759865.1	Bradyrhizobium_pachyrhizi	GPL21_13485	267907..271452	280333	BREX-system-P-loop-protein-BrxC-[Bradyrhizobium-pachyrhizi]
WP_212056797.1	Type1.BREX	<-SF1-SF2-Helicase<-MORC+DUF3684+REase<-HHH_4+SF1-SF2-Helicase+REase<-AbiEi_4<-SIGMA+OB-Fold+AAA-ATPase+HKD-DNase-BrxL<-PglZ<-N6-METHYLASE<-DUF6079*<-BrxB-BrxF-iSTAND<-BrxA-DNA-binding<-wHTH+WYL+WCX||35Exo+REase->SF1-SF2-Helicase+REase->Small-B-barrel+SF1-SF2-Helicase+RdgC+REase-><-?||GDResolvase+Recombinase+ZnR->	1180	Bacteria>Pseudomonadota>Alphaproteobacteria>Hyphomicrobiales>Nitrobacteraceae>Bradyrhizobium	GCF_018131045.1	Bradyrhizobium_elkanii	JQ569_RS15725	68040..71582	29448	BREX-system-P-loop-protein-BrxC-[Bradyrhizobium-elkanii]
WP_225161486.1	Type1.BREX	<-ParB+HNH+Schlafen<-?<-SIGMA+OB-Fold+AAA-ATPase+HKD-DNase-BrxL<-PglZ<-N6-METHYLASE<-DUF6079*<-BrxB-BrxF-iSTAND<-BrxA-DNA-binding<-wHTH+WYL+WCX||?->35Exo+REase->SF1-SF2-Helicase+REase->35exo+SF1-SF2-Helicase-><-Phage_integrase	1183	Bacteria>Pseudomonadota>Alphaproteobacteria>Hyphomicrobiales>Nitrobacteraceae>Bradyrhizobium	GCF_020194035.1	Bradyrhizobium_sp._BRP56	I6F38_19500	73459..77010	2793819	BREX-system-P-loop-protein-BrxC-[Bradyrhizobium-sp.-BRP56]
WP_247808194.1	Type1.BREX	<-TOPRIM<-SAM-METHYLASE+SF1-SF2-Helicase||?-><-ParB<-DUF932||XIS-HTH-><-SIGMA+OB-Fold+AAA-ATPase+Lon-Protease-BrxL<-PglZ<-N6-METHYLASE<-DUF6079*<-BrxB-BrxF-iSTAND<-BrxA-DNA-binding||Calcineurin-SbcD->SbcC+CCDC158->SIGMA-HTH->?->Peptidase+SF1-SF2-Helicase+REase->	1149	Bacteria>Pseudomonadota>Alphaproteobacteria>Hyphomicrobiales>Nitrobacteraceae>Bradyrhizobium	GCF_023101025.1	unclassified_Bradyrhizobium	IVB17_RS35670	7402145..7405594	2631580	MULTISPECIES:-BREX-system-P-loop-protein-BrxC-[unclassified-Bradyrhizobium]
WP_260373497.1	Type1.BREX	<-ParB+HNH+Schlafen<-?<-SIGMA+OB-Fold+AAA-ATPase+HKD-DNase-BrxL<-PglZ<-N6-METHYLASE<-DUF6079*<-BrxB-BrxF-iSTAND<-BrxA-DNA-binding<-wHTH+WYL+WCX||?->35Exo+REase->SF1-SF2-Helicase+REase->35exo+SF1-SF2-Helicase-><-Phage_integrase	1183	Bacteria>Pseudomonadota>Alphaproteobacteria>Hyphomicrobiales>Nitrobacteraceae>Bradyrhizobium	GCF_025200925.1	Bradyrhizobium_sp._CB1015	N2604_RS01610	347161..350712	2976822	BREX-system-P-loop-protein-BrxC-[Bradyrhizobium-sp.-CB1015]
WP_275169502.1	Type1.BREX	<-Trypsin+REase||?-><-?||?-><-SIGMA+OB-Fold+AAA-ATPase+Lon-Protease-BrxL<-PglZ<-N6-METHYLASE<-DUF6079*<-BrxB-BrxF-iSTAND<-BrxA-DNA-binding||<-?<-tRNA<-?<-METHYLASE<-?||?->?->HTH_Tnp_2->	1145	Bacteria>Pseudomonadota>Alphaproteobacteria>Hyphomicrobiales>Nitrobacteraceae>Bradyrhizobium	GCF_029051725.1	Bradyrhizobium_sp._CSS354	GWG67_RS11755	378554..381991	2699172	BREX-system-P-loop-protein-BrxC-[Bradyrhizobium-sp.-CSS354]
WP_110780661.1	Type1.BREX	<-DOC+HTH<-SF1-SF2-Helicase<-HKD-DNase+HKD-DNase<-wHTH<-SF1-SF2-Helicase+REase<-SF1-SF2-Helicase+REase<-SIGMA+OB-Fold+AAA-ATPase+HKD-DNase-BrxL<-PglZ<-N6-METHYLASE<-DUF6079*<-BrxB-BrxF-iSTAND<-BrxA-DNA-binding<-wHTH+WYL+WCX<-MODE-HTH+PBPII||TetR-HTH-><-RHH<-VirB10<-VirB9<-VirB8	1179	Bacteria>Pseudomonadota>Alphaproteobacteria>Hyphomicrobiales>Nitrobacteraceae>Rhodopseudomonas	GCF_003217325.1	Rhodopseudomonas_faecalis	BJ122_RS08545	91514..95053	99655	BREX-system-P-loop-protein-BrxC-[Rhodopseudomonas-faecalis]
WP_184518066.1	Type1.BREX	HTH+MPTase-><-SIGMA+OB-Fold+AAA-ATPase+Lon-Protease-BrxL<-PglZ<-N6-METHYLASE-p<-N6-METHYLASE<-DUF6079*<-BrxB-BrxF-iSTAND<-BrxA-DNA-binding<-CopG/RHH<-TraI-VirD2-RCRE<-?<-?<-?<-ABhydrolase<-HISKIN	1196	Bacteria>Pseudomonadota>Alphaproteobacteria>Hyphomicrobiales>Nitrobacteraceae>Tardiphaga	GCF_014189415.1	Tardiphaga_robiniae	HB776_RS12405	2595278..2598868	943830	BREX-system-P-loop-protein-BrxC-[Tardiphaga-robiniae]
MAM94519.1	Type1.BREX	VirB10->RHH-><-TetR-HTH||MODE-HTH+PBPII-><-SbcC+SbcC<-Calcineurin+Calcineurin-SbcD||wHTH+WYL+WCX->BrxA-DNA-binding->BrxB-BrxF-iSTAND->DUF6079*->N6-METHYLASE->PglZ->SIGMA+OB-Fold+AAA-ATPase+HKD-DNase-BrxL->OLD-ABC+TOPRIM->SF1-SF2-Helicase-><-Arm-DNA-bind_3+Phage_int_SAM_3+Phage_integrase<-?<-?<-MerR-HTH	1179	Bacteria>Pseudomonadota>Alphaproteobacteria>Hyphomicrobiales>Parvibaculaceae>Parvibaculum	GCA_002694985.1	Parvibaculum_sp.	CMI61_07805	145242..148781	2024848	hypothetical-protein-[Parvibaculum-sp.]
WP_110030326.1	Type1.BREX	RAMPs->RAMPs->?->RVT->CRISPR_Cas6->?-><-?||BrxA-DNA-binding->BrxB-BrxF-iSTAND->DUF6079*->N6-METHYLASE->PglZ->SIGMA+OB-Fold+AAA-ATPase+HKD-DNase-BrxL->?->HKD-DNase+HKD-DNase->SF1-SF2-Helicase->MORC+DUF3684+REase->REase+SF1-SF2-Helicase->	1173	Bacteria>Pseudomonadota>Alphaproteobacteria>Hyphomicrobiales>Phyllobacteriaceae>Hoeflea	GCF_003182275.1	Hoeflea_marina	DFR52_RS02490	542434..545955	274592	BREX-system-P-loop-protein-BrxC-[Hoeflea-marina]
ESX29129.1	Type1.BREX	<-SIGMA+OB-Fold+AAA-ATPase+HKD-DNase-BrxL<-PglZ<-N6-METHYLASE<-DUF6079*<-BrxB-BrxF-iSTAND<-BrxA-DNA-binding<-?||REase->HIN-HTH->?->LZ_Tnp_IS66+zf-IS66+DDE_Tnp_IS66+DDE_Tnp_IS66_C->	1186	Bacteria>Pseudomonadota>Alphaproteobacteria>Hyphomicrobiales>Phyllobacteriaceae>Mesorhizobium	GCA_000502755.1	Mesorhizobium_sp._LSJC264A00	X767_13205	150936..154496	1287321	hypothetical-protein-X767_13205-[Mesorhizobium-sp.-LSJC264A00]
ESX84801.1	Type1.BREX	PIWI->HIN-HTH->HTH_21+rve+rve_3->Y1_Tnp-><-SIGMA+OB-Fold+AAA-ATPase+HKD-DNase-BrxL<-PglZ<-N6-METHYLASE<-NERD<-OLD-ABC<-DUF6079*<-BrxB-BrxF-iSTAND<-BrxA-DNA-binding||?->?-><-HEPN<-HTH_37	1177	Bacteria>Pseudomonadota>Alphaproteobacteria>Hyphomicrobiales>Phyllobacteriaceae>Mesorhizobium	GCA_000503035.1	Mesorhizobium_sp._LSHC412B00	X756_23895	63877..67410	1287285	hypothetical-protein-X756_23895-[Mesorhizobium-sp.-LSHC412B00]
RWC29997.1	Type1.BREX	SbcC+CCDC158->?->REase+PNTD1+PNTD2->PIWI-><-SIGMA+OB-Fold+AAA-ATPase+HKD-DNase-BrxL<-PglZ<-HNH<-OLD-ABC<-N6-METHYLASE<-DUF6079*<-BrxB-BrxF-iSTAND<-BrxA-DNA-binding||REase->?-><-?||REase->?->rve->	1181	Bacteria>Pseudomonadota>Alphaproteobacteria>Hyphomicrobiales>Phyllobacteriaceae>Mesorhizobium	GCA_004017395.1	Mesorhizobium_sp.	EOS27_14215	67406..70951	1871066	BREX-system-P-loop-protein-BrxC-[Mesorhizobium-sp.]
RWE30575.1	Type1.BREX	HTH_35->N6-METHYLASE->REase-><-?||SbcC-><-SbcC+CCDC158<-Calcineurin-SbcD||BrxA-DNA-binding->BrxB-BrxF-iSTAND->DUF6079*->	1034	Bacteria>Pseudomonadota>Alphaproteobacteria>Hyphomicrobiales>Phyllobacteriaceae>Mesorhizobium	GCA_004017865.1	Mesorhizobium_sp.	EOS77_19540	17552..20653	1871066	BREX-system-P-loop-protein-BrxC,-partial-[Mesorhizobium-sp.]
RWI14537.1	Type1.BREX	<-ArdCN+MPTase<-?<-SIGMA+OB-Fold+AAA-ATPase+Lon-Protease-BrxL<-PglZ<-N6-METHYLASE<-DUF6079*<-BrxB-BrxF-iSTAND<-BrxA-DNA-binding<-?<-Phage_integrase	1202	Bacteria>Pseudomonadota>Alphaproteobacteria>Hyphomicrobiales>Phyllobacteriaceae>Mesorhizobium	GCA_004018995.1	Mesorhizobium_sp.	EOQ92_28755	60098..63706	1871066	BREX-system-P-loop-protein-BrxC-[Mesorhizobium-sp.]
RWK37907.1	Type1.BREX	HTH_3->?->?->?->?-><-SIGMA+OB-Fold+AAA-ATPase+Lon-Protease-BrxL<-PglZ<-N6-METHYLASE<-DUF6079*<-BrxB-BrxF-iSTAND<-BrxA-DNA-binding||?->GDResolvase->?-><-MORC+REase-p||?-><-?<-N6-METHYLASE	1137	Bacteria>Pseudomonadota>Alphaproteobacteria>Hyphomicrobiales>Phyllobacteriaceae>Mesorhizobium	GCA_004019695.1	Mesorhizobium_sp.	EOR46_25140	24164..27577	1871066	BREX-system-P-loop-protein-BrxC-[Mesorhizobium-sp.]
RWM07956.1	Type1.BREX	CHTH->Phage_integrase-><-?<-TnsC-ATPase<-rve+Mu-transpos_C<-?||?->BrxA-DNA-binding->BrxB-BrxF-iSTAND->DUF6079*->N6-METHYLASE->PglZ->SIGMA+OB-Fold+AAA-ATPase+HKD-DNase-BrxL->GreA_GreB+SF1-SF2-Helicase-><-wHTH+WYL+WCX||?->?->?-><-HEPN	1184	Bacteria>Pseudomonadota>Alphaproteobacteria>Hyphomicrobiales>Phyllobacteriaceae>Mesorhizobium	GCA_004020545.1	Mesorhizobium_sp.	EOR71_14460	11856..15410	1871066	BREX-system-P-loop-protein-BrxC-[Mesorhizobium-sp.]
RWO20271.1	Type1.BREX	AbiEi_4-><-DEDD_Tnp_IS110+Transposase_20<-SIGMA+OB-Fold+AAA-ATPase+HKD-DNase-BrxL<-PglZ<-N6-METHYLASE<-OLD-ABC<-DUF6079*<-BrxB-BrxF-iSTAND<-BrxA-DNA-binding<-wHTH+WYL+WCX||35Exo+REase->SF1-SF2-Helicase+REase->Small-B-barrel+SF1-SF2-Helicase+RdgC+REase->NUDIX-><-?||NACHT+SF1-SF2-Helicase+HKD-DNase->	1178	Bacteria>Pseudomonadota>Alphaproteobacteria>Hyphomicrobiales>Phyllobacteriaceae>Mesorhizobium	GCA_004021465.1	Mesorhizobium_sp.	EOS09_27860	18398..21934	1871066	BREX-system-P-loop-protein-BrxC-[Mesorhizobium-sp.]
TIL41012.1	Type1.BREX	Peptidase_S11->tRNA->||RNaseH->BrxA-DNA-binding->BrxB-BrxF-iSTAND->DUF6079-p*->?-><-?<-MarR-HTH||?-><-ABC-ATPase+SbcC	845	Bacteria>Pseudomonadota>Alphaproteobacteria>Hyphomicrobiales>Phyllobacteriaceae>Mesorhizobium	GCA_004961205.1	Mesorhizobium_sp.	E5Y82_03575	245762..248299	1871066	BREX-system-P-loop-protein-BrxC-[Mesorhizobium-sp.]
TIV88924.1	Type1.BREX	<-35Exo+REase||wHTH+WYL+WCX->BrxA-DNA-binding->BrxB-BrxF-iSTAND->DUF6079*->OLD-ABC->NERD->N6-METHYLASE->PglZ->SIGMA+OB-Fold+AAA-ATPase+HKD-DNase-BrxL-><-HEPN<-RVT<-AbiEi_4	1179	Bacteria>Pseudomonadota>Alphaproteobacteria>Hyphomicrobiales>Phyllobacteriaceae>Mesorhizobium	GCA_004963965.1	Mesorhizobium_sp.	E5V78_03705	4955..8494	1871066	BREX-system-P-loop-protein-BrxC-[Mesorhizobium-sp.]
WP_156951459.1	Type1.BREX	<-SIGMA+OB-Fold+AAA-ATPase+HKD-DNase-BrxL<-PglZ<-N6-METHYLASE<-DUF6079*<-BrxB-BrxF-iSTAND<-BrxA-DNA-binding<-?||REase->HIN-HTH->?->LZ_Tnp_IS66+zf-IS66+DDE_Tnp_IS66+DDE_Tnp_IS66_C->	1188	Bacteria>Pseudomonadota>Alphaproteobacteria>Hyphomicrobiales>Phyllobacteriaceae>Mesorhizobium	GCF_000502755.1	Mesorhizobium_sp._LSJC264A00	X768_RS154485	150936..154502	1287321	BREX-system-P-loop-protein-BrxC-[Mesorhizobium-sp.-LSJC264A00]
WP_023809232.1	Type1.BREX	<-ParB+HNH<-?<-ParB+HNH-p<-N6-METHYLASE||BrxA-DNA-binding->BrxB-BrxF-iSTAND->DUF6079*->N6-METHYLASE->PglZ->SIGMA+OB-Fold+AAA-ATPase+Lon-Protease-BrxL-><-GDResolvase+Recombinase<-ParB<-?<-MULE-TRANSPOSASE	1150	Bacteria>Pseudomonadota>Alphaproteobacteria>Hyphomicrobiales>Phyllobacteriaceae>Mesorhizobium	GCF_000502875.1	Mesorhizobium_sp._L2C084A000	X735_RS161770	861519..864971	1287116	BREX-system-P-loop-protein-BrxC-[Mesorhizobium-sp.-L2C084A000]
WP_156933469.1	Type1.BREX	REase+PNTD1+PNTD2->PIWI->Y1_Tnp-><-SIGMA+OB-Fold+AAA-ATPase+HKD-DNase-BrxL<-PglZ<-N6-METHYLASE<-NERD<-OLD-ABC<-DUF6079*<-BrxB-BrxF-iSTAND<-BrxA-DNA-binding||?->RVT-><-HEPN<-HTH_37	1179	Bacteria>Pseudomonadota>Alphaproteobacteria>Hyphomicrobiales>Phyllobacteriaceae>Mesorhizobium	GCF_000503035.1	Mesorhizobium_sp._LSHC412B00	X757_RS142950	63877..67416	1287285	BREX-system-P-loop-protein-BrxC-[Mesorhizobium-sp.-LSHC412B00]
WP_071036304.1	Type1.BREX	<-SAM-METHYLASE||?->?-><-?||tRNA->BrxA-DNA-binding->BrxB-BrxF-iSTAND->DUF6079*->N6-METHYLASE->PglZ->SIGMA+OB-Fold+AAA-ATPase+Lon-Protease-BrxL->?->?->GDResolvase+HIN-HTH-><-Primpol	1137	Bacteria>Pseudomonadota>Alphaproteobacteria>Hyphomicrobiales>Phyllobacteriaceae>Mesorhizobium	GCF_001854815.1	Mesorhizobium_sp._LCM_4576	LCM4576_RS31865	30762..34175	1848289	BREX-system-P-loop-protein-BrxC-[Mesorhizobium-sp.-LCM-4576]
WP_095085237.1	Type1.BREX	<-DOC+HTH<-?<-DDE_Tnp_IS66<-?<-?<-NERD+Pkinase+Pkinase+SF1-SF2-Helicase<-SIGMA+OB-Fold+AAA-ATPase+Lon-Protease-BrxL<-PglZ<-N6-METHYLASE<-DUF6079*<-BrxB-BrxF-iSTAND<-BrxA-DNA-binding<-XIS-HTH<-Phage_integrase<-?<-?<-TraI-VirD2-RCRE<-TraJ-RHH<-ANK	1138	Bacteria>Pseudomonadota>Alphaproteobacteria>Hyphomicrobiales>Phyllobacteriaceae>Mesorhizobium	GCF_002270415.1	Mesorhizobium_sophorae	CGZ66_RS16485	77748..81164	1300294	BREX-system-P-loop-protein-BrxC-[Mesorhizobium-sophorae]
WP_128286158.1	Type1.BREX	ParB+HNH-><-SIGMA+OB-Fold+AAA-ATPase+HKD-DNase-BrxL<-PglZ<-HNH<-OLD-ABC<-N6-METHYLASE<-DUF6079*<-BrxB-BrxF-iSTAND<-BrxA-DNA-binding<-RNaseH	1181	Bacteria>Pseudomonadota>Alphaproteobacteria>Hyphomicrobiales>Phyllobacteriaceae>Mesorhizobium	GCF_004023115.1	Mesorhizobium_sp._M6A.T.Cr.TU.017.01.1.1	EOB36_RS28010	14606..18151	2496774	BREX-system-P-loop-protein-BrxC-[Mesorhizobium-sp.-M6A.T.Cr.TU.017.01.1.1]
WP_136091391.1	Type1.BREX	REase+PNTD1+PNTD2->PIWI->Y1_Tnp-><-SIGMA+OB-Fold+AAA-ATPase+HKD-DNase-BrxL<-PglZ<-HNH<-OLD-ABC<-N6-METHYLASE<-DUF6079*<-BrxB-BrxF-iSTAND<-BrxA-DNA-binding||REase->?-><-?||REase->rve->TnsC-ATPase->TnsC-ATPase->	1181	Bacteria>Pseudomonadota>Alphaproteobacteria>Hyphomicrobiales>Phyllobacteriaceae>Mesorhizobium	GCF_004794715.1	Mesorhizobium_sp._M8A.F.Ca.ET.207.01.1.1	EN850_RS12645	56072..59617	2563968	BREX-system-P-loop-protein-BrxC-[Mesorhizobium-sp.-M8A.F.Ca.ET.207.01.1.1]
WP_199202187.1	Type1.BREX	DOC+HTH-><-?||?-><-Small-B-barrel+SF1-SF2-Helicase+RdgC+REase<-SF1-SF2-Helicase+REase<-35Exo+REase||wHTH+WYL+WCX->BrxA-DNA-binding->BrxB-BrxF-iSTAND->DUF6079*->N6-METHYLASE->PglZ->SIGMA+OB-Fold+AAA-ATPase+HKD-DNase-BrxL->REase+PNTD1+PNTD2->PIWI-><-?<-?||RADICAL-SAM->	1185	Bacteria>Pseudomonadota>Alphaproteobacteria>Hyphomicrobiales>Phyllobacteriaceae>Mesorhizobium	GCF_016756495.1	Mesorhizobium_sp._113-3-3	MesoLj113b_71250	318356..321913	2744516	BREX-system-P-loop-protein-BrxC-[Mesorhizobium-sp.-113-3-3]
WP_092958042.1	Type1.BREX	<-Aminotran_1_2<-?||?->?-><-tRNA||Arm-DNA-bind_3+Phage_int_SAM_3+Phage_integrase-><-SF1-SF2-Helicase<-SIGMA+OB-Fold+AAA-ATPase+HKD-DNase-BrxL<-PglZ<-N6-METHYLASE<-DUF6079*<-BrxB-BrxF-iSTAND<-BrxA-DNA-binding<-wHTH+WYL+WCX<-MODE-HTH+PBPII||TetR-HTH-><-RHH<-VirB10<-VirB9<-VirB8	1179	Bacteria>Pseudomonadota>Alphaproteobacteria>Hyphomicrobiales>Phyllobacteriaceae>Mesorhizobium	GCF_020164785.1	Mesorhizobium_sp._ES1-1	LB513_RS22695	51971..55510	2876629	BREX-system-P-loop-protein-BrxC-[Mesorhizobium-sp.-ES1-1]
WP_256745318.1	Type1.BREX	<-D5ATPase<-XIS-HTH||MerR-HTH->?->?->HKD-DNase+HKD-Rease->?->BrxA-DNA-binding->BrxB-BrxF-iSTAND->DUF6079*->N6-METHYLASE->N6-METHYLASE->PglZ->SIGMA+OB-Fold+AAA-ATPase+HKD-DNase-BrxL-><-?<-CHTH	1211	Bacteria>Pseudomonadota>Alphaproteobacteria>Hyphomicrobiales>Phyllobacteriaceae>Mesorhizobium	GCF_024518695.1	Mesorhizobium_sp._LMG17149	NP945_RS00755	156415..160050	2968497	BREX-system-P-loop-protein-BrxC-[Mesorhizobium-sp.-LMG17149]
WP_009450523.1	Type1.BREX	ACET-><-SF1-SF2-Helicase<-HKD-DNase+HKD-DNase<-wHTH<-SF1-SF2-Helicase+REase<-SF1-SF2-Helicase+REase<-SIGMA+OB-Fold+AAA-ATPase+HKD-DNase-BrxL<-PglZ<-N6-METHYLASE<-DUF6079*<-BrxB-BrxF-iSTAND<-BrxA-DNA-binding<-wHTH+WYL+WCX<-MODE-HTH+PBPII||TetR-HTH-><-RHH<-VirB10<-VirB9<-VirB8	1179	Bacteria>Pseudomonadota>Alphaproteobacteria>Hyphomicrobiales>Phyllobacteriaceae>Nitratireductor	GCF_000300515.1	Nitratireductor_indicus	NA8A_RS11845	137447..140986	721133	BREX-system-P-loop-protein-BrxC-[Nitratireductor-indicus]
WP_080921652.1	Type1.BREX	HEPN->HEPN->?-><-DEDD_Tnp_IS110+Transposase_20||<-?<-HEPN<-SIGMA+OB-Fold+AAA-ATPase+HKD-DNase-BrxL<-PglZ<-N6-METHYLASE<-DUF6079*<-BrxB-BrxF-iSTAND<-BrxA-DNA-binding<-wHTH+WYL+WCX||Calcineurin-SbcD->SbcC+CCDC158->?->HTH_3->Peptidase+SF1-SF2-Helicase+REase->	1179	Bacteria>Pseudomonadota>Alphaproteobacteria>Hyphomicrobiales>Phyllobacteriaceae>Pseudaminobacter	GCF_002075885.1	Pseudaminobacter_manganicus	BFN67_RS22220	17195..20734	1873176	BREX-system-P-loop-protein-BrxC-[Pseudaminobacter-manganicus]
WP_150288342.1	Type1.BREX	Glyco_hydro_53+Phage-tail_3->?->?-><-URI-DNase+RAMA<-SIGMA+OB-Fold+AAA-ATPase+HKD-DNase-BrxL<-PglZ<-N6-METHYLASE<-DUF6079*<-BrxB-BrxF-iSTAND<-BrxA-DNA-binding<-wHTH+WYL+WCX<-XIS-HTH	1173	Bacteria>Pseudomonadota>Alphaproteobacteria>Hyphomicrobiales>Rhabdaerophilaceae>Rhabdaerophilum	GCF_008641065.1	Rhabdaerophilum_calidifontis	FWJ24_RS13435	109797..113318	2604328	BREX-system-P-loop-protein-BrxC-[Rhabdaerophilum-calidifontis]
WP_154722158.1	Type1.BREX	<-Classical-AAA<-?<-SMODS<-SF1-SF2-Helicase+REase<-35Exo+REase<-?||wHTH+WYL+WCX->BrxA-DNA-binding->BrxB-BrxF-iSTAND->DUF6079*->N6-METHYLASE->PglZ->SIGMA+OB-Fold+AAA-ATPase+HKD-DNase-BrxL-><-REase<-REase||?->35exo->	1181	Bacteria>Pseudomonadota>Alphaproteobacteria>Hyphomicrobiales>Rhizobiaceae>Ciceribacter	GCF_900492205.1	Ciceribacter_naphthalenivorans	DYZ14_RS19075	12704..16249	1118451	BREX-system-P-loop-protein-BrxC-[Ciceribacter-naphthalenivorans]
WP_225246472.1	Type1.BREX	<-DDE_Tnp_1_4<-GDResolvase+HIN-HTH<-?<-?<-?<-?||wHTH+WYL+WCX->BrxA-DNA-binding->BrxB-BrxF-iSTAND->DUF6079*->N6-METHYLASE->PglZ->SIGMA+OB-Fold+AAA-ATPase+HKD-DNase-BrxL->?-><-?<-LexA-protease||?-><-?<-HTH_3	1175	Bacteria>Pseudomonadota>Alphaproteobacteria>Hyphomicrobiales>Rhizobiaceae>Rhizobium/Agrobacterium_group	-	Agrobacterium_tumefaciens_complex	HW573_RS24905	44719..48246	1183400	MULTISPECIES:-BREX-system-P-loop-protein-BrxC-[Agrobacterium-tumefaciens-complex]
WP_174060722.1	Type1.BREX	DEDD_Tnp_IS110+Transposase_20-><-HTH_3||HTH_3-><-DEDD_Tnp_IS110+Transposase_20||Relaxase+SF1-SF2-Helicase->||EVE+HNH->REase->BrxA-DNA-binding->BrxB-BrxF-iSTAND->DUF6079*->N6-METHYLASE->PglZ->SIGMA+OB-Fold+AAA-ATPase+HKD-DNase-BrxL-><-?<-?<-REase<-MORC+DUF3684+DUF3883||?->||HIN-HTH->	1175	Bacteria>Pseudomonadota>Alphaproteobacteria>Hyphomicrobiales>Rhizobiaceae>Rhizobium/Agrobacterium_group	-	Agrobacterium_tumefaciens	G6M83_RS06120	12437..15964	358	BREX-system-P-loop-protein-BrxC-[Agrobacterium-tumefaciens]
WP_156535776.1	Type1.BREX	<-ParB<-ArdCN+MPTase<-Small-B-barrel+SF1-SF2-Helicase+RdgC+REase<-SF1-SF2-Helicase+REase<-35Exo+REase||wHTH+WYL+WCX->BrxA-DNA-binding->BrxB-BrxF-iSTAND->DUF6079*->N6-METHYLASE->PglZ->SIGMA+OB-Fold+AAA-ATPase+HKD-DNase-BrxL->?->Calcineurin+Calcineurin-SbcD->SbcC+CCDC158->?->?-><-HTH+REase	1181	Bacteria>Pseudomonadota>Alphaproteobacteria>Hyphomicrobiales>Rhizobiaceae>Rhizobium/Agrobacterium_group	-	Agrobacterium_vitis	GDZ98_21320	20620..24165	373	BREX-system-P-loop-protein-BrxC-[Agrobacterium-vitis]
OJF91479.1	Type1.BREX	<-TOPRIM+Topoisom_bac<-?<-SWIM||Arm-DNA-bind_3+Phage_int_SAM_3+Phage_integrase->BrxA-DNA-binding->DUF6079*->N6-METHYLASE->PglZ->SIGMA+OB-Fold+AAA-ATPase+Lon-Protease-BrxL->URI-DNase->URI-DNase+RAMA->	1163	Bacteria>Pseudomonadota>Alphaproteobacteria>Hyphomicrobiales>Rhizobiaceae>Rhizobium/Agrobacterium_group	GCA_001885575.1	Rhizobium_sp._58	AX761_22345	8315..11806	1798804	hypothetical-protein-AX761_22345-[Rhizobium-sp.-58]
PZU76796.1	Type1.BREX	XIS-HTH->?->?->?->TraJ-RHH->TraI-VirD2-RCRE->?->BrxA-DNA-binding->BrxB-BrxF-iSTAND->DUF6079*->N6-METHYLASE->PglZ->SIGMA+OB-Fold+AAA-ATPase+HKD-DNase-BrxL-><-tRNA<-?||?-><-?||PAS+HISKIN->	1188	Bacteria>Pseudomonadota>Alphaproteobacteria>Hyphomicrobiales>Rhizobiaceae>Rhizobium/Agrobacterium_group	GCA_003248675.1	Rhizobium_sp.	DI546_06985	39524..43090	391	BREX-system-P-loop-protein-BrxC-[Rhizobium-sp.]
TQN59072.1	Type1.BREX	<-SF1-SF2-Helicase<-HKD-DNase+HKD-DNase<-wHTH<-SF1-SF2-Helicase+REase<-SIGMA+OB-Fold+AAA-ATPase+HKD-DNase-BrxL<-PglZ<-N6-METHYLASE<-DUF6079*<-BrxB-BrxF-iSTAND<-BrxA-DNA-binding<-wHTH+WYL+WCX<-MODE-HTH+PBPII||TetR-HTH-><-RHH<-VirB10<-VirB9<-VirB8	1179	Bacteria>Pseudomonadota>Alphaproteobacteria>Hyphomicrobiales>Rhizobiaceae>Rhizobium/Agrobacterium_group	GCA_006757705.1	Agrobacterium_tumefaciens	FLX27_23795	22501..26040	358	BREX-system-P-loop-protein-BrxC-[Agrobacterium-tumefaciens]
NEH61390.1	Type1.BREX	<-REase<-Classical-AAA<-Classical-AAA<-REase+ZnR<-ParB||?->?->BrxA-DNA-binding->BrxB-BrxF-iSTAND->DUF6079*->N6-METHYLASE->PglZ->SIGMA+OB-Fold+AAA-ATPase+HKD-DNase-BrxL-><-SbcC<-ABC-3C<-?||NACHT-><-ParB+HNH<-ParB+HNH	1174	Bacteria>Pseudomonadota>Alphaproteobacteria>Hyphomicrobiales>Rhizobiaceae>Rhizobium/Agrobacterium_group	GCA_010668215.1	Rhizobium_leguminosarum	GR207_02220	29199..32723	384	BREX-system-P-loop-protein-BrxC-[Rhizobium-leguminosarum]
MBY3093307.1	Type1.BREX	Phage_integrase-><-HTH+Phage_integrase+Phage_integrase<-?<-Classical-AAA<-Classical-AAA<-REase+ZnR<-ParB||BrxA-DNA-binding->BrxB-BrxF-iSTAND->DUF6079*->N6-METHYLASE->PglZ->SIGMA+OB-Fold+AAA-ATPase+HKD-DNase-BrxL->||HIN-HTH->?->LZ_Tnp_IS66+zf-IS66+DDE_Tnp_IS66+DDE_Tnp_IS66_C->?->?-><-SbcC	1174	Bacteria>Pseudomonadota>Alphaproteobacteria>Hyphomicrobiales>Rhizobiaceae>Rhizobium/Agrobacterium_group	GCA_019778505.1	Rhizobium_laguerreae	HFD72_21270	353189..356713	1076926	BREX-system-P-loop-protein-BrxC-[Rhizobium-laguerreae]
MBY5873817.1	Type1.BREX	DnaB+McrB-AAA->McrC-NTD+REase+ZnR->?->OLD-ABC+TOPRIM->SF1-SF2-Helicase->ParB+HNH+Schlafen-><-SIGMA+OB-Fold+AAA-ATPase+HKD-DNase-BrxL<-PglZ<-N6-METHYLASE<-DUF6079*<-BrxB-BrxF-iSTAND<-BrxA-DNA-binding||ParB->REase+ZnR->?->Classical-AAA->Classical-AAA->REase->	1174	Bacteria>Pseudomonadota>Alphaproteobacteria>Hyphomicrobiales>Rhizobiaceae>Rhizobium/Agrobacterium_group	GCA_019799745.1	Rhizobium_leguminosarum	HFN53_17500	401377..404901	384	BREX-system-P-loop-protein-BrxC-[Rhizobium-leguminosarum]
UIJ87239.1	Type1.BREX	APATPase->?-><-MIT_C<-MIT_C<-SIGMA+OB-Fold+AAA-ATPase+Lon-Protease-BrxL<-PglZ<-N6-METHYLASE<-DUF6079*<-BrxB-BrxF-iSTAND<-BrxA-DNA-binding<-?||ParB->REase+ZnR-><-?||Classical-AAA->Classical-AAA->REase->	1174	Bacteria>Pseudomonadota>Alphaproteobacteria>Hyphomicrobiales>Rhizobiaceae>Rhizobium/Agrobacterium_group	GCA_021391615.1	Rhizobium_leguminosarum	LZK77_04465	905701..909225	384	BREX-system-P-loop-protein-BrxC-[Rhizobium-leguminosarum]
UIK00356.1	Type1.BREX	<-ABhydrolase<-?<-?||tRNA->?->BrxA-DNA-binding->BrxB-BrxF-iSTAND->DUF6079*->N6-METHYLASE->PglZ->?->SIGMA+OB-Fold+AAA-ATPase+Lon-Protease-BrxL->	1137	Bacteria>Pseudomonadota>Alphaproteobacteria>Hyphomicrobiales>Rhizobiaceae>Rhizobium/Agrobacterium_group	GCA_021391655.1	Rhizobium_leguminosarum	LZK82_09445	1889801..1893214	384	BREX-system-P-loop-protein-BrxC-[Rhizobium-leguminosarum]
UIK12452.1	Type1.BREX	<-ABhydrolase<-?<-?||tRNA->?->BrxA-DNA-binding->BrxB-BrxF-iSTAND->DUF6079-p*->DUF6079-p->DUF6079-p->N6-METHYLASE-p->N6-METHYLASE->N6-METHYLASE-p->PglZ->PglZ-p->?->SIGMA+OB-Fold+AAA-ATPase+Lon-Protease-BrxL->	292	Bacteria>Pseudomonadota>Alphaproteobacteria>Hyphomicrobiales>Rhizobiaceae>Rhizobium/Agrobacterium_group	GCA_021391695.1	Rhizobium_leguminosarum	LZK80_09475	1889966..1890844	384	hypothetical-protein-LZK80_09475-[Rhizobium-leguminosarum]
UIK12453.1	Type1.BREX	<-ABhydrolase<-?<-?||tRNA->?->BrxA-DNA-binding->BrxB-BrxF-iSTAND->DUF6079-p*->DUF6079-p->DUF6079-p->N6-METHYLASE-p->N6-METHYLASE->N6-METHYLASE-p->PglZ->PglZ-p->?->SIGMA+OB-Fold+AAA-ATPase+Lon-Protease-BrxL->SIGMA+OB-Fold+AAA-ATPase+Lon-Protease-BrxL->	533	Bacteria>Pseudomonadota>Alphaproteobacteria>Hyphomicrobiales>Rhizobiaceae>Rhizobium/Agrobacterium_group	GCA_021391695.1	Rhizobium_leguminosarum	LZK80_09480	1891035..1892636	384	hypothetical-protein-LZK80_09480-[Rhizobium-leguminosarum]
UIL29451.1	Type1.BREX	BrxA-DNA-binding->BrxB-BrxF-iSTAND->DUF6079-p*->DUF6079-p->N6-METHYLASE-p->PglZ->?->SIGMA+OB-Fold+AAA-ATPase+Lon-Protease-BrxL->	410	Bacteria>Pseudomonadota>Alphaproteobacteria>Hyphomicrobiales>Rhizobiaceae>Rhizobium/Agrobacterium_group	GCA_021397605.1	Rhizobium_leguminosarum	LZK75_09525	1890231..1891463	384	hypothetical-protein-LZK75_09525-[Rhizobium-leguminosarum]
UIL29452.1	Type1.BREX	BrxA-DNA-binding->BrxB-BrxF-iSTAND->DUF6079-p->DUF6079-p*->N6-METHYLASE-p->PglZ->?->SIGMA+OB-Fold+AAA-ATPase+Lon-Protease-BrxL->	662	Bacteria>Pseudomonadota>Alphaproteobacteria>Hyphomicrobiales>Rhizobiaceae>Rhizobium/Agrobacterium_group	GCA_021397605.1	Rhizobium_leguminosarum	LZK75_09530	1891654..1893642	384	hypothetical-protein-LZK75_09530-[Rhizobium-leguminosarum]
WP_131614693.1	Type1.BREX	HKD-DNase->SF1-SF2-Helicase->?->?-><-SIGMA+OB-Fold+AAA-ATPase+HKD-DNase-BrxL<-PglZ<-N6-METHYLASE<-NERD<-OLD-ABC<-DUF6079*<-BrxB-BrxF-iSTAND<-BrxA-DNA-binding<-?||ParB->REase+ZnR->Classical-AAA->Classical-AAA->REase->	1174	Bacteria>Pseudomonadota>Alphaproteobacteria>Hyphomicrobiales>Rhizobiaceae>Rhizobium/Agrobacterium_group	GCA_030820635.1	Rhizobium_leguminosarum	HFN52_RS21030	83963..87487	384	BREX-system-P-loop-protein-BrxC-[Rhizobium-leguminosarum]
CAD6617282.1	Type1.BREX	<-XIS-HTH<-Arm-DNA-bind_3+Phage_integrase<-SIGMA+OB-Fold+AAA-ATPase+HKD-DNase-BrxL<-PglZ<-N6-METHYLASE<-REase<-OLD-ABC<-DUF6079*<-BrxB-BrxF-iSTAND<-BrxA-DNA-binding<-wHTH+WYL+WCX||GDResolvase+HIN-HTH->	1172	Bacteria>Pseudomonadota>Alphaproteobacteria>Hyphomicrobiales>Rhizobiaceae>Rhizobium/Agrobacterium_group	GCA_902502895.2	Rhizobium_sp._Khangiran2	RKHAN_03345	3387102..3390620	1435607	BREX-system-P-loop-protein-BrxC-[Rhizobium-sp.-Khangiran2]
WP_018481320.1	Type1.BREX	<-AbiGi+seqA+RAMA<-SIGMA+OB-Fold+AAA-ATPase+Lon-Protease-BrxL<-PglZ<-N6-METHYLASE<-DUF6079*<-BrxB-BrxF-iSTAND<-BrxA-DNA-binding<-DDE_Tnp	1207	Bacteria>Pseudomonadota>Alphaproteobacteria>Hyphomicrobiales>Rhizobiaceae>Rhizobium/Agrobacterium_group	GCF_000373285.1	Rhizobium_leguminosarum	B052_RS0106775	169147..172770	384	BREX-system-P-loop-protein-BrxC-[Rhizobium-leguminosarum]
WP_027666511.1	Type1.BREX	<-SbcC<-ABC-3C<-?<-SIGMA+OB-Fold+AAA-ATPase+HKD-DNase-BrxL<-PglZ<-N6-METHYLASE<-DUF6079*<-BrxB-BrxF-iSTAND<-BrxA-DNA-binding<-?<-?||ParB->REase+ZnR->?->Classical-AAA->Classical-AAA->	1174	Bacteria>Pseudomonadota>Alphaproteobacteria>Hyphomicrobiales>Rhizobiaceae>Rhizobium/Agrobacterium_group	GCF_000427945.1	Rhizobium_leguminosarum	A19Q_RS0117655	62384..65908	384	BREX-system-P-loop-protein-BrxC-[Rhizobium-leguminosarum]
WP_046669699.1	Type1.BREX	<-ParB<-?<-?||?->wHTH+WYL+WCX->BrxA-DNA-binding->BrxB-BrxF-iSTAND->DUF6079*->	1182	Bacteria>Pseudomonadota>Alphaproteobacteria>Hyphomicrobiales>Rhizobiaceae>Rhizobium/Agrobacterium_group	GCF_000985915.1	Neorhizobium_galegae	AAW20_RS29810	5236..8784	399	BREX-system-P-loop-protein-BrxC-[Neorhizobium-galegae]
WP_088677288.1	Type1.BREX	<-SPOUT||tRNA->tRNA->?->?->?->tRNA->?->Classical-AAA+S2P->HEPN->BrxA-DNA-binding->BrxB-BrxF-iSTAND->DUF6079*->N6-METHYLASE->PglZ->SIGMA+OB-Fold+AAA-ATPase+HKD-DNase-BrxL-><-?||?->?->?->MORC+REase->RNase->	1194	Bacteria>Pseudomonadota>Alphaproteobacteria>Hyphomicrobiales>Rhizobiaceae>Rhizobium/Agrobacterium_group	GCF_002211395.1	Rhizobium_sp._R339	ATY76_RS24585	353019..356603	1764273	BREX-system-P-loop-protein-BrxC-[Rhizobium-sp.-R339]
WP_126920114.1	Type1.BREX	<-ABC-3C<-?||35exo+SF1-SF2-Helicase+REase||MORC+SF1-SF2-Helicase-><-HHH_4+HTH+wHTH+SF1-SF2-Helicase+REase-><-?<-?<-SIGMA+OB-Fold+AAA-ATPase+HKD-DNase-BrxL<-PglZ<-N6-METHYLASE<-DUF6079*<-BrxB-BrxF-iSTAND<-BrxA-DNA-binding<-nSTAND1+HNH||ParB->REase+ZnR->?->Classical-AAA->Classical-AAA->REase->	1175	Bacteria>Pseudomonadota>Alphaproteobacteria>Hyphomicrobiales>Rhizobiaceae>Rhizobium/Agrobacterium_group	GCF_003985155.1	Rhizobium_vallis	EFQ99_RS06935	372119..375646	634290	BREX-system-P-loop-protein-BrxC-[Rhizobium-vallis]
WP_128407956.1	Type1.BREX	<-MODE-HTH+PBPII||CHTH-><-?||BrxA-DNA-binding->BrxB-BrxF-iSTAND->DUF6079*->N6-METHYLASE->PglZ->SIGMA+OB-Fold+AAA-ATPase+Lon-Protease-BrxL->HTH_3-><-RNasePH<-DDE_Tnp_IS66+DDE_Tnp_IS66_C	1137	Bacteria>Pseudomonadota>Alphaproteobacteria>Hyphomicrobiales>Rhizobiaceae>Rhizobium/Agrobacterium_group	GCF_004028055.1	Rhizobium_leguminosarum	EHI43_RS33385	4123..7536	384	BREX-system-P-loop-protein-BrxC-[Rhizobium-leguminosarum]
WP_130741058.1	Type1.BREX	<-CHTH||Classical-AAA->HTH->Calcineurin->HTH->SbcC-><-?||BrxA-DNA-binding->BrxB-BrxF-iSTAND->DUF6079*->N6-METHYLASE->PglZ->SIGMA+OB-Fold+AAA-ATPase+Lon-Protease-BrxL-><-Radical-SAM<-METHYLASE||?->Phage_integrase->	1207	Bacteria>Pseudomonadota>Alphaproteobacteria>Hyphomicrobiales>Rhizobiaceae>Rhizobium/Agrobacterium_group	GCF_004303625.1	Rhizobium_leguminosarum	ELI01_RS08965	1760640..1764263	384	BREX-system-P-loop-protein-BrxC-[Rhizobium-leguminosarum]
WP_130755622.1	Type1.BREX	35exo+SF1-SF2-Helicase->MarR-HTH->Polbeta->REase->SbcC-><-Phage_int_SAM_4<-SIGMA+OB-Fold+AAA-ATPase+HKD-DNase-BrxL<-PglZ<-N6-METHYLASE<-DUF6079*<-BrxB-BrxF-iSTAND<-BrxA-DNA-binding<-?||ParB->REase+ZnR->Classical-AAA->Classical-AAA->REase->	1174	Bacteria>Pseudomonadota>Alphaproteobacteria>Hyphomicrobiales>Rhizobiaceae>Rhizobium/Agrobacterium_group	GCF_004304705.1	Rhizobium_leguminosarum	ELH79_RS02355	502725..506249	384	BREX-system-P-loop-protein-BrxC-[Rhizobium-leguminosarum]
WP_130805164.1	Type1.BREX	<-CHTH||Classical-AAA->HTH->Calcineurin->HTH->SbcC-><-?||BrxA-DNA-binding->BrxB-BrxF-iSTAND->DUF6079*->N6-METHYLASE->PglZ->SIGMA+OB-Fold+AAA-ATPase+Lon-Protease-BrxL-><-Radical-SAM<-METHYLASE||?-><-HEPN	1207	Bacteria>Pseudomonadota>Alphaproteobacteria>Hyphomicrobiales>Rhizobiaceae>Rhizobium/Agrobacterium_group	GCF_004305935.1	Rhizobium_leguminosarum	ELG76_RS08485	1639412..1643035	384	BREX-system-P-loop-protein-BrxC-[Rhizobium-leguminosarum]
WP_130816714.1	Type1.BREX	Aminotran_1_2->NAD_binding_3+Homoserine_dh+ACT->?->?-><-tRNA<-HTH_3||SF1-SF2-Helicase->tRNA-><-Glyoxalase+ZnR||BrxA-DNA-binding->BrxB-BrxF-iSTAND->DUF6079*->N6-METHYLASE->PglZ->SIGMA+OB-Fold+AAA-ATPase+Lon-Protease-BrxL-><-?<-ANK+ANK||SIR2->REase->SbcC-><-Phage_integrase+Phage_integrase	1137	Bacteria>Pseudomonadota>Alphaproteobacteria>Hyphomicrobiales>Rhizobiaceae>Rhizobium/Agrobacterium_group	GCF_004306815.1	Rhizobium_leguminosarum	ELG72_RS09600	1900026..1903439	384	BREX-system-P-loop-protein-BrxC-[Rhizobium-leguminosarum]
WP_131718742.1	Type1.BREX	URI-DNase->REase->?-><-SbcC+SbcC<-Calcineurin+Calcineurin-SbcD||wHTH+WYL+WCX->BrxA-DNA-binding->BrxB-BrxF-iSTAND->DUF6079*->N6-METHYLASE->PglZ->SIGMA+OB-Fold+AAA-ATPase+HKD-DNase-BrxL-><-?<-GDResolvase<-?<-?<-tRNA||?->SAM-METHYLASE->	1178	Bacteria>Pseudomonadota>Alphaproteobacteria>Hyphomicrobiales>Rhizobiaceae>Rhizobium/Agrobacterium_group	GCF_004330655.1	Rhizobium_leguminosarum	EOH57_RS05710	350884..354420	384	BREX-system-P-loop-protein-BrxC-[Rhizobium-leguminosarum]
WP_164009387.1	Type1.BREX	Aminotran_1_2->NAD_binding_3+Homoserine_dh+ACT->?->?-><-tRNA<-HTH_3||SF1-SF2-Helicase->tRNA-><-Glyoxalase+ZnR||BrxA-DNA-binding->BrxB-BrxF-iSTAND->DUF6079*->N6-METHYLASE->PglZ->SIGMA+OB-Fold+AAA-ATPase+Lon-Protease-BrxL-><-?<-ANK+ANK||SIR2->REase->SbcC-><-Phage_integrase+Phage_integrase	1137	Bacteria>Pseudomonadota>Alphaproteobacteria>Hyphomicrobiales>Rhizobiaceae>Rhizobium/Agrobacterium_group	GCF_010668805.1	Rhizobium_phaseoli	GR197_RS11280	144008..147421	396	BREX-system-P-loop-protein-BrxC-[Rhizobium-phaseoli]
WP_168337993.1	Type1.BREX	<-SIGMA+OB-Fold+AAA-ATPase+HKD-DNase-BrxL<-PglZ<-N6-METHYLASE<-DUF6079*<-BrxB-BrxF-iSTAND<-BrxA-DNA-binding||?->ParB->REase+ZnR->?->Classical-AAA->Classical-AAA->REase->	1174	Bacteria>Pseudomonadota>Alphaproteobacteria>Hyphomicrobiales>Rhizobiaceae>Rhizobium/Agrobacterium_group	GCF_012275885.1	Rhizobium_laguerreae	GFL43_RS28845	8439..11963	1076926	BREX-system-P-loop-protein-BrxC-[Rhizobium-laguerreae]
WP_168349960.1	Type1.BREX	<-SIGMA+OB-Fold+AAA-ATPase+HKD-DNase-BrxL<-PglZ<-ParB+HNH<-N6-METHYLASE<-N6-METHYLASE<-DUF6079-p*<-Classical-AAA<-REase+wHTH||?->PIN-><-ParB<-ParB<-GDResolvase+PPP1-HTH||CHTH->	190	Bacteria>Pseudomonadota>Alphaproteobacteria>Hyphomicrobiales>Rhizobiaceae>Rhizobium/Agrobacterium_group	GCF_012276085.1	Rhizobium_leguminosarum	GFM28_RS29310	8641..9213	384	hypothetical-protein-[Rhizobium-leguminosarum]
WP_182828337.1	Type1.BREX	<-PIN<-PhdYeFM_antitox||?-><-BrxL<-PglZ<-N6-METHYLASE<-DUF6079*<-BrxB-BrxF-iSTAND<-?<-BrxA-DNA-binding-p<-BrxA-DNA-binding<-HTH||Phage_integrase-><-tRNA||SAM-METHYLASE->Glyoxalase->	1150	Bacteria>Pseudomonadota>Alphaproteobacteria>Hyphomicrobiales>Rhizobiaceae>Rhizobium/Agrobacterium_group	GCF_014158035.1	Rhizobium_sp._G21	H2D14_RS15370	3060977..3064429	2758439	BREX-system-P-loop-protein-BrxC-[Rhizobium-sp.-G21]
WP_136542766.1	Type1.BREX	ACET-><-SF1-SF2-Helicase<-HKD-DNase+HKD-DNase<-wHTH<-SF1-SF2-Helicase+REase<-SF1-SF2-Helicase+REase<-SIGMA+OB-Fold+AAA-ATPase+HKD-DNase-BrxL<-PglZ<-N6-METHYLASE<-DUF6079*<-BrxB-BrxF-iSTAND<-BrxA-DNA-binding<-wHTH+WYL+WCX<-MODE-HTH+PBPII||TetR-HTH-><-RHH<-VirB10<-VirB9<-VirB8	1179	Bacteria>Pseudomonadota>Alphaproteobacteria>Hyphomicrobiales>Rhizobiaceae>Rhizobium/Agrobacterium_group	GCF_014202175.1	Rhizobium_rosettiformans	FAA86_RS19555	64899..68438	1368430	BREX-system-P-loop-protein-BrxC-[Rhizobium-rosettiformans]
WP_192117159.1	Type1.BREX	<-REase<-Classical-AAA<-Classical-AAA<-?<-REase+ZnR<-ParB||nSTAND1+HNH->BrxA-DNA-binding->BrxB-BrxF-iSTAND->DUF6079*->N6-METHYLASE->PglZ->SIGMA+OB-Fold+AAA-ATPase+HKD-DNase-BrxL->?->SF1-SF2-Helicase-><-?|||HTH_Tnp_1+HTH_21+rve+rve_3->?->HKD-DNase+HKD-Rease->	1174	Bacteria>Pseudomonadota>Alphaproteobacteria>Hyphomicrobiales>Rhizobiaceae>Rhizobium/Agrobacterium_group	GCF_014841565.1	Rhizobium_sp._CFBP_8762	IFT84_RS03115	659482..663006	2775279	BREX-system-P-loop-protein-BrxC-[Rhizobium-sp.-CFBP-8762]
WP_215570403.1	Type1.BREX	HTH_3->?-><-?||LexA-protease->?-><-?<-SIGMA+OB-Fold+AAA-ATPase+HKD-DNase-BrxL<-PglZ<-N6-METHYLASE<-DUF6079*<-BrxB-BrxF-iSTAND<-BrxA-DNA-binding<-wHTH+WYL+WCX||?->?->?->?->GDResolvase+HIN-HTH-><-DUF772+DDE_Tnp_1+DDE_Tnp_1_6	1175	Bacteria>Pseudomonadota>Alphaproteobacteria>Hyphomicrobiales>Rhizobiaceae>Rhizobium/Agrobacterium_group	GCF_018760785.1	Rhizobium_sp._CSW-27	KLP42_RS24160	30886..34413	2839985	BREX-system-P-loop-protein-BrxC-[Rhizobium-sp.-CSW-27]
WP_246638929.1	Type1.BREX	<-REase<-Classical-AAA<-Classical-AAA<-REase+ZnR<-ParB||?->BrxA-DNA-binding->BrxB-BrxF-iSTAND->DUF6079*->N6-METHYLASE->PglZ->SIGMA+OB-Fold+AAA-ATPase+HKD-DNase-BrxL->||HIN-HTH->?->LZ_Tnp_IS66+zf-IS66+DDE_Tnp_IS66+DDE_Tnp_IS66_C->?->?-><-SbcC	1170	Bacteria>Pseudomonadota>Alphaproteobacteria>Hyphomicrobiales>Rhizobiaceae>Rhizobium/Agrobacterium_group	GCF_019778505.1	Rhizobium_laguerreae	HFD72_RS21050	353201..356713	1076926	BREX-system-P-loop-protein-BrxC-[Rhizobium-laguerreae]
WP_222012036.1	Type1.BREX	<-Thioredoxin<-?||tRNA-><-SIGMA+OB-Fold+AAA-ATPase+Lon-Protease-BrxL<-PglZ<-N6-METHYLASE<-DUF6079*<-BrxB-BrxF-iSTAND<-BrxA-DNA-binding<-MetJarc-HTH<-?<-Couple_hipA+HipA_C<-HTH_3	1141	Bacteria>Pseudomonadota>Alphaproteobacteria>Hyphomicrobiales>Rhizobiaceae>Rhizobium/Agrobacterium_group	GCF_019778925.1	Rhizobium_laguerreae	HFD18_RS33045	24769..28194	1076926	BREX-system-P-loop-protein-BrxC-[Rhizobium-laguerreae]
WP_222013551.1	Type1.BREX	<-SIGMA+OB-Fold+AAA-ATPase+Lon-Protease-BrxL<-PglZ<-N6-METHYLASE<-DUF6079*<-BrxB-BrxF-iSTAND<-BrxA-DNA-binding<-HEPN<-?<-PIWI<-SIR2<-HARE-HTH||Phage_integrase->Phage_Integr_2+Phage_integrase->	1137	Bacteria>Pseudomonadota>Alphaproteobacteria>Hyphomicrobiales>Rhizobiaceae>Rhizobium/Agrobacterium_group	GCF_019779115.1	Rhizobium_laguerreae	HFD05_RS34495	7944..11357	1076926	BREX-system-P-loop-protein-BrxC-[Rhizobium-laguerreae]
WP_222362949.1	Type1.BREX	<-SF1-SF2-Helicase<-OLD-ABC+TOPRIM<-SIGMA+OB-Fold+AAA-ATPase+HKD-DNase-BrxL<-PglZ<-N6-METHYLASE<-DUF6079*<-BrxB-BrxF-iSTAND<-BrxA-DNA-binding<-?<-?||ParB->REase+ZnR->?->Classical-AAA->Classical-AAA->	1174	Bacteria>Pseudomonadota>Alphaproteobacteria>Hyphomicrobiales>Rhizobiaceae>Rhizobium/Agrobacterium_group	GCF_019798305.1	Rhizobium_leguminosarum	HFD99_RS19360	191490..195014	384	BREX-system-P-loop-protein-BrxC-[Rhizobium-leguminosarum]
WP_131591195.1	Type1.BREX	<-HTH_3||SF1-SF2-Helicase->tRNA-><-Glyoxalase+ZnR||BrxA-DNA-binding->BrxB-BrxF-iSTAND->DUF6079*->N6-METHYLASE->PglZ->SIGMA+OB-Fold+AAA-ATPase+Lon-Protease-BrxL-><-ANK+ANK||SIR2->REase->SbcC-><-Phage_integrase+Phage_integrase	1137	Bacteria>Pseudomonadota>Alphaproteobacteria>Hyphomicrobiales>Rhizobiaceae>Rhizobium/Agrobacterium_group	GCF_019798685.1	Rhizobium_leguminosarum	EOH55_RS21895	87979..91392	384	BREX-system-P-loop-protein-BrxC-[Rhizobium-leguminosarum]
WP_222263630.1	Type1.BREX	HTH_3->Couple_hipA+HipA_C->?->MetJarc-HTH->BrxA-DNA-binding->BrxB-BrxF-iSTAND->DUF6079*->N6-METHYLASE->PglZ->SIGMA+OB-Fold+AAA-ATPase+Lon-Protease-BrxL-><-?<-?<-tRNA||?->Thioredoxin->	1141	Bacteria>Pseudomonadota>Alphaproteobacteria>Hyphomicrobiales>Rhizobiaceae>Rhizobium/Agrobacterium_group	GCF_019798865.1	Rhizobium_leguminosarum	HFO42_RS34260	4774..8199	384	BREX-system-P-loop-protein-BrxC-[Rhizobium-leguminosarum]
WP_222338071.1	Type1.BREX	<-ACET<-?||DDE_Tnp_1->HTH_3->Couple_hipA+HipA_C->?->MetJarc-HTH->BrxA-DNA-binding->BrxB-BrxF-iSTAND->DUF6079*->N6-METHYLASE->PglZ->SIGMA+OB-Fold+AAA-ATPase+Lon-Protease-BrxL-><-?<-?<-tRNA<-tRNA||?->Thioredoxin->	1141	Bacteria>Pseudomonadota>Alphaproteobacteria>Hyphomicrobiales>Rhizobiaceae>Rhizobium/Agrobacterium_group	GCF_019799025.1	Rhizobium_leguminosarum	HFO50_RS18885	16648..20073	384	BREX-system-P-loop-protein-BrxC-[Rhizobium-leguminosarum]
WP_222342569.1	Type1.BREX	<-REase<-Classical-AAA<-Classical-AAA||?-><-REase+ZnR<-ParB||BrxA-DNA-binding->BrxB-BrxF-iSTAND->DUF6079*->N6-METHYLASE->PglZ->SIGMA+OB-Fold+AAA-ATPase+HKD-DNase-BrxL-><-SbcC<-ABC-3C	1174	Bacteria>Pseudomonadota>Alphaproteobacteria>Hyphomicrobiales>Rhizobiaceae>Rhizobium/Agrobacterium_group	GCF_019799465.1	Rhizobium_leguminosarum	HFO12_RS18165	9414..12938	384	BREX-system-P-loop-protein-BrxC-[Rhizobium-leguminosarum]
WP_229574330.1	Type1.BREX	<-MarR-HTH||tRNA-><-GDResolvase+Recombinase+ZnR||BrxA-DNA-binding->BrxB-BrxF-iSTAND->DUF6079*->N6-METHYLASE->PglZ->SIGMA+OB-Fold+AAA-ATPase+Lon-Protease-BrxL->HTH_3->	1137	Bacteria>Pseudomonadota>Alphaproteobacteria>Hyphomicrobiales>Rhizobiaceae>Rhizobium/Agrobacterium_group	GCF_020889605.1	Rhizobium_sp._'Codium_1'	LJE98_RS06515	266612..270025	2940484	BREX-system-P-loop-protein-BrxC-[Rhizobium-sp.-'Codium-1']
WP_231283598.1	Type1.BREX	HNH->SF1-SF2-Helicase->TPR-><-wHTH+REase||BrxA-DNA-binding->BrxB-BrxF-iSTAND->DUF6079*->N6-METHYLASE->PglZ->SIGMA+OB-Fold+AAA-ATPase+Lon-Protease-BrxL-><-LZ_Tnp_IS66+zf-IS66+DDE_Tnp_IS66+DDE_Tnp_IS66_C<-?<-HIN-HTH<-?<-?<-Phage_integrase+Phage_integrase	1207	Bacteria>Pseudomonadota>Alphaproteobacteria>Hyphomicrobiales>Rhizobiaceae>Rhizobium/Agrobacterium_group	GCF_021052345.1	Rhizobium_leguminosarum	RlegSU303_RS06785	1350799..1354422	384	BREX-system-P-loop-protein-BrxC-[Rhizobium-leguminosarum]
WP_221976415.1	Type1.BREX	ParB+HNH->?-><-NACHT||?->ABC-3C->SbcC-><-SIGMA+OB-Fold+AAA-ATPase+HKD-DNase-BrxL<-PglZ<-N6-METHYLASE<-DUF6079*<-BrxB-BrxF-iSTAND<-BrxA-DNA-binding<-?||ParB->REase+ZnR-><-?||Classical-AAA->Classical-AAA->REase->	1174	Bacteria>Pseudomonadota>Alphaproteobacteria>Hyphomicrobiales>Rhizobiaceae>Rhizobium/Agrobacterium_group	GCF_021391715.1	Rhizobium	LZK79_RS04540	933670..937194	379	MULTISPECIES:-BREX-system-P-loop-protein-BrxC-[Rhizobium]
WP_239619846.1	Type1.BREX	<-Classical-AAA||?->?-><-REase+ZnR<-ParB<-?||BrxA-DNA-binding->BrxB-BrxF-iSTAND->DUF6079*->N6-METHYLASE->PglZ-p->PglZ->SIGMA+OB-Fold+AAA-ATPase+HKD-DNase-BrxL->?->ABC-3C->SbcC-><-?||HIN-HTH->AAA-ATPase->	1174	Bacteria>Pseudomonadota>Alphaproteobacteria>Hyphomicrobiales>Rhizobiaceae>Rhizobium/Agrobacterium_group	GCF_022354505.1	Rhizobium_sp._C104	MF410_RS14985	2774142..2777666	2917727	BREX-system-P-loop-protein-BrxC-[Rhizobium-sp.-C104]
WP_245134299.1	Type1.BREX	<-Peptidase_M29<-?||?-><-SIGMA+OB-Fold+AAA-ATPase+HKD-DNase-BrxL<-PglZ<-N6-METHYLASE<-DUF6079*<-BrxB-BrxF-iSTAND<-BrxA-DNA-binding<-wHTH+WYL+WCX||35Exo+REase->SF1-SF2-Helicase+REase->Small-B-barrel+SF1-SF2-Helicase+RdgC+REase->?->HKD-DNase->SF1-SF2-Helicase->	1177	Bacteria>Pseudomonadota>Alphaproteobacteria>Hyphomicrobiales>Rhizobiaceae>Rhizobium/Agrobacterium_group	GCF_030369675.1	Rhizobium_sp._SSM4.3	MKJ03_RS01550	316527..320060	2919917	BREX-system-P-loop-protein-BrxC-[Rhizobium-sp.-SSM4.3]
WP_077987199.1	Type1.BREX	HTH_3->Couple_hipA+HipA_C-><-?||OLD-ABC+TOPRIM->SF1-SF2-Helicase-><-SIGMA+OB-Fold+AAA-ATPase+HKD-DNase-BrxL<-PglZ<-N6-METHYLASE<-DUF6079*<-BrxB-BrxF-iSTAND<-BrxA-DNA-binding<-REase||ParB->REase+ZnR->REase+ZnR->?->Classical-AAA->Classical-AAA->	1175	Bacteria>Pseudomonadota>Alphaproteobacteria>Hyphomicrobiales>Rhizobiaceae>Rhizobium/Agrobacterium_group	GCF_030440405.1	Rhizobium_pusense	BTE56_RS17195	18928..22455	648995	BREX-system-P-loop-protein-BrxC-[Rhizobium-pusense]
WP_149748055.1	Type1.BREX	nSTAND1+HNH->REase->BrxA-DNA-binding->BrxB-BrxF-iSTAND->DUF6079*->N6-METHYLASE->PglZ->SIGMA+OB-Fold+AAA-ATPase+HKD-DNase-BrxL->?->SF1-SF2-Helicase+DUF1998->	1175	Bacteria>Pseudomonadota>Alphaproteobacteria>Hyphomicrobiales>Rhizobiaceae>Rhizobium/Agrobacterium_group	GCF_900156005.1	Rhizobium_sp._RU35A	F3H95_RS25745	3768..7295	1907414	BREX-system-P-loop-protein-BrxC-[Rhizobium-sp.-RU35A]
WP_245278427.1	Type1.BREX	<-REase<-Classical-AAA<-Classical-AAA<-REase+ZnR<-ParB||?->BrxA-DNA-binding->BrxB-BrxF-iSTAND->DUF6079*->N6-METHYLASE->PglZ->SIGMA+OB-Fold+AAA-ATPase+HKD-DNase-BrxL-><-?<-?<-?||?-><-McrC-NTD+REase+ZnR<-DnaB+McrB-AAA	1170	Bacteria>Pseudomonadota>Alphaproteobacteria>Hyphomicrobiales>Rhizobiaceae>Rhizobium/Agrobacterium_group	-	Rhizobium_leguminosarum	A3A3_RS0116705	192694..196206	384	BREX-system-P-loop-protein-BrxC-[Rhizobium-leguminosarum]
WP_131707092.1	Type1.BREX	<-REase<-Classical-AAA<-Classical-AAA<-REase+ZnR<-ParB||?->BrxA-DNA-binding->BrxB-BrxF-iSTAND->DUF6079*->N6-METHYLASE->PglZ->SIGMA+OB-Fold+AAA-ATPase+HKD-DNase-BrxL-><-?<-?<-SF1-SF2-Helicase<-HKD-DNase<-?<-Peptidase_S8	1174	Bacteria>Pseudomonadota>Alphaproteobacteria>Hyphomicrobiales>Rhizobiaceae>Rhizobium/Agrobacterium_group	-	Rhizobium_leguminosarum	EOH59_RS18255	300646..304170	384	BREX-system-P-loop-protein-BrxC-[Rhizobium-leguminosarum]
WP_024321352.1	Type1.BREX	DnaB+McrB-AAA->McrC-NTD+REase+ZnR-><-?||?->?->?-><-SIGMA+OB-Fold+AAA-ATPase+HKD-DNase-BrxL<-PglZ<-N6-METHYLASE<-DUF6079*<-BrxB-BrxF-iSTAND<-BrxA-DNA-binding<-?||ParB->REase+ZnR->Classical-AAA->Classical-AAA->REase->	1174	Bacteria>Pseudomonadota>Alphaproteobacteria>Hyphomicrobiales>Rhizobiaceae>Rhizobium/Agrobacterium_group	-	Rhizobium_leguminosarum	EOJ20_RS04835	93871..97395	384	BREX-system-P-loop-protein-BrxC-[Rhizobium-leguminosarum]
WP_130784075.1	Type1.BREX	<-LZ_Tnp_IS66+zf-IS66+DDE_Tnp_IS66+DDE_Tnp_IS66_C<-?<-HIN-HTH<-SIGMA+OB-Fold+AAA-ATPase+HKD-DNase-BrxL<-PglZ<-N6-METHYLASE<-DUF6079*<-BrxB-BrxF-iSTAND<-BrxA-DNA-binding<-wHTH+WYL+WCX||Calcineurin+Calcineurin-SbcD->SbcC+SbcC-><-?<-REase<-URI-DNase	1178	Bacteria>Pseudomonadota>Alphaproteobacteria>Hyphomicrobiales>Rhizobiaceae>Rhizobium/Agrobacterium_group	-	Rhizobium_leguminosarum	ELG96_RS28915	11184..14720	384	BREX-system-P-loop-protein-BrxC-[Rhizobium-leguminosarum]
WP_018070454.1	Type1.BREX	HNH->SF1-SF2-Helicase->TPR-><-wHTH+REase||BrxA-DNA-binding->BrxB-BrxF-iSTAND->DUF6079*->N6-METHYLASE->PglZ->SIGMA+OB-Fold+AAA-ATPase+Lon-Protease-BrxL-><-?<-?<-Phage_integrase+Phage_integrase<-Phage_int_SAM_5<-Phage_integrase	1207	Bacteria>Pseudomonadota>Alphaproteobacteria>Hyphomicrobiales>Rhizobiaceae>Rhizobium/Agrobacterium_group	-	Rhizobium_leguminosarum	ELH14_RS07055	1387374..1390997	384	BREX-system-P-loop-protein-BrxC-[Rhizobium-leguminosarum]
WP_130823534.1	Type1.BREX	Aminotran_1_2->NAD_binding_3+Homoserine_dh+ACT->?->?-><-tRNA<-HTH_3||SF1-SF2-Helicase->tRNA-><-Glyoxalase+ZnR||BrxA-DNA-binding->BrxB-BrxF-iSTAND->DUF6079*->N6-METHYLASE->PglZ->SIGMA+OB-Fold+AAA-ATPase+Lon-Protease-BrxL-><-?<-LZ_Tnp_IS66+zf-IS66+DDE_Tnp_IS66+DDE_Tnp_IS66_C<-?<-HIN-HTH<-?<-ANK+ANK	1137	Bacteria>Pseudomonadota>Alphaproteobacteria>Hyphomicrobiales>Rhizobiaceae>Rhizobium/Agrobacterium_group	-	Rhizobium_leguminosarum	ELH15_RS08895	1753674..1757087	384	BREX-system-P-loop-protein-BrxC-[Rhizobium-leguminosarum]
WP_130764093.1	Type1.BREX	<-REase<-Classical-AAA<-Classical-AAA||?-><-REase+ZnR<-ParB||<-?||BrxA-DNA-binding->BrxB-BrxF-iSTAND->DUF6079*->N6-METHYLASE->PglZ->SIGMA+OB-Fold+AAA-ATPase+HKD-DNase-BrxL->?->ABC-3C->SbcC-><-?<-McrC-NTD+REase+ZnR	1174	Bacteria>Pseudomonadota>Alphaproteobacteria>Hyphomicrobiales>Rhizobiaceae>Rhizobium/Agrobacterium_group	-	Rhizobium_leguminosarum	ELI00_RS29085	22850..26374	384	BREX-system-P-loop-protein-BrxC-[Rhizobium-leguminosarum]
WP_245488667.1	Type1.BREX	DADA-Ligase->||?->?->SbcC-><-SIGMA+OB-Fold+AAA-ATPase+HKD-DNase-BrxL<-PglZ<-N6-METHYLASE<-DUF6079*<-BrxB-BrxF-iSTAND<-BrxA-DNA-binding<-?||ParB->REase+ZnR->?->Classical-AAA->Classical-AAA->REase->	1170	Bacteria>Pseudomonadota>Alphaproteobacteria>Hyphomicrobiales>Rhizobiaceae>Rhizobium/Agrobacterium_group	-	Rhizobium_leguminosarum	ELI09_RS02630	538702..542214	384	BREX-system-P-loop-protein-BrxC-[Rhizobium-leguminosarum]
WP_130672003.1	Type1.BREX	DADA-Ligase->||?->?->SbcC-><-SIGMA+OB-Fold+AAA-ATPase+HKD-DNase-BrxL<-PglZ<-N6-METHYLASE<-DUF6079*<-BrxB-BrxF-iSTAND<-BrxA-DNA-binding<-?||ParB->REase+ZnR->?->Classical-AAA->Classical-AAA->REase->	1174	Bacteria>Pseudomonadota>Alphaproteobacteria>Hyphomicrobiales>Rhizobiaceae>Rhizobium/Agrobacterium_group	-	Rhizobium_leguminosarum	ELI10_RS02625	537164..540688	384	BREX-system-P-loop-protein-BrxC-[Rhizobium-leguminosarum]
WP_165586283.1	Type1.BREX	<-CR-ATPase5+REase||?->?->?->PIWI->BrxA-DNA-binding->BrxB-BrxF-iSTAND->DUF6079*->N6-METHYLASE->PglZ->SIGMA+OB-Fold+AAA-ATPase+Lon-Protease-BrxL-><-Radical-SAM<-METHYLASE||?-><-HEPN||?->Phage_integrase->	1207	Bacteria>Pseudomonadota>Alphaproteobacteria>Hyphomicrobiales>Rhizobiaceae>Rhizobium/Agrobacterium_group	-	Rhizobium_leguminosarum	G7039_RS08625	1692346..1695969	384	BREX-system-P-loop-protein-BrxC-[Rhizobium-leguminosarum]
WP_168316672.1	Type1.BREX	<-Small-B-barrel+SF1-SF2-Helicase+RdgC+REase<-SF1-SF2-Helicase+REase<-35Exo+REase<-?||wHTH+WYL+WCX->BrxA-DNA-binding->BrxB-BrxF-iSTAND->DUF6079*->N6-METHYLASE->PglZ->SIGMA+OB-Fold+AAA-ATPase+HKD-DNase-BrxL->	1181	Bacteria>Pseudomonadota>Alphaproteobacteria>Hyphomicrobiales>Rhizobiaceae>Rhizobium/Agrobacterium_group	-	Rhizobium_leguminosarum	GFL49_RS25835	227535..231080	384	BREX-system-P-loop-protein-BrxC-[Rhizobium-leguminosarum]
WP_168308740.1	Type1.BREX	<-ParB<-Small-B-barrel+SF1-SF2-Helicase+RdgC+REase<-SF1-SF2-Helicase+REase<-35Exo+REase<-?||wHTH+WYL+WCX->BrxA-DNA-binding->BrxB-BrxF-iSTAND->DUF6079*->N6-METHYLASE->PglZ->SIGMA+OB-Fold+AAA-ATPase+HKD-DNase-BrxL-><-SIR2<-HTH+REase<-Arm-DNA-bind_3+Phage_integrase||?->Peptidase->	1181	Bacteria>Pseudomonadota>Alphaproteobacteria>Hyphomicrobiales>Rhizobiaceae>Rhizobium/Agrobacterium_group	-	Rhizobium_leguminosarum	GFM13_RS06520	103411..106956	384	BREX-system-P-loop-protein-BrxC-[Rhizobium-leguminosarum]
WP_246708550.1	Type1.BREX	<-Classical-AAA<-Classical-AAA||?-><-REase+ZnR<-ParB||?->?->BrxA-DNA-binding->BrxB-BrxF-iSTAND->DUF6079*->N6-METHYLASE->PglZ->SIGMA+OB-Fold+AAA-ATPase+HKD-DNase-BrxL-><-SbcC<-ABC-3C<-?||NACHT-><-ParB+HNH<-ParB+HNH	1170	Bacteria>Pseudomonadota>Alphaproteobacteria>Hyphomicrobiales>Rhizobiaceae>Rhizobium/Agrobacterium_group	-	Rhizobium_leguminosarum	GR207_RS02230	29211..32723	384	BREX-system-P-loop-protein-BrxC-[Rhizobium-leguminosarum]
PZU19146.1	Type1.BREX	HTH_3->?-><-?||LexA-protease->?-><-SIGMA+OB-Fold+AAA-ATPase+HKD-DNase-BrxL<-PglZ<-N6-METHYLASE<-DUF6079*<-BrxB-BrxF-iSTAND<-BrxA-DNA-binding<-wHTH+WYL+WCX||?->?->?->?->GDResolvase+HIN-HTH->	1175	Bacteria>Pseudomonadota>Alphaproteobacteria>Hyphomicrobiales>Rhizobiaceae>Shinella	GCA_003248545.1	Shinella_sp.	DI589_22170	12827..16354	1870904	MAG:-BREX-system-P-loop-protein-BrxC-[Shinella-sp.]
WP_004434288.1	Type1.BREX	<-PIN<-PhdYeFM_antitox<-SIGMA+OB-Fold+AAA-ATPase+HKD-DNase-BrxL<-PglZ<-N6-METHYLASE<-REase<-OLD-ABC<-DUF6079*<-BrxB-BrxF-iSTAND<-BrxA-DNA-binding<-wHTH+WYL+WCX||35Exo+REase->SF1-SF2-Helicase+REase->Small-B-barrel+SF1-SF2-Helicase+RdgC+REase-><-SF1-SF2-Helicase<-HKD-DNase	1177	Bacteria>Pseudomonadota>Alphaproteobacteria>Hyphomicrobiales>Rhizobiaceae>Sinorhizobium/Ensifer_group	GCF_000236945.1	Sinorhizobium_meliloti	SM0020_RS29775	166866..170399	382	BREX-system-P-loop-protein-BrxC-[Sinorhizobium-meliloti]
WP_037424884.1	Type1.BREX	<-GDResolvase+HIN-HTH<-Small-B-barrel+SF1-SF2-Helicase+RdgC+REase<-35Exo+REase<-?||wHTH+WYL+WCX->BrxA-DNA-binding->BrxB-BrxF-iSTAND->DUF6079*->N6-METHYLASE->PglZ->SIGMA+OB-Fold+AAA-ATPase+HKD-DNase-BrxL-><-REase||?->?->?-><-?||URI-DNase->	1181	Bacteria>Pseudomonadota>Alphaproteobacteria>Hyphomicrobiales>Rhizobiaceae>Sinorhizobium/Ensifer_group	GCF_000261005.1	Sinorhizobium_sp._CCBAU_05631	SS05631_RS30325	18322..21867	794846	BREX-system-P-loop-protein-BrxC-[Sinorhizobium-sp.-CCBAU-05631]
WP_244425598.1	Type1.BREX	<-PglZ-p<-N6-METHYLASE<-N6-METHYLASE-p<-DUF6079-p<-DUF6079-p*<-BrxB-BrxF-iSTAND<-BrxA-DNA-binding	705	Bacteria>Pseudomonadota>Alphaproteobacteria>Hyphomicrobiales>Rhizobiaceae>Sinorhizobium/Ensifer_group	GCF_000261865.1	Sinorhizobium_fredii	IC47_RS36510	5889..8006	380	BREX-system-P-loop-protein-BrxC-[Sinorhizobium-fredii]
WP_028054052.1	Type1.BREX	<-PIN+OST-HTH<-?||REase-><-SIGMA+OB-Fold+AAA-ATPase+Lon-Protease-BrxL<-PglZ<-N6-METHYLASE<-DUF6079*<-BrxB-BrxF-iSTAND<-BrxA-DNA-binding||?-><-HTH||HTH_3->?-><-?<-Phage_int_SAM_5+Phage_integrase	1150	Bacteria>Pseudomonadota>Alphaproteobacteria>Hyphomicrobiales>Rhizobiaceae>Sinorhizobium/Ensifer_group	GCF_000427485.1	Sinorhizobium_medicae	SINMEL_RS0108765	254501..257953	110321	BREX-system-P-loop-protein-BrxC-[Sinorhizobium-medicae]
WP_164817971.1	Type1.BREX	HTH_3->?->?->?->MetJarc-HTH->BrxA-DNA-binding->BrxB-BrxF-iSTAND->DUF6079*->N6-METHYLASE->PglZ->SIGMA+OB-Fold+AAA-ATPase+Lon-Protease-BrxL->?->?-><-?||?->HTH_38+rve->	1141	Bacteria>Pseudomonadota>Alphaproteobacteria>Hyphomicrobiales>Rhizobiaceae>Sinorhizobium/Ensifer_group	GCF_004001385.1	Sinorhizobium_meliloti	CN238_RS30780	8247..11672	382	BREX-system-P-loop-protein-BrxC-[Sinorhizobium-meliloti]
WP_127638472.1	Type1.BREX	BrxA-DNA-binding->BrxB-BrxF-iSTAND->DUF6079*->N6-METHYLASE->PglZ->SIGMA+OB-Fold+AAA-ATPase+HKD-DNase-BrxL-><-REase+HTH+35exo+Small-B-barrel+SF1-SF2-Helicase<-EVE+REase||?->?->HEPN+RES-><-Thermonuclease	1188	Bacteria>Pseudomonadota>Alphaproteobacteria>Hyphomicrobiales>Rhizobiaceae>Sinorhizobium/Ensifer_group	GCF_004003915.1	Sinorhizobium_meliloti	CN087_RS12760	106891..110457	382	BREX-system-P-loop-protein-BrxC-[Sinorhizobium-meliloti]
CAG0989916.1	Type1.BREX	Arm-DNA-bind_3+Phage_int_SAM_3+Phage_integrase->AbiEi_4->AbiEii-><-SIGMA+OB-Fold+AAA-ATPase+Lon-Protease-BrxL<-PglZ<-N6-METHYLASE<-DUF6079*<-BrxB-BrxF-iSTAND<-BrxA-DNA-binding<-?<-wHTH+WYL+WCX||?->35Exo+REase->SF1-SF2-Helicase+REase->HisKin->	1140	Bacteria>Pseudomonadota>Alphaproteobacteria>Hyphomicrobiales>Rhizobiaceae>unclassified_Rhizobiaceae	GCA_905339215.1	Rhizobiaceae_bacterium	RHIZO_02194	11543..14965	1913961	hypothetical-protein-RHIZO_02194-[Rhizobiaceae-bacterium]
WP_027288600.1	Type1.BREX	TPR+TPR+TPR->?->tRNA->Phage_integrase->BrxA-DNA-binding->BrxB-BrxF-iSTAND->DUF6079*->N6-METHYLASE->PglZ->SIGMA+OB-Fold+AAA-ATPase+Lon-Protease-BrxL-><-ParB+HNH||?->||?->?->SbcC->	1150	Bacteria>Pseudomonadota>Alphaproteobacteria>Hyphomicrobiales>Rhodovibrionaceae>Rhodovibrio	GCF_000515255.1	Rhodovibrio_salinarum	RHOSA_RS0110320	2222437..2225889	1087	BREX-system-P-loop-protein-BrxC-[Rhodovibrio-salinarum]
WP_074444907.1	Type1.BREX	<-CHTH||?->?->?-><-SIGMA+OB-Fold+AAA-ATPase+Lon-Protease-BrxL<-PglZ<-REase<-N6-METHYLASE<-N6-METHYLASE-p<-DUF6079-p*<-BrxB-BrxF-iSTAND<-BrxA-DNA-binding<-ABC-ATPase+SbcC<-?<-?<-?||AraC-HTH+AraC-HTH->	1148	Bacteria>Pseudomonadota>Alphaproteobacteria>Hyphomicrobiales>Salinarimonadaceae>Saliniramus	GCF_900094735.1	Saliniramus_fredricksonii	GA0071312_RS10335	795252..798698	1653334	BREX-system-P-loop-protein-BrxC-[Saliniramus-fredricksonii]
WP_035540926.1	Type1.BREX	<-SbcC+SbcC||?-><-HTH_3||SIGMA-HTH->SIGMA-HTH->?-><-SIGMA+OB-Fold+AAA-ATPase+HKD-DNase-BrxL<-PglZ<-N6-METHYLASE<-DUF6079*<-BrxB-BrxF-iSTAND<-BrxA-DNA-binding||ArdA->	1157	Bacteria>Pseudomonadota>Alphaproteobacteria>Hyphomonadales>Hyphomonadaceae>Hyphomonas	GCF_000685295.1	Hyphomonas_oceanitis	HOC_RS17370	29720..33193	81033	BREX-system-P-loop-protein-BrxC-[Hyphomonas-oceanitis]
MCE2892634.1	Type1.BREX	<-SIGMA+OB-Fold+AAA-ATPase+HKD-DNase-BrxL<-PglZ<-N6-METHYLASE<-DUF6079*<-BrxB-BrxF-iSTAND<-BrxA-DNA-binding<-HTH_3<-?<-?<-Peptidase<-VirB10<-VirB9<-VirB8	1195	Bacteria>Pseudomonadota>Alphaproteobacteria>Hyphomonadales>Hyphomonadaceae>unclassified_Hyphomonadaceae	GCA_021299265.1	Hyphomonadaceae_bacterium	LW848_16090	9073..12660	2026748	BREX-system-P-loop-protein-BrxC-[Hyphomonadaceae-bacterium]
WP_130470524.1	Type1.BREX	Primpol+PriCT->SF1-SF2-Helicase->?-><-GDResolvase+Recombinase||?->?->BrxA-DNA-binding->BrxB-BrxF-iSTAND->DUF6079*->N6-METHYLASE-p->	1193	Bacteria>Pseudomonadota>Alphaproteobacteria>Magnetococcales>Candidatus_Magnetaquicoccaceae>Candidatus_Magnetaquicoccus	GCF_004217665.1	Candidatus_Magnetaquicoccus_inordinatus	EJP87_RS03730	21320..24901	2496818	BREX-system-P-loop-protein-BrxC-[Candidatus-Magnetaquicoccus-inordinatus]
MBF0098005.1	Type1.BREX	<-PglZ<-Schlafen+HATPase+wHTH<-PHP+SbcC<-HNH<-OLD-ABC<-REase+AIPR<-N6-METHYLASE<-HicA_toxin<-HicB_lk_antitox<-DUF6079*<-BrxB-BrxF-iSTAND<-BrxA-DNA-binding<-Radical_SAM	1144	Bacteria>Pseudomonadota>Alphaproteobacteria>Magnetococcales>unclassified_Magnetococcales	GCA_015228825.1	Magnetococcales_bacterium	HQM05_11780	14372..17806	2026759	BREX-system-P-loop-protein-BrxC-[Magnetococcales-bacterium]
MBF0399382.1	Type1.BREX	BrxA-DNA-binding->BrxB-BrxF-iSTAND->DUF6079*->HicB_lk_antitox->HicA_toxin->N6-METHYLASE->OLD-ABC->HNH->PglZ->SIGMA+OB-Fold+AAA-ATPase+Lon-Protease-BrxL->?->STAS	1144	Bacteria>Pseudomonadota>Alphaproteobacteria>Magnetococcales>unclassified_Magnetococcales	GCA_015232635.1	Magnetococcales_bacterium	HQL90_01290	18375..21809	2026759	BREX-system-P-loop-protein-BrxC-[Magnetococcales-bacterium]
ADM09203.1	Type1.BREX	DUF736->?->XIS-HTH->Peptidase_S26->TraI-VirD2-RCRE-><-SIGMA+OB-Fold+AAA-ATPase+HKD-DNase-BrxL<-PglZ<-N6-METHYLASE<-DUF6079*<-BrxB-BrxF-iSTAND<-BrxA-DNA-binding<-wHTH+WYL+WCX||VirD4-TraG+VirD4-FtsK->?->GreA_GreB+SF1-SF2-Helicase->HTH_21+rve+rve_3->VirB11+GTPase-AIG->VirB2->	1171	Bacteria>Pseudomonadota>Alphaproteobacteria>Parvularculales>Parvularculaceae>Parvularcula	GCA_000152825.2	Parvularcula_bermudensis_HTCC2503	PB2503_05652	1076655..1080170	314260	hypothetical-protein-PB2503_05652-[Parvularcula-bermudensis-HTCC2503]
WP_238525821.1	Type1.BREX	ParB->DUF736->?->XIS-HTH->Peptidase_S26->TraI-VirD2-RCRE-><-SIGMA+OB-Fold+AAA-ATPase+HKD-DNase-BrxL<-PglZ<-N6-METHYLASE<-DUF6079*<-BrxB-BrxF-iSTAND<-BrxA-DNA-binding<-wHTH+WYL+WCX||VirD4-TraG+VirD4-FtsK->?->GreA_GreB+SF1-SF2-Helicase->HIN-HTH->VirB11+GTPase-AIG->VirB2->	1168	Bacteria>Pseudomonadota>Alphaproteobacteria>Parvularculales>Parvularculaceae>Parvularcula	GCF_000152825.2	Parvularcula_bermudensis	PB2503_RS05170	1076655..1080161	208216	BREX-system-P-loop-protein-BrxC-[Parvularcula-bermudensis]
WP_217361198.1	Type1.BREX	<-HATPase_c||Arm-DNA-bind_3+Phage_integrase->LON-AAA->?->?-><-?||wHTH+WYL+WCX->BrxA-DNA-binding->BrxB-BrxF-iSTAND->DUF6079*->N6-METHYLASE->PglZ->SIGMA+OB-Fold+AAA-ATPase+HKD-DNase-BrxL->?->HEPN+PAS+DUF4011+SF1-SF2-Helicase+REase->ParB+HNH+Schlafen->	1168	Bacteria>Pseudomonadota>Alphaproteobacteria>Rhodobacterales>Paracoccaceae>Aliiroseovarius	GCF_013283115.1	unclassified_Aliiroseovarius	XMV209_RS00060	11614..15120	2623558	MULTISPECIES:-BREX-system-P-loop-protein-BrxC-[unclassified-Aliiroseovarius]
WP_173486905.1	Type1.BREX	<-REase+iREase<-SIGMA+OB-Fold+AAA-ATPase+HKD-DNase-BrxL<-PglZ<-N6-METHYLASE<-DUF6079*<-BrxB-BrxF-iSTAND<-BrxA-DNA-binding<-wHTH+WYL+WCX||?->?->?-><-?<-ANK+ANK+ANK	1173	Bacteria>Pseudomonadota>Alphaproteobacteria>Rhodobacterales>Paracoccaceae>Aliiroseovarius	GCF_013283525.1	unclassified_Aliiroseovarius	XMM309_RS11965	259901..263422	2623558	MULTISPECIES:-BREX-system-P-loop-protein-BrxC-[unclassified-Aliiroseovarius]
WP_035840066.1	Type1.BREX	Phage_integrase-><-XIS-HTH<-?||?->?->Phage_integrase->BrxA-DNA-binding->BrxB-BrxF-iSTAND->DUF6079*->N6-METHYLASE->PglZ->SIGMA+OB-Fold+AAA-ATPase+Lon-Protease-BrxL-><-DOC+HTH||ArdCN+MPTase-><-HEPN	1208	Bacteria>Pseudomonadota>Alphaproteobacteria>Rhodobacterales>Paracoccaceae>Allgaiera	GCF_014653075.1	Allgaiera_indica	IE192_RS04280	311977..315603	765699	BREX-system-P-loop-protein-BrxC-[Allgaiera-indica]
WP_245513828.1	Type1.BREX	HTH_3->?-><-TrbM<-Thermonuclease<-?||wHTH+WYL+WCX->BrxA-DNA-binding->BrxB-BrxF-iSTAND->DUF6079-p*->	659	Bacteria>Pseudomonadota>Alphaproteobacteria>Rhodobacterales>Paracoccaceae>Antarcticimicrobium	GCF_004358185.1	Antarcticimicrobium_luteum	E1832_RS04855	61315..63294	2547397	BREX-system-P-loop-protein-BrxC-[Antarcticimicrobium-luteum]
WP_146284416.1	Type1.BREX	Glyco_hydro_53+Phage-tail_3->?-><-SIGMA+OB-Fold+AAA-ATPase+HKD-DNase-BrxL<-PglZ<-N6-METHYLASE<-DUF6079*<-BrxB-BrxF-iSTAND<-BrxA-DNA-binding<-wHTH+WYL+WCX<-Arm-DNA-bind_3+Phage_int_SAM_3+Phage_integrase||SWIM-><-HTH_21+rve+rve_3	1173	Bacteria>Pseudomonadota>Alphaproteobacteria>Rhodobacterales>Paracoccaceae>Gemmobacter	GCF_007859075.1	Gemmobacter_aquaticus	FQU74_RS00760	54180..57701	490185	BREX-system-P-loop-protein-BrxC-[Gemmobacter-aquaticus]
WP_076528450.1	Type1.BREX	Arm-DNA-bind_3+Phage_int_SAM_3+Phage_integrase-><-SF1-SF2-Helicase<-OLD-ABC+TOPRIM<-SIGMA+OB-Fold+AAA-ATPase+HKD-DNase-BrxL<-PglZ<-N6-METHYLASE<-DUF6079*<-BrxB-BrxF-iSTAND<-BrxA-DNA-binding<-wHTH+WYL+WCX||Calcineurin+Calcineurin-SbcD->SbcC+SbcC-><-MODE-HTH+PBPII||TetR-HTH-><-RHH<-VirB10	1179	Bacteria>Pseudomonadota>Alphaproteobacteria>Rhodobacterales>Paracoccaceae>Gemmobacter	GCF_014636395.1	Gemmobacter_megaterium	IEV79_RS02805	604470..608009	1086013	BREX-system-P-loop-protein-BrxC-[Gemmobacter-megaterium]
WP_229704463.1	Type1.BREX	<-SWIM||Arm-DNA-bind_3+Phage_int_SAM_3+Phage_integrase->wHTH+WYL+WCX->BrxA-DNA-binding->BrxB-BrxF-iSTAND->DUF6079*->N6-METHYLASE->PglZ->SIGMA+OB-Fold+AAA-ATPase+HKD-DNase-BrxL-><-?<-Glyco_hydro_53+Phage-tail_3	1169	Bacteria>Pseudomonadota>Alphaproteobacteria>Rhodobacterales>Paracoccaceae>Gemmobacter	GCF_014645295.1	Gemmobacter_aquaticus	IEX45_RS17475	6610..10119	490185	BREX-system-P-loop-protein-BrxC-[Gemmobacter-aquaticus]
WP_131389735.1	Type1.BREX	VirB8->VirB9->VirB10->RHH-><-TetR-HTH||MODE-HTH+PBPII->wHTH+WYL+WCX->BrxA-DNA-binding->BrxB-BrxF-iSTAND->DUF6079*->N6-METHYLASE->PglZ->SIGMA+OB-Fold+AAA-ATPase+HKD-DNase-BrxL->SF1-SF2-Helicase+REase->SF1-SF2-Helicase+REase->wHTH->HKD-DNase+HKD-DNase->SF1-SF2-Helicase->	1179	Bacteria>Pseudomonadota>Alphaproteobacteria>Rhodobacterales>Paracoccaceae>Haematobacter	GCF_004328555.1	Haematobacter_massiliensis	HmaOT1_RS15910	65338..68877	195105	BREX-system-P-loop-protein-BrxC-[Haematobacter-massiliensis]
WP_039019257.1	Type1.BREX	<-VirD4-TraG+VirD4-FtsK<-TraI-VirD2-RCRE<-HIN-HTH+TraJ-RHH<-?<-FurR-HTH<-ArdCN+MPTase||wHTH+WYL+WCX->BrxA-DNA-binding->BrxB-BrxF-iSTAND->DUF6079*->N6-METHYLASE->PglZ->SIGMA+OB-Fold+AAA-ATPase+HKD-DNase-BrxL->REase+iREase-><-?<-PIN<-XIS-HTH<-HTH_36<-Phage_integrase	1172	Bacteria>Pseudomonadota>Alphaproteobacteria>Rhodobacterales>Paracoccaceae>Halocynthiibacter	GCF_000812685.1	Halocynthiibacter_namhaensis	RJ38_RS12670	13538..17056	1290553	BREX-system-P-loop-protein-BrxC-[Halocynthiibacter-namhaensis]
WP_263953721.1	Type1.BREX	<-PAS||wHTH+WYL+WCX->BrxA-DNA-binding->BrxB-BrxF-iSTAND->DUF6079*->N6-METHYLASE->PglZ->SIGMA+OB-Fold+AAA-ATPase+HKD-DNase-BrxL-><-SLOG<-HAD<-GP+REase||Phage_integrase->	1172	Bacteria>Pseudomonadota>Alphaproteobacteria>Rhodobacterales>Paracoccaceae>Halocynthiibacter	GCF_025802015.1	unclassified_Halocynthiibacter	OH136_RS09920	459600..463118	2640401	MULTISPECIES:-BREX-system-P-loop-protein-BrxC-[unclassified-Halocynthiibacter]
WP_274653192.1	Type1.BREX	<-ParB+HNH+Schlafen<-?<-SIGMA+OB-Fold+AAA-ATPase+HKD-DNase-BrxL<-PglZ<-N6-METHYLASE<-NERD<-OLD-ABC<-DUF6079*<-BrxB-BrxF-iSTAND<-BrxA-DNA-binding<-wHTH+WYL+WCX||ArdCN+MPTase->?->?->FurR-HTH->?->HIN-HTH+TraJ-RHH->	1172	Bacteria>Pseudomonadota>Alphaproteobacteria>Rhodobacterales>Paracoccaceae>Halocynthiibacter	GCF_028823805.1	Halocynthiibacter_sp._C4	OU789_RS03095	642215..645733	2992758	BREX-system-P-loop-protein-BrxC-[Halocynthiibacter-sp.-C4]
WP_269398497.1	Type1.BREX	<-Integrase_DNA+Phage_integrase||?->SF1-SF2-Helicase+DUF1998-><-SIGMA+OB-Fold+AAA-ATPase+HKD-DNase-BrxL<-PglZ<-N6-METHYLASE<-DUF6079*<-BrxB-BrxF-iSTAND<-BrxA-DNA-binding<-?<-DnaB||XIS-HTH->XIS-HTH->Phage_integrase->Phage_integrase->	1171	Bacteria>Pseudomonadota>Alphaproteobacteria>Rhodobacterales>Paracoccaceae>Limimaricola	GCF_027213785.1	Limimaricola_sp._G21655-S1	O4G76_RS17300	20007..23522	3014768	BREX-system-P-loop-protein-BrxC-[Limimaricola-sp.-G21655-S1]
WP_094023246.1	Type1.BREX	<-Phage_integrase<-XIS-HTH||HIN-HTH->AAA-ATPase->DEOR-HTH->wHTH+WYL+WCX-p->wHTH+WYL+WCX->BrxA-DNA-binding->BrxB-BrxF-iSTAND->DUF6079*->N6-METHYLASE->PglZ->?->SIGMA+OB-Fold+AAA-ATPase+HKD-DNase-BrxL->SF1-SF2-Helicase->	1175	Bacteria>Pseudomonadota>Alphaproteobacteria>Rhodobacterales>Paracoccaceae>Maliponia	GCF_900184945.1	Maliponia_aquimaris	MAA8898_RS22355	47898..51425	1673631	BREX-system-P-loop-protein-BrxC-[Maliponia-aquimaris]
MBO6774806.1	Type1.BREX	<-HHH_5+SF1-SF2-Helicase<-Thermonuclease<-DOC+HTH||RNase_T+35exo-><-?<-?||wHTH+WYL+WCX->BrxA-DNA-binding->BrxB-BrxF-iSTAND->DUF6079*->N6-METHYLASE->PglZ->SIGMA+OB-Fold+AAA-ATPase+HKD-DNase-BrxL->REase+iREase->?->?-><-RADICAL-SAM	1172	Bacteria>Pseudomonadota>Alphaproteobacteria>Rhodobacterales>Paracoccaceae>Marinibacterium	GCA_017643305.1	Marinibacterium_sp.	JJ897_04735	11284..14802	1931201	BREX-system-P-loop-protein-BrxC-[Marinibacterium-sp.]
APG08218.1	Type1.BREX	<-HEPN+PAS+DUF4011+SF1-SF2-Helicase+REase<-?<-SIGMA+OB-Fold+AAA-ATPase+HKD-DNase-BrxL<-PglZ<-N6-METHYLASE<-DUF6079*<-BrxB-BrxF-iSTAND<-BrxA-DNA-binding<-wHTH+WYL+WCX||?-><-LON-AAA||<-tRNA||PRTase->?->REC+OmpR-HTH->HPT+HISKIN+CheW+REC->	1166	Bacteria>Pseudomonadota>Alphaproteobacteria>Rhodobacterales>Paracoccaceae>Marivivens_group	GCA_001908835.1	Marivivens_sp._JLT3646	BSK21_01620	313072..316572	1920883	hypothetical-protein-BSK21_01620-[Marivivens-sp.-JLT3646]
WP_017998950.1	Type1.BREX	<-HIN-HTH<-RNasePH<-LZ_Tnp_IS66+zf-IS66+DDE_Tnp_IS66+DDE_Tnp_IS66_C<-?<-HIN-HTH<-SIGMA+OB-Fold+AAA-ATPase+HKD-DNase-BrxL<-PglZ<-N6-METHYLASE<-DUF6079*<-BrxB-BrxF-iSTAND<-BrxA-DNA-binding<-wHTH+WYL+WCX<-AAA+HTH<-?||?->?->Classical-AAA->	1177	Bacteria>Pseudomonadota>Alphaproteobacteria>Rhodobacterales>Paracoccaceae>Paracoccus	GCF_000371965.1	Paracoccus_sp._N5	PARN5_RS0106405	1284773..1288306	1101189	BREX-system-P-loop-protein-BrxC-[Paracoccus-sp.-N5]

Protein-ID	System	Gene-Neighbourhoods	Length	Taxonomy	Assembly-ID	Strain	Locus-Tag	Genomic-coordinates	TaxID	Fasta-define
WP_042245816.1	Type1.BREX	DDE_Tnp->DDE_Tnp-><-HKD-DNase+Preader14<-SIGMA+OB-Fold+AAA-ATPase+HKD-DNase-BrxL<-PglZ<-PglZ-p<-N6-METHYLASE-p<-N6-METHYLASE<-DUF6079*<-BrxB-BrxF-iSTAND<-BrxA-DNA-binding<-35exo<-RelE-ParE<-MetJarc-HTH||?-><-DDE_3	1174	Bacteria>Pseudomonadota>Alphaproteobacteria>Rhodobacterales>Paracoccaceae>Paracoccus	GCF_000787695.1	Paracoccus_sp._PAMC_22219	TY51_RS03260	14723..18247	1569209	BREX-system-P-loop-protein-BrxC-[Paracoccus-sp.-PAMC-22219]
WP_046001801.1	Type1.BREX	TarH+HAMP+MA-><-Glyoxalase<-?<-ARG||WXG-><-PIN<-SIGMA+OB-Fold+AAA-ATPase+HKD-DNase-BrxL<-PglZ<-N6-METHYLASE<-DUF6079*<-BrxB-BrxF-iSTAND<-BrxA-DNA-binding<-35exo<-RelE-ParE<-MetJarc-HTH||HKD-DNase+DUF3150->HTH_Tnp_1->	1174	Bacteria>Pseudomonadota>Alphaproteobacteria>Rhodobacterales>Paracoccaceae>Paracoccus	GCF_000967825.1	Paracoccus_sp._228	SY26_RS18685	30060..33584	1192054	BREX-system-P-loop-protein-BrxC-[Paracoccus-sp.-228]
WP_199257821.1	Type1.BREX	GDResolvase+Recombinase+ZnR-><-SF1-SF2-Helicase<-HKD-DNase+HKD-DNase<-wHTH<-SF1-SF2-Helicase+REase<-SF1-SF2-Helicase+REase<-SIGMA+OB-Fold+AAA-ATPase+HKD-DNase-BrxL<-PglZ<-N6-METHYLASE<-DUF6079*<-BrxB-BrxF-iSTAND<-BrxA-DNA-binding<-wHTH+WYL+WCX<-MODE-HTH+PBPII||TetR-HTH-><-RHH<-VirB10<-VirB9<-VirB8	1179	Bacteria>Pseudomonadota>Alphaproteobacteria>Rhodobacterales>Paracoccaceae>Paracoccus	GCF_016446475.1	Paracoccus_binzhouensis	I9P22_RS02450	23714..27253	2796149	BREX-system-P-loop-protein-BrxC-[Paracoccus-binzhouensis]
WP_199258165.1	Type1.BREX	<-N6-METHYLASE-p<-REase<-OLD-ABC<-DUF6079*<-BrxB-BrxF-iSTAND<-BrxA-DNA-binding<-wHTH+WYL+WCX||?->Thermonuclease->TrbM-><-?<-HTH_3	1173	Bacteria>Pseudomonadota>Alphaproteobacteria>Rhodobacterales>Paracoccaceae>Paracoccus	GCF_016446475.1	Paracoccus_binzhouensis	I9P22_RS04365	3344..6865	2796149	BREX-system-P-loop-protein-BrxC-[Paracoccus-binzhouensis]
WP_260276663.1	Type1.BREX	URI-DNase+RAMA->XIS-HTH->wHTH+WYL+WCX->BrxA-DNA-binding->BrxB-BrxF-iSTAND->DUF6079*->N6-METHYLASE->PglZ->SIGMA+OB-Fold+AAA-ATPase+HKD-DNase-BrxL->URI-DNase+RAMA-><-?<-Glyco_hydro_53+Phage-tail_3	1173	Bacteria>Pseudomonadota>Alphaproteobacteria>Rhodobacterales>Paracoccaceae>Paracoccus	GCF_025195095.1	Paracoccus_sp._YLB-12	MU516_RS07900	40235..43756	2933292	BREX-system-P-loop-protein-BrxC-[Paracoccus-sp.-YLB-12]
WP_271109779.1	Type1.BREX	MerR-HTH-><-HTH_Tnp_1+HTH_28+HTH_32+rve+rve_3||BrxB-BrxF-iSTAND->DUF6079*->DDE_Tnp_ISL3->	1173	Bacteria>Pseudomonadota>Alphaproteobacteria>Rhodobacterales>Paracoccaceae>Paracoccus	GCF_027912675.1	Paracoccus_sp._SCSIO_75233	PAF12_17375	77006..80527	3017782	BREX-system-P-loop-protein-BrxC-[Paracoccus-sp.-SCSIO-75233]
WP_271118256.1	Type1.BREX	HTH_3->?-><-TrbM<-Thermonuclease<-?||wHTH+WYL+WCX->BrxA-DNA-binding->BrxB-BrxF-iSTAND->DUF6079*->N6-METHYLASE->PglZ->SIGMA+OB-Fold+AAA-ATPase+HKD-DNase-BrxL->HIN-HTH->AAA-ATPase->	1173	Bacteria>Pseudomonadota>Alphaproteobacteria>Rhodobacterales>Paracoccaceae>Paracoccus	GCF_027912835.1	Paracoccus_sp._SCSIO_76264	PAF18_RS16795	74012..77533	3017783	BREX-system-P-loop-protein-BrxC-[Paracoccus-sp.-SCSIO-76264]
WP_149766716.1	Type1.BREX	<-HTH<-Classical-AAA<-?<-?||?->AAA+HTH->wHTH+WYL+WCX->BrxA-DNA-binding->BrxB-BrxF-iSTAND->DUF6079*->PglZ->SIGMA+OB-Fold+AAA-ATPase+HKD-DNase-BrxL->?->ParB->	1177	Bacteria>Pseudomonadota>Alphaproteobacteria>Rhodobacterales>Paracoccaceae>Paracoccus	GCF_900156255.1	Paracoccus_thiocyanatus	F3H32_RS16120	13661..17194	34006	BREX-system-P-loop-protein-BrxC-[Paracoccus-thiocyanatus]
WP_085502591.1	Type1.BREX	<-ABhydrolase+ABhydrolase<-?||MODE-HTH+PBPII-><-SbcC+SbcC<-Calcineurin+Calcineurin-SbcD||wHTH+WYL+WCX->BrxA-DNA-binding->BrxB-BrxF-iSTAND->DUF6079*->N6-METHYLASE->PglZ->SIGMA+OB-Fold+AAA-ATPase+HKD-DNase-BrxL->?-><-?<-?||SF1-SF2-Helicase+DUF1998->?->RelE-ParE->	1179	Bacteria>Pseudomonadota>Alphaproteobacteria>Rhodobacterales>Paracoccaceae>Paracoccus	GCF_900177635.1	Paracoccus_sp._J56	B9Y79_RS14640	24785..28324	935850	BREX-system-P-loop-protein-BrxC-[Paracoccus-sp.-J56]
WP_113913307.1	Type1.BREX	SIR2-><-?<-REase<-Immank+ANK+ANK<-GreA_GreB+SF1-SF2-Helicase<-SIGMA+OB-Fold+AAA-ATPase+HKD-DNase-BrxL<-PglZ<-N6-METHYLASE<-DUF6079*<-BrxB-BrxF-iSTAND<-BrxA-DNA-binding<-wHTH+WYL+WCX||Calcineurin+Calcineurin-SbcD->SbcC+SbcC->	1179	Bacteria>Pseudomonadota>Alphaproteobacteria>Rhodobacterales>Paracoccaceae>Pelagivirga	GCF_003316635.1	Pelagivirga_dicentrarchi	DQ230_RS14395	19757..23296	2250573	BREX-system-P-loop-protein-BrxC-[Pelagivirga-dicentrarchi]
NYS26401.1	Type1.BREX	<-N6-METHYLASE-p<-DUF6079*<-DUF6079-p<-BrxB-BrxF-iSTAND<-BrxA-DNA-binding<-MetJarc-HTH<-?<-GDResolvase+ZnR<-RAMA	1025	Bacteria>Pseudomonadota>Alphaproteobacteria>Rhodobacterales>Paracoccaceae>Rhabdonatronobacter	GCA_013415485.1	Rhabdonatronobacter_sediminivivens	HUK65_15550	1855..4932	2743469	BREX-system-P-loop-protein-BrxC-[Rhabdonatronobacter-sediminivivens]
WP_268839433.1	Type1.BREX	<-N6-METHYLASE-p<-DUF6079*<-DUF6079-p<-BrxB-BrxF-iSTAND<-BrxA-DNA-binding<-?<-GDResolvase+ZnR<-RAMA	1018	Bacteria>Pseudomonadota>Alphaproteobacteria>Rhodobacterales>Paracoccaceae>Rhabdonatronobacter	GCF_013415485.1	Rhabdonatronobacter_sediminivivens	HUK65_RS15575	1855..4911	2743469	BREX-system-P-loop-protein-BrxC-[Rhabdonatronobacter-sediminivivens]
WP_179907198.1	Type1.BREX	<-N6-METHYLASE-p<-DUF6079<-DUF6079-p*<-BrxB-BrxF-iSTAND<-BrxA-DNA-binding<-?<-GDResolvase+ZnR<-RAMA	105	Bacteria>Pseudomonadota>Alphaproteobacteria>Rhodobacterales>Paracoccaceae>Rhabdonatronobacter	GCF_013415485.1	Rhabdonatronobacter_sediminivivens	HUK65_RS15580	4960..5277	2743469	hypothetical-protein-[Rhabdonatronobacter-sediminivivens]
RBP80165.1	Type1.BREX	XIS-HTH->wHTH+WYL+WCX->BrxA-DNA-binding->BrxB-BrxF-iSTAND->DUF6079*->OLD-ABC->N6-METHYLASE->PglZ->SIGMA+OB-Fold+AAA-ATPase+HKD-DNase-BrxL-><-?<-Glyco_hydro_53+Phage-tail_3	1172	Bacteria>Pseudomonadota>Alphaproteobacteria>Rhodobacterales>Paracoccaceae>Rhodobacter	GCA_003315475.1	Rhodobacter_sp._140A	DFO80_1418	4623..8141	2183910	hypothetical-protein-DFO80_1418-[Rhodobacter-sp.-140A]
WP_223637900.1	Type1.BREX	MMR_HSR1+sGTP+sGTP->?-><-?||?->?->SF1-SF2-Helicase+DUF1998->BrxA-DNA-binding->BrxB-BrxF-iSTAND->DUF6079*->N6-METHYLASE->PglZ->SIGMA+OB-Fold+AAA-ATPase+HKD-DNase-BrxL->	1175	Bacteria>Pseudomonadota>Alphaproteobacteria>Rhodobacterales>Paracoccaceae>Rhodobacter	GCF_020023995.1	Rhodobacter_sp._TJ_12	CKO11_RS10405	86480..90007	2029399	BREX-system-P-loop-protein-BrxC-[Rhodobacter-sp.-TJ_12]
WP_243695875.1	Type1.BREX	HTH_3->?-><-TrbM<-Thermonuclease<-?||wHTH+WYL+WCX->BrxA-DNA-binding->BrxB-BrxF-iSTAND->DUF6079-p*->DDE_Tnp_1_5+DDE_Tnp_1->GDResolvase+HIN-HTH-><-TetR-HTH||?->ABC-ATPase+SbcC->?->ABhydrolase->	633	Bacteria>Pseudomonadota>Alphaproteobacteria>Rhodobacterales>Paracoccaceae>Rhodovulum	GCF_004343075.1	Rhodovulum_marinum	EV662_RS15600	23147..25048	320662	BREX-system-P-loop-protein-BrxC-[Rhodovulum-marinum]
WP_227427259.1	Type1.BREX	<-EVE<-?<-?<-Peptidase_M22||?->?->?->tRNA-><-SIGMA+OB-Fold+AAA-ATPase+Lon-Protease-BrxL<-N6-METHYLASE<-DUF6079*<-BrxB-BrxF-iSTAND<-BrxA-DNA-binding<-MetJarc-HTH||RNase_T+35exo-><-Primpol<-XIS-HTH<-?<-Arm-DNA-bind_3+Phage_integrase	1140	Bacteria>Pseudomonadota>Alphaproteobacteria>Rhodobacterales>Paracoccaceae>Roseibaca	GCF_020667835.1	Roseibaca_sp._YO-43	J1C52_RS07230	292113..295535	2816854	BREX-system-P-loop-protein-BrxC-[Roseibaca-sp.-YO-43]
WP_121096034.1	Type1.BREX	Glyco_hydro_53+Phage-tail_3->?->?->?-><-SIGMA+OB-Fold+AAA-ATPase+HKD-DNase-BrxL<-PglZ<-N6-METHYLASE<-DUF6079*<-BrxB-BrxF-iSTAND<-BrxA-DNA-binding<-wHTH+WYL+WCX<-RNase_T+35exo<-TPR<-Couple_hipA+HipA_C<-HTH_3||Arm-DNA-bind_3+Phage_int_SAM_3+Phage_integrase->	1173	Bacteria>Pseudomonadota>Alphaproteobacteria>Rhodobacterales>Paracoccaceae>Roseibaca	GCF_900499075.1	Roseibaca_ekhonensis	ROE7235_RS13515	24046..27567	254356	BREX-system-P-loop-protein-BrxC-[Roseibaca-ekhonensis]
WP_245964025.1	Type1.BREX	<-DUF6079-p*<-BrxB-BrxF-iSTAND<-BrxA-DNA-binding<-wHTH+WYL+WCX||?->Thermonuclease->TrbM->	637	Bacteria>Pseudomonadota>Alphaproteobacteria>Rhodobacterales>Paracoccaceae>Roseibaca	GCF_900499075.1	Roseibaca_ekhonensis	ROE7235_RS18160	88..2001	254356	BREX-system-P-loop-protein-BrxC-[Roseibaca-ekhonensis]
WP_218631289.1	Type1.BREX	<-SIGMA+OB-Fold+AAA-ATPase+HKD-DNase-BrxL<-PglZ<-N6-METHYLASE<-DUF6079*<-BrxB-BrxF-iSTAND<-BrxA-DNA-binding<-?<-ZnR+TOPRIM+AAA-helicase+wHTH<-TOPRIM<-Phage_integrase<-tRNA||?-><-?<-PALP	1173	Bacteria>Pseudomonadota>Alphaproteobacteria>Rhodobacterales>Paracoccaceae>Sedimentimonas	GCF_019264375.1	Sedimentimonas_flavescens	KTQ37_RS14800	8221..11742	2851012	BREX-system-P-loop-protein-BrxC-[Sedimentimonas-flavescens]
WP_108641830.1	Type1.BREX	<-Schlafen<-?<-ParB+HNH+Schlafen<-?<-SIGMA+OB-Fold+AAA-ATPase+HKD-DNase-BrxL<-PglZ<-N6-METHYLASE<-DUF6079*<-BrxB-BrxF-iSTAND<-BrxA-DNA-binding<-wHTH+WYL+WCX||?->Thermonuclease->TrbM-><-?<-HTH_3	1173	Bacteria>Pseudomonadota>Alphaproteobacteria>Rhodobacterales>Paracoccaceae>Thalassorhabdomicrobium	GCF_003072065.1	Thalassorhabdomicrobium_marinisediminis	DC363_RS14250	78584..82105	2170577	BREX-system-P-loop-protein-BrxC-[Thalassorhabdomicrobium-marinisediminis]
WP_058289795.1	Type1.BREX	<-TraI-VirD2-RCRE<-HIN-HTH+TraJ-RHH<-?<-FurR-HTH<-?<-ArdCN+MPTase||wHTH+WYL+WCX->BrxA-DNA-binding->BrxB-BrxF-iSTAND->DUF6079*->OLD-ABC->NERD->N6-METHYLASE->PglZ->SIGMA+OB-Fold+AAA-ATPase+HKD-DNase-BrxL->ParB+HNH+Schlafen-><-?<-Couple_hipA+HipA_C<-HTH_3	1172	Bacteria>Pseudomonadota>Alphaproteobacteria>Rhodobacterales>Paracoccaceae>Tritonibacter	GCF_001458415.1	Tritonibacter_multivorans	PGB32_RS12460	15411..18929	928856	BREX-system-P-loop-protein-BrxC-[Tritonibacter-multivorans]
WP_065322029.1	Type1.BREX	XIS-HTH->PIN-><-AbiEi_4||HTH_3->?-><-REase+iREase<-SIGMA+OB-Fold+AAA-ATPase+HKD-DNase-BrxL<-PglZ<-N6-METHYLASE<-DUF6079*<-BrxB-BrxF-iSTAND<-BrxA-DNA-binding<-wHTH+WYL+WCX||ArdCN+MPTase->?->?->FurR-HTH-><-?||Phage_integrase->	1172	Bacteria>Pseudomonadota>Alphaproteobacteria>Rhodobacterales>Paracoccaceae>Tritonibacter	GCF_001681255.1	Tritonibacter_mobilis	UB66_RS09365	554629..558147	379347	BREX-system-P-loop-protein-BrxC-[Tritonibacter-mobilis]
WP_225262956.1	Type1.BREX	<-HEPN+OST-HTH<-ParB+HNH<-Phage_integrase+Phage_integrase<-?<-Phage_int_SAM_1+Phage_integrase<-ArdCN+MPTase||wHTH+WYL+WCX->BrxA-DNA-binding->BrxB-BrxF-iSTAND->DUF6079*->N6-METHYLASE->PglZ->SIGMA+OB-Fold+AAA-ATPase+HKD-DNase-BrxL->S1COLD->?-><-AAA-ATPase<-HIN-HTH	1172	Bacteria>Pseudomonadota>Alphaproteobacteria>Rhodobacterales>Paracoccaceae>Tritonibacter	GCF_020216085.1	Tritonibacter_mobilis	LC038_RS07430	133716..137234	379347	BREX-system-P-loop-protein-BrxC-[Tritonibacter-mobilis]
WP_065331859.1	Type1.BREX	TraJ-RHH->TraI-VirD2-RCRE-><-AbiEii<-AbiEi_3_N+AbiEi_3||BrxA-DNA-binding->BrxB-BrxF-iSTAND->DUF6079*->N6-METHYLASE->PglZ->SIGMA+OB-Fold+AAA-ATPase+Lon-Protease-BrxL->SbcC->Arm-DNA-bind_3-><-ARSR-HTH||?->?->ArsR-HTH->	1204	Bacteria>Pseudomonadota>Alphaproteobacteria>Rhodobacterales>Paracoccaceae>Tritonibacter	GCF_030159715.1	Tritonibacter_mobilis	BLU61_RS13075	124334..127948	379347	BREX-system-P-loop-protein-BrxC-[Tritonibacter-mobilis]
PHO04850.1	Type1.BREX	<-SIGMA+OB-Fold+AAA-ATPase+HKD-DNase-BrxL<-PglZ<-N6-METHYLASE<-DUF6079*<-BrxB-BrxF-iSTAND<-BrxA-DNA-binding||?->Classical-AAA->?-><-?<-?<-TraI-VirD2-RCRE<-CopG/RHH	1166	Bacteria>Pseudomonadota>Alphaproteobacteria>Rhodobacterales>Paracoccaceae>unclassified_Paracoccaceae	GCA_002700045.1	Rhodobacteraceae_bacterium_4F10	CSC82_04805	8196..11696	2045445	hypothetical-protein-CSC82_04805-[Rhodobacteraceae-bacterium-4F10]
NKX29277.1	Type1.BREX	<-McrC-NTD+REase<-HTH+McrB-AAA<-SbcC<-SbcC-p<-Calcineurin-SbcD<-SIGMA+OB-Fold+AAA-ATPase+HKD-DNase-BrxL<-PglZ<-N6-METHYLASE<-DUF6079*<-BrxB-BrxF-iSTAND<-BrxA-DNA-binding<-wHTH+WYL+WCX||ArdCN+MPTase->Phage_integrase->	1168	Bacteria>Pseudomonadota>Alphaproteobacteria>Rhodobacterales>Paracoccaceae>unclassified_Paracoccaceae	GCA_012395685.1	Rhodobacteraceae_bacterium_R_SAG6	HGE68_05220	14041..17547	2719008	BREX-system-P-loop-protein-BrxC-[Rhodobacteraceae-bacterium-R_SAG6]
MBD1206697.1	Type1.BREX	<-SIGMA-HTH+HIN-HTH<-SIGMA+OB-Fold+AAA-ATPase+HKD-DNase-BrxL<-PglZ<-N6-METHYLASE<-DUF6079*<-BrxB-BrxF-iSTAND<-BrxA-DNA-binding<-REase<-wHTH+WYL+WCX<-GDResolvase+HIN-HTH	1181	Bacteria>Pseudomonadota>Alphaproteobacteria>Rhodobacterales>Paracoccaceae>unclassified_Paracoccaceae	GCA_014654775.1	Paracoccaceae_bacterium	H9533_21535	9124..12669	1904441	BREX-system-P-loop-protein-BrxC-[Paracoccaceae-bacterium]
MBL4542464.1	Type1.BREX	Glyco_hydro_53+Phage-tail_3->?-><-URI-DNase+RAMA<-SIGMA+OB-Fold+AAA-ATPase+HKD-DNase-BrxL<-PglZ<-N6-METHYLASE<-DUF6079*<-BrxB-BrxF-iSTAND<-BrxA-DNA-binding<-wHTH+WYL+WCX<-Arm-DNA-bind_3+Phage_int_SAM_3+Phage_integrase||<-?||?->ABC-ATPase+SbcC->	1173	Bacteria>Pseudomonadota>Alphaproteobacteria>Rhodobacterales>Paracoccaceae>unclassified_Paracoccaceae	GCA_016762795.1	Paracoccaceae_bacterium	JKP97_11775	15111..18632	1904441	BREX-system-P-loop-protein-BrxC-[Paracoccaceae-bacterium]
MBL4767704.1	Type1.BREX	Exonuc_V_gamma+REase->SF1-SF2-Helicase+REase-><-ABhydrolase<-?||?-><-?||BrxA-DNA-binding->BrxB-BrxF-iSTAND->DUF6079-p*->	663	Bacteria>Pseudomonadota>Alphaproteobacteria>Rhodobacterales>Paracoccaceae>unclassified_Paracoccaceae	GCA_016764515.1	Paracoccaceae_bacterium	JKY94_08320	11908..13895	1904441	BREX-system-P-loop-protein-BrxC-[Paracoccaceae-bacterium]
MCR9111160.1	Type1.BREX	DDE_Tnp_IS66+DDE_Tnp_IS66_C-><-?||<-HTH_3||?->HKD-DNase+DUF3150->wHTH+WYL+WCX->BrxA-DNA-binding->BrxB-BrxF-iSTAND->DUF6079*->N6-METHYLASE->PglZ->SIGMA+OB-Fold+AAA-ATPase+HKD-DNase-BrxL->?->CHTH->?-><-Arm-DNA-bind_3+Phage_integrase	1176	Bacteria>Pseudomonadota>Alphaproteobacteria>Rhodobacterales>Paracoccaceae>unclassified_Paracoccaceae	GCA_024742895.1	Paracoccaceae_bacterium	NXH94_20060	7296..10826	1904441	BREX-system-P-loop-protein-BrxC-[Paracoccaceae-bacterium]
MBD3677805.1	Type1.BREX	<-SbcC+SbcC<-Calcineurin||wHTH+WYL+WCX->?->?-><-SIGMA+OB-Fold+AAA-ATPase+HKD-DNase-BrxL<-PglZ<-N6-METHYLASE<-DUF6079*<-BrxB-BrxF-iSTAND<-BrxA-DNA-binding<-wHTH+WYL+WCX||?->Thermonuclease->TrbM-><-?<-HTH_3	1173	Bacteria>Pseudomonadota>Alphaproteobacteria>Rhodobacterales>Paracoccaceae>unclassified_Paracoccaceae	-	Paracoccaceae_bacterium	HUJ27_05300	15046..18567	1904441	BREX-system-P-loop-protein-BrxC-[Paracoccaceae-bacterium]
WP_089279695.1	Type1.BREX	Glyco_hydro_53+Phage-tail_3->?->?->?-><-SIGMA+OB-Fold+AAA-ATPase+HKD-DNase-BrxL<-PglZ<-N6-METHYLASE<-DUF6079*<-BrxB-BrxF-iSTAND<-BrxA-DNA-binding<-wHTH+WYL+WCX<-35exo<-TPR<-Couple_hipA+HipA_C<-HTH_3	1173	Bacteria>Pseudomonadota>Alphaproteobacteria>Rhodobacterales>Roseobacteraceae>Antarctobacter	GCF_900188425.1	Antarctobacter_heliothermus	CHB90_RS20470	19687..23208	74033	BREX-system-P-loop-protein-BrxC-[Antarctobacter-heliothermus]
WP_089279817.1	Type1.BREX	<-RelE-ParE<-ParD_antitoxin<-CHTH||?->GDResolvase+PPP1-HTH->ParB->wHTH+WYL+WCX->BrxA-DNA-binding->BrxB-BrxF-iSTAND->DUF6079*->N6-METHYLASE->PglZ->SIGMA+OB-Fold+AAA-ATPase+Lon-Protease-BrxL->ParB+HNH-><-Peptidase-M15<-?<-?<-?<-Glyco_hydro_53+Phage-tail_3	1147	Bacteria>Pseudomonadota>Alphaproteobacteria>Rhodobacterales>Roseobacteraceae>Antarctobacter	GCF_900188425.1	Antarctobacter_heliothermus	CHB90_RS21205	7452..10895	74033	BREX-system-P-loop-protein-BrxC-[Antarctobacter-heliothermus]
MAN15901.1	Type1.BREX	<-TatD_DNase<-?<-Thymidylate_kin<-Peptidase_S11+PBP5_C<-?||tRNA-><-DUF6079-p*	265	Bacteria>Pseudomonadota>Alphaproteobacteria>Rhodobacterales>Roseobacteraceae>Dinoroseobacter	GCA_002693265.1	Dinoroseobacter_sp.	CL945_14875	7316..8113	2003366	ATPase-[Dinoroseobacter-sp.]
WP_106262543.1	Type1.BREX	<-Phage_int_SAM_1+Phage_integrase<-?<-?||SKI->?-><-HEPN||wHTH+WYL+WCX->BrxA-DNA-binding->BrxB-BrxF-iSTAND->DUF6079*->OLD-ABC->REase->N6-METHYLASE->PglZ->SIGMA+OB-Fold+AAA-ATPase+HKD-DNase-BrxL-><-?||HTH_Tnp_1->HTH_Tnp_1+HTH_21+rve+rve_3-><-HIN-HTH	1172	Bacteria>Pseudomonadota>Alphaproteobacteria>Rhodobacterales>Roseobacteraceae>Donghicola	GCF_003003355.1	Donghicola_tyrosinivorans	CLV74_RS02110	436162..439680	1652492	BREX-system-P-loop-protein-BrxC-[Donghicola-tyrosinivorans]
WP_027259561.1	Type1.BREX	<-TraI-VirD2-RCRE<-HIN-HTH+TraJ-RHH<-?<-FurR-HTH<-?<-ArdCN+MPTase||wHTH+WYL+WCX->BrxA-DNA-binding->BrxB-BrxF-iSTAND->DUF6079*->N6-METHYLASE->PglZ->SIGMA+OB-Fold+AAA-ATPase+HKD-DNase-BrxL->REase+iREase-><-?<-HTH_3||AbiEi_4->AbiEii-><-HTH_36	1172	Bacteria>Pseudomonadota>Alphaproteobacteria>Rhodobacterales>Roseobacteraceae>Leisingera	GCF_000473165.1	Leisingera_aquimarina	AQUI_RS0122640	3713027..3716545	476529	BREX-system-P-loop-protein-BrxC-[Leisingera-aquimarina]
WP_263764345.1	Type1.BREX	<-HTH_3<-?||DEDD_Tnp_IS110+Transposase_20-><-?<-?||wHTH+WYL+WCX->BrxA-DNA-binding->BrxB-BrxF-iSTAND->DUF6079*->N6-METHYLASE->PglZ->SIGMA+OB-Fold+AAA-ATPase+HKD-DNase-BrxL-><-?||HNH->	1175	Bacteria>Pseudomonadota>Alphaproteobacteria>Rhodobacterales>Roseobacteraceae>Lentibacter	GCF_025751535.1	Lentibacter_sp._XHP0401	OE750_RS11445	67112..70639	2984334	BREX-system-P-loop-protein-BrxC-[Lentibacter-sp.-XHP0401]
WP_263765218.1	Type1.BREX	<-REase<-Immank+ANK+ANK<-GreA_GreB+SF1-SF2-Helicase<-SIGMA+OB-Fold+AAA-ATPase+HKD-DNase-BrxL<-PglZ<-N6-METHYLASE<-DUF6079*<-BrxB-BrxF-iSTAND<-BrxA-DNA-binding<-wHTH+WYL+WCX||Calcineurin+Calcineurin-SbcD->SbcC+SbcC->?->AAA+HTH-><-?||LON-AAA->	1167	Bacteria>Pseudomonadota>Alphaproteobacteria>Rhodobacterales>Roseobacteraceae>Lentibacter	GCF_025751535.1	Lentibacter_sp._XHP0401	OE750_RS15480	13619..17122	2984334	BREX-system-P-loop-protein-BrxC-[Lentibacter-sp.-XHP0401]
WP_088668915.1	Type1.BREX	VirB10->RHH-><-TetR-HTH||MODE-HTH+PBPII-><-SbcC+SbcC<-Calcineurin+Calcineurin-SbcD||wHTH+WYL+WCX->BrxA-DNA-binding->BrxB-BrxF-iSTAND->DUF6079*->N6-METHYLASE->PglZ->SIGMA+OB-Fold+AAA-ATPase+HKD-DNase-BrxL-><-Zn_ribbon_recom||?->ParA-Soj-PloopNTPase->	1179	Bacteria>Pseudomonadota>Alphaproteobacteria>Rhodobacterales>Roseobacteraceae>Mameliella	GCF_002211215.1	Mameliella_alba	CDZ95_RS03685	62213..65752	561184	BREX-system-P-loop-protein-BrxC-[Mameliella-alba]
WP_167646419.1	Type1.BREX	BrxA-DNA-binding->BrxB-BrxF-iSTAND->DUF6079*->N6-METHYLASE->PglZ->SIGMA+OB-Fold+AAA-ATPase+HKD-DNase-BrxL->?->DUF4096+DDE_Tnp_1+DDE_Tnp_1_2->	1175	Bacteria>Pseudomonadota>Alphaproteobacteria>Rhodobacterales>Roseobacteraceae>Mameliella	GCF_011858205.1	Mameliella_alba	DAA47_RS08650	638377..641904	561184	BREX-system-P-loop-protein-BrxC-[Mameliella-alba]
WP_091845786.1	Type1.BREX	<-Phage_integrase<-?<-ArdCN+MPTase||wHTH+WYL+WCX->BrxA-DNA-binding->BrxB-BrxF-iSTAND->DUF6079*->N6-METHYLASE->PglZ->SIGMA+OB-Fold+AAA-ATPase+HKD-DNase-BrxL->REase+iREase-><-?<-?<-HORMA<-STYKinase-like<-TRPR-HTH	1172	Bacteria>Pseudomonadota>Alphaproteobacteria>Rhodobacterales>Roseobacteraceae>Maribius	GCF_900110115.1	Maribius_pelagius	BMX96_RS12425	183654..187172	387096	BREX-system-P-loop-protein-BrxC-[Maribius-pelagius]
WP_274591702.1	Type1.BREX	XIS-HTH->wHTH+WYL+WCX->BrxA-DNA-binding->BrxB-BrxF-iSTAND->DUF6079*->OLD-ABC->REase->N6-METHYLASE->PglZ->SIGMA+OB-Fold+AAA-ATPase+HKD-DNase-BrxL->	1173	Bacteria>Pseudomonadota>Alphaproteobacteria>Rhodobacterales>Roseobacteraceae>Marinovum	GCF_028807375.1	Marinovum_sp._PR37	PVW49_RS22970	3287..6808	3028382	BREX-system-P-loop-protein-BrxC-[Marinovum-sp.-PR37]
WP_123792783.1	Type1.BREX	<-ParB+HNH+Schlafen<-SIGMA+OB-Fold+AAA-ATPase+HKD-DNase-BrxL<-PglZ<-N6-METHYLASE<-REase<-OLD-ABC<-N6-METHYLASE<-DUF6079*<-BrxB-BrxF-iSTAND<-BrxA-DNA-binding<-HIN-HTH<-HTH_36<-?<-?<-Arm-DNA-bind_3+Phage_integrase	1172	Bacteria>Pseudomonadota>Alphaproteobacteria>Rhodobacterales>Roseobacteraceae>Pacificibacter	GCF_003814915.1	Pacificibacter_maritimus	EDD53_RS08420	394668..398186	762213	BREX-system-P-loop-protein-BrxC-[Pacificibacter-maritimus]
MAE92616.1	Type1.BREX	<-N6-METHYLASE<-DUF6079*<-BrxB-BrxF-iSTAND<-BrxA-DNA-binding<-wHTH+WYL+WCX||?->?->?->?-><-TraG_N	1210	Bacteria>Pseudomonadota>Alphaproteobacteria>Rhodobacterales>Roseobacteraceae>Pelagibaca	GCA_002687285.1	Pelagibaca_sp.	CMI67_24080	2344..5976	1979400	ATPase-[Pelagibaca-sp.]
MAE92765.1	Type1.BREX	<-Thioredoxin_4||wHTH+WYL+WCX->BrxA-DNA-binding->BrxB-BrxF-iSTAND->DUF6079*->N6-METHYLASE->ParB+HNH->PglZ->SIGMA+OB-Fold+AAA-ATPase+Lon-Protease-BrxL->REase+PNTD1+PNTD2->PIWI->REase->	1219	Bacteria>Pseudomonadota>Alphaproteobacteria>Rhodobacterales>Roseobacteraceae>Pelagibaca	GCA_002687285.1	Pelagibaca_sp.	CMI67_24850	4770..8429	1979400	ATPase-[Pelagibaca-sp.]
WP_244686757.1	Type1.BREX	SSB-><-?||?-><-35exo+HRDC<-?<-?||tRNA->wHTH+WYL+WCX->BrxA-DNA-binding->BrxB-BrxF-iSTAND->DUF6079*->N6-METHYLASE-p->N6-METHYLASE->PglZ->MIT_C-><-?<-REase<-McrB-AAA<-?<-SF1-SF2-Helicase	1173	Bacteria>Pseudomonadota>Alphaproteobacteria>Rhodobacterales>Roseobacteraceae>Phaeobacter	GCF_022867865.1	Phaeobacter_sp._J2-8	MUB49_RS06760	203466..206987	2931394	BREX-system-P-loop-protein-BrxC-[Phaeobacter-sp.-J2-8]
WP_260112390.1	Type1.BREX	<-TraI-VirD2-RCRE<-HIN-HTH+TraJ-RHH<-?<-FurR-HTH<-?<-ArdCN+MPTase||wHTH+WYL+WCX->BrxA-DNA-binding->BrxB-BrxF-iSTAND->DUF6079*->OLD-ABC->NERD->N6-METHYLASE->PglZ->SIGMA+OB-Fold+AAA-ATPase+HKD-DNase-BrxL->REase+iREase-><-?<-HTH_3||AbiEi_4->	1172	Bacteria>Pseudomonadota>Alphaproteobacteria>Rhodobacterales>Roseobacteraceae>Phaeobacter	GCF_025142595.1	Phaeobacter_inhibens	K4F86_RS11215	2363506..2367024	221822	BREX-system-P-loop-protein-BrxC-[Phaeobacter-inhibens]
WP_009810044.1	Type1.BREX	<-HARE-HTH+REase<-SIGMA+OB-Fold+AAA-ATPase+HKD-DNase-BrxL<-PglZ<-N6-METHYLASE<-DUF6079*<-BrxB-BrxF-iSTAND<-BrxA-DNA-binding<-wHTH+WYL+WCX||?->?->?-><-RNase_T+35exo<-?||DOC+HTH->	1172	Bacteria>Pseudomonadota>Alphaproteobacteria>Rhodobacterales>Roseobacteraceae>Roseobacter	GCF_000152965.1	Roseobacter_sp._MED193	MED193_RS12240	2047846..2051364	314262	BREX-system-P-loop-protein-BrxC-[Roseobacter-sp.-MED193]
KJS41393.1	Type1.BREX	<-SbcC+SbcC<-Calcineurin+Calcineurin-SbcD||wHTH+WYL+WCX->BrxA-DNA-binding->BrxB-BrxF-iSTAND->DUF6079*->N6-METHYLASE->PglZ->SIGMA+OB-Fold+AAA-ATPase+HKD-DNase-BrxL->?->SF1-SF2-Helicase+DUF1998->CR-ATPase9->HEPN-><-DOC+HTH	1178	Bacteria>Pseudomonadota>Alphaproteobacteria>Rhodobacterales>Roseobacteraceae>Roseovarius	GCA_000961715.1	Roseovarius_sp._BRH_c41	VR71_18660	26779..30315	1629709	hypothetical-protein-VR71_18660-[Roseovarius-sp.-BRH_c41]
WP_160325893.1	Type1.BREX	Arm-DNA-bind_3+Phage_int_SAM_3+Phage_integrase->XIS-HTH->Primpol+PriCT1->PriCT1+D5N+D5ATPase->XIS-HTH->?->MetJarc-HTH->BrxA-DNA-binding->BrxB-BrxF-iSTAND->DUF6079*->	1110	Bacteria>Pseudomonadota>Alphaproteobacteria>Rhodobacterales>Roseobacteraceae>Roseovarius	GCF_001441635.1	Roseovarius_indicus	XM52_RS23920	112621..115950	540747	BREX-system-P-loop-protein-BrxC,-partial-[Roseovarius-indicus]
WP_074940632.1	Type1.BREX	XIS-HTH->Primpol+PriCT1->PriCT1+D5N+D5ATPase->XIS-HTH->?->?->MetJarc-HTH->BrxA-DNA-binding->BrxB-BrxF-iSTAND->DUF6079*-><-AAA-ATPase<-SIGMA-HTH+HIN-HTH||N6-METHYLASE->PglZ-><-AAA-ATPase<-HTH+rve||SIGMA+OB-Fold+AAA-ATPase+Lon-Protease-BrxL->	1119	Bacteria>Pseudomonadota>Alphaproteobacteria>Rhodobacterales>Roseobacteraceae>Roseovarius	GCF_008728195.1	Roseovarius_indicus	RIdsm_RS22200	4630317..4633676	540747	BREX-system-P-loop-protein-BrxC-[Roseovarius-indicus]
WP_139076871.1	Type1.BREX	WXG-><-?<-?||RAMA->?->?->BrxA-DNA-binding->BrxB-BrxF-iSTAND->DUF6079*->N6-METHYLASE->PglZ->SIGMA+OB-Fold+AAA-ATPase+HKD-DNase-BrxL->	1175	Bacteria>Pseudomonadota>Alphaproteobacteria>Rhodobacterales>Roseobacteraceae>Rubellimicrobium	GCF_006152115.1	Rubellimicrobium_rubrum	FHG66_11370	28349..31876	2585369	BREX-system-P-loop-protein-BrxC-[Rubellimicrobium-rubrum]
WP_068340009.1	Type1.BREX	<-VirD4-TraG+VirD4-FtsK<-?<-?<-?||?->BrxA-DNA-binding->BrxB-BrxF-iSTAND->DUF6079*->N6-METHYLASE->PglZ->SIGMA+OB-Fold+AAA-ATPase+Lon-Protease-BrxL->?->ArdCN+MPTase->	1203	Bacteria>Pseudomonadota>Alphaproteobacteria>Rhodobacterales>Roseobacteraceae>Ruegeria	GCF_001507545.1	Ruegeria_profundi	AVO44_RS17790	11048..14659	1685378	BREX-system-P-loop-protein-BrxC-[Ruegeria-profundi]
WP_254429641.1	Type1.BREX	HORMA-><-?||CopG/RHH->TraI-VirD2-RCRE-><-Classical-AAA||?->BrxA-DNA-binding->BrxB-BrxF-iSTAND->DUF6079*->N6-METHYLASE->PglZ->SIGMA+OB-Fold+AAA-ATPase+HKD-DNase-BrxL-><-?||?-><-tRNA<-?<-RNASE-EG	1170	Bacteria>Pseudomonadota>Alphaproteobacteria>Rhodobacterales>Roseobacteraceae>Ruegeria	GCF_013031165.1	Ruegeria_sp._HKCCD7251	GOX75_RS12475	203228..206740	2683011	BREX-system-P-loop-protein-BrxC-[Ruegeria-sp.-HKCCD7251]
WP_171123550.1	Type1.BREX	RNASE-EG->?->tRNA-><-?||?-><-SIGMA+OB-Fold+AAA-ATPase+HKD-DNase-BrxL<-PglZ<-N6-METHYLASE<-DUF6079*<-BrxB-BrxF-iSTAND<-BrxA-DNA-binding<-?||Classical-AAA-><-TraI-VirD2-RCRE<-CopG/RHH||?-><-HORMA	1174	Bacteria>Pseudomonadota>Alphaproteobacteria>Rhodobacterales>Roseobacteraceae>Ruegeria	GCF_013031185.1	Ruegeria_sp._HKCCD7239	GOX74_RS13410	208876..212400	2683010	BREX-system-P-loop-protein-BrxC-[Ruegeria-sp.-HKCCD7239]
WP_170339699.1	Type1.BREX	TraI-VirD2-RCRE->?->?-><-Glyco_hydro_3<-Classical-AAA<-?||?->BrxA-DNA-binding->BrxB-BrxF-iSTAND->DUF6079*->N6-METHYLASE->PglZ->SIGMA+OB-Fold+AAA-ATPase+HKD-DNase-BrxL->?-><-RVT<-?<-CR-ATPase4	1173	Bacteria>Pseudomonadota>Alphaproteobacteria>Rhodobacterales>Roseobacteraceae>Ruegeria	GCF_013031325.1	Ruegeria_arenilitoris	GOX56_RS07985	128142..131663	1173585	BREX-system-P-loop-protein-BrxC-[Ruegeria-arenilitoris]
WP_170340900.1	Type1.BREX	SF1-SF2-Helicase->?->?-><-35exo||MetJarc-HTH->BrxA-DNA-binding->BrxB-BrxF-iSTAND->DUF6079*->N6-METHYLASE->SIGMA+OB-Fold+AAA-ATPase+Lon-Protease-BrxL-><-tRNA||?->?->?-><-Phage_integrase<-Arm-DNA-bind_3+Phage_int_SAM_3+Phage_integrase	1140	Bacteria>Pseudomonadota>Alphaproteobacteria>Rhodobacterales>Roseobacteraceae>Ruegeria	GCF_013031325.1	Ruegeria_arenilitoris	GOX56_RS11935	236782..240204	1173585	BREX-system-P-loop-protein-BrxC-[Ruegeria-arenilitoris]
WP_254433969.1	Type1.BREX	CopG/RHH-><-?<-?<-?<-Classical-AAA||?->BrxA-DNA-binding->BrxB-BrxF-iSTAND->DUF6079*->N6-METHYLASE->PglZ->SIGMA+OB-Fold+AAA-ATPase+HKD-DNase-BrxL-><-?<-tRNA<-?<-RNASE-EG<-?<-?<-CN_hydrolase	1170	Bacteria>Pseudomonadota>Alphaproteobacteria>Rhodobacterales>Roseobacteraceae>Ruegeria	GCF_013032735.1	Ruegeria_arenilitoris	GOW80_RS09865	349565..353077	1173585	BREX-system-P-loop-protein-BrxC-[Ruegeria-arenilitoris]
WP_170509651.1	Type1.BREX	<-Classical-AAA||?->BrxA-DNA-binding->BrxB-BrxF-iSTAND->DUF6079*->N6-METHYLASE->PglZ->SIGMA+OB-Fold+AAA-ATPase+HKD-DNase-BrxL-><-?<-tRNA<-?<-RNASE-EG<-?<-?<-CN_hydrolase	1174	Bacteria>Pseudomonadota>Alphaproteobacteria>Rhodobacterales>Roseobacteraceae>Ruegeria	GCF_013032765.1	Ruegeria_arenilitoris	GOW79_RS10365	805076..808600	1173585	BREX-system-P-loop-protein-BrxC-[Ruegeria-arenilitoris]
WP_170364516.1	Type1.BREX	<-wHTH+HNH||?->Arm-DNA-bind_3+Phage_integrase->XIS-HTH->Primpol+PriCT-><-RNase_T+35exo||MetJarc-HTH->BrxA-DNA-binding->BrxB-BrxF-iSTAND->DUF6079*->N6-METHYLASE->PglZ->SIGMA+OB-Fold+AAA-ATPase+Lon-Protease-BrxL-><-tRNA<-?<-?<-?<-?<-REC+OmpR-HTH<-MarR-HTH	1140	Bacteria>Pseudomonadota>Alphaproteobacteria>Rhodobacterales>Roseobacteraceae>Ruegeria	GCF_013032855.1	Ruegeria_arenilitoris	GOW72_RS01075	235735..239157	1173585	BREX-system-P-loop-protein-BrxC-[Ruegeria-arenilitoris]
WP_209509473.1	Type1.BREX	TPR-><-?<-SIGMA+OB-Fold+AAA-ATPase+Lon-Protease-BrxL<-PglZ<-N6-METHYLASE<-DUF6079*<-BrxB-BrxF-iSTAND<-BrxA-DNA-binding<-?<-TOPRIM+DnaB<-?<-?<-XIS-HTH	1147	Bacteria>Pseudomonadota>Alphaproteobacteria>Rhodobacterales>Roseobacteraceae>Ruegeria	GCF_017872615.1	Ruegeria_sp._HKCCE4150	I9183_RS11345	408329..411772	2794828	BREX-system-P-loop-protein-BrxC-[Ruegeria-sp.-HKCCE4150]
WP_222457132.1	Type1.BREX	CopG/RHH-><-?<-?<-?<-Classical-AAA||?->BrxA-DNA-binding->BrxB-BrxF-iSTAND->DUF6079*->N6-METHYLASE->PglZ->SIGMA+OB-Fold+AAA-ATPase+HKD-DNase-BrxL-><-?<-?<-tRNA<-?<-RNASE-EG	1174	Bacteria>Pseudomonadota>Alphaproteobacteria>Rhodobacterales>Roseobacteraceae>Ruegeria	GCF_019801065.1	Ruegeria_arenilitoris	KUW05_RS05645	1133713..1137237	1173585	BREX-system-P-loop-protein-BrxC-[Ruegeria-arenilitoris]
WP_234170466.1	Type1.BREX	ABC-ATPase+SbcC-><-?<-DDE_Tnp_IS240||?->BrxB-BrxF-iSTAND-p->DUF6079-p*->?-><-?<-?||?->?->?->?->?->ABhydrolase+ABhydrolase->	899	Bacteria>Pseudomonadota>Alphaproteobacteria>Rhodobacterales>Roseobacteraceae>Ruegeria	GCF_021405425.1	Ruegeria_pomeroyi	KBY28_RS08200	126596..129295	89184	BREX-system-P-loop-protein-BrxC-[Ruegeria-pomeroyi]
WP_150282504.1	Type1.BREX	MPTase-><-HTH+MPTase<-?<-SIGMA+OB-Fold+AAA-ATPase+HKD-DNase-BrxL<-PglZ<-N6-METHYLASE<-REase<-OLD-ABC<-DUF6079*<-BrxB-BrxF-iSTAND<-BrxA-DNA-binding<-wHTH+WYL+WCX||?->Thermonuclease->TrbM->	1173	Bacteria>Pseudomonadota>Alphaproteobacteria>Rhodobacterales>Roseobacteraceae>Salipiger	GCF_008692285.1	Salipiger_aestuarii	AL036_RS18780	13068..16589	568098	BREX-system-P-loop-protein-BrxC-[Salipiger-aestuarii]
WP_089852186.1	Type1.BREX	GDResolvase+HIN-HTH->HTH->ParA-Soj-PloopNTPase->?->?->?->BrxA-DNA-binding->BrxB-BrxF-iSTAND->DUF6079*->N6-METHYLASE->PglZ->SIGMA+OB-Fold+AAA-ATPase+HKD-DNase-BrxL->	1173	Bacteria>Pseudomonadota>Alphaproteobacteria>Rhodobacterales>Roseobacteraceae>Salipiger	GCF_900100085.1	Salipiger_marinus	BLR51_RS25320	13015..16536	555512	BREX-system-P-loop-protein-BrxC-[Salipiger-marinus]
WP_025045421.1	Type1.BREX	<-Ntox49<-?||GDResolvase+HIN-HTH->ArdCN+MPTase-><-?<-?<-SIGMA+OB-Fold+AAA-ATPase+Lon-Protease-BrxL<-PglZ<-N6-METHYLASE<-DUF6079*<-BrxB-BrxF-iSTAND<-BrxA-DNA-binding<-wHTH+WYL+WCX<-?<-?||tRNA->ABC-ATPase+SbcC->?->?->ABhydrolase->	1147	Bacteria>Pseudomonadota>Alphaproteobacteria>Rhodobacterales>Roseobacteraceae>Sulfitobacter	GCF_013407805.1	Sulfitobacter_geojensis	GGR94_RS09535	1982054..1985497	1342299	BREX-system-P-loop-protein-BrxC-[Sulfitobacter-geojensis]
WP_203210578.1	Type1.BREX	STYKinase-like->?-><-McrC-NTD+REase<-HTH+McrB-AAA<-SbcC+CCDC158<-Calcineurin-SbcD<-SIGMA+OB-Fold+AAA-ATPase+HKD-DNase-BrxL<-PglZ<-N6-METHYLASE<-DUF6079*<-BrxB-BrxF-iSTAND<-BrxA-DNA-binding<-wHTH+WYL+WCX||ArdCN+MPTase->?->FurR-HTH->?->HIN-HTH+TraJ-RHH->TraI-VirD2-RCRE->	1172	Bacteria>Pseudomonadota>Alphaproteobacteria>Rhodobacterales>Roseobacteraceae>Sulfitobacter	GCF_016801775.1	Sulfitobacter_mediterraneus	JNX03_RS00635	149782..153300	83219	BREX-system-P-loop-protein-BrxC-[Sulfitobacter-mediterraneus]
WP_259953659.1	Type1.BREX	XIS-HTH->?-><-?<-?||BrxA-DNA-binding->BrxB-BrxF-iSTAND->DUF6079*->N6-METHYLASE->PglZ->SIGMA+OB-Fold+AAA-ATPase+HKD-DNase-BrxL->REase+iREase->Calcineurin-SbcD->SbcC+CCDC158->	1174	Bacteria>Pseudomonadota>Alphaproteobacteria>Rhodobacterales>Roseobacteraceae>Sulfitobacter	GCF_025140475.1	Sulfitobacter_mediterraneus	K3753_RS16990	3485906..3489430	83219	BREX-system-P-loop-protein-BrxC-[Sulfitobacter-mediterraneus]
WP_267167638.1	Type1.BREX	Phage_integrase->Arm-DNA-bind_3+Phage_integrase-><-?<-HAD+PRTase+PRTase+REase+SF1-SF2-Helicase||?->?->?->BrxA-DNA-binding->BrxB-BrxF-iSTAND->DUF6079*->N6-METHYLASE->PglZ->SIGMA+OB-Fold+AAA-ATPase+HKD-DNase-BrxL->TraJ-RHH-><-?||?-><-?<-ZnR	1173	Bacteria>Pseudomonadota>Alphaproteobacteria>Rhodobacterales>Roseobacteraceae>Sulfitobacter	GCF_026410285.1	Sulfitobacter_sp._F26169L	OS189_RS12380	41979..45500	2996015	BREX-system-P-loop-protein-BrxC-[Sulfitobacter-sp.-F26169L]
WP_271692167.1	Type1.BREX	ACET-><-?<-?<-?<-?||MULE-TRANSPOSASE-><-?||MULE-TRANSPOSASE-><-N6-METHYLASE-p<-DUF6079*<-BrxB-BrxF-iSTAND<-BrxA-DNA-binding<-wHTH+WYL+WCX<-?||OLD-ABC+TOPRIM->?-><-LON-AAA	1174	Bacteria>Pseudomonadota>Alphaproteobacteria>Rhodobacterales>Roseobacteraceae>Sulfitobacter	GCF_028201715.1	Sulfitobacter_faviae	PL335_RS12830	2584668..2588192	1775881	BREX-system-P-loop-protein-BrxC-[Sulfitobacter-faviae]
WP_223427796.1	Type1.BREX	TPR+TPR+TPR+TPR->?-><-RNase<-?<-Peptidase_M20||ParB->||BrxA-DNA-binding->BrxB-BrxF-iSTAND->DUF6079*->N6-METHYLASE-p->	1179	Bacteria>Pseudomonadota>Alphaproteobacteria>Rhodobacterales>Roseobacteraceae>Tateyamaria	GCF_019966495.1	Tateyamaria_pelophila	LC354_RS21665	9757..13296	328415	BREX-system-P-loop-protein-BrxC-[Tateyamaria-pelophila]
WP_058242644.1	Type1.BREX	wHTH+WYL+WCX->BrxA-DNA-binding->BrxB-BrxF-iSTAND->DUF6079*->N6-METHYLASE-p-><-DDE_Tnp_IS66+DDE_Tnp_IS66_C	1173	Bacteria>Pseudomonadota>Alphaproteobacteria>Rhodobacterales>Roseobacteraceae>Thalassobius	GCF_001458255.1	Thalassobius_autumnalis	TL5120_RS05495	4804..8325	2072972	BREX-system-P-loop-protein-BrxC-[Thalassobius-autumnalis]
WP_058243041.1	Type1.BREX	<-AbiEii<-AbiEi_4||HTH_3->?->?-><-REase+iREase<-SIGMA+OB-Fold+AAA-ATPase+HKD-DNase-BrxL<-PglZ<-N6-METHYLASE<-DUF6079*<-BrxB-BrxF-iSTAND<-BrxA-DNA-binding<-wHTH+WYL+WCX||ArdCN+MPTase->?->FurR-HTH->?->HIN-HTH+TraJ-RHH->TraI-VirD2-RCRE->	1172	Bacteria>Pseudomonadota>Alphaproteobacteria>Rhodobacterales>Roseobacteraceae>Thalassobius	GCF_001458275.1	Thalassobius_autumnalis	TL5120_RS07660	24794..28312	2072972	BREX-system-P-loop-protein-BrxC-[Thalassobius-autumnalis]
OHC57392.1	Type1.BREX	HIN-HTH->?->Calcineurin+Calcineurin-SbcD->SbcC+SbcC-><-?<-?||wHTH+WYL+WCX->BrxA-DNA-binding->BrxB-BrxF-iSTAND->DUF6079*->N6-METHYLASE->PglZ->SIGMA+OB-Fold+AAA-ATPase+HKD-DNase-BrxL->	1173	Bacteria>Pseudomonadota>Alphaproteobacteria>Rhodobacterales>unclassified_Rhodobacterales	GCA_001828855.1	Rhodobacterales_bacterium_RIFCSPHIGHO2_02_FULL_62_130	A3D16_23615	14989..18510	1802014	hypothetical-protein-A3D16_23615-[Rhodobacterales-bacterium-RIFCSPHIGHO2_02_FULL_62_130]
OUS21258.1	Type1.BREX	HTH_3->?-><-TrbM<-Thermonuclease<-?||wHTH+WYL+WCX->BrxA-DNA-binding->BrxB-BrxF-iSTAND->DUF6079*->OLD-ABC->REase->N6-METHYLASE->PglZ->SIGMA+OB-Fold+AAA-ATPase+HKD-DNase-BrxL->REase+iREase->	1173	Bacteria>Pseudomonadota>Alphaproteobacteria>Rhodobacterales>unclassified_Rhodobacterales	GCA_002162885.1	Rhodobacterales_bacterium_59_46_T64	A9Q95_07015	5961..9482	1856300	hypothetical-protein-A9Q95_07015-[Rhodobacterales-bacterium-59_46_T64]
WP_061508432.1	Type1.BREX	<-sGTP<-SF1-SF2-Helicase+REase<-35Exo+REase||wHTH+WYL+WCX->BrxA-DNA-binding->BrxB-BrxF-iSTAND->DUF6079*->N6-METHYLASE->PglZ->SIGMA+OB-Fold+AAA-ATPase+HKD-DNase-BrxL-><-DOC+HTH<-?<-RNase	1177	Bacteria>Pseudomonadota>Alphaproteobacteria>Rhodospirillales>Acetobacteraceae>Acetobacter	GCF_001580695.1	Acetobacter_malorum	AD933_RS09485	34256..37789	178901	BREX-system-P-loop-protein-BrxC-[Acetobacter-malorum]
WP_212335128.1	Type1.BREX	DOC+HTH-><-SIGMA+OB-Fold+AAA-ATPase+HKD-DNase-BrxL<-PglZ<-N6-METHYLASE<-DUF6079*<-BrxB-BrxF-iSTAND<-BrxA-DNA-binding<-wHTH+WYL+WCX||35Exo+REase->	1177	Bacteria>Pseudomonadota>Alphaproteobacteria>Rhodospirillales>Acetobacteraceae>Acetobacter	GCF_018256855.1	Acetobacter_thailandicus	JK172_RS08930	62967..66500	1502842	BREX-system-P-loop-protein-BrxC-[Acetobacter-thailandicus]
OYV89048.1	Type1.BREX	<-DUF6079*<-BrxB-BrxF-iSTAND<-BrxA-DNA-binding<-?<-Phage_integrase<-XIS-HTH	1173	Bacteria>Pseudomonadota>Alphaproteobacteria>Rhodospirillales>Acetobacteraceae>Acidiphilium	GCA_002279355.1	Acidiphilium_sp._37-60-79	B7Z57_13830	139..3660	1970298	MAG:-hypothetical-protein-B7Z57_13830-[Acidiphilium-sp.-37-60-79]
CAG4901729.1	Type1.BREX	RHH->TraJ-RHH->ACET+ACET->BrxA-DNA-binding-p->DUF6079->HIN-HTH->?->LZ_Tnp_IS66+zf-IS66+DDE_Tnp_IS66+DDE_Tnp_IS66_C->?->DUF6079-p*->N6-METHYLASE->N6-METHYLASE->HTH_29+HTH_32+HTH_33+DDE_3->PglZ->SIGMA+OB-Fold+AAA-ATPase+HKD-DNase-BrxL-><-Arm-DNA-bind_3+Phage_int_SAM_3+Phage_integrase<-?||DUF4096+DDE_Tnp_1+DDE_Tnp_1_2->	459	Bacteria>Pseudomonadota>Alphaproteobacteria>Rhodospirillales>Acetobacteraceae>Acidocella	GCA_907164655.1	Acidocella_sp._C78	ACIDOCELLA78_258	233780..235159	1671486	unnamed-protein-product,-partial-[Acidocella-sp.-C78]
WP_164698243.1	Type1.BREX	<-SIGMA+OB-Fold+AAA-ATPase+Lon-Protease-BrxL<-PglZ<-N6-METHYLASE<-DUF6079*<-BrxB-BrxF-iSTAND<-BrxA-DNA-binding||MazE_antitoxin->PemK_toxin->?->GDResolvase-><-ABhydrolase	1143	Bacteria>Pseudomonadota>Alphaproteobacteria>Rhodospirillales>Acetobacteraceae>Falsiroseomonas	GCF_011040385.1	Falsiroseomonas_algicola	G3576_RS30405	8811..12242	2716930	BREX-system-P-loop-protein-BrxC-[Falsiroseomonas-algicola]
WP_211586184.1	Type1.BREX	SF1-SF2-Helicase-><-?<-DSBH<-ADH_N+ADH_zinc_N_2||YTCD-HTH-><-SIGMA+OB-Fold+AAA-ATPase+Lon-Protease-BrxL<-PglZ<-N6-METHYLASE<-DUF6079*<-BrxB-BrxF-iSTAND<-BrxA-DNA-binding<-N6-METHYLASE<-?||HTH+LexA-protease->	1211	Bacteria>Pseudomonadota>Alphaproteobacteria>Rhodospirillales>Acetobacteraceae>Gluconobacter	GCF_018256215.1	Gluconobacter_sp._Dm-73	JK176_RS02065	37036..40671	2799802	BREX-system-P-loop-protein-BrxC-[Gluconobacter-sp.-Dm-73]
WP_226610978.1	Type1.BREX	<-SIGMA+OB-Fold+AAA-ATPase+Lon-Protease-BrxL<-PglZ<-ArdCN+MPTase<-N6-METHYLASE<-DUF6079*<-BrxB-BrxF-iSTAND<-BrxA-DNA-binding<-tRNA||?-><-?<-?<-ABC-ATPase+SbcC<-?<-Glycos_transf_1+Glyco_trans_1_2<-Glycos_transf	1144	Bacteria>Pseudomonadota>Alphaproteobacteria>Rhodospirillales>Acetobacteraceae>Roseicella	GCF_020523605.1	Roseicella_aerolata	LHA35_RS18965	9480..12914	2883479	BREX-system-P-loop-protein-BrxC-[Roseicella-aerolata]
MCA3353385.1	Type1.BREX	<-SIGMA+OB-Fold+AAA-ATPase+HKD-DNase-BrxL<-PglZ<-N6-METHYLASE<-NERD<-OLD-ABC<-DUF6079*<-BrxB-BrxF-iSTAND<-BrxA-DNA-binding<-wHTH+WYL+WCX||35Exo+REase->SF1-SF2-Helicase+REase->Small-B-barrel+SF1-SF2-Helicase+RdgC+REase-><-SF1-SF2-Helicase<-HKD-DNase	1178	Bacteria>Pseudomonadota>Alphaproteobacteria>Rhodospirillales>Acetobacteraceae>Roseomonas	GCA_020249745.1	Roseomonas_sp.	INF80_03760	34583..38119	1869190	BREX-system-P-loop-protein-BrxC-[Roseomonas-sp.]
WP_209351251.1	Type1.BREX	HKD-DNase->SF1-SF2-Helicase-><-Small-B-barrel+SF1-SF2-Helicase+RdgC+REase<-SF1-SF2-Helicase+REase<-35Exo+REase||wHTH+WYL+WCX->BrxA-DNA-binding->BrxB-BrxF-iSTAND->DUF6079*->OLD-ABC->NERD->N6-METHYLASE->PglZ->SIGMA+OB-Fold+AAA-ATPase+HKD-DNase-BrxL->?->?-><-?<-Glyoxalase	1177	Bacteria>Pseudomonadota>Alphaproteobacteria>Rhodospirillales>Acetobacteraceae>Roseomonas	GCF_017813355.1	Roseomonas_sp._PWR1	J5Y09_RS08145	78258..81791	2820810	BREX-system-P-loop-protein-BrxC-[Roseomonas-sp.-PWR1]
WP_239419500.1	Type1.BREX	SbcC-><-PIN<-?||ParA-Soj-PloopNTPase->?->HTH_36-><-?||BrxA-DNA-binding->BrxB-BrxF-iSTAND->DUF6079*->N6-METHYLASE->PglZ->SIGMA+OB-Fold+AAA-ATPase+HKD-DNase-BrxL-><-?<-MerR-HTH<-Relaxase+SF1-SF2-Helicase||?->VirD4-TraG+VirD4-FtsK->	1170	Bacteria>Pseudomonadota>Alphaproteobacteria>Rhodospirillales>Acetobacteraceae>Roseomonas	-	Roseomonas_sp._ACRSG	MHZ93_RS20975	7121..10633	2918212	BREX-system-P-loop-protein-BrxC-[Roseomonas-sp.-ACRSG]
PWC40000.1	Type1.BREX	<-MarR-HTH||CN_hydrolase-><-?<-?||?-><-SIGMA+OB-Fold+AAA-ATPase+Lon-Protease-BrxL<-PglZ<-AIPR<-N6-METHYLASE<-DUF6079*<-BrxB-BrxF-iSTAND<-BrxA-DNA-binding<-HTH_23+HTH_32+HTH_33||?->TetR-HTH->	1148	Bacteria>Pseudomonadota>Alphaproteobacteria>Rhodospirillales>Azospirillaceae>Azospirillum	GCA_003116095.1	Azospirillum_sp._TSO35-2	TSO352_06450	1114896..1118342	716796	hypothetical-protein-TSO352_06450-[Azospirillum-sp.-TSO35-2]
WP_014187927.1	Type1.BREX	NERD+Pkinase-p->Pkinase->Cas4+SF1-SF2-Helicase->?->?-><-SIGMA+OB-Fold+AAA-ATPase+Lon-Protease-BrxL<-PglZ<-AIPR<-N6-METHYLASE<-DUF6079*<-BrxB-BrxF-iSTAND<-BrxA-DNA-binding||Phage_integrase->?-><-?||?-><-Transposase_20<-HTH_Tnp_1+HTH_21+rve+rve_3	1148	Bacteria>Pseudomonadota>Alphaproteobacteria>Rhodospirillales>Azospirillaceae>Azospirillum	GCF_000283655.1	Azospirillum_lipoferum	AZOLI_RS14530	158802..162248	193	BREX-system-P-loop-protein-BrxC-[Azospirillum-lipoferum]
WP_094302078.1	Type1.BREX	Phage_integrase-><-GDResolvase+HIN-HTH||?-><-N6-N4-METHYLASE<-SIGMA+OB-Fold+AAA-ATPase+Lon-Protease-BrxL<-PglZ<-N6-METHYLASE<-DUF6079*<-BrxB-BrxF-iSTAND<-BrxA-DNA-binding<-?||?->?-><-?||?-><-?<-TatD_DNase	1215	Bacteria>Pseudomonadota>Alphaproteobacteria>Rhodospirillales>Azospirillaceae>Azospirillum	GCF_002245955.1	Azospirillum_brasilense	CHT98_04470	49941..53588	192	BREX-system-P-loop-protein-BrxC-[Azospirillum-brasilense]
WP_109096920.1	Type1.BREX	ART-DarT->NADAR-><-N6-N4-METHYLASE||?-><-?||?-><-SIGMA+OB-Fold+AAA-ATPase+Lon-Protease-BrxL<-PglZ<-N6-METHYLASE<-DUF6079*<-BrxB-BrxF-iSTAND<-BrxA-DNA-binding||?->Phage_integrase->EAL-><-?||PAS+HISKIN+REC->	1215	Bacteria>Pseudomonadota>Alphaproteobacteria>Rhodospirillales>Azospirillaceae>Azospirillum	GCF_003116035.1	Azospirillum_sp._TSH64	TSH64_RS11970	2295743..2299390	652740	BREX-system-P-loop-protein-BrxC-[Azospirillum-sp.-TSH64]
WP_247871928.1	Type1.BREX	<-MarR-HTH||CN_hydrolase-><-?<-?||?-><-SIGMA+OB-Fold+AAA-ATPase+Lon-Protease-BrxL<-PglZ<-AIPR<-N6-METHYLASE<-DUF6079*<-BrxB-BrxF-iSTAND<-BrxA-DNA-binding||?->?->TetR-HTH->	1149	Bacteria>Pseudomonadota>Alphaproteobacteria>Rhodospirillales>Azospirillaceae>Azospirillum	GCF_003116095.1	Azospirillum_sp._TSO35-2	TSO352_RS06555	1114896..1118345	716796	BREX-system-P-loop-protein-BrxC-[Azospirillum-sp.-TSO35-2]
WP_137104425.1	Type1.BREX	<-McrC-NTD+REase<-wHTH+McrB-AAA<-SIGMA+OB-Fold+AAA-ATPase+Lon-Protease-BrxL<-PglZ<-AIPR<-N6-METHYLASE<-TOPRIM<-OLD-ABC<-DUF6079*<-BrxB-BrxF-iSTAND<-BrxA-DNA-binding||?->?-><-?<-?<-MODE-HTH+PBPII	1148	Bacteria>Pseudomonadota>Alphaproteobacteria>Rhodospirillales>Azospirillaceae>Azospirillum	GCF_005222185.1	Azospirillum_argentinense	D3867_15345	176394..179840	2970906	BREX-system-P-loop-protein-BrxC-[Azospirillum-argentinense]
WP_089290578.1	Type1.BREX	<-ABhydrolase||?-><-?<-TetR-HTH||?->?->BrxA-DNA-binding->BrxB-BrxF-iSTAND->DUF6079*->N6-METHYLASE->PglZ->SIGMA+OB-Fold+AAA-ATPase+HKD-DNase-BrxL->	1202	Bacteria>Pseudomonadota>Alphaproteobacteria>Rhodospirillales>Azospirillaceae>Azospirillum	GCF_900188385.1	unclassified_Azospirillum	CHB97_RS24120	34901..38509	2630922	MULTISPECIES:-BREX-system-P-loop-protein-BrxC-[unclassified-Azospirillum]
MAD36284.1	Type1.BREX	DUF6079-p*->N6-METHYLASE->PglZ->SIGMA+OB-Fold+AAA-ATPase+Lon-Protease-BrxL->Exonuc_V_gamma+REase->SF1-SF2-Helicase+REase->HKD-DNase+OGG-like->	971	Bacteria>Pseudomonadota>Alphaproteobacteria>Rhodospirillales>Geminicoccaceae>Tistrella	GCA_002684955.1	Tistrella_sp.	CMO28_06235	72..2987	2024861	hypothetical-protein-[Tistrella-sp.]
MBA78722.1	Type1.BREX	DUF6079-p*->N6-METHYLASE->PglZ->SIGMA+OB-Fold+AAA-ATPase+Lon-Protease-BrxL->Exonuc_V_gamma+REase->SF1-SF2-Helicase+REase->HKD-DNase+OGG-like->	971	Bacteria>Pseudomonadota>Alphaproteobacteria>Rhodospirillales>Geminicoccaceae>Tistrella	GCA_002709065.1	Tistrella_sp.	CMO30_25940	72..2987	2024861	hypothetical-protein-[Tistrella-sp.]
BAE50844.1	Type1.BREX	<-CBS+CBS<-?<-ZnR<-?<-?||?->BrxA-DNA-binding->BrxB-BrxF-iSTAND->DUF6079-p*-><-HTH_Tnp_1+HTH_21+rve+rve_3<-HTH_Tnp_1||DUF6079-p->N6-METHYLASE-><-DUF4096+DDE_Tnp_1+DDE_Tnp_1_2||PglZ->SIGMA+OB-Fold+AAA-ATPase+Lon-Protease-BrxL->?->35exo->	794	Bacteria>Pseudomonadota>Alphaproteobacteria>Rhodospirillales>Rhodospirillaceae>Magnetospirillum	GCA_000009985.1	Magnetospirillum_magneticum_AMB-1	amb2040	2216694..2219078	342108	hypothetical-protein-amb2040-[Magnetospirillum-magneticum-AMB-1]
WP_043746510.1	Type1.BREX	<-CBS+CBS||Phage_integrase->?-><-?<-?<-?||BrxA-DNA-binding->BrxB-BrxF-iSTAND->DUF6079-p*-><-HTH_Tnp_1+HTH_21+rve+rve_3||DUF6079-p->N6-METHYLASE->?-><-DUF4096+DDE_Tnp_1+DDE_Tnp_1_2||PglZ->SIGMA+OB-Fold+AAA-ATPase+Lon-Protease-BrxL->?->35exo->	790	Bacteria>Pseudomonadota>Alphaproteobacteria>Rhodospirillales>Rhodospirillaceae>Magnetospirillum	GCF_000009985.1	Magnetospirillum_magneticum	AMB_RS10300	2216706..2219078	84159	BREX-system-P-loop-protein-BrxC,-partial-[Magnetospirillum-magneticum]
WP_008620174.1	Type1.BREX	REC->HISKIN-><-HKD-DNase<-?<-35exo<-?<-SIGMA+OB-Fold+AAA-ATPase+Lon-Protease-BrxL<-PglZ<-N6-METHYLASE<-DUF6079*<-BrxB-BrxF-iSTAND<-BrxA-DNA-binding	1205	Bacteria>Pseudomonadota>Alphaproteobacteria>Rhodospirillales>Rhodospirillaceae>Magnetospirillum	GCF_000342045.1	Magnetospirillum_caucaseum	H261_RS17615	20424..24041	1244869	BREX-system-P-loop-protein-BrxC-[Magnetospirillum-caucaseum]
WP_008621107.1	Type1.BREX	<-SIR2+SIR2<-SIGMA+OB-Fold+AAA-ATPase+Lon-Protease-BrxL<-PglZ<-N6-METHYLASE-p<-N6-METHYLASE-p<-DUF6079*<-BrxB-BrxF-iSTAND<-BrxA-DNA-binding||ZnR->?->?-><-N6-N4-METHYLASE	1208	Bacteria>Pseudomonadota>Alphaproteobacteria>Rhodospirillales>Rhodospirillaceae>Magnetospirillum	GCF_000342045.1	Magnetospirillum_caucaseum	H261_RS19775	10510..14136	1244869	BREX-system-P-loop-protein-BrxC-[Magnetospirillum-caucaseum]
WP_068504545.1	Type1.BREX	<-HKD-DNase<-?<-?<-SIGMA+OB-Fold+AAA-ATPase+Lon-Protease-BrxL<-PglZ<-N6-METHYLASE<-DUF6079*<-BrxB-BrxF-iSTAND<-BrxA-DNA-binding||?->?->?->?->ZnR-><-Phage_integrase||ParA-Soj-PloopNTPase->	1205	Bacteria>Pseudomonadota>Alphaproteobacteria>Rhodospirillales>Rhodospirillaceae>Magnetospirillum	GCF_001650635.1	Magnetospirillum_moscoviense	A6A05_RS18805	69930..73547	1437059	BREX-system-P-loop-protein-BrxC-[Magnetospirillum-moscoviense]
MBA4095641.1	Type1.BREX	HamA->SF1-SF2-Helicase->?->ParB+HNH-><-?<-SIR2||BrxA-DNA-binding->BrxB-BrxF-iSTAND->DUF6079*->N6-METHYLASE->ArdCN+MPTase->PglZ->SIGMA+OB-Fold+AAA-ATPase+Lon-Protease-BrxL->?-><-?<-?||HTH_3->DOC+HTH->	1149	Bacteria>Pseudomonadota>Alphaproteobacteria>Rhodospirillales>Rhodospirillaceae>Rhodospirillum	GCA_013822765.1	Rhodospirillum_sp.	C0484_02575	552126..555575	37792	BREX-system-P-loop-protein-BrxC-[Rhodospirillum-sp.]
WP_086466635.1	Type1.BREX	ParB-><-?<-HKD-DNase+OGG-like<-SF1-SF2-Helicase+REase<-Exonuc_V_gamma+REase<-SIGMA+OB-Fold+AAA-ATPase+Lon-Protease-BrxL<-PglZ<-N6-METHYLASE<-DUF6079*<-BrxB-BrxF-iSTAND<-BrxA-DNA-binding||Classical-AAA->Peptidase_S8-><-?<-AlcRN+AraC-HTH+AraC-HTH||?->Thermonuclease->PAPS-reduct->	1149	Bacteria>Pseudomonadota>Alphaproteobacteria>Rhodospirillales>Thalassobaculaceae>Oceanibaculum	GCF_002148795.1	Oceanibaculum_nanhaiense	BKM74_RS15615	60819..64268	1909734	BREX-system-P-loop-protein-BrxC-[Oceanibaculum-nanhaiense]
WP_069959207.1	Type1.BREX	<-GDResolvase+HIN-HTH||STYKinase-like->?-><-?<-SIGMA+OB-Fold+AAA-ATPase+HKD-DNase-BrxL<-PglZ<-N6-METHYLASE<-DUF6079*<-BrxB-BrxF-iSTAND<-BrxA-DNA-binding<-wHTH+WYL+WCX<-?<-N6-N4-METHYLASE<-N6-METHYLASE<-?||?->Phage_integrase->	1190	Bacteria>Pseudomonadota>Alphaproteobacteria>Rhodospirillales>Thalassospiraceae>Magnetovibrio	GCF_001746755.1	Magnetovibrio_blakemorei	BEN30_RS15365	12021..15593	28181	BREX-system-P-loop-protein-BrxC-[Magnetovibrio-blakemorei]
MBL4843424.1	Type1.BREX	<-SF1-SF2-Helicase<-HKD-DNase+HKD-DNase<-wHTH<-SF1-SF2-Helicase+REase<-SF1-SF2-Helicase+REase<-SIGMA+OB-Fold+AAA-ATPase+HKD-DNase-BrxL<-PglZ<-N6-METHYLASE<-DUF6079*<-BrxB-BrxF-iSTAND<-BrxA-DNA-binding<-wHTH+WYL+WCX||Calcineurin+Calcineurin-SbcD->SbcC+SbcC-><-MODE-HTH+PBPII||TetR-HTH-><-RHH||HTH_Tnp_1+HTH_21+rve+rve_3->	1179	Bacteria>Pseudomonadota>Alphaproteobacteria>Rhodospirillales>Thalassospiraceae>Thalassospira	GCA_016765055.1	Thalassospira_sp.	JKY47_21635	20483..24022	1912094	BREX-system-P-loop-protein-BrxC-[Thalassospira-sp.]
WP_085595207.1	Type1.BREX	XIS-HTH->BrxA-DNA-binding->BrxB-BrxF-iSTAND->DUF6079*->OLD-ABC->REase->N6-METHYLASE->HTH+REase->PglZ->SIGMA+OB-Fold+AAA-ATPase+HKD-DNase-BrxL-><-SF1-SF2-Helicase	1182	Bacteria>Pseudomonadota>Alphaproteobacteria>Rhodospirillales>Thalassospiraceae>Thalassospira	GCF_002115825.1	Thalassospira_sp._MCCC_1A01428	THS27_RS23975	1729..5277	1470575	BREX-system-P-loop-protein-BrxC-[Thalassospira-sp.-MCCC-1A01428]
SEP16519.1	Type1.BREX	PTSEIIA1-><-?<-HIN-HTH||BrxA-DNA-binding->BrxB-BrxF-iSTAND->DUF6079*->N6-METHYLASE->N6-METHYLASE-p->PglZ->SIGMA+OB-Fold+AAA-ATPase+Lon-Protease-BrxL->?-><-?<-AAA-ATPase<-RADICAL-SAM<-CNMP+CRP-HTH	1142	Bacteria>Pseudomonadota>Alphaproteobacteria>Rhodospirillales>unclassified_Rhodospirillales	GCA_900110395.1	Rhodospirillales_bacterium_URHD0017	SAMN02990966_04223	161142..164570	1380357	hypothetical-protein-SAMN02990966_04223-[Rhodospirillales-bacterium-URHD0017]
MBY0534082.1	Type1.BREX	<-GDResolvase+Recombinase||?->?-><-HTH_36||HTH_3->BrxA-DNA-binding->BrxB-BrxF-iSTAND->DUF6079*->N6-METHYLASE-p->	1197	Bacteria>Pseudomonadota>Alphaproteobacteria>Rickettsiales>Rickettsiaceae>unclassified_Rickettsiaceae	GCA_019752735.1	Rickettsiaceae_bacterium	K2P31_03860	4925..8518	2023360	BREX-system-P-loop-protein-BrxC-[Rickettsiaceae-bacterium]
WP_143251171.1	Type1.BREX	GGDEF->?->?-><-?<-?||?-><-SIGMA+OB-Fold+AAA-ATPase+Lon-Protease-BrxL<-PglZ<-N6-METHYLASE<-DUF6079*<-BrxB-BrxF-iSTAND<-AIPR<-?<-METHYLASE<-HTH_3||DUF932->HD+PspB-><-HTH_3<-D5ATPase	1141	Bacteria>Pseudomonadota>Alphaproteobacteria>Sphingomonadales>Erythrobacteraceae>Altererythrobacter	GCF_007065885.1	Altererythrobacter_sp._TH136	C0V74_RS07000	1448696..1452121	2067415	BREX-system-P-loop-protein-BrxC-[Altererythrobacter-sp.-TH136]
WP_160484443.1	Type1.BREX	TraJ-RHH->VirD4-TraG+VirD4-FtsK->MobF-><-LexA-protease||BrxA-DNA-binding->BrxB-BrxF-iSTAND->DUF6079*->OLD-ABC->NERD->N6-METHYLASE->PglZ->SIGMA+OB-Fold+AAA-ATPase+HKD-DNase-BrxL->REase+iREase-><-Couple_hipA+HipA_C<-HTH_3	1172	Bacteria>Pseudomonadota>Alphaproteobacteria>Sphingomonadales>Erythrobacteraceae>Aurantiacibacter	GCF_009807005.1	Aurantiacibacter_rhizosphaerae	GRF63_RS02615	556566..560084	2691582	BREX-system-P-loop-protein-BrxC-[Aurantiacibacter-rhizosphaerae]
RNJ61798.1	Type1.BREX	TraN->Thioredoxin->TraH->?-><-?||wHTH+WYL+WCX->BrxA-DNA-binding->BrxB-BrxF-iSTAND->DUF6079*->N6-METHYLASE->PglZ->SIGMA+OB-Fold+AAA-ATPase+HKD-DNase-BrxL-><-?<-SF1-SF2-Helicase+DUF1998<-?<-SWIM+DUF3670+SF1-SF2-Helicase	1173	Bacteria>Pseudomonadota>Alphaproteobacteria>Sphingomonadales>Erythrobacteraceae>Erythrobacter/Porphyrobacter_group	GCA_003724185.1	Porphyrobacter_sp._IPPAS_B-1204	EDM03_09690	47855..51376	2483793	BREX-system-P-loop-protein-BrxC-[Porphyrobacter-sp.-IPPAS-B-1204]
WP_011415860.1	Type1.BREX	SSB-><-?||?->TraJ-RHH->VirD4-TraG+VirD4-FtsK->MobF-><-LexA-protease||BrxA-DNA-binding->BrxB-BrxF-iSTAND->DUF6079*->OLD-ABC->REase->N6-METHYLASE->PglZ->SIGMA+OB-Fold+AAA-ATPase+HKD-DNase-BrxL->REase+iREase-><-Couple_hipA+HipA_C<-HTH_3	1172	Bacteria>Pseudomonadota>Alphaproteobacteria>Sphingomonadales>Erythrobacteraceae>Erythrobacter/Porphyrobacter_group	GCF_000013005.1	Erythrobacter_litoralis	EL2594_RS14475	2964416..2967934	39960	BREX-system-P-loop-protein-BrxC-[Erythrobacter-litoralis]
WP_196847550.1	Type1.BREX	SWIM+DUF3670+SF1-SF2-Helicase->?->?->SF1-SF2-Helicase+DUF1998->BrxA-DNA-binding->BrxB-BrxF-iSTAND->DUF6079*->N6-METHYLASE->PglZ->SIGMA+OB-Fold+AAA-ATPase+HKD-DNase-BrxL->REase+iREase-><-Couple_hipA+HipA_C<-HTH_3||?-><-?<-TraH	1172	Bacteria>Pseudomonadota>Alphaproteobacteria>Sphingomonadales>Erythrobacteraceae>Erythrobacter/Porphyrobacter_group	GCF_015767595.1	Erythrobacter_sp._A30-3	IT881_RS05450	1101811..1105329	2785907	BREX-system-P-loop-protein-BrxC-[Erythrobacter-sp.-A30-3]
WP_226699433.1	Type1.BREX	<-Cysteine-Desulfurase<-ADH_N+ADH_zinc_N||?->GDResolvase+HIN-HTH-><-?||BrxA-DNA-binding->BrxB-BrxF-iSTAND->DUF6079*->N6-METHYLASE->PglZ->SIGMA+OB-Fold+AAA-ATPase+HKD-DNase-BrxL-><-?<-Phage_int_SAM_5+Phage_integrase	1170	Bacteria>Pseudomonadota>Alphaproteobacteria>Sphingomonadales>Erythrobacteraceae>Qipengyuania	GCF_020524765.1	Qipengyuania_gaetbuli	LC083_RS04940	959412..962924	266952	BREX-system-P-loop-protein-BrxC-[Qipengyuania-gaetbuli]
MBK9012577.1	Type1.BREX	VirB2->VirB3->VirB4-FtsK+A32->TrwJ->?->VirB6->VirB10->RHH->||BrxA-DNA-binding->DUF6079*->?->?->?->?-><-?<-?<-SbcC	1212	Bacteria>Pseudomonadota>Alphaproteobacteria>Sphingomonadales>Sphingomonadaceae>Novosphingobium	GCA_016719755.1	Novosphingobium_sp.	IPM87_18255	676827..680465	1874826	BREX-system-P-loop-protein-BrxC-[Novosphingobium-sp.]
WP_054107369.1	Type1.BREX	wHTH+WYL+WCX->?->XIS-HTH-><-Arm-DNA-bind_3+Phage_integrase<-?<-LexA-protease||?->BrxA-DNA-binding->BrxB-BrxF-iSTAND->DUF6079*->N6-METHYLASE->PglZ->SIGMA+OB-Fold+AAA-ATPase+Lon-Protease-BrxL->URI-DNase+RAMA-><-?<-?||?->TPR+TPR+TPR+TPR->	1214	Bacteria>Pseudomonadota>Alphaproteobacteria>Sphingomonadales>Sphingomonadaceae>Novosphingobium	GCF_001295795.1	Novosphingobium_sp._AAP83	IP81_RS07510	164945..168589	1523425	BREX-system-P-loop-protein-BrxC-[Novosphingobium-sp.-AAP83]
WP_240530317.1	Type1.BREX	N6-METHYLASE->PIN-><-?<-GreA_GreB<-SF1-SF2-Helicase<-SF1-SF2-Helicase+REase<-35Exo+REase||wHTH+WYL+WCX->BrxA-DNA-binding->BrxB-BrxF-iSTAND->DUF6079-p*->	735	Bacteria>Pseudomonadota>Alphaproteobacteria>Sphingomonadales>Sphingomonadaceae>Novosphingobium	GCF_002556635.1	Novosphingobium_sp._PC22D	B2G71_RS22885	26272..28479	1962403	BREX-system-P-loop-protein-BrxC-[Novosphingobium-sp.-PC22D]
WP_160987331.1	Type1.BREX	TraN->TraH->?-><-?||GNTR-HTH-><-?<-?||BrxA-DNA-binding->BrxB-BrxF-iSTAND->DUF6079-p*->HTH_Tnp_1+HTH_28+HTH_32+rve+rve_3-><-DUF772+DDE_Tnp_1<-LZ_Tnp_IS66+zf-IS66+DDE_Tnp_IS66+DDE_Tnp_IS66_C<-?<-HIN-HTH<-?||MarR-HTH->?-><-GDResolvase+HIN-HTH	675	Bacteria>Pseudomonadota>Alphaproteobacteria>Sphingomonadales>Sphingomonadaceae>Novosphingobium	GCF_009856825.1	Novosphingobium_silvae	GR702_RS19995	24258..26285	2692619	BREX-system-P-loop-protein-BrxC-[Novosphingobium-silvae]
WP_243992543.1	Type1.BREX	<-SIGMA-HTH<-SIGMA+OB-Fold+AAA-ATPase+HKD-DNase-BrxL<-PglZ<-N6-METHYLASE<-NERD<-OLD-ABC<-DUF6079*<-BrxB-BrxF-iSTAND<-BrxA-DNA-binding<-?<-XIS-HTH<-?<-Arm-DNA-bind_3+Phage_integrase	1189	Bacteria>Pseudomonadota>Alphaproteobacteria>Sphingomonadales>Sphingomonadaceae>Novosphingobium	GCF_022832385.1	Novosphingobium_sp._B2580	MTR64_RS07820	57412..60981	2930093	BREX-system-P-loop-protein-BrxC-[Novosphingobium-sp.-B2580]
WP_243995499.1	Type1.BREX	<-PIN<-?<-?<-?<-SIGMA+OB-Fold+AAA-ATPase+HKD-DNase-BrxL<-PglZ<-N6-METHYLASE<-DUF6079*<-BrxB-BrxF-iSTAND<-BrxA-DNA-binding<-wHTH+WYL+WCX||35Exo+REase->SF1-SF2-Helicase+REase->GreA_GreB+SF1-SF2-Helicase->?-><-PIN<-N6-METHYLASE	1179	Bacteria>Pseudomonadota>Alphaproteobacteria>Sphingomonadales>Sphingomonadaceae>Novosphingobium	GCF_022832385.1	Novosphingobium_sp._B2580	MTR64_RS16290	56132..59671	2930093	BREX-system-P-loop-protein-BrxC-[Novosphingobium-sp.-B2580]
WP_159983052.1	Type1.BREX	<-SIGMA+OB-Fold+AAA-ATPase+Lon-Protease-BrxL<-PglZ<-N6-METHYLASE<-DUF6079*<-BrxB-BrxF-iSTAND<-BrxA-DNA-binding<-SbcC+CCDC158<-Calcineurin<-REase+HTH+35exo+Small-B-barrel+SF1-SF2-Helicase||wHTH+WYL+WCX-><-PAS+PAS+GAF+SHELIX+HISKIN+MORC+REC<-Phage_int_SAM_1+Phage_integrase<-RelE-ParE	1212	Bacteria>Pseudomonadota>Alphaproteobacteria>Sphingomonadales>Sphingomonadaceae>Novosphingobium	GCF_902706195.1	unclassified_Novosphingobium	GVL61_RS22205	8858..12496	2644732	MULTISPECIES:-BREX-system-P-loop-protein-BrxC-[unclassified-Novosphingobium]
WP_022673601.1	Type1.BREX	HTH_3->Couple_hipA+HipA_C->HNH-><-REase+HTH+35exo+Small-B-barrel+SF1-SF2-Helicase<-SIGMA+OB-Fold+AAA-ATPase+HKD-DNase-BrxL<-PglZ<-N6-METHYLASE<-REase<-OLD-ABC<-DUF6079*<-BrxB-BrxF-iSTAND<-BrxA-DNA-binding<-wHTH+WYL+WCX<-HTH||LexA-protease-><-EVE+HNH<-MobF<-VirD4-TraG+VirD4-FtsK<-TraJ-RHH	1189	Bacteria>Pseudomonadota>Alphaproteobacteria>Sphingomonadales>Sphingomonadaceae>Novosphingopyxis	GCF_000420305.1	Novosphingopyxis_baekryungensis	G410_RS0110420	254465..258034	279369	BREX-system-P-loop-protein-BrxC-[Novosphingopyxis-baekryungensis]
ARR56525.1	Type1.BREX	<-OBFOLD-NUCLEASE<-LexA-protease||HTH->wHTH+WYL+WCX->BrxA-DNA-binding->BrxB-BrxF-iSTAND->DUF6079*->OLD-ABC->NERD->Zn_Tnp_IS1595+DDE_Tnp_IS1595->N6-METHYLASE-p->PglZ->SIGMA+OB-Fold+AAA-ATPase+HKD-DNase-BrxL-><-?||DDE_Tnp_1_4->HTH+HTH+SF1-SF2-Helicase-p->	1189	Bacteria>Pseudomonadota>Alphaproteobacteria>Sphingomonadales>Sphingomonadaceae>Rhizorhabdus	GCA_000715175.2	Rhizorhabdus_wittichii_DC-6	HY78_25365	5371615..5375184	1283312	hypothetical-protein-HY78_25365-[Rhizorhabdus-wittichii-DC-6]
WP_209047668.1	Type1.BREX	MODE-HTH+PBPII-><-?<-HTH_3<-SIGMA+OB-Fold+AAA-ATPase+HKD-DNase-BrxL<-PglZ<-N6-METHYLASE<-DUF6079*<-BrxB-BrxF-iSTAND<-BrxA-DNA-binding<-wHTH+WYL+WCX||35Exo+REase->SF1-SF2-Helicase+REase->GreA_GreB+SF1-SF2-Helicase->?-><-PIN<-N6-METHYLASE	1179	Bacteria>Pseudomonadota>Alphaproteobacteria>Sphingomonadales>Sphingomonadaceae>Rhizorhabdus	GCF_029543145.1	Rhizorhabdus_histidinilytica	P7B04_RS17610	19171..22710	439228	MULTISPECIES:-BREX-system-P-loop-protein-BrxC-[Sphingomonadaceae]
WP_010336176.1	Type1.BREX	VirB8->VirB9->VirB10->RHH-><-TetR-HTH||MODE-HTH+PBPII->wHTH+WYL+WCX->BrxA-DNA-binding->BrxB-BrxF-iSTAND->DUF6079*->N6-METHYLASE->PglZ->SIGMA+OB-Fold+AAA-ATPase+HKD-DNase-BrxL->SF1-SF2-Helicase+REase->SF1-SF2-Helicase+REase->wHTH->HKD-DNase+HKD-DNase->SF1-SF2-Helicase->	1179	Bacteria>Pseudomonadota>Alphaproteobacteria>Sphingomonadales>Sphingomonadaceae>Sphingobium	GCF_000224695.1	Sphingobium_yanoikuyae	SYA_RS0104815	38393..41932	13690	BREX-system-P-loop-protein-BrxC-[Sphingobium-yanoikuyae]
WP_007688323.1	Type1.BREX	MobF->?-><-?<-?<-OBFOLD-NUCLEASE<-LexA-protease||wHTH+WYL+WCX->BrxA-DNA-binding->BrxB-BrxF-iSTAND->DUF6079*->OLD-ABC->NERD->N6-METHYLASE->PglZ->SIGMA+OB-Fold+AAA-ATPase+HKD-DNase-BrxL->MORC+SF1-SF2-Helicase->?-><-?<-TraH	1189	Bacteria>Pseudomonadota>Alphaproteobacteria>Sphingomonadales>Sphingomonadaceae>Sphingobium	GCF_000264945.2	Sphingobium_indicum	SIDU_RS03565	700467..704036	332055	BREX-system-P-loop-protein-BrxC-[Sphingobium-indicum]
WP_021240096.1	Type1.BREX	HKD-DNase->SF1-SF2-Helicase-><-Small-B-barrel+SF1-SF2-Helicase+RdgC+REase<-SF1-SF2-Helicase+REase<-35Exo+REase<-?||wHTH+WYL+WCX->BrxA-DNA-binding->BrxB-BrxF-iSTAND->DUF6079*->OLD-ABC->REase->N6-METHYLASE->PglZ->SIGMA+OB-Fold+AAA-ATPase+HKD-DNase-BrxL-><-?<-Arm-DNA-bind_3+Phage_integrase	1177	Bacteria>Pseudomonadota>Alphaproteobacteria>Sphingomonadales>Sphingomonadaceae>Sphingobium	GCF_000445065.1	Sphingobium_quisquiliarum	L288_RS41575	32905..36438	538379	BREX-system-P-loop-protein-BrxC-[Sphingobium-quisquiliarum]
WP_130029930.1	Type1.BREX	XIS-HTH-><-SIGMA+OB-Fold+AAA-ATPase+Lon-Protease-BrxL<-PglZ<-N6-METHYLASE<-DUF6079*<-BrxB-BrxF-iSTAND<-BrxA-DNA-binding<-XIS-HTH<-Arm-DNA-bind_3+Phage_integrase	1140	Bacteria>Pseudomonadota>Alphaproteobacteria>Sphingomonadales>Sphingomonadaceae>Sphingobium	GCF_014636045.1	Sphingobium_fuliginis	EWH10_RS07560	584587..588009	336203	BREX-system-P-loop-protein-BrxC-[Sphingobium-fuliginis]
WP_264583946.1	Type1.BREX	ParB->HipA_C-><-ParB+HNH+Schlafen<-GreA_GreB+SF1-SF2-Helicase<-SF1-SF2-Helicase+REase<-35Exo+REase||wHTH+WYL+WCX->BrxA-DNA-binding->BrxB-BrxF-iSTAND->DUF6079*->N6-METHYLASE->PglZ->SIGMA+OB-Fold+AAA-ATPase+HKD-DNase-BrxL-><-?<-?<-?<-NUDIX	1179	Bacteria>Pseudomonadota>Alphaproteobacteria>Sphingomonadales>Sphingomonadaceae>Sphingobium	GCF_025961895.1	Sphingobium_sp._B7D2B	M2341_RS01365	293056..296595	2940583	BREX-system-P-loop-protein-BrxC-[Sphingobium-sp.-B7D2B]
EJU13065.1	Type1.BREX	<-N6-METHYLASE-p<-DUF6079*<-BrxB-BrxF-iSTAND<-BrxA-DNA-binding<-wHTH+WYL+WCX||35Exo+REase->GreA_GreB+SF1-SF2-Helicase->	1179	Bacteria>Pseudomonadota>Alphaproteobacteria>Sphingomonadales>Sphingomonadaceae>Sphingomonas	GCA_000293195.2	Sphingomonas_sp._LH128	LH128_10616	1306..4845	473781	hypothetical-protein-LH128_10616-[Sphingomonas-sp.-LH128]
MBY0305717.1	Type1.BREX	VirB6->VirB8->VirB9->VirB10->RHH-><-MODE-HTH+PBPII||BrxA-DNA-binding->BrxB-BrxF-iSTAND->DUF6079*->N6-METHYLASE->PglZ->SIGMA+OB-Fold+AAA-ATPase+Lon-Protease-BrxL->ArdCN+MPTase->?->ParB->	1212	Bacteria>Pseudomonadota>Alphaproteobacteria>Sphingomonadales>Sphingomonadaceae>Sphingomonas	GCA_019750015.1	Sphingomonas_sp.	K2W86_11290	121509..125147	28214	BREX-system-P-loop-protein-BrxC-[Sphingomonas-sp.]
WP_056486438.1	Type1.BREX	<-SIGMA+OB-Fold+AAA-ATPase+Lon-Protease-BrxL<-PglZ<-N6-METHYLASE<-DUF6079*<-BrxB-BrxF-iSTAND<-BrxA-DNA-binding<-?<-?||?-><-?<-?<-?||GDResolvase+HIN-HTH->	1214	Bacteria>Pseudomonadota>Alphaproteobacteria>Sphingomonadales>Sphingomonadaceae>Sphingomonas	GCF_001421415.1	Sphingomonas_sp._Leaf208	ASE69_07675	294901..298545	1735679	BREX-system-P-loop-protein-BrxC-[Sphingomonas-sp.-Leaf208]
WP_066478880.1	Type1.BREX	Peptidase_S26->DUF736->?->TraI-VirD2-RCRE->||MODE-HTH+PBPII->wHTH+WYL+WCX->BrxA-DNA-binding->BrxB-BrxF-iSTAND->DUF6079*->N6-METHYLASE->PglZ->SIGMA+OB-Fold+AAA-ATPase+HKD-DNase-BrxL->SF1-SF2-Helicase+REase->SF1-SF2-Helicase+REase->wHTH->HKD-DNase+HKD-DNase->SF1-SF2-Helicase->	1179	Bacteria>Pseudomonadota>Alphaproteobacteria>Sphingomonadales>Sphingomonadaceae>Sphingomonas	GCF_001555785.1	unclassified_Sphingomonas	AX667_RS15610	42451..45990	196159	MULTISPECIES:-BREX-system-P-loop-protein-BrxC-[unclassified-Sphingomonas]
WP_107968397.1	Type1.BREX	<-OBFOLD-NUCLEASE<-LexA-protease||HTH->wHTH+WYL+WCX->BrxA-DNA-binding->BrxB-BrxF-iSTAND->DUF6079*->N6-METHYLASE->PglZ->SIGMA+OB-Fold+AAA-ATPase+HKD-DNase-BrxL->MORC+DUF3684+REase->SF1-SF2-Helicase->?-><-?<-TraH<-Thioredoxin	1186	Bacteria>Pseudomonadota>Alphaproteobacteria>Sphingomonadales>Sphingomonadaceae>Sphingomonas	GCF_003050615.1	Sphingomonas_oleivorans	CLG96_RS13035	363272..366832	1735121	BREX-system-P-loop-protein-BrxC-[Sphingomonas-oleivorans]
WP_192976950.1	Type1.BREX	Phage_integrase->HEPN-><-HKD-DNase+Preader14<-?||PIWI->REase-><-SIGMA+OB-Fold+AAA-ATPase+HKD-DNase-BrxL<-PglZ<-N6-METHYLASE<-DUF6079*<-BrxB-BrxF-iSTAND<-BrxA-DNA-binding<-wHTH+WYL+WCX<-?<-SWACOS||OLD-ABC+TOPRIM->SF1-SF2-Helicase-><-SbcC+CCDC158<-Calcineurin	1177	Bacteria>Pseudomonadota>Alphaproteobacteria>Sphingomonadales>Sphingomonadaceae>Sphingomonas	GCF_014892665.1	Sphingomonas_sp._CFBP_13603	IFR23_RS17335	87025..90558	2774040	BREX-system-P-loop-protein-BrxC-[Sphingomonas-sp.-CFBP-13603]
WP_089221081.1	Type1.BREX	<-Small-B-barrel+SF1-SF2-Helicase+RdgC+REase<-SF1-SF2-Helicase+REase<-35Exo+REase||wHTH+WYL+WCX->BrxA-DNA-binding->BrxB-BrxF-iSTAND->DUF6079*->N6-METHYLASE->PglZ->SIGMA+OB-Fold+AAA-ATPase+HKD-DNase-BrxL->	1179	Bacteria>Pseudomonadota>Alphaproteobacteria>Sphingomonadales>Sphingomonadaceae>Sphingomonas	GCF_900188165.1	Sphingomonas_laterariae	CHB74_RS18985	16083..19622	861865	BREX-system-P-loop-protein-BrxC-[Sphingomonas-laterariae]
WP_079639881.1	Type1.BREX	GDResolvase+HIN-HTH->?->DUF4158+DDE_Tnp_Tn3-><-?<-SF1-SF2-Helicase+REase<-35Exo+REase||wHTH+WYL+WCX->BrxA-DNA-binding->BrxB-BrxF-iSTAND->DUF6079*->N6-METHYLASE->	1179	Bacteria>Pseudomonadota>Alphaproteobacteria>Sphingomonadales>Sphingomonadaceae>Sphingopyxis	GCF_900168005.1	Sphingopyxis_flava	B5X69_RS17550	26842..30381	1507287	BREX-system-P-loop-protein-BrxC-[Sphingopyxis-flava]
WP_133494816.1	Type1.BREX	MobF->?-><-?<-OBFOLD-NUCLEASE<-LexA-protease||wHTH+WYL+WCX->BrxA-DNA-binding->BrxB-BrxF-iSTAND->DUF6079*->OLD-ABC->NERD->N6-METHYLASE->PglZ->SIGMA+OB-Fold+AAA-ATPase+HKD-DNase-BrxL->MORC+SF1-SF2-Helicase->?-><-?<-TraH	1189	Bacteria>Pseudomonadota>Alphaproteobacteria>Sphingomonadales>Sphingomonadaceae>Stakelama	GCF_014645395.1	Stakelama_pacifica	IEX20_RS07270	305060..308629	517720	BREX-system-P-loop-protein-BrxC-[Stakelama-pacifica]
WP_121052748.1	Type1.BREX	<-GDResolvase+Recombinase+ZnR<-?||XIS-HTH->Tox-RES-><-SIGMA+OB-Fold+AAA-ATPase+HKD-DNase-BrxL<-PglZ<-N6-METHYLASE<-NERD<-OLD-ABC<-DUF6079*<-BrxB-BrxF-iSTAND<-BrxA-DNA-binding<-wHTH+WYL+WCX||?->35Exo+REase->SF1-SF2-Helicase+REase->Small-B-barrel+SF1-SF2-Helicase+RdgC+REase->ArdCN+MPTase->SAM-METHYLASE+SF1-SF2-Helicase->	1179	Bacteria>Pseudomonadota>Alphaproteobacteria>Sphingomonadales>Sphingosinicellaceae>Sphingosinicella	GCF_003967095.1	Sphingosinicella_microcystinivorans	SmB9_RS16135	3426933..3430472	335406	BREX-system-P-loop-protein-BrxC-[Sphingosinicella-microcystinivorans]
OYY96513.1	Type1.BREX	wHTH+WYL+WCX->BrxA-DNA-binding->BrxB-BrxF-iSTAND->DUF6079*->N6-METHYLASE->PglZ->SIGMA+OB-Fold+AAA-ATPase+HKD-DNase-BrxL->REase+iREase->	1189	Bacteria>Pseudomonadota>Alphaproteobacteria>Sphingomonadales>unclassified_Sphingomonadales	GCA_002281585.1	Sphingomonadales_bacterium_28-56-43	B7Y38_10875	3957..7526	1970590	MAG:-hypothetical-protein-B7Y38_10875-[Sphingomonadales-bacterium-28-56-43]
TNE62528.1	Type1.BREX	REase+wHTH->rve->TnsC-ATPase->LON-AAA->?-><-Schlafen+REase<-SIGMA+OB-Fold+AAA-ATPase+HKD-DNase-BrxL<-PglZ<-N6-METHYLASE<-DUF6079*<-BrxB-BrxF-iSTAND<-BrxA-DNA-binding<-wHTH+WYL+WCX||REase+wHTH->HTH_28+rve+Mu-transpos_C->	1175	Bacteria>Pseudomonadota>Alphaproteobacteria>Sphingomonadales>unclassified_Sphingomonadales	GCA_006226385.1	Sphingomonadales_bacterium	EP341_00225	33441..36968	1978525	MAG:-BREX-system-P-loop-protein-BrxC-[Sphingomonadales-bacterium]
WP_015740180.1	Type1.BREX	VirB10->RHH-><-TetR-HTH||MODE-HTH+PBPII-><-SbcC+SbcC<-Calcineurin+Calcineurin-SbcD||wHTH+WYL+WCX->BrxA-DNA-binding->BrxB-BrxF-iSTAND->DUF6079*->N6-METHYLASE->PglZ->SIGMA+OB-Fold+AAA-ATPase+HKD-DNase-BrxL->SF1-SF2-Helicase+REase->?->?-><-MODE-HTH+PBPII<-ArdCN||GNTR-HTH+Aminotran_1_2->	1179	Bacteria>Pseudomonadota>Alphaproteobacteria>Sphingomonadales>Zymomonadaceae>Zymomonas	GCF_000024245.1	Zymomonas_mobilis	Za10_1374	1591366..1594905	542	BREX-system-P-loop-protein-BrxC-[Zymomonas-mobilis]
OQC17755.1	Type1.BREX	RHH_5->BrxA-DNA-binding->BrxB-BrxF-iSTAND->DUF6079*->N6-METHYLASE->ArdCN+MPTase->PglZ->SIGMA+OB-Fold+AAA-ATPase+Lon-Protease-BrxL->ABhydrolase-><-REC+LuxR-HTH	1145	Bacteria>Pseudomonadota>Alphaproteobacteria>unclassified_Alphaproteobacteria	GCA_002070795.1	Alphaproteobacteria_bacterium_ADurb.Bin100	BWX79_00187	2015..5452	1852791	hypothetical-protein-BWX79_00187-[Alphaproteobacteria-bacterium-ADurb.Bin100]
OYU71768.1	Type1.BREX	TrwC+SF1-SF2-Helicase+TOPRIM-><-?||?->?->ANK+ANK-><-SIGMA+OB-Fold+AAA-ATPase+Lon-Protease-BrxL<-PglZ<-N6-METHYLASE<-DUF6079*<-BrxB-BrxF-iSTAND<-BrxA-DNA-binding<-Couple_hipA+HipA_C<-HTH_3<-Phage_int_SAM_1+Phage_integrase	1219	Bacteria>Pseudomonadota>Alphaproteobacteria>unclassified_Alphaproteobacteria	GCA_002256425.1	Alphaproteobacteria_bacterium_PA2	CFE28_16215	3389137..3392796	2015570	hypothetical-protein-CFE28_16215-[Alphaproteobacteria-bacterium-PA2]

<table>
<tr><th>Protein-ID</th><th>System</th><th>Gene-Neighbourhoods</th><th>Length</th><th>Taxonomy</th><th>Assembly-ID</th><th>Strain</th><th>Locus-Tag</th><th>Genomic-coordinates</th><th>TaxID</th><th>Fasta-defline</th></tr>
<tr><td>RYH00367.1</td><td>Type1.BREX</td><td><-HTH_3<-XIS-HTH<-?<-ParB+HNH||?->Preader17-><-SIGMA+OB-Fold+AAA-ATPase+Lon-Protease-BrxL<-PglZ<-N6-METHYLASE<-DUF6079*<-BrxB-BrxF-iSTAND<-BrxA-DNA-binding||?->?->?->?->35exo+SF1-SF2-Helicase->HORMA-></td><td>1212</td><td>Bacteria>Pseudomonadota>Alphaproteobacteria>unclassified_Alphaproteobacteria</td><td>GCA_004145575.1</td><td>Alphaproteobacteria_bacterium</td><td>EON58_00375</td><td>13524..17162</td><td>1913988</td><td>BREX-system-P-loop-protein-BrxC-[Alphaproteobacteria-bacterium]</td></tr>
<tr><td>MBN9530327.1</td><td>Type1.BREX</td><td><-ICLR-HTH<-?||?->HTH_21+rve+rve_3-><-SIGMA+OB-Fold+AAA-ATPase+HKD-DNase-BrxL<-PglZ<-N6-METHYLASE<-DUF6079*<-BrxB-BrxF-iSTAND<-BrxA-DNA-binding<-wHTH+WYL+WCX||35Exo+REase->SF1-SF2-Helicase+REase->Small-B-barrel+SF1-SF2-Helicase+RdgC+REase-><-?||?-><-HTH_3</td><td>1180</td><td>Bacteria>Pseudomonadota>Alphaproteobacteria>unclassified_Alphaproteobacteria</td><td>GCA_017307375.1</td><td>Alphaproteobacteria_bacterium</td><td>JOH82_29215</td><td>188733..192275</td><td>1913988</td><td>BREX-system-P-loop-protein-BrxC-[Alphaproteobacteria-bacterium]</td></tr>
<tr><td>MBN9589014.1</td><td>Type1.BREX</td><td><-S4+PSYN||?->?-><-?||tRNA-><-Peptidase_M29+DUF2971||?-><-SIGMA+OB-Fold+AAA-ATPase+Lon-Protease-BrxL<-PglZ<-N6-METHYLASE<-DUF6079*<-BrxB-BrxF-iSTAND<-BrxA-DNA-binding<-?||REase+DUF5655->REase+SF1-SF2-Helicase-><-REase+HTH+35exo+Small-B-barrel+SF1-SF2-Helicase||HTH_3-></td><td>1155</td><td>Bacteria>Pseudomonadota>Alphaproteobacteria>unclassified_Alphaproteobacteria</td><td>GCA_017308475.1</td><td>Alphaproteobacteria_bacterium</td><td>JOG99_08395</td><td>190029..193496</td><td>1913988</td><td>BREX-system-P-loop-protein-BrxC-[Alphaproteobacteria-bacterium]</td></tr>
<tr><td>MBQ2845004.1</td><td>Type1.BREX</td><td>Peptidase-M15->?-><-HKD-DNase<-?||BrxA-DNA-binding->BrxB-BrxF-iSTAND->DUF6079*->N6-METHYLASE->PglZ->SIGMA+OB-Fold+AAA-ATPase+Lon-Protease-BrxL->ANK+ANK+ANK->?->Small-B-barrel+SF1-SF2-Helicase+RdgC+REase-></td><td>1173</td><td>Bacteria>Pseudomonadota>Alphaproteobacteria>unclassified_Alphaproteobacteria</td><td>GCA_017409215.1</td><td>Alphaproteobacteria_bacterium</td><td>IJE79_03245</td><td>53916..57437</td><td>1913988</td><td>BREX-system-P-loop-protein-BrxC-[Alphaproteobacteria-bacterium]</td></tr>
<tr><td>MBQ2886030.1</td><td>Type1.BREX</td><td>AbiEi_4->AbiEii-><-Response_reg||HTH_3->BrxA-DNA-binding->BrxB-BrxF-iSTAND->DUF6079*-></td><td>1131</td><td>Bacteria>Pseudomonadota>Alphaproteobacteria>unclassified_Alphaproteobacteria</td><td>GCA_017409945.1</td><td>Alphaproteobacteria_bacterium</td><td>IJE43_20100</td><td>7355..10747</td><td>1913988</td><td>hypothetical-protein-[Alphaproteobacteria-bacterium]</td></tr>
<tr><td>MBQ2886288.1</td><td>Type1.BREX</td><td><-wHTH+WYL<-REase<-SIGMA+OB-Fold+AAA-ATPase+Lon-Protease-BrxL<-PglZ<-Schlafen+HATPase+wHTH<-N6-METHYLASE<-PIN<-OLD-ABC<-DUF6079*<-BrxB-BrxF-iSTAND<-BrxA-DNA-binding<-HTH_3</td><td>1180</td><td>Bacteria>Pseudomonadota>Alphaproteobacteria>unclassified_Alphaproteobacteria</td><td>GCA_017409945.1</td><td>Alphaproteobacteria_bacterium</td><td>IJE43_21410</td><td>13139..16681</td><td>1913988</td><td>BREX-system-P-loop-protein-BrxC-[Alphaproteobacteria-bacterium]</td></tr>
<tr><td>MBQ8347028.1</td><td>Type1.BREX</td><td>VirD4-TraG+VirD4-FtsK->?-><-TraI-VirD2-RCRE<-?<-?<-?<-SIGMA+OB-Fold+AAA-ATPase+HKD-DNase-BrxL<-PglZ<-N6-METHYLASE<-DUF6079*<-BrxB-BrxF-iSTAND<-BrxA-DNA-binding||?-><-?<-ArdCN+MPTase||SF1-SF2-Helicase-></td><td>1150</td><td>Bacteria>Pseudomonadota>Alphaproteobacteria>unclassified_Alphaproteobacteria</td><td>GCA_017462585.1</td><td>Alphaproteobacteria_bacterium</td><td>IJY17_05445</td><td>46698..50150</td><td>1913988</td><td>BREX-system-P-loop-protein-BrxC-[Alphaproteobacteria-bacterium]</td></tr>
<tr><td>MBT3788616.1</td><td>Type1.BREX</td><td>ParA-Soj-PloopNTPase->?-><-?<-?||DEDD_Tnp_IS110+Transposase_20->?->HTH_Tnp_1+HTH_21+rve+rve_3-><-DUF6079*</td><td>1185</td><td>Bacteria>Pseudomonadota>Alphaproteobacteria>unclassified_Alphaproteobacteria</td><td>GCA_018654745.1</td><td>Alphaproteobacteria_bacterium</td><td>HOF94_09595</td><td>7711..11268</td><td>1913988</td><td>BREX-system-P-loop-protein-BrxC-[Alphaproteobacteria-bacterium]</td></tr>
<tr><td>EXI84705.1</td><td>Type1.BREX</td><td>DDE_Tnp_1-><-?<-MqsA_antitoxin<-HTH<-?<-Arm-DNA-bind_3+Phage_integrase||BrxA-DNA-binding->BrxB-BrxF-iSTAND->DUF6079*->N6-METHYLASE->HTH+REase->PglZ->SIGMA+OB-Fold+AAA-ATPase+Lon-Protease-BrxL->PIN+HTH-></td><td>1204</td><td>Bacteria>Pseudomonadota>Betaproteobacteria>Betaproteobacteria_incertae_sedis>Candidatus_Accumulibacter</td><td>GCA_000585075.1</td><td>Candidatus_Accumulibacter_regalis</td><td>AW11_03760</td><td>6873..10487</td><td>522306</td><td>MAG:-hypothetical-protein-AW11_03760-[Candidatus-Accumulibacter-regalis]</td></tr>
<tr><td>RDE50960.1</td><td>Type1.BREX</td><td><-Phage_int_SAM_4+Phage_integrase||?->TPR+TPR+TPR+TPR+TPR->BrxA-DNA-binding->BrxB-BrxF-iSTAND->DUF6079*->HTH_3->N6-METHYLASE->SF1-SF2-Helicase+PRTase->SLOG+BirA-HTH->PglZ->SIGMA+OB-Fold+AAA-ATPase+Lon-Protease-BrxL->SIGMA-HTH->?->Sulfatase+DUF4994-></td><td>1154</td><td>Bacteria>Pseudomonadota>Betaproteobacteria>Betaproteobacteria_incertae_sedis>Candidatus_Accumulibacter</td><td>GCA_003332265.1</td><td>Candidatus_Accumulibacter_meliphilus</td><td>DVS81_08175</td><td>4944..8408</td><td>2211374</td><td>MAG:-BREX-system-P-loop-protein-BrxC-[Candidatus-Accumulibacter-meliphilus]</td></tr>
<tr><td>RDE49751.1</td><td>Type1.BREX</td><td><-PIN+HTH<-SIGMA+OB-Fold+AAA-ATPase+Lon-Protease-BrxL<-PglZ<-TOPRIM<-OLD-ABC<-DUF6079*<-BrxB-BrxF-iSTAND<-BrxA-DNA-binding||?->?-><-?<-?||?-><-?<-Zn_Tnp_IS91+Y2_Tnp</td><td>1071</td><td>Bacteria>Pseudomonadota>Betaproteobacteria>Betaproteobacteria_incertae_sedis>Candidatus_Accumulibacter</td><td>GCA_003332265.1</td><td>Candidatus_Accumulibacter_meliphilus</td><td>DVS81_14920</td><td>17642..20857</td><td>2211374</td><td>MAG:-BREX-system-P-loop-protein-BrxC-[Candidatus-Accumulibacter-meliphilus]</td></tr>
<tr><td>MQM35874.1</td><td>Type1.BREX</td><td><-MqsA_antitoxin<-HTH<-?<-Arm-DNA-bind_3+Phage_integrase||BrxA-DNA-binding->BrxB-BrxF-iSTAND->DUF6079-p*-></td><td>732</td><td>Bacteria>Pseudomonadota>Betaproteobacteria>Betaproteobacteria_incertae_sedis>Candidatus_Accumulibacter</td><td>GCA_009467855.1</td><td>Candidatus_Accumulibacter_phosphatis</td><td>CRU72_16025</td><td>5196..7392</td><td>327160</td><td>ATPase-[Candidatus-Accumulibacter-phosphatis]</td></tr>
<tr><td>MQM30738.1</td><td>Type1.BREX</td><td>PolB-><-SIGMA+OB-Fold+AAA-ATPase+Lon-Protease-BrxL<-PglZ<-?<-SbcC<-N6-METHYLASE<-HTH_3<-DUF6079*<-BrxB-BrxF-iSTAND<-BrxA-DNA-binding<-TPR+TPR+TPR+TPR+TPR<-?||?->?->?->NUDIX-></td><td>1154</td><td>Bacteria>Pseudomonadota>Betaproteobacteria>Betaproteobacteria_incertae_sedis>Candidatus_Accumulibacter</td><td>GCA_009467885.1</td><td>Candidatus_Accumulibacter_phosphatis</td><td>CRU78_09485</td><td>11967..15431</td><td>327160</td><td>hypothetical-protein-[Candidatus-Accumulibacter-phosphatis]</td></tr>
<tr><td>WP_169070206.1</td><td>Type1.BREX</td><td>BrxA-DNA-binding->BrxB-BrxF-iSTAND->DUF6079*->OLD-ABC->?->OLD-ABC->TOPRIM->HTH+REase->?->HTH+REase->?->SIGMA+OB-Fold+AAA-ATPase+Lon-Protease-BrxL-></td><td>1204</td><td>Bacteria>Pseudomonadota>Betaproteobacteria>Betaproteobacteria_incertae_sedis>Candidatus_Accumulibacter</td><td>GCF_012940005.1</td><td>Candidatus_Accumulibacter_contiguus</td><td>E4Q08_RS09460</td><td>34303..37917</td><td>2954381</td><td>BREX-system-P-loop-protein-BrxC-[Candidatus-Accumulibacter-contiguus]</td></tr>
<tr><td>EXI72915.1</td><td>Type1.BREX</td><td><-PIN+OST-HTH<-?||?->?->?-><-?||BrxA-DNA-binding->BrxB-BrxF-iSTAND->DUF6079*->HTH_3->DOC+HTH->N6-METHYLASE-><-PIN<-MazE_antitoxin||SF1-SF2-Helicase+PRTase-><-?||BrxL-></td><td>1155</td><td>Bacteria>Pseudomonadota>Betaproteobacteria>Betaproteobacteria_incertae_sedis>Candidatus_Accumulibacter>unclassified_Candidatus_Accumulibacter</td><td>GCA_000584995.1</td><td>Candidatus_Accumulibacter_sp._SK-11</td><td>AW07_03051</td><td>55405..58872</td><td>1454000</td><td>MAG:-hypothetical-protein-AW07_03051-[Candidatus-Accumulibacter-sp.-SK-11]</td></tr>
<tr><td>HAY27737.1</td><td>Type1.BREX</td><td>BrxA-DNA-binding->DUF6079*->HTH_3->DOC+HTH->N6-METHYLASE-><-PIN<-MazE_antitoxin||SF1-SF2-Helicase+PRTase->SLOG+BirA-HTH-></td><td>1286</td><td>Bacteria>Pseudomonadota>Betaproteobacteria>Betaproteobacteria_incertae_sedis>Candidatus_Accumulibacter>unclassified_Candidatus_Accumulibacter</td><td>GCA_003487685.1</td><td>Candidatus_Accumulibacter_sp.</td><td>DCY47_09605</td><td>6303..10163</td><td>2053492</td><td>BREX-system-P-loop-protein-BrxC-[Candidatus-Accumulibacter-sp.]</td></tr>
<tr><td>[illegible]</td><td>[illegible]</td><td>[illegible]</td><td>[illegible]</td><td>[illegible]</td><td>[illegible]</td><td>[illegible]</td><td>[illegible]</td><td>[illegible]</td><td>[illegible]</td><td>[illegible]</td></tr>
</table>

Protein-ID	System	Gene-Neighbourhoods	Length	Taxonomy	Assembly-ID	Strain	Locus-Tag	Genomic-coordinates	TaxID	Fasta-define
MBK6612622.1	Type1.BREX	TraJ-RHH-><-?<-?<-SIGMA+OB-Fold+AAA-ATPase+Lon-Protease-BrxL<-PglZ<-?<-viral-capsid<-ZnR<-N6-METHYLASE<-DUF6079*<-BrxB-BrxF-iSTAND<-BrxA-DNA-binding<-RHH_5<-MULE-TRANSPOSASE<-?<-Phage [illegible]	1153	Bacteria>Pseudomonadota>Betaproteobacteria>Burkholderiales>Comamonadaceae>Ottowia	GCA_016703855.1	Ottowia_sp.	IPG27_00505	113652..117113	1898956	BREX-system-P-loop-protein-BrxC-[Ottowia-sp.]
OOG58024.1	Type1.BREX	<-HTH_3<-?<-?||?-><-SIGMA+OB-Fold+AAA-ATPase+Lon-Protease-BrxL<-PglZ<-HEPN<-N6-METHYLASE<-DUF6079*<-BrxB-BrxF-iSTAND<-BrxA-DNA-binding||?->HD+PspB->	1207	Bacteria>Pseudomonadota>Betaproteobacteria>Burkholderiales>Comamonadaceae>Polaromonas	GCA_002002705.1	Polaromonas_sp._C04	BOE49_04110	64714..68337	1945857	ATPase-[Polaromonas-sp.-C04]
TAG33475.1	Type1.BREX	<-DUF6079*<-BrxB-BrxF-iSTAND<-BrxA-DNA-binding<-HTH_26<-?<-Peptidase_M20||?->?->?->ABC-ATPase+SbcC->	1139	Bacteria>Pseudomonadota>Betaproteobacteria>Burkholderiales>Comamonadaceae>Polaromonas	GCA_004293655.1	Polaromonas_sp.	EAZ34_07410	1..3418	1869339	MAG:-BREX-system-P-loop-protein-BrxC,-partial-[Polaromonas-sp.]
MBG6075367.1	Type1.BREX	<-SWIM<-SIGMA+OB-Fold+AAA-ATPase+Lon-Protease-BrxL<-PglZ<-HTH+REase<-DOC+HTH<-?<-N6-METHYLASE<-Couple_hipA+HipA_C<-HTH_3<-DUF6079-p*||?-><-BrxB-BrxF-iSTAND<-BrxA-DNA-binding<-?<-?<-Ph [illegible]	921	Bacteria>Pseudomonadota>Betaproteobacteria>Burkholderiales>Comamonadaceae>Polaromonas	GCA_015752225.1	Polaromonas_sp._CG_9.11	IWX85_001192	30062..32827	2787730	hypothetical-protein-[Polaromonas-sp.-CG_9.11]
WP_011485879.1	Type1.BREX	N6-METHYLASE-><-?<-?<-?<-REC<-HISKIN||HTH_3->BrxA-DNA-binding->BrxB-BrxF-iSTAND->DUF6079*->CHTH->Tox-RES->N6-METHYLASE->HEPN->NERD+Pkinase+Pkinase+SF1-SF2-Helicase->?->PglZ->SIGMA+OB- [illegible]	1202	Bacteria>Pseudomonadota>Betaproteobacteria>Burkholderiales>Comamonadaceae>Polaromonas	GCF_000013865.1	Polaromonas_sp._JS666	BPRO_RS25145	121747..125355	296591	BREX-system-P-loop-protein-BrxC-[Polaromonas-sp.-JS666]
WP_231379566.1	Type1.BREX	<-SWIM<-SIGMA+OB-Fold+AAA-ATPase+Lon-Protease-BrxL<-PglZ<-HTH+REase<-DOC+HTH<-?<-N6-METHYLASE<-Couple_hipA+HipA_C<-HTH_3<-DUF6079*<-BrxB-BrxF-iSTAND<-BrxA-DNA-binding<-?<-?<-Phage_int [illegible]	1220	Bacteria>Pseudomonadota>Betaproteobacteria>Burkholderiales>Comamonadaceae>Polaromonas	GCF_015752225.1	Polaromonas_sp._CG_9.11	IWX85_RS05685	30062..33724	2787730	BREX-system-P-loop-protein-BrxC-[Polaromonas-sp.-CG_9.11]
WP_169420224.1	Type1.BREX	<-SIGMA+OB-Fold+AAA-ATPase+Lon-Protease-BrxL<-PglZ<-ParB+HNH<-?<-TPR+REase<-?<-?<-N6-METHYLASE<-DUF6079*<-BrxB-BrxF-iSTAND<-BrxA-DNA-binding<-RHH_5<-HAD+CHTH<-?<-ParA-Soj-PloopNTPase| [illegible]	1153	Bacteria>Pseudomonadota>Betaproteobacteria>Burkholderiales>Comamonadaceae>Ramlibacter	GCF_012927085.1	Ramlibacter_agri	HHL11_RS19385	189581..193042	2728837	BREX-system-P-loop-protein-BrxC-[Ramlibacter-agri]
APW46516.1	Type1.BREX	Glyco_transf_9-><-?||?->?->?->TPR+TPR+TPR->?->tRNA-><-?||DUF6079-p*->MarR-HTH+Polbeta->HEPN->HEPN->N6-METHYLASE-p->N6-METHYLASE->?->?->?-><-DDE_Tnp_1+DDE_Tnp_1	269	Bacteria>Pseudomonadota>Betaproteobacteria>Burkholderiales>Comamonadaceae>Rhodoferax	GCA_001955735.1	Rhodoferax_antarcticus	RA876_09190	1894413..1895222	81479	hypothetical-protein-RA876_09190-[Rhodoferax-antarcticus]
MBP9058688.1	Type1.BREX	<-PglZ<-SLOG+BirA-HTH<-SF1-SF2-Helicase+PRTase<-?<-TPR+REase<-?<-N6-METHYLASE<-HEPN<-MarR-HTH+Polbeta<-DUF6079*<-BrxB-BrxF-iSTAND<-BrxA-DNA-binding<-RHH_5||N6-METHYLASE->ORC-CDC6+ZnR- [illegible]	1153	Bacteria>Pseudomonadota>Betaproteobacteria>Burkholderiales>Comamonadaceae>Rhodoferax	GCA_018057375.1	Rhodoferax_sp.	KBF98_00065	15882..19343	50421	BREX-system-P-loop-protein-BrxC-[Rhodoferax-sp.]
MCF8208673.1	Type1.BREX	<-DUF6079-p*<-BrxB-BrxF-iSTAND<-BrxA-DNA-binding<-Peptidase_M3_N+Peptidase_M3||NTP_transf->dCache_1+HisKA+HATPase_c->TPR+TPR+TPR+TPR+TPR+TPR+HISKIN+REC-><-REC+GGDEF+EAL<-REC+OmpR-HTH [illegible]	609	Bacteria>Pseudomonadota>Betaproteobacteria>Burkholderiales>Comamonadaceae>Rhodoferax	GCA_021738345.1	Rhodoferax_sp.	K9K38_04605	1..1828	50421	BREX-system-P-loop-protein-BrxC-[Rhodoferax-sp.]
MCF8211220.1	Type1.BREX	BrxA-DNA-binding->BrxB-BrxF-iSTAND->DUF6079*->MarR-HTH+Polbeta->HEPN->N6-METHYLASE->	1153	Bacteria>Pseudomonadota>Betaproteobacteria>Burkholderiales>Comamonadaceae>Rhodoferax	GCA_021738345.1	Rhodoferax_sp.	K9K38_17735	1925..5386	50421	BREX-system-P-loop-protein-BrxC-[Rhodoferax-sp.]
WP_139313405.1	Type1.BREX	ABC-ATPase+SbcC->?->?->Glyco_transf_9-><-?||?->?->TPR+TPR+TPR->?->tRNA->||DUF6079-p*->MarR-HTH+Polbeta->HEPN->?->HEPN->N6-METHYLASE-p->N6-METHYLASE->?->?->RAMA->	323	Bacteria>Pseudomonadota>Betaproteobacteria>Burkholderiales>Comamonadaceae>Rhodoferax	GCF_001955735.1	Rhodoferax_antarcticus	RA876_RS09190	1894251..1895222	81479	hypothetical-protein-[Rhodoferax-antarcticus]
WP_264569449.1	Type1.BREX	<-Glyco_transf_9||?->?->?->TPR+TPR+TPR->?->tRNA->||DUF6079-p*->MarR-HTH+Polbeta->HEPN->?->HEPN->N6-METHYLASE-p->N6-METHYLASE-p->N6-METHYLASE-p->	323	Bacteria>Pseudomonadota>Betaproteobacteria>Burkholderiales>Comamonadaceae>Rhodoferax	GCF_025961535.1	Rhodoferax_antarcticus	M2244_RS19480	9793..10764	81479	hypothetical-protein-[Rhodoferax-antarcticus]
PIQ51624.1	Type1.BREX	<-OLD-ABC<-HEPN<-?<-N6-METHYLASE<-?<-?<-N6-METHYLASE-p<-AbiEii+ZnR<-AbiEi_4+AbiEi_1<-DUF6079*<-BrxB-BrxF-iSTAND<-BrxA-DNA-binding<-wHTH+WYL+WCX<-?<-RADICAL-SAM	1204	Bacteria>Pseudomonadota>Betaproteobacteria>Burkholderiales>Comamonadaceae>unclassified_Comamonadaceae	GCA_002786915.1	Comamonadaceae_bacterium_CG12_big_fil_rev_8_21_14_0_65_59_15	COW02_13880	31766..35380	1973952	ATPase-[Comamonadaceae-bacterium-CG12_big_fil_rev_8_21_14_0_65_59_15]
PIQ50876.1	Type1.BREX	dCache_1+HisKA+HATPase_c-><-SIGMA+OB-Fold+AAA-ATPase+Lon-Protease-BrxL<-PglZ<-Schlafen+HATPase+wHTH<-SbcC<-N6-METHYLASE<-PIN<-MazE_antitoxin<-DUF6079*<-BrxB-BrxF-iSTAND<-BrxA-DNA-bind [illegible]	1153	Bacteria>Pseudomonadota>Betaproteobacteria>Burkholderiales>Comamonadaceae>unclassified_Comamonadaceae	GCA_002786915.1	Comamonadaceae_bacterium_CG12_big_fil_rev_8_21_14_0_65_59_15	COW02_17785	48153..51614	1973952	hypothetical-protein-COW02_17785-[Comamonadaceae-bacterium-CG12_big_fil_rev_8_21_14_0_65_59_15]
MBH2043138.1	Type1.BREX	RHH_5->BrxA-DNA-binding->BrxB-BrxF-iSTAND->DUF6079*->MarR-HTH+Polbeta->HEPN->OLD-ABC->HNH->PglZ->SIGMA+OB-Fold+AAA-ATPase+Lon-Protease-BrxL->	1153	Bacteria>Pseudomonadota>Betaproteobacteria>Burkholderiales>Comamonadaceae>unclassified_Comamonadaceae	GCA_016000285.1	Comamonadaceae_bacterium	I8H87_06315	1878..5339	1871071	BREX-system-P-loop-protein-BrxC-[Comamonadaceae-bacterium]
WP_097197978.1	Type1.BREX	<-DOC+HTH||Phage_integrase->HIN-HTH->?->LZ_Tnp_IS66+zf-IS66+DDE_Tnp_IS66+DDE_Tnp_IS66_C->?->||BrxA-DNA-binding->BrxB-BrxF-iSTAND->DUF6079*->?->?->N6-METHYLASE->PHP+SbcC->PglZ->SIGMA+OB-F [illegible]	1153	Bacteria>Pseudomonadota>Betaproteobacteria>Burkholderiales>Comamonadaceae>Variovorax	GCF_900215425.1	Variovorax_sp._YR752	CRP32_RS05955	1262049..1265510	1884383	BREX-system-P-loop-protein-BrxC-[Variovorax-sp.-YR752]
WP_186915942.1	Type1.BREX	wHTH+WYL+WCX->?->?->wHTH+WYL+WCX-p->ANK->XIS-HTH->BrxA-DNA-binding->BrxB-BrxF-iSTAND->DUF6079*->OLD-ABC->REase->N6-METHYLASE->ParB+HNH->PglZ->SIGMA+OB-Fold+AAA-ATPase+HKD-DNase-BrxL-> [illegible]	1183	Bacteria>Pseudomonadota>Betaproteobacteria>Burkholderiales>Oxalobacteraceae	GCF_014284115.1	Undibacterium	H8K35_RS08325	157325..160876	401469	MULTISPECIES:-BREX-system-P-loop-protein-BrxC-[Undibacterium]
WP_253548067.1	Type1.BREX	<-SF1-SF2-Helicase<-OLD-ABC+TOPRIM||GDResolvase+HIN-HTH-><-SIGMA+OB-Fold+AAA-ATPase+Lon-Protease-BrxL<-PglZ<-N6-METHYLASE<-N6-METHYLASE-p<-DUF6079*<-BrxB-BrxF-iSTAND<-BrxA-DNA-binding [illegible]	1153	Bacteria>Pseudomonadota>Betaproteobacteria>Burkholderiales>Oxalobacteraceae>Herbaspirillum	GCF_024170025.1	Herbaspirillum_rubrisubalbicans	J2S30_RS12610	2752801..2756262	80842	BREX-system-P-loop-protein-BrxC-[Herbaspirillum-rubrisubalbicans]
PHV04095.1	Type1.BREX	BrxA-DNA-binding->BrxB-BrxF-iSTAND->DUF6079*->PglZ->SIGMA+OB-Fold+AAA-ATPase+Lon-Protease-BrxL->SMODS->?->JAB->SAVED->AAA-ATPase-p->Zn_Tnp_IS1595+DUF2089+DDE_Tnp_IS1595->	1205	Bacteria>Pseudomonadota>Betaproteobacteria>Burkholderiales>Oxalobacteraceae>Janthinobacterium	GCA_002735605.1	Janthinobacterium_sp._BJB412	CSQ96_27685	29262..32879	1871178	ATPase-[Janthinobacterium-sp.-BJB412]
WP_071078524.1	Type1.BREX	Phage_integrase-><-?<-SIGMA+OB-Fold+AAA-ATPase+Lon-Protease-BrxL<-PglZ<-HNH<-OLD-ABC<-N6-METHYLASE<-DUF6079*<-BrxB-BrxF-iSTAND<-BrxA-DNA-binding<-RHH_5<-?<-HEPN+OST-HTH	1153	Bacteria>Pseudomonadota>Betaproteobacteria>Burkholderiales>Oxalobacteraceae>Janthinobacterium	GCF_001854915.1	Janthinobacterium_lividum	AKG95_RS19180	51113..54574	29581	BREX-system-P-loop-protein-BrxC-[Janthinobacterium-lividum]
WP_096234088.1	Type1.BREX	HTH_3->?->?->?->BrxA-DNA-binding->BrxB-BrxF-iSTAND->DUF6079*->N6-METHYLASE->OLD-ABC->TOPRIM->PglZ->SIGMA+OB-Fold+AAA-ATPase+Lon-Protease-BrxL->HEPN-><-TPR	1204	Bacteria>Pseudomonadota>Betaproteobacteria>Burkholderiales>Oxalobacteraceae>Janthinobacterium	GCF_002327145.1	Janthinobacterium_svalbardensis	CNX70_RS06880	1564323..1567937	368607	BREX-system-P-loop-protein-BrxC-[Janthinobacterium-svalbardensis]
WP_099389566.1	Type1.BREX	[illegible]	1153	Bacteria>Pseudomonadota>Betaproteobacteria>Burkholderiales>Oxalobacteraceae>Janthinobacterium	GCF_002735685.1	Janthinobacterium_sp._BJB301	CSQ91_RS21655	521004..524465	1560195	BREX-system-P-loop-protein-BrxC-[Janthinobacterium-sp.-BJB301]
WP_219136384.1	Type1.BREX	[illegible]	1153	Bacteria>Pseudomonadota>Betaproteobacteria>Burkholderiales>Oxalobacteraceae>Janthinobacterium	GCF_900095235.1	Janthinobacterium_sp._UMAB-60	KOF86_RS22385	15275..18736	1365365	BREX-system-P-loop-protein-BrxC-[Janthinobacterium-sp.-UMAB-60]
WP_106758218.1	Type1.BREX	[illegible]	1154	Bacteria>Pseudomonadota>Betaproteobacteria>Burkholderiales>Oxalobacteraceae>Massilia_group	GCF_003011895.2	Massilia_glaciei	C7C56_RS15200	27208..30672	1524097	BREX-system-P-loop-protein-BrxC-[Massilia-glaciei]
WP_183556379.1	Type1.BREX	[illegible]	1153	Bacteria>Pseudomonadota>Betaproteobacteria>Burkholderiales>Oxalobacteraceae>Massilia_group	GCF_014200505.1	Massilia_aurea	HD842_RS19385	505200..508661	373040	BREX-system-P-loop-protein-BrxC-[Massilia-aurea]
WP_223466304.1	Type1.BREX	[illegible]	1205	Bacteria>Pseudomonadota>Betaproteobacteria>Burkholderiales>Oxalobacteraceae>Massilia_group	GCF_016809835.1	Massilia_soli	I4X03_RS04425	2147..5764	2792854	BREX-system-P-loop-protein-BrxC-[Massilia-soli]
WP_258819858.1	Type1.BREX	[illegible]	1186	Bacteria>Pseudomonadota>Betaproteobacteria>Burkholderiales>Oxalobacteraceae>Massilia_group	GCF_024753285.1	Massilia_pinisoli	NX784_RS27510	42185..45745	1772194	BREX-system-P-loop-protein-BrxC-[Massilia-pinisoli]
WP_090328302.1	Type1.BREX	[illegible]	1204	Bacteria>Pseudomonadota>Betaproteobacteria>Burkholderiales>Oxalobacteraceae>Massilia_group	GCF_900110275.1	Duganella_sp._CF517	BMX85_RS29475	9688..13302	1881038	BREX-system-P-loop-protein-BrxC-[Duganella-sp.-CF517]
WP_146328427.1	Type1.BREX	[illegible]	1153	Bacteria>Pseudomonadota>Betaproteobacteria>Burkholderiales>Oxalobacteraceae>Noviherbaspirillum	GCF_007874125.1	Noviherbaspirillum_sp._UKPF54	FAY22_RS00565	114292..117753	2601898	BREX-system-P-loop-protein-BrxC-[Noviherbaspirillum-sp.-UKPF54]
WP_182219587.1	Type1.BREX	[illegible]	1186	Bacteria>Pseudomonadota>Betaproteobacteria>Burkholderiales>Oxalobacteraceae>Rugamonas	GCF_014042365.1	Rugamonas_fusca	H3H36_RS18580	34208..37768	2758568	BREX-system-P-loop-protein-BrxC-[Rugamonas-fusca]
WP_151122046.1	Type1.BREX	[illegible]	1153	Bacteria>Pseudomonadota>Betaproteobacteria>Burkholderiales>Sphaerotilaceae>Ideonella	GCF_021049305.1	Ideonella_dechloratans	LRM40_RS14105	3039018..3042479	36863	BREX-system-P-loop-protein-BrxC-[Ideonella-dechloratans]
EGJ09025.1	Type1.BREX	[illegible]	280	Bacteria>Pseudomonadota>Betaproteobacteria>Burkholderiales>Sphaerotilaceae>Rubrivivax	GCA_000190375.2	Rubrivivax_benzoatilyticus_JA2_=_ATCC_BAA-35	RBXJA2T_01790	1..841	987059	hypothetical-protein-RBXJA2T_01790,-partial-[Rubrivivax-benzoatilyticus-JA2-=-ATCC-BAA-35]
WP_009855692.1	Type1.BREX	[illegible]	874	Bacteria>Pseudomonadota>Betaproteobacteria>Burkholderiales>Sphaerotilaceae>Rubrivivax	GCF_000190375.1	Rubrivivax_benzoatilyticus	RBXJA2T_RS02255	28686..31310	316997	BREX-system-P-loop-protein-BrxC,-partial-[Rubrivivax-benzoatilyticus]
WP_029719002.1	Type1.BREX	[illegible]	1153	Bacteria>Pseudomonadota>Betaproteobacteria>Burkholderiales>Sphaerotilaceae>Rubrivivax	GCF_000420125.1	Rubrivivax_benzoatilyticus	F799_RS0117210	9638..13099	316997	BREX-system-P-loop-protein-BrxC-[Rubrivivax-benzoatilyticus]
WP_230248250.1	Type1.BREX	[illegible]	1207	Bacteria>Pseudomonadota>Betaproteobacteria>Burkholderiales>Sphaerotilaceae>Rubrivivax	GCF_020966545.1	unclassified_Rubrivivax	LOC64_RS00220	46220..49843	2649762	MULTISPECIES:-BREX-system-P-loop-protein-BrxC-[unclassified-Rubrivivax]
MAG79942.1	Type1.BREX	[illegible]	1155	Bacteria>Pseudomonadota>Betaproteobacteria>Burkholderiales>Sutterellaceae>unclassified_Sutterellaceae	GCA_002690025.1	Sutterellaceae_bacterium	CMN88_03370	89998..93465	2026794	hypothetical-protein-[Sutterellaceae-bacterium]
RIK89376.1	Type1.BREX	[illegible]	1146	Bacteria>Pseudomonadota>Betaproteobacteria>Burkholderiales>unclassified_Burkholderiales	GCA_003577305.1	Burkholderiales_bacterium	DCC70_08090	13043..16483	1891238	BREX-system-P-loop-protein-BrxC-[Burkholderiales-bacterium]
MBL8529773.1	Type1.BREX	[illegible]	1244	Bacteria>Pseudomonadota>Betaproteobacteria>Burkholderiales>unclassified_Burkholderiales	GCA_016791285.1	Burkholderiales_bacterium	JNL68_18995	11051..14785	1891238	BREX-system-P-loop-protein-BrxC-[Burkholderiales-bacterium]
MBN8486519.1	Type1.BREX	[illegible]	1186	Bacteria>Pseudomonadota>Betaproteobacteria>Burkholderiales>unclassified_Burkholderiales	GCA_017302485.1	Burkholderiales_bacterium	JOM20_02125	4400..7960	1891238	BREX-system-P-loop-protein-BrxC-[Burkholderiales-bacterium]
MBP7846551.1	Type1.BREX	[illegible]	1193	Bacteria>Pseudomonadota>Betaproteobacteria>Burkholderiales>unclassified_Burkholderiales	GCA_018001735.1	Burkholderiales_bacterium	KAZ94_00285	68005..71586	1891238	BREX-system-P-loop-protein-BrxC-[Burkholderiales-bacterium]
MBX9598966.1	Type1.BREX	[illegible]	1193	Bacteria>Pseudomonadota>Betaproteobacteria>Burkholderiales>unclassified_Burkholderiales	GCA_019748155.1	Burkholderiales_bacterium	K2X04_10405	1415..4996	1891238	BREX-system-P-loop-protein-BrxC-[Burkholderiales-bacterium]
MCP5252671.1	Type1.BREX	[illegible]	1144	Bacteria>Pseudomonadota>Betaproteobacteria>Burkholderiales>unclassified_Burkholderiales	GCA_024235735.1	Burkholderiales_bacterium	H6939_13335	16624..20058	1891238	BREX-system-P-loop-protein-BrxC-[Burkholderiales-bacterium]
MCP5292583.1	Type1.BREX	[illegible]	1144	Bacteria>Pseudomonadota>Betaproteobacteria>Burkholderiales>unclassified_Burkholderiales	GCA_024235775.1	Burkholderiales_bacterium	H6938_08650	46881..50315	1891238	BREX-system-P-loop-protein-BrxC-[Burkholderiales-bacterium]
MCP5275500.1	Type1.BREX	[illegible]	1176	Bacteria>Pseudomonadota>Betaproteobacteria>Burkholderiales>unclassified_Burkholderiales	GCA_024235795.1	Burkholderiales_bacterium	H6936_11775	653459..656989	1891238	BREX-system-P-loop-protein-BrxC-[Burkholderiales-bacterium]
MCP5275800.1	Type1.BREX	[illegible]	1177	Bacteria>Pseudomonadota>Betaproteobacteria>Burkholderiales>unclassified_Burkholderiales	GCA_024235795.1	Burkholderiales_bacterium	H6936_13310	1016712..1020245	1891238	BREX-system-P-loop-protein-BrxC-[Burkholderiales-bacterium]
WP_131354865.1	Type1.BREX	[illegible]	1224	Bacteria>Pseudomonadota>Betaproteobacteria>Neisseriales>Chromobacteriaceae>Aquitalea	GCF_004328945.1	Aquitalea_sp._USM4	DKK66_RS01410	305665..309339	1590041	BREX-system-P-loop-protein-BrxC-[Aquitalea-sp.-USM4]
WP_205677506.1	Type1.BREX	[illegible]	1165	Bacteria>Pseudomonadota>Betaproteobacteria>Neisseriales>Chromobacteriaceae>Aquitalea	GCF_009856625.1	Aquitalea_denitrificans	GSR16_RS06460	1403398..1406895	519081	BREX-system-P-loop-protein-BrxC-[Aquitalea-denitrificans]
WP_046167751.1	Type1.BREX	[illegible]	1153	Bacteria>Pseudomonadota>Betaproteobacteria>Neisseriales>Chromobacteriaceae>Chromobacterium_group	GCF_000971355.1	Chromobacterium_vaccinii	VL68_RS13365	2032..5493	1108595	BREX-system-P-loop-protein-BrxC-[Chromobacterium-vaccinii]
WP_047248551.1	Type1.BREX	[illegible]	1228	Bacteria>Pseudomonadota>Betaproteobacteria>Neisseriales>Chromobacteriaceae>Chromobacterium_group	GCF_001020585.1	Chromobacterium_subtsugae	AA094_RS04310	55231..58917	251747	BREX-system-P-loop-protein-BrxC-[Chromobacterium-subtsugae]
WP_199155307.1	Type1.BREX	[illegible]	1225	Bacteria>Pseudomonadota>Betaproteobacteria>Neisseriales>Chromobacteriaceae>Chromobacterium_group	GCF_016428945.1	Chromobacterium_sp._ASV23	JHD59_RS18460	1242..4919	2795110	BREX-system-P-loop-protein-BrxC-[Chromobacterium-sp.-ASV23]
WP_203569835.1	Type1.BREX	[illegible]	1156	Bacteria>Pseudomonadota>Betaproteobacteria>Neisseriales>Chromobacteriaceae	GCF_014042245.2	Deefgea	H1229_RS02985	646302..649772	400947	MULTISPECIES:-BREX-system-P-loop-protein-BrxC-[Deefgea]
MCG9052529.1	Type1.BREX	[illegible]	1224	Bacteria>Pseudomonadota>Betaproteobacteria>Neisseriales>Chromobacteriaceae>Laribacter	GCA_022394235.1	Laribacter_hongkongensis	LH447_05335	18538..22212	168471	BREX-system-P-loop-protein-BrxC-[Laribacter-hongkongensis]
MCG9059785.1	Type1.BREX	[illegible]	1217	Bacteria>Pseudomonadota>Betaproteobacteria>Neisseriales>Chromobacteriaceae>Laribacter	GCA_022394335.1	Laribacter_hongkongensis	LH452_12755	30373..34026	168471	BREX-system-P-loop-protein-BrxC-[Laribacter-hongkongensis]
MCG9118882.1	Type1.BREX	[illegible]	1221	Bacteria>Pseudomonadota>Betaproteobacteria>Neisseriales>Chromobacteriaceae>Laribacter	GCA_022394685.1	Laribacter_hongkongensis	LH456_09760	59894..63559	168471	BREX-system-P-loop-protein-BrxC-[Laribacter-hongkongensis]
WP_275663441.1	Type1.BREX	[illegible]	1229	Bacteria>Pseudomonadota>Betaproteobacteria>Neisseriales>Chromobacteriaceae>Laribacter	GCF_022394235.1	Laribacter_hongkongensis	LH442_RS13325	2333..6022	168471	BREX-system-P-loop-protein-BrxC-[Laribacter-hongkongensis]
WP_239907373.1	Type1.BREX	[illegible]	1185	Bacteria>Pseudomonadota>Betaproteobacteria>Neisseriales>Chromobacteriaceae>Laribacter	GCF_022394255.1	Laribacter_hongkongensis	LH425_05855	38844..42401	168471	BREX-system-P-loop-protein-BrxC-[Laribacter-hongkongensis]
WP_239898705.1	Type1.BREX	[illegible]	1222	Bacteria>Pseudomonadota>Betaproteobacteria>Neisseriales>Chromobacteriaceae>Laribacter	GCF_022394335.1	Laribacter_hongkongensis	LH452_RS12745	30373..34041	168471	BREX-system-P-loop-protein-BrxC-[Laribacter-hongkongensis]
WP_239902638.1	Type1.BREX	[illegible]	1153	Bacteria>Pseudomonadota>Betaproteobacteria>Neisseriales>Chromobacteriaceae>Laribacter	GCF_022394475.1	Laribacter_hongkongensis	LH449_RS00385	78525..81986	168471	BREX-system-P-loop-protein-BrxC-[Laribacter-hongkongensis]
WP_239909460.1	Type1.BREX	[illegible]	1186	Bacteria>Pseudomonadota>Betaproteobacteria>Neisseriales>Chromobacteriaceae>Laribacter	GCF_022394655.1	Laribacter_hongkongensis	LH423_RS01205	99182..102742	168471	BREX-system-P-loop-protein-BrxC-[Laribacter-hongkongensis]
WP_275667170.1	Type1.BREX	[illegible]	1216	Bacteria>Pseudomonadota>Betaproteobacteria>Neisseriales>Chromobacteriaceae>Laribacter	GCF_022394685.1	Laribacter_hongkongensis	LH456_RS09740	59894..63544	168471	BREX-system-P-loop-protein-BrxC-[Laribacter-hongkongensis]
WP_239900332.1	Type1.BREX	[illegible]	1224	Bacteria>Pseudomonadota>Betaproteobacteria>Neisseriales>Chromobacteriaceae>Laribacter	-	Laribacter_hongkongensis	LH447_05335	18538..22212	168471	BREX-system-P-loop-protein-BrxC-[Laribacter-hongkongensis]
WP_239891840.1	Type1.BREX	[illegible]	1217	Bacteria>Pseudomonadota>Betaproteobacteria>Neisseriales>Chromobacteriaceae>Laribacter	-	Laribacter_hongkongensis	LH452_12755	30373..34026	168471	BREX-system-P-loop-protein-BrxC-[Laribacter-hongkongensis]
WP_239900962.1	Type1.BREX	[illegible]	1224	Bacteria>Pseudomonadota>Betaproteobacteria>Neisseriales>Chromobacteriaceae>Laribacter	-	Laribacter_hongkongensis	LH454_02040	72475..76149	168471	BREX-system-P-loop-protein-BrxC-[Laribacter-hongkongensis]
WP_239901116.1	Type1.BREX	[illegible]	1221	Bacteria>Pseudomonadota>Betaproteobacteria>Neisseriales>Chromobacteriaceae>Laribacter	-	Laribacter_hongkongensis	LH456_09760	59894..63559	168471	BREX-system-P-loop-protein-BrxC-[Laribacter-hongkongensis]
WP_089417825.1	Type1.BREX	[illegible]	1153	Bacteria>Pseudomonadota>Betaproteobacteria>Neisseriales>Neisseriaceae>Vitreoscilla	GCF_002222655.1	Vitreoscilla_filiformis	VITFI_RS15935	3397543..3401004	63	BREX-system-P-loop-protein-BrxC-[Vitreoscilla-filiformis]
NCN89398.1	Type1.BREX	[illegible]	1207	Bacteria>Pseudomonadota>Betaproteobacteria>Nitrosomonadales>Gallionellaceae>Gallionella	GCA_009992105.1	Gallionella_sp.	GW936_08180	1399..5022	2052161	BREX-system-P-loop-protein-BrxC-[Gallionella-sp.]
WP_013292857.1	Type1.BREX	[illegible]	1207	Bacteria>Pseudomonadota>Betaproteobacteria>Nitrosomonadales>Gallionellaceae>Gallionella	GCF_000145255.1	Gallionella_capsiferriformans	GALF_RS04415	928777..932400	370405	BREX-system-P-loop-protein-BrxC-[Gallionella-capsiferriformans]
WP_013293480.1	Type1.BREX	[illegible]	1161	Bacteria>Pseudomonadota>Betaproteobacteria>Nitrosomonadales>Gallionellaceae>Gallionella	GCF_000145255.1	Gallionella_capsiferriformans	GALF_RS07565	1653086..1656571	370405	BREX-system-P-loop-protein-BrxC-[Gallionella-capsiferriformans]
TXI44178.1	Type1.BREX	[illegible]	1198	Bacteria>Pseudomonadota>Betaproteobacteria>Nitrosomonadales>Methylophilaceae>Methylophilus	GCA_008015755.1	Methylophilus_sp.	E6Q52_09965	12844..16440	29541	MAG:-BREX-system-P-loop-protein-BrxC-[Methylophilus-sp.]
ROH85757.1	Type1.BREX	[illegible]	1146	Bacteria>Pseudomonadota>Betaproteobacteria>Nitrosomonadales>Methylophilaceae>Pseudomethylobacillus	GCA_003730115.1	Pseudomethylobacillus_aquaticus	ED236_08405	68439..71879	2676064	BREX-system-P-loop-protein-BrxC-[Pseudomethylobacillus-aquaticus]
WP_205125664.1	Type1.BREX	[illegible]	1150	Bacteria>Pseudomonadota>Betaproteobacteria>Nitrosomonadales>Methylophilaceae>Pseudomethylobacillus	GCF_003730115.1	Pseudomethylobacillus_aquaticus	ED236_RS08405	68439..71891	2676064	BREX-system-P-loop-protein-BrxC-[Pseudomethylobacillus-aquaticus]
PTQ77404.1	Type1.BREX	[illegible]	1208	Bacteria>Pseudomonadota>Betaproteobacteria>Nitrosomonadales>Nitrosomonadaceae>Nitrosomonas	GCA_003050805.1	Nitrosomonas_oligotropha	C8R26_10850	43938..47564	42354	hypothetical-protein-C8R26_10850-[Nitrosomonas-oligotropha]
MBK7364915.1	Type1.BREX	[illegible]	1187	Bacteria>Pseudomonadota>Betaproteobacteria>Nitrosomonadales>Nitrosomonadaceae>Nitrosomonas	GCA_016705625.1	Nitrosomonas_sp.	IPI97_07925	1671776..1675339	42353	BREX-system-P-loop-protein-BrxC-[Nitrosomonas-sp.]
MBK7491769.1	Type1.BREX	[illegible]	1203	Bacteria>Pseudomonadota>Betaproteobacteria>Nitrosomonadales>Nitrosomonadaceae>Nitrosomonas	GCA_016707165.1	Nitrosomonas_sp.	IPI17_07005	52164..55775	42353	BREX-system-P-loop-protein-BrxC-[Nitrosomonas-sp.]
MBK7352718.1	Type1.BREX	[illegible]	705	Bacteria>Pseudomonadota>Betaproteobacteria>Nitrosomonadales>Nitrosomonadaceae>Nitrosomonas	GCA_016710135.1	Nitrosomonas_sp.	IPJ05_03020	595038..597155	42353	BREX-system-P-loop-protein-BrxC-[Nitrosomonas-sp.]
MBV6446594.1	Type1.BREX	[illegible]	1182	Bacteria>Pseudomonadota>Betaproteobacteria>Nitrosomonadales>Nitrosomonadaceae>Nitrosomonas	GCA_019187415.1	Nitrosomonas_sp.	FCKEOINB_00187	17095..20643	42353	hypothetical-protein-[Nitrosomonas-sp.]
MBX3639968.1	Type1.BREX	[illegible]	1182	Bacteria>Pseudomonadota>Betaproteobacteria>Nitrosomonadales>Nitrosomonadaceae>Nitrosomonas	GCA_019635175.1	Nitrosomonas_sp.	KF888_05565	120382..123930	42353	BREX-system-P-loop-protein-BrxC-[Nitrosomonas-sp.]
MBX9637025.1	Type1.BREX	[illegible]	1212	Bacteria>Pseudomonadota>Betaproteobacteria>Nitrosomonadales>Nitrosomonadaceae>Nitrosomonas	GCA_019748355.1	Nitrosomonas_sp.	K2Q45_05685	2429..6067	42353	BREX-system-P-loop-protein-BrxC-[Nitrosomonas-sp.]
MCB1936159.1	Type1.BREX	[illegible]	1174	Bacteria>Pseudomonadota>Betaproteobacteria>Nitrosomonadales>Nitrosomonadaceae>Nitrosomonas	GCA_020446045.1	Nitrosomonas_sp.	KDF59_09470	5848..9372	42353	BREX-system-P-loop-protein-BrxC-[Nitrosomonas-sp.]
SDW37048.1	Type1.BREX	[illegible]	1145	Bacteria>Pseudomonadota>Betaproteobacteria>Nitrosomonadales>Nitrosomonadaceae>Nitrosomonas	GCA_900106545.1	Nitrosomonas_communis	SAMN05421882_100921	53532..56969	44574	hypothetical-protein-SAMN05421882_100921-[Nitrosomonas-communis]
WP_181258202.1	Type1.BREX	[illegible]	1179	Bacteria>Pseudomonadota>Betaproteobacteria>Nitrosomonadales>Nitrosomonadaceae>Nitrosomonas	GCF_003046585.1	Nitrosomonas_aestuarii	C8R11_107137	171439..174978	52441	BREX-system-P-loop-protein-BrxC-[Nitrosomonas-aestuarii]
WP_107802981.1	Type1.BREX	[illegible]	1208	Bacteria>Pseudomonadota>Betaproteobacteria>Nitrosomonadales>Nitrosomonadaceae>Nitrosomonas	GCF_003050805.1	Nitrosomonas_oligotropha	C8R26_RS06410	43938..47564	42354	BREX-system-P-loop-protein-BrxC-[Nitrosomonas-oligotropha]
WP_107790392.1	Type1.BREX	[illegible]	1180	Bacteria>Pseudomonadota>Betaproteobacteria>Nitrosomonadales>Nitrosomonadaceae>Nitrosomonas	GCF_003051105.1	Nitrosomonas_nitrosa	C8R30_RS13785	18563..22105	52442	BREX-system-P-loop-protein-BrxC-[Nitrosomonas-nitrosa]
WP_211752264.1	Type1.BREX	[illegible]	1146	Bacteria>Pseudomonadota>Betaproteobacteria>Nitrosomonadales>Nitrosomonadaceae>Nitrosomonas	GCF_900106545.1	Nitrosomonas_communis	BLV21_RS05435	53532..56972	44574	BREX-system-P-loop-protein-BrxC-[Nitrosomonas-communis]
WP_090415704.1	Type1.BREX	[illegible]	1182	Bacteria>Pseudomonadota>Betaproteobacteria>Nitrosomonadales>Nitrosomonadaceae>Nitrosomonas	GCF_900107165.1	Nitrosomonas_halophila	BLV85_RS13200	2958..6506	44576	BREX-system-P-loop-protein-BrxC-[Nitrosomonas-halophila]
WP_204800510.1	Type1.BREX	[illegible]	1180	Bacteria>Pseudomonadota>Betaproteobacteria>Nitrosomonadales>Nitrosomonadaceae>Nitrosomonas	GCF_905220645.1	Nitrosomonas_nitrosa	NUZ5A_RS15530	137140..140682	52442	BREX-system-P-loop-protein-BrxC-[Nitrosomonas-nitrosa]
NOT17080.1	Type1.BREX	[illegible]	1202	Bacteria>Pseudomonadota>Betaproteobacteria>Nitrosomonadales>Sulfuricellaceae>Sulfuriferula	GCA_013140765.1	Sulfuriferula_sp.	HOP20_03285	21073..24681	2025307	BREX-system-P-loop-protein-BrxC-[Sulfuriferula-sp.]
ODV10849.1	Type1.BREX	[illegible]	451	Bacteria>Pseudomonadota>Betaproteobacteria>Nitrosomonadales>Thiobacillaceae>Thiobacillus	GCA_001725535.1	Thiobacillus_sp._SCN_64-317	ABT22_10740	12066..13421	1660143	MAG:-hypothetical-protein-ABT22_10740-[Thiobacillus-sp.-SCN-64-317]
MBN8763784.1	Type1.BREX	[illegible]	865	Bacteria>Pseudomonadota>Betaproteobacteria>Nitrosomonadales>Thiobacillaceae>Thiobacillus	GCA_017309235.1	Thiobacillus_sp.	JOJ02_08935	6734..9331	924	hypothetical-protein-[Thiobacillus-sp.]
OGS68620.1	Type1.BREX	[illegible]	1207	Bacteria>Pseudomonadota>Betaproteobacteria>Nitrosomonadales>unclassified_Nitrosomonadales	GCA_001801025.1	Gallionellales_bacterium_GWA2_54_124	A2Z87_08865	78784..82407	1798243	ATPase-[Gallionellales-bacterium-GWA2_54_124]
OGT18472.1	Type1.BREX	[illegible]	1154	Bacteria>Pseudomonadota>Betaproteobacteria>Nitrosomonadales>unclassified_Nitrosomonadales	GCA_001801155.1	Gallionellales_bacterium_RIFCSPHIGHO2_02_FULL_57_16	A3J49_13100	5649..9113	1798251	hypothetical-protein-A3J49_13100-[Gallionellales-bacterium-RIFCSPHIGHO2_02_FULL_57_16]
OGS98638.1	Type1.BREX	[illegible]	1207	Bacteria>Pseudomonadota>Betaproteobacteria>Nitrosomonadales>unclassified_Nitrosomonadales	GCA_001801215.1	Gallionellales_bacterium_RIFCSPLOWO2_02_FULL_59_110	A3H99_12810	1693..5316	1798255	ATPase-[Gallionellales-bacterium-RIFCSPLOWO2_02_FULL_59_110]
PIX05110.1	Type1.BREX	[illegible]	340	Bacteria>Pseudomonadota>Betaproteobacteria>Nitrosomonadales>unclassified_Nitrosomonadales	GCA_002782465.1	Gallionellales_bacterium_CG_4_8_14_3_um_filter_54_18	COZ77_02910	1..1019	1974014	BREX-system-P-loop-protein-BrxC,-partial-[Gallionellales-bacterium-CG_4_8_14_3_um_filter_54_18]
MBK9161130.1	Type1.BREX	[illegible]	1146	Bacteria>Pseudomonadota>Betaproteobacteria>Nitrosomonadales>unclassified_Nitrosomonadales	GCA_016716325.1	Nitrosomonadales_bacterium	IPM27_06155	1292283..1295723	2026761	BREX-system-P-loop-protein-BrxC-[Nitrosomonadales-bacterium]
MBL8410223.1	Type1.BREX	[illegible]	1154	Bacteria>Pseudomonadota>Betaproteobacteria>Rhodocyclales>Azonexaceae>Dechloromonas	GCA_016791065.1	Dechloromonas_sp.	JNM15_02850	32710..36174	1917218	BREX-system-P-loop-protein-BrxC-[Dechloromonas-sp.]
MBT9520412.1	Type1.BREX	[illegible]	1208	Bacteria>Pseudomonadota>Betaproteobacteria>Rhodocyclales>Azonexaceae>Dechloromonas	GCA_018780195.1	Dechloromonas_sp.	IV101_05910	54359..57985	1917218	BREX-system-P-loop-protein-BrxC-[Dechloromonas-sp.]
MCK0506222.1	Type1.BREX	[illegible]	1186	Bacteria>Pseudomonadota>Betaproteobacteria>Rhodocyclales>Rhodocyclaceae>Aromatoleum	GCA_012910705.2	Aromatoleum_anaerobium	GO606_004945	104237..107797	182180	BREX-system-P-loop-protein-BrxC-[Aromatoleum-anaerobium]
NMG55067.1	Type1.BREX	[illegible]	1189	Bacteria>Pseudomonadota>Betaproteobacteria>Rhodocyclales>Rhodocyclaceae>Aromatoleum	GCA_012910915.1	Aromatoleum_aromaticum	GPA23_10435	14208..17777	551760	BREX-system-P-loop-protein-BrxC-[Aromatoleum-aromaticum]
WP_011238768.1	Type1.BREX	[illegible]	1203	Bacteria>Pseudomonadota>Betaproteobacteria>Rhodocyclales>Rhodocyclaceae>Aromatoleum	GCF_000025965.1	Aromatoleum_aromaticum	EBN1_RS14750	3092506..3096117	551760	BREX-system-P-loop-protein-BrxC-[Aromatoleum-aromaticum]
WP_169117250.1	Type1.BREX	[illegible]	1203	Bacteria>Pseudomonadota>Betaproteobacteria>Rhodocyclales>Rhodocyclaceae>Aromatoleum	GCF_012910705.2	Aromatoleum_anaerobium	GO606_RS04945	104237..107848	182180	BREX-system-P-loop-protein-BrxC-[Aromatoleum-anaerobium]
WP_168953997.1	Type1.BREX	[illegible]	1188	Bacteria>Pseudomonadota>Betaproteobacteria>Rhodocyclales>Rhodocyclaceae>Aromatoleum	GCF_023704195.1	Aromatoleum_aromaticum	L8M16_RS08665	1808924..1812490	551760	BREX-system-P-loop-protein-BrxC-[Aromatoleum-aromaticum]
WP_205230204.1	Type1.BREX	[illegible]	1153	Bacteria>Pseudomonadota>Betaproteobacteria>Rhodocyclales>Rhodocyclaceae>Azospira	GCF_905120365.1	Azospira_oryzae	JYJ48_RS05820	17001..20462	146939	BREX-system-P-loop-protein-BrxC-[Azospira-oryzae]
MBP6421402.1	Type1.BREX	[illegible]	923	Bacteria>Pseudomonadota>Betaproteobacteria>Rhodocyclales>Rhodocyclaceae>Propionivibrio	GCA_017988215.1	Propionivibrio_sp.	KA271_00765	12654..15427	2212460	BREX-system-P-loop-protein-BrxC-[Propionivibrio-sp.]
MCQ3924618.1	Type1.BREX	[illegible]	1164	Bacteria>Pseudomonadota>Betaproteobacteria>Rhodocyclales>Rhodocyclaceae>unclassified_Rhodocyclaceae	GCA_024434155.1	Rhodocyclaceae_bacterium	DPW12_10550	16269..19763	1898103	BREX-system-P-loop-protein-BrxC-[Rhodocyclaceae-bacterium]
OHC72318.1	Type1.BREX	[illegible]	1152	Bacteria>Pseudomonadota>Betaproteobacteria>Rhodocyclales>unclassified_Rhodocyclales	GCA_001828935.1	Rhodocyclales_bacterium_RIFCSPLOWO2_02_FULL_63_24	A3H93_14585	39152..42610	1802018	MAG:-hypothetical-protein-A3H93_14585-[Rhodocyclales-bacterium-RIFCSPLOWO2_02_FULL_63_24]
WP_191660805.1	Type1.BREX	[illegible]	1204	Bacteria>Pseudomonadota>Betaproteobacteria>Rhodocyclales>Zoogloeaceae>Azoarcus	-	Azoarcus_sp._Aa7	AZOA_39690	55571..59185	2303328	BREX-system-P-loop-protein-BrxC-[Azoarcus-sp.-Aa7]
NMG49986.1	Type1.BREX	[illegible]	1189	Bacteria>Pseudomonadota>Betaproteobacteria>Rhodocyclales>Zoogloeaceae>Parazoarcus	GCA_012910825.1	Parazoarcus_communis	GO613_17970	21745..25314	41977	BREX-system-P-loop-protein-BrxC-[Parazoarcus-communis]
WP_108973925.1	Type1.BREX	[illegible]	1215	Bacteria>Pseudomonadota>Betaproteobacteria>Rhodocyclales>Zoogloeaceae>Parazoarcus	GCF_003111665.1	Parazoarcus_communis	CEW87_RS14060	3075229..3078876	41977	BREX-system-P-loop-protein-BrxC-[Parazoarcus-communis]
WP_168942691.1	Type1.BREX	[illegible]	1188	Bacteria>Pseudomonadota>Betaproteobacteria>Rhodocyclales>Zoogloeaceae>Parazoarcus	GCF_012910825.1	Parazoarcus_communis	GO613_RS17995	21748..25314	41977	BREX-system-P-loop-protein-BrxC-[Parazoarcus-communis]
TXH82461.1	Type1.BREX	[illegible]	1153	Bacteria>Pseudomonadota>Betaproteobacteria>Rhodocyclales>Zoogloeaceae>Thauera	GCA_008015255.1	Thauera_aminoaromatica	E6Q80_15435	2474..5935	164330	BREX-system-P-loop-protein-BrxC-[Thauera-aminoaromatica]
MBP6132433.1	Type1.BREX	[illegible]	1153	Bacteria>Pseudomonadota>Betaproteobacteria>Rhodocyclales>Zoogloeaceae>Thauera	GCA_017985115.1	Thauera_sp.	KA496_12935	35100..38561	1905334	BREX-system-P-loop-protein-BrxC-[Thauera-sp.]
CAH1748211.1	Type1.BREX	[illegible]	1203	Bacteria>Pseudomonadota>Betaproteobacteria>Rhodocyclales>Zoogloeaceae>Thauera	GCA_932126575.1	Thauera_sp._PIV-1	C4PIVTH_3071	3140142..3143753	204461	ATPase-like-protein-[Thauera-sp.-PIV-1]
WP_037984790.1	Type1.BREX	[illegible]	1203	Bacteria>Pseudomonadota>Betaproteobacteria>Rhodocyclales>Zoogloeaceae>Thauera	GCF_000310145.2	Thauera_sp._28	C662_RS14985	3144..6755	303682	BREX-system-P-loop-protein-BrxC-[Thauera-sp.-28]
WP_043743640.1	Type1.BREX	[illegible]	1153	Bacteria>Pseudomonadota>Betaproteobacteria>Rhodocyclales>Zoogloeaceae>Thauera	GCF_000831325.1	Thauera_sp._SWB20	PO78_RS10565	31733..35194	1572758	BREX-system-P-loop-protein-BrxC-[Thauera-sp.-SWB20]
WP_068639549.1	Type1.BREX	[illegible]	1188	Bacteria>Pseudomonadota>Betaproteobacteria>Rhodocyclales>Zoogloeaceae>Thauera	GCF_001591165.1	Thauera_butanivorans	TBU01S_RS21545	4844..8410	86174	BREX-system-P-loop-protein-BrxC-[Thauera-butanivorans]
WP_068803137.1	Type1.BREX	[illegible]	1153	Bacteria>Pseudomonadota>Betaproteobacteria>Rhodocyclales>Zoogloeaceae>Thauera	GCF_001696715.1	Thauera_phenolivorans	BB334_RS00200	55015..58476	1792543	BREX-system-P-loop-protein-BrxC-[Thauera-phenolivorans]
WP_094269325.1	Type1.BREX	[illegible]	1153	Bacteria>Pseudomonadota>Betaproteobacteria>Rhodocyclales>Zoogloeaceae>Thauera	GCF_002245655.1	Thauera_propionica	CGK74_RS15430	17351..20812	2019431	BREX-system-P-loop-protein-BrxC-[Thauera-propionica]
WP_096448538.1	Type1.BREX	[illegible]	1153	Bacteria>Pseudomonadota>Betaproteobacteria>Rhodocyclales>Zoogloeaceae>Thauera	GCF_002354895.1	Thauera_sp._K11	CCZ27_12255	2842153..2845614	2005884	BREX-system-P-loop-protein-BrxC-[Thauera-sp.-K11]
WP_107221110.1	Type1.BREX	[illegible]	1153	Bacteria>Pseudomonadota>Betaproteobacteria>Rhodocyclales>Zoogloeaceae>Thauera	GCF_003030465.1	Thauera_aromatica	Tharo_RS10350	2186270..2189731	59405	BREX-system-P-loop-protein-BrxC-[Thauera-aromatica]
WP_247724881.1	Type1.BREX	[illegible]	1153	Bacteria>Pseudomonadota>Betaproteobacteria>Rhodocyclales>Zoogloeaceae>Thauera	GCF_023093595.1	Thauera_aromatica	MXC99_RS07615	6533..9994	59405	BREX-system-P-loop-protein-BrxC-[Thauera-aromatica]
WP_247725480.1	Type1.BREX	[illegible]	1203	Bacteria>Pseudomonadota>Betaproteobacteria>Rhodocyclales>Zoogloeaceae>Thauera	GCF_023093595.1	Thauera_aromatica	MXC99_RS10910	53320..56931	59405	BREX-system-P-loop-protein-BrxC-[Thauera-aromatica]
WP_247737217.1	Type1.BREX	[illegible]	1153	Bacteria>Pseudomonadota>Betaproteobacteria>Rhodocyclales>Zoogloeaceae>Thauera	-	Thauera_aromatica	MX653_RS17175	1332..4793	59405	BREX-system-P-loop-protein-BrxC-[Thauera-aromatica]
MBK6655412.1	Type1.BREX	[illegible]	1153	Bacteria>Pseudomonadota>Betaproteobacteria>Rhodocyclales>Zoogloeaceae>Zoogloea	GCA_016703645.1	Zoogloea_sp.	IPG40_16580	748210..751671	49181	BREX-system-P-loop-protein-BrxC-[Zoogloea-sp.]
MBK7848388.1	Type1.BREX	[illegible]	1153	Bacteria>Pseudomonadota>Betaproteobacteria>Rhodocyclales>Zoogloeaceae>Zoogloea	GCA_016714095.1	Zoogloea_sp.	IPJ73_15250	34710..38171	49181	BREX-system-P-loop-protein-BrxC-[Zoogloea-sp.]
WP_141354347.1	Type1.BREX	[illegible]	1203	Bacteria>Pseudomonadota>Betaproteobacteria>Rhodocyclales>Zoogloeaceae>Zoogloea	GCF_006539865.1	Zoogloea_ramigera	ZRA01_RS16820	2482..6093	350	BREX-system-P-loop-protein-BrxC-[Zoogloea-ramigera]
WP_148578091.1	Type1.BREX	[illegible]	1186	Bacteria>Pseudomonadota>Betaproteobacteria>Rhodocyclales>Zoogloeaceae>Zoogloea	GCF_008107625.1	Zoogloea_oleivorans	ETQ85_RS05700	193093..196653	1552750	BREX-system-P-loop-protein-BrxC-[Zoogloea-oleivorans]
WP_148578351.1	Type1.BREX	[illegible]	1146	Bacteria>Pseudomonadota>Betaproteobacteria>Rhodocyclales>Zoogloeaceae>Zoogloea	GCF_008107625.1	Zoogloea_oleivorans	ETQ85_RS07180	242245..245685	1552750	BREX-system-P-loop-protein-BrxC-[Zoogloea-oleivorans]
OQA32553.1	Type1.BREX	[illegible]	1140	Bacteria>Pseudomonadota>Betaproteobacteria>unclassified_Betaproteobacteria	GCA_002068855.1	Betaproteobacteria_bacterium_ADurb.Bin341	BWY57_01861	6243..9665	1852821	hypothetical-protein-BWY57_01861-[Betaproteobacteria-bacterium-ADurb.Bin341]
PKO80619.1	Type1.BREX	[illegible]	1153	Bacteria>Pseudomonadota>Betaproteobacteria>unclassified_Betaproteobacteria	GCA_002842325.1	Betaproteobacteria_bacterium_HGW-Betaproteobacteria-13	CVU19_11375	4671..8132	2013704	BREX-system-P-loop-protein-BrxC-[Betaproteobacteria-bacterium-HGW-Betaproteobacteria-13]
PKO87722.1	Type1.BREX	[illegible]	1149	Bacteria>Pseudomonadota>Betaproteobacteria>unclassified_Betaproteobacteria	GCA_002842415.1	Betaproteobacteria_bacterium_HGW-Betaproteobacteria-10	CVU16_15240	6333..9782	2013701	BREX-system-P-loop-protein-BrxC-[Betaproteobacteria-bacterium-HGW-Betaproteobacteria-10]
MBI3372694.1	Type1.BREX	[illegible]	1148	Bacteria>Pseudomonadota>Betaproteobacteria>unclassified_Betaproteobacteria	GCA_016195915.1	Betaproteobacteria_bacterium	HY017_13195	52819..56265	1891241	BREX-system-P-loop-protein-BrxC-[Betaproteobacteria-bacterium]
WP_219254923.1	Type1.BREX	[illegible]	1226	Bacteria>Pseudomonadota>Gammaproteobacteria>Aeromonadales>Aeromonadaceae>Aeromonas	-	Aeromonas_hydrophila	GPV00_14065	286470..290150	644	BREX-system-P-loop-protein-BrxC-[Aeromonas-hydrophila]
AUZ77883.1	Type1.BREX	[illegible]	1183	Bacteria>Pseudomonadota>Gammaproteobacteria>Aeromonadales>Aeromonadaceae>Aeromonas	GCA_002934425.1	Aeromonas_sp._ASNIH4	C2U40_16585	3601638..3605189	1636609	BREX-system-P-loop-protein-BrxC-[Aeromonas-sp.-ASNIH4]
PZQ93954.1	Type1.BREX	[illegible]	1218	Bacteria>Pseudomonadota>Gammaproteobacteria>Aeromonadales>Aeromonadaceae>Aeromonas	GCA_003243215.1	Aeromonas_media	DI541_18955	3274..6930	651	BREX-system-P-loop-protein-BrxC-[Aeromonas-media]
BBR09393.1	Type1.BREX	[illegible]	1226	Bacteria>Pseudomonadota>Gammaproteobacteria>Aeromonadales>Aeromonadaceae>Aeromonas	GCA_014158455.1	Aeromonas_caviae	WP3S18E02_10540	1118346..1122026	648	hypothetical-protein-WP3S18E02_10540-[Aeromonas-caviae]
HAU4888102.1	Type1.BREX	[illegible]	1231	Bacteria>Pseudomonadota>Gammaproteobacteria>Aeromonadales>Aeromonadaceae>Aeromonas	GCA_016067875.1	Aeromonas_hydrophila	OD804_RS12725	3923..7618	644	BREX-system-P-loop-protein-BrxC-[Aeromonas-hydrophila]
EGX6956813.1	Type1.BREX	[illegible]	1218	Bacteria>Pseudomonadota>Gammaproteobacteria>Aeromonadales>Aeromonadaceae>Aeromonas	GCA_016414985.1	Aeromonas_hydrophila	JFQ85_000577	650804..654460	644	BREX-system-P-loop-protein-BrxC-[Aeromonas-hydrophila]
MBS4697267.1	Type1.BREX	[illegible]	1226	Bacteria>Pseudomonadota>Gammaproteobacteria>Aeromonadales>Aeromonadaceae>Aeromonas	GCA_018359965.1	Aeromonas_allosaccharophila	J4G65_17610	87511..91191	656	BREX-system-P-loop-protein-BrxC-[Aeromonas-allosaccharophila]
MBW3845327.1	Type1.BREX	[illegible]	1226	Bacteria>Pseudomonadota>Gammaproteobacteria>Aeromonadales>Aeromonadaceae>Aeromonas	GCA_019348915.1	Aeromonas_hydrophila	GPV00_14065	286470..290150	644	BREX-system-P-loop-protein-BrxC-[Aeromonas-hydrophila]
MBX9561629.1	Type1.BREX	[illegible]	1157	Bacteria>Pseudomonadota>Gammaproteobacteria>Aeromonadales>Aeromonadaceae>Aeromonas	GCA_019742455.1	Aeromonas_hydrophila	FKF73_02860	531067..534540	644	BREX-system-P-loop-protein-BrxC-[Aeromonas-hydrophila]
GJA32139.1	Type1.BREX	[illegible]	1227	Bacteria>Pseudomonadota>Gammaproteobacteria>Aeromonadales>Aeromonadaceae>Aeromonas	GCA_019971155.1	Aeromonas_caviae	KAM347_17720	32751..36434	648	hypothetical-protein-KAM341_18170-[Aeromonas-caviae]
GJA65768.1	Type1.BREX	[illegible]	927	Bacteria>Pseudomonadota>Gammaproteobacteria>Aeromonadales>Aeromonadaceae>Aeromonas	GCA_019971515.1	Aeromonas_caviae	KAM351_43790	4448..7231	648	hypothetical-protein-KAM351_43790-[Aeromonas-caviae]

Protein-ID	System	Gene-Neighbourhoods	Length	Taxonomy	Assembly-ID	Strain	Locus-Tag	Genomic-coordinates	TaxID	Fasta-define
BDO08311.1	Type1.BREX	[illegible]	1089	Bacteria>Pseudomonadota>Gammaproteobacteria>Aeromonadales>Aeromonadaceae>Aeromonas	GCA_024347775.1	Aeromonas_caviae	KAM643c_18840	2028863..2032132	648	hypothetical-protein-KAM643c_18840-[Aeromonas-caviae]
EJN6954191.1	Type1.BREX	[illegible]	1157	Bacteria>Pseudomonadota>Gammaproteobacteria>Aeromonadales>Aeromonadaceae>Aeromonas	GCA_024506355.1	Aeromonas_hydrophila	NRD12_000732	206979..210452	644	BREX-system-P-loop-protein-BrxC-[Aeromonas-hydrophila]
WP_010634561.1	Type1.BREX	[illegible]	1157	Bacteria>Pseudomonadota>Gammaproteobacteria>Aeromonadales>Aeromonadaceae>Aeromonas	GCF_000315195.1	Aeromonas_dhakensis	AQU_RS10070	136801..140274	196024	BREX-system-P-loop-protein-BrxC-[Aeromonas-dhakensis]
WP_043124789.1	Type1.BREX	[illegible]	1157	Bacteria>Pseudomonadota>Gammaproteobacteria>Aeromonadales>Aeromonadaceae>Aeromonas	GCF_000724965.1	Aeromonas_hydrophila	HR52_RS13935	3102820..3106293	644	BREX-system-P-loop-protein-BrxC-[Aeromonas-hydrophila]
WP_043557612.1	Type1.BREX	[illegible]	1182	Bacteria>Pseudomonadota>Gammaproteobacteria>Aeromonadales>Aeromonadaceae>Aeromonas	GCF_000819745.1	Aeromonas_bestiarum	BN1118_RS19650	69736..73284	105751	BREX-system-P-loop-protein-BrxC-[Aeromonas-bestiarum]
WP_042011559.1	Type1.BREX	[illegible]	1239	Bacteria>Pseudomonadota>Gammaproteobacteria>Aeromonadales>Aeromonadaceae>Aeromonas	GCF_000819885.1	Aeromonas_fluvialis	BN1125_RS07645	79425..83144	591962	BREX-system-P-loop-protein-BrxC-[Aeromonas-fluvialis]
WP_059169685.1	Type1.BREX	[illegible]	1240	Bacteria>Pseudomonadota>Gammaproteobacteria>Aeromonadales>Aeromonadaceae>Aeromonas	GCF_001481535.2	Aeromonas_salmonicida	VO70_RS19205	18975..22697	645	BREX-system-P-loop-protein-BrxC-[Aeromonas-salmonicida]
WP_087756130.1	Type1.BREX	[illegible]	1231	Bacteria>Pseudomonadota>Gammaproteobacteria>Aeromonadales>Aeromonadaceae>Aeromonas	GCF_002180335.1	Aeromonas_salmonicida	O23A_RS10590	2337193..2340888	645	BREX-system-P-loop-protein-BrxC-[Aeromonas-salmonicida]
WP_095593101.1	Type1.BREX	[illegible]	1231	Bacteria>Pseudomonadota>Gammaproteobacteria>Aeromonadales>Aeromonadaceae>Aeromonas	GCF_002285935.1	Aeromonas_dhakensis	CK627_RS18865	4072109..4075804	196024	BREX-system-P-loop-protein-BrxC-[Aeromonas-dhakensis]
WP_101617899.1	Type1.BREX	[illegible]	1231	Bacteria>Pseudomonadota>Gammaproteobacteria>Aeromonadales>Aeromonadaceae>Aeromonas	GCF_002850695.3	Aeromonas_hydrophila	C1A23_RS07645	1215070..1218765	644	BREX-system-P-loop-protein-BrxC-[Aeromonas-hydrophila]
WP_113721905.1	Type1.BREX	[illegible]	1157	Bacteria>Pseudomonadota>Gammaproteobacteria>Aeromonadales>Aeromonadaceae>Aeromonas	GCF_003265515.1	Aeromonas_salmonicida	CPA05_RS19945	20643..24116	645	BREX-system-P-loop-protein-BrxC-[Aeromonas-salmonicida]
WP_113995752.1	Type1.BREX	[illegible]	1157	Bacteria>Pseudomonadota>Gammaproteobacteria>Aeromonadales>Aeromonadaceae>Aeromonas	GCF_003323285.1	Aeromonas_hydrophila	C6C11_RS19255	2250..5723	644	BREX-system-P-loop-protein-BrxC-[Aeromonas-hydrophila]
WP_114523382.1	Type1.BREX	[illegible]	1157	Bacteria>Pseudomonadota>Gammaproteobacteria>Aeromonadales>Aeromonadaceae>Aeromonas	GCF_003344485.1	Aeromonas_sp._ARM81	ASJ36_RS21100	4447..7920	1747384	BREX-system-P-loop-protein-BrxC-[Aeromonas-sp.-ARM81]
WP_120415687.1	Type1.BREX	[illegible]	1231	Bacteria>Pseudomonadota>Gammaproteobacteria>Aeromonadales>Aeromonadaceae>Aeromonas	GCF_003611985.1	Aeromonas_veronii	D6R50_RS16910	827539..831234	654	BREX-system-P-loop-protein-BrxC-[Aeromonas-veronii]
WP_123173885.1	Type1.BREX	[illegible]	1231	Bacteria>Pseudomonadota>Gammaproteobacteria>Aeromonadales>Aeromonadaceae>Aeromonas	GCF_003722175.1	Aeromonas_veronii	EFI48_RS19250	4187979..4191674	654	BREX-system-P-loop-protein-BrxC-[Aeromonas-veronii]
WP_128297388.1	Type1.BREX	[illegible]	1225	Bacteria>Pseudomonadota>Gammaproteobacteria>Aeromonadales>Aeromonadaceae>Aeromonas	GCF_004024195.1	Aeromonas_caviae	DN612_RS19420	2423..6100	648	BREX-system-P-loop-protein-BrxC-[Aeromonas-caviae]
WP_240039238.1	Type1.BREX	[illegible]	1280	Bacteria>Pseudomonadota>Gammaproteobacteria>Aeromonadales>Aeromonadaceae>Aeromonas	GCF_006243275.1	Aeromonas_hydrophila	CF138_RS14425	159803..163645	644	BREX-system-P-loop-protein-BrxC-[Aeromonas-hydrophila]
WP_160837717.1	Type1.BREX	[illegible]	1231	Bacteria>Pseudomonadota>Gammaproteobacteria>Aeromonadales>Aeromonadaceae>Aeromonas	GCF_009831085.1	Aeromonas_caviae	GR302_RS08560	7464..11159	648	BREX-system-P-loop-protein-BrxC-[Aeromonas-caviae]
WP_159144201.1	Type1.BREX	[illegible]	1231	Bacteria>Pseudomonadota>Gammaproteobacteria>Aeromonadales>Aeromonadaceae>Aeromonas	GCF_009834065.1	Aeromonas_veronii	GRF56_RS18105	3933894..3937589	654	BREX-system-P-loop-protein-BrxC-[Aeromonas-veronii]
WP_171280306.1	Type1.BREX	[illegible]	1157	Bacteria>Pseudomonadota>Gammaproteobacteria>Aeromonadales>Aeromonadaceae>Aeromonas	GCF_013085505.1	Aeromonas_sp._2692-1	E5E97_RS12155	2531425..2534898	2560029	BREX-system-P-loop-protein-BrxC-[Aeromonas-sp.-2692-1]
WP_232090117.1	Type1.BREX	[illegible]	1281	Bacteria>Pseudomonadota>Gammaproteobacteria>Aeromonadales>Aeromonadaceae>Aeromonas	GCF_014158455.1	Aeromonas_caviae	H7X73_RS05535	1118181..1122026	648	BREX-system-P-loop-protein-BrxC-[Aeromonas-caviae]
WP_182951579.1	Type1.BREX	[illegible]	1157	Bacteria>Pseudomonadota>Gammaproteobacteria>Aeromonadales>Aeromonadaceae>Aeromonas	GCF_014168615.1	Aeromonas_sp._WP2-W18-CRE-05	WP2W18C05_13360	1426228..1429701	2675707	BREX-system-P-loop-protein-BrxC-[Aeromonas-sp.-WP2-W18-CRE-05]
WP_190283730.1	Type1.BREX	[illegible]	1226	Bacteria>Pseudomonadota>Gammaproteobacteria>Aeromonadales>Aeromonadaceae>Aeromonas	GCF_014679585.1	Aeromonas_hydrophila	IFA22_RS17960	3928101..3931781	644	BREX-system-P-loop-protein-BrxC-[Aeromonas-hydrophila]
WP_193000848.1	Type1.BREX	[illegible]	1226	Bacteria>Pseudomonadota>Gammaproteobacteria>Aeromonadales>Aeromonadaceae>Aeromonas	GCF_014892695.1	Aeromonas_simiae	FE240_RS10745	2272968..2276648	218936	BREX-system-P-loop-protein-BrxC-[Aeromonas-simiae]
WP_201964470.1	Type1.BREX	[illegible]	432	Bacteria>Pseudomonadota>Gammaproteobacteria>Aeromonadales>Aeromonadaceae>Aeromonas	GCF_016729105.1	Aeromonas_caviae	JD512_RS20845	12456..13754	648	BREX-system-P-loop-protein-BrxC-[Aeromonas-caviae]
WP_201944276.1	Type1.BREX	[illegible]	1231	Bacteria>Pseudomonadota>Gammaproteobacteria>Aeromonadales>Aeromonadaceae>Aeromonas	GCF_016729595.1	Aeromonas_veronii	JD494_RS16545	22676..26371	654	BREX-system-P-loop-protein-BrxC-[Aeromonas-veronii]
WP_201932781.1	Type1.BREX	[illegible]	1226	Bacteria>Pseudomonadota>Gammaproteobacteria>Aeromonadales>Aeromonadaceae>Aeromonas	GCF_016729935.1	Aeromonas_caviae	JD546_RS01995	64762..68442	648	BREX-system-P-loop-protein-BrxC-[Aeromonas-caviae]
WP_202047008.1	Type1.BREX	[illegible]	1226	Bacteria>Pseudomonadota>Gammaproteobacteria>Aeromonadales>Aeromonadaceae>Aeromonas	GCF_016730065.1	Aeromonas_media	JD497_RS12375	531279..534959	651	BREX-system-P-loop-protein-BrxC-[Aeromonas-media]
WP_203130882.1	Type1.BREX	[illegible]	935	Bacteria>Pseudomonadota>Gammaproteobacteria>Aeromonadales>Aeromonadaceae>Aeromonas	GCF_016805325.1	Aeromonas_jandaei	GNZ06_RS06060	150251..153058	650	BREX-system-P-loop-protein-BrxC-[Aeromonas-jandaei]
WP_203130883.1	Type1.BREX	[illegible]	226	Bacteria>Pseudomonadota>Gammaproteobacteria>Aeromonadales>Aeromonadaceae>Aeromonas	GCF_016805325.1	Aeromonas_jandaei	GNZ06_RS06065	153058..153738	650	hypothetical-protein-[Aeromonas-jandaei]
WP_203133261.1	Type1.BREX	[illegible]	1157	Bacteria>Pseudomonadota>Gammaproteobacteria>Aeromonadales>Aeromonadaceae>Aeromonas	GCF_016805365.1	Aeromonas_jandaei	G4224_RS08640	376570..380043	650	BREX-system-P-loop-protein-BrxC-[Aeromonas-jandaei]
WP_210557479.1	Type1.BREX	[illegible]	1218	Bacteria>Pseudomonadota>Gammaproteobacteria>Aeromonadales>Aeromonadaceae>Aeromonas	GCF_017939245.1	Aeromonas_sp._PrichA-15	J9889_RS17255	4362..8018	2823360	BREX-system-P-loop-protein-BrxC-[Aeromonas-sp.-PrichA-15]
WP_213387487.1	Type1.BREX	[illegible]	1157	Bacteria>Pseudomonadota>Gammaproteobacteria>Aeromonadales>Aeromonadaceae>Aeromonas	GCF_018359765.1	Aeromonas_veronii	J4G64_RS11995	290568..294041	654	BREX-system-P-loop-protein-BrxC-[Aeromonas-veronii]
WP_244842130.1	Type1.BREX	[illegible]	1281	Bacteria>Pseudomonadota>Gammaproteobacteria>Aeromonadales>Aeromonadaceae>Aeromonas	GCF_018359965.1	Aeromonas_allosaccharophila	J4G65_RS17605	87511..91356	656	BREX-system-P-loop-protein-BrxC-[Aeromonas-allosaccharophila]
WP_215805224.1	Type1.BREX	[illegible]	1157	Bacteria>Pseudomonadota>Gammaproteobacteria>Aeromonadales>Aeromonadaceae>Aeromonas	GCF_018802365.1	Aeromonas_jandaei	HQ398_RS10760	2353585..2357058	650	BREX-system-P-loop-protein-BrxC-[Aeromonas-jandaei]
WP_216986928.1	Type1.BREX	[illegible]	1157	Bacteria>Pseudomonadota>Gammaproteobacteria>Aeromonadales>Aeromonadaceae>Aeromonas	GCF_019048565.1	Aeromonas_sp._FDAARGOS_1406	I6L36_RS00705	137070..140543	2778055	BREX-system-P-loop-protein-BrxC-[Aeromonas-sp.-FDAARGOS-1406]
WP_216990395.1	Type1.BREX	[illegible]	1157	Bacteria>Pseudomonadota>Gammaproteobacteria>Aeromonadales>Aeromonadaceae>Aeromonas	GCF_019048705.1	Aeromonas_sp._FDAARGOS_1409	I6L39_RS17660	3801324..3804797	2778058	BREX-system-P-loop-protein-BrxC-[Aeromonas-sp.-FDAARGOS-1409]
WP_216992842.1	Type1.BREX	[illegible]	1157	Bacteria>Pseudomonadota>Gammaproteobacteria>Aeromonadales>Aeromonadaceae>Aeromonas	GCF_019048725.1	Aeromonas_sp._FDAARGOS_1407	I6L37_20730	4426496..4429969	2778056	BREX-system-P-loop-protein-BrxC-[Aeromonas-sp.-FDAARGOS-1407]
WP_203133976.1	Type1.BREX	[illegible]	1156	Bacteria>Pseudomonadota>Gammaproteobacteria>Aeromonadales>Aeromonadaceae>Aeromonas	GCF_019348745.1	Aeromonas_hydrophila	PVL97_RS11265	2527654..2531124	644	BREX-system-P-loop-protein-BrxC-[Aeromonas-hydrophila]
WP_257621860.1	Type1.BREX	[illegible]	1281	Bacteria>Pseudomonadota>Gammaproteobacteria>Aeromonadales>Aeromonadaceae>Aeromonas	GCF_019348915.1	Aeromonas_hydrophila	GPV00_RS14000	286470..290315	644	BREX-system-P-loop-protein-BrxC-[Aeromonas-hydrophila]
WP_223938541.1	Type1.BREX	[illegible]	1157	Bacteria>Pseudomonadota>Gammaproteobacteria>Aeromonadales>Aeromonadaceae>Aeromonas	GCF_019971155.1	Aeromonas_caviae	LDN24_RS08690	33954..37427	648	BREX-system-P-loop-protein-BrxC-[Aeromonas-caviae]
WP_223931722.1	Type1.BREX	[illegible]	1231	Bacteria>Pseudomonadota>Gammaproteobacteria>Aeromonadales>Aeromonadaceae>Aeromonas	GCF_019972495.1	Aeromonas_caviae	LDN23_RS02850	18313..22008	648	BREX-system-P-loop-protein-BrxC-[Aeromonas-caviae]
WP_234578020.1	Type1.BREX	[illegible]	1231	Bacteria>Pseudomonadota>Gammaproteobacteria>Aeromonadales>Aeromonadaceae>Aeromonas	GCF_021440865.1	Aeromonas_salmonicida	LZ632_RS15270	23400..27095	645	BREX-system-P-loop-protein-BrxC-[Aeromonas-salmonicida]
WP_235462874.1	Type1.BREX	[illegible]	1157	Bacteria>Pseudomonadota>Gammaproteobacteria>Aeromonadales>Aeromonadaceae>Aeromonas	GCF_021538905.1	Aeromonas_australiensis	EHZ86_RS12350	8704..12177	1114880	BREX-system-P-loop-protein-BrxC-[Aeromonas-australiensis]
WP_236274804.1	Type1.BREX	[illegible]	1231	Bacteria>Pseudomonadota>Gammaproteobacteria>Aeromonadales>Aeromonadaceae>Aeromonas	GCF_021609985.1	Aeromonas_caviae	L1871_RS17025	3638140..3641835	648	BREX-system-P-loop-protein-BrxC-[Aeromonas-caviae]
WP_139392567.1	Type1.BREX	[illegible]	1225	Bacteria>Pseudomonadota>Gammaproteobacteria>Aeromonadales>Aeromonadaceae>Aeromonas	GCF_021654355.1	Aeromonas_hydrophila	L6424_RS03515	743150..746827	644	BREX-system-P-loop-protein-BrxC-[Aeromonas-hydrophila]
WP_241325388.1	Type1.BREX	[illegible]	1157	Bacteria>Pseudomonadota>Gammaproteobacteria>Aeromonadales>Aeromonadaceae>Aeromonas	GCF_022549295.1	Aeromonas_sp._MR7	MM192_RS09020	75622..79095	2923419	BREX-system-P-loop-protein-BrxC-[Aeromonas-sp.-MR7]
WP_241871100.1	Type1.BREX	[illegible]	1157	Bacteria>Pseudomonadota>Gammaproteobacteria>Aeromonadales>Aeromonadaceae>Aeromonas	GCF_022631175.1	Aeromonas_hydrophila	MOO40_RS17050	3752340..3755813	644	BREX-system-P-loop-protein-BrxC-[Aeromonas-hydrophila]
WP_247824022.1	Type1.BREX	[illegible]	1157	Bacteria>Pseudomonadota>Gammaproteobacteria>Aeromonadales>Aeromonadaceae>Aeromonas	GCF_023100745.1	Aeromonas_veronii	MYF86_09055	1896227..1899700	654	BREX-system-P-loop-protein-BrxC-[Aeromonas-veronii]
WP_252657151.1	Type1.BREX	[illegible]	1231	Bacteria>Pseudomonadota>Gammaproteobacteria>Aeromonadales>Aeromonadaceae>Aeromonas	GCF_023958325.1	Aeromonas_veronii	NG896_RS17505	21137..24832	654	BREX-system-P-loop-protein-BrxC-[Aeromonas-veronii]
WP_261925329.1	Type1.BREX	[illegible]	1089	Bacteria>Pseudomonadota>Gammaproteobacteria>Aeromonadales>Aeromonadaceae>Aeromonas	GCF_024347775.1	Aeromonas_caviae	OCX50_RS09760	2028863..2032132	648	BREX-system-P-loop-protein-BrxC-[Aeromonas-caviae]
WP_256683847.1	Type1.BREX	[illegible]	991	Bacteria>Pseudomonadota>Gammaproteobacteria>Aeromonadales>Aeromonadaceae>Aeromonas	GCF_024508255.1	Aeromonas_salmonicida	NP805_RS12070	2625523..2628498	645	BREX-system-P-loop-protein-BrxC-[Aeromonas-salmonicida]
WP_257706959.1	Type1.BREX	[illegible]	1182	Bacteria>Pseudomonadota>Gammaproteobacteria>Aeromonadales>Aeromonadaceae>Aeromonas	GCF_024649325.1	Aeromonas_caviae	NUK33_RS01495	150155..153703	648	BREX-system-P-loop-protein-BrxC-[Aeromonas-caviae]
WP_257688091.1	Type1.BREX	[illegible]	1231	Bacteria>Pseudomonadota>Gammaproteobacteria>Aeromonadales>Aeromonadaceae>Aeromonas	GCF_024649335.1	Aeromonas_veronii	NUK45_RS19510	3671..7366	654	BREX-system-P-loop-protein-BrxC-[Aeromonas-veronii]
WP_257712004.1	Type1.BREX	[illegible]	1281	Bacteria>Pseudomonadota>Gammaproteobacteria>Aeromonadales>Aeromonadaceae>Aeromonas	GCF_024649525.1	Aeromonas_caviae	NUK31_RS14230	17564..21409	648	BREX-system-P-loop-protein-BrxC-[Aeromonas-caviae]
WP_264787731.1	Type1.BREX	[illegible]	1157	Bacteria>Pseudomonadota>Gammaproteobacteria>Aeromonadales>Aeromonadaceae>Aeromonas	GCF_026000055.1	Aeromonas_caviae	OPI30_RS10860	2213781..2217254	648	BREX-system-P-loop-protein-BrxC-[Aeromonas-caviae]
WP_265455065.1	Type1.BREX	[illegible]	1231	Bacteria>Pseudomonadota>Gammaproteobacteria>Aeromonadales>Aeromonadaceae>Aeromonas	GCF_026174125.1	Aeromonas_salmonicida	ORM31_RS04470	132358..136053	645	BREX-system-P-loop-protein-BrxC-[Aeromonas-salmonicida]
WP_265776110.1	Type1.BREX	[illegible]	1231	Bacteria>Pseudomonadota>Gammaproteobacteria>Aeromonadales>Aeromonadaceae>Aeromonas	GCF_026230455.1	Aeromonas_veronii	OD804_RS12725	3923..7618	654	BREX-system-P-loop-protein-BrxC-[Aeromonas-veronii]
WP_265783524.1	Type1.BREX	[illegible]	1235	Bacteria>Pseudomonadota>Gammaproteobacteria>Aeromonadales>Aeromonadaceae>Aeromonas	GCF_026230585.1	Aeromonas_veronii	OD810_RS03320	107717..111424	654	BREX-system-P-loop-protein-BrxC-[Aeromonas-veronii]
WP_270670318.1	Type1.BREX	[illegible]	1157	Bacteria>Pseudomonadota>Gammaproteobacteria>Aeromonadales>Aeromonadaceae>Aeromonas	GCF_027705165.1	Aeromonas_sp._QDB04	OKC37_RS13865	9577..13050	2990477	BREX-system-P-loop-protein-BrxC-[Aeromonas-sp.-QDB04]
WP_270698108.1	Type1.BREX	[illegible]	1280	Bacteria>Pseudomonadota>Gammaproteobacteria>Aeromonadales>Aeromonadaceae>Aeromonas	GCF_027705185.1	Aeromonas_sp._QDB05	OKC32_RS09690	4766..8608	2990478	BREX-system-P-loop-protein-BrxC-[Aeromonas-sp.-QDB05]
WP_270661186.1	Type1.BREX	[illegible]	1182	Bacteria>Pseudomonadota>Gammaproteobacteria>Aeromonadales>Aeromonadaceae>Aeromonas	GCF_027705205.1	unclassified_Aeromonas	OKC30_RS15245	6003..9551	257493	MULTISPECIES:-BREX-system-P-loop-protein-BrxC-[unclassified-Aeromonas]
WP_270793601.1	Type1.BREX	[illegible]	1226	Bacteria>Pseudomonadota>Gammaproteobacteria>Aeromonadales>Aeromonadaceae>Aeromonas	GCF_027705385.1	unclassified_Aeromonas	OKB76_RS13245	1893..5573	257493	MULTISPECIES:-BREX-system-P-loop-protein-BrxC-[unclassified-Aeromonas]
WP_270805301.1	Type1.BREX	[illegible]	1240	Bacteria>Pseudomonadota>Gammaproteobacteria>Aeromonadales>Aeromonadaceae>Aeromonas	GCF_027705505.1	Aeromonas_sp._QDB56	OKB81_RS14905	8131..11853	2990495	BREX-system-P-loop-protein-BrxC-[Aeromonas-sp.-QDB56]
WP_270809144.1	Type1.BREX	[illegible]	1157	Bacteria>Pseudomonadota>Gammaproteobacteria>Aeromonadales>Aeromonadaceae>Aeromonas	GCF_027705685.1	Aeromonas_sp._QDB08	OKB79_RS12620	11744..15217	2990480	BREX-system-P-loop-protein-BrxC-[Aeromonas-sp.-QDB08]
WP_270659881.1	Type1.BREX	[illegible]	1157	Bacteria>Pseudomonadota>Gammaproteobacteria>Aeromonadales>Aeromonadaceae>Aeromonas	GCF_027705965.1	Aeromonas_sp._Y301-2	OKB83_RS15925	143279..146752	2990505	BREX-system-P-loop-protein-BrxC-[Aeromonas-sp.-Y301-2]
WP_275056847.1	Type1.BREX	[illegible]	1231	Bacteria>Pseudomonadota>Gammaproteobacteria>Aeromonadales>Aeromonadaceae>Aeromonas	GCF_029024985.1	Aeromonas_allosaccharophila	PYU98_RS18280	3929296..3932991	656	BREX-system-P-loop-protein-BrxC-[Aeromonas-allosaccharophila]
WP_076362312.1	Type1.BREX	[illegible]	1231	Bacteria>Pseudomonadota>Gammaproteobacteria>Aeromonadales>Aeromonadaceae>Aeromonas	GCF_900155975.1	Aeromonas_hydrophila	BW963_RS17620	135140..138835	644	BREX-system-P-loop-protein-BrxC-[Aeromonas-hydrophila]
WP_050664996.1	Type1.BREX	[illegible]	1231	Bacteria>Pseudomonadota>Gammaproteobacteria>Aeromonadales>Aeromonadaceae	GCF_000820105.1	Aeromonas	BN1133_RS02615	38531..42226	642	MULTISPECIES:-BREX-system-P-loop-protein-BrxC-[Aeromonas]
WP_181014924.1	Type1.BREX	[illegible]	1182	Bacteria>Pseudomonadota>Gammaproteobacteria>Aeromonadales>Aeromonadaceae	GCF_002934425.1	Aeromonas	C2U40_RS16580	3601638..3605186	642	MULTISPECIES:-BREX-system-P-loop-protein-BrxC-[Aeromonas]
WP_182788683.1	Type1.BREX	[illegible]	1236	Bacteria>Pseudomonadota>Gammaproteobacteria>Aeromonadales>Aeromonadaceae	GCF_014161425.1	Aeromonas	H7K58_RS09750	2083810..2087520	642	MULTISPECIES:-BREX-system-P-loop-protein-BrxC-[Aeromonas]
WP_223915718.1	Type1.BREX	[illegible]	1218	Bacteria>Pseudomonadota>Gammaproteobacteria>Aeromonadales>Aeromonadaceae	GCF_019972515.1	Aeromonas	LDN24_RS09390	22833..26489	642	MULTISPECIES:-BREX-system-P-loop-protein-BrxC-[Aeromonas]
WP_201934227.1	Type1.BREX	[illegible]	1231	Bacteria>Pseudomonadota>Gammaproteobacteria>Aeromonadales>Aeromonadaceae	GCF_019972675.1	Aeromonas	MWG22_RS17445	1818..5513	642	MULTISPECIES:-BREX-system-P-loop-protein-BrxC-[Aeromonas]
WP_171273737.1	Type1.BREX	[illegible]	1231	Bacteria>Pseudomonadota>Gammaproteobacteria>Aeromonadales>Aeromonadaceae	GCF_021654375.1	Aeromonas	E4185_RS15845	3355159..3358854	642	MULTISPECIES:-BREX-system-P-loop-protein-BrxC-[Aeromonas]
WP_234605103.1	Type1.BREX	[illegible]	1231	Bacteria>Pseudomonadota>Gammaproteobacteria>Aeromonadales>Aeromonadaceae	GCF_026173065.1	Aeromonas	ORM39_RS00835	21547..25242	642	BREX-system-P-loop-protein-BrxC-[Aeromonas-salmonicida]
WP_270796656.1	Type1.BREX	[illegible]	1157	Bacteria>Pseudomonadota>Gammaproteobacteria>Aeromonadales>Aeromonadaceae	GCF_029024985.1	Aeromonas	OKC40_RS19420	7580..11053	642	MULTISPECIES:-BREX-system-P-loop-protein-BrxC-[Aeromonas]
WP_069785631.1	Type1.BREX	[illegible]	1157	Bacteria>Pseudomonadota>Gammaproteobacteria>Aeromonadales>Aeromonadaceae	GCF_029773025.1	Aeromonas	QBS29_RS17430	3038195..3041668	642	MULTISPECIES:-BREX-system-P-loop-protein-BrxC-[Aeromonas]
WP_019935798.1	Type1.BREX	[illegible]	1146	Bacteria>Pseudomonadota>Gammaproteobacteria>Aeromonadales>Aeromonadaceae>Oceanimonas	GCF_000381965.1	Oceanimonas_smirnovii	G364_RS0114130	4273..7713	264574	BREX-system-P-loop-protein-BrxC-[Oceanimonas-smirnovii]
WP_228212351.1	Type1.BREX	[illegible]	1221	Bacteria>Pseudomonadota>Gammaproteobacteria>Aeromonadales>Aeromonadaceae>Oceanimonas	GCF_020781865.1	Oceanimonas_baumannii	LL240_RS06775	52784..56449	129578	BREX-system-P-loop-protein-BrxC-[Oceanimonas-baumannii]
MBP8039255.1	Type1.BREX	[illegible]	1043	Bacteria>Pseudomonadota>Gammaproteobacteria>Aeromonadales>Aeromonadaceae>unclassified_Aeromonadaceae	GCA_018004635.1	Aeromonadaceae_bacterium	KAY67_01800	1..3131	1948889	BREX-system-P-loop-protein-BrxC-[Aeromonadaceae-bacterium]
WP_019002750.1	Type1.BREX	[illegible]	1210	Bacteria>Pseudomonadota>Gammaproteobacteria>Aeromonadales>Succinivibrionaceae>Succinimonas	GCF_000378405.1	Succinimonas_amylolytica	F557_RS20035	169312..172944	83769	BREX-system-P-loop-protein-BrxC-[Succinimonas-amylolytica]
WP_219938189.1	Type1.BREX	[illegible]	1210	Bacteria>Pseudomonadota>Gammaproteobacteria>Aeromonadales>Succinivibrionaceae>Succinivibrio	GCF_019448115.1	Succinivibrio_faecicola	J5V48_RS08685	2494..6126	2820300	BREX-system-P-loop-protein-BrxC-[Succinivibrio-faecicola]
MCR4553833.1	Type1.BREX	[illegible]	1184	Bacteria>Pseudomonadota>Gammaproteobacteria>Aeromonadales>Succinivibrionaceae>unclassified_Succinivibrionaceae	GCA_024683965.1	Succinivibrionaceae_bacterium	K5752_02815	10310..13864	2053620	BREX-system-P-loop-protein-BrxC-[Succinivibrionaceae-bacterium]
WP_163133170.1	Type1.BREX	[illegible]	1160	Bacteria>Pseudomonadota>Gammaproteobacteria>Alteromonadales>Alteromonadaceae>Agarivorans	GCF_900537085.1	Agarivorans_sp._Alg241-V36	G6R11_RS11270	302012..305494	2305992	BREX-system-P-loop-protein-BrxC-[Agarivorans-sp.-Alg241-V36]
WP_008844769.1	Type1.BREX	[illegible]	1229	Bacteria>Pseudomonadota>Gammaproteobacteria>Alteromonadales>Alteromonadaceae>Aliiglaciecola	GCF_000314975.1	Aliiglaciecola_lipolytica	GLIP_RS11310	7346..11035	477689	BREX-system-P-loop-protein-BrxC-[Aliiglaciecola-lipolytica]
OZB42971.1	Type1.BREX	[illegible]	1224	Bacteria>Pseudomonadota>Gammaproteobacteria>Alteromonadales>Alteromonadaceae>Alishewanella	GCA_002278995.1	Alishewanella_sp._34-51-39	B7X50_02430	10903..14577	1970316	MAG:-hypothetical-protein-B7X50_02430-[Alishewanella-sp.-34-51-39]
GGW57088.1	Type1.BREX	[illegible]	1114	Bacteria>Pseudomonadota>Gammaproteobacteria>Alteromonadales>Alteromonadaceae>Alishewanella	GCA_014652375.1	Alishewanella_tabrizica	GCM10008111_11430	80462..83806	671278	hypothetical-protein-GCM10008111_11430-[Alishewanella-tabrizica]
WP_057786312.1	Type1.BREX	[illegible]	1157	Bacteria>Pseudomonadota>Gammaproteobacteria>Alteromonadales>Alteromonadaceae>Alishewanella	GCF_001441695.1	Alishewanella_sp._WH16-1	AAY72_RS06860	5881..9354	1651088	BREX-system-P-loop-protein-BrxC-[Alishewanella-sp.-WH16-1]
WP_229796961.1	Type1.BREX	[illegible]	1125	Bacteria>Pseudomonadota>Gammaproteobacteria>Alteromonadales>Alteromonadaceae>Alishewanella	GCF_014652375.1	Alishewanella_tabrizica	IE070_RS05825	80429..83806	671278	BREX-system-P-loop-protein-BrxC-[Alishewanella-tabrizica]
WP_265045927.1	Type1.BREX	[illegible]	1194	Bacteria>Pseudomonadota>Gammaproteobacteria>Alteromonadales>Alteromonadaceae>Alkalimarinus	GCF_026016465.1	Alkalimarinus_sp._A2M4	NKI27_RS10060	2208861..2212445	2961619	BREX-system-P-loop-protein-BrxC-[Alkalimarinus-sp.-A2M4]
WP_014998461.1	Type1.BREX	[illegible]	1157	Bacteria>Pseudomonadota>Gammaproteobacteria>Alteromonadales>Alteromonadaceae>Alteromonas/Salinimonas_group	-	Alteromonas_macleodii	AMBLS11_RS09820	2297331..2300804	28108	BREX-system-P-loop-protein-BrxC-[Alteromonas-macleodii]
WP_100972015.1	Type1.BREX	[illegible]	1157	Bacteria>Pseudomonadota>Gammaproteobacteria>Alteromonadales>Alteromonadaceae>Alteromonas/Salinimonas_group	GCF_002831605.1	Alteromonas_sp._MB-3u-76	CW735_RS10480	2338685..2342158	2058133	BREX-system-P-loop-protein-BrxC-[Alteromonas-sp.-MB-3u-76]
WP_189403478.1	Type1.BREX	[illegible]	1215	Bacteria>Pseudomonadota>Gammaproteobacteria>Alteromonadales>Alteromonadaceae>Alteromonas/Salinimonas_group	GCF_014651815.1	Alteromonas_halophila	IE054_RS02435	519507..523154	516698	BREX-system-P-loop-protein-BrxC-[Alteromonas-halophila]
WP_207611650.1	Type1.BREX	[illegible]	1157	Bacteria>Pseudomonadota>Gammaproteobacteria>Alteromonadales>Alteromonadaceae>Alteromonas/Salinimonas_group	GCF_017368475.1	Alteromonas_sp._5E99-2	J3L16_RS04210	79358..82831	2817683	BREX-system-P-loop-protein-BrxC-[Alteromonas-sp.-5E99-2]
WP_273638258.1	Type1.BREX	[illegible]	1215	Bacteria>Pseudomonadota>Gammaproteobacteria>Alteromonadales>Alteromonadaceae>Alteromonas/Salinimonas_group	GCF_028595265.1	Alteromonas_sp._chi3	OIK42_RS02990	673231..676878	2987522	BREX-system-P-loop-protein-BrxC-[Alteromonas-sp.-chi3]
WP_006006589.1	Type1.BREX	[illegible]	1189	Bacteria>Pseudomonadota>Gammaproteobacteria>Alteromonadales>Alteromonadaceae>Glaciecola	GCF_000252165.1	Glaciecola_punicea	GPUN_RS10800	142812..146381	56804	BREX-system-P-loop-protein-BrxC-[Glaciecola-punicea]
WP_136550441.1	Type1.BREX	[illegible]	1193	Bacteria>Pseudomonadota>Gammaproteobacteria>Alteromonadales>Alteromonadaceae>Hydrocarboniclastica	GCF_004851605.1	Hydrocarboniclastica_marina	soil367_RS18210	4131404..4134985	2259620	BREX-system-P-loop-protein-BrxC-[Hydrocarboniclastica-marina]
PKG98979.1	Type1.BREX	[illegible]	1231	Bacteria>Pseudomonadota>Gammaproteobacteria>Alteromonadales>Alteromonadaceae>Paraglaciecola	GCA_002835935.1	Paraglaciecola_sp._MB-3u-78	CXF95_12105	233468..237163	2058332	BREX-system-P-loop-protein-BrxC-[Paraglaciecola-sp.-MB-3u-78]
WP_007105022.1	Type1.BREX	[illegible]	1229	Bacteria>Pseudomonadota>Gammaproteobacteria>Alteromonadales>Alteromonadaceae>Paraglaciecola	GCF_000315055.1	Paraglaciecola_polaris	GPLA_RS11155	47408..51097	222814	BREX-system-P-loop-protein-BrxC-[Paraglaciecola-polaris]
WP_232787809.1	Type1.BREX	[illegible]	1241	Bacteria>Pseudomonadota>Gammaproteobacteria>Alteromonadales>Alteromonadaceae>Paraglaciecola	GCF_002835935.1	Paraglaciecola_sp._MB-3u-78	CXF95_RS12105	233438..237163	2058332	BREX-system-P-loop-protein-BrxC-[Paraglaciecola-sp.-MB-3u-78]
WP_158768857.1	Type1.BREX	[illegible]	1222	Bacteria>Pseudomonadota>Gammaproteobacteria>Alteromonadales>Alteromonadaceae>Paraglaciecola	GCF_009796745.1	Paraglaciecola_sp._L1A13	GQR89_RS03920	955654..959322	2686359	BREX-system-P-loop-protein-BrxC-[Paraglaciecola-sp.-L1A13]
WP_240919947.1	Type1.BREX	[illegible]	1180	Bacteria>Pseudomonadota>Gammaproteobacteria>Alteromonadales>Alteromonadaceae>Paraglaciecola	GCF_011378905.1	Paraglaciecola_sp._20A4	GQS54_RS13935	91436..94978	2687288	BREX-system-P-loop-protein-BrxC-[Paraglaciecola-sp.-20A4]
NQY33786.1	Type1.BREX	[illegible]	1200	Bacteria>Pseudomonadota>Gammaproteobacteria>Alteromonadales>Alteromonadaceae>unclassified_Alteromonadaceae	GCA_013216015.1	Alteromonadaceae_bacterium	HRT37_02225	108462..112064	1916082	BREX-system-P-loop-protein-BrxC-[Alteromonadaceae-bacterium]
ASP49913.1	Type1.BREX	[illegible]	1229	Bacteria>Pseudomonadota>Gammaproteobacteria>Alteromonadales>Colwelliaceae>Cognaticolwellia	GCA_002076895.1	Cognaticolwellia_beringensis	B5D82_06290	1500021..1503710	1967665	DNA-repair-protein-[Cognaticolwellia-beringensis]
WP_245807568.1	Type1.BREX	[illegible]	1232	Bacteria>Pseudomonadota>Gammaproteobacteria>Alteromonadales>Colwelliaceae>Cognaticolwellia	GCF_002076895.1	Cognaticolwellia_beringensis	B5D82_RS06290	1500012..1503710	1967665	BREX-system-P-loop-protein-BrxC-[Cognaticolwellia-beringensis]
AWB59509.1	Type1.BREX	[illegible]	1232	Bacteria>Pseudomonadota>Gammaproteobacteria>Alteromonadales>Colwelliaceae>Colwellia	GCA_003061515.1	Colwellia_sp._Arc7-D	DB093_09830	2239405..2243103	2161872	BREX-system-P-loop-protein-BrxC-[Colwellia-sp.-Arc7-D]
WP_239058953.1	Type1.BREX	[illegible]	1235	Bacteria>Pseudomonadota>Gammaproteobacteria>Alteromonadales>Colwelliaceae>Colwellia	GCF_003061515.1	Colwellia_sp._Arc7-D	DB093_RS09830	2239405..2243112	2161872	BREX-system-P-loop-protein-BrxC-[Colwellia-sp.-Arc7-D]
WP_146799747.1	Type1.BREX	[illegible]	1165	Bacteria>Pseudomonadota>Gammaproteobacteria>Alteromonadales>Colwelliaceae>Colwellia	GCF_007954305.1	Colwellia_hornerae	ESZ26_RS11985	115858..119355	89402	BREX-system-P-loop-protein-BrxC-[Colwellia-hornerae]
WP_085284213.1	Type1.BREX	[illegible]	1243	Bacteria>Pseudomonadota>Gammaproteobacteria>Alteromonadales>Colwelliaceae>Colwellia	GCF_900109795.1	Colwellia_chukchiensis	B6K83_RS06980	108494..112225	641665	BREX-system-P-loop-protein-BrxC-[Colwellia-chukchiensis]
WP_130603913.1	Type1.BREX	[illegible]	1188	Bacteria>Pseudomonadota>Gammaproteobacteria>Alteromonadales>Colwelliaceae>Litorilituus	GCF_004295665.1	Litorilituus_sediminis	EMK97_RS16655	3755554..3759120	718192	BREX-system-P-loop-protein-BrxC-[Litorilituus-sediminis]
WP_189376285.1	Type1.BREX	[illegible]	1235	Bacteria>Pseudomonadota>Gammaproteobacteria>Alteromonadales>Colwelliaceae>Thalassotalea	GCF_014653195.1	Thalassotalea_profundi	IE155_RS01345	297657..301364	2036687	BREX-system-P-loop-protein-BrxC-[Thalassotalea-profundi]
KXS36208.1	Type1.BREX	[illegible]	1150	Bacteria>Pseudomonadota>Gammaproteobacteria>Alteromonadales>Idiomarinaceae>Idiomarina	GCA_001577655.1	Idiomarina_sp._T82-3	AWU56_323	88359..91811	1794809	hypothetical-protein-AWU56_323-[Idiomarina-sp.-T82-3]
WP_258240870.1	Type1.BREX	[illegible]	1150	Bacteria>Pseudomonadota>Gammaproteobacteria>Alteromonadales>Idiomarinaceae>Pseudidiomarina	GCF_024723895.1	Pseudidiomarina_homiensis	NV376_RS09715	149869..153321	364198	BREX-system-P-loop-protein-BrxC-[Pseudidiomarina-homiensis]
WP_092590479.1	Type1.BREX	[illegible]	1224	Bacteria>Pseudomonadota>Gammaproteobacteria>Alteromonadales>Idiomarinaceae>Pseudidiomarina	GCF_900104245.1	Pseudidiomarina_indica	BLP64_RS00065	10133..13807	1159017	BREX-system-P-loop-protein-BrxC-[Pseudidiomarina-indica]
WP_112714387.1	Type1.BREX	[illegible]	1199	Bacteria>Pseudomonadota>Gammaproteobacteria>Alteromonadales>Moritellaceae>Moritella	GCF_900465055.1	Moritella_yayanosii	MORIYA_RS08605	1893116..1896715	69539	BREX-system-P-loop-protein-BrxC-[Moritella-yayanosii]
WP_010607320.1	Type1.BREX	[illegible]	1188	Bacteria>Pseudomonadota>Gammaproteobacteria>Alteromonadales>Pseudoalteromonadaceae	GCF_000276645.1	Pseudoalteromonas	UY7_RS0121200	90697..94263	53246	MULTISPECIES:-BREX-system-P-loop-protein-BrxC-[Pseudoalteromonas]
CCQ10850.1	Type1.BREX	[illegible]	1237	Bacteria>Pseudomonadota>Gammaproteobacteria>Alteromonadales>Pseudoalteromonadaceae>Pseudoalteromonas	GCA_000333235.1	Pseudoalteromonas_luteoviolacea_B_=_ATCC_29581	PALB_17240	21761..25474	1268239	ATPase-like-protein-[Pseudoalteromonas-luteoviolacea-B-=-ATCC-29581]
HDY93539.1	Type1.BREX	[illegible]	1159	Bacteria>Pseudomonadota>Gammaproteobacteria>Alteromonadales>Pseudoalteromonadaceae>Pseudoalteromonas	GCA_011050215.1	Pseudoalteromonas_sp.	ENH79_15755	24321..27800	53249	BREX-system-P-loop-protein-BrxC-[Pseudoalteromonas-sp.]
WP_024611453.1	Type1.BREX	[illegible]	1193	Bacteria>Pseudomonadota>Gammaproteobacteria>Alteromonadales>Pseudoalteromonadaceae>Pseudoalteromonas	GCF_000498095.1	Pseudoalteromonas_sp._TB64	L647_RS0112855	131987..135568	1938600	BREX-system-P-loop-protein-BrxC-[Pseudoalteromonas-sp.-TB64]
WP_054983666.1	Type1.BREX	[illegible]	1159	Bacteria>Pseudomonadota>Gammaproteobacteria>Alteromonadales>Pseudoalteromonadaceae>Pseudoalteromonas	GCF_001400005.1	Pseudoalteromonas_sp._P1-30	AN395_RS16640	61256..64735	1723760	BREX-system-P-loop-protein-BrxC-[Pseudoalteromonas-sp.-P1-30]
WP_058373560.1	Type1.BREX	[illegible]	1229	Bacteria>Pseudomonadota>Gammaproteobacteria>Alteromonadales>Pseudoalteromonadaceae>Pseudoalteromonas	GCF_001465295.1	Pseudoalteromonas_translucida	PTRA_a2157	1987847..1991536	166935	BREX-system-P-loop-protein-BrxC-[Pseudoalteromonas-translucida]
WP_064663367.1	Type1.BREX	[illegible]	1242	Bacteria>Pseudomonadota>Gammaproteobacteria>Alteromonadales>Pseudoalteromonadaceae>Pseudoalteromonas	GCF_001661605.1	Pseudoalteromonas_sp._MQS005	A9479_RS10285	16730..20458	1854052	BREX-system-P-loop-protein-BrxC-[Pseudoalteromonas-sp.-MQS005]
WP_076923084.1	Type1.BREX	[illegible]	1229	Bacteria>Pseudomonadota>Gammaproteobacteria>Alteromonadales>Pseudoalteromonadaceae>Pseudoalteromonas	GCF_001974845.1	Pseudoalteromonas_sp._SK20	BW140_RS17330	3309..6998	1938367	BREX-system-P-loop-protein-BrxC-[Pseudoalteromonas-sp.-SK20]
WP_121955020.1	Type1.BREX	[illegible]	1027	Bacteria>Pseudomonadota>Gammaproteobacteria>Alteromonadales>Pseudoalteromonadaceae>Pseudoalteromonas	GCF_002863625.1	Pseudoalteromonas_sp._MelDa3	CXF89_RS14715	1..3085	888435	BREX-system-P-loop-protein-BrxC,-partial-[Pseudoalteromonas-sp.-MelDa3]
WP_234000034.1	Type1.BREX	[illegible]	1252	Bacteria>Pseudomonadota>Gammaproteobacteria>Alteromonadales>Pseudoalteromonadaceae>Pseudoalteromonas	GCF_002964725.1	Pseudoalteromonas_sp._T1lg76	C2M35_RS06890	48486..52244	2077103	BREX-system-P-loop-protein-BrxC-[Pseudoalteromonas-sp.-T1lg76]
WP_233998422.1	Type1.BREX	[illegible]	1252	Bacteria>Pseudomonadota>Gammaproteobacteria>Alteromonadales>Pseudoalteromonadaceae>Pseudoalteromonas	GCF_002964785.1	Pseudoalteromonas_sp._T1lg48	C2M29_RS12220	655771..659529	2077100	BREX-system-P-loop-protein-BrxC-[Pseudoalteromonas-sp.-T1lg48]
WP_130052623.1	Type1.BREX	[illegible]	1244	Bacteria>Pseudomonadota>Gammaproteobacteria>Alteromonadales>Pseudoalteromonadaceae>Pseudoalteromonas	GCF_004195145.1	Pseudoalteromonas_sp._MEBiC_03485	EVU92_RS18430	351830..355564	2571103	BREX-system-P-loop-protein-BrxC-[Pseudoalteromonas-sp.-MEBiC-03485]
WP_130146693.1	Type1.BREX	[illegible]	1225	Bacteria>Pseudomonadota>Gammaproteobacteria>Alteromonadales>Pseudoalteromonadaceae>Pseudoalteromonas	GCF_004208955.1	Pseudoalteromonas_sp._CO325X	EXT46_RS08650	70759..74436	1777262	BREX-system-P-loop-protein-BrxC-[Pseudoalteromonas-sp.-CO325X]
WP_138480410.1	Type1.BREX	[illegible]	1233	Bacteria>Pseudomonadota>Gammaproteobacteria>Alteromonadales>Pseudoalteromonadaceae>Pseudoalteromonas	GCF_005876835.1	Pseudoalteromonas_phenolica	C1E24_RS08165	13273..16974	161398	BREX-system-P-loop-protein-BrxC-[Pseudoalteromonas-phenolica]
WP_096038338.1	Type1.BREX	[illegible]	1159	Bacteria>Pseudomonadota>Gammaproteobacteria>Alteromonadales>Pseudoalteromonadaceae>Pseudoalteromonas	GCF_007991235.1	Pseudoalteromonas_tetraodonis	PTE01_RS15665	26152..29631	43659	BREX-system-P-loop-protein-BrxC-[Pseudoalteromonas-tetraodonis]
WP_089368387.1	Type1.BREX	[illegible]	1229	Bacteria>Pseudomonadota>Gammaproteobacteria>Alteromonadales>Pseudoalteromonadaceae>Pseudoalteromonas	GCF_007991255.1	Pseudoalteromonas_nigrifaciens	PNIG_RS09950	2060327..2064016	28109	BREX-system-P-loop-protein-BrxC-[Pseudoalteromonas-nigrifaciens]
WP_089368479.1	Type1.BREX	[illegible]	1169	Bacteria>Pseudomonadota>Gammaproteobacteria>Alteromonadales>Pseudoalteromonadaceae>Pseudoalteromonas	GCF_007991255.1	Pseudoalteromonas_nigrifaciens	PNIG_RS10730	2243615..2247124	28109	BREX-system-P-loop-protein-BrxC-[Pseudoalteromonas-nigrifaciens]
WP_167658112.1	Type1.BREX	[illegible]	1188	Bacteria>Pseudomonadota>Gammaproteobacteria>Alteromonadales>Pseudoalteromonadaceae>Pseudoalteromonas	GCF_011806445.1	Pseudoalteromonas_sp._HF66	HCZ11_RS07660	212811..216377	2721559	BREX-system-P-loop-protein-BrxC-[Pseudoalteromonas-sp.-HF66]
WP_182739649.1	Type1.BREX	[illegible]	1235	Bacteria>Pseudomonadota>Gammaproteobacteria>Alteromonadales>Pseudoalteromonadaceae>Pseudoalteromonas	GCF_014163545.1	Pseudoalteromonas_sp._SR45-6	H5158_RS05620	135410..139117	2760927	BREX-system-P-loop-protein-BrxC-[Pseudoalteromonas-sp.-SR45-6]
WP_182752860.1	Type1.BREX	[illegible]	1159	Bacteria>Pseudomonadota>Gammaproteobacteria>Alteromonadales>Pseudoalteromonadaceae>Pseudoalteromonas	GCF_014163875.1	Pseudoalteromonas_sp._SR43-3	H5088_RS10925	129286..132765	2760943	BREX-system-P-loop-protein-BrxC-[Pseudoalteromonas-sp.-SR43-3]
WP_193038371.1	Type1.BREX	[illegible]	1159	Bacteria>Pseudomonadota>Gammaproteobacteria>Alteromonadales>Pseudoalteromonadaceae>Pseudoalteromonas	GCF_014897925.1	Pseudoalteromonas_nigrifaciens	INT11_RS00030	7982..11461	28109	BREX-system-P-loop-protein-BrxC-[Pseudoalteromonas-nigrifaciens]
WP_074988793.1	Type1.BREX	[illegible]	1242	Bacteria>Pseudomonadota>Gammaproteobacteria>Alteromonadales>Pseudoalteromonadaceae>Pseudoalteromonas	GCF_014925285.1	Pseudoalteromonas_lipolytica	PLIP_RS19215	618195..621923	570156	BREX-system-P-loop-protein-BrxC-[Pseudoalteromonas-lipolytica]
WP_197117904.1	Type1.BREX	[illegible]	1229	Bacteria>Pseudomonadota>Gammaproteobacteria>Alteromonadales>Pseudoalteromonadaceae>Pseudoalteromonas	GCF_015864765.1	Pseudoalteromonas_sp._NZS11	I6E82_RS11050	319401..323090	2792049	BREX-system-P-loop-protein-BrxC-[Pseudoalteromonas-sp.-NZS11]
WP_200220630.1	Type1.BREX	[illegible]	1193	Bacteria>Pseudomonadota>Gammaproteobacteria>Alteromonadales>Pseudoalteromonadaceae>Pseudoalteromonas	GCF_016598755.1	Pseudoalteromonas_sp._LC2018020214	JG479_RS17805	301190..304771	2799564	BREX-system-P-loop-protein-BrxC-[Pseudoalteromonas-sp.-LC2018020214]
WP_248533819.1	Type1.BREX	[illegible]	1229	Bacteria>Pseudomonadota>Gammaproteobacteria>Alteromonadales>Pseudoalteromonadaceae>Pseudoalteromonas	GCF_023161935.1	Pseudoalteromonas_sp._2CM28B	MTN96_RS29910	10677..14366	2929851	BREX-system-P-loop-protein-BrxC-[Pseudoalteromonas-sp.-2CM28B]
WP_165723736.1	Type1.BREX	[illegible]	1225	Bacteria>Pseudomonadota>Gammaproteobacteria>Alteromonadales>Pseudoalteromonadaceae>Pseudoalteromonas	GCF_943789085.1	Pseudoalteromonas_sp._Z1A2	QDP19_RS17640	3065370..3069047	2686350	MULTISPECIES:-BREX-system-P-loop-protein-BrxC-[Pseudoalteromonas]
WP_197236686.1	Type1.BREX	[illegible]	1234	Bacteria>Pseudomonadota>Gammaproteobacteria>Alteromonadales>Pseudoalteromonadaceae>Pseudoalteromonas	-	Pseudoalteromonas_sp._NZS71	I6E85_05230	196559..200263	2792052	BREX-system-P-loop-protein-BrxC-[Pseudoalteromonas-sp.-NZS71]
MCK5817827.1	Type1.BREX	[illegible]	582	Bacteria>Pseudomonadota>Gammaproteobacteria>Alteromonadales>Psychromonadaceae>Psychromonas	GCA_023145575.1	Psychromonas_sp.	KAH18_00955	1..1748	1884585	BREX-system-P-loop-protein-BrxC-[Psychromonas-sp.]

Protein-ID	System	Gene-Neighbourhoods	Length	Taxonomy	Assembly-ID	Strain	Locus-Tag	Genomic-coordinates	TaxID	Fasta-define
WP_015465415.1	Type1.BREX	wHTH+WYL+WCX->FGase-><-?||SF1-SF2-Helicase+REase->SF1-SF2-Helicase+REase-><-TPR<-HEPN+SF1-SF2-Helicase+RNase+RNase<-PglZ<-N6-METHYLASE<-DUF6079*<-BrxB-BrxF-iSTAND<-BrxA-DNA-binding<-wHTH+WYL+WCX<-XIS-HTH||?-><-tRNA||?-><-?||?->35exo+HRDC->	1159	Bacteria>Pseudomonadota>Gammaproteobacteria>Alteromonadales>Psychromonadaceae>Psychromonas	GCF_000153405.2	Psychromonas_sp._CNPT3	PCNPT3_RS08205	1892696..1896175	314282	BREX-system-P-loop-protein-BrxC-[Psychromonas-sp.-CNPT3]
WP_037429580.1	Type1.BREX	<-PglZ<-ParB+HNH<-N6-METHYLASE<-DUF6079*<-BrxB-BrxF-iSTAND<-BrxA-DNA-binding<-?<-HEPN<-?<-MoxR-AAA<-NtrC-AAA+FIS-HTH<-XIS-HTH	1157	Bacteria>Pseudomonadota>Gammaproteobacteria>Alteromonadales>Shewanellaceae	GCF_000282755.1	Shewanella	SHEWPOL2_RS15310	7136..10609	22	MULTISPECIES:-BREX-system-P-loop-protein-BrxC-[Shewanella]
PKG74712.1	Type1.BREX	<-SIGMA+OB-Fold+AAA-ATPase+HKD-DNase-BrxL<-PglZ<-ParB+HNH<-N6-METHYLASE<-REase<-OLD-ABC<-DUF6079*<-BrxB-BrxF-iSTAND<-BrxA-DNA-binding<-XIS-HTH<-?<-TnsC-ATPase<-rve+Mu-transpos_C<-REase+wHTH	1191	Bacteria>Pseudomonadota>Gammaproteobacteria>Alteromonadales>Shewanellaceae>Shewanella	GCA_002836135.1	Shewanella_sp._GutCb	CXF86_11690	122253..125828	2058315	BREX-system-P-loop-protein-BrxC-[Shewanella-sp.-GutCb]
HAY94623.1	Type1.BREX	<-DUF2787<-CbeA_antitoxin<-?<-?||XIS-HTH->wHTH+WYL+WCX-p->wHTH+WYL+WCX->BrxA-DNA-binding->BrxB-BrxF-iSTAND->DUF6079*->HNH->?->?->ParB+HNH->N6-METHYLASE->HEPN->N6-METHYLASE-p->	1224	Bacteria>Pseudomonadota>Gammaproteobacteria>Alteromonadales>Shewanellaceae>Shewanella	GCA_003486485.1	Shewanella_sp.	DCY70_12125	4933..8607	50422	BREX-system-P-loop-protein-BrxC-[Shewanella-sp.]
MBB1438394.1	Type1.BREX	<-CHTH||XIS-HTH->BrxA-DNA-binding->BrxB-BrxF-iSTAND->DUF6079*->N6-METHYLASE->PglZ->SIGMA+OB-Fold+AAA-ATPase+HKD-DNase-BrxL-><-?<-MerR-HTH<-?<-HTH_26||Phage_AlpA->Arm-DNA-bind_2+Phage_int_SAM_3+Phage_integrase->	1182	Bacteria>Pseudomonadota>Gammaproteobacteria>Alteromonadales>Shewanellaceae>Shewanella	GCA_014164505.1	Shewanella_sp._SG41-4	H5202_06785	3542..7090	2760976	BREX-system-P-loop-protein-BrxC-[Shewanella-sp.-SG41-4]
MBO2546554.1	Type1.BREX	<-SIGMA+OB-Fold+AAA-ATPase+Lon-Protease-BrxL<-PglZ<-?<-N6-METHYLASE-p<-RVT<-N6-METHYLASE<-?<-HNH<-DUF6079*<-BrxB-BrxF-iSTAND<-BrxA-DNA-binding<-wHTH+WYL+WCX<-?<-?<-XIS-HTH<-XIS-HTH<-?<-Arm-DNA-bind_3+Phage_integrase	1225	Bacteria>Pseudomonadota>Gammaproteobacteria>Alteromonadales>Shewanellaceae>Shewanella	GCA_017580215.1	Shewanella_algae	I6M36_02045	117246..120923	38313	BREX-system-P-loop-protein-BrxC-[Shewanella-algae]
MCD8561045.1	Type1.BREX	<-N6-METHYLASE-p<-HEPN<-N6-METHYLASE-p<-N6-METHYLASE-p<-N6-METHYLASE<-?<-HNH<-DUF6079*<-BrxB-BrxF-iSTAND<-BrxA-DNA-binding<-wHTH+WYL+WCX<-XIS-HTH	1231	Bacteria>Pseudomonadota>Gammaproteobacteria>Alteromonadales>Shewanellaceae>Shewanella	GCA_021209305.1	Shewanella_xiamenensis	LRY75_19995	6155..9850	332186	BREX-system-P-loop-protein-BrxC-[Shewanella-xiamenensis]
VEE64276.1	Type1.BREX	<-Cas10||Cas_NE0113-><-?<-HDRnase<-Arm-DNA-bind_2+Phage_int_SAM_3+Phage_integrase<-Phage_AlpA||wHTH+WYL+WCX->BrxA-DNA-binding->BrxB-BrxF-iSTAND->DUF6079*->N6-METHYLASE->N6-METHYLASE-p->RVT->HEPN->PglZ->PglZ->SIGMA+OB-Fold+AAA-ATPase+Lon-Protease-BrxL->REase->CHTH->	1268	Bacteria>Pseudomonadota>Gammaproteobacteria>Alteromonadales>Shewanellaceae>Shewanella	GCA_900636665.1	Shewanella_putrefaciens	NCTC12093_04119	4646571..4650377	24	Uncharacterised-protein-[Shewanella-putrefaciens]
WP_011622483.1	Type1.BREX	XIS-HTH->NtrC-AAA+FIS-HTH->MoxR-AAA->?->HEPN->?->BrxA-DNA-binding->BrxB-BrxF-iSTAND->DUF6079*->N6-METHYLASE->ParB+HNH->PglZ->ZetaToxin->?-><-SF1-SF2-Helicase+REase<-SF1-SF2-Helicase+REase||HerA/FtsK->	1157	Bacteria>Pseudomonadota>Gammaproteobacteria>Alteromonadales>Shewanellaceae>Shewanella	GCF_000014685.1	Shewanella_sp._MR-4	SHEWMR4_RS09030	2019364..2022837	60480	BREX-system-P-loop-protein-BrxC-[Shewanella-sp.-MR-4]
WP_011716828.1	Type1.BREX	XIS-HTH->NtrC-AAA+FIS-HTH->MoxR-AAA->?->HEPN->?->BrxA-DNA-binding->BrxB-BrxF-iSTAND->DUF6079*->N6-METHYLASE->ParB+HNH->PglZ->ZetaToxin->?-><-SF1-SF2-Helicase+REase<-SF1-SF2-Helicase+REase||HerA/FtsK->	1157	Bacteria>Pseudomonadota>Gammaproteobacteria>Alteromonadales>Shewanellaceae>Shewanella	GCF_000203935.1	Shewanella_sp._ANA-3	SHEWANA3_RS09590	2144277..2147750	94122	BREX-system-P-loop-protein-BrxC-[Shewanella-sp.-ANA-3]
WP_006086465.1	Type1.BREX	<-TraI-VirD2-RCRE||?-><-?||?->wHTH+WYL+WCX->Peptidase_C47->wHTH+WYL+WCX->BrxA-DNA-binding->BrxB-BrxF-iSTAND->DUF6079*->ParB+HNH->OLD-ABC->TOPRIM->N6-METHYLASE->PglZ->SIGMA+OB-Fold+AAA-ATPase+Lon-Protease-BrxL->SF1-SF2-Helicase->?-><-DDE_Tnp_1_assoc+DDE_Tnp_1	1229	Bacteria>Pseudomonadota>Gammaproteobacteria>Alteromonadales>Shewanellaceae>Shewanella	GCF_000231345.1	Shewanella_baltica	SBAL625DRAFT_RS15525	212954..216643	62322	BREX-system-P-loop-protein-BrxC-[Shewanella-baltica]
WP_028764576.1	Type1.BREX	BrxA-DNA-binding->BrxB-BrxF-iSTAND->DUF6079*->N6-METHYLASE->ParB+HNH->PglZ->HEPN+Sel1+SEC-C-><-SF1-SF2-Helicase+REase<-SF1-SF2-Helicase+REase<-?<-MerR-HTH	1157	Bacteria>Pseudomonadota>Gammaproteobacteria>Alteromonadales>Shewanellaceae>Shewanella	GCF_000518705.1	Shewanella_colwelliana	L876_RS0115155	4861..8334	23	BREX-system-P-loop-protein-BrxC-[Shewanella-colwelliana]
WP_231570521.1	Type1.BREX	TetR-HTH-><-?<-?<-?||?->?-><-PglZ<-ParB+HNH<-N6-METHYLASE<-DUF6079-p*||?->?-><-?<-PDZ+Peptidase<-PTSEIIA1	941	Bacteria>Pseudomonadota>Gammaproteobacteria>Alteromonadales>Shewanellaceae>Shewanella	GCF_000798835.1	Shewanella_sp._ZOR0012	L976_RS20155	4209796..4212621	1339231	BREX-system-P-loop-protein-BrxC-[Shewanella-sp.-ZOR0012]
WP_055648628.1	Type1.BREX	<-PVC-AAA<-?||SF1-SF2-Helicase+REase->SF1-SF2-Helicase+REase-><-?<-ZetaToxin<-PglZ<-ParB+HNH<-N6-METHYLASE<-DUF6079*<-BrxB-BrxF-iSTAND<-BrxA-DNA-binding<-?<-HEPN	1157	Bacteria>Pseudomonadota>Gammaproteobacteria>Alteromonadales>Shewanellaceae>Shewanella	GCF_001308045.1	Shewanella_sp._Sh95	AEA42_RS19845	36695..40168	1689868	BREX-system-P-loop-protein-BrxC-[Shewanella-sp.-Sh95]
WP_244159705.1	Type1.BREX	<-HDRnase<-DDE_Tnp_1<-Arm-DNA-bind_2+Phage_int_SAM_3+Phage_integrase<-Phage_AlpA||HTH_26->wHTH+WYL+WCX->BrxA-DNA-binding->BrxB-BrxF-iSTAND->DUF6079*->HNH->?->N6-METHYLASE->N6-METHYLASE-p->HEPN->N6-METHYLASE-p->MULE-TRANSPOSASE->N6-METHYLASE-p->	1254	Bacteria>Pseudomonadota>Gammaproteobacteria>Alteromonadales>Shewanellaceae>Shewanella	GCF_001723195.1	Shewanella_xiamenensis	ABT47_RS18205	4085889..4089653	332186	BREX-system-P-loop-protein-BrxC-[Shewanella-xiamenensis]
WP_069454895.1	Type1.BREX	<-HDRnase<-Arm-DNA-bind_2+Phage_int_SAM_3+Phage_integrase<-Phage_AlpA||HTH_26->wHTH+WYL+WCX->BrxA-DNA-binding->BrxB-BrxF-iSTAND->DUF6079*->HNH->?->N6-METHYLASE->N6-METHYLASE-p->HEPN->N6-METHYLASE-p->MULE-TRANSPOSASE->N6-METHYLASE-p->	1240	Bacteria>Pseudomonadota>Gammaproteobacteria>Alteromonadales>Shewanellaceae>Shewanella	GCF_002074855.1	Shewanella_xiamenensis	VO00_RS12940	4702..8424	332186	BREX-system-P-loop-protein-BrxC-[Shewanella-xiamenensis]
WP_080916542.1	Type1.BREX	<-Pkinase+STYKIN||?->?->?->wHTH+WYL+WCX->BrxA-DNA-binding->BrxB-BrxF-iSTAND->DUF6079-p->DUF6079*->ParB+HNH->ParB+HNH-p->HEPN->PglZ->SIGMA+OB-Fold+AAA-ATPase+Lon-Protease-BrxL-><-MarR-HTH||?->?->Phage_int_SAM_4->	1234	Bacteria>Pseudomonadota>Gammaproteobacteria>Alteromonadales>Shewanellaceae>Shewanella	GCF_002075795.1	Shewanella_japonica	SJ2017_RS16905	3936240..3939944	93973	BREX-system-P-loop-protein-BrxC-[Shewanella-japonica]
WP_088585687.1	Type1.BREX	ZnR-><-HEPN-p<-HEPN-p<-SIGMA+OB-Fold+AAA-ATPase+Lon-Protease-BrxL<-PglZ<-N6-METHYLASE-p||DDE_Tnp_1-><-N6-METHYLASE<-DUF6079*<-BrxB-BrxF-iSTAND<-BrxA-DNA-binding<-DDE_Tnp_1<-Prok-JAB<-wHTH+WYL+WCX<-?<-XIS-HTH	1229	Bacteria>Pseudomonadota>Gammaproteobacteria>Alteromonadales>Shewanellaceae>Shewanella	GCF_002209245.2	Shewanella_sp._FDAARGOS_354	CEQ32_RS05910	1298859..1302548	1930557	BREX-system-P-loop-protein-BrxC-[Shewanella-sp.-FDAARGOS_354]
WP_257985331.1	Type1.BREX	<-SIGMA+OB-Fold+AAA-ATPase+HKD-DNase-BrxL<-PglZ<-ParB+HNH<-N6-METHYLASE<-REase<-OLD-ABC<-DUF6079*<-BrxB-BrxF-iSTAND<-BrxA-DNA-binding<-XIS-HTH<-?<-TnsC-ATPase<-rve+Mu-transpos_C<-REase+wHTH	1193	Bacteria>Pseudomonadota>Gammaproteobacteria>Alteromonadales>Shewanellaceae>Shewanella	GCF_002836135.1	Shewanella_sp._GutCb	CXF86_RS11730	122253..125834	2058315	BREX-system-P-loop-protein-BrxC-[Shewanella-sp.-GutCb]
WP_107947297.1	Type1.BREX	<-Phage_integrase<-DUF2787<-DUF2787<-CbeA_antitoxin<-?<-?||wHTH+WYL+WCX->BrxA-DNA-binding->BrxB-BrxF-iSTAND->DUF6079*->HNH->?->N6-METHYLASE->SIR2->HerA/FtsK->N6-METHYLASE-p->PglZ->SIGMA+OB-Fold+AAA-ATPase+Lon-Protease-BrxL->	1246	Bacteria>Pseudomonadota>Gammaproteobacteria>Alteromonadales>Shewanellaceae>Shewanella	GCF_003052765.1	Shewanella_baltica	C7T95_RS09475	2180761..2184501	62322	BREX-system-P-loop-protein-BrxC-[Shewanella-baltica]
WP_108947326.1	Type1.BREX	<-Phage_integrase<-Phage_integrase<-?||35exo->?->wHTH+WYL+WCX->BrxA-DNA-binding->BrxB-BrxF-iSTAND->DUF6079*->ParB+HNH->N6-METHYLASE->PglZ->SIGMA+OB-Fold+AAA-ATPase+Lon-Protease-BrxL->SbcC->HDRnase->VirD4-TraG+VirB4-FtsK->	1225	Bacteria>Pseudomonadota>Gammaproteobacteria>Alteromonadales>Shewanellaceae>Shewanella	GCF_003112715.1	Shewanella_halifaxensis	C6V78_RS24360	248833..252510	271098	BREX-system-P-loop-protein-BrxC-[Shewanella-halifaxensis]
WP_173294709.1	Type1.BREX	wHTH(SBD)+HNH->BrxA-DNA-binding->BrxB-BrxF-iSTAND->DUF6079*->N6-METHYLASE->PglZ-><-SF1-SF2-Helicase+REase<-SF1-SF2-Helicase+REase||?-><-PAIREDC-HTH	1157	Bacteria>Pseudomonadota>Gammaproteobacteria>Alteromonadales>Shewanellaceae>Shewanella	GCF_013248905.1	Shewanella_sp._VB17	HQQ94_RS12350	2876249..2879722	2739432	BREX-system-P-loop-protein-BrxC-[Shewanella-sp.-VB17]
WP_252977707.1	Type1.BREX	XIS-HTH->NtrC-AAA+FIS-HTH->MoxR-AAA->?->?->?->BrxA-DNA-binding->BrxB-BrxF-iSTAND->DUF6079-p*-><-DEDD_Tnp_IS110+Transposase_20||DUF6079-p->DUF6079-p->?->N6-METHYLASE-p->N6-METHYLASE-p->?->N6-METHYLASE-p->ParB+HNH->	261	Bacteria>Pseudomonadota>Gammaproteobacteria>Alteromonadales>Shewanellaceae>Shewanella	GCF_013307415.1	Shewanella_sp._ZOR0012	HT094_RS07805	1783246..1784031	1339231	hypothetical-protein-[Shewanella-sp.-ZOR0012]
WP_252977708.1	Type1.BREX	NtrC-AAA+FIS-HTH->MoxR-AAA->?->?->?->BrxA-DNA-binding->BrxB-BrxF-iSTAND->DUF6079-p-><-DEDD_Tnp_IS110+Transposase_20||DUF6079-p*->DUF6079-p->?->N6-METHYLASE-p->?->N6-METHYLASE-p->ParB+HNH->PglZ->ZetaToxin->	746	Bacteria>Pseudomonadota>Gammaproteobacteria>Alteromonadales>Shewanellaceae>Shewanella	GCF_013307415.1	Shewanella_sp._ZOR0012	HT094_RS07815	1785170..1787410	1339231	BREX-system-P-loop-protein-BrxC-[Shewanella-sp.-ZOR0012]
WP_259397220.1	Type1.BREX	<-CHTH||XIS-HTH->BrxA-DNA-binding->BrxB-BrxF-iSTAND->DUF6079*->N6-METHYLASE->PglZ->SIGMA+OB-Fold+AAA-ATPase+HKD-DNase-BrxL-><-?<-MerR-HTH<-?<-HTH_26||Phage_AlpA->Arm-DNA-bind_2+Phage_int_SAM_3+Phage_integrase->	1180	Bacteria>Pseudomonadota>Gammaproteobacteria>Alteromonadales>Shewanellaceae>Shewanella	GCF_014164505.1	Shewanella_sp._SG41-4	H5202_RS06785	3548..7090	2760976	BREX-system-P-loop-protein-BrxC-[Shewanella-sp.-SG41-4]
WP_203431812.1	Type1.BREX	<-LexA-protease<-SIGMA+OB-Fold+AAA-ATPase+Lon-Protease-BrxL<-PglZ<-N6-METHYLASE<-DUF6079*<-BrxB-BrxF-iSTAND<-BrxA-DNA-binding<-wHTH+WYL+WCX<-Prok-JAB<-wHTH+WYL+WCX<-?<-XIS-HTH	1224	Bacteria>Pseudomonadota>Gammaproteobacteria>Alteromonadales>Shewanellaceae>Shewanella	GCF_016859145.1	Shewanella_sp._LZH-2	JM642_RS11340	2554148..2557822	2806008	BREX-system-P-loop-protein-BrxC-[Shewanella-sp.-LZH-2]
WP_208124435.1	Type1.BREX	XIS-HTH->NtrC-AAA+FIS-HTH->MoxR-AAA->?->HEPN->BrxA-DNA-binding->BrxB-BrxF-iSTAND->DUF6079*->N6-METHYLASE->ParB+HNH->PglZ->ZetaToxin-><-SF1-SF2-Helicase+REase<-SF1-SF2-Helicase+REase||REase->HIN-HTH-><-GDResolvase+HIN-HTH	1157	Bacteria>Pseudomonadota>Gammaproteobacteria>Alteromonadales>Shewanellaceae>Shewanella	GCF_017570045.1	Shewanella_algae	JKK45_RS07515	1709365..1712838	38313	BREX-system-P-loop-protein-BrxC-[Shewanella-algae]
WP_208251007.1	Type1.BREX	<-DUF2787<-?<-?||XIS-HTH-><-?||HTH_3->wHTH+WYL+WCX->BrxA-DNA-binding->BrxB-BrxF-iSTAND->DUF6079*->OLD-ABC->TOPRIM->N6-METHYLASE->PglZ->SIGMA+OB-Fold+AAA-ATPase+Lon-Protease-BrxL->?->?->?->ANK+ANK+ANK->	1225	Bacteria>Pseudomonadota>Gammaproteobacteria>Alteromonadales>Shewanellaceae>Shewanella	GCF_017580085.1	Shewanella_algae	I6M29_RS02105	9413..13090	38313	BREX-system-P-loop-protein-BrxC-[Shewanella-algae]
WP_243891830.1	Type1.BREX	<-SIGMA+OB-Fold+AAA-ATPase+Lon-Protease-BrxL<-PglZ<-?<-N6-METHYLASE-p<-RVT<-N6-METHYLASE<-?<-HNH<-DUF6079*<-BrxB-BrxF-iSTAND<-BrxA-DNA-binding<-wHTH+WYL+WCX<-?<-?<-XIS-HTH<-?<-Arm-DNA-bind_3+Phage_integrase	1226	Bacteria>Pseudomonadota>Gammaproteobacteria>Alteromonadales>Shewanellaceae>Shewanella	GCF_017580215.1	Shewanella_algae	I6M36_RS02045	117246..120926	38313	BREX-system-P-loop-protein-BrxC-[Shewanella-algae]
WP_144225094.1	Type1.BREX	<-Phage_integrase<-DUF2787<-?<-?||XIS-HTH->XIS-HTH->BrxA-DNA-binding->BrxB-BrxF-iSTAND-p->DUF6079*->OLD-ABC->PIN->N6-METHYLASE->PglZ->SIGMA+OB-Fold+AAA-ATPase+Lon-Protease-BrxL->?-><-Arm-DNA-bind_3+Phage_integrase<-?||RNasePH->	1145	Bacteria>Pseudomonadota>Gammaproteobacteria>Alteromonadales>Shewanellaceae>Shewanella	GCF_017580345.1	Shewanella_algae	I6M43_RS01845	49386..52823	38313	BREX-system-P-loop-protein-BrxC-[Shewanella-algae]
WP_101059463.1	Type1.BREX	HTH_21+rve+rve_3->BrxA-DNA-binding->BrxB-BrxF-iSTAND->DUF6079*->N6-METHYLASE->ParB+HNH->PglZ->ZetaToxin->?-><-SF1-SF2-Helicase+REase<-SF1-SF2-Helicase+REase	1157	Bacteria>Pseudomonadota>Gammaproteobacteria>Alteromonadales>Shewanellaceae>Shewanella	GCF_017580385.1	Shewanella_algae	CX652_RS12220	2312..5785	38313	BREX-system-P-loop-protein-BrxC-[Shewanella-algae]
WP_144191359.1	Type1.BREX	XIS-HTH->NtrC-AAA+FIS-HTH->MoxR-AAA->?->HEPN->BrxA-DNA-binding->BrxB-BrxF-iSTAND->DUF6079*->N6-METHYLASE->ParB+HNH->PglZ->ZetaToxin->?-><-SF1-SF2-Helicase+REase<-SF1-SF2-Helicase+REase	1157	Bacteria>Pseudomonadota>Gammaproteobacteria>Alteromonadales>Shewanellaceae>Shewanella	GCF_017580415.1	Shewanella_algae	I6M45_RS07395	495996..499469	38313	BREX-system-P-loop-protein-BrxC-[Shewanella-algae]
WP_208223521.1	Type1.BREX	rve->BrxA-DNA-binding->BrxB-BrxF-iSTAND->DUF6079*->N6-METHYLASE->ParB+HNH->PglZ->ZetaToxin->?-><-SF1-SF2-Helicase+REase<-SF1-SF2-Helicase+REase||REase-><-GDResolvase+HIN-HTH	1157	Bacteria>Pseudomonadota>Gammaproteobacteria>Alteromonadales>Shewanellaceae>Shewanella	GCF_017580425.1	Shewanella_algae	I6M46_RS07445	1788..5261	38313	BREX-system-P-loop-protein-BrxC-[Shewanella-algae]
WP_208162985.1	Type1.BREX	HTH_3->wHTH+WYL+WCX->BrxA-DNA-binding->BrxB-BrxF-iSTAND->DUF6079*->OLD-ABC->TOPRIM->N6-METHYLASE->PglZ->SIGMA+OB-Fold+AAA-ATPase+Lon-Protease-BrxL->	1225	Bacteria>Pseudomonadota>Gammaproteobacteria>Alteromonadales>Shewanellaceae>Shewanella	GCF_017580535.1	Shewanella_algae	I6M49_RS22460	3510..7187	38313	BREX-system-P-loop-protein-BrxC-[Shewanella-algae]
WP_220062217.1	Type1.BREX	<-wHTH||MerR-HTH->?->SF1-SF2-Helicase+REase->SF1-SF2-Helicase+REase-><-HEPN+Sel1+SEC-C<-PglZ<-ParB+HNH<-N6-METHYLASE<-DUF6079*<-BrxB-BrxF-iSTAND<-BrxA-DNA-binding||sGTP->sGTP->?->sGTP->HEPN-><-XIS-HTH	1157	Bacteria>Pseudomonadota>Gammaproteobacteria>Alteromonadales>Shewanellaceae>Shewanella	GCF_019457515.1	Shewanella_sp._FJAT-53870	KOI73_RS17055	3880275..3883748	2864208	BREX-system-P-loop-protein-BrxC-[Shewanella-sp.-FJAT-53870]
WP_220768073.1	Type1.BREX	MobI-><-?<-LexA-protease||35exo->?->wHTH+WYL+WCX->BrxA-DNA-binding->BrxB-BrxF-iSTAND->DUF6079*->ParB+HNH->N6-METHYLASE->PglZ->SIGMA+OB-Fold+AAA-ATPase+Lon-Protease-BrxL->NACHT+HEAT+REase->HDRnase->VirD4-TraG+VirB4-FtsK->	1225	Bacteria>Pseudomonadota>Gammaproteobacteria>Alteromonadales>Shewanellaceae>Shewanella	GCF_019654915.1	Shewanella_sp._MBTL60-007	K5Q92_RS05655	54742..58419	2815911	BREX-system-P-loop-protein-BrxC-[Shewanella-sp.-MBTL60-007]
WP_221058246.1	Type1.BREX	<-ANK+ANK+ANK<-?<-?<-?<-SIGMA+OB-Fold+AAA-ATPase+Lon-Protease-BrxL<-PglZ<-N6-METHYLASE<-ParB+HNH<-DUF6079*<-BrxB-BrxF-iSTAND<-BrxA-DNA-binding<-wHTH+WYL+WCX<-HTH_3||?-><-XIS-HTH||?->?->DUF2787->	1199	Bacteria>Pseudomonadota>Gammaproteobacteria>Alteromonadales>Shewanellaceae>Shewanella	GCF_019670685.1	Shewanella_algae	K5631_RS12960	2926333..2929932	38313	BREX-system-P-loop-protein-BrxC-[Shewanella-algae]
WP_224020606.1	Type1.BREX	<-HerA/FtsK||SF1-SF2-Helicase+REase->SF1-SF2-Helicase+REase-><-?<-ZetaToxin<-PglZ<-ParB+HNH<-N6-METHYLASE<-DUF6079*<-BrxB-BrxF-iSTAND<-BrxA-DNA-binding<-?<-HEPN<-?<-MoxR-AAA<-NtrC-AAA+FIS-HTH<-XIS-HTH	1157	Bacteria>Pseudomonadota>Gammaproteobacteria>Alteromonadales>Shewanellaceae>Shewanella	GCF_019973655.1	Shewanella_xiamenensis	LDO18_RS12250	2746465..2749938	332186	BREX-system-P-loop-protein-BrxC-[Shewanella-xiamenensis]
WP_248084385.1	Type1.BREX	SF1-SF2-Helicase-><-DUF2787<-CbeA_antitoxin<-?<-?||XIS-HTH->wHTH+WYL+WCX->BrxA-DNA-binding->BrxB-BrxF-iSTAND->DUF6079*->HNH->?->ParB+HNH->N6-METHYLASE->N6-METHYLASE-p->HEPN->N6-METHYLASE-p->	1231	Bacteria>Pseudomonadota>Gammaproteobacteria>Alteromonadales>Shewanellaceae>Shewanella	GCF_023159095.1	unclassified_Shewanella	MOD77_RS02670	216752..220447	196818	MULTISPECIES:-BREX-system-P-loop-protein-BrxC-[unclassified-Shewanella]
WP_249554920.1	Type1.BREX	TetR-HTH->ABhydrolase->?-><-PRTase<-RNasePH||?->Arm-DNA-bind_3+Phage_integrase->BrxA-DNA-binding->BrxB-BrxF-iSTAND->DUF6079*->N6-METHYLASE->PglZ->SIGMA+OB-Fold+AAA-ATPase+HKD-DNase-BrxL->MORC+DUF3684+REase-><-XIS-HTH||?->?->DUF2787->Phage_integrase->	1188	Bacteria>Pseudomonadota>Gammaproteobacteria>Alteromonadales>Shewanellaceae>Shewanella	GCF_023475945.1	Shewanella_sp._8A	M0413_RS16385	25717..29283	2943323	BREX-system-P-loop-protein-BrxC-[Shewanella-sp.-8A]
WP_249553736.1	Type1.BREX	<-SIGMA+OB-Fold+AAA-ATPase+Lon-Protease-BrxL<-PglZ<-?<-N6-METHYLASE<-HEPN<-RVT<-N6-METHYLASE-p<-N6-METHYLASE<-DUF6079*<-BrxB-BrxF-iSTAND<-BrxA-DNA-binding<-wHTH+WYL+WCX||?->?->CbeA_antitoxin->DUF2787->DUF2787->Phage_integrase->	1268	Bacteria>Pseudomonadota>Gammaproteobacteria>Alteromonadales>Shewanellaceae>Shewanella	GCF_023475945.1	Shewanella_sp._8A	M2897_RS12020	2756867..2760673	2943323	BREX-system-P-loop-protein-BrxC-[Shewanella-sp.-8A]
WP_259476695.1	Type1.BREX	XIS-HTH->?->wHTH+WYL+WCX->Prok-JAB->wHTH+WYL+WCX->BrxA-DNA-binding->BrxB-BrxF-iSTAND->DUF6079*->ParB+HNH->N6-METHYLASE->PglZ->SIGMA+OB-Fold+AAA-ATPase+Lon-Protease-BrxL->?->HerA/FtsK->	1225	Bacteria>Pseudomonadota>Gammaproteobacteria>Alteromonadales>Shewanellaceae>Shewanella	GCF_024972795.1	Shewanella_xiamenensis	K5M76_RS09815	2212502..2216179	332186	BREX-system-P-loop-protein-BrxC-[Shewanella-xiamenensis]
WP_259573703.1	Type1.BREX	<-CHTH<-REase<-SIGMA+OB-Fold+AAA-ATPase+Lon-Protease-BrxL<-PglZ<-N6-METHYLASE-p<-RVT<-N6-METHYLASE<-DUF6079*<-BrxB-BrxF-iSTAND<-BrxA-DNA-binding<-wHTH+WYL+WCX<-HTH_26||Phage_AlpA->Arm-DNA-bind_2+Phage_int_SAM_3+Phage_integrase->HDRnase->?-><-SF1-SF2-Helicase	1240	Bacteria>Pseudomonadota>Gammaproteobacteria>Alteromonadales>Shewanellaceae>Shewanella	GCF_024999165.1	Shewanella_baltica	G7A68_RS06675	25637..29359	62322	BREX-system-P-loop-protein-BrxC-[Shewanella-baltica]
WP_259506670.1	Type1.BREX	<-Herpes_LMP1+REase<-?<-?||SF1-SF2-Helicase+REase->SF1-SF2-Helicase+REase-><-HEPN+Sel1+SEC-C<-PglZ<-ParB+HNH<-N6-METHYLASE<-DUF6079*<-BrxB-BrxF-iSTAND<-BrxA-DNA-binding||?->?->CbeA_antitoxin->DUF2787->Phage_integrase->?->RHH_1->	1157	Bacteria>Pseudomonadota>Gammaproteobacteria>Alteromonadales>Shewanellaceae>Shewanella	GCF_024999425.1	Shewanella_baltica	G3479_RS14595	93624..97097	62322	BREX-system-P-loop-protein-BrxC-[Shewanella-baltica]
WP_261753251.1	Type1.BREX	Arm-DNA-bind_3+Phage_integrase->SF1-SF2-Helicase+REase->SF1-SF2-Helicase+REase-><-?<-ZetaToxin<-PglZ<-N6-METHYLASE<-DUF6079*<-BrxB-BrxF-iSTAND<-BrxA-DNA-binding	1157	Bacteria>Pseudomonadota>Gammaproteobacteria>Alteromonadales>Shewanellaceae>Shewanella	GCF_025398225.1	Shewanella_algae	N4S66_RS19550	70548..74021	38313	BREX-system-P-loop-protein-BrxC-[Shewanella-algae]
WP_261732933.1	Type1.BREX	Peptidase_M2-><-?||<-HDRnase<-Arm-DNA-bind_2+Phage_int_SAM_3+Phage_integrase<-Phage_AlpA||HTH_26->BrxA-DNA-binding->BrxB-BrxF-iSTAND->DUF6079*->N6-METHYLASE->N6-METHYLASE-p->OLD-ABC->?->TOPRIM->PglZ-p->PglZ->HEPN+Sel1+SEC-C->	1157	Bacteria>Pseudomonadota>Gammaproteobacteria>Alteromonadales>Shewanellaceae>Shewanella	GCF_025398315.1	Shewanella_sp._KJ10-1	N4T56_RS08695	1854721..1858194	2978345	BREX-system-P-loop-protein-BrxC-[Shewanella-sp.-KJ10-1]
WP_263145911.1	Type1.BREX	<-N6-METHYLASE<-HerA/FtsK<-SIR2<-N6-METHYLASE<-ParB+HNH<-?<-HNH<-DUF6079*<-BrxB-BrxF-iSTAND<-BrxA-DNA-binding<-wHTH+WYL+WCX<-XIS-HTH||?->?->CbeA_antitoxin->DUF2787->DUF2787->	1224	Bacteria>Pseudomonadota>Gammaproteobacteria>Alteromonadales>Shewanellaceae>Shewanella	GCF_025653395.1	Shewanella_sp._SW24	L5M17_RS05810	187685..191359	2912815	BREX-system-P-loop-protein-BrxC-[Shewanella-sp.-SW24]
WP_263212792.1	Type1.BREX	<-McrC-NTD+REase<-Preader17<-SIGMA+OB-Fold+AAA-ATPase+Lon-Protease-BrxL<-PglZ<-N6-METHYLASE<-DUF6079*<-BrxB-BrxF-iSTAND<-BrxA-DNA-binding<-wHTH+WYL+WCX<-Peptidase_C47<-wHTH+WYL+WCX<-?<-XIS-HTH	1224	Bacteria>Pseudomonadota>Gammaproteobacteria>Alteromonadales>Shewanellaceae>Shewanella	GCF_025653775.1	Shewanella_sp._SM72	L5M36_RS22725	11851..15525	2912805	BREX-system-P-loop-protein-BrxC-[Shewanella-sp.-SM72]
WP_263171306.1	Type1.BREX	<-Phage_integrase<-DUF2787<-DUF2787<-CbeA_antitoxin<-?<-?||wHTH+WYL+WCX->BrxA-DNA-binding->BrxB-BrxF-iSTAND->DUF6079*->HNH->?->N6-METHYLASE->SIR2->HerA/FtsK->N6-METHYLASE->PglZ->SIGMA+OB-Fold+AAA-ATPase+Lon-Protease-BrxL->	1235	Bacteria>Pseudomonadota>Gammaproteobacteria>Alteromonadales>Shewanellaceae>Shewanella	GCF_025653795.1	Shewanella_sp._SM73	L5M51_RS01900	416485..420192	2912806	BREX-system-P-loop-protein-BrxC-[Shewanella-sp.-SM73]
WP_263177453.1	Type1.BREX	<-SIGMA+OB-Fold+AAA-ATPase+Lon-Protease-BrxL<-PglZ<-N6-METHYLASE<-ParB+HNH<-DUF6079*<-BrxB-BrxF-iSTAND<-BrxA-DNA-binding<-wHTH+WYL+WCX<-?<-wHTH+WYL+WCX<-?<-XIS-HTH||<-?||TraI-VirD2-RCRE->	1225	Bacteria>Pseudomonadota>Gammaproteobacteria>Alteromonadales>Shewanellaceae>Shewanella	GCF_025654415.1	unclassified_Shewanella	L5M16_RS21425	11352..15029	196818	MULTISPECIES:-BREX-system-P-loop-protein-BrxC-[unclassified-Shewanella]
WP_263197229.1	Type1.BREX	<-SIGMA+OB-Fold+AAA-ATPase+Lon-Protease-BrxL<-PglZ<-N6-METHYLASE-p<-HEPN<-N6-METHYLASE-p<-N6-METHYLASE<-?<-HNH<-DUF6079*<-BrxB-BrxF-iSTAND<-BrxA-DNA-binding<-wHTH+WYL+WCX<-HTH_26||Phage_AlpA->Arm-DNA-bind_2+Phage_int_SAM_3+Phage_integrase->HDRnase->	1246	Bacteria>Pseudomonadota>Gammaproteobacteria>Alteromonadales>Shewanellaceae>Shewanella	GCF_025654545.1	Shewanella_sp._SM20	L5M18_RS13095	42119..45859	2912792	BREX-system-P-loop-protein-BrxC-[Shewanella-sp.-SM20]
WP_264921054.1	Type1.BREX	XIS-HTH->NtrC-AAA+FIS-HTH->MoxR-AAA->?->HEPN->HIN-HTH->BrxA-DNA-binding->BrxB-BrxF-iSTAND->DUF6079*->N6-METHYLASE->ParB+HNH->PglZ->ZetaToxin->?-><-SF1-SF2-Helicase+REase<-SF1-SF2-Helicase+REase<-ZnR+HTH_Tnp_ISL3+DDE_Tnp_ISL3||N6-N4-METHYLASE->	1157	Bacteria>Pseudomonadota>Gammaproteobacteria>Alteromonadales>Shewanellaceae>Shewanella	GCF_026013945.1	Shewanella_algae	OLL83_RS07695	1703466..1706939	38313	BREX-system-P-loop-protein-BrxC-[Shewanella-algae]
WP_275482470.1	Type1.BREX	MobI-><-?<-LexA-protease||35exo->?->wHTH+WYL+WCX->BrxA-DNA-binding->BrxB-BrxF-iSTAND->DUF6079*->ParB+HNH->N6-METHYLASE->PglZ->SIGMA+OB-Fold+AAA-ATPase+Lon-Protease-BrxL->CARF+NtrC-AAA+FIS-HTH->OBFOLD-NUCLEASE->	1225	Bacteria>Pseudomonadota>Gammaproteobacteria>Alteromonadales>Shewanellaceae>Shewanella	GCF_029167175.1	Shewanella_sp._A32	PY479_RS00080	16338..20015	3031327	BREX-system-P-loop-protein-BrxC-[Shewanella-sp.-A32]
WP_076500079.1	Type1.BREX	SF1-SF2-Helicase+REase->SF1-SF2-Helicase+REase-><-REase+SF1-SF2-Helicase<-REase<-PglZ<-TOPRIM<-OLD-ABC<-N6-METHYLASE<-DUF6079*<-BrxB-BrxF-iSTAND<-BrxA-DNA-binding||?->?->CbeA_antitoxin->DUF2787->DUF2787->Phage_integrase->	1157	Bacteria>Pseudomonadota>Gammaproteobacteria>Alteromonadales>Shewanellaceae>Shewanella	GCF_900156405.1	Shewanella_morhuae	BW947_RS12490	20495..23968	365591	BREX-system-P-loop-protein-BrxC-[Shewanella-morhuae]
WP_112352819.1	Type1.BREX	MerR-HTH->?-><-SIGMA+OB-Fold+AAA-ATPase+HKD-DNase-BrxL<-PglZ<-N6-METHYLASE<-REase<-OLD-ABC<-DUF6079*<-BrxB-BrxF-iSTAND<-BrxA-DNA-binding<-XIS-HTH<-HNH+NERD+ZnR<-SAM-METHYLASE+wHTH+HNH<-?||URI-DNase-><-MarR-HTH	1197	Bacteria>Pseudomonadota>Gammaproteobacteria>Alteromonadales>Shewanellaceae>Shewanella	GCF_900476435.1	Shewanella_benthica	SHEWBE_RS12055	2634220..2637813	43661	BREX-system-P-loop-protein-BrxC-[Shewanella-benthica]
WP_126513302.1	Type1.BREX	Cas_NE0113-><-?<-HDRnase<-Arm-DNA-bind_2+Phage_int_SAM_3+Phage_integrase<-Phage_AlpA||HTH_26->wHTH+WYL+WCX->BrxA-DNA-binding->BrxB-BrxF-iSTAND->DUF6079*->N6-METHYLASE->N6-METHYLASE-p->RVT->HEPN->N6-METHYLASE-p->PglZ->SIGMA+OB-Fold+AAA-ATPase+Lon-Protease-BrxL->REase->CHTH->	1254	Bacteria>Pseudomonadota>Gammaproteobacteria>Alteromonadales>Shewanellaceae>Shewanella	-	Shewanella_putrefaciens	EL161_RS20440	4646613..4650377	24	BREX-system-P-loop-protein-BrxC-[Shewanella-putrefaciens]
WP_102039083.1	Type1.BREX	<-SIGMA+OB-Fold+AAA-ATPase+HKD-DNase-BrxL<-PglZ<-ParB+HNH<-N6-METHYLASE<-REase<-OLD-ABC<-DUF6079*<-BrxB-BrxF-iSTAND<-BrxA-DNA-binding<-XIS-HTH<-?<-TnsC-ATPase<-rve+Mu-transpos_C<-REase+wHTH	1191	Bacteria>Pseudomonadota>Gammaproteobacteria>Alteromonadales>Shewanellaceae>Shewanella	-	Shewanella_sp._GutCb	CXF86_11690	122253..125828	2058315	BREX-system-P-loop-protein-BrxC-[Shewanella-sp.-GutCb]
WP_182771402.1	Type1.BREX	<-CHTH||XIS-HTH->BrxA-DNA-binding->BrxB-BrxF-iSTAND->DUF6079*->N6-METHYLASE->PglZ->SIGMA+OB-Fold+AAA-ATPase+HKD-DNase-BrxL-><-?<-MerR-HTH<-?<-HTH_26||Phage_AlpA->Arm-DNA-bind_2+Phage_int_SAM_3+Phage_integrase->	1182	Bacteria>Pseudomonadota>Gammaproteobacteria>Alteromonadales>Shewanellaceae>Shewanella	-	Shewanella_sp._SG41-4	H5202_06785	3542..7090	2760976	BREX-system-P-loop-protein-BrxC-[Shewanella-sp.-SG41-4]
MBV7434872.1	Type1.BREX	<-SIGMA+OB-Fold+AAA-ATPase+HKD-DNase-BrxL<-PglZ<-ParB+HNH<-N6-METHYLASE<-DUF6079*<-BrxB-BrxF-iSTAND<-BrxA-DNA-binding<-?<-?<-?<-?<-Peptidase_M20	1175	Bacteria>Pseudomonadota>Gammaproteobacteria>Cardiobacteriales>Cardiobacteriaceae>unclassified_Cardiobacteriaceae	GCA_019218315.1	Cardiobacteriaceae_bacterium_TAE3-ERU3	KRX19_07510	264796..268323	2856340	BREX-system-P-loop-protein-BrxC-[Cardiobacteriaceae-bacterium-TAE3-ERU3]
WP_096087095.1	Type1.BREX	<-SbcC<-SIGMA+OB-Fold+AAA-ATPase+Lon-Protease-BrxL<-PglZ<-N6-METHYLASE<-TOPRIM<-OLD-ABC<-DUF6079*<-BrxB-BrxF-iSTAND<-BrxA-DNA-binding<-wHTH+WYL+WCX<-?<-35exo||LexA-protease->?-><-MobI	1225	Bacteria>Pseudomonadota>Gammaproteobacteria>Cellvibrionales>Cellvibrionaceae>Agaribacterium	GCA_002312815.1	Agaribacterium_haliotis	CFK45_RS14065	11991..15668	2013869	BREX-system-P-loop-protein-BrxC-[Agaribacterium-haliotis]
WP_007642692.1	Type1.BREX	<-SIGMA+OB-Fold+AAA-ATPase+Lon-Protease-BrxL<-PglZ<-TOPRIM<-OLD-ABC<-N6-METHYLASE-p<-RVT<-N6-METHYLASE<-HATPase+wHTH<-DUF6079*<-BrxB-BrxF-iSTAND<-BrxA-DNA-binding<-wHTH+WYL+WCX<-REase||?-><-?||?-><-MPTase	1220	Bacteria>Pseudomonadota>Gammaproteobacteria>Cellvibrionales>Cellvibrionaceae>Cellvibrio	GCF_000263355.1	Cellvibrio_sp._BR	O59_RS11305	237548..241210	1134474	BREX-system-P-loop-protein-BrxC-[Cellvibrio-sp.-BR]
WP_054114051.1	Type1.BREX	DDE_Tnp_IS66+DDE_Tnp_IS66_C-><-SIGMA+OB-Fold+AAA-ATPase+Lon-Protease-BrxL<-PglZ<-N6-METHYLASE<-TOPRIM<-OLD-ABC<-DUF6079*<-BrxB-BrxF-iSTAND<-BrxA-DNA-binding<-wHTH+WYL+WCX<-?||DDE_Tnp_IS66+DDE_Tnp_IS66_C->	1229	Bacteria>Pseudomonadota>Gammaproteobacteria>Cellvibrionales>Cellvibrionaceae>Marinagarivorans	GCF_001296225.1	Marinagarivorans_algicola	AC526_RS14090	30886..34575	1513270	BREX-system-P-loop-protein-BrxC-[Marinagarivorans-algicola]
WP_265366287.1	Type1.BREX	Arm-DNA-bind_3+Phage_integrase-><-XIS-HTH<-AAA+HTH<-XIS-HTH||MobI->XIS-HTH->BrxA-DNA-binding->BrxB-BrxF-iSTAND->DUF6079*->Schlafen+HATPase+wHTH+HTH->OLD-ABC->REase->N6-METHYLASE->ParB+HNH->PglZ->SIGMA+OB-Fold+AAA-ATPase+HKD-DNase-BrxL->SbcC+APATPase+SbcC->	1193	Bacteria>Pseudomonadota>Gammaproteobacteria>Cellvibrionales>Cellvibrionaceae>Marinimicrobium	GCF_026153455.1	Marinimicrobium_koreense	OOT55_RS13025	3024461..3028042	306545	BREX-system-P-loop-protein-BrxC-[Marinimicrobium-sp.-C6131]
NHN36564.1	Type1.BREX	<-SIGMA+OB-Fold+AAA-ATPase+Lon-Protease-BrxL<-PglZ<-Polbeta<-MarR-HTH<-N6-METHYLASE<-?<-DUF6079*<-BrxB-BrxF-iSTAND<-BrxA-DNA-binding<-tRNA||?-><-MerR-HTH	1151	Bacteria>Pseudomonadota>Gammaproteobacteria>Cellvibrionales>Cellvibrionaceae>Pseudomaricurvus	GCA_011440395.1	Pseudomaricurvus_alcaniphilus	G8764_04575	1080165..1083620	1166482	BREX-system-P-loop-protein-BrxC-[Pseudomaricurvus-alcaniphilus]
WP_011469234.1	Type1.BREX	HNH-><-TraJ-RHH<-RelE-ParE<-PhdYeFM_antitox<-SIGMA+OB-Fold+AAA-ATPase+Lon-Protease-BrxL<-PglZ<-TOPRIM<-OLD-ABC<-N6-METHYLASE<-DUF6079*<-BrxB-BrxF-iSTAND<-BrxA-DNA-binding<-XIS-HTH||?->GDResolvase+ZnR->?-><-ACET	1145	Bacteria>Pseudomonadota>Gammaproteobacteria>Cellvibrionales>Cellvibrionaceae>Saccharophagus	GCF_000013665.1	Saccharophagus_degradans	SDE_RS14425	3492242..3495679	86304	BREX-system-P-loop-protein-BrxC-[Saccharophagus-degradans]
WP_187410606.1	Type1.BREX	Phage_AlpA->DnaB->?->TraJ-RHH->?->PIN->||wHTH+WYL+WCX->BrxA-DNA-binding->BrxB-BrxF-iSTAND->DUF6079*->N6-METHYLASE->PHP+SbcC->ParB+HNH->PglZ->SIGMA+OB-Fold+AAA-ATPase+Lon-Protease-BrxL->?->ABC-ATPase+SbcC->	1146	Bacteria>Pseudomonadota>Gammaproteobacteria>Cellvibrionales>Cellvibrionaceae>Saccharophagus	GCF_014336955.1	Saccharophagus_sp._K07	DWB84_RS14900	71821..75261	2283636	BREX-system-P-loop-protein-BrxC-[Saccharophagus-sp.-K07]
WP_009576096.1	Type1.BREX	Calcineurin-><-?<-?<-?<-HD<-SLATT||nSTAND1+HNH->BrxA-DNA-binding->BrxB-BrxF-iSTAND->DUF6079*->N6-METHYLASE->PglZ->SIGMA+OB-Fold+AAA-ATPase+HKD-DNase-BrxL->URI-DNase+RAMA->?->?-><-?<-?<-RADICAL-SAM	1178	Bacteria>Pseudomonadota>Gammaproteobacteria>Cellvibrionales>Halieaceae>Aequoribacter	GCF_000204315.1	Aequoribacter_fuscus	EYZ66_RS04240	942285..945821	2518989	BREX-system-P-loop-protein-BrxC-[Aequoribacter-fuscus]
WP_084199902.1	Type1.BREX	CHTH->Calcineurin-><-?<-TnsC-ATPase<-?<-?||XIS-HTH->BrxA-DNA-binding->BrxB-BrxF-iSTAND->DUF6079*->OLD-ABC->REase->N6-METHYLASE->PglZ->SIGMA+OB-Fold+AAA-ATPase+HKD-DNase-BrxL->	1184	Bacteria>Pseudomonadota>Gammaproteobacteria>Cellvibrionales>Halieaceae>Halioglobus	GCF_014652255.1	Halioglobus_japonicus	C0029_RS05530	1195751..1199305	930805	BREX-system-P-loop-protein-BrxC-[Halioglobus-japonicus]
WP_101520955.1	Type1.BREX	HEPN->HNH+Polbeta->?->Peptidase_M29+DUF2971->XIS-HTH->BrxA-DNA-binding->BrxB-BrxF-iSTAND->DUF6079*->N6-METHYLASE->MarR-HTH->Polbeta->PglZ->SIGMA+OB-Fold+AAA-ATPase+Lon-Protease-BrxL->HEPN+SF1-SF2-Helicase+RNase+RNase->TPR-><-?<-Arm-DNA-bind_3+Phage_integrase	1151	Bacteria>Pseudomonadota>Gammaproteobacteria>Cellvibrionales>Halieaceae>Kineobactrum	GCF_002868715.1	Kineobactrum_sediminis	CWI75_RS08140	627029..630484	1905677	BREX-system-P-loop-protein-BrxC-[Kineobactrum-sediminis]
WP_148062597.1	Type1.BREX	XIS-HTH->TOPRIM->AAA+wHTH->?->?->XIS-HTH->BrxA-DNA-binding->BrxB-BrxF-iSTAND->DUF6079*->SF1-SF2-Helicase+PRTase->SLOG+BirA-HTH->N6-METHYLASE->RVT->PglZ->Immank+ANK+ANK->REase->SIGMA+OB-Fold+AAA-ATPase+HKD-DNase-BrxL->	1180	Bacteria>Pseudomonadota>Gammaproteobacteria>Cellvibrionales>Halieaceae>Parahaliea	GCF_008064635.1	Parahaliea_aestuarii	FVW59_RS02260	491803..495345	1852021	BREX-system-P-loop-protein-BrxC-[Parahaliea-aestuarii]
WP_043319021.1	Type1.BREX	<-TetR-HTH<-?||?-><-SIGMA+OB-Fold+AAA-ATPase+Lon-Protease-BrxL<-PglZ<-ParB+HNH<-N6-METHYLASE<-Schlafen+HATPase+wHTH<-DUF6079*<-BrxB-BrxF-iSTAND<-BrxA-DNA-binding<-tRNA<-?<-?<-?<-Classical-AAA<-SAM-METHYLASE	1145	Bacteria>Pseudomonadota>Gammaproteobacteria>Cellvibrionales>Microbulbiferaceae>Microbulbifer	GCF_000708675.1	Microbulbifer_sp._HZ11	AU11_RS09640	451911..455348	1453501	BREX-system-P-loop-protein-BrxC-[Microbulbifer-sp.-HZ11]
WP_265965539.1	Type1.BREX	ABhydrolase->MORC+DNA_gyrase+TOPRIM->HTH_3->?->BrxA-DNA-binding->BrxB-BrxF-iSTAND->DUF6079*->N6-METHYLASE->SIR2->HerA/FtsK->N6-METHYLASE-p->?->PglZ->SIGMA+OB-Fold+AAA-ATPase+Lon-Protease-BrxL-><-?<-ACET+ACET	1223	Bacteria>Pseudomonadota>Gammaproteobacteria>Cellvibrionales>Microbulbiferaceae>Microbulbifer	GCF_026241645.1	Microbulbifer_thermotolerans	OQJ62_RS10425	90626..94297	252514	BREX-system-P-loop-protein-BrxC-[Microbulbifer-thermotolerans]
WP_036859475.1	Type1.BREX	Classical-AAA->?->?->?->?->tRNA->Arm-DNA-bind_3+Phage_integrase->XIS-HTH->BrxA-DNA-binding->BrxB-BrxF-iSTAND->DUF6079*->SF1-SF2-Helicase+PRTase->SLOG+BirA-HTH->N6-METHYLASE->ParB+HNH->PglZ->SIGMA+OB-Fold+AAA-ATPase+HKD-DNase-BrxL->?->XIS-HTH->	1187	Bacteria>Pseudomonadota>Gammaproteobacteria>Cellvibrionales>Porticoccaceae>Porticoccus	GCF_000744735.1	Porticoccus_hydrocarbonoclasticus	U740_RS05135	1073957..1077520	1073414	BREX-system-P-loop-protein-BrxC-[Porticoccus-hydrocarbonoclasticus]
MAT50826.1	Type1.BREX	<-N6-METHYLASE<-SLOG+BirA-HTH<-SF1-SF2-Helicase+PRTase<-Schlafen+HATPase+wHTH+HTH<-DUF6079*<-BrxB-BrxF-iSTAND<-BrxA-DNA-binding<-XIS-HTH||?->?-><-GDResolvase	1182	Bacteria>Pseudomonadota>Gammaproteobacteria>Cellvibrionales>Porticoccaceae>unclassified_Porticoccaceae	GCA_002702725.1	Porticoccaceae_bacterium	CMK32_06560	7929..11477	2026782	hypothetical-protein-[Porticoccaceae-bacterium]
WP_019531408.1	Type1.BREX	<-DUF6079*<-BrxB-BrxF-iSTAND<-BrxA-DNA-binding<-SF1-SF2-Helicase+REase<-Exonuc_V_gamma+REase<-MULE-TRANSPOSASE<-?<-TraJ-RHH||?->Peptidase_M50+PDZ_2+Lon_C->	1156	Bacteria>Pseudomonadota>Gammaproteobacteria>Cellvibrionales>Spongiibacteraceae>Dasania	GCF_000373485.1	Dasania_marina	B067_RS0117580	1458..4928	471499	BREX-system-P-loop-protein-BrxC-[Dasania-marina]
WP_268905143.1	Type1.BREX	<-PHP+SbcC<-REase+HTH+35exo+Small-B-barrel+SF1-SF2-Helicase<-REase+iREase<-PIWI<-REase+PNTD1+PNTD2<-SIGMA+OB-Fold+AAA-ATPase+Lon-Protease-BrxL<-PglZ<-N6-METHYLASE<-DUF6079*<-BrxB-BrxF-iSTAND<-BrxA-DNA-binding<-wHTH+WYL+WCX||Thioredoxin_4->	1219	Bacteria>Pseudomonadota>Gammaproteobacteria>Cellvibrionales>Spongiibacteraceae>Dasania	GCF_026891035.1	Dasania_sp._GY-19	OOV09_RS14670	22695..26354	2965066	BREX-system-P-loop-protein-BrxC-[Dasania-sp.-GY-19]
WP_268905395.1	Type1.BREX	Phage_integrase-><-?<-SMODS<-SLATT<-SIGMA+OB-Fold+AAA-ATPase+Lon-Protease-BrxL<-PglZ<-ParB+HNH<-N6-METHYLASE<-Schlafen+HATPase+wHTH<-DUF6079*<-BrxB-BrxF-iSTAND<-BrxA-DNA-binding<-wHTH+WYL+WCX||GGDEF+EAL-><-HTH_3	1220	Bacteria>Pseudomonadota>Gammaproteobacteria>Cellvibrionales>Spongiibacteraceae>Dasania	GCF_026891035.1	Dasania_sp._GY-19	OOV09_RS18305	21121..24783	2965066	BREX-system-P-loop-protein-BrxC-[Dasania-sp.-GY-19]
WP_269621356.1	Type1.BREX	wHTH+WYL+WCX->BrxA-DNA-binding->BrxB-BrxF-iSTAND->DUF6079*->MarR-HTH->AbiEii->?->N6-METHYLASE->DUF4158+DDE_Tnp_Tn3->	1219	Bacteria>Pseudomonadota>Gammaproteobacteria>Cellvibrionales>Spongiibacteraceae>Marortus	GCF_027257955.1	Marortus_sp._BJYM1	O9X78_RS19220	4639..8298	2975599	BREX-system-P-loop-protein-BrxC-[Marortus-sp.-BJYM1]
MBU73272.1	Type1.BREX	<-N6-METHYLASE-p<-DUF6079*<-BrxB-BrxF-iSTAND<-BrxA-DNA-binding<-?<-?<-TraI-VirD2-RCRE<-TraJ-RHH	1221	Bacteria>Pseudomonadota>Gammaproteobacteria>Cellvibrionales>Spongiibacteraceae>Spongiibacter	GCA_002730815.1	Spongiibacter_sp.	CMN81_14305	814..4479	2024860	ATPase-[Spongiibacter-sp.]
WP_190766547.1	Type1.BREX	ARG-><-SIGMA+OB-Fold+AAA-ATPase+Lon-Protease-BrxL<-DOC+HTH<-?<-ZNR+RmuC<-?<-?<-N6-METHYLASE<-ParB+HNH<-DUF6079*<-BrxB-BrxF-iSTAND<-BrxA-DNA-binding||wHTH+WYL+WCX-><-?<-SF1-SF2-Helicase	1192	Bacteria>Pseudomonadota>Gammaproteobacteria>Cellvibrionales>Spongiibacteraceae>Spongiibacter	GCF_014705705.1	Spongiibacter_pelagi	IB286_RS13890	27960..31538	2760804	BREX-system-P-loop-protein-BrxC-[Spongiibacter-pelagi]
WP_273033648.1	Type1.BREX	<-SIGMA+OB-Fold+AAA-ATPase+HKD-DNase-BrxL<-PglZ<-ParB+HNH<-N6-METHYLASE-p<-HerA/FtsK<-?<-CR-ATPase3<-N6-METHYLASE<-Schlafen+HATPase+wHTH+HTH<-DUF6079*<-BrxB-BrxF-iSTAND<-BrxA-DNA-binding<-XIS-HTH<-MobI||XIS-HTH->AAA+HTH->XIS-HTH-><-Arm-DNA-bind_3+Phage_integrase<-SIGMA-HTH	1193	Bacteria>Pseudomonadota>Gammaproteobacteria>Cellvibrionales>Spongiibacteraceae>Spongiibacter	GCF_950061765.1	Spongiibacter_tropicus	B4C78_RS03795	102796..106377	454602	BREX-system-P-loop-protein-BrxC-[Spongiibacter-tropicus]
EIF42662.1	Type1.BREX	wHTH+WYL+WCX->BrxA-DNA-binding->BrxB-BrxF-iSTAND->DUF6079*->MarR-HTH->AbiEii->?->N6-METHYLASE->PglZ->SIGMA+OB-Fold+AAA-ATPase+Lon-Protease-BrxL->SbcC->REase+iREase->HEPN->	1220	Bacteria>Pseudomonadota>Gammaproteobacteria>Cellvibrionales>Spongiibacteraceae>unclassified_Spongiibacteraceae	GCA_000259575.1	gamma_proteobacterium_BDW918	DOK_12571	2372..6034	1168065	putative-ATPase-like-protein-[gamma-proteobacterium-BDW918]
MBN49711.1	Type1.BREX	<-SIGMA+OB-Fold+AAA-ATPase+Lon-Protease-BrxL<-PglZ<-DOC+HTH<-?<-ZNR+RmuC<-HEPN<-?<-N6-METHYLASE<-?<-DUF6079*<-BrxB-BrxF-iSTAND<-BrxA-DNA-binding||wHTH+WYL+WCX->?-><-?<-SF1-SF2-Helicase||?->?->Phage_integrase->	1193	Bacteria>Pseudomonadota>Gammaproteobacteria>Cellvibrionales>Spongiibacteraceae>unclassified_Spongiibacteraceae	GCA_002723235.1	Spongiibacteraceae_bacterium	CMN85_09225	177592..181173	2026793	hypothetical-protein-[Spongiibacteraceae-bacterium]
MTW21824.1	Type1.BREX	<-SAM-METHYLASE<-SIGMA+OB-Fold+AAA-ATPase+HKD-DNase-BrxL<-PglZ<-ParB+HNH<-Schlafen+HATPase+wHTH<-PhdYeFM_antitox<-N6-METHYLASE<-REase<-OLD-ABC<-DUF6079*<-BrxB-BrxF-iSTAND<-BrxA-DNA-binding<-DnaB+HINT+DnaB+HINT	1173	Bacteria>Pseudomonadota>Gammaproteobacteria>Chromatiales>Chromatiaceae>Allochromatium	GCA_009720725.1	Allochromatium_palmeri	GJ668_12065	37225..40746	231048	BREX-system-P-loop-protein-BrxC-[Allochromatium-palmeri]
WP_186343071.1	Type1.BREX	<-SAM-METHYLASE<-SIGMA+OB-Fold+AAA-ATPase+HKD-DNase-BrxL<-PglZ<-ParB+HNH<-Schlafen+HATPase+wHTH<-PhdYeFM_antitox<-N6-METHYLASE<-REase<-OLD-ABC<-DUF6079*<-BrxB-BrxF-iSTAND<-BrxA-DNA-binding<-DnaB+HINT+DnaB+HINT	1172	Bacteria>Pseudomonadota>Gammaproteobacteria>Chromatiales>Chromatiaceae>Allochromatium	GCF_009720725.1	Allochromatium_palmeri	GJ668_RS12065	37225..40743	231048	BREX-system-P-loop-protein-BrxC-[Allochromatium-palmeri]
WP_176976536.1	Type1.BREX	<-SF1-SF2-Helicase<-?<-HD+HD_assoc<-SIGMA+OB-Fold+AAA-ATPase+Lon-Protease-BrxL<-PglZ<-TOPRIM<-OLD-ABC<-N6-METHYLASE<-DUF6079*<-BrxB-BrxF-iSTAND<-BrxA-DNA-binding<-?<-Arm-DNA-bind_3+Phage_integrase<-CHTH<-RelE	1198	Bacteria>Pseudomonadota>Gammaproteobacteria>Chromatiales>Chromatiaceae>Allochromatium	GCF_013385175.1	Allochromatium_humboldtianum	HW932_RS10995	221965..225561	504901	BREX-system-P-loop-protein-BrxC-[Allochromatium-humboldtianum]
WP_200158841.1	Type1.BREX	<-SIGMA+OB-Fold+AAA-ATPase+Lon-Protease-BrxL<-PglZ<-HNH<-OLD-ABC<-SF1-SF2-Helicase<-OLD-ABC+TOPRIM<-N6-METHYLASE<-PIN<-OLD-ABC<-DUF6079*<-BrxB-BrxF-iSTAND<-BrxA-DNA-binding<-GDResolvase+HIN-HTH<-ZnR	1154	Bacteria>Pseudomonadota>Gammaproteobacteria>Chromatiales>Chromatiaceae>Allochromatium	GCF_016584135.1	Allochromatium_vinosum	CKO29_RS16480	18892..22356	1049	BREX-system-P-loop-protein-BrxC-[Allochromatium-vinosum]
NEX15944.1	Type1.BREX	<-NTP_transf<-?<-SIGMA+OB-Fold+AAA-ATPase+HKD-DNase-BrxL<-PglZ-p<-PglZ<-ParB+HNH<-N6-METHYLASE<-REase<-OLD-ABC<-DUF6079*<-BrxB-BrxF-iSTAND<-BrxA-DNA-binding<-?<-?<-ABC-ATPase+SbcC<-?<-?||PALP->	1173	Bacteria>Pseudomonadota>Gammaproteobacteria>Chromatiales>Chromatiaceae>Halochromatium	GCA_010912705.1	Halochromatium_sp.	C1943_04720	67001..70522	2049430	BREX-system-P-loop-protein-BrxC-[Halochromatium-sp.]
WP_200346460.1	Type1.BREX	CR-ATPase5+REase->MazE_antitoxin->PemK_toxin->VapB_antitoxin->BrxA-DNA-binding->BrxB-BrxF-iSTAND->DUF6079*->RHH_6->N6-METHYLASE->OLD-ABC+TOPRIM->SF1-SF2-Helicase->Schlafen+HATPase+wHTH->MerR-HTH+GDResolvase->HTH_OrfB_IS605+OrfB_IS605+OrfB_Zn_ribbon->HTH_3->PhdYeFM_antitox->	1152	Bacteria>Pseudomonadota>Gammaproteobacteria>Chromatiales>Chromatiaceae>Halochromatium	GCF_016584085.1	Halochromatium_glycolicum	CKO40_RS12030	27931..31389	85075	BREX-system-P-loop-protein-BrxC-[Halochromatium-glycolicum]
WP_201220083.1	Type1.BREX	<-OLD-ABC<-SF1-SF2-Helicase<-OLD-ABC+TOPRIM<-N6-METHYLASE<-VapB_antitoxin<-PemK_toxin<-MazE_antitoxin<-PemK_toxin<-RHH<-DUF6079*<-BrxB-BrxF-iSTAND<-BrxA-DNA-binding<-?<-?<-?||ABhydrolase->MlaE->ABC-ATPase+SbcC->	1154	Bacteria>Pseudomonadota>Gammaproteobacteria>Chromatiales>Chromatiaceae>Halochromatium	GCF_016653315.1	Halochromatium_roseum	CCR96_RS10495	53560..57024	391920	BREX-system-P-loop-protein-BrxC-[Halochromatium-roseum]
WP_201222419.1	Type1.BREX	<-PCMT+METHYLASE<-?<-?<-?||?->tRNA->?->BrxA-DNA-binding->BrxB-BrxF-iSTAND->DUF6079*->OLD-ABC->REase->N6-METHYLASE->Schlafen+HATPase+wHTH->ParB+HNH->PglZ->REase->MIT_C->	1174	Bacteria>Pseudomonadota>Gammaproteobacteria>Chromatiales>Chromatiaceae>Halochromatium	GCF_016653315.1	Halochromatium_roseum	CCR96_RS19410	137258..140782	391920	BREX-system-P-loop-protein-BrxC-[Halochromatium-roseum]
WP_200250838.1	Type1.BREX	<-ParB+HNH<-HNH<-OLD-ABC<-N6-METHYLASE<-REase<-OLD-ABC<-DUF6079*<-BrxB-BrxF-iSTAND<-BrxA-DNA-binding	1173	Bacteria>Pseudomonadota>Gammaproteobacteria>Chromatiales>Chromatiaceae>Lamprobacter	GCF_016583905.1	Lamprobacter_modestohalophilus	CKO42_RS24315	8724..12245	1064514	BREX-system-P-loop-protein-BrxC-[Lamprobacter-modestohalophilus]
MBV5272983.1	Type1.BREX	<-PglZ<-?<-REase+AIPR<-N6-METHYLASE-p<-N6-METHYLASE-p<-N6-METHYLASE-p<-PIN<-MazE_antitoxin<-DUF6079*<-BrxB-BrxF-iSTAND<-BrxA-DNA-binding<-Aminotran_3<-FURR-HTH||?-><-?<-?<-?<-Classical-AAA	1143	Bacteria>Pseudomonadota>Gammaproteobacteria>Chromatiales>Chromatiaceae>Lamprocystis	GCA_019163055.1	Lamprocystis_purpurea	JZU52_04840	9550..12981	61598	BREX-system-P-loop-protein-BrxC-[Lamprocystis-purpurea]
WP_020505452.1	Type1.BREX	RNase-><-?<-?||BrxA-DNA-binding->BrxB-BrxF-iSTAND->DUF6079*-><-?||N6-METHYLASE->Schlafen+HATPase+wHTH->PglZ->SIGMA+OB-Fold+AAA-ATPase+Lon-Protease-BrxL->	1146	Bacteria>Pseudomonadota>Gammaproteobacteria>Chromatiales>Chromatiaceae>Lamprocystis	GCF_000379525.1	Lamprocystis_purpurea	A39O_RS0114850	122427..125867	61598	BREX-system-P-loop-protein-BrxC-[Lamprocystis-purpurea]
WP_020507234.1	Type1.BREX	<-PIN<-MazE_antitoxin<-DUF6079*<-BrxB-BrxF-iSTAND<-BrxA-DNA-binding<-Aminotran_3<-FURR-HTH	1153	Bacteria>Pseudomonadota>Gammaproteobacteria>Chromatiales>Chromatiaceae>Lamprocystis	GCF_000379525.1	Lamprocystis_purpurea	A39O_RS0124120	1671..5132	61598	BREX-system-P-loop-protein-BrxC-[Lamprocystis-purpurea]
WP_062271501.1	Type1.BREX	<-OLD-ABC<-RHH<-SF1-SF2-Helicase<-OLD-ABC+TOPRIM<-N6-METHYLASE<-PIN<-OLD-ABC<-PIN<-?<-DUF6079*<-BrxB-BrxF-iSTAND<-BrxA-DNA-binding<-tRNA<-Aminotran_1_2||SF1-SF2-Helicase->	1153	Bacteria>Pseudomonadota>Gammaproteobacteria>Chromatiales>Chromatiaceae>Marichromatium	GCF_001583505.1	Marichromatium_gracile	AY586_RS00820	23071..26532	1048	BREX-system-P-loop-protein-BrxC-[Marichromatium-gracile]
WP_134356376.1	Type1.BREX	<-DUF488-1<-DUF4326||?-><-?||?->DDE_Tnp_1_assoc+DDE_Tnp_1-><-?||BrxA-DNA-binding->BrxB-BrxF-iSTAND->DUF6079*->HicB_lk_antitox->HicA_toxin->N6-METHYLASE->PglZ->SIGMA+OB-Fold+AAA-ATPase+Lon-Protease-BrxL-><-?<-DDE_3<-HTH_23+HTH_32+HTH_33	1147	Bacteria>Pseudomonadota>Gammaproteobacteria>Chromatiales>Chromatiaceae>Nitrosococcus	GCF_004421105.1	Nitrosococcus_wardiae	E3U44_RS01695	339932..343375	1814290	BREX-system-P-loop-protein-BrxC-[Nitrosococcus-wardiae]
TXH98297.1	Type1.BREX	<-DUF6079*<-BrxB-BrxF-iSTAND<-BrxA-DNA-binding<-tRNA<-?||?->?-><-Glyco_hydro_3+Glyco_hydro_3_C+Fn3-like	1197	Bacteria>Pseudomonadota>Gammaproteobacteria>Chromatiales>Chromatiaceae>Rheinheimera	GCA_008015325.1	Rheinheimera_sp.	E6Q75_01850	1..3593	1869214	BREX-system-P-loop-protein-BrxC,-partial-[Rheinheimera-sp.]
WP_264346732.1	Type1.BREX	HEPN->?->PVC-AAA->?->?->?->GreA_GreB+SF1-SF2-Helicase->BrxA-DNA-binding->BrxB-BrxF-iSTAND->DUF6079*->N6-METHYLASE->OLD-ABC->HNH->ParB+HNH->PglZ->HEPN+Sel1+SEC-C-><-NERD+SF1-SF2-Helicase<-?<-SF1-SF2-Helicase+REase	1157	Bacteria>Pseudomonadota>Gammaproteobacteria>Chromatiales>Chromatiaceae>Rheinheimera	GCF_947090785.1	Rheinheimera_sp.	OM978_RS09960	2096767..2100240	1869214	BREX-system-P-loop-protein-BrxC-[Rheinheimera-sp.-MM224]
QVL48954.1	Type1.BREX	<-PglZ<-PIN<-ParD_antitoxin<-wHTH<-TOPRIM<-OLD-ABC<-N6-METHYLASE<-ACET+ACET<-TraJ-RHH<-DUF6079*<-BrxB-BrxF-iSTAND<-BrxA-DNA-binding<-tRNA||RADICAL-SAM->Glycos_transf_2-><-?||?-><-tRNA||NTP_transf+HDRnase->	1136	Bacteria>Pseudomonadota>Gammaproteobacteria>Chromatiales>Chromatiaceae>Thiocapsa	GCA_018399035.1	Thiocapsa_sp.	KFB96_26095	5699625..5703035	2024551	MAG:-BREX-system-P-loop-protein-BrxC-[Thiocapsa-sp.]
WP_120797099.1	Type1.BREX	<-HTH_3<-DDE_Tnp||?-><-?||?->?->BrxA-DNA-binding->BrxB-BrxF-iSTAND->DUF6079*->N6-METHYLASE-p-><-DDE_Tnp||ParB+HNH->PglZ->?->SIGMA+OB-Fold+AAA-ATPase+HKD-DNase-BrxL->	1173	Bacteria>Pseudomonadota>Gammaproteobacteria>Chromatiales>Chromatiaceae>Thiocapsa	GCF_003634315.1	Thiocapsa_rosea	BDD21_RS10290	2320412..2323933	69360	BREX-system-P-loop-protein-BrxC-[Thiocapsa-rosea]
WP_200385867.1	Type1.BREX	DUF6079*->?->PIN-><-?||N6-METHYLASE->OLD-ABC->HNH->PglZ->AbrB-HTH->RHH->	1179	Bacteria>Pseudomonadota>Gammaproteobacteria>Chromatiales>Chromatiaceae>Thiocapsa	GCF_016583985.1	Thiocapsa_imhoffii	CKO25_RS00005	26..3565	382777	BREX-system-P-loop-protein-BrxC-[Thiocapsa-imhoffii]
WP_200387145.1	Type1.BREX	<-SIGMA+OB-Fold+AAA-ATPase+Lon-Protease-BrxL<-PglZ<-wHTH<-TOPRIM<-OLD-ABC<-N6-METHYLASE<-ACET+ACET<-TraJ-RHH<-DUF6079*<-BrxB-BrxF-iSTAND<-BrxA-DNA-binding<-tRNA||RADICAL-SAM->Glycos_transf_2-><-?||?-><-tRNA||NTP_transf+HDRnase->	1136	Bacteria>Pseudomonadota>Gammaproteobacteria>Chromatiales>Chromatiaceae>Thiocapsa	GCF_016583985.1	Thiocapsa_imhoffii	CKO25_RS06645	47680..51090	382777	BREX-system-P-loop-protein-BrxC-[Thiocapsa-imhoffii]
WP_200389392.1	Type1.BREX	<-PAS+PAS+PAS+GGDEF<-?<-RuvB-AAA+RuvB-HTH<-?||tRNA->BrxA-DNA-binding->BrxB-BrxF-iSTAND->DUF6079*->MazE_antitoxin->MazE_antitoxin->AbrB-HTH->PIN->?->?->?->OLD-ABC->TOPRIM->	1193	Bacteria>Pseudomonadota>Gammaproteobacteria>Chromatiales>Chromatiaceae>Thiocapsa	GCF_016583985.1	Thiocapsa_imhoffii	CKO25_RS18260	8675..12256	382777	BREX-system-P-loop-protein-BrxC-[Thiocapsa-imhoffii]
WP_232409231.1	Type1.BREX	<-Tox-RelE<-TOPRIM<-OLD-ABC<-?<-PIN<-VapB_antitoxin<-VapB_antitoxin<-PemK_toxin<-MazE_antitoxin<-DUF6079-p*||DDE_Tnp_1_4-><-DUF6079-p<-BrxB-BrxF-iSTAND<-BrxA-DNA-binding<-?<-HEPN<-Ntox45<-HTH_Tnp_1+HTH_21+rve+rve_3	909	Bacteria>Pseudomonadota>Gammaproteobacteria>Chromatiales>Chromatiaceae>Thiocapsa	GCF_021228795.1	Thiocapsa_bogorovii	LT988_RS05545	1229820..1232549	521689	BREX-system-P-loop-protein-BrxC-[Thiocapsa-bogorovii]
WP_232409232.1	Type1.BREX	<-OLD-ABC<-?<-PIN<-VapB_antitoxin<-VapB_antitoxin<-PemK_toxin<-MazE_antitoxin<-DUF6079-p||DDE_Tnp_1_4-><-DUF6079-p*<-BrxB-BrxF-iSTAND<-BrxA-DNA-binding<-?<-HEPN<-Ntox45<-HTH_Tnp_1+HTH_21+rve+rve_3<-?||ICLR-HTH->MoxR-AAA->	245	Bacteria>Pseudomonadota>Gammaproteobacteria>Chromatiales>Chromatiaceae>Thiocapsa	GCF_021228795.1	Thiocapsa_bogorovii	LT988_RS05555	1234280..1235017	521689	hypothetical-protein-[Thiocapsa-bogorovii]
WP_100919677.1	Type1.BREX	<-PspA||REC+OmpR-HTH->HISKIN->?-><-CASPASE||BrxA-DNA-binding->BrxB-BrxF-iSTAND->DUF6079*->?->TraJ-RHH->N6-METHYLASE->OLD-ABC->TOPRIM->wHTH->PglZ->SIGMA+OB-Fold+AAA-ATPase+Lon-Protease-BrxL-><-ROK-HTH	1156	Bacteria>Pseudomonadota>Gammaproteobacteria>Chromatiales>Chromatiaceae>Thiodictyon	GCF_002813775.1	Candidatus_Thiodictyon_syntrophicum	THSYN_RS13895	3302661..3306131	1166950	BREX-system-P-loop-protein-BrxC-[Candidatus-Thiodictyon-syntrophicum]
WP_100920747.1	Type1.BREX	BrxA-DNA-binding->BrxB-BrxF-iSTAND->?->DUF6079*->PhdYeFM_antitox->PIN->MqsA_antitoxin->N6-METHYLASE-p->N6-METHYLASE->Transposase_31->OLD-ABC->Polbeta->PglZ->	1194	Bacteria>Pseudomonadota>Gammaproteobacteria>Chromatiales>Chromatiaceae>Thiodictyon	GCF_002813775.1	Candidatus_Thiodictyon_syntrophicum	THSYN_RS20415	4833326..4836910	1166950	BREX-system-P-loop-protein-BrxC-[Candidatus-Thiodictyon-syntrophicum]
MBK1629582.1	Type1.BREX	<-SbcC<-?<-RFC-AAA+DNAA-HTH||?->RNaseP->?->sGTP->BrxA-DNA-binding->BrxB-BrxF-iSTAND->DUF6079*->MazE_antitoxin->PemK_toxin->DOC+HTH->N6-METHYLASE-p->PHP+SbcC->Schlafen+HATPase+wHTH->PglZ->SIGMA+OB-Fold+AAA-ATPase+Lon-Protease-BrxL->	1152	Bacteria>Pseudomonadota>Gammaproteobacteria>Chromatiales>Chromatiaceae>Thiohalocapsa	GCA_016583825.1	Thiohalocapsa_halophila	CKO31_02265	100295..103753	69359	hypothetical-protein-[Thiohalocapsa-halophila]
MCG6940983.1	Type1.BREX	<-N6-METHYLASE-p<-PIN<-VapB_antitoxin<-DUF6079-p*<-tRNA<-?<-AA_kinase+ACT+ACT_7	251	Bacteria>Pseudomonadota>Gammaproteobacteria>Chromatiales>Chromatiaceae>Thiohalocapsa	GCA_022340605.1	Thiohalocapsa_sp.	LJE69_07005	1995..2750	2497641	hypothetical-protein-[Thiohalocapsa-sp.]
WP_058554877.1	Type1.BREX	DDE_Tnp_1->?->?->?->?->AA_kinase+ACT+ACT_7->?->tRNA->BrxA-DNA-binding->BrxB-BrxF-iSTAND->DUF6079*->VapB_antitoxin->VapB_antitoxin->PIN-><-?||N6-METHYLASE->	1152	Bacteria>Pseudomonadota>Gammaproteobacteria>Chromatiales>Chromatiaceae>Thiohalocapsa	GCF_001469165.1	Thiohalocapsa_sp._ML1	ML01_RS08025	156057..159515	1431688	BREX-system-P-loop-protein-BrxC-[Thiohalocapsa-sp.-ML1]
WP_242475077.1	Type1.BREX	HAD+HAD-><-MORC+DNA_gyrase+TOPRIM<-SbcC<-RFC-AAA+DNAA-HTH||?->?->sGTP->BrxA-DNA-binding->BrxB-BrxF-iSTAND->DUF6079*->MazE_antitoxin->PemK_toxin->RHH_6-><-?||N6-METHYLASE->DOC+HTH->N6-METHYLASE-p->PHP+SbcC->Schlafen+HATPase+wHTH->	1154	Bacteria>Pseudomonadota>Gammaproteobacteria>Chromatiales>Chromatiaceae>Thiohalocapsa	GCF_016583825.1	Thiohalocapsa_halophila	CKO31_RS02315	100289..103753	69359	BREX-system-P-loop-protein-BrxC-[Thiohalocapsa-halophila]
WP_200239121.1	Type1.BREX	<-PglZ<-PIN<-PhdYeFM_antitox<-HicA_toxin<-HicB_lk_antitox<-MazE_antitoxin<-PIN<-MazE_antitoxin<-DUF6079*<-BrxB-BrxF-iSTAND<-BrxA-DNA-binding<-?<-ABhydrolase<-?<-?||?->?-><-REase	1197	Bacteria>Pseudomonadota>Gammaproteobacteria>Chromatiales>Chromatiaceae>Thiohalocapsa	GCF_016583825.1	Thiohalocapsa_halophila	CKO31_RS15145	13443..17036	69359	BREX-system-P-loop-protein-BrxC-[Thiohalocapsa-halophila]
EIC19850.1	Type1.BREX	<-SIGMA+OB-Fold+AAA-ATPase+Lon-Protease-BrxL<-PglZ<-RelE-ParE<-PhdYeFM_antitox<-Schlafen+HATPase+wHTH<-N6-METHYLASE<-PIN<-VapB_antitoxin<-DUF6079*<-BrxB-BrxF-iSTAND<-BrxA-DNA-binding<-?<-HISKIN<-?<-?<-?<-Thioredoxin	1152	Bacteria>Pseudomonadota>Gammaproteobacteria>Chromatiales>Chromatiaceae>Thiorhodovibrio	GCA_000228725.3	Thiorhodovibrio_sp._970	Thi970DRAFT_03455	369329..372787	631362	hypothetical-protein-Thi970DRAFT_03455-[Thiorhodovibrio-sp.-970]
WP_223296802.1	Type1.BREX	<-SIGMA+OB-Fold+AAA-ATPase+Lon-Protease-BrxL<-PglZ<-RelE-ParE<-PhdYeFM_antitox<-Schlafen+HATPase+wHTH<-N6-METHYLASE<-PIN<-VapB_antitoxin<-DUF6079*<-BrxB-BrxF-iSTAND<-BrxA-DNA-binding<-?<-HISKIN<-?<-?<-Thioredoxin||?->REC+GGDEF+EAL->	1154	Bacteria>Pseudomonadota>Gammaproteobacteria>Chromatiales>Chromatiaceae>Thiorhodovibrio	GCF_000228725.2	Thiorhodovibrio_sp._970	THI970DRAFT_RS16355	369329..372793	631362	BREX-system-P-loop-protein-BrxC-[Thiorhodovibrio-sp.-970]
WP_201066887.1	Type1.BREX	<-wHTH<-?<-PIN<-?<-N6-METHYLASE<-HicB_lk_antitox<-MazE_antitoxin<-?<-PIN<-DUF6079*<-BrxB-BrxF-iSTAND<-BrxA-DNA-binding<-tRNA<-Sulfate_transp+STAS	1203	Bacteria>Pseudomonadota>Gammaproteobacteria>Chromatiales>Chromatiaceae>Thiorhodovibrio	GCF_016653415.1	Thiorhodovibrio_winogradskyi	CCR91_RS10560	25140..28751	77007	BREX-system-P-loop-protein-BrxC-[Thiorhodovibrio-winogradskyi]
MBK8638067.1	Type1.BREX	AAA-ATPase->?->ZnR->?->?->DUF6079*->PhdYeFM_antitox->AbrB-HTH->PIN->N6-METHYLASE->PIN->?->PglZ->SIGMA+OB-Fold+AAA-ATPase+Lon-Protease-BrxL-><-SIR2	1067	Bacteria>Pseudomonadota>Gammaproteobacteria>Chromatiales>Chromatiaceae>unclassified_Chromatiaceae	GCA_016714645.1	Chromatiaceae_bacterium	IPN92_07155	42706..45909	2049432	BREX-system-P-loop-protein-BrxC-[Chromatiaceae-bacterium]
MBP6581931.1	Type1.BREX	<-SIGMA+OB-Fold+AAA-ATPase+Lon-Protease-BrxL<-PglZ<-?<-?<-PglZ-p<-SbcC<-CCDC158<-Calcineurin-SbcD<-N6-METHYLASE<-DUF6079*<-BrxB-BrxF-iSTAND<-BrxA-DNA-binding<-tRNA||?-><-?<-?<-?<-?<-?<-ABC-ATPase+SbcC	1144	Bacteria>Pseudomonadota>Gammaproteobacteria>Chromatiales>Chromatiaceae>unclassified_Chromatiaceae	GCA_017989555.1	Chromatiaceae_bacterium	KA204_00415	17018..20452	2049432	BREX-system-P-loop-protein-BrxC-[Chromatiaceae-bacterium]
MBP6733459.1	Type1.BREX	BrxA-DNA-binding->BrxB-BrxF-iSTAND->DUF6079*-><-BrxB-BrxF-iSTAND||?->PhdYeFM_antitox->PIN->N6-METHYLASE->HTH+REase->REase->N6-METHYLASE-p->	1171	Bacteria>Pseudomonadota>Gammaproteobacteria>Chromatiales>Chromatiaceae>unclassified_Chromatiaceae	GCA_017990755.1	Chromatiaceae_bacterium	KA142_01990	1791..5306	2049432	BREX-system-P-loop-protein-BrxC-[Chromatiaceae-bacterium]
MBP6806725.1	Type1.BREX	<-SIGMA+OB-Fold+AAA-ATPase+Lon-Protease-BrxL<-PglZ<-?<-?<-PglZ-p<-SbcC<-CCDC158<-Calcineurin-SbcD<-N6-METHYLASE<-DUF6079-p*	782	Bacteria>Pseudomonadota>Gammaproteobacteria>Chromatiales>Chromatiaceae>unclassified_Chromatiaceae	GCA_017991215.1	Chromatiaceae_bacterium	KA125_00340	14653..17002	2049432	BREX-system-P-loop-protein-BrxC-[Chromatiaceae-bacterium]
MBP6807296.1	Type1.BREX	BrxA-DNA-binding->BrxB-BrxF-iSTAND->DUF6079*->MazE_antitoxin->PIN->N6-METHYLASE-p->AbrB-HTH->	1194	Bacteria>Pseudomonadota>Gammaproteobacteria>Chromatiales>Chromatiaceae>unclassified_Chromatiaceae	GCA_017991215.1	Chromatiaceae_bacterium	KA125_03275	984..4568	2049432	BREX-system-P-loop-protein-BrxC-[Chromatiaceae-bacterium]
MCF7978332.1	Type1.BREX	<-AbrB-HTH<-PIN<-AbrB-HTH<-N6-METHYLASE<-DOC<-MazE_antitoxin<-MqsA_antitoxin<-?<-PIN<-DUF6079*	1223	Bacteria>Pseudomonadota>Gammaproteobacteria>Chromatiales>Chromatiaceae>unclassified_Chromatiaceae	GCA_021736685.1	Chromatiaceae_bacterium	K9L82_09985	9865..13536	2049432	BREX-system-P-loop-protein-BrxC-[Chromatiaceae-bacterium]
MCF8004221.1	Type1.BREX	<-DUF6079-p*<-BrxB-BrxF-iSTAND<-BrxA-DNA-binding<-ABC-ATPase+SbcC<-?<-?<-?||PALP->	370	Bacteria>Pseudomonadota>Gammaproteobacteria>Chromatiales>Chromatiaceae>unclassified_Chromatiaceae	GCA_021736845.1	Chromatiaceae_bacterium	K9L32_08450	1..1111	2049432	BREX-system-P-loop-protein-BrxC-[Chromatiaceae-bacterium]
MCF8016509.1	Type1.BREX	<-AbrB-HTH<-PIN<-AbrB-HTH<-N6-METHYLASE<-DOC<-MazE_antitoxin<-MqsA_antitoxin<-?<-PIN<-DUF6079-p*	835	Bacteria>Pseudomonadota>Gammaproteobacteria>Chromatiales>Chromatiaceae>unclassified_Chromatiaceae	GCA_021736865.1	Chromatiaceae_bacterium	K9L65_12595	6416..8923	2049432	BREX-system-P-loop-protein-BrxC-[Chromatiaceae-bacterium]
MCP5407916.1	Type1.BREX	ABhydrolase->?->TraU->?->TraG_N-><-?||BrxA-DNA-binding->BrxB-BrxF-iSTAND->DUF6079*->MazE_antitoxin->PIN->N6-METHYLASE->SIR2->?->PglZ->SIGMA+OB-Fold+AAA-ATPase+Lon-Protease-BrxL->XIS-HTH->	1194	Bacteria>Pseudomonadota>Gammaproteobacteria>Chromatiales>Chromatiaceae>unclassified_Chromatiaceae	GCA_024235335.1	Chromatiaceae_bacterium	H6963_01295	248686..252270	2049432	BREX-system-P-loop-protein-BrxC-[Chromatiaceae-bacterium]
WP_018716649.1	Type1.BREX	HerA/FtsK->Relaxase+SF1-SF2-Helicase->?->XIS-HTH->BrxA-DNA-binding->BrxB-BrxF-iSTAND->DUF6079*->Schlafen+HATPase+wHTH->N6-METHYLASE->ParB+HNH->PglZ->SIGMA+OB-Fold+AAA-ATPase+Lon-Protease-BrxL->	1144	Bacteria>Pseudomonadota>Gammaproteobacteria>Chromatiales>Ectothiorhodospiraceae>Arhodomonas	GCF_000374645.1	Arhodomonas_aquaeolei	C516_RS0101825	422257..425691	2369	BREX-system-P-loop-protein-BrxC-[Arhodomonas-aquaeolei]
WP_063464066.1	Type1.BREX	ZnR->RADICAL-SAM->?->?->?->GGDEF->XIS-HTH->BrxA-DNA-binding->BrxB-BrxF-iSTAND->DUF6079*->Schlafen+HATPase+wHTH+HTH->N6-METHYLASE-p->OLD-ABC->REase->SIR2->HerA/FtsK->N6-METHYLASE-p->OLD-ABC->HNH->	1189	Bacteria>Pseudomonadota>Gammaproteobacteria>Chromatiales>Ectothiorhodospiraceae>Ectothiorhodospira	GCF_001632845.1	Ectothiorhodospira_sp._BSL-9	ECTOBSL9_RS04500	914827..918396	1442136	BREX-system-P-loop-protein-BrxC-[Ectothiorhodospira-sp.-BSL-9]
WP_238627878.1	Type1.BREX	<-DUF6079-p*<-BrxB-BrxF-iSTAND<-BrxA-DNA-binding<-XIS-HTH<-?<-?<-?<-RADICAL-SAM<-ZnR||SWACOS->	688	Bacteria>Pseudomonadota>Gammaproteobacteria>Chromatiales>Ectothiorhodospiraceae>Ectothiorhodospira	GCF_022227565.1	Ectothiorhodospira_sp._9100	LPW29_RS09995	1..2064	2897388	BREX-system-P-loop-protein-BrxC,-partial-[Ectothiorhodospira-sp.-9100]
WP_238631208.1	Type1.BREX	<-PglZ<-TOPRIM<-OLD-ABC<-N6-METHYLASE-p<-HerA/FtsK<-?<-CR-ATPase3<-N6-METHYLASE<-Schlafen+HATPase+wHTH+HTH<-DUF6079*<-BrxB-BrxF-iSTAND<-BrxA-DNA-binding<-XIS-HTH<-?<-?<-?<-RADICAL-SAM<-ZnR||SWACOS->	1189	Bacteria>Pseudomonadota>Gammaproteobacteria>Chromatiales>Ectothiorhodospiraceae>Ectothiorhodospira	GCF_022227695.1	Ectothiorhodospira_sp._9905	LPW28_RS03070	43824..47393	2897387	BREX-system-P-loop-protein-BrxC-[Ectothiorhodospira-sp.-9905]
WP_238620557.1	Type1.BREX	sGTP-><-35exo+T7DNApol||?->?->BrxA-DNA-binding->BrxB-BrxF-iSTAND->DUF6079*->N6-METHYLASE->OLD-ABC->HNH->OLD-ABC->HNH->PglZ->SIGMA+OB-Fold+AAA-ATPase+Lon-Protease-BrxL-><-Schlafen+HATPase+wHTH<-Schlafen+HATPase+wHTH	1154	Bacteria>Pseudomonadota>Gammaproteobacteria>Chromatiales>Ectothiorhodospiraceae>Ectothiorhodospira	GCF_022227785.1	Ectothiorhodospira_sp._B14B	LQ952_00335	78448..81912	2899127	BREX-system-P-loop-protein-BrxC-[Ectothiorhodospira-sp.-B14B]
WP_090207826.1	Type1.BREX	SbcC->REase->OrfB_IS605+OrfB_Zn_ribbon-><-SIGMA+OB-Fold+AAA-ATPase+Lon-Protease-BrxL<-p<-Phage_AlpA<-PglZ<-TOPRIM<-OLD-ABC<-N6-METHYLASE<-DUF6079*<-BrxB-BrxF-iSTAND<-BrxA-DNA-binding||tRNA-><-?<-REC+REC<-tRNA<-tRNA<-?<-?<-?<-?<-GAF+PAS+GGDEF+EAL	1231	Bacteria>Pseudomonadota>Gammaproteobacteria>Chromatiales>Ectothiorhodospiraceae>Ectothiorhodospira	GCF_900110965.1	Ectothiorhodospira_magna	BMX62_RS09270	49857..53552	867345	BREX-system-P-loop-protein-BrxC-[Ectothiorhodospira-magna]
MBK5942672.1	Type1.BREX	<-ParB+HNH<-?<-N6-METHYLASE<-DUF6079*<-BrxB-BrxF-iSTAND<-BrxA-DNA-binding<-SbcC+CCDC158<-Calcineurin<-?<-STAS<-HDRnase<-?<-Arm-DNA-bind_2+Phage_int_SAM_3+Phage_integrase	1146	Bacteria>Pseudomonadota>Gammaproteobacteria>Chromatiales>Ectothiorhodospiraceae>Halorhodospira	GCA_016653405.1	Halorhodospira_halophila	CCR79_02095	7976..11416	1053	hypothetical-protein-[Halorhodospira-halophila]
WP_242510795.1	Type1.BREX	<-ParB+HNH<-?<-N6-METHYLASE<-DUF6079*<-BrxB-BrxF-iSTAND<-BrxA-DNA-binding<-Calcineurin<-?<-STAS<-HDRnase<-?<-Arm-DNA-bind_2+Phage_int_SAM_3+Phage_integrase<-Phage_AlpA	1148	Bacteria>Pseudomonadota>Gammaproteobacteria>Chromatiales>Ectothiorhodospiraceae>Halorhodospira	GCF_016653405.1	Halorhodospira_halophila	CCR79_RS02135	7976..11422	1053	BREX-system-P-loop-protein-BrxC-[Halorhodospira-halophila]
AGM41053.1	Type1.BREX	PhdYeFM_antitox->PIN-><-?<-nSTAND1+HNH||?-><-ParB+HNH<-SIGMA+OB-Fold+AAA-ATPase+Lon-Protease-BrxL<-PglZ<-N6-METHYLASE<-DUF6079*<-BrxB-BrxF-iSTAND<-BrxA-DNA-binding<-CopG/RHH<-?||?->?->SAM-METHYLASE-><-MerR-HTH	1145	Bacteria>Pseudomonadota>Gammaproteobacteria>Chromatiales>Ectothiorhodospiraceae>Spiribacter	GCA_000319575.2	Spiribacter_salinus_M19-40	SPISAL_04795	979011..982448	1260251	hypothetical-protein-SPISAL_04795-[Spiribacter-salinus-M19-40]
TQE98798.1	Type1.BREX	BrxA-DNA-binding->BrxB-BrxF-iSTAND->DUF6079*->Schlafen+HATPase+wHTH->N6-METHYLASE-p->	1193	Bacteria>Pseudomonadota>Gammaproteobacteria>Chromatiales>Ectothiorhodospiraceae>Spiribacter	GCA_006569195.1	Spiribacter_salinus	FKY71_11930	1820..5401	1335746	BREX-system-P-loop-protein-BrxC-[Spiribacter-salinus]
KAF0285942.1	Type1.BREX	<-Phage_AlpA||?->?->?->TPR->?->CopG/RHH->BrxA-DNA-binding->BrxB-BrxF-iSTAND->DUF6079*->N6-METHYLASE->PglZ->SIGMA+OB-Fold+AAA-ATPase+Lon-Protease-BrxL->ParB+HNH->	1145	Bacteria>Pseudomonadota>Gammaproteobacteria>Chromatiales>Ectothiorhodospiraceae>Spiribacter	GCA_009805925.1	Spiribacter_sp._SSL99	BA899_01280	102096..105533	1866884	hypothetical-protein-BA899_01280-[Spiribacter-sp.-SSL99]
WP_245539889.1	Type1.BREX	PhdYeFM_antitox->PIN-><-?<-nSTAND1+HNH<-?<-ParB+HNH<-SIGMA+OB-Fold+AAA-ATPase+Lon-Protease-BrxL<-PglZ<-N6-METHYLASE<-DUF6079*<-BrxB-BrxF-iSTAND<-BrxA-DNA-binding<-CopG/RHH<-?||?->?->SAM-METHYLASE->?-><-MerR-HTH	1151	Bacteria>Pseudomonadota>Gammaproteobacteria>Chromatiales>Ectothiorhodospiraceae>Spiribacter	GCF_000319575.2	Spiribacter_salinus	SPISAL_RS04875	979011..982466	1335746	BREX-system-P-loop-protein-BrxC-[Spiribacter-salinus]
WP_154297058.1	Type1.BREX	<-ParB+HNH<-SIGMA+OB-Fold+AAA-ATPase+Lon-Protease-BrxL<-PglZ<-Polbeta<-MarR-HTH<-N6-METHYLASE<-DUF6079*<-BrxB-BrxF-iSTAND<-BrxA-DNA-binding||SIGMA-HTH+HIN-HTH-><-?<-MerR-HTH<-HTH_Tnp_1+HTH_21+rve+rve_3<-?<-DNA_METHYLASE	1145	Bacteria>Pseudomonadota>Gammaproteobacteria>Chromatiales>Ectothiorhodospiraceae>Spiribacter	GCF_009676705.1	Spiribacter_sp._2438	GJ672_RS05635	1128117..1131554	2666185	BREX-system-P-loop-protein-BrxC-[Spiribacter-sp.-2438]
WP_235013293.1	Type1.BREX	TPR->?->CopG/RHH->BrxA-DNA-binding->BrxB-BrxF-iSTAND->DUF6079*->N6-METHYLASE->PglZ->SIGMA+OB-Fold+AAA-ATPase+Lon-Protease-BrxL->ParB+HNH->	1151	Bacteria>Pseudomonadota>Gammaproteobacteria>Chromatiales>Ectothiorhodospiraceae>Spiribacter	GCF_009805925.1	Spiribacter_sp._SSL99	BA899_RS04165	102078..105533	1866884	BREX-system-P-loop-protein-BrxC-[Spiribacter-sp.-SSL99]
WP_244406389.1	Type1.BREX	<-PVC-AAA+Classical-AAA<-?||wHTH+WYL+WCX->?->?->?->BrxA-DNA-binding->BrxB-BrxF-iSTAND->DUF6079*->MULE-TRANSPOSASE->DUF6079-p->REase->N6-METHYLASE->MarR-HTH->Polbeta->PglZ->SIGMA+OB-Fold+AAA-ATPase+Lon-Protease-BrxL->HIN-HTH->	1041	Bacteria>Pseudomonadota>Gammaproteobacteria>Chromatiales>Ectothiorhodospiraceae>Thioalkalivibrio	GCF_000025545.1	Thioalkalivibrio_sp._K90mix	TK90_RS05670	1196495..1199620	396595	BREX-system-P-loop-protein-BrxC-[Thioalkalivibrio-sp.-K90mix]
WP_019570731.1	Type1.BREX	BrxA-DNA-binding->BrxB-BrxF-iSTAND->DUF6079*->?->N6-METHYLASE->MarR-HTH->Polbeta->PglZ->SIGMA+OB-Fold+AAA-ATPase+Lon-Protease-BrxL-><-AAA-ToxA+Classical-AAA	1145	Bacteria>Pseudomonadota>Gammaproteobacteria>Chromatiales>Ectothiorhodospiraceae>Thioalkalivibrio	GCF_000381205.1	Thioalkalivibrio_sp._ALE11	F620_RS0111965	2244..5681	1265494	BREX-system-P-loop-protein-BrxC-[Thioalkalivibrio-sp.-ALE11]
WP_019570748.1	Type1.BREX	PglZ->SIGMA+OB-Fold+AAA-ATPase+Lon-Protease-BrxL-><-AAA-ToxA+Classical-AAA||?->?->?->wHTH+WYL+WCX->BrxA-DNA-binding->BrxB-BrxF-iSTAND->DUF6079*->SF1-SF2-Helicase+PRTase->SLOG+BirA-HTH->N6-METHYLASE->ParB+HNH->PglZ->SIGMA+OB-Fold+AAA-ATPase+Lon-Protease-BrxL->?->Phage_integrase->	1225	Bacteria>Pseudomonadota>Gammaproteobacteria>Chromatiales>Ectothiorhodospiraceae>Thioalkalivibrio	GCF_000381205.1	Thioalkalivibrio_sp._ALE11	F620_RS0112040	26049..29726	1265494	BREX-system-P-loop-protein-BrxC-[Thioalkalivibrio-sp.-ALE11]
QEP43020.1	Type1.BREX	<-DDE_Tnp_1<-HTH_Tnp_1+HTH_21+rve+rve_3<-HTH_Tnp_1<-TOPRIM<-OLD-ABC<-RNase<-N6-METHYLASE<-HNH<-OLD-ABC<-DUF6079*<-BrxB-BrxF-iSTAND<-BrxA-DNA-binding<-Phage_holin<-?<-?||GDResolvase+ZnR->?->DDE_Tnp_IS66->HD+SF1-SF2-Helicase->	1145	Bacteria>Pseudomonadota>Gammaproteobacteria>Chromatiales>Ectothiorhodospiraceae>unclassified_Ectothiorhodospiraceae	GCA_008375315.1	Ectothiorhodospiraceae_bacterium_BW-2	D5085_07760	1613185..1616622	947515	BREX-system-P-loop-protein-BrxC-[Ectothiorhodospiraceae-bacterium-BW-2]

Protein-ID	System	Gene-Neighbourhoods	Length	Taxonomy	Assembly-ID	Strain	Locus-Tag	Genomic-coordinates	TaxID	Fasta-define
MCB1864346.1	Type1.BREX	<-DNA_METHYLASE||?->TraU->TraH->?->TraG_N-><-?||BrxA-DNA-binding->BrxB-BrxF-iSTAND->DUF6079*->N6-METHYLASE->PglZ->SIGMA+OB-Fold+AAA-ATPase+Lon-Protease-BrxL->REase+TIR->PIWI->PIWI-> [illegible]	1147	Bacteria>Pseudomonadota>Gammaproteobacteria>Chromatiales>unclassified_Chromatiales	GCA_020445605.1	Chromatiales_bacterium	KDG50_02880	27912..31355	2026725	BREX-system-P-loop-protein-BrxC-[Chromatiales-bacterium]
MCK7578720.1	Type1.BREX	BrxA-DNA-binding->BrxB-BrxF-iSTAND->DUF6079-p->DUF6079-p*->DUF6079-p->PhdYeFM_antitox->RelE-ParE->N6-METHYLASE-p->N6-METHYLASE-p->N6-METHYLASE-p->HicA_toxin->HicB-><-PemK_toxin	647	Bacteria>Pseudomonadota>Gammaproteobacteria>Chromatiales>unclassified_Chromatiales	GCA_023156155.1	Chromatiales_bacterium	MZV65_25270	40289..42232	2026725	BREX-system-P-loop-protein-BrxC-[Chromatiales-bacterium]
MCK7579547.1	Type1.BREX	<-OLD-ABC+TOPRIM<-?<-?<-N6-METHYLASE<-PIN<-OLD-ABC||?-><-DUF6079-p<-DUF6079-p*<-?<-DUF6079-p<-BrxB-BrxF-iSTAND<-BrxA-DNA-binding-p<-ZnR	299	Bacteria>Pseudomonadota>Gammaproteobacteria>Chromatiales>unclassified_Chromatiales	GCA_023156155.1	Chromatiales_bacterium	MZV65_30115	14491..15390	2026725	hypothetical-protein-[Chromatiales-bacterium]
MCK7579549.1	Type1.BREX	<-N6-METHYLASE<-PIN<-OLD-ABC||?-><-DUF6079-p<-DUF6079-p<-?<-DUF6079-p*<-BrxB-BrxF-iSTAND<-BrxA-DNA-binding-p<-ZnR	648	Bacteria>Pseudomonadota>Gammaproteobacteria>Chromatiales>unclassified_Chromatiales	GCA_023156155.1	Chromatiales_bacterium	MZV65_30125	15595..17541	2026725	BREX-system-P-loop-protein-BrxC-[Chromatiales-bacterium]
RFF27207.1	Type1.BREX	<-SbcC+SbcC<-?<-HEPN<-?||Integrase_1-><-?<-SIGMA+OB-Fold+AAA-ATPase+Lon-Protease-BrxL<-PglZ<-N6-METHYLASE<-DUF6079*<-BrxB-BrxF-iSTAND<-BrxA-DNA-binding<-?<-REase|| [illegible]	1166	Bacteria>Pseudomonadota>Gammaproteobacteria>Chromatiales>Wenzhouxiangellaceae>Wenzhouxiangella	GCA_003410035.1	Wenzhouxiangella_sp._15181	DZK25_09525	145045..148545	2301224	BREX-system-P-loop-protein-BrxC-[Wenzhouxiangella-sp.-15181]
WP_181918413.1	Type1.BREX	<-SbcC+SbcC<-?<-HEPN<-?||Integrase_1-><-?<-SIGMA+OB-Fold+AAA-ATPase+Lon-Protease-BrxL<-PglZ<-N6-METHYLASE<-DUF6079*<-BrxB-BrxF-iSTAND<-BrxA-DNA-binding<-?<-REase|| [illegible]	1149	Bacteria>Pseudomonadota>Gammaproteobacteria>Chromatiales>Wenzhouxiangellaceae>Wenzhouxiangella	GCF_003410035.1	unclassified_Wenzhouxiangella	DZK25_RS09530	145045..148494	2613841	MULTISPECIES:-BREX-system-P-loop-protein-BrxC-[unclassified-Wenzhouxiangella]
RJT19525.1	Type1.BREX	<-PglZ<-RVT<-HEPN<-N6-METHYLASE<-ParB+HNH<-DUF6079*<-BrxB-BrxF-iSTAND<-BrxA-DNA-binding<-?<-XIS-HTH||?->?->DUF932->?->wHTH+WYL->	1233	Bacteria>Pseudomonadota>Gammaproteobacteria>Enterobacterales>Enterobacteriaceae>Buttiauxella	GCA_003601925.1	Buttiauxella_izardii	D6029_18525	11062..14763	82991	BREX-system-P-loop-protein-BrxC-[Buttiauxella-izardii]
WP_244212427.1	Type1.BREX	<-PglZ<-N6-METHYLASE-p<-N6-METHYLASE-p<-RVT<-HEPN<-N6-METHYLASE<-ParB+HNH<-DUF6079*<-BrxB-BrxF-iSTAND<-BrxA-DNA-binding<-?<-XIS-HTH||?->?->DUF932->?->wHTH+WYL->	1237	Bacteria>Pseudomonadota>Gammaproteobacteria>Enterobacterales>Enterobacteriaceae>Buttiauxella	GCF_003601925.1	Buttiauxella_izardii	D6029_RS18475	11062..14775	82991	BREX-system-P-loop-protein-BrxC-[Buttiauxella-izardii]
WP_124023337.1	Type1.BREX	<-sGTP<-wHTH+WYL<-NACHT+SF1-SF2-Helicase+HKD-DNase||SF1-SF2-Helicase+REase->SF1-SF2-Helicase+REase-><-PglZ<-ParB+HNH<-N6-METHYLASE<-DUF6079*<-BrxB-BrxF-iSTAND<-BrxA-DNA-binding [illegible]	1157	Bacteria>Pseudomonadota>Gammaproteobacteria>Enterobacterales>Enterobacteriaceae>Buttiauxella	GCF_003818135.1	Buttiauxella_warmboldiae	EHN07_RS06350	61248..64721	82993	BREX-system-P-loop-protein-BrxC-[Buttiauxella-warmboldiae]
WP_167614622.1	Type1.BREX	<-HerA/FtsK+A32||?-><-?<-SAM-METHYLASE||tRNA->Arm-DNA-bind_3+Phage_integrase-><-?<-SIGMA+OB-Fold+AAA-ATPase+Lon-Protease-BrxL<-PglZ<-N6-METHYLASE<-DUF6079*<-BrxB-BrxF-iSTAND [illegible]	1213	Bacteria>Pseudomonadota>Gammaproteobacteria>Enterobacterales>Enterobacteriaceae>Cedecea	GCF_011808225.1	Cedecea_colo	E2L00_RS19595	113572..117213	2552946	BREX-system-P-loop-protein-BrxC-[Cedecea-colo]
WP_094760992.1	Type1.BREX	XIS-HTH->?-><-Sulfatase<-?<-?<-?<-PHP+SbcC||BrxA-DNA-binding->BrxB-BrxF-iSTAND->DUF6079*->ParB+HNH->N6-METHYLASE->PglZ->SIGMA+OB-Fold+AAA-ATPase+Lon-Protease-BrxL-> [illegible]	1233	Bacteria>Pseudomonadota>Gammaproteobacteria>Enterobacterales>Enterobacteriaceae>Citrobacter	-	Citrobacter_braakii	B9P84_05295	62196..65897	57706	MULTISPECIES:-BREX-system-P-loop-protein-BrxC-[Citrobacter]
WP_181218785.1	Type1.BREX	CbeA_antitoxin->?->METHYLASE->HTH_3->?->REase-><-SIGMA+OB-Fold+AAA-ATPase+Lon-Protease-BrxL<-PglZ<-N6-METHYLASE<-DUF6079*<-BrxB-BrxF-iSTAND<-BrxA-DNA-binding||Glyoxalase_8-> [illegible]	1213	Bacteria>Pseudomonadota>Gammaproteobacteria>Enterobacterales>Enterobacteriaceae>Citrobacter	-	Citrobacter_freundii	HV183_02915	608608..612249	546	BREX-system-P-loop-protein-BrxC-[Citrobacter-freundii]
EJM8694624.1	Type1.BREX	DOC+HTH-><-?||Phage_Cox->XIS-HTH->AAA+wHTH->?-><-SIGMA+OB-Fold+AAA-ATPase+Lon-Protease-BrxL<-PglZ<-N6-METHYLASE<-DUF6079*<-BrxB-BrxF-iSTAND<-BrxA-DNA-binding||GTPase-AIG->DUF932-> [illegible]	1213	Bacteria>Pseudomonadota>Gammaproteobacteria>Enterobacterales>Enterobacteriaceae>Citrobacter	-	Citrobacter_freundii	NO746_003414	23548..27189	546	BREX-system-P-loop-protein-BrxC-[Citrobacter-freundii]
WP_182347709.1	Type1.BREX	<-Schlafen+HATPase+wHTH<-PHP+SbcC<-?||BrxA-DNA-binding->BrxB-BrxF-iSTAND->DUF6079*->ParB+HNH->N6-METHYLASE->PglZ->SIGMA+OB-Fold+AAA-ATPase+Lon-Protease-BrxL-> [illegible]	1233	Bacteria>Pseudomonadota>Gammaproteobacteria>Enterobacterales>Enterobacteriaceae>Citrobacter	-	Citrobacter_sp._RHBSTW-00013	HV034_17810	3734345..3738046	2742628	BREX-system-P-loop-protein-BrxC-[Citrobacter-sp.-RHBSTW-00013]
WP_181630678.1	Type1.BREX	<-Sulfatase<-?<-?<-?<-Schlafen+HATPase+wHTH<-PHP+SbcC<-?||BrxA-DNA-binding->BrxB-BrxF-iSTAND->DUF6079*->ParB+HNH->N6-METHYLASE->HEPN-><-HIN-HTH||N6-METHYLASE-p->N6-METHYLASE-p->PglZ-> [illegible]	1233	Bacteria>Pseudomonadota>Gammaproteobacteria>Enterobacterales>Enterobacteriaceae>Citrobacter	-	Citrobacter_sp._RHBSTW-01013	HV350_21470	4470372..4474073	2742677	BREX-system-P-loop-protein-BrxC-[Citrobacter-sp.-RHBSTW-01013]
OXU12831.1	Type1.BREX	XIS-HTH->?-><-Sulfatase<-?<-?<-?<-PHP+SbcC||BrxA-DNA-binding->BrxB-BrxF-iSTAND->DUF6079*->ParB+HNH->N6-METHYLASE->PglZ->SIGMA+OB-Fold+AAA-ATPase+Lon-Protease-BrxL-> [illegible]	1233	Bacteria>Pseudomonadota>Gammaproteobacteria>Enterobacterales>Enterobacteriaceae>Citrobacter	GCA_002239605.1	Citrobacter_braakii	B9P84_05295	62196..65897	57706	DNA-repair-protein-[Citrobacter-braakii]
QLR26611.1	Type1.BREX	[illegible]	1233	Bacteria>Pseudomonadota>Gammaproteobacteria>Enterobacterales>Enterobacteriaceae>Citrobacter	GCA_013781985.1	Citrobacter_sp._RHBSTW-01013	HV350_21470	4470372..4474073	2742677	BREX-system-P-loop-protein-BrxC-[Citrobacter-sp.-RHBSTW-01013]
MBA8131541.1	Type1.BREX	[illegible]	1233	Bacteria>Pseudomonadota>Gammaproteobacteria>Enterobacterales>Enterobacteriaceae>Citrobacter	GCA_014104375.1	Citrobacter_sp._RHBSTW-00013	HV034_17810	3734345..3738046	2742628	BREX-system-P-loop-protein-BrxC-[Citrobacter-sp.-RHBSTW-00013]
MBM9568541.1	Type1.BREX	[illegible]	1233	Bacteria>Pseudomonadota>Gammaproteobacteria>Enterobacterales>Enterobacteriaceae>Citrobacter	GCA_014931625.2	Citrobacter_sedlakii	INP82_14060	158441..162142	67826	BREX-system-P-loop-protein-BrxC-[Citrobacter-sedlakii]
EGT0661408.1	Type1.BREX	[illegible]	1233	Bacteria>Pseudomonadota>Gammaproteobacteria>Enterobacterales>Enterobacteriaceae>Citrobacter	GCA_015942525.1	Citrobacter_werkmanii	JAF83_001575	366383..370084	67827	BREX-system-P-loop-protein-BrxC-[Citrobacter-werkmanii]
HAT7499716.1	Type1.BREX	[illegible]	1157	Bacteria>Pseudomonadota>Gammaproteobacteria>Enterobacterales>Enterobacteriaceae>Citrobacter	GCA_015956985.1	Citrobacter_freundii	JAV71_002653	147091..150564	546	BREX-system-P-loop-protein-BrxC-[Citrobacter-freundii]
HAU4366733.1	Type1.BREX	[illegible]	1213	Bacteria>Pseudomonadota>Gammaproteobacteria>Enterobacterales>Enterobacteriaceae>Citrobacter	GCA_016081455.1	Citrobacter_amalonaticus	F6W26_02175	132901..136542	35703	BREX-system-P-loop-protein-BrxC-[Citrobacter-amalonaticus]
HBC2890663.1	Type1.BREX	[illegible]	1204	Bacteria>Pseudomonadota>Gammaproteobacteria>Enterobacterales>Enterobacteriaceae>Citrobacter	GCA_018089985.1	Citrobacter_freundii	JD321_004755	1401..5015	546	BREX-system-P-loop-protein-BrxC-[Citrobacter-freundii]
HCL6754282.1	Type1.BREX	[illegible]	1213	Bacteria>Pseudomonadota>Gammaproteobacteria>Enterobacterales>Enterobacteriaceae>Citrobacter	GCA_025039155.1	Citrobacter_freundii	N2R09_003052	112932..116573	546	BREX-system-P-loop-protein-BrxC-[Citrobacter-freundii]
SYX30363.1	Type1.BREX	[illegible]	1233	Bacteria>Pseudomonadota>Gammaproteobacteria>Enterobacterales>Enterobacteriaceae>Citrobacter	GCA_900520375.1	Citrobacter_freundii	CFU2785_04435	4465123..4468824	546	Uncharacterised-protein-[Citrobacter-freundii]
WP_044264596.1	Type1.BREX	[illegible]	1213	Bacteria>Pseudomonadota>Gammaproteobacteria>Enterobacterales>Enterobacteriaceae>Citrobacter	GCF_000731055.1	Citrobacter_amalonaticus	DQ02_RS05315	398614..402255	35703	BREX-system-P-loop-protein-BrxC-[Citrobacter-amalonaticus]
WP_046487315.1	Type1.BREX	[illegible]	1213	Bacteria>Pseudomonadota>Gammaproteobacteria>Enterobacterales>Enterobacteriaceae>Citrobacter	GCF_000981805.1	Citrobacter_amalonaticus	F384_RS16675	3635289..3638930	35703	BREX-system-P-loop-protein-BrxC-[Citrobacter-amalonaticus]
WP_231587318.1	Type1.BREX	[illegible]	1240	Bacteria>Pseudomonadota>Gammaproteobacteria>Enterobacterales>Enterobacteriaceae>Citrobacter	GCF_001037515.1	Citrobacter_sp._MGH110	SK38_RS14780	3143856..3147578	1686383	BREX-system-P-loop-protein-BrxC-[Citrobacter-sp.-MGH110]
WP_121581750.1	Type1.BREX	[illegible]	1204	Bacteria>Pseudomonadota>Gammaproteobacteria>Enterobacterales>Enterobacteriaceae>Citrobacter	GCF_003665675.1	Citrobacter_freundii	CUC48_RS18615	3736317..3739931	546	BREX-system-P-loop-protein-BrxC-[Citrobacter-freundii]
WP_135912646.1	Type1.BREX	[illegible]	1213	Bacteria>Pseudomonadota>Gammaproteobacteria>Enterobacterales>Enterobacteriaceae>Citrobacter	GCF_004792475.1	Citrobacter_freundii	E5284_RS20140	4206675..4210316	546	BREX-system-P-loop-protein-BrxC-[Citrobacter-freundii]
WP_149691146.1	Type1.BREX	[illegible]	1204	Bacteria>Pseudomonadota>Gammaproteobacteria>Enterobacterales>Enterobacteriaceae>Citrobacter	GCF_008373795.1	Citrobacter_pasteurii	DXF85_RS00930	181362..184976	1563222	BREX-system-P-loop-protein-BrxC-[Citrobacter-pasteurii]
WP_129695037.1	Type1.BREX	[illegible]	1213	Bacteria>Pseudomonadota>Gammaproteobacteria>Enterobacterales>Enterobacteriaceae>Citrobacter	GCF_013618825.1	Citrobacter_werkmanii	EVY00_RS24285	8302..11943	67827	BREX-system-P-loop-protein-BrxC-[Citrobacter-werkmanii]
WP_249418019.1	Type1.BREX	[illegible]	1240	Bacteria>Pseudomonadota>Gammaproteobacteria>Enterobacterales>Enterobacteriaceae>Citrobacter	GCF_013781985.1	Citrobacter_sp._RHBSTW-01013	HV350_RS21465	4470351..4474073	2742677	BREX-system-P-loop-protein-BrxC-[Citrobacter-sp.-RHBSTW-01013]
WP_181621180.1	Type1.BREX	[illegible]	1204	Bacteria>Pseudomonadota>Gammaproteobacteria>Enterobacterales>Enterobacteriaceae>Citrobacter	GCF_013791615.1	Citrobacter_freundii	HV083_RS19895	4141726..4145340	546	BREX-system-P-loop-protein-BrxC-[Citrobacter-freundii]
WP_249424276.1	Type1.BREX	[illegible]	1240	Bacteria>Pseudomonadota>Gammaproteobacteria>Enterobacterales>Enterobacteriaceae>Citrobacter	GCF_014104375.1	Citrobacter_sp._RHBSTW-00013	HV034_RS17810	3734324..3738046	2742628	BREX-system-P-loop-protein-BrxC-[Citrobacter-sp.-RHBSTW-00013]
WP_192466357.1	Type1.BREX	[illegible]	1213	Bacteria>Pseudomonadota>Gammaproteobacteria>Enterobacterales>Enterobacteriaceae>Citrobacter	GCF_014855915.1	Citrobacter_koseri	FOT44_RS18785	338007..341648	545	BREX-system-P-loop-protein-BrxC-[Citrobacter-koseri]
WP_200025358.1	Type1.BREX	[illegible]	1213	Bacteria>Pseudomonadota>Gammaproteobacteria>Enterobacterales>Enterobacteriaceae>Citrobacter	GCF_016500865.1	Citrobacter_freundii	I5388_RS15310	359245..362886	546	BREX-system-P-loop-protein-BrxC-[Citrobacter-freundii]
WP_200031143.1	Type1.BREX	[illegible]	1213	Bacteria>Pseudomonadota>Gammaproteobacteria>Enterobacterales>Enterobacteriaceae>Citrobacter	GCF_016501985.1	Citrobacter_koseri	I5424_RS14480	22464..26105	545	BREX-system-P-loop-protein-BrxC-[Citrobacter-koseri]
WP_200054816.1	Type1.BREX	[illegible]	1157	Bacteria>Pseudomonadota>Gammaproteobacteria>Enterobacterales>Enterobacteriaceae>Citrobacter	GCF_016504925.1	Citrobacter_freundii	GHT45_RS09240	237227..240700	546	BREX-system-P-loop-protein-BrxC-[Citrobacter-freundii]
WP_203457741.1	Type1.BREX	[illegible]	1204	Bacteria>Pseudomonadota>Gammaproteobacteria>Enterobacterales>Enterobacteriaceae>Citrobacter	GCF_016859695.1	Citrobacter_braakii	GF710_RS09095	85023..88637	57706	BREX-system-P-loop-protein-BrxC-[Citrobacter-braakii]
WP_205688206.1	Type1.BREX	[illegible]	1157	Bacteria>Pseudomonadota>Gammaproteobacteria>Enterobacterales>Enterobacteriaceae>Citrobacter	GCF_017086505.1	Citrobacter_freundii	GHT30_RS20265	4262904..4266377	546	BREX-system-P-loop-protein-BrxC-[Citrobacter-freundii]
WP_241511105.1	Type1.BREX	[illegible]	1197	Bacteria>Pseudomonadota>Gammaproteobacteria>Enterobacterales>Enterobacteriaceae>Citrobacter	GCF_022585175.1	Citrobacter_freundii	MMQ01_RS10405	144077..147670	546	BREX-system-P-loop-protein-BrxC-[Citrobacter-freundii]
WP_252170698.1	Type1.BREX	[illegible]	1204	Bacteria>Pseudomonadota>Gammaproteobacteria>Enterobacterales>Enterobacteriaceae>Citrobacter	GCF_023821945.1	Citrobacter_cronae	M4W13_RS12100	107491..111105	1748967	BREX-system-P-loop-protein-BrxC-[Citrobacter-cronae]
WP_275379687.1	Type1.BREX	[illegible]	1240	Bacteria>Pseudomonadota>Gammaproteobacteria>Enterobacterales>Enterobacteriaceae>Citrobacter	GCF_029104295.1	Citrobacter_freundii	L2W90_RS09965	166217..169939	546	BREX-system-P-loop-protein-BrxC-[Citrobacter-freundii]
WP_275399266.1	Type1.BREX	[illegible]	1095	Bacteria>Pseudomonadota>Gammaproteobacteria>Enterobacterales>Enterobacteriaceae>Citrobacter	GCF_029104415.1	Citrobacter_freundii	L2W96_RS12535	133812..137099	546	BREX-system-P-loop-protein-BrxC-[Citrobacter-freundii]
WP_196010428.1	Type1.BREX	[illegible]	1213	Bacteria>Pseudomonadota>Gammaproteobacteria>Enterobacterales>Enterobacteriaceae>Citrobacter	GCF_029717585.1	Citrobacter_amalonaticus	NFJ54_RS22965	4694172..4697813	35703	MULTISPECIES:-BREX-system-P-loop-protein-BrxC-[Enterobacteriaceae]
WP_148977713.1	Type1.BREX	[illegible]	1204	Bacteria>Pseudomonadota>Gammaproteobacteria>Enterobacterales>Enterobacteriaceae>Citrobacter	GCF_902807175.1	Citrobacter_freundii	FYK57_RS19440	6782..10396	546	BREX-system-P-loop-protein-BrxC-[Citrobacter-freundii]
PUV47226.1	Type1.BREX	[illegible]	1233	Bacteria>Pseudomonadota>Gammaproteobacteria>Enterobacterales>Enterobacteriaceae>Cronobacter	GCA_002532375.1	Cronobacter_sakazakii	CDU02_01545	336773..340474	28141	BREX-system-P-loop-protein-BrxC-[Cronobacter-sakazakii]
KAB0815149.1	Type1.BREX	[illegible]	1233	Bacteria>Pseudomonadota>Gammaproteobacteria>Enterobacterales>Enterobacteriaceae>Cronobacter	GCA_008805175.1	Cronobacter_sakazakii	FZI24_17905	37972..41673	28141	BREX-system-P-loop-protein-BrxC-[Cronobacter-sakazakii]
EGT4327973.1	Type1.BREX	[illegible]	1213	Bacteria>Pseudomonadota>Gammaproteobacteria>Enterobacterales>Enterobacteriaceae>Cronobacter	GCA_016076655.1	Cronobacter_sakazakii	AUM89_20300	1368..5009	28141	BREX-system-P-loop-protein-BrxC-[Cronobacter-sakazakii]
EGT4509729.1	Type1.BREX	[illegible]	1213	Bacteria>Pseudomonadota>Gammaproteobacteria>Enterobacterales>Enterobacteriaceae>Cronobacter	GCA_016077415.1	Cronobacter_sakazakii	AUN08_13950	41596..45237	28141	BREX-system-P-loop-protein-BrxC-[Cronobacter-sakazakii]
WP_007729758.1	Type1.BREX	[illegible]	1213	Bacteria>Pseudomonadota>Gammaproteobacteria>Enterobacterales>Enterobacteriaceae>Cronobacter	GCF_000409225.1	Cronobacter_dublinensis	AFK67_RS02785	592165..595806	413497	BREX-system-P-loop-protein-BrxC-[Cronobacter-dublinensis]
WP_075184066.1	Type1.BREX	[illegible]	1213	Bacteria>Pseudomonadota>Gammaproteobacteria>Enterobacterales>Enterobacteriaceae>Cronobacter	GCF_001922925.1	Cronobacter_sakazakii	BS410_RS19680	61425..65066	28141	BREX-system-P-loop-protein-BrxC-[Cronobacter-sakazakii]
WP_239984508.1	Type1.BREX	[illegible]	1240	Bacteria>Pseudomonadota>Gammaproteobacteria>Enterobacterales>Enterobacteriaceae>Cronobacter	GCF_002942265.1	Cronobacter_sakazakii	C3D67_RS10955	271432..275154	28141	BREX-system-P-loop-protein-BrxC-[Cronobacter-sakazakii]
WP_105547575.1	Type1.BREX	[illegible]	1213	Bacteria>Pseudomonadota>Gammaproteobacteria>Enterobacterales>Enterobacteriaceae>Cronobacter	GCF_002974775.1	Cronobacter_sakazakii	C5959_RS02070	327806..331447	28141	BREX-system-P-loop-protein-BrxC-[Cronobacter-sakazakii]
WP_105579035.1	Type1.BREX	[illegible]	1213	Bacteria>Pseudomonadota>Gammaproteobacteria>Enterobacterales>Enterobacteriaceae>Cronobacter	GCF_002977115.1	Cronobacter_sakazakii	CI818_RS20655	12068..15709	28141	BREX-system-P-loop-protein-BrxC-[Cronobacter-sakazakii]
WP_110812233.1	Type1.BREX	[illegible]	1213	Bacteria>Pseudomonadota>Gammaproteobacteria>Enterobacterales>Enterobacteriaceae>Cronobacter	GCF_003206925.1	Cronobacter_sakazakii	DOM37_RS13070	492836..496477	28141	BREX-system-P-loop-protein-BrxC-[Cronobacter-sakazakii]
WP_161575571.1	Type1.BREX	[illegible]	1157	Bacteria>Pseudomonadota>Gammaproteobacteria>Enterobacterales>Enterobacteriaceae>Cronobacter	GCF_009938355.1	Cronobacter_sakazakii	EHJ08_RS11755	205127..208600	28141	BREX-system-P-loop-protein-BrxC-[Cronobacter-sakazakii]
WP_166500158.1	Type1.BREX	[illegible]	1213	Bacteria>Pseudomonadota>Gammaproteobacteria>Enterobacterales>Enterobacteriaceae>Cronobacter	GCF_011605595.1	Cronobacter_sakazakii	G9X52_RS02005	2082..5723	28141	BREX-system-P-loop-protein-BrxC-[Cronobacter-sakazakii]
WP_166501056.1	Type1.BREX	[illegible]	1213	Bacteria>Pseudomonadota>Gammaproteobacteria>Enterobacterales>Enterobacteriaceae>Cronobacter	GCF_011605635.1	Cronobacter_dublinensis	G9X53_RS16040	318383..322024	413497	BREX-system-P-loop-protein-BrxC-[Cronobacter-dublinensis]
WP_239978191.1	Type1.BREX	[illegible]	1240	Bacteria>Pseudomonadota>Gammaproteobacteria>Enterobacterales>Enterobacteriaceae>Cronobacter	GCF_017845055.1	Cronobacter_sakazakii	KAR72_RS09245	217726..221448	28141	BREX-system-P-loop-protein-BrxC-[Cronobacter-sakazakii]
WP_269506681.1	Type1.BREX	[illegible]	1204	Bacteria>Pseudomonadota>Gammaproteobacteria>Enterobacterales>Enterobacteriaceae>Cronobacter	GCF_027277125.1	Cronobacter_sakazakii	O6D31_RS12675	364123..367737	28141	BREX-system-P-loop-protein-BrxC-[Cronobacter-sakazakii]
CAF2998376.1	Type1.BREX	[illegible]	1240	Bacteria>Pseudomonadota>Gammaproteobacteria>Enterobacterales>Enterobacteriaceae>Enterobacter	-	Enterobacter_cloacae	AI2972V1_0508	557695..561417	550	hypothetical-protein-AI2972V1_0508-[Enterobacter-cloacae]
WP_129258922.1	Type1.BREX	[illegible]	1157	Bacteria>Pseudomonadota>Gammaproteobacteria>Enterobacterales>Enterobacteriaceae>Enterobacter	-	Enterobacter_cloacae_complex_sp._GF14B	DD606_17375	24450..27923	2511982	BREX-system-P-loop-protein-BrxC-[Enterobacter-cloacae-complex-sp.-GF14B]
WP_163363286.1	Type1.BREX	[illegible]	1197	Bacteria>Pseudomonadota>Gammaproteobacteria>Enterobacterales>Enterobacteriaceae>Enterobacter	-	Enterobacter_cloacae	G4556_RS12910	16910..20503	550	BREX-system-P-loop-protein-BrxC-[Enterobacter-cloacae]
WP_203465577.1	Type1.BREX	[illegible]	1213	Bacteria>Pseudomonadota>Gammaproteobacteria>Enterobacterales>Enterobacteriaceae>Enterobacter	-	Enterobacter_cloacae	GEA59_RS02615	551852..555493	550	BREX-system-P-loop-protein-BrxC-[Enterobacter-cloacae]
WP_039268698.1	Type1.BREX	[illegible]	1233	Bacteria>Pseudomonadota>Gammaproteobacteria>Enterobacterales>Enterobacteriaceae>Enterobacter	-	Enterobacter_hormaechei	LI66_02640	557969..561670	158836	MULTISPECIES:-BREX-system-P-loop-protein-BrxC-[Enterobacter]
WP_181507052.1	Type1.BREX	[illegible]	1233	Bacteria>Pseudomonadota>Gammaproteobacteria>Enterobacterales>Enterobacteriaceae>Enterobacter	-	Enterobacter_roggenkampii	JAF83_001575	366383..370084	1812935	MULTISPECIES:-BREX-system-P-loop-protein-BrxC-[Enterobacteriaceae]
WP_212478308.1	Type1.BREX	[illegible]	1213	Bacteria>Pseudomonadota>Gammaproteobacteria>Enterobacterales>Enterobacteriaceae>Enterobacter	-	Enterobacter_sp._JGM127	JK158_RS17050	116414..120055	2799790	BREX-system-P-loop-protein-BrxC-[Enterobacter-sp.-JGM127]
AFP68425.1	Type1.BREX	[illegible]	1233	Bacteria>Pseudomonadota>Gammaproteobacteria>Enterobacterales>Enterobacteriaceae>Enterobacter	GCA_000286275.1	Enterobacter_kobei	ECENHK_02660	581253..584954	208224	hypothetical-protein-ECENHK_02660-[Enterobacter-kobei]
ESM83248.1	Type1.BREX	[illegible]	611	Bacteria>Pseudomonadota>Gammaproteobacteria>Enterobacterales>Enterobacteriaceae>Enterobacter	GCA_000492895.1	Enterobacter_roggenkampii_MGH_34	L380_02580	2783361..2785196	1329832	hypothetical-protein-L380_02580-[Enterobacter-roggenkampii-MGH-34]
AJB80307.1	Type1.BREX	[illegible]	1233	Bacteria>Pseudomonadota>Gammaproteobacteria>Enterobacterales>Enterobacteriaceae>Enterobacter	GCA_000814225.1	Enterobacter_hormaechei_subsp._xiangfangensis	LI66_02640	557969..561670	1296536	DNA-repair-protein-[Enterobacter-hormaechei-subsp.-xiangfangensis]
KZR28106.1	Type1.BREX	[illegible]	1233	Bacteria>Pseudomonadota>Gammaproteobacteria>Enterobacterales>Enterobacteriaceae>Enterobacter	GCA_001631925.1	Enterobacter_hormaechei_subsp._steigerwaltii	A3N67_04030	129053..132754	299766	DNA-repair-protein-[Enterobacter-hormaechei-subsp.-steigerwaltii]
AWF32098.1	Type1.BREX	[illegible]	1233	Bacteria>Pseudomonadota>Gammaproteobacteria>Enterobacterales>Enterobacteriaceae>Enterobacter	GCA_003073995.1	Enterobacter_hormaechei	CSC19_3711	3849441..3853142	158836	hypothetical-protein-CSC19_3711-[Enterobacter-hormaechei]
PWG69505.1	Type1.BREX	[illegible]	1213	Bacteria>Pseudomonadota>Gammaproteobacteria>Enterobacterales>Enterobacteriaceae>Enterobacter	GCA_003129945.1	Enterobacter_mori	DEM28_19810	168758..172399	539813	BREX-system-P-loop-protein-BrxC-[Enterobacter-mori]
RVR09283.1	Type1.BREX	[illegible]	859	Bacteria>Pseudomonadota>Gammaproteobacteria>Enterobacterales>Enterobacteriaceae>Enterobacter	GCA_004004525.1	Enterobacter_hormaechei	EOL34_23625	1..2579	158836	BREX-system-P-loop-protein-BrxC,-partial-[Enterobacter-hormaechei]
MBG0523800.1	Type1.BREX	[illegible]	1233	Bacteria>Pseudomonadota>Gammaproteobacteria>Enterobacterales>Enterobacteriaceae>Enterobacter	GCA_015704905.1	Enterobacter_cloacae	IOR24_17915	3774454..3778155	550	BREX-system-P-loop-protein-BrxC-[Enterobacter-cloacae]
HAV2283855.1	Type1.BREX	[illegible]	932	Bacteria>Pseudomonadota>Gammaproteobacteria>Enterobacterales>Enterobacteriaceae>Enterobacter	GCA_016445985.1	Enterobacter_cloacae	JHK37_004627	1..2796	550	BREX-system-P-loop-protein-BrxC-[Enterobacter-cloacae]
MBK3145473.1	Type1.BREX	[illegible]	1233	Bacteria>Pseudomonadota>Gammaproteobacteria>Enterobacterales>Enterobacteriaceae>Enterobacter	GCA_016616795.1	Enterobacter_hormaechei	FKK11_12155	273388..277089	158836	BREX-system-P-loop-protein-BrxC-[Enterobacter-hormaechei]
MBT2005130.1	Type1.BREX	[illegible]	1204	Bacteria>Pseudomonadota>Gammaproteobacteria>Enterobacterales>Enterobacteriaceae>Enterobacter	GCA_018597745.1	Enterobacter_hormaechei_subsp._xiangfangensis	KKZ39_07415	340677..344291	1296536	BREX-system-P-loop-protein-BrxC-[Enterobacter-hormaechei-subsp.-xiangfangensis]
MBX8934563.1	Type1.BREX	[illegible]	1225	Bacteria>Pseudomonadota>Gammaproteobacteria>Enterobacterales>Enterobacteriaceae>Enterobacter	GCA_019731165.1	Enterobacter_sp._K62_1	HCH30_08625	10503..14180	2718933	BREX-system-P-loop-protein-BrxC-[Enterobacter-sp.-K62_1]
HBL8846881.1	Type1.BREX	[illegible]	1089	Bacteria>Pseudomonadota>Gammaproteobacteria>Enterobacterales>Enterobacteriaceae>Enterobacter	GCA_021095225.1	Enterobacter_hormaechei	LTW48_004335	1..3271	158836	BREX-system-P-loop-protein-BrxC-[Enterobacter-hormaechei]
HBM2523095.1	Type1.BREX	[illegible]	599	Bacteria>Pseudomonadota>Gammaproteobacteria>Enterobacterales>Enterobacteriaceae>Enterobacter	GCA_021175225.1	Enterobacter_hormaechei	LUX61_004833	1..1799	158836	BREX-system-P-loop-protein-BrxC-[Enterobacter-hormaechei]
HBM2647612.1	Type1.BREX	[illegible]	586	Bacteria>Pseudomonadota>Gammaproteobacteria>Enterobacterales>Enterobacteriaceae>Enterobacter	GCA_021176145.1	Enterobacter_hormaechei	LUX77_003527	1..1758	158836	BREX-system-P-loop-protein-BrxC-[Enterobacter-hormaechei]
MCE1563408.1	Type1.BREX	[illegible]	1204	Bacteria>Pseudomonadota>Gammaproteobacteria>Enterobacterales>Enterobacteriaceae>Enterobacter	GCA_021285765.1	Enterobacter_hormaechei	LWT81_04630	337849..341463	158836	BREX-system-P-loop-protein-BrxC-[Enterobacter-hormaechei]
MCI9498943.1	Type1.BREX	[illegible]	1204	Bacteria>Pseudomonadota>Gammaproteobacteria>Enterobacterales>Enterobacteriaceae>Enterobacter	GCA_022800955.1	Enterobacter_hormaechei_subsp._steigerwaltii	HFE69_15020	342834..346448	299766	BREX-system-P-loop-protein-BrxC-[Enterobacter-hormaechei-subsp.-steigerwaltii]
HCM9270259.1	Type1.BREX	[illegible]	1157	Bacteria>Pseudomonadota>Gammaproteobacteria>Enterobacterales>Enterobacteriaceae>Enterobacter	GCA_025212085.1	Enterobacter_cloacae_subsp._cloacae	N5L33_003913	155329..158802	336306	BREX-system-P-loop-protein-BrxC-[Enterobacter-cloacae-subsp.-cloacae]
HCM9559898.1	Type1.BREX	[illegible]	1157	Bacteria>Pseudomonadota>Gammaproteobacteria>Enterobacterales>Enterobacteriaceae>Enterobacter	GCA_025214055.1	Enterobacter_cloacae_subsp._cloacae	N5O13_002966	176473..179946	336306	BREX-system-P-loop-protein-BrxC-[Enterobacter-cloacae-subsp.-cloacae]
WP_228371847.1	Type1.BREX	[illegible]	1240	Bacteria>Pseudomonadota>Gammaproteobacteria>Enterobacterales>Enterobacteriaceae>Enterobacter	GCF_000286275.1	Enterobacter_kobei	ECENHK_RS02760	581253..584975	208224	BREX-system-P-loop-protein-BrxC-[Enterobacter-kobei]
WP_021240656.1	Type1.BREX	[illegible]	1213	Bacteria>Pseudomonadota>Gammaproteobacteria>Enterobacterales>Enterobacteriaceae>Enterobacter	GCF_000467655.2	Enterobacter_roggenkampii	L799_RS145635	328329..331970	1812935	BREX-system-P-loop-protein-BrxC-[Enterobacter-roggenkampii]
WP_234704204.1	Type1.BREX	[illegible]	1240	Bacteria>Pseudomonadota>Gammaproteobacteria>Enterobacterales>Enterobacteriaceae>Enterobacter	GCF_000814225.1	Enterobacter_cloacae_complex	LI66_RS02640	557969..561691	354276	MULTISPECIES:-BREX-system-P-loop-protein-BrxC-[Enterobacter-cloacae-complex]
WP_058675376.1	Type1.BREX	[illegible]	1204	Bacteria>Pseudomonadota>Gammaproteobacteria>Enterobacterales>Enterobacteriaceae>Enterobacter	GCF_001473175.1	Enterobacter_hormaechei	ASV30_RS20360	113168..116782	158836	BREX-system-P-loop-protein-BrxC-[Enterobacter-hormaechei]
WP_059514247.1	Type1.BREX	[illegible]	1204	Bacteria>Pseudomonadota>Gammaproteobacteria>Enterobacterales>Enterobacteriaceae>Enterobacter	GCF_001525475.1	Enterobacter_hormaechei	CI630_RS14005	1978..5592	158836	BREX-system-P-loop-protein-BrxC-[Enterobacter-hormaechei]
WP_230373248.1	Type1.BREX	[illegible]	1240	Bacteria>Pseudomonadota>Gammaproteobacteria>Enterobacterales>Enterobacteriaceae>Enterobacter	GCF_001631925.1	Enterobacter_hormaechei	A3N67_RS02925	129032..132754	158836	BREX-system-P-loop-protein-BrxC-[Enterobacter-hormaechei]
WP_069597812.1	Type1.BREX	[illegible]	1204	Bacteria>Pseudomonadota>Gammaproteobacteria>Enterobacterales>Enterobacteriaceae>Enterobacter	GCF_001729805.1	Enterobacter_roggenkampii	BFV67_RS02540	543370..546984	1812935	BREX-system-P-loop-protein-BrxC-[Enterobacter-roggenkampii]
WP_095453563.1	Type1.BREX	[illegible]	1213	Bacteria>Pseudomonadota>Gammaproteobacteria>Enterobacterales>Enterobacteriaceae>Enterobacter	GCF_002278355.1	Enterobacter_cloacae	CIW57_RS16410	135960..139601	550	BREX-system-P-loop-protein-BrxC-[Enterobacter-cloacae]
WP_100165592.1	Type1.BREX	[illegible]	1204	Bacteria>Pseudomonadota>Gammaproteobacteria>Enterobacterales>Enterobacteriaceae>Enterobacter	GCF_002785605.1	Enterobacter_mori	B9Q20_RS04890	379505..383119	539813	BREX-system-P-loop-protein-BrxC-[Enterobacter-mori]
WP_237764241.1	Type1.BREX	[illegible]	1240	Bacteria>Pseudomonadota>Gammaproteobacteria>Enterobacterales>Enterobacteriaceae>Enterobacter	GCF_003073995.1	Enterobacter_hormaechei	CSC19_RS19005	3849441..3853163	158836	BREX-system-P-loop-protein-BrxC-[Enterobacter-hormaechei]
WP_110108109.1	Type1.BREX	[illegible]	1197	Bacteria>Pseudomonadota>Gammaproteobacteria>Enterobacterales>Enterobacteriaceae>Enterobacter	GCF_003186415.1	Enterobacter_hormaechei	BET69_RS02790	559852..563445	158836	BREX-system-P-loop-protein-BrxC-[Enterobacter-hormaechei]
WP_110875386.1	Type1.BREX	[illegible]	1157	Bacteria>Pseudomonadota>Gammaproteobacteria>Enterobacterales>Enterobacteriaceae>Enterobacter	GCF_003207215.1	Enterobacter_hormaechei	DOM70_RS06250	341163..344636	158836	BREX-system-P-loop-protein-BrxC-[Enterobacter-hormaechei]
WP_123923007.1	Type1.BREX	[illegible]	1204	Bacteria>Pseudomonadota>Gammaproteobacteria>Enterobacterales>Enterobacteriaceae>Enterobacter	GCF_003812145.1	Enterobacter_roggenkampii	EGY04_RS13705	2428639..2432253	1812935	BREX-system-P-loop-protein-BrxC-[Enterobacter-roggenkampii]
WP_125357895.1	Type1.BREX	[illegible]	1225	Bacteria>Pseudomonadota>Gammaproteobacteria>Enterobacterales>Enterobacteriaceae>Enterobacter	GCF_003937295.2	Enterobacter_hormaechei	EGK38_RS19825	4108658..4112335	158836	BREX-system-P-loop-protein-BrxC-[Enterobacter-hormaechei]
WP_126349337.1	Type1.BREX	[illegible]	1157	Bacteria>Pseudomonadota>Gammaproteobacteria>Enterobacterales>Enterobacteriaceae>Enterobacter	GCF_003965695.1	Enterobacter_roggenkampii	EKN52_RS20255	23415..26888	1812935	BREX-system-P-loop-protein-BrxC-[Enterobacter-roggenkampii]
WP_126800556.1	Type1.BREX	[illegible]	1157	Bacteria>Pseudomonadota>Gammaproteobacteria>Enterobacterales>Enterobacteriaceae>Enterobacter	GCF_003986875.1	Enterobacter_sp._WCHEn090032	EKN83_RS06700	53011..56484	2497435	BREX-system-P-loop-protein-BrxC-[Enterobacter-sp.-WCHEn090032]
WP_241243342.1	Type1.BREX	[illegible]	866	Bacteria>Pseudomonadota>Gammaproteobacteria>Enterobacterales>Enterobacteriaceae>Enterobacter	GCF_004004525.1	Enterobacter_hormaechei	EOL34_RS23625	1..2600	158836	BREX-system-P-loop-protein-BrxC,-partial-[Enterobacter-hormaechei]
WP_129328058.1	Type1.BREX	[illegible]	1204	Bacteria>Pseudomonadota>Gammaproteobacteria>Enterobacterales>Enterobacteriaceae>Enterobacter	GCF_004127365.1	Enterobacter_cloacae	DD604_RS20965	29802..33416	550	BREX-system-P-loop-protein-BrxC-[Enterobacter-cloacae]
WP_129694025.1	Type1.BREX	[illegible]	1157	Bacteria>Pseudomonadota>Gammaproteobacteria>Enterobacterales>Enterobacteriaceae>Enterobacter	GCF_004138605.1	Enterobacter_roggenkampii	D8675_RS06390	1085829..1089302	1812935	BREX-system-P-loop-protein-BrxC-[Enterobacter-roggenkampii]
WP_148769092.1	Type1.BREX	[illegible]	1213	Bacteria>Pseudomonadota>Gammaproteobacteria>Enterobacterales>Enterobacteriaceae>Enterobacter	GCF_008122455.1	Enterobacter_sp._C6	DOU50_RS06770	7671..11312	1299469	BREX-system-P-loop-protein-BrxC-[Enterobacter-sp.-C6]
WP_164474334.1	Type1.BREX	[illegible]	1157	Bacteria>Pseudomonadota>Gammaproteobacteria>Enterobacterales>Enterobacteriaceae>Enterobacter	GCF_011045335.1	Enterobacter_hormaechei	G5C52_RS10105	2135134..2138607	158836	BREX-system-P-loop-protein-BrxC-[Enterobacter-hormaechei]
WP_168980132.1	Type1.BREX	[illegible]	1213	Bacteria>Pseudomonadota>Gammaproteobacteria>Enterobacterales>Enterobacteriaceae>Enterobacter	GCF_012843805.1	Enterobacter_asburiae	HF856_RS13430	88040..91681	61645	BREX-system-P-loop-protein-BrxC-[Enterobacter-asburiae]
WP_181236548.1	Type1.BREX	[illegible]	1204	Bacteria>Pseudomonadota>Gammaproteobacteria>Enterobacterales>Enterobacteriaceae>Enterobacter	GCF_013624605.1	Enterobacter_hormaechei	HV094_RS19965	4127702..4131316	158836	BREX-system-P-loop-protein-BrxC-[Enterobacter-hormaechei]
WP_182062523.1	Type1.BREX	[illegible]	1202	Bacteria>Pseudomonadota>Gammaproteobacteria>Enterobacterales>Enterobacteriaceae>Enterobacter	GCF_013927005.1	Enterobacter_sp._RHBSTW-00175	HV107_RS06435	1368672..1372280	2742639	BREX-system-P-loop-protein-BrxC-[Enterobacter-sp.-RHBSTW-00175]
WP_182014101.1	Type1.BREX	[illegible]	1204	Bacteria>Pseudomonadota>Gammaproteobacteria>Enterobacterales>Enterobacteriaceae>Enterobacter	GCF_013927185.1	Enterobacter_roggenkampii	HV097_RS13200	2641536..2645150	1812935	BREX-system-P-loop-protein-BrxC-[Enterobacter-roggenkampii]
WP_182278818.1	Type1.BREX	[illegible]	1213	Bacteria>Pseudomonadota>Gammaproteobacteria>Enterobacterales>Enterobacteriaceae>Enterobacter	GCF_014101255.1	Enterobacter_roggenkampii	HV177_RS13220	2740666..2744307	1812935	BREX-system-P-loop-protein-BrxC-[Enterobacter-roggenkampii]
WP_100126890.1	Type1.BREX	[illegible]	1204	Bacteria>Pseudomonadota>Gammaproteobacteria>Enterobacterales>Enterobacteriaceae>Enterobacter	GCF_014103055.1	Enterobacter_cloacae_complex	HV354_RS20740	74806..78420	354276	MULTISPECIES:-BREX-system-P-loop-protein-BrxC-[Enterobacter-cloacae-complex]
WP_057055966.1	Type1.BREX	[illegible]	1197	Bacteria>Pseudomonadota>Gammaproteobacteria>Enterobacterales>Enterobacteriaceae>Enterobacter	GCF_014169295.1	Enterobacter_cloacae_complex	H7R61_RS03370	684757..688350	354276	MULTISPECIES:-BREX-system-P-loop-protein-BrxC-[Enterobacter-cloacae-complex]
WP_230863208.1	Type1.BREX	[illegible]	1240	Bacteria>Pseudomonadota>Gammaproteobacteria>Enterobacterales>Enterobacteriaceae>Enterobacter	GCF_015704905.1	Enterobacter_cloacae	IOR24_RS17915	3774454..3778176	550	BREX-system-P-loop-protein-BrxC-[Enterobacter-cloacae]
WP_234450174.1	Type1.BREX	[illegible]	1240	Bacteria>Pseudomonadota>Gammaproteobacteria>Enterobacterales>Enterobacteriaceae>Enterobacter	GCF_016616795.1	Enterobacter_hormaechei	FKK11_RS12010	273367..277089	158836	BREX-system-P-loop-protein-BrxC-[Enterobacter-hormaechei]
WP_211449384.1	Type1.BREX	[illegible]	1157	Bacteria>Pseudomonadota>Gammaproteobacteria>Enterobacterales>Enterobacteriaceae>Enterobacter	GCF_018299605.1	Enterobacter_mori	GUY16_RS21845	58984..62457	539813	BREX-system-P-loop-protein-BrxC-[Enterobacter-mori]
WP_006812019.1	Type1.BREX	[illegible]	1204	Bacteria>Pseudomonadota>Gammaproteobacteria>Enterobacterales>Enterobacteriaceae>Enterobacter	GCF_019048245.1	Enterobacter_hormaechei	I6L63_RS02015	410151..413765	158836	BREX-system-P-loop-protein-BrxC-[Enterobacter-hormaechei]
WP_219280521.1	Type1.BREX	[illegible]	1197	Bacteria>Pseudomonadota>Gammaproteobacteria>Enterobacterales>Enterobacteriaceae>Enterobacter	GCF_019351595.1	Enterobacter_cloacae	JW319_RS20560	173870..177463	550	BREX-system-P-loop-protein-BrxC-[Enterobacter-cloacae]
WP_222665203.1	Type1.BREX	[illegible]	1204	Bacteria>Pseudomonadota>Gammaproteobacteria>Enterobacterales>Enterobacteriaceae>Enterobacter	GCF_019839635.1	Enterobacter_roggenkampii	K4X45_RS16780	84245..87859	1812935	BREX-system-P-loop-protein-BrxC-[Enterobacter-roggenkampii]
WP_032634492.1	Type1.BREX	[illegible]	1204	Bacteria>Pseudomonadota>Gammaproteobacteria>Enterobacterales>Enterobacteriaceae>Enterobacter	GCF_019974415.1	Enterobacter_cloacae_complex	DDA68_RS23710	1545..5159	354276	MULTISPECIES:-BREX-system-P-loop-protein-BrxC-[Enterobacter-cloacae-complex]
WP_230238963.1	Type1.BREX	[illegible]	1157	Bacteria>Pseudomonadota>Gammaproteobacteria>Enterobacterales>Enterobacteriaceae>Enterobacter	GCF_020971765.1	Enterobacter_sp._AN-K1	LOC22_RS13480	2816173..2819646	2894203	BREX-system-P-loop-protein-BrxC-[Enterobacter-sp.-AN-K1]
WP_241512123.1	Type1.BREX	[illegible]	1240	Bacteria>Pseudomonadota>Gammaproteobacteria>Enterobacterales>Enterobacteriaceae>Enterobacter	GCF_022585395.1	Enterobacter_hormaechei	MMO50_RS10500	125167..128889	158836	BREX-system-P-loop-protein-BrxC-[Enterobacter-hormaechei]
WP_248201800.1	Type1.BREX	[illegible]	1213	Bacteria>Pseudomonadota>Gammaproteobacteria>Enterobacterales>Enterobacteriaceae>Enterobacter	GCF_023151575.1	Enterobacter_asburiae	L8T09_RS18420	27571..31212	61645	BREX-system-P-loop-protein-BrxC-[Enterobacter-asburiae]
WP_248198186.1	Type1.BREX	[illegible]	1213	Bacteria>Pseudomonadota>Gammaproteobacteria>Enterobacterales>Enterobacteriaceae>Enterobacter	GCF_023151985.1	Enterobacter_bugandensis	L8S35_RS17040	181002..184643	881260	BREX-system-P-loop-protein-BrxC-[Enterobacter-bugandensis]
WP_248241159.1	Type1.BREX	[illegible]	1204	Bacteria>Pseudomonadota>Gammaproteobacteria>Enterobacterales>Enterobacteriaceae>Enterobacter	GCF_023153155.1	Enterobacter_roggenkampii	L8Q49_RS17945	130096..133710	1812935	BREX-system-P-loop-protein-BrxC-[Enterobacter-roggenkampii]
WP_248076370.1	Type1.BREX	[illegible]	1240	Bacteria>Pseudomonadota>Gammaproteobacteria>Enterobacterales>Enterobacteriaceae>Enterobacter	GCF_023153855.1	Enterobacter_cloacae	L8P05_RS09265	348723..352445	550	BREX-system-P-loop-protein-BrxC-[Enterobacter-cloacae]
WP_248053563.1	Type1.BREX	[illegible]	1197	Bacteria>Pseudomonadota>Gammaproteobacteria>Enterobacterales>Enterobacteriaceae>Enterobacter	GCF_023154935.1	Enterobacter_chengduensis	L8P32_RS20960	6466..10059	2494701	BREX-system-P-loop-protein-BrxC-[Enterobacter-chengduensis]
WP_032621388.1	Type1.BREX	[illegible]	1204	Bacteria>Pseudomonadota>Gammaproteobacteria>Enterobacterales>Enterobacteriaceae>Enterobacter	GCF_023734955.1	Enterobacter_hormaechei	NVR78_RS04785	326171..329785	158836	BREX-system-P-loop-protein-BrxC-[Enterobacter-hormaechei]
WP_192468115.1	Type1.BREX	[illegible]	1213	Bacteria>Pseudomonadota>Gammaproteobacteria>Enterobacterales>Enterobacteriaceae>Enterobacter	GCF_023749245.1	Enterobacter_asburiae	M8N38_RS13285	26000..29641	61645	BREX-system-P-loop-protein-BrxC-[Enterobacter-asburiae]
WP_252070049.1	Type1.BREX	[illegible]	1213	Bacteria>Pseudomonadota>Gammaproteobacteria>Enterobacterales>Enterobacteriaceae>Enterobacter	GCF_023750345.1	Enterobacter_ludwigii	M8O29_RS15875	136440..140081	299767	BREX-system-P-loop-protein-BrxC-[Enterobacter-ludwigii]
WP_253179320.1	Type1.BREX	[illegible]	1204	Bacteria>Pseudomonadota>Gammaproteobacteria>Enterobacterales>Enterobacteriaceae>Enterobacter	GCF_024128585.1	Enterobacter_mori	NJA11_RS17930	132016..135630	539813	BREX-system-P-loop-protein-BrxC-[Enterobacter-mori]
WP_257273059.1	Type1.BREX	[illegible]	398	Bacteria>Pseudomonadota>Gammaproteobacteria>Enterobacterales>Enterobacteriaceae>Enterobacter	GCF_024607735.1	Enterobacter_soli	NR352_RS15925	54991..56187	885040	BREX-system-P-loop-protein-BrxC-[Enterobacter-soli]
WP_257246766.1	Type1.BREX	[illegible]	1197	Bacteria>Pseudomonadota>Gammaproteobacteria>Enterobacterales>Enterobacteriaceae>Enterobacter	GCF_024607805.1	unclassified_Enterobacter	NR293_RS15365	69785..73378	2608935	MULTISPECIES:-BREX-system-P-loop-protein-BrxC-[unclassified-Enterobacter]
WP_058688926.1	Type1.BREX	[illegible]	1213	Bacteria>Pseudomonadota>Gammaproteobacteria>Enterobacterales>Enterobacteriaceae>Enterobacter	GCF_024655335.1	Enterobacter_hormaechei	ASU86_RS07715	183720..187361	158836	BREX-system-P-loop-protein-BrxC-[Enterobacter-hormaechei]
WP_258247898.1	Type1.BREX	[illegible]	1204	Bacteria>Pseudomonadota>Gammaproteobacteria>Enterobacterales>Enterobacteriaceae>Enterobacter	GCF_024733415.1	Enterobacter_hormaechei	NUJ48_RS00150	29183..32797	158836	BREX-system-P-loop-protein-BrxC-[Enterobacter-hormaechei]
WP_265188117.1	Type1.BREX	[illegible]	1204	Bacteria>Pseudomonadota>Gammaproteobacteria>Enterobacterales>Enterobacteriaceae>Enterobacter	GCF_026115025.1	Enterobacter_hormaechei	M7778_RS15260	340339..343953	158836	BREX-system-P-loop-protein-BrxC-[Enterobacter-hormaechei]
WP_271139057.1	Type1.BREX	[illegible]	1204	Bacteria>Pseudomonadota>Gammaproteobacteria>Enterobacterales>Enterobacteriaceae>Enterobacter	GCF_027920365.1	Enterobacter_hormaechei	PBS85_RS02750	569121..572735	158836	BREX-system-P-loop-protein-BrxC-[Enterobacter-hormaechei]
WP_274416986.1	Type1.BREX	[illegible]	1204	Bacteria>Pseudomonadota>Gammaproteobacteria>Enterobacterales>Enterobacteriaceae>Enterobacter	GCF_028765355.1	Enterobacter_kobei	PVM09_RS00190	32375..35989	208224	BREX-system-P-loop-protein-BrxC-[Enterobacter-kobei]
WP_111946923.1	Type1.BREX	[illegible]	1204	Bacteria>Pseudomonadota>Gammaproteobacteria>Enterobacterales>Enterobacteriaceae>Enterobacter	GCF_030324195.1	Enterobacter_hormaechei	J9785_RS10685	292266..295880	158836	BREX-system-P-loop-protein-BrxC-[Enterobacter-hormaechei]
WP_180264800.1	Type1.BREX	[illegible]	1204	Bacteria>Pseudomonadota>Gammaproteobacteria>Enterobacterales>Enterobacteriaceae>Enterobacter	GCF_900558725.1	Enterobacter_hormaechei	H0848_RS18555	27730..31344	158836	BREX-system-P-loop-protein-BrxC-[Enterobacter-hormaechei]

Protein-ID	System	Gene-Neighbourhoods	Length	Taxonomy	Assembly-ID	Strain	Locus-Tag	Genomic-coordinates	TaxID	Fasta-defline
WP_063851018.1	Type1.BREX	<-HTH_36<-HTH+HTH<-ParB+BAM<-XIS-HTH<-wHTH<-ZnR||BrxA-DNA-binding->BrxB-BrxF-iSTAND->DUF6079*->N6-METHYLASE->OLD-ABC->TOPRIM->PglZ->SIGMA+OB-Fold+AAA-ATPase+Lon-Protease-BrxL-><-wHTH(SBD)+HNH||SymE_toxin->	1204	Bacteria>Pseudomonadota>Gammaproteobacteria>Enterobacterales>Enterobacteriaceae>Enterobacter	GCF_902160625.1	Enterobacter_hormaechei	FN214_RS22220	26549..30163	158836	BREX-system-P-loop-protein-BrxC-[Enterobacter-hormaechei]
WP_112781824.1	Type1.BREX	<-SIGMA+OB-Fold+AAA-ATPase+Lon-Protease-BrxL<-PglZ<-TOPRIM<-OLD-ABC<-N6-METHYLASE<-DUF6079*<-BrxB-BrxF-iSTAND<-BrxA-DNA-binding<-XIS-HTH<-?||CopG/RHH->DUF932->ArdB->	1204	Bacteria>Pseudomonadota>Gammaproteobacteria>Enterobacterales>Enterobacteriaceae>Enterobacter	GCF_902387755.1	Enterobacter_cloacae	FYC20_RS23065	9489..13103	550	MULTISPECIES:-BREX-system-P-loop-protein-BrxC-[Enterobacteriaceae]
EHX5558535.1	Type1.BREX	<-HerA/FtsK+A32<-?||tRNA->Arm-DNA-bind_3+Phage_integrase->EVE+HNH-><-REase+iREase||BrxA-DNA-binding->BrxB-BrxF-iSTAND->DUF6079*->N6-METHYLASE->PglZ->SIGMA+OB-Fold+AAA-ATPase+Lon-Protease-BrxL-><-ZnR<-ZnR<-?<-?||RHH_antitoxin->XIS-HTH->	1213	Bacteria>Pseudomonadota>Gammaproteobacteria>Enterobacterales>Enterobacteriaceae>Escherichia	-	Escherichia_albertii	K2D51_002305	108873..112514	208962	BREX-system-P-loop-protein-BrxC-[Escherichia-albertii]
WP_095526333.1	Type1.BREX	<-35exo<-SIGMA+OB-Fold+AAA-ATPase+Lon-Protease-BrxL<-PglZ<-N6-METHYLASE<-ParB+HNH<-DUF6079*<-BrxB-BrxF-iSTAND<-BrxA-DNA-binding<-wHTH+WYL+WCX<-XIS-HTH<-ZnR||?-><-?||?->ArdB->	1225	Bacteria>Pseudomonadota>Gammaproteobacteria>Enterobacterales>Enterobacteriaceae>Escherichia	-	Escherichia_coli	CDN88_RS21130	10739..14416	562	BREX-system-P-loop-protein-BrxC-[Escherichia-coli]
WP_121868993.1	Type1.BREX	<-HerA/FtsK+A32<-?||tRNA->Arm-DNA-bind_3+Phage_integrase-><-SIGMA+OB-Fold+AAA-ATPase+Lon-Protease-BrxL<-PglZ<-N6-METHYLASE-p<-N6-METHYLASE<-ParB+HNH<-DUF6079*<-BrxB-BrxF-iSTAND<-BrxA-DNA-binding<-?<-?<-XIS-HTH||?->nSTAND1+sGTP->wHTH+WYL->	1233	Bacteria>Pseudomonadota>Gammaproteobacteria>Enterobacterales>Enterobacteriaceae>Escherichia	-	Escherichia_coli	EAI39_15065	84115..87816	562	BREX-system-P-loop-protein-BrxC-[Escherichia-coli]
EFH4523723.1	Type1.BREX	<-METHYLASE||SKI-><-?||tRNA->Arm-DNA-bind_3+Phage_integrase-><-REase+iREase||BrxA-DNA-binding->BrxB-BrxF-iSTAND->DUF6079*->N6-METHYLASE->PglZ->SIGMA+OB-Fold+AAA-ATPase+Lon-Protease-BrxL-><-ZnR<-ZnR	1213	Bacteria>Pseudomonadota>Gammaproteobacteria>Enterobacterales>Enterobacteriaceae>Escherichia	-	Escherichia_coli	F9608_003902	25000..28641	562	BREX-system-P-loop-protein-BrxC-[Escherichia-coli]
WP_181716349.1	Type1.BREX	XIS-HTH->PIWI-><-DDE_Tnp_1_5+DDE_Tnp_1||PIWI-><-?<-Phage_integrase||Arm-DNA-bind_3+Phage_integrase->BrxA-DNA-binding->BrxB-BrxF-iSTAND->DUF6079*->N6-METHYLASE->OLD-ABC->TOPRIM->SIGMA+OB-Fold+AAA-ATPase+Lon-Protease-BrxL-><-DDE_Tnp_1_5+DDE_Tnp_1<-?||DDE_Tnp_1->RNase-><-HKD-DNase+MED12	1204	Bacteria>Pseudomonadota>Gammaproteobacteria>Enterobacterales>Enterobacteriaceae>Escherichia	-	Escherichia_coli	H5E40_RS01425	278751..282365	562	BREX-system-P-loop-protein-BrxC-[Escherichia-coli]
WP_174580485.1	Type1.BREX	DDE_Tnp_IS1->?->ZnR->BrxA-DNA-binding->BrxB-BrxF-iSTAND->DUF6079*->ParB+HNH->N6-METHYLASE->HEPN->RVT->N6-METHYLASE-p->PglZ->SIGMA+OB-Fold+AAA-ATPase+Lon-Protease-BrxL-><-Arm-DNA-bind_3+Phage_integrase||Phage_integrase->	1233	Bacteria>Pseudomonadota>Gammaproteobacteria>Enterobacterales>Enterobacteriaceae>Escherichia	-	Escherichia_coli	HUC40_15465	2945..6646	562	BREX-system-P-loop-protein-BrxC-[Escherichia-coli]
WP_227303300.1	Type1.BREX	<-Phage_integrase<-Phage_integrase||?->?->?->||wHTH+SAD+HNH-><-REase+iREase||BrxA-DNA-binding->BrxB-BrxF-iSTAND->DUF6079*->N6-METHYLASE->PglZ->SIGMA+OB-Fold+AAA-ATPase+Lon-Protease-BrxL->HKD-DNase+OGG-like->||AbiEi_3_N+AbiEi_3->AbiEii->?-><-DUF6216	1213	Bacteria>Pseudomonadota>Gammaproteobacteria>Enterobacterales>Enterobacteriaceae>Escherichia	-	Escherichia_coli	LJD42_RS04270	217680..221321	562	BREX-system-P-loop-protein-BrxC-[Escherichia-coli]
MCN1699418.1	Type1.BREX	<-LACI-HTH+PBPI<-?<-?<-METHYLASE||SKI-><-?||tRNA->Arm-DNA-bind_3+Phage_integrase->BrxA-DNA-binding->BrxB-BrxF-iSTAND->DUF6079*->N6-METHYLASE->OLD-ABC->TOPRIM->PglZ->SIGMA+OB-Fold+AAA-ATPase+Lon-Protease-BrxL-><-URI-DNase+RAMA	1204	Bacteria>Pseudomonadota>Gammaproteobacteria>Enterobacterales>Enterobacteriaceae>Escherichia	-	Escherichia_coli	MLI78_16455	116212..119826	562	BREX-system-P-loop-protein-BrxC-[Escherichia-coli]
MCN1334053.1	Type1.BREX	<-LACI-HTH+PBPI||?->?-><-Zn_Tnp_IS1+HTH_Tnp_IS1+DDE_Tnp_IS1<-REase<-REase||BrxA-DNA-binding->BrxB-BrxF-iSTAND->DUF6079*->N6-METHYLASE->PglZ->SIGMA+OB-Fold+AAA-ATPase+Lon-Protease-BrxL-><-ZnR<-HTH_Tnp_1+HTH_21+rve+rve_3	1213	Bacteria>Pseudomonadota>Gammaproteobacteria>Enterobacterales>Enterobacteriaceae>Escherichia	-	Escherichia_coli	MLR45_18135	8747..12388	562	BREX-system-P-loop-protein-BrxC-[Escherichia-coli]
MCN3919819.1	Type1.BREX	<-METHYLASE||SKI-><-?||tRNA->Arm-DNA-bind_3+Phage_integrase-><-SIGMA+OB-Fold+AAA-ATPase+Lon-Protease-BrxL<-PglZ<-N6-METHYLASE<-ParB+HNH<-DUF6079*<-BrxB-BrxF-iSTAND<-BrxA-DNA-binding<-?<-?<-XIS-HTH||?->wHTH+WYL->	1240	Bacteria>Pseudomonadota>Gammaproteobacteria>Enterobacterales>Enterobacteriaceae>Escherichia	-	Escherichia_coli	MLZ57_18615	66742..70464	562	BREX-system-P-loop-protein-BrxC-[Escherichia-coli]
EIH98049.1	Type1.BREX	REase+N6_METHYLASE->?->ParB->?-><-?<-SIGMA+OB-Fold+AAA-ATPase+Lon-Protease-BrxL<-PglZ<-N6-METHYLASE<-DUF6079-p*<-DUF6079-p<-BrxB-BrxF-iSTAND<-BrxA-DNA-binding||REase+iREase-><-wHTH+SAD+HNH||HNH->	804	Bacteria>Pseudomonadota>Gammaproteobacteria>Enterobacterales>Enterobacteriaceae>Escherichia	GCA_000215245.2	Escherichia_coli_96.154	EC96154_4669	254732..257146	869683	hypothetical-protein-EC96154_4669-[Escherichia-coli-96.154]
EHW21311.1	Type1.BREX	<-AbiEii<-AbiEi_3_N+AbiEi_3<-HKD-DNase+OGG-like||?-><-SIGMA+OB-Fold+AAA-ATPase+Lon-Protease-BrxL<-Zn_Tnp_IS91+Y2_Tnp<-?||?-><-N6-METHYLASE<-DUF6079*||HIN-HTH->HTH_Tnp_1+HTH_21+rve+rve_3-><-BrxA-DNA-binding||REase+iREase-><-wHTH+SAD+HNH||HNH->	1001	Bacteria>Pseudomonadota>Gammaproteobacteria>Enterobacterales>Enterobacteriaceae>Escherichia	GCA_000249875.2	Escherichia_coli_DEC8E	ECDEC8E_5320	144024..147029	868174	putative-DNA-repair-ATPase-[Escherichia-coli-DEC8E]
EHX52156.1	Type1.BREX	DUF6216-><-?<-AbiEii<-AbiEi_3_N+AbiEi_3<-HKD-DNase+OGG-like<-SIGMA+OB-Fold+AAA-ATPase+Lon-Protease-BrxL<-PglZ<-N6-METHYLASE-p<-N6-METHYLASE<-DUF6079-p*<-DUF6079-p<-BrxB-BrxF-iSTAND<-BrxA-DNA-binding||?->REase+iREase-><-wHTH+SAD+HNH||HNH->	788	Bacteria>Pseudomonadota>Gammaproteobacteria>Enterobacterales>Enterobacteriaceae>Escherichia	GCA_000250315.2	Escherichia_coli_DEC13A	ECDEC13A_0072	76997..79363	868196	putative-DNA-repair-ATPase-[Escherichia-coli-DEC13A]
EHX55090.1	Type1.BREX	<-AbiEii<-AbiEi_3_N+AbiEi_3<-HKD-DNase+OGG-like<-SIGMA+OB-Fold+AAA-ATPase+Lon-Protease-BrxL<-PglZ<-N6-METHYLASE-p<-N6-METHYLASE-p<-N6-METHYLASE<-DUF6079-p*<-DUF6079-p<-BrxB-BrxF-iSTAND<-BrxA-DNA-binding||REase+iREase-><-wHTH+SAD+HNH||HNH->	804	Bacteria>Pseudomonadota>Gammaproteobacteria>Enterobacterales>Enterobacteriaceae>Escherichia	GCA_000250335.2	Escherichia_coli_DEC13B	ECDEC13B_4632	100761..103175	868197	putative-DNA-repair-ATPase-[Escherichia-coli-DEC13B]
ESD29615.1	Type1.BREX	ZnR-><-SIGMA+OB-Fold+AAA-ATPase+Lon-Protease-BrxL<-PglZ<-N6-METHYLASE<-DUF6079*<-BrxB-BrxF-iSTAND<-BrxA-DNA-binding<-?||REase->REase->	1218	Bacteria>Pseudomonadota>Gammaproteobacteria>Enterobacterales>Enterobacteriaceae>Escherichia	GCA_000488435.1	Escherichia_coli_907715	HMPREF1600_01130	9186..12842	1268987	hypothetical-protein-HMPREF1600_01130-[Escherichia-coli-907715]
ESD66367.1	Type1.BREX	wHTH+SAD+HNH-><-REase+iREase||?->BrxA-DNA-binding->BrxB-BrxF-iSTAND->DUF6079*->N6-METHYLASE->	1218	Bacteria>Pseudomonadota>Gammaproteobacteria>Enterobacterales>Enterobacteriaceae>Escherichia	GCA_000488595.1	Escherichia_coli_908541	HMPREF1609_05038	9764..13420	1268996	hypothetical-protein-HMPREF1609_05038-[Escherichia-coli-908541]
CDL44502.1	Type1.BREX	<-DDE_Tnp_IS1<-?<-REase<-REase<-?||BrxA-DNA-binding->BrxB-BrxF-iSTAND->DUF6079*->DUF6079-p->N6-METHYLASE->N6-METHYLASE-p->PglZ->SIGMA+OB-Fold+AAA-ATPase+Lon-Protease-BrxL-><-ZnR<-HTH_Tnp_1+HTH_21+rve+rve_3<-RVT	1116	Bacteria>Pseudomonadota>Gammaproteobacteria>Enterobacterales>Enterobacteriaceae>Escherichia	GCA_000529555.1	Escherichia_coli_ISC41	-	3336..6686	1432559	putative-DNA-repair-ATPase-[Escherichia-coli-ISC41]
KEM88219.1	Type1.BREX	<-HTH_Tnp_1+HTH_21+rve+rve_3<-HTH_Tnp_1||wHTH+WYL+WCX->BrxA-DNA-binding->BrxB-BrxF-iSTAND->DUF6079-p->DUF6079-p*->ParB+HNH->N6-METHYLASE->PglZ->SIGMA+OB-Fold+AAA-ATPase+Lon-Protease-BrxL->35exo-><-?->HTH_21+rve+rve_3<-HIN-HTH||DDE_Tnp_IS1->	947	Bacteria>Pseudomonadota>Gammaproteobacteria>Enterobacterales>Enterobacteriaceae>Escherichia	GCA_000713255.1	Escherichia_coli_6-537-08_S4_C1	AC92_4028	7171..10014	1444227	putative-aTPase-like-protein-[Escherichia-coli-6-537-08_S4_C1]
KGM58490.1	Type1.BREX	<-DDE_Tnp_IS1<-TetR-HTH<-?<-?<-?<-REase+iREase<-?||BrxA-DNA-binding->BrxB-BrxF-iSTAND->DUF6079*->N6-METHYLASE->PglZ->SIGMA+OB-Fold+AAA-ATPase+Lon-Protease-BrxL-><-LZ_Tnp_IS66+zf-IS66+DDE_Tnp_IS66_C<-?<-HIN-HTH<-DUF772+DDE_Tnp_1<-DDE_Tnp_IS1	1218	Bacteria>Pseudomonadota>Gammaproteobacteria>Enterobacterales>Enterobacteriaceae>Escherichia	GCA_000768425.1	Escherichia_coli_G3/10	EL76_4293	581374..585030	1455601	hypothetical-protein-EL76_4293-[Escherichia-coli-G3/10]
CTT26978.1	Type1.BREX	DUF6079*->N6-METHYLASE->N6-METHYLASE-p->PglZ->SIGMA+OB-Fold+AAA-ATPase+Lon-Protease-BrxL->HKD-DNase+OGG-like->AbiEi_3_N+AbiEi_3->AbiEii-><-DUF6216<-Arm-DNA-bind_3	1088	Bacteria>Pseudomonadota>Gammaproteobacteria>Enterobacterales>Enterobacteriaceae>Escherichia	GCA_001283645.1	Escherichia_coli	ERS085343_03077	214..3480	562	ATPase-like-protein-[Escherichia-coli]
CTW38723.1	Type1.BREX	DUF6079-p*->N6-METHYLASE->PglZ->SIGMA+OB-Fold+AAA-ATPase+Lon-Protease-BrxL->HKD-DNase+OGG-like->AbiEi_3_N+AbiEi_3->AbiEii-><-DUF6216<-Arm-DNA-bind_3	788	Bacteria>Pseudomonadota>Gammaproteobacteria>Enterobacterales>Enterobacteriaceae>Escherichia	GCA_001283765.1	Escherichia_coli	ERS085413_04497	243..2609	562	ATPase-like-protein-[Escherichia-coli]
CUA46950.1	Type1.BREX	DUF6079*->N6-METHYLASE->PglZ->SIGMA+OB-Fold+AAA-ATPase+Lon-Protease-BrxL->HKD-DNase+OGG-like->AbiEi_3_N+AbiEi_3->AbiEii-><-DUF6216<-Arm-DNA-bind_3	1088	Bacteria>Pseudomonadota>Gammaproteobacteria>Enterobacterales>Enterobacteriaceae>Escherichia	GCA_001286505.1	Escherichia_coli	ERS139261_03500	135..3401	562	ATPase-like-protein-[Escherichia-coli]
OWF25643.1	Type1.BREX	DUF6079-p*->ParB+HNH->N6-METHYLASE-p->PglZ->SIGMA+OB-Fold+AAA-ATPase+Lon-Protease-BrxL-><-HTH_Tnp_1+HTH_21+rve+rve_3	902	Bacteria>Pseudomonadota>Gammaproteobacteria>Enterobacterales>Enterobacteriaceae>Escherichia	GCA_002190095.1	Escherichia_coli	A8M76_10400	1..2709	562	DNA-repair-protein,-partial-[Escherichia-coli]
POU04217.1	Type1.BREX	HNH-><-?||?->XIS-HTH->SF1-SF2-Helicase+REase->SF1-SF2-Helicase+REase-><-PglZ<-ParB+HNH<-N6-METHYLASE<-DUF6079*<-BrxB-BrxF-iSTAND<-BrxA-DNA-binding<-Arm-DNA-bind_3+Phage_integrase<-tRNA||wHTH(SBD)+HNH->SAM-METHYLASE->	1157	Bacteria>Pseudomonadota>Gammaproteobacteria>Enterobacterales>Enterobacteriaceae>Escherichia	GCA_002918705.1	Escherichia_sp._ESNIH1	C3369_02380	500495..503968	1985876	BREX-system-P-loop-protein-BrxC-[Escherichia-sp.-ESNIH1]
AUZ10893.1	Type1.BREX	<-35exo||?->?->?-><-HNH||wHTH+SAD+HNH-><-REase+iREase||BrxA-DNA-binding->BrxB-BrxF-iSTAND->||DUF6079-p*->N6-METHYLASE->PglZ->SIGMA+OB-Fold+AAA-ATPase+Lon-Protease-BrxL->?-><-?<-ParB<-?||<-REase+N6_METHYLASE<-TRD	788	Bacteria>Pseudomonadota>Gammaproteobacteria>Enterobacterales>Enterobacteriaceae>Escherichia	GCA_002968935.1	Escherichia_coli	BWI88_05955	1182148..1184514	562	DNA-repair-ATPase-[Escherichia-coli]
RLY30110.1	Type1.BREX	<-HerA/FtsK+A32<-?||tRNA->Arm-DNA-bind_3+Phage_integrase-><-SIGMA+OB-Fold+AAA-ATPase+Lon-Protease-BrxL<-PglZ<-N6-METHYLASE-p<-N6-METHYLASE<-ParB+HNH<-DUF6079*<-BrxB-BrxF-iSTAND<-BrxA-DNA-binding<-?<-?<-XIS-HTH||?->nSTAND1+sGTP->wHTH+WYL->	1233	Bacteria>Pseudomonadota>Gammaproteobacteria>Enterobacterales>Enterobacteriaceae>Escherichia	GCA_003687125.1	Escherichia_coli	EAI39_15065	84115..87816	562	BREX-system-P-loop-protein-BrxC-[Escherichia-coli]
EEG9579271.1	Type1.BREX	DUF6079*->N6-METHYLASE->PglZ->SIGMA+OB-Fold+AAA-ATPase+Lon-Protease-BrxL->HKD-DNase+OGG-like->||AbiEi_3_N+AbiEi_3->AbiEii->?-><-DUF6216<-Arm-DNA-bind_3	1111	Bacteria>Pseudomonadota>Gammaproteobacteria>Enterobacterales>Enterobacteriaceae>Escherichia	GCA_003736085.2	Escherichia_coli	EB576_RS15540	1..3336	562	BREX-system-P-loop-protein-BrxC-[Escherichia-coli]
EED0771052.1	Type1.BREX	BrxA-DNA-binding->BrxB-BrxF-iSTAND->DUF6079*->N6-METHYLASE->PglZ->SIGMA+OB-Fold+AAA-ATPase+Lon-Protease-BrxL->HKD-DNase+OGG-like->||AbiEi_3_N+AbiEi_3->AbiEii-><-DUF6216<-Arm-DNA-bind_3	1213	Bacteria>Pseudomonadota>Gammaproteobacteria>Enterobacterales>Enterobacteriaceae>Escherichia	GCA_003747865.2	Escherichia_coli	ED219_RS15230	935..4576	562	BREX-system-P-loop-protein-BrxC-[Escherichia-coli]
EED1762068.1	Type1.BREX	DUF6079*->PglZ->SIGMA+OB-Fold+AAA-ATPase+Lon-Protease-BrxL->HKD-DNase+OGG-like->||AbiEi_3_N+AbiEi_3->AbiEii-><-DUF6216<-Arm-DNA-bind_3<-tRNA||?->HerA/FtsK+A32->	1073	Bacteria>Pseudomonadota>Gammaproteobacteria>Enterobacterales>Enterobacteriaceae>Escherichia	GCA_003786715.2	Escherichia_coli	EEP34_RS13370	1..3223	562	BREX-system-P-loop-protein-BrxC-[Escherichia-coli]
EEC9685943.1	Type1.BREX	DUF6079-p*->N6-METHYLASE->PglZ->SIGMA+OB-Fold+AAA-ATPase+Lon-Protease-BrxL->HKD-DNase+OGG-like->||AbiEi_3_N+AbiEi_3->AbiEii-><-DUF6216<-Arm-DNA-bind_3	989	Bacteria>Pseudomonadota>Gammaproteobacteria>Enterobacterales>Enterobacteriaceae>Escherichia	GCA_004231765.2	Escherichia_coli	D8K54_RS17535	1..2971	562	BREX-system-P-loop-protein-BrxC-[Escherichia-coli]
EEC7405250.1	Type1.BREX	<-Phage_integrase||?->?->?-><-HNH||wHTH+SAD+HNH-><-REase+iREase||BrxA-DNA-binding->BrxB-BrxF-iSTAND->DUF6079*->N6-METHYLASE->PglZ->SIGMA+OB-Fold+AAA-ATPase+Lon-Protease-BrxL->HKD-DNase+OGG-like->||AbiEi_3_N+AbiEi_3->AbiEii-><-DUF6216<-Arm-DNA-bind_3	1213	Bacteria>Pseudomonadota>Gammaproteobacteria>Enterobacterales>Enterobacteriaceae>Escherichia	GCA_004272605.2	Escherichia_coli	D4L89_RS08005	71644..75285	562	BREX-system-P-loop-protein-BrxC-[Escherichia-coli]
GCK20492.1	Type1.BREX	<-Fimbrial<-Phage_integrase<-Phage_integrase||?->?->?->wHTH(SBD)+HNH->BrxA-DNA-binding->BrxB-BrxF-iSTAND->DUF6079-p*->	621	Bacteria>Pseudomonadota>Gammaproteobacteria>Enterobacterales>Enterobacteriaceae>Escherichia	GCA_005401165.1	Escherichia_coli	BvCmsA58A_02120	111143..113008	562	ATPase-like-protein-[Escherichia-coli]
TZF09961.1	Type1.BREX	RVT->DEDD_Tnp_IS110+Transposase_20-><-SIGMA+OB-Fold+AAA-ATPase+Lon-Protease-BrxL<-PglZ<-N6-METHYLASE<-ParB+HNH<-DUF6079*	1149	Bacteria>Pseudomonadota>Gammaproteobacteria>Enterobacterales>Enterobacteriaceae>Escherichia	GCA_008273745.1	Escherichia_coli	EOI42_22665	16149..19600	562	BREX-system-P-loop-protein-BrxC,-partial-[Escherichia-coli]
TZE19045.1	Type1.BREX	RVT->DEDD_Tnp_IS110+Transposase_20-><-SIGMA+OB-Fold+AAA-ATPase+Lon-Protease-BrxL<-PglZ<-N6-METHYLASE<-DUF6079*<-BrxB-BrxF-iSTAND<-BrxA-DNA-binding<-wHTH+WYL+WCX<-?||RHH->||CopG/RHH->?-><-DDE_Tnp_IS1	1225	Bacteria>Pseudomonadota>Gammaproteobacteria>Enterobacterales>Enterobacteriaceae>Escherichia	GCA_008273875.1	Escherichia_coli	EOI13_22465	16132..19809	562	BREX-system-P-loop-protein-BrxC-[Escherichia-coli]
TZE05560.1	Type1.BREX	<-CopG/RHH||<-RHH||?->wHTH+WYL+WCX->BrxA-DNA-binding->BrxB-BrxF-iSTAND->DUF6079*->ParB+HNH->N6-METHYLASE->PglZ->SIGMA+OB-Fold+AAA-ATPase+Lon-Protease-BrxL-><-DEDD_Tnp_IS110+Transposase_20<-RVT	1225	Bacteria>Pseudomonadota>Gammaproteobacteria>Enterobacterales>Enterobacteriaceae>Escherichia	GCA_008273905.1	Escherichia_coli	EOI14_22605	9953..13630	562	BREX-system-P-loop-protein-BrxC-[Escherichia-coli]
KAA9812768.1	Type1.BREX	DUF6079-p*->N6-METHYLASE->PglZ->SIGMA+OB-Fold+AAA-ATPase+Lon-Protease-BrxL->HKD-DNase+OGG-like->||AbiEi_3_N+AbiEi_3->AbiEii-><-DUF6216<-Arm-DNA-bind_3	891	Bacteria>Pseudomonadota>Gammaproteobacteria>Enterobacterales>Enterobacteriaceae>Escherichia	GCA_008755545.1	Escherichia_coli	F7F73_23785	1..2676	562	BREX-system-P-loop-protein-BrxC,-partial-[Escherichia-coli]
KAA9812739.1	Type1.BREX	DUF6079-p*->N6-METHYLASE->PglZ->SIGMA+OB-Fold+AAA-ATPase+Lon-Protease-BrxL-><-ZnR<-ZnR<-?||HIN-HTH+HIN-HTH->	891	Bacteria>Pseudomonadota>Gammaproteobacteria>Enterobacterales>Enterobacteriaceae>Escherichia	GCA_008755545.1	Escherichia_coli	F7F73_23930	1..2676	562	BREX-system-P-loop-protein-BrxC,-partial-[Escherichia-coli]
KAB2983059.1	Type1.BREX	<-Phage_integrase||?->?->?-><-HNH||wHTH+SAD+HNH-><-REase+iREase||BrxA-DNA-binding->BrxB-BrxF-iSTAND->DUF6079*->N6-METHYLASE->PglZ->SIGMA+OB-Fold+AAA-ATPase+Lon-Protease-BrxL->?-><-?<-ParB<-?||<-REase+N6_METHYLASE<-TRD	1213	Bacteria>Pseudomonadota>Gammaproteobacteria>Enterobacterales>Enterobacteriaceae>Escherichia	GCA_009014785.1	Escherichia_coli	F9179_13170	9843..13484	562	BREX-system-P-loop-protein-BrxC-[Escherichia-coli]
EEQ1797639.1	Type1.BREX	DUF6079-p*->N6-METHYLASE->PglZ->SIGMA+OB-Fold+AAA-ATPase+Lon-Protease-BrxL->HKD-DNase+OGG-like->||AbiEi_3_N+AbiEi_3->AbiEii-><-DUF6216<-Arm-DNA-bind_3	861	Bacteria>Pseudomonadota>Gammaproteobacteria>Enterobacterales>Enterobacteriaceae>Escherichia	GCA_011802225.1	Escherichia_coli	GUY43_002901	1..2587	562	BREX-system-P-loop-protein-BrxC-[Escherichia-coli]
EEQ2509736.1	Type1.BREX	BrxA-DNA-binding->BrxB-BrxF-iSTAND->DUF6079*->N6-METHYLASE->PglZ->SIGMA+OB-Fold+AAA-ATPase+Lon-Protease-BrxL->HKD-DNase+OGG-like->||AbiEi_3_N+AbiEi_3->AbiEii-><-DUF6216<-Arm-DNA-bind_3	1213	Bacteria>Pseudomonadota>Gammaproteobacteria>Enterobacterales>Enterobacteriaceae>Escherichia	GCA_011806325.1	Escherichia_coli	GSE75_002859	1085..4726	562	BREX-system-P-loop-protein-BrxC-[Escherichia-coli]
EEQ6465042.1	Type1.BREX	<-XIS-HTH<-RHH_antitoxin||?->?->ZnR->ZnR-><-SIGMA+OB-Fold+AAA-ATPase+Lon-Protease-BrxL<-PglZ<-N6-METHYLASE<-DUF6079*<-BrxB-BrxF-iSTAND<-BrxA-DNA-binding||<-HTH_Tnp_1+HTH_21+rve+rve_3	1213	Bacteria>Pseudomonadota>Gammaproteobacteria>Enterobacterales>Enterobacteriaceae>Escherichia	GCA_011825175.1	Escherichia_coli	GMM94_000612	221240..224881	562	BREX-system-P-loop-protein-BrxC-[Escherichia-coli]
EEQ7780471.1	Type1.BREX	<-HNH||wHTH+SAD+HNH-><-REase+iREase||BrxA-DNA-binding->BrxB-BrxF-iSTAND->DUF6079*->N6-METHYLASE->PglZ->SIGMA+OB-Fold+AAA-ATPase+Lon-Protease-BrxL->HKD-DNase+OGG-like->||AbiEi_3_N+AbiEi_3->AbiEii-><-DUF6216<-Arm-DNA-bind_3	1213	Bacteria>Pseudomonadota>Gammaproteobacteria>Enterobacterales>Enterobacteriaceae>Escherichia	GCA_011830195.1	Escherichia_coli	GLS89_002414	7714..11355	562	BREX-system-P-loop-protein-BrxC-[Escherichia-coli]
EER7097196.1	Type1.BREX	DUF6079*->N6-METHYLASE->PglZ->SIGMA+OB-Fold+AAA-ATPase+Lon-Protease-BrxL->HKD-DNase+OGG-like->||AbiEi_3_N+AbiEi_3->AbiEii-><-DUF6216<-Arm-DNA-bind_3	1064	Bacteria>Pseudomonadota>Gammaproteobacteria>Enterobacterales>Enterobacteriaceae>Escherichia	GCA_011867305.1	Escherichia_coli	F2Z67_003270	1..3197	562	BREX-system-P-loop-protein-BrxC-[Escherichia-coli]
EER7518149.1	Type1.BREX	DUF6079-p*->N6-METHYLASE->PglZ->SIGMA+OB-Fold+AAA-ATPase+Lon-Protease-BrxL->HKD-DNase+OGG-like->||AbiEi_3_N+AbiEi_3->AbiEii-><-DUF6216<-Arm-DNA-bind_3	990	Bacteria>Pseudomonadota>Gammaproteobacteria>Enterobacterales>Enterobacteriaceae>Escherichia	GCA_011869725.1	Escherichia_coli	F1Z98_003251	1..2974	562	BREX-system-P-loop-protein-BrxC-[Escherichia-coli]
EER8632679.1	Type1.BREX	DUF6079*->N6-METHYLASE->PglZ->SIGMA+OB-Fold+AAA-ATPase+Lon-Protease-BrxL->HKD-DNase+OGG-like->||AbiEi_3_N+AbiEi_3->AbiEii-><-DUF6216<-Arm-DNA-bind_3	1080	Bacteria>Pseudomonadota>Gammaproteobacteria>Enterobacterales>Enterobacteriaceae>Escherichia	GCA_011873745.1	Escherichia_coli	F0E18_004248	1..3245	562	BREX-system-P-loop-protein-BrxC-[Escherichia-coli]
MBC1090136.1	Type1.BREX	<-HerA/FtsK+A32<-?||tRNA->Arm-DNA-bind_3+Phage_integrase->EVE+HNH-><-REase+iREase||BrxA-DNA-binding->BrxB-BrxF-iSTAND->DUF6079*->N6-METHYLASE->PglZ->SIGMA+OB-Fold+AAA-ATPase+Lon-Protease-BrxL-><-ZnR<-ZnR<-?<-?||RHH_antitoxin->XIS-HTH->	1213	Bacteria>Pseudomonadota>Gammaproteobacteria>Enterobacterales>Enterobacteriaceae>Escherichia	GCA_011890865.2	Escherichia_coli	FSG28_003850	139502..143143	562	BREX-system-P-loop-protein-BrxC-[Escherichia-coli]
EES3817132.1	Type1.BREX	DUF6079-p*->N6-METHYLASE->PglZ->SIGMA+OB-Fold+AAA-ATPase+Lon-Protease-BrxL->HKD-DNase+OGG-like->||AbiEi_3_N+AbiEi_3->AbiEii-><-DUF6216<-Arm-DNA-bind_3	952	Bacteria>Pseudomonadota>Gammaproteobacteria>Enterobacterales>Enterobacteriaceae>Escherichia	GCA_011896505.1	Escherichia_coli	FS433_003751	1..2860	562	BREX-system-P-loop-protein-BrxC-[Escherichia-coli]
EES8430772.1	Type1.BREX	DUF6079-p*->N6-METHYLASE->PglZ->SIGMA+OB-Fold+AAA-ATPase+Lon-Protease-BrxL->HKD-DNase+OGG-like->||AbiEi_3_N+AbiEi_3->AbiEii-><-DUF6216<-Arm-DNA-bind_3	988	Bacteria>Pseudomonadota>Gammaproteobacteria>Enterobacterales>Enterobacteriaceae>Escherichia	GCA_011915225.1	Escherichia_coli	FU162_003513	1..2968	562	BREX-system-P-loop-protein-BrxC-[Escherichia-coli]
EET5616501.1	Type1.BREX	<-RHH_antitoxin||?->?->ZnR->ZnR-><-SIGMA+OB-Fold+AAA-ATPase+Lon-Protease-BrxL<-PglZ<-N6-METHYLASE<-DUF6079*<-BrxB-BrxF-iSTAND<-BrxA-DNA-binding||<-EVE+HNH<-Arm-DNA-bind_3+Phage_integrase<-tRNA||?->HerA/FtsK+A32-><-?<-?||Peptidase_M17_N+Peptidase_M17->	1213	Bacteria>Pseudomonadota>Gammaproteobacteria>Enterobacterales>Enterobacteriaceae>Escherichia	GCA_011940945.1	Escherichia_coli	FFE61_003335	12923..16564	562	BREX-system-P-loop-protein-BrxC-[Escherichia-coli]
EET5967869.1	Type1.BREX	DUF6079*->N6-METHYLASE->PglZ->SIGMA+OB-Fold+AAA-ATPase+Lon-Protease-BrxL->HKD-DNase+OGG-like->||AbiEi_3_N+AbiEi_3->AbiEii-><-DUF6216<-Arm-DNA-bind_3	1065	Bacteria>Pseudomonadota>Gammaproteobacteria>Enterobacterales>Enterobacteriaceae>Escherichia	GCA_011942865.1	Escherichia_coli	FE570_001648	1..3198	562	BREX-system-P-loop-protein-BrxC-[Escherichia-coli]
EEU4094847.1	Type1.BREX	DUF6079-p*->N6-METHYLASE->PglZ->SIGMA+OB-Fold+AAA-ATPase+Lon-Protease-BrxL->HKD-DNase+OGG-like->||AbiEi_3_N+AbiEi_3->AbiEii-><-DUF6216<-Arm-DNA-bind_3	779	Bacteria>Pseudomonadota>Gammaproteobacteria>Enterobacterales>Enterobacteriaceae>Escherichia	GCA_011974935.1	Escherichia_coli	E8H53_003141	1..2340	562	BREX-system-P-loop-protein-BrxC-[Escherichia-coli]
EEU4096206.1	Type1.BREX	<-DUF6079-p*<-BrxB-BrxF-iSTAND<-BrxA-DNA-binding||<-wHTH(SBD)+HNH||HNH-><-?<-?<-?||Phage_integrase->Phage_integrase->	433	Bacteria>Pseudomonadota>Gammaproteobacteria>Enterobacterales>Enterobacteriaceae>Escherichia	GCA_011974935.1	Escherichia_coli	E8H53_004589	1..1299	562	BREX-system-P-loop-protein-BrxC-[Escherichia-coli]
EEV0352856.1	Type1.BREX	DUF6079*->N6-METHYLASE->PglZ->SIGMA+OB-Fold+AAA-ATPase+Lon-Protease-BrxL->HKD-DNase+OGG-like->||AbiEi_3_N+AbiEi_3->AbiEii-><-DUF6216<-Arm-DNA-bind_3	1194	Bacteria>Pseudomonadota>Gammaproteobacteria>Enterobacterales>Enterobacteriaceae>Escherichia	GCA_012011485.1	Escherichia_coli	EYE11_003099	1..3587	562	BREX-system-P-loop-protein-BrxC-[Escherichia-coli]
EEV1146578.1	Type1.BREX	<-Phage_integrase||?->?->?-><-HNH||wHTH(SBD)+HNH->||BrxA-DNA-binding->BrxB-BrxF-iSTAND->DUF6079*->N6-METHYLASE->	1213	Bacteria>Pseudomonadota>Gammaproteobacteria>Enterobacterales>Enterobacteriaceae>Escherichia	GCA_012013845.1	Escherichia_coli	EUB49_004354	8713..12354	562	BREX-system-P-loop-protein-BrxC-[Escherichia-coli]
EEV8721794.1	Type1.BREX	<-DUF6079-p*<-BrxB-BrxF-iSTAND<-BrxA-DNA-binding||REase+iREase-><-wHTH+SAD+HNH||HNH-><-?<-?<-?||Phage_integrase->	634	Bacteria>Pseudomonadota>Gammaproteobacteria>Enterobacterales>Enterobacteriaceae>Escherichia	GCA_012057805.1	Escherichia_coli	EHU66_22975	1..1904	562	BREX-system-P-loop-protein-BrxC-[Escherichia-coli]
EEV8657615.1	Type1.BREX	DUF6079*->N6-METHYLASE->PglZ->SIGMA+OB-Fold+AAA-ATPase+Lon-Protease-BrxL->HKD-DNase+OGG-like->||AbiEi_3_N+AbiEi_3->AbiEii-><-DUF6216<-Arm-DNA-bind_3	1180	Bacteria>Pseudomonadota>Gammaproteobacteria>Enterobacterales>Enterobacteriaceae>Escherichia	GCA_012058765.1	Escherichia_coli	EHJ50_001183	1..3543	562	BREX-system-P-loop-protein-BrxC-[Escherichia-coli]
EEW9926442.1	Type1.BREX	DUF772+DDE_Tnp_1-><-REase+iREase||BrxA-DNA-binding->BrxB-BrxF-iSTAND->DUF6079*->N6-METHYLASE->PglZ->SIGMA+OB-Fold+AAA-ATPase+Lon-Protease-BrxL-><-ZnR<-ZnR<-?<-?<-?||RHH_antitoxin->	1213	Bacteria>Pseudomonadota>Gammaproteobacteria>Enterobacterales>Enterobacteriaceae>Escherichia	GCA_012061085.1	Escherichia_coli	EG211_22240	4333..7974	562	BREX-system-P-loop-protein-BrxC-[Escherichia-coli]
EEW0980710.1	Type1.BREX	<-Phage_integrase||?->?->?-><-HNH||wHTH+SAD+HNH-><-REase+iREase||BrxA-DNA-binding->BrxB-BrxF-iSTAND->DUF6079*->N6-METHYLASE->PglZ->SIGMA+OB-Fold+AAA-ATPase+Lon-Protease-BrxL->HKD-DNase+OGG-like->||AbiEi_3_N+AbiEi_3->AbiEii-><-DUF6216<-Arm-DNA-bind_3	1213	Bacteria>Pseudomonadota>Gammaproteobacteria>Enterobacterales>Enterobacteriaceae>Escherichia	GCA_012068555.1	Escherichia_coli	ED289_06180	111258..114899	562	BREX-system-P-loop-protein-BrxC-[Escherichia-coli]
EEW0945476.1	Type1.BREX	<-Phage_integrase||?->?->?-><-HNH||wHTH+SAD+HNH-><-REase+iREase||BrxA-DNA-binding->BrxB-BrxF-iSTAND->DUF6079*->N6-METHYLASE->PglZ->SIGMA+OB-Fold+AAA-ATPase+Lon-Protease-BrxL->?-><-?<-ParB<-?||<-REase+N6_METHYLASE<-TRD<-REase	1213	Bacteria>Pseudomonadota>Gammaproteobacteria>Enterobacterales>Enterobacteriaceae>Escherichia	GCA_012068805.1	Escherichia_coli	BCB75_000479	153764..157405	562	BREX-system-P-loop-protein-BrxC-[Escherichia-coli]
EEW0874914.1	Type1.BREX	<-Phage_integrase||?->?->?-><-HNH||wHTH+SAD+HNH-><-REase+iREase||BrxA-DNA-binding->BrxB-BrxF-iSTAND->DUF6079*->PglZ->SIGMA+OB-Fold+AAA-ATPase+Lon-Protease-BrxL->?-><-?<-ParB	1213	Bacteria>Pseudomonadota>Gammaproteobacteria>Enterobacterales>Enterobacteriaceae>Escherichia	GCA_012068885.1	Escherichia_coli	ED728_000829	111230..114871	562	BREX-system-P-loop-protein-BrxC-[Escherichia-coli]
EEW2565584.1	Type1.BREX	Arm-DNA-bind_3+Phage_integrase->N6-METHYLASE->ORC-CDC6+ZnR->HEPN-><-REase<-REase||BrxA-DNA-binding->BrxB-BrxF-iSTAND->DUF6079*->N6-METHYLASE->PglZ->SIGMA+OB-Fold+AAA-ATPase+Lon-Protease-BrxL-><-ZnR<-ZnR<-?<-?||RHH_antitoxin->XIS-HTH->	1213	Bacteria>Pseudomonadota>Gammaproteobacteria>Enterobacterales>Enterobacteriaceae>Escherichia	GCA_012076045.1	Escherichia_coli	D9E67_18610	72989..76630	562	BREX-system-P-loop-protein-BrxC-[Escherichia-coli]
EEW3089662.1	Type1.BREX	<-Phage_integrase<-Phage_integrase||?->?->?-><-HNH||wHTH(SBD)+HNH->||BrxA-DNA-binding->BrxB-BrxF-iSTAND->DUF6079*->N6-METHYLASE->PglZ->SIGMA+OB-Fold+AAA-ATPase+Lon-Protease-BrxL->HKD-DNase+OGG-like->||AbiEi_3_N+AbiEi_3->AbiEii-><-DUF6216<-Arm-DNA-bind_3	1213	Bacteria>Pseudomonadota>Gammaproteobacteria>Enterobacterales>Enterobacteriaceae>Escherichia	GCA_012078325.1	Escherichia_coli	EBA98_002639	14697..18338	562	BREX-system-P-loop-protein-BrxC-[Escherichia-coli]
EEW3885152.1	Type1.BREX	<-HNH||wHTH(SBD)+HNH->||BrxA-DNA-binding->BrxB-BrxF-iSTAND->DUF6079*->N6-METHYLASE->PglZ->SIGMA+OB-Fold+AAA-ATPase+Lon-Protease-BrxL->HKD-DNase+OGG-like->||AbiEi_3_N+AbiEi_3->AbiEii-><-DUF6216<-Arm-DNA-bind_3	1213	Bacteria>Pseudomonadota>Gammaproteobacteria>Enterobacterales>Enterobacteriaceae>Escherichia	GCA_012080795.1	Escherichia_coli	D9Y07_001392	7166..10807	562	BREX-system-P-loop-protein-BrxC-[Escherichia-coli]
EEW4281070.1	Type1.BREX	DUF6079*->N6-METHYLASE->PglZ->SIGMA+OB-Fold+AAA-ATPase+Lon-Protease-BrxL->HKD-DNase+OGG-like->||AbiEi_3_N+AbiEi_3->AbiEii-><-DUF6216<-Arm-DNA-bind_3	1085	Bacteria>Pseudomonadota>Gammaproteobacteria>Enterobacterales>Enterobacteriaceae>Escherichia	GCA_012082755.1	Escherichia_coli	D9T01_23345	1..3259	562	BREX-system-P-loop-protein-BrxC-[Escherichia-coli]
EEW6030677.1	Type1.BREX	DUF6079*->PglZ->SIGMA+OB-Fold+AAA-ATPase+Lon-Protease-BrxL->HKD-DNase+OGG-like->||AbiEi_3_N+AbiEi_3->AbiEii-><-DUF6216<-Arm-DNA-bind_3<-tRNA||?->HerA/FtsK+A32->	1178	Bacteria>Pseudomonadota>Gammaproteobacteria>Enterobacterales>Enterobacteriaceae>Escherichia	GCA_012088895.1	Escherichia_coli	D7V62_16715	1..3539	562	BREX-system-P-loop-protein-BrxC-[Escherichia-coli]
EEW5973241.1	Type1.BREX	DUF6079*->N6-METHYLASE->PglZ->SIGMA+OB-Fold+AAA-ATPase+Lon-Protease-BrxL-><-ZnR<-ZnR<-?||RHH_antitoxin->XIS-HTH->	1050	Bacteria>Pseudomonadota>Gammaproteobacteria>Enterobacterales>Enterobacteriaceae>Escherichia	GCA_012088995.1	Escherichia_coli	D7W18_24725	1..3153	562	BREX-system-P-loop-protein-BrxC-[Escherichia-coli]
EEW8735903.1	Type1.BREX	<-HIN-HTH||<-35exo<-SIGMA+OB-Fold+AAA-ATPase+Lon-Protease-BrxL<-PglZ<-N6-METHYLASE<-ParB+HNH<-DUF6079*<-BrxB-BrxF-iSTAND<-BrxA-DNA-binding<-wHTH+WYL+WCX<-ZnR	1221	Bacteria>Pseudomonadota>Gammaproteobacteria>Enterobacterales>Enterobacteriaceae>Escherichia	GCA_012099745.1	Escherichia_coli	D5Q61_02350	121702..125367	562	BREX-system-P-loop-protein-BrxC-[Escherichia-coli]
EEX1719682.1	Type1.BREX	<-HNH||wHTH(SBD)+HNH->||BrxA-DNA-binding->BrxB-BrxF-iSTAND->DUF6079*->N6-METHYLASE->PglZ->SIGMA+OB-Fold+AAA-ATPase+Lon-Protease-BrxL->HKD-DNase+OGG-like->||AbiEi_3_N+AbiEi_3->AbiEii-><-DUF6216<-Arm-DNA-bind_3	1213	Bacteria>Pseudomonadota>Gammaproteobacteria>Enterobacterales>Enterobacteriaceae>Escherichia	GCA_012115095.1	Escherichia_coli	D3S79_003407	6685..10326	562	BREX-system-P-loop-protein-BrxC-[Escherichia-coli]
EEX4954053.1	Type1.BREX	BrxB-BrxF-iSTAND->DUF6079*->N6-METHYLASE->PglZ->SIGMA+OB-Fold+AAA-ATPase+Lon-Protease-BrxL->HKD-DNase+OGG-like->||AbiEi_3_N+AbiEi_3->AbiEii->	1213	Bacteria>Pseudomonadota>Gammaproteobacteria>Enterobacterales>Enterobacteriaceae>Escherichia	GCA_012126795.1	Escherichia_coli	D0791_003833	606..4247	562	BREX-system-P-loop-protein-BrxC-[Escherichia-coli]
EEX5072170.1	Type1.BREX	<-N6-METHYLASE<-DUF6079*<-BrxB-BrxF-iSTAND<-BrxA-DNA-binding||REase+iREase-><-wHTH+SAD+HNH||HNH-><-?<-?<-?||Phage_integrase->	1213	Bacteria>Pseudomonadota>Gammaproteobacteria>Enterobacterales>Enterobacteriaceae>Escherichia	GCA_012127315.1	Escherichia_coli	DYU47_004374	2562..6203	562	BREX-system-P-loop-protein-BrxC-[Escherichia-coli]
EEX6773445.1	Type1.BREX	DUF6079-p*->N6-METHYLASE->PglZ->SIGMA+OB-Fold+AAA-ATPase+Lon-Protease-BrxL->HKD-DNase+OGG-like->||AbiEi_3_N+AbiEi_3->AbiEii-><-DUF6216<-Arm-DNA-bind_3	987	Bacteria>Pseudomonadota>Gammaproteobacteria>Enterobacterales>Enterobacteriaceae>Escherichia	GCA_012133985.1	Escherichia_coli	DYL55_002403	1..2965	562	BREX-system-P-loop-protein-BrxC-[Escherichia-coli]
EEX6733972.1	Type1.BREX	<-DUF6079*<-BrxB-BrxF-iSTAND<-BrxA-DNA-binding||<-wHTH(SBD)+HNH||HNH-><-?<-?<-?||Phage_integrase->Phage_integrase->	1175	Bacteria>Pseudomonadota>Gammaproteobacteria>Enterobacterales>Enterobacteriaceae>Escherichia	GCA_012135265.1	Escherichia_coli	L0765_004693	1..3524	562	BREX-system-P-loop-protein-BrxC-[Escherichia-coli]
EEX7673116.1	Type1.BREX	DUF6079*->N6-METHYLASE->PglZ->SIGMA+OB-Fold+AAA-ATPase+Lon-Protease-BrxL->HKD-DNase+OGG-like->||AbiEi_3_N+AbiEi_3->AbiEii-><-DUF6216<-Arm-DNA-bind_3	1156	Bacteria>Pseudomonadota>Gammaproteobacteria>Enterobacterales>Enterobacteriaceae>Escherichia	GCA_012137685.1	Escherichia_coli	DX034_003680	1..3471	562	BREX-system-P-loop-protein-BrxC-[Escherichia-coli]
EEY1277168.1	Type1.BREX	DUF6079*->N6-METHYLASE->PglZ->SIGMA+OB-Fold+AAA-ATPase+Lon-Protease-BrxL->HKD-DNase+OGG-like->||AbiEi_3_N+AbiEi_3->AbiEii->?-><-DUF6216<-Arm-DNA-bind_3	1189	Bacteria>Pseudomonadota>Gammaproteobacteria>Enterobacterales>Enterobacteriaceae>Escherichia	GCA_012153105.1	Escherichia_coli	BFU70_002920	1..3572	562	BREX-system-P-loop-protein-BrxC-[Escherichia-coli]
EEY1600790.1	Type1.BREX	<-Phage_integrase||?->?->?-><-HNH||wHTH+SAD+HNH-><-REase+iREase||BrxA-DNA-binding->BrxB-BrxF-iSTAND->DUF6079*->N6-METHYLASE->PglZ->SIGMA+OB-Fold+AAA-ATPase+Lon-Protease-BrxL->HKD-DNase+OGG-like->||AbiEi_3_N+AbiEi_3->AbiEii-><-DUF6216<-Arm-DNA-bind_3	1213	Bacteria>Pseudomonadota>Gammaproteobacteria>Enterobacterales>Enterobacteriaceae>Escherichia	GCA_012154025.1	Escherichia_coli	DP662_003727	9743..13384	562	BREX-system-P-loop-protein-BrxC-[Escherichia-coli]
EEY2596885.1	Type1.BREX	DUF6079*->N6-METHYLASE->PglZ->SIGMA+OB-Fold+AAA-ATPase+Lon-Protease-BrxL->HKD-DNase+OGG-like->||AbiEi_3_N+AbiEi_3->AbiEii-><-DUF6216<-tRNA||?->HerA/FtsK+A32->	1032	Bacteria>Pseudomonadota>Gammaproteobacteria>Enterobacterales>Enterobacteriaceae>Escherichia	GCA_012158085.1	Escherichia_coli	E9W20_002908	1..3101	562	BREX-system-P-loop-protein-BrxC-[Escherichia-coli]
EEY3542786.1	Type1.BREX	<-DUF6079*<-BrxB-BrxF-iSTAND<-BrxA-DNA-binding||REase+iREase-><-wHTH+SAD+HNH||HNH-><-?<-?<-?||Phage_integrase->	1157	Bacteria>Pseudomonadota>Gammaproteobacteria>Enterobacterales>Enterobacteriaceae>Escherichia	GCA_012162105.1	Escherichia_coli	EQJ55_17955	1..3471	562	BREX-system-P-loop-protein-BrxC-[Escherichia-coli]
EEY3544735.1	Type1.BREX	<-Phage_integrase||?->?->?-><-HNH||wHTH+SAD+HNH-><-REase+iREase||BrxA-DNA-binding->BrxB-BrxF-iSTAND->DUF6079*->N6-METHYLASE->PglZ->SIGMA+OB-Fold+AAA-ATPase+Lon-Protease-BrxL->?-><-?<-ParB<-?||<-REase+N6_METHYLASE<-TRD	1213	Bacteria>Pseudomonadota>Gammaproteobacteria>Enterobacterales>Enterobacteriaceae>Escherichia	GCA_012162185.1	Escherichia_coli	EQJ57_01200	8758..12399	562	BREX-system-P-loop-protein-BrxC-[Escherichia-coli]
EEY3950320.1	Type1.BREX	<-RHH_antitoxin||?->?->ZnR->ZnR-><-SIGMA+OB-Fold+AAA-ATPase+Lon-Protease-BrxL<-PglZ<-N6-METHYLASE<-DUF6079*<-BrxB-BrxF-iSTAND<-BrxA-DNA-binding||REase+iREase-><-EVE+HNH<-Arm-DNA-bind_3+Phage_integrase<-tRNA||?->HerA/FtsK+A32->	1213	Bacteria>Pseudomonadota>Gammaproteobacteria>Enterobacterales>Enterobacteriaceae>Escherichia	GCA_012163455.1	Escherichia_coli	DFX87_15375	24900..28541	562	BREX-system-P-loop-protein-BrxC-[Escherichia-coli]
EEY5880446.1	Type1.BREX	<-Phage_integrase||?->?->?-><-HNH||wHTH+SAD+HNH-><-REase+iREase||BrxA-DNA-binding->BrxB-BrxF-iSTAND->DUF6079*->N6-METHYLASE->PglZ->SIGMA+OB-Fold+AAA-ATPase+Lon-Protease-BrxL->HKD-DNase+OGG-like->||AbiEi_3_N+AbiEi_3->AbiEii-><-DUF6216<-Arm-DNA-bind_3	1213	Bacteria>Pseudomonadota>Gammaproteobacteria>Enterobacterales>Enterobacteriaceae>Escherichia	GCA_012171595.1	Escherichia_coli	EU637_10820	71590..75231	562	BREX-system-P-loop-protein-BrxC-[Escherichia-coli]
EEZ0357268.1	Type1.BREX	<-Phage_integrase||?->?->?-><-HNH||wHTH+SAD+HNH-><-REase+iREase||BrxA-DNA-binding->BrxB-BrxF-iSTAND->DUF6079*->N6-METHYLASE->PglZ->SIGMA+OB-Fold+AAA-ATPase+Lon-Protease-BrxL->?-><-?<-ParB<-?||<-REase+N6_METHYLASE<-TRD	1213	Bacteria>Pseudomonadota>Gammaproteobacteria>Enterobacterales>Enterobacteriaceae>Escherichia	GCA_012192205.1	Escherichia_coli	E9E86_001508	71741..75382	562	BREX-system-P-loop-protein-BrxC-[Escherichia-coli]
EEZ3165536.1	Type1.BREX	DUF6079*->N6-METHYLASE->PglZ->SIGMA+OB-Fold+AAA-ATPase+Lon-Protease-BrxL->HKD-DNase+OGG-like->||AbiEi_3_N+AbiEi_3->AbiEii-><-DUF6216<-Arm-DNA-bind_3	1136	Bacteria>Pseudomonadota>Gammaproteobacteria>Enterobacterales>Enterobacteriaceae>Escherichia	GCA_012200425.1	Escherichia_coli	DIN68_003954	1..3411	562	BREX-system-P-loop-protein-BrxC-[Escherichia-coli]
EEZ2582139.1	Type1.BREX	<-DUF6079-p*<-BrxB-BrxF-iSTAND<-BrxA-DNA-binding||<-wHTH(SBD)+HNH||HNH-><-?<-?<-?||Phage_integrase->Phage_integrase->	800	Bacteria>Pseudomonadota>Gammaproteobacteria>Enterobacterales>Enterobacteriaceae>Escherichia	GCA_012201865.1	Escherichia_coli	DMH54_003958	1..2402	562	BREX-system-P-loop-protein-BrxC-[Escherichia-coli]
EEZ3215399.1	Type1.BREX	<-HNH||wHTH(SBD)+HNH->||BrxA-DNA-binding->BrxB-BrxF-iSTAND->DUF6079*->N6-METHYLASE->	1213	Bacteria>Pseudomonadota>Gammaproteobacteria>Enterobacterales>Enterobacteriaceae>Escherichia	GCA_012202545.1	Escherichia_coli	DKV48_004298	6695..10336	562	BREX-system-P-loop-protein-BrxC-[Escherichia-coli]
EEZ5289151.1	Type1.BREX	<-Phage_integrase||?->?->?-><-HNH||wHTH+SAD+HNH-><-REase+iREase||BrxA-DNA-binding->BrxB-BrxF-iSTAND->DUF6079*->N6-METHYLASE->PglZ->SIGMA+OB-Fold+AAA-ATPase+Lon-Protease-BrxL->HKD-DNase+OGG-like->||AbiEi_3_N+AbiEi_3->AbiEii-><-DUF6216<-Arm-DNA-bind_3	1213	Bacteria>Pseudomonadota>Gammaproteobacteria>Enterobacterales>Enterobacteriaceae>Escherichia	GCA_012210255.1	Escherichia_coli	DDL85_000894	111149..114790	562	BREX-system-P-loop-protein-BrxC-[Escherichia-coli]
EEZ5688036.1	Type1.BREX	<-Phage_integrase||?->?->?-><-HNH||wHTH+SAD+HNH-><-REase+iREase||BrxA-DNA-binding->BrxB-BrxF-iSTAND->DUF6079*->N6-METHYLASE->PglZ->SIGMA+OB-Fold+AAA-ATPase+Lon-Protease-BrxL->HKD-DNase+OGG-like->||AbiEi_3_N+AbiEi_3->AbiEii-><-DUF6216<-Arm-DNA-bind_3	1213	Bacteria>Pseudomonadota>Gammaproteobacteria>Enterobacterales>Enterobacteriaceae>Escherichia	GCA_012215265.1	Escherichia_coli_O25	DBU11_001474	113366..117007	1095709	BREX-system-P-loop-protein-BrxC-[Escherichia-coli-O25]
EEZ5714529.1	Type1.BREX	<-wHTH+WYL<-nSTAND1+sGTP<-?||XIS-HTH->?->?->ZnR->BrxA-DNA-binding->BrxB-BrxF-iSTAND->DUF6079*->ParB+HNH->N6-METHYLASE->HEPN->RVT->N6-METHYLASE-p->PglZ->SIGMA+OB-Fold+AAA-ATPase+Lon-Protease-BrxL-><-Arm-DNA-bind_3+Phage_integrase	1233	Bacteria>Pseudomonadota>Gammaproteobacteria>Enterobacterales>Enterobacteriaceae>Escherichia	GCA_012215525.1	Escherichia_coli_O25	DBU27_000026	20065..23766	1095709	BREX-system-P-loop-protein-BrxC-[Escherichia-coli-O25]
EEZ7212406.1	Type1.BREX	<-REase+iREase||BrxA-DNA-binding->BrxB-BrxF-iSTAND->DUF6079*->N6-METHYLASE->PglZ->SIGMA+OB-Fold+AAA-ATPase+Lon-Protease-BrxL->HKD-DNase+OGG-like->||AbiEi_3_N+AbiEi_3->AbiEii-><-DUF6216<-Arm-DNA-bind_3	1213	Bacteria>Pseudomonadota>Gammaproteobacteria>Enterobacterales>Enterobacteriaceae>Escherichia	GCA_012218605.1	Escherichia_coli	DAB40_000420	2198..5839	562	BREX-system-P-loop-protein-BrxC-[Escherichia-coli]
EEZ7669684.1	Type1.BREX	<-N6-METHYLASE<-DUF6079*<-BrxB-BrxF-iSTAND<-BrxA-DNA-binding||REase+iREase-><-wHTH+SAD+HNH||HNH-><-?<-?<-?||Phage_integrase->	1213	Bacteria>Pseudomonadota>Gammaproteobacteria>Enterobacterales>Enterobacteriaceae>Escherichia	GCA_012222835.1	Escherichia_coli	C7P55_004830	2589..6230	562	BREX-system-P-loop-protein-BrxC-[Escherichia-coli]
EEZ9804432.1	Type1.BREX	<-Phage_integrase||?->?->?-><-HNH||wHTH+SAD+HNH-><-REase+iREase||BrxA-DNA-binding->BrxB-BrxF-iSTAND->DUF6079*->N6-METHYLASE->PglZ->SIGMA+OB-Fold+AAA-ATPase+Lon-Protease-BrxL->?-><-?<-ParB<-?||<-REase+N6_METHYLASE<-TRD	1213	Bacteria>Pseudomonadota>Gammaproteobacteria>Enterobacterales>Enterobacteriaceae>Escherichia	GCA_012226505.1	Escherichia_coli_O25	DF264_000712	59918..63559	1095709	BREX-system-P-loop-protein-BrxC-[Escherichia-coli-O25]
EEZ9156819.1	Type1.BREX	DUF6079*->N6-METHYLASE->PglZ->SIGMA+OB-Fold+AAA-ATPase+Lon-Protease-BrxL->HKD-DNase+OGG-like->||AbiEi_3_N+AbiEi_3->AbiEii-><-DUF6216<-Arm-DNA-bind_3	1010	Bacteria>Pseudomonadota>Gammaproteobacteria>Enterobacterales>Enterobacteriaceae>Escherichia	GCA_012228465.1	Escherichia_coli	DDB94_002970	1..3035	562	BREX-system-P-loop-protein-BrxC-[Escherichia-coli]
EEZ8952063.1	Type1.BREX	<-Phage_integrase||?->?->?-><-HNH||wHTH+SAD+HNH-><-REase+iREase||BrxA-DNA-binding->BrxB-BrxF-iSTAND->DUF6079*->PglZ->SIGMA+OB-Fold+AAA-ATPase+Lon-Protease-BrxL->HKD-DNase+OGG-like->||AbiEi_3_N+AbiEi_3->AbiEii-><-DUF6216<-Arm-DNA-bind_3<-TOPRIM-DNAG+D5N+D5ATPase+P4PRIMASE-HTH	1171	Bacteria>Pseudomonadota>Gammaproteobacteria>Enterobacterales>Enterobacteriaceae>Escherichia	GCA_012229475.1	Escherichia_coli_O35	C2D51_003183	9758..13273	2072460	BREX-system-P-loop-protein-BrxC-[Escherichia-coli-O35]
EFA3420990.1	Type1.BREX	DUF6079*->N6-METHYLASE->PglZ->SIGMA+OB-Fold+AAA-ATPase+Lon-Protease-BrxL-><-ZnR	1196	Bacteria>Pseudomonadota>Gammaproteobacteria>Enterobacterales>Enterobacteriaceae>Escherichia	GCA_012244525.1	Escherichia_coli	E2K00_23170	1..3593	562	BREX-system-P-loop-protein-BrxC-[Escherichia-coli]
EFA4240016.1	Type1.BREX	<-HNH||wHTH+SAD+HNH-><-REase+iREase||BrxA-DNA-binding->BrxB-BrxF-iSTAND->DUF6079*->N6-METHYLASE->PglZ->SIGMA+OB-Fold+AAA-ATPase+Lon-Protease-BrxL->?-><-?<-ParB<-?||<-REase+N6_METHYLASE<-TRD	1213	Bacteria>Pseudomonadota>Gammaproteobacteria>Enterobacterales>Enterobacteriaceae>Escherichia	GCA_012246545.1	Escherichia_coli_O36:H5	C5879_000979	4688..8329	2100480	BREX-system-P-loop-protein-BrxC-[Escherichia-coli-O36:H5]
EFA5158822.1	Type1.BREX	<-Phage_integrase||?->?->?-><-HNH||wHTH+SAD+HNH-><-REase+iREase||BrxA-DNA-binding->BrxB-BrxF-iSTAND->DUF6079*->N6-METHYLASE->PglZ->SIGMA+OB-Fold+AAA-ATPase+Lon-Protease-BrxL->HKD-DNase+OGG-like->||AbiEi_3_N+AbiEi_3->AbiEii-><-DUF6216<-Arm-DNA-bind_3	1213	Bacteria>Pseudomonadota>Gammaproteobacteria>Enterobacterales>Enterobacteriaceae>Escherichia	GCA_012250845.1	Escherichia_coli	E2930_09975	121051..124692	562	BREX-system-P-loop-protein-BrxC-[Escherichia-coli]
EFA5480296.1	Type1.BREX	<-XIS-HTH<-RHH_antitoxin||?->ZnR->ZnR-><-SIGMA+OB-Fold+AAA-ATPase+Lon-Protease-BrxL<-PglZ<-N6-METHYLASE<-DUF6079*<-BrxB-BrxF-iSTAND<-BrxA-DNA-binding||REase->REase-><-HEPN<-ORC-CDC6+ZnR<-N6-METHYLASE<-Arm-DNA-bind_3+Phage_integrase	1213	Bacteria>Pseudomonadota>Gammaproteobacteria>Enterobacterales>Enterobacteriaceae>Escherichia	GCA_012252925.1	Escherichia_coli_O8	EIG07_17100	60763..64404	1010796	BREX-system-P-loop-protein-BrxC-[Escherichia-coli-O8]
EFA6389818.1	Type1.BREX	<-nSTAND1+GTPase-AIG+sGTP<-wHTH+WYL||ZnR->ZnR-><-SIGMA+OB-Fold+AAA-ATPase+Lon-Protease-BrxL<-PglZ<-TOPRIM<-OLD-ABC<-N6-METHYLASE<-DUF6079*<-BrxB-BrxF-iSTAND<-BrxA-DNA-binding<-Arm-DNA-bind_3+Phage_integrase<-tRNA||?->HerA/FtsK+A32-><-?<-?||Peptidase_M17_N+Peptidase_M17->	1204	Bacteria>Pseudomonadota>Gammaproteobacteria>Enterobacterales>Enterobacteriaceae>Escherichia	GCA_012256985.1	Escherichia_coli	CIW09_001013	351923..355537	562	BREX-system-P-loop-protein-BrxC-[Escherichia-coli]
EFA8105886.1	Type1.BREX	<-REase+iREase||BrxA-DNA-binding->BrxB-BrxF-iSTAND->DUF6079*->N6-METHYLASE->PglZ->SIGMA+OB-Fold+AAA-ATPase+Lon-Protease-BrxL-><-ZnR<-AAA-ATPase<-HIN-HTH<-?<-?||XIS-HTH->	1213	Bacteria>Pseudomonadota>Gammaproteobacteria>Enterobacterales>Enterobacteriaceae>Escherichia	GCA_012266535.1	Escherichia_coli	C3Z38_001327	2540..6181	562	BREX-system-P-loop-protein-BrxC-[Escherichia-coli]
EFA9203627.1	Type1.BREX	DUF6079*->N6-METHYLASE->SIGMA+OB-Fold+AAA-ATPase+Lon-Protease-BrxL->?-><-?<-ParB<-?||<-REase+N6_METHYLASE<-TRD<-REase	1112	Bacteria>Pseudomonadota>Gammaproteobacteria>Enterobacterales>Enterobacteriaceae>Escherichia	GCA_012268365.1	Escherichia_coli	C2702_002033	1..3339	562	BREX-system-P-loop-protein-BrxC-[Escherichia-coli]
EFB1479888.1	Type1.BREX	<-DUF6079-p*<-BrxB-BrxF-iSTAND<-BrxA-DNA-binding||REase+iREase-><-wHTH+SAD+HNH||HNH-><-?<-?<-?||Phage_integrase->	428	Bacteria>Pseudomonadota>Gammaproteobacteria>Enterobacterales>Enterobacteriaceae>Escherichia	GCA_012288905.1	Escherichia_coli	FJ807_23450	1..1284	562	BREX-system-P-loop-protein-BrxC-[Escherichia-coli]
EFB2626049.1	Type1.BREX	<-HKD-DNase+OGG-like<-SIGMA+OB-Fold+AAA-ATPase+Lon-Protease-BrxL<-PglZ<-N6-METHYLASE<-DUF6079*<-BrxB-BrxF-iSTAND<-BrxA-DNA-binding||REase+iREase-><-wHTH+SAD+HNH||HNH-><-?<-?<-?||Phage_integrase->	1213	Bacteria>Pseudomonadota>Gammaproteobacteria>Enterobacterales>Enterobacteriaceae>Escherichia	GCA_012293805.1	Escherichia_coli	FHL63_20525	10185..13826	562	BREX-system-P-loop-protein-BrxC-[Escherichia-coli]
HAG7757106.1	Type1.BREX	<-wHTH+WYL||ZnR->ZnR-><-?<-SIGMA+OB-Fold+AAA-ATPase+Lon-Protease-BrxL<-PglZ<-TOPRIM<-OLD-ABC<-N6-METHYLASE<-DUF6079*<-BrxB-BrxF-iSTAND<-BrxA-DNA-binding<-Arm-DNA-bind_3+Phage_integrase<-tRNA||?->HerA/FtsK+A32-><-?<-?||Peptidase_M17_N+Peptidase_M17->	1204	Bacteria>Pseudomonadota>Gammaproteobacteria>Enterobacterales>Enterobacteriaceae>Escherichia	GCA_012297605.1	Escherichia_coli	HGR18_09675	351902..355516	562	BREX-system-P-loop-protein-BrxC-[Escherichia-coli]
EFB3218130.1	Type1.BREX	<-Zn_Tnp_IS1+HTH_Tnp_IS1||wHTH+WYL+WCX->BrxA-DNA-binding->BrxB-BrxF-iSTAND->DUF6079*->ParB+HNH->N6-METHYLASE->PglZ->SIGMA+OB-Fold+AAA-ATPase+Lon-Protease-BrxL-><-HTH_Tnp_1+HTH_21+rve+rve_3<-HIN-HTH<-PIN<-MazE_antitoxin	1225	Bacteria>Pseudomonadota>Gammaproteobacteria>Enterobacterales>Enterobacteriaceae>Escherichia	GCA_012299345.1	Escherichia_coli	FLO28_20235	2574..6251	562	BREX-system-P-loop-protein-BrxC-[Escherichia-coli]
EFB4448575.1	Type1.BREX	<-SIGMA+OB-Fold+AAA-ATPase+Lon-Protease-BrxL<-PglZ<-N6-METHYLASE<-DUF6079*<-BrxB-BrxF-iSTAND<-BrxA-DNA-binding||?->?->TetR-HTH-><-Arm-DNA-bind_3+Phage_integrase	1213	Bacteria>Pseudomonadota>Gammaproteobacteria>Enterobacterales>Enterobacteriaceae>Escherichia	GCA_012307385.1	Escherichia_coli	FQB94_07765	8545..12186	562	BREX-system-P-loop-protein-BrxC-[Escherichia-coli]
EFB4439448.1	Type1.BREX	Arm-DNA-bind_3+Phage_integrase-><-?<-?<-REase+iREase||BrxA-DNA-binding->BrxB-BrxF-iSTAND->DUF6079*->N6-METHYLASE->PglZ->SIGMA+OB-Fold+AAA-ATPase+Lon-Protease-BrxL-><-ZnR<-ZnR<-?<-?||RHH_antitoxin->	1213	Bacteria>Pseudomonadota>Gammaproteobacteria>Enterobacterales>Enterobacteriaceae>Escherichia	GCA_012309125.1	Escherichia_coli	COW91_001589	30727..34368	562	BREX-system-P-loop-protein-BrxC-[Escherichia-coli]
EFB6629151.1	Type1.BREX	<-SIGMA-HTH<-?<-ArsR-HTH||?-><-DDE_Tnp_1_5+DDE_Tnp_1||||wHTH+WYL+WCX->BrxA-DNA-binding->BrxB-BrxF-iSTAND->DUF6079*->ParB+HNH->N6-METHYLASE->PglZ->SIGMA+OB-Fold+AAA-ATPase+Lon-Protease-BrxL-><-HTH_Tnp_1+HTH_21+rve+rve_3<-HIN-HTH<-PIN<-MazE_antitoxin	1225	Bacteria>Pseudomonadota>Gammaproteobacteria>Enterobacterales>Enterobacteriaceae>Escherichia	GCA_012316585.1	Escherichia_coli	FQF26_18830	72071..75748	562	BREX-system-P-loop-protein-BrxC-[Escherichia-coli]
EFB7539253.1	Type1.BREX	<-Phage_integrase||?->?->?-><-HNH||wHTH+SAD+HNH-><-REase+iREase||BrxA-DNA-binding->BrxB-BrxF-iSTAND->DUF6079*->N6-METHYLASE->PglZ->SIGMA+OB-Fold+AAA-ATPase+Lon-Protease-BrxL->?-><-?<-ParB<-?||<-REase+N6_METHYLASE<-TRD	1213	Bacteria>Pseudomonadota>Gammaproteobacteria>Enterobacterales>Enterobacteriaceae>Escherichia	GCA_012318925.1	Escherichia_coli	E4J12_05355	111245..114886	562	BREX-system-P-loop-protein-BrxC-[Escherichia-coli]
EFB9770829.1	Type1.BREX	<-REase<-REase||BrxA-DNA-binding->BrxB-BrxF-iSTAND->DUF6079*->N6-METHYLASE->PglZ->SIGMA+OB-Fold+AAA-ATPase+Lon-Protease-BrxL-><-ZnR<-ZnR<-?||RHH_antitoxin->XIS-HTH->	1213	Bacteria>Pseudomonadota>Gammaproteobacteria>Enterobacterales>Enterobacteriaceae>Escherichia	GCA_012329995.1	Escherichia_coli	FAD49_21945	2845..6486	562	BREX-system-P-loop-protein-BrxC-[Escherichia-coli]
EFB9701597.1	Type1.BREX	ParB->?-><-?<-SIGMA+OB-Fold+AAA-ATPase+Lon-Protease-BrxL<-DUF6079*<-BrxB-BrxF-iSTAND<-BrxA-DNA-binding||REase+iREase->	1385	Bacteria>Pseudomonadota>Gammaproteobacteria>Enterobacterales>Enterobacteriaceae>Escherichia	GCA_012330505.1	Escherichia_coli	E5H11_23300	6377..10534	562	BREX-system-P-loop-protein-BrxC-[Escherichia-coli]
EFC0631243.1	Type1.BREX	<-Peptidase_M17_N+Peptidase_M17||?-><-?<-HerA/FtsK+A32<-?||tRNA->Arm-DNA-bind_3+Phage_integrase->BrxA-DNA-binding->BrxB-BrxF-iSTAND->DUF6079*->N6-METHYLASE->OLD-ABC->TOPRIM->PglZ->SIGMA+OB-Fold+AAA-ATPase+Lon-Protease-BrxL-><-?<-ZnR<-ZnR<-wHTH	1204	Bacteria>Pseudomonadota>Gammaproteobacteria>Enterobacterales>Enterobacteriaceae>Escherichia	GCA_012334465.1	Escherichia_coli	FA817_13710	286581..290195	562	BREX-system-P-loop-protein-BrxC-[Escherichia-coli]
EFC1641769.1	Type1.BREX	<-HerA/FtsK+A32<-?||tRNA->DUF6216-><-AbiEii<-AbiEi_3_N+AbiEi_3||<-HKD-DNase+OGG-like<-SIGMA+OB-Fold+AAA-ATPase+Lon-Protease-BrxL<-N6-METHYLASE<-DUF6079*	1140	Bacteria>Pseudomonadota>Gammaproteobacteria>Enterobacterales>Enterobacteriaceae>Escherichia	GCA_012339895.1	Escherichia_coli	F2I36_20020	26288..29710	562	BREX-system-P-loop-protein-BrxC-[Escherichia-coli]
EFC2245710.1	Type1.BREX	<-Phage_integrase||?->?->?-><-HNH||wHTH+SAD+HNH-><-REase+iREase||BrxA-DNA-binding->BrxB-BrxF-iSTAND->DUF6079*->SIGMA+OB-Fold+AAA-ATPase+Lon-Protease-BrxL->?-><-?<-ParB<-?||<-REase+N6_METHYLASE<-TRD<-REase	1385	Bacteria>Pseudomonadota>Gammaproteobacteria>Enterobacterales>Enterobacteriaceae>Escherichia	GCA_012340135.1	Escherichia_coli	E5H86_07830	9758..13915	562	BREX-system-P-loop-protein-BrxC-[Escherichia-coli]
EFC3446201.1	Type1.BREX	<-DUF6079-p*<-BrxB-BrxF-iSTAND<-BrxA-DNA-binding||<-wHTH(SBD)+HNH||HNH-><-?<-?<-?||Phage_integrase->Phage_integrase->	844	Bacteria>Pseudomonadota>Gammaproteobacteria>Enterobacterales>Enterobacteriaceae>Escherichia	GCA_012346015.1	Escherichia_coli	CO751_004598	1..2534	562	BREX-system-P-loop-protein-BrxC-[Escherichia-coli]
MBB9587758.1	Type1.BREX	TRD->REase+N6_METHYLASE->||?->ParB->?-><-?<-SIGMA+OB-Fold+AAA-ATPase+Lon-Protease-BrxL<-PglZ<-N6-METHYLASE<-DUF6079*<-BrxB-BrxF-iSTAND<-BrxA-DNA-binding||REase+iREase-><-wHTH+SAD+HNH||HNH-><-?<-?<-?||Phage_integrase->	1213	Bacteria>Pseudomonadota>Gammaproteobacteria>Enterobacterales>Enterobacteriaceae>Escherichia	GCA_012370635.2	Escherichia_coli	FSJ86_010580	16354..19995	562	BREX-system-P-loop-protein-BrxC-[Escherichia-coli]
EFD0914260.1	Type1.BREX	<-DUF6079*<-BrxB-BrxF-iSTAND<-BrxA-DNA-binding||REase+iREase-><-wHTH+SAD+HNH||HNH->	1198	Bacteria>Pseudomonadota>Gammaproteobacteria>Enterobacterales>Enterobacteriaceae>Escherichia	GCA_012379395.1	Escherichia_coli	KO979_004413	1..3594	562	BREX-system-P-loop-protein-BrxC-[Escherichia-coli]
EFD0312067.1	Type1.BREX	GDResolvase+HIN-HTH-><-SIGMA+OB-Fold+AAA-ATPase+Lon-Protease-BrxL<-PglZ<-N6-METHYLASE-p<-RVT<-HEPN<-N6-METHYLASE<-ParB+HNH<-DUF6079*<-BrxB-BrxF-iSTAND<-BrxA-DNA-binding<-?<-?<-XIS-HTH<-?||?->nSTAND1+GTPase-AIG+sGTP->DUF932->	1233	Bacteria>Pseudomonadota>Gammaproteobacteria>Enterobacterales>Enterobacteriaceae>Escherichia	GCA_012380475.1	Escherichia_coli	E6D44_20535	19474..23175	562	BREX-system-P-loop-protein-BrxC-[Escherichia-coli]
MBC0786087.1	Type1.BREX	<-Phage_integrase||?->?->?-><-HNH||wHTH+SAD+HNH-><-REase+iREase||BrxA-DNA-binding->BrxB-BrxF-iSTAND->DUF6079*->N6-METHYLASE->PglZ->SIGMA+OB-Fold+AAA-ATPase+Lon-Protease-BrxL->HKD-DNase+OGG-like->||AbiEi_3_N+AbiEi_3->AbiEii->?-><-DUF6216<-Arm-DNA-bind_3	1213	Bacteria>Pseudomonadota>Gammaproteobacteria>Enterobacterales>Enterobacteriaceae>Escherichia	GCA_012382335.2	Escherichia_coli	FSG57_002365	111024..114665	562	BREX-system-P-loop-protein-BrxC-[Escherichia-coli]
EFD1659139.1	Type1.BREX	<-Phage_integrase||?->?->?-><-HNH||wHTH+SAD+HNH-><-REase+iREase||BrxA-DNA-binding->BrxB-BrxF-iSTAND->DUF6079*->N6-METHYLASE->PglZ->SIGMA+OB-Fold+AAA-ATPase+Lon-Protease-BrxL->HKD-DNase+OGG-like->||AbiEi_3_N+AbiEi_3->AbiEii-><-DUF6216<-Arm-DNA-bind_3	1213	Bacteria>Pseudomonadota>Gammaproteobacteria>Enterobacterales>Enterobacteriaceae>Escherichia	GCA_012382975.1	Escherichia_coli	FGU32_03035	69619..73260	562	BREX-system-P-loop-protein-BrxC-[Escherichia-coli]
EFD3459874.1	Type1.BREX	<-HNH||wHTH(SBD)+HNH->||BrxA-DNA-binding->BrxB-BrxF-iSTAND->DUF6079*->N6-METHYLASE->PglZ->SIGMA+OB-Fold+AAA-ATPase+Lon-Protease-BrxL->	1213	Bacteria>Pseudomonadota>Gammaproteobacteria>Enterobacterales>Enterobacteriaceae>Escherichia	GCA_012389335.1	Escherichia_coli	CL211_004302	6759..10400	562	BREX-system-P-loop-protein-BrxC-[Escherichia-coli]
EFE1965719.1	Type1.BREX	<-Phage_integrase<-Phage_integrase||?->?->?-><-HNH||wHTH(SBD)+HNH->||BrxA-DNA-binding->BrxB-BrxF-iSTAND->DUF6079*->N6-METHYLASE->PglZ->SIGMA+OB-Fold+AAA-ATPase+Lon-Protease-BrxL->HKD-DNase+OGG-like->||AbiEi_3_N+AbiEi_3->AbiEii-><-DUF6216<-Arm-DNA-bind_3	1213	Bacteria>Pseudomonadota>Gammaproteobacteria>Enterobacterales>Enterobacteriaceae>Escherichia	GCA_012428445.1	Escherichia_coli	CJV81_002667	14696..18337	562	BREX-system-P-loop-protein-BrxC-[Escherichia-coli]
EFE8676226.1	Type1.BREX	BrxA-DNA-binding->BrxB-BrxF-iSTAND->DUF6079*->N6-METHYLASE->PglZ->SIGMA+OB-Fold+AAA-ATPase+Lon-Protease-BrxL-><-ZnR<-ZnR<-?<-?||RHH_antitoxin->	1213	Bacteria>Pseudomonadota>Gammaproteobacteria>Enterobacterales>Enterobacteriaceae>Escherichia	GCA_012457165.1	Escherichia_coli	CAA49_004703	1709..5350	562	BREX-system-P-loop-protein-BrxC-[Escherichia-coli]
EFF2616657.1	Type1.BREX	<-Phage_integrase||?->?->?-><-HNH||wHTH+SAD+HNH-><-REase+iREase||BrxA-DNA-binding->BrxB-BrxF-iSTAND->DUF6079*->N6-METHYLASE->PglZ->SIGMA+OB-Fold+AAA-ATPase+Lon-Protease-BrxL->?-><-?<-ParB<-?||<-REase+N6_METHYLASE<-TRD	1213	Bacteria>Pseudomonadota>Gammaproteobacteria>Enterobacterales>Enterobacteriaceae>Escherichia	GCA_012476875.1	Escherichia_coli	B9L79_001313	111240..114881	562	BREX-system-P-loop-protein-BrxC-[Escherichia-coli]
EFF3077603.1	Type1.BREX	<-Phage_integrase||?->?->?-><-HNH||wHTH+SAD+HNH-><-REase+iREase||BrxA-DNA-binding->BrxB-BrxF-iSTAND->DUF6079*->N6-METHYLASE->PglZ->SIGMA+OB-Fold+AAA-ATPase+Lon-Protease-BrxL->?-><-?<-ParB<-?||<-REase+N6_METHYLASE<-TRD	1213	Bacteria>Pseudomonadota>Gammaproteobacteria>Enterobacterales>Enterobacteriaceae>Escherichia	GCA_012480115.1	Escherichia_coli	BOR91_001555	57086..60727	562	BREX-system-P-loop-protein-BrxC-[Escherichia-coli]
EFF3363917.1	Type1.BREX	<-Phage_integrase||?->?->?-><-HNH||wHTH+SAD+HNH-><-REase+iREase||BrxA-DNA-binding->BrxB-BrxF-iSTAND->DUF6079*->N6-METHYLASE->PglZ->SIGMA+OB-Fold+AAA-ATPase+Lon-Protease-BrxL->?-><-?<-ParB<-?||<-REase+N6_METHYLASE<-TRD	1213	Bacteria>Pseudomonadota>Gammaproteobacteria>Enterobacterales>Enterobacteriaceae>Escherichia	GCA_012481335.1	Escherichia_coli	B7M15_000791	9758..13399	562	BREX-system-P-loop-protein-BrxC-[Escherichia-coli]
EFF3839116.1	Type1.BREX	<-Phage_integrase||?->?->?-><-HNH||wHTH+SAD+HNH-><-REase+iREase||BrxA-DNA-binding->BrxB-BrxF-iSTAND->DUF6079-p*->	473	Bacteria>Pseudomonadota>Gammaproteobacteria>Enterobacterales>Enterobacteriaceae>Escherichia	GCA_012482765.1	Escherichia_coli	B6310_003036	111336..112756	562	BREX-system-P-loop-protein-BrxC-[Escherichia-coli]
EFF3839660.1	Type1.BREX	DUF6079-p*->N6-METHYLASE->PglZ->SIGMA+OB-Fold+AAA-ATPase+Lon-Protease-BrxL->HKD-DNase+OGG-like->||AbiEi_3_N+AbiEi_3->AbiEii-><-DUF6216<-Arm-DNA-bind_3	739	Bacteria>Pseudomonadota>Gammaproteobacteria>Enterobacterales>Enterobacteriaceae>Escherichia	GCA_012482765.1	Escherichia_coli	B6310_003615	1..2221	562	BREX-system-P-loop-protein-BrxC-[Escherichia-coli]
EFF7960190.1	Type1.BREX	<-Phage_integrase||?->?->?-><-HNH||wHTH+SAD+HNH-><-REase+iREase||BrxA-DNA-binding->BrxB-BrxF-iSTAND->DUF6079*->N6-METHYLASE->PglZ->SIGMA+OB-Fold+AAA-ATPase+Lon-Protease-BrxL->?-><-?<-ParB<-?||<-REase+N6_METHYLASE<-TRD	1213	Bacteria>Pseudomonadota>Gammaproteobacteria>Enterobacterales>Enterobacteriaceae>Escherichia	GCA_012505155.1	Escherichia_coli	BUX12_001709	111293..114934	562	BREX-system-P-loop-protein-BrxC-[Escherichia-coli]
HAH2520952.1	Type1.BREX	<-Phage_integrase||?->?->?-><-HNH||wHTH+SAD+HNH-><-REase+iREase||BrxA-DNA-binding->BrxB-BrxF-iSTAND->DUF6079*->PglZ->SIGMA+OB-Fold+AAA-ATPase+Lon-Protease-BrxL->HKD-DNase+OGG-like->||AbiEi_3_N+AbiEi_3->AbiEii->	1162	Bacteria>Pseudomonadota>Gammaproteobacteria>Enterobacterales>Enterobacteriaceae>Escherichia	GCA_012530295.1	Escherichia_coli	GF659_15740	71446..74934	562	BREX-system-P-loop-protein-BrxC-[Escherichia-coli]
EFG1482424.1	Type1.BREX	<-Zn_Tnp_IS1+HTH_Tnp_IS1<-REase<-REase||BrxA-DNA-binding->BrxB-BrxF-iSTAND->DUF6079*->N6-METHYLASE->PglZ->SIGMA+OB-Fold+AAA-ATPase+Lon-Protease-BrxL-><-ZnR<-ZnR	1213	Bacteria>Pseudomonadota>Gammaproteobacteria>Enterobacterales>Enterobacteriaceae>Escherichia	GCA_012544495.1	Escherichia_coli	BRV97_004207	3158..6799	562	BREX-system-P-loop-protein-BrxC-[Escherichia-coli]
EFG3005928.1	Type1.BREX	DUF6079-p*->N6-METHYLASE->PglZ->SIGMA+OB-Fold+AAA-ATPase+Lon-Protease-BrxL->HKD-DNase+OGG-like->||AbiEi_3_N+AbiEi_3->AbiEii-><-DUF6216<-Arm-DNA-bind_3	897	Bacteria>Pseudomonadota>Gammaproteobacteria>Enterobacterales>Enterobacteriaceae>Escherichia	GCA_012550755.1	Escherichia_coli	BOZ89_003087	1..2694	562	BREX-system-P-loop-protein-BrxC-[Escherichia-coli]
EFG4404338.1	Type1.BREX	<-Phage_integrase<-Phage_integrase||?->?->?-><-HNH||wHTH(SBD)+HNH->||BrxA-DNA-binding->BrxB-BrxF-iSTAND->DUF6079*->N6-METHYLASE->PglZ->SIGMA+OB-Fold+AAA-ATPase+Lon-Protease-BrxL->HKD-DNase+OGG-like->||AbiEi_3_N+AbiEi_3->AbiEii-><-DUF6216<-Arm-DNA-bind_3	1213	Bacteria>Pseudomonadota>Gammaproteobacteria>Enterobacterales>Enterobacteriaceae>Escherichia	GCA_012556825.1	Escherichia_coli	BM772_002472	14697..18338	562	BREX-system-P-loop-protein-BrxC-[Escherichia-coli]
EFH0548056.1	Type1.BREX	DUF6079-p*->N6-METHYLASE->PglZ->SIGMA+OB-Fold+AAA-ATPase+Lon-Protease-BrxL->HKD-DNase+OGG-like->||AbiEi_3_N+AbiEi_3->AbiEii-><-DUF6216<-Arm-DNA-bind_3	789	Bacteria>Pseudomonadota>Gammaproteobacteria>Enterobacterales>Enterobacteriaceae>Escherichia	GCA_012582395.1	Escherichia_coli	BJE23_002820	1..2370	562	BREX-system-P-loop-protein-BrxC-[Escherichia-coli]
EFH0549024.1	Type1.BREX	<-Phage_integrase<-Phage_integrase||?->?->?-><-HNH||wHTH(SBD)+HNH->||BrxA-DNA-binding->BrxB-BrxF-iSTAND->DUF6079-p*->	422	Bacteria>Pseudomonadota>Gammaproteobacteria>Enterobacterales>Enterobacteriaceae>Escherichia	GCA_012582395.1	Escherichia_coli	BJE23_003837	39992..41258	562	BREX-system-P-loop-protein-BrxC-[Escherichia-coli]
EFH3554397.1	Type1.BREX	<-Phage_integrase||?->?->?-><-HNH||wHTH+SAD+HNH-><-REase+iREase||BrxA-DNA-binding->BrxB-BrxF-iSTAND->DUF6079*->N6-METHYLASE-p->	1213	Bacteria>Pseudomonadota>Gammaproteobacteria>Enterobacterales>Enterobacteriaceae>Escherichia	GCA_012596955.1	Escherichia_coli	GQW80_16270	69288..72929	562	BREX-system-P-loop-protein-BrxC-[Escherichia-coli]
EFH3789843.1	Type1.BREX	<-TetR-HTH<-?<-?||BrxA-DNA-binding->BrxB-BrxF-iSTAND->DUF6079*->N6-METHYLASE->PglZ->SIGMA+OB-Fold+AAA-ATPase+Lon-Protease-BrxL->	1213	Bacteria>Pseudomonadota>Gammaproteobacteria>Enterobacterales>Enterobacteriaceae>Escherichia	GCA_012598785.1	Escherichia_coli	F9418_21630	6934..10575	562	BREX-system-P-loop-protein-BrxC-[Escherichia-coli]
EFH4793016.1	Type1.BREX	BrxA-DNA-binding->BrxB-BrxF-iSTAND->DUF6079*->N6-METHYLASE->PglZ->SIGMA+OB-Fold+AAA-ATPase+Lon-Protease-BrxL->	1213	Bacteria>Pseudomonadota>Gammaproteobacteria>Enterobacterales>Enterobacteriaceae>Escherichia	GCA_012602795.1	Escherichia_coli	F9381_22830	5106..8747	562	BREX-system-P-loop-protein-BrxC-[Escherichia-coli]
EFH4936891.1	Type1.BREX	<-Phage_integrase||?->?->?-><-HNH||wHTH+SAD+HNH-><-REase+iREase||BrxA-DNA-binding->BrxB-BrxF-iSTAND->DUF6079*->N6-METHYLASE->PglZ->SIGMA+OB-Fold+AAA-ATPase+Lon-Protease-BrxL->HKD-DNase+OGG-like->||AbiEi_3_N+AbiEi_3->AbiEii-><-DUF6216<-Arm-DNA-bind_3	1213	Bacteria>Pseudomonadota>Gammaproteobacteria>Enterobacterales>Enterobacteriaceae>Escherichia	GCA_012603315.1	Escherichia_coli	F9408_05585	110160..113801	562	BREX-system-P-loop-protein-BrxC-[Escherichia-coli]
EFH7369082.1	Type1.BREX	<-RelE-ParE<-CopG/RHH||<-RHH||?->wHTH+WYL+WCX->BrxA-DNA-binding->BrxB-BrxF-iSTAND->DUF6079*->ParB+HNH->N6-METHYLASE-p->	1225	Bacteria>Pseudomonadota>Gammaproteobacteria>Enterobacterales>Enterobacteriaceae>Escherichia	GCA_012614485.1	Escherichia_coli	GXQ19_21210	5665..9342	562	BREX-system-P-loop-protein-BrxC-[Escherichia-coli]
EFH8432482.1	Type1.BREX	ParB->?-><-?<-SIGMA+OB-Fold+AAA-ATPase+Lon-Protease-BrxL<-PglZ<-N6-METHYLASE<-DUF6079*<-BrxB-BrxF-iSTAND<-BrxA-DNA-binding||REase+iREase->	1213	Bacteria>Pseudomonadota>Gammaproteobacteria>Enterobacterales>Enterobacteriaceae>Escherichia	GCA_012619365.1	Escherichia_coli	GSM49_21410	10956..14597	562	BREX-system-P-loop-protein-BrxC-[Escherichia-coli]
EFH9688867.1	Type1.BREX	ZnR->ZnR-><-SIGMA+OB-Fold+AAA-ATPase+Lon-Protease-BrxL<-PglZ<-N6-METHYLASE<-DUF6079*<-BrxB-BrxF-iSTAND<-BrxA-DNA-binding||REase->REase-><-HEPN<-ORC-CDC6+ZnR<-N6-METHYLASE<-Arm-DNA-bind_3+Phage_integrase	1213	Bacteria>Pseudomonadota>Gammaproteobacteria>Enterobacterales>Enterobacteriaceae>Escherichia	GCA_012624895.1	Escherichia_coli	F8Z35_13525	11254..14895	562	BREX-system-P-loop-protein-BrxC-[Escherichia-coli]
EFH9748630.1	Type1.BREX	<-Phage_integrase||?->?->?-><-HNH||wHTH+SAD+HNH-><-REase+iREase||BrxA-DNA-binding->BrxB-BrxF-iSTAND->DUF6079*->N6-METHYLASE->PglZ->SIGMA+OB-Fold+AAA-ATPase+Lon-Protease-BrxL->?-><-?<-ParB<-?||<-REase+N6_METHYLASE<-TRD	1213	Bacteria>Pseudomonadota>Gammaproteobacteria>Enterobacterales>Enterobacteriaceae>Escherichia	GCA_012625865.1	Escherichia_coli	F7N48_00500	90362..94003	562	BREX-system-P-loop-protein-BrxC-[Escherichia-coli]

Protein-ID	System	Gene-Neighbourhoods	Length	Taxonomy	Assembly-ID	Strain	Locus-Tag	Genomic-coordinates	TaxID	Fasta-define
EFI1580322.1	Type1.BREX	DUF6079*->N6-METHYLASE->PglZ->SIGMA+OB-Fold+AAA-ATPase+Lon-Protease-BrxL->HKD-DNase+OGG-like->||AbiEi_3_N+AbiEi_3->AbiEii-><-DUF6216<-Arm-DNA-bind_3	1071	Bacteria>Pseudomonadota>Gammaproteobacteria>Enterobacterales>Enterobacteriaceae>Escherichia	GCA_012634445.1	Escherichia_coli	BFN79_002999	1..3216	562	BREX-system-P-loop-protein-BrxC-[Escherichia-coli]
EFI3320066.1	Type1.BREX	DDE_3-><-HKD-DNase+OGG-like<-SIGMA+OB-Fold+AAA-ATPase+Lon-Protease-BrxL<-PglZ<-N6-METHYLASE<-DUF6079*<-BrxB-BrxF-iSTAND<-BrxA-DNA-binding||REase+iREase-><-wHTH+SAD+HNH||HNH-><-?<-?<-?||Phage_integrase->	1213	Bacteria>Pseudomonadota>Gammaproteobacteria>Enterobacterales>Enterobacteriaceae>Escherichia	GCA_012643725.1	Escherichia_coli	BCC72_001616	10313..13954	562	BREX-system-P-loop-protein-BrxC-[Escherichia-coli]
EFI8237360.1	Type1.BREX	DUF6079*->N6-METHYLASE->PglZ->SIGMA+OB-Fold+AAA-ATPase+Lon-Protease-BrxL-><-DDE_Tnp_IS66+DDE_Tnp_IS66_C	1179	Bacteria>Pseudomonadota>Gammaproteobacteria>Enterobacterales>Enterobacteriaceae>Escherichia	GCA_012674225.1	Escherichia_coli	HIB78_004536	1..3541	562	BREX-system-P-loop-protein-BrxC-[Escherichia-coli]
EFI9515006.1	Type1.BREX	<-XIS-HTH<-RHH_antitoxin||?->?->ZnR->ZnR-><-SIGMA+OB-Fold+AAA-ATPase+Lon-Protease-BrxL<-PglZ<-N6-METHYLASE<-DUF6079*<-BrxB-BrxF-iSTAND<-BrxA-DNA-binding||REase+iREase->	1213	Bacteria>Pseudomonadota>Gammaproteobacteria>Enterobacterales>Enterobacteriaceae>Escherichia	GCA_012683285.1	Escherichia_coli	G4U18_004344	13351..16992	562	BREX-system-P-loop-protein-BrxC-[Escherichia-coli]
EFJ2123507.1	Type1.BREX	DDE_Tnp_1_5+DDE_Tnp_1-><-?||DDE_Tnp_1_5+DDE_Tnp_1->||?-><-RHH||?->wHTH+WYL+WCX->BrxA-DNA-binding->BrxB-BrxF-iSTAND->DUF6079-p*->	422	Bacteria>Pseudomonadota>Gammaproteobacteria>Enterobacterales>Enterobacteriaceae>Escherichia	GCA_012696185.1	Escherichia_coli	G6K59_004265	24578..25844	562	BREX-system-P-loop-protein-BrxC-[Escherichia-coli]
EFJ5167524.1	Type1.BREX	DUF6079*->N6-METHYLASE->PglZ->SIGMA+OB-Fold+AAA-ATPase+Lon-Protease-BrxL->HKD-DNase+OGG-like->||AbiEi_3_N+AbiEi_3->AbiEii-><-DUF6216<-Arm-DNA-bind_3	1181	Bacteria>Pseudomonadota>Gammaproteobacteria>Enterobacterales>Enterobacteriaceae>Escherichia	GCA_012707625.1	Escherichia_coli	A5T96_003401	1..3546	562	BREX-system-P-loop-protein-BrxC-[Escherichia-coli]
EFJ6798268.1	Type1.BREX	<-XIS-HTH<-RHH_antitoxin||?->?->ZnR->ZnR-><-SIGMA+OB-Fold+AAA-ATPase+Lon-Protease-BrxL<-PglZ<-N6-METHYLASE<-DUF6079*<-BrxB-BrxF-iSTAND<-BrxA-DNA-binding||REase+iREase-><-EVE+HNH<-Arm-DNA-bind_3+Phage_integrase<-tRNA||?->HerA/FtsK+A32->	1213	Bacteria>Pseudomonadota>Gammaproteobacteria>Enterobacterales>Enterobacteriaceae>Escherichia	GCA_012714605.1	Escherichia_coli	A2G00_000895	22019..25660	562	BREX-system-P-loop-protein-BrxC-[Escherichia-coli]
HAH8430180.1	Type1.BREX	DUF6079-p*->N6-METHYLASE->PglZ->SIGMA+OB-Fold+AAA-ATPase+Lon-Protease-BrxL->HKD-DNase+OGG-like->||AbiEi_3_N+AbiEi_3->AbiEii-><-DUF6216<-Arm-DNA-bind_3	846	Bacteria>Pseudomonadota>Gammaproteobacteria>Enterobacterales>Enterobacteriaceae>Escherichia	GCA_012716735.1	Escherichia_coli	HIR43_003168	1..2542	562	BREX-system-P-loop-protein-BrxC-[Escherichia-coli]
EFK2991875.1	Type1.BREX	<-REase<-REase||BrxA-DNA-binding->BrxB-BrxF-iSTAND->DUF6079*->N6-METHYLASE->PglZ->SIGMA+OB-Fold+AAA-ATPase+Lon-Protease-BrxL-><-ZnR<-ZnR<-?||RHH_antitoxin->XIS-HTH->	1213	Bacteria>Pseudomonadota>Gammaproteobacteria>Enterobacterales>Enterobacteriaceae>Escherichia	GCA_012752375.1	Escherichia_coli	A8514_004288	2939..6580	562	BREX-system-P-loop-protein-BrxC-[Escherichia-coli]
HAH9940666.1	Type1.BREX	<-Phage_integrase||?->?->?-><-HNH||wHTH+SAD+HNH-><-REase+iREase||BrxA-DNA-binding->BrxB-BrxF-iSTAND->DUF6079*->N6-METHYLASE->PglZ->SIGMA+OB-Fold+AAA-ATPase+Lon-Protease-BrxL->HKD-DNase+OGG-like->||AbiEi_3_N+AbiEi_3->AbiEii-><-DUF6216<-Arm-DNA-bind_3<-REase	1213	Bacteria>Pseudomonadota>Gammaproteobacteria>Enterobacterales>Enterobacteriaceae>Escherichia	GCA_012753595.1	Escherichia_coli	HIF13_004044	9744..13385	562	BREX-system-P-loop-protein-BrxC-[Escherichia-coli]
EFK7079585.1	Type1.BREX	<-Phage_integrase||?->?->?-><-HNH||wHTH+SAD+HNH-><-REase+iREase||BrxA-DNA-binding->BrxB-BrxF-iSTAND->DUF6079*->PglZ->SIGMA+OB-Fold+AAA-ATPase+Lon-Protease-BrxL->?-><-?<-?||<-REase+N6_METHYLASE<-TRD<-REase	1213	Bacteria>Pseudomonadota>Gammaproteobacteria>Enterobacterales>Enterobacteriaceae>Escherichia	GCA_012771275.1	Escherichia_coli	ASO76_001188	111240..114881	562	BREX-system-P-loop-protein-BrxC-[Escherichia-coli]
EFK7446827.1	Type1.BREX	DDE_Tnp_IS1-><-SIGMA+OB-Fold+AAA-ATPase+Lon-Protease-BrxL<-PglZ<-N6-METHYLASE<-DUF6079*<-BrxB-BrxF-iSTAND<-BrxA-DNA-binding||REase->?->||<-Arm-DNA-bind_3+Phage_integrase<-tRNA||?->HerA/FtsK+A32->	1213	Bacteria>Pseudomonadota>Gammaproteobacteria>Enterobacterales>Enterobacteriaceae>Escherichia	GCA_012772735.1	Escherichia_coli	BBQ60_004521	8855..12496	562	BREX-system-P-loop-protein-BrxC-[Escherichia-coli]
EFK8341906.1	Type1.BREX	<-Phage_integrase<-Phage_integrase||?->?->?->wHTH+SAD+HNH-><-REase+iREase||BrxA-DNA-binding->BrxB-BrxF-iSTAND->DUF6079-p*->	800	Bacteria>Pseudomonadota>Gammaproteobacteria>Enterobacterales>Enterobacteriaceae>Escherichia	GCA_012777335.1	Escherichia_coli	AO793_004522	36430..38831	562	BREX-system-P-loop-protein-BrxC-[Escherichia-coli]
EFL0019973.1	Type1.BREX	<-Phage_integrase||?->?->?-><-HNH||wHTH+SAD+HNH-><-REase+iREase||BrxA-DNA-binding->BrxB-BrxF-iSTAND->DUF6079*->N6-METHYLASE->PglZ->SIGMA+OB-Fold+AAA-ATPase+Lon-Protease-BrxL->?-><-?<-ParB<-?||<-REase+N6_METHYLASE<-TRD	1213	Bacteria>Pseudomonadota>Gammaproteobacteria>Enterobacterales>Enterobacteriaceae>Escherichia	GCA_012786995.1	Escherichia_coli	ACG50_000782	71932..75573	562	BREX-system-P-loop-protein-BrxC-[Escherichia-coli]
EFL0544862.1	Type1.BREX	DUF6079-p*->N6-METHYLASE->PglZ->SIGMA+OB-Fold+AAA-ATPase+Lon-Protease-BrxL->HKD-DNase+OGG-like->||AbiEi_3_N+AbiEi_3->AbiEii-><-DUF6216<-Arm-DNA-bind_3<-TOPRIM-DNAG+D5N+D5ATPase+P4PRIMASE-HTH	803	Bacteria>Pseudomonadota>Gammaproteobacteria>Enterobacterales>Enterobacteriaceae>Escherichia	GCA_012789875.1	Escherichia_coli	HIZ25_002081	1..2412	562	BREX-system-P-loop-protein-BrxC-[Escherichia-coli]
EFL4011089.1	Type1.BREX	<-Phage_integrase||?->?->?-><-HNH||wHTH+SAD+HNH-><-REase+iREase||BrxA-DNA-binding->BrxB-BrxF-iSTAND->DUF6079*->N6-METHYLASE->PglZ->SIGMA+OB-Fold+AAA-ATPase+Lon-Protease-BrxL->HKD-DNase+OGG-like->||AbiEi_3_N+AbiEi_3->AbiEii-><-DUF6216<-Arm-DNA-bind_3	1213	Bacteria>Pseudomonadota>Gammaproteobacteria>Enterobacterales>Enterobacteriaceae>Escherichia	GCA_012809955.1	Escherichia_coli	AC312_001861	91319..94960	562	BREX-system-P-loop-protein-BrxC-[Escherichia-coli]
EFL7364898.1	Type1.BREX	DUF6079*->N6-METHYLASE->PglZ->SIGMA+OB-Fold+AAA-ATPase+Lon-Protease-BrxL->?-><-?<-ParB<-?||<-REase+N6_METHYLASE<-TRD	1211	Bacteria>Pseudomonadota>Gammaproteobacteria>Enterobacterales>Enterobacteriaceae>Escherichia	GCA_012822795.1	Escherichia_coli	VT16_000316	1..3636	562	BREX-system-P-loop-protein-BrxC-[Escherichia-coli]
EFL7041959.1	Type1.BREX	<-XIS-HTH<-RHH_antitoxin||?->?->ZnR->ZnR-><-SIGMA+OB-Fold+AAA-ATPase+Lon-Protease-BrxL<-PglZ<-N6-METHYLASE<-DUF6079*<-BrxB-BrxF-iSTAND<-BrxA-DNA-binding||<-EVE+HNH<-Arm-DNA-bind_3+Phage_integrase<-tRNA||?->HerA/FtsK+A32-><-?<-?||Peptidase_M17_N+Peptidase_M17->	1213	Bacteria>Pseudomonadota>Gammaproteobacteria>Enterobacterales>Enterobacteriaceae>Escherichia	GCA_012822855.1	Escherichia_coli	XO77_001368	13786..17427	562	BREX-system-P-loop-protein-BrxC-[Escherichia-coli]
EFM0326192.1	Type1.BREX	<-Phage_integrase||?->?->?-><-HNH||wHTH+SAD+HNH-><-REase+iREase||BrxA-DNA-binding->BrxB-BrxF-iSTAND->DUF6079*->N6-METHYLASE->PglZ->SIGMA+OB-Fold+AAA-ATPase+Lon-Protease-BrxL->?-><-?<-ParB<-?||<-REase+N6_METHYLASE<-TRD	1213	Bacteria>Pseudomonadota>Gammaproteobacteria>Enterobacterales>Enterobacteriaceae>Escherichia	GCA_012835895.1	Escherichia_coli	CM47_002122	16867..20508	562	BREX-system-P-loop-protein-BrxC-[Escherichia-coli]
MBB7947572.1	Type1.BREX	BrxA-DNA-binding->BrxB-BrxF-iSTAND->DUF6079*->N6-METHYLASE->PglZ->SIGMA+OB-Fold+AAA-ATPase+Lon-Protease-BrxL-><-ZnR<-AAA-ATPase<-HIN-HTH	1213	Bacteria>Pseudomonadota>Gammaproteobacteria>Enterobacterales>Enterobacteriaceae>Escherichia	GCA_012847135.2	Escherichia_coli	HEL38_020820	1537..5178	562	BREX-system-P-loop-protein-BrxC-[Escherichia-coli]
HAI2941917.1	Type1.BREX	<-METHYLASE||SKI-><-?||tRNA->Arm-DNA-bind_3+Phage_integrase->EVE+HNH-><-REase+iREase||BrxA-DNA-binding->BrxB-BrxF-iSTAND->DUF6079*->N6-METHYLASE->PglZ->SIGMA+OB-Fold+AAA-ATPase+Lon-Protease-BrxL-><-ZnR<-ZnR<-?<-?||XIS-HTH->	1213	Bacteria>Pseudomonadota>Gammaproteobacteria>Enterobacterales>Enterobacteriaceae>Escherichia	GCA_012856395.1	Escherichia_coli	HJL49_003091	149607..153248	562	BREX-system-P-loop-protein-BrxC-[Escherichia-coli]
HAI3030635.1	Type1.BREX	<-Zn_Tnp_IS1+HTH_Tnp_IS1<-TetR-HTH-><-?<-?||BrxA-DNA-binding->BrxB-BrxF-iSTAND->DUF6079*->N6-METHYLASE->PglZ->SIGMA+OB-Fold+AAA-ATPase+Lon-Protease-BrxL-><-?<-HIN-HTH<-ParB+HNH<-ParB+HNH||RVT->DEDD_Tnp_IS110+Transposase_20->	1213	Bacteria>Pseudomonadota>Gammaproteobacteria>Enterobacterales>Enterobacteriaceae>Escherichia	GCA_012856755.1	Escherichia_coli	HJL73_003515	6743..10384	562	BREX-system-P-loop-protein-BrxC-[Escherichia-coli]
HAI3857478.1	Type1.BREX	DEDD_Tnp_IS110+Transposase_20-><-RHH||?->wHTH+WYL+WCX->BrxA-DNA-binding->BrxB-BrxF-iSTAND->DUF6079*->ParB+HNH->N6-METHYLASE->PglZ->SIGMA+OB-Fold+AAA-ATPase+Lon-Protease-BrxL-><-DEDD_Tnp_IS110+Transposase_20<-PIN<-MazE_antitoxin	1225	Bacteria>Pseudomonadota>Gammaproteobacteria>Enterobacterales>Enterobacteriaceae>Escherichia	GCA_012860255.1	Escherichia_coli	HJN25_004326	5261..8938	562	BREX-system-P-loop-protein-BrxC-[Escherichia-coli]
HAI4157988.1	Type1.BREX	<-METHYLASE||SKI-><-?||tRNA->Arm-DNA-bind_3+Phage_integrase->EVE+HNH-><-REase+iREase||BrxA-DNA-binding->BrxB-BrxF-iSTAND->DUF6079*->N6-METHYLASE->PglZ->SIGMA+OB-Fold+AAA-ATPase+Lon-Protease-BrxL-><-ZnR<-ZnR<-?||XIS-HTH->	1213	Bacteria>Pseudomonadota>Gammaproteobacteria>Enterobacterales>Enterobacteriaceae>Escherichia	GCA_012863435.1	Escherichia_coli	HJO09_003308	150572..154213	562	BREX-system-P-loop-protein-BrxC-[Escherichia-coli]
EFM7055934.1	Type1.BREX	DUF6079*->N6-METHYLASE->PglZ->SIGMA+OB-Fold+AAA-ATPase+Lon-Protease-BrxL->HKD-DNase+OGG-like->||AbiEi_3_N+AbiEi_3->AbiEii-><-DUF6216<-Arm-DNA-bind_3	1050	Bacteria>Pseudomonadota>Gammaproteobacteria>Enterobacterales>Enterobacteriaceae>Escherichia	GCA_012894495.1	Escherichia_coli	BAU66_002360	1..3155	562	BREX-system-P-loop-protein-BrxC-[Escherichia-coli]
EFN0218458.1	Type1.BREX	<-Zn_Tnp_IS1+HTH_Tnp_IS1<-REase+iREase||BrxA-DNA-binding->BrxB-BrxF-iSTAND->DUF6079*->N6-METHYLASE->PglZ->SIGMA+OB-Fold+AAA-ATPase+Lon-Protease-BrxL-><-ZnR<-ZnR<-?<-?||RHH_antitoxin->XIS-HTH->	1213	Bacteria>Pseudomonadota>Gammaproteobacteria>Enterobacterales>Enterobacteriaceae>Escherichia	GCA_012909925.1	Escherichia_fergusonii	HNH53_002980	3165..6806	564	BREX-system-P-loop-protein-BrxC-[Escherichia-fergusonii]
EFN0586557.1	Type1.BREX	<-REase<-REase||BrxA-DNA-binding->BrxB-BrxF-iSTAND->DUF6079*->N6-METHYLASE->PglZ->SIGMA+OB-Fold+AAA-ATPase+Lon-Protease-BrxL->	1213	Bacteria>Pseudomonadota>Gammaproteobacteria>Enterobacterales>Enterobacteriaceae>Escherichia	GCA_012914285.1	Escherichia_coli	HIK38_004176	2784..6425	562	BREX-system-P-loop-protein-BrxC-[Escherichia-coli]
MBB7654234.1	Type1.BREX	<-XIS-HTH<-RHH_antitoxin||?->?->ZnR->ZnR-><-SIGMA+OB-Fold+AAA-ATPase+Lon-Protease-BrxL<-PglZ<-N6-METHYLASE<-DUF6079*<-BrxB-BrxF-iSTAND<-BrxA-DNA-binding||REase+iREase-><-EVE+HNH<-Arm-DNA-bind_3+Phage_integrase<-tRNA||?->HerA/FtsK+A32->	1213	Bacteria>Pseudomonadota>Gammaproteobacteria>Enterobacterales>Enterobacteriaceae>Escherichia	GCA_012916875.2	Escherichia_coli	HEM24_008890	24657..28298	562	BREX-system-P-loop-protein-BrxC-[Escherichia-coli]
EFN2130505.1	Type1.BREX	ParB->?-><-?<-SIGMA+OB-Fold+AAA-ATPase+Lon-Protease-BrxL<-PglZ<-N6-METHYLASE<-DUF6079*<-BrxB-BrxF-iSTAND<-BrxA-DNA-binding||REase+iREase-><-wHTH+SAD+HNH||MAD-NTD+HNH->	1213	Bacteria>Pseudomonadota>Gammaproteobacteria>Enterobacterales>Enterobacteriaceae>Escherichia	GCA_012923035.1	Escherichia_coli	HG367_004308	11483..15124	562	BREX-system-P-loop-protein-BrxC-[Escherichia-coli]
HAI9090450.1	Type1.BREX	DUF6079*->N6-METHYLASE->PglZ->SIGMA+OB-Fold+AAA-ATPase+Lon-Protease-BrxL->HKD-DNase+OGG-like->||AbiEi_3_N+AbiEi_3->AbiEii-><-DUF6216<-Arm-DNA-bind_3	1180	Bacteria>Pseudomonadota>Gammaproteobacteria>Enterobacterales>Enterobacteriaceae>Escherichia	GCA_012925565.1	Escherichia_coli	HKC72_003106	1..3544	562	BREX-system-P-loop-protein-BrxC-[Escherichia-coli]
EFN3304096.1	Type1.BREX	ZnR-><-SIGMA+OB-Fold+AAA-ATPase+Lon-Protease-BrxL<-PglZ<-N6-METHYLASE<-DUF6079*<-BrxB-BrxF-iSTAND<-BrxA-DNA-binding||REase+iREase-><-EVE+HNH	1213	Bacteria>Pseudomonadota>Gammaproteobacteria>Enterobacterales>Enterobacteriaceae>Escherichia	GCA_012969905.1	Escherichia_coli	HKH56_004291	12014..15655	562	BREX-system-P-loop-protein-BrxC-[Escherichia-coli]
EFN3922650.1	Type1.BREX	<-Phage_integrase||?->?->?-><-HNH||wHTH+SAD+HNH-><-REase+iREase||BrxA-DNA-binding->BrxB-BrxF-iSTAND->DUF6079*->N6-METHYLASE->PglZ->SIGMA+OB-Fold+AAA-ATPase+Lon-Protease-BrxL->?-><-?<-ParB<-?||<-REase+N6_METHYLASE<-TRD	1213	Bacteria>Pseudomonadota>Gammaproteobacteria>Enterobacterales>Enterobacteriaceae>Escherichia	GCA_012987865.1	Escherichia_coli	FQJ23_02905	87001..90642	562	BREX-system-P-loop-protein-BrxC-[Escherichia-coli]
EFN5198232.1	Type1.BREX	<-TIR||BrxA-DNA-binding->BrxB-BrxF-iSTAND->DUF6079*->ParB+HNH->N6-METHYLASE->HEPN->RVT->N6-METHYLASE-p->PglZ->SIGMA+OB-Fold+AAA-ATPase+Lon-Protease-BrxL->wHTH+WYL+WCX->	1233	Bacteria>Pseudomonadota>Gammaproteobacteria>Enterobacterales>Enterobacteriaceae>Escherichia	GCA_012993025.1	Escherichia_coli	FQG10_00765	2767..6468	562	BREX-system-P-loop-protein-BrxC-[Escherichia-coli]
EFN5705755.1	Type1.BREX	<-RHH_antitoxin||?->?->?->ZnR->ZnR-><-SIGMA+OB-Fold+AAA-ATPase+Lon-Protease-BrxL<-PglZ<-N6-METHYLASE<-DUF6079*<-BrxB-BrxF-iSTAND<-BrxA-DNA-binding||REase+iREase-><-EVE+HNH<-Arm-DNA-bind_3+Phage_integrase<-tRNA||?->HerA/FtsK+A32->	1213	Bacteria>Pseudomonadota>Gammaproteobacteria>Enterobacterales>Enterobacteriaceae>Escherichia	GCA_012995165.1	Escherichia_coli	FQH62_10005	24813..28454	562	BREX-system-P-loop-protein-BrxC-[Escherichia-coli]
EFN5734792.1	Type1.BREX	<-Phage_integrase||?->?->?-><-HNH||wHTH+SAD+HNH-><-REase+iREase||BrxA-DNA-binding->BrxB-BrxF-iSTAND->DUF6079*->N6-METHYLASE->PglZ->SIGMA+OB-Fold+AAA-ATPase+Lon-Protease-BrxL->HKD-DNase+OGG-like->||AbiEi_3_N+AbiEi_3->AbiEii-><-DUF6216<-Arm-DNA-bind_3	1213	Bacteria>Pseudomonadota>Gammaproteobacteria>Enterobacterales>Enterobacteriaceae>Escherichia	GCA_012995705.1	Escherichia_coli	FQJ28_11185	111254..114895	562	BREX-system-P-loop-protein-BrxC-[Escherichia-coli]
HAJ1412079.1	Type1.BREX	DUF6079*->PglZ->SIGMA+OB-Fold+AAA-ATPase+Lon-Protease-BrxL->HKD-DNase+OGG-like->||AbiEi_3_N+AbiEi_3->AbiEii->	1046	Bacteria>Pseudomonadota>Gammaproteobacteria>Enterobacterales>Enterobacteriaceae>Escherichia	GCA_013000285.1	Escherichia_coli	HMG43_22065	1..3142	562	BREX-system-P-loop-protein-BrxC-[Escherichia-coli]
HAJ4265401.1	Type1.BREX	BrxA-DNA-binding->BrxB-BrxF-iSTAND->DUF6079*->N6-METHYLASE->PglZ->SIGMA+OB-Fold+AAA-ATPase+Lon-Protease-BrxL->	1213	Bacteria>Pseudomonadota>Gammaproteobacteria>Enterobacterales>Enterobacteriaceae>Escherichia	GCA_013021445.1	Escherichia_coli	HLU44_19895	1490..5131	562	BREX-system-P-loop-protein-BrxC-[Escherichia-coli]
HAJ6497178.1	Type1.BREX	DsbC+DsbD+Thioredoxin_7->TetR-HTH->tRNA->Arm-DNA-bind_3+Phage_integrase-><-Sulfatase<-?||DDE_Tnp_1_5+DDE_Tnp_1-><-REase+iREase||BrxA-DNA-binding->BrxB-BrxF-iSTAND->DUF6079*->N6-METHYLASE->PglZ->SIGMA+OB-Fold+AAA-ATPase+Lon-Protease-BrxL-><-ZnR<-ZnR	1213	Bacteria>Pseudomonadota>Gammaproteobacteria>Enterobacterales>Enterobacteriaceae>Escherichia	GCA_013038245.1	Escherichia_coli	HNC37_02435	510930..514571	562	BREX-system-P-loop-protein-BrxC-[Escherichia-coli]
HAJ6661538.1	Type1.BREX	<-RHH_antitoxin||?->?->?->ZnR->ZnR-><-SIGMA+OB-Fold+AAA-ATPase+Lon-Protease-BrxL<-PglZ<-N6-METHYLASE<-DUF6079*<-BrxB-BrxF-iSTAND<-BrxA-DNA-binding||REase+iREase->	1213	Bacteria>Pseudomonadota>Gammaproteobacteria>Enterobacterales>Enterobacteriaceae>Escherichia	GCA_013038825.1	Escherichia_coli	HNC51_24215	13272..16913	562	BREX-system-P-loop-protein-BrxC-[Escherichia-coli]
HAJ6872573.1	Type1.BREX	<-Phage_integrase||?->?->?-><-HNH||wHTH+SAD+HNH-><-REase+iREase||BrxA-DNA-binding->BrxB-BrxF-iSTAND->DUF6079-p*->	576	Bacteria>Pseudomonadota>Gammaproteobacteria>Enterobacterales>Enterobacteriaceae>Escherichia	GCA_013039665.1	Escherichia_coli	HND09_18460	71676..73402	562	BREX-system-P-loop-protein-BrxC-[Escherichia-coli]
EFN7556473.1	Type1.BREX	<-REase+iREase||BrxA-DNA-binding->BrxB-BrxF-iSTAND->DUF6079*->N6-METHYLASE->PglZ->SIGMA+OB-Fold+AAA-ATPase+Lon-Protease-BrxL-><-ZnR<-ZnR<-?<-?<-?||RHH_antitoxin->	1213	Bacteria>Pseudomonadota>Gammaproteobacteria>Enterobacterales>Enterobacteriaceae>Escherichia	GCA_013041485.1	Escherichia_coli	EHH76_16080	2195..5836	562	BREX-system-P-loop-protein-BrxC-[Escherichia-coli]
EFO0007667.1	Type1.BREX	<-N6-METHYLASE<-DUF6079*<-BrxB-BrxF-iSTAND<-BrxA-DNA-binding||REase+iREase->	1213	Bacteria>Pseudomonadota>Gammaproteobacteria>Enterobacterales>Enterobacteriaceae>Escherichia	GCA_013057425.1	Escherichia_coli	D1356_22460	2492..6133	562	BREX-system-P-loop-protein-BrxC-[Escherichia-coli]
EFO0695969.1	Type1.BREX	<-Phage_integrase||?->?->?-><-HNH||wHTH+SAD+HNH-><-REase+iREase||BrxA-DNA-binding->BrxB-BrxF-iSTAND->DUF6079*->N6-METHYLASE->PglZ->SIGMA+OB-Fold+AAA-ATPase+Lon-Protease-BrxL->	1213	Bacteria>Pseudomonadota>Gammaproteobacteria>Enterobacterales>Enterobacteriaceae>Escherichia	GCA_013059045.1	Escherichia_coli	KI087_004400	8657..12298	562	BREX-system-P-loop-protein-BrxC-[Escherichia-coli]
EFO0712882.1	Type1.BREX	<-METHYLASE||SKI-><-?||tRNA->Arm-DNA-bind_3+Phage_integrase->EVE+HNH-><-REase+iREase||BrxA-DNA-binding->BrxB-BrxF-iSTAND->DUF6079*->N6-METHYLASE->PglZ->SIGMA+OB-Fold+AAA-ATPase+Lon-Protease-BrxL-><-ZnR<-ZnR<-?<-?<-?||RHH_antitoxin->	1213	Bacteria>Pseudomonadota>Gammaproteobacteria>Enterobacterales>Enterobacteriaceae>Escherichia	GCA_013059225.1	Escherichia_coli	DYR48_06815	148369..152010	562	BREX-system-P-loop-protein-BrxC-[Escherichia-coli]
EFO1505998.1	Type1.BREX	<-DUF6079-p*<-BrxB-BrxF-iSTAND<-BrxA-DNA-binding||REase+iREase-><-wHTH+SAD+HNH||HNH-><-?<-?<-?||35exo->	919	Bacteria>Pseudomonadota>Gammaproteobacteria>Enterobacterales>Enterobacteriaceae>Escherichia	GCA_013063585.1	Escherichia_coli	DWG87_23060	1..2758	562	BREX-system-P-loop-protein-BrxC-[Escherichia-coli]
EFO2091244.1	Type1.BREX	<-35exo||?->?->?-><-HNH||wHTH+SAD+HNH-><-REase+iREase||BrxA-DNA-binding->BrxB-BrxF-iSTAND->DUF6079*->N6-METHYLASE->PglZ->SIGMA+OB-Fold+AAA-ATPase+Lon-Protease-BrxL->HKD-DNase+OGG-like->||AbiEi_3_N+AbiEi_3->AbiEii-><-DUF6216<-Arm-DNA-bind_3	1213	Bacteria>Pseudomonadota>Gammaproteobacteria>Enterobacterales>Enterobacteriaceae>Escherichia	GCA_013066245.1	Escherichia_coli_O54	DP047_19880	9767..13408	2184075	BREX-system-P-loop-protein-BrxC-[Escherichia-coli-O54]
EFO2510526.1	Type1.BREX	ZnR->ZnR-><-SIGMA+OB-Fold+AAA-ATPase+Lon-Protease-BrxL<-PglZ<-N6-METHYLASE<-DUF6079*<-BrxB-BrxF-iSTAND<-BrxA-DNA-binding||REase->REase-><-HEPN<-ORC-CDC6+ZnR<-N6-METHYLASE<-Arm-DNA-bind_3+Phage_integrase	1213	Bacteria>Pseudomonadota>Gammaproteobacteria>Enterobacterales>Enterobacteriaceae>Escherichia	GCA_013067425.1	Escherichia_coli	B6Q63_13725	11254..14895	562	BREX-system-P-loop-protein-BrxC-[Escherichia-coli]
HAJ7467856.1	Type1.BREX	<-RelE-ParE<-CopG/RHH||<-RHH||?->wHTH+WYL+WCX->BrxA-DNA-binding->BrxB-BrxF-iSTAND->DUF6079*->ParB+HNH->N6-METHYLASE->PglZ->SIGMA+OB-Fold+AAA-ATPase+Lon-Protease-BrxL-><-RVT<-PIN<-MazE_antitoxin||?->Phage_holin->	1225	Bacteria>Pseudomonadota>Gammaproteobacteria>Enterobacterales>Enterobacteriaceae>Escherichia	GCA_013077315.1	Escherichia_coli	HNV92_21025	13565..17242	562	BREX-system-P-loop-protein-BrxC-[Escherichia-coli]
EFP0188320.1	Type1.BREX	DUF772+DDE_Tnp_1->BrxA-DNA-binding->BrxB-BrxF-iSTAND->DUF6079*->N6-METHYLASE->PglZ->SIGMA+OB-Fold+AAA-ATPase+Lon-Protease-BrxL-><-ZnR<-ZnR||||nSTAND1+sGTP->	1213	Bacteria>Pseudomonadota>Gammaproteobacteria>Enterobacterales>Enterobacteriaceae>Escherichia	GCA_013129375.1	Escherichia_coli	HLX13_004311	1679..5320	562	BREX-system-P-loop-protein-BrxC-[Escherichia-coli]
EFP2415668.1	Type1.BREX	<-Phage_integrase||?->?->?-><-HNH||wHTH+SAD+HNH-><-REase+iREase||BrxA-DNA-binding->BrxB-BrxF-iSTAND->DUF6079*->ZnR->ZnR-><-wHTH+WYL||?->?->?->HIN-HTH->	1160	Bacteria>Pseudomonadota>Gammaproteobacteria>Enterobacterales>Enterobacteriaceae>Escherichia	GCA_013157045.1	Escherichia_coli	HP898_000185	8665..12147	562	BREX-system-P-loop-protein-BrxC-[Escherichia-coli]
NUC65042.1	Type1.BREX	DDE_Tnp_IS1->?->ZnR->BrxA-DNA-binding->BrxB-BrxF-iSTAND->DUF6079*->N6-METHYLASE->HEPN->RVT->N6-METHYLASE-p->PglZ->SIGMA+OB-Fold+AAA-ATPase+Lon-Protease-BrxL-><-Arm-DNA-bind_3+Phage_integrase||Phage_integrase->	1233	Bacteria>Pseudomonadota>Gammaproteobacteria>Enterobacterales>Enterobacteriaceae>Escherichia	GCA_013342565.1	Escherichia_coli	HUC40_15465	2945..6646	562	BREX-system-P-loop-protein-BrxC-[Escherichia-coli]
EFR2049109.1	Type1.BREX	DUF6079-p*->N6-METHYLASE->PglZ->SIGMA+OB-Fold+AAA-ATPase+Lon-Protease-BrxL->HKD-DNase+OGG-like->||AbiEi_3_N+AbiEi_3->AbiEii-><-DUF6216<-Arm-DNA-bind_3	884	Bacteria>Pseudomonadota>Gammaproteobacteria>Enterobacterales>Enterobacteriaceae>Escherichia	GCA_013546455.1	Escherichia_coli	HZO98_003737	1..2655	562	BREX-system-P-loop-protein-BrxC-[Escherichia-coli]
EFR9656371.1	Type1.BREX	DUF6079-p*->N6-METHYLASE->PglZ->SIGMA+OB-Fold+AAA-ATPase+Lon-Protease-BrxL->HKD-DNase+OGG-like->||AbiEi_3_N+AbiEi_3->AbiEii-><-DUF6216<-Arm-DNA-bind_3	991	Bacteria>Pseudomonadota>Gammaproteobacteria>Enterobacterales>Enterobacteriaceae>Escherichia	GCA_013588925.1	Escherichia_coli	H2N11_003246	1..2976	562	BREX-system-P-loop-protein-BrxC-[Escherichia-coli]
EFS8463113.1	Type1.BREX	<-Phage_integrase||?->?->?-><-HNH||wHTH+SAD+HNH-><-REase+iREase||BrxA-DNA-binding->BrxB-BrxF-iSTAND->DUF6079*->N6-METHYLASE->PglZ->SIGMA+OB-Fold+AAA-ATPase+Lon-Protease-BrxL->HKD-DNase+OGG-like->||AbiEi_3_N+AbiEi_3->AbiEii-><-DUF6216<-Arm-DNA-bind_3	1213	Bacteria>Pseudomonadota>Gammaproteobacteria>Enterobacterales>Enterobacteriaceae>Escherichia	GCA_013669455.1	Escherichia_coli	HWT39_002319	97196..100837	562	BREX-system-P-loop-protein-BrxC-[Escherichia-coli]
EFT2808154.1	Type1.BREX	ZnR->wHTH+WYL+WCX->BrxA-DNA-binding->BrxB-BrxF-iSTAND->DUF6079*->ParB+HNH->N6-METHYLASE->PglZ->SIGMA+OB-Fold+AAA-ATPase+Lon-Protease-BrxL->35exo->	1221	Bacteria>Pseudomonadota>Gammaproteobacteria>Enterobacterales>Enterobacteriaceae>Escherichia	GCA_013688275.1	Escherichia_coli	HVL53_003849	3457..7122	562	BREX-system-P-loop-protein-BrxC-[Escherichia-coli]
EFT2742093.1	Type1.BREX	TRD->REase+N6_METHYLASE->||?->ParB->?-><-?<-SIGMA+OB-Fold+AAA-ATPase+Lon-Protease-BrxL<-PglZ<-N6-METHYLASE<-DUF6079*<-BrxB-BrxF-iSTAND<-BrxA-DNA-binding||REase+iREase-><-wHTH+SAD+HNH||HNH-><-?<-?<-?||Phage_integrase->	1213	Bacteria>Pseudomonadota>Gammaproteobacteria>Enterobacterales>Enterobacteriaceae>Escherichia	GCA_013688615.1	Escherichia_coli	HVL39_003223	27812..31453	562	BREX-system-P-loop-protein-BrxC-[Escherichia-coli]
EFU0098527.1	Type1.BREX	DUF6079*->N6-METHYLASE->PglZ->SIGMA+OB-Fold+AAA-ATPase+Lon-Protease-BrxL->HKD-DNase+OGG-like->||AbiEi_3_N+AbiEi_3->AbiEii->?-><-DUF6216<-Arm-DNA-bind_3	1009	Bacteria>Pseudomonadota>Gammaproteobacteria>Enterobacterales>Enterobacteriaceae>Escherichia	GCA_013728855.1	Escherichia_coli	HVV04_003689	1..3030	562	BREX-system-P-loop-protein-BrxC-[Escherichia-coli]
HAL0559649.1	Type1.BREX	DUF6079*->N6-METHYLASE->PglZ->SIGMA+OB-Fold+AAA-ATPase+Lon-Protease-BrxL-><-ZnR<-HTH_Tnp_1+HTH_21+rve+rve_3	1111	Bacteria>Pseudomonadota>Gammaproteobacteria>Enterobacterales>Enterobacteriaceae>Escherichia	GCA_013967645.1	Escherichia_coli	H4D61_004677	1..3338	562	BREX-system-P-loop-protein-BrxC-[Escherichia-coli]
HAL0969191.1	Type1.BREX	DUF6079*->N6-METHYLASE->PglZ->SIGMA+OB-Fold+AAA-ATPase+Lon-Protease-BrxL-><-ZnR<-HTH_Tnp_1+HTH_21+rve+rve_3	1186	Bacteria>Pseudomonadota>Gammaproteobacteria>Enterobacterales>Enterobacteriaceae>Escherichia	GCA_013984825.1	Escherichia_coli	H4H65_005028	1..3562	562	BREX-system-P-loop-protein-BrxC-[Escherichia-coli]
HAL9997354.1	Type1.BREX	DUF6079*->N6-METHYLASE->PglZ->SIGMA+OB-Fold+AAA-ATPase+Lon-Protease-BrxL->HKD-DNase+OGG-like->||AbiEi_3_N+AbiEi_3->AbiEii-><-DUF6216<-Arm-DNA-bind_3	1081	Bacteria>Pseudomonadota>Gammaproteobacteria>Enterobacterales>Enterobacteriaceae>Escherichia	GCA_014189205.1	Escherichia_coli	H7W48_003266	1..3246	562	BREX-system-P-loop-protein-BrxC-[Escherichia-coli]
EGC7303784.1	Type1.BREX	HIN-HTH->AAA-ATPase->?->ZnR-><-SIGMA+OB-Fold+AAA-ATPase+Lon-Protease-BrxL<-PglZ<-N6-METHYLASE<-DUF6079*<-BrxB-BrxF-iSTAND<-BrxA-DNA-binding||REase+iREase-><-EVE+HNH<-Arm-DNA-bind_3+Phage_integrase<-tRNA||?->HerA/FtsK+A32->	1213	Bacteria>Pseudomonadota>Gammaproteobacteria>Enterobacterales>Enterobacteriaceae>Escherichia	GCA_014304595.1	Escherichia_coli	JTG03_003848	38080..41721	562	BREX-system-P-loop-protein-BrxC-[Escherichia-coli]
EGD5155863.1	Type1.BREX	<-35exo||?->?->?-><-HNH||wHTH+SAD+HNH-><-REase+iREase||BrxA-DNA-binding->BrxB-BrxF-iSTAND->DUF6079*->N6-METHYLASE->PglZ->SIGMA+OB-Fold+AAA-ATPase+Lon-Protease-BrxL->HKD-DNase+OGG-like->||AbiEi_3_N+AbiEi_3->AbiEii-><-DUF6216<-Arm-DNA-bind_3	1213	Bacteria>Pseudomonadota>Gammaproteobacteria>Enterobacterales>Enterobacteriaceae>Escherichia	GCA_014387005.1	Escherichia_coli	DTY31_07100	71746..75387	562	BREX-system-P-loop-protein-BrxC-[Escherichia-coli]
HAM5125585.1	Type1.BREX	DUF6079-p*->N6-METHYLASE->PglZ->SIGMA+OB-Fold+AAA-ATPase+Lon-Protease-BrxL-><-ZnR<-RVT	770	Bacteria>Pseudomonadota>Gammaproteobacteria>Enterobacterales>Enterobacteriaceae>Escherichia	GCA_014468575.1	Escherichia_coli	IB005_004014	1..2313	562	BREX-system-P-loop-protein-BrxC-[Escherichia-coli]
EGF7347429.1	Type1.BREX	DUF6079*->HKD-DNase+OGG-like->||AbiEi_3_N+AbiEi_3->AbiEii-><-DUF6216<-tRNA||?->HerA/FtsK+A32-><-?<-?||Peptidase_M17_N+Peptidase_M17->	1213	Bacteria>Pseudomonadota>Gammaproteobacteria>Enterobacterales>Enterobacteriaceae>Escherichia	GCA_014521725.1	Escherichia_coli	HJV04_003560	44..3685	562	BREX-system-P-loop-protein-BrxC-[Escherichia-coli]
HAN3626817.1	Type1.BREX	<-N6-METHYLASE<-DUF6079*<-BrxB-BrxF-iSTAND<-BrxA-DNA-binding||REase+iREase-><-wHTH+SAD+HNH||HNH-><-?<-?<-?||Phage_integrase->	1213	Bacteria>Pseudomonadota>Gammaproteobacteria>Enterobacterales>Enterobacteriaceae>Escherichia	GCA_014691525.1	Escherichia_coli	IE966_004457	2731..6372	562	BREX-system-P-loop-protein-BrxC-[Escherichia-coli]
HAN6349201.1	Type1.BREX	<-Zn_Tnp_IS1+HTH_Tnp_IS1<-REase||BrxA-DNA-binding->BrxB-BrxF-iSTAND->DUF6079*->N6-METHYLASE->PglZ->SIGMA+OB-Fold+AAA-ATPase+Lon-Protease-BrxL-><-ZnR<-ZnR<-?||XIS-HTH->	1213	Bacteria>Pseudomonadota>Gammaproteobacteria>Enterobacterales>Enterobacteriaceae>Escherichia	GCA_014709895.1	Escherichia_coli	IF023_004329	2523..6164	562	BREX-system-P-loop-protein-BrxC-[Escherichia-coli]
EGI2318416.1	Type1.BREX	<-Phage_integrase||?->?->?-><-HNH||wHTH+SAD+HNH-><-REase+iREase||BrxA-DNA-binding->BrxB-BrxF-iSTAND->DUF6079*->	1027	Bacteria>Pseudomonadota>Gammaproteobacteria>Enterobacterales>Enterobacteriaceae>Escherichia	GCA_014736875.1	Escherichia_coli	IGA36_003675	97131..100211	562	BREX-system-P-loop-protein-BrxC-[Escherichia-coli]
EGI6859711.1	Type1.BREX	DUF6079*->N6-METHYLASE->PglZ->SIGMA+OB-Fold+AAA-ATPase+Lon-Protease-BrxL-><-ZnR<-ZnR<-?||RHH_antitoxin->XIS-HTH->	1173	Bacteria>Pseudomonadota>Gammaproteobacteria>Enterobacterales>Enterobacteriaceae>Escherichia	GCA_014766135.1	Escherichia_coli	IGX90_003813	1..3522	562	BREX-system-P-loop-protein-BrxC-[Escherichia-coli]
HAO0324358.1	Type1.BREX	<-HNH||wHTH+SAD+HNH-><-REase||BrxA-DNA-binding->BrxB-BrxF-iSTAND->DUF6079*->N6-METHYLASE-p->	1213	Bacteria>Pseudomonadota>Gammaproteobacteria>Enterobacterales>Enterobacteriaceae>Escherichia	GCA_014771515.1	Escherichia_coli	IGZ64_004245	6773..10414	562	BREX-system-P-loop-protein-BrxC-[Escherichia-coli]
HAO2821705.1	Type1.BREX	DUF6079*->PglZ->SIGMA+OB-Fold+AAA-ATPase+Lon-Protease-BrxL->HKD-DNase+OGG-like->||AbiEi_3_N+AbiEi_3->AbiEii-><-DUF6216<-Arm-DNA-bind_3<-tRNA||?->HerA/FtsK+A32->	1111	Bacteria>Pseudomonadota>Gammaproteobacteria>Enterobacterales>Enterobacteriaceae>Escherichia	GCA_014823275.1	Escherichia_coli	IHL91_001055	1..3336	562	BREX-system-P-loop-protein-BrxC-[Escherichia-coli]
HAO3163381.1	Type1.BREX	<-HIN-HTH<-RelE-ParE<-CopG/RHH||<-RHH||?->wHTH+WYL+WCX->BrxA-DNA-binding->BrxB-BrxF-iSTAND->DUF6079*->	1136	Bacteria>Pseudomonadota>Gammaproteobacteria>Enterobacterales>Enterobacteriaceae>Escherichia	GCA_014826135.1	Escherichia_coli	IHN66_004302	5400..8808	562	BREX-system-P-loop-protein-BrxC-[Escherichia-coli]
EGK3607993.1	Type1.BREX	Arm-DNA-bind_3+Phage_integrase->XIS-HTH->?->?->?->BrxA-DNA-binding->BrxB-BrxF-iSTAND->DUF6079*->ParB+HNH->N6-METHYLASE->SIR2->HerA/FtsK->N6-METHYLASE-p->	1238	Bacteria>Pseudomonadota>Gammaproteobacteria>Enterobacterales>Enterobacteriaceae>Escherichia	GCA_014957385.1	Escherichia_coli	IOT31_003182	92049..95765	562	BREX-system-P-loop-protein-BrxC-[Escherichia-coli]
EGK3603428.1	Type1.BREX	DUF6079*->PglZ->SIGMA+OB-Fold+AAA-ATPase+Lon-Protease-BrxL->HKD-DNase+OGG-like->||AbiEi_3_N+AbiEi_3->AbiEii-><-DUF6216<-Arm-DNA-bind_3<-tRNA||?->HerA/FtsK+A32->	1179	Bacteria>Pseudomonadota>Gammaproteobacteria>Enterobacterales>Enterobacteriaceae>Escherichia	GCA_014957425.1	Escherichia_coli	IOT28_003449	1..3540	562	BREX-system-P-loop-protein-BrxC-[Escherichia-coli]
EGJ9762745.1	Type1.BREX	<-Phage_integrase<-Phage_integrase||?->?->?->||wHTH+SAD+HNH-><-REase+iREase||BrxA-DNA-binding->BrxB-BrxF-iSTAND->DUF6079*->N6-METHYLASE->PglZ->SIGMA+OB-Fold+AAA-ATPase+Lon-Protease-BrxL->?-><-?<-ParB<-?||<-REase+N6_METHYLASE<-TRD	1213	Bacteria>Pseudomonadota>Gammaproteobacteria>Enterobacterales>Enterobacteriaceae>Escherichia	GCA_014978025.1	Escherichia_coli	LZF81_002887	55534..59175	562	BREX-system-P-loop-protein-BrxC-[Escherichia-coli]
HAO5539965.1	Type1.BREX	<-REase<-REase||BrxA-DNA-binding->BrxB-BrxF-iSTAND->DUF6079*->N6-METHYLASE->PglZ->SIGMA+OB-Fold+AAA-ATPase+Lon-Protease-BrxL-><-ZnR<-RVT	1213	Bacteria>Pseudomonadota>Gammaproteobacteria>Enterobacterales>Enterobacteriaceae>Escherichia	GCA_015013895.1	Escherichia_coli	IP613_004381	2370..6011	562	BREX-system-P-loop-protein-BrxC-[Escherichia-coli]
EGN4319649.1	Type1.BREX	DUF6079*->N6-METHYLASE->PglZ->SIGMA+OB-Fold+AAA-ATPase+Lon-Protease-BrxL->?-><-?<-ParB<-?||<-REase+N6_METHYLASE	1081	Bacteria>Pseudomonadota>Gammaproteobacteria>Enterobacterales>Enterobacteriaceae>Escherichia	GCA_015200385.1	Escherichia_coli	IH255_004211	1..3247	562	BREX-system-P-loop-protein-BrxC-[Escherichia-coli]
HAU8224518.1	Type1.BREX	<-Phage_integrase||?->?->?-><-HNH||wHTH+SAD+HNH-><-REase+iREase||BrxA-DNA-binding->BrxB-BrxF-iSTAND->DUF6079*->N6-METHYLASE->PglZ->SIGMA+OB-Fold+AAA-ATPase+Lon-Protease-BrxL->?-><-?<-ParB<-?||<-REase+N6_METHYLASE<-TRD	1213	Bacteria>Pseudomonadota>Gammaproteobacteria>Enterobacterales>Enterobacteriaceae>Escherichia	GCA_016109325.1	Escherichia_coli	IG138_000798	111239..114880	562	BREX-system-P-loop-protein-BrxC-[Escherichia-coli]
HAU8802979.1	Type1.BREX	DDE_Tnp_IS1-><-SIGMA+OB-Fold+AAA-ATPase+Lon-Protease-BrxL<-PglZ<-N6-METHYLASE<-DUF6079*<-BrxB-BrxF-iSTAND<-BrxA-DNA-binding||REase->?->||<-Arm-DNA-bind_3+Phage_integrase<-tRNA||?->HerA/FtsK+A32->	1213	Bacteria>Pseudomonadota>Gammaproteobacteria>Enterobacterales>Enterobacteriaceae>Escherichia	GCA_016115265.1	Escherichia_coli	JEW39_004409	9027..12668	562	BREX-system-P-loop-protein-BrxC-[Escherichia-coli]
EGV9472870.1	Type1.BREX	<-Phage_integrase||?->?->?-><-HNH||wHTH+SAD+HNH-><-REase+iREase||BrxA-DNA-binding->BrxB-BrxF-iSTAND->DUF6079*->N6-METHYLASE->PglZ->SIGMA+OB-Fold+AAA-ATPase+Lon-Protease-BrxL->?-><-?<-ParB<-?||<-REase+N6_METHYLASE<-TRD	1213	Bacteria>Pseudomonadota>Gammaproteobacteria>Enterobacterales>Enterobacteriaceae>Escherichia	GCA_016298225.1	Escherichia_coli	JF871_001476	111292..114933	562	BREX-system-P-loop-protein-BrxC-[Escherichia-coli]
EGY5636086.1	Type1.BREX	DUF6079*->N6-METHYLASE->PglZ->SIGMA+OB-Fold+AAA-ATPase+Lon-Protease-BrxL->HKD-DNase+OGG-like->||AbiEi_3_N+AbiEi_3->AbiEii-><-DUF6216<-Arm-DNA-bind_3	1171	Bacteria>Pseudomonadota>Gammaproteobacteria>Enterobacterales>Enterobacteriaceae>Escherichia	GCA_016519105.1	Escherichia_coli	JI450_003022	1..3517	562	BREX-system-P-loop-protein-BrxC-[Escherichia-coli]
EGY5620863.1	Type1.BREX	<-Phage_integrase<-Phage_integrase||?->?->?-><-HNH||wHTH(SBD)+HNH->||BrxA-DNA-binding->BrxB-BrxF-iSTAND->DUF6079*->N6-METHYLASE->PglZ->SIGMA+OB-Fold+AAA-ATPase+Lon-Protease-BrxL->HKD-DNase+OGG-like->||AbiEi_3_N+AbiEi_3->AbiEii-><-DUF6216<-Arm-DNA-bind_3	1213	Bacteria>Pseudomonadota>Gammaproteobacteria>Enterobacterales>Enterobacteriaceae>Escherichia	GCA_016519195.1	Escherichia_coli	JI433_002890	14696..18337	562	BREX-system-P-loop-protein-BrxC-[Escherichia-coli]
EGY7451904.1	Type1.BREX	<-DUF6079-p*<-BrxB-BrxF-iSTAND<-BrxA-DNA-binding||<-wHTH(SBD)+HNH||HNH-><-?<-?<-?||Phage_integrase->Phage_integrase->	968	Bacteria>Pseudomonadota>Gammaproteobacteria>Enterobacterales>Enterobacteriaceae>Escherichia	GCA_016533805.1	Escherichia_coli	JI519_004377	1..2904	562	BREX-system-P-loop-protein-BrxC-[Escherichia-coli]
EGY8947056.1	Type1.BREX	DUF6079-p*->N6-METHYLASE->PglZ->SIGMA+OB-Fold+AAA-ATPase+Lon-Protease-BrxL->HKD-DNase+OGG-like->||AbiEi_3_N+AbiEi_3->AbiEii-><-DUF6216<-Arm-DNA-bind_3	763	Bacteria>Pseudomonadota>Gammaproteobacteria>Enterobacterales>Enterobacteriaceae>Escherichia	GCA_016541865.1	Escherichia_coli	JI613_002965	1..2292	562	BREX-system-P-loop-protein-BrxC-[Escherichia-coli]
EGY8948619.1	Type1.BREX	<-DUF6079-p*<-BrxB-BrxF-iSTAND<-BrxA-DNA-binding||<-wHTH(SBD)+HNH||HNH-><-?<-?<-?||Phage_integrase->Phage_integrase->	450	Bacteria>Pseudomonadota>Gammaproteobacteria>Enterobacterales>Enterobacteriaceae>Escherichia	GCA_016541865.1	Escherichia_coli	JI613_004635	1..1349	562	BREX-system-P-loop-protein-BrxC-[Escherichia-coli]
EGY9082468.1	Type1.BREX	DUF6079*->N6-METHYLASE->PglZ->SIGMA+OB-Fold+AAA-ATPase+Lon-Protease-BrxL->HKD-DNase+OGG-like->||AbiEi_3_N+AbiEi_3->AbiEii-><-DUF6216<-Arm-DNA-bind_3	1175	Bacteria>Pseudomonadota>Gammaproteobacteria>Enterobacterales>Enterobacteriaceae>Escherichia	GCA_016542525.1	Escherichia_coli	JI646_002303	1..3528	562	BREX-system-P-loop-protein-BrxC-[Escherichia-coli]
EGY9306082.1	Type1.BREX	<-HNH||wHTH(SBD)+HNH->||BrxA-DNA-binding->BrxB-BrxF-iSTAND->DUF6079*->N6-METHYLASE->PglZ->	1213	Bacteria>Pseudomonadota>Gammaproteobacteria>Enterobacterales>Enterobacteriaceae>Escherichia	GCA_016543505.1	Escherichia_coli	HZO96_004640	4089..7730	562	BREX-system-P-loop-protein-BrxC-[Escherichia-coli]
EGY9336114.1	Type1.BREX	DUF6079*->N6-METHYLASE->PglZ->SIGMA+OB-Fold+AAA-ATPase+Lon-Protease-BrxL->HKD-DNase+OGG-like->||AbiEi_3_N+AbiEi_3->AbiEii-><-DUF6216<-Arm-DNA-bind_3	1177	Bacteria>Pseudomonadota>Gammaproteobacteria>Enterobacterales>Enterobacteriaceae>Escherichia	GCA_016543525.1	Escherichia_coli	HZO78_003642	1..3535	562	BREX-system-P-loop-protein-BrxC-[Escherichia-coli]
EGY9928133.1	Type1.BREX	DUF6079-p*->N6-METHYLASE->PglZ->SIGMA+OB-Fold+AAA-ATPase+Lon-Protease-BrxL-><-ZnR<-?||HIN-HTH+HIN-HTH->	865	Bacteria>Pseudomonadota>Gammaproteobacteria>Enterobacterales>Enterobacteriaceae>Escherichia	GCA_016546565.1	Escherichia_coli	HVV03_004451	1..2599	562	BREX-system-P-loop-protein-BrxC-[Escherichia-coli]
EGZ0051356.1	Type1.BREX	DUF6079-p*->N6-METHYLASE->PglZ->SIGMA+OB-Fold+AAA-ATPase+Lon-Protease-BrxL->HKD-DNase+OGG-like->||AbiEi_3_N+AbiEi_3->AbiEii-><-DUF6216<-Arm-DNA-bind_3	834	Bacteria>Pseudomonadota>Gammaproteobacteria>Enterobacterales>Enterobacteriaceae>Escherichia	GCA_016546855.1	Escherichia_coli	JJA24_002218	1..2505	562	BREX-system-P-loop-protein-BrxC-[Escherichia-coli]
EGZ1085523.1	Type1.BREX	DUF6079-p*->N6-METHYLASE->PglZ->SIGMA+OB-Fold+AAA-ATPase+Lon-Protease-BrxL-><-ZnR<-ZnR	964	Bacteria>Pseudomonadota>Gammaproteobacteria>Enterobacterales>Enterobacteriaceae>Escherichia	GCA_016551725.1	Escherichia_coli	FOI46_004816	1..2896	562	BREX-system-P-loop-protein-BrxC-[Escherichia-coli]
EGZ1391640.1	Type1.BREX	DUF6079-p*->N6-METHYLASE->PglZ->SIGMA+OB-Fold+AAA-ATPase+Lon-Protease-BrxL->HKD-DNase+OGG-like->||AbiEi_3_N+AbiEi_3->AbiEii-><-DUF6216<-Arm-DNA-bind_3	789	Bacteria>Pseudomonadota>Gammaproteobacteria>Enterobacterales>Enterobacteriaceae>Escherichia	GCA_016552825.1	Escherichia_coli	FG160_002854	1..2370	562	BREX-system-P-loop-protein-BrxC-[Escherichia-coli]
EGZ1415768.1	Type1.BREX	Arm-DNA-bind_3->DUF6216-><-AbiEii<-AbiEi_3_N+AbiEi_3||<-HKD-DNase+OGG-like<-SIGMA+OB-Fold+AAA-ATPase+Lon-Protease-BrxL<-PglZ<-N6-METHYLASE<-DUF6079*<-BrxB-BrxF-iSTAND<-BrxA-DNA-binding||<-wHTH+SAD+HNH||HNH->	1213	Bacteria>Pseudomonadota>Gammaproteobacteria>Enterobacterales>Enterobacteriaceae>Escherichia	GCA_016552885.1	Escherichia_coli	FFU33_001578	154175..157816	562	BREX-system-P-loop-protein-BrxC-[Escherichia-coli]
EGZ1559467.1	Type1.BREX	DUF6079-p*->N6-METHYLASE->PglZ->SIGMA+OB-Fold+AAA-ATPase+Lon-Protease-BrxL->HKD-DNase+OGG-like->||AbiEi_3_N+AbiEi_3->AbiEii-><-DUF6216<-Arm-DNA-bind_3	768	Bacteria>Pseudomonadota>Gammaproteobacteria>Enterobacterales>Enterobacteriaceae>Escherichia	GCA_016553465.1	Escherichia_coli	FAV16_003764	1..2307	562	BREX-system-P-loop-protein-BrxC-[Escherichia-coli]
EGZ1560300.1	Type1.BREX	<-DUF6079-p*<-BrxB-BrxF-iSTAND<-BrxA-DNA-binding||<-wHTH(SBD)+HNH||HNH-><-?<-?<-?||Phage_integrase->Phage_integrase->	444	Bacteria>Pseudomonadota>Gammaproteobacteria>Enterobacterales>Enterobacteriaceae>Escherichia	GCA_016553465.1	Escherichia_coli	FAV16_004661	1..1332	562	BREX-system-P-loop-protein-BrxC-[Escherichia-coli]
EGZ1869331.1	Type1.BREX	DUF6079-p*->N6-METHYLASE->PglZ->SIGMA+OB-Fold+AAA-ATPase+Lon-Protease-BrxL->HKD-DNase+OGG-like->||AbiEi_3_N+AbiEi_3->AbiEii-><-DUF6216<-Arm-DNA-bind_3	796	Bacteria>Pseudomonadota>Gammaproteobacteria>Enterobacterales>Enterobacteriaceae>Escherichia	GCA_016554585.1	Escherichia_coli	EYE54_001992	1..2391	562	BREX-system-P-loop-protein-BrxC-[Escherichia-coli]
EGZ1871551.1	Type1.BREX	<-DUF6079-p*<-BrxB-BrxF-iSTAND<-BrxA-DNA-binding||<-wHTH(SBD)+HNH||HNH-><-?<-?<-?||Phage_integrase->Phage_integrase->	416	Bacteria>Pseudomonadota>Gammaproteobacteria>Enterobacterales>Enterobacteriaceae>Escherichia	GCA_016554585.1	Escherichia_coli	EYE54_004341	1..1248	562	BREX-system-P-loop-protein-BrxC-[Escherichia-coli]
EGZ2189929.1	Type1.BREX	<-DUF6079-p*<-BrxB-BrxF-iSTAND<-BrxA-DNA-binding||<-wHTH(SBD)+HNH||HNH-><-?<-?<-?||Phage_integrase->	628	Bacteria>Pseudomonadota>Gammaproteobacteria>Enterobacterales>Enterobacteriaceae>Escherichia	GCA_016555885.1	Escherichia_coli	ED609_004465	1..1884	562	BREX-system-P-loop-protein-BrxC-[Escherichia-coli]
EGZ2354229.1	Type1.BREX	<-Phage_integrase<-Phage_integrase||?->?->?-><-HNH||wHTH(SBD)+HNH->||BrxA-DNA-binding->BrxB-BrxF-iSTAND->DUF6079*->N6-METHYLASE->PglZ->SIGMA+OB-Fold+AAA-ATPase+Lon-Protease-BrxL->HKD-DNase+OGG-like->||AbiEi_3_N+AbiEi_3->AbiEii-><-DUF6216<-Arm-DNA-bind_3	1213	Bacteria>Pseudomonadota>Gammaproteobacteria>Enterobacterales>Enterobacteriaceae>Escherichia	GCA_016556665.1	Escherichia_coli	D7J47_002044	14697..18338	562	BREX-system-P-loop-protein-BrxC-[Escherichia-coli]
EGZ2397683.1	Type1.BREX	DUF6079-p*->N6-METHYLASE->PglZ->SIGMA+OB-Fold+AAA-ATPase+Lon-Protease-BrxL->HKD-DNase+OGG-like->||AbiEi_3_N+AbiEi_3->AbiEii-><-DUF6216<-Arm-DNA-bind_3	969	Bacteria>Pseudomonadota>Gammaproteobacteria>Enterobacterales>Enterobacteriaceae>Escherichia	GCA_016557105.1	Escherichia_coli	D6W78_003631	1..2912	562	BREX-system-P-loop-protein-BrxC-[Escherichia-coli]
EGZ2497894.1	Type1.BREX	DUF6079*->N6-METHYLASE->PglZ->SIGMA+OB-Fold+AAA-ATPase+Lon-Protease-BrxL-><-ZnR<-ZnR<-?||HIN-HTH+PAIREDC-HTH->||RHH_antitoxin->XIS-HTH->	1012	Bacteria>Pseudomonadota>Gammaproteobacteria>Enterobacterales>Enterobacteriaceae>Escherichia	GCA_016557105.1	Escherichia_coli	D6W78_003856	1..3041	562	BREX-system-P-loop-protein-BrxC-[Escherichia-coli]
EGZ2674120.1	Type1.BREX	DUF6079*->N6-METHYLASE->PglZ->SIGMA+OB-Fold+AAA-ATPase+Lon-Protease-BrxL->HKD-DNase+OGG-like->||AbiEi_3_N+AbiEi_3->AbiEii-><-DUF6216<-Arm-DNA-bind_3	1096	Bacteria>Pseudomonadota>Gammaproteobacteria>Enterobacterales>Enterobacteriaceae>Escherichia	GCA_016557745.1	Escherichia_coli	D1H08_002340	1..3291	562	BREX-system-P-loop-protein-BrxC-[Escherichia-coli]
EGZ2777080.1	Type1.BREX	<-Phage_integrase<-Phage_integrase||?->?->?-><-HNH||wHTH(SBD)+HNH->||BrxA-DNA-binding->BrxB-BrxF-iSTAND->DUF6079*->N6-METHYLASE->PglZ->SIGMA+OB-Fold+AAA-ATPase+Lon-Protease-BrxL->HKD-DNase+OGG-like->||AbiEi_3_N+AbiEi_3->AbiEii-><-DUF6216<-Arm-DNA-bind_3	1213	Bacteria>Pseudomonadota>Gammaproteobacteria>Enterobacterales>Enterobacteriaceae>Escherichia	GCA_016558225.1	Escherichia_coli	DVO41_002389	14697..18338	562	BREX-system-P-loop-protein-BrxC-[Escherichia-coli]
EGZ3242782.1	Type1.BREX	<-Phage_integrase<-Phage_integrase||?->?->?-><-HNH||wHTH(SBD)+HNH->||BrxA-DNA-binding->BrxB-BrxF-iSTAND->DUF6079*->N6-METHYLASE->PglZ->SIGMA+OB-Fold+AAA-ATPase+Lon-Protease-BrxL->HKD-DNase+OGG-like->||AbiEi_3_N+AbiEi_3->AbiEii-><-DUF6216<-Arm-DNA-bind_3	1213	Bacteria>Pseudomonadota>Gammaproteobacteria>Enterobacterales>Enterobacteriaceae>Escherichia	GCA_016560025.1	Escherichia_coli	C5N70_002404	39991..43632	562	BREX-system-P-loop-protein-BrxC-[Escherichia-coli]
EGZ4145352.1	Type1.BREX	DUF6079*->N6-METHYLASE->PglZ->SIGMA+OB-Fold+AAA-ATPase+Lon-Protease-BrxL->HKD-DNase+OGG-like->||AbiEi_3_N+AbiEi_3->AbiEii-><-DUF6216<-Arm-DNA-bind_3	1095	Bacteria>Pseudomonadota>Gammaproteobacteria>Enterobacterales>Enterobacteriaceae>Escherichia	GCA_016563925.1	Escherichia_coli	CKY95_002785	1..3290	562	BREX-system-P-loop-protein-BrxC-[Escherichia-coli]
EGZ5166758.1	Type1.BREX	<-DUF6079-p*<-BrxB-BrxF-iSTAND<-BrxA-DNA-binding||<-wHTH(SBD)+HNH||HNH-><-?<-?<-?||Phage_integrase->Phage_integrase->	624	Bacteria>Pseudomonadota>Gammaproteobacteria>Enterobacterales>Enterobacteriaceae>Escherichia	GCA_016568175.1	Escherichia_coli	CDS35_004366	1..1874	562	BREX-system-P-loop-protein-BrxC-[Escherichia-coli]
EGZ5520004.1	Type1.BREX	DUF6079-p*->N6-METHYLASE->PglZ->SIGMA+OB-Fold+AAA-ATPase+Lon-Protease-BrxL->HKD-DNase+OGG-like->||AbiEi_3_N+AbiEi_3->AbiEii->?-><-DUF6216<-Arm-DNA-bind_3	815	Bacteria>Pseudomonadota>Gammaproteobacteria>Enterobacterales>Enterobacteriaceae>Escherichia	GCA_016569715.1	Escherichia_coli	LF100_002181	1..2450	562	BREX-system-P-loop-protein-BrxC-[Escherichia-coli]
EGZ5591347.1	Type1.BREX	<-DUF6079-p*<-BrxB-BrxF-iSTAND<-BrxA-DNA-binding||<-wHTH(SBD)+HNH||HNH-><-?<-?<-?||Phage_integrase->Phage_integrase->	707	Bacteria>Pseudomonadota>Gammaproteobacteria>Enterobacterales>Enterobacteriaceae>Escherichia	GCA_016569835.1	Escherichia_coli	BZZ56_004473	1..2121	562	BREX-system-P-loop-protein-BrxC-[Escherichia-coli]
EGZ5731917.1	Type1.BREX	<-DUF6079-p*<-BrxB-BrxF-iSTAND<-BrxA-DNA-binding||<-wHTH(SBD)+HNH||HNH-><-?<-?<-?||Phage_integrase->Phage_integrase->	885	Bacteria>Pseudomonadota>Gammaproteobacteria>Enterobacterales>Enterobacteriaceae>Escherichia	GCA_016570515.1	Escherichia_coli	BL334_004670	1..2655	562	BREX-system-P-loop-protein-BrxC-[Escherichia-coli]
EGZ5978150.1	Type1.BREX	<-Phage_integrase<-Phage_integrase||?->?->?-><-HNH||wHTH(SBD)+HNH->||BrxA-DNA-binding->BrxB-BrxF-iSTAND->DUF6079-p*->	421	Bacteria>Pseudomonadota>Gammaproteobacteria>Enterobacterales>Enterobacteriaceae>Escherichia	GCA_016571515.1	Escherichia_coli	BIB10_004104	39992..41255	562	BREX-system-P-loop-protein-BrxC-[Escherichia-coli]
EGZ7391807.1	Type1.BREX	DUF6079-p*->N6-METHYLASE->PglZ->SIGMA+OB-Fold+AAA-ATPase+Lon-Protease-BrxL->HKD-DNase+OGG-like->||AbiEi_3_N+AbiEi_3->AbiEii-><-DUF6216<-Arm-DNA-bind_3	970	Bacteria>Pseudomonadota>Gammaproteobacteria>Enterobacterales>Enterobacteriaceae>Escherichia	GCA_016578925.1	Escherichia_coli	BAU88_004031	1..2913	562	BREX-system-P-loop-protein-BrxC-[Escherichia-coli]
EGZ7392043.1	Type1.BREX	DUF6079-p*->N6-METHYLASE->PglZ->SIGMA+OB-Fold+AAA-ATPase+Lon-Protease-BrxL-><-ZnR<-ZnR<-?||HIN-HTH+PAIREDC-HTH->||RHH_antitoxin->XIS-HTH->	928	Bacteria>Pseudomonadota>Gammaproteobacteria>Enterobacterales>Enterobacteriaceae>Escherichia	GCA_016578925.1	Escherichia_coli	BAU88_004280	1..2788	562	BREX-system-P-loop-protein-BrxC-[Escherichia-coli]
EGZ7370259.1	Type1.BREX	DUF6079-p*->N6-METHYLASE->PglZ->SIGMA+OB-Fold+AAA-ATPase+Lon-Protease-BrxL->HKD-DNase+OGG-like->||AbiEi_3_N+AbiEi_3->AbiEii-><-DUF6216<-Arm-DNA-bind_3	880	Bacteria>Pseudomonadota>Gammaproteobacteria>Enterobacterales>Enterobacteriaceae>Escherichia	GCA_016578935.1	Escherichia_coli	BAU61_002957	1..2643	562	BREX-system-P-loop-protein-BrxC-[Escherichia-coli]
EGZ7468561.1	Type1.BREX	DUF6079-p*->N6-METHYLASE->PglZ->SIGMA+OB-Fold+AAA-ATPase+Lon-Protease-BrxL->HKD-DNase+OGG-like->||AbiEi_3_N+AbiEi_3->AbiEii-><-DUF6216<-Arm-DNA-bind_3	931	Bacteria>Pseudomonadota>Gammaproteobacteria>Enterobacterales>Enterobacteriaceae>Escherichia	GCA_016579325.1	Escherichia_coli	A6F02_004042	1..2798	562	BREX-system-P-loop-protein-BrxC-[Escherichia-coli]
EGZ7468749.1	Type1.BREX	DUF6079-p*->N6-METHYLASE->PglZ->SIGMA+OB-Fold+AAA-ATPase+Lon-Protease-BrxL-><-ZnR<-ZnR<-?||HIN-HTH+PAIREDC-HTH->||RHH_antitoxin->XIS-HTH->	931	Bacteria>Pseudomonadota>Gammaproteobacteria>Enterobacterales>Enterobacteriaceae>Escherichia	GCA_016579325.1	Escherichia_coli	A6F02_004243	1..2798	562	BREX-system-P-loop-protein-BrxC-[Escherichia-coli]
EGZ7821797.1	Type1.BREX	DUF6079-p*->N6-METHYLASE->PglZ->SIGMA+OB-Fold+AAA-ATPase+Lon-Protease-BrxL->HKD-DNase+OGG-like->||AbiEi_3_N+AbiEi_3->AbiEii-><-DUF6216<-Arm-DNA-bind_3	729	Bacteria>Pseudomonadota>Gammaproteobacteria>Enterobacterales>Enterobacteriaceae>Escherichia	GCA_016581105.1	Escherichia_coli	AWC87_001490	1..2190	562	BREX-system-P-loop-protein-BrxC-[Escherichia-coli]
EGZ8838308.1	Type1.BREX	<-Phage_integrase||?->?->?-><-HNH||wHTH(SBD)+HNH->||BrxA-DNA-binding->BrxB-BrxF-iSTAND->DUF6079*->N6-METHYLASE->PglZ->SIGMA+OB-Fold+AAA-ATPase+Lon-Protease-BrxL->HKD-DNase+OGG-like->||AbiEi_3_N+AbiEi_3->AbiEii-><-DUF6216<-Arm-DNA-bind_3	1213	Bacteria>Pseudomonadota>Gammaproteobacteria>Enterobacterales>Enterobacteriaceae>Escherichia	GCA_016590495.1	Escherichia_coli	AT823_000493	8719..12360	562	BREX-system-P-loop-protein-BrxC-[Escherichia-coli]
EGZ9024440.1	Type1.BREX	DUF6079-p*->N6-METHYLASE->PglZ->SIGMA+OB-Fold+AAA-ATPase+Lon-Protease-BrxL->HKD-DNase+OGG-like->||AbiEi_3_N+AbiEi_3->AbiEii-><-DUF6216<-Arm-DNA-bind_3	963	Bacteria>Pseudomonadota>Gammaproteobacteria>Enterobacterales>Enterobacteriaceae>Escherichia	GCA_016591155.1	Escherichia_coli	AFE50_004176	1..2892	562	BREX-system-P-loop-protein-BrxC-[Escherichia-coli]
EGZ9024631.1	Type1.BREX	DUF6079-p*->N6-METHYLASE->PglZ->SIGMA+OB-Fold+AAA-ATPase+Lon-Protease-BrxL-><-ZnR<-ZnR<-?||HIN-HTH+PAIREDC-HTH->||RHH_antitoxin->XIS-HTH->	980	Bacteria>Pseudomonadota>Gammaproteobacteria>Enterobacterales>Enterobacteriaceae>Escherichia	GCA_016591155.1	Escherichia_coli	AFE50_004379	1..2945	562	BREX-system-P-loop-protein-BrxC-[Escherichia-coli]
EHA0949388.1	Type1.BREX	DUF6079-p*->N6-METHYLASE->PglZ->SIGMA+OB-Fold+AAA-ATPase+Lon-Protease-BrxL->HKD-DNase+OGG-like->||AbiEi_3_N+AbiEi_3->AbiEii-><-DUF6216<-Arm-DNA-bind_3	762	Bacteria>Pseudomonadota>Gammaproteobacteria>Enterobacterales>Enterobacteriaceae>Escherichia	GCA_016610545.1	Escherichia_coli	JI650_002930	1..2291	562	BREX-system-P-loop-protein-BrxC-[Escherichia-coli]
HAW1448903.1	Type1.BREX	<-Phage_integrase||?->?->?-><-HNH||wHTH+SAD+HNH-><-REase+iREase||BrxA-DNA-binding->BrxB-BrxF-iSTAND->DUF6079*->N6-METHYLASE->PglZ->SIGMA+OB-Fold+AAA-ATPase+Lon-Protease-BrxL->HKD-DNase+OGG-like->||AbiEi_3_N+AbiEi_3->AbiEii-><-DUF6216<-Arm-DNA-bind_3	1213	Bacteria>Pseudomonadota>Gammaproteobacteria>Enterobacterales>Enterobacteriaceae>Escherichia	GCA_016674355.1	Escherichia_coli	JLM49_000126	135704..139345	562	BREX-system-P-loop-protein-BrxC-[Escherichia-coli]
HAW2754029.1	Type1.BREX	<-HerA/FtsK+A32<-?||tRNA->Arm-DNA-bind_3+Phage_integrase->EVE+HNH-><-REase+iREase||BrxA-DNA-binding->BrxB-BrxF-iSTAND->DUF6079*->N6-METHYLASE->PglZ->SIGMA+OB-Fold+AAA-ATPase+Lon-Protease-BrxL-><-ZnR<-ZnR	1213	Bacteria>Pseudomonadota>Gammaproteobacteria>Enterobacterales>Enterobacteriaceae>Escherichia	GCA_016680635.1	Escherichia_coli	JLQ48_003685	19788..23429	562	BREX-system-P-loop-protein-BrxC-[Escherichia-coli]
HAW6060333.1	Type1.BREX	<-DUF6079-p*<-BrxB-BrxF-iSTAND<-BrxA-DNA-binding||REase+iREase-><-wHTH+SAD+HNH	613	Bacteria>Pseudomonadota>Gammaproteobacteria>Enterobacterales>Enterobacteriaceae>Escherichia	GCA_016697785.1	Escherichia_coli	JL413_004248	1..1839	562	BREX-system-P-loop-protein-BrxC-[Escherichia-coli]
HAW6308706.1	Type1.BREX	ZnR->ZnR-><-SIGMA+OB-Fold+AAA-ATPase+Lon-Protease-BrxL<-PglZ<-N6-METHYLASE<-DUF6079*<-BrxB-BrxF-iSTAND<-BrxA-DNA-binding||REase+iREase->?->?-><-DDE_Tnp_IS1	1213	Bacteria>Pseudomonadota>Gammaproteobacteria>Enterobacterales>Enterobacteriaceae>Escherichia	GCA_016734185.1	Escherichia_coli	JLP42_004227	9916..13557	562	BREX-system-P-loop-protein-BrxC-[Escherichia-coli]
EHA9087556.1	Type1.BREX	<-Phage_integrase<-Phage_integrase||?->?->?-><-HNH||wHTH(SBD)+HNH->||BrxA-DNA-binding->BrxB-BrxF-iSTAND->DUF6079*->N6-METHYLASE->PglZ->SIGMA+OB-Fold+AAA-ATPase+Lon-Protease-BrxL->HKD-DNase+OGG-like->||AbiEi_3_N+AbiEi_3->AbiEii-><-DUF6216<-Arm-DNA-bind_3	1213	Bacteria>Pseudomonadota>Gammaproteobacteria>Enterobacterales>Enterobacteriaceae>Escherichia	GCA_016846225.1	Escherichia_coli	D4094_002230	14699..18340	562	BREX-system-P-loop-protein-BrxC-[Escherichia-coli]
HAW9526082.1	Type1.BREX	<-N6-METHYLASE<-DUF6079*<-BrxB-BrxF-iSTAND<-BrxA-DNA-binding||REase+iREase-><-wHTH+SAD+HNH||HNH-><-?<-?<-?||Phage_integrase->	1213	Bacteria>Pseudomonadota>Gammaproteobacteria>Enterobacterales>Enterobacteriaceae>Escherichia	GCA_016946495.1	Escherichia_coli	JY346_004480	2578..6219	562	BREX-system-P-loop-protein-BrxC-[Escherichia-coli]
HAX1741345.1	Type1.BREX	MobI->?-><-?<-LexA-protease||35exo->?->wHTH+WYL+WCX->BrxA-DNA-binding->BrxB-BrxF-iSTAND->DUF6079-p*->	414	Bacteria>Pseudomonadota>Gammaproteobacteria>Enterobacterales>Enterobacteriaceae>Escherichia	GCA_016984655.1	Escherichia_coli	JXS76_004269	14153..15393	562	BREX-system-P-loop-protein-BrxC-[Escherichia-coli]
HAX1741551.1	Type1.BREX	DUF6079-p*->ParB+HNH->N6-METHYLASE->PglZ->SIGMA+OB-Fold+AAA-ATPase+Lon-Protease-BrxL->SbcC->	793	Bacteria>Pseudomonadota>Gammaproteobacteria>Enterobacterales>Enterobacteriaceae>Escherichia	GCA_016984655.1	Escherichia_coli	JXS76_004485	1..2383	562	BREX-system-P-loop-protein-BrxC-[Escherichia-coli]
HAX2048855.1	Type1.BREX	ZnR->ZnR-><-SIGMA+OB-Fold+AAA-ATPase+Lon-Protease-BrxL<-PglZ<-N6-METHYLASE<-DUF6079*<-BrxB-BrxF-iSTAND<-BrxA-DNA-binding||REase+iREase->	1213	Bacteria>Pseudomonadota>Gammaproteobacteria>Enterobacterales>Enterobacteriaceae>Escherichia	GCA_016988055.1	Escherichia_coli	JW528_004279	9399..13040	562	BREX-system-P-loop-protein-BrxC-[Escherichia-coli]
HAX3725917.1	Type1.BREX	Glyoxalase_8-><-?<-?<-VirD4-TraG+VirB4-FtsK<-HDRnase<-SbcC<-SIGMA+OB-Fold+AAA-ATPase+Lon-Protease-BrxL<-PglZ<-N6-METHYLASE<-DUF6079-p*	928	Bacteria>Pseudomonadota>Gammaproteobacteria>Enterobacterales>Enterobacteriaceae>Escherichia	GCA_017002415.1	Escherichia_coli	JV380_003192	66035..68823	562	BREX-system-P-loop-protein-BrxC-[Escherichia-coli]
HAX3800328.1	Type1.BREX	<-REase<-REase||BrxA-DNA-binding->BrxB-BrxF-iSTAND->DUF6079*->N6-METHYLASE->PglZ->SIGMA+OB-Fold+AAA-ATPase+Lon-Protease-BrxL-><-ZnR<-ZnR<-?<-?||RHH_antitoxin->XIS-HTH->	1213	Bacteria>Pseudomonadota>Gammaproteobacteria>Enterobacterales>Enterobacteriaceae>Escherichia	GCA_017002675.1	Escherichia_coli	JV366_004401	2784..6425	562	BREX-system-P-loop-protein-BrxC-[Escherichia-coli]
HAX4031531.1	Type1.BREX	<-Phage_integrase||?->?->?-><-HNH||wHTH+SAD+HNH-><-REase+iREase||BrxA-DNA-binding->BrxB-BrxF-iSTAND->DUF6079*->N6-METHYLASE->PglZ->SIGMA+OB-Fold+AAA-ATPase+Lon-Protease-BrxL->HKD-DNase+OGG-like->||AbiEi_3_N+AbiEi_3->AbiEii-><-DUF6216<-Arm-DNA-bind_3	1213	Bacteria>Pseudomonadota>Gammaproteobacteria>Enterobacterales>Enterobacteriaceae>Escherichia	GCA_017003755.1	Escherichia_coli	JV397_001890	160694..164335	562	BREX-system-P-loop-protein-BrxC-[Escherichia-coli]
HAX4880267.1	Type1.BREX	<-wHTH+WYL||wHTH->ZnR->ZnR-><-SIGMA+OB-Fold+AAA-ATPase+Lon-Protease-BrxL<-PglZ<-TOPRIM<-OLD-ABC<-N6-METHYLASE<-DUF6079*<-BrxB-BrxF-iSTAND<-BrxA-DNA-binding<-tRNA||?-><-SKI||METHYLASE->?->?->LACI-HTH+PBPI->HerA/FtsK+A32->	1204	Bacteria>Pseudomonadota>Gammaproteobacteria>Enterobacterales>Enterobacteriaceae>Escherichia	GCA_017016975.1	Escherichia_coli	JZF83_001472	109249..112863	562	BREX-system-P-loop-protein-BrxC-[Escherichia-coli]
EHC1662562.1	Type1.BREX	<-N6-METHYLASE<-DUF6079*<-BrxB-BrxF-iSTAND<-BrxA-DNA-binding||REase+iREase-><-wHTH+SAD+HNH||HNH->	1213	Bacteria>Pseudomonadota>Gammaproteobacteria>Enterobacterales>Enterobacteriaceae>Escherichia	GCA_017030065.1	Escherichia_coli	JTZ37_004160	2115..5756	562	BREX-system-P-loop-protein-BrxC-[Escherichia-coli]
EHC2076257.1	Type1.BREX	<-Phage_integrase||?->?->?-><-HNH||wHTH+SAD+HNH-><-REase+iREase||BrxA-DNA-binding->BrxB-BrxF-iSTAND->DUF6079*->N6-METHYLASE->PglZ->SIGMA+OB-Fold+AAA-ATPase+Lon-Protease-BrxL->HKD-DNase+OGG-like->||AbiEi_3_N+AbiEi_3->AbiEii-><-DUF6216<-Arm-DNA-bind_3	1213	Bacteria>Pseudomonadota>Gammaproteobacteria>Enterobacterales>Enterobacteriaceae>Escherichia	GCA_017031715.1	Escherichia_coli	JT651_000259	8773..12414	562	BREX-system-P-loop-protein-BrxC-[Escherichia-coli]
EHC2846675.1	Type1.BREX	Arm-DNA-bind_3+Phage_integrase-><-Sulfatase<-?||DDE_Tnp_1_5+DDE_Tnp_1-><-REase+iREase||BrxA-DNA-binding->BrxB-BrxF-iSTAND->DUF6079*->N6-METHYLASE->PglZ->SIGMA+OB-Fold+AAA-ATPase+Lon-Protease-BrxL-><-ZnR<-ZnR<-?<-?||RHH_antitoxin->XIS-HTH->	1213	Bacteria>Pseudomonadota>Gammaproteobacteria>Enterobacterales>Enterobacteriaceae>Escherichia	GCA_017035095.1	Escherichia_coli	JTY00_000601	175379..179020	562	BREX-system-P-loop-protein-BrxC-[Escherichia-coli]
HAX6962711.1	Type1.BREX	<-XIS-HTH<-RHH_antitoxin||?->ZnR->ZnR-><-SIGMA+OB-Fold+AAA-ATPase+Lon-Protease-BrxL<-PglZ<-N6-METHYLASE<-DUF6079*<-BrxB-BrxF-iSTAND<-BrxA-DNA-binding||REase+iREase-><-EVE+HNH<-Arm-DNA-bind_3+Phage_integrase<-tRNA||?->HerA/FtsK+A32->	1213	Bacteria>Pseudomonadota>Gammaproteobacteria>Enterobacterales>Enterobacteriaceae>Escherichia	GCA_017045635.1	Escherichia_coli	JTN21_000929	60484..64125	562	BREX-system-P-loop-protein-BrxC-[Escherichia-coli]
HAX7126694.1	Type1.BREX	<-TetR-HTH<-?<-?<-REase+iREase||BrxA-DNA-binding->BrxB-BrxF-iSTAND->DUF6079*->N6-METHYLASE->PglZ->SIGMA+OB-Fold+AAA-ATPase+Lon-Protease-BrxL-><-ZnR<-ZnR<-?||RHH_antitoxin->XIS-HTH->	1213	Bacteria>Pseudomonadota>Gammaproteobacteria>Enterobacterales>Enterobacteriaceae>Escherichia	GCA_017046015.1	Escherichia_coli	JTN94_003485	7595..11236	562	BREX-system-P-loop-protein-BrxC-[Escherichia-coli]
EHC4928901.1	Type1.BREX	BrxA-DNA-binding->BrxB-BrxF-iSTAND->DUF6079*->N6-METHYLASE->PglZ->SIGMA+OB-Fold+AAA-ATPase+Lon-Protease-BrxL-><-ZnR<-ZnR	1213	Bacteria>Pseudomonadota>Gammaproteobacteria>Enterobacterales>Enterobacteriaceae>Escherichia	GCA_017057795.1	Escherichia_coli	JS667_004217	1769..5410	562	BREX-system-P-loop-protein-BrxC-[Escherichia-coli]
HAX9732773.1	Type1.BREX	<-Phage_integrase||?->?->?-><-HNH||wHTH+SAD+HNH-><-REase+iREase||BrxA-DNA-binding->BrxB-BrxF-iSTAND->DUF6079*->N6-METHYLASE->PglZ->SIGMA+OB-Fold+AAA-ATPase+Lon-Protease-BrxL->HKD-DNase+OGG-like->||AbiEi_3_N+AbiEi_3->AbiEii-><-DUF6216<-Arm-DNA-bind_3	1213	Bacteria>Pseudomonadota>Gammaproteobacteria>Enterobacterales>Enterobacteriaceae>Escherichia	GCA_017073235.1	Escherichia_coli	JS163_001163	104185..107826	562	BREX-system-P-loop-protein-BrxC-[Escherichia-coli]
HAZ7757191.1	Type1.BREX	Glyoxalase_8-><-?<-?<-VirD4-TraG+VirB4-FtsK<-HDRnase<-SbcC<-SIGMA+OB-Fold+AAA-ATPase+Lon-Protease-BrxL<-PglZ<-HTH_Tnp_1+HTH_21+rve+rve_3<-DUF6079*<-BrxB-BrxF-iSTAND<-BrxA-DNA-binding||DDE_Tnp_IS240-><-wHTH+WYL+WCX<-?<-35exo||LexA-protease->?-><-MobI	1225	Bacteria>Pseudomonadota>Gammaproteobacteria>Enterobacterales>Enterobacteriaceae>Escherichia	GCA_017680345.1	Escherichia_coli	J7089_002325	28074..31751	562	BREX-system-P-loop-protein-BrxC-[Escherichia-coli]
HBA3405375.1	Type1.BREX	ZnR->ZnR-><-SIGMA+OB-Fold+AAA-ATPase+Lon-Protease-BrxL<-PglZ<-N6-METHYLASE<-DUF6079*<-BrxB-BrxF-iSTAND<-BrxA-DNA-binding||REase+iREase->?->?->TetR-HTH->	1213	Bacteria>Pseudomonadota>Gammaproteobacteria>Enterobacterales>Enterobacteriaceae>Escherichia	GCA_017728455.1	Escherichia_coli	J5T69_004101	10657..14298	562	BREX-system-P-loop-protein-BrxC-[Escherichia-coli]
HBA3375039.1	Type1.BREX	<-Phage_integrase||?->?->?-><-HNH||wHTH+SAD+HNH-><-REase+iREase||BrxA-DNA-binding->BrxB-BrxF-iSTAND->DUF6079*->N6-METHYLASE->PglZ->SIGMA+OB-Fold+AAA-ATPase+Lon-Protease-BrxL->HKD-DNase+OGG-like->||AbiEi_3_N+AbiEi_3->AbiEii-><-DUF6216<-Arm-DNA-bind_3	1213	Bacteria>Pseudomonadota>Gammaproteobacteria>Enterobacterales>Enterobacteriaceae>Escherichia	GCA_017728515.1	Escherichia_coli	J8Z50_001951	111486..115127	562	BREX-system-P-loop-protein-BrxC-[Escherichia-coli]
HBA6912638.1	Type1.BREX	<-Phage_integrase||?->?->?-><-HNH||wHTH+SAD+HNH-><-REase+iREase||BrxA-DNA-binding->BrxB-BrxF-iSTAND->DUF6079*->N6-METHYLASE->PglZ->SIGMA+OB-Fold+AAA-ATPase+Lon-Protease-BrxL->HKD-DNase+OGG-like->||AbiEi_3_N+AbiEi_3->AbiEii-><-DUF6216<-Arm-DNA-bind_3	1213	Bacteria>Pseudomonadota>Gammaproteobacteria>Enterobacterales>Enterobacteriaceae>Escherichia	GCA_017762865.1	Escherichia_coli	J1V61_002563	8737..12378	562	BREX-system-P-loop-protein-BrxC-[Escherichia-coli]

<table>
<tr><th>Protein-ID</th><th>System</th><th>Gene-Neighbourhoods</th><th>Length</th><th>Taxonomy</th><th>Assembly-ID</th><th>Strain</th><th>Locus-Tag</th><th>Genomic-coordinates</th><th>TaxID</th><th>Fasta-defline</th></tr>
<tr><td>HBA9095564.1</td><td>Type1.BREX</td><td>AraC-HTH+AraC-HTH-><-DDE_Tnp_IS240||?->ZnR->ZnR-><-SIGMA+OB-Fold+AAA-ATPase+Lon-Protease-BrxL<-PglZ<-N6-METHYLASE<-DUF6079*<-BrxB-BrxF-iSTAND<-BrxA-DNA-binding||REase->REase-><-HEPN<-ORC-CDC6+ZnR<-N6-METHYLASE<-Arm-DNA-bind_3+Phage_integrase</td><td>1213</td><td>Bacteria>Pseudomonadota>Gammaproteobacteria>Enterobacterales>Enterobacteriaceae>Escherichia</td><td>GCA_017772965.1</td><td>Escherichia_coli</td><td>J1298_002132</td><td>65423..69064</td><td>562</td><td>BREX-system-P-loop-protein-BrxC-[Escherichia-coli]</td></tr>
<tr><td>HBB1458964.1</td><td>Type1.BREX</td><td><-XIS-HTH||?->?->ZnR->ZnR-><-SIGMA+OB-Fold+AAA-ATPase+Lon-Protease-BrxL<-PglZ<-N6-METHYLASE<-DUF6079*<-BrxB-BrxF-iSTAND<-BrxA-DNA-binding||REase+iREase-><-EVE+HNH<-Arm-DNA-bind_3+Phage_integrase<-tRNA||?->HerA/FtsK+A32-></td><td>1213</td><td>Bacteria>Pseudomonadota>Gammaproteobacteria>Enterobacterales>Enterobacteriaceae>Escherichia</td><td>GCA_017785795.1</td><td>Escherichia_coli</td><td>J8712_001728</td><td>57793..61434</td><td>562</td><td>BREX-system-P-loop-protein-BrxC-[Escherichia-coli]</td></tr>
<tr><td>HBB1702364.1</td><td>Type1.BREX</td><td><-Phage_integrase||?->?->?-><-HNH||wHTH+SAD+HNH-><-REase+iREase||BrxA-DNA-binding->BrxB-BrxF-iSTAND->DUF6079*-><-?<-?<-?||HTH_6+SIS->||?->?->?->DndB-></td><td>1213</td><td>Bacteria>Pseudomonadota>Gammaproteobacteria>Enterobacterales>Enterobacteriaceae>Escherichia</td><td>GCA_017786745.1</td><td>Escherichia_coli</td><td>J0606_001985</td><td>110955..114596</td><td>562</td><td>BREX-system-P-loop-protein-BrxC-[Escherichia-coli]</td></tr>
<tr><td>HBB3467020.1</td><td>Type1.BREX</td><td>DUF6079-p*->N6-METHYLASE->PglZ->SIGMA+OB-Fold+AAA-ATPase+Lon-Protease-BrxL-><-ZnR<-HTH_Tnp_1+HTH_21+rve+rve_3</td><td>834</td><td>Bacteria>Pseudomonadota>Gammaproteobacteria>Enterobacterales>Enterobacteriaceae>Escherichia</td><td>GCA_017841335.1</td><td>Escherichia_coli</td><td>KAD37_004247</td><td>1..2507</td><td>562</td><td>BREX-system-P-loop-protein-BrxC-[Escherichia-coli]</td></tr>
<tr><td>HBB4280095.1</td><td>Type1.BREX</td><td>DUF6079*->N6-METHYLASE->PglZ->SIGMA+OB-Fold+AAA-ATPase+Lon-Protease-BrxL->HKD-DNase+OGG-like-></td><td>1060</td><td>Bacteria>Pseudomonadota>Gammaproteobacteria>Enterobacterales>Enterobacteriaceae>Escherichia</td><td>GCA_017899135.1</td><td>Escherichia_coli</td><td>KAW34_004647</td><td>1..3185</td><td>562</td><td>BREX-system-P-loop-protein-BrxC-[Escherichia-coli]</td></tr>
<tr><td>EHJ8640897.1</td><td>Type1.BREX</td><td><-Phage_integrase<-Phage_integrase||?->?->?-><-HNH||wHTH(SBD)+HNH->||BrxA-DNA-binding->BrxB-BrxF-iSTAND->DUF6079*->N6-METHYLASE->PglZ->SIGMA+OB-Fold+AAA-ATPase+Lon-Protease-BrxL->HKD-DNase+OGG-like->||AbiEi_3_N+AbiEi_3->AbiEii->?-><-DUF6216<-Arm-DNA-bind_3</td><td>1213</td><td>Bacteria>Pseudomonadota>Gammaproteobacteria>Enterobacterales>Enterobacteriaceae>Escherichia</td><td>GCA_017945505.1</td><td>Escherichia_coli</td><td>KB366_000307</td><td>330961..334602</td><td>562</td><td>BREX-system-P-loop-protein-BrxC-[Escherichia-coli]</td></tr>
<tr><td>EHK0633846.1</td><td>Type1.BREX</td><td><-Phage_integrase||?->?->?-><-HNH||wHTH+SAD+HNH-><-REase+iREase||BrxA-DNA-binding->BrxB-BrxF-iSTAND->DUF6079*->N6-METHYLASE->PglZ->SIGMA+OB-Fold+AAA-ATPase+Lon-Protease-BrxL->HKD-DNase+OGG-like->||AbiEi_3_N+AbiEi_3->AbiEii->?-><-DUF6216<-Arm-DNA-bind_3</td><td>1213</td><td>Bacteria>Pseudomonadota>Gammaproteobacteria>Enterobacterales>Enterobacteriaceae>Escherichia</td><td>GCA_017971565.1</td><td>Escherichia_coli</td><td>KCG30_002478</td><td>111267..114908</td><td>562</td><td>BREX-system-P-loop-protein-BrxC-[Escherichia-coli]</td></tr>
<tr><td>EHK3416136.1</td><td>Type1.BREX</td><td>DUF6079*->N6-METHYLASE->PglZ->SIGMA+OB-Fold+AAA-ATPase+Lon-Protease-BrxL->HKD-DNase+OGG-like->||AbiEi_3_N+AbiEi_3->AbiEii->?-><-DUF6216<-Arm-DNA-bind_3</td><td>1126</td><td>Bacteria>Pseudomonadota>Gammaproteobacteria>Enterobacterales>Enterobacteriaceae>Escherichia</td><td>GCA_018020775.1</td><td>Escherichia_coli</td><td>JYE89_003262</td><td>1..3383</td><td>562</td><td>BREX-system-P-loop-protein-BrxC-[Escherichia-coli]</td></tr>
<tr><td>EHK3567501.1</td><td>Type1.BREX</td><td>AAA+wHTH->?-><-SWIM+DUF3670+SF1-SF2-Helicase<-?<-?<-?||BrxA-DNA-binding->BrxB-BrxF-iSTAND->DUF6079*->N6-METHYLASE->PglZ->SIGMA+OB-Fold+AAA-ATPase+Lon-Protease-BrxL-><-DDE_Tnp_IS1</td><td>1213</td><td>Bacteria>Pseudomonadota>Gammaproteobacteria>Enterobacterales>Enterobacteriaceae>Escherichia</td><td>GCA_018022115.1</td><td>Escherichia_coli</td><td>JT624_003007</td><td>41653..45294</td><td>562</td><td>BREX-system-P-loop-protein-BrxC-[Escherichia-coli]</td></tr>
<tr><td>EHK3830299.1</td><td>Type1.BREX</td><td>HTH_Tnp_1+HTH_21+rve+rve_3->wHTH+WYL+WCX->BrxA-DNA-binding->BrxB-BrxF-iSTAND->DUF6079*->ParB+HNH->N6-METHYLASE->PglZ->SIGMA+OB-Fold+AAA-ATPase+Lon-Protease-BrxL-><-DEDD_Tnp_IS110+Transposase_20</td><td>1225</td><td>Bacteria>Pseudomonadota>Gammaproteobacteria>Enterobacterales>Enterobacteriaceae>Escherichia</td><td>GCA_018024135.1</td><td>Escherichia_coli</td><td>JR760_003915</td><td>2875..6552</td><td>562</td><td>BREX-system-P-loop-protein-BrxC-[Escherichia-coli]</td></tr>
<tr><td>EHL0175571.1</td><td>Type1.BREX</td><td><-DUF6079*<-BrxB-BrxF-iSTAND<-BrxA-DNA-binding||<-wHTH(SBD)+HNH||HNH-><-?<-?<-?||Phage_integrase->Phage_integrase-></td><td>1016</td><td>Bacteria>Pseudomonadota>Gammaproteobacteria>Enterobacterales>Enterobacteriaceae>Escherichia</td><td>GCA_018090545.1</td><td>Escherichia_coli</td><td>D7K57_004368</td><td>1..3050</td><td>562</td><td>BREX-system-P-loop-protein-BrxC-[Escherichia-coli]</td></tr>
<tr><td>EHL0363096.1</td><td>Type1.BREX</td><td>DUF6079-p*->N6-METHYLASE->PglZ->SIGMA+OB-Fold+AAA-ATPase+Lon-Protease-BrxL->HKD-DNase+OGG-like->||AbiEi_3_N+AbiEi_3->AbiEii->?-><-DUF6216<-Arm-DNA-bind_3</td><td>921</td><td>Bacteria>Pseudomonadota>Gammaproteobacteria>Enterobacterales>Enterobacteriaceae>Escherichia</td><td>GCA_018090865.1</td><td>Escherichia_coli</td><td>D1H14_003269</td><td>1..2766</td><td>562</td><td>BREX-system-P-loop-protein-BrxC-[Escherichia-coli]</td></tr>
<tr><td>EHL0676199.1</td><td>Type1.BREX</td><td><-Phage_integrase<-Phage_integrase||?->?->?-><-HNH||wHTH(SBD)+HNH->||BrxA-DNA-binding->BrxB-BrxF-iSTAND->DUF6079*->N6-METHYLASE-></td><td>1213</td><td>Bacteria>Pseudomonadota>Gammaproteobacteria>Enterobacterales>Enterobacteriaceae>Escherichia</td><td>GCA_018092345.1</td><td>Escherichia_coli</td><td>CP667_004253</td><td>9829..13470</td><td>562</td><td>BREX-system-P-loop-protein-BrxC-[Escherichia-coli]</td></tr>
<tr><td>EHL0920537.1</td><td>Type1.BREX</td><td>DUF6079-p*->N6-METHYLASE->PglZ->SIGMA+OB-Fold+AAA-ATPase+Lon-Protease-BrxL->HKD-DNase+OGG-like->||AbiEi_3_N+AbiEi_3->AbiEii->?-><-DUF6216<-Arm-DNA-bind_3</td><td>767</td><td>Bacteria>Pseudomonadota>Gammaproteobacteria>Enterobacterales>Enterobacteriaceae>Escherichia</td><td>GCA_018093285.1</td><td>Escherichia_coli</td><td>CFF49_003158</td><td>1..2306</td><td>562</td><td>BREX-system-P-loop-protein-BrxC-[Escherichia-coli]</td></tr>
<tr><td>EHL1132628.1</td><td>Type1.BREX</td><td>DUF6079-p*->N6-METHYLASE->PglZ->SIGMA+OB-Fold+AAA-ATPase+Lon-Protease-BrxL->HKD-DNase+OGG-like->||AbiEi_3_N+AbiEi_3->AbiEii->?-><-DUF6216<-Arm-DNA-bind_3</td><td>752</td><td>Bacteria>Pseudomonadota>Gammaproteobacteria>Enterobacterales>Enterobacteriaceae>Escherichia</td><td>GCA_018094045.1</td><td>Escherichia_coli</td><td>BIB01_002904</td><td>1..2259</td><td>562</td><td>BREX-system-P-loop-protein-BrxC-[Escherichia-coli]</td></tr>
<tr><td>EHL1134570.1</td><td>Type1.BREX</td><td><-DUF6079-p*<-BrxB-BrxF-iSTAND<-BrxA-DNA-binding||<-wHTH(SBD)+HNH||HNH-><-?<-?<-?||Phage_integrase->Phage_integrase-></td><td>460</td><td>Bacteria>Pseudomonadota>Gammaproteobacteria>Enterobacterales>Enterobacteriaceae>Escherichia</td><td>GCA_018094045.1</td><td>Escherichia_coli</td><td>BIB01_004961</td><td>1..1380</td><td>562</td><td>BREX-system-P-loop-protein-BrxC-[Escherichia-coli]</td></tr>
<tr><td>EHL1162648.1</td><td>Type1.BREX</td><td><-Phage_integrase<-Phage_integrase||?->?->?-><-HNH||wHTH(SBD)+HNH->||BrxA-DNA-binding->BrxB-BrxF-iSTAND->DUF6079*->N6-METHYLASE->PglZ->SIGMA+OB-Fold+AAA-ATPase+Lon-Protease-BrxL->HKD-DNase+OGG-like->||AbiEi_3_N+AbiEi_3->AbiEii->?-><-DUF6216<-Arm-DNA-bind_3</td><td>1213</td><td>Bacteria>Pseudomonadota>Gammaproteobacteria>Enterobacterales>Enterobacteriaceae>Escherichia</td><td>GCA_018094265.1</td><td>Escherichia_coli</td><td>BE892_002471</td><td>14698..18339</td><td>562</td><td>BREX-system-P-loop-protein-BrxC-[Escherichia-coli]</td></tr>
<tr><td>EHL3508970.1</td><td>Type1.BREX</td><td>DUF6079*->N6-METHYLASE->PglZ->SIGMA+OB-Fold+AAA-ATPase+Lon-Protease-BrxL->?-><-?<-ParB<-?||<-REase+N6_METHYLASE<-TRD</td><td>1212</td><td>Bacteria>Pseudomonadota>Gammaproteobacteria>Enterobacterales>Enterobacteriaceae>Escherichia</td><td>GCA_018111065.1</td><td>Escherichia_coli</td><td>KDM69_001438</td><td>1..3639</td><td>562</td><td>BREX-system-P-loop-protein-BrxC-[Escherichia-coli]</td></tr>
<tr><td>EHL6853247.1</td><td>Type1.BREX</td><td><-Phage_integrase||?->?->?-><-HNH||wHTH+SAD+HNH-><-REase+iREase||BrxA-DNA-binding->BrxB-BrxF-iSTAND->DUF6079*->N6-METHYLASE-p-></td><td>1213</td><td>Bacteria>Pseudomonadota>Gammaproteobacteria>Enterobacterales>Enterobacteriaceae>Escherichia</td><td>GCA_018143355.1</td><td>Escherichia_coli</td><td>KEN32_003203</td><td>111251..114892</td><td>562</td><td>BREX-system-P-loop-protein-BrxC-[Escherichia-coli]</td></tr>
<tr><td>HBC6897266.1</td><td>Type1.BREX</td><td>DEDD_Tnp_IS110+Transposase_20-><-RHH||?->wHTH+WYL+WCX->BrxA-DNA-binding->BrxB-BrxF-iSTAND->DUF6079*->ParB+HNH->N6-METHYLASE->PglZ-p-></td><td>1225</td><td>Bacteria>Pseudomonadota>Gammaproteobacteria>Enterobacterales>Enterobacteriaceae>Escherichia</td><td>GCA_018163035.1</td><td>Escherichia_coli</td><td>KEZ94_003915</td><td>5261..8938</td><td>562</td><td>BREX-system-P-loop-protein-BrxC-[Escherichia-coli]</td></tr>
<tr><td>HBC7950668.1</td><td>Type1.BREX</td><td><-Phage_integrase||?->?->?-><-HNH||wHTH+SAD+HNH-><-REase+iREase||BrxA-DNA-binding->BrxB-BrxF-iSTAND->DUF6079*->N6-METHYLASE->PglZ->SIGMA+OB-Fold+AAA-ATPase+Lon-Protease-BrxL->?-><-?<-ParB<-?||<-REase+N6_METHYLASE<-TRD</td><td>1213</td><td>Bacteria>Pseudomonadota>Gammaproteobacteria>Enterobacterales>Enterobacteriaceae>Escherichia</td><td>GCA_018168015.1</td><td>Escherichia_coli</td><td>KEZ88_001190</td><td>111008..114649</td><td>562</td><td>BREX-system-P-loop-protein-BrxC-[Escherichia-coli]</td></tr>
<tr><td>EHM5922455.1</td><td>Type1.BREX</td><td><-HNH||wHTH+SAD+HNH-><-REase+iREase||BrxA-DNA-binding->BrxB-BrxF-iSTAND->DUF6079*->N6-METHYLASE->PglZ->SIGMA+OB-Fold+AAA-ATPase+Lon-Protease-BrxL->HKD-DNase+OGG-like->||AbiEi_3_N+AbiEi_3->AbiEii->?-><-DUF6216<-Arm-DNA-bind_3</td><td>1213</td><td>Bacteria>Pseudomonadota>Gammaproteobacteria>Enterobacterales>Enterobacteriaceae>Escherichia</td><td>GCA_018270935.1</td><td>Escherichia_coli</td><td>KGR67_002708</td><td>6748..10389</td><td>562</td><td>BREX-system-P-loop-protein-BrxC-[Escherichia-coli]</td></tr>
<tr><td>HBD2985690.1</td><td>Type1.BREX</td><td><-REase+iREase||BrxA-DNA-binding->BrxB-BrxF-iSTAND->DUF6079*->N6-METHYLASE->PglZ->SIGMA+OB-Fold+AAA-ATPase+Lon-Protease-BrxL-><-ZnR<-ZnR<-?<-?||RHH_antitoxin->XIS-HTH-></td><td>1213</td><td>Bacteria>Pseudomonadota>Gammaproteobacteria>Enterobacterales>Enterobacteriaceae>Escherichia</td><td>GCA_018351255.1</td><td>Escherichia_coli</td><td>NP403_001237</td><td>2672..6313</td><td>562</td><td>BREX-system-P-loop-protein-BrxC-[Escherichia-coli]</td></tr>
<tr><td>EHN3342070.1</td><td>Type1.BREX</td><td><-Phage_integrase<-Phage_integrase||?->?->?-><-HNH||wHTH(SBD)+HNH->||BrxA-DNA-binding->BrxB-BrxF-iSTAND->DUF6079*->N6-METHYLASE->PglZ->SIGMA+OB-Fold+AAA-ATPase+Lon-Protease-BrxL->HKD-DNase+OGG-like->||AbiEi_3_N+AbiEi_3->AbiEii->?-><-DUF6216<-Arm-DNA-bind_3</td><td>1213</td><td>Bacteria>Pseudomonadota>Gammaproteobacteria>Enterobacterales>Enterobacteriaceae>Escherichia</td><td>GCA_018356405.1</td><td>Escherichia_coli</td><td>KIR71_003047</td><td>14697..18338</td><td>562</td><td>BREX-system-P-loop-protein-BrxC-[Escherichia-coli]</td></tr>
<tr><td>HBD3449422.1</td><td>Type1.BREX</td><td><-N6-METHYLASE-p<-ParB+HNH<-DUF6079*<-BrxB-BrxF-iSTAND<-BrxA-DNA-binding<-wHTH+WYL+WCX<-Arm-DNA-bind_3+Phage_integrase||?-><-?||?->METHYLASE-></td><td>1225</td><td>Bacteria>Pseudomonadota>Gammaproteobacteria>Enterobacterales>Enterobacteriaceae>Escherichia</td><td>GCA_018390165.1</td><td>Escherichia_coli</td><td>KMB60_001824</td><td>3370..7047</td><td>562</td><td>BREX-system-P-loop-protein-BrxC-[Escherichia-coli]</td></tr>
<tr><td>GIP82493.1</td><td>Type1.BREX</td><td><-Peptidase_M17_N+Peptidase_M17||?->?-><-HerA/FtsK+A32||tRNA->Arm-DNA-bind_3+Phage_integrase->EVE+HNH-><-REase+iREase||BrxA-DNA-binding->BrxB-BrxF-iSTAND->DUF6079*->N6-METHYLASE->PglZ->SIGMA+OB-Fold+AAA-ATPase+Lon-Protease-BrxL-><-ZnR<-ZnR<-?||RHH_antitoxin->XIS-HTH-></td><td>1213</td><td>Bacteria>Pseudomonadota>Gammaproteobacteria>Enterobacterales>Enterobacteriaceae>Escherichia</td><td>GCA_018403555.1</td><td>Escherichia_coli</td><td>pm083_35810</td><td>54245..57886</td><td>562</td><td>ATPase-like-protein-[Escherichia-coli]</td></tr>
<tr><td>HBD4982009.1</td><td>Type1.BREX</td><td><-XIS-HTH<-RHH_antitoxin||?->?->ZnR->ZnR-><-SIGMA+OB-Fold+AAA-ATPase+Lon-Protease-BrxL<-PglZ<-N6-METHYLASE<-DUF6079*<-BrxB-BrxF-iSTAND<-BrxA-DNA-binding||REase+iREase-></td><td>1213</td><td>Bacteria>Pseudomonadota>Gammaproteobacteria>Enterobacterales>Enterobacteriaceae>Escherichia</td><td>GCA_018458945.1</td><td>Escherichia_coli</td><td>KKK67_004276</td><td>14430..18071</td><td>562</td><td>BREX-system-P-loop-protein-BrxC-[Escherichia-coli]</td></tr>
<tr><td>HBD5250199.1</td><td>Type1.BREX</td><td><-Phage_integrase||Arm-DNA-bind_3->DUF6216-><-AbiEii<-AbiEi_3_N+AbiEi_3||<-HKD-DNase+OGG-like<-SIGMA+OB-Fold+AAA-ATPase+Lon-Protease-BrxL<-PglZ<-N6-METHYLASE<-DUF6079*</td><td>1060</td><td>Bacteria>Pseudomonadota>Gammaproteobacteria>Enterobacterales>Enterobacteriaceae>Escherichia</td><td>GCA_018460085.1</td><td>Escherichia_coli</td><td>KKL26_003784</td><td>28719..31903</td><td>562</td><td>BREX-system-P-loop-protein-BrxC-[Escherichia-coli]</td></tr>
<tr><td>HBD5388086.1</td><td>Type1.BREX</td><td><-HIN-HTH<-RHH_antitoxin||?->?->ZnR->ZnR-><-SIGMA+OB-Fold+AAA-ATPase+Lon-Protease-BrxL<-PglZ<-N6-METHYLASE<-DUF6079*<-BrxB-BrxF-iSTAND<-BrxA-DNA-binding||REase->REase-></td><td>1213</td><td>Bacteria>Pseudomonadota>Gammaproteobacteria>Enterobacterales>Enterobacteriaceae>Escherichia</td><td>GCA_018460465.1</td><td>Escherichia_coli</td><td>KKL44_003112</td><td>108799..112440</td><td>562</td><td>BREX-system-P-loop-protein-BrxC-[Escherichia-coli]</td></tr>
<tr><td>HBE5478594.1</td><td>Type1.BREX</td><td>DUF6079*->N6-METHYLASE->PglZ->SIGMA+OB-Fold+AAA-ATPase+Lon-Protease-BrxL-></td><td>1064</td><td>Bacteria>Pseudomonadota>Gammaproteobacteria>Enterobacterales>Enterobacteriaceae>Escherichia</td><td>GCA_018566785.1</td><td>Escherichia_coli</td><td>KMB17_004111</td><td>1..3196</td><td>562</td><td>BREX-system-P-loop-protein-BrxC-[Escherichia-coli]</td></tr>
<tr><td>HBE6046090.1</td><td>Type1.BREX</td><td><-Phage_integrase||?->?->?-><-HNH||wHTH+SAD+HNH-><-REase+iREase||BrxA-DNA-binding->BrxB-BrxF-iSTAND->DUF6079*->N6-METHYLASE->PglZ->SIGMA+OB-Fold+AAA-ATPase+Lon-Protease-BrxL->?-><-?<-ParB<-?||<-REase+N6_METHYLASE<-TRD</td><td>1213</td><td>Bacteria>Pseudomonadota>Gammaproteobacteria>Enterobacterales>Enterobacteriaceae>Escherichia</td><td>GCA_018577025.1</td><td>Escherichia_coli</td><td>KMG52_000552</td><td>111009..114650</td><td>562</td><td>BREX-system-P-loop-protein-BrxC-[Escherichia-coli]</td></tr>
<tr><td>EHQ7823790.1</td><td>Type1.BREX</td><td>DUF6079-p*->N6-METHYLASE->PglZ->SIGMA+OB-Fold+AAA-ATPase+Lon-Protease-BrxL->HKD-DNase+OGG-like->||AbiEi_3_N+AbiEi_3->AbiEii->?-><-DUF6216<-Arm-DNA-bind_3</td><td>927</td><td>Bacteria>Pseudomonadota>Gammaproteobacteria>Enterobacterales>Enterobacteriaceae>Escherichia</td><td>GCA_018975885.1</td><td>Escherichia_coli</td><td>KSS40_002990</td><td>1..2786</td><td>562</td><td>BREX-system-P-loop-protein-BrxC-[Escherichia-coli]</td></tr>
<tr><td>HBH4475334.1</td><td>Type1.BREX</td><td><-Zn_Tnp_IS1+HTH_Tnp_IS1||BrxA-DNA-binding->BrxB-BrxF-iSTAND->DUF6079*->PglZ->SIGMA+OB-Fold+AAA-ATPase+Lon-Protease-BrxL-></td><td>1213</td><td>Bacteria>Pseudomonadota>Gammaproteobacteria>Enterobacterales>Enterobacteriaceae>Escherichia</td><td>GCA_019001705.1</td><td>Escherichia_coli</td><td>KTE98_004875</td><td>1931..5572</td><td>562</td><td>BREX-system-P-loop-protein-BrxC-[Escherichia-coli]</td></tr>
<tr><td>EHR8027111.1</td><td>Type1.BREX</td><td><-REase+iREase||BrxA-DNA-binding->BrxB-BrxF-iSTAND->DUF6079*->N6-METHYLASE->PglZ->SIGMA+OB-Fold+AAA-ATPase+Lon-Protease-BrxL-></td><td>1213</td><td>Bacteria>Pseudomonadota>Gammaproteobacteria>Enterobacterales>Enterobacteriaceae>Escherichia</td><td>GCA_019077505.1</td><td>Escherichia_coli</td><td>KUT12_004099</td><td>2676..6317</td><td>562</td><td>BREX-system-P-loop-protein-BrxC-[Escherichia-coli]</td></tr>
<tr><td>HBH7337791.1</td><td>Type1.BREX</td><td>BrxA-DNA-binding->BrxB-BrxF-iSTAND->DUF6079*->N6-METHYLASE->PglZ->SIGMA+OB-Fold+AAA-ATPase+Lon-Protease-BrxL-><-ZnR<-ZnR<-HIN-HTH<-?<-?||RHH_antitoxin-></td><td>1213</td><td>Bacteria>Pseudomonadota>Gammaproteobacteria>Enterobacterales>Enterobacteriaceae>Escherichia</td><td>GCA_019178095.1</td><td>Escherichia_coli</td><td>KWY89_004355</td><td>1906..5547</td><td>562</td><td>BREX-system-P-loop-protein-BrxC-[Escherichia-coli]</td></tr>
<tr><td>EHW5636696.1</td><td>Type1.BREX</td><td><-Phage_integrase||?->?->?-><-HNH||wHTH+SAD+HNH-><-REase+iREase||BrxA-DNA-binding->BrxB-BrxF-iSTAND->DUF6079*->N6-METHYLASE->PglZ->SIGMA+OB-Fold+AAA-ATPase+Lon-Protease-BrxL->HKD-DNase+OGG-like->||AbiEi_3_N+AbiEi_3->AbiEii->?-><-DUF6216<-Arm-DNA-bind_3</td><td>1213</td><td>Bacteria>Pseudomonadota>Gammaproteobacteria>Enterobacterales>Enterobacteriaceae>Escherichia</td><td>GCA_019482305.1</td><td>Escherichia_coli</td><td>K2955_003127</td><td>35708..39349</td><td>562</td><td>BREX-system-P-loop-protein-BrxC-[Escherichia-coli]</td></tr>
<tr><td>EHX2153194.1</td><td>Type1.BREX</td><td>BrxA-DNA-binding->BrxB-BrxF-iSTAND->DUF6079*->N6-METHYLASE->PglZ->SIGMA+OB-Fold+AAA-ATPase+Lon-Protease-BrxL->HKD-DNase+OGG-like->||AbiEi_3_N+AbiEi_3->AbiEii->?-><-DUF6216<-Arm-DNA-bind_3</td><td>1213</td><td>Bacteria>Pseudomonadota>Gammaproteobacteria>Enterobacterales>Enterobacteriaceae>Escherichia</td><td>GCA_019516635.1</td><td>Escherichia_coli</td><td>K3P68_001530</td><td>1396..5037</td><td>562</td><td>BREX-system-P-loop-protein-BrxC-[Escherichia-coli]</td></tr>
<tr><td>EHX8392339.1</td><td>Type1.BREX</td><td>Arm-DNA-bind_3->DUF6216-><-?<-AbiEii<-AbiEi_3_N+AbiEi_3||<-HKD-DNase+OGG-like<-SIGMA+OB-Fold+AAA-ATPase+Lon-Protease-BrxL<-PglZ<-DUF6079*<-BrxB-BrxF-iSTAND<-BrxA-DNA-binding||REase+iREase-><-wHTH+SAD+HNH||HNH-><-?<-?<-?||Phage_integrase-></td><td>1213</td><td>Bacteria>Pseudomonadota>Gammaproteobacteria>Enterobacterales>Enterobacteriaceae>Escherichia</td><td>GCA_019550135.1</td><td>Escherichia_coli</td><td>K3368_004098</td><td>15146..18787</td><td>562</td><td>BREX-system-P-loop-protein-BrxC-[Escherichia-coli]</td></tr>
<tr><td>EHX8476217.1</td><td>Type1.BREX</td><td><-Phage_integrase||?->?->?-><-HNH||wHTH+SAD+HNH-><-REase+iREase||BrxA-DNA-binding->BrxB-BrxF-iSTAND->DUF6079*->N6-METHYLASE->PglZ->SIGMA+OB-Fold+AAA-ATPase+Lon-Protease-BrxL->HKD-DNase+OGG-like->||AbiEi_3_N+AbiEi_3->AbiEii->?-><-DUF6216<-Arm-DNA-bind_3</td><td>1213</td><td>Bacteria>Pseudomonadota>Gammaproteobacteria>Enterobacterales>Enterobacteriaceae>Escherichia</td><td>GCA_019550495.1</td><td>Escherichia_coli</td><td>K3324_002225</td><td>23193..26834</td><td>562</td><td>BREX-system-P-loop-protein-BrxC-[Escherichia-coli]</td></tr>
<tr><td>EHY5030619.1</td><td>Type1.BREX</td><td><-wHTH+WYL<-?||XIS-HTH->?->BrxA-DNA-binding->BrxB-BrxF-iSTAND->DUF6079*->ParB+HNH->N6-METHYLASE->PglZ->SIGMA+OB-Fold+AAA-ATPase+Lon-Protease-BrxL-><-Arm-DNA-bind_3+Phage_integrase||Phage_integrase-></td><td>1233</td><td>Bacteria>Pseudomonadota>Gammaproteobacteria>Enterobacterales>Enterobacteriaceae>Escherichia</td><td>GCA_019582855.1</td><td>Escherichia_coli</td><td>K4I20_002754</td><td>15002..18703</td><td>562</td><td>BREX-system-P-loop-protein-BrxC-[Escherichia-coli]</td></tr>
<tr><td>EHY5298658.1</td><td>Type1.BREX</td><td>DUF6079-p*->N6-METHYLASE->PglZ->SIGMA+OB-Fold+AAA-ATPase+Lon-Protease-BrxL->HKD-DNase+OGG-like->||AbiEi_3_N+AbiEi_3->AbiEii->?-><-DUF6216<-Arm-DNA-bind_3</td><td>861</td><td>Bacteria>Pseudomonadota>Gammaproteobacteria>Enterobacterales>Enterobacteriaceae>Escherichia</td><td>GCA_019588505.1</td><td>Escherichia_coli</td><td>K4I57_002534</td><td>1..2588</td><td>562</td><td>BREX-system-P-loop-protein-BrxC-[Escherichia-coli]</td></tr>
<tr><td>EHY5690081.1</td><td>Type1.BREX</td><td><-Phage_integrase<-Phage_integrase||?->?->?-><-HNH||wHTH(SBD)+HNH->||BrxA-DNA-binding->BrxB-BrxF-iSTAND->DUF6079*->N6-METHYLASE->PglZ->SIGMA+OB-Fold+AAA-ATPase+Lon-Protease-BrxL->HKD-DNase+OGG-like->||AbiEi_3_N+AbiEi_3->AbiEii->?-><-DUF6216<-Arm-DNA-bind_3</td><td>1213</td><td>Bacteria>Pseudomonadota>Gammaproteobacteria>Enterobacterales>Enterobacteriaceae>Escherichia</td><td>GCA_019590565.1</td><td>Escherichia_coli</td><td>K4J54_002559</td><td>14696..18337</td><td>562</td><td>BREX-system-P-loop-protein-BrxC-[Escherichia-coli]</td></tr>
<tr><td>EHZ2564701.1</td><td>Type1.BREX</td><td>DUF6079*->N6-METHYLASE->PglZ->SIGMA+OB-Fold+AAA-ATPase+Lon-Protease-BrxL->HKD-DNase+OGG-like->||AbiEi_3_N+AbiEi_3->AbiEii->?-><-DUF6216<-Arm-DNA-bind_3</td><td>1130</td><td>Bacteria>Pseudomonadota>Gammaproteobacteria>Enterobacterales>Enterobacteriaceae>Escherichia</td><td>GCA_019681855.1</td><td>Escherichia_coli</td><td>K5N16_003075</td><td>1..3394</td><td>562</td><td>BREX-system-P-loop-protein-BrxC-[Escherichia-coli]</td></tr>
<tr><td>EHZ4708255.1</td><td>Type1.BREX</td><td><-Phage_integrase<-Phage_integrase||?->?->?->||wHTH+SAD+HNH-><-REase+iREase||BrxA-DNA-binding->BrxB-BrxF-iSTAND->DUF6079*->N6-METHYLASE->PglZ->SIGMA+OB-Fold+AAA-ATPase+Lon-Protease-BrxL-></td><td>1213</td><td>Bacteria>Pseudomonadota>Gammaproteobacteria>Enterobacterales>Enterobacteriaceae>Escherichia</td><td>GCA_019701545.1</td><td>Escherichia_coli</td><td>K5X72_003965</td><td>35776..39417</td><td>562</td><td>BREX-system-P-loop-protein-BrxC-[Escherichia-coli]</td></tr>
<tr><td>EIB0293606.1</td><td>Type1.BREX</td><td><-DUF6079-p*<-BrxB-BrxF-iSTAND<-BrxA-DNA-binding||<-wHTH(SBD)+HNH||HNH-><-?<-?<-?||Phage_integrase->Phage_integrase-></td><td>529</td><td>Bacteria>Pseudomonadota>Gammaproteobacteria>Enterobacterales>Enterobacteriaceae>Escherichia</td><td>GCA_019848435.1</td><td>Escherichia_coli</td><td>K7644_004610</td><td>1..1589</td><td>562</td><td>BREX-system-P-loop-protein-BrxC-[Escherichia-coli]</td></tr>
<tr><td>HBI8276592.1</td><td>Type1.BREX</td><td><-METHYLASE||SKI-><-?||tRNA->Arm-DNA-bind_3+Phage_integrase->EVE+HNH-><-REase<-REase||BrxA-DNA-binding->BrxB-BrxF-iSTAND->DUF6079*->N6-METHYLASE->PglZ->SIGMA+OB-Fold+AAA-ATPase+Lon-Protease-BrxL-><-ZnR<-ZnR<-?<-?||RHH_antitoxin->XIS-HTH-></td><td>1213</td><td>Bacteria>Pseudomonadota>Gammaproteobacteria>Enterobacterales>Enterobacteriaceae>Escherichia</td><td>GCA_019886465.1</td><td>Escherichia_coli</td><td>K8321_003292</td><td>148369..152010</td><td>562</td><td>BREX-system-P-loop-protein-BrxC-[Escherichia-coli]</td></tr>
<tr><td>HBJ0235562.1</td><td>Type1.BREX</td><td><-HKD-DNase+OGG-like<-SIGMA+OB-Fold+AAA-ATPase+Lon-Protease-BrxL<-PglZ<-N6-METHYLASE<-DUF6079*<-BrxB-BrxF-iSTAND<-BrxA-DNA-binding||REase+iREase-><-wHTH+SAD+HNH||HNH-></td><td>1213</td><td>Bacteria>Pseudomonadota>Gammaproteobacteria>Enterobacterales>Enterobacteriaceae>Escherichia</td><td>GCA_019980105.1</td><td>Escherichia_coli</td><td>LAM28_003686</td><td>10586..14227</td><td>562</td><td>BREX-system-P-loop-protein-BrxC-[Escherichia-coli]</td></tr>
<tr><td>HBJ1150970.1</td><td>Type1.BREX</td><td>BrxA-DNA-binding->BrxB-BrxF-iSTAND->DUF6079*->N6-METHYLASE->PglZ-><-ZnR<-ZnR<-?<-?||RHH_antitoxin->XIS-HTH-></td><td>1213</td><td>Bacteria>Pseudomonadota>Gammaproteobacteria>Enterobacterales>Enterobacteriaceae>Escherichia</td><td>GCA_019988955.1</td><td>Escherichia_coli</td><td>LAQ35_004277</td><td>1344..4985</td><td>562</td><td>BREX-system-P-loop-protein-BrxC-[Escherichia-coli]</td></tr>
<tr><td>HBJ1306075.1</td><td>Type1.BREX</td><td><-METHYLASE||SKI-><-?||tRNA->Arm-DNA-bind_3+Phage_integrase->EVE+HNH-><-REase+iREase||BrxA-DNA-binding->BrxB-BrxF-iSTAND->DUF6079*->N6-METHYLASE->PglZ->SIGMA+OB-Fold+AAA-ATPase+Lon-Protease-BrxL-><-ZnR</td><td>1213</td><td>Bacteria>Pseudomonadota>Gammaproteobacteria>Enterobacterales>Enterobacteriaceae>Escherichia</td><td>GCA_019996045.1</td><td>Escherichia_coli</td><td>LAS09_001693</td><td>95554..99195</td><td>562</td><td>BREX-system-P-loop-protein-BrxC-[Escherichia-coli]</td></tr>
<tr><td>EID3638522.1</td><td>Type1.BREX</td><td><-Phage_integrase||?->?->?-><-HNH||wHTH+SAD+HNH-><-REase+iREase||BrxA-DNA-binding->BrxB-BrxF-iSTAND->DUF6079*->N6-METHYLASE->PglZ->SIGMA+OB-Fold+AAA-ATPase+Lon-Protease-BrxL->HKD-DNase+OGG-like->||AbiEi_3_N+AbiEi_3->AbiEii->?-><-DUF6216<-Arm-DNA-bind_3</td><td>1213</td><td>Bacteria>Pseudomonadota>Gammaproteobacteria>Enterobacterales>Enterobacteriaceae>Escherichia</td><td>GCA_020041725.1</td><td>Escherichia_coli</td><td>LB206_001712</td><td>80689..84330</td><td>562</td><td>BREX-system-P-loop-protein-BrxC-[Escherichia-coli]</td></tr>
<tr><td>EID8685857.1</td><td>Type1.BREX</td><td>Arm-DNA-bind_3->DUF6216-><-?<-AbiEii<-AbiEi_3_N+AbiEi_3||<-HKD-DNase+OGG-like<-SIGMA+OB-Fold+AAA-ATPase+Lon-Protease-BrxL<-PglZ<-N6-METHYLASE<-DUF6079-p*</td><td>949</td><td>Bacteria>Pseudomonadota>Gammaproteobacteria>Enterobacterales>Enterobacteriaceae>Escherichia</td><td>GCA_020094635.1</td><td>Escherichia_coli</td><td>LCI88_002092</td><td>151835..154685</td><td>562</td><td>BREX-system-P-loop-protein-BrxC-[Escherichia-coli]</td></tr>
<tr><td>EID8792051.1</td><td>Type1.BREX</td><td>wHTH+WYL+WCX->BrxA-DNA-binding->BrxB-BrxF-iSTAND->DUF6079*->ParB+HNH->N6-METHYLASE->PglZ->SIGMA+OB-Fold+AAA-ATPase+Lon-Protease-BrxL-><-HTH_Tnp_1+HTH_21+rve+rve_3<-HIN-HTH<-PIN<-MazE_antitoxin</td><td>1225</td><td>Bacteria>Pseudomonadota>Gammaproteobacteria>Enterobacterales>Enterobacteriaceae>Escherichia</td><td>GCA_020095335.1</td><td>Escherichia_coli</td><td>LCJ17_004431</td><td>3670..7347</td><td>562</td><td>BREX-system-P-loop-protein-BrxC-[Escherichia-coli]</td></tr>
<tr><td>MBZ8679488.1</td><td>Type1.BREX</td><td><-Phage_integrase||?->?->?-><-HNH||wHTH+SAD+HNH-><-REase+iREase||BrxA-DNA-binding->BrxB-BrxF-iSTAND->DUF6079*->N6-METHYLASE->PglZ->SIGMA+OB-Fold+AAA-ATPase+Lon-Protease-BrxL->?-><-?<-ParB<-?||<-REase+N6_METHYLASE<-TRD</td><td>1213</td><td>Bacteria>Pseudomonadota>Gammaproteobacteria>Enterobacterales>Enterobacteriaceae>Escherichia</td><td>GCA_020145545.1</td><td>Escherichia_coli</td><td>H5S60_20510</td><td>9779..13420</td><td>562</td><td>BREX-system-P-loop-protein-BrxC-[Escherichia-coli]</td></tr>
<tr><td>EIF2649115.1</td><td>Type1.BREX</td><td>DUF6079*->N6-METHYLASE->PglZ->SIGMA+OB-Fold+AAA-ATPase+Lon-Protease-BrxL-><-ZnR<-ZnR<-?||HIN-HTH+PAIREDC-HTH->||RHH_antitoxin->XIS-HTH-></td><td>1005</td><td>Bacteria>Pseudomonadota>Gammaproteobacteria>Enterobacterales>Enterobacteriaceae>Escherichia</td><td>GCA_020238205.1</td><td>Escherichia_coli</td><td>LFR91_004210</td><td>1..3019</td><td>562</td><td>BREX-system-P-loop-protein-BrxC-[Escherichia-coli]</td></tr>
<tr><td>EIG2625391.1</td><td>Type1.BREX</td><td>DUF6079-p*->N6-METHYLASE->PglZ->SIGMA+OB-Fold+AAA-ATPase+Lon-Protease-BrxL->HKD-DNase+OGG-like->||AbiEi_3_N+AbiEi_3->AbiEii-><-DUF6216<-Arm-DNA-bind_3</td><td>771</td><td>Bacteria>Pseudomonadota>Gammaproteobacteria>Enterobacterales>Enterobacteriaceae>Escherichia</td><td>GCA_020303335.1</td><td>Escherichia_coli</td><td>GLS16_002890</td><td>1..2318</td><td>562</td><td>BREX-system-P-loop-protein-BrxC-[Escherichia-coli]</td></tr>
<tr><td>EIG4139450.1</td><td>Type1.BREX</td><td><-REase+iREase||BrxA-DNA-binding->BrxB-BrxF-iSTAND->DUF6079*->N6-METHYLASE->PglZ->SIGMA+OB-Fold+AAA-ATPase+Lon-Protease-BrxL-><-ZnR<-ZnR<-?<-?||XIS-HTH-></td><td>1213</td><td>Bacteria>Pseudomonadota>Gammaproteobacteria>Enterobacterales>Enterobacteriaceae>Escherichia</td><td>GCA_020309325.1</td><td>Escherichia_coli</td><td>FPR33_004782</td><td>2597..6238</td><td>562</td><td>BREX-system-P-loop-protein-BrxC-[Escherichia-coli]</td></tr>
<tr><td>EIG6470759.1</td><td>Type1.BREX</td><td><-DUF6079-p*<-BrxB-BrxF-iSTAND<-BrxA-DNA-binding||<-wHTH(SBD)+HNH||HNH-><-?<-?<-?||Phage_integrase->Phage_integrase-></td><td>634</td><td>Bacteria>Pseudomonadota>Gammaproteobacteria>Enterobacterales>Enterobacteriaceae>Escherichia</td><td>GCA_020319175.1</td><td>Escherichia_coli</td><td>EG665_005071</td><td>1..1903</td><td>562</td><td>BREX-system-P-loop-protein-BrxC-[Escherichia-coli]</td></tr>
<tr><td>EIG7860374.1</td><td>Type1.BREX</td><td>DUF6079*->N6-METHYLASE->PglZ->SIGMA+OB-Fold+AAA-ATPase+Lon-Protease-BrxL->HKD-DNase+OGG-like->||AbiEi_3_N+AbiEi_3->AbiEii->?-><-DUF6216<-Arm-DNA-bind_3</td><td>1077</td><td>Bacteria>Pseudomonadota>Gammaproteobacteria>Enterobacterales>Enterobacteriaceae>Escherichia</td><td>GCA_020324895.1</td><td>Escherichia_coli</td><td>D0584_004201</td><td>1..3234</td><td>562</td><td>BREX-system-P-loop-protein-BrxC-[Escherichia-coli]</td></tr>
<tr><td>HBK2401145.1</td><td>Type1.BREX</td><td><-Phage_integrase||?->?->?-><-HNH||wHTH+SAD+HNH-><-REase+iREase||BrxA-DNA-binding->BrxB-BrxF-iSTAND->DUF6079*-></td><td>1149</td><td>Bacteria>Pseudomonadota>Gammaproteobacteria>Enterobacterales>Enterobacteriaceae>Escherichia</td><td>GCA_020359375.1</td><td>Escherichia_coli</td><td>LGJ14_002758</td><td>111240..114688</td><td>562</td><td>BREX-system-P-loop-protein-BrxC-[Escherichia-coli]</td></tr>
<tr><td>HBK2662992.1</td><td>Type1.BREX</td><td><-Phage_integrase||?->?->?-><-HNH||wHTH+SAD+HNH-><-REase+iREase||BrxA-DNA-binding->BrxB-BrxF-iSTAND->DUF6079-p*-></td><td>626</td><td>Bacteria>Pseudomonadota>Gammaproteobacteria>Enterobacterales>Enterobacteriaceae>Escherichia</td><td>GCA_020362085.1</td><td>Escherichia_coli</td><td>LGK41_002196</td><td>111240..113117</td><td>562</td><td>BREX-system-P-loop-protein-BrxC-[Escherichia-coli]</td></tr>
<tr><td>EIH7117202.1</td><td>Type1.BREX</td><td>DUF6079*->N6-METHYLASE->PglZ->SIGMA+OB-Fold+AAA-ATPase+Lon-Protease-BrxL->HKD-DNase+OGG-like->||AbiEi_3_N+AbiEi_3->AbiEii->?-><-DUF6216<-Arm-DNA-bind_3</td><td>1174</td><td>Bacteria>Pseudomonadota>Gammaproteobacteria>Enterobacterales>Enterobacteriaceae>Escherichia</td><td>GCA_020376505.1</td><td>Escherichia_coli</td><td>BB077_003959</td><td>1..3527</td><td>562</td><td>BREX-system-P-loop-protein-BrxC-[Escherichia-coli]</td></tr>
<tr><td>MCA7636345.1</td><td>Type1.BREX</td><td><-XIS-HTH<-RHH_antitoxin||?->?->ZnR->ZnR-><-SIGMA+OB-Fold+AAA-ATPase+Lon-Protease-BrxL<-PglZ<-N6-METHYLASE<-DUF6079*</td><td>1132</td><td>Bacteria>Pseudomonadota>Gammaproteobacteria>Enterobacterales>Enterobacteriaceae>Escherichia</td><td>GCA_020418475.1</td><td>Escherichia_coli</td><td>LEZ98_21685</td><td>17948..21346</td><td>562</td><td>BREX-system-P-loop-protein-BrxC-[Escherichia-coli]</td></tr>
<tr><td>MCA7852868.1</td><td>Type1.BREX</td><td><-Phage_integrase<-Phage_integrase||?->?->?->||wHTH+SAD+HNH-><-REase+iREase||BrxA-DNA-binding->BrxB-BrxF-iSTAND->DUF6079-p*-></td><td>920</td><td>Bacteria>Pseudomonadota>Gammaproteobacteria>Enterobacterales>Enterobacteriaceae>Escherichia</td><td>GCA_020420865.1</td><td>Escherichia_coli</td><td>LE030_10780</td><td>111187..113946</td><td>562</td><td>BREX-system-P-loop-protein-BrxC-[Escherichia-coli]</td></tr>
<tr><td>EIL3113271.1</td><td>Type1.BREX</td><td><-HerA/FtsK+A32<-?||tRNA->Arm-DNA-bind_3+Phage_integrase->EVE+HNH-><-REase+iREase||BrxA-DNA-binding->BrxB-BrxF-iSTAND->DUF6079*->N6-METHYLASE->PglZ->SIGMA+OB-Fold+AAA-ATPase+Lon-Protease-BrxL-><-ZnR<-ZnR<-?<-?||RHH_antitoxin->XIS-HTH-></td><td>1213</td><td>Bacteria>Pseudomonadota>Gammaproteobacteria>Enterobacterales>Enterobacteriaceae>Escherichia</td><td>GCA_020689665.1</td><td>Escherichia_coli</td><td>LLT94_003844</td><td>18999..22640</td><td>562</td><td>BREX-system-P-loop-protein-BrxC-[Escherichia-coli]</td></tr>
<tr><td>EIN0528288.1</td><td>Type1.BREX</td><td><-GDResolvase+HIN-HTH<-?<-RelE-ParE<-CopG/RHH||<-RHH||?->wHTH+WYL+WCX->BrxA-DNA-binding->BrxB-BrxF-iSTAND->DUF6079-p*-></td><td>671</td><td>Bacteria>Pseudomonadota>Gammaproteobacteria>Enterobacterales>Enterobacteriaceae>Escherichia</td><td>GCA_020842825.1</td><td>Escherichia_coli</td><td>LMH12_004589</td><td>11532..13543</td><td>562</td><td>BREX-system-P-loop-protein-BrxC-[Escherichia-coli]</td></tr>
<tr><td>EIN2019349.1</td><td>Type1.BREX</td><td><-Phage_integrase||?->?->?-><-HNH||wHTH+SAD+HNH-><-REase+iREase||BrxA-DNA-binding->BrxB-BrxF-iSTAND->DUF6079*->N6-METHYLASE->PglZ->SIGMA+OB-Fold+AAA-ATPase+Lon-Protease-BrxL->?-><-?<-ParB<-?||<-REase+N6_METHYLASE<-TRD</td><td>1213</td><td>Bacteria>Pseudomonadota>Gammaproteobacteria>Enterobacterales>Enterobacteriaceae>Escherichia</td><td>GCA_020867105.1</td><td>Escherichia_coli</td><td>LOJ72_000111</td><td>111240..114881</td><td>562</td><td>BREX-system-P-loop-protein-BrxC-[Escherichia-coli]</td></tr>
<tr><td>EIO0019318.1</td><td>Type1.BREX</td><td>DUF6079-p*->N6-METHYLASE->PglZ->SIGMA+OB-Fold+AAA-ATPase+Lon-Protease-BrxL->HKD-DNase+OGG-like->||AbiEi_3_N+AbiEi_3->AbiEii->?-><-DUF6216<-Arm-DNA-bind_3</td><td>816</td><td>Bacteria>Pseudomonadota>Gammaproteobacteria>Enterobacterales>Enterobacteriaceae>Escherichia</td><td>GCA_020946425.1</td><td>Escherichia_coli</td><td>LO099_004443</td><td>1..2453</td><td>562</td><td>BREX-system-P-loop-protein-BrxC-[Escherichia-coli]</td></tr>
<tr><td>EIO1138690.1</td><td>Type1.BREX</td><td><-Phage_integrase||?->?->?-><-HNH||wHTH+SAD+HNH-><-REase+iREase||BrxA-DNA-binding->BrxB-BrxF-iSTAND->DUF6079*->N6-METHYLASE->PglZ->SIGMA+OB-Fold+AAA-ATPase+Lon-Protease-BrxL->?-><-?<-ParB<-?||<-REase+N6_METHYLASE<-TRD</td><td>1213</td><td>Bacteria>Pseudomonadota>Gammaproteobacteria>Enterobacterales>Enterobacteriaceae>Escherichia</td><td>GCA_020952215.1</td><td>Escherichia_coli</td><td>LQD99_001531</td><td>111293..114934</td><td>562</td><td>BREX-system-P-loop-protein-BrxC-[Escherichia-coli]</td></tr>
<tr><td>HBL7859045.1</td><td>Type1.BREX</td><td>DUF6079*->N6-METHYLASE->PglZ->SIGMA+OB-Fold+AAA-ATPase+Lon-Protease-BrxL-><-ZnR<-HTH_Tnp_1+HTH_21+rve+rve_3</td><td>1097</td><td>Bacteria>Pseudomonadota>Gammaproteobacteria>Enterobacterales>Enterobacteriaceae>Escherichia</td><td>GCA_021087345.1</td><td>Escherichia_coli</td><td>LTB56_004870</td><td>1..3294</td><td>562</td><td>BREX-system-P-loop-protein-BrxC-[Escherichia-coli]</td></tr>
<tr><td>EIS3583963.1</td><td>Type1.BREX</td><td><-N6-METHYLASE<-DUF6079*<-BrxB-BrxF-iSTAND<-BrxA-DNA-binding||REase+iREase-><-wHTH+SAD+HNH||HNH-><-?<-?<-?||Phage_integrase-></td><td>1213</td><td>Bacteria>Pseudomonadota>Gammaproteobacteria>Enterobacterales>Enterobacteriaceae>Escherichia</td><td>GCA_021351055.1</td><td>Escherichia_coli</td><td>LZ081_004279</td><td>2281..5922</td><td>562</td><td>BREX-system-P-loop-protein-BrxC-[Escherichia-coli]</td></tr>
<tr><td>EIS4879897.1</td><td>Type1.BREX</td><td>Arm-DNA-bind_3+Phage_integrase->N6-METHYLASE->ORC-CDC6+ZnR->HEPN-><-REase<-REase||BrxA-DNA-binding->BrxB-BrxF-iSTAND->DUF6079*->N6-METHYLASE->PglZ->SIGMA+OB-Fold+AAA-ATPase+Lon-Protease-BrxL-><-ZnR<-ZnR<-?||RHH_antitoxin->XIS-HTH-></td><td>1213</td><td>Bacteria>Pseudomonadota>Gammaproteobacteria>Enterobacterales>Enterobacteriaceae>Escherichia</td><td>GCA_021362895.1</td><td>Escherichia_coli</td><td>LZX86_002736</td><td>36303..39944</td><td>562</td><td>BREX-system-P-loop-protein-BrxC-[Escherichia-coli]</td></tr>
<tr><td>HBM7577416.1</td><td>Type1.BREX</td><td><-REase+iREase||BrxA-DNA-binding->BrxB-BrxF-iSTAND->DUF6079*->N6-METHYLASE->PglZ->SIGMA+OB-Fold+AAA-ATPase+Lon-Protease-BrxL-></td><td>1213</td><td>Bacteria>Pseudomonadota>Gammaproteobacteria>Enterobacterales>Enterobacteriaceae>Escherichia</td><td>GCA_021378865.1</td><td>Escherichia_coli</td><td>LZR97_004339</td><td>2197..5838</td><td>562</td><td>BREX-system-P-loop-protein-BrxC-[Escherichia-coli]</td></tr>
<tr><td>HBM8747261.1</td><td>Type1.BREX</td><td><-Phage_integrase||?->?->?-><-HNH||wHTH+SAD+HNH-><-REase+iREase||BrxA-DNA-binding->BrxB-BrxF-iSTAND->DUF6079*->N6-METHYLASE->PglZ->SIGMA+OB-Fold+AAA-ATPase+Lon-Protease-BrxL->HKD-DNase+OGG-like->||AbiEi_3_N+AbiEi_3->AbiEii->?-><-DUF6216<-Arm-DNA-bind_3</td><td>1213</td><td>Bacteria>Pseudomonadota>Gammaproteobacteria>Enterobacterales>Enterobacteriaceae>Escherichia</td><td>GCA_021417505.1</td><td>Escherichia_coli</td><td>LOV31_003260</td><td>39874..43515</td><td>562</td><td>BREX-system-P-loop-protein-BrxC-[Escherichia-coli]</td></tr>
<tr><td>HBM8228633.1</td><td>Type1.BREX</td><td>TRD->REase+N6_METHYLASE-><-?||?->ParB->?-><-?<-SIGMA+OB-Fold+AAA-ATPase+Lon-Protease-BrxL<-PglZ<-N6-METHYLASE<-DUF6079*<-BrxB-BrxF-iSTAND<-BrxA-DNA-binding||REase+iREase-><-wHTH+SAD+HNH||HNH-><-?<-?<-?||Phage_integrase-></td><td>1213</td><td>Bacteria>Pseudomonadota>Gammaproteobacteria>Enterobacterales>Enterobacteriaceae>Escherichia</td><td>GCA_021429145.1</td><td>Escherichia_coli</td><td>LOW06_003442</td><td>20140..23781</td><td>562</td><td>BREX-system-P-loop-protein-BrxC-[Escherichia-coli]</td></tr>
<tr><td>HBN0250039.1</td><td>Type1.BREX</td><td><-DUF932<-ArdB<-?||?->XIS-HTH->ZnR->wHTH+WYL+WCX->BrxA-DNA-binding->BrxB-BrxF-iSTAND->DUF6079*->ParB+HNH->N6-METHYLASE->PglZ->SIGMA+OB-Fold+AAA-ATPase+Lon-Protease-BrxL->35exo->DUF772+DDE_Tnp_1->OBFOLD-NUCLEASE-></td><td>1225</td><td>Bacteria>Pseudomonadota>Gammaproteobacteria>Enterobacterales>Enterobacteriaceae>Escherichia</td><td>GCA_021454405.1</td><td>Escherichia_coli</td><td>L1L95_003624</td><td>57124..60801</td><td>562</td><td>BREX-system-P-loop-protein-BrxC-[Escherichia-coli]</td></tr>
<tr><td>HBN0573063.1</td><td>Type1.BREX</td><td>Arm-DNA-bind_3->DUF6216-><-?<-AbiEii<-AbiEi_3_N+AbiEi_3||<-HKD-DNase+OGG-like<-SIGMA+OB-Fold+AAA-ATPase+Lon-Protease-BrxL<-PglZ<-N6-METHYLASE<-DUF6079*<-BrxB-BrxF-iSTAND<-BrxA-DNA-binding||REase+iREase-><-wHTH+SAD+HNH||HNH-><-?<-?<-?||Phage_integrase-></td><td>1213</td><td>Bacteria>Pseudomonadota>Gammaproteobacteria>Enterobacterales>Enterobacteriaceae>Escherichia</td><td>GCA_021454505.1</td><td>Escherichia_coli</td><td>L1N12_002093</td><td>35071..38712</td><td>562</td><td>BREX-system-P-loop-protein-BrxC-[Escherichia-coli]</td></tr>
<tr><td>HBN0877260.1</td><td>Type1.BREX</td><td><-XIS-HTH||?->?->ZnR->ZnR-><-SIGMA+OB-Fold+AAA-ATPase+Lon-Protease-BrxL<-PglZ<-N6-METHYLASE<-DUF6079*<-BrxB-BrxF-iSTAND<-BrxA-DNA-binding||REase+iREase-></td><td>1213</td><td>Bacteria>Pseudomonadota>Gammaproteobacteria>Enterobacterales>Enterobacteriaceae>Escherichia</td><td>GCA_021468605.1</td><td>Escherichia_coli</td><td>L1V71_000763</td><td>365927..369568</td><td>562</td><td>BREX-system-P-loop-protein-BrxC-[Escherichia-coli]</td></tr>
<tr><td>HBN1947859.1</td><td>Type1.BREX</td><td>BrxA-DNA-binding->BrxB-BrxF-iSTAND->DUF6079*->N6-METHYLASE->PglZ->SIGMA+OB-Fold+AAA-ATPase+Lon-Protease-BrxL-></td><td>1213</td><td>Bacteria>Pseudomonadota>Gammaproteobacteria>Enterobacterales>Enterobacteriaceae>Escherichia</td><td>GCA_021489855.1</td><td>Escherichia_coli</td><td>L1401_003657</td><td>2289..5930</td><td>562</td><td>BREX-system-P-loop-protein-BrxC-[Escherichia-coli]</td></tr>
<tr><td>HBP9919082.1</td><td>Type1.BREX</td><td><-Phage_integrase<-Phage_integrase||?->?->?-><-HNH||wHTH(SBD)+HNH->||BrxA-DNA-binding->BrxB-BrxF-iSTAND->DUF6079*->N6-METHYLASE->PglZ->SIGMA+OB-Fold+AAA-ATPase+Lon-Protease-BrxL->HKD-DNase+OGG-like->||AbiEi_3_N+AbiEi_3->AbiEii->?-><-DUF6216<-Arm-DNA-bind_3</td><td>1213</td><td>Bacteria>Pseudomonadota>Gammaproteobacteria>Enterobacterales>Enterobacteriaceae>Escherichia</td><td>GCA_021766545.1</td><td>Escherichia_coli</td><td>L7G70_002290</td><td>14696..18337</td><td>562</td><td>BREX-system-P-loop-protein-BrxC-[Escherichia-coli]</td></tr>
<tr><td>HBQ4241594.1</td><td>Type1.BREX</td><td><-N6-METHYLASE<-DUF6079*<-BrxB-BrxF-iSTAND<-BrxA-DNA-binding||REase+iREase-><-EVE+HNH<-Arm-DNA-bind_3+Phage_integrase<-tRNA||?-><-SKI||METHYLASE-></td><td>1213</td><td>Bacteria>Pseudomonadota>Gammaproteobacteria>Enterobacterales>Enterobacteriaceae>Escherichia</td><td>GCA_021857785.1</td><td>Escherichia_coli</td><td>L7222_003680</td><td>2636..6277</td><td>562</td><td>BREX-system-P-loop-protein-BrxC-[Escherichia-coli]</td></tr>
<tr><td>HBV0129404.1</td><td>Type1.BREX</td><td><-DUF6079-p*<-BrxB-BrxF-iSTAND<-BrxA-DNA-binding||REase+iREase->?->?->TetR-HTH-><-AAA-ATPase<-HIN-HTH<-AraC-HTH</td><td>804</td><td>Bacteria>Pseudomonadota>Gammaproteobacteria>Enterobacterales>Enterobacteriaceae>Escherichia</td><td>GCA_022118015.1</td><td>Escherichia_coli</td><td>L4R27_004066</td><td>1..2413</td><td>562</td><td>BREX-system-P-loop-protein-BrxC-[Escherichia-coli]</td></tr>
<tr><td>EIW5817588.1</td><td>Type1.BREX</td><td><-Phage_integrase||?->?->?-><-HNH||wHTH+SAD+HNH-><-REase+iREase||BrxA-DNA-binding->BrxB-BrxF-iSTAND->DUF6079*->N6-METHYLASE->PglZ->SIGMA+OB-Fold+AAA-ATPase+Lon-Protease-BrxL->?-><-?<-ParB<-?||<-REase+N6_METHYLASE<-TRD</td><td>1213</td><td>Bacteria>Pseudomonadota>Gammaproteobacteria>Enterobacterales>Enterobacteriaceae>Escherichia</td><td>GCA_022185325.1</td><td>Escherichia_coli</td><td>ME061_001720</td><td>111299..114940</td><td>562</td><td>BREX-system-P-loop-protein-BrxC-[Escherichia-coli]</td></tr>
<tr><td>EIX9838476.1</td><td>Type1.BREX</td><td>DUF6079*->N6-METHYLASE->PglZ->SIGMA+OB-Fold+AAA-ATPase+Lon-Protease-BrxL->HKD-DNase+OGG-like->||AbiEi_3_N+AbiEi_3->AbiEii->?-><-DUF6216<-Arm-DNA-bind_3</td><td>1208</td><td>Bacteria>Pseudomonadota>Gammaproteobacteria>Enterobacterales>Enterobacteriaceae>Escherichia</td><td>GCA_022433775.1</td><td>Escherichia_coli</td><td>ML439_002959</td><td>1..3628</td><td>562</td><td>BREX-system-P-loop-protein-BrxC-[Escherichia-coli]</td></tr>
<tr><td>EIZ4505608.1</td><td>Type1.BREX</td><td><-XIS-HTH<-RHH_antitoxin||?->?->ZnR->ZnR-><-SIGMA+OB-Fold+AAA-ATPase+Lon-Protease-BrxL<-PglZ<-N6-METHYLASE<-DUF6079*<-BrxB-BrxF-iSTAND<-BrxA-DNA-binding||REase+iREase->?->?->TetR-HTH-></td><td>1213</td><td>Bacteria>Pseudomonadota>Gammaproteobacteria>Enterobacterales>Enterobacteriaceae>Escherichia</td><td>GCA_022636095.1</td><td>Escherichia_coli</td><td>MPU38_003973</td><td>70705..74346</td><td>562</td><td>BREX-system-P-loop-protein-BrxC-[Escherichia-coli]</td></tr>
<tr><td>EJA4563483.1</td><td>Type1.BREX</td><td><-Phage_integrase||?->?->?-><-HNH||wHTH+SAD+HNH-><-REase+iREase||BrxA-DNA-binding->BrxB-BrxF-iSTAND->DUF6079*->N6-METHYLASE->PglZ->SIGMA+OB-Fold+AAA-ATPase+Lon-Protease-BrxL->HKD-DNase+OGG-like->||AbiEi_3_N+AbiEi_3->AbiEii->?-><-DUF6216<-Arm-DNA-bind_3</td><td>1213</td><td>Bacteria>Pseudomonadota>Gammaproteobacteria>Enterobacterales>Enterobacteriaceae>Escherichia</td><td>GCA_022888875.1</td><td>Escherichia_coli</td><td>MVR17_003830</td><td>10074..13715</td><td>562</td><td>BREX-system-P-loop-protein-BrxC-[Escherichia-coli]</td></tr>
<tr><td>EJB1753906.1</td><td>Type1.BREX</td><td>ZnR->ZnR-><-SIGMA+OB-Fold+AAA-ATPase+Lon-Protease-BrxL<-PglZ<-N6-METHYLASE<-DUF6079*<-BrxB-BrxF-iSTAND<-BrxA-DNA-binding||REase+iREase-><-EVE+HNH<-Arm-DNA-bind_3+Phage_integrase<-tRNA||?->HerA/FtsK+A32-></td><td>1213</td><td>Bacteria>Pseudomonadota>Gammaproteobacteria>Enterobacterales>Enterobacteriaceae>Escherichia</td><td>GCA_022927665.1</td><td>Escherichia_coli</td><td>MWZ83_002631</td><td>9550..13191</td><td>562</td><td>BREX-system-P-loop-protein-BrxC-[Escherichia-coli]</td></tr>
<tr><td>EJB4937501.1</td><td>Type1.BREX</td><td><-XIS-HTH<-RHH_antitoxin||?->?->ZnR->ZnR-><-SIGMA+OB-Fold+AAA-ATPase+Lon-Protease-BrxL<-PglZ<-N6-METHYLASE<-DUF6079*<-BrxB-BrxF-iSTAND<-BrxA-DNA-binding||REase+iREase->?->?->TetR-HTH-><-AAA-ATPase</td><td>1213</td><td>Bacteria>Pseudomonadota>Gammaproteobacteria>Enterobacterales>Enterobacteriaceae>Escherichia</td><td>GCA_022947155.1</td><td>Escherichia_coli</td><td>MTZ30_000457</td><td>137240..140881</td><td>562</td><td>BREX-system-P-loop-protein-BrxC-[Escherichia-coli]</td></tr>
<tr><td>HCB2140406.1</td><td>Type1.BREX</td><td><-Zn_Tnp_IS91+Y2_Tnp||<-?<-Phage_integrase||Arm-DNA-bind_3+Phage_integrase->BrxA-DNA-binding->BrxB-BrxF-iSTAND->DUF6079*->N6-METHYLASE-></td><td>1204</td><td>Bacteria>Pseudomonadota>Gammaproteobacteria>Enterobacterales>Enterobacteriaceae>Escherichia</td><td>GCA_023036775.1</td><td>Escherichia_coli</td><td>MYQ63_004000</td><td>17767..21381</td><td>562</td><td>BREX-system-P-loop-protein-BrxC-[Escherichia-coli]</td></tr>
<tr><td>EJC4972778.1</td><td>Type1.BREX</td><td><-XIS-HTH<-RHH_antitoxin||?->?->ZnR->ZnR-><-SIGMA+OB-Fold+AAA-ATPase+Lon-Protease-BrxL<-PglZ<-N6-METHYLASE<-DUF6079*<-BrxB-BrxF-iSTAND<-BrxA-DNA-binding||Zn_Tnp_IS1+HTH_Tnp_IS1-></td><td>1213</td><td>Bacteria>Pseudomonadota>Gammaproteobacteria>Enterobacterales>Enterobacteriaceae>Escherichia</td><td>GCA_023055825.1</td><td>Escherichia_coli</td><td>MZE40_000805</td><td>401924..405565</td><td>562</td><td>BREX-system-P-loop-protein-BrxC-[Escherichia-coli]</td></tr>
<tr><td>MCK3244657.1</td><td>Type1.BREX</td><td><-HIN-HTH<-RelE-ParE<-CopG/RHH||<-RHH||?->wHTH+WYL+WCX->BrxA-DNA-binding->BrxB-BrxF-iSTAND->DUF6079*->ParB+HNH-></td><td>1225</td><td>Bacteria>Pseudomonadota>Gammaproteobacteria>Enterobacterales>Enterobacteriaceae>Escherichia</td><td>GCA_023098955.1</td><td>Escherichia_coli</td><td>MZH07_24520</td><td>5688..9365</td><td>562</td><td>BREX-system-P-loop-protein-BrxC-[Escherichia-coli]</td></tr>
<tr><td>EJD7925982.1</td><td>Type1.BREX</td><td><-REC+CITB-HTH<-HIN-HTH||DDE_Tnp_1_5+DDE_Tnp_1->?-><-REase+iREase||BrxA-DNA-binding->BrxB-BrxF-iSTAND->DUF6079*->N6-METHYLASE->PglZ->SIGMA+OB-Fold+AAA-ATPase+Lon-Protease-BrxL-><-ZnR<-ZnR<-?||RHH_antitoxin->XIS-HTH-></td><td>1213</td><td>Bacteria>Pseudomonadota>Gammaproteobacteria>Enterobacterales>Enterobacteriaceae>Escherichia</td><td>GCA_023216415.1</td><td>Escherichia_coli</td><td>M2Y98_004402</td><td>13269..16910</td><td>562</td><td>BREX-system-P-loop-protein-BrxC-[Escherichia-coli]</td></tr>
<tr><td>EJE3980761.1</td><td>Type1.BREX</td><td><-RelE-ParE<-CopG/RHH||<-RHH||?->wHTH+WYL+WCX->BrxA-DNA-binding->BrxB-BrxF-iSTAND->DUF6079*->ParB+HNH->N6-METHYLASE->PglZ->SIGMA+OB-Fold+AAA-ATPase+Lon-Protease-BrxL-><-DEDD_Tnp_IS110+Transposase_20<-RVT</td><td>1225</td><td>Bacteria>Pseudomonadota>Gammaproteobacteria>Enterobacterales>Enterobacteriaceae>Escherichia</td><td>GCA_023310495.1</td><td>Escherichia_coli</td><td>M4B16_004238</td><td>5667..9344</td><td>562</td><td>BREX-system-P-loop-protein-BrxC-[Escherichia-coli]</td></tr>
<tr><td>EJE7557557.1</td><td>Type1.BREX</td><td><-DUF6079-p*<-BrxB-BrxF-iSTAND<-BrxA-DNA-binding||REase+iREase->?->?->TetR-HTH-><-AAA-ATPase<-HIN-HTH</td><td>625</td><td>Bacteria>Pseudomonadota>Gammaproteobacteria>Enterobacterales>Enterobacteriaceae>Escherichia</td><td>GCA_023344655.1</td><td>Escherichia_coli</td><td>M5073_003716</td><td>1..1876</td><td>562</td><td>BREX-system-P-loop-protein-BrxC-[Escherichia-coli]</td></tr>
<tr><td>EJE7788338.1</td><td>Type1.BREX</td><td><-Phage_integrase||?->?->?-><-HNH||wHTH+SAD+HNH-><-REase+iREase||BrxA-DNA-binding->BrxB-BrxF-iSTAND->DUF6079-p*-></td><td>859</td><td>Bacteria>Pseudomonadota>Gammaproteobacteria>Enterobacterales>Enterobacteriaceae>Escherichia</td><td>GCA_023345825.1</td><td>Escherichia_coli</td><td>M5R54_003441</td><td>111186..113763</td><td>562</td><td>BREX-system-P-loop-protein-BrxC-[Escherichia-coli]</td></tr>
<tr><td>EJH0789763.1</td><td>Type1.BREX</td><td>DUF6079*->N6-METHYLASE->PglZ->SIGMA+OB-Fold+AAA-ATPase+Lon-Protease-BrxL->HKD-DNase+OGG-like->||AbiEi_3_N+AbiEi_3->AbiEii->?-><-DUF6216<-Arm-DNA-bind_3</td><td>1107</td><td>Bacteria>Pseudomonadota>Gammaproteobacteria>Enterobacterales>Enterobacteriaceae>Escherichia</td><td>GCA_023637565.1</td><td>Escherichia_coli</td><td>NCI57_002418</td><td>1..3324</td><td>562</td><td>BREX-system-P-loop-protein-BrxC-[Escherichia-coli]</td></tr>
<tr><td>MCM4369403.1</td><td>Type1.BREX</td><td><-HerA/FtsK+A32<-?||tRNA->Arm-DNA-bind_3+Phage_integrase->EVE+HNH-><-REase+iREase||BrxA-DNA-binding->BrxB-BrxF-iSTAND->DUF6079*->N6-METHYLASE->PglZ->SIGMA+OB-Fold+AAA-ATPase+Lon-Protease-BrxL-><-ZnR<-ZnR<-?||?-><-?||XIS-HTH-></td><td>1213</td><td>Bacteria>Pseudomonadota>Gammaproteobacteria>Enterobacterales>Enterobacteriaceae>Escherichia</td><td>GCA_023723445.1</td><td>Escherichia_coli</td><td>MMS14_20555</td><td>25622..29263</td><td>562</td><td>BREX-system-P-loop-protein-BrxC-[Escherichia-coli]</td></tr>
<tr><td>EJH6809378.1</td><td>Type1.BREX</td><td><-DUF6079-p*<-BrxB-BrxF-iSTAND<-BrxA-DNA-binding||REase+iREase-><-wHTH+SAD+HNH||HNH-></td><td>851</td><td>Bacteria>Pseudomonadota>Gammaproteobacteria>Enterobacterales>Enterobacteriaceae>Escherichia</td><td>GCA_023728085.1</td><td>Escherichia_coli</td><td>NFZ65_004801</td><td>1..2554</td><td>562</td><td>BREX-system-P-loop-protein-BrxC-[Escherichia-coli]</td></tr>
<tr><td>EJH8598536.1</td><td>Type1.BREX</td><td><-XIS-HTH<-RHH_antitoxin||?->?->ZnR->ZnR-><-SIGMA+OB-Fold+AAA-ATPase+Lon-Protease-BrxL<-PglZ<-N6-METHYLASE<-DUF6079*<-BrxB-BrxF-iSTAND<-BrxA-DNA-binding||REase+iREase-><-EVE+HNH<-Arm-DNA-bind_3+Phage_integrase<-tRNA||?->HerA/FtsK+A32-></td><td>1213</td><td>Bacteria>Pseudomonadota>Gammaproteobacteria>Enterobacterales>Enterobacteriaceae>Escherichia</td><td>GCA_023738335.1</td><td>Escherichia_coli</td><td>NF498_001054</td><td>22625..26266</td><td>562</td><td>BREX-system-P-loop-protein-BrxC-[Escherichia-coli]</td></tr>
<tr><td>HCE0292887.1</td><td>Type1.BREX</td><td><-REase+iREase||BrxA-DNA-binding->BrxB-BrxF-iSTAND->DUF6079*->N6-METHYLASE->PglZ->SIGMA+OB-Fold+AAA-ATPase+Lon-Protease-BrxL-><-ZnR<-XIS-HTH<-?||?-><-?||CopG/RHH-></td><td>1213</td><td>Bacteria>Pseudomonadota>Gammaproteobacteria>Enterobacterales>Enterobacteriaceae>Escherichia</td><td>GCA_023779205.1</td><td>Escherichia_coli</td><td>NE028_002917</td><td>2558..6199</td><td>562</td><td>BREX-system-P-loop-protein-BrxC-[Escherichia-coli]</td></tr>
<tr><td>EJI6887231.1</td><td>Type1.BREX</td><td><-REase+iREase||BrxA-DNA-binding->BrxB-BrxF-iSTAND->DUF6079*->N6-METHYLASE->PglZ->SIGMA+OB-Fold+AAA-ATPase+Lon-Protease-BrxL-><-ZnR<-XIS-HTH</td><td>1213</td><td>Bacteria>Pseudomonadota>Gammaproteobacteria>Enterobacterales>Enterobacteriaceae>Escherichia</td><td>GCA_023803175.1</td><td>Escherichia_coli</td><td>NFV42_004498</td><td>5105..8746</td><td>562</td><td>BREX-system-P-loop-protein-BrxC-[Escherichia-coli]</td></tr>
<tr><td>HCE4109215.1</td><td>Type1.BREX</td><td><-XIS-HTH||?->?->ZnR->ZnR-><-SIGMA+OB-Fold+AAA-ATPase+Lon-Protease-BrxL<-PglZ<-N6-METHYLASE<-DUF6079*</td><td>1120</td><td>Bacteria>Pseudomonadota>Gammaproteobacteria>Enterobacterales>Enterobacteriaceae>Escherichia</td><td>GCA_023826315.1</td><td>Escherichia_coli</td><td>NES30_001638</td><td>158765..162129</td><td>562</td><td>BREX-system-P-loop-protein-BrxC-[Escherichia-coli]</td></tr>
<tr><td>MCN5345816.1</td><td>Type1.BREX</td><td><-DUF932<-nSTAND1+GTPase-AIG+sGTP<-?||?->XIS-HTH-><-?<-ZnR||BrxA-DNA-binding->BrxB-BrxF-iSTAND->DUF6079*->N6-METHYLASE->OLD-ABC->TOPRIM->PglZ->SIGMA+OB-Fold+AAA-ATPase+Lon-Protease-BrxL->Zn_Tnp_IS1+HTH_Tnp_IS1-></td><td>1204</td><td>Bacteria>Pseudomonadota>Gammaproteobacteria>Enterobacterales>Enterobacteriaceae>Escherichia</td><td>GCA_023869305.1</td><td>Escherichia_coli</td><td>MLU97_13810</td><td>69828..73442</td><td>562</td><td>BREX-system-P-loop-protein-BrxC-[Escherichia-coli]</td></tr>
<tr><td>EJM6441909.1</td><td>Type1.BREX</td><td>DUF6079*->N6-METHYLASE->PglZ->SIGMA+OB-Fold+AAA-ATPase+Lon-Protease-BrxL->HKD-DNase+OGG-like->||AbiEi_3_N+AbiEi_3->AbiEii->?-><-DUF6216<-Arm-DNA-bind_3</td><td>1006</td><td>Bacteria>Pseudomonadota>Gammaproteobacteria>Enterobacterales>Enterobacteriaceae>Escherichia</td><td>GCA_024340725.1</td><td>Escherichia_coli</td><td>NOR68_003993</td><td>1..3023</td><td>562</td><td>BREX-system-P-loop-protein-BrxC-[Escherichia-coli]</td></tr>
<tr><td>EJN1844348.1</td><td>Type1.BREX</td><td><-RHH||?->wHTH+WYL+WCX->BrxA-DNA-binding->BrxB-BrxF-iSTAND->DUF6079*->ParB+HNH->N6-METHYLASE->PglZ->SIGMA+OB-Fold+AAA-ATPase+Lon-Protease-BrxL-><-DEDD_Tnp_IS110+Transposase_20</td><td>1225</td><td>Bacteria>Pseudomonadota>Gammaproteobacteria>Enterobacterales>Enterobacteriaceae>Escherichia</td><td>GCA_024422675.1</td><td>Escherichia_coli</td><td>NPJ45_004376</td><td>3256..6933</td><td>562</td><td>BREX-system-P-loop-protein-BrxC-[Escherichia-coli]</td></tr>
<tr><td>MCQ4517261.1</td><td>Type1.BREX</td><td>DUF6216-><-?<-AbiEii<-AbiEi_3_N+AbiEi_3||<-HKD-DNase+OGG-like<-SIGMA+OB-Fold+AAA-ATPase+Lon-Protease-BrxL<-PglZ<-N6-METHYLASE<-DUF6079*<-BrxB-BrxF-iSTAND<-BrxA-DNA-binding||REase+iREase-></td><td>1213</td><td>Bacteria>Pseudomonadota>Gammaproteobacteria>Enterobacterales>Enterobacteriaceae>Escherichia</td><td>GCA_024450325.1</td><td>Escherichia_coli</td><td>NO030_20220</td><td>28424..32065</td><td>562</td><td>BREX-system-P-loop-protein-BrxC-[Escherichia-coli]</td></tr>
<tr><td>HCJ2602063.1</td><td>Type1.BREX</td><td><-TetR-HTH<-?<-?<-REase+iREase||BrxA-DNA-binding->BrxB-BrxF-iSTAND->DUF6079*->N6-METHYLASE->PglZ->SIGMA+OB-Fold+AAA-ATPase+Lon-Protease-BrxL-></td><td>1213</td><td>Bacteria>Pseudomonadota>Gammaproteobacteria>Enterobacterales>Enterobacteriaceae>Escherichia</td><td>GCA_024493375.1</td><td>Escherichia_coli</td><td>NQS58_004405</td><td>6692..10333</td><td>562</td><td>BREX-system-P-loop-protein-BrxC-[Escherichia-coli]</td></tr>
<tr><td>MCQ6644900.1</td><td>Type1.BREX</td><td><-CbeA_antitoxin<-?<-?<-DUF932<-nSTAND1+sGTP<-?<-XIS-HTH||||Zn_Tnp_IS1+HTH_Tnp_IS1+DDE_Tnp_IS1-><-N6-METHYLASE-p<-DUF6079*<-BrxB-BrxF-iSTAND<-BrxA-DNA-binding||REase+iREase-><-Zn_Tnp_IS1+HTH_Tnp_IS1+DDE_Tnp_IS1||HIN-HTH->?->?->?->PhdYeFM_antitox-></td><td>1213</td><td>Bacteria>Pseudomonadota>Gammaproteobacteria>Enterobacterales>Enterobacteriaceae>Escherichia</td><td>GCA_024496475.1</td><td>Escherichia_coli</td><td>NO371_23560</td><td>27231..30872</td><td>562</td><td>BREX-system-P-loop-protein-BrxC-[Escherichia-coli]</td></tr>
<tr><td>EJN8021038.1</td><td>Type1.BREX</td><td><-REase+iREase||BrxA-DNA-binding->BrxB-BrxF-iSTAND->DUF6079*->N6-METHYLASE->PglZ->SIGMA+OB-Fold+AAA-ATPase+Lon-Protease-BrxL->HKD-DNase+OGG-like->||AbiEi_3_N+AbiEi_3->AbiEii->?-><-DUF6216<-Arm-DNA-bind_3</td><td>1213</td><td>Bacteria>Pseudomonadota>Gammaproteobacteria>Enterobacterales>Enterobacteriaceae>Escherichia</td><td>GCA_024524615.1</td><td>Escherichia_coli</td><td>NRW59_000779</td><td>2207..5848</td><td>562</td><td>BREX-system-P-loop-protein-BrxC-[Escherichia-coli]</td></tr>
<tr><td>EJO9498073.1</td><td>Type1.BREX</td><td><-HNH||wHTH+SAD+HNH-><-REase+iREase||BrxA-DNA-binding->BrxB-BrxF-iSTAND->DUF6079*->N6-METHYLASE->PglZ->SIGMA+OB-Fold+AAA-ATPase+Lon-Protease-BrxL->HKD-DNase+OGG-like->||AbiEi_3_N+AbiEi_3->AbiEii->?-><-DUF6216<-Arm-DNA-bind_3</td><td>1213</td><td>Bacteria>Pseudomonadota>Gammaproteobacteria>Enterobacterales>Enterobacteriaceae>Escherichia</td><td>GCA_024612475.1</td><td>Escherichia_coli</td><td>NUZ09_002875</td><td>7894..11535</td><td>562</td><td>BREX-system-P-loop-protein-BrxC-[Escherichia-coli]</td></tr>
<tr><td>HCJ9453756.1</td><td>Type1.BREX</td><td><-DDE_Tnp_IS240<-?||XIS-HTH->PIWI-><-?<-Phage_integrase||Arm-DNA-bind_3+Phage_integrase->BrxA-DNA-binding->BrxB-BrxF-iSTAND->DUF6079*->N6-METHYLASE->OLD-ABC->TOPRIM->PglZ->SIGMA+OB-Fold+AAA-ATPase+Lon-Protease-BrxL-></td><td>1204</td><td>Bacteria>Pseudomonadota>Gammaproteobacteria>Enterobacterales>Enterobacteriaceae>Escherichia</td><td>GCA_024682335.1</td><td>Escherichia_coli</td><td>NV934_004004</td><td>9250..12864</td><td>562</td><td>BREX-system-P-loop-protein-BrxC-[Escherichia-coli]</td></tr>
<tr><td>HCJ9689412.1</td><td>Type1.BREX</td><td><-SWIM+DUF3670+SF1-SF2-Helicase<-?<-?<-?||BrxA-DNA-binding->BrxB-BrxF-iSTAND->DUF6079*->N6-METHYLASE->PglZ->SIGMA+OB-Fold+AAA-ATPase+Lon-Protease-BrxL-></td><td>1213</td><td>Bacteria>Pseudomonadota>Gammaproteobacteria>Enterobacterales>Enterobacteriaceae>Escherichia</td><td>GCA_024685835.1</td><td>Escherichia_coli</td><td>NV971_003963</td><td>13318..16959</td><td>562</td><td>BREX-system-P-loop-protein-BrxC-[Escherichia-coli]</td></tr>
<tr><td>MCS2047788.1</td><td>Type1.BREX</td><td><-GntR-HTH+TrmB+ZDNA-HTH<-?<-?||?-><-?<-Fimbrial<-Fimbrial<-Zn_Tnp_IS1+HTH_Tnp_IS1+DDE_Tnp_IS1||DUF6079*->N6-METHYLASE->PglZ->SIGMA+OB-Fold+AAA-ATPase+Lon-Protease-BrxL->?-><-?<-ParB<-?<-?||<-REase+N6_METHYLASE</td><td>1213</td><td>Bacteria>Pseudomonadota>Gammaproteobacteria>Enterobacterales>Enterobacteriaceae>Escherichia</td><td>GCA_024768515.1</td><td>Escherichia_coli</td><td>MWK73_15380</td><td>3165783..3169424</td><td>562</td><td>BREX-system-P-loop-protein-BrxC-[Escherichia-coli]</td></tr>
<tr><td>EJQ5102253.1</td><td>Type1.BREX</td><td>DUF6079-p*->N6-METHYLASE->PglZ->SIGMA+OB-Fold+AAA-ATPase+Lon-Protease-BrxL-><-ZnR<-ZnR<-?||HIN-HTH+HIN-HTH-></td><td>973</td><td>Bacteria>Pseudomonadota>Gammaproteobacteria>Enterobacterales>Enterobacteriaceae>Escherichia</td><td>GCA_024841725.1</td><td>Escherichia_coli</td><td>NOK27_004784</td><td>1..2924</td><td>562</td><td>BREX-system-P-loop-protein-BrxC-[Escherichia-coli]</td></tr>
<tr><td>EJQ6147017.1</td><td>Type1.BREX</td><td><-XIS-HTH<-RHH_antitoxin||?->?->ZnR->ZnR-><-SIGMA+OB-Fold+AAA-ATPase+Lon-Protease-BrxL<-PglZ<-N6-METHYLASE<-DUF6079*<-BrxB-BrxF-iSTAND<-BrxA-DNA-binding||REase+iREase-><-EVE+HNH<-Arm-DNA-bind_3+Phage_integrase<-tRNA||?->HerA/FtsK+A32-></td><td>1213</td><td>Bacteria>Pseudomonadota>Gammaproteobacteria>Enterobacterales>Enterobacteriaceae>Escherichia</td><td>GCA_024846805.1</td><td>Escherichia_albertii</td><td>NZZ21_002663</td><td>93437..97078</td><td>208962</td><td>BREX-system-P-loop-protein-BrxC-[Escherichia-albertii]</td></tr>
<tr><td>HCL8518718.1</td><td>Type1.BREX</td><td><-Phage_integrase||?->?->?-><-HNH||wHTH+SAD+HNH-><-REase+iREase||BrxA-DNA-binding->BrxB-BrxF-iSTAND->DUF6079-p*-></td><td>605</td><td>Bacteria>Pseudomonadota>Gammaproteobacteria>Enterobacterales>Enterobacteriaceae>Escherichia</td><td>GCA_025048635.1</td><td>Escherichia_coli</td><td>N2025_002882</td><td>80925..82740</td><td>562</td><td>BREX-system-P-loop-protein-BrxC-[Escherichia-coli]</td></tr>
<tr><td>EJT7594528.1</td><td>Type1.BREX</td><td><-N6-METHYLASE<-DUF6079*<-BrxB-BrxF-iSTAND<-BrxA-DNA-binding||REase+iREase-><-wHTH+SAD+HNH||HNH-><-?<-?<-?||Phage_integrase-></td><td>1213</td><td>Bacteria>Pseudomonadota>Gammaproteobacteria>Enterobacterales>Enterobacteriaceae>Escherichia</td><td>GCA_025102805.1</td><td>Escherichia_coli</td><td>N3T17_004670</td><td>1985..5626</td><td>562</td><td>BREX-system-P-loop-protein-BrxC-[Escherichia-coli]</td></tr>
<tr><td>EJU9647232.1</td><td>Type1.BREX</td><td><-DDE_Tnp_1_5+DDE_Tnp_1||||wHTH+WYL+WCX->BrxA-DNA-binding->BrxB-BrxF-iSTAND->DUF6079*->ParB+HNH->N6-METHYLASE->PglZ->SIGMA+OB-Fold+AAA-ATPase+Lon-Protease-BrxL-><-HTH_Tnp_1+HTH_21+rve+rve_3<-HIN-HTH<-PIN<-MazE_antitoxin</td><td>1225</td><td>Bacteria>Pseudomonadota>Gammaproteobacteria>Enterobacterales>Enterobacteriaceae>Escherichia</td><td>GCA_025224895.1</td><td>Escherichia_coli</td><td>N5N25_003986</td><td>5462..9139</td><td>562</td><td>BREX-system-P-loop-protein-BrxC-[Escherichia-coli]</td></tr>
<tr><td>HCN4580231.1</td><td>Type1.BREX</td><td><-Phage_integrase||?->?->?-><-HNH||wHTH+SAD+HNH-><-REase+iREase||BrxA-DNA-binding->BrxB-BrxF-iSTAND->DUF6079*-><-wHTH+WYL</td><td>1213</td><td>Bacteria>Pseudomonadota>Gammaproteobacteria>Enterobacterales>Enterobacteriaceae>Escherichia</td><td>GCA_025275895.1</td><td>Escherichia_coli</td><td>N6V60_002026</td><td>111191..114832</td><td>562</td><td>BREX-system-P-loop-protein-BrxC-[Escherichia-coli]</td></tr>
<tr><td>HCN4662824.1</td><td>Type1.BREX</td><td><-REase+iREase||BrxA-DNA-binding->BrxB-BrxF-iSTAND->DUF6079*->N6-METHYLASE->PglZ->SIGMA+OB-Fold+AAA-ATPase+Lon-Protease-BrxL->HKD-DNase+OGG-like->||AbiEi_3_N+AbiEi_3->AbiEii->?-><-DUF6216<-Arm-DNA-bind_3</td><td>1213</td><td>Bacteria>Pseudomonadota>Gammaproteobacteria>Enterobacterales>Enterobacteriaceae>Escherichia</td><td>GCA_025276155.1</td><td>Escherichia_coli</td><td>N6W02_002919</td><td>2176..5817</td><td>562</td><td>BREX-system-P-loop-protein-BrxC-[Escherichia-coli]</td></tr>
<tr><td>HCN5442922.1</td><td>Type1.BREX</td><td>DUF6079*->N6-METHYLASE->PglZ->SIGMA+OB-Fold+AAA-ATPase+Lon-Protease-BrxL->?-><-?<-ParB<-?||<-REase+N6_METHYLASE<-TRD</td><td>1088</td><td>Bacteria>Pseudomonadota>Gammaproteobacteria>Enterobacterales>Enterobacteriaceae>Escherichia</td><td>GCA_025279415.1</td><td>Escherichia_coli</td><td>N6X60_000278</td><td>1..3269</td><td>562</td><td>BREX-system-P-loop-protein-BrxC-[Escherichia-coli]</td></tr>
<tr><td>HCN5870003.1</td><td>Type1.BREX</td><td>DUF6079*->N6-METHYLASE->PglZ->SIGMA+OB-Fold+AAA-ATPase+Lon-Protease-BrxL-><-ZnR<-ZnR<-?||RHH_antitoxin->XIS-HTH-></td><td>1016</td><td>Bacteria>Pseudomonadota>Gammaproteobacteria>Enterobacterales>Enterobacteriaceae>Escherichia</td><td>GCA_025281175.1</td><td>Escherichia_coli</td><td>N6Y80_004176</td><td>1..3051</td><td>562</td><td>BREX-system-P-loop-protein-BrxC-[Escherichia-coli]</td></tr>
<tr><td>HCN7834282.1</td><td>Type1.BREX</td><td><-N6-METHYLASE<-DUF6079*<-BrxB-BrxF-iSTAND<-BrxA-DNA-binding<-tRNA||?-><-SKI||METHYLASE->?->?->LACI-HTH+PBPI->HerA/FtsK+A32-></td><td>1204</td><td>Bacteria>Pseudomonadota>Gammaproteobacteria>Enterobacterales>Enterobacteriaceae>Escherichia</td><td>GCA_025289555.1</td><td>Escherichia_coli</td><td>N6366_003464</td><td>2753..6367</td><td>562</td><td>BREX-system-P-loop-protein-BrxC-[Escherichia-coli]</td></tr>
<tr><td>HCO1420187.1</td><td>Type1.BREX</td><td><-MAD-NTD+HNH||wHTH+SAD+HNH-><-REase+iREase||BrxA-DNA-binding->BrxB-BrxF-iSTAND->DUF6079*->N6-METHYLASE-></td><td>1213</td><td>Bacteria>Pseudomonadota>Gammaproteobacteria>Enterobacterales>Enterobacteriaceae>Escherichia</td><td>GCA_025325225.1</td><td>Escherichia_coli</td><td>N7L77_004122</td><td>7756..11397</td><td>562</td><td>BREX-system-P-loop-protein-BrxC-[Escherichia-coli]</td></tr>
<tr><td>EJV7137460.1</td><td>Type1.BREX</td><td><-Phage_integrase||?->?->?-><-HNH||wHTH+SAD+HNH-><-REase+iREase||BrxA-DNA-binding->BrxB-BrxF-iSTAND->DUF6079*->N6-METHYLASE->PglZ->SIGMA+OB-Fold+AAA-ATPase+Lon-Protease-BrxL->?-><-?<-ParB<-?||<-REase+N6_METHYLASE<-TRD</td><td>1213</td><td>Bacteria>Pseudomonadota>Gammaproteobacteria>Enterobacterales>Enterobacteriaceae>Escherichia</td><td>GCA_025337425.1</td><td>Escherichia_coli</td><td>FHP62_000281</td><td>290632..294273</td><td>562</td><td>BREX-system-P-loop-protein-BrxC-[Escherichia-coli]</td></tr>
<tr><td>HCO5071633.1</td><td>Type1.BREX</td><td><-RelE-ParE<-CopG/RHH||<-RHH||?->wHTH+WYL+WCX->BrxA-DNA-binding->BrxB-BrxF-iSTAND->DUF6079*->ParB+HNH-></td><td>1225</td><td>Bacteria>Pseudomonadota>Gammaproteobacteria>Enterobacterales>Enterobacteriaceae>Escherichia</td><td>GCA_025390635.1</td><td>Escherichia_coli</td><td>N8039_004383</td><td>5665..9342</td><td>562</td><td>BREX-system-P-loop-protein-BrxC-[Escherichia-coli]</td></tr>
<tr><td>HCO6583160.1</td><td>Type1.BREX</td><td><-Phage_integrase||?->?->?-><-HNH||wHTH+SAD+HNH-><-REase+iREase||BrxA-DNA-binding->BrxB-BrxF-iSTAND->DUF6079*->N6-METHYLASE->PglZ->SIGMA+OB-Fold+AAA-ATPase+Lon-Protease-BrxL->HKD-DNase+OGG-like->||AbiEi_3_N+AbiEi_3->AbiEii->?-><-DUF6216<-Arm-DNA-bind_3</td><td>1213</td><td>Bacteria>Pseudomonadota>Gammaproteobacteria>Enterobacterales>Enterobacteriaceae>Escherichia</td><td>GCA_025412985.1</td><td>Escherichia_coli</td><td>OBR12_001368</td><td>111262..114903</td><td>562</td><td>BREX-system-P-loop-protein-BrxC-[Escherichia-coli]</td></tr>
<tr><td>HCO7023289.1</td><td>Type1.BREX</td><td><-Phage_integrase||?->?->?-><-HNH||wHTH+SAD+HNH-><-REase+iREase||BrxA-DNA-binding->BrxB-BrxF-iSTAND->DUF6079-p*-></td><td>886</td><td>Bacteria>Pseudomonadota>Gammaproteobacteria>Enterobacterales>Enterobacteriaceae>Escherichia</td><td>GCA_025415015.1</td><td>Escherichia_coli</td><td>OBS61_002730</td><td>83196..85855</td><td>562</td><td>BREX-system-P-loop-protein-BrxC-[Escherichia-coli]</td></tr>
<tr><td>EJW3212697.1</td><td>Type1.BREX</td><td><-DUF6079-p*<-BrxB-BrxF-iSTAND<-BrxA-DNA-binding||<-wHTH(SBD)+HNH||HNH-><-?<-?<-?||Phage_integrase->Phage_integrase-></td><td>418</td><td>Bacteria>Pseudomonadota>Gammaproteobacteria>Enterobacterales>Enterobacteriaceae>Escherichia</td><td>GCA_025418235.1</td><td>Escherichia_coli</td><td>OBZ04_004372</td><td>1..1254</td><td>562</td><td>BREX-system-P-loop-protein-BrxC-[Escherichia-coli]</td></tr>
<tr><td>HCP0312404.1</td><td>Type1.BREX</td><td><-METHYLASE||SKI-><-?||tRNA->Arm-DNA-bind_3+Phage_integrase->EVE+HNH-><-REase+iREase||BrxA-DNA-binding->BrxB-BrxF-iSTAND->DUF6079*->N6-METHYLASE->PglZ->SIGMA+OB-Fold+AAA-ATPase+Lon-Protease-BrxL-><-ZnR<-ZnR<-?<-?||XIS-HTH-></td><td>1213</td><td>Bacteria>Pseudomonadota>Gammaproteobacteria>Enterobacterales>Enterobacteriaceae>Escherichia</td><td>GCA_025467455.1</td><td>Escherichia_coli</td><td>OCM84_003824</td><td>150572..154213</td><td>562</td><td>BREX-system-P-loop-protein-BrxC-[Escherichia-coli]</td></tr>
<tr><td>HCP1392420.1</td><td>Type1.BREX</td><td>Arm-DNA-bind_3->DUF6216-><-AbiEii<-AbiEi_3_N+AbiEi_3||<-HKD-DNase+OGG-like<-SIGMA+OB-Fold+AAA-ATPase+Lon-Protease-BrxL<-PglZ<-N6-METHYLASE<-DUF6079*<-BrxB-BrxF-iSTAND<-BrxA-DNA-binding||REase+iREase-><-wHTH+SAD+HNH||HNH-><-?<-?<-?||Phage_integrase-></td><td>1213</td><td>Bacteria>Pseudomonadota>Gammaproteobacteria>Enterobacterales>Enterobacteriaceae>Escherichia</td><td>GCA_025491965.1</td><td>Escherichia_coli</td><td>OCX38_000210</td><td>218312..221953</td><td>562</td><td>BREX-system-P-loop-protein-BrxC-[Escherichia-coli]</td></tr>
<tr><td>WP_250907975.1</td><td>Type1.BREX</td><td>DDE_Tnp_IS1->?->ZnR->BrxA-DNA-binding->BrxB-BrxF-iSTAND->DUF6079*->ParB+HNH->N6-METHYLASE->HEPN->RVT->N6-METHYLASE-p->PglZ->SIGMA+OB-Fold+AAA-ATPase+Lon-Protease-BrxL-><-Arm-DNA-bind_3+Phage_integrase||Phage_integrase-></td><td>1240</td><td>Bacteria>Pseudomonadota>Gammaproteobacteria>Enterobacterales>Enterobacteriaceae>Escherichia</td><td>GCA_030820635.1</td><td>Escherichia_coli</td><td>HUC40_RS15465</td><td>2924..6646</td><td>562</td><td>BREX-system-P-loop-protein-BrxC-[Escherichia-coli]</td></tr>
<tr><td>CEK08497.1</td><td>Type1.BREX</td><td>DUF6216-><-?<-AbiEii<-AbiEi_3_N+AbiEi_3<-HKD-DNase+OGG-like<-SIGMA+OB-Fold+AAA-ATPase+Lon-Protease-BrxL<-PglZ<-N6-METHYLASE<-N6-METHYLASE-p<-DUF6079*<-BrxB-BrxF-iSTAND<-BrxA-DNA-binding||REase+iREase-><-wHTH+SAD+HNH<-?<-?<-?||Phage_integrase-></td><td>1218</td><td>Bacteria>Pseudomonadota>Gammaproteobacteria>Enterobacterales>Enterobacteriaceae>Escherichia</td><td>GCA_900000205.1</td><td>Escherichia_coli_O26:H11</td><td>ECO26H__800033</td><td>35711..39367</td><td>244319</td><td>conserved-hypothetical-protein-[Escherichia-coli-O26:H11]</td></tr>
<tr><td>STH24241.1</td><td>Type1.BREX</td><td><-DUF772+DDE_Tnp_1||?->?->DiS_P_DiS+Peptidase_A24->?-><-DDE_Tnp_IS1<-DUF6079-p*<-BrxB-BrxF-iSTAND<-BrxA-DNA-binding||REase+iREase->?->?->?-><-DUF772+DDE_Tnp_1||?->TetR-HTH-></td><td>778</td><td>Bacteria>Pseudomonadota>Gammaproteobacteria>Enterobacterales>Enterobacteriaceae>Escherichia</td><td>GCA_900448945.1</td><td>Escherichia_coli</td><td>NCTC11122_00780</td><td>798799..801135</td><td>562</td><td>ATPase-like-protein-[Escherichia-coli]</td></tr>
<tr><td>STI20016.1</td><td>Type1.BREX</td><td><-Phage_integrase||?->?->?->wHTH+SAD+HNH-><-REase+iREase||BrxA-DNA-binding->BrxB-BrxF-iSTAND->DUF6079-p*->DUF6079-p->DUF6079-p->N6-METHYLASE-p->N6-METHYLASE-p->N6-METHYLASE-p->PglZ-></td><td>488</td><td>Bacteria>Pseudomonadota>Gammaproteobacteria>Enterobacterales>Enterobacteriaceae>Escherichia</td><td>GCA_900448955.1</td><td>Escherichia_coli</td><td>NCTC10865_05411</td><td>4206168..4207634</td><td>562</td><td>ATPase-like-protein-[Escherichia-coli]</td></tr>
<tr><td>STN20139.1</td><td>Type1.BREX</td><td>DDE_Tnp_1_5+DDE_Tnp_1-><-?||HTH_21+rve+rve_3-><-RHH||?->wHTH+WYL+WCX->BrxA-DNA-binding->BrxB-BrxF-iSTAND->DUF6079-p*->DUF6079-p->DUF6079-p->ParB+HNH->ParB+HNH->N6-METHYLASE->?->PglZ-p-></td><td>353</td><td>Bacteria>Pseudomonadota>Gammaproteobacteria>Enterobacterales>Enterobacteriaceae>Escherichia</td><td>GCA_900450565.1</td><td>Escherichia_fergusonii</td><td>NCTC12128_00054</td><td>38373..39434</td><td>564</td><td>ATPase-(modular-protein)-[Escherichia-fergusonii]</td></tr>
<tr><td>WP_001019654.1</td><td>Type1.BREX</td><td>LexA-protease->||Arm-DNA-bind_3+Phage_integrase->N6-METHYLASE->ORC-CDC6+ZnR->HEPN-><-Zn_Tnp_IS1+HTH_Tnp_IS1+DDE_Tnp_IS1<-REase||BrxA-DNA-binding->BrxB-BrxF-iSTAND->DUF6079*->N6-METHYLASE->PglZ->SIGMA+OB-Fold+AAA-ATPase+Lon-Protease-BrxL-><-ZnR<-ZnR<-?||HTH_Tnp_1+HTH_21+rve+rve_3-><-LuxR-HTH</td><td>1213</td><td>Bacteria>Pseudomonadota>Gammaproteobacteria>Enterobacterales>Enterobacteriaceae>Escherichia</td><td>GCF_000017765.1</td><td>Escherichia_coli</td><td>ECHS_RS01710</td><td>342111..345752</td><td>562</td><td>BREX-system-P-loop-protein-BrxC-[Escherichia-coli]</td></tr>
<tr><td>WP_001019642.1</td><td>Type1.BREX</td><td>DUF6216-><-?<-AbiEii<-AbiEi_3_N+AbiEi_3||<-HKD-DNase+OGG-like<-SIGMA+OB-Fold+AAA-ATPase+Lon-Protease-BrxL<-PglZ<-N6-METHYLASE||HTH_Tnp_1+HTH_21+rve+rve_3-><-DUF6079*<-BrxB-BrxF-iSTAND<-BrxA-DNA-binding||<-wHTH+SAD+HNH||<-?<-?<-?||Phage_integrase->Phage_integrase-></td><td>1188</td><td>Bacteria>Pseudomonadota>Gammaproteobacteria>Enterobacterales>Enterobacteriaceae>Escherichia</td><td>GCF_000194355.1</td><td>Escherichia_coli</td><td>ECJB195_RS15905</td><td>80241..83807</td><td>562</td><td>BREX-system-P-loop-protein-BrxC-[Escherichia-coli]</td></tr>
<tr><td>WP_001019646.1</td><td>Type1.BREX</td><td>REase+N6_METHYLASE-><-?||?->ParB->?-><-?<-SIGMA+OB-Fold+AAA-ATPase+Lon-Protease-BrxL<-PglZ<-N6-METHYLASE<-DUF6079*<-BrxB-BrxF-iSTAND<-BrxA-DNA-binding||REase+iREase-><-wHTH+SAD+HNH||<-?<-?<-?||Phage_integrase->Phage_integrase-></td><td>1213</td><td>Bacteria>Pseudomonadota>Gammaproteobacteria>Enterobacterales>Enterobacteriaceae>Escherichia</td><td>GCF_000234235.1</td><td>Escherichia_coli</td><td>KEM_RS0123875</td><td>120239..123880</td><td>562</td><td>BREX-system-P-loop-protein-BrxC-[Escherichia-coli]</td></tr>
<tr><td>WP_001019647.1</td><td>Type1.BREX</td><td>Arm-DNA-bind_3+Phage_integrase->N6-METHYLASE->ORC-CDC6+ZnR->HEPN-><-Zn_Tnp_IS1+HTH_Tnp_IS1+DDE_Tnp_IS1<-REase<-REase||BrxA-DNA-binding->BrxB-BrxF-iSTAND->DUF6079*->N6-METHYLASE->PglZ->SIGMA+OB-Fold+AAA-ATPase+Lon-Protease-BrxL-><-ZnR<-ZnR<-?||RHH_antitoxin->XIS-HTH-><-HTH_Tnp_1+HTH_21+rve+rve_3</td><td>1213</td><td>Bacteria>Pseudomonadota>Gammaproteobacteria>Enterobacterales>Enterobacteriaceae>Escherichia</td><td>GCF_000249715.1</td><td>Escherichia_coli</td><td>ECDEC7B_RS22125</td><td>12544..16185</td><td>562</td><td>BREX-system-P-loop-protein-BrxC-[Escherichia-coli]</td></tr>
<tr><td>WP_001019640.1</td><td>Type1.BREX</td><td>TRD->REase+N6_METHYLASE-><-?||?->ParB->?-><-?<-SIGMA+OB-Fold+AAA-ATPase+Lon-Protease-BrxL<-PglZ<-DUF6079*<-BrxB-BrxF-iSTAND<-BrxA-DNA-binding||REase+iREase-><-wHTH+SAD+HNH||<-?<-?<-?||Phage_integrase->Fimbrial-></td><td>1213</td><td>Bacteria>Pseudomonadota>Gammaproteobacteria>Enterobacterales>Enterobacteriaceae>Escherichia</td><td>GCF_000250075.1</td><td>Escherichia_coli</td><td>BE83_RS00870</td><td>326860..330501</td><td>562</td><td>BREX-system-P-loop-protein-BrxC-[Escherichia-coli]</td></tr>
<tr><td>WP_001019639.1</td><td>Type1.BREX</td><td>REase+N6_METHYLASE-><-?||?->ParB->?-><-?<-SIGMA+OB-Fold+AAA-ATPase+Lon-Protease-BrxL<-PglZ<-DUF6079*<-BrxB-BrxF-iSTAND<-BrxA-DNA-binding||REase+iREase-><-wHTH+SAD+HNH||<-?<-?<-?||Phage_integrase->Phage_integrase-></td><td>1213</td><td>Bacteria>Pseudomonadota>Gammaproteobacteria>Enterobacterales>Enterobacteriaceae>Escherichia</td><td>GCF_000250475.1</td><td>Escherichia_coli</td><td>BE27_RS00915</td><td>17908..21549</td><td>562</td><td>BREX-system-P-loop-protein-BrxC-[Escherichia-coli]</td></tr>
<tr><td>WP_001019650.1</td><td>Type1.BREX</td><td>wHTH->ZnR->ZnR->URI-DNase+RAMA-><-SIGMA+OB-Fold+AAA-ATPase+Lon-Protease-BrxL<-PglZ<-TOPRIM<-OLD-ABC<-N6-METHYLASE<-DUF6079*<-BrxB-BrxF-iSTAND<-BrxA-DNA-binding</td><td>1204</td><td>Bacteria>Pseudomonadota>Gammaproteobacteria>Enterobacterales>Enterobacteriaceae>Escherichia</td><td>GCF_000264195.1</td><td>Escherichia_coli</td><td>ECKD2_RS09380</td><td>24128..27742</td><td>562</td><td>BREX-system-P-loop-protein-BrxC-[Escherichia-coli]</td></tr>
<tr><td>WP_001019643.1</td><td>Type1.BREX</td><td>ZnR-><-SIGMA+OB-Fold+AAA-ATPase+Lon-Protease-BrxL<-PglZ<-N6-METHYLASE<-DUF6079*<-BrxB-BrxF-iSTAND<-BrxA-DNA-binding||REase->REase-></td><td>1213</td><td>Bacteria>Pseudomonadota>Gammaproteobacteria>Enterobacterales>Enterobacteriaceae>Escherichia</td><td>GCF_000326345.1</td><td>Escherichia_coli</td><td>WK3_RS01055</td><td>9311..12952</td><td>562</td><td>BREX-system-P-loop-protein-BrxC-[Escherichia-coli]</td></tr>
</table>

Protein-ID	System	Gene-Neighbourhoods	Length	Taxonomy	Assembly-ID	Strain	Locus-Tag	Genomic-coordinates	TaxID	Fasta-defline
WP_001698869.1	Type1.BREX	[illegible]	1225	Bacteria>Pseudomonadota>Gammaproteobacteria>Enterobacterales>Enterobacteriaceae>Escherichia	GCF_000340255.1	Escherichia_coli	C201_RS01205	14823..18500	562	BREX-system-P-loop-protein-BrxC-[Escherichia-coli]
WP_001704019.1	Type1.BREX	[illegible]	1213	Bacteria>Pseudomonadota>Gammaproteobacteria>Enterobacterales>Enterobacteriaceae>Escherichia	GCF_000354275.2	Escherichia_coli	EC2875000_RS00745	314498..318139	562	BREX-system-P-loop-protein-BrxC-[Escherichia-coli]
WP_001704755.1	Type1.BREX	[illegible]	1213	Bacteria>Pseudomonadota>Gammaproteobacteria>Enterobacterales>Enterobacteriaceae>Escherichia	GCF_000354455.1	Escherichia_coli	EC2872800_RS00650	314338..317979	562	BREX-system-P-loop-protein-BrxC-[Escherichia-coli]
WP_001707406.1	Type1.BREX	[illegible]	1213	Bacteria>Pseudomonadota>Gammaproteobacteria>Enterobacterales>Enterobacteriaceae>Escherichia	GCF_000354815.2	Escherichia_coli	EC2867750_RS24725	314336..317977	562	BREX-system-P-loop-protein-BrxC-[Escherichia-coli]
WP_004019909.1	Type1.BREX	[illegible]	1213	Bacteria>Pseudomonadota>Gammaproteobacteria>Enterobacterales>Enterobacteriaceae>Escherichia	GCF_000354915.1	Escherichia_coli	EC174750_RS00780	7732..11373	562	BREX-system-P-loop-protein-BrxC-[Escherichia-coli]
WP_003969750.1	Type1.BREX	[illegible]	1213	Bacteria>Pseudomonadota>Gammaproteobacteria>Enterobacterales>Enterobacteriaceae>Escherichia	GCF_000354935.2	Escherichia_coli	EC2866450_RS25180	218299..221940	562	BREX-system-P-loop-protein-BrxC-[Escherichia-coli]
WP_001709521.1	Type1.BREX	[illegible]	1213	Bacteria>Pseudomonadota>Gammaproteobacteria>Enterobacterales>Enterobacteriaceae>Escherichia	GCF_000354955.1	Escherichia_coli	EC2866550_RS00550	55545..59186	562	BREX-system-P-loop-protein-BrxC-[Escherichia-coli]
WP_001713779.1	Type1.BREX	[illegible]	1213	Bacteria>Pseudomonadota>Gammaproteobacteria>Enterobacterales>Enterobacteriaceae>Escherichia	GCF_000355015.2	Escherichia_coli	EC2851500_RS25090	314221..317862	562	BREX-system-P-loop-protein-BrxC-[Escherichia-coli]
WP_001715918.1	Type1.BREX	[illegible]	1213	Bacteria>Pseudomonadota>Gammaproteobacteria>Enterobacterales>Enterobacteriaceae>Escherichia	GCF_000355055.1	Escherichia_coli	EC2850400_RS01425	314375..318016	562	BREX-system-P-loop-protein-BrxC-[Escherichia-coli]
WP_001714681.1	Type1.BREX	[illegible]	1213	Bacteria>Pseudomonadota>Gammaproteobacteria>Enterobacterales>Enterobacteriaceae>Escherichia	GCF_000355335.2	Escherichia_coli	EC2850750_RS25235	55686..59327	562	BREX-system-P-loop-protein-BrxC-[Escherichia-coli]
WP_001462984.1	Type1.BREX	[illegible]	1213	Bacteria>Pseudomonadota>Gammaproteobacteria>Enterobacterales>Enterobacteriaceae>Escherichia	GCF_000357005.2	Escherichia_coli	ECP02994383_RS24020	76495..80136	562	BREX-system-P-loop-protein-BrxC-[Escherichia-coli]
WP_001464881.1	Type1.BREX	[illegible]	1213	Bacteria>Pseudomonadota>Gammaproteobacteria>Enterobacterales>Enterobacteriaceae>Escherichia	GCF_000357045.2	Escherichia_coli	ECP02994386_RS23895	144466..148107	562	BREX-system-P-loop-protein-BrxC-[Escherichia-coli]
WP_001466204.1	Type1.BREX	[illegible]	1213	Bacteria>Pseudomonadota>Gammaproteobacteria>Enterobacterales>Enterobacteriaceae>Escherichia	GCF_000357245.1	Escherichia_coli	ECP02994387_RS14340	10081..13722	562	BREX-system-P-loop-protein-BrxC-[Escherichia-coli]
WP_001505004.1	Type1.BREX	[illegible]	1213	Bacteria>Pseudomonadota>Gammaproteobacteria>Enterobacterales>Enterobacteriaceae>Escherichia	GCF_000446625.2	Escherichia_coli	ECC52794_RS10870	64245..67886	562	BREX-system-P-loop-protein-BrxC-[Escherichia-coli]
WP_024222704.1	Type1.BREX	[illegible]	1213	Bacteria>Pseudomonadota>Gammaproteobacteria>Enterobacterales>Enterobacteriaceae>Escherichia	GCF_000446725.2	Escherichia_coli	CTH77_RS23215	13991..17632	562	BREX-system-P-loop-protein-BrxC-[Escherichia-coli]
WP_024236748.1	Type1.BREX	[illegible]	1129	Bacteria>Pseudomonadota>Gammaproteobacteria>Enterobacterales>Enterobacteriaceae>Escherichia	GCF_000446745.2	Escherichia_coli	ECC71710_RS03330	1..3392	562	BREX-system-P-loop-protein-BrxC,-partial-[Escherichia-coli]
WP_024243520.1	Type1.BREX	[illegible]	1213	Bacteria>Pseudomonadota>Gammaproteobacteria>Enterobacterales>Enterobacteriaceae>Escherichia	GCF_000446965.2	Escherichia_coli	C3I80_RS17935	35165..38806	562	BREX-system-P-loop-protein-BrxC-[Escherichia-coli]
WP_001019649.1	Type1.BREX	[illegible]	1204	Bacteria>Pseudomonadota>Gammaproteobacteria>Enterobacterales>Enterobacteriaceae>Escherichia	GCF_000503455.1	Escherichia_coli	U109_RS0110040	13723..17337	562	BREX-system-P-loop-protein-BrxC-[Escherichia-coli]
WP_025670435.1	Type1.BREX	[illegible]	1213	Bacteria>Pseudomonadota>Gammaproteobacteria>Enterobacterales>Enterobacteriaceae>Escherichia	GCF_000511525.1	Escherichia_coli	A7216_RS07695	16662..20303	562	BREX-system-P-loop-protein-BrxC-[Escherichia-coli]
WP_029488486.1	Type1.BREX	[illegible]	1213	Bacteria>Pseudomonadota>Gammaproteobacteria>Enterobacterales>Enterobacteriaceae>Escherichia	GCF_000511545.1	Escherichia_coli	U124_RS0103885	150968..154609	562	BREX-system-P-loop-protein-BrxC-[Escherichia-coli]
WP_032271764.1	Type1.BREX	[illegible]	996	Bacteria>Pseudomonadota>Gammaproteobacteria>Enterobacterales>Enterobacteriaceae>Escherichia	GCF_000614035.2	Escherichia_coli	BX93_RS13740	78498..81489	562	BREX-system-P-loop-protein-BrxC,-partial-[Escherichia-coli]
WP_032272074.1	Type1.BREX	[illegible]	1188	Bacteria>Pseudomonadota>Gammaproteobacteria>Enterobacterales>Enterobacteriaceae>Escherichia	GCF_000615605.2	Escherichia_coli	BX59_RS22390	1..3568	562	BREX-system-P-loop-protein-BrxC,-partial-[Escherichia-coli]
WP_032353829.1	Type1.BREX	[illegible]	1213	Bacteria>Pseudomonadota>Gammaproteobacteria>Enterobacterales>Enterobacteriaceae>Escherichia	GCF_000632675.2	Escherichia_coli	BX99_RS21625	78780..82421	562	BREX-system-P-loop-protein-BrxC-[Escherichia-coli]
WP_032216088.1	Type1.BREX	[illegible]	1225	Bacteria>Pseudomonadota>Gammaproteobacteria>Enterobacterales>Enterobacteriaceae>Escherichia	GCF_000713175.1	Escherichia_coli	AD46_RS16460	5265..8942	562	BREX-system-P-loop-protein-BrxC-[Escherichia-coli]
WP_032305083.1	Type1.BREX	[illegible]	1225	Bacteria>Pseudomonadota>Gammaproteobacteria>Enterobacterales>Enterobacteriaceae>Escherichia	GCF_000714055.1	Escherichia_coli	AC36_RS01690	6338..10015	562	BREX-system-P-loop-protein-BrxC-[Escherichia-coli]
WP_032222683.1	Type1.BREX	[illegible]	1225	Bacteria>Pseudomonadota>Gammaproteobacteria>Enterobacterales>Enterobacteriaceae>Escherichia	GCF_000714385.1	Escherichia_coli	AC91_RS16385	5217..8894	562	BREX-system-P-loop-protein-BrxC-[Escherichia-coli]
WP_033804924.1	Type1.BREX	[illegible]	862	Bacteria>Pseudomonadota>Gammaproteobacteria>Enterobacterales>Enterobacteriaceae>Escherichia	GCF_000752535.1	Escherichia_coli	MY85_RS14095	76836..79424	562	BREX-system-P-loop-protein-BrxC,-partial-[Escherichia-coli]
WP_042106733.1	Type1.BREX	[illegible]	895	Bacteria>Pseudomonadota>Gammaproteobacteria>Enterobacterales>Enterobacteriaceae>Escherichia	GCF_000777265.1	Escherichia_coli	PP93_RS24150	26949..29632	562	BREX-system-P-loop-protein-BrxC,-partial-[Escherichia-coli]
WP_033566562.1	Type1.BREX	[illegible]	781	Bacteria>Pseudomonadota>Gammaproteobacteria>Enterobacterales>Enterobacteriaceae>Escherichia	GCF_000783335.1	Escherichia_coli	LW92_RS03930	20620..22963	562	BREX-system-P-loop-protein-BrxC,-partial-[Escherichia-coli]
WP_034167068.1	Type1.BREX	[illegible]	1225	Bacteria>Pseudomonadota>Gammaproteobacteria>Enterobacterales>Enterobacteriaceae>Escherichia	GCF_000804325.1	Escherichia_coli	RK25_RS05450	34844..38521	562	BREX-system-P-loop-protein-BrxC-[Escherichia-coli]
WP_038989008.1	Type1.BREX	[illegible]	1204	Bacteria>Pseudomonadota>Gammaproteobacteria>Enterobacterales>Enterobacteriaceae>Escherichia	GCF_000812725.1	Escherichia_coli	RK29_RS16985	15030..18644	562	BREX-system-P-loop-protein-BrxC-[Escherichia-coli]
WP_040091953.1	Type1.BREX	[illegible]	837	Bacteria>Pseudomonadota>Gammaproteobacteria>Enterobacterales>Enterobacteriaceae>Escherichia	GCF_000819205.1	Escherichia_coli	PU45_RS22665	1..2514	562	BREX-system-P-loop-protein-BrxC,-partial-[Escherichia-coli]
WP_050867845.1	Type1.BREX	[illegible]	1213	Bacteria>Pseudomonadota>Gammaproteobacteria>Enterobacterales>Enterobacteriaceae>Escherichia	GCF_001191345.1	Escherichia_coli	WQ87_RS09225	9779..13420	562	BREX-system-P-loop-protein-BrxC-[Escherichia-coli]
WP_052909519.1	Type1.BREX	[illegible]	1213	Bacteria>Pseudomonadota>Gammaproteobacteria>Enterobacterales>Enterobacteriaceae>Escherichia	GCF_001262895.1	Escherichia_coli	AGA27_RS05565	9779..13420	562	BREX-system-P-loop-protein-BrxC-[Escherichia-coli]
WP_053916754.1	Type1.BREX	[illegible]	1213	Bacteria>Pseudomonadota>Gammaproteobacteria>Enterobacterales>Enterobacteriaceae>Escherichia	GCF_001283285.1	Escherichia_coli	AOH25_RS20055	35212..38853	562	BREX-system-P-loop-protein-BrxC-[Escherichia-coli]
WP_053892086.1	Type1.BREX	[illegible]	1213	Bacteria>Pseudomonadota>Gammaproteobacteria>Enterobacterales>Enterobacteriaceae>Escherichia	GCF_001285285.1	Escherichia_coli	AOH78_RS17135	37162..40803	562	BREX-system-P-loop-protein-BrxC-[Escherichia-coli]
WP_021568966.1	Type1.BREX	[illegible]	1213	Bacteria>Pseudomonadota>Gammaproteobacteria>Enterobacterales>Enterobacteriaceae>Escherichia	GCF_001285965.1	Escherichia_coli	AOJ37_RS12845	35181..38822	562	BREX-system-P-loop-protein-BrxC-[Escherichia-coli]
WP_032257286.1	Type1.BREX	[illegible]	1213	Bacteria>Pseudomonadota>Gammaproteobacteria>Enterobacterales>Enterobacteriaceae>Escherichia	GCF_001286385.1	Escherichia_coli	AOJ18_RS01745	37207..40848	562	BREX-system-P-loop-protein-BrxC-[Escherichia-coli]
WP_053897162.1	Type1.BREX	[illegible]	1132	Bacteria>Pseudomonadota>Gammaproteobacteria>Enterobacterales>Enterobacteriaceae>Escherichia	GCF_001286505.1	Escherichia_coli	AOJ58_RS17615	1..3401	562	BREX-system-P-loop-protein-BrxC,-partial-[Escherichia-coli]
WP_060616116.1	Type1.BREX	[illegible]	1204	Bacteria>Pseudomonadota>Gammaproteobacteria>Enterobacterales>Enterobacteriaceae>Escherichia	GCF_001484475.1	Escherichia_coli	DT570_RS09710	10713..14327	562	BREX-system-P-loop-protein-BrxC-[Escherichia-coli]
WP_059338855.1	Type1.BREX	[illegible]	991	Bacteria>Pseudomonadota>Gammaproteobacteria>Enterobacterales>Enterobacteriaceae>Escherichia	GCF_001519915.1	Escherichia_coli	AWF46_RS03555	9408..12384	562	BREX-system-P-loop-protein-BrxC,-partial-[Escherichia-coli]
WP_059322911.1	Type1.BREX	[illegible]	992	Bacteria>Pseudomonadota>Gammaproteobacteria>Enterobacterales>Enterobacteriaceae>Escherichia	GCF_001520675.1	Escherichia_coli	AWF39_RS02795	1..2981	562	BREX-system-P-loop-protein-BrxC,-partial-[Escherichia-coli]
WP_061359766.1	Type1.BREX	[illegible]	1213	Bacteria>Pseudomonadota>Gammaproteobacteria>Enterobacterales>Enterobacteriaceae>Escherichia	GCF_001575355.1	Escherichia_coli	AUP84_RS21650	2336..5977	562	BREX-system-P-loop-protein-BrxC-[Escherichia-coli]
WP_062863778.1	Type1.BREX	[illegible]	998	Bacteria>Pseudomonadota>Gammaproteobacteria>Enterobacterales>Enterobacteriaceae>Escherichia	GCF_001607105.1	Escherichia_coli	AML45_RS08440	1..2999	562	BREX-system-P-loop-protein-BrxC,-partial-[Escherichia-coli]
WP_063100938.1	Type1.BREX	[illegible]	1213	Bacteria>Pseudomonadota>Gammaproteobacteria>Enterobacterales>Enterobacteriaceae>Escherichia	GCF_001615735.1	Escherichia_coli	AVN29_RS04305	18241..21882	562	BREX-system-P-loop-protein-BrxC-[Escherichia-coli]
WP_069924879.1	Type1.BREX	[illegible]	1213	Bacteria>Pseudomonadota>Gammaproteobacteria>Enterobacterales>Enterobacteriaceae>Escherichia	GCF_001748825.1	Escherichia_coli	BHF18_RS13015	262302..265943	562	BREX-system-P-loop-protein-BrxC-[Escherichia-coli]
WP_072679154.1	Type1.BREX	[illegible]	1213	Bacteria>Pseudomonadota>Gammaproteobacteria>Enterobacterales>Enterobacteriaceae>Escherichia	GCF_001894085.1	Escherichia_coli	BK377_RS08410	37601..41242	562	BREX-system-P-loop-protein-BrxC-[Escherichia-coli]
WP_072752451.1	Type1.BREX	[illegible]	1225	Bacteria>Pseudomonadota>Gammaproteobacteria>Enterobacterales>Enterobacteriaceae>Escherichia	GCF_001894415.1	Escherichia_coli	BK380_RS19185	23163..26840	562	BREX-system-P-loop-protein-BrxC-[Escherichia-coli]
WP_072748589.1	Type1.BREX	[illegible]	1213	Bacteria>Pseudomonadota>Gammaproteobacteria>Enterobacterales>Enterobacteriaceae>Escherichia	GCF_001894585.1	Escherichia_coli	BK398_RS07575	128185..131826	562	BREX-system-P-loop-protein-BrxC-[Escherichia-coli]
WP_074525756.1	Type1.BREX	[illegible]	1213	Bacteria>Pseudomonadota>Gammaproteobacteria>Enterobacterales>Enterobacteriaceae>Escherichia	GCF_001911555.1	Escherichia_coli	ACN68_RS20915	50623..54264	562	BREX-system-P-loop-protein-BrxC-[Escherichia-coli]
WP_085314183.1	Type1.BREX	[illegible]	1078	Bacteria>Pseudomonadota>Gammaproteobacteria>Enterobacterales>Enterobacteriaceae>Escherichia	GCF_002104645.1	Escherichia_coli	B8A27_RS24555	59111..62345	562	BREX-system-P-loop-protein-BrxC,-partial-[Escherichia-coli]
WP_244571630.1	Type1.BREX	[illegible]	673	Bacteria>Pseudomonadota>Gammaproteobacteria>Enterobacterales>Enterobacteriaceae>Escherichia	GCF_002231255.1	Escherichia_coli	ATB30_RS25725	4607..6624	562	BREX-system-P-loop-protein-BrxC,-partial-[Escherichia-coli]
WP_089613509.1	Type1.BREX	[illegible]	1213	Bacteria>Pseudomonadota>Gammaproteobacteria>Enterobacterales>Enterobacteriaceae>Escherichia	GCF_002231555.1	Escherichia_coli	ATB14_RS09425	78080..81721	562	BREX-system-P-loop-protein-BrxC-[Escherichia-coli]
WP_089592460.1	Type1.BREX	[illegible]	1213	Bacteria>Pseudomonadota>Gammaproteobacteria>Enterobacterales>Enterobacteriaceae>Escherichia	GCF_002232915.1	Escherichia_coli	AS998_RS25490	17146..20787	562	BREX-system-P-loop-protein-BrxC-[Escherichia-coli]
WP_096933204.1	Type1.BREX	[illegible]	1213	Bacteria>Pseudomonadota>Gammaproteobacteria>Enterobacterales>Enterobacteriaceae>Escherichia	GCF_002442365.1	Escherichia_coli	BMR57_RS14870	5774..9415	562	BREX-system-P-loop-protein-BrxC-[Escherichia-coli]
WP_096939573.1	Type1.BREX	[illegible]	1213	Bacteria>Pseudomonadota>Gammaproteobacteria>Enterobacterales>Enterobacteriaceae>Escherichia	GCF_002464005.1	Escherichia_coli	A7578_RS18845	20590..24231	562	BREX-system-P-loop-protein-BrxC-[Escherichia-coli]
WP_096969144.1	Type1.BREX	[illegible]	1225	Bacteria>Pseudomonadota>Gammaproteobacteria>Enterobacterales>Enterobacteriaceae>Escherichia	GCF_002466035.1	Escherichia_coli	BSN44_RS10850	73237..76914	562	BREX-system-P-loop-protein-BrxC-[Escherichia-coli]
WP_097339933.1	Type1.BREX	[illegible]	1213	Bacteria>Pseudomonadota>Gammaproteobacteria>Enterobacterales>Enterobacteriaceae>Escherichia	GCF_002484685.1	Escherichia_coli	A7197_RS22885	8895..12536	562	BREX-system-P-loop-protein-BrxC-[Escherichia-coli]
WP_097426405.1	Type1.BREX	[illegible]	1213	Bacteria>Pseudomonadota>Gammaproteobacteria>Enterobacterales>Enterobacteriaceae>Escherichia	GCF_002510325.1	Escherichia_coli	A7660_RS13795	13148..16789	562	BREX-system-P-loop-protein-BrxC-[Escherichia-coli]
WP_097429067.1	Type1.BREX	[illegible]	1213	Bacteria>Pseudomonadota>Gammaproteobacteria>Enterobacterales>Enterobacteriaceae>Escherichia	GCF_002510645.1	Escherichia_coli	A7614_RS02560	133569..137210	562	BREX-system-P-loop-protein-BrxC-[Escherichia-coli]
WP_244587252.1	Type1.BREX	[illegible]	1137	Bacteria>Pseudomonadota>Gammaproteobacteria>Enterobacterales>Enterobacteriaceae>Escherichia	GCF_002519325.1	Escherichia_coli	A7034_RS17685	1..3416	562	BREX-system-P-loop-protein-BrxC,-partial-[Escherichia-coli]
WP_097760627.1	Type1.BREX	[illegible]	1213	Bacteria>Pseudomonadota>Gammaproteobacteria>Enterobacterales>Enterobacteriaceae>Escherichia	GCF_002534675.1	Escherichia_coli	BEA63_RS10320	9803..13444	562	BREX-system-P-loop-protein-BrxC-[Escherichia-coli]
WP_099139404.1	Type1.BREX	[illegible]	1213	Bacteria>Pseudomonadota>Gammaproteobacteria>Enterobacterales>Enterobacteriaceae>Escherichia	GCF_002631765.2	Escherichia_coli	CQR85_RS13645	71476..75117	562	BREX-system-P-loop-protein-BrxC-[Escherichia-coli]
WP_096974508.1	Type1.BREX	[illegible]	1213	Bacteria>Pseudomonadota>Gammaproteobacteria>Enterobacterales>Enterobacteriaceae>Escherichia	GCF_002741355.1	Escherichia_coli	BFZ27_RS06875	15053..18694	562	BREX-system-P-loop-protein-BrxC-[Escherichia-coli]
WP_102812546.1	Type1.BREX	[illegible]	1213	Bacteria>Pseudomonadota>Gammaproteobacteria>Enterobacterales>Enterobacteriaceae>Escherichia	GCF_002888475.1	Escherichia_coli	C1T15_RS24495	11055..14696	562	BREX-system-P-loop-protein-BrxC-[Escherichia-coli]
WP_148116075.1	Type1.BREX	[illegible]	1213	Bacteria>Pseudomonadota>Gammaproteobacteria>Enterobacterales>Enterobacteriaceae>Escherichia	GCF_002951795.1	Escherichia_coli	BWI87_RS06400	1249242..1252883	562	BREX-system-P-loop-protein-BrxC-[Escherichia-coli]
WP_106879201.1	Type1.BREX	[illegible]	1213	Bacteria>Pseudomonadota>Gammaproteobacteria>Enterobacterales>Enterobacteriaceae>Escherichia	GCF_003018215.1	Escherichia_coli	C6P67_RS24165	4665409..4669050	562	BREX-system-P-loop-protein-BrxC-[Escherichia-coli]
WP_044805810.1	Type1.BREX	[illegible]	1213	Bacteria>Pseudomonadota>Gammaproteobacteria>Enterobacterales>Enterobacteriaceae>Escherichia	GCF_003018495.1	Escherichia_coli	C6953_RS06925	1324145..1327786	562	BREX-system-P-loop-protein-BrxC-[Escherichia-coli]
WP_109548991.1	Type1.BREX	[illegible]	1213	Bacteria>Pseudomonadota>Gammaproteobacteria>Enterobacterales>Enterobacteriaceae>Escherichia	GCF_003145675.1	Escherichia_coli	B6S34_RS22850	50490..54131	562	BREX-system-P-loop-protein-BrxC-[Escherichia-coli]
WP_097502528.1	Type1.BREX	[illegible]	1213	Bacteria>Pseudomonadota>Gammaproteobacteria>Enterobacterales>Enterobacteriaceae>Escherichia	GCF_003176235.1	Escherichia_coli	DDA49_RS07225	8727..12368	562	BREX-system-P-loop-protein-BrxC-[Escherichia-coli]
WP_113316937.1	Type1.BREX	[illegible]	1213	Bacteria>Pseudomonadota>Gammaproteobacteria>Enterobacterales>Enterobacteriaceae>Escherichia	GCF_003299815.1	Escherichia_coli	DUK12_RS25275	2498..6139	562	BREX-system-P-loop-protein-BrxC-[Escherichia-coli]
WP_121351411.1	Type1.BREX	[illegible]	1213	Bacteria>Pseudomonadota>Gammaproteobacteria>Enterobacterales>Enterobacteriaceae>Escherichia	GCF_003651285.1	Escherichia_coli	EAO63_RS07885	97180..100821	562	BREX-system-P-loop-protein-BrxC-[Escherichia-coli]
WP_252363318.1	Type1.BREX	[illegible]	1240	Bacteria>Pseudomonadota>Gammaproteobacteria>Enterobacterales>Enterobacteriaceae>Escherichia	GCF_003687125.1	Escherichia_coli	EAI39_RS15070	84115..87837	562	BREX-system-P-loop-protein-BrxC-[Escherichia-coli]
WP_122927386.1	Type1.BREX	[illegible]	1213	Bacteria>Pseudomonadota>Gammaproteobacteria>Enterobacterales>Enterobacteriaceae>Escherichia	GCF_003711005.1	Escherichia_coli	EB670_RS21355	4330120..4333761	562	BREX-system-P-loop-protein-BrxC-[Escherichia-coli]
WP_128448083.1	Type1.BREX	[illegible]	1213	Bacteria>Pseudomonadota>Gammaproteobacteria>Enterobacterales>Enterobacteriaceae>Escherichia	GCF_004054205.1	Escherichia_coli	EQH54_15620	68233..71874	562	BREX-system-P-loop-protein-BrxC-[Escherichia-coli]
WP_135129151.1	Type1.BREX	[illegible]	1204	Bacteria>Pseudomonadota>Gammaproteobacteria>Enterobacterales>Enterobacteriaceae>Escherichia	GCF_004566475.1	Escherichia_coli	ELY39_RS16045	71744..75358	562	BREX-system-P-loop-protein-BrxC-[Escherichia-coli]
WP_135497797.1	Type1.BREX	[illegible]	1213	Bacteria>Pseudomonadota>Gammaproteobacteria>Enterobacterales>Enterobacteriaceae>Escherichia	GCF_004767105.1	Escherichia_coli	DAH29_RS19080	36143..39784	562	BREX-system-P-loop-protein-BrxC-[Escherichia-coli]
WP_016246797.1	Type1.BREX	[illegible]	1213	Bacteria>Pseudomonadota>Gammaproteobacteria>Enterobacterales>Enterobacteriaceae>Escherichia	GCF_005384525.1	Escherichia_coli	FFB03_RS10735	79495..83136	562	BREX-system-P-loop-protein-BrxC-[Escherichia-coli]
WP_044782573.1	Type1.BREX	[illegible]	1213	Bacteria>Pseudomonadota>Gammaproteobacteria>Enterobacterales>Enterobacteriaceae>Escherichia	GCF_005390485.1	Escherichia_coli	FFG15_RS13655	95922..99563	562	BREX-system-P-loop-protein-BrxC-[Escherichia-coli]
WP_136724038.1	Type1.BREX	[illegible]	1213	Bacteria>Pseudomonadota>Gammaproteobacteria>Enterobacterales>Enterobacteriaceae>Escherichia	GCF_005390625.1	Escherichia_coli	FFG35_RS20705	9778..13419	562	BREX-system-P-loop-protein-BrxC-[Escherichia-coli]
WP_097512272.1	Type1.BREX	[illegible]	1213	Bacteria>Pseudomonadota>Gammaproteobacteria>Enterobacterales>Enterobacteriaceae>Escherichia	GCF_005391145.1	Escherichia_coli	A7301_RS09385	138963..142604	562	BREX-system-P-loop-protein-BrxC-[Escherichia-coli]
WP_137536909.1	Type1.BREX	[illegible]	1213	Bacteria>Pseudomonadota>Gammaproteobacteria>Enterobacterales>Enterobacteriaceae>Escherichia	GCF_005392025.1	Escherichia_coli	FFI00_RS00495	95917..99558	562	BREX-system-P-loop-protein-BrxC-[Escherichia-coli]
WP_063115883.1	Type1.BREX	[illegible]	1213	Bacteria>Pseudomonadota>Gammaproteobacteria>Enterobacterales>Enterobacteriaceae>Escherichia	GCF_005393265.1	Escherichia_coli	MMU60_RS13225	76316..79957	562	BREX-system-P-loop-protein-BrxC-[Escherichia-coli]
WP_137551577.1	Type1.BREX	[illegible]	1213	Bacteria>Pseudomonadota>Gammaproteobacteria>Enterobacterales>Enterobacteriaceae>Escherichia	GCF_005394845.1	Escherichia_coli	FFJ65_RS02650	22335..25976	562	BREX-system-P-loop-protein-BrxC-[Escherichia-coli]
WP_137573648.1	Type1.BREX	[illegible]	1213	Bacteria>Pseudomonadota>Gammaproteobacteria>Enterobacterales>Enterobacteriaceae>Escherichia	GCF_005397085.1	Escherichia_coli	FFK68_RS25060	21797..25438	562	BREX-system-P-loop-protein-BrxC-[Escherichia-coli]
WP_137585959.1	Type1.BREX	[illegible]	1213	Bacteria>Pseudomonadota>Gammaproteobacteria>Enterobacterales>Enterobacteriaceae>Escherichia	GCF_005399505.1	Escherichia_coli	FFL73_RS10880	97640..101281	562	BREX-system-P-loop-protein-BrxC-[Escherichia-coli]
WP_137590371.1	Type1.BREX	[illegible]	1213	Bacteria>Pseudomonadota>Gammaproteobacteria>Enterobacterales>Enterobacteriaceae>Escherichia	GCF_005399685.1	Escherichia_coli	FFM00_RS14520	19836..23477	562	BREX-system-P-loop-protein-BrxC-[Escherichia-coli]
WP_139060210.1	Type1.BREX	[illegible]	635	Bacteria>Pseudomonadota>Gammaproteobacteria>Enterobacterales>Enterobacteriaceae>Escherichia	GCF_006151715.1	Escherichia_coli	FD592_RS22960	1..1905	562	BREX-system-P-loop-protein-BrxC,-partial-[Escherichia-coli]
WP_147697439.1	Type1.BREX	[illegible]	1225	Bacteria>Pseudomonadota>Gammaproteobacteria>Enterobacterales>Enterobacteriaceae>Escherichia	GCF_008041475.1	Escherichia_coli	FV285_RS21415	12602..16279	562	BREX-system-P-loop-protein-BrxC-[Escherichia-coli]
WP_032192228.1	Type1.BREX	[illegible]	1213	Bacteria>Pseudomonadota>Gammaproteobacteria>Enterobacterales>Enterobacteriaceae>Escherichia	GCF_008326465.1	Escherichia_coli	F1555_RS25960	8938..12579	562	BREX-system-P-loop-protein-BrxC-[Escherichia-coli]
WP_149488190.1	Type1.BREX	[illegible]	1225	Bacteria>Pseudomonadota>Gammaproteobacteria>Enterobacterales>Enterobacteriaceae>Escherichia	GCF_008364435.1	Escherichia_coli	F0318_RS09425	23572..27249	562	BREX-system-P-loop-protein-BrxC-[Escherichia-coli]
WP_023063795.1	Type1.BREX	[illegible]	1225	Bacteria>Pseudomonadota>Gammaproteobacteria>Enterobacterales>Enterobacteriaceae>Escherichia	GCF_008370755.1	Escherichia_coli	L913_RS00645	11831..15508	562	BREX-system-P-loop-protein-BrxC-[Escherichia-coli]
WP_050939406.1	Type1.BREX	[illegible]	1213	Bacteria>Pseudomonadota>Gammaproteobacteria>Enterobacterales>Enterobacteriaceae>Escherichia	GCF_009618015.1	Escherichia_coli	DTB71_RS01510	297715..301356	562	BREX-system-P-loop-protein-BrxC-[Escherichia-coli]
WP_154139479.1	Type1.BREX	[illegible]	1213	Bacteria>Pseudomonadota>Gammaproteobacteria>Enterobacterales>Enterobacteriaceae>Escherichia	GCF_009671045.1	Escherichia_coli	ML316_RS21570	8749..12390	562	BREX-system-P-loop-protein-BrxC-[Escherichia-coli]
WP_160518859.1	Type1.BREX	[illegible]	1213	Bacteria>Pseudomonadota>Gammaproteobacteria>Enterobacterales>Enterobacteriaceae>Escherichia	GCF_009820915.1	Escherichia_coli	FQ032_RS06170	111352..114993	562	BREX-system-P-loop-protein-BrxC-[Escherichia-coli]
WP_163451335.1	Type1.BREX	[illegible]	1204	Bacteria>Pseudomonadota>Gammaproteobacteria>Enterobacterales>Enterobacteriaceae>Escherichia	GCF_011008705.1	Escherichia_coli	G4V04_RS14425	2939..6553	562	BREX-system-P-loop-protein-BrxC-[Escherichia-coli]
WP_164476769.1	Type1.BREX	[illegible]	1213	Bacteria>Pseudomonadota>Gammaproteobacteria>Enterobacterales>Enterobacteriaceae>Escherichia	GCF_011045235.1	Escherichia_coli	G6Z93_RS19880	4188029..4191670	562	BREX-system-P-loop-protein-BrxC-[Escherichia-coli]
WP_136516625.1	Type1.BREX	[illegible]	1225	Bacteria>Pseudomonadota>Gammaproteobacteria>Enterobacterales>Enterobacteriaceae>Escherichia	GCF_011045875.1	Escherichia_coli	EIZ78_RS19775	10945..14622	562	BREX-system-P-loop-protein-BrxC-[Escherichia-coli]
WP_072657276.1	Type1.BREX	[illegible]	1225	Bacteria>Pseudomonadota>Gammaproteobacteria>Enterobacterales>Enterobacteriaceae>Escherichia	GCF_011077045.1	Escherichia_coli	G6Q90_RS23590	2674..6351	562	BREX-system-P-loop-protein-BrxC-[Escherichia-coli]
WP_152922120.1	Type1.BREX	[illegible]	1213	Bacteria>Pseudomonadota>Gammaproteobacteria>Enterobacterales>Enterobacteriaceae>Escherichia	GCF_011404475.1	Escherichia_coli	HRR20_RS19820	4109090..4112731	562	BREX-system-P-loop-protein-BrxC-[Escherichia-coli]
WP_167824001.1	Type1.BREX	[illegible]	1213	Bacteria>Pseudomonadota>Gammaproteobacteria>Enterobacterales>Enterobacteriaceae>Escherichia	GCF_012063235.1	Escherichia_coli	GTJ82_RS11140	125491..129132	562	BREX-system-P-loop-protein-BrxC-[Escherichia-coli]
WP_250854521.1	Type1.BREX	[illegible]	887	Bacteria>Pseudomonadota>Gammaproteobacteria>Enterobacterales>Enterobacteriaceae>Escherichia	GCF_012556305.1	Escherichia_coli	MLIZ_RS15475	1..2666	562	BREX-system-P-loop-protein-BrxC,-partial-[Escherichia-coli]
WP_170969386.1	Type1.BREX	[illegible]	1213	Bacteria>Pseudomonadota>Gammaproteobacteria>Enterobacterales>Enterobacteriaceae>Escherichia	GCF_013085185.1	Escherichia_coli	HHJ24_09500	1928934..1932575	562	BREX-system-P-loop-protein-BrxC-[Escherichia-coli]
WP_174563320.1	Type1.BREX	[illegible]	1213	Bacteria>Pseudomonadota>Gammaproteobacteria>Enterobacterales>Enterobacteriaceae>Escherichia	GCF_013341485.1	Escherichia_coli	HUC04_RS15605	6608..10249	562	BREX-system-P-loop-protein-BrxC-[Escherichia-coli]
WP_097472690.1	Type1.BREX	[illegible]	1213	Bacteria>Pseudomonadota>Gammaproteobacteria>Enterobacterales>Enterobacteriaceae>Escherichia	GCF_013341925.1	Escherichia_coli	MMN47_RS21975	16744..20385	562	BREX-system-P-loop-protein-BrxC-[Escherichia-coli]
WP_032218952.1	Type1.BREX	[illegible]	1204	Bacteria>Pseudomonadota>Gammaproteobacteria>Enterobacterales>Enterobacteriaceae>Escherichia	GCF_013370905.1	Escherichia_coli	DNN97_RS07945	182755..186369	562	BREX-system-P-loop-protein-BrxC-[Escherichia-coli]
WP_176670934.1	Type1.BREX	[illegible]	1213	Bacteria>Pseudomonadota>Gammaproteobacteria>Enterobacterales>Enterobacteriaceae>Escherichia	GCF_013376895.1	Escherichia_coli	A7A13_RS22790	4759790..4763431	562	BREX-system-P-loop-protein-BrxC-[Escherichia-coli]
WP_179778150.1	Type1.BREX	[illegible]	1213	Bacteria>Pseudomonadota>Gammaproteobacteria>Enterobacterales>Enterobacteriaceae>Escherichia	GCF_013413745.1	Escherichia_coli	HZT14_RS03845	97447..101088	562	BREX-system-P-loop-protein-BrxC-[Escherichia-coli]
WP_032228177.1	Type1.BREX	[illegible]	1225	Bacteria>Pseudomonadota>Gammaproteobacteria>Enterobacterales>Enterobacteriaceae>Escherichia	GCF_013424245.1	Escherichia_coli	H0P07_RS08595	2755..6432	562	BREX-system-P-loop-protein-BrxC-[Escherichia-coli]
WP_180489728.1	Type1.BREX	[illegible]	1213	Bacteria>Pseudomonadota>Gammaproteobacteria>Enterobacterales>Enterobacteriaceae>Escherichia	GCF_013424415.1	Escherichia_coli	H0O79_RS20980	3102..6743	562	BREX-system-P-loop-protein-BrxC-[Escherichia-coli]
WP_181678095.1	Type1.BREX	[illegible]	1213	Bacteria>Pseudomonadota>Gammaproteobacteria>Enterobacterales>Enterobacteriaceae>Escherichia	GCF_013851105.1	Escherichia_coli	H4962_RS13940	2269..5910	562	BREX-system-P-loop-protein-BrxC-[Escherichia-coli]
WP_182913085.1	Type1.BREX	[illegible]	1213	Bacteria>Pseudomonadota>Gammaproteobacteria>Enterobacterales>Enterobacteriaceae>Escherichia	GCF_014168755.1	Escherichia_coli	WP2S18E08_08260	876105..879746	562	BREX-system-P-loop-protein-BrxC-[Escherichia-coli]
WP_025653408.1	Type1.BREX	[illegible]	1213	Bacteria>Pseudomonadota>Gammaproteobacteria>Enterobacterales>Enterobacteriaceae>Escherichia	GCF_014169595.1	Escherichia_coli	H7R13_RS19900	4165197..4168838	562	BREX-system-P-loop-protein-BrxC-[Escherichia-coli]
WP_192481452.1	Type1.BREX	[illegible]	1213	Bacteria>Pseudomonadota>Gammaproteobacteria>Enterobacterales>Enterobacteriaceae>Escherichia	GCF_014858085.1	Escherichia_coli	IHV13_RS01935	12078..15719	562	BREX-system-P-loop-protein-BrxC-[Escherichia-coli]
WP_192960469.1	Type1.BREX	[illegible]	1213	Bacteria>Pseudomonadota>Gammaproteobacteria>Enterobacterales>Enterobacteriaceae>Escherichia	GCF_014892435.1	Escherichia_coli	INS74_RS22970	2229..5870	562	BREX-system-P-loop-protein-BrxC-[Escherichia-coli]
WP_193098973.1	Type1.BREX	[illegible]	684	Bacteria>Pseudomonadota>Gammaproteobacteria>Enterobacterales>Enterobacteriaceae>Escherichia	GCF_014900935.1	Escherichia_coli	ICK29_RS00005	1..2052	562	BREX-system-P-loop-protein-BrxC,-partial-[Escherichia-coli]
WP_193097043.1	Type1.BREX	[illegible]	1213	Bacteria>Pseudomonadota>Gammaproteobacteria>Enterobacterales>Enterobacteriaceae>Escherichia	GCF_014900955.1	Escherichia_coli	ICK28_RS00010	345..3986	562	BREX-system-P-loop-protein-BrxC-[Escherichia-coli]
WP_087888065.1	Type1.BREX	[illegible]	1225	Bacteria>Pseudomonadota>Gammaproteobacteria>Enterobacterales>Enterobacteriaceae>Escherichia	GCF_015229345.1	Escherichia_coli	GVI22_RS02065	20896..24573	562	BREX-system-P-loop-protein-BrxC-[Escherichia-coli]
WP_097450867.1	Type1.BREX	[illegible]	1213	Bacteria>Pseudomonadota>Gammaproteobacteria>Enterobacterales>Enterobacteriaceae>Escherichia	GCF_015476745.1	Escherichia_coli	I0B98_RS17950	606603..610244	562	BREX-system-P-loop-protein-BrxC-[Escherichia-coli]
WP_195390166.1	Type1.BREX	[illegible]	1213	Bacteria>Pseudomonadota>Gammaproteobacteria>Enterobacterales>Enterobacteriaceae>Escherichia	GCF_015553825.1	Escherichia_coli	I2D81_RS15750	15887..19528	562	BREX-system-P-loop-protein-BrxC-[Escherichia-coli]
WP_195745416.1	Type1.BREX	[illegible]	1213	Bacteria>Pseudomonadota>Gammaproteobacteria>Enterobacterales>Enterobacteriaceae>Escherichia	GCF_015620345.1	Escherichia_coli	I3C17_RS21905	2642..6283	562	BREX-system-P-loop-protein-BrxC-[Escherichia-coli]
WP_197364841.1	Type1.BREX	[illegible]	1213	Bacteria>Pseudomonadota>Gammaproteobacteria>Enterobacterales>Enterobacteriaceae>Escherichia	GCF_015912555.1	Escherichia_coli	F2X22_RS13815	740..4381	562	BREX-system-P-loop-protein-BrxC-[Escherichia-coli]
WP_201358321.1	Type1.BREX	[illegible]	1213	Bacteria>Pseudomonadota>Gammaproteobacteria>Enterobacterales>Enterobacteriaceae>Escherichia	GCF_016587155.1	Escherichia_coli	JMH48_RS18845	518753..522394	562	BREX-system-P-loop-protein-BrxC-[Escherichia-coli]
WP_202845940.1	Type1.BREX	[illegible]	1213	Bacteria>Pseudomonadota>Gammaproteobacteria>Enterobacterales>Enterobacteriaceae>Escherichia	GCF_016776325.1	Escherichia_coli	JNN51_RS16875	3525018..3528659	562	BREX-system-P-loop-protein-BrxC-[Escherichia-coli]
WP_032343442.1	Type1.BREX	[illegible]	1213	Bacteria>Pseudomonadota>Gammaproteobacteria>Enterobacterales>Enterobacteriaceae>Escherichia	GCF_016858785.1	Escherichia_coli	BEA16_RS14875	18248..21889	562	BREX-system-P-loop-protein-BrxC-[Escherichia-coli]
WP_208634821.1	Type1.BREX	[illegible]	1213	Bacteria>Pseudomonadota>Gammaproteobacteria>Enterobacterales>Enterobacteriaceae>Escherichia	GCF_016904795.1	Escherichia_albertii	JRC43_RS04070	837246..840887	208962	BREX-system-P-loop-protein-BrxC-[Escherichia-albertii]
WP_072666953.1	Type1.BREX	[illegible]	1213	Bacteria>Pseudomonadota>Gammaproteobacteria>Enterobacterales>Enterobacteriaceae>Escherichia	GCF_017088485.1	Escherichia_coli	JR883_RS20005	4162845..4166486	562	BREX-system-P-loop-protein-BrxC-[Escherichia-coli]
WP_205937296.1	Type1.BREX	[illegible]	1213	Bacteria>Pseudomonadota>Gammaproteobacteria>Enterobacterales>Enterobacteriaceae>Escherichia	GCF_017117165.1	Escherichia_coli	C3E82_RS10505	78550..82191	562	BREX-system-P-loop-protein-BrxC-[Escherichia-coli]
WP_207516242.1	Type1.BREX	[illegible]	1213	Bacteria>Pseudomonadota>Gammaproteobacteria>Enterobacterales>Enterobacteriaceae>Escherichia	GCF_017356105.1	Escherichia_coli	J4Q20_RS14625	71539..75180	562	BREX-system-P-loop-protein-BrxC-[Escherichia-coli]
WP_208482710.1	Type1.BREX	[illegible]	1213	Bacteria>Pseudomonadota>Gammaproteobacteria>Enterobacterales>Enterobacteriaceae>Escherichia	GCF_017598785.1	Escherichia_coli	J7R96_RS22835	18064..21705	562	BREX-system-P-loop-protein-BrxC-[Escherichia-coli]
WP_210173219.1	Type1.BREX	[illegible]	1213	Bacteria>Pseudomonadota>Gammaproteobacteria>Enterobacterales>Enterobacteriaceae>Escherichia	GCF_017897155.1	Escherichia_coli	J8352_RS05700	22726..26367	562	BREX-system-P-loop-protein-BrxC-[Escherichia-coli]
WP_186240487.1	Type1.BREX	[illegible]	1213	Bacteria>Pseudomonadota>Gammaproteobacteria>Enterobacterales>Enterobacteriaceae>Escherichia	GCF_018141145.1	Escherichia_coli	HUS69_RS19530	4048381..4052022	562	BREX-system-P-loop-protein-BrxC-[Escherichia-coli]
WP_097470372.1	Type1.BREX	[illegible]	1204	Bacteria>Pseudomonadota>Gammaproteobacteria>Enterobacterales>Enterobacteriaceae>Escherichia	GCF_018682695.1	Escherichia_coli	BEG75_RS10330	67748..71362	562	BREX-system-P-loop-protein-BrxC-[Escherichia-coli]
WP_216317187.1	Type1.BREX	[illegible]	1213	Bacteria>Pseudomonadota>Gammaproteobacteria>Enterobacterales>Enterobacteriaceae>Escherichia	GCF_018884345.1	Escherichia_coli	EXA04_RS21875	4479425..4483066	562	BREX-system-P-loop-protein-BrxC-[Escherichia-coli]
WP_217800889.1	Type1.BREX	[illegible]	1213	Bacteria>Pseudomonadota>Gammaproteobacteria>Enterobacterales>Enterobacteriaceae>Escherichia	GCF_019120875.1	Escherichia_coli	KR452_RS02525	132614..136255	562	BREX-system-P-loop-protein-BrxC-[Escherichia-coli]
WP_220298575.1	Type1.BREX	[illegible]	1213	Bacteria>Pseudomonadota>Gammaproteobacteria>Enterobacterales>Enterobacteriaceae>Escherichia	GCF_019509425.1	Escherichia_coli	KZA94_RS20415	22821..26462	562	BREX-system-P-loop-protein-BrxC-[Escherichia-coli]
WP_222694585.1	Type1.BREX	[illegible]	849	Bacteria>Pseudomonadota>Gammaproteobacteria>Enterobacterales>Enterobacteriaceae>Escherichia	GCF_019839475.1	Escherichia_coli	K6681_RS00785	145766..148312	562	BREX-system-P-loop-protein-BrxC,-partial-[Escherichia-coli]
WP_222687482.1	Type1.BREX	[illegible]	1213	Bacteria>Pseudomonadota>Gammaproteobacteria>Enterobacterales>Enterobacteriaceae>Escherichia	GCF_019840005.1	Escherichia_coli	K6684_RS15390	1591..5232	562	BREX-system-P-loop-protein-BrxC-[Escherichia-coli]
WP_222698798.1	Type1.BREX	[illegible]	1213	Bacteria>Pseudomonadota>Gammaproteobacteria>Enterobacterales>Enterobacteriaceae>Escherichia	GCF_019840195.1	Escherichia_coli	K6666_RS17905	2229..5870	562	BREX-system-P-loop-protein-BrxC-[Escherichia-coli]
WP_220680248.1	Type1.BREX	[illegible]	1213	Bacteria>Pseudomonadota>Gammaproteobacteria>Enterobacterales>Enterobacteriaceae>Escherichia	GCF_019926805.1	Escherichia_coli	JOW43_RS22215	13140..16781	562	BREX-system-P-loop-protein-BrxC-[Escherichia-coli]
WP_219370674.1	Type1.BREX	[illegible]	1213	Bacteria>Pseudomonadota>Gammaproteobacteria>Enterobacterales>Enterobacteriaceae>Escherichia	GCF_020002285.1	Escherichia_fergusonii	KYC43_RS19615	4024013..4027654	564	BREX-system-P-loop-protein-BrxC-[Escherichia-fergusonii]
WP_224452732.1	Type1.BREX	[illegible]	1225	Bacteria>Pseudomonadota>Gammaproteobacteria>Enterobacterales>Enterobacteriaceae>Escherichia	GCF_020162075.2	Escherichia_coli	KD159_RS16650	41518..45195	562	BREX-system-P-loop-protein-BrxC-[Escherichia-coli]
WP_089559427.1	Type1.BREX	[illegible]	1213	Bacteria>Pseudomonadota>Gammaproteobacteria>Enterobacterales>Enterobacteriaceae>Escherichia	GCF_020230375.1	Escherichia_coli	LF808_RS18060	3738120..3741761	562	BREX-system-P-loop-protein-BrxC-[Escherichia-coli]

Protein-ID	System	Gene-Neighbourhoods	Length	Taxonomy	Assembly-ID	Strain	Locus-Tag	Genomic-coordinates	TaxID	Fasta-defline
WP_063122485.1	Type1.BREX	<-RHH_antitoxin||?->?->ZnR->ZnR-><-SIGMA+OB-Fold+AAA-ATPase+Lon-Protease-BrxL<-PglZ<-N6-METHYLASE<-DUF6079*<-BrxB-BrxF-iSTAND<-BrxA-DNA-binding||REase+iREase->?->?-><-Arm-DNA-bind_3+ [illegible]	1213	Bacteria>Pseudomonadota>Gammaproteobacteria>Enterobacterales>Enterobacteriaceae>Escherichia	GCF_020230435.1	Escherichia_coli	LF812_RS09505	1992870..1996511	562	BREX-system-P-loop-protein-BrxC-[Escherichia-coli]
WP_130560426.1	Type1.BREX	<-XIS-HTH||?->?->ZnR->ZnR-><-SIGMA+OB-Fold+AAA-ATPase+Lon-Protease-BrxL<-PglZ<-N6-METHYLASE<-DUF6079*<-BrxB-BrxF-iSTAND<-BrxA-DNA-binding<-DDE_Tnp_IS1	1213	Bacteria>Pseudomonadota>Gammaproteobacteria>Enterobacterales>Enterobacteriaceae>Escherichia	GCF_020282105.1	Escherichia_coli	IHV37_RS24260	16243..19884	562	BREX-system-P-loop-protein-BrxC-[Escherichia-coli]
WP_227262353.1	Type1.BREX	<-Peptidase_M17_N+Peptidase_M17||?->?-><-HerA/FtsK+A32||tRNA->Arm-DNA-bind_3+Phage_integrase->EVE+HNH-><-REase+iREase||BrxA-DNA-binding->BrxB-BrxF-iSTAND->DUF6079*->N6-METHYLASE-> [illegible]	1213	Bacteria>Pseudomonadota>Gammaproteobacteria>Enterobacterales>Enterobacteriaceae>Escherichia	GCF_020594775.1	Escherichia_coli	LJD39_RS10205	66062..69703	562	BREX-system-P-loop-protein-BrxC-[Escherichia-coli]
WP_229322431.1	Type1.BREX	<-HerA/FtsK+A32<-?||tRNA->Arm-DNA-bind_3+Phage_integrase->EVE+HNH-><-REase+iREase||BrxA-DNA-binding->BrxB-BrxF-iSTAND->DUF6079*->N6-METHYLASE->PglZ->SIGMA+OB-Fold+AAA-ATPase+ [illegible]	1213	Bacteria>Pseudomonadota>Gammaproteobacteria>Enterobacterales>Enterobacteriaceae>Escherichia	GCF_020883515.1	Escherichia_coli	L2W85_RS01370	290770..294411	562	BREX-system-P-loop-protein-BrxC-[Escherichia-coli]
WP_231833501.1	Type1.BREX	<-ParB+HNH<-DUF6079*<-BrxB-BrxF-iSTAND<-BrxA-DNA-binding<-wHTH+WYL+WCX||DUF4158+DDE_Tnp_Tn3->	1225	Bacteria>Pseudomonadota>Gammaproteobacteria>Enterobacterales>Enterobacteriaceae>Escherichia	GCF_021168085.1	Escherichia_coli	LTT78_RS21790	2950..6627	562	BREX-system-P-loop-protein-BrxC-[Escherichia-coli]
WP_097495602.1	Type1.BREX	<-TetR-HTH<-?<-?<-REase<-REase||BrxA-DNA-binding->BrxB-BrxF-iSTAND->DUF6079*->N6-METHYLASE->PglZ->SIGMA+OB-Fold+AAA-ATPase+Lon-Protease-BrxL->	1213	Bacteria>Pseudomonadota>Gammaproteobacteria>Enterobacterales>Enterobacteriaceae>Escherichia	GCF_021547795.1	Escherichia_coli	BEC04_RS22745	6957..10598	562	BREX-system-P-loop-protein-BrxC-[Escherichia-coli]
WP_236265313.1	Type1.BREX	<-XIS-HTH<-RHH_antitoxin||?->?->ZnR->ZnR-><-SIGMA+OB-Fold+AAA-ATPase+Lon-Protease-BrxL<-PglZ<-N6-METHYLASE<-DUF6079*<-BrxB-BrxF-iSTAND<-BrxA-DNA-binding||REase+iREase->	1213	Bacteria>Pseudomonadota>Gammaproteobacteria>Enterobacterales>Enterobacteriaceae>Escherichia	GCF_021647095.1	Escherichia_coli	L6H61_RS22580	19345..22986	562	BREX-system-P-loop-protein-BrxC-[Escherichia-coli]
WP_236486706.1	Type1.BREX	BrxA-DNA-binding->BrxB-BrxF-iSTAND->DUF6079*->N6-METHYLASE->PglZ->SIGMA+OB-Fold+AAA-ATPase+Lon-Protease-BrxL-><-ZnR<-ZnR	1213	Bacteria>Pseudomonadota>Gammaproteobacteria>Enterobacterales>Enterobacteriaceae>Escherichia	GCF_021648945.1	Escherichia_coli	L6E82_RS22710	1925..5566	562	BREX-system-P-loop-protein-BrxC-[Escherichia-coli]
WP_112885923.1	Type1.BREX	<-HerA/FtsK+A32<-?||tRNA->Arm-DNA-bind_3+Phage_integrase->EVE+HNH-><-REase+iREase||BrxA-DNA-binding->BrxB-BrxF-iSTAND->DUF6079*->N6-METHYLASE->PglZ->SIGMA+OB-Fold+AAA-ATPase+ [illegible]	1213	Bacteria>Pseudomonadota>Gammaproteobacteria>Enterobacterales>Enterobacteriaceae>Escherichia	GCF_021697115.1	Escherichia_coli	IHV65_RS22435	19394..23035	562	BREX-system-P-loop-protein-BrxC-[Escherichia-coli]
WP_236936469.1	Type1.BREX	<-Phage_integrase<-Phage_integrase||?->?->?->||wHTH+SAD+HNH-><-REase+iREase||BrxA-DNA-binding->BrxB-BrxF-iSTAND->DUF6079*->N6-METHYLASE->PglZ->SIGMA+OB-Fold+AAA-ATPase+Lon-Protease- [illegible]	1213	Bacteria>Pseudomonadota>Gammaproteobacteria>Enterobacterales>Enterobacteriaceae>Escherichia	GCF_021698335.1	Escherichia_coli	IHV39_RS14485	12915..16556	562	BREX-system-P-loop-protein-BrxC-[Escherichia-coli]
WP_089663046.1	Type1.BREX	<-Peptidase_M17_N+Peptidase_M17||?->?-><-HerA/FtsK+A32<-?||tRNA->Arm-DNA-bind_3+Phage_integrase->BrxA-DNA-binding->BrxB-BrxF-iSTAND->DUF6079*->N6-METHYLASE->OLD-ABC->TOPRIM-> [illegible]	1204	Bacteria>Pseudomonadota>Gammaproteobacteria>Enterobacterales>Enterobacteriaceae>Escherichia	GCF_022489725.1	Escherichia_coli	ML322_RS02965	174957..178571	562	BREX-system-P-loop-protein-BrxC-[Escherichia-coli]
WP_213838985.1	Type1.BREX	<-RHH_antitoxin||?->?->?->ZnR->ZnR-><-SIGMA+OB-Fold+AAA-ATPase+Lon-Protease-BrxL<-PglZ<-N6-METHYLASE<-DUF6079*<-BrxB-BrxF-iSTAND<-BrxA-DNA-binding||REase+iREase->?->?-> [illegible]	1213	Bacteria>Pseudomonadota>Gammaproteobacteria>Enterobacterales>Enterobacteriaceae>Escherichia	GCF_022493455.1	Escherichia_coli	AOY76_RS04040	833620..837261	562	BREX-system-P-loop-protein-BrxC-[Escherichia-coli]
WP_241227056.1	Type1.BREX	XIS-HTH->AAA+wHTH->?-><-SWIM+DUF3670+SF1-SF2-Helicase<-?<-?||BrxA-DNA-binding->DUF6079*->N6-METHYLASE->PglZ->SIGMA+OB-Fold+AAA-ATPase+Lon-Protease-BrxL-><-DDE_Tnp_IS1	1213	Bacteria>Pseudomonadota>Gammaproteobacteria>Enterobacterales>Enterobacteriaceae>Escherichia	GCF_022547975.1	Escherichia_coli	EFK08_RS14245	31309..34950	562	BREX-system-P-loop-protein-BrxC-[Escherichia-coli]
WP_109114033.1	Type1.BREX	ZnR->ZnR-><-SIGMA+OB-Fold+AAA-ATPase+Lon-Protease-BrxL<-PglZ<-N6-METHYLASE<-DUF6079*<-BrxB-BrxF-iSTAND<-BrxA-DNA-binding	1213	Bacteria>Pseudomonadota>Gammaproteobacteria>Enterobacterales>Enterobacteriaceae>Escherichia	GCF_022602835.1	Escherichia_coli	JEU19_RS23720	11740..15381	562	BREX-system-P-loop-protein-BrxC-[Escherichia-coli]
WP_247153716.1	Type1.BREX	<-Phage_integrase<-Phage_integrase||?->?->?->||wHTH+SAD+HNH-><-REase+iREase||BrxA-DNA-binding->BrxB-BrxF-iSTAND->DUF6079-p*->	427	Bacteria>Pseudomonadota>Gammaproteobacteria>Enterobacterales>Enterobacteriaceae>Escherichia	GCF_022968215.1	Escherichia_coli	MWG73_RS16615	71483..72763	562	BREX-system-P-loop-protein-BrxC,-partial-[Escherichia-coli]
WP_247178533.1	Type1.BREX	<-Phage_integrase<-Phage_integrase||?->?->?->||wHTH+SAD+HNH-><-REase+iREase||BrxA-DNA-binding->BrxB-BrxF-iSTAND->DUF6079-p*->	625	Bacteria>Pseudomonadota>Gammaproteobacteria>Enterobacterales>Enterobacteriaceae>Escherichia	GCF_022970855.1	Escherichia_coli	MZQ30_RS16040	111241..113117	562	BREX-system-P-loop-protein-BrxC,-partial-[Escherichia-coli]
WP_069898759.1	Type1.BREX	<-Phage_integrase<-Phage_integrase||?->?->?->||wHTH+SAD+HNH-><-REase+iREase||BrxA-DNA-binding->BrxB-BrxF-iSTAND->DUF6079*->N6-METHYLASE->PglZ->SIGMA+OB-Fold+AAA-ATPase+Lon-Protease- [illegible]	1213	Bacteria>Pseudomonadota>Gammaproteobacteria>Enterobacterales>Enterobacteriaceae>Escherichia	GCF_022971155.1	Escherichia_coli	EAI54_RS17715	71532..75173	562	BREX-system-P-loop-protein-BrxC-[Escherichia-coli]
WP_247149149.1	Type1.BREX	<-METHYLASE<-?||?-><-?||<-AAA-ATPase<-HIN-HTH||wHTH+WYL+WCX->BrxA-DNA-binding->BrxB-BrxF-iSTAND->DUF6079*->ParB+HNH->N6-METHYLASE->PglZ->SIGMA+OB-Fold+AAA-ATPase+Lon-Protease-BrxL-> [illegible]	1225	Bacteria>Pseudomonadota>Gammaproteobacteria>Enterobacterales>Enterobacteriaceae>Escherichia	GCF_022975115.1	Escherichia_coli	MZS66_RS10030	63457..67134	562	BREX-system-P-loop-protein-BrxC-[Escherichia-coli]
WP_247168067.1	Type1.BREX	<-XIS-HTH<-RHH_antitoxin||?->?->ZnR->ZnR-><-SIGMA+OB-Fold+AAA-ATPase+Lon-Protease-BrxL<-PglZ<-N6-METHYLASE<-DUF6079*<-BrxB-BrxF-iSTAND<-BrxA-DNA-binding	1213	Bacteria>Pseudomonadota>Gammaproteobacteria>Enterobacterales>Enterobacteriaceae>Escherichia	GCF_022975475.1	Escherichia_coli	MZT24_RS24480	15782..19423	562	BREX-system-P-loop-protein-BrxC-[Escherichia-coli]
WP_247151822.1	Type1.BREX	<-SIGMA+OB-Fold+AAA-ATPase+Lon-Protease-BrxL<-PglZ<-N6-METHYLASE<-DUF6079*<-BrxB-BrxF-iSTAND<-BrxA-DNA-binding||REase+iREase-><-wHTH+SAD+HNH||<-?<-?||Phage_integrase-> [illegible]	1213	Bacteria>Pseudomonadota>Gammaproteobacteria>Enterobacterales>Enterobacteriaceae>Escherichia	GCF_022976225.1	Escherichia_coli	MZU12_RS18965	11408..15049	562	BREX-system-P-loop-protein-BrxC-[Escherichia-coli]
WP_247169646.1	Type1.BREX	DUF6079-p*->N6-METHYLASE->PglZ->SIGMA+OB-Fold+AAA-ATPase+Lon-Protease-BrxL-><-ZnR<-ZnR<-?<-?||RHH_antitoxin->XIS-HTH->	956	Bacteria>Pseudomonadota>Gammaproteobacteria>Enterobacterales>Enterobacteriaceae>Escherichia	GCF_022977435.1	Escherichia_coli	MZU72_RS23215	1..2871	562	BREX-system-P-loop-protein-BrxC,-partial-[Escherichia-coli]
WP_152933333.1	Type1.BREX	BrxA-DNA-binding->BrxB-BrxF-iSTAND->DUF6079*->N6-METHYLASE->PglZ->SIGMA+OB-Fold+AAA-ATPase+Lon-Protease-BrxL-><-ZnR<-ZnR	1213	Bacteria>Pseudomonadota>Gammaproteobacteria>Enterobacterales>Enterobacteriaceae>Escherichia	GCF_023482315.1	Escherichia_coli	M5X22_RS20110	1744..5385	562	BREX-system-P-loop-protein-BrxC-[Escherichia-coli]
WP_249573915.1	Type1.BREX	wHTH+WYL+WCX->BrxA-DNA-binding->BrxB-BrxF-iSTAND->DUF6079*->ParB+HNH->N6-METHYLASE->PglZ->SIGMA+OB-Fold+AAA-ATPase+Lon-Protease-BrxL->35exo->	1225	Bacteria>Pseudomonadota>Gammaproteobacteria>Enterobacterales>Enterobacteriaceae>Escherichia	GCF_023483525.1	Escherichia_coli	M5X65_RS22540	2590..6267	562	BREX-system-P-loop-protein-BrxC-[Escherichia-coli]
WP_250239414.1	Type1.BREX	MobI-><-?<- [illegible]	1225	Bacteria>Pseudomonadota>Gammaproteobacteria>Enterobacterales>Enterobacteriaceae>Escherichia	GCF_023599595.1	Escherichia_coli	NFF73_RS04910	234938..238615	562	MULTISPECIES:-BREX-system-P-loop-protein-BrxC-[Enterobacterales]
WP_097733507.1	Type1.BREX	<-HerA/FtsK+A32<-?||tRNA [illegible]	1213	Bacteria>Pseudomonadota>Gammaproteobacteria>Enterobacterales>Enterobacteriaceae>Escherichia	GCF_023600485.1	Escherichia_coli	LL309_RS17435	16120..19761	562	BREX-system-P-loop-protein-BrxC-[Escherichia-coli]
WP_089551103.1	Type1.BREX	<-XIS-HTH<-RHH_antitoxin [illegible]	1213	Bacteria>Pseudomonadota>Gammaproteobacteria>Enterobacterales>Enterobacteriaceae>Escherichia	GCF_023953245.1	Escherichia_coli	M1V37_RS24515	16803..20444	562	BREX-system-P-loop-protein-BrxC-[Escherichia-coli]
WP_254825569.1	Type1.BREX	XIS-HTH->PIWI-><-?<- [illegible]	1204	Bacteria>Pseudomonadota>Gammaproteobacteria>Enterobacterales>Enterobacteriaceae>Escherichia	GCF_024298945.1	Escherichia_coli	LCR13_RS23605	136646..140260	562	BREX-system-P-loop-protein-BrxC-[Escherichia-coli]
WP_255397240.1	Type1.BREX	<-Phage_integrase<- [illegible]	919	Bacteria>Pseudomonadota>Gammaproteobacteria>Enterobacterales>Enterobacteriaceae>Escherichia	GCF_024380595.1	Escherichia_coli	NOE18_RS13095	111191..113948	562	BREX-system-P-loop-protein-BrxC,-partial-[Escherichia-coli]
WP_255944649.1	Type1.BREX	DUF6216-><-?<-AbiEii<- [illegible]	1213	Bacteria>Pseudomonadota>Gammaproteobacteria>Enterobacterales>Enterobacteriaceae>Escherichia	GCF_024450325.1	Escherichia_coli	NO030_RS20220	28424..32065	562	BREX-system-P-loop-protein-BrxC-[Escherichia-coli]
WP_256168548.1	Type1.BREX	DsbC+DsbD+Thioredoxin_7 [illegible]	1213	Bacteria>Pseudomonadota>Gammaproteobacteria>Enterobacterales>Enterobacteriaceae>Escherichia	GCF_024467035.1	Escherichia_coli	NO473_RS12000	2442717..2446358	562	BREX-system-P-loop-protein-BrxC-[Escherichia-coli]
WP_256876221.1	Type1.BREX	AraC-HTH->?-><-TetR-HTH [illegible]	1213	Bacteria>Pseudomonadota>Gammaproteobacteria>Enterobacterales>Enterobacteriaceae>Escherichia	GCF_024532055.1	Escherichia_albertii	NHH32_RS22595	12425..16066	208962	BREX-system-P-loop-protein-BrxC-[Escherichia-albertii]
WP_262043070.1	Type1.BREX	RVT->ZnR-><-SIGMA+OB-Fold [illegible]	1213	Bacteria>Pseudomonadota>Gammaproteobacteria>Enterobacterales>Enterobacteriaceae>Escherichia	GCF_024546885.1	Escherichia_coli	NO383_RS24480	18816..22457	562	BREX-system-P-loop-protein-BrxC-[Escherichia-coli]
WP_257249285.1	Type1.BREX	HNH->?->?->||wHTH+SAD+HNH [illegible]	1213	Bacteria>Pseudomonadota>Gammaproteobacteria>Enterobacterales>Enterobacteriaceae>Escherichia	GCF_024608215.1	Escherichia_coli	NO078_RS04965	41836..45477	562	BREX-system-P-loop-protein-BrxC-[Escherichia-coli]
WP_257766119.1	Type1.BREX	Arm-DNA-bind_3+Phage_in [illegible]	1213	Bacteria>Pseudomonadota>Gammaproteobacteria>Enterobacterales>Enterobacteriaceae>Escherichia	GCF_024655425.1	Escherichia_coli	NUU44_RS01945	122932..126573	562	BREX-system-P-loop-protein-BrxC-[Escherichia-coli]
WP_257797852.1	Type1.BREX	<-XIS-HTH<-RHH_antitoxin [illegible]	1213	Bacteria>Pseudomonadota>Gammaproteobacteria>Enterobacterales>Enterobacteriaceae>Escherichia	GCF_024700395.1	Escherichia_coli	NU286_RS11325	47264..50905	562	BREX-system-P-loop-protein-BrxC-[Escherichia-coli]
WP_260253294.1	Type1.BREX	Zn_Tnp_IS1+HTH_Tnp_IS1+ [illegible]	1213	Bacteria>Pseudomonadota>Gammaproteobacteria>Enterobacterales>Enterobacteriaceae>Escherichia	GCF_025200615.1	Escherichia_coli	N1707_RS18325	3745633..3749274	562	BREX-system-P-loop-protein-BrxC-[Escherichia-coli]
WP_261629031.1	Type1.BREX	<-HIN-HTH<-RelE-ParE<- [illegible]	1225	Bacteria>Pseudomonadota>Gammaproteobacteria>Enterobacterales>Enterobacteriaceae>Escherichia	GCF_025404235.1	Escherichia_coli	J0W24_RS20355	5362..9039	562	BREX-system-P-loop-protein-BrxC-[Escherichia-coli]
WP_262931584.1	Type1.BREX	ZnR->ZnR-><-SIGMA+OB-Fold [illegible]	1213	Bacteria>Pseudomonadota>Gammaproteobacteria>Enterobacterales>Enterobacteriaceae>Escherichia	GCF_025599895.1	Escherichia_albertii	N6N71_RS13920	11398..15039	208962	BREX-system-P-loop-protein-BrxC-[Escherichia-albertii]
WP_263767786.1	Type1.BREX	DUF6216-><-?<-AbiEii<- [illegible]	1072	Bacteria>Pseudomonadota>Gammaproteobacteria>Enterobacterales>Enterobacteriaceae>Escherichia	GCF_025750965.1	Escherichia_coli	OHF01_RS16785	78632..81850	562	BREX-system-P-loop-protein-BrxC,-partial-[Escherichia-coli]
WP_171854687.1	Type1.BREX	<-XIS-HTH<-RHH_antitoxin [illegible]	1213	Bacteria>Pseudomonadota>Gammaproteobacteria>Enterobacterales>Enterobacteriaceae>Escherichia	GCF_025946525.1	Escherichia_coli	N7L43_RS20535	4233126..4236767	562	BREX-system-P-loop-protein-BrxC-[Escherichia-coli]
WP_265188955.1	Type1.BREX	<-XIS-HTH<-RHH_antitoxin [illegible]	1213	Bacteria>Pseudomonadota>Gammaproteobacteria>Enterobacterales>Enterobacteriaceae>Escherichia	GCF_026134335.1	Escherichia_coli	OHD11_RS10990	2114745..2118386	562	BREX-system-P-loop-protein-BrxC-[Escherichia-coli]
WP_265199179.1	Type1.BREX	Arm-DNA-bind_3+Phage_in [illegible]	1213	Bacteria>Pseudomonadota>Gammaproteobacteria>Enterobacterales>Enterobacteriaceae>Escherichia	GCF_026135205.1	Escherichia_coli	OHD45_RS08935	1859993..1863634	562	BREX-system-P-loop-protein-BrxC-[Escherichia-coli]
WP_265209809.1	Type1.BREX	TPR-><-LACI-HTH+PBPI||? [illegible]	1213	Bacteria>Pseudomonadota>Gammaproteobacteria>Enterobacterales>Enterobacteriaceae>Escherichia	GCF_026135825.1	Escherichia_coli	NYV54_RS21460	4297326..4300967	562	BREX-system-P-loop-protein-BrxC-[Escherichia-coli]
WP_265931224.1	Type1.BREX	<-Peptidase_M17_N+Pepti [illegible]	1204	Bacteria>Pseudomonadota>Gammaproteobacteria>Enterobacterales>Enterobacteriaceae>Escherichia	GCF_026228105.1	Escherichia_coli	NMW20_RS04715	287437..291051	562	BREX-system-P-loop-protein-BrxC-[Escherichia-coli]
WP_267833950.1	Type1.BREX	BrxA-DNA-binding->BrxB- [illegible]	1213	Bacteria>Pseudomonadota>Gammaproteobacteria>Enterobacterales>Enterobacteriaceae>Escherichia	GCF_026612595.1	Escherichia_coli	IGP49_RS06410	1748..5389	562	BREX-system-P-loop-protein-BrxC-[Escherichia-coli]
WP_268646541.1	Type1.BREX	<-VirD4-TraG+VirB4-FtsK [illegible]	1225	Bacteria>Pseudomonadota>Gammaproteobacteria>Enterobacterales>Enterobacteriaceae>Escherichia	GCF_026801415.1	Escherichia_coli	OZI01_RS14450	44846..48523	562	BREX-system-P-loop-protein-BrxC-[Escherichia-coli]
WP_270658579.1	Type1.BREX	<-Fimbrial<-Phage_integ [illegible]	1213	Bacteria>Pseudomonadota>Gammaproteobacteria>Enterobacterales>Enterobacteriaceae>Escherichia	GCF_027696005.1	Escherichia_coli	PG805_RS04695	85075..88716	562	BREX-system-P-loop-protein-BrxC-[Escherichia-coli]
WP_272434363.1	Type1.BREX	<-DUF772+DDE_Tnp_1<-HIN [illegible]	1225	Bacteria>Pseudomonadota>Gammaproteobacteria>Enterobacterales>Enterobacteriaceae>Escherichia	GCF_028436275.1	Escherichia_coli	IXW67_RS19930	12892..16569	562	BREX-system-P-loop-protein-BrxC-[Escherichia-coli]
WP_071702268.1	Type1.BREX	<-TetR-HTH<-?<-?<-REase [illegible]	1213	Bacteria>Pseudomonadota>Gammaproteobacteria>Enterobacterales>Enterobacteriaceae>Escherichia	GCF_029351265.1	Escherichia_coli	P3474_RS20935	8656..12297	562	BREX-system-P-loop-protein-BrxC-[Escherichia-coli]
WP_115723901.1	Type1.BREX	ZnR->ZnR->ZnR->URI-DNas [illegible]	1204	Bacteria>Pseudomonadota>Gammaproteobacteria>Enterobacterales>Enterobacteriaceae>Escherichia	GCF_029542125.1	Escherichia_coli	OM087_RS11620	2419674..2423288	562	BREX-system-P-loop-protein-BrxC-[Escherichia-coli]
WP_023157617.1	Type1.BREX	Phage_Cox->||XIS-HTH->A [illegible]	1204	Bacteria>Pseudomonadota>Gammaproteobacteria>Enterobacterales>Enterobacteriaceae>Escherichia	GCF_030163335.1	Escherichia_coli	HMPREF1620_RS03645	19303..22917	562	BREX-system-P-loop-protein-BrxC-[Escherichia-coli]
WP_112915232.1	Type1.BREX	<-SIGMA+OB-Fold+AAA-ATP [illegible]	1213	Bacteria>Pseudomonadota>Gammaproteobacteria>Enterobacterales>Enterobacteriaceae>Escherichia	GCF_030324795.1	Escherichia_coli	EFJ90_RS23235	8334..11975	562	BREX-system-P-loop-protein-BrxC-[Escherichia-coli]
WP_087894339.1	Type1.BREX	<-35exo<-SIGMA+OB-Fold+ [illegible]	1225	Bacteria>Pseudomonadota>Gammaproteobacteria>Enterobacterales>Enterobacteriaceae>Escherichia	GCF_030716655.1	Escherichia_coli	A8C62_RS24175	11745..15422	562	BREX-system-P-loop-protein-BrxC-[Escherichia-coli]
WP_096309279.1	Type1.BREX	<-DUF6079-p*<-BrxB-BrxF [illegible]	918	Bacteria>Pseudomonadota>Gammaproteobacteria>Enterobacterales>Enterobacteriaceae>Escherichia	GCF_900195535.1	Escherichia_coli	CPH42_RS29515	1..2754	562	BREX-system-P-loop-protein-BrxC,-partial-[Escherichia-coli]
WP_096262508.1	Type1.BREX	<-XIS-HTH<-RHH_antitoxi [illegible]	1213	Bacteria>Pseudomonadota>Gammaproteobacteria>Enterobacterales>Enterobacteriaceae>Escherichia	GCF_900196515.1	Escherichia_coli	CPH64_RS18250	76258..79899	562	BREX-system-P-loop-protein-BrxC-[Escherichia-coli]
WP_096317297.1	Type1.BREX	HTH_21+rve+rve_3->||?-> [illegible]	1225	Bacteria>Pseudomonadota>Gammaproteobacteria>Enterobacterales>Enterobacteriaceae>Escherichia	GCF_900196545.1	Escherichia_coli	LLQ47_RS24465	3245..6922	562	BREX-system-P-loop-protein-BrxC-[Escherichia-coli]
WP_110093138.1	Type1.BREX	BrxA-DNA-binding->BrxB- [illegible]	1213	Bacteria>Pseudomonadota>Gammaproteobacteria>Enterobacterales>Enterobacteriaceae>Escherichia	GCF_900196585.1	Escherichia_coli	CPH25_RS23340	1849..5490	562	BREX-system-P-loop-protein-BrxC-[Escherichia-coli]
WP_115738447.1	Type1.BREX	REase+N6_METHYLASE-><-? [illegible]	1213	Bacteria>Pseudomonadota>Gammaproteobacteria>Enterobacterales>Enterobacteriaceae>Escherichia	GCF_900499355.1	Escherichia_coli	DY383_RS04210	180625..184266	562	BREX-system-P-loop-protein-BrxC-[Escherichia-coli]
WP_126509145.1	Type1.BREX	<-XIS-HTH<-RHH_antitoxi [illegible]	1213	Bacteria>Pseudomonadota>Gammaproteobacteria>Enterobacterales>Enterobacteriaceae>Escherichia	GCF_900636125.1	Escherichia_coli	EL118_RS04180	856584..860225	562	BREX-system-P-loop-protein-BrxC-[Escherichia-coli]
WP_001548950.1	Type1.BREX	TetR-HTH->tRNA->Arm-DNA [illegible]	1213	Bacteria>Pseudomonadota>Gammaproteobacteria>Enterobacterales>Enterobacteriaceae>Escherichia	GCF_900706885.1	Escherichia_coli	LF679_RS14520	38542..42183	562	BREX-system-P-loop-protein-BrxC-[Escherichia-coli]
WP_152072595.1	Type1.BREX	<-Phage_integrase<-Phag [illegible]	1213	Bacteria>Pseudomonadota>Gammaproteobacteria>Enterobacterales>Enterobacteriaceae>Escherichia	GCF_902501575.1	Escherichia_coli	GDK01_RS16655	24181..27822	562	BREX-system-P-loop-protein-BrxC-[Escherichia-coli]
WP_001552307.1	Type1.BREX	wHTH->ZnR->ZnR->URI-DNa [illegible]	1204	Bacteria>Pseudomonadota>Gammaproteobacteria>Enterobacterales>Enterobacteriaceae>Escherichia	GCF_902505225.1	Escherichia_coli	GDK13_RS03595	266015..269629	562	BREX-system-P-loop-protein-BrxC-[Escherichia-coli]
WP_096955310.1	Type1.BREX	DUF6216-><-?<-AbiEii<-A [illegible]	1213	Bacteria>Pseudomonadota>Gammaproteobacteria>Enterobacterales>Enterobacteriaceae>Escherichia	GCF_904831825.1	Escherichia_coli	A7198_RS13140	15059..18700	562	BREX-system-P-loop-protein-BrxC-[Escherichia-coli]
WP_001702945.1	Type1.BREX	Arm-DNA-bind_3+Phage_in [illegible]	1213	Bacteria>Pseudomonadota>Gammaproteobacteria>Enterobacterales>Enterobacteriaceae>Escherichia	GCF_907176105.1	Escherichia_coli	A7133_RS15540	52191..55832	562	BREX-system-P-loop-protein-BrxC-[Escherichia-coli]
WP_187798258.1	Type1.BREX	<-Sulfatase<-?||DDE_Tnp [illegible]	1213	Bacteria>Pseudomonadota>Gammaproteobacteria>Enterobacterales>Enterobacteriaceae>Escherichia	GCF_929617055.1	Escherichia_coli	MJ085_RS02910	12212..15853	562	BREX-system-P-loop-protein-BrxC-[Escherichia-coli]
WP_088209948.1	Type1.BREX	<-RNase<-DEOR-HTH||?->D [illegible]	1204	Bacteria>Pseudomonadota>Gammaproteobacteria>Enterobacterales>Enterobacteriaceae>Escherichia	GCF_943733315.1	Escherichia_coli	OVQ79_RS01305	107612..111226	562	BREX-system-P-loop-protein-BrxC-[Escherichia-coli]
WP_096964704.1	Type1.BREX	DUF6216-><-?<-AbiEii<-A [illegible]	1213	Bacteria>Pseudomonadota>Gammaproteobacteria>Enterobacterales>Enterobacteriaceae>Escherichia	GCF_948144475.1	Escherichia_coli	A7257_RS18210	50280..53921	562	BREX-system-P-loop-protein-BrxC-[Escherichia-coli]
WP_052920517.1	Type1.BREX	ZnR->ZnR->URI-DNase+RAM [illegible]	1204	Bacteria>Pseudomonadota>Gammaproteobacteria>Enterobacterales>Enterobacteriaceae>Escherichia	GCF_948144575.1	Escherichia_coli	AGA26_RS17910	13907..17521	562	BREX-system-P-loop-protein-BrxC-[Escherichia-coli]
WP_244586021.1	Type1.BREX	<-wHTH+WYL<-?||XIS-HTH- [illegible]	1240	Bacteria>Pseudomonadota>Gammaproteobacteria>Enterobacterales>Enterobacteriaceae>Escherichia	GCF_948144585.1	Escherichia_coli	A7Y91_RS22675	15059..18781	562	BREX-system-P-loop-protein-BrxC-[Escherichia-coli]
WP_160450150.1	Type1.BREX	<-ParB+HNH<-?||GNTR-HTH [illegible]	1213	Bacteria>Pseudomonadota>Gammaproteobacteria>Enterobacterales>Enterobacteriaceae	-	Escherichia	H1X95_RS06210	181594..185235	561	MULTISPECIES:-BREX-system-P-loop-protein-BrxC-[Escherichia]
WP_024559724.1	Type1.BREX	<-SF1-SF2-Helicase<-SF1 [illegible]	1213	Bacteria>Pseudomonadota>Gammaproteobacteria>Enterobacterales>Enterobacteriaceae>Franconibacter	GCF_000485985.1	Franconibacter_pulveris	U709_RS0101725	38623..42264	435910	BREX-system-P-loop-protein-BrxC-[Franconibacter-pulveris]
WP_001019652.1	Type1.BREX	Arm-DNA-bind_3->NACHT+S [illegible]	1213	Bacteria>Pseudomonadota>Gammaproteobacteria>Enterobacterales>Enterobacteriaceae	GCF_000006945.2	Salmonella	SL1344_RS23060	4767568..4771209	590	MULTISPECIES:-BREX-system-P-loop-protein-BrxC-[Salmonella]
WP_008501367.1	Type1.BREX	<-SymE_toxin<-?<-?<-?|| [illegible]	1213	Bacteria>Pseudomonadota>Gammaproteobacteria>Enterobacterales>Enterobacteriaceae	GCF_000286655.1	Enterobacter	B498_RS11105	63158..66799	547	MULTISPECIES:-BREX-system-P-loop-protein-BrxC-[Enterobacter]
WP_052939500.1	Type1.BREX	<-HEPN<-?||YjhX_toxin-> [illegible]	1204	Bacteria>Pseudomonadota>Gammaproteobacteria>Enterobacterales>Enterobacteriaceae	GCF_001569545.1	Salmonella	MA624_RS20100	4127586..4131200	590	MULTISPECIES:-BREX-system-P-loop-protein-BrxC-[Salmonella]
WP_063308429.1	Type1.BREX	Glyoxalase_8->URI-DNase [illegible]	1213	Bacteria>Pseudomonadota>Gammaproteobacteria>Enterobacterales>Enterobacteriaceae	GCF_001623605.1	Enterobacter	A4308_RS00205	35188..38829	547	MULTISPECIES:-BREX-system-P-loop-protein-BrxC-[Enterobacter]
WP_241755053.1	Type1.BREX	<-METHYLASE||SKI-><-?|| [illegible]	1240	Bacteria>Pseudomonadota>Gammaproteobacteria>Enterobacterales>Enterobacteriaceae	GCF_013791465.1	Citrobacter	HV084_RS03275	673516..677238	544	MULTISPECIES:-BREX-system-P-loop-protein-BrxC-[Citrobacter]
WP_233470897.1	Type1.BREX	<-DUF932<-nSTAND1+GTPas [illegible]	1240	Bacteria>Pseudomonadota>Gammaproteobacteria>Enterobacterales>Enterobacteriaceae	GCF_014931625.2	Citrobacter	INP82_RS14360	158420..162142	544	MULTISPECIES:-BREX-system-P-loop-protein-BrxC-[Citrobacter]
WP_129336016.1	Type1.BREX	<-HEPN<-REase+iREase<-? [illegible]	1213	Bacteria>Pseudomonadota>Gammaproteobacteria>Enterobacterales>Enterobacteriaceae	GCF_019670325.1	Salmonella	K5676_RS20565	4193364..4197005	590	MULTISPECIES:-BREX-system-P-loop-protein-BrxC-[Salmonella]
WP_080247850.1	Type1.BREX	<-HEPN<-REase+iREase<-? [illegible]	1213	Bacteria>Pseudomonadota>Gammaproteobacteria>Enterobacterales>Enterobacteriaceae	GCF_020221795.1	Salmonella	LDO68_RS20280	4140919..4144560	590	MULTISPECIES:-BREX-system-P-loop-protein-BrxC-[Salmonella]
WP_070534016.1	Type1.BREX	LACI-HTH+PBPI-><-ASCH+G [illegible]	1213	Bacteria>Pseudomonadota>Gammaproteobacteria>Enterobacterales>Enterobacteriaceae	GCF_024104775.1	Enterobacter	KGY84_RS21865	326949..330590	547	MULTISPECIES:-BREX-system-P-loop-protein-BrxC-[Enterobacter]
WP_047724721.1	Type1.BREX	<-LuxR-HTH<-?|||BrxA-D [illegible]	1213	Bacteria>Pseudomonadota>Gammaproteobacteria>Enterobacterales>Enterobacteriaceae	GCF_026008675.1	Klebsiella/Raoultella_group	OPC88_RS09775	105880..109521	2890311	MULTISPECIES:-BREX-system-P-loop-protein-BrxC-[Klebsiella/Raoultella-group]
WP_192856165.1	Type1.BREX	TetR-HTH->tRNA->Arm-DNA [illegible]	1213	Bacteria>Pseudomonadota>Gammaproteobacteria>Enterobacterales>Enterobacteriaceae	GCF_029962465.1	Escherichia	H5I61_RS19040	73585..77226	561	MULTISPECIES:-BREX-system-P-loop-protein-BrxC-[Escherichia]
WP_050858669.1	Type1.BREX	<-Peptidase_M17_N+Pepti [illegible]	1204	Bacteria>Pseudomonadota>Gammaproteobacteria>Enterobacterales>Enterobacteriaceae	GCF_030295055.1	Escherichia	AST78_RS16735	106796..110410	561	MULTISPECIES:-BREX-system-P-loop-protein-BrxC-[Escherichia]
WP_048999825.1	Type1.BREX	<-NACHT+SF1-SF2-Helicas [illegible]	1204	Bacteria>Pseudomonadota>Gammaproteobacteria>Enterobacterales>Enterobacteriaceae	GCF_900775105.1	Klebsiella/Raoultella_group	E7V87_RS13635	63633..67247	2890311	MULTISPECIES:-BREX-system-P-loop-protein-BrxC-[Klebsiella/Raoultella-group]
WP_225990544.1	Type1.BREX	Glyoxalase->ACET->YjhX_ [illegible]	1240	Bacteria>Pseudomonadota>Gammaproteobacteria>Enterobacterales>Enterobacteriaceae	GCF_954736865.1	Enterobacter	CU081_RS00925	173954..177676	547	MULTISPECIES:-BREX-system-P-loop-protein-BrxC-[Enterobacter]
CDL15658.1	Type1.BREX	Arm-DNA-bind_3+Phage_in [illegible]	950	Bacteria>Pseudomonadota>Gammaproteobacteria>Enterobacterales>Enterobacteriaceae>Klebsiella/Raoultella_group	GCA_000529945.1	Klebsiella_pneumoniae_IS46	.	6252..9104	1432553	putative-DNA-repair-ATPase-[Klebsiella-pneumoniae-IS46]
PLL44193.1	Type1.BREX	<-N6-METHYLASE-p<-N6-ME [illegible]	1233	Bacteria>Pseudomonadota>Gammaproteobacteria>Enterobacterales>Enterobacteriaceae>Klebsiella/Raoultella_group	GCA_002856085.1	Klebsiella_michiganensis	CWN50_01600	4530..8231	1134687	BREX-system-P-loop-protein-BrxC-[Klebsiella-michiganensis]
AUN75366.1	Type1.BREX	Arm-DNA-bind_3+Phage_in [illegible]	1233	Bacteria>Pseudomonadota>Gammaproteobacteria>Enterobacterales>Enterobacteriaceae>Klebsiella/Raoultella_group	GCA_002870985.1	Klebsiella_pneumoniae	C0077_01580	326913..330614	573	BREX-system-P-loop-protein-BrxC-[Klebsiella-pneumoniae]
QHX00137.1	Type1.BREX	YjhX_toxin->ACET->METHY [illegible]	1233	Bacteria>Pseudomonadota>Gammaproteobacteria>Enterobacterales>Enterobacteriaceae>Klebsiella/Raoultella_group	GCA_010365565.1	Klebsiella_variicola	GZS05_24295	4949707..4953408	244366	BREX-system-P-loop-protein-BrxC-[Klebsiella-variicola]
QJK24913.1	Type1.BREX	DUF2754-><-?<-NUDIX||Co [illegible]	1213	Bacteria>Pseudomonadota>Gammaproteobacteria>Enterobacterales>Enterobacteriaceae>Klebsiella/Raoultella_group	GCA_012967125.1	Klebsiella_pneumoniae	HJX28_24000	4748106..4751747	573	BREX-system-P-loop-protein-BrxC-[Klebsiella-pneumoniae]
QJM45197.1	Type1.BREX	<-wHTH+WYL<-sGTP<-?||XI [illegible]	1233	Bacteria>Pseudomonadota>Gammaproteobacteria>Enterobacterales>Enterobacteriaceae>Klebsiella/Raoultella_group	GCA_012970665.1	Klebsiella_pneumoniae	HJW71_23035	4620464..4624165	573	BREX-system-P-loop-protein-BrxC-[Klebsiella-pneumoniae]
HAT1683007.1	Type1.BREX	Peptidase_M20->?->?->DD [illegible]	1213	Bacteria>Pseudomonadota>Gammaproteobacteria>Enterobacterales>Enterobacteriaceae>Klebsiella/Raoultella_group	GCA_015893305.1	Klebsiella_oxytoca	I8Y21_003722	15957..19598	571	BREX-system-P-loop-protein-BrxC-[Klebsiella-oxytoca]
EGT0044780.1	Type1.BREX	<-DUF905<-DUF932<-CopG/ [illegible]	1233	Bacteria>Pseudomonadota>Gammaproteobacteria>Enterobacterales>Enterobacteriaceae>Klebsiella/Raoultella_group	GCA_015929385.1	Klebsiella_oxytoca	I9Y61_001542	37432..41133	571	BREX-system-P-loop-protein-BrxC-[Klebsiella-oxytoca]
EGT0068692.1	Type1.BREX	HIN-HTH->?->LZ_Tnp_IS66 [illegible]	1213	Bacteria>Pseudomonadota>Gammaproteobacteria>Enterobacterales>Enterobacteriaceae>Klebsiella/Raoultella_group	GCA_015929475.1	Klebsiella_michiganensis	I9Y62_005522	4489..8130	1134687	BREX-system-P-loop-protein-BrxC-[Klebsiella-michiganensis]
EJG2380286.1	Type1.BREX	<-DUF905<-DUF932<-CopG/ [illegible]	1233	Bacteria>Pseudomonadota>Gammaproteobacteria>Enterobacterales>Enterobacteriaceae>Klebsiella/Raoultella_group	GCA_016088515.2	Raoultella_ornithinolytica	AM381_RS08835	19475..23176	54291	BREX-system-P-loop-protein-BrxC-[Raoultella-ornithinolytica]
HAV2047149.1	Type1.BREX	<-Calcineurin<-?<-?<-?| [illegible]	1233	Bacteria>Pseudomonadota>Gammaproteobacteria>Enterobacterales>Enterobacteriaceae>Klebsiella/Raoultella_group	GCA_016445365.1	Raoultella_ornithinolytica	JHJ87_003574	41911..45612	54291	BREX-system-P-loop-protein-BrxC-[Raoultella-ornithinolytica]
MBL3233196.1	Type1.BREX	<-HKD-DNase+OGG-like<-S [illegible]	1213	Bacteria>Pseudomonadota>Gammaproteobacteria>Enterobacterales>Enterobacteriaceae>Klebsiella/Raoultella_group	GCA_016755095.1	Klebsiella_pneumoniae	ELP31_24695	10226..13867	573	BREX-system-P-loop-protein-BrxC-[Klebsiella-pneumoniae]
MBN7733855.1	Type1.BREX	<-wHTH+WYL<-sGTP<-?||XI [illegible]	1233	Bacteria>Pseudomonadota>Gammaproteobacteria>Enterobacterales>Enterobacteriaceae>Klebsiella/Raoultella_group	GCA_017255495.1	Klebsiella_pneumoniae	JX616_18220	31914..35615	573	BREX-system-P-loop-protein-BrxC-[Klebsiella-pneumoniae]
MBZ6545107.1	Type1.BREX	<-wHTH+WYL<-nSTAND1+sGT [illegible]	1233	Bacteria>Pseudomonadota>Gammaproteobacteria>Enterobacterales>Enterobacteriaceae>Klebsiella/Raoultella_group	GCA_020115175.1	Klebsiella_pneumoniae	FMJ66_23200	19841..23542	573	BREX-system-P-loop-protein-BrxC-[Klebsiella-pneumoniae]
MBZ7196032.1	Type1.BREX	ACET->?->YjhX_toxin->AC [illegible]	1233	Bacteria>Pseudomonadota>Gammaproteobacteria>Enterobacterales>Enterobacteriaceae>Klebsiella/Raoultella_group	GCA_020118595.1	Klebsiella_oxytoca	FMK61_04485	39995..43696	571	BREX-system-P-loop-protein-BrxC-[Klebsiella-oxytoca]
MCB3335667.1	Type1.BREX	<-DDE_Tnp_1_5+DDE_Tnp_1 [illegible]	1213	Bacteria>Pseudomonadota>Gammaproteobacteria>Enterobacterales>Enterobacteriaceae>Klebsiella/Raoultella_group	GCA_020478865.1	Klebsiella_pneumoniae	H5849_24975	11785..15426	573	BREX-system-P-loop-protein-BrxC-[Klebsiella-pneumoniae]
HBQ2245107.1	Type1.BREX	<-DUF6079*<-BrxB-BrxF-i [illegible]	1164	Bacteria>Pseudomonadota>Gammaproteobacteria>Enterobacterales>Enterobacteriaceae>Klebsiella/Raoultella_group	GCA_021823445.1	Klebsiella_pneumoniae	L7O82_005482	1..3492	573	BREX-system-P-loop-protein-BrxC-[Klebsiella-pneumoniae]
HBQ2805341.1	Type1.BREX	<-DUF6079-p*<-BrxB-BrxF [illegible]	606	Bacteria>Pseudomonadota>Gammaproteobacteria>Enterobacterales>Enterobacteriaceae>Klebsiella/Raoultella_group	GCA_021848835.1	Klebsiella_pneumoniae	L7V14_005501	1..1820	573	BREX-system-P-loop-protein-BrxC-[Klebsiella-pneumoniae]
EIV3098057.1	Type1.BREX	<-wHTH+WYL<-nSTAND1+sGT [illegible]	1233	Bacteria>Pseudomonadota>Gammaproteobacteria>Enterobacterales>Enterobacteriaceae>Klebsiella/Raoultella_group	GCA_021854435.1	Klebsiella_pneumoniae	L7W77_004838	45573..49274	573	BREX-system-P-loop-protein-BrxC-[Klebsiella-pneumoniae]
HBQ4025835.1	Type1.BREX	<-ABC-ATPase+SbcC<-?<-? [illegible]	1213	Bacteria>Pseudomonadota>Gammaproteobacteria>Enterobacterales>Enterobacteriaceae>Klebsiella/Raoultella_group	GCA_021857085.1	Klebsiella_pneumoniae	L7127_004405	18391..22032	573	BREX-system-P-loop-protein-BrxC-[Klebsiella-pneumoniae]
HBQ4939450.1	Type1.BREX	XIS-HTH->SF1-SF2-Helica [illegible]	1157	Bacteria>Pseudomonadota>Gammaproteobacteria>Enterobacterales>Enterobacteriaceae>Klebsiella/Raoultella_group	GCA_021861305.1	Klebsiella_pneumoniae	L7366_003904	52372..55845	573	BREX-system-P-loop-protein-BrxC-[Klebsiella-pneumoniae]
EIV3902456.1	Type1.BREX	Arm-DNA-bind_3+Phage_in [illegible]	1204	Bacteria>Pseudomonadota>Gammaproteobacteria>Enterobacterales>Enterobacteriaceae>Klebsiella/Raoultella_group	GCA_021871195.1	Klebsiella_pneumoniae	L8E75_001739	326019..329633	573	BREX-system-P-loop-protein-BrxC-[Klebsiella-pneumoniae]
HBQ6953848.1	Type1.BREX	XIS-HTH->XIS-HTH->?->RE [illegible]	1043	Bacteria>Pseudomonadota>Gammaproteobacteria>Enterobacterales>Enterobacteriaceae>Klebsiella/Raoultella_group	GCA_021875055.1	Klebsiella_pneumoniae	L8O79_001073	40057..43187	573	BREX-system-P-loop-protein-BrxC-[Klebsiella-pneumoniae]
HBQ7765849.1	Type1.BREX	<-DUF6079-p*<-BrxB-BrxF [illegible]	741	Bacteria>Pseudomonadota>Gammaproteobacteria>Enterobacterales>Enterobacteriaceae>Klebsiella/Raoultella_group	GCA_021878555.1	Klebsiella_pneumoniae	L8R48_006010	1..2224	573	BREX-system-P-loop-protein-BrxC-[Klebsiella-pneumoniae]
HBQ7782691.1	Type1.BREX	<-DUF6079*<-BrxB-BrxF-i [illegible]	1042	Bacteria>Pseudomonadota>Gammaproteobacteria>Enterobacterales>Enterobacteriaceae>Klebsiella/Raoultella_group	GCA_021878575.1	Klebsiella_pneumoniae	L8R50_005941	1..3128	573	BREX-system-P-loop-protein-BrxC-[Klebsiella-pneumoniae]
HBR1207023.1	Type1.BREX	<-DUF905<-?<-?<-?<-wHTH [illegible]	1204	Bacteria>Pseudomonadota>Gammaproteobacteria>Enterobacterales>Enterobacteriaceae>Klebsiella/Raoultella_group	GCA_021909055.1	Klebsiella_pneumoniae	L9K92_004097	80517..84131	573	BREX-system-P-loop-protein-BrxC-[Klebsiella-pneumoniae]
HBR1207682.1	Type1.BREX	Glyco_hydro_43+GH43_C2- [illegible]	1204	Bacteria>Pseudomonadota>Gammaproteobacteria>Enterobacterales>Enterobacteriaceae>Klebsiella/Raoultella_group	GCA_021909055.1	Klebsiella_pneumoniae	L9K92_004777	40693..44307	573	BREX-system-P-loop-protein-BrxC-[Klebsiella-pneumoniae]
HBR1265003.1	Type1.BREX	<-AP_endonuc_2||?->Glyc [illegible]	1213	Bacteria>Pseudomonadota>Gammaproteobacteria>Enterobacterales>Enterobacteriaceae>Klebsiella/Raoultella_group	GCA_021909255.1	Klebsiella_quasipneumoniae_subsp._quasipneumoniae	L9L24_003166	12201..15842	1667327	BREX-system-P-loop-protein-BrxC-[Klebsiella-quasipneumoniae-subsp.-quasipneumoniae]
HBR1743014.1	Type1.BREX	wHTH+SAD+HNH-><-?||?->D [illegible]	1213	Bacteria>Pseudomonadota>Gammaproteobacteria>Enterobacterales>Enterobacteriaceae>Klebsiella/Raoultella_group	GCA_021911065.1	Klebsiella_pneumoniae	L9M23_004127	37623..41264	573	BREX-system-P-loop-protein-BrxC-[Klebsiella-pneumoniae]
HBR1749211.1	Type1.BREX	REase+iREase-><-RHH||?- [illegible]	1225	Bacteria>Pseudomonadota>Gammaproteobacteria>Enterobacterales>Enterobacteriaceae>Klebsiella/Raoultella_group	GCA_021911085.1	Klebsiella_pneumoniae	L9M14_005022	6747..10424	573	BREX-system-P-loop-protein-BrxC-[Klebsiella-pneumoniae]
HBR1784209.1	Type1.BREX	<-wHTH+WYL<-sGTP<-?||XI [illegible]	1233	Bacteria>Pseudomonadota>Gammaproteobacteria>Enterobacterales>Enterobacteriaceae>Klebsiella/Raoultella_group	GCA_021911385.1	Klebsiella_quasipneumoniae_subsp._quasipneumoniae	L9M28_003408	20306..24007	1667327	BREX-system-P-loop-protein-BrxC-[Klebsiella-quasipneumoniae-subsp.-quasipneumoniae]
HBR3944992.1	Type1.BREX	<-HTH_36<-HTH+HTH<-ParB [illegible]	1157	Bacteria>Pseudomonadota>Gammaproteobacteria>Enterobacterales>Enterobacteriaceae>Klebsiella/Raoultella_group	GCA_021923985.1	Klebsiella_pneumoniae	L9T85_005193	16003..19476	573	BREX-system-P-loop-protein-BrxC-[Klebsiella-pneumoniae]
HBR4416044.1	Type1.BREX	wHTH+SAD+HNH-><-?||?->D [illegible]	1213	Bacteria>Pseudomonadota>Gammaproteobacteria>Enterobacterales>Enterobacteriaceae>Klebsiella/Raoultella_group	GCA_021925925.1	Klebsiella_pneumoniae	L9V39_001889	16089..19730	573	BREX-system-P-loop-protein-BrxC-[Klebsiella-pneumoniae]
HBR4497442.1	Type1.BREX	REase-><-SIGMA+OB-Fold+ [illegible]	1213	Bacteria>Pseudomonadota>Gammaproteobacteria>Enterobacterales>Enterobacteriaceae>Klebsiella/Raoultella_group	GCA_021926505.1	Klebsiella_pneumoniae	L9V75_004938	11616..15257	573	BREX-system-P-loop-protein-BrxC-[Klebsiella-pneumoniae]
HBR5241810.1	Type1.BREX	DUF2754-><-?<-NUDIX||Co [illegible]	1213	Bacteria>Pseudomonadota>Gammaproteobacteria>Enterobacterales>Enterobacteriaceae>Klebsiella/Raoultella_group	GCA_021929495.1	Klebsiella_pneumoniae	L9Y21_004664	37685..41326	573	BREX-system-P-loop-protein-BrxC-[Klebsiella-pneumoniae]
HBR6062602.1	Type1.BREX	ColE7+HNH->?->BrxA-DNA- [illegible]	1213	Bacteria>Pseudomonadota>Gammaproteobacteria>Enterobacterales>Enterobacteriaceae>Klebsiella/Raoultella_group	GCA_021932975.1	Klebsiella_pneumoniae	L9O24_005225	4087..7728	573	BREX-system-P-loop-protein-BrxC-[Klebsiella-pneumoniae]
HBR8923219.1	Type1.BREX	DUF6079*->N6-METHYLASE- [illegible]	1005	Bacteria>Pseudomonadota>Gammaproteobacteria>Enterobacterales>Enterobacteriaceae>Klebsiella/Raoultella_group	GCA_021945235.1	Klebsiella_pneumoniae	L9729_005179	1..3020	573	BREX-system-P-loop-protein-BrxC-[Klebsiella-pneumoniae]
HBS2746978.1	Type1.BREX	<-wHTH+WYL<-nSTAND1+sGT [illegible]	1233	Bacteria>Pseudomonadota>Gammaproteobacteria>Enterobacterales>Enterobacteriaceae>Klebsiella/Raoultella_group	GCA_021960165.1	Klebsiella_quasipneumoniae_subsp._quasipneumoniae	MAE70_005431	15539..19240	1667327	BREX-system-P-loop-protein-BrxC-[Klebsiella-quasipneumoniae-subsp.-quasipneumoniae]
HBS5818882.1	Type1.BREX	Arm-DNA-bind_3+Phage_in [illegible]	1157	Bacteria>Pseudomonadota>Gammaproteobacteria>Enterobacterales>Enterobacteriaceae>Klebsiella/Raoultella_group	GCA_021971955.1	Klebsiella_pneumoniae	MAK71_003676	3132..6605	573	BREX-system-P-loop-protein-BrxC-[Klebsiella-pneumoniae]
HBS6040887.1	Type1.BREX	Glyoxalase_8->URI-DNase [illegible]	1213	Bacteria>Pseudomonadota>Gammaproteobacteria>Enterobacterales>Enterobacteriaceae>Klebsiella/Raoultella_group	GCA_021973155.1	Klebsiella_aerogenes	MAL38_002165	35308..38949	548	BREX-system-P-loop-protein-BrxC-[Klebsiella-aerogenes]
HBS6654372.1	Type1.BREX	<-HNH-p||wHTH+SAD+HNH-> [illegible]	1204	Bacteria>Pseudomonadota>Gammaproteobacteria>Enterobacterales>Enterobacteriaceae>Klebsiella/Raoultella_group	GCA_021975495.1	Klebsiella_pneumoniae	MAN10_004114	53582..57196	573	BREX-system-P-loop-protein-BrxC-[Klebsiella-pneumoniae]
HBS6827429.1	Type1.BREX	<-DUF932<-nSTAND1+GTPas [illegible]	1021	Bacteria>Pseudomonadota>Gammaproteobacteria>Enterobacterales>Enterobacteriaceae>Klebsiella/Raoultella_group	GCA_021976135.1	Klebsiella_pneumoniae	MAN94_004909	32044..35105	573	BREX-system-P-loop-protein-BrxC-[Klebsiella-pneumoniae]
HBS7652074.1	Type1.BREX	<-Calcineurin<-?<-?<-?< [illegible]	1204	Bacteria>Pseudomonadota>Gammaproteobacteria>Enterobacterales>Enterobacteriaceae>Klebsiella/Raoultella_group	GCA_021979555.1	Klebsiella_pneumoniae	MAR21_002848	317524..321138	573	BREX-system-P-loop-protein-BrxC-[Klebsiella-pneumoniae]
HBS9599072.1	Type1.BREX	<-HNH-p||wHTH+SAD+HNH-> [illegible]	1204	Bacteria>Pseudomonadota>Gammaproteobacteria>Enterobacterales>Enterobacteriaceae>Klebsiella/Raoultella_group	GCA_021986995.1	Klebsiella_pneumoniae	MAW98_001666	53581..57195	573	BREX-system-P-loop-protein-BrxC-[Klebsiella-pneumoniae]
EIV7918660.1	Type1.BREX	DUF2754-><-HTH_Tnp_1+HT [illegible]	1213	Bacteria>Pseudomonadota>Gammaproteobacteria>Enterobacterales>Enterobacteriaceae>Klebsiella/Raoultella_group	GCA_022009395.1	Klebsiella_pneumoniae	MA315_005123	14682..18323	573	BREX-system-P-loop-protein-BrxC-[Klebsiella-pneumoniae]
EJD7072427.1	Type1.BREX	Arm-DNA-bind_3+Phage_in [illegible]	584	Bacteria>Pseudomonadota>Gammaproteobacteria>Enterobacterales>Enterobacteriaceae>Klebsiella/Raoultella_group	GCA_022024375.2	Klebsiella_pneumoniae	M0J36_RS17010	155077..156830	573	BREX-system-P-loop-protein-BrxC-[Klebsiella-pneumoniae]
HBT6124508.1	Type1.BREX	<-DUF6079-p*<-BrxB-BrxF [illegible]	662	Bacteria>Pseudomonadota>Gammaproteobacteria>Enterobacterales>Enterobacteriaceae>Klebsiella/Raoultella_group	GCA_022055945.1	Klebsiella_pneumoniae	MCD08_005761	1..1988	573	BREX-system-P-loop-protein-BrxC-[Klebsiella-pneumoniae]
HBU3475135.1	Type1.BREX	DUF2754->||ColE7+HNH->? [illegible]	1213	Bacteria>Pseudomonadota>Gammaproteobacteria>Enterobacterales>Enterobacteriaceae>Klebsiella/Raoultella_group	GCA_022085545.1	Klebsiella_pneumoniae	MCW21_004534	37275..40916	573	BREX-system-P-loop-protein-BrxC-[Klebsiella-pneumoniae]
HBV1956328.1	Type1.BREX	URI-DNase->BrxA-DNA-bin [illegible]	1213	Bacteria>Pseudomonadota>Gammaproteobacteria>Enterobacterales>Enterobacteriaceae>Klebsiella/Raoultella_group	GCA_022126105.1	Klebsiella_pneumoniae	MDQ85_004847	2358..5999	573	BREX-system-P-loop-protein-BrxC-[Klebsiella-pneumoniae]
HBV3572283.1	Type1.BREX	DUF6079*->N6-METHYLASE- [illegible]	1061	Bacteria>Pseudomonadota>Gammaproteobacteria>Enterobacterales>Enterobacteriaceae>Klebsiella/Raoultella_group	GCA_022140925.1	Klebsiella_pneumoniae	MDV19_005297	1..3186	573	BREX-system-P-loop-protein-BrxC-[Klebsiella-pneumoniae]
HBV3618888.1	Type1.BREX	DUF6079*->N6-METHYLASE- [illegible]	1060	Bacteria>Pseudomonadota>Gammaproteobacteria>Enterobacterales>Enterobacteriaceae>Klebsiella/Raoultella_group	GCA_022141165.1	Klebsiella_pneumoniae	MDV14_005210	1..3185	573	BREX-system-P-loop-protein-BrxC-[Klebsiella-pneumoniae]
HBV8971411.1	Type1.BREX	<-LACI-HTH+PBPI<-?||HNH [illegible]	1213	Bacteria>Pseudomonadota>Gammaproteobacteria>Enterobacterales>Enterobacteriaceae>Klebsiella/Raoultella_group	GCA_022162485.1	Klebsiella_oxytoca	MD887_005073	22560..26201	571	BREX-system-P-loop-protein-BrxC-[Klebsiella-oxytoca]
HBW3476175.1	Type1.BREX	nSTAND1+HNH->RNaseH->NA [illegible]	1162	Bacteria>Pseudomonadota>Gammaproteobacteria>Enterobacterales>Enterobacteriaceae>Klebsiella/Raoultella_group	GCA_022184305.1	Klebsiella_pneumoniae	MEZ45_004510	80809..84297	573	BREX-system-P-loop-protein-BrxC-[Klebsiella-pneumoniae]
HBW3608486.1	Type1.BREX	DUF2754-><-?||ColE7+HNH [illegible]	1213	Bacteria>Pseudomonadota>Gammaproteobacteria>Enterobacterales>Enterobacteriaceae>Klebsiella/Raoultella_group	GCA_022184865.1	Klebsiella_pneumoniae	MEZ81_004956	16088..19729	573	BREX-system-P-loop-protein-BrxC-[Klebsiella-pneumoniae]
HBW4624689.1	Type1.BREX	ZnR->ZnR-><-SIGMA+OB-Fo [illegible]	1213	Bacteria>Pseudomonadota>Gammaproteobacteria>Enterobacterales>Enterobacteriaceae>Klebsiella/Raoultella_group	GCA_022189205.1	Klebsiella_pneumoniae	ME566_005166	11368..15009	573	BREX-system-P-loop-protein-BrxC-[Klebsiella-pneumoniae]
HBW6426293.1	Type1.BREX	BrxA-DNA-binding->BrxB- [illegible]	1213	Bacteria>Pseudomonadota>Gammaproteobacteria>Enterobacterales>Enterobacteriaceae>Klebsiella/Raoultella_group	GCA_022195805.1	Klebsiella_pneumoniae	MFA43_005255	1622..5263	573	BREX-system-P-loop-protein-BrxC-[Klebsiella-pneumoniae]
HBW7740799.1	Type1.BREX	DUF2754-><-?||ColE7+HNH [illegible]	1213	Bacteria>Pseudomonadota>Gammaproteobacteria>Enterobacterales>Enterobacteriaceae>Klebsiella/Raoultella_group	GCA_022200625.1	Klebsiella_pneumoniae	MFE66_003732	16104..19745	573	BREX-system-P-loop-protein-BrxC-[Klebsiella-pneumoniae]
HBW8426142.1	Type1.BREX	wHTH+SAD+HNH-><-?||?->D [illegible]	1213	Bacteria>Pseudomonadota>Gammaproteobacteria>Enterobacterales>Enterobacteriaceae>Klebsiella/Raoultella_group	GCA_022203225.1	Klebsiella_pneumoniae	MFL89_004730	37523..41164	573	BREX-system-P-loop-protein-BrxC-[Klebsiella-pneumoniae]

Protein-ID	System	Gene-Neighbourhoods	Length	Taxonomy	Assembly-ID	Strain	Locus-Tag	Genomic-coordinates	TaxID	Fasta-define
HBW9808854.1	Type1.BREX	<-DUF6079*<-BrxB-BrxF-iSTAND<-BrxA-DNA-binding||REase+iREase-><-EVE+HNH<-Arm-DNA-bind_3+Phage_integrase	1056	Bacteria>Pseudomonadota>Gammaproteobacteria>Enterobacterales>Enterobacteriaceae>Klebsiella/Raoultella_group	GCA_022208425.1	Klebsiella_pneumoniae	MFX42_22985	1..3169	573	BREX-system-P-loop-protein-BrxC-[Klebsiella-pneumoniae]
HBX0790077.1	Type1.BREX	wHTH+SAD+HNH-><-?<-?||?->DUF2754->||ColE7+HNH->?->BrxA-DNA-binding->BrxB-BrxF-iSTAND->DUF6079*->N6-METHYLASE->PglZ->SIGMA+OB-Fold+AAA-ATPase+Lon-Protease-BrxL->HKD-DNase+OGG-like-><-REase<-NACHT+SF1-SF2-Helicase+HKD-DNase<-RNaseH<-nSTAND1+HNH	1213	Bacteria>Pseudomonadota>Gammaproteobacteria>Enterobacterales>Enterobacteriaceae>Klebsiella/Raoultella_group	GCA_022212165.1	Klebsiella_pneumoniae	MF115_18880	50016..53657	573	BREX-system-P-loop-protein-BrxC-[Klebsiella-pneumoniae]
HBX5767913.1	Type1.BREX	MULE-TRANSPOSASE-><-AAA-ATPase<-rve||<-SF1-SF2-Helicase<-HKD-DNase+HKD-DNase<-?<-?||BrxA-DNA-binding->BrxB-BrxF-iSTAND->DUF6079*->N6-METHYLASE->PglZ->SIGMA+OB-Fold+AAA-ATPase+Lon-Protease-BrxL-><-RVT||?-><-TPR||<-?<-DnaJ	1213	Bacteria>Pseudomonadota>Gammaproteobacteria>Enterobacterales>Enterobacteriaceae>Klebsiella/Raoultella_group	GCA_022248915.1	Klebsiella_pneumoniae	MH553_25875	12227..15868	573	BREX-system-P-loop-protein-BrxC-[Klebsiella-pneumoniae]
HBX5876367.1	Type1.BREX	<-SF1-SF2-Helicase||BrxA-DNA-binding->BrxB-BrxF-iSTAND->DUF6079*->N6-METHYLASE->OLD-ABC->TOPRIM->PglZ->SIGMA+OB-Fold+AAA-ATPase+Lon-Protease-BrxL-><-Arm-DNA-bind_3+Phage_integrase	1204	Bacteria>Pseudomonadota>Gammaproteobacteria>Enterobacterales>Enterobacteriaceae>Klebsiella/Raoultella_group	GCA_022249175.1	Klebsiella_pneumoniae	MH570_26215	1979..5593	573	BREX-system-P-loop-protein-BrxC-[Klebsiella-pneumoniae]
HBX7751797.1	Type1.BREX	ColE7+HNH->?-><-SIGMA+OB-Fold+AAA-ATPase+Lon-Protease-BrxL<-PglZ<-TOPRIM<-OLD-ABC<-N6-METHYLASE<-DUF6079*<-BrxB-BrxF-iSTAND<-BrxA-DNA-binding||ZnR->	1204	Bacteria>Pseudomonadota>Gammaproteobacteria>Enterobacterales>Enterobacteriaceae>Klebsiella/Raoultella_group	GCA_022258155.1	Klebsiella_pneumoniae	MIG23_15250	14841..18455	573	BREX-system-P-loop-protein-BrxC-[Klebsiella-pneumoniae]
HBY0194703.1	Type1.BREX	<-DUF6079-p*<-BrxB-BrxF-iSTAND<-BrxA-DNA-binding<-URI-DNase||ASCH+Glyoxalase->Phage_integrase->	704	Bacteria>Pseudomonadota>Gammaproteobacteria>Enterobacterales>Enterobacteriaceae>Klebsiella/Raoultella_group	GCA_022267575.1	Klebsiella_pneumoniae	MIN90_27480	1..2112	573	BREX-system-P-loop-protein-BrxC-[Klebsiella-pneumoniae]
HBY1687637.1	Type1.BREX	Glyoxalase_8->URI-DNase-><-RHH_antitoxin||?->XIS-HTH->?->ZnR->BrxA-DNA-binding->BrxB-BrxF-iSTAND->DUF6079*->N6-METHYLASE->PglZ->SIGMA+OB-Fold+AAA-ATPase+Lon-Protease-BrxL->HKD-DNase+OGG-like-><-Arm-DNA-bind_3+Phage_integrase	1213	Bacteria>Pseudomonadota>Gammaproteobacteria>Enterobacterales>Enterobacteriaceae>Klebsiella/Raoultella_group	GCA_022272455.1	Klebsiella_pneumoniae	MIQ44_23625	54198..57839	573	BREX-system-P-loop-protein-BrxC-[Klebsiella-pneumoniae]
HBY1974060.1	Type1.BREX	<-DUF6079*<-BrxB-BrxF-iSTAND<-BrxA-DNA-binding||REase+iREase-><-EVE+HNH<-Arm-DNA-bind_3+Phage_integrase	1065	Bacteria>Pseudomonadota>Gammaproteobacteria>Enterobacterales>Enterobacteriaceae>Klebsiella/Raoultella_group	GCA_022274115.1	Klebsiella_pneumoniae	MIR32_28085	1..3194	573	BREX-system-P-loop-protein-BrxC-[Klebsiella-pneumoniae]
HBY5777257.1	Type1.BREX	<-DUF6079*<-BrxB-BrxF-iSTAND<-BrxA-DNA-binding<-URI-DNase||ASCH+Glyoxalase->Phage_integrase->	1075	Bacteria>Pseudomonadota>Gammaproteobacteria>Enterobacterales>Enterobacteriaceae>Klebsiella/Raoultella_group	GCA_022292055.1	Klebsiella_pneumoniae	MIZ42_28895	1..3225	573	BREX-system-P-loop-protein-BrxC-[Klebsiella-pneumoniae]
HBY8309079.1	Type1.BREX	DUF6079*->ParB+HNH->N6-METHYLASE->PglZ->SIGMA+OB-Fold+AAA-ATPase+Lon-Protease-BrxL-><-HTH_Tnp_1+HTH_21+rve+rve_3	1167	Bacteria>Pseudomonadota>Gammaproteobacteria>Enterobacterales>Enterobacteriaceae>Klebsiella/Raoultella_group	GCA_022301875.1	Klebsiella_pneumoniae	MI837_25465	1..3506	573	BREX-system-P-loop-protein-BrxC-[Klebsiella-pneumoniae]
HBZ4474772.1	Type1.BREX	<-DUF6079-p*<-BrxB-BrxF-iSTAND<-BrxA-DNA-binding<-URI-DNase||Phage_integrase->	737	Bacteria>Pseudomonadota>Gammaproteobacteria>Enterobacterales>Enterobacteriaceae>Klebsiella/Raoultella_group	GCA_022336725.1	Klebsiella_pneumoniae	L8R61_005966	1..2211	573	BREX-system-P-loop-protein-BrxC-[Klebsiella-pneumoniae]
EIX9162689.1	Type1.BREX	<-MODE-HTH+PBPII||ANK+ANK+ANK->?->?->?->?->ACET->||BrxA-DNA-binding->BrxB-BrxF-iSTAND->DUF6079*->N6-METHYLASE->PglZ->SIGMA+OB-Fold+AAA-ATPase+Lon-Protease-BrxL-><-ZnR<-ZnR<-?<-?||RHH_antitoxin->XIS-HTH->	1213	Bacteria>Pseudomonadota>Gammaproteobacteria>Enterobacterales>Enterobacteriaceae>Klebsiella/Raoultella_group	GCA_022430005.1	Klebsiella_pneumoniae	MLJ99_002334	77875..81516	573	BREX-system-P-loop-protein-BrxC-[Klebsiella-pneumoniae]
EIX9370752.1	Type1.BREX	<-wHTH+WYL<-nSTAND1+sGTP<-?||XIS-HTH->?->BrxA-DNA-binding->BrxB-BrxF-iSTAND->DUF6079*->ParB+HNH->N6-METHYLASE->PglZ->SIGMA+OB-Fold+AAA-ATPase+Lon-Protease-BrxL-><-Arm-DNA-bind_3+Phage_integrase	1233	Bacteria>Pseudomonadota>Gammaproteobacteria>Enterobacterales>Enterobacteriaceae>Klebsiella/Raoultella_group	GCA_022430905.1	Klebsiella_pneumoniae	L7649_004474	53291..56992	573	BREX-system-P-loop-protein-BrxC-[Klebsiella-pneumoniae]
EIX9551660.1	Type1.BREX	<-MAD-NTD+HNH||<-?||?->||?->?-><-Phage_integrase||URI-DNase->BrxA-DNA-binding->BrxB-BrxF-iSTAND->DUF6079*->N6-METHYLASE->PglZ->SIGMA+OB-Fold+AAA-ATPase+Lon-Protease-BrxL->HKD-DNase+OGG-like-><-REase	1213	Bacteria>Pseudomonadota>Gammaproteobacteria>Enterobacterales>Enterobacteriaceae>Klebsiella/Raoultella_group	GCA_022431205.1	Klebsiella_pneumoniae	ML061_004130	17308..20949	573	BREX-system-P-loop-protein-BrxC-[Klebsiella-pneumoniae]
EIX9552648.1	Type1.BREX	<-DUF6079-p*<-BrxB-BrxF-iSTAND<-BrxA-DNA-binding<-URI-DNase||Phage_integrase->	891	Bacteria>Pseudomonadota>Gammaproteobacteria>Enterobacterales>Enterobacteriaceae>Klebsiella/Raoultella_group	GCA_022431205.1	Klebsiella_pneumoniae	ML061_005170	1..2675	573	BREX-system-P-loop-protein-BrxC-[Klebsiella-pneumoniae]
HBZ8012775.1	Type1.BREX	<-MAD-NTD+HNH||wHTH+SAD+HNH->||?->DUF2754-><-?||ColE7+HNH->?->BrxA-DNA-binding->BrxB-BrxF-iSTAND->DUF6079*->N6-METHYLASE->PglZ->SIGMA+OB-Fold+AAA-ATPase+Lon-Protease-BrxL->HKD-DNase+OGG-like-><-REase<-NACHT+SF1-SF2-Helicase+HKD-DNase<-RNaseH<-nSTAND1+HNH	1213	Bacteria>Pseudomonadota>Gammaproteobacteria>Enterobacterales>Enterobacteriaceae>Klebsiella/Raoultella_group	GCA_022500545.1	Klebsiella_pneumoniae	MM158_002941	16412..20053	573	BREX-system-P-loop-protein-BrxC-[Klebsiella-pneumoniae]
EIY5117082.1	Type1.BREX	<-Calcineurin<-?<-?<-?<-?<-?||tRNA-><-ZnR||BrxA-DNA-binding->BrxB-BrxF-iSTAND->DUF6079*->N6-METHYLASE->OLD-ABC->TOPRIM->PglZ->SIGMA+OB-Fold+AAA-ATPase+Lon-Protease-BrxL-><-wHTH(SBD)+HNH||MAD-NTD+HNH-><-Glyco_hydro_43+GH43_C2	1197	Bacteria>Pseudomonadota>Gammaproteobacteria>Enterobacterales>Enterobacteriaceae>Klebsiella/Raoultella_group	GCA_022503905.1	Klebsiella_quasipneumoniae	ML076_004996	20031..23624	1463165	BREX-system-P-loop-protein-BrxC-[Klebsiella-quasipneumoniae]
HCA0942642.1	Type1.BREX	Glyco_hydro_43+GH43_C2-><-MAD-NTD+HNH||<-?||||?->?-><-Phage_integrase||URI-DNase->BrxA-DNA-binding->BrxB-BrxF-iSTAND->DUF6079*->N6-METHYLASE->PglZ->SIGMA+OB-Fold+AAA-ATPase+Lon-Protease-BrxL->HKD-DNase+OGG-like-><-REase	1213	Bacteria>Pseudomonadota>Gammaproteobacteria>Enterobacterales>Enterobacteriaceae>Klebsiella/Raoultella_group	GCA_022528725.1	Klebsiella_pneumoniae	MNJ11_004335	17307..20948	573	BREX-system-P-loop-protein-BrxC-[Klebsiella-pneumoniae]
HCA3733838.1	Type1.BREX	DUF6079*->N6-METHYLASE->PglZ->SIGMA+OB-Fold+AAA-ATPase+Lon-Protease-BrxL-><-ZnR<-ZnR	1102	Bacteria>Pseudomonadota>Gammaproteobacteria>Enterobacterales>Enterobacteriaceae>Klebsiella/Raoultella_group	GCA_022599315.1	Klebsiella_pneumoniae	MPI41_005488	1..3309	573	BREX-system-P-loop-protein-BrxC-[Klebsiella-pneumoniae]
HCB0434648.1	Type1.BREX	DUF2754-><-?||ColE7+HNH->?->BrxA-DNA-binding->BrxB-BrxF-iSTAND->DUF6079*->N6-METHYLASE->PglZ->SIGMA+OB-Fold+AAA-ATPase+Lon-Protease-BrxL->HKD-DNase+OGG-like-><-REase<-NACHT+SF1-SF2-Helicase+HKD-DNase<-RNaseH<-nSTAND1+HNH	1213	Bacteria>Pseudomonadota>Gammaproteobacteria>Enterobacterales>Enterobacteriaceae>Klebsiella/Raoultella_group	GCA_023030065.1	Klebsiella_variicola_subsp._variicola	MYM82_004336	18617..22258	2590157	BREX-system-P-loop-protein-BrxC-[Klebsiella-variicola-subsp.-variicola]
HCB0569860.1	Type1.BREX	YjhX_toxin->ACET->METHYLASE->ZnR->Glyoxalase->BrxA-DNA-binding->BrxB-BrxF-iSTAND->DUF6079*->OLD-ABC->TOPRIM->N6-METHYLASE->PglZ->SIGMA+OB-Fold+AAA-ATPase+Lon-Protease-BrxL-><-Arm-DNA-bind_3+Phage_integrase	1233	Bacteria>Pseudomonadota>Gammaproteobacteria>Enterobacterales>Enterobacteriaceae>Klebsiella/Raoultella_group	GCA_023030625.1	Klebsiella_quasipneumoniae_subsp._similipneumoniae	MYL58_003257	37179..40880	1463164	BREX-system-P-loop-protein-BrxC-[Klebsiella-quasipneumoniae-subsp.-similipneumoniae]
HCD1392381.1	Type1.BREX	<-wHTH+WYL+WCX<-SIGMA+OB-Fold+AAA-ATPase+Lon-Protease-BrxL<-PglZ<-N6-METHYLASE-p<-RVT<-HEPN<-N6-METHYLASE<-ParB+HNH<-DUF6079*<-BrxB-BrxF-iSTAND<-BrxA-DNA-binding||TIR->TIR-><-XIS-HTH<-?||CopG/RHH->DUF932->DUF905->	1233	Bacteria>Pseudomonadota>Gammaproteobacteria>Enterobacterales>Enterobacteriaceae>Klebsiella/Raoultella_group	GCA_023530865.1	Klebsiella_pneumoniae_subsp._pneumoniae	M9022_004645	23383..27084	72407	BREX-system-P-loop-protein-BrxC-[Klebsiella-pneumoniae-subsp.-pneumoniae]
HCD5426239.1	Type1.BREX	<-DUF4400<-XIS-HTH<-wHTH<-ZnR||BrxA-DNA-binding->BrxB-BrxF-iSTAND->DUF6079*->N6-METHYLASE->	1204	Bacteria>Pseudomonadota>Gammaproteobacteria>Enterobacterales>Enterobacteriaceae>Klebsiella/Raoultella_group	GCA_023613715.1	Klebsiella_aerogenes	NBX34_004630	15041..18655	548	BREX-system-P-loop-protein-BrxC-[Klebsiella-aerogenes]
MCM6196114.1	Type1.BREX	<-Calcineurin<-?<-?<-?<-?<-?||tRNA->Arm-DNA-bind_3+Phage_integrase->BrxA-DNA-binding->BrxB-BrxF-iSTAND->DUF6079*->N6-METHYLASE->ParB+HNH->PglZ-><-SF1-SF2-Helicase+REase<-SF1-SF2-Helicase+REase<-XIS-HTH	1157	Bacteria>Pseudomonadota>Gammaproteobacteria>Enterobacterales>Enterobacteriaceae>Klebsiella/Raoultella_group	GCA_023741115.1	Klebsiella_pneumoniae	M4C47_13300	309323..312796	573	BREX-system-P-loop-protein-BrxC-[Klebsiella-pneumoniae]
HCF8566127.1	Type1.BREX	<-HNH-p||wHTH+SAD+HNH-><-HIN-HTH<-SIGMA+OB-Fold+AAA-ATPase+Lon-Protease-BrxL<-PglZ<-TOPRIM<-OLD-ABC<-N6-METHYLASE<-DUF6079*<-BrxB-BrxF-iSTAND<-BrxA-DNA-binding||ZnR-><-tRNA||?->?->?->?->?->Calcineurin->	1204	Bacteria>Pseudomonadota>Gammaproteobacteria>Enterobacterales>Enterobacteriaceae>Klebsiella/Raoultella_group	GCA_023980665.1	Klebsiella_pneumoniae	NIV99_001798	32314..35928	573	BREX-system-P-loop-protein-BrxC-[Klebsiella-pneumoniae]
MCP6627038.1	Type1.BREX	nSTAND1+HNH->RNaseH->NACHT+SF1-SF2-Helicase+HKD-DNase->REase-><-HKD-DNase+OGG-like<-SIGMA+OB-Fold+AAA-ATPase+Lon-Protease-BrxL<-PglZ<-N6-METHYLASE<-DUF6079*<-BrxB-BrxF-iSTAND<-BrxA-DNA-binding<-?<-ColE7+HNH<-EVE+HNH||NUDIX->?-><-DUF2754	1213	Bacteria>Pseudomonadota>Gammaproteobacteria>Enterobacterales>Enterobacteriaceae>Klebsiella/Raoultella_group	GCA_024240675.1	Klebsiella_pneumoniae	NL507_17490	188287..191928	573	BREX-system-P-loop-protein-BrxC-[Klebsiella-pneumoniae]
HCI4589554.1	Type1.BREX	XIS-HTH->PIWI->?-><-Phage_integrase<-ZnR||BrxA-DNA-binding->BrxB-BrxF-iSTAND->DUF6079*->N6-METHYLASE->OLD-ABC->TOPRIM->PglZ->SIGMA+OB-Fold+AAA-ATPase+Lon-Protease-BrxL-><-SF1-SF2-Helicase<-?||HIN-HTH->URI-DNase->	1204	Bacteria>Pseudomonadota>Gammaproteobacteria>Enterobacterales>Enterobacteriaceae>Klebsiella/Raoultella_group	GCA_024379455.1	Klebsiella_quasipneumoniae_subsp._quasipneumoniae	NO899_004582	16188..19802	1667327	BREX-system-P-loop-protein-BrxC-[Klebsiella-quasipneumoniae-subsp.-quasipneumoniae]
HCI5816641.1	Type1.BREX	XIS-HTH->?->REase+SPF-cons-><-SF1-SF2-Helicase<-HKD-DNase+HKD-DNase<-?||BrxA-DNA-binding->BrxB-BrxF-iSTAND->DUF6079*->N6-METHYLASE->PglZ->SIGMA+OB-Fold+AAA-ATPase+Lon-Protease-BrxL-><-RVT<-TPR<-?<-DnaJ<-Arm-DNA-bind_3+Phage_integrase	1213	Bacteria>Pseudomonadota>Gammaproteobacteria>Enterobacterales>Enterobacteriaceae>Klebsiella/Raoultella_group	GCA_024431185.1	Klebsiella_pneumoniae	NPN32_003719	27788..31429	573	BREX-system-P-loop-protein-BrxC-[Klebsiella-pneumoniae]
HCI5938461.1	Type1.BREX	<-ZnR||BrxA-DNA-binding->BrxB-BrxF-iSTAND->DUF6079*->N6-METHYLASE->OLD-ABC->TOPRIM->PglZ->SIGMA+OB-Fold+AAA-ATPase+Lon-Protease-BrxL->	1197	Bacteria>Pseudomonadota>Gammaproteobacteria>Enterobacterales>Enterobacteriaceae>Klebsiella/Raoultella_group	GCA_024431715.1	Klebsiella_quasipneumoniae_subsp._quasipneumoniae	NPN45_004672	2243..5836	1667327	BREX-system-P-loop-protein-BrxC-[Klebsiella-quasipneumoniae-subsp.-quasipneumoniae]
MCS5967892.1	Type1.BREX	<-nSTAND1+sGTP<-?<-?||?->BrxA-DNA-binding->BrxB-BrxF-iSTAND->DUF6079-p*->DUF6079-p->?->DUF6079-p->ParB+HNH->ParB+HNH->N6-METHYLASE->HEPN->?->RVT->	389	Bacteria>Pseudomonadota>Gammaproteobacteria>Enterobacterales>Enterobacteriaceae>Klebsiella/Raoultella_group	GCA_024990005.1	Klebsiella_variicola_subsp._variicola	LNP26_07865	1516930..1518099	2590157	BREX-system-P-loop-protein-BrxC-[Klebsiella-variicola-subsp.-variicola]
GKN17816.1	Type1.BREX	Arm-DNA-bind_3+Phage_integrase-><-HKD-DNase+OGG-like<-SIGMA+OB-Fold+AAA-ATPase+Lon-Protease-BrxL<-PglZ<-N6-METHYLASE<-DUF6079-p*<-DUF6079-p<-BrxB-BrxF-iSTAND<-BrxA-DNA-binding<-?<-XIS-HTH<-?||RHH_antitoxin-><-URI-DNase<-Glyoxalase_8	725	Bacteria>Pseudomonadota>Gammaproteobacteria>Enterobacterales>Enterobacteriaceae>Klebsiella/Raoultella_group	GCA_026006635.1	Klebsiella_pneumoniae	NUBL17184_45010	12144..14321	573	hypothetical-protein-NUBL17184_45010-[Klebsiella-pneumoniae]
GKN17819.1	Type1.BREX	Arm-DNA-bind_3+Phage_integrase-><-HKD-DNase+OGG-like<-SIGMA+OB-Fold+AAA-ATPase+Lon-Protease-BrxL<-PglZ<-N6-METHYLASE<-DUF6079-p*<-DUF6079-p*<-BrxB-BrxF-iSTAND<-BrxA-DNA-binding<-?<-XIS-HTH<-?||RHH_antitoxin-><-URI-DNase<-Glyoxalase_8<-RNase	453	Bacteria>Pseudomonadota>Gammaproteobacteria>Enterobacterales>Enterobacteriaceae>Klebsiella/Raoultella_group	GCA_026006635.1	Klebsiella_pneumoniae	NUBL17184_45020	14424..15785	573	hypothetical-protein-NUBL17184_45020-[Klebsiella-pneumoniae]
GKP62583.1	Type1.BREX	<-HIN-HTH<-HIN-HTH||HIN-HTH->?->LZ_Tnp_IS66+zf-IS66+DDE_Tnp_IS66+DDE_Tnp_IS66_C->BrxA-DNA-binding->BrxB-BrxF-iSTAND->DUF6079*->N6-METHYLASE->OLD-ABC->TOPRIM->PglZ->SIGMA+OB-Fold+AAA-ATPase+Lon-Protease-BrxL-><-?<-?<-Arm-DNA-bind_3+Phage_integrase	1204	Bacteria>Pseudomonadota>Gammaproteobacteria>Enterobacterales>Enterobacteriaceae>Klebsiella/Raoultella_group	GCA_026008315.1	Klebsiella_quasipneumoniae	NUKP47_17630	173713..177327	1463165	hypothetical-protein-NUKP47_17630-[Klebsiella-quasipneumoniae]
WEJ89011.1	Type1.BREX	<-wHTH(SBD)+HNH||tRNA->Arm-DNA-bind_3+Phage_integrase-><-SIGMA+OB-Fold+AAA-ATPase+Lon-Protease-BrxL<-PglZ<-N6-METHYLASE-p<-RVT<-HEPN<-N6-METHYLASE<-ParB+HNH<-DUF6079*<-BrxB-BrxF-iSTAND<-BrxA-DNA-binding<-ZnR<-XIS-HTH<-?||HIN-HTH->?->LZ_Tnp_IS66+zf-IS66+DDE_Tnp_IS66+DDE_Tnp_IS66_C->	1240	Bacteria>Pseudomonadota>Gammaproteobacteria>Enterobacterales>Enterobacteriaceae>Klebsiella/Raoultella_group	GCA_029202365.1	Klebsiella_huaxiensis	POY63_27795	5833350..5837072	2153354	MAG:-BREX-system-P-loop-protein-BrxC-[Klebsiella-huaxiensis]
SAY23702.1	Type1.BREX	RVT->HTH_Tnp_1+HTH_21+rve+rve_3-><-SIGMA+OB-Fold+AAA-ATPase+Lon-Protease-BrxL<-PglZ||?->?-><-DUF6079*<-BrxB-BrxF-iSTAND<-BrxA-DNA-binding	1052	Bacteria>Pseudomonadota>Gammaproteobacteria>Enterobacterales>Enterobacteriaceae>Klebsiella/Raoultella_group	GCA_900086305.1	Klebsiella_quasipneumoniae	SAMEA2273696_05187	11957..15115	1463165	Uncharacterised-protein-[Klebsiella-quasipneumoniae]
STR42120.1	Type1.BREX	<-SF1-SF2-Helicase<-HKD-DNase+HKD-DNase<-?<-?<-?<-?<-?||BrxA-DNA-binding->BrxB-BrxF-iSTAND->DUF6079-p*->DUF6079-p->DUF6079-p->N6-METHYLASE-p->?->ACET->N6-METHYLASE-p->N6-METHYLASE-p->N6-METHYLASE-p->PglZ-p->	800	Bacteria>Pseudomonadota>Gammaproteobacteria>Enterobacterales>Enterobacteriaceae>Klebsiella/Raoultella_group	GCA_900451375.1	Klebsiella_michiganensis	NCTC11694_03329	2640700..2643102	1134687	Uncharacterised-protein-[Klebsiella-michiganensis]
SYJ62179.1	Type1.BREX	<-DUF6079*<-BrxB-BrxF-iSTAND<-BrxA-DNA-binding<-ZnR<-?<-XIS-HTH<-?||RHH_antitoxin-><-URI-DNase<-Glyoxalase_8	1186	Bacteria>Pseudomonadota>Gammaproteobacteria>Enterobacterales>Enterobacteriaceae>Klebsiella/Raoultella_group	GCA_900515475.1	Klebsiella_pneumoniae	SAMEA3538642_04756	88..3648	573	Uncharacterised-protein-[Klebsiella-pneumoniae]
VAO27126.1	Type1.BREX	DUF2754-><-?<-?||ACET->ColE7+HNH->?->BrxA-DNA-binding->BrxB-BrxF-iSTAND->DUF6079*->N6-METHYLASE->PglZ->SIGMA+OB-Fold+AAA-ATPase+Lon-Protease-BrxL-><-DDE_Tnp_1_5+DDE_Tnp_1<-?<-DnaJ<-Arm-DNA-bind_3+Phage_integrase	1213	Bacteria>Pseudomonadota>Gammaproteobacteria>Enterobacterales>Enterobacteriaceae>Klebsiella/Raoultella_group	GCA_900559435.1	Klebsiella_pneumoniae	SAMEA2053954_04091	16530..20171	573	Uncharacterised-protein-[Klebsiella-pneumoniae]
VTO17843.1	Type1.BREX	RNaseH->NACHT+SF1-SF2-Helicase+HKD-DNase->REase-><-HKD-DNase+OGG-like<-HKD-DNase+OGG-like<-SIGMA+OB-Fold+AAA-ATPase+Lon-Protease-BrxL<-SIGMA+OB-Fold+AAA-ATPase+Lon-Protease-BrxL<-PglZ<-N6-METHYLASE<-DUF6079-p*<-DUF6079-p<-BrxB-BrxF-iSTAND<-BrxA-DNA-binding<-?<-ColE7+HNH<-ACET||?->?-><-DUF2754	864	Bacteria>Pseudomonadota>Gammaproteobacteria>Enterobacterales>Enterobacteriaceae>Klebsiella/Raoultella_group	GCA_901421915.1	Klebsiella_variicola	NCTC9178_02481	2086491..2089085	244366	Uncharacterised-protein-[Klebsiella-variicola]
WP_014837241.1	Type1.BREX	<-wHTH+HNH||tRNA->Arm-DNA-bind_3+Phage_integrase->DnaJ->?->TPR-><-DEDD_Tnp_IS110+Transposase_20||RVT-><-SIGMA+OB-Fold+AAA-ATPase+Lon-Protease-BrxL<-PglZ<-DUF6079*<-BrxB-BrxF-iSTAND<-BrxA-DNA-binding<-DDE_Tnp_1_5+DDE_Tnp_1||?-><-?<-MODE-HTH+PBPII	1213	Bacteria>Pseudomonadota>Gammaproteobacteria>Enterobacterales>Enterobacteriaceae>Klebsiella/Raoultella_group	GCF_000276705.2	Klebsiella	A225_RS02820	612873..616514	570	MULTISPECIES:-BREX-system-P-loop-protein-BrxC-[Klebsiella]
WP_228134609.1	Type1.BREX	Glyoxalase->ACET->YjhX_toxin->METHYLASE->ZnR->Glyoxalase->BrxA-DNA-binding->BrxB-BrxF-iSTAND->DUF6079*->ParB+HNH->N6-METHYLASE->PglZ->SIGMA+OB-Fold+AAA-ATPase+Lon-Protease-BrxL-><-Arm-DNA-bind_3+Phage_integrase<-tRNA||SAM-METHYLASE->	1240	Bacteria>Pseudomonadota>Gammaproteobacteria>Enterobacterales>Enterobacteriaceae>Klebsiella/Raoultella_group	GCF_000632415.1	Klebsiella_michiganensis	J415_RS00260	45760..49482	1134687	BREX-system-P-loop-protein-BrxC-[Klebsiella-michiganensis]
WP_038432196.1	Type1.BREX	KAP-NTPase->?->?->TatD_DNase->REase-><-HKD-DNase+OGG-like<-SIGMA+OB-Fold+AAA-ATPase+Lon-Protease-BrxL<-PglZ<-N6-METHYLASE<-DUF6079*<-BrxB-BrxF-iSTAND<-BrxA-DNA-binding<-URI-DNase||<-?<-?||<-?||?->||MAD-NTD+HNH-><-Glyco_hydro_43+GH43_C2	1213	Bacteria>Pseudomonadota>Gammaproteobacteria>Enterobacterales>Enterobacteriaceae>Klebsiella/Raoultella_group	GCF_000742755.1	Klebsiella_pneumoniae	PF543_RS25520	27597..31238	573	BREX-system-P-loop-protein-BrxC-[Klebsiella-pneumoniae]
WP_047066027.1	Type1.BREX	ColE7+HNH->?->NACHT+SF1-SF2-Helicase+HKD-DNase->REase-><-SIGMA+OB-Fold+AAA-ATPase+Lon-Protease-BrxL<-PglZ<-N6-METHYLASE<-DUF6079*<-BrxB-BrxF-iSTAND<-BrxA-DNA-binding<-wHTH+SAD+HNH||REase+iREase-><-?<-HNH	1213	Bacteria>Pseudomonadota>Gammaproteobacteria>Enterobacterales>Enterobacteriaceae>Klebsiella/Raoultella_group	GCF_001011795.1	Klebsiella_aerogenes	G4873_RS08800	147034..150675	548	BREX-system-P-loop-protein-BrxC-[Klebsiella-aerogenes]
WP_064406990.1	Type1.BREX	<-HTH_23+HTH_32+HTH_33+DDE_3<-RHH_antitoxin||?->XIS-HTH->?->ZnR->BrxA-DNA-binding->BrxB-BrxF-iSTAND->DUF6079*->N6-METHYLASE->PglZ->SIGMA+OB-Fold+AAA-ATPase+Lon-Protease-BrxL->HKD-DNase+OGG-like-><-REase<-Arm-DNA-bind_3+Phage_integrase	1213	Bacteria>Pseudomonadota>Gammaproteobacteria>Enterobacterales>Enterobacteriaceae>Klebsiella/Raoultella_group	GCF_001598695.1	Klebsiella_oxytoca	KO1_RS15010	40025..43666	571	BREX-system-P-loop-protein-BrxC-[Klebsiella-oxytoca]
WP_094487773.1	Type1.BREX	DUF2754-><-?<-NUDIX||ACET->ColE7+HNH->?->BrxA-DNA-binding->BrxB-BrxF-iSTAND->DUF6079*->N6-METHYLASE->PglZ->SIGMA+OB-Fold+AAA-ATPase+Lon-Protease-BrxL->HKD-DNase+OGG-like-><-REase<-NACHT+SF1-SF2-Helicase+HKD-DNase<-nSTAND1+HNH	1213	Bacteria>Pseudomonadota>Gammaproteobacteria>Enterobacterales>Enterobacteriaceae>Klebsiella/Raoultella_group	GCF_002251875.2	Klebsiella_pneumoniae	CI667_RS22335	4584289..4587930	573	BREX-system-P-loop-protein-BrxC-[Klebsiella-pneumoniae]
WP_256991583.1	Type1.BREX	<-DUF905<-DUF932<-CopG/RHH||?->XIS-HTH->ZnR-><-TIR||BrxA-DNA-binding->BrxB-BrxF-iSTAND->DUF6079*->ParB+HNH->N6-METHYLASE->HEPN->RVT->N6-METHYLASE-p->PglZ->SIGMA+OB-Fold+AAA-ATPase+Lon-Protease-BrxL->wHTH+WYL+WCX->	1240	Bacteria>Pseudomonadota>Gammaproteobacteria>Enterobacterales>Enterobacteriaceae>Klebsiella/Raoultella_group	GCF_002264145.1	Raoultella_planticola	CIG23_RS11860	59081..62803	575	BREX-system-P-loop-protein-BrxC-[Raoultella-planticola]
WP_096925291.1	Type1.BREX	URI-DNase->BrxA-DNA-binding->BrxB-BrxF-iSTAND->DUF6079*->N6-METHYLASE->PglZ->SIGMA+OB-Fold+AAA-ATPase+Lon-Protease-BrxL->HKD-DNase+OGG-like-><-REase	1213	Bacteria>Pseudomonadota>Gammaproteobacteria>Enterobacterales>Enterobacteriaceae>Klebsiella/Raoultella_group	GCF_002411925.1	Klebsiella_pneumoniae	CP905_RS22210	5854..9495	573	BREX-system-P-loop-protein-BrxC-[Klebsiella-pneumoniae]
WP_096927069.1	Type1.BREX	ZnR->ZnR-><-SIGMA+OB-Fold+AAA-ATPase+Lon-Protease-BrxL<-PglZ<-N6-METHYLASE<-DUF6079*<-BrxB-BrxF-iSTAND<-BrxA-DNA-binding||REase+iREase-><-Arm-DNA-bind_3+Phage_integrase	1213	Bacteria>Pseudomonadota>Gammaproteobacteria>Enterobacterales>Enterobacteriaceae>Klebsiella/Raoultella_group	GCF_002417545.1	Klebsiella_pneumoniae	CQA60_RS24305	11428..15069	573	BREX-system-P-loop-protein-BrxC-[Klebsiella-pneumoniae]
WP_102012827.1	Type1.BREX	<-DUF6079-p*<-BrxB-BrxF-iSTAND<-BrxA-DNA-binding<-?<-ColE7+HNH<-ACET||NUDIX->	986	Bacteria>Pseudomonadota>Gammaproteobacteria>Enterobacterales>Enterobacteriaceae>Klebsiella/Raoultella_group	GCF_002856315.1	Klebsiella_pneumoniae	CWN62_RS18100	1..2959	573	BREX-system-P-loop-protein-BrxC,-partial-[Klebsiella-pneumoniae]
WP_105178007.1	Type1.BREX	DUF2754-><-?<-NUDIX||EVE+HNH->ColE7+HNH->?->BrxA-DNA-binding->BrxB-BrxF-iSTAND->DUF6079*->N6-METHYLASE->PglZ->SIGMA+OB-Fold+AAA-ATPase+Lon-Protease-BrxL->HKD-DNase+OGG-like-><-REase<-NACHT+SF1-SF2-Helicase+HKD-DNase<-RNaseH<-nSTAND1+HNH	1213	Bacteria>Pseudomonadota>Gammaproteobacteria>Enterobacterales>Enterobacteriaceae>Klebsiella/Raoultella_group	GCF_002934865.1	Klebsiella_pneumoniae	CSC91_RS11390	28681..32322	573	BREX-system-P-loop-protein-BrxC-[Klebsiella-pneumoniae]
WP_104443227.1	Type1.BREX	<-ZnR||BrxA-DNA-binding->BrxB-BrxF-iSTAND->DUF6079*->N6-METHYLASE->OLD-ABC->TOPRIM->PglZ->SIGMA+OB-Fold+AAA-ATPase+Lon-Protease-BrxL-><-?<-ColE7+HNH	1204	Bacteria>Pseudomonadota>Gammaproteobacteria>Enterobacterales>Enterobacteriaceae>Klebsiella/Raoultella_group	GCF_002934985.1	Klebsiella_pneumoniae	CSC89_RS24445	2230..5844	573	BREX-system-P-loop-protein-BrxC-[Klebsiella-pneumoniae]
WP_108704264.1	Type1.BREX	MPTase->?-><-REase||BrxA-DNA-binding->BrxB-BrxF-iSTAND->DUF6079*->N6-METHYLASE->PglZ->SIGMA+OB-Fold+AAA-ATPase+Lon-Protease-BrxL-><-TPR<-?<-DnaJ<-?<-Arm-DNA-bind_3+Phage_integrase<-tRNA||wHTH+HNH->	1213	Bacteria>Pseudomonadota>Gammaproteobacteria>Enterobacterales>Enterobacteriaceae>Klebsiella/Raoultella_group	GCF_003073975.1	Klebsiella_oxytoca	CSC17_RS12015	2422340..2425981	571	BREX-system-P-loop-protein-BrxC-[Klebsiella-oxytoca]
WP_108986419.1	Type1.BREX	<-DUF6079*<-BrxB-BrxF-iSTAND<-BrxA-DNA-binding||REase+iREase-><-Arm-DNA-bind_3+Phage_integrase	1064	Bacteria>Pseudomonadota>Gammaproteobacteria>Enterobacterales>Enterobacteriaceae>Klebsiella/Raoultella_group	GCF_003115315.1	Klebsiella_pneumoniae	DB703_RS28015	1..3192	573	BREX-system-P-loop-protein-BrxC,-partial-[Klebsiella-pneumoniae]
WP_023313883.1	Type1.BREX	Glyco_hydro_43+GH43_C2-><-MAD-NTD+HNH||<-?||?->||?->?->||URI-DNase->BrxA-DNA-binding->BrxB-BrxF-iSTAND->DUF6079*->N6-METHYLASE->PglZ->SIGMA+OB-Fold+AAA-ATPase+Lon-Protease-BrxL->HKD-DNase+OGG-like-><-REase	1213	Bacteria>Pseudomonadota>Gammaproteobacteria>Enterobacterales>Enterobacteriaceae>Klebsiella/Raoultella_group	GCF_003427195.1	Klebsiella_pneumoniae	D2N15_RS14250	17171..20812	573	BREX-system-P-loop-protein-BrxC-[Klebsiella-pneumoniae]
WP_087652989.1	Type1.BREX	DUF2754-><-?<-DUF772+DDE_Tnp_1<-?||ColE7+HNH->?->BrxA-DNA-binding->BrxB-BrxF-iSTAND->DUF6079*->N6-METHYLASE->PglZ->SIGMA+OB-Fold+AAA-ATPase+Lon-Protease-BrxL->HKD-DNase+OGG-like-><-REase<-NACHT+SF1-SF2-Helicase+HKD-DNase<-RNaseH<-nSTAND1+HNH	1213	Bacteria>Pseudomonadota>Gammaproteobacteria>Enterobacterales>Enterobacteriaceae>Klebsiella/Raoultella_group	GCF_003429625.1	Klebsiella_variicola	BC497_RS18300	3693589..3697230	244366	BREX-system-P-loop-protein-BrxC-[Klebsiella-variicola]
WP_264757344.1	Type1.BREX	<-wHTH+WYL<-nSTAND1+sGTP<-?||XIS-HTH->?->?->BrxA-DNA-binding->BrxB-BrxF-iSTAND-><-DDE_Tnp_1_5+DDE_Tnp_1||DUF6079-p*->ParB+HNH->N6-METHYLASE->HEPN->RVT->N6-METHYLASE-p->PglZ->SIGMA+OB-Fold+AAA-ATPase+Lon-Protease-BrxL-><-Arm-DNA-bind_3+Phage_integrase	995	Bacteria>Pseudomonadota>Gammaproteobacteria>Enterobacterales>Enterobacteriaceae>Klebsiella/Raoultella_group	GCF_003660185.1	Klebsiella_pneumoniae	D9K63_RS23205	4667642..4670629	573	BREX-system-P-loop-protein-BrxC-[Klebsiella-pneumoniae]
WP_049088891.1	Type1.BREX	Arm-DNA-bind_3+Phage_integrase->DnaJ->?->TPR-><-SIGMA+OB-Fold+AAA-ATPase+Lon-Protease-BrxL<-PglZ<-N6-METHYLASE<-DUF6079*<-BrxB-BrxF-iSTAND<-BrxA-DNA-binding||?->HKD-DNase+HKD-DNase->SF1-SF2-Helicase-><-REase+SPF-cons<-?<-XIS-HTH	1213	Bacteria>Pseudomonadota>Gammaproteobacteria>Enterobacterales>Enterobacteriaceae>Klebsiella/Raoultella_group	GCF_003851825.1	Klebsiella	KPPSC5_RS01070	215107..218748	570	MULTISPECIES:-BREX-system-P-loop-protein-BrxC-[Klebsiella]
WP_206615409.1	Type1.BREX	<-ABC-ATPase+SbcC<-Calcineurin<-?<-?<-?||tRNA->Arm-DNA-bind_3+Phage_integrase->wHTH+WYL+WCX->BrxA-DNA-binding->BrxB-BrxF-iSTAND->DUF6079-p*->	129	Bacteria>Pseudomonadota>Gammaproteobacteria>Enterobacterales>Enterobacteriaceae>Klebsiella/Raoultella_group	GCF_004024155.1	Raoultella_planticola	DN603_RS10420	221178..221567	575	DUF6079-family-protein-[Raoultella-planticola]
WP_087867252.1	Type1.BREX	TOPRIM-DNAG+D5N+D5ATPase+P4PRIMASE-HTH->nSTAND1+HNH->RNaseH->NACHT+SF1-SF2-Helicase+HKD-DNase->REase-><-HKD-DNase+OGG-like<-SIGMA+OB-Fold+AAA-ATPase+Lon-Protease-BrxL<-PglZ<-N6-METHYLASE<-DUF6079*<-BrxB-BrxF-iSTAND<-BrxA-DNA-binding<-?<-ColE7+HNH<-ACET||?-><-DUF2754	1213	Bacteria>Pseudomonadota>Gammaproteobacteria>Enterobacterales>Enterobacteriaceae>Klebsiella/Raoultella_group	GCF_004311705.1	Klebsiella_pneumoniae	E0M06_RS18340	199992..203633	573	BREX-system-P-loop-protein-BrxC-[Klebsiella-pneumoniae]
WP_130973675.1	Type1.BREX	<-SIGMA+OB-Fold+AAA-ATPase+Lon-Protease-BrxL<-PglZ<-N6-METHYLASE<-ParB+HNH<-DUF6079*<-BrxB-BrxF-iSTAND<-BrxA-DNA-binding<-wHTH+WYL+WCX<-?||RHH->	1225	Bacteria>Pseudomonadota>Gammaproteobacteria>Enterobacterales>Enterobacteriaceae>Klebsiella/Raoultella_group	GCF_004311945.1	Klebsiella_pneumoniae	E0M14_RS25420	10722..14399	573	BREX-system-P-loop-protein-BrxC-[Klebsiella-pneumoniae]
WP_131033948.1	Type1.BREX	nSTAND1+HNH->RNaseH->NACHT+SF1-SF2-Helicase+HKD-DNase->REase-><-HKD-DNase+OGG-like<-SIGMA+OB-Fold+AAA-ATPase+Lon-Protease-BrxL<-PglZ<-N6-METHYLASE<-DUF6079*<-BrxB-BrxF-iSTAND<-BrxA-DNA-binding<-?<-ColE7+HNH||NUDIX->?-><-DUF2754	1213	Bacteria>Pseudomonadota>Gammaproteobacteria>Enterobacterales>Enterobacteriaceae>Klebsiella/Raoultella_group	GCF_004312565.1	Klebsiella_variicola	E0N46_RS24005	106854..110495	244366	BREX-system-P-loop-protein-BrxC-[Klebsiella-variicola]
WP_032410729.1	Type1.BREX	Glyoxalase_8->URI-DNase-><-RHH_antitoxin||?->XIS-HTH->?->ZnR->BrxA-DNA-binding->BrxB-BrxF-iSTAND->DUF6079*->N6-METHYLASE->PglZ->SIGMA+OB-Fold+AAA-ATPase+Lon-Protease-BrxL->HKD-DNase+OGG-like-><-Arm-DNA-bind_3+Phage_integrase	1213	Bacteria>Pseudomonadota>Gammaproteobacteria>Enterobacterales>Enterobacteriaceae>Klebsiella/Raoultella_group	GCF_004312665.1	Klebsiella_pneumoniae	E0N31_RS20140	33140..36781	573	BREX-system-P-loop-protein-BrxC-[Klebsiella-pneumoniae]
WP_223814682.1	Type1.BREX	<-wHTH+WYL<-nSTAND1+sGTP<-?||XIS-HTH->?->BrxA-DNA-binding->BrxB-BrxF-iSTAND->DUF6079*->ParB+HNH->N6-METHYLASE->HEPN->RVT->N6-METHYLASE-p->PglZ->SIGMA+OB-Fold+AAA-ATPase+Lon-Protease-BrxL-><-Arm-DNA-bind_3+Phage_integrase	1240	Bacteria>Pseudomonadota>Gammaproteobacteria>Enterobacterales>Enterobacteriaceae>Klebsiella/Raoultella_group	GCF_004313365.1	Klebsiella	E0O18_RS24630	24494..28216	570	MULTISPECIES:-BREX-system-P-loop-protein-BrxC-[Klebsiella]
WP_048334010.1	Type1.BREX	DUF2754-><-?<-NUDIX||EVE+HNH->ColE7+HNH->?->BrxA-DNA-binding->BrxB-BrxF-iSTAND->DUF6079*->N6-METHYLASE->PglZ->SIGMA+OB-Fold+AAA-ATPase+Lon-Protease-BrxL->HKD-DNase+OGG-like-><-REase	1213	Bacteria>Pseudomonadota>Gammaproteobacteria>Enterobacterales>Enterobacteriaceae>Klebsiella/Raoultella_group	GCF_004313665.1	Klebsiella_pneumoniae	E0O19_RS25945	16153..19794	573	BREX-system-P-loop-protein-BrxC-[Klebsiella-pneumoniae]
WP_131930858.1	Type1.BREX	MPTase->?-><-REase<-HTH_23+HTH_32+HTH_33+DDE_3||BrxA-DNA-binding->BrxB-BrxF-iSTAND->DUF6079*->N6-METHYLASE->PglZ->SIGMA+OB-Fold+AAA-ATPase+Lon-Protease-BrxL-><-?<-DnaJ<-Arm-DNA-bind_3+Phage_integrase<-tRNA||wHTH+HNH->SAM-METHYLASE->	1213	Bacteria>Pseudomonadota>Gammaproteobacteria>Enterobacterales>Enterobacteriaceae>Klebsiella/Raoultella_group	GCF_004343645.1	Klebsiella_grimontii	E0D83_RS25820	59532..63173	2058152	BREX-system-P-loop-protein-BrxC-[Klebsiella-grimontii]
WP_147075822.1	Type1.BREX	<-HNH-p||wHTH+SAD+HNH->wHTH+HNH-><-HIN-HTH<-SIGMA+OB-Fold+AAA-ATPase+Lon-Protease-BrxL<-PglZ<-TOPRIM<-OLD-ABC<-N6-METHYLASE<-DUF6079*<-BrxB-BrxF-iSTAND<-BrxA-DNA-binding||ZnR-><-tRNA||?->?->?->?->?->Calcineurin->	1204	Bacteria>Pseudomonadota>Gammaproteobacteria>Enterobacterales>Enterobacteriaceae>Klebsiella/Raoultella_group	GCF_007955295.1	Klebsiella_pneumoniae	FTO41_25020	32326..35940	573	BREX-system-P-loop-protein-BrxC-[Klebsiella-pneumoniae]
WP_096812545.1	Type1.BREX	Arm-DNA-bind_3+Phage_integrase->BrxA-DNA-binding->BrxB-BrxF-iSTAND->DUF6079*->N6-METHYLASE->ParB+HNH->PglZ-><-SF1-SF2-Helicase+REase<-SF1-SF2-Helicase+REase<-XIS-HTH	1157	Bacteria>Pseudomonadota>Gammaproteobacteria>Enterobacterales>Enterobacteriaceae>Klebsiella/Raoultella_group	GCF_009907625.1	Klebsiella_pneumoniae	GV742_RS00800	3194..6667	573	BREX-system-P-loop-protein-BrxC-[Klebsiella-pneumoniae]
WP_227665788.1	Type1.BREX	XIS-HTH->ZnR-><-TIR<-TIR||BrxA-DNA-binding->BrxB-BrxF-iSTAND->DUF6079*->ParB+HNH->N6-METHYLASE->?->N6-METHYLASE-p->PglZ->SIGMA+OB-Fold+AAA-ATPase+Lon-Protease-BrxL-><-Arm-DNA-bind_3+Phage_integrase	1240	Bacteria>Pseudomonadota>Gammaproteobacteria>Enterobacterales>Enterobacteriaceae>Klebsiella/Raoultella_group	GCF_010602405.1	Klebsiella_variicola	G4552_RS17215	23636..27358	244366	BREX-system-P-loop-protein-BrxC-[Klebsiella-variicola]
WP_181250259.1	Type1.BREX	<-LuxR-HTH<-?||||BrxA-DNA-binding->BrxB-BrxF-iSTAND->DUF6079*->N6-METHYLASE->PglZ->SIGMA+OB-Fold+AAA-ATPase+Lon-Protease-BrxL-><-?<-DnaJ<-?<-Arm-DNA-bind_3+Phage_integrase	1213	Bacteria>Pseudomonadota>Gammaproteobacteria>Enterobacterales>Enterobacteriaceae>Klebsiella/Raoultella_group	GCF_013624375.1	Klebsiella_michiganensis	HV105_RS26495	5508481..5512122	1134687	BREX-system-P-loop-protein-BrxC-[Klebsiella-michiganensis]
WP_032425511.1	Type1.BREX	DUF2754-><-?<-?<-NUDIX||EVE+HNH->ColE7+HNH->?->BrxA-DNA-binding->BrxB-BrxF-iSTAND->DUF6079*->N6-METHYLASE->PglZ->SIGMA+OB-Fold+AAA-ATPase+Lon-Protease-BrxL->HKD-DNase+OGG-like-><-REase<-NACHT+SF1-SF2-Helicase+HKD-DNase<-RNaseH<-nSTAND1+HNH	1213	Bacteria>Pseudomonadota>Gammaproteobacteria>Enterobacterales>Enterobacteriaceae>Klebsiella/Raoultella_group	GCF_013735155.1	Klebsiella_pneumoniae	HV247_RS22800	4632201..4635842	573	BREX-system-P-loop-protein-BrxC-[Klebsiella-pneumoniae]
WP_182012819.1	Type1.BREX	HIN-HTH-><-SIGMA+OB-Fold+AAA-ATPase+Lon-Protease-BrxL<-PglZ<-N6-METHYLASE<-ParB+HNH<-DUF6079*<-BrxB-BrxF-iSTAND<-BrxA-DNA-binding<-wHTH+WYL+WCX<-Phage_int_SAM_4+Phage_integrase<-?<-ArdCN+MPTase<-?||HEPN-><-Phage_int_SAM_2+Integrase_1	1225	Bacteria>Pseudomonadota>Gammaproteobacteria>Enterobacterales>Enterobacteriaceae>Klebsiella/Raoultella_group	GCF_013926025.1	Klebsiella_michiganensis	HV264_RS11965	2488431..2492108	1134687	BREX-system-P-loop-protein-BrxC-[Klebsiella-michiganensis]
WP_182409424.1	Type1.BREX	HerA/FtsK->HNH->?-><-SIGMA+OB-Fold+AAA-ATPase+Lon-Protease-BrxL<-PglZ<-TOPRIM<-OLD-ABC<-N6-METHYLASE<-DUF6079*<-BrxB-BrxF-iSTAND<-BrxA-DNA-binding<-XIS-HTH<-?||CopG/RHH->DUF932->ArdB->	1204	Bacteria>Pseudomonadota>Gammaproteobacteria>Enterobacterales>Enterobacteriaceae>Klebsiella/Raoultella_group	GCF_014129405.1	Klebsiella_michiganensis	H0753_RS11150	98138..101752	1134687	BREX-system-P-loop-protein-BrxC-[Klebsiella-michiganensis]
WP_088607455.1	Type1.BREX	<-XIS-HTH<-RHH_antitoxin||?->?->ZnR->ZnR-><-SIGMA+OB-Fold+AAA-ATPase+Lon-Protease-BrxL<-PglZ<-N6-METHYLASE<-DUF6079*<-BrxB-BrxF-iSTAND<-BrxA-DNA-binding||REase+iREase-><-Arm-DNA-bind_3+Phage_integrase	1213	Bacteria>Pseudomonadota>Gammaproteobacteria>Enterobacterales>Enterobacteriaceae>Klebsiella/Raoultella_group	GCF_014169335.1	Klebsiella_pneumoniae	H7R18_RS22570	4678469..4682110	573	BREX-system-P-loop-protein-BrxC-[Klebsiella-pneumoniae]
WP_187950301.1	Type1.BREX	wHTH+WYL+WCX->BrxA-DNA-binding->BrxB-BrxF-iSTAND->DUF6079*->ParB+HNH->N6-METHYLASE->PglZ->SIGMA+OB-Fold+AAA-ATPase+Lon-Protease-BrxL->	1225	Bacteria>Pseudomonadota>Gammaproteobacteria>Enterobacterales>Enterobacteriaceae>Klebsiella/Raoultella_group	GCF_014500865.1	Klebsiella_pneumoniae	HKB44_RS26575	2539..6216	573	BREX-system-P-loop-protein-BrxC-[Klebsiella-pneumoniae]
WP_196942032.1	Type1.BREX	HNH->SymE_toxin->Glyoxalase->?->||URI-DNase->?->BrxA-DNA-binding->BrxB-BrxF-iSTAND->DUF6079*->N6-METHYLASE->PglZ->SIGMA+OB-Fold+AAA-ATPase+Lon-Protease-BrxL->HKD-DNase+OGG-like-><-REase<-NACHT+SF1-SF2-Helicase+HKD-DNase<-nSTAND1+HNH||wHTH->XIS-HTH->	1213	Bacteria>Pseudomonadota>Gammaproteobacteria>Enterobacterales>Enterobacteriaceae>Klebsiella/Raoultella_group	GCF_015773165.1	Klebsiella_michiganensis	I5M64_RS05730	22758..26399	1134687	BREX-system-P-loop-protein-BrxC-[Klebsiella-michiganensis]
WP_200794272.1	Type1.BREX	DUF2754-><-?<-NUDIX||EVE+HNH->ColE7+HNH->?->BrxA-DNA-binding->BrxB-BrxF-iSTAND->DUF6079*->N6-METHYLASE->PglZ->SIGMA+OB-Fold+AAA-ATPase+Lon-Protease-BrxL->HKD-DNase+OGG-like-><-REase<-NACHT+SF1-SF2-Helicase+HKD-DNase<-nSTAND1+HNH||RVT->	1213	Bacteria>Pseudomonadota>Gammaproteobacteria>Enterobacterales>Enterobacteriaceae>Klebsiella/Raoultella_group	GCF_016635835.1	Klebsiella_pneumoniae	HZ910_RS23410	16106..19747	573	BREX-system-P-loop-protein-BrxC-[Klebsiella-pneumoniae]
WP_202707962.1	Type1.BREX	wHTH+WYL+WCX->BrxA-DNA-binding->BrxB-BrxF-iSTAND->DUF6079*->ParB+HNH->N6-METHYLASE->PglZ->SIGMA+OB-Fold+AAA-ATPase+Lon-Protease-BrxL->	1225	Bacteria>Pseudomonadota>Gammaproteobacteria>Enterobacterales>Enterobacteriaceae>Klebsiella/Raoultella_group	GCF_016761975.1	Klebsiella_pneumoniae	H9R49_RS24845	3004..6681	573	BREX-system-P-loop-protein-BrxC-[Klebsiella-pneumoniae]
WP_117118908.1	Type1.BREX	HTH_Tnp_1+HTH_21+rve+rve_3-><-?||?->MPTase->?-><-REase<-HTH_23+HTH_32+HTH_33+DDE_3||BrxA-DNA-binding->BrxB-BrxF-iSTAND->DUF6079*->N6-METHYLASE->PglZ->SIGMA+OB-Fold+AAA-ATPase+Lon-Protease-BrxL-><-?<-DnaJ<-?<-Arm-DNA-bind_3+Phage_integrase	1213	Bacteria>Pseudomonadota>Gammaproteobacteria>Enterobacterales>Enterobacteriaceae>Klebsiella/Raoultella_group	GCF_017310385.1	Klebsiella_pneumoniae	H4I72_RS22460	4645304..4648945	573	BREX-system-P-loop-protein-BrxC-[Klebsiella-pneumoniae]
WP_211540916.1	Type1.BREX	<-SIGMA+OB-Fold+AAA-ATPase+Lon-Protease-BrxL<-PglZ<-N6-METHYLASE<-DUF6079*<-BrxB-BrxF-iSTAND	1213	Bacteria>Pseudomonadota>Gammaproteobacteria>Enterobacterales>Enterobacteriaceae>Klebsiella/Raoultella_group	GCF_018108615.1	Klebsiella_pneumoniae	FD796_RS26745	8816..12457	573	BREX-system-P-loop-protein-BrxC-[Klebsiella-pneumoniae]
WP_099719181.1	Type1.BREX	nSTAND1+HNH->RNaseH->NACHT+SF1-SF2-Helicase+HKD-DNase->REase-><-HKD-DNase+OGG-like<-SIGMA+OB-Fold+AAA-ATPase+Lon-Protease-BrxL<-PglZ<-N6-METHYLASE<-DUF6079*<-BrxB-BrxF-iSTAND<-BrxA-DNA-binding<-?<-HNH<-ACET||?-><-DUF2754	1213	Bacteria>Pseudomonadota>Gammaproteobacteria>Enterobacterales>Enterobacteriaceae>Klebsiella/Raoultella_group	GCF_018137755.1	Klebsiella_pneumoniae	KFB18_RS20840	34480..38121	573	BREX-system-P-loop-protein-BrxC-[Klebsiella-pneumoniae]
WP_211586105.1	Type1.BREX	DUF2754-><-?||ACET->ColE7+HNH->?->BrxA-DNA-binding->BrxB-BrxF-iSTAND->DUF6079*->N6-METHYLASE->PglZ->SIGMA+OB-Fold+AAA-ATPase+Lon-Protease-BrxL->HKD-DNase+OGG-like-><-REase<-NACHT+SF1-SF2-Helicase+HKD-DNase<-RNaseH<-nSTAND1+HNH	1213	Bacteria>Pseudomonadota>Gammaproteobacteria>Enterobacterales>Enterobacteriaceae>Klebsiella/Raoultella_group	GCF_018137855.1	Klebsiella_pneumoniae	G3W03_RS23040	37519..41160	573	BREX-system-P-loop-protein-BrxC-[Klebsiella-pneumoniae]
WP_211776395.1	Type1.BREX	<-ZnR||BrxA-DNA-binding->BrxB-BrxF-iSTAND->DUF6079*->N6-METHYLASE->OLD-ABC->TOPRIM->PglZ->SIGMA+OB-Fold+AAA-ATPase+Lon-Protease-BrxL-><-?<-ColE7+HNH<-ACET	1204	Bacteria>Pseudomonadota>Gammaproteobacteria>Enterobacterales>Enterobacteriaceae>Klebsiella/Raoultella_group	GCF_018156605.1	Klebsiella_pneumoniae	J5D66_RS24885	2349..5963	573	BREX-system-P-loop-protein-BrxC-[Klebsiella-pneumoniae]
WP_049123912.1	Type1.BREX	Arm-DNA-bind_3+Phage_integrase->BrxA-DNA-binding->BrxB-BrxF-iSTAND->DUF6079*->N6-METHYLASE->ParB+HNH->PglZ-><-?<-XIS-HTH||CopG/RHH->nSTAND1+sGTP->?->DUF932->	1157	Bacteria>Pseudomonadota>Gammaproteobacteria>Enterobacterales>Enterobacteriaceae>Klebsiella/Raoultella_group	GCF_018223925.1	Klebsiella	KHQ26_RS17845	3287..6760	570	MULTISPECIES:-BREX-system-P-loop-protein-BrxC-[Klebsiella]
WP_217046681.1	Type1.BREX	<-XIS-HTH<-RHH_antitoxin||?->ZnR->ZnR-><-SIGMA+OB-Fold+AAA-ATPase+Lon-Protease-BrxL<-PglZ<-N6-METHYLASE<-DUF6079*<-BrxB-BrxF-iSTAND<-BrxA-DNA-binding||Zn_Tnp_IS1+HTH_Tnp_IS1+DDE_Tnp_IS1-><-ACET<-?<-?<-?<-?<-ANK+ANK+ANK	1213	Bacteria>Pseudomonadota>Gammaproteobacteria>Enterobacterales>Enterobacteriaceae>Klebsiella/Raoultella_group	GCF_019038575.1	Klebsiella_pneumoniae	KRP25_RS23525	4832898..4836539	573	BREX-system-P-loop-protein-BrxC-[Klebsiella-pneumoniae]
WP_217074485.1	Type1.BREX	<-REase+iREase||wHTH(SBD)+HNH-><-?<-?<-SIGMA+OB-Fold+AAA-ATPase+Lon-Protease-BrxL<-PglZ<-TOPRIM<-OLD-ABC<-N6-METHYLASE<-DUF6079*<-BrxB-BrxF-iSTAND<-BrxA-DNA-binding||ZnR->||?->?->?->ParB+HNH-><-Arm-DNA-bind_3+Phage_integrase	1204	Bacteria>Pseudomonadota>Gammaproteobacteria>Enterobacterales>Enterobacteriaceae>Klebsiella/Raoultella_group	GCF_019048885.1	Klebsiella_aerogenes	KSL85_RS22185	4652934..4656548	548	BREX-system-P-loop-protein-BrxC-[Klebsiella-aerogenes]
WP_219665441.1	Type1.BREX	<-Phage_holin<-?||MazE_antitoxin->PIN->DEDD_Tnp_IS110+Transposase_20-><-SIGMA+OB-Fold+AAA-ATPase+Lon-Protease-BrxL<-PglZ<-N6-METHYLASE<-ParB+HNH<-DUF6079*<-BrxB-BrxF-iSTAND<-BrxA-DNA-binding<-wHTH+WYL+WCX	1225	Bacteria>Pseudomonadota>Gammaproteobacteria>Enterobacterales>Enterobacteriaceae>Klebsiella/Raoultella_group	GCF_019401235.1	Klebsiella_pneumoniae	GQQ64_RS25415	25134..28811	573	BREX-system-P-loop-protein-BrxC-[Klebsiella-pneumoniae]
WP_219810043.1	Type1.BREX	<-Zn_Tnp_IS1+HTH_Tnp_IS1<-SIGMA+OB-Fold+AAA-ATPase+Lon-Protease-BrxL<-PglZ<-TOPRIM<-OLD-ABC<-N6-METHYLASE<-DUF6079*<-BrxB-BrxF-iSTAND<-BrxA-DNA-binding||<-DEDD_Tnp_IS110+Transposase_20||Phage_integrase->Arm-DNA-bind_3+Phage_integrase->XIS-HTH->DnaB->?->HTH_37->	1189	Bacteria>Pseudomonadota>Gammaproteobacteria>Enterobacterales>Enterobacteriaceae>Klebsiella/Raoultella_group	GCF_019401565.1	Klebsiella_pneumoniae	GQQ45_RS24745	10483..14052	573	BREX-system-P-loop-protein-BrxC-[Klebsiella-pneumoniae]
WP_219712976.1	Type1.BREX	DUF2754-><-?||ACET->ColE7+HNH->?->BrxA-DNA-binding->BrxB-BrxF-iSTAND->DUF6079*->N6-METHYLASE->	1213	Bacteria>Pseudomonadota>Gammaproteobacteria>Enterobacterales>Enterobacteriaceae>Klebsiella/Raoultella_group	GCF_019426525.1	Klebsiella_pneumoniae	CFM93_RS25160	7914..11555	573	BREX-system-P-loop-protein-BrxC-[Klebsiella-pneumoniae]
WP_221013059.1	Type1.BREX	ColE7+HNH->?->BrxA-DNA-binding->BrxB-BrxF-iSTAND->DUF6079*->N6-METHYLASE->	1213	Bacteria>Pseudomonadota>Gammaproteobacteria>Enterobacterales>Enterobacteriaceae>Klebsiella/Raoultella_group	GCF_019679075.1	Klebsiella_pneumoniae	C6952_RS26355	3277..6918	573	BREX-system-P-loop-protein-BrxC-[Klebsiella-pneumoniae]
WP_223098448.1	Type1.BREX	DUF2754-><-?||EVE+HNH->ColE7+HNH->?->BrxA-DNA-binding->BrxB-BrxF-iSTAND->DUF6079*->N6-METHYLASE->PglZ->SIGMA+OB-Fold+AAA-ATPase+Lon-Protease-BrxL->HKD-DNase+OGG-like-><-REase<-NACHT+SF1-SF2-Helicase+HKD-DNase<-RNaseH<-nSTAND1+HNH	1213	Bacteria>Pseudomonadota>Gammaproteobacteria>Enterobacterales>Enterobacteriaceae>Klebsiella/Raoultella_group	GCF_019900805.2	Klebsiella_pneumoniae	KZ657_RS22900	4647952..4651593	573	BREX-system-P-loop-protein-BrxC-[Klebsiella-pneumoniae]
WP_223065518.1	Type1.BREX	<-AP_endonuc_2||?->Glyco_hydro_43+GH43_C2-><-MAD-NTD+HNH||wHTH+SAD+HNH-><-AbiEii<-AbiEi_4||DDE_Tnp_1_5+DDE_Tnp_1->BrxB-BrxF-iSTAND->DUF6079*->N6-METHYLASE->PglZ->SIGMA+OB-Fold+AAA-ATPase+Lon-Protease-BrxL->?-><-Arm-DNA-bind_3+Phage_integrase	1213	Bacteria>Pseudomonadota>Gammaproteobacteria>Enterobacterales>Enterobacteriaceae>Klebsiella/Raoultella_group	GCF_019900825.1	Klebsiella_quasipneumoniae	KZ658_RS22630	4681396..4685037	1463165	BREX-system-P-loop-protein-BrxC-[Klebsiella-quasipneumoniae]
WP_053064557.1	Type1.BREX	Arm-DNA-bind_3+Phage_integrase-><-HKD-DNase+OGG-like<-SIGMA+OB-Fold+AAA-ATPase+Lon-Protease-BrxL<-PglZ<-N6-METHYLASE<-DUF6079*<-BrxB-BrxF-iSTAND<-BrxA-DNA-binding<-ZnR<-?<-XIS-HTH<-?||RHH_antitoxin-><-URI-DNase<-Glyoxalase_8	1213	Bacteria>Pseudomonadota>Gammaproteobacteria>Enterobacterales>Enterobacteriaceae>Klebsiella/Raoultella_group	GCF_019930185.1	Klebsiella	KFB82_RS19625	81811..85452	570	MULTISPECIES:-BREX-system-P-loop-protein-BrxC-[Klebsiella]
WP_194863478.1	Type1.BREX	MPTase->?-><-REase||DEDD_Tnp_IS110+Transposase_20->BrxA-DNA-binding->BrxB-BrxF-iSTAND->DUF6079*->N6-METHYLASE->	1213	Bacteria>Pseudomonadota>Gammaproteobacteria>Enterobacterales>Enterobacteriaceae>Klebsiella/Raoultella_group	GCF_020119055.1	Klebsiella_michiganensis	FMK49_RS27050	40834..44475	1134687	BREX-system-P-loop-protein-BrxC-[Klebsiella-michiganensis]
WP_101858977.1	Type1.BREX	DUF2754-><-?<-NUDIX||ColE7+HNH->?->BrxA-DNA-binding->BrxB-BrxF-iSTAND->DUF6079*->N6-METHYLASE->PglZ->SIGMA+OB-Fold+AAA-ATPase+Lon-Protease-BrxL->HKD-DNase+OGG-like-><-NACHT+SF1-SF2-Helicase+HKD-DNase<-RNaseH<-nSTAND1+HNH	1213	Bacteria>Pseudomonadota>Gammaproteobacteria>Enterobacterales>Enterobacteriaceae>Klebsiella/Raoultella_group	GCF_020479405.1	Klebsiella_variicola	B6I40_RS13715	18030..21671	244366	BREX-system-P-loop-protein-BrxC-[Klebsiella-variicola]
WP_116956119.1	Type1.BREX	<-XIS-HTH<-RHH_antitoxin||?->ZnR->ZnR-><-SIGMA+OB-Fold+AAA-ATPase+Lon-Protease-BrxL<-PglZ<-N6-METHYLASE<-DUF6079*<-BrxB-BrxF-iSTAND<-BrxA-DNA-binding||REase+iREase->?->?->TetR-HTH-><-?<-AAA-ATPase<-HIN-HTH	1213	Bacteria>Pseudomonadota>Gammaproteobacteria>Enterobacterales>Enterobacteriaceae>Klebsiella/Raoultella_group	GCF_020576615.1	Klebsiella_pneumoniae	KAF60_RS20580	68875..72516	573	BREX-system-P-loop-protein-BrxC-[Klebsiella-pneumoniae]
WP_211639658.1	Type1.BREX	<-wHTH+WYL<-nSTAND1+GTPase-AIG+sGTP||HIN-HTH->?->XIS-HTH-><-AAA+wHTH||BrxA-DNA-binding->BrxB-BrxF-iSTAND->DUF6079*->N6-METHYLASE->PglZ->SIGMA+OB-Fold+AAA-ATPase+Lon-Protease-BrxL->	1213	Bacteria>Pseudomonadota>Gammaproteobacteria>Enterobacterales>Enterobacteriaceae>Klebsiella/Raoultella_group	GCF_020576935.1	Klebsiella_pneumoniae	KAF51_RS13395	161239..164880	573	BREX-system-P-loop-protein-BrxC-[Klebsiella-pneumoniae]
WP_100751493.1	Type1.BREX	nSTAND1+HNH->RNaseH->NACHT+SF1-SF2-Helicase+HKD-DNase->REase-><-HKD-DNase+OGG-like<-SIGMA+OB-Fold+AAA-ATPase+Lon-Protease-BrxL<-PglZ<-N6-METHYLASE<-DUF6079*<-BrxB-BrxF-iSTAND<-BrxA-DNA-binding<-?<-ColE7+HNH||?-><-DUF2754	1213	Bacteria>Pseudomonadota>Gammaproteobacteria>Enterobacterales>Enterobacteriaceae>Klebsiella/Raoultella_group	GCF_021261545.1	Klebsiella	CWM56_RS26125	34746..38387	570	MULTISPECIES:-BREX-system-P-loop-protein-BrxC-[Klebsiella]
WP_023329155.1	Type1.BREX	<-SAM-METHYLASE<-wHTH(SBD)+HNH||tRNA->Arm-DNA-bind_3+Phage_integrase->wHTH+WYL+WCX->BrxA-DNA-binding->BrxB-BrxF-iSTAND->DUF6079*->N6-METHYLASE->ParB+HNH->PglZ-><-SF1-SF2-Helicase+REase<-SF1-SF2-Helicase+REase||NACHT+SF1-SF2-Helicase+HKD-DNase->	1157	Bacteria>Pseudomonadota>Gammaproteobacteria>Enterobacterales>Enterobacteriaceae>Klebsiella/Raoultella_group	GCF_021264605.1	Klebsiella_oxytoca	LVW29_RS13105	76515..79988	571	BREX-system-P-loop-protein-BrxC-[Klebsiella-oxytoca]
WP_234580820.1	Type1.BREX	<-DUF932<-CopG/RHH||?->XIS-HTH-><-TIR<-TIR||BrxA-DNA-binding->BrxB-BrxF-iSTAND->DUF6079-p*->	744	Bacteria>Pseudomonadota>Gammaproteobacteria>Enterobacterales>Enterobacteriaceae>Klebsiella/Raoultella_group	GCF_021440775.1	Raoultella_ornithinolytica	LZ676_RS28690	5491..7723	54291	BREX-system-P-loop-protein-BrxC,-partial-[Raoultella-ornithinolytica]
WP_234545002.1	Type1.BREX	<-DUF6079-p*<-BrxB-BrxF-iSTAND<-BrxA-DNA-binding||TIR->TIR-><-XIS-HTH<-?||CopG/RHH->DUF932->DUF905->	741	Bacteria>Pseudomonadota>Gammaproteobacteria>Enterobacterales>Enterobacteriaceae>Klebsiella/Raoultella_group	GCF_021441325.1	Raoultella_ornithinolytica	LZ678_RS28780	1..2223	54291	BREX-system-P-loop-protein-BrxC,-partial-[Raoultella-ornithinolytica]
WP_235410418.1	Type1.BREX	<-wHTH+WYL<-sGTP<-?||XIS-HTH->?->BrxA-DNA-binding->BrxB-BrxF-iSTAND->DUF6079*->ParB+HNH->N6-METHYLASE->HEPN->RVT->N6-METHYLASE-p->PglZ->SIGMA+OB-Fold+AAA-ATPase+Lon-Protease-BrxL-><-Arm-DNA-bind_3+Phage_integrase	1240	Bacteria>Pseudomonadota>Gammaproteobacteria>Enterobacterales>Enterobacteriaceae>Klebsiella/Raoultella_group	GCF_021532695.1	Klebsiella_pneumoniae	LZ602_RS12600	31915..35637	573	BREX-system-P-loop-protein-BrxC-[Klebsiella-pneumoniae]
WP_233435617.1	Type1.BREX	PIWI-><-Phage_integrase||tRNA-><-SIGMA+OB-Fold+AAA-ATPase+Lon-Protease-BrxL<-PglZ<-N6-METHYLASE-p<-ParB+HNH<-DUF6079*<-BrxB-BrxF-iSTAND<-BrxA-DNA-binding||?-><-Glyco_hydro_43+GH43_C2<-?||AP_endonuc_2->DDE_Tnp_1_5+DDE_Tnp_1->	1240	Bacteria>Pseudomonadota>Gammaproteobacteria>Enterobacterales>Enterobacteriaceae>Klebsiella/Raoultella_group	GCF_021699015.1	Raoultella_ornithinolytica	INS59_RS15575	205995..209717	54291	BREX-system-P-loop-protein-BrxC-[Raoultella-ornithinolytica]
WP_238640446.1	Type1.BREX	Arm-DNA-bind_3+Phage_integrase-><-SIGMA+OB-Fold+AAA-ATPase+Lon-Protease-BrxL<-PglZ<-N6-METHYLASE<-ParB+HNH<-DUF6079*<-BrxB-BrxF-iSTAND<-BrxA-DNA-binding<-?<-XIS-HTH||?->nSTAND1+sGTP->wHTH+WYL->	1240	Bacteria>Pseudomonadota>Gammaproteobacteria>Enterobacterales>Enterobacteriaceae>Klebsiella/Raoultella_group	GCF_022229165.1	Klebsiella_pneumoniae	LLG23_RS24005	30146..33868	573	BREX-system-P-loop-protein-BrxC-[Klebsiella-pneumoniae]
WP_223339928.1	Type1.BREX	<-wHTH+WYL<-sGTP<-?||XIS-HTH->?->BrxA-DNA-binding->BrxB-BrxF-iSTAND->DUF6079*->ParB+HNH->N6-METHYLASE->HEPN->RVT->N6-METHYLASE-p->PglZ->SIGMA+OB-Fold+AAA-ATPase+Lon-Protease-BrxL-><-Arm-DNA-bind_3+Phage_integrase	1240	Bacteria>Pseudomonadota>Gammaproteobacteria>Enterobacterales>Enterobacteriaceae>Klebsiella/Raoultella_group	GCF_022353805.1	Klebsiella_pneumoniae	JX616_RS18220	31893..35615	573	BREX-system-P-loop-protein-BrxC-[Klebsiella-pneumoniae]
WP_241187802.1	Type1.BREX	<-XIS-HTH<-wHTH||nSTAND1+HNH->NACHT+SF1-SF2-Helicase+HKD-DNase->REase-><-HKD-DNase+OGG-like<-SIGMA+OB-Fold+AAA-ATPase+Lon-Protease-BrxL<-PglZ<-N6-METHYLASE<-DUF6079*<-BrxB-BrxF-iSTAND<-BrxA-DNA-binding<-URI-DNase||<-?<-Glyoxalase<-SymE_toxin<-HNH||?->LACI-HTH+PBPI->	1213	Bacteria>Pseudomonadota>Gammaproteobacteria>Enterobacterales>Enterobacteriaceae>Klebsiella/Raoultella_group	GCF_022543825.1	Klebsiella_michiganensis	MOA98_RS25025	250948..254589	1134687	BREX-system-P-loop-protein-BrxC-[Klebsiella-michiganensis]
WP_241506431.1	Type1.BREX	nSTAND1+HNH->RNaseH->NACHT+SF1-SF2-Helicase+HKD-DNase->REase-><-HKD-DNase+OGG-like<-SIGMA+OB-Fold+AAA-ATPase+Lon-Protease-BrxL<-PglZ<-N6-METHYLASE<-DUF6079*<-BrxB-BrxF-iSTAND<-BrxA-DNA-binding<-URI-DNase||<-?<-?||<-?||?->||MAD-NTD+HNH-><-Glyco_hydro_43+GH43_C2	1213	Bacteria>Pseudomonadota>Gammaproteobacteria>Enterobacterales>Enterobacteriaceae>Klebsiella/Raoultella_group	GCF_022585755.1	Klebsiella_pneumoniae	MMP84_RS15875	154032..157673	573	BREX-system-P-loop-protein-BrxC-[Klebsiella-pneumoniae]
WP_157261982.1	Type1.BREX	Arm-DNA-bind_3+Phage_integrase->?->DnaJ->?-><-SIGMA+OB-Fold+AAA-ATPase+Lon-Protease-BrxL<-PglZ<-N6-METHYLASE<-DUF6079*<-BrxB-BrxF-iSTAND<-BrxA-DNA-binding||?->HKD-DNase+HKD-DNase->SF1-SF2-Helicase-><-REase+SPF-cons<-?<-XIS-HTH	1213	Bacteria>Pseudomonadota>Gammaproteobacteria>Enterobacterales>Enterobacteriaceae>Klebsiella/Raoultella_group	GCF_022759445.1	Klebsiella	GSE35_RS20710	46310..49951	570	MULTISPECIES:-BREX-system-P-loop-protein-BrxC-[Klebsiella]
WP_202181851.1	Type1.BREX	<-XIS-HTH||nSTAND1+HNH->RNaseH->NACHT+SF1-SF2-Helicase+HKD-DNase->REase-><-HKD-DNase+OGG-like<-SIGMA+OB-Fold+AAA-ATPase+Lon-Protease-BrxL<-PglZ<-N6-METHYLASE<-DUF6079*<-BrxB-BrxF-iSTAND<-BrxA-DNA-binding<-?<-ColE7+HNH<-ACET||?-><-DUF2754	1213	Bacteria>Pseudomonadota>Gammaproteobacteria>Enterobacterales>Enterobacteriaceae>Klebsiella/Raoultella_group	GCF_023703955.1	Klebsiella_pneumoniae	KU670_RS11590	2422249..2425890	573	BREX-system-P-loop-protein-BrxC-[Klebsiella-pneumoniae]
WP_254541266.1	Type1.BREX	<-Calcineurin<-?<-?<-?<-?<-?||tRNA->Arm-DNA-bind_3+Phage_integrase->BrxA-DNA-binding->BrxB-BrxF-iSTAND->DUF6079*->N6-METHYLASE->ParB+HNH->PglZ-><-SF1-SF2-Helicase+REase<-SF1-SF2-Helicase+REase||HIN-HTH-><-XIS-HTH	1157	Bacteria>Pseudomonadota>Gammaproteobacteria>Enterobacterales>Enterobacteriaceae>Klebsiella/Raoultella_group	GCF_024224875.1	Klebsiella_pneumoniae	NLZ12_RS02805	582600..586073	573	BREX-system-P-loop-protein-BrxC-[Klebsiella-pneumoniae]
WP_255075067.1	Type1.BREX	nSTAND1+HNH->RNaseH->NACHT+SF1-SF2-Helicase+HKD-DNase->REase-><-HKD-DNase+OGG-like<-SIGMA+OB-Fold+AAA-ATPase+Lon-Protease-BrxL<-PglZ<-N6-METHYLASE<-DUF6079*<-BrxB-BrxF-iSTAND<-BrxA-DNA-binding<-?<-ColE7+HNH	1213	Bacteria>Pseudomonadota>Gammaproteobacteria>Enterobacterales>Enterobacteriaceae>Klebsiella/Raoultella_group	GCF_024357365.1	Klebsiella_pneumoniae	L1C80_RS20005	166541..170182	573	BREX-system-P-loop-protein-BrxC-[Klebsiella-pneumoniae]
WP_250099520.1	Type1.BREX	Zn_Tnp_IS1+HTH_Tnp_IS1+DDE_Tnp_IS1->BrxA-DNA-binding->BrxB-BrxF-iSTAND->DUF6079*->N6-METHYLASE->PglZ->SIGMA+OB-Fold+AAA-ATPase+Lon-Protease-BrxL-><-ZnR	1213	Bacteria>Pseudomonadota>Gammaproteobacteria>Enterobacterales>Enterobacteriaceae>Klebsiella/Raoultella_group	GCF_024516295.1	Klebsiella_pneumoniae	JEK46_RS25555	2056..5697	573	BREX-system-P-loop-protein-BrxC-[Klebsiella-pneumoniae]
WP_256768592.1	Type1.BREX	<-MAD-NTD+HNH||wHTH+SAD+HNH->wHTH+HNH-><-HIN-HTH<-SIGMA+OB-Fold+AAA-ATPase+Lon-Protease-BrxL<-PglZ<-TOPRIM<-OLD-ABC<-N6-METHYLASE<-DUF6079*<-BrxB-BrxF-iSTAND<-BrxA-DNA-binding||ZnR-><-tRNA||?->?->?->?->?->Calcineurin->	1204	Bacteria>Pseudomonadota>Gammaproteobacteria>Enterobacterales>Enterobacteriaceae>Klebsiella/Raoultella_group	GCF_024517905.1	Klebsiella_pneumoniae	JEK00_RS18440	19794..23408	573	BREX-system-P-loop-protein-BrxC-[Klebsiella-pneumoniae]
WP_101881469.1	Type1.BREX	Arm-DNA-bind_3+Phage_integrase->?->DnaJ->?-><-SIGMA+OB-Fold+AAA-ATPase+Lon-Protease-BrxL<-PglZ<-N6-METHYLASE<-DUF6079*<-BrxB-BrxF-iSTAND<-BrxA-DNA-binding||?->HKD-DNase+HKD-DNase->SF1-SF2-Helicase-><-REase+SPF-cons<-?<-XIS-HTH<-XIS-HTH	1213	Bacteria>Pseudomonadota>Gammaproteobacteria>Enterobacterales>Enterobacteriaceae>Klebsiella/Raoultella_group	GCF_024813835.1	Klebsiella_quasipneumoniae	B6J71_RS18320	31229..34870	1463165	BREX-system-P-loop-protein-BrxC-[Klebsiella-quasipneumoniae]
WP_196588765.1	Type1.BREX	<-ArdB<-DUF932<-CopG/RHH||?->XIS-HTH->BrxA-DNA-binding->BrxB-BrxF-iSTAND->DUF6079*->N6-METHYLASE->OLD-ABC->TOPRIM->PglZ->SIGMA+OB-Fold+AAA-ATPase+Lon-Protease-BrxL-><-?<-HNH<-HerA/FtsK	1204	Bacteria>Pseudomonadota>Gammaproteobacteria>Enterobacterales>Enterobacteriaceae>Klebsiella/Raoultella_group	GCF_024917295.1	Klebsiella	M5R35_RS24840	5265325..5268939	570	MULTISPECIES:-BREX-system-P-loop-protein-BrxC-[Klebsiella]
WP_259574830.1	Type1.BREX	REase-><-HKD-DNase+OGG-like<-SIGMA+OB-Fold+AAA-ATPase+Lon-Protease-BrxL<-PglZ<-N6-METHYLASE<-DUF6079*<-BrxB-BrxF-iSTAND<-BrxA-DNA-binding<-URI-DNase	1213	Bacteria>Pseudomonadota>Gammaproteobacteria>Enterobacterales>Enterobacteriaceae>Klebsiella/Raoultella_group	GCF_024998145.1	Klebsiella_pneumoniae	KQ276_RS10525	179571..183212	573	BREX-system-P-loop-protein-BrxC-[Klebsiella-pneumoniae]
WP_064144681.1	Type1.BREX	<-N6-METHYLASE<-DUF6079*<-BrxB-BrxF-iSTAND<-BrxA-DNA-binding<-?<-ColE7+HNH	1213	Bacteria>Pseudomonadota>Gammaproteobacteria>Enterobacterales>Enterobacteriaceae>Klebsiella/Raoultella_group	GCF_026007595.1	Klebsiella_variicola	OPB18_RS25725	2398..6039	244366	BREX-system-P-loop-protein-BrxC-[Klebsiella-variicola]
WP_264975991.1	Type1.BREX	HIN-HTH->?->LZ_Tnp_IS66+zf-IS66+DDE_Tnp_IS66+DDE_Tnp_IS66_C->BrxA-DNA-binding->BrxB-BrxF-iSTAND->DUF6079*->N6-METHYLASE->OLD-ABC->TOPRIM->PglZ->SIGMA+OB-Fold+AAA-ATPase+Lon-Protease-BrxL-><-?<-Arm-DNA-bind_3+Phage_integrase<-tRNA<-?<-TetR-HTH	1204	Bacteria>Pseudomonadota>Gammaproteobacteria>Enterobacterales>Enterobacteriaceae>Klebsiella/Raoultella_group	GCF_026008315.1	Klebsiella_quasipneumoniae	OPC12_RS09035	173713..177327	1463165	BREX-system-P-loop-protein-BrxC-[Klebsiella-quasipneumoniae]
WP_265815522.1	Type1.BREX	<-wHTH||nSTAND1+HNH->RNaseH->NACHT+SF1-SF2-Helicase+HKD-DNase->REase-><-HKD-DNase+OGG-like<-SIGMA+OB-Fold+AAA-ATPase+Lon-Protease-BrxL<-PglZ<-N6-METHYLASE<-DUF6079*<-BrxB-BrxF-iSTAND<-BrxA-DNA-binding<-URI-DNase||<-?<-Glyoxalase<-SymE_toxin<-HNH||?->LACI-HTH+PBPI->	1213	Bacteria>Pseudomonadota>Gammaproteobacteria>Enterobacterales>Enterobacteriaceae>Klebsiella/Raoultella_group	GCF_026224285.1	Klebsiella_oxytoca	ORM16_RS17370	107113..110754	571	BREX-system-P-loop-protein-BrxC-[Klebsiella-oxytoca]
WP_224260387.1	Type1.BREX	<-RelE-ParE<-RHH<-DUF4400<-ZnR||BrxA-DNA-binding->BrxB-BrxF-iSTAND->DUF6079*->N6-METHYLASE-><-?<-Glyoxalase<-SymE_toxin<-HNH	1204	Bacteria>Pseudomonadota>Gammaproteobacteria>Enterobacterales>Enterobacteriaceae>Klebsiella/Raoultella_group	GCF_026224455.1	Klebsiella_michiganensis	ORL69_RS11440	318057..321671	1134687	BREX-system-P-loop-protein-BrxC-[Klebsiella-michiganensis]
WP_064161342.1	Type1.BREX	DUF4158+DDE_Tnp_Tn3->DEDD_Tnp_IS110+Transposase_20->||DEDD_Tnp_IS110+Transposase_20-><-REase+ZnR||DUF772+DDE_Tnp_1-><-Zn_Tnp_IS1+HTH_Tnp_IS1+DDE_Tnp_IS1||wHTH+WYL+WCX->BrxA-DNA-binding->BrxB-BrxF-iSTAND->DUF6079*->ParB+HNH->N6-METHYLASE->PglZ->SIGMA+OB-Fold+AAA-ATPase+Lon-Protease-BrxL->||GDResolvase+HIN-HTH->?->?->DDE_Tnp_1_5+DDE_Tnp_1->	1225	Bacteria>Pseudomonadota>Gammaproteobacteria>Enterobacterales>Enterobacteriaceae>Klebsiella/Raoultella_group	GCF_027577885.1	Klebsiella	OEE45_RS29300	43964..47641	570	MULTISPECIES:-BREX-system-P-loop-protein-BrxC-[Klebsiella]
WP_274901739.1	Type1.BREX	nSTAND1+HNH->RNaseH->NACHT+SF1-SF2-Helicase+HKD-DNase->REase-><-HKD-DNase+OGG-like<-SIGMA+OB-Fold+AAA-ATPase+Lon-Protease-BrxL<-PglZ<-N6-METHYLASE<-DUF6079*<-BrxB-BrxF-iSTAND<-BrxA-DNA-binding<-?<-ColE7+HNH||HTH_Tnp_1+HTH_21+rve+rve_3-><-DUF2754	1213	Bacteria>Pseudomonadota>Gammaproteobacteria>Enterobacterales>Enterobacteriaceae>Klebsiella/Raoultella_group	GCF_028894275.1	Klebsiella_pneumoniae	PXW84_RS25015	34732..38373	573	BREX-system-P-loop-protein-BrxC-[Klebsiella-pneumoniae]
WP_275268565.1	Type1.BREX	DUF6079*->N6-METHYLASE->PglZ->SIGMA+OB-Fold+AAA-ATPase+Lon-Protease-BrxL->HKD-DNase+OGG-like-><-NACHT+SF1-SF2-Helicase+HKD-DNase<-RNaseH<-nSTAND1+HNH	1196	Bacteria>Pseudomonadota>Gammaproteobacteria>Enterobacterales>Enterobacteriaceae>Klebsiella/Raoultella_group	GCF_029079245.1	Klebsiella_variicola	PZY30_RS10230	1..3591	244366	BREX-system-P-loop-protein-BrxC,-partial-[Klebsiella-variicola]
WP_064171377.1	Type1.BREX	DUF2754-><-?||ColE7+HNH->?->BrxA-DNA-binding->BrxB-BrxF-iSTAND->DUF6079*->N6-METHYLASE->PglZ->SIGMA+OB-Fold+AAA-ATPase+Lon-Protease-BrxL->HKD-DNase+OGG-like-><-REase<-NACHT+SF1-SF2-Helicase+HKD-DNase<-RNaseH<-nSTAND1+HNH<-ParB+HNH	1213	Bacteria>Pseudomonadota>Gammaproteobacteria>Enterobacterales>Enterobacteriaceae>Klebsiella/Raoultella_group	GCF_900084795.1	Klebsiella_variicola	GJ687_RS21830	17638..21279	244366	BREX-system-P-loop-protein-BrxC-[Klebsiella-variicola]
WP_064182275.1	Type1.BREX	MazE_antitoxin->PIN->DEDD_Tnp_IS110+Transposase_20->DEDD_Tnp_IS110+Transposase_20-><-SIGMA+OB-Fold+AAA-ATPase+Lon-Protease-BrxL<-PglZ<-N6-METHYLASE<-ParB+HNH<-DUF6079*<-BrxB-BrxF-iSTAND<-BrxA-DNA-binding<-wHTH+WYL+WCX||Zn_Tnp_IS1+HTH_Tnp_IS1+DDE_Tnp_IS1->DDE_Tnp-><-HEPN<-RelB||HTH_3->	1225	Bacteria>Pseudomonadota>Gammaproteobacteria>Enterobacterales>Enterobacteriaceae>Klebsiella/Raoultella_group	GCF_900084835.1	Klebsiella_pneumoniae	A8U42_RS24700	50900..54577	573	BREX-system-P-loop-protein-BrxC-[Klebsiella-pneumoniae]
WP_064143068.1	Type1.BREX	<-Calcineurin<-?<-?<-?<-?<-?||tRNA-><-ZnR||BrxA-DNA-binding->BrxB-BrxF-iSTAND->DUF6079*->N6-METHYLASE->OLD-ABC->TOPRIM->PglZ->SIGMA+OB-Fold+AAA-ATPase+Lon-Protease-BrxL->	1204	Bacteria>Pseudomonadota>Gammaproteobacteria>Enterobacterales>Enterobacteriaceae>Klebsiella/Raoultella_group	GCF_900085185.1	Klebsiella_variicola	A8S52_RS23955	19840..23454	244366	BREX-system-P-loop-protein-BrxC-[Klebsiella-variicola]
WP_064158594.1	Type1.BREX	RVT-><-SIGMA+OB-Fold+AAA-ATPase+Lon-Protease-BrxL<-PglZ<-N6-METHYLASE<-DUF6079*<-DUF6079-p<-BrxB-BrxF-iSTAND<-BrxA-DNA-binding	1058	Bacteria>Pseudomonadota>Gammaproteobacteria>Enterobacterales>Enterobacteriaceae>Klebsiella/Raoultella_group	GCF_900086305.1	Klebsiella_quasipneumoniae	A8T30_RS26420	11957..15133	1463165	BREX-system-P-loop-protein-BrxC,-partial-[Klebsiella-quasipneumoniae]
WP_065808708.1	Type1.BREX	DUF2754-><-?||ACET->ColE7+HNH->?->BrxA-DNA-binding->BrxB-BrxF-iSTAND->DUF6079*->N6-METHYLASE->PglZ->SIGMA+OB-Fold+AAA-ATPase+Lon-Protease-BrxL->HKD-DNase+OGG-like-><-REase<-NACHT+SF1-SF2-Helicase+HKD-DNase<-RNaseH<-nSTAND1+HNH	1213	Bacteria>Pseudomonadota>Gammaproteobacteria>Enterobacterales>Enterobacteriaceae>Klebsiella/Raoultella_group	GCF_900093245.1	Klebsiella_pneumoniae	BC433_RS15565	37858..41499	573	BREX-system-P-loop-protein-BrxC-[Klebsiella-pneumoniae]
WP_080916293.1	Type1.BREX	<-HNH+NERD+ZnR<-Peptidase_S49+Peptidase_S49<-?<-DDE_Tnp_1_5+DDE_Tnp_1||?->?->||URI-DNase->BrxA-DNA-binding->BrxB-BrxF-iSTAND->DUF6079*->N6-METHYLASE->PglZ->SIGMA+OB-Fold+AAA-ATPase+Lon-Protease-BrxL->HKD-DNase+OGG-like-><-REase	1213	Bacteria>Pseudomonadota>Gammaproteobacteria>Enterobacterales>Enterobacteriaceae>Klebsiella/Raoultella_group	GCF_900172605.1	Klebsiella_pneumoniae	B7F35_RS00110	14289..17930	573	BREX-system-P-loop-protein-BrxC-[Klebsiella-pneumoniae]
WP_227643422.1	Type1.BREX	Arm-DNA-bind_3+Phage_integrase-><-SIGMA+OB-Fold+AAA-ATPase+Lon-Protease-BrxL<-PglZ<-N6-METHYLASE-p<-RVT<-HEPN<-N6-METHYLASE<-ParB+HNH<-DUF6079*<-BrxB-BrxF-iSTAND<-BrxA-DNA-binding<-?<-XIS-HTH<-?||CopG/RHH->nSTAND1+GTPase-AIG+sGTP->DUF932->	1240	Bacteria>Pseudomonadota>Gammaproteobacteria>Enterobacterales>Enterobacteriaceae>Klebsiella/Raoultella_group	GCF_900173735.1	Klebsiella_pneumoniae	B7E22_RS15515	109572..113294	573	BREX-system-P-loop-protein-BrxC-[Klebsiella-pneumoniae]
WP_109220871.1	Type1.BREX	Glyoxalase_8->URI-DNase-><-RHH_antitoxin||?->XIS-HTH->?->ZnR->BrxA-DNA-binding->BrxB-BrxF-iSTAND->DUF6079*->PglZ->SIGMA+OB-Fold+AAA-ATPase+Lon-Protease-BrxL->HKD-DNase+OGG-like-><-Arm-DNA-bind_3+Phage_integrase<-tRNA||wHTH(SBD)+HNH->SAM-METHYLASE->	1213	Bacteria>Pseudomonadota>Gammaproteobacteria>Enterobacterales>Enterobacteriaceae>Klebsiella/Raoultella_group	GCF_900407115.1	Klebsiella_oxytoca	DK336_RS07845	45020..48661	571	BREX-system-P-loop-protein-BrxC-[Klebsiella-oxytoca]
WP_115658225.1	Type1.BREX	nSTAND1+HNH->RNaseH->NACHT+SF1-SF2-Helicase+HKD-DNase->REase-><-HKD-DNase+OGG-like<-SIGMA+OB-Fold+AAA-ATPase+Lon-Protease-BrxL<-PglZ<-N6-METHYLASE<-DUF6079*<-BrxB-BrxF-iSTAND<-BrxA-DNA-binding<-?<-ColE7+HNH<-ACET||?-><-DUF2754	1213	Bacteria>Pseudomonadota>Gammaproteobacteria>Enterobacterales>Enterobacteriaceae>Klebsiella/Raoultella_group	GCF_900498955.1	Klebsiella_pneumoniae	DY137_RS22260	323150..326791	573	BREX-system-P-loop-protein-BrxC-[Klebsiella-pneumoniae]
WP_117104235.1	Type1.BREX	nSTAND1+HNH->RNaseH->NACHT+SF1-SF2-Helicase+HKD-DNase->REase-><-HKD-DNase+OGG-like<-SIGMA+OB-Fold+AAA-ATPase+Lon-Protease-BrxL<-PglZ<-N6-METHYLASE<-DUF6079*<-BrxB-BrxF-iSTAND<-BrxA-DNA-binding<-?<-ColE7+HNH||?-><-DUF2754||?-><-wHTH+SAD+HNH||MAD-NTD+HNH->	1213	Bacteria>Pseudomonadota>Gammaproteobacteria>Enterobacterales>Enterobacteriaceae>Klebsiella/Raoultella_group	GCF_900502405.1	Klebsiella_pneumoniae	D2Z44_RS21380	236554..240195	573	BREX-system-P-loop-protein-BrxC-[Klebsiella-pneumoniae]
WP_117044181.1	Type1.BREX	DUF2754-><-?<-?||EVE+HNH->ColE7+HNH->?->BrxA-DNA-binding->BrxB-BrxF-iSTAND->DUF6079*->N6-METHYLASE->PglZ->SIGMA+OB-Fold+AAA-ATPase+Lon-Protease-BrxL->HKD-DNase+OGG-like-><-REase<-NACHT+SF1-SF2-Helicase+HKD-DNase<-RNaseH<-nSTAND1+HNH	1213	Bacteria>Pseudomonadota>Gammaproteobacteria>Enterobacterales>Enterobacteriaceae>Klebsiella/Raoultella_group	GCF_900503165.1	Klebsiella_pneumoniae	D2094_RS08665	16725..20366	573	BREX-system-P-loop-protein-BrxC-[Klebsiella-pneumoniae]
WP_117268636.1	Type1.BREX	HNH->?->HIN-HTH->?->LZ_Tnp_IS66+zf-IS66+DDE_Tnp_IS66+DDE_Tnp_IS66_C-><-SIGMA+OB-Fold+AAA-ATPase+Lon-Protease-BrxL<-PglZ<-TOPRIM<-OLD-ABC<-DUF6079*<-BrxB-BrxF-iSTAND<-BrxA-DNA-binding<-XIS-HTH<-?||CopG/RHH->DUF932->ArdB->	1204	Bacteria>Pseudomonadota>Gammaproteobacteria>Enterobacterales>Enterobacteriaceae>Klebsiella/Raoultella_group	GCF_900509065.1	Klebsiella_pneumoniae	D2728_RS15300	268407..272021	573	BREX-system-P-loop-protein-BrxC-[Klebsiella-pneumoniae]
WP_117133599.1	Type1.BREX	nSTAND1+HNH->RNaseH->NACHT+SF1-SF2-Helicase+HKD-DNase->REase-><-HKD-DNase+OGG-like<-SIGMA+OB-Fold+AAA-ATPase+Lon-Protease-BrxL<-PglZ<-N6-METHYLASE<-DUF6079*<-BrxB-BrxF-iSTAND<-BrxA-DNA-binding<-?<-ColE7+HNH||?-><-DUF2754	1213	Bacteria>Pseudomonadota>Gammaproteobacteria>Enterobacterales>Enterobacteriaceae>Klebsiella/Raoultella_group	GCF_900509525.1	Klebsiella_variicola	D2R55_RS21945	231466..235107	244366	BREX-system-P-loop-protein-BrxC-[Klebsiella-variicola]
WP_117082329.1	Type1.BREX	REase-><-HKD-DNase+OGG-like<-SIGMA+OB-Fold+AAA-ATPase+Lon-Protease-BrxL<-PglZ<-N6-METHYLASE<-DUF6079*<-BrxB-BrxF-iSTAND<-BrxA-DNA-binding||?-><-URI-DNase<-HIN-HTH	1213	Bacteria>Pseudomonadota>Gammaproteobacteria>Enterobacterales>Enterobacteriaceae>Klebsiella/Raoultella_group	GCF_900514375.1	Klebsiella_pneumoniae	SAMEA3515241_04816	29985..33626	573	BREX-system-P-loop-protein-BrxC-[Klebsiella-pneumoniae]
WP_117102453.1	Type1.BREX	Arm-DNA-bind_3+Phage_integrase->?->DnaJ->?-><-SIGMA+OB-Fold+AAA-ATPase+Lon-Protease-BrxL<-PglZ<-N6-METHYLASE<-DUF6079*<-BrxB-BrxF-iSTAND<-BrxA-DNA-binding||HTH_23+HTH_32+HTH_33+DDE_3->REase-><-?<-MPTase<-?||||?->HKD-DNase+HKD-DNase->	1213	Bacteria>Pseudomonadota>Gammaproteobacteria>Enterobacterales>Enterobacteriaceae>Klebsiella/Raoultella_group	GCF_900517475.1	Klebsiella_pneumoniae	D2Y27_RS13960	242329..245970	573	BREX-system-P-loop-protein-BrxC-[Klebsiella-pneumoniae]
WP_122533088.1	Type1.BREX	DUF2754-><-?||ColE7+HNH->?->BrxA-DNA-binding->BrxB-BrxF-iSTAND->DUF6079*->N6-METHYLASE->PglZ->SIGMA+OB-Fold+AAA-ATPase+Lon-Protease-BrxL->HKD-DNase+OGG-like-><-REase<-NACHT+SF1-SF2-Helicase+HKD-DNase<-RNaseH<-nSTAND1+HNH	1213	Bacteria>Pseudomonadota>Gammaproteobacteria>Enterobacterales>Enterobacteriaceae>Klebsiella/Raoultella_group	GCF_900576735.1	Klebsiella_variicola	EES49_RS00110	18527..22168	244366	BREX-system-P-loop-protein-BrxC-[Klebsiella-variicola]
WP_085803383.1	Type1.BREX	nSTAND1+HNH->RNaseH->NACHT+SF1-SF2-Helicase+HKD-DNase->REase-><-HKD-DNase+OGG-like<-SIGMA+OB-Fold+AAA-ATPase+Lon-Protease-BrxL<-PglZ<-N6-METHYLASE<-DUF6079*<-BrxB-BrxF-iSTAND<-BrxA-DNA-binding<-?<-ColE7+HNH<-EVE+HNH||?-><-DUF2754	1213	Bacteria>Pseudomonadota>Gammaproteobacteria>Enterobacterales>Enterobacteriaceae>Klebsiella/Raoultella_group	GCF_900774735.1	Klebsiella_pneumoniae	EF470_RS14885	16936..20577	573	BREX-system-P-loop-protein-BrxC-[Klebsiella-pneumoniae]
WP_058836894.1	Type1.BREX	XIS-HTH-><-AAA+wHTH<-Zn_Tnp_IS1+HTH_Tnp_IS1+DDE_Tnp_IS1<-?<-?<-DUF772+DDE_Tnp_1||BrxA-DNA-binding->BrxB-BrxF-iSTAND->DUF6079*->N6-METHYLASE->PglZ->SIGMA+OB-Fold+AAA-ATPase+Lon-Protease-BrxL-><-?<-DnaJ<-?<-Arm-DNA-bind_3+Phage_integrase	1213	Bacteria>Pseudomonadota>Gammaproteobacteria>Enterobacterales>Enterobacteriaceae>Klebsiella/Raoultella_group	GCF_900774805.1	Klebsiella	E7U83_RS18910	22123..25764	570	MULTISPECIES:-BREX-system-P-loop-protein-BrxC-[Klebsiella]
WP_031591770.1	Type1.BREX	DUF2754-><-?||ACET->ColE7+HNH->?->BrxA-DNA-binding->BrxB-BrxF-iSTAND->DUF6079*->N6-METHYLASE->PglZ->SIGMA+OB-Fold+AAA-ATPase+Lon-Protease-BrxL->HKD-DNase+OGG-like-><-REase<-NACHT+SF1-SF2-Helicase+HKD-DNase<-RNaseH<-nSTAND1+HNH	1213	Bacteria>Pseudomonadota>Gammaproteobacteria>Enterobacterales>Enterobacteriaceae>Klebsiella/Raoultella_group	GCF_900774955.1	Klebsiella_pneumoniae	P3L21_RS14210	37429..41070	573	BREX-system-P-loop-protein-BrxC-[Klebsiella-pneumoniae]
WP_135729167.1	Type1.BREX	wHTH+SAD+HNH->wHTH+HNH-><-HIN-HTH<-SIGMA+OB-Fold+AAA-ATPase+Lon-Protease-BrxL<-PglZ<-TOPRIM<-OLD-ABC<-N6-METHYLASE<-DUF6079*<-BrxB-BrxF-iSTAND<-BrxA-DNA-binding||ZnR-><-tRNA||?->?->?->?->?->Calcineurin->	1204	Bacteria>Pseudomonadota>Gammaproteobacteria>Enterobacterales>Enterobacteriaceae>Klebsiella/Raoultella_group	GCF_900775295.1	Klebsiella_pneumoniae	E7W89_RS19490	55176..58790	573	BREX-system-P-loop-protein-BrxC-[Klebsiella-pneumoniae]
WP_114690464.1	Type1.BREX	nSTAND1+HNH->RNaseH->NACHT+SF1-SF2-Helicase+HKD-DNase->REase-><-HKD-DNase+OGG-like<-SIGMA+OB-Fold+AAA-ATPase+Lon-Protease-BrxL<-PglZ<-N6-METHYLASE<-DUF6079*<-BrxB-BrxF-iSTAND<-BrxA-DNA-binding<-?<-ColE7+HNH<-ACET||NUDIX->?-><-DUF2754	1213	Bacteria>Pseudomonadota>Gammaproteobacteria>Enterobacterales>Enterobacteriaceae>Klebsiella/Raoultella_group	GCF_900775385.1	Klebsiella_pneumoniae	MC274_RS12705	209352..212993	573	BREX-system-P-loop-protein-BrxC-[Klebsiella-pneumoniae]

Protein-ID	System	Gene-Neighbourhoods	Length	Taxonomy	Assembly-ID	Strain	Locus-Tag	Genomic-coordinates	TaxID	Fasta-define
WP_023285784.1	Type1.BREX	<-Calcineurin<-?<-?<-?<-?<-?||tRNA-><-ZnR||BrxA-DNA-binding->BrxB-BrxF-iSTAND->DUF6079*->N6-METHYLASE->OLD-ABC->TOPRIM->PglZ->SIGMA+OB-Fold+AAA-ATPase+Lon-Protease-BrxL->HIN-HTH-><-wHTH+HNH<-wHTH+SAD+HNH||<-Glyco_hydro_43+GH43_C2	1204	Bacteria>Pseudomonadota>Gammaproteobacteria>Enterobacterales>Enterobacteriaceae>Klebsiella/Raoultella_group	GCF_900775395.1	Klebsiella_pneumoniae	PXW47_RS20390	324730..328344	573	BREX-system-P-loop-protein-BrxC-[Klebsiella-pneumoniae]
WP_064153723.1	Type1.BREX	nSTAND1+HNH->RNaseH->NACHT+SF1-SF2-Helicase+HKD-DNase->REase-><-HKD-DNase+OGG-like<-SIGMA+OB-Fold+AAA-ATPase+Lon-Protease-BrxL<-PglZ<-N6-METHYLASE<-DUF6079*<-BrxB-BrxF-iSTAND<-BrxA-DNA-binding	1213	Bacteria>Pseudomonadota>Gammaproteobacteria>Enterobacterales>Enterobacteriaceae>Klebsiella/Raoultella_group	GCF_900775555.1	Klebsiella_pneumoniae	E7W23_RS23250	82036..85677	573	BREX-system-P-loop-protein-BrxC-[Klebsiella-pneumoniae]
WP_135732751.1	Type1.BREX	XIS-HTH->?->?->?->TOPRIM-DNAG+D5N+D5ATPase+P4PRIMASE-HTH-><-ZnR||BrxA-DNA-binding->BrxB-BrxF-iSTAND->DUF6079*->N6-METHYLASE->OLD-ABC->TOPRIM->PglZ->SIGMA+OB-Fold+AAA-ATPase+Lon-Protease-BrxL->	1204	Bacteria>Pseudomonadota>Gammaproteobacteria>Enterobacterales>Enterobacteriaceae>Klebsiella/Raoultella_group	GCF_900775595.1	Klebsiella_quasipneumoniae	ICC30_RS25220	11695..15309	1463165	BREX-system-P-loop-protein-BrxC-[Klebsiella-quasipneumoniae]
WP_135723013.1	Type1.BREX	Arm-DNA-bind_3+Phage_integrase-><-HKD-DNase+OGG-like<-HKD-DNase+OGG-like<-SIGMA+OB-Fold+AAA-ATPase+Lon-Protease-BrxL<-PglZ<-TOPRIM<-OLD-ABC<-N6-METHYLASE<-DUF6079*<-BrxB-BrxF-iSTAND<-BrxA-DNA-binding||NACHT+SF1-SF2-Helicase+HKD-DNase->	1204	Bacteria>Pseudomonadota>Gammaproteobacteria>Enterobacterales>Enterobacteriaceae>Klebsiella/Raoultella_group	GCF_900775875.1	Klebsiella_pneumoniae	E7X40_RS22910	78837..82451	573	BREX-system-P-loop-protein-BrxC-[Klebsiella-pneumoniae]
WP_136086191.1	Type1.BREX	nSTAND1+HNH->RNaseH->NACHT+SF1-SF2-Helicase+HKD-DNase->REase-><-HKD-DNase+OGG-like<-SIGMA+OB-Fold+AAA-ATPase+Lon-Protease-BrxL<-PglZ<-N6-METHYLASE<-DUF6079*<-BrxB-BrxF-iSTAND<-BrxA-DNA-binding<-?<-ColE7+HNH||?-><-DUF2754	1213	Bacteria>Pseudomonadota>Gammaproteobacteria>Enterobacterales>Enterobacteriaceae>Klebsiella/Raoultella_group	GCF_900978435.1	Klebsiella_variicola	E9559_RS11315	84067..87708	244366	BREX-system-P-loop-protein-BrxC-[Klebsiella-variicola]
WP_142470399.1	Type1.BREX	<-SAM-METHYLASE<-wHTH+HNH||tRNA->Arm-DNA-bind_3+Phage_integrase-><-HKD-DNase+OGG-like<-SIGMA+OB-Fold+AAA-ATPase+Lon-Protease-BrxL<-PglZ<-N6-METHYLASE<-DUF6079*<-BrxB-BrxF-iSTAND<-BrxA-DNA-binding<-ZnR<-?<-XIS-HTH<-?||?->RHH_antitoxin-><-URI-DNase	1213	Bacteria>Pseudomonadota>Gammaproteobacteria>Enterobacterales>Enterobacteriaceae>Klebsiella/Raoultella_group	GCF_902158685.1	Klebsiella_pasteurii	SB6424_04698	319733..323374	2587529	BREX-system-P-loop-protein-BrxC-[Klebsiella-pasteurii]
WP_224385469.1	Type1.BREX	<-HTH_Tnp_1+HTH_21+rve+rve_3<-PglZ<-N6-METHYLASE-p<-N6-METHYLASE<-ParB+HNH<-DUF6079*<-BrxB-BrxF-iSTAND<-BrxA-DNA-binding<-Glyoxalase<-ZnR<-METHYLASE<-YjhX_toxin<-ACET<-Glyoxalase	1240	Bacteria>Pseudomonadota>Gammaproteobacteria>Enterobacterales>Enterobacteriaceae>Klebsiella/Raoultella_group	GCF_902158715.1	Klebsiella	FN110_RS17010	11054..14776	570	MULTISPECIES:-BREX-system-P-loop-protein-BrxC-[Klebsiella]
WP_256682067.1	Type1.BREX	<-DUF905<-DUF932<-CopG/RHH||?->XIS-HTH-><-TIR<-TIR||BrxA-DNA-binding->BrxB-BrxF-iSTAND->DUF6079*->ParB+HNH->N6-METHYLASE->?->N6-METHYLASE-p->PglZ->SIGMA+OB-Fold+AAA-ATPase+Lon-Protease-BrxL->wHTH+WYL+WCX->?->HNH->	1240	Bacteria>Pseudomonadota>Gammaproteobacteria>Enterobacterales>Enterobacteriaceae>Klebsiella/Raoultella_group	GCF_902158845.1	Klebsiella_michiganensis	FN096_RS13265	22370..26092	1134687	BREX-system-P-loop-protein-BrxC-[Klebsiella-michiganensis]
WP_154062666.1	Type1.BREX	<-HTH_36<-HTH+HTH<-ParB+BAM<-XIS-HTH<-wHTH<-ZnR||BrxA-DNA-binding->BrxB-BrxF-iSTAND->DUF6079*->N6-METHYLASE->OLD-ABC->TOPRIM->PglZ->SIGMA+OB-Fold+AAA-ATPase+Lon-Protease-BrxL-><-?<-Glyoxalase<-SymE_toxin	1204	Bacteria>Pseudomonadota>Gammaproteobacteria>Enterobacterales>Enterobacteriaceae>Klebsiella/Raoultella_group	GCF_902159535.1	Klebsiella_michiganensis	FN635_RS25500	5342283..5345897	1134687	BREX-system-P-loop-protein-BrxC-[Klebsiella-michiganensis]
WP_227635281.1	Type1.BREX	XIS-HTH-><-TIR<-TIR||BrxA-DNA-binding->BrxB-BrxF-iSTAND->DUF6079*->ParB+HNH->N6-METHYLASE->HEPN->RVT->N6-METHYLASE-p->PglZ->SIGMA+OB-Fold+AAA-ATPase+Lon-Protease-BrxL->wHTH+WYL+WCX->	1240	Bacteria>Pseudomonadota>Gammaproteobacteria>Enterobacterales>Enterobacteriaceae>Klebsiella/Raoultella_group	GCF_902159715.1	Klebsiella	PIK69_RS17535	2737..6459	570	MULTISPECIES:-BREX-system-P-loop-protein-BrxC-[Klebsiella]
WP_048266247.1	Type1.BREX	REase-><-HKD-DNase+OGG-like<-SIGMA+OB-Fold+AAA-ATPase+Lon-Protease-BrxL<-PglZ<-N6-METHYLASE<-DUF6079*<-BrxB-BrxF-iSTAND<-BrxA-DNA-binding<-URI-DNase	1213	Bacteria>Pseudomonadota>Gammaproteobacteria>Enterobacterales>Enterobacteriaceae>Klebsiella/Raoultella_group	GCF_902161325.1	Klebsiella_pneumoniae	FN306_RS22675	100097..103738	573	BREX-system-P-loop-protein-BrxC-[Klebsiella-pneumoniae]
WP_085820485.1	Type1.BREX	REase-><-HKD-DNase+OGG-like<-SIGMA+OB-Fold+AAA-ATPase+Lon-Protease-BrxL<-PglZ<-N6-METHYLASE<-DUF6079*<-BrxB-BrxF-iSTAND<-BrxA-DNA-binding<-URI-DNase||<-?<-?||<-?||?->||MAD-NTD+HNH-><-Glyco_hydro_43+GH43_C2	1213	Bacteria>Pseudomonadota>Gammaproteobacteria>Enterobacterales>Enterobacteriaceae>Klebsiella/Raoultella_group	GCF_902508285.1	Klebsiella_pneumoniae	GVM87_RS22970	29420..33061	573	BREX-system-P-loop-protein-BrxC-[Klebsiella-pneumoniae]
WP_177340625.1	Type1.BREX	<-OBFOLD-NUCLEASE<-CARF+NtrC-AAA+FIS-HTH<-SIGMA+OB-Fold+AAA-ATPase+Lon-Protease-BrxL<-PglZ<-N6-METHYLASE<-DUF6079*<-BrxB-BrxF-iSTAND<-BrxA-DNA-binding<-wHTH+WYL+WCX<-?<-35exo<-RVT||LexA-protease->?-><-MobI	1225	Bacteria>Pseudomonadota>Gammaproteobacteria>Enterobacterales>Enterobacteriaceae>Klebsiella/Raoultella_group	GCF_902754085.1	Klebsiella_michiganensis	HV187_RS24210	5087544..5091221	1134687	BREX-system-P-loop-protein-BrxC-[Klebsiella-michiganensis]
WP_224423598.1	Type1.BREX	DUF2754-><-?||ACET->ColE7+HNH->?->BrxA-DNA-binding->BrxB-BrxF-iSTAND->DUF6079*->N6-METHYLASE->PglZ->SIGMA+OB-Fold+AAA-ATPase+Lon-Protease-BrxL->?-><-Arm-DNA-bind_3+Phage_integrase||ParA-Soj-PloopNTPase->?-><-AbiGi+seqA+RAMA||RelB->	1213	Bacteria>Pseudomonadota>Gammaproteobacteria>Enterobacterales>Enterobacteriaceae>Klebsiella/Raoultella_group	GCF_903166255.1	Klebsiella_quasivariicola	QJU95_RS24650	36202..39843	2026240	BREX-system-P-loop-protein-BrxC-[Klebsiella-quasivariicola]
WP_225377085.1	Type1.BREX	<-NACHT+SF1-SF2-Helicase+HKD-DNase||BrxA-DNA-binding->BrxB-BrxF-iSTAND->DUF6079*->N6-METHYLASE->OLD-ABC->TOPRIM->PglZ->SIGMA+OB-Fold+AAA-ATPase+Lon-Protease-BrxL->HKD-DNase+OGG-like->HKD-DNase+OGG-like-><-Arm-DNA-bind_3+Phage_integrase	1204	Bacteria>Pseudomonadota>Gammaproteobacteria>Enterobacterales>Enterobacteriaceae>Klebsiella/Raoultella_group	GCF_916172235.1	Klebsiella_pneumoniae	LF666_RS16195	10713..14327	573	BREX-system-P-loop-protein-BrxC-[Klebsiella-pneumoniae]
WP_225386868.1	Type1.BREX	DUF2754-><-?<-?||ColE7+HNH->?->BrxA-DNA-binding->BrxB-BrxF-iSTAND->DUF6079*->N6-METHYLASE->PglZ->SIGMA+OB-Fold+AAA-ATPase+Lon-Protease-BrxL->HKD-DNase+OGG-like-><-REase<-NACHT+SF1-SF2-Helicase+HKD-DNase<-RNaseH<-nSTAND1+HNH	1213	Bacteria>Pseudomonadota>Gammaproteobacteria>Enterobacterales>Enterobacteriaceae>Klebsiella/Raoultella_group	GCF_916173145.1	Klebsiella_variicola	LF736_RS25380	17904..21545	244366	BREX-system-P-loop-protein-BrxC-[Klebsiella-variicola]
WP_048289717.1	Type1.BREX	<-NUDIX||EVE+HNH->ColE7+HNH->?-><-SIGMA+OB-Fold+AAA-ATPase+Lon-Protease-BrxL<-PglZ<-TOPRIM<-OLD-ABC<-N6-METHYLASE<-DUF6079*<-BrxB-BrxF-iSTAND<-BrxA-DNA-binding||ZnR->	1204	Bacteria>Pseudomonadota>Gammaproteobacteria>Enterobacterales>Enterobacteriaceae>Klebsiella/Raoultella_group	GCF_934247105.1	Klebsiella_pneumoniae	A8T29_RS25610	25582..29196	573	BREX-system-P-loop-protein-BrxC-[Klebsiella-pneumoniae]
WP_101867996.1	Type1.BREX	<-SAM-METHYLASE<-wHTH(SBD)+HNH||tRNA->Arm-DNA-bind_3+Phage_integrase->wHTH+WYL+WCX->BrxA-DNA-binding->BrxB-BrxF-iSTAND->DUF6079*->N6-METHYLASE->ParB+HNH->	1157	Bacteria>Pseudomonadota>Gammaproteobacteria>Enterobacterales>Enterobacteriaceae>Klebsiella/Raoultella_group	-	Klebsiella_michiganensis	CWN04_RS12955	12917..16390	1134687	BREX-system-P-loop-protein-BrxC-[Klebsiella-michiganensis]
WP_154904474.1	Type1.BREX	<-N6-METHYLASE-p<-N6-METHYLASE<-ParB+HNH<-DUF6079*<-BrxB-BrxF-iSTAND<-BrxA-DNA-binding||<-XIS-HTH	1233	Bacteria>Pseudomonadota>Gammaproteobacteria>Enterobacterales>Enterobacteriaceae>Klebsiella/Raoultella_group	-	Klebsiella_michiganensis	CWN50_01600	4530..8231	1134687	BREX-system-P-loop-protein-BrxC-[Klebsiella-michiganensis]
WP_227627429.1	Type1.BREX	<-wHTH+WYL+WCX<-?<-SIGMA+OB-Fold+AAA-ATPase+Lon-Protease-BrxL<-PglZ<-N6-METHYLASE-p<-RVT<-HEPN<-N6-METHYLASE<-ParB+HNH<-DUF6079*<-BrxB-BrxF-iSTAND<-BrxA-DNA-binding||TIR-><-ZnR<-XIS-HTH<-?||?->DUF932->DUF905->	1240	Bacteria>Pseudomonadota>Gammaproteobacteria>Enterobacterales>Enterobacteriaceae>Klebsiella/Raoultella_group	-	Klebsiella_michiganensis	G4403_RS10440	98242..101964	1134687	BREX-system-P-loop-protein-BrxC-[Klebsiella-michiganensis]
WP_097365540.1	Type1.BREX	<-DUF905<-DUF932<-CopG/RHH||?->XIS-HTH-><-TIR<-TIR||BrxA-DNA-binding->BrxB-BrxF-iSTAND->DUF6079*->ParB+HNH->N6-METHYLASE->N6-METHYLASE-p->PglZ->SIGMA+OB-Fold+AAA-ATPase+Lon-Protease-BrxL->wHTH+WYL+WCX->?->HNH->	1233	Bacteria>Pseudomonadota>Gammaproteobacteria>Enterobacterales>Enterobacteriaceae>Klebsiella/Raoultella_group	-	Klebsiella_oxytoca	I9Y61_001542	37432..41133	571	MULTISPECIES:-BREX-system-P-loop-protein-BrxC-[Klebsiella]
ADJ51152.1	Type1.BREX	<-DUF6079-p*<-BrxB-BrxF-iSTAND<-BrxA-DNA-binding<-wHTH+WYL+WCX||Zn_Tnp_IS1+HTH_Tnp_IS1->DDE_Tnp_IS1-><-?||DDE_Tnp_1_5+DDE_Tnp_1->HIN-HTH->HTH_21+rve+rve_3->	484	Bacteria>Pseudomonadota>Gammaproteobacteria>Enterobacterales>Enterobacteriaceae>Klebsiella/Raoultella_group	-	Klebsiella_pneumoniae	.	1..1453	573	ATPase-like-protein,-partial-[Klebsiella-pneumoniae]
WP_117042583.1	Type1.BREX	<-DUF905<-DUF932<-CopG/RHH||?->XIS-HTH-><-TIR<-TIR||BrxA-DNA-binding->BrxB-BrxF-iSTAND->DUF6079*->ParB+HNH->N6-METHYLASE->HEPN->RVT->N6-METHYLASE-p->PglZ->SIGMA+OB-Fold+AAA-ATPase+Lon-Protease-BrxL->wHTH+WYL+WCX->	1233	Bacteria>Pseudomonadota>Gammaproteobacteria>Enterobacterales>Enterobacteriaceae>Klebsiella/Raoultella_group	-	Klebsiella_pneumoniae	AM381_RS08835	19475..23176	573	MULTISPECIES:-BREX-system-P-loop-protein-BrxC-[Klebsiella]
WP_114263049.1	Type1.BREX	<-DUF932<-GspA-ATPase+GTPase<-CopG/RHH||?->XIS-HTH->?->?->BrxA-DNA-binding->BrxB-BrxF-iSTAND->DUF6079*->ParB+HNH->N6-METHYLASE->HEPN->RVT->N6-METHYLASE-p->PglZ->SIGMA+OB-Fold+AAA-ATPase+Lon-Protease-BrxL-><-Arm-DNA-bind_3+Phage_integrase	1233	Bacteria>Pseudomonadota>Gammaproteobacteria>Enterobacterales>Enterobacteriaceae>Klebsiella/Raoultella_group	-	Klebsiella_pneumoniae	AN2353V1_3685	3967252..3970953	573	MULTISPECIES:-BREX-system-P-loop-protein-BrxC-[Enterobacteriaceae]
WP_062729754.1	Type1.BREX	Arm-DNA-bind_3+Phage_integrase-><-SIGMA+OB-Fold+AAA-ATPase+Lon-Protease-BrxL<-PglZ<-N6-METHYLASE-p<-RVT<-HEPN<-N6-METHYLASE<-ParB+HNH<-DUF6079*<-BrxB-BrxF-iSTAND<-BrxA-DNA-binding<-?<-?<-XIS-HTH||?->nSTAND1+sGTP->wHTH+WYL->	1233	Bacteria>Pseudomonadota>Gammaproteobacteria>Enterobacterales>Enterobacteriaceae>Klebsiella/Raoultella_group	-	Klebsiella_pneumoniae	C0077_01580	326913..330614	573	MULTISPECIES:-BREX-system-P-loop-protein-BrxC-[Enterobacteriaceae]
WP_073511310.1	Type1.BREX	<-wHTH+WYL<-sGTP<-?||XIS-HTH->?->BrxA-DNA-binding->BrxB-BrxF-iSTAND->DUF6079*->ParB+HNH->N6-METHYLASE->HEPN->RVT->N6-METHYLASE-p->PglZ->SIGMA+OB-Fold+AAA-ATPase+Lon-Protease-BrxL-><-Arm-DNA-bind_3+Phage_integrase	1233	Bacteria>Pseudomonadota>Gammaproteobacteria>Enterobacterales>Enterobacteriaceae>Klebsiella/Raoultella_group	-	Klebsiella_pneumoniae	L7V03_004584	32186..35887	573	BREX-system-P-loop-protein-BrxC-[Klebsiella-pneumoniae]
WP_117247358.1	Type1.BREX	<-wHTH+WYL<-nSTAND1+sGTP<-?||XIS-HTH->?->?->BrxA-DNA-binding->BrxB-BrxF-iSTAND->DUF6079*->ParB+HNH->N6-METHYLASE->HEPN->RVT->N6-METHYLASE-p->PglZ->SIGMA+OB-Fold+AAA-ATPase+Lon-Protease-BrxL-><-Arm-DNA-bind_3+Phage_integrase	1233	Bacteria>Pseudomonadota>Gammaproteobacteria>Enterobacterales>Enterobacteriaceae>Klebsiella/Raoultella_group	-	Klebsiella_pneumoniae	L7W77_004838	45573..49274	573	MULTISPECIES:-BREX-system-P-loop-protein-BrxC-[Klebsiella]
EIV5715275.1	Type1.BREX	METHYLASE->nSTAND1+HNH->RNaseH->NACHT+SF1-SF2-Helicase+HKD-DNase->REase-><-HKD-DNase+OGG-like<-SIGMA+OB-Fold+AAA-ATPase+Lon-Protease-BrxL<-PglZ<-N6-METHYLASE<-DUF6079*<-BrxB-BrxF-iSTAND<-BrxA-DNA-binding<-?<-ColE7+HNH||NUDIX->?-><-DUF2754	1213	Bacteria>Pseudomonadota>Gammaproteobacteria>Enterobacterales>Enterobacteriaceae>Klebsiella/Raoultella_group	-	Klebsiella_pneumoniae	L9F37_004806	56067..59708	573	BREX-system-P-loop-protein-BrxC-[Klebsiella-pneumoniae]
HCB8952633.1	Type1.BREX	DUF2754-><-?<-NUDIX||ColE7+HNH->?->BrxA-DNA-binding->BrxB-BrxF-iSTAND->DUF6079*->N6-METHYLASE-p->	1213	Bacteria>Pseudomonadota>Gammaproteobacteria>Enterobacterales>Enterobacteriaceae>Klebsiella/Raoultella_group	-	Klebsiella_pneumoniae	M2O84_005147	37298..40939	573	BREX-system-P-loop-protein-BrxC-[Klebsiella-pneumoniae]
HCC8216583.1	Type1.BREX	GDResolvase+HIN-HTH->ZnR-><-SIGMA+OB-Fold+AAA-ATPase+Lon-Protease-BrxL<-PglZ<-N6-METHYLASE<-DUF6079*<-BrxB-BrxF-iSTAND<-BrxA-DNA-binding||REase+iREase-><-DDE_Tnp_IS240	1213	Bacteria>Pseudomonadota>Gammaproteobacteria>Enterobacterales>Enterobacteriaceae>Klebsiella/Raoultella_group	-	Klebsiella_pneumoniae	M2P48_005497	10102..13743	573	BREX-system-P-loop-protein-BrxC-[Klebsiella-pneumoniae]
WP_117076897.1	Type1.BREX	<-wHTH+WYL<-nSTAND1+sGTP<-?||XIS-HTH->?->BrxA-DNA-binding->BrxB-BrxF-iSTAND->DUF6079*->ParB+HNH->N6-METHYLASE->PglZ->SIGMA+OB-Fold+AAA-ATPase+Lon-Protease-BrxL-><-Arm-DNA-bind_3+Phage_integrase	1233	Bacteria>Pseudomonadota>Gammaproteobacteria>Enterobacterales>Enterobacteriaceae>Klebsiella/Raoultella_group	-	Klebsiella_pneumoniae	ML041_004566	31807..35508	573	BREX-system-P-loop-protein-BrxC-[Klebsiella-pneumoniae]
HCA5579377.1	Type1.BREX	XIS-HTH->?->REase+SPF-cons-><-SF1-SF2-Helicase<-HKD-DNase+HKD-DNase<-?||BrxA-DNA-binding->BrxB-BrxF-iSTAND->DUF6079*->N6-METHYLASE->PglZ->SIGMA+OB-Fold+AAA-ATPase+Lon-Protease-BrxL-><-RVT<-TPR<-?<-DnaJ<-Arm-DNA-bind_3+Phage_integrase	1213	Bacteria>Pseudomonadota>Gammaproteobacteria>Enterobacterales>Enterobacteriaceae>Klebsiella/Raoultella_group	-	Klebsiella_pneumoniae	MXD08_004593	70758..74399	573	BREX-system-P-loop-protein-BrxC-[Klebsiella-pneumoniae]
WP_168234029.1	Type1.BREX	ParB+HNH->nSTAND1+HNH->RNaseH->NACHT+SF1-SF2-Helicase+HKD-DNase->REase-><-HKD-DNase+OGG-like<-SIGMA+OB-Fold+AAA-ATPase+Lon-Protease-BrxL<-PglZ<-N6-METHYLASE<-DUF6079*<-BrxB-BrxF-iSTAND<-BrxA-DNA-binding<-?<-ColE7+HNH	1213	Bacteria>Pseudomonadota>Gammaproteobacteria>Enterobacterales>Enterobacteriaceae>Klebsiella/Raoultella_group	-	Klebsiella_variicola	HGK55_RS15775	337116..340757	244366	BREX-system-P-loop-protein-BrxC-[Klebsiella-variicola]
EJN1576646.1	Type1.BREX	Glyoxalase->ACET->YjhX_toxin->ACET->METHYLASE->ZnR->Glyoxalase->BrxA-DNA-binding->BrxB-BrxF-iSTAND->DUF6079*->ParB+HNH->N6-METHYLASE->N6-METHYLASE-p->PglZ->SIGMA+OB-Fold+AAA-ATPase+Lon-Protease-BrxL-><-Arm-DNA-bind_3+Phage_integrase	1233	Bacteria>Pseudomonadota>Gammaproteobacteria>Enterobacterales>Enterobacteriaceae>Klebsiella/Raoultella_group	-	Klebsiella_variicola	NPH96_004932	32414..36115	244366	BREX-system-P-loop-protein-BrxC-[Klebsiella-variicola]
URQ56457.1	Type1.BREX	HTH_21+rve+rve_3-><-RHH||?->wHTH+WYL+WCX->BrxA-DNA-binding->BrxB-BrxF-iSTAND->DUF6079-p*->DUF6079-p->ParB+HNH->N6-METHYLASE->PglZ->SIGMA+OB-Fold+AAA-ATPase+Lon-Protease-BrxL-><-DEDD_Tnp_IS110+Transposase_20<-HIN-HTH||DEDD_Tnp_IS110+Transposase_20-><-PIN	769	Bacteria>Pseudomonadota>Gammaproteobacteria>Enterobacterales>Enterobacteriaceae>Klebsiella/Raoultella_group	-	Raoultella_ornithinolytica	.	79835..82144	54291	Hypothetical-protein-[Raoultella-ornithinolytica]
EHT9899814.1	Type1.BREX	<-Calcineurin<-?<-?<-?||tRNA-><-SIGMA+OB-Fold+AAA-ATPase+Lon-Protease-BrxL<-PglZ<-N6-METHYLASE-p<-N6-METHYLASE<-ParB+HNH<-DUF6079*<-BrxB-BrxF-iSTAND<-BrxA-DNA-binding||?-><-Glyco_hydro_43+GH43_C2<-?||AP_endonuc_2->?-><-METHYLASE<-YjhX_toxin	1233	Bacteria>Pseudomonadota>Gammaproteobacteria>Enterobacterales>Enterobacteriaceae>Klebsiella/Raoultella_group	-	Raoultella_ornithinolytica	KY209_001651	277271..280972	54291	BREX-system-P-loop-protein-BrxC-[Raoultella-ornithinolytica]
WP_094898295.1	Type1.BREX	<-TIR||BrxA-DNA-binding->BrxB-BrxF-iSTAND->DUF6079*->ParB+HNH->N6-METHYLASE->HEPN->RVT->N6-METHYLASE-p->PglZ->SIGMA+OB-Fold+AAA-ATPase+Lon-Protease-BrxL->wHTH+WYL+WCX->	1233	Bacteria>Pseudomonadota>Gammaproteobacteria>Enterobacterales>Enterobacteriaceae>Klebsiella/Raoultella_group	-	Raoultella_planticola	FQG10_00765	2767..6468	575	BREX-system-P-loop-protein-BrxC-[Raoultella-planticola]
HAT1572918.1	Type1.BREX	MarR-HTH->?->?->BZIP+SF1-SF2-Helicase->||HIN-HTH->HTH_Tnp_1+HTH_21+rve+rve_3->||wHTH+WYL+WCX->BrxA-DNA-binding->BrxB-BrxF-iSTAND->DUF6079*->ParB+HNH->N6-METHYLASE->PglZ->SIGMA+OB-Fold+AAA-ATPase+Lon-Protease-BrxL->HIN-HTH->	1225	Bacteria>Pseudomonadota>Gammaproteobacteria>Enterobacterales>Enterobacteriaceae>Kluyvera	GCA_015892965.1	Kluyvera_cryocrescens	I8Y10_004331	41743..45420	580	BREX-system-P-loop-protein-BrxC-[Kluyvera-cryocrescens]
WP_271458914.1	Type1.BREX	<-N6-METHYLASE<-DUF6079*<-BrxB-BrxF-iSTAND<-BrxA-DNA-binding||ZnR->wHTH->XIS-HTH->ParB+BAM->HTH+HTH->HTH_36->	1204	Bacteria>Pseudomonadota>Gammaproteobacteria>Enterobacterales>Enterobacteriaceae>Kluyvera	GCF_028069715.1	Kluyvera_sp._Awk_3	NNN71_RS16325	2505..6119	2963956	BREX-system-P-loop-protein-BrxC-[Kluyvera-sp.-Awk-3]
WP_112204418.1	Type1.BREX	GDResolvase+HIN-HTH-><-REase<-?||HTH_3->Arm-DNA-bind_3+Phage_integrase-><-?<-PglZ<-N6-METHYLASE<-DUF6079*<-BrxB-BrxF-iSTAND<-BrxA-DNA-binding||ArdB->?->CbeA_antitoxin->?->REase->	1213	Bacteria>Pseudomonadota>Gammaproteobacteria>Enterobacterales>Enterobacteriaceae>Kluyvera	GCF_900452275.1	Kluyvera_cryocrescens	DS850_RS16305	27927..31568	580	BREX-system-P-loop-protein-BrxC-[Kluyvera-cryocrescens]
WP_064568621.1	Type1.BREX	ABC-ATPase+SbcC->?-><-DSBH+AraC-HTH+AraC-HTH||?->?->TarH+HAMP+MA->BrxA-DNA-binding->BrxB-BrxF-iSTAND->DUF6079*->N6-METHYLASE->PglZ->SIGMA+OB-Fold+AAA-ATPase+Lon-Protease-BrxL->?-><-tRNA||REC+LuxR-HTH-><-?<-?<-?<-Phage_lysozyme	1216	Bacteria>Pseudomonadota>Gammaproteobacteria>Enterobacterales>Enterobacteriaceae>Kosakonia	GCF_001658025.2	Kosakonia_oryzae	AWR26_RS22590	4759672..4763322	497725	BREX-system-P-loop-protein-BrxC-[Kosakonia-oryzae]
WP_144838631.1	Type1.BREX	Aminotran_3->GNTR-HTH->SymE_toxin-><-?||wHTH+HNH->SAM-METHYLASE->BrxA-DNA-binding->BrxB-BrxF-iSTAND->DUF6079*->N6-METHYLASE->PglZ->SIGMA+OB-Fold+AAA-ATPase+Lon-Protease-BrxL->HKD-DNase+OGG-like-><-REase<-NACHT+SF1-SF2-Helicase+HKD-DNase<-RNaseH<-nSTAND1+HNH	1213	Bacteria>Pseudomonadota>Gammaproteobacteria>Enterobacterales>Enterobacteriaceae>Leclercia	GCF_007680555.1	Leclercia_adecarboxylata	FS784_RS12375	119273..122914	83655	BREX-system-P-loop-protein-BrxC-[Leclercia-adecarboxylata]
QXB23949.1	Type1.BREX	XIS-HTH->?->ZnR->ZnR->?->||BrxA-DNA-binding->BrxB-BrxF-iSTAND->DUF6079*->ParB+HNH->N6-METHYLASE->HEPN->RVT->N6-METHYLASE-><-HIN-HTH||PglZ->SIGMA+OB-Fold+AAA-ATPase+Lon-Protease-BrxL->	1233	Bacteria>Pseudomonadota>Gammaproteobacteria>Enterobacterales>Enterobacteriaceae>Lelliottia	GCA_019048185.1	Lelliottia_amnigena	I6L76_15755	3242307..3246008	61646	BREX-system-P-loop-protein-BrxC-[Lelliottia-amnigena]
WP_102104498.1	Type1.BREX	<-HKD-DNase+OGG-like<-SIGMA+OB-Fold+AAA-ATPase+Lon-Protease-BrxL<-PglZ<-TOPRIM<-OLD-ABC<-N6-METHYLASE<-DUF6079*<-BrxB-BrxF-iSTAND<-BrxA-DNA-binding||NACHT+SF1-SF2-Helicase+HKD-DNase-><-Glyoxalase<-ZnR<-SAM-METHYLASE<-YjhX_toxin<-LACI-HTH+PBPI	1204	Bacteria>Pseudomonadota>Gammaproteobacteria>Enterobacterales>Enterobacteriaceae>Lelliottia	GCF_002870005.1	unclassified_Lelliottia	F159L0C_RS08780	168198..171812	2642424	MULTISPECIES:-BREX-system-P-loop-protein-BrxC-[unclassified-Lelliottia]
WP_254208058.1	Type1.BREX	XIS-HTH->?->ZnR->ZnR->?->||BrxA-DNA-binding->BrxB-BrxF-iSTAND->DUF6079*->ParB+HNH->N6-METHYLASE->HEPN->RVT->N6-METHYLASE-><-HIN-HTH||PglZ->SIGMA+OB-Fold+AAA-ATPase+Lon-Protease-BrxL->	1237	Bacteria>Pseudomonadota>Gammaproteobacteria>Enterobacterales>Enterobacteriaceae>Lelliottia	GCF_019048185.1	Lelliottia_amnigena	I6L76_RS15755	3242295..3246008	61646	BREX-system-P-loop-protein-BrxC-[Lelliottia-amnigena]
WP_216981896.1	Type1.BREX	XIS-HTH->?->ZnR->ZnR->?->||BrxA-DNA-binding->BrxB-BrxF-iSTAND->DUF6079*->ParB+HNH->N6-METHYLASE->HEPN->RVT->N6-METHYLASE-><-HIN-HTH||PglZ->SIGMA+OB-Fold+AAA-ATPase+Lon-Protease-BrxL->	1233	Bacteria>Pseudomonadota>Gammaproteobacteria>Enterobacterales>Enterobacteriaceae>Lelliottia	-	Lelliottia_amnigena	I6L76_15755	3242307..3246008	61646	BREX-system-P-loop-protein-BrxC-[Lelliottia-amnigena]
WP_077737804.1	Type1.BREX	ZnR->ZnR-><-SIGMA+OB-Fold+AAA-ATPase+Lon-Protease-BrxL<-PglZ<-N6-METHYLASE<-DUF6079*<-BrxB-BrxF-iSTAND<-BrxA-DNA-binding||REase+iREase->Zn_Tnp_IS1+HTH_Tnp_IS1+DDE_Tnp_IS1-><-?<-DDE_Tnp_1_5+DDE_Tnp_1||REC+CITB-HTH->	1213	Bacteria>Pseudomonadota>Gammaproteobacteria>Enterobacterales	-	Enterobacteriaceae	NF083_RS22575	9834..13475	543	MULTISPECIES:-BREX-system-P-loop-protein-BrxC-[Enterobacteriaceae]
WP_233672193.1	Type1.BREX	<-SIGMA+OB-Fold+AAA-ATPase+Lon-Protease-BrxL<-PglZ<-TOPRIM<-OLD-ABC<-N6-METHYLASE-p<-TIR<-N6-METHYLASE<-TOPRIM<-OLD-ABC<-DUF6079*<-BrxB-BrxF-iSTAND<-BrxA-DNA-binding<-?<-?<-?<-XIS-HTH<-Arm-DNA-bind_3+Phage_integrase||MODE-HTH+PBPII->	1238	Bacteria>Pseudomonadota>Gammaproteobacteria>Enterobacterales>Enterobacteriaceae>Plesiomonas	GCF_021368255.1	Plesiomonas_sp._PI-19	LQ901_RS09600	2145541..2149257	2898798	BREX-system-P-loop-protein-BrxC-[Plesiomonas-sp.-PI-19]
WP_265846293.1	Type1.BREX	<-RAMPs<-Cas10||GTPase-AIG->GTPase-AIG->XIS-HTH->?->?->BrxA-DNA-binding->BrxB-BrxF-iSTAND->DUF6079*->ParB+HNH->TIR->N6-METHYLASE-p->?->PglZ->	1238	Bacteria>Pseudomonadota>Gammaproteobacteria>Enterobacterales>Enterobacteriaceae>Plesiomonas	GCF_026240155.1	Plesiomonas_shigelloides	OQ486_RS15170	39648..43364	703	BREX-system-P-loop-protein-BrxC-[Plesiomonas-shigelloides]
EHC84522.1	Type1.BREX	<-SIGMA+OB-Fold+AAA-ATPase+Lon-Protease-BrxL<-PglZ<-N6-METHYLASE-p<-N6-METHYLASE<-ParB+HNH<-DUF6079*<-BrxB-BrxF-iSTAND<-BrxA-DNA-binding<-ZnR<-Glyoxalase<-METHYLASE	1247	Bacteria>Pseudomonadota>Gammaproteobacteria>Enterobacterales>Enterobacteriaceae>Salmonella	GCA_000231705.2	Salmonella_enterica_subsp._enterica_serovar_Uganda_str._R8-3404	LTSEUGA_5994	10868..14611	913083	hypothetical-protein-LTSEUGA_5994-[Salmonella-enterica-subsp.-enterica-serovar-Uganda-str.-R8-3404]
ESG75014.1	Type1.BREX	<-HEPN<-?||YjhX_toxin->METHYLASE->?-><-wHTH+SAD+HNH||?->BrxA-DNA-binding->DUF6079*->?->N6-METHYLASE->OLD-ABC->TOPRIM->PglZ->SIGMA+OB-Fold+AAA-ATPase+Lon-Protease-BrxL->HKD-DNase+OGG-like-><-REase<-NACHT+SF1-SF2-Helicase+HKD-DNase	1142	Bacteria>Pseudomonadota>Gammaproteobacteria>Enterobacterales>Enterobacteriaceae>Salmonella	GCA_000487055.1	Salmonella_enterica_subsp._enterica_serovar_Kentucky_str._ATCC_9263	SEEK9263_16857	19218..22646	984236	ATPase-like-protein-[Salmonella-enterica-subsp.-enterica-serovar-Kentucky-str.-ATCC-9263]
APV85542.1	Type1.BREX	Glyoxalase->ACET->YjhX_toxin->ACET->METHYLASE->ZnR->Glyoxalase->BrxA-DNA-binding->BrxB-BrxF-iSTAND->DUF6079*->ParB+HNH->N6-METHYLASE->PglZ->SIGMA+OB-Fold+AAA-ATPase+Lon-Protease-BrxL-><-Arm-DNA-bind_3+Phage_integrase<-?<-tRNA||?-><-SKI||METHYLASE->	1233	Bacteria>Pseudomonadota>Gammaproteobacteria>Enterobacterales>Enterobacteriaceae>Salmonella	GCA_000487155.2	Salmonella_enterica_subsp._enterica_serovar_Inverness_str._ATCC_10720	SEEI0720_021515	4182897..4186598	941187	DNA-repair-protein-[Salmonella-enterica-subsp.-enterica-serovar-Inverness-str.-ATCC-10720]
AXD95438.1	Type1.BREX	<-METHYLASE||SKI-><-?||tRNA->Arm-DNA-bind_3+Phage_integrase-><-SIGMA+OB-Fold+AAA-ATPase+Lon-Protease-BrxL<-PglZ<-N6-METHYLASE<-ParB+HNH<-DUF6079*<-BrxB-BrxF-iSTAND<-BrxA-DNA-binding<-Glyoxalase<-ZnR<-METHYLASE<-ACET<-YjhX_toxin||<-ACET<-Glyoxalase	1233	Bacteria>Pseudomonadota>Gammaproteobacteria>Enterobacterales>Enterobacteriaceae>Salmonella	GCA_003325015.1	Salmonella_enterica	CHC75_12745	2557798..2561499	28901	BREX-system-P-loop-protein-BrxC-[Salmonella-enterica]
MDG46363.1	Type1.BREX	<-HEPN<-REase+iREase<-?||YjhX_toxin->SAM-METHYLASE->?->BrxA-DNA-binding->BrxB-BrxF-iSTAND->DUF6079*->N6-METHYLASE->OLD-ABC->TOPRIM->PglZ->SIGMA+OB-Fold+AAA-ATPase+Lon-Protease-BrxL-><-REase<-NACHT+SF1-SF2-Helicase+HKD-DNase<-Arm-DNA-bind_3	1213	Bacteria>Pseudomonadota>Gammaproteobacteria>Enterobacterales>Enterobacteriaceae>Salmonella	GCA_003733245.1	Salmonella_enterica	EB289_07920	38672..42313	28901	BREX-system-P-loop-protein-BrxC-[Salmonella-enterica]
MIX28204.1	Type1.BREX	YjhX_toxin->SAM-METHYLASE-><-?||?->?->BrxA-DNA-binding->BrxB-BrxF-iSTAND->DUF6079*->N6-METHYLASE->OLD-ABC->TOPRIM->PglZ->SIGMA+OB-Fold+AAA-ATPase+Lon-Protease-BrxL-><-REase<-NACHT+SF1-SF2-Helicase+HKD-DNase	1213	Bacteria>Pseudomonadota>Gammaproteobacteria>Enterobacterales>Enterobacteriaceae>Salmonella	GCA_003876625.1	Salmonella_enterica_subsp._enterica_serovar_Livingstone	CRQ34_16010	159179..162820	260680	BREX-system-P-loop-protein-BrxC-[Salmonella-enterica-subsp.-enterica-serovar-Livingstone]
MLQ35368.1	Type1.BREX	<-MORC+HISKIN<-DNA_METHYLASE<-?||HIN-HTH-><-SIGMA+OB-Fold+AAA-ATPase+Lon-Protease-BrxL<-PglZ<-TOPRIM<-OLD-ABC<-N6-METHYLASE<-DUF6079*<-BrxB-BrxF-iSTAND<-BrxA-DNA-binding<-?<-?<-SAM-METHYLASE<-YjhX_toxin||<-?||?->REase+iREase->	1213	Bacteria>Pseudomonadota>Gammaproteobacteria>Enterobacterales>Enterobacteriaceae>Salmonella	GCA_003910355.1	Salmonella_enterica_subsp._enterica	DRM12_04740	261903..265544	59201	BREX-system-P-loop-protein-BrxC-[Salmonella-enterica-subsp.-enterica]
MLX95744.1	Type1.BREX	<-HEPN<-REase+iREase<-?||||YjhX_toxin->SAM-METHYLASE->?->?->BrxA-DNA-binding->BrxB-BrxF-iSTAND->DUF6079-p*->	669	Bacteria>Pseudomonadota>Gammaproteobacteria>Enterobacterales>Enterobacteriaceae>Salmonella	GCA_003913195.1	Salmonella_enterica_subsp._enterica	DSF42_09165	170394..172402	59201	BREX-system-P-loop-protein-BrxC-[Salmonella-enterica-subsp.-enterica]
AZT73891.1	Type1.BREX	<-wHTH||wHTH+WYL+WCX-><-MarR-wHTH<-SIGMA-HTH+REase<-wHTH+SAD+HNH||?-><-ZnR||BrxA-DNA-binding->BrxB-BrxF-iSTAND->DUF6079*->N6-METHYLASE->OLD-ABC->TOPRIM->PglZ->SIGMA+OB-Fold+AAA-ATPase+Lon-Protease-BrxL->HKD-DNase+OGG-like-><-REase<-NACHT+SF1-SF2-Helicase+HKD-DNase<-Arm-DNA-bind_3	1204	Bacteria>Pseudomonadota>Gammaproteobacteria>Enterobacterales>Enterobacteriaceae>Salmonella	GCA_003994735.1	Salmonella_enterica_subsp._enterica_serovar_Waycross	ELZ91_19520	3951400..3955014	58101	BREX-system-P-loop-protein-BrxC-[Salmonella-enterica-subsp.-enterica-serovar-Waycross]
EAA2289696.1	Type1.BREX	<-HEPN<-REase+iREase<-?||||YjhX_toxin->SAM-METHYLASE->?->?->BrxA-DNA-binding->BrxB-BrxF-iSTAND->DUF6079*->	1103	Bacteria>Pseudomonadota>Gammaproteobacteria>Enterobacterales>Enterobacteriaceae>Salmonella	GCA_004165775.1	Salmonella_enterica_subsp._enterica	DNI00_14480	60001..63308	59201	BREX-system-P-loop-protein-BrxC-[Salmonella-enterica-subsp.-enterica]
EAA3385003.1	Type1.BREX	<-HEPN<-?||||YjhX_toxin->SAM-METHYLASE-><-?||?->?->BrxA-DNA-binding->BrxB-BrxF-iSTAND->DUF6079*->N6-METHYLASE->OLD-ABC->TOPRIM->PglZ->SIGMA+OB-Fold+AAA-ATPase+Lon-Protease-BrxL-><-REase<-NACHT+SF1-SF2-Helicase+HKD-DNase<-Arm-DNA-bind_3	1213	Bacteria>Pseudomonadota>Gammaproteobacteria>Enterobacterales>Enterobacteriaceae>Salmonella	GCA_004174495.1	Salmonella_enterica	DMV54_10710	131940..135581	28901	BREX-system-P-loop-protein-BrxC-[Salmonella-enterica]
EAA3988072.1	Type1.BREX	Arm-DNA-bind_3->NACHT+SF1-SF2-Helicase+HKD-DNase->REase-><-SIGMA+OB-Fold+AAA-ATPase+Lon-Protease-BrxL<-PglZ<-TOPRIM<-OLD-ABC<-N6-METHYLASE<-DUF6079*<-BrxB-BrxF-iSTAND<-BrxA-DNA-binding<-?<-?<-SAM-METHYLASE<-YjhX_toxin||||?->REase+iREase->HEPN->	1213	Bacteria>Pseudomonadota>Gammaproteobacteria>Enterobacterales>Enterobacteriaceae>Salmonella	GCA_004177075.1	Salmonella_enterica_subsp._enterica	DO557_10295	132678..136319	59201	BREX-system-P-loop-protein-BrxC-[Salmonella-enterica-subsp.-enterica]
EAA4435938.1	Type1.BREX	XIS-HTH->?-><-?<-ZnR||BrxA-DNA-binding->BrxB-BrxF-iSTAND->DUF6079*->ParB+HNH->N6-METHYLASE->HEPN->RVT->N6-METHYLASE-p->PglZ->SIGMA+OB-Fold+AAA-ATPase+Lon-Protease-BrxL-><-tRNA||?-><-SKI	1233	Bacteria>Pseudomonadota>Gammaproteobacteria>Enterobacterales>Enterobacteriaceae>Salmonella	GCA_004179155.1	Salmonella_enterica_subsp._salamae	DPE44_05505	71371..75072	59202	BREX-system-P-loop-protein-BrxC-[Salmonella-enterica-subsp.-salamae]
EAA4290852.1	Type1.BREX	<-HEPN<-REase+iREase<-?||||YjhX_toxin->SAM-METHYLASE->?->?->BrxA-DNA-binding->BrxB-BrxF-iSTAND->DUF6079-p*->	989	Bacteria>Pseudomonadota>Gammaproteobacteria>Enterobacterales>Enterobacteriaceae>Salmonella	GCA_004180125.1	Salmonella_enterica_subsp._enterica	DO981_06670	47529..50496	59201	BREX-system-P-loop-protein-BrxC-[Salmonella-enterica-subsp.-enterica]
EAA4442641.1	Type1.BREX	<-HEPN<-REase+iREase<-?||||YjhX_toxin->SAM-METHYLASE->?->?->BrxA-DNA-binding->BrxB-BrxF-iSTAND->DUF6079-p*->	833	Bacteria>Pseudomonadota>Gammaproteobacteria>Enterobacterales>Enterobacteriaceae>Salmonella	GCA_004180805.1	Salmonella_enterica_subsp._enterica	DPE76_16250	60221..62721	59201	BREX-system-P-loop-protein-BrxC-[Salmonella-enterica-subsp.-enterica]
EAA4605168.1	Type1.BREX	<-HEPN<-REase+iREase<-?||||YjhX_toxin->SAM-METHYLASE-><-?||?->||BrxA-DNA-binding->BrxB-BrxF-iSTAND->DUF6079*->N6-METHYLASE->PglZ->SIGMA+OB-Fold+AAA-ATPase+Lon-Protease-BrxL->	1213	Bacteria>Pseudomonadota>Gammaproteobacteria>Enterobacterales>Enterobacteriaceae>Salmonella	GCA_004181295.1	Salmonella_enterica_subsp._enterica	DPJ21_07760	164877..168518	59201	BREX-system-P-loop-protein-BrxC-[Salmonella-enterica-subsp.-enterica]
EAA4842519.1	Type1.BREX	<-HEPN<-REase+iREase<-?||||YjhX_toxin->SAM-METHYLASE->?->?->BrxA-DNA-binding->BrxB-BrxF-iSTAND->DUF6079*->OLD-ABC->TOPRIM->PglZ->SIGMA+OB-Fold+AAA-ATPase+Lon-Protease-BrxL-><-REase<-NACHT+SF1-SF2-Helicase+HKD-DNase<-Arm-DNA-bind_3<-tRNA||?-><-SKI	1213	Bacteria>Pseudomonadota>Gammaproteobacteria>Enterobacterales>Enterobacteriaceae>Salmonella	GCA_004183005.1	Salmonella_enterica_subsp._enterica	DPP49_10705	60116..63757	59201	BREX-system-P-loop-protein-BrxC-[Salmonella-enterica-subsp.-enterica]
EAA5451231.1	Type1.BREX	<-REase+iREase<-?||||YjhX_toxin->SAM-METHYLASE-><-?||?->?->BrxA-DNA-binding->BrxB-BrxF-iSTAND->DUF6079*->N6-METHYLASE->OLD-ABC->TOPRIM->PglZ->SIGMA+OB-Fold+AAA-ATPase+Lon-Protease-BrxL-><-REase<-NACHT+SF1-SF2-Helicase+HKD-DNase<-Arm-DNA-bind_3	1213	Bacteria>Pseudomonadota>Gammaproteobacteria>Enterobacterales>Enterobacteriaceae>Salmonella	GCA_004184965.1	Salmonella_enterica_subsp._enterica	DQC86_00800	160528..164169	59201	BREX-system-P-loop-protein-BrxC-[Salmonella-enterica-subsp.-enterica]
EAA5642506.1	Type1.BREX	<-HEPN<-?||?->||YjhX_toxin->SAM-METHYLASE->?->?->BrxA-DNA-binding->BrxB-BrxF-iSTAND->DUF6079*->N6-METHYLASE->OLD-ABC->TOPRIM->PglZ->SIGMA+OB-Fold+AAA-ATPase+Lon-Protease-BrxL->?-><-REase	1213	Bacteria>Pseudomonadota>Gammaproteobacteria>Enterobacterales>Enterobacteriaceae>Salmonella	GCA_004185395.1	Salmonella_enterica_subsp._enterica	DQJ71_10390	149103..152744	59201	BREX-system-P-loop-protein-BrxC-[Salmonella-enterica-subsp.-enterica]
EAA5580610.1	Type1.BREX	<-HEPN<-?||?->||YjhX_toxin->SAM-METHYLASE->?->?->BrxA-DNA-binding->BrxB-BrxF-iSTAND->DUF6079*->N6-METHYLASE->OLD-ABC->TOPRIM->PglZ->SIGMA+OB-Fold+AAA-ATPase+Lon-Protease-BrxL->HKD-DNase+OGG-like-><-REase<-NACHT+SF1-SF2-Helicase+HKD-DNase	1213	Bacteria>Pseudomonadota>Gammaproteobacteria>Enterobacterales>Enterobacteriaceae>Salmonella	GCA_004185805.1	Salmonella_enterica_subsp._enterica	DQD90_06840	58937..62578	59201	BREX-system-P-loop-protein-BrxC-[Salmonella-enterica-subsp.-enterica]
EAA6071870.1	Type1.BREX	YjhX_toxin->SAM-METHYLASE-><-?||?->?->BrxA-DNA-binding->BrxB-BrxF-iSTAND->DUF6079*->N6-METHYLASE->OLD-ABC->TOPRIM->PglZ->SIGMA+OB-Fold+AAA-ATPase+Lon-Protease-BrxL-><-REase<-Couple_hipA+HipA_C<-HTH_3	1213	Bacteria>Pseudomonadota>Gammaproteobacteria>Enterobacterales>Enterobacteriaceae>Salmonella	GCA_004187475.1	Salmonella_enterica_subsp._enterica	DQZ25_06110	158264..161905	59201	BREX-system-P-loop-protein-BrxC-[Salmonella-enterica-subsp.-enterica]
EAA6168130.1	Type1.BREX	HTH_3->Couple_hipA+HipA_C->REase-><-SIGMA+OB-Fold+AAA-ATPase+Lon-Protease-BrxL<-PglZ<-TOPRIM<-OLD-ABC<-N6-METHYLASE<-DUF6079*<-BrxB-BrxF-iSTAND<-BrxA-DNA-binding<-?<-?||?-><-SAM-METHYLASE<-YjhX_toxin||||?->REase+iREase->	1213	Bacteria>Pseudomonadota>Gammaproteobacteria>Enterobacterales>Enterobacteriaceae>Salmonella	GCA_004188635.1	Salmonella_enterica_subsp._enterica	DQZ68_14465	24635..28276	59201	BREX-system-P-loop-protein-BrxC-[Salmonella-enterica-subsp.-enterica]
EAA7005308.1	Type1.BREX	<-HEPN<-?||||YjhX_toxin->SAM-METHYLASE-><-?||?->?->BrxA-DNA-binding->BrxB-BrxF-iSTAND->DUF6079*->N6-METHYLASE->	1213	Bacteria>Pseudomonadota>Gammaproteobacteria>Enterobacterales>Enterobacteriaceae>Salmonella	GCA_004191915.1	Salmonella_enterica	AVW22_12590	47439..51080	28901	BREX-system-P-loop-protein-BrxC-[Salmonella-enterica]
EAA7109615.1	Type1.BREX	YjhX_toxin->SAM-METHYLASE-><-?||?->?->BrxA-DNA-binding->BrxB-BrxF-iSTAND->DUF6079*->N6-METHYLASE->OLD-ABC->TOPRIM->PglZ->SIGMA+OB-Fold+AAA-ATPase+Lon-Protease-BrxL-><-REase	1213	Bacteria>Pseudomonadota>Gammaproteobacteria>Enterobacterales>Enterobacteriaceae>Salmonella	GCA_004192185.1	Salmonella_enterica_subsp._enterica	DSE94_16630	52127..55768	59201	BREX-system-P-loop-protein-BrxC-[Salmonella-enterica-subsp.-enterica]
EAA7310017.1	Type1.BREX	<-REase+iREase<-?||||YjhX_toxin->SAM-METHYLASE-><-?||?->?->BrxA-DNA-binding->BrxB-BrxF-iSTAND->DUF6079*->N6-METHYLASE->OLD-ABC->TOPRIM->PglZ->SIGMA+OB-Fold+AAA-ATPase+Lon-Protease-BrxL-><-REase<-Couple_hipA+HipA_C<-HTH_3	1213	Bacteria>Pseudomonadota>Gammaproteobacteria>Enterobacterales>Enterobacteriaceae>Salmonella	GCA_004192645.1	Salmonella_enterica_subsp._enterica	TL61_10425	156005..159646	59201	BREX-system-P-loop-protein-BrxC-[Salmonella-enterica-subsp.-enterica]
EAA7604354.1	Type1.BREX	<-HEPN<-?||||YjhX_toxin->SAM-METHYLASE->?->?->BrxA-DNA-binding->BrxB-BrxF-iSTAND->DUF6079*->N6-METHYLASE->OLD-ABC->TOPRIM->PglZ->SIGMA+OB-Fold+AAA-ATPase+Lon-Protease-BrxL->	1213	Bacteria>Pseudomonadota>Gammaproteobacteria>Enterobacterales>Enterobacteriaceae>Salmonella	GCA_004218255.1	Salmonella_enterica	ADQ66_03555	130477..134118	28901	BREX-system-P-loop-protein-BrxC-[Salmonella-enterica]
EAA7639151.1	Type1.BREX	BrxB-BrxF-iSTAND->ACET->||?-><-Phage_integrase||BrxA-DNA-binding->BrxB-BrxF-iSTAND->DUF6079*->N6-METHYLASE->PglZ->SIGMA+OB-Fold+AAA-ATPase+Lon-Protease-BrxL-><-AAA+HTH<-?<-XIS-HTH	1213	Bacteria>Pseudomonadota>Gammaproteobacteria>Enterobacterales>Enterobacteriaceae>Salmonella	GCA_004218325.1	Salmonella_enterica_subsp._enterica	AAV76_19225	212134..215775	59201	BREX-system-P-loop-protein-BrxC-[Salmonella-enterica-subsp.-enterica]
EAA8317754.1	Type1.BREX	<-HEPN<-?||?->||YjhX_toxin->SAM-METHYLASE->?->?->BrxA-DNA-binding->BrxB-BrxF-iSTAND->DUF6079*->N6-METHYLASE->OLD-ABC->TOPRIM->PglZ->SIGMA+OB-Fold+AAA-ATPase+Lon-Protease-BrxL->?-><-REase<-NACHT+SF1-SF2-Helicase+HKD-DNase	1213	Bacteria>Pseudomonadota>Gammaproteobacteria>Enterobacterales>Enterobacteriaceae>Salmonella	GCA_004221325.1	Salmonella_enterica	AOS09_04965	162610..166251	28901	BREX-system-P-loop-protein-BrxC-[Salmonella-enterica]
EAA8523361.1	Type1.BREX	<-HEPN<-?||||YjhX_toxin->SAM-METHYLASE-><-?||?->?->BrxA-DNA-binding->BrxB-BrxF-iSTAND->DUF6079*->N6-METHYLASE->OLD-ABC->TOPRIM->PglZ->SIGMA+OB-Fold+AAA-ATPase+Lon-Protease-BrxL-><-REase<-NACHT+SF1-SF2-Helicase+HKD-DNase<-Arm-DNA-bind_3	1213	Bacteria>Pseudomonadota>Gammaproteobacteria>Enterobacterales>Enterobacteriaceae>Salmonella	GCA_004222265.1	Salmonella_enterica_subsp._enterica	ADQ53_00815	161051..164692	59201	BREX-system-P-loop-protein-BrxC-[Salmonella-enterica-subsp.-enterica]
EAA8875705.1	Type1.BREX	<-HEPN<-?||YjhX_toxin->SAM-METHYLASE->?-><-wHTH+SAD+HNH||URI-DNase->BrxA-DNA-binding->BrxB-BrxF-iSTAND->DUF6079*->N6-METHYLASE->OLD-ABC->TOPRIM->PglZ->SIGMA+OB-Fold+AAA-ATPase+Lon-Protease-BrxL->HKD-DNase+OGG-like-><-REase<-NACHT+SF1-SF2-Helicase+HKD-DNase<-nSTAND1+HNH	1213	Bacteria>Pseudomonadota>Gammaproteobacteria>Enterobacterales>Enterobacteriaceae>Salmonella	GCA_004223005.1	Salmonella_enterica	CIU45_09625	381857..385498	28901	BREX-system-P-loop-protein-BrxC-[Salmonella-enterica]
EAA8954969.1	Type1.BREX	YjhX_toxin->SAM-METHYLASE-><-?||?->?->BrxA-DNA-binding->BrxB-BrxF-iSTAND->DUF6079*->N6-METHYLASE->PglZ->SIGMA+OB-Fold+AAA-ATPase+Lon-Protease-BrxL->||PIN-><-HTH_21+rve+rve_3	1213	Bacteria>Pseudomonadota>Gammaproteobacteria>Enterobacterales>Enterobacteriaceae>Salmonella	GCA_004224545.1	Salmonella_enterica	CBJ10_12720	157324..160965	28901	BREX-system-P-loop-protein-BrxC-[Salmonella-enterica]
EAA9319736.1	Type1.BREX	YjhX_toxin->SAM-METHYLASE-><-?||?->?->BrxA-DNA-binding->BrxB-BrxF-iSTAND->DUF6079*->N6-METHYLASE->OLD-ABC->TOPRIM->PglZ->SIGMA+OB-Fold+AAA-ATPase+Lon-Protease-BrxL-><-REase<-Couple_hipA+HipA_C<-HTH_3<-HIN-HTH	1213	Bacteria>Pseudomonadota>Gammaproteobacteria>Enterobacterales>Enterobacteriaceae>Salmonella	GCA_004226405.1	Salmonella_enterica	CRQ39_09900	167436..171077	28901	BREX-system-P-loop-protein-BrxC-[Salmonella-enterica]
EAA9627982.1	Type1.BREX	<-HEPN<-REase+iREase<-?||||YjhX_toxin->SAM-METHYLASE->?->?->BrxA-DNA-binding->BrxB-BrxF-iSTAND->DUF6079*->N6-METHYLASE->OLD-ABC->TOPRIM->PglZ->SIGMA+OB-Fold+AAA-ATPase+Lon-Protease-BrxL-><-REase<-NACHT+SF1-SF2-Helicase+HKD-DNase<-Arm-DNA-bind_3	1213	Bacteria>Pseudomonadota>Gammaproteobacteria>Enterobacterales>Enterobacteriaceae>Salmonella	GCA_004227565.1	Salmonella_enterica_subsp._enterica	DV01_07255	170919..174560	59201	BREX-system-P-loop-protein-BrxC-[Salmonella-enterica-subsp.-enterica]
EAB1768963.1	Type1.BREX	<-HEPN<-REase+iREase<-?||YjhX_toxin->SAM-METHYLASE->?->BrxA-DNA-binding->BrxB-BrxF-iSTAND->DUF6079*->N6-METHYLASE->OLD-ABC->TOPRIM->PglZ->SIGMA+OB-Fold+AAA-ATPase+Lon-Protease-BrxL-><-REase<-NACHT+SF1-SF2-Helicase+HKD-DNase<-Arm-DNA-bind_3	1213	Bacteria>Pseudomonadota>Gammaproteobacteria>Enterobacterales>Enterobacteriaceae>Salmonella	GCA_004236005.1	Salmonella_enterica	EIS13_11220	18269..21910	28901	BREX-system-P-loop-protein-BrxC-[Salmonella-enterica]
EAB2294236.1	Type1.BREX	<-HEPN<-?||?->YjhX_toxin->SAM-METHYLASE->?->||BrxA-DNA-binding->BrxB-BrxF-iSTAND->DUF6079*->N6-METHYLASE-p->	1213	Bacteria>Pseudomonadota>Gammaproteobacteria>Enterobacterales>Enterobacteriaceae>Salmonella	GCA_004236865.1	Salmonella_enterica	EHO06_05115	132073..135714	28901	BREX-system-P-loop-protein-BrxC-[Salmonella-enterica]
EAB2013162.1	Type1.BREX	XIS-HTH->?->?-><-ZnR||?->BrxA-DNA-binding->BrxB-BrxF-iSTAND->DUF6079*->N6-METHYLASE->OLD-ABC->TOPRIM->PglZ->SIGMA+OB-Fold+AAA-ATPase+Lon-Protease-BrxL->	1204	Bacteria>Pseudomonadota>Gammaproteobacteria>Enterobacterales>Enterobacteriaceae>Salmonella	GCA_004236885.1	Salmonella_enterica	D7J64_23090	5108..8722	28901	BREX-system-P-loop-protein-BrxC-[Salmonella-enterica]
EAB2831578.1	Type1.BREX	<-REase+iREase<-?||?->||YjhX_toxin->SAM-METHYLASE->?->?->BrxA-DNA-binding->BrxB-BrxF-iSTAND->DUF6079*->N6-METHYLASE->OLD-ABC->TOPRIM->PglZ->SIGMA+OB-Fold+AAA-ATPase+Lon-Protease-BrxL->HKD-DNase+OGG-like-><-REase<-NACHT+SF1-SF2-Helicase+HKD-DNase	1213	Bacteria>Pseudomonadota>Gammaproteobacteria>Enterobacterales>Enterobacteriaceae>Salmonella	GCA_004240605.1	Salmonella_enterica	EAV48_05270	157994..161635	28901	BREX-system-P-loop-protein-BrxC-[Salmonella-enterica]
EAB2910642.1	Type1.BREX	Arm-DNA-bind_3->NACHT+SF1-SF2-Helicase+HKD-DNase->REase-><-SIGMA+OB-Fold+AAA-ATPase+Lon-Protease-BrxL<-PglZ<-TOPRIM<-OLD-ABC<-N6-METHYLASE<-DUF6079*<-BrxB-BrxF-iSTAND<-BrxA-DNA-binding<-?<-SAM-METHYLASE<-YjhX_toxin||?->REase+iREase->HEPN->	1213	Bacteria>Pseudomonadota>Gammaproteobacteria>Enterobacterales>Enterobacteriaceae>Salmonella	GCA_004240965.1	Salmonella_enterica	EHO38_04055	132608..136249	28901	BREX-system-P-loop-protein-BrxC-[Salmonella-enterica]
EAB6380447.1	Type1.BREX	HTH_3->Couple_hipA+HipA_C->REase-><-SIGMA+OB-Fold+AAA-ATPase+Lon-Protease-BrxL<-PglZ<-TOPRIM<-OLD-ABC<-N6-METHYLASE<-DUF6079*<-BrxB-BrxF-iSTAND<-BrxA-DNA-binding<-?<-?||?-><-SAM-METHYLASE<-YjhX_toxin||||?->REase+iREase->	1213	Bacteria>Pseudomonadota>Gammaproteobacteria>Enterobacterales>Enterobacteriaceae>Salmonella	GCA_004255205.1	Salmonella_enterica_subsp._enterica	CWG97_20720	30069..33710	59201	BREX-system-P-loop-protein-BrxC-[Salmonella-enterica-subsp.-enterica]
EAB6416573.1	Type1.BREX	YjhX_toxin->SAM-METHYLASE-><-?||?->||?->BrxA-DNA-binding->BrxB-BrxF-iSTAND->DUF6079*->N6-METHYLASE->OLD-ABC->TOPRIM->PglZ->SIGMA+OB-Fold+AAA-ATPase+Lon-Protease-BrxL-><-REase<-NACHT+SF1-SF2-Helicase+HKD-DNase<-Arm-DNA-bind_3||ParB+HNH->	1213	Bacteria>Pseudomonadota>Gammaproteobacteria>Enterobacterales>Enterobacteriaceae>Salmonella	GCA_004256685.1	Salmonella_enterica_subsp._enterica	D6S13_10910	52351..55992	59201	BREX-system-P-loop-protein-BrxC-[Salmonella-enterica-subsp.-enterica]
EAB6711531.1	Type1.BREX	HTH_3->Couple_hipA+HipA_C->REase-><-SIGMA+OB-Fold+AAA-ATPase+Lon-Protease-BrxL<-PglZ<-N6-METHYLASE<-DUF6079*<-BrxB-BrxF-iSTAND<-BrxA-DNA-binding<-?<-?||?-><-SAM-METHYLASE<-YjhX_toxin||||?->HEPN->	1213	Bacteria>Pseudomonadota>Gammaproteobacteria>Enterobacterales>Enterobacteriaceae>Salmonella	GCA_004258665.1	Salmonella_enterica_subsp._enterica	DU754_17280	25213..28854	59201	BREX-system-P-loop-protein-BrxC-[Salmonella-enterica-subsp.-enterica]
EAB6843525.1	Type1.BREX	<-GntR-HTH++TrmB+ZDNA-HTH||<-?<-HEPN<-?||YjhX_toxin->METHYLASE->?->BrxA-DNA-binding->BrxB-BrxF-iSTAND->DUF6079*->N6-METHYLASE->OLD-ABC->TOPRIM->PglZ->SIGMA+OB-Fold+AAA-ATPase+Lon-Protease-BrxL->HKD-DNase+OGG-like-><-REase<-NACHT+SF1-SF2-Helicase+HKD-DNase||ParB+HNH->	1213	Bacteria>Pseudomonadota>Gammaproteobacteria>Enterobacterales>Enterobacteriaceae>Salmonella	GCA_004258725.1	Salmonella_enterica_subsp._salamae	XT66_14185	50997..54638	59202	BREX-system-P-loop-protein-BrxC-[Salmonella-enterica-subsp.-salamae]
EAB7363624.1	Type1.BREX	<-HEPN<-REase+iREase<-?||YjhX_toxin->SAM-METHYLASE->?->BrxA-DNA-binding->BrxB-BrxF-iSTAND->DUF6079*->N6-METHYLASE->OLD-ABC->TOPRIM->PglZ->SIGMA+OB-Fold+AAA-ATPase+Lon-Protease-BrxL-><-REase<-NACHT+SF1-SF2-Helicase+HKD-DNase<-Arm-DNA-bind_3	1213	Bacteria>Pseudomonadota>Gammaproteobacteria>Enterobacterales>Enterobacteriaceae>Salmonella	GCA_004260605.1	Salmonella_enterica_subsp._enterica	EBO89_06925	58191..61832	59201	BREX-system-P-loop-protein-BrxC-[Salmonella-enterica-subsp.-enterica]
EAB9078796.1	Type1.BREX	YjhX_toxin->SAM-METHYLASE-><-?||?->?->BrxA-DNA-binding->BrxB-BrxF-iSTAND->DUF6079*->N6-METHYLASE->OLD-ABC->TOPRIM->PglZ->SIGMA+OB-Fold+AAA-ATPase+Lon-Protease-BrxL->HKD-DNase+OGG-like-><-REase<-NACHT+SF1-SF2-Helicase+HKD-DNase	1213	Bacteria>Pseudomonadota>Gammaproteobacteria>Enterobacterales>Enterobacteriaceae>Salmonella	GCA_004265565.1	Salmonella_enterica_subsp._enterica	AAL03_01510	52372..56013	59201	BREX-system-P-loop-protein-BrxC-[Salmonella-enterica-subsp.-enterica]
EAB9374475.1	Type1.BREX	<-REase+iREase<-?||?->||YjhX_toxin->SAM-METHYLASE->?->?->BrxA-DNA-binding->BrxB-BrxF-iSTAND->DUF6079*->N6-METHYLASE->OLD-ABC->TOPRIM->PglZ->SIGMA+OB-Fold+AAA-ATPase+Lon-Protease-BrxL-><-HIN-HTH||?->DNA_METHYLASE->MORC+HISKIN->	1213	Bacteria>Pseudomonadota>Gammaproteobacteria>Enterobacterales>Enterobacteriaceae>Salmonella	GCA_004270415.1	Salmonella_enterica_subsp._enterica	D7N03_15200	60704..64345	59201	BREX-system-P-loop-protein-BrxC-[Salmonella-enterica-subsp.-enterica]
EAC1009595.1	Type1.BREX	YjhX_toxin->SAM-METHYLASE-><-?||?->?->BrxA-DNA-binding->BrxB-BrxF-iSTAND->DUF6079*->N6-METHYLASE->OLD-ABC->TOPRIM->PglZ->SIGMA+OB-Fold+AAA-ATPase+Lon-Protease-BrxL->?-><-REase<-NACHT+SF1-SF2-Helicase+HKD-DNase<-Arm-DNA-bind_3	1213	Bacteria>Pseudomonadota>Gammaproteobacteria>Enterobacterales>Enterobacteriaceae>Salmonella	GCA_004275715.1	Salmonella_enterica_subsp._enterica	YR14_13800	150440..154081	59201	BREX-system-P-loop-protein-BrxC-[Salmonella-enterica-subsp.-enterica]
EAC1236045.1	Type1.BREX	<-REase+iREase<-?||?->||YjhX_toxin->SAM-METHYLASE->?->?->BrxA-DNA-binding->BrxB-BrxF-iSTAND->DUF6079*->N6-METHYLASE->OLD-ABC->TOPRIM->PglZ->SIGMA+OB-Fold+AAA-ATPase+Lon-Protease-BrxL-><-REase<-Couple_hipA+HipA_C<-HTH_3	1213	Bacteria>Pseudomonadota>Gammaproteobacteria>Enterobacterales>Enterobacteriaceae>Salmonella	GCA_004276135.1	Salmonella_enterica_subsp._enterica	DTG84_00810	157931..161572	59201	BREX-system-P-loop-protein-BrxC-[Salmonella-enterica-subsp.-enterica]
EAM1956840.1	Type1.BREX	<-HEPN<-REase+iREase<-?||||YjhX_toxin->?->?->BrxA-DNA-binding->BrxB-BrxF-iSTAND->DUF6079*->N6-METHYLASE->	1213	Bacteria>Pseudomonadota>Gammaproteobacteria>Enterobacterales>Enterobacteriaceae>Salmonella	GCA_005406505.1	Salmonella_enterica	D7U14_11535	170827..174468	28901	BREX-system-P-loop-protein-BrxC-[Salmonella-enterica]
EAM3013482.1	Type1.BREX	<-HEPN<-?||?->||YjhX_toxin->SAM-METHYLASE->?->?->BrxA-DNA-binding->BrxB-BrxF-iSTAND->DUF6079*->N6-METHYLASE->OLD-ABC->TOPRIM->PglZ->SIGMA+OB-Fold+AAA-ATPase+Lon-Protease-BrxL->HKD-DNase+OGG-like-><-REase<-NACHT+SF1-SF2-Helicase+HKD-DNase	1213	Bacteria>Pseudomonadota>Gammaproteobacteria>Enterobacterales>Enterobacteriaceae>Salmonella	GCA_005412095.1	Salmonella_enterica	CBJ93_01865	394707..398348	28901	BREX-system-P-loop-protein-BrxC-[Salmonella-enterica]
EAM3141100.1	Type1.BREX	Arm-DNA-bind_3->NACHT+SF1-SF2-Helicase+HKD-DNase->REase-><-HKD-DNase+OGG-like<-SIGMA+OB-Fold+AAA-ATPase+Lon-Protease-BrxL<-PglZ<-OLD-ABC<-N6-METHYLASE<-DUF6079*<-BrxB-BrxF-iSTAND<-BrxA-DNA-binding<-?||wHTH+SAD+HNH->SIGMA-HTH+REase->MarR-wHTH-><-wHTH+WYL+WCX||wHTH->MukE->	1204	Bacteria>Pseudomonadota>Gammaproteobacteria>Enterobacterales>Enterobacteriaceae>Salmonella	GCA_005412435.1	Salmonella_enterica	DMT78_01470	307802..311416	28901	BREX-system-P-loop-protein-BrxC-[Salmonella-enterica]
EAM5567470.1	Type1.BREX	<-SIGMA+OB-Fold+AAA-ATPase+Lon-Protease-BrxL<-PglZ<-TOPRIM<-OLD-ABC<-N6-METHYLASE<-DUF6079*<-BrxB-BrxF-iSTAND<-BrxA-DNA-binding	1213	Bacteria>Pseudomonadota>Gammaproteobacteria>Enterobacterales>Enterobacteriaceae>Salmonella	GCA_005423155.1	Salmonella_enterica	EK556_21780	11069..14710	28901	BREX-system-P-loop-protein-BrxC-[Salmonella-enterica]
EAM6081349.1	Type1.BREX	Arm-DNA-bind_3->NACHT+SF1-SF2-Helicase+HKD-DNase->REase-><-?<-SIGMA+OB-Fold+AAA-ATPase+Lon-Protease-BrxL<-PglZ<-TOPRIM<-OLD-ABC<-N6-METHYLASE<-DUF6079*<-BrxB-BrxF-iSTAND<-BrxA-DNA-binding<-?<-SAM-METHYLASE<-YjhX_toxin||?->HEPN->	1213	Bacteria>Pseudomonadota>Gammaproteobacteria>Enterobacterales>Enterobacteriaceae>Salmonella	GCA_005424535.1	Salmonella_enterica	ERR94_15350	17401..21042	28901	BREX-system-P-loop-protein-BrxC-[Salmonella-enterica]
EAM5877058.1	Type1.BREX	Arm-DNA-bind_3->NACHT+SF1-SF2-Helicase+HKD-DNase->REase-><-SIGMA+OB-Fold+AAA-ATPase+Lon-Protease-BrxL<-PglZ<-TOPRIM<-OLD-ABC<-N6-METHYLASE<-DUF6079*<-BrxB-BrxF-iSTAND<-BrxA-DNA-binding<-?<-SAM-METHYLASE<-YjhX_toxin||?->REase+iREase->HEPN->	1213	Bacteria>Pseudomonadota>Gammaproteobacteria>Enterobacterales>Enterobacteriaceae>Salmonella	GCA_005424775.1	Salmonella_enterica	EPF42_07745	346357..349998	28901	BREX-system-P-loop-protein-BrxC-[Salmonella-enterica]
EAM9976205.1	Type1.BREX	<-HEPN<-REase+iREase<-?||YjhX_toxin->SAM-METHYLASE->?->BrxA-DNA-binding->BrxB-BrxF-iSTAND->DUF6079*->N6-METHYLASE-p->	1213	Bacteria>Pseudomonadota>Gammaproteobacteria>Enterobacterales>Enterobacteriaceae>Salmonella	GCA_005442735.1	Salmonella_enterica	EU643_14555	35351..38992	28901	BREX-system-P-loop-protein-BrxC-[Salmonella-enterica]
EAN1187097.1	Type1.BREX	<-HEPN<-REase+iREase<-?||||YjhX_toxin->SAM-METHYLASE->?->?->BrxA-DNA-binding->BrxB-BrxF-iSTAND->DUF6079-p*->	797	Bacteria>Pseudomonadota>Gammaproteobacteria>Enterobacterales>Enterobacteriaceae>Salmonella	GCA_005448045.1	Salmonella_enterica	D7G02_16655	60001..62392	28901	BREX-system-P-loop-protein-BrxC-[Salmonella-enterica]
EAN9691870.1	Type1.BREX	<-HEPN<-?||?->YjhX_toxin->SAM-METHYLASE->?->BrxA-DNA-binding->BrxB-BrxF-iSTAND->DUF6079*->N6-METHYLASE->	1213	Bacteria>Pseudomonadota>Gammaproteobacteria>Enterobacterales>Enterobacteriaceae>Salmonella	GCA_005483095.1	Salmonella_enterica	ETI19_18710	73016..76657	28901	BREX-system-P-loop-protein-BrxC-[Salmonella-enterica]
EAO1947720.1	Type1.BREX	<-HEPN<-REase+iREase<-?||YjhX_toxin->SAM-METHYLASE->?->BrxA-DNA-binding->BrxB-BrxF-iSTAND->DUF6079*->N6-METHYLASE->OLD-ABC->TOPRIM->PglZ->SIGMA+OB-Fold+AAA-ATPase+Lon-Protease-BrxL-><-REase<-NACHT+SF1-SF2-Helicase+HKD-DNase<-Arm-DNA-bind_3	1213	Bacteria>Pseudomonadota>Gammaproteobacteria>Enterobacterales>Enterobacteriaceae>Salmonella	GCA_005492965.1	Salmonella_enterica	E2X64_08235	25607..29248	28901	BREX-system-P-loop-protein-BrxC-[Salmonella-enterica]
EAO2684614.1	Type1.BREX	<-GntR-HTH++TrmB+ZDNA-HTH||<-?<-HEPN<-?||YjhX_toxin->SAM-METHYLASE->?->||BrxA-DNA-binding->BrxB-BrxF-iSTAND->DUF6079*->N6-METHYLASE->OLD-ABC->TOPRIM->PglZ->SIGMA+OB-Fold+AAA-ATPase+Lon-Protease-BrxL-><-tRNA||?-><-SKI||METHYLASE->	1213	Bacteria>Pseudomonadota>Gammaproteobacteria>Enterobacterales>Enterobacteriaceae>Salmonella	GCA_005496785.1	Salmonella_enterica	E2X65_05650	67584..71225	28901	BREX-system-P-loop-protein-BrxC-[Salmonella-enterica]
EAO4363249.1	Type1.BREX	<-HEPN<-REase+iREase<-?||?->YjhX_toxin->SAM-METHYLASE->?->||BrxA-DNA-binding->BrxB-BrxF-iSTAND->DUF6079*->N6-METHYLASE->OLD-ABC->TOPRIM->PglZ->SIGMA+OB-Fold+AAA-ATPase+Lon-Protease-BrxL->||HIN-HTH->	1213	Bacteria>Pseudomonadota>Gammaproteobacteria>Enterobacterales>Enterobacteriaceae>Salmonella	GCA_005505735.1	Salmonella_enterica	E5197_09130	175421..179062	28901	BREX-system-P-loop-protein-BrxC-[Salmonella-enterica]
EAO8797691.1	Type1.BREX	<-HEPN<-REase+iREase<-?||YjhX_toxin->SAM-METHYLASE->?->BrxA-DNA-binding->BrxB-BrxF-iSTAND->DUF6079*->N6-METHYLASE->OLD-ABC->TOPRIM->PglZ->SIGMA+OB-Fold+AAA-ATPase+Lon-Protease-BrxL-><-REase<-NACHT+SF1-SF2-Helicase+HKD-DNase<-Arm-DNA-bind_3	1213	Bacteria>Pseudomonadota>Gammaproteobacteria>Enterobacterales>Enterobacteriaceae>Salmonella	GCA_005530015.1	Salmonella_enterica	E0391_14535	25872..29513	28901	BREX-system-P-loop-protein-BrxC-[Salmonella-enterica]
EGG4120336.1	Type1.BREX	<-HEPN<-REase+iREase<-?||YjhX_toxin->SAM-METHYLASE->?->BrxA-DNA-binding->BrxB-BrxF-iSTAND->DUF6079*->N6-METHYLASE->OLD-ABC->TOPRIM->PglZ->SIGMA+OB-Fold+AAA-ATPase+Lon-Protease-BrxL-><-REase<-Couple_hipA+HipA_C<-HTH_3	1213	Bacteria>Pseudomonadota>Gammaproteobacteria>Enterobacterales>Enterobacteriaceae>Salmonella	GCA_005532775.2	Salmonella_enterica	SI35_20740	157325..160966	28901	BREX-system-P-loop-protein-BrxC-[Salmonella-enterica]
EAP0128501.1	Type1.BREX	YjhX_toxin->SAM-METHYLASE-><-?||?->?->BrxA-DNA-binding->BrxB-BrxF-iSTAND->DUF6079*->N6-METHYLASE->OLD-ABC->TOPRIM->PglZ->SIGMA+OB-Fold+AAA-ATPase+Lon-Protease-BrxL->?-><-REase<-NACHT+SF1-SF2-Helicase+HKD-DNase	1213	Bacteria>Pseudomonadota>Gammaproteobacteria>Enterobacterales>Enterobacteriaceae>Salmonella	GCA_005535515.1	Salmonella_enterica	DKP73_00815	161390..165031	28901	BREX-system-P-loop-protein-BrxC-[Salmonella-enterica]
EAP0644643.1	Type1.BREX	Arm-DNA-bind_3->NACHT+SF1-SF2-Helicase+HKD-DNase->REase-><-SIGMA+OB-Fold+AAA-ATPase+Lon-Protease-BrxL<-PglZ<-TOPRIM<-OLD-ABC<-N6-METHYLASE<-DUF6079*<-BrxB-BrxF-iSTAND<-BrxA-DNA-binding<-?<-?<-SAM-METHYLASE<-YjhX_toxin||||?->REase+iREase->HEPN->	1213	Bacteria>Pseudomonadota>Gammaproteobacteria>Enterobacterales>Enterobacteriaceae>Salmonella	GCA_005538295.1	Salmonella_enterica	C6277_11605	108063..111704	28901	BREX-system-P-loop-protein-BrxC-[Salmonella-enterica]
EAP5601609.1	Type1.BREX	wHTH+WYL+WCX-><-MarR-wHTH<-SIGMA-HTH+REase<-wHTH+SAD+HNH||?-><-?<-ZnR||BrxA-DNA-binding->BrxB-BrxF-iSTAND->DUF6079*->N6-METHYLASE->	1204	Bacteria>Pseudomonadota>Gammaproteobacteria>Enterobacterales>Enterobacteriaceae>Salmonella	GCA_005559495.1	Salmonella_enterica	EHT80_06400	135199..138813	28901	BREX-system-P-loop-protein-BrxC-[Salmonella-enterica]
EAP9950624.1	Type1.BREX	XIS-HTH->?->?-><-ZnR||?->BrxA-DNA-binding->BrxB-BrxF-iSTAND->DUF6079*->N6-METHYLASE->OLD-ABC->TOPRIM->PglZ->SIGMA+OB-Fold+AAA-ATPase+Lon-Protease-BrxL->	1204	Bacteria>Pseudomonadota>Gammaproteobacteria>Enterobacterales>Enterobacteriaceae>Salmonella	GCA_005576875.1	Salmonella_enterica	D3G27_06335	176089..179703	28901	BREX-system-P-loop-protein-BrxC-[Salmonella-enterica]
EAQ0497027.1	Type1.BREX	<-HEPN<-REase+iREase<-?||YjhX_toxin->SAM-METHYLASE->?->BrxA-DNA-binding->BrxB-BrxF-iSTAND->DUF6079*->N6-METHYLASE->OLD-ABC->TOPRIM->PglZ->SIGMA+OB-Fold+AAA-ATPase+Lon-Protease-BrxL-><-REase<-NACHT+SF1-SF2-Helicase+HKD-DNase<-Arm-DNA-bind_3	1213	Bacteria>Pseudomonadota>Gammaproteobacteria>Enterobacterales>Enterobacteriaceae>Salmonella	GCA_005582475.1	Salmonella_enterica	EW683_07755	36904..40545	28901	BREX-system-P-loop-protein-BrxC-[Salmonella-enterica]
EAQ5906792.1	Type1.BREX	<-HEPN<-REase+iREase<-?||YjhX_toxin->SAM-METHYLASE->?->BrxA-DNA-binding->BrxB-BrxF-iSTAND->DUF6079*->N6-METHYLASE->	1213	Bacteria>Pseudomonadota>Gammaproteobacteria>Enterobacterales>Enterobacteriaceae>Salmonella	GCA_005605705.1	Salmonella_enterica	FEF66_01945	118005..121646	28901	BREX-system-P-loop-protein-BrxC-[Salmonella-enterica]
EAQ6130543.1	Type1.BREX	<-HEPN<-?||||YjhX_toxin->SAM-METHYLASE-><-?||?->?->BrxA-DNA-binding->BrxB-BrxF-iSTAND->DUF6079*->N6-METHYLASE->OLD-ABC->TOPRIM->PglZ->SIGMA+OB-Fold+AAA-ATPase+Lon-Protease-BrxL->?-><-REase<-NACHT+SF1-SF2-Helicase+HKD-DNase	1213	Bacteria>Pseudomonadota>Gammaproteobacteria>Enterobacterales>Enterobacteriaceae>Salmonella	GCA_005607075.1	Salmonella_enterica	AXA91_18730	62340..65981	28901	BREX-system-P-loop-protein-BrxC-[Salmonella-enterica]
EAQ7293575.1	Type1.BREX	YjhX_toxin->SAM-METHYLASE-><-?||?->?->BrxA-DNA-binding->BrxB-BrxF-iSTAND->DUF6079*->N6-METHYLASE->OLD-ABC->TOPRIM->PglZ->SIGMA+OB-Fold+AAA-ATPase+Lon-Protease-BrxL-><-REase<-Couple_hipA+HipA_C<-HTH_3	1213	Bacteria>Pseudomonadota>Gammaproteobacteria>Enterobacterales>Enterobacteriaceae>Salmonella	GCA_005612115.1	Salmonella_enterica	CRQ46_08445	406739..410380	28901	BREX-system-P-loop-protein-BrxC-[Salmonella-enterica]
EAS1883622.1	Type1.BREX	<-REase+iREase<-?||||YjhX_toxin->SAM-METHYLASE-><-?||?->?->BrxA-DNA-binding->BrxB-BrxF-iSTAND->DUF6079*->N6-METHYLASE->PglZ->SIGMA+OB-Fold+AAA-ATPase+Lon-Protease-BrxL-><-REase<-Couple_hipA+HipA_C<-HTH_3	1213	Bacteria>Pseudomonadota>Gammaproteobacteria>Enterobacterales>Enterobacteriaceae>Salmonella	GCA_005675995.1	Salmonella_enterica	AIH17_08775	402397..406038	28901	BREX-system-P-loop-protein-BrxC-[Salmonella-enterica]
EAS9365410.1	Type1.BREX	<-HEPN<-REase+iREase<-?||YjhX_toxin->SAM-METHYLASE->?->BrxA-DNA-binding->BrxB-BrxF-iSTAND->DUF6079*->N6-METHYLASE->OLD-ABC->TOPRIM->PglZ->SIGMA+OB-Fold+AAA-ATPase+Lon-Protease-BrxL-><-REase<-NACHT+SF1-SF2-Helicase+HKD-DNase<-Arm-DNA-bind_3	1213	Bacteria>Pseudomonadota>Gammaproteobacteria>Enterobacterales>Enterobacteriaceae>Salmonella	GCA_005712675.1	Salmonella_enterica	EPA30_12270	170842..174483	28901	BREX-system-P-loop-protein-BrxC-[Salmonella-enterica]
EAT1838141.1	Type1.BREX	<-HEPN<-REase+iREase<-?||YjhX_toxin->SAM-METHYLASE->?->BrxA-DNA-binding->BrxB-BrxF-iSTAND->DUF6079*->N6-METHYLASE->OLD-ABC->TOPRIM->PglZ->SIGMA+OB-Fold+AAA-ATPase+Lon-Protease-BrxL-><-REase<-NACHT+SF1-SF2-Helicase+HKD-DNase<-Arm-DNA-bind_3	1213	Bacteria>Pseudomonadota>Gammaproteobacteria>Enterobacterales>Enterobacteriaceae>Salmonella	GCA_005717435.1	Salmonella_enterica	EJR21_08315	102985..106626	28901	BREX-system-P-loop-protein-BrxC-[Salmonella-enterica]
EAT1639063.1	Type1.BREX	<-HEPN<-?||?->YjhX_toxin->SAM-METHYLASE->?->BrxA-DNA-binding->BrxB-BrxF-iSTAND->DUF6079*->N6-METHYLASE->OLD-ABC->TOPRIM->PglZ->SIGMA+OB-Fold+AAA-ATPase+Lon-Protease-BrxL->HKD-DNase+OGG-like-><-REase<-NACHT+SF1-SF2-Helicase+HKD-DNase	1213	Bacteria>Pseudomonadota>Gammaproteobacteria>Enterobacterales>Enterobacteriaceae>Salmonella	GCA_005720135.1	Salmonella_enterica	EKF56_11740	25006..28647	28901	BREX-system-P-loop-protein-BrxC-[Salmonella-enterica]
EAT2430707.1	Type1.BREX	<-HEPN<-REase+iREase<-?||YjhX_toxin->SAM-METHYLASE->?->BrxA-DNA-binding->BrxB-BrxF-iSTAND->DUF6079*->N6-METHYLASE->OLD-ABC->TOPRIM->PglZ->SIGMA+OB-Fold+AAA-ATPase+Lon-Protease-BrxL->HKD-DNase+OGG-like-><-REase<-NACHT+SF1-SF2-Helicase+HKD-DNase<-Arm-DNA-bind_3	1213	Bacteria>Pseudomonadota>Gammaproteobacteria>Enterobacterales>Enterobacteriaceae>Salmonella	GCA_005726115.1	Salmonella_enterica	EI207_04325	177142..180783	28901	BREX-system-P-loop-protein-BrxC-[Salmonella-enterica]
EAU0116267.1	Type1.BREX	YjhX_toxin->SAM-METHYLASE-><-?||?->?->BrxA-DNA-binding->BrxB-BrxF-iSTAND->DUF6079*->N6-METHYLASE->OLD-ABC->TOPRIM->PglZ->SIGMA+OB-Fold+AAA-ATPase+Lon-Protease-BrxL->?-><-REase<-NACHT+SF1-SF2-Helicase+HKD-DNase<-Arm-DNA-bind_3	1213	Bacteria>Pseudomonadota>Gammaproteobacteria>Enterobacterales>Enterobacteriaceae>Salmonella	GCA_005756415.1	Salmonella_enterica	ASR58_09430	25917..29558	28901	BREX-system-P-loop-protein-BrxC-[Salmonella-enterica]
EAU0152691.1	Type1.BREX	YjhX_toxin->SAM-METHYLASE-><-?||?->?->BrxA-DNA-binding->BrxB-BrxF-iSTAND->DUF6079*->N6-METHYLASE->OLD-ABC->TOPRIM->PglZ->SIGMA+OB-Fold+AAA-ATPase+Lon-Protease-BrxL->?-><-REase<-NACHT+SF1-SF2-Helicase+HKD-DNase<-Arm-DNA-bind_3	1213	Bacteria>Pseudomonadota>Gammaproteobacteria>Enterobacterales>Enterobacteriaceae>Salmonella	GCA_005756695.1	Salmonella_enterica	AZG01_01865	390716..394357	28901	BREX-system-P-loop-protein-BrxC-[Salmonella-enterica]
EAU0181515.1	Type1.BREX	Glyoxalase->ACET->||YjhX_toxin->ACET->METHYLASE->ZnR->Glyoxalase->BrxA-DNA-binding->BrxB-BrxF-iSTAND->DUF6079*->ParB+HNH->N6-METHYLASE->PglZ->SIGMA+OB-Fold+AAA-ATPase+Lon-Protease-BrxL-><-Arm-DNA-bind_3+Phage_integrase<-tRNA||?-><-SKI||METHYLASE->	1233	Bacteria>Pseudomonadota>Gammaproteobacteria>Enterobacterales>Enterobacteriaceae>Salmonella	GCA_005756755.1	Salmonella_enterica	A4I02_04465	211497..215198	28901	BREX-system-P-loop-protein-BrxC-[Salmonella-enterica]
EAV4850774.1	Type1.BREX	Response_reg->NACHT+SF1-SF2-Helicase+HKD-DNase->REase-><-?<-SIGMA+OB-Fold+AAA-ATPase+Lon-Protease-BrxL<-PglZ<-TOPRIM<-OLD-ABC<-N6-METHYLASE<-DUF6079*<-BrxB-BrxF-iSTAND<-BrxA-DNA-binding||<-?<-SAM-METHYLASE<-YjhX_toxin||?->REase+iREase->HEPN->	1213	Bacteria>Pseudomonadota>Gammaproteobacteria>Enterobacterales>Enterobacteriaceae>Salmonella	GCA_005847515.1	Salmonella_enterica	EGE73_10390	37027..40668	28901	BREX-system-P-loop-protein-BrxC-[Salmonella-enterica]
EAW0893908.1	Type1.BREX	<-HEPN<-?||?->||YjhX_toxin->SAM-METHYLASE->?->BrxA-DNA-binding->BrxB-BrxF-iSTAND->DUF6079*->N6-METHYLASE->OLD-ABC->TOPRIM->PglZ->SIGMA+OB-Fold+AAA-ATPase+Lon-Protease-BrxL->?-><-REase<-NACHT+SF1-SF2-Helicase+HKD-DNase<-Arm-DNA-bind_3	1213	Bacteria>Pseudomonadota>Gammaproteobacteria>Enterobacterales>Enterobacteriaceae>Salmonella	GCA_005897045.1	Salmonella_enterica	EA086_11660	157064..160705	28901	BREX-system-P-loop-protein-BrxC-[Salmonella-enterica]
EAW3206019.1	Type1.BREX	Arm-DNA-bind_3->NACHT+SF1-SF2-Helicase+HKD-DNase->REase-><-HKD-DNase+OGG-like<-SIGMA+OB-Fold+AAA-ATPase+Lon-Protease-BrxL<-PglZ<-TOPRIM<-OLD-ABC<-N6-METHYLASE<-DUF6079*<-BrxB-BrxF-iSTAND<-BrxA-DNA-binding||ZnR->ZnR->?-><-?||SIGMA-HTH+REase->?->MarR-wHTH->	1204	Bacteria>Pseudomonadota>Gammaproteobacteria>Enterobacterales>Enterobacteriaceae>Salmonella	GCA_005905825.1	Salmonella_enterica	FEL99_04750	232863..236477	28901	BREX-system-P-loop-protein-BrxC-[Salmonella-enterica]
EAW5830197.1	Type1.BREX	<-HEPN<-REase+iREase<-?||YjhX_toxin->SAM-METHYLASE->?->BrxA-DNA-binding->BrxB-BrxF-iSTAND->DUF6079*->N6-METHYLASE->OLD-ABC->TOPRIM->PglZ->SIGMA+OB-Fold+AAA-ATPase+Lon-Protease-BrxL-><-REase<-Arm-DNA-bind_3<-tRNA||?-><-SKI	1213	Bacteria>Pseudomonadota>Gammaproteobacteria>Enterobacterales>Enterobacteriaceae>Salmonella	GCA_005917905.1	Salmonella_enterica	FF074_06105	170712..174353	28901	BREX-system-P-loop-protein-BrxC-[Salmonella-enterica]
EAW8145855.1	Type1.BREX	<-HEPN<-?||YjhX_toxin->SAM-METHYLASE->?->BrxA-DNA-binding->BrxB-BrxF-iSTAND->DUF6079*->N6-METHYLASE->OLD-ABC->TOPRIM->PglZ->SIGMA+OB-Fold+AAA-ATPase+Lon-Protease-BrxL->?->||<-tRNA||?-><-SKI||METHYLASE->	1213	Bacteria>Pseudomonadota>Gammaproteobacteria>Enterobacterales>Enterobacteriaceae>Salmonella	GCA_005934015.1	Salmonella_enterica	HM10_03825	163517..167158	28901	BREX-system-P-loop-protein-BrxC-[Salmonella-enterica]
EAW9396387.1	Type1.BREX	<-wHTH||wHTH+WYL+WCX-><-MarR-wHTH<-SIGMA-HTH+REase<-wHTH+SAD+HNH||?-><-ZnR||BrxA-DNA-binding->BrxB-BrxF-iSTAND->DUF6079*->N6-METHYLASE->OLD-ABC->TOPRIM->PglZ->SIGMA+OB-Fold+AAA-ATPase+Lon-Protease-BrxL->HKD-DNase+OGG-like-><-REase<-NACHT+SF1-SF2-Helicase+HKD-DNase<-Arm-DNA-bind_3	1204	Bacteria>Pseudomonadota>Gammaproteobacteria>Enterobacterales>Enterobacteriaceae>Salmonella	GCA_005940865.1	Salmonella_enterica	QE38_19735	48409..52023	28901	BREX-system-P-loop-protein-BrxC-[Salmonella-enterica]
EAW9404815.1	Type1.BREX	NACHT+SF1-SF2-Helicase+HKD-DNase->REase-><-?<-SIGMA+OB-Fold+AAA-ATPase+Lon-Protease-BrxL<-PglZ<-TOPRIM<-OLD-ABC<-N6-METHYLASE<-DUF6079*<-BrxB-BrxF-iSTAND<-BrxA-DNA-binding<-?<-SAM-METHYLASE<-YjhX_toxin||?->HEPN->	1213	Bacteria>Pseudomonadota>Gammaproteobacteria>Enterobacterales>Enterobacteriaceae>Salmonella	GCA_005940875.1	Salmonella_enterica	QD58_16790	37524..41165	28901	BREX-system-P-loop-protein-BrxC-[Salmonella-enterica]
EAX4456710.1	Type1.BREX	<-HEPN<-REase+iREase<-?||YjhX_toxin->SAM-METHYLASE->?->BrxA-DNA-binding->BrxB-BrxF-iSTAND->DUF6079*->N6-METHYLASE->OLD-ABC->TOPRIM->PglZ->SIGMA+OB-Fold+AAA-ATPase+Lon-Protease-BrxL-><-REase<-NACHT+SF1-SF2-Helicase+HKD-DNase<-Arm-DNA-bind_3	1213	Bacteria>Pseudomonadota>Gammaproteobacteria>Enterobacterales>Enterobacteriaceae>Salmonella	GCA_005963445.1	Salmonella_enterica	AC467_14820	18038..21679	28901	BREX-system-P-loop-protein-BrxC-[Salmonella-enterica]
EAX8471827.1	Type1.BREX	<-GntR-HTH++TrmB+ZDNA-HTH||<-?<-HEPN<-?||YjhX_toxin->SAM-METHYLASE->?->BrxA-DNA-binding->BrxB-BrxF-iSTAND->DUF6079*->N6-METHYLASE->OLD-ABC->TOPRIM->PglZ->SIGMA+OB-Fold+AAA-ATPase+Lon-Protease-BrxL->	1213	Bacteria>Pseudomonadota>Gammaproteobacteria>Enterobacterales>Enterobacteriaceae>Salmonella	GCA_005980785.1	Salmonella_enterica	AH421_04140	246530..250171	28901	BREX-system-P-loop-protein-BrxC-[Salmonella-enterica]
EAY3206374.1	Type1.BREX	<-HEPN<-?||YjhX_toxin->SAM-METHYLASE->?->BrxA-DNA-binding->BrxB-BrxF-iSTAND->DUF6079*->N6-METHYLASE->	1213	Bacteria>Pseudomonadota>Gammaproteobacteria>Enterobacterales>Enterobacteriaceae>Salmonella	GCA_006001705.1	Salmonella_enterica	EAO91_18695	11389..15030	28901	BREX-system-P-loop-protein-BrxC-[Salmonella-enterica]
EAY5330993.1	Type1.BREX	ACET->METHYLASE->ZnR->Glyoxalase->BrxA-DNA-binding->BrxB-BrxF-iSTAND->DUF6079*->ParB+HNH->N6-METHYLASE->PglZ->SIGMA+OB-Fold+AAA-ATPase+Lon-Protease-BrxL-><-Arm-DNA-bind_3+Phage_integrase<-tRNA||?-><-SKI||METHYLASE->	1233	Bacteria>Pseudomonadota>Gammaproteobacteria>Enterobacterales>Enterobacteriaceae>Salmonella	GCA_006011505.1	Salmonella_enterica	LS33_08700	4073..7774	28901	BREX-system-P-loop-protein-BrxC-[Salmonella-enterica]
EAY5508618.1	Type1.BREX	<-wHTH||wHTH+WYL+WCX-><-MarR-wHTH<-?<-SIGMA-HTH+REase<-wHTH(SBD)+HNH||?->BrxA-DNA-binding->BrxB-BrxF-iSTAND->DUF6079*->N6-METHYLASE->OLD-ABC->TOPRIM->PglZ->SIGMA+OB-Fold+AAA-ATPase+Lon-Protease-BrxL->HKD-DNase+OGG-like-><-REase<-NACHT+SF1-SF2-Helicase+HKD-DNase<-Arm-DNA-bind_3	1204	Bacteria>Pseudomonadota>Gammaproteobacteria>Enterobacterales>Enterobacteriaceae>Salmonella	GCA_006012725.1	Salmonella_enterica	NE47_11015	49392..53006	28901	BREX-system-P-loop-protein-BrxC-[Salmonella-enterica]
EAY8201055.1	Type1.BREX	Glyoxalase->ACET->YjhX_toxin->ACET->METHYLASE->ZnR->Glyoxalase->BrxA-DNA-binding->BrxB-BrxF-iSTAND->DUF6079*->ParB+HNH->N6-METHYLASE->PglZ->SIGMA+OB-Fold+AAA-ATPase+Lon-Protease-BrxL-><-Arm-DNA-bind_3+Phage_integrase<-tRNA||?-><-SKI||METHYLASE->	1233	Bacteria>Pseudomonadota>Gammaproteobacteria>Enterobacterales>Enterobacteriaceae>Salmonella	GCA_006024175.1	Salmonella_enterica	ATQ84_14875	10975..14676	28901	BREX-system-P-loop-protein-BrxC-[Salmonella-enterica]
EAZ0388355.1	Type1.BREX	<-HEPN<-REase+iREase<-?||YjhX_toxin->SAM-METHYLASE->?->BrxA-DNA-binding->BrxB-BrxF-iSTAND->DUF6079*->	1023	Bacteria>Pseudomonadota>Gammaproteobacteria>Enterobacterales>Enterobacteriaceae>Salmonella	GCA_006033355.1	Salmonella_enterica	A7762_09675	30532..33600	28901	BREX-system-P-loop-protein-BrxC-[Salmonella-enterica]
EAZ5773634.1	Type1.BREX	Arm-DNA-bind_3->NACHT+SF1-SF2-Helicase+HKD-DNase->REase-><-SIGMA+OB-Fold+AAA-ATPase+Lon-Protease-BrxL<-PglZ<-TOPRIM<-OLD-ABC<-N6-METHYLASE<-DUF6079*<-BrxB-BrxF-iSTAND<-BrxA-DNA-binding<-?<-SAM-METHYLASE<-YjhX_toxin||?->REase+iREase->HEPN->	1213	Bacteria>Pseudomonadota>Gammaproteobacteria>Enterobacterales>Enterobacteriaceae>Salmonella	GCA_006057755.1	Salmonella_enterica	CCZ51_07205	108064..111705	28901	BREX-system-P-loop-protein-BrxC-[Salmonella-enterica]
EAZ9311961.1	Type1.BREX	<-HEPN<-?||YjhX_toxin->SAM-METHYLASE->?->BrxA-DNA-binding->BrxB-BrxF-iSTAND->DUF6079*->N6-METHYLASE->OLD-ABC->TOPRIM->PglZ->SIGMA+OB-Fold+AAA-ATPase+Lon-Protease-BrxL->HKD-DNase+OGG-like-><-REase<-NACHT+SF1-SF2-Helicase+HKD-DNase<-Arm-DNA-bind_3	1213	Bacteria>Pseudomonadota>Gammaproteobacteria>Enterobacterales>Enterobacteriaceae>Salmonella	GCA_006073635.1	Salmonella_enterica	CBJ28_03025	158675..162316	28901	BREX-system-P-loop-protein-BrxC-[Salmonella-enterica]
EAZ9943132.1	Type1.BREX	<-HEPN<-REase+iREase<-?||YjhX_toxin->SAM-METHYLASE->?->BrxA-DNA-binding->BrxB-BrxF-iSTAND->DUF6079*->N6-METHYLASE->	1213	Bacteria>Pseudomonadota>Gammaproteobacteria>Enterobacterales>Enterobacteriaceae>Salmonella	GCA_006074375.1	Salmonella_enterica_subsp._enterica_serovar_Typhimurium	EP721_03820	139949..143590	90371	BREX-system-P-loop-protein-BrxC-[Salmonella-enterica-subsp.-enterica-serovar-Typhimurium]

Protein-ID	System	Gene-Neighbourhoods	Length	Taxonomy	Assembly-ID	Strain	Locus-Tag	Genomic-coordinates	TaxID	Fasta-define
EBA4497717.1	Type1.BREX	[illegible]	1213	Bacteria>Pseudomonadota>Gammaproteobacteria>Enterobacterales>Enterobacteriaceae>Salmonella	GCA_006097275.1	Salmonella_enterica	A7G53_12020	208894..212535	28901	BREX-system-P-loop-protein-BrxC-[Salmonella-enterica]
EBB0684615.1	Type1.BREX	[illegible]	1213	Bacteria>Pseudomonadota>Gammaproteobacteria>Enterobacterales>Enterobacteriaceae>Salmonella	GCA_006125875.1	Salmonella_enterica	CG859_01610	345657..349298	28901	BREX-system-P-loop-protein-BrxC-[Salmonella-enterica]
EBB1941184.1	Type1.BREX	[illegible]	1213	Bacteria>Pseudomonadota>Gammaproteobacteria>Enterobacterales>Enterobacteriaceae>Salmonella	GCA_006131775.1	Salmonella_enterica	CJP21_07730	38679..42320	28901	BREX-system-P-loop-protein-BrxC-[Salmonella-enterica]
EBB3832829.1	Type1.BREX	[illegible]	1213	Bacteria>Pseudomonadota>Gammaproteobacteria>Enterobacterales>Enterobacteriaceae>Salmonella	GCA_006140415.1	Salmonella_enterica	CJ140_05520	149724..153365	28901	BREX-system-P-loop-protein-BrxC-[Salmonella-enterica]
EBB4495255.1	Type1.BREX	[illegible]	1213	Bacteria>Pseudomonadota>Gammaproteobacteria>Enterobacterales>Enterobacteriaceae>Salmonella	GCA_006143655.1	Salmonella_enterica_subsp._enterica_serovar_Typhimurium	CET45_10230	170918..174559	90371	BREX-system-P-loop-protein-BrxC-[Salmonella-enterica-subsp.-enterica-serovar-Typhimurium]
EBB5938894.1	Type1.BREX	[illegible]	1213	Bacteria>Pseudomonadota>Gammaproteobacteria>Enterobacterales>Enterobacteriaceae>Salmonella	GCA_006151465.1	Salmonella_enterica	CMR97_09925	156823..160464	28901	BREX-system-P-loop-protein-BrxC-[Salmonella-enterica]
EBC0774151.1	Type1.BREX	[illegible]	1213	Bacteria>Pseudomonadota>Gammaproteobacteria>Enterobacterales>Enterobacteriaceae>Salmonella	GCA_006172705.1	Salmonella_enterica	CS696_13045	23540..27181	28901	BREX-system-P-loop-protein-BrxC-[Salmonella-enterica]
EBC4250051.1	Type1.BREX	[illegible]	1213	Bacteria>Pseudomonadota>Gammaproteobacteria>Enterobacterales>Enterobacteriaceae>Salmonella	GCA_006188785.1	Salmonella_enterica	C2B00_07690	213870..217511	28901	BREX-system-P-loop-protein-BrxC-[Salmonella-enterica]
EBD9210544.1	Type1.BREX	[illegible]	1213	Bacteria>Pseudomonadota>Gammaproteobacteria>Enterobacterales>Enterobacteriaceae>Salmonella	GCA_006272795.1	Salmonella_enterica	DA198_05755	187002..190643	28901	BREX-system-P-loop-protein-BrxC-[Salmonella-enterica]
EBE1252189.1	Type1.BREX	[illegible]	1213	Bacteria>Pseudomonadota>Gammaproteobacteria>Enterobacterales>Enterobacteriaceae>Salmonella	GCA_006282055.1	Salmonella_enterica	DDQ39_02640	124417..128058	28901	BREX-system-P-loop-protein-BrxC-[Salmonella-enterica]
EBE3451720.1	Type1.BREX	[illegible]	1011	Bacteria>Pseudomonadota>Gammaproteobacteria>Enterobacterales>Enterobacteriaceae>Salmonella	GCA_006291715.1	Salmonella_enterica	DKG97_14175	60236..63269	28901	BREX-system-P-loop-protein-BrxC-[Salmonella-enterica]
EBE5553332.1	Type1.BREX	[illegible]	1213	Bacteria>Pseudomonadota>Gammaproteobacteria>Enterobacterales>Enterobacteriaceae>Salmonella	GCA_006301715.1	Salmonella_enterica	CP597_01400	309928..313569	28901	BREX-system-P-loop-protein-BrxC-[Salmonella-enterica]
EBE9623448.1	Type1.BREX	[illegible]	1213	Bacteria>Pseudomonadota>Gammaproteobacteria>Enterobacterales>Enterobacteriaceae>Salmonella	GCA_006320265.1	Salmonella_enterica	DA967_03425	170963..174604	28901	BREX-system-P-loop-protein-BrxC-[Salmonella-enterica]
EBF0840956.1	Type1.BREX	[illegible]	1213	Bacteria>Pseudomonadota>Gammaproteobacteria>Enterobacterales>Enterobacteriaceae>Salmonella	GCA_006325425.1	Salmonella_enterica	DDQ86_07950	150304..153945	28901	BREX-system-P-loop-protein-BrxC-[Salmonella-enterica]
EBF3214239.1	Type1.BREX	[illegible]	1233	Bacteria>Pseudomonadota>Gammaproteobacteria>Enterobacterales>Enterobacteriaceae>Salmonella	GCA_006336125.1	Salmonella_enterica	FHK34_03265	183037..186738	28901	BREX-system-P-loop-protein-BrxC-[Salmonella-enterica]
EBF4455779.1	Type1.BREX	[illegible]	1204	Bacteria>Pseudomonadota>Gammaproteobacteria>Enterobacterales>Enterobacteriaceae>Salmonella	GCA_006343165.1	Salmonella_enterica	FH443_09905	412115..415729	28901	BREX-system-P-loop-protein-BrxC-[Salmonella-enterica]
EBF8311517.1	Type1.BREX	[illegible]	1213	Bacteria>Pseudomonadota>Gammaproteobacteria>Enterobacterales>Enterobacteriaceae>Salmonella	GCA_006394955.1	Salmonella_enterica_subsp._enterica	FGE90_18580	53285..56926	59201	BREX-system-P-loop-protein-BrxC-[Salmonella-enterica-subsp.-enterica]
EBG0770112.1	Type1.BREX	[illegible]	1213	Bacteria>Pseudomonadota>Gammaproteobacteria>Enterobacterales>Enterobacteriaceae>Salmonella	GCA_006406395.1	Salmonella_enterica	FH890_08325	3073..6714	28901	BREX-system-P-loop-protein-BrxC-[Salmonella-enterica]
EBG3411773.1	Type1.BREX	[illegible]	1213	Bacteria>Pseudomonadota>Gammaproteobacteria>Enterobacterales>Enterobacteriaceae>Salmonella	GCA_006423935.1	Salmonella_enterica	FIW39_06205	171028..174669	28901	BREX-system-P-loop-protein-BrxC-[Salmonella-enterica]
EBG5655091.1	Type1.BREX	[illegible]	1213	Bacteria>Pseudomonadota>Gammaproteobacteria>Enterobacterales>Enterobacteriaceae>Salmonella	GCA_006430455.1	Salmonella_enterica_subsp._enterica	FI236_11975	108064..111705	59201	BREX-system-P-loop-protein-BrxC-[Salmonella-enterica-subsp.-enterica]
EBG7129666.1	Type1.BREX	[illegible]	1213	Bacteria>Pseudomonadota>Gammaproteobacteria>Enterobacterales>Enterobacteriaceae>Salmonella	GCA_006457905.1	Salmonella_enterica_subsp._enterica	FJA05_19325	25635..29276	59201	BREX-system-P-loop-protein-BrxC-[Salmonella-enterica-subsp.-enterica]
EBH0811989.1	Type1.BREX	[illegible]	1213	Bacteria>Pseudomonadota>Gammaproteobacteria>Enterobacterales>Enterobacteriaceae>Salmonella	GCA_006475165.1	Salmonella_enterica	FJ896_02670	170932..174573	28901	BREX-system-P-loop-protein-BrxC-[Salmonella-enterica]
EBH4970956.1	Type1.BREX	[illegible]	1213	Bacteria>Pseudomonadota>Gammaproteobacteria>Enterobacterales>Enterobacteriaceae>Salmonella	GCA_006499395.1	Salmonella_enterica	CKR35_08925	170347..173988	28901	BREX-system-P-loop-protein-BrxC-[Salmonella-enterica]
EBH5838355.1	Type1.BREX	[illegible]	1204	Bacteria>Pseudomonadota>Gammaproteobacteria>Enterobacterales>Enterobacteriaceae>Salmonella	GCA_006503215.1	Salmonella_enterica	CQL58_06375	305201..308815	28901	BREX-system-P-loop-protein-BrxC-[Salmonella-enterica]
EBI6018519.1	Type1.BREX	[illegible]	1213	Bacteria>Pseudomonadota>Gammaproteobacteria>Enterobacterales>Enterobacteriaceae>Salmonella	GCA_006562665.1	Salmonella_enterica	DW438_24035	31966..35607	28901	BREX-system-P-loop-protein-BrxC-[Salmonella-enterica]
EBI8027930.1	Type1.BREX	[illegible]	1213	Bacteria>Pseudomonadota>Gammaproteobacteria>Enterobacterales>Enterobacteriaceae>Salmonella	GCA_006571625.1	Salmonella_enterica	CHM23_07390	170919..174560	28901	BREX-system-P-loop-protein-BrxC-[Salmonella-enterica]
EBK5803035.1	Type1.BREX	[illegible]	1213	Bacteria>Pseudomonadota>Gammaproteobacteria>Enterobacterales>Enterobacteriaceae>Salmonella	GCA_006648525.1	Salmonella_enterica	DPM88_04065	158403..162044	28901	BREX-system-P-loop-protein-BrxC-[Salmonella-enterica]
EBK7108450.1	Type1.BREX	[illegible]	1213	Bacteria>Pseudomonadota>Gammaproteobacteria>Enterobacterales>Enterobacteriaceae>Salmonella	GCA_006653645.1	Salmonella_enterica	DSC93_06685	170929..174570	28901	BREX-system-P-loop-protein-BrxC-[Salmonella-enterica]
EBK9871839.1	Type1.BREX	[illegible]	1213	Bacteria>Pseudomonadota>Gammaproteobacteria>Enterobacterales>Enterobacteriaceae>Salmonella	GCA_006668805.1	Salmonella_enterica	DYD89_19150	25917..29558	28901	BREX-system-P-loop-protein-BrxC-[Salmonella-enterica]
EBL1260729.1	Type1.BREX	[illegible]	1213	Bacteria>Pseudomonadota>Gammaproteobacteria>Enterobacterales>Enterobacteriaceae>Salmonella	GCA_006672445.1	Salmonella_enterica	DO790_07470	171130..174771	28901	BREX-system-P-loop-protein-BrxC-[Salmonella-enterica]
EBL2857392.1	Type1.BREX	[illegible]	1213	Bacteria>Pseudomonadota>Gammaproteobacteria>Enterobacterales>Enterobacteriaceae>Salmonella	GCA_006679365.1	Salmonella_enterica	D3Q95_12500	132391..136032	28901	BREX-system-P-loop-protein-BrxC-[Salmonella-enterica]
EBL4178053.1	Type1.BREX	[illegible]	1213	Bacteria>Pseudomonadota>Gammaproteobacteria>Enterobacterales>Enterobacteriaceae>Salmonella	GCA_006685525.1	Salmonella_enterica_subsp._enterica_serovar_Typhimurium	E5L78_02825	18164..21805	90371	BREX-system-P-loop-protein-BrxC-[Salmonella-enterica-subsp.-enterica-serovar-Typhimurium]
EBL6294791.1	Type1.BREX	[illegible]	846	Bacteria>Pseudomonadota>Gammaproteobacteria>Enterobacterales>Enterobacteriaceae>Salmonella	GCA_006694585.1	Salmonella_enterica_subsp._enterica_serovar_Typhimurium	AC864_13995	181813..184355	90371	BREX-system-P-loop-protein-BrxC-[Salmonella-enterica-subsp.-enterica-serovar-Typhimurium]
EBL7150924.1	Type1.BREX	[illegible]	1213	Bacteria>Pseudomonadota>Gammaproteobacteria>Enterobacterales>Enterobacteriaceae>Salmonella	GCA_006698245.1	Salmonella_enterica	CFL44_11230	80176..83817	28901	BREX-system-P-loop-protein-BrxC-[Salmonella-enterica]
EBM2234242.1	Type1.BREX	[illegible]	1213	Bacteria>Pseudomonadota>Gammaproteobacteria>Enterobacterales>Enterobacteriaceae>Salmonella	GCA_006721345.1	Salmonella_enterica	DWD21_19365	25859..29500	28901	BREX-system-P-loop-protein-BrxC-[Salmonella-enterica]
EBM6187520.1	Type1.BREX	[illegible]	1213	Bacteria>Pseudomonadota>Gammaproteobacteria>Enterobacterales>Enterobacteriaceae>Salmonella	GCA_006738745.1	Salmonella_enterica	D1943_04230	100576..104217	28901	BREX-system-P-loop-protein-BrxC-[Salmonella-enterica]
EBN5015145.1	Type1.BREX	[illegible]	1213	Bacteria>Pseudomonadota>Gammaproteobacteria>Enterobacterales>Enterobacteriaceae>Salmonella	GCA_006773125.1	Salmonella_enterica	DT736_14590	144280..147921	28901	BREX-system-P-loop-protein-BrxC-[Salmonella-enterica]
EBQ4102575.1	Type1.BREX	[illegible]	1213	Bacteria>Pseudomonadota>Gammaproteobacteria>Enterobacterales>Enterobacteriaceae>Salmonella	GCA_006909045.1	Salmonella_enterica	BFN90_18795	2722..6363	28901	BREX-system-P-loop-protein-BrxC-[Salmonella-enterica]
EBQ6490464.1	Type1.BREX	[illegible]	1213	Bacteria>Pseudomonadota>Gammaproteobacteria>Enterobacterales>Enterobacteriaceae>Salmonella	GCA_006919175.1	Salmonella_enterica	BU548_03115	149047..152688	28901	BREX-system-P-loop-protein-BrxC-[Salmonella-enterica]
EBR0226079.1	Type1.BREX	[illegible]	1204	Bacteria>Pseudomonadota>Gammaproteobacteria>Enterobacterales>Enterobacteriaceae>Salmonella	GCA_006936115.1	Salmonella_enterica_subsp._enterica_serovar_Monschaui	GPN34_002360	410647..414261	29480	BREX-system-P-loop-protein-BrxC-[Salmonella-enterica-subsp.-enterica-serovar-Monschaui]
EBR4438819.1	Type1.BREX	[illegible]	1213	Bacteria>Pseudomonadota>Gammaproteobacteria>Enterobacterales>Enterobacteriaceae>Salmonella	GCA_006955825.1	Salmonella_enterica	BSE18_11505	108064..111705	28901	BREX-system-P-loop-protein-BrxC-[Salmonella-enterica]
EBR5149893.1	Type1.BREX	[illegible]	1204	Bacteria>Pseudomonadota>Gammaproteobacteria>Enterobacterales>Enterobacteriaceae>Salmonella	GCA_006958505.1	Salmonella_enterica	B5W64_09845	350811..354425	28901	BREX-system-P-loop-protein-BrxC-[Salmonella-enterica]
EBR9945161.1	Type1.BREX	[illegible]	1213	Bacteria>Pseudomonadota>Gammaproteobacteria>Enterobacterales>Enterobacteriaceae>Salmonella	GCA_006983125.1	Salmonella_enterica_subsp._enterica_serovar_Krefeld	DTF03_11145	153391..157032	1242084	BREX-system-P-loop-protein-BrxC-[Salmonella-enterica-subsp.-enterica-serovar-Krefeld]
EBS4429082.1	Type1.BREX	[illegible]	1213	Bacteria>Pseudomonadota>Gammaproteobacteria>Enterobacterales>Enterobacteriaceae>Salmonella	GCA_007008305.1	Salmonella_enterica_subsp._enterica_serovar_Poona	DQJ97_07365	25927..29568	436295	BREX-system-P-loop-protein-BrxC-[Salmonella-enterica-subsp.-enterica-serovar-Poona]
EBS7702178.1	Type1.BREX	[illegible]	1213	Bacteria>Pseudomonadota>Gammaproteobacteria>Enterobacterales>Enterobacteriaceae>Salmonella	GCA_007022165.1	Salmonella_enterica	CD603_08310	64360..68001	28901	BREX-system-P-loop-protein-BrxC-[Salmonella-enterica]
EBS7807126.1	Type1.BREX	[illegible]	1233	Bacteria>Pseudomonadota>Gammaproteobacteria>Enterobacterales>Enterobacteriaceae>Salmonella	GCA_007023485.1	Salmonella_enterica	CD664_12540	43837..47538	28901	BREX-system-P-loop-protein-BrxC-[Salmonella-enterica]
EBS9243151.1	Type1.BREX	[illegible]	1213	Bacteria>Pseudomonadota>Gammaproteobacteria>Enterobacterales>Enterobacteriaceae>Salmonella	GCA_007029675.1	Salmonella_enterica	CF713_13020	212136..215777	28901	BREX-system-P-loop-protein-BrxC-[Salmonella-enterica]
EBT4520742.1	Type1.BREX	[illegible]	962	Bacteria>Pseudomonadota>Gammaproteobacteria>Enterobacterales>Enterobacteriaceae>Salmonella	GCA_007056145.1	Salmonella_enterica	CKQ08_17680	108064..110954	28901	BREX-system-P-loop-protein-BrxC-[Salmonella-enterica]
EBU3113248.1	Type1.BREX	[illegible]	778	Bacteria>Pseudomonadota>Gammaproteobacteria>Enterobacterales>Enterobacteriaceae>Salmonella	GCA_007098435.1	Salmonella_enterica_subsp._enterica	CSQ72_21675	1..2335	59201	BREX-system-P-loop-protein-BrxC-[Salmonella-enterica-subsp.-enterica]
EBU3750854.1	Type1.BREX	[illegible]	1213	Bacteria>Pseudomonadota>Gammaproteobacteria>Enterobacterales>Enterobacteriaceae>Salmonella	GCA_007101645.1	Salmonella_enterica	CVX24_10925	25914..29555	28901	BREX-system-P-loop-protein-BrxC-[Salmonella-enterica]
EBU6515329.1	Type1.BREX	[illegible]	1213	Bacteria>Pseudomonadota>Gammaproteobacteria>Enterobacterales>Enterobacteriaceae>Salmonella	GCA_007137465.1	Salmonella_enterica_subsp._enterica_serovar_Typhimurium	DKJ21_01470	4814..8455	90371	BREX-system-P-loop-protein-BrxC-[Salmonella-enterica-subsp.-enterica-serovar-Typhimurium]
EBU6749119.1	Type1.BREX	[illegible]	874	Bacteria>Pseudomonadota>Gammaproteobacteria>Enterobacterales>Enterobacteriaceae>Salmonella	GCA_007137985.1	Salmonella_enterica_subsp._enterica_serovar_Typhimurium	DK355_01035	1..2627	90371	BREX-system-P-loop-protein-BrxC-[Salmonella-enterica-subsp.-enterica-serovar-Typhimurium]
EBU7641088.1	Type1.BREX	[illegible]	1213	Bacteria>Pseudomonadota>Gammaproteobacteria>Enterobacterales>Enterobacteriaceae>Salmonella	GCA_007142065.1	Salmonella_enterica_subsp._enterica_serovar_Brevik	DK709_13715	59395..63036	2564275	BREX-system-P-loop-protein-BrxC-[Salmonella-enterica-subsp.-enterica-serovar-Brevik]
EBU8677660.1	Type1.BREX	[illegible]	1213	Bacteria>Pseudomonadota>Gammaproteobacteria>Enterobacterales>Enterobacteriaceae>Salmonella	GCA_007146445.1	Salmonella_enterica_subsp._enterica_serovar_Parkroyal	DLR30_20715	39960..43601	2564909	BREX-system-P-loop-protein-BrxC-[Salmonella-enterica-subsp.-enterica-serovar-Parkroyal]
EBU9347433.1	Type1.BREX	[illegible]	1204	Bacteria>Pseudomonadota>Gammaproteobacteria>Enterobacterales>Enterobacteriaceae>Salmonella	GCA_007149485.1	Salmonella_enterica_subsp._enterica_serovar_Angoda	DMU95_04690	163241..166855	2564159	BREX-system-P-loop-protein-BrxC-[Salmonella-enterica-subsp.-enterica-serovar-Angoda]
EBU9954738.1	Type1.BREX	[illegible]	1213	Bacteria>Pseudomonadota>Gammaproteobacteria>Enterobacterales>Enterobacteriaceae>Salmonella	GCA_007151145.1	Salmonella_enterica_subsp._enterica_serovar_Tamberma	DSB42_14900	163634..167275	2565079	BREX-system-P-loop-protein-BrxC-[Salmonella-enterica-subsp.-enterica-serovar-Tamberma]
EBU9775078.1	Type1.BREX	[illegible]	1213	Bacteria>Pseudomonadota>Gammaproteobacteria>Enterobacterales>Enterobacteriaceae>Salmonella	GCA_007151525.1	Salmonella_enterica_subsp._enterica_serovar_Kumasi	DM304_21645	3074..6715	2493647	BREX-system-P-loop-protein-BrxC-[Salmonella-enterica-subsp.-enterica-serovar-Kumasi]
EBU9878489.1	Type1.BREX	[illegible]	1213	Bacteria>Pseudomonadota>Gammaproteobacteria>Enterobacterales>Enterobacteriaceae>Salmonella	GCA_007152025.1	Salmonella_enterica_subsp._enterica_serovar_Typhimurium	DM361_04630	60215..63856	90371	BREX-system-P-loop-protein-BrxC-[Salmonella-enterica-subsp.-enterica-serovar-Typhimurium]
EBV0268830.1	Type1.BREX	[illegible]	1213	Bacteria>Pseudomonadota>Gammaproteobacteria>Enterobacterales>Enterobacteriaceae>Salmonella	GCA_007152865.1	Salmonella_enterica_subsp._enterica_serovar_Typhimurium	DNH74_05345	60001..63642	90371	BREX-system-P-loop-protein-BrxC-[Salmonella-enterica-subsp.-enterica-serovar-Typhimurium]
EBV2735692.1	Type1.BREX	[illegible]	1213	Bacteria>Pseudomonadota>Gammaproteobacteria>Enterobacterales>Enterobacteriaceae>Salmonella	GCA_007164145.1	Salmonella_enterica_subsp._enterica_serovar_Stourbridge	DOA65_09445	58944..62585	1160766	BREX-system-P-loop-protein-BrxC-[Salmonella-enterica-subsp.-enterica-serovar-Stourbridge]
EBV4609756.1	Type1.BREX	[illegible]	1213	Bacteria>Pseudomonadota>Gammaproteobacteria>Enterobacterales>Enterobacteriaceae>Salmonella	GCA_007170635.1	Salmonella_enterica_subsp._enterica_serovar_Solt	DOX24_02175	3073..6714	2565040	BREX-system-P-loop-protein-BrxC-[Salmonella-enterica-subsp.-enterica-serovar-Solt]
EBV4672043.1	Type1.BREX	[illegible]	1213	Bacteria>Pseudomonadota>Gammaproteobacteria>Enterobacterales>Enterobacteriaceae>Salmonella	GCA_007170935.1	Salmonella_enterica_subsp._enterica_serovar_Tamberma	DOW22_12520	149175..152816	2565079	BREX-system-P-loop-protein-BrxC-[Salmonella-enterica-subsp.-enterica-serovar-Tamberma]
EBV5450231.1	Type1.BREX	[illegible]	1213	Bacteria>Pseudomonadota>Gammaproteobacteria>Enterobacterales>Enterobacteriaceae>Salmonella	GCA_007175875.1	Salmonella_enterica_subsp._enterica_serovar_Typhimurium	DO810_06270	60219..63860	90371	BREX-system-P-loop-protein-BrxC-[Salmonella-enterica-subsp.-enterica-serovar-Typhimurium]
EBV7110754.1	Type1.BREX	[illegible]	1213	Bacteria>Pseudomonadota>Gammaproteobacteria>Enterobacterales>Enterobacteriaceae>Salmonella	GCA_007180535.1	Salmonella_enterica_subsp._enterica_serovar_Ohio	YY60_02790	154724..158365	117541	BREX-system-P-loop-protein-BrxC-[Salmonella-enterica-subsp.-enterica-serovar-Ohio]
EBW0065987.1	Type1.BREX	[illegible]	1213	Bacteria>Pseudomonadota>Gammaproteobacteria>Enterobacterales>Enterobacteriaceae>Salmonella	GCA_007195055.1	Salmonella_enterica_subsp._enterica_serovar_Typhimurium_var._5-	AR716_20745	18317..21958	1620419	BREX-system-P-loop-protein-BrxC-[Salmonella-enterica-subsp.-enterica-serovar-Typhimurium-var.-5-]
EBW2326418.1	Type1.BREX	[illegible]	1213	Bacteria>Pseudomonadota>Gammaproteobacteria>Enterobacterales>Enterobacteriaceae>Salmonella	GCA_007201245.1	Salmonella_enterica_subsp._enterica_serovar_Agoueve	CBJ63_000885	165096..168737	1505961	BREX-system-P-loop-protein-BrxC-[Salmonella-enterica-subsp.-enterica-serovar-Agoueve]
EBW1953456.1	Type1.BREX	[illegible]	1213	Bacteria>Pseudomonadota>Gammaproteobacteria>Enterobacterales>Enterobacteriaceae>Salmonella	GCA_007201375.1	Salmonella_enterica_subsp._enterica_serovar_Cerro	C3N96_11410	44119..47760	340188	BREX-system-P-loop-protein-BrxC-[Salmonella-enterica-subsp.-enterica-serovar-Cerro]
EBW2731416.1	Type1.BREX	[illegible]	1213	Bacteria>Pseudomonadota>Gammaproteobacteria>Enterobacterales>Enterobacteriaceae>Salmonella	GCA_007205375.1	Salmonella_enterica_subsp._enterica_serovar_Typhimurium	DPA65_05335	170935..174576	90371	BREX-system-P-loop-protein-BrxC-[Salmonella-enterica-subsp.-enterica-serovar-Typhimurium]
EBW3295108.1	Type1.BREX	[illegible]	1213	Bacteria>Pseudomonadota>Gammaproteobacteria>Enterobacterales>Enterobacteriaceae>Salmonella	GCA_007207035.1	Salmonella_enterica_subsp._enterica_serovar_Cerro	DPE09_02360	44118..47759	340188	BREX-system-P-loop-protein-BrxC-[Salmonella-enterica-subsp.-enterica-serovar-Cerro]
EBW4591360.1	Type1.BREX	[illegible]	1144	Bacteria>Pseudomonadota>Gammaproteobacteria>Enterobacterales>Enterobacteriaceae>Salmonella	GCA_007212435.1	Salmonella_enterica_subsp._enterica_serovar_Typhimurium	DPP13_05415	60001..63431	90371	BREX-system-P-loop-protein-BrxC-[Salmonella-enterica-subsp.-enterica-serovar-Typhimurium]
EBW5570928.1	Type1.BREX	[illegible]	1213	Bacteria>Pseudomonadota>Gammaproteobacteria>Enterobacterales>Enterobacteriaceae>Salmonella	GCA_007216555.1	Salmonella_enterica_subsp._enterica_serovar_Emek	DPY54_19345	30061..33702	286784	BREX-system-P-loop-protein-BrxC-[Salmonella-enterica-subsp.-enterica-serovar-Emek]
EBW6592762.1	Type1.BREX	[illegible]	907	Bacteria>Pseudomonadota>Gammaproteobacteria>Enterobacterales>Enterobacteriaceae>Salmonella	GCA_007223235.1	Salmonella_enterica_subsp._enterica_serovar_Kentucky	DP741_24395	20426..23147	192955	BREX-system-P-loop-protein-BrxC-[Salmonella-enterica-subsp.-enterica-serovar-Kentucky]
EBW8181804.1	Type1.BREX	[illegible]	1233	Bacteria>Pseudomonadota>Gammaproteobacteria>Enterobacterales>Enterobacteriaceae>Salmonella	GCA_007227235.1	Salmonella_enterica_subsp._enterica_serovar_Uganda	AR874_12565	406191..409892	487004	BREX-system-P-loop-protein-BrxC-[Salmonella-enterica-subsp.-enterica-serovar-Uganda]
EBW8394988.1	Type1.BREX	[illegible]	1213	Bacteria>Pseudomonadota>Gammaproteobacteria>Enterobacterales>Enterobacteriaceae>Salmonella	GCA_007228915.1	Salmonella_enterica_subsp._enterica_serovar_Florida	A3X38_19915	3074..6715	2564436	BREX-system-P-loop-protein-BrxC-[Salmonella-enterica-subsp.-enterica-serovar-Florida]
EBX0519400.1	Type1.BREX	[illegible]	1213	Bacteria>Pseudomonadota>Gammaproteobacteria>Enterobacterales>Enterobacteriaceae>Salmonella	GCA_007236875.1	Salmonella_enterica_subsp._enterica_serovar_Saintpaul	AHL42_03320	144059..147700	90105	BREX-system-P-loop-protein-BrxC-[Salmonella-enterica-subsp.-enterica-serovar-Saintpaul]
EBX6018293.1	Type1.BREX	[illegible]	1213	Bacteria>Pseudomonadota>Gammaproteobacteria>Enterobacterales>Enterobacteriaceae>Salmonella	GCA_007258805.1	Salmonella_enterica_subsp._enterica_serovar_Dortmund	DSR74_22245	12037..15678	2564368	BREX-system-P-loop-protein-BrxC-[Salmonella-enterica-subsp.-enterica-serovar-Dortmund]
EBX6302551.1	Type1.BREX	[illegible]	1213	Bacteria>Pseudomonadota>Gammaproteobacteria>Enterobacterales>Enterobacteriaceae>Salmonella	GCA_007260765.1	Salmonella_enterica_subsp._enterica_serovar_Enugu	DS191_04270	25805..29446	2564400	BREX-system-P-loop-protein-BrxC-[Salmonella-enterica-subsp.-enterica-serovar-Enugu]
EBX8836172.1	Type1.BREX	[illegible]	1213	Bacteria>Pseudomonadota>Gammaproteobacteria>Enterobacterales>Enterobacteriaceae>Salmonella	GCA_007270555.1	Salmonella_enterica_subsp._enterica_serovar_Urbana	DTU76_05370	52211..55852	260677	BREX-system-P-loop-protein-BrxC-[Salmonella-enterica-subsp.-enterica-serovar-Urbana]
EBY1390424.1	Type1.BREX	[illegible]	1213	Bacteria>Pseudomonadota>Gammaproteobacteria>Enterobacterales>Enterobacteriaceae>Salmonella	GCA_007281935.1	Salmonella_enterica_subsp._enterica_serovar_Wagenia	DUW06_08905	152900..156541	322833	BREX-system-P-loop-protein-BrxC-[Salmonella-enterica-subsp.-enterica-serovar-Wagenia]
EBY1767815.1	Type1.BREX	[illegible]	1213	Bacteria>Pseudomonadota>Gammaproteobacteria>Enterobacterales>Enterobacteriaceae>Salmonella	GCA_007283275.1	Salmonella_enterica_subsp._enterica_serovar_Georgia	DU102_16105	50651..54292	2564467	BREX-system-P-loop-protein-BrxC-[Salmonella-enterica-subsp.-enterica-serovar-Georgia]
EBY2665620.1	Type1.BREX	[illegible]	1213	Bacteria>Pseudomonadota>Gammaproteobacteria>Enterobacterales>Enterobacteriaceae>Salmonella	GCA_007287115.1	Salmonella_enterica_subsp._enterica_serovar_Teko	DVF23_02315	17137..20778	2079198	BREX-system-P-loop-protein-BrxC-[Salmonella-enterica-subsp.-enterica-serovar-Teko]
EBY3147217.1	Type1.BREX	[illegible]	1213	Bacteria>Pseudomonadota>Gammaproteobacteria>Enterobacterales>Enterobacteriaceae>Salmonella	GCA_007287795.1	Salmonella_enterica_subsp._enterica_serovar_Teshie	KZE80_000349	384366..388007	2565098	BREX-system-P-loop-protein-BrxC-[Salmonella-enterica-subsp.-enterica-serovar-Teshie]
EBY3006707.1	Type1.BREX	[illegible]	1213	Bacteria>Pseudomonadota>Gammaproteobacteria>Enterobacterales>Enterobacteriaceae>Salmonella	GCA_007287955.1	Salmonella_enterica_subsp._enterica_serovar_Ekpoui	DVG04_05655	135015..138656	2564392	BREX-system-P-loop-protein-BrxC-[Salmonella-enterica-subsp.-enterica-serovar-Ekpoui]
EBZ6440064.1	Type1.BREX	[illegible]	897	Bacteria>Pseudomonadota>Gammaproteobacteria>Enterobacterales>Enterobacteriaceae>Salmonella	GCA_007344875.1	Salmonella_enterica_subsp._enterica_serovar_Typhimurium	ED482_12665	1..2696	90371	BREX-system-P-loop-protein-BrxC-[Salmonella-enterica-subsp.-enterica-serovar-Typhimurium]
EBZ6491792.1	Type1.BREX	[illegible]	1213	Bacteria>Pseudomonadota>Gammaproteobacteria>Enterobacterales>Enterobacteriaceae>Salmonella	GCA_007345015.1	Salmonella_enterica_subsp._enterica_serovar_Dugbe	ED480_20060	16934..20575	2564376	BREX-system-P-loop-protein-BrxC-[Salmonella-enterica-subsp.-enterica-serovar-Dugbe]
EBZ8208146.1	Type1.BREX	[illegible]	1233	Bacteria>Pseudomonadota>Gammaproteobacteria>Enterobacterales>Enterobacteriaceae>Salmonella	GCA_007351255.1	Salmonella_enterica_subsp._enterica_serovar_Zanzibar	EGK94_05800	411162..414863	399588	BREX-system-P-loop-protein-BrxC-[Salmonella-enterica-subsp.-enterica-serovar-Zanzibar]
EBZ9408825.1	Type1.BREX	[illegible]	1213	Bacteria>Pseudomonadota>Gammaproteobacteria>Enterobacterales>Enterobacteriaceae>Salmonella	GCA_007357915.1	Salmonella_enterica_subsp._enterica_serovar_Saintpaul	EHQ15_16500	82377..86018	90105	BREX-system-P-loop-protein-BrxC-[Salmonella-enterica-subsp.-enterica-serovar-Saintpaul]
ECA8969862.1	Type1.BREX	[illegible]	1213	Bacteria>Pseudomonadota>Gammaproteobacteria>Enterobacterales>Enterobacteriaceae>Salmonella	GCA_007399965.1	Salmonella_enterica_subsp._enterica_serovar_Omuna	ETB55_05725	57828..61469	2564887	BREX-system-P-loop-protein-BrxC-[Salmonella-enterica-subsp.-enterica-serovar-Omuna]
ECB3141175.1	Type1.BREX	[illegible]	876	Bacteria>Pseudomonadota>Gammaproteobacteria>Enterobacterales>Enterobacteriaceae>Salmonella	GCA_007419865.1	Salmonella_enterica_subsp._enterica_serovar_Typhimurium	EWE34_19370	60221..62849	90371	BREX-system-P-loop-protein-BrxC-[Salmonella-enterica-subsp.-enterica-serovar-Typhimurium]
ECB3805264.1	Type1.BREX	[illegible]	1213	Bacteria>Pseudomonadota>Gammaproteobacteria>Enterobacterales>Enterobacteriaceae>Salmonella	GCA_007422765.1	Salmonella_enterica_subsp._enterica_serovar_Fufu	EW367_11360	52142..55783	2564449	BREX-system-P-loop-protein-BrxC-[Salmonella-enterica-subsp.-enterica-serovar-Fufu]
ECB4380457.1	Type1.BREX	[illegible]	685	Bacteria>Pseudomonadota>Gammaproteobacteria>Enterobacterales>Enterobacteriaceae>Salmonella	GCA_007426465.1	Salmonella_enterica_subsp._enterica_serovar_Typhimurium	EXA28_15315	1..2055	90371	BREX-system-P-loop-protein-BrxC-[Salmonella-enterica-subsp.-enterica-serovar-Typhimurium]
ECC0743076.1	Type1.BREX	[illegible]	1213	Bacteria>Pseudomonadota>Gammaproteobacteria>Enterobacterales>Enterobacteriaceae>Salmonella	GCA_007457215.1	Salmonella_enterica_subsp._enterica	FMV06_07395	108064..111705	59201	BREX-system-P-loop-protein-BrxC-[Salmonella-enterica-subsp.-enterica]
ECC1667299.1	Type1.BREX	[illegible]	1213	Bacteria>Pseudomonadota>Gammaproteobacteria>Enterobacterales>Enterobacteriaceae>Salmonella	GCA_007463295.1	Salmonella_enterica_subsp._salamae	FNN95_01255	267827..271468	59202	BREX-system-P-loop-protein-BrxC-[Salmonella-enterica-subsp.-salamae]
ECC3171797.1	Type1.BREX	[illegible]	1213	Bacteria>Pseudomonadota>Gammaproteobacteria>Enterobacterales>Enterobacteriaceae>Salmonella	GCA_007472855.1	Salmonella_enterica_subsp._enterica	R914_02595	77607..81248	59201	BREX-system-P-loop-protein-BrxC-[Salmonella-enterica-subsp.-enterica]
ECC8603900.1	Type1.BREX	[illegible]	987	Bacteria>Pseudomonadota>Gammaproteobacteria>Enterobacterales>Enterobacteriaceae>Salmonella	GCA_007497185.1	Salmonella_enterica_subsp._enterica	DK044_12730	60001..62961	59201	BREX-system-P-loop-protein-BrxC-[Salmonella-enterica-subsp.-enterica]
ECD1705587.1	Type1.BREX	[illegible]	1213	Bacteria>Pseudomonadota>Gammaproteobacteria>Enterobacterales>Enterobacteriaceae>Salmonella	GCA_007509785.1	Salmonella_enterica_subsp._enterica	EWD17_08950	108064..111705	59201	BREX-system-P-loop-protein-BrxC-[Salmonella-enterica-subsp.-enterica]
ECD2821735.1	Type1.BREX	[illegible]	1213	Bacteria>Pseudomonadota>Gammaproteobacteria>Enterobacterales>Enterobacteriaceae>Salmonella	GCA_007513645.1	Salmonella_enterica_subsp._enterica_serovar_Fanti	EXA45_11150	52318..55959	2564418	BREX-system-P-loop-protein-BrxC-[Salmonella-enterica-subsp.-enterica-serovar-Fanti]
ECD3895174.1	Type1.BREX	[illegible]	1213	Bacteria>Pseudomonadota>Gammaproteobacteria>Enterobacterales>Enterobacteriaceae>Salmonella	GCA_007520185.1	Salmonella_enterica_subsp._enterica_serovar_Gloucester	EOU70_05950	164775..168416	2564474	BREX-system-P-loop-protein-BrxC-[Salmonella-enterica-subsp.-enterica-serovar-Gloucester]
ECD4420808.1	Type1.BREX	[illegible]	1233	Bacteria>Pseudomonadota>Gammaproteobacteria>Enterobacterales>Enterobacteriaceae>Salmonella	GCA_007522325.1	Salmonella_enterica_subsp._enterica_serovar_Berkeley	EO910_10430	8115..11816	1965103	BREX-system-P-loop-protein-BrxC-[Salmonella-enterica-subsp.-enterica-serovar-Berkeley]
ECD9608662.1	Type1.BREX	[illegible]	1213	Bacteria>Pseudomonadota>Gammaproteobacteria>Enterobacterales>Enterobacteriaceae>Salmonella	GCA_007542335.1	Salmonella_enterica_subsp._salamae	FNZ18_04560	256517..260158	59202	BREX-system-P-loop-protein-BrxC-[Salmonella-enterica-subsp.-salamae]
ECE0383737.1	Type1.BREX	[illegible]	1213	Bacteria>Pseudomonadota>Gammaproteobacteria>Enterobacterales>Enterobacteriaceae>Salmonella	GCA_007548695.1	Salmonella_enterica_subsp._enterica	XZ45_12010	25704..29345	59201	BREX-system-P-loop-protein-BrxC-[Salmonella-enterica-subsp.-enterica]
ECE6154798.1	Type1.BREX	[illegible]	1213	Bacteria>Pseudomonadota>Gammaproteobacteria>Enterobacterales>Enterobacteriaceae>Salmonella	GCA_007577415.1	Salmonella_enterica_subsp._enterica	DPK65_21905	11265..14906	59201	BREX-system-P-loop-protein-BrxC-[Salmonella-enterica-subsp.-enterica]
ECE6725605.1	Type1.BREX	[illegible]	1226	Bacteria>Pseudomonadota>Gammaproteobacteria>Enterobacterales>Enterobacteriaceae>Salmonella	GCA_007580505.1	Salmonella_enterica_subsp._enterica	DSQ87_23555	10655..14335	59201	BREX-system-P-loop-protein-BrxC-[Salmonella-enterica-subsp.-enterica]
ECE7382814.1	Type1.BREX	[illegible]	693	Bacteria>Pseudomonadota>Gammaproteobacteria>Enterobacterales>Enterobacteriaceae>Salmonella	GCA_007582365.1	Salmonella_enterica_subsp._enterica	D7M99_18000	1..2079	59201	BREX-system-P-loop-protein-BrxC-[Salmonella-enterica-subsp.-enterica]
ECF0142189.1	Type1.BREX	[illegible]	1204	Bacteria>Pseudomonadota>Gammaproteobacteria>Enterobacterales>Enterobacteriaceae>Salmonella	GCA_007595955.1	Salmonella_enterica_subsp._enterica_serovar_Monschaui	EX931_14155	69682..73296	29480	BREX-system-P-loop-protein-BrxC-[Salmonella-enterica-subsp.-enterica-serovar-Monschaui]
ECF4176041.1	Type1.BREX	[illegible]	864	Bacteria>Pseudomonadota>Gammaproteobacteria>Enterobacterales>Enterobacteriaceae>Salmonella	GCA_007613175.1	Salmonella_enterica_subsp._enterica_serovar_Typhimurium	E5U03_12955	108064..110659	90371	BREX-system-P-loop-protein-BrxC-[Salmonella-enterica-subsp.-enterica-serovar-Typhimurium]
ECF4172399.1	Type1.BREX	[illegible]	1076	Bacteria>Pseudomonadota>Gammaproteobacteria>Enterobacterales>Enterobacteriaceae>Salmonella	GCA_007613215.1	Salmonella_enterica_subsp._enterica	E5100_18995	60001..63229	59201	BREX-system-P-loop-protein-BrxC-[Salmonella-enterica-subsp.-enterica]
ECG1483122.1	Type1.BREX	[illegible]	1213	Bacteria>Pseudomonadota>Gammaproteobacteria>Enterobacterales>Enterobacteriaceae>Salmonella	GCA_007649655.1	Salmonella_enterica_subsp._enterica	DT144_20820	52449..56090	59201	BREX-system-P-loop-protein-BrxC-[Salmonella-enterica-subsp.-enterica]
ECG1893117.1	Type1.BREX	[illegible]	1213	Bacteria>Pseudomonadota>Gammaproteobacteria>Enterobacterales>Enterobacteriaceae>Salmonella	GCA_007650535.1	Salmonella_enterica_subsp._enterica	EI460_05105	155639..159280	59201	BREX-system-P-loop-protein-BrxC-[Salmonella-enterica-subsp.-enterica]
ECG3781073.1	Type1.BREX	[illegible]	1213	Bacteria>Pseudomonadota>Gammaproteobacteria>Enterobacterales>Enterobacteriaceae>Salmonella	GCA_007660485.1	Salmonella_enterica_subsp._enterica_serovar_Breukelen	EXA83_17820	52108..55749	2564274	BREX-system-P-loop-protein-BrxC-[Salmonella-enterica-subsp.-enterica-serovar-Breukelen]
ECG5099189.1	Type1.BREX	[illegible]	1213	Bacteria>Pseudomonadota>Gammaproteobacteria>Enterobacterales>Enterobacteriaceae>Salmonella	GCA_007668905.1	Salmonella_enterica_subsp._enterica	E0547_20045	59933..63574	59201	BREX-system-P-loop-protein-BrxC-[Salmonella-enterica-subsp.-enterica]
ECG6954637.1	Type1.BREX	[illegible]	1213	Bacteria>Pseudomonadota>Gammaproteobacteria>Enterobacterales>Enterobacteriaceae>Salmonella	GCA_007687065.1	Salmonella_enterica_subsp._enterica_serovar_Typhimurium	E5150_19655	60001..63642	90371	BREX-system-P-loop-protein-BrxC-[Salmonella-enterica-subsp.-enterica-serovar-Typhimurium]
ECG8301814.1	Type1.BREX	[illegible]	1213	Bacteria>Pseudomonadota>Gammaproteobacteria>Enterobacterales>Enterobacteriaceae>Salmonella	GCA_007694625.1	Salmonella_enterica_subsp._enterica_serovar_Glostrup	E1853_02135	58935..62576	1151180	BREX-system-P-loop-protein-BrxC-[Salmonella-enterica-subsp.-enterica-serovar-Glostrup]
ECG9327787.1	Type1.BREX	[illegible]	1213	Bacteria>Pseudomonadota>Gammaproteobacteria>Enterobacterales>Enterobacteriaceae>Salmonella	GCA_007700105.1	Salmonella_enterica	FO199_03415	131756..135397	28901	BREX-system-P-loop-protein-BrxC-[Salmonella-enterica]
ECH3013710.1	Type1.BREX	[illegible]	1213	Bacteria>Pseudomonadota>Gammaproteobacteria>Enterobacterales>Enterobacteriaceae>Salmonella	GCA_007716945.1	Salmonella_enterica	FDV18_07420	343098..346739	28901	BREX-system-P-loop-protein-BrxC-[Salmonella-enterica]
ECI2073658.1	Type1.BREX	[illegible]	851	Bacteria>Pseudomonadota>Gammaproteobacteria>Enterobacterales>Enterobacteriaceae>Salmonella	GCA_007762095.1	Salmonella_enterica_subsp._enterica_serovar_Kentucky	AUD11_09545	108064..110621	192955	BREX-system-P-loop-protein-BrxC-[Salmonella-enterica-subsp.-enterica-serovar-Kentucky]
ECI3716680.1	Type1.BREX	[illegible]	1213	Bacteria>Pseudomonadota>Gammaproteobacteria>Enterobacterales>Enterobacteriaceae>Salmonella	GCA_007768905.1	Salmonella_enterica_subsp._enterica	DK077_11320	44493..48134	59201	BREX-system-P-loop-protein-BrxC-[Salmonella-enterica-subsp.-enterica]
ECI5247229.1	Type1.BREX	[illegible]	849	Bacteria>Pseudomonadota>Gammaproteobacteria>Enterobacterales>Enterobacteriaceae>Salmonella	GCA_007773965.1	Salmonella_enterica_subsp._enterica	DUU01_16740	60001..62549	59201	BREX-system-P-loop-protein-BrxC-[Salmonella-enterica-subsp.-enterica]
ECI6492267.1	Type1.BREX	[illegible]	1213	Bacteria>Pseudomonadota>Gammaproteobacteria>Enterobacterales>Enterobacteriaceae>Salmonella	GCA_007781245.1	Salmonella_enterica_subsp._enterica	EIL18_20695	55793..59434	59201	BREX-system-P-loop-protein-BrxC-[Salmonella-enterica-subsp.-enterica]
ECI6612371.1	Type1.BREX	[illegible]	1213	Bacteria>Pseudomonadota>Gammaproteobacteria>Enterobacterales>Enterobacteriaceae>Salmonella	GCA_007781585.1	Salmonella_enterica_subsp._enterica	EU408_19955	62734..66375	59201	BREX-system-P-loop-protein-BrxC-[Salmonella-enterica-subsp.-enterica]
ECI6683601.1	Type1.BREX	[illegible]	1213	Bacteria>Pseudomonadota>Gammaproteobacteria>Enterobacterales>Enterobacteriaceae>Salmonella	GCA_007781825.1	Salmonella_enterica_subsp._enterica	EHE65_20725	52107..55748	59201	BREX-system-P-loop-protein-BrxC-[Salmonella-enterica-subsp.-enterica]
ECI6882011.1	Type1.BREX	[illegible]	929	Bacteria>Pseudomonadota>Gammaproteobacteria>Enterobacterales>Enterobacteriaceae>Salmonella	GCA_007782565.1	Salmonella_enterica_subsp._enterica	EWD50_16145	60001..62787	59201	BREX-system-P-loop-protein-BrxC-[Salmonella-enterica-subsp.-enterica]
ECI6944166.1	Type1.BREX	[illegible]	979	Bacteria>Pseudomonadota>Gammaproteobacteria>Enterobacterales>Enterobacteriaceae>Salmonella	GCA_007783905.1	Salmonella_enterica_subsp._enterica	EWD54_17660	60227..63165	59201	BREX-system-P-loop-protein-BrxC-[Salmonella-enterica-subsp.-enterica]
ECI7333736.1	Type1.BREX	[illegible]	1213	Bacteria>Pseudomonadota>Gammaproteobacteria>Enterobacterales>Enterobacteriaceae>Salmonella	GCA_007783945.1	Salmonella_enterica_subsp._enterica	EZ611_10915	108064..111705	59201	BREX-system-P-loop-protein-BrxC-[Salmonella-enterica-subsp.-enterica]
ECJ2539588.1	Type1.BREX	[illegible]	1157	Bacteria>Pseudomonadota>Gammaproteobacteria>Enterobacterales>Enterobacteriaceae>Salmonella	GCA_007808255.1	Salmonella_enterica_subsp._salamae	FN164_03990	384975..388448	59202	BREX-system-P-loop-protein-BrxC-[Salmonella-enterica-subsp.-salamae]
ECJ4523530.1	Type1.BREX	[illegible]	599	Bacteria>Pseudomonadota>Gammaproteobacteria>Enterobacterales>Enterobacteriaceae>Salmonella	GCA_007819955.1	Salmonella_enterica_subsp._enterica	DPQ09_03955	170822..172620	59201	BREX-system-P-loop-protein-BrxC-[Salmonella-enterica-subsp.-enterica]
ECJ6092446.1	Type1.BREX	[illegible]	1213	Bacteria>Pseudomonadota>Gammaproteobacteria>Enterobacterales>Enterobacteriaceae>Salmonella	GCA_007834095.1	Salmonella_enterica	GR78_06760	372350..375991	28901	BREX-system-P-loop-protein-BrxC-[Salmonella-enterica]
ECJ9595725.1	Type1.BREX	[illegible]	1213	Bacteria>Pseudomonadota>Gammaproteobacteria>Enterobacterales>Enterobacteriaceae>Salmonella	GCA_007852735.1	Salmonella_enterica	FQP47_21215	18123..21764	28901	BREX-system-P-loop-protein-BrxC-[Salmonella-enterica]
ECK3339581.1	Type1.BREX	[illegible]	1233	Bacteria>Pseudomonadota>Gammaproteobacteria>Enterobacterales>Enterobacteriaceae>Salmonella	GCA_007873945.1	Salmonella_enterica	FRH68_09850	220275..223976	28901	BREX-system-P-loop-protein-BrxC-[Salmonella-enterica]
ECK5773200.1	Type1.BREX	[illegible]	1213	Bacteria>Pseudomonadota>Gammaproteobacteria>Enterobacterales>Enterobacteriaceae>Salmonella	GCA_007886645.1	Salmonella_enterica_subsp._enterica_serovar_Typhimurium	AXS32_00815	172577..176218	90371	BREX-system-P-loop-protein-BrxC-[Salmonella-enterica-subsp.-enterica-serovar-Typhimurium]
ECM2518301.1	Type1.BREX	[illegible]	636	Bacteria>Pseudomonadota>Gammaproteobacteria>Enterobacterales>Enterobacteriaceae>Salmonella	GCA_008018705.1	Salmonella_enterica_subsp._enterica_serovar_Typhimurium	PZ44_21595	1..1907	90371	BREX-system-P-loop-protein-BrxC-[Salmonella-enterica-subsp.-enterica-serovar-Typhimurium]
ECM3029728.1	Type1.BREX	[illegible]	1213	Bacteria>Pseudomonadota>Gammaproteobacteria>Enterobacterales>Enterobacteriaceae>Salmonella	GCA_008020915.1	Salmonella_enterica_subsp._enterica_serovar_Typhimurium	QB31_08405	60227..63868	90371	BREX-system-P-loop-protein-BrxC-[Salmonella-enterica-subsp.-enterica-serovar-Typhimurium]
ECM3363968.1	Type1.BREX	[illegible]	1213	Bacteria>Pseudomonadota>Gammaproteobacteria>Enterobacterales>Enterobacteriaceae>Salmonella	GCA_008021815.1	Salmonella_enterica_subsp._enterica_serovar_Typhimurium	PW85_07020	108063..111704	90371	BREX-system-P-loop-protein-BrxC-[Salmonella-enterica-subsp.-enterica-serovar-Typhimurium]
ECN0902565.1	Type1.BREX	[illegible]	1213	Bacteria>Pseudomonadota>Gammaproteobacteria>Enterobacterales>Enterobacteriaceae>Salmonella	GCA_008061715.1	Salmonella_enterica_subsp._enterica_serovar_Typhimurium	YR82_02405	60002..63643	90371	BREX-system-P-loop-protein-BrxC-[Salmonella-enterica-subsp.-enterica-serovar-Typhimurium]
ECO0332142.1	Type1.BREX	[illegible]	1213	Bacteria>Pseudomonadota>Gammaproteobacteria>Enterobacterales>Enterobacteriaceae>Salmonella	GCA_008112305.1	Salmonella_enterica_subsp._enterica_serovar_Typhimurium	ZS66_00475	108064..111705	90371	BREX-system-P-loop-protein-BrxC-[Salmonella-enterica-subsp.-enterica-serovar-Typhimurium]
ECO1322281.1	Type1.BREX	[illegible]	631	Bacteria>Pseudomonadota>Gammaproteobacteria>Enterobacterales>Enterobacteriaceae>Salmonella	GCA_008116905.1	Salmonella_enterica_subsp._enterica_serovar_Typhimurium	AC815_12045	38663..40557	90371	BREX-system-P-loop-protein-BrxC-[Salmonella-enterica-subsp.-enterica-serovar-Typhimurium]
ECO5284314.1	Type1.BREX	[illegible]	1213	Bacteria>Pseudomonadota>Gammaproteobacteria>Enterobacterales>Enterobacteriaceae>Salmonella	GCA_008153185.1	Salmonella_enterica	FYE58_11845	398014..401655	28901	BREX-system-P-loop-protein-BrxC-[Salmonella-enterica]
ECO7189041.1	Type1.BREX	[illegible]	1213	Bacteria>Pseudomonadota>Gammaproteobacteria>Enterobacterales>Enterobacteriaceae>Salmonella	GCA_008162045.1	Salmonella_enterica	F6029_01875	16689..20330	28901	BREX-system-P-loop-protein-BrxC-[Salmonella-enterica]
ECQ9432768.1	Type1.BREX	[illegible]	1213	Bacteria>Pseudomonadota>Gammaproteobacteria>Enterobacterales>Enterobacteriaceae>Salmonella	GCA_008312495.1	Salmonella_enterica	F0371_05165	2795..6436	28901	BREX-system-P-loop-protein-BrxC-[Salmonella-enterica]
ECR2528674.1	Type1.BREX	[illegible]	1213	Bacteria>Pseudomonadota>Gammaproteobacteria>Enterobacterales>Enterobacteriaceae>Salmonella	GCA_008341925.1	Salmonella_enterica_subsp._enterica	F1J42_15865	108064..111705	59201	BREX-system-P-loop-protein-BrxC-[Salmonella-enterica-subsp.-enterica]
ECS4740325.1	Type1.BREX	[illegible]	907	Bacteria>Pseudomonadota>Gammaproteobacteria>Enterobacterales>Enterobacteriaceae>Salmonella	GCA_008423865.1	Salmonella_enterica_subsp._enterica_serovar_Typhimurium_var._5-	A9R80_15960	85998..88723	1620419	BREX-system-P-loop-protein-BrxC-[Salmonella-enterica-subsp.-enterica-serovar-Typhimurium-var.-5-]
ECT8081314.1	Type1.BREX	[illegible]	1213	Bacteria>Pseudomonadota>Gammaproteobacteria>Enterobacterales>Enterobacteriaceae>Salmonella	GCA_008481645.1	Salmonella_enterica_subsp._enterica_serovar_Carrau	BLY96_09860	150076..153717	1160739	BREX-system-P-loop-protein-BrxC-[Salmonella-enterica-subsp.-enterica-serovar-Carrau]
ECU0288387.1	Type1.BREX	[illegible]	1213	Bacteria>Pseudomonadota>Gammaproteobacteria>Enterobacterales>Enterobacteriaceae>Salmonella	GCA_008492025.1	Salmonella_enterica_subsp._enterica_serovar_Typhimurium	C7B88_00710	140025..143666	90371	BREX-system-P-loop-protein-BrxC-[Salmonella-enterica-subsp.-enterica-serovar-Typhimurium]

Protein-ID	System	Gene-Neighbourhoods	Length	Taxonomy	Assembly-ID	Strain	Locus-Tag	Genomic-coordinates	TaxID	Fasta-define
ECU9351200.1	Type1.BREX	<-HEPN<-REase+iREase<-?||||YjhX_toxin->SAM-METHYLASE->?->?->BrxA-DNA-binding->BrxB-BrxF-iSTAND->DUF6079*->N6-METHYLASE->OLD-ABC->TOPRIM->PglZ->SIGMA+OB-Fold+AAA-ATPase+Lon-Protease-BrxL-><-REase<-NACHT+SF1-SF2-Helicase+HKD-DNase<-Arm-DNA-bind_3	1213	Bacteria>Pseudomonadota>Gammaproteobacteria>Enterobacterales>Enterobacteriaceae>Salmonella	GCA_008530195.1	Salmonella_enterica_subsp._enterica_serovar_Typhimurium	CIH13_12040	171029..174670	90371	BREX-system-P-loop-protein-BrxC-[Salmonella-enterica-subsp.-enterica-serovar-Typhimurium]
ECV7778489.1	Type1.BREX	<-HEPN<-REase+iREase<-?||YjhX_toxin->SAM-METHYLASE->?->BrxA-DNA-binding->BrxB-BrxF-iSTAND->DUF6079*->N6-METHYLASE->OLD-ABC->TOPRIM->PglZ->SIGMA+OB-Fold+AAA-ATPase+Lon-Protease-BrxL-><-REase<-NACHT+SF1-SF2-Helicase+HKD-DNase<-Arm-DNA-bind_3	1213	Bacteria>Pseudomonadota>Gammaproteobacteria>Enterobacterales>Enterobacteriaceae>Salmonella	GCA_008576505.1	Salmonella_enterica_subsp._enterica_serovar_Typhimurium	ZX52_02590	60002..63643	90371	BREX-system-P-loop-protein-BrxC-[Salmonella-enterica-subsp.-enterica-serovar-Typhimurium]
ECY3255615.1	Type1.BREX	<-HEPN<-?||YjhX_toxin->SAM-METHYLASE->?->||?-><-ZnR||BrxA-DNA-binding->BrxB-BrxF-iSTAND->DUF6079*->N6-METHYLASE->OLD-ABC->TOPRIM->PglZ->SIGMA+OB-Fold+AAA-ATPase+Lon-Protease-BrxL->HKD-DNase+OGG-like-><-REase<-NACHT+SF1-SF2-Helicase+HKD-DNase<-Arm-DNA-bind_3	1204	Bacteria>Pseudomonadota>Gammaproteobacteria>Enterobacterales>Enterobacteriaceae>Salmonella	GCA_008731355.1	Salmonella_enterica_subsp._enterica_serovar_Alachua	AU577_03140	17059..20673	913240	BREX-system-P-loop-protein-BrxC-[Salmonella-enterica-subsp.-enterica-serovar-Alachua]
ECY8229161.1	Type1.BREX	<-HEPN<-?||YjhX_toxin->SAM-METHYLASE->?->BrxA-DNA-binding->BrxB-BrxF-iSTAND->DUF6079*->N6-METHYLASE->OLD-ABC->TOPRIM->PglZ->SIGMA+OB-Fold+AAA-ATPase+Lon-Protease-BrxL-><-REase<-SF1-SF2-Helicase+HKD-DNase	1213	Bacteria>Pseudomonadota>Gammaproteobacteria>Enterobacterales>Enterobacteriaceae>Salmonella	GCA_008752395.1	Salmonella_enterica_subsp._enterica_serovar_Stanley	AU695_08280	160733..164374	192953	BREX-system-P-loop-protein-BrxC-[Salmonella-enterica-subsp.-enterica-serovar-Stanley]
ECZ9372117.1	Type1.BREX	Arm-DNA-bind_3->NACHT+SF1-SF2-Helicase+HKD-DNase->REase-><-SIGMA+OB-Fold+AAA-ATPase+Lon-Protease-BrxL<-PglZ<-TOPRIM<-OLD-ABC<-N6-METHYLASE<-DUF6079*<-BrxB-BrxF-iSTAND<-BrxA-DNA-binding<-?<-SAM-METHYLASE<-YjhX_toxin||?->REase+iREase->HEPN->	1213	Bacteria>Pseudomonadota>Gammaproteobacteria>Enterobacterales>Enterobacteriaceae>Salmonella	GCA_008834045.1	Salmonella_enterica_subsp._enterica_serovar_Typhimurium	A3U95_10245	108064..111705	90371	BREX-system-P-loop-protein-BrxC-[Salmonella-enterica-subsp.-enterica-serovar-Typhimurium]
EDA5898036.1	Type1.BREX	<-HEPN<-?||YjhX_toxin->SAM-METHYLASE->?->||BrxA-DNA-binding->BrxB-BrxF-iSTAND->DUF6079*->N6-METHYLASE->OLD-ABC->TOPRIM->PglZ->SIGMA+OB-Fold+AAA-ATPase+Lon-Protease-BrxL-><-REase<-NACHT+SF1-SF2-Helicase+HKD-DNase	1213	Bacteria>Pseudomonadota>Gammaproteobacteria>Enterobacterales>Enterobacteriaceae>Salmonella	GCA_008864045.1	Salmonella_enterica_subsp._enterica	F9X73_05935	25917..29558	59201	BREX-system-P-loop-protein-BrxC-[Salmonella-enterica-subsp.-enterica]
EDA7420100.1	Type1.BREX	<-HEPN<-REase+iREase<-?||YjhX_toxin->SAM-METHYLASE->?->BrxA-DNA-binding->BrxB-BrxF-iSTAND->DUF6079*->N6-METHYLASE->OLD-ABC->TOPRIM->PglZ->SIGMA+OB-Fold+AAA-ATPase+Lon-Protease-BrxL-><-REase<-NACHT+SF1-SF2-Helicase+HKD-DNase<-Arm-DNA-bind_3	1213	Bacteria>Pseudomonadota>Gammaproteobacteria>Enterobacterales>Enterobacteriaceae>Salmonella	GCA_008872905.1	Salmonella_enterica_subsp._enterica_serovar_Typhimurium	A3U84_08760	60219..63860	90371	BREX-system-P-loop-protein-BrxC-[Salmonella-enterica-subsp.-enterica-serovar-Typhimurium]
EDB0845244.1	Type1.BREX	<-HEPN<-?||YjhX_toxin->SAM-METHYLASE->?->||BrxA-DNA-binding->BrxB-BrxF-iSTAND->DUF6079*->N6-METHYLASE->PglZ->SIGMA+OB-Fold+AAA-ATPase+Lon-Protease-BrxL->HKD-DNase+OGG-like-><-REase<-NACHT+SF1-SF2-Helicase+HKD-DNase<-Arm-DNA-bind_3<-tRNA||?-><-SKI	1213	Bacteria>Pseudomonadota>Gammaproteobacteria>Enterobacterales>Enterobacteriaceae>Salmonella	GCA_008888525.1	Salmonella_enterica	F9C59_12685	144464..148105	28901	BREX-system-P-loop-protein-BrxC-[Salmonella-enterica]
EDB2044587.1	Type1.BREX	Glyoxalase->ACET->YjhX_toxin->ACET->METHYLASE->ZnR->Glyoxalase->BrxA-DNA-binding->BrxB-BrxF-iSTAND->DUF6079*->ParB+HNH->N6-METHYLASE->PglZ->SIGMA+OB-Fold+AAA-ATPase+Lon-Protease-BrxL-><-Arm-DNA-bind_3+Phage_integrase<-tRNA||?-><-SKI||METHYLASE->	1233	Bacteria>Pseudomonadota>Gammaproteobacteria>Enterobacterales>Enterobacteriaceae>Salmonella	GCA_008894145.1	Salmonella_enterica_subsp._enterica	F9P84_01850	405984..409685	59201	BREX-system-P-loop-protein-BrxC-[Salmonella-enterica-subsp.-enterica]
EDB9656968.1	Type1.BREX	<-HEPN<-REase+iREase<-?||YjhX_toxin->SAM-METHYLASE->?->BrxA-DNA-binding->BrxB-BrxF-iSTAND->DUF6079*->N6-METHYLASE->OLD-ABC->TOPRIM->PglZ->SIGMA+OB-Fold+AAA-ATPase+Lon-Protease-BrxL-><-REase<-Arm-DNA-bind_3<-tRNA||?-><-SKI	1213	Bacteria>Pseudomonadota>Gammaproteobacteria>Enterobacterales>Enterobacteriaceae>Salmonella	GCA_008938335.1	Salmonella_enterica_subsp._enterica_serovar_Typhimurium	BE039_09545	27455..31096	90371	BREX-system-P-loop-protein-BrxC-[Salmonella-enterica-subsp.-enterica-serovar-Typhimurium]
EDC2707334.1	Type1.BREX	<-HEPN<-REase+iREase<-?||YjhX_toxin->SAM-METHYLASE->?->BrxA-DNA-binding->BrxB-BrxF-iSTAND->DUF6079*->N6-METHYLASE->	1213	Bacteria>Pseudomonadota>Gammaproteobacteria>Enterobacterales>Enterobacteriaceae>Salmonella	GCA_008954065.1	Salmonella_enterica	GAD24_16260	80632..84273	28901	BREX-system-P-loop-protein-BrxC-[Salmonella-enterica]
EDC2377951.1	Type1.BREX	<-HEPN<-?||YjhX_toxin->SAM-METHYLASE->?->||BrxA-DNA-binding->BrxB-BrxF-iSTAND->DUF6079*->N6-METHYLASE->OLD-ABC->TOPRIM->PglZ->SIGMA+OB-Fold+AAA-ATPase+Lon-Protease-BrxL->?-><-REase<-NACHT+SF1-SF2-Helicase+HKD-DNase<-Arm-DNA-bind_3	1213	Bacteria>Pseudomonadota>Gammaproteobacteria>Enterobacterales>Enterobacteriaceae>Salmonella	GCA_008954485.1	Salmonella_enterica	GAA61_09305	25917..29558	28901	BREX-system-P-loop-protein-BrxC-[Salmonella-enterica]
EDE2293304.1	Type1.BREX	Arm-DNA-bind_3->NACHT+SF1-SF2-Helicase+HKD-DNase->REase-><-SIGMA+OB-Fold+AAA-ATPase+Lon-Protease-BrxL<-PglZ<-TOPRIM<-OLD-ABC<-N6-METHYLASE<-DUF6079*<-BrxB-BrxF-iSTAND<-BrxA-DNA-binding<-?<-SAM-METHYLASE<-YjhX_toxin||?->REase+iREase->HEPN->	1213	Bacteria>Pseudomonadota>Gammaproteobacteria>Enterobacterales>Enterobacteriaceae>Salmonella	GCA_009089805.1	Salmonella_enterica	GBE85_07255	108064..111705	28901	BREX-system-P-loop-protein-BrxC-[Salmonella-enterica]
EDH2237970.1	Type1.BREX	<-HEPN<-REase+iREase<-?||YjhX_toxin->SAM-METHYLASE->?->BrxA-DNA-binding->BrxB-BrxF-iSTAND->DUF6079*->N6-METHYLASE->OLD-ABC->TOPRIM->PglZ->SIGMA+OB-Fold+AAA-ATPase+Lon-Protease-BrxL-><-REase<-NACHT+SF1-SF2-Helicase+HKD-DNase<-Arm-DNA-bind_3	1213	Bacteria>Pseudomonadota>Gammaproteobacteria>Enterobacterales>Enterobacteriaceae>Salmonella	GCA_009231185.1	Salmonella_enterica	GC580_06310	170513..174154	28901	BREX-system-P-loop-protein-BrxC-[Salmonella-enterica]
EDH5631745.1	Type1.BREX	<-HEPN<-?||YjhX_toxin->SAM-METHYLASE->?->||BrxA-DNA-binding->BrxB-BrxF-iSTAND->DUF6079*->N6-METHYLASE->OLD-ABC->TOPRIM->PglZ->SIGMA+OB-Fold+AAA-ATPase+Lon-Protease-BrxL->	1213	Bacteria>Pseudomonadota>Gammaproteobacteria>Enterobacterales>Enterobacteriaceae>Salmonella	GCA_009247085.1	Salmonella_enterica_subsp._enterica_serovar_Claibornei	CB172_15080	158451..162092	2572709	BREX-system-P-loop-protein-BrxC-[Salmonella-enterica-subsp.-enterica-serovar-Claibornei]
EDH5972513.1	Type1.BREX	<-HEPN<-REase+iREase<-?||YjhX_toxin->SAM-METHYLASE->?->BrxA-DNA-binding->BrxB-BrxF-iSTAND->DUF6079*->	1176	Bacteria>Pseudomonadota>Gammaproteobacteria>Enterobacterales>Enterobacteriaceae>Salmonella	GCA_009248125.1	Salmonella_enterica_subsp._enterica_serovar_Typhimurium	CB276_16685	60001..63528	90371	BREX-system-P-loop-protein-BrxC-[Salmonella-enterica-subsp.-enterica-serovar-Typhimurium]
EDH6190230.1	Type1.BREX	<-nSTAND1+GTPase-AIG+sGTP<-?||?->XIS-HTH-><-ZnR-><-NtrC-AAA+FIS-HTH||<-SIGMA+OB-Fold+AAA-ATPase+Lon-Protease-BrxL<-PglZ<-N6-METHYLASE<-DUF6079*<-BrxB-BrxF-iSTAND<-BrxA-DNA-binding||REase->?->||<-?<-Arm-DNA-bind_3+Phage_integrase	1213	Bacteria>Pseudomonadota>Gammaproteobacteria>Enterobacterales>Enterobacteriaceae>Salmonella	GCA_009249585.1	Salmonella_enterica_subsp._enterica_serovar_Typhi	CB347_02510	200361..204002	90370	BREX-system-P-loop-protein-BrxC-[Salmonella-enterica-subsp.-enterica-serovar-Typhi]
EDH5995080.1	Type1.BREX	<-HEPN<-REase+iREase<-?||YjhX_toxin->SAM-METHYLASE->?->BrxA-DNA-binding->BrxB-BrxF-iSTAND->DUF6079-p*->	958	Bacteria>Pseudomonadota>Gammaproteobacteria>Enterobacterales>Enterobacteriaceae>Salmonella	GCA_009250025.1	Salmonella_enterica_subsp._enterica_serovar_Typhimurium	CB280_15355	60001..62876	90371	BREX-system-P-loop-protein-BrxC-[Salmonella-enterica-subsp.-enterica-serovar-Typhimurium]
EDH6721610.1	Type1.BREX	<-Phage_integrase<-?<-LexA-protease||?->35exo->?->wHTH+WYL+WCX->BrxA-DNA-binding->BrxB-BrxF-iSTAND->DUF6079-p*->	691	Bacteria>Pseudomonadota>Gammaproteobacteria>Enterobacterales>Enterobacteriaceae>Salmonella	GCA_009251865.1	Salmonella_enterica_subsp._enterica_serovar_Heidelberg	CB494_23570	5530..7603	611	BREX-system-P-loop-protein-BrxC-[Salmonella-enterica-subsp.-enterica-serovar-Heidelberg]
EDH9429027.1	Type1.BREX	<-HEPN<-REase+iREase<-?||YjhX_toxin->SAM-METHYLASE->?->BrxA-DNA-binding->BrxB-BrxF-iSTAND->DUF6079*->	1069	Bacteria>Pseudomonadota>Gammaproteobacteria>Enterobacterales>Enterobacteriaceae>Salmonella	GCA_009288565.1	Salmonella_enterica_subsp._enterica_serovar_Typhimurium	CC452_16960	59990..63196	90371	BREX-system-P-loop-protein-BrxC-[Salmonella-enterica-subsp.-enterica-serovar-Typhimurium]
EDH9929876.1	Type1.BREX	<-HEPN<-REase+iREase<-?||YjhX_toxin->SAM-METHYLASE->?->BrxA-DNA-binding->BrxB-BrxF-iSTAND->DUF6079*->	1213	Bacteria>Pseudomonadota>Gammaproteobacteria>Enterobacterales>Enterobacteriaceae>Salmonella	GCA_009291205.1	Salmonella_enterica_subsp._enterica_serovar_Typhimurium	CC611_10195	170364..174005	90371	BREX-system-P-loop-protein-BrxC-[Salmonella-enterica-subsp.-enterica-serovar-Typhimurium]
EDH9999209.1	Type1.BREX	<-HEPN<-REase+iREase<-?||YjhX_toxin->SAM-METHYLASE->?->BrxA-DNA-binding->BrxB-BrxF-iSTAND->DUF6079-p*->	978	Bacteria>Pseudomonadota>Gammaproteobacteria>Enterobacterales>Enterobacteriaceae>Salmonella	GCA_009291525.1	Salmonella_enterica_subsp._enterica_serovar_Typhimurium	CC636_17605	60002..62935	90371	BREX-system-P-loop-protein-BrxC-[Salmonella-enterica-subsp.-enterica-serovar-Typhimurium]
EDH9995453.1	Type1.BREX	DUF6079-p*->N6-METHYLASE->OLD-ABC->TOPRIM->PglZ->SIGMA+OB-Fold+AAA-ATPase+Lon-Protease-BrxL-><-REase	837	Bacteria>Pseudomonadota>Gammaproteobacteria>Enterobacterales>Enterobacteriaceae>Salmonella	GCA_009292885.1	Salmonella_enterica_subsp._enterica_serovar_Java	CC627_22205	1..2514	224729	BREX-system-P-loop-protein-BrxC-[Salmonella-enterica-subsp.-enterica-serovar-Java]
EDI1182989.1	Type1.BREX	<-HEPN<-REase+iREase<-?||YjhX_toxin->SAM-METHYLASE->?->BrxA-DNA-binding->BrxB-BrxF-iSTAND->DUF6079*->	1060	Bacteria>Pseudomonadota>Gammaproteobacteria>Enterobacterales>Enterobacteriaceae>Salmonella	GCA_009300115.1	Salmonella_enterica_subsp._enterica_serovar_Typhimurium	CC995_04970	170835..174015	90371	BREX-system-P-loop-protein-BrxC-[Salmonella-enterica-subsp.-enterica-serovar-Typhimurium]
EDI5281730.1	Type1.BREX	<-HEPN<-REase+iREase<-?||YjhX_toxin->SAM-METHYLASE->?->BrxA-DNA-binding->BrxB-BrxF-iSTAND->DUF6079*->N6-METHYLASE->OLD-ABC->TOPRIM->SIGMA+OB-Fold+AAA-ATPase+Lon-Protease-BrxL-><-REase<-NACHT+SF1-SF2-Helicase+HKD-DNase<-Arm-DNA-bind_3<-tRNA||?-><-SKI	1213	Bacteria>Pseudomonadota>Gammaproteobacteria>Enterobacterales>Enterobacteriaceae>Salmonella	GCA_009327915.1	Salmonella_enterica_subsp._enterica_serovar_Typhimurium	CEM90_10175	170818..174459	90371	BREX-system-P-loop-protein-BrxC-[Salmonella-enterica-subsp.-enterica-serovar-Typhimurium]
EDI9669678.1	Type1.BREX	<-HEPN<-REase+iREase<-?||YjhX_toxin->SAM-METHYLASE->?->BrxA-DNA-binding->BrxB-BrxF-iSTAND->DUF6079*->OLD-ABC->TOPRIM->PglZ->SIGMA+OB-Fold+AAA-ATPase+Lon-Protease-BrxL-><-REase<-NACHT+SF1-SF2-Helicase+HKD-DNase<-Arm-DNA-bind_3<-tRNA||?-><-SKI	1213	Bacteria>Pseudomonadota>Gammaproteobacteria>Enterobacterales>Enterobacteriaceae>Salmonella	GCA_009349595.1	Salmonella_enterica	GEV75_12805	38669..42310	28901	BREX-system-P-loop-protein-BrxC-[Salmonella-enterica]
EDL1929631.1	Type1.BREX	Arm-DNA-bind_3->NACHT+SF1-SF2-Helicase+HKD-DNase->REase-><-SIGMA+OB-Fold+AAA-ATPase+Lon-Protease-BrxL<-PglZ<-TOPRIM<-OLD-ABC<-N6-METHYLASE<-DUF6079*<-BrxB-BrxF-iSTAND<-BrxA-DNA-binding<-?<-SAM-METHYLASE<-YjhX_toxin||?->REase+iREase->HEPN->	1213	Bacteria>Pseudomonadota>Gammaproteobacteria>Enterobacterales>Enterobacteriaceae>Salmonella	GCA_009508775.1	Salmonella_enterica_subsp._enterica_serovar_Typhimurium	CQD92_11450	108064..111705	90371	BREX-system-P-loop-protein-BrxC-[Salmonella-enterica-subsp.-enterica-serovar-Typhimurium]
EDL2092523.1	Type1.BREX	Arm-DNA-bind_3->NACHT+SF1-SF2-Helicase+HKD-DNase->REase-><-SIGMA+OB-Fold+AAA-ATPase+Lon-Protease-BrxL<-PglZ<-TOPRIM<-OLD-ABC<-N6-METHYLASE<-DUF6079*<-BrxB-BrxF-iSTAND<-BrxA-DNA-binding<-?<-SAM-METHYLASE<-YjhX_toxin||?->REase+iREase->HEPN->	1213	Bacteria>Pseudomonadota>Gammaproteobacteria>Enterobacterales>Enterobacteriaceae>Salmonella	GCA_009509795.1	Salmonella_enterica_subsp._enterica_serovar_Typhimurium	CQF25_02855	72413..76054	90371	BREX-system-P-loop-protein-BrxC-[Salmonella-enterica-subsp.-enterica-serovar-Typhimurium]
EDL6685014.1	Type1.BREX	<-HEPN<-REase+iREase<-?||YjhX_toxin->SAM-METHYLASE->?->BrxA-DNA-binding->BrxB-BrxF-iSTAND->DUF6079*->N6-METHYLASE->OLD-ABC->TOPRIM->PglZ->SIGMA+OB-Fold+AAA-ATPase+Lon-Protease-BrxL-><-REase<-NACHT+SF1-SF2-Helicase+HKD-DNase<-Arm-DNA-bind_3	1213	Bacteria>Pseudomonadota>Gammaproteobacteria>Enterobacterales>Enterobacteriaceae>Salmonella	GCA_009531865.1	Salmonella_enterica	CXC07_02670	170913..174554	28901	BREX-system-P-loop-protein-BrxC-[Salmonella-enterica]
EDL7421472.1	Type1.BREX	Arm-DNA-bind_3->NACHT+SF1-SF2-Helicase+HKD-DNase->REase-><-?<-SIGMA+OB-Fold+AAA-ATPase+Lon-Protease-BrxL<-PglZ<-TOPRIM<-OLD-ABC<-N6-METHYLASE<-DUF6079*<-BrxB-BrxF-iSTAND<-BrxA-DNA-binding<-?<-SAM-METHYLASE<-YjhX_toxin||?->HEPN->	1213	Bacteria>Pseudomonadota>Gammaproteobacteria>Enterobacterales>Enterobacteriaceae>Salmonella	GCA_009534895.1	Salmonella_enterica	CU840_19785	23186..26827	28901	BREX-system-P-loop-protein-BrxC-[Salmonella-enterica]
EDM4126305.1	Type1.BREX	<-HEPN<-REase+iREase<-?||YjhX_toxin->SAM-METHYLASE->?->BrxA-DNA-binding->BrxB-BrxF-iSTAND->DUF6079*->N6-METHYLASE->OLD-ABC->TOPRIM->PglZ->SIGMA+OB-Fold+AAA-ATPase+Lon-Protease-BrxL-><-REase<-NACHT+SF1-SF2-Helicase+HKD-DNase<-Arm-DNA-bind_3	1213	Bacteria>Pseudomonadota>Gammaproteobacteria>Enterobacterales>Enterobacteriaceae>Salmonella	GCA_009562515.1	Salmonella_enterica_subsp._enterica_serovar_Typhimurium	CR810_06735	172578..176219	90371	BREX-system-P-loop-protein-BrxC-[Salmonella-enterica-subsp.-enterica-serovar-Typhimurium]
EDM6640280.1	Type1.BREX	<-HEPN<-REase+iREase<-?||YjhX_toxin->SAM-METHYLASE->?->BrxA-DNA-binding->BrxB-BrxF-iSTAND->DUF6079*->N6-METHYLASE->OLD-ABC->TOPRIM->PglZ->SIGMA+OB-Fold+AAA-ATPase+Lon-Protease-BrxL-><-REase<-NACHT+SF1-SF2-Helicase+HKD-DNase<-Arm-DNA-bind_3	1213	Bacteria>Pseudomonadota>Gammaproteobacteria>Enterobacterales>Enterobacteriaceae>Salmonella	GCA_009573765.1	Salmonella_enterica_subsp._enterica_serovar_Typhimurium	CUM00_00810	170918..174559	90371	BREX-system-P-loop-protein-BrxC-[Salmonella-enterica-subsp.-enterica-serovar-Typhimurium]
EDN6133533.1	Type1.BREX	<-HEPN<-?||YjhX_toxin->SAM-METHYLASE->?->||?-><-ZnR||BrxA-DNA-binding->BrxB-BrxF-iSTAND->DUF6079*->N6-METHYLASE->OLD-ABC->TOPRIM->PglZ->SIGMA+OB-Fold+AAA-ATPase+Lon-Protease-BrxL->HKD-DNase+OGG-like-><-REase<-NACHT+SF1-SF2-Helicase+HKD-DNase<-Arm-DNA-bind_3	1204	Bacteria>Pseudomonadota>Gammaproteobacteria>Enterobacterales>Enterobacteriaceae>Salmonella	GCA_010008765.1	Salmonella_enterica_subsp._enterica_serovar_Kentucky	GJQ78_12610	62378..65992	192955	BREX-system-P-loop-protein-BrxC-[Salmonella-enterica-subsp.-enterica-serovar-Kentucky]
HAC8921685.1	Type1.BREX	Arm-DNA-bind_3->NACHT+SF1-SF2-Helicase+HKD-DNase->REase-><-SIGMA+OB-Fold+AAA-ATPase+Lon-Protease-BrxL<-PglZ<-TOPRIM<-OLD-ABC<-N6-METHYLASE<-DUF6079-p*	778	Bacteria>Pseudomonadota>Gammaproteobacteria>Enterobacterales>Enterobacteriaceae>Salmonella	GCA_010188005.1	Salmonella_enterica	GOJ09_01290	58631..60969	28901	BREX-system-P-loop-protein-BrxC-[Salmonella-enterica]
HAD0448194.1	Type1.BREX	Arm-DNA-bind_3->NACHT+SF1-SF2-Helicase+HKD-DNase->REase-><-SIGMA+OB-Fold+AAA-ATPase+Lon-Protease-BrxL<-PglZ<-TOPRIM<-OLD-ABC<-N6-METHYLASE<-DUF6079*<-BrxB-BrxF-iSTAND<-BrxA-DNA-binding<-?<-SAM-METHYLASE<-YjhX_toxin||?->REase+iREase->HEPN->	1213	Bacteria>Pseudomonadota>Gammaproteobacteria>Enterobacterales>Enterobacteriaceae>Salmonella	GCA_010193865.1	Salmonella_enterica_subsp._enterica_serovar_Typhimurium	GON57_00710	150026..153667	90371	BREX-system-P-loop-protein-BrxC-[Salmonella-enterica-subsp.-enterica-serovar-Typhimurium]
HAD1283638.1	Type1.BREX	Arm-DNA-bind_3->NACHT+SF1-SF2-Helicase+HKD-DNase->REase-><-SIGMA+OB-Fold+AAA-ATPase+Lon-Protease-BrxL<-PglZ<-TOPRIM<-OLD-ABC<-N6-METHYLASE<-DUF6079*	1107	Bacteria>Pseudomonadota>Gammaproteobacteria>Enterobacterales>Enterobacteriaceae>Salmonella	GCA_010197825.1	Salmonella_enterica_subsp._enterica_serovar_Typhimurium	GTH76_16775	108064..111389	90371	BREX-system-P-loop-protein-BrxC-[Salmonella-enterica-subsp.-enterica-serovar-Typhimurium]
EDP8828062.1	Type1.BREX	<-DUF6079-p*<-BrxB-BrxF-iSTAND<-BrxA-DNA-binding||<-?<-SAM-METHYLASE<-YjhX_toxin||?->HEPN->	793	Bacteria>Pseudomonadota>Gammaproteobacteria>Enterobacterales>Enterobacteriaceae>Salmonella	GCA_010369285.1	Salmonella_enterica_subsp._enterica	S669_001969	1..2380	59201	BREX-system-P-loop-protein-BrxC-[Salmonella-enterica-subsp.-enterica]
EDQ1995876.1	Type1.BREX	<-RelE-ParE<-CopG/RHH||<-RHH||?->wHTH+WYL+WCX->BrxA-DNA-binding->BrxB-BrxF-iSTAND->DUF6079-p*->	509	Bacteria>Pseudomonadota>Gammaproteobacteria>Enterobacterales>Enterobacteriaceae>Salmonella	GCA_010387725.1	Salmonella_enterica	GT143_004731	5665..7190	28901	BREX-system-P-loop-protein-BrxC-[Salmonella-enterica]
EDQ2665795.1	Type1.BREX	<-HEPN<-?||YjhX_toxin->SAM-METHYLASE->?->BrxA-DNA-binding->BrxB-BrxF-iSTAND->DUF6079*->N6-METHYLASE->PglZ->SIGMA+OB-Fold+AAA-ATPase+Lon-Protease-BrxL->||PIN-><-HTH_21+rve+rve_3	1213	Bacteria>Pseudomonadota>Gammaproteobacteria>Enterobacterales>Enterobacteriaceae>Salmonella	GCA_010390975.1	Salmonella_enterica_subsp._enterica	ZH92_003658	52353..55994	59201	BREX-system-P-loop-protein-BrxC-[Salmonella-enterica-subsp.-enterica]
EDQ2405063.1	Type1.BREX	<-HEPN<-?||YjhX_toxin->SAM-METHYLASE->?->||BrxA-DNA-binding->BrxB-BrxF-iSTAND->DUF6079*->N6-METHYLASE->PglZ->SIGMA+OB-Fold+AAA-ATPase+Lon-Protease-BrxL->HKD-DNase+OGG-like-><-REase<-NACHT+SF1-SF2-Helicase+HKD-DNase<-Arm-DNA-bind_3<-tRNA||?-><-SKI	1213	Bacteria>Pseudomonadota>Gammaproteobacteria>Enterobacterales>Enterobacteriaceae>Salmonella	GCA_010391095.1	Salmonella_enterica_subsp._enterica_serovar_Saintpaul	DQ96_001225	144464..148105	90105	BREX-system-P-loop-protein-BrxC-[Salmonella-enterica-subsp.-enterica-serovar-Saintpaul]
EDR5087378.1	Type1.BREX	HTH_3->Couple_hipA+HipA_C->REase-><-SIGMA+OB-Fold+AAA-ATPase+Lon-Protease-BrxL<-PglZ<-TOPRIM<-OLD-ABC<-N6-METHYLASE<-DUF6079*<-BrxB-BrxF-iSTAND<-BrxA-DNA-binding<-?<-SAM-METHYLASE<-YjhX_toxin||?->HEPN->	1213	Bacteria>Pseudomonadota>Gammaproteobacteria>Enterobacterales>Enterobacteriaceae>Salmonella	GCA_010449355.1	Salmonella_enterica_subsp._enterica	GRZ33_003685	38268..41909	59201	BREX-system-P-loop-protein-BrxC-[Salmonella-enterica-subsp.-enterica]
HAE3790823.1	Type1.BREX	<-HEPN<-REase+iREase<-?||YjhX_toxin->SAM-METHYLASE->?->BrxA-DNA-binding->BrxB-BrxF-iSTAND->DUF6079*->N6-METHYLASE->OLD-ABC->TOPRIM->PglZ->SIGMA+OB-Fold+AAA-ATPase+Lon-Protease-BrxL-><-REase<-NACHT+SF1-SF2-Helicase+HKD-DNase<-Arm-DNA-bind_3	1213	Bacteria>Pseudomonadota>Gammaproteobacteria>Enterobacterales>Enterobacteriaceae>Salmonella	GCA_010485735.1	Salmonella_enterica_subsp._enterica_serovar_Typhimurium	G4A23_000143	146702..150343	90371	BREX-system-P-loop-protein-BrxC-[Salmonella-enterica-subsp.-enterica-serovar-Typhimurium]
EDS3575399.1	Type1.BREX	<-ZnR<-ZnR||BrxA-DNA-binding->BrxB-BrxF-iSTAND->DUF6079*->N6-METHYLASE->OLD-ABC->TOPRIM->PglZ->SIGMA+OB-Fold+AAA-ATPase+Lon-Protease-BrxL->HKD-DNase+OGG-like-><-REase<-NACHT+SF1-SF2-Helicase+HKD-DNase<-Arm-DNA-bind_3	1204	Bacteria>Pseudomonadota>Gammaproteobacteria>Enterobacterales>Enterobacteriaceae>Salmonella	GCA_010490095.1	Salmonella_enterica_subsp._enterica	GR179_002518	3402..7016	59201	BREX-system-P-loop-protein-BrxC-[Salmonella-enterica-subsp.-enterica]
EDS5421358.1	Type1.BREX	<-HEPN<-REase+iREase<-?||YjhX_toxin->SAM-METHYLASE->?->BrxA-DNA-binding->BrxB-BrxF-iSTAND->DUF6079*->N6-METHYLASE->OLD-ABC->TOPRIM->PglZ->SIGMA+OB-Fold+AAA-ATPase+Lon-Protease-BrxL-><-REase<-Couple_hipA+HipA_C<-HTH_3	1213	Bacteria>Pseudomonadota>Gammaproteobacteria>Enterobacterales>Enterobacteriaceae>Salmonella	GCA_010498755.1	Salmonella_enterica_subsp._enterica	ZH17_000156	158281..161922	59201	BREX-system-P-loop-protein-BrxC-[Salmonella-enterica-subsp.-enterica]
EDT1456935.1	Type1.BREX	<-HEPN<-?||YjhX_toxin->SAM-METHYLASE->?->BrxA-DNA-binding->BrxB-BrxF-iSTAND->DUF6079*->N6-METHYLASE->OLD-ABC->TOPRIM->PglZ->SIGMA+OB-Fold+AAA-ATPase+Lon-Protease-BrxL->?-><-REase<-NACHT+SF1-SF2-Helicase+HKD-DNase<-REase	1213	Bacteria>Pseudomonadota>Gammaproteobacteria>Enterobacterales>Enterobacteriaceae>Salmonella	GCA_010529175.1	Salmonella_enterica_subsp._enterica_serovar_Ramatgan	CE894_001670	153160..156801	2564942	BREX-system-P-loop-protein-BrxC-[Salmonella-enterica-subsp.-enterica-serovar-Ramatgan]
EDT2838946.1	Type1.BREX	HTH_3->Couple_hipA+HipA_C->REase-><-SIGMA+OB-Fold+AAA-ATPase+Lon-Protease-BrxL<-PglZ<-TOPRIM<-OLD-ABC<-N6-METHYLASE<-DUF6079*<-BrxB-BrxF-iSTAND<-BrxA-DNA-binding||<-?<-SAM-METHYLASE<-YjhX_toxin||?->REase+iREase->HEPN->	1213	Bacteria>Pseudomonadota>Gammaproteobacteria>Enterobacterales>Enterobacteriaceae>Salmonella	GCA_010536715.1	Salmonella_enterica_subsp._enterica	S958_003413	14802..18443	59201	BREX-system-P-loop-protein-BrxC-[Salmonella-enterica-subsp.-enterica]
EDT5820088.1	Type1.BREX	Arm-DNA-bind_3->NACHT+SF1-SF2-Helicase+HKD-DNase->REase-><-SIGMA+OB-Fold+AAA-ATPase+Lon-Protease-BrxL<-PglZ<-TOPRIM<-OLD-ABC<-N6-METHYLASE<-DUF6079*<-BrxB-BrxF-iSTAND<-BrxA-DNA-binding<-?<-SAM-METHYLASE<-YjhX_toxin||?->REase+iREase->HEPN->	1213	Bacteria>Pseudomonadota>Gammaproteobacteria>Enterobacterales>Enterobacteriaceae>Salmonella	GCA_010551565.1	Salmonella_enterica_subsp._enterica_serovar_4,[5],12:i:-	B7921_002250	108064..111705	440524	BREX-system-P-loop-protein-BrxC-[Salmonella-enterica-subsp.-enterica-serovar-4,[5]
EDT6691760.1	Type1.BREX	<-GntR-HTH++TrmB+ZDNA-HTH||<-?<-HEPN<-?||YjhX_toxin->SAM-METHYLASE->?->BrxA-DNA-binding->BrxB-BrxF-iSTAND->DUF6079*->N6-METHYLASE->OLD-ABC->TOPRIM->PglZ->SIGMA+OB-Fold+AAA-ATPase+Lon-Protease-BrxL-><-REase<-NACHT+SF1-SF2-Helicase+HKD-DNase<-Arm-DNA-bind_3	1213	Bacteria>Pseudomonadota>Gammaproteobacteria>Enterobacterales>Enterobacteriaceae>Salmonella	GCA_010555745.1	Salmonella_enterica_subsp._enterica	H2V91_002192	153095..156736	59201	BREX-system-P-loop-protein-BrxC-[Salmonella-enterica-subsp.-enterica]
EDT7239824.1	Type1.BREX	<-HEPN<-?||YjhX_toxin->SAM-METHYLASE->?->BrxA-DNA-binding->BrxB-BrxF-iSTAND->DUF6079*->N6-METHYLASE->OLD-ABC->TOPRIM->PglZ->SIGMA+OB-Fold+AAA-ATPase+Lon-Protease-BrxL-><-REase<-NACHT+SF1-SF2-Helicase+HKD-DNase<-Arm-DNA-bind_3	1213	Bacteria>Pseudomonadota>Gammaproteobacteria>Enterobacterales>Enterobacteriaceae>Salmonella	GCA_010557585.1	Salmonella_enterica_subsp._enterica	AHZ96_001967	93382..97023	59201	BREX-system-P-loop-protein-BrxC-[Salmonella-enterica-subsp.-enterica]
EDT7223673.1	Type1.BREX	<-MukE<-wHTH||wHTH+WYL+WCX-><-MarR-wHTH<-SIGMA-HTH+REase<-wHTH+SAD+HNH||<-ZnR||BrxA-DNA-binding->BrxB-BrxF-iSTAND->DUF6079*->N6-METHYLASE->OLD-ABC->TOPRIM->PglZ->SIGMA+OB-Fold+AAA-ATPase+Lon-Protease-BrxL->HKD-DNase+OGG-like-><-REase<-NACHT+SF1-SF2-Helicase+HKD-DNase<-Arm-DNA-bind_3	1204	Bacteria>Pseudomonadota>Gammaproteobacteria>Enterobacterales>Enterobacteriaceae>Salmonella	GCA_010559045.1	Salmonella_enterica_subsp._enterica	AH268_003334	169288..172902	59201	BREX-system-P-loop-protein-BrxC-[Salmonella-enterica-subsp.-enterica]
EDT8878002.1	Type1.BREX	<-HEPN<-?||YjhX_toxin->SAM-METHYLASE->?->BrxA-DNA-binding->BrxB-BrxF-iSTAND->DUF6079*->OLD-ABC->PglZ->SIGMA+OB-Fold+AAA-ATPase+Lon-Protease-BrxL->?-><-REase<-HKD-DNase	1213	Bacteria>Pseudomonadota>Gammaproteobacteria>Enterobacterales>Enterobacteriaceae>Salmonella	GCA_010565975.1	Salmonella_enterica_subsp._enterica	EK51_003288	149002..152643	59201	BREX-system-P-loop-protein-BrxC-[Salmonella-enterica-subsp.-enterica]
EDT9358547.1	Type1.BREX	<-HEPN<-?||YjhX_toxin->SAM-METHYLASE->?->BrxA-DNA-binding->BrxB-BrxF-iSTAND->DUF6079*->N6-METHYLASE-p->	1213	Bacteria>Pseudomonadota>Gammaproteobacteria>Enterobacterales>Enterobacteriaceae>Salmonella	GCA_010568345.1	Salmonella_enterica_subsp._enterica_serovar_Miami	A9571_000728	158750..162391	286780	BREX-system-P-loop-protein-BrxC-[Salmonella-enterica-subsp.-enterica-serovar-Miami]
EDU3766992.1	Type1.BREX	<-HEPN<-?||YjhX_toxin->SAM-METHYLASE->?->BrxA-DNA-binding->BrxB-BrxF-iSTAND->DUF6079*->N6-METHYLASE->OLD-ABC->TOPRIM->PglZ->SIGMA+OB-Fold+AAA-ATPase+Lon-Protease-BrxL->?-><-REase	1213	Bacteria>Pseudomonadota>Gammaproteobacteria>Enterobacterales>Enterobacteriaceae>Salmonella	GCA_010595665.1	Salmonella_enterica_subsp._enterica_serovar_Minnesota	C4D09_000357	390744..394385	70803	BREX-system-P-loop-protein-BrxC-[Salmonella-enterica-subsp.-enterica-serovar-Minnesota]
EDU7470137.1	Type1.BREX	Arm-DNA-bind_3->NACHT+SF1-SF2-Helicase+HKD-DNase->REase-><-SIGMA+OB-Fold+AAA-ATPase+Lon-Protease-BrxL<-PglZ<-TOPRIM<-OLD-ABC<-N6-METHYLASE<-DUF6079*<-BrxB-BrxF-iSTAND<-BrxA-DNA-binding<-?<-SAM-METHYLASE<-YjhX_toxin||?->REase+iREase->HEPN->	1213	Bacteria>Pseudomonadota>Gammaproteobacteria>Enterobacterales>Enterobacteriaceae>Salmonella	GCA_010625705.1	Salmonella_enterica_subsp._enterica	CQD18_002340	108064..111705	59201	BREX-system-P-loop-protein-BrxC-[Salmonella-enterica-subsp.-enterica]
EDU9161212.1	Type1.BREX	<-HIN-HTH||<-SIGMA+OB-Fold+AAA-ATPase+Lon-Protease-BrxL<-PglZ<-TOPRIM<-OLD-ABC<-N6-METHYLASE<-DUF6079*<-BrxB-BrxF-iSTAND<-BrxA-DNA-binding<-?<-SAM-METHYLASE<-YjhX_toxin||?->REase+iREase->HEPN->	1213	Bacteria>Pseudomonadota>Gammaproteobacteria>Enterobacterales>Enterobacteriaceae>Salmonella	GCA_010634945.1	Salmonella_enterica_subsp._enterica	IW54_004329	11544..15185	59201	BREX-system-P-loop-protein-BrxC-[Salmonella-enterica-subsp.-enterica]
EDU9624695.1	Type1.BREX	<-HEPN<-REase+iREase<-?||YjhX_toxin->SAM-METHYLASE->?->BrxA-DNA-binding->BrxB-BrxF-iSTAND->DUF6079*->N6-METHYLASE->OLD-ABC->TOPRIM->PglZ->SIGMA+OB-Fold+AAA-ATPase+Lon-Protease-BrxL-><-REase<-Couple_hipA+HipA_C<-HTH_3	1213	Bacteria>Pseudomonadota>Gammaproteobacteria>Enterobacterales>Enterobacteriaceae>Salmonella	GCA_010637145.1	Salmonella_enterica_subsp._enterica	XW65_002877	59802..63443	59201	BREX-system-P-loop-protein-BrxC-[Salmonella-enterica-subsp.-enterica]
EDV2155932.1	Type1.BREX	<-HEPN<-?||YjhX_toxin->SAM-METHYLASE->?->BrxA-DNA-binding->BrxB-BrxF-iSTAND->DUF6079*->N6-METHYLASE->OLD-ABC->TOPRIM->PglZ->SIGMA+OB-Fold+AAA-ATPase+Lon-Protease-BrxL-><-REase<-Couple_hipA+HipA_C<-HTH_3	1213	Bacteria>Pseudomonadota>Gammaproteobacteria>Enterobacterales>Enterobacteriaceae>Salmonella	GCA_010649605.1	Salmonella_enterica	JR65_004155	25186..28827	28901	BREX-system-P-loop-protein-BrxC-[Salmonella-enterica]
EDV2418012.1	Type1.BREX	HTH_3->Couple_hipA+HipA_C->REase-><-SIGMA+OB-Fold+AAA-ATPase+Lon-Protease-BrxL<-PglZ<-TOPRIM<-OLD-ABC<-N6-METHYLASE<-DUF6079*<-BrxB-BrxF-iSTAND<-BrxA-DNA-binding<-?<-SAM-METHYLASE<-YjhX_toxin||?->REase+iREase->HEPN->	1213	Bacteria>Pseudomonadota>Gammaproteobacteria>Enterobacterales>Enterobacteriaceae>Salmonella	GCA_010650645.1	Salmonella_enterica_subsp._enterica_serovar_Ohio	CQ222_000233	225731..229372	117541	BREX-system-P-loop-protein-BrxC-[Salmonella-enterica-subsp.-enterica-serovar-Ohio]
HAE7521102.1	Type1.BREX	<-HEPN<-?||YjhX_toxin->SAM-METHYLASE->?->BrxA-DNA-binding->BrxB-BrxF-iSTAND->DUF6079*->N6-METHYLASE->PglZ->SIGMA+OB-Fold+AAA-ATPase+Lon-Protease-BrxL->?-><-Arm-DNA-bind_3+Phage_integrase<-tRNA||?-><-SKI||METHYLASE->	1213	Bacteria>Pseudomonadota>Gammaproteobacteria>Enterobacterales>Enterobacteriaceae>Salmonella	GCA_010655205.1	Salmonella_enterica_subsp._enterica_serovar_Carrau	G4P13_000881	149047..152688	1160739	BREX-system-P-loop-protein-BrxC-[Salmonella-enterica-subsp.-enterica-serovar-Carrau]
EDV4604931.1	Type1.BREX	Arm-DNA-bind_3->NACHT+SF1-SF2-Helicase+HKD-DNase->REase-><-SIGMA+OB-Fold+AAA-ATPase+Lon-Protease-BrxL<-PglZ<-TOPRIM<-OLD-ABC<-N6-METHYLASE<-DUF6079*<-BrxB-BrxF-iSTAND<-BrxA-DNA-binding<-?<-SAM-METHYLASE<-YjhX_toxin||?->REase+iREase->HEPN->	1213	Bacteria>Pseudomonadota>Gammaproteobacteria>Enterobacterales>Enterobacteriaceae>Salmonella	GCA_010662535.1	Salmonella_enterica_subsp._enterica	ZP62_000973	72422..76063	59201	BREX-system-P-loop-protein-BrxC-[Salmonella-enterica-subsp.-enterica]
EDV5116990.1	Type1.BREX	<-HEPN<-?||YjhX_toxin->SAM-METHYLASE->?->BrxA-DNA-binding->BrxB-BrxF-iSTAND->DUF6079*->N6-METHYLASE->OLD-ABC->TOPRIM->PglZ->SIGMA+OB-Fold+AAA-ATPase+Lon-Protease-BrxL->HKD-DNase+OGG-like-><-REase<-NACHT+SF1-SF2-Helicase+HKD-DNase<-Arm-DNA-bind_3	1213	Bacteria>Pseudomonadota>Gammaproteobacteria>Enterobacterales>Enterobacteriaceae>Salmonella	GCA_010665025.1	Salmonella_enterica_subsp._enterica	ZJ36_000825	28924..32565	59201	BREX-system-P-loop-protein-BrxC-[Salmonella-enterica-subsp.-enterica]
EDV6600966.1	Type1.BREX	Arm-DNA-bind_3->NACHT+SF1-SF2-Helicase+HKD-DNase->REase-><-SIGMA+OB-Fold+AAA-ATPase+Lon-Protease-BrxL<-PglZ<-TOPRIM<-OLD-ABC<-N6-METHYLASE<-DUF6079*<-BrxB-BrxF-iSTAND<-BrxA-DNA-binding<-?<-SAM-METHYLASE<-YjhX_toxin||?->REase+iREase->HEPN->	1213	Bacteria>Pseudomonadota>Gammaproteobacteria>Enterobacterales>Enterobacteriaceae>Salmonella	GCA_010670385.1	Salmonella_enterica_subsp._enterica_serovar_Typhimurium_var._5-	BH535_001152	81343..84984	1620419	BREX-system-P-loop-protein-BrxC-[Salmonella-enterica-subsp.-enterica-serovar-Typhimurium-var.-5-]
EDV9644204.1	Type1.BREX	<-HEPN<-REase+iREase<-?||YjhX_toxin->SAM-METHYLASE->?->||BrxA-DNA-binding->BrxB-BrxF-iSTAND->DUF6079*->N6-METHYLASE->OLD-ABC->TOPRIM->PglZ->SIGMA+OB-Fold+AAA-ATPase+Lon-Protease-BrxL-><-REase<-Couple_hipA+HipA_C<-HTH_3	1213	Bacteria>Pseudomonadota>Gammaproteobacteria>Enterobacterales>Enterobacteriaceae>Salmonella	GCA_010680145.1	Salmonella_enterica_subsp._enterica_serovar_Carno	HH25_003997	164513..168154	2024268	BREX-system-P-loop-protein-BrxC-[Salmonella-enterica-subsp.-enterica-serovar-Carno]
EDV9109398.1	Type1.BREX	<-HEPN<-?||YjhX_toxin->SAM-METHYLASE->?->BrxA-DNA-binding->BrxB-BrxF-iSTAND->DUF6079*->N6-METHYLASE->OLD-ABC->TOPRIM->PglZ->SIGMA+OB-Fold+AAA-ATPase+Lon-Protease-BrxL->HKD-DNase+OGG-like-><-REase<-NACHT+SF1-SF2-Helicase+HKD-DNase<-Arm-DNA-bind_3	1213	Bacteria>Pseudomonadota>Gammaproteobacteria>Enterobacterales>Enterobacteriaceae>Salmonella	GCA_010691445.1	Salmonella_enterica_subsp._enterica	ZX68_001600	51998..55639	59201	BREX-system-P-loop-protein-BrxC-[Salmonella-enterica-subsp.-enterica]
EDW0696245.1	Type1.BREX	<-HEPN<-?||YjhX_toxin->SAM-METHYLASE->?->BrxA-DNA-binding->BrxB-BrxF-iSTAND->DUF6079*->N6-METHYLASE->OLD-ABC->TOPRIM->PglZ->SIGMA+OB-Fold+AAA-ATPase+Lon-Protease-BrxL-><-REase<-NACHT+SF1-SF2-Helicase+HKD-DNase	1213	Bacteria>Pseudomonadota>Gammaproteobacteria>Enterobacterales>Enterobacteriaceae>Salmonella	GCA_010695935.1	Salmonella_enterica_subsp._enterica	KV01_000535	150350..153991	59201	BREX-system-P-loop-protein-BrxC-[Salmonella-enterica-subsp.-enterica]
EDW2626492.1	Type1.BREX	<-HEPN<-REase+iREase<-?||YjhX_toxin->SAM-METHYLASE->?->BrxA-DNA-binding->BrxB-BrxF-iSTAND->DUF6079*->N6-METHYLASE->OLD-ABC->TOPRIM->PglZ->SIGMA+OB-Fold+AAA-ATPase+Lon-Protease-BrxL-><-REase<-Couple_hipA+HipA_C<-HTH_3	1213	Bacteria>Pseudomonadota>Gammaproteobacteria>Enterobacterales>Enterobacteriaceae>Salmonella	GCA_010704865.1	Salmonella_enterica_subsp._enterica	XX60_002382	156709..160350	59201	BREX-system-P-loop-protein-BrxC-[Salmonella-enterica-subsp.-enterica]
EDW4488615.1	Type1.BREX	<-HEPN<-?||YjhX_toxin->SAM-METHYLASE->?->BrxA-DNA-binding->BrxB-BrxF-iSTAND->DUF6079*->N6-METHYLASE->OLD-ABC->TOPRIM->PglZ->SIGMA+OB-Fold+AAA-ATPase+Lon-Protease-BrxL->HKD-DNase+OGG-like-><-REase<-NACHT+SF1-SF2-Helicase+HKD-DNase	1213	Bacteria>Pseudomonadota>Gammaproteobacteria>Enterobacterales>Enterobacteriaceae>Salmonella	GCA_010711495.1	Salmonella_enterica_subsp._enterica	YV39_000487	166105..169746	59201	BREX-system-P-loop-protein-BrxC-[Salmonella-enterica-subsp.-enterica]
EDW7722488.1	Type1.BREX	<-HEPN<-REase+iREase<-?||YjhX_toxin->SAM-METHYLASE->?->BrxA-DNA-binding->BrxB-BrxF-iSTAND->DUF6079*->N6-METHYLASE->OLD-ABC->TOPRIM->PglZ->SIGMA+OB-Fold+AAA-ATPase+Lon-Protease-BrxL-><-REase<-Couple_hipA+HipA_C<-HTH_3	1213	Bacteria>Pseudomonadota>Gammaproteobacteria>Enterobacterales>Enterobacteriaceae>Salmonella	GCA_010729405.1	Salmonella_enterica	GTR06_001871	397975..401616	28901	BREX-system-P-loop-protein-BrxC-[Salmonella-enterica]
EDX0369803.1	Type1.BREX	<-HEPN<-REase+iREase<-?||YjhX_toxin->SAM-METHYLASE->?->||BrxA-DNA-binding->BrxB-BrxF-iSTAND->DUF6079*->N6-METHYLASE->OLD-ABC->TOPRIM->PglZ->SIGMA+OB-Fold+AAA-ATPase+Lon-Protease-BrxL->HKD-DNase+OGG-like-><-REase<-NACHT+SF1-SF2-Helicase+HKD-DNase<-Arm-DNA-bind_3	1213	Bacteria>Pseudomonadota>Gammaproteobacteria>Enterobacterales>Enterobacteriaceae>Salmonella	GCA_010742735.1	Salmonella_enterica	GTL21_000361	386606..390247	28901	BREX-system-P-loop-protein-BrxC-[Salmonella-enterica]
EDX7895849.1	Type1.BREX	<-HEPN<-REase+iREase<-?||YjhX_toxin->SAM-METHYLASE->?->BrxA-DNA-binding->BrxB-BrxF-iSTAND->DUF6079*->N6-METHYLASE->OLD-ABC->TOPRIM->PglZ->SIGMA+OB-Fold+AAA-ATPase+Lon-Protease-BrxL-><-REase<-Arm-DNA-bind_3<-tRNA||?-><-SKI	1213	Bacteria>Pseudomonadota>Gammaproteobacteria>Enterobacterales>Enterobacteriaceae>Salmonella	GCA_010782335.1	Salmonella_enterica_subsp._enterica_serovar_4,[5],12:i:-	C2Y98_001768	38662..42303	440524	BREX-system-P-loop-protein-BrxC-[Salmonella-enterica-subsp.-enterica-serovar-4,[5]
EDZ7084220.1	Type1.BREX	BrxA-DNA-binding->BrxB-BrxF-iSTAND->DUF6079*->N6-METHYLASE->OLD-ABC->TOPRIM->PglZ->SIGMA+OB-Fold+AAA-ATPase+Lon-Protease-BrxL-><-REase<-NACHT+SF1-SF2-Helicase+HKD-DNase<-Arm-DNA-bind_3	1213	Bacteria>Pseudomonadota>Gammaproteobacteria>Enterobacterales>Enterobacteriaceae>Salmonella	GCA_010862365.1	Salmonella_enterica	GS086_003556	2815..6456	28901	BREX-system-P-loop-protein-BrxC-[Salmonella-enterica]
EEB8473036.1	Type1.BREX	<-HEPN<-REase+iREase<-?||YjhX_toxin->SAM-METHYLASE->?->BrxA-DNA-binding->BrxB-BrxF-iSTAND->DUF6079*->N6-METHYLASE->OLD-ABC->TOPRIM->PglZ->SIGMA+OB-Fold+AAA-ATPase+Lon-Protease-BrxL-><-REase<-Arm-DNA-bind_3<-tRNA||?-><-SKI	1213	Bacteria>Pseudomonadota>Gammaproteobacteria>Enterobacterales>Enterobacteriaceae>Salmonella	GCA_010964635.1	Salmonella_enterica	GN471_06045	170718..174359	28901	BREX-system-P-loop-protein-BrxC-[Salmonella-enterica]
EEC0292871.1	Type1.BREX	<-GntR-HTH++TrmB+ZDNA-HTH||<-?<-HEPN<-?||YjhX_toxin->SAM-METHYLASE->?->||BrxA-DNA-binding->BrxB-BrxF-iSTAND->DUF6079*->N6-METHYLASE->OLD-ABC->TOPRIM->PglZ->SIGMA+OB-Fold+AAA-ATPase+Lon-Protease-BrxL->?->||HIN-HTH-><-tRNA||?-><-SKI	1213	Bacteria>Pseudomonadota>Gammaproteobacteria>Enterobacterales>Enterobacteriaceae>Salmonella	GCA_010970835.1	Salmonella_enterica_subsp._enterica	YV76_000179	186389..190030	59201	BREX-system-P-loop-protein-BrxC-[Salmonella-enterica-subsp.-enterica]
EEC1070526.1	Type1.BREX	<-HEPN<-REase+iREase<-?||YjhX_toxin->SAM-METHYLASE->?->BrxA-DNA-binding->BrxB-BrxF-iSTAND->DUF6079*->N6-METHYLASE->OLD-ABC->TOPRIM->PglZ->SIGMA+OB-Fold+AAA-ATPase+Lon-Protease-BrxL-><-REase<-NACHT+SF1-SF2-Helicase+HKD-DNase<-Arm-DNA-bind_3	1213	Bacteria>Pseudomonadota>Gammaproteobacteria>Enterobacterales>Enterobacteriaceae>Salmonella	GCA_010973255.1	Salmonella_enterica_subsp._enterica	AH545_000138	139989..143630	59201	BREX-system-P-loop-protein-BrxC-[Salmonella-enterica-subsp.-enterica]
EEC4816191.1	Type1.BREX	<-HEPN<-?||YjhX_toxin->SAM-METHYLASE->?->BrxA-DNA-binding->BrxB-BrxF-iSTAND->DUF6079*->OLD-ABC-><-?<-?<-?<-?<-?<-?<-?<-XIS-HTH	1213	Bacteria>Pseudomonadota>Gammaproteobacteria>Enterobacterales>Enterobacteriaceae>Salmonella	GCA_010993355.1	Salmonella_enterica	G4L83_001633	157032..160673	28901	BREX-system-P-loop-protein-BrxC-[Salmonella-enterica]
EED3636775.1	Type1.BREX	<-HEPN<-?||YjhX_toxin->SAM-METHYLASE->?->BrxA-DNA-binding->BrxB-BrxF-iSTAND->DUF6079*->N6-METHYLASE->OLD-ABC->TOPRIM->PglZ->SIGMA+OB-Fold+AAA-ATPase+Lon-Protease-BrxL->?-><-REase<-NACHT+SF1-SF2-Helicase+HKD-DNase<-Arm-DNA-bind_3	1213	Bacteria>Pseudomonadota>Gammaproteobacteria>Enterobacterales>Enterobacteriaceae>Salmonella	GCA_011081015.1	Salmonella_enterica_subsp._enterica	S719_003281	157579..161220	59201	BREX-system-P-loop-protein-BrxC-[Salmonella-enterica-subsp.-enterica]
EEE1152714.1	Type1.BREX	REase->NACHT+SF1-SF2-Helicase+HKD-DNase->REase-><-HKD-DNase+OGG-like<-SIGMA+OB-Fold+AAA-ATPase+Lon-Protease-BrxL<-PglZ<-TOPRIM<-OLD-ABC<-N6-METHYLASE<-DUF6079*<-BrxB-BrxF-iSTAND<-BrxA-DNA-binding<-?<-SAM-METHYLASE<-YjhX_toxin||?->HEPN->	1213	Bacteria>Pseudomonadota>Gammaproteobacteria>Enterobacterales>Enterobacteriaceae>Salmonella	GCA_011117415.1	Salmonella_enterica_subsp._enterica_serovar_Teko	CAE49_001975	330756..334397	2079198	BREX-system-P-loop-protein-BrxC-[Salmonella-enterica-subsp.-enterica-serovar-Teko]
HAF0812803.1	Type1.BREX	Arm-DNA-bind_3->NACHT+SF1-SF2-Helicase+HKD-DNase->REase-><-SIGMA+OB-Fold+AAA-ATPase+Lon-Protease-BrxL<-PglZ<-TOPRIM<-OLD-ABC<-N6-METHYLASE<-DUF6079*<-BrxB-BrxF-iSTAND<-BrxA-DNA-binding<-?<-SAM-METHYLASE<-YjhX_toxin||?->REase+iREase->HEPN->	1213	Bacteria>Pseudomonadota>Gammaproteobacteria>Enterobacterales>Enterobacteriaceae>Salmonella	GCA_011125695.1	Salmonella_enterica	G9G17_001783	108064..111705	28901	BREX-system-P-loop-protein-BrxC-[Salmonella-enterica]
EEE6742962.1	Type1.BREX	<-HEPN<-?||YjhX_toxin->SAM-METHYLASE->?->BrxA-DNA-binding->BrxB-BrxF-iSTAND->DUF6079*->N6-METHYLASE->OLD-ABC->TOPRIM->PglZ->SIGMA+OB-Fold+AAA-ATPase+Lon-Protease-BrxL->HKD-DNase+OGG-like-><-REase<-NACHT+SF1-SF2-Helicase+HKD-DNase	1213	Bacteria>Pseudomonadota>Gammaproteobacteria>Enterobacterales>Enterobacteriaceae>Salmonella	GCA_011144815.1	Salmonella_enterica_subsp._enterica_serovar_Westhampton	CBJ05_002020	408792..412433	356621	BREX-system-P-loop-protein-BrxC-[Salmonella-enterica-subsp.-enterica-serovar-Westhampton]
EEE9192570.1	Type1.BREX	<-HEPN<-REase+iREase<-?||YjhX_toxin->SAM-METHYLASE->?->BrxA-DNA-binding->BrxB-BrxF-iSTAND->DUF6079-p*->	555	Bacteria>Pseudomonadota>Gammaproteobacteria>Enterobacterales>Enterobacteriaceae>Salmonella	GCA_011156215.1	Salmonella_enterica_subsp._enterica_serovar_Typhimurium	DA558_21335	48506..50170	90371	BREX-system-P-loop-protein-BrxC-[Salmonella-enterica-subsp.-enterica-serovar-Typhimurium]
EEH0007757.1	Type1.BREX	HTH_3->Couple_hipA+HipA_C->REase-><-SIGMA+OB-Fold+AAA-ATPase+Lon-Protease-BrxL<-PglZ<-TOPRIM<-OLD-ABC<-N6-METHYLASE<-DUF6079*<-BrxB-BrxF-iSTAND<-BrxA-DNA-binding<-?<-SAM-METHYLASE<-YjhX_toxin||?->REase+iREase->HEPN->	1213	Bacteria>Pseudomonadota>Gammaproteobacteria>Enterobacterales>Enterobacteriaceae>Salmonella	GCA_011260745.1	Salmonella_enterica	G3P26_001654	368950..372591	28901	BREX-system-P-loop-protein-BrxC-[Salmonella-enterica]
EEH2707246.1	Type1.BREX	<-HEPN<-?||YjhX_toxin->SAM-METHYLASE->?->BrxA-DNA-binding->BrxB-BrxF-iSTAND->DUF6079*->N6-METHYLASE->OLD-ABC->TOPRIM->PglZ->SIGMA+OB-Fold+AAA-ATPase+Lon-Protease-BrxL->HKD-DNase+OGG-like-><-REase<-NACHT+SF1-SF2-Helicase+HKD-DNase	1213	Bacteria>Pseudomonadota>Gammaproteobacteria>Enterobacterales>Enterobacteriaceae>Salmonella	GCA_011274245.1	Salmonella_enterica_subsp._enterica	GYE71_000437	54012..57653	59201	BREX-system-P-loop-protein-BrxC-[Salmonella-enterica-subsp.-enterica]
EEH3158636.1	Type1.BREX	Arm-DNA-bind_3->NACHT+SF1-SF2-Helicase+HKD-DNase->REase-><-SIGMA+OB-Fold+AAA-ATPase+Lon-Protease-BrxL<-PglZ<-TOPRIM<-OLD-ABC<-N6-METHYLASE<-DUF6079*<-BrxB-BrxF-iSTAND<-BrxA-DNA-binding<-?<-SAM-METHYLASE<-YjhX_toxin||?->REase+iREase->HEPN->	1213	Bacteria>Pseudomonadota>Gammaproteobacteria>Enterobacterales>Enterobacteriaceae>Salmonella	GCA_011275545.1	Salmonella_enterica	GZB32_002496	108064..111705	28901	BREX-system-P-loop-protein-BrxC-[Salmonella-enterica]
EEH9995094.1	Type1.BREX	<-HEPN<-REase+iREase<-?||YjhX_toxin->SAM-METHYLASE->?->BrxA-DNA-binding->BrxB-BrxF-iSTAND->DUF6079*->N6-METHYLASE->OLD-ABC->TOPRIM->PglZ->SIGMA+OB-Fold+AAA-ATPase+Lon-Protease-BrxL-><-REase<-Arm-DNA-bind_3<-tRNA||?-><-SKI	1213	Bacteria>Pseudomonadota>Gammaproteobacteria>Enterobacterales>Enterobacteriaceae>Salmonella	GCA_011315605.1	Salmonella_enterica_subsp._enterica_serovar_4,[5],12:i:-	G7H85_000159	170419..174060	440524	BREX-system-P-loop-protein-BrxC-[Salmonella-enterica-subsp.-enterica-serovar-4,[5]
EEI0202361.1	Type1.BREX	Arm-DNA-bind_3->NACHT+SF1-SF2-Helicase+HKD-DNase->REase-><-SIGMA+OB-Fold+AAA-ATPase+Lon-Protease-BrxL<-PglZ<-TOPRIM<-OLD-ABC<-N6-METHYLASE<-DUF6079-p*	908	Bacteria>Pseudomonadota>Gammaproteobacteria>Enterobacterales>Enterobacteriaceae>Salmonella	GCA_011321375.1	Salmonella_enterica	GNM15_17120	108064..110791	28901	BREX-system-P-loop-protein-BrxC-[Salmonella-enterica]
EEI5362885.1	Type1.BREX	YjhX_toxin->SAM-METHYLASE->?->||?-><-ZnR<-ZnR||BrxA-DNA-binding->BrxB-BrxF-iSTAND->DUF6079*->N6-METHYLASE->OLD-ABC->TOPRIM->PglZ->SIGMA+OB-Fold+AAA-ATPase+Lon-Protease-BrxL->HKD-DNase+OGG-like-><-REase<-NACHT+SF1-SF2-Helicase+HKD-DNase<-Arm-DNA-bind_3	1204	Bacteria>Pseudomonadota>Gammaproteobacteria>Enterobacterales>Enterobacteriaceae>Salmonella	GCA_011357245.1	Salmonella_enterica_subsp._enterica_serovar_Kentucky	GXM95_001563	173373..176987	192955	BREX-system-P-loop-protein-BrxC-[Salmonella-enterica-subsp.-enterica-serovar-Kentucky]
EEJ3565315.1	Type1.BREX	<-HEPN<-?||YjhX_toxin->SAM-METHYLASE->?->BrxA-DNA-binding->BrxB-BrxF-iSTAND->DUF6079*->N6-METHYLASE->OLD-ABC->TOPRIM->PglZ->SIGMA+OB-Fold+AAA-ATPase+Lon-Protease-BrxL->?-><-REase<-NACHT+SF1-SF2-Helicase+HKD-DNase<-REase	1213	Bacteria>Pseudomonadota>Gammaproteobacteria>Enterobacterales>Enterobacteriaceae>Salmonella	GCA_011417115.1	Salmonella_enterica_subsp._enterica	AA437_001844	125694..129335	59201	BREX-system-P-loop-protein-BrxC-[Salmonella-enterica-subsp.-enterica]
EEJ7235528.1	Type1.BREX	<-HEPN<-?||YjhX_toxin->SAM-METHYLASE->?->BrxA-DNA-binding->BrxB-BrxF-iSTAND->DUF6079*->N6-METHYLASE->OLD-ABC->TOPRIM->PglZ->SIGMA+OB-Fold+AAA-ATPase+Lon-Protease-BrxL->	1213	Bacteria>Pseudomonadota>Gammaproteobacteria>Enterobacterales>Enterobacteriaceae>Salmonella	GCA_011437855.1	Salmonella_enterica_subsp._salamae	S483_003519	152399..156040	59202	BREX-system-P-loop-protein-BrxC-[Salmonella-enterica-subsp.-salamae]
EEJ7298184.1	Type1.BREX	HTH_3->Couple_hipA+HipA_C->REase-><-SIGMA+OB-Fold+AAA-ATPase+Lon-Protease-BrxL<-PglZ<-TOPRIM<-OLD-ABC<-N6-METHYLASE<-DUF6079*<-BrxB-BrxF-iSTAND<-BrxA-DNA-binding<-?<-SAM-METHYLASE<-YjhX_toxin||?->REase+iREase->HEPN->	1213	Bacteria>Pseudomonadota>Gammaproteobacteria>Enterobacterales>Enterobacteriaceae>Salmonella	GCA_011438135.1	Salmonella_enterica_subsp._enterica_serovar_Livingstone	DV24_002305	312113..315754	260680	BREX-system-P-loop-protein-BrxC-[Salmonella-enterica-subsp.-enterica-serovar-Livingstone]
EEJ9771966.1	Type1.BREX	<-HEPN<-REase+iREase<-?||YjhX_toxin->SAM-METHYLASE->?->BrxA-DNA-binding->BrxB-BrxF-iSTAND->DUF6079*->N6-METHYLASE->OLD-ABC->TOPRIM->PglZ->SIGMA+OB-Fold+AAA-ATPase+Lon-Protease-BrxL-><-REase<-NACHT+SF1-SF2-Helicase+HKD-DNase<-Arm-DNA-bind_3	1213	Bacteria>Pseudomonadota>Gammaproteobacteria>Enterobacterales>Enterobacteriaceae>Salmonella	GCA_011448615.1	Salmonella_enterica_subsp._enterica_serovar_Typhimurium	HL79_09630	170931..174572	90371	BREX-system-P-loop-protein-BrxC-[Salmonella-enterica-subsp.-enterica-serovar-Typhimurium]
EEK0538631.1	Type1.BREX	Arm-DNA-bind_3->NACHT+SF1-SF2-Helicase+HKD-DNase->REase-><-SIGMA+OB-Fold+AAA-ATPase+Lon-Protease-BrxL<-PglZ<-TOPRIM<-OLD-ABC<-N6-METHYLASE<-DUF6079*<-BrxB-BrxF-iSTAND<-BrxA-DNA-binding<-?<-SAM-METHYLASE<-YjhX_toxin||?->REase+iREase->HEPN->	1213	Bacteria>Pseudomonadota>Gammaproteobacteria>Enterobacterales>Enterobacteriaceae>Salmonella	GCA_011452795.1	Salmonella_enterica_subsp._enterica_serovar_Typhimurium	IH96_05510	150023..153664	90371	BREX-system-P-loop-protein-BrxC-[Salmonella-enterica-subsp.-enterica-serovar-Typhimurium]
EEK5480526.1	Type1.BREX	<-HEPN<-REase+iREase<-?||YjhX_toxin->SAM-METHYLASE->?->BrxA-DNA-binding->BrxB-BrxF-iSTAND->DUF6079*->N6-METHYLASE->OLD-ABC->TOPRIM->PglZ->SIGMA+OB-Fold+AAA-ATPase+Lon-Protease-BrxL-><-REase<-NACHT+SF1-SF2-Helicase+HKD-DNase<-Arm-DNA-bind_3	1213	Bacteria>Pseudomonadota>Gammaproteobacteria>Enterobacterales>Enterobacteriaceae>Salmonella	GCA_011476295.1	Salmonella_enterica_subsp._enterica_serovar_Typhimurium	K6Z50_000356	395891..399532	90371	BREX-system-P-loop-protein-BrxC-[Salmonella-enterica-subsp.-enterica-serovar-Typhimurium]
EEL0135869.1	Type1.BREX	Arm-DNA-bind_3->NACHT+SF1-SF2-Helicase+HKD-DNase->REase-><-SIGMA+OB-Fold+AAA-ATPase+Lon-Protease-BrxL<-PglZ<-TOPRIM<-OLD-ABC<-N6-METHYLASE<-DUF6079*<-BrxB-BrxF-iSTAND<-BrxA-DNA-binding<-?<-SAM-METHYLASE<-YjhX_toxin||?->REase+iREase->HEPN->	1213	Bacteria>Pseudomonadota>Gammaproteobacteria>Enterobacterales>Enterobacteriaceae>Salmonella	GCA_011496945.1	Salmonella_enterica	G4L63_001980	126120..129761	28901	BREX-system-P-loop-protein-BrxC-[Salmonella-enterica]
EEL6995865.1	Type1.BREX	<-HEPN<-REase+iREase<-?||YjhX_toxin->SAM-METHYLASE->?->BrxA-DNA-binding->BrxB-BrxF-iSTAND->DUF6079*->N6-METHYLASE->	1213	Bacteria>Pseudomonadota>Gammaproteobacteria>Enterobacterales>Enterobacteriaceae>Salmonella	GCA_011533325.1	Salmonella_enterica	FPY45_19715	27496..31137	28901	BREX-system-P-loop-protein-BrxC-[Salmonella-enterica]
EEL7097503.1	Type1.BREX	<-HEPN<-?||YjhX_toxin->SAM-METHYLASE->?->BrxA-DNA-binding->BrxB-BrxF-iSTAND->DUF6079*->N6-METHYLASE->OLD-ABC->TOPRIM->PglZ->SIGMA+OB-Fold+AAA-ATPase+Lon-Protease-BrxL->?-><-REase<-NACHT+SF1-SF2-Helicase+HKD-DNase<-Arm-DNA-bind_3	1213	Bacteria>Pseudomonadota>Gammaproteobacteria>Enterobacterales>Enterobacteriaceae>Salmonella	GCA_011533985.1	Salmonella_enterica	GKH84_08575	158871..162512	28901	BREX-system-P-loop-protein-BrxC-[Salmonella-enterica]
EEM2455575.1	Type1.BREX	<-HEPN<-?||YjhX_toxin->SAM-METHYLASE->?->||?-><-ZnR||BrxA-DNA-binding->BrxB-BrxF-iSTAND->DUF6079*->N6-METHYLASE->OLD-ABC->TOPRIM->PglZ->SIGMA+OB-Fold+AAA-ATPase+Lon-Protease-BrxL->HKD-DNase+OGG-like-><-REase<-NACHT+SF1-SF2-Helicase+HKD-DNase<-Arm-DNA-bind_3	1204	Bacteria>Pseudomonadota>Gammaproteobacteria>Enterobacterales>Enterobacteriaceae>Salmonella	GCA_011558015.1	Salmonella_enterica_subsp._enterica_serovar_Kentucky	CWG70_02775	173706..177320	192955	BREX-system-P-loop-protein-BrxC-[Salmonella-enterica-subsp.-enterica-serovar-Kentucky]
EED3564960.1	Type1.BREX	<-HEPN<-?||YjhX_toxin->SAM-METHYLASE->?->BrxA-DNA-binding->BrxB-BrxF-iSTAND->DUF6079*->N6-METHYLASE->OLD-ABC->TOPRIM->PglZ->SIGMA+OB-Fold+AAA-ATPase+Lon-Protease-BrxL->HKD-DNase+OGG-like-><-REase<-NACHT+SF1-SF2-Helicase+HKD-DNase	1213	Bacteria>Pseudomonadota>Gammaproteobacteria>Enterobacterales>Enterobacteriaceae>Salmonella	GCA_011665275.1	Salmonella_enterica_subsp._enterica_serovar_Poona	DEI68_08995	52368..56009	436295	BREX-system-P-loop-protein-BrxC-[Salmonella-enterica-subsp.-enterica-serovar-Poona]
EEP2127992.1	Type1.BREX	<-HEPN<-REase+iREase<-?||YjhX_toxin->SAM-METHYLASE->?->BrxA-DNA-binding->BrxB-BrxF-iSTAND->DUF6079*->N6-METHYLASE->OLD-ABC->TOPRIM->PglZ->SIGMA+OB-Fold+AAA-ATPase+Lon-Protease-BrxL->||PIN-><-HIN-HTH	1213	Bacteria>Pseudomonadota>Gammaproteobacteria>Enterobacterales>Enterobacteriaceae>Salmonella	GCA_011714235.1	Salmonella_enterica	HA780_002410	111301..114942	28901	BREX-system-P-loop-protein-BrxC-[Salmonella-enterica]
EEP5746981.1	Type1.BREX	Arm-DNA-bind_3->NACHT+SF1-SF2-Helicase+HKD-DNase->REase-><-SIGMA+OB-Fold+AAA-ATPase+Lon-Protease-BrxL<-PglZ<-TOPRIM<-OLD-ABC<-N6-METHYLASE<-DUF6079*<-BrxB-BrxF-iSTAND<-BrxA-DNA-binding<-?<-SAM-METHYLASE<-YjhX_toxin||?->REase+iREase->HEPN->	1213	Bacteria>Pseudomonadota>Gammaproteobacteria>Enterobacterales>Enterobacteriaceae>Salmonella	GCA_011732675.1	Salmonella_enterica	HBB29_002275	108064..111705	28901	BREX-system-P-loop-protein-BrxC-[Salmonella-enterica]
EEP8318663.1	Type1.BREX	<-HEPN<-REase+iREase<-?||YjhX_toxin->SAM-METHYLASE->?->BrxA-DNA-binding->BrxB-BrxF-iSTAND->DUF6079*->N6-METHYLASE->OLD-ABC->TOPRIM->PglZ->SIGMA+OB-Fold+AAA-ATPase+Lon-Protease-BrxL-><-REase<-Couple_hipA+HipA_C<-HTH_3	1213	Bacteria>Pseudomonadota>Gammaproteobacteria>Enterobacterales>Enterobacteriaceae>Salmonella	GCA_011746585.1	Salmonella_enterica_subsp._enterica	HCM12_000954	52440..56081	59201	BREX-system-P-loop-protein-BrxC-[Salmonella-enterica-subsp.-enterica]
EEP9788441.1	Type1.BREX	<-HEPN<-REase+iREase<-?||YjhX_toxin->SAM-METHYLASE->?->BrxA-DNA-binding->BrxB-BrxF-iSTAND->DUF6079*->N6-METHYLASE->OLD-ABC->TOPRIM->PglZ->SIGMA+OB-Fold+AAA-ATPase+Lon-Protease-BrxL-><-REase<-NACHT+SF1-SF2-Helicase+HKD-DNase<-Arm-DNA-bind_3	1213	Bacteria>Pseudomonadota>Gammaproteobacteria>Enterobacterales>Enterobacteriaceae>Salmonella	GCA_011756305.1	Salmonella_enterica_subsp._enterica_serovar_Saintpaul	HCP67_002105	64937..68578	90105	BREX-system-P-loop-protein-BrxC-[Salmonella-enterica-subsp.-enterica-serovar-Saintpaul]
HAF9991656.1	Type1.BREX	<-HEPN<-REase+iREase<-?||YjhX_toxin->SAM-METHYLASE->?->BrxA-DNA-binding->BrxB-BrxF-iSTAND->DUF6079*->N6-METHYLASE->OLD-ABC->TOPRIM->PglZ->SIGMA+OB-Fold+AAA-ATPase+Lon-Protease-BrxL-><-REase<-NACHT+SF1-SF2-Helicase+HKD-DNase<-Arm-DNA-bind_3	1213	Bacteria>Pseudomonadota>Gammaproteobacteria>Enterobacterales>Enterobacteriaceae>Salmonella	GCA_011779145.1	Salmonella_enterica	G8Q07_002648	170744..174385	28901	BREX-system-P-loop-protein-BrxC-[Salmonella-enterica]
HAG2842216.1	Type1.BREX	Arm-DNA-bind_3->NACHT+SF1-SF2-Helicase+HKD-DNase->REase-><-SIGMA+OB-Fold+AAA-ATPase+Lon-Protease-BrxL<-PglZ<-TOPRIM<-OLD-ABC<-N6-METHYLASE<-DUF6079*<-BrxB-BrxF-iSTAND<-BrxA-DNA-binding<-?<-SAM-METHYLASE<-YjhX_toxin||?->REase+iREase->HEPN->	1213	Bacteria>Pseudomonadota>Gammaproteobacteria>Enterobacterales>Enterobacteriaceae>Salmonella	GCA_011791385.1	Salmonella_enterica	G8Z41_002592	114973..118614	28901	BREX-system-P-loop-protein-BrxC-[Salmonella-enterica]
HAG5552356.1	Type1.BREX	Arm-DNA-bind_3->NACHT+SF1-SF2-Helicase+HKD-DNase->REase-><-SIGMA+OB-Fold+AAA-ATPase+Lon-Protease-BrxL<-PglZ<-TOPRIM<-OLD-ABC<-N6-METHYLASE<-DUF6079*<-BrxB-BrxF-iSTAND<-BrxA-DNA-binding<-?<-SAM-METHYLASE<-YjhX_toxin||?->REase+iREase->HEPN->	1213	Bacteria>Pseudomonadota>Gammaproteobacteria>Enterobacterales>Enterobacteriaceae>Salmonella	GCA_011798725.1	Salmonella_enterica	G8Q25_001512	186770..190411	28901	BREX-system-P-loop-protein-BrxC-[Salmonella-enterica]
EFP5236617.1	Type1.BREX	<-HEPN<-REase+iREase<-?||YjhX_toxin->SAM-METHYLASE->?->BrxA-DNA-binding->BrxB-BrxF-iSTAND->DUF6079*->N6-METHYLASE->OLD-ABC->TOPRIM->PglZ->SIGMA+OB-Fold+AAA-ATPase+Lon-Protease-BrxL-><-REase<-NACHT+SF1-SF2-Helicase+HKD-DNase<-Arm-DNA-bind_3	1213	Bacteria>Pseudomonadota>Gammaproteobacteria>Enterobacterales>Enterobacteriaceae>Salmonella	GCA_013442935.1	Salmonella_enterica	H1F05_002027	170348..173989	28901	BREX-system-P-loop-protein-BrxC-[Salmonella-enterica]
EFP6288854.1	Type1.BREX	<-HEPN<-REase+iREase<-?||YjhX_toxin->SAM-METHYLASE->?->BrxA-DNA-binding->BrxB-BrxF-iSTAND->DUF6079-p*->	755	Bacteria>Pseudomonadota>Gammaproteobacteria>Enterobacterales>Enterobacteriaceae>Salmonella	GCA_013446435.1	Salmonella_enterica	H1E36_001915	171036..173302	28901	BREX-system-P-loop-protein-BrxC-[Salmonella-enterica]
EFQ1578017.1	Type1.BREX	<-HEPN<-REase+iREase<-?||YjhX_toxin->SAM-METHYLASE->?->BrxA-DNA-binding->BrxB-BrxF-iSTAND->DUF6079*->N6-METHYLASE->OLD-ABC->TOPRIM->PglZ->SIGMA+OB-Fold+AAA-ATPase+Lon-Protease-BrxL-><-REase<-NACHT+SF1-SF2-Helicase+HKD-DNase<-Arm-DNA-bind_3	1213	Bacteria>Pseudomonadota>Gammaproteobacteria>Enterobacterales>Enterobacteriaceae>Salmonella	GCA_013479125.1	Salmonella_enterica	H1L81_001832	38672..42313	28901	BREX-system-P-loop-protein-BrxC-[Salmonella-enterica]
HAK0222817.1	Type1.BREX	<-ParB<-MerR-HTH+ParA-Soj-PloopNTPase||?->DEDD_Tnp_IS110+Transposase_20-><-RHH||?->wHTH+WYL+WCX->BrxA-DNA-binding->BrxB-BrxF-iSTAND->DUF6079-p*->	805	Bacteria>Pseudomonadota>Gammaproteobacteria>Enterobacterales>Enterobacteriaceae>Salmonella	GCA_013485925.1	Salmonella_enterica	H1O47_004541	10945..13359	28901	BREX-system-P-loop-protein-BrxC-[Salmonella-enterica]
HAK0744369.1	Type1.BREX	<-HEPN<-REase+iREase<-?||YjhX_toxin->SAM-METHYLASE->?->BrxA-DNA-binding->BrxB-BrxF-iSTAND->DUF6079-p*->	982	Bacteria>Pseudomonadota>Gammaproteobacteria>Enterobacterales>Enterobacteriaceae>Salmonella	GCA_013487565.1	Salmonella_enterica	H1P65_002115	170411..173355	28901	BREX-system-P-loop-protein-BrxC-[Salmonella-enterica]
EFQ3650906.1	Type1.BREX	<-HEPN<-?||YjhX_toxin->SAM-METHYLASE->?->BrxA-DNA-binding->BrxB-BrxF-iSTAND->DUF6079*->N6-METHYLASE->PglZ->SIGMA+OB-Fold+AAA-ATPase+Lon-Protease-BrxL-><-Arm-DNA-bind_3+Phage_integrase<-tRNA||?-><-SKI||METHYLASE->	1213	Bacteria>Pseudomonadota>Gammaproteobacteria>Enterobacterales>Enterobacteriaceae>Salmonella	GCA_013488685.1	Salmonella_enterica	HZ593_001598	149045..152686	28901	BREX-system-P-loop-protein-BrxC-[Salmonella-enterica]
EFR0716065.1	Type1.BREX	DUF6079-p*->N6-METHYLASE->OLD-ABC->TOPRIM->PglZ->SIGMA+OB-Fold+AAA-ATPase+Lon-Protease-BrxL-><-REase<-HKD-DNase	720	Bacteria>Pseudomonadota>Gammaproteobacteria>Enterobacterales>Enterobacteriaceae>Salmonella	GCA_013531855.1	Salmonella_enterica	HYQ91_004636	1..2164	28901	BREX-system-P-loop-protein-BrxC-[Salmonella-enterica]
EFQ9845222.1	Type1.BREX	Arm-DNA-bind_3->NACHT+SF1-SF2-Helicase+HKD-DNase->REase-><-SIGMA+OB-Fold+AAA-ATPase+Lon-Protease-BrxL<-PglZ<-TOPRIM<-OLD-ABC<-N6-METHYLASE<-DUF6079*<-BrxB-BrxF-iSTAND<-BrxA-DNA-binding<-?<-SAM-METHYLASE<-YjhX_toxin||?->REase+iREase->HEPN->	1213	Bacteria>Pseudomonadota>Gammaproteobacteria>Enterobacterales>Enterobacteriaceae>Salmonella	GCA_013535435.1	Salmonella_enterica	HZL63_001579	150017..153658	28901	BREX-system-P-loop-protein-BrxC-[Salmonella-enterica]
EFS3421932.1	Type1.BREX	<-HEPN<-?||YjhX_toxin->SAM-METHYLASE->?->BrxA-DNA-binding->BrxB-BrxF-iSTAND->DUF6079*->N6-METHYLASE->OLD-ABC->TOPRIM->PglZ->SIGMA+OB-Fold+AAA-ATPase+Lon-Protease-BrxL->?-><-REase	1213	Bacteria>Pseudomonadota>Gammaproteobacteria>Enterobacterales>Enterobacteriaceae>Salmonella	GCA_013632355.1	Salmonella_enterica	HX201_001605	150173..153814	28901	BREX-system-P-loop-protein-BrxC-[Salmonella-enterica]
HAK8810847.1	Type1.BREX	<-GntR-HTH++TrmB+ZDNA-HTH||<-?<-HEPN||YjhX_toxin->SAM-METHYLASE->?->BrxA-DNA-binding->BrxB-BrxF-iSTAND->DUF6079*->N6-METHYLASE->OLD-ABC->TOPRIM->PglZ->SIGMA+OB-Fold+AAA-ATPase+Lon-Protease-BrxL-><-REase<-NACHT+SF1-SF2-Helicase+HKD-DNase	1213	Bacteria>Pseudomonadota>Gammaproteobacteria>Enterobacterales>Enterobacteriaceae>Salmonella	GCA_013646795.1	Salmonella_enterica	H2X42_001962	158401..162042	28901	BREX-system-P-loop-protein-BrxC-[Salmonella-enterica]
EFS5965938.1	Type1.BREX	<-GntR-HTH++TrmB+ZDNA-HTH||<-?<-HEPN<-?||YjhX_toxin->SAM-METHYLASE->?->BrxA-DNA-binding->BrxB-BrxF-iSTAND->DUF6079*->N6-METHYLASE->OLD-ABC->TOPRIM->PglZ->SIGMA+OB-Fold+AAA-ATPase+Lon-Protease-BrxL->?-><-REase<-NACHT+SF1-SF2-Helicase+HKD-DNase<-REase	1213	Bacteria>Pseudomonadota>Gammaproteobacteria>Enterobacterales>Enterobacteriaceae>Salmonella	GCA_013657295.1	Salmonella_enterica	HW654_000972	195304..198945	28901	BREX-system-P-loop-protein-BrxC-[Salmonella-enterica]
EFS7669868.1	Type1.BREX	DUF6079*->N6-METHYLASE->OLD-ABC->TOPRIM->PglZ->SIGMA+OB-Fold+AAA-ATPase+Lon-Protease-BrxL-><-REase<-NACHT+SF1-SF2-Helicase+HKD-DNase<-Arm-DNA-bind_3	1078	Bacteria>Pseudomonadota>Gammaproteobacteria>Enterobacterales>Enterobacteriaceae>Salmonella	GCA_013666175.1	Salmonella_enterica	HWZ11_001253	1..3239	28901	BREX-system-P-loop-protein-BrxC-[Salmonella-enterica]
EFU4335446.1	Type1.BREX	<-HEPN<-REase+iREase<-?||YjhX_toxin->SAM-METHYLASE->?->BrxA-DNA-binding->BrxB-BrxF-iSTAND->DUF6079*->N6-METHYLASE->OLD-ABC->TOPRIM->PglZ->SIGMA+OB-Fold+AAA-ATPase+Lon-Protease-BrxL-><-REase<-NACHT+SF1-SF2-Helicase+HKD-DNase<-Arm-DNA-bind_3	1213	Bacteria>Pseudomonadota>Gammaproteobacteria>Enterobacterales>Enterobacteriaceae>Salmonella	GCA_013751665.1	Salmonella_enterica	HUC58_001813	170349..173990	28901	BREX-system-P-loop-protein-BrxC-[Salmonella-enterica]
EGA9157240.1	Type1.BREX	<-HEPN<-?||YjhX_toxin->SAM-METHYLASE->?->BrxA-DNA-binding->BrxB-BrxF-iSTAND->DUF6079*->N6-METHYLASE->OLD-ABC->TOPRIM->PglZ->SIGMA+OB-Fold+AAA-ATPase+Lon-Protease-BrxL-><-REase<-Arm-DNA-bind_3<-tRNA||?-><-SKI	1213	Bacteria>Pseudomonadota>Gammaproteobacteria>Enterobacterales>Enterobacteriaceae>Salmonella	GCA_014125285.1	Salmonella_enterica_subsp._enterica_serovar_Kirkee	H6M98_002631	52334..55975	2564624	BREX-system-P-loop-protein-BrxC-[Salmonella-enterica-subsp.-enterica-serovar-Kirkee]
EGB5523462.1	Type1.BREX	<-HEPN<-?||YjhX_toxin->SAM-METHYLASE->?->BrxA-DNA-binding->BrxB-BrxF-iSTAND->DUF6079*->N6-METHYLASE->OLD-ABC->TOPRIM->PglZ->SIGMA+OB-Fold+AAA-ATPase+Lon-Protease-BrxL->?-><-REase<-NACHT+SF1-SF2-Helicase+HKD-DNase<-Arm-DNA-bind_3	1213	Bacteria>Pseudomonadota>Gammaproteobacteria>Enterobacterales>Enterobacteriaceae>Salmonella	GCA_014209965.1	Salmonella_enterica	H7074_002104	159039..162680	28901	BREX-system-P-loop-protein-BrxC-[Salmonella-enterica]
EGB5697881.1	Type1.BREX	Arm-DNA-bind_3->NACHT+SF1-SF2-Helicase+HKD-DNase->REase-><-HKD-DNase+OGG-like<-SIGMA+OB-Fold+AAA-ATPase+Lon-Protease-BrxL<-PglZ<-TOPRIM<-OLD-ABC<-N6-METHYLASE<-DUF6079*<-BrxB-BrxF-iSTAND<-BrxA-DNA-binding||SIGMA-HTH+REase->MarR-wHTH-><-wHTH+WYL+WCX||wHTH->MukE->MukB->	1204	Bacteria>Pseudomonadota>Gammaproteobacteria>Enterobacterales>Enterobacteriaceae>Salmonella	GCA_014213495.1	Salmonella_enterica_subsp._enterica	H7531_003071	17313..20927	59201	BREX-system-P-loop-protein-BrxC-[Salmonella-enterica-subsp.-enterica]
EGC3767042.1	Type1.BREX	<-HEPN<-REase+iREase<-?||YjhX_toxin->SAM-METHYLASE->?->BrxA-DNA-binding->BrxB-BrxF-iSTAND->DUF6079*->N6-METHYLASE->OLD-ABC->TOPRIM->PglZ->SIGMA+OB-Fold+AAA-ATPase+Lon-Protease-BrxL-><-REase<-Arm-DNA-bind_3<-tRNA||?-><-SKI	1213	Bacteria>Pseudomonadota>Gammaproteobacteria>Enterobacterales>Enterobacteriaceae>Salmonella	GCA_014295145.1	Salmonella_enterica	H9A68_002330	38672..42313	28901	BREX-system-P-loop-protein-BrxC-[Salmonella-enterica]
EGC8584468.1	Type1.BREX	<-N6-METHYLASE<-DUF6079*<-BrxB-BrxF-iSTAND<-BrxA-DNA-binding<-?<-SAM-METHYLASE<-YjhX_toxin||?->REase+iREase->HEPN->	1213	Bacteria>Pseudomonadota>Gammaproteobacteria>Enterobacterales>Enterobacteriaceae>Salmonella	GCA_014317295.1	Salmonella_enterica	H9103_003699	1964..5605	28901	BREX-system-P-loop-protein-BrxC-[Salmonella-enterica]
EGD1472167.1	Type1.BREX	Arm-DNA-bind_3->NACHT+SF1-SF2-Helicase+HKD-DNase->REase-><-SIGMA+OB-Fold+AAA-ATPase+Lon-Protease-BrxL<-PglZ<-TOPRIM<-OLD-ABC<-N6-METHYLASE<-DUF6079*<-BrxB-BrxF-iSTAND<-BrxA-DNA-binding<-?<-SAM-METHYLASE<-YjhX_toxin||?->REase+iREase->HEPN->	1213	Bacteria>Pseudomonadota>Gammaproteobacteria>Enterobacterales>Enterobacteriaceae>Salmonella	GCA_014351875.1	Salmonella_enterica	IAV68_002850	108064..111705	28901	BREX-system-P-loop-protein-BrxC-[Salmonella-enterica]
EGF1249453.1	Type1.BREX	<-HEPN<-REase+iREase<-?||YjhX_toxin->SAM-METHYLASE->?->BrxA-DNA-binding->BrxB-BrxF-iSTAND->DUF6079*->N6-METHYLASE->OLD-ABC->TOPRIM->PglZ->SIGMA+OB-Fold+AAA-ATPase+Lon-Protease-BrxL-><-REase<-NACHT+SF1-SF2-Helicase+HKD-DNase<-Arm-DNA-bind_3	1213	Bacteria>Pseudomonadota>Gammaproteobacteria>Enterobacterales>Enterobacteriaceae>Salmonella	GCA_014468995.1	Salmonella_enterica	IB037_001321	170919..174560	28901	BREX-system-P-loop-protein-BrxC-[Salmonella-enterica]
EGH1765314.1	Type1.BREX	<-HEPN<-REase+iREase<-?||YjhX_toxin->SAM-METHYLASE->?->BrxA-DNA-binding->BrxB-BrxF-iSTAND->DUF6079*->N6-METHYLASE->OLD-ABC->TOPRIM->PglZ->SIGMA+OB-Fold+AAA-ATPase+Lon-Protease-BrxL-><-NACHT+SF1-SF2-Helicase+HKD-DNase<-Arm-DNA-bind_3<-tRNA||?-><-SKI	1213	Bacteria>Pseudomonadota>Gammaproteobacteria>Enterobacterales>Enterobacteriaceae>Salmonella	GCA_014604855.1	Salmonella_enterica	ID459_000689	170687..174328	28901	BREX-system-P-loop-protein-BrxC-[Salmonella-enterica]
EGH2238321.1	Type1.BREX	<-HEPN<-?||YjhX_toxin->SAM-METHYLASE->?->BrxA-DNA-binding->BrxB-BrxF-iSTAND->DUF6079*->N6-METHYLASE->OLD-ABC->TOPRIM->PglZ->SIGMA+OB-Fold+AAA-ATPase+Lon-Protease-BrxL-><-REase<-Couple_hipA+HipA_C<-HTH_3	1213	Bacteria>Pseudomonadota>Gammaproteobacteria>Enterobacterales>Enterobacteriaceae>Salmonella	GCA_014610595.1	Salmonella_enterica	ID764_000354	390871..394512	28901	BREX-system-P-loop-protein-BrxC-[Salmonella-enterica]
EGH8508053.1	Type1.BREX	<-HEPN<-?||YjhX_toxin->SAM-METHYLASE->?->BrxA-DNA-binding->BrxB-BrxF-iSTAND->DUF6079*->N6-METHYLASE->OLD-ABC->TOPRIM->PglZ->SIGMA+OB-Fold+AAA-ATPase+Lon-Protease-BrxL-><-NACHT+SF1-SF2-Helicase+HKD-DNase<-Arm-DNA-bind_3<-tRNA||?-><-SKI	1213	Bacteria>Pseudomonadota>Gammaproteobacteria>Enterobacterales>Enterobacteriaceae>Salmonella	GCA_014678145.1	Salmonella_enterica	IFE76_001736	26096..29737	28901	BREX-system-P-loop-protein-BrxC-[Salmonella-enterica]
EGH9456388.1	Type1.BREX	<-HEPN<-?||YjhX_toxin->SAM-METHYLASE->?->BrxA-DNA-binding->BrxB-BrxF-iSTAND->DUF6079*->N6-METHYLASE->OLD-ABC->TOPRIM->PglZ->SIGMA+OB-Fold+AAA-ATPase+Lon-Protease-BrxL->?-><-REase<-NACHT+SF1-SF2-Helicase+HKD-DNase<-Arm-DNA-bind_3	1213	Bacteria>Pseudomonadota>Gammaproteobacteria>Enterobacterales>Enterobacteriaceae>Salmonella	GCA_014692265.1	Salmonella_enterica_subsp._enterica_serovar_Leeuwarden	HKU92_000347	389340..392981	2564678	BREX-system-P-loop-protein-BrxC-[Salmonella-enterica-subsp.-enterica-serovar-Leeuwarden]
EGI8941862.1	Type1.BREX	<-HEPN<-?||YjhX_toxin->SAM-METHYLASE->?->BrxA-DNA-binding->BrxB-BrxF-iSTAND->DUF6079*->N6-METHYLASE->OLD-ABC->TOPRIM->PglZ->SIGMA+OB-Fold+AAA-ATPase+Lon-Protease-BrxL->?-><-REase<-NACHT+SF1-SF2-Helicase+HKD-DNase<-Arm-DNA-bind_3	1213	Bacteria>Pseudomonadota>Gammaproteobacteria>Enterobacterales>Enterobacteriaceae>Salmonella	GCA_014780755.1	Salmonella_enterica	IG687_002353	272986..276627	28901	BREX-system-P-loop-protein-BrxC-[Salmonella-enterica]
EGJ1340019.1	Type1.BREX	<-HEPN<-REase+iREase<-?||YjhX_toxin->SAM-METHYLASE->?->BrxA-DNA-binding->BrxB-BrxF-iSTAND->DUF6079*->N6-METHYLASE->	1213	Bacteria>Pseudomonadota>Gammaproteobacteria>Enterobacterales>Enterobacteriaceae>Salmonella	GCA_014793405.1	Salmonella_enterica_subsp._enterica_serovar_Typhimurium	IHB80_004023	18317..21958	90371	BREX-system-P-loop-protein-BrxC-[Salmonella-enterica-subsp.-enterica-serovar-Typhimurium]
EGK3406186.1	Type1.BREX	HTH_3->Couple_hipA+HipA_C->REase-><-SIGMA+OB-Fold+AAA-ATPase+Lon-Protease-BrxL<-PglZ<-TOPRIM<-OLD-ABC<-N6-METHYLASE<-DUF6079*<-BrxB-BrxF-iSTAND<-BrxA-DNA-binding<-?<-SAM-METHYLASE<-YjhX_toxin||?->REase+iREase->HEPN->	1213	Bacteria>Pseudomonadota>Gammaproteobacteria>Enterobacterales>Enterobacteriaceae>Salmonella	GCA_014949985.1	Salmonella_enterica	IOS83_004393	25212..28853	28901	BREX-system-P-loop-protein-BrxC-[Salmonella-enterica]
EGL7108184.1	Type1.BREX	<-HEPN<-REase+iREase<-?||YjhX_toxin->?->BrxA-DNA-binding->BrxB-BrxF-iSTAND->DUF6079*->N6-METHYLASE->OLD-ABC->TOPRIM->PglZ->SIGMA+OB-Fold+AAA-ATPase+Lon-Protease-BrxL-><-REase<-NACHT+SF1-SF2-Helicase+HKD-DNase<-Arm-DNA-bind_3	1213	Bacteria>Pseudomonadota>Gammaproteobacteria>Enterobacterales>Enterobacteriaceae>Salmonella	GCA_015048385.1	Salmonella_enterica	IMT80_001261	170929..174570	28901	BREX-system-P-loop-protein-BrxC-[Salmonella-enterica]
EGP1242555.1	Type1.BREX	<-HEPN<-REase+iREase<-?||YjhX_toxin->SAM-METHYLASE->?->BrxA-DNA-binding->BrxB-BrxF-iSTAND->DUF6079*->N6-METHYLASE->OLD-ABC->TOPRIM->PglZ->SIGMA+OB-Fold+AAA-ATPase+Lon-Protease-BrxL-><-REase<-NACHT+SF1-SF2-Helicase+HKD-DNase<-Arm-DNA-bind_3	1213	Bacteria>Pseudomonadota>Gammaproteobacteria>Enterobacterales>Enterobacteriaceae>Salmonella	GCA_015377445.1	Salmonella_enterica	IV995_001915	60140..63781	28901	BREX-system-P-loop-protein-BrxC-[Salmonella-enterica]
HAS9574207.1	Type1.BREX	Arm-DNA-bind_3->NACHT+SF1-SF2-Helicase+HKD-DNase->REase-><-SIGMA+OB-Fold+AAA-ATPase+Lon-Protease-BrxL<-PglZ<-TOPRIM<-OLD-ABC<-N6-METHYLASE<-DUF6079*<-BrxB-BrxF-iSTAND<-BrxA-DNA-binding<-?<-SAM-METHYLASE<-YjhX_toxin||?->REase+iREase->HEPN->	1213	Bacteria>Pseudomonadota>Gammaproteobacteria>Enterobacterales>Enterobacteriaceae>Salmonella	GCA_015871885.1	Salmonella_enterica_subsp._enterica_serovar_Typhimurium	I8M64_002289	108065..111706	90371	BREX-system-P-loop-protein-BrxC-[Salmonella-enterica-subsp.-enterica-serovar-Typhimurium]
EGS3682914.1	Type1.BREX	<-HEPN<-REase+iREase<-?||YjhX_toxin->SAM-METHYLASE->?->BrxA-DNA-binding->BrxB-BrxF-iSTAND->DUF6079-p*->	912	Bacteria>Pseudomonadota>Gammaproteobacteria>Enterobacterales>Enterobacteriaceae>Salmonella	GCA_015875385.1	Salmonella_enterica	I5X77_001841	392372..396108	28901	BREX-system-P-loop-protein-BrxC-[Salmonella-enterica]
HAT0208696.1	Type1.BREX	Arm-DNA-bind_3->NACHT+SF1-SF2-Helicase+HKD-DNase->REase-><-SIGMA+OB-Fold+AAA-ATPase+Lon-Protease-BrxL<-PglZ<-TOPRIM<-OLD-ABC<-N6-METHYLASE<-DUF6079-p*	773	Bacteria>Pseudomonadota>Gammaproteobacteria>Enterobacterales>Enterobacteriaceae>Salmonella	GCA_015882745.1	Salmonella_enterica_subsp._enterica	I8Q75_000460	75065..77386	59201	BREX-system-P-loop-protein-BrxC-[Salmonella-enterica-subsp.-enterica]
HAT0211425.1	Type1.BREX	<-DUF6079-p*<-BrxB-BrxF-iSTAND<-BrxA-DNA-binding<-?<-SAM-METHYLASE<-YjhX_toxin||?->REase+iREase->HEPN->	408	Bacteria>Pseudomonadota>Gammaproteobacteria>Enterobacterales>Enterobacteriaceae>Salmonella	GCA_015882745.1	Salmonella_enterica_subsp._enterica	I8Q75_003248	1..1225	59201	BREX-system-P-loop-protein-BrxC-[Salmonella-enterica-subsp.-enterica]
EGT0819117.1	Type1.BREX	HTH_3->Couple_hipA+HipA_C->REase-><-SIGMA+OB-Fold+AAA-ATPase+Lon-Protease-BrxL<-PglZ<-TOPRIM<-OLD-ABC<-N6-METHYLASE<-DUF6079*<-BrxB-BrxF-iSTAND<-BrxA-DNA-binding<-?<-SAM-METHYLASE<-YjhX_toxin||?->REase+iREase->HEPN->	1213	Bacteria>Pseudomonadota>Gammaproteobacteria>Enterobacterales>Enterobacteriaceae>Salmonella	GCA_015953925.1	Salmonella_enterica_subsp._enterica_serovar_Ohio	I3I53_003218	25100..28741	117541	BREX-system-P-loop-protein-BrxC-[Salmonella-enterica-subsp.-enterica-serovar-Ohio]
EGT1190058.1	Type1.BREX	Arm-DNA-bind_3->NACHT+SF1-SF2-Helicase+HKD-DNase->REase-><-SIGMA+OB-Fold+AAA-ATPase+Lon-Protease-BrxL<-PglZ<-TOPRIM<-OLD-ABC<-N6-METHYLASE<-DUF6079*<-BrxB-BrxF-iSTAND<-BrxA-DNA-binding<-?<-SAM-METHYLASE<-YjhX_toxin||?->REase+iREase->HEPN->	1213	Bacteria>Pseudomonadota>Gammaproteobacteria>Enterobacterales>Enterobacteriaceae>Salmonella	GCA_015955585.1	Salmonella_enterica	I3G58_002375	108064..111705	28901	BREX-system-P-loop-protein-BrxC-[Salmonella-enterica]
MBH5277084.1	Type1.BREX	Arm-DNA-bind_3->NACHT+SF1-SF2-Helicase+HKD-DNase->REase-><-HKD-DNase+OGG-like<-SIGMA+OB-Fold+AAA-ATPase+Lon-Protease-BrxL<-PglZ<-TOPRIM<-OLD-ABC<-N6-METHYLASE<-DUF6079*<-BrxB-BrxF-iSTAND<-BrxA-DNA-binding<-?<-SAM-METHYLASE<-YjhX_toxin||?->HEPN->	1213	Bacteria>Pseudomonadota>Gammaproteobacteria>Enterobacterales>Enterobacteriaceae>Salmonella	GCA_016019715.1	Salmonella_enterica	I3A81_01650	358597..362238	28901	BREX-system-P-loop-protein-BrxC-[Salmonella-enterica]
HAU6657706.1	Type1.BREX	<-HEPN<-REase+iREase<-?||YjhX_toxin->SAM-METHYLASE->?->BrxA-DNA-binding->BrxB-BrxF-iSTAND->DUF6079*->	1157	Bacteria>Pseudomonadota>Gammaproteobacteria>Enterobacterales>Enterobacteriaceae>Salmonella	GCA_016095655.1	Salmonella_enterica	JEL20_001876	30497..33967	28901	BREX-system-P-loop-protein-BrxC-[Salmonella-enterica]
EGU3680633.1	Type1.BREX	<-HEPN<-REase+iREase<-?||YjhX_toxin->SAM-METHYLASE->?->BrxA-DNA-binding->BrxB-BrxF-iSTAND->DUF6079*->N6-METHYLASE->OLD-ABC->TOPRIM->PglZ->SIGMA+OB-Fold+AAA-ATPase+Lon-Protease-BrxL-><-REase<-NACHT+SF1-SF2-Helicase+HKD-DNase<-Arm-DNA-bind_3	1213	Bacteria>Pseudomonadota>Gammaproteobacteria>Enterobacterales>Enterobacteriaceae>Salmonella	GCA_016165525.1	Salmonella_enterica	HOR40_002395	27396..31037	28901	BREX-system-P-loop-protein-BrxC-[Salmonella-enterica]
EGU5502543.1	Type1.BREX	Arm-DNA-bind_3->NACHT+SF1-SF2-Helicase+HKD-DNase->REase-><-SIGMA+OB-Fold+AAA-ATPase+Lon-Protease-BrxL<-PglZ<-TOPRIM<-OLD-ABC<-N6-METHYLASE<-DUF6079*<-BrxB-BrxF-iSTAND<-BrxA-DNA-binding<-?<-SAM-METHYLASE<-YjhX_toxin||?->REase+iREase->HEPN->	1213	Bacteria>Pseudomonadota>Gammaproteobacteria>Enterobacterales>Enterobacteriaceae>Salmonella	GCA_016174335.1	Salmonella_enterica	HGK88_001891	343296..346937	28901	BREX-system-P-loop-protein-BrxC-[Salmonella-enterica]

Protein-ID	System	Gene-Neighbourhoods	Length	Taxonomy	Assembly-ID	Strain	Locus-Tag	Genomic-coordinates	TaxID	Fasta-defline
HAV0565027.1	Type1.BREX	Arm-DNA-bind_3->NACHT+SF1-SF2-Helicase+HKD-DNase->REase-><-SIGMA+OB-Fold+AAA-ATPase+Lon-Protease-BrxL<-PglZ<-TOPRIM<-OLD-ABC<-N6-METHYLASE<-DUF6079*<-BrxB-BrxF-iSTAND<-[illegible]	1213	Bacteria>Pseudomonadota>Gammaproteobacteria>Enterobacterales>Enterobacteriaceae>Salmonella	GCA_016249375.1	Salmonella_enterica_subsp._enterica	JF300_000748	89373..93014	59201	BREX-system-P-loop-protein-BrxC-[Salmonella-enterica-subsp.-enterica]
EGV8384248.1	Type1.BREX	<-HEPN<-REase+iREase<-?||YjhX_toxin->SAM-METHYLASE->?->BrxA-DNA-binding->BrxB-BrxF-iSTAND->DUF6079*->N6-METHYLASE->OLD-ABC->TOPRIM->PglZ->SIGMA+OB-Fold+AAA-ATPase+Lon-Protease-BrxL->[illegible]	1214	Bacteria>Pseudomonadota>Gammaproteobacteria>Enterobacterales>Enterobacteriaceae>Salmonella	GCA_016279875.1	Salmonella_enterica	EHN43_00830	170919..174563	28901	BREX-system-P-loop-protein-BrxC-[Salmonella-enterica]
EGZ8568475.1	Type1.BREX	<-HEPN<-REase+iREase<-?||YjhX_toxin->SAM-METHYLASE->?->BrxA-DNA-binding->BrxB-BrxF-iSTAND->DUF6079*->N6-METHYLASE->OLD-ABC->TOPRIM->PglZ->SIGMA+OB-Fold+AAA-ATPase+Lon-Protease-BrxL->[illegible]	1213	Bacteria>Pseudomonadota>Gammaproteobacteria>Enterobacterales>Enterobacteriaceae>Salmonella	GCA_016588995.1	Salmonella_enterica_subsp._enterica_serovar_Typhimurium	JJM06_003392	12585..16226	90371	BREX-system-P-loop-protein-BrxC-[Salmonella-enterica-subsp.-enterica-serovar-Typhimurium]
EHA2523073.1	Type1.BREX	<-HEPN<-?||YjhX_toxin->SAM-METHYLASE->?->BrxA-DNA-binding->BrxB-BrxF-iSTAND->DUF6079*->N6-METHYLASE->OLD-ABC->TOPRIM->PglZ->SIGMA+OB-Fold+AAA-ATPase+Lon-Protease-BrxL-><-REase<-[illegible]	1213	Bacteria>Pseudomonadota>Gammaproteobacteria>Enterobacterales>Enterobacteriaceae>Salmonella	GCA_016628375.1	Salmonella_enterica	JKI82_003469	165981..169622	28901	BREX-system-P-loop-protein-BrxC-[Salmonella-enterica]
EHA8894993.1	Type1.BREX	<-HEPN<-?||YjhX_toxin->SAM-METHYLASE->?->BrxA-DNA-binding->BrxB-BrxF-iSTAND->DUF6079*->N6-METHYLASE->OLD-ABC->TOPRIM->PglZ->SIGMA+OB-Fold+AAA-ATPase+Lon-Protease-BrxL-><-REase<-[illegible]	1213	Bacteria>Pseudomonadota>Gammaproteobacteria>Enterobacterales>Enterobacteriaceae>Salmonella	GCA_016843845.1	Salmonella_enterica_subsp._enterica	JHW15_001254	44404..48045	59201	BREX-system-P-loop-protein-BrxC-[Salmonella-enterica-subsp.-enterica]
EHB1109136.1	Type1.BREX	<-HEPN<-REase+iREase<-?||YjhX_toxin->SAM-METHYLASE->?->BrxA-DNA-binding->BrxB-BrxF-iSTAND->DUF6079*->N6-METHYLASE->	1213	Bacteria>Pseudomonadota>Gammaproteobacteria>Enterobacterales>Enterobacteriaceae>Salmonella	GCA_016950725.1	Salmonella_enterica_subsp._enterica_serovar_Typhimurium	JYP19_004114	18317..21958	90371	BREX-system-P-loop-protein-BrxC-[Salmonella-enterica-subsp.-enterica-serovar-Typhimurium]
EHC3781321.1	Type1.BREX	<-HEPN<-REase+iREase<-?||YjhX_toxin->SAM-METHYLASE->?->BrxA-DNA-binding->BrxB-BrxF-iSTAND->DUF6079*->N6-METHYLASE->OLD-ABC->TOPRIM->PglZ->SIGMA+OB-Fold+AAA-ATPase+Lon-Protease-BrxL->[illegible]	1213	Bacteria>Pseudomonadota>Gammaproteobacteria>Enterobacterales>Enterobacteriaceae>Salmonella	GCA_017044955.1	Salmonella_enterica_subsp._enterica_serovar_Typhimurium	JS923_000750	173044..176685	90371	BREX-system-P-loop-protein-BrxC-[Salmonella-enterica-subsp.-enterica-serovar-Typhimurium]
QSG17817.1	Type1.BREX	<-Peptidase_M17_N+Peptidase_M17||?-><-?||SKI-><-?||tRNA->Arm-DNA-bind_3->NACHT+SF1-SF2-Helicase+HKD-DNase-><-TOPRIM<-DUF6079*<-BrxA-DNA-binding<-?<-YjhX_toxin||REase+iREase->?-><-?||[illegible]	1213	Bacteria>Pseudomonadota>Gammaproteobacteria>Enterobacterales>Enterobacteriaceae>Salmonella	GCA_017094545.1	Salmonella_enterica_subsp._enterica_serovar_Typhimurium	IRY60_23155	4744141..4747782	90371	BREX-system-P-loop-protein-BrxC-[Salmonella-enterica-subsp.-enterica-serovar-Typhimurium]
QSG23651.1	Type1.BREX	<-LACI-HTH+PBPI||SKI-><-?||tRNA->Arm-DNA-bind_3->REase-><-SIGMA+OB-Fold+AAA-ATPase+Lon-Protease-BrxL<-TOPRIM<-OLD-ABC<-DUF6079*<-?<-YjhX_toxin||REase+iREase->?-><-?||<-?||<-MODE-HTH[illegible]	1213	Bacteria>Pseudomonadota>Gammaproteobacteria>Enterobacterales>Enterobacteriaceae>Salmonella	GCA_017094585.1	Salmonella_enterica_subsp._enterica_serovar_Typhimurium	IRY58_23115	4749695..4753336	90371	BREX-system-P-loop-protein-BrxC-[Salmonella-enterica-subsp.-enterica-serovar-Typhimurium]
EHD7670518.1	Type1.BREX	<-HEPN<-REase+iREase<-?||YjhX_toxin->SAM-METHYLASE->?->BrxA-DNA-binding->BrxB-BrxF-iSTAND->DUF6079*->N6-METHYLASE->OLD-ABC->TOPRIM->PglZ->SIGMA+OB-Fold+AAA-ATPase+Lon-Protease-BrxL->[illegible]	1213	Bacteria>Pseudomonadota>Gammaproteobacteria>Enterobacterales>Enterobacteriaceae>Salmonella	GCA_017183075.1	Salmonella_enterica	JQD35_001754	170582..174223	28901	BREX-system-P-loop-protein-BrxC-[Salmonella-enterica]
EHD9459645.1	Type1.BREX	<-HEPN<-REase+iREase<-?||YjhX_toxin->SAM-METHYLASE->?->BrxA-DNA-binding->BrxB-BrxF-iSTAND->DUF6079*->N6-METHYLASE->OLD-ABC->TOPRIM->PglZ->SIGMA+OB-Fold+AAA-ATPase+Lon-Protease-BrxL->[illegible]	1213	Bacteria>Pseudomonadota>Gammaproteobacteria>Enterobacterales>Enterobacteriaceae>Salmonella	GCA_017198155.1	Salmonella_enterica_subsp._enterica_serovar_Typhimurium	JYL48_000531	170932..174573	90371	BREX-system-P-loop-protein-BrxC-[Salmonella-enterica-subsp.-enterica-serovar-Typhimurium]
EHE5127202.1	Type1.BREX	ACET->YjhX_toxin->ACET->METHYLASE->ZnR->Glyoxalase->ZnR->BrxA-DNA-binding->BrxB-BrxF-iSTAND->DUF6079*->ParB+HNH->N6-METHYLASE->PglZ->SIGMA+OB-Fold+AAA-ATPase+Lon-Protease-BrxL-><-[illegible]	1233	Bacteria>Pseudomonadota>Gammaproteobacteria>Enterobacterales>Enterobacteriaceae>Salmonella	GCA_017241415.1	Salmonella_enterica	JMY69_002120	181649..185350	28901	BREX-system-P-loop-protein-BrxC-[Salmonella-enterica]
EHH9023834.1	Type1.BREX	<-HEPN<-REase+iREase<-?||YjhX_toxin->SAM-METHYLASE->?->BrxA-DNA-binding->BrxB-BrxF-iSTAND->DUF6079*->N6-METHYLASE->OLD-ABC->TOPRIM->PglZ->SIGMA+OB-Fold+AAA-ATPase+Lon-Protease-BrxL->[illegible]	1213	Bacteria>Pseudomonadota>Gammaproteobacteria>Enterobacterales>Enterobacteriaceae>Salmonella	GCA_017748765.1	Salmonella_enterica	J8439_003629	18317..21958	28901	BREX-system-P-loop-protein-BrxC-[Salmonella-enterica]
EHI1197052.1	Type1.BREX	[illegible]	1213	Bacteria>Pseudomonadota>Gammaproteobacteria>Enterobacterales>Enterobacteriaceae>Salmonella	GCA_017785955.1	Salmonella_enterica	J8551_000470	108064..111705	28901	BREX-system-P-loop-protein-BrxC-[Salmonella-enterica]
EHI4209832.1	Type1.BREX	[illegible]	1213	Bacteria>Pseudomonadota>Gammaproteobacteria>Enterobacterales>Enterobacteriaceae>Salmonella	GCA_017802295.1	Salmonella_enterica	J9Y57_000957	124173..127814	28901	BREX-system-P-loop-protein-BrxC-[Salmonella-enterica]
EHJ0877869.1	Type1.BREX	[illegible]	1213	Bacteria>Pseudomonadota>Gammaproteobacteria>Enterobacterales>Enterobacteriaceae>Salmonella	GCA_017846115.1	Salmonella_enterica	KAB51_002697	18125..21766	28901	BREX-system-P-loop-protein-BrxC-[Salmonella-enterica]
EHJ1242128.1	Type1.BREX	[illegible]	1213	Bacteria>Pseudomonadota>Gammaproteobacteria>Enterobacterales>Enterobacteriaceae>Salmonella	GCA_017851875.1	Salmonella_enterica_subsp._enterica_serovar_Typhimurium	KAC06_001466	81358..84999	90371	BREX-system-P-loop-protein-BrxC-[Salmonella-enterica-subsp.-enterica-serovar-Typhimurium]
EHJ7117733.1	Type1.BREX	[illegible]	1204	Bacteria>Pseudomonadota>Gammaproteobacteria>Enterobacterales>Enterobacteriaceae>Salmonella	GCA_017926475.1	Salmonella_enterica_subsp._enterica_serovar_Kentucky	KAP12_002965	412329..415943	192955	BREX-system-P-loop-protein-BrxC-[Salmonella-enterica-subsp.-enterica-serovar-Kentucky]
EHJ9839635.1	Type1.BREX	[illegible]	1213	Bacteria>Pseudomonadota>Gammaproteobacteria>Enterobacterales>Enterobacteriaceae>Salmonella	GCA_017967145.1	Salmonella_enterica	KEH66_000998	398114..401755	28901	BREX-system-P-loop-protein-BrxC-[Salmonella-enterica]
EHN4465874.1	Type1.BREX	[illegible]	1213	Bacteria>Pseudomonadota>Gammaproteobacteria>Enterobacterales>Enterobacteriaceae>Salmonella	GCA_018387865.1	Salmonella_enterica	KIZ21_001044	348400..352041	28901	BREX-system-P-loop-protein-BrxC-[Salmonella-enterica]
EHN4525913.1	Type1.BREX	[illegible]	1213	Bacteria>Pseudomonadota>Gammaproteobacteria>Enterobacterales>Enterobacteriaceae>Salmonella	GCA_018388245.1	Salmonella_enterica_subsp._enterica_serovar_Ohio	KI018_001081	92040..95681	117541	BREX-system-P-loop-protein-BrxC-[Salmonella-enterica-subsp.-enterica-serovar-Ohio]
EHO4506760.1	Type1.BREX	[illegible]	1213	Bacteria>Pseudomonadota>Gammaproteobacteria>Enterobacterales>Enterobacteriaceae>Salmonella	GCA_018524125.1	Salmonella_enterica_subsp._enterica_serovar_Carrau	KD666_001066	149262..152903	1160739	BREX-system-P-loop-protein-BrxC-[Salmonella-enterica-subsp.-enterica-serovar-Carrau]
MBT0981085.1	Type1.BREX	[illegible]	1213	Bacteria>Pseudomonadota>Gammaproteobacteria>Enterobacterales>Enterobacteriaceae>Salmonella	GCA_018531305.1	Salmonella_enterica_subsp._enterica_serovar_Typhimurium	KJ032_08020	170949..174590	90371	BREX-system-P-loop-protein-BrxC-[Salmonella-enterica-subsp.-enterica-serovar-Typhimurium]
EHO5101500.1	Type1.BREX	[illegible]	1213	Bacteria>Pseudomonadota>Gammaproteobacteria>Enterobacterales>Enterobacteriaceae>Salmonella	GCA_018532585.1	Salmonella_enterica	KLP74_002745	12418..16059	28901	BREX-system-P-loop-protein-BrxC-[Salmonella-enterica]
EHO5062413.1	Type1.BREX	[illegible]	1213	Bacteria>Pseudomonadota>Gammaproteobacteria>Enterobacterales>Enterobacteriaceae>Salmonella	GCA_018532705.1	Salmonella_enterica	KLP81_000353	388533..392174	28901	BREX-system-P-loop-protein-BrxC-[Salmonella-enterica]
EHO5648342.1	Type1.BREX	[illegible]	1213	Bacteria>Pseudomonadota>Gammaproteobacteria>Enterobacterales>Enterobacteriaceae>Salmonella	GCA_018535125.1	Salmonella_enterica	KLR08_000690	144464..148105	28901	BREX-system-P-loop-protein-BrxC-[Salmonella-enterica]
EHO5627401.1	Type1.BREX	[illegible]	1213	Bacteria>Pseudomonadota>Gammaproteobacteria>Enterobacterales>Enterobacteriaceae>Salmonella	GCA_018535225.1	Salmonella_enterica	KLQ90_002307	30800..34441	28901	BREX-system-P-loop-protein-BrxC-[Salmonella-enterica]
EHP4799940.1	Type1.BREX	[illegible]	1213	Bacteria>Pseudomonadota>Gammaproteobacteria>Enterobacterales>Enterobacteriaceae>Salmonella	GCA_018776965.1	Salmonella_enterica_subsp._enterica_serovar_Sangera	KM018_002015	44674..48315	1173574	BREX-system-P-loop-protein-BrxC-[Salmonella-enterica-subsp.-enterica-serovar-Sangera]
EHQ7935671.1	Type1.BREX	[illegible]	1213	Bacteria>Pseudomonadota>Gammaproteobacteria>Enterobacterales>Enterobacteriaceae>Salmonella	GCA_018976465.1	Salmonella_enterica	KSM73_002317	108064..111705	28901	BREX-system-P-loop-protein-BrxC-[Salmonella-enterica]
EHS1468535.1	Type1.BREX	[illegible]	1213	Bacteria>Pseudomonadota>Gammaproteobacteria>Enterobacterales>Enterobacteriaceae>Salmonella	GCA_019106545.1	Salmonella_enterica_subsp._enterica_serovar_Typhimurium	KVU04_001403	171297..174938	90371	BREX-system-P-loop-protein-BrxC-[Salmonella-enterica-subsp.-enterica-serovar-Typhimurium]
EHS3976100.1	Type1.BREX	[illegible]	1213	Bacteria>Pseudomonadota>Gammaproteobacteria>Enterobacterales>Enterobacteriaceae>Salmonella	GCA_019140515.1	Salmonella_enterica_subsp._enterica_serovar_Poona	KV430_003532	2009..5650	436295	BREX-system-P-loop-protein-BrxC-[Salmonella-enterica-subsp.-enterica-serovar-Poona]
EHS4662101.1	Type1.BREX	[illegible]	1213	Bacteria>Pseudomonadota>Gammaproteobacteria>Enterobacterales>Enterobacteriaceae>Salmonella	GCA_019144395.1	Salmonella_enterica	KV604_003674	73553..77194	28901	BREX-system-P-loop-protein-BrxC-[Salmonella-enterica]
EHS5456435.1	Type1.BREX	[illegible]	1213	Bacteria>Pseudomonadota>Gammaproteobacteria>Enterobacterales>Enterobacteriaceae>Salmonella	GCA_019151305.1	Salmonella_enterica_subsp._enterica_serovar_Corvallis	KV802_001386	165237..168878	593905	BREX-system-P-loop-protein-BrxC-[Salmonella-enterica-subsp.-enterica-serovar-Corvallis]
EHT1034281.1	Type1.BREX	[illegible]	1213	Bacteria>Pseudomonadota>Gammaproteobacteria>Enterobacterales>Enterobacteriaceae>Salmonella	GCA_019186465.1	Salmonella_enterica	KW750_001284	149102..152743	28901	BREX-system-P-loop-protein-BrxC-[Salmonella-enterica]
EHT6941866.1	Type1.BREX	[illegible]	1213	Bacteria>Pseudomonadota>Gammaproteobacteria>Enterobacterales>Enterobacteriaceae>Salmonella	GCA_019250575.1	Salmonella_enterica	KYH33_001187	170918..174559	28901	BREX-system-P-loop-protein-BrxC-[Salmonella-enterica]
EHU5044814.1	Type1.BREX	[illegible]	1213	Bacteria>Pseudomonadota>Gammaproteobacteria>Enterobacterales>Enterobacteriaceae>Salmonella	GCA_019291225.1	Salmonella_enterica	KY920_001891	99712..103353	28901	BREX-system-P-loop-protein-BrxC-[Salmonella-enterica]
EHU7518552.1	Type1.BREX	[illegible]	1213	Bacteria>Pseudomonadota>Gammaproteobacteria>Enterobacterales>Enterobacteriaceae>Salmonella	GCA_019318345.1	Salmonella_enterica	KZL07_001983	273071..276712	28901	BREX-system-P-loop-protein-BrxC-[Salmonella-enterica]
EHV2030459.1	Type1.BREX	[illegible]	1213	Bacteria>Pseudomonadota>Gammaproteobacteria>Enterobacterales>Enterobacteriaceae>Salmonella	GCA_019356775.1	Salmonella_enterica	KZ138_001531	389696..393337	28901	BREX-system-P-loop-protein-BrxC-[Salmonella-enterica]
EHW6237841.1	Type1.BREX	[illegible]	1213	Bacteria>Pseudomonadota>Gammaproteobacteria>Enterobacterales>Enterobacteriaceae>Salmonella	GCA_019485205.1	Salmonella_enterica	K2495_002337	30739..34380	28901	BREX-system-P-loop-protein-BrxC-[Salmonella-enterica]
EHW8503808.1	Type1.BREX	[illegible]	1213	Bacteria>Pseudomonadota>Gammaproteobacteria>Enterobacterales>Enterobacteriaceae>Salmonella	GCA_019496265.1	Salmonella_enterica	K3U91_002209	149047..152688	28901	BREX-system-P-loop-protein-BrxC-[Salmonella-enterica]
EHX4782365.1	Type1.BREX	[illegible]	1213	Bacteria>Pseudomonadota>Gammaproteobacteria>Enterobacterales>Enterobacteriaceae>Salmonella	GCA_019533715.1	Salmonella_enterica	K3Y02_002640	144286..147927	28901	BREX-system-P-loop-protein-BrxC-[Salmonella-enterica]
EHZ3116575.1	Type1.BREX	[illegible]	957	Bacteria>Pseudomonadota>Gammaproteobacteria>Enterobacterales>Enterobacteriaceae>Salmonella	GCA_019692095.1	Salmonella_enterica_subsp._enterica_serovar_Typhimurium	K5O64_003315	60042..62911	90371	BREX-system-P-loop-protein-BrxC-[Salmonella-enterica-subsp.-enterica-serovar-Typhimurium]
EHZ3783988.1	Type1.BREX	[illegible]	1213	Bacteria>Pseudomonadota>Gammaproteobacteria>Enterobacterales>Enterobacteriaceae>Salmonella	GCA_019697575.1	Salmonella_enterica	K5U30_001272	170919..174560	28901	BREX-system-P-loop-protein-BrxC-[Salmonella-enterica]
EIA3194373.1	Type1.BREX	[illegible]	1213	Bacteria>Pseudomonadota>Gammaproteobacteria>Enterobacterales>Enterobacteriaceae>Salmonella	GCA_019788365.1	Salmonella_enterica	K7D38_001961	187001..190642	28901	BREX-system-P-loop-protein-BrxC-[Salmonella-enterica]
EIA3335811.1	Type1.BREX	[illegible]	1213	Bacteria>Pseudomonadota>Gammaproteobacteria>Enterobacterales>Enterobacteriaceae>Salmonella	GCA_019789135.1	Salmonella_enterica	K7D78_001298	400174..403815	28901	BREX-system-P-loop-protein-BrxC-[Salmonella-enterica]
EIB1064378.1	Type1.BREX	[illegible]	1213	Bacteria>Pseudomonadota>Gammaproteobacteria>Enterobacterales>Enterobacteriaceae>Salmonella	GCA_019855255.1	Salmonella_enterica	K7907_001965	108064..111705	28901	BREX-system-P-loop-protein-BrxC-[Salmonella-enterica]
EIB7346154.1	Type1.BREX	[illegible]	1213	Bacteria>Pseudomonadota>Gammaproteobacteria>Enterobacterales>Enterobacteriaceae>Salmonella	GCA_019904735.1	Salmonella_enterica	K9H00_003649	2808..6449	28901	BREX-system-P-loop-protein-BrxC-[Salmonella-enterica]
EIB7574746.1	Type1.BREX	[illegible]	1233	Bacteria>Pseudomonadota>Gammaproteobacteria>Enterobacterales>Enterobacteriaceae>Salmonella	GCA_019905755.1	Salmonella_enterica	K9I12_000373	406371..410072	28901	BREX-system-P-loop-protein-BrxC-[Salmonella-enterica]
EIC4097432.1	Type1.BREX	[illegible]	1213	Bacteria>Pseudomonadota>Gammaproteobacteria>Enterobacterales>Enterobacteriaceae>Salmonella	GCA_019951695.1	Salmonella_enterica	K9796_003003	22191..25832	28901	BREX-system-P-loop-protein-BrxC-[Salmonella-enterica]
EIC8525255.1	Type1.BREX	[illegible]	1204	Bacteria>Pseudomonadota>Gammaproteobacteria>Enterobacterales>Enterobacteriaceae>Salmonella	GCA_019993965.1	Salmonella_enterica	LAR10_002215	30447..34061	28901	BREX-system-P-loop-protein-BrxC-[Salmonella-enterica]
HBJ3502651.1	Type1.BREX	[illegible]	1213	Bacteria>Pseudomonadota>Gammaproteobacteria>Enterobacterales>Enterobacteriaceae>Salmonella	GCA_020048005.1	Salmonella_enterica_subsp._enterica_serovar_Typhimurium	LB282_001657	170703..174344	90371	BREX-system-P-loop-protein-BrxC-[Salmonella-enterica-subsp.-enterica-serovar-Typhimurium]
MBZ4773899.1	Type1.BREX	[illegible]	1129	Bacteria>Pseudomonadota>Gammaproteobacteria>Enterobacterales>Enterobacteriaceae>Salmonella	GCA_020070065.1	Salmonella_enterica_subsp._enterica_serovar_Typhimurium	HU130_03520	1..3389	90371	BREX-system-P-loop-protein-BrxC-[Salmonella-enterica-subsp.-enterica-serovar-Typhimurium]
MBZ4755449.1	Type1.BREX	[illegible]	1174	Bacteria>Pseudomonadota>Gammaproteobacteria>Enterobacterales>Enterobacteriaceae>Salmonella	GCA_020070115.1	Salmonella_enterica_subsp._enterica_serovar_Typhimurium	HU026_04360	60999..64520	90371	BREX-system-P-loop-protein-BrxC-[Salmonella-enterica-subsp.-enterica-serovar-Typhimurium]
EID5777526.1	Type1.BREX	[illegible]	1213	Bacteria>Pseudomonadota>Gammaproteobacteria>Enterobacterales>Enterobacteriaceae>Salmonella	GCA_020070325.1	Salmonella_enterica	LB861_002368	82497..86138	28901	BREX-system-P-loop-protein-BrxC-[Salmonella-enterica]
MBZ4898657.1	Type1.BREX	[illegible]	1213	Bacteria>Pseudomonadota>Gammaproteobacteria>Enterobacterales>Enterobacteriaceae>Salmonella	GCA_020070625.1	Salmonella_enterica_subsp._enterica_serovar_Typhimurium	HU117_01215	58948..62589	90371	BREX-system-P-loop-protein-BrxC-[Salmonella-enterica-subsp.-enterica-serovar-Typhimurium]
MBZ5058250.1	Type1.BREX	[illegible]	1213	Bacteria>Pseudomonadota>Gammaproteobacteria>Enterobacterales>Enterobacteriaceae>Salmonella	GCA_020071235.1	Salmonella_enterica_subsp._enterica_serovar_Typhimurium	HU058_13730	15756..19397	90371	BREX-system-P-loop-protein-BrxC-[Salmonella-enterica-subsp.-enterica-serovar-Typhimurium]
MBZ5141240.1	Type1.BREX	[illegible]	601	Bacteria>Pseudomonadota>Gammaproteobacteria>Enterobacterales>Enterobacteriaceae>Salmonella	GCA_020071745.1	Salmonella_enterica_subsp._enterica_serovar_Typhimurium	HU084_13180	16232..18035	90371	BREX-system-P-loop-protein-BrxC-[Salmonella-enterica-subsp.-enterica-serovar-Typhimurium]
EIE3342236.1	Type1.BREX	[illegible]	1213	Bacteria>Pseudomonadota>Gammaproteobacteria>Enterobacterales>Enterobacteriaceae>Salmonella	GCA_020155375.1	Salmonella_enterica_subsp._enterica_serovar_Typhimurium	LDC84_002298	60209..63850	90371	BREX-system-P-loop-protein-BrxC-[Salmonella-enterica-subsp.-enterica-serovar-Typhimurium]
EIF4998474.1	Type1.BREX	[illegible]	848	Bacteria>Pseudomonadota>Gammaproteobacteria>Enterobacterales>Enterobacteriaceae>Salmonella	GCA_020260065.1	Salmonella_enterica_subsp._enterica_serovar_Typhimurium	LFO03_001522	60001..62543	90371	BREX-system-P-loop-protein-BrxC-[Salmonella-enterica-subsp.-enterica-serovar-Typhimurium]
EIF5096942.1	Type1.BREX	[illegible]	899	Bacteria>Pseudomonadota>Gammaproteobacteria>Enterobacterales>Enterobacteriaceae>Salmonella	GCA_020260525.1	Salmonella_enterica_subsp._enterica_serovar_Typhimurium	LFF62_002381	60001..62698	90371	BREX-system-P-loop-protein-BrxC-[Salmonella-enterica-subsp.-enterica-serovar-Typhimurium]
EIF5593860.1	Type1.BREX	[illegible]	1058	Bacteria>Pseudomonadota>Gammaproteobacteria>Enterobacterales>Enterobacteriaceae>Salmonella	GCA_020262845.1	Salmonella_enterica_subsp._enterica_serovar_Typhimurium	LFF21_002838	60001..63175	90371	BREX-system-P-loop-protein-BrxC-[Salmonella-enterica-subsp.-enterica-serovar-Typhimurium]
EIF9427095.1	Type1.BREX	[illegible]	1213	Bacteria>Pseudomonadota>Gammaproteobacteria>Enterobacterales>Enterobacteriaceae>Salmonella	GCA_020283115.1	Salmonella_enterica	HW154_002103	25917..29558	28901	BREX-system-P-loop-protein-BrxC-[Salmonella-enterica]
EIF9623260.1	Type1.BREX	[illegible]	1213	Bacteria>Pseudomonadota>Gammaproteobacteria>Enterobacterales>Enterobacteriaceae>Salmonella	GCA_020284415.1	Salmonella_enterica	HQ981_002275	25917..29558	28901	BREX-system-P-loop-protein-BrxC-[Salmonella-enterica]
EII7462484.1	Type1.BREX	[illegible]	865	Bacteria>Pseudomonadota>Gammaproteobacteria>Enterobacterales>Enterobacteriaceae>Salmonella	GCA_020462505.1	Salmonella_enterica_subsp._enterica_serovar_Typhimurium	LHR57_000982	1..2599	90371	BREX-system-P-loop-protein-BrxC-[Salmonella-enterica-subsp.-enterica-serovar-Typhimurium]
EIJ0372099.1	Type1.BREX	[illegible]	1213	Bacteria>Pseudomonadota>Gammaproteobacteria>Enterobacterales>Enterobacteriaceae>Salmonella	GCA_020490715.1	Salmonella_enterica_subsp._enterica_serovar_Typhimurium	LH081_001559	170919..174560	90371	BREX-system-P-loop-protein-BrxC-[Salmonella-enterica-subsp.-enterica-serovar-Typhimurium]
EIJ0643398.1	Type1.BREX	[illegible]	1101	Bacteria>Pseudomonadota>Gammaproteobacteria>Enterobacterales>Enterobacteriaceae>Salmonella	GCA_020492735.1	Salmonella_enterica	LH177_001939	72298..75605	28901	BREX-system-P-loop-protein-BrxC-[Salmonella-enterica]
EIJ2988432.1	Type1.BREX	[illegible]	1213	Bacteria>Pseudomonadota>Gammaproteobacteria>Enterobacterales>Enterobacteriaceae>Salmonella	GCA_020513955.1	Salmonella_enterica	LIS40_000161	170919..174560	28901	BREX-system-P-loop-protein-BrxC-[Salmonella-enterica]
EIJ6143992.1	Type1.BREX	[illegible]	1213	Bacteria>Pseudomonadota>Gammaproteobacteria>Enterobacterales>Enterobacteriaceae>Salmonella	GCA_020548845.1	Salmonella_enterica_subsp._enterica_serovar_4:a:-	LI678_001014	394746..398387	2885150	BREX-system-P-loop-protein-BrxC-[Salmonella-enterica-subsp.-enterica-serovar-4:a:-]
EIM3792263.1	Type1.BREX	[illegible]	1213	Bacteria>Pseudomonadota>Gammaproteobacteria>Enterobacterales>Enterobacteriaceae>Salmonella	GCA_020780095.1	Salmonella_enterica	LM721_003554	27348..30989	28901	BREX-system-P-loop-protein-BrxC-[Salmonella-enterica]
EIN7178581.1	Type1.BREX	[illegible]	1137	Bacteria>Pseudomonadota>Gammaproteobacteria>Enterobacterales>Enterobacteriaceae>Salmonella	GCA_020912625.1	Salmonella_enterica_subsp._enterica_serovar_Typhimurium	LPQ66_002313	59987..63398	90371	BREX-system-P-loop-protein-BrxC-[Salmonella-enterica-subsp.-enterica-serovar-Typhimurium]
EIO7765743.1	Type1.BREX	[illegible]	1213	Bacteria>Pseudomonadota>Gammaproteobacteria>Enterobacterales>Enterobacteriaceae>Salmonella	GCA_020997775.1	Salmonella_enterica	LQR80_000699	170919..174560	28901	BREX-system-P-loop-protein-BrxC-[Salmonella-enterica]
HBL9882169.1	Type1.BREX	[illegible]	1213	Bacteria>Pseudomonadota>Gammaproteobacteria>Enterobacterales>Enterobacteriaceae>Salmonella	GCA_021103155.1	Salmonella_enterica_subsp._enterica_serovar_Jangwani	LT029_003490	155244..158885	2564577	BREX-system-P-loop-protein-BrxC-[Salmonella-enterica-subsp.-enterica-serovar-Jangwani]
HBM1480881.1	Type1.BREX	[illegible]	700	Bacteria>Pseudomonadota>Gammaproteobacteria>Enterobacterales>Enterobacteriaceae>Salmonella	GCA_021143585.1	Salmonella_enterica_subsp._enterica	LUI79_002478	1..2100	59201	BREX-system-P-loop-protein-BrxC-[Salmonella-enterica-subsp.-enterica]
EIQ4281221.1	Type1.BREX	[illegible]	1213	Bacteria>Pseudomonadota>Gammaproteobacteria>Enterobacterales>Enterobacteriaceae>Salmonella	GCA_021189835.1	Salmonella_enterica	LU454_001105	102830..106471	28901	BREX-system-P-loop-protein-BrxC-[Salmonella-enterica]
EIQ7941123.1	Type1.BREX	[illegible]	1180	Bacteria>Pseudomonadota>Gammaproteobacteria>Enterobacterales>Enterobacteriaceae>Salmonella	GCA_021224035.1	Salmonella_enterica_subsp._enterica_serovar_Typhimurium	LVZ53_003070	1..3541	90371	BREX-system-P-loop-protein-BrxC-[Salmonella-enterica-subsp.-enterica-serovar-Typhimurium]
EIR7569906.1	Type1.BREX	[illegible]	873	Bacteria>Pseudomonadota>Gammaproteobacteria>Enterobacterales>Enterobacteriaceae>Salmonella	GCA_021281445.1	Salmonella_enterica_subsp._enterica_serovar_Agbeni	LXI61_003348	30366..32989	1967642	BREX-system-P-loop-protein-BrxC-[Salmonella-enterica-subsp.-enterica-serovar-Agbeni]
EIR8875059.1	Type1.BREX	[illegible]	1213	Bacteria>Pseudomonadota>Gammaproteobacteria>Enterobacterales>Enterobacteriaceae>Salmonella	GCA_021305935.1	Salmonella_enterica	LXE31_003492	2812..6453	28901	BREX-system-P-loop-protein-BrxC-[Salmonella-enterica]
EIS9096680.1	Type1.BREX	[illegible]	1213	Bacteria>Pseudomonadota>Gammaproteobacteria>Enterobacterales>Enterobacteriaceae>Salmonella	GCA_021435525.1	Salmonella_enterica	L0705_004818	2605..6246	28901	BREX-system-P-loop-protein-BrxC-[Salmonella-enterica]
HBN2343839.1	Type1.BREX	[illegible]	1083	Bacteria>Pseudomonadota>Gammaproteobacteria>Enterobacterales>Enterobacteriaceae>Salmonella	GCA_021498555.1	Salmonella_enterica_subsp._enterica_serovar_Typhimurium	L2A29_001994	59989..63237	90371	BREX-system-P-loop-protein-BrxC-[Salmonella-enterica-subsp.-enterica-serovar-Typhimurium]
EIT9287885.1	Type1.BREX	[illegible]	960	Bacteria>Pseudomonadota>Gammaproteobacteria>Enterobacterales>Enterobacteriaceae>Salmonella	GCA_021558855.1	Salmonella_enterica_subsp._enterica_serovar_Derby	L3N85_002601	53297..56175	28144	BREX-system-P-loop-protein-BrxC-[Salmonella-enterica-subsp.-enterica-serovar-Derby]
EIU6597714.1	Type1.BREX	[illegible]	1213	Bacteria>Pseudomonadota>Gammaproteobacteria>Enterobacterales>Enterobacteriaceae>Salmonella	GCA_021692515.1	Salmonella_enterica	L5W02_001568	377290..380931	28901	BREX-system-P-loop-protein-BrxC-[Salmonella-enterica]
HBZ6150869.1	Type1.BREX	[illegible]	1213	Bacteria>Pseudomonadota>Gammaproteobacteria>Enterobacterales>Enterobacteriaceae>Salmonella	GCA_022432985.1	Salmonella_enterica	ML330_001731	63563..67204	28901	BREX-system-P-loop-protein-BrxC-[Salmonella-enterica]
EJB4514208.1	Type1.BREX	[illegible]	1213	Bacteria>Pseudomonadota>Gammaproteobacteria>Enterobacterales>Enterobacteriaceae>Salmonella	GCA_022945175.1	Salmonella_enterica	MT088_002306	380173..383814	28901	BREX-system-P-loop-protein-BrxC-[Salmonella-enterica]
EJC3638306.1	Type1.BREX	[illegible]	1213	Bacteria>Pseudomonadota>Gammaproteobacteria>Enterobacterales>Enterobacteriaceae>Salmonella	GCA_023047235.1	Salmonella_enterica	MYX88_003393	151139..154780	28901	BREX-system-P-loop-protein-BrxC-[Salmonella-enterica]
HCB4841064.1	Type1.BREX	[illegible]	1213	Bacteria>Pseudomonadota>Gammaproteobacteria>Enterobacterales>Enterobacteriaceae>Salmonella	GCA_023082205.1	Salmonella_enterica_subsp._enterica_serovar_Bredeney	M0F44_000351	390385..394026	134047	BREX-system-P-loop-protein-BrxC-[Salmonella-enterica-subsp.-enterica-serovar-Bredeney]
EJF4683727.1	Type1.BREX	[illegible]	1213	Bacteria>Pseudomonadota>Gammaproteobacteria>Enterobacterales>Enterobacteriaceae>Salmonella	GCA_023476405.1	Salmonella_enterica	M8N25_002793	108064..111705	28901	BREX-system-P-loop-protein-BrxC-[Salmonella-enterica]
MCL8965752.1	Type1.BREX	[illegible]	1213	Bacteria>Pseudomonadota>Gammaproteobacteria>Enterobacterales>Enterobacteriaceae>Salmonella	GCA_023608175.1	Salmonella_enterica_subsp._enterica_serovar_Enteritidis	MU637_15175	170549..174190	149539	BREX-system-P-loop-protein-BrxC-[Salmonella-enterica-subsp.-enterica-serovar-Enteritidis]
MCM9949031.1	Type1.BREX	[illegible]	1213	Bacteria>Pseudomonadota>Gammaproteobacteria>Enterobacterales>Enterobacteriaceae>Salmonella	GCA_023843835.1	Salmonella_enterica	M5G05_07280	348323..351964	28901	BREX-system-P-loop-protein-BrxC-[Salmonella-enterica]
EJK0228892.1	Type1.BREX	[illegible]	1213	Bacteria>Pseudomonadota>Gammaproteobacteria>Enterobacterales>Enterobacteriaceae>Salmonella	GCA_024068175.1	Salmonella_enterica	NKK90_001819	323151..326792	28901	BREX-system-P-loop-protein-BrxC-[Salmonella-enterica]
HCH7501034.1	Type1.BREX	[illegible]	633	Bacteria>Pseudomonadota>Gammaproteobacteria>Enterobacterales>Enterobacteriaceae>Salmonella	GCA_024265105.1	Salmonella_enterica	NM874_003883	7331..9230	28901	BREX-system-P-loop-protein-BrxC-[Salmonella-enterica]
EJM7985883.1	Type1.BREX	[illegible]	1213	Bacteria>Pseudomonadota>Gammaproteobacteria>Enterobacterales>Enterobacteriaceae>Salmonella	GCA_024373005.1	Salmonella_enterica	N0486_001112	369092..372733	28901	BREX-system-P-loop-protein-BrxC-[Salmonella-enterica]
EJN2869328.1	Type1.BREX	[illegible]	1213	Bacteria>Pseudomonadota>Gammaproteobacteria>Enterobacterales>Enterobacteriaceae>Salmonella	GCA_024442015.1	Salmonella_enterica_subsp._enterica_serovar_Derby	NPT71_003578	165015..168656	28144	BREX-system-P-loop-protein-BrxC-[Salmonella-enterica-subsp.-enterica-serovar-Derby]
UTZ95782.1	Type1.BREX	[illegible]	1204	Bacteria>Pseudomonadota>Gammaproteobacteria>Enterobacterales>Enterobacteriaceae>Salmonella	GCA_024442275.1	Salmonella_enterica_subsp._enterica_serovar_Kentucky	NOQ95_21560	4448475..4452089	192955	BREX-system-P-loop-protein-BrxC-[Salmonella-enterica-subsp.-enterica-serovar-Kentucky]
EJN9121676.1	Type1.BREX	[illegible]	1213	Bacteria>Pseudomonadota>Gammaproteobacteria>Enterobacterales>Enterobacteriaceae>Salmonella	GCA_024535515.1	Salmonella_enterica	NR521_001803	170929..174570	28901	BREX-system-P-loop-protein-BrxC-[Salmonella-enterica]
EJO5979497.1	Type1.BREX	[illegible]	1213	Bacteria>Pseudomonadota>Gammaproteobacteria>Enterobacterales>Enterobacteriaceae>Salmonella	GCA_024573615.1	Salmonella_enterica	NRR85_002625	159039..162680	28901	BREX-system-P-loop-protein-BrxC-[Salmonella-enterica]
EJS8182320.1	Type1.BREX	[illegible]	1213	Bacteria>Pseudomonadota>Gammaproteobacteria>Enterobacterales>Enterobacteriaceae>Salmonella	GCA_024988615.1	Salmonella_enterica	NWJ04_001330	25917..29558	28901	BREX-system-P-loop-protein-BrxC-[Salmonella-enterica]
HCL5270538.1	Type1.BREX	[illegible]	1213	Bacteria>Pseudomonadota>Gammaproteobacteria>Enterobacterales>Enterobacteriaceae>Salmonella	GCA_025012985.1	Salmonella_enterica	N2G33_002753	3666..7307	28901	BREX-system-P-loop-protein-BrxC-[Salmonella-enterica]
HCM2417898.1	Type1.BREX	[illegible]	1213	Bacteria>Pseudomonadota>Gammaproteobacteria>Enterobacterales>Enterobacteriaceae>Salmonella	GCA_025074775.1	Salmonella_enterica_subsp._enterica_serovar_Typhimurium_var._monophasic_4,[5],12:i:-	N3D22_002716	108064..111705	1461749	BREX-system-P-loop-protein-BrxC-[Salmonella-enterica-subsp.-enterica-serovar-Typhimurium-[illegible]
HCM2697231.1	Type1.BREX	[illegible]	1213	Bacteria>Pseudomonadota>Gammaproteobacteria>Enterobacterales>Enterobacteriaceae>Salmonella	GCA_025075935.1	Salmonella_enterica_subsp._enterica_serovar_Yaba	N3D89_002099	63778..67419	2036205	BREX-system-P-loop-protein-BrxC-[Salmonella-enterica-subsp.-enterica-serovar-Yaba]
EJU7772063.1	Type1.BREX	[illegible]	1225	Bacteria>Pseudomonadota>Gammaproteobacteria>Enterobacterales>Enterobacteriaceae>Salmonella	GCA_025208445.1	Salmonella_enterica_subsp._salamae_serovar_4,12:e,n,x:1,6	KAB52_003008	279421..283062	2978379	BREX-system-P-loop-protein-BrxC-[Salmonella-enterica-subsp.-salamae-serovar-4,12:e,n,x:1,[illegible]
EJV9378859.1	Type1.BREX	[illegible]	1213	Bacteria>Pseudomonadota>Gammaproteobacteria>Enterobacterales>Enterobacteriaceae>Salmonella	GCA_025367915.1	Salmonella_enterica	N7887_004537	2923..6600	28901	BREX-system-P-loop-protein-BrxC-[Salmonella-enterica]
EJW7283568.1	Type1.BREX	[illegible]	1166	Bacteria>Pseudomonadota>Gammaproteobacteria>Enterobacterales>Enterobacteriaceae>Salmonella	GCA_025474975.1	Salmonella_enterica_subsp._enterica_serovar_Typhimurium	OCR56_001321	81267..84908	90371	BREX-system-P-loop-protein-BrxC-[Salmonella-enterica-subsp.-enterica-serovar-Typhimurium]
WP_096117035.1	Type1.BREX	[illegible]	1213	Bacteria>Pseudomonadota>Gammaproteobacteria>Enterobacterales>Enterobacteriaceae>Salmonella	GCA_030820635.1	Salmonella_enterica	CDC58_RS01455	1..3498	28901	BREX-system-P-loop-protein-BrxC,-partial-[Salmonella-enterica]
WP_111761031.1	Type1.BREX	[illegible]	1213	Bacteria>Pseudomonadota>Gammaproteobacteria>Enterobacterales>Enterobacteriaceae>Salmonella	GCA_030820635.1	Salmonella_enterica	KXD81_RS02520	21979..25620	28901	BREX-system-P-loop-protein-BrxC-[Salmonella-enterica]
WP_256863860.1	Type1.BREX	[illegible]	840	Bacteria>Pseudomonadota>Gammaproteobacteria>Enterobacterales>Enterobacteriaceae>Salmonella	GCA_030820635.1	Salmonella_enterica	SO19_RS21855	1..2520	28901	BREX-system-P-loop-protein-BrxC,-partial-[Salmonella-enterica]
SUG73766.1	Type1.BREX	[illegible]	900	Bacteria>Pseudomonadota>Gammaproteobacteria>Enterobacterales>Enterobacteriaceae>Salmonella	GCA_900456435.1	Salmonella_enterica_subsp._enterica	NCTC5798_05055	4086397..4089099	59201	DNA-repair-ATPase-[Salmonella-enterica-subsp.-enterica]
SUI30166.1	Type1.BREX	[illegible]	864	Bacteria>Pseudomonadota>Gammaproteobacteria>Enterobacterales>Enterobacteriaceae>Salmonella	GCA_900456825.1	Salmonella_enterica_subsp._enterica_serovar_Derby	NCTC5722_04070	4063548..4066142	28144	DNA-repair-ATPase-[Salmonella-enterica-subsp.-enterica-serovar-Derby]
SUH94769.1	Type1.BREX	[illegible]	530	Bacteria>Pseudomonadota>Gammaproteobacteria>Enterobacterales>Enterobacteriaceae>Salmonella	GCA_900456925.1	Salmonella_enterica_subsp._enterica_serovar_Typhimurium	NCTC13347_04212	4172999..4174591	90371	DNA-repair-ATPase-[Salmonella-enterica-subsp.-enterica-serovar-Typhimurium]
VUC90716.1	Type1.BREX	[illegible]	532	Bacteria>Pseudomonadota>Gammaproteobacteria>Enterobacterales>Enterobacteriaceae>Salmonella	GCA_902109495.1	Salmonella_sp._NCTC_11881	NCTC11881_05559	4058427..4060025	2583579	DNA-repair-ATPase-[Salmonella-sp.-NCTC-11881]
WP_001523601.1	Type1.BREX	[illegible]	1213	Bacteria>Pseudomonadota>Gammaproteobacteria>Enterobacterales>Enterobacteriaceae>Salmonella	GCF_000170235.1	Salmonella_enterica	SESPB_RS10230	40505..44146	28901	BREX-system-P-loop-protein-BrxC-[Salmonella-enterica]
WP_001019653.1	Type1.BREX	[illegible]	1213	Bacteria>Pseudomonadota>Gammaproteobacteria>Enterobacterales>Enterobacteriaceae>Salmonella	GCF_000230875.1	Salmonella_enterica	SEENINOB_RS28275	4768987..4772628	28901	BREX-system-P-loop-protein-BrxC-[Salmonella-enterica]
WP_001019651.1	Type1.BREX	[illegible]	1213	Bacteria>Pseudomonadota>Gammaproteobacteria>Enterobacterales>Enterobacteriaceae>Salmonella	GCF_000252875.1	Salmonella_enterica	JM982_RS03985	854415..858056	28901	BREX-system-P-loop-protein-BrxC-[Salmonella-enterica]
WP_024154967.1	Type1.BREX	[illegible]	1213	Bacteria>Pseudomonadota>Gammaproteobacteria>Enterobacterales>Enterobacteriaceae>Salmonella	GCF_000412855.1	Salmonella_enterica	AP074_RS03115	646291..649932	28901	BREX-system-P-loop-protein-BrxC-[Salmonella-enterica]
WP_071055527.1	Type1.BREX	[illegible]	1213	Bacteria>Pseudomonadota>Gammaproteobacteria>Enterobacterales>Enterobacteriaceae>Salmonella	GCF_000486165.2	Salmonella_enterica	SES26_RS19315	4009469..4013110	28901	BREX-system-P-loop-protein-BrxC-[Salmonella-enterica]
WP_023217629.1	Type1.BREX	[illegible]	1213	Bacteria>Pseudomonadota>Gammaproteobacteria>Enterobacterales>Enterobacteriaceae>Salmonella	GCF_000486855.2	Salmonella_enterica	SEEM9284_RS19920	3931825..3935466	28901	BREX-system-P-loop-protein-BrxC-[Salmonella-enterica]
WP_077956997.1	Type1.BREX	[illegible]	1240	Bacteria>Pseudomonadota>Gammaproteobacteria>Enterobacterales>Enterobacteriaceae>Salmonella	GCF_000487155.2	Salmonella_enterica	A7T10_RS09345	418937..422659	28901	BREX-system-P-loop-protein-BrxC-[Salmonella-enterica]
WP_023213578.1	Type1.BREX	[illegible]	1213	Bacteria>Pseudomonadota>Gammaproteobacteria>Enterobacterales>Enterobacteriaceae>Salmonella	GCF_000493295.2	Salmonella_enterica	SEEP1673_RS21585	4142040..4145681	28901	BREX-system-P-loop-protein-BrxC-[Salmonella-enterica]
WP_050956242.1	Type1.BREX	[illegible]	1213	Bacteria>Pseudomonadota>Gammaproteobacteria>Enterobacterales>Enterobacteriaceae>Salmonella	GCF_001217085.1	Salmonella_enterica	AYW49_RS05955	256224..259865	28901	BREX-system-P-loop-protein-BrxC-[Salmonella-enterica]
WP_052925311.1	Type1.BREX	[illegible]	1213	Bacteria>Pseudomonadota>Gammaproteobacteria>Enterobacterales>Enterobacteriaceae>Salmonella	GCF_001243555.1	Salmonella_enterica	AEV74_RS03660	108085..111726	28901	BREX-system-P-loop-protein-BrxC-[Salmonella-enterica]
WP_057523065.1	Type1.BREX	[illegible]	1213	Bacteria>Pseudomonadota>Gammaproteobacteria>Enterobacterales>Enterobacteriaceae>Salmonella	GCF_001326235.1	Salmonella_enterica	HG82_RS07610	171678..175319	28901	BREX-system-P-loop-protein-BrxC-[Salmonella-enterica]
WP_058799135.1	Type1.BREX	[illegible]	1213	Bacteria>Pseudomonadota>Gammaproteobacteria>Enterobacterales>Enterobacteriaceae>Salmonella	GCF_001478065.1	Salmonella_enterica	DD74_RS03635	38743..42384	28901	BREX-system-P-loop-protein-BrxC-[Salmonella-enterica]
WP_061383284.1	Type1.BREX	[illegible]	1204	Bacteria>Pseudomonadota>Gammaproteobacteria>Enterobacterales>Enterobacteriaceae>Salmonella	GCF_001570045.1	Salmonella_enterica	AW140_RS06880	353892..357506	28901	BREX-system-P-loop-protein-BrxC-[Salmonella-enterica]
WP_061380447.1	Type1.BREX	[illegible]	1213	Bacteria>Pseudomonadota>Gammaproteobacteria>Enterobacterales>Enterobacteriaceae>Salmonella	GCF_001570285.1	Salmonella_enterica	AW153_RS18915	158422..162063	28901	BREX-system-P-loop-protein-BrxC-[Salmonella-enterica]
WP_061584452.1	Type1.BREX	[illegible]	1213	Bacteria>Pseudomonadota>Gammaproteobacteria>Enterobacterales>Enterobacteriaceae>Salmonella	GCF_001588495.1	Salmonella_enterica	AF388_RS19360	33009..36650	28901	BREX-system-P-loop-protein-BrxC-[Salmonella-enterica]
WP_064013329.1	Type1.BREX	[illegible]	1204	Bacteria>Pseudomonadota>Gammaproteobacteria>Enterobacterales>Enterobacteriaceae>Salmonella	GCF_001643815.1	Salmonella_enterica	IJ96_RS19570	3320..6934	28901	BREX-system-P-loop-protein-BrxC-[Salmonella-enterica]
WP_069106895.1	Type1.BREX	[illegible]	1213	Bacteria>Pseudomonadota>Gammaproteobacteria>Enterobacterales>Enterobacteriaceae>Salmonella	GCF_001714545.1	Salmonella_enterica	AHW33_RS11985	172706..176347	28901	BREX-system-P-loop-protein-BrxC-[Salmonella-enterica]
WP_070132690.1	Type1.BREX	[illegible]	1213	Bacteria>Pseudomonadota>Gammaproteobacteria>Enterobacterales>Enterobacteriaceae>Salmonella	GCF_001756155.1	Salmonella_enterica	BG484_RS05620	12664..16305	28901	BREX-system-P-loop-protein-BrxC-[Salmonella-enterica]
WP_070134780.1	Type1.BREX	[illegible]	1201	Bacteria>Pseudomonadota>Gammaproteobacteria>Enterobacterales>Enterobacteriaceae>Salmonella	GCF_001756205.1	Salmonella_enterica	BG487_15355	1..3606	28901	BREX-system-P-loop-protein-BrxC,-partial-[Salmonella-enterica]
WP_070793518.1	Type1.BREX	[illegible]	1213	Bacteria>Pseudomonadota>Gammaproteobacteria>Enterobacterales>Enterobacteriaceae>Salmonella	GCF_001832595.1	Salmonella_enterica	A7T54_RS11885	259865..263506	28901	BREX-system-P-loop-protein-BrxC-[Salmonella-enterica]
WP_076937571.1	Type1.BREX	[illegible]	1213	Bacteria>Pseudomonadota>Gammaproteobacteria>Enterobacterales>Enterobacteriaceae>Salmonella	GCF_001975325.1	Salmonella_enterica	LFZ3_RS17405	3406419..3410060	28901	BREX-system-P-loop-protein-BrxC-[Salmonella-enterica]
WP_076736168.1	Type1.BREX	[illegible]	1213	Bacteria>Pseudomonadota>Gammaproteobacteria>Enterobacterales>Enterobacteriaceae>Salmonella	GCF_001975445.1	Salmonella_enterica	LFZ24_RS24380	4843050..4846691	28901	BREX-system-P-loop-protein-BrxC-[Salmonella-enterica]
WP_078367156.1	Type1.BREX	[illegible]	1213	Bacteria>Pseudomonadota>Gammaproteobacteria>Enterobacterales>Enterobacteriaceae>Salmonella	GCF_002018325.1	Salmonella_enterica	B1S11_RS05200	170490..174131	28901	BREX-system-P-loop-protein-BrxC-[Salmonella-enterica]
WP_078351828.1	Type1.BREX	[illegible]	1213	Bacteria>Pseudomonadota>Gammaproteobacteria>Enterobacterales>Enterobacteriaceae>Salmonella	GCF_002018355.1	Salmonella_enterica	B1211_RS18830	150004..153645	28901	BREX-system-P-loop-protein-BrxC-[Salmonella-enterica]
WP_001729064.1	Type1.BREX	[illegible]	1213	Bacteria>Pseudomonadota>Gammaproteobacteria>Enterobacterales>Enterobacteriaceae>Salmonella	GCF_002034005.1	Salmonella_enterica	AJH59_RS23280	9917..13558	28901	BREX-system-P-loop-protein-BrxC-[Salmonella-enterica]
WP_079808688.1	Type1.BREX	[illegible]	1213	Bacteria>Pseudomonadota>Gammaproteobacteria>Enterobacterales>Enterobacteriaceae>Salmonella	GCF_002035875.1	Salmonella_enterica	Y985_RS08730	120043..123684	28901	BREX-system-P-loop-protein-BrxC-[Salmonella-enterica]
WP_079814448.1	Type1.BREX	[illegible]	1204	Bacteria>Pseudomonadota>Gammaproteobacteria>Enterobacterales>Enterobacteriaceae>Salmonella	GCF_002036095.1	Salmonella_enterica	S481_13075	121983..125597	28901	BREX-system-P-loop-protein-BrxC-[Salmonella-enterica]
WP_079897969.1	Type1.BREX	[illegible]	1204	Bacteria>Pseudomonadota>Gammaproteobacteria>Enterobacterales>Enterobacteriaceae>Salmonella	GCF_002045805.1	Salmonella_enterica	VY62_RS13065	181728..185342	28901	BREX-system-P-loop-protein-BrxC-[Salmonella-enterica]
WP_079972900.1	Type1.BREX	[illegible]	1213	Bacteria>Pseudomonadota>Gammaproteobacteria>Enterobacterales>Enterobacteriaceae>Salmonella	GCF_002046615.1	Salmonella_enterica	VX95_RS09560	163568..167209	28901	BREX-system-P-loop-protein-BrxC-[Salmonella-enterica]
WP_079965457.1	Type1.BREX	[illegible]	1213	Bacteria>Pseudomonadota>Gammaproteobacteria>Enterobacterales>Enterobacteriaceae>Salmonella	GCF_002046745.1	Salmonella_enterica	VX54_RS10650	52174..55815	28901	BREX-system-P-loop-protein-BrxC-[Salmonella-enterica]
WP_079902376.1	Type1.BREX	[illegible]	1213	Bacteria>Pseudomonadota>Gammaproteobacteria>Enterobacterales>Enterobacteriaceae>Salmonella	GCF_002048195.1	Salmonella_enterica	VX33_RS03275	157928..161569	28901	BREX-system-P-loop-protein-BrxC-[Salmonella-enterica]
WP_080094962.1	Type1.BREX	[illegible]	1213	Bacteria>Pseudomonadota>Gammaproteobacteria>Enterobacterales>Enterobacteriaceae>Salmonella	GCF_002052895.1	Salmonella_enterica	DL104_05730	29230..32871	28901	BREX-system-P-loop-protein-BrxC-[Salmonella-enterica]
WP_079944402.1	Type1.BREX	[illegible]	1213	Bacteria>Pseudomonadota>Gammaproteobacteria>Enterobacterales>Enterobacteriaceae>Salmonella	GCF_002058485.1	Salmonella_enterica	AJM56_RS05775	30789..34430	28901	BREX-system-P-loop-protein-BrxC-[Salmonella-enterica]
WP_080162863.1	Type1.BREX	[illegible]	1213	Bacteria>Pseudomonadota>Gammaproteobacteria>Enterobacterales>Enterobacteriaceae>Salmonella	GCF_002062115.1	Salmonella_enterica	SO66_RS04055	158804..162445	28901	BREX-system-P-loop-protein-BrxC-[Salmonella-enterica]

Protein-ID	System	Gene-Neighbourhoods	Length	Taxonomy	Assembly-ID	Strain	Locus-Tag	Genomic-coordinates	TaxID	Fasta-define
WP_139780228.1	Type1.BREX	<-DUF6079*<-BrxB-BrxF-iSTAND<-BrxA-DNA-binding<-?<-SAM-METHYLASE<-YjhX_toxin||?->HEPN->	1015	Bacteria>Pseudomonadota>Gammaproteobacteria>Enterobacterales>Enterobacteriaceae>Salmonella	GCF_002064225.1	Salmonella_enterica	SN68_RS12600	1..3045	28901	BREX-system-P-loop-protein-BrxC,-partial-[Salmonella-enterica]
WP_079905548.1	Type1.BREX	<-MukE<-wHTH||wHTH+WYL+WCX-><-HKD-DNase<-MarR-wHTH<-SIGMA-HTH+REase||?->BrxA-DNA-binding->BrxB-BrxF-iSTAND->DUF6079*->N6-METHYLASE->OLD-ABC->TOPRIM->PglZ->SIGMA+OB-Fold+AAA-ATPase+[illegible]	1204	Bacteria>Pseudomonadota>Gammaproteobacteria>Enterobacterales>Enterobacteriaceae>Salmonella	GCF_002065435.1	Salmonella_enterica	SN48_RS13425	18385..21999	28901	BREX-system-P-loop-protein-BrxC-[Salmonella-enterica]
WP_080250285.1	Type1.BREX	<-HEPN<-?||YjhX_toxin->SAM-METHYLASE->?->BrxA-DNA-binding->BrxB-BrxF-iSTAND->DUF6079*->N6-METHYLASE->OLD-ABC->TOPRIM->PglZ->SIGMA+OB-Fold+AAA-ATPase+Lon-Protease-BrxL->HKD-DNase+[illegible]	1213	Bacteria>Pseudomonadota>Gammaproteobacteria>Enterobacterales>Enterobacteriaceae>Salmonella	GCF_002066965.1	Salmonella_enterica	RZ13_RS02295	164882..168523	28901	BREX-system-P-loop-protein-BrxC-[Salmonella-enterica]
WP_111769566.1	Type1.BREX	NACHT+SF1-SF2-Helicase+HKD-DNase->REase-><-?<-SIGMA+OB-Fold+AAA-ATPase+Lon-Protease-BrxL<-PglZ<-TOPRIM<-OLD-ABC<-N6-METHYLASE<-DUF6079*<-BrxB-BrxF-iSTAND<-BrxA-DNA-binding<-?<-[illegible]	1213	Bacteria>Pseudomonadota>Gammaproteobacteria>Enterobacterales>Enterobacteriaceae>Salmonella	GCF_002106425.1	Salmonella_enterica	R537_RS06030	154441..158082	28901	BREX-system-P-loop-protein-BrxC-[Salmonella-enterica]
WP_086805524.1	Type1.BREX	NACHT+SF1-SF2-Helicase+HKD-DNase->REase-><-HKD-DNase+OGG-like<-SIGMA+OB-Fold+AAA-ATPase+Lon-Protease-BrxL<-PglZ<-TOPRIM<-OLD-ABC<-N6-METHYLASE<-DUF6079*<-BrxB-BrxF-iSTAND<-[illegible]	1204	Bacteria>Pseudomonadota>Gammaproteobacteria>Enterobacterales>Enterobacteriaceae>Salmonella	GCF_002156225.1	Salmonella_enterica	CCL33_RS10730	52147..55761	28901	BREX-system-P-loop-protein-BrxC-[Salmonella-enterica]
WP_065348243.1	Type1.BREX	<-HEPN<-?||YjhX_toxin->SAM-METHYLASE->?->BrxA-DNA-binding->BrxB-BrxF-iSTAND->DUF6079*->N6-METHYLASE->OLD-ABC->TOPRIM->PglZ->SIGMA+OB-Fold+AAA-ATPase+Lon-Protease-BrxL->?-><-REase<-[illegible]	1213	Bacteria>Pseudomonadota>Gammaproteobacteria>Enterobacterales>Enterobacteriaceae>Salmonella	GCF_002196535.1	Salmonella_enterica	GX95_RS20905	4055067..4058708	28901	BREX-system-P-loop-protein-BrxC-[Salmonella-enterica]
WP_088546528.1	Type1.BREX	<-GntR-HTH++TrmB+ZDNA-HTH||?-><-?<-HEPN<-?||YjhX_toxin->?->BrxA-DNA-binding->BrxB-BrxF-iSTAND->DUF6079*->N6-METHYLASE->OLD-ABC->TOPRIM->PglZ->SIGMA+OB-Fold+AAA-ATPase+Lon-Protease-[illegible]	1213	Bacteria>Pseudomonadota>Gammaproteobacteria>Enterobacterales>Enterobacteriaceae>Salmonella	GCF_002208255.1	Salmonella_enterica	LFZ35_RS21090	4061970..4065611	28901	BREX-system-P-loop-protein-BrxC-[Salmonella-enterica]
WP_088759215.1	Type1.BREX	<-MukB<-MukE<-wHTH||wHTH+WYL+WCX-><-MarR-wHTH<-SIGMA-HTH+REase||?->BrxA-DNA-binding->BrxB-BrxF-iSTAND->DUF6079*->N6-METHYLASE->OLD-ABC->TOPRIM->PglZ->SIGMA+OB-Fold+AAA-ATPase+[illegible]	1204	Bacteria>Pseudomonadota>Gammaproteobacteria>Enterobacterales>Enterobacteriaceae>Salmonella	GCF_002211845.1	Salmonella_enterica	CE137_RS21050	4095775..4099389	28901	BREX-system-P-loop-protein-BrxC-[Salmonella-enterica]
WP_080090697.1	Type1.BREX	<-HEPN<-REase+iREase<-?||YjhX_toxin->SAM-METHYLASE->?->BrxA-DNA-binding->BrxB-BrxF-iSTAND->DUF6079*->N6-METHYLASE->OLD-ABC->TOPRIM->PglZ->SIGMA+OB-Fold+AAA-ATPase+Lon-Protease-BrxL->[illegible]	1213	Bacteria>Pseudomonadota>Gammaproteobacteria>Enterobacterales>Enterobacteriaceae>Salmonella	GCF_002234755.2	Salmonella_enterica	CHD02_RS05505	1125404..1129045	28901	BREX-system-P-loop-protein-BrxC-[Salmonella-enterica]
WP_053445538.1	Type1.BREX	<-HEPN<-?||YjhX_toxin->SAM-METHYLASE->?->BrxA-DNA-binding->BrxB-BrxF-iSTAND->DUF6079*->N6-METHYLASE->OLD-ABC->TOPRIM->PglZ->SIGMA+OB-Fold+AAA-ATPase+Lon-Protease-BrxL-><-REase	1213	Bacteria>Pseudomonadota>Gammaproteobacteria>Enterobacterales>Enterobacteriaceae>Salmonella	GCF_002238275.1	Salmonella_enterica	F1E41_RS17160	150695..154336	28901	BREX-system-P-loop-protein-BrxC-[Salmonella-enterica]
WP_094797764.1	Type1.BREX	<-HEPN<-REase+iREase<-?||YjhX_toxin->SAM-METHYLASE->?->BrxA-DNA-binding->BrxB-BrxF-iSTAND->DUF6079*->N6-METHYLASE->OLD-ABC->TOPRIM->PglZ->SIGMA+OB-Fold+AAA-ATPase+Lon-Protease-BrxL->[illegible]	1213	Bacteria>Pseudomonadota>Gammaproteobacteria>Enterobacterales>Enterobacteriaceae>Salmonella	GCF_002260565.1	Salmonella_enterica	CFB03_RS03285	15872..19513	28901	BREX-system-P-loop-protein-BrxC-[Salmonella-enterica]
WP_094887773.1	Type1.BREX	REase-><-SIGMA+OB-Fold+AAA-ATPase+Lon-Protease-BrxL<-PglZ<-TOPRIM<-OLD-ABC<-N6-METHYLASE<-DUF6079*<-BrxB-BrxF-iSTAND<-BrxA-DNA-binding	1213	Bacteria>Pseudomonadota>Gammaproteobacteria>Enterobacterales>Enterobacteriaceae>Salmonella	GCF_002265975.1	Salmonella_enterica	CC043_RS23255	12854..16495	28901	BREX-system-P-loop-protein-BrxC-[Salmonella-enterica]
WP_094964979.1	Type1.BREX	<-HEPN<-?||YjhX_toxin->SAM-METHYLASE->?->BrxA-DNA-binding->BrxB-BrxF-iSTAND->DUF6079*->N6-METHYLASE->OLD-ABC->TOPRIM->PglZ->SIGMA+OB-Fold+AAA-ATPase+Lon-Protease-BrxL->||<-HTH_Tnp_1+[illegible]	1213	Bacteria>Pseudomonadota>Gammaproteobacteria>Enterobacterales>Enterobacteriaceae>Salmonella	GCF_002266055.1	Salmonella_enterica	CC049_RS14335	399067..402708	28901	BREX-system-P-loop-protein-BrxC-[Salmonella-enterica]
WP_094946975.1	Type1.BREX	<-HEPN<-?||YjhX_toxin->SAM-METHYLASE->?->BrxA-DNA-binding->BrxB-BrxF-iSTAND->DUF6079*->N6-METHYLASE->OLD-ABC->TOPRIM->PglZ->SIGMA+OB-Fold+AAA-ATPase+Lon-Protease-BrxL-><-REase<-NACHT+[illegible]	1213	Bacteria>Pseudomonadota>Gammaproteobacteria>Enterobacterales>Enterobacteriaceae>Salmonella	GCF_002266315.1	Salmonella_enterica	CC036_RS01880	387042..390683	28901	BREX-system-P-loop-protein-BrxC-[Salmonella-enterica]
WP_097369131.1	Type1.BREX	<-HEPN<-REase+iREase<-?||YjhX_toxin->SAM-METHYLASE->?->BrxA-DNA-binding->BrxB-BrxF-iSTAND->DUF6079*->N6-METHYLASE->OLD-ABC->TOPRIM->PglZ->SIGMA+OB-Fold+AAA-ATPase+Lon-Protease-BrxL->[illegible]	1213	Bacteria>Pseudomonadota>Gammaproteobacteria>Enterobacterales>Enterobacteriaceae>Salmonella	GCF_002507575.1	Salmonella_enterica	CBI65_RS08825	170892..174533	28901	BREX-system-P-loop-protein-BrxC-[Salmonella-enterica]
WP_097368036.1	Type1.BREX	<-HEPN<-REase+iREase<-?||YjhX_toxin->SAM-METHYLASE->?->BrxA-DNA-binding->BrxB-BrxF-iSTAND->DUF6079*->N6-METHYLASE->OLD-ABC->TOPRIM->PglZ->SIGMA+OB-Fold+AAA-ATPase+Lon-Protease-BrxL->[illegible]	1213	Bacteria>Pseudomonadota>Gammaproteobacteria>Enterobacterales>Enterobacteriaceae>Salmonella	GCF_002507625.1	Salmonella_enterica	CBI66_RS11180	171059..174700	28901	BREX-system-P-loop-protein-BrxC-[Salmonella-enterica]
WP_097375567.1	Type1.BREX	<-HEPN<-REase+iREase<-?||[illegible]	1213	Bacteria>Pseudomonadota>Gammaproteobacteria>Enterobacterales>Enterobacteriaceae>Salmonella	GCF_002507635.1	Salmonella_enterica	CBI67_RS08835	170640..174281	28901	BREX-system-P-loop-protein-BrxC-[Salmonella-enterica]
WP_107326618.1	Type1.BREX	Arm-DNA-bind_3->[illegible]	1213	Bacteria>Pseudomonadota>Gammaproteobacteria>Enterobacterales>Enterobacteriaceae>Salmonella	GCF_003032235.1	Salmonella_enterica	C5I04_RS23495	346379..350020	28901	BREX-system-P-loop-protein-BrxC-[Salmonella-enterica]
WP_116574272.1	Type1.BREX	TOPRIM-DNAG+D5N+D5ATPase+[illegible]	1213	Bacteria>Pseudomonadota>Gammaproteobacteria>Enterobacterales>Enterobacteriaceae>Salmonella	GCF_003077035.1	Salmonella_enterica	C4874_RS10725	43609..47250	28901	BREX-system-P-loop-protein-BrxC-[Salmonella-enterica]
WP_058114204.1	Type1.BREX	<-GntR-HTH++TrmB+ZDNA-HTH||[illegible]	1213	Bacteria>Pseudomonadota>Gammaproteobacteria>Enterobacterales>Enterobacteriaceae>Salmonella	GCF_003324795.1	Salmonella_enterica	D0E60_RS06770	1364315..1367956	28901	BREX-system-P-loop-protein-BrxC-[Salmonella-enterica]
WP_079798429.1	Type1.BREX	Glyoxalase->ACET->YjhX_toxin->[illegible]	1240	Bacteria>Pseudomonadota>Gammaproteobacteria>Enterobacterales>Enterobacteriaceae>Salmonella	GCF_003325015.1	Salmonella_enterica	HFU03_RS19925	4101513..4105235	28901	BREX-system-P-loop-protein-BrxC-[Salmonella-enterica]
WP_114051212.1	Type1.BREX	HTH_3->Couple_hipA+HipA_C->[illegible]	1213	Bacteria>Pseudomonadota>Gammaproteobacteria>Enterobacterales>Enterobacteriaceae>Salmonella	GCF_003325275.1	Salmonella_enterica	CHE40_RS23180	4543503..4547144	28901	BREX-system-P-loop-protein-BrxC-[Salmonella-enterica]
WP_114052981.1	Type1.BREX	<-GntR-HTH++TrmB+ZDNA-HTH||?->[illegible]	1213	Bacteria>Pseudomonadota>Gammaproteobacteria>Enterobacterales>Enterobacteriaceae>Salmonella	GCF_003325295.1	Salmonella_enterica	CHD06_RS08895	1727033..1730674	28901	BREX-system-P-loop-protein-BrxC-[Salmonella-enterica]
WP_133207218.1	Type1.BREX	XIS-HTH->?->AAA+HTH-><-[illegible]	1213	Bacteria>Pseudomonadota>Gammaproteobacteria>Enterobacterales>Enterobacteriaceae>Salmonella	GCF_004354245.1	Salmonella_enterica	EIM31_RS05595	1133023..1136664	28901	BREX-system-P-loop-protein-BrxC-[Salmonella-enterica]
WP_140237021.1	Type1.BREX	BrxA-DNA-binding->[illegible]	1213	Bacteria>Pseudomonadota>Gammaproteobacteria>Enterobacterales>Enterobacteriaceae>Salmonella	GCF_006379925.2	Salmonella_enterica	FG677_RS21910	2844..6485	28901	BREX-system-P-loop-protein-BrxC-[Salmonella-enterica]
WP_080166838.1	Type1.BREX	<-HEPN<-?||YjhX_toxin->[illegible]	1213	Bacteria>Pseudomonadota>Gammaproteobacteria>Enterobacterales>Enterobacteriaceae>Salmonella	GCF_012273475.1	Salmonella_enterica	FOC17_RS02975	512521..516162	28901	BREX-system-P-loop-protein-BrxC-[Salmonella-enterica]
WP_181299515.1	Type1.BREX	<-HEPN<-?||||YjhX_toxin->[illegible]	1213	Bacteria>Pseudomonadota>Gammaproteobacteria>Enterobacterales>Enterobacteriaceae>Salmonella	GCF_013624525.1	Salmonella_enterica	D6413_08715	52330..55971	28901	BREX-system-P-loop-protein-BrxC-[Salmonella-enterica]
WP_080213758.1	Type1.BREX	<-GntR-HTH++TrmB+ZDNA-HTH||?->[illegible]	1213	Bacteria>Pseudomonadota>Gammaproteobacteria>Enterobacterales>Enterobacteriaceae>Salmonella	GCF_015180625.1	Salmonella_enterica	AS04_RS02250	44431..48072	28901	BREX-system-P-loop-protein-BrxC-[Salmonella-enterica]
WP_079788274.1	Type1.BREX	<-HEPN<-REase+iREase<-?||[illegible]	1213	Bacteria>Pseudomonadota>Gammaproteobacteria>Enterobacterales>Enterobacteriaceae>Salmonella	GCF_016772815.1	Salmonella_enterica	JM978_RS19965	4115108..4118749	28901	BREX-system-P-loop-protein-BrxC-[Salmonella-enterica]
WP_079805580.1	Type1.BREX	SLOG->HAD-><-[illegible]	1240	Bacteria>Pseudomonadota>Gammaproteobacteria>Enterobacterales>Enterobacteriaceae>Salmonella	GCF_016903075.1	Salmonella_enterica	AJH70_RS01330	291078..294800	28901	BREX-system-P-loop-protein-BrxC-[Salmonella-enterica]
WP_210138875.1	Type1.BREX	SKI-><-?||tRNA->[illegible]	1213	Bacteria>Pseudomonadota>Gammaproteobacteria>Enterobacterales>Enterobacteriaceae>Salmonella	GCF_017893505.1	Salmonella_enterica	JJB80_RS22605	4685033..4688674	28901	BREX-system-P-loop-protein-BrxC-[Salmonella-enterica]
WP_241575978.1	Type1.BREX	Arm-DNA-bind_3->[illegible]	1213	Bacteria>Pseudomonadota>Gammaproteobacteria>Enterobacterales>Enterobacteriaceae>Salmonella	GCF_022603615.1	Salmonella_enterica	K5800_RS10145	108176..111817	28901	BREX-system-P-loop-protein-BrxC-[Salmonella-enterica]
WP_247258912.1	Type1.BREX	NACHT+SF1-SF2-Helicase+[illegible]	1204	Bacteria>Pseudomonadota>Gammaproteobacteria>Enterobacterales>Enterobacteriaceae>Salmonella	GCF_023007725.1	Salmonella_enterica	MA804_RS14880	357716..361330	28901	BREX-system-P-loop-protein-BrxC-[Salmonella-enterica]
WP_247223752.1	Type1.BREX	<-HEPN<-REase+iREase<-?||[illegible]	1213	Bacteria>Pseudomonadota>Gammaproteobacteria>Enterobacterales>Enterobacteriaceae>Salmonella	GCF_023016865.1	Salmonella_sp._L-S2638	MVT37_RS15260	170948..174589	2933299	BREX-system-P-loop-protein-BrxC-[Salmonella-sp.-L-S2638]
WP_198899489.1	Type1.BREX	<-HEPN<-REase+iREase<-?||[illegible]	1213	Bacteria>Pseudomonadota>Gammaproteobacteria>Enterobacterales>Enterobacteriaceae>Salmonella	GCF_023017625.1	Salmonella_sp._14ESS1484	MVA85_RS11375	170949..174590	2933314	BREX-system-P-loop-protein-BrxC-[Salmonella-sp.-14ESS1484]
WP_254499948.1	Type1.BREX	Arm-DNA-bind_3->[illegible]	1213	Bacteria>Pseudomonadota>Gammaproteobacteria>Enterobacterales>Enterobacteriaceae>Salmonella	GCF_024261625.1	Salmonella_enterica	NNJ27_RS08465	108147..111788	28901	BREX-system-P-loop-protein-BrxC-[Salmonella-enterica]
WP_255223204.1	Type1.BREX	<-HEPN<-REase+iREase<-?<-[illegible]	1213	Bacteria>Pseudomonadota>Gammaproteobacteria>Enterobacterales>Enterobacteriaceae>Salmonella	GCF_024364985.1	Salmonella_enterica	NMU37_RS20780	4227543..4231184	28901	BREX-system-P-loop-protein-BrxC-[Salmonella-enterica]
WP_255929194.1	Type1.BREX	<-HEPN<-?||YjhX_toxin->[illegible]	1204	Bacteria>Pseudomonadota>Gammaproteobacteria>Enterobacterales>Enterobacteriaceae>Salmonella	GCF_024442275.1	Salmonella_enterica	NOQ95_RS21560	4448475..4452089	28901	BREX-system-P-loop-protein-BrxC-[Salmonella-enterica]
WP_259423574.1	Type1.BREX	<-METHYLASE||SKI-><-?||tRNA->[illegible]	921	Bacteria>Pseudomonadota>Gammaproteobacteria>Enterobacterales>Enterobacteriaceae>Salmonella	GCF_024919285.1	Salmonella_enterica	NXM87_RS15320	123096..125861	28901	BREX-system-P-loop-protein-BrxC-[Salmonella-enterica]
WP_260776303.1	Type1.BREX	<-HEPN<-?||YjhX_toxin->[illegible]	1213	Bacteria>Pseudomonadota>Gammaproteobacteria>Enterobacterales>Enterobacteriaceae>Salmonella	GCF_025291975.1	Salmonella_enterica	M1709_RS12435	17008..20649	28901	BREX-system-P-loop-protein-BrxC-[Salmonella-enterica]
WP_080092317.1	Type1.BREX	<-HEPN<-?||YjhX_toxin->[illegible]	1213	Bacteria>Pseudomonadota>Gammaproteobacteria>Enterobacterales>Enterobacteriaceae>Salmonella	GCF_025594515.1	Salmonella_enterica	J3T40_RS14905	380423..384064	28901	BREX-system-P-loop-protein-BrxC-[Salmonella-enterica]
WP_267272757.1	Type1.BREX	<-SIGMA+OB-Fold+AAA-ATPase+[illegible]	1213	Bacteria>Pseudomonadota>Gammaproteobacteria>Enterobacterales>Enterobacteriaceae>Salmonella	GCF_026460795.1	Salmonella_enterica	OWO74_RS14195	11359..15000	28901	BREX-system-P-loop-protein-BrxC-[Salmonella-enterica]
WP_274877667.1	Type1.BREX	Glyoxalase->ACET->YjhX_toxin->[illegible]	806	Bacteria>Pseudomonadota>Gammaproteobacteria>Enterobacterales>Enterobacteriaceae>Salmonella	GCF_028892685.1	Salmonella_enterica	PQP98_RS20365	4148316..4150736	28901	BREX-system-P-loop-protein-BrxC-[Salmonella-enterica]
WP_274894925.1	Type1.BREX	Glyoxalase->ACET->YjhX_toxin->[illegible]	1240	Bacteria>Pseudomonadota>Gammaproteobacteria>Enterobacterales>Enterobacteriaceae>Salmonella	GCF_028892745.1	Salmonella_enterica	PQQ04_RS20380	4148528..4152250	28901	BREX-system-P-loop-protein-BrxC-[Salmonella-enterica]
WP_097173203.1	Type1.BREX	Arm-DNA-bind_3->[illegible]	1213	Bacteria>Pseudomonadota>Gammaproteobacteria>Enterobacterales>Enterobacteriaceae>Salmonella	GCF_900003195.1	Salmonella_enterica	CRP09_RS12230	81403..85044	28901	BREX-system-P-loop-protein-BrxC-[Salmonella-enterica]
WP_261983090.1	Type1.BREX	<-METHYLASE||SKI-><-?||tRNA->[illegible]	1240	Bacteria>Pseudomonadota>Gammaproteobacteria>Enterobacterales>Enterobacteriaceae>Salmonella	GCF_901212535.1	Salmonella_enterica	ECOMC1_RS14180	234231..237953	28901	BREX-system-P-loop-protein-BrxC-[Salmonella-enterica]
WP_094914772.1	Type1.BREX	<-HEPN<-?||YjhX_toxin->[illegible]	1213	Bacteria>Pseudomonadota>Gammaproteobacteria>Enterobacterales>Enterobacteriaceae>Salmonella	GCF_903930985.1	Salmonella_enterica	DQN83_RS20180	3946680..3950321	28901	BREX-system-P-loop-protein-BrxC-[Salmonella-enterica]
WP_088524188.1	Type1.BREX	NACHT+SF1-SF2-Helicase+[illegible]	1204	Bacteria>Pseudomonadota>Gammaproteobacteria>Enterobacterales>Enterobacteriaceae>Salmonella	GCF_903931065.1	Salmonella_enterica	LFZ40_19410	3810154..3813768	28901	BREX-system-P-loop-protein-BrxC-[Salmonella-enterica]
WP_183072251.1	Type1.BREX	<-HEPN<-REase+iREase<-?||[illegible]	1213	Bacteria>Pseudomonadota>Gammaproteobacteria>Enterobacterales>Enterobacteriaceae>Salmonella	GCF_903931105.1	Salmonella_enterica	H7T17_RS07610	54171..57812	28901	BREX-system-P-loop-protein-BrxC-[Salmonella-enterica]
WP_058685106.1	Type1.BREX	Arm-DNA-bind_3->[illegible]	1213	Bacteria>Pseudomonadota>Gammaproteobacteria>Enterobacterales>Enterobacteriaceae>Salmonella	GCF_940677185.1	Salmonella_enterica	NBW86_RS10200	108141..111782	28901	BREX-system-P-loop-protein-BrxC-[Salmonella-enterica]
WP_080205562.1	Type1.BREX	<-HEPN<-REase+iREase<-?||[illegible]	1213	Bacteria>Pseudomonadota>Gammaproteobacteria>Enterobacterales>Enterobacteriaceae>Salmonella	GCF_943114325.1	Salmonella_enterica	OCU44_RS00985	212044..215685	28901	BREX-system-P-loop-protein-BrxC-[Salmonella-enterica]
WP_039590210.1	Type1.BREX	<-HEPN<-REase+iREase<-?||[illegible]	1213	Bacteria>Pseudomonadota>Gammaproteobacteria>Enterobacterales>Enterobacteriaceae>Salmonella	GCF_943119195.1	Salmonella_enterica	OCU40_RS05645	1155326..1158967	28901	BREX-system-P-loop-protein-BrxC-[Salmonella-enterica]
WP_154023496.1	Type1.BREX	Glyoxalase->ACET->||[illegible]	1233	Bacteria>Pseudomonadota>Gammaproteobacteria>Enterobacterales>Enterobacteriaceae>Salmonella	-	Salmonella_enterica	A4I02_04465	211497..215198	28901	BREX-system-P-loop-protein-BrxC-[Salmonella-enterica]
WP_097363546.1	Type1.BREX	<-METHYLASE||SKI-><-?||tRNA->[illegible]	1233	Bacteria>Pseudomonadota>Gammaproteobacteria>Enterobacterales>Enterobacteriaceae>Salmonella	-	Salmonella_enterica	CHC75_12745	2557798..2561499	28901	BREX-system-P-loop-protein-BrxC-[Salmonella-enterica]
EJF5860346.1	Type1.BREX	<-HEPN<-REase+iREase<-?||[illegible]	1213	Bacteria>Pseudomonadota>Gammaproteobacteria>Enterobacterales>Enterobacteriaceae>Salmonella	-	Salmonella_enterica	M8X04_001881	170514..174155	28901	BREX-system-P-loop-protein-BrxC-[Salmonella-enterica]
WP_023227805.1	Type1.BREX	Glyoxalase->ACET->YjhX_toxin->[illegible]	1233	Bacteria>Pseudomonadota>Gammaproteobacteria>Enterobacterales>Enterobacteriaceae>Salmonella	-	Salmonella_enterica	SEEI0720_021515	4182897..4186598	28901	BREX-system-P-loop-protein-BrxC-[Salmonella-enterica]
ECG0705015.1	Type1.BREX	<-HEPN<-REase+iREase<-?||||[illegible]	1213	Bacteria>Pseudomonadota>Gammaproteobacteria>Enterobacterales>Enterobacteriaceae>Salmonella	-	Salmonella_enterica_subsp._salamae	DL122_04700	35596..39237	59202	BREX-system-P-loop-protein-BrxC-[Salmonella-enterica-subsp.-salamae]
HCC77786.1	Type1.BREX	AAA+HTH-><-[illegible]	1216	Bacteria>Pseudomonadota>Gammaproteobacteria>Enterobacterales>Enterobacteriaceae>Shigella	GCA_003517755.1	Shigella_sp.	DEQ42_19255	11300..14950	625	BREX-system-P-loop-protein-BrxC-[Shigella-sp.]
MRS92356.1	Type1.BREX	DUF932-><-?||ArdB->?->?->[illegible]	1157	Bacteria>Pseudomonadota>Gammaproteobacteria>Enterobacterales>Enterobacteriaceae>unclassified_Enterobacteriaceae	GCA_009668015.1	Enterobacteriaceae_bacterium_RIT714	GJV04_20305	30577..34050	2666193	BREX-system-P-loop-protein-BrxC-[Enterobacteriaceae-bacterium-RIT714]
NUU66316.1	Type1.BREX	BrxA-DNA-binding->[illegible]	1213	Bacteria>Pseudomonadota>Gammaproteobacteria>Enterobacterales>Enterobacteriaceae>unclassified_Enterobacteriaceae	GCA_013359925.1	Enterobacteriaceae_bacterium_BIT-123	HQN64_09360	363314..366955	2742797	BREX-system-P-loop-protein-BrxC-[Enterobacteriaceae-bacterium-BIT-123]
UCB31815.1	Type1.BREX	<-wHTH+WYL<-?||XIS-HTH->?->[illegible]	1240	Bacteria>Pseudomonadota>Gammaproteobacteria>Enterobacterales>Erwiniaceae>Duffyella	GCA_020342335.1	Duffyella_gerundensis	J9874_02366	2569295..2573017	1619313	hypothetical-protein-J9874_02366-[Duffyella-gerundensis]
WP_225700910.1	Type1.BREX	<-wHTH+WYL<-?||XIS-HTH->?->[illegible]	1237	Bacteria>Pseudomonadota>Gammaproteobacteria>Enterobacterales>Erwiniaceae>Duffyella	GCF_020342335.1	Duffyella_gerundensis	J9874_RS11820	2569304..2573017	1619313	BREX-system-P-loop-protein-BrxC-[Duffyella-gerundensis]
WP_012669046.1	Type1.BREX	HTH_Tnp_1+HTH_21+rve+rve_3->[illegible]	1214	Bacteria>Pseudomonadota>Gammaproteobacteria>Enterobacterales>Erwiniaceae>Erwinia	GCF_000027265.1	Erwinia_pyrifoliae	EPC_RS14330	3076453..3080097	79967	BREX-system-P-loop-protein-BrxC-[Erwinia-pyrifoliae]
WP_014543466.1	Type1.BREX	PCMT+METHYLASE->LysM+PTSEIIA1-[illegible]	1214	Bacteria>Pseudomonadota>Gammaproteobacteria>Enterobacterales>Erwiniaceae>Erwinia	GCF_000165815.1	Erwinia_sp._Ejp617	EJP617_RS09645	2138156..2141800	215689	BREX-system-P-loop-protein-BrxC-[Erwinia-sp.-Ejp617]
WP_253458939.1	Type1.BREX	XIS-HTH->?->AAA+HTH-><-[illegible]	1216	Bacteria>Pseudomonadota>Gammaproteobacteria>Enterobacterales>Erwiniaceae>Erwinia	GCF_024168495.1	Erwinia_persicina	J3D56_RS16965	3646326..3649976	55211	BREX-system-P-loop-protein-BrxC-[Erwinia-persicina]
WP_259816994.1	Type1.BREX	SIGMA-HTH+SIGMA-HTH-><-?<-[illegible]	1214	Bacteria>Pseudomonadota>Gammaproteobacteria>Enterobacterales>Erwiniaceae>Erwinia	GCF_025153555.1	Erwinia_pyrifoliae	NYP82_RS06320	1343257..1346901	79967	BREX-system-P-loop-protein-BrxC-[Erwinia-pyrifoliae]
WP_259826113.1	Type1.BREX	PCMT+METHYLASE->LysM+PTSEIIA1-[illegible]	1214	Bacteria>Pseudomonadota>Gammaproteobacteria>Enterobacterales>Erwiniaceae>Erwinia	GCF_025159015.1	Erwinia_pyrifoliae	NYP84_RS03425	759232..762876	79967	BREX-system-P-loop-protein-BrxC-[Erwinia-pyrifoliae]
WP_261642253.1	Type1.BREX	<-SIGMA+OB-Fold+AAA-ATPase+[illegible]	1213	Bacteria>Pseudomonadota>Gammaproteobacteria>Enterobacterales>Erwiniaceae>Erwinia	GCF_025388925.1	Erwinia_mallotivora	OCH37_RS12855	149371..153012	69222	BREX-system-P-loop-protein-BrxC-[Erwinia-mallotivora]
WP_159378885.1	Type1.BREX	HTH_Tnp_1+HTH_21+rve+rve_3-><-[illegible]	1213	Bacteria>Pseudomonadota>Gammaproteobacteria>Enterobacterales>Erwiniaceae>Mixta	GCF_009866505.1	Mixta_calida	Y888_RS19575	9519..13160	665913	BREX-system-P-loop-protein-BrxC-[Mixta-calida]
HCP27301.1	Type1.BREX	Phage_integrase-><-[illegible]	1233	Bacteria>Pseudomonadota>Gammaproteobacteria>Enterobacterales>Erwiniaceae>Pantoea	GCA_003533915.1	Pantoea_ananatis	DIT85_12780	28453..32154	553	BREX-system-P-loop-protein-BrxC-[Pantoea-ananatis]
RZK06926.1	Type1.BREX	<-DEDD_Tnp_IS110<-?||?->?->?->[illegible]	1213	Bacteria>Pseudomonadota>Gammaproteobacteria>Enterobacterales>Erwiniaceae>Pantoea	GCA_004295855.1	Pantoea_sp.	EOO84_12150	162361..166002	69393	MAG:-BREX-system-P-loop-protein-BrxC-[Pantoea-sp.]
NEG80386.1	Type1.BREX	<-wHTH(SBD)+HNH||?->?->?->[illegible]	1233	Bacteria>Pseudomonadota>Gammaproteobacteria>Enterobacterales>Erwiniaceae>Pantoea	GCA_010667945.1	Pantoea_agglomerans	GQQ17_05160	300399..304100	549	BREX-system-P-loop-protein-BrxC-[Pantoea-agglomerans]
WP_007890763.1	Type1.BREX	ABhydrolase+METHYLASE->?->?->[illegible]	1202	Bacteria>Pseudomonadota>Gammaproteobacteria>Enterobacterales>Erwiniaceae>Pantoea	GCF_000282675.1	Pantoea_sp._GMO1	PMI17_RS16905	182397..186005	1144320	BREX-system-P-loop-protein-BrxC-[Pantoea-sp.-GMO1]
WP_137241123.1	Type1.BREX	<-SymE_toxin<-CHTH<-RelE||[illegible]	1202	Bacteria>Pseudomonadota>Gammaproteobacteria>Enterobacterales>Erwiniaceae>Pantoea	GCF_005233495.1	Pantoea_agglomerans	PagCFBP13505_RS08740	40890..44498	549	BREX-system-P-loop-protein-BrxC-[Pantoea-agglomerans]
WP_145889319.1	Type1.BREX	<-SAM-METHYLASE<-?<-?||[illegible]	1213	Bacteria>Pseudomonadota>Gammaproteobacteria>Enterobacterales>Erwiniaceae>Pantoea	GCF_007833795.1	Pantoea_dispersa	D8B20_RS13380	2861795..2865436	59814	BREX-system-P-loop-protein-BrxC-[Pantoea-dispersa]
WP_154209858.1	Type1.BREX	<-SIGMA+OB-Fold+AAA-ATPase+[illegible]	1202	Bacteria>Pseudomonadota>Gammaproteobacteria>Enterobacterales>Erwiniaceae>Pantoea	GCF_009668105.1	Pantoea_agglomerans	GJV05_RS04780	299589..303197	549	BREX-system-P-loop-protein-BrxC-[Pantoea-agglomerans]
WP_158149594.1	Type1.BREX	<-CHTH||ColE7+HNH->MarR-HTH-><-[illegible]	1157	Bacteria>Pseudomonadota>Gammaproteobacteria>Enterobacterales>Erwiniaceae>Pantoea	GCF_009765475.1	Pantoea_agglomerans	GSQ69_RS16285	3495115..3498588	549	BREX-system-P-loop-protein-BrxC-[Pantoea-agglomerans]
WP_163841538.1	Type1.BREX	<-DEDD_Tnp_IS110<-?||?->?->?->[illegible]	1213	Bacteria>Pseudomonadota>Gammaproteobacteria>Enterobacterales>Erwiniaceae>Pantoea	GCF_010667775.1	Pantoea_agglomerans	GQQ23_RS01345	302336..305977	549	BREX-system-P-loop-protein-BrxC-[Pantoea-agglomerans]
WP_242462783.1	Type1.BREX	<-wHTH(SBD)+HNH||?->?->?->[illegible]	1240	Bacteria>Pseudomonadota>Gammaproteobacteria>Enterobacterales>Erwiniaceae>Pantoea	GCF_010667945.1	Pantoea_agglomerans	GQQ17_RS05165	300399..304121	549	BREX-system-P-loop-protein-BrxC-[Pantoea-agglomerans]
WP_167070220.1	Type1.BREX	<-wHTH+WYL+WCX||?->ZnR-><-[illegible]	1184	Bacteria>Pseudomonadota>Gammaproteobacteria>Enterobacterales>Erwiniaceae>Pantoea	GCF_011752565.1	Pantoea_sp._Ap-967	F3J45_27210	36650..40204	2608362	BREX-system-P-loop-protein-BrxC-[Pantoea-sp.-Ap-967]
WP_182687314.1	Type1.BREX	<-DEDD_Tnp_IS110<-?||?->?->?->[illegible]	1213	Bacteria>Pseudomonadota>Gammaproteobacteria>Enterobacterales>Erwiniaceae>Pantoea	GCF_014156615.1	Pantoea_pleuroti	EPG66_RS14530	313158..316799	1592631	BREX-system-P-loop-protein-BrxC-[Pantoea-pleuroti]
WP_187492467.1	Type1.BREX	<-SymE_toxin||?-><-[illegible]	1157	Bacteria>Pseudomonadota>Gammaproteobacteria>Enterobacterales>Erwiniaceae>Pantoea	GCF_014353895.1	Pantoea_agglomerans	IBU46_RS01740	23077..26550	549	BREX-system-P-loop-protein-BrxC-[Pantoea-agglomerans]
WP_191919036.1	Type1.BREX	<-SymE_toxin<-CHTH||HIN-HTH->[illegible]	1157	Bacteria>Pseudomonadota>Gammaproteobacteria>Enterobacterales>Erwiniaceae>Pantoea	GCF_014838975.1	Pantoea_agglomerans	IFU26_RS12730	289507..292980	549	BREX-system-P-loop-protein-BrxC-[Pantoea-agglomerans]
WP_202940066.1	Type1.BREX	<-DEDD_Tnp_IS110<-?||?->?->?->[illegible]	1021	Bacteria>Pseudomonadota>Gammaproteobacteria>Enterobacterales>Erwiniaceae>Pantoea	GCF_014839325.1	Pantoea_agglomerans	IFT41_RS17295	170215..173280	549	BREX-system-P-loop-protein-BrxC-[Pantoea-agglomerans]
WP_222233110.1	Type1.BREX	<-HKD-DNase+OGG-like<-[illegible]	1202	Bacteria>Pseudomonadota>Gammaproteobacteria>Enterobacterales>Erwiniaceae>Pantoea	GCF_019795975.1	Pantoea_sp._DY-17	K5Y33_RS01225	252070..255678	2871490	BREX-system-P-loop-protein-BrxC-[Pantoea-sp.-DY-17]
WP_245115608.1	Type1.BREX	<-SymE_toxin<-CHTH||ColE7+HNH-[illegible]	1204	Bacteria>Pseudomonadota>Gammaproteobacteria>Enterobacterales>Erwiniaceae>Pantoea	GCF_022966105.1	Pantoea_agglomerans	LZ609_RS16055	2632558..2636172	549	BREX-system-P-loop-protein-BrxC-[Pantoea-agglomerans]
WP_264214894.1	Type1.BREX	<-DEDD_Tnp_IS110<-?||tRNA->[illegible]	1213	Bacteria>Pseudomonadota>Gammaproteobacteria>Enterobacterales>Erwiniaceae>Pantoea	GCF_025881995.1	Pantoea_ananatis	NB695_RS01430	332354..335995	553	BREX-system-P-loop-protein-BrxC-[Pantoea-ananatis]
WP_272844016.1	Type1.BREX	CbeA_antitoxin->?->METHYLASE->[illegible]	1157	Bacteria>Pseudomonadota>Gammaproteobacteria>Enterobacterales>Erwiniaceae>Pantoea	GCF_028555165.1	Pantoea_ananatis	SZ66_RS15300	342532..346005	553	BREX-system-P-loop-protein-BrxC-[Pantoea-ananatis]
PIJ55036.1	Type1.BREX	<-N6-METHYLASE<-DUF6079*<-[illegible]	1202	Bacteria>Pseudomonadota>Gammaproteobacteria>Enterobacterales>Erwiniaceae>unclassified_Erwiniaceae	GCA_002752575.1	Erwinia_sp._OLMDLW33	BOM23_20680	2080..5688	1928656	DNA-repair-protein-[Erwinia-sp.-OLMDLW33]
WP_001019641.1	Type1.BREX	<-Phage_integrase<-[illegible]	1213	Bacteria>Pseudomonadota>Gammaproteobacteria>Enterobacterales	GCF_000010765.1	Enterobacteriaceae	PYB84_RS07565	16979..20620	543	MULTISPECIES:-BREX-system-P-loop-protein-BrxC-[Enterobacteriaceae]
WP_000687847.1	Type1.BREX	<-LexA-protease||[illegible]	1225	Bacteria>Pseudomonadota>Gammaproteobacteria>Enterobacterales	GCF_000026225.1	Enterobacteriaceae	EFER_RS00085	15017..18694	543	MULTISPECIES:-BREX-system-P-loop-protein-BrxC-[Enterobacteriaceae]
WP_001019638.1	Type1.BREX	<-Phage_integrase<-[illegible]	1213	Bacteria>Pseudomonadota>Gammaproteobacteria>Enterobacterales	GCF_000166575.1	Enterobacteriaceae	EC33884_RS23090	1218224..1221865	543	MULTISPECIES:-BREX-system-P-loop-protein-BrxC-[Enterobacteriaceae]
WP_001019648.1	Type1.BREX	Arm-DNA-bind_3+Phage_integrase[illegible]	1213	Bacteria>Pseudomonadota>Gammaproteobacteria>Enterobacterales	GCF_000194705.1	Enterobacteriaceae	ECB41_RS05715	178680..182321	543	MULTISPECIES:-BREX-system-P-loop-protein-BrxC-[Enterobacteriaceae]
WP_001019636.1	Type1.BREX	<-METHYLASE||SKI-><-?||tRNA->[illegible]	1213	Bacteria>Pseudomonadota>Gammaproteobacteria>Enterobacterales	GCF_000233875.1	Enterobacteriaceae	I02_RS24015	4889707..4893348	543	MULTISPECIES:-BREX-system-P-loop-protein-BrxC-[Enterobacteriaceae]
WP_001019644.1	Type1.BREX	<-Fimbrial<-Fimbrial<-[illegible]	1213	Bacteria>Pseudomonadota>Gammaproteobacteria>Enterobacterales	GCF_000282855.1	Enterobacteriaceae	DTB66_RS23695	24279..27920	543	MULTISPECIES:-BREX-system-P-loop-protein-BrxC-[Enterobacteriaceae]
WP_006788201.1	Type1.BREX	<-SIGMA+OB-Fold+AAA-ATPase+[illegible]	1225	Bacteria>Pseudomonadota>Gammaproteobacteria>Enterobacterales	GCF_000388555.1	Enterobacteriaceae	MUG00_RS24960	10799..14476	543	MULTISPECIES:-BREX-system-P-loop-protein-BrxC-[Enterobacteriaceae]
WP_032187199.1	Type1.BREX	HEPN+SF1-SF2-Helicase+RNase+[illegible]	1225	Bacteria>Pseudomonadota>Gammaproteobacteria>Enterobacterales	GCF_000703445.1	Enterobacteriaceae	AD25_RS20800	23140..26817	543	MULTISPECIES:-BREX-system-P-loop-protein-BrxC-[Enterobacteriaceae]
WP_053287615.1	Type1.BREX	<-Zn_Tnp_IS1+HTH_Tnp_IS1<-?<-[illegible]	1213	Bacteria>Pseudomonadota>Gammaproteobacteria>Enterobacterales	GCF_001268905.1	Enterobacteriaceae	AMM63_RS24455	5812..9453	543	MULTISPECIES:-BREX-system-P-loop-protein-BrxC-[Enterobacteriaceae]
WP_054528932.1	Type1.BREX	DDE_Tnp_1_5+DDE_Tnp_1-><-McrC-[illegible]	1225	Bacteria>Pseudomonadota>Gammaproteobacteria>Enterobacterales	GCF_002788295.1	Enterobacteriaceae	AM338_RS26100	11174..14851	543	MULTISPECIES:-BREX-system-P-loop-protein-BrxC-[Enterobacteriaceae]
WP_032408831.1	Type1.BREX	Arm-DNA-bind_3+Phage_integrase[illegible]	1213	Bacteria>Pseudomonadota>Gammaproteobacteria>Enterobacterales	GCF_002897415.1	Enterobacteriaceae	HED41_RS19555	135079..138720	543	MULTISPECIES:-BREX-system-P-loop-protein-BrxC-[Enterobacteriaceae]
WP_097333547.1	Type1.BREX	<-HIN-HTH||?->?->ZnR->ZnR-><-[illegible]	1213	Bacteria>Pseudomonadota>Gammaproteobacteria>Enterobacterales	GCF_004194615.2	Enterobacteriaceae	DBZ52_RS03520	719980..723621	543	MULTISPECIES:-BREX-system-P-loop-protein-BrxC-[Enterobacteriaceae]
WP_223177211.1	Type1.BREX	<-wHTH+WYL<-nSTAND1+sGTP<-?||[illegible]	1240	Bacteria>Pseudomonadota>Gammaproteobacteria>Enterobacterales	GCF_004312085.1	Enterobacteriaceae	EOM53_RS14900	53286..57008	543	MULTISPECIES:-BREX-system-P-loop-protein-BrxC-[Enterobacteriaceae]
WP_004249342.1	Type1.BREX	<-N6-METHYLASE<-DUF6079*<-[illegible]	1225	Bacteria>Pseudomonadota>Gammaproteobacteria>Enterobacterales	GCF_008041895.1	Morganellaceae	E4V60_RS00205	3632..7309	1903414	MULTISPECIES:-BREX-system-P-loop-protein-BrxC-[Morganellaceae]
WP_019078269.1	Type1.BREX	<-HerA/FtsK+A32<-?||tRNA->[illegible]	1213	Bacteria>Pseudomonadota>Gammaproteobacteria>Enterobacterales	GCF_011030295.1	Enterobacteriaceae	QFD02_RS07350	209926..213567	543	MULTISPECIES:-BREX-system-P-loop-protein-BrxC-[Enterobacteriaceae]
WP_080865889.1	Type1.BREX	GDResolvase+HIN-HTH->?-><-?||[illegible]	1213	Bacteria>Pseudomonadota>Gammaproteobacteria>Enterobacterales	GCF_011604705.1	Enterobacteriaceae	HA514_RS19415	4027238..4030879	543	MULTISPECIES:-BREX-system-P-loop-protein-BrxC-[Enterobacteriaceae]
WP_049194743.1	Type1.BREX	MobI-><-?<-LexA-protease||[illegible]	1225	Bacteria>Pseudomonadota>Gammaproteobacteria>Enterobacterales	GCF_013357405.1	Morganellaceae	HO271_RS14475	3106185..3109862	1903414	MULTISPECIES:-BREX-system-P-loop-protein-BrxC-[Morganellaceae]
WP_117259785.1	Type1.BREX	<-Peptidase_M17_N+[illegible]	1157	Bacteria>Pseudomonadota>Gammaproteobacteria>Enterobacterales	GCF_013395115.1	Enterobacteriaceae	HYG96_RS13000	239421..242894	543	MULTISPECIES:-BREX-system-P-loop-protein-BrxC-[Enterobacteriaceae]
WP_115625931.1	Type1.BREX	HIN-HTH->||?->[illegible]	1204	Bacteria>Pseudomonadota>Gammaproteobacteria>Enterobacterales	GCF_013635755.1	Enterobacteriaceae	HV027_RS18510	3797222..3800836	543	MULTISPECIES:-BREX-system-P-loop-protein-BrxC-[Enterobacteriaceae]
WP_242461228.1	Type1.BREX	Zn_Tnp_IS1+HTH_Tnp_IS1+[illegible]	1240	Bacteria>Pseudomonadota>Gammaproteobacteria>Enterobacterales	GCF_013705425.1	Enterobacteriaceae	HV198_RS20030	4234756..4238478	543	MULTISPECIES:-BREX-system-P-loop-protein-BrxC-[Enterobacteriaceae]
WP_232051962.1	Type1.BREX	<-DUF932<-nSTAND1+GTPase-AIG+[illegible]	1240	Bacteria>Pseudomonadota>Gammaproteobacteria>Enterobacterales	GCF_018421715.1	Enterobacteriaceae	L6492_RS02235	461320..465042	543	MULTISPECIES:-BREX-system-P-loop-protein-BrxC-[Enterobacteriaceae]
WP_222691531.1	Type1.BREX	BrxA-DNA-binding->[illegible]	1204	Bacteria>Pseudomonadota>Gammaproteobacteria>Enterobacterales	GCF_019841255.1	Enterobacteriaceae	K7A35_RS15405	1428..5042	543	MULTISPECIES:-BREX-system-P-loop-protein-BrxC-[Enterobacteriaceae]
WP_200541075.1	Type1.BREX	<-SIGMA+OB-Fold+AAA-ATPase+[illegible]	1213	Bacteria>Pseudomonadota>Gammaproteobacteria>Enterobacterales	GCF_019927965.1	Enterobacteriaceae	KFB01_RS26200	8731..12372	543	MULTISPECIES:-BREX-system-P-loop-protein-BrxC-[Enterobacteriaceae]
WP_224243997.1	Type1.BREX	<-SymE_toxin<-Glyoxalase_8||[illegible]	1157	Bacteria>Pseudomonadota>Gammaproteobacteria>Enterobacterales	GCF_023752605.1	Enterobacteriaceae	M8S83_RS11470	342574..346047	543	MULTISPECIES:-BREX-system-P-loop-protein-BrxC-[Enterobacteriaceae]
WP_088474912.1	Type1.BREX	Phage_integrase-><-Sulfatase<-[illegible]	1213	Bacteria>Pseudomonadota>Gammaproteobacteria>Enterobacterales	GCF_024713235.1	Enterobacteriaceae	CD912_RS19430	9686..13327	543	MULTISPECIES:-BREX-system-P-loop-protein-BrxC-[Enterobacteriaceae]
WP_080816097.1	Type1.BREX	Phage_integrase-><-?<-?<-?||[illegible]	1213	Bacteria>Pseudomonadota>Gammaproteobacteria>Enterobacterales	GCF_026460795.1	Enterobacteriaceae	OWO74_RS12055	662264..665905	543	MULTISPECIES:-BREX-system-P-loop-protein-BrxC-[Enterobacteriaceae]
WP_224293199.1	Type1.BREX	<-DUF905<-DUF932<-CopG/RHH||[illegible]	1240	Bacteria>Pseudomonadota>Gammaproteobacteria>Enterobacterales	GCF_029226505.1	Enterobacteriaceae	PVK22_RS25565	5368784..5372506	543	MULTISPECIES:-BREX-system-P-loop-protein-BrxC-[Enterobacteriaceae]
WP_047644001.1	Type1.BREX	<-XIS-HTH<-RHH_antitoxin||?->[illegible]	1213	Bacteria>Pseudomonadota>Gammaproteobacteria>Enterobacterales	GCF_030296855.1	Enterobacteriaceae	CPH82_RS06655	82494..86135	543	MULTISPECIES:-BREX-system-P-loop-protein-BrxC-[Enterobacteriaceae]
WP_063149682.1	Type1.BREX	<-SIGMA+OB-Fold+AAA-ATPase+[illegible]	1225	Bacteria>Pseudomonadota>Gammaproteobacteria>Enterobacterales	GCF_900075035.1	Enterobacteriaceae	K1J36_RS23220	10587..14264	543	MULTISPECIES:-BREX-system-P-loop-protein-BrxC-[Enterobacteriaceae]
WP_072662972.1	Type1.BREX	DDE_Tnp_IS1-><-SIGMA+OB-Fold+[illegible]	1213	Bacteria>Pseudomonadota>Gammaproteobacteria>Enterobacterales	GCF_900775105.1	Enterobacteriaceae	B5I96_RS21955	8761..12402	543	MULTISPECIES:-BREX-system-P-loop-protein-BrxC-[Enterobacteriaceae]
WP_045345932.1	Type1.BREX	XIS-HTH->?->?->SF1-SF2-Helicase[illegible]	1157	Bacteria>Pseudomonadota>Gammaproteobacteria>Enterobacterales	GCF_902160145.1	Enterobacteriaceae	FN179_RS14130	343492..346965	543	MULTISPECIES:-BREX-system-P-loop-protein-BrxC-[Enterobacteriaceae]
WP_044349975.1	Type1.BREX	DEDD_Tnp_IS110+Transposase_20-[illegible]	1213	Bacteria>Pseudomonadota>Gammaproteobacteria>Enterobacterales	GCF_902162255.1	Enterobacteriaceae	FN407_RS22090	106116..109757	543	MULTISPECIES:-BREX-system-P-loop-protein-BrxC-[Enterobacteriaceae]
WP_110225552.1	Type1.BREX	BrxA-DNA-binding->[illegible]	1213	Bacteria>Pseudomonadota>Gammaproteobacteria>Enterobacterales	GCF_902499505.1	Enterobacteriaceae	G4V40_RS25520	1772..5413	543	MULTISPECIES:-BREX-system-P-loop-protein-BrxC-[Enterobacteriaceae]
WP_117108864.1	Type1.BREX	<-METHYLASE||SKI-><-?||tRNA-><-[illegible]	1189	Bacteria>Pseudomonadota>Gammaproteobacteria>Enterobacterales	GCF_903684385.1	Enterobacteriaceae	MJ801_RS10050	169597..173166	543	MULTISPECIES:-BREX-system-P-loop-protein-BrxC-[Enterobacteriaceae]
WP_039021203.1	Type1.BREX	rve-><-RHH_antitoxin||?->ZnR->[illegible]	1213	Bacteria>Pseudomonadota>Gammaproteobacteria>Enterobacterales	GCF_904387335.1	Enterobacteriaceae	G3M52_RS00045	12929..16570	543	MULTISPECIES:-BREX-system-P-loop-protein-BrxC-[Enterobacteriaceae]
WP_047929056.1	Type1.BREX	MazE_antitoxin->PIN->[illegible]	1225	Bacteria>Pseudomonadota>Gammaproteobacteria>Enterobacterales	GCF_904799765.1	Enterobacteriaceae	PCN061_RS24650	104442..108119	543	MULTISPECIES:-BREX-system-P-loop-protein-BrxC-[Enterobacteriaceae]
WP_064143490.1	Type1.BREX	<-Phage_integrase<-[illegible]	1213	Bacteria>Pseudomonadota>Gammaproteobacteria>Enterobacterales	GCF_910592795.1	Enterobacteriaceae	KXI98_RS20085	95010..98651	543	MULTISPECIES:-BREX-system-P-loop-protein-BrxC-[Enterobacteriaceae]
WP_175320436.1	Type1.BREX	<-DUF932<-nSTAND1+GTPase-AIG+[illegible]	1233	Bacteria>Pseudomonadota>Gammaproteobacteria>Enterobacterales>Hafniaceae>Hafnia	GCF_013360205.1	Hafnia_paralvei	G4F99_RS14350	55379..59080	546367	BREX-system-P-loop-protein-BrxC-[Hafnia-paralvei]
WP_226228481.1	Type1.BREX	GNTR-HTH+Aminotran_1_2->?-><-[illegible]	1202	Bacteria>Pseudomonadota>Gammaproteobacteria>Enterobacterales>Hafniaceae>Hafnia	GCF_020450285.1	Hafnia_paralvei	LA328_RS17860	3833328..3836936	546367	BREX-system-P-loop-protein-BrxC-[Hafnia-paralvei]
WP_234619586.1	Type1.BREX	<-nSTAND1+GTPase-AIG+sGTP<-[illegible]	1237	Bacteria>Pseudomonadota>Gammaproteobacteria>Enterobacterales>Hafniaceae>Hafnia	GCF_021440285.1	Hafnia_alvei	LZ667_RS17495	58169..61882	569	BREX-system-P-loop-protein-BrxC-[Hafnia-alvei]
WP_108479632.1	Type1.BREX	MobI-><-?<-LexA-protease||[illegible]	1225	Bacteria>Pseudomonadota>Gammaproteobacteria>Enterobacterales>Morganellaceae	GCF_003057415.1	Providencia	C6581_RS17855	39720..43397	586	MULTISPECIES:-BREX-system-P-loop-protein-BrxC-[Providencia]
WP_241543889.1	Type1.BREX	<-HTH_37<-?||GDResolvase+[illegible]	1225	Bacteria>Pseudomonadota>Gammaproteobacteria>Enterobacterales>Morganellaceae>Moellerella	GCF_022591895.1	Moellerella_wisconsensis	MNY64_RS16755	120814..124491	158849	BREX-system-P-loop-protein-BrxC-[Moellerella-wisconsensis]
WP_241542954.1	Type1.BREX	<-SIGMA+OB-Fold+AAA-ATPase+[illegible]	1225	Bacteria>Pseudomonadota>Gammaproteobacteria>Enterobacterales>Morganellaceae>Moellerella	GCF_022592015.1	Moellerella_wisconsensis	MNY72_RS15945	14916..18593	158849	BREX-system-P-loop-protein-BrxC-[Moellerella-wisconsensis]
WP_241501907.1	Type1.BREX	<-HTH_37<-?||GDResolvase+[illegible]	1225	Bacteria>Pseudomonadota>Gammaproteobacteria>Enterobacterales>Morganellaceae>Moellerella	GCF_022592395.1	Moellerella_wisconsensis	MNY66_RS17045	197034..200711	158849	BREX-system-P-loop-protein-BrxC-[Moellerella-wisconsensis]
AUR32853.1	Type1.BREX	HTH->Pertactin+Autotransporter[illegible]	1233	Bacteria>Pseudomonadota>Gammaproteobacteria>Enterobacterales>Morganellaceae>Morganella	GCA_002891475.1	Morganella_morganii	C1O70_15730	3342273..3345974	582	BREX-system-P-loop-protein-BrxC-[Morganella-morganii]
MBS5193172.1	Type1.BREX	REase->wHTH+WYL+WCX->?->?->[illegible]	1157	Bacteria>Pseudomonadota>Gammaproteobacteria>Enterobacterales>Morganellaceae>Morganella	GCA_018366325.1	Morganella_morganii	KHY92_05180	18454..21927	582	BREX-system-P-loop-protein-BrxC-[Morganella-morganii]
WP_225973345.1	Type1.BREX	HTH->Pertactin+Autotransporter[illegible]	1240	Bacteria>Pseudomonadota>Gammaproteobacteria>Enterobacterales>Morganellaceae>Morganella	GCF_002891475.1	Morganella_morganii	C1O70_RS15730	3342252..3345974	582	BREX-system-P-loop-protein-BrxC-[Morganella-morganii]
WP_214179407.1	Type1.BREX	XIS-HTH->BrxA-DNA-binding->[illegible]	1225	Bacteria>Pseudomonadota>Gammaproteobacteria>Enterobacterales>Morganellaceae>Morganella	GCF_018474625.1	Morganella_morganii	IS084_RS18050	5738..9415	582	BREX-system-P-loop-protein-BrxC-[Morganella-morganii]
WP_274765494.1	Type1.BREX	<-Peptidase_M17_N+[illegible]	1157	Bacteria>Pseudomonadota>Gammaproteobacteria>Enterobacterales>Morganellaceae>Morganella	GCF_028867915.1	Morganella_morganii	PS169_RS13665	158962..162435	582	BREX-system-P-loop-protein-BrxC-[Morganella-morganii]
WP_081119725.1	Type1.BREX	<-wHTH+WYL<-?<-?||?->XIS-HTH->[illegible]	1225	Bacteria>Pseudomonadota>Gammaproteobacteria>Enterobacterales>Morganellaceae>Morganella	-	Morganella_morganii	B2H99_RS12750	8810..12487	582	BREX-system-P-loop-protein-BrxC-[Morganella-morganii]
CAQ82523.1	Type1.BREX	Peptidase_M28->ABhydrolase+[illegible]	1216	Bacteria>Pseudomonadota>Gammaproteobacteria>Enterobacterales>Morganellaceae>Photorhabdus	GCA_000196475.1	Photorhabdus_asymbiotica	PAU_00430	471573..475223	291112	putative-atpase-involved-in-dna-repair-[Photorhabdus-asymbiotica]
WP_041381498.1	Type1.BREX	Peptidase_M28->ABhydrolase+[illegible]	1215	Bacteria>Pseudomonadota>Gammaproteobacteria>Enterobacterales>Morganellaceae>Photorhabdus	GCF_000196475.1	Photorhabdus_asymbiotica	PAU_RS02125	471576..475223	291112	BREX-system-P-loop-protein-BrxC-[Photorhabdus-asymbiotica]
WP_036771207.1	Type1.BREX	ABhydrolase+ABhydrolase->?->?-[illegible]	1213	Bacteria>Pseudomonadota>Gammaproteobacteria>Enterobacterales>Morganellaceae>Photorhabdus	GCF_000711895.1	Photorhabdus_australis	BO26_RS10370	20693..24334	286156	BREX-system-P-loop-protein-BrxC-[Photorhabdus-australis]
WP_054478994.1	Type1.BREX	<-PglZ<-N6-METHYLASE<-DUF6079*[illegible]	1213	Bacteria>Pseudomonadota>Gammaproteobacteria>Enterobacterales>Morganellaceae>Photorhabdus	GCF_001280945.1	Photorhabdus_heterorhabditis	AM629_RS11280	27281..30922	880156	BREX-system-P-loop-protein-BrxC-[Photorhabdus-heterorhabditis]

Protein-ID	System	Gene-Neighbourhoods	Length	Taxonomy	Assembly-ID	Strain	Locus-Tag	Genomic-coordinates	TaxID	Fasta-define
WP_065824091.1	Type1.BREX	<-METHYLASE||TetR-HTH->REase+N6_Mtase->N6-METHYLASE->?-><-PglZ<-N6-METHYLASE<-DUF6079*<-BrxB-BrxF-iSTAND<-BrxA-DNA-binding<-GNAT<-Aminotran_3<-?<-?<-?<-ABhydrolase+ABhydrolase<-[illegible]	1213	Bacteria>Pseudomonadota>Gammaproteobacteria>Enterobacterales>Morganellaceae>Photorhabdus	GCF_001696805.1	Photorhabdus_australis	Ppb6_RS16855	33737..37378	286156	BREX-system-P-loop-protein-BrxC-[Photorhabdus-australis]
WP_149617320.1	Type1.BREX	<-Fimbrial||?-><-?<-?<-?<-?||BrxA-DNA-binding->BrxB-BrxF-iSTAND->DUF6079*->N6-METHYLASE->PglZ->	1213	Bacteria>Pseudomonadota>Gammaproteobacteria>Enterobacterales>Morganellaceae>Photorhabdus	GCF_008370335.1	Photorhabdus_heterorhabditis	FOL16_RS17495	23615..27256	880156	BREX-system-P-loop-protein-BrxC-[Photorhabdus-heterorhabditis]
WP_166309887.1	Type1.BREX	<-Fimbrial||?-><-?<-?<-?<-?||BrxA-DNA-binding->BrxB-BrxF-iSTAND->DUF6079*->N6-METHYLASE->PglZ->	1213	Bacteria>Pseudomonadota>Gammaproteobacteria>Enterobacterales>Morganellaceae>Photorhabdus	GCF_011189575.1	Photorhabdus_cinerea	C5469_RS18920	23326..26967	471575	BREX-system-P-loop-protein-BrxC-[Photorhabdus-cinerea]
WP_172909759.1	Type1.BREX	BrxA-DNA-binding->BrxB-BrxF-iSTAND->DUF6079*->N6-METHYLASE->PglZ->	1213	Bacteria>Pseudomonadota>Gammaproteobacteria>Enterobacterales>Morganellaceae>Photorhabdus	GCF_013277735.1	Photorhabdus_heterorhabditis	HHJ84_RS11995	18661..22302	880156	BREX-system-P-loop-protein-BrxC-[Photorhabdus-heterorhabditis]
WP_214086333.1	Type1.BREX	<-PglZ<-N6-METHYLASE<-DUF6079*<-BrxB-BrxF-iSTAND<-BrxA-DNA-binding||?->?->?->?->Fimbrial->	1213	Bacteria>Pseudomonadota>Gammaproteobacteria>Enterobacterales>Morganellaceae>Photorhabdus	GCF_018448905.1	Photorhabdus_heterorhabditis	EAE89_RS17790	25052..28693	880156	BREX-system-P-loop-protein-BrxC-[Photorhabdus-heterorhabditis]
QIG07578.1	Type1.BREX	HTH_8->?->XIS-HTH->XIS-HTH->?->?->BrxA-DNA-binding->BrxB-BrxF-iSTAND->DUF6079*->OLD-ABC->REase->N6-METHYLASE->PglZ->SIGMA+OB-Fold+AAA-ATPase+HKD-DNase-BrxL-><-[illegible]	1183	Bacteria>Pseudomonadota>Gammaproteobacteria>Enterobacterales>Morganellaceae>Proteus	GCA_011046025.1	Proteus_sp._ZN5	GTK47_19170	4181966..4185517	2697019	BREX-system-P-loop-protein-BrxC-[Proteus-sp.-ZN5]
HAT2164885.1	Type1.BREX	<-MULE-TRANSPOSASE<-?<-LexA-protease||?->35exo->?->wHTH+WYL+WCX->BrxA-DNA-binding->BrxB-BrxF-iSTAND->DUF6079*->ParB+HNH->N6-METHYLASE->OLD-ABC->TOPRIM->PglZ->[illegible]	1225	Bacteria>Pseudomonadota>Gammaproteobacteria>Enterobacterales>Morganellaceae>Proteus	GCA_015896365.1	Proteus_mirabilis	I8052_002910	10079..13756	584	BREX-system-P-loop-protein-BrxC-[Proteus-mirabilis]
MBI6417160.1	Type1.BREX	<-CARF+NtrC-AAA+FIS-HTH<-SIGMA+OB-Fold+AAA-ATPase+Lon-Protease-BrxL<-PglZ<-TOPRIM<-OLD-ABC<-N6-METHYLASE<-ParB+HNH<-DUF6079*<-BrxB-BrxF-iSTAND<-BrxA-DNA-binding<-wHTH+WYL+WCX<-[illegible]	1225	Bacteria>Pseudomonadota>Gammaproteobacteria>Enterobacterales>Morganellaceae>Proteus	GCA_016238675.1	Proteus_mirabilis	JEP37_15655	69055..72732	584	BREX-system-P-loop-protein-BrxC-[Proteus-mirabilis]
HBM9710663.1	Type1.BREX	wHTH+WYL+WCX->BrxA-DNA-binding->BrxB-BrxF-iSTAND->DUF6079*->N6-METHYLASE->PglZ->SIGMA+OB-Fold+AAA-ATPase+Lon-Protease-BrxL->SbcC->HDRnase->VirD4-TraG+VirB4-FtsK->?->?-><-[illegible]	1225	Bacteria>Pseudomonadota>Gammaproteobacteria>Enterobacterales>Morganellaceae>Proteus	GCA_021439885.1	Proteus_mirabilis	L1C35_000984	2327..6004	584	BREX-system-P-loop-protein-BrxC-[Proteus-mirabilis]
WP_087726352.1	Type1.BREX	MobI-><-?<-LexA-protease||35exo->?->wHTH+WYL+WCX->BrxA-DNA-binding->BrxB-BrxF-iSTAND->DUF6079*->N6-METHYLASE->PglZ->SIGMA+OB-Fold+AAA-ATPase+Lon-Protease-BrxL->MarR-HTH->HEPN->[illegible]	1229	Bacteria>Pseudomonadota>Gammaproteobacteria>Enterobacterales>Morganellaceae>Proteus	GCF_002180235.1	Proteus_mirabilis	NFG13_RS03655	19477..23166	584	BREX-system-P-loop-protein-BrxC-[Proteus-mirabilis]
WP_152118569.1	Type1.BREX	<-Peptidase_S8<-Classical-AAA<-?<-LexA-protease||35exo->?->wHTH+WYL+WCX->BrxA-DNA-binding->BrxB-BrxF-iSTAND->DUF6079*->ParB+HNH->N6-METHYLASE->PglZ->[illegible]	1225	Bacteria>Pseudomonadota>Gammaproteobacteria>Enterobacterales>Morganellaceae>Proteus	GCF_009183735.1	Proteus_mirabilis	GBN13_RS03935	69067..72744	584	BREX-system-P-loop-protein-BrxC-[Proteus-mirabilis]
WP_206535871.1	Type1.BREX	HTH_8->?->XIS-HTH->XIS-HTH->?->?->BrxA-DNA-binding->BrxB-BrxF-iSTAND->DUF6079*->OLD-ABC->REase->N6-METHYLASE->PglZ->SIGMA+OB-Fold+AAA-ATPase+HKD-DNase-BrxL-><-[illegible]	1185	Bacteria>Pseudomonadota>Gammaproteobacteria>Enterobacterales>Morganellaceae>Proteus	GCF_011046025.1	Proteus_sp._ZN5	GTK47_RS19230	4181960..4185517	2697019	BREX-system-P-loop-protein-BrxC-[Proteus-sp.-ZN5]
WP_165544872.1	Type1.BREX	<-DDE_Tnp_1||wHTH+WYL+WCX->BrxA-DNA-binding->BrxB-BrxF-iSTAND->DUF6079*->N6-METHYLASE->PglZ->SIGMA+OB-Fold+AAA-ATPase+Lon-Protease-BrxL->SbcC->HDRnase->VirD4-TraG+VirB4-FtsK->?->?->[illegible]	1225	Bacteria>Pseudomonadota>Gammaproteobacteria>Enterobacterales>Morganellaceae>Proteus	GCF_011149675.1	Proteus_mirabilis	G9C79_RS07990	1716006..1719683	584	BREX-system-P-loop-protein-BrxC-[Proteus-mirabilis]
WP_209124420.1	Type1.BREX	<-DUF6079-p*<-BrxB-BrxF-iSTAND<-BrxA-DNA-binding<-XIS-HTH<-?||?->?->wHTH+WYL->	761	Bacteria>Pseudomonadota>Gammaproteobacteria>Enterobacterales>Morganellaceae>Proteus	GCF_017693315.1	Proteus_mirabilis	IS513_RS13265	1..2284	584	BREX-system-P-loop-protein-BrxC,-partial-[Proteus-mirabilis]
WP_220699168.1	Type1.BREX	<-Phage_integrase<-LexA-protease||?->35exo->?->wHTH+WYL+WCX->BrxA-DNA-binding->BrxB-BrxF-iSTAND->DUF6079*->ParB+HNH->N6-METHYLASE->PglZ->SIGMA+OB-Fold+AAA-ATPase+Lon-Protease-BrxL->[illegible]	1225	Bacteria>Pseudomonadota>Gammaproteobacteria>Enterobacterales>Morganellaceae>Proteus	GCF_019660825.1	Proteus_mirabilis	K2V98_RS14125	5608..9285	584	BREX-system-P-loop-protein-BrxC-[Proteus-mirabilis]
WP_210849185.1	Type1.BREX	<-OBFOLD-NUCLEASE<-CARF+NtrC-AAA+FIS-HTH<-SIGMA+OB-Fold+AAA-ATPase+Lon-Protease-BrxL<-PglZ<-TOPRIM<-OLD-ABC<-N6-METHYLASE<-ParB+HNH<-DUF6079*<-BrxB-BrxF-iSTAND<-BrxA-DNA-binding<-[illegible]	1225	Bacteria>Pseudomonadota>Gammaproteobacteria>Enterobacterales>Morganellaceae>Proteus	GCF_022349905.1	Proteus_mirabilis	MJ636_RS02915	47390..51067	584	BREX-system-P-loop-protein-BrxC-[Proteus-mirabilis]
WP_243213747.1	Type1.BREX	[illegible]	1225	Bacteria>Pseudomonadota>Gammaproteobacteria>Enterobacterales>Morganellaceae>Proteus	GCF_022802355.1	Proteus_mirabilis	JNG69_RS16550	64025..67702	584	BREX-system-P-loop-protein-BrxC-[Proteus-mirabilis]
WP_261229609.1	Type1.BREX	[illegible]	1225	Bacteria>Pseudomonadota>Gammaproteobacteria>Enterobacterales>Morganellaceae>Proteus	GCF_025379495.1	Proteus_mirabilis	NFG18_RS15790	23870..27547	584	BREX-system-P-loop-protein-BrxC-[Proteus-mirabilis]
WP_261598777.1	Type1.BREX	[illegible]	1225	Bacteria>Pseudomonadota>Gammaproteobacteria>Enterobacterales>Morganellaceae>Proteus	GCF_025399735.1	Proteus_mirabilis	NMC40_RS18460	15597..19274	584	BREX-system-P-loop-protein-BrxC-[Proteus-mirabilis]
WP_271972693.1	Type1.BREX	[illegible]	1225	Bacteria>Pseudomonadota>Gammaproteobacteria>Enterobacterales>Morganellaceae>Proteus	GCF_028335785.1	Proteus_terrae	ONR71_RS01795	360053..363730	1574161	BREX-system-P-loop-protein-BrxC-[Proteus-terrae]
WP_209036130.1	Type1.BREX	[illegible]	1225	Bacteria>Pseudomonadota>Gammaproteobacteria>Enterobacterales>Morganellaceae>Proteus	GCF_029542405.1	Proteus_mirabilis	IGB04_RS13130	82611..86288	584	BREX-system-P-loop-protein-BrxC-[Proteus-mirabilis]
UVX22921.1	Type1.BREX	[illegible]	789	Bacteria>Pseudomonadota>Gammaproteobacteria>Enterobacterales>Morganellaceae>Proteus	-	Proteus_terrae_subsp._cibarius	.	73397..75766	626774	hypothetical-protein-[Proteus-terrae-subsp.-cibarius]
OBY34180.1	Type1.BREX	[illegible]	1225	Bacteria>Pseudomonadota>Gammaproteobacteria>Enterobacterales>Morganellaceae>Providencia	GCA_001680125.1	Providencia_rettgeri	PR729_03900	44936..48613	587	DNA-repair-protein-[Providencia-rettgeri]
EJD6671300.1	Type1.BREX	[illegible]	1225	Bacteria>Pseudomonadota>Gammaproteobacteria>Enterobacterales>Morganellaceae>Providencia	GCA_020680725.2	Providencia_rettgeri	MOK80_RS10540	473799..477476	587	BREX-system-P-loop-protein-BrxC-[Providencia-rettgeri]
MCL0017528.1	Type1.BREX	[illegible]	1225	Bacteria>Pseudomonadota>Gammaproteobacteria>Enterobacterales>Morganellaceae>Providencia	GCA_022387615.2	Providencia_rettgeri	MKT11_000360	78084..81761	587	BREX-system-P-loop-protein-BrxC-[Providencia-rettgeri]
MCK9997621.1	Type1.BREX	[illegible]	1225	Bacteria>Pseudomonadota>Gammaproteobacteria>Enterobacterales>Morganellaceae>Providencia	GCA_022387885.2	Providencia_rettgeri	MKT39_000560	19282..22959	587	BREX-system-P-loop-protein-BrxC-[Providencia-rettgeri]
MCL0006618.1	Type1.BREX	[illegible]	1225	Bacteria>Pseudomonadota>Gammaproteobacteria>Enterobacterales>Morganellaceae>Providencia	GCA_022388475.2	Providencia_rettgeri	MKT60_007090	64410..68087	587	BREX-system-P-loop-protein-BrxC-[Providencia-rettgeri]
WP_137022260.1	Type1.BREX	[illegible]	1204	Bacteria>Pseudomonadota>Gammaproteobacteria>Enterobacterales>Morganellaceae>Providencia	GCF_005157325.1	Providencia_heimbachae	C9446_RS17890	3853703..3857317	333962	BREX-system-P-loop-protein-BrxC-[Providencia-heimbachae]
WP_166185547.1	Type1.BREX	[illegible]	1225	Bacteria>Pseudomonadota>Gammaproteobacteria>Enterobacterales>Morganellaceae>Providencia	GCF_011078165.1	Providencia_sp._M-8	G6Q30_RS18775	21019..24696	2713151	BREX-system-P-loop-protein-BrxC-[Providencia-sp.-M-8]
WP_265497329.1	Type1.BREX	[illegible]	1225	Bacteria>Pseudomonadota>Gammaproteobacteria>Enterobacterales>Morganellaceae>Providencia	GCF_026173365.1	Providencia_rustigianii	ORM28_RS01805	9364..13041	158850	BREX-system-P-loop-protein-BrxC-[Providencia-rustigianii]
WP_269723554.1	Type1.BREX	[illegible]	1225	Bacteria>Pseudomonadota>Gammaproteobacteria>Enterobacterales>Morganellaceae>Providencia	GCF_027362595.1	Providencia_sp._210H12SH02B-Prov	O7C57_RS02370	500730..504407	3015951	BREX-system-P-loop-protein-BrxC-[Providencia-sp.-210H12SH02B-Prov]
WP_272572142.1	Type1.BREX	[illegible]	1225	Bacteria>Pseudomonadota>Gammaproteobacteria>Enterobacterales>Morganellaceae>Providencia	GCF_028477905.1	unclassified_Providencia	PR658_RS19440	8800..12477	2633465	MULTISPECIES:-BREX-system-P-loop-protein-BrxC-[unclassified-Providencia]
WP_272670012.1	Type1.BREX	[illegible]	1185	Bacteria>Pseudomonadota>Gammaproteobacteria>Enterobacterales>Morganellaceae>Providencia	GCF_028480605.1	unclassified_Providencia	PSE59_RS21125	8138..11695	2633465	MULTISPECIES:-BREX-system-P-loop-protein-BrxC-[unclassified-Providencia]
WP_272668877.1	Type1.BREX	[illegible]	1225	Bacteria>Pseudomonadota>Gammaproteobacteria>Enterobacterales>Morganellaceae>Providencia	GCF_028481005.1	Providencia_sp._PROV151	PSE52_RS00990	220703..224380	2949861	BREX-system-P-loop-protein-BrxC-[Providencia-sp.-PROV151]
WP_272687949.1	Type1.BREX	[illegible]	1225	Bacteria>Pseudomonadota>Gammaproteobacteria>Enterobacterales>Morganellaceae>Providencia	GCF_028484835.1	Providencia_sp._PROV035	PSF71_RS08200	5027..8704	2949766	BREX-system-P-loop-protein-BrxC-[Providencia-sp.-PROV035]
AAM07998.1	Type1.BREX	[illegible]	947	Bacteria>Pseudomonadota>Gammaproteobacteria>Enterobacterales>Morganellaceae>Providencia	-	Providencia_rettgeri	.	15799..18642	587	putative-ATPase-[Providencia-rettgeri]
CAD27664.1	Type1.BREX	[illegible]	485	Bacteria>Pseudomonadota>Gammaproteobacteria>Enterobacterales>Morganellaceae>Providencia	-	Providencia_rettgeri	.	6214..7668	587	hypothetical-protein,-partial-[Providencia-rettgeri]
EIP8110719.1	Type1.BREX	[illegible]	1225	Bacteria>Pseudomonadota>Gammaproteobacteria>Enterobacterales>Morganellaceae>Providencia	-	Providencia_rettgeri	LT185_001364	559434..563111	587	BREX-system-P-loop-protein-BrxC-[Providencia-rettgeri]
WP_045967689.1	Type1.BREX	[illegible]	1212	Bacteria>Pseudomonadota>Gammaproteobacteria>Enterobacterales>Morganellaceae>Xenorhabdus	GCF_000968195.1	Xenorhabdus_doucetiae	LY16_RS12985	24233..27871	351671	BREX-system-P-loop-protein-BrxC-[Xenorhabdus-doucetiae]
WP_121588635.1	Type1.BREX	[illegible]	1213	Bacteria>Pseudomonadota>Gammaproteobacteria>Enterobacterales>Pectobacteriaceae>Brenneria	GCF_022358645.1	Brenneria_goodwinii	JMY82_RS09645	95315..98956	1109412	BREX-system-P-loop-protein-BrxC-[Brenneria-goodwinii]
WP_102802217.1	Type1.BREX	[illegible]	1213	Bacteria>Pseudomonadota>Gammaproteobacteria>Enterobacterales>Pectobacteriaceae>Dickeya	GCF_002887555.1	Dickeya_zeae	C1030_RS16230	3581616..3585257	204042	BREX-system-P-loop-protein-BrxC-[Dickeya-zeae]
WP_107760228.1	Type1.BREX	[illegible]	1213	Bacteria>Pseudomonadota>Gammaproteobacteria>Enterobacterales>Pectobacteriaceae>Dickeya	GCF_003049805.1	Dickeya_sp._Secpp_1600	CPA59_RS17580	3939970..3943611	2037915	BREX-system-P-loop-protein-BrxC-[Dickeya-sp.-Secpp-1600]
WP_040031759.1	Type1.BREX	[illegible]	1202	Bacteria>Pseudomonadota>Gammaproteobacteria>Enterobacterales>Pectobacteriaceae	GCF_026723725.1	Pectobacterium	OWC53_RS08230	1782306..1785914	122277	MULTISPECIES:-BREX-system-P-loop-protein-BrxC-[Pectobacterium]
GKV76958.1	Type1.BREX	[illegible]	1157	Bacteria>Pseudomonadota>Gammaproteobacteria>Enterobacterales>Pectobacteriaceae>Pectobacterium	GCA_024342785.1	Pectobacterium_carotovorum_subsp._carotovorum	PEC106568_21320	196499..199972	555	hypothetical-protein-PEC106568_21320-[Pectobacterium-carotovorum-subsp.-carotovorum]
GKX39595.1	Type1.BREX	[illegible]	1202	Bacteria>Pseudomonadota>Gammaproteobacteria>Enterobacterales>Pectobacteriaceae>Pectobacterium	GCA_024343445.1	Pectobacterium_carotovorum_subsp._carotovorum	SOASR014_33340	124830..128438	555	hypothetical-protein-SOASR014_33340-[Pectobacterium-carotovorum-subsp.-carotovorum]
WP_005974434.1	Type1.BREX	[illegible]	1204	Bacteria>Pseudomonadota>Gammaproteobacteria>Enterobacterales>Pectobacteriaceae>Pectobacterium	GCF_000291725.1	Pectobacterium_wasabiae	Y17_RS17690	22336..25950	55208	BREX-system-P-loop-protein-BrxC-[Pectobacterium-wasabiae]
WP_014916385.1	Type1.BREX	[illegible]	1213	Bacteria>Pseudomonadota>Gammaproteobacteria>Enterobacterales>Pectobacteriaceae>Pectobacterium	GCF_000294535.1	Pectobacterium_carotovorum	PCC21_RS16225	3704007..3707648	554	BREX-system-P-loop-protein-BrxC-[Pectobacterium-carotovorum]
WP_103160989.1	Type1.BREX	[illegible]	1202	Bacteria>Pseudomonadota>Gammaproteobacteria>Enterobacterales>Pectobacteriaceae>Pectobacterium	GCF_002904045.1	Pectobacterium_odoriferum	BVY05_RS19610	74039..77647	78398	BREX-system-P-loop-protein-BrxC-[Pectobacterium-odoriferum]
WP_103199124.1	Type1.BREX	[illegible]	1202	Bacteria>Pseudomonadota>Gammaproteobacteria>Enterobacterales>Pectobacteriaceae>Pectobacterium	GCF_002904085.1	Pectobacterium_odoriferum	BV921_RS15390	113976..117584	78398	BREX-system-P-loop-protein-BrxC-[Pectobacterium-odoriferum]
WP_116155987.1	Type1.BREX	[illegible]	1202	Bacteria>Pseudomonadota>Gammaproteobacteria>Enterobacterales>Pectobacteriaceae>Pectobacterium	GCF_003382585.2	Pectobacterium_aquaticum	DMB81_RS10735	4900..8508	2204145	BREX-system-P-loop-protein-BrxC-[Pectobacterium-aquaticum]
WP_181847885.1	Type1.BREX	[illegible]	1204	Bacteria>Pseudomonadota>Gammaproteobacteria>Enterobacterales>Pectobacteriaceae>Pectobacterium	GCF_013913645.1	Pectobacterium_aroidearum	H2Y54_RS11285	236602..240216	1201031	BREX-system-P-loop-protein-BrxC-[Pectobacterium-aroidearum]
WP_211024717.1	Type1.BREX	[illegible]	1157	Bacteria>Pseudomonadota>Gammaproteobacteria>Enterobacterales>Pectobacteriaceae>Pectobacterium	GCF_018094745.1	Pectobacterium_versatile	F9U42_RS09295	89992..93465	2488639	BREX-system-P-loop-protein-BrxC-[Pectobacterium-versatile]
WP_249681848.1	Type1.BREX	[illegible]	1157	Bacteria>Pseudomonadota>Gammaproteobacteria>Enterobacterales>Pectobacteriaceae>Pectobacterium	-	Pectobacterium_polaris	EXT50_03115	82893..86366	2042057	BREX-system-P-loop-protein-BrxC-[Pectobacterium-polaris]
WP_060426782.1	Type1.BREX	[illegible]	1213	Bacteria>Pseudomonadota>Gammaproteobacteria>Enterobacterales>Yersiniaceae	GCF_001537885.1	Serratia	AWX06_RS15845	182718..186359	613	MULTISPECIES:-BREX-system-P-loop-protein-BrxC-[Serratia]
WP_050135880.1	Type1.BREX	[illegible]	1225	Bacteria>Pseudomonadota>Gammaproteobacteria>Enterobacterales>Yersiniaceae	GCF_025758435.1	Yersinia	N4W04_RS19100	4124889..4128566	629	MULTISPECIES:-BREX-system-P-loop-protein-BrxC-[Yersinia]
WP_138775748.1	Type1.BREX	[illegible]	1202	Bacteria>Pseudomonadota>Gammaproteobacteria>Enterobacterales>Yersiniaceae	GCF_902168625.1	Yersinia	FFE93_018510	3893626..3897234	629	MULTISPECIES:-BREX-system-P-loop-protein-BrxC-[Yersinia]
WP_112197427.1	Type1.BREX	[illegible]	1204	Bacteria>Pseudomonadota>Gammaproteobacteria>Enterobacterales>Yersiniaceae>Rahnella	GCF_003263525.1	Rahnella_sp._NRRL_B-41462	UC14_RS06670	31474..35088	1610579	BREX-system-P-loop-protein-BrxC-[Rahnella-sp.-NRRL-B-41462]
WP_217147154.1	Type1.BREX	[illegible]	1157	Bacteria>Pseudomonadota>Gammaproteobacteria>Enterobacterales>Yersiniaceae>Rahnella	GCF_019049515.1	Rahnella_aceris	J1789_RS04120	11901..15374	2703885	BREX-system-P-loop-protein-BrxC-[Rahnella-aceris]
WP_232770162.1	Type1.BREX	[illegible]	1157	Bacteria>Pseudomonadota>Gammaproteobacteria>Enterobacterales>Yersiniaceae>Rahnella	GCF_021276285.1	Rahnella_victoriana	J9880_RS04875	1051789..1055262	1510570	BREX-system-P-loop-protein-BrxC-[Rahnella-victoriana]
WP_266057125.1	Type1.BREX	[illegible]	1157	Bacteria>Pseudomonadota>Gammaproteobacteria>Enterobacterales>Yersiniaceae>Rahnella	GCF_026241995.1	Rahnella_perminowiae	ORG37_RS00070	21089..24562	2816244	BREX-system-P-loop-protein-BrxC-[Rahnella-perminowiae]
NYA44684.1	Type1.BREX	[illegible]	1158	Bacteria>Pseudomonadota>Gammaproteobacteria>Enterobacterales>Yersiniaceae>Serratia	GCA_013401715.1	Serratia_fonticola	HZI31_15400	48979..52455	47917	BREX-system-P-loop-protein-BrxC-[Serratia-fonticola]
EGS9996488.1	Type1.BREX	[illegible]	1233	Bacteria>Pseudomonadota>Gammaproteobacteria>Enterobacterales>Yersiniaceae>Serratia	GCA_015929585.1	Serratia_marcescens	I9Y15_003567	178923..182624	615	BREX-system-P-loop-protein-BrxC-[Serratia-marcescens]
HBE9089660.1	Type1.BREX	[illegible]	1204	Bacteria>Pseudomonadota>Gammaproteobacteria>Enterobacterales>Yersiniaceae>Serratia	GCA_018627075.1	Serratia_fonticola	KNG84_001684	34672..38286	47917	BREX-system-P-loop-protein-BrxC-[Serratia-fonticola]
HBK4794038.1	Type1.BREX	[illegible]	1233	Bacteria>Pseudomonadota>Gammaproteobacteria>Enterobacterales>Yersiniaceae>Serratia	GCA_020682225.1	Serratia_marcescens	LLT15_004323	35577..39278	615	BREX-system-P-loop-protein-BrxC-[Serratia-marcescens]
WP_089185250.1	Type1.BREX	[illegible]	1213	Bacteria>Pseudomonadota>Gammaproteobacteria>Enterobacterales>Yersiniaceae>Serratia	GCF_002220535.1	Serratia_marcescens	BVG90_RS23850	5002559..5006200	615	BREX-system-P-loop-protein-BrxC-[Serratia-marcescens]
WP_218833283.1	Type1.BREX	[illegible]	1197	Bacteria>Pseudomonadota>Gammaproteobacteria>Enterobacterales>Yersiniaceae>Serratia	GCF_013401715.1	Serratia_fonticola	HZI31_RS15400	48979..52572	47917	BREX-system-P-loop-protein-BrxC-[Serratia-fonticola]
WP_197785670.1	Type1.BREX	[illegible]	1213	Bacteria>Pseudomonadota>Gammaproteobacteria>Enterobacterales>Yersiniaceae>Serratia	GCF_016007555.1	Serratia_ureilytica	I5P92_RS12860	24369..28010	300181	BREX-system-P-loop-protein-BrxC-[Serratia-ureilytica]
WP_206196473.1	Type1.BREX	[illegible]	1157	Bacteria>Pseudomonadota>Gammaproteobacteria>Enterobacterales>Yersiniaceae>Serratia	GCF_017152235.1	Serratia_ureilytica	JY544_RS23375	37448..40921	300181	BREX-system-P-loop-protein-BrxC-[Serratia-ureilytica]
WP_212602139.1	Type1.BREX	[illegible]	1213	Bacteria>Pseudomonadota>Gammaproteobacteria>Enterobacterales>Yersiniaceae>Serratia	GCF_018257315.1	Serratia_rubidaea	JK228_RS02460	34162..37803	61652	BREX-system-P-loop-protein-BrxC-[Serratia-rubidaea]
WP_222398282.1	Type1.BREX	[illegible]	1213	Bacteria>Pseudomonadota>Gammaproteobacteria>Enterobacterales>Yersiniaceae>Serratia	GCF_019795995.1	Serratia_marcescens	L8N14_16510	93200..96841	615	BREX-system-P-loop-protein-BrxC-[Serratia-marcescens]
WP_223536794.1	Type1.BREX	[illegible]	1158	Bacteria>Pseudomonadota>Gammaproteobacteria>Enterobacterales>Yersiniaceae>Serratia	GCF_019968705.1	unclassified_Serratia_(in:_enterobacteria)	KGP26_RS00385	95452..98928	2647522	MULTISPECIES:-BREX-system-P-loop-protein-BrxC-[unclassified-Serratia-(in:-enterobacteria)]
WP_244757920.1	Type1.BREX	[illegible]	1213	Bacteria>Pseudomonadota>Gammaproteobacteria>Enterobacterales>Yersiniaceae>Serratia	GCF_022870585.1	Serratia_marcescens	HW877_RS02460	535877..539518	615	BREX-system-P-loop-protein-BrxC-[Serratia-marcescens]
WP_260965787.1	Type1.BREX	[illegible]	1240	Bacteria>Pseudomonadota>Gammaproteobacteria>Enterobacterales>Yersiniaceae>Serratia	GCF_025336405.1	Serratia_marcescens	N7751_RS05105	1087639..1091361	615	BREX-system-P-loop-protein-BrxC-[Serratia-marcescens]
WP_273866014.1	Type1.BREX	[illegible]	1157	Bacteria>Pseudomonadota>Gammaproteobacteria>Enterobacterales>Yersiniaceae>Serratia	GCF_028616515.1	Serratia_ureilytica	PVU68_RS17145	216123..219596	300181	BREX-system-P-loop-protein-BrxC-[Serratia-ureilytica]
WP_261139451.1	Type1.BREX	[illegible]	1213	Bacteria>Pseudomonadota>Gammaproteobacteria>Enterobacterales>Yersiniaceae>Serratia	GCF_946226675.1	Serratia_liquefaciens	N8M82_RS16050	322435..326076	614	BREX-system-P-loop-protein-BrxC-[Serratia-liquefaciens]
WP_261135874.1	Type1.BREX	[illegible]	1213	Bacteria>Pseudomonadota>Gammaproteobacteria>Enterobacterales>Yersiniaceae>Serratia	GCF_946226775.1	Serratia_liquefaciens	N8M94_RS14775	322685..326326	614	BREX-system-P-loop-protein-BrxC-[Serratia-liquefaciens]
WP_261427666.1	Type1.BREX	[illegible]	1213	Bacteria>Pseudomonadota>Gammaproteobacteria>Enterobacterales>Yersiniaceae>Serratia	GCF_946227925.1	Serratia_proteamaculans	OCH17_RS14295	357572..361213	28151	BREX-system-P-loop-protein-BrxC-[Serratia-proteamaculans]
WP_262254721.1	Type1.BREX	[illegible]	1240	Bacteria>Pseudomonadota>Gammaproteobacteria>Enterobacterales>Yersiniaceae>Serratia	GCF_946228755.1	Serratia_proteamaculans	OC575_RS10145	2133622..2137344	28151	BREX-system-P-loop-protein-BrxC-[Serratia-proteamaculans]
WP_195869502.1	Type1.BREX	[illegible]	1202	Bacteria>Pseudomonadota>Gammaproteobacteria>Enterobacterales>Yersiniaceae>Serratia	-	Serratia_liquefaciens	I1A46_RS14725	213767..217375	614	BREX-system-P-loop-protein-BrxC-[Serratia-liquefaciens]
WP_004722527.1	Type1.BREX	[illegible]	1202	Bacteria>Pseudomonadota>Gammaproteobacteria>Enterobacterales>Yersiniaceae>Yersinia	GCF_000173755.1	Yersinia_ruckeri	YRUCK0001_RS14080	15308..18916	29486	BREX-system-P-loop-protein-BrxC-[Yersinia-ruckeri]
WP_046051668.1	Type1.BREX	[illegible]	1157	Bacteria>Pseudomonadota>Gammaproteobacteria>Enterobacterales>Yersiniaceae>Yersinia	GCF_000968115.1	Yersinia_enterocolitica	YE5303_RS19630	4225906..4229379	630	BREX-system-P-loop-protein-BrxC-[Yersinia-enterocolitica]
WP_050073003.1	Type1.BREX	[illegible]	1157	Bacteria>Pseudomonadota>Gammaproteobacteria>Enterobacterales>Yersiniaceae>Yersinia	GCF_001088505.1	Yersinia_intermedia	AE858_RS05265	31386..34859	631	BREX-system-P-loop-protein-BrxC-[Yersinia-intermedia]
WP_050124850.1	Type1.BREX	[illegible]	1202	Bacteria>Pseudomonadota>Gammaproteobacteria>Enterobacterales>Yersiniaceae>Yersinia	GCF_001115185.1	Yersinia_aleksiciae	AEB18_RS00865	216657..220265	263819	BREX-system-P-loop-protein-BrxC-[Yersinia-aleksiciae]
WP_053011787.1	Type1.BREX	[illegible]	1202	Bacteria>Pseudomonadota>Gammaproteobacteria>Enterobacterales>Yersiniaceae>Yersinia	GCF_001244675.1	Yersinia_intermedia	ALC37_RS11575	459968..463576	631	BREX-system-P-loop-protein-BrxC-[Yersinia-intermedia]
WP_128451204.1	Type1.BREX	[illegible]	1202	Bacteria>Pseudomonadota>Gammaproteobacteria>Enterobacterales>Yersiniaceae>Yersinia	GCF_004100405.1	Yersinia_sp._2105_StPb_PI	EQP49_RS07200	292011..295619	2507058	BREX-system-P-loop-protein-BrxC-[Yersinia-sp.-2105-StPb-PI]
WP_145931270.1	Type1.BREX	[illegible]	1202	Bacteria>Pseudomonadota>Gammaproteobacteria>Enterobacterales>Yersiniaceae>Yersinia	GCF_902168565.1	Yersinia_bercovieri	FS908_RS01775	53708..57316	634	BREX-system-P-loop-protein-BrxC-[Yersinia-bercovieri]
WP_145532653.1	Type1.BREX	[illegible]	1202	Bacteria>Pseudomonadota>Gammaproteobacteria>Enterobacterales>Yersiniaceae>Yersinia	GCF_902170345.1	Yersinia_alsatica	FTB07_RS01240	22698..26306	2890317	BREX-system-P-loop-protein-BrxC-[Yersinia-alsatica]
WP_145580439.1	Type1.BREX	[illegible]	1202	Bacteria>Pseudomonadota>Gammaproteobacteria>Enterobacterales>Yersiniaceae>Yersinia	GCF_902170795.1	Yersinia_mollaretii	FTB33_RS16505	6738..10346	33060	BREX-system-P-loop-protein-BrxC-[Yersinia-mollaretii]
MBZ4194014.1	Type1.BREX	[illegible]	1138	Bacteria>Pseudomonadota>Gammaproteobacteria>Gammaproteobacteria_incertae_sedis>Candidatus_Competibacteraceae>Candidatus_Contendobacter	GCA_020035205.1	Candidatus_Contendobacter_sp.	LAE24_06875	5150..8566	2651161	BREX-system-P-loop-protein-BrxC-[Candidatus-Contendobacter-sp.]
WP_034430846.1	Type1.BREX	[illegible]	1138	Bacteria>Pseudomonadota>Gammaproteobacteria>Gammaproteobacteria_incertae_sedis>Candidatus_Competibacteraceae>Candidatus_Contendobacter	GCF_000531125.1	Candidatus_Contendobacter_odensis	BN874_RS03435	764..4180	1400860	BREX-system-P-loop-protein-BrxC-[Candidatus-Contendobacter-odensis]
WP_132542053.1	Type1.BREX	[illegible]	1153	Bacteria>Pseudomonadota>Gammaproteobacteria>Gammaproteobacteria_incertae_sedis>Candidatus_Competibacteraceae>Plasticicumulans	GCF_004341245.1	Plasticicumulans_lactativorans	EV699_RS09620	184945..188406	1133106	BREX-system-P-loop-protein-BrxC-[Plasticicumulans-lactativorans]
MBK8752263.1	Type1.BREX	[illegible]	1139	Bacteria>Pseudomonadota>Gammaproteobacteria>Gammaproteobacteria_incertae_sedis>Candidatus_Competibacteraceae>unclassified_Candidatus_Competibacteraceae	GCA_016710535.1	Candidatus_Competibacteraceae_bacterium	IPL99_11760	9123..12542	2053538	BREX-system-P-loop-protein-BrxC-[Candidatus-Competibacteraceae-bacterium]
MBK8753015.1	Type1.BREX	[illegible]	1154	Bacteria>Pseudomonadota>Gammaproteobacteria>Gammaproteobacteria_incertae_sedis>Candidatus_Competibacteraceae>unclassified_Candidatus_Competibacteraceae	GCA_016710535.1	Candidatus_Competibacteraceae_bacterium	IPL99_15935	14692..18156	2053538	BREX-system-P-loop-protein-BrxC-[Candidatus-Competibacteraceae-bacterium]
MBK8536094.1	Type1.BREX	[illegible]	491	Bacteria>Pseudomonadota>Gammaproteobacteria>Gammaproteobacteria_incertae_sedis>Candidatus_Competibacteraceae>unclassified_Candidatus_Competibacteraceae	GCA_016710825.1	Candidatus_Competibacteraceae_bacterium	IPL59_13760	11734..13209	2053538	BREX-system-P-loop-protein-BrxC-[Candidatus-Competibacteraceae-bacterium]
MCB1769904.1	Type1.BREX	[illegible]	760	Bacteria>Pseudomonadota>Gammaproteobacteria>Gammaproteobacteria_incertae_sedis>Candidatus_Competibacteraceae>unclassified_Candidatus_Competibacteraceae	GCA_020445005.1	Candidatus_Competibacteraceae_bacterium	KDJ31_09455	11685..13969	2053538	BREX-system-P-loop-protein-BrxC-[Candidatus-Competibacteraceae-bacterium]
WP_271768605.1	Type1.BREX	[illegible]	1154	Bacteria>Pseudomonadota>Gammaproteobacteria>Gammaproteobacteria_incertae_sedis>Candidatus_Pseudothioglobus	GCF_028273125.1	Candidatus_Pseudothioglobus_singularis	OAH81_RS01105	228598..232062	1427364	BREX-system-P-loop-protein-BrxC-[Candidatus-Pseudothioglobus-singularis]
WP_248566606.1	Type1.BREX	[illegible]	1145	Bacteria>Pseudomonadota>Gammaproteobacteria>Gammaproteobacteria_incertae_sedis>Methylonatrum	GCF_023195885.1	Methylonatrum_kenyense	MO534_RS01350	290769..294206	455253	BREX-system-P-loop-protein-BrxC-[Methylonatrum-kenyense]
HDZ09174.1	Type1.BREX	[illegible]	1208	Bacteria>Pseudomonadota>Gammaproteobacteria>Gammaproteobacteria_incertae_sedis>Pseudohongiella>unclassified_Pseudohongiella	GCA_011050365.1	Pseudohongiella_sp.	ENH71_08845	47068..50694	1979412	BREX-system-P-loop-protein-BrxC-[Pseudohongiella-sp.]
WP_029131813.1	Type1.BREX	[illegible]	1147	Bacteria>Pseudomonadota>Gammaproteobacteria>Gammaproteobacteria_incertae_sedis>Sedimenticola	GCF_000428045.1	Sedimenticola_selenatireducens	A3GO_RS0101005	196433..199876	191960	BREX-system-P-loop-protein-BrxC-[Sedimenticola-selenatireducens]
MBT4669056.1	Type1.BREX	[illegible]	1167	Bacteria>Pseudomonadota>Gammaproteobacteria>Gammaproteobacteria_incertae_sedis>sulfur-oxidizing_symbionts>Candidatus_Ruthia	GCA_018657745.1	Candidatus_Ruthia_sp.	HN615_06440	8657..12160	2735566	BREX-system-P-loop-protein-BrxC-[Candidatus-Ruthturnera-sp.]
MCG7867606.1	Type1.BREX	[illegible]	1172	Bacteria>Pseudomonadota>Gammaproteobacteria>Gammaproteobacteria_incertae_sedis>sulfur-oxidizing_symbionts>Candidatus_Thiodiazotropha	GCA_016842585.1	Candidatus_Thiodiazotropha_taylori	JAY74_14755	23622..27140	2792791	BREX-system-P-loop-protein-BrxC-[Candidatus-Thiodiazotropha-taylori]
MCG8047516.1	Type1.BREX	[illegible]	1147	Bacteria>Pseudomonadota>Gammaproteobacteria>Gammaproteobacteria_incertae_sedis>sulfur-oxidizing_symbionts>Candidatus_Thiodiazotropha	GCA_016843075.1	Candidatus_Thiodiazotropha_taylori	JAY66_17825	23755..27198	2792791	BREX-system-P-loop-protein-BrxC-[Candidatus-Thiodiazotropha-taylori]
MCG7871843.1	Type1.BREX	[illegible]	1174	Bacteria>Pseudomonadota>Gammaproteobacteria>Gammaproteobacteria_incertae_sedis>sulfur-oxidizing_symbionts>Candidatus_Thiodiazotropha	GCA_016848925.1	Candidatus_Thiodiazotropha_lotti	JAZ11_07035	23024..26548	2792787	BREX-system-P-loop-protein-BrxC-[Candidatus-Thiodiazotropha-lotti]
MCG7872379.1	Type1.BREX	[illegible]	1170	Bacteria>Pseudomonadota>Gammaproteobacteria>Gammaproteobacteria_incertae_sedis>sulfur-oxidizing_symbionts>Candidatus_Thiodiazotropha	GCA_016848925.1	Candidatus_Thiodiazotropha_lotti	JAZ11_09800	178889..182401	2792787	BREX-system-P-loop-protein-BrxC-[Candidatus-Thiodiazotropha-lotti]
MCG7872685.1	Type1.BREX	[illegible]	1151	Bacteria>Pseudomonadota>Gammaproteobacteria>Gammaproteobacteria_incertae_sedis>sulfur-oxidizing_symbionts>Candidatus_Thiodiazotropha	GCA_016848925.1	Candidatus_Thiodiazotropha_lotti	JAZ11_11345	100935..104390	2792787	BREX-system-P-loop-protein-BrxC-[Candidatus-Thiodiazotropha-lotti]
WP_177417917.1	Type1.BREX	[illegible]	1188	Bacteria>Pseudomonadota>Gammaproteobacteria>Gammaproteobacteria_incertae_sedis>sulfur-oxidizing_symbionts	GCF_011947365.2	endosymbiont_of_Lamellibrachia_barhami	G9Q73_RS00010	458..4024	205975	BREX-system-P-loop-protein-BrxC-[endosymbiont-of-Lamellibrachia-barhami]
WP_044865655.1	Type1.BREX	[illegible]	1213	Bacteria>Pseudomonadota>Gammaproteobacteria	GCF_001540705.1	Enterobacterales	TJ11_RS03570	21952..25593	91347	MULTISPECIES:-BREX-system-P-loop-protein-BrxC-[Enterobacterales]
WP_223374968.1	Type1.BREX	[illegible]	1240	Bacteria>Pseudomonadota>Gammaproteobacteria	GCF_003051945.2	Enterobacterales	N5D94_RS08195	82368..86090	91347	MULTISPECIES:-BREX-system-P-loop-protein-BrxC-[Enterobacterales]
WP_060554888.1	Type1.BREX	[illegible]	1225	Bacteria>Pseudomonadota>Gammaproteobacteria	GCF_003176495.1	Enterobacterales	DDA63_RS11920	2661..6338	91347	MULTISPECIES:-BREX-system-P-loop-protein-BrxC-[Enterobacterales]
WP_052921406.1	Type1.BREX	[illegible]	1225	Bacteria>Pseudomonadota>Gammaproteobacteria	GCF_003402955.1	Enterobacterales	DS732_RS04425	849664..853341	91347	MULTISPECIES:-BREX-system-P-loop-protein-BrxC-[Enterobacterales]
WP_112020499.1	Type1.BREX	[illegible]	1204	Bacteria>Pseudomonadota>Gammaproteobacteria	GCF_013927185.1	Enterobacterales	DP180_RS12665	36252..39866	91347	MULTISPECIES:-BREX-system-P-loop-protein-BrxC-[Enterobacterales]
WP_099433763.1	Type1.BREX	[illegible]	1233	Bacteria>Pseudomonadota>Gammaproteobacteria	GCF_014168815.1	Enterobacterales	H7R56_RS21645	4484852..4488553	91347	MULTISPECIES:-BREX-system-P-loop-protein-BrxC-[Enterobacterales]
WP_192176748.1	Type1.BREX	[illegible]	1225	Bacteria>Pseudomonadota>Gammaproteobacteria	GCF_014843115.1	Enterobacterales	H6P66_RS01965	387207..390884	91347	MULTISPECIES:-BREX-system-P-loop-protein-BrxC-[Enterobacterales]
MAZ66828.1	Type1.BREX	[illegible]	1234	Bacteria>Pseudomonadota>Gammaproteobacteria>Kangiellales>Kangiellaceae>unclassified_Kangiellaceae	GCA_002707725.1	Kangiellaceae_bacterium	CMF25_06935	12477..16181	2026751	DNA-repair-protein-[Kangiellaceae-bacterium]
WP_207387236.1	Type1.BREX	[illegible]	1194	Bacteria>Pseudomonadota>Gammaproteobacteria>Legionellales>Legionellaceae>Fluoribacter	GCF_900640105.1	Fluoribacter_gormanii	E1P13_RS01655	70546..74130	464	BREX-system-P-loop-protein-BrxC-[Fluoribacter-gormanii]
TIG67286.1	Type1.BREX	[illegible]	1185	Bacteria>Pseudomonadota>Gammaproteobacteria>Legionellales>Legionellaceae>Legionella	GCA_004921705.1	Legionella_pneumophila	JAN10_12325	31417..34974	446	BREX-system-P-loop-protein-BrxC-[Legionella-pneumophila]
HAT1978996.1	Type1.BREX	[illegible]	1184	Bacteria>Pseudomonadota>Gammaproteobacteria>Legionellales>Legionellaceae>Legionella	GCA_015895665.1	Legionella_pneumophila	I8Z83_002252	23760..27314	446	BREX-system-P-loop-protein-BrxC-[Legionella-pneumophila]
HAT6348316.1	Type1.BREX	[illegible]	1185	Bacteria>Pseudomonadota>Gammaproteobacteria>Legionellales>Legionellaceae>Legionella	GCA_015945075.1	Legionella_pneumophila	JAJ33_001368	159147..162704	446	BREX-system-P-loop-protein-BrxC-[Legionella-pneumophila]
HAT6334489.1	Type1.BREX	[illegible]	1185	Bacteria>Pseudomonadota>Gammaproteobacteria>Legionellales>Legionellaceae>Legionella	GCA_015946305.1	Legionella_pneumophila	JAJ38_001743	27953..31510	446	BREX-system-P-loop-protein-BrxC-[Legionella-pneumophila]
HAT9211453.1	Type1.BREX	[illegible]	1185	Bacteria>Pseudomonadota>Gammaproteobacteria>Legionellales>Legionellaceae>Legionella	GCA_015973865.1	Legionella_pneumophila_subsp._pneumophila	JBB81_06050	33487..37044	91891	BREX-system-P-loop-protein-BrxC-[Legionella-pneumophila-subsp.-pneumophila]
HAU0774874.1	Type1.BREX	[illegible]	1185	Bacteria>Pseudomonadota>Gammaproteobacteria>Legionellales>Legionellaceae>Legionella	GCA_015983005.1	Legionella_pneumophila	JBH09_07400	2447..6004	446	BREX-system-P-loop-protein-BrxC-[Legionella-pneumophila]
HAU2155475.1	Type1.BREX	[illegible]	1185	Bacteria>Pseudomonadota>Gammaproteobacteria>Legionellales>Legionellaceae>Legionella	GCA_016020315.1	Legionella_pneumophila	JBK36_03075	164784..168341	446	BREX-system-P-loop-protein-BrxC-[Legionella-pneumophila]
HAU3861597.1	Type1.BREX	[illegible]	1185	Bacteria>Pseudomonadota>Gammaproteobacteria>Legionellales>Legionellaceae>Legionella	GCA_016062615.1	Legionella_pneumophila	F7Z80_07285	265287..268844	446	BREX-system-P-loop-protein-BrxC-[Legionella-pneumophila]
HCJ1122636.1	Type1.BREX	[illegible]	1184	Bacteria>Pseudomonadota>Gammaproteobacteria>Legionellales>Legionellaceae>Legionella	GCA_024486645.1	Legionella_pneumophila	NQN52_000248	14141..17695	446	BREX-system-P-loop-protein-BrxC-[Legionella-pneumophila]
HCJ1069247.1	Type1.BREX	[illegible]	1225	Bacteria>Pseudomonadota>Gammaproteobacteria>Legionellales>Legionellaceae>Legionella	GCA_024486765.1	Legionella_pneumophila	NQN29_000516	40432..44109	446	BREX-system-P-loop-protein-BrxC-[Legionella-pneumophila]
WP_061693758.1	Type1.BREX	[illegible]	1184	Bacteria>Pseudomonadota>Gammaproteobacteria>Legionellales>Legionellaceae>Legionella	GCF_002002625.1	Legionella_pneumophila	LPM_RS06580	16051..19605	446	BREX-system-P-loop-protein-BrxC-[Legionella-pneumophila]
WP_192875104.1	Type1.BREX	[illegible]	1184	Bacteria>Pseudomonadota>Gammaproteobacteria>Legionellales>Legionellaceae>Legionella	GCF_003004175.1	Legionella_pneumophila	CA922_RS00900	198017..201571	446	BREX-system-P-loop-protein-BrxC-[Legionella-pneumophila]
WP_061721013.1	Type1.BREX	[illegible]	1184	Bacteria>Pseudomonadota>Gammaproteobacteria>Legionellales>Legionellaceae>Legionella	GCF_900054435.1	Legionella_pneumophila	A2E62_RS14865	12190..15744	446	BREX-system-P-loop-protein-BrxC-[Legionella-pneumophila]
WP_061637858.1	Type1.BREX	[illegible]	1184	Bacteria>Pseudomonadota>Gammaproteobacteria>Legionellales>Legionellaceae>Legionella	GCF_900061645.1	Legionella_pneumophila	A1182_RS06820	180647..184201	446	BREX-system-P-loop-protein-BrxC-[Legionella-pneumophila]
WP_027222687.1	Type1.BREX	[illegible]	1184	Bacteria>Pseudomonadota>Gammaproteobacteria>Legionellales>Legionellaceae>Legionella	GCF_900639945.1	Legionella_pneumophila	E1076_RS05405	246005..249559	446	BREX-system-P-loop-protein-BrxC-[Legionella-pneumophila]
WP_165474921.1	Type1.BREX	[illegible]	1184	Bacteria>Pseudomonadota>Gammaproteobacteria>Legionellales>Legionellaceae>Legionella	GCF_900640175.1	Legionella_sp._W10-070	E1065_RS06915	46772..50326	1117709	BREX-system-P-loop-protein-BrxC-[Legionella-sp.-W10-070]
MCD2450192.1	Type1.BREX	[illegible]	1213	Bacteria>Pseudomonadota>Gammaproteobacteria>Methylococcales>Methylococcaceae>Methylicorpusculum	GCA_009828925.2	Methylicorpusculum_oleiharenae	GO003_007310	1557698..1561339	1338687	BREX-system-P-loop-protein-BrxC-[Methylicorpusculum-oleiharenae]
WP_159653266.1	Type1.BREX	[illegible]	1184	Bacteria>Pseudomonadota>Gammaproteobacteria>Methylococcales>Methylococcaceae>Methylicorpusculum	GCF_009828925.2	Methylicorpusculum_oleiharenae	GO003_RS20515	4601128..4604682	1338687	BREX-system-P-loop-protein-BrxC-[Methylicorpusculum-oleiharenae]
WP_159652892.1	Type1.BREX	[illegible]	1213	Bacteria>Pseudomonadota>Gammaproteobacteria>Methylococcales>Methylococcaceae>Methylicorpusculum	-	Methylicorpusculum_oleiharenae	GO003_007310	1557698..1561339	1338687	BREX-system-P-loop-protein-BrxC-[Methylicorpusculum-oleiharenae]
MCL7421787.1	Type1.BREX	[illegible]	1146	Bacteria>Pseudomonadota>Gammaproteobacteria>Methylococcales>Methylococcaceae>Methylobacter	GCA_023544625.1	Methylobacter_sp.	M8364_12865	42290..45730	2051955	BREX-system-P-loop-protein-BrxC-[Methylobacter-sp.]
WP_031438510.1	Type1.BREX	[illegible]	1218	Bacteria>Pseudomonadota>Gammaproteobacteria>Methylococcales>Methylococcaceae>Methylobacter	GCF_000733835.1	Methylobacter_tundripaludum	T449_RS0121480	4638570..4642226	173365	BREX-system-P-loop-protein-BrxC-[Methylobacter-tundripaludum]
WP_262965888.1	Type1.BREX	[illegible]	1212	Bacteria>Pseudomonadota>Gammaproteobacteria>Methylococcales>Methylococcaceae>Methylobacter	GCF_025583945.1	Methylobacter_psychrophilus	N7974_RS12230	10707..14345	96941	BREX-system-P-loop-protein-BrxC-[Methylobacter-psychrophilus]
WP_174625954.1	Type1.BREX	[illegible]	1149	Bacteria>Pseudomonadota>Gammaproteobacteria>Methylococcales>Methylococcaceae>Methylobacter	GCF_902806695.1	Candidatus_Methylobacter_favarea	HUX64_RS09295	14302..17751	2707345	BREX-system-P-loop-protein-BrxC-[Candidatus-Methylobacter-favarea]
WP_210024304.1	Type1.BREX	[illegible]	1199	Bacteria>Pseudomonadota>Gammaproteobacteria>Methylococcales>Methylococcaceae>Methylocaldum	GCF_017832375.1	Methylocaldum_sp._RMAD-M	JOD69_RS22430	17805..21404	2806557	BREX-system-P-loop-protein-BrxC-[Methylocaldum-sp.-RMAD-M]
MBE0434438.1	Type1.BREX	[illegible]	1177	Bacteria>Pseudomonadota>Gammaproteobacteria>Methylococcales>Methylococcaceae>Methylomicrobium	GCA_014859425.1	Methylomicrobium_sp.	IBX56_01390	2720..6253	2303549	BREX-system-P-loop-protein-BrxC-[Methylomicrobium-sp.]
MBE0436274.1	Type1.BREX	[illegible]	251	Bacteria>Pseudomonadota>Gammaproteobacteria>Methylococcales>Methylococcaceae>Methylomicrobium	GCA_014859425.1	Methylomicrobium_sp.	IBX56_10780	2140..2891	2303549	BREX-system-P-loop-protein-BrxC-[Methylomicrobium-sp.]
MBS3952115.1	Type1.BREX	[illegible]	1206	Bacteria>Pseudomonadota>Gammaproteobacteria>Methylococcales>Methylococcaceae>Methylomicrobium	GCA_018333785.1	Methylomicrobium_sp.	KGZ88_04105	40122..43742	2303549	BREX-system-P-loop-protein-BrxC-[Methylomicrobium-sp.]
MBS3952424.1	Type1.BREX	[illegible]	1178	Bacteria>Pseudomonadota>Gammaproteobacteria>Methylococcales>Methylococcaceae>Methylomicrobium	GCA_018333785.1	Methylomicrobium_sp.	KGZ88_05695	2093..5629	2303549	BREX-system-P-loop-protein-BrxC-[Methylomicrobium-sp.]

Protein-ID	System	Gene-Neighbourhoods	Length	Taxonomy	Assembly-ID	Strain	Locus-Tag	Genomic-coordinates	TaxID	Fasta-define
WP_024296674.1	Type1.BREX	GDResolvase+HIN-HTH->?->BrxA-DNA-binding->BrxB-BrxF-iSTAND->DUF6079*->ParD_antitoxin->PIN->N6-METHYLASE->SbcC->Schlafen+HATPase+wHTH->PglZ->SIGMA+OB-Fold+AAA-ATPase+Lon-Protease-BrxL->	1202	Bacteria>Pseudomonadota>Gammaproteobacteria>Methylococcales>Methylococcaceae>Methylomicrobium	GCF_000527095.1	Methylomicrobium_lacus	METLA_RS0100460	95992..99600	136992	BREX-system-P-loop-protein-BrxC-[Methylomicrobium-lacus]
MBS3964846.1	Type1.BREX	HTH_Tnp_1+HTH_21+rve+rve_3->BrxA-DNA-binding->BrxB-BrxF-iSTAND->DUF6079*->Calcineurin->SbcC+SbcC->SIR2->N6-METHYLASE->OLD-ABC->TOPRIM->DOC+HTH->PglZ->SIGMA+OB-Fold+AAA-ATPase+Lon-Protease-BrxL->	1209	Bacteria>Pseudomonadota>Gammaproteobacteria>Methylococcales>Methylococcaceae>Methylomonas	GCA_018333915.1	Methylomonas_sp.	KGZ80_10205	1968..5597	418	BREX-system-P-loop-protein-BrxC-[Methylomonas-sp.]
WP_256360610.1	Type1.BREX	<-SIGMA+OB-Fold+AAA-ATPase+HKD-DNase-BrxL<-PglZ<-REase<-REase<-HTH+REase<-N6-METHYLASE-p<-N6-METHYLASE-p<-N6-METHYLASE-p<-DUF6079*	1145	Bacteria>Pseudomonadota>Gammaproteobacteria>Methylococcales>Methylococcaceae>Methylomonas	GCF_001312005.1	Methylomonas_koyamae	JCM16701_RS06940	49293..52730	702114	BREX-system-P-loop-protein-BrxC-[Methylomonas-koyamae]
WP_071159674.1	Type1.BREX	<-SIGMA+OB-Fold+AAA-ATPase+Lon-Protease-BrxL<-PglZ<-HNH<-OLD-ABC<-N6-METHYLASE<-DUF6079*<-BrxB-BrxF-iSTAND<-BrxA-DNA-binding<-XIS-HTH||?-><-?<-?<-?<-?<-JAB+sGTP	1153	Bacteria>Pseudomonadota>Gammaproteobacteria>Methylococcales>Methylococcaceae>Methylomonas	GCF_001856095.1	Methylomonas_sp._LWB	BJL95_RS17210	210511..213972	1905845	BREX-system-P-loop-protein-BrxC-[Methylomonas-sp.-LWB]
WP_096876853.1	Type1.BREX	GDResolvase+HIN-HTH->?->XIS-HTH->BrxA-DNA-binding->BrxB-BrxF-iSTAND->DUF6079*->OLD-ABC->REase->N6-METHYLASE->HTH+REase->PglZ->SIGMA+OB-Fold+AAA-ATPase+HKD-DNase-BrxL->Calcineurin-SbcD->SbcC+CCDC158->SbcC->	1180	Bacteria>Pseudomonadota>Gammaproteobacteria>Methylococcales>Methylococcaceae>Methylomonas	GCF_002441955.1	Methylomonas_koyamae	MKLM6_RS22285	58076..61618	702114	BREX-system-P-loop-protein-BrxC-[Methylomonas-koyamae]
WP_221054675.1	Type1.BREX	DUF6079*->N6-METHYLASE->HTH+REase->REase->PglZ->SIGMA+OB-Fold+AAA-ATPase+HKD-DNase-BrxL->Calcineurin-SbcD->SbcC+CCDC158->OLD-ABC+TOPRIM->SF1-SF2-Helicase->	1176	Bacteria>Pseudomonadota>Gammaproteobacteria>Methylococcales>Methylococcaceae>Methylomonas	GCF_019669905.1	Methylomonas_koyamae	MKFW12EY_RS21895	239254..1338	702114	BREX-system-P-loop-protein-BrxC-[Methylomonas-koyamae]
WP_255188315.1	Type1.BREX	<-PIN<-PIN<-SIGMA+OB-Fold+AAA-ATPase+Lon-Protease-BrxL<-PglZ<-TOPRIM<-OLD-ABC<-N6-METHYLASE<-PIN<-ParD_antitoxin<-DUF6079*<-BrxB-BrxF-iSTAND<-BrxA-DNA-binding||?-><-?<-HIN-HTH||?-><-ACET+ACET<-TraJ-RHH	1206	Bacteria>Pseudomonadota>Gammaproteobacteria>Methylococcales>Methylococcaceae>Methylomonas	GCF_024360925.2	Methylomonas_rapida	NM686_RS13235	2825288..2828908	2963939	BREX-system-P-loop-protein-BrxC-[Methylomonas-rapida]
WP_255189595.1	Type1.BREX	<-DDE_Tnp_1_4||?-><-zf-ISL3+HTH_Tnp_ISL3+DDE_Tnp_ISL3||?->DDE_Tnp_1_4->Phage_integrase+Phage_integrase->35exo->wHTH+WYL+WCX->BrxB-BrxF-iSTAND->DUF6079*->Calcineurin->SbcC+SbcC->N6-METHYLASE-p->zf-ISL3+HTH_Tnp_ISL3+DDE_Tnp_ISL3->N6-METHYLASE->OLD-ABC->TOPRIM->DOC+HTH->PglZ->	1209	Bacteria>Pseudomonadota>Gammaproteobacteria>Methylococcales>Methylococcaceae>Methylomonas	GCF_024360925.2	Methylomonas_rapida	NM686_RS20105	4241783..4245412	2963939	BREX-system-P-loop-protein-BrxC-[Methylomonas-rapida]
WP_192375581.1	Type1.BREX	<-KAP-glycosylase<-KAP-NTPase<-PIN<-SIGMA+OB-Fold+AAA-ATPase+Lon-Protease-BrxL<-SIGMA+OB-Fold+AAA-ATPase+Lon-Protease-BrxL<-PglZ<-N6-METHYLASE<-PIN<-ParD_antitoxin<-DUF6079*<-BrxB-BrxF-iSTAND<-BrxA-DNA-binding||RNase-><-?<-GDResolvase+HIN-HTH	1207	Bacteria>Pseudomonadota>Gammaproteobacteria>Methylococcales>Methylococcaceae>Methylomonas	GCF_903064715.1	Methylomonas_albis	EBA_RS15675	3420343..3423966	1854563	BREX-system-P-loop-protein-BrxC-[Methylomonas-albis]
MBE0471466.1	Type1.BREX	<-N6-METHYLASE-p||SbcC->TOPRIM-><-DUF6079*<-BrxB-BrxF-iSTAND<-BrxA-DNA-binding	1214	Bacteria>Pseudomonadota>Gammaproteobacteria>Methylococcales>Methylococcaceae>Methyloprofundus	GCA_014859465.1	Methyloprofundus_sp.	IBX55_18395	2520..6164	2020875	BREX-system-P-loop-protein-BrxC-[Methyloprofundus-sp.]
WP_080523593.1	Type1.BREX	<-N6-METHYLASE-p||HTH_Tnp_1+HTH_21+rve+rve_3-><-N6-METHYLASE<-PIN<-?<-ACET+ACET<-BrxB-BrxF-iSTAND<-DUF6079*<-BrxB-BrxF-iSTAND<-BrxA-DNA-binding<-?<-?||GSDH-><-?<-BrxB-BrxF-iSTAND<-?<-PIN	1206	Bacteria>Pseudomonadota>Gammaproteobacteria>Methylococcales>Methylococcaceae>Methyloprofundus	GCF_002072955.1	Methyloprofundus_sedimenti	AU255_RS14210	277762..281382	1420851	BREX-system-P-loop-protein-BrxC-[Methyloprofundus-sedimenti]
MBZ4201977.1	Type1.BREX	<-DUF6079-p*<-BrxB-BrxF-iSTAND<-BrxA-DNA-binding||?-><-tRNA<-?<-RADICAL-SAM<-TPR+TPR+TPR	909	Bacteria>Pseudomonadota>Gammaproteobacteria>Methylococcales>Methylococcaceae>Methylovulum	GCA_020035215.1	Methylovulum_sp.	LAC70_02510	1..2727	1916980	BREX-system-P-loop-protein-BrxC-[Methylovulum-sp.]
MCF8007296.1	Type1.BREX	BrxA-DNA-binding->BrxB-BrxF-iSTAND->DUF6079-p*->N6-METHYLASE-p->?->PIN->PglZ-p-><-Aminotran_1_2	781	Bacteria>Pseudomonadota>Gammaproteobacteria>Methylococcales>Methylococcaceae>Methylovulum	GCA_021736885.1	Methylovulum_sp.	K9K84_07785	12351..14696	1916980	BREX-system-P-loop-protein-BrxC-[Methylovulum-sp.]
WP_088619247.1	Type1.BREX	<-Glycos_transf<-?<-SIGMA+OB-Fold+AAA-ATPase+Lon-Protease-BrxL<-PglZ<-Schlafen+HATPase+wHTH<-PHP+SbcC<-N6-METHYLASE<-PIN<-ParD_antitoxin<-DUF6079*<-BrxB-BrxF-iSTAND<-BrxA-DNA-binding<-wHTH+WYL+WCX	1201	Bacteria>Pseudomonadota>Gammaproteobacteria>Methylococcales>Methylococcaceae>Methylovulum	GCF_002209385.1	Methylovulum_psychrotolerans	CEK71_RS09965	2189788..2193393	1704499	BREX-system-P-loop-protein-BrxC-[Methylovulum-psychrotolerans]
WP_103975698.1	Type1.BREX	<-SIGMA+OB-Fold+AAA-ATPase+Lon-Protease-BrxL<-PglZ<-Schlafen+HATPase+wHTH<-PHP+SbcC<-?<-?<-N6-METHYLASE<-PIN<-ParD_antitoxin<-DUF6079*<-BrxB-BrxF-iSTAND<-BrxA-DNA-binding<-wHTH+WYL+WCX||?->SAM-METHYLASE->?->SbcC->SAM-METHYLASE->SAM-METHYLASE->	1201	Bacteria>Pseudomonadota>Gammaproteobacteria>Methylococcales>Methylococcaceae>Methylovulum	GCF_002923755.1	Methylovulum_psychrotolerans	AADEFJLK_RS21345	25888..29493	1704499	BREX-system-P-loop-protein-BrxC-[Methylovulum-psychrotolerans]
WP_215479507.1	Type1.BREX	wHTH+WYL+WCX-p->wHTH+WYL+WCX->BrxA-DNA-binding->BrxB-BrxF-iSTAND->DUF6079*->ParD_antitoxin->PIN->N6-METHYLASE->?->?->PHP+SbcC->Schlafen+HATPase+wHTH->PglZ->SIGMA+OB-Fold+AAA-ATPase+Lon-Protease-BrxL->	1201	Bacteria>Pseudomonadota>Gammaproteobacteria>Methylococcales>Methylococcaceae>Methylovulum	GCF_018722605.1	Methylovulum_psychrotolerans	KFZ76_RS21360	105012..108617	1704499	BREX-system-P-loop-protein-BrxC-[Methylovulum-psychrotolerans]
TSA38613.1	Type1.BREX	BrxA-DNA-binding->BrxB-BrxF-iSTAND->DUF6079*->PRTase->?->PIN->?->N6-METHYLASE-p->?->?->PIN->N6-METHYLASE-p->	1218	Bacteria>Pseudomonadota>Gammaproteobacteria>Methylococcales>Methylococcaceae>unclassified_Methylococcaceae	GCA_007280895.1	Methylococcaceae_bacterium	D4R63_10680	1521..5177	1933926	BREX-system-P-loop-protein-BrxC-[Methylococcaceae-bacterium]
MCF7971355.1	Type1.BREX	<-N6-METHYLASE-p<-ACET<-BrxB-BrxF-iSTAND<-REase<-DUF6079*<-BrxB-BrxF-iSTAND<-BrxA-DNA-binding<-XIS-HTH<-Phage_integrase<-tRNA<-ABC-ATPase+SbcC+ABC-ATPase+SbcC||REC->	1151	Bacteria>Pseudomonadota>Gammaproteobacteria>Methylococcales>Methylococcaceae>unclassified_Methylococcaceae	GCA_021736705.1	Methylococcaceae_bacterium	K9L22_09360	3141..6596	1933926	BREX-system-P-loop-protein-BrxC-[Methylococcaceae-bacterium]
RIZ69585.1	Type1.BREX	<-Pkinase||?->SLATT->ORC-CDC6->?-><-N6-METHYLASE-p<-?<-SIR2<-DUF6079-p*	799	Bacteria>Pseudomonadota>Gammaproteobacteria>Methylococcales>unclassified_Methylococcales	GCA_003584865.1	Methylococcales_bacterium	D0528_03735	16784..19183	2304002	BREX-system-P-loop-protein-BrxC-[Methylococcales-bacterium]
WP_104129248.1	Type1.BREX	<-DUF6079-p*<-BrxB-BrxF-iSTAND<-BrxA-DNA-binding<-wHTH+WYL+WCX||TetR-HTH-><-Cap4_nuclease<-TRD	739	Bacteria>Pseudomonadota>Gammaproteobacteria>Moraxellales>Moraxellaceae>Acinetobacter	-	Acinetobacter_baumannii	AbaMCR10126_RS06085	1..2216	470	BREX-system-P-loop-protein-BrxC,-partial-[Acinetobacter-baumannii]
EGY2544509.1	Type1.BREX	CR-ATPase5+REase-><-DUF4236||wHTH+WYL+WCX->BrxA-DNA-binding->BrxB-BrxF-iSTAND->DUF6079-p*->	942	Bacteria>Pseudomonadota>Gammaproteobacteria>Moraxellales>Moraxellaceae>Acinetobacter	-	Acinetobacter_baumannii	JHZ71_003464	4150..6977	470	BREX-system-P-loop-protein-BrxC-[Acinetobacter-baumannii]
EHN8630147.1	Type1.BREX	<-AbiGi+seqA+RAMA<-CHTH<-RelE<-DDE_Tnp_1_5+DDE_Tnp_1||CR-ATPase5+REase-><-DUF4236||wHTH+WYL+WCX->BrxA-DNA-binding->BrxB-BrxF-iSTAND->DUF6079-p*->	561	Bacteria>Pseudomonadota>Gammaproteobacteria>Moraxellales>Moraxellaceae>Acinetobacter	-	Acinetobacter_baumannii	KKI61_003746	8069..9753	470	BREX-system-P-loop-protein-BrxC-[Acinetobacter-baumannii]
EJC5774375.1	Type1.BREX	<-DUF6079-p*<-BrxB-BrxF-iSTAND<-BrxA-DNA-binding<-wHTH+WYL+WCX||DUF4236-><-DOC+HTH<-?<-ParB+HNH<-Phage_integrase+Phage_integrase<-Phage_integrase+Phage_integrase	881	Bacteria>Pseudomonadota>Gammaproteobacteria>Moraxellales>Moraxellaceae>Acinetobacter	-	Acinetobacter_baumannii	MZL57_003344	1..2643	470	BREX-system-P-loop-protein-BrxC-[Acinetobacter-baumannii]
WP_099476278.1	Type1.BREX	Phage_integrase+Phage_integrase->Phage_integrase+Phage_integrase->ParB+HNH->?->DOC+HTH-><-DUF4236||wHTH+WYL+WCX->BrxA-DNA-binding->BrxB-BrxF-iSTAND->DUF6079*->N6-METHYLASE->ParB+HNH->PglZ->SIGMA+OB-Fold+AAA-ATPase+Lon-Protease-BrxL->?->GDResolvase+HIN-HTH->	1226	Bacteria>Pseudomonadota>Gammaproteobacteria>Moraxellales>Moraxellaceae>Acinetobacter	-	Acinetobacter_pittii	HPG40_RS00205	37613..41293	48296	BREX-system-P-loop-protein-BrxC-[Acinetobacter-pittii]
WP_252316424.1	Type1.BREX	<-DDE_Tnp_1_5+DDE_Tnp_1||?->DOC+HTH->||wHTH+WYL+WCX->BrxA-DNA-binding->BrxB-BrxF-iSTAND->DUF6079*->N6-METHYLASE->?->PglZ->SIGMA+OB-Fold+AAA-ATPase+Lon-Protease-BrxL->SF1-SF2-Helicase+REase->OLD-ABC+TOPRIM-><-PhdYeFM_antitox	1212	Bacteria>Pseudomonadota>Gammaproteobacteria>Moraxellales>Moraxellaceae>Acinetobacter	-	Acinetobacter_sp._225588	J687_RS15310	12712..16350	1310788	BREX-system-P-loop-protein-BrxC-[Acinetobacter-sp.-225588]
WP_019836725.1	Type1.BREX	RelB-><-TPR+TPR<-?||<-SIGMA+OB-Fold+AAA-ATPase+Lon-Protease-BrxL<-PglZ<-ParB+HNH<-N6-METHYLASE<-DUF6079*<-BrxB-BrxF-iSTAND<-BrxA-DNA-binding<-wHTH+WYL+WCX||DUF4236-><-CR-ATPase5+REase<-ParB+HNH||PAIREDC-HTH->	1226	Bacteria>Pseudomonadota>Gammaproteobacteria>Moraxellales>Moraxellaceae>Acinetobacter	-	Acinetobacter_sp._MDS7A	H048_RS0104285	13386..17066	1209355	BREX-system-P-loop-protein-BrxC-[Acinetobacter-sp.-MDS7A]
WP_005303500.1	Type1.BREX	ZnR+ZnR->DUF932->?->?-><-?<-TetR-HTH||wHTH+WYL+WCX->BrxA-DNA-binding->BrxB-BrxF-iSTAND->DUF6079*->N6-METHYLASE->ParB+HNH->PglZ->SIGMA+OB-Fold+AAA-ATPase+Lon-Protease-BrxL->SF1-SF2-Helicase+REase->DarT+Peptidase->MACRODOMAIN->?-><-Peptidase_M48	1226	Bacteria>Pseudomonadota>Gammaproteobacteria>Moraxellales>Moraxellaceae>Acinetobacter	-	Acinetobacter_sp._NIPH_542	F886_RS07830	464704..468384	1217688	BREX-system-P-loop-protein-BrxC-[Acinetobacter-sp.-NIPH-542]
CAM84763.1	Type1.BREX	<-OLD-ABC+TOPRIM<-HIN-HTH<-SF1-SF2-Helicase+REase+HTH<-SIGMA+OB-Fold+AAA-ATPase+Lon-Protease-BrxL<-PglZ<-?<-N6-METHYLASE<-DUF6079*<-BrxB-BrxF-iSTAND<-BrxA-DNA-binding<-wHTH+WYL+WCX||<-?||DDE_Tnp_1_5+DDE_Tnp_1-><-?<-?<-?||TRPR-HTH->	1229	Bacteria>Pseudomonadota>Gammaproteobacteria>Moraxellales>Moraxellaceae>Acinetobacter	GCA_000069245.1	Acinetobacter_baumannii_AYE	p3ABAYE0070	63476..67165	509173	putative-ATPase-like-protein-[Acinetobacter-baumannii-AYE]
EEY88530.1	Type1.BREX	HTH_Tnp_1+HTH_21+rve+rve_3->ParB+HNH->CR-ATPase5+REase->ParB+HNH-p->?-><-DUF4236||wHTH+WYL+WCX->BrxA-DNA-binding->BrxB-BrxF-iSTAND->DUF6079*->N6-METHYLASE-p->N6-METHYLASE->PglZ->SIGMA+OB-Fold+AAA-ATPase+Lon-Protease-BrxL->	793	Bacteria>Pseudomonadota>Gammaproteobacteria>Moraxellales>Moraxellaceae>Acinetobacter	GCA_000162095.1	Acinetobacter_lwoffii_SH145	HMPREF0017_02853	41683..44064	575588	hypothetical-protein-HMPREF0017_02853-[Acinetobacter-lwoffii-SH145]
ENX11167.1	Type1.BREX	CR-ATPase5+REase-><-DUF4236||wHTH+WYL+WCX->BrxA-DNA-binding->BrxB-BrxF-iSTAND->DUF6079-p-><-HTH_Tnp_1+HTH_21+rve+rve_3<-HIN-HTH||DUF6079-p*->N6-METHYLASE->ParB+HNH->PglZ->SIGMA+OB-Fold+AAA-ATPase+Lon-Protease-BrxL-><-?<-AbiEii<-AbiEii<-MarR-HTH<-DEOR-HTH	911	Bacteria>Pseudomonadota>Gammaproteobacteria>Moraxellales>Moraxellaceae>Acinetobacter	GCA_000369645.1	Acinetobacter_sp._CIP_64.2	F895_03860	13889..16624	1217694	hypothetical-protein-F895_03860-[Acinetobacter-sp.-CIP-64.2]
EXS00102.1	Type1.BREX	<-DDE_Tnp_1_5+DDE_Tnp_1||?->DOC+HTH->wHTH+WYL+WCX->BrxA-DNA-binding->BrxB-BrxF-iSTAND->DUF6079*->N6-METHYLASE->?->PglZ->SIGMA+OB-Fold+AAA-ATPase+Lon-Protease-BrxL->SF1-SF2-Helicase+REase->HIN-HTH->HTH_Tnp_1+HTH_21+rve+rve_3->OLD-ABC+TOPRIM-><-PhdYeFM_antitox	1190	Bacteria>Pseudomonadota>Gammaproteobacteria>Moraxellales>Moraxellaceae>Acinetobacter	GCA_000588415.1	Acinetobacter_sp._225588	J687_2205	12778..16350	1310788	hypothetical-protein-J687_2205-[Acinetobacter-sp.-225588]
AZB97741.1	Type1.BREX	HTH_37-><-DEOR-HTH||?-><-?||<-DUF6079-p<-DUF6079-p<-DUF6079-p<-DUF6079-p*	728	Bacteria>Pseudomonadota>Gammaproteobacteria>Moraxellales>Moraxellaceae>Acinetobacter	GCA_003359275.2	Acinetobacter_pittii	DKE42_019155	44169..46355	48296	BREX-system-P-loop-protein-BrxC-[Acinetobacter-pittii]
HAV3580163.1	Type1.BREX	<-MACRODOMAIN<-DarT+Peptidase<-SF1-SF2-Helicase+REase<-SIGMA+OB-Fold+AAA-ATPase+Lon-Protease-BrxL<-PglZ<-ParB+HNH<-N6-METHYLASE<-DUF6079*<-BrxB-BrxF-iSTAND<-BrxA-DNA-binding<-wHTH+WYL+WCX||TetR-HTH->?-><-?<-?<-?<-DUF932	1226	Bacteria>Pseudomonadota>Gammaproteobacteria>Moraxellales>Moraxellaceae>Acinetobacter	GCA_016485765.1	Acinetobacter_baumannii	JIE63_001443	180845..184525	470	BREX-system-P-loop-protein-BrxC-[Acinetobacter-baumannii]
HAV4485227.1	Type1.BREX	<-HIN-HTH<-DUF4158+DDE_Tnp_Tn3||GDResolvase+HIN-HTH->CR-ATPase5+REase-><-DUF4236||wHTH+WYL+WCX->BrxA-DNA-binding->BrxB-BrxF-iSTAND->DUF6079-p*->	759	Bacteria>Pseudomonadota>Gammaproteobacteria>Moraxellales>Moraxellaceae>Acinetobacter	GCA_016498165.1	Acinetobacter_baumannii	JIR97_15920	19224..21501	470	BREX-system-P-loop-protein-BrxC-[Acinetobacter-baumannii]
HAV4527427.1	Type1.BREX	<-HIN-HTH<-DUF4158+DDE_Tnp_Tn3||GDResolvase+HIN-HTH->CR-ATPase5+REase-><-DUF4236||wHTH+WYL+WCX->BrxA-DNA-binding->BrxB-BrxF-iSTAND->DUF6079-p*->	759	Bacteria>Pseudomonadota>Gammaproteobacteria>Moraxellales>Moraxellaceae>Acinetobacter	GCA_016498325.1	Acinetobacter_baumannii	JIR72_17730	19224..21501	470	BREX-system-P-loop-protein-BrxC-[Acinetobacter-baumannii]
HAV4540658.1	Type1.BREX	Phage_integrase+Phage_integrase->Phage_integrase+Phage_integrase->ParB+HNH->?->DOC+HTH-><-DUF4236||wHTH+WYL+WCX->BrxA-DNA-binding->BrxB-BrxF-iSTAND->DUF6079*->N6-METHYLASE->PglZ->SIGMA+OB-Fold+AAA-ATPase+Lon-Protease-BrxL-><-?<-ABhydrolase<-Classical-AAA<-?<-SMODS	1226	Bacteria>Pseudomonadota>Gammaproteobacteria>Moraxellales>Moraxellaceae>Acinetobacter	GCA_016498465.1	Acinetobacter_baumannii	JIS19_16850	25447..29127	470	BREX-system-P-loop-protein-BrxC-[Acinetobacter-baumannii]
HAV4891449.1	Type1.BREX	<-HIN-HTH<-DUF4158+DDE_Tnp_Tn3||GDResolvase+HIN-HTH->CR-ATPase5+REase-><-DUF4236||wHTH+WYL+WCX->BrxA-DNA-binding->BrxB-BrxF-iSTAND->DUF6079-p*->	676	Bacteria>Pseudomonadota>Gammaproteobacteria>Moraxellales>Moraxellaceae>Acinetobacter	GCA_016503425.1	Acinetobacter_baumannii	JIU86_16385	19224..21252	470	BREX-system-P-loop-protein-BrxC-[Acinetobacter-baumannii]
HAV4979216.1	Type1.BREX	<-HTH_Tnp_1+HTH_21+rve+rve_3||<-HTH_3<-HigB_toxin||CR-ATPase5+REase-><-DUF4236||wHTH+WYL+WCX->BrxA-DNA-binding->BrxB-BrxF-iSTAND->DUF6079*->N6-METHYLASE->SF1-SF2-Helicase+PRTase->SLOG+BirA-HTH->ParB+HNH->PglZ->DDE_Tnp_IS240-><-?<-PAS+NtrC-AAA+FIS-HTH	1226	Bacteria>Pseudomonadota>Gammaproteobacteria>Moraxellales>Moraxellaceae>Acinetobacter	GCA_016507565.1	Acinetobacter_baumannii	JIZ05_14385	7251..10931	470	BREX-system-P-loop-protein-BrxC-[Acinetobacter-baumannii]
HAV5318348.1	Type1.BREX	Cap4_nuclease->||wHTH+WYL+WCX->BrxA-DNA-binding->BrxB-BrxF-iSTAND->DUF6079*->DarT+Peptidase->MACRODOMAIN-><-?<-Peptidase_M48<-?||?-><-?||TetR-HTH->Phage_GPD+Phage_base_V+T6SS_Vgr+DUF2345->	1226	Bacteria>Pseudomonadota>Gammaproteobacteria>Moraxellales>Moraxellaceae>Acinetobacter	GCA_016510035.1	Acinetobacter_baumannii	JI043_16030	3866..7546	470	BREX-system-P-loop-protein-BrxC-[Acinetobacter-baumannii]
HAV6134262.1	Type1.BREX	ZnR+DUF3482+ZnR-><-PIN+OST-HTH||DUF932->?->?->?->||wHTH+WYL+WCX->BrxA-DNA-binding->BrxB-BrxF-iSTAND->DUF6079*->N6-METHYLASE->SF1-SF2-Helicase+PRTase->SLOG+BirA-HTH->ParB+HNH->PglZ->SIGMA+OB-Fold+AAA-ATPase+Lon-Protease-BrxL->SF1-SF2-Helicase+REase->DarT+Peptidase->MACRODOMAIN->	1226	Bacteria>Pseudomonadota>Gammaproteobacteria>Moraxellales>Moraxellaceae>Acinetobacter	GCA_016514395.1	Acinetobacter_baumannii	JI295_12625	9155..12835	470	BREX-system-P-loop-protein-BrxC-[Acinetobacter-baumannii]
EHU1604380.1	Type1.BREX	HTH_Tnp_1+HTH_21+rve+rve_3->ParB+HNH->CR-ATPase5+REase-><-DUF4236||wHTH+WYL+WCX->BrxA-DNA-binding->BrxB-BrxF-iSTAND->DUF6079*->N6-METHYLASE->PglZ->SIGMA+OB-Fold+AAA-ATPase+Lon-Protease-BrxL-><-DDE_Tnp_IS240	1226	Bacteria>Pseudomonadota>Gammaproteobacteria>Moraxellales>Moraxellaceae>Acinetobacter	GCA_016522135.2	Acinetobacter_baumannii	A1Z38_RS18510	4859..8539	470	BREX-system-P-loop-protein-BrxC-[Acinetobacter-baumannii]
EHU2434196.1	Type1.BREX	DDE_Tnp_IS240-><-SIGMA+OB-Fold+AAA-ATPase+Lon-Protease-BrxL<-PglZ<-?<-N6-METHYLASE<-DUF6079*<-BrxB-BrxF-iSTAND<-BrxA-DNA-binding<-wHTH+WYL+WCX||DUF4236->||DDE_Tnp_1_5+DDE_Tnp_1->RelE->CHTH->AbiGi+seqA+RAMA->	1226	Bacteria>Pseudomonadota>Gammaproteobacteria>Moraxellales>Moraxellaceae>Acinetobacter	GCA_016527835.2	Acinetobacter_baumannii	A0165_RS18865	10464..14144	470	BREX-system-P-loop-protein-BrxC-[Acinetobacter-baumannii]
GIT84604.1	Type1.BREX	<-N6-METHYLASE-p<-DUF6079*<-BrxB-BrxF-iSTAND<-BrxA-DNA-binding<-wHTH+WYL+WCX||DUF4236->	1224	Bacteria>Pseudomonadota>Gammaproteobacteria>Moraxellales>Moraxellaceae>Acinetobacter	GCA_018403785.1	Acinetobacter_seohaensis	DSM16313_23860	1741..5415	281376	hypothetical-protein-DSM16313_23860-[Acinetobacter-seohaensis]
BCZ16231.1	Type1.BREX	<-HTH_21+rve+rve_3<-SIGMA+OB-Fold+AAA-ATPase+Lon-Protease-BrxL<-HTH_Tnp_1+HTH_21+rve+rve_3<-HIN-HTH<-SIGMA+OB-Fold+AAA-ATPase+Lon-Protease-BrxL-p<-PglZ<-ParB+HNH<-N6-METHYLASE<-DUF6079*<-HTH_Tnp_1+HTH_21+rve+rve_3<-HIN-HTH<-DUF6079-p<-BrxB-BrxF-iSTAND<-BrxA-DNA-binding<-wHTH+WYL+WCX||DUF4236-><-CR-ATPase5+REase	1142	Bacteria>Pseudomonadota>Gammaproteobacteria>Moraxellales>Moraxellaceae>Acinetobacter	GCA_019703285.1	Acinetobacter_baumannii	OCUAc18_37710	57364..60792	470	hypothetical-protein-OCUAc18_37710-[Acinetobacter-baumannii]
UDM39811.1	Type1.BREX	DDE_Tnp_1->?->ABC-ATPase+SbcC->?->?->wHTH+WYL+WCX->BrxA-DNA-binding->BrxB-BrxF-iSTAND->DUF6079*->N6-METHYLASE->||?->MACRODOMAIN-><-DOC<-?<-?||DDE_Tnp_1->	1226	Bacteria>Pseudomonadota>Gammaproteobacteria>Moraxellales>Moraxellaceae>Acinetobacter	GCA_020616635.1	Acinetobacter_haemolyticus	LIS44_16805	284403..288083	29430	BREX-system-P-loop-protein-BrxC-[Acinetobacter-haemolyticus]
MCE1270820.1	Type1.BREX	CR-ATPase5+REase-><-DUF4236||wHTH+WYL+WCX->BrxA-DNA-binding->BrxB-BrxF-iSTAND->DUF6079*->N6-METHYLASE->PglZ->SIGMA+OB-Fold+AAA-ATPase+Lon-Protease-BrxL->?-><-?||SIGMA-HTH->HTH_3->Peptidase+SF1-SF2-Helicase+REase->	1226	Bacteria>Pseudomonadota>Gammaproteobacteria>Moraxellales>Moraxellaceae>Acinetobacter	GCA_021287125.1	Acinetobacter_sp.	LWW88_04505	5070..8750	472	BREX-system-P-loop-protein-BrxC-[Acinetobacter-sp.]
HCA4923662.1	Type1.BREX	Peptidase_M48-><-HIN-HTH<-?||<-SIGMA+OB-Fold+AAA-ATPase+Lon-Protease-BrxL<-PglZ<-ParB+HNH<-N6-METHYLASE<-DUF6079*<-BrxB-BrxF-iSTAND<-BrxA-DNA-binding<-wHTH+WYL+WCX||TetR-HTH-><-?<-?<-Cap4_nuclease<-TRD	1226	Bacteria>Pseudomonadota>Gammaproteobacteria>Moraxellales>Moraxellaceae>Acinetobacter	GCA_022931145.1	Acinetobacter_baumannii	MW112_001902	190856..194536	470	BREX-system-P-loop-protein-BrxC-[Acinetobacter-baumannii]
USI88235.1	Type1.BREX	<-ParB<-ParA-Soj-PloopNTPase||?->CR-ATPase5+REase-><-DUF4236||wHTH+WYL+WCX->BrxA-DNA-binding->BrxB-BrxF-iSTAND->DUF6079*->N6-METHYLASE->ParB+HNH->PglZ->SIGMA+OB-Fold+AAA-ATPase+Lon-Protease-BrxL->SF1-SF2-Helicase+REase-><-?<-AA_permease	1226	Bacteria>Pseudomonadota>Gammaproteobacteria>Moraxellales>Moraxellaceae>Acinetobacter	GCA_023822225.1	Acinetobacter_johnsonii	LZ086_18665	10226..13906	40214	BREX-system-P-loop-protein-BrxC-[Acinetobacter-johnsonii]
WP_136143645.1	Type1.BREX	SMODS->?->Classical-AAA->ABhydrolase->?-><-SIGMA+OB-Fold+AAA-ATPase+Lon-Protease-BrxL<-PglZ<-HKD-DNase+DUF3150<-N6-METHYLASE<-DUF6079*<-BrxB-BrxF-iSTAND<-BrxA-DNA-binding<-wHTH+WYL+WCX||DUF4236-><-DOC+HTH<-?<-ParB+HNH<-Phage_integrase+Phage_integrase<-Phage_integrase+Phage_integrase	1226	Bacteria>Pseudomonadota>Gammaproteobacteria>Moraxellales>Moraxellaceae>Acinetobacter	GCA_030820635.1	Acinetobacter_towneri	EQQ52_RS02450	14374..18054	202956	BREX-system-P-loop-protein-BrxC-[Acinetobacter-towneri]
CAI3142244.1	Type1.BREX	<-AA_permease<-?<-?||GNTR-HTH+Aminotran_1_2-><-HTH_Tnp_1||?-><-?<-TraI-VirD2-RCRE||DUF6079-p*->HIN-HTH->HTH_Tnp_1+HTH_21+rve+rve_3->DDE_Tnp_1_5+DDE_Tnp_1->	416	Bacteria>Pseudomonadota>Gammaproteobacteria>Moraxellales>Moraxellaceae>Acinetobacter	GCA_947045015.1	Acinetobacter_baumannii	MWMV16_MWMV16_03034	31325..32575	470	hypothetical-protein-MWMV16_MWMV16_03034-[Acinetobacter-baumannii]
WP_001021844.1	Type1.BREX	PhdYeFM_antitox-><-OLD-ABC+TOPRIM<-SF1-SF2-Helicase+REase<-SIGMA+OB-Fold+AAA-ATPase+Lon-Protease-BrxL<-PglZ<-?<-N6-METHYLASE<-DUF6079*<-BrxB-BrxF-iSTAND<-BrxA-DNA-binding<-wHTH+WYL+WCX||<-DOC+HTH<-?||DDE_Tnp_1_5+DDE_Tnp_1->	1226	Bacteria>Pseudomonadota>Gammaproteobacteria>Moraxellales>Moraxellaceae>Acinetobacter	GCF_000069245.1	Acinetobacter_calcoaceticus/baumannii_complex	ABAYE_RS00380	63476..67156	909768	MULTISPECIES:-BREX-system-P-loop-protein-BrxC-[Acinetobacter-calcoaceticus/baumannii-complex]
WP_005401825.1	Type1.BREX	<-SIGMA+OB-Fold+AAA-ATPase+Lon-Protease-BrxL<-PglZ<-ParB+HNH<-N6-METHYLASE<-DUF6079*<-BrxB-BrxF-iSTAND<-BrxA-DNA-binding<-wHTH+WYL+WCX||DUF4236-><-?<-ParB+HNH-p<-CR-ATPase5+REase||HicA->HicB->	1226	Bacteria>Pseudomonadota>Gammaproteobacteria>Moraxellales>Moraxellaceae>Acinetobacter	GCF_000162055.1	Acinetobacter_johnsonii	HMPREF0016_RS15525	47644..51324	40214	BREX-system-P-loop-protein-BrxC-[Acinetobacter-johnsonii]
WP_010116441.1	Type1.BREX	<-SIGMA+OB-Fold+AAA-ATPase+Lon-Protease-BrxL<-PglZ<-ParB+HNH<-N6-METHYLASE<-DUF6079*<-BrxB-BrxF-iSTAND<-BrxA-DNA-binding<-wHTH+WYL+WCX||DUF4236-><-CR-ATPase5+REase<-ParB+HNH	1226	Bacteria>Pseudomonadota>Gammaproteobacteria>Moraxellales>Moraxellaceae>Acinetobacter	GCF_000214135.1	Acinetobacter_sp._P8-3-8	GGG_RS0115320	12069..15749	1029823	BREX-system-P-loop-protein-BrxC-[Acinetobacter-sp.-P8-3-8]
WP_017400109.1	Type1.BREX	Peptidase_A8->DDE_Tnp_1_5+DDE_Tnp_1->?->ParB+HNH->CR-ATPase5+REase-><-DUF4236||wHTH+WYL+WCX->BrxA-DNA-binding->BrxB-BrxF-iSTAND->DUF6079*->DUF4096+DDE_Tnp_1+DDE_Tnp_1_2->?->PglZ->SIGMA+OB-Fold+AAA-ATPase+Lon-Protease-BrxL->||DDE_Tnp_IS240-><-?<-ParA-Soj-PloopNTPase	1226	Bacteria>Pseudomonadota>Gammaproteobacteria>Moraxellales>Moraxellaceae>Acinetobacter	GCF_000302375.1	Acinetobacter_calcoaceticus/baumannii_complex	E8E07_RS19610	55428..59108	909768	MULTISPECIES:-BREX-system-P-loop-protein-BrxC-[Acinetobacter-calcoaceticus/baumannii-complex]
WP_001021843.1	Type1.BREX	<-MACRODOMAIN<-DarT+Peptidase<-SF1-SF2-Helicase+REase<-SIGMA+OB-Fold+AAA-ATPase+Lon-Protease-BrxL<-PglZ<-ParB+HNH<-SLOG+BirA-HTH<-SF1-SF2-Helicase+PRTase<-N6-METHYLASE<-DUF6079*<-BrxB-BrxF-iSTAND<-BrxA-DNA-binding<-wHTH+WYL+WCX||TetR-HTH-><-Cap4_nuclease<-TRD	1226	Bacteria>Pseudomonadota>Gammaproteobacteria>Moraxellales>Moraxellaceae>Acinetobacter	GCF_000305235.1	Acinetobacter_baumannii	ACIN5098_RS09930	47279..50959	470	BREX-system-P-loop-protein-BrxC-[Acinetobacter-baumannii]
WP_019383579.1	Type1.BREX	DDE_Tnp_1_5+DDE_Tnp_1->CR-ATPase5+REase-><-DUF4236||wHTH+WYL+WCX->BrxA-DNA-binding->BrxB-BrxF-iSTAND->DUF6079*->N6-METHYLASE->PglZ->SIGMA+OB-Fold+AAA-ATPase+Lon-Protease-BrxL->?->SMODS-><-?||<-DDE_Tnp_1_3||GDResolvase+HIN-HTH->	1226	Bacteria>Pseudomonadota>Gammaproteobacteria>Moraxellales>Moraxellaceae>Acinetobacter	GCF_000308235.1	Acinetobacter_venetianus	A489_RS0100450	77471..81151	52133	BREX-system-P-loop-protein-BrxC-[Acinetobacter-venetianus]
WP_019384245.1	Type1.BREX	<-Calcineurin||ParB+HNH->SAM-METHYLASE->?->SF1-SF2-Helicase->wHTH+HNH->wHTH+WYL+WCX->BrxA-DNA-binding->BrxB-BrxF-iSTAND->DUF6079*->N6-METHYLASE->PglZ->SIGMA+OB-Fold+AAA-ATPase+Lon-Protease-BrxL->?->?->NERD+Pkinase+Pkinase+SF1-SF2-Helicase->	1225	Bacteria>Pseudomonadota>Gammaproteobacteria>Moraxellales>Moraxellaceae>Acinetobacter	GCF_000308235.1	Acinetobacter_venetianus	A489_RS0105135	855128..858805	52133	BREX-system-P-loop-protein-BrxC-[Acinetobacter-venetianus]
WP_004758928.1	Type1.BREX	<-DDE_Tnp_1<-HIN-HTH<-SIGMA+OB-Fold+AAA-ATPase+Lon-Protease-BrxL<-PglZ<-N6-METHYLASE<-DUF6079*<-BrxB-BrxF-iSTAND<-BrxA-DNA-binding<-wHTH+WYL+WCX	1226	Bacteria>Pseudomonadota>Gammaproteobacteria>Moraxellales>Moraxellaceae>Acinetobacter	GCF_000368305.1	Acinetobacter_sp._CIP_102129	F973_RS14130	48127..51807	1144664	BREX-system-P-loop-protein-BrxC-[Acinetobacter-sp.-CIP-102129]
WP_004811070.1	Type1.BREX	HTH_Tnp_4+DDE_Tnp_4-><-?<-SF1-SF2-Helicase+REase<-SIGMA+OB-Fold+AAA-ATPase+Lon-Protease-BrxL<-PglZ<-ParB+HNH<-SLOG+BirA-HTH<-SF1-SF2-Helicase+PRTase<-N6-METHYLASE<-DUF6079*<-BrxB-BrxF-iSTAND<-BrxA-DNA-binding<-wHTH+WYL+WCX<-wHTH(SBD)+HNH<-SAM-METHYLASE<-XIS-HTH<-?<-?<-DUF932	1225	Bacteria>Pseudomonadota>Gammaproteobacteria>Moraxellales>Moraxellaceae>Acinetobacter	GCF_000368465.1	Acinetobacter_schindleri	F965_RS04725	393977..397654	108981	BREX-system-P-loop-protein-BrxC-[Acinetobacter-schindleri]
WP_004827026.1	Type1.BREX	<-HIN-HTH||MarR-HTH->AbiEii->?->HIN-HTH-><-SIGMA+OB-Fold+AAA-ATPase+Lon-Protease-BrxL<-PglZ<-N6-METHYLASE<-DUF6079*<-BrxB-BrxF-iSTAND<-BrxA-DNA-binding<-wHTH+WYL+WCX||DUF4236-><-CR-ATPase5+REase||?-><-ParB+HNH<-ParB	1226	Bacteria>Pseudomonadota>Gammaproteobacteria>Moraxellales>Moraxellaceae>Acinetobacter	GCF_000368505.1	Acinetobacter_bereziniae	F963_00144	48426..52106	106648	BREX-system-P-loop-protein-BrxC-[Acinetobacter-bereziniae]
WP_004984466.1	Type1.BREX	ZnR+ZnR->DUF932->?->?->XIS-HTH->BrxA-DNA-binding->BrxB-BrxF-iSTAND->DUF6079*->OLD-ABC->REase->N6-METHYLASE->PglZ->SIGMA+OB-Fold+AAA-ATPase+HKD-DNase-BrxL->?->?->ABC-ATPase+SbcC-><-ArsR-HTH	1190	Bacteria>Pseudomonadota>Gammaproteobacteria>Moraxellales>Moraxellaceae>Acinetobacter	GCF_000368805.1	Acinetobacter_johnsonii	F946_RS15710	148915..152487	40214	BREX-system-P-loop-protein-BrxC-[Acinetobacter-johnsonii]
WP_005245153.1	Type1.BREX	<-GDResolvase+HIN-HTH||?->BrnT_toxin->BrnA_antitoxin-><-?||?-><-SIGMA+OB-Fold+AAA-ATPase+Lon-Protease-BrxL<-PglZ<-N6-METHYLASE<-DUF6079*<-BrxB-BrxF-iSTAND<-BrxA-DNA-binding<-wHTH+WYL+WCX||DUF4236-><-CR-ATPase5+REase<-ParB+HNH<-?||RelB->RelE-ParE->	1226	Bacteria>Pseudomonadota>Gammaproteobacteria>Moraxellales>Moraxellaceae>Acinetobacter	GCF_000369665.1	Acinetobacter_sp._CIP_51.11	F894_RS00155	31081..34761	1144670	BREX-system-P-loop-protein-BrxC-[Acinetobacter-sp.-CIP-51.11]
WP_005247073.1	Type1.BREX	<-DsbC+DsbD+Thioredoxin_7||REC+OmpR-HTH->HISKIN-><-SF1-SF2-Helicase+REase<-SIGMA+OB-Fold+AAA-ATPase+Lon-Protease-BrxL<-PglZ<-N6-METHYLASE<-DUF6079*<-BrxB-BrxF-iSTAND<-BrxA-DNA-binding<-wHTH+WYL+WCX<-wHTH+HNH<-SAM-METHYLASE<-XIS-HTH<-?<-?<-DUF932	1226	Bacteria>Pseudomonadota>Gammaproteobacteria>Moraxellales>Moraxellaceae>Acinetobacter	GCF_000369665.1	Acinetobacter_sp._CIP_51.11	F894_RS08220	945028..948708	1144670	BREX-system-P-loop-protein-BrxC-[Acinetobacter-sp.-CIP-51.11]
WP_005253593.1	Type1.BREX	HTH_Tnp_1+HTH_21+rve+rve_3->ParB+HNH->CR-ATPase5+REase->ParB+HNH-p->?-><-DUF4236||wHTH+WYL+WCX->BrxA-DNA-binding->BrxB-BrxF-iSTAND->DUF6079*->N6-METHYLASE->PglZ->SIGMA+OB-Fold+AAA-ATPase+Lon-Protease-BrxL->||?-><-DDE_Tnp_IS240<-DDE_Tnp_1_3	1226	Bacteria>Pseudomonadota>Gammaproteobacteria>Moraxellales>Moraxellaceae>Acinetobacter	GCF_000369685.1	Acinetobacter_sp._CIP_102136	F893_RS16420	115413..119093	1144665	BREX-system-P-loop-protein-BrxC-[Acinetobacter-sp.-CIP-102136]
WP_005264232.1	Type1.BREX	DEOR-HTH-><-?<-?<-ABhydrolase||MerR-HTH-><-SIGMA+OB-Fold+AAA-ATPase+Lon-Protease-BrxL<-PglZ<-SbcC<-N6-METHYLASE<-DUF6079*<-BrxB-BrxF-iSTAND<-BrxA-DNA-binding<-wHTH+WYL+WCX||DUF4236-><-?<-ParB+HNH-p<-CR-ATPase5+REase<-ParB+HNH<-HTH_Tnp_1+HTH_21+rve+rve_3	1226	Bacteria>Pseudomonadota>Gammaproteobacteria>Moraxellales>Moraxellaceae>Acinetobacter	GCF_000369725.1	Acinetobacter_sp._CIP_101966	F891_RS14635	23007..26687	1144662	BREX-system-P-loop-protein-BrxC-[Acinetobacter-sp.-CIP-101966]
WP_016539314.1	Type1.BREX	<-wHTH+WYL+WCX<-HAD+HAD<-?||S1+RNase->?->tRNA->tRNA->tRNA->tRNA-><-DOC+HTH<-PglZ<-N6-METHYLASE<-DUF6079*<-BrxB-BrxF-iSTAND<-BrxA-DNA-binding<-?<-?<-DUF932<-ZnR+ZnR||?->?->ANK+ANK->	1248	Bacteria>Pseudomonadota>Gammaproteobacteria>Moraxellales>Moraxellaceae>Acinetobacter	GCF_000414055.1	Acinetobacter_guillouiae	Agu01S_RS05845	113550..117296	106649	BREX-system-P-loop-protein-BrxC-[Acinetobacter-guillouiae]
WP_023013360.1	Type1.BREX	<-SIGMA+OB-Fold+AAA-ATPase+Lon-Protease-BrxL<-PglZ<-HKD-DNase+DUF3150<-N6-METHYLASE<-DUF6079*<-BrxB-BrxF-iSTAND<-BrxA-DNA-binding<-wHTH+WYL+WCX<-wHTH+HNH<-SF1-SF2-Helicase<-?<-SAM-METHYLASE<-JAB	1226	Bacteria>Pseudomonadota>Gammaproteobacteria>Moraxellales>Moraxellaceae>Acinetobacter	GCF_000475335.1	Acinetobacter_sp._COS3	ID68_RS25385	11690..15370	1397525	BREX-system-P-loop-protein-BrxC-[Acinetobacter-sp.-COS3]
WP_031946304.1	Type1.BREX	DUF932->?->?->?-><-?<-TetR-HTH||wHTH+WYL+WCX->BrxA-DNA-binding->BrxB-BrxF-iSTAND->DUF6079*->N6-METHYLASE->ParB+HNH->PglZ->SIGMA+OB-Fold+AAA-ATPase+Lon-Protease-BrxL->SF1-SF2-Helicase+REase->DarT+Peptidase->MACRODOMAIN->	1226	Bacteria>Pseudomonadota>Gammaproteobacteria>Moraxellales>Moraxellaceae>Acinetobacter	GCF_000516575.2	Acinetobacter_baumannii	UN97_RS12380	246428..250108	470	BREX-system-P-loop-protein-BrxC-[Acinetobacter-baumannii]
WP_034686569.1	Type1.BREX	CR-ATPase5+REase-><-DUF4236||wHTH+WYL+WCX->BrxA-DNA-binding->BrxB-BrxF-iSTAND->DUF6079*->N6-METHYLASE->ParB+HNH->	1226	Bacteria>Pseudomonadota>Gammaproteobacteria>Moraxellales>Moraxellaceae>Acinetobacter	GCF_000581035.1	Acinetobacter_sp._869535	J520_RS12320	4406..8086	1310621	BREX-system-P-loop-protein-BrxC-[Acinetobacter-sp.-869535]
WP_034680334.1	Type1.BREX	Phage_integrase+Phage_integrase->Phage_integrase+Phage_integrase->ParB+HNH->CR-ATPase5+REase-><-DUF4236||wHTH+WYL+WCX->BrxA-DNA-binding->BrxB-BrxF-iSTAND->DUF6079*->N6-METHYLASE->PglZ->SIGMA+OB-Fold+AAA-ATPase+Lon-Protease-BrxL->||<-?<-DDE_Tnp_IS240	1226	Bacteria>Pseudomonadota>Gammaproteobacteria>Moraxellales>Moraxellaceae>Acinetobacter	GCF_000582135.1	Acinetobacter_sp._983759	J559_RS13455	17998..21678	1310660	BREX-system-P-loop-protein-BrxC-[Acinetobacter-sp.-983759]
WP_032016259.1	Type1.BREX	<-Phage_GPD+Phage_base_V+T6SS_Vgr+DUF2345<-TetR-HTH<-?||?->Peptidase_M48-><-MACRODOMAIN<-DarT+Peptidase<-DUF6079*<-BrxB-BrxF-iSTAND<-BrxA-DNA-binding<-wHTH+WYL+WCX||TetR-HTH-><-Cap4_nuclease<-TRD	1226	Bacteria>Pseudomonadota>Gammaproteobacteria>Moraxellales>Moraxellaceae>Acinetobacter	GCF_000584335.1	Acinetobacter_baumannii	J609_RS12655	131163..134843	470	BREX-system-P-loop-protein-BrxC-[Acinetobacter-baumannii]
WP_025095052.1	Type1.BREX	<-RelE-ParE<-PhdYeFM_antitox||wHTH+WYL+WCX->BrxA-DNA-binding->BrxB-BrxF-iSTAND->DUF6079*->N6-METHYLASE->SF1-SF2-Helicase+PRTase->SLOG+BirA-HTH->ParB+HNH->PglZ->SIGMA+OB-Fold+AAA-ATPase+Lon-Protease-BrxL->SF1-SF2-Helicase+REase->SF1-SF2-Helicase->PIN->	1226	Bacteria>Pseudomonadota>Gammaproteobacteria>Moraxellales>Moraxellaceae>Acinetobacter	GCF_000633005.1	Acinetobacter_soli	EG58_RS0106955	8205..11885	487316	BREX-system-P-loop-protein-BrxC-[Acinetobacter-soli]
WP_034584422.1	Type1.BREX	ParA-Soj-PloopNTPase->ParB->||ParB+HNH->CR-ATPase5+REase-><-DUF4236||wHTH+WYL+WCX->BrxA-DNA-binding->BrxB-BrxF-iSTAND->DUF6079*->N6-METHYLASE->PglZ->SIGMA+OB-Fold+AAA-ATPase+Lon-Protease-BrxL-><-ARG	1226	Bacteria>Pseudomonadota>Gammaproteobacteria>Moraxellales>Moraxellaceae>Acinetobacter	GCF_000773685.1	Acinetobacter_sp._HR7	GW12_RS05240	10375..14055	1509403	BREX-system-P-loop-protein-BrxC-[Acinetobacter-sp.-HR7]
WP_039048213.1	Type1.BREX	<-GDResolvase+HIN-HTH||<-SbcC+ABC-ATPase<-?<-?<-?||<-SIGMA+OB-Fold+AAA-ATPase+Lon-Protease-BrxL<-PglZ<-HKD-DNase+DUF3150<-N6-METHYLASE<-DUF6079*<-BrxB-BrxF-iSTAND<-BrxA-DNA-binding<-wHTH+WYL+WCX||DUF4236-><-CR-ATPase5+REase<-ParB+HNH<-Phage_integrase+Phage_integrase<-Phage_integrase+Phage_integrase	1226	Bacteria>Pseudomonadota>Gammaproteobacteria>Moraxellales>Moraxellaceae>Acinetobacter	GCF_000813495.1	Acinetobacter_junii	RP26_RS13815	17614..21294	40215	BREX-system-P-loop-protein-BrxC-[Acinetobacter-junii]
WP_045544683.1	Type1.BREX	DDE_Tnp_IS240-><-ADH_N+ADH_zinc_N+METHYLASE<-?<-?||DDE_Tnp_IS240-><-SIGMA+OB-Fold+AAA-ATPase+Lon-Protease-BrxL<-PglZ<-ParB+HNH<-N6-METHYLASE<-DUF6079*<-BrxB-BrxF-iSTAND<-BrxA-DNA-binding<-wHTH+WYL+WCX||DUF4236-><-CR-ATPase5+REase<-GDResolvase+HIN-HTH||DUF4158+DDE_Tnp_Tn3->	1226	Bacteria>Pseudomonadota>Gammaproteobacteria>Moraxellales>Moraxellaceae>Acinetobacter	GCF_000828935.1	Acinetobacter_baumannii	IOMTU433_RS19345	66852..70532	470	BREX-system-P-loop-protein-BrxC-[Acinetobacter-baumannii]
WP_016658783.1	Type1.BREX	ParB+HNH->CR-ATPase5+REase-><-DUF4236||wHTH+WYL+WCX->BrxA-DNA-binding->BrxB-BrxF-iSTAND->DUF6079*->N6-METHYLASE->PglZ->SIGMA+OB-Fold+AAA-ATPase+Lon-Protease-BrxL->	1226	Bacteria>Pseudomonadota>Gammaproteobacteria>Moraxellales>Moraxellaceae>Acinetobacter	GCF_000830155.1	Acinetobacter_indicus	AIN01_RS11690	13692..17372	756892	BREX-system-P-loop-protein-BrxC-[Acinetobacter-indicus]
WP_043972752.1	Type1.BREX	ParB->ParB+HNH->CR-ATPase5+REase-><-DUF4236||wHTH+WYL+WCX->BrxA-DNA-binding->BrxB-BrxF-iSTAND->DUF6079*->N6-METHYLASE->SF1-SF2-Helicase+PRTase->SLOG+BirA-HTH->ParB+HNH->PglZ->SIGMA+OB-Fold+AAA-ATPase+Lon-Protease-BrxL->SF1-SF2-Helicase+REase-><-SbcC+ABC-ATPase	1226	Bacteria>Pseudomonadota>Gammaproteobacteria>Moraxellales>Moraxellaceae>Acinetobacter	GCF_000876115.1	Acinetobacter_sp._NBRC_110496	Aba263_RS13795	7444..11124	1550715	BREX-system-P-loop-protein-BrxC-[Acinetobacter-sp.-NBRC-110496]
WP_228136291.1	Type1.BREX	ZnR+ZnR->DUF932->?->DUF6079-p*->N6-METHYLASE-p->N6-METHYLASE-p->?->?->?->CR-Kinase+Classical-AAA-><-?<-REase+SPF-cons	613	Bacteria>Pseudomonadota>Gammaproteobacteria>Moraxellales>Moraxellaceae>Acinetobacter	GCF_000949675.1	Acinetobacter_nosocomialis	Aba28S_RS17090	3475..5316	106654	hypothetical-protein-[Acinetobacter-nosocomialis]
WP_032059948.1	Type1.BREX	DUF932->?->?->?-><-?<-TetR-HTH||wHTH+WYL+WCX->BrxA-DNA-binding->BrxB-BrxF-iSTAND->DUF6079*->N6-METHYLASE->ParB+HNH->PglZ->SIGMA+OB-Fold+AAA-ATPase+Lon-Protease-BrxL->HIN-HTH->?->||?->HIN-HTH->	1226	Bacteria>Pseudomonadota>Gammaproteobacteria>Moraxellales>Moraxellaceae>Acinetobacter	GCF_001077555.2	Acinetobacter_baumannii	AB895_RS13070	2715747..2719427	470	BREX-system-P-loop-protein-BrxC-[Acinetobacter-baumannii]
WP_058952662.1	Type1.BREX	<-Phage_integrase<-?||<-ParA-Soj-PloopNTPase||?->CR-ATPase5+REase-><-DUF4236||wHTH+WYL+WCX->BrxA-DNA-binding->BrxB-BrxF-iSTAND->DUF6079*->N6-METHYLASE->ParB+HNH->PglZ->SIGMA+OB-Fold+AAA-ATPase+Lon-Protease-BrxL->||?->?->?->SbcC+ABC-ATPase->||GDResolvase+HIN-HTH->	1220	Bacteria>Pseudomonadota>Gammaproteobacteria>Moraxellales>Moraxellaceae>Acinetobacter	GCF_001484935.1	Acinetobacter_johnsonii	EHS42_RS19740	21243..24905	40214	BREX-system-P-loop-protein-BrxC-[Acinetobacter-johnsonii]
WP_058971759.1	Type1.BREX	DDE_Tnp_1_5+DDE_Tnp_1->?->ParB->ParB+HNH->CR-ATPase5+REase-><-DUF4236||wHTH+WYL+WCX->BrxA-DNA-binding->BrxB-BrxF-iSTAND->DUF6079*->N6-METHYLASE->PglZ->SIGMA+OB-Fold+AAA-ATPase+Lon-Protease-BrxL-><-?<-AbiEii<-MarR-HTH<-CHTH<-RelE	1226	Bacteria>Pseudomonadota>Gammaproteobacteria>Moraxellales>Moraxellaceae>Acinetobacter	GCF_001500155.2	Acinetobacter_bereziniae	NDM229_RS00350	66308..69988	106648	BREX-system-P-loop-protein-BrxC-[Acinetobacter-bereziniae]
WP_065184483.1	Type1.BREX	<-Phage_GPD+Phage_base_V<-TetR-HTH<-?<-SIGMA+OB-Fold+AAA-ATPase+Lon-Protease-BrxL<-PglZ<-ParB+HNH<-N6-METHYLASE<-DUF6079*<-BrxB-BrxF-iSTAND<-BrxA-DNA-binding<-wHTH+WYL+WCX<-DUF932||PIN+OST-HTH-><-ZnR+DUF3482+ZnR	1226	Bacteria>Pseudomonadota>Gammaproteobacteria>Moraxellales>Moraxellaceae>Acinetobacter	GCF_001674695.1	Acinetobacter_baumannii	GKP36_RS06470	16105..19785	470	BREX-system-P-loop-protein-BrxC-[Acinetobacter-baumannii]
WP_068540167.1	Type1.BREX	<-DDE_Tnp_1_5+DDE_Tnp_1||?->DOC+HTH->||wHTH+WYL+WCX->BrxA-DNA-binding->BrxB-BrxF-iSTAND->DUF6079*->N6-METHYLASE->?->PglZ->SIGMA+OB-Fold+AAA-ATPase+Lon-Protease-BrxL->	1226	Bacteria>Pseudomonadota>Gammaproteobacteria>Moraxellales>Moraxellaceae>Acinetobacter	GCF_001706415.2	Acinetobacter_pittii	BFR83_RS18935	77132..80812	48296	BREX-system-P-loop-protein-BrxC-[Acinetobacter-pittii]
WP_069122561.1	Type1.BREX	ZnR+DUF3482+ZnR-><-PIN+OST-HTH||DUF932->wHTH+WYL+WCX->BrxA-DNA-binding->BrxB-BrxF-iSTAND->DUF6079*->N6-METHYLASE->ParB+HNH->PglZ->SIGMA+OB-Fold+AAA-ATPase+Lon-Protease-BrxL-><-TetR-HTH<-DSBH<-RNase<-?<-MODE-HTH+PBPII	1226	Bacteria>Pseudomonadota>Gammaproteobacteria>Moraxellales>Moraxellaceae>Acinetobacter	GCF_001716945.1	Acinetobacter_pittii	AXH23_RS05625	247550..251230	48296	BREX-system-P-loop-protein-BrxC-[Acinetobacter-pittii]
WP_075040289.1	Type1.BREX	DUF932->?->?->XIS-HTH->SAM-METHYLASE->wHTH(SBD)+HNH->wHTH+WYL+WCX->BrxA-DNA-binding->BrxB-BrxF-iSTAND->DUF6079*->N6-METHYLASE->ParB+HNH->PglZ->SIGMA+OB-Fold+AAA-ATPase+Lon-Protease-BrxL->SF1-SF2-Helicase+REase->wHTH+WYL+WCX->GTPase-AIG->sGTP-><-DOC+HTH	1226	Bacteria>Pseudomonadota>Gammaproteobacteria>Moraxellales>Moraxellaceae>Acinetobacter	GCF_001917365.1	Acinetobacter_radioresistens	SA188iCDA_RS03905	54372..58052	40216	BREX-system-P-loop-protein-BrxC-[Acinetobacter-radioresistens]
WP_078390063.1	Type1.BREX	<-RelE<-GDResolvase+HIN-HTH<-RNase<-?<-?<-?<-SIGMA+OB-Fold+AAA-ATPase+Lon-Protease-BrxL<-PglZ<-N6-METHYLASE<-DUF6079*<-BrxB-BrxF-iSTAND<-BrxA-DNA-binding<-wHTH+WYL+WCX||DUF4236-><-CR-ATPase5+REase	1224	Bacteria>Pseudomonadota>Gammaproteobacteria>Moraxellales>Moraxellaceae>Acinetobacter	GCF_002018895.1	Acinetobacter_sp._MF4640	MF4640_RS14775	20084..23758	1960826	BREX-system-P-loop-protein-BrxC-[Acinetobacter-sp.-MF4640]
WP_086231303.1	Type1.BREX	<-SIGMA+OB-Fold+AAA-ATPase+Lon-Protease-BrxL<-PglZ<-ParB+HNH<-SLOG+BirA-HTH<-SF1-SF2-Helicase+PRTase<-N6-METHYLASE<-DUF6079*<-BrxB-BrxF-iSTAND<-BrxA-DNA-binding<-wHTH+WYL+WCX<-wHTH(SBD)+HNH<-SF1-SF2-Helicase	1226	Bacteria>Pseudomonadota>Gammaproteobacteria>Moraxellales>Moraxellaceae>Acinetobacter	GCF_002137255.1	Acinetobacter_baumannii	B9Y01_RS02195	14367..18047	470	BREX-system-P-loop-protein-BrxC-[Acinetobacter-baumannii]
WP_143224472.1	Type1.BREX	<-DDE_Tnp_1_5+DDE_Tnp_1||?->DOC+HTH->||wHTH+WYL+WCX->BrxA-DNA-binding->BrxB-BrxF-iSTAND->DUF6079-p*->	593	Bacteria>Pseudomonadota>Gammaproteobacteria>Moraxellales>Moraxellaceae>Acinetobacter	GCF_002145235.1	Acinetobacter_pittii	CAT65_RS01675	11536..13316	48296	BREX-system-P-loop-protein-BrxC,-partial-[Acinetobacter-pittii]
WP_087537459.1	Type1.BREX	DUF4372+DDE_Tnp_1-><-HTH_37<-?||ParB+HNH->CR-ATPase5+REase-><-DUF4236||wHTH+WYL+WCX->BrxA-DNA-binding->BrxB-BrxF-iSTAND->DUF6079*->N6-METHYLASE->PglZ->SIGMA+OB-Fold+AAA-ATPase+Lon-Protease-BrxL-><-?<-ABhydrolase<-Classical-AAA<-?<-SMODS||GDResolvase+HIN-HTH->	1226	Bacteria>Pseudomonadota>Gammaproteobacteria>Moraxellales>Moraxellaceae>Acinetobacter	GCF_002165255.2	Acinetobacter_sp._WCHA45	CDG55_RS00865	132887..136567	2004644	BREX-system-P-loop-protein-BrxC-[Acinetobacter-sp.-WCHA45]
WP_087528528.1	Type1.BREX	<-NADAR<-ART-DarT||TPR+TPR-><-?<-SIGMA+OB-Fold+AAA-ATPase+Lon-Protease-BrxL<-PglZ<-TOPRIM<-OLD-ABC<-N6-METHYLASE<-DUF6079*<-BrxB-BrxF-iSTAND<-BrxA-DNA-binding<-wHTH+WYL+WCX||DUF4236-><-CR-ATPase5+REase<-ParB+HNH<-HIN-HTH+PAIREDC-HTH||<-Zn_Tnp_IS91+Y2_Tnp	1226	Bacteria>Pseudomonadota>Gammaproteobacteria>Moraxellales>Moraxellaceae>Acinetobacter	GCF_002165305.2	Acinetobacter_sp._WCHA55	CDG62_RS00815	35375..39055	2004646	BREX-system-P-loop-protein-BrxC-[Acinetobacter-sp.-WCHA55]
WP_087554207.1	Type1.BREX	Phage_integrase+Phage_integrase->Phage_integrase+Phage_integrase->ParB+HNH->?->DOC+HTH-><-DUF4236||wHTH+WYL+WCX->BrxA-DNA-binding->BrxB-BrxF-iSTAND->DUF6079*->N6-METHYLASE->PglZ->SIGMA+OB-Fold+AAA-ATPase+Lon-Protease-BrxL-><-DDE_Tnp_1<-Phage_int_SAM_4+Phage_integrase	1226	Bacteria>Pseudomonadota>Gammaproteobacteria>Moraxellales>Moraxellaceae>Acinetobacter	GCF_002165345.2	Acinetobacter_wuhouensis	CDG68_RS02780	35949..39629	1879050	BREX-system-P-loop-protein-BrxC-[Acinetobacter-wuhouensis]
WP_096902245.1	Type1.BREX	<-ACET<-?||<-SIGMA+OB-Fold+AAA-ATPase+Lon-Protease-BrxL<-PglZ<-ParB+HNH<-N6-METHYLASE<-DUF6079*<-BrxB-BrxF-iSTAND<-BrxA-DNA-binding<-wHTH+WYL+WCX||<-?<-ParB+HNH-p<-CR-ATPase5+REase||HigB_toxin->HTH_3->||Zn_Tnp_IS91+Y2_Tnp->	1222	Bacteria>Pseudomonadota>Gammaproteobacteria>Moraxellales>Moraxellaceae>Acinetobacter	GCF_002412355.1	Acinetobacter_sp._YT-02	CF596_RS10765	15331..18999	2018564	BREX-system-P-loop-protein-BrxC-[Acinetobacter-sp.-YT-02]
WP_099349802.1	Type1.BREX	<-AbiGi+seqA+RAMA<-CHTH<-RelE<-DDE_Tnp_1_5+DDE_Tnp_1||CR-ATPase5+REase-><-DUF4236||wHTH+WYL+WCX->BrxA-DNA-binding->BrxB-BrxF-iSTAND->DUF6079*->N6-METHYLASE->PglZ->SIGMA+OB-Fold+AAA-ATPase+Lon-Protease-BrxL->	1226	Bacteria>Pseudomonadota>Gammaproteobacteria>Moraxellales>Moraxellaceae>Acinetobacter	GCF_002727055.2	Acinetobacter_baumannii	CPI82_RS19405	19969..23649	470	BREX-system-P-loop-protein-BrxC-[Acinetobacter-baumannii]
WP_099475573.1	Type1.BREX	Phage_integrase+Phage_integrase->Phage_integrase+Phage_integrase->ParB+HNH->?->DOC+HTH-><-DUF4236||wHTH+WYL+WCX->BrxA-DNA-binding->BrxB-BrxF-iSTAND->DUF6079*->N6-METHYLASE->PglZ->SIGMA+OB-Fold+AAA-ATPase+Lon-Protease-BrxL->?->GDResolvase+HIN-HTH-><-DDE_Tnp_IS240	1226	Bacteria>Pseudomonadota>Gammaproteobacteria>Moraxellales>Moraxellaceae>Acinetobacter	GCF_002740745.1	Acinetobacter_pittii	CT134_RS17990	28430..32110	48296	BREX-system-P-loop-protein-BrxC-[Acinetobacter-pittii]
WP_100834299.1	Type1.BREX	ParB+HNH-p-><-HTH_Tnp_1+HTH_21+rve+rve_3|||?->Phage_integrase->||?-><-DUF4236||wHTH+WYL+WCX->BrxA-DNA-binding->BrxB-BrxF-iSTAND->DUF6079*->N6-METHYLASE->PglZ->SIGMA+OB-Fold+AAA-ATPase+Lon-Protease-BrxL-><-?<-AbiEii<-MarR-HTH<-?||?->RNase->	1226	Bacteria>Pseudomonadota>Gammaproteobacteria>Moraxellales>Moraxellaceae>Acinetobacter	GCF_002813065.1	Acinetobacter_haemolyticus	BSR56_RS16115	29817..33497	29430	BREX-system-P-loop-protein-BrxC-[Acinetobacter-haemolyticus]
WP_104123635.1	Type1.BREX	TRD->Cap4_nuclease-><-TetR-HTH||wHTH+WYL+WCX->BrxA-DNA-binding->BrxB-BrxF-iSTAND->DUF6079*->DarT+Peptidase->MACRODOMAIN-><-Peptidase_M48<-?||?->Phage_GPD+Phage_base_V+T6SS_Vgr+DUF2345->	1226	Bacteria>Pseudomonadota>Gammaproteobacteria>Moraxellales>Moraxellaceae>Acinetobacter	GCF_002927775.1	Acinetobacter_baumannii	AbaMCR9238_RS08400	60585..64265	470	BREX-system-P-loop-protein-BrxC-[Acinetobacter-baumannii]
WP_104442411.1	Type1.BREX	<-DDE_Tnp_1||<-?<-HTH_3<-?<-SIGMA+OB-Fold+AAA-ATPase+Lon-Protease-BrxL<-PglZ<-ParB+HNH<-N6-METHYLASE<-DUF6079*<-BrxB-BrxF-iSTAND<-BrxA-DNA-binding<-wHTH+WYL+WCX||DUF4236-><-?<-CR-ATPase5+REase||<-DDE_Tnp_IS240||?->?->ArsR-HTH->	1226	Bacteria>Pseudomonadota>Gammaproteobacteria>Moraxellales>Moraxellaceae>Acinetobacter	GCF_002934965.1	Acinetobacter_sp._ACNIH2	C3F34_RS18690	72475..76155	1758189	BREX-system-P-loop-protein-BrxC-[Acinetobacter-sp.-ACNIH2]
WP_104500994.1	Type1.BREX	<-SIGMA+OB-Fold+AAA-ATPase+Lon-Protease-BrxL<-PglZ<-ParB+HNH<-SLOG+BirA-HTH<-SF1-SF2-Helicase+PRTase<-N6-METHYLASE<-DUF6079*<-BrxB-BrxF-iSTAND<-BrxA-DNA-binding<-wHTH+WYL+WCX||DUF4236-><-CR-ATPase5+REase<-ParB+HNH<-Phage_integrase+Phage_integrase<-Phage_integrase+Phage_integrase	1226	Bacteria>Pseudomonadota>Gammaproteobacteria>Moraxellales>Moraxellaceae>Acinetobacter	GCF_002938745.1	Acinetobacter_indicus	C5505_RS10055	19022..22702	756892	BREX-system-P-loop-protein-BrxC-[Acinetobacter-indicus]
WP_104852217.1	Type1.BREX	<-AAA-ATPase<-HIN-HTH||DDE_Tnp_1_3-><-?<-?||HIN-HTH-><-PglZ<-N6-METHYLASE<-DUF6079*<-BrxB-BrxF-iSTAND<-BrxA-DNA-binding<-wHTH(SBD)+HNH<-SAM-METHYLASE<-XIS-HTH<-?<-?<-DUF932	1247	Bacteria>Pseudomonadota>Gammaproteobacteria>Moraxellales>Moraxellaceae>Acinetobacter	GCF_002946595.1	Acinetobacter_sp._SWBY1	C3Y93_RS04635	951564..955307	2079596	BREX-system-P-loop-protein-BrxC-[Acinetobacter-sp.-SWBY1]
WP_109291743.1	Type1.BREX	<-DDE_Tnp_1_5+DDE_Tnp_1||CR-ATPase5+REase-><-DUF4236||wHTH+WYL+WCX->BrxA-DNA-binding->BrxB-BrxF-iSTAND->DUF6079*->N6-METHYLASE->ParB+HNH->PglZ->SIGMA+OB-Fold+AAA-ATPase+Lon-Protease-BrxL->SF1-SF2-Helicase+REase-><-?<-CHTH<-RelE	1226	Bacteria>Pseudomonadota>Gammaproteobacteria>Moraxellales>Moraxellaceae>Acinetobacter	GCF_003131095.1	Acinetobacter_baumannii	DF172_RS10505	4712..8392	470	BREX-system-P-loop-protein-BrxC-[Acinetobacter-baumannii]
WP_110975939.1	Type1.BREX	<-DUF4236||wHTH+WYL+WCX->BrxA-DNA-binding->BrxB-BrxF-iSTAND->DUF6079*->N6-METHYLASE->SbcC->	1226	Bacteria>Pseudomonadota>Gammaproteobacteria>Moraxellales>Moraxellaceae>Acinetobacter	GCF_003231195.1	Acinetobacter_sp._WCHAc060042	DPG01_RS17630	3221..6901	2213016	BREX-system-P-loop-protein-BrxC-[Acinetobacter-sp.-WCHAc060042]
WP_111281982.1	Type1.BREX	DUF932->?->XIS-HTH->SAM-METHYLASE->wHTH(SBD)+HNH->wHTH+WYL+WCX->BrxA-DNA-binding->BrxB-BrxF-iSTAND->DUF6079*->N6-METHYLASE->SF1-SF2-Helicase+PRTase->SLOG+BirA-HTH->ParB+HNH->PglZ->SIGMA+OB-Fold+AAA-ATPase+Lon-Protease-BrxL->SF1-SF2-Helicase+REase->wHTH+WYL+WCX->GTPase-AIG->	1226	Bacteria>Pseudomonadota>Gammaproteobacteria>Moraxellales>Moraxellaceae>Acinetobacter	GCF_003258335.1	Acinetobacter_radioresistens	NVV25_RS10895	13399..17079	40216	BREX-system-P-loop-protein-BrxC-[Acinetobacter-radioresistens]
WP_119025213.1	Type1.BREX	<-PhdYeFM_antitox||wHTH+WYL+WCX->BrxA-DNA-binding->BrxB-BrxF-iSTAND->DUF6079*->N6-METHYLASE->ParB+HNH->PglZ->SIGMA+OB-Fold+AAA-ATPase+Lon-Protease-BrxL->SF1-SF2-Helicase+REase-><-HTH_Tnp_1+HTH_21+rve+rve_3<-nSTAND1+HNH	1226	Bacteria>Pseudomonadota>Gammaproteobacteria>Moraxellales>Moraxellaceae>Acinetobacter	GCF_003569585.1	Acinetobacter_soli	D5I70_RS01315	8436..12116	487316	BREX-system-P-loop-protein-BrxC-[Acinetobacter-soli]
WP_119047908.1	Type1.BREX	ZnR+DUF3482+ZnR-><-PIN+OST-HTH||DUF932->?-><-TetR-HTH||wHTH+WYL+WCX->BrxA-DNA-binding->BrxB-BrxF-iSTAND->DUF6079*->N6-METHYLASE->SF1-SF2-Helicase+PRTase->SLOG+BirA-HTH->ParB+HNH->PglZ->SIGMA+OB-Fold+AAA-ATPase+Lon-Protease-BrxL->SF1-SF2-Helicase+REase->DarT+Peptidase->MACRODOMAIN->	1226	Bacteria>Pseudomonadota>Gammaproteobacteria>Moraxellales>Moraxellaceae>Acinetobacter	GCF_003569645.1	Acinetobacter_baumannii	D5I76_RS05685	52847..56527	470	BREX-system-P-loop-protein-BrxC-[Acinetobacter-baumannii]
WP_119518033.1	Type1.BREX	<-DUF6079-p*<-BrxB-BrxF-iSTAND<-BrxA-DNA-binding<-wHTH+WYL+WCX||DUF4236-><-CR-ATPase5+REase-p	817	Bacteria>Pseudomonadota>Gammaproteobacteria>Moraxellales>Moraxellaceae>Acinetobacter	GCF_003583605.1	Acinetobacter_baumannii	D3X29_RS17285	1..2451	470	BREX-system-P-loop-protein-BrxC,-partial-[Acinetobacter-baumannii]
WP_119879428.1	Type1.BREX	<-SIGMA+OB-Fold+AAA-ATPase+Lon-Protease-BrxL<-PglZ<-SbcC<-N6-METHYLASE<-DUF6079*<-BrxB-BrxF-iSTAND<-BrxA-DNA-binding<-wHTH+WYL+WCX||DUF4236-><-CR-ATPase5+REase<-HTH_Tnp_1+HTH_21+rve+rve_3<-ParB+HNH<-Phage_integrase	1226	Bacteria>Pseudomonadota>Gammaproteobacteria>Moraxellales>Moraxellaceae>Acinetobacter	GCF_003594775.1	Acinetobacter_radioresistens	D5055_RS12040	10913..14593	40216	BREX-system-P-loop-protein-BrxC-[Acinetobacter-radioresistens]
WP_120366206.1	Type1.BREX	HTH_Tnp_1+HTH_21+rve+rve_3->?-><-HTH_26||?-><-HicB<-Arm-DNA-bind_3+Phage_integrase||<-SIGMA+OB-Fold+AAA-ATPase+Lon-Protease-BrxL<-PglZ<-N6-METHYLASE<-DUF6079*<-BrxB-BrxF-iSTAND<-BrxA-DNA-binding	1226	Bacteria>Pseudomonadota>Gammaproteobacteria>Moraxellales>Moraxellaceae>Acinetobacter	GCF_003611535.1	Acinetobacter_cumulans	D7V68_RS14625	28683..32363	2136182	BREX-system-P-loop-protein-BrxC-[Acinetobacter-cumulans]
WP_121523424.1	Type1.BREX	ParB+HNH-><-?||SAM-METHYLASE->?->SF1-SF2-Helicase->wHTH+HNH->wHTH+WYL+WCX->BrxA-DNA-binding->BrxB-BrxF-iSTAND->DUF6079*->N6-METHYLASE->PglZ->SIGMA+OB-Fold+AAA-ATPase+Lon-Protease-BrxL->SF1-SF2-Helicase+REase->	1226	Bacteria>Pseudomonadota>Gammaproteobacteria>Moraxellales>Moraxellaceae>Acinetobacter	GCF_003664645.1	Acinetobacter_chengduensis	D9K81_RS12845	89350..93030	2420890	BREX-system-P-loop-protein-BrxC-[Acinetobacter-chengduensis]
WP_121533276.1	Type1.BREX	CR-ATPase5+REase-><-DUF4236||wHTH+WYL+WCX->BrxA-DNA-binding->BrxB-BrxF-iSTAND->DUF6079*->N6-METHYLASE->PglZ->SIGMA+OB-Fold+AAA-ATPase+Lon-Protease-BrxL->?->HTH_3->?-><-?<-?<-CHTH	1226	Bacteria>Pseudomonadota>Gammaproteobacteria>Moraxellales>Moraxellaceae>Acinetobacter	GCF_003664655.1	Acinetobacter_cumulans	D9K79_RS15510	4801..8481	2136182	BREX-system-P-loop-protein-BrxC-[Acinetobacter-cumulans]
WP_123785432.1	Type1.BREX	<-DDE_Tnp_IS240||?-><-?<-SIGMA+OB-Fold+AAA-ATPase+Lon-Protease-BrxL<-PglZ<-ParB+HNH<-N6-METHYLASE<-DUF6079*<-BrxB-BrxF-iSTAND<-BrxA-DNA-binding<-wHTH+WYL+WCX||DUF4236-><-CR-ATPase5+REase	1226	Bacteria>Pseudomonadota>Gammaproteobacteria>Moraxellales>Moraxellaceae>Acinetobacter	GCF_003812065.1	Acinetobacter_baumannii	LH406_RS10280	68046..71726	470	BREX-system-P-loop-protein-BrxC-[Acinetobacter-baumannii]
WP_125269804.1	Type1.BREX	<-HTH+HEPN<-SF1-SF2-Helicase<-STYKIN<-?<-?<-?<-?<-Cas_NE0113<-SIGMA+OB-Fold+AAA-ATPase+Lon-Protease-BrxL<-DUF6079*<-BrxB-BrxF-iSTAND<-BrxA-DNA-binding<-HTH_Tnp_1+HTH_21+rve+rve_3||<-?<-SAM-METHYLASE<-ParB+HNH||Calcineurin->	1512	Bacteria>Pseudomonadota>Gammaproteobacteria>Moraxellales>Moraxellaceae>Acinetobacter	GCF_003938865.1	Acinetobacter_sp._FDAARGOS_515	EGS47_RS18730	3064192..3068730	2420307	BREX-system-P-loop-protein-BrxC-[Acinetobacter-sp.-FDAARGOS_515]
WP_125274900.1	Type1.BREX	<-DUF4372+DDE_Tnp_1<-SIGMA+OB-Fold+AAA-ATPase+Lon-Protease-BrxL<-PglZ<-N6-METHYLASE<-DUF6079*<-BrxB-BrxF-iSTAND<-BrxA-DNA-binding<-wHTH+WYL+WCX||DUF4236-><-CR-ATPase5+REase	1226	Bacteria>Pseudomonadota>Gammaproteobacteria>Moraxellales>Moraxellaceae>Acinetobacter	GCF_003940285.1	Acinetobacter_johnsonii	EGT73_RS16195	9004..12684	40214	BREX-system-P-loop-protein-BrxC-[Acinetobacter-johnsonii]
WP_125318492.1	Type1.BREX	ParB+HNH->CR-ATPase5+REase-><-DUF4236||wHTH+WYL+WCX->BrxA-DNA-binding->BrxB-BrxF-iSTAND->DUF6079*->N6-METHYLASE->PglZ->SIGMA+OB-Fold+AAA-ATPase+Lon-Protease-BrxL-><-?<-ABhydrolase<-Classical-AAA<-?<-SMODS||Thioredoxin->	1226	Bacteria>Pseudomonadota>Gammaproteobacteria>Moraxellales>Moraxellaceae>Acinetobacter	GCF_003940305.1	Acinetobacter_junii	EGT64_RS07935	8553..12233	40215	BREX-system-P-loop-protein-BrxC-[Acinetobacter-junii]
WP_125525774.1	Type1.BREX	<-DDE_Tnp_1_5+DDE_Tnp_1||?->DOC+HTH->||wHTH+WYL+WCX->BrxA-DNA-binding->BrxB-BrxF-iSTAND->DUF6079*->N6-METHYLASE-p->	1226	Bacteria>Pseudomonadota>Gammaproteobacteria>Moraxellales>Moraxellaceae>Acinetobacter	GCF_003947765.1	Acinetobacter_pittii	EA755_RS16870	59824..63504	48296	BREX-system-P-loop-protein-BrxC-[Acinetobacter-pittii]
WP_127800367.1	Type1.BREX	<-SIGMA+OB-Fold+AAA-ATPase+Lon-Protease-BrxL<-PglZ<-N6-METHYLASE<-DUF6079*<-BrxB-BrxF-iSTAND<-BrxA-DNA-binding<-wHTH+WYL+WCX||DUF4236-><-CR-ATPase5+REase||?->?->HATPase+wHTH->	1226	Bacteria>Pseudomonadota>Gammaproteobacteria>Moraxellales>Moraxellaceae>Acinetobacter	GCF_004005445.1	Acinetobacter_indicus	ENC21_RS11140	10000..13680	756892	BREX-system-P-loop-protein-BrxC-[Acinetobacter-indicus]
WP_129260966.1	Type1.BREX	DDE_Tnp_1-><-?||MarR-HTH->AbiEii->?-><-SIGMA+OB-Fold+AAA-ATPase+Lon-Protease-BrxL<-PglZ<-N6-METHYLASE<-DUF6079*<-BrxB-BrxF-iSTAND<-BrxA-DNA-binding<-wHTH+WYL+WCX||DUF4236-><-CR-ATPase5+REase<-DDE_Tnp_1_5+DDE_Tnp_1	1226	Bacteria>Pseudomonadota>Gammaproteobacteria>Moraxellales>Moraxellaceae>Acinetobacter	GCF_004123275.1	Acinetobacter_junii	ETZ13_RS00160	34893..38573	40215	BREX-system-P-loop-protein-BrxC-[Acinetobacter-junii]
WP_131273640.1	Type1.BREX	<-SIGMA+OB-Fold+AAA-ATPase+Lon-Protease-BrxL<-PglZ<-ParB+HNH<-DUF932<-N6-METHYLASE<-DUF6079*<-BrxB-BrxF-iSTAND<-BrxA-DNA-binding<-wHTH(SBD)+HNH<-SAM-METHYLASE<-XIS-HTH<-?<-?<-DUF932<-ZnR+ZnR	1154	Bacteria>Pseudomonadota>Gammaproteobacteria>Moraxellales>Moraxellaceae>Acinetobacter	GCF_004331195.1	Acinetobacter_sp._ANC_4216	EOH88_RS07250	81102..84566	2529840	BREX-system-P-loop-protein-BrxC-[Acinetobacter-sp.-ANC-4216]
WP_131214141.1	Type1.BREX	ABC-ATPase+SbcC+ABC-ATPase+SbcC->?-><-SIGMA+OB-Fold+AAA-ATPase+HKD-DNase-BrxL<-PglZ<-N6-METHYLASE<-REase<-OLD-ABC<-DUF6079*<-BrxB-BrxF-iSTAND<-BrxA-DNA-binding<-XIS-HTH<-XIS-HTH<-?<-?<-DUF932<-ZnR+ZnR	1190	Bacteria>Pseudomonadota>Gammaproteobacteria>Moraxellales>Moraxellaceae>Acinetobacter	GCF_004331285.1	Acinetobacter_sp._ANC_3781	EOH89_RS01980	72363..75935	2529835	BREX-system-P-loop-protein-BrxC-[Acinetobacter-sp.-ANC-3781]
WP_138000997.1	Type1.BREX	<-PglZ<-ParB+HNH<-N6-METHYLASE<-DUF6079*<-BrxB-BrxF-iSTAND<-BrxA-DNA-binding<-wHTH+WYL+WCX||DUF4236-><-CR-ATPase5+REase<-ParB+HNH<-Phage_integrase+Phage_integrase<-Phage_integrase+Phage_integrase	1226	Bacteria>Pseudomonadota>Gammaproteobacteria>Moraxellales>Moraxellaceae>Acinetobacter	GCF_005519305.1	Acinetobacter_radioresistens	E3H47_RS14125	6771..10451	40216	BREX-system-P-loop-protein-BrxC-[Acinetobacter-radioresistens]
WP_140948309.1	Type1.BREX	<-MACRODOMAIN<-DarT+Peptidase<-SF1-SF2-Helicase+REase<-SIGMA+OB-Fold+AAA-ATPase+Lon-Protease-BrxL<-PglZ<-ParB+HNH<-N6-METHYLASE<-DUF6079*<-BrxB-BrxF-iSTAND<-BrxA-DNA-binding<-wHTH+WYL+WCX||TetR-HTH->?-><-?<-?<-?<-DUF932	1226	Bacteria>Pseudomonadota>Gammaproteobacteria>Moraxellales>Moraxellaceae>Acinetobacter	GCF_006491975.1	Acinetobacter_baumannii	FJV17_RS05945	24470..28150	470	BREX-system-P-loop-protein-BrxC-[Acinetobacter-baumannii]
WP_140967408.1	Type1.BREX	<-GDResolvase+HIN-HTH<-?<-?||ARG-><-SIGMA+OB-Fold+AAA-ATPase+Lon-Protease-BrxL<-PglZ<-N6-METHYLASE<-DUF6079*<-BrxB-BrxF-iSTAND<-BrxA-DNA-binding<-wHTH+WYL+WCX||DUF4236-><-Phage_integrase	1226	Bacteria>Pseudomonadota>Gammaproteobacteria>Moraxellales>Moraxellaceae>Acinetobacter	GCF_006492985.1	Acinetobacter_baumannii	FJU65_RS09860	11803..15483	470	BREX-system-P-loop-protein-BrxC-[Acinetobacter-baumannii]
WP_005021608.1	Type1.BREX	Phage_integrase+Phage_integrase->Phage_integrase+Phage_integrase->DDE_Tnp_1_5+DDE_Tnp_1->ParB+HNH->CR-ATPase5+REase-><-DUF4236||wHTH+WYL+WCX->BrxA-DNA-binding->BrxB-BrxF-iSTAND->DUF6079*->N6-METHYLASE->PglZ->SIGMA+OB-Fold+AAA-ATPase+Lon-Protease-BrxL->||<-?<-?<-?<-?<-?<-ArsR-HTH	1226	Bacteria>Pseudomonadota>Gammaproteobacteria>Moraxellales>Moraxellaceae>Acinetobacter	GCF_006757745.1	Acinetobacter_radioresistens	ACRAD_RS15795	43375..47055	40216	BREX-system-P-loop-protein-BrxC-[Acinetobacter-radioresistens]
WP_144583841.1	Type1.BREX	<-HTH_Tnp_1<-DUF4236||wHTH+WYL+WCX->BrxA-DNA-binding->BrxB-BrxF-iSTAND->DUF6079*->N6-METHYLASE->ParB+HNH->PglZ->SIGMA+OB-Fold+AAA-ATPase+Lon-Protease-BrxL->||PRTase->	1226	Bacteria>Pseudomonadota>Gammaproteobacteria>Moraxellales>Moraxellaceae>Acinetobacter	GCF_007713425.1	Acinetobacter_colistiniresistens	FPV60_RS17750	3413..7093	280145	BREX-system-P-loop-protein-BrxC-[Acinetobacter-colistiniresistens]
WP_148728183.1	Type1.BREX	<-DDE_Tnp_1_5+DDE_Tnp_1||?->DOC+HTH->||wHTH+WYL+WCX->BrxA-DNA-binding->BrxB-BrxF-iSTAND->DUF6079*->N6-METHYLASE->	1226	Bacteria>Pseudomonadota>Gammaproteobacteria>Moraxellales>Moraxellaceae>Acinetobacter	GCF_008121475.1	Acinetobacter_pittii	FXO17_RS20770	81542..85222	48296	BREX-system-P-loop-protein-BrxC-[Acinetobacter-pittii]
WP_149931258.1	Type1.BREX	<-DUF6079*<-BrxB-BrxF-iSTAND<-BrxA-DNA-binding<-wHTH+WYL+WCX||DUF4236-><-CR-ATPase5+REase<-HTH_26||?-><-HIN-HTH+PAIREDC-HTH<-HicB	1220	Bacteria>Pseudomonadota>Gammaproteobacteria>Moraxellales>Moraxellaceae>Acinetobacter	GCF_008580825.1	Acinetobacter_soli	F6C27_RS15450	2605..6267	487316	BREX-system-P-loop-protein-BrxC-[Acinetobacter-soli]
WP_151708311.1	Type1.BREX	ParB+HNH-p->CR-ATPase5+REase-><-DUF4236||wHTH+WYL+WCX->BrxA-DNA-binding->BrxB-BrxF-iSTAND->DUF6079*->N6-METHYLASE->SbcC->PglZ->SIGMA+OB-Fold+AAA-ATPase+Lon-Protease-BrxL-><-?<-AbiEii	1226	Bacteria>Pseudomonadota>Gammaproteobacteria>Moraxellales>Moraxellaceae>Acinetobacter	GCF_008979805.1	Acinetobacter_junii	GBZ45_RS13505	5536..9216	40215	BREX-system-P-loop-protein-BrxC-[Acinetobacter-junii]
WP_151725173.1	Type1.BREX	<-N6-METHYLASE-p<-DUF6079*<-BrxB-BrxF-iSTAND<-BrxA-DNA-binding<-wHTH+WYL+WCX||DUF4236-><-CR-ATPase5+REase<-ParB+HNH<-ParB	1226	Bacteria>Pseudomonadota>Gammaproteobacteria>Moraxellales>Moraxellaceae>Acinetobacter	GCF_008981805.1	Acinetobacter_ursingii	GBJ57_RS12485	1752..5432	108980	BREX-system-P-loop-protein-BrxC-[Acinetobacter-ursingii]
WP_151726277.1	Type1.BREX	<-SIGMA+OB-Fold+AAA-ATPase+Lon-Protease-BrxL<-PglZ<-N6-METHYLASE<-DUF6079*<-BrxB-BrxF-iSTAND<-BrxA-DNA-binding<-wHTH+WYL+WCX||DUF4236-><-CR-ATPase5+REase<-ParB+HNH	1226	Bacteria>Pseudomonadota>Gammaproteobacteria>Moraxellales>Moraxellaceae>Acinetobacter	GCF_008982065.1	Acinetobacter_ursingii	GBJ55_RS11105	8766..12446	108980	BREX-system-P-loop-protein-BrxC-[Acinetobacter-ursingii]
WP_151732002.1	Type1.BREX	<-GNTR-HTH+Aminotran_1_2||?->?->AA_permease->?-><-SF1-SF2-Helicase+REase<-SIGMA+OB-Fold+AAA-ATPase+Lon-Protease-BrxL<-PglZ<-N6-METHYLASE<-DUF6079*<-BrxB-BrxF-iSTAND<-BrxA-DNA-binding<-wHTH+WYL+WCX||<-Phage_integrase<-Phage_integrase+Phage_integrase	1226	Bacteria>Pseudomonadota>Gammaproteobacteria>Moraxellales>Moraxellaceae>Acinetobacter	GCF_008982325.1	Acinetobacter_ursingii	GBJ63_RS14825	20101..23781	108980	BREX-system-P-loop-protein-BrxC-[Acinetobacter-ursingii]
WP_151742861.1	Type1.BREX	<-N6-METHYLASE<-DUF6079*<-BrxB-BrxF-iSTAND<-BrxA-DNA-binding<-wHTH+WYL+WCX||DUF4236-><-CR-ATPase5+REase	1226	Bacteria>Pseudomonadota>Gammaproteobacteria>Moraxellales>Moraxellaceae>Acinetobacter	GCF_008982465.1	unclassified_Acinetobacter	GBL32_RS17905	1894..5574	196816	MULTISPECIES:-BREX-system-P-loop-protein-BrxC-[unclassified-Acinetobacter]
WP_151792238.1	Type1.BREX	<-TatD_DNase<-?<-?<-sGTP-MglA+sGTP<-DOC+HTH<-PglZ<-N6-METHYLASE<-DUF6079*<-BrxB-BrxF-iSTAND<-BrxA-DNA-binding<-?<-?<-DUF932<-ZnR+ZnR<-TPR+REase	1249	Bacteria>Pseudomonadota>Gammaproteobacteria>Moraxellales>Moraxellaceae>Acinetobacter	GCF_008995635.1	Acinetobacter_seifertii	IC766_RS15995	3376176..3379925	1530123	BREX-system-P-loop-protein-BrxC-[Acinetobacter-seifertii]
WP_151817454.1	Type1.BREX	<-SIGMA+OB-Fold+AAA-ATPase+Lon-Protease-BrxL<-PglZ<-N6-METHYLASE<-DUF6079*<-BrxB-BrxF-iSTAND<-BrxA-DNA-binding<-wHTH+WYL+WCX||DUF4236-><-CR-ATPase5+REase<-ParB+HNH	1226	Bacteria>Pseudomonadota>Gammaproteobacteria>Moraxellales>Moraxellaceae>Acinetobacter	GCF_009004075.1	Acinetobacter_ursingii	GBR91_RS15640	8514..12194	108980	BREX-system-P-loop-protein-BrxC-[Acinetobacter-ursingii]
WP_151834043.1	Type1.BREX	XIS-HTH->BrxA-DNA-binding->BrxB-BrxF-iSTAND->DUF6079*->OLD-ABC->REase->N6-METHYLASE->PglZ->SIGMA+OB-Fold+AAA-ATPase+HKD-DNase-BrxL->?->HerA/FtsK->ParB+HNH->ParB+HNH->	1190	Bacteria>Pseudomonadota>Gammaproteobacteria>Moraxellales>Moraxellaceae>Acinetobacter	GCF_009012055.1	Acinetobacter_ursingii	GBX89_RS12550	5375..8947	108980	BREX-system-P-loop-protein-BrxC-[Acinetobacter-ursingii]
WP_151837873.1	Type1.BREX	CR-ATPase5+REase-><-DUF4236||wHTH+WYL+WCX->BrxA-DNA-binding->BrxB-BrxF-iSTAND->DUF6079*->N6-METHYLASE-p->	1226	Bacteria>Pseudomonadota>Gammaproteobacteria>Moraxellales>Moraxellaceae>Acinetobacter	GCF_009013055.1	unclassified_Acinetobacter	GBZ97_RS11405	21380..25060	196816	MULTISPECIES:-BREX-system-P-loop-protein-BrxC-[unclassified-Acinetobacter]
WP_151959064.1	Type1.BREX	ParB+HNH->CR-ATPase5+REase-><-DUF4236||wHTH+WYL+WCX->BrxA-DNA-binding->BrxB-BrxF-iSTAND->DUF6079*->	1226	Bacteria>Pseudomonadota>Gammaproteobacteria>Moraxellales>Moraxellaceae>Acinetobacter	GCF_009013895.1	Acinetobacter_junii	GB010_RS14460	6225..9905	40215	BREX-system-P-loop-protein-BrxC-[Acinetobacter-junii]
WP_153389475.1	Type1.BREX	HTH_3-><-?<-?<-?<-?<-SIGMA+OB-Fold+AAA-ATPase+Lon-Protease-BrxL<-PglZ<-ParB+HNH<-N6-METHYLASE<-DUF6079*<-BrxB-BrxF-iSTAND<-BrxA-DNA-binding<-wHTH+WYL+WCX	1224	Bacteria>Pseudomonadota>Gammaproteobacteria>Moraxellales>Moraxellaceae>Acinetobacter	GCF_009601085.1	Acinetobacter_wanghuae	GHJ48_RS13160	17934..21608	2662362	BREX-system-P-loop-protein-BrxC-[Acinetobacter-wanghuae]
WP_153569296.1	Type1.BREX	Amidohydro_1->35exo->tRNA-><-?<-DDE_Tnp_1_5+DDE_Tnp_1||?->DOC+HTH->||wHTH+WYL+WCX->BrxA-DNA-binding->BrxB-BrxF-iSTAND->DUF6079*->N6-METHYLASE->?->PglZ->SIGMA+OB-Fold+AAA-ATPase+Lon-Protease-BrxL->SF1-SF2-Helicase+REase->OLD-ABC+TOPRIM-><-PhdYeFM_antitox	1226	Bacteria>Pseudomonadota>Gammaproteobacteria>Moraxellales>Moraxellaceae>Acinetobacter	GCF_009645555.1	Acinetobacter_nosocomialis	F4T96_RS05690	68625..72305	106654	BREX-system-P-loop-protein-BrxC-[Acinetobacter-nosocomialis]
WP_159516812.1	Type1.BREX	<-SIGMA+OB-Fold+AAA-ATPase+Lon-Protease-BrxL<-PglZ<-N6-METHYLASE<-DUF6079*<-BrxB-BrxF-iSTAND<-BrxA-DNA-binding<-wHTH+WYL+WCX||DUF4236-><-CR-ATPase5+REase<-HTH_26||?-><-HicB<-Arm-DNA-bind_3+Phage_integrase	1226	Bacteria>Pseudomonadota>Gammaproteobacteria>Moraxellales>Moraxellaceae>Acinetobacter	GCF_009829025.1	Acinetobacter_sp._18QD2AZ41W	GVV58_RS14230	9426..13106	2692137	BREX-system-P-loop-protein-BrxC-[Acinetobacter-sp.-18QD2AZ41W]
WP_160126737.1	Type1.BREX	DDE_Tnp_1_5+DDE_Tnp_1->?->ParB->ParB+HNH->CR-ATPase5+REase-><-DUF4236||wHTH+WYL+WCX->BrxA-DNA-binding->BrxB-BrxF-iSTAND->DUF6079*->N6-METHYLASE->PglZ->SIGMA+OB-Fold+AAA-ATPase+Lon-Protease-BrxL-><-?<-ABhydrolase<-Classical-AAA<-?<-SMODS<-HTH_Tnp_1+HTH_21+rve+rve_3	1226	Bacteria>Pseudomonadota>Gammaproteobacteria>Moraxellales>Moraxellaceae>Acinetobacter	GCF_009892245.1	Acinetobacter_haemolyticus	AhaeAN59_RS17345	50610..54290	29430	BREX-system-P-loop-protein-BrxC-[Acinetobacter-haemolyticus]
WP_160255370.1	Type1.BREX	<-SIGMA+OB-Fold+AAA-ATPase+Lon-Protease-BrxL<-PglZ<-ParB+HNH<-N6-METHYLASE<-DUF6079*<-BrxB-BrxF-iSTAND<-BrxA-DNA-binding<-wHTH+WYL+WCX||DUF4236-><-CR-ATPase5+REase||?-><-Zn_Tnp_IS91+Y2_Tnp	1226	Bacteria>Pseudomonadota>Gammaproteobacteria>Moraxellales>Moraxellaceae>Acinetobacter	GCF_009914395.1	Acinetobacter_indicus	GA646_RS00530	100361..104041	756892	BREX-system-P-loop-protein-BrxC-[Acinetobacter-indicus]
WP_163071913.1	Type1.BREX	<-SIGMA+OB-Fold+AAA-ATPase+Lon-Protease-BrxL<-PglZ<-ParB+HNH<-N6-METHYLASE<-DUF6079*<-BrxB-BrxF-iSTAND<-BrxA-DNA-binding<-wHTH+WYL+WCX||DUF4236-><-CR-ATPase5+REase<-GDResolvase+HIN-HTH||DUF4158+DDE_Tnp_Tn3->	1226	Bacteria>Pseudomonadota>Gammaproteobacteria>Moraxellales>Moraxellaceae>Acinetobacter	GCF_010499775.1	Acinetobacter_baumannii	G3N14_RS16470	10817..14497	470	BREX-system-P-loop-protein-BrxC-[Acinetobacter-baumannii]
WP_163146588.1	Type1.BREX	Zn_Tnp_IS91+Y2_Tnp->?->?-><-HATPase+wHTH<-?||CR-ATPase5+REase-><-DUF4236||wHTH+WYL+WCX->BrxA-DNA-binding->BrxB-BrxF-iSTAND->DUF6079*->N6-METHYLASE->?->PglZ->SIGMA+OB-Fold+AAA-ATPase+Lon-Protease-BrxL->	1226	Bacteria>Pseudomonadota>Gammaproteobacteria>Moraxellales>Moraxellaceae>Acinetobacter	GCF_010918375.1	Acinetobacter_indicus	FSC09_RS16655	95015..98695	756892	BREX-system-P-loop-protein-BrxC-[Acinetobacter-indicus]
WP_164543101.1	Type1.BREX	ParB+HNH->CR-ATPase5+REase->ParB+HNH-p->?-><-DUF4236||wHTH+WYL+WCX->BrxA-DNA-binding->BrxB-BrxF-iSTAND->DUF6079*->N6-METHYLASE->PglZ->SIGMA+OB-Fold+AAA-ATPase+Lon-Protease-BrxL->||?->?->?->?->?->Sulfate_transp+STAS->	1224	Bacteria>Pseudomonadota>Gammaproteobacteria>Moraxellales>Moraxellaceae>Acinetobacter	GCF_011058205.1	Acinetobacter_lwoffii	G4V72_RS07960	7784..11458	28090	BREX-system-P-loop-protein-BrxC-[Acinetobacter-lwoffii]

Protein-ID	System	Gene-Neighbourhoods	Length	Taxonomy	Assembly-ID	Strain	Locus-Tag	Genomic-coordinates	TaxID	Fasta-defline
WP_166137070.1	Type1.BREX	<-N6-METHYLASE<-DUF6079*<-BrxB-BrxF-iSTAND<-BrxA-DNA-binding<-wHTH+WYL+WCX||DUF4236-><-CR-ATPase5+REase<-ParB+HNH<-Phage_integrase<-?<-SbcC+SbcC+ABC-ATPase+SbcC	1226	Bacteria>Pseudomonadota>Gammaproteobacteria>Moraxellales>Moraxellaceae>Acinetobacter	GCF_011192225.1	unclassified_Acinetobacter	G5T41_RS07850	4541..8221	196816	MULTISPECIES:-BREX-system-P-loop-protein-BrxC-[unclassified-Acinetobacter]
WP_168370063.1	Type1.BREX	DUF932->?->?->XIS-HTH->SAM-METHYLASE->wHTH(SBD)+HNH->wHTH+WYL+WCX->BrxA-DNA-binding->BrxB-BrxF-iSTAND->DUF6079*->N6-METHYLASE->ParB+HNH->PglZ->SIGMA+OB-Fold+AAA-ATPase+Lon-Protease-Br [illegible]	1224	Bacteria>Pseudomonadota>Gammaproteobacteria>Moraxellales>Moraxellaceae>Acinetobacter	GCF_012278715.1	Acinetobacter_indicus	HHC64_RS10945	2259038..2262712	756892	BREX-system-P-loop-protein-BrxC-[Acinetobacter-indicus]
WP_168382103.1	Type1.BREX	Phage_integrase+Phage_integrase->Phage_integrase+Phage_integrase->?->ParB+HNH->CR-ATPase5+REase-><-DUF4236||wHTH+WYL+WCX->BrxA-DNA-binding->BrxB-BrxF-iSTAND->DUF6079*->N6-METHYLASE->P [illegible]	1226	Bacteria>Pseudomonadota>Gammaproteobacteria>Moraxellales>Moraxellaceae>Acinetobacter	GCF_012366635.1	Acinetobacter_indicus	HHC47_RS11970	17406..21086	756892	BREX-system-P-loop-protein-BrxC-[Acinetobacter-indicus]
WP_168396480.1	Type1.BREX	Phage_integrase+Phage_integrase->Phage_integrase+Phage_integrase->?->ParB+HNH->CR-ATPase5+REase-><-DUF4236||wHTH+WYL+WCX->BrxA-DNA-binding->BrxB-BrxF-iSTAND->DUF6079*->N6-METHYLASE->S [illegible]	1224	Bacteria>Pseudomonadota>Gammaproteobacteria>Moraxellales>Moraxellaceae>Acinetobacter	GCF_012366875.1	Acinetobacter_indicus	HG607_RS04385	142310..145984	756892	BREX-system-P-loop-protein-BrxC-[Acinetobacter-indicus]
WP_168386656.1	Type1.BREX	HTH_Tnp_1+HTH_21+rve+rve_3-><-?<-ABhydrolase||MerR-HTH->DUF4096+DDE_Tnp_1+DDE_Tnp_1_2->MerR-HTH-><-SIGMA+OB-Fold+AAA-ATPase+Lon-Protease-BrxL<-PglZ<-N6-METHYLASE<-DUF6079*<-BrxB-BrxF- [illegible]	1226	Bacteria>Pseudomonadota>Gammaproteobacteria>Moraxellales>Moraxellaceae>Acinetobacter	GCF_012393445.1	Acinetobacter_lwoffii	FDB28_RS00230	39022..42702	28090	BREX-system-P-loop-protein-BrxC-[Acinetobacter-lwoffii]
WP_169025139.1	Type1.BREX	Phage_integrase+Phage_integrase->Phage_integrase->HTH_Tnp_1+HTH_21+rve+rve_3->CR-ATPase5+REase-><-DUF4236||wHTH+WYL+WCX->BrxA-DNA-binding->BrxB-BrxF-iSTAND->DUF6079*->N6-METHYLASE->Pa [illegible]	1226	Bacteria>Pseudomonadota>Gammaproteobacteria>Moraxellales>Moraxellaceae>Acinetobacter	GCF_012931665.1	Acinetobacter_baumannii	HIN88_RS18590	12016..15696	470	BREX-system-P-loop-protein-BrxC-[Acinetobacter-baumannii]
WP_171530296.1	Type1.BREX	<-ACET<-?||<-SIGMA+OB-Fold+AAA-ATPase+Lon-Protease-BrxL<-PglZ<-ParB+HNH<-N6-METHYLASE<-DUF6079*<-BrxB-BrxF-iSTAND<-BrxA-DNA-binding<-wHTH+WYL+WCX||DUF4236-><-CR-ATPase5+REase<-ParB+HN [illegible]	1226	Bacteria>Pseudomonadota>Gammaproteobacteria>Moraxellales>Moraxellaceae>Acinetobacter	GCF_013072655.1	Acinetobacter_defluvii	A7P53_RS01880	14398..18078	1871111	BREX-system-P-loop-protein-BrxC-[Acinetobacter-defluvii]
WP_171431587.1	Type1.BREX	ParB+HNH->CR-ATPase5+REase-><-DUF4236||wHTH+WYL+WCX->BrxA-DNA-binding->BrxB-BrxF-iSTAND->DUF6079*->N6-METHYLASE->PglZ->SIGMA+OB-Fold+AAA-ATPase+Lon-Protease-BrxL->||?->?->?->?->?->AAA [illegible]	1228	Bacteria>Pseudomonadota>Gammaproteobacteria>Moraxellales>Moraxellaceae>Acinetobacter	GCF_013116605.1	Acinetobacter_indicus	HMI53_RS01195	262958..266644	756892	BREX-system-P-loop-protein-BrxC-[Acinetobacter-indicus]
WP_174660656.1	Type1.BREX	<-GDResolvase+HIN-HTH<-DDE_Tnp_1_5+DDE_Tnp_1||?->?->?-><-ICLR-HTH<-SIGMA+OB-Fold+AAA-ATPase+Lon-Protease-BrxL<-PglZ<-N6-METHYLASE<-DUF6079*<-BrxB-BrxF-iSTAND<-BrxA-DNA-binding<-wHTH+W [illegible]	1226	Bacteria>Pseudomonadota>Gammaproteobacteria>Moraxellales>Moraxellaceae>Acinetobacter	GCF_013343215.1	Acinetobacter_sp._10FS3-1	E5Y90_RS16040	29443..33123	2563897	BREX-system-P-loop-protein-BrxC-[Acinetobacter-sp.-10FS3-1]
WP_174660691.1	Type1.BREX	<-ACET<-?||TRD+TRD-><-?<-SIGMA+OB-Fold+AAA-ATPase+Lon-Protease-BrxL<-PglZ<-SbcC<-N6-METHYLASE<-DUF6079*<-BrxB-BrxF-iSTAND<-BrxA-DNA-binding<-wHTH+WYL+WCX||DUF4236-><-DOC+HTH<-?<-ParB+ [illegible]	1226	Bacteria>Pseudomonadota>Gammaproteobacteria>Moraxellales>Moraxellaceae>Acinetobacter	GCF_013343215.1	Acinetobacter_sp._10FS3-1	E5Y90_RS16730	19958..23638	2563897	BREX-system-P-loop-protein-BrxC-[Acinetobacter-sp.-10FS3-1]
WP_174727826.1	Type1.BREX	BrxA-DNA-binding->BrxB-BrxF-iSTAND->DUF6079*->N6-METHYLASE->ParB+HNH->PglZ->?->?->?->?->?->ZnR+ZnR->	1224	Bacteria>Pseudomonadota>Gammaproteobacteria>Moraxellales>Moraxellaceae>Acinetobacter	GCF_013344765.1	Acinetobacter_lactucae	HUN22_RS07895	1711..5385	1785128	BREX-system-P-loop-protein-BrxC-[Acinetobacter-lactucae]
WP_174761284.1	Type1.BREX	<-N6-METHYLASE<-DUF6079*<-BrxB-BrxF-iSTAND<-BrxA-DNA-binding<-wHTH+WYL+WCX||DUF4236->	1226	Bacteria>Pseudomonadota>Gammaproteobacteria>Moraxellales>Moraxellaceae>Acinetobacter	GCF_013346575.1	Acinetobacter_bereziniae	HUN33_RS09130	1857..5537	106648	BREX-system-P-loop-protein-BrxC-[Acinetobacter-bereziniae]
WP_176538601.1	Type1.BREX	<-DUF4372+DDE_Tnp_1<-LZ_Tnp_IS66+zf-IS66+DDE_Tnp_IS66+DDE_Tnp_IS66_C<-?<-HIN-HTH<-SIGMA+OB-Fold+AAA-ATPase+Lon-Protease-BrxL<-PglZ<-N6-METHYLASE<-DUF6079*<-BrxB-BrxF-iSTAND<-BrxA-DNA- [illegible]	1226	Bacteria>Pseudomonadota>Gammaproteobacteria>Moraxellales>Moraxellaceae>Acinetobacter	GCF_013374795.1	Acinetobacter_sp._NEB_394	HUK62_RS18295	47703..51383	2743575	BREX-system-P-loop-protein-BrxC-[Acinetobacter-sp.-NEB-394]
WP_179994566.1	Type1.BREX	Arm-DNA-bind_3+Phage_integrase-><-?<-?<-?<-TetR-HTH||wHTH+WYL+WCX->BrxA-DNA-binding->BrxB-BrxF-iSTAND->DUF6079*->N6-METHYLASE->ParB+HNH->PglZ->SIGMA+OB-Fold+AAA-ATPase+Lon-Protease [illegible]	1226	Bacteria>Pseudomonadota>Gammaproteobacteria>Moraxellales>Moraxellaceae>Acinetobacter	GCF_013416255.1	Acinetobacter_baumannii	HLG75_RS04210	863150..866830	470	BREX-system-P-loop-protein-BrxC-[Acinetobacter-baumannii]
WP_180070068.1	Type1.BREX	Phage_integrase->||Zn_Tnp_IS1+DDE_Tnp_IS1->||Y1_Tnp->ParB+HNH->CR-ATPase5+REase-><-DUF4236||wHTH+WYL+WCX->BrxA-DNA-binding->BrxB-BrxF-iSTAND->DUF6079*->N6-METHYLASE-p->	1226	Bacteria>Pseudomonadota>Gammaproteobacteria>Moraxellales>Moraxellaceae>Acinetobacter	GCF_013417275.1	Acinetobacter_sp._YH16038	FTV34_RS10085	73238..76918	2601183	BREX-system-P-loop-protein-BrxC-[Acinetobacter-sp.-YH16038]
WP_180001763.1	Type1.BREX	<-SIGMA+OB-Fold+AAA-ATPase+Lon-Protease-BrxL<-PglZ<-N6-METHYLASE<-DUF6079*<-BrxB-BrxF-iSTAND<-BrxA-DNA-binding<-wHTH+WYL+WCX<-ABC-ATPase+SbcC+ABC-ATPase+SbcC	1226	Bacteria>Pseudomonadota>Gammaproteobacteria>Moraxellales>Moraxellaceae>Acinetobacter	GCF_013417355.1	Acinetobacter_sp._YH12255	FTV30_RS13425	9473..13153	2601179	BREX-system-P-loop-protein-BrxC-[Acinetobacter-sp.-YH12255]
WP_180012151.1	Type1.BREX	CR-ATPase5+REase-><-DUF4236||wHTH+WYL+WCX->BrxA-DNA-binding->BrxB-BrxF-iSTAND->DUF6079*->N6-METHYLASE->PglZ->SIGMA+OB-Fold+AAA-ATPase+Lon-Protease-BrxL->||?->?->?->?->?->GDResolvase+H [illegible]	1226	Bacteria>Pseudomonadota>Gammaproteobacteria>Moraxellales>Moraxellaceae>Acinetobacter	GCF_013417705.1	Acinetobacter_sp._YH12235	FTV13_RS12105	5418..9098	2601162	BREX-system-P-loop-protein-BrxC-[Acinetobacter-sp.-YH12235]
WP_180058343.1	Type1.BREX	<-HTH_3<-HigB_toxin||<-SF1-SF2-Helicase+REase<-SIGMA+OB-Fold+AAA-ATPase+Lon-Protease-BrxL<-PglZ<-N6-METHYLASE<-DUF6079*<-BrxB-BrxF-iSTAND<-BrxA-DNA-binding<-wHTH+WYL+WCX<-wHTH+HNH||<- [illegible]	1223	Bacteria>Pseudomonadota>Gammaproteobacteria>Moraxellales>Moraxellaceae>Acinetobacter	GCF_013417785.1	Acinetobacter_sp._YH12227	FTV09_RS11090	15051..18722	2601158	BREX-system-P-loop-protein-BrxC-[Acinetobacter-sp.-YH12227]
WP_180041457.1	Type1.BREX	ParB+HNH-><-?||SAM-METHYLASE->?->SF1-SF2-Helicase->wHTH+HNH->wHTH+WYL+WCX->BrxA-DNA-binding->BrxB-BrxF-iSTAND->DUF6079*->N6-METHYLASE->HKD-DNase+DUF3150->PglZ->SIGMA+OB-Fold+AAA-ATPas [illegible]	1221	Bacteria>Pseudomonadota>Gammaproteobacteria>Moraxellales>Moraxellaceae>Acinetobacter	GCF_013417925.1	Acinetobacter_sp._YH12218	FTV03_RS11390	13993..17658	2601152	BREX-system-P-loop-protein-BrxC-[Acinetobacter-sp.-YH12218]
WP_180082253.1	Type1.BREX	DEOR-HTH-><-SIGMA+OB-Fold+AAA-ATPase+Lon-Protease-BrxL<-PglZ<-?<-N6-METHYLASE<-DUF6079*<-BrxB-BrxF-iSTAND<-BrxA-DNA-binding<-wHTH+WYL+WCX||DUF4236-><-CR-ATPase5+REase	1226	Bacteria>Pseudomonadota>Gammaproteobacteria>Moraxellales>Moraxellaceae>Acinetobacter	GCF_013418145.1	Acinetobacter_sp._YH12201	FTU91_RS11740	12103..15783	2601140	BREX-system-P-loop-protein-BrxC-[Acinetobacter-sp.-YH12201]
WP_180106519.1	Type1.BREX	MarR-HTH->AbiEii->?-><-SIGMA+OB-Fold+AAA-ATPase+Lon-Protease-BrxL<-PglZ<-N6-METHYLASE<-DUF6079*<-BrxB-BrxF-iSTAND<-BrxA-DNA-binding<-wHTH+WYL+WCX	1226	Bacteria>Pseudomonadota>Gammaproteobacteria>Moraxellales>Moraxellaceae>Acinetobacter	GCF_013418285.1	unclassified_Acinetobacter	FTU83_RS11060	37089..40769	196816	MULTISPECIES:-BREX-system-P-loop-protein-BrxC-[unclassified-Acinetobacter]
WP_180056096.1	Type1.BREX	CR-ATPase5+REase-><-DUF4236||wHTH+WYL+WCX->BrxA-DNA-binding->BrxB-BrxF-iSTAND->DUF6079*->N6-METHYLASE->PglZ->SIGMA+OB-Fold+AAA-ATPase+Lon-Protease-BrxL-><-?<-AbiEii<-MarR-HTH	1226	Bacteria>Pseudomonadota>Gammaproteobacteria>Moraxellales>Moraxellaceae>Acinetobacter	GCF_013418435.1	Acinetobacter_sp._YH12142	FTU77_RS14435	5429..9109	2601126	BREX-system-P-loop-protein-BrxC-[Acinetobacter-sp.-YH12142]
WP_180058089.1	Type1.BREX	wHTH+WYL+WCX->BrxA-DNA-binding->BrxB-BrxF-iSTAND->DUF6079*->N6-METHYLASE->PglZ->	1226	Bacteria>Pseudomonadota>Gammaproteobacteria>Moraxellales>Moraxellaceae>Acinetobacter	GCF_013418465.1	Acinetobacter_sp._YH12141	FTU76_RS14840	1801..5481	2601125	BREX-system-P-loop-protein-BrxC-[Acinetobacter-sp.-YH12141]
WP_180052746.1	Type1.BREX	<-SIGMA+OB-Fold+AAA-ATPase+Lon-Protease-BrxL<-PglZ<-?<-N6-METHYLASE<-DUF6079*<-BrxB-BrxF-iSTAND<-BrxA-DNA-binding<-wHTH+WYL+WCX||DUF4236-><-CR-ATPase5+REase<-ParB+HNH<-Y1_Tnp||HTH_Orf [illegible]	1226	Bacteria>Pseudomonadota>Gammaproteobacteria>Moraxellales>Moraxellaceae>Acinetobacter	GCF_013418535.1	unclassified_Acinetobacter	FTU72_RS09265	17103..20783	196816	MULTISPECIES:-BREX-system-P-loop-protein-BrxC-[unclassified-Acinetobacter]
WP_180045992.1	Type1.BREX	LZ_Tnp_IS66+zf-IS66+DDE_Tnp_IS66+DDE_Tnp_IS66_C->ParB+HNH->SAM-METHYLASE->LexA-protease->?->wHTH(SBD)+HNH->wHTH+WYL+WCX->BrxA-DNA-binding->BrxB-BrxF-iSTAND->DUF6079*->N6-METHYLASE->Pa [illegible]	1218	Bacteria>Pseudomonadota>Gammaproteobacteria>Moraxellales>Moraxellaceae>Acinetobacter	GCF_013418865.1	Acinetobacter_sp._YH12117	FTU55_RS01900	159420..163076	2601104	BREX-system-P-loop-protein-BrxC-[Acinetobacter-sp.-YH12117]
WP_180047260.1	Type1.BREX	CR-ATPase5+REase-><-DUF4236||wHTH+WYL+WCX->BrxA-DNA-binding->BrxB-BrxF-iSTAND->DUF6079*->N6-METHYLASE->PglZ->SIGMA+OB-Fold+AAA-ATPase+Lon-Protease-BrxL->||SbcC-><-HTH_3<-RelE-ParE||?- [illegible]	1226	Bacteria>Pseudomonadota>Gammaproteobacteria>Moraxellales>Moraxellaceae>Acinetobacter	GCF_013418865.1	Acinetobacter_sp._YH12117	FTU55_RS12625	5420..9100	2601104	BREX-system-P-loop-protein-BrxC-[Acinetobacter-sp.-YH12117]
WP_180074827.1	Type1.BREX	<-SIGMA+OB-Fold+AAA-ATPase+Lon-Protease-BrxL<-PglZ<-N6-METHYLASE<-DUF6079*<-BrxB-BrxF-iSTAND<-BrxA-DNA-binding<-wHTH+WYL+WCX||DUF4236-><-CR-ATPase5+REase<-ParB+HNH	1226	Bacteria>Pseudomonadota>Gammaproteobacteria>Moraxellales>Moraxellaceae>Acinetobacter	GCF_013418895.1	Acinetobacter_sp._YH12116	FTU54_RS13385	8809..12489	2601103	BREX-system-P-loop-protein-BrxC-[Acinetobacter-sp.-YH12116]
WP_180076555.1	Type1.BREX	Phage_integrase+Phage_integrase->?->ParB+HNH->?->DOC+HTH-><-DUF4236||wHTH+WYL+WCX->BrxA-DNA-binding->BrxB-BrxF-iSTAND->DUF6079*->N6-METHYLASE->PglZ->SIGMA+OB-Fold+AAA-ATPase+Lon-Prote [illegible]	1224	Bacteria>Pseudomonadota>Gammaproteobacteria>Moraxellales>Moraxellaceae>Acinetobacter	GCF_013418995.1	Acinetobacter_sp._YH12106	FTU45_RS13255	18925..22599	2601094	BREX-system-P-loop-protein-BrxC-[Acinetobacter-sp.-YH12106]
WP_180086491.1	Type1.BREX	<-DUF6079*<-BrxB-BrxF-iSTAND<-BrxA-DNA-binding<-wHTH+WYL+WCX||DUF4236-><-DOC+HTH<-?<-?||?-><-ParB	1224	Bacteria>Pseudomonadota>Gammaproteobacteria>Moraxellales>Moraxellaceae>Acinetobacter	GCF_013419115.1	Acinetobacter_sp._YH12103	FTU43_RS13620	1696..5370	2601092	BREX-system-P-loop-protein-BrxC-[Acinetobacter-sp.-YH12103]
WP_180036170.1	Type1.BREX	<-SIGMA+OB-Fold+AAA-ATPase+Lon-Protease-BrxL<-PglZ<-N6-METHYLASE<-DUF6079*<-BrxB-BrxF-iSTAND<-BrxA-DNA-binding<-wHTH+WYL+WCX||DUF4236->	1226	Bacteria>Pseudomonadota>Gammaproteobacteria>Moraxellales>Moraxellaceae>Acinetobacter	GCF_013419295.1	unclassified_Acinetobacter	FTU34_RS16295	9372..13052	196816	MULTISPECIES:-BREX-system-P-loop-protein-BrxC-[unclassified-Acinetobacter]
WP_180108075.1	Type1.BREX	<-SIGMA+OB-Fold+AAA-ATPase+HKD-DNase-BrxL<-PglZ<-N6-METHYLASE<-REase<-OLD-ABC<-DUF6079*<-BrxB-BrxF-iSTAND<-XIS-HTH<-?<-wHTH+HNH<-SF1-SF2-Helicase<-?<-SAM-METHYLASE||?-><-ParB+HNH	1190	Bacteria>Pseudomonadota>Gammaproteobacteria>Moraxellales>Moraxellaceae>Acinetobacter	GCF_013419415.1	Acinetobacter_sp._YH12085	FTU28_RS13915	49013..52585	2601077	BREX-system-P-loop-protein-BrxC-[Acinetobacter-sp.-YH12085]
WP_180131186.1	Type1.BREX	Phage_integrase-><-HTH_OrfB_IS605+OrfB_IS605+OrfB_Zn_ribbon||Y1_Tnp->ParB+HNH->CR-ATPase5+REase-><-DUF4236||wHTH+WYL+WCX->BrxA-DNA-binding->BrxB-BrxF-iSTAND->DUF6079*->N6-METHYLASE->H [illegible]	1226	Bacteria>Pseudomonadota>Gammaproteobacteria>Moraxellales>Moraxellaceae>Acinetobacter	GCF_013419565.1	Acinetobacter_sp._YH12072	FTU19_RS11080	35259..38939	2601068	BREX-system-P-loop-protein-BrxC-[Acinetobacter-sp.-YH12072]
WP_180114868.1	Type1.BREX	CR-ATPase5+REase-><-DUF4236||wHTH+WYL+WCX->BrxA-DNA-binding->BrxB-BrxF-iSTAND->DUF6079*->N6-METHYLASE->PglZ->SIGMA+OB-Fold+AAA-ATPase+Lon-Protease-BrxL->||?->?->?->?-><-DDE_Tnp_IS240	1226	Bacteria>Pseudomonadota>Gammaproteobacteria>Moraxellales>Moraxellaceae>Acinetobacter	GCF_013419715.1	Acinetobacter_sp._YH12063	FTU12_RS15130	5418..9098	2601061	BREX-system-P-loop-protein-BrxC-[Acinetobacter-sp.-YH12063]
WP_180160550.1	Type1.BREX	<-SIGMA+OB-Fold+AAA-ATPase+Lon-Protease-BrxL<-PglZ<-?<-N6-METHYLASE<-DUF6079*<-BrxB-BrxF-iSTAND<-BrxA-DNA-binding<-wHTH+WYL+WCX||DUF4236-><-CR-ATPase5+REase	1226	Bacteria>Pseudomonadota>Gammaproteobacteria>Moraxellales>Moraxellaceae>Acinetobacter	GCF_013419795.1	Acinetobacter_sp._YH12054	FTU07_RS15240	20642..24322	2601056	BREX-system-P-loop-protein-BrxC-[Acinetobacter-sp.-YH12054]
WP_180165638.1	Type1.BREX	<-SIGMA+OB-Fold+AAA-ATPase+Lon-Protease-BrxL<-PglZ<-?<-N6-METHYLASE<-DUF6079*<-BrxB-BrxF-iSTAND<-BrxA-DNA-binding<-wHTH+WYL+WCX||DUF4236-><-CR-ATPase5+REase	1226	Bacteria>Pseudomonadota>Gammaproteobacteria>Moraxellales>Moraxellaceae>Acinetobacter	GCF_013419875.1	Acinetobacter_sp._YH12049	FTU05_RS13040	31963..35643	2601054	BREX-system-P-loop-protein-BrxC-[Acinetobacter-sp.-YH12049]
WP_180174851.1	Type1.BREX	<-DUF4096+DDE_Tnp_1+DDE_Tnp_1_2<-?||?-><-MACRODOMAIN<-REase||<-SF1-SF2-Helicase+REase<-SIGMA+OB-Fold+AAA-ATPase+Lon-Protease-BrxL<-PglZ<-N6-METHYLASE<-DUF6079*<-BrxB-BrxF-iSTAND<-BrxA [illegible]	1226	Bacteria>Pseudomonadota>Gammaproteobacteria>Moraxellales>Moraxellaceae>Acinetobacter	GCF_013420235.1	Acinetobacter_sp._YH01022	FTT87_RS10805	36696..40376	2601036	BREX-system-P-loop-protein-BrxC-[Acinetobacter-sp.-YH01022]
WP_180087504.1	Type1.BREX	[illegible]	1226	Bacteria>Pseudomonadota>Gammaproteobacteria>Moraxellales>Moraxellaceae>Acinetobacter	GCF_013420255.1	unclassified_Acinetobacter	FTT86_RS12000	35844..39524	196816	MULTISPECIES:-BREX-system-P-loop-protein-BrxC-[unclassified-Acinetobacter]
WP_180178840.1	Type1.BREX	[illegible]	1226	Bacteria>Pseudomonadota>Gammaproteobacteria>Moraxellales>Moraxellaceae>Acinetobacter	GCF_013420795.1	Acinetobacter_sp._YH01013	FTT80_RS13290	20368..24048	2601029	BREX-system-P-loop-protein-BrxC-[Acinetobacter-sp.-YH01013]
WP_180006134.1	Type1.BREX	[illegible]	1226	Bacteria>Pseudomonadota>Gammaproteobacteria>Moraxellales>Moraxellaceae>Acinetobacter	GCF_013420805.1	unclassified_Acinetobacter	FTT79_RS10555	27794..31474	196816	MULTISPECIES:-BREX-system-P-loop-protein-BrxC-[unclassified-Acinetobacter]
WP_182925313.1	Type1.BREX	[illegible]	1226	Bacteria>Pseudomonadota>Gammaproteobacteria>Moraxellales>Moraxellaceae>Acinetobacter	GCF_014169655.1	Acinetobacter_baumannii	H7R41_RS13110	2778553..2782233	470	BREX-system-P-loop-protein-BrxC-[Acinetobacter-baumannii]
WP_185218776.1	Type1.BREX	[illegible]	1226	Bacteria>Pseudomonadota>Gammaproteobacteria>Moraxellales>Moraxellaceae>Acinetobacter	GCF_014218985.1	Acinetobacter_baumannii	H6W49_RS17735	6511..10191	470	BREX-system-P-loop-protein-BrxC-[Acinetobacter-baumannii]
WP_188199768.1	Type1.BREX	[illegible]	1226	Bacteria>Pseudomonadota>Gammaproteobacteria>Moraxellales>Moraxellaceae>Acinetobacter	GCF_014560035.1	Acinetobacter_baumannii	HPJ37_RS18500	29485..33165	470	BREX-system-P-loop-protein-BrxC-[Acinetobacter-baumannii]
WP_193893631.1	Type1.BREX	[illegible]	1226	Bacteria>Pseudomonadota>Gammaproteobacteria>Moraxellales>Moraxellaceae>Acinetobacter	GCF_015209685.1	Acinetobacter_albensis	IQR32_RS12760	7183..10863	1673609	BREX-system-P-loop-protein-BrxC-[Acinetobacter-albensis]
WP_180025627.1	Type1.BREX	[illegible]	1226	Bacteria>Pseudomonadota>Gammaproteobacteria>Moraxellales>Moraxellaceae>Acinetobacter	GCF_015218525.1	unclassified_Acinetobacter	GO029_RS17570	86789..90469	196816	MULTISPECIES:-BREX-system-P-loop-protein-BrxC-[unclassified-Acinetobacter]
WP_136512845.1	Type1.BREX	[illegible]	1226	Bacteria>Pseudomonadota>Gammaproteobacteria>Moraxellales>Moraxellaceae>Acinetobacter	GCF_015265775.1	Acinetobacter_baumannii	O9X70_RS06810	133723..137403	470	BREX-system-P-loop-protein-BrxC-[Acinetobacter-baumannii]
WP_196075355.1	Type1.BREX	[illegible]	1204	Bacteria>Pseudomonadota>Gammaproteobacteria>Moraxellales>Moraxellaceae>Acinetobacter	GCF_015676315.1	unclassified_Acinetobacter	IMO39_RS19270	9799..13413	196816	MULTISPECIES:-BREX-system-P-loop-protein-BrxC-[unclassified-Acinetobacter]
WP_198080379.1	Type1.BREX	[illegible]	1222	Bacteria>Pseudomonadota>Gammaproteobacteria>Moraxellales>Moraxellaceae>Acinetobacter	GCF_016082245.1	Acinetobacter_calcoaceticus	BL242_RS20750	15792..19460	471	BREX-system-P-loop-protein-BrxC-[Acinetobacter-calcoaceticus]
WP_199973361.1	Type1.BREX	[illegible]	1249	Bacteria>Pseudomonadota>Gammaproteobacteria>Moraxellales>Moraxellaceae>Acinetobacter	GCF_016502305.1	Acinetobacter_courvalinii	I6M62_RS06800	311191..314940	280147	BREX-system-P-loop-protein-BrxC-[Acinetobacter-courvalinii]
WP_032001825.1	Type1.BREX	[illegible]	1226	Bacteria>Pseudomonadota>Gammaproteobacteria>Moraxellales>Moraxellaceae>Acinetobacter	GCF_016502765.1	Acinetobacter_calcoaceticus/baumannii_complex	I6M92_RS14775	66667..70347	909768	MULTISPECIES:-BREX-system-P-loop-protein-BrxC-[Acinetobacter-calcoaceticus/baumannii-complex]
WP_199939733.1	Type1.BREX	[illegible]	1226	Bacteria>Pseudomonadota>Gammaproteobacteria>Moraxellales>Moraxellaceae>Acinetobacter	GCF_016506315.1	Acinetobacter_baumannii	I5555_RS13010	139911..143591	470	BREX-system-P-loop-protein-BrxC-[Acinetobacter-baumannii]
WP_200230916.1	Type1.BREX	[illegible]	1226	Bacteria>Pseudomonadota>Gammaproteobacteria>Moraxellales>Moraxellaceae>Acinetobacter	GCF_016607565.1	Acinetobacter_variabilis	IAQ69_RS15330	32669..36349	70346	BREX-system-P-loop-protein-BrxC-[Acinetobacter-variabilis]
WP_201704335.1	Type1.BREX	[illegible]	1223	Bacteria>Pseudomonadota>Gammaproteobacteria>Moraxellales>Moraxellaceae>Acinetobacter	GCF_016726625.1	Acinetobacter_johnsonii	I6I51_RS17025	51852..55523	40214	BREX-system-P-loop-protein-BrxC-[Acinetobacter-johnsonii]
WP_203080037.1	Type1.BREX	[illegible]	1226	Bacteria>Pseudomonadota>Gammaproteobacteria>Moraxellales>Moraxellaceae>Acinetobacter	GCF_016804045.1	Acinetobacter_pittii	HRJ47_RS04290	884929..888609	48296	BREX-system-P-loop-protein-BrxC-[Acinetobacter-pittii]
WP_204086201.1	Type1.BREX	[illegible]	1226	Bacteria>Pseudomonadota>Gammaproteobacteria>Moraxellales>Moraxellaceae>Acinetobacter	GCF_016889825.1	Acinetobacter_pittii	I6J44_RS19355	45796..49476	48296	BREX-system-P-loop-protein-BrxC-[Acinetobacter-pittii]
WP_205779680.1	Type1.BREX	[illegible] 66+DDE_Tnp_IS66_C	1226	Bacteria>Pseudomonadota>Gammaproteobacteria>Moraxellales>Moraxellaceae>Acinetobacter	GCF_017132735.1	Acinetobacter_indicus	JYB86_RS06365	1326455..1330135	756892	BREX-system-P-loop-protein-BrxC-[Acinetobacter-indicus]
WP_206863034.1	Type1.BREX	[illegible]	1226	Bacteria>Pseudomonadota>Gammaproteobacteria>Moraxellales>Moraxellaceae>Acinetobacter	GCF_017309425.1	Acinetobacter_indicus	JOW32_RS14080	4215..7895	756892	BREX-system-P-loop-protein-BrxC-[Acinetobacter-indicus]
WP_206896797.1	Type1.BREX	[illegible]	1226	Bacteria>Pseudomonadota>Gammaproteobacteria>Moraxellales>Moraxellaceae>Acinetobacter	GCF_017309445.1	Acinetobacter_indicus	JOW33_RS13795	2887588..2891268	756892	BREX-system-P-loop-protein-BrxC-[Acinetobacter-indicus]
WP_208464845.1	Type1.BREX	[illegible]	537	Bacteria>Pseudomonadota>Gammaproteobacteria>Moraxellales>Moraxellaceae>Acinetobacter	GCF_017592465.1	Acinetobacter_haemolyticus	J5N55_RS15595	1..1612	29430	BREX-system-P-loop-protein-BrxC,-partial-[Acinetobacter-haemolyticus]
WP_109426631.1	Type1.BREX	[illegible]	1226	Bacteria>Pseudomonadota>Gammaproteobacteria>Moraxellales>Moraxellaceae>Acinetobacter	GCF_017726575.1	Acinetobacter_baumannii	J6I88_RS04260	873693..877373	470	BREX-system-P-loop-protein-BrxC-[Acinetobacter-baumannii]
WP_212272673.1	Type1.BREX	[illegible]	1226	Bacteria>Pseudomonadota>Gammaproteobacteria>Moraxellales>Moraxellaceae>Acinetobacter	GCF_018139165.1	Acinetobacter_nosocomialis	KC103_RS15085	69521..73201	106654	BREX-system-P-loop-protein-BrxC-[Acinetobacter-nosocomialis]
WP_213705174.1	Type1.BREX	[illegible]	1226	Bacteria>Pseudomonadota>Gammaproteobacteria>Moraxellales>Moraxellaceae>Acinetobacter	GCF_018446675.1	Acinetobacter_baumannii	KKV18_RS14925	28314..31994	470	BREX-system-P-loop-protein-BrxC-[Acinetobacter-baumannii]
WP_215276195.1	Type1.BREX	[illegible]	1226	Bacteria>Pseudomonadota>Gammaproteobacteria>Moraxellales>Moraxellaceae>Acinetobacter	GCF_018678125.1	Acinetobacter_baumannii	KMP96_RS12165	16275..19955	470	BREX-system-P-loop-protein-BrxC-[Acinetobacter-baumannii]
WP_216174886.1	Type1.BREX	[illegible]	1226	Bacteria>Pseudomonadota>Gammaproteobacteria>Moraxellales>Moraxellaceae>Acinetobacter	GCF_018863255.1	Acinetobacter_baumannii	KPE97_RS15710	83323..87003	470	BREX-system-P-loop-protein-BrxC-[Acinetobacter-baumannii]
WP_216116819.1	Type1.BREX	[illegible]	1226	Bacteria>Pseudomonadota>Gammaproteobacteria>Moraxellales>Moraxellaceae>Acinetobacter	GCF_018883565.1	Acinetobacter_baumannii	KPF69_RS05265	35520..39200	470	BREX-system-P-loop-protein-BrxC-[Acinetobacter-baumannii]
WP_216936951.1	Type1.BREX	[illegible]	1190	Bacteria>Pseudomonadota>Gammaproteobacteria>Moraxellales>Moraxellaceae>Acinetobacter	GCF_019038255.1	unclassified_Acinetobacter	KUG40_RS10660	10626..14198	196816	MULTISPECIES:-BREX-system-P-loop-protein-BrxC-[unclassified-Acinetobacter]
WP_218698253.1	Type1.BREX	[illegible]	1224	Bacteria>Pseudomonadota>Gammaproteobacteria>Moraxellales>Moraxellaceae>Acinetobacter	GCF_019279015.1	Acinetobacter_harbinensis	KZA13_RS08660	34272..37946	1353941	BREX-system-P-loop-protein-BrxC-[Acinetobacter-harbinensis]
WP_221260378.1	Type1.BREX	[illegible]	1226	Bacteria>Pseudomonadota>Gammaproteobacteria>Moraxellales>Moraxellaceae>Acinetobacter	GCF_019702905.1	Acinetobacter_sp._Tol_5	TOL5_RS12615	2696813..2700493	710648	BREX-system-P-loop-protein-BrxC-[Acinetobacter-sp.-Tol-5]
WP_223106928.1	Type1.BREX	[illegible]	1226	Bacteria>Pseudomonadota>Gammaproteobacteria>Moraxellales>Moraxellaceae>Acinetobacter	GCF_019903235.1	Acinetobacter_baumannii	H2787_RS17960	80252..83932	470	BREX-system-P-loop-protein-BrxC-[Acinetobacter-baumannii]
WP_223239187.1	Type1.BREX	[illegible]	1226	Bacteria>Pseudomonadota>Gammaproteobacteria>Moraxellales>Moraxellaceae>Acinetobacter	GCF_019920865.1	Acinetobacter_baumannii	HLI16_RS17115	2695..6375	470	BREX-system-P-loop-protein-BrxC-[Acinetobacter-baumannii]
WP_207272916.1	Type1.BREX	[illegible]	1226	Bacteria>Pseudomonadota>Gammaproteobacteria>Moraxellales>Moraxellaceae>Acinetobacter	GCF_019921105.1	Acinetobacter_baumannii	HLH91_RS14165	18027..21707	470	BREX-system-P-loop-protein-BrxC-[Acinetobacter-baumannii]
WP_005236280.1	Type1.BREX	[illegible]	1226	Bacteria>Pseudomonadota>Gammaproteobacteria>Moraxellales>Moraxellaceae>Acinetobacter	GCF_020099375.1	Acinetobacter_variabilis	LA331_RS04655	963646..967326	70346	BREX-system-P-loop-protein-BrxC-[Acinetobacter-variabilis]
WP_225554349.1	Type1.BREX	[illegible]	1226	Bacteria>Pseudomonadota>Gammaproteobacteria>Moraxellales>Moraxellaceae>Acinetobacter	GCF_020271765.1	Acinetobacter_pseudolwoffii	LDO52_RS14245	193616..1867	2053287	BREX-system-P-loop-protein-BrxC-[Acinetobacter-pseudolwoffii]
WP_225554403.1	Type1.BREX	[illegible]	1226	Bacteria>Pseudomonadota>Gammaproteobacteria>Moraxellales>Moraxellaceae>Acinetobacter	GCF_020271765.1	Acinetobacter_pseudolwoffii	LDO52_RS15430	47806..51486	2053287	BREX-system-P-loop-protein-BrxC-[Acinetobacter-pseudolwoffii]
WP_228414014.1	Type1.BREX	[illegible]	1226	Bacteria>Pseudomonadota>Gammaproteobacteria>Moraxellales>Moraxellaceae>Acinetobacter	GCF_020810655.1	Acinetobacter_baumannii	NLHDIDDJ_RS06545	1352098..1355778	470	BREX-system-P-loop-protein-BrxC-[Acinetobacter-baumannii]
WP_230245698.1	Type1.BREX	[illegible]	1226	Bacteria>Pseudomonadota>Gammaproteobacteria>Moraxellales>Moraxellaceae>Acinetobacter	GCF_020982655.1	Acinetobacter_sp._PW68	K1T76_RS14000	121130..124810	2865162	BREX-system-P-loop-protein-BrxC-[Acinetobacter-sp.-PW68]
WP_233751176.1	Type1.BREX	[illegible]	1226	Bacteria>Pseudomonadota>Gammaproteobacteria>Moraxellales>Moraxellaceae>Acinetobacter	GCF_021386775.1	Acinetobacter_pittii	KR570_RS16050	9603..13283	48296	BREX-system-P-loop-protein-BrxC-[Acinetobacter-pittii]
WP_233945509.1	Type1.BREX	[illegible]	1226	Bacteria>Pseudomonadota>Gammaproteobacteria>Moraxellales>Moraxellaceae>Acinetobacter	GCF_021391555.1	Acinetobacter_sp._SH20PTE14	LXF01_RS17045	120215..123895	2905879	BREX-system-P-loop-protein-BrxC-[Acinetobacter-sp.-SH20PTE14]
WP_234115082.1	Type1.BREX	[illegible]	1226	Bacteria>Pseudomonadota>Gammaproteobacteria>Moraxellales>Moraxellaceae>Acinetobacter	GCF_021432045.1	Acinetobacter_towneri	LZG54_RS14455	23931..27611	202956	BREX-system-P-loop-protein-BrxC-[Acinetobacter-towneri]
WP_234693498.1	Type1.BREX	[illegible]	1226	Bacteria>Pseudomonadota>Gammaproteobacteria>Moraxellales>Moraxellaceae>Acinetobacter	GCF_021460555.1	Acinetobacter_baumannii	LH676_RS19735	26787..30467	470	BREX-system-P-loop-protein-BrxC-[Acinetobacter-baumannii]
WP_236095241.1	Type1.BREX	[illegible]	1226	Bacteria>Pseudomonadota>Gammaproteobacteria>Moraxellales>Moraxellaceae>Acinetobacter	GCF_021560715.1	Acinetobacter_sp._SCLZS86	LZP46_RS14905	6362..10042	2908637	BREX-system-P-loop-protein-BrxC-[Acinetobacter-sp.-SCLZS86]
WP_236163089.1	Type1.BREX	[illegible]	1226	Bacteria>Pseudomonadota>Gammaproteobacteria>Moraxellales>Moraxellaceae>Acinetobacter	GCF_021602685.1	unclassified_Acinetobacter	L6B80_RS11000	14595..18275	196816	MULTISPECIES:-BREX-system-P-loop-protein-BrxC-[unclassified-Acinetobacter]
WP_239046817.1	Type1.BREX	[illegible]	1226	Bacteria>Pseudomonadota>Gammaproteobacteria>Moraxellales>Moraxellaceae>Acinetobacter	GCF_022343575.1	Acinetobacter_sp._AG3	L3V46_RS22595	25482..29162	2912245	BREX-system-P-loop-protein-BrxC-[Acinetobacter-sp.-AG3]
WP_241305487.1	Type1.BREX	[illegible]	1248	Bacteria>Pseudomonadota>Gammaproteobacteria>Moraxellales>Moraxellaceae>Acinetobacter	GCF_022549325.1	Acinetobacter_sp._NIPH_605	MMP73_RS14775	133059..136805	2923434	BREX-system-P-loop-protein-BrxC-[Acinetobacter-sp.-NIPH-605]
WP_241338465.1	Type1.BREX	[illegible]	1226	Bacteria>Pseudomonadota>Gammaproteobacteria>Moraxellales>Moraxellaceae>Acinetobacter	GCF_022549435.1	Acinetobacter_sp._ANC_5659	MMP64_RS09425	122256..125936	2923427	BREX-system-P-loop-protein-BrxC-[Acinetobacter-sp.-ANC-5659]
WP_243475070.1	Type1.BREX	[illegible]	1226	Bacteria>Pseudomonadota>Gammaproteobacteria>Moraxellales>Moraxellaceae>Acinetobacter	GCF_022809915.1	Acinetobacter_lwoffii	MTX11_RS10505	45498..49178	28090	BREX-system-P-loop-protein-BrxC-[Acinetobacter-lwoffii]
WP_248102847.1	Type1.BREX	[illegible]	1226	Bacteria>Pseudomonadota>Gammaproteobacteria>Moraxellales>Moraxellaceae>Acinetobacter	GCF_023158935.1	Acinetobacter_portensis	MZD21_RS15025	45372..49052	1839785	BREX-system-P-loop-protein-BrxC-[Acinetobacter-portensis]
WP_249468201.1	Type1.BREX	[illegible]	1226	Bacteria>Pseudomonadota>Gammaproteobacteria>Moraxellales>Moraxellaceae>Acinetobacter	GCF_023371125.1	Acinetobacter_baumannii	M4045_RS18565	26114..29794	470	BREX-system-P-loop-protein-BrxC-[Acinetobacter-baumannii]
WP_249571539.1	Type1.BREX	[illegible]	1226	Bacteria>Pseudomonadota>Gammaproteobacteria>Moraxellales>Moraxellaceae>Acinetobacter	GCF_023488045.1	Acinetobacter_sp._ANC5681	M5121_RS14220	11775..15455	2929504	BREX-system-P-loop-protein-BrxC-[Acinetobacter-sp.-ANC5681]
WP_250180441.1	Type1.BREX	[illegible]	386	Bacteria>Pseudomonadota>Gammaproteobacteria>Moraxellales>Moraxellaceae>Acinetobacter	GCF_023572305.1	Acinetobacter_baumannii	M9Y19_RS19145	4860..6017	470	BREX-system-P-loop-protein-BrxC,-partial-[Acinetobacter-baumannii]
WP_128202440.1	Type1.BREX	[illegible]	1226	Bacteria>Pseudomonadota>Gammaproteobacteria>Moraxellales>Moraxellaceae>Acinetobacter	GCF_023896505.1	Acinetobacter_baumannii	O9X56_RS14280	107190..110870	470	BREX-system-P-loop-protein-BrxC-[Acinetobacter-baumannii]
WP_253153041.1	Type1.BREX	[illegible]	1226	Bacteria>Pseudomonadota>Gammaproteobacteria>Moraxellales>Moraxellaceae>Acinetobacter	GCF_024129285.1	Acinetobacter_sp._S4397-1	NI467_RS11480	6279..9959	2972915	BREX-system-P-loop-protein-BrxC-[Acinetobacter-sp.-S4397-1]
WP_253097522.1	Type1.BREX	[illegible]	1226	Bacteria>Pseudomonadota>Gammaproteobacteria>Moraxellales>Moraxellaceae>Acinetobacter	GCF_024129755.1	Acinetobacter_indicus	NI393_RS13245	9324..13004	756892	BREX-system-P-loop-protein-BrxC-[Acinetobacter-indicus]
WP_253167193.1	Type1.BREX	[illegible]	1226	Bacteria>Pseudomonadota>Gammaproteobacteria>Moraxellales>Moraxellaceae>Acinetobacter	GCF_024129795.1	Acinetobacter_indicus	NI451_RS01020	208268..211948	756892	BREX-system-P-loop-protein-BrxC-[Acinetobacter-indicus]
WP_253133338.1	Type1.BREX	[illegible]	1224	Bacteria>Pseudomonadota>Gammaproteobacteria>Moraxellales>Moraxellaceae>Acinetobacter	GCF_024129835.1	Acinetobacter_indicus	NI394_RS03575	37848..41522	756892	BREX-system-P-loop-protein-BrxC-[Acinetobacter-indicus]
WP_253145790.1	Type1.BREX	[illegible]	1222	Bacteria>Pseudomonadota>Gammaproteobacteria>Moraxellales>Moraxellaceae>Acinetobacter	GCF_024129855.1	Acinetobacter_lwoffii	NI465_RS14465	11210..14878	28090	BREX-system-P-loop-protein-BrxC-[Acinetobacter-lwoffii]
WP_257259040.1	Type1.BREX	[illegible]	1226	Bacteria>Pseudomonadota>Gammaproteobacteria>Moraxellales>Moraxellaceae>Acinetobacter	GCF_024589115.1	Acinetobacter_baumannii	L2615_RS07865	180845..184525	470	BREX-system-P-loop-protein-BrxC-[Acinetobacter-baumannii]
WP_180004195.1	Type1.BREX	[illegible]	1226	Bacteria>Pseudomonadota>Gammaproteobacteria>Moraxellales>Moraxellaceae>Acinetobacter	GCF_024605785.1	unclassified_Acinetobacter	MST16_RS17335	65383..69063	196816	MULTISPECIES:-BREX-system-P-loop-protein-BrxC-[unclassified-Acinetobacter]
WP_257935145.1	Type1.BREX	[illegible]	1226	Bacteria>Pseudomonadota>Gammaproteobacteria>Moraxellales>Moraxellaceae>Acinetobacter	GCF_024705725.1	Acinetobacter_baumannii	MSH04_RS15115	32588..36268	470	BREX-system-P-loop-protein-BrxC-[Acinetobacter-baumannii]
WP_238640059.1	Type1.BREX	[illegible]	1226	Bacteria>Pseudomonadota>Gammaproteobacteria>Moraxellales>Moraxellaceae>Acinetobacter	GCF_025264005.1	Acinetobacter_baumannii	NT399_RS17670	10742..14422	470	BREX-system-P-loop-protein-BrxC-[Acinetobacter-baumannii]
WP_262463188.1	Type1.BREX	[illegible]	1226	Bacteria>Pseudomonadota>Gammaproteobacteria>Moraxellales>Moraxellaceae>Acinetobacter	GCF_025513035.1	Acinetobacter_pittii	KTG74_RS19040	10083..13763	48296	BREX-system-P-loop-protein-BrxC-[Acinetobacter-pittii]
WP_262456128.1	Type1.BREX	[illegible]	1226	Bacteria>Pseudomonadota>Gammaproteobacteria>Moraxellales>Moraxellaceae>Acinetobacter	GCF_025513465.1	Acinetobacter_bereziniae	KTH44_RS20855	4679..8359	106648	BREX-system-P-loop-protein-BrxC-[Acinetobacter-bereziniae]
WP_262468026.1	Type1.BREX	[illegible]	1226	Bacteria>Pseudomonadota>Gammaproteobacteria>Moraxellales>Moraxellaceae>Acinetobacter	GCF_025513595.1	Acinetobacter_schindleri	KTH34_RS14665	11459..15139	108981	BREX-system-P-loop-protein-BrxC-[Acinetobacter-schindleri]
WP_262737085.1	Type1.BREX	[illegible]	1226	Bacteria>Pseudomonadota>Gammaproteobacteria>Moraxellales>Moraxellaceae>Acinetobacter	GCF_025514155.1	Acinetobacter_pittii	KTI11_RS17510	13692..17372	48296	BREX-system-P-loop-protein-BrxC-[Acinetobacter-pittii]
WP_086355030.1	Type1.BREX	[illegible]	1226	Bacteria>Pseudomonadota>Gammaproteobacteria>Moraxellales>Moraxellaceae>Acinetobacter	GCF_025514625.1	Acinetobacter_calcoaceticus/baumannii_complex	KTI87_RS17630	13118..16798	909768	MULTISPECIES:-BREX-system-P-loop-protein-BrxC-[Acinetobacter-calcoaceticus/baumannii-complex]
WP_262737771.1	Type1.BREX	[illegible]	1226	Bacteria>Pseudomonadota>Gammaproteobacteria>Moraxellales>Moraxellaceae>Acinetobacter	GCF_025515095.1	Acinetobacter_variabilis	KTJ53_RS03615	103263..106943	70346	BREX-system-P-loop-protein-BrxC-[Acinetobacter-variabilis]
WP_262579272.1	Type1.BREX	[illegible]	1226	Bacteria>Pseudomonadota>Gammaproteobacteria>Moraxellales>Moraxellaceae>Acinetobacter	GCF_025562355.1	Acinetobacter_junii	KTH64_RS13010	10561..14241	40215	BREX-system-P-loop-protein-BrxC-[Acinetobacter-junii]
WP_262558821.1	Type1.BREX	[illegible]	1224	Bacteria>Pseudomonadota>Gammaproteobacteria>Moraxellales>Moraxellaceae>Acinetobacter	GCF_025562515.1	Acinetobacter_bereziniae	KTH91_RS20770	1047..4721	106648	BREX-system-P-loop-protein-BrxC-[Acinetobacter-bereziniae]
WP_263513455.1	Type1.BREX	[illegible]	1226	Bacteria>Pseudomonadota>Gammaproteobacteria>Moraxellales>Moraxellaceae>Acinetobacter	GCF_025723145.1	Acinetobacter_ursingii	LSO60_RS18250	102062..105742	108980	BREX-system-P-loop-protein-BrxC-[Acinetobacter-ursingii]
WP_265466015.1	Type1.BREX	[illegible]	1226	Bacteria>Pseudomonadota>Gammaproteobacteria>Moraxellales>Moraxellaceae>Acinetobacter	GCF_026168575.1	Acinetobacter_sp._BIT-DXN8	OKC24_RS16365	19397..23077	2989714	BREX-system-P-loop-protein-BrxC-[Acinetobacter-sp.-BIT-DXN8]
WP_267982262.1	Type1.BREX	[illegible]	1226	Bacteria>Pseudomonadota>Gammaproteobacteria>Moraxellales>Moraxellaceae>Acinetobacter	GCF_026736615.1	Acinetobacter_sp._VNH17	OYK78_RS17990	7578..11258	2998078	BREX-system-P-loop-protein-BrxC-[Acinetobacter-sp.-VNH17]
WP_071225618.1	Type1.BREX	[illegible]	1226	Bacteria>Pseudomonadota>Gammaproteobacteria>Moraxellales>Moraxellaceae>Acinetobacter	GCF_027854895.1	Acinetobacter_baumannii	MJO31_RS19545	43789..47469	470	BREX-system-P-loop-protein-BrxC-[Acinetobacter-baumannii]
WP_274579352.1	Type1.BREX	[illegible]	1226	Bacteria>Pseudomonadota>Gammaproteobacteria>Moraxellales>Moraxellaceae>Acinetobacter	GCF_028774645.1	Acinetobacter_lactucae	MOO54_RS17975	24360..28040	1785128	BREX-system-P-loop-protein-BrxC-[Acinetobacter-lactucae]
WP_275290158.1	Type1.BREX	[illegible]	1226	Bacteria>Pseudomonadota>Gammaproteobacteria>Moraxellales>Moraxellaceae>Acinetobacter	GCF_029100495.1	Acinetobacter_nosocomialis	JIZ05_14385	7251..10931	106654	BREX-system-P-loop-protein-BrxC-[Acinetobacter-nosocomialis]
WP_161409476.1	Type1.BREX	[illegible]	1226	Bacteria>Pseudomonadota>Gammaproteobacteria>Moraxellales>Moraxellaceae>Acinetobacter	GCF_030329445.1	Acinetobacter_haemolyticus	GPS61_RS07925	49241..52921	29430	BREX-system-P-loop-protein-BrxC-[Acinetobacter-haemolyticus]
WP_005115116.1	Type1.BREX	[illegible]	1226	Bacteria>Pseudomonadota>Gammaproteobacteria>Moraxellales>Moraxellaceae>Acinetobacter	GCF_030413705.1	Acinetobacter_baumannii	AWM09_RS06130	10901..14581	470	BREX-system-P-loop-protein-BrxC-[Acinetobacter-baumannii]
WP_112987434.1	Type1.BREX	[illegible]	1226	Bacteria>Pseudomonadota>Gammaproteobacteria>Moraxellales>Moraxellaceae>Acinetobacter	GCF_030665425.1	Acinetobacter_junii	DDF86_RS15435	8518..12198	40215	BREX-system-P-loop-protein-BrxC-[Acinetobacter-junii]
WP_092692667.1	Type1.BREX	[illegible]	1224	Bacteria>Pseudomonadota>Gammaproteobacteria>Moraxellales>Moraxellaceae>Acinetobacter	GCF_900107285.1	Acinetobacter_kyonggiensis	BLV99_RS16485	12458..16132	595670	BREX-system-P-loop-protein-BrxC-[Acinetobacter-kyonggiensis]
WP_074947383.1	Type1.BREX	[illegible]	1226	Bacteria>Pseudomonadota>Gammaproteobacteria>Moraxellales>Moraxellaceae>Acinetobacter	GCF_900116265.1	Acinetobacter_bohemicus	F7P73_RS14505	5974..9654	1435036	BREX-system-P-loop-protein-BrxC-[Acinetobacter-bohemicus]
WP_005403809.1	Type1.BREX	[illegible]	1226	Bacteria>Pseudomonadota>Gammaproteobacteria>Moraxellales>Moraxellaceae>Acinetobacter	GCF_902374105.1	Acinetobacter_junii	FXX57_RS02410	133086..136766	40215	BREX-system-P-loop-protein-BrxC-[Acinetobacter-junii]
WP_002120185.1	Type1.BREX	[illegible]	1226	Bacteria>Pseudomonadota>Gammaproteobacteria>Moraxellales>Moraxellaceae	-	Acinetobacter	LG653_RS19250	36203..39883	469	MULTISPECIES:-BREX-system-P-loop-protein-BrxC-[Acinetobacter]
WP_115736701.1	Type1.BREX	[illegible]	1226	Bacteria>Pseudomonadota>Gammaproteobacteria>Moraxellales>Moraxellaceae	-	Acinetobacter	MK935_RS00110	25389..29069	469	MULTISPECIES:-BREX-system-P-loop-protein-BrxC-[Acinetobacter]
WP_180169606.1	Type1.BREX	[illegible]	1226	Bacteria>Pseudomonadota>Gammaproteobacteria>Moraxellales>Moraxellaceae>Acinetobacter	-	unclassified_Acinetobacter	H5J21_RS00665	124577..128257	196816	MULTISPECIES:-BREX-system-P-loop-protein-BrxC-[unclassified-Acinetobacter]
WP_180172593.1	Type1.BREX	[illegible]	1226	Bacteria>Pseudomonadota>Gammaproteobacteria>Moraxellales>Moraxellaceae>Acinetobacter	-	unclassified_Acinetobacter	LW346_RS00160	40718..44398	196816	MULTISPECIES:-BREX-system-P-loop-protein-BrxC-[unclassified-Acinetobacter]
WP_107866915.1	Type1.BREX	[illegible]	1216	Bacteria>Pseudomonadota>Gammaproteobacteria>Moraxellales>Moraxellaceae>Agitococcus	GCF_003051055.1	Agitococcus_lubricus	C8N29_RS15175	21839..25489	1077255	BREX-system-P-loop-protein-BrxC-[Agitococcus-lubricus]
WP_134244556.1	Type1.BREX	[illegible]	1226	Bacteria>Pseudomonadota>Gammaproteobacteria>Moraxellales>Moraxellaceae>Alkanindiges	GCF_004377485.1	Alkanindiges_illinoisensis	E2B99_RS08410	8100..11780	197183	BREX-system-P-loop-protein-BrxC-[Alkanindiges-illinoisensis]
WP_002010901.1	Type1.BREX	[illegible]	1226	Bacteria>Pseudomonadota>Gammaproteobacteria>Moraxellales>Moraxellaceae	GCF_000214965.1	Acinetobacter	ACINNAV18_RS19255	65599..69279	469	MULTISPECIES:-BREX-system-P-loop-protein-BrxC-[Acinetobacter]
WP_001021842.1	Type1.BREX	[illegible]	1226	Bacteria>Pseudomonadota>Gammaproteobacteria>Moraxellales>Moraxellaceae	GCF_000241705.1	Acinetobacter	HO22_RS0119175	11899..15579	469	MULTISPECIES:-BREX-system-P-loop-protein-BrxC-[Acinetobacter]
WP_111885889.1	Type1.BREX	[illegible]	1226	Bacteria>Pseudomonadota>Gammaproteobacteria>Moraxellales>Moraxellaceae	GCF_002339435.1	Acinetobacter	NVV26_RS12530	20211..23891	469	MULTISPECIES:-BREX-system-P-loop-protein-BrxC-[Acinetobacter]
WP_108675357.1	Type1.BREX	[illegible]	1225	Bacteria>Pseudomonadota>Gammaproteobacteria>Moraxellales>Moraxellaceae	GCF_003072585.2	Acinetobacter	CO119_RS05575	1118908..1122585	469	MULTISPECIES:-BREX-system-P-loop-protein-BrxC-[Acinetobacter]
WP_071320614.1	Type1.BREX	[illegible]	1224	Bacteria>Pseudomonadota>Gammaproteobacteria>Moraxellales>Moraxellaceae	GCF_007833615.1	Acinetobacter	DPR03_RS15465	213962..217636	469	MULTISPECIES:-BREX-system-P-loop-protein-BrxC-[Acinetobacter]
WP_075316556.1	Type1.BREX	[illegible]	1226	Bacteria>Pseudomonadota>Gammaproteobacteria>Moraxellales>Moraxellaceae	GCF_008993855.1	Acinetobacter	GBN82_RS18495	2355..6035	469	MULTISPECIES:-BREX-system-P-loop-protein-BrxC-[Acinetobacter]
WP_152630429.1	Type1.BREX	[illegible]	1226	Bacteria>Pseudomonadota>Gammaproteobacteria>Moraxellales>Moraxellaceae	GCF_009014155.1	Acinetobacter	QJ110_RS14020	22608..26288	469	MULTISPECIES:-BREX-system-P-loop-protein-BrxC-[Acinetobacter]
WP_152342636.1	Type1.BREX	[illegible]	1226	Bacteria>Pseudomonadota>Gammaproteobacteria>Moraxellales>Moraxellaceae	GCF_009296145.1	Acinetobacter	MOW12_RS15415	73736..77416	469	MULTISPECIES:-BREX-system-P-loop-protein-BrxC-[Acinetobacter]
WP_100355920.1	Type1.BREX	[illegible]	1226	Bacteria>Pseudomonadota>Gammaproteobacteria>Moraxellales>Moraxellaceae	GCF_009914395.1	Acinetobacter	GA646_RS15620	96338..100018	469	MULTISPECIES:-BREX-system-P-loop-protein-BrxC-[Acinetobacter]
WP_163167826.1	Type1.BREX	[illegible]	1226	Bacteria>Pseudomonadota>Gammaproteobacteria>Moraxellales>Moraxellaceae	GCF_010917815.1	Acinetobacter	FSC02_RS15715	99085..102765	469	MULTISPECIES:-BREX-system-P-loop-protein-BrxC-[Acinetobacter]
WP_178319334.1	Type1.BREX	[illegible]	1226	Bacteria>Pseudomonadota>Gammaproteobacteria>Moraxellales>Moraxellaceae	GCF_013390985.1	Acinetobacter	HX743_RS11980	35327..39007	469	MULTISPECIES:-BREX-system-P-loop-protein-BrxC-[Acinetobacter]
WP_159138903.1	Type1.BREX	[illegible]	1226	Bacteria>Pseudomonadota>Gammaproteobacteria>Moraxellales>Moraxellaceae	GCF_013417415.1	Acinetobacter	FTV29_RS13965	21709..25389	469	MULTISPECIES:-BREX-system-P-loop-protein-BrxC-[Acinetobacter]
WP_159153679.1	Type1.BREX	[illegible]	1226	Bacteria>Pseudomonadota>Gammaproteobacteria>Moraxellales>Moraxellaceae	GCF_013420015.1	Acinetobacter	FTT98_RS13935	5674..9354	469	MULTISPECIES:-BREX-system-P-loop-protein-BrxC-[Acinetobacter]
WP_180192273.1	Type1.BREX	[illegible]	1224	Bacteria>Pseudomonadota>Gammaproteobacteria>Moraxellales>Moraxellaceae	GCF_015217905.1	Acinetobacter	GO027_RS16200	28364..32038	469	MULTISPECIES:-BREX-system-P-loop-protein-BrxC-[Acinetobacter]
WP_180047180.1	Type1.BREX	[illegible]	1226	Bacteria>Pseudomonadota>Gammaproteobacteria>Moraxellales>Moraxellaceae	GCF_015218165.1	Acinetobacter	GO028_RS19990	40652..44332	469	MULTISPECIES:-BREX-system-P-loop-protein-BrxC-[Acinetobacter]
WP_199980844.1	Type1.BREX	[illegible]	1225	Bacteria>Pseudomonadota>Gammaproteobacteria>Moraxellales>Moraxellaceae	GCF_019702905.1	Acinetobacter	TOL5_RS22165	83634..87311	469	MULTISPECIES:-BREX-system-P-loop-protein-BrxC-[Acinetobacter]
WP_252172191.1	Type1.BREX	[illegible]	1226	Bacteria>Pseudomonadota>Gammaproteobacteria>Moraxellales>Moraxellaceae	GCF_023809625.1	Acinetobacter	N5980_RS16655	54910..58590	469	MULTISPECIES:-BREX-system-P-loop-protein-BrxC-[Acinetobacter]
WP_180000666.1	Type1.BREX	[illegible]	1224	Bacteria>Pseudomonadota>Gammaproteobacteria>Moraxellales>Moraxellaceae	GCF_024159345.1	Acinetobacter	MUB06_RS13820	43929..47603	469	MULTISPECIES:-BREX-system-P-loop-protein-BrxC-[Acinetobacter]

Protein-ID	System	Gene-Neighbourhoods	Length	Taxonomy	Assembly-ID	Strain	Locus-Tag	Genomic-coordinates	TaxID	Fasta-define
ABE75386.1	Type1.BREX	[illegible]	1201	[illegible]	[illegible]	[illegible]	Pcryo_1609	1979457..1983062	335284	[illegible]
GAF52839.1	Type1.BREX	[illegible]	1200	[illegible]	[illegible]	[illegible]	JCM18900_11381	174807..178411	1298608	[illegible]
OLF41388.1	Type1.BREX	[illegible]	1154	[illegible]	[illegible]	[illegible]	BTV99_04785	263905..267369	1926479	[illegible]
HCI75257.1	Type1.BREX	[illegible]	1201	[illegible]	[illegible]	[illegible]	DHV27_03225	245755..249360	56811	[illegible]
MCG3880112.1	Type1.BREX	[illegible]	1154	[illegible]	[illegible]	[illegible]	I3255_11455	48655..52119	2790960	[illegible]
WP_226939352.1	Type1.BREX	[illegible]	1221	[illegible]	[illegible]	[illegible]	CEP87_RS02910	676881..680546	330922	[illegible]
WP_261863080.1	Type1.BREX	[illegible]	801	[illegible]	[illegible]	[illegible]	TT10_RS07330	174747..177152	1298608	[illegible]
WP_261869530.1	Type1.BREX	[illegible]	642	[illegible]	[illegible]	[illegible]	TT11_RS05300	11820..13748	1298609	[illegible]
WP_218607358.1	Type1.BREX	[illegible]	1162	[illegible]	[illegible]	[illegible]	BTV99_RS04780	263881..267369	1926479	[illegible]
WP_089554755.1	Type1.BREX	[illegible]	1223	[illegible]	[illegible]	[illegible]	CAN34_RS05290	13543..17214	1028414	[illegible]
WP_226950212.1	Type1.BREX	[illegible]	1221	[illegible]	[illegible]	[illegible]	INS97_RS03560	152962..156627	196806	[illegible]
WP_191691193.1	Type1.BREX	[illegible]	1222	[illegible]	[illegible]	[illegible]	H9653_RS05990	24340..28008	2762238	[illegible]
WP_192529491.1	Type1.BREX	[illegible]	1218	[illegible]	[illegible]	[illegible]	EI161_09970	10554..14210	2487706	[illegible]
WP_207991848.1	Type1.BREX	[illegible]	1221	[illegible]	[illegible]	[illegible]	J3492_RS09680	48612..52277	2818440	[illegible]
WP_238038933.1	Type1.BREX	[illegible]	1162	[illegible]	[illegible]	[illegible]	I3255_RS11455	48631..52119	2790960	[illegible]
WP_241878421.1	Type1.BREX	[illegible]	709	[illegible]	[illegible]	[illegible]	MN210_RS12710	2340484..2342613	2565531	[illegible]
WP_199774478.1	Type1.BREX	[illegible]	1164	[illegible]	[illegible]	[illegible]	C6W44_RS10120	2338090..2341584	2086574	[illegible]
WP_198336379.1	Type1.BREX	[illegible]	1201	[illegible]	[illegible]	[illegible]	KF936_RS04990	111156..114761	306572	[illegible]
WP_201558729.1	Type1.BREX	[illegible]	1201	[illegible]	[illegible]	[illegible]	JMW74_RS07725	23770..27375	498	[illegible]
WP_201567145.1	Type1.BREX	[illegible]	1221	[illegible]	[illegible]	[illegible]	JMW93_RS11045	31356..35021	498	[illegible]
WP_201625912.1	Type1.BREX	[illegible]	1146	[illegible]	[illegible]	[illegible]	JMX31_RS11870	11441..14881	498	[illegible]
WP_201583149.1	Type1.BREX	[illegible]	1201	[illegible]	[illegible]	[illegible]	JMX18_RS01805	438947..442552	179010	[illegible]
WP_198334111.1	Type1.BREX	[illegible]	1210	[illegible]	[illegible]	[illegible]	JMX36_RS06945	21523..25155	292734	[illegible]
WP_201608697.1	Type1.BREX	[illegible]	1221	[illegible]	[illegible]	[illegible]	JMX84_RS13335	11608..15273	212403	[illegible]
WP_201591398.1	Type1.BREX	[illegible]	1221	[illegible]	[illegible]	[illegible]	JMX70_RS09025	249655..253320	228654	[illegible]
WP_201542137.1	Type1.BREX	[illegible]	1210	[illegible]	[illegible]	[illegible]	LK453_RS13670	3248348..3251980	861445	[illegible]
WP_265088975.1	Type1.BREX	[illegible]	1221	[illegible]	[illegible]	[illegible]	JMX96_RS01730	439208..442873	1298608	[illegible]
WP_201579699.1	Type1.BREX	[illegible]	1223	[illegible]	[illegible]	[illegible]	JMY00_RS07535	19023..22694	2774133	[illegible]
WP_011513935.1	Type1.BREX	[illegible]	1201	[illegible]	[illegible]	[illegible]	Pcryo_1609	1979457..1983062	330922	[illegible]
TQC95078.1	Type1.BREX	[illegible]	1192	[illegible]	[illegible]	[illegible]	FK216_14950	12152..15730	2591038	[illegible]
MBC7753680.1	Type1.BREX	[illegible]	1163	[illegible]	[illegible]	[illegible]	H7Z71_05545	8991..12482	1889775	[illegible]
MBL0229728.1	Type1.BREX	[illegible]	1147	[illegible]	[illegible]	[illegible]	IPP76_02595	6399..9842	1889775	[illegible]
MCP5177609.1	Type1.BREX	[illegible]	1192	[illegible]	[illegible]	[illegible]	H6997_08670	33703..37281	1889775	[illegible]
WP_010129447.1	Type1.BREX	[illegible]	1225	Bacteria>Pseudomonadota	[illegible]	[illegible]	MTT88_RS00175	38411..42088	1236	[illegible]
WP_228349567.1	Type1.BREX	[illegible]	1203	[illegible]	[illegible]	[illegible]	JN531_RS14475	2992880..2996491	2806210	[illegible]
WP_022975319.1	Type1.BREX	[illegible]	1223	[illegible]	[illegible]	[illegible]	G513_RS0102845	457016..460687	64002	[illegible]
MAY26112.1	Type1.BREX	[illegible]	1203	[illegible]	[illegible]	[illegible]	CMK02_07435	15345..18956	2024852	[illegible]
WP_063140235.1	Type1.BREX	[illegible]	1145	[illegible]	[illegible]	[illegible]	A3Q32_03160	117678..121115	1817793	[illegible]
WP_161382580.1	Type1.BREX	[illegible]	1193	[illegible]	[illegible]	[illegible]	GN155_RS00640	132435..136016	2695858	[illegible]
WP_161384457.1	Type1.BREX	[illegible]	1192	[illegible]	[illegible]	[illegible]	GN155_RS10555	118232..121810	2695858	[illegible]
EDX89607.1	Type1.BREX	[illegible]	1187	[illegible]	[illegible]	[illegible]	ADG881_1709	3122242..3125805	236097	[illegible]
MAD69915.1	Type1.BREX	[illegible]	1193	[illegible]	[illegible]	[illegible]	CL538_05260	26266..29847	1872427	[illegible]
KAF0808603.1	Type1.BREX	[illegible]	1165	[illegible]	[illegible]	[illegible]	A167_02568	1..3498	1177159	[illegible]
MBA3979207.1	Type1.BREX	[illegible]	1145	[illegible]	[illegible]	[illegible]	C0462_01260	7976..11413	1872427	[illegible]
HIK75733.1	Type1.BREX	[illegible]	1193	[illegible]	[illegible]	[illegible]	EYG00_13690	18356..21937	1872427	[illegible]
MCH2557814.1	Type1.BREX	[illegible]	1193	[illegible]	[illegible]	[illegible]	MK005_11000	47715..51296	1872427	[illegible]
WP_039926861.1	Type1.BREX	[illegible]	1192	[illegible]	[illegible]	[illegible]	ADG881_RS14175	3122227..3125805	236097	[illegible]
WP_272975234.1	Type1.BREX	[illegible]	1192	[illegible]	[illegible]	[illegible]	B2Z32_RS11825	47111..50689	64988	[illegible]
WP_159559845.1	Type1.BREX	[illegible]	1165	[illegible]	[illegible]	[illegible]	A167_RS13005	1..3498	1177159	[illegible]
WP_103916700.1	Type1.BREX	[illegible]	1193	[illegible]	[illegible]	[illegible]	C4S63_RS03510	780383..783964	1798238	[illegible]
MBF5052669.1	Type1.BREX	[illegible]	1187	[illegible]	[illegible]	[illegible]	ISO4_01271	133562..137125	1177184	[illegible]
WP_145953601.1	Type1.BREX	[illegible]	1192	[illegible]	[illegible]	[illegible]	P40_RS14805	3223957..3227535	1094342	[illegible]
WP_194855579.1	Type1.BREX	[illegible]	1192	[illegible]	[illegible]	[illegible]	ISO4_RS06450	133547..137125	172371	[illegible]
WP_204429962.1	Type1.BREX	[illegible]	1193	[illegible]	[illegible]	[illegible]	IM760_RS12910	16365..19946	1177169	[illegible]
WP_228232948.1	Type1.BREX	[illegible]	1193	[illegible]	[illegible]	[illegible]	LL252_RS03465	287759..291340	1177169	[illegible]
WP_262731202.1	Type1.BREX	[illegible]	1145	[illegible]	[illegible]	[illegible]	B27N_RS13560	6066..9503	1177169	[illegible]
WP_008734404.1	Type1.BREX	[illegible]	1192	[illegible]	[illegible]	[illegible]	S7S_RS18615	4158589..4162167	1306787	[illegible]
RLP52821.1	Type1.BREX	[illegible]	1193	[illegible]	[illegible]	[illegible]	D6160_18810	15503..19084	2083498	[illegible]
WP_101745536.1	Type1.BREX	[illegible]	1220	[illegible]	[illegible]	[illegible]	CX664_RS04280	14964..18626	1701104	[illegible]
WP_153766763.1	Type1.BREX	[illegible]	1223	[illegible]	[illegible]	[illegible]	EOPP23_14760	167593..171264	2072845	[illegible]
WP_163371285.1	Type1.BREX	[illegible]	1220	[illegible]	[illegible]	[illegible]	EO230_RS03420	3343..7005	1701104	[illegible]
WP_257265108.1	Type1.BREX	[illegible]	1183	[illegible]	[illegible]	[illegible]	KDK14_RS10760	1444..4995	2828741	[illegible]
WP_257266232.1	Type1.BREX	[illegible]	1223	[illegible]	[illegible]	[illegible]	KDK14_RS16285	8032..11703	2828741	[illegible]
WP_262567770.1	Type1.BREX	[illegible]	1221	[illegible]	[illegible]	[illegible]	NX722_RS09445	2085702..2089367	1234144	[illegible]
WP_262597505.1	Type1.BREX	[illegible]	1227	[illegible]	[illegible]	[illegible]	NX720_RS21900	5400416..5404099	1234143	[illegible]
WP_269676957.1	Type1.BREX	[illegible]	1218	[illegible]	[illegible]	[illegible]	O2T12_RS06825	1602761..1606417	3009078	[illegible]
MBE8233358.1	Type1.BREX	[illegible]	1234	[illegible]	[illegible]	[illegible]	HAW67_06440	1819..5523	2590577	[illegible]
MCK5893416.1	Type1.BREX	[illegible]	578	[illegible]	[illegible]	[illegible]	KAG53_03165	4085..5821	2590577	[illegible]
AZZ95337.1	Type1.BREX	[illegible]	1183	[illegible]	[illegible]	[illegible]	ENC22_30655	6892256..6895807	1628392	[illegible]
WP_164887423.1	Type1.BREX	[illegible]	1182	[illegible]	[illegible]	[illegible]	EUZ85_RS02365	526761..530309	1628392	[illegible]
MCP5206320.1	Type1.BREX	[illegible]	910	[illegible]	[illegible]	[illegible]	H7A01_03865	876629..879361	2048971	[illegible]
MCP5206322.1	Type1.BREX	[illegible]	261	[illegible]	[illegible]	[illegible]	H7A01_03875	880608..881393	2048971	[illegible]
MCP5213002.1	Type1.BREX	[illegible]	1218	[illegible]	[illegible]	[illegible]	H6998_18960	8319..11975	2048971	[illegible]
WP_040239271.1	Type1.BREX	[illegible]	1144	[illegible]	[illegible]	[illegible]	BN995_RS00415	38131..41565	223900	[illegible]
WP_075368269.1	Type1.BREX	[illegible]	1232	[illegible]	[illegible]	[illegible]	BTW10_RS03965	313854..317552	223900	[illegible]
WP_062359357.1	Type1.BREX	[illegible]	1225	[illegible]	[illegible]	[illegible]	TK80_RS02225	5404..9081	2745	[illegible]
WP_041158482.1	Type1.BREX	[illegible]	1227	[illegible]	[illegible]	[illegible]	BLR10_RS07715	76580..80263	2745	[illegible]
PHR03208.1	Type1.BREX	[illegible]	1144	[illegible]	[illegible]	[illegible]	COB32_05665	22826..26260	1486246	[illegible]
PHR03139.1	Type1.BREX	[illegible]	1227	[illegible]	[illegible]	[illegible]	COB32_06215	64469..68152	1486246	[illegible]
MBL1268350.1	Type1.BREX	[illegible]	1236	[illegible]	[illegible]	[illegible]	COA87_011520	231341..235051	1486246	[illegible]
HBS18545.1	Type1.BREX	[illegible]	380	[illegible]	[illegible]	[illegible]	DD835_12090	760..1900	1486246	[illegible]
HBQ05713.1	Type1.BREX	[illegible]	642	[illegible]	[illegible]	[illegible]	DD676_03555	4448..6375	1486246	[illegible]
NGO89106.1	Type1.BREX	[illegible]	1225	[illegible]	[illegible]	[illegible]	GBQ79_05545	39516..43193	1486246	[illegible]
NQY75752.1	Type1.BREX	[illegible]	1227	[illegible]	[illegible]	[illegible]	HRT78_01215	164704..168387	1486246	[illegible]
WP_008956851.1	Type1.BREX	[illegible]	1230	[illegible]	[illegible]	[illegible]	HAL1_RS04490	19261..22953	550984	[illegible]
WP_009095648.1	Type1.BREX	[illegible]	1225	[illegible]	[illegible]	[illegible]	BB497_RS11605	2566793..2570470	1118153	[illegible]
WP_017428908.1	Type1.BREX	[illegible]	1247	[illegible]	[illegible]	[illegible]	C793_RS0100125	27130..30873	553386	[illegible]
WP_027962108.1	Type1.BREX	[illegible]	1144	[illegible]	[illegible]	[illegible]	Q358_RS0111245	19354..22788	28252	[illegible]
WP_027961732.1	Type1.BREX	[illegible]	1239	[illegible]	[illegible]	[illegible]	Q358_RS0113555	784..4503	28252	[illegible]
WP_031385055.1	Type1.BREX	[illegible]	1193	[illegible]	[illegible]	[illegible]	HALZIN_RS0115275	3254805..3258386	1178777	[illegible]
WP_035558509.1	Type1.BREX	[illegible]	1236	[illegible]	[illegible]	[illegible]	KO116_RS11715	2523126..2526836	1504981	[illegible]
WP_253272941.1	Type1.BREX	[illegible]	525	[illegible]	[illegible]	[illegible]	CH74_RS19345	4877..6454	1471202	[illegible]
WP_062374360.1	Type1.BREX	[illegible]	1227	[illegible]	[illegible]	[illegible]	A3220_RS08340	63501..67184	1819251	[illegible]
WP_078087405.1	Type1.BREX	[illegible]	1225	[illegible]	[illegible]	[illegible]	BV504_RS06350	1459939..1463616	2609666	[illegible]
WP_083023778.1	Type1.BREX	[illegible]	1227	[illegible]	[illegible]	[illegible]	B4967_RS04180	22810..26493	1144478	[illegible]
WP_273192082.1	Type1.BREX	[illegible]	1236	[illegible]	[illegible]	[illegible]	COA87_011520	231341..235051	1486246	[illegible]
WP_101147206.1	Type1.BREX	[illegible]	1230	[illegible]	[illegible]	[illegible]	CXF87_RS15340	100201..103893	2058321	[illegible]
WP_106232068.1	Type1.BREX	[illegible]	1239	[illegible]	[illegible]	[illegible]	BCL64_RS14665	13662..17381	229007	[illegible]
WP_107181354.1	Type1.BREX	[illegible]	1147	[illegible]	[illegible]	[illegible]	C9J49_RS09500	199072..202515	2137478	[illegible]
WP_114487751.1	Type1.BREX	[illegible]	1222	[illegible]	[illegible]	[illegible]	DU506_RS15210	37022..40690	2282306	[illegible]
WP_125748596.1	Type1.BREX	[illegible]	1227	[illegible]	[illegible]	[illegible]	DDR56_RS08110	77879..81562	44935	[illegible]
WP_133481548.1	Type1.BREX	[illegible]	1193	[illegible]	[illegible]	[illegible]	DFO68_RS01985	17598..21179	229007	[illegible]
WP_133481656.1	Type1.BREX	[illegible]	1144	[illegible]	[illegible]	[illegible]	DFO68_RS02360	102047..105481	229007	[illegible]
WP_133482273.1	Type1.BREX	[illegible]	1231	[illegible]	[illegible]	[illegible]	DFO68_RS03945	156725..160420	229007	[illegible]
WP_074210658.1	Type1.BREX	[illegible]	1227	[illegible]	[illegible]	[illegible]	HME01_RS04155	107071..110754	29570	[illegible]
WP_159343441.1	Type1.BREX	[illegible]	1193	[illegible]	[illegible]	[illegible]	CTT34_RS16815	3563093..3566674	29570	[illegible]
WP_161430980.1	Type1.BREX	[illegible]	1239	[illegible]	[illegible]	[illegible]	GRB96_RS04755	991195..994914	147248	[illegible]
WP_163558376.1	Type1.BREX	[illegible]	1144	[illegible]	[illegible]	[illegible]	FEF15_RS06635	15883..19317	2484813	[illegible]
WP_172420975.1	Type1.BREX	[illegible]	1227	[illegible]	[illegible]	[illegible]	HHSLTHF2_RS10320	2155809..2159492	115561	[illegible]
WP_172514441.1	Type1.BREX	[illegible]	1227	[illegible]	[illegible]	[illegible]	HMEPL2_RS01760	381650..385333	29570	[illegible]
WP_172515133.1	Type1.BREX	[illegible]	1193	[illegible]	[illegible]	[illegible]	HMEPL2_RS11780	2496852..2500433	29570	[illegible]
WP_153844139.1	Type1.BREX	[illegible]	1227	[illegible]	[illegible]	[illegible]	GYM47_RS09720	2110719..2114402	2609667	[illegible]
WP_169956446.1	Type1.BREX	[illegible]	1193	[illegible]	[illegible]	[illegible]	HIR79_RS02110	421842..425423	2730360	[illegible]
WP_179915949.1	Type1.BREX	[illegible]	1227	[illegible]	[illegible]	[illegible]	HZS80_RS09710	42867..46550	186761	[illegible]
WP_180091726.1	Type1.BREX	[illegible]	1227	[illegible]	[illegible]	[illegible]	HZU72_RS10350	63822..67505	2729618	[illegible]
WP_183333607.1	Type1.BREX	[illegible]	1144	[illegible]	[illegible]	[illegible]	BDK63_RS15585	2230..5664	213554	[illegible]
WP_188638083.1	Type1.BREX	[illegible]	1154	[illegible]	[illegible]	[illegible]	IEW65_RS03380	721900..725364	1602943	[illegible]
WP_019020105.1	Type1.BREX	[illegible]	1193	[illegible]	[illegible]	[illegible]	IE067_RS16235	61170..64751	453962	[illegible]
WP_192538995.1	Type1.BREX	[illegible]	1227	[illegible]	[illegible]	[illegible]	EI547_RS13715	35008..38691	2487703	[illegible]
WP_193059740.1	Type1.BREX	[illegible]	1227	[illegible]	[illegible]	[illegible]	INT28_RS17780	3434..7117	2609666	[illegible]
WP_218314905.1	Type1.BREX	[illegible]	1227	[illegible]	[illegible]	[illegible]	KVY02_RS07715	1661242..1664925	2855441	[illegible]
WP_231664595.1	Type1.BREX	[illegible]	1153	[illegible]	[illegible]	[illegible]	K7I12_RS15025	26514..29975	2876584	[illegible]
WP_239296660.1	Type1.BREX	[illegible]	1193	[illegible]	[illegible]	[illegible]	MHM35_RS10400	116133..119714	2609666	[illegible]
WP_240567170.1	Type1.BREX	[illegible]	1232	[illegible]	[illegible]	[illegible]	MKP05_RS04205	52453..56151	2920385	[illegible]
WP_253557196.1	Type1.BREX	[illegible]	1225	[illegible]	[illegible]	[illegible]	BIS08_RS08535	5439..9116	2609666	[illegible]
WP_263591023.1	Type1.BREX	[illegible]	1144	[illegible]	[illegible]	[illegible]	OCT48_RS01560	327087..330521	2982692	[illegible]
WP_089705394.1	Type1.BREX	[illegible]	1189	[illegible]	[illegible]	[illegible]	BLR97_RS09380	247223..250792	416873	[illegible]
WP_089676470.1	Type1.BREX	[illegible]	1239	[illegible]	[illegible]	[illegible]	BLP95_RS01590	346471..350190	419597	[illegible]
WP_103969332.1	Type1.BREX	[illegible]	1193	[illegible]	[illegible]	[illegible]	C4S71_RS12465	87857..91438	52021	[illegible]
WP_089675693.1	Type1.BREX	[illegible]	1227	[illegible]	[illegible]	[illegible]	BMY49_RS12195	13754..17437	77097	[illegible]
WP_079550430.1	Type1.BREX	[illegible]	1240	[illegible]	[illegible]	[illegible]	B5495_RS00605	132715..136437	29571	[illegible]
WP_159177832.1	Type1.BREX	[illegible]	1193	[illegible]	[illegible]	[illegible]	JMX33_RS24540	5309589..5313170	2609666	[illegible]
WP_179915499.1	Type1.BREX	[illegible]	1144	[illegible]	[illegible]	[illegible]	HZS80_RS06355	4022..7456	186761	[illegible]
WP_213229339.1	Type1.BREX	[illegible]	1241	[illegible]	[illegible]	[illegible]	DYI26_RS12555	49628..53353	2109328	[illegible]
WP_254893719.1	Type1.BREX	[illegible]	1210	[illegible]	[illegible]	[illegible]	B7L72_RS00095	19375..23007	1670448	[illegible]
WP_081193131.1	Type1.BREX	[illegible]	1154	[illegible]	[illegible]	[illegible]	B7L72_RS05665	10416..13880	1670448	[illegible]
WP_249273207.1	Type1.BREX	[illegible]	530	[illegible]	[illegible]	[illegible]	B7L71_RS08255	397144..398734	1670449	[illegible]
WP_086901154.1	Type1.BREX	[illegible]	1237	[illegible]	[illegible]	[illegible]	B9H00_RS13900	3054889..3058602	157779	[illegible]
WP_189514712.1	Type1.BREX	[illegible]	1144	[illegible]	[illegible]	[illegible]	IE123_RS02380	531699..535133	1508770	[illegible]
WP_163650581.1	Type1.BREX	[illegible]	1144	[illegible]	[illegible]	[illegible]	G7A98_RS15465	3721..7155	2679914	[illegible]
WP_224416377.1	Type1.BREX	[illegible]	1144	[illegible]	[illegible]	[illegible]	KGQ90_RS13860	2977413..2980847	390637	[illegible]
WP_087719602.1	Type1.BREX	[illegible]	1145	[illegible]	[illegible]	[illegible]	CCD76_RS11235	10059..13496	430457	[illegible]
WP_110649043.1	Type1.BREX	[illegible]	1153	[illegible]	[illegible]	[illegible]	DOM57_RS09900	59337..62798	1267424	[illegible]

Protein-ID	System	Gene-Neighbourhoods	Length	Taxonomy	Assembly-ID	Strain	Locus-Tag	Genomic-coordinates	TaxID	Fasta-defline
WP_027706208.1	Type1.BREX	<-METHYLASE<-SAM-METHYLASE||tRNA-><-?||?-><-SIGMA+OB-Fold+AAA-ATPase+Lon-Protease-BrxL->PglZ<-N6-METHYLASE<-DUF6079*<-BrxB-BrxF-iSTAND<-BrxA-DNA-binding||?->?->?->ZnR->	1213	Bacteria>Pseudomonadota>Gammaproteobacteria>Oceanospirillales>Halomonadaceae>Zymobacter_group	GCF_003610015.1	Zymobacter_palmae	ZBT109_RS10990	2474695..2478336	33074	BREX-system-P-loop-protein-BrxC-[Zymobacter-palmae]
WP_261843643.1	Type1.BREX	<-LexA-protease||?->35exo->?->wHTH+WYL+WCX->BrxA-DNA-binding->BrxB-BrxF-iSTAND->DUF6079*->ParB+HNH->N6-METHYLASE->PglZ->SIGMA+OB-Fold+AAA-ATPase+Lon-Protease-BrxL->SbcC->HDRnase->VirD4-TraG+VirB4-FtsK->	1225	Bacteria>Pseudomonadota>Gammaproteobacteria>Oceanospirillales>Oceanospirillaceae>Aliamphritea	GCF_024347215.1	Aliamphritea_ceti	OCU49_RS03510	764184..767861	1524258	BREX-system-P-loop-protein-BrxC-[Aliamphritea-ceti]
WP_027853089.1	Type1.BREX	<-SIGMA+OB-Fold+AAA-ATPase+Lon-Protease-BrxL<-PglZ<-HNH<-OLD-ABC<-TOPRIM<-OLD-ABC<-ParB+HNH<-N6-METHYLASE<-DUF6079*<-BrxB-BrxF-iSTAND<-BrxA-DNA-binding<-wHTH+WYL+WCX<-TIR<-?<-?<-wHTH+WYL+WCX	1154	Bacteria>Pseudomonadota>Gammaproteobacteria>Oceanospirillales>Oceanospirillaceae>Marinobacterium	GCF_000428985.1	Marinobacterium_litorale	G403_RS0105385	191863..195327	404770	BREX-system-P-loop-protein-BrxC-[Marinobacterium-litorale]
WP_027854332.1	Type1.BREX	GDResolvase+HIN-HTH->?-><-?-><-SIGMA+OB-Fold+AAA-ATPase+HKD-DNase-BrxL<-PglZ<-N6-METHYLASE<-REase<-OLD-ABC<-Schlafen+HATPase+wHTH+HTH<-DUF6079*<-BrxB-BrxF-iSTAND<-BrxA-DNA-binding<-XIS-HTH<-MobI||XIS-HTH->AAA+HTH->XIS-HTH-><-Arm-DNA-bind_3+Phage_integrase	1193	Bacteria>Pseudomonadota>Gammaproteobacteria>Oceanospirillales>Oceanospirillaceae>Marinobacterium	GCF_000428985.1	Marinobacterium_litorale	G403_RS0112740	74962..78543	404770	BREX-system-P-loop-protein-BrxC-[Marinobacterium-litorale]
WP_181736357.1	Type1.BREX	XIS-HTH->?->?->TIR->wHTH+WYL+WCX->BrxA-DNA-binding->BrxB-BrxF-iSTAND->DUF6079*->N6-METHYLASE->ParB+HNH->OLD-ABC->HNH->PglZ->SIGMA+OB-Fold+AAA-ATPase+Lon-Protease-BrxL->ParB+HNH->	1154	Bacteria>Pseudomonadota>Gammaproteobacteria>Oceanospirillales>Oceanospirillaceae>Marinobacterium	GCF_013868415.1	Marinobacterium_sp._3-1745	H1S06_RS00670	30655..34119	2756129	BREX-system-P-loop-protein-BrxC-[Marinobacterium-sp.-3-1745]
WP_206838922.1	Type1.BREX	MODE-HTH+PBPII-><-ADH_N+ADH_zinc_N<-?||?->?->wHTH+WYL+WCX->BrxA-DNA-binding->BrxB-BrxF-iSTAND->DUF6079*->N6-METHYLASE->DUF932->OLD-ABC->TOPRIM->OLD-ABC->HNH->PglZ->SIGMA+OB-Fold+AAA-ATPase+Lon-Protease-BrxL->Integrase_1->	1154	Bacteria>Pseudomonadota>Gammaproteobacteria>Oceanospirillales>Oceanospirillaceae>Marinobacterium	GCF_017310015.1	Marinobacterium_georgiense	CFI10_RS02405	480710..484174	48076	BREX-system-P-loop-protein-BrxC-[Marinobacterium-iners]
WP_217336191.1	Type1.BREX	DUF772+DDE_Tnp_1+DDE_Tnp_1_6->wHTH+WYL+WCX->BrxA-DNA-binding->BrxB-BrxF-iSTAND->DUF6079*->N6-METHYLASE->DUF932->OLD-ABC->HNH->OLD-ABC->HNH->PglZ->SIGMA+OB-Fold+AAA-ATPase+Lon-Protease-BrxL->	1154	Bacteria>Pseudomonadota>Gammaproteobacteria>Oceanospirillales>Oceanospirillaceae>Marinobacterium	GCF_019061305.1	Marinobacterium_sp._A346	KTN04_RS15750	3266..6730	2851016	BREX-system-P-loop-protein-BrxC-[Marinobacterium-sp.-A346]
WP_239040425.1	Type1.BREX	<-SF1-SF2-Helicase<-ParB+HNH<-SF1-SF2-Helicase<-N6-METHYLASE-p<-RVT<-N6-METHYLASE<-?<-AbiEii<-MarR-HTH<-DUF6079*<-BrxB-BrxF-iSTAND<-BrxA-DNA-binding<-?<-?<-wHTH+WYL+WCX<-?<-?<-?<-HTH_26	1223	Bacteria>Pseudomonadota>Gammaproteobacteria>Oceanospirillales>Oceanospirillaceae>Marinobacterium	GCF_022343685.1	Marinobacterium_sediminicola	LN244_RS14165	3103586..3107257	518898	BREX-system-P-loop-protein-BrxC-[Marinobacterium-sediminicola]
AWY02455.1	Type1.BREX	<-HTH_26||Phage_AlpA->Arm-DNA-bind_2+Phage_int_SAM_3+Phage_integrase->?-><-SIGMA+OB-Fold+AAA-ATPase+HKD-DNase-BrxL<-PglZ<-ParB+HNH<-N6-METHYLASE<-REase<-OLD-ABC<-DUF6079*<-BrxB-BrxF-iSTAND<-BrxA-DNA-binding<-HTH_26||DUF772+DDE_Tnp_1->DUF772+DDE_Tnp_1-><-HTH_Tnp_1+HTH_21+rve+rve_3<-HIN-HTH||?->SF1-SF2-Helicase->	1197	Bacteria>Pseudomonadota>Gammaproteobacteria>Oceanospirillales>Oceanospirillaceae>Marinomonas	GCA_003285205.1	Marinomonas_primoryensis	A8139_14855	3171018..3174611	178399	hypothetical-protein-A8139_14855-[Marinomonas-primoryensis]
RUM53671.1	Type1.BREX	wHTH+WYL+WCX->AAA-ToxA+Classical-AAA-><-?<-?<-?<-?<-SIGMA+OB-Fold+AAA-ATPase+Lon-Protease-BrxL<-PglZ<-N6-METHYLASE<-DUF6079*<-BrxB-BrxF-iSTAND<-BrxA-DNA-binding<-HTH_26||Phage_AlpA->Arm-DNA-bind_2+Phage_int_SAM_3+Phage_integrase->	1229	Bacteria>Pseudomonadota>Gammaproteobacteria>Oceanospirillales>Oceanospirillaceae>Marinomonas	GCA_003978655.1	Marinomonas_sp.	DSY85_08610	29236..32925	1904862	BREX-system-P-loop-protein-BrxC-[Marinomonas-sp.]
WP_036163554.1	Type1.BREX	<-SIGMA+OB-Fold+AAA-ATPase+Lon-Protease-BrxL<-PglZ<-GP+REase<-McrC-NTD+REase<-McrB-AAA<-N6-METHYLASE<-SLOG+BirA-HTH<-SF1-SF2-Helicase+PRTase<-DUF6079*<-BrxB-BrxF-iSTAND<-BrxA-DNA-binding<-ABhydrolase<-HTH_26||Phage_AlpA->Arm-DNA-bind_2+Phage_int_SAM_3+Phage_integrase->?-><-?<-LexA-protease	1246	Bacteria>Pseudomonadota>Gammaproteobacteria>Oceanospirillales>Oceanospirillaceae>Marinomonas	GCF_000521805.1	Marinomonas_ushuaiensis	MUS1_RS14035	47481..51221	263818	BREX-system-P-loop-protein-BrxC-[Marinomonas-ushuaiensis]
WP_100635909.1	Type1.BREX	XIS-HTH->?->BrxA-DNA-binding->BrxB-BrxF-iSTAND->DUF6079*->N6-METHYLASE->ParB+HNH->PglZ->ZetaToxin->?-><-SF1-SF2-Helicase+REase<-SF1-SF2-Helicase+REase<-URI-DNase+RAMA	1157	Bacteria>Pseudomonadota>Gammaproteobacteria>Oceanospirillales>Oceanospirillaceae>Marinomonas	GCF_002806845.1	Marinomonas_sp._ef1	CCS02_RS04315	398110..401583	2005043	BREX-system-P-loop-protein-BrxC-[Marinomonas-sp.-ef1]
WP_239495510.1	Type1.BREX	Phage_AlpA->Arm-DNA-bind_2+Phage_int_SAM_3+Phage_integrase->?-><-SIGMA+OB-Fold+AAA-ATPase+HKD-DNase-BrxL<-PglZ<-ParB+HNH<-N6-METHYLASE<-REase<-OLD-ABC<-DUF6079*<-BrxB-BrxF-iSTAND<-BrxA-DNA-binding<-HTH_26||<-HTH_Tnp_1+HTH_21+rve+rve_3<-HTH_19||?->SF1-SF2-Helicase->TPR+HEPN-><-SF1-SF2-Helicase+wHTH+Small-B-barrel+DUF1998	1199	Bacteria>Pseudomonadota>Gammaproteobacteria>Oceanospirillales>Oceanospirillaceae>Marinomonas	GCF_003285205.1	Marinomonas_primoryensis	A8139_RS14980	3171018..3174617	178399	BREX-system-P-loop-protein-BrxC-[Marinomonas-primoryensis]
WP_205113926.1	Type1.BREX	TPR+TPR+TPR+TPR->?->?-><-Sulfate_transp+STAS||SF1-SF2-Helicase->?->tRNA->BrxA-DNA-binding->BrxB-BrxF-iSTAND->DUF6079*->ABC-ATPase->REase->N6-METHYLASE->PglZ->SIGMA+OB-Fold+AAA-ATPase+HKD-DNase-BrxL-><-HTH_26||Phage_AlpA->Arm-DNA-bind_2+Phage_int_SAM_3+Phage_integrase->	1191	Bacteria>Pseudomonadota>Gammaproteobacteria>Oceanospirillales>Oceanospirillaceae>Marinomonas	GCF_016906065.1	Marinomonas_foliarum	JSY38_RS15135	3246777..3250352	491950	BREX-system-P-loop-protein-BrxC-[Marinomonas-foliarum]
WP_226752736.1	Type1.BREX	<-SIGMA+OB-Fold+AAA-ATPase+Lon-Protease-BrxL<-PglZ<-Peptidase_S8<-Classical-AAA<-SbcC<-N6-METHYLASE<-TOPRIM<-OLD-ABC<-DUF6079*<-BrxB-BrxF-iSTAND<-BrxA-DNA-binding<-HTH_3||wHTH+WYL->SF1-SF2-Helicase->Phage_integrase-><-CACHE+GGDEF	1237	Bacteria>Pseudomonadota>Gammaproteobacteria>Oceanospirillales>Oceanospirillaceae>Marinomonas	GCF_020532605.1	Marinomonas_algarum	LG368_RS00225	46423..50136	2883105	BREX-system-P-loop-protein-BrxC-[Marinomonas-algarum]
WP_067206359.1	Type1.BREX	<-VirD4-TraG+VirB4-FtsK<-HDRnase<-SbcC<-SIGMA+OB-Fold+AAA-ATPase+Lon-Protease-BrxL<-PglZ<-N6-METHYLASE<-TOPRIM<-OLD-ABC<-DUF6079*<-BrxB-BrxF-iSTAND<-BrxA-DNA-binding<-wHTH+WYL+WCX<-?<-35exo||LexA-protease->?-><-MobI	1225	Bacteria>Pseudomonadota>Gammaproteobacteria>Oceanospirillales>Oceanospirillaceae>Marinomonas	GCF_900089755.1	Marinomonas_aquimarina	MAQ5080_RS04935	83930..87607	295068	BREX-system-P-loop-protein-BrxC-[Marinomonas-aquimarina]
MBE0507005.1	Type1.BREX	BrxA-DNA-binding->BrxB-BrxF-iSTAND->DUF6079*->OLD-ABC->REase->N6-METHYLASE->HTH+REase->REase->PglZ->SIGMA+OB-Fold+AAA-ATPase+Lon-Protease-BrxL->	1183	Bacteria>Pseudomonadota>Gammaproteobacteria>Oceanospirillales>Oceanospirillaceae>Marinospirillum	GCA_014859565.1	Marinospirillum_sp.	IBX50_09850	1716..5267	2183934	BREX-system-P-loop-protein-BrxC-[Marinospirillum-sp.]
MBE0507394.1	Type1.BREX	<-N6-METHYLASE<-DUF6079*<-BrxB-BrxF-iSTAND<-BrxA-DNA-binding<-wHTH+WYL+WCX||?-><-Mrr-N||LexA-protease->	1159	Bacteria>Pseudomonadota>Gammaproteobacteria>Oceanospirillales>Oceanospirillaceae>Marinospirillum	GCA_014859565.1	Marinospirillum_sp.	IBX50_11860	2713..6192	2183934	BREX-system-P-loop-protein-BrxC-[Marinospirillum-sp.]
WP_138986811.1	Type1.BREX	<-SIGMA+OB-Fold+AAA-ATPase+HKD-DNase-BrxL<-PglZ<-HTH+REase<-N6-METHYLASE<-DUF6079*<-BrxB-BrxF-iSTAND<-BrxA-DNA-binding<-XIS-HTH	1190	Bacteria>Pseudomonadota>Gammaproteobacteria>Oceanospirillales>Oceanospirillaceae>Neptunomonas	GCF_008630635.1	Neptunomonas_concharum	F0U83_RS12940	2741645..2745217	1031538	BREX-system-P-loop-protein-BrxC-[Neptunomonas-concharum]
WP_202806908.1	Type1.BREX	<-PglZ<-TOPRIM<-OLD-ABC<-SbcC<-N6-METHYLASE-p<-N6-METHYLASE-p<-PIN<-OLD-ABC<-DUF6079-p*<-DUF6079-p<-BrxB-BrxF-iSTAND<-BrxA-DNA-binding<-XIS-HTH<-?<-HDRnase<-Phage_int_SAM<-Integrase_1||XIS-HTH->	460	Bacteria>Pseudomonadota>Gammaproteobacteria>Oceanospirillales>Oceanospirillaceae>Nitrincola	GCF_000585235.1	Nitrincola_nitratireducens	D791_RS20495	12347..13729	1229521	hypothetical-protein-[Nitrincola-nitratireducens]
WP_202806909.1	Type1.BREX	<-TOPRIM<-OLD-ABC<-SbcC<-N6-METHYLASE-p<-N6-METHYLASE-p<-PIN<-OLD-ABC<-DUF6079-p*<-DUF6079-p<-BrxB-BrxF-iSTAND<-BrxA-DNA-binding<-XIS-HTH<-?<-HDRnase<-Phage_int_SAM<-Integrase_1||XIS-HTH->Phage_integrase->	697	Bacteria>Pseudomonadota>Gammaproteobacteria>Oceanospirillales>Oceanospirillaceae>Nitrincola	GCF_000585235.1	Nitrincola_nitratireducens	D791_RS20500	13689..15782	1229521	BREX-system-P-loop-protein-BrxC-[Nitrincola-nitratireducens]
MBL4799472.1	Type1.BREX	<-N6-METHYLASE<-DUF6079*<-BrxB-BrxF-iSTAND<-BrxA-DNA-binding<-wHTH+WYL+WCX<-SF1-SF2-Helicase<-SbcC<-?<-SIR2	1239	Bacteria>Pseudomonadota>Gammaproteobacteria>Oceanospirillales>Oceanospirillaceae>Oleispira	GCA_016764675.1	Oleispira_sp.	JXX82_09395	2259..5978	2024846	BREX-system-P-loop-protein-BrxC-[Oleispira-sp.]
WP_225677254.1	Type1.BREX	URI-DNase-><-AAA-ATPase||HEPN-><-UDG-conflict<-REase+iREase<-SIGMA+OB-Fold+AAA-ATPase+Lon-Protease-BrxL<-PglZ<-N6-METHYLASE<-DUF6079*<-BrxB-BrxF-iSTAND<-BrxA-DNA-binding<-wHTH+WYL+WCX	1219	Bacteria>Pseudomonadota>Gammaproteobacteria>Oceanospirillales>Oceanospirillaceae>Thalassolituus	GCF_020320395.1	Thalassolituus_marinus	I9W95_RS17495	45926..49585	671053	BREX-system-P-loop-protein-BrxC-[Thalassolituus-marinus]
WP_260997651.1	Type1.BREX	<-Arm-DNA-bind_2+Phage_int_SAM_3+Phage_integrase<-Phage_AlpA||HTH_26-><-SIGMA+OB-Fold+AAA-ATPase+Lon-Protease-BrxL<-PglZ<-TOPRIM<-OLD-ABC<-ParB+HNH<-N6-METHYLASE<-DUF6079*<-BrxB-BrxF-iSTAND<-BrxA-DNA-binding<-wHTH+WYL+WCX	1156	Bacteria>Pseudomonadota>Gammaproteobacteria>Oceanospirillales>Oceanospirillaceae>Thalassolituus	GCF_025345565.1	Thalassolituus_hydrocarbonoclasticus	HUF19_RS16715	3723205..3726675	2742796	BREX-system-P-loop-protein-BrxC-[Thalassolituus-hydrocarbonoclasticus]
EPJ48519.1	Type1.BREX	<-DUF772+DDE_Tnp_1<-DUF772+DDE_Tnp_1||?->MULE-TRANSPOSASE->wHTH+WYL+WCX->BrxA-DNA-binding-><-?||BrxB-BrxF-iSTAND->DUF6079*->MarR-HTH->AbiEii->?->N6-METHYLASE->TIR->?->DOC+HTH->PglZ->SIGMA+OB-Fold+AAA-ATPase+Lon-Protease-BrxL->	1226	Bacteria>Pseudomonadota>Gammaproteobacteria>Oceanospirillales>Oceanospirillaceae>unclassified_Oceanospirillaceae	GCA_000416275.1	Osedax_symbiont_Rs1	OFPII_05770	35450..39130	1330036	MAG:-putative-ATPase-like-protein-[Osedax-symbiont-Rs1]
MAD46040.1	Type1.BREX	<-SIGMA+OB-Fold+AAA-ATPase+HKD-DNase-BrxL<-PglZ<-HTH+REase<-N6-METHYLASE<-DUF6079*<-BrxB-BrxF-iSTAND<-BrxA-DNA-binding	1189	Bacteria>Pseudomonadota>Gammaproteobacteria>Oceanospirillales>Oceanospirillaceae>unclassified_Oceanospirillaceae	GCA_002685055.1	Oceanospirillaceae_bacterium	CMH98_13650	9512..13081	1899355	hypothetical-protein-[Oceanospirillaceae-bacterium]
MAS25385.1	Type1.BREX	<-SIGMA+OB-Fold+AAA-ATPase+Lon-Protease-BrxL<-PglZ<-ParB+HNH<-PHP+SbcC<-N6-METHYLASE<-Schlafen+HATPase+wHTH<-DUF6079*<-BrxB-BrxF-iSTAND<-BrxA-DNA-binding<-XIS-HTH	1145	Bacteria>Pseudomonadota>Gammaproteobacteria>Oceanospirillales>Oceanospirillaceae>unclassified_Oceanospirillaceae	GCA_002698675.1	Oceanospirillaceae_bacterium	CMI08_10750	15208..18645	1899355	hypothetical-protein-[Oceanospirillaceae-bacterium]
MAR01289.1	Type1.BREX	BrxB-BrxF-iSTAND-><-AbiEii<-wHTH+AbiEi_2||?-><-SIGMA+OB-Fold+AAA-ATPase+HKD-DNase-BrxL<-REase<-Immank+ANK+ANK<-PglZ<-N6-METHYLASE<-DUF6079*<-BrxB-BrxF-iSTAND<-BrxA-DNA-binding||HTH+REase-><-?<-?<-?<-PBPI	1185	Bacteria>Pseudomonadota>Gammaproteobacteria>Oceanospirillales>Oceanospirillaceae>unclassified_Oceanospirillaceae	GCA_002701445.1	Oceanospirillaceae_bacterium	CMI00_12230	13782..17339	1899355	hypothetical-protein-[Oceanospirillaceae-bacterium]
MBM94539.1	Type1.BREX	CRISPR_Cas6->?-><-SIGMA+OB-Fold+AAA-ATPase+Lon-Protease-BrxL<-PglZ<-TOPRIM<-OLD-ABC<-N6-METHYLASE<-TOPRIM<-OLD-ABC<-DUF6079*<-BrxB-BrxF-iSTAND<-BrxA-DNA-binding||?-><-?<-?<-Arm-DNA-bind_2+Phage_int_SAM_3+Phage_integrase<-Phage_AlpA||HTH_26->	1225	Bacteria>Pseudomonadota>Gammaproteobacteria>Oceanospirillales>Oceanospirillaceae>unclassified_Oceanospirillaceae	GCA_002723385.1	Oceanospirillaceae_bacterium	CMI09_01635	47843..51520	1899355	DNA-repair-protein-[Oceanospirillaceae-bacterium]
MBN56612.1	Type1.BREX	<-PglZ<-?<-N6-METHYLASE<-?<-AbiEii<-MarR-HTH<-RNase<-DUF3584+DUF5401<-MukF<-DUF6079*<-BrxB-BrxF-iSTAND<-BrxA-DNA-binding<-wHTH+WYL+WCX||Thioredoxin_4->	1216	Bacteria>Pseudomonadota>Gammaproteobacteria>Oceanospirillales>Oceanospirillaceae>unclassified_Oceanospirillaceae	GCA_002724925.1	Oceanospirillaceae_bacterium	CMI04_02830	19450..23100	1899355	ATPase-[Oceanospirillaceae-bacterium]
NVK73702.1	Type1.BREX	DUF4158+DDE_Tnp_Tn3->wHTH+WYL+WCX->BrxA-DNA-binding->BrxB-BrxF-iSTAND->DUF6079*->N6-METHYLASE->PglZ->SIGMA+OB-Fold+AAA-ATPase+Lon-Protease-BrxL->SbcC->REase+iREase->REase+HTH+35exo+Small-B-barrel+SF1-SF2-Helicase-><-Phage_integrase||?-><-GGDEF	1219	Bacteria>Pseudomonadota>Gammaproteobacteria>Oceanospirillales>Oceanospirillaceae>unclassified_Oceanospirillaceae	GCA_013373235.1	Oceanospirillaceae_bacterium	HWE24_09495	6489..10148	1899355	BREX-system-P-loop-protein-BrxC-[Oceanospirillaceae-bacterium]
WP_116688140.1	Type1.BREX	DDE_Tnp-><-HTH_3||?-><-?||?->DUF4096+DDE_Tnp_1+DDE_Tnp_1_2->HTH_26->BrxA-DNA-binding->BrxB-BrxF-iSTAND->DUF6079*->ParB+HNH->N6-METHYLASE->OLD-ABC->TOPRIM->OLD-ABC->TOPRIM->PglZ->SIGMA+OB-Fold+AAA-ATPase+Lon-Protease-BrxL-><-ANK+ANK	1236	Bacteria>Pseudomonadota>Gammaproteobacteria>Oceanospirillales>Oceanospirillales_incertae_sedis>Pelagibaculum	GCF_003097315.1	Pelagibaculum_spongiae	DC094_RS16040	158438..162148	2080658	BREX-system-P-loop-protein-BrxC-[Pelagibaculum-spongiae]
WP_100257386.1	Type1.BREX	<-NERD+SF1-SF2-Helicase<-SIGMA+OB-Fold+AAA-ATPase+Lon-Protease-BrxL<-PglZ<-ParB+HNH<-N6-METHYLASE<-DUF6079*<-BrxB-BrxF-iSTAND<-BrxA-DNA-binding<-tRNA<-MerR-HTH	1215	Bacteria>Pseudomonadota>Gammaproteobacteria>Oceanospirillales>Saccharospirillaceae>Reinekea	GCF_002795845.1	Reinekea_forsetii	REIFOR_RS09825	2117568..2121215	1336806	BREX-system-P-loop-protein-BrxC-[Reinekea-forsetii]
WP_119395059.1	Type1.BREX	SF1-SF2-Helicase->HTH+HEPN->McrB-AAA->wHTH+REase+ZnR->BrxA-DNA-binding->BrxB-BrxF-iSTAND->DUF6079*->NHH->N6-METHYLASE->sGTP->sGTP->?->HerA/FtsK->HNH->?->PglZ->	1155	Bacteria>Pseudomonadota>Gammaproteobacteria>Oceanospirillales>Saccharospirillaceae>Salinibius	GCF_003577475.1	Salinibius_halmophilus	DUW71_RS09715	145555..149022	1853216	BREX-system-P-loop-protein-BrxC-[Salinibius-halmophilus]
NRB78692.1	Type1.BREX	<-sGTP<-sGTP<-?<-SIGMA+OB-Fold+AAA-ATPase+Lon-Protease-BrxL<-PglZ<-N6-METHYLASE<-DUF6079*<-BrxB-BrxF-iSTAND<-BrxA-DNA-binding<-HNH<-?||?->?->?-><-SF1-SF2-Helicase<-HKD-DNase+HKD-Rease	1233	Bacteria>Pseudomonadota>Gammaproteobacteria>Oceanospirillales>Saccharospirillaceae>unclassified_Saccharospirillaceae	GCA_013214075.1	Saccharospirillaceae_bacterium	HRU38_08495	18482..22183	2497634	BREX-system-P-loop-protein-BrxC-[Saccharospirillaceae-bacterium]
NRB80667.1	Type1.BREX	<-N6-METHYLASE<-DUF6079*<-BrxB-BrxF-iSTAND<-BrxA-DNA-binding<-wHTH+WYL+WCX<-HTH_19<-TnsC-ATPase<-Tn7_Tnp_TnsA_N+rve+Mu-transpos_C<-ncRNA<-GGDEF+EAL	1225	Bacteria>Pseudomonadota>Gammaproteobacteria>Oceanospirillales>Saccharospirillaceae>unclassified_Saccharospirillaceae	GCA_013214075.1	Saccharospirillaceae_bacterium	HRU38_18705	4651..8328	2497634	BREX-system-P-loop-protein-BrxC-[Saccharospirillaceae-bacterium]
MBR9867112.1	Type1.BREX	<-SbcC<-SIGMA+OB-Fold+AAA-ATPase+Lon-Protease-BrxL<-PglZ<-TOPRIM<-OLD-ABC<-N6-METHYLASE<-ParB+HNH<-DUF6079*<-BrxB-BrxF-iSTAND<-BrxA-DNA-binding<-wHTH+WYL+WCX<-?<-SLOG+BirA-HTH<-SF1-SF2-Helicase+PRTase||DnaJ->	1225	Bacteria>Pseudomonadota>Gammaproteobacteria>Oceanospirillales>unclassified_Oceanospirillales	GCA_018223265.1	Oceanospirillales_bacterium	GYB18_08415	21169..24846	1914300	BREX-system-P-loop-protein-BrxC-[Oceanospirillales-bacterium]
WP_241468927.1	Type1.BREX	<-VirD4-TraG+VirB4-FtsK<-HDRnase<-SbcC<-SIGMA+OB-Fold+AAA-ATPase+Lon-Protease-BrxL<-PglZ<-N6-METHYLASE<-ParB+HNH<-DUF6079*<-BrxB-BrxF-iSTAND<-BrxA-DNA-binding<-wHTH+WYL+WCX<-?<-35exo<-?||LexA-protease->?->Phage_integrase->	1225	Bacteria>Pseudomonadota>Gammaproteobacteria>Pasteurellales>Pasteurellaceae>Pasteurella	GCF_022559605.1	Pasteurella_multocida	MMZ64_RS09105	1895707..1899384	747	BREX-system-P-loop-protein-BrxC-[Pasteurella-multocida]
WP_119518392.1	Type1.BREX	Arm-DNA-bind_3+Phage_integrase->REase-><-SIGMA+OB-Fold+AAA-ATPase+Lon-Protease-BrxL<-PglZ<-ParB+HNH<-?<-HNH+GCN1<-?<-N6-METHYLASE<-DUF6079*<-BrxB-BrxF-iSTAND<-BrxA-DNA-binding<-?<-Peptidase_M15<-?||wHTH+WYL+WCX->?-><-PhdYeFM_antitox<-NERD	1154	Bacteria>Pseudomonadota>Gammaproteobacteria>Pseudomonadales	GCF_021610005.1	Pseudomonadaceae	L1776_RS13375	2929669..2933133	135621	MULTISPECIES:-BREX-system-P-loop-protein-BrxC-[Pseudomonadaceae]
KXS54417.1	Type1.BREX	BrxA-DNA-binding->BrxB-BrxF-iSTAND->DUF6079*->DOC+HTH->N6-METHYLASE->OLD-ABC+TOPRIM->OLD-ABC->	1221	Bacteria>Pseudomonadota>Gammaproteobacteria>Pseudomonadales>Marinobacteraceae>Marinobacter	GCA_001577575.1	Marinobacter_sp._T13-3	AWU57_1199	1913..5578	1794906	hypothetical-protein-AWU57_1199-[Marinobacter-sp.-T13-3]
KXS53697.1	Type1.BREX	<-HNH<-?<-OLD-ABC<-N6-METHYLASE<-HTH+HTH<-Schlafen+HATPase+wHTH<-DUF6079-p||?-><-DUF6079-p*<-BrxB-BrxF-iSTAND<-BrxA-DNA-binding<-XIS-HTH<-MobI||XIS-HTH->AAA+HTH->XIS-HTH-><-Arm-DNA-bind_3+Phage_integrase	539	Bacteria>Pseudomonadota>Gammaproteobacteria>Pseudomonadales>Marinobacteraceae>Marinobacter	GCA_001577575.1	Marinobacter_sp._T13-3	AWU57_1918	9032..10651	1794906	hypothetical-protein-AWU57_1918-[Marinobacter-sp.-T13-3]
MAH31343.1	Type1.BREX	<-Schlafen+HATPase+wHTH<-DUF6079*<-BrxB-BrxF-iSTAND<-BrxA-DNA-binding<-XIS-HTH<-MobI||XIS-HTH->AAA+HTH->XIS-HTH-><-Arm-DNA-bind_3+Phage_integrase	1193	Bacteria>Pseudomonadota>Gammaproteobacteria>Pseudomonadales>Marinobacteraceae>Marinobacter	GCA_002690365.1	Marinobacter_sp.	CMG78_04665	1378..4959	50741	hypothetical-protein-[Marinobacter-sp.]
MBE95557.1	Type1.BREX	BrxB-BrxF-iSTAND->DUF6079*->Schlafen+HATPase+wHTH+HTH->SF1-SF2-Helicase+PRTase->SLOG+BirA-HTH->N6-METHYLASE->ParB+HNH->PglZ->SIGMA+OB-Fold+AAA-ATPase+HKD-DNase-BrxL-><-?<-IstB_IS21	1193	Bacteria>Pseudomonadota>Gammaproteobacteria>Pseudomonadales>Marinobacteraceae>Marinobacter	GCA_002714765.1	Marinobacter_sp.	CMG89_11180	696..4277	50741	hypothetical-protein-[Marinobacter-sp.]
PTB94103.1	Type1.BREX	<-Phage_integrase||BrxA-DNA-binding->BrxB-BrxF-iSTAND->DUF6079*->DOC+HTH->N6-METHYLASE->	1221	Bacteria>Pseudomonadota>Gammaproteobacteria>Pseudomonadales>Marinobacteraceae>Marinobacter	GCA_003030785.1	Marinobacter_sp._B9-2	C9974_06180	29766..33431	2137198	BREX-system-P-loop-protein-BrxC-[Marinobacter-sp.-B9-2]
HBC35650.1	Type1.BREX	BrxB-BrxF-iSTAND->BrxB-BrxF-iSTAND-p->DUF6079-p->DUF6079-p*->DUF6079-p->DOC+HTH->DOC+HTH->N6-METHYLASE-p->	726	Bacteria>Pseudomonadota>Gammaproteobacteria>Pseudomonadales>Marinobacteraceae>Marinobacter	GCA_003487885.1	Marinobacter_adhaerens	DC045_15360	1248..3427	1033846	BREX-system-P-loop-protein-BrxC-[Marinobacter-adhaerens]
TVT30739.1	Type1.BREX	GDResolvase+HIN-HTH-><-?<-REase<-SIGMA+OB-Fold+AAA-ATPase+Lon-Protease-BrxL<-PglZ<-Schlafen+HATPase+wHTH<-PHP+SbcC<-N6-METHYLASE<-Schlafen+HATPase+wHTH<-DUF6079*<-BrxB-BrxF-iSTAND<-BrxA-DNA-binding<-XIS-HTH||XIS-HTH->APATPase->?-><-Phage_int_SAM_5+Phage_integrase	1145	Bacteria>Pseudomonadota>Gammaproteobacteria>Pseudomonadales>Marinobacteraceae>Marinobacter	GCA_007713625.1	Marinobacter_vinifirmus	FHK81_16390	18158..21595	355591	BREX-system-P-loop-protein-BrxC-[Marinobacter-vinifirmus]
MBC7192650.1	Type1.BREX	<-SIGMA+OB-Fold+AAA-ATPase+Lon-Protease-BrxL<-PglZ<-HNH<-OLD-ABC<-TOPRIM<-OLD-ABC<-HTH+REase<-N6-METHYLASE<-Schlafen+HATPase+wHTH<-DUF6079*<-BrxB-BrxF-iSTAND<-BrxA-DNA-binding<-XIS-HTH||HTH_Tnp_1+HTH_21+rve+rve_3->XIS-HTH->DnaB->?-><-Phage_int_SAM_5+Phage_integrase<-sGTP	1145	Bacteria>Pseudomonadota>Gammaproteobacteria>Pseudomonadales>Marinobacteraceae>Marinobacter	GCA_014359475.1	Marinobacter_sp.	H5U31_09890	22634..26071	50741	BREX-system-P-loop-protein-BrxC-[Marinobacter-sp.]
WBU42642.1	Type1.BREX	<-Mrr-N||?->?->?->SWIM+DUF3670+SF1-SF2-Helicase->HTH_3->BrxA-DNA-binding->BrxB-BrxF-iSTAND->DUF6079*->DOC+HTH->N6-METHYLASE->?->N6-METHYLASE-p->HEPN->?->TIR->?->TIR->	1221	Bacteria>Pseudomonadota>Gammaproteobacteria>Pseudomonadales>Marinobacteraceae>Marinobacter	GCA_027912435.1	Marinobacter_alkaliphilus	PBN92_07055	1547680..1551345	254719	BREX-system-P-loop-protein-BrxC-[Marinobacter-alkaliphilus]
WP_011784101.1	Type1.BREX	Arm-DNA-bind_3+Phage_integrase-><-XIS-HTH<-AAA+HTH<-XIS-HTH||MobI->XIS-HTH->SF1-SF2-Helicase->BrxA-DNA-binding->BrxB-BrxF-iSTAND->DUF6079*->Schlafen+HATPase+wHTH+HTH->SF1-SF2-Helicase+PRTase->SLOG+BirA-HTH->N6-METHYLASE->OLD-ABC->TOPRIM->PglZ->SIGMA+OB-Fold+AAA-ATPase+HKD-DNase-BrxL->SbcC->	1193	Bacteria>Pseudomonadota>Gammaproteobacteria>Pseudomonadales>Marinobacteraceae>Marinobacter	GCF_000015365.1	Marinobacter_nauticus	MAQU_RS02805	628948..632529	2743	BREX-system-P-loop-protein-BrxC-[Marinobacter-nauticus]
WP_007349110.1	Type1.BREX	<-Phage_int_SAM_1+Phage_integrase<-REase+N6_Mtase<-HTH_26<-TetR-HTH<-Phage_integrase+Phage_integrase<-Phage_integrase+Phage_integrase<-Phage_integrase<-N6-METHYLASE-p<-Schlafen+HATPase+wHTH<-DUF6079*<-BrxB-BrxF-iSTAND<-BrxA-DNA-binding<-XIS-HTH||Arm-DNA-bind_3->?->DDE_Tnp_1_4-><-?<-?<-DDE_Tnp_IS66	1145	Bacteria>Pseudomonadota>Gammaproteobacteria>Pseudomonadales>Marinobacteraceae>Marinobacter	GCF_000169375.1	Marinobacter_sp._ELB17	MELB17_RS05280	142493..145930	270374	BREX-system-P-loop-protein-BrxC-[Marinobacter-sp.-ELB17]
WP_008174228.1	Type1.BREX	Arm-DNA-bind_3+Phage_integrase-><-XIS-HTH<-AAA+HTH<-XIS-HTH||MobI->XIS-HTH->BrxA-DNA-binding->BrxB-BrxF-iSTAND->DUF6079*->Schlafen+HATPase+wHTH+HTH->N6-METHYLASE->OLD-ABC->TOPRIM->	1193	Bacteria>Pseudomonadota>Gammaproteobacteria>Pseudomonadales>Marinobacteraceae>Marinobacter	GCF_000235625.1	Marinobacter_manganoxydans	KYE_13690	42273..45854	1150997	BREX-system-P-loop-protein-BrxC-[Marinobacter-manganoxydans]
WP_018403235.1	Type1.BREX	CHTH->GDResolvase->ZnR-><-GDResolvase+HIN-HTH<-SIGMA+OB-Fold+AAA-ATPase+Lon-Protease-BrxL<-PglZ<-REase+AIPR<-N6-METHYLASE<-Schlafen+HATPase+wHTH<-DUF6079*<-BrxB-BrxF-iSTAND<-BrxA-DNA-binding<-XIS-HTH<-tRNA<-HAD+HAD<-HDRnase<-?<-?<-DNA_METHYLASE	1145	Bacteria>Pseudomonadota>Gammaproteobacteria>Pseudomonadales>Marinobacteraceae>Marinobacter	GCF_000372805.1	Marinobacter_gelidimuriae	A3OI_RS0105075	19872..23309	2739064	BREX-system-P-loop-protein-BrxC-[Marinobacter-gelidimuriae]
WP_018404435.1	Type1.BREX	OrfB_Zn_ribbon->BrxA-DNA-binding->BrxB-BrxF-iSTAND->DUF6079*->Schlafen+HATPase+wHTH+HTH->N6-METHYLASE->OLD-ABC->HNH->PglZ->SIGMA+OB-Fold+AAA-ATPase+HKD-DNase-BrxL->N6-METHYLASE->	1194	Bacteria>Pseudomonadota>Gammaproteobacteria>Pseudomonadales>Marinobacteraceae>Marinobacter	GCF_000372805.1	Marinobacter_gelidimuriae	A3OI_RS0111615	2203..5787	2739064	BREX-system-P-loop-protein-BrxC-[Marinobacter-gelidimuriae]
WP_022988850.1	Type1.BREX	<-PglZ<-TOPRIM<-OLD-ABC<-HNH<-OLD-ABC<-N6-METHYLASE<-REase<-OLD-ABC<-Schlafen+HATPase+wHTH+HTH<-DUF6079*<-BrxB-BrxF-iSTAND<-BrxA-DNA-binding<-XIS-HTH<-MobI	1193	Bacteria>Pseudomonadota>Gammaproteobacteria>Pseudomonadales>Marinobacteraceae>Marinobacter	GCF_000475255.1	Marinobacter_sp._ES-1	Q668_RS06800	88554..92135	1396858	BREX-system-P-loop-protein-BrxC-[Marinobacter-sp.-ES-1]
WP_022990203.1	Type1.BREX	<-DnaB<-XIS-HTH||XIS-HTH->BrxA-DNA-binding->BrxB-BrxF-iSTAND->DUF6079*->Schlafen+HATPase+wHTH+HTH->N6-METHYLASE->REase->SIR2->HerA/FtsK->N6-METHYLASE-p->PglZ->Immank+ANK+ANK->REase->	1189	Bacteria>Pseudomonadota>Gammaproteobacteria>Pseudomonadales>Marinobacteraceae>Marinobacter	GCF_000475255.1	Marinobacter_sp._ES-1	Q668_RS13780	2583..6152	1396858	BREX-system-P-loop-protein-BrxC-[Marinobacter-sp.-ES-1]
WP_023011723.1	Type1.BREX	<-OLD-ABC<-N6-METHYLASE-p<-HerA/FtsK<-SIR2<-REase<-N6-METHYLASE<-SLOG+BirA-HTH<-SF1-SF2-Helicase+PRTase<-Schlafen+HATPase+wHTH+HTH<-DUF6079*<-BrxB-BrxF-iSTAND<-BrxA-DNA-binding<-XIS-HTH<-MobI||XIS-HTH->AAA+HTH->XIS-HTH-><-Arm-DNA-bind_3+Phage_integrase	1193	Bacteria>Pseudomonadota>Gammaproteobacteria>Pseudomonadales>Marinobacteraceae>Marinobacter	GCF_000475315.1	Marinobacter_sp._EN3	Q675_RS32885	62164..65745	1397533	BREX-system-P-loop-protein-BrxC-[Marinobacter-sp.-EN3]
WP_022992169.1	Type1.BREX	Arm-DNA-bind_3+Phage_integrase-><-XIS-HTH<-AAA+HTH<-XIS-HTH||MobI->XIS-HTH->BrxA-DNA-binding->BrxB-BrxF-iSTAND->DUF6079*->Schlafen+HATPase+wHTH+HTH->N6-METHYLASE->OLD-ABC->HNH->OLD-ABC->TOPRIM->PglZ->SIGMA+OB-Fold+AAA-ATPase+HKD-DNase-BrxL->	1193	Bacteria>Pseudomonadota>Gammaproteobacteria>Pseudomonadales>Marinobacteraceae>Marinobacter	GCF_000475355.1	Marinobacter_sp._C1S70	Q669_RS10410	149187..152768	1396859	BREX-system-P-loop-protein-BrxC-[Marinobacter-sp.-C1S70]
WP_023007675.1	Type1.BREX	sGTP->Phage_int_SAM_5+Phage_integrase->?-><-DnaB<-XIS-HTH||XIS-HTH->BrxA-DNA-binding->BrxB-BrxF-iSTAND->DUF6079*->Schlafen+HATPase+wHTH+HTH->N6-METHYLASE-p->	1145	Bacteria>Pseudomonadota>Gammaproteobacteria>Pseudomonadales>Marinobacteraceae>Marinobacter	GCF_000475375.1	Marinobacter_sp._EVN1	Q674_RS21825	56461..59898	1397532	BREX-system-P-loop-protein-BrxC-[Marinobacter-sp.-EVN1]
WP_023008352.1	Type1.BREX	<-SbcC<-SIGMA+OB-Fold+AAA-ATPase+HKD-DNase-BrxL<-PglZ<-TOPRIM<-OLD-ABC<-HNH<-OLD-ABC<-N6-METHYLASE<-Schlafen+HATPase+wHTH+HTH<-DUF6079*<-BrxB-BrxF-iSTAND<-BrxA-DNA-binding<-XIS-HTH<-MobI||XIS-HTH->AAA+HTH->XIS-HTH-><-Arm-DNA-bind_3+Phage_integrase	1193	Bacteria>Pseudomonadota>Gammaproteobacteria>Pseudomonadales>Marinobacteraceae>Marinobacter	GCF_000475375.1	Marinobacter_sp._EVN1	Q674_RS26680	52773..56354	1397532	BREX-system-P-loop-protein-BrxC-[Marinobacter-sp.-EVN1]
WP_048384700.1	Type1.BREX	Arm-DNA-bind_3+Phage_integrase-><-AbiGi||?->CHTH->XIS-HTH->?->XIS-HTH->BrxA-DNA-binding->BrxB-BrxF-iSTAND->DUF6079*->Schlafen+HATPase+wHTH+HTH->N6-METHYLASE->ParB+HNH->?->DUF3644->PglZ->SIGMA+OB-Fold+AAA-ATPase+Lon-Protease-BrxL->Integrase_1-><-CHTH	1145	Bacteria>Pseudomonadota>Gammaproteobacteria>Pseudomonadales>Marinobacteraceae>Marinobacter	GCF_001043175.1	Marinobacter_psychrophilus	ABA45_RS05800	1298938..1302375	330734	BREX-system-P-loop-protein-BrxC-[Marinobacter-psychrophilus]
WP_058091394.1	Type1.BREX	wHTH+WYL+WCX->?-><-Mrr-N||HTH_3->BrxA-DNA-binding->BrxB-BrxF-iSTAND->DUF6079*->DOC+HTH->SF1-SF2-Helicase->N6-METHYLASE->HEPN->?->TIR->OLD-ABC->TOPRIM->	1221	Bacteria>Pseudomonadota>Gammaproteobacteria>Pseudomonadales>Marinobacteraceae>Marinobacter	GCF_001447155.2	Marinobacter_sp._LQ44	ASQ50_RS18570	4052781..4056446	1749259	BREX-system-P-loop-protein-BrxC-[Marinobacter-sp.-LQ44]
WP_071262841.1	Type1.BREX	<-DrsE<-?||tRNA->BrxA-DNA-binding->BrxB-BrxF-iSTAND->DUF6079*->MarR-HTH->AbiEii->AbiEii->?->N6-METHYLASE-p->ParB+HNH->HKD-DNase->DUF3150->	1217	Bacteria>Pseudomonadota>Gammaproteobacteria>Pseudomonadales>Marinobacteraceae>Marinobacter	GCF_001858325.1	Marinobacter_sp._AC-23	BCA33_RS00460	104149..107802	1879031	BREX-system-P-loop-protein-BrxC-[Marinobacter-sp.-AC-23]
WP_104416052.1	Type1.BREX	Arm-DNA-bind_3+Phage_integrase-><-XIS-HTH<-AAA+HTH<-XIS-HTH||MobI->XIS-HTH->BrxA-DNA-binding->BrxB-BrxF-iSTAND->DUF6079*->Schlafen+HATPase+wHTH+HTH->N6-METHYLASE->OLD-ABC->HNH->OLD-ABC->TOPRIM->PglZ->SIGMA+OB-Fold+AAA-ATPase+HKD-DNase-BrxL->	1193	Bacteria>Pseudomonadota>Gammaproteobacteria>Pseudomonadales>Marinobacteraceae>Marinobacter	GCF_002934485.1	Marinobacter_persicus	BY455_RS08980	65339..68920	930118	BREX-system-P-loop-protein-BrxC-[Marinobacter-persicus]
WP_113863603.1	Type1.BREX	Arm-DNA-bind_3+Phage_integrase-><-XIS-HTH<-AAA+HTH<-XIS-HTH||MobI->XIS-HTH->BrxA-DNA-binding->BrxB-BrxF-iSTAND->DUF6079*->Schlafen+HATPase+wHTH+HTH->N6-METHYLASE->OLD-ABC->HNH->OLD-ABC->TOPRIM->PglZ->SIGMA+OB-Fold+AAA-ATPase+HKD-DNase-BrxL->	1193	Bacteria>Pseudomonadota>Gammaproteobacteria>Pseudomonadales>Marinobacteraceae>Marinobacter	GCF_003315345.1	Marinobacter_pelagius	DET50_RS14950	40642..44223	379482	BREX-system-P-loop-protein-BrxC-[Marinobacter-pelagius]
WP_113879482.1	Type1.BREX	<-N6-METHYLASE<-Schlafen+HATPase+wHTH+HTH<-DUF6079*<-BrxB-BrxF-iSTAND<-BrxA-DNA-binding<-XIS-HTH<-MobI||XIS-HTH->AAA+HTH->XIS-HTH-><-Arm-DNA-bind_3+Phage_integrase	1193	Bacteria>Pseudomonadota>Gammaproteobacteria>Pseudomonadales>Marinobacteraceae>Marinobacter	GCF_003315555.1	Marinobacter_nauticus	DET64_RS06865	4494..8075	2743	BREX-system-P-loop-protein-BrxC-[Marinobacter-nauticus]
WP_114434424.1	Type1.BREX	<-ParB<-HerA/FtsK<-?<-LZ_Tnp_IS66+DDE_Tnp_IS66+DDE_Tnp_IS66_C<-?<-HIN-HTH<-SIR2<-N6-METHYLASE<-SF1-SF2-Helicase<-DUF6079*<-BrxB-BrxF-iSTAND<-BrxA-DNA-binding||ANK+ANK->SF1-SF2-Helicase+REase->PIWI-><-?||?-><-TraI-VirD2-RCRE<-TraJ-RHH	1217	Bacteria>Pseudomonadota>Gammaproteobacteria>Pseudomonadales>Marinobacteraceae>Marinobacter	GCF_003337515.1	Marinobacter_nauticus	DET61_RS09095	118513..122166	2743	BREX-system-P-loop-protein-BrxC-[Marinobacter-nauticus]
WP_141068072.1	Type1.BREX	Arm-DNA-bind_3+Phage_integrase-><-XIS-HTH<-AAA+HTH<-XIS-HTH||MobI->XIS-HTH->BrxA-DNA-binding->BrxB-BrxF-iSTAND->DUF6079*->Schlafen+HATPase+wHTH+HTH->OLD-ABC->REase->N6-METHYLASE->ParB+HNH->PglZ->SIGMA+OB-Fold+AAA-ATPase+HKD-DNase-BrxL->SbcC+APATPase+SbcC->	1193	Bacteria>Pseudomonadota>Gammaproteobacteria>Pseudomonadales>Marinobacteraceae>Marinobacter	GCF_006516615.1	Marinobacter_nauticus	FH712_RS17655	33566..37147	2743	BREX-system-P-loop-protein-BrxC-[Marinobacter-nauticus]
WP_273135111.1	Type1.BREX	GDResolvase+HIN-HTH-><-?<-REase<-SIGMA+OB-Fold+AAA-ATPase+Lon-Protease-BrxL<-PglZ<-Schlafen+HATPase+wHTH<-PHP+SbcC<-N6-METHYLASE<-Schlafen+HATPase+wHTH<-DUF6079*<-BrxB-BrxF-iSTAND<-BrxA-DNA-binding<-XIS-HTH||XIS-HTH->APATPase->?-><-Phage_int_SAM_5+Phage_integrase	1145	Bacteria>Pseudomonadota>Gammaproteobacteria>Pseudomonadales>Marinobacteraceae>Marinobacter	GCF_007713625.1	Marinobacter_vinifirmus	FHK81_RS16410	18158..21595	355591	BREX-system-P-loop-protein-BrxC-[Marinobacter-vinifirmus]
WP_213480159.1	Type1.BREX	<-Couple_hipA<-SIGMA+OB-Fold+AAA-ATPase+Lon-Protease-BrxL<-PglZ<-ParB+HNH<-PHP+SbcC<-N6-METHYLASE<-DUF6079*<-BrxB-BrxF-iSTAND<-BrxA-DNA-binding<-XIS-HTH||ANK+ANK->TPR-><-wHTH+WYL+WCX||?->SbcC->	1145	Bacteria>Pseudomonadota>Gammaproteobacteria>Pseudomonadales>Marinobacteraceae>Marinobacter	GCF_018424725.1	Marinobacter_salarius	DYI41_RS19390	15297..18734	1420917	BREX-system-P-loop-protein-BrxC-[Marinobacter-salarius]
WP_216008134.1	Type1.BREX	<-PglZ<-TIR<-?<-ParB+HNH<-?<-N6-METHYLASE-p<-RVT<-N6-METHYLASE<-SF1-SF2-Helicase<-DUF6079*<-BrxB-BrxF-iSTAND<-BrxA-DNA-binding||ANK+ANK->?->?->?->?->?->JetB->	1217	Bacteria>Pseudomonadota>Gammaproteobacteria>Pseudomonadales>Marinobacteraceae>Marinobacter	GCF_018860765.1	Marinobacter_salexigens	K0508_RS09770	175903..179556	1925763	BREX-system-P-loop-protein-BrxC-[Marinobacter-salexigens]
WP_228224785.1	Type1.BREX	TraJ-RHH->TraI-VirD2-RCRE-><-?||?-><-PIWI<-SF1-SF2-Helicase+REase<-ANK+ANK||BrxA-DNA-binding->BrxB-BrxF-iSTAND->DUF6079*->MarR-HTH->AbiEii->?->N6-METHYLASE->RVT->N6-METHYLASE-p->?->GP+REase->	1217	Bacteria>Pseudomonadota>Gammaproteobacteria>Pseudomonadales>Marinobacteraceae>Marinobacter	GCF_020781935.1	Marinobacter_nauticus	LL254_RS15890	125400..129053	2743	BREX-system-P-loop-protein-BrxC-[Marinobacter-nauticus]
WP_247116152.1	Type1.BREX	<-SIGMA+OB-Fold+AAA-ATPase+HKD-DNase-BrxL<-REase<-Immank+ANK+ANK<-PglZ<-?<-HIN-HTH<-ABC<-ParB+HNH<-N6-METHYLASE<-DUF6079*<-BrxB-BrxF-iSTAND<-BrxA-DNA-binding||?-><-?||?-><-?||?->||?->Arm-DNA-bind_2+Phage_integrase->	1189	Bacteria>Pseudomonadota>Gammaproteobacteria>Pseudomonadales>Marinobacteraceae>Marinobacter	GCF_023016045.1	Marinobacter_sp._S6332	MWU62_RS17920	19915..23484	2926403	BREX-system-P-loop-protein-BrxC-[Marinobacter-sp.-S6332]
WP_248185657.1	Type1.BREX	<-OLD-ABC<-Schlafen+HATPase+wHTH+HTH<-DUF6079*<-BrxB-BrxF-iSTAND<-BrxA-DNA-binding<-XIS-HTH<-MobI<-?||XIS-HTH->AAA+HTH->XIS-HTH-><-Phage_integrase	1193	Bacteria>Pseudomonadota>Gammaproteobacteria>Pseudomonadales>Marinobacteraceae>Marinobacter	GCF_023156235.1	Marinobacter_goseongensis	MLC70_RS17480	2864..6445	453838	BREX-system-P-loop-protein-BrxC-[Marinobacter-goseongensis]
WP_248236652.1	Type1.BREX	<-Integrase_1<-SIGMA+OB-Fold+AAA-ATPase+Lon-Protease-BrxL<-PglZ<-DUF3644<-?<-ParB+HNH<-N6-METHYLASE<-Schlafen+HATPase+wHTH<-DUF6079*<-BrxB-BrxF-iSTAND<-BrxA-DNA-binding<-XIS-HTH<-Phage_AlpA<-Arm-DNA-bind_3+Phage_integrase	1145	Bacteria>Pseudomonadota>Gammaproteobacteria>Pseudomonadales>Marinobacteraceae>Marinobacter	GCF_023156245.1	Marinobacter_bryozoorum	MLC59_RS17300	17294..20731	256324	BREX-system-P-loop-protein-BrxC-[Marinobacter-bryozoorum]
WP_249013861.1	Type1.BREX	<-TIR<-?<-ParB+HNH<-?<-N6-METHYLASE-p<-RVT<-N6-METHYLASE<-DOC+HTH<-DUF6079*<-BrxB-BrxF-iSTAND<-BrxA-DNA-binding<-?||?->?->DUF4096+DDE_Tnp_1+DDE_Tnp_1_2->?->?->TOPRIM->JetC->	1217	Bacteria>Pseudomonadota>Gammaproteobacteria>Pseudomonadales>Marinobacteraceae>Marinobacter	GCF_023311895.1	Marinobacter_sp._M3C	MIH18_RS02775	634198..637851	2917715	BREX-system-P-loop-protein-BrxC-[Marinobacter-sp.-M3C]
WP_092033189.1	Type1.BREX	Arm-DNA-bind_3+Phage_integrase-><-XIS-HTH<-AAA+HTH<-XIS-HTH||MobI->XIS-HTH->BrxA-DNA-binding->BrxB-BrxF-iSTAND->DUF6079*->Schlafen+HATPase+wHTH+HTH->OLD-ABC->REase->N6-METHYLASE->PglZ->SIGMA+OB-Fold+AAA-ATPase+HKD-DNase-BrxL->SbcC->Calcineurin+Calcineurin-SbcD->SbcC->	1193	Bacteria>Pseudomonadota>Gammaproteobacteria>Pseudomonadales>Marinobacteraceae>Marinobacter	GCF_900112835.1	Marinobacter_sp._DSM_26671	BM035_RS16335	75345..78926	1761793	BREX-system-P-loop-protein-BrxC-[Marinobacter-sp.-DSM-26671]
WP_092021229.1	Type1.BREX	DUF932->?->?->?-><-HTH_3||?->?->wHTH+WYL+WCX->BrxA-DNA-binding->BrxB-BrxF-iSTAND->DUF6079*->N6-METHYLASE->ParB+HNH->PglZ->SIGMA+OB-Fold+AAA-ATPase+Lon-Protease-BrxL->REase+PNTD1+PNTD2->PIWI->TOPRIM->	1219	Bacteria>Pseudomonadota>Gammaproteobacteria>Pseudomonadales>Marinobacteraceae>Marinobacter	GCF_900114775.1	Marinobacter_zhejiangensis	BMZ67_RS06955	334318..337977	488535	BREX-system-P-loop-protein-BrxC-[Marinobacter-zhejiangensis]
BBJ05051.1	Type1.BREX	Arm-DNA-bind_3+Phage_integrase-><-XIS-HTH<-AAA+HTH<-XIS-HTH||MobI->XIS-HTH->BrxA-DNA-binding->BrxB-BrxF-iSTAND->DUF6079*->Schlafen+HATPase+wHTH+HTH->N6-METHYLASE->RVT->HerA/FtsK->N6-METHYLASE-p->OLD-ABC->TOPRIM->PglZ->SIGMA+OB-Fold+AAA-ATPase+HKD-DNase-BrxL->	1193	Bacteria>Pseudomonadota>Gammaproteobacteria>Pseudomonadales>Marinobacteraceae>Marinobacter	-	Marinobacter_nauticus	YBY_29000	3185607..3189188	2743	hypothetical-protein-YBY_29000-[Marinobacter-nauticus]
WP_116918937.1	Type1.BREX	<-XIS-HTH<-?<-?<-Arm-DNA-bind_2+Phage_int_SAM_3+Phage_integrase<-Phage_AlpA||HTH_26->BrxA-DNA-binding->BrxB-BrxF-iSTAND->DUF6079*->Schlafen+HATPase+wHTH+HTH->OLD-ABC->REase->N6-METHYLASE->PglZ->Immank+ANK+ANK->REase->SIGMA+OB-Fold+AAA-ATPase+HKD-DNase-BrxL->	1193	Bacteria>Pseudomonadota>Gammaproteobacteria>Pseudomonadales>Marinobacteraceae>Tamilnaduibacter	GCF_003096655.1	Tamilnaduibacter_salinus	C8D92_104115	123558..127139	1484056	BREX-system-P-loop-protein-BrxC-[Tamilnaduibacter-salinus]
WP_152390158.1	Type1.BREX	VirB10->?-><-SIGMA+OB-Fold+AAA-ATPase+Lon-Protease-BrxL<-PglZ<-TOPRIM<-OLD-ABC<-N6-METHYLASE<-Schlafen+HATPase+wHTH<-DUF6079*<-BrxB-BrxF-iSTAND<-BrxA-DNA-binding<-wHTH+WYL+WCX<-Tox-RES<-CHTH||?->?->DnaB+HerA/FtsK->	1224	Bacteria>Pseudomonadota>Gammaproteobacteria>Pseudomonadales>Pseudomonadaceae>Azotobacter_group	GCF_009363155.1	Azotobacter_salinestris	GCU53_RS24425	150496..154170	69964	BREX-system-P-loop-protein-BrxC-[Azotobacter-salinestris]
WP_022966741.1	Type1.BREX	DDE_Tnp_IS66+DDE_Tnp_IS66_C->PVC-AAA-><-?<-?<-NACHT<-SIGMA+OB-Fold+AAA-ATPase+HKD-DNase-BrxL<-PglZ<-N6-METHYLASE<-DUF6079*<-BrxB-BrxF-iSTAND<-BrxA-DNA-binding<-?<-AbiEii<-AbiEi_3_N+AbiEi_3<-35exo<-?<-?<-LexA-protease	1184	Bacteria>Pseudomonadota>Gammaproteobacteria>Pseudomonadales>Pseudomonadaceae>Denitrificimonas	GCF_000421765.1	Denitrificimonas_caeni	H591_RS0109630	17243..20797	521720	BREX-system-P-loop-protein-BrxC-[Denitrificimonas-caeni]
WP_003439533.1	Type1.BREX	<-PglZ||ZnR-><-TPR<-NERD+GspA-ATPase<-?<-?<-?<-N6-METHYLASE<-DOC+HTH<-DUF6079*<-BrxB-BrxF-iSTAND<-BrxA-DNA-binding<-wHTH+WYL+WCX<-?<-Phage_AlpA<-?<-Arm-DNA-bind_3+Phage_integrase	1225	Bacteria>Pseudomonadota>Gammaproteobacteria>Pseudomonadales>Pseudomonadaceae	GCF_000346225.2	Pseudomonas	HBN87_RS22825	103169..106846	286	MULTISPECIES:-BREX-system-P-loop-protein-BrxC-[Pseudomonas]
WP_033898350.1	Type1.BREX	<-wHTH+WYL+WCX||?->ARG->XIS-HTH->BrxA-DNA-binding->BrxB-BrxF-iSTAND->DUF6079*->N6-METHYLASE-p->?-><-?||N6-METHYLASE-p->OLD-ABC->HNH->SIGMA+OB-Fold+AAA-ATPase+HKD-DNase-BrxL->	1185	Bacteria>Pseudomonadota>Gammaproteobacteria>Pseudomonadales>Pseudomonadaceae	GCF_000510895.2	Pseudomonas	GIW75_RS00865	8119..11676	286	MULTISPECIES:-BREX-system-P-loop-protein-BrxC-[Pseudomonas]
WP_057711677.1	Type1.BREX	<-Ntox49<-SF1-SF2-Helicase<-HKD-DNase+HKD-DNase<-?||wHTH+WYL+WCX->?->||wHTH+WYL+WCX->BrxA-DNA-binding->BrxB-BrxF-iSTAND->DUF6079*->N6-METHYLASE->NERD+GspA-ATPase->TPR->PglZ->SIGMA+OB-Fold+AAA-ATPase+Lon-Protease-BrxL->?->?-><-tRNA<-MerR-HTH	1222	Bacteria>Pseudomonadota>Gammaproteobacteria>Pseudomonadales>Pseudomonadaceae	GCF_001439845.1	Pseudomonas	TX24_RS25940	84684..88352	286	MULTISPECIES:-BREX-system-P-loop-protein-BrxC-[Pseudomonas]
WP_057024539.1	Type1.BREX	ABhydrolase->?->?-><-?||XIS-HTH->?->wHTH+WYL+WCX->BrxA-DNA-binding->BrxB-BrxF-iSTAND->DUF6079*->N6-METHYLASE->ParB+HNH->PglZ->SIGMA+OB-Fold+AAA-ATPase+Lon-Protease-BrxL-><-DUF4969	1154	Bacteria>Pseudomonadota>Gammaproteobacteria>Pseudomonadales>Pseudomonadaceae	GCF_002091795.1	Pseudomonas	TU77_RS22280	179418..182882	286	MULTISPECIES:-BREX-system-P-loop-protein-BrxC-[Pseudomonas]
WP_096426782.1	Type1.BREX	ZnR-><-MA+PspA+PspA+sGTP<-sGTP<-sGTP||?->||XIS-HTH->BrxA-DNA-binding->BrxB-BrxF-iSTAND->DUF6079*->N6-METHYLASE->ParB+HNH->PglZ->SIGMA+OB-Fold+AAA-ATPase+HKD-DNase-BrxL-><-?<-?<-DUF932	1185	Bacteria>Pseudomonadota>Gammaproteobacteria>Pseudomonadales>Pseudomonadaceae	GCF_002356095.1	Pseudomonas	J3A71_RS03870	842777..846334	286	MULTISPECIES:-BREX-system-P-loop-protein-BrxC-[Pseudomonas]
WP_105523318.1	Type1.BREX	<-SIGMA+OB-Fold+AAA-ATPase+HKD-DNase-BrxL<-PglZ<-N6-METHYLASE-p<-?<-REase<-OLD-ABC<-N6-METHYLASE<-DUF6079*<-BrxB-BrxF-iSTAND<-BrxA-DNA-binding<-XIS-HTH<-ARG<-?||wHTH+WYL+WCX->sGTP<-sGTP	1185	Bacteria>Pseudomonadota>Gammaproteobacteria>Pseudomonadales>Pseudomonadaceae	GCF_002975295.1	Pseudomonas	CQ048_RS15780	97047..100604	286	MULTISPECIES:-BREX-system-P-loop-protein-BrxC-[Pseudomonas]
WP_106120190.1	Type1.BREX	LZ_Tnp_IS66+zf-IS66+DDE_Tnp_IS66+DDE_Tnp_IS66_C-><-?<-GAF+PAS+GGDEF+EAL||<-SIGMA+OB-Fold+AAA-ATPase+Lon-Protease-BrxL<-PglZ<-TPR<-NERD+GspA-ATPase<-N6-METHYLASE<-DUF6079*<-BrxB-BrxF-iSTAND<-BrxA-DNA-binding<-wHTH+WYL+WCX||SF1-SF2-Helicase->?->SF1-SF2-Helicase+REase->?->MORC+REase->	1223	Bacteria>Pseudomonadota>Gammaproteobacteria>Pseudomonadales>Pseudomonadaceae	GCF_003001535.1	Pseudomonas	FHL09_RS12740	68451..72122	286	MULTISPECIES:-BREX-system-P-loop-protein-BrxC-[Pseudomonas]
WP_170037970.1	Type1.BREX	GAF+PAS+GGDEF+EAL->?-><-LZ_Tnp_IS66+zf-IS66+DDE_Tnp_IS66+DDE_Tnp_IS66_C<-?||?-><-SIGMA+OB-Fold+AAA-ATPase+HKD-DNase-BrxL<-PglZ<-?<-N6-METHYLASE<-DUF6079*<-BrxB-BrxF-iSTAND<-BrxA-DNA-binding<-XIS-HTH<-ARG<-?||wHTH+WYL+WCX->sGTP<-sGTP	1185	Bacteria>Pseudomonadota>Gammaproteobacteria>Pseudomonadales>Pseudomonadaceae	GCF_012985445.1	Pseudomonas	HBD05_RS12455	196790..200347	286	MULTISPECIES:-BREX-system-P-loop-protein-BrxC-[Pseudomonas]
WP_176516884.1	Type1.BREX	<-SIGMA+OB-Fold+AAA-ATPase+Lon-Protease-BrxL<-PglZ<-REase<-N6-METHYLASE-p<-?<-HerA/FtsK<-SIR2<-N6-METHYLASE<-DUF6079*<-BrxB-BrxF-iSTAND<-BrxA-DNA-binding<-wHTH+WYL+WCX||?->RVT->	1223	Bacteria>Pseudomonadota>Gammaproteobacteria>Pseudomonadales>Pseudomonadaceae	GCF_013373895.1	Pseudomonas	HXS67_RS28935	22447..26118	286	MULTISPECIES:-BREX-system-P-loop-protein-BrxC-[Pseudomonas]
WP_196184087.1	Type1.BREX	DUF932->?->?->?->DUF4969-><-SIGMA+OB-Fold+AAA-ATPase+Lon-Protease-BrxL<-PglZ<-ParB+HNH<-N6-METHYLASE<-DUF6079*<-BrxB-BrxF-iSTAND<-BrxA-DNA-binding<-wHTH+WYL+WCX<-?<-XIS-HTH||RHH-><-tRNA<-MerR-HTH	1154	Bacteria>Pseudomonadota>Gammaproteobacteria>Pseudomonadales>Pseudomonadaceae	GCF_015680805.1	Pseudomonas	IR013_RS15060	51443..54907	286	MULTISPECIES:-BREX-system-P-loop-protein-BrxC-[Pseudomonas]
WP_198830848.1	Type1.BREX	HNH->?->JetC-><-SIGMA+OB-Fold+AAA-ATPase+Lon-Protease-BrxL<-PglZ<-?<-?<-?<-N6-METHYLASE<-DUF6079*<-BrxB-BrxF-iSTAND<-BrxA-DNA-binding<-wHTH+WYL+WCX||?-><-wHTH+WYL+WCX||?->ARG->	1223	Bacteria>Pseudomonadota>Gammaproteobacteria>Pseudomonadales>Pseudomonadaceae	GCF_016887925.1	Pseudomonas	JQF37_RS21260	352403..356074	286	MULTISPECIES:-BREX-system-P-loop-protein-BrxC-[Pseudomonas]
WP_218430053.1	Type1.BREX	Phage_integrase->?->?->TraH-><-?<-SIGMA+OB-Fold+AAA-ATPase+HKD-DNase-BrxL<-PglZ<-N6-METHYLASE<-DUF6079*<-BrxB-BrxF-iSTAND<-BrxA-DNA-binding<-XIS-HTH||DUF772+DDE_Tnp_1+DDE_Tnp_1_6-><-?<-?||?->?-><-ParB	1189	Bacteria>Pseudomonadota>Gammaproteobacteria>Pseudomonadales>Pseudomonadaceae	GCF_019219185.1	Pseudomonas	KW849_RS14820	325397..328966	286	MULTISPECIES:-BREX-system-P-loop-protein-BrxC-[Pseudomonas]
WP_064314265.1	Type1.BREX	<-RHH||XIS-HTH->?->wHTH+WYL+WCX->BrxA-DNA-binding->BrxB-BrxF-iSTAND->DUF6079*->N6-METHYLASE->ParB+HNH->OLD-ABC->HNH->PglZ->SIGMA+OB-Fold+AAA-ATPase+Lon-Protease-BrxL-><-DUF4969	1154	Bacteria>Pseudomonadota>Gammaproteobacteria>Pseudomonadales>Pseudomonadaceae	GCF_021601465.1	Pseudomonas	L6D68_RS06035	31411..34875	286	MULTISPECIES:-BREX-system-P-loop-protein-BrxC-[Pseudomonas]
WP_021219096.1	Type1.BREX	<-SIGMA+OB-Fold+AAA-ATPase+Lon-Protease-BrxL<-PglZ<-AbiEii<-AbiEi_4<-?<-?<-N6-METHYLASE<-DNA-glycosylase<-ParB+HNH<-DUF6079*<-BrxB-BrxF-iSTAND<-BrxA-DNA-binding<-?||wHTH+WYL+WCX->	1220	Bacteria>Pseudomonadota>Gammaproteobacteria>Pseudomonadales>Pseudomonadaceae	GCF_023093875.1	Pseudomonas	MY055_RS15275	3320590..3324252	286	MULTISPECIES:-BREX-system-P-loop-protein-BrxC-[Pseudomonas]
WP_060765715.1	Type1.BREX	<-HKD-DNase+HKD-DNase<-?||||<-SIGMA+OB-Fold+AAA-ATPase+HKD-DNase-BrxL<-PglZ<-ParB+HNH<-N6-METHYLASE-p<-REase<-OLD-ABC<-N6-METHYLASE-p<-DUF6079*<-BrxB-BrxF-iSTAND<-BrxA-DNA-binding<-XIS-HTH<-?<-?<-XIS-HTH||RHH-><-tRNA<-MerR-HTH	1186	Bacteria>Pseudomonadota>Gammaproteobacteria>Pseudomonadales>Pseudomonadaceae	GCF_029959765.1	Pseudomonas	PFL603g_RS03160	383988..387548	286	MULTISPECIES:-BREX-system-P-loop-protein-BrxC-[Pseudomonas]
WP_034126881.1	Type1.BREX	RHH_5->BrxA-DNA-binding->BrxB-BrxF-iSTAND->DUF6079*->N6-METHYLASE->SbcC->Schlafen+HATPase+wHTH->PglZ->SIGMA+OB-Fold+AAA-ATPase+Lon-Protease-BrxL->	1153	Bacteria>Pseudomonadota>Gammaproteobacteria>Pseudomonadales>Pseudomonadaceae	GCF_030505435.1	Pseudomonas	FHL14_RS24020	3439..6900	286	MULTISPECIES:-BREX-system-P-loop-protein-BrxC-[Pseudomonas]
WP_023115268.1	Type1.BREX	<-wHTH+WYL+WCX||?->Peptidase_M15->?->BrxA-DNA-binding->BrxB-BrxF-iSTAND->DUF6079*->N6-METHYLASE->?->HNH+GCN1->?->ParB+HNH->PglZ->SIGMA+OB-Fold+AAA-ATPase+Lon-Protease-BrxL->REase->	1154	Bacteria>Pseudomonadota>Gammaproteobacteria>Pseudomonadales>Pseudomonadaceae	GCF_900707185.1	Pseudomonas	E4Y87_RS04340	6868..10332	286	MULTISPECIES:-BREX-system-P-loop-protein-BrxC-[Pseudomonas]
WP_025297447.1	Type1.BREX	<-DUF6079*<-BrxB-BrxF-iSTAND<-BrxA-DNA-binding<-XIS-HTH<-?<-Peptidase_M15	1184	Bacteria>Pseudomonadota>Gammaproteobacteria>Pseudomonadales>Pseudomonadaceae	GCF_900707275.1	Pseudomonas	E4Z27_RS28920	187..3741	286	MULTISPECIES:-BREX-system-P-loop-protein-BrxC-[Pseudomonas]
WP_198417420.1	Type1.BREX	<-NERD<-?<-Integrase_1<-?||XIS-HTH->?->wHTH+WYL+WCX->BrxA-DNA-binding->BrxB-BrxF-iSTAND->DUF6079*->N6-METHYLASE->ParB+HNH->PglZ->SIGMA+OB-Fold+AAA-ATPase+Lon-Protease-BrxL-><-DUF4969<-?<-?<-HIN-HTH+rve+rve_3	1159	Bacteria>Pseudomonadota>Gammaproteobacteria>Pseudomonadales>Pseudomonadaceae	GCF_902329585.1	Pseudomonas	PPTH39_RS22285	114113..117592	286	MULTISPECIES:-BREX-system-P-loop-protein-BrxC-[Pseudomonas]
WP_104739193.1	Type1.BREX	<-wHTH+WYL+WCX<-DUF772+DDE_Tnp_1+DDE_Tnp_1_6||?->DUF6361->HKD-DNase->SF1-SF2-Helicase->||XIS-HTH->BrxA-DNA-binding->BrxB-BrxF-iSTAND->DUF6079*->N6-METHYLASE->SbcC-p->REase->RVT->N6-METHYLASE-p->ParB+HNH->PglZ->SIGMA+OB-Fold+AAA-ATPase+HKD-DNase-BrxL-><-NtrC-AAA	1180	Bacteria>Pseudomonadota>Gammaproteobacteria>Pseudomonadales>Pseudomonadaceae>Halopseudomonas	GCF_014641295.1	Halopseudomonas_oceani	IEX06_RS08390	366595..370137	1708783	BREX-system-P-loop-protein-BrxC-[Halopseudomonas-oceani]
WP_269686934.1	Type1.BREX	SF1-SF2-Helicase->SF1-SF2-Helicase->?-><-RADICAL-SAM<-?<-MazE_antitoxin||wHTH+WYL+WCX->BrxA-DNA-binding->BrxB-BrxF-iSTAND->DUF6079*->?->OLD-ABC->HNH->PglZ->SIGMA+OB-Fold+AAA-ATPase+Lon-Protease-BrxL-><-HTH_3||PadR-HTH->	1223	Bacteria>Pseudomonadota>Gammaproteobacteria>Pseudomonadales>Pseudomonadaceae>Halopseudomonas	GCF_030295685.1	Halopseudomonas_sp._MFKK-1	O5D48_RS10320	2203460..2207131	3015994	BREX-system-P-loop-protein-BrxC-[Halopseudomonas-sp.-MFKK-1]
AUB77630.1	Type1.BREX	Arm-DNA-bind_3+Phage_integrase->?->Phage_AlpA->?->wHTH+WYL+WCX->BrxA-DNA-binding->BrxB-BrxF-iSTAND->DUF6079*->DOC+HTH->N6-METHYLASE->?->?->?->NERD+GspA-ATPase->TPR->PglZ->SIGMA+OB-Fold+AAA-ATPase+Lon-Protease-BrxL->	1223	Bacteria>Pseudomonadota>Gammaproteobacteria>Pseudomonadales>Pseudomonadaceae>Pseudomonas	GCA_000346225.2	Pseudomonas_sp._Lz4W	B195_019235	4255709..4259380	1206777	ATPase-[Pseudomonas-sp.-Lz4W]
ERV97546.1	Type1.BREX	LZ_Tnp_IS66+zf-IS66+DDE_Tnp_IS66_C->?->DDE_Tnp_1->?->?<-N6-METHYLASE<-DOC+HTH<-?<-?<-DUF6079*<-BrxB-BrxF-iSTAND<-BrxA-DNA-binding<-wHTH+WYL+WCX||OLD-ABC+TOPRIM->SF1-SF2-Helicase->SF1-SF2-Helicase->?-><-Phage_integrase	1223	Bacteria>Pseudomonadota>Gammaproteobacteria>Pseudomonadales>Pseudomonadaceae>Pseudomonas	GCA_000481225.1	Pseudomonas_aeruginosa_BWHPSA024	Q037_05156	13246..16917	1402524	hypothetical-protein-Q037_05156-[Pseudomonas-aeruginosa-BWHPSA024]
KPX73888.1	Type1.BREX	PIN+OST-HTH->HNH->?->?->DUF6079-p*->PglZ->SIGMA+OB-Fold+AAA-ATPase+Lon-Protease-BrxL->HIN-HTH->HTH_Tnp_1+HTH_21+rve+rve_3->	889	Bacteria>Pseudomonadota>Gammaproteobacteria>Pseudomonadales>Pseudomonadaceae>Pseudomonas	GCA_001400855.1	Pseudomonas_syringae_pv._maculicola	ALO84_01403	56181..58850	59511	Uncharacterized-protein-ALO84_01403-[Pseudomonas-syringae-pv.-maculicola]
KSG52894.1	Type1.BREX	<-ABC-ATPase+SbcC<-?<-TIR<-?<-N6-METHYLASE-p<-RVT<-N6-METHYLASE<-DOC+HTH<-DUF6079*<-BrxB-BrxF-iSTAND<-BrxA-DNA-binding<-wHTH+WYL+WCX<-?<-Phage_AlpA<-?||PAIREDC-HTH->	1223	Bacteria>Pseudomonadota>Gammaproteobacteria>Pseudomonadales>Pseudomonadaceae>Pseudomonas	GCA_001450115.1	Pseudomonas_aeruginosa	AO953_18465	48360..52031	287	ATPase-[Pseudomonas-aeruginosa]
ARQ76275.1	Type1.BREX	DUF4969-><-SIGMA+OB-Fold+AAA-ATPase+Lon-Protease-BrxL<-PglZ<-TOPRIM<-OLD-ABC<-ParB+HNH<-N6-METHYLASE<-DUF6079*<-BrxB-BrxF-iSTAND<-BrxA-DNA-binding<-?<-XIS-HTH||?-><-?<-?<-?||Aminotran_1_2->	1156	Bacteria>Pseudomonadota>Gammaproteobacteria>Pseudomonadales>Pseudomonadaceae>Pseudomonas	GCA_002128325.1	Pseudomonas_fragi	B6D87_19590	4227178..4230648	296	hypothetical-protein-B6D87_19590-[Pseudomonas-fragi]
PAA41060.1	Type1.BREX	HIN-HTH->AAA-ATPase-><-?||XIS-HTH->?->BrxA-DNA-binding->BrxB-BrxF-iSTAND->DUF6079*->N6-METHYLASE->ParB+HNH->PglZ->SIGMA+OB-Fold+AAA-ATPase+Lon-Protease-BrxL-><-DUF4969<-?<-?<-HIN-HTH+rve+rve_3	1156	Bacteria>Pseudomonadota>Gammaproteobacteria>Pseudomonadales>Pseudomonadaceae>Pseudomonas	GCA_002269445.1	Pseudomonas_fragi	CJU80_12345	107560..111030	296	hypothetical-protein-CJU80_12345-[Pseudomonas-fragi]
ATN11139.1	Type1.BREX	<-TraI-VirD2-RCRE<-ParD_antitoxin||HNH->?->||BrxA-DNA-binding->BrxB-BrxF-iSTAND->DUF6079*->DNA-glycosylase->N6-METHYLASE->ParB+HNH->AbiEi_3_N+AbiEi_3->AbiEii->PglZ->SIGMA+OB-Fold+AAA-ATPase+Lon-Protease-BrxL->	1222	Bacteria>Pseudomonadota>Gammaproteobacteria>Pseudomonadales>Pseudomonadaceae>Pseudomonas	GCA_002591235.1	Pseudomonas_sp._FDAARGOS_380	CRN80_16460	3507093..3510761	2018067	hypothetical-protein-CRN80_16460-[Pseudomonas-sp.-FDAARGOS_380]
MBB59705.1	Type1.BREX	<-DUF3584+DUF5401<-MukF<-SIGMA+OB-Fold+AAA-ATPase+Lon-Protease-BrxL<-PglZ<-TOPRIM<-OLD-ABC<-ParB+HNH<-N6-METHYLASE<-DUF932<-N6-METHYLASE<-DUF6079*<-BrxB-BrxF-iSTAND<-BrxA-DNA-binding<-wHTH+WYL+WCX<-?<-ARG||wHTH+WYL+WCX->LexA-protease->	1154	Bacteria>Pseudomonadota>Gammaproteobacteria>Pseudomonadales>Pseudomonadaceae>Pseudomonas	GCA_002709345.1	Pseudomonas_sp.	CML00_00850	39770..43234	306	hypothetical-protein-[Pseudomonas-sp.]
PPB00131.1	Type1.BREX	<-TraE<-TraM<-TraM<-DUF3262<-?||HipA_C->XIS-HTH->BrxA-DNA-binding->BrxB-BrxF-iSTAND->DUF6079*->N6-METHYLASE->OLD-ABC->REase->SIR2->HerA/FtsK->N6-METHYLASE->PglZ->SIGMA+OB-Fold+AAA-ATPase+HKD-DNase-BrxL->SbcC+SbcC->	1194	Bacteria>Pseudomonadota>Gammaproteobacteria>Pseudomonadales>Pseudomonadaceae>Pseudomonas	GCA_002927135.1	Pseudomonas_aeruginosa	HV84_25295	2273516..2277100	287	BREX-system-P-loop-protein-BrxC-[Pseudomonas-aeruginosa]
PPV34665.1	Type1.BREX	<-RVT<-N6-METHYLASE<-DOC+HTH<-DUF6079*<-BrxB-BrxF-iSTAND<-BrxA-DNA-binding<-wHTH+WYL+WCX	1223	Bacteria>Pseudomonadota>Gammaproteobacteria>Pseudomonadales>Pseudomonadaceae>Pseudomonas	GCA_002941105.1	Pseudomonas_oleovorans	C5L43_18395	7326..10997	301	BREX-system-P-loop-protein-BrxC-[Pseudomonas-oleovorans]
HBC00740.1	Type1.BREX	TOPRIM+Primase->?-><-?<-SIGMA+OB-Fold+AAA-ATPase+Lon-Protease-BrxL<-PglZ<-Schlafen+HATPase+wHTH<-SbcC<-N6-METHYLASE<-?<-DUF6079*<-BrxB-BrxF-iSTAND<-BrxA-DNA-binding<-RHH_5<-?<-?<-?||DEDD_Tnp_IS110+Transposase_20-><-Arm-DNA-bind_3+Phage_integrase<-tRNA<-N6-METHYLASE	1153	Bacteria>Pseudomonadota>Gammaproteobacteria>Pseudomonadales>Pseudomonadaceae>Pseudomonas	GCA_003488145.1	Pseudomonas_sp.	DC032_09195	21916..25377	306	BREX-system-P-loop-protein-BrxC-[Pseudomonas-sp.]
HBM65158.1	Type1.BREX	<-ANK+ANK+ANK||RADICAL-SAM->HicA_toxin->BrxA-DNA-binding->BrxB-BrxF-iSTAND->DUF6079*->N6-METHYLASE-p->	1154	Bacteria>Pseudomonadota>Gammaproteobacteria>Pseudomonadales>Pseudomonadaceae>Pseudomonas	GCA_003504465.1	Pseudomonas_sp.	DD418_16300	5931..9395	306	BREX-system-P-loop-protein-BrxC-[Pseudomonas-sp.]
RPD95236.1	Type1.BREX	ZnR-><-wHTH+WYL+WCX||XIS-HTH->BrxA-DNA-binding->BrxB-BrxF-iSTAND->DUF6079*->N6-METHYLASE-p->PglZ->SIGMA+OB-Fold+AAA-ATPase+HKD-DNase-BrxL-><-?<-Arm-DNA-bind_3+Phage_integrase<-?<-?<-?||GNTR-HTH+LRP-HTH->	1191	Bacteria>Pseudomonadota>Gammaproteobacteria>Pseudomonadales>Pseudomonadaceae>Pseudomonas	GCA_003813925.1	Pseudomonas_monteilii	EGN69_04400	958521..962096	76759	BREX-system-P-loop-protein-BrxC-[Pseudomonas-monteilii]
RPM42463.1	Type1.BREX	<-ABhydrolase<-TetR-HTH||?->Arm-DNA-bind_3+Phage_integrase->Phage_AlpA->?->wHTH+WYL+WCX->BrxA-DNA-binding->BrxB-BrxF-iSTAND->DUF6079*->DOC+HTH->N6-METHYLASE->RVT->N6-METHYLASE-p->?-><-Y1_Tnp||TIR->?->ABC-ATPase+SbcC->	1223	Bacteria>Pseudomonadota>Gammaproteobacteria>Pseudomonadales>Pseudomonadaceae>Pseudomonas	GCA_003833645.1	Pseudomonas_aeruginosa	IPC1294_03635	745407..749078	287	ATPase-[Pseudomonas-aeruginosa]
TKK14312.1	Type1.BREX	<-TraI-VirD2-RCRE<-ParD_antitoxin||HNH->?->||BrxA-DNA-binding->BrxB-BrxF-iSTAND->DUF6079*->DNA-glycosylase->N6-METHYLASE->ParB+HNH->AbiEi_3_N+AbiEi_3->AbiEii->PglZ->SIGMA+OB-Fold+AAA-ATPase+Lon-Protease-BrxL->	1222	Bacteria>Pseudomonadota>Gammaproteobacteria>Pseudomonadales>Pseudomonadaceae>Pseudomonas	GCA_005233695.1	Pseudomonas_fluorescens	Pf1CFBP13510_04710	495926..499594	294	BREX-system-P-loop-protein-BrxC-[Pseudomonas-fluorescens]
TXH92079.1	Type1.BREX	<-RelE-ParE<-RHH_1<-PglZ<-HNH<-OLD-ABC<-N6-METHYLASE-p<-NERD<-OLD-ABC<-N6-METHYLASE<-DUF6079*<-BrxB-BrxF-iSTAND<-BrxA-DNA-binding<-XIS-HTH<-?||wHTH+WYL+WCX->	1186	Bacteria>Pseudomonadota>Gammaproteobacteria>Pseudomonadales>Pseudomonadaceae>Pseudomonas	GCA_008015405.1	Pseudomonas_sp.	E6Q71_01680	13269..16829	306	BREX-system-P-loop-protein-BrxC-[Pseudomonas-sp.]
MRJ36970.1	Type1.BREX	<-XIS-HTH||?->DUF932->?->?->wHTH+WYL+WCX->BrxA-DNA-binding->BrxB-BrxF-iSTAND->DUF6079*->N6-METHYLASE->NERD+GspA-ATPase->TPR->PglZ->SIGMA+OB-Fold+AAA-ATPase+Lon-Protease-BrxL->	1223	Bacteria>Pseudomonadota>Gammaproteobacteria>Pseudomonadales>Pseudomonadaceae>Pseudomonas	GCA_009659625.1	Pseudomonas_haemolytica	FRT59_08305	447566..451237	2600065	BREX-system-P-loop-protein-BrxC-[Pseudomonas-haemolytica]
MVW77192.1	Type1.BREX	<-PglZ<-AbiEii<-AbiEi_3_N+AbiEi_3<-?<-?<-ParB+HNH<-N6-METHYLASE<-DNA-glycosylase<-ParB+HNH<-DUF6079*<-BrxB-BrxF-iSTAND<-BrxA-DNA-binding<-?||wHTH+WYL+WCX->?-><-?||Arm-DNA-bind_2+Phage_int_SAM_3+Phage_integrase-><-REase||ZnR->	1221	Bacteria>Pseudomonadota>Gammaproteobacteria>Pseudomonadales>Pseudomonadaceae>Pseudomonas	GCA_009763245.1	Pseudomonas_xionganensis	GJV18_17880	24794..28459	2654845	BREX-system-P-loop-protein-BrxC-[Pseudomonas-xionganensis]
NLU01326.1	Type1.BREX	<-XIS-HTH||?->DUF932->?->?->?->wHTH+WYL+WCX->BrxA-DNA-binding->BrxB-BrxF-iSTAND->DUF6079*->N6-METHYLASE->ParB+HNH->PglZ->SIGMA+OB-Fold+AAA-ATPase+Lon-Protease-BrxL->?-><-tRNA<-MerR-HTH	1222	Bacteria>Pseudomonadota>Gammaproteobacteria>Pseudomonadales>Pseudomonadaceae>Pseudomonas	GCA_012720005.1	Pseudomonas_lundensis	GXW88_11975	7910..11578	86185	BREX-system-P-loop-protein-BrxC-[Pseudomonas-lundensis]
NMY25069.1	Type1.BREX	PIN+OST-HTH->HNH->?->?-><-wHTH+WYL+WCX||?->ARG->BrxA-DNA-binding->BrxB-BrxF-iSTAND->DUF6079*->DNA-glycosylase->N6-METHYLASE->ParB+HNH->AbiEi_3_N+AbiEi_3->AbiEii->PglZ->SIGMA+OB-Fold+AAA-ATPase+Lon-Protease-BrxL->JAB-><-MerR-HTH	1222	Bacteria>Pseudomonadota>Gammaproteobacteria>Pseudomonadales>Pseudomonadaceae>Pseudomonas	GCA_012985535.1	Pseudomonas_sp._WS_5021	HBO19_03685	794090..797758	2717490	BREX-system-P-loop-protein-BrxC-[Pseudomonas-sp.-WS-5021]
NMY20845.1	Type1.BREX	<-SIGMA+OB-Fold+AAA-ATPase+Lon-Protease-BrxL<-PglZ<-TPR<-NERD+GspA-ATPase<-N6-METHYLASE<-DUF6079*<-BrxB-BrxF-iSTAND<-BrxA-DNA-binding<-wHTH+WYL+WCX||?->HKD-DNase+HKD-DNase->SF1-SF2-Helicase->?->SF1-SF2-Helicase+REase->	1223	Bacteria>Pseudomonadota>Gammaproteobacteria>Pseudomonadales>Pseudomonadaceae>Pseudomonas	GCA_012985645.1	Pseudomonas_sp._WS_5410	HBO24_11430	134586..138257	2717485	BREX-system-P-loop-protein-BrxC-[Pseudomonas-sp.-WS-5410]
NMY53287.1	Type1.BREX	<-N6-METHYLASE<-?<-DUF6079*<-BrxB-BrxF-iSTAND<-BrxA-DNA-binding<-?||wHTH+WYL+WCX-><-SF1-SF2-Helicase||?->?-><-Phage_integrase	1222	Bacteria>Pseudomonadota>Gammaproteobacteria>Pseudomonadales>Pseudomonadaceae>Pseudomonas	GCA_012985775.1	Pseudomonas_sp._WS_5011	HBN63_20215	4647..8315	2717477	BREX-system-P-loop-protein-BrxC-[Pseudomonas-sp.-WS-5011]
NWF08290.1	Type1.BREX	<-SIGMA+OB-Fold+AAA-ATPase+Lon-Protease-BrxL<-PglZ<-AbiEii<-AbiEi_3_N+AbiEi_3<-ParB+HNH<-N6-METHYLASE<-DNA-glycosylase<-DUF6079*<-BrxB-BrxF-iSTAND<-BrxA-DNA-binding<-?||wHTH+WYL+WCX-><-tRNA<-MerR-HTH	1222	Bacteria>Pseudomonadota>Gammaproteobacteria>Pseudomonadales>Pseudomonadaceae>Pseudomonas	GCA_013386885.1	Pseudomonas_salomonii	HXB10_11520	509017..512685	191391	BREX-system-P-loop-protein-BrxC-[Pseudomonas-salomonii]
MBD9399119.1	Type1.BREX	<-wHTH+WYL+WCX||?->JetB->?->RNase->?->BrxA-DNA-binding->BrxB-BrxF-iSTAND->DUF6079*->?->N6-METHYLASE->?->AbiEi_4->AbiEii->PglZ->SIGMA+OB-Fold+AAA-ATPase+Lon-Protease-BrxL->	1222	Bacteria>Pseudomonadota>Gammaproteobacteria>Pseudomonadales>Pseudomonadaceae>Pseudomonas	GCA_014851505.1	Pseudomonas_sp._PDM11	IB224_16075	119844..123512	2769309	BREX-system-P-loop-protein-BrxC-[Pseudomonas-sp.-PDM11]
MBF9242733.1	Type1.BREX	SSB->Phage_integrase->Phage_integrase->Phage_integrase->?-><-HEPN||BrxA-DNA-binding->BrxB-BrxF-iSTAND->DUF6079*->ParB+HNH->DNA-glycosylase->N6-METHYLASE->PglZ->	1222	Bacteria>Pseudomonadota>Gammaproteobacteria>Pseudomonadales>Pseudomonadaceae>Pseudomonas	GCA_015694365.1	Pseudomonas_syringae_pv._tomato	I4I80_00295	46308..49976	323	BREX-system-P-loop-protein-BrxC-[Pseudomonas-syringae-pv.-tomato]
MBG4373384.1	Type1.BREX	<-HIN-HTH<-REase<-wHTH+WYL+WCX||?->Peptidase_M15->?->Ntox49->BrxA-DNA-binding->BrxB-BrxF-iSTAND->DUF6079*->N6-METHYLASE->?->HNH+GCN1->?->ParB+HNH->PglZ->SIGMA+OB-Fold+AAA-ATPase+Lon-Protease-BrxL-><-GreA_GreB+SF1-SF2-Helicase<-Arm-DNA-bind_3+Phage_integrase	1154	Bacteria>Pseudomonadota>Gammaproteobacteria>Pseudomonadales>Pseudomonadaceae>Pseudomonas	GCA_015734445.1	Pseudomonas_aeruginosa	I5F50_23085	8082..11546	287	BREX-system-P-loop-protein-BrxC-[Pseudomonas-aeruginosa]
MBG4395801.1	Type1.BREX	<-wHTH+WYL+WCX||?->Peptidase_M15->?->Ntox49->BrxA-DNA-binding->BrxB-BrxF-iSTAND->DUF6079*->N6-METHYLASE->?->HNH+GCN1->?->ParB+HNH->PglZ->SIGMA+OB-Fold+AAA-ATPase+Lon-Protease-BrxL-><-GreA_GreB+SF1-SF2-Helicase->Arm-DNA-bind_3+Phage_integrase	1154	Bacteria>Pseudomonadota>Gammaproteobacteria>Pseudomonadales>Pseudomonadaceae>Pseudomonas	GCA_015734545.1	Pseudomonas_aeruginosa	GHT73_12640	7113..10577	287	BREX-system-P-loop-protein-BrxC-[Pseudomonas-aeruginosa]
MBG4463093.1	Type1.BREX	<-HIN-HTH<-REase<-wHTH+WYL+WCX||?->Peptidase_M15->?->Ntox49->BrxA-DNA-binding->BrxB-BrxF-iSTAND->DUF6079*->N6-METHYLASE->?->HNH+GCN1->?->ParB+HNH->PglZ->SIGMA+OB-Fold+AAA-ATPase+Lon-Protease-BrxL-><-GreA_GreB+SF1-SF2-Helicase<-Arm-DNA-bind_3+Phage_integrase	1154	Bacteria>Pseudomonadota>Gammaproteobacteria>Pseudomonadales>Pseudomonadaceae>Pseudomonas	GCA_015734755.1	Pseudomonas_aeruginosa	GHU50_17045	10342..13806	287	BREX-system-P-loop-protein-BrxC-[Pseudomonas-aeruginosa]
MBG4530231.1	Type1.BREX	MerR-HTH->tRNA->XIS-HTH->BrxA-DNA-binding->BrxB-BrxF-iSTAND->DUF6079*->N6-METHYLASE->OLD-ABC->REase->SIR2->HerA/FtsK->N6-METHYLASE->PglZ->SIGMA+OB-Fold+AAA-ATPase+HKD-DNase-BrxL->XIS-HTH->	1181	Bacteria>Pseudomonadota>Gammaproteobacteria>Pseudomonadales>Pseudomonadaceae>Pseudomonas	GCA_015734885.1	Pseudomonas_aeruginosa	I4952_20115	27108..30653	287	BREX-system-P-loop-protein-BrxC-[Pseudomonas-aeruginosa]
MBG4863325.1	Type1.BREX	<-HIN-HTH||XIS-HTH->BrxA-DNA-binding->BrxB-BrxF-iSTAND->DUF6079*->N6-METHYLASE->OLD-ABC->NERD->N6-METHYLASE-p->OLD-ABC->TOPRIM->PglZ->SIGMA+OB-Fold+AAA-ATPase+HKD-DNase-BrxL->HIN-HTH->	1184	Bacteria>Pseudomonadota>Gammaproteobacteria>Pseudomonadales>Pseudomonadaceae>Pseudomonas	GCA_015735985.1	Pseudomonas_aeruginosa	GHU23_28970	1726..5280	287	BREX-system-P-loop-protein-BrxC-[Pseudomonas-aeruginosa]
MBG5216128.1	Type1.BREX	<-wHTH+WYL+WCX||?->Peptidase_M15->?->BrxA-DNA-binding->BrxB-BrxF-iSTAND->DUF6079*->N6-METHYLASE->?->HNH+GCN1->?->ParB+HNH->PglZ->SIGMA+OB-Fold+AAA-ATPase+Lon-Protease-BrxL->REase->	1154	Bacteria>Pseudomonadota>Gammaproteobacteria>Pseudomonadales>Pseudomonadaceae>Pseudomonas	GCA_015737185.1	Pseudomonas_aeruginosa	GHT75_27125	7080..10544	287	BREX-system-P-loop-protein-BrxC-[Pseudomonas-aeruginosa]
MBG6730505.1	Type1.BREX	<-LexA-protease<-wHTH+WYL+WCX||?->XIS-HTH->BrxA-DNA-binding->BrxB-BrxF-iSTAND->DUF6079*->N6-METHYLASE->SbcC-p->REase->N6-METHYLASE-p->OLD-ABC->TOPRIM->PglZ->SIGMA+OB-Fold+AAA-ATPase+HKD-DNase-BrxL-><-DUF4969	1184	Bacteria>Pseudomonadota>Gammaproteobacteria>Pseudomonadales>Pseudomonadaceae>Pseudomonas	GCA_015764285.1	Pseudomonas_aeruginosa	I5I92_11320	4371..7925	287	BREX-system-P-loop-protein-BrxC-[Pseudomonas-aeruginosa]
MBH9015057.1	Type1.BREX	<-TetR-HTH||?->Arm-DNA-bind_3+Phage_integrase->?->?||Phage_AlpA->?->wHTH+WYL+WCX->BrxA-DNA-binding->BrxB-BrxF-iSTAND->DUF6079*->?->?->DOC+HTH->N6-METHYLASE->?->||MODE-HTH+PBPII->	1223	Bacteria>Pseudomonadota>Gammaproteobacteria>Pseudomonadales>Pseudomonadaceae>Pseudomonas	GCA_016090735.1	Pseudomonas_aeruginosa	I5K34_26080	19802..23473	287	BREX-system-P-loop-protein-BrxC-[Pseudomonas-aeruginosa]
MBI7168904.1	Type1.BREX	DDE_Tnp_1-><-?<-N6-METHYLASE<-DOC+HTH<-?<-?<-DUF6079*<-BrxB-BrxF-iSTAND<-BrxA-DNA-binding<-wHTH+WYL+WCX	1223	Bacteria>Pseudomonadota>Gammaproteobacteria>Pseudomonadales>Pseudomonadaceae>Pseudomonas	GCA_016338125.1	Pseudomonas_aeruginosa	JEU09_31365	9152..12823	287	BREX-system-P-loop-protein-BrxC-[Pseudomonas-aeruginosa]
MBI8737743.1	Type1.BREX	RelE-ParE-><-?<-wHTH+WYL+WCX||?->Peptidase_M15->?->Ntox49->BrxA-DNA-binding->BrxB-BrxF-iSTAND->DUF6079*->N6-METHYLASE->?->HNH+GCN1->?->ParB+HNH->PglZ->SIGMA+OB-Fold+AAA-ATPase+Lon-Protease-BrxL->	1154	Bacteria>Pseudomonadota>Gammaproteobacteria>Pseudomonadales>Pseudomonadaceae>Pseudomonas	GCA_016342945.1	Pseudomonas_aeruginosa	JEZ70_02820	55146..58610	287	BREX-system-P-loop-protein-BrxC-[Pseudomonas-aeruginosa]
MBM1201834.1	Type1.BREX	XIS-HTH->?->wHTH+WYL+WCX->BrxA-DNA-binding->BrxB-BrxF-iSTAND->DUF6079*->N6-METHYLASE->ParB+HNH->PglZ->SIGMA+OB-Fold+AAA-ATPase+Lon-Protease-BrxL-><-DUF4969<-?<-?<-DUF932	1154	Bacteria>Pseudomonadota>Gammaproteobacteria>Pseudomonadales>Pseudomonadaceae>Pseudomonas	GCA_016812215.1	Pseudomonas_fragi	GYM96_18820	91266..94730	296	BREX-system-P-loop-protein-BrxC-[Pseudomonas-fragi]
MBM2633269.1	Type1.BREX	<-ABC-ATPase+SbcC<-?<-?<-MODE-HTH+PBPII||<-?<-N6-METHYLASE<-DOC+HTH<-?<-?<-DUF6079*<-BrxB-BrxF-iSTAND<-BrxA-DNA-binding<-wHTH+WYL+WCX||OLD-ABC+TOPRIM->SF1-SF2-Helicase->SF1-SF2-Helicase-><-Phage_integrase	1223	Bacteria>Pseudomonadota>Gammaproteobacteria>Pseudomonadales>Pseudomonadaceae>Pseudomonas	GCA_016836445.1	Pseudomonas_aeruginosa	JQ480_07705	1618759..1622430	287	BREX-system-P-loop-protein-BrxC-[Pseudomonas-aeruginosa]
BCR26098.1	Type1.BREX	Phage_integrase-><-?<-SF1-SF2-Helicase<-?<-SbcC||?->wHTH+WYL+WCX->BrxA-DNA-binding->BrxB-BrxF-iSTAND->DUF6079*->?->?->DOC+HTH->N6-METHYLASE->RVT->N6-METHYLASE-p-><-DUF772+DDE_Tnp_1||N6-METHYLASE-p->	1223	Bacteria>Pseudomonadota>Gammaproteobacteria>Pseudomonadales>Pseudomonadaceae>Pseudomonas	GCA_016861485.1	Pseudomonas_alcaligenes	KAM426_36250	3797295..3800966	43263	hypothetical-protein-KAM426_36250-[Pseudomonas-alcaligenes]
MBN0979708.1	Type1.BREX	<-DNA-glycosylase<-DUF6079*<-BrxB-BrxF-iSTAND<-BrxA-DNA-binding||<-?<-GTPase-AIG<-GTPase-AIG	1222	Bacteria>Pseudomonadota>Gammaproteobacteria>Pseudomonadales>Pseudomonadaceae>Pseudomonas	GCA_016924785.1	Pseudomonas_sp._SDM007_2	JV174_20995	2384..6052	2812898	BREX-system-P-loop-protein-BrxC-[Pseudomonas-sp.-SDM007_2]
MBV5724753.1	Type1.BREX	DUF4969-><-SIGMA+OB-Fold+AAA-ATPase+HKD-DNase-BrxL<-PglZ<-ParB+HNH<-N6-METHYLASE-p<-RVT<-NERD<-OLD-ABC<-N6-METHYLASE<-DUF6079*<-BrxB-BrxF-iSTAND<-BrxA-DNA-binding<-XIS-HTH<-?||wHTH+WYL+WCX-><-DUF4158+DDE_Tnp_Tn3-><-GDResolvase+HIN-HTH<-GDResolvase+HIN-HTH<-Tox-RelE	1184	Bacteria>Pseudomonadota>Gammaproteobacteria>Pseudomonadales>Pseudomonadaceae>Pseudomonas	GCA_019166825.1	Pseudomonas_aeruginosa	KUT37_26485	44544..48098	287	BREX-system-P-loop-protein-BrxC-[Pseudomonas-aeruginosa]
GIZ69010.1	Type1.BREX	<-DUF6079*<-BrxB-BrxF-iSTAND<-BrxA-DNA-binding<-wHTH+WYL+WCX<-?||SbcC->?->SF1-SF2-Helicase->?-><-Phage_integrase	1226	Bacteria>Pseudomonadota>Gammaproteobacteria>Pseudomonadales>Pseudomonadaceae>Pseudomonas	GCA_019656515.1	Pseudomonas_alcaligenes	KAM428_40950	2224..5904	43263	hypothetical-protein-KAM428_40950-[Pseudomonas-alcaligenes]

Protein-ID	System	Gene-Neighbourhoods	Length	Taxonomy	Assembly-ID	Strain	Locus-Tag	Genomic-coordinates	TaxID	Fasta-define
MBX6150345.1	Type1.BREX	<-TetR-HTH||?-><-SIGMA+OB-Fold+AAA-ATPase+Lon-Protease-BrxL<-PglZ<-ParB+HNH<-?<-HNH+GCN1<-?<-N6-METHYLASE<-DUF6079*<-BrxB-BrxF-iSTAND<-BrxA-DNA-binding<-?<-Peptidase_M15<-?||wHTH+WYL+[illegible]	1154	Bacteria>Pseudomonadota>Gammaproteobacteria>Pseudomonadales>Pseudomonadaceae>Pseudomonas	GCA_019686265.1	Pseudomonas_aeruginosa	ISD36_12550	248185..251649	287	BREX-system-P-loop-protein-BrxC-[Pseudomonas-aeruginosa]
MBX5763959.1	Type1.BREX	Phage_integrase-><-?<-SIGMA+OB-Fold+AAA-ATPase+Lon-Protease-BrxL<-PglZ<-HNH<-OLD-ABC<-?<-?<-N6-METHYLASE<-DUF6079*<-BrxB-BrxF-iSTAND<-BrxA-DNA-binding<-wHTH+WYL+WCX||REase+NACHT->?-><[illegible]	1223	Bacteria>Pseudomonadota>Gammaproteobacteria>Pseudomonadales>Pseudomonadaceae>Pseudomonas	GCA_019687095.1	Pseudomonas_aeruginosa	ISD89_03975	862812..866483	287	BREX-system-P-loop-protein-BrxC-[Pseudomonas-aeruginosa]
MBX5915342.1	Type1.BREX	<-wHTH+WYL+WCX||?->Peptidase_M15->?->BrxA-DNA-binding->BrxB-BrxF-iSTAND->DUF6079*->N6-METHYLASE->?->HNH+GCN1->?->ParB+HNH->PglZ->SIGMA+OB-Fold+AAA-ATPase+Lon-Protease-BrxL->REase->	1154	Bacteria>Pseudomonadota>Gammaproteobacteria>Pseudomonadales>Pseudomonadaceae>Pseudomonas	GCA_019688355.1	Pseudomonas_aeruginosa	ISE50_29115	6954..10418	287	BREX-system-P-loop-protein-BrxC-[Pseudomonas-aeruginosa]
MBY8973665.1	Type1.BREX	<-SIGMA+OB-Fold+AAA-ATPase+Lon-Protease-BrxL<-PglZ<-AbiEii<-AbiEi_3_N+AbiEi_3<-ParB+HNH<-N6-METHYLASE<-DNA-glycosylase<-DUF6079*<-BrxB-BrxF-iSTAND<-BrxA-DNA-binding<-ARG<-?||wHTH+WYL+[illegible]	1222	Bacteria>Pseudomonadota>Gammaproteobacteria>Pseudomonadales>Pseudomonadaceae>Pseudomonas	GCA_019891455.1	Pseudomonas_sp._P867	J1G33_25110	13708..17376	2816050	BREX-system-P-loop-protein-BrxC-[Pseudomonas-sp.-P867]
HBN8454461.1	Type1.BREX	XIS-HTH-><-?<-tRNA||HTH_36->Phage_int_SAM_2+Integrase_1-><-SIGMA+OB-Fold+AAA-ATPase+Lon-Protease-BrxL<-PglZ<-HNH<-OLD-ABC<-N6-METHYLASE<-DUF6079*<-BrxB-BrxF-iSTAND<-BrxA-DNA-binding<-[illegible]	1146	Bacteria>Pseudomonadota>Gammaproteobacteria>Pseudomonadales>Pseudomonadaceae>Pseudomonas	GCA_021587585.1	Pseudomonas_aeruginosa	L3711_005718	39513..42953	287	BREX-system-P-loop-protein-BrxC-[Pseudomonas-aeruginosa]
HBN9708921.1	Type1.BREX	PhdYeFM_antitox->RelE-ParE-><-?<-wHTH+WYL+WCX||?->Peptidase_M15->?->BrxA-DNA-binding->BrxB-BrxF-iSTAND->DUF6079*->N6-METHYLASE->MNS+ORC-CDC6->MarR-HTH->?->ParB+HNH->PglZ->SIGMA+OB-Fol[illegible]	1154	Bacteria>Pseudomonadota>Gammaproteobacteria>Pseudomonadales>Pseudomonadaceae>Pseudomonas	GCA_021591785.1	Pseudomonas_aeruginosa	L3920_001787	275283..278747	287	BREX-system-P-loop-protein-BrxC-[Pseudomonas-aeruginosa]
HBO1619179.1	Type1.BREX	<-DUF3584+DUF5401<-JetB<-MukF<-SIGMA+OB-Fold+AAA-ATPase+Lon-Protease-BrxL<-PglZ<-TOPRIM<-OLD-ABC<-ParB+HNH<-N6-METHYLASE<-DUF6079*<-BrxB-BrxF-iSTAND<-BrxA-DNA-binding<-wHTH+WYL+WCX<-?[illegible]	1154	Bacteria>Pseudomonadota>Gammaproteobacteria>Pseudomonadales>Pseudomonadaceae>Pseudomonas	GCA_021598085.1	Pseudomonas_aeruginosa	L4F62_005712	16995..20459	287	BREX-system-P-loop-protein-BrxC-[Pseudomonas-aeruginosa]
EIU3464059.1	Type1.BREX	<-DUF3584+DUF5401<-JetB<-MukF<-SIGMA+OB-Fold+AAA-ATPase+Lon-Protease-BrxL<-PglZ<-HNH<-OLD-ABC<-ParB+HNH<-N6-METHYLASE<-DUF6079*<-BrxB-BrxF-iSTAND<-BrxA-DNA-binding<-wHTH+WYL+WCX<-?<-A[illegible]	1154	Bacteria>Pseudomonadota>Gammaproteobacteria>Pseudomonadales>Pseudomonadaceae>Pseudomonas	GCA_021644165.1	Pseudomonas_aeruginosa	L4P80_001287	38375..41839	287	BREX-system-P-loop-protein-BrxC-[Pseudomonas-aeruginosa]
HBO3135817.1	Type1.BREX	<-OLD-ABC<-N6-METHYLASE-p<-RVT<-NERD<-OLD-ABC<-SbcC-p<-N6-METHYLASE<-REase<-ABC-ATPase<-DUF6079*<-BrxB-BrxF-iSTAND<-BrxA-DNA-binding<-XIS-HTH	1184	Bacteria>Pseudomonadota>Gammaproteobacteria>Pseudomonadales>Pseudomonadaceae>Pseudomonas	GCA_021651475.1	Pseudomonas_aeruginosa	L4Q96_001363	40663..44217	287	BREX-system-P-loop-protein-BrxC-[Pseudomonas-aeruginosa]
HBO3110869.1	Type1.BREX	Tox-RelE->HTH_3-><-tRNA||HTH_36->Phage_int_SAM_2+Integrase_1-><-SIGMA+OB-Fold+AAA-ATPase+Lon-Protease-BrxL<-PglZ<-HNH<-OLD-ABC<-N6-METHYLASE<-DUF6079*<-BrxB-BrxF-iSTAND<-BrxA-DNA-bind[illegible]	1146	Bacteria>Pseudomonadota>Gammaproteobacteria>Pseudomonadales>Pseudomonadaceae>Pseudomonas	GCA_021651555.1	Pseudomonas_aeruginosa	L4R01_001445	31462..34902	287	BREX-system-P-loop-protein-BrxC-[Pseudomonas-aeruginosa]
HBO2945375.1	Type1.BREX	REase+SF1-SF2-Helicase->Arm-DNA-bind_3+Phage_integrase-><-SIGMA+OB-Fold+AAA-ATPase+HKD-DNase-BrxL<-PglZ<-N6-METHYLASE-p<-RVT<-REase<-SbcC-p<-N6-METHYLASE<-DUF6079*<-BrxB-BrxF-iSTAND<-[illegible]	1184	Bacteria>Pseudomonadota>Gammaproteobacteria>Pseudomonadales>Pseudomonadaceae>Pseudomonas	GCA_021651875.1	Pseudomonas_aeruginosa	L4R51_003866	17556..21110	287	BREX-system-P-loop-protein-BrxC-[Pseudomonas-aeruginosa]
HBO6336105.1	Type1.BREX	<-wHTH+WYL+WCX||?->Peptidase_M15->?->BrxA-DNA-binding->BrxB-BrxF-iSTAND->DUF6079*->N6-METHYLASE->?->HNH+GCN1->?->ParB+HNH->PglZ->SIGMA+OB-Fold+AAA-ATPase+Lon-Protease-BrxL->REase->	1154	Bacteria>Pseudomonadota>Gammaproteobacteria>Pseudomonadales>Pseudomonadaceae>Pseudomonas	GCA_021670735.1	Pseudomonas_aeruginosa	L4747_005548	6948..10412	287	BREX-system-P-loop-protein-BrxC-[Pseudomonas-aeruginosa]
HBO7186446.1	Type1.BREX	DUF6079-p*->N6-METHYLASE->SbcC-p->REase->N6-METHYLASE-p->PglZ->SIGMA+OB-Fold+AAA-ATPase+HKD-DNase-BrxL-><-Arm-DNA-bind_3+Phage_integrase<-REase+SF1-SF2-Helicase<-MazG	978	Bacteria>Pseudomonadota>Gammaproteobacteria>Pseudomonadales>Pseudomonadaceae>Pseudomonas	GCA_021674675.1	Pseudomonas_aeruginosa	L4968_002122	1..2938	287	BREX-system-P-loop-protein-BrxC-[Pseudomonas-aeruginosa]
HBO6991083.1	Type1.BREX	PhdYeFM_antitox->RelE-ParE-><-?<-wHTH+WYL+WCX||?->?->XIS-HTH->BrxA-DNA-binding->BrxB-BrxF-iSTAND->DUF6079-p*->	567	Bacteria>Pseudomonadota>Gammaproteobacteria>Pseudomonadales>Pseudomonadaceae>Pseudomonas	GCA_021674875.1	Pseudomonas_aeruginosa	L4955_003120	106733..108434	287	BREX-system-P-loop-protein-BrxC-[Pseudomonas-aeruginosa]
HBO7921132.1	Type1.BREX	ParB+HNH-><-LexA-protease<-wHTH+WYL+WCX||?->XIS-HTH->BrxA-DNA-binding->BrxB-BrxF-iSTAND->DUF6079*->N6-METHYLASE->OLD-ABC->REase->SIR2->HerA/FtsK->N6-METHYLASE->OLD-ABC->TOPRIM->PglZ->[illegible]	1184	Bacteria>Pseudomonadota>Gammaproteobacteria>Pseudomonadales>Pseudomonadaceae>Pseudomonas	GCA_021678155.1	Pseudomonas_aeruginosa	L5C37_005426	13663..17217	287	BREX-system-P-loop-protein-BrxC-[Pseudomonas-aeruginosa]
HBP6330513.1	Type1.BREX	<-RHH_1||XIS-HTH-><-SIGMA+OB-Fold+AAA-ATPase+Lon-Protease-BrxL<-PglZ<-HNH<-OLD-ABC<-ParB+HNH<-DUF932<-N6-METHYLASE<-DUF6079*<-BrxB-BrxF-iSTAND<-BrxA-DNA-binding<-wHTH+WYL+WCX||REase->[illegible]	1154	Bacteria>Pseudomonadota>Gammaproteobacteria>Pseudomonadales>Pseudomonadaceae>Pseudomonas	GCA_021710255.1	Pseudomonas_aeruginosa	L6A37_21805	72354..75818	287	BREX-system-P-loop-protein-BrxC-[Pseudomonas-aeruginosa]
HBP6726062.1	Type1.BREX	[illegible]	1223	Bacteria>Pseudomonadota>Gammaproteobacteria>Pseudomonadales>Pseudomonadaceae>Pseudomonas	GCA_021711795.1	Pseudomonas_aeruginosa	L6B36_03955	73272..76943	287	BREX-system-P-loop-protein-BrxC-[Pseudomonas-aeruginosa]
MCH2341807.1	Type1.BREX	[illegible]	1186	Bacteria>Pseudomonadota>Gammaproteobacteria>Pseudomonadales>Pseudomonadaceae>Pseudomonas	GCA_022448005.1	Pseudomonas_sp.	MK363_18230	17987..21547	306	BREX-system-P-loop-protein-BrxC-[Pseudomonas-sp.]
HCA6672119.1	Type1.BREX	[illegible]	1088	Bacteria>Pseudomonadota>Gammaproteobacteria>Pseudomonadales>Pseudomonadaceae>Pseudomonas	GCA_022988605.1	Pseudomonas_aeruginosa	MXV24_006573	2961..6226	287	BREX-system-P-loop-protein-BrxC-[Pseudomonas-aeruginosa]
MCK9534930.1	Type1.BREX	[illegible]	1154	Bacteria>Pseudomonadota>Gammaproteobacteria>Pseudomonadales>Pseudomonadaceae>Pseudomonas	GCA_023228505.1	Pseudomonas_sp.	MOQ98_09595	5981..9445	306	BREX-system-P-loop-protein-BrxC-[Pseudomonas-sp.]
MCO1735128.1	Type1.BREX	[illegible]	1189	Bacteria>Pseudomonadota>Gammaproteobacteria>Pseudomonadales>Pseudomonadaceae>Pseudomonas	GCA_023905245.1	Pseudomonas_aeruginosa	FA456_28435	3288..6857	287	BREX-system-P-loop-protein-BrxC-[Pseudomonas-aeruginosa]
MCO2467177.1	Type1.BREX	[illegible]	1146	Bacteria>Pseudomonadota>Gammaproteobacteria>Pseudomonadales>Pseudomonadaceae>Pseudomonas	GCA_023907445.1	Pseudomonas_aeruginosa	FA343_15170	67465..70905	287	BREX-system-P-loop-protein-BrxC-[Pseudomonas-aeruginosa]
MCO2679293.1	Type1.BREX	[illegible]	638	Bacteria>Pseudomonadota>Gammaproteobacteria>Pseudomonadales>Pseudomonadaceae>Pseudomonas	GCA_023908095.1	Pseudomonas_aeruginosa	FA310_30470	1..1913	287	BREX-system-P-loop-protein-BrxC-[Pseudomonas-aeruginosa]
MCO2922446.1	Type1.BREX	[illegible]	1189	Bacteria>Pseudomonadota>Gammaproteobacteria>Pseudomonadales>Pseudomonadaceae>Pseudomonas	GCA_023909145.1	Pseudomonas_aeruginosa	FA260_04940	145579..149148	287	BREX-system-P-loop-protein-BrxC-[Pseudomonas-aeruginosa]
HCE7036149.1	Type1.BREX	[illegible]	945	Bacteria>Pseudomonadota>Gammaproteobacteria>Pseudomonadales>Pseudomonadaceae>Pseudomonas	GCA_023918585.1	Pseudomonas_aeruginosa	NHP64_005580	1..2836	287	BREX-system-P-loop-protein-BrxC-[Pseudomonas-aeruginosa]
HCE7261574.1	Type1.BREX	[illegible]	1154	Bacteria>Pseudomonadota>Gammaproteobacteria>Pseudomonadales>Pseudomonadaceae>Pseudomonas	GCA_023919205.1	Pseudomonas_aeruginosa	NHQ13_005985	6842..10306	287	BREX-system-P-loop-protein-BrxC-[Pseudomonas-aeruginosa]
HCF1762884.1	Type1.BREX	[illegible]	1168	Bacteria>Pseudomonadota>Gammaproteobacteria>Pseudomonadales>Pseudomonadaceae>Pseudomonas	GCA_023941915.1	Pseudomonas_aeruginosa	NH889_002155	1..3508	287	BREX-system-P-loop-protein-BrxC-[Pseudomonas-aeruginosa]
HCF5867680.1	Type1.BREX	[illegible]	1181	Bacteria>Pseudomonadota>Gammaproteobacteria>Pseudomonadales>Pseudomonadaceae>Pseudomonas	GCA_023959235.1	Pseudomonas_aeruginosa	NIH49_002287	171515..175060	287	BREX-system-P-loop-protein-BrxC-[Pseudomonas-aeruginosa]
HCF9860952.1	Type1.BREX	[illegible]	1223	Bacteria>Pseudomonadota>Gammaproteobacteria>Pseudomonadales>Pseudomonadaceae>Pseudomonas	GCA_023993215.1	Pseudomonas_aeruginosa	NI798_001205	427888..431559	287	BREX-system-P-loop-protein-BrxC-[Pseudomonas-aeruginosa]
HCH0453160.1	Type1.BREX	[illegible]	1154	Bacteria>Pseudomonadota>Gammaproteobacteria>Pseudomonadales>Pseudomonadaceae>Pseudomonas	GCA_024072695.1	Pseudomonas_aeruginosa	NKO07_001951	228071..231535	287	BREX-system-P-loop-protein-BrxC-[Pseudomonas-aeruginosa]
BDM24922.1	Type1.BREX	[illegible]	1154	Bacteria>Pseudomonadota>Gammaproteobacteria>Pseudomonadales>Pseudomonadaceae>Pseudomonas	GCA_024349685.1	Pseudomonas_sp._LRP2-20	KMS_R46790	5328463..5331927	2944234	BREX-system-P-loop-protein-BrxC-[Pseudomonas-sp.-LRP2-20]
WP_132538218.1	Type1.BREX	[illegible]	1154	Bacteria>Pseudomonadota>Gammaproteobacteria>Pseudomonadales>Pseudomonadaceae>Pseudomonas	GCA_030820635.1	Pseudomonas_aeruginosa	EQ548_RS01360	6947..10411	287	BREX-system-P-loop-protein-BrxC-[Pseudomonas-aeruginosa]
WP_257648015.1	Type1.BREX	[illegible]	1073	Bacteria>Pseudomonadota>Gammaproteobacteria>Pseudomonadales>Pseudomonadaceae>Pseudomonas	GCA_030820635.1	Pseudomonas_aeruginosa	K3Z95_RS14360	10117..13340	287	BREX-system-P-loop-protein-BrxC,-partial-[Pseudomonas-aeruginosa]
WP_230691811.1	Type1.BREX	[illegible]	1225	Bacteria>Pseudomonadota>Gammaproteobacteria>Pseudomonadales>Pseudomonadaceae>Pseudomonas	GCA_030820635.1	Pseudomonas_syringae_group_genomosp._3	I4I80_RS00295	46299..49976	251701	BREX-system-P-loop-protein-BrxC-[Pseudomonas-syringae-group-genomosp.-3]
WP_241090907.1	Type1.BREX	[illegible]	1225	Bacteria>Pseudomonadota>Gammaproteobacteria>Pseudomonadales>Pseudomonadaceae>Pseudomonas	GCA_030820635.1	Pseudomonas_viridiflava	EE220_RS21960	64975..68652	33069	BREX-system-P-loop-protein-BrxC-[Pseudomonas-viridiflava]
SBW85146.1	Type1.BREX	[illegible]	1154	Bacteria>Pseudomonadota>Gammaproteobacteria>Pseudomonadales>Pseudomonadaceae>Pseudomonas	GCA_900092355.1	Pseudomonas_veronii_1YdBTEX2	PVE_P0103	88310..91774	1295141	hypothetical-protein-PVE_P0103-[Pseudomonas-veronii-1YdBTEX2]
CAB5551672.1	Type1.BREX	[illegible]	1154	Bacteria>Pseudomonadota>Gammaproteobacteria>Pseudomonadales>Pseudomonadaceae>Pseudomonas	GCA_903684265.1	Pseudomonas_putida	GHA_03446	127342..130806	303	Uncharacterised-protein-[Pseudomonas-putida]
CAB5576233.1	Type1.BREX	[illegible]	1223	Bacteria>Pseudomonadota>Gammaproteobacteria>Pseudomonadales>Pseudomonadaceae>Pseudomonas	GCA_903684465.1	Pseudomonas_putida	GHA_00144	159878..163549	303	ATPase-involved-in-DNA-repair-[Pseudomonas-putida]
WP_010463595.1	Type1.BREX	[illegible]	1185	Bacteria>Pseudomonadota>Gammaproteobacteria>Pseudomonadales>Pseudomonadaceae>Pseudomonas	GCF_000257545.3	Pseudomonas_mandelii	OU5_RS13450	3003724..3007281	75612	BREX-system-P-loop-protein-BrxC-[Pseudomonas-mandelii]
WP_019485333.1	Type1.BREX	[illegible]	1193	Bacteria>Pseudomonadota>Gammaproteobacteria>Pseudomonadales>Pseudomonadaceae>Pseudomonas	GCF_000290555.2	Pseudomonas_aeruginosa	E4028_RS01350	3998..7579	287	BREX-system-P-loop-protein-BrxC-[Pseudomonas-aeruginosa]
WP_228382933.1	Type1.BREX	[illegible]	1225	Bacteria>Pseudomonadota>Gammaproteobacteria>Pseudomonadales>Pseudomonadaceae>Pseudomonas	GCF_000481225.1	Pseudomonas_aeruginosa	Q037_RS32345	325963..329640	287	BREX-system-P-loop-protein-BrxC-[Pseudomonas-aeruginosa]
WP_023113968.1	Type1.BREX	[illegible]	1146	Bacteria>Pseudomonadota>Gammaproteobacteria>Pseudomonadales>Pseudomonadaceae>Pseudomonas	GCF_000481245.1	Pseudomonas_aeruginosa	Q036_RS16830	2151526..2154966	287	BREX-system-P-loop-protein-BrxC-[Pseudomonas-aeruginosa]
WP_042135808.1	Type1.BREX	[illegible]	1184	Bacteria>Pseudomonadota>Gammaproteobacteria>Pseudomonadales>Pseudomonadaceae>Pseudomonas	GCF_000730625.1	Pseudomonas_oryzihabitans	POR01S_RS10925	121811..125365	47885	BREX-system-P-loop-protein-BrxC-[Pseudomonas-oryzihabitans]
WP_238579794.1	Type1.BREX	[illegible]	1150	Bacteria>Pseudomonadota>Gammaproteobacteria>Pseudomonadales>Pseudomonadaceae>Pseudomonas	GCF_000789745.1	Pseudomonas_aeruginosa	NR64_RS21585	177..3629	287	BREX-system-P-loop-protein-BrxC-[Pseudomonas-aeruginosa]
WP_034081877.1	Type1.BREX	[illegible]	1184	Bacteria>Pseudomonadota>Gammaproteobacteria>Pseudomonadales>Pseudomonadaceae>Pseudomonas	GCF_000793185.1	Pseudomonas_aeruginosa	NT71_RS08285	193065..196619	287	BREX-system-P-loop-protein-BrxC-[Pseudomonas-aeruginosa]
WP_033954069.1	Type1.BREX	[illegible]	1154	Bacteria>Pseudomonadota>Gammaproteobacteria>Pseudomonadales>Pseudomonadaceae>Pseudomonas	GCF_000793385.1	Pseudomonas_aeruginosa	NT88_RS29575	15006..18470	287	BREX-system-P-loop-protein-BrxC-[Pseudomonas-aeruginosa]
WP_043106421.1	Type1.BREX	[illegible]	1223	Bacteria>Pseudomonadota>Gammaproteobacteria>Pseudomonadales>Pseudomonadaceae>Pseudomonas	GCF_000794515.1	Pseudomonas_aeruginosa	NQ84_RS27950	382..4053	287	BREX-system-P-loop-protein-BrxC-[Pseudomonas-aeruginosa]
WP_202808417.1	Type1.BREX	[illegible]	937	Bacteria>Pseudomonadota>Gammaproteobacteria>Pseudomonadales>Pseudomonadaceae>Pseudomonas	GCF_000794515.1	Pseudomonas_aeruginosa	NQ84_RS28370	16579..19392	287	BREX-system-P-loop-protein-BrxC-[Pseudomonas-aeruginosa]
WP_228480203.1	Type1.BREX	[illegible]	619	Bacteria>Pseudomonadota>Gammaproteobacteria>Pseudomonadales>Pseudomonadaceae>Pseudomonas	GCF_000829885.1	Pseudomonas_aeruginosa	EG09_RS07505	1715440..1717299	287	BREX-system-P-loop-protein-BrxC-[Pseudomonas-aeruginosa]
WP_041506104.1	Type1.BREX	[illegible]	1184	Bacteria>Pseudomonadota>Gammaproteobacteria>Pseudomonadales>Pseudomonadaceae>Pseudomonas	GCF_000831585.1	Pseudomonas_plecoglossicida	RK21_RS13525	2976546..2980100	70775	BREX-system-P-loop-protein-BrxC-[Pseudomonas-plecoglossicida]
WP_052507173.1	Type1.BREX	[illegible]	886	Bacteria>Pseudomonadota>Gammaproteobacteria>Pseudomonadales>Pseudomonadaceae>Pseudomonas	GCF_000935725.1	Pseudomonas_syringae_group_genomosp._3	AC506_RS17885	8350..11010	251701	BREX-system-P-loop-protein-BrxC-[Pseudomonas-syringae-group-genomosp.-3]
WP_044727109.1	Type1.BREX	[illegible]	1184	Bacteria>Pseudomonadota>Gammaproteobacteria>Pseudomonadales>Pseudomonadaceae>Pseudomonas	GCF_000937465.2	Pseudomonas_aeruginosa	TN45_RS09710	568754..572308	287	BREX-system-P-loop-protein-BrxC-[Pseudomonas-aeruginosa]
WP_047303167.1	Type1.BREX	[illegible]	1223	Bacteria>Pseudomonadota>Gammaproteobacteria>Pseudomonadales>Pseudomonadaceae>Pseudomonas	GCF_001020715.1	Pseudomonas_fluorescens	AA061_RS10335	96304..99975	294	BREX-system-P-loop-protein-BrxC-[Pseudomonas-fluorescens]
WP_047306816.1	Type1.BREX	[illegible]	1185	Bacteria>Pseudomonadota>Gammaproteobacteria>Pseudomonadales>Pseudomonadaceae>Pseudomonas	GCF_001020715.1	Pseudomonas_fluorescens	AA061_RS32150	16461..20018	294	BREX-system-P-loop-protein-BrxC-[Pseudomonas-fluorescens]
WP_047283135.1	Type1.BREX	[illegible]	1154	Bacteria>Pseudomonadota>Gammaproteobacteria>Pseudomonadales>Pseudomonadaceae>Pseudomonas	GCF_001020725.2	Pseudomonas_lundensis	AA042_RS07115	1582722..1586186	86185	BREX-system-P-loop-protein-BrxC-[Pseudomonas-lundensis]
WP_048385719.1	Type1.BREX	[illegible]	1185	Bacteria>Pseudomonadota>Gammaproteobacteria>Pseudomonadales>Pseudomonadaceae>Pseudomonas	GCF_001043065.1	Pseudomonas_helleri	TU85_RS02975	88982..92539	1608996	BREX-system-P-loop-protein-BrxC-[Pseudomonas-helleri]
WP_053154299.1	Type1.BREX	[illegible]	1154	Bacteria>Pseudomonadota>Gammaproteobacteria>Pseudomonadales>Pseudomonadaceae>Pseudomonas	GCF_001269805.1	Pseudomonas_sp._P1.8	AD668_RS02725	49573..53037	1699310	BREX-system-P-loop-protein-BrxC-[Pseudomonas-sp.-P1.8]
WP_057391032.1	Type1.BREX	[illegible]	1154	Bacteria>Pseudomonadota>Gammaproteobacteria>Pseudomonadales>Pseudomonadaceae>Pseudomonas	GCF_001374115.1	Pseudomonas_aeruginosa	AYU10_RS17350	11442..14906	287	BREX-system-P-loop-protein-BrxC-[Pseudomonas-aeruginosa]
WP_057429193.1	Type1.BREX	[illegible]	1156	Bacteria>Pseudomonadota>Gammaproteobacteria>Pseudomonadales>Pseudomonadaceae>Pseudomonas	GCF_001401385.1	Pseudomonas_syringae_group_genomosp._3	AL087_RS04320	127987..131457	251701	BREX-system-P-loop-protein-BrxC-[Pseudomonas-syringae-group-genomosp.-3]
WP_057978570.1	Type1.BREX	[illegible]	1223	Bacteria>Pseudomonadota>Gammaproteobacteria>Pseudomonadales>Pseudomonadaceae>Pseudomonas	GCF_001439745.1	Pseudomonas_lactis	TX25_RS28475	27732..31403	1615674	BREX-system-P-loop-protein-BrxC-[Pseudomonas-lactis]
WP_235565439.1	Type1.BREX	[illegible]	1225	Bacteria>Pseudomonadota>Gammaproteobacteria>Pseudomonadales>Pseudomonadaceae>Pseudomonas	GCF_001450115.1	Pseudomonas_aeruginosa	AO953_RS18460	48360..52037	287	BREX-system-P-loop-protein-BrxC-[Pseudomonas-aeruginosa]
WP_058174899.1	Type1.BREX	[illegible]	1184	Bacteria>Pseudomonadota>Gammaproteobacteria>Pseudomonadales>Pseudomonadaceae>Pseudomonas	GCF_001452485.1	Pseudomonas_aeruginosa	APA89_RS03075	652482..656036	287	BREX-system-P-loop-protein-BrxC-[Pseudomonas-aeruginosa]
WP_059319043.1	Type1.BREX	[illegible]	1154	Bacteria>Pseudomonadota>Gammaproteobacteria>Pseudomonadales>Pseudomonadaceae>Pseudomonas	GCF_001516265.1	Pseudomonas_aeruginosa	HV84_RS13920	329043..332507	287	BREX-system-P-loop-protein-BrxC-[Pseudomonas-aeruginosa]
WP_031639867.1	Type1.BREX	[illegible]	1184	Bacteria>Pseudomonadota>Gammaproteobacteria>Pseudomonadales>Pseudomonadaceae>Pseudomonas	GCF_001548335.1	Pseudomonas_aeruginosa	IOMTU133_RS23320	5069423..5072977	287	BREX-system-P-loop-protein-BrxC-[Pseudomonas-aeruginosa]
WP_065615729.1	Type1.BREX	[illegible]	1223	Bacteria>Pseudomonadota>Gammaproteobacteria>Pseudomonadales>Pseudomonadaceae>Pseudomonas	GCF_001691585.1	Pseudomonas_moraviensis	BBA88_RS08910	1046415..1050086	321662	BREX-system-P-loop-protein-BrxC-[Pseudomonas-moraviensis]
WP_069953193.1	Type1.BREX	[illegible]	828	Bacteria>Pseudomonadota>Gammaproteobacteria>Pseudomonadales>Pseudomonadaceae>Pseudomonas	GCF_001750225.1	Pseudomonas_aeruginosa	A7R77_RS00815	21562..24049	287	BREX-system-P-loop-protein-BrxC,-partial-[Pseudomonas-aeruginosa]
WP_070166840.1	Type1.BREX	[illegible]	1182	Bacteria>Pseudomonadota>Gammaproteobacteria>Pseudomonadales>Pseudomonadaceae>Pseudomonas	GCF_001756585.1	Pseudomonas_aeruginosa	AN464_RS32885	78098..81646	287	BREX-system-P-loop-protein-BrxC-[Pseudomonas-aeruginosa]
WP_070624003.1	Type1.BREX	[illegible]	1154	Bacteria>Pseudomonadota>Gammaproteobacteria>Pseudomonadales>Pseudomonadaceae>Pseudomonas	GCF_001810465.1	Pseudomonas_aeruginosa	HMPREF2746_RS21475	11855..15319	287	BREX-system-P-loop-protein-BrxC-[Pseudomonas-aeruginosa]
WP_073844625.1	Type1.BREX	[illegible]	1217	Bacteria>Pseudomonadota>Gammaproteobacteria>Pseudomonadales>Pseudomonadaceae>Pseudomonas	GCF_001907715.1	Pseudomonas_sp._BTN1	BMH52_RS18610	18591..22244	1750647	BREX-system-P-loop-protein-BrxC-[Pseudomonas-sp.-BTN1]
WP_079783554.1	Type1.BREX	[illegible]	1154	Bacteria>Pseudomonadota>Gammaproteobacteria>Pseudomonadales>Pseudomonadaceae>Pseudomonas	GCF_002037565.1	Pseudomonas_sp._LPH1	BHQ29_RS09350	2029984..2033448	1898684	BREX-system-P-loop-protein-BrxC-[Pseudomonas-sp.-LPH1]
WP_084308437.1	Type1.BREX	[illegible]	1224	Bacteria>Pseudomonadota>Gammaproteobacteria>Pseudomonadales>Pseudomonadaceae>Pseudomonas	GCF_002091575.1	Pseudomonas_flavescens	PFL01S_RS23715	11165..14839	29435	BREX-system-P-loop-protein-BrxC-[Pseudomonas-flavescens]
WP_074859114.1	Type1.BREX	[illegible]	1184	Bacteria>Pseudomonadota>Gammaproteobacteria>Pseudomonadales>Pseudomonadaceae>Pseudomonas	GCF_002091815.1	Pseudomonas_oleovorans	POL01S_RS18595	25960..29514	301	BREX-system-P-loop-protein-BrxC-[Pseudomonas-oleovorans]
WP_084929168.1	Type1.BREX	[illegible]	1184	Bacteria>Pseudomonadota>Gammaproteobacteria>Pseudomonadales>Pseudomonadaceae>Pseudomonas	GCF_002094805.1	Pseudomonas_aeruginosa	B7H20_RS24080	14270..17824	287	BREX-system-P-loop-protein-BrxC-[Pseudomonas-aeruginosa]
WP_205881761.1	Type1.BREX	[illegible]	1159	Bacteria>Pseudomonadota>Gammaproteobacteria>Pseudomonadales>Pseudomonadaceae>Pseudomonas	GCF_002128325.1	Pseudomonas_fragi	B6D87_RS19610	4227178..4230657	296	BREX-system-P-loop-protein-BrxC-[Pseudomonas-fragi]
WP_086230732.1	Type1.BREX	[illegible]	1184	Bacteria>Pseudomonadota>Gammaproteobacteria>Pseudomonadales>Pseudomonadaceae>Pseudomonas	GCF_002135805.1	Pseudomonas_aeruginosa	CAZ03_RS13145	128061..131615	287	BREX-system-P-loop-protein-BrxC-[Pseudomonas-aeruginosa]
WP_088135363.1	Type1.BREX	[illegible]	1154	Bacteria>Pseudomonadota>Gammaproteobacteria>Pseudomonadales>Pseudomonadaceae>Pseudomonas	GCF_002193745.1	Pseudomonas_aeruginosa	GNQ21_RS29160	3852..7316	287	BREX-system-P-loop-protein-BrxC-[Pseudomonas-aeruginosa]
WP_095025594.1	Type1.BREX	[illegible]	1154	Bacteria>Pseudomonadota>Gammaproteobacteria>Pseudomonadales>Pseudomonadaceae>Pseudomonas	GCF_002269515.1	Pseudomonas_fragi	CJU79_RS17655	17310..20774	296	BREX-system-P-loop-protein-BrxC-[Pseudomonas-fragi]
WP_095626344.1	Type1.BREX	[illegible]	1154	Bacteria>Pseudomonadota>Gammaproteobacteria>Pseudomonadales>Pseudomonadaceae>Pseudomonas	GCF_002287035.1	Pseudomonas_sp._WN033	CK507_RS15715	130328..133792	2032629	BREX-system-P-loop-protein-BrxC-[Pseudomonas-sp.-WN033]
WP_256588486.1	Type1.BREX	[illegible]	1225	Bacteria>Pseudomonadota>Gammaproteobacteria>Pseudomonadales>Pseudomonadaceae>Pseudomonas	GCF_002591235.1	Pseudomonas_sp._FDAARGOS_380	CRN80_RS16460	3507084..3510761	2018067	BREX-system-P-loop-protein-BrxC-[Pseudomonas-sp.-FDAARGOS_380]
WP_101196767.1	Type1.BREX	[illegible]	1180	Bacteria>Pseudomonadota>Gammaproteobacteria>Pseudomonadales>Pseudomonadaceae>Pseudomonas	GCF_002835905.1	Pseudomonas_monteilii	CXB65_RS25690	173341..176883	76759	BREX-system-P-loop-protein-BrxC-[Pseudomonas-monteilii]
WP_102082564.1	Type1.BREX	[illegible]	1184	Bacteria>Pseudomonadota>Gammaproteobacteria>Pseudomonadales>Pseudomonadaceae>Pseudomonas	GCF_002864915.1	Pseudomonas_guariconensis	CXG53_RS23735	60064..63618	1288410	BREX-system-P-loop-protein-BrxC-[Pseudomonas-guariconensis]
WP_102368059.1	Type1.BREX	[illegible]	1188	Bacteria>Pseudomonadota>Gammaproteobacteria>Pseudomonadales>Pseudomonadaceae>Pseudomonas	GCF_002874965.1	Pseudomonas_sp._NCO2	C0058_RS03795	845324..848890	2067572	BREX-system-P-loop-protein-BrxC-[Pseudomonas-sp.-NCO2]
WP_102684491.1	Type1.BREX	[illegible]	1222	Bacteria>Pseudomonadota>Gammaproteobacteria>Pseudomonadales>Pseudomonadaceae>Pseudomonas	GCF_002883975.1	Pseudomonas_sp._FW305-42	C1X79_RS19065	47194..50862	2070677	BREX-system-P-loop-protein-BrxC-[Pseudomonas-sp.-FW305-42]
WP_102886089.1	Type1.BREX	[illegible]	1184	Bacteria>Pseudomonadota>Gammaproteobacteria>Pseudomonadales>Pseudomonadaceae>Pseudomonas	GCF_002891885.1	Pseudomonas_putida	CBL13_04161	1040459..1044013	303	BREX-system-P-loop-protein-BrxC-[Pseudomonas-putida]
WP_104727392.1	Type1.BREX	[illegible]	1222	Bacteria>Pseudomonadota>Gammaproteobacteria>Pseudomonadales>Pseudomonadaceae>Pseudomonas	GCF_002941105.1	Pseudomonas_oleovorans	C5L43_RS04465	3008..6676	301	BREX-system-P-loop-protein-BrxC-[Pseudomonas-oleovorans]
WP_242445006.1	Type1.BREX	[illegible]	1225	Bacteria>Pseudomonadota>Gammaproteobacteria>Pseudomonadales>Pseudomonadaceae>Pseudomonas	GCF_002941105.1	Pseudomonas_oleovorans	C5L43_RS18405	7326..11003	301	BREX-system-P-loop-protein-BrxC-[Pseudomonas-oleovorans]
WP_106118416.1	Type1.BREX	[illegible]	1223	Bacteria>Pseudomonadota>Gammaproteobacteria>Pseudomonadales>Pseudomonadaceae>Pseudomonas	GCF_003001555.1	Pseudomonas_fluorescens	C7A10_RS23710	130630..134301	294	BREX-system-P-loop-protein-BrxC-[Pseudomonas-fluorescens]
WP_109933363.1	Type1.BREX	[illegible]	1154	Bacteria>Pseudomonadota>Gammaproteobacteria>Pseudomonadales>Pseudomonadaceae>Pseudomonas	GCF_003176655.1	Pseudomonas_sp._RW407	DK254_RS08115	332506..335970	2202894	BREX-system-P-loop-protein-BrxC-[Pseudomonas-sp.-RW407]
WP_116801381.1	Type1.BREX	[illegible]	1184	Bacteria>Pseudomonadota>Gammaproteobacteria>Pseudomonadales>Pseudomonadaceae>Pseudomonas	GCF_003410555.2	Pseudomonas_aeruginosa	DZ982_RS28380	35413..38967	287	BREX-system-P-loop-protein-BrxC-[Pseudomonas-aeruginosa]
WP_116843887.1	Type1.BREX	[illegible]	549	Bacteria>Pseudomonadota>Gammaproteobacteria>Pseudomonadales>Pseudomonadaceae>Pseudomonas	GCF_003411125.2	Pseudomonas_aeruginosa	DZ918_RS18285	1..1646	287	BREX-system-P-loop-protein-BrxC,-partial-[Pseudomonas-aeruginosa]
WP_116805555.1	Type1.BREX	[illegible]	1154	Bacteria>Pseudomonadota>Gammaproteobacteria>Pseudomonadales>Pseudomonadaceae>Pseudomonas	GCF_003412045.2	Pseudomonas_aeruginosa	L4E78_005908	8239..11703	287	BREX-system-P-loop-protein-BrxC-[Pseudomonas-aeruginosa]
WP_119519035.1	Type1.BREX	[illegible]	1220	Bacteria>Pseudomonadota>Gammaproteobacteria>Pseudomonadales>Pseudomonadaceae>Pseudomonas	GCF_003585225.1	Pseudomonas_aeruginosa	AXX04_RS18775	17983..21645	287	BREX-system-P-loop-protein-BrxC-[Pseudomonas-aeruginosa]
WP_121398877.1	Type1.BREX	[illegible]	1180	Bacteria>Pseudomonadota>Gammaproteobacteria>Pseudomonadales>Pseudomonadaceae>Pseudomonas	GCF_003631245.1	Pseudomonas_aeruginosa	DQH61_RS02890	22908..26450	287	BREX-system-P-loop-protein-BrxC-[Pseudomonas-aeruginosa]
WP_128551186.1	Type1.BREX	[illegible]	1184	Bacteria>Pseudomonadota>Gammaproteobacteria>Pseudomonadales>Pseudomonadaceae>Pseudomonas	GCF_003698635.1	Pseudomonas_aeruginosa	IPC82_RS03385	45130..48684	287	BREX-system-P-loop-protein-BrxC-[Pseudomonas-aeruginosa]
WP_236079235.1	Type1.BREX	[illegible]	1225	Bacteria>Pseudomonadota>Gammaproteobacteria>Pseudomonadales>Pseudomonadaceae>Pseudomonas	GCF_003833645.1	Pseudomonas_aeruginosa	IPC1294_RS03655	745401..749078	287	BREX-system-P-loop-protein-BrxC-[Pseudomonas-aeruginosa]
WP_124138810.1	Type1.BREX	[illegible]	1223	Bacteria>Pseudomonadota>Gammaproteobacteria>Pseudomonadales>Pseudomonadaceae>Pseudomonas	GCF_003833965.1	Pseudomonas_aeruginosa	IPC1352_RS13795	491665..495336	287	BREX-system-P-loop-protein-BrxC-[Pseudomonas-aeruginosa]
WP_124127279.1	Type1.BREX	[illegible]	1223	Bacteria>Pseudomonadota>Gammaproteobacteria>Pseudomonadales>Pseudomonadaceae>Pseudomonas	GCF_003836525.1	Pseudomonas_aeruginosa	IPC776_RS25325	73240..76911	287	BREX-system-P-loop-protein-BrxC-[Pseudomonas-aeruginosa]
WP_201271146.1	Type1.BREX	[illegible]	599	Bacteria>Pseudomonadota>Gammaproteobacteria>Pseudomonadales>Pseudomonadaceae>Pseudomonas	GCF_003838365.1	Pseudomonas_aeruginosa	IPC642_RS23000	207947..209746	287	BREX-system-P-loop-protein-BrxC-[Pseudomonas-aeruginosa]
WP_124187492.1	Type1.BREX	[illegible]	1154	Bacteria>Pseudomonadota>Gammaproteobacteria>Pseudomonadales>Pseudomonadaceae>Pseudomonas	GCF_003840445.1	Pseudomonas_aeruginosa	IPC460_RS14135	71105..74569	287	BREX-system-P-loop-protein-BrxC-[Pseudomonas-aeruginosa]
WP_125881186.1	Type1.BREX	[illegible]	1154	Bacteria>Pseudomonadota>Gammaproteobacteria>Pseudomonadales>Pseudomonadaceae>Pseudomonas	GCF_003935455.1	Pseudomonas_sp._o96-267	EGJ23_RS18845	351..3815	2479853	BREX-system-P-loop-protein-BrxC-[Pseudomonas-sp.-o96-267]
WP_126190092.1	Type1.BREX	[illegible]	1185	Bacteria>Pseudomonadota>Gammaproteobacteria>Pseudomonadales>Pseudomonadaceae>Pseudomonas	GCF_003957495.3	Pseudomonas_songnenensis	EJA06_RS16615	27316..30873	1176259	BREX-system-P-loop-protein-BrxC-[Pseudomonas-songnenensis]
WP_126525533.1	Type1.BREX	[illegible]	1154	Bacteria>Pseudomonadota>Gammaproteobacteria>Pseudomonadales>Pseudomonadaceae>Pseudomonas	GCF_003969495.1	Pseudomonas_aeruginosa	DZA06_RS08835	36272..39736	287	BREX-system-P-loop-protein-BrxC-[Pseudomonas-aeruginosa]
WP_126532550.1	Type1.BREX	[illegible]	1223	Bacteria>Pseudomonadota>Gammaproteobacteria>Pseudomonadales>Pseudomonadaceae>Pseudomonas	GCF_003969645.1	Pseudomonas_aeruginosa	DY969_RS10905	72707..76378	287	BREX-system-P-loop-protein-BrxC-[Pseudomonas-aeruginosa]
WP_126588230.1	Type1.BREX	[illegible]	1223	Bacteria>Pseudomonadota>Gammaproteobacteria>Pseudomonadales>Pseudomonadaceae>Pseudomonas	GCF_003970115.1	Pseudomonas_veronii	EXA83_RS07390	206636..210307	76761	BREX-system-P-loop-protein-BrxC-[Pseudomonas-veronii]
WP_126634432.1	Type1.BREX	[illegible]	904	Bacteria>Pseudomonadota>Gammaproteobacteria>Pseudomonadales>Pseudomonadaceae>Pseudomonas	GCF_003970625.1	Pseudomonas_aeruginosa	DZA20_RS16095	55154..57868	287	BREX-system-P-loop-protein-BrxC-[Pseudomonas-aeruginosa]
WP_260478239.1	Type1.BREX	[illegible]	1225	Bacteria>Pseudomonadota>Gammaproteobacteria>Pseudomonadales>Pseudomonadaceae>Pseudomonas	GCF_003973785.1	Pseudomonas_aeruginosa	IPC1623_RS31675	17836..21513	287	BREX-system-P-loop-protein-BrxC-[Pseudomonas-aeruginosa]
WP_128704208.1	Type1.BREX	[illegible]	1184	Bacteria>Pseudomonadota>Gammaproteobacteria>Pseudomonadales>Pseudomonadaceae>Pseudomonas	GCF_003974605.1	Pseudomonas_aeruginosa	IPC1390_RS17360	217632..221186	287	BREX-system-P-loop-protein-BrxC-[Pseudomonas-aeruginosa]
WP_129388875.1	Type1.BREX	[illegible]	1156	Bacteria>Pseudomonadota>Gammaproteobacteria>Pseudomonadales>Pseudomonadaceae>Pseudomonas	GCF_004124275.1	Pseudomonas_sp._DTU12.3	C2E19_RS05070	1143497..1146967	2073078	BREX-system-P-loop-protein-BrxC-[Pseudomonas-sp.-DTU12.3]
WP_130899395.1	Type1.BREX	[illegible]	1154	Bacteria>Pseudomonadota>Gammaproteobacteria>Pseudomonadales>Pseudomonadaceae>Pseudomonas	GCF_004307505.1	Pseudomonas_sp._Sample_11	D8U60_RS02310	26340..29804	2448261	BREX-system-P-loop-protein-BrxC-[Pseudomonas-sp.-Sample_11]
WP_134242485.1	Type1.BREX	[illegible]	1154	Bacteria>Pseudomonadota>Gammaproteobacteria>Pseudomonadales>Pseudomonadaceae>Pseudomonas	GCF_004370105.1	Pseudomonas_aeruginosa	IPC1592_RS30135	15141..18605	287	BREX-system-P-loop-protein-BrxC-[Pseudomonas-aeruginosa]
WP_202876546.1	Type1.BREX	[illegible]	531	Bacteria>Pseudomonadota>Gammaproteobacteria>Pseudomonadales>Pseudomonadaceae>Pseudomonas	GCF_004373095.1	Pseudomonas_aeruginosa	IPC174_RS30040	16693..18288	287	BREX-system-P-loop-protein-BrxC-[Pseudomonas-aeruginosa]
WP_202876713.1	Type1.BREX	[illegible]	539	Bacteria>Pseudomonadota>Gammaproteobacteria>Pseudomonadales>Pseudomonadaceae>Pseudomonas	GCF_004373095.1	Pseudomonas_aeruginosa	IPC175_RS30340	15284..16903	287	hypothetical-protein-[Pseudomonas-aeruginosa]
WP_134282343.1	Type1.BREX	[illegible]	1154	Bacteria>Pseudomonadota>Gammaproteobacteria>Pseudomonadales>Pseudomonadaceae>Pseudomonas	GCF_004373575.1	Pseudomonas_aeruginosa	IPC135_RS30160	15285..18749	287	BREX-system-P-loop-protein-BrxC-[Pseudomonas-aeruginosa]
WP_202876545.1	Type1.BREX	[illegible]	624	Bacteria>Pseudomonadota>Gammaproteobacteria>Pseudomonadales>Pseudomonadaceae>Pseudomonas	GCF_004373665.1	Pseudomonas_aeruginosa	IPC174_RS30035	14826..16700	287	hypothetical-protein-[Pseudomonas-aeruginosa]
WP_136489742.1	Type1.BREX	[illegible]	1154	Bacteria>Pseudomonadota>Gammaproteobacteria>Pseudomonadales>Pseudomonadaceae>Pseudomonas	GCF_004801855.1	Pseudomonas_sp._A-1	E5198_RS09425	20467..23931	1821274	BREX-system-P-loop-protein-BrxC-[Pseudomonas-sp.-A-1]
WP_155677773.1	Type1.BREX	[illegible]	1155	Bacteria>Pseudomonadota>Gammaproteobacteria>Pseudomonadales>Pseudomonadaceae>Pseudomonas	GCF_004919535.4	Pseudomonas_veronii	E4167_12595	2651456..2654923	76761	BREX-system-P-loop-protein-BrxC-[Pseudomonas-veronii]
WP_155678129.1	Type1.BREX	[illegible]	1221	Bacteria>Pseudomonadota>Gammaproteobacteria>Pseudomonadales>Pseudomonadaceae>Pseudomonas	GCF_004919535.4	Pseudomonas_veronii	E4167_RS17855	3896046..3899711	76761	BREX-system-P-loop-protein-BrxC-[Pseudomonas-veronii]
WP_249351844.1	Type1.BREX	[illegible]	1225	Bacteria>Pseudomonadota>Gammaproteobacteria>Pseudomonadales>Pseudomonadaceae>Pseudomonas	GCF_005223285.1	Pseudomonas_asiatica	FD498_RS21455	9358..13035	2219225	BREX-system-P-loop-protein-BrxC-[Pseudomonas-asiatica]
WP_256658147.1	Type1.BREX	[illegible]	1220	Bacteria>Pseudomonadota>Gammaproteobacteria>Pseudomonadales>Pseudomonadaceae>Pseudomonas	GCF_005502555.1	Pseudomonas_sp._D(2018)	FFS60_RS25250	8931..12593	2502238	BREX-system-P-loop-protein-BrxC-[Pseudomonas-sp.-D(2018)]
WP_240721840.1	Type1.BREX	[illegible]	947	Bacteria>Pseudomonadota>Gammaproteobacteria>Pseudomonadales>Pseudomonadaceae>Pseudomonas	GCF_005863515.1	Pseudomonas_aeruginosa	FEM26_RS29610	9..2852	287	BREX-system-P-loop-protein-BrxC-[Pseudomonas-aeruginosa]
WP_144958058.1	Type1.BREX	[illegible]	1223	Bacteria>Pseudomonadota>Gammaproteobacteria>Pseudomonadales>Pseudomonadaceae>Pseudomonas	GCF_007680465.1	Pseudomonas_oryzihabitans	FS730_RS21810	185497..189168	47885	BREX-system-P-loop-protein-BrxC-[Pseudomonas-oryzihabitans]
WP_115288849.1	Type1.BREX	[illegible]	1184	Bacteria>Pseudomonadota>Gammaproteobacteria>Pseudomonadales>Pseudomonadaceae>Pseudomonas	GCF_007833895.1	Pseudomonas_aeruginosa	FF962_RS31550	6589881..6593435	287	BREX-system-P-loop-protein-BrxC-[Pseudomonas-aeruginosa]
WP_150073350.1	Type1.BREX	[illegible]	1186	Bacteria>Pseudomonadota>Gammaproteobacteria>Pseudomonadales>Pseudomonadaceae>Pseudomonas	GCF_008632325.1	Pseudomonas_lactis	F3K52_RS19340	4156..7716	1615674	BREX-system-P-loop-protein-BrxC-[Pseudomonas-lactis]
WP_151183841.1	Type1.BREX	[illegible]	1181	Bacteria>Pseudomonadota>Gammaproteobacteria>Pseudomonadales>Pseudomonadaceae>Pseudomonas	GCF_008807395.1	Pseudomonas_sp._C27(2019)	FXF61_RS02795	588989..592534	2604941	BREX-system-P-loop-protein-BrxC-[Pseudomonas-sp.-C27(2019)]
WP_152745526.1	Type1.BREX	[illegible]	1183	Bacteria>Pseudomonadota>Gammaproteobacteria>Pseudomonadales>Pseudomonadaceae>Pseudomonas	GCF_009296165.1	Pseudomonas_kitaguniensis	F0169_RS03300	21652..25203	2607908	BREX-system-P-loop-protein-BrxC-[Pseudomonas-kitaguniensis]
WP_153329486.1	Type1.BREX	[illegible]	1184	Bacteria>Pseudomonadota>Gammaproteobacteria>Pseudomonadales>Pseudomonadaceae>Pseudomonas	GCF_009600225.1	Pseudomonas_helleri	GHO39_RS16765	34385..37939	1608996	BREX-system-P-loop-protein-BrxC-[Pseudomonas-helleri]
WP_153386820.1	Type1.BREX	[illegible]	1183	Bacteria>Pseudomonadota>Gammaproteobacteria>Pseudomonadales>Pseudomonadaceae>Pseudomonas	GCF_009600315.1	Pseudomonas_helleri	GHN86_RS14975	48582..52133	1608996	BREX-system-P-loop-protein-BrxC-[Pseudomonas-helleri]
WP_153350777.1	Type1.BREX	[illegible]	1146	Bacteria>Pseudomonadota>Gammaproteobacteria>Pseudomonadales>Pseudomonadaceae>Pseudomonas	GCF_009600425.1	Pseudomonas_helleri	GHO30_RS02550	6514..9954	1608996	BREX-system-P-loop-protein-BrxC-[Pseudomonas-helleri]
WP_153384138.1	Type1.BREX	[illegible]	1186	Bacteria>Pseudomonadota>Gammaproteobacteria>Pseudomonadales>Pseudomonadaceae>Pseudomonas	GCF_009600565.1	unclassified_Pseudomonas	GHO24_RS06625	35218..38778	196821	MULTISPECIES:-BREX-system-P-loop-protein-BrxC-[unclassified-Pseudomonas]
WP_233280221.1	Type1.BREX	[illegible]	1216	Bacteria>Pseudomonadota>Gammaproteobacteria>Pseudomonadales>Pseudomonadaceae>Pseudomonas	GCF_009763245.1	Pseudomonas_xionganensis	GJV18_RS17875	24794..28444	2654845	BREX-system-P-loop-protein-BrxC-[Pseudomonas-xionganensis]
WP_166590589.1	Type1.BREX	[illegible]	1223	Bacteria>Pseudomonadota>Gammaproteobacteria>Pseudomonadales>Pseudomonadaceae>Pseudomonas	GCF_009905775.1	Pseudomonas_sp._BC115LW	GVN25_RS11920	39380..43051	2683267	BREX-system-P-loop-protein-BrxC-[Pseudomonas-sp.-BC115LW]
WP_161892243.1	Type1.BREX	[illegible]	1154	Bacteria>Pseudomonadota>Gammaproteobacteria>Pseudomonadales>Pseudomonadaceae>Pseudomonas	GCF_009932375.1	Pseudomonas_juntendi	GYA94_RS10075	73827..77291	2666183	BREX-system-P-loop-protein-BrxC-[Pseudomonas-juntendi]
WP_262377456.1	Type1.BREX	[illegible]	787	Bacteria>Pseudomonadota>Gammaproteobacteria>Pseudomonadales>Pseudomonadaceae>Pseudomonas	GCF_012985365.1	Pseudomonas_veronii	HBO38_RS22345	78028..80391	76761	BREX-system-P-loop-protein-BrxC-[Pseudomonas-veronii]
WP_262386226.1	Type1.BREX	[illegible]	1225	Bacteria>Pseudomonadota>Gammaproteobacteria>Pseudomonadales>Pseudomonadaceae>Pseudomonas	GCF_012985535.1	Pseudomonas_sp._WS_5021	HBO19_RS03675	794081..797758	2717490	BREX-system-P-loop-protein-BrxC-[Pseudomonas-sp.-WS-5021]
WP_169989748.1	Type1.BREX	[illegible]	1186	Bacteria>Pseudomonadota>Gammaproteobacteria>Pseudomonadales>Pseudomonadaceae>Pseudomonas	GCF_012985575.1	Pseudomonas_sp._WS_5413	HBO21_RS11490	97465..101025	2717488	BREX-system-P-loop-protein-BrxC-[Pseudomonas-sp.-WS-5413]
WP_170050473.1	Type1.BREX	[illegible]	1154	Bacteria>Pseudomonadota>Gammaproteobacteria>Pseudomonadales>Pseudomonadaceae>Pseudomonas	GCF_012985775.1	Pseudomonas_sp._WS_5011	HBN63_RS18875	35814..39278	2717477	BREX-system-P-loop-protein-BrxC-[Pseudomonas-sp.-WS-5011]
WP_262499066.1	Type1.BREX	[illegible]	1217	Bacteria>Pseudomonadota>Gammaproteobacteria>Pseudomonadales>Pseudomonadaceae>Pseudomonas	GCF_012985775.1	Pseudomonas_sp._WS_5011	HBN63_RS20190	4647..8300	2717477	BREX-system-P-loop-protein-BrxC-[Pseudomonas-sp.-WS-5011]
WP_094987826.1	Type1.BREX	[illegible]	1154	Bacteria>Pseudomonadota>Gammaproteobacteria>Pseudomonadales>Pseudomonadaceae>Pseudomonas	GCF_012986325.1	Pseudomonas_lundensis	HBN80_RS14475	101799..105263	86185	BREX-system-P-loop-protein-BrxC-[Pseudomonas-lundensis]
WP_169839752.1	Type1.BREX	[illegible]	1154	Bacteria>Pseudomonadota>Gammaproteobacteria>Pseudomonadales>Pseudomonadaceae>Pseudomonas	GCF_012986505.1	Pseudomonas_lundensis	HBN72_RS14625	129337..132801	86185	BREX-system-P-loop-protein-BrxC-[Pseudomonas-lundensis]
WP_223184021.1	Type1.BREX	[illegible]	122	Bacteria>Pseudomonadota>Gammaproteobacteria>Pseudomonadales>Pseudomonadaceae>Pseudomonas	GCF_012986825.1	Pseudomonas_fragi	HBN89_RS04420	39782..40150	296	hypothetical-protein-[Pseudomonas-fragi]
WP_172792831.1	Type1.BREX	[illegible]	1154	Bacteria>Pseudomonadota>Gammaproteobacteria>Pseudomonadales>Pseudomonadaceae>Pseudomonas	GCF_013260445.1	Pseudomonas_aeruginosa	HQN50_RS11915	2493948..2497412	287	BREX-system-P-loop-protein-BrxC-[Pseudomonas-aeruginosa]
WP_173406509.1	Type1.BREX	[illegible]	1154	Bacteria>Pseudomonadota>Gammaproteobacteria>Pseudomonadales>Pseudomonadaceae>Pseudomonas	GCF_013283895.1	Pseudomonas_sp._MPDS	HQ912_RS04605	1048127..1051591	2762896	BREX-system-P-loop-protein-BrxC-[Pseudomonas-sp.-MPDS]
WP_177031785.1	Type1.BREX	[illegible]	1185	Bacteria>Pseudomonadota>Gammaproteobacteria>Pseudomonadales>Pseudomonadaceae>Pseudomonas	GCF_013386345.1	Pseudomonas_veronii	HX878_RS08640	6154..9711	76761	BREX-system-P-loop-protein-BrxC-[Pseudomonas-veronii]

Protein-ID	System	Gene-Neighbourhoods	Length	Taxonomy	Assembly-ID	Strain	Locus-Tag	Genomic-coordinates	TaxID	Fasta-define
WP_178910878.1	Type1.BREX	<-SIGMA+OB-Fold+AAA-ATPase+HKD-DNase-BrxL<-PglZ<-N6-METHYLASE-p<-RVT<-NERD<-OLD-ABC<-N6-METHYLASE<-DUF6079*<-BrxB-BrxF-iSTAND<-BrxA-DNA-binding<-XIS-HTH<-?||?->?->||Aminotran_1_2->	1186	Bacteria>Pseudomonadota>Gammaproteobacteria>Pseudomonadales>Pseudomonadaceae>Pseudomonas	GCF_013386495.1	Pseudomonas_tolaasii	HX799_RS03165	122791..126351	29442	BREX-system-P-loop-protein-BrxC-[Pseudomonas-tolaasii]
WP_244972677.1	Type1.BREX	<-SIGMA+OB-Fold+AAA-ATPase+Lon-Protease-BrxL<-PglZ<-AbiEii<-AbiEi_3_N+AbiEi_3<-HNH<-N6-METHYLASE<-DNA-glycosylase<-DUF6079*<-BrxB-BrxF-iSTAND<-BrxA-DNA-binding<-?||wHTH+WYL+WCX-><-tRNA<-MerR-HTH	1225	Bacteria>Pseudomonadota>Gammaproteobacteria>Pseudomonadales>Pseudomonadaceae>Pseudomonas	GCF_013386885.1	Pseudomonas_salomonii	HX810_RS11525	509017..512694	191391	BREX-system-P-loop-protein-BrxC-[Pseudomonas-salomonii]
WP_177095197.1	Type1.BREX	<-N6-METHYLASE-p<-REase<-OLD-ABC<-N6-METHYLASE<-DUF6079*<-BrxB-BrxF-iSTAND<-BrxA-DNA-binding<-XIS-HTH<-ARG<-?||wHTH+WYL+WCX-><-sGTP<-sGTP	1185	Bacteria>Pseudomonadota>Gammaproteobacteria>Pseudomonadales>Pseudomonadaceae>Pseudomonas	GCF_013387065.1	Pseudomonas_reactans	HX796_RS11550	4461..8018	117680	BREX-system-P-loop-protein-BrxC-[Pseudomonas-reactans]
WP_180689220.1	Type1.BREX	ABC-ATPase+SbcC->?->?->?->?->DUF772+DDE_Tnp_1->XIS-HTH->BrxA-DNA-binding->BrxB-BrxF-iSTAND->DUF6079*->N6-METHYLASE->ParB+HNH->PglZ->SIGMA+OB-Fold+AAA-ATPase+HKD-DNase-BrxL-><-Phage_integrase<-Integrase_1<-wHTH<-AAA+wHTH<-Arm-DNA-bind_3+Phage_integrase	1185	Bacteria>Pseudomonadota>Gammaproteobacteria>Pseudomonadales>Pseudomonadaceae>Pseudomonas	GCF_013425825.1	Pseudomonas_putida	H0H12_RS03375	759269..762826	303	BREX-system-P-loop-protein-BrxC-[Pseudomonas-putida]
WP_182367101.1	Type1.BREX	Arm-DNA-bind_3+Phage_integrase->GreA_GreB+SF1-SF2-Helicase-><-SIGMA+OB-Fold+AAA-ATPase+Lon-Protease-BrxL<-PglZ<-ParB+HNH<-?<-MarR-HTH<-MNS+ORC-CDC6<-N6-METHYLASE<-DUF6079*<-BrxB-BrxF-iSTAND<-BrxA-DNA-binding<-Ntox49<-?<-Peptidase_M15<-?||wHTH+WYL+WCX-><-?<-RelE-ParE	1154	Bacteria>Pseudomonadota>Gammaproteobacteria>Pseudomonadales>Pseudomonadaceae>Pseudomonas	GCF_014109765.1	Pseudomonas_sp._MSPm1	HS968_RS15945	3439076..3442540	2726956	BREX-system-P-loop-protein-BrxC-[Pseudomonas-sp.-MSPm1]
WP_192985630.1	Type1.BREX	HTH_19-><-SIGMA+OB-Fold+AAA-ATPase+Lon-Protease-BrxL<-PglZ<-AbiEii<-AbiEi_4<-?<-N6-METHYLASE<-DNA-glycosylase<-ParB+HNH<-DUF6079*<-BrxB-BrxF-iSTAND<-BrxA-DNA-binding	1220	Bacteria>Pseudomonadota>Gammaproteobacteria>Pseudomonadales>Pseudomonadaceae>Pseudomonas	GCF_014892995.1	Pseudomonas_sp._EggHat1	INS93_RS22765	65820..69482	2761624	BREX-system-P-loop-protein-BrxC-[Pseudomonas-sp.-EggHat1]
WP_230091331.1	Type1.BREX	<-SIGMA+OB-Fold+AAA-ATPase+Lon-Protease-BrxL<-PglZ<-AbiEii<-AbiEi_4<-?<-?<-N6-METHYLASE<-DNA-glycosylase<-ParB+HNH<-DUF6079*<-BrxB-BrxF-iSTAND<-BrxA-DNA-binding	1215	Bacteria>Pseudomonadota>Gammaproteobacteria>Pseudomonadales>Pseudomonadaceae>Pseudomonas	GCF_015666575.1	Pseudomonas_aeruginosa	I1957_RS22560	16962..20609	287	BREX-system-P-loop-protein-BrxC-[Pseudomonas-aeruginosa]
WP_196169752.1	Type1.BREX	DUF4969-><-SIGMA+OB-Fold+AAA-ATPase+Lon-Protease-BrxL<-PglZ<-HNH<-OLD-ABC<-DUF932<-N6-METHYLASE<-DUF6079*<-BrxB-BrxF-iSTAND<-BrxA-DNA-binding<-wHTH+WYL+WCX<-?<-XIS-HTH||CopG/RHH-><-MODE-HTH+PBPII	1154	Bacteria>Pseudomonadota>Gammaproteobacteria>Pseudomonadales>Pseudomonadaceae>Pseudomonas	GCF_015680295.1	Pseudomonas_guariconensis	IRZ59_RS14965	185250..188714	1288410	BREX-system-P-loop-protein-BrxC-[Pseudomonas-guariconensis]
WP_196476665.1	Type1.BREX	PhdYeFM_antitox->RelE-ParE-><-?<-wHTH+WYL+WCX||?->Peptidase_M15->?->BrxA-DNA-binding->BrxB-BrxF-iSTAND->DUF6079*->N6-METHYLASE->?->HNH+GCN1->?->ParB+HNH->PglZ->SIGMA+OB-Fold+AAA-ATPase+Lon-Protease-BrxL->REase->SF1-SF2-Helicase->	1154	Bacteria>Pseudomonadota>Gammaproteobacteria>Pseudomonadales>Pseudomonadaceae>Pseudomonas	GCF_015712105.2	Pseudomonas_sp._LMG_31766	H3221_RS24535	30166..33630	2758695	BREX-system-P-loop-protein-BrxC-[Pseudomonas-chaetocerotis]
WP_198726217.1	Type1.BREX	<-TetR-HTH<-wHTH+WYL+WCX||?->ARG->BrxA-DNA-binding->BrxB-BrxF-iSTAND->DUF6079*->N6-METHYLASE->MNS+ORC-CDC6->?->ParB+HNH->PglZ-><-Phage_integrase<-Integrase_1<-wHTH<-AAA+wHTH	1154	Bacteria>Pseudomonadota>Gammaproteobacteria>Pseudomonadales>Pseudomonadaceae>Pseudomonas	GCF_016307475.1	Pseudomonas_corrugata	YA0788_RS19970	31466..34930	47879	BREX-system-P-loop-protein-BrxC-[Pseudomonas-corrugata]
WP_198790924.1	Type1.BREX	<-CopG/RHH||XIS-HTH->?->?->XIS-HTH->BrxA-DNA-binding->BrxB-BrxF-iSTAND->DUF6079*->N6-METHYLASE-p->OLD-ABC->REase->?->N6-METHYLASE-p->ParB+HNH->PglZ->SIGMA+OB-Fold+AAA-ATPase+HKD-DNase-BrxL->	1185	Bacteria>Pseudomonadota>Gammaproteobacteria>Pseudomonadales>Pseudomonadaceae>Pseudomonas	GCF_016405175.1	Pseudomonas_sp._MF7451	JFT65_RS10900	194530..198087	2797538	BREX-system-P-loop-protein-BrxC-[Pseudomonas-sp.-MF7451]
WP_198832884.1	Type1.BREX	<-SIGMA+OB-Fold+AAA-ATPase+HKD-DNase-BrxL<-PglZ<-?<-N6-METHYLASE<-DUF6079*<-BrxB-BrxF-iSTAND<-BrxA-DNA-binding<-XIS-HTH<-ARG<-?||wHTH+WYL+WCX-><-sGTP	1185	Bacteria>Pseudomonadota>Gammaproteobacteria>Pseudomonadales>Pseudomonadaceae>Pseudomonas	GCF_016405445.1	Pseudomonas_haemolytica	JFT42_RS28230	10084..13641	2600065	BREX-system-P-loop-protein-BrxC-[Pseudomonas-haemolytica]
WP_198849248.1	Type1.BREX	wHTH+WYL+WCX-><-SIGMA+OB-Fold+AAA-ATPase+HKD-DNase-BrxL<-HNH<-OLD-ABC<-N6-METHYLASE-p<-HerA/FtsK<-SIR2<-N6-METHYLASE-p<-DUF6079*<-BrxB-BrxF-iSTAND<-BrxA-DNA-binding<-XIS-HTH<-?<-tRNA<-MerR-HTH	1186	Bacteria>Pseudomonadota>Gammaproteobacteria>Pseudomonadales>Pseudomonadaceae>Pseudomonas	GCF_016405505.1	Pseudomonas_sp._MF6772	JFT60_RS22275	24346..27906	2797533	BREX-system-P-loop-protein-BrxC-[Pseudomonas-sp.-MF6772]
WP_203327703.1	Type1.BREX	XIS-HTH->?->wHTH+WYL+WCX->BrxA-DNA-binding->BrxB-BrxF-iSTAND->DUF6079*->N6-METHYLASE->ParB+HNH->PglZ->SIGMA+OB-Fold+AAA-ATPase+Lon-Protease-BrxL-><-DUF4969<-?<-?<-?<-DUF932	1157	Bacteria>Pseudomonadota>Gammaproteobacteria>Pseudomonadales>Pseudomonadaceae>Pseudomonas	GCF_016812215.1	Pseudomonas_fragi	GYM96_RS18895	91257..94730	296	BREX-system-P-loop-protein-BrxC-[Pseudomonas-fragi]
WP_108110611.1	Type1.BREX	<-HIN-HTH<-REase<-?<-wHTH+WYL+WCX<-?||Peptidase_M15->?->BrxA-DNA-binding->BrxB-BrxF-iSTAND->DUF6079*->N6-METHYLASE->MNS+ORC-CDC6->MarR-HTH->?->ParB+HNH->PglZ->SIGMA+OB-Fold+AAA-ATPase+Lon-Protease-BrxL-><-GreA_GreB+SF1-SF2-Helicase<-HTH_Tnp_1+HTH_21+rve+rve_3	1154	Bacteria>Pseudomonadota>Gammaproteobacteria>Pseudomonadales>Pseudomonadaceae>Pseudomonas	GCF_016820125.1	Pseudomonas_aeruginosa	JQS52_RS11310	2388468..2391932	287	BREX-system-P-loop-protein-BrxC-[Pseudomonas-aeruginosa]
WP_239000709.1	Type1.BREX	<-ABC-ATPase+SbcC<-?<-?<-MODE-HTH+PBPII||<-?<-N6-METHYLASE<-DOC+HTH<-?<-?<-DUF6079*<-BrxB-BrxF-iSTAND<-BrxA-DNA-binding<-wHTH+WYL+WCX||OLD-ABC+TOPRIM->SF1-SF2-Helicase->?->SF1-SF2-Helicase-><-Phage_integrase	1225	Bacteria>Pseudomonadota>Gammaproteobacteria>Pseudomonadales>Pseudomonadaceae>Pseudomonas	GCF_016836415.1	Pseudomonas_aeruginosa	JQ482_RS07395	1563823..1567500	287	BREX-system-P-loop-protein-BrxC-[Pseudomonas-aeruginosa]
WP_239068292.1	Type1.BREX	Phage_integrase->Phage_integrase->?->Phage_integrase-><-SF1-SF2-Helicase<-?||wHTH+WYL+WCX->BrxA-DNA-binding->BrxB-BrxF-iSTAND->DUF6079*->?->?-><-?<-AAA-ATPase<-HTH+rve||DOC+HTH->N6-METHYLASE->RVT->N6-METHYLASE-p->	1225	Bacteria>Pseudomonadota>Gammaproteobacteria>Pseudomonadales>Pseudomonadaceae>Pseudomonas	GCF_016861485.1	Pseudomonas_alcaligenes	JUH08_RS19055	3949819..3953496	43263	BREX-system-P-loop-protein-BrxC-[Pseudomonas-alcaligenes]
WP_203542487.1	Type1.BREX	RHH_5->SbcC->Schlafen+HATPase+wHTH-><-?||NERD->wHTH+WYL+WCX->BrxA-DNA-binding->BrxB-BrxF-iSTAND->DUF6079*->N6-METHYLASE->ParB+HNH->PglZ->SIGMA+OB-Fold+AAA-ATPase+Lon-Protease-BrxL->	1146	Bacteria>Pseudomonadota>Gammaproteobacteria>Pseudomonadales>Pseudomonadaceae>Pseudomonas	GCF_016865555.1	Pseudomonas_arcuscaelestis	IIE18_RS12195	346577..350017	2710591	BREX-system-P-loop-protein-BrxC-[Pseudomonas-arcuscaelestis]
WP_240344493.1	Type1.BREX	<-N6-METHYLASE-p<-DNA-glycosylase<-DUF6079*<-BrxB-BrxF-iSTAND<-BrxA-DNA-binding||<-?<-GTPase-AIG<-GTPase-AIG	1225	Bacteria>Pseudomonadota>Gammaproteobacteria>Pseudomonadales>Pseudomonadaceae>Pseudomonas	GCF_016924785.1	Pseudomonas_sp._SDM007_2	JV174_RS20995	2384..6061	2812898	BREX-system-P-loop-protein-BrxC-[Pseudomonas-sp.-SDM007_2]
WP_205518899.1	Type1.BREX	<-PglZ<-AbiEii<-AbiEi_3_N+AbiEi_3<-HNH<-ParB+HNH<-N6-METHYLASE||HIN-HTH+PAIREDC-HTH-><-N6-METHYLASE-p<-DNA-glycosylase<-DUF6079*<-DUF6079-p<-BrxB-BrxF-iSTAND<-BrxA-DNA-binding||<-?<-GTPase-AIG<-GTPase-AIG	1107	Bacteria>Pseudomonadota>Gammaproteobacteria>Pseudomonadales>Pseudomonadaceae>Pseudomonas	GCF_016925675.1	Pseudomonas_sp._SDM007	JTY93_RS19480	4366691..4370014	2812000	BREX-system-P-loop-protein-BrxC-[Pseudomonas-sp.-SDM007]
WP_206408616.1	Type1.BREX	ARG->?->wHTH+WYL+WCX->BrxA-DNA-binding->BrxB-BrxF-iSTAND->DUF6079*->N6-METHYLASE->OLD-ABC->HNH->ParB+HNH->OLD-ABC->TOPRIM->PglZ->SIGMA+OB-Fold+AAA-ATPase+Lon-Protease-BrxL->MukF->	1154	Bacteria>Pseudomonadota>Gammaproteobacteria>Pseudomonadales>Pseudomonadaceae>Pseudomonas	GCF_017168035.1	Pseudomonas_oleovorans	ACP89_RS12320	3896..7360	301	BREX-system-P-loop-protein-BrxC-[Pseudomonas-oleovorans]
WP_219730400.1	Type1.BREX	METHYLASE->BrxA-DNA-binding->BrxB-BrxF-iSTAND->DUF6079*->ParB+HNH->DNA-glycosylase->N6-METHYLASE->||PglZ->SIGMA+OB-Fold+AAA-ATPase+Lon-Protease-BrxL-><-Phage_integrase||DUF4096+DDE_Tnp_1+DDE_Tnp_1_2-><-wHTH<-AAA+wHTH	1225	Bacteria>Pseudomonadota>Gammaproteobacteria>Pseudomonadales>Pseudomonadaceae>Pseudomonas	GCF_017350895.1	unclassified_Pseudomonas	J4E12_RS18510	3053..6730	196821	MULTISPECIES:-BREX-system-P-loop-protein-BrxC-[unclassified-Pseudomonas]
WP_210224970.1	Type1.BREX	SF1-SF2-Helicase-><-HTH_3||?->CHTH-><-?<-REase+NACHT||wHTH+WYL+WCX->BrxA-DNA-binding->BrxB-BrxF-iSTAND->DUF6079*->N6-METHYLASE->SIR2->HerA/FtsK->?->N6-METHYLASE-p->sGTP->?->PglZ->SIGMA+OB-Fold+AAA-ATPase+Lon-Protease-BrxL->	1225	Bacteria>Pseudomonadota>Gammaproteobacteria>Pseudomonadales>Pseudomonadaceae>Pseudomonas	GCF_017900915.1	Pseudomonas_aeruginosa	J9247_RS05115	1084735..1088412	287	BREX-system-P-loop-protein-BrxC-[Pseudomonas-aeruginosa]
WP_125970731.1	Type1.BREX	DUF4969-><-SLOG+BirA-HTH<-SF1-SF2-Helicase+PRTase<-SIGMA+OB-Fold+AAA-ATPase+Lon-Protease-BrxL<-PglZ<-ParB+HNH<-DUF932<-N6-METHYLASE<-DUF6079*<-BrxB-BrxF-iSTAND<-BrxA-DNA-binding<-wHTH+WYL+WCX<-?<-XIS-HTH<-BirA-HTH||CopG/RHH->	1154	Bacteria>Pseudomonadota>Gammaproteobacteria>Pseudomonadales>Pseudomonadaceae>Pseudomonas	GCF_018336155.1	Pseudomonas_fluorescens_group	EJJ21_RS24495	5255523..5258987	136843	MULTISPECIES:-BREX-system-P-loop-protein-BrxC-[Pseudomonas-fluorescens-group]
WP_213936039.1	Type1.BREX	DUF4969-><-SIGMA+OB-Fold+AAA-ATPase+Lon-Protease-BrxL<-PglZ<-TOPRIM<-OLD-ABC<-ParB+HNH<-N6-METHYLASE<-DUF6079*<-BrxB-BrxF-iSTAND<-BrxA-DNA-binding<-wHTH+WYL+WCX<-?<-XIS-HTH<-BirA-HTH||CopG/RHH-><-Pkinase+CR-Kinase	1157	Bacteria>Pseudomonadota>Gammaproteobacteria>Pseudomonadales>Pseudomonadaceae>Pseudomonas	GCF_018449005.1	Pseudomonas_lundensis	KIY13_RS15530	3400509..3403982	86185	BREX-system-P-loop-protein-BrxC-[Pseudomonas-lundensis]
WP_198712541.1	Type1.BREX	<-MORC+REase<-?<-SF1-SF2-Helicase+REase<-?||?->wHTH+WYL+WCX->BrxA-DNA-binding->BrxB-BrxF-iSTAND->DUF6079*->N6-METHYLASE->NERD+GspA-ATPase->TPR->PglZ->SIGMA+OB-Fold+AAA-ATPase+Lon-Protease-BrxL-><-JetC<-?<-HNH<-tRNA<-MerR-HTH	1217	Bacteria>Pseudomonadota>Gammaproteobacteria>Pseudomonadales>Pseudomonadaceae>Pseudomonas	GCF_019464415.1	Pseudomonas_sp._So3.2b	KIX80_RS19120	4293365..4297018	2864101	BREX-system-P-loop-protein-BrxC-[Pseudomonas-sp.-So3.2b]
WP_222018162.1	Type1.BREX	N6-METHYLASE->?-><-SIGMA+OB-Fold+AAA-ATPase+Lon-Protease-BrxL<-PglZ||?-><-N6-METHYLASE<-TOPRIM<-OLD-ABC<-DUF6079*<-BrxB-BrxF-iSTAND<-BrxA-DNA-binding<-wHTH+WYL+WCX<-?<-?<-DUF932	1223	Bacteria>Pseudomonadota>Gammaproteobacteria>Pseudomonadales>Pseudomonadaceae>Pseudomonas	GCF_019775695.1	Pseudomonas_sp._AO-1	KVQ82_RS04465	959176..962847	2855434	BREX-system-P-loop-protein-BrxC-[Pseudomonas-sp.-AO-1]
WP_223023201.1	Type1.BREX	<-REase||DNA_METHYLASE-><-CopG/RHH||XIS-HTH->?->XIS-HTH->BrxA-DNA-binding->BrxB-BrxF-iSTAND->DUF6079*->OLD-ABC->REase->N6-METHYLASE->PglZ->SIGMA+OB-Fold+AAA-ATPase+HKD-DNase-BrxL-><-?<-?<-?||HTH_3->	1183	Bacteria>Pseudomonadota>Gammaproteobacteria>Pseudomonadales>Pseudomonadaceae>Pseudomonas	GCF_019891425.1	Pseudomonas_carnis	J1G36_06600	89435..92986	2487355	BREX-system-P-loop-protein-BrxC-[Pseudomonas-carnis]
WP_262416944.1	Type1.BREX	<-SIGMA+OB-Fold+AAA-ATPase+Lon-Protease-BrxL<-PglZ<-AbiEii<-AbiEi_3_N+AbiEi_3<-ParB+HNH<-N6-METHYLASE<-DNA-glycosylase<-DUF6079*<-BrxB-BrxF-iSTAND<-BrxA-DNA-binding<-?||wHTH+WYL+WCX-><-tRNA<-MerR-HTH	1225	Bacteria>Pseudomonadota>Gammaproteobacteria>Pseudomonadales>Pseudomonadaceae>Pseudomonas	GCF_019891455.1	Pseudomonas_sp._P867	J1G33_RS25105	13708..17385	2816050	BREX-system-P-loop-protein-BrxC-[Pseudomonas-sp.-P867]
WP_231661141.1	Type1.BREX	<-RHH||XIS-HTH->?->wHTH+WYL+WCX->BrxA-DNA-binding->BrxB-BrxF-iSTAND->DUF6079*->N6-METHYLASE->ParB+HNH->PglZ->SIGMA+OB-Fold+AAA-ATPase+Lon-Protease-BrxL-><-DUF4969<-?<-?||ABhydrolase-><-RNase	1156	Bacteria>Pseudomonadota>Gammaproteobacteria>Pseudomonadales>Pseudomonadaceae>Pseudomonas	GCF_021147865.1	Pseudomonas_sp._CDFA_610	KDX39_RS16865	240745..244215	2829825	BREX-system-P-loop-protein-BrxC-[Pseudomonas-sp.-CDFA-610]
WP_196706085.1	Type1.BREX	<-SIGMA+OB-Fold+AAA-ATPase+HKD-DNase-BrxL<-PglZ<-N6-METHYLASE-p<-HerA/FtsK<-SIR2<-REase<-OLD-ABC<-N6-METHYLASE<-DUF6079*<-BrxB-BrxF-iSTAND<-BrxA-DNA-binding<-XIS-HTH<-?||<-?||sGTP->sGTP->MA+PspA+PspA+sGTP-><-ARG	1184	Bacteria>Pseudomonadota>Gammaproteobacteria>Pseudomonadales>Pseudomonadaceae>Pseudomonas	GCF_021460775.1	Pseudomonas_putida	LH705_RS23300	5093270..5096824	303	BREX-system-P-loop-protein-BrxC-[Pseudomonas-putida]
WP_236166438.1	Type1.BREX	DUF4969-><-SIGMA+OB-Fold+AAA-ATPase+Lon-Protease-BrxL<-PglZ<-HNH<-OLD-ABC<-ParB+HNH<-N6-METHYLASE<-DUF6079*<-BrxB-BrxF-iSTAND<-BrxA-DNA-binding<-wHTH+WYL+WCX<-?<-XIS-HTH	1154	Bacteria>Pseudomonadota>Gammaproteobacteria>Pseudomonadales>Pseudomonadaceae>Pseudomonas	GCF_021601865.1	Pseudomonas_juntendi	L6D32_RS09730	148894..152358	2666183	BREX-system-P-loop-protein-BrxC-[Pseudomonas-juntendi]
WP_236238652.1	Type1.BREX	<-HIN-HTH<-SIGMA+OB-Fold+AAA-ATPase+HKD-DNase-BrxL<-PglZ<-ParB+HNH<-N6-METHYLASE-p<-REase<-OLD-ABC<-N6-METHYLASE-p<-DUF6079*<-BrxB-BrxF-iSTAND<-BrxA-DNA-binding<-XIS-HTH	1185	Bacteria>Pseudomonadota>Gammaproteobacteria>Pseudomonadales>Pseudomonadaceae>Pseudomonas	GCF_021603345.1	Pseudomonas_rhodesiae	L6E14_RS13730	266364..269921	76760	BREX-system-P-loop-protein-BrxC-[Pseudomonas-rhodesiae]
WP_236310112.1	Type1.BREX	<-HTH+rve<-?||XIS-HTH->?->wHTH+WYL+WCX->BrxA-DNA-binding->BrxB-BrxF-iSTAND->DUF6079*->N6-METHYLASE->DUF932->ParB+HNH->OLD-ABC->TOPRIM->PglZ->SIGMA+OB-Fold+AAA-ATPase+Lon-Protease-BrxL-><-DUF4969	1154	Bacteria>Pseudomonadota>Gammaproteobacteria>Pseudomonadales>Pseudomonadaceae>Pseudomonas	GCF_021605645.1	Pseudomonas_gessardii	GIW56_RS19725	10406..13870	78544	BREX-system-P-loop-protein-BrxC-[Pseudomonas-gessardii]
WP_236471270.1	Type1.BREX	XIS-HTH->?-><-REase+ZnR||NERD->NERD->wHTH+WYL+WCX->BrxA-DNA-binding->BrxB-BrxF-iSTAND->DUF6079*->N6-METHYLASE->ParB+HNH->PglZ->SIGMA+OB-Fold+AAA-ATPase+Lon-Protease-BrxL-><-DUF4969<-?<-?<-?<-DUF932	1156	Bacteria>Pseudomonadota>Gammaproteobacteria>Pseudomonadales>Pseudomonadaceae>Pseudomonas	GCF_021606115.1	Pseudomonas_syringae	GIW32_RS04305	7736..11206	317	BREX-system-P-loop-protein-BrxC-[Pseudomonas-syringae]
WP_243242998.1	Type1.BREX	Amidohydro_2->ABhydrolase->AraC-HTH->?->ABC-ATPase+SbcC->?->?->BrxA-DNA-binding->BrxB-BrxF-iSTAND->DUF6079*->N6-METHYLASE->MNS+ORC-CDC6->MarR-HTH->?->ParB+HNH->PglZ->SIGMA+OB-Fold+AAA-ATPase+Lon-Protease-BrxL-><-Integrase_1<-Arm-DNA-bind_3+Phage_integrase	1154	Bacteria>Pseudomonadota>Gammaproteobacteria>Pseudomonadales>Pseudomonadaceae>Pseudomonas	GCF_022802935.1	Pseudomonas_atacamensis	PSD_RS21520	46330..49794	2565368	BREX-system-P-loop-protein-BrxC-[Pseudomonas-atacamensis]
WP_253132516.1	Type1.BREX	XIS-HTH->?-><-?<-LexA-protease<-wHTH+WYL+WCX||?->XIS-HTH->BrxA-DNA-binding->BrxB-BrxF-iSTAND->DUF6079*->N6-METHYLASE->OLD-ABC->REase->SIR2->HerA/FtsK->?->N6-METHYLASE-p->ParB+HNH->PglZ->	1184	Bacteria>Pseudomonadota>Gammaproteobacteria>Pseudomonadales>Pseudomonadaceae>Pseudomonas	GCF_024128115.1	Pseudomonas_guariconensis	NJC29_RS17685	128726..132280	1288410	BREX-system-P-loop-protein-BrxC-[Pseudomonas-guariconensis]
WP_043087434.1	Type1.BREX	<-HIN-HTH<-wHTH+WYL+WCX<-?||?->Peptidase_M15->?->BrxA-DNA-binding->BrxB-BrxF-iSTAND->DUF6079*->N6-METHYLASE->MNS+ORC-CDC6->MarR-HTH->?->ParB+HNH->PglZ->SIGMA+OB-Fold+AAA-ATPase+Lon-Protease-BrxL-><-GreA_GreB+SF1-SF2-Helicase<-HTH_Tnp_1+HTH_21+rve+rve_3	1154	Bacteria>Pseudomonadota>Gammaproteobacteria>Pseudomonadales>Pseudomonadaceae>Pseudomonas	GCF_024300845.1	Pseudomonas_aeruginosa	MUG61_RS11500	2420957..2424421	287	BREX-system-P-loop-protein-BrxC-[Pseudomonas-aeruginosa]
WP_261941654.1	Type1.BREX	DUF932->?->?->?->DUF4969-><-SIGMA+OB-Fold+AAA-ATPase+Lon-Protease-BrxL<-PglZ<-ParB+HNH<-N6-METHYLASE<-DUF6079*<-BrxB-BrxF-iSTAND<-BrxA-DNA-binding<-wHTH+WYL+WCX<-?<-XIS-HTH||RHH->?->?-><-AraC-HTH+AraC-HTH	1154	Bacteria>Pseudomonadota>Gammaproteobacteria>Pseudomonadales>Pseudomonadaceae>Pseudomonas	GCF_024349685.1	Pseudomonas_sp._LRP2-20	OCX61_RS23895	5328463..5331927	2944234	BREX-system-P-loop-protein-BrxC-[Pseudomonas-sp.-LRP2-20]
WP_010445526.1	Type1.BREX	<-MULE-TRANSPOSASE<-?<-?||HEPN-><-SIGMA+OB-Fold+AAA-ATPase+Lon-Protease-BrxL<-PglZ<-?<-sGTP<-N6-METHYLASE<-DUF6079*<-BrxB-BrxF-iSTAND<-BrxA-DNA-binding<-wHTH+WYL+WCX<-HTH+DOC<-PadR-HTH||?-><-ABhydrolase	1223	Bacteria>Pseudomonadota>Gammaproteobacteria>Pseudomonadales>Pseudomonadaceae>Pseudomonas	GCF_024585345.2	Pseudomonas_fuscovaginae	NLK61_RS17300	3830482..3834153	50340	BREX-system-P-loop-protein-BrxC-[Pseudomonas-fuscovaginae]
WP_257780136.1	Type1.BREX	<-LZ_Tnp_IS66+zf-IS66+DDE_Tnp_IS66_C<-?<-HIN-HTH<-SIGMA+OB-Fold+AAA-ATPase+Lon-Protease-BrxL<-PglZ<-N6-METHYLASE<-DNA-glycosylase<-ParB+HNH<-DUF6079*<-BrxB-BrxF-iSTAND<-BrxA-DNA-binding<-?<-?<-?<-SSB<-?||nSTAND1+SbcC+ABC-ATPase+SbcC->	1225	Bacteria>Pseudomonadota>Gammaproteobacteria>Pseudomonadales>Pseudomonadaceae>Pseudomonas	GCF_024655625.1	Pseudomonas_sp._18.1.10	NUV89_RS02215	18654..22331	2969302	BREX-system-P-loop-protein-BrxC-[Pseudomonas-sp.-18.1.10]
WP_258640408.1	Type1.BREX	ABC-ATPase+SbcC->?->?-><-?<-RHH||XIS-HTH->?->wHTH+WYL+WCX->BrxA-DNA-binding->BrxB-BrxF-iSTAND->DUF6079*->N6-METHYLASE-p->N6-METHYLASE->ParB+HNH->PglZ->SIGMA+OB-Fold+AAA-ATPase+Lon-Protease-BrxL-><-DUF4969<-?<-?||ABhydrolase->	1154	Bacteria>Pseudomonadota>Gammaproteobacteria>Pseudomonadales>Pseudomonadaceae>Pseudomonas	GCF_024748715.1	Pseudomonas_moraviensis	LOY57_RS03675	870131..873595	321662	BREX-system-P-loop-protein-BrxC-[Pseudomonas-moraviensis]
WP_258626742.1	Type1.BREX	<-SIGMA+OB-Fold+AAA-ATPase+Lon-Protease-BrxL<-PglZ<-AbiEii<-AbiEi_3_N+AbiEi_3<-ParB+HNH<-N6-METHYLASE<-DNA-glycosylase<-DUF6079*<-BrxB-BrxF-iSTAND<-BrxA-DNA-binding<-ARG<-?||wHTH+WYL+WCX-><-?<-GTPase-AIG<-GTPase-AIG	1225	Bacteria>Pseudomonadota>Gammaproteobacteria>Pseudomonadales>Pseudomonadaceae>Pseudomonas	GCF_024749045.1	Pseudomonas_sp._B21-028	LOY35_RS21325	4957302..4960979	2895480	BREX-system-P-loop-protein-BrxC-[Pseudomonas-sp.-B21-028]
WP_259155464.1	Type1.BREX	DUF932->?->?->?->DUF4969-><-SIGMA+OB-Fold+AAA-ATPase+Lon-Protease-BrxL<-PglZ<-ParB+HNH<-N6-METHYLASE<-DUF6079*<-BrxB-BrxF-iSTAND<-BrxA-DNA-binding<-wHTH+WYL+WCX<-?<-XIS-HTH||?-><-Cysteine-Desulfurase	1154	Bacteria>Pseudomonadota>Gammaproteobacteria>Pseudomonadales>Pseudomonadaceae>Pseudomonas	GCF_024809425.1	Pseudomonas_sp._JAI111	HNR03_RS20420	285382..288846	2735913	BREX-system-P-loop-protein-BrxC-[Pseudomonas-sp.-JAI111]
WP_260092499.1	Type1.BREX	<-SF1-SF2-Helicase<-REase<-SIGMA+OB-Fold+AAA-ATPase+Lon-Protease-BrxL<-PglZ<-ParB+HNH<-?<-HNH+GCN1<-?<-N6-METHYLASE<-DUF6079*<-BrxB-BrxF-iSTAND<-BrxA-DNA-binding<-Ntox49<-?<-Peptidase_M15<-?||wHTH+WYL+WCX->?-><-RelE-ParE	1154	Bacteria>Pseudomonadota>Gammaproteobacteria>Pseudomonadales>Pseudomonadaceae>Pseudomonas	GCF_025152895.1	Pseudomonas_aeruginosa	MNU23_RS16440	110369..113833	287	BREX-system-P-loop-protein-BrxC-[Pseudomonas-aeruginosa]
WP_261753794.1	Type1.BREX	<-SIGMA+OB-Fold+AAA-ATPase+Lon-Protease-BrxL<-PglZ||ZnR-><-TOPRIM<-OLD-ABC<-N6-METHYLASE<-DOC+HTH<-DUF6079*<-BrxB-BrxF-iSTAND<-BrxA-DNA-binding<-wHTH+WYL+WCX<-?<-Phage_AlpA<-?<-Arm-DNA-bind_3+Phage_integrase	1225	Bacteria>Pseudomonadota>Gammaproteobacteria>Pseudomonadales>Pseudomonadaceae>Pseudomonas	GCF_025397875.1	Pseudomonas_veronii	MN113_RS13530	508981..512658	76761	BREX-system-P-loop-protein-BrxC-[Pseudomonas-veronii]
WP_261744726.1	Type1.BREX	HISKIN->REC+PAS+HISKIN+REC-><-?<-PglZ<-?<-sGTP<-SIGMA+OB-Fold+AAA-ATPase+Lon-Protease-BrxL<-DUF6079*<-BrxB-BrxF-iSTAND<-BrxA-DNA-binding<-wHTH+WYL+WCX<-Phage_integrase<-Integrase_1<-wHTH<-AAA+wHTH	1223	Bacteria>Pseudomonadota>Gammaproteobacteria>Pseudomonadales>Pseudomonadaceae>Pseudomonas	GCF_025398015.1	Pseudomonas_promysalinigenes	N5C08_RS03555	755414..759085	485898	BREX-system-P-loop-protein-BrxC-[Pseudomonas-promysalinigenes]
WP_170059731.1	Type1.BREX	<-SF1-SF2-Helicase+REase<-?<-SF1-SF2-Helicase<-HKD-DNase+HKD-DNase<-?||wHTH+WYL+WCX->BrxA-DNA-binding->BrxB-BrxF-iSTAND->DUF6079*->N6-METHYLASE->NERD+GspA-ATPase->TPR->PglZ->SIGMA+OB-Fold+AAA-ATPase+Lon-Protease-BrxL->?-><-tRNA<-MerR-HTH	1223	Bacteria>Pseudomonadota>Gammaproteobacteria>Pseudomonadales>Pseudomonadaceae>Pseudomonas	GCF_025447475.1	Pseudomonas_shahriarae	N8A88_RS04290	981426..985097	2745512	BREX-system-P-loop-protein-BrxC-[Pseudomonas-shahriarae]
WP_023127678.1	Type1.BREX	NUDIX-><-?<-wHTH+WYL+WCX||ARG->?->wHTH+WYL+WCX->BrxA-DNA-binding->BrxB-BrxF-iSTAND->DUF6079*->N6-METHYLASE->DUF932->ParB+HNH->OLD-ABC->TOPRIM->PglZ->SIGMA+OB-Fold+AAA-ATPase+Lon-Protease-BrxL->MukF->JetB->	1154	Bacteria>Pseudomonadota>Gammaproteobacteria>Pseudomonadales>Pseudomonadaceae>Pseudomonas	GCF_025696785.1	Pseudomonas_aeruginosa	KUC66_RS22970	76351..79815	287	BREX-system-P-loop-protein-BrxC-[Pseudomonas-aeruginosa]
WP_124171099.1	Type1.BREX	RelE-ParE-><-?<-wHTH+WYL+WCX||?->Peptidase_M15->?->XIS-HTH->BrxA-DNA-binding->BrxB-BrxF-iSTAND->DUF6079*->N6-METHYLASE->SbcC-p->REase->RVT->N6-METHYLASE-p->PglZ->SIGMA+OB-Fold+AAA-ATPase+HKD-DNase-BrxL-><-Arm-DNA-bind_3+Phage_integrase<-REase+SF1-SF2-Helicase	1184	Bacteria>Pseudomonadota>Gammaproteobacteria>Pseudomonadales>Pseudomonadaceae>Pseudomonas	GCF_025697325.1	Pseudomonas_aeruginosa	IPC368_RS11155	506506..510060	287	BREX-system-P-loop-protein-BrxC-[Pseudomonas-aeruginosa]
WP_265072431.1	Type1.BREX	<-SIGMA+OB-Fold+AAA-ATPase+HKD-DNase-BrxL<-PglZ<-ParB+HNH<-N6-METHYLASE-p<-REase<-OLD-ABC<-N6-METHYLASE-p<-DUF6079*<-BrxB-BrxF-iSTAND<-BrxA-DNA-binding<-XIS-HTH||HTH+REase-><-?<-?<-?<-Glyco_hydro_68	1186	Bacteria>Pseudomonadota>Gammaproteobacteria>Pseudomonadales>Pseudomonadaceae>Pseudomonas	GCF_026016265.1	Pseudomonas_viciae	LOY66_RS25465	5798638..5802198	2505979	BREX-system-P-loop-protein-BrxC-[Pseudomonas-viciae]
WP_265535081.1	Type1.BREX	<-SIGMA+OB-Fold+AAA-ATPase+HKD-DNase-BrxL<-PglZ<-N6-METHYLASE-p<-HerA/FtsK<-SIR2<-REase<-OLD-ABC<-N6-METHYLASE<-DUF6079*<-BrxB-BrxF-iSTAND<-BrxA-DNA-binding<-XIS-HTH<-?||<-?||sGTP->sGTP->MA+PspA+PspA+sGTP-><-ZnR	1184	Bacteria>Pseudomonadota>Gammaproteobacteria>Pseudomonadales>Pseudomonadaceae>Pseudomonas	GCF_026173695.1	Pseudomonas_saponiphila	ORM81_RS30140	14715..18269	556534	BREX-system-P-loop-protein-BrxC-[Pseudomonas-saponiphila]
WP_265980027.1	Type1.BREX	Phage_integrase-><-SIGMA+OB-Fold+AAA-ATPase+HKD-DNase-BrxL<-PglZ<-ParB+HNH<-N6-METHYLASE-p<-?<-NERD<-OLD-ABC<-N6-METHYLASE<-DUF6079*<-BrxB-BrxF-iSTAND<-BrxA-DNA-binding<-XIS-HTH||<-?<-?||wHTH+WYL+WCX-><-JAB	1184	Bacteria>Pseudomonadota>Gammaproteobacteria>Pseudomonadales>Pseudomonadaceae>Pseudomonas	GCF_026241005.1	Pseudomonas_sp._DCB_BG	OO254_RS23990	64105..67659	2993595	BREX-system-P-loop-protein-BrxC-[Pseudomonas-sp.-DCB_BG]
WP_270945767.1	Type1.BREX	MerR-HTH->||?->?->DUF772+DDE_Tnp_1-><-?||wHTH+WYL+WCX->BrxA-DNA-binding->BrxB-BrxF-iSTAND->DUF6079*->N6-METHYLASE->?->?->OLD-ABC->HNH->PglZ->SIGMA+OB-Fold+AAA-ATPase+Lon-Protease-BrxL->?->HTH_3->	1223	Bacteria>Pseudomonadota>Gammaproteobacteria>Pseudomonadales>Pseudomonadaceae>Pseudomonas	GCF_027856115.1	Pseudomonas_aeruginosa	KKQ36_RS16450	3486481..3490152	287	BREX-system-P-loop-protein-BrxC-[Pseudomonas-aeruginosa]
WP_196620444.1	Type1.BREX	<-XIS-HTH<-SIGMA+OB-Fold+AAA-ATPase+HKD-DNase-BrxL<-PglZ<-N6-METHYLASE-p<-HerA/FtsK<-SIR2<-REase<-OLD-ABC<-N6-METHYLASE<-DUF6079*<-BrxB-BrxF-iSTAND<-BrxA-DNA-binding<-XIS-HTH<-tRNA<-MerR-HTH	1181	Bacteria>Pseudomonadota>Gammaproteobacteria>Pseudomonadales>Pseudomonadaceae>Pseudomonas	GCF_027857235.1	Pseudomonas_aeruginosa	OFL78_RS02500	39831..43376	287	BREX-system-P-loop-protein-BrxC-[Pseudomonas-aeruginosa]
WP_271674697.1	Type1.BREX	GTPase-AIG->GTPase-AIG->?-><-wHTH+WYL+WCX||?->ARG->BrxA-DNA-binding->BrxB-BrxF-iSTAND->DUF6079*->DNA-glycosylase->N6-METHYLASE-p->	1225	Bacteria>Pseudomonadota>Gammaproteobacteria>Pseudomonadales>Pseudomonadaceae>Pseudomonas	GCF_028200675.1	Pseudomonas_extremaustralis	PLO26_RS31670	20252..23929	359110	BREX-system-P-loop-protein-BrxC-[Pseudomonas-extremaustralis]
WP_272218090.1	Type1.BREX	RelE-ParE-><-?<-wHTH+WYL+WCX||?->Peptidase_M15->Peptidase_M15->?-><-AAA-ATPase<-HIN-HTH||DUF6079-p*->HIN-HTH->?->LZ_Tnp_IS66+zf-IS66+DDE_Tnp_IS66+DDE_Tnp_IS66_C->N6-METHYLASE-p->OLD-ABC->NERD->RVT->N6-METHYLASE-p->ParB+HNH->	932	Bacteria>Pseudomonadota>Gammaproteobacteria>Pseudomonadales>Pseudomonadaceae>Pseudomonas	GCF_028404085.1	Pseudomonas_aeruginosa	PMJ90_RS12960	2696481..2699279	287	BREX-system-P-loop-protein-BrxC-[Pseudomonas-aeruginosa]
WP_273917578.1	Type1.BREX	<-SIGMA+OB-Fold+AAA-ATPase+Lon-Protease-BrxL<-PglZ<-HEPN<-TPR<-NERD+GspA-ATPase<-N6-METHYLASE<-N6-METHYLASE<-TOPRIM<-OLD-ABC<-DUF6079*<-BrxB-BrxF-iSTAND<-BrxA-DNA-binding<-wHTH+WYL+WCX<-wHTH+WYL+WCX||?->ARG->	1223	Bacteria>Pseudomonadota>Gammaproteobacteria>Pseudomonadales>Pseudomonadaceae>Pseudomonas	GCF_028656895.1	Pseudomonas_sp._TNT2022_ID1044	M5G20_RS01570	347595..351266	2942636	BREX-system-P-loop-protein-BrxC-[Pseudomonas-sp.-TNT2022-ID1044]
WP_273916456.1	Type1.BREX	XIS-HTH->BrxA-DNA-binding->BrxB-BrxF-iSTAND->DUF6079*->N6-METHYLASE->OLD-ABC->NERD->?->?->N6-METHYLASE-p->PglZ->SIGMA+OB-Fold+AAA-ATPase+HKD-DNase-BrxL->	1186	Bacteria>Pseudomonadota>Gammaproteobacteria>Pseudomonadales>Pseudomonadaceae>Pseudomonas	GCF_028656995.1	Pseudomonas_sp._TNT2022_ID642	M5G26_RS21745	58505..62065	2942632	BREX-system-P-loop-protein-BrxC-[Pseudomonas-sp.-TNT2022-ID642]
WP_274102063.1	Type1.BREX	Arm-DNA-bind_3+Phage_integrase->AAA+wHTH->wHTH->Integrase_1->Phage_integrase->wHTH+WYL+WCX->BrxA-DNA-binding->BrxB-BrxF-iSTAND->DUF6079*->N6-METHYLASE->PglZ->SIGMA+OB-Fold+AAA-ATPase+Lon-Protease-BrxL-><-HTH+REase<-ANK||sGTP->?->sGTP->sGTP->	1222	Bacteria>Pseudomonadota>Gammaproteobacteria>Pseudomonadales>Pseudomonadaceae>Pseudomonas	GCF_028682345.1	Pseudomonas_putida	NPS33_RS09465	85396..89064	303	BREX-system-P-loop-protein-BrxC-[Pseudomonas-putida]
WP_274151131.1	Type1.BREX	Glyoxalase+Sel1+Sel1-><-NERD+ZnR<-REase+SF1-SF2-Helicase<-SIGMA+OB-Fold+AAA-ATPase+Lon-Protease-BrxL<-PglZ<-N6-METHYLASE<-DUF6079*<-BrxB-BrxF-iSTAND<-BrxA-DNA-binding<-wHTH+WYL+WCX<-Phage_integrase<-Integrase_1<-wHTH<-AAA+wHTH<-Arm-DNA-bind_3+Phage_integrase	1223	Bacteria>Pseudomonadota>Gammaproteobacteria>Pseudomonadales>Pseudomonadaceae>Pseudomonas	GCF_028682495.1	Pseudomonas_sp._25571	NP527_RS02645	592231..595902	2967216	BREX-system-P-loop-protein-BrxC-[Pseudomonas-sp.-25571]
WP_023131618.1	Type1.BREX	<-wHTH+WYL+WCX||?->BrxA-DNA-binding->BrxB-BrxF-iSTAND->DUF6079*->ParB+HNH->DNA-glycosylase->N6-METHYLASE->?->?->AbiEi_4->AbiEii->PglZ->SIGMA+OB-Fold+AAA-ATPase+Lon-Protease-BrxL->	1223	Bacteria>Pseudomonadota>Gammaproteobacteria>Pseudomonadales>Pseudomonadaceae>Pseudomonas	GCF_029842935.1	Pseudomonas_aeruginosa	N5I19_RS16015	16585..20256	287	MULTISPECIES:-BREX-system-P-loop-protein-BrxC-[Pseudomonas]
WP_121324126.1	Type1.BREX	<-REase<-wHTH+WYL+WCX||?->Peptidase_M15->?->Ntox49->BrxA-DNA-binding->BrxB-BrxF-iSTAND->DUF6079*->N6-METHYLASE->?->HNH+GCN1->?->ParB+HNH->PglZ->SIGMA+OB-Fold+AAA-ATPase+Lon-Protease-BrxL-><-GreA_GreB+SF1-SF2-Helicase<-Arm-DNA-bind_3+Phage_integrase	1154	Bacteria>Pseudomonadota>Gammaproteobacteria>Pseudomonadales>Pseudomonadaceae>Pseudomonas	GCF_030121855.1	Pseudomonas_aeruginosa	DQH07_RS08025	7041..10505	287	BREX-system-P-loop-protein-BrxC-[Pseudomonas-aeruginosa]
WP_153334718.1	Type1.BREX	<-SF1-SF2-Helicase+REase<-?<-?<-HKD-DNase+HKD-DNase<-?||wHTH+WYL+WCX->BrxA-DNA-binding->BrxB-BrxF-iSTAND->DUF6079*->N6-METHYLASE->NERD+GspA-ATPase->TPR->PglZ->SIGMA+OB-Fold+AAA-ATPase+Lon-Protease-BrxL->	1223	Bacteria>Pseudomonadota>Gammaproteobacteria>Pseudomonadales>Pseudomonadaceae>Pseudomonas	GCF_030144705.1	Pseudomonas_sp._FSL_R10-2398	GH036_RS09020	68086..71757	2662201	BREX-system-P-loop-protein-BrxC-[Pseudomonas-sp.-FSL-R10-2398]
WP_065945478.1	Type1.BREX	<-AAA+wHTH<-?<-SIGMA+OB-Fold+AAA-ATPase+Lon-Protease-BrxL<-PglZ<-ParB+HNH<-?<-MarR-HTH<-MNS+ORC-CDC6<-N6-METHYLASE<-DUF6079*<-BrxB-BrxF-iSTAND<-BrxA-DNA-binding<-ARG<-?||wHTH+WYL+WCX->	1154	Bacteria>Pseudomonadota>Gammaproteobacteria>Pseudomonadales>Pseudomonadaceae>Pseudomonas	GCF_900004795.1	Pseudomonas_sp._24_R_17	BFP98_RS06365	99601..103065	1844096	BREX-system-P-loop-protein-BrxC-[Pseudomonas-sp.-24-R-17]
WP_176947249.1	Type1.BREX	<-TraK<-TraE<-TraM<-TraM<-DUF3262<-?||XIS-HTH->BrxA-DNA-binding->BrxB-BrxF-iSTAND->DUF6079*->N6-METHYLASE->OLD-ABC->REase->SIR2->HerA/FtsK->N6-METHYLASE-p->PglZ->SIGMA+OB-Fold+AAA-ATPase+HKD-DNase-BrxL->Phage-HTH->	1188	Bacteria>Pseudomonadota>Gammaproteobacteria>Pseudomonadales>Pseudomonadaceae>Pseudomonas	GCF_900100495.1	Pseudomonas_benzenivorans	BLS63_RS10310	86902..90468	556533	BREX-system-P-loop-protein-BrxC-[Pseudomonas-benzenivorans]
WP_092146146.1	Type1.BREX	<-AAA+wHTH<-?<-SIGMA+OB-Fold+AAA-ATPase+Lon-Protease-BrxL<-PglZ<-ParB+HNH<-?<-MarR-HTH<-MNS+ORC-CDC6<-N6-METHYLASE<-DUF6079*<-BrxB-BrxF-iSTAND<-BrxA-DNA-binding<-ARG<-?||wHTH+WYL+WCX->?->sGTP->sGTP->sGTP->	1154	Bacteria>Pseudomonadota>Gammaproteobacteria>Pseudomonadales>Pseudomonadaceae>Pseudomonas	GCF_900102655.1	unclassified_Pseudomonas	BLP71_RS14905	103193..106657	196821	MULTISPECIES:-BREX-system-P-loop-protein-BrxC-[unclassified-Pseudomonas]
WP_090347579.1	Type1.BREX	<-wHTH+WYL+WCX<-?||ARG-><-ANK+ANK+ANK||RADICAL-SAM->HicA_toxin->BrxA-DNA-binding->BrxB-BrxF-iSTAND->DUF6079*->N6-METHYLASE->?->HNH+GCN1->?->?->?->?->ParB+HNH->OLD-ABC->	1154	Bacteria>Pseudomonadota>Gammaproteobacteria>Pseudomonadales>Pseudomonadaceae>Pseudomonas	GCF_900104805.1	Pseudomonas_oryzae	BLT78_RS03155	660554..664018	1392877	BREX-system-P-loop-protein-BrxC-[Pseudomonas-oryzae]
WP_090214592.1	Type1.BREX	REC+PP2C->STAS-><-?<-?<-HTH_Tnp_1+HTH_21+rve+rve_3||?->BrxA-DNA-binding->BrxB-BrxF-iSTAND->DUF6079*->ParB+HNH->?->N6-METHYLASE->?->?->?-><-ZnR||REase->	1218	Bacteria>Pseudomonadota>Gammaproteobacteria>Pseudomonadales>Pseudomonadaceae>Pseudomonas	GCF_900105885.1	Pseudomonas_guangdongensis	BLU22_RS11505	2422793..2426449	1245526	BREX-system-P-loop-protein-BrxC-[Pseudomonas-guangdongensis]
WP_090192550.1	Type1.BREX	<-35exo+SF1-SF2-Helicase||XIS-HTH->BrxA-DNA-binding->BrxB-BrxF-iSTAND->DUF6079*->N6-METHYLASE->?->TPR+REase->?->SF1-SF2-Helicase+PRTase->SLOG+BirA-HTH->PglZ->SIGMA+OB-Fold+AAA-ATPase+Lon-Protease-BrxL->	1153	Bacteria>Pseudomonadota>Gammaproteobacteria>Pseudomonadales>Pseudomonadaceae>Pseudomonas	GCF_900105995.1	Pseudomonas_pohangensis	BLT89_RS00315	68625..72086	364197	BREX-system-P-loop-protein-BrxC-[Pseudomonas-pohangensis]
WP_076583447.1	Type1.BREX	DUF4969-><-SIGMA+OB-Fold+AAA-ATPase+HKD-DNase-BrxL<-PglZ<-ParB+HNH<-N6-METHYLASE-p<-RVT<-REase<-OLD-ABC<-N6-METHYLASE<-DUF6079*<-BrxB-BrxF-iSTAND<-BrxA-DNA-binding<-XIS-HTH<-?||wHTH+WYL+WCX-><-?<-?<-KAP-NTPase||LexA-protease->	1184	Bacteria>Pseudomonadota>Gammaproteobacteria>Pseudomonadales>Pseudomonadaceae>Pseudomonas	GCF_900156545.1	Pseudomonas_alcaligenes	BW984_RS17050	273058..276612	43263	BREX-system-P-loop-protein-BrxC-[Pseudomonas-alcaligenes]
WP_095181954.1	Type1.BREX	<-SIGMA+OB-Fold+AAA-ATPase+Lon-Protease-BrxL<-PglZ<-N6-METHYLASE<-DNA-glycosylase<-ParB+HNH<-DUF6079*<-BrxB-BrxF-iSTAND<-BrxA-DNA-binding||?->Phage_GPD+Phage_base_V+DUF2345->	1222	Bacteria>Pseudomonadota>Gammaproteobacteria>Pseudomonadales>Pseudomonadaceae>Pseudomonas	GCF_900187595.1	Pseudomonas_sp._Irchel_3F6	CKW30_RS22375	287368..291036	2009003	BREX-system-P-loop-protein-BrxC-[Pseudomonas-sp.-Irchel-3F6]
WP_115284295.1	Type1.BREX	AAA-ATPase->?->?->DUF4969-><-SIGMA+OB-Fold+AAA-ATPase+Lon-Protease-BrxL<-PglZ<-ParB+HNH<-DUF932<-N6-METHYLASE<-DUF6079*<-BrxB-BrxF-iSTAND<-BrxA-DNA-binding<-wHTH+WYL+WCX||<-?<-HKD-DNase<-?||?-><-?<-NADAR	1154	Bacteria>Pseudomonadota>Gammaproteobacteria>Pseudomonadales>Pseudomonadaceae>Pseudomonas	GCF_900455465.1	Pseudomonas_fluorescens	DYC60_RS18930	4157779..4161243	294	BREX-system-P-loop-protein-BrxC-[Pseudomonas-fluorescens]
WP_122521120.1	Type1.BREX	<-RHH||XIS-HTH->?->wHTH+WYL+WCX->BrxA-DNA-binding->BrxB-BrxF-iSTAND->DUF6079*->N6-METHYLASE->ParB+HNH->PglZ->SIGMA+OB-Fold+AAA-ATPase+Lon-Protease-BrxL-><-DUF4969<-?<-?||ABhydrolase->SAM-METHYLASE->	1154	Bacteria>Pseudomonadota>Gammaproteobacteria>Pseudomonadales>Pseudomonadaceae>Pseudomonas	GCF_900576635.1	Pseudomonas_viridiflava	EET02_RS10950	375629..379093	33069	BREX-system-P-loop-protein-BrxC-[Pseudomonas-viridiflava]
WP_127881958.1	Type1.BREX	DUF932->?->?->?-><-SIGMA+OB-Fold+AAA-ATPase+Lon-Protease-BrxL<-PglZ<-ParB+HNH<-N6-METHYLASE<-DUF6079*<-BrxB-BrxF-iSTAND<-BrxA-DNA-binding<-wHTH+WYL+WCX	1146	Bacteria>Pseudomonadota>Gammaproteobacteria>Pseudomonadales>Pseudomonadaceae>Pseudomonas	GCF_900618835.1	Pseudomonas_carnis	PCSC_RS23730	94995..98435	2487355	BREX-system-P-loop-protein-BrxC-[Pseudomonas-carnis]
WP_150549236.1	Type1.BREX	<-DUF3584+DUF5401<-JetB<-MukF<-SIGMA+OB-Fold+AAA-ATPase+Lon-Protease-BrxL<-PglZ<-TOPRIM<-OLD-ABC<-ParB+HNH<-N6-METHYLASE<-DUF6079*<-BrxB-BrxF-iSTAND<-BrxA-DNA-binding<-wHTH+WYL+WCX<-?<-?<-?<-XIS-HTH||?-><-tRNA<-MerR-HTH	1154	Bacteria>Pseudomonadota>Gammaproteobacteria>Pseudomonadales>Pseudomonadaceae>Pseudomonas	GCF_900682675.2	Pseudomonas_marincola	PMYSY11_RS11215	2416219..2419683	437900	BREX-system-P-loop-protein-BrxC-[Pseudomonas-marincola]
WP_043107115.1	Type1.BREX	GreA_GreB+SF1-SF2-Helicase-><-SIGMA+OB-Fold+AAA-ATPase+Lon-Protease-BrxL<-PglZ<-ParB+HNH<-?<-HNH+GCN1<-?<-N6-METHYLASE<-DUF6079*<-BrxB-BrxF-iSTAND<-BrxA-DNA-binding<-Ntox49<-?<-Peptidase_M15<-?||wHTH+WYL+WCX->REase->HIN-HTH->	1154	Bacteria>Pseudomonadota>Gammaproteobacteria>Pseudomonadales>Pseudomonadaceae>Pseudomonas	GCF_900706985.1	Pseudomonas_aeruginosa_group	E4Z08_RS29645	1460204..1463668	136841	MULTISPECIES:-BREX-system-P-loop-protein-BrxC-[Pseudomonas-aeruginosa-group]
WP_134576235.1	Type1.BREX	<-AbiEi_4<-?<-?<-N6-METHYLASE<-DNA-glycosylase<-ParB+HNH<-DUF6079-p*	769	Bacteria>Pseudomonadota>Gammaproteobacteria>Pseudomonadales>Pseudomonadaceae>Pseudomonas	GCF_900707355.1	Pseudomonas_aeruginosa	E4Z10_RS26780	10282..12591	287	BREX-system-P-loop-protein-BrxC,-partial-[Pseudomonas-aeruginosa]
WP_134454974.1	Type1.BREX	<-DUF6079-p*<-BrxB-BrxF-iSTAND<-BrxA-DNA-binding<-?<-Peptidase_M15<-?||wHTH+WYL+WCX->	704	Bacteria>Pseudomonadota>Gammaproteobacteria>Pseudomonadales>Pseudomonadaceae>Pseudomonas	GCF_900707495.1	Pseudomonas_aeruginosa	E4Y88_RS09690	1..2111	287	BREX-system-P-loop-protein-BrxC,-partial-[Pseudomonas-aeruginosa]
WP_176605727.1	Type1.BREX	<-MODE-HTH+PBPII||<-?<-N6-METHYLASE<-DOC+HTH<-?<-?<-DUF6079*<-BrxB-BrxF-iSTAND<-BrxA-DNA-binding<-wHTH+WYL+WCX||OLD-ABC+TOPRIM->SF1-SF2-Helicase->?->SF1-SF2-Helicase-><-Phage_integrase	1225	Bacteria>Pseudomonadota>Gammaproteobacteria>Pseudomonadales>Pseudomonadaceae>Pseudomonas	GCF_900708415.1	Pseudomonas_aeruginosa	BUG32_RS23730	10964..14641	287	BREX-system-P-loop-protein-BrxC-[Pseudomonas-aeruginosa]
WP_031684509.1	Type1.BREX	PhdYeFM_antitox->?-><-wHTH+WYL+WCX||?->Peptidase_M15->?->XIS-HTH->BrxA-DNA-binding->BrxB-BrxF-iSTAND->DUF6079*->N6-METHYLASE->SbcC-p->REase->?->N6-METHYLASE-p->PglZ->SIGMA+OB-Fold+AAA-ATPase+HKD-DNase-BrxL-><-Arm-DNA-bind_3+Phage_integrase||SF1-SF2-Helicase->	1184	Bacteria>Pseudomonadota>Gammaproteobacteria>Pseudomonadales>Pseudomonadaceae>Pseudomonas	GCF_900708485.1	Pseudomonas_aeruginosa	ID019_RS13300	2848972..2852526	287	BREX-system-P-loop-protein-BrxC-[Pseudomonas-aeruginosa]
WP_150597441.1	Type1.BREX	DUF4969-><-SIGMA+OB-Fold+AAA-ATPase+Lon-Protease-BrxL<-PglZ<-ParB+HNH<-N6-METHYLASE<-DUF6079*<-BrxB-BrxF-iSTAND<-BrxA-DNA-binding<-wHTH+WYL+WCX<-?<-TPR<-SF1-SF2-Helicase+RNase+RNase<-XIS-HTH||RHH->	1154	Bacteria>Pseudomonadota>Gammaproteobacteria>Pseudomonadales>Pseudomonadaceae>Pseudomonas	GCF_902497985.1	Pseudomonas_fluorescens	F8N16_RS17980	132187..135651	294	BREX-system-P-loop-protein-BrxC-[Pseudomonas-fluorescens]
WP_236582582.1	Type1.BREX	<-TetR-HTH||?->Arm-DNA-bind_3+Phage_integrase->?->Phage_AlpA->?->wHTH+WYL+WCX->BrxA-DNA-binding->BrxB-BrxF-iSTAND->DUF6079*->?->?->DOC+HTH->N6-METHYLASE->NERD+GspA-ATPase->TPR-><-ZnR||PglZ->SIGMA+OB-Fold+AAA-ATPase+Lon-Protease-BrxL->	1225	Bacteria>Pseudomonadota>Gammaproteobacteria>Pseudomonadales>Pseudomonadaceae>Pseudomonas	GCF_905071885.1	Pseudomonas_aeruginosa	JMX72_RS23520	4985087..4988764	287	BREX-system-P-loop-protein-BrxC-[Pseudomonas-aeruginosa]
WP_229999483.1	Type1.BREX	XIS-HTH->?->URI-DNase+RAMA->Schlafen+HATPase+wHTH->DnaB+McrB-AAA->McrC-NTD+REase+ZnR->wHTH+WYL+WCX->BrxA-DNA-binding->BrxB-BrxF-iSTAND->DUF6079*->N6-METHYLASE->ParB+HNH->OLD-ABC->TOPRIM->PglZ->SIGMA+OB-Fold+AAA-ATPase+Lon-Protease-BrxL-><-DUF4969	1156	Bacteria>Pseudomonadota>Gammaproteobacteria>Pseudomonadales>Pseudomonadaceae>Pseudomonas	GCF_918697895.1	Pseudomonas_carnis	LQ224_RS08485	110382..113852	2487355	BREX-system-P-loop-protein-BrxC-[Pseudomonas-carnis]
WP_033991202.1	Type1.BREX	<-HNH<-SIGMA+OB-Fold+AAA-ATPase+Lon-Protease-BrxL<-MNS+REase<-?<-HNH+GCN1<-?<-N6-METHYLASE<-DUF6079*<-BrxB-BrxF-iSTAND<-BrxA-DNA-binding<-RNase<-?<-JetB<-?<-Peptidase_M15<-?||wHTH+WYL+WCX->	1154	Bacteria>Pseudomonadota>Gammaproteobacteria>Pseudomonadales>Pseudomonadaceae>Pseudomonas	GCF_929614685.1	Pseudomonas_aeruginosa	MJP43_RS27275	28461..31925	287	BREX-system-P-loop-protein-BrxC-[Pseudomonas-aeruginosa]
WP_033944490.1	Type1.BREX	Arm-DNA-bind_3+Phage_integrase-><-SIGMA+OB-Fold+AAA-ATPase+HKD-DNase-BrxL<-PglZ<-ParB+HNH<-N6-METHYLASE-p<-RVT<-NERD<-OLD-ABC<-N6-METHYLASE<-DUF6079*<-BrxB-BrxF-iSTAND<-BrxA-DNA-binding<-XIS-HTH<-?<-?||?->?->?->HIN-HTH->	1184	Bacteria>Pseudomonadota>Gammaproteobacteria>Pseudomonadales>Pseudomonadaceae>Pseudomonas	GCF_947387505.1	Pseudomonas_aeruginosa	GPV34_RS03115	17071..20625	287	BREX-system-P-loop-protein-BrxC-[Pseudomonas-aeruginosa]
WP_128722024.1	Type1.BREX	<-TetR-HTH||?->Arm-DNA-bind_3+Phage_integrase->?->||Phage_AlpA->?->wHTH+WYL+WCX->BrxA-DNA-binding->BrxB-BrxF-iSTAND->DUF6079*->?->?->DOC+HTH->N6-METHYLASE->?->||MODE-HTH+PBPII->	1223	Bacteria>Pseudomonadota>Gammaproteobacteria>Pseudomonadales>Pseudomonadaceae>Pseudomonas	-	Pseudomonas_aeruginosa	ISK34_26080	19802..23473	287	BREX-system-P-loop-protein-BrxC-[Pseudomonas-aeruginosa]
EIU5588796.1	Type1.BREX	PhdYeFM_antitox->RelE-ParE-><-?<-wHTH+WYL+WCX||?->Peptidase_M15->?->BrxA-DNA-binding->BrxB-BrxF-iSTAND->DUF6079*->N6-METHYLASE->?->HNH+GCN1->?->ParB+HNH->PglZ->SIGMA+OB-Fold+AAA-ATPase+Lon-Protease-BrxL->REase->SF1-SF2-Helicase->	1154	Bacteria>Pseudomonadota>Gammaproteobacteria>Pseudomonadales>Pseudomonadaceae>Pseudomonas	-	Pseudomonas_aeruginosa	L4689_001050	82930..86394	287	BREX-system-P-loop-protein-BrxC-[Pseudomonas-aeruginosa]
EIU3152871.1	Type1.BREX	Phage_integrase->DDE_Tnp_1-><-SF1-SF2-Helicase<-?<-SF1-SF2-Helicase<-OLD-ABC+TOPRIM||wHTH+WYL+WCX->BrxA-DNA-binding->BrxB-BrxF-iSTAND->DUF6079*->?->?->DOC+HTH->N6-METHYLASE-p->	1223	Bacteria>Pseudomonadota>Gammaproteobacteria>Pseudomonadales>Pseudomonadaceae>Pseudomonas	-	Pseudomonas_aeruginosa	L4P27_006182	20932..24603	287	BREX-system-P-loop-protein-BrxC-[Pseudomonas-aeruginosa]
WP_023124092.1	Type1.BREX	Phage_integrase->DDE_Tnp_1-><-SF1-SF2-Helicase<-?<-SF1-SF2-Helicase<-OLD-ABC+TOPRIM||wHTH+WYL+WCX->BrxA-DNA-binding->BrxB-BrxF-iSTAND->DUF6079*->?->?->DOC+HTH->N6-METHYLASE-p->	1223	Bacteria>Pseudomonadota>Gammaproteobacteria>Pseudomonadales>Pseudomonadaceae>Pseudomonas	-	Pseudomonas_aeruginosa	L4P27_006182	20932..24603	287	BREX-system-P-loop-protein-BrxC-[Pseudomonas-aeruginosa]
EIU3153299.1	Type1.BREX	GreA_GreB+SF1-SF2-Helicase-p-><-SIGMA+OB-Fold+AAA-ATPase+Lon-Protease-BrxL<-PglZ<-ParB+HNH<-?<-HNH+GCN1<-?<-N6-METHYLASE<-DUF6079-p*	512	Bacteria>Pseudomonadota>Gammaproteobacteria>Pseudomonadales>Pseudomonadaceae>Pseudomonas	-	Pseudomonas_aeruginosa	L4P27_006629	14238..15778	287	hypothetical-protein-[Pseudomonas-aeruginosa]
EIU3153568.1	Type1.BREX	<-wHTH+WYL+WCX||?->Peptidase_M15->?->Ntox49->BrxA-DNA-binding->BrxB-BrxF-iSTAND->DUF6079-p*->	641	Bacteria>Pseudomonadota>Gammaproteobacteria>Pseudomonadales>Pseudomonadaceae>Pseudomonas	-	Pseudomonas_aeruginosa	L4P27_006902	6274..8196	287	BREX-system-P-loop-protein-BrxC-[Pseudomonas-aeruginosa]
EIU4198817.1	Type1.BREX	DUF932->?->?->?-><-SIGMA+OB-Fold+AAA-ATPase+HKD-DNase-BrxL<-PglZ<-ParB+HNH<-N6-METHYLASE<-DUF6079*<-BrxB-BrxF-iSTAND<-BrxA-DNA-binding<-XIS-HTH||<-?||sGTP->sGTP->MA+PspA+PspA+sGTP-><-ZnR<-ARG	1180	Bacteria>Pseudomonadota>Gammaproteobacteria>Pseudomonadales>Pseudomonadaceae>Pseudomonas	-	Pseudomonas_aeruginosa	L4V49_002912	27945..31487	287	BREX-system-P-loop-protein-BrxC-[Pseudomonas-aeruginosa]
WP_203788563.1	Type1.BREX	Phage_integrase-><-?<-SF1-SF2-Helicase<-?<-SbcC||?->wHTH+WYL+WCX->BrxA-DNA-binding->BrxB-BrxF-iSTAND->DUF6079*->?->?->DOC+HTH->N6-METHYLASE->RVT->N6-METHYLASE-p-><-DUF772+DDE_Tnp_1||N6-METHYLASE-p->	1223	Bacteria>Pseudomonadota>Gammaproteobacteria>Pseudomonadales>Pseudomonadaceae>Pseudomonas	-	Pseudomonas_alcaligenes	KAM426_36250	3797295..3800966	43263	BREX-system-P-loop-protein-BrxC-[Pseudomonas-alcaligenes]
WP_154949571.1	Type1.BREX	DUF932->?->?->DUF4969-><-SIGMA+OB-Fold+AAA-ATPase+Lon-Protease-BrxL<-PglZ<-ParB+HNH<-N6-METHYLASE<-DUF6079*<-BrxB-BrxF-iSTAND<-BrxA-DNA-binding<-wHTH+WYL+WCX<-?<-XIS-HTH||RHH-><-tRNA<-MerR-HTH	1154	Bacteria>Pseudomonadota>Gammaproteobacteria>Pseudomonadales>Pseudomonadaceae>Pseudomonas	-	Pseudomonas_fluorescens	F8N22_RS18775	172273..175737	294	BREX-system-P-loop-protein-BrxC-[Pseudomonas-fluorescens]
WP_154851174.1	Type1.BREX	<-SF1-SF2-Helicase+REase<-?<-SF1-SF2-Helicase<-HKD-DNase+HKD-DNase<-?||wHTH+WYL+WCX->BrxA-DNA-binding->BrxB-BrxF-iSTAND->DUF6079*->?->?->N6-METHYLASE->NERD+GspA-ATPase->TPR->PglZ->SIGMA+OB-Fold+AAA-ATPase+Lon-Protease-BrxL-><-XIS-HTH	1224	Bacteria>Pseudomonadota>Gammaproteobacteria>Pseudomonadales>Pseudomonadaceae>Pseudomonas	-	Pseudomonas_fluorescens	F8N48_RS14725	264016..267690	294	BREX-system-P-loop-protein-BrxC-[Pseudomonas-fluorescens]
WP_032902483.1	Type1.BREX	<-AAA+wHTH<-?<-SIGMA+OB-Fold+AAA-ATPase+Lon-Protease-BrxL<-PglZ<-ParB+HNH<-?<-MarR-HTH<-MNS+ORC-CDC6<-N6-METHYLASE<-DUF6079*<-BrxB-BrxF-iSTAND<-BrxA-DNA-binding<-ARG<-?||wHTH+WYL+WCX->Arm-DNA-bind_3+Phage_integrase->SF1-SF2-Helicase+REase->XIS-HTH->TOPRIM->	1154	Bacteria>Pseudomonadota>Gammaproteobacteria>Pseudomonadales>Pseudomonadaceae>Pseudomonas	-	Pseudomonas_fluorescens	H098_RS08890	14970..18434	294	BREX-system-P-loop-protein-BrxC-[Pseudomonas-fluorescens]
WP_241666011.1	Type1.BREX	<-TraI-VirD2-RCRE<-ParD_antitoxin||HNH->?-><-wHTH+WYL||ARG->BrxA-DNA-binding->BrxB-BrxF-iSTAND->DUF6079*->DNA-glycosylase->N6-METHYLASE->ParB+HNH->AbiEi_3_N+AbiEi_3->AbiEii->PglZ->SIGMA+OB-Fold+AAA-ATPase+Lon-Protease-BrxL->	1225	Bacteria>Pseudomonadota>Gammaproteobacteria>Pseudomonadales>Pseudomonadaceae>Pseudomonas	-	Pseudomonas_fluorescens	PflCFBP13510_RS04715	495917..499594	294	BREX-system-P-loop-protein-BrxC-[Pseudomonas-fluorescens]
WP_010656375.1	Type1.BREX	<-SF1-SF2-Helicase+REase<-?<-Ntox49<-SF1-SF2-Helicase<-HKD-DNase+HKD-DNase<-?||wHTH+WYL+WCX->BrxA-DNA-binding->BrxB-BrxF-iSTAND->DUF6079*->N6-METHYLASE->sGTP->?->PglZ->SIGMA+OB-Fold+AAA-ATPase+Lon-Protease-BrxL->?-><-tRNA<-MerR-HTH	1223	Bacteria>Pseudomonadota>Gammaproteobacteria>Pseudomonadales>Pseudomonadaceae>Pseudomonas	-	Pseudomonas_fragi	AV641_RS14580	3283222..3286893	296	BREX-system-P-loop-protein-BrxC-[Pseudomonas-fragi]
WP_112249959.1	Type1.BREX	wHTH+WYL+WCX->BrxA-DNA-binding->BrxB-BrxF-iSTAND->DUF6079*->N6-METHYLASE->ParB+HNH->PglZ->SIGMA+OB-Fold+AAA-ATPase+Lon-Protease-BrxL-><-DUF4969<-?<-?<-?<-DUF932	1154	Bacteria>Pseudomonadota>Gammaproteobacteria>Pseudomonadales>Pseudomonadaceae>Pseudomonas	-	Pseudomonas_putida	GT37_RS03100	3180..6644	303	BREX-system-P-loop-protein-BrxC-[Pseudomonas-putida]
WP_046164318.1	Type1.BREX	<-SIGMA+OB-Fold+AAA-ATPase+HKD-DNase-BrxL<-PglZ<-ParB+HNH<-N6-METHYLASE-p<-HerA/FtsK<-SIR2<-REase<-OLD-ABC<-N6-METHYLASE<-DUF6079*<-BrxB-BrxF-iSTAND<-BrxA-DNA-binding<-XIS-HTH<-?||wHTH+WYL+WCX->sGTP->sGTP->sGTP->	1186	Bacteria>Pseudomonadota>Gammaproteobacteria>Pseudomonadales>Pseudomonadaceae>Pseudomonas	-	Pseudomonas_sp._10B238	RT21_RS21355	15645..19205	1586417	BREX-system-P-loop-protein-BrxC-[Pseudomonas-sp.-10B238]
WP_099493679.1	Type1.BREX	<-SIGMA+OB-Fold+AAA-ATPase+Lon-Protease-BrxL<-PglZ<-AbiEii<-AbiEi_3_N+AbiEi_3<-ParB+HNH<-N6-METHYLASE<-DNA-glycosylase<-DUF6079*<-BrxB-BrxF-iSTAND<-BrxA-DNA-binding<-ARG<-?||wHTH+WYL+WCX-><-?<-?||HIN-HTH->	1222	Bacteria>Pseudomonadota>Gammaproteobacteria>Pseudomonadales>Pseudomonadaceae>Pseudomonas	-	Pseudomonas_sp._2588-5	AOA57_22445	341678..345346	1712676	BREX-system-P-loop-protein-BrxC-[Pseudomonas-sp.-2588-5]
WP_098950596.1	Type1.BREX	<-TraI-VirD2-RCRE<-ParD_antitoxin||HNH->?->||BrxA-DNA-binding->BrxB-BrxF-iSTAND->DUF6079*->DNA-glycosylase->N6-METHYLASE->ParB+HNH->AbiEi_3_N+AbiEi_3->AbiEii->PglZ->SIGMA+OB-Fold+AAA-ATPase+Lon-Protease-BrxL->	1222	Bacteria>Pseudomonadota>Gammaproteobacteria>Pseudomonadales>Pseudomonadaceae>Pseudomonas	-	Pseudomonas_sp._FDAARGOS_380	CRN80_16460	3507093..3510761	2018067	BREX-system-P-loop-protein-BrxC-[Pseudomonas-sp.-FDAARGOS_380]
WP_223019012.1	Type1.BREX	<-SIGMA+OB-Fold+AAA-ATPase+Lon-Protease-BrxL<-PglZ<-AbiEii<-AbiEi_3_N+AbiEi_3<-ParB+HNH<-N6-METHYLASE<-DNA-glycosylase<-DUF6079*<-BrxB-BrxF-iSTAND<-BrxA-DNA-binding<-ARG<-?||wHTH+WYL+WCX-><-tRNA<-MerR-HTH	1222	Bacteria>Pseudomonadota>Gammaproteobacteria>Pseudomonadales>Pseudomonadaceae>Pseudomonas	-	Pseudomonas_sp._P867	J1G33_25110	13708..17376	2816050	BREX-system-P-loop-protein-BrxC-[Pseudomonas-sp.-P867]
WP_225609641.1	Type1.BREX	<-wHTH+WYL+WCX||?->JetB->?->RNase->?->BrxA-DNA-binding->BrxB-BrxF-iSTAND->DUF6079*->?->N6-METHYLASE->?->AbiEi_4->AbiEii->PglZ->SIGMA+OB-Fold+AAA-ATPase+Lon-Protease-BrxL->	1225	Bacteria>Pseudomonadota>Gammaproteobacteria>Pseudomonadales>Pseudomonadaceae>Pseudomonas	-	Pseudomonas_sp._PDM11	IB224_RS16070	119835..123512	2769309	BREX-system-P-loop-protein-BrxC-[Pseudomonas-sp.-PDM11]
WP_170056913.1	Type1.BREX	PIN+OST-HTH->HNH->?->?-><-wHTH+WYL+WCX||?->ARG->BrxA-DNA-binding->BrxB-BrxF-iSTAND->DUF6079*->DNA-glycosylase->N6-METHYLASE->ParB+HNH->AbiEi_3_N+AbiEi_3->AbiEii->PglZ->SIGMA+OB-Fold+AAA-ATPase+Lon-Protease-BrxL->JAB-><-MerR-HTH	1222	Bacteria>Pseudomonadota>Gammaproteobacteria>Pseudomonadales>Pseudomonadaceae>Pseudomonas	-	Pseudomonas_sp._WS_5021	HBO19_03685	794090..797758	2717490	BREX-system-P-loop-protein-BrxC-[Pseudomonas-sp.-WS-5021]
WP_240033455.1	Type1.BREX	<-wHTH+WYL+WCX||?->ARG->BrxA-DNA-binding->BrxB-BrxF-iSTAND->DUF6079*->DNA-glycosylase->N6-METHYLASE->ParB+HNH->AbiEi_3_N+AbiEi_3->AbiEii->PglZ->SIGMA+OB-Fold+AAA-ATPase+Lon-Protease-BrxL->?->SMODS->	1225	Bacteria>Pseudomonadota>Gammaproteobacteria>Pseudomonadales>Pseudomonadaceae>Pseudomonas	-	Pseudomonas_viridiflava	EE173_RS11530	5062..8739	33069	BREX-system-P-loop-protein-BrxC-[Pseudomonas-viridiflava]
WP_240043383.1	Type1.BREX	<-Thymidylate_kin<-?||ZnR-><-?<-?<-TOPRIM<-OLD-ABC<-N6-METHYLASE<-DOC+HTH<-DUF6079*<-BrxB-BrxF-iSTAND<-BrxA-DNA-binding<-wHTH+WYL+WCX	1225	Bacteria>Pseudomonadota>Gammaproteobacteria>Pseudomonadales>Pseudomonadaceae>Pseudomonas	-	Pseudomonas_viridiflava	EFA20_RS09810	85838..89515	33069	BREX-system-P-loop-protein-BrxC-[Pseudomonas-viridiflava]
AEJ06724.1	Type1.BREX	HTH+DOC-><-?||Arm-DNA-bind_3+Phage_integrase->wHTH+WYL+WCX->BrxA-DNA-binding->BrxB-BrxF-iSTAND->DUF6079*->?-><-AAA-ATPase<-HIN-HTH||REC-><-ABC-ATPase+SbcC	1223	Bacteria>Pseudomonadota>Gammaproteobacteria>Pseudomonadales>Pseudomonadaceae>Stutzerimonas	GCA_000219605.1	Stutzerimonas_stutzeri	PSTAB_3443	3706589..3710260	316	conserved-hypothetical-protein-[Stutzerimonas-stutzeri]
EPL61008.1	Type1.BREX	<-Phage-HTH<-SIGMA+OB-Fold+AAA-ATPase+HKD-DNase-BrxL<-PglZ<-N6-METHYLASE-p<-HerA/FtsK<-SIR2<-REase<-OLD-ABC<-N6-METHYLASE<-DUF6079*<-BrxB-BrxF-iSTAND<-BrxA-DNA-binding<-XIS-HTH||?->DUF3262->TraM->TraM->TraE->TraK->	1188	Bacteria>Pseudomonadota>Gammaproteobacteria>Pseudomonadales>Pseudomonadaceae>Stutzerimonas	GCA_000416345.1	Stutzerimonas_stutzeri_B1SMN1	B382_18428	65079..68645	1212549	hypothetical-protein-B382_18428-[Stutzerimonas-stutzeri-B1SMN1]
OWG36865.1	Type1.BREX	<-RNase<-?<-JetB<-?<-wHTH+WYL+WCX||XIS-HTH->BrxA-DNA-binding->BrxB-BrxF-iSTAND->DUF6079*->N6-METHYLASE->ParB+HNH->PglZ->SIGMA+OB-Fold+AAA-ATPase+HKD-DNase-BrxL-><-?<-?<-?<-DUF932	1185	Bacteria>Pseudomonadota>Gammaproteobacteria>Pseudomonadales>Pseudomonadaceae>Stutzerimonas	GCA_002193135.1	Stutzerimonas_stutzeri	CAQ69_18770	20186..23743	316	hypothetical-protein-CAQ69_18770-[Stutzerimonas-stutzeri]
PZR87477.1	Type1.BREX	RHH-><-TetR-HTH||MODE-HTH+PBPII->wHTH+WYL+WCX->BrxA-DNA-binding->BrxB-BrxF-iSTAND->DUF6079*->PglZ->SIGMA+OB-Fold+AAA-ATPase+HKD-DNase-BrxL-><-?<-HAD+HAD	1179	Bacteria>Pseudomonadota>Gammaproteobacteria>Pseudomonadales>Pseudomonadaceae>Stutzerimonas	GCA_003243385.1	Stutzerimonas_stutzeri	DI537_26065	4408..7947	316	MAG:-BREX-system-P-loop-protein-BrxC-[Stutzerimonas-stutzeri]
QOZ94737.1	Type1.BREX	<-HIRAN||HIN-HTH+PAIREDC-HTH-><-?<-N6-METHYLASE-p<-N6-METHYLASE<-DOC+HTH<-?<-?<-DUF6079*<-BrxB-BrxF-iSTAND<-BrxA-DNA-binding<-wHTH+WYL+WCX<-?<-Phage_AlpA<-?<-Arm-DNA-bind_3+Phage_integrase	1223	Bacteria>Pseudomonadota>Gammaproteobacteria>Pseudomonadales>Pseudomonadaceae>Stutzerimonas	GCA_015291885.1	Stutzerimonas_stutzeri	Pstu14405_04940	1078194..1081865	316	BREX-system-P-loop-protein-BrxC-[Stutzerimonas-stutzeri]
MCQ2029875.1	Type1.BREX	<-sGTP<-sGTP<-sGTP<-wHTH+WYL+WCX||?-><-?||XIS-HTH->BrxA-DNA-binding->BrxB-BrxF-iSTAND->DUF6079*->N6-METHYLASE->OLD-ABC->NERD->RVT->N6-METHYLASE-p->ParB+HNH->PglZ->SIGMA+OB-Fold+AAA-ATPase+HKD-DNase-BrxL->RHH_1->	1186	Bacteria>Pseudomonadota>Gammaproteobacteria>Pseudomonadales>Pseudomonadaceae>Stutzerimonas	GCA_024397535.1	[Pseudomonas]_zhaodongensis	NA613_09780	17231..20791	1176257	BREX-system-P-loop-protein-BrxC-[[Pseudomonas]
MCQ4256509.1	Type1.BREX	<-SF1-SF2-Helicase<-REase<-SIGMA+OB-Fold+AAA-ATPase+Lon-Protease-BrxL<-PglZ<-ParB+HNH<-?<-MarR-HTH<-MNS+ORC-CDC6<-N6-METHYLASE<-DUF6079*<-BrxB-BrxF-iSTAND<-BrxA-DNA-binding<-REase<-NERD||?->DUF932->	1154	Bacteria>Pseudomonadota>Gammaproteobacteria>Pseudomonadales>Pseudomonadaceae>Stutzerimonas	GCA_024448895.1	Stutzerimonas_stutzeri	NA619_00645	147157..150621	316	BREX-system-P-loop-protein-BrxC-[Stutzerimonas-stutzeri]
MCQ4319996.1	Type1.BREX	Phage_integrase->?->?->?-><-?<-?||wHTH+WYL+WCX->BrxA-DNA-binding->BrxB-BrxF-iSTAND->DUF6079*->N6-METHYLASE->sGTP->?->PglZ->SIGMA+OB-Fold+AAA-ATPase+Lon-Protease-BrxL->	1223	Bacteria>Pseudomonadota>Gammaproteobacteria>Pseudomonadales>Pseudomonadaceae>Stutzerimonas	GCA_024448935.1	Stutzerimonas_stutzeri	NA637_07215	17580..21251	316	BREX-system-P-loop-protein-BrxC-[Stutzerimonas-stutzeri]
WP_232244016.1	Type1.BREX	MazE_antitoxin->Arm-DNA-bind_3+Phage_integrase->?->Phage_AlpA->?->wHTH+WYL+WCX->BrxA-DNA-binding->BrxB-BrxF-iSTAND->DUF6079*->?-><-AAA-ATPase<-HIN-HTH||REC-><-ABC-ATPase+SbcC	1225	Bacteria>Pseudomonadota>Gammaproteobacteria>Pseudomonadales>Pseudomonadaceae>Stutzerimonas	GCF_000219605.1	Stutzerimonas_stutzeri	I6G32_RS07530	1635442..1639119	316	BREX-system-P-loop-protein-BrxC-[Stutzerimonas-stutzeri]
WP_003283611.1	Type1.BREX	<-N6-METHYLASE-p<-RVT<-N6-METHYLASE<-DOC+HTH<-?<-DUF6079*<-BrxB-BrxF-iSTAND<-BrxA-DNA-binding<-wHTH+WYL+WCX<-?<-Phage_AlpA	1225	Bacteria>Pseudomonadota>Gammaproteobacteria>Pseudomonadales>Pseudomonadaceae>Stutzerimonas	GCF_000237885.1	Stutzerimonas_stutzeri	PSTZOBELL_RS12590	11831..15508	316	BREX-system-P-loop-protein-BrxC-[Stutzerimonas-stutzeri]
WP_014819601.1	Type1.BREX	<-Phage-HTH<-SIGMA+OB-Fold+AAA-ATPase+HKD-DNase-BrxL<-PglZ<-N6-METHYLASE-p<-HerA/FtsK<-SIR2<-REase<-OLD-ABC<-N6-METHYLASE<-DUF6079*<-BrxB-BrxF-iSTAND<-BrxA-DNA-binding<-XIS-HTH||?->DUF3262->TraM->TraM->TraE->TraK->	1188	Bacteria>Pseudomonadota>Gammaproteobacteria>Pseudomonadales>Pseudomonadaceae>Stutzerimonas	GCF_000267545.1	Stutzerimonas_stutzeri_subgroup	A458_RS28085	1395712..1399278	578833	MULTISPECIES:-BREX-system-P-loop-protein-BrxC-[Stutzerimonas-stutzeri-subgroup]
WP_017245161.1	Type1.BREX	<-PglZ<-AbiEii<-AbiEi_4<-?<-?<-ParB+HNH<-N6-METHYLASE<-DNA-glycosylase<-ParB+HNH<-DUF6079*<-BrxB-BrxF-iSTAND<-BrxA-DNA-binding||?->Arm-DNA-bind_2+Phage_int_SAM_3+Phage_integrase-><-?<-?||HTH_3->XIS-HTH->	1220	Bacteria>Pseudomonadota>Gammaproteobacteria>Pseudomonadales>Pseudomonadaceae>Stutzerimonas	GCF_000280555.1	Stutzerimonas_stutzeri	A598_RS0110895	41008..44670	316	BREX-system-P-loop-protein-BrxC-[Stutzerimonas-stutzeri]
WP_053526945.1	Type1.BREX	<-HTH_3<-?<-SIGMA+OB-Fold+AAA-ATPase+Lon-Protease-BrxL<-PglZ<-HNH<-OLD-ABC<-?<-?<-N6-METHYLASE<-DUF6079*<-BrxB-BrxF-iSTAND<-BrxA-DNA-binding<-wHTH+WYL+WCX||?->RVT->?-><-SF1-SF2-Helicase	1223	Bacteria>Pseudomonadota>Gammaproteobacteria>Pseudomonadales>Pseudomonadaceae>Stutzerimonas	GCF_001276475.1	Stutzerimonas_stutzeri	ABW53_RS00980	14361..18032	316	BREX-system-P-loop-protein-BrxC-[Stutzerimonas-stutzeri]
WP_245890967.1	Type1.BREX	JetB->?->RNase->?->BrxA-DNA-binding->BrxB-BrxF-iSTAND->DUF6079*->AbiGi+seqA+RAMA->N6-METHYLASE->?->REase+Bacteriocin_IIc->?->AbiEi_3_N+AbiEi_3->AbiEii->PglZ->SIGMA+OB-Fold+AAA-ATPase+Lon-Protease-BrxL->	1225	Bacteria>Pseudomonadota>Gammaproteobacteria>Pseudomonadales>Pseudomonadaceae>Stutzerimonas	GCF_002929225.1	Stutzerimonas_kunmingensis	CW305_RS04090	634964..638641	1211807	BREX-system-P-loop-protein-BrxC-[Stutzerimonas-kunmingensis]
WP_125460588.1	Type1.BREX	<-PglZ<-AbiEii<-AbiEi_3_N+AbiEi_3<-SF1-SF2-Helicase<-HKD-DNase+HKD-DNase<-REase<-?<-N6-METHYLASE<-AbiGi+seqA+RAMA<-DUF6079*<-BrxB-BrxF-iSTAND<-BrxA-DNA-binding<-DUF772+DDE_Tnp_1||?->Arm-DNA-bind_2+Phage_int_SAM_3+Phage_integrase->	1220	Bacteria>Pseudomonadota>Gammaproteobacteria>Pseudomonadales>Pseudomonadaceae>Stutzerimonas	GCF_003936745.1	Stutzerimonas_stutzeri	EGJ43_RS19065	24827..28489	316	BREX-system-P-loop-protein-BrxC-[Stutzerimonas-stutzeri]
WP_125856771.1	Type1.BREX	<-LexA-protease<-wHTH+WYL+WCX||ARG->?->wHTH+WYL+WCX->BrxA-DNA-binding->BrxB-BrxF-iSTAND->DUF6079*->N6-METHYLASE->ParB+HNH->OLD-ABC->HNH->PglZ->SIGMA+OB-Fold+AAA-ATPase+Lon-Protease-BrxL->MukF->JetB->DUF3584+DUF5401->	1154	Bacteria>Pseudomonadota>Gammaproteobacteria>Pseudomonadales>Pseudomonadaceae>Stutzerimonas	GCF_003940515.1	Stutzerimonas_stutzeri	EGJ36_RS18105	6035..9499	316	BREX-system-P-loop-protein-BrxC-[Stutzerimonas-stutzeri]
WP_200291399.1	Type1.BREX	Peptidase_A8-><-?<-?||LexA-protease->?-><-Phage_int_SAM<-Integrase_1||BrxA-DNA-binding->BrxB-BrxF-iSTAND->DUF6079*->CR-ATPase5+REase->N6-METHYLASE->GP+REase->N6-METHYLASE-p->SIR2->HerA/FtsK->?->N6-METHYLASE-p->S1COLD->	1223	Bacteria>Pseudomonadota>Gammaproteobacteria>Pseudomonadales>Pseudomonadaceae>Stutzerimonas	GCF_016599775.1	Stutzerimonas_balearica	I6H70_RS19230	4083458..4087129	74829	BREX-system-P-loop-protein-BrxC-[Stutzerimonas-balearica]
WP_218422415.1	Type1.BREX	<-HTH_Tnp_1+HTH_21+rve+rve_3<-?||?-><-ANK+ANK+ANK||RADICAL-SAM->HicA_toxin->BrxA-DNA-binding->BrxB-BrxF-iSTAND->DUF6079*->N6-METHYLASE->?->HNH+GCN1->?->?->?->?->ParB+HNH->MNS+REase->	1154	Bacteria>Pseudomonadota>Gammaproteobacteria>Pseudomonadales>Pseudomonadaceae>Stutzerimonas	GCF_019203165.1	Stutzerimonas_stutzeri	KUD99_RS07220	1491661..1495125	316	BREX-system-P-loop-protein-BrxC-[Stutzerimonas-stutzeri]
WP_218421912.1	Type1.BREX	<-SIGMA+OB-Fold+AAA-ATPase+Lon-Protease-BrxL<-PglZ<-REase<-N6-METHYLASE-p<-?<-HerA/FtsK<-SIR2<-N6-METHYLASE<-DUF6079*<-BrxB-BrxF-iSTAND<-BrxA-DNA-binding<-wHTH+WYL+WCX||?->MORC->RVT->	1223	Bacteria>Pseudomonadota>Gammaproteobacteria>Pseudomonadales>Pseudomonadaceae>Stutzerimonas	GCF_019203165.1	Stutzerimonas_stutzeri	KUD99_RS18220	3895806..3899477	316	BREX-system-P-loop-protein-BrxC-[Stutzerimonas-stutzeri]
WP_125870426.1	Type1.BREX	<-DUF932<-?||NERD->REase->BrxA-DNA-binding->BrxB-BrxF-iSTAND->DUF6079*->?->HNH+GCN1->?->PglZ->SIGMA+OB-Fold+AAA-ATPase+Lon-Protease-BrxL->REase->SF1-SF2-Helicase-><-Glycos_transf	1154	Bacteria>Pseudomonadota>Gammaproteobacteria>Pseudomonadales>Pseudomonadaceae>Stutzerimonas	GCF_024199075.1	Stutzerimonas_stutzeri	NLG33_RS12680	207505..210969	316	BREX-system-P-loop-protein-BrxC-[Stutzerimonas-stutzeri]
WP_255830664.1	Type1.BREX	<-sGTP<-sGTP<-sGTP<-wHTH+WYL+WCX||?-><-?||XIS-HTH->BrxA-DNA-binding->BrxB-BrxF-iSTAND->DUF6079*->N6-METHYLASE->OLD-ABC->NERD->RVT->N6-METHYLASE-p->ParB+HNH->PglZ->SIGMA+OB-Fold+AAA-ATPase+HKD-DNase-BrxL->RHH_1->	1186	Bacteria>Pseudomonadota>Gammaproteobacteria>Pseudomonadales>Pseudomonadaceae>Stutzerimonas	GCF_024397535.1	[Pseudomonas]_zhaodongensis	NA613_RS09805	17231..20791	1176257	BREX-system-P-loop-protein-BrxC-[[Pseudomonas]
WP_255958979.1	Type1.BREX	<-SF1-SF2-Helicase<-REase<-SIGMA+OB-Fold+AAA-ATPase+Lon-Protease-BrxL<-PglZ<-ParB+HNH<-?<-MarR-HTH<-MNS+ORC-CDC6<-N6-METHYLASE<-DUF6079*<-BrxB-BrxF-iSTAND<-BrxA-DNA-binding<-REase<-NERD||?->DUF932->	1154	Bacteria>Pseudomonadota>Gammaproteobacteria>Pseudomonadales>Pseudomonadaceae>Stutzerimonas	GCF_024448895.1	Stutzerimonas_stutzeri	NA619_RS00645	147157..150621	316	BREX-system-P-loop-protein-BrxC-[Stutzerimonas-stutzeri]
WP_269263428.1	Type1.BREX	<-HerA/FtsK<-SIR2<-N6-METHYLASE-p<-GP+REase<-N6-METHYLASE<-DOC+HTH<-?<-?<-DUF6079*<-BrxB-BrxF-iSTAND<-BrxA-DNA-binding||Integrase_1->Phage_int_SAM-><-?||DSBH->?-><-Phage_integrase||DUF772+DDE_Tnp_1->	1223	Bacteria>Pseudomonadota>Gammaproteobacteria>Pseudomonadales>Pseudomonadaceae>Stutzerimonas	GCF_027158265.1	Stutzerimonas_balearica	O3V18_RS18735	22059..25730	74829	BREX-system-P-loop-protein-BrxC-[Stutzerimonas-balearica]
WP_238586968.1	Type1.BREX	<-TraK<-TraE<-TraM<-TraM<-DUF3262<-?||XIS-HTH->BrxA-DNA-binding->BrxB-BrxF-iSTAND->DUF6079-p*->PglZ->SIGMA+OB-Fold+AAA-ATPase+HKD-DNase-BrxL->Phage-HTH-><-DUF4400<-VirD4-TraG	718	Bacteria>Pseudomonadota>Gammaproteobacteria>Pseudomonadales>Pseudomonadaceae>Stutzerimonas	-	Stutzerimonas_degradans	.	57411..59567	2968968	BREX-system-P-loop-protein-BrxC-[Stutzerimonas-degradans]
BBJ02099.1	Type1.BREX	<-TraK<-TraE<-TraM<-TraM<-DUF3262<-?||XIS-HTH->BrxA-DNA-binding->BrxB-BrxF-iSTAND->DUF6079-p*->PglZ->SIGMA+OB-Fold+AAA-ATPase+HKD-DNase-BrxL->Phage-HTH-><-DUF4400<-VirD4-TraG	718	Bacteria>Pseudomonadota>Gammaproteobacteria>Pseudomonadales>Pseudomonadaceae>Stutzerimonas	-	Stutzerimonas_degradans	.	57411..59567	2968968	uncharacterized-protein-sporadically-distributed-in-bacteria-and-archaea-[Stutzerimonas-degradans]
WP_020308058.1	Type1.BREX	<-Phage-HTH<-SIGMA+OB-Fold+AAA-ATPase+HKD-DNase-BrxL<-PglZ<-N6-METHYLASE-p<-HerA/FtsK<-SIR2<-REase<-OLD-ABC<-N6-METHYLASE<-DUF6079*<-BrxB-BrxF-iSTAND<-BrxA-DNA-binding<-XIS-HTH||?->DUF3262->TraM->TraM->TraE->TraK->	1188	Bacteria>Pseudomonadota>Gammaproteobacteria>Pseudomonadales>Pseudomonadaceae>Stutzerimonas	-	Stutzerimonas_stutzeri	B382_18428	65079..68645	316	BREX-system-P-loop-protein-BrxC-[Pseudomonas-stutzeri]
WP_095461351.1	Type1.BREX	<-wHTH+WYL+WCX-p||?-><-?<-wHTH+WYL+WCX||XIS-HTH->BrxA-DNA-binding->BrxB-BrxF-iSTAND->DUF6079*->N6-METHYLASE->OLD-ABC->TOPRIM->PglZ->SIGMA+OB-Fold+AAA-ATPase+Lon-Protease-BrxL->MukF->JetB->	1154	Bacteria>Pseudomonadota>Gammaproteobacteria>Pseudomonadales>Pseudomonadaceae>Stutzerimonas	-	Stutzerimonas_stutzeri	BV581_21185	4303..7767	316	BREX-system-P-loop-protein-BrxC-[Stutzerimonas-stutzeri]
WP_045633496.1	Type1.BREX	<-RNase<-?<-JetB<-?<-wHTH+WYL+WCX||XIS-HTH->BrxA-DNA-binding->BrxB-BrxF-iSTAND->DUF6079*->N6-METHYLASE->ParB+HNH->PglZ->SIGMA+OB-Fold+AAA-ATPase+HKD-DNase-BrxL-><-?<-?<-?<-DUF932	1185	Bacteria>Pseudomonadota>Gammaproteobacteria>Pseudomonadales>Pseudomonadaceae>Stutzerimonas	-	Stutzerimonas_stutzeri	CAQ69_18770	20186..23743	316	MULTISPECIES:-BREX-system-P-loop-protein-BrxC-[Gammaproteobacteria]
WP_053105412.1	Type1.BREX	METHYLASE->?->?-><-?<-?<-?||wHTH+WYL+WCX->BrxA-DNA-binding->BrxB-BrxF-iSTAND->DUF6079*->N6-METHYLASE->ParB+HNH->OLD-ABC->TOPRIM->HEPN->PglZ->SIGMA+OB-Fold+AAA-ATPase+Lon-Protease-BrxL->Arm-DNA-bind_3+Phage_integrase-><-SIGMA+OB-Fold+AAA-ATPase+Lon-Protease-BrxL	1155	Bacteria>Pseudomonadota>Gammaproteobacteria>Pseudomonadales>Pseudomonadaceae>Thiopseudomonas	GCF_001267275.1	Thiopseudomonas_alkaliphila	AKN90_RS00615	123321..126788	1697053	BREX-system-P-loop-protein-BrxC-[Thiopseudomonas-alkaliphila]
MBX9914998.1	Type1.BREX	<-N6-METHYLASE<-Schlafen+HATPase+wHTH<-DUF6079*<-BrxB-BrxF-iSTAND<-BrxA-DNA-binding<-wHTH+WYL+WCX	1220	Bacteria>Pseudomonadota>Gammaproteobacteria>Pseudomonadales>Pseudomonadaceae>unclassified_Pseudomonadaceae	GCA_019745275.1	Pseudomonadaceae_bacterium	K2Y25_13980	5013..8675	1914538	BREX-system-P-loop-protein-BrxC-[Pseudomonadaceae-bacterium]
MBX9762031.1	Type1.BREX	<-N6-METHYLASE<-DUF6079*<-BrxB-BrxF-iSTAND<-BrxA-DNA-binding<-wHTH+WYL+WCX||?->MORC->	1223	Bacteria>Pseudomonadota>Gammaproteobacteria>Pseudomonadales>Pseudomonadaceae>unclassified_Pseudomonadaceae	GCA_019745805.1	Pseudomonadaceae_bacterium	K2Y24_03255	4991..8662	1914538	BREX-system-P-loop-protein-BrxC-[Pseudomonadaceae-bacterium]
MBX9763927.1	Type1.BREX	<-LexA-protease<-SF1-SF2-Helicase<-?<-wHTH+WYL+WCX||ARG->?->wHTH+WYL+WCX->BrxA-DNA-binding->BrxB-BrxF-iSTAND->DUF6079*->N6-METHYLASE->	1154	Bacteria>Pseudomonadota>Gammaproteobacteria>Pseudomonadales>Pseudomonadaceae>unclassified_Pseudomonadaceae	GCA_019745805.1	Pseudomonadaceae_bacterium	K2Y24_13005	12458..15922	1914538	BREX-system-P-loop-protein-BrxC-[Pseudomonadaceae-bacterium]
MAB42111.1	Type1.BREX	<-SIGMA+OB-Fold+AAA-ATPase+Lon-Protease-BrxL<-PglZ<-TOPRIM<-OLD-ABC<-ParB+HNH<-N6-METHYLASE<-DUF6079*<-BrxB-BrxF-iSTAND<-BrxA-DNA-binding||wHTH+WYL+WCX->	1154	Bacteria>Pseudomonadota>Gammaproteobacteria>Pseudomonadales>unclassified_Pseudomonadales	GCA_002684155.1	Pseudomonadales_bacterium	CMK77_07490	13688..17152	1891229	hypothetical-protein-[Pseudomonadales-bacterium]
MAQ23221.1	Type1.BREX	<-Calcineurin+Calcineurin-SbcD<-PglZ<-ParB+HNH<-N6-METHYLASE<-REase<-OLD-ABC<-Schlafen+HATPase+wHTH+HTH<-DUF6079*<-BrxB-BrxF-iSTAND<-BrxA-DNA-binding<-XIS-HTH<-MobI||XIS-HTH->AAA+HTH->XIS-HTH-><-Arm-DNA-bind_3+Phage_integrase	1193	Bacteria>Pseudomonadota>Gammaproteobacteria>Pseudomonadales>unclassified_Pseudomonadales	GCA_002701315.1	Pseudomonadales_bacterium	CMK83_03295	18015..21596	1891229	hypothetical-protein-[Pseudomonadales-bacterium]
MBI27695.1	Type1.BREX	Arm-DNA-bind_3+Phage_integrase-><-XIS-HTH<-AAA+HTH<-XIS-HTH||MobI->XIS-HTH->BrxA-DNA-binding->BrxB-BrxF-iSTAND->DUF6079*->	1009	Bacteria>Pseudomonadota>Gammaproteobacteria>Pseudomonadales>unclassified_Pseudomonadales	GCA_002718075.1	Pseudomonadales_bacterium	CMK90_12665	612690..615718	1891229	hypothetical-protein-[Pseudomonadales-bacterium]

Protein-ID	System	Gene-Neighbourhoods	Length	Taxonomy	Assembly-ID	Strain	Locus-Tag	Genomic-coordinates	TaxID	Fasta-define
MBV1884607.1	Type1.BREX	<-N6-METHYLASE<-?<-AbiEii<-MarR-HTH<-DUF6079*<-BrxB-BrxF-iSTAND<-BrxA-DNA-binding<-wHTH+WYL+WCX	1218	Bacteria>Pseudomonadota>Gammaproteobacteria>Pseudomonadales>unclassified_Pseudomonadales	GCA_019090455.1	Pseudomonadales_bacterium	KUG82_23410	5916..9572	1891229	BREX-system-P-loop-protein-BrxC-[Pseudomonadales-bacterium]
MCB1656877.1	Type1.BREX	<-N6-METHYLASE-p<-Couple_hipA+HipA_C<-HTH_3<-DUF6079*<-BrxB-BrxF-iSTAND<-BrxA-DNA-binding<-wHTH+WYL+WCX<-?<-HTH_Tnp_1+HTH_21+rve+rve_3	1216	Bacteria>Pseudomonadota>Gammaproteobacteria>Pseudomonadales>unclassified_Pseudomonadales	GCA_020443205.1	Pseudomonadales_bacterium	KDI39_01465	3417..7067	1891229	BREX-system-P-loop-protein-BrxC-[Pseudomonadales-bacterium]
MCG8312832.1	Type1.BREX	GDResolvase+HIN-HTH-><-?||?->?->XIS-HTH->BrxA-DNA-binding->BrxB-BrxF-iSTAND->DUF6079-p*->	468	Bacteria>Pseudomonadota>Gammaproteobacteria>Pseudomonadales>unclassified_Pseudomonadales	GCA_022360035.1	Pseudomonadales_bacterium	MI976_06400	7277..8681	1891229	BREX-system-P-loop-protein-BrxC-[Pseudomonadales-bacterium]
MCG8610925.1	Type1.BREX	<-N6-METHYLASE<-HTH+REase<-DUF6079*<-BrxB-BrxF-iSTAND<-BrxA-DNA-binding<-wHTH+WYL+WCX||ParA-Soj-PloopNTPase->	1229	Bacteria>Pseudomonadota>Gammaproteobacteria>Pseudomonadales>unclassified_Pseudomonadales	GCA_022361325.1	Pseudomonadales_bacterium	MI864_10365	4826..8515	1891229	BREX-system-P-loop-protein-BrxC-[Pseudomonadales-bacterium]
MCJ8311755.1	Type1.BREX	<-sGTP<-sGTP<-?<-SIGMA+OB-Fold+AAA-ATPase+Lon-Protease-BrxL<-PglZ<-N6-METHYLASE<-DUF6079*<-BrxB-BrxF-iSTAND<-BrxA-DNA-binding<-HNH<-?||?->?-><-SF1-SF2-Helicase<-HKD-DNase+HKD-Rease	1233	Bacteria>Pseudomonadota>Gammaproteobacteria>Pseudomonadales>unclassified_Pseudomonadales	GCA_022968985.1	Pseudomonadales_bacterium	HRU38_08495	18482..22183	1891229	BREX-system-P-loop-protein-BrxC-[Pseudomonadales-bacterium]
MCP5320940.1	Type1.BREX	[illegible]	1148	Bacteria>Pseudomonadota>Gammaproteobacteria>Pseudomonadales>unclassified_Pseudomonadales	GCA_024234165.1	Pseudomonadales_bacterium	H7A12_08980	738054..741500	1891229	BREX-system-P-loop-protein-BrxC-[Pseudomonadales-bacterium]
MCP5344826.1	Type1.BREX	<-PALP<-SIGMA+OB-Fold+AAA-ATPase+Lon-Protease-BrxL<-PglZ<-PHP+SbcC<-N6-METHYLASE<-REase<-OLD-ABC<-DUF6079-p*	834	Bacteria>Pseudomonadota>Gammaproteobacteria>Pseudomonadales>unclassified_Pseudomonadales	GCA_024234345.1	Pseudomonadales_bacterium	H7A05_09410	50993..53498	1891229	BREX-system-P-loop-protein-BrxC-[Pseudomonadales-bacterium]
MCP5331432.1	Type1.BREX	[illegible]	1145	Bacteria>Pseudomonadota>Gammaproteobacteria>Pseudomonadales>unclassified_Pseudomonadales	GCA_024234365.1	Pseudomonadales_bacterium	H7A07_10830	172054..175491	1891229	BREX-system-P-loop-protein-BrxC-[Pseudomonadales-bacterium]
MCP5171278.1	Type1.BREX	TraG_N-><-?<-?<-?<-?<-?||wHTH+WYL+WCX->BrxA-DNA-binding->BrxB-BrxF-iSTAND->DUF6079*->MukF->JetB->DUF3584+DUF5401->RNase->MarR-HTH->AbiEii->?->N6-METHYLASE->ParB+HNH->	1216	Bacteria>Pseudomonadota>Gammaproteobacteria>Pseudomonadales>unclassified_Pseudomonadales	GCA_024234435.1	Pseudomonadales_bacterium	H7A02_03290	695235..698885	1891229	BREX-system-P-loop-protein-BrxC-[Pseudomonadales-bacterium]
MCP5179014.1	Type1.BREX	[illegible]	1153	Bacteria>Pseudomonadota>Gammaproteobacteria>Pseudomonadales>unclassified_Pseudomonadales	-	Pseudomonadales_bacterium	H6994_03785	340860..344321	1891229	BREX-system-P-loop-protein-BrxC-[Pseudomonadales-bacterium]
WP_101840169.1	Type1.BREX	ZnR+TOPRIM+TOPRIM->SIGMA-HTH+SIGMA-HTH+SIGMA-HTH-><-?<-wHTH+WYL+WCX||?->HEPN->BrxA-DNA-binding->BrxB-BrxF-iSTAND->DUF6079*->N6-METHYLASE->	1210	Bacteria>Pseudomonadota>Gammaproteobacteria>Thiotrichales>Piscirickettsiaceae>Hydrogenovibrio	GCF_002848135.1	Hydrogenovibrio_sp._SC-1	CYQ88_RS04565	151062..154694	2065820	BREX-system-P-loop-protein-BrxC-[Hydrogenovibrio-sp.-SC-1]
WP_007146327.1	Type1.BREX	[illegible]	1179	Bacteria>Pseudomonadota>Gammaproteobacteria>Thiotrichales>Piscirickettsiaceae>Methylophaga	GCF_000214595.1	Methylophaga_aminisulfidivorans	MAMP_RS12010	87136..90675	230105	BREX-system-P-loop-protein-BrxC-[Methylophaga-aminisulfidivorans]
CAA6806547.1	Type1.BREX	<-ParB+HNH<-N6-METHYLASE<-N6-METHYLASE-p<-?<-REase<-ABC-ATPase<-DUF6079-p*	849	Bacteria>Pseudomonadota>Gammaproteobacteria>Thiotrichales>Thiotrichaceae>environmental_samples	-	uncultured_Thiotrichaceae_bacterium	HELGO_WM31114	6431..8980	298394	FIG00607287:-hypothetical-protein,-partial-[uncultured-Thiotrichaceae-bacterium]
WP_022954802.1	Type1.BREX	CR-ATPase5+REase-><-?||wHTH+WYL+WCX->BrxA-DNA-binding->BrxB-BrxF-iSTAND->DUF6079*->N6-METHYLASE->OLD-ABC->TOPRIM->PglZ->SIGMA+OB-Fold+AAA-ATPase+Lon-Protease-BrxL->	1222	Bacteria>Pseudomonadota>Gammaproteobacteria>Thiotrichales>Thiotrichaceae>Leucothrix	GCF_000419525.1	Leucothrix_mucor	LEUMU_RS0123780	65328..68996	45248	BREX-system-P-loop-protein-BrxC-[Leucothrix-mucor]
OQX14377.1	Type1.BREX	[illegible]	1145	Bacteria>Pseudomonadota>Gammaproteobacteria>Thiotrichales>Thiotrichaceae>Thiothrix	GCA_002083875.1	Thiothrix_lacustris	BWK73_09690	20114..23551	525917	hypothetical-protein-BWK73_09690-[Thiothrix-lacustris]
QLQ31990.1	Type1.BREX	[illegible]	1209	Bacteria>Pseudomonadota>Gammaproteobacteria>Thiotrichales>Thiotrichaceae>Thiothrix	GCA_013693955.1	Candidatus_Thiothrix_singaporensis	HZT40_10770	2133663..2137292	2799669	BREX-system-P-loop-protein-BrxC-[Candidatus-Thiothrix-singaporensis]
WP_002710590.1	Type1.BREX	[illegible]	1209	Bacteria>Pseudomonadota>Gammaproteobacteria>Thiotrichales>Thiotrichaceae>Thiothrix	GCF_000260135.1	Thiothrix_nivea	THINI_RS21415	4300442..4304071	1031	BREX-system-P-loop-protein-BrxC-[Thiothrix-nivea]
WP_210225661.1	Type1.BREX	[illegible]	1173	Bacteria>Pseudomonadota>Gammaproteobacteria>Thiotrichales>Thiotrichaceae>Thiothrix	GCF_017901155.1	Candidatus_Thiothrix_anitrata	J8380_RS10820	2094889..2098410	2823902	BREX-system-P-loop-protein-BrxC-[Candidatus-Thiothrix-anitrata]
MBL1259972.1	Type1.BREX	[illegible]	1219	Bacteria>Pseudomonadota>Gammaproteobacteria>Thiotrichales>Thiotrichaceae>unclassified_Thiotrichaceae	GCA_002401765.2	Thiotrichaceae_bacterium	COB33_005510	47175..50834	2030882	BREX-system-P-loop-protein-BrxC-[Thiotrichaceae-bacterium]
PCJ88241.1	Type1.BREX	[illegible]	1177	Bacteria>Pseudomonadota>Gammaproteobacteria>Thiotrichales>Thiotrichaceae>unclassified_Thiotrichaceae	GCA_002746365.1	Thiotrichaceae_bacterium	COA54_02955	15853..19386	2030882	hypothetical-protein-COA54_02955-[Thiotrichaceae-bacterium]
MBD3820859.1	Type1.BREX	[illegible]	1191	Bacteria>Pseudomonadota>Gammaproteobacteria>Thiotrichales>unclassified_Thiotrichales	GCA_014764155.1	Thiotrichales_bacterium	IE914_01145	1632..5207	2026796	BREX-system-P-loop-protein-BrxC-[Thiotrichales-bacterium]
MBT3348501.1	Type1.BREX	[illegible]	1170	Bacteria>Pseudomonadota>Gammaproteobacteria>Thiotrichales>unclassified_Thiotrichales	GCA_018648495.1	Thiotrichales_bacterium	HN398_08700	13875..17387	2026796	BREX-system-P-loop-protein-BrxC-[Thiotrichales-bacterium]
MBK85426.1	Type1.BREX	[illegible]	1217	Bacteria>Pseudomonadota>Gammaproteobacteria>unclassified_Gammaproteobacteria	GCA_002721025.1	Gammaproteobacteria_bacterium	CMD81_16515	7659..11312	1913989	ATPase-[Gammaproteobacteria-bacterium]
HAG71474.1	Type1.BREX	[illegible]	416	Bacteria>Pseudomonadota>Gammaproteobacteria>unclassified_Gammaproteobacteria	GCA_003450175.1	Gammaproteobacteria_bacterium	DCL66_04645	1..1249	1913989	BREX-system-P-loop-protein-BrxC-[Gammaproteobacteria-bacterium]
RLA52302.1	Type1.BREX	[illegible]	1210	Bacteria>Pseudomonadota>Gammaproteobacteria>unclassified_Gammaproteobacteria	GCA_003646365.1	Gammaproteobacteria_bacterium	DRR42_07940	3455..7087	1913989	BREX-system-P-loop-protein-BrxC-[Gammaproteobacteria-bacterium]
TXH19452.1	Type1.BREX	[illegible]	1027	Bacteria>Pseudomonadota>Gammaproteobacteria>unclassified_Gammaproteobacteria	GCA_008012265.1	Gammaproteobacteria_bacterium	E6R00_01845	1..3080	1913989	BREX-system-P-loop-protein-BrxC,-partial-[Gammaproteobacteria-bacterium]
QFY90721.2	Type1.BREX	[illegible]	1179	Bacteria>Pseudomonadota>Gammaproteobacteria>unclassified_Gammaproteobacteria	GCA_009497875.2	gamma_proteobacterium_SS-5	D5125_15330	2185771..2189310	947516	BREX-system-P-loop-protein-BrxC-[gamma-proteobacterium-SS-5]
MXY51046.1	Type1.BREX	[illegible]	1251	Bacteria>Pseudomonadota>Gammaproteobacteria>unclassified_Gammaproteobacteria	GCA_009837725.1	Gammaproteobacteria_bacterium	F4Y86_00740	6119..9874	1913989	BREX-system-P-loop-protein-BrxC-[Gammaproteobacteria-bacterium]
MYB36894.1	Type1.BREX	[illegible]	1146	Bacteria>Pseudomonadota>Gammaproteobacteria>unclassified_Gammaproteobacteria	GCA_009838905.1	Gammaproteobacteria_bacterium	F4Y26_05795	15436..18876	1913989	BREX-system-P-loop-protein-BrxC-[Gammaproteobacteria-bacterium]
NCA89504.1	Type1.BREX	[illegible]	1152	Bacteria>Pseudomonadota>Gammaproteobacteria>unclassified_Gammaproteobacteria	GCA_009928645.1	Gammaproteobacteria_bacterium	EOM92_11510	6384..9842	1913989	BREX-system-P-loop-protein-BrxC-[Gammaproteobacteria-bacterium]
HFD80332.1	Type1.BREX	[illegible]	1156	Bacteria>Pseudomonadota>Gammaproteobacteria>unclassified_Gammaproteobacteria	GCA_011321775.1	Gammaproteobacteria_bacterium	ENK05_08090	12367..15837	1913989	BREX-system-P-loop-protein-BrxC-[Gammaproteobacteria-bacterium]
NLY14765.1	Type1.BREX	[illegible]	438	Bacteria>Pseudomonadota>Gammaproteobacteria>unclassified_Gammaproteobacteria	GCA_012728415.1	Gammaproteobacteria_bacterium	GXZ05_00075	12795..14111	1913989	hypothetical-protein-[Gammaproteobacteria-bacterium]
HIJ47516.1	Type1.BREX	[illegible]	470	Bacteria>Pseudomonadota>Gammaproteobacteria>unclassified_Gammaproteobacteria	GCA_013349775.1	Gammaproteobacteria_bacterium	HPP72_05790	9159..10569	1913989	BREX-system-P-loop-protein-BrxC-[Gammaproteobacteria-bacterium]
HIJ32929.1	Type1.BREX	[illegible]	1200	Bacteria>Pseudomonadota>Gammaproteobacteria>unclassified_Gammaproteobacteria	GCA_013349805.1	Gammaproteobacteria_bacterium	HPP65_00765	772..4374	1913989	BREX-system-P-loop-protein-BrxC-[Gammaproteobacteria-bacterium]
HIJ28180.1	Type1.BREX	[illegible]	466	Bacteria>Pseudomonadota>Gammaproteobacteria>unclassified_Gammaproteobacteria	GCA_013349855.1	Gammaproteobacteria_bacterium	HPP91_06560	8328..9724	1913989	BREX-system-P-loop-protein-BrxC-[Gammaproteobacteria-bacterium]
NVK29385.1	Type1.BREX	[illegible]	1180	Bacteria>Pseudomonadota>Gammaproteobacteria>unclassified_Gammaproteobacteria	GCA_013373515.1	Gammaproteobacteria_bacterium	HWE20_00180	17762..21304	1913989	BREX-system-P-loop-protein-BrxC-[Gammaproteobacteria-bacterium]
MBK7728002.1	Type1.BREX	[illegible]	1048	Bacteria>Pseudomonadota>Gammaproteobacteria>unclassified_Gammaproteobacteria	GCA_016709565.1	Gammaproteobacteria_bacterium	IPJ33_05745	303360..306506	1913989	BREX-system-P-loop-protein-BrxC-[Gammaproteobacteria-bacterium]
MBR9924470.1	Type1.BREX	[illegible]	1227	Bacteria>Pseudomonadota>Gammaproteobacteria>unclassified_Gammaproteobacteria	GCA_018223005.1	Gammaproteobacteria_bacterium	GYB28_05690	375823..379506	1913989	BREX-system-P-loop-protein-BrxC-[Gammaproteobacteria-bacterium]
MBT3489237.1	Type1.BREX	[illegible]	499	Bacteria>Pseudomonadota>Gammaproteobacteria>unclassified_Gammaproteobacteria	GCA_018647365.1	Gammaproteobacteria_bacterium	HN455_06145	1..1498	1913989	BREX-system-P-loop-protein-BrxC-[Gammaproteobacteria-bacterium]
MBT3403047.1	Type1.BREX	[illegible]	701	Bacteria>Pseudomonadota>Gammaproteobacteria>unclassified_Gammaproteobacteria	GCA_018648065.1	Gammaproteobacteria_bacterium	HN421_04975	34194..36297	1913989	BREX-system-P-loop-protein-BrxC-[Gammaproteobacteria-bacterium]
MBT3892721.1	Type1.BREX	[illegible]	742	Bacteria>Pseudomonadota>Gammaproteobacteria>unclassified_Gammaproteobacteria	GCA_018649125.1	Gammaproteobacteria_bacterium	HOC39_05580	1..2226	1913989	BREX-system-P-loop-protein-BrxC-[Gammaproteobacteria-bacterium]
MBT4196271.1	Type1.BREX	[illegible]	1178	Bacteria>Pseudomonadota>Gammaproteobacteria>unclassified_Gammaproteobacteria	GCA_018651435.1	Gammaproteobacteria_bacterium	HOE12_18240	31044..34580	1913989	BREX-system-P-loop-protein-BrxC-[Gammaproteobacteria-bacterium]
MBT3718524.1	Type1.BREX	[illegible]	570	Bacteria>Pseudomonadota>Gammaproteobacteria>unclassified_Gammaproteobacteria	GCA_018653885.1	Gammaproteobacteria_bacterium	HOG37_06305	1..1711	1913989	BREX-system-P-loop-protein-BrxC-[Gammaproteobacteria-bacterium]
MBT3846012.1	Type1.BREX	[illegible]	730	Bacteria>Pseudomonadota>Gammaproteobacteria>unclassified_Gammaproteobacteria	GCA_018655435.1	Gammaproteobacteria_bacterium	HOF59_12730	1..2190	1913989	BREX-system-P-loop-protein-BrxC-[Gammaproteobacteria-bacterium]
MBT5223355.1	Type1.BREX	[illegible]	1192	Bacteria>Pseudomonadota>Gammaproteobacteria>unclassified_Gammaproteobacteria	GCA_018661125.1	Gammaproteobacteria_bacterium	HOM15_11165	1377..4955	1913989	BREX-system-P-loop-protein-BrxC-[Gammaproteobacteria-bacterium]
MBT5688340.1	Type1.BREX	[illegible]	960	Bacteria>Pseudomonadota>Gammaproteobacteria>unclassified_Gammaproteobacteria	GCA_018667295.1	Gammaproteobacteria_bacterium	HOI61_07455	1..2880	1913989	BREX-system-P-loop-protein-BrxC-[Gammaproteobacteria-bacterium]
MBT7480622.1	Type1.BREX	[illegible]	580	Bacteria>Pseudomonadota>Gammaproteobacteria>unclassified_Gammaproteobacteria	GCA_018691505.1	Gammaproteobacteria_bacterium	HN661_14095	1..1740	1913989	BREX-system-P-loop-protein-BrxC-[Gammaproteobacteria-bacterium]
MBT6879379.1	Type1.BREX	[illegible]	757	Bacteria>Pseudomonadota>Gammaproteobacteria>unclassified_Gammaproteobacteria	GCA_018695175.1	Gammaproteobacteria_bacterium	HOA22_07610	1..2271	1913989	BREX-system-P-loop-protein-BrxC-[Gammaproteobacteria-bacterium]
MBU0810851.1	Type1.BREX	[illegible]	1184	Bacteria>Pseudomonadota>Gammaproteobacteria>unclassified_Gammaproteobacteria	GCA_018815345.1	Gammaproteobacteria_bacterium	KKH69_04595	28957..32511	1913989	BREX-system-P-loop-protein-BrxC-[Gammaproteobacteria-bacterium]
MBU1286063.1	Type1.BREX	[illegible]	1222	Bacteria>Pseudomonadota>Gammaproteobacteria>unclassified_Gammaproteobacteria	GCA_018819505.1	Gammaproteobacteria_bacterium	KJ794_21910	1033..4701	1913989	BREX-system-P-loop-protein-BrxC-[Gammaproteobacteria-bacterium]
MBZ0070158.1	Type1.BREX	[illegible]	1147	Bacteria>Pseudomonadota>Gammaproteobacteria>unclassified_Gammaproteobacteria	GCA_019911805.1	Gammaproteobacteria_bacterium	K8I04_00285	50684..54127	1913989	BREX-system-P-loop-protein-BrxC-[Gammaproteobacteria-bacterium]
MCG3145056.1	Type1.BREX	[illegible]	1220	Bacteria>Pseudomonadota>Gammaproteobacteria>unclassified_Gammaproteobacteria	GCA_022072255.1	Gammaproteobacteria_bacterium	HONDAALG_02597	52539..56201	1913989	hypothetical-protein-[Gammaproteobacteria-bacterium]
MCP4410534.1	Type1.BREX	[illegible]	791	Bacteria>Pseudomonadota>Gammaproteobacteria>unclassified_Gammaproteobacteria	GCA_024222475.1	Gammaproteobacteria_bacterium	GY807_22880	5382..7757	1913989	BREX-system-P-loop-protein-BrxC-[Gammaproteobacteria-bacterium]
MCP5451255.1	Type1.BREX	[illegible]	1154	Bacteria>Pseudomonadota>Gammaproteobacteria>unclassified_Gammaproteobacteria	GCA_024235115.1	Gammaproteobacteria_bacterium	H6972_12070	41829..45293	1913989	BREX-system-P-loop-protein-BrxC-[Gammaproteobacteria-bacterium]
WP_245395138.1	Type1.BREX	[illegible]	1115	Bacteria>Pseudomonadota>Gammaproteobacteria>unclassified_Gammaproteobacteria	GCF_016097405.1	methane-oxidizing_endosymbiont_of_Gigantopelta_aegis	JEU20_RS06805	1356880..1360227	2794938	BREX-system-P-loop-protein-BrxC-[methane-oxidizing-endosymbiont-of-Gigantopelta-aegis]
WP_005419548.1	Type1.BREX	[illegible]	1157	Bacteria>Pseudomonadota>Gammaproteobacteria>Vibrionales>Vibrionaceae>Aliivibrio	GCF_000241785.1	Aliivibrio_fischeri	VFSR5_RS07520	1604629..1608102	668	BREX-system-P-loop-protein-BrxC-[Aliivibrio-fischeri]
WP_065641602.1	Type1.BREX	[illegible]	1227	Bacteria>Pseudomonadota>Gammaproteobacteria>Vibrionales>Vibrionaceae>Aliivibrio	GCF_001690915.1	Aliivibrio_fischeri	A6E13_RS16565	253649..257332	668	BREX-system-P-loop-protein-BrxC-[Aliivibrio-fischeri]
WP_130049303.1	Type1.BREX	[illegible]	1142	Bacteria>Pseudomonadota>Gammaproteobacteria>Vibrionales>Vibrionaceae>Aliivibrio	GCF_004168445.1	Aliivibrio_finisterrensis	ERW57_RS17830	13668..17098	511998	BREX-system-P-loop-protein-BrxC,-partial-[Aliivibrio-finisterrensis]
WP_133416081.1	Type1.BREX	[illegible]	1227	Bacteria>Pseudomonadota>Gammaproteobacteria>Vibrionales>Vibrionaceae>Aliivibrio	GCF_004359415.1	Aliivibrio_fischeri	VFFQA001_RS16045	453579..457262	668	BREX-system-P-loop-protein-BrxC-[Aliivibrio-fischeri]
WP_155683402.1	Type1.BREX	[illegible]	1233	Bacteria>Pseudomonadota>Gammaproteobacteria>Vibrionales>Vibrionaceae>Aliivibrio	GCF_009727645.1	Aliivibrio_fischeri	GNP73_RS13855	802390..806091	668	BREX-system-P-loop-protein-BrxC-[Aliivibrio-fischeri]
WP_188863624.1	Type1.BREX	[illegible]	1227	Bacteria>Pseudomonadota>Gammaproteobacteria>Vibrionales>Vibrionaceae>Aliivibrio	GCF_014647395.1	Aliivibrio_fischeri	IEY04_RS10160	4332..8015	668	BREX-system-P-loop-protein-BrxC-[Aliivibrio-fischeri]
WP_190395653.1	Type1.BREX	[illegible]	1157	Bacteria>Pseudomonadota>Gammaproteobacteria>Vibrionales>Vibrionaceae>Aliivibrio	GCF_014694495.1	Aliivibrio_sp._S10_S31	HC728_RS13680	57939..61412	2720224	BREX-system-P-loop-protein-BrxC-[Aliivibrio-sp.-S10_S31]
WP_199414880.1	Type1.BREX	[illegible]	1157	Bacteria>Pseudomonadota>Gammaproteobacteria>Vibrionales>Vibrionaceae>Aliivibrio	GCF_023983475.1	Aliivibrio_fischeri	AVFI_RS07680	1646065..1649538	668	BREX-system-P-loop-protein-BrxC-[Aliivibrio-fischeri]
WP_102260244.1	Type1.BREX	[illegible]	1157	Bacteria>Pseudomonadota>Gammaproteobacteria>Vibrionales>Vibrionaceae>Enterovibrio	GCF_002874375.1	Enterovibrio_norvegicus	BCU62_16195	25204..28677	188144	BREX-system-P-loop-protein-BrxC-[Enterovibrio-norvegicus]
WP_150138110.1	Type1.BREX	[illegible]	1159	Bacteria>Pseudomonadota>Gammaproteobacteria>Vibrionales>Vibrionaceae>Enterovibrio	GCF_008692425.1	Candidatus_Enterovibrio_escacola	EDM10_RS06800	2982..6461	1927127	BREX-system-P-loop-protein-BrxC-[Candidatus-Enterovibrio-escacola]
WP_006071674.1	Type1.BREX	[illegible]	1225	Bacteria>Pseudomonadota>Gammaproteobacteria>Vibrionales>Vibrionaceae	GCF_000181535.1	Vibrio	VSAK1_RS11985	54730..58407	662	MULTISPECIES:-BREX-system-P-loop-protein-BrxC-[Vibrio]
WP_025612391.1	Type1.BREX	[illegible]	1157	Bacteria>Pseudomonadota>Gammaproteobacteria>Vibrionales>Vibrionaceae	GCF_000489615.1	Vibrio	DX35_RS0111130	15561..19034	662	MULTISPECIES:-BREX-system-P-loop-protein-BrxC-[Vibrio]
WP_103201396.1	Type1.BREX	[illegible]	1225	Bacteria>Pseudomonadota>Gammaproteobacteria>Vibrionales>Vibrionaceae	GCF_003047085.1	Vibrio	DA442_RS13700	2691122..2694799	662	MULTISPECIES:-BREX-system-P-loop-protein-BrxC-[Vibrio]
WP_159406353.1	Type1.BREX	[illegible]	1157	Bacteria>Pseudomonadota>Gammaproteobacteria>Vibrionales>Vibrionaceae	GCF_009883895.1	Vibrio	O8X55_RS08175	512098..515571	662	MULTISPECIES:-BREX-system-P-loop-protein-BrxC-[Vibrio]
WP_206969396.1	Type1.BREX	[illegible]	1225	Bacteria>Pseudomonadota>Gammaproteobacteria>Vibrionales>Vibrionaceae	GCF_017313425.1	Vibrio	J0673_RS01390	328257..331934	662	MULTISPECIES:-BREX-system-P-loop-protein-BrxC-[Vibrio]
WP_228075537.1	Type1.BREX	[illegible]	1248	Bacteria>Pseudomonadota>Gammaproteobacteria>Vibrionales>Vibrionaceae	GCF_020740925.1	Vibrio	IB290_RS12355	161430..165176	662	MULTISPECIES:-BREX-system-P-loop-protein-BrxC-[Vibrio]
WP_180208765.1	Type1.BREX	[illegible]	1157	Bacteria>Pseudomonadota>Gammaproteobacteria>Vibrionales>Vibrionaceae	GCF_021725735.1	Vibrio	L3V27_RS03750	866566..870039	662	MULTISPECIES:-BREX-system-P-loop-protein-BrxC-[Vibrio]
WP_086713494.1	Type1.BREX	[illegible]	1157	Bacteria>Pseudomonadota>Gammaproteobacteria>Vibrionales>Vibrionaceae	GCF_024347515.1	Vibrio	EDB41_RS15380	67742..71215	662	MULTISPECIES:-BREX-system-P-loop-protein-BrxC-[Vibrio]
WP_198107480.1	Type1.BREX	[illegible]	1157	Bacteria>Pseudomonadota>Gammaproteobacteria>Vibrionales>Vibrionaceae	GCF_028615395.1	Vibrio	O8Z82_RS06535	628630..632103	662	MULTISPECIES:-BREX-system-P-loop-protein-BrxC-[Vibrio]
WP_130066623.1	Type1.BREX	[illegible]	1227	Bacteria>Pseudomonadota>Gammaproteobacteria>Vibrionales>Vibrionaceae	GCF_028765545.1	Aliivibrio	ERW52_RS16080	26875..30558	511678	MULTISPECIES:-BREX-system-P-loop-protein-BrxC-[Aliivibrio]
PSU45803.1	Type1.BREX	[illegible]	1185	Bacteria>Pseudomonadota>Gammaproteobacteria>Vibrionales>Vibrionaceae>Photobacterium	GCA_003025615.1	Photobacterium_frigidiphilum	C9J12_20970	5140..8697	264736	BREX-system-P-loop-protein-BrxC-[Photobacterium-frigidiphilum]
NAW85774.1	Type1.BREX	[illegible]	1154	Bacteria>Pseudomonadota>Gammaproteobacteria>Vibrionales>Vibrionaceae>Photobacterium	GCA_009904625.1	Photobacterium_halotolerans	CAG71_04765	1726..5190	265726	BREX-system-P-loop-protein-BrxC-[Photobacterium-halotolerans]
QOQ70156.1	Type1.BREX	[illegible]	1154	Bacteria>Pseudomonadota>Gammaproteobacteria>Vibrionales>Vibrionaceae>Photobacterium	GCA_014930975.1	Photobacterium_damselae_subsp._damselae	IL982_10195	2142038..2145502	85581	BREX-system-P-loop-protein-BrxC-[Photobacterium-damselae-subsp.-damselae]
WP_006647488.1	Type1.BREX	[illegible]	1193	Bacteria>Pseudomonadota>Gammaproteobacteria>Vibrionales>Vibrionaceae>Photobacterium	GCF_000153325.1	Photobacterium_sp._SKA34	SKA34_RS21260	87534..91115	121723	BREX-system-P-loop-protein-BrxC-[Photobacterium-sp.-SKA34]
WP_227876424.1	Type1.BREX	[illegible]	1187	Bacteria>Pseudomonadota>Gammaproteobacteria>Vibrionales>Vibrionaceae>Photobacterium	GCF_003025615.1	Photobacterium_frigidiphilum	C9J12_RS20995	5134..8697	264736	BREX-system-P-loop-protein-BrxC-[Photobacterium-frigidiphilum]
WP_115426815.1	Type1.BREX	[illegible]	1232	Bacteria>Pseudomonadota>Gammaproteobacteria>Vibrionales>Vibrionaceae>Photobacterium	GCF_003355625.1	Photobacterium_damselae	BC461_RS14900	129759..133457	38293	BREX-system-P-loop-protein-BrxC-[Photobacterium-damselae]
WP_138222247.1	Type1.BREX	[illegible]	1232	Bacteria>Pseudomonadota>Gammaproteobacteria>Vibrionales>Vibrionaceae>Photobacterium	GCF_005819835.1	Photobacterium_damselae	FD717_RS16650	68908..72606	38293	BREX-system-P-loop-protein-BrxC-[Photobacterium-damselae]
WP_138976661.1	Type1.BREX	[illegible]	1232	Bacteria>Pseudomonadota>Gammaproteobacteria>Vibrionales>Vibrionaceae>Photobacterium	GCF_006124915.1	Photobacterium_damselae	DA092_RS00785	169698..173396	38293	BREX-system-P-loop-protein-BrxC-[Photobacterium-damselae]
WP_176285150.1	Type1.BREX	[illegible]	1157	Bacteria>Pseudomonadota>Gammaproteobacteria>Vibrionales>Vibrionaceae>Photobacterium	GCF_013371045.1	Photobacterium_damselae	HU831_RS17605	57170..60643	38293	BREX-system-P-loop-protein-BrxC-[Photobacterium-damselae]
WP_232092144.1	Type1.BREX	[illegible]	1153	Bacteria>Pseudomonadota>Gammaproteobacteria>Vibrionales>Vibrionaceae>Photobacterium	GCF_014930975.1	Photobacterium_damselae	IL982_RS10195	2142038..2145499	38293	BREX-system-P-loop-protein-BrxC-[Photobacterium-damselae]
WP_207103965.1	Type1.BREX	[illegible]	1225	Bacteria>Pseudomonadota>Gammaproteobacteria>Vibrionales>Vibrionaceae>Photobacterium	GCF_017329545.1	Photobacterium_ganghwense	FH974_RS17035	241897..245574	320778	BREX-system-P-loop-protein-BrxC-[Photobacterium-ganghwense]
WP_232606822.1	Type1.BREX	[illegible]	1157	Bacteria>Pseudomonadota>Gammaproteobacteria>Vibrionales>Vibrionaceae>Photobacterium	GCF_021237045.1	Photobacterium_phosphoreum	CJF26_RS18915	30954..34427	659	BREX-system-P-loop-protein-BrxC-[Photobacterium-phosphoreum]
WP_232580803.1	Type1.BREX	[illegible]	1238	Bacteria>Pseudomonadota>Gammaproteobacteria>Vibrionales>Vibrionaceae>Photobacterium	GCF_021504545.1	Photobacterium_phosphoreum	GLP29_RS03805	135359..139075	659	BREX-system-P-loop-protein-BrxC-[Photobacterium-phosphoreum]
WP_239972118.1	Type1.BREX	[illegible]	1232	Bacteria>Pseudomonadota>Gammaproteobacteria>Vibrionales>Vibrionaceae>Photobacterium	GCF_022398535.1	Photobacterium_damselae	L1D45_RS07310	258079..261777	38293	BREX-system-P-loop-protein-BrxC-[Photobacterium-damselae]
WP_101770358.1	Type1.BREX	[illegible]	1223	Bacteria>Pseudomonadota>Gammaproteobacteria>Vibrionales>Vibrionaceae>Photobacterium	GCF_027923345.1	Photobacterium_carnosum	CIK00_20185	17344..21015	2023717	BREX-system-P-loop-protein-BrxC-[Photobacterium-carnosum]
WP_101767576.1	Type1.BREX	[illegible]	1238	Bacteria>Pseudomonadota>Gammaproteobacteria>Vibrionales>Vibrionaceae>Photobacterium	GCF_027923345.1	Photobacterium_carnosum	CIK00_RS03640	195656..199372	2023717	BREX-system-P-loop-protein-BrxC-[Photobacterium-carnosum]
WP_087820331.1	Type1.BREX	[illegible]	1238	Bacteria>Pseudomonadota>Gammaproteobacteria>Vibrionales>Vibrionaceae>Photobacterium	GCF_900185565.1	Photobacterium_aquimaris	PAQU9191_RS07280	444867..448583	512643	BREX-system-P-loop-protein-BrxC-[Photobacterium-aquimaris]
CAQ34953.1	Type1.BREX	[illegible]	1225	Bacteria>Pseudomonadota>Gammaproteobacteria>Vibrionales>Vibrionaceae>Photobacterium	-	Photobacterium_damselae_subsp._piscicida	.	21165..24842	38294	hypothetical-protein-[Photobacterium-damselae-subsp.-piscicida]
WP_077485085.1	Type1.BREX	[illegible]	1157	Bacteria>Pseudomonadota>Gammaproteobacteria>Vibrionales>Vibrionaceae>Salinivibrio	GCF_001995745.1	Salinivibrio_sp._ML323	BZG13_RS03555	125341..128814	1909474	BREX-system-P-loop-protein-BrxC-[Salinivibrio-sp.-ML323]
WP_077678370.1	Type1.BREX	[illegible]	1229	Bacteria>Pseudomonadota>Gammaproteobacteria>Vibrionales>Vibrionaceae>Salinivibrio	GCF_001996125.1	Salinivibrio_sp._PR932	BZG84_RS14215	25023..28712	1909492	BREX-system-P-loop-protein-BrxC-[Salinivibrio-sp.-PR932]
WP_077677746.1	Type1.BREX	[illegible]	1232	Bacteria>Pseudomonadota>Gammaproteobacteria>Vibrionales>Vibrionaceae>Salinivibrio	GCF_001996245.1	Salinivibrio_sp._MA607	BZG81_RS14680	10387..14085	1909457	BREX-system-P-loop-protein-BrxC-[Salinivibrio-sp.-MA607]
TNF18455.1	Type1.BREX	[illegible]	1145	Bacteria>Pseudomonadota>Gammaproteobacteria>Vibrionales>Vibrionaceae>unclassified_Vibrionaceae	GCA_006226665.1	Vibrionaceae_bacterium	EP325_05025	51838..55275	1889773	BREX-system-P-loop-protein-BrxC-[Vibrionaceae-bacterium]
EKK94843.1	Type1.BREX	[illegible]	1151	Bacteria>Pseudomonadota>Gammaproteobacteria>Vibrionales>Vibrionaceae>Vibrio	GCA_000304775.1	Vibrio_cholerae_HC-1A2	VCHC1A2_1591	58635..62090	991937	hypothetical-protein-VCHC1A2_1591-[Vibrio-cholerae-HC-1A2]
GAJ78762.1	Type1.BREX	[illegible]	800	Bacteria>Pseudomonadota>Gammaproteobacteria>Vibrionales>Vibrionaceae>Vibrio	GCA_000695055.1	Vibrio_sp._JCM_18905	JCM18905_4752	16842..19244	1298600	hypothetical-protein-JCM18905_4752-[Vibrio-sp.-JCM-18905]
GAJ78763.1	Type1.BREX	[illegible]	425	Bacteria>Pseudomonadota>Gammaproteobacteria>Vibrionales>Vibrionaceae>Vibrio	GCA_000695055.1	Vibrio_sp._JCM_18905	JCM18905_4753	19241..20518	1298600	hypothetical-protein-JCM18905_4753-[Vibrio-sp.-JCM-18905]
AJZ99161.1	Type1.BREX	[illegible]	1151	Bacteria>Pseudomonadota>Gammaproteobacteria>Vibrionales>Vibrionaceae>Vibrio	GCA_000963555.1	Vibrio_cholerae_O1_biovar_El_Tor	IR04_08395	718003..721458	686	hypothetical-protein-IR04_08395-[Vibrio-cholerae-O1-biovar-El-Tor]
CSC57771.1	Type1.BREX	[illegible]	1237	Bacteria>Pseudomonadota>Gammaproteobacteria>Vibrionales>Vibrionaceae>Vibrio	GCA_001247885.1	Vibrio_cholerae	ERS013147_02837	124661..128374	666	Uncharacterised-protein-[Vibrio-cholerae]
CSC91133.1	Type1.BREX	[illegible]	268	Bacteria>Pseudomonadota>Gammaproteobacteria>Vibrionales>Vibrionaceae>Vibrio	GCA_001254955.1	Vibrio_cholerae	ERS013216_03026	6147..6953	666	Uncharacterised-protein-[Vibrio-cholerae]
CSC91159.1	Type1.BREX	[illegible]	933	Bacteria>Pseudomonadota>Gammaproteobacteria>Vibrionales>Vibrionaceae>Vibrio	GCA_001254955.1	Vibrio_cholerae	ERS013216_03027	7037..9838	666	Uncharacterised-protein-[Vibrio-cholerae]
CSA58950.1	Type1.BREX	[illegible]	324	Bacteria>Pseudomonadota>Gammaproteobacteria>Vibrionales>Vibrionaceae>Vibrio	GCA_001257255.1	Vibrio_cholerae	ERS013131_02346	7278..8252	666	Uncharacterised-protein-[Vibrio-cholerae]
CSA58983.1	Type1.BREX	[illegible]	820	Bacteria>Pseudomonadota>Gammaproteobacteria>Vibrionales>Vibrionaceae>Vibrio	GCA_001257255.1	Vibrio_cholerae	ERS013131_02347	8501..10963	666	Uncharacterised-protein-[Vibrio-cholerae]
CSD13814.1	Type1.BREX	[illegible]	327	Bacteria>Pseudomonadota>Gammaproteobacteria>Vibrionales>Vibrionaceae>Vibrio	GCA_001258535.1	Vibrio_cholerae	ERS013225_02589	127512..128495	666	Uncharacterised-protein-[Vibrio-cholerae]
KOY19349.1	Type1.BREX	[illegible]	1154	Bacteria>Pseudomonadota>Gammaproteobacteria>Vibrionales>Vibrionaceae>Vibrio	GCA_001280635.1	Vibrio_parahaemolyticus	ACX06_21960	167502..170966	670	hypothetical-protein-ACX06_21960-[Vibrio-parahaemolyticus]
ODY54465.1	Type1.BREX	[illegible]	1154	Bacteria>Pseudomonadota>Gammaproteobacteria>Vibrionales>Vibrionaceae>Vibrio	GCA_001727265.1	Vibrio_parahaemolyticus	BBM25_06240	3424..6888	670	hypothetical-protein-BBM25_06240-[Vibrio-parahaemolyticus]
ASG04983.1	Type1.BREX	[illegible]	1185	Bacteria>Pseudomonadota>Gammaproteobacteria>Vibrionales>Vibrionaceae>Vibrio	GCA_002211985.1	Vibrio_anguillarum	CEJ46_04105	862780..866337	55601	hypothetical-protein-CEJ46_04105-[Vibrio-anguillarum]
ASI98027.1	Type1.BREX	[illegible]	1183	Bacteria>Pseudomonadota>Gammaproteobacteria>Vibrionales>Vibrionaceae>Vibrio	GCA_002214395.1	Vibrio_rotiferianus	BSZ04_17745	1918666..1922217	190895	hypothetical-protein-BSZ04_17745-[Vibrio-rotiferianus]
MVB73516.1	Type1.BREX	[illegible]	1225	Bacteria>Pseudomonadota>Gammaproteobacteria>Vibrionales>Vibrionaceae>Vibrio	GCA_009747185.1	Vibrio_cholerae	D6U90_04200	143741..147418	666	BREX-system-P-loop-protein-BrxC-[Vibrio-cholerae]
MVC74394.1	Type1.BREX	[illegible]	1225	Bacteria>Pseudomonadota>Gammaproteobacteria>Vibrionales>Vibrionaceae>Vibrio	GCA_009747245.1	Vibrio_cholerae	D6U22_06265	184143..187820	666	BREX-system-P-loop-protein-BrxC-[Vibrio-cholerae]
NAZ69809.1	Type1.BREX	[illegible]	1185	Bacteria>Pseudomonadota>Gammaproteobacteria>Vibrionales>Vibrionaceae>Vibrio	GCA_009906175.1	Vibrio_toranzoniae	GL180_08045	10464..14021	1194427	BREX-system-P-loop-protein-BrxC-[Vibrio-toranzoniae]
MBE3824873.1	Type1.BREX	[illegible]	1225	Bacteria>Pseudomonadota>Gammaproteobacteria>Vibrionales>Vibrionaceae>Vibrio	GCA_014918955.1	Vibrio_parahaemolyticus	HJ179_16470	255963..259640	670	BREX-system-P-loop-protein-BrxC-[Vibrio-parahaemolyticus]
MBE3881970.1	Type1.BREX	[illegible]	1225	Bacteria>Pseudomonadota>Gammaproteobacteria>Vibrionales>Vibrionaceae>Vibrio	GCA_014919165.1	Vibrio_parahaemolyticus	HJ168_09865	226253..229930	670	BREX-system-P-loop-protein-BrxC-[Vibrio-parahaemolyticus]
MBE4310404.1	Type1.BREX	[illegible]	1157	Bacteria>Pseudomonadota>Gammaproteobacteria>Vibrionales>Vibrionaceae>Vibrio	GCA_014921165.1	Vibrio_parahaemolyticus	HJ064_04290	963533..967006	670	BREX-system-P-loop-protein-BrxC-[Vibrio-parahaemolyticus]
MBE4432200.1	Type1.BREX	[illegible]	1225	Bacteria>Pseudomonadota>Gammaproteobacteria>Vibrionales>Vibrionaceae>Vibrio	GCA_014921655.1	Vibrio_parahaemolyticus	HJ022_02625	614361..618038	670	BREX-system-P-loop-protein-BrxC-[Vibrio-parahaemolyticus]
MBE5192784.1	Type1.BREX	[illegible]	1157	Bacteria>Pseudomonadota>Gammaproteobacteria>Vibrionales>Vibrionaceae>Vibrio	GCA_014963905.1	Vibrio_parahaemolyticus	HJ051_04870	1092166..1095639	670	BREX-system-P-loop-protein-BrxC-[Vibrio-parahaemolyticus]
EGQ7646156.1	Type1.BREX	[illegible]	1225	Bacteria>Pseudomonadota>Gammaproteobacteria>Vibrionales>Vibrionaceae>Vibrio	GCA_015741395.1	Vibrio_alginolyticus	I2703_000228	284534..288211	663	BREX-system-P-loop-protein-BrxC-[Vibrio-alginolyticus]
EGQ7901736.1	Type1.BREX	[illegible]	1225	Bacteria>Pseudomonadota>Gammaproteobacteria>Vibrionales>Vibrionaceae>Vibrio	GCA_015746405.1	Vibrio_alginolyticus	H5028_000763	854695..858372	663	BREX-system-P-loop-protein-BrxC-[Vibrio-alginolyticus]
HAS3953742.1	Type1.BREX	[illegible]	1210	Bacteria>Pseudomonadota>Gammaproteobacteria>Vibrionales>Vibrionaceae>Vibrio	GCA_015750645.1	Vibrio_cholerae	I6306_000460	124735..128369	666	BREX-system-P-loop-protein-BrxC-[Vibrio-cholerae]
QPL56152.1	Type1.BREX	[illegible]	1225	Bacteria>Pseudomonadota>Gammaproteobacteria>Vibrionales>Vibrionaceae>Vibrio	GCA_015767675.1	Vibrio_navarrensis	I3X05_18825	378934..382611	29495	BREX-system-P-loop-protein-BrxC-[Vibrio-navarrensis]
EGQ8448095.1	Type1.BREX	[illegible]	1185	Bacteria>Pseudomonadota>Gammaproteobacteria>Vibrionales>Vibrionaceae>Vibrio	GCA_015775815.1	Vibrio_alginolyticus	GTP59_14930	64166..67723	663	BREX-system-P-loop-protein-BrxC-[Vibrio-alginolyticus]
HAS6096890.1	Type1.BREX	[illegible]	1229	Bacteria>Pseudomonadota>Gammaproteobacteria>Vibrionales>Vibrionaceae>Vibrio	GCA_015776315.1	Vibrio_vulnificus	I7145_19865	48069..51758	672	BREX-system-P-loop-protein-BrxC-[Vibrio-vulnificus]
HAS6061420.1	Type1.BREX	[illegible]	1232	Bacteria>Pseudomonadota>Gammaproteobacteria>Vibrionales>Vibrionaceae>Vibrio	GCA_015776335.1	Vibrio_vulnificus	I7135_04535	448..4146	672	BREX-system-P-loop-protein-BrxC-[Vibrio-vulnificus]
HAS6187654.1	Type1.BREX	[illegible]	1225	Bacteria>Pseudomonadota>Gammaproteobacteria>Vibrionales>Vibrionaceae>Vibrio	GCA_015777055.1	Vibrio_vulnificus	I7170_07515	257971..261648	672	BREX-system-P-loop-protein-BrxC-[Vibrio-vulnificus]
HAS6259595.1	Type1.BREX	[illegible]	1225	Bacteria>Pseudomonadota>Gammaproteobacteria>Vibrionales>Vibrionaceae>Vibrio	GCA_015777345.1	Vibrio_vulnificus	I7226_03370	263913..267590	672	BREX-system-P-loop-protein-BrxC-[Vibrio-vulnificus]
HAS6362912.1	Type1.BREX	[illegible]	1248	Bacteria>Pseudomonadota>Gammaproteobacteria>Vibrionales>Vibrionaceae>Vibrio	GCA_015777565.1	Vibrio_vulnificus	I7230_15365	52785..56531	672	BREX-system-P-loop-protein-BrxC-[Vibrio-vulnificus]
HAS6390651.1	Type1.BREX	[illegible]	1157	Bacteria>Pseudomonadota>Gammaproteobacteria>Vibrionales>Vibrionaceae>Vibrio	GCA_015777605.1	Vibrio_vulnificus	I7251_16610	90517..93990	672	BREX-system-P-loop-protein-BrxC-[Vibrio-vulnificus]
HAS6345170.1	Type1.BREX	[illegible]	1232	Bacteria>Pseudomonadota>Gammaproteobacteria>Vibrionales>Vibrionaceae>Vibrio	GCA_015778705.1	Vibrio_vulnificus	I7243_17845	83102..86800	672	BREX-system-P-loop-protein-BrxC-[Vibrio-vulnificus]
HAS6333998.1	Type1.BREX	[illegible]	1248	Bacteria>Pseudomonadota>Gammaproteobacteria>Vibrionales>Vibrionaceae>Vibrio	GCA_015778755.1	Vibrio_vulnificus	I7245_08190	199206..202952	672	BREX-system-P-loop-protein-BrxC-[Vibrio-vulnificus]
HAS6310370.1	Type1.BREX	[illegible]	1157	Bacteria>Pseudomonadota>Gammaproteobacteria>Vibrionales>Vibrionaceae>Vibrio	GCA_015778905.1	Vibrio_vulnificus	I7207_04135	43135..46608	672	BREX-system-P-loop-protein-BrxC-[Vibrio-vulnificus]
EGQ8649255.1	Type1.BREX	[illegible]	1225	Bacteria>Pseudomonadota>Gammaproteobacteria>Vibrionales>Vibrionaceae>Vibrio	GCA_015781355.1	Vibrio_cholerae	GNS53_08430	57189..60866	666	BREX-system-P-loop-protein-BrxC-[Vibrio-cholerae]
EGQ8730608.1	Type1.BREX	[illegible]	1225	Bacteria>Pseudomonadota>Gammaproteobacteria>Vibrionales>Vibrionaceae>Vibrio	GCA_015781475.1	Vibrio_parahaemolyticus	GKA80_01950	455980..459657	670	BREX-system-P-loop-protein-BrxC-[Vibrio-parahaemolyticus]
EGQ8898350.1	Type1.BREX	[illegible]	1225	Bacteria>Pseudomonadota>Gammaproteobacteria>Vibrionales>Vibrionaceae>Vibrio	GCA_015781875.1	Vibrio_parahaemolyticus	DMW33_24570	2854..6531	670	BREX-system-P-loop-protein-BrxC-[Vibrio-parahaemolyticus]
EGQ9599053.1	Type1.BREX	[illegible]	1157	Bacteria>Pseudomonadota>Gammaproteobacteria>Vibrionales>Vibrionaceae>Vibrio	GCA_015785155.1	Vibrio_parahaemolyticus	F3R80_21565	8289..11762	670	BREX-system-P-loop-protein-BrxC-[Vibrio-parahaemolyticus]
EGR0072241.1	Type1.BREX	[illegible]	1248	Bacteria>Pseudomonadota>Gammaproteobacteria>Vibrionales>Vibrionaceae>Vibrio	GCA_015787395.1	Vibrio_vulnificus	FR269_18510	2637..6383	672	BREX-system-P-loop-protein-BrxC-[Vibrio-vulnificus]
EGR0489752.1	Type1.BREX	[illegible]	1157	Bacteria>Pseudomonadota>Gammaproteobacteria>Vibrionales>Vibrionaceae>Vibrio	GCA_015788475.1	Vibrio_cholerae	FG089_09200	79189..82662	666	BREX-system-P-loop-protein-BrxC-[Vibrio-cholerae]
EGR0719595.1	Type1.BREX	[illegible]	1157	Bacteria>Pseudomonadota>Gammaproteobacteria>Vibrionales>Vibrionaceae>Vibrio	GCA_015789455.1	Vibrio_alginolyticus	EX464_05795	8586..12059	663	BREX-system-P-loop-protein-BrxC-[Vibrio-alginolyticus]
EGR0286978.1	Type1.BREX	[illegible]	1154	Bacteria>Pseudomonadota>Gammaproteobacteria>Vibrionales>Vibrionaceae>Vibrio	GCA_015789595.1	Vibrio_cholerae	FK206_11700	85283..88747	666	BREX-system-P-loop-protein-BrxC-[Vibrio-cholerae]
EGR0983685.1	Type1.BREX	[illegible]	1225	Bacteria>Pseudomonadota>Gammaproteobacteria>Vibrionales>Vibrionaceae>Vibrio	GCA_015797355.1	Vibrio_parahaemolyticus	EH301_01885	134210..137887	670	BREX-system-P-loop-protein-BrxC-[Vibrio-parahaemolyticus]
EGR1144248.1	Type1.BREX	[illegible]	1154	Bacteria>Pseudomonadota>Gammaproteobacteria>Vibrionales>Vibrionaceae>Vibrio	GCA_015798095.1	Vibrio_parahaemolyticus	EC562_15505	41063..44527	670	BREX-system-P-loop-protein-BrxC-[Vibrio-parahaemolyticus]

Protein-ID	System	Gene-Neighbourhoods	Length	Taxonomy	Assembly-ID	Strain	Locus-Tag	Genomic-coordinates	TaxID	Fasta-define
EGR1548466.1	Type1.BREX	Arm-DNA-bind_3+Phage_integrase->SF1-SF2-Helicase+REase->SF1-SF2-Helicase+REase-><-PglZ<-TOPRIM<-OLD-ABC<-N6-METHYLASE<-DUF6079*<-BrxB-BrxF-iSTAND<-BrxA-DNA-binding<-wHTH+WYL+WCX<-XIS-HTH	1154	Bacteria>Pseudomonadota>Gammaproteobacteria>Vibrionales>Vibrionaceae>Vibrio	GCA_015798755.1	Vibrio_parahaemolyticus	D6Y11_16950	161246..164710	670	BREX-system-P-loop-protein-BrxC-[Vibrio-parahaemolyticus]
EGR2430327.1	Type1.BREX	<-wHTH+WYL+WCX<-HTH<-DUF2787<-?<-?||XIS-HTH->wHTH+WYL+WCX->BrxA-DNA-binding->BrxB-BrxF-iSTAND->DUF6079*->N6-METHYLASE->OLD-ABC->TOPRIM->PglZ-><-SF1-SF2-Helicase+REase<-SF1-SF2-Helicase+REase<-Arm-DNA-bind_3+Phage_integrase	1154	Bacteria>Pseudomonadota>Gammaproteobacteria>Vibrionales>Vibrionaceae>Vibrio	GCA_015802095.1	Vibrio_cholerae	DYB83_10140	10866..14330	666	BREX-system-P-loop-protein-BrxC-[Vibrio-cholerae]
EGR3302280.1	Type1.BREX	XIS-HTH->wHTH+WYL+WCX->BrxA-DNA-binding->BrxB-BrxF-iSTAND->DUF6079*->N6-METHYLASE->OLD-ABC->TOPRIM->PglZ-><-SF1-SF2-Helicase+REase<-SF1-SF2-Helicase+REase<-Arm-DNA-bind_3+Phage_integrase<-?||GDResolvase+HIN-HTH->	1154	Bacteria>Pseudomonadota>Gammaproteobacteria>Vibrionales>Vibrionaceae>Vibrio	GCA_015805535.1	Vibrio_parahaemolyticus	DMJ21_13130	8098..11562	670	BREX-system-P-loop-protein-BrxC-[Vibrio-parahaemolyticus]
EGR3003176.1	Type1.BREX	viral-protein->SF1-SF2-Helicase+REase->SF1-SF2-Helicase+REase-><-ZetaToxin<-PglZ<-ParB+HNH<-N6-METHYLASE<-DUF6079*<-BrxB-BrxF-iSTAND<-BrxA-DNA-binding<-wHTH+WYL+WCX||<-?<-CR-ATPase4	1157	Bacteria>Pseudomonadota>Gammaproteobacteria>Vibrionales>Vibrionaceae>Vibrio	GCA_015807135.1	Vibrio_parahaemolyticus	DMW06_23030	12898..16371	670	BREX-system-P-loop-protein-BrxC-[Vibrio-parahaemolyticus]
EGR4188113.1	Type1.BREX	AbiEi->viral-protein->SF1-SF2-Helicase+REase->SF1-SF2-Helicase+REase-><-TPR<-HEPN+SF1-SF2-Helicase+RNase+RNase<-PglZ<-ParB+HNH<-N6-METHYLASE<-DUF6079*<-BrxB-BrxF-iSTAND<-BrxA-DNA-binding<-wHTH+WYL+WCX||DDE_Tnp_1_5+DDE_Tnp_1->PIN+OST-HTH-><-?<-HTH_20	1157	Bacteria>Pseudomonadota>Gammaproteobacteria>Vibrionales>Vibrionaceae>Vibrio	GCA_015809855.1	Vibrio_cholerae	DDM99_15775	19819..23292	666	BREX-system-P-loop-protein-BrxC-[Vibrio-cholerae]
EJG0966117.1	Type1.BREX	<-ZnR<-?||?->SF1-SF2-Helicase->AbiEi->viral-protein->wHTH+WYL+WCX->BrxA-DNA-binding->BrxB-BrxF-iSTAND->DUF6079*->N6-METHYLASE->ParB+HNH->GrpE->?->PglZ->SIGMA+OB-Fold+AAA-ATPase+Lon-Protease-BrxL-><-wHTH||DUF4113->	1225	Bacteria>Pseudomonadota>Gammaproteobacteria>Vibrionales>Vibrionaceae>Vibrio	GCA_015813265.2	Vibrio_parahaemolyticus	C4G65_RS20915	24307..27984	670	BREX-system-P-loop-protein-BrxC-[Vibrio-parahaemolyticus]
EJG0997619.1	Type1.BREX	AbiEi->viral-protein->SF1-SF2-Helicase+REase->SF1-SF2-Helicase+REase-><-?<-ZetaToxin<-PglZ<-ParB+HNH<-N6-METHYLASE<-DUF6079*<-BrxB-BrxF-iSTAND<-BrxA-DNA-binding<-wHTH+WYL+WCX||DUF4113-><-?<-CR-ATPase4||SbcC->HEPN-><-HTH_20	1157	Bacteria>Pseudomonadota>Gammaproteobacteria>Vibrionales>Vibrionaceae>Vibrio	GCA_015813305.2	Vibrio_parahaemolyticus	C4G52_RS11760	21329..24802	670	BREX-system-P-loop-protein-BrxC-[Vibrio-parahaemolyticus]
EJG0998764.1	Type1.BREX	<-ZnR<-?||?->SF1-SF2-Helicase->AbiEi->viral-protein->wHTH+WYL+WCX->BrxA-DNA-binding->BrxB-BrxF-iSTAND->DUF6079*->N6-METHYLASE->?->?->PglZ->SIGMA+OB-Fold+AAA-ATPase+Lon-Protease-BrxL-><-wHTH||DUF4113-><-?<-CR-ATPase4	1225	Bacteria>Pseudomonadota>Gammaproteobacteria>Vibrionales>Vibrionaceae>Vibrio	GCA_015813305.2	Vibrio_parahaemolyticus	C4G52_RS17670	119644..123321	670	BREX-system-P-loop-protein-BrxC-[Vibrio-parahaemolyticus]
EJG0981293.1	Type1.BREX	Arm-DNA-bind_3+Phage_integrase->SF1-SF2-Helicase+REase->SF1-SF2-Helicase+REase-><-PglZ<-TOPRIM<-OLD-ABC<-N6-METHYLASE<-DUF6079*<-BrxB-BrxF-iSTAND<-BrxA-DNA-binding<-wHTH+WYL+WCX<-XIS-HTH	1154	Bacteria>Pseudomonadota>Gammaproteobacteria>Vibrionales>Vibrionaceae>Vibrio	GCA_015813375.2	Vibrio_parahaemolyticus	C4G85_RS03500	776707..780171	670	BREX-system-P-loop-protein-BrxC-[Vibrio-parahaemolyticus]
EJG0638704.1	Type1.BREX	<-ZnR<-?||?->SF1-SF2-Helicase->AbiEi->viral-protein->wHTH+WYL+WCX->BrxA-DNA-binding->BrxB-BrxF-iSTAND->DUF6079*->N6-METHYLASE->ParB+HNH->GrpE->?->PglZ->SIGMA+OB-Fold+AAA-ATPase+Lon-Protease-BrxL-><-wHTH||DUF4113->	1225	Bacteria>Pseudomonadota>Gammaproteobacteria>Vibrionales>Vibrionaceae>Vibrio	GCA_015813785.2	Vibrio_parahaemolyticus	C4G37_RS13070	147789..151466	670	BREX-system-P-loop-protein-BrxC-[Vibrio-parahaemolyticus]
EJG0936611.1	Type1.BREX	<-GDResolvase+HIN-HTH<-?||Arm-DNA-bind_3+Phage_integrase->SF1-SF2-Helicase+REase->SF1-SF2-Helicase+REase-><-PglZ<-TOPRIM<-OLD-ABC<-N6-METHYLASE<-DUF6079*<-BrxB-BrxF-iSTAND<-BrxA-DNA-binding<-wHTH+WYL+WCX||?->?->DUF2787->?->||<-?||MODE-HTH+PBPII->	1154	Bacteria>Pseudomonadota>Gammaproteobacteria>Vibrionales>Vibrionaceae>Vibrio	GCA_015814185.2	Vibrio_parahaemolyticus	C4G97_RS14675	118301..121765	670	BREX-system-P-loop-protein-BrxC-[Vibrio-parahaemolyticus]
EGR4671255.1	Type1.BREX	AbiEi->viral-protein->SF1-SF2-Helicase+REase->SF1-SF2-Helicase+REase-><-TPR<-HEPN+SF1-SF2-Helicase+RNase+RNase<-PglZ<-ParB+HNH<-N6-METHYLASE<-DUF6079*<-BrxB-BrxF-iSTAND<-BrxA-DNA-binding<-wHTH+WYL+WCX||DUF4113-><-?<-CR-ATPase4||SbcC->	1157	Bacteria>Pseudomonadota>Gammaproteobacteria>Vibrionales>Vibrionaceae>Vibrio	GCA_015814545.1	Vibrio_parahaemolyticus	C4G45_02530	555237..558710	670	BREX-system-P-loop-protein-BrxC-[Vibrio-parahaemolyticus]
EJG1873897.1	Type1.BREX	AbiEi->viral-protein->SF1-SF2-Helicase+REase->SF1-SF2-Helicase+REase-><-?<-ZetaToxin<-PglZ<-ParB+HNH<-N6-METHYLASE<-DUF6079*<-BrxB-BrxF-iSTAND<-BrxA-DNA-binding<-wHTH+WYL+WCX||DUF4113-><-?<-CR-ATPase4	1157	Bacteria>Pseudomonadota>Gammaproteobacteria>Vibrionales>Vibrionaceae>Vibrio	GCA_015817955.2	Vibrio_parahaemolyticus	BS053_RS09090	170796..174269	670	BREX-system-P-loop-protein-BrxC-[Vibrio-parahaemolyticus]
HAS8442986.1	Type1.BREX	MobI-><-LexA-protease||?->35exo->?->wHTH+WYL+WCX->BrxA-DNA-binding->BrxB-BrxF-iSTAND->DUF6079*->ParB+HNH->N6-METHYLASE->PglZ->SIGMA+OB-Fold+AAA-ATPase+Lon-Protease-BrxL->NACHT+HEAT+REase->HDRnase->VirD4-TraG+VirB4-FtsK->	1225	Bacteria>Pseudomonadota>Gammaproteobacteria>Vibrionales>Vibrionaceae>Vibrio	GCA_015820485.1	Vibrio_vulnificus	I7740_20130	17039..20716	672	BREX-system-P-loop-protein-BrxC-[Vibrio-vulnificus]
HAS8502529.1	Type1.BREX	<-ZnR<-?||?->SF1-SF2-Helicase->AbiEi->viral-protein->wHTH+WYL+WCX->BrxA-DNA-binding->BrxB-BrxF-iSTAND->DUF6079*->N6-METHYLASE->ParB+HNH->GrpE->?->PglZ->SIGMA+OB-Fold+AAA-ATPase+Lon-Protease-BrxL-><-wHTH	1225	Bacteria>Pseudomonadota>Gammaproteobacteria>Vibrionales>Vibrionaceae>Vibrio	GCA_015820675.1	Vibrio_vulnificus	I7764_01770	430591..434268	672	BREX-system-P-loop-protein-BrxC-[Vibrio-vulnificus]
HAT7627508.1	Type1.BREX	<-VirD4-TraG+VirB4-FtsK<-HDRnase<-HEPN<-MarR-HTH<-SIGMA+OB-Fold+AAA-ATPase+Lon-Protease-BrxL<-PglZ<-N6-METHYLASE<-DUF6079*	1216	Bacteria>Pseudomonadota>Gammaproteobacteria>Vibrionales>Vibrionaceae>Vibrio	GCA_015958965.1	Vibrio_cholerae_O1	JAW37_002070	69756..73407	127906	BREX-system-P-loop-protein-BrxC-[Vibrio-cholerae-O1]
MBH9740666.1	Type1.BREX	SF1-SF2-Helicase+HTH->MukF->JetB->XIS-HTH->BrxA-DNA-binding->BrxB-BrxF-iSTAND->DUF6079*->OLD-ABC->REase->N6-METHYLASE->PglZ->SIGMA+OB-Fold+AAA-ATPase+HKD-DNase-BrxL->	1185	Bacteria>Pseudomonadota>Gammaproteobacteria>Vibrionales>Vibrionaceae>Vibrio	GCA_016094345.1	Vibrio_navarrensis	CJ419_13180	152917..156474	29495	hypothetical-protein-[Vibrio-navarrensis]
MBM4838763.1	Type1.BREX	AbiEi->viral-protein->SF1-SF2-Helicase+REase->SF1-SF2-Helicase+REase-><-TPR<-HEPN+SF1-SF2-Helicase+RNase+RNase<-PglZ<-ParB+HNH<-N6-METHYLASE<-DUF6079*<-BrxB-BrxF-iSTAND<-BrxA-DNA-binding<-wHTH+WYL+WCX||HTH_Tnp_1+HTH_21+rve+rve_3-><-wHTH||DUF4113-><-?<-CR-ATPase4||PIN+OST-HTH->	1157	Bacteria>Pseudomonadota>Gammaproteobacteria>Vibrionales>Vibrionaceae>Vibrio	GCA_016876235.1	Vibrio_parahaemolyticus	HYO55_06530	138658..142131	670	BREX-system-P-loop-protein-BrxC-[Vibrio-parahaemolyticus]
MBM4875012.1	Type1.BREX	<-AAA-ATPase-p<-HEPN||CR-ATPase4->?->||wHTH+WYL+WCX->BrxA-DNA-binding->BrxB-BrxF-iSTAND->DUF6079*->N6-METHYLASE->ParB+HNH->PglZ->HEPN+SF1-SF2-Helicase+RNase+RNase->TPR-><-SF1-SF2-Helicase+REase<-SF1-SF2-Helicase+REase<-viral-protein<-AbiEi	1157	Bacteria>Pseudomonadota>Gammaproteobacteria>Vibrionales>Vibrionaceae>Vibrio	GCA_016876415.1	Vibrio_parahaemolyticus	HYO45_05440	517620..521093	670	BREX-system-P-loop-protein-BrxC-[Vibrio-parahaemolyticus]
EHD2253057.1	Type1.BREX	viral-protein->wHTH+WYL+WCX->BrxA-DNA-binding->BrxB-BrxF-iSTAND->DUF6079*->N6-METHYLASE->ParB+HNH->GrpE->?->PglZ->SIGMA+OB-Fold+AAA-ATPase+Lon-Protease-BrxL-><-wHTH||DUF4113->	1225	Bacteria>Pseudomonadota>Gammaproteobacteria>Vibrionales>Vibrionaceae>Vibrio	GCA_017126235.1	Vibrio_vulnificus	JRT24_004016	2833..6510	672	BREX-system-P-loop-protein-BrxC-[Vibrio-vulnificus]
EHH1264392.1	Type1.BREX	<-MORC<-PIN+OST-HTH<-CARF+NtrC-AAA<-HKD-DNase+HKD-DNase<-MarR-HTH<-SF1-SF2-Helicase||wHTH+WYL+WCX->BrxA-DNA-binding->BrxB-BrxF-iSTAND->DUF6079*->N6-METHYLASE->ParB+HNH->?->?->PglZ->SIGMA+OB-Fold+AAA-ATPase+Lon-Protease-BrxL-><-?<-CR-ATPase4	1248	Bacteria>Pseudomonadota>Gammaproteobacteria>Vibrionales>Vibrionaceae>Vibrio	GCA_017604425.1	Vibrio_parahaemolyticus	J7I02_003660	14110..17856	670	BREX-system-P-loop-protein-BrxC-[Vibrio-parahaemolyticus]
EHH2507631.1	Type1.BREX	Arm-DNA-bind_3+Phage_integrase->SF1-SF2-Helicase+REase->SF1-SF2-Helicase+REase-><-PglZ<-TOPRIM<-OLD-ABC<-N6-METHYLASE<-DUF6079*<-BrxB-BrxF-iSTAND<-BrxA-DNA-binding<-wHTH+WYL+WCX<-XIS-HTH	1154	Bacteria>Pseudomonadota>Gammaproteobacteria>Vibrionales>Vibrionaceae>Vibrio	GCA_017637525.1	Vibrio_parahaemolyticus	J7X10_003794	109543..113007	670	BREX-system-P-loop-protein-BrxC-[Vibrio-parahaemolyticus]
EHK0048698.1	Type1.BREX	<-DUF4113||wHTH-><-SIGMA+OB-Fold+AAA-ATPase+Lon-Protease-BrxL<-PglZ<-?<-GrpE<-ParB+HNH<-N6-METHYLASE<-DUF6079*<-BrxB-BrxF-iSTAND<-BrxA-DNA-binding<-wHTH+WYL+WCX<-viral-protein<-AbiEi<-SF1-SF2-Helicase<-?||?->ZnR->	1225	Bacteria>Pseudomonadota>Gammaproteobacteria>Vibrionales>Vibrionaceae>Vibrio	GCA_017968185.1	Vibrio_parahaemolyticus	KCA08_003292	85751..89428	670	BREX-system-P-loop-protein-BrxC-[Vibrio-parahaemolyticus]
HBC2045456.1	Type1.BREX	<-VirD4-TraG+VirB4-FtsK<-HDRnase<-HEPN<-MarR-HTH<-SIGMA+OB-Fold+AAA-ATPase+Lon-Protease-BrxL<-PglZ<-N6-METHYLASE<-DUF6079*	1184	Bacteria>Pseudomonadota>Gammaproteobacteria>Vibrionales>Vibrionaceae>Vibrio	GCA_018073665.1	Vibrio_cholerae	I6Z89_000440	124693..128249	666	BREX-system-P-loop-protein-BrxC-[Vibrio-cholerae]
HBC2117287.1	Type1.BREX	DUF6079*->N6-METHYLASE->PglZ->SIGMA+OB-Fold+AAA-ATPase+Lon-Protease-BrxL->MarR-HTH->HEPN->HDRnase->VirD4-TraG+VirB4-FtsK->	1191	Bacteria>Pseudomonadota>Gammaproteobacteria>Vibrionales>Vibrionaceae>Vibrio	GCA_018074265.1	Vibrio_cholerae	I6207_001264	1..3578	666	BREX-system-P-loop-protein-BrxC-[Vibrio-cholerae]
EHK9043270.1	Type1.BREX	<-MORC<-PIN+OST-HTH<-CARF+NtrC-AAA<-HKD-DNase+HKD-DNase<-MarR-HTH<-SF1-SF2-Helicase||wHTH+WYL+WCX->BrxA-DNA-binding->BrxB-BrxF-iSTAND->DUF6079*->N6-METHYLASE->ParB+HNH->ZnR+3C-ABC+TPR->?->?->?->?->PglZ->SIGMA+OB-Fold+AAA-ATPase+Lon-Protease-BrxL->	1248	Bacteria>Pseudomonadota>Gammaproteobacteria>Vibrionales>Vibrionaceae>Vibrio	GCA_018080905.1	Vibrio_vulnificus	KCU37_001412	195245..198991	672	BREX-system-P-loop-protein-BrxC-[Vibrio-vulnificus]
HBC3376470.1	Type1.BREX	<-ZnR<-?||?->SF1-SF2-Helicase->AbiEi->viral-protein->wHTH+WYL+WCX->BrxA-DNA-binding->BrxB-BrxF-iSTAND->DUF6079*->N6-METHYLASE->ParB+HNH->GrpE->?->PglZ->SIGMA+OB-Fold+AAA-ATPase+Lon-Protease-BrxL-><-TPR<-HEPN+SF1-SF2-Helicase+RNase+RNase||LexA-protease->	1225	Bacteria>Pseudomonadota>Gammaproteobacteria>Vibrionales>Vibrionaceae>Vibrio	GCA_018118685.1	Vibrio_cholerae	KDX67_000916	90395..94072	666	BREX-system-P-loop-protein-BrxC-[Vibrio-cholerae]
HBC3399177.1	Type1.BREX	<-LexA-protease||HEPN+SF1-SF2-Helicase+RNase+RNase->TPR-><-SIGMA+OB-Fold+AAA-ATPase+Lon-Protease-BrxL<-PglZ<-?<-GrpE<-ParB+HNH<-N6-METHYLASE<-DUF6079*<-BrxB-BrxF-iSTAND<-BrxA-DNA-binding<-wHTH+WYL+WCX<-viral-protein<-AbiEi<-SF1-SF2-Helicase||?->ZnR->	1225	Bacteria>Pseudomonadota>Gammaproteobacteria>Vibrionales>Vibrionaceae>Vibrio	GCA_018118805.1	Vibrio_parahaemolyticus	KDX79_000987	173788..177465	670	BREX-system-P-loop-protein-BrxC-[Vibrio-parahaemolyticus]
HBC3428133.1	Type1.BREX	<-PglZ<-?<-?<-?<-?<-ZnR+3C-ABC+TPR<-ParB+HNH<-DUF932<-N6-METHYLASE<-DUF6079*<-BrxB-BrxF-iSTAND<-BrxA-DNA-binding<-wHTH+WYL+WCX||SF1-SF2-Helicase->MarR-HTH->HKD-DNase+HKD-DNase->CARF+NtrC-AAA->PIN+OST-HTH->||HTH_Tnp_1->	1248	Bacteria>Pseudomonadota>Gammaproteobacteria>Vibrionales>Vibrionaceae>Vibrio	GCA_018118885.1	Vibrio_parahaemolyticus	KDY17_000940	500367..504113	670	BREX-system-P-loop-protein-BrxC-[Vibrio-parahaemolyticus]
HBC3445072.1	Type1.BREX	<-ZnR<-?||AbiEi->viral-protein->wHTH+WYL+WCX->BrxA-DNA-binding->BrxB-BrxF-iSTAND->DUF6079*->N6-METHYLASE->?->?->PglZ->SIGMA+OB-Fold+AAA-ATPase+Lon-Protease-BrxL-><-TPR<-HEPN+SF1-SF2-Helicase+RNase+RNase||<-?<-CR-ATPase4	1225	Bacteria>Pseudomonadota>Gammaproteobacteria>Vibrionales>Vibrionaceae>Vibrio	GCA_018119025.1	Vibrio_parahaemolyticus	KDY48_003788	6147..9824	670	BREX-system-P-loop-protein-BrxC-[Vibrio-parahaemolyticus]
HBC3481459.1	Type1.BREX	REase+SPF-cons->?-><-PIN+OST-HTH||CR-ATPase4->?-><-DUF4113||wHTH+WYL+WCX->BrxA-DNA-binding->BrxB-BrxF-iSTAND->DUF6079*->N6-METHYLASE->ParB+HNH->PglZ->ZetaToxin->?-><-SF1-SF2-Helicase+REase<-SF1-SF2-Helicase+REase<-viral-protein<-AbiEi	1157	Bacteria>Pseudomonadota>Gammaproteobacteria>Vibrionales>Vibrionaceae>Vibrio	GCA_018119205.1	Vibrio_parahaemolyticus	KDY88_002916	65183..68656	670	BREX-system-P-loop-protein-BrxC-[Vibrio-parahaemolyticus]
HBC3540120.1	Type1.BREX	<-ZnR+3C-ABC+TPR<-ParB+HNH<-?<-N6-METHYLASE<-DUF6079*<-BrxB-BrxF-iSTAND<-BrxA-DNA-binding<-wHTH+WYL+WCX||SF1-SF2-Helicase->MarR-HTH->HKD-DNase+HKD-DNase->CARF+NtrC-AAA->PIN+OST-HTH->||HTH_Tnp_1->	1248	Bacteria>Pseudomonadota>Gammaproteobacteria>Vibrionales>Vibrionaceae>Vibrio	GCA_018119555.1	Vibrio_parahaemolyticus	KDZ97_004679	8277..12023	670	BREX-system-P-loop-protein-BrxC-[Vibrio-parahaemolyticus]
HBC3921009.1	Type1.BREX	<-HigA||CR-ATPase4->?-><-?<-LexA-protease||wHTH+WYL+WCX->BrxA-DNA-binding->BrxB-BrxF-iSTAND->DUF6079*->N6-METHYLASE->ParB+HNH->PglZ->ZetaToxin->?-><-SF1-SF2-Helicase+REase<-SF1-SF2-Helicase+REase<-viral-protein<-AbiEi	1157	Bacteria>Pseudomonadota>Gammaproteobacteria>Vibrionales>Vibrionaceae>Vibrio	GCA_018126325.1	Vibrio_parahaemolyticus	KD834_003320	38197..41670	670	BREX-system-P-loop-protein-BrxC-[Vibrio-parahaemolyticus]
HBC3861549.1	Type1.BREX	<-VirD4-TraG+VirB4-FtsK<-HDRnase<-SbcC<-SIGMA+OB-Fold+AAA-ATPase+Lon-Protease-BrxL<-PglZ<-N6-METHYLASE<-ParB+HNH<-DUF6079*<-BrxB-BrxF-iSTAND<-BrxA-DNA-binding<-wHTH+WYL+WCX<-?<-35exo||LexA-protease->?-><-MobI	1225	Bacteria>Pseudomonadota>Gammaproteobacteria>Vibrionales>Vibrionaceae>Vibrio	GCA_018126725.1	Vibrio_parahaemolyticus	KD871_001930	256177..259854	670	BREX-system-P-loop-protein-BrxC-[Vibrio-parahaemolyticus]
HBC3986622.1	Type1.BREX	<-DUF4113||wHTH-><-SIGMA+OB-Fold+AAA-ATPase+Lon-Protease-BrxL<-PglZ<-?<-GrpE<-ParB+HNH<-N6-METHYLASE<-DUF6079*<-BrxB-BrxF-iSTAND<-BrxA-DNA-binding<-wHTH+WYL+WCX<-viral-protein<-AbiEi||?->ZnR->	1225	Bacteria>Pseudomonadota>Gammaproteobacteria>Vibrionales>Vibrionaceae>Vibrio	GCA_018126825.1	Vibrio_parahaemolyticus	KD867_003188	79006..82683	670	BREX-system-P-loop-protein-BrxC-[Vibrio-parahaemolyticus]
HBC3940910.1	Type1.BREX	<-HigA||CR-ATPase4->?-><-?<-LexA-protease||wHTH+WYL+WCX->BrxA-DNA-binding->BrxB-BrxF-iSTAND->DUF6079*->N6-METHYLASE->ParB+HNH->PglZ->ZetaToxin->?-><-SF1-SF2-Helicase+REase<-SF1-SF2-Helicase+REase<-viral-protein<-AbiEi	1157	Bacteria>Pseudomonadota>Gammaproteobacteria>Vibrionales>Vibrionaceae>Vibrio	GCA_018127045.1	Vibrio_parahaemolyticus	KD880_004471	11650..15123	670	BREX-system-P-loop-protein-BrxC-[Vibrio-parahaemolyticus]
MBT0011870.1	Type1.BREX	<-SIGMA+OB-Fold+AAA-ATPase+HKD-DNase-BrxL<-PglZ<-N6-METHYLASE<-REase<-OLD-ABC<-DUF6079*<-BrxB-BrxF-iSTAND<-BrxA-DNA-binding<-XIS-HTH<-HNH<-XIS-HTH||?->?->DUF2787->wHTH+WYL+WCX->	1187	Bacteria>Pseudomonadota>Gammaproteobacteria>Vibrionales>Vibrionaceae>Vibrio	GCA_018457755.1	Vibrio_alginolyticus	N7I24_002738	64007..67570	663	BREX-system-P-loop-protein-BrxC-[Vibrio-alginolyticus]
EHW0635156.1	Type1.BREX	wHTH-><-SIGMA+OB-Fold+AAA-ATPase+Lon-Protease-BrxL<-PglZ<-?<-GrpE<-ParB+HNH<-N6-METHYLASE<-DUF6079*<-BrxB-BrxF-iSTAND<-BrxA-DNA-binding<-wHTH+WYL+WCX<-viral-protein<-AbiEi<-SF1-SF2-Helicase<-?||?->ZnR->	1225	Bacteria>Pseudomonadota>Gammaproteobacteria>Vibrionales>Vibrionaceae>Vibrio	GCA_019442565.1	Vibrio_vulnificus	K1B29_001386	71922..75599	672	BREX-system-P-loop-protein-BrxC-[Vibrio-vulnificus]
EHW0654650.1	Type1.BREX	HTH_20-><-HEPN<-SbcC||CR-ATPase4->?-><-DUF4113||wHTH+WYL+WCX->BrxA-DNA-binding->BrxB-BrxF-iSTAND->DUF6079*->N6-METHYLASE->ParB+HNH->PglZ->ZetaToxin->?-><-SF1-SF2-Helicase+REase<-SF1-SF2-Helicase+REase<-viral-protein<-AbiEi	1156	Bacteria>Pseudomonadota>Gammaproteobacteria>Vibrionales>Vibrionaceae>Vibrio	GCA_019442625.1	Vibrio_parahaemolyticus	K1B36_003414	61374..64844	670	BREX-system-P-loop-protein-BrxC-[Vibrio-parahaemolyticus]
EHZ2755060.1	Type1.BREX	<-ZnR<-?||?->SF1-SF2-Helicase->AbiEi->viral-protein->wHTH+WYL+WCX->BrxA-DNA-binding->BrxB-BrxF-iSTAND->DUF6079*->N6-METHYLASE->SbcC->Schlafen+HATPase+wHTH->PglZ->SIGMA+OB-Fold+AAA-ATPase+Lon-Protease-BrxL-><-wHTH<-?<-CR-ATPase4	1225	Bacteria>Pseudomonadota>Gammaproteobacteria>Vibrionales>Vibrionaceae>Vibrio	GCA_019682715.1	Vibrio_vulnificus	K5N53_003177	34547..38224	672	BREX-system-P-loop-protein-BrxC-[Vibrio-vulnificus]
MBY7947240.1	Type1.BREX	CR-ATPase4->?->||?->wHTH-><-SIGMA+OB-Fold+AAA-ATPase+Lon-Protease-BrxL<-PglZ<-Schlafen+HATPase+wHTH<-SbcC<-N6-METHYLASE<-DUF6079*<-BrxB-BrxF-iSTAND<-BrxA-DNA-binding<-wHTH+WYL+WCX<-viral-protein<-AbiEi||?->ZnR->	1225	Bacteria>Pseudomonadota>Gammaproteobacteria>Vibrionales>Vibrionaceae>Vibrio	GCA_019853335.1	Vibrio_fluvialis	KW461_01980	454560..458237	676	BREX-system-P-loop-protein-BrxC-[Vibrio-fluvialis]
MBY8044957.1	Type1.BREX	<-HTH_Tnp_1+HTH_21+rve+rve_3||<-TPR<-HEPN+SF1-SF2-Helicase+RNase+RNase<-PglZ<-ParB+HNH<-N6-METHYLASE<-DUF6079*<-BrxB-BrxF-iSTAND<-BrxA-DNA-binding<-wHTH+WYL+WCX||LexA-protease->?-><-CR-ATPase4||<-RVT||HigA->	1157	Bacteria>Pseudomonadota>Gammaproteobacteria>Vibrionales>Vibrionaceae>Vibrio	GCA_019853775.1	Vibrio_fluvialis	KW486_21175	14278..17751	676	BREX-system-P-loop-protein-BrxC-[Vibrio-fluvialis]
MBY8148861.1	Type1.BREX	AbiEi->viral-protein->SF1-SF2-Helicase+REase->SF1-SF2-Helicase+REase-><-?<-ZetaToxin<-PglZ<-ParB+HNH<-N6-METHYLASE<-DUF6079*<-BrxB-BrxF-iSTAND<-BrxA-DNA-binding<-wHTH+WYL+WCX||LexA-protease->?-><-?<-CR-ATPase4||HigA-><-Phage_integrase	1157	Bacteria>Pseudomonadota>Gammaproteobacteria>Vibrionales>Vibrionaceae>Vibrio	GCA_019854195.1	Vibrio_fluvialis	KW504_05155	39947..43420	676	BREX-system-P-loop-protein-BrxC-[Vibrio-fluvialis]
MBY8207859.1	Type1.BREX	<-DUF4113||wHTH-><-SIGMA+OB-Fold+AAA-ATPase+Lon-Protease-BrxL<-PglZ<-?<-GrpE<-ParB+HNH<-N6-METHYLASE<-DUF6079*<-BrxB-BrxF-iSTAND<-BrxA-DNA-binding<-wHTH+WYL+WCX<-viral-protein<-AbiEi||?->ZnR->	1225	Bacteria>Pseudomonadota>Gammaproteobacteria>Vibrionales>Vibrionaceae>Vibrio	GCA_019854355.1	Vibrio_fluvialis	KW531_20835	16453..20130	676	BREX-system-P-loop-protein-BrxC-[Vibrio-fluvialis]
EIE1271992.1	Type1.BREX	<-ZnR<-?||?->SF1-SF2-Helicase->AbiEi->viral-protein->wHTH+WYL+WCX->BrxA-DNA-binding->BrxB-BrxF-iSTAND->DUF6079*->N6-METHYLASE->?->?->PglZ->SIGMA+OB-Fold+AAA-ATPase+Lon-Protease-BrxL-><-TPR<-HEPN+SF1-SF2-Helicase+RNase+RNase||LexA-protease->	1225	Bacteria>Pseudomonadota>Gammaproteobacteria>Vibrionales>Vibrionaceae>Vibrio	GCA_020124895.1	Vibrio_parahaemolyticus	LCX75_001329	151524..155201	670	BREX-system-P-loop-protein-BrxC-[Vibrio-parahaemolyticus]
EII2403787.1	Type1.BREX	<-GDResolvase+HIN-HTH<-?||Arm-DNA-bind_3+Phage_integrase->SF1-SF2-Helicase+REase->SF1-SF2-Helicase+REase-><-PglZ<-TOPRIM<-OLD-ABC<-N6-METHYLASE<-DUF6079*<-BrxB-BrxF-iSTAND<-BrxA-DNA-binding<-wHTH+WYL+WCX<-XIS-HTH	1154	Bacteria>Pseudomonadota>Gammaproteobacteria>Vibrionales>Vibrionaceae>Vibrio	GCA_020407245.1	Vibrio_parahaemolyticus	LG159_002760	116849..120313	670	BREX-system-P-loop-protein-BrxC-[Vibrio-parahaemolyticus]
EII3725635.1	Type1.BREX	<-VirD4-TraG+VirB4-FtsK<-HDRnase<-SbcC<-SIGMA+OB-Fold+AAA-ATPase+Lon-Protease-BrxL<-PglZ<-TOPRIM<-OLD-ABC<-N6-METHYLASE<-ParB+HNH<-DUF6079*<-BrxB-BrxF-iSTAND<-BrxA-DNA-binding<-wHTH+WYL+WCX<-?<-35exo<-?||LexA-protease->?-><-MobI	1225	Bacteria>Pseudomonadota>Gammaproteobacteria>Vibrionales>Vibrionaceae>Vibrio	GCA_020414365.1	Vibrio_cholerae	LG468_000348	129198..132875	666	BREX-system-P-loop-protein-BrxC-[Vibrio-cholerae]
HBK3330408.1	Type1.BREX	<-TPR+HEPN||<-PIN+OST-HTH<-CARF+NtrC-AAA<-HKD-DNase+HKD-DNase<-MarR-HTH<-SF1-SF2-Helicase||wHTH+WYL+WCX->BrxA-DNA-binding->BrxB-BrxF-iSTAND->DUF6079*->N6-METHYLASE->ParB+HNH->ZnR+3C-ABC+TPR->?->?->PglZ->SIGMA+OB-Fold+AAA-ATPase+Lon-Protease-BrxL-><-?<-CR-ATPase4	1248	Bacteria>Pseudomonadota>Gammaproteobacteria>Vibrionales>Vibrionaceae>Vibrio	GCA_020473965.1	Vibrio_parahaemolyticus	KD833_003429	17712..21458	670	BREX-system-P-loop-protein-BrxC-[Vibrio-parahaemolyticus]
EIT6975184.1	Type1.BREX	Couple_hipA+HipA_C->SF1-SF2-Helicase-><-?||viral-protein-><-?<-?||wHTH+WYL+WCX->BrxA-DNA-binding->BrxB-BrxF-iSTAND->DUF6079*->	1232	Bacteria>Pseudomonadota>Gammaproteobacteria>Vibrionales>Vibrionaceae>Vibrio	GCA_021537035.1	Vibrio_vulnificus	L2596_001303	248208..251906	672	BREX-system-P-loop-protein-BrxC-[Vibrio-vulnificus]
EIU6866063.1	Type1.BREX	CR-ATPase4->?-><-DUF4113||wHTH-><-SIGMA+OB-Fold+AAA-ATPase+Lon-Protease-BrxL<-PglZ<-?<-?<-N6-METHYLASE<-DUF6079*<-BrxB-BrxF-iSTAND<-BrxA-DNA-binding<-wHTH+WYL+WCX<-viral-protein<-AbiEi<-SF1-SF2-Helicase<-?||?->ZnR->	1225	Bacteria>Pseudomonadota>Gammaproteobacteria>Vibrionales>Vibrionaceae>Vibrio	GCA_021704015.1	Vibrio_parahaemolyticus	L5169_000073	92916..96593	670	BREX-system-P-loop-protein-BrxC-[Vibrio-parahaemolyticus]
EIU7849849.1	Type1.BREX	AbiEi->viral-protein->SF1-SF2-Helicase+REase->SF1-SF2-Helicase+REase-><-?<-ZetaToxin<-PglZ<-ParB+HNH<-N6-METHYLASE<-DUF6079*<-BrxB-BrxF-iSTAND<-BrxA-DNA-binding<-wHTH+WYL+WCX||<-?<-CR-ATPase4||?->HEPN-><-sGTP	1157	Bacteria>Pseudomonadota>Gammaproteobacteria>Vibrionales>Vibrionaceae>Vibrio	GCA_021716735.1	Vibrio_parahaemolyticus	L3I74_001636	175648..179121	670	BREX-system-P-loop-protein-BrxC-[Vibrio-parahaemolyticus]
EIV8505581.1	Type1.BREX	<-DUF4113||wHTH-><-SIGMA+OB-Fold+AAA-ATPase+Lon-Protease-BrxL<-PglZ<-Schlafen+HATPase+wHTH<-SbcC<-N6-METHYLASE<-DUF6079*<-BrxB-BrxF-iSTAND<-BrxA-DNA-binding<-wHTH+WYL+WCX<-viral-protein<-AbiEi||?->ZnR->	1225	Bacteria>Pseudomonadota>Gammaproteobacteria>Vibrionales>Vibrionaceae>Vibrio	GCA_022019715.1	Vibrio_parahaemolyticus	M5229_003413	80224..83901	670	BREX-system-P-loop-protein-BrxC-[Vibrio-parahaemolyticus]
MCG9593371.1	Type1.BREX	CR-ATPase4->?-><-DUF4113||wHTH-><-SIGMA+OB-Fold+AAA-ATPase+Lon-Protease-BrxL<-PglZ<-?<-?<-N6-METHYLASE<-DUF6079*	1171	Bacteria>Pseudomonadota>Gammaproteobacteria>Vibrionales>Vibrionaceae>Vibrio	GCA_022398055.1	Vibrio_diabolicus	L1D58_17270	13232..16749	50719	BREX-system-P-loop-protein-BrxC-[Vibrio-diabolicus]
EIY9800890.1	Type1.BREX	<-ZnR<-?||?->SF1-SF2-Helicase->AbiEi->viral-protein->wHTH+WYL+WCX->BrxA-DNA-binding->BrxB-BrxF-iSTAND->DUF6079*->N6-METHYLASE->ParB+HNH->GrpE->?->PglZ->SIGMA+OB-Fold+AAA-ATPase+Lon-Protease-BrxL-><-wHTH	1225	Bacteria>Pseudomonadota>Gammaproteobacteria>Vibrionales>Vibrionaceae>Vibrio	GCA_022594155.1	Vibrio_parahaemolyticus	MNO17_002467	349786..353463	670	BREX-system-P-loop-protein-BrxC-[Vibrio-parahaemolyticus]
EIZ1010574.1	Type1.BREX	HTH_3->Couple_hipA+HipA_C->SAM-METHYLASE->HKD-DNase+SF1-SF2-Helicase+HTH+HTH+SRA-><-SIGMA+OB-Fold+AAA-ATPase+HKD-DNase-BrxL<-PglZ<-N6-METHYLASE<-REase<-OLD-ABC<-DUF6079*<-BrxB-BrxF-iSTAND<-BrxA-DNA-binding<-XIS-HTH<-RNase<-DUF3584+DUF5401<-JetB<-MukF||wHTH+WYL+WCX-><-TPR	1187	Bacteria>Pseudomonadota>Gammaproteobacteria>Vibrionales>Vibrionaceae>Vibrio	GCA_022609845.1	Vibrio_vulnificus	MOS09_002154	288384..291947	672	BREX-system-P-loop-protein-BrxC-[Vibrio-vulnificus]
EJB1796336.1	Type1.BREX	SbcC+SbcC->?-><-?<-?||?->Arm-DNA-bind_3+Phage_integrase-><-SIGMA+OB-Fold+AAA-ATPase+Lon-Protease-BrxL<-PglZ<-N6-METHYLASE<-DUF6079*<-BrxB-BrxF-iSTAND<-BrxA-DNA-binding<-XIS-HTH<-XIS-HTH||?->?->DUF2787-><-RNase_H||HTH->	1221	Bacteria>Pseudomonadota>Gammaproteobacteria>Vibrionales>Vibrionaceae>Vibrio	GCA_022927965.1	Vibrio_parahaemolyticus	MUE05_003019	106094..109759	670	BREX-system-P-loop-protein-BrxC-[Vibrio-parahaemolyticus]
EJB8347940.1	Type1.BREX	<-VirD4-TraG+VirB4-FtsK<-HDRnase<-NACHT+HEAT+REase<-SIGMA+OB-Fold+AAA-ATPase+Lon-Protease-BrxL<-PglZ<-N6-METHYLASE<-TOPRIM<-OLD-ABC<-DUF6079*<-BrxB-BrxF-iSTAND<-BrxA-DNA-binding<-wHTH+WYL+WCX<-?<-35exo||LexA-protease->?-><-MobI	1225	Bacteria>Pseudomonadota>Gammaproteobacteria>Vibrionales>Vibrionaceae>Vibrio	GCA_022966375.1	Vibrio_cholerae	MW328_000282	40595..44272	666	BREX-system-P-loop-protein-BrxC-[Vibrio-cholerae]
EJB8581804.1	Type1.BREX	<-VirD4-TraG+VirB4-FtsK<-HDRnase<-SbcC<-SIGMA+OB-Fold+AAA-ATPase+Lon-Protease-BrxL<-PglZ<-N6-METHYLASE<-TOPRIM<-OLD-ABC<-DUF6079*<-BrxB-BrxF-iSTAND<-BrxA-DNA-binding<-wHTH+WYL+WCX<-?<-35exo||LexA-protease->?->Glyoxalase_8-><-MobI	1229	Bacteria>Pseudomonadota>Gammaproteobacteria>Vibrionales>Vibrionaceae>Vibrio	GCA_022967455.1	Vibrio_cholerae	MW361_003123	34970..38659	666	BREX-system-P-loop-protein-BrxC-[Vibrio-cholerae]
EJC1210117.1	Type1.BREX	<-DUF2787<-?<-?||XIS-HTH->wHTH+WYL+WCX->BrxA-DNA-binding->BrxB-BrxF-iSTAND->DUF6079*->N6-METHYLASE->OLD-ABC->TOPRIM->PglZ-><-SF1-SF2-Helicase+REase<-SF1-SF2-Helicase+REase<-Arm-DNA-bind_3+Phage_integrase||?->GDResolvase+HIN-HTH->	1154	Bacteria>Pseudomonadota>Gammaproteobacteria>Vibrionales>Vibrionaceae>Vibrio	GCA_022997325.1	Vibrio_parahaemolyticus	MX350_002594	5689..9153	670	BREX-system-P-loop-protein-BrxC-[Vibrio-parahaemolyticus]
EJC6937227.1	Type1.BREX	SF1-SF2-Helicase->AbiEi->viral-protein->BrxA-DNA-binding->BrxB-BrxF-iSTAND->DUF6079*->N6-METHYLASE->?->?->PglZ->SIGMA+OB-Fold+AAA-ATPase+Lon-Protease-BrxL-><-wHTH||DUF4113->	1225	Bacteria>Pseudomonadota>Gammaproteobacteria>Vibrionales>Vibrionaceae>Vibrio	GCA_023080515.1	Vibrio_parahaemolyticus	MX856_004599	4649..8326	670	BREX-system-P-loop-protein-BrxC-[Vibrio-parahaemolyticus]
GHW31175.1	Type1.BREX	<-HTH_Tnp_1||wHTH-><-SIGMA+OB-Fold+AAA-ATPase+Lon-Protease-BrxL<-PglZ<-?<-GrpE<-ParB+HNH<-N6-METHYLASE<-DUF6079*<-BrxB-BrxF-iSTAND<-BrxA-DNA-binding<-wHTH+WYL+WCX<-viral-protein<-AbiEi<-SF1-SF2-Helicase<-?||?->ZnR->	1225	Bacteria>Pseudomonadota>Gammaproteobacteria>Vibrionales>Vibrionaceae>Vibrio	GCA_023163105.1	Vibrio_cholerae	VCSRO150_3322	15322..18999	666	DNA-repair-protein-[Vibrio-cholerae]
EJE4207362.1	Type1.BREX	<-VirD4-TraG+VirB4-FtsK<-HDRnase<-SbcC<-SIGMA+OB-Fold+AAA-ATPase+Lon-Protease-BrxL<-PglZ<-N6-METHYLASE<-TOPRIM<-OLD-ABC<-DUF6079*<-BrxB-BrxF-iSTAND<-BrxA-DNA-binding<-wHTH+WYL+WCX<-?<-35exo||LexA-protease->?-><-MobI	1225	Bacteria>Pseudomonadota>Gammaproteobacteria>Vibrionales>Vibrionaceae>Vibrio	GCA_023313315.1	Vibrio_parahaemolyticus	M3899_001773	267109..270786	670	BREX-system-P-loop-protein-BrxC-[Vibrio-parahaemolyticus]
EJE8514353.1	Type1.BREX	CR-ATPase4->?-><-DUF4113||wHTH-><-SIGMA+OB-Fold+AAA-ATPase+Lon-Protease-BrxL<-PglZ<-Schlafen+HATPase+wHTH<-SbcC<-N6-METHYLASE<-DUF6079*<-BrxB-BrxF-iSTAND<-BrxA-DNA-binding<-wHTH+WYL+WCX<-viral-protein<-AbiEi<-SF1-SF2-Helicase<-?||?->ZnR->	1225	Bacteria>Pseudomonadota>Gammaproteobacteria>Vibrionales>Vibrionaceae>Vibrio	GCA_023352155.1	Vibrio_parahaemolyticus	M5244_002608	72363..76040	670	BREX-system-P-loop-protein-BrxC-[Vibrio-parahaemolyticus]
EJE8545413.1	Type1.BREX	<-SIGMA+OB-Fold+AAA-ATPase+Lon-Protease-BrxL<-PglZ||RADICAL-SAM->?-><-?<-?<-ParB+HNH<-N6-METHYLASE<-DUF6079*<-BrxB-BrxF-iSTAND<-BrxA-DNA-binding<-wHTH+WYL+WCX<-viral-protein||?-><-SF1-SF2-Helicase<-Couple_hipA+HipA_C	1232	Bacteria>Pseudomonadota>Gammaproteobacteria>Vibrionales>Vibrionaceae>Vibrio	GCA_023352275.1	Vibrio_vulnificus	M5238_000046	65248..68946	672	BREX-system-P-loop-protein-BrxC-[Vibrio-vulnificus]
EJE8564157.1	Type1.BREX	AbiEi->viral-protein->SF1-SF2-Helicase+REase->SF1-SF2-Helicase+REase-><-?<-ZetaToxin<-PglZ<-ParB+HNH<-N6-METHYLASE<-DUF6079*<-BrxB-BrxF-iSTAND<-BrxA-DNA-binding<-wHTH+WYL+WCX||DUF4113-><-?<-CR-ATPase4||SbcC->HEPN-><-HTH_20	1157	Bacteria>Pseudomonadota>Gammaproteobacteria>Vibrionales>Vibrionaceae>Vibrio	GCA_023352375.1	Vibrio_parahaemolyticus	M5224_000723	57760..61233	670	BREX-system-P-loop-protein-BrxC-[Vibrio-parahaemolyticus]
EJF4458718.1	Type1.BREX	<-DUF4113||wHTH-><-SIGMA+OB-Fold+AAA-ATPase+Lon-Protease-BrxL<-PglZ<-?<-GrpE<-ParB+HNH<-N6-METHYLASE<-DUF6079*<-BrxB-BrxF-iSTAND<-BrxA-DNA-binding<-wHTH+WYL+WCX<-viral-protein<-AbiEi<-SF1-SF2-Helicase<-?||ZnR->	1225	Bacteria>Pseudomonadota>Gammaproteobacteria>Vibrionales>Vibrionaceae>Vibrio	GCA_023466975.1	Vibrio_parahaemolyticus	MT303_002826	138363..142040	670	BREX-system-P-loop-protein-BrxC-[Vibrio-parahaemolyticus]
HCD5120999.1	Type1.BREX	AbiEi->viral-protein->wHTH+WYL+WCX->BrxA-DNA-binding->BrxB-BrxF-iSTAND->DUF6079*->N6-METHYLASE->ParB+HNH->GrpE->?->PglZ->SIGMA+OB-Fold+AAA-ATPase+Lon-Protease-BrxL-><-wHTH	1225	Bacteria>Pseudomonadota>Gammaproteobacteria>Vibrionales>Vibrionaceae>Vibrio	GCA_023611065.1	Vibrio_parahaemolyticus	NB556_01245	3462..7139	670	BREX-system-P-loop-protein-BrxC-[Vibrio-parahaemolyticus]
HCE1978782.1	Type1.BREX	<-ZnR<-?||?->SF1-SF2-Helicase->AbiEi->viral-protein->wHTH+WYL+WCX->BrxA-DNA-binding->BrxB-BrxF-iSTAND->DUF6079*->N6-METHYLASE->	1225	Bacteria>Pseudomonadota>Gammaproteobacteria>Vibrionales>Vibrionaceae>Vibrio	GCA_023810845.1	Vibrio_parahaemolyticus	NGS68_002079	171773..175450	670	BREX-system-P-loop-protein-BrxC-[Vibrio-parahaemolyticus]
HCE2271447.1	Type1.BREX	<-HEPN||CR-ATPase4->?->||wHTH-><-SIGMA+OB-Fold+AAA-ATPase+Lon-Protease-BrxL<-PglZ<-?<-?<-N6-METHYLASE<-DUF6079*<-BrxB-BrxF-iSTAND<-BrxA-DNA-binding<-wHTH+WYL+WCX<-viral-protein<-AbiEi||?->ZnR->?-><-PAS+GGDEF	1225	Bacteria>Pseudomonadota>Gammaproteobacteria>Vibrionales>Vibrionaceae>Vibrio	GCA_023811985.1	Vibrio_parahaemolyticus	NGT56_000627	721824..725501	670	BREX-system-P-loop-protein-BrxC-[Vibrio-parahaemolyticus]
HCE2679243.1	Type1.BREX	<-SIGMA+OB-Fold+AAA-ATPase+Lon-Protease-BrxL<-PglZ<-?<-?<-?<-?<-ZnR+3C-ABC+TPR<-ParB+HNH<-N6-METHYLASE<-DUF6079*<-BrxB-BrxF-iSTAND<-BrxA-DNA-binding<-wHTH+WYL+WCX||SF1-SF2-Helicase->MarR-HTH->HKD-DNase+HKD-DNase->CARF+NtrC-AAA->PIN+OST-HTH->MORC->	1248	Bacteria>Pseudomonadota>Gammaproteobacteria>Vibrionales>Vibrionaceae>Vibrio	GCA_023813525.1	Vibrio_parahaemolyticus	NGU92_000683	148376..152122	670	BREX-system-P-loop-protein-BrxC-[Vibrio-parahaemolyticus]
HCE3362112.1	Type1.BREX	<-VirD4-TraG+VirB4-FtsK<-HDRnase<-SbcC<-SIGMA+OB-Fold+AAA-ATPase+Lon-Protease-BrxL<-PglZ<-N6-METHYLASE<-TOPRIM<-OLD-ABC<-DUF6079*<-BrxB-BrxF-iSTAND<-BrxA-DNA-binding<-wHTH+WYL+WCX<-?<-35exo<-?||LexA-protease->?->Glyoxalase_8->	1225	Bacteria>Pseudomonadota>Gammaproteobacteria>Vibrionales>Vibrionaceae>Vibrio	GCA_023821665.1	Vibrio_parahaemolyticus	NG589_000244	272459..276136	670	BREX-system-P-loop-protein-BrxC-[Vibrio-parahaemolyticus]
HCG5108676.1	Type1.BREX	AbiEi->viral-protein->SF1-SF2-Helicase+REase->SF1-SF2-Helicase+REase-><-?<-ZetaToxin<-PglZ<-ParB+HNH<-N6-METHYLASE<-DUF6079*<-BrxB-BrxF-iSTAND<-BrxA-DNA-binding<-wHTH+WYL+WCX	1157	Bacteria>Pseudomonadota>Gammaproteobacteria>Vibrionales>Vibrionaceae>Vibrio	GCA_024034975.1	Vibrio_parahaemolyticus	NJY97_002344	346649..350122	670	BREX-system-P-loop-protein-BrxC-[Vibrio-parahaemolyticus]
HCG6132052.1	Type1.BREX	viral-protein->SF1-SF2-Helicase+REase->SF1-SF2-Helicase+REase-><-?<-?<-sGTP<-ParB+HNH<-N6-METHYLASE<-DUF6079*<-BrxB-BrxF-iSTAND<-BrxA-DNA-binding<-wHTH+WYL+WCX||<-?<-CR-ATPase4||?->HEPN-><-?<-DEOR-HTH	1157	Bacteria>Pseudomonadota>Gammaproteobacteria>Vibrionales>Vibrionaceae>Vibrio	GCA_024038995.1	Vibrio_parahaemolyticus	NJ068_002907	67806..71279	670	BREX-system-P-loop-protein-BrxC-[Vibrio-parahaemolyticus]
HCG7048824.1	Type1.BREX	<-VirD4-TraG+VirB4-FtsK<-HDRnase<-SbcC<-SIGMA+OB-Fold+AAA-ATPase+Lon-Protease-BrxL<-PglZ<-N6-METHYLASE<-ParB+HNH<-DUF6079*<-BrxB-BrxF-iSTAND<-BrxA-DNA-binding<-wHTH+WYL+WCX<-?<-35exo||LexA-protease->?-><-MobI	1225	Bacteria>Pseudomonadota>Gammaproteobacteria>Vibrionales>Vibrionaceae>Vibrio	GCA_024042595.1	Vibrio_parahaemolyticus	NJ298_000555	545999..549676	670	BREX-system-P-loop-protein-BrxC-[Vibrio-parahaemolyticus]
HCG7393723.1	Type1.BREX	McrB-AAA->McrC-NTD+REase->?->HNH->?-><-HEPN||XIS-HTH->BrxA-DNA-binding->BrxB-BrxF-iSTAND->DUF6079*->OLD-ABC->REase->N6-METHYLASE->PglZ->SIGMA+OB-Fold+AAA-ATPase+HKD-DNase-BrxL->Phage_integrase-><-ParB<-GDResolvase+HIN-HTH||OLD-ABC->	1187	Bacteria>Pseudomonadota>Gammaproteobacteria>Vibrionales>Vibrionaceae>Vibrio	GCA_024044075.1	Vibrio_parahaemolyticus	NJ383_004129	13694..17257	670	BREX-system-P-loop-protein-BrxC-[Vibrio-parahaemolyticus]
HCG7769728.1	Type1.BREX	<-SIGMA+OB-Fold+AAA-ATPase+Lon-Protease-BrxL<-PglZ<-?<-?<-ParB+HNH<-N6-METHYLASE<-DUF6079*<-BrxB-BrxF-iSTAND<-BrxA-DNA-binding<-wHTH+WYL+WCX||?->?->?->?->?-><-HTH_3	1248	Bacteria>Pseudomonadota>Gammaproteobacteria>Vibrionales>Vibrionaceae>Vibrio	GCA_024045435.1	Vibrio_parahaemolyticus	NJ450_003037	12821..16567	670	BREX-system-P-loop-protein-BrxC-[Vibrio-parahaemolyticus]
HCG8025121.1	Type1.BREX	<-ZnR<-?||?->SF1-SF2-Helicase->AbiEi->viral-protein->wHTH+WYL+WCX->BrxA-DNA-binding->BrxB-BrxF-iSTAND->DUF6079*->N6-METHYLASE->ParB+HNH->GrpE->?->PglZ->SIGMA+OB-Fold+AAA-ATPase+Lon-Protease-BrxL-><-wHTH||DUF4113->	1225	Bacteria>Pseudomonadota>Gammaproteobacteria>Vibrionales>Vibrionaceae>Vibrio	GCA_024046515.1	Vibrio_parahaemolyticus	NJ909_002125	181233..184910	670	BREX-system-P-loop-protein-BrxC-[Vibrio-parahaemolyticus]
HCG8164047.1	Type1.BREX	<-PIN+OST-HTH||CR-ATPase4->?->||wHTH-><-SIGMA+OB-Fold+AAA-ATPase+Lon-Protease-BrxL<-PglZ<-?<-?<-N6-METHYLASE<-DUF6079*<-BrxB-BrxF-iSTAND<-BrxA-DNA-binding<-wHTH+WYL+WCX<-viral-protein<-AbiEi||?->ZnR->	1225	Bacteria>Pseudomonadota>Gammaproteobacteria>Vibrionales>Vibrionaceae>Vibrio	GCA_024047235.1	Vibrio_parahaemolyticus	NJ496_004031	74363..78040	670	BREX-system-P-loop-protein-BrxC-[Vibrio-parahaemolyticus]
HCG8269540.1	Type1.BREX	<-ZnR<-?||?->SF1-SF2-Helicase->AbiEi->viral-protein->wHTH+WYL+WCX->BrxA-DNA-binding->BrxB-BrxF-iSTAND->DUF6079*->N6-METHYLASE->ParB+HNH->GrpE->?->PglZ->SIGMA+OB-Fold+AAA-ATPase+Lon-Protease-BrxL-><-wHTH||<-?<-CR-ATPase4	1225	Bacteria>Pseudomonadota>Gammaproteobacteria>Vibrionales>Vibrionaceae>Vibrio	GCA_024047655.1	Vibrio_parahaemolyticus	NJ590_004433	15397..19074	670	BREX-system-P-loop-protein-BrxC-[Vibrio-parahaemolyticus]
HCH5919840.1	Type1.BREX	<-ZnR<-?||?->SF1-SF2-Helicase->AbiEi->viral-protein->wHTH+WYL+WCX->BrxA-DNA-binding->BrxB-BrxF-iSTAND->DUF6079*->N6-METHYLASE->ParB+HNH->GrpE->?->PglZ->SIGMA+OB-Fold+AAA-ATPase+Lon-Protease-BrxL-><-wHTH||DUF4113->	1225	Bacteria>Pseudomonadota>Gammaproteobacteria>Vibrionales>Vibrionaceae>Vibrio	GCA_024097015.1	Vibrio_parahaemolyticus	NK048_004352	19128..22805	670	BREX-system-P-loop-protein-BrxC-[Vibrio-parahaemolyticus]
EJK2098984.1	Type1.BREX	<-VirD4-TraG+VirB4-FtsK<-HDRnase<-SbcC<-SIGMA+OB-Fold+AAA-ATPase+Lon-Protease-BrxL<-PglZ<-N6-METHYLASE<-TOPRIM<-OLD-ABC<-DUF6079*<-BrxB-BrxF-iSTAND<-BrxA-DNA-binding<-wHTH+WYL+WCX<-?<-HTH_23+HTH_32<-35exo||LexA-protease->	1225	Bacteria>Pseudomonadota>Gammaproteobacteria>Vibrionales>Vibrionaceae>Vibrio	GCA_024103575.1	Vibrio_cholerae	NK421_001793	214069..217746	666	BREX-system-P-loop-protein-BrxC-[Vibrio-cholerae]
EJL6432478.1	Type1.BREX	<-VirD4-TraG+VirB4-FtsK<-HDRnase<-SbcC<-SIGMA+OB-Fold+AAA-ATPase+Lon-Protease-BrxL<-PglZ<-N6-METHYLASE<-ParB+HNH<-DUF6079*<-BrxB-BrxF-iSTAND<-BrxA-DNA-binding<-wHTH+WYL+WCX<-?<-35exo<-?||LexA-protease->?-><-MobI	1225	Bacteria>Pseudomonadota>Gammaproteobacteria>Vibrionales>Vibrionaceae>Vibrio	GCA_024216615.1	Vibrio_cholerae	NMR72_000938	126901..130578	666	BREX-system-P-loop-protein-BrxC-[Vibrio-cholerae]
EJL6578344.1	Type1.BREX	Arm-DNA-bind_3+Phage_integrase->SF1-SF2-Helicase+REase->SF1-SF2-Helicase+REase-><-PglZ<-TOPRIM<-OLD-ABC<-N6-METHYLASE<-DUF6079*<-BrxB-BrxF-iSTAND<-BrxA-DNA-binding<-wHTH+WYL+WCX<-XIS-HTH||?->?->DUF2787->GTPase-AIG->	1154	Bacteria>Pseudomonadota>Gammaproteobacteria>Vibrionales>Vibrionaceae>Vibrio	GCA_024217525.1	Vibrio_cholerae	NMS50_002630	71340..74804	666	BREX-system-P-loop-protein-BrxC-[Vibrio-cholerae]
EJL6824798.1	Type1.BREX	<-VirD4-TraG+VirB4-FtsK<-HDRnase<-HEPN<-MarR-HTH<-SIGMA+OB-Fold+AAA-ATPase+Lon-Protease-BrxL<-PglZ<-N6-METHYLASE<-DUF6079*	1202	Bacteria>Pseudomonadota>Gammaproteobacteria>Vibrionales>Vibrionaceae>Vibrio	GCA_024218935.1	Vibrio_cholerae	NMT01_000642	124775..128383	666	BREX-system-P-loop-protein-BrxC-[Vibrio-cholerae]
UTZ28051.1	Type1.BREX	ABhydrolase-><-HTH_18+HTH_AraC<-?<-SIGMA+OB-Fold+AAA-ATPase+HKD-DNase-BrxL<-PglZ<-N6-METHYLASE<-NERD<-OLD-ABC<-DUF6079*<-BrxB-BrxF-iSTAND<-BrxA-DNA-binding<-XIS-HTH<-RNase<-JetB<-MukF||wHTH+WYL+WCX-><-tRNA<-?<-IONCHANNEL+CACHE+HISKIN+REC	1183	Bacteria>Pseudomonadota>Gammaproteobacteria>Vibrionales>Vibrionaceae>Vibrio	GCA_024442175.1	Vibrio_campbellii	HB761_02515	550346..553897	680	BREX-system-P-loop-protein-BrxC-[Vibrio-campbellii]
EJO3860683.1	Type1.BREX	AbiEi->viral-protein->SF1-SF2-Helicase+REase->SF1-SF2-Helicase+REase-><-?<-ZetaToxin<-PglZ<-ParB+HNH<-N6-METHYLASE<-DUF6079*<-BrxB-BrxF-iSTAND<-BrxA-DNA-binding<-wHTH+WYL+WCX||DUF4113-><-?<-CR-ATPase4||?-><-HTH_20	1157	Bacteria>Pseudomonadota>Gammaproteobacteria>Vibrionales>Vibrionaceae>Vibrio	GCA_024563785.1	Vibrio_parahaemolyticus	NRI58_000919	1072634..1076107	670	BREX-system-P-loop-protein-BrxC-[Vibrio-parahaemolyticus]
HCJ6872077.1	Type1.BREX	<-OBFOLD-NUCLEASE<-CARF+NtrC-AAA+FIS-HTH<-SIGMA+OB-Fold+AAA-ATPase+Lon-Protease-BrxL<-PglZ<-N6-METHYLASE<-ParB+HNH<-DUF6079*<-BrxB-BrxF-iSTAND<-BrxA-DNA-binding<-wHTH+WYL+WCX<-?<-35exo||LexA-protease->?-><-MobI	1225	Bacteria>Pseudomonadota>Gammaproteobacteria>Vibrionales>Vibrionaceae>Vibrio	GCA_024621265.1	Vibrio_cholerae	NU929_001221	142702..146379	666	BREX-system-P-loop-protein-BrxC-[Vibrio-cholerae]
HCJ7299597.1	Type1.BREX	<-OBFOLD-NUCLEASE<-CARF+NtrC-AAA+FIS-HTH<-SIGMA+OB-Fold+AAA-ATPase+Lon-Protease-BrxL<-PglZ<-N6-METHYLASE<-ParB+HNH<-DUF6079*<-BrxB-BrxF-iSTAND<-BrxA-DNA-binding<-wHTH+WYL+WCX<-?<-35exo||LexA-protease->?-><-MobI	1225	Bacteria>Pseudomonadota>Gammaproteobacteria>Vibrionales>Vibrionaceae>Vibrio	GCA_024632775.1	Vibrio_cholerae	NVH34_000348	35814..39491	666	BREX-system-P-loop-protein-BrxC-[Vibrio-cholerae]
MCR9580722.1	Type1.BREX	viral-protein->wHTH+WYL+WCX->BrxA-DNA-binding->BrxB-BrxF-iSTAND->DUF6079*->N6-METHYLASE->?->?->PglZ->SIGMA+OB-Fold+AAA-ATPase+Lon-Protease-BrxL-><-wHTH||DUF4113-><-?<-CR-ATPase4	1225	Bacteria>Pseudomonadota>Gammaproteobacteria>Vibrionales>Vibrionaceae>Vibrio	GCA_024744095.1	Vibrio_antiquarius	NB519_11830	2837..6514	150340	BREX-system-P-loop-protein-BrxC-[Vibrio-antiquarius]
HCM0920431.1	Type1.BREX	<-wHTH+WYL+WCX<-HTH||<-DUF2787<-?<-?||XIS-HTH->wHTH+WYL+WCX->BrxA-DNA-binding->BrxB-BrxF-iSTAND->DUF6079*->N6-METHYLASE->OLD-ABC->TOPRIM->PglZ-><-SF1-SF2-Helicase+REase<-SF1-SF2-Helicase+REase	1154	Bacteria>Pseudomonadota>Gammaproteobacteria>Vibrionales>Vibrionaceae>Vibrio	GCA_025068375.1	Vibrio_parahaemolyticus	N2828_003369	93414..96878	670	BREX-system-P-loop-protein-BrxC-[Vibrio-parahaemolyticus]
EJU9535832.1	Type1.BREX	AbiEi->viral-protein->SF1-SF2-Helicase+REase->SF1-SF2-Helicase+REase-><-?<-ZetaToxin<-PglZ<-ParB+HNH<-N6-METHYLASE<-DUF6079*<-BrxB-BrxF-iSTAND<-BrxA-DNA-binding<-wHTH+WYL+WCX||<-?<-CR-ATPase4||?->HEPN-><-?<-DEOR-HTH	1157	Bacteria>Pseudomonadota>Gammaproteobacteria>Vibrionales>Vibrionaceae>Vibrio	GCA_025224335.1	Vibrio_alginolyticus	N5M46_001148	14652..18125	663	BREX-system-P-loop-protein-BrxC-[Vibrio-alginolyticus]
EJV5950193.1	Type1.BREX	<-SIGMA+OB-Fold+AAA-ATPase+HKD-DNase-BrxL<-PglZ<-N6-METHYLASE<-REase<-OLD-ABC<-DUF6079*<-BrxB-BrxF-iSTAND<-BrxA-DNA-binding<-XIS-HTH<-HNH<-XIS-HTH||?->?->DUF2787->wHTH+WYL+WCX->	1187	Bacteria>Pseudomonadota>Gammaproteobacteria>Vibrionales>Vibrionaceae>Vibrio	GCA_025320405.1	Vibrio_alginolyticus	N7I24_002738	64007..67570	663	BREX-system-P-loop-protein-BrxC-[Vibrio-alginolyticus]
GLT15886.1	Type1.BREX	<-wHTH+WYL+WCX||MukF->JetB->DUF3584+DUF5401->RNase->XIS-HTH->BrxA-DNA-binding->BrxB-BrxF-iSTAND->DUF6079*->OLD-ABC->REase->N6-METHYLASE->PglZ->SIGMA+OB-Fold+AAA-ATPase+HKD-DNase-BrxL->?->HTH_3->Couple_hipA+HipA_C->	1186	Bacteria>Pseudomonadota>Gammaproteobacteria>Vibrionales>Vibrionaceae>Vibrio	GCA_030161215.1	Vibrio_algivorus	GCM10007931_28610	496776..500336	1667024	hypothetical-protein-GCM10007931_28610-[Vibrio-algivorus]
WP_053056240.1	Type1.BREX	<-VirD4-TraG+VirB4-FtsK<-HDRnase<-HEPN<-MarR-HTH<-SIGMA+OB-Fold+AAA-ATPase+Lon-Protease-BrxL<-PglZ<-N6-METHYLASE<-N6-METHYLASE-p<-DUF6079-p*<-wHTH+WYL+WCX<-?<-35exo<-?||LexA-protease->?->Phage_integrase->	721	Bacteria>Pseudomonadota>Gammaproteobacteria>Vibrionales>Vibrionaceae>Vibrio	GCA_030820635.1	Vibrio_cholerae	ALE43_RS03710	109450..111613	666	BREX-system-P-loop-protein-BrxC,-partial-[Vibrio-cholerae]
WP_242567369.1	Type1.BREX	<-Phage_integrase<-?<-LexA-protease||?->35exo->?->wHTH+WYL+WCX->BrxB-BrxF-iSTAND->DUF6079-p*->N6-METHYLASE->PglZ->	994	Bacteria>Pseudomonadota>Gammaproteobacteria>Vibrionales>Vibrionaceae>Vibrio	GCA_030820635.1	Vibrio_cholerae	ALG09_RS16130	6852..9838	666	BREX-system-P-loop-protein-BrxC,-partial-[Vibrio-cholerae]
WP_114867813.1	Type1.BREX	<-SbcC<-SIGMA+OB-Fold+AAA-ATPase+Lon-Protease-BrxL<-PglZ<-N6-METHYLASE<-ParB+HNH<-DUF6079*<-BrxB-BrxF-iSTAND<-BrxA-DNA-binding<-wHTH+WYL+WCX<-?<-35exo||LexA-protease->?-><-MobI	1225	Bacteria>Pseudomonadota>Gammaproteobacteria>Vibrionales>Vibrionaceae>Vibrio	GCA_030820635.1	Vibrio_parahaemolyticus	CFH89_RS14230	2819482..2823159	670	BREX-system-P-loop-protein-BrxC-[Vibrio-parahaemolyticus]
WP_178326691.1	Type1.BREX	CR-ATPase4->?->||wHTH-><-SIGMA+OB-Fold+AAA-ATPase+Lon-Protease-BrxL<-PglZ<-?<-GrpE<-ParB+HNH<-N6-METHYLASE<-DUF6079-p*	952	Bacteria>Pseudomonadota>Gammaproteobacteria>Vibrionales>Vibrionaceae>Vibrio	GCA_030820635.1	Vibrio_parahaemolyticus	HX770_RS05795	37869..40727	670	BREX-system-P-loop-protein-BrxC,-partial-[Vibrio-parahaemolyticus]
WP_005376166.1	Type1.BREX	sGTP-><-HEPN||CR-ATPase4->?->||wHTH+WYL+WCX->BrxA-DNA-binding->BrxB-BrxF-iSTAND->DUF6079*->N6-METHYLASE->ParB+HNH->PglZ->ZetaToxin->?-><-SF1-SF2-Helicase+REase<-SF1-SF2-Helicase+REase<-viral-protein<-AbiEi	1157	Bacteria>Pseudomonadota>Gammaproteobacteria>Vibrionales>Vibrionaceae>Vibrio	GCF_000153505.1	Vibrio_alginolyticus	V12G01_RS31815	1598723..1602196	663	BREX-system-P-loop-protein-BrxC-[Vibrio-alginolyticus]
WP_000152592.1	Type1.BREX	<-MULE-TRANSPOSASE<-Phage_integrase<-LexA-protease||?->35exo->?->wHTH+WYL+WCX->BrxA-DNA-binding->BrxB-BrxF-iSTAND->DUF6079*->N6-METHYLASE->PglZ->SIGMA+OB-Fold+AAA-ATPase+Lon-Protease-BrxL->MarR-HTH->HEPN->HDRnase->VirD4-TraG+VirB4-FtsK->	1229	Bacteria>Pseudomonadota>Gammaproteobacteria>Vibrionales>Vibrionaceae>Vibrio	GCF_000166455.1	Vibrio_cholerae	HJ37_RS00485	111831..115520	666	BREX-system-P-loop-protein-BrxC-[Vibrio-cholerae]
WP_000934396.1	Type1.BREX	TraI-VirD2-RCRE->?->?->CHTH->||SbcC-><-?||wHTH+WYL+WCX->BrxA-DNA-binding->BrxB-BrxF-iSTAND->DUF6079*->N6-METHYLASE->ParB+HNH->PglZ->sGTP->?->?-><-SF1-SF2-Helicase+REase<-SF1-SF2-Helicase+REase<-viral-protein	1157	Bacteria>Pseudomonadota>Gammaproteobacteria>Vibrionales>Vibrionaceae>Vibrio	GCF_000220785.1	Vibrio_cholerae	VCHE48_RS12985	863732..867205	666	BREX-system-P-loop-protein-BrxC-[Vibrio-cholerae]
WP_004748886.1	Type1.BREX	<-Thioredoxin_4||wHTH+WYL+WCX->BrxA-DNA-binding->BrxB-BrxF-iSTAND->DUF6079*->N6-METHYLASE->PglZ->SIGMA+OB-Fold+AAA-ATPase+Lon-Protease-BrxL->REase+PNTD1+PNTD2->PIWI->REase+iREase->REase+HTH+35exo+Small-B-barrel+SF1-SF2-Helicase->PHP+SbcC->	1219	Bacteria>Pseudomonadota>Gammaproteobacteria>Vibrionales>Vibrionaceae>Vibrio	GCF_000222665.1	Vibrio_tubiashii	RH87_RS24955	79516..83175	29498	BREX-system-P-loop-protein-BrxC-[Vibrio-tubiashii]
WP_016790631.1	Type1.BREX	XIS-HTH->?->SF1-SF2-Helicase+HTH->HNH->wHTH+WYL+WCX->BrxA-DNA-binding->BrxB-BrxF-iSTAND->DUF6079*->N6-METHYLASE->ParB+HNH->PglZ->SF1-SF2-Helicase+PRTase->SLOG+BirA-HTH-><-SF1-SF2-Helicase+REase<-SF1-SF2-Helicase+REase<-?||MODE-HTH+PBPII->	1157	Bacteria>Pseudomonadota>Gammaproteobacteria>Vibrionales>Vibrionaceae>Vibrio	GCF_000256605.2	Vibrio_cyclitrophicus	OAI_RS19775	259622..263095	47951	BREX-system-P-loop-protein-BrxC-[Vibrio-cyclitrophicus]
WP_005444576.1	Type1.BREX	AbiEi->viral-protein->wHTH+WYL+WCX->BrxA-DNA-binding->BrxB-BrxF-iSTAND->DUF6079*->N6-METHYLASE->ParB+HNH->GrpE->?->PglZ->SIGMA+OB-Fold+AAA-ATPase+Lon-Protease-BrxL-><-TPR<-HEPN+SF1-SF2-Helicase+RNase+RNase||LexA-protease->	1225	Bacteria>Pseudomonadota>Gammaproteobacteria>Vibrionales>Vibrionaceae>Vibrio	GCF_000259935.1	Vibrio_harveyi	MUQ_RS25205	3434..7111	669	BREX-system-P-loop-protein-BrxC-[Vibrio-harveyi]
WP_017044128.1	Type1.BREX	HTH_19-><-ParE<-MobI<-MobI<-SIGMA+OB-Fold+AAA-ATPase+Lon-Protease-BrxL<-PglZ<-N6-METHYLASE<-TOPRIM<-OLD-ABC<-DUF6079*<-BrxB-BrxF-iSTAND<-BrxA-DNA-binding<-wHTH+WYL+WCX||XIS-HTH->AAA+HTH->XIS-HTH-><-Phage_int_SAM_5+Phage_integrase<-sGTP	1225	Bacteria>Pseudomonadota>Gammaproteobacteria>Vibrionales>Vibrionaceae>Vibrio	GCF_000287075.2	Vibrio_ordalii	A1QQ_08605	37533..41210	28174	BREX-system-P-loop-protein-BrxC-[Vibrio-ordalii]
WP_000152596.1	Type1.BREX	MobI-><-?<-LexA-protease||35exo->?->wHTH+WYL+WCX->BrxA-DNA-binding->BrxB-BrxF-iSTAND->DUF6079*->N6-METHYLASE->PglZ->SIGMA+OB-Fold+AAA-ATPase+Lon-Protease-BrxL->MarR-HTH->HEPN->HDRnase->VirD4-TraG+VirB4-FtsK->	1229	Bacteria>Pseudomonadota>Gammaproteobacteria>Vibrionales>Vibrionaceae>Vibrio	GCF_000304775.1	Vibrio_cholerae	VCHC1A2_RS09815	58401..62090	666	BREX-system-P-loop-protein-BrxC-[Vibrio-cholerae]
WP_001963844.1	Type1.BREX	MobI-><-?<-LexA-protease||35exo->?->wHTH+WYL+WCX->BrxA-DNA-binding->BrxB-BrxF-iSTAND->DUF6079*->ParB+HNH->N6-METHYLASE->PglZ->SIGMA+OB-Fold+AAA-ATPase+Lon-Protease-BrxL->CARF+NtrC-AAA+FIS-HTH->OBFOLD-NUCLEASE->	1225	Bacteria>Pseudomonadota>Gammaproteobacteria>Vibrionales>Vibrionaceae>Vibrio	GCF_000304915.2	Vibrio_cholerae	VCCP1035_RS13195	133177..136854	666	BREX-system-P-loop-protein-BrxC-[Vibrio-cholerae]
WP_023585137.1	Type1.BREX	CR-ATPase4->?-><-SIGMA+OB-Fold+AAA-ATPase+Lon-Protease-BrxL<-PglZ<-?<-?<-ZnR+3C-ABC+TPR<-ParB+HNH<-N6-METHYLASE<-DUF6079*<-BrxB-BrxF-iSTAND<-BrxA-DNA-binding<-wHTH+WYL+WCX||?->?->?->?->?->HTH_Tnp_1->	1248	Bacteria>Pseudomonadota>Gammaproteobacteria>Vibrionales>Vibrionaceae>Vibrio	GCF_000347495.1	Vibrio_parahaemolyticus	VPPCV08_RS0113765	243193..246939	670	BREX-system-P-loop-protein-BrxC-[Vibrio-parahaemolyticus]
WP_021709035.1	Type1.BREX	<-Thioredoxin<-?<-Phage_integrase||?->?->?-><-?||wHTH+WYL+WCX->BrxA-DNA-binding->BrxB-BrxF-iSTAND->DUF6079*->N6-METHYLASE->HEPN->PglZ->SIGMA+OB-Fold+AAA-ATPase+Lon-Protease-BrxL->GNAT-><-Peptidase	1229	Bacteria>Pseudomonadota>Gammaproteobacteria>Vibrionales>Vibrionaceae>Vibrio	GCF_000400265.1	Vibrio_azureus	VAN_RS15510	14465..18154	512649	BREX-system-P-loop-protein-BrxC-[Vibrio-azureus]
WP_025605138.1	Type1.BREX	<-ZnR<-?||?->SF1-SF2-Helicase->AbiEi->viral-protein->wHTH+WYL+WCX->BrxA-DNA-binding->BrxB-BrxF-iSTAND->DUF6079*->N6-METHYLASE->ParB+HNH->GrpE->?->PglZ->SIGMA+OB-Fold+AAA-ATPase+Lon-Protease-BrxL-><-wHTH||DUF4113->	1225	Bacteria>Pseudomonadota>Gammaproteobacteria>Vibrionales>Vibrionaceae>Vibrio	GCF_000489075.1	Vibrio_parahaemolyticus	DX10_RS0106510	11492..15169	670	BREX-system-P-loop-protein-BrxC-[Vibrio-parahaemolyticus]
WP_025525716.1	Type1.BREX	Calcineurin->?-><-ZnR<-?||AbiEi->viral-protein->wHTH+WYL+WCX->BrxA-DNA-binding->BrxB-BrxF-iSTAND->DUF6079*->N6-METHYLASE->ParB+HNH->GrpE->?->PglZ->SIGMA+OB-Fold+AAA-ATPase+Lon-Protease-BrxL-><-wHTH||DUF4113->	1225	Bacteria>Pseudomonadota>Gammaproteobacteria>Vibrionales>Vibrionaceae>Vibrio	GCF_000489535.1	Vibrio_parahaemolyticus	DX89_RS0109235	6826..10503	670	BREX-system-P-loop-protein-BrxC-[Vibrio-parahaemolyticus]
WP_029810016.1	Type1.BREX	Calcineurin->?-><-ZnR<-?||AbiEi->viral-protein->wHTH+WYL+WCX->BrxA-DNA-binding->BrxB-BrxF-iSTAND->DUF6079*->N6-METHYLASE->ParB+HNH->GrpE->?->PglZ->SIGMA+OB-Fold+AAA-ATPase+Lon-Protease-BrxL-><-TPR<-HEPN+SF1-SF2-Helicase+RNase+RNase	1225	Bacteria>Pseudomonadota>Gammaproteobacteria>Vibrionales>Vibrionaceae>Vibrio	GCF_000490615.1	Vibrio_parahaemolyticus	DX93_RS0104660	6873..10550	670	BREX-system-P-loop-protein-BrxC-[Vibrio-parahaemolyticus]
WP_029847728.1	Type1.BREX	ANK+ANK->XIS-HTH-><-?<-?<-?||XIS-HTH->wHTH+WYL+WCX->BrxA-DNA-binding->BrxB-BrxF-iSTAND->DUF6079-p*->	621	Bacteria>Pseudomonadota>Gammaproteobacteria>Vibrionales>Vibrionaceae>Vibrio	GCF_000491375.1	Vibrio_parahaemolyticus	DY29_RS0118160	8133..9996	670	BREX-system-P-loop-protein-BrxC,-partial-[Vibrio-parahaemolyticus]
WP_029830481.1	Type1.BREX	wHTH-><-SIGMA+OB-Fold+AAA-ATPase+Lon-Protease-BrxL<-PglZ<-?<-GrpE<-ParB+HNH<-N6-METHYLASE<-DUF6079*<-BrxB-BrxF-iSTAND<-BrxA-DNA-binding<-wHTH+WYL+WCX<-viral-protein	1225	Bacteria>Pseudomonadota>Gammaproteobacteria>Vibrionales>Vibrionaceae>Vibrio	GCF_000491395.1	Vibrio_parahaemolyticus	DY37_RS0104880	15763..19440	670	BREX-system-P-loop-protein-BrxC-[Vibrio-parahaemolyticus]
WP_029805255.1	Type1.BREX	<-ZnR<-?||SF1-SF2-Helicase->AbiEi->viral-protein->wHTH+WYL+WCX->BrxA-DNA-binding->BrxB-BrxF-iSTAND->DUF6079*->N6-METHYLASE->ParB+HNH->GrpE->?->PglZ->SIGMA+OB-Fold+AAA-ATPase+Lon-Protease-BrxL-><-wHTH||DUF4113->	1225	Bacteria>Pseudomonadota>Gammaproteobacteria>Vibrionales>Vibrionaceae>Vibrio	GCF_000492075.1	Vibrio_parahaemolyticus	UF29_RS13050	359665..363342	670	BREX-system-P-loop-protein-BrxC-[Vibrio-parahaemolyticus]
WP_024701189.1	Type1.BREX	CR-ATPase4->?->||wHTH+WYL+WCX->BrxA-DNA-binding->BrxB-BrxF-iSTAND->DUF6079*->N6-METHYLASE->ParB+HNH->PglZ->ZetaToxin->?-><-SF1-SF2-Helicase+REase<-SF1-SF2-Helicase+REase<-viral-protein<-AbiEi	1157	Bacteria>Pseudomonadota>Gammaproteobacteria>Vibrionales>Vibrionaceae>Vibrio	GCF_000500485.1	Vibrio_parahaemolyticus	T247_RS0121000	24076..27549	670	BREX-system-P-loop-protein-BrxC-[Vibrio-parahaemolyticus]
WP_025796292.1	Type1.BREX	AbiEi->viral-protein->SF1-SF2-Helicase+REase->SF1-SF2-Helicase+REase-><-?<-ZetaToxin<-PglZ<-ParB+HNH<-N6-METHYLASE<-DUF6079*<-BrxB-BrxF-iSTAND<-BrxA-DNA-binding<-wHTH+WYL+WCX||DUF4113-><-?<-CR-ATPase4	1157	Bacteria>Pseudomonadota>Gammaproteobacteria>Vibrionales>Vibrionaceae>Vibrio	GCF_000591555.1	Vibrio_parahaemolyticus	T6V_RS20315	13981..17454	670	BREX-system-P-loop-protein-BrxC-[Vibrio-parahaemolyticus]
WP_032482144.1	Type1.BREX	<-LexA-protease||35exo->?->wHTH+WYL+WCX->BrxA-DNA-binding->BrxB-BrxF-iSTAND->DUF6079*->ParB+HNH->N6-METHYLASE->OLD-ABC->TOPRIM->PglZ->SIGMA+OB-Fold+AAA-ATPase+Lon-Protease-BrxL->SbcC->HDRnase->VirD4-TraG+VirB4-FtsK->	1225	Bacteria>Pseudomonadota>Gammaproteobacteria>Vibrionales>Vibrionaceae>Vibrio	GCF_000736875.1	Vibrio_cholerae	DN38_RS05545	8784..12461	666	BREX-system-P-loop-protein-BrxC-[Vibrio-cholerae]
WP_033931157.1	Type1.BREX	<-VirD4-TraG+VirB4-FtsK<-HDRnase<-NACHT+HEAT+REase<-SIGMA+OB-Fold+AAA-ATPase+Lon-Protease-BrxL<-PglZ<-N6-METHYLASE<-ParB+HNH<-DUF6079*<-BrxB-BrxF-iSTAND<-BrxA-DNA-binding<-wHTH+WYL+WCX<-?<-35exo<-?||LexA-protease->?-><-MobI	1225	Bacteria>Pseudomonadota>Gammaproteobacteria>Vibrionales>Vibrionaceae>Vibrio	GCF_000788515.1	Vibrio_cholerae	NH59_RS10860	62787..66464	666	BREX-system-P-loop-protein-BrxC-[Vibrio-cholerae]
WP_045378182.1	Type1.BREX	<-SIGMA+OB-Fold+AAA-ATPase+Lon-Protease-BrxL<-PglZ<-?<-GrpE<-ANK+ANK+ANK<-ParB+HNH<-?<-N6-METHYLASE<-?<-DUF6079*<-BrxB-BrxF-iSTAND<-BrxA-DNA-binding<-wHTH+WYL+WCX<-?||AbiEii-><-REase<-?<-TraI-VirD2-RCRE<-TraJ-RHH	1248	Bacteria>Pseudomonadota>Gammaproteobacteria>Vibrionales>Vibrionaceae>Vibrio	GCF_000818335.1	Vibrio_campbellii	TY56_RS07080	532127..535873	680	BREX-system-P-loop-protein-BrxC-[Vibrio-campbellii]
WP_041154134.1	Type1.BREX	MobI-><-?<-LexA-protease||35exo->HTH_OrfB_IS605+OrfB_IS605+OrfB_Zn_ribbon-><-?||BrxA-DNA-binding->BrxB-BrxF-iSTAND->DUF6079*->N6-METHYLASE->MarR-HTH->HEPN->HDRnase->?->?-><-HTH_3||TraE->	1227	Bacteria>Pseudomonadota>Gammaproteobacteria>Vibrionales>Vibrionaceae>Vibrio	GCF_000830505.1	Vibrio_mytili	SU60_RS02235	11952..15635	50718	BREX-system-P-loop-protein-BrxC-[Vibrio-mytili]
WP_253277773.1	Type1.BREX	<-wHTH+WYL+WCX<-DUF2787<-?<-?||wHTH+WYL+WCX->BrxA-DNA-binding->BrxB-BrxF-iSTAND->DUF6079*->N6-METHYLASE->OLD-ABC->TOPRIM->PglZ-><-SF1-SF2-Helicase+REase<-SF1-SF2-Helicase+REase<-Arm-DNA-bind_3+Phage_integrase	1153	Bacteria>Pseudomonadota>Gammaproteobacteria>Vibrionales>Vibrionaceae>Vibrio	GCF_000959745.1	Vibrio_vulnificus	WG98_RS00450	91740..95201	672	BREX-system-P-loop-protein-BrxC-[Vibrio-vulnificus]
WP_047110486.1	Type1.BREX	sGTP-><-HEPN||?->?-><-?<-SIGMA+OB-Fold+AAA-ATPase+Lon-Protease-BrxL<-PglZ<-ParB+HNH<-DUF932<-N6-METHYLASE<-DUF6079*<-BrxB-BrxF-iSTAND<-BrxA-DNA-binding<-wHTH+WYL+WCX<-viral-protein||?->ZnR-><-SF1-SF2-Helicase<-Couple_hipA+HipA_C<-HTH_3	1232	Bacteria>Pseudomonadota>Gammaproteobacteria>Vibrionales>Vibrionaceae>Vibrio	GCF_001013325.1	Vibrio_vulnificus	VVYB158_12225	27343..31041	672	BREX-system-P-loop-protein-BrxC-[Vibrio-vulnificus]
WP_050646850.1	Type1.BREX	SF1-SF2-Helicase+REase->SF1-SF2-Helicase+REase-><-?<-ZetaToxin<-PglZ<-ParB+HNH<-N6-METHYLASE<-DUF6079*<-BrxB-BrxF-iSTAND<-BrxA-DNA-binding<-wHTH+WYL+WCX<-?<-?<-SF1-SF2-Helicase<-Arm-DNA-bind_3	1157	Bacteria>Pseudomonadota>Gammaproteobacteria>Vibrionales>Vibrionaceae>Vibrio	GCF_001243675.1	Vibrio_sp._J2-4	VCR4J2_RS00160	35844..39317	1507977	BREX-system-P-loop-protein-BrxC-[Vibrio-sp.-J2-4]
WP_240182084.1	Type1.BREX	<-VirD4-TraG+VirB4-FtsK<-HDRnase<-HEPN<-MarR-HTH<-SIGMA+OB-Fold+AAA-ATPase+Lon-Protease-BrxL<-PglZ<-N6-METHYLASE<-DUF6079*<-BrxB-BrxF-iSTAND<-BrxA-DNA-binding<-wHTH+WYL+WCX<-?<-35exo<-?||LexA-protease->	1072	Bacteria>Pseudomonadota>Gammaproteobacteria>Vibrionales>Vibrionaceae>Vibrio	GCF_001247885.1	Vibrio_cholerae	ALC59_RS14100	125159..128374	666	BREX-system-P-loop-protein-BrxC,-partial-[Vibrio-cholerae]
WP_053044233.1	Type1.BREX	BrxB-BrxF-iSTAND->DUF6079-p*->DUF6079-p->N6-METHYLASE->PglZ->SIGMA+OB-Fold+AAA-ATPase+Lon-Protease-BrxL->MarR-HTH->HEPN->HDRnase->VirD4-TraG+VirB4-FtsK->	822	Bacteria>Pseudomonadota>Gammaproteobacteria>Vibrionales>Vibrionaceae>Vibrio	GCF_001248645.1	Vibrio_cholerae	ALC75_RS05685	604..3070	666	BREX-system-P-loop-protein-BrxC,-partial-[Vibrio-cholerae]
WP_114025876.1	Type1.BREX	DUF6079*->N6-METHYLASE->PglZ->SIGMA+OB-Fold+AAA-ATPase+Lon-Protease-BrxL->MarR-HTH->HEPN->HDRnase->VirD4-TraG+VirB4-FtsK->	1166	Bacteria>Pseudomonadota>Gammaproteobacteria>Vibrionales>Vibrionaceae>Vibrio	GCF_001254815.1	Vibrio_cholerae	ALE22_RS06100	1004..4506	666	BREX-system-P-loop-protein-BrxC,-partial-[Vibrio-cholerae]
WP_053027640.1	Type1.BREX	DUF6079-p*->N6-METHYLASE->PglZ->SIGMA+OB-Fold+AAA-ATPase+Lon-Protease-BrxL->HEPN->HDRnase->VirD4-TraG+VirB4-FtsK->?->?-><-Glyoxalase_8	848	Bacteria>Pseudomonadota>Gammaproteobacteria>Vibrionales>Vibrionaceae>Vibrio	GCF_001259875.1	Vibrio_cholerae	ALV71_RS07765	696..3240	666	BREX-system-P-loop-protein-BrxC,-partial-[Vibrio-cholerae]
WP_241772680.1	Type1.BREX	<-GDResolvase+HIN-HTH||?->Arm-DNA-bind_3+Phage_integrase->SF1-SF2-Helicase+REase->SF1-SF2-Helicase+REase-><-PglZ<-TOPRIM<-OLD-ABC<-N6-METHYLASE<-DUF6079*<-BrxB-BrxF-iSTAND<-BrxA-DNA-binding<-wHTH+WYL+WCX<-XIS-HTH	1153	Bacteria>Pseudomonadota>Gammaproteobacteria>Vibrionales>Vibrionaceae>Vibrio	GCF_001280635.1	Vibrio_parahaemolyticus	ACX06_RS21960	167502..170963	670	BREX-system-P-loop-protein-BrxC-[Vibrio-parahaemolyticus]
WP_054541287.1	Type1.BREX	BPD_transp_2->?->?->?->SF1-SF2-Helicase+HTH->HNH->XIS-HTH->BrxA-DNA-binding->BrxB-BrxF-iSTAND->DUF6079*->N6-METHYLASE->OLD-ABC->TOPRIM->N6-METHYLASE->PglZ->SIGMA+OB-Fold+AAA-ATPase+Lon-Protease-BrxL-><-?||?-><-MODE-HTH+PBPII	1235	Bacteria>Pseudomonadota>Gammaproteobacteria>Vibrionales>Vibrionaceae>Vibrio	GCF_001306215.1	Vibrio_splendidus	AN167_RS02930	103766..107473	29497	BREX-system-P-loop-protein-BrxC-[Vibrio-splendidus]
WP_061039701.1	Type1.BREX	<-SLOG+BirA-HTH<-SF1-SF2-Helicase+PRTase<-?<-GrpE<-ANK+ANK+ANK<-ParB+HNH<-HEPN+Sel1+SEC-C<-N6-METHYLASE<-?<-DUF6079*<-BrxB-BrxF-iSTAND<-BrxA-DNA-binding<-wHTH+WYL+WCX||SF1-SF2-Helicase+HTH-><-TraI-VirD2-RCRE<-?<-Peptidase_M32	1247	Bacteria>Pseudomonadota>Gammaproteobacteria>Vibrionales>Vibrionaceae>Vibrio	GCF_001557855.1	Vibrio_coralliirubri	ATB73_RS08125	549280..553023	1516159	BREX-system-P-loop-protein-BrxC-[Vibrio-coralliirubri]
WP_061065627.1	Type1.BREX	<-wHTH+WYL+WCX<-DUF2787<-?<-?||?->XIS-HTH->wHTH+WYL+WCX->BrxA-DNA-binding->BrxB-BrxF-iSTAND->DUF6079*->N6-METHYLASE->ParB+HNH->PglZ-><-SF1-SF2-Helicase+REase<-SF1-SF2-Helicase+REase<-Arm-DNA-bind_3+Phage_integrase	1157	Bacteria>Pseudomonadota>Gammaproteobacteria>Vibrionales>Vibrionaceae>Vibrio	GCF_001558435.2	Vibrio_harveyi	AL538_RS13470	2675377..2678850	669	BREX-system-P-loop-protein-BrxC-[Vibrio-harveyi]
WP_241782177.1	Type1.BREX	<-HTH||RNase-><-DUF2787<-?<-?||XIS-HTH->wHTH+WYL+WCX->BrxA-DNA-binding->BrxB-BrxF-iSTAND->DUF6079*->N6-METHYLASE->OLD-ABC->TOPRIM->PglZ-><-SF1-SF2-Helicase+REase<-SF1-SF2-Helicase+REase||?->SF1-SF2-Helicase-><-Mob_Pre	1153	Bacteria>Pseudomonadota>Gammaproteobacteria>Vibrionales>Vibrionaceae>Vibrio	GCF_001608855.1	Vibrio_parahaemolyticus	AWQ17_RS02375	93824..97285	670	BREX-system-P-loop-protein-BrxC-[Vibrio-parahaemolyticus]
WP_065090484.1	Type1.BREX	AbiEi->viral-protein->SF1-SF2-Helicase+REase->SF1-SF2-Helicase+REase-><-?<-ZetaToxin<-PglZ<-ParB+HNH<-N6-METHYLASE<-DUF6079*<-BrxB-BrxF-iSTAND<-BrxA-DNA-binding<-wHTH+WYL+WCX||<-?<-CR-ATPase4||PIN+OST-HTH-><-REase+SPF-cons	1157	Bacteria>Pseudomonadota>Gammaproteobacteria>Vibrionales>Vibrionaceae>Vibrio	GCF_001675245.1	Vibrio_vulnificus	FORC17_RS08380	1790096..1793569	672	BREX-system-P-loop-protein-BrxC-[Vibrio-vulnificus]
WP_065091082.1	Type1.BREX	CR-ATPase4->?-><-DUF4113||wHTH-><-SIGMA+OB-Fold+AAA-ATPase+Lon-Protease-BrxL<-PglZ<-Schlafen+HATPase+wHTH<-SbcC<-N6-METHYLASE<-DUF6079*<-BrxB-BrxF-iSTAND<-BrxA-DNA-binding<-wHTH+WYL+WCX<-viral-protein<-AbiEi||?->ZnR-><-?<-Calcineurin	1225	Bacteria>Pseudomonadota>Gammaproteobacteria>Vibrionales>Vibrionaceae>Vibrio	GCF_001675245.1	Vibrio_vulnificus	FORC17_RS18340	777696..781373	672	BREX-system-P-loop-protein-BrxC-[Vibrio-vulnificus]
WP_256093930.1	Type1.BREX	XIS-HTH->wHTH+WYL+WCX->BrxA-DNA-binding->BrxB-BrxF-iSTAND->DUF6079*->N6-METHYLASE->OLD-ABC->TOPRIM->PglZ-><-SF1-SF2-Helicase+REase<-SF1-SF2-Helicase+REase<-Arm-DNA-bind_3+Phage_integrase	1153	Bacteria>Pseudomonadota>Gammaproteobacteria>Vibrionales>Vibrionaceae>Vibrio	GCF_001727265.1	Vibrio_parahaemolyticus	BBM25_RS06240	3427..6888	670	BREX-system-P-loop-protein-BrxC-[Vibrio-parahaemolyticus]
WP_069530140.1	Type1.BREX	wHTH-><-SIGMA+OB-Fold+AAA-ATPase+Lon-Protease-BrxL<-PglZ<-?<-GrpE<-ParB+HNH<-N6-METHYLASE<-DUF6079*<-BrxB-BrxF-iSTAND<-BrxA-DNA-binding<-wHTH+WYL+WCX<-viral-protein<-AbiEi||?->ZnR-><-?<-Calcineurin	1225	Bacteria>Pseudomonadota>Gammaproteobacteria>Vibrionales>Vibrionaceae>Vibrio	GCF_001728565.1	Vibrio_parahaemolyticus	BBN06_RS08190	27008..30685	670	BREX-system-P-loop-protein-BrxC-[Vibrio-parahaemolyticus]
WP_074189993.1	Type1.BREX	<-DUF4113||wHTH-><-SIGMA+OB-Fold+AAA-ATPase+Lon-Protease-BrxL<-PglZ<-?<-GrpE<-ParB+HNH<-N6-METHYLASE<-DUF6079*<-BrxB-BrxF-iSTAND<-BrxA-DNA-binding<-wHTH+WYL+WCX<-viral-protein<-AbiEi||?->ZnR-><-?<-Calcineurin	1225	Bacteria>Pseudomonadota>Gammaproteobacteria>Vibrionales>Vibrionaceae>Vibrio	GCF_001909305.1	Vibrio_harveyi_group	H058_RS04230	543659..547336	717610	MULTISPECIES:-BREX-system-P-loop-protein-BrxC-[Vibrio-harveyi-group]
WP_215796802.1	Type1.BREX	wHTH+WYL+WCX->BrxA-DNA-binding->BrxB-BrxF-iSTAND->DUF6079*->N6-METHYLASE->SbcC->Schlafen+HATPase+wHTH->PglZ->SIGMA+OB-Fold+AAA-ATPase+Lon-Protease-BrxL->	1229	Bacteria>Pseudomonadota>Gammaproteobacteria>Vibrionales>Vibrionaceae>Vibrio	GCF_001989775.2	Vibrio_anguillarum	PN39_RS13080	3578..7267	55601	BREX-system-P-loop-protein-BrxC-[Vibrio-anguillarum]
WP_080540649.1	Type1.BREX	HTH_3->?->?-><-SIGMA+OB-Fold+AAA-ATPase+Lon-Protease-BrxL<-PglZ<-ParB+HNH<-N6-METHYLASE<-DUF6079*<-BrxB-BrxF-iSTAND<-BrxA-DNA-binding<-wHTH+WYL+WCX<-viral-protein||?->ZnR-><-SF1-SF2-Helicase<-Couple_hipA+HipA_C<-HTH_3	1232	Bacteria>Pseudomonadota>Gammaproteobacteria>Vibrionales>Vibrionaceae>Vibrio	GCF_002073135.1	Vibrio_vulnificus	XM77_RS07295	72638..76336	672	BREX-system-P-loop-protein-BrxC-[Vibrio-vulnificus]
WP_139834421.1	Type1.BREX	HTH_19-><-ParE<-MobI<-MobI<-SIGMA+OB-Fold+AAA-ATPase+Lon-Protease-BrxL<-PglZ<-N6-METHYLASE<-TOPRIM<-OLD-ABC<-DUF6079*	1085	Bacteria>Pseudomonadota>Gammaproteobacteria>Vibrionales>Vibrionaceae>Vibrio	GCF_002114605.1	Vibrio_parahaemolyticus	B5C31_RS21495	50871..54130	670	BREX-system-P-loop-protein-BrxC,-partial-[Vibrio-parahaemolyticus]
WP_198300681.1	Type1.BREX	<-DeoR-HTH+ISOCOT<-DeoR-HTH+ISOCOT<-?<-?||XIS-HTH->XIS-HTH->BrxA-DNA-binding->BrxB-BrxF-iSTAND->DUF6079*->OLD-ABC->REase->N6-METHYLASE->PglZ->SIGMA+OB-Fold+AAA-ATPase+HKD-DNase-BrxL-><-Arm-DNA-bind_3+Phage_integrase	1187	Bacteria>Pseudomonadota>Gammaproteobacteria>Vibrionales>Vibrionaceae>Vibrio	GCF_002211985.1	Vibrio_anguillarum	CEJ46_RS04105	862774..866337	55601	BREX-system-P-loop-protein-BrxC-[Vibrio-anguillarum]
WP_088731321.1	Type1.BREX	HTH_19-><-ParE<-MobI<-MobI<-SIGMA+OB-Fold+AAA-ATPase+Lon-Protease-BrxL<-PglZ<-N6-METHYLASE<-ParB+HNH<-DUF6079*<-BrxB-BrxF-iSTAND<-BrxA-DNA-binding<-wHTH+WYL+WCX||AAA+HTH->XIS-HTH-><-Phage_int_SAM_5+Phage_integrase<-sGTP	1225	Bacteria>Pseudomonadota>Gammaproteobacteria>Vibrionales>Vibrionaceae>Vibrio	GCF_002212025.1	Vibrio_anguillarum	CEQ50_RS00060	15615..19292	55601	BREX-system-P-loop-protein-BrxC-[Vibrio-anguillarum]
WP_197697128.1	Type1.BREX	<-SIGMA+OB-Fold+AAA-ATPase+HKD-DNase-BrxL<-PglZ<-N6-METHYLASE<-NERD<-OLD-ABC<-DUF6079*<-BrxB-BrxF-iSTAND<-BrxA-DNA-binding<-XIS-HTH<-RNase<-DUF3584+DUF5401<-JetB<-MukF||wHTH+WYL+WCX->	1185	Bacteria>Pseudomonadota>Gammaproteobacteria>Vibrionales>Vibrionaceae>Vibrio	GCF_002214395.1	Vibrio_rotiferianus	BSZ04_RS17965	1918666..1922223	190895	BREX-system-P-loop-protein-BrxC-[Vibrio-rotiferianus]

Protein-ID	System	Gene-Neighbourhoods	Length	Taxonomy	Assembly-ID	Strain	Locus-Tag	Genomic-coordinates	TaxID	Fasta-defline
WP_089123599.1	Type1.BREX	<-Couple_hipA+HipA_C<-HTH_3<-?<-SIGMA+OB-Fold+AAA-ATPase+HKD-DNase-BrxL<-PglZ<-N6-METHYLASE<-REase<-OLD-ABC<-DUF6079*<-BrxB-BrxF-iSTAND<-BrxA-DNA-binding<-XIS-HTH<-RNase<-DUF3584+DUF5401<-JetB<-MukF||wHTH+WYL+WCX->	1184	Bacteria>Pseudomonadota>Gammaproteobacteria>Vibrionales>Vibrionaceae>Vibrio	GCF_002218005.2	Vibrio_algivorus	AVR_RS07075	1502603..1506157	1667024	BREX-system-P-loop-protein-BrxC-[Vibrio-algivorus]
WP_095475146.1	Type1.BREX	Arm-DNA-bind_3+Phage_integrase->SF1-SF2-Helicase+REase->SF1-SF2-Helicase+REase-><-?<-ZetaToxin<-PglZ<-ParB+HNH<-N6-METHYLASE<-DUF6079*<-BrxB-BrxF-iSTAND<-BrxA-DNA-binding<-wHTH+WYL+WCX<-?<-SF1-SF2-Helicase+REase||wHTH+WYL+WCX->	1157	Bacteria>Pseudomonadota>Gammaproteobacteria>Vibrionales>Vibrionaceae>Vibrio	GCF_002283835.1	Vibrio_metoecus	CGT99_RS14700	71972..75445	1481663	BREX-system-P-loop-protein-BrxC-[Vibrio-metoecus]
WP_095470311.1	Type1.BREX	Arm-DNA-bind_3+Phage_integrase->SF1-SF2-Helicase+REase->SF1-SF2-Helicase+REase-><-?<-ZetaToxin<-PglZ<-ParB+HNH<-N6-METHYLASE<-DUF6079*<-BrxB-BrxF-iSTAND<-BrxA-DNA-binding<-wHTH+WYL+WCX<-?<-SF1-SF2-Helicase+REase||wHTH+WYL+WCX->	1157	Bacteria>Pseudomonadota>Gammaproteobacteria>Vibrionales>Vibrionaceae>Vibrio	GCF_002283955.1	Vibrio_metoecus	CGT93_RS13890	71728..75201	1481663	BREX-system-P-loop-protein-BrxC-[Vibrio-metoecus]
WP_096070868.1	Type1.BREX	HTH_20->CR-ATPase4->?-><-?<-LexA-protease||wHTH+WYL+WCX->BrxA-DNA-binding->BrxB-BrxF-iSTAND->DUF6079*->N6-METHYLASE->ParB+HNH->PglZ->HEPN+SF1-SF2-Helicase+RNase+RNase->TPR-><-LZ_Tnp_IS66+zf-IS66+DDE_Tnp_IS66+DDE_Tnp_IS66_C<-?<-HTH_Tnp_1<-AAA-ATPase	1157	Bacteria>Pseudomonadota>Gammaproteobacteria>Vibrionales>Vibrionaceae>Vibrio	GCF_002313025.1	Vibrio_cholerae	FORC55_RS15555	276972..280445	666	BREX-system-P-loop-protein-BrxC-[Vibrio-cholerae]
WP_100267429.1	Type1.BREX	MobI-><-?<-LexA-protease||35exo->?->wHTH+WYL+WCX->BrxA-DNA-binding->BrxB-BrxF-iSTAND->DUF6079*->ParB+HNH->N6-METHYLASE->PglZ->SIGMA+OB-Fold+AAA-ATPase+Lon-Protease-BrxL->SbcC->HDRnase->VirD4-TraG+VirB4-FtsK->	1225	Bacteria>Pseudomonadota>Gammaproteobacteria>Vibrionales>Vibrionaceae>Vibrio	GCF_002796765.1	Vibrio_fluvialis	CV024_RS02745	224853..228530	676	BREX-system-P-loop-protein-BrxC-[Vibrio-fluvialis]
WP_100754281.1	Type1.BREX	<-ParB+HNH<-N6-METHYLASE<-DUF6079*<-BrxB-BrxF-iSTAND<-BrxA-DNA-binding<-wHTH+WYL+WCX||?-><-ParB||RNase->?-><-?<-Phage_integrase+Phage_integrase	1248	Bacteria>Pseudomonadota>Gammaproteobacteria>Vibrionales>Vibrionaceae>Vibrio	GCF_002811245.1	Vibrio_salilacus	CDZ87_RS15130	5916..9662	1323749	BREX-system-P-loop-protein-BrxC-[Vibrio-salilacus]
WP_102465509.1	Type1.BREX	<-BrxD<-SIGMA+OB-Fold+AAA-ATPase+Lon-Protease-BrxL<-PglZ<-?<-?<-ZnR+3C-ABC+TPR<-ANK+ANK+ANK<-ParB+HNH<-N6-METHYLASE<-DUF6079*<-BrxB-BrxF-iSTAND<-BrxA-DNA-binding<-wHTH+WYL+WCX||SF1-SF2-Helicase+HTH-><-?<-?||?-><-Calcineurin-SbcD<-REase	1242	Bacteria>Pseudomonadota>Gammaproteobacteria>Vibrionales>Vibrionaceae>Vibrio	GCF_002873515.1	Vibrio_cyclitrophicus	BCU82_RS15555	51743..55471	47951	BREX-system-P-loop-protein-BrxC-[Vibrio-cyclitrophicus]
WP_102402203.1	Type1.BREX	<-SF1-SF2-Helicase+HTH||wHTH+WYL+WCX->BrxA-DNA-binding->BrxB-BrxF-iSTAND->DUF6079*->N6-METHYLASE->ParB+HNH->ANK+ANK+ANK->ZnR+3C-ABC+TPR->?->?->PglZ->SIGMA+OB-Fold+AAA-ATPase+Lon-Protease-BrxL->	1243	Bacteria>Pseudomonadota>Gammaproteobacteria>Vibrionales>Vibrionaceae>Vibrio	GCF_002873825.1	Vibrio_cyclitrophicus	BCV09_RS12225	6080..9811	47951	BREX-system-P-loop-protein-BrxC-[Vibrio-cyclitrophicus]
WP_102420196.1	Type1.BREX	TraI-VirD2-RCRE-><-?<-SF1-SF2-Helicase+HTH||wHTH+WYL+WCX->BrxA-DNA-binding->BrxB-BrxF-iSTAND->DUF6079*->N6-METHYLASE->?->ParB+HNH->?->?->PglZ->SIGMA+OB-Fold+AAA-ATPase+Lon-Protease-BrxL->	1247	Bacteria>Pseudomonadota>Gammaproteobacteria>Vibrionales>Vibrionaceae>Vibrio	GCF_002875525.1	Vibrio_sp._10N.222.55.C12	BCT20_08060	8620..12363	1884470	BREX-system-P-loop-protein-BrxC-[Vibrio-sp.-10N.222.55.C12]
WP_102376359.1	Type1.BREX	ParB-><-SIGMA+OB-Fold+AAA-ATPase+Lon-Protease-BrxL<-PglZ<-TOPRIM<-OLD-ABC<-?<-?<-N6-METHYLASE<-ParB+HNH<-DUF6079*<-BrxB-BrxF-iSTAND<-BrxA-DNA-binding<-wHTH+WYL+WCX<-?<-XIS-HTH<-Arm-DNA-bind_3+Phage_integrase	1233	Bacteria>Pseudomonadota>Gammaproteobacteria>Vibrionales>Vibrionaceae>Vibrio	GCF_002876115.1	Vibrio_breoganii	BCT61_RS03015	196035..199736	553239	BREX-system-P-loop-protein-BrxC-[Vibrio-breoganii]
WP_102582639.1	Type1.BREX	REase->Calcineurin-SbcD-><-?||?->?-><-SF1-SF2-Helicase+HTH||wHTH+WYL+WCX->BrxA-DNA-binding->BrxB-BrxF-iSTAND->DUF6079*->N6-METHYLASE->ParB+HNH->ANK+ANK+ANK->ZnR+3C-ABC+TPR->?->?->PglZ->SIGMA+OB-Fold+AAA-ATPase+Lon-Protease-BrxL->BrxD->	1242	Bacteria>Pseudomonadota>Gammaproteobacteria>Vibrionales>Vibrionaceae>Vibrio	GCF_002876245.1	Vibrio_tasmaniensis	BCT76_RS12975	27484..31212	212663	BREX-system-P-loop-protein-BrxC-[Vibrio-tasmaniensis]
WP_102250444.1	Type1.BREX	<-SF1-SF2-Helicase+PRTase-p<-?<-GrpE<-ANK+ANK+ANK<-ParB+HNH<-HEPN+Sel1+SEC-C<-N6-METHYLASE<-?<-DUF6079*<-BrxB-BrxF-iSTAND<-BrxA-DNA-binding<-wHTH+WYL+WCX||SF1-SF2-Helicase+HTH->	1247	Bacteria>Pseudomonadota>Gammaproteobacteria>Vibrionales>Vibrionaceae>Vibrio	GCF_002876335.1	Vibrio_tasmaniensis	BCT83_RS20570	15143..18886	212663	BREX-system-P-loop-protein-BrxC-[Vibrio-tasmaniensis]
WP_102573392.1	Type1.BREX	REase->Calcineurin-SbcD-><-?||?->?-><-SF1-SF2-Helicase+HTH||wHTH+WYL+WCX->BrxA-DNA-binding->BrxB-BrxF-iSTAND->DUF6079*->N6-METHYLASE->ParB+HNH->ANK+ANK+ANK->GrpE->?->?->?->PglZ->SIGMA+OB-Fold+AAA-ATPase+Lon-Protease-BrxL->	1247	Bacteria>Pseudomonadota>Gammaproteobacteria>Vibrionales>Vibrionaceae>Vibrio	GCF_002876905.1	Vibrio_splendidus	BCT62_RS21755	9396..13139	29497	BREX-system-P-loop-protein-BrxC-[Vibrio-splendidus]
WP_102456769.1	Type1.BREX	<-SIGMA+OB-Fold+AAA-ATPase+Lon-Protease-BrxL<-PglZ<-?<-?<-ZnR+3C-ABC+TPR<-ANK+ANK+ANK<-ParB+HNH<-N6-METHYLASE<-DUF6079*<-BrxB-BrxF-iSTAND<-BrxA-DNA-binding<-wHTH+WYL+WCX||SF1-SF2-Helicase+HTH->?-><-TraI-VirD2-RCRE<-?<-?||PALP->	1247	Bacteria>Pseudomonadota>Gammaproteobacteria>Vibrionales>Vibrionaceae>Vibrio	GCF_002877315.1	Vibrio_splendidus	BCU08_RS20225	19418..23161	29497	BREX-system-P-loop-protein-BrxC-[Vibrio-splendidus]
WP_102457574.1	Type1.BREX	<-SF1-SF2-Helicase+HTH||wHTH+WYL+WCX->BrxA-DNA-binding->BrxB-BrxF-iSTAND->DUF6079-p*->	468	Bacteria>Pseudomonadota>Gammaproteobacteria>Vibrionales>Vibrionaceae>Vibrio	GCF_002877525.1	Vibrio_splendidus	BCU23_RS04325	6659..8062	29497	BREX-system-P-loop-protein-BrxC,-partial-[Vibrio-splendidus]
WP_102887270.1	Type1.BREX	MobI->?-><-?<-LexA-protease||35exo->?->wHTH+WYL+WCX->BrxA-DNA-binding->BrxB-BrxF-iSTAND->DUF6079*->ParB+HNH->N6-METHYLASE->PglZ->SIGMA+OB-Fold+AAA-ATPase+Lon-Protease-BrxL->SbcC->HDRnase->VirD4-TraG+VirB4-FtsK->	1225	Bacteria>Pseudomonadota>Gammaproteobacteria>Vibrionales>Vibrionaceae>Vibrio	GCF_002891755.1	Vibrio_vulnificus	TI06_RS01685	47134..50811	672	BREX-system-P-loop-protein-BrxC-[Vibrio-vulnificus]
WP_104040811.1	Type1.BREX	viral-protein-><-?<-?<-?<-?||wHTH+WYL+WCX->BrxA-DNA-binding->BrxB-BrxF-iSTAND->DUF6079*->SF1-SF2-Helicase+PRTase->SLOG+BirA-HTH->?->?->N6-METHYLASE->?->ParB+HNH->ANK+ANK+ANK->GrpE->	1232	Bacteria>Pseudomonadota>Gammaproteobacteria>Vibrionales>Vibrionaceae>Vibrio	GCF_002929885.1	Vibrio_hyugaensis	C0Q55_RS11480	5964..9662	1534743	BREX-system-P-loop-protein-BrxC-[Vibrio-hyugaensis]
WP_104969987.1	Type1.BREX	sGTP->Phage_int_SAM_5+Phage_integrase-><-XIS-HTH<-AAA+HTH<-XIS-HTH||wHTH+WYL+WCX->BrxA-DNA-binding->BrxB-BrxF-iSTAND->DUF6079*->OLD-ABC->TOPRIM->N6-METHYLASE->PglZ->SIGMA+OB-Fold+AAA-ATPase+Lon-Protease-BrxL->MobI->MobI->	1225	Bacteria>Pseudomonadota>Gammaproteobacteria>Vibrionales>Vibrionaceae>Vibrio	GCF_002953335.1	Vibrio_diabolicus	AL552_RS16285	3102231..3105908	50719	BREX-system-P-loop-protein-BrxC-[Vibrio-diabolicus]
WP_040122842.1	Type1.BREX	AbiEi->viral-protein->SF1-SF2-Helicase+REase->SF1-SF2-Helicase+REase-><-?<-ZetaToxin<-PglZ<-ParB+HNH<-N6-METHYLASE<-DUF6079*<-BrxB-BrxF-iSTAND<-BrxA-DNA-binding<-wHTH+WYL+WCX||LexA-protease->?-><-?<-CR-ATPase4	1157	Bacteria>Pseudomonadota>Gammaproteobacteria>Vibrionales>Vibrionaceae>Vibrio	GCF_003031205.1	Vibrio_anguillarum	CEA93_RS18125	702795..706268	55601	BREX-system-P-loop-protein-BrxC-[Vibrio-anguillarum]
WP_108187618.1	Type1.BREX	<-SIGMA+OB-Fold+AAA-ATPase+Lon-Protease-BrxL<-PglZ<-TOPRIM<-OLD-ABC<-?<-?<-?<-N6-METHYLASE<-ParB+HNH<-DUF6079*<-BrxB-BrxF-iSTAND<-BrxA-DNA-binding<-wHTH+WYL+WCX||SF1-SF2-Helicase->MarR-HTH->HKD-DNase+HKD-DNase->CARF+NtrC-AAA->PIN+OST-HTH->HIN-HTH->	1233	Bacteria>Pseudomonadota>Gammaproteobacteria>Vibrionales>Vibrionaceae>Vibrio	GCF_003049965.1	Vibrio_splendidus	CW007_RS09240	50503..54204	29497	BREX-system-P-loop-protein-BrxC-[Vibrio-splendidus]
WP_108693859.1	Type1.BREX	<-ZnR<-?||AbiEi->viral-protein->wHTH+WYL+WCX->BrxA-DNA-binding->BrxB-BrxF-iSTAND->DUF6079*->N6-METHYLASE->SbcC->Schlafen+HATPase+wHTH->PglZ->SIGMA+OB-Fold+AAA-ATPase+Lon-Protease-BrxL-><-wHTH||DUF4113->	1225	Bacteria>Pseudomonadota>Gammaproteobacteria>Vibrionales>Vibrionaceae>Vibrio	GCF_003072005.1	Vibrio_vulnificus	DC364_RS00725	56102..59779	672	BREX-system-P-loop-protein-BrxC-[Vibrio-vulnificus]
WP_108745199.1	Type1.BREX	<-DUF4113||wHTH-><-SIGMA+OB-Fold+AAA-ATPase+Lon-Protease-BrxL<-PglZ<-?<-?<-N6-METHYLASE<-DUF6079*<-BrxB-BrxF-iSTAND<-BrxA-DNA-binding<-wHTH+WYL+WCX<-viral-protein<-AbiEi<-SF1-SF2-Helicase<-?||?->ZnR->	1225	Bacteria>Pseudomonadota>Gammaproteobacteria>Vibrionales>Vibrionaceae>Vibrio	GCF_003076895.1	Vibrio_parahaemolyticus	C9I78_RS18225	191992..195669	670	BREX-system-P-loop-protein-BrxC-[Vibrio-parahaemolyticus]
WP_113603075.1	Type1.BREX	<-VirD4-TraG+VirB4-FtsK<-HDRnase<-SbcC<-SIGMA+OB-Fold+AAA-ATPase+Lon-Protease-BrxL<-PglZ<-N6-METHYLASE<-ParB+HNH<-DUF6079*<-BrxB-BrxF-iSTAND<-BrxA-DNA-binding<-wHTH+WYL+WCX<-?<-35exo<-?||LexA-protease->?-><-MobI	1225	Bacteria>Pseudomonadota>Gammaproteobacteria>Vibrionales>Vibrionaceae>Vibrio	GCF_003311885.1	Vibrio_tarriae	DLR65_RS02205	60103..63780	2014742	BREX-system-P-loop-protein-BrxC-[Vibrio-tarriae]
WP_235868160.1	Type1.BREX	Arm-DNA-bind_3+Phage_integrase->SF1-SF2-Helicase+REase->SF1-SF2-Helicase+REase-><-PglZ<-TOPRIM<-OLD-ABC<-N6-METHYLASE<-DUF6079*<-BrxB-BrxF-iSTAND<-BrxA-DNA-binding<-wHTH+WYL+WCX<-XIS-HTH||?->?->DUF2787->HTH->wHTH+WYL+WCX->	1153	Bacteria>Pseudomonadota>Gammaproteobacteria>Vibrionales>Vibrionaceae>Vibrio	GCF_003311905.1	Vibrio_paracholerae	DLR66_RS03590	70731..74192	650003	BREX-system-P-loop-protein-BrxC-[Vibrio-paracholerae]
WP_116869874.1	Type1.BREX	sGTP->Phage_int_SAM_5+Phage_integrase-><-XIS-HTH<-AAA+HTH<-XIS-HTH||wHTH+WYL+WCX->BrxA-DNA-binding->BrxB-BrxF-iSTAND->DUF6079*->OLD-ABC->TOPRIM->N6-METHYLASE->PglZ->SIGMA+OB-Fold+AAA-ATPase+Lon-Protease-BrxL->MobI->MobI->ParE-><-HTH_19	1225	Bacteria>Pseudomonadota>Gammaproteobacteria>Vibrionales>Vibrionaceae>Vibrio	GCF_003408655.1	Vibrio_splendidus	D0583_RS01655	344855..348532	29497	BREX-system-P-loop-protein-BrxC-[Vibrio-splendidus]
WP_119471206.1	Type1.BREX	<-LexA-protease||?->35exo->?->wHTH+WYL+WCX->BrxA-DNA-binding->BrxB-BrxF-iSTAND->DUF6079*->N6-METHYLASE->PglZ->	1229	Bacteria>Pseudomonadota>Gammaproteobacteria>Vibrionales>Vibrionaceae>Vibrio	GCF_003585465.1	Vibrio_cholerae	CG489_RS12450	5990..9679	666	BREX-system-P-loop-protein-BrxC-[Vibrio-cholerae]
WP_119561011.1	Type1.BREX	DUF6079*->N6-METHYLASE->PglZ->MarR-HTH->HEPN->HDRnase->VirD4-TraG+VirB4-FtsK->?->?-><-Glyoxalase_8	1071	Bacteria>Pseudomonadota>Gammaproteobacteria>Vibrionales>Vibrionaceae>Vibrio	GCF_003585485.1	Vibrio_cholerae	CG490_RS09140	1..3217	666	BREX-system-P-loop-protein-BrxC,-partial-[Vibrio-cholerae]
WP_122015998.1	Type1.BREX	CR-ATPase4->?-><-SIGMA+OB-Fold+AAA-ATPase+Lon-Protease-BrxL<-PglZ<-?<-?<-ParB+HNH<-N6-METHYLASE<-DUF6079*<-BrxB-BrxF-iSTAND<-BrxA-DNA-binding<-wHTH+WYL+WCX||SF1-SF2-Helicase->MarR-HTH->HKD-DNase+HKD-DNase->CARF+NtrC-AAA->PIN+OST-HTH->	1248	Bacteria>Pseudomonadota>Gammaproteobacteria>Vibrionales>Vibrionaceae>Vibrio	GCF_003691525.1	Vibrio_parahaemolyticus	D0871_RS08265	1554661..1558407	670	BREX-system-P-loop-protein-BrxC-[Vibrio-parahaemolyticus]
WP_130200308.1	Type1.BREX	AbiEi->viral-protein->SF1-SF2-Helicase+REase->SF1-SF2-Helicase+REase-><-?<-ZetaToxin<-PglZ<-ParB+HNH<-N6-METHYLASE<-DUF6079*<-BrxB-BrxF-iSTAND<-BrxA-DNA-binding<-wHTH+WYL+WCX<-DDE_Tnp_1_5+DDE_Tnp_1||<-?<-CR-ATPase4||SbcC->	1157	Bacteria>Pseudomonadota>Gammaproteobacteria>Vibrionales>Vibrionaceae>Vibrio	GCF_004214625.1	Vibrio_vulnificus	D8T51_RS18945	13637..17110	672	BREX-system-P-loop-protein-BrxC-[Vibrio-vulnificus]
WP_130241041.1	Type1.BREX	<-DDE_Tnp_1||viral-protein->wHTH+WYL+WCX->BrxA-DNA-binding->BrxB-BrxF-iSTAND->DUF6079-p*-><-Zn_Tnp_IS1+HTH_Tnp_IS1+DDE_Tnp_IS1<-?<-?||ADA-DBD+AraC-HTH+O6MGT-HTH-><-TraI-VirD2-RCRE<-TraJ-RHH	803	Bacteria>Pseudomonadota>Gammaproteobacteria>Vibrionales>Vibrionaceae>Vibrio	GCF_004214665.1	Vibrio_vulnificus	D8T54_RS22380	4485..6896	672	BREX-system-P-loop-protein-BrxC-[Vibrio-vulnificus]
WP_130241697.1	Type1.BREX	MobI-><-?<-LexA-protease||35exo->?->wHTH+WYL+WCX->BrxA-DNA-binding->BrxB-BrxF-iSTAND->DUF6079*->N6-METHYLASE->PglZ->SIGMA+OB-Fold+AAA-ATPase+Lon-Protease-BrxL->MarR-HTH->HEPN->HDRnase->VirD4-TraG+VirB4-FtsK->?-><-Glyoxalase_8	1231	Bacteria>Pseudomonadota>Gammaproteobacteria>Vibrionales>Vibrionaceae>Vibrio	GCF_004214765.1	Vibrio_vulnificus	D8T65_RS00475	112647..116342	672	BREX-system-P-loop-protein-BrxC-[Vibrio-vulnificus]
WP_130361357.1	Type1.BREX	<-N6-METHYLASE<-?<-DUF6079*<-BrxB-BrxF-iSTAND<-BrxA-DNA-binding<-wHTH+WYL+WCX<-viral-protein	1231	Bacteria>Pseudomonadota>Gammaproteobacteria>Vibrionales>Vibrionaceae>Vibrio	GCF_004215315.1	Vibrio_vulnificus	D8T58_RS22585	4068..7763	672	BREX-system-P-loop-protein-BrxC-[Vibrio-vulnificus]
WP_137027181.1	Type1.BREX	SF1-SF2-Helicase->AbiEi->viral-protein->wHTH+WYL+WCX->BrxA-DNA-binding->BrxB-BrxF-iSTAND->DUF6079*->N6-METHYLASE->ParB+HNH->GrpE->?->PglZ->SIGMA+OB-Fold+AAA-ATPase+Lon-Protease-BrxL-><-wHTH||DUF4113->	1225	Bacteria>Pseudomonadota>Gammaproteobacteria>Vibrionales>Vibrionaceae>Vibrio	GCF_005145785.1	Vibrio_kanaloae	FCV44_20755	6017..9694	170673	BREX-system-P-loop-protein-BrxC-[Vibrio-kanaloae]
WP_136997941.1	Type1.BREX	SF1-SF2-Helicase->AbiEi->viral-protein->wHTH+WYL+WCX->BrxA-DNA-binding->BrxB-BrxF-iSTAND->DUF6079*->N6-METHYLASE->?->?->PglZ->SIGMA+OB-Fold+AAA-ATPase+Lon-Protease-BrxL-><-wHTH||DUF4113-><-?<-CR-ATPase4	1225	Bacteria>Pseudomonadota>Gammaproteobacteria>Vibrionales>Vibrionaceae>Vibrio	GCF_005146415.1	Vibrio_kanaloae	FCV52_RS09450	5982..9659	170673	BREX-system-P-loop-protein-BrxC-[Vibrio-kanaloae]
WP_140128967.1	Type1.BREX	AbiEi->viral-protein->wHTH+WYL+WCX->BrxA-DNA-binding->BrxB-BrxF-iSTAND->DUF6079*->N6-METHYLASE->ParB+HNH->GrpE->?->PglZ->SIGMA+OB-Fold+AAA-ATPase+Lon-Protease-BrxL-><-wHTH<-?<-CR-ATPase4	1225	Bacteria>Pseudomonadota>Gammaproteobacteria>Vibrionales>Vibrionaceae>Vibrio	GCF_006368845.1	Vibrio_parahaemolyticus	CGJ25_RS23760	3581..7258	670	BREX-system-P-loop-protein-BrxC-[Vibrio-parahaemolyticus]
WP_140244484.1	Type1.BREX	RNase_H->XIS-HTH->TPR-><-wHTH+WYL+WCX||?->XIS-HTH->BrxA-DNA-binding->BrxB-BrxF-iSTAND->DUF6079*->N6-METHYLASE->PglZ->SIGMA+OB-Fold+AAA-ATPase+Lon-Protease-BrxL->Phage_integrase-><-ParB<-GDResolvase+HIN-HTH<-SIR2||HAMP+MA+MA->	1221	Bacteria>Pseudomonadota>Gammaproteobacteria>Vibrionales>Vibrionaceae>Vibrio	GCF_006371105.1	Vibrio_parahaemolyticus	CGJ91_RS11715	157645..161310	670	BREX-system-P-loop-protein-BrxC-[Vibrio-parahaemolyticus]
WP_140244622.1	Type1.BREX	CR-ATPase4->?->wHTH-><-SIGMA+OB-Fold+AAA-ATPase+Lon-Protease-BrxL<-PglZ<-?<-?<-N6-METHYLASE<-DUF6079*<-BrxB-BrxF-iSTAND<-BrxA-DNA-binding<-wHTH+WYL+WCX<-viral-protein<-AbiEi<-SF1-SF2-Helicase<-?||?->ZnR->	1225	Bacteria>Pseudomonadota>Gammaproteobacteria>Vibrionales>Vibrionaceae>Vibrio	GCF_006371105.1	Vibrio_parahaemolyticus	CGJ91_RS14635	88177..91854	670	BREX-system-P-loop-protein-BrxC-[Vibrio-parahaemolyticus]
WP_140247448.1	Type1.BREX	AbiEi->viral-protein->SF1-SF2-Helicase+REase->SF1-SF2-Helicase+REase-><-?<-ZetaToxin<-PglZ<-ParB+HNH<-N6-METHYLASE<-DUF6079*<-BrxB-BrxF-iSTAND<-BrxA-DNA-binding<-wHTH+WYL+WCX||<-?<-CR-ATPase4||PIN+OST-HTH-><-REase+SPF-cons	1157	Bacteria>Pseudomonadota>Gammaproteobacteria>Vibrionales>Vibrionaceae>Vibrio	GCF_006371495.1	Vibrio_parahaemolyticus	CGJ41_RS23680	13693..17166	670	BREX-system-P-loop-protein-BrxC-[Vibrio-parahaemolyticus]
WP_140344322.1	Type1.BREX	CR-ATPase4->?->||wHTH+WYL+WCX->BrxA-DNA-binding->BrxB-BrxF-iSTAND->DUF6079*->N6-METHYLASE->ParB+HNH->PglZ->SF1-SF2-Helicase->	1157	Bacteria>Pseudomonadota>Gammaproteobacteria>Vibrionales>Vibrionaceae>Vibrio	GCF_006372045.1	Vibrio_parahaemolyticus	CGJ19_RS21455	3824..7297	670	BREX-system-P-loop-protein-BrxC-[Vibrio-parahaemolyticus]
WP_140256559.1	Type1.BREX	MobI-><-?<-LexA-protease||35exo->?->wHTH+WYL+WCX->BrxA-DNA-binding->BrxB-BrxF-iSTAND->DUF6079*->ParB+HNH->N6-METHYLASE->PglZ->SIGMA+OB-Fold+AAA-ATPase+Lon-Protease-BrxL->SbcC->HDRnase->VirD4-TraG+VirB4-FtsK->	1225	Bacteria>Pseudomonadota>Gammaproteobacteria>Vibrionales>Vibrionaceae>Vibrio	GCF_006372155.1	Vibrio_parahaemolyticus	D9A28_03775	149461..153138	670	BREX-system-P-loop-protein-BrxC-[Vibrio-parahaemolyticus]
WP_140272583.1	Type1.BREX	CR-ATPase4->?-><-SIGMA+OB-Fold+AAA-ATPase+Lon-Protease-BrxL<-PglZ<-?<-?<-ParB+HNH<-N6-METHYLASE<-DUF6079*<-BrxB-BrxF-iSTAND<-BrxA-DNA-binding<-wHTH+WYL+WCX||SF1-SF2-Helicase->MarR-HTH->HKD-DNase+HKD-DNase->CARF+NtrC-AAA->PIN+OST-HTH->	1248	Bacteria>Pseudomonadota>Gammaproteobacteria>Vibrionales>Vibrionaceae>Vibrio	GCF_006372715.1	Vibrio_parahaemolyticus	CGI87_RS22525	16069..19815	670	BREX-system-P-loop-protein-BrxC-[Vibrio-parahaemolyticus]
WP_148491305.1	Type1.BREX	MobI-><-?<-LexA-protease||35exo->?->wHTH+WYL+WCX->BrxA-DNA-binding->BrxB-BrxF-iSTAND->DUF6079*->N6-METHYLASE->PglZ->SIGMA+OB-Fold+AAA-ATPase+Lon-Protease-BrxL->MarR-HTH->HEPN->HDRnase->?->?->DOC+HTH->	1229	Bacteria>Pseudomonadota>Gammaproteobacteria>Vibrionales>Vibrionaceae>Vibrio	GCF_008083215.1	Vibrio_cholerae	FXF11_RS16935	299548..303237	666	BREX-system-P-loop-protein-BrxC-[Vibrio-cholerae]
WP_148513618.1	Type1.BREX	AbiEi->viral-protein->SF1-SF2-Helicase+REase->SF1-SF2-Helicase+REase-><-TPR<-HEPN+SF1-SF2-Helicase+RNase+RNase<-PglZ<-ParB+HNH<-N6-METHYLASE<-DUF6079*<-BrxB-BrxF-iSTAND<-BrxA-DNA-binding<-wHTH+WYL+WCX||DDE_Tnp_1_5+DDE_Tnp_1->	1157	Bacteria>Pseudomonadota>Gammaproteobacteria>Vibrionales>Vibrionaceae>Vibrio	GCF_008083315.1	Vibrio_cholerae	FXF10_RS00340	29414..32887	666	BREX-system-P-loop-protein-BrxC-[Vibrio-cholerae]
WP_148532770.1	Type1.BREX	<-VirD4-TraG+VirB4-FtsK<-HDRnase<-SbcC<-SIGMA+OB-Fold+AAA-ATPase+Lon-Protease-BrxL<-PglZ<-N6-METHYLASE<-ParB+HNH<-DUF6079*<-BrxB-BrxF-iSTAND<-BrxA-DNA-binding<-wHTH+WYL+WCX<-?<-35exo<-?||LexA-protease->	1225	Bacteria>Pseudomonadota>Gammaproteobacteria>Vibrionales>Vibrionaceae>Vibrio	GCF_008084025.1	Vibrio_cholerae	FXE74_RS08220	132887..136564	666	BREX-system-P-loop-protein-BrxC-[Vibrio-cholerae]
WP_148490856.1	Type1.BREX	<-DDE_Tnp_1_5+DDE_Tnp_1||HTH_Tnp_1+HTH_21+rve+rve_3-><-SbcC||CR-ATPase4->?-><-DUF4113||wHTH+WYL+WCX->BrxA-DNA-binding->BrxB-BrxF-iSTAND->DUF6079*->N6-METHYLASE->ParB+HNH->PglZ->HEPN+SF1-SF2-Helicase+RNase+RNase->TPR-><-SF1-SF2-Helicase+REase<-SF1-SF2-Helicase+REase<-viral-protein<-AbiEi	1157	Bacteria>Pseudomonadota>Gammaproteobacteria>Vibrionales>Vibrionaceae>Vibrio	GCF_008084645.1	Vibrio_cholerae	FXE54_RS16715	26974..30447	666	BREX-system-P-loop-protein-BrxC-[Vibrio-cholerae]
WP_148536810.1	Type1.BREX	<-CARF+NtrC-AAA+FIS-HTH<-SIGMA+OB-Fold+AAA-ATPase+Lon-Protease-BrxL<-PglZ<-N6-METHYLASE<-ParB+HNH<-DUF6079*<-BrxB-BrxF-iSTAND<-BrxA-DNA-binding<-wHTH+WYL+WCX<-?<-35exo||LexA-protease->?-><-MobI	1225	Bacteria>Pseudomonadota>Gammaproteobacteria>Vibrionales>Vibrionaceae>Vibrio	GCF_008085615.1	Vibrio_cholerae	FXE25_RS13680	35779..39456	666	BREX-system-P-loop-protein-BrxC-[Vibrio-cholerae]
WP_152820636.1	Type1.BREX	<-SIGMA+OB-Fold+AAA-ATPase+Lon-Protease-BrxL<-PglZ<-SLOG+BirA-HTH<-SF1-SF2-Helicase+PRTase<-ParB+HNH<-HEPN+Sel1+SEC-C<-N6-METHYLASE<-DUF6079*<-BrxB-BrxF-iSTAND<-BrxA-DNA-binding<-wHTH+WYL+WCX	1247	Bacteria>Pseudomonadota>Gammaproteobacteria>Vibrionales>Vibrionaceae>Vibrio	GCF_009372095.1	Vibrio_sp._B1Z05	GCU72_RS07750	793260..797003	2654980	BREX-system-P-loop-protein-BrxC-[Vibrio-sp.-B1Z05]
WP_158108820.1	Type1.BREX	<-ZnR<-?||?->SF1-SF2-Helicase->AbiEi->viral-protein->wHTH+WYL+WCX->BrxA-DNA-binding->BrxB-BrxF-iSTAND->DUF6079*->N6-METHYLASE->?->?->PglZ->SIGMA+OB-Fold+AAA-ATPase+Lon-Protease-BrxL-><-wHTH||DUF4113-><-?<-CR-ATPase4	1225	Bacteria>Pseudomonadota>Gammaproteobacteria>Vibrionales>Vibrionaceae>Vibrio	GCF_009763225.1	Vibrio_furnissii	GPY43_RS22260	1428276..1431953	29494	BREX-system-P-loop-protein-BrxC-[Vibrio-furnissii]
WP_123161578.1	Type1.BREX	MobI-><-?<-LexA-protease||35exo->?->wHTH+WYL+WCX->BrxA-DNA-binding->BrxB-BrxF-iSTAND->DUF6079*->ParB+HNH->N6-METHYLASE->PglZ->SIGMA+OB-Fold+AAA-ATPase+Lon-Protease-BrxL->SbcC->HDRnase->VirD4-TraG+VirB4-FtsK->	1225	Bacteria>Pseudomonadota>Gammaproteobacteria>Vibrionales>Vibrionaceae>Vibrio	GCF_009763665.1	Vibrio_cholerae	GPY01_RS14950	2265324..2269001	666	BREX-system-P-loop-protein-BrxC-[Vibrio-cholerae]
WP_158155131.1	Type1.BREX	Calcineurin->?-><-ZnR<-?||AbiEi->viral-protein->wHTH+WYL+WCX->BrxA-DNA-binding->BrxB-BrxF-iSTAND->DUF6079*->N6-METHYLASE-p->?->?->PglZ->SIGMA+OB-Fold+AAA-ATPase+Lon-Protease-BrxL-><-wHTH<-?<-CR-ATPase4	1225	Bacteria>Pseudomonadota>Gammaproteobacteria>Vibrionales>Vibrionaceae>Vibrio	GCF_009763745.1	Vibrio_alginolyticus	GPY47_RS00180	30700..34377	663	BREX-system-P-loop-protein-BrxC-[Vibrio-alginolyticus]
WP_159408537.1	Type1.BREX	DUF6079*->SbcC->Schlafen+HATPase+wHTH->PglZ->SIGMA+OB-Fold+AAA-ATPase+Lon-Protease-BrxL-><-REase+NACHT||HTH_Tnp_1+HTH_21+rve+rve_3-><-BrxB-BrxF-iSTAND<-?<-RADICAL-SAM	1229	Bacteria>Pseudomonadota>Gammaproteobacteria>Vibrionales>Vibrionaceae>Vibrio	GCF_009883875.1	Vibrio_parahaemolyticus	EHC69_RS27010	189487..53	670	BREX-system-P-loop-protein-BrxC-[Vibrio-parahaemolyticus]
WP_202601719.1	Type1.BREX	<-SIGMA+OB-Fold+AAA-ATPase+HKD-DNase-BrxL<-PglZ<-N6-METHYLASE<-REase<-OLD-ABC<-DUF6079*<-BrxB-BrxF-iSTAND<-BrxA-DNA-binding<-XIS-HTH<-HNH<-SF1-SF2-Helicase+HTH<-SF1-SF2-Helicase<-?<-XIS-HTH	1187	Bacteria>Pseudomonadota>Gammaproteobacteria>Vibrionales>Vibrionaceae>Vibrio	GCF_009906175.1	Vibrio_toranzoniae	GL180_RS08040	10464..14027	1194427	BREX-system-P-loop-protein-BrxC-[Vibrio-toranzoniae]
WP_171981218.1	Type1.BREX	CR-ATPase4->?-><-?<-LexA-protease||?->wHTH+WYL+WCX->BrxA-DNA-binding->BrxB-BrxF-iSTAND->DUF6079*->	1157	Bacteria>Pseudomonadota>Gammaproteobacteria>Vibrionales>Vibrionaceae>Vibrio	GCF_011090175.1	Vibrio_aestuarianus	G6Z92_RS15410	17195..20668	28171	BREX-system-P-loop-protein-BrxC-[Vibrio-aestuarianus]
WP_171981504.1	Type1.BREX	<-N6-METHYLASE-p<-DUF6079*<-BrxB-BrxF-iSTAND<-BrxA-DNA-binding<-wHTH+WYL+WCX<-viral-protein<-AbiEi<-SF1-SF2-Helicase	1225	Bacteria>Pseudomonadota>Gammaproteobacteria>Vibrionales>Vibrionaceae>Vibrio	GCF_011090175.1	Vibrio_aestuarianus	G6Z92_RS17720	1620..5297	28171	BREX-system-P-loop-protein-BrxC-[Vibrio-aestuarianus]
WP_176314025.1	Type1.BREX	<-DUF6079*<-BrxB-BrxF-iSTAND<-BrxA-DNA-binding<-wHTH+WYL+WCX<-?||LexA-protease->?-><-?<-CR-ATPase4	1157	Bacteria>Pseudomonadota>Gammaproteobacteria>Vibrionales>Vibrionaceae>Vibrio	GCF_011090275.1	Vibrio_aestuarianus	G6Z96_RS18110	255..3728	28171	BREX-system-P-loop-protein-BrxC-[Vibrio-aestuarianus]
WP_172566988.1	Type1.BREX	CR-ATPase4->?-><-?<-LexA-protease||wHTH+WYL+WCX->BrxA-DNA-binding->BrxB-BrxF-iSTAND->DUF6079*->N6-METHYLASE->ParB+HNH->PglZ->ZetaToxin->?-><-SF1-SF2-Helicase+REase<-SF1-SF2-Helicase+REase<-viral-protein<-AbiEi	1157	Bacteria>Pseudomonadota>Gammaproteobacteria>Vibrionales>Vibrionaceae>Vibrio	GCF_012274945.1	Vibrio_fluvialis	HF303_RS09150	434217..437690	676	BREX-system-P-loop-protein-BrxC-[Vibrio-fluvialis]
WP_169592914.1	Type1.BREX	MobI-><-Glyoxalase_8<-?<-LexA-protease||35exo->?->wHTH+WYL+WCX->BrxA-DNA-binding->BrxB-BrxF-iSTAND->DUF6079*->ParB+HNH->N6-METHYLASE->PglZ->SIGMA+OB-Fold+AAA-ATPase+Lon-Protease-BrxL->SbcC->HDRnase->VirD4-TraG+VirB4-FtsK->	1225	Bacteria>Pseudomonadota>Gammaproteobacteria>Vibrionales>Vibrionaceae>Vibrio	GCF_012956215.1	Vibrio_parahaemolyticus	HKB29_RS26535	221026..224703	670	BREX-system-P-loop-protein-BrxC-[Vibrio-parahaemolyticus]
WP_170894125.1	Type1.BREX	HTH_19-><-ParE<-MobI<-MobI<-SIGMA+OB-Fold+AAA-ATPase+Lon-Protease-BrxL<-PglZ<-N6-METHYLASE<-TOPRIM<-OLD-ABC<-DUF6079*<-BrxB-BrxF-iSTAND<-BrxA-DNA-binding<-wHTH+WYL+WCX||XIS-HTH->AAA+HTH->XIS-HTH-><-Phage_int_SAM_5+Phage_integrase<-sGTP	1225	Bacteria>Pseudomonadota>Gammaproteobacteria>Vibrionales>Vibrionaceae>Vibrio	GCF_013048535.1	unclassified_Vibrio	FKN13_RS12350	70010..73687	2614977	MULTISPECIES:-BREX-system-P-loop-protein-BrxC-[unclassified-Vibrio]
WP_171375390.1	Type1.BREX	<-ZnR<-?||?->SF1-SF2-Helicase->AbiEi->viral-protein->wHTH+WYL+WCX->BrxA-DNA-binding->BrxB-BrxF-iSTAND->DUF6079*->N6-METHYLASE->ParB+HNH->GrpE->?->PglZ->SIGMA+OB-Fold+AAA-ATPase+Lon-Protease-BrxL-><-TPR<-HEPN+SF1-SF2-Helicase+RNase+RNase||LexA-protease->	1225	Bacteria>Pseudomonadota>Gammaproteobacteria>Vibrionales>Vibrionaceae>Vibrio	GCF_013114495.1	Vibrio_aestuarianus	F0230_RS00325	78960..82637	28171	BREX-system-P-loop-protein-BrxC-[Vibrio-aestuarianus]
WP_179877426.1	Type1.BREX	<-VirD4-TraG+VirB4-FtsK<-HDRnase<-HEPN<-MarR-HTH<-SIGMA+OB-Fold+AAA-ATPase+Lon-Protease-BrxL<-PglZ<-N6-METHYLASE<-DUF6079*	1169	Bacteria>Pseudomonadota>Gammaproteobacteria>Vibrionales>Vibrionaceae>Vibrio	GCF_013414485.1	Vibrio_cholerae	EYV61_RS05685	69787..73298	666	BREX-system-P-loop-protein-BrxC,-partial-[Vibrio-cholerae]
WP_182291541.1	Type1.BREX	REase+SPF-cons-><-PIN+OST-HTH||CR-ATPase4->?->||wHTH+WYL+WCX->BrxA-DNA-binding->BrxB-BrxF-iSTAND->DUF6079*->N6-METHYLASE->ParB+HNH->PglZ->ZetaToxin->?-><-SF1-SF2-Helicase+REase<-SF1-SF2-Helicase+REase<-viral-protein<-AbiEi	1157	Bacteria>Pseudomonadota>Gammaproteobacteria>Vibrionales>Vibrionaceae>Vibrio	GCF_014107515.1	Vibrio_vulnificus	F6X00_RS18205	373031..376504	672	BREX-system-P-loop-protein-BrxC-[Vibrio-vulnificus]
WP_238930029.1	Type1.BREX	HTH_20-><-SbcC||CR-ATPase4->?->||wHTH+WYL+WCX->BrxA-DNA-binding->BrxB-BrxF-iSTAND->DUF6079-p*->DUF6079-p->N6-METHYLASE->ParB+HNH->PglZ->ZetaToxin->?-><-SF1-SF2-Helicase+REase<-SF1-SF2-Helicase+REase<-Tnp_DNA_bind+DDE_Tnp_1+Dimer_Tnp_Tn5	779	Bacteria>Pseudomonadota>Gammaproteobacteria>Vibrionales>Vibrionaceae>Vibrio	GCF_014525155.1	Vibrio_vulnificus	G0186_RS04075	575963..578302	672	BREX-system-P-loop-protein-BrxC-[Vibrio-vulnificus]
WP_238930031.1	Type1.BREX	HTH_20-><-SbcC||CR-ATPase4->?->||wHTH+WYL+WCX->BrxA-DNA-binding->BrxB-BrxF-iSTAND->DUF6079-p->DUF6079-p*->N6-METHYLASE->ParB+HNH->PglZ->ZetaToxin->?-><-SF1-SF2-Helicase+REase<-SF1-SF2-Helicase+REase<-Tnp_DNA_bind+DDE_Tnp_1+Dimer_Tnp_Tn5<-AbiEi	366	Bacteria>Pseudomonadota>Gammaproteobacteria>Vibrionales>Vibrionaceae>Vibrio	GCF_014525155.1	Vibrio_vulnificus	G0186_RS25670	578332..579432	672	hypothetical-protein-[Vibrio-vulnificus]
WP_191116552.1	Type1.BREX	CR-ATPase4->?-><-SIGMA+OB-Fold+AAA-ATPase+Lon-Protease-BrxL<-PglZ<-?<-?<-ParB+HNH<-N6-METHYLASE<-DUF6079*<-BrxB-BrxF-iSTAND<-BrxA-DNA-binding<-wHTH+WYL+WCX||SF1-SF2-Helicase->MarR-HTH->HKD-DNase+HKD-DNase->CARF+NtrC-AAA->PIN+OST-HTH-><-AAA-ATPase	1248	Bacteria>Pseudomonadota>Gammaproteobacteria>Vibrionales>Vibrionaceae>Vibrio	GCF_014769095.1	Vibrio_parahaemolyticus	EUV06_RS14935	117914..121660	670	BREX-system-P-loop-protein-BrxC-[Vibrio-parahaemolyticus]
WP_054579585.1	Type1.BREX	AbiEi->viral-protein->SF1-SF2-Helicase+REase->SF1-SF2-Helicase+REase-><-?<-ZetaToxin<-PglZ<-ParB+HNH<-N6-METHYLASE<-DUF6079*<-BrxB-BrxF-iSTAND<-BrxA-DNA-binding<-wHTH+WYL+WCX||<-?<-CR-ATPase4||HEPN-><-sGTP	1157	Bacteria>Pseudomonadota>Gammaproteobacteria>Vibrionales>Vibrionaceae>Vibrio	GCF_014769135.1	Vibrio_alginolyticus	A0R09_RS18625	85180..88653	663	BREX-system-P-loop-protein-BrxC-[Vibrio-alginolyticus]
WP_191786376.1	Type1.BREX	HTH_19-><-ParE<-MobI<-MobI<-SIGMA+OB-Fold+AAA-ATPase+Lon-Protease-BrxL<-PglZ<-N6-METHYLASE<-TOPRIM<-OLD-ABC<-DUF6079*<-BrxB-BrxF-iSTAND<-BrxA-DNA-binding<-wHTH+WYL+WCX||XIS-HTH->AAA+HTH->XIS-HTH-><-Phage_int_SAM_5+Phage_integrase<-sGTP	1225	Bacteria>Pseudomonadota>Gammaproteobacteria>Vibrionales>Vibrionaceae>Vibrio	GCF_014836305.1	Vibrio_parahaemolyticus	G3220_RS19475	66422..70099	670	BREX-system-P-loop-protein-BrxC-[Vibrio-parahaemolyticus]
WP_194436055.1	Type1.BREX	MODE-HTH+PBPII->tRNA-><-HTH_26||Phage_AlpA->Arm-DNA-bind_2+Phage_int_SAM_3+Phage_integrase->?-><-?<-SIGMA+OB-Fold+AAA-ATPase+Lon-Protease-BrxL<-PglZ<-N6-METHYLASE<-DUF6079*<-BrxB-BrxF-iSTAND||OmpR-HTH->?-><-?<-OmpR-HTH	1219	Bacteria>Pseudomonadota>Gammaproteobacteria>Vibrionales>Vibrionaceae>Vibrio	GCF_015278125.1	Vibrio_sp._A621	IRQ95_RS05790	62806..66465	2783614	BREX-system-P-loop-protein-BrxC-[Vibrio-sp.-A621]
WP_198106729.1	Type1.BREX	SF1-SF2-Helicase+HTH->MukF->JetB->DUF3584+DUF5401->RNase->XIS-HTH->BrxA-DNA-binding->BrxB-BrxF-iSTAND->DUF6079*->OLD-ABC->REase->N6-METHYLASE->PglZ->SIGMA+OB-Fold+AAA-ATPase+HKD-DNase-BrxL->Phage_integrase->	1187	Bacteria>Pseudomonadota>Gammaproteobacteria>Vibrionales>Vibrionaceae>Vibrio	GCF_016094345.1	Vibrio_navarrensis	CJ419_RS12980	152911..156474	29495	BREX-system-P-loop-protein-BrxC-[Vibrio-navarrensis]
WP_206630456.1	Type1.BREX	GTPase-AIG->GTPase-AIG-><-HEPN||?->?-><-SIGMA+OB-Fold+AAA-ATPase+Lon-Protease-BrxL<-PglZ<-ParB+HNH<-N6-METHYLASE<-DUF6079*<-BrxB-BrxF-iSTAND<-BrxA-DNA-binding<-wHTH+WYL+WCX<-viral-protein||?->ZnR-><-SF1-SF2-Helicase<-Couple_hipA+HipA_C<-HTH_3	1232	Bacteria>Pseudomonadota>Gammaproteobacteria>Vibrionales>Vibrionaceae>Vibrio	GCF_017291155.1	Vibrio_vulnificus	J0J25_RS14690	90356..94054	672	BREX-system-P-loop-protein-BrxC-[Vibrio-vulnificus]
WP_206946493.1	Type1.BREX	sGTP-><-HEPN<-?||CR-ATPase4->?->||wHTH+WYL+WCX->BrxA-DNA-binding->BrxB-BrxF-iSTAND->DUF6079*->N6-METHYLASE->ParB+HNH->PglZ->ZetaToxin->?-><-SF1-SF2-Helicase+REase<-SF1-SF2-Helicase+REase<-viral-protein<-AbiEi	1157	Bacteria>Pseudomonadota>Gammaproteobacteria>Vibrionales>Vibrionaceae>Vibrio	GCF_017313505.1	Vibrio_sp._Vb2424	J0679_RS01025	227908..231381	2816074	BREX-system-P-loop-protein-BrxC-[Vibrio-sp.-Vb2424]
WP_210499744.1	Type1.BREX	NUDIX->SF1-SF2-Helicase+HTH->HNH->XIS-HTH->BrxA-DNA-binding->BrxB-BrxF-iSTAND->DUF6079*->OLD-ABC->NERD->N6-METHYLASE->PglZ->SIGMA+OB-Fold+AAA-ATPase+HKD-DNase-BrxL-><-wHTH+WYL+WCX||TOPRIM->JetC->	1185	Bacteria>Pseudomonadota>Gammaproteobacteria>Vibrionales>Vibrionaceae>Vibrio	GCF_017916775.1	Vibrio_crassostreae	KB594_RS01450	322447..326004	246167	BREX-system-P-loop-protein-BrxC-[Vibrio-crassostreae]
WP_210447442.1	Type1.BREX	XIS-HTH-><-DUF2787<-?<-?||XIS-HTH->?->wHTH+WYL+WCX->BrxA-DNA-binding->BrxB-BrxF-iSTAND->DUF6079*->N6-METHYLASE->OLD-ABC->TOPRIM->PglZ->SF1-SF2-Helicase+PRTase->SLOG+BirA-HTH->?->URI-DNase->sGTP->	1157	Bacteria>Pseudomonadota>Gammaproteobacteria>Vibrionales>Vibrionaceae>Vibrio	GCF_017917465.1	Vibrio_crassostreae	KB624_RS13660	42369..45842	246167	BREX-system-P-loop-protein-BrxC-[Vibrio-crassostreae]
WP_210942219.1	Type1.BREX	<-wHTH+WYL+WCX||SF1-SF2-Helicase+REase->?->XIS-HTH->wHTH+WYL+WCX->BrxA-DNA-binding->BrxB-BrxF-iSTAND->DUF6079*->N6-METHYLASE->ParB+HNH->PglZ->ZetaToxin->?-><-SF1-SF2-Helicase+REase<-SF1-SF2-Helicase+REase<-Arm-DNA-bind_3+Phage_integrase	1157	Bacteria>Pseudomonadota>Gammaproteobacteria>Vibrionales>Vibrionaceae>Vibrio	GCF_018035165.1	Vibrio_paracholerae	GSG51_RS14590	9402..12875	650003	BREX-system-P-loop-protein-BrxC-[Vibrio-paracholerae]
WP_250156418.1	Type1.BREX	<-wHTH+WYL+WCX<-DUF2787<-?<-?||XIS-HTH->HNH->XIS-HTH->BrxA-DNA-binding->BrxB-BrxF-iSTAND->DUF6079*->OLD-ABC->REase->N6-METHYLASE->PglZ->SIGMA+OB-Fold+AAA-ATPase+HKD-DNase-BrxL->	1185	Bacteria>Pseudomonadota>Gammaproteobacteria>Vibrionales>Vibrionaceae>Vibrio	GCF_018457845.1	Vibrio_alginolyticus	J4H60_RS12980	321648..325205	663	BREX-system-P-loop-protein-BrxC-[Vibrio-alginolyticus]
WP_213895854.1	Type1.BREX	Calcineurin->?-><-ZnR<-?||AbiEi->viral-protein->wHTH+WYL+WCX->BrxA-DNA-binding->BrxB-BrxF-iSTAND->DUF6079*->N6-METHYLASE->?->?->PglZ->SIGMA+OB-Fold+AAA-ATPase+Lon-Protease-BrxL-><-wHTH<-?<-CR-ATPase4||DDE_Tnp_1_5+DDE_Tnp_1->	1225	Bacteria>Pseudomonadota>Gammaproteobacteria>Vibrionales>Vibrionaceae>Vibrio	GCF_018458125.1	Vibrio_alginolyticus	J4H64_RS03750	118729..122406	663	BREX-system-P-loop-protein-BrxC-[Vibrio-alginolyticus]
WP_213968024.1	Type1.BREX	<-PIN+OST-HTH<-CARF+NtrC-AAA<-HKD-DNase+HKD-DNase<-MarR-HTH<-SF1-SF2-Helicase||wHTH+WYL+WCX->BrxA-DNA-binding->BrxB-BrxF-iSTAND->DUF6079*->N6-METHYLASE->ParB+HNH->ZnR+3C-ABC+TPR->?->?->PglZ->SIGMA+OB-Fold+AAA-ATPase+Lon-Protease-BrxL-><-?<-CR-ATPase4	1248	Bacteria>Pseudomonadota>Gammaproteobacteria>Vibrionales>Vibrionaceae>Vibrio	GCF_018458215.1	Vibrio_alginolyticus	J4H41_RS21340	12665..16411	663	BREX-system-P-loop-protein-BrxC-[Vibrio-alginolyticus]
WP_140279956.1	Type1.BREX	<-ZnR<-?||?->SF1-SF2-Helicase->AbiEi->viral-protein->wHTH+WYL+WCX->BrxA-DNA-binding->BrxB-BrxF-iSTAND->DUF6079*->N6-METHYLASE->?->?->PglZ->SIGMA+OB-Fold+AAA-ATPase+Lon-Protease-BrxL-><-wHTH<-?<-CR-ATPase4	1225	Bacteria>Pseudomonadota>Gammaproteobacteria>Vibrionales>Vibrionaceae>Vibrio	GCF_018458225.1	Vibrio_harveyi_group	O8042_RS07565	631131..634808	717610	MULTISPECIES:-BREX-system-P-loop-protein-BrxC-[Vibrio-harveyi-group]
WP_213970810.1	Type1.BREX	AbiEi->viral-protein->SF1-SF2-Helicase+REase->SF1-SF2-Helicase+REase-><-?<-ZetaToxin<-PglZ<-ParB+HNH<-N6-METHYLASE<-DUF6079*<-BrxB-BrxF-iSTAND<-BrxA-DNA-binding<-wHTH+WYL+WCX||DUF4113-><-?<-CR-ATPase4	1157	Bacteria>Pseudomonadota>Gammaproteobacteria>Vibrionales>Vibrionaceae>Vibrio	GCF_018458405.1	Vibrio_alginolyticus	J4H58_RS14535	108443..111916	663	BREX-system-P-loop-protein-BrxC-[Vibrio-alginolyticus]
WP_213899843.1	Type1.BREX	AbiEi->viral-protein->SF1-SF2-Helicase+REase->SF1-SF2-Helicase+REase-><-TPR<-HEPN+SF1-SF2-Helicase+RNase+RNase<-PglZ<-ParB+HNH<-N6-METHYLASE<-DUF6079*<-BrxB-BrxF-iSTAND<-BrxA-DNA-binding<-wHTH+WYL+WCX||<-?<-CR-ATPase4||SbcC->HEPN-><-HTH_20	1157	Bacteria>Pseudomonadota>Gammaproteobacteria>Vibrionales>Vibrionaceae>Vibrio	GCF_018458525.1	Vibrio_alginolyticus	J4H25_RS19085	22843..26316	663	BREX-system-P-loop-protein-BrxC-[Vibrio-alginolyticus]
WP_213874127.1	Type1.BREX	sGTP-><-HEPN<-?||CR-ATPase4->?->||wHTH+WYL+WCX->BrxA-DNA-binding->BrxB-BrxF-iSTAND->DUF6079*->N6-METHYLASE->ParB+HNH->PglZ->ZetaToxin->?-><-SF1-SF2-Helicase+REase<-SF1-SF2-Helicase+REase<-viral-protein<-AbiEi	1157	Bacteria>Pseudomonadota>Gammaproteobacteria>Vibrionales>Vibrionaceae>Vibrio	GCF_018458545.1	Vibrio_alginolyticus	J4H28_RS04435	1018248..1021721	663	BREX-system-P-loop-protein-BrxC-[Vibrio-alginolyticus]
WP_216506285.1	Type1.BREX	<-N6-METHYLASE<-DUF6079*<-BrxB-BrxF-iSTAND<-BrxA-DNA-binding<-wHTH+WYL+WCX<-?<-35exo	1229	Bacteria>Pseudomonadota>Gammaproteobacteria>Vibrionales>Vibrionaceae>Vibrio	GCF_018920395.1	Vibrio_cholerae	IN947_RS12990	4223..7912	666	BREX-system-P-loop-protein-BrxC-[Vibrio-cholerae]
WP_217555808.1	Type1.BREX	AbiEi->viral-protein->SF1-SF2-Helicase+REase->SF1-SF2-Helicase+REase-><-?<-ZetaToxin<-PglZ<-ParB+HNH<-N6-METHYLASE<-DUF6079*<-BrxB-BrxF-iSTAND<-BrxA-DNA-binding<-wHTH+WYL+WCX||LexA-protease->	1157	Bacteria>Pseudomonadota>Gammaproteobacteria>Vibrionales>Vibrionaceae>Vibrio	GCF_019091765.1	Vibrio_metschnikovii	KVT01_RS15810	26769..30242	28172	BREX-system-P-loop-protein-BrxC-[Vibrio-metschnikovii]
WP_222133201.1	Type1.BREX	<-PIN+OST-HTH<-CARF+NtrC-AAA<-HKD-DNase+HKD-DNase<-MarR-HTH<-SF1-SF2-Helicase||wHTH+WYL+WCX->BrxA-DNA-binding->BrxB-BrxF-iSTAND->DUF6079*->N6-METHYLASE->ParB+HNH->?->?->PglZ->SIGMA+OB-Fold+AAA-ATPase+Lon-Protease-BrxL->	1248	Bacteria>Pseudomonadota>Gammaproteobacteria>Vibrionales>Vibrionaceae>Vibrio	GCF_019793245.1	Vibrio_alginolyticus	K6L11_RS14305	27139..30885	663	BREX-system-P-loop-protein-BrxC-[Vibrio-alginolyticus]
WP_222134563.1	Type1.BREX	<-VirD4-TraG+VirB4-FtsK<-HDRnase<-NACHT+HEAT+REase<-SIGMA+OB-Fold+AAA-ATPase+Lon-Protease-BrxL<-PglZ<-N6-METHYLASE<-ParB+HNH<-DUF6079*<-BrxB-BrxF-iSTAND<-BrxA-DNA-binding<-wHTH+WYL+WCX<-?<-35exo||LexA-protease->?-><-MobI	1225	Bacteria>Pseudomonadota>Gammaproteobacteria>Vibrionales>Vibrionaceae>Vibrio	GCF_019793275.1	Vibrio_cholerae	NB536_RS17220	131235..134912	666	BREX-system-P-loop-protein-BrxC-[Vibrio-cholerae]
WP_224214086.1	Type1.BREX	<-ZnR<-?||?->SF1-SF2-Helicase->AbiEi->viral-protein->wHTH+WYL+WCX->BrxA-DNA-binding->BrxB-BrxF-iSTAND->DUF6079*->N6-METHYLASE->?->?->PglZ->SIGMA+OB-Fold+AAA-ATPase+Lon-Protease-BrxL->?-><-?<-CR-ATPase4||SbcC->	1225	Bacteria>Pseudomonadota>Gammaproteobacteria>Vibrionales>Vibrionaceae>Vibrio	GCF_020103255.1	Vibrio_metschnikovii	LDQ06_RS03825	838008..841685	28172	BREX-system-P-loop-protein-BrxC-[Vibrio-metschnikovii]
WP_224640811.1	Type1.BREX	MobI-><-?<-LexA-protease||?->35exo->?->wHTH+WYL+WCX->BrxA-DNA-binding->BrxB-BrxF-iSTAND->DUF6079*->ParB+HNH->N6-METHYLASE->PglZ->SIGMA+OB-Fold+AAA-ATPase+Lon-Protease-BrxL->NACHT+HEAT+REase->HDRnase->VirD4-TraG+VirB4-FtsK->	1225	Bacteria>Pseudomonadota>Gammaproteobacteria>Vibrionales>Vibrionaceae>Vibrio	GCF_020169625.1	Vibrio_vulnificus	KU011_RS09480	205814..209491	672	BREX-system-P-loop-protein-BrxC-[Vibrio-vulnificus]
WP_225491794.1	Type1.BREX	CR-ATPase4->?-><-DUF4113||wHTH-><-SIGMA+OB-Fold+AAA-ATPase+Lon-Protease-BrxL<-PglZ<-?<-?<-N6-METHYLASE<-DUF6079*<-BrxB-BrxF-iSTAND<-BrxA-DNA-binding<-wHTH+WYL+WCX<-viral-protein<-AbiEi<-SF1-SF2-Helicase||ZnR->?->CACHE+GGDEF->	1225	Bacteria>Pseudomonadota>Gammaproteobacteria>Vibrionales>Vibrionaceae>Vibrio	GCF_020223715.1	Vibrio_alginolyticus	LAZ44_RS12495	20548..24225	663	BREX-system-P-loop-protein-BrxC-[Vibrio-alginolyticus]
WP_225482458.1	Type1.BREX	<-SIGMA+OB-Fold+AAA-ATPase+Lon-Protease-BrxL<-PglZ||RADICAL-SAM-><-?<-?<-ParB+HNH<-N6-METHYLASE<-?<-DUF6079*<-BrxB-BrxF-iSTAND<-BrxA-DNA-binding<-wHTH+WYL+WCX||?->?-><-viral-protein||?-><-SF1-SF2-Helicase<-Couple_hipA+HipA_C	1231	Bacteria>Pseudomonadota>Gammaproteobacteria>Vibrionales>Vibrionaceae>Vibrio	GCF_020251885.1	Vibrio_vulnificus	JKP23_RS02685	250869..254564	672	BREX-system-P-loop-protein-BrxC-[Vibrio-vulnificus]
WP_225454267.1	Type1.BREX	TraJ-RHH->TraI-VirD2-RCRE->REase-><-AbiEi_4||?->wHTH+WYL+WCX->BrxA-DNA-binding->BrxB-BrxF-iSTAND->DUF6079*->N6-METHYLASE->ParB+HNH->ANK+ANK+ANK->ZnR+3C-ABC+TPR->?->?->PglZ->SIGMA+OB-Fold+AAA-ATPase+Lon-Protease-BrxL->	1248	Bacteria>Pseudomonadota>Gammaproteobacteria>Vibrionales>Vibrionaceae>Vibrio	GCF_020252125.1	Vibrio_vulnificus	JKP08_RS08925	222135..225881	672	BREX-system-P-loop-protein-BrxC-[Vibrio-vulnificus]
WP_228096077.1	Type1.BREX	<-HTH_Tnp_1||SF1-SF2-Helicase+REase->SF1-SF2-Helicase+REase-><-?<-ZetaToxin<-PglZ<-ParB+HNH<-N6-METHYLASE<-DUF6079*<-BrxB-BrxF-iSTAND<-BrxA-DNA-binding<-wHTH+WYL+WCX||DUF4113-><-?<-CR-ATPase4||SbcC->HEPN-><-HTH_20	1157	Bacteria>Pseudomonadota>Gammaproteobacteria>Vibrionales>Vibrionaceae>Vibrio	GCF_020741035.1	Vibrio_parahaemolyticus	IB294_RS01075	246074..249547	670	BREX-system-P-loop-protein-BrxC-[Vibrio-parahaemolyticus]
WP_228070153.1	Type1.BREX	<-ZnR<-?||AbiEi->viral-protein->wHTH+WYL+WCX->BrxA-DNA-binding->BrxB-BrxF-iSTAND->DUF6079*->N6-METHYLASE->?->?->PglZ->SIGMA+OB-Fold+AAA-ATPase+Lon-Protease-BrxL-><-wHTH||DUF4113->	1225	Bacteria>Pseudomonadota>Gammaproteobacteria>Vibrionales>Vibrionaceae>Vibrio	GCF_020741165.1	Vibrio_parahaemolyticus	IB301_RS18305	37667..41344	670	BREX-system-P-loop-protein-BrxC-[Vibrio-parahaemolyticus]
WP_236110294.1	Type1.BREX	<-GspA-ATPase||SF1-SF2-Helicase->?->HKD-DNase+HKD-DNase->CARF+NtrC-AAA->wHTH+WYL+WCX->BrxA-DNA-binding->BrxB-BrxF-iSTAND->DUF6079*->N6-METHYLASE->ParB+HNH->PglZ->ZetaToxin->?-><-SF1-SF2-Helicase+REase<-SF1-SF2-Helicase+REase	1157	Bacteria>Pseudomonadota>Gammaproteobacteria>Vibrionales>Vibrionaceae>Vibrio	GCF_021568915.1	Vibrio_sp._McD22-P3	L2W36_RS13785	194928..198401	2724880	BREX-system-P-loop-protein-BrxC-[Vibrio-sp.-McD22-P3]
WP_238795743.1	Type1.BREX	MobI-><-?<-LexA-protease||35exo->?->wHTH+WYL+WCX->BrxA-DNA-binding->BrxB-BrxF-iSTAND->DUF6079*->ParB+HNH->N6-METHYLASE->PglZ->SIGMA+OB-Fold+AAA-ATPase+Lon-Protease-BrxL->CARF+NtrC-AAA+FIS-HTH->OBFOLD-NUCLEASE->	1225	Bacteria>Pseudomonadota>Gammaproteobacteria>Vibrionales>Vibrionaceae>Vibrio	GCF_022289115.1	Vibrio_furnissii	K6U56_RS11800	143910..147587	29494	BREX-system-P-loop-protein-BrxC-[Vibrio-furnissii]
WP_238812493.1	Type1.BREX	<-CopG/RHH||<-?<-DUF2787<-?<-?||wHTH+WYL+WCX->BrxA-DNA-binding->BrxB-BrxF-iSTAND->DUF6079*->N6-METHYLASE->OLD-ABC->TOPRIM->PglZ->	1153	Bacteria>Pseudomonadota>Gammaproteobacteria>Vibrionales>Vibrionaceae>Vibrio	GCF_022290755.1	Vibrio_parahaemolyticus	K6U44_RS14230	7820..11281	670	BREX-system-P-loop-protein-BrxC-[Vibrio-parahaemolyticus]
WP_140273790.1	Type1.BREX	<-AAA-ATPase-p<-HEPN||CR-ATPase4->?->||wHTH+WYL+WCX->BrxA-DNA-binding->BrxB-BrxF-iSTAND->DUF6079*->N6-METHYLASE->ParB+HNH->PglZ->ZetaToxin->?-><-SF1-SF2-Helicase+REase<-SF1-SF2-Helicase+REase<-viral-protein<-AbiEi	1157	Bacteria>Pseudomonadota>Gammaproteobacteria>Vibrionales>Vibrionaceae>Vibrio	GCF_022291015.1	Vibrio_parahaemolyticus	K6U15_RS27495	32395..35868	670	BREX-system-P-loop-protein-BrxC-[Vibrio-parahaemolyticus]
WP_238967171.1	Type1.BREX	sGTP-><-HEPN<-?||CR-ATPase4->?->||wHTH+WYL+WCX->BrxA-DNA-binding->BrxB-BrxF-iSTAND->DUF6079*->N6-METHYLASE->ParB+HNH->PglZ->ZetaToxin->?-><-SF1-SF2-Helicase+REase<-SF1-SF2-Helicase+REase<-viral-protein<-AbiEi	1157	Bacteria>Pseudomonadota>Gammaproteobacteria>Vibrionales>Vibrionaceae>Vibrio	GCF_022343145.1	Vibrio_alginolyticus	K6750_RS21880	1365022..1368495	663	BREX-system-P-loop-protein-BrxC-[Vibrio-alginolyticus]
WP_256963697.1	Type1.BREX	CR-ATPase4->?-><-DUF4113||wHTH-><-SIGMA+OB-Fold+AAA-ATPase+Lon-Protease-BrxL<-PglZ<-?<-?<-N6-METHYLASE<-DUF6079*	1171	Bacteria>Pseudomonadota>Gammaproteobacteria>Vibrionales>Vibrionaceae>Vibrio	GCF_022398055.1	Vibrio_diabolicus	L1D58_RS17270	13232..16749	50719	BREX-system-P-loop-protein-BrxC,-partial-[Vibrio-diabolicus]
WP_243579240.1	Type1.BREX	<-BrxD<-SIGMA+OB-Fold+AAA-ATPase+Lon-Protease-BrxL<-PglZ<-?<-?<-ZnR+3C-ABC+TPR<-ANK+ANK+ANK<-ParB+HNH<-N6-METHYLASE<-DUF6079*<-BrxB-BrxF-iSTAND<-BrxA-DNA-binding<-wHTH+WYL+WCX||SF1-SF2-Helicase+HTH-><-?<-?||?-><-Calcineurin-SbcD<-REase	1247	Bacteria>Pseudomonadota>Gammaproteobacteria>Vibrionales>Vibrionaceae>Vibrio	GCF_022812045.1	Vibrio_splendidus	LTQ02_RS02805	621543..625286	29497	BREX-system-P-loop-protein-BrxC-[Vibrio-splendidus]
WP_253644856.1	Type1.BREX	CR-ATPase4->?-><-SIGMA+OB-Fold+AAA-ATPase+Lon-Protease-BrxL<-PglZ<-?<-ParB+HNH<-N6-METHYLASE<-DUF6079*<-BrxB-BrxF-iSTAND<-BrxA-DNA-binding<-wHTH+WYL+WCX	1248	Bacteria>Pseudomonadota>Gammaproteobacteria>Vibrionales>Vibrionaceae>Vibrio	GCF_024181785.1	Vibrio_sp._Y42_MX_L11	NL643_RS16680	61256..65002	2957765	BREX-system-P-loop-protein-BrxC-[Vibrio-sp.-Y42_MX_L11]
WP_253658043.1	Type1.BREX	<-SIGMA+OB-Fold+AAA-ATPase+Lon-Protease-BrxL<-PglZ<-?<-?<-?<-?<-ZnR+3C-ABC+TPR<-ParB+HNH<-N6-METHYLASE<-DUF6079*<-BrxB-BrxF-iSTAND<-BrxA-DNA-binding<-wHTH+WYL+WCX||SF1-SF2-Helicase->MarR-HTH->HKD-DNase+HKD-DNase->CARF+NtrC-AAA->PIN+OST-HTH->MORC->	1248	Bacteria>Pseudomonadota>Gammaproteobacteria>Vibrionales>Vibrionaceae>Vibrio	GCF_024181795.1	unclassified_Vibrio	NL644_RS04175	947816..951562	2614977	MULTISPECIES:-BREX-system-P-loop-protein-BrxC-[unclassified-Vibrio]
WP_261872478.1	Type1.BREX	<-ABC-ATPase+SbcC<-?<-?||?-><-?||Peptidase_M32->wHTH+WYL+WCX->BrxA-DNA-binding->BrxB-BrxF-iSTAND->DUF6079*->N6-METHYLASE->OLD-ABC->TOPRIM->PglZ->SIGMA+OB-Fold+AAA-ATPase+Lon-Protease-BrxL-><-HIN-HTH	1239	Bacteria>Pseudomonadota>Gammaproteobacteria>Vibrionales>Vibrionaceae>Vibrio	GCF_024347075.1	Vibrio_rarus	OCU56_RS06785	1441704..1445423	413403	BREX-system-P-loop-protein-BrxC-[Vibrio-rarus]
WP_261872676.1	Type1.BREX	<-wHTH+WYL+WCX||SF1-SF2-Helicase+REase->SF1-SF2-Helicase+REase-><-?<-ZetaToxin<-PglZ<-ParB+HNH<-N6-METHYLASE<-N6-METHYLASE<-DUF6079*<-BrxB-BrxF-iSTAND<-BrxA-DNA-binding<-tRNA||?-><-?<-TPR+TPR+TPR	1151	Bacteria>Pseudomonadota>Gammaproteobacteria>Vibrionales>Vibrionaceae>Vibrio	GCF_024347075.1	Vibrio_rarus	OCU56_RS07815	1705601..1709056	413403	BREX-system-P-loop-protein-BrxC-[Vibrio-rarus]
WP_086712895.1	Type1.BREX	<-AAA-ATPase-p<-HEPN||CR-ATPase4->?->||wHTH+WYL+WCX->BrxA-DNA-binding->BrxB-BrxF-iSTAND->DUF6079*->N6-METHYLASE->	1157	Bacteria>Pseudomonadota>Gammaproteobacteria>Vibrionales>Vibrionaceae>Vibrio	GCF_024347515.1	Vibrio_gigantis	B1026_RS08100	182974..186447	296199	BREX-system-P-loop-protein-BrxC-[Vibrio-gigantis]
WP_255937424.1	Type1.BREX	ABhydrolase-><-HTH_18+HTH_AraC<-?<-SIGMA+OB-Fold+AAA-ATPase+HKD-DNase-BrxL<-PglZ<-N6-METHYLASE<-NERD<-OLD-ABC<-DUF6079*<-BrxB-BrxF-iSTAND<-BrxA-DNA-binding<-XIS-HTH<-RNase<-?<-DUF3584+DUF5401<-JetB<-MukF||wHTH+WYL+WCX->	1185	Bacteria>Pseudomonadota>Gammaproteobacteria>Vibrionales>Vibrionaceae>Vibrio	GCF_024442175.1	Vibrio_campbellii	HB761_RS02525	550346..553903	680	BREX-system-P-loop-protein-BrxC-[Vibrio-campbellii]
WP_256897643.1	Type1.BREX	<-TrkH<-REC+OmpR-HTH<-HISKIN<-?<-?<-?||wHTH+WYL+WCX->BrxA-DNA-binding->BrxB-BrxF-iSTAND->DUF6079*->N6-METHYLASE->ParB+HNH->?->?->PglZ->SIGMA+OB-Fold+AAA-ATPase+Lon-Protease-BrxL-><-?<-CR-ATPase4	1248	Bacteria>Pseudomonadota>Gammaproteobacteria>Vibrionales>Vibrionaceae>Vibrio	GCF_024530955.1	Vibrio_parahaemolyticus	H2C82_RS04830	209368..213114	670	BREX-system-P-loop-protein-BrxC-[Vibrio-parahaemolyticus]
WP_258495358.1	Type1.BREX	AbiEi->viral-protein->SF1-SF2-Helicase+REase->SF1-SF2-Helicase+REase-><-?<-ZetaToxin<-PglZ<-ParB+HNH<-N6-METHYLASE<-DUF6079*<-BrxB-BrxF-iSTAND<-BrxA-DNA-binding<-wHTH+WYL+WCX||<-?<-CR-ATPase4||HEPN-><-sGTP	1157	Bacteria>Pseudomonadota>Gammaproteobacteria>Vibrionales>Vibrionaceae>Vibrio	GCF_024744595.1	Vibrio_alginolyticus	NB477_RS23070	13774..17247	663	BREX-system-P-loop-protein-BrxC-[Vibrio-alginolyticus]
WP_248354663.1	Type1.BREX	AbiEi->viral-protein->SF1-SF2-Helicase+REase->SF1-SF2-Helicase+REase-><-TPR<-HEPN+SF1-SF2-Helicase+RNase+RNase<-PglZ<-ParB+HNH<-N6-METHYLASE<-DUF6079*<-BrxB-BrxF-iSTAND<-BrxA-DNA-binding<-wHTH+WYL+WCX||DDE_Tnp_1_5+DDE_Tnp_1->PIN+OST-HTH-><-?<-HTH_20	1157	Bacteria>Pseudomonadota>Gammaproteobacteria>Vibrionales>Vibrionaceae>Vibrio	GCF_024745175.1	Vibrio_parahaemolyticus	DDM99_15775	19819..23292	670	BREX-system-P-loop-protein-BrxC-[Vibrio-parahaemolyticus]
WP_258484886.1	Type1.BREX	MobI-><-?<-LexA-protease||35exo->?->wHTH+WYL+WCX->BrxA-DNA-binding->BrxB-BrxF-iSTAND->DUF6079*->ParB+HNH->N6-METHYLASE->PglZ->SIGMA+OB-Fold+AAA-ATPase+Lon-Protease-BrxL->CARF+NtrC-AAA+FIS-HTH->OBFOLD-NUCLEASE->	1225	Bacteria>Pseudomonadota>Gammaproteobacteria>Vibrionales>Vibrionaceae>Vibrio	GCF_024745305.1	Vibrio_parahaemolyticus	NB589_RS16770	223237..226914	670	BREX-system-P-loop-protein-BrxC-[Vibrio-parahaemolyticus]
WP_258517485.1	Type1.BREX	AbiEi->viral-protein->wHTH+WYL+WCX->BrxA-DNA-binding->BrxB-BrxF-iSTAND->DUF6079*->N6-METHYLASE->ParB+HNH->GrpE->?->PglZ->SIGMA+OB-Fold+AAA-ATPase+Lon-Protease-BrxL-><-wHTH	1225	Bacteria>Pseudomonadota>Gammaproteobacteria>Vibrionales>Vibrionaceae>Vibrio	GCF_024745395.1	Vibrio_parahaemolyticus	NB556_RS01245	3462..7139	670	BREX-system-P-loop-protein-BrxC-[Vibrio-parahaemolyticus]
WP_140132901.1	Type1.BREX	<-ZnR<-?||?->SF1-SF2-Helicase->AbiEi->viral-protein->wHTH+WYL+WCX->BrxA-DNA-binding->BrxB-BrxF-iSTAND->DUF6079*->N6-METHYLASE->?->?->PglZ->SIGMA+OB-Fold+AAA-ATPase+Lon-Protease-BrxL-><-wHTH<-?<-CR-ATPase4	1225	Bacteria>Pseudomonadota>Gammaproteobacteria>Vibrionales>Vibrionaceae>Vibrio	GCF_024748295.1	Vibrio_harveyi_group	ND930_RS02780	146702..150379	717610	MULTISPECIES:-BREX-system-P-loop-protein-BrxC-[Vibrio-harveyi-group]
WP_264912148.1	Type1.BREX	<-SIGMA+OB-Fold+AAA-ATPase+Lon-Protease-BrxL<-PglZ<-?<-?<-?<-?<-ZnR+3C-ABC+TPR<-ParB+HNH<-N6-METHYLASE<-DUF6079*<-BrxB-BrxF-iSTAND<-BrxA-DNA-binding<-wHTH+WYL+WCX||SF1-SF2-Helicase->MarR-HTH->HKD-DNase+HKD-DNase->CARF+NtrC-AAA->PIN+OST-HTH->MORC->	1248	Bacteria>Pseudomonadota>Gammaproteobacteria>Vibrionales>Vibrionaceae>Vibrio	GCF_026000495.1	Vibrio_sp._STUT-A16	OO778_RS24845	1838056..1841802	2976237	BREX-system-P-loop-protein-BrxC-[Vibrio-sp.-STUT-A16]
WP_267365291.1	Type1.BREX	<-ACET<-ZnR<-?||wHTH+WYL+WCX->BrxA-DNA-binding->BrxB-BrxF-iSTAND->DUF6079*->N6-METHYLASE->ParB+HNH->ANK+ANK+ANK->ZnR+3C-ABC+TPR->?->?->?->?->?->PglZ->	1248	Bacteria>Pseudomonadota>Gammaproteobacteria>Vibrionales>Vibrionaceae>Vibrio	GCF_026537625.1	Vibrio_parahaemolyticus	NOL37_RS23780	19146..22892	670	BREX-system-P-loop-protein-BrxC-[Vibrio-parahaemolyticus]
WP_267363047.1	Type1.BREX	<-XIS-HTH<-Arm-DNA-bind_3+Phage_int_SAM_3+Phage_integrase||?->?->||?-><-HEPN||XIS-HTH->BrxA-DNA-binding->BrxB-BrxF-iSTAND->DUF6079*->OLD-ABC->REase->N6-METHYLASE->PglZ->SIGMA+OB-Fold+AAA-ATPase+HKD-DNase-BrxL->	1187	Bacteria>Pseudomonadota>Gammaproteobacteria>Vibrionales>Vibrionaceae>Vibrio	GCF_026537625.1	Vibrio_parahaemolyticus	NOL37_RS24175	9284..12847	670	BREX-system-P-loop-protein-BrxC-[Vibrio-parahaemolyticus]
WP_140298951.1	Type1.BREX	<-ZnR<-?||?->SF1-SF2-Helicase->AbiEi->viral-protein->wHTH+WYL+WCX->BrxA-DNA-binding->BrxB-BrxF-iSTAND->DUF6079*->N6-METHYLASE->?->?->PglZ->SIGMA+OB-Fold+AAA-ATPase+Lon-Protease-BrxL-><-wHTH||DUF4113-><-?<-CR-ATPase4	1225	Bacteria>Pseudomonadota>Gammaproteobacteria>Vibrionales>Vibrionaceae>Vibrio	GCF_026538205.1	Vibrio_parahaemolyticus	CGI26_02815	152399..156076	670	BREX-system-P-loop-protein-BrxC-[Vibrio-parahaemolyticus]
WP_268670710.1	Type1.BREX	MODE-HTH+PBPII-><-?||XIS-HTH->?->SF1-SF2-Helicase+HTH->HNH->XIS-HTH->BrxA-DNA-binding->BrxB-BrxF-iSTAND->DUF6079*->OLD-ABC->REase->N6-METHYLASE->PglZ->SIGMA+OB-Fold+AAA-ATPase+HKD-DNase-BrxL-><-PALP<-?<-MODE-HTH+PBPII	1185	Bacteria>Pseudomonadota>Gammaproteobacteria>Vibrionales>Vibrionaceae>Vibrio	GCF_026798035.1	Vibrio_coralliirubri	OTK49_RS06670	229636..233193	1516159	BREX-system-P-loop-protein-BrxC-[Vibrio-coralliirubri]
WP_269646266.1	Type1.BREX	MobI-><-?<-LexA-protease||35exo->?->wHTH+WYL+WCX->BrxA-DNA-binding->BrxB-BrxF-iSTAND->DUF6079*->ParB+HNH->N6-METHYLASE->PglZ->SIGMA+OB-Fold+AAA-ATPase+Lon-Protease-BrxL->SbcC->HDRnase->VirD4-TraG+VirB4-FtsK->	1225	Bacteria>Pseudomonadota>Gammaproteobacteria>Vibrionales>Vibrionaceae>Vibrio	GCF_027277845.1	Vibrio_parahaemolyticus	O4N70_RS12810	220363..224040	670	BREX-system-P-loop-protein-BrxC-[Vibrio-parahaemolyticus]
WP_269665659.1	Type1.BREX	MobI-><-?<-LexA-protease||35exo->?->wHTH+WYL+WCX->BrxA-DNA-binding->BrxB-BrxF-iSTAND->DUF6079*->ParB+HNH->N6-METHYLASE->PglZ->SIGMA+OB-Fold+AAA-ATPase+Lon-Protease-BrxL->NACHT+HEAT+REase->HDRnase->VirD4-TraG+VirB4-FtsK->	1225	Bacteria>Pseudomonadota>Gammaproteobacteria>Vibrionales>Vibrionaceae>Vibrio	GCF_027277905.1	Vibrio_parahaemolyticus	O4M94_RS09585	134179..137856	670	BREX-system-P-loop-protein-BrxC-[Vibrio-parahaemolyticus]
WP_272236739.1	Type1.BREX	TraJ-RHH->TraI-VirD2-RCRE->REase-><-AbiEi_4<-?||?->wHTH+WYL+WCX->BrxA-DNA-binding->BrxB-BrxF-iSTAND->DUF6079*->N6-METHYLASE->HEPN->PglZ->SIGMA+OB-Fold+AAA-ATPase+Lon-Protease-BrxL-><-?<-?<-SF1-SF2-Helicase<-?<-Arm-DNA-bind_3	1229	Bacteria>Pseudomonadota>Gammaproteobacteria>Vibrionales>Vibrionaceae>Vibrio	GCF_028446685.1	Vibrio_europaeus	OPW18_RS21520	351695..355384	300876	BREX-system-P-loop-protein-BrxC-[Vibrio-europaeus]
WP_272245950.1	Type1.BREX	wHTH+WYL+WCX->BrxA-DNA-binding->BrxB-BrxF-iSTAND->DUF6079*->N6-METHYLASE->SbcC->Schlafen+HATPase+wHTH->PglZ->SIGMA+OB-Fold+AAA-ATPase+Lon-Protease-BrxL-><-?<-?<-?<-SF1-SF2-Helicase	1229	Bacteria>Pseudomonadota>Gammaproteobacteria>Vibrionales>Vibrionaceae>Vibrio	GCF_028446755.1	Vibrio_europaeus	OPW38_RS04390	2959..6648	300876	BREX-system-P-loop-protein-BrxC-[Vibrio-europaeus]
WP_274056407.1	Type1.BREX	HTH_19-><-ParE<-MobI<-MobI<-SIGMA+OB-Fold+AAA-ATPase+Lon-Protease-BrxL<-PglZ<-N6-METHYLASE<-TOPRIM<-OLD-ABC<-DUF6079*<-BrxB-BrxF-iSTAND<-BrxA-DNA-binding<-wHTH+WYL+WCX||XIS-HTH->AAA+HTH->XIS-HTH-><-Phage_int_SAM_5+Phage_integrase<-sGTP	1225	Bacteria>Pseudomonadota>Gammaproteobacteria>Vibrionales>Vibrionaceae>Vibrio	GCF_028616275.1	Vibrio_parahaemolyticus	O8051_RS20180	64443..68120	670	BREX-system-P-loop-protein-BrxC-[Vibrio-parahaemolyticus]
WP_274020835.1	Type1.BREX	<-PglZ<-?<-?<-?<-?<-ZnR+3C-ABC+TPR<-ParB+HNH<-N6-METHYLASE-p<-N6-METHYLASE<-DUF6079*<-BrxB-BrxF-iSTAND<-BrxA-DNA-binding<-wHTH+WYL+WCX||SF1-SF2-Helicase->MarR-HTH->HKD-DNase+HKD-DNase->CARF+NtrC-AAA->PIN+OST-HTH->MORC->	1248	Bacteria>Pseudomonadota>Gammaproteobacteria>Vibrionales>Vibrionaceae>Vibrio	GCF_028617275.1	Vibrio_parahaemolyticus	O8254_RS13605	106668..110414	670	BREX-system-P-loop-protein-BrxC-[Vibrio-parahaemolyticus]
WP_274674022.1	Type1.BREX	<-HAMP+GGDEF<-?<-SIGMA+OB-Fold+AAA-ATPase+HKD-DNase-BrxL<-PglZ<-N6-METHYLASE<-REase<-OLD-ABC<-DUF6079*<-BrxB-BrxF-iSTAND<-BrxA-DNA-binding<-XIS-HTH	1187	Bacteria>Pseudomonadota>Gammaproteobacteria>Vibrionales>Vibrionaceae>Vibrio	GCF_028857845.1	Vibrio_aestuarianus	L9W73_RS08980	42250..45813	28171	BREX-system-P-loop-protein-BrxC-[Vibrio-aestuarianus]
WP_274695775.1	Type1.BREX	sGTP->Phage_int_SAM_5+Phage_integrase-><-XIS-HTH<-AAA+HTH<-XIS-HTH||wHTH+WYL+WCX->BrxA-DNA-binding->BrxB-BrxF-iSTAND->DUF6079*->OLD-ABC->TOPRIM->N6-METHYLASE->PglZ->SIGMA+OB-Fold+AAA-ATPase+Lon-Protease-BrxL->MobI->MobI->ParE->ParE->	1225	Bacteria>Pseudomonadota>Gammaproteobacteria>Vibrionales>Vibrionaceae>Vibrio	GCF_028858205.1	Vibrio_aestuarianus	MEG05_RS14945	3088024..3091701	28171	BREX-system-P-loop-protein-BrxC-[Vibrio-aestuarianus]
WP_274702484.1	Type1.BREX	<-DDE_Tnp_IS66<-SLOG+BirA-HTH<-SF1-SF2-Helicase+PRTase<-?<-DUF6079*<-HTH_23+HTH_32<-BrxB-BrxF-iSTAND||SF1-SF2-Helicase->	1228	Bacteria>Pseudomonadota>Gammaproteobacteria>Vibrionales>Vibrionaceae>Vibrio	GCF_028858475.1	Vibrio_aestuarianus	MCT07_RS16955	5869..9555	28171	BREX-system-P-loop-protein-BrxC-[Vibrio-aestuarianus]
WP_253254707.1	Type1.BREX	<-wHTH+WYL+WCX<-?<-DUF2787<-?<-?||wHTH+WYL+WCX->BrxA-DNA-binding->BrxB-BrxF-iSTAND->DUF6079*->N6-METHYLASE->OLD-ABC->TOPRIM->PglZ-><-SF1-SF2-Helicase+REase<-SF1-SF2-Helicase+REase<-Arm-DNA-bind_3+Phage_integrase	1153	Bacteria>Pseudomonadota>Gammaproteobacteria>Vibrionales>Vibrionaceae>Vibrio	GCF_030238805.1	Vibrio_parahaemolyticus	CSR03_RS22350	121618..125079	670	BREX-system-P-loop-protein-BrxC-[Vibrio-parahaemolyticus]
WP_182025746.1	Type1.BREX	HNH-><-wHTH+WYL+WCX||MukF->JetB->DUF3584+DUF5401->RNase->BrxA-DNA-binding->BrxB-BrxF-iSTAND->DUF6079*->N6-METHYLASE->DUF6079->N6-METHYLASE->PglZ->SIGMA+OB-Fold+AAA-ATPase+Lon-Protease-BrxL->	1233	Bacteria>Pseudomonadota>Gammaproteobacteria>Vibrionales>Vibrionaceae>Vibrio	GCF_903986985.1	Vibrio_rotiferianus	H7A65_RS05210	396251..399952	190895	BREX-system-P-loop-protein-BrxC-[Vibrio-rotiferianus]
WP_069535824.1	Type1.BREX	REase+SPF-cons-><-PIN+OST-HTH||CR-ATPase4->?->||wHTH+WYL+WCX->BrxA-DNA-binding->BrxB-BrxF-iSTAND->DUF6079*->N6-METHYLASE->ParB+HNH->PglZ->ZetaToxin->?-><-SF1-SF2-Helicase+REase<-SF1-SF2-Helicase+REase<-viral-protein<-AbiEi	1157	Bacteria>Pseudomonadota>Gammaproteobacteria>Vibrionales>Vibrionaceae>Vibrio	GCF_905331795.1	Vibrio_parahaemolyticus	LPR62_RS04710	409258..412731	670	BREX-system-P-loop-protein-BrxC-[Vibrio-parahaemolyticus]
WP_229611425.1	Type1.BREX	REase+SPF-cons-><-PIN+OST-HTH||CR-ATPase4->?->||wHTH+WYL+WCX->BrxA-DNA-binding->BrxB-BrxF-iSTAND->DUF6079*->N6-METHYLASE->ParB+HNH->PglZ->HEPN+SF1-SF2-Helicase+RNase+RNase->TPR-><-SF1-SF2-Helicase+REase<-SF1-SF2-Helicase+REase<-viral-protein<-AbiEi	1157	Bacteria>Pseudomonadota>Gammaproteobacteria>Vibrionales>Vibrionaceae>Vibrio	GCF_905331955.1	Vibrio_parahaemolyticus	LPR99_RS16740	18511..21984	670	BREX-system-P-loop-protein-BrxC-[Vibrio-parahaemolyticus]
WP_237468615.1	Type1.BREX	wHTH+WYL+WCX->BrxA-DNA-binding->BrxB-BrxF-iSTAND->DUF6079*->N6-METHYLASE->ParB+HNH->ParB+HNH->PglZ->SIGMA+OB-Fold+AAA-ATPase+Lon-Protease-BrxL-><-Acetate_kinase	1231	Bacteria>Pseudomonadota>Gammaproteobacteria>Vibrionales>Vibrionaceae>Vibrio	GCF_921293875.1	Vibrio_stylophorae	L9P36_RS15955	492812..496507	659351	BREX-system-P-loop-protein-BrxC-[Vibrio-stylophorae]
WP_168780466.1	Type1.BREX	<-SIGMA+OB-Fold+AAA-ATPase+Lon-Protease-BrxL<-PglZ<-?<-?<-?<-GrpE<-ANK+ANK+ANK<-ParB+HNH<-N6-METHYLASE<-DUF6079*<-BrxB-BrxF-iSTAND<-BrxA-DNA-binding<-wHTH+WYL+WCX<-?||AbiEi_4-><-REase<-TraI-VirD2-RCRE<-TraJ-RHH<-SbcC	1248	Bacteria>Pseudomonadota>Gammaproteobacteria>Vibrionales>Vibrionaceae>Vibrio	GCF_944738875.1	Vibrio_aestuarianus	OCX59_RS00150	25401..29147	28171	BREX-system-P-loop-protein-BrxC-[Vibrio-aestuarianus]
WP_272166069.1	Type1.BREX	CR-ATPase4->?-><-SIGMA+OB-Fold+AAA-ATPase+Lon-Protease-BrxL<-PglZ<-?<-?<-ParB+HNH<-N6-METHYLASE<-DUF6079*<-BrxB-BrxF-iSTAND<-BrxA-DNA-binding<-wHTH+WYL+WCX||SF1-SF2-Helicase->MarR-HTH->HKD-DNase+HKD-DNase->CARF+NtrC-AAA->Vrr-REase->HEPN+PAS+DUF4011+SF1-SF2-Helicase+REase->	1248	Bacteria>Pseudomonadota>Gammaproteobacteria>Vibrionales>Vibrionaceae>Vibrio	GCF_947503745.1	Vibrio_diabolicus	PQF93_RS21015	28188..31934	50719	BREX-system-P-loop-protein-BrxC-[Vibrio-diabolicus]
WP_241766700.1	Type1.BREX	<-GDResolvase+HIN-HTH||?->Arm-DNA-bind_3+Phage_integrase->SF1-SF2-Helicase+REase->SF1-SF2-Helicase+REase-><-PglZ<-TOPRIM<-OLD-ABC<-N6-METHYLASE<-DUF6079*<-BrxB-BrxF-iSTAND<-BrxA-DNA-binding<-wHTH+WYL+WCX<-XIS-HTH||?->?->?-><-XIS-HTH<-ANK+ANK	1153	Bacteria>Pseudomonadota>Gammaproteobacteria>Vibrionales>Vibrionaceae>Vibrio	GCF_948193335.1	Vibrio_parahaemolyticus	PO77_RS19450	167850..171311	670	BREX-system-P-loop-protein-BrxC-[Vibrio-parahaemolyticus]
WP_213868350.1	Type1.BREX	<-SIGMA+OB-Fold+AAA-ATPase+HKD-DNase-BrxL<-PglZ<-N6-METHYLASE<-REase<-OLD-ABC<-DUF6079*<-BrxB-BrxF-iSTAND<-BrxA-DNA-binding<-XIS-HTH<-HNH<-XIS-HTH||?->?->DUF2787->wHTH+WYL+WCX->	1187	Bacteria>Pseudomonadota>Gammaproteobacteria>Vibrionales>Vibrionaceae>Vibrio	-	Vibrio_alginolyticus	N7I24_002738	64007..67570	663	BREX-system-P-loop-protein-BrxC-[Vibrio-alginolyticus]
WP_222717221.1	Type1.BREX	<-DUF6079*<-BrxB-BrxF-iSTAND<-BrxA-DNA-binding<-wHTH+WYL+WCX||SF1-SF2-Helicase->MarR-HTH->HKD-DNase+HKD-DNase->CARF+NtrC-AAA->PIN+OST-HTH->wHTH+WYL+WCX->	1233	Bacteria>Pseudomonadota>Gammaproteobacteria>Vibrionales>Vibrionaceae>Vibrio	-	Vibrio_atlanticus	JFJ08_RS20295	521..4222	693153	BREX-system-P-loop-protein-BrxC-[Vibrio-atlanticus]
AAL59733.1	Type1.BREX	DUF4158-><-Phage_integrase<-?<-LexA-protease||35exo->?->wHTH+WYL+WCX->BrxA-DNA-binding->BrxB-BrxF-iSTAND->DUF6079-p*->DUF6079-p->OLD-ABC->OLD-ABC->TOPRIM->N6-METHYLASE-p->N6-METHYLASE-p->N6-METHYLASE-p->?->PglZ->	631	Bacteria>Pseudomonadota>Gammaproteobacteria>Vibrionales>Vibrionaceae>Vibrio	-	Vibrio_cholerae	.	28378..30273	666	unknown-[Vibrio-cholerae]
WP_142738823.1	Type1.BREX	MobI-><-?<-LexA-protease||?->wHTH+WYL+WCX->BrxA-DNA-binding->BrxB-BrxF-iSTAND->DUF6079*->ParB+HNH->N6-METHYLASE->TOPRIM->PglZ->SIGMA+OB-Fold+AAA-ATPase+Lon-Protease-BrxL->SbcC->HDRnase->VirD4-TraG+VirB4-FtsK->	1224	Bacteria>Pseudomonadota>Gammaproteobacteria>Vibrionales>Vibrionaceae>Vibrio	-	Vibrio_cholerae	FLL79_RS01410	36024..39698	666	BREX-system-P-loop-protein-BrxC-[Vibrio-cholerae]
WP_069490026.1	Type1.BREX	<-HTH_Tnp_1||XIS-HTH->wHTH+WYL+WCX->BrxA-DNA-binding->BrxB-BrxF-iSTAND->DUF6079*->N6-METHYLASE->OLD-ABC->TOPRIM->PglZ-><-SF1-SF2-Helicase+REase<-SF1-SF2-Helicase+REase<-Arm-DNA-bind_3+Phage_integrase	1154	Bacteria>Pseudomonadota>Gammaproteobacteria>Vibrionales>Vibrionaceae>Vibrio	-	Vibrio_parahaemolyticus	BBM25_06240	3424..6888	670	BREX-system-P-loop-protein-BrxC-[Vibrio-parahaemolyticus]
WP_254664663.1	Type1.BREX	XIS-HTH->wHTH+WYL+WCX->BrxA-DNA-binding->BrxB-BrxF-iSTAND->DUF6079*->N6-METHYLASE->OLD-ABC->TOPRIM->PglZ-><-SF1-SF2-Helicase+REase<-SF1-SF2-Helicase+REase<-Arm-DNA-bind_3+Phage_integrase	1153	Bacteria>Pseudomonadota>Gammaproteobacteria>Vibrionales>Vibrionaceae>Vibrio	-	Vibrio_parahaemolyticus	BED52_RS16125	3457..6918	670	BREX-system-P-loop-protein-BrxC-[Vibrio-parahaemolyticus]
WP_047508417.1	Type1.BREX	XIS-HTH->wHTH+WYL+WCX->BrxA-DNA-binding->BrxB-BrxF-iSTAND->DUF6079*->N6-METHYLASE->OLD-ABC->TOPRIM->PglZ-><-SF1-SF2-Helicase+REase<-SF1-SF2-Helicase+REase<-Arm-DNA-bind_3+Phage_integrase<-?||GDResolvase+HIN-HTH->	1154	Bacteria>Pseudomonadota>Gammaproteobacteria>Vibrionales>Vibrionaceae>Vibrio	-	Vibrio_parahaemolyticus	DMJ21_13130	8098..11562	670	BREX-system-P-loop-protein-BrxC-[Vibrio-parahaemolyticus]
WP_062854373.1	Type1.BREX	<-wHTH+WYL+WCX<-HTH||<-DUF2787<-?<-?||XIS-HTH->wHTH+WYL+WCX->BrxA-DNA-binding->BrxB-BrxF-iSTAND->DUF6079*->N6-METHYLASE->OLD-ABC->TOPRIM->PglZ-><-SF1-SF2-Helicase+REase<-SF1-SF2-Helicase+REase	1154	Bacteria>Pseudomonadota>Gammaproteobacteria>Vibrionales>Vibrionaceae>Vibrio	-	Vibrio_parahaemolyticus	LZX80_001782	93413..96877	670	BREX-system-P-loop-protein-BrxC-[Vibrio-parahaemolyticus]
WP_226420703.1	Type1.BREX	<-MORC<-PIN+OST-HTH<-CARF+NtrC-AAA<-HKD-DNase+HKD-DNase<-MarR-HTH<-SF1-SF2-Helicase||wHTH+WYL+WCX->BrxA-DNA-binding->BrxB-BrxF-iSTAND->DUF6079*->N6-METHYLASE->?->ParB+HNH->ZnR+3C-ABC+TPR->?->?->?->?->PglZ->	1248	Bacteria>Pseudomonadota>Gammaproteobacteria>Vibrionales>Vibrionaceae>Vibrio	-	Vibrio_sp._E14	LIX84_RS04085	940499..944245	2849869	BREX-system-P-loop-protein-BrxC-[Vibrio-sp.-E14]
WP_211003808.1	Type1.BREX	<-SIGMA+OB-Fold+AAA-ATPase+Lon-Protease-BrxL<-PglZ<-N6-METHYLASE<-DUF6079*<-BrxB-BrxF-iSTAND<-BrxA-DNA-binding<-wHTH+WYL+WCX||GDResolvase+PPP1-HTH->REase-><-?||?->REase+wHTH->HTH_Tnp+DDE_Tnp->	1215	Bacteria>Pseudomonadota>Gammaproteobacteria>Xanthomonadales>Rhodanobacteraceae>Rhodanobacter	GCF_018101315.1	Rhodanobacter_sp._B2A1Ga4	IMW82_RS02800	624703..628350	2778647	BREX-system-P-loop-protein-BrxC-[Rhodanobacter-sp.-B2A1Ga4]
MBK7210551.1	Type1.BREX	<-HDRnase<-Arm-DNA-bind_2+Phage_int_SAM_3+Phage_integrase<-Phage_AlpA||HTH_26->SLOG+BirA-HTH->ParB+HNH-><-?||BrxA-DNA-binding->BrxB-BrxF-iSTAND->DUF6079*->?->PglZ->SIGMA+OB-Fold+AAA-ATPase+Lon-Protease-BrxL-><-tRNA||NUDIX->?->?-><-HISKIN+REC+HPT<-REC+GGDEF+EAL	1144	Bacteria>Pseudomonadota>Gammaproteobacteria>Xanthomonadales>unclassified_Xanthomonadales	GCA_016707485.1	Xanthomonadales_bacterium	IPH99_09390	1022709..1026143	2006849	BREX-system-P-loop-protein-BrxC-[Xanthomonadales-bacterium]

Protein-ID	System	Gene-Neighbourhoods	Length	Taxonomy	Assembly-ID	Strain	Locus-Tag	Genomic-coordinates	TaxID	Fasta-define
MBK9494691.1	Type1.BREX	<-RVT<-HTH_3<-SIGMA+OB-Fold+AAA-ATPase+Lon-Protease-BrxL<-PglZ<-DUF932<-DUF6079*<-BrxB-BrxF-iSTAND<-BrxA-DNA-binding<-?<-?<-?<-?<-?<-?||XIS-HTH->	1145	Bacteria>Pseudomonadota>Gammaproteobacteria>Xanthomonadales>unclassified_Xanthomonadales	GCA_016718605.1	Xanthomonadales_bacterium	IP008_09120	1864889..1868326	2006849	BREX-system-P-loop-protein-BrxC-[Xanthomonadales-bacterium]
WP_194339884.1	Type1.BREX	35exo->REase-><-SIGMA+OB-Fold+AAA-ATPase+HKD-DNase-BrxL<-PglZ<-N6-METHYLASE-p<-NERD<-OLD-ABC<-N6-METHYLASE-p<-?<-DUF6079*<-BrxB-BrxF-iSTAND<-BrxA-DNA-binding<-XIS-HTH<-?<-SbcC+SbcC<-Calcineurin+Calcineurin-SbcD	1190	Bacteria>Pseudomonadota>Gammaproteobacteria>Xanthomonadales>Xanthomonadaceae>Lysobacter	GCF_015277535.1	Lysobacter_sp._H21R4	INQ40_RS07385	1620041..1623613	2781021	BREX-system-P-loop-protein-BrxC-[Lysobacter-sp.-H21R4]
WP_162376315.1	Type1.BREX	<-NTP_transf_3+Hexapep+Hexapep||?-><-?<-SIGMA+OB-Fold+AAA-ATPase+Lon-Protease-BrxL<-PglZ<-Polbeta<-MarR-HTH<-ParB+HNH<-N6-METHYLASE<-DUF6079*<-BrxB-BrxF-iSTAND<-BrxA-DNA-binding||REase-><-?<-?<-TOPRIM<-XIS-HTH	1148	Bacteria>Pseudomonadota>Gammaproteobacteria>Xanthomonadales>Xanthomonadaceae>Pseudoxanthomonas	GCF_010211765.1	Pseudoxanthomonas_kaohsiungensis	CSC66_RS05120	191406..194852	283923	BREX-system-P-loop-protein-BrxC-[Pseudoxanthomonas-kaohsiungensis]
WP_271250641.1	Type1.BREX	<-SIGMA+OB-Fold+AAA-ATPase+Lon-Protease-BrxL<-PglZ<-Polbeta<-MarR-HTH<-ParB+HNH<-N6-METHYLASE<-DUF6079*<-BrxB-BrxF-iSTAND<-BrxA-DNA-binding||REase-><-?<-?<-?<-TOPRIM<-?<-XIS-HTH	1148	Bacteria>Pseudomonadota>Gammaproteobacteria>Xanthomonadales>Xanthomonadaceae>Pseudoxanthomonas	GCF_027941875.1	Pseudoxanthomonas_mexicana	PE064_RS03375	689805..693251	128785	BREX-system-P-loop-protein-BrxC-[Pseudoxanthomonas-mexicana]
MBH1702634.1	Type1.BREX	<-RFC-AAA+RuvB-AAA||tRNA->?->AAA+wHTH-><-SIGMA+OB-Fold+AAA-ATPase+Lon-Protease-BrxL<-PglZ<-HNH<-OLD-ABC<-ParB+HNH<-N6-METHYLASE<-DUF6079*<-BrxB-BrxF-iSTAND<-BrxA-DNA-binding<-wHTH+WYL+WCX<-?<-?<-?<-DUF932||XIS-HTH->	1146	Bacteria>Pseudomonadota>Gammaproteobacteria>Xanthomonadales>Xanthomonadaceae>Stenotrophomonas	GCA_015998095.1	Stenotrophomonas_maltophilia	I5U93_05090	24102..27542	40324	BREX-system-P-loop-protein-BrxC-[Stenotrophomonas-maltophilia]
WP_125428654.1	Type1.BREX	<-RFC-AAA+RuvB-AAA||tRNA->?->AAA+wHTH-><-SIGMA+OB-Fold+AAA-ATPase+Lon-Protease-BrxL<-PglZ<-HNH<-OLD-ABC<-ParB+HNH<-N6-METHYLASE<-DUF6079*<-BrxB-BrxF-iSTAND<-BrxA-DNA-binding<-wHTH+WYL+WCX<-?<-?<-?<-DUF932||XIS-HTH->	1146	Bacteria>Pseudomonadota>Gammaproteobacteria>Xanthomonadales>Xanthomonadaceae>Stenotrophomonas	GCF_003935615.1	Stenotrophomonas_maltophilia	EGJ89_RS09210	127004..130444	40324	BREX-system-P-loop-protein-BrxC-[Stenotrophomonas-maltophilia]
WP_192288052.1	Type1.BREX	SF1-SF2-Helicase->tRNA->tRNA->BrxA-DNA-binding->BrxB-BrxF-iSTAND->DUF6079*->N6-METHYLASE->ParB+HNH->MarR-HTH->Polbeta->PglZ->SIGMA+OB-Fold+AAA-ATPase+Lon-Protease-BrxL->?-><-HTH_3||TPR->	1148	Bacteria>Pseudomonadota>Gammaproteobacteria>Xanthomonadales>Xanthomonadaceae>Stenotrophomonas	GCF_014852135.1	Stenotrophomonas_sp._STM01	IB227_RS14890	150260..153706	2769278	BREX-system-P-loop-protein-BrxC-[Stenotrophomonas-sp.-STM01]
WP_256643585.1	Type1.BREX	<-STYKIN+Pkinase+CR-Kinase||Glyco_transf_9-><-?<-?<-SIGMA+OB-Fold+AAA-ATPase+Lon-Protease-BrxL<-PglZ<-N6-METHYLASE<-DUF6079*<-BrxB-BrxF-iSTAND<-BrxA-DNA-binding<-?<-tRNA||?-><-35exo<-RNaseH<-METHYLASE	1219	Bacteria>Pseudomonadota>Gammaproteobacteria>Xanthomonadales>Xanthomonadaceae>Stenotrophomonas	GCF_024499815.1	Stenotrophomonas_sp._9(2022)	NQ204_RS08610	96146..99805	2950153	BREX-system-P-loop-protein-BrxC-[Stenotrophomonas-sp.-9(2022)]
WP_225763940.1	Type1.BREX	<-TRPR-HTH||?->PRTase->HTH_Tnp_1+HTH_21+rve+rve_3->XIS-HTH->BrxA-DNA-binding->BrxB-BrxF-iSTAND->DUF6079*->?->N6-METHYLASE->OLD-ABC->REase->?->N6-METHYLASE-p->PglZ->SIGMA+OB-Fold+AAA-ATPase+HKD-DNase-BrxL->	1181	Bacteria>Pseudomonadota>Gammaproteobacteria>Xanthomonadales>Xanthomonadaceae>Stenotrophomonas	GCF_916618915.1	Stenotrophomonas_sp._Marseille-Q4652	LG380_RS05370	1171221..1174766	2866595	BREX-system-P-loop-protein-BrxC-[Stenotrophomonas-sp.-Marseille-Q4652]
WP_139716037.1	Type1.BREX	ARG-><-REase||HIN-HTH->?->ZnR+TOPRIM+AAA-helicase+wHTH->XIS-HTH->BrxA-DNA-binding->BrxB-BrxF-iSTAND->DUF6079*->N6-METHYLASE->PglZ->SIGMA+OB-Fold+AAA-ATPase+Lon-Protease-BrxL->?-><-?<-?<-?<-?<-DUF932	1205	Bacteria>Pseudomonadota>Gammaproteobacteria>Xanthomonadales>Xanthomonadaceae>Thermomonas	GCF_006337105.1	Thermomonas_aquatica	FHQ07_RS06475	1333468..1337085	2202149	BREX-system-P-loop-protein-BrxC-[Thermomonas-aquatica]
ODU42543.1	Type1.BREX	<-SIGMA+OB-Fold+AAA-ATPase+Lon-Protease-BrxL<-PglZ<-Polbeta<-MarR-HTH<-HNH<-OLD-ABC<-N6-METHYLASE<-DUF6079*<-BrxB-BrxF-iSTAND<-BrxA-DNA-binding||?-><-?||HKD-DNase->?->HNH->N6-METHYLASE->	1148	Bacteria>Pseudomonadota>Gammaproteobacteria>Xanthomonadales>Xanthomonadaceae>unclassified_Xanthomonadaceae	GCA_001725645.1	Xanthomonadaceae_bacterium_SCN_69-123	ABS96_27585	191713..195159	1660170	hypothetical-protein-ABS96_27585-[Xanthomonadaceae-bacterium-SCN-69-123]
RYD14825.1	Type1.BREX	<-35exo<-SF1-SF2-Helicase+wHTH+Small-B-barrel+DUF1998||HTH_3->Couple_hipA+HipA_C->BrxA-DNA-binding->BrxB-BrxF-iSTAND->DUF6079*->N6-METHYLASE->PglZ->SIGMA+OB-Fold+AAA-ATPase+Lon-Protease-BrxL->REase-><-tRNA||NUDIX->?->?-><-REC+GGDEF+EAL	1207	Bacteria>Pseudomonadota>Gammaproteobacteria>Xanthomonadales>Xanthomonadaceae>unclassified_Xanthomonadaceae	GCA_004143945.1	Xanthomonadaceae_bacterium	EOP90_11180	76143..79766	1926873	BREX-system-P-loop-protein-BrxC-[Xanthomonadaceae-bacterium]
EGD07603.1	Type1.BREX	<-RFC-AAA+RuvB-AAA||tRNA->?->AAA+wHTH-><-SIGMA+OB-Fold+AAA-ATPase+Lon-Protease-BrxL<-PglZ<-ParB+HNH<-N6-METHYLASE<-DUF6079*<-BrxB-BrxF-iSTAND<-BrxA-DNA-binding<-wHTH+WYL+WCX<-DUF772+DDE_Tnp_1||XIS-HTH->	1152	Bacteria>Pseudomonadota>Gammaproteobacteria>Xanthomonadales>Xanthomonadaceae>Xanthomonas	GCA_000192025.2	Xanthomonas_vesicatoria_ATCC_35937	XVE_4193	13272..16730	925775	hypothetical-protein-XVE_4193-[Xanthomonas-vesicatoria-ATCC-35937]
WP_042828568.1	Type1.BREX	<-RFC-AAA+RuvB-AAA||tRNA->?->AAA+wHTH-><-SIGMA+OB-Fold+AAA-ATPase+Lon-Protease-BrxL<-PglZ<-ParB+HNH<-N6-METHYLASE<-DUF6079*<-BrxB-BrxF-iSTAND<-BrxA-DNA-binding<-wHTH+WYL+WCX||<-DUF772+DDE_Tnp_1||XIS-HTH->	1146	Bacteria>Pseudomonadota>Gammaproteobacteria>Xanthomonadales>Xanthomonadaceae>Xanthomonas	GCF_000192025.1	Xanthomonas_vesicatoria	XVE_RS20000	13272..16712	56460	BREX-system-P-loop-protein-BrxC-[Xanthomonas-vesicatoria]
WP_068573669.1	Type1.BREX	<-STYKIN+Pkinase+CR-Kinase||Glyco_transf_9-><-?||Arm-DNA-bind_3+Phage_integrase->?-><-SIGMA+OB-Fold+AAA-ATPase+Lon-Protease-BrxL<-PglZ<-N6-METHYLASE<-DUF6079*<-BrxB-BrxF-iSTAND<-BrxA-DNA-binding<-tRNA||?-><-35exo<-RNaseH<-METHYLASE	1215	Bacteria>Pseudomonadota>Gammaproteobacteria>Xanthomonadales>Xanthomonadaceae>Xanthomonas	GCF_000589895.1	Xanthomonas_campestris	XCCCFBP1712_RS16305	3805862..3809509	339	BREX-system-P-loop-protein-BrxC-[Xanthomonas-campestris]
WP_212590115.1	Type1.BREX	<-STYKIN+Pkinase+CR-Kinase||Glyco_transf_9-><-?||Arm-DNA-bind_3+Phage_integrase-><-SIGMA+OB-Fold+AAA-ATPase+Lon-Protease-BrxL<-PglZ<-N6-METHYLASE<-N6-METHYLASE-p<-DUF6079*<-BrxB-BrxF-iSTAND<-BrxA-DNA-binding<-tRNA||?-><-35exo<-RNaseH<-METHYLASE	1219	Bacteria>Pseudomonadota>Gammaproteobacteria>Xanthomonadales>Xanthomonadaceae>Xanthomonas	GCF_000589955.1	Xanthomonas_campestris	XCCCFBP4955_RS16075	3750413..3754072	339	BREX-system-P-loop-protein-BrxC-[Xanthomonas-campestris]
WP_218196661.1	Type1.BREX	Arm-DNA-bind_3+Phage_integrase-><-SIGMA+OB-Fold+AAA-ATPase+HKD-DNase-BrxL<-PglZ<-N6-METHYLASE-p<-?<-REase<-OLD-ABC<-N6-METHYLASE<-?<-DUF6079*<-BrxB-BrxF-iSTAND<-BrxA-DNA-binding<-XIS-HTH<-Phage_int_SAM_2+Integrase_1<-Phage_rep	1182	Bacteria>Pseudomonadota>Gammaproteobacteria>Xanthomonadales>Xanthomonadaceae>Xanthomonas	GCF_019193005.1	Xanthomonas_euvesicatoria	KV691_RS15985	182541..186089	456327	BREX-system-P-loop-protein-BrxC-[Xanthomonas-euvesicatoria]
WP_227971952.1	Type1.BREX	<-N6-METHYLASE<-DUF6079*<-BrxB-BrxF-iSTAND<-BrxA-DNA-binding<-tRNA||?-><-35exo<-RNaseH<-METHYLASE	1219	Bacteria>Pseudomonadota>Gammaproteobacteria>Xanthomonadales>Xanthomonadaceae>Xanthomonas	GCF_020731405.1	Xanthomonas_campestris	LLE63_RS17440	2847..6506	339	BREX-system-P-loop-protein-BrxC-[Xanthomonas-campestris]
WP_228944544.1	Type1.BREX	HTH_Tnp_1+HTH_21+rve+rve_3-><-SIGMA+OB-Fold+AAA-ATPase+Lon-Protease-BrxL<-PglZ<-N6-METHYLASE<-DUF6079*<-BrxB-BrxF-iSTAND<-BrxA-DNA-binding<-Arm-DNA-bind_3+Phage_integrase<-tRNA<-SIGMA-HTH+SIGMA-HTH+SIGMA-HTH<-?||?-><-ACET	1217	Bacteria>Pseudomonadota>Gammaproteobacteria>Xanthomonadales>Xanthomonadaceae>Xanthomonas	GCF_020879405.1	Xanthomonas_campestris	LN454_RS12865	9123..12776	339	BREX-system-P-loop-protein-BrxC-[Xanthomonas-campestris]
WP_260816965.1	Type1.BREX	DnaJ->?->?->?-><-?<-XIS-HTH||wHTH+WYL+WCX->BrxA-DNA-binding->BrxB-BrxF-iSTAND->DUF6079*->N6-METHYLASE->ParB+HNH->PglZ->SIGMA+OB-Fold+AAA-ATPase+Lon-Protease-BrxL-><-AAA+wHTH<-?<-tRNA||RFC-AAA+RuvB-AAA->?->HHH_5+RecR+TOPRIM->	1146	Bacteria>Pseudomonadota>Gammaproteobacteria>Xanthomonadales>Xanthomonadaceae>Xanthomonas	GCF_025263745.1	Xanthomonas_prunicola	MOD45_RS16865	3940549..3943989	2053930	BREX-system-P-loop-protein-BrxC-[Xanthomonas-prunicola]
WP_274376279.1	Type1.BREX	METHYLASE->RNaseH->35exo-><-?||tRNA->BrxA-DNA-binding->BrxB-BrxF-iSTAND->DUF6079*->N6-METHYLASE-><-DUF772+DDE_Tnp_1+DDE_Tnp_1_6||N6-METHYLASE-p->PglZ->SIGMA+OB-Fold+AAA-ATPase+Lon-Protease-BrxL-><-?||?-><-HIN-HTH	1219	Bacteria>Pseudomonadota>Gammaproteobacteria>Xanthomonadales>Xanthomonadaceae>Xanthomonas	GCF_028749825.1	Xanthomonas_campestris	JH286_RS05255	1188606..1192265	339	BREX-system-P-loop-protein-BrxC-[Xanthomonas-campestris]
WP_274375850.1	Type1.BREX	[illegible]	1218	Bacteria>Pseudomonadota>Gammaproteobacteria>Xanthomonadales>Xanthomonadaceae>Xanthomonas	GCF_028749885.1	Xanthomonas_campestris	JH293_RS16145	3775277..3778933	339	BREX-system-P-loop-protein-BrxC-[Xanthomonas-campestris]
WP_274507195.1	Type1.BREX	[illegible]	482	Bacteria>Pseudomonadota>Gammaproteobacteria>Xanthomonadales>Xanthomonadaceae>Xanthomonas	GCF_028750015.1	Xanthomonas_campestris	JH282_RS16380	3813238..3814686	339	BREX-system-P-loop-protein-BrxC-[Xanthomonas-campestris]
WP_000687848.1	Type1.BREX	[illegible]	1225	Bacteria>Pseudomonadota	GCF_000022585.1	Gammaproteobacteria	ALD23_RS14425	130597..134274	1236	MULTISPECIES:-BREX-system-P-loop-protein-BrxC-[Gammaproteobacteria]
WP_000687849.1	Type1.BREX	[illegible]	1225	Bacteria>Pseudomonadota	GCF_000299535.2	Gammaproteobacteria	B934_RS0100870	5534..9211	1236	MULTISPECIES:-BREX-system-P-loop-protein-BrxC-[Gammaproteobacteria]
WP_069709528.1	Type1.BREX	[illegible]	1179	Bacteria>Pseudomonadota	GCF_001742225.1	Alphaproteobacteria	BES08_RS24075	1524283..1527822	28211	MULTISPECIES:-BREX-system-P-loop-protein-BrxC-[Alphaproteobacteria]
WP_107394298.1	Type1.BREX	[illegible]	1140	Bacteria>Pseudomonadota	GCF_003034225.1	Alphaproteobacteria	CV103_RS05945	27177..30599	28211	MULTISPECIES:-BREX-system-P-loop-protein-BrxC-[Alphaproteobacteria]
WP_066700609.1	Type1.BREX	[illegible]	1179	Bacteria>Pseudomonadota	GCF_003967075.1	Alphaproteobacteria	SAMIE_RS17570	3625371..3628910	28211	MULTISPECIES:-BREX-system-P-loop-protein-BrxC-[Alphaproteobacteria]
WP_156734107.1	Type1.BREX	[illegible]	1225	Bacteria>Pseudomonadota	GCF_009740265.1	Gammaproteobacteria	GTH23_RS00050	5219..8896	1236	MULTISPECIES:-BREX-system-P-loop-protein-BrxC-[Gammaproteobacteria]
WP_119567031.1	Type1.BREX	[illegible]	1225	Bacteria>Pseudomonadota	GCF_013602615.1	Gammaproteobacteria	G9G97_RS25355	26642..30319	1236	MULTISPECIES:-BREX-system-P-loop-protein-BrxC-[Gammaproteobacteria]
WP_125941663.1	Type1.BREX	[illegible]	1154	Bacteria>Pseudomonadota	GCF_019046925.1	Gammaproteobacteria	I6L32_RS07295	1524921..1528385	1236	MULTISPECIES:-BREX-system-P-loop-protein-BrxC-[Gammaproteobacteria]
WP_110076276.1	Type1.BREX	[illegible]	1225	Bacteria>Pseudomonadota	GCF_021431945.1	Gammaproteobacteria	LY388_RS16340	2424979..2428656	1236	MULTISPECIES:-BREX-system-P-loop-protein-BrxC-[Gammaproteobacteria]
WP_111805008.1	Type1.BREX	[illegible]	1225	Bacteria>Pseudomonadota	GCF_028482435.1	Gammaproteobacteria	DR130_RS11140	144958..148635	1236	MULTISPECIES:-BREX-system-P-loop-protein-BrxC-[Gammaproteobacteria]
OYW40486.1	Type1.BREX	[illegible]	1200	Bacteria>Pseudomonadota>Hydrogenophilia>Hydrogenophilales>unclassified_Hydrogenophilales	GCA_002279795.1	Hydrogenophilales_bacterium_12-61-10	B7Z35_01010	8163..11765	1970515	ATPase-[Hydrogenophilales-bacterium-12-61-10]
OYX29919.1	Type1.BREX	[illegible]	1200	Bacteria>Pseudomonadota>Hydrogenophilia>Hydrogenophilales>unclassified_Hydrogenophilales	GCA_002280425.1	Hydrogenophilales_bacterium_32-62-9	B7Z03_07680	35609..39211	1970531	ATPase-[Hydrogenophilales-bacterium-32-62-9]
PIQ12823.1	Type1.BREX	[illegible]	1146	Bacteria>Pseudomonadota>Hydrogenophilia>Hydrogenophilales>unclassified_Hydrogenophilales	GCA_002770925.1	Hydrogenophilales_bacterium_CG18_big_fil_WC_8_21_14_2_50_58_12	COW70_07940	10882..14322	1974036	hypothetical-protein-COW70_07940-[Hydrogenophilales-bacterium-CG18_big_fil_WC_8_21_14_2_50_58_12]
PIY00903.1	Type1.BREX	[illegible]	1207	Bacteria>Pseudomonadota>Hydrogenophilia>Hydrogenophilales>unclassified_Hydrogenophilales	GCA_002784065.1	Hydrogenophilales_bacterium_CG_4_10_14_3_um_filter_58_23	COZ23_06050	25268..28891	1974027	BREX-system-P-loop-protein-BrxC-[Hydrogenophilales-bacterium-CG_4_10_14_3_um_filter_58_23]
KAA0218648.1	Type1.BREX	[illegible]	1148	Bacteria>Pseudomonadota>unclassified_Pseudomonadota	GCA_008363105.1	Pseudomonadota_bacterium	EDM78_03970	14965..18411	1977087	BREX-system-P-loop-protein-BrxC-[Pseudomonadota-bacterium]
MYJ95828.1	Type1.BREX	[illegible]	751	Bacteria>Pseudomonadota>unclassified_Pseudomonadota	GCA_009845555.1	Pseudomonadota_bacterium	F4053_09660	8125..10376	1977087	BREX-system-P-loop-protein-BrxC-[Pseudomonadota-bacterium]
MBK8482432.1	Type1.BREX	[illegible]	896	Bacteria>Pseudomonadota>unclassified_Pseudomonadota	GCA_016711215.1	Pseudomonadota_bacterium	IPL40_14915	141091..143781	1977087	hypothetical-protein-[Pseudomonadota-bacterium]
MBQ1925249.1	Type1.BREX	[illegible]	984	Bacteria>Pseudomonadota>unclassified_Pseudomonadota	GCA_017392365.1	Pseudomonadota_bacterium	II180_03915	15942..18894	1977087	BREX-system-P-loop-protein-BrxC-[Pseudomonadota-bacterium]
MBQ9395285.1	Type1.BREX	[illegible]	1183	Bacteria>Pseudomonadota>unclassified_Pseudomonadota	GCA_017455165.1	Pseudomonadota_bacterium	IJU23_07180	2296..5847	1977087	BREX-system-P-loop-protein-BrxC-[Pseudomonadota-bacterium]
MBS0598081.1	Type1.BREX	[illegible]	1146	Bacteria>Pseudomonadota>unclassified_Pseudomonadota	GCA_018240335.1	Pseudomonadota_bacterium	JSR92_12305	1723..5163	1977087	BREX-system-P-loop-protein-BrxC-[Pseudomonadota-bacterium]
MBS0349076.1	Type1.BREX	[illegible]	1153	Bacteria>Pseudomonadota>unclassified_Pseudomonadota	GCA_018241665.1	Pseudomonadota_bacterium	JSR69_21735	2072..5533	1977087	BREX-system-P-loop-protein-BrxC-[Pseudomonadota-bacterium]
MBS1228526.1	Type1.BREX	[illegible]	1213	Bacteria>Pseudomonadota>unclassified_Pseudomonadota	GCA_018262095.1	Pseudomonadota_bacterium	H6R17_1803	75999..79640	1977087	ATPase-like-protein-[Pseudomonadota-bacterium]
MBU0673651.1	Type1.BREX	[illegible]	1167	Bacteria>Pseudomonadota>unclassified_Pseudomonadota	GCA_018814305.1	Pseudomonadota_bacterium	KJ950_03300	48865..52368	1977087	BREX-system-P-loop-protein-BrxC-[Pseudomonadota-bacterium]
MBU1208417.1	Type1.BREX	[illegible]	1147	Bacteria>Pseudomonadota>unclassified_Pseudomonadota	GCA_018819135.1	Pseudomonadota_bacterium	KKH04_16070	11316..14759	1977087	BREX-system-P-loop-protein-BrxC-[Pseudomonadota-bacterium]
MBU1744600.1	Type1.BREX	[illegible]	992	Bacteria>Pseudomonadota>unclassified_Pseudomonadota	GCA_018822765.1	Pseudomonadota_bacterium	KJ649_06870	12750..15730	1977087	BREX-system-P-loop-protein-BrxC-[Pseudomonadota-bacterium]
MBU2055247.1	Type1.BREX	[illegible]	1139	Bacteria>Pseudomonadota>unclassified_Pseudomonadota	GCA_018825885.1	Pseudomonadota_bacterium	KKC25_09760	17486..20905	1977087	BREX-system-P-loop-protein-BrxC-[Pseudomonadota-bacterium]
MBU2228449.1	Type1.BREX	[illegible]	1139	Bacteria>Pseudomonadota>unclassified_Pseudomonadota	GCA_018827585.1	Pseudomonadota_bacterium	KJ936_12455	147..3566	1977087	BREX-system-P-loop-protein-BrxC-[Pseudomonadota-bacterium]
MBU2261623.1	Type1.BREX	[illegible]	120	Bacteria>Pseudomonadota>unclassified_Pseudomonadota	GCA_018827665.1	Pseudomonadota_bacterium	KKF02_06820	1..360	1977087	BREX-system-P-loop-protein-BrxC-[Pseudomonadota-bacterium]
MBU4371661.1	Type1.BREX	[illegible]	1146	Bacteria>Pseudomonadota>unclassified_Pseudomonadota	GCA_018896355.1	Pseudomonadota_bacterium	KKD68_07180	16992..20432	1977087	BREX-system-P-loop-protein-BrxC-[Pseudomonadota-bacterium]
MBU4294441.1	Type1.BREX	[illegible]	1176	Bacteria>Pseudomonadota>unclassified_Pseudomonadota	GCA_018897105.1	Pseudomonadota_bacterium	KJ862_00965	66976..70506	1977087	BREX-system-P-loop-protein-BrxC-[Pseudomonadota-bacterium]
MBU4235493.1	Type1.BREX	[illegible]	1147	Bacteria>Pseudomonadota>unclassified_Pseudomonadota	GCA_018897885.1	Pseudomonadota_bacterium	KKI01_04080	7059..10502	1977087	BREX-system-P-loop-protein-BrxC-[Pseudomonadota-bacterium]
MBU3982443.1	Type1.BREX	[illegible]	851	Bacteria>Pseudomonadota>unclassified_Pseudomonadota	GCA_018900775.1	Pseudomonadota_bacterium	KJ985_02955	1..2554	1977087	BREX-system-P-loop-protein-BrxC-[Pseudomonadota-bacterium]
MCG8416875.1	Type1.BREX	[illegible]	1253	Bacteria>Pseudomonadota>unclassified_Pseudomonadota	GCA_022360315.1	Pseudomonadota_bacterium	MJE77_02885	60070..63831	1977087	BREX-system-P-loop-protein-BrxC-[Pseudomonadota-bacterium]
MCG8417945.1	Type1.BREX	[illegible]	1203	Bacteria>Pseudomonadota>unclassified_Pseudomonadota	GCA_022360315.1	Pseudomonadota_bacterium	MJE77_08390	23306..26917	1977087	BREX-system-P-loop-protein-BrxC-[Pseudomonadota-bacterium]
WP_053322564.1	Type1.BREX	[illegible]	1181	Bacteria>Pseudomonadota>Zetaproteobacteria>Mariprofundales>Mariprofundaceae>Mariprofundus	GCF_001273615.1	Mariprofundus_ferrooxydans	AL013_RS02410	88551..92096	314344	BREX-system-P-loop-protein-BrxC-[Mariprofundus-ferrooxydans]
MBN2327413.1	Type1.BREX	[illegible]	1173	Bacteria>PVC_group>Candidatus_Omnitrophica>unclassified_Candidatus_Omnitrophica	GCA_016934335.1	Candidatus_Omnitrophica_bacterium	JXR73_09680	2054..5575	2035772	BREX-system-P-loop-protein-BrxC-[Candidatus-Omnitrophota-bacterium]
MBU1810879.1	Type1.BREX	[illegible]	1179	Bacteria>PVC_group>Candidatus_Omnitrophica>unclassified_Candidatus_Omnitrophica	GCA_018823915.1	Candidatus_Omnitrophica_bacterium	KJ629_07045	3409..6948	2035772	BREX-system-P-loop-protein-BrxC-[Candidatus-Omnitrophota-bacterium]
MBU4477606.1	Type1.BREX	[illegible]	1181	Bacteria>PVC_group>Candidatus_Omnitrophica>unclassified_Candidatus_Omnitrophica	GCA_018895275.1	Candidatus_Omnitrophica_bacterium	KKH34_00715	10161..13706	2035772	BREX-system-P-loop-protein-BrxC-[Candidatus-Omnitrophota-bacterium]
MCG2712035.1	Type1.BREX	[illegible]	1181	Bacteria>PVC_group>Candidatus_Omnitrophica>unclassified_Candidatus_Omnitrophica	GCA_022013205.1	Candidatus_Omnitrophica_bacterium	L6416_06895	3795..7340	2035772	BREX-system-P-loop-protein-BrxC-[Candidatus-Omnitrophota-bacterium]
MBQ6007751.1	Type1.BREX	[illegible]	1146	Bacteria>PVC_group>Kiritimatiellota>Kiritimatiellia>unclassified_Kiritimatiellia	GCA_017427485.1	Kiritimatiellia_bacterium	IJL17_04385	19209..22649	2212473	BREX-system-P-loop-protein-BrxC-[Kiritimatiellia-bacterium]
MBQ6248038.1	Type1.BREX	[illegible]	1069	Bacteria>PVC_group>Kiritimatiellota>Kiritimatiellia>unclassified_Kiritimatiellia	GCA_017429725.1	Kiritimatiellia_bacterium	IJK04_14290	11611..14817	2212473	BREX-system-P-loop-protein-BrxC-[Kiritimatiellia-bacterium]
MBQ6141563.1	Type1.BREX	[illegible]	1188	Bacteria>PVC_group>Kiritimatiellota>Kiritimatiellia>unclassified_Kiritimatiellia	GCA_017432735.1	Kiritimatiellia_bacterium	IJI54_09780	25512..29078	2212473	BREX-system-P-loop-protein-BrxC-[Kiritimatiellia-bacterium]
MBQ7190494.1	Type1.BREX	[illegible]	1172	Bacteria>PVC_group>Kiritimatiellota>Kiritimatiellia>unclassified_Kiritimatiellia	GCA_017450695.1	Kiritimatiellia_bacterium	IJR99_13895	2360..5878	2212473	BREX-system-P-loop-protein-BrxC-[Kiritimatiellia-bacterium]
MBQ9739436.1	Type1.BREX	[illegible]	1148	Bacteria>PVC_group>Kiritimatiellota>Kiritimatiellia>unclassified_Kiritimatiellia	GCA_017457135.1	Kiritimatiellia_bacterium	IJV91_00715	10471..13917	2212473	BREX-system-P-loop-protein-BrxC-[Kiritimatiellia-bacterium]
MBR4615907.1	Type1.BREX	[illegible]	1178	Bacteria>PVC_group>Kiritimatiellota>Kiritimatiellia>unclassified_Kiritimatiellia	GCA_017525665.1	Kiritimatiellia_bacterium	IKO55_09905	7558..11094	2212473	BREX-system-P-loop-protein-BrxC-[Kiritimatiellia-bacterium]
MBQ9367250.1	Type1.BREX	[illegible]	1177	Bacteria>PVC_group>Lentisphaerota>Lentisphaeria>Victivallales>unclassified_Victivallales	GCA_017458805.1	Victivallales_bacterium	IJT83_05670	3304..6837	2212475	BREX-system-P-loop-protein-BrxC-[Victivallales-bacterium]
MBR6059872.1	Type1.BREX	[illegible]	1178	Bacteria>PVC_group>Lentisphaerota>Lentisphaeria>Victivallales>unclassified_Victivallales	GCA_017542865.1	Victivallales_bacterium	IKP58_17025	13303..16839	2212475	BREX-system-P-loop-protein-BrxC-[Victivallales-bacterium]
MCF7790541.1	Type1.BREX	[illegible]	1176	Bacteria>PVC_group>Lentisphaerota>Lentisphaeria>Victivallales>unclassified_Victivallales	GCA_021733985.1	Victivallales_bacterium	K9M56_00960	14578..18108	2212475	BREX-system-P-loop-protein-BrxC-[Victivallales-bacterium]
OGV31172.1	Type1.BREX	[illegible]	1201	Bacteria>PVC_group>Lentisphaerota>unclassified_Lentisphaerota	GCA_001804785.1	Lentisphaerae_bacterium_GWF2_45_14	A2020_01685	2431..6036	1798572	MAG:-hypothetical-protein-A2020_01685-[Lentisphaerae-bacterium-GWF2_45_14]
CAJ71903.1	Type1.BREX	[illegible]	1220	Bacteria>PVC_group>Planctomycetota>Candidatus_Brocadiia>Candidatus_Brocadiales>Candidatus_Brocadiaceae	-	Candidatus_Kuenenia_stuttgartiensis	kustc1158	789101..792763	174633	hypothetical-protein-kustc1158-[Candidatus-Kuenenia-stuttgartiensis]
TVL98972.1	Type1.BREX	[illegible]	1144	Bacteria>PVC_group>Planctomycetota>Candidatus_Brocadiia>Candidatus_Brocadiales>Candidatus_Brocadiaceae	GCA_007618135.1	Candidatus_Brocadia_sp._WS118	CV087_19775	5481..8915	2040625	MAG:-BREX-system-P-loop-protein-BrxC-[Candidatus-Brocadia-sp.-WS118]
WP_164995431.1	Type1.BREX	[illegible]	1217	Bacteria>PVC_group>Planctomycetota>Candidatus_Brocadiia>Candidatus_Brocadiales>Candidatus_Brocadiaceae	GCF_011066545.1	Candidatus_Kuenenia_stuttgartiensis	KsCSTR_RS16670	3735232..3738885	174633	BREX-system-P-loop-protein-BrxC-[Candidatus-Kuenenia-stuttgartiensis]
WP_099325977.1	Type1.BREX	[illegible]	1217	Bacteria>PVC_group>Planctomycetota>Candidatus_Brocadiia>Candidatus_Brocadiales>Candidatus_Brocadiaceae	GCF_027493735.1	Candidatus_Kuenenia_stuttgartiensis	KSMBR1_RS14595	3124365..3128018	174633	BREX-system-P-loop-protein-BrxC-[Candidatus-Kuenenia-stuttgartiensis]
WP_169703663.1	Type1.BREX	[illegible]	1217	Bacteria>PVC_group>Planctomycetota>Candidatus_Brocadiia>Candidatus_Brocadiales>Candidatus_Brocadiaceae	GCF_900232175.1	Candidatus_Kuenenia_stuttgartiensis	HL046_RS19030	82646..86299	174633	BREX-system-P-loop-protein-BrxC-[Candidatus-Kuenenia-stuttgartiensis]
ODS31202.1	Type1.BREX	[illegible]	986	Bacteria>PVC_group>Planctomycetota>Candidatus_Brocadiia>Candidatus_Brocadiales>Candidatus_Scalinduaceae	GCA_001723765.1	Candidatus_Scalindua_rubra	SCARUB_03673	2450..5410	1872076	hypothetical-protein-SCARUB_03673-[Candidatus-Scalindua-rubra]
MBC8551961.1	Type1.BREX	[illegible]	1206	Bacteria>PVC_group>Planctomycetota>Candidatus_Brocadiia>Candidatus_Brocadiales>unclassified_Candidatus_Brocadiales	GCA_014384865.1	Candidatus_Brocadiales_bacterium	H8D23_20125	1912..5532	2760054	BREX-system-P-loop-protein-BrxC-[Candidatus-Brocadiales-bacterium]
MBX3384511.1	Type1.BREX	[illegible]	1151	Bacteria>PVC_group>Planctomycetota>Phycisphaerae>Phycisphaerales>Phycisphaeraceae	GCA_019636615.1	Phycisphaeraceae_bacterium	KF864_13495	1423896..1427351	2026777	BREX-system-P-loop-protein-BrxC-[Phycisphaeraceae-bacterium]
MCC6426045.1	Type1.BREX	[illegible]	1159	Bacteria>PVC_group>Planctomycetota>Phycisphaerae>Phycisphaerales>unclassified_Phycisphaerales	GCA_020847535.1	Phycisphaerales_bacterium	IT435_04420	30643..34122	2052180	BREX-system-P-loop-protein-BrxC-[Phycisphaerales-bacterium]
MCL4743298.1	Type1.BREX	[illegible]	1150	Bacteria>PVC_group>Planctomycetota>Phycisphaerae>Phycisphaerales>unclassified_Phycisphaerales	GCA_023384195.1	Phycisphaerales_bacterium	KJZ54_13970	8587..12039	2052180	BREX-system-P-loop-protein-BrxC-[Phycisphaerales-bacterium]
WP_206292407.1	Type1.BREX	[illegible]	1147	Bacteria>PVC_group>Planctomycetota>Phycisphaerae>Tepidisphaerales>Tepidisphaeraceae	GCF_015169395.1	Humisphaera_borealis	IPV69_RS24760	6565905..6569348	2807512	BREX-system-P-loop-protein-BrxC-[Humisphaera-borealis]
QDV91803.1	Type1.BREX	[illegible]	1149	Bacteria>PVC_group>Planctomycetota>Phycisphaerae>unclassified_Phycisphaerae	GCA_007753915.1	Phycisphaerae_bacterium_RAS2	RAS2_29090	3426719..3430168	2528035	hypothetical-protein-RAS2_29090-[Phycisphaerae-bacterium-RAS2]
MBI9018588.1	Type1.BREX	[illegible]	1191	Bacteria>PVC_group>Planctomycetota>Phycisphaerae>unclassified_Phycisphaerae	GCA_016342495.1	Phycisphaerae_bacterium	JEZ07_15150	1153..4728	2026778	BREX-system-P-loop-protein-BrxC-[Phycisphaerae-bacterium]
MBP7937491.1	Type1.BREX	[illegible]	1149	Bacteria>PVC_group>Planctomycetota>Phycisphaerae>unclassified_Phycisphaerae	GCA_018003015.1	Phycisphaerae_bacterium	KA354_22855	13009..16458	2026778	BREX-system-P-loop-protein-BrxC-[Phycisphaerae-bacterium]
MBP8911432.1	Type1.BREX	[illegible]	1214	Bacteria>PVC_group>Planctomycetota>Phycisphaerae>unclassified_Phycisphaerae	GCA_018055905.1	Phycisphaerae_bacterium	KBI32_08005	5154..8798	2026778	BREX-system-P-loop-protein-BrxC-[Phycisphaerae-bacterium]
MCS7166358.1	Type1.BREX	[illegible]	483	Bacteria>PVC_group>Planctomycetota>Planctomycetia>Gemmatales>unclassified_Gemmatales	GCA_025060195.1	Gemmatales_bacterium	NZ914_01855	14978..16429	2838789	hypothetical-protein-[Gemmatales-bacterium]
WP_145275550.1	Type1.BREX	[illegible]	1197	Bacteria>PVC_group>Planctomycetota>Planctomycetia>Isosphaerales>Isosphaeraceae	GCF_007752535.1	Tautonia_plasticadhaerens	ElP_RS26955	6757809..6761402	2527974	BREX-system-P-loop-protein-BrxC-[Tautonia-plasticadhaerens]
WP_152053060.1	Type1.BREX	[illegible]	1200	Bacteria>PVC_group>Planctomycetota>Planctomycetia>Isosphaerales>Isosphaeraceae	GCF_009177065.1	Tautonia_marina	GA615_RS19855	162087..165689	2653855	BREX-system-P-loop-protein-BrxC-[Tautonia-marina]
WP_146571387.1	Type1.BREX	[illegible]	1144	Bacteria>PVC_group>Planctomycetota>Planctomycetia>Pirellulales>Lacipirellulaceae	GCF_007859815.1	Botrimarina_hoheduenensis	Pla111_RS03510	890428..893862	2528000	BREX-system-P-loop-protein-BrxC-[Botrimarina-hoheduenensis]
NLF73010.1	Type1.BREX	[illegible]	1145	Bacteria>PVC_group>Planctomycetota>Planctomycetia>Pirellulales>Pirellulaceae	GCA_012515235.1	Candidatus_Anammoximicrobium_sp.	GX575_28570	2105..5542	2699742	BREX-system-P-loop-protein-BrxC-[Candidatus-Anammoximicrobium-sp.]
NLY01510.1	Type1.BREX	[illegible]	1215	Bacteria>PVC_group>Planctomycetota>Planctomycetia>Pirellulales>Pirellulaceae	GCA_012728855.1	Rhodopirellula_sp.	GXY83_35915	2404..6051	2024855	BREX-system-P-loop-protein-BrxC-[Rhodopirellula-sp.]
MCC7337722.1	Type1.BREX	[illegible]	1171	Bacteria>PVC_group>Planctomycetota>Planctomycetia>Pirellulales>Pirellulaceae	GCA_020852965.1	Pirellulaceae_bacterium	IT422_21750	7905..11420	2699754	BREX-system-P-loop-protein-BrxC-[Pirellulaceae-bacterium]
WP_145350724.1	Type1.BREX	[illegible]	1169	Bacteria>PVC_group>Planctomycetota>Planctomycetia>Pirellulales>Pirellulaceae	GCF_007741495.1	Roseimaritima_multifibrata	FF011L_RS05780	1542279..1545788	1930274	BREX-system-P-loop-protein-BrxC-[Roseimaritima-multifibrata]
WP_145095869.1	Type1.BREX	[illegible]	1192	Bacteria>PVC_group>Planctomycetota>Planctomycetia>Pirellulales>Pirellulaceae	GCF_007753095.1	Rosistilla_carotiformis	Poly24_RS13225	3653187..3656765	2528017	BREX-system-P-loop-protein-BrxC-[Rosistilla-carotiformis]
WP_145388328.1	Type1.BREX	[illegible]	1202	Bacteria>PVC_group>Planctomycetota>Planctomycetia>Pirellulales>Pirellulaceae	GCF_007754155.1	Stieleria_neptunia	Enr13x_RS17960	5195275..5198883	2527979	BREX-system-P-loop-protein-BrxC-[Stieleria-neptunia]
WP_197454902.1	Type1.BREX	[illegible]	1149	Bacteria>PVC_group>Planctomycetota>Planctomycetia>Pirellulales>Pirellulaceae	GCF_007860045.1	Stieleria_varia	Pla52n_RS23260	441169..444618	2528005	BREX-system-P-loop-protein-BrxC-[Stieleria-varia]
WP_197171628.1	Type1.BREX	[illegible]	1149	Bacteria>PVC_group>Planctomycetota>Planctomycetia>Pirellulales>Pirellulaceae	GCF_007860185.1	Novipirellula_aureliae	Q31b_RS15730	214901..218350	2527966	BREX-system-P-loop-protein-BrxC-[Novipirellula-aureliae]
WP_068129828.1	Type1.BREX	[illegible]	1169	Bacteria>PVC_group>Planctomycetota>Planctomycetia>Pirellulales>Pirellulaceae	GCF_008065135.1	Roseimaritima_ulvae	UC8_RS27055	7584147..7587656	980254	BREX-system-P-loop-protein-BrxC-[Roseimaritima-ulvae]
WP_153558877.1	Type1.BREX	[illegible]	1174	Bacteria>PVC_group>Planctomycetota>Planctomycetia>Pirellulales>Pirellulaceae	GCF_009618275.1	Roseimaritima_sediminicola	GE027_RS19390	33307..36831	2662066	BREX-system-P-loop-protein-BrxC-[Roseimaritima-sediminicola]
WP_207395558.1	Type1.BREX	[illegible]	1152	Bacteria>PVC_group>Planctomycetota>Planctomycetia>Pirellulales>Pirellulaceae	GCF_013618625.1	Bremerella_alba	HOV93_RS05900	45922..49380	980252	BREX-system-P-loop-protein-BrxC-[Bremerella-alba]
WP_207398110.1	Type1.BREX	[illegible]	1192	Bacteria>PVC_group>Planctomycetota>Planctomycetia>Pirellulales>Pirellulaceae	GCF_013618625.1	Bremerella_alba	HOV93_RS18975	110238..113816	980252	BREX-system-P-loop-protein-BrxC-[Bremerella-alba]
WP_184301662.1	Type1.BREX	[illegible]	1169	Bacteria>PVC_group>Planctomycetota>Planctomycetia>Pirellulales>Pirellulaceae	GCF_014192335.1	Rhodopirellula_rubra	FHS27_RS03210	253910..257419	980271	BREX-system-P-loop-protein-BrxC-[Rhodopirellula-rubra]
WP_164102234.1	Type1.BREX	[illegible]	1196	Bacteria>PVC_group>Planctomycetota>Planctomycetia>Pirellulales>Pirellulaceae	GCF_016837145.1	Candidatus_Laterigemmans_baculatus	ICJ56_RS09300	149679..153269	2770505	BREX-system-P-loop-protein-BrxC-[Candidatus-Laterigemmans-baculatus]
WP_259631594.1	Type1.BREX	[illegible]	1147	Bacteria>PVC_group>Planctomycetota>Planctomycetia>Pirellulales>Pirellulaceae	GCF_025060895.1	Stieleria_sedimenti	NZK35_RS05520	42893..46336	2976331	BREX-system-P-loop-protein-BrxC-[Stieleria-sedimenti]
WP_274403450.1	Type1.BREX	[illegible]	1149	Bacteria>PVC_group>Planctomycetota>Planctomycetia>Pirellulales>Pirellulaceae	GCF_028768465.1	Rhodopirellula_sp._P2	PSR62_RS13060	3699964..3703413	2127060	BREX-system-P-loop-protein-BrxC-[Rhodopirellula-sp.-P2]
MBQ9812620.1	Type1.BREX	[illegible]	596	Bacteria>PVC_group>Planctomycetota>Planctomycetia>Pirellulales>Thermoguttaceae	GCA_017441335.1	Thermoguttaceae_bacterium	IJM54_04830	4203..5993	2691551	BREX-system-P-loop-protein-BrxC-[Thermoguttaceae-bacterium]
MBP3694027.1	Type1.BREX	[illegible]	1201	Bacteria>PVC_group>Planctomycetota>Planctomycetia>Pirellulales>Thermoguttaceae	GCA_017939795.1	Thermoguttaceae_bacterium	J6J31_06290	52..3657	2691551	BREX-system-P-loop-protein-BrxC-[Thermoguttaceae-bacterium]
MBV11173.1	Type1.BREX	[illegible]	800	Bacteria>PVC_group>Planctomycetota>Planctomycetia>Planctomycetales>Planctomycetaceae	GCA_002731545.1	Rubinisphaera_sp.	CMN21_18375	8990..11389	2024857	hypothetical-protein-[Rubinisphaera-sp.]
HBJ35663.1	Type1.BREX	[illegible]	1188	Bacteria>PVC_group>Planctomycetota>Planctomycetia>Planctomycetales>Planctomycetaceae	GCA_003506095.1	Planctomycetaceae_bacterium	DDZ51_13145	16532..20098	2026779	BREX-system-P-loop-protein-BrxC-[Planctomycetaceae-bacterium]
HCS52258.1	Type1.BREX	[illegible]	340	Bacteria>PVC_group>Planctomycetota>Planctomycetia>Planctomycetales>Planctomycetaceae	GCA_003531135.1	Planctomycetaceae_bacterium	DIW81_11810	12228..13250	2026779	BREX-system-P-loop-protein-BrxC-[Planctomycetaceae-bacterium]
HCS52259.1	Type1.BREX	[illegible]	393	Bacteria>PVC_group>Planctomycetota>Planctomycetia>Planctomycetales>Planctomycetaceae	GCA_003531135.1	Planctomycetaceae_bacterium	DIW81_11815	13400..14582	2026779	BREX-system-P-loop-protein-BrxC-[Planctomycetaceae-bacterium]
HCS52260.1	Type1.BREX	[illegible]	395	Bacteria>PVC_group>Planctomycetota>Planctomycetia>Planctomycetales>Planctomycetaceae	GCA_003531135.1	Planctomycetaceae_bacterium	DIW81_11820	14698..15884	2026779	BREX-system-P-loop-protein-BrxC-[Planctomycetaceae-bacterium]
MBA4188926.1	Type1.BREX	[illegible]	1142	Bacteria>PVC_group>Planctomycetota>Planctomycetia>Planctomycetales>Planctomycetaceae	GCA_013823135.1	Planctomycetaceae_bacterium	C0467_13090	97209..100637	2026779	BREX-system-P-loop-protein-BrxC-[Planctomycetaceae-bacterium]
MCA9037354.1	Type1.BREX	[illegible]	1154	Bacteria>PVC_group>Planctomycetota>Planctomycetia>Planctomycetales>Planctomycetaceae	GCA_020426345.1	Planctomycetaceae_bacterium	KDA91_19595	2587..6051	2026779	BREX-system-P-loop-protein-BrxC-[Planctomycetaceae-bacterium]
MCR9202528.1	Type1.BREX	[illegible]	1218	Bacteria>PVC_group>Planctomycetota>Planctomycetia>Planctomycetales>Planctomycetaceae	GCA_024742875.1	Planctomycetaceae_bacterium	NXI04_28130	14549..18205	2026779	BREX-system-P-loop-protein-BrxC-[Planctomycetaceae-bacterium]
GIX04293.1	Type1.BREX	[illegible]	1221	Bacteria>PVC_group>Planctomycetota>Planctomycetia>Planctomycetales>Planctomycetaceae	GCA_026004855.1	Planctomycetaceae_bacterium	KatS3mg114_0162	186824..190489	2026779	MAG:-hypothetical-protein-KatS3mg114_0162-[Planctomycetaceae-bacterium]
WP_145192991.1	Type1.BREX	[illegible]	1220	Bacteria>PVC_group>Planctomycetota>Planctomycetia>Planctomycetales>Planctomycetaceae	GCF_007744075.1	Gimesia_chilikensis	HG66A1_RS30060	7903021..7906683	2605989	BREX-system-P-loop-protein-BrxC-[Gimesia-chilikensis]
HBI47071.1	Type1.BREX	[illegible]	1147	Bacteria>PVC_group>Planctomycetota>Planctomycetia>Planctomycetales>unclassified_Planctomycetales	GCA_003508275.1	Planctomycetales_bacterium	DDY78_30090	20346..23789	2053591	BREX-system-P-loop-protein-BrxC-[Planctomycetales-bacterium]
MCA9126638.1	Type1.BREX	[illegible]	1149	Bacteria>PVC_group>Planctomycetota>Planctomycetia>Planctomycetales>unclassified_Planctomycetales	GCA_020426625.1	Planctomycetales_bacterium	KDB22_06125	7854..11303	2053591	BREX-system-P-loop-protein-BrxC-[Planctomycetales-bacterium]
GDX95758.1	Type1.BREX	[illegible]	1161	Bacteria>PVC_group>Planctomycetota>Planctomycetia>unclassified_Planctomycetia	GCA_014192425.1	Planctomycetia_bacterium	LBMAG47_14220	155405..158890	2052181	hypothetical-protein-LBMAG47_14220-[Planctomycetia-bacterium]
MBE7444731.1	Type1.BREX	[illegible]	1144	Bacteria>PVC_group>Planctomycetota>Planctomycetia>unclassified_Planctomycetia	GCA_015075005.1	Planctomycetia_bacterium	HS132_05580	1084624..1088058	2052181	BREX-system-P-loop-protein-BrxC-[Planctomycetia-bacterium]
MBL8798552.1	Type1.BREX	[illegible]	1139	Bacteria>PVC_group>Planctomycetota>Planctomycetia>unclassified_Planctomycetia	GCA_016795185.1	Planctomycetia_bacterium	JNM56_31945	4217..7633	2052181	BREX-system-P-loop-protein-BrxC-[Planctomycetia-bacterium]
OQA03646.1	Type1.BREX	[illegible]	1145	Bacteria>PVC_group>Planctomycetota>unclassified_Planctomycetota	GCA_002068635.1	Planctomycetes_bacterium_ADurb.Bin401	BWY69_00331	3976..7413	1852904	hypothetical-protein-BWY69_00331-[Planctomycetes-bacterium-ADurb.Bin401]
RIK67118.1	Type1.BREX	[illegible]	1149	Bacteria>PVC_group>Planctomycetota>unclassified_Planctomycetota	GCA_003576905.1	Planctomycetota_bacterium	DCC65_07820	141257..144706	2026780	MAG:-BREX-system-P-loop-protein-BrxC-[Planctomycetota-bacterium]
TET34868.1	Type1.BREX	[illegible]	1347	Bacteria>PVC_group>Planctomycetota>unclassified_Planctomycetota	GCA_004376375.1	Planctomycetota_bacterium	E3J72_12085	41890..45933	2026780	MAG:-hypothetical-protein-E3J72_12085-[Planctomycetota-bacterium]
QDV07299.1	Type1.BREX	[illegible]	1155	Bacteria>PVC_group>Planctomycetota>unclassified_Planctomycetota	GCA_007751475.1	Planctomycetes_bacterium_Poly30	Poly30_28220	3862242..3865709	2528018	hypothetical-protein-Poly30_28220-[Planctomycetes-bacterium-Poly30]
TWU10529.1	Type1.BREX	[illegible]	765	Bacteria>PVC_group>Planctomycetota>unclassified_Planctomycetota	GCA_007860105.1	Planctomycetes_bacterium_Poly21	Poly21_44340	182735..185032	2528014	hypothetical-protein-Poly21_44340-[Planctomycetes-bacterium-Poly21]
TWU09954.1	Type1.BREX	[illegible]	1205	Bacteria>PVC_group>Planctomycetota>unclassified_Planctomycetota	GCA_007860105.1	Planctomycetes_bacterium_Poly21	Poly21_52830	31284..34901	2528014	hypothetical-protein-Poly21_52830-[Planctomycetes-bacterium-Poly21]
MBI3097943.1	Type1.BREX	[illegible]	1104	Bacteria>PVC_group>Planctomycetota>unclassified_Planctomycetota	GCA_016192525.1	Planctomycetota_bacterium	HYY93_06795	72529..75843	2026780	BREX-system-P-loop-protein-BrxC-[Planctomycetota-bacterium]
MBI3468946.1	Type1.BREX	[illegible]	1222	Bacteria>PVC_group>Planctomycetota>unclassified_Planctomycetota	GCA_016196255.1	Planctomycetota_bacterium	HY000_38570	1768..5436	2026780	BREX-system-P-loop-protein-BrxC-[Planctomycetota-bacterium]
MBI4584108.1	Type1.BREX	[illegible]	1148	Bacteria>PVC_group>Planctomycetota>unclassified_Planctomycetota	GCA_016209605.1	Planctomycetota_bacterium	HY717_08805	43440..46886	2026780	BREX-system-P-loop-protein-BrxC-[Planctomycetota-bacterium]
MBI4586093.1	Type1.BREX	[illegible]	1226	Bacteria>PVC_group>Planctomycetota>unclassified_Planctomycetota	GCA_016209605.1	Planctomycetota_bacterium	HY717_18950	18652..22332	2026780	BREX-system-P-loop-protein-BrxC-[Planctomycetota-bacterium]
MBK9385761.1	Type1.BREX	[illegible]	1149	Bacteria>PVC_group>Planctomycetota>unclassified_Planctomycetota	GCA_016719295.1	Planctomycetota_bacterium	IPN34_13205	3513017..3516466	2026780	BREX-system-P-loop-protein-BrxC-[Planctomycetota-bacterium]
MBL1217602.1	Type1.BREX	[illegible]	1165	Bacteria>PVC_group>Planctomycetota>unclassified_Planctomycetota	GCA_016743455.1	Planctomycetota_bacterium	D8M59_08910	1591..5088	2026780	BREX-system-P-loop-protein-BrxC-[Planctomycetota-bacterium]
MBL4771871.1	Type1.BREX	[illegible]	1192	Bacteria>PVC_group>Planctomycetota>unclassified_Planctomycetota	GCA_016764635.1	Planctomycetota_bacterium	JKY61_12205	4696..8274	2026780	BREX-system-P-loop-protein-BrxC-[Planctomycetota-bacterium]
MBL8695658.1	Type1.BREX	[illegible]	1150	Bacteria>PVC_group>Planctomycetota>unclassified_Planctomycetota	GCA_016792805.1	Planctomycetota_bacterium	JNJ88_16320	208992..212444	2026780	BREX-system-P-loop-protein-BrxC-[Planctomycetota-bacterium]
MBM4067775.1	Type1.BREX	[illegible]	1193	Bacteria>PVC_group>Planctomycetota>unclassified_Planctomycetota	GCA_016873095.1	Planctomycetota_bacterium	FJ271_02350	79784..83365	2026780	BREX-system-P-loop-protein-BrxC-[Planctomycetota-bacterium]
MBW3538721.1	Type1.BREX	[illegible]	1230	Bacteria>PVC_group>Planctomycetota>unclassified_Planctomycetota	GCA_019347905.1	Planctomycetota_bacterium	KY476_00475	36012..39705	2026780	BREX-system-P-loop-protein-BrxC-[Planctomycetota-bacterium]
MBW8002871.1	Type1.BREX	[illegible]	1214	Bacteria>PVC_group>Planctomycetota>unclassified_Planctomycetota	GCA_019456255.1	Planctomycetota_bacterium	FVQ80_12760	16380..20024	2026780	BREX-system-P-loop-protein-BrxC-[Planctomycetota-bacterium]
MBW8002946.1	Type1.BREX	[illegible]	1164	Bacteria>PVC_group>Planctomycetota>unclassified_Planctomycetota	GCA_019456255.1	Planctomycetota_bacterium	FVQ80_13155	4626..8120	2026780	BREX-system-P-loop-protein-BrxC-[Planctomycetota-bacterium]
MCC6785237.1	Type1.BREX	[illegible]	1150	Bacteria>PVC_group>Planctomycetota>unclassified_Planctomycetota	GCA_020848735.1	Planctomycetota_bacterium	IT457_20475	22318..25770	2026780	BREX-system-P-loop-protein-BrxC-[Planctomycetota-bacterium]

Protein-ID	System	Gene-Neighbourhoods	Length	Taxonomy	Assembly-ID	Strain	Locus-Tag	Genomic-coordinates	TaxID	Fasta-define
MCC7315424.1	Type1.BREX	<-SIGMA+OB-Fold+AAA-ATPase+Lon-Protease-BrxL<-PglZ<-ArdCN+MPTase<-N6-METHYLASE<-?<-TIR<-DUF6079*<-BrxB-BrxF-iSTAND<-BrxA-DNA-binding||HTH_23+HTH_32-><-Peptidase_S8<-Classical-AAA<-BirA-HTH<-?||MarR-HTH->Peptidase_S74->	1147	Bacteria>PVC_group>Planctomycetota>unclassified_Planctomycetota	GCA_020853195.1	Planctomycetota_bacterium	IT419_11445	15860..19303	2026780	BREX-system-P-loop-protein-BrxC-[Planctomycetota-bacterium]
MCD6405191.1	Type1.BREX	<-Schlafen+HATPase+wHTH-p<-PHP+SbcC<-SIGMA+OB-Fold+AAA-ATPase+Lon-Protease-BrxL<-PglZ<-ParB+HNH<-N6-METHYLASE<-DUF6079*<-BrxB-BrxF-iSTAND<-BrxA-DNA-binding<-XIS-HTH	1143	Bacteria>PVC_group>Planctomycetota>unclassified_Planctomycetota	GCA_021160625.1	Planctomycetota_bacterium	J7M19_05155	12563..15994	2026780	BREX-system-P-loop-protein-BrxC-[Planctomycetota-bacterium]
MCE9589095.1	Type1.BREX	<-XIS-HTH<-XIS-HTH<-?<-URI-DNase+RAMA<-SIGMA+OB-Fold+AAA-ATPase+Lon-Protease-BrxL<-PglZ<-REase+AIPR<-N6-METHYLASE<-DUF6079*<-BrxB-BrxF-iSTAND<-BrxA-DNA-binding<-DDE_Tnp	1146	Bacteria>PVC_group>Planctomycetota>unclassified_Planctomycetota	GCA_021413865.1	Planctomycetota_bacterium	K8S99_01060	75553..78993	2026780	BREX-system-P-loop-protein-BrxC-[Planctomycetota-bacterium]
MCL5279432.1	Type1.BREX	DUF6079-p*->N6-METHYLASE->AbiEi_4->AbiEii->?->PglZ->SIGMA+OB-Fold+AAA-ATPase+Lon-Protease-BrxL->TIR->	661	Bacteria>PVC_group>Planctomycetota>unclassified_Planctomycetota	GCA_023481165.1	Planctomycetota_bacterium	M1376_05960	1..1987	2026780	BREX-system-P-loop-protein-BrxC-[Planctomycetota-bacterium]
AHF94674.1	Type1.BREX	<-PglZ<-TOPRIM<-OLD-ABC<-PIWI<-REase+PNTD1+PNTD2<-N6-METHYLASE-p<-DOC+HTH<-N6-METHYLASE<-?<-DUF6079*<-BrxB-BrxF-iSTAND<-BrxA-DNA-binding<-?||TrwC+SF1-SF2-Helicase->?->?->ClpABC-AAA->	1248	Bacteria>PVC_group>Verrucomicrobiota>Opitutae>Opitutales>Opitutaceae	GCA_000242935.3	Opitutaceae_bacterium_TAV5	OPIT5_23275	5485357..5489103	794903	hypothetical-protein-OPIT5_23275-[Opitutaceae-bacterium-TAV5]
MBC7388090.1	Type1.BREX	DUF6079-p*->REase->N6-METHYLASE->?->PglZ->SIGMA+OB-Fold+AAA-ATPase+Lon-Protease-BrxL->	828	Bacteria>PVC_group>Verrucomicrobiota>Opitutae>Opitutales>Opitutaceae	GCA_014376535.1	Opitutaceae_bacterium	H7329_02660	1..2487	2006848	hypothetical-protein-[Opitutaceae-bacterium]
MBX3736102.1	Type1.BREX	GDResolvase+Recombinase+ZnR-><-HTH_3<-Couple_hipA+HipA_C<-HTH_3||BrxA-DNA-binding->BrxB-BrxF-iSTAND->DUF6079*->TIR->N6-METHYLASE->?->TIR->TIR->PglZ->SIGMA+OB-Fold+AAA-ATPase+Lon-Protease-BrxL->	1149	Bacteria>PVC_group>Verrucomicrobiota>Opitutae>Opitutales>Opitutaceae	GCA_019634575.1	Candidatus_Didemnitutus_sp.	KF715_05395	1290269..1293718	2814259	BREX-system-P-loop-protein-BrxC-[Candidatus-Didemnitutus-sp.]
MCC5808698.1	Type1.BREX	Phage_integrase-><-SIGMA+OB-Fold+AAA-ATPase+HKD-DNase-BrxL<-PglZ<-HNH<-OLD-ABC<-N6-METHYLASE<-REase<-OLD-ABC<-DUF6079*<-BrxB-BrxF-iSTAND<-BrxA-DNA-binding<-XIS-HTH	1183	Bacteria>PVC_group>Verrucomicrobiota>Opitutae>Opitutales>unclassified_Opitutales	GCA_020831525.1	Opitutales_bacterium	JJU00_20390	12178..15729	2026772	BREX-system-P-loop-protein-BrxC-[Opitutales-bacterium]
MCH8474945.1	Type1.BREX	<-DUF4236+TPR||tRNA->BrxA-DNA-binding->BrxB-BrxF-iSTAND->DUF6079*->SF1-SF2-Helicase->?->?->N6-METHYLASE->N6-METHYLASE-p->SbcC->Schlafen+HATPase+wHTH->MarR-HTH->Polbeta->	1147	Bacteria>PVC_group>Verrucomicrobiota>Opitutae>Opitutales>unclassified_Opitutales	GCA_022565335.1	Opitutales_bacterium	LAT55_06930	5092..8535	2026772	BREX-system-P-loop-protein-BrxC-[Opitutales-bacterium]
MBT62430.1	Type1.BREX	<-KAP-NTPase<-N6-METHYLASE-p<-DDE_Tnp<-N6-METHYLASE-p<-RVT<-N6-METHYLASE-p<-?||?-><-Schlafen+HATPase+wHTH<-DUF6079*<-BrxB-BrxF-iSTAND<-BrxA-DNA-binding<-HTH_3	1165	Bacteria>PVC_group>Verrucomicrobiota>Opitutae>Puniceicoccales>Puniceicoccaceae	GCA_002729725.1	Puniceicoccaceae_bacterium	CML13_04370	15109..18606	2026784	hypothetical-protein-[Puniceicoccaceae-bacterium]
MBU3665708.1	Type1.BREX	ABhydrolase-><-?<-?<-SIGMA+OB-Fold+AAA-ATPase+HKD-DNase-BrxL<-PglZ<-KAP-NTPase<-N6-METHYLASE<-Polbeta<-MarR-HTH<-DUF6079*<-BrxB-BrxF-iSTAND<-BrxA-DNA-binding<-ZnR<-?<-?<-?<-?<-VIT+VWA<-MerR-HTH	1169	Bacteria>PVC_group>Verrucomicrobiota>Spartobacteria>Chthoniobacterales>unclassified_Chthoniobacterales	GCA_018883245.1	Chthoniobacterales_bacterium	FGM15_07520	29744..33253	2201154	BREX-system-P-loop-protein-BrxC-[Chthoniobacterales-bacterium]
PTY02769.1	Type1.BREX	RAMA->GDResolvase+ZnR->PglZ->SIGMA+OB-Fold+AAA-ATPase+HKD-DNase-BrxL-><-N6-METHYLASE<-Polbeta<-MarR-HTH<-DUF6079*<-BrxB-BrxF-iSTAND<-BrxA-DNA-binding||DUF4096+DDE_Tnp_1+DDE_Tnp_1_2->	1172	Bacteria>PVC_group>Verrucomicrobiota>unclassified_Verrucomicrobiota	GCA_003054695.1	Verrucomicrobia_bacterium_LW23	DB346_08320	46382..49900	2161867	BREX-system-P-loop-protein-BrxC-[Verrucomicrobia-bacterium-LW23]
HCF96075.1	Type1.BREX	<-N6-METHYLASE-p<-N6-METHYLASE<-REase<-OLD-ABC<-DUF6079-p<-DUF6079-p*<-DUF6079-p<-DUF6079-p<-DUF6079-p<-BrxB-BrxF-iSTAND<-BrxA-DNA-binding	268	Bacteria>PVC_group>Verrucomicrobiota>unclassified_Verrucomicrobiota	GCA_003534995.1	Verrucomicrobiota_bacterium	DEW46_13535	6770..7576	2026799	BREX-system-P-loop-protein-BrxC-[Verrucomicrobiota-bacterium]
HCF96078.1	Type1.BREX	<-N6-METHYLASE-p<-N6-METHYLASE<-REase<-OLD-ABC<-DUF6079-p<-DUF6079-p<-DUF6079-p<-DUF6079-p<-DUF6079-p*<-BrxB-BrxF-iSTAND<-BrxA-DNA-binding	531	Bacteria>PVC_group>Verrucomicrobiota>unclassified_Verrucomicrobiota	GCA_003534995.1	Verrucomicrobiota_bacterium	DEW46_13550	8732..10325	2026799	BREX-system-P-loop-protein-BrxC-[Verrucomicrobiota-bacterium]
KAB2639241.1	Type1.BREX	<-Y1_Tnp<-N6-METHYLASE-p<-Four-helical-protein<-Schlafen+HATPase+wHTH<-DUF6079*<-BrxB-BrxF-iSTAND<-BrxA-DNA-binding	1168	Bacteria>PVC_group>Verrucomicrobiota>unclassified_Verrucomicrobiota	GCA_008932935.1	Verrucomicrobiota_bacterium	DVB25_06025	8272..11778	2026799	BREX-system-P-loop-protein-BrxC-[Verrucomicrobiota-bacterium]
NBB80453.1	Type1.BREX	HTH_3->BrxA-DNA-binding->BrxB-BrxF-iSTAND->DUF6079*->OLD-ABC->PIN->N6-METHYLASE->OLD-ABC->HNH->ParB+HNH->Schlafen+HATPase+wHTH->PglZ->SIGMA+OB-Fold+AAA-ATPase+Lon-Protease-BrxL->	1147	Bacteria>PVC_group>Verrucomicrobiota>unclassified_Verrucomicrobiota	GCA_009908325.1	Verrucomicrobiota_bacterium	GVY36_13595	2227..5670	2026799	BREX-system-P-loop-protein-BrxC-[Verrucomicrobiota-bacterium]
MBI4023965.1	Type1.BREX	CBS->BrxA-DNA-binding->BrxB-BrxF-iSTAND->DUF6079*->HEPN->N6-METHYLASE->REase+AIPR->PglZ->SIGMA+OB-Fold+AAA-ATPase+Lon-Protease-BrxL->	1148	Bacteria>PVC_group>Verrucomicrobiota>unclassified_Verrucomicrobiota	GCA_016198745.1	Verrucomicrobiota_bacterium	HY360_03230	10391..13837	2026799	BREX-system-P-loop-protein-BrxC-[Verrucomicrobiota-bacterium]
MBI4624648.1	Type1.BREX	<-PadR-HTH||?->?->?->?->HTH_Tnp->BrxA-DNA-binding->BrxB-BrxF-iSTAND->DUF6079*->OLD-ABC->TOPRIM->	1155	Bacteria>PVC_group>Verrucomicrobiota>unclassified_Verrucomicrobiota	GCA_016209255.1	Verrucomicrobiota_bacterium	HY736_15695	9824..13291	2026799	BREX-system-P-loop-protein-BrxC-[Verrucomicrobiota-bacterium]
MBM3880197.1	Type1.BREX	XIS-HTH->BrxA-DNA-binding->BrxB-BrxF-iSTAND->DUF6079*->N6-METHYLASE->SbcC->PIN->?->DOC+HTH->N6-METHYLASE-p->OLD-ABC->TOPRIM->PglZ->	1246	Bacteria>PVC_group>Verrucomicrobiota>unclassified_Verrucomicrobiota	GCA_016871715.1	Verrucomicrobiota_bacterium	FJ387_10840	14385..18125	2026799	BREX-system-P-loop-protein-BrxC-[Verrucomicrobiota-bacterium]
MBN9688605.1	Type1.BREX	<-SIGMA+OB-Fold+AAA-ATPase+Lon-Protease-BrxL<-PglZ<-Schlafen+HATPase+wHTH<-PHP+SbcC<-N6-METHYLASE<-TIR<-?<-SMODS<-DUF6079*<-BrxB-BrxF-iSTAND<-BrxA-DNA-binding<-TPR<-Peptidase||?-><-wHTH+HNH<-DOC+HTH<-?<-HTH+REase	1142	Bacteria>PVC_group>Verrucomicrobiota>unclassified_Verrucomicrobiota	GCA_017309115.1	Verrucomicrobiota_bacterium	JOM24_00070	18647..22075	2026799	BREX-system-P-loop-protein-BrxC-[Verrucomicrobiota-bacterium]
MBP7946078.1	Type1.BREX	<-N6-METHYLASE<-Schlafen<-DUF6079*<-BrxB-BrxF-iSTAND<-BrxA-DNA-binding<-?<-?<-?<-?||?->?-><-TPR+TPR+TPR+TPR+TPR	1195	Bacteria>PVC_group>Verrucomicrobiota>unclassified_Verrucomicrobiota	GCA_018003215.1	Verrucomicrobiota_bacterium	KA191_08410	4464..8051	2026799	BREX-system-P-loop-protein-BrxC-[Verrucomicrobiota-bacterium]
MBP7946757.1	Type1.BREX	AbiEi_4+AbiEi_1->AbiEii+ZnR->?-><-DDE_Tnp||?->?->?->BrxA-DNA-binding->BrxB-BrxF-iSTAND->DUF6079*->N6-METHYLASE->	1214	Bacteria>PVC_group>Verrucomicrobiota>unclassified_Verrucomicrobiota	GCA_018003215.1	Verrucomicrobiota_bacterium	KA191_11880	29346..32990	2026799	BREX-system-P-loop-protein-BrxC-[Verrucomicrobiota-bacterium]
MCC5847925.1	Type1.BREX	XIS-HTH->AAA-ATPase->?-><-SIGMA+OB-Fold+AAA-ATPase+Lon-Protease-BrxL<-PglZ<-nSTAND<-N6-METHYLASE<-TIR<-Schlafen+HATPase+wHTH<-DUF6079*<-BrxB-BrxF-iSTAND<-BrxA-DNA-binding||?-><-?<-Phage_integrase||Peptidase->	1154	Bacteria>PVC_group>Verrucomicrobiota>unclassified_Verrucomicrobiota	GCA_020831635.1	Verrucomicrobiota_bacterium	JJU29_07500	21995..25459	2026799	BREX-system-P-loop-protein-BrxC-[Verrucomicrobiota-bacterium]
MCF7764832.1	Type1.BREX	<-REC+NtrC-AAA+FIS-HTH<-HISKIN||?->?-><-?<-?||tRNA->?->BrxA-DNA-binding->BrxB-BrxF-iSTAND->DUF6079*->MarR-HTH->Polbeta->N6-METHYLASE-p->	1167	Bacteria>PVC_group>Verrucomicrobiota>unclassified_Verrucomicrobiota	GCA_021733945.1	Verrucomicrobiota_bacterium	K9N62_14270	13767..17270	2026799	BREX-system-P-loop-protein-BrxC-[Verrucomicrobiota-bacterium]
MCU0772111.1	Type1.BREX	<-SIGMA+OB-Fold+AAA-ATPase+Lon-Protease-BrxL<-PglZ<-?<-N6-METHYLASE<-RVT<-DUF6079*<-BrxB-BrxF-iSTAND<-BrxA-DNA-binding||?->?-><-NACHT<-?<-?||?->S1+GTPase-AIG->	1144	Bacteria>PVC_group>Verrucomicrobiota>unclassified_Verrucomicrobiota	GCA_025457615.1	Verrucomicrobiota_bacterium	MUE94_10150	10422..13856	2026799	BREX-system-P-loop-protein-BrxC-[Verrucomicrobiota-bacterium]
SPE51781.1	Type1.BREX	<-SIGMA+OB-Fold+AAA-ATPase+Lon-Protease-BrxL<-PglZ-p<-PglZ<-HTH+REase<-N6-METHYLASE<-?<-SMODS<-DUF6079*<-BrxB-BrxF-iSTAND<-BrxB-BrxF-iSTAND<-BrxA-DNA-binding	1149	Bacteria>PVC_group>Verrucomicrobiota>unclassified_Verrucomicrobiota	GCA_900290355.1	Verrucomicrobiota_bacterium	SBV1_1420061	72835..76284	2026799	putative-P-loop-containing-nucleoside-triphosphate-hydrolase-[Verrucomicrobiota-bacterium]
PAW64461.1	Type1.BREX	BrxB-BrxF-iSTAND->DUF6079*->N6-METHYLASE->HTH+REase->Calcineurin-SbcD->SbcC+CCDC158->	1145	Bacteria>PVC_group>Verrucomicrobiota>Verrucomicrobiae>unclassified_Verrucomicrobiae	GCA_002304315.1	Verrucomicrobiae_bacterium_Tous-C4TDCM	B9S38_16345	386..3823	1982332	MAG:-hypothetical-protein-B9S38_16345-[Verrucomicrobiae-bacterium-Tous-C4TDCM]
MCB1062255.1	Type1.BREX	<-N6-METHYLASE-p<-DUF6079*<-BrxB-BrxF-iSTAND<-BrxA-DNA-binding<-BrxA-DNA-binding<-?||MODE-HTH+PBPII->	1172	Bacteria>PVC_group>Verrucomicrobiota>Verrucomicrobiae>unclassified_Verrucomicrobiae	GCA_020440025.1	Verrucomicrobiae_bacterium	KDN20_04945	1016..4534	2499141	BREX-system-P-loop-protein-BrxC-[Verrucomicrobiae-bacterium]
MBE6418907.1	Type1.BREX	<-PglZ<-N6-METHYLASE<-DUF6079*<-BrxB-BrxF-iSTAND<-BrxA-DNA-binding||?-><-ACET||BirA-HTH->?->Glycos_transf_1+Glyco_trans_1_2->	1189	Bacteria>PVC_group>Verrucomicrobiota>Verrucomicrobiae>Verrucomicrobiales>Akkermansiaceae	GCA_015062055.1	Akkermansiaceae_bacterium	E7031_02125	17805..21374	2562705	BREX-system-P-loop-protein-BrxC-[Akkermansiaceae-bacterium]
MBQ8375884.1	Type1.BREX	<-SF1-SF2-Helicase+DUF3418||?->Calcineurin->?->HAD+HAD->tRNA->TRPR-HTH->STYKinase-like->BrxA-DNA-binding->BrxB-BrxF-iSTAND->DUF6079*->N6-METHYLASE->PglZ->SIGMA+OB-Fold+AAA-ATPase+Lon-Protease-BrxL->Tox-ART-HYD3-><-?<-?<-BirA-HTH<-Phage_integrase	1185	Bacteria>PVC_group>Verrucomicrobiota>Verrucomicrobiae>Verrucomicrobiales>Akkermansiaceae	GCA_017451815.1	Akkermansia_sp.	IJX33_02385	35942..39499	1872421	BREX-system-P-loop-protein-BrxC-[Akkermansia-sp.]
WP_215427518.1	Type1.BREX	ANK+ANK+ANK->HNH->HTH_3->Couple_hipA->HipA_C->?->XIS-HTH->BrxA-DNA-binding->BrxB-BrxF-iSTAND->DUF6079*->N6-METHYLASE->PglZ->SIGMA+OB-Fold+AAA-ATPase+Lon-Protease-BrxL->HNH-><-ParB+HNH||DUF932->PadR-HTH->	1141	Bacteria>PVC_group>Verrucomicrobiota>Verrucomicrobiae>Verrucomicrobiales>Akkermansiaceae	GCF_018709685.1	Akkermansia_muciniphila	HHJ06_RS03145	88477..91902	239935	BREX-system-P-loop-protein-BrxC-[Akkermansia-muciniphila]
MBR01191.1	Type1.BREX	BrxA-DNA-binding->BrxB-BrxF-iSTAND->DUF6079*->N6-METHYLASE->PglZ->REase+iREase-><-SF1-SF2-Helicase	1162	Bacteria>PVC_group>Verrucomicrobiota>Verrucomicrobiae>Verrucomicrobiales>unclassified_Verrucomicrobiales	GCA_002729295.1	Verrucomicrobiales_bacterium	CMD60_10085	1798..5286	2026801	hypothetical-protein-[Verrucomicrobiales-bacterium]
MBL9158389.1	Type1.BREX	<-SIGMA+OB-Fold+AAA-ATPase+Lon-Protease-BrxL<-PglZ<-HTH+REase<-N6-METHYLASE-p<-SbcC+ABC-ATPase<-N6-METHYLASE<-TIR<-DUF6079*<-BrxB-BrxF-iSTAND<-BrxA-DNA-binding||?->?->?->?->XIS-HTH->RVT->	1152	Bacteria>PVC_group>Verrucomicrobiota>Verrucomicrobiae>Verrucomicrobiales>unclassified_Verrucomicrobiales	GCA_016795385.1	Verrucomicrobiales_bacterium	JNJ70_13005	50985..54443	2026801	BREX-system-P-loop-protein-BrxC-[Verrucomicrobiales-bacterium]
MCI0536692.1	Type1.BREX	TIR-><-?||BrxA-DNA-binding->BrxB-BrxF-iSTAND->DUF6079*->OLD-ABC->TOPRIM->N6-METHYLASE-p->	1149	Bacteria>PVC_group>Verrucomicrobiota>Verrucomicrobiae>Verrucomicrobiales>unclassified_Verrucomicrobiales	GCA_022615925.1	Verrucomicrobiales_bacterium	LOZ50_15855	3424..6873	2026801	BREX-system-P-loop-protein-BrxC-[Verrucomicrobiales-bacterium]
MBL9184319.1	Type1.BREX	DUF932->BrxA-DNA-binding->BrxB-BrxF-iSTAND->DUF6079*->PIN->TIR->N6-METHYLASE->?->PglZ->SIGMA+OB-Fold+AAA-ATPase+Lon-Protease-BrxL->REase+PNTD1+PNTD2->PIWI->	1153	Bacteria>PVC_group>Verrucomicrobiota>Verrucomicrobiae>Verrucomicrobiales>Verrucomicrobiaceae	GCA_016795465.1	Verrucomicrobiaceae_bacterium	JNN17_19395	2113..5574	2026800	BREX-system-P-loop-protein-BrxC-[Verrucomicrobiaceae-bacterium]
MCF7788313.1	Type1.BREX	BrxA-DNA-binding->BrxB-BrxF-iSTAND->DUF6079*->HKD-DNase+DUF3150->MarR-HTH->Polbeta->N6-METHYLASE->?->?->SbcC->PglZ-p->REase->	1167	Bacteria>PVC_group>Verrucomicrobiota>Verrucomicrobiae>Verrucomicrobiales>Verrucomicrobiaceae	GCA_021733925.1	Prosthecobacter_sp.	K9N47_19475	2530..6033	1965333	BREX-system-P-loop-protein-BrxC-[Prosthecobacter-sp.]
MCH7225050.1	Type1.BREX	<-SIGMA+OB-Fold+AAA-ATPase+HKD-DNase-BrxL<-PglZ<-URI-DNase<-ZnR<-?<-N6-METHYLASE<-?<-DUF6079*<-BrxB-BrxF-iSTAND<-BrxA-DNA-binding<-TPR<-Peptidase||?->?->TPR+TPR+TPR+TPR+TPR->	1166	Bacteria>PVC_group>Verrucomicrobiota>Verrucomicrobiae>Verrucomicrobiales>Verrucomicrobiaceae	GCA_022548915.1	Verrucomicrobiaceae_bacterium_E54	MLD59_02825	188898..192398	2922395	BREX-system-P-loop-protein-BrxC-[Verrucomicrobiaceae-bacterium-E54]
WP_035610953.1	Type1.BREX	<-SIGMA+OB-Fold+AAA-ATPase+Lon-Protease-BrxL<-PglZ<-N6-METHYLASE<-DUF6079*<-BrxB-BrxF-iSTAND<-BrxA-DNA-binding<-tRNA||N6-METHYLASE->HTH_3->	1145	Bacteria>PVC_group>Verrucomicrobiota>Verrucomicrobiae>Verrucomicrobiales>Verrucomicrobiaceae	GCF_000739615.1	Haloferula_sp._BvORR071	BV071_RS19610	47391..50828	1396141	BREX-system-P-loop-protein-BrxC-[Haloferula-sp.-BvORR071]
TGL58697.1	Type1.BREX	<-N6-METHYLASE<-DUF6079*<-BrxB-BrxF-iSTAND<-BrxA-DNA-binding<-?<-Classical-AAA<-?<-?<-?||?->ParA-Soj-PloopNTPase->	1155	Bacteria>Spirochaetota>Spirochaetia>Leptospirales>Leptospiraceae>Leptospira	GCA_004770745.1	Leptospira_ognonensis	EHQ58_10020	3017..6484	2484945	BREX-system-P-loop-protein-BrxC-[Leptospira-ognonensis]
WP_210412803.1	Type1.BREX	<-N6-METHYLASE<-DUF6079*<-BrxB-BrxF-iSTAND<-BrxA-DNA-binding<-?<-Classical-AAA<-?<-?<-?||?->ParA-Soj-PloopNTPase->	1157	Bacteria>Spirochaetota>Spirochaetia>Leptospirales>Leptospiraceae>Leptospira	GCF_004770745.1	Leptospira_ognonensis	EHQ58_RS10025	3017..6490	2484945	BREX-system-P-loop-protein-BrxC-[Leptospira-ognonensis]
WP_135627473.1	Type1.BREX	DUF6079*->N6-METHYLASE->ParB+HNH->PglZ->SIGMA+OB-Fold+AAA-ATPase+Lon-Protease-BrxL->	1122	Bacteria>Spirochaetota>Spirochaetia>Leptospirales>Leptospiraceae>Leptospira	GCF_004770835.1	Leptospira_selangorensis	EHQ82_RS10885	1..3369	2484982	BREX-system-P-loop-protein-BrxC,-partial-[Leptospira-selangorensis]
MCB1192727.1	Type1.BREX	<-SecA+SF1-SF2-Helicase<-ArdCN||BrxA-DNA-binding->BrxB-BrxF-iSTAND->DUF6079*->REase+HTH->N6-METHYLASE->PglZ->	1148	Bacteria>Spirochaetota>Spirochaetia>Leptospirales>Leptospiraceae>unclassified_Leptospiraceae	GCA_020440835.1	Leptospiraceae_bacterium	KDK90_19935	18936..22382	2026757	BREX-system-P-loop-protein-BrxC-[Leptospiraceae-bacterium]
MCP5494239.1	Type1.BREX	CR-ATPase8+REase-><-SIGMA+OB-Fold+AAA-ATPase+Lon-Protease-BrxL<-?<-?<-?<-PglZ<-N6-METHYLASE<-REase+HTH<-DUF6079*<-BrxB-BrxF-iSTAND<-BrxA-DNA-binding||ArdCN->SecA+SF1-SF2-Helicase->	1148	Bacteria>Spirochaetota>Spirochaetia>Leptospirales>Leptospiraceae>unclassified_Leptospiraceae	GCA_024233865.1	Leptospiraceae_bacterium	H7A23_06750	342984..346430	2026757	BREX-system-P-loop-protein-BrxC-[Leptospiraceae-bacterium]
MCH3907669.1	Type1.BREX	GspA-ATPase+REase-><-Peptidase_S58<-RHH<-?<-?<-?||XIS-HTH->BrxA-DNA-binding->BrxB-BrxF-iSTAND->DUF6079*->N6-METHYLASE->Schlafen+HATPase+wHTH->PglZ->SIGMA+OB-Fold+AAA-ATPase+HKD-DNase-BrxL-><-SF1-SF2-Helicase<-SbcC<-DDE_Tnp_1	1157	Bacteria>Spirochaetota>Spirochaetia>Spirochaetales>Sphaerochaetaceae>Sphaerochaeta	GCA_022482495.1	Sphaerochaeta_sp.	LKE28_05345	253773..257246	1972642	BREX-system-P-loop-protein-BrxC-[Sphaerochaeta-sp.]
WP_114628929.1	Type1.BREX	<-Thymidylate_kin||ParB+HNH->ParB+HNH-><-?<-D5N+D5ATPase||XIS-HTH->BrxA-DNA-binding->BrxB-BrxF-iSTAND->DUF6079*->OLD-ABC->REase->N6-METHYLASE->SIGMA+OB-Fold+AAA-ATPase+HKD-DNase-BrxL-><-AbiEii<-AbiEi_3_N+AbiEi_3	1173	Bacteria>Spirochaetota>Spirochaetia>Spirochaetales>Spirochaetaceae>Oceanispirochaeta	GCF_013166645.2	unclassified_Oceanispirochaeta	HNV09_RS06050	178318..181839	2635722	MULTISPECIES:-BREX-system-P-loop-protein-BrxC-[unclassified-Oceanispirochaeta]
OQY41116.1	Type1.BREX	REase->BrxA-DNA-binding->BrxB-BrxF-iSTAND->DUF6079*->OLD-ABC->TOPRIM->N6-METHYLASE->ParB+HNH->SIGMA+OB-Fold+AAA-ATPase+Lon-Protease-BrxL->	1207	Bacteria>Spirochaetota>Spirochaetia>Spirochaetales>Spirochaetaceae>unclassified_Spirochaetaceae	GCA_002084785.1	Spirochaetaceae_bacterium_4572_7	B6229_00020	2034..5657	1972456	hypothetical-protein-B6229_00020-[Spirochaetaceae-bacterium-4572_7]
OQY33741.1	Type1.BREX	<-REase<-OLD-ABC<-DUF6079*<-BrxB-BrxF-iSTAND<-BrxA-DNA-binding<-?<-?||NtrC-AAA+FIS-HTH->HAMP+HisKin+HATPase_c->REC+AraC-HTH+AraC-HTH->?->ABC-ATPase+SbcC+ABC-ATPase+SbcC->	1173	Bacteria>Spirochaetota>Spirochaetia>Spirochaetales>Spirochaetaceae>unclassified_Spirochaetaceae	GCA_002084805.1	Spirochaetaceae_bacterium_4572_59	B6241_06720	4557..8078	1971727	hypothetical-protein-B6241_06720-[Spirochaetaceae-bacterium-4572_59]
TVQ28583.1	Type1.BREX	DSBH+AraC-HTH+AraC-HTH-><-YTCD-HTH||?-><-?<-Tox-RelE<-?||BrxA-DNA-binding->BrxB-BrxF-iSTAND->DUF6079*->N6-METHYLASE-p->	1185	Bacteria>Spirochaetota>Spirochaetia>Spirochaetales>Spirochaetaceae>unclassified_Spirochaetaceae	GCA_007694005.1	Spirochaetaceae_bacterium	EA383_00220	6436..9993	1898206	BREX-system-P-loop-protein-BrxC-[Spirochaetaceae-bacterium]
MCP5452900.1	Type1.BREX	<-DUF6079*<-BrxB-BrxF-iSTAND<-BrxA-DNA-binding<-XIS-HTH<-?<-?<-?||?->MoxR-AAA->	1147	Bacteria>Spirochaetota>Spirochaetia>Spirochaetales>Spirochaetaceae>unclassified_Spirochaetaceae	-	Spirochaetaceae_bacterium	H7A27_02910	204..3647	1898206	BREX-system-P-loop-protein-BrxC-[Spirochaetaceae-bacterium]
MBQ5908482.1	Type1.BREX	<-TPR+TPR<-?||BrxA-DNA-binding->BrxB-BrxF-iSTAND->DUF6079*->Schlafen+HATPase+wHTH->N6-METHYLASE->?->PglZ->SIGMA+OB-Fold+AAA-ATPase+Lon-Protease-BrxL->HTH_19->	1172	Bacteria>Spirochaetota>Spirochaetia>Spirochaetales>Treponemataceae>Treponema	GCA_017381365.1	Treponema_sp.	IIW80_10580	5234..8752	166	BREX-system-P-loop-protein-BrxC-[Treponema-sp.]
MCR5123681.1	Type1.BREX	<-N6-METHYLASE<-Schlafen+HATPase+wHTH<-DUF6079*<-BrxB-BrxF-iSTAND<-BrxA-DNA-binding<-HTH_26||?->?-><-ZetaToxin<-ABC-3<-ABC-ATPase+SbcC	1172	Bacteria>Spirochaetota>Spirochaetia>Spirochaetales>Treponemataceae>Treponema	GCA_024697755.1	Treponema_sp.	K6B43_00615	5680..9198	166	BREX-system-P-loop-protein-BrxC-[Treponema-sp.]
NLK13330.1	Type1.BREX	<-PglZ-p<-N6-METHYLASE<-DUF6079*<-BrxB-BrxF-iSTAND<-BrxA-DNA-binding<-?<-?||SF1-SF2-Helicase->?->sGTP->	1160	Bacteria>Spirochaetota>Spirochaetia>Spirochaetales>unclassified_Spirochaetales	GCA_012519115.1	Spirochaetales_bacterium	GX313_01060	4407..7889	2026792	BREX-system-P-loop-protein-BrxC-[Spirochaetales-bacterium]
NLV85368.1	Type1.BREX	DUF6079*->N6-METHYLASE->HEPN->N6-METHYLASE-p->PglZ->SIGMA+OB-Fold+AAA-ATPase+Lon-Protease-BrxL->	1160	Bacteria>Spirochaetota>Spirochaetia>Spirochaetales>unclassified_Spirochaetales	GCA_012729295.1	Spirochaetales_bacterium	GXY60_12460	48..3530	2026792	BREX-system-P-loop-protein-BrxC-[Spirochaetales-bacterium]
MBR5915376.1	Type1.BREX	<-SH3||Peptidase_M20-><-?||?->REase-><-SIGMA+OB-Fold+AAA-ATPase+Lon-Protease-BrxL<-PglZ<-Schlafen+HATPase+wHTH<-N6-METHYLASE<-DUF6079*<-BrxB-BrxF-iSTAND<-BrxA-DNA-binding<-CR-ATPase5+REase<-ACET<-?<-?<-?<-HD	1170	Bacteria>Spirochaetota>Spirochaetia>unclassified_Spirochaetia	GCA_017554185.1	Spirochaetia_bacterium	IKZ57_02880	16752..20264	2053615	BREX-system-P-loop-protein-BrxC-[Spirochaetia-bacterium]
OQB04958.1	Type1.BREX	<-DUF6079*<-BrxB-BrxF-iSTAND<-BrxA-DNA-binding<-XIS-HTH<-?<-wHTH+WYL+WCX||?->Mrr_N+ACET->	1147	Bacteria>Spirochaetota>unclassified_Spirochaetota	GCA_002070025.1	Spirochaetes_bacterium_ADurb.Bin215	BWY20_00149	30..3473	1852910	hypothetical-protein-BWY20_00149-[Spirochaetes-bacterium-ADurb.Bin215]
RKX87882.1	Type1.BREX	<-MarR-HTH||?->tRNA->BrxA-DNA-binding->BrxB-BrxF-iSTAND->DUF6079*->PolB->HEPN->Polbeta-><-?||N6-METHYLASE-p->	1168	Bacteria>Spirochaetota>unclassified_Spirochaetota	GCA_003644105.1	Spirochaetota_bacterium	DRP70_07570	3107..6613	2202144	BREX-system-P-loop-protein-BrxC-[Spirochaetota-bacterium]
TXT43486.1	Type1.BREX	<-SIGMA+OB-Fold+AAA-ATPase+HKD-DNase-BrxL<-?||?-><-HTH+REase<-N6-METHYLASE-p<-N6-METHYLASE-p<-Schlafen+HATPase+wHTH-p<-DUF6079*<-BrxB-BrxF-iSTAND<-BrxA-DNA-binding<-XIS-HTH	1162	Bacteria>Spirochaetota>unclassified_Spirochaetota	GCA_008080685.1	Spirochaetota_bacterium	FD137_1824	16028..19516	2202144	hypothetical-protein-FD137_1824-[Spirochaetota-bacterium]
MCI5863824.1	Type1.BREX	<-REC+OmpR-HTH<-?<-?||?-><-MACRODOMAIN||?->ANK+ANK+ANK->BrxA-DNA-binding->BrxB-BrxF-iSTAND->DUF6079-p*->DUF6079-p->N6-METHYLASE->?->PglZ->SIGMA+OB-Fold+AAA-ATPase+Lon-Protease-BrxL->	875	Bacteria>Synergistota>Synergistia>Synergistales>Synergistaceae>Cloacibacillus	GCA_022771785.1	Cloacibacillus_porcorum	MR000_01150	159552..162179	1197717	BREX-system-P-loop-protein-BrxC-[Cloacibacillus-porcorum]
WP_051485152.1	Type1.BREX	MODE-HTH+PBPII->?->?-><-?<-MULE-TRANSPOSASE||BrxA-DNA-binding->BrxB-BrxF-iSTAND->DUF6079*->N6-METHYLASE->SbcC->Schlafen+HATPase+wHTH->PglZ->SIGMA+OB-Fold+AAA-ATPase+Lon-Protease-BrxL->?->?->transposase-><-GNTR-HTH	1216	Bacteria>Synergistota>Synergistia>Synergistales>Synergistaceae>Cloacibacillus	GCF_000585335.1	Cloacibacillus_evryensis	CLOEV_RS14965	3354177..3357827	508460	BREX-system-P-loop-protein-BrxC-[Cloacibacillus-evryensis]
WP_273308638.1	Type1.BREX	<-REC+OmpR-HTH<-?<-?||?-><-MACRODOMAIN||?->ANK+ANK+ANK->BrxA-DNA-binding->BrxB-BrxF-iSTAND->DUF6079-p*->DUF6079-p->N6-METHYLASE->?->PglZ->SIGMA+OB-Fold+AAA-ATPase+Lon-Protease-BrxL->	875	Bacteria>Synergistota>Synergistia>Synergistales>Synergistaceae>Cloacibacillus	GCF_022771785.1	Cloacibacillus_porcorum	MR000_RS01170	159552..162179	1197717	BREX-system-P-loop-protein-BrxC-[Cloacibacillus-porcorum]
WP_006582937.1	Type1.BREX	Amidohydro_2->?->ABhydrolase->?->?->tRNA->tRNA->tRNA->tRNA->tRNA->XIS-HTH->BrxB-BrxF-iSTAND->DUF6079-p*->REase+N6_METHYLASE->SIR2->HerA/FtsK->TRD+TRD->DOC+HTH->REase+SF1-SF2-Helicase->PglZ-p->BrxA-DNA-binding->	532	Bacteria>Synergistota>Synergistia>Synergistales>Synergistaceae>Thermanaerovibrio	GCF_000237825.1	Thermanaerovibrio_velox	THEVEDRAFT_RS01385	298726..300324	108007	DUF6079-family-protein-[Thermanaerovibrio-velox]
MBL3593011.1	Type1.BREX	<-CHTH||DUF488-1->DUF488-s->?->SF1-SF2-Helicase->?->XIS-HTH->BrxA-DNA-binding->BrxB-BrxF-iSTAND->DUF6079*->Schlafen+HATPase+wHTH->N6-METHYLASE->PglZ->SIGMA+OB-Fold+AAA-ATPase+Lon-Protease-BrxL->HARE-HTH+REase->?->GGDEF->Aminotran_4->DUF488->	1149	Bacteria>Synergistota>Synergistia>Synergistales>Synergistaceae>unclassified_Synergistaceae	GCA_016756725.1	Synergistaceae_bacterium	JMJ93_05810	374580..378029	2053624	BREX-system-P-loop-protein-BrxC-[Synergistaceae-bacterium]
MCK9436263.1	Type1.BREX	<-SIGMA+OB-Fold+AAA-ATPase+Lon-Protease-BrxL<-PglZ<-N6-METHYLASE<-DUF6079-p*	474	Bacteria>Synergistota>Synergistia>Synergistales>Synergistaceae>unclassified_Synergistaceae	GCA_023229345.1	Synergistaceae_bacterium	MOQ12_03525	7446..8870	2053624	hypothetical-protein-[Synergistaceae-bacterium]
GHV28822.1	Type1.BREX	<-HicA<-?<-REase<-?<-?<-N6-METHYLASE<-N6-METHYLASE-p<-NERD<-OLD-ABC<-DUF6079*<-BrxB-BrxF-iSTAND<-BrxA-DNA-binding	1195	Bacteria>Synergistota>Synergistia>Synergistales>unclassified_Synergistales	GCA_026002695.1	Synergistales_bacterium	FACS1894167_06880	20211..23798	2268201	hypothetical-protein-FACS1894167_06880-[Synergistales-bacterium]
BCM94553.1	Type1.BREX	<-HTH_28+rve<-HTH_3||HTH+REase-><-GDResolvase+Recombinase+Zn_ribbon_recom<-?<-ArdCN<-SIGMA+OB-Fold+AAA-ATPase-BrxL<-PglZ<-DUF6079*<-BrxB-BrxF-iSTAND<-DUF4007<-N6-METHYLASE<-SIGMA-HTH	1371	Bacteria>Terrabacteria_group>Abditibacteriota>unclassified_Abditibacteriota	GCA_023189805.1	Abditibacteriota_bacterium	IAD21_06460	92337..96452	2783868	hypothetical-protein-IAD21_06460-[Abditibacteriota-bacterium]
MCB0987052.1	Type1.BREX	<-N6-METHYLASE-p<-DUF6079*<-BrxB-BrxF-iSTAND<-BrxA-DNA-binding<-?||?-><-Phage_integrase<-PIN<-HTH+MPTase<-?<-PolB	1154	Bacteria>Terrabacteria_group>Actinomycetota>Acidimicrobiia>Acidimicrobiales>unclassified_Acidimicrobiales	GCA_020439505.1	Acidimicrobiales_bacterium	KDB09_04685	1462..4926	2201156	BREX-system-P-loop-protein-BrxC-[Acidimicrobiales-bacterium]
WP_075374702.1	Type1.BREX	TetR-HTH->ABC-ATPase+SbcC->?->?-><-SIGMA+OB-Fold+AAA-ATPase+HKD-DNase-BrxL<-PglZ<-Schlafen+HATPase+wHTH<-SbcC<-N6-METHYLASE<-DUF6079*<-BrxB-BrxF-iSTAND<-BrxA-DNA-binding||PhoH->?->?->sGTP->?->MetJarc-HTH->PemK_toxin->	1172	Bacteria>Terrabacteria_group>Actinomycetota>Actinomycetes>Actinomycetales>Actinomycetaceae	-	Actinomyces_oris	BKH18_06090	96462..99980	544580	BREX-system-P-loop-protein-BrxC-[Actinomyces-oris]
WP_075392896.1	Type1.BREX	TetR-HTH->ABC-ATPase+SbcC->?->?-><-SIGMA+OB-Fold+AAA-ATPase+HKD-DNase-BrxL<-PglZ<-Schlafen+HATPase+wHTH<-N6-METHYLASE<-DUF6079*<-BrxB-BrxF-iSTAND<-BrxA-DNA-binding||PhoH->?->?->sGTP->?->MetJarc-HTH->PemK_toxin->	1172	Bacteria>Terrabacteria_group>Actinomycetota>Actinomycetes>Actinomycetales>Actinomycetaceae	-	Actinomyces_oris	BKH22_09300	69704..73222	544580	BREX-system-P-loop-protein-BrxC-[Actinomyces-oris]
WP_075371911.1	Type1.BREX	TetR-HTH->ABC-ATPase+SbcC->?->?-><-SIGMA+OB-Fold+AAA-ATPase+HKD-DNase-BrxL<-PglZ<-Schlafen+HATPase+wHTH<-SbcC<-N6-METHYLASE<-DUF6079*<-BrxB-BrxF-iSTAND<-BrxA-DNA-binding||PhoH->?->?->sGTP->?->MetJarc-HTH->PemK_toxin->	1172	Bacteria>Terrabacteria_group>Actinomycetota>Actinomycetes>Actinomycetales>Actinomycetaceae	-	Actinomyces_oris	BKH23_02505	283433..286951	544580	BREX-system-P-loop-protein-BrxC-[Actinomyces-oris]
WP_075400926.1	Type1.BREX	<-PemK_toxin<-MetJarc-HTH<-?<-sGTP<-?<-?<-PhoH||BrxA-DNA-binding->BrxB-BrxF-iSTAND->DUF6079*->N6-METHYLASE->SbcC->Schlafen+HATPase+wHTH->PglZ->SIGMA+OB-Fold+AAA-ATPase+HKD-DNase-BrxL-><-?<-?<-ABC-ATPase+SbcC<-TetR-HTH	1171	Bacteria>Terrabacteria_group>Actinomycetota>Actinomycetes>Actinomycetales>Actinomycetaceae	-	Actinomyces_oris	BKH24_00995	75611..79126	544580	BREX-system-P-loop-protein-BrxC-[Actinomyces-oris]
WP_075550941.1	Type1.BREX	TetR-HTH->ABC-ATPase+SbcC->?->?-><-SIGMA+OB-Fold+AAA-ATPase+HKD-DNase-BrxL<-PglZ<-Schlafen+HATPase+wHTH<-SbcC<-N6-METHYLASE<-DUF6079*<-BrxB-BrxF-iSTAND<-BrxA-DNA-binding||PhoH->?->?->sGTP->?->MetJarc-HTH->PemK_toxin->	1172	Bacteria>Terrabacteria_group>Actinomycetota>Actinomycetes>Actinomycetales>Actinomycetaceae	-	Actinomyces_oris	BKH26_09165	68268..71786	544580	BREX-system-P-loop-protein-BrxC-[Actinomyces-oris]
WP_075411517.1	Type1.BREX	TetR-HTH->ABC-ATPase+SbcC->?->?-><-SIGMA+OB-Fold+AAA-ATPase+HKD-DNase-BrxL<-PglZ<-Schlafen+HATPase+wHTH<-SbcC<-N6-METHYLASE<-DUF6079*<-BrxB-BrxF-iSTAND<-BrxA-DNA-binding||PhoH->?->?->sGTP->?->MetJarc-HTH->PemK_toxin->	1172	Bacteria>Terrabacteria_group>Actinomycetota>Actinomycetes>Actinomycetales>Actinomycetaceae	-	Actinomyces_oris	BKH31_05960	55043..58561	544580	BREX-system-P-loop-protein-BrxC-[Actinomyces-oris]
WP_141407194.1	Type1.BREX	HTH_3-><-PemK_toxin<-?<-sGTP<-?<-?<-PhoH||BrxA-DNA-binding->BrxB-BrxF-iSTAND->DUF6079*->N6-METHYLASE->SbcC->Schlafen+HATPase+wHTH->PglZ->SIGMA+OB-Fold+AAA-ATPase+HKD-DNase-BrxL-><-?<-?<-ABC-ATPase+SbcC<-TetR-HTH	1172	Bacteria>Terrabacteria_group>Actinomycetota>Actinomycetes>Actinomycetales>Actinomycetaceae	-	Actinomyces_oris	I6I08_06235	1496012..1499530	544580	BREX-system-P-loop-protein-BrxC-[Actinomyces-oris]
WP_119835580.1	Type1.BREX	HMA+HAD+HAD-><-MarR-HTH+PADR-HTH<-?||?->BrxA-DNA-binding->BrxB-BrxF-iSTAND->DUF6079*->N6-METHYLASE->SbcC->Schlafen+HATPase+wHTH->PglZ->SIGMA+OB-Fold+AAA-ATPase+HKD-DNase-BrxL->ABC-ATPase+SbcC->	1171	Bacteria>Terrabacteria_group>Actinomycetota>Actinomycetes>Actinomycetales>Actinomycetaceae	-	Actinomyces_sp._2119	D4740_02085	49121..52636	2321393	BREX-system-P-loop-protein-BrxC-[Actinomyces-sp.-2119]
AOS47539.1	Type1.BREX	<-LACI-HTH+PBPI<-?<-?||?->DOC+HTH->BrxA-DNA-binding->BrxB-BrxF-iSTAND->DUF6079*->N6-METHYLASE->OLD-ABC->TOPRIM->PglZ->SIGMA+OB-Fold+AAA-ATPase+HKD-DNase-BrxL-><-ABC-3<-ABC-ATPase+SbcC	1176	Bacteria>Terrabacteria_group>Actinomycetota>Actinomycetes>Actinomycetales>Actinomycetaceae	GCA_001746855.1	Pauljensenia_hongkongensis	BH719_06520	1584234..1587764	178339	hypothetical-protein-BH719_06520-[Pauljensenia-hongkongensis]
OFQ22233.1	Type1.BREX	<-ABC-ATPase+SbcC<-ABC-ATPase+SbcC<-?<-SIGMA+OB-Fold+AAA-ATPase+HKD-DNase-BrxL<-PglZ<-?<-OLD-ABC<-N6-METHYLASE<-DUF6079*<-BrxB-BrxF-iSTAND<-BrxA-DNA-binding	1173	Bacteria>Terrabacteria_group>Actinomycetota>Actinomycetes>Actinomycetales>Actinomycetaceae	GCA_001813985.1	Actinomyces_sp._HMSC062G12	HMPREF2946_04765	260177..263698	1739450	hypothetical-protein-HMPREF2946_04765-[Actinomyces-sp.-HMSC062G12]
OLO46937.1	Type1.BREX	TetR-HTH->ABC-ATPase+SbcC->?->?-><-SIGMA+OB-Fold+AAA-ATPase+HKD-DNase-BrxL<-PglZ<-Schlafen+HATPase+wHTH<-SbcC<-N6-METHYLASE<-DUF6079*<-BrxB-BrxF-iSTAND<-BrxA-DNA-binding||PhoH->?->?->sGTP->?->MetJarc-HTH->PemK_toxin->	1172	Bacteria>Terrabacteria_group>Actinomycetota>Actinomycetes>Actinomycetales>Actinomycetaceae	GCA_001937365.1	Actinomyces_oris	BKH31_05960	55043..58561	544580	hypothetical-protein-BKH31_05960-[Actinomyces-oris]
OLO54967.1	Type1.BREX	TetR-HTH->ABC-ATPase+SbcC->?->?-><-SIGMA+OB-Fold+AAA-ATPase+HKD-DNase-BrxL<-PglZ<-Schlafen+HATPase+wHTH<-SbcC<-N6-METHYLASE<-DUF6079*<-BrxB-BrxF-iSTAND<-BrxA-DNA-binding||PhoH->?->?->sGTP->?->MetJarc-HTH->PemK_toxin->	1172	Bacteria>Terrabacteria_group>Actinomycetota>Actinomycetes>Actinomycetales>Actinomycetaceae	GCA_001937415.1	Actinomyces_oris	BKH26_09165	68268..71786	544580	hypothetical-protein-BKH26_09165-[Actinomyces-oris]
OLO62906.1	Type1.BREX	<-PemK_toxin<-MetJarc-HTH<-?<-sGTP<-?<-?<-PhoH||BrxA-DNA-binding->BrxB-BrxF-iSTAND->DUF6079*->N6-METHYLASE->SbcC->Schlafen+HATPase+wHTH->PglZ->SIGMA+OB-Fold+AAA-ATPase+HKD-DNase-BrxL-><-?<-?<-ABC-ATPase+SbcC<-TetR-HTH	1171	Bacteria>Terrabacteria_group>Actinomycetota>Actinomycetes>Actinomycetales>Actinomycetaceae	GCA_001937425.1	Actinomyces_oris	BKH24_00995	75611..79126	544580	hypothetical-protein-BKH24_00995-[Actinomyces-oris]
OLO63895.1	Type1.BREX	TetR-HTH->ABC-ATPase+SbcC->?->?-><-SIGMA+OB-Fold+AAA-ATPase+HKD-DNase-BrxL<-PglZ<-Schlafen+HATPase+wHTH<-SbcC<-N6-METHYLASE<-DUF6079*<-BrxB-BrxF-iSTAND<-BrxA-DNA-binding||PhoH->?->?->sGTP->?->MetJarc-HTH->PemK_toxin->	1172	Bacteria>Terrabacteria_group>Actinomycetota>Actinomycetes>Actinomycetales>Actinomycetaceae	GCA_001937485.1	Actinomyces_oris	BKH23_02505	283433..286951	544580	hypothetical-protein-BKH23_02505-[Actinomyces-oris]
OLO77585.1	Type1.BREX	TetR-HTH->ABC-ATPase+SbcC->?->?-><-SIGMA+OB-Fold+AAA-ATPase+HKD-DNase-BrxL<-PglZ<-Schlafen+HATPase+wHTH<-SbcC<-N6-METHYLASE<-DUF6079*<-BrxB-BrxF-iSTAND<-BrxA-DNA-binding||PhoH->?->?->sGTP->?->MetJarc-HTH->PemK_toxin->	1172	Bacteria>Terrabacteria_group>Actinomycetota>Actinomycetes>Actinomycetales>Actinomycetaceae	GCA_001937535.1	Actinomyces_oris	BKH18_06090	96462..99980	544580	hypothetical-protein-BKH18_06090-[Actinomyces-oris]
OLO63014.1	Type1.BREX	TetR-HTH->ABC-ATPase+SbcC->?->?-><-SIGMA+OB-Fold+AAA-ATPase+HKD-DNase-BrxL<-PglZ<-Schlafen+HATPase+wHTH<-N6-METHYLASE<-DUF6079*<-BrxB-BrxF-iSTAND<-BrxA-DNA-binding||PhoH->?->?->sGTP->?->MetJarc-HTH->PemK_toxin->	1172	Bacteria>Terrabacteria_group>Actinomycetota>Actinomycetes>Actinomycetales>Actinomycetaceae	GCA_001937725.1	Actinomyces_oris	BKH22_09300	69704..73222	544580	hypothetical-protein-BKH22_09300-[Actinomyces-oris]
RJF44065.1	Type1.BREX	HMA+HAD+HAD-><-MarR-HTH+PADR-HTH<-?||?->BrxA-DNA-binding->BrxB-BrxF-iSTAND->DUF6079*->N6-METHYLASE->SbcC->Schlafen+HATPase+wHTH->PglZ->SIGMA+OB-Fold+AAA-ATPase+HKD-DNase-BrxL->ABC-ATPase+SbcC->	1171	Bacteria>Terrabacteria_group>Actinomycetota>Actinomycetes>Actinomycetales>Actinomycetaceae	GCA_003591215.1	Actinomyces_sp._2119	D4740_02085	49121..52636	2321393	BREX-system-P-loop-protein-BrxC-[Actinomyces-sp.-2119]
MBF0967807.1	Type1.BREX	BrxB-BrxF-iSTAND->DUF6079*->N6-METHYLASE->OLD-ABC->TOPRIM->PglZ->SIGMA+OB-Fold+AAA-ATPase+HKD-DNase-BrxL-><-?||?->ABC-ATPase+SbcC+ABC-ATPase+SbcC->	1169	Bacteria>Terrabacteria_group>Actinomycetota>Actinomycetes>Actinomycetales>Actinomycetaceae	GCA_015259445.1	Actinomyces_sp.	HXK08_00790	718..4227	29317	BREX-system-P-loop-protein-BrxC-[Actinomyces-sp.]
QQC41011.1	Type1.BREX	HTH_3-><-PemK_toxin<-?<-sGTP<-?<-?<-PhoH||BrxA-DNA-binding->BrxB-BrxF-iSTAND->DUF6079*->N6-METHYLASE->SbcC->Schlafen+HATPase+wHTH->PglZ->SIGMA+OB-Fold+AAA-ATPase+HKD-DNase-BrxL-><-?<-?<-ABC-ATPase+SbcC<-TetR-HTH	1172	Bacteria>Terrabacteria_group>Actinomycetota>Actinomycetes>Actinomycetales>Actinomycetaceae	GCA_016127955.1	Actinomyces_oris	I6I08_06235	1496012..1499530	544580	BREX-system-P-loop-protein-BrxC-[Actinomyces-oris]
MBS6968676.1	Type1.BREX	DEOR-HTH+ISOCOT->?-><-SIGMA+OB-Fold+AAA-ATPase+HKD-DNase-BrxL<-PglZ<-TOPRIM<-OLD-ABC<-N6-METHYLASE<-DUF6079*<-BrxB-BrxF-iSTAND<-BrxA-DNA-binding	1169	Bacteria>Terrabacteria_group>Actinomycetota>Actinomycetes>Actinomycetales>Actinomycetaceae	GCA_018381115.1	Actinomyces_sp.	KH140_06985	13628..17137	29317	BREX-system-P-loop-protein-BrxC-[Actinomyces-sp.]
MCI1675766.1	Type1.BREX	ABhydrolase-><-?<-?<-?||?->Calcineurin->tRNA-><-MarR-HTH+TipAS||BrxA-DNA-binding->BrxB-BrxF-iSTAND->DUF6079*->N6-METHYLASE->	1164	Bacteria>Terrabacteria_group>Actinomycetota>Actinomycetes>Actinomycetales>Actinomycetaceae	GCA_022648165.1	Ancrocorticia_sp.	LKI28_05730	185696..189190	2593684	BREX-system-P-loop-protein-BrxC-[Ancrocorticia-sp.]
MCI1641321.1	Type1.BREX	sGTP->?->?->?->His_Phos_1->tRNA->BrxA-DNA-binding->BrxB-BrxF-iSTAND->DUF6079*->?->N6-METHYLASE-p->	1167	Bacteria>Terrabacteria_group>Actinomycetota>Actinomycetes>Actinomycetales>Actinomycetaceae	GCA_022648365.1	Actinomyces_sp.	LKI38_02150	177491..180994	29317	BREX-system-P-loop-protein-BrxC-[Actinomyces-sp.]
MCI6557834.1	Type1.BREX	ABhydrolase-><-?<-SIGMA+OB-Fold+AAA-ATPase+HKD-DNase-BrxL<-PglZ<-?<-N6-METHYLASE-p<-Phage_int_SAM_3+Phage_integrase||N6-METHYLASE-p-><-N6-METHYLASE<-DUF6079*<-BrxB-BrxF-iSTAND<-BrxA-DNA-binding||SF1-SF2-Helicase+DUF3418->	1160	Bacteria>Terrabacteria_group>Actinomycetota>Actinomycetes>Actinomycetales>Actinomycetaceae	GCA_022780845.1	Schaalia_hyovaginalis	MR422_09445	69432..72914	29316	BREX-system-P-loop-protein-BrxC-[Schaalia-hyovaginalis]
MCI7671412.1	Type1.BREX	<-SIGMA+OB-Fold+AAA-ATPase+HKD-DNase-BrxL<-PglZ<-?<-N6-METHYLASE-p<-Phage_int_SAM_3+Phage_integrase||N6-METHYLASE-p->DDE_Tnp_IS240->ParA-Soj-PloopNTPase-><-N6-METHYLASE-p<-DUF6079*<-BrxB-BrxF-iSTAND<-BrxA-DNA-binding||SF1-SF2-Helicase+DUF3418-><-Glyco_hydro_36N+Melibiase<-?||?->?->?->LACI-HTH+PBPI->	1161	Bacteria>Terrabacteria_group>Actinomycetota>Actinomycetes>Actinomycetales>Actinomycetaceae	GCA_022786785.1	Schaalia_hyovaginalis	MSS20_03495	24103..27588	29316	BREX-system-P-loop-protein-BrxC-[Schaalia-hyovaginalis]
WP_009648402.1	Type1.BREX	REase-><-?<-DDE_Tnp<-DDE_Tnp<-?||?->BrxA-DNA-binding->BrxB-BrxF-iSTAND->DUF6079*->N6-METHYLASE->RVT->PglZ->SIGMA+OB-Fold+AAA-ATPase+HKD-DNase-BrxL->HEPN->	1169	Bacteria>Terrabacteria_group>Actinomycetota>Actinomycetes>Actinomycetales>Actinomycetaceae	GCF_000278725.1	Actinomyces_sp._ICM47	HMPREF1136_RS05845	15295..18804	936548	BREX-system-P-loop-protein-BrxC-[Actinomyces-sp.-ICM47]
WP_024332155.1	Type1.BREX	HTH+REase->?->35exo->BrxA-DNA-binding->BrxB-BrxF-iSTAND->DUF6079*->N6-METHYLASE->PglZ->SIGMA+OB-Fold+AAA-ATPase+HKD-DNase-BrxL->?->?->?-><-?||?-><-ABhydrolase	1184	Bacteria>Terrabacteria_group>Actinomycetota>Actinomycetes>Actinomycetales>Actinomycetaceae	GCF_000420405.1	Winkia_neuii	G437_RS0108590	20470..24024	33007	BREX-system-P-loop-protein-BrxC-[Winkia-neuii]
WP_026406950.1	Type1.BREX	TetR-HTH->ABC-ATPase+SbcC->?->?-><-SIGMA+OB-Fold+AAA-ATPase+HKD-DNase-BrxL<-PglZ<-Schlafen+HATPase+wHTH<-SbcC<-N6-METHYLASE<-DUF6079*<-BrxB-BrxF-iSTAND<-BrxA-DNA-binding||PhoH->?->?->sGTP->Peptidase_S9->	1174	Bacteria>Terrabacteria_group>Actinomycetota>Actinomycetes>Actinomycetales>Actinomycetaceae	GCF_000429265.1	Actinomyces_gerencseriae	G448_RS0104040	223321..226845	52769	BREX-system-P-loop-protein-BrxC-[Actinomyces-gerencseriae]
WP_173647185.1	Type1.BREX	<-LACI-HTH+PBPI<-?<-?||?->DOC+HTH->BrxA-DNA-binding->BrxB-BrxF-iSTAND->DUF6079*->N6-METHYLASE->OLD-ABC->TOPRIM->PglZ->SIGMA+OB-Fold+AAA-ATPase+HKD-DNase-BrxL-><-ABC-3<-ABC-ATPase+SbcC	1174	Bacteria>Terrabacteria_group>Actinomycetota>Actinomycetes>Actinomycetales>Actinomycetaceae	GCF_001746855.1	Pauljensenia_hongkongensis	BH719_RS06520	1584240..1587764	178339	BREX-system-P-loop-protein-BrxC-[Pauljensenia-hongkongensis]
WP_256693306.1	Type1.BREX	TetR-HTH->ABC-ATPase+SbcC->?->?-><-SIGMA+OB-Fold+AAA-ATPase+HKD-DNase-BrxL<-PglZ<-Schlafen+HATPase+wHTH<-SbcC<-N6-METHYLASE<-DUF6079*<-BrxB-BrxF-iSTAND<-BrxA-DNA-binding||PhoH->?->?->sGTP->?->PemK_toxin-><-HTH_3	1175	Bacteria>Terrabacteria_group>Actinomycetota>Actinomycetes>Actinomycetales>Actinomycetaceae	GCF_001937365.1	Actinomyces_oris	BKH31_RS05960	55043..58570	544580	BREX-system-P-loop-protein-BrxC-[Actinomyces-oris]
WP_256693847.1	Type1.BREX	TetR-HTH->ABC-ATPase+SbcC->?->?-><-SIGMA+OB-Fold+AAA-ATPase+HKD-DNase-BrxL<-PglZ<-Schlafen+HATPase+wHTH<-SbcC<-N6-METHYLASE<-DUF6079*<-BrxB-BrxF-iSTAND<-BrxA-DNA-binding||PhoH->?->?->sGTP->?->MetJarc-HTH->PemK_toxin->	1175	Bacteria>Terrabacteria_group>Actinomycetota>Actinomycetes>Actinomycetales>Actinomycetaceae	GCF_001937415.1	Actinomyces_oris	BKH26_RS09145	68268..71795	544580	BREX-system-P-loop-protein-BrxC-[Actinomyces-oris]
WP_256699678.1	Type1.BREX	HTH_3-><-PemK_toxin<-?<-sGTP<-?<-?<-PhoH||BrxA-DNA-binding->BrxB-BrxF-iSTAND->DUF6079*->N6-METHYLASE->SbcC->Schlafen+HATPase+wHTH->PglZ->SIGMA+OB-Fold+AAA-ATPase+HKD-DNase-BrxL-><-?<-?<-ABC-ATPase+SbcC<-TetR-HTH	1174	Bacteria>Terrabacteria_group>Actinomycetota>Actinomycetes>Actinomycetales>Actinomycetaceae	GCF_001937425.1	Actinomyces_oris	BKH24_RS00995	75602..79126	544580	BREX-system-P-loop-protein-BrxC-[Actinomyces-oris]
WP_256699054.1	Type1.BREX	TetR-HTH->ABC-ATPase+SbcC->?->?-><-SIGMA+OB-Fold+AAA-ATPase+HKD-DNase-BrxL<-PglZ<-Schlafen+HATPase+wHTH<-SbcC<-N6-METHYLASE<-DUF6079*<-BrxB-BrxF-iSTAND<-BrxA-DNA-binding||PhoH->?->?->sGTP->?->PemK_toxin-><-HTH_3	1175	Bacteria>Terrabacteria_group>Actinomycetota>Actinomycetes>Actinomycetales>Actinomycetaceae	GCF_001937485.1	Actinomyces_oris	BKH23_RS02505	283433..286960	544580	BREX-system-P-loop-protein-BrxC-[Actinomyces-oris]
WP_256694375.1	Type1.BREX	TetR-HTH->ABC-ATPase+SbcC->?->?-><-SIGMA+OB-Fold+AAA-ATPase+HKD-DNase-BrxL<-PglZ<-Schlafen+HATPase+wHTH<-SbcC<-N6-METHYLASE<-DUF6079*<-BrxB-BrxF-iSTAND<-BrxA-DNA-binding||PhoH->?->?->sGTP->?->MetJarc-HTH->PemK_toxin->	1175	Bacteria>Terrabacteria_group>Actinomycetota>Actinomycetes>Actinomycetales>Actinomycetaceae	GCF_001937535.1	Actinomyces_oris	BKH18_RS06085	96462..99989	544580	BREX-system-P-loop-protein-BrxC-[Actinomyces-oris]
WP_256697766.1	Type1.BREX	<-MetJarc-HTH||TetR-HTH->ABC-ATPase+SbcC->?->?-><-SIGMA+OB-Fold+AAA-ATPase+HKD-DNase-BrxL<-PglZ<-Schlafen+HATPase+wHTH<-N6-METHYLASE<-DUF6079*<-BrxB-BrxF-iSTAND<-BrxA-DNA-binding||PhoH->?->?->sGTP->?->PemK_toxin-><-HTH_3	1175	Bacteria>Terrabacteria_group>Actinomycetota>Actinomycetes>Actinomycetales>Actinomycetaceae	GCF_001937725.1	Actinomyces_oris	BKH22_RS09310	69704..73231	544580	BREX-system-P-loop-protein-BrxC-[Actinomyces-oris]
WP_076066523.1	Type1.BREX	<-AraC-HTH+AraC-HTH||?->NAD_binding_10-><-SIGMA+OB-Fold+AAA-ATPase+HKD-DNase-BrxL<-PglZ<-Schlafen+HATPase+wHTH<-SbcC<-N6-METHYLASE<-DUF6079*<-BrxB-BrxF-iSTAND<-BrxA-DNA-binding||PhoH->?->?->sGTP->?-><-?||TetR-HTH->	1174	Bacteria>Terrabacteria_group>Actinomycetota>Actinomycetes>Actinomycetales>Actinomycetaceae	GCF_001956485.1	Actinomyces_naeslundii	BKH36_RS03225	74556..78080	1655	BREX-system-P-loop-protein-BrxC-[Actinomyces-naeslundii]
WP_108716712.1	Type1.BREX	<-SIGMA+OB-Fold+AAA-ATPase+HKD-DNase-BrxL<-PglZ<-?<-Schlafen-p<-SbcC<-N6-METHYLASE-p<-N6-METHYLASE<-DUF6079*<-BrxB-BrxF-iSTAND<-BrxA-DNA-binding||Schlafen+HATPase+wHTH-p->Schlafen+HATPase+wHTH-p-><-tRNA<-?<-?<-ABC-ATPase+SbcC||Sensor+HisKA_3+HATPase_c->REC+LuxR-HTH->	1174	Bacteria>Terrabacteria_group>Actinomycetota>Actinomycetes>Actinomycetales>Actinomycetaceae	GCF_003073475.1	Actinobaculum_sp._313	DDD63_RS08615	1982694..1986218	2495645	BREX-system-P-loop-protein-BrxC-[Actinobaculum-sp.-313]
WP_109093701.1	Type1.BREX	<-PIN<-PhdYeFM_antitox||?->REase-><-ParB+HNH<-SIGMA+OB-Fold+AAA-ATPase+HKD-DNase-BrxL<-PglZ<-Schlafen+HATPase+wHTH<-N6-METHYLASE<-DUF6079*<-BrxB-BrxF-iSTAND<-BrxA-DNA-binding||?-><-?<-?<-LACI-HTH+PBPI<-Glyco_hydro_1	1170	Bacteria>Terrabacteria_group>Actinomycetota>Actinomycetes>Actinomycetales>Actinomycetaceae	GCF_003123745.1	Ancrocorticia_populi	DD236_RS06730	275849..279361	2175228	BREX-system-P-loop-protein-BrxC-[Ancrocorticia-populi]
WP_259390601.1	Type1.BREX	HMA+HAD+HAD-><-MarR-HTH+PADR-HTH<-?||?->BrxA-DNA-binding->BrxB-BrxF-iSTAND->DUF6079*->N6-METHYLASE->SbcC->Schlafen+HATPase+wHTH->PglZ->SIGMA+OB-Fold+AAA-ATPase+HKD-DNase-BrxL->ABC-ATPase+SbcC->	1174	Bacteria>Terrabacteria_group>Actinomycetota>Actinomycetes>Actinomycetales>Actinomycetaceae	GCF_003591215.1	Actinomyces_sp._2119	D4740_RS02085	49112..52636	2321393	BREX-system-P-loop-protein-BrxC-[Actinomyces-sp.-2119]
WP_124851247.1	Type1.BREX	<-SIGMA+OB-Fold+AAA-ATPase+HKD-DNase-BrxL<-PglZ<-HTH+REase<-N6-METHYLASE-p<-Phage_int_SAM_3+Phage_integrase||N6-METHYLASE-p-><-N6-METHYLASE<-DUF6079*<-BrxB-BrxF-iSTAND<-BrxA-DNA-binding<-?||?->?->?->SbcC+SbcC->	1170	Bacteria>Terrabacteria_group>Actinomycetota>Actinomycetes>Actinomycetales>Actinomycetaceae	GCF_003858495.1	Buchananella_hordeovulneris	EII12_RS02040	65131..68643	52770	BREX-system-P-loop-protein-BrxC-[Buchananella-hordeovulneris]
WP_126145915.1	Type1.BREX	<-SIGMA+OB-Fold+AAA-ATPase+HKD-DNase-BrxL<-PglZ<-?<-SbcC<-N6-METHYLASE<-DUF6079*<-BrxB-BrxF-iSTAND<-BrxA-DNA-binding||Schlafen+HATPase+wHTH-><-MPTase<-HTH_3||HTH_3->	1174	Bacteria>Terrabacteria_group>Actinomycetota>Actinomycetes>Actinomycetales>Actinomycetaceae	GCF_003957265.1	Actinobaculum_sp._352	EXN07_RS06065	220063..223587	2490946	BREX-system-P-loop-protein-BrxC-[Actinobaculum-sp.-352]
WP_243118374.1	Type1.BREX	<-ABC-ATPase+SbcC<-SIGMA+OB-Fold+AAA-ATPase+HKD-DNase-BrxL<-PglZ<-Schlafen+HATPase+wHTH<-?<-N6-METHYLASE<-DUF6079*<-BrxB-BrxF-iSTAND<-BrxA-DNA-binding<-?||?->MarR-HTH+PADR-HTH-><-HMA+HAD+HAD	1174	Bacteria>Terrabacteria_group>Actinomycetota>Actinomycetes>Actinomycetales>Actinomycetaceae	GCF_004010955.1	Actinomyces_wuliandei	CWS50_RS00410	93588..97112	2057743	BREX-system-P-loop-protein-BrxC-[Actinomyces-wuliandei]
WP_247595282.1	Type1.BREX	ADH_N+ADH_zinc_N->TetR-HTH->ABC-ATPase+SbcC->?->?-><-SIGMA+OB-Fold+AAA-ATPase+HKD-DNase-BrxL<-PglZ<-Schlafen+HATPase+wHTH<-REase<-N6-METHYLASE<-DUF6079*<-BrxB-BrxF-iSTAND<-BrxA-DNA-binding||?->PhoH->?->?->sGTP->Peptidase_S9->	1174	Bacteria>Terrabacteria_group>Actinomycetota>Actinomycetes>Actinomycetales>Actinomycetaceae	GCF_004796555.1	Actinomyces_procaprae	FAQ94_RS07530	102106..105630	2560010	BREX-system-P-loop-protein-BrxC-[Actinomyces-procaprae]
WP_167202365.1	Type1.BREX	<-GNTR-HTH+UTRA||?->?->?->SIR2->BrxA-DNA-binding->BrxB-BrxF-iSTAND->DUF6079*->N6-METHYLASE->SbcC->Schlafen+HATPase+wHTH->PglZ->SIGMA+OB-Fold+AAA-ATPase+HKD-DNase-BrxL->	1171	Bacteria>Terrabacteria_group>Actinomycetota>Actinomycetes>Actinomycetales>Actinomycetaceae	GCF_011751985.1	Actinomyces_respiraculi	HCR20_RS03210	129007..132522	2744574	BREX-system-P-loop-protein-BrxC-[Actinomyces-respiraculi]
WP_182050348.1	Type1.BREX	HTH->Arm-DNA-bind_4+Phage_int_SAM_3+Phage_integrase->TraJ-RHH-><-?<-ZnR+ZnR<-SIGMA+OB-Fold+AAA-ATPase+Lon-Protease-BrxL<-PglZ<-DOC+HTH<-N6-METHYLASE<-DUF6079*<-BrxB-BrxF-iSTAND<-BrxA-DNA-binding<-TraJ-RHH||?->?->XIS-HTH-><-REase+Bacteriocin_IIc<-REase	1193	Bacteria>Terrabacteria_group>Actinomycetota>Actinomycetes>Actinomycetales>Actinomycetaceae	GCF_014041995.1	Changpingibacter_yushuensis	H2017_RS03430	798134..801715	2758440	BREX-system-P-loop-protein-BrxC-[Changpingibacter-yushuensis]
WP_184451418.1	Type1.BREX	Glyco_hydro_36N+Melibiase-><-SF1-SF2-Helicase+DUF3418||?->BrxA-DNA-binding->BrxB-BrxF-iSTAND->DUF6079*->N6-METHYLASE-><-N6-METHYLASE-p||Phage_int_SAM_3+Phage_integrase->N6-METHYLASE-p->?->PglZ->SIGMA+OB-Fold+AAA-ATPase+HKD-DNase-BrxL->	1160	Bacteria>Terrabacteria_group>Actinomycetota>Actinomycetes>Actinomycetales>Actinomycetaceae	GCF_014208035.1	Schaalia_hyovaginalis	HD592_RS01490	338165..341647	29316	BREX-system-P-loop-protein-BrxC-[Schaalia-hyovaginalis]
WP_187994004.1	Type1.BREX	<-NAD_binding_10||?->ADH_N+ADH_zinc_N-><-?||TetR-HTH-><-HipA_C<-?||BrxA-DNA-binding->BrxB-BrxF-iSTAND->DUF6079*->N6-METHYLASE-><-N6-METHYLASE-p||Phage_int_SAM_3+Phage_integrase->N6-METHYLASE-p->Schlafen+HATPase+wHTH->HATPase+wHTH->PglZ->SIGMA+OB-Fold+AAA-ATPase+HKD-DNase-BrxL->	1173	Bacteria>Terrabacteria_group>Actinomycetota>Actinomycetes>Actinomycetales>Actinomycetaceae	GCF_014525425.1	Schaalia_sp._JY-X159	IDL90_RS00760	170975..174496	2758575	BREX-system-P-loop-protein-BrxC-[Schaalia-sp.-JY-X159]
WP_166855918.1	Type1.BREX	<-SIGMA+OB-Fold+AAA-ATPase+HKD-DNase-BrxL<-PglZ<-Schlafen+HATPase+wHTH<-SbcC<-N6-METHYLASE<-DUF6079*<-BrxB-BrxF-iSTAND<-BrxA-DNA-binding<-SIR2<-?<-?<-?||GNTR-HTH+UTRA->	1171	Bacteria>Terrabacteria_group>Actinomycetota>Actinomycetes>Actinomycetales>Actinomycetaceae	GCF_014595995.2	Actinomyces	ID810_RS09115	2190565..2194080	1654	MULTISPECIES:-BREX-system-P-loop-protein-BrxC-[Actinomyces]
WP_232622595.1	Type1.BREX	HTH_3-><-PemK_toxin<-?<-sGTP<-?<-?<-PhoH||BrxA-DNA-binding->BrxB-BrxF-iSTAND->DUF6079*->N6-METHYLASE->SbcC->Schlafen+HATPase+wHTH->PglZ->SIGMA+OB-Fold+AAA-ATPase+HKD-DNase-BrxL-><-?<-?<-ABC-ATPase+SbcC<-TetR-HTH	1175	Bacteria>Terrabacteria_group>Actinomycetota>Actinomycetes>Actinomycetales>Actinomycetaceae	GCF_016127955.1	Actinomyces_oris	I6I08_RS06235	1496003..1499530	544580	BREX-system-P-loop-protein-BrxC-[Actinomyces-oris]
WP_271289309.1	Type1.BREX	TetR-HTH->ABC-ATPase+SbcC->?->?-><-SIGMA+OB-Fold+AAA-ATPase+HKD-DNase-BrxL<-PglZ<-Schlafen+HATPase+wHTH<-?<-N6-METHYLASE<-DUF6079*<-BrxB-BrxF-iSTAND<-BrxA-DNA-binding||PhoH->?->?->sGTP->?->MetJarc-HTH->PemK_toxin->	1175	Bacteria>Terrabacteria_group>Actinomycetota>Actinomycetes>Actinomycetales>Actinomycetaceae	GCF_027945475.1	Actinomyces_oris	PGE45_RS05470	1261783..1265310	544580	BREX-system-P-loop-protein-BrxC-[Actinomyces-oris]
WP_272432709.1	Type1.BREX	ABhydrolase-><-?<-SIGMA+OB-Fold+AAA-ATPase+HKD-DNase-BrxL<-PglZ<-?<-N6-METHYLASE-p<-Phage_int_SAM_3+Phage_integrase||N6-METHYLASE-p-><-N6-METHYLASE<-DUF6079*<-BrxB-BrxF-iSTAND<-BrxA-DNA-binding||SF1-SF2-Helicase+DUF3418-><-Glyco_hydro_36N+Melibiase<-?||?->?->?->LACI-HTH+PBPI->	1160	Bacteria>Terrabacteria_group>Actinomycetota>Actinomycetes>Actinomycetales>Actinomycetaceae	GCF_028437565.1	Actinomyces_sp._B33	M3T53_RS01725	406019..409501	2942131	BREX-system-P-loop-protein-BrxC-[Actinomyces-sp.-B33]
WP_126382192.1	Type1.BREX	<-SIGMA+OB-Fold+AAA-ATPase+HKD-DNase-BrxL<-PglZ<-Schlafen+HATPase+wHTH<-REase<-N6-METHYLASE<-DUF6079*<-BrxB-BrxF-iSTAND<-BrxA-DNA-binding<-SIR2<-?<-?<-?||GNTR-HTH+UTRA->	1170	Bacteria>Terrabacteria_group>Actinomycetota>Actinomycetes>Actinomycetales>Actinomycetaceae	GCF_900637165.1	Actinomyces_howellii	GYB74_RS04555	58123..61635	52771	BREX-system-P-loop-protein-BrxC-[Actinomyces-howellii]
WP_004808447.1	Type1.BREX	<-RNase<-?||?->?->?->?->HTH+REase->BrxA-DNA-binding->BrxB-BrxF-iSTAND->DUF6079*->N6-METHYLASE->PglZ->SIGMA+OB-Fold+AAA-ATPase+HKD-DNase-BrxL-><-?<-?<-ABhydrolase<-DOC+HTH<-?||ZnR->	1184	Bacteria>Terrabacteria_group>Actinomycetota>Actinomycetes>Actinomycetales	GCF_028201595.1	Actinomycetaceae	PIG85_RS08980	1926978..1930532	2049	MULTISPECIES:-BREX-system-P-loop-protein-BrxC-[Actinomycetaceae]
MBK6301657.1	Type1.BREX	<-VirB11+MuB-ATPase||?-><-?<-Phage_integrase+Phage_integrase<-HTH_26<-Phage_integrase<-PHP+SbcC<-SIGMA+OB-Fold+AAA-ATPase+Lon-Protease-BrxL<-PglZ<-N6-METHYLASE<-DUF6079*<-BrxB-BrxF-iSTAND<-BrxA-DNA-binding<-XIS-HTH<-ABhydrolase<-?<-MPTase<-HTH||ArdCN+MPTase->Primpol+PriCT1->	1152	Bacteria>Terrabacteria_group>Actinomycetota>Actinomycetes>Actinomycetales>unclassified_Actinomycetales	GCA_016705565.1	Actinomycetales_bacterium	IPF40_11645	85880..89338	1911520	BREX-system-P-loop-protein-BrxC-[Actinomycetales-bacterium]
MBK6956672.1	Type1.BREX	<-PglZ<-N6-METHYLASE<-DUF6079-p*<-DUF6079-p<-BrxB-BrxF-iSTAND<-BrxA-DNA-binding<-tRNA||?->REC+OmpR-HTH->HAMP+HISKIN+ST-Hiskin->	557	Bacteria>Terrabacteria_group>Actinomycetota>Actinomycetes>Actinomycetales>unclassified_Actinomycetales	GCA_016708855.1	Actinomycetales_bacterium	IPH27_14930	7874..9547	1911520	hypothetical-protein-[Actinomycetales-bacterium]
MBK6956673.1	Type1.BREX	<-PglZ<-N6-METHYLASE<-DUF6079-p<-DUF6079-p*<-BrxB-BrxF-iSTAND<-BrxA-DNA-binding<-tRNA||?->REC+OmpR-HTH->HAMP+HISKIN+ST-Hiskin->	595	Bacteria>Terrabacteria_group>Actinomycetota>Actinomycetes>Actinomycetales>unclassified_Actinomycetales	GCA_016708855.1	Actinomycetales_bacterium	IPH27_14935	9553..11340	1911520	BREX-system-P-loop-protein-BrxC-[Actinomycetales-bacterium]
WP_129869215.1	Type1.BREX	MODE-HTH+PBPII->YTCD-HTH-><-HTH_3<-RelE-ParE<-ACET+ACET<-ZnR<-ZnR||BrxA-DNA-binding->BrxB-BrxF-iSTAND->DUF6079*->N6-METHYLASE->PglZ->SIGMA+OB-Fold+AAA-ATPase+HKD-DNase-BrxL-><-PRTase	1214	Bacteria>Terrabacteria_group>Actinomycetota>Actinomycetes>Bifidobacteriales>Bifidobacteriaceae	-	Bifidobacterium_pseudolongum	PG1791B_RS04610	183618..187262	1694	BREX-system-P-loop-protein-BrxC-[Bifidobacterium-pseudolongum]
KFI62072.1	Type1.BREX	<-XIS-HTH+GDResolvase||REase->?->BrxA-DNA-binding->Schlafen+HATPase+wHTH+HTH-><-XIS-HTH||BrxA-DNA-binding->BrxB-BrxF-iSTAND->DUF6079*->HEPN->N6-METHYLASE->PglZ->SIGMA+OB-Fold+AAA-ATPase+HKD-DNase-BrxL-><-?<-ZnR+GP<-?<-MPTase	1219	Bacteria>Terrabacteria_group>Actinomycetota>Actinomycetes>Bifidobacteriales>Bifidobacteriaceae	GCA_000741575.1	Bifidobacterium_cuniculi	BCUN_1388	51093..54752	1688	putative-ATP-binding-protein-[Bifidobacterium-cuniculi]
OQM55032.1	Type1.BREX	<-HIN-HTH<-N6-METHYLASE-p<-DUF6079*<-DUF6079-p<-BrxB-BrxF-iSTAND<-BrxA-DNA-binding<-?||ACET-><-?||HTH-><-35exo+BRCT	1059	Bacteria>Terrabacteria_group>Actinomycetota>Actinomycetes>Bifidobacteriales>Bifidobacteriaceae	GCA_002075955.1	Bifidobacterium_dentium	B5790_1954	1122..4301	1689	hypothetical-protein-B5790_1954-[Bifidobacterium-dentium]
PJM72971.1	Type1.BREX	MACRODOMAIN-><-?<-SIGMA+OB-Fold+AAA-ATPase+HKD-DNase-BrxL<-PglZ<-?||OLD-ABC->TOPRIM-><-N6-METHYLASE<-DUF6079*<-BrxB-BrxF-iSTAND<-BrxA-DNA-binding||?->?-><-?<-?<-JetB	1243	Bacteria>Terrabacteria_group>Actinomycetota>Actinomycetes>Bifidobacteriales>Bifidobacteriaceae	GCA_002802875.1	Bifidobacterium_primatium	CS006_06875	170776..174507	2045438	hypothetical-protein-CS006_06875-[Bifidobacterium-primatium]
PKC83363.1	Type1.BREX	<-HTH_Tnp_1||RelB->N6-METHYLASE->CR-ATPase5+REase-p-><-CR-Kinase<-?||BrxA-DNA-binding->BrxB-BrxF-iSTAND->DUF6079-p->DUF6079-p*->N6-METHYLASE->N6-METHYLASE->SLATT->SMODS->Schlafen+wHTH->PglZ->Lon_2->SIGMA+OB-Fold+AAA-ATPase+HKD-DNase-BrxL->TIR->	977	Bacteria>Terrabacteria_group>Actinomycetota>Actinomycetes>Bifidobacteriales>Bifidobacteriaceae	GCA_002832945.1	Bifidobacterium_longum	DPC6317_0876	237584..240517	216816	hypothetical-protein-DPC6317_0876-[Bifidobacterium-longum]
PKV03451.1	Type1.BREX	<-DUF6079-p||DUF6079-p*->N6-METHYLASE->PglZ->SIGMA+OB-Fold+AAA-ATPase+HKD-DNase-BrxL-><-HEPN<-PRTase	839	Bacteria>Terrabacteria_group>Actinomycetota>Actinomycetes>Bifidobacteriales>Bifidobacteriaceae	GCA_002846775.1	Bifidobacterium_pseudolongum_subsp._globosum	CQR50_1159	592..3111	1690	hypothetical-protein-CQR50_1159-[Bifidobacterium-pseudolongum-subsp.-globosum]
PLS30252.1	Type1.BREX	<-ABC-ATPase+SbcC<-?<-?<-SIGMA+OB-Fold+AAA-ATPase+HKD-DNase-BrxL<-PglZ<-Schlafen+HATPase+wHTH<-N6-METHYLASE<-?<-DUF6079*<-BrxB-BrxF-iSTAND<-BrxA-DNA-binding<-?<-?<-?||Peptidase_A24->	1244	Bacteria>Terrabacteria_group>Actinomycetota>Actinomycetes>Bifidobacteriales>Bifidobacteriaceae	GCA_002860355.1	Bifidobacterium_margollesii	Uis1B_1922	37676..41410	2020964	hypothetical-protein-Uis1B_1922-[Bifidobacterium-margollesii]
RDX32761.1	Type1.BREX	RelB->N6-METHYLASE->REase-><-CR-Kinase<-?||BrxA-DNA-binding->BrxB-BrxF-iSTAND->DUF6079-p->DUF6079*->N6-METHYLASE->PglZ->PglZ-p->SIGMA+OB-Fold+AAA-ATPase+HKD-DNase-BrxL->TIR->	727	Bacteria>Terrabacteria_group>Actinomycetota>Actinomycetes>Bifidobacteriales>Bifidobacteriaceae	GCA_003370195.1	Bifidobacterium_breve	CE167_03785	16097..18282	1685	hypothetical-protein-CE167_03785,-partial-[Bifidobacterium-breve]
RGY77464.1	Type1.BREX	ABC-ATPase+SbcC->Pkinase->?->REC+LuxR-HTH->HISKIN-><-SbcD||BrxA-DNA-binding->DUF6079*->N6-METHYLASE->SIR2->HerA/FtsK->N6-METHYLASE->SIR2->PglZ->SIGMA+OB-Fold+AAA-ATPase+HKD-DNase-BrxL->ZnR+GP->ZnR->	1219	Bacteria>Terrabacteria_group>Actinomycetota>Actinomycetes>Bifidobacteriales>Bifidobacteriaceae	GCA_003463615.1	Bifidobacterium_pseudocatenulatum	DXA22_03235	61960..65619	28026	BREX-system-P-loop-protein-BrxC-[Bifidobacterium-pseudocatenulatum]
KAB5743305.1	Type1.BREX	<-Abhydrolase<-ParB+HNH+RAMA<-ZnR+GP<-SIGMA+OB-Fold+AAA-ATPase+HKD-DNase-BrxL<-PglZ<-Schlafen+HATPase+wHTH<-SIR2<-N6-METHYLASE<-DUF6079*<-BrxB-BrxF-iSTAND<-BrxA-DNA-binding<-Pentapeptide+Pentapeptide<-RelE-ParE<-HAD||SbcD-><-?<-?<-ACET+ACET	1218	Bacteria>Terrabacteria_group>Actinomycetota>Actinomycetes>Bifidobacteriales>Bifidobacteriaceae	GCA_009075895.1	Bifidobacterium_adolescentis	GA749_07780	122966..126622	1680	BREX-system-P-loop-protein-BrxC-[Bifidobacterium-adolescentis]
KAB5930818.1	Type1.BREX	PemK_toxin->PolB->HEPN-><-?<-?||?->||Pentapeptide+Pentapeptide+Pentapeptide->BrxA-DNA-binding->BrxB-BrxF-iSTAND->DUF6079*->N6-METHYLASE->SIR2->Schlafen+HATPase+wHTH->PglZ->SIGMA+OB-Fold+AAA-ATPase+HKD-DNase-BrxL->?->?-><-REase	1219	Bacteria>Terrabacteria_group>Actinomycetota>Actinomycetes>Bifidobacteriales>Bifidobacteriaceae	GCA_009094485.1	Bifidobacterium_adolescentis	GA595_06005	91928..95587	1680	BREX-system-P-loop-protein-BrxC-[Bifidobacterium-adolescentis]
KAB6780790.1	Type1.BREX	HTH+MPTase-><-?<-HEPN<-?||?->BrxA-DNA-binding->BrxB-BrxF-iSTAND->DUF6079*->EVE+McrB-AAA->McrC-NTD+REase->METHYLASE-p->N6-METHYLASE-p->	1229	Bacteria>Terrabacteria_group>Actinomycetota>Actinomycetes>Bifidobacteriales>Bifidobacteriaceae	GCA_009153345.1	Bifidobacterium_longum	GBL14_01920	66538..70227	216816	BREX-system-P-loop-protein-BrxC-[Bifidobacterium-longum]
KAB6790527.1	Type1.BREX	<-N6-METHYLASE-p<-METHYLASE-p<-McrC-NTD+REase<-EVE+McrB-AAA<-DUF6079*<-BrxB-BrxF-iSTAND<-BrxA-DNA-binding	1229	Bacteria>Terrabacteria_group>Actinomycetota>Actinomycetes>Bifidobacteriales>Bifidobacteriaceae	GCA_009153545.1	Bifidobacterium_longum	GBK34_07935	4820..8509	216816	BREX-system-P-loop-protein-BrxC-[Bifidobacterium-longum]
[truncated: 6,563,705 more chars]
