# Supplementary material for: Expanding the landscape of BREX diversity: uncovering multi-layered functional frameworks and identification of novel BREX-related defense systems
Source: Nucleic Acids Res. 2026 Jan 27;54(3):gkag035. doi: 10.1093/nar/gkag035 (PMC12839542; doi:10.1093/nar/gkag035)
Supplement: gkag035_Supplemental_Files [file gkag035_supplemental_files.zip › Supplementary_Data_S2.pdf]

## Supplementary Data S2

This PDF presents hit lists from structural similarity searches (primarily via the DALI server) along with their corresponding structure-guided sequence alignments, which we used to annotate multiple previously uncharacterized domains associated with various components of BREX and their related systems.
Amino acid residues in the alignments are shown in fluorescent colors against a black background for clarity, with conserved blocks and secondary structural elements highlighted in uppercase.


1. BrxC-ATPase; N-terminal ORC/CDC6 AAA+ ATPase….3
2. BrxC-ATPase; Canonical wHTH1 immediately following ORC/CDC6….5
3. BrxC-ATPase; Alpha+Beta-Domain….7
4. BrxC-ATPase; wHTH2….8-9
5. BrxC-ATPase; C-terminal Coiled-coil like extension.…10-11
6. BrxX-MTase; N-terminal domain….12
7. BrxX-MTase; Target-recognition-domain….13
8. BrxX-MTase; C-terminal CC-like helical extension….13-15
9. Type-1 BREX; BREX-MTase wHTH….16
10. PglZ; N-terminal inactive P-loop of Swi2/Snf2….17-19
11. Structure-based Sequence Alignment of "PglZ N-terminal Inactive P-loop Swi2/Snf2" with various inactive P-loop domains from Swi2/Snf2-Helicases….19-20
12. PglZ; (Third Tri-helical HTH-like element)….20-22
13. PglZ; core enzymatic phosphatase (NPP superfamily)….23
14. PglZ; C-terminal β-Sandwich (Immunoglobulin-like)….24-25
15. PglZ; C-terminal wHTH (Type-2 BREX-specific)….26-27
16. PglZ; N-terminal inactive STAND (Type-4 BREX-specific)….29-30
17. BrxL; Core MCM AAA+ ATPase.…31-32
18. BrxL; N-terminal SIGMA-HTH like….33-34
19. BrxL; N-terminal OB-fold….34-36
20. BrxL; C-terminal HKD-EndoDNase….36-37
21. BrxA; N-Terminal HTH1 (found in a subset of Type-1 BrxA, and in all Type-3 BrxA)….37-38
22. BrxA; HTH2….39-40
23. BrxA; C-terminal wHTH.…41-42
24. BrxB; inactive STAND NTPase….42-44
25. BrxF; inactive STAND NTPase (Type-3 BREX)….44-46
26. PglW; wHTH1 (encoded in the C-terminal half of protein)….47-49
27. PglW; wHTH2….50-52
28. PglW; wHTH3….53-54
29. PglW; C-terminal inactive STAND….55-57
30. BrxHI-Helicase; wHTH (Type-2 BREX)….57-59
31. BrxHI-Helicase; HAS-β barrel (Small β-barrel assemblage)….59-60
32. BrxHI-Helicase; Lhr-helicase CTD….60-61
33. BrxD-ATPase; standalone ORC/CDC6 AAA+ ATPase.…61-63
34. Type-3 BREX; MTase C-terminal HTH1….64-67
35. Type-3 BREX; MTase C-terminal HTH2….67-72
36. Type-3 BREX; MTase C-terminal wHTH….72-75
37. Type-3 BREX; BrxHII-Helicase N-terminal TUDOR….75-77
38. Type-3 BREX; BrxHII-Helicase C-terminal inactive PDDEXK-REase fold….77-78
39. Structural search anchored on "*C-terminal Inactive PDDEXK-REase Domain*" found in RapA-Helicase….78-80
40. Type-3 BREX; BrxHII-Helicase C-terminal α+β domain….80-81
41. BREX-Related systems; DUF499-ATPase C-terminal RRM/Ferredoxin 1….82-84
42. BREX-Related systems; DUF499-ATPase C-terminal RRM/Ferredoxin 2….84-87

43. BREX-Related systems; DUF499-ATPase C-terminal Fn-III-like domain….87-89
44. BREX-Related systems; DUF3780….89-90
45. BREX-Related systems (Type-3); Inactive STAND-NTPase….91-92
46. Type-4 BREX; PAPS-Reductase α+β domain….92-93
47. Type-4 BREX; PAPS-Reductase Fe-S cluster binding Fer4_7….94-95
48. BREX-related Capture systems; HerA/FtsK….95-96


***Note: Titles are internally hyperlinked. Click on the titles to access the material***

```
Job: Type-1 BREX; BrxC-ATPase N-terminal ORC/CDC6 AAA+ ATPase
Query: s001A
No: Chain Z rmsd lali nres id PDB Description
1: 2qby-B 14.1 4.5 249 368 11 MOLECULE: CELL DIVISION CONTROL PROTEIN 6 HOMOLOG 1;
2: 1w5s-B 13.2 3.7 228 396 12 MOLECULE: ORIGIN RECOGNITION COMPLEX SUBUNIT 2 ORC2;
3: 3bos-B 12.5 4.7 188 231 11 MOLECULE: PUTATIVE DNA REPLICATION FACTOR;
4: 5zr1-A 11.7 4.5 251 494 14 MOLECULE: ORIGIN RECOGNITION COMPLEX SUBUNIT 1;
5: 5zr1-E 11.5 5.6 247 460 11 MOLECULE: ORIGIN RECOGNITION COMPLEX SUBUNIT 1;
6: 7rzy-1 11.4 4.4 248 311 14 MOLECULE: TN6677 VIBRIO CHOLERAE TRANSPOSON TNSC (VCHTNSC);
7: 8c0v-A 11.3 4.4 218 823 10 MOLECULE: PEROXISOMAL ATPASE PEX1;
8: 6p8v-C 11.3 4.4 225 303 9 MOLECULE: ATPASE, AAA FAMILY;
9: 7jgr-D 11.2 4.8 263 441 10 MOLECULE: ORIGIN RECOGNITION COMPLEX SUBUNIT 2;
10: 8btg-B 11.1 5.8 211 335 11 MOLECULE: CHROMOSOMAL REPLICATION INITIATOR PROTEIN DNAA;
12: 4rh7-A 11.1 4.6 236 3005 13 MOLECULE: GREEN FLUORESCENT PROTEIN/CYTOPLASMIC DYNEIN 2 HE
13: 8fcv-R 11.0 4.1 238 343 13 MOLECULE: DNA (60-MER);
14: 9emc-D 11.0 4.6 244 337 14 MOLECULE: RUVB-LIKE 1;
15: 8xvb-A 11.0 4.5 223 246 12 MOLECULE: DNA (5'-
16: 8hra-C 10.8 4.3 273 852 13 MOLECULE: ARCHAEAL ATPASE;
17: 7plh-A 10.5 4.2 225 260 11 MOLECULE: SHTNSC;
18: 8fnu-A 10.4 4.1 245 796 8 MOLECULE: KAP NTPASE DOMAIN-CONTAINING PROTEIN;
20: 2fna-A 10.4 3.5 215 352 11 MOLECULE: CONSERVED HYPOTHETICAL PROTEIN;
21: 7vcs-A 10.2 4.9 216 766 13 MOLECULE: TRANSITIONAL ENDOPLASMIC RETICULUM ATPASE;
22: 6fhs-C 10.1 5.5 265 459 12 MOLECULE: RUVB-LIKE HELICASE;
23: 8bob-C 10.1 6.1 205 412 9 MOLECULE: PROTEIN MALY;
24: 9cru-C 10.1 3.7 199 741 9 MOLECULE: VESICULAR-FUSION PROTEIN SEC18;
25: 5e7p-A 10.0 4.1 218 719 11 MOLECULE: CELL DIVISION CONTROL PROTEIN CDC48;
26: 8umy-A 9.9 4.3 205 546 9 MOLECULE: CHROMOSOME TRANSMISSION FIDELITY PROTEIN 18 HOMOL
27: 7uqj-A 9.9 4.6 225 585 9 MOLECULE: ATPASE HISTONE CHAPERONE YTA7;
28: 7st9-A 9.8 6.1 214 522 11 MOLECULE: CHECKPOINT PROTEIN RAD24;
29: 8tw7-2 9.8 4.1 216 339 10 MOLECULE: REPLICATION FACTOR C SUBUNIT 5;
30: 7crc-B 9.6 6.4 231 1112 10 MOLECULE: NAD+ HYDROLASE (NADASE);
31: 8dr5-A 9.6 4.0 197 646 13 MOLECULE: REPLICATION FACTOR C SUBUNIT 1;
32: 5kzf-K 9.6 5.2 233 498 13 MOLECULE: PROTEASOME-ASSOCIATED ATPASE;
33: 6f0x-B 9.5 3.9 208 398 12 MOLECULE: PACHYTENE CHECKPOINT PROTEIN 2 HOMOLOG;
34: 9e22-A 9.5 4.3 229 2925 13 MOLECULE: CYTOPLASMIC DYNEIN 1 HEAVY CHAIN 1;
35: 7st9-E 9.4 3.9 209 354 11 MOLECULE: CHECKPOINT PROTEIN RAD24;
36: 7ykk-A 9.4 4.4 215 735 12 MOLECULE: ATPASE FAMILY GENE 2 PROTEIN;
37: 9bv1-A 9.3 4.4 201 405 13 MOLECULE: 26S PROTEASOME NON-ATPASE REGULATORY SUBUNIT 1;
38: 6qem-G 9.3 3.4 146 239 15 MOLECULE: REPLICATIVE DNA HELICASE;
39: 8q3w-A 9.3 3.2 163 246 15 MOLECULE: INSERTION SEQUENCE IS5376 PUTATIVE ATP-BINDING PR
40: 8glw-A 9.3 3.9 233 483 14 MOLECULE: TRANSPOSON TN7 TRANSPOSITION PROTEIN TNSC;
41: 6l1q-A 9.1 4.7 206 267 12 MOLECULE: CBBQ PROTEIN;
42: 8val-E 9.1 7.2 209 337 9 MOLECULE: DNA POLYMERASE III SUBUNIT DELTA;
43: 5j1s-A 9.1 4.8 194 277 9 MOLECULE: TORSIN-1A;
44: 6z1f-3 9.0 4.2 210 284 12 MOLECULE: RIBULOSE BISPHOSPHATE CARBOXYLASE/OXYGENASE ACTIV
45: 8c0v-B 9.0 3.8 178 1030 13 MOLECULE: PEROXISOMAL ATPASE PEX1;
46: 8xku-A 9.0 4.8 234 730 12 MOLECULE: PROBABLE INACTIVE ATP-DEPENDENT ZINC METALLOPROTE
47: 6pxk-K 8.9 4.8 230 429 13 MOLECULE: ATP-DEPENDENT PROTEASE ATPASE SUBUNIT HSLU;
48: 5zr1-B 8.9 7.3 177 374 11 MOLECULE: ORIGIN RECOGNITION COMPLEX SUBUNIT 1;
49: 4m9s-B 8.8 5.2 215 511 7 MOLECULE: CELL DEATH PROTEIN 4;
50: 3glf-G 8.8 5.0 212 378 12 MOLECULE: DNA POLYMERASE III SUBUNIT DELTA;
51: 8xks-D 8.8 4.3 216 971 14 MOLECULE: CTAP1;
52: 2gno-A 8.7 4.2 165 296 10 MOLECULE: DNA POLYMERASE III, GAMMA SUBUNIT-RELATED PROTEIN
53: 7w42-B 8.7 4.2 197 402 11 MOLECULE: UNCHARACTERIZED ATPASE YJOB;
54: 8xks-B 8.7 18.2 188 603 12 MOLECULE: CTAP1;
55: 7mi8-A 8.7 15.0 175 868 12 MOLECULE: FUSION PROTEIN OF DYNEIN AND ENDOLYSIN;
56: 1g4a-E 8.6 7.9 248 356 13 MOLECULE: ATP-DEPENDENT HSL PROTEASE ATP-BINDING SUBUNIT
57: 8xku-C 8.6 4.8 196 921 11 MOLECULE: PROBABLE INACTIVE ATP-DEPENDENT ZINC METALLOPROTE
58: 5j1s-B 8.5 3.9 157 235 12 MOLECULE: TORSIN-1A;
59: 7qo4-M 8.5 5.1 207 421 13 MOLECULE: 26S PROTEASOME REGULATORY SUBUNIT RPN1;

0001 s001A   MINEYVFTV-DIVNGLYRTL-EAIK-NG-S--VNH-NGIWVSGYFGSGKSHFLKYVSYCLRNWFASES-VRTIMFNMGEAG-FSNAKEG-FDFLMVFWRKFNEMRGYGRNLAIAYFLEVHNRVP-L--S--VADFMADVKEWLDAQ-G--KNCR-LVFCVDEIS-QYI-N-NR-GNLLLQLQEIVTAFHNAGGAKVWLVCTAQ-QDMSESASASFGKIMGR-FP-IMVQLSAASTEYITRKRVL-DGEADRDDFIAT-YPFLGCHF-RLMGQVLRAFVKDNARSVLTLTLDVAKATRDCEA-GKKFI-AFDEFFS-QSL-NDG
0002 2qbyB   -SVFEIPF---REDILRDAA-IAIR-YF-N--EVK-FSNLFLGLTGTGKTFVSKYIFNEIEEVKKEKD-VKQAYVNCREVG----------TPQAVLSSLAGKTGFS-VPKH----------GI-N--L--G-EYIDKIKNGTR-------nIR-AIIYLDEVD-TLV-K--R-RGGDIVLYQLL---RSDA--NISVIMISN-DI--NVRDYMEPRVLSS-LG-PSVIFKPYQLKFILSKYAE-Y-----------kgTYDDSYI-AAISAKE----HGDARKAVNLLFRAAQLASGG-----GII-RKEHVDI-VDY-EQE
0003 1w5sB   -PPELRV----RRGEAEALA-RIYL-NR--sgaglsDVNMIYGRVGIGKTTLAKFTVKRVSEAAAKLT-VKQAYVNAFNAP-----------NLYTILSLIVRTGYP-iQVRG----------A-P--A--L-DILKALVDNLYVE-N----HY-LLVILDEFQ-SML-S-SP--IAAEDLYTLLR-VHEE-VNRIGFLLVAS-DVALSYMREKiPQVESQ-IG-FKLHLPAYELYTILEQRAE-------------vWEPR-LIS-DVYG-------dgSARRAIVALKMACEM---------aerdsEDLVRV-SENe---
0004 3bosB   -TTSYYPAA-GNDELIGALK-SAGD-GV--------QAIYLWGPVKSGRTHLIHAACARANE----LE-RRSFYIPLGIHA-------S-IST-ALLEGLE-----------------------------------------------------qfDLICIDDVD-A-V-A-GH-PLWEEAIFDLYNRVAE--QKRGSLIVSAS-ASPXEAGFV-LPDLVSR-XG-LTYQLQPXXDDE-KLAALQ-R--aaxrglQLP-EDV----------------rarDLRTLFD---vldrlkaHQRK---LTI-PKEXLRL--------
0005 5zr1A   ---YYLPA---RENEFASIY-LSAY-SA--esdsA-TTIYVAGTPGVGKTLTVREVVKELLSSSAQPD-FLYVEINGLKMV-K------pTDCYETLWNKVS-----GERL------------twaE--S--LEFYFKR--VPKNKK-------T-IVVLLDELD-AMV-T-KS-QDIMYNFFNWTT--YENA--KLIVIAVAN-TM--DLPERQNKITSRI-GF-TRIMFTGYELKNIIDL-RL-K-----------lrMSADEIA-SRKV------VSGDARRALKVCKRAAEIAEK---hgVQTV-HITHVMK-aLN-ETL
0006 5zr1E   ---mttVAF-R-EYQTNCLA-SYISaDP-D--ItP-SNLILQGYSGTGKTYTLKKYFNANPN-------LHAVWLEPVELV-S------wKPLLQAIARTVQYKLK-pniptTDYD----pLQV-E-fL--LVKTLHNIFVQ-YES-L-qEKTC-LFLILDGFD-SLqdL-DA-A-LFNKYIKL---NELLPKiNIKFIYTML-ET--SFLQR-YSTHC----I-PTVMFPRYEVSTILVMSRC-G-----fQNVAA-NF--HLIV-QAFHSYTG----NDIFALNDLIDFKWPKVSRINI-FEPLksAIKLFLS-TDD-NL-
0007 7rzy1    ---AFVSTP-SVRKILSYMD-RCRD-LS-DlesEP-TCMMVYGASGVGKTTVIKKYLNQNRR-----I-IPVLHIELPD--------nakpVDAARELLVEM--GDPLLYET----------dlA-R--L--TKRLTELIPAVG----------V-KLIIIDE-F-QHL-V-VL-TQVGNWLKMILNKTK------CPIVIFGM-PYSKVVLQA-NSQLHGR-FS-IQVELRPFVFKTFLEYLDL-P-------QAGL----LQKKL-YAFSQ-------GNMRSLRNLIYQASIEAIDNQH-titEE-DFVFASK-LTS-GdK
0008 8c0vA   --DDFITVN----SIKKEMV-NYLT-SP-I--IAT-PAIILDGKQGIGKTRLLKELINEVEK----HH-IFVKYADCE-TL-HEsnlDK-TQLIMEWC---------------------------------------sfcYWYG----------P-SLIVLDNVE-ALF-G-KA-SKLLNFFINQVTKIFNKDNKRIRVLFSGK-QK-TQIN----PLLFHF-VS-ETWSLRAPARAKLLEYFFS----klnrdLQFS-----SLET-E----------gfSPLDLEIFTEKIFYDLqDCDN-vtrEL-FSKSLSA--------
0009 6p8vC   -LA-RLVG---LDEHKDRLS-KILG-LL-L--RRP-PLVVLAGDVGSGKTELAETIGDAVAR-QEDID-ITLYPL-SLATR-GQGRVGE-XLVSAAFDY-------------------------------------tiEAADKLK---K--ARGA-VLLLIDQAD-ALA-Q-SD-RAGVNAFIRGIDRIANQKL-PAAVLXCTN-RL-KALD----PAVQRR-AA-EILTFSRPQRHYLLHS-KL-T-----lgLNST---AVEELV-RLTGP---RD-gftFSDITRLIPSIILAAYPY---vsvHS-ALQVVNK--------
0010 7jgrD   ----TLRG---YAKERSNVR-LLLQ-RT--aemgesNSLLLLGPRGSGKTTLINSVLADLLPSFG-EN-TLIVHLDGNLHT-------ddRVALKSITVQMQL-enADGKVF----------GS-F--AenLAFLLQCLKAGGKHS------KS-VIFILEEFD-LFC-A--H-HNQ-TLLYNLFDVSQSAQ-aPICVLGVTC-RL--DVIEL-KRVKSRF-SH-RQVFLFPSLYVDLCRD-LL-S-----qiCKVL-A--ARSTL-QALHDFD-----iSEAYLKNFLFRLVA-HLRPQ--itaEK-MAAVGS--QFE-G--
0011 8btgB   -TDTFVIGR-FAHAASLAVA-EAPA-KA-------yNPLFIYGGVGLGKTHLMHAIGHYVID-HNPS--AKVVYLSSEKFT-NiIRDNK--afRNRYR---------------------------------------------------------nvDVLLIDDIQ--FL-A-GK-EQTQEEFFHTFNTLHEE---SKQIVISSD-RPPKEIPT--EDRLRSR-FG-LITDITPPDrIAILR-KKA-K-----------dIPNELYIA-NQID--------SNIRELEGALIRVVAYSSINKD---INA-DAEALKD--------
0012 5v8fD   --KIIFLQD-CQQEIDRIIK-QSII-QK------esHSVILVGPRQSYKTYLLDYELSLLQQ-SYKEQ-FITIRLNGFIHS--------eQTAINGIATQLEQQLQ-htISSG-------slTE-V--F---EKILLLLDS------I--TKIT-VVFIFDEID--TF-A-GP-VR-QTLLYNLFDMVEHSRV-PVCIFGCTT-KL--NILEY-EKRVKSF-SQ-RVIYMPQIDMVDAVRNL-L-T---------------LNRHI-RMNFETF-----rSLPTLKNSIIPLVATS-KNFG-lcTAI-KCSFLD--IYN-KNQ
0013 4rh7A   -EANYEIIP-NQIKKALELY-EQLC-Q--------R-MGVVIVGPSGAGKSTLWRMLRAALCK--TGKV-VKQYTMNPKAMP--------rYQLLGVLTNSA------------------------------------RQVV-REPQ------DVS-SWIICDGDI---------D-PEWIESLNSVLDDNRLQFGPNVNFVFETH-D--LSCA---SPATISR-M--GMIFLETDL-NSLI-KSWLrN----eyrNNLE-NWIGDYF--EKALQWVLKQ-TSLVGTVMNGLSHLH-GCRD---------hdIINLIR--------
0014 8fcvR   --NYTVAHP-RLKEVYEILM-RTIA-EP----AGA-SFIFVYGASGVGKTTLRLRVEQKLTE-ALPKLrVPVVGIEA--IA-PESR--Y-FNWKEYYTRALITLEE-kfdygvrGISRveSKVV-A--PalRRALENALIHRH----------P-DVFFVDEA--QHF-G-KL-QDQLDCLKSLANMTG------ILHCLLGT-YELLTFR---SGQLSRR-SV--DIHFRRYAFKSVLLTFQQ-H-----------eTPNLVDH--WEYFYER----iGTLKDWLKRVLSDALDREA--------I-TLKDLQK--------
0015 9emcD   -RQGMVGQL-AARRAAGVVL-EMIR-EG-K--IAG-RAVLIAGQPGTGKTAIAMGMAQALGP----D-TPFTAIAGS-EI-FSLE-MS-KTLTQAFRR----SLHE--iDVINSRT-dtgeIK-S--E----vreQINAKVAEW--A--EIIP-GVLFIDEVH-ML-----D-IESFSFLNRALE---SDMA--PVLIMATN-RGtPHGI---PIDLLDR-L--LIVSTTPYDTKQILRIRCE-------------vEMSEYTVL-TRIGLET------SLRYAIQLITAASLVCRKR------kV-QVDDIYS-LFL-esR
0016 8xvbA   ---RFVETQ-TVKQIWTSMR-FASL---------teSIAVVCGNPGVGKTEAAREYRRTNN---------NVWMITITPSC------asvLECLTELAFELG-----MNDAP----------RR-K--GplSRALRRRLE-----------gTQ-GLVIIDEAD-HLG------AEVLEELRLLQESTR------IGLVLMGN-HRVYSNMT--FARLFSR-IA-KRTAINKTKVKAI-ADAW------------QI-NG-ELELL-QQIA------KPGALRILNHSLRLAAMTAHGKGE-----nedRQAFRE---L-DLD
0017 8hraC   --EDDLI----QKSLRDQLV-ESIR-NS---iaGR-NVFFVDGTRGAGKTTFINSVVKSLNS--DQDD-VKVNIKCLPTID-PT----K-LPRHEPILVTVTARLNKdklkgywasndqlQRGLhdkdL--SEIFEELVKRACEIL-D---cKA-ILITFDD-I-DTQ-F-DG-WDVLESIRKF-FNSR-----KLVVVATGD----LRLYSQLQQYLLKL-FPqKRIQLKTML------------dv---------------YVN-E----------kQPVRLLMQVLQDFYTKKYHAT-----SV-PNRNALY-GSM-LSS
0018 7plhA   --IVPLQ---QVKTLHDWLD-GKRK-AR---------KSCRVVGESRTGKTVACDAYRYRHK-----pT-VPVVYIRPHQ------------KCGPKDLFKKITEYLKY-----RVTK------GT-V--S----DFRDRTIEVLKGC------GV-EMLIIDEAD-R---------lKPETF-ADVRDIAEDLG--IAVVLVGT-DRLDAVIKR-DEQVLER-FR-AHLRFGKLSFKNTVEMWEQ-M--klpvsSNLK-S--MLRIL-TSAT-------egYIGRLDEILREAAIRLSRG----lkKI-DKAVLQE--VA-KEY
0019 8fnuA   -----NYIF---SSQLDKVN-LILE-HM-DggiHS-RNIAITGD--rgktSFIETLKLVLEK----QN-YYVFDIVSPTVL-S----sH-LNILEIVISSIYREIDQ-----------iqhlKK-V--MflDEEIKELFCYFKKVL-NneVIKD-LVLIIDDLD-L-V-E-NNvYDLLRDIQHYLDSQ-------LIVIFAYK-E---GQLEQSERFLTKL-VPsNRIPLFKELLNKtIGEFL----------nKNN-L--EWFYesifyrSRLM---lREMVQLCEE-------------------lhSMQV----------
0020 8rwvE   -LENVVCRE-SQVSILQSLF-G---hHF-S--F---PSIFIYGHTASGKTYVTQTLLKTLE--------LPHVFVNCVEC-----------FTLRLL-LEQILNKLNHedgcsTEIT-------C-E--T--FNDFVRLFKQVTTAE-N-lKDQT-VYIVLDKA--EYL-R--D-MEA-NLLPGFLR-LQELADRNVTVLFLSE-IVWEKFR---NTGCFE---P-FVLYFPDYNLQKILSHD---H----ySADFYA-A-YINILL-GVFYTVC-----RDLKELRHLAVLNFPKYCEPAS-ERDT---------vyLR-EI-
0021 2fnaA   -RKDFFD----REKEIEKLK-GLR----------A-PITLVLGLRRTGKSSIIKIGINELN--------LPYIYLDLRK----enyisY-KDFLLELQKEINKLVKrEIKFN------------R-L--S--FANLLESFEQAS--------KDN-VIIVLDEAQ--EL-V-KL-RGV--NLLPALAYAYDNLK-RIKFIXSGS-EXLYDYL---plFGRA---F-STVELKPFEAIEFLRRGFQ-E-----------iDFKDYEVV-YEKIG------pGWLTYFGFIYLDNK-----------------NLDF--------
0022 7vcsA   -YDDIGGCR-KQLAQIKEMVeLPLR-HP--gvKPP-RGILLYGPPGTGKTLIARAVANETG--------AFFFLINGPEIM-SKL--AG-ES-ESNLRKAFEEA----------------------------------------------eknAP-AIIFIDELD-AIA-P-KE-RRIVSQLLTLMDGL--KQRAHVIVMAATN-RP-NSID----PALRRR-FD-REVDIGIPGRLEILQ-IHT-K-----------ddVDLEQVA-NETHG-------hVGADLAALCSEAALQAIRkdleavTMD-D-RWALS-QSN----
0023 6fhsC   -KAGFVGQC-AAREACGVVV-DLIK-AH-K--MAG-RGVLLAGGPGTGKTALALAISQELGT-----K-IPFCPITGSEIY-ST---EV-KKT-EVLMENFRRTLHD--lDVANARP-pkmtEI-T--D---kLRMEINKVVQKYI-A--ELIP-GVLFIDEAH-MLD------IECFTYLNKALE---SPIA--PIVVLASN-RGtAHGI---PPDFLQR-L--LIIPTHPYEIRRIVRIRAQ-T-----------qLTDADRVA--EHGVRI------SLRYCLQLLAPASILARNGRT--qVDV-Q-AEAEE-LFL-DaR
```

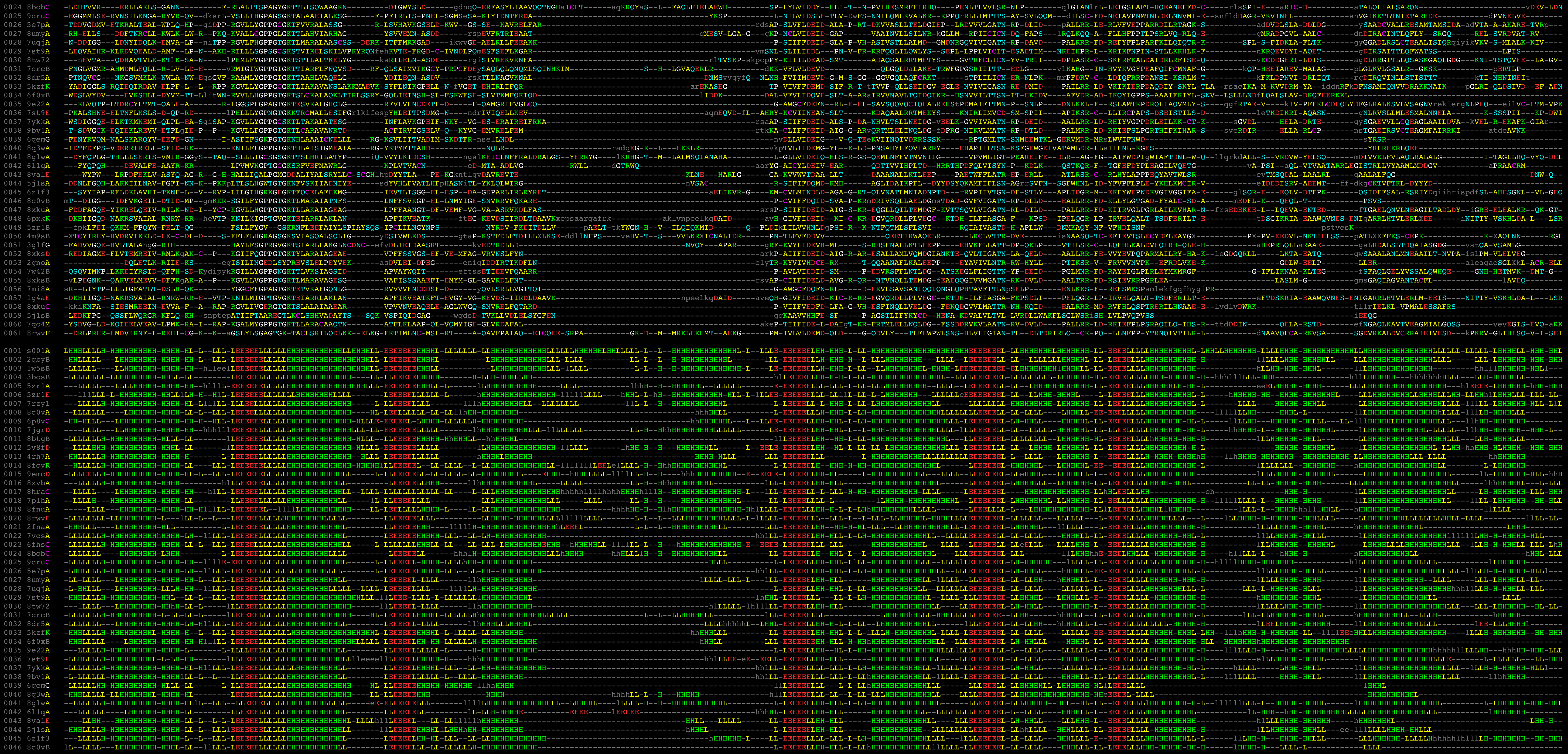

```
0047 8xkuA   -LLLLLLLH-HHHHHHHHHHH-HHHH-LH-L--LLL-LEEEEELLLLLHHHHHHHHHHHHHL--------LLEEEEEHH-HL-LLLL-LL-HH-HHHHHHHHHHH----------------------------------------------hhhL-EEEEEELLL-LLL-L-LH-HHHHHHHHHHHHHLL-LLLLLLEEEEEELL-LL-LLLL----LLLLLL-LL-EEEELLLLHHHHHHHHHLL-L--lllHHHHHH-H--HHHHH-HLLLL-------lLHHHHHHHHHHHHHHHHHLLLL--lLHH-HHHHHHH--HL-LL-
0048 6pxkK   -LLLLLLLH-HHHHHHHHHHH-HHHH-HH--hhLLL-LLEEEELLLLLHHHHHHHHHHHLL--------LLEEEEEHHH------hlLL-LLLLHHHHHHHHHHHHhllllhhhhhhh-----------hhhllhhhhhhHHHH-------hhH-LEEEEELHH--HH-L-LH-HHHHHHHHHHHHLLL--ELLL-LEEEEEEL-L---LHHH--LHHHHLL-LL-EEEELLLLL-HHHHHHHHH-L-----------lHHHHHHHH-HHHHHHHHH-LLLhHHHHHHHHHHHHHHH------hEEEEH-HHHHLHH-H---HHH
0049 5zr1B   --lhhHHHH-LLLL-HHHHH-HHHL-LL--------LEEEEELL--LLLHHHHHHHHLHHHHHHHLL-LLEEEEELLLLL----------LHHHH-HHHHHHHHL------lLLLL-llLLLL-H--H--HHHHHHHHH-------L--LLLLlEEEEEELLLlLLL-L--L-HHHHHHHHHHHLL------LLEEEEEELL-L-HHHHL---LHHHHHH-LE-EEEELLLL-------------------------------------llllllL--------------------------------------------
0050 4m9sB   -LLLLLLHH-HHHHHHHHHHH-LL-L-LL-L----L-EEEEEELLLLLHHHHHHHHLLLLLL------lLEEEEEELL-------lllL-LHHHHHHHHHHHHHLLL-llllLLLL----llLL-L--H---HHHHHHHHHHLLLL------LL-EEEEEEEEL-------------LHHHHHHHHHLL------LEEEEEEL-LHH-------hhHHLLL-LL-EELLLLLLHHHHHHHHLLL-----------LL-LL-HHHHH-HHHHHHLL---hHHHHHHHHL-LLLL-------------H-HHHHHH------LHH
0051 3glfG   -LLLLLLLH-HHHHHHHhhL-LLL------------LEEEEELLLLLHHHHHHHHHHHLLLHHH--hllLEEEEELLLLL-------lhHHHHHHHH-----------------------------------HLLL---LLLL------lLL-EEEEEELHH-HL------L-HHHHHHHHHHHHHLLL----LLEEEEEELL-LH-HHLL----HHHHLL-L--EEEELLLLLHHHHHH-HHH-H-----------lLLHHHHHHhHHLL--------llHHHHHHHHHHHHHHHLLL----llhHH-HHHHHHH---------
0052 8xksD   -LLLLLLLH-HHHHHHHHHHH-HHHLlLL-L--L---LEEEEELLLLLHHHHHHHHHHHHL--------LLEEEEEHH-HH-LL-LLLL-HHHHHHHHH--------------------------------------------------hhhL-LEEEEELLH-HHH-L-LH-HHHHHHHHHHHHHHHHLLL-LLEEEEEEL-LH-HHLL----LLLLLL-LL-EEEELLLLHHHHHHHH-HL-L--llLLHHHHH-----HHHH-HLLL-------llLHHHHHHHHHHHHHHLL-LLLL-llhHH-HHHHHHH--------
0053 2gnoA   -------------LHHHHHH-HHHH-LL------llEEEEEELLLLHHHHHHHHHHHHHHL------llLEEEE-LLLL------llllHHHHHHHHHHHHL----------------------------------------------lllLL-EEEEELLHH-HL------L-HHHHHHLHHHHHLLL----LLEEEEEEEL-LH-HHLL----HHHHLL-L--EEEELLLLH--HHHHHHHH-H----------------LLHH-HHLH--------HHHL-------------------hhhhhhHHHHhH-HHH-HHH
0054 7w42B   -LHHLLLLHhHHHHHHHHHHH-HHHH-LL-LlllllEEEEEELLLLLHHHHHHHHHHHLL--------LLEEEEELL-------llllhHHHHHHHHHHHHH--------------------------------------------------L-EEEEELLHH-HL------L-HHHHHHHHHHHHHL--LLLLLLEEEEEEEL-LH-HHLL----HHHLLL-LL-EEEELLLLHHHHHHHHHHLH------------H-HLLHHHHH-HHLLL-------lLHHHHHHHHHHHHHHHHHHH-----LLL-LHHHHH--HHH-L--
0055 8xksB   -lLLLLLL--LHHHHHHHHH-HHHHlLL-L--L---LEEEEELLLLLHHHHHHHHHHHHL--------LLEELLLLLLLL-LLLL-LH-HHHHHHHHH-------------------------------------------------hhhLL-LEEEEELHH-HLL-L-LL--HHHHHHHHHHLL-lLLLLLLLEEEEEEL-LL-LLLL----LLLLLL-LL-LLLLLLLLHHHHL-----------------------HHHHL-L----------lllHHHHHHHHHHHHHHL---------------hHHHH--------
0056 7mi8A   lL--LLLLH-HHHHHHHHHHH-HHHH-LL--------LEEEEELLLLLHHHHHHHHHHHLL--------LLLEEELLLLLL-L------hHHHHHHHHHHHHHH-------------------------------------------------L-LEEEELLLL-LL-----L-HHHHHHHHHHHHHHHHHHHLLLEEEEEEELlLLLL-------HHHLLL-E--EEEELLLLhhhhhhhhhllllLH--------------------------------------------------------------------------
0057 1g4aE   -HHHLLLLH-HHHHHHHHHHH-HHHH-HH-L--LLL-LLEEEELLLLLHHHHHHHHHHHHL--------LLEEEEELLHHH-LLLL-LL-LLHHH-HHHHHLHHHH--------------------------lllhhhhLLLL-----hhhHL-LEEEEELLL-LLL-L-LH-HHHHHLLHHHHHLL--EHHH-LEEEELLL-LLHHHLL----HHHHHL-LL-EEEELLLLL-HHHHHHHHH-L-----------lLLHHHHHH-HHHHHHHHH-LLLLLHHHHHHHHHHLL-HHHH----LLLLH-HHHHLHH-H---LHH
0058 8xkuC   -lllLLLL--LLLLLHHHHH-HHHH-H--L--LLL-LEEEEEELLLLHHHHHHHHHHHHHL--------LLEEEELHHHHL-LLLLLLH-HHHHHHHHHHHHL--------------------------------------------------L-LEEEELLLL-LLL-L-LH-HHHHHHHHHHHHLL--LLLLLLLEEEEEEL-LL-LLLL----HHLLL-LL-EEEEELLLLHHHHHHHHHH-H--hllllLHHH---------------------llllHHHHH-HHHHHHHHHHLL-----------------------
0059 5jlsB   -HHHHLLL--LLHHHHHHHHH-HHHH-HH--llllllEEEEEEELHHHHHHHHHHHHHHHHLL---LLL-LLLEEEELLL-----lllhH-HHHHHHHHHHHHHHHHHL----------------------------------------------llLEEEEELHH-HL------L-HHHHHHHHHHHHL--LLLL-LLLEEEEEEEE-LHHHHHHHHHHHHHHHHhLL-LEEELLLLHH------------------------------------------hHHHL---------------------------------------
0060 7qo4M   -LLLLL-LH-HHHHHHHHHHH-HHHH-LH-L--LLL-LLEEEELLLLLHHHHHHHHHHHHL--------LEEEEEEHH-HH-LLLLLLL-LHHHHHHHHH--------------------------------------------------hhhL-EEEEEEL-L-LLLlL-LL-LHHHHHHHHLLLL--LLLLLLEEEEEELL-LL-LLLL----HHHHLL-LL-EEEELLLLHHHHHHH-HHH-H--lllLLLL-------HHHH-HHLL-------llLHHHHHHHHHHHHHHHHHLLLL-------llhHHHH-HLL-lLL
0061 8rwvF   --LLLLLLL-LLHHHHHHH-H-HHHH-HL-L--L--lLLLEEEELLLLL-HHHHHHHHHHHHHH--HLLL-LLEELLLH-HHL-LL----L-LLLLLLLLL--LLLLLL-LLLL----------LL-L--L--LLLHHHHHL--LLLL------LL-EEEEEELHH-HHL----L-LHHHL---LLLLHHHLLLL-LEEEEEEEE-LL--LLLLLLLLL--LL-LL--EEELLL-LLHHHHHHHHH-H-----------lLLLHHHHH-HHHHH------LLLHHHHHHHHHHHHHHHHHH---lLLLL-LLHHHHL-L-L-HHH
```

```
Job: Type-1 BREX; BrxC-ATPase MBR4615907 Canonical wHTH1
Query: s001A
No: Chain Z rmsd lali nres id PDB Description
1: 4ad9-A 9.1 1.9 70 288 13 MOLECULE: BETA-LACTAMASE-LIKE PROTEIN 2;
2: 6j0e-B 8.8 1.7 60 125 22 MOLECULE: ARSENIC RESPONSIVE REPRESSOR ARSR;
3: 6qpq-D 8.8 3.0 70 82 9 MOLECULE: STRUCTURAL MAINTENANCE OF CHROMOSOMES PROTEIN,STR
4: 5j6x-A 8.8 1.4 60 66 10 MOLECULE: Z-DNA BINDING PROTEIN KINASE;
5: 3r0a-B 8.7 1.8 62 123 16 MOLECULE: PUTATIVE TRANSCRIPTIONAL REGULATOR;
6: 2jt1-A 8.5 2.6 65 71 14 MOLECULE: PEFI PROTEIN;
7: 6uvu-B 8.5 1.9 59 114 12 MOLECULE: ARSR FAMILY TRANSCRIPTIONAL REGULATOR;
8: 1sfx-A 8.4 1.7 60 109 12 MOLECULE: CONSERVED HYPOTHETICAL PROTEIN AF2008;
9: 7z1n-O 8.4 1.8 62 570 10 MOLECULE: DNA-DIRECTED RNA POLYMERASE III SUBUNIT RPC1;
10: 8ffz-B 8.3 1.2 58 905 21 MOLECULE: TRANSCRIPTION FACTOR IIIA;
11: 7a6h-O 8.1 1.6 60 512 5 MOLECULE: DNA-DIRECTED RNA POLYMERASE III SUBUNIT RPC1;
12: 6qfd-B 8.0 2.3 65 116 11 MOLECULE: DNA-BINDING PROTEIN;
13: 2obp-A 8.0 3.1 67 81 9 MOLECULE: PUTATIVE DNA-BINDING PROTEIN;
14: 4ru7-B 8.0 1.2 58 99 12 MOLECULE: ASPA;
15: 4ija-A 8.0 1.9 59 363 14 MOLECULE: XYLR PROTEIN;
16: 1stz-A 8.0 2.2 62 323 10 MOLECULE: HEAT-INDUCIBLE TRANSCRIPTION REPRESSOR HRCA HOMOL
17: 3f8f-A 8.0 2.2 67 114 10 MOLECULE: TRANSCRIPTIONAL REGULATOR, PADR-LIKE FAMILY;
18: 8iue-P 8.0 1.6 62 303 10 MOLECULE: DNA-DIRECTED RNA POLYMERASE III SUBUNIT RPC1;
19: 1ku9-A 8.0 1.8 62 151 15 MOLECULE: HYPOTHETICAL PROTEIN MJ223;
20: 4i99-C 8.0 2.8 66 70 9 MOLECULE: CHROMOSOME PARTITION PROTEIN SMC;
21: 5y6i-B 7.9 1.7 59 234 10 MOLECULE: TRANSCRIPTIONAL REGULATOR KDGR;
22: 3ov8-A 7.9 1.9 64 91 13 MOLECULE: PROTEIN AF_1382;
23: 1sfu-A 7.9 2.0 61 70 15 MOLECULE: 5'-D(*T*CP*GP*CP*GP*CP*G)-3';
24: 4ets-B 7.8 1.8 59 149 10 MOLECULE: FERRIC UPTAKE REGULATION PROTEIN;
25: 6pco-B 7.8 1.7 58 135 10 MOLECULE: MARR-FAMILY TRANSCRIPTIONAL REGULATOR;
26: 5vyv-A 7.8 3.0 70 173 10 MOLECULE: UPF0502 PROTEIN YCEH;
27: 3lmm-D 7.8 2.3 60 556 12 MOLECULE: UNCHARACTERIZED PROTEIN;
28: 2zkz-C 7.7 1.7 58 87 10 MOLECULE: TRANSCRIPTIONAL REPRESSOR PAGR;
29: 1xsd-A 7.7 2.0 63 125 10 MOLECULE: 5'-
30: 2nyx-B 7.7 1.8 60 147 15 MOLECULE: PROBABLE TRANSCRIPTIONAL REGULATORY PROTEIN, RV14
31: 8jxk-D 7.6 2.1 66 166 6 MOLECULE: CONSERVED PROTEIN;
32: 7rhe-A 7.6 1.3 55 368 15 MOLECULE: ROK FAMILY PROTEIN;
33: 3cuq-A 7.6 2.0 60 219 15 MOLECULE: VACUOLAR-SORTING PROTEIN SNF8;
34: 6j05-A 7.6 2.1 61 101 8 MOLECULE: TRANSCRIPTIONAL REGULATOR ARSR;
35: 7nyw-E 7.6 2.9 72 212 13 MOLECULE: CHROMOSOME PARTITION PROTEIN MUKB;
36: 4o5v-A 7.6 1.6 57 213 11 MOLECULE: IRON-DEPENDENT TRANSCRIPTION REPRESSOR RELATED PR
37: 1cf7-B 7.6 1.9 64 82 11 MOLECULE: DNA (5'-
38: 6kf4-G 7.6 1.4 61 166 16 MOLECULE: DNA-DIRECTED RNA POLYMERASE SUBUNIT;
39: 5a31-N 7.6 2.8 62 703 2 MOLECULE: ANAPHASE-PROMOTING COMPLEX SUBUNIT 1;
40: 5u8o-B 7.6 2.0 69 360 17 MOLECULE: ZN-DEPENDENT HYDROLASE;
41: 9gm7-C 7.6 3.2 74 440 12 MOLECULE: CHROMOSOME PARTITION PROTEIN MUKF;
42: 1ylf-C 7.6 2.4 63 119 8 MOLECULE: RRF2 FAMILY PROTEIN;
43: 5zqh-A 7.6 1.9 63 101 11 MOLECULE: PADR FAMILY TRANSCRIPTIONAL REGULATOR;
44: 4fht-B 7.5 1.8 61 142 13 MOLECULE: PCAV TRANSCRIPTIONAL REGULATOR;
45: 2p4w-B 7.5 1.7 60 198 10 MOLECULE: TRANSCRIPTIONAL REGULATORY PROTEIN ARSR FAMILY;
46: 2oqg-A 7.5 1.7 59 109 8 MOLECULE: POSSIBLE TRANSCRIPTIONAL REGULATOR, ARSR FAMILY P
47: 7xjg-J 7.5 1.8 62 306 13 MOLECULE: RNA-DIRECTED DNA POLYMERASE FROM RETRON EC86;
48: 2cwe-A 7.5 2.1 60 191 15 MOLECULE: HYPOTHETICAL TRANSCRIPTION REGULATOR PROTEIN, PH1
49: 2rdp-A 7.5 1.5 58 140 14 MOLECULE: PUTATIVE TRANSCRIPTIONAL REGULATOR MARR;
50: 2xrn-B 7.5 1.7 58 241 14 MOLECULE: HTH-TYPE TRANSCRIPTIONAL REGULATOR TTGV;
```

```
0001 s001A   ------DKAFATRVLNTLF-MIC-HLDYKDAQAFP--ATIGNLTSLM--M---TDITT---SRKELKERIEAEVKYLCDESVLMR-ET-S-D--S-----G--V-EYFKFY-----------------------------
0002 4ad9A   qqyishRNIREQQILTLFR-ENF-EK--------S--FTVMELVKII-yK---NTPEN---LHEMAKHNLLLHLKKLEKEGKIFS-NT-D----------P--D-KKWKAHl----------------------------
0003 6j0eB   dlfkvlGDPVRLRILSQLAaGGC-G-----------pVSVNELTDLM--G--------------lSQPTISHHLKKMTEAGFLDR-VP-E----G-----R--V-VLHRVRpelfaelrtvlqigsmellehhhhhh----
0004 6qpqD   --------KAIVQMAKILR-KEL-SE------ekE--VIFTDVLKSQ--A---NTEEN---ITKREASRGFFDILSLATEGCIGL-SQ-T-E--A----fG--N-IKIDAKpalferfi---------------------
0005 5j6xA   ---msaENEIEMRICDYLR-RHG-------------rSTVQDIFKEL--K--------------lEKSTVNRHLYSLQASKQVFK-TV-E-D--N-----K--R-PVWDLVe----------------------------
0006 3r0aB   ikcalnLTKADLNVXKSFL-NEP-D--------rW--IDTDALSKSL--K--------------lDVSTVQRSVKKLHEKEILQR-SQ-Q-N--L-dgggY--V-YIYKIYsknqirniiqkivqswadrlgqelkeweng
0007 2jt1A   -----msESIVTKIISIVQ-ERQnMDDG-----AP--VKTRDIADAA--G--------------lSIYQVRLYLEQLHDVGVLEK-VN-A-G--K-----G--VpGLWRLLe----------------------------
0008 6uvuB   vlfkvlANDTRLRLLHALA-RSG-------------gLCVTDLAAAV--G--------------mKPQAVSNQLQRLADRRILRA-AR-C----G-----N--N-IHYRIVdpcvlrmlelglclieeaeqqagg------
0009 1sfxA   aleklsFKPSDVRIYSLLL-ERG-------------gXRVSEIAREL--D--------------lSARFVRDRLKVLLKRGFVRR-EI-V-E--K---gwV--G-YIYSAEkpekvlkefkssilgeieriekxftdgs--
0010 7z1nO   elvkahLGERAASVIGMLV-ALG-----------R--LSVRELVEKI--D---G----------mDVDSVKTTLVSLTQLRCVKY-LQ-E-T--A-isgkK--T-TYYYYNeegihillysgliideiitqmrvndeeehk
0011 8ffzB   sirpniWKQVVTMVVNEII-FHP-----------G--ITLSRLQSRC--R---E---------vlSLHEISEICKWLLERQVLIT-TD---------------F-DGYWVNhnwysiyest-------------------
0012 7a6hO   svvqerFGSRCARIFRLVL-QKK-------------hIEQKQVEDFA--M--------------iPAKEAKDMLYKMLSENFMSL-QE-I-PksR-----T--F-YLYTVNilsaarmllhrcyksianlierrqfetken
0013 6qfdB   rsdardLTAFQKNILTVLG-EEA--------------RYGLAIKREL--E---E--GE---EV--NHGRLYPNLDDLVNKGLVEK-SE-L-D--K-----R--T-NEYALTnegfdavvddlewtlskfvadadrrervet
0014 2obpA   -----gIDPAIVEVLLVLR-EA--GIENGAT---P--WSLPKIAKRA--Q--------------lPXSVLRRVLTQLQAAGLADV-SV-E-A--D-----G--R-GHASLTqegaalaaqlfp-----------------
0015 4ru7B   stdkyifLTPRAYIIVHLL-KVG-------------kAKASEISENT--Q--------------iPYQTVIQNIRWLLAEGYVVK-EQ-K-G--------E--E-IYYKLTdkgkqlataelekirklvevvqhhhhhh-
```

```
0016 4ijaA   -----pMNDNEKRVLREIY-NHH------------N--ISRTQISKNL--E--------------iNKATISSILNKLKYKSLVNE-VG-----gR-----K--P-ILLKVNhlygyfisldltyssvevmynyfdgnvikh
0017 1stzA   -----kLNDRQRKVLYCIV-REY-IE-----nkKP--VSSQRVLEVS--N-------------ieFSSATIRNDMKKLEYLGYIYQ-PH-T-------------S-AGRIPTdkglrfyyeemlkisketseadlavetfks
0018 3f8fA   ipkemlRAQTNVILLNVLK-QGD--------------NYVYGIIKQVkeA---SNG-E---MEL-NEATLYTIFKRLEKDGIISS-YW-G-D--E---ggR--R-KYYRLTeighenmrlafeswsrvdkiienleankks
0019 8iueP   agkmkgSDNQEKLVYQIIE-DAG-NK----------gIWSRDIRYKS--N--------------lPLTEINKILKNLESKKLIKA-VK-SvA--A-----S--KkKVYMLYnlqpdrsvtggawysdqdfesefvevlnqq
0020 1ku9A   lakihgLNKSVGAVYAILY-LSD-K---------P--LTISDIXEEL--K--------------iSKGNVSXSLKKLEELGFVRK-VW-I-K--G-----E-rK-NYYEAVdgfssikdiakrkhdliaktyedlkkleek
0021 4i99C   -----dIEKYVEELYKVVK-KIY-EKT-----gTP--IKFWDLVP---------------dVEPKIIARTFLYLLFLENMGRVEI-IQ-E-E--P----fG--E-ILVVPM---------------------------
0022 5y6iB   ----vrSAEVGTDILKALA-ELS-P---------A--TSLSRLAEHV--G--------------mPASKVHRYLQALIASGFAVQ-DA-S-------------T-NHYSLGrealrvglaaldsmdvlksaaaplaelrdv
0023 3ov8A   erikllFKEKALEILMTIY-YES-LGG------ND--VYIQYIASKV--N--------------sPHSYVWLIIKKFEEAKMVEC-EL-E----G-----R--T-KIIRLTdkgqkiaqqiksiidimend----------
0024 1sfuA   --ctvnDAEIFSLVKKEVL-S-L-NTN------dY--TTAISLSNRL--K--------------iNKKKINQQLYKLQKEDTVKM-VP-S----------N--P-PKWFKNync-------------------------
0025 4etsB   llerfkYTKQREVLLKTLY-HS----------dTH--YTPESLYMEI--K---Q----aepdlnvGIATVYRTLNLLEEAEMVTS-IS-F----------gsaG-KKYELAnkphhdhmickncgkiiefenpiierqqal
0026 6pcoB   tipdskLTAAQFVVLCALR-DQG-------------aCSLVDVVKAT--A--------------iDQATVRGVIERLKARKLLAV-SH------R-----R--K-VLVTLTpdgralveemvpfaeqitqstfgglnpaer
0027 5vyvA   --mkyqLTALEARVIGCLL-EKQ-VT----TPEQY-pLSVNGVVTAC--N---QKT--repvmnlSESEVQEQLDNLVKRHYLRT-VS-G-F-gN-----R--V-TKYEQRfcnsefgdlklsaaevalittlllrgaqtp
0028 3lmmD   fspvrylsTDQAELTNAAX-LWL-SE------vgD--LATSDLXAXC--G--------------vSRGTAKACVDGLVDEERVVA-VG-G----G-----R--S-RRYRLVe-----------------------------
0029 2zkzC   ellktxAHPXRLKIVNELY-KHK-------------aLNVTQIIQIL--K--------------lPQSTVSQHLCKXR-GKVLKR-NR-Q----G-----L--E-IYYSINnpkvegiiklln-----------------
0030 1xsdA   tnkqveiSMAEWDVMNIIW-DKK-------------sVSANEIVVEI--Q---KY--K---EVS--DKTIRTLITRLYKKEIIKR-YK-S----E-----N--I-YFYSSNikeddikmktaktflnklyggdmkslvlnf
0031 2nyxB   aqvdenITIPQFRTLVILS-NHG-------------pINLATLATLL--G--------------vQPSATGRXVDRLVGAELIDR-LPhP-T--S-----R-rE-LLAALTkrgrdvvrqvtehrrteiariveqxapaer
0032 8jxkD   ------EFMLELAILGLLI-ESP-------------MHGYELRKRL--T-glLGAFR---AF--SYGSLYPALRRMQADGLIAE-NA-A-P--A-----G---rRVYQLTdkgrrrfgelvadtgphnytddgfgvhlaf
0033 7rheA   ----nvRRYNERLLLKTLR-RAG-------------sASKADLARLA--N--------------mTGTAVGSIIASLADAKLIEF-A-----------------A-SLIRLDprgafgigvhldrmrietalvnfagdvlgr
0034 3cuqA   qsvpaeLNMDHTVVLQLAE-KNG-------------yVTVSEIKASL--K--------------wETERARQVLEHLLKEGLAWL-DL-Q-A--P-----G--E-AHYWLPalftdlysqeitaee--------------
0035 6j05A   arlealASPVRLEIFRLLV-EQE-PT----------gLVSGDIAEHL--G--------------qPHNGISFHLKNLQHAGLVTV-QR-E----G-----R--Y-QRYRAAmpvvralvayltencchgtrdcals-----
0036 7nywE   liprsvLSELDMMVGKILC-YLY-LSP--ERLANqgiFTSQELYEEL--IslaDEGKlsdlDKQKLQEKVRTTNLRLRRLGMVYF-LP-N-------------nnNKFTITeavfrfgadvrsgddpreiqlrmirdgeam
0037 4o5vA   -----irsVTEEDYLKIIQ-ELV-LY------kgY--ATLADISRSL--N--------------vKRQSVRDEINHLISLSXAEK-IE----------------R-GKYRLTpsgdreanrflrkhrtaeillsrcigipwe
0038 1cf7B   ---gkgLRHFSMKVCEKVQ-RKG-------------tTSYNEVADELvsE---FTNAY---DQKNIRRRVYDALNVLMAMNIISK-EK-----------------KEIKWIglp-----------------------
0039 6kf4G   eiaqeiGGEEAVEVVKALE-KMK-----------E--ATDEELAEAT--G--------------iRVNTVRRILYMLNDEGLADF-KR-I-R--DpetgwY--Y-YYWRLEtkklpeiirsrkmaelkklkemleeetsei
0040 5a31N   dnmvliKEEELLLFWTYIQ-AML-TN------leS--LSLDRIYNML--RmfvVTGP---alaeiDLQELQGYLQKKVRDQQLVY-SA-----------------GVYRLP--------------------------
0041 5u8oB   aqlrahHDARLDEVRVACA-EQP-------------MSAADIVPIM--F---RRRELdihQMTFALGEAIAHLNLLWLAGELVR-EQ-G-D--D-----G--V-LRFIAr---------------------------
0042 9gm7C   ndfsisLPVERLAFLMAIA-VLN-SE-RLDG---E--MSEGELIDAF--R---E--EQ---TAESVAVRANNAINDMVRQKLLNR-FT-S-E--L-----AdgN-AIYRLTplgisisdyyirqrefstlrlsmqlsivan
0043 1ylfC   -------ISSRFSIAVHIL-SIL-KN----NPSSL--CTSDYXAESV--N--------------tNPVVIRKIXSYLKQAGFVYV-NR-G-------------P-GGAGLLkdlheitlldvyhavnvganiqavleiili
0044 5zqhA   -etqllKGVLEGCVLDMIG-QKE-------------RYGYELVQTL--R---E--fD---TIV---GTIYPLLQKLEKNQWIRG-DM-R-P-gP-----D--R-KYFSLMkegeervsvfwqqwddlsqkvegikn----
0045 4fhtB   tmvseeTTSPQYAVLNALV-AEP-----------G--LDQRTVGERV--G--------------lDRSTIAEVVSRLGRRGLLDK-VR-D-P-dG-----R--R-SLLRLTdeglrvhrrlgvriarmnqvflaplaadeq
0046 2p4wB   rlldvlGNETRRRILFLLT-KRP-------------YFVSELSREL--G--------------vGQKAVLEHLRILEEAGLIES-RV-E-K-gR-----P--R-KYYMIKkglrleilltptlfgsemyeakgvrkspey
0047 2oggA   svfaalSDETRWEILTELG-RAD-------------QSASSLATRL--P--------------vSRQAIAKHLNALQACGLVES-VK-V-G--------R--E-IRYRALgaelnktartlerigaewdrrlaaikqiae
0048 7xjgJ   skdignILYAERFLLPCIY-LLD-----------S--VNYRTLCELA--F---K--kQ---DDVLSKIIVRSVVSRLINERKILQ-MT-----------------DGYQVTalgasyvrsvfdrktldrlrleimnfenrr
0049 2cweA   evikvmLEDTRRKILKLLR-NKE-------------MTISQLSEIL--G--------------kTPQTIYHHIEKLKEAGLVEVkRT-E-M--K--gnlV--E-KYYGRTadvfyinlylgdeelryiarsrlktkidif
0050 2rdpA   iltnypiTPPQFVALQWLL-EEG-------------dLTVGELSNKX--Y--------------lACSTTTDLVDRXERNGLVAR-VR-D-E--H--------V-VRIRLLekgeriieeviekrqrdlanvlesfsdeei
0051 2xrnB   ---siqVIARAASIMRALG-SHP-H-----------gLSLAAIAQLV--G--------------lPRSTVQRIINALEEEFLVEA-LG-P-------------A-GGFRLGpalgqlinqaqtdilslvkpylrslaeeld

0001 s001A   ------LHHHHHHHHHHHH-HHH-LLLHHHHHHLL--LLHHHHHHHH--L---LLLLL---LHHHHHHHHHHHHHHHHHLLLEEE-EE-L-L--L-----L--L-EEEEEL-----------------------------
0002 4ad9A   hhhhhhHHHHHHHHHHHHH-HLL-LL--------L--LLHHHHHHHH-lL---LLLHH---HHHHHHHHHHHHHHHHHHLLLEEE-LL-L-----------L--L-LLEEELl----------------------------
0003 6j0eB   hhhhhhLLHHHHHHHHHHhLLL-L-----------lEEHHHHHHHH--L--------------lLHHHHHHHHHHHHHHLLLEEE-EE-E----L-----L--E-EEEEELhhhhhhhhhhllleeeeeeeeeell----
0004 6qpqD   --------LHHHHHHHHHH-HHH-LL------llL--EEHHHHHHHH--H---LLLHH---LLHHHHHHHHHHHHHHHHLLLEEE-EL-L-L--L----lL--L-LEEEELhhhhllll---------------------
0005 5j6xA   ---llhHHHHHHHHHHHHH-HHL-------------lLLHHHHHHHL--L--------------lLHHHHHHHHHHHHHHLLLEEE-EL-L-L--L-----L--L-LEEEELl----------------------------
0006 3r0aB   hhhhhlLLHHHHHHHHHHH-HLL-L---------lL--LLHHHHHHHL--L--------------lLHHHHHHHHHHHHHHLLLEEE-EE-E-E--L-llllE--E-EEEEELlhhhhhhhhhhhhhhhhhhhhhhhhhhhll
0007 2jt1A   -----llLLHHHHHHHHHH-HHHhHHLL-----LL--EEHHHHHHHH--L--------------lLHHHHHHHHHHHHHHLLLEEE-EL-L-L--L-----L--LlLEEEELl----------------------------
0008 6uvuB   hhhhhhLLHHHHHHHHHHH-HHL-------------lLLHHHHHHHL--L--------------lLHHHHHHHHHHHHHHLLLEEE-EE-E----L-----L--E-EEEEELlhhhhhhhhhhhhhhhhhhhhlll------
0009 1sfxA   hhhhllLLHHHHHHHHHHH-HHL-------------lLLHHHHHHHH--L--------------lLHHHHHHHHHHHHHHLLLEEE-EE-E-E--L---llE--E-EEEEELlhhhhhhhhhhhhhhhhhhhlllllll--
0010 7z1nO   hhhhhhLLHHHHHHHHHHH-HHL-----------L--EEHHHHHHHL--L---L----------lLHHHHHHHHHHHHHHLLLLE-EE-E-E--L-llllE--E-EEELLLhhhhhhhhlhhhhhhhhhhhhhhlllllllhh
0011 8ffzB   lllhhhHHHHHHHHHHHHH-HLL-----------L--EEHHHHHHHL--L---L---------llLHHHHHHHHHHHHHHLLLEEE-EL----------------L-LEEEELllllllllll------------------
0012 7a6hO   hhhhhhHLHHHHHHHHHHH-HLL-------------lLLHHHHHHHH--L--------------lLLLLHHHHHHHHHLLLLLL-LL-E-ElhH-----E--E-LLLLLLhhhhhhhhhhhhhhhhhhhhhhhhhhhhhhhl
0013 6qfdB   hhhhllLLHHHHHHHHHHH-HLL--------------EEHHHHHHHH--H---H--LL---LL--LHHHHHHHHHHHHHHLLLEEE-EE-E-E--L-----L--E-EEEEELhhhhhhhhhhhhhhhhhhhlllhhhhhhhhh
0014 2obpA   -----lLLHHHHHHHHHHH-HH--LLLLLLL---L--LLHHHHHHHH--L--------------lLHHHHHHHHHHHHHHLLLEEE-EE-L-L--L-----L--L-EEEEELhhhhhhhhhhll-----------------
0015 4ru7B   lllllllLLHHHHHHHHHH-LLL-------------eELHHHHHHHH--L--------------lLHHHHHHHHHHHHHHHLLEEL-LL-L-L--------L--L-LLEEELhhhhhhhhhhhhhhhhhhhhhhhlllllll-
0016 4ijaA   -----lLHHHHHHHHHHHH-HLL-----------L--LLHHHHHHHH--L--------------lLHHHHHHHHHHHHHHLLLEEE-LL-----lL-----L--L-LLEEELhhhleeeeeeelllleeeeeeeellllleeee
0017 1stzA   -----lLLHHHHHHHHHHH-HHH-HH-----hlLL--LLHHHHHHHL--L------------llLLHHHHHHHHHHHHHHLLLEEL-LL-L-------------L-LLLEELhhhhhhhhhhhhhhlllllllllhhhhllll
0018 3f8fA   llhhhhHHHHHHHHHHHHH-HLL--------------LLHHHHHHHHhhH---LLL-L---LLL-LHHHHHHHHHHHHHHLLLEEE-EE-L-L--L---llL--L-EEEEELhhhhhhhhhhhhhhhhhhhhhhhhhhhhhhh
0019 8iueP   llllllLLHHHHHHHHHHH-HHH-HH----------lLLHHHHHHHH--L--------------lLHHHHHHHHHHHHHHLLLEEE-EE-LlL--L-----L--LlEEEEELlllllllllllleelleelhhhhhhhhhh
0020 1ku9A   hhhhllLLHHHHHHHHHHH-HLL-L---------L--EEHHHHHHHH--L--------------lLHHHHHHHHHHHHHHLLLEEE-EL-L-L--L-----L-lL-LEEEELlhhhhhhhhhhhhhhhhhhhhhhhhhhhhhh
0021 4i99C   -----lHHHHHHHHHHHHH-HHH-HHH-----lLL--EEHHHHLL---------------lLLHHHHHHHHHHHHHHHHHLLLEEE-EL-L-L--L----lL--L-LEEEEL-----------------------------
0022 5y6iB   ----llHHHHHHHHHHHHH-HLL-L---------L--EEHHHHHHHH--L--------------lLHHHHHHHHHHHHHHLLEEE-LL-L-------------L-LEEEELhhhhhhhhhhhllllhhhhhhhhhhhhhh
0023 3ov8A   lhhhhhLLHHHHHHHHHHH-HHH-HLL------LL--EEHHHHHHHL--L--------------lLHHHHHHHHHHHHHHLLLEEE-EE-E----L-----L--E-EEEEELhhhhhhhhhhhhhhhhhhhll----------
0024 1sfuA   --llllLHHHHHHHHHHHH-L-L-LLL------lE--ELHHHHHHHL--L--------------lLHHHHHHHHHHHHHHLLLEEE-EL-L----------L--L-LEEEELlll--------------------------
0025 4etsB   hhhhhlLLHHHHHHHHHHH-HL----------lLL--LLHHHHHHHH--H---H----hlhhhllLHHHHHHHHHHLLLLLLEEL-LL-E----------ellE-ELLEELlllllleeeeellllleeeellhhhhhhhhh
0026 6pcoB   hlhhhlLLHHHHHHHHHHH-HHL-------------lLLHHHHHHHH--L--------------lLHHHHHHHHHHHHHHLLLEEE-EL------L-----L--L-LEEEELhhhhhhhhhhhhhhhhhhhhhhhhhhllllhhhh
0027 5vyvA   --llllLLHHHHHHHHHHH-HHH-HH----LHHHL-lEEHHHHHHHH--L---LLL--llllllLLHHHHHHHHHHHHHHLLLEEE-EL-L-L-lL-----L--L-LEEEELlllllllllllhhhhhhhhhhhhhhlllllh
0028 3lmmD   llllllllLLHHHHHHHHH-HHH-HH------llL--EEHHHHHHHL--L--------------lLHHHHHHHHHHHHHHLLLEEE-EL-L----L-----L--L-LEEEELl----------------------------
0029 2zkzC   hhhhhhLLHHHHHHHHHHH-HHL-------------lEEHHHHHHHH--L--------------lLHHHHHHHHHHHL-LLLEEE-EE-E----L-----L--E-EEEEELlhhhhhhhhhhl-----------------
0030 1xsdA   lllllllLLLLHHHHHHHH-HHL-------------lEEHHHHHHHH--H---LL--L---LLL--HHHHHHHHHHHHHHLLLEEE-EE-L----L-----L--L-EEEEELllhhhhhhhhhhhhhhhhhhlllhhhhhhhhh
0031 2nyxB   hhhlllLLHHHHHHHHHHH-HHL-------------lEEHHHHHHHH--L--------------lLHHHHHHHHHHHHHHLLLEEE-ELlL-L--L-----L-lL-LEEEELhhhhhhhhhhhhhhhhhhhhhhhhhllllhhhl
0032 8jxkD   ------LHHHHHHHHHHHH-HLL--------------EEHHHHHHHH--H-hhLLLLL---LL--LLLLHHHHHHHHHHLLLEEE-LL-L-L--L-----L---lLEEEELhhhhhhhhhhhhlllhhhllhhhhhhhhhh
0033 7rheA   ----lhHHHHHHHHHHHHH-HHL-------------lEEHHHHHHHH--H--------------lLHHHHHHHHHHHHHHLLLEEE-L----------------L-LEEEELllllleeeeeeeelleeeeeeeellllleeee
0034 3cuqA   elllllLLHHHHHHHHHHL-LLL-------------eELHHHHHHHH--L--------------lLHHHHHHHHHHHHHHHLLLEE-EL-L-L--L-----L--L-LEEELLlllllllllllhhhl--------------
0035 6j05A   hhhhhhLLHHHHHHHHHHH-HLL-LL----------lEEHHHHHHHH--L--------------lLHHHHHHHHHHHHHHLLLEEE-EE-E----L-----L--E-EEEEELhhhhhhhhhhlllllllllllll-----
0036 7nywE   llleeeLLHHHHHHHHHHH-HHH-HLL--HHHHHlleEEHHHHHHHH--HhhlLHHHhlhhHHHHHHHHHHHHHHHHHHLLLEEE-LL-L-------------llLEEEELhhhhhhlllllllhhhhhhhhhhhhllee
0037 4o5vA   -----lllLLHHHHHHHHH-HHH-HH------hlL--LLHHHHHHHH--L--------------lLHHHHHHHHHHHHHHLLLEEE-EE----------------L-LEEEELhhhhhhhhhhhhhhhhhhhhhhhhhhhlllll
0038 1cf7B   ---llhHHHHHHHHHHHHH-HHL-------------eELHHHHHHHHhhH---HHLHH---HHHHHHHHHHHHHHHHHHLLLLLL-LL-----------------LLEELLlll-------------------------
0039 6kf4G   hhhhhhLLHHHHHHHHHHH-HHL-----------L--LLHHHHHHLL--L--------------lLHHHHHHHHHHHHHHLLLEEE-EE-E-E--LlllllE--E-EEEEELlllhhhhhhhhhhhhhhhhhhhhhhhhlllll
0040 5a31N   llllllLHHHHHHHHHHHH-HHH-HH------hlL--LLHHHHHHHH--HhhlLLLL---lllllLHHHHHHHHHHHHHHLLLLEE-EL-----------------LEEELL--------------------------
0041 5u8oB   hhhhhhHHHHHHHHHHHHH-HLL-------------LLHHHHHHHH--L---LLLLLlhhHHHHHHHHHHHHHHHHHLLLEEE-EE-L-L--L-----L--L-EEEEELl----------------------------
0042 9gm7C   hllleeELHHHHHHHHHHH-HHH-HL-LLLL---L--EEHHHHHHHH--H---H--LL---LLLLHHHHHHHHHHHHHHLLLEEE-EE-L-H--H-----HllL-EEEEELhhhhhhhhhhhlllllhhhhhhhhhhhhh
0043 1ylfC   -------LLHHHHHHHHHH-HHH-HH----LLLLL--LLHHHHHHHL--L--------------lLHHHHHHHHHHHHHHHLLEEE-LL-L-------------L-LLEEELllhhhllhhhhhhhhlllllhhhhhhhhhh
0044 5zqhA   -llhhhHHHHHHHHHHHHH-HLL--------------EEHHHHHHHH--H---H--lL---LLL---LLLHHHHHHHHHLLLEEE-EE-E-E-lL-----E--E-EEEEELhhhhhhhhhhhhhhhhhhhhhhhhhhhl----
0045 4fhtB   hhllllLLHHHHHHHHHHH-HLL-----------L--LLHHHHHHHH--L--------------lLHHHHHHHHHHHHHHHLLEEE-EE-L-L-lL-----L--L-EEEEELhhhhhhhhhhhhhhhhhhhhhhhhllllhhhh
0046 2p4wB   hhhhhlLLHHHHHHHHHHL-LLL--------------EEHHHHHHHH--L--------------lLHHHHHHHHHHHHHHLLLEEE-EE-L-L-lL-----L--L-EEEEELlleeeeeeeelleeeeeeeellllllhhh
0047 2oggA   hhhhhlLLHHHHHHHHHHH-HLL--------------LLHHHHHHHL--L--------------lLHHHHHHHHHHHHHHLLLEEE-EE-E-L--------L--E-EEEEELlhhhhhhhhhhhhhhhhhhhhhhhhhhhhhhl
0048 7xjgJ   llllllLLLLHHHHHHHHH-HLL-----------L--LLHHHHHHHH--H---H--lL---LLHHHHHHHHHHHHHHHHLLLEEE-EL-----------------LEEEELhhhhhhhhhhllhhhhhhhhhhhhhhhhll
```

```
0049 2cweA   hhhhhhLLHHHHHHHHHHHL-LLL--------------LLHHHHHHHH--L--------------lLHHHHHHHHHHHHHLLLEEEeEE-E-E--E--lleE--E-EEEEELlleeeellllllhhhhhhhhhhhhhhhhhh
0050 2rdpA   hhllllLHHHHHHHHHHH-HHL-------------lLLHHHHHHHH--L--------------lLHHHHHHHHHHHHHLLLEEE-EE-L-L--L--------L-EEEEELhhhhhhhhhhhhhhhhhhhhhhhhhhllhhhh
0051 2xrnB   ---llhHHHHHHHHHHHHH-LLL-L-----------lEEHHHHHHHL--L--------------lLHHHHHHHHHHHHLLLLEEE-LH-H-------------H-LEEEELlhhhhhhhhhhhhhhhhhhhhhhhhhhhhhl
```

```
Job: Type-1 BREX; BrxC-ATPase Alpha+Beta domain
Query: s001A
No: Chain Z rmsd lali nres id PDB Description
1: 1yvp-B 5.2 11.4 73 532 12 MOLECULE: Y RNA SEQUENCE, FIRST STRAND;
2: 6vdq-A 5.1 7.7 81 305 14 MOLECULE: 3-METHYL-L-TYROSINE PEROXYGENASE;
3: 3kkz-A 5.1 13.5 84 257 8 MOLECULE: UNCHARACTERIZED PROTEIN Q5LES9;
4: 6thk-A 5.0 5.1 96 466 9 MOLECULE: PYOCIN S5;
5: 1u7z-B 4.8 4.1 108 218 5 MOLECULE: COENZYME A BIOSYNTHESIS BIFUNCTIONAL PROTEIN
6: 5b86-A 4.7 4.2 75 579 8 MOLECULE: TUMOR NECROSIS FACTOR ALPHA-INDUCED PROTEIN 2;
7: 8yho-C 4.6 9.1 78 264 6 MOLECULE: DUF87 DOMAIN-CONTAINING PROTEIN;
8: 3iuk-A 4.6 9.7 87 552 6 MOLECULE: UNCHARACTERIZED PROTEIN;
9: 6s8f-F 4.5 10.9 79 2571 11 MOLECULE: SERINE/THREONINE-PROTEIN KINASE TEL1,SERINE/THREO
10: 3trc-A 4.5 3.5 59 168 7 MOLECULE: PHOSPHOENOLPYRUVATE-PROTEIN PHOSPHOTRANSFERASE;
11: 7eld-A 4.5 8.6 77 1137 10 MOLECULE: ENDORIBONUCLEASE DICER HOMOLOG 1;
12: 8g7m-G 4.4 3.5 60 343 5 MOLECULE: 60 KDA HEAT SHOCK PROTEIN, MITOCHONDRIAL;
13: 4hm9-A 4.4 6.8 79 511 4 MOLECULE: BETA-CATENIN-LIKE PROTEIN 1;
14: 7sjr-A 4.4 13.9 58 911 12 MOLECULE: DNA HELICASE;
15: 9jm7-B 4.4 3.7 55 65 9 MOLECULE: LRD-2A;
16: 6vjp-A 4.4 3.7 91 155 7 MOLECULE: ACETYLTRANSFERASE;
17: 3cit-A 4.3 3.6 63 155 5 MOLECULE: SENSOR HISTIDINE KINASE;
18: 2ooc-B 4.3 3.0 50 105 2 MOLECULE: HISTIDINE PHOSPHOTRANSFERASE;
19: 5ipx-A 4.3 3.3 68 282 7 MOLECULE: ORF49 PROTEIN;
20: 7tjl-A 4.3 2.1 52 85 6 MOLECULE: DE NOVO DESIGNED PROTEIN, SEWN0.1;
21: 6icz-A 4.3 4.4 77 2253 12 MOLECULE: PROTEIN MAGO NASHI HOMOLOG 2;
22: 5v8z-C 4.3 3.7 54 98 7 MOLECULE: ENDOPLASMIC RETICULUM RESIDENT PROTEIN 29;
23: 1xkp-A 4.3 5.4 74 230 11 MOLECULE: PUTATIVE MEMBRANE-BOUND YOP TARGETING PROTEIN YOP
24: 5j4a-B 4.1 2.8 65 104 15 MOLECULE: TRNA NUCLEASE CDIA;
25: 8eki-C 4.1 7.4 87 701 7 MOLECULE: PROTEIN TRANSPORT PROTEIN SEC20;
26: 4zmk-A 4.1 2.2 49 71 8 MOLECULE: TELOMERE LENGTH REGULATOR TAZ1;
27: 8usp-A 4.1 3.0 53 982 6 MOLECULE: DNA REPAIR/TRANSCRIPTION PROTEIN MET18/MMS19;
28: 6tgv-C 4.1 9.6 102 389 14 MOLECULE: PHOSPHOPANTOTHENATE-CYSTEINE
29: 7c0q-A 4.1 3.3 55 279 5 MOLECULE: EFFECTOR LPG2505;
30: 8ye6-B 4.1 2.9 55 88 9 MOLECULE: A32;
31: 5eqz-A 4.1 4.3 70 138 11 MOLECULE: REV PROTEIN;
32: 8iqi-C 4.1 5.9 70 916 7 MOLECULE: PUTATIVE PRIMASE C962R;
33: 6cjd-A 4.1 9.2 78 128 5 MOLECULE: PUTATIVE CYTOPLASMIC PROTEIN;
34: 6hd5-t 4.1 8.4 69 838 12 MOLECULE: N-TERMINAL ACETYLTRANSFERASE A COMPLEX SUBUNIT NA
35: 8r33-A 4.1 3.4 53 489 2 MOLECULE: PHO90 ISOFORM 1;
36: 3exe-A 4.1 4.3 100 363 9 MOLECULE: PYRUVATE DEHYDROGENASE E1 COMPONENT SUBUNIT ALPHA
37: 3u24-A 4.1 7.4 84 546 10 MOLECULE: PUTATIVE LIPOPROTEIN;
38: 9b85-N 4.0 6.0 82 286 11 MOLECULE: ALPHA-CENTRACTIN;
39: 4hhx-A 4.0 2.4 52 104 10 MOLECULE: TOXIN COREGULATED PILUS BIOSYNTHESIS PROTEIN E;
40: 7dqk-B 4.0 4.7 87 479 3 MOLECULE: PROTEIN DETOXIFICATION;
41: 6dmp-B 3.9 3.7 74 82 11 MOLECULE: DESIGNED ORTHOGONAL PROTEIN DHD13_XAAA_A;
42: 7yqh-A 3.9 9.2 91 1069 7 MOLECULE: STRUCTURAL MAINTENANCE OF CHROMOSOMES PROTEIN 5;
43: 4xng-D 3.9 3.6 54 144 6 MOLECULE: UNCHARACTERIZED PROTEIN MG218.1;
44: 4g9p-A 3.9 10.5 84 405 10 MOLECULE: 4-HYDROXY-3-METHYLBUT-2-EN-1-YL DIPHOSPHATE SYNTH
45: 8f5d-A 3.9 13.9 80 772 9 MOLECULE: MULTIFUNCTIONAL FUSION PROTEIN;
46: 9iz4-A 3.9 4.1 89 165 7 MOLECULE: PUTATIVE PHOSPHONOPYRUVATE DECARBOXYLASE ALPHA SU
47: 7bc4-B 3.9 5.2 87 2054 5 MOLECULE: FATTY ACID SYNTHASE SUBUNIT ALPHA;
48: 5cos-D 3.9 2.9 46 61 13 MOLECULE: SIDEROPHORE-INTERACTING PROTEIN;
49: 6wkr-A 3.9 3.3 59 439 8 MOLECULE: UBIQUITIN;
50: 7xad-C 3.9 5.8 72 96 7 MOLECULE: PROGRAMMED CELL DEATH 1 LIGAND 1;
```

```
0001 s001A   -YTKVEAEIDNRIKNLIPGDLADTWAELLSDCLG---GAL--KPKIEFHKVRLSVGMDVLGRQLLNHKA-DVNIRVHV--NSGGV-TLDQFRLGNADASLVFFFC-DLYTADTK-FR-DAL-YSYCKFSMF-GKQSALN---S-Q-EEKDAF-EAFRERARKLRT-T-CLLPT---V---E--KF--IR--DAPV----V-SGQGE-IPKSSA-----SS-AAKR-YEEV-L-AKH-LERL-Y-
0002 1yvpB   kpaNEGLTMVAKYVSK--------------------------------------------------------------------------------------------------------------GWKEV-QEAKEKE--lS-P-ETEKVL-KYLEATERVKRT-KehLLTI---H---L--KS--K--taDSV----L-APAS----------------lvalnkLRW-I---P-DTSI-Ve
0003 6vdqA   aaVSARAGEAVRGLNRPG-------------------------------------------------------------------------------------------------artDLA-ALLAATERT-RAALAPV-rgP-V-AELLVD-AALAALAGILEV---AVDRG---SdgpA--GP--P------------------------vfmlyagD-ANRE-QYNE-Q-IR---PTL-Mp
0004 3kkzA   tIHDFELNLICDFFSNXErpaeindfwxdaypeI---DTIpnQVAK------------------------pVATF--------------------------------ilPENCW-TDhYFT-PKVAAQKIF-LTKYAG----------NKIA-EEFSXLQSIEEE-L--YHKY---K---E--YY-------------------------------------------------fiaKKIR-Ll
0005 6thkA   ekKEINKKVSDQVDGLQIT----------------------------------------------------------------------------------------------gkreaTRQ-ERVDVMSAV-LHKSDLE-ykQ-D-SLSEAV-KVLRQELNKQKA---LKEKE---D---L--SQ--LE--RDYRtrkaN-LQMK-------agstplEK-QKAI-YNGE-L-LVD-EIAS-Lq
0006 1u7zB   ------------DPVRYIS-DHSS--GKMGFAIA---AAA--A-avnasvqQQNIFIGCAAV--lkdhR-PYVVGFAA--ET-NN-VEEYARQKRINLDLICAND---VSQPT------------------------------------------------------gfNSDN---N--------------ALHL----F-WQDGD-KVLP----erkeL-LGQL-LLDE-I-VTR-YDEK-Nr
0007 5b86A   wrGVVAEVAAERLDAQP------------------------------------------------------------------------------------------------G-RS-EAE-SRFLHXGRT-XKELEVV-nvV-R-TYAESY-HYHFASHLCALA-----RDT---Y-----aXF--LS------------------------------N-EVTS-VkqlwtqDIL-Q------
0008 8yhoC   pSELVQLPILQMGLKYAIE-----------------------------------------------------------------------------nmdsntekkfftlseviekakynvS-SFNKLLEGL-NY-LLEE-kgN-N-QARSYS-ATLETRIKNVQT-R-F-SNL---F-------------dTELE------------DKSIVY-----SV--------------kkmKLED---
0009 3iukA   rpkSAIDAVADAYTE-------------------------------------------------------lielnpsfattlHET-E-------------------yqDYSPAG-AA-AHA-EATRLALEA-LAGLEPS---D-D-VDAVTL-DAXRERLGLELE-I--HQSG--wD---A--AD--L--aSPAQivieQ-TGRY----------------agtslgAFL-R-D----------
0010 6s8fF   kvvdSKAKGLYYAIKNLP---------------------------------------------------------------------------------------------------qDVG-FAEKSLEKS-LLTFDSR-isQ-T-EWMDTL-NAIIEFIKIAA----IPQDVtvkC---S--KY----------gsiiqlanlmsvKNIAKL---------yDD-PSVV-S-Q----QNE-Kn
0011 3trcA   snAXNXLKILRQITQE------------------------------------------------------------------------------------------------vnaaP-NLE-QALKLVVVR-LCEALpF---A-E-EEEAFC-VTLAIHLAAEIA-H-A-RAK---Ga------------------------------------------------------------------
0012 7eldA   qaSSGAANLIHKLRA-------------------------------------------------------------------------------------------nytlaELG-QW-CAY-KVGQSFLSA-LQSDVNF---Q-V-DVKFQE-SYLSEVVSLLQC---ELLEG---A---S--L-----------lllkyqhTADF-RAI-----------hvtlfdlakaR-KPG-S------
0013 8g7mG   lmLQGVDLLADAVAVVIIEVT----------------------------------------------------------------------------------------------------kDGVTVAKSI-----dLK---D-K-YKNIGA-KLIQDVANNTNE-E-AGD-----------aelkgvI--TVK--------------------------------------------------
0014 4hm9A   fdANKLYCSE------------------------------------------------------------------------------------------------llqdndenrEL-LGelDGIDVLLQQ-LSVFNPS---T-A-EEQEMM-ENLFDSLCSCLM-LsSNRE--gpE---G--TD-nchkrNLRGqrtrL-LNKF----------------hicY-IMAE-I-CNA--NVP-Qi
0015 7sjrA   gRQPAAAALLTVLDVTAtgSDA------------------------------------------------------------------------------------------------------RYTLWQA-WHASGLQ-gaqaD-RDLDAV-TTLFDVADQYVN-R-----------------------------------------------------------------------GTQ-Hl
0016 9jm7B   gvEAAKKEIKKLKEE-------------------------------------------------------------------------------------------vlkkykKG-EI-NEE-EAIKEFVEK-ALKLAVG---D-E-AVKKFA-IEEAKALVEEL--------------------------------------------------------------------------------
0017 6vjpA   -------------------liGDSVMVDIGNVFTkkiPNA--Q-qykdyaaaGQKVVVEL----dsFGK-ADIYLVSI--RvEGR-INKLIYEAARSNVHLVDWY-KASAGHPEyFA-YDG-I-------------------------------------------hlEYAG---S---K--AL--TD------------------------------------lIVKT-M-ETHa-------
0018 3citA   qsRAARLRLLVDTGQ-------------------------------------------------------------------------------------------------eliqlPPE-AXRKCVLQR-ACAFV-P---D-G-EDIESL-QLLATLLAAHLE---NNRLL---E---A--LV--AR--D----------------------------------------------------
0019 2oocB   ---GAVD--FAYLEGFAA-------------------------------------------------------------------------------------------------------gDFAVVDEVL-ALFRE-A---L-D-AALLDI-AAYAHEQALRSL-K-G---------------------------------------------------------------------------
0020 5ipxA   yhyPSWDQILQELDTLS------------------------------------------------------------------------------------------vatLNPD-CH-VPA-LNVEKTLYL-AKTQILVqqkL-V-HIISTS-LTLLKSTARSFF-A--WYDL---Y---R--PNls---------------------------------------------------------
0021 7tjlA   akKEIEQAMYADALI---------------------------------------------------------------------------------------------------------------NPIRF-TEKAKYI-tyG-F-RGQEAY-DQVKKEMFEKLY---KYFME---K---L---------------------------------------------------------------
0022 6iczA   vdDESMQRFHNRVRQI---------------------------------------------------------------------------------------------lmasG-ST-TFT-KIVNKWNTA-LIGLM-N---T-Q-ELLDLL-VKCENKIQTRIK---IGLNeyrvrtqalgiPT--LK--IS---------------lIQIF--------akimlrERI-R------SSE-Pt
0023 5v8zC   ----SLPVYDALAGE------------------------------------------------------------------------------------------------firaS-GVE-ARQALLKQG-QDNLSVK---E-T--QKKWA-EQYLKIMGKILD-Q-GED-------------------------------------------------------------------------
0024 1xkpA   spNISLSQLXAYLEGXS--------------------------------------------------------------------------------------------------------eEPSEQFXML-CGLDALX-peL-A-HLSHLV-EQALVSMAEEQG-E-T---R---D---A--V------xALSA-------------dlQSQ-----QS-GSGR-EXLGf-------------
0025 5j4aB   vSTSQFDYLVSRIGDQFHS---------------------------------------------------------------------------------dmwikdfisteSLN-SS-GLS-IFLATVMRA-RAASAEE---S----FPLYE-NVWNQLVEKLRQdA-RLG-------------------------------------------------------------------------
0026 8ekiC   wiNYEVEMANRQFINI-----------------------------------------------------------------------------------------------tkN-PE-DFP-KSSQNFVKL-INKYDYL-ryK-L-MTCSLIfMNLTSSYLDYIL-T-V--YQ---T---M-----kslsvhRIQ----KllkdensinVLRR---liNK-LDsmkVKNE-L-LNV-IVNY-Ft
0027 4zmkA   -DTFSERTLG------------------------------------------------------------------------------------------------------lnsidnTE-ISEVVSLGL-VSSDKIT-sqA-R-DFSHTL-SKSLKSRAKSLS-Q-K---------------------------------------------------------------------------
```

```
0028 8uspA   [illegible]
0029 6tgvC   [illegible]
0030 7c0gA   [illegible]
0031 8ye6B   [illegible]
0032 5eqzA   [illegible]
0033 8iqiC   [illegible]
0034 6cjdA   [illegible]
0035 6hd5t   [illegible]
0036 8r33A   [illegible]
0037 3exeA   [illegible]
0038 3u24A   [illegible]
0039 9b85N   [illegible]
0040 4hhxA   [illegible]
0041 7dqkB   [illegible]

0001 s001A   [illegible]
0002 1yvpB   [illegible]
0003 6vdgA   [illegible]
0004 3kkzA   [illegible]
0005 6thkA   [illegible]
0006 1u7zB   [illegible]
0007 5b86A   [illegible]
0008 8yhoC   [illegible]
0009 3iukA   [illegible]
0010 6s8fF   [illegible]
0011 3trcA   [illegible]
0012 7eldA   [illegible]
0013 8g7mG   [illegible]
0014 4hm9A   [illegible]
0015 7sjrA   [illegible]
0016 9jm7B   [illegible]
0017 6vjpA   [illegible]
0018 3citA   [illegible]
0019 2oocB   [illegible]
0020 5ipxA   [illegible]
0021 7tjlA   [illegible]
0022 6iczA   [illegible]
0023 5v8zC   [illegible]
0024 1xkpA   [illegible]
0025 5j4aB   [illegible]
0026 8ekiC   [illegible]
0027 4zmkA   [illegible]
0028 8uspA   [illegible]
0029 6tgvC   [illegible]
0030 7c0gA   [illegible]
0031 8ye6B   [illegible]
0032 5eqzA   [illegible]
0033 8iqiC   [illegible]
0034 6cjdA   [illegible]
0035 6hd5t   [illegible]
0036 8r33A   [illegible]
0037 3exeA   [illegible]
0038 3u24A   [illegible]
0039 9b85N   [illegible]
0040 4hhxA   lLHHHHHHHHHHHHHHHH[illegible]
0041 7dqkB   hHHHHHHHHHHHHHHHHHHH[illegible]
```

```
Job: Type-1 BREX; BrxC-ATPase MBR4615907 wHTH2
Query: s001A
No: Chain Z rmsd lali nres id PDB Description
1: 1xsd-A 8.0 2.6 72 125 10 MOLECULE: 5'-
2: 4mtd-D 7.9 3.3 74 151 12 MOLECULE: ZINC UPTAKE REGULATION PROTEIN;
3: 2mh2-A 7.8 1.8 63 64 14 MOLECULE: HOMOLOGOUS-PAIRING PROTEIN 2 HOMOLOG;
4: 3s2w-A 7.7 1.9 66 146 14 MOLECULE: TRANSCRIPTIONAL REGULATOR, MARR FAMILY;
5: 5vyv-A 7.7 1.9 69 173 17 MOLECULE: UPF0502 PROTEIN YCEH;
6: 1ucr-B 7.5 1.6 63 75 25 MOLECULE: PROTEIN DSVD;
7: 6j0e-B 7.5 2.3 70 125 9 MOLECULE: ARSENIC RESPONSIVE REPRESSOR ARSR;
8: 2k4b-A 7.5 2.0 66 69 18 MOLECULE: TRANSCRIPTIONAL REGULATOR;
9: 2eth-A 7.5 2.4 67 141 6 MOLECULE: TRANSCRIPTIONAL REGULATOR, PUTATIVE, MAR FAMILY;
10: 2g9w-A 7.4 2.1 68 119 16 MOLECULE: CONSERVED HYPOTHETICAL PROTEIN;
11: 5hs5-A 7.4 2.0 69 116 12 MOLECULE: HTH-TYPE TRANSCRIPTIONAL REGULATOR SARX;
12: 5l0p-A 7.3 2.7 74 311 5 MOLECULE: TRANSCRIPTION FACTOR ETV6, TRANSCRIPTION FACTOR E
13: 6j05-A 7.3 1.9 67 101 7 MOLECULE: TRANSCRIPTIONAL REGULATOR ARSR;
14: 4yif-B 7.3 2.5 66 141 9 MOLECULE: MARR FAMILY PROTEIN RV0880;
15: 3f72-B 7.2 2.8 70 108 10 MOLECULE: CADMIUM EFFLUX SYSTEM ACCESSORY PROTEIN;
16: 1p4x-A 7.2 2.2 67 250 10 MOLECULE: STAPHYLOCOCCAL ACCESSORY REGULATOR A HOMOLOGUE;
17: 8xt8-B 7.2 1.9 66 145 12 MOLECULE: MARR FAMILY TRANSCRIPTIONAL REGULATOR;
18: 9mj5-B 7.2 2.3 65 208 12 MOLECULE: REPLICATION PROTEIN A 14 KDA SUBUNIT;
19: 6qpq-D 7.2 2.4 65 82 11 MOLECULE: STRUCTURAL MAINTENANCE OF CHROMOSOMES PROTEIN,STR
20: 3bpx-A 7.2 1.9 66 147 12 MOLECULE: TRANSCRIPTIONAL REGULATOR;
21: 8gcc-A 7.1 2.6 79 707 11 MOLECULE: DNA TOPOISOMERASE 2;
22: 2ns0-A 7.1 2.4 72 85 17 MOLECULE: HYPOTHETICAL PROTEIN;
23: 6juv-A 7.1 1.9 62 192 6 MOLECULE: SEGREGATION AND CONDENSATION PROTEIN B;
24: 6qfd-B 7.1 2.8 72 116 10 MOLECULE: DNA-BINDING PROTEIN;
25: 2rdp-A 7.1 2.2 64 140 9 MOLECULE: PUTATIVE TRANSCRIPTIONAL REGULATOR MARR;
```

```
26: 8ylg-A 7.0 2.2 67 161 18 MOLECULE: MARR FAMILY TRANSCRIPTIONAL REGULATOR;
27: 8iue-P 7.0 1.9 63 303 11 MOLECULE: DNA-DIRECTED RNA POLYMERASE III SUBUNIT RPC1;
28: 7wze-A 7.0 2.1 65 161 12 MOLECULE: UNCHARACTERIZED HTH-TYPE TRANSCRIPTIONAL REGULATO
29: 4hqe-B 7.0 2.2 68 108 10 MOLECULE: TRANSCRIPTIONAL REGULATOR QSRR;
30: 3r0a-B 7.0 2.7 67 123 10 MOLECULE: PUTATIVE TRANSCRIPTIONAL REGULATOR;
31: 5eri-A 7.0 2.0 66 153 12 MOLECULE: MARR FAMILY TRANSCRIPTIONAL REGULATOR;
32: 1sfu-A 7.0 1.9 62 70 10 MOLECULE: 5'-D(*T*CP*GP*CP*GP*CP*G)-3';
33: 4asn-A 6.9 2.3 69 90 13 MOLECULE: TUBR;
34: 4gyi-A 6.9 2.7 71 339 10 MOLECULE: RIO2 KINASE;
35: 5jbr-A 6.9 2.3 66 149 11 MOLECULE: UNCHARACTERIZED PROTEIN BCAV_2135;
36: 5j6x-A 6.9 1.8 60 66 15 MOLECULE: Z-DNA BINDING PROTEIN KINASE;
37: 2d1h-A 6.9 1.7 65 102 9 MOLECULE: 109AA LONG HYPOTHETICAL TRANSCRIPTIONAL REGULATOR
38: 2nyx-B 6.9 2.8 67 147 10 MOLECULE: PROBABLE TRANSCRIPTIONAL REGULATORY PROTEIN, RV14
39: 7bzh-A 6.9 1.7 59 59 12 MOLECULE: SUL7S;
40: 1jhf-A 6.9 2.5 66 197 12 MOLECULE: LEXA REPRESSOR;
41: 6pco-B 6.9 2.5 65 135 20 MOLECULE: MARR-FAMILY TRANSCRIPTIONAL REGULATOR;
42: 4ija-A 6.8 2.3 62 363 10 MOLECULE: XYLR PROTEIN;
43: 2p4w-B 6.8 2.4 67 198 12 MOLECULE: TRANSCRIPTIONAL REGULATORY PROTEIN ARSR FAMILY;
44: 5n35-A 6.8 1.7 58 60 10 MOLECULE: POLB1 BINDING PROTEIN 2 (PBP2);
45: 5y6i-B 6.8 1.8 61 234 10 MOLECULE: TRANSCRIPTIONAL REGULATOR KDGR;
46: 7dg2-A 6.8 2.3 68 231 10 MOLECULE: NON-STRUCTURAL MAINTENANCE OF CHROMOSOMES ELEMENT
47: 7z1n-O 6.8 1.8 64 570 19 MOLECULE: DNA-DIRECTED RNA POLYMERASE III SUBUNIT RPC1;
48: 1ku9-A 6.8 2.8 66 151 9 MOLECULE: HYPOTHETICAL PROTEIN MJ223;
49: 4i99-C 6.8 2.6 61 70 11 MOLECULE: CHROMOSOME PARTITION PROTEIN SMC;
50: 1lva-A 6.7 1.8 60 258 7 MOLECULE: SELENOCYSTEINE-SPECIFIC ELONGATION FACTOR;
51: 8zwa-A 6.7 3.0 77 414 9 MOLECULE: DISEASE RESISTANCE PROTEIN ADR1;
52: 1qgp-A 6.7 1.9 63 76 8 MOLECULE: PROTEIN (DOUBLE STRANDED RNA ADENOSINE
53: 8a5y-T 6.7 1.9 64 650 5 MOLECULE: ANAPHASE-PROMOTING COMPLEX SUBUNIT CDC27;
54: 4nb5-B 6.7 2.3 65 149 9 MOLECULE: DNA BINDING PROTEIN;
55: 4ad9-A 6.7 2.7 71 288 13 MOLECULE: BETA-LACTAMASE-LIKE PROTEIN 2;
56: 8ffz-B 6.7 3.0 67 905 3 MOLECULE: TRANSCRIPTION FACTOR IIIA;
57: 3cuq-A 6.7 2.4 67 219 12 MOLECULE: VACUOLAR-SORTING PROTEIN SNF8;
58: 1yyv-A 6.7 2.6 68 114 9 MOLECULE: PUTATIVE TRANSCRIPTIONAL REGULATOR;
59: 5nl9-A 6.7 3.1 74 146 7 MOLECULE: TRANSCRIPTIONAL REGULATOR (FUR FAMILY);
60: 2xco-A 6.6 2.5 79 636 10 MOLECULE: DNA GYRASE SUBUNIT B, DNA GYRASE SUBUNIT A;

0001 s001A   --TGYHPPL-PG-S---PLAEAEQEVEGYLNG---N---P------S--VCPVVNVVDYFKN-R-----PYG----WDEYSTLAALCRLVAG--ERREFHYNGAPNPSRDVVAANLAKNQAYFSIAT-KA-L----
0002 1xsdA   -------tN-KQ-V---EISMAEWDVMNIIWD---K----------K--SVSANEIVVEIQK-Y------KE----VSDKTIRTLITRLYKK--EIIKRYKSEN-----------------IYFYSS-NI-Keddi
0003 4mtdD   laqaeKICAqRN-V---RLTPQRLEVLRLMSL---Q---D------G--AISAYDLLDLLR--E-----AEP---qAKPPTVYRALDFLLEQ--GFVHKVEST-------------------NSYVL-CH-Lfdqp
0004 2mh2A   --------------------GAPGIILRYLQE---Q---N------R--PYSAQDVFGNLQK-E------HG----LGKAAVVKALDQLAQE--GKIKEKTYG-----------------kQKIYFA-D-------
0005 3s2wA   ygqiyigkkieP-Y---GIGSGQFPFLXRLYR---E----------D--GINQESLSDYLK---------------IDKGTTARAIQKLVDE--GYVFRQRDE-----------------RSYRVFL-TE-Kgkkl
0006 5vyvA   ----------MK-Y---QLTALEARVIGCLLE---K---QvttpeqY--PLSVNGVVTACNQ-KtnrepVMN----LSESEVQEQLDNLVKR--HYLRTVSG---------------fgnrVTKYEQ-R-------
0007 1ucrB   ------------------MEEAKQKVVDFLNSksgS---K------S--KFYFNDFTDLFP----------D----MKQREVKKILTALVND--EVLEYWSSG-----------------sTTMYGL-KGa-----
0008 6j0eB   sdesehyAD-LF-K---VLDPVRLRILSQLAA---G---G-----cG--PVSVNELTDLMG---------------LSQPTISHHLKKMTEA--GFLDRVPEGR-----------------VVLHRV-RP-Elfae
0009 2k4bA   -----------fnV---SNAE--LIVMRVIWS---L---G---------EARVDEIYAQI-P-Q-----ELE----WSLATVKTLLGRLVKK--EMLSTEKEG-----------------rKFVYRP-LM-E----
0010 2ethA   fssylpsnE-EI-S---DXKTTELYAFLYVAL---F---G---------PKKXKEIAEFLS---------------TTKSNVTNVVDSLEKR--GLVVREXDP--------------vdrrTYRVVL-TE-Kgkei
0011 2g9wA   ----------KL-T---RLGDLERAVXDHLWS---R---T------E--PQTVRQVHEALSA-------RRD----LAYTTVXAVLQRLAKK--NLVLQIR--------------------AHRYAP-VHg-----
0012 5hs5A   qykalseyidkK-Y---KLSLNDLAVLDLTXK---H---C----kdE--KVLXQSFLKTAXD-E------LD----LSRTKLLVSIRRLIEK--ERLSKVRS---------------kderKIYIYL-NN-Ddisk
0013 5l0pA   qdnntalKK-AG-L---KVTLPRLKILEVLQE--pD---N------H--HVSAEDLYKRLID-M-----GEE----IGLATVYRVLNQFDDA--GIVTRHNF---------------EGGK-SVFEL-T-------
0014 6j05A   -----qdpA-QI-V----ASPVRLEIFRLLVE---Q---E-----pT--GLVSGDIAEHLG---------------QPHNGISFHLKNLQHA--GLVTVQRE-----------------gRYQRYRA-AM-Pvvra
0015 4yifB   rlsrqlrfrnPS-S---PVSLSQLSALTTLAN---E----------G--AMTPGALAIRER---------------VRPPSMTRVIASLADM--GFVDRAPHP--------------idgrQVLVSV-SE-Sgael
0016 3f72B   lqtvdisGV-SQ-I---LADENRAKITYALCQ---D----------E--ELCVCDIANILG---------------VTIANASHHLRTLYKQ--GVVNFRKEG-----------------KLALYSL-GG-E----
0017 1p4xA   ytmyfkniikkH-L---TLSFVEFTILAIITS---Q--nK------N--IVLLKDLIETIH---------------HKYPQTVRALNNLKKQ--GYLIKERST--------------edeRKILIHM-DD-Aqqdh
0018 8xt8B   kddlkkmfkqnG-Y---DITTDHYALLRFLWE---Q----------D--GISQIDLCEKSC---------------KDKSNTTRILDVMKNK--GLIVRKVDV--------------kdrRKFQIFL-TD-Lgrel
0019 9mj5B   ansqpsasfmpA-N---GLTVAQNQVLNLIKA---C---P------RpeGLNFQDLKNQLK----------H----MSVSSIKQAVDFLSNE--GHIYSTVD-------------------DDHFKS-TDa-----
0020 6qpqD   --------------------KAIVQMAKILRK---ElseE------K--EVIFTDVLKSQAN-T-----EpenitkrEASRGFFDILSLATE--GCIGLSQT---------------AFGN-IKIDA-KP-Alfer
0021 3bpxA   shrvfigrelgH-L---NLTDAQVACLLRIHR---E----------P--GIKQDELATFFH---------------VDKGTIARTLRRLEES--GFIEREQDP--------------enrRRYILEV-TR-Rgeei
0022 8gccA   vadcersipsvI-D---GLKPGQRKIIFSSFK---R-rlT------R--SIKVVQLAGYVSE-----haAYH----HGEQSLVQTIVGLAQnnvPLLQQDG----qFGTRlGKDHA--AGRYIFTRL-TN-Iaryi
0023 2ns0A   -------------------RELEECIRALLDA---R---A----dsA--SICPSDVARAVAP-D---------dwrpLXEPVREAAGRLADA--GEVEVTQKGAVVD----RSAR-----gPIRIRW-TR-T----
0024 6juvA   eysqkvihlxpK-P---ELRAGELKTLALIAY---L----------Q--PVEQSKIIKLR------------------GSQAYEHIKKLLEX--GLIYAEPYE-----------------rTKLLGT-TQ-Kfael
0025 6qfdB   -----pdAR-SDaR---DLTAFQKNILTVLGE---E-------------ARYGLAIKRELEEyY-----GEE----VNHGRLYPNLDDLVNK--GLVEKSELD----------------kRTNEYAL-TNe-----
0026 2rdpA   nlkqrgreiltN-Y---PITPPQFVALQWLLE---E---G---------DLTVGELSNKXY---------------LACSTTTDLVDRXERN--GLVARVRD-----------------ehVVRIRL-LE-Kgeri
0027 8ylgA   iardrlnplfaR-Y---GLQPGEFDVLATLRR---S-gaP------Y--ALTPTALYDAAM---------------ISSGSMTNRIDRLEKA--GWVERRANP--------------adgRGTLVAL-TS-Agral
0028 8iueP   -------------------VEIENRIIELCHQ---F---P------H--GITDQVIQNEMP----------H----IEAQQRAVAINRLLSM--GQLDLLRSN-----------------TGLLYRI-KDs-----
0029 7wzeA   kxnhvxehyfaG-R---GLSEGKFKILXLLFD---A---K-----dH--RLSPTELAKRSN---------------VTKATITGLLDGLARD--GFVSRRHH------------------rKISIEL-TT-Egkar
0030 4hqeB   --evcpylE-ET-F---KIRSWNGLIINYLSR---C---N-----dC--SAHFSDMKRDLK----------T----ITPRALSLKLSELAQW--ELVEKQII---------------tspvQIIYVL-TE-Kgkal
0031 3r0aB   kefkvedvikcA-L---NLTKADLNVXKSFLN---E--pD------R--WIDTDALSKSLK---------------LDVSTVQRSVKKLHEK--EILQRSQQN--------------gggyVYIYKI-YS-K----
0032 5eriA   avnsindikykE-L---KLQKGQFTFLTRICE---N----------P--GINLVELSNMLK---------------VDKATTTKAIQKLIKA--GYVDKKQDK--------------fdkRGYNLTP-TD-Kslev
0033 1sfuA   -------------------AEIFSLVKKEVLS---L---N----tnD--YTTAISLSNRLK---------------INKKKINQQLYKLQKE--DTVKMVPS------------------NPPKWFK-NY-Nc---
0034 4asnA   ---sdyfEE-VX-R---KLTIEDVSILGWLFQ---N---E------AfkAIKKSSIADELE---------------YSTANFRKTLNKLEAI--HFIGTVTGG-----------------kEHKLYL-TE-Ygqqa
0035 4gyiA   ----kldTR-AM-R---HLTAEDWRVLTAVEM---G---S---knhE--IVPTPLIEKIARL-R------GG----SS--GVHKSIATLAKA--GLIARMKEA-----------------kYDGYRL-TY-Ggldy
0036 5jbrA   tfsqaxgdxlaT-W---QLPRTTGRTYGYLLL---Q---S------E--ATSFQEIGADLG---------------LSPGAVSTSVRELVAW--GLARTIPQ---------------pgsRRLLVEA-AG-Gfeql
0037 5j6xA   ------------------ENEIEMRICDYLRR---H---G---------RSTVQDIFKELK---------------LEKSTVNRHLYSLQAS--KQVFKTVE----------------dnkRPVWDL-VE------
0038 2d1hA   kleskkdeircC-Y---KITDTDVAVLLKXVE---I---E------K--PITSEELADIFK---------------LSKTTVENSLKKLIEL--GLVVRTKT------------------pKYYYSI-SS-Nilek
0039 2nyxB   aisahsiaQ-VD-E---NITIPQFRTLVILSN---H---G---------PINLATLATLLG---------------VQPSATGRXVDRLVGA--ELIDRLPHP--------------tsrrELLAAL-TK-Rgrdv
0040 7bzhA   ------------------MEDVKQSVEKIIKD---R----------E--WVTFNDLLKYIP---------------YPAPEVYDALSQLIKE--NKVGRRG---------------------RYFYY-IK-R----
0041 1jhfA   -------------K---ALTARQQEVFDLIRD---H---I--sqtgM--PPTRAEIAQRLG--------------fRSPNAAEEHLKALARK--GVIEIVSGA------------------SRGIRL-LQ-E----
0042 6pcoB   rhvaifqqtipD-S---KLTAAQFVVLCALRD---Q---G---------ACSLVDVVKATA---------------IDQATVRGVIERLKAR--KLLAVSHRR------------------KVLVTL-TP-Dgral
0043 4ijaA   -----------------PMNDNEKRVLREIYN---H---H---------NISRTQISKNLE---------------INKATISSILNKLKYK--SLVNEVG----------------ggrKPILLKV-NH-Lygyf
0044 2p4wB   ---mgeeLN-RL-L---DVNETRRRILFLLTK---R-------------PYFVSELSRELG---------------VGQKAVLEHLRILEEA--GLIESRVEK--------------rgrpRKYYMI-KK-Glrle
0045 5n35A   -------------------QKEIEIAIEYFKN---Y----------I--SVGEIVATMDLKA--------RG----IS--NPQAVISKLIEM--GIIEKGE---------------------GCYNL-VR------
0046 5y6iB   -------------------AEVGTDILKALAE---L---S------P--ATSLSRLAEHVG---------------MPASKVHRYLQALIAS--GFAVQDAS-------------------TNHYSL-GR-Ealrv
0047 7dg2A   vnrvenditkmA-S---DYAENELELFRKTME---L---I-ilsdnG--FATSISILNLADElQ-----SKK----MKKKEVEQLLQSFVQE--KWLIGRN---------------------GEYTL-HT-Rcime
0048 7z1nO   lnpdlflykelvkA---HLGERAASVIGMLVA---L---G---------RLSVRELVEKI----------DG----MDVDSVKTTLVSLTQL--RCVKYLQE--------------tsgkkTTYYYY-NE-Egihi
0049 1ku9A   liielfselakI-H---GLNKSVGAVYAILYL---S---D------K--PLTISDIXEELK---------------ISKGNVSXSLKKLEEL--GFVRKVWI---------------kgerKNYYEA-VD-G----
```

```
0050 4i99C   ------------------IEKYVEELYKVVKK---I---Y--ektgT--PIKFWDLV--PDV-E-----------pKIIARTFLYLLFLENM--GRVEIIQEE---------------pfgEILVVP-M-------
0051 1lvaA   -------------------gSPEKILAQIIQE---H---R------E--GLDWQEAATRAS---------------LSLEETRKLLQSXAAA--GQVTLLRV------------------eNDLYAIS-TEr----
0052 8zwaA   ------faH-ME-EsleNLDPKIRDCFLDMGA---F---P------EdkKIPLDLLTSVWVE-------rHD----IDEETAFSFVLRLADK--NLLTIVN----------NPRFgdIGYYDVFVTQ-HD-Vlrdl
0053 1qgpA   -------------------QDQEQRILKFLEE---L---G------EgkATTAHDLSGKLG---------------TPKKEINRVLYSLAKK--GKLQKEAG------------------TPPLWKI-AV-S----
0054 8a5yT   iqlafkdgrkLV-L---DVSLEQCSVINQFDS---pndeP------I--CLSLEQLSESLN---------------IAPPRLTHLLDFWIQK--GVLLKEN---------------------GTYSV-IE-H----
0055 4nb5B   mefveqmggyfesR---SLTRLAGRLLGWLLV---C--dP------E--RQSSEELATALA---------------ASSGGISTNARMLIQF--GFIERLAV---------------agdRRTYFRL-RP-Nafaa
0056 4ad9A   -yDYMNslkellkikadinNIREQQILTLFRE---N--fE------K--SFTVMELVKIIYK-N-----TpenlhemAKHNLLLHLKKLEKE--GKIFSNTDP------------------DKKWKA-HL------
0057 8ffzB   rkSAVKVplgkpfsrlwvWKQVVTMVVNEIIF---H----------P--GITLSRLQSRCRE---------V----LSLHEISEICKWLLER--QVLITTD--------------------FDGYWV-NH-Nwysi
0058 3cuqA   vggtyliqS-VP-A---ELNMDHTVVLQLAE----K---N------G--YVTVSEIKASLK---------------WETERARQVLEHLLKE--GLAWLDLQA----------------pGEAHYWL-PAl-----
0059 1yyvA   lfaeqcpSR-EV-L---KTSRWGVLILVALRD---G-------------THRFSDLRRXX----------GG----VSEXXLAQSLQALEQD--GFLNRVSYP--------------vvppHVEYSL-TP-Lgeqv
0060 5nl9A   erskkilED-AG-I---NVTVQRLQMANLLLS---K---P------Q--HLTADQVFQLINE-H-----MPN----ASRATIFNNLKLFAEK--GIVNLLEL----------------ksgITLYDSnVI-H----
0061 2xcoA   svivaralpdvR-D---GLKPVHRRILYGLNE---Q---G------MksYKKSARIVGDVMG-K-----YHP----HGDSSIYEAMVRMAQ-ryPLVDGQG-----NFGS-gDGAA--AMRYTEARM-TK-Itlel

0001 s001A   --HHLLLLL-LL-L---LLLHHHHHHHHHHHL---L---L------L--EEEHHHHHHHHHL-L-----LLL----LLHHHHHHHHHHHHHL--LLEEEEELLEELLLHHHHHHHLLLLHHHEEEEE-LL-L----
0002 1xsdA   -------lL-LL-L---LLLLLLHHHHHHHHH---H----------L--LEEHHHHHHHHHL-L------LL----LLHHHHHHHHHHHHHL--LLEEEEELLL-----------------LEEEEE-LL-Lhhhh
0003 4mtdD   hhhhhHHHHhLL-L---LLLHHHHHHHHHHHL---L---L------L--LEEHHHHHHHHH--H-----HLL---lLLHHHHHHHHHHHHHL--LLEEEELLL-------------------LEEEE-LL-Lllll
0004 2mh2A   --------------------LHHHHHHHHHHH---H---L------L--LEEHHHHHHHHHL-L------LL----LLHHHHHHHHHHHHHH--LLEEEEEEL-----------------lEEEEEE-L-------
0005 3s2wA   hhhhhhhhhhhH-H---LLLLLLHHHHHHHHH---L----------L--LEEHHHHHHHHHL--------------LLHHHHHHHHHHHHHL--LLEEEEELL-----------------LLEEEEE-LH-Hhhhh
0006 5vyvA   ----------LL-L---LLLHHHHHHHHHHHH---H---HhhlhhhL--LEEHHHHHHHHLL-LlllllLLL----LLHHHHHHHHHHHHHL--LLEEEELL---------------llllLLEEEE-L-------
0007 1ucrB   ------------------LLHHHHHHHHHHLLlllL---L------L--LEEHHHHHHHHHLL--------L----LLHHHHHHHHHHHHHL--LLEEEEEEL------------------lEEEEEE-LLh----
0008 6j0eB   lllhhhhHH-HH-H---HHLHHHHHHHHHHHH---L---L-----lL--LEEHHHHHHHHHL--------------LLHHHHHHHHHHHHHL--LLEEEEEELL-----------------EEEEEE-LH-Hhhhh
0009 2k4bA   -----------llL---LLLL--LHHHHHHHH---H---L---------LEEHHHHHHLL-L-H-----HHL----LLHHHHHHHHHHHHHL--LLLEEEEEL-----------------lEEEEEL-LL-L----
0010 2ethA   hhllllllH-HH-H---HLLHHHHHHHHHHHH---H---L---------LLLHHHHHHHHLL--------------LLHHHHHHHHHHHHHL--LLEEEEELL--------------llllLEEEEE-LH-Hhhhh
0011 2g9wA   ----------LH-H---HLLHHHHHHHHHHHL---L---L------L--LEEHHHHHHHHHLL------LLL----LLHHHHHHHHHHHHHL--LLEEEEL--------------------LLEEEE-LLl-----
0012 5hs5A   hhhhhhhhhhhH-H---LLLHHHHHHHHHHHH---H---H----llL--LEEHHHHHHHHHH-H------HL----LLHHHHHHHHHHHHHL--LLLEEELL---------------llllLLEEEL-LH-Hhhhh
0013 5l0pA   llhhhhhHH-LL-L---LLLHHHHHHHHHHLL--lL---L------L--EEEHHHHHHHHHH-L-----LLL----LLHHHHHHHHHHHHHL--LLEEEELL---------------LLLL-LEEEE-L-------
0014 6j05A   -----llhH-HH-H----LLHHHHHHHHHHHH---L---L-----lL--LEEHHHHHHHHHL--------------LLHHHHHHHHHHHHHL--LLEEEEEE------------------lEEEEEE-LH-Hhhhh
0015 4yifB   hhhhhhhlllLL-L---LLLHHHHHHHHHHHH---H----------L--LLLHHHHHHHHHL--------------LLHHHHHHHHHHHHHL--LLEEEEELL--------------llllLEEEEE-LH-Hhhhh
0016 3f72B   hhlllhhHH-HH-H---HHLHHHHHHHHHLLL---L----------L--LEEHHHHHHHHHL--------------LLHHHHHHHHHHHHHL--LLEEEEELL-----------------LLEEEEE-LL-H----
0017 1p4xA   hhhhhhhhhhhH-L---LLLHHHHHHHHHHHL---L--lL------L--LEEHHHHHHHHLL--------------LLHHHHHHHHHHHHHH--LLLEEEELL--------------lllLLEEEEL-LH-Hhhhh
0018 8xt8B   hhhhhhhhhhlL-L---LLLHHHHHHHHHHHH---L----------L--LLLHHHHHHHHHL--------------LLLHHHHHHHHHHHLL--LLEEEEELL--------------lllLLEEEEE-LH-Hhhhl
0019 9mj5B   hhhllllllllL-L---LLLHHHHHHHHHHHH---L---L------LllLLLHHHHHHHHLL---------L----LLHHHHHHHHHHHHHH--LLEELLLL-------------------LLLLEE-LLl-----
0020 6qpqD   --------------------LHHHHHHHHHHH---HhllL------L--LEEHHHHHHHHHL-L-----LhhhllhhHHHHHHHHHHHHHHL--LLEEEELL---------------LLLL-LEEEE-LH-Hhhll
0021 3bpxA   hhhhhhhhhhhH-H---LLLHHHHHHHHHHHL---L----------L--LLLHHHHHHHHHL--------------LLHHHHHHHHHHHHHL--LLEEEEEEL--------------leeEEEEEEE-LH-Hhhhh
0022 8gccA   hhhhhhhllllL-L---LLLHHHHHHHHHHHH---H-llL------L--LEEHHHHHHHHHH-----hlLLL----LLHHHHHHHHHHHHLlllLLEEEEL----lLLLLlLLLLL--LLLLLEEEE-LL-Lhhhh
0023 2ns0A   -------------------HHHHHHHHHHHHH---L---L----llL--LLLHHHHHHHHLL-L---------llhhHHHHHHHHHHHHHHL--LLEEEEELLEELL----LLLL-----lLLEEEE-LL-L----
0024 6juvA   hhhlllhhhllL-L---LLLHHHHHHHHHHHH---H----------L--LEEHHHHHHLL------------------HHHHHHHHHHHHHL--LLEEEEEEL-----------------lEEEEEE-LH-Hhhhh
0025 6qfdB   -----llLH-HHhL---LLLHHHHHHHHHHHH---L-------------LEEHHHHHHHHHHhH-----LLL----LLHHHHHHHHHHHHHL--LLEEEEEEE-----------------lLEEEEEE-LHh----
0026 2rdpA   hhhhhhhhhhlL-L---LLLHHHHHHHHHHHH---H---L---------LLLHHHHHHHHHL--------------LLHHHHHHHHHHHHHL--LLEEEEEL-----------------llLEEEEE-LH-Hhhhh
0027 8ylgA   hhhhlhhhhhhH-L---LLLHHHHHHHHHHHH---L-llL------L--EELHHHHHHHHHL--------------LLHHHHHHHHHHHHHL--LLEEEEELL--------------lllLLEEEEE-LH-Hhhhh
0028 8iueP   -------------------HHHHHHHHHHHHH---L---L------L--LLLHHHHHHHHLL---------L----LLHHHHHHHHHHHHHH--LLEEEEEEL-----------------LEEEEEE-LHh-----
0029 7wzeA   hhhhhhhhhhhL-L---LLLHHHHHHHHHHHH---L---H-----hH--EELHHHHHHHHLL--------------LLHHHHHHHHHHHHHH--LLEEEEEL------------------lLEEEEE-LH-Hhhhh
0030 4hqeB   --lllhhhH-HH-H---HHLLLHHHHHHHHLL---L---H-----hH--EELHHHHHHHHHL---------L----LLHHHHHHHHHHHHHL--LLEEEEEE---------------llllEEEEEE-LH-Hhhhh
0031 3r0aB   eeeehhhhhhhH-H---LLLHHHHHHHHHHHH---L--lL------L--LLLHHHHHHHHLL--------------LLHHHHHHHHHHHHHL--LLEEEEEEE--------------llleEEEEEE-LL-H----
0032 5eriA   hhhhhhhhhllL-L---LLLLLHHHHHHHHHH---L----------L--LEEHHHHHHHHHL--------------LLHHHHHHHHHHHHHL--LLEEEEELL--------------lllLLEEEEE-LH-Hhhhh
0033 1sfuA   -------------------HHHHHHHHHHHHL---L---L----llL--EELHHHHHHHHLL--------------LLHHHHHHHHHHHHHL--LLEEEELL------------------LLLEEEE-LL-Ll---
0034 4asnA   ---lhhhHH-HH-H---LLLHHHHHHHHHHHH---L---L------LhhLEEHHHHHLLLL---------------LLLHHHHHHHHHHHHL--LLEEEELLL-----------------lLLEEEE-LH-Hhhhh
0035 4gyiA   ----lllLL-HH-H---LLLHHHHHHHHHHHH---H---L---lllL--LEEHHHHHHHHLL-L------LL----LL--LHHHHHHHHHHL--LLEEEEELL-----------------lLEEEEE-LH-Hhhhh
0036 5jbrA   hhhhhhhhhhhH-H---LLLHHHHHHHHHHHL---L---L------L--LEEHHHHHHHHHL--------------LLHHHHHHHHHHHHHL--LLEEEEEE---------------lleEEEEEEE-LL-Llhhh
0037 5j6xA   ------------------HHHHHHHHHHHHHH---H---L---------LLLHHHHHHHHLL--------------LLHHHHHHHHHHHHHL--LLEEEELL----------------lllLLEEEE-LL------
0038 2d1hA   hhhhhhhhhhhH-H---LLLHHHHHHHHHHHH---H---L------L--LEEHHHHHHHHHL--------------LLHHHHHHHHHHHHHL--LLEEEEEL------------------lLEEEEE-LL-Lhhhh
0039 2nyxB   hhhhhhhhH-HL-L---LLLHHHHHHHHHHHH---H---L---------LEEHHHHHHHHHL--------------LLHHHHHHHHHHHHHL--LLEEEELLL--------------llllLLEEEE-LH-Hhhhh
0040 7bzhA   ------------------LLLHHHHHHHHHHH---H----------L--EEEHHHHHHHLL---------------LLHHHHHHHHHHHHHL--LLEEEEL---------------------LEEEE-LL-L----
0041 1jhfA   -------------L---LLLHHHHHHHHHHHH---H---H--hhhlL--LLLHHHHHHHLL--------------lLLHHHHHHHHHHHHHL--LLEEELLLL------------------LLLEEE-LL-L----
0042 6pcoB   hhhhhhhhhlhH-H---LLLHHHHHHHHHHHH---H---L---------LLLHHHHHHHHHL--------------LLHHHHHHHHHHHHHL--LLEEEELLL------------------LLEEEE-LH-Hhhhh
0043 4ijaA   -----------------LLHHHHHHHHHHHHH---L---L---------LLLHHHHHHHHHL--------------LLHHHHHHHHHHHHHL--LLEEELL----------------lllLLLLEEE-LH-Hhlee
0044 2p4wB   ---llhhHH-HH-H---HHLHHHHHHHHHHLL---L-------------LEEHHHHHHHHHL--------------LLHHHHHHHHHHHHHL--LLEEEEELL--------------llllLEEEEE-LL-Leeee
0045 5n35A   -------------------HHHHHHHHHHHHH---L----------L--EEEHHHHHHHHHH--------LL----LL--LHHHHHHHHHHL--LLEEELL---------------------LEEEE-LL------
0046 5y6iB   -------------------HHHHHHHHHHHHH---L---L------L--LEEHHHHHHHHHL--------------LLHHHHHHHHHHHHHH--LLEEELLL-------------------LLEEEE-LH-Hhhhh
0047 7dg2A   eellllllhhhL-L---LLLHHHHHHHHHHHH---H---H-hhlllL--LEEHHHHHHHHHHlL-----LLL----LLHHHHHHHHHHHHHL--LLEEEEL---------------------LEEEE-LH-Hhhhh
0048 7z1nO   llhhhhhhhhhhhH---HLLHHHHHHHHHHHH---H---L---------LEEHHHHHHHL----------LL----LLHHHHHHHHHHHHHL--LLLLEEEE---------------elllEEEELL-LH-Hhhhh
0049 1ku9A   hhhhhhhhhhhH-L---LLLHHHHHHHHHHHH---L---L------L--LEEHHHHHHHHHL--------------LLHHHHHHHHHHHHHL--LLEEEELL---------------llllLLEEEE-LL-H----
0050 4i99C   ------------------HHHHHHHHHHHHHH---H---H--hhhlL--LEEHHHHL--LLL-L-----------hHHHHHHHHHHHHHHHL--LLEEEELLL---------------lllLLEEEE-L-------
0051 1lvaA   -------------------lLHHHHHHHHHHL---L---L------L--LEEHHHHHHHHHL--------------LLHHHHHHHHHHHHHL--LLEEEEEE-----------------lLEEEEEE-HHh-----
0052 8zwaA   ------lhH-HH-HhhhHLLHHHHHHHHHHLL---L---L------LllLLLHHHHHHHHHH-------hHL----LLHHHHHHHHHHHHHL--LLLEELL----------LHHHhlLLLLLLLEEL-LH-Hhhhh
0053 1qgpA   -------------------HHHHHHHHHHHHH---H---L------LllLEEHHHHHHHHHL--------------LLHHHHHHHHHHHHHH--LLEEEELL------------------LLLEEEE-LL-L----
0054 8a5yT   eeeellllleEE-E---EEEHHHHHHHHLLLL---llllL------L--LEEHHHHHHHHHL--------------LLHHHHHHHHHHHHHL--LLEEEEL---------------------LEEEE-LL-L----
0055 4nb5B   hhhhhhhhhhhhhL---LLLHHHHHHHHHHHH---L--lL------L--LEEHHHHHHHHLL--------------LLHHHHHHHHHHHHHH--LLEEEELL---------------lllLLLEEEE-LL-Lhhhh
0056 4ad9A   -hHHHHhhhhhhhhlllelHHHHHHHHHHHHH---L--lL------L--LLLHHHHHHHHLL-L-----LlhhhhhhHHHHHHHHHHHHHHL--LLEEELLLL------------------LLLEEE-LL------
0057 8ffzB   llLLLLLlllllllllllHHHHHHHHHHHHHH---L----------L--LEEHHHHHHHLLL---------L----LLHHHHHHHHHHHHHL--LLEEEEL--------------------LLEEEE-LL-Lllll
0058 3cuqA   elleeeeeL-LL-L---LLLHHHHHHHHHHL----L---L------L--EELHHHHHHHHHL--------------LLHHHHHHHHHHHHHH--LLLEEELLL----------------lLLLEEEL-LLl-----
0059 1yyvA   lllllllHH-HH-H---HHLHHHHHHHHHHHH---L-------------LEEHHHHHHHL----------LL----LLHHHHHHHHHHHHHH--LLEEEEEEL--------------llllEEEEEE-LH-Hhhhh
0060 5nl9A   hhhhhhhHH-LL-L---LLLHHHHHHHHHHHL---L---L------L--EELHHHHHHHHHH-H-----LLL----LLHHHHHHHHHHHHHL--LLLEEEEL----------------lllLEEEELlLL-L----
0061 2xcoA   hhhhhllllllL-L---LLLHHHHHHHHHHHH---L---L------LllLEELHHHHHHHHH-H-----LLL----LLHHHHHHHHHHHLL-llLLEEEEL-----LLLL-lLLLL--LLLLLEEEE-LH-Hhhll
```

Job: Type-1 BREX; BrxC-ATPase C-terminal Coiled-Coil Extension
Query: s001A
No: Chain Z rmsd lali nres id PDB Description
1: 6hyd-A 7.5 22.1 91 1574 9 MOLECULE: MIDASIN,MIDASIN,MIDASIN;
2: 7kfu-C 7.3 24.2 105 909 10 MOLECULE: CAS2;
3: 8r5s-A 6.9 3.2 92 237 12 MOLECULE: CHAIN A, B, C AND H OF THE SOLUBLE HOMOTETRAMER;
4: 3rfy-A 6.7 11.1 96 356 7 MOLECULE: PEPTIDYL-PROLYL CIS-TRANS ISOMERASE CYP38, CHLORO
5: 9u78-A 6.7 2.3 78 118 10 MOLECULE: SINGLE CHAIN PROTEIN OF DE NOVO DESIGNED HELIX BU
6: 8x9p-C 6.7 3.0 77 107 9 MOLECULE: TUBULIN ALPHA CHAIN;
7: 5a7d-R 6.5 14.7 125 311 8 MOLECULE: PINS;

```
8: 6qv7-A 6.4 2.6 78 316 14 MOLECULE: UNCHARACTERIZED PROTEIN;
9: 8dt0-A 6.3 3.1 83 140 13 MOLECULE: SCAFFOLDING PROTEIN FUNCTIONAL SITES;
10: 8wcn-A 6.3 8.5 104 379 6 MOLECULE: DIGUANYLATE CYCLASE;
11: 6r6n-A 6.3 2.9 77 111 4 MOLECULE: SMALL SOLUBLE CYT C;
12: 6dfk-I 6.2 3.7 80 236 13 MOLECULE: SUBUNIT OF PROTEASEOME ACTIVATOR COMPLEX,PUTATIVE
13: 3rgu-A 6.2 3.1 66 88 6 MOLECULE: FIMBRIAE-ASSOCIATED PROTEIN FAP1;
14: 9ri9-B 6.2 9.1 105 868 14 MOLECULE: SLNRC3;
15: 6n1l-A 6.1 2.9 80 141 13 MOLECULE: FIBRONECTIN-BINDING PROTEIN BBK32;
16: 2ot4-A 5.9 10.6 122 520 7 MOLECULE: EIGHT-HEME NITRITE REDUCTASE;
17: 8vdq-A 5.9 13.7 134 2192 8 MOLECULE: GREEN FLUORESCENT PROTEIN,TALIN-1;
18: 6poo-A 5.9 9.8 172 273 9 MOLECULE: BIBA;
19: 7nd2-A 5.8 22.7 87 330 5 MOLECULE: PROTEIN PHOSPHATASE 1 REGULATORY SUBUNIT 21;
20: 9b8o-U 5.8 3.3 92 214 4 MOLECULE: ATPASE H+-TRANSPORTING V1 SUBUNIT D;
21: 9ivk-A 5.7 7.1 108 278 3 MOLECULE: HBC599 MEMBRANE PROTEIN BINDER;
22: 8a6t-B 5.6 3.2 73 630 14 MOLECULE: ELECTRON BIFURCATING HYDROGENASE SUBUNIT HYDA1;
23: 9n0i-E 5.6 2.9 82 133 7 MOLECULE: DE NOVO DESIGNED KABLE PROTEIN;
24: 2win-M 5.6 3.3 73 84 8 MOLECULE: COMPLEMENT C3 BETA CHAIN;
25: 9dbd-A 5.6 3.2 78 326 9 MOLECULE: TET4 VARIANT;
26: 7et3-1 5.6 2.7 67 285 6 MOLECULE: TRIPLEX CAPSID PROTEIN 2;
27: 5m0i-C 5.6 4.6 112 240 7 MOLECULE: SWI5-DEPENDENT HO EXPRESSION PROTEIN 2;
28: 8tn1-A 5.6 2.9 85 147 12 MOLECULE: DE NOVO DESIGNED 4 HELIX BUNDLES;
29: 8ka6-B 5.6 3.1 85 148 11 MOLECULE: DE NOVO DESIGN PROTEIN -NA7;
30: 4avm-A 5.6 4.0 80 230 11 MOLECULE: BRIDGING INTEGRATOR 2;
31: 9bia-A 5.5 2.6 76 110 8 MOLECULE: NINJURIN-1;
32: 7m95-B 5.5 4.0 81 167 5 MOLECULE: SIGMA INTRACELLULAR RECEPTOR 2;
33: 2jx0-A 5.5 3.4 86 131 10 MOLECULE: ARF GTPASE-ACTIVATING PROTEIN GIT1;
34: 7d6v-C 5.5 3.0 84 264 7 MOLECULE: SUCCINATE DEHYDROGENASE SUBUNIT A;
35: 6u4o-A 5.5 5.5 119 479 7 MOLECULE: FUMARATE HYDRATASE;
36: 8xi9-A 5.5 9.0 85 201 8 MOLECULE: FRB-FKBP FUSION PROTEIN;
37: 6s18-A 5.5 2.9 85 143 8 MOLECULE: AROMATIC ACID CHEMORECEPTOR;
38: 4od4-A 5.5 4.2 80 275 5 MOLECULE: 4-HYDROXYBENZOATE OCTAPRENYLTRANSFERASE;
39: 8ccr-A 5.4 2.5 72 105 7 MOLECULE: 4D2 (MUTANT T19D);
40: 1a7d-A 5.4 3.0 80 118 9 MOLECULE: MYOHEMERYTHRIN;
41: 8oyy-A 5.4 4.7 98 233 12 MOLECULE: DE NOVO DESIGNED SOLUBLE GPCR-LIKE PROTEIN;
42: 1rj1-A 5.4 2.9 75 148 7 MOLECULE: INVERTASE INHIBITOR;
43: 5ziy-A 5.4 5.2 101 195 5 MOLECULE: FLAGELLAR HOOK-ASSOCIATED PROTEIN 3;
44: 4kbq-C 5.4 7.1 81 91 10 MOLECULE: E3 UBIQUITIN-PROTEIN LIGASE CHIP;
45: 3lmf-A 5.4 2.4 74 109 4 MOLECULE: UNCHARACTERIZED PROTEIN;
46: 8w6e-B 5.4 3.3 89 161 13 MOLECULE: HBC599 IN COMPLEX WITH WFAP1.1;
47: 9had-C 5.4 3.1 83 108 6 MOLECULE: MITE ALLERGEN DER F 21.0101;
48: 7lsv-B 5.3 5.7 85 158 15 MOLECULE: CALMODULIN-DEPENDENT PROTEIN KINASE;
49: 3qwe-A 5.3 4.4 100 260 6 MOLECULE: GEM-INTERACTING PROTEIN;
50: 3nrh-A 5.3 2.6 83 158 10 MOLECULE: UNCHARACTERIZED PROTEIN BF1032;
```

```
0001 s001A   --HIRAVKDVIRE---F-FG-KTIT--S--D-DQDTLMRDYR-SYAQTKKSAIENMNSR-L-PG-R-RILE-EA-LR-LIDDTV-K--I--N--DPI-SF-YKRVNE-LCD-DFIEVGL-D-MG-DLNSF-L-EGVQ--KDKF-LHGVKAF-KPYT-L--IPKLE-EYDK--KLMGAIM--ELLDHDTKRIEPDIYADWKMVKTSIA-RPYS-QRLLDRIDPKFEELLDKLK-SV-T-DIAALNGIPSES--NALLQNCLR-
0002 6hydA   -----------------------------------SIPEFQKAG-DIIETVLKSVLKL-KQ-W-P-eH-ATLK-EL-YR-VSQEFL-N--Y--P--IKT-P--LARQLQ-KIE-QIYTYLA-E-WE-K-YAS-S-E-----------------------------------------------------------------------------------------mdsylekispnfanlaniapfnneylnldllpklrsaav
0004 8r5sA   --AIKELLELLKK---A-LEkskAK--G--IeAFELACEVLK-KTLEAIKAVADDKAAI-LaAQ-Q-KALA-ET-NA-LCTDSL-IavE--G--VEK-GlkIKLAKE-SVE-KNLEKLK-A----------------------------------------------------------------------------------------------------------------------------------------------
0005 3rfyA   irevqkplEDIT------------dslK--I-AGVKALDSVE-RNVRQASRTLQQGKIV-A-GG-N-EMIE-KL-EA-GMQDML-K--I--V--EDR-KR-DAVAP--KQK-EILKYVG-G-IE-EDMV-------------------------DG-F--P--YE-VPeeyseltPSNS--------------------------------------------------niL-DG-Ry------------------------
0006 9u78A   --------------------------------G-GIEELLRKII-EDEVRHIAELEDIWLK-D-VI-E-ELLR-KI-IE-DEVRHI-A--E-lE--DIE----eELLRK-IIE-DEVRHIA-E-LE-DIEKW-L-KDP------------------------------------------------------------------------------------------------------------------------------
0007 8x9pC   --------------------------hgV--P-YFRNILQSET-EKLTSHCFEWDRKELD-I-PA-K-DLIR-TA-VG-QTRLLM-K--ErfK--QFE----WDMVSF-QIE-DVIHKFN-N-LI-KLEES-G----------------------------------------------------------------------------------------------------------------------------------
0008 5a7dR   ---------------vrqlLQ-VHlQ--A--S-SLPEALAGAT-QLLSHLDDFTATERRG----Y-E-QHLE-QI-RT-VSKDTR-Y--S---rqHYI-N--LESLLD-DVQ-LLKRHTL-I-TL-RLIFE-R--LVR--VLVI--------lsDAF-VqnEAVRT-LLVV--VLDHkqssaawhgSEHRVPGLRDCAESLVAGLAA--------------------------------------------------------
0009 6qv7A   ---------------rkA-LK-K------gsE-TSDAELHELR-IDCKKLRYLLEFFAS--L-FP-P-KATA-QV-LR-QMKTLQ-D--NlgtekVRE-HF-HEIFSG-FDSnETGELFD-E-L---LTGL-A----------------------------------------------------------------------------------------------------------------------------------
0010 8dt0A   kelekilrDEEG-----------------daE-EAAELFRAES-IDEMKHAEELAKLKKG-G-LL-R-ELLE-EL-AE-LELVAI-N-qY--R--EAR-EA-LEEALA-LEL-DGAKLAR-A-AL-EAVEK-L-L--------------------------------------------------------------------------------------------------------------------------------
0012 6r6nA   qsmrAVTG---------------------------TSGQCTQLS-KDAAAILAGVKESSIS-V-DY-K-VLND-EV-AY-HAANID-A--A-aK--AND----LEEVQV-QFR-RMTIACR-N-CH-KIYKTe-----------------------------------------------------------------------------------------------------------------------------------
0013 6dfkI   ---------------kydiSL-RE-----elE-KIKTYASELI-EIIGNIKLWIQLNVPRnN-FG-V-GIQE-EA-IQ-ELARVE-E--SafN--LYD----IVDMRN-NYI-MLYDLLY-K-NW-EKvvK-P-KN-------------------------------------------------------------------------------------------------------------------------------
0015 9ri9B   ------------------------------giQ-GEVENLLTDL-NYFNAFLKEAAKSR------E-N-EVLK-EL-VK-KIRKVV-N--D---adEIK-SI-RERVKE-IRD-NDAYGLQ-A-IT--LDDN----------------EERK-------------------------------eddvvgfddeaktviDRLIG--GSDY-ykiykdpkveYEFFTrreiflnII-SKfvlmttrqsnvakrcltkdeswellek
0016 6n1lA   ynvdsaiqTITK-----------------akF-TKEDLEKNFK-TLLNYIQVSVKTANFVdT-HA-K-RKLE-NI-EA-EIKTLIaK--I--K--ELY-EA-YKAIVT-SIL-LMRDSLK-E-VQ-GIID-------------------------------------------------------------------------------------------------------------------------------------
0018 8vdqA   aqsrlneaaaglnqkaI-AV-T------snT-SPE-ELGPLA-NQLTSDYGRLASAKPA-A--I-K-HRVQ-EL-GH-GCSALV-T--KagA--LQCvyT-KKELIE-CAR-RVSEKVS-H-VL-AALQA-G----N--RGTQ-AC-----vqnaagS--QEKLA-QAAQ--SSVATIT--RLADVVKLGAASLGAEDPetqvvlinaAVAAgNSCRQeDVIA---------------------------------------
0020 7nd2A   ------------------------------alA-FVQD-LVTAL-LNFHTYTEQRIQIFP----TI-S-PLNQ-KF-SQ-YLHENA-S--Y-vR--PLE----lTSYIC-FLR-KILPYQL-K-SL-EEECE-S-SLC----TSA-L---------------------------------------------------------------------------------------------------------------------r
0021 9b8oU   -------------------------frvvK--E-PLGFVKVLQW-VFAIFAFATCGSYTltvG-DY-S-SSAE-FF-VT-VAVFAF-L--YsmG--ALA----fLATAV-FAF-MWLVSSS-A-WA-KGLSD-V-KM-A--TDP--------------------nII-KEM---PMCRQTGn----------------------------------------------------------------------------------
0022 9ivkA   ---------------lkklLN-DNeK--A--D-NAAQVKDALT-KMRAAALDAQKATPP-----E-M-KDFR-HG-FD-ILVGQI-DdaL--K--LAK-EA-QAAAEQ-LKT-TRNAYIQ-K-YL-EKAKE-T--MEK--RKEI-RrelekI--------llkEIL-G-----------------------------------------------------------------------------------------------
0023 8a6tB   ---------------kfkasG-GCVP---vvV-DEDT--CMVD-FAKFFTNFIVEESCGK-C-IPcR-EGNK-KM-LE-ILERIT-E--G-------------kGKEG-DIE-LLEELGD-V-II-SA-SL-C-G--------------------------------------------------------------------------------------------------------------------------------
0024 9n0iE   ---------------eaI-GP-R------dlA-RVANLLAELK-ELFKKDLNLANAMAAA-V-ALlA-EQLE-RL-KK-IQAMFA-A-aV--N--AFE-AF-GALLEA-IIN-EGKALLP-L-VE-AIKEA-I----------------------------------------------------------------------------------------------------------------------------------
0026 9dbdA   adweaamlTLLM-----------viekA--D-NAAQVLTALW-FMALAAHMAALATSPA-S-EM-I-DFRV-GF-EE-LATEIG-K-aI--K--LAK-EA-QAAAEQ-LKT-TVNAHWQ-K-YR-S----------------------------------------------------------------------------------------------------------------------------------------
0027 7et31   ---------------ekrlfN-ELMlwlgweE-LERKCLARIQ-ERCKLLVKELRMC--------lS-FDSN-YC-RN-ILKHA---------------veN-GDSADT-LLE-LLIEDFD-I-YV-DSFPQs-----------------------------------------------------------------------------------------------------------------------------------
0028 5m0iC   --TSELVEQIL-A---L-LSsVLSYnaS--E-FIDKVILPIA-SMFVKSVETFDLYYLTsI-LA-IdDTYN-HF-VK-FSQWMI-E--S--L--R------eEEFQTlSAA-WHSILDG-K-LS-ALDEE-F--DVV--ATK------------------WHDKF---GK---LK---------------------------------------------------------------------------------------
0029 8tn1A   ---------------lsrlAW-KT-----dlA-TYLDQAASYQ-SQVDQYAVETVRAELKaD-RA-L-QIAE-KL-LK-TVQEAS-KtlD--T--AVA-KA-FNQFVS-LGN-QADTLFT-Q-LQ-RTLTN-L-NKK------------------------------------------------------------------------------------------------------------------------------
0030 8ka6B   ---------------lskvLV-EEIE--G--R-DPKAAVEGLI-ETMRRAADLLIELELNlS-EA-G-RVLA-EA-AV-AMREAA-D--RlrA--ELA----SAAADE-ALA-VFVEAVR-R-VA-AALLE-H-HH-------------------------------------------------------------------------------------------------------------------------------
0031 4avmA   ------------------------------lyK-DLKNFLSAVK-VMHESSKRVSETLIYS-SeEL-K-AIVW-NN-DL-LWEDYE-E--KlaD--QAVqni-SNLRDV-FYR-EMSKLNH-N-LY-EVMSK-L-ERQHsn---------------------------------------------------------------------------------------------------------------------------
0032 9biaA   ------------------------------vnH-YANKQSAAES-MLDIALLMANASKAVVgndPL-V-VLIS-IS-LV-LQIGVG-V--LliF--LVK----dFLNNL-ATG-LVFIIVV-V-NI-FITAF-G-VQ-------------------------------------------------------------------------------------------------------------------------------
0033 7m95B   --------------------------gtL--G-ARRGLEWFLG-FYFLSHIPITLLLQGVfK-SF-L-FCELvFQ-LP-FFPIAA-Y--A---ffKGG-C---KWIRT-PAI-IYSVHTM-T-TL-I-------TLLL---dfskaSHFRGQ---------------------------------------------------------------------------------------------------------------
0034 2jx0A   ilkteqvtKNIQ-----------raaqE--F-KHDSFVPCSE-KIHLAVTEMASLFPPA-L-EPvR-SSLR-LL-NA-SAYRLQ-S--E-cR--KTVqLL-TQQVIQ-CAY-DIAKAAK-Q-LV-TITTR---EKKQ-----------------------------------------------------------------------------------------------------------------------------
0035 7d6vC   --YYR----------------avaplilQ--N-IHRYFFYAAV-LISLVNTYDAITAFHF-G-LG-N-VILT-GN-VI-LLWVYT-L--S----cHSC-R--ntRHML-FAW-ITLGTLV-L-TD-FYIML-V-ASGT--I--------------------------------------------------------------------------------------------------------------------------
0036 6u4oA   --PTAMNLSIAME---TalnlqqCH--L--A-pALAGHDALL-QLSGCFNTTATARLSNcE-SL-R-MVCL-QI-MG-NHFTTS-M-aA--S--QGN-VC-KPLI----AA-NLLHTCE-L-LT-DSTRC-F-AD-K--CVRD-LQ----L--------------------------------------------------------------------------------------elnllcgekFDEIhn----------
0037 8xi9A   -----gwhemwhE---G-LE-E-FG--E--R-----NVKGMF-EVLEPLHAMMERGPQE-T-SFnQ-AYGR-DL-ME-AQEWCR-K-yM--K--SGN----VKDLTQ-AWD-LYYHVFR-R-IS-K----------------------------------------------------------------------------------------------------------------------------------------
0038 6s18A   ---------------nnA-LL-M------agK-RMALSVELNN-KSQALVDEFVENARE--P-AL-R-GLAT-EL-QA-TFAEYA-KavA--G--QRE----nSDAGN-AMG-RLQTLRQ-Q-LV-TTLSE-R-GQ-Q--IMLEs----------------------------------------------------------------------------------------------------------------------
0039 4od4A   -------------------------sipaL--L-GPKGALAASL-AMHAAAVALFIAGAYG-L-GA-T-VSTA-LT-AL-VIILVQ-A--M-aW--LGR----VKESFN-LNL-AVPIIIG-A-GI-I--------------------------DML-----------------------------------------------------------------------------------------------------------
0040 8ccrA   ---------------rekhVY-AD-----------SNSPELRE-KHRALAEQVYATGQ--rE-KH-R-ALAE-QV-YA-TGQEML-K--N--T----------SNSPE-LRE-KHRALAE-Q-VY-ATGQE-M-L--------------------------------------------------------------------------------------------------------------------------------
0041 1a7dA   ---------------rvfyHK-KI-----irD-NSAPNLATLV-KVTTNHFTHEEAMAAK-Y-SV-V-PHKK-MH-KD-FLEKIG-G--L--S--APV-D--aKNVDY-CKE-WLVNHIKgT-DF-K-YKG-K-L--------------------------------------------------------------------------------------------------------------------------------
0042 8oyyA   --LIKLLKSMV-G---I-LAvkEFE--E--L-VLRELLEFLR-ELAEKTLEAAEEYDPD-D-EL-A-KGLL-EAaRR-ILEALE-R-aL--R--AMD-LA-IAEA----AV-EIAEAAI-ElVI-KPVVE---KLKE-----------------------------------------------------------------------------------------------------------------------------
0043 1rj1A   ---------------------clKTL--LitT-LALIMVDAIK-AKANQAAVTISKLNPP-A-AW-K-GPLK-NC-AF-SYKVIL-TasL--P--EAE-DG-MVGSSG-DAQ-ECEEYfS-K-SPf-----------------------------------------------------------------------------------------------------------------------------------------
0044 5ziyA   ---------------lkS-LT-R------neK-ELQAIGVEID-QILKQVVYLANTIFGGfR-NG-E-ALLS-PV-IK-TLKQMS-E--A-mQ--NGD----QKALKP-LLE-ENKQNLD-G-II-NRTTE-V-GSTM-------------------nT--METFK-TILS--EQNVALQ-----------------------------------------------------------------------------------
0045 4kbqC   -----------------------------------DPFTEFSLE-SYAFNMKATVEDELQG-K-IN-D-EDKQ-KI-LD-KCNEII-NwlD--K--NQT-A--KEEFEH-QQK-ELEKVCN-P-II-TKLYQ-S-AG------------------------------pPTIE--------------------------------------------------------------------------------------------
0046 3lmfA   -----------------------------------TDQNLQACI-DACNHCYRTCLRXHCL-E--H-L-RLXX-NC-AE-ICQTSL-N--FxlS--GSR-FS-PKVCGV-CAE-ICDACAK-S-CE-Q-LDG-Xe---------------------------------------------------------------------------------------------------------------------------------
0047 8w6eB   ieevkrllEKDE-----------RE--F--Y-EFRRVIKLDE-EVMKRSEELLKRALLR-ElrL-K-ELYE-EA-KR-LMEKAK-E-lT--K--RIR-EW-YEIVRE-LLE-RAKEIIE-E-IE-RLLRR-----------------------LLE-I--LGle--------------------------------------------------------------------------------------------------
```

```
0048 9hadC   deeklklldtllT---M-VE-W-eesvE--K-NSRMRHIRAV-MWAEYMLEIARSL--------eD-EKIL-EI-AE-KLEKAL-P--E--K-sKMF-T--KEEYEK-LME-VLEELEE-V-LE-EKKEE-V-EE-R--IEG-----------------------------------------------------------------------------------------------------------------------
0049 7lsvB   --------------------------------KNQMSKQQLL-GEIQGFKENYWNMKDL-L-TN-R-HHLR-VF-LE-YLDNIC-SafK--D--DK--TD-EKSARA-A----YDFLNA-Q-IN-KLFED-N----S--KNS---------------K--PSFesfNPEQs------------------------------------------------------------------------------------------
0050 3qweA   ----------------------------alE-YAKTWSRYAK-ELLAWTEKRASYEFAKlG-TL-AmETVA-QQ-KRdYYQPLA-A-kR--T--EIF-QG-DEVLRR-VTL-SLFGLRG-A-Q---------------------------eRGPR-A--FAALA-EC-C--APF--EP--GQRYQEFVRAL---------------------------------------------------------------------
0051 3nrhA   ---------------------------------FDKNVIEFV-TVAAEFCAFLERALAS-I-LI-S-EDLA-DI-YQ-DIKDFI-F--V--FneTXN-DS-LAICQE-NFGlLWGQKLV-N-TX-RALHD-V-KYS----------------------pKARL-------------------------------------------------------------------------------------------------

0001 s001A   [illegible]
0002 6hydA   [illegible]
0004 8r5sA   [illegible]
0005 3rfyA   [illegible]
0006 9u78A   [illegible]
0007 8x9pC   [illegible]
0008 5a7dR   [illegible]
0009 6qv7A   [illegible]
0010 8dt0A   [illegible]
0012 6r6nA   [illegible]
0013 6dfkI   [illegible]
0015 9ri9B   [illegible]
0016 6n11A   [illegible]
0018 8vdqA   [illegible]
0020 7nd2A   [illegible]
0021 9b8oU   [illegible]
0022 9ivkA   [illegible]
0023 8a6tB   [illegible]
0024 9n0iE   [illegible]
0026 9dbdA   [illegible]
0027 7et31   [illegible]
0028 5m0iC   [illegible]
0029 8tn1A   [illegible]
0030 8ka6B   [illegible]
0031 4avmA   [illegible]
0032 9biaA   [illegible]
0033 7m95B   [illegible]
0034 2jx0A   [illegible]
0035 7d6vC   [illegible]
0036 6u4oA   [illegible]
0037 8xi9A   [illegible]
0038 6s18A   [illegible]
0039 4od4A   [illegible]
0040 8ccrA   [illegible]
0041 1a7dA   [illegible]
0042 8oyyA   [illegible]
0043 1rj1A   [illegible]
0044 5zjyA   [illegible]
0045 4kbgC   [illegible]
0046 3lmfA   [illegible]
0047 8w6eB   [illegible]
0048 9hadC   [illegible]
0049 7lsvB   [illegible]
0050 3qweA   [illegible]
0051 3nrhA   [illegible]

########################################################################################################################################################################################################################################################################################################################################################################
########################################################################################################################################################################################################################################################################################################################################################################
########################################################################################################################################################################################################################################################################################################################################################################

Job: Type-1 BREX; BrxX-MTase MBZ5596028 N-terminal Domain
Query: s001A
No: Chain Z rmsd lali nres id PDB Description
1: 8c45-A 17.8 2.4 192 1219 23 MOLECULE: SITE-SPECIFIC DNA-METHYLTRANSFERASE (ADENINE-SPEC
2: 3khk-A 7.7 4.0 122 488 12 MOLECULE: TYPE I RESTRICTION-MODIFICATION SYSTEM
3: 2y7h-B 7.4 3.5 123 530 11 MOLECULE: TYPE-1 RESTRICTION ENZYME ECOKI SPECIFICITY PROTE
4: 5ybb-A 6.8 4.0 127 488 15 MOLECULE: TYPE I RESTRICTION-MODIFICATION SYSTEM METHYLTRAN
5: 3ufb-A 6.7 3.8 123 484 10 MOLECULE: TYPE I RESTRICTION-MODIFICATION SYSTEM METHYLTRAN
6: 2okc-B 6.6 3.5 121 427 15 MOLECULE: TYPE I RESTRICTION ENZYME STYSJI M PROTEIN;
9: 7vru-A 5.9 3.8 120 498 11 MOLECULE: SITE-SPECIFIC DNA-METHYLTRANSFERASE (ADENINE-SPEC
10: 3lkd-B 5.9 3.6 118 472 10 MOLECULE: TYPE I RESTRICTION-MODIFICATION SYSTEM
11: 5hr4-J 5.6 3.5 126 752 13 MOLECULE: MMEI;
12: 7eew-A 4.3 4.0 111 611 12 MOLECULE: TYPE I RESTRICTION-MODIFICATION SYSTEM METHYLTRAN
13: 8w0p-A 3.9 4.4 118 917 9 MOLECULE: RM.BSAXI;
16: 2f8l-A 3.6 3.5 84 324 8 MOLECULE: HYPOTHETICAL PROTEIN LMO1582;
18: 3s1s-A 3.4 4.0 109 871 8 MOLECULE: RESTRICTION ENDONUCLEASE BPUSI;

0001 s001A   MILGKALRNEIRKMVGAIRDLLNEDIRIQLEGTFRILREIAPQEAVEHFTREVGFTSLNRLAALKMMEARGLIQESVS-RGQESSGSSQPDGGYRR-YLELLFDDAAVELRVLFDRALPHSRIFPRP-DALNRALKLLNESCPAEVWKSDETIGWIYQYFTPKEDREALRGGAQ
0002 3khkA   ---qQFLNDLDNQLWRAADKL-RSNL------------------------DAANYKHVVLGLIFLKYVSDAFEigARWNkLRDV---TLPT--SVSW-LIDNAFDDIEKLKGILN------SQYQLDA-DKLIGLINEFS----KDIL--GHVYEYFLGQFALeGKQGgTQIPAC
0003 2y7hB   -----mnNNDLVAKLWKLCDNLRDGG-----------------------vSYQNYVNELASLLFLKMCKETGQEAyRWD-DLKS-----riGQEQLQ-FYRKMLVHLGELVQAVFHN----vSTTIEP-KQITALVSNMDSLDWyngaKSRDFGDMYEGLLKNANETiFYAQGVK
0004 5ybbA   ----PQTRESLANEIWRACDIMRRDNN---------------------cTGIMEYVEHLAWLLFLRFLDAQE-sEYRWRhWATK---dwPADEAFVHgRLIPYLRS--ETIRSLFSER---NVIVCSG-YNLKDVIQIVNE-INFHS---iFTVSQVYEELLLGNE-fSKGNPIQ
0005 3ufbA   adqpmtTAQQLGAIVKSSRQIMRKDKG---------------------lNGDLDRLPMLTWIMFLKFLDDLEQwRDWAA-IEGGitg------------giGLFAYLRSVIATVFKG----MQNRMNG-YLLRDVVDKING-IHFN---MHTLSRLYETMLMRDAGDSGaPYTDI
0006 2okcB   ------TEQSLTKKVWNLATTLAGQG-----------------------iGFTDYITQLTYLLFLKXDAENSAIPYQWAdLIAF------dGLDLVK-QYEETLKLLSELIGTIYTK----aQNKIKP-VYLKKVITXIDEE----QWLIXDvKGAIYESILNGQD---tLATNK
0008 7vruA   -xtlinLKDLEAHLW-HAAHIITGPI-----------------------DASD-YKTYIFPILFFKRICDVYvGCHW-ddVFAK-------NHDIGK-ALKDAFLGIEQPLHGIFG-----DASWLPD-ELLATLLNHFNQ-VNLG---XGRAYEYLIKRFADK-ANKKrAQNTL
0009 3lkdB   ------TSQSLYQALWNSADVLRSKX------------------------DANDYKSYLLGXVFYKYLSDKXtpDLTFTaLVER----vndGSFQLE-DLAQGFRDIEQLYENLFE-----DIDLgQN-QTVAAVXKELAVL----DVAGGDAYEYLIGQFAT----GKNQNIX-
0010 5hr4J   ----kpqdpINIKAAERMGKLHDTLKLVG--------------------yEGHALELYLVRLLFCLFAEDTTIFELFQE-YIETK--tleDGSDLAH-HINTLFYVLNTINGKLFEEP---LPPAQFDkAMREALLD-LCSL----DWSR--ISPAIFGSLFQaKKRRNLGPFVG
0011 7eewA   ------------NKLMNIIELIRKDTGI---------------------NNAIDAVEQLALLLLVRYTHEVASdSFKN---------lffdlNVID---FYTLRDKLnEKIENILDQ----IPFRIST-KILDLVIHRLE-ELDL----SEGI-EIDFDHLLNMVK-LAPYSKVN
0012 8w0pA   VRIDREEIEGLGRNLHNVLWG-------------------------gggtNDSEIFYSLVNIILAKIQDEYEKqEYDF--QVYQ---nvESPQKLFD-RINALYKRALREQLnvtdeqkiaNRNKFPL-NKLVYTVQALES----LSFLLGDFFESIIRDGFtfttpKKGEKPMP
0013 2f8lA   ---ANEATQELFQVLDNTAIILQNELE----------------------iSYLEAVYETGENLFQK----------------------------------------------------------QKE-EKQLKLQASYES-IELEN-FSNEIRKGLQLALKGXKH---FKSQAR
0014 3s1sA   leIIDFfkhdpnSSTANLVDILEN------------------------rkDNSTSWHQALVVAGYEYIRGVLVPTRDA---------------yFKS-RPGRLLEEGRKDFNVLFSE----pepNTND-NDIWN-VNLLSS--lnDLGRILTG-DELAELIHDaTRGRGHEPYVS
```

```
0001 s001A   [illegible]
0002 3khkA   [illegible]
0003 2y7hB   [illegible]
0004 5ybbA   [illegible]
0005 3ufbA   [illegible]
0006 2okcB   [illegible]
0008 7vruA   [illegible]
0009 3lkdB   [illegible]
0010 5hr4J   [illegible]
0011 7eewA   [illegible]
0012 8w0pA   [illegible]
0013 2f8lA   [illegible]
0014 3s1sA   [illegible]

Job: Type-1 BREX; BrxX-MTase MBZ5596028 (TRD)
Query: s001A
No: Chain Z rmsd lali nres id PDB Description
1: 7lt5-B 18.2 2.9 209 577 12 MOLECULE: SITE-SPECIFIC DNA-METHYLTRANSFERASE (ADENINE-SPEC
2: 7qw8-A 17.6 3.0 208 525 18 MOLECULE: MODIFICATION METHYLASE BSECI;
3: 3s1s-A 13.5 3.5 193 871 12 MOLECULE: RESTRICTION ENDONUCLEASE BPUSI;
4: 7vs4-C 12.2 3.8 181 374 9 MOLECULE: SITE-SPECIFIC DNA-METHYLTRANSFERASE (ADENINE-SPEC
5: 1yf2-A 11.0 4.2 182 425 10 MOLECULE: TYPE I RESTRICTION-MODIFICATION ENZYME, S SUBUNIT
6: 1ydx-A 10.9 3.4 176 374 7 MOLECULE: TYPE I RESTRICTION ENZYME SPECIFICITY PROTEIN MG4
7: 5hr4-J 9.9 3.6 201 752 12 MOLECULE: MMEI;
8: 2y7c-A 9.2 4.2 192 464 9 MOLECULE: TYPE-1 RESTRICTION ENZYME ECOKI SPECIFICITY PROTE
9: 3okg-B 9.2 3.3 141 391 11 MOLECULE: RESTRICTION ENDONUCLEASE S SUBUNITS;
10: 4xqk-A 8.9 4.0 182 1517 8 MOLECULE: LLABIII;
11: 7eew-A 8.6 4.8 147 611 8 MOLECULE: TYPE I RESTRICTION-MODIFICATION SYSTEM METHYLTRAN
12: 7bst-A 7.8 3.8 164 384 10 MOLECULE: TYPE I RESTRICTION ENZYME R PROTEIN;

0001 s001A   [illegible]
0002 7lt5B   [illegible]
0003 7qw8A   [illegible]
0004 3s1sA   [illegible]
0005 7vs4C   [illegible]
0006 1yf2A   [illegible]
0007 1ydxA   [illegible]
0008 5hr4J   [illegible]
0009 2y7cA   [illegible]
0010 3okgB   [illegible]
0011 4xqkA   [illegible]
0012 7eewA   [illegible]
0013 7bstA   [illegible]
0014 2np6A   [illegible]

0001 s001A   [illegible]
0002 7lt5B   [illegible]
0003 7qw8A   [illegible]
0004 3s1sA   [illegible]
0005 7vs4C   [illegible]
0006 1yf2A   [illegible]
0007 1ydxA   [illegible]
0008 5hr4J   [illegible]
0009 2y7cA   [illegible]
0010 3okgB   [illegible]
0011 4xqkA   [illegible]
0012 7eewA   [illegible]
0013 7bstA   [illegible]
0014 2np6A   [illegible]

Job: Type-1 BREX; BrxX-MTase_MBZ5596028 (C-terminal CC-like Helical Extension)
Query: s001A
No: Chain Z rmsd lali nres id PDB Description
1: 6cnn-A 7.7 13.6 122 360 7 MOLECULE: INTERMEDIATE CONDUCTANCE CALCIUM-ACTIVATED POTASS
2: 5xg2-A 7.6 2.3 69 237 16 MOLECULE: CHROMOSOME PARTITION PROTEIN SMC;
3: 1lrz-A 7.6 8.6 114 400 5 MOLECULE: FACTOR ESSENTIAL FOR EXPRESSION OF METHICILLIN
4: 2qup-A 7.6 5.0 73 119 3 MOLECULE: BH1478 PROTEIN;
5: 4l0r-A 7.4 1.4 67 74 13 MOLECULE: CENTROSOMAL PROTEIN OF 57 KDA;
6: 5j10-A 7.3 2.1 69 70 10 MOLECULE: PEPTIDE DESIGN 2L4HC2_24;
7: 8ap9-G 7.3 3.8 76 279 8 MOLECULE: ATP SYNTHASE GAMMA SUBUNIT;
8: 6njp-G 7.2 1.1 64 64 9 MOLECULE: TRANSLOCATOR ESCN;
9: 5xbj-A 7.2 2.8 68 500 7 MOLECULE: FLAGELLAR HOOK-ASSOCIATED PROTEIN FLGK;
10: 5h69-A 7.2 1.7 68 252 9 MOLECULE: CHROMOSOME PARTITION PROTEIN SMC;
11: 2x0l-A 7.2 9.6 91 670 15 MOLECULE: LYSINE-SPECIFIC HISTONE DEMETHYLASE 1;
12: 8fbi-A 7.2 4.0 103 274 8 MOLECULE: KWOCA_39;
13: 5c21-A 7.2 13.5 95 267 9 MOLECULE: CHROMOSOMAL HEMOLYSIN D;
14: 6msr-A 7.1 2.1 71 76 8 MOLECULE: PRO-2.5;
15: 5nik-D 7.1 13.2 97 340 10 MOLECULE: OUTER MEMBRANE PROTEIN TOLC;
16: 5i6r-A 7.1 9.6 96 419 10 MOLECULE: SLIT-ROBO RHO GTPASE-ACTIVATING PROTEIN 2;
17: 4b6x-A 7.1 2.1 62 69 8 MOLECULE: AVIRULENCE PROTEIN;
18: 8ij9-C 7.0 2.7 69 71 10 MOLECULE: RAS-RELATED PROTEIN RAB-6B;
19: 7w1m-D 7.0 7.5 109 967 7 MOLECULE: STRUCTURAL MAINTENANCE OF CHROMOSOMES PROTEIN 1A;
20: 8fih-A 7.0 2.7 75 105 19 MOLECULE: 3HB05;
```

```
0001 s001A   ----YHRL-----TR-DTLFHLLRNYIEPKIEHEEGRRHELRRLYKAGA-----D---ERRLAKEVDAQETLVGELADFRAKIEAVA-P-GW---D--PD----LNDG--
0002 6cnnA   ----NKAE-----KH-VHNFMMDI-QYTKEMKESAARVLQEAWMFYKHT---shA---ARRHQRKLLAAINAFRQVRLKHRKLREQV-N-SM---V--------------
0003 5xg2A   ------TK-----GAiVRWGKRKE-KLIEEIRAREEERNALVVRLGEID---npE---ARELTEKIRAVEKEIAALREELSRVEGKL-E-GL------------------
0004 1lrzA   ----RF--lsevplayiNFDEYIK-ELNEERDILNKDLNKALKDIEKRP-----E---NKKAHNKRDNLQQQLDANEQKIEEGKRLQ-E-EH---G--------------
0005 2qupA   ----gVSF-----SE-VMGKQRDE-KAYERLQALMSKIDDQGKLLSE-T-----R---TIEELRKYKELVKEFVGDAVELGLRLEER-----------------------
0006 4l0rA   -------G-----IN-EELSEVLQ-TLQDEFGQMSFDHQQLAKLIQES----veL---KDKLECELEALVGRMEAKANQITKVRKYQ-A-QLekq---------------
0007 5j10A   ------TD-----EL-LRLAKEQA-ELLKEIKKLVEEIARLVKEIQEDP---------SDELLKTLAELVRKLKELVEDMERSMKEQ-L-YI---Ik-------------
0008 8ap9G   ------SG-----KL-RLYKEKLE-GYNRFYSIVKTIKMVTLAKYRAAQgrienE---LSEQAARLVAVEGQLTNISSLQQRTSSLY-N-KT---RqfGI----TAALie
0009 6njpG   ----------------HMLDRILS-IRKSRANRLRESMAKINSQIKEV------E---KRSLLDSQKRTKENLQHVNKSVEKLSFAI-K-EH------------------
0010 5xbjA   --------------D-EYSYYKLK-GASTQLEYTKYMASTLQEIAQRFP-----DlqlTGKIASDGENNNVVNSSNETLYNSVYSEY-Q-SK---S--G-----------
0011 5h69A   -------------HM-AAQKTELE-QHEALLHQARQYRQQTKARQQWLE---nrE---LEMLSAKLQEMDETIARLERAVAAKRHEL-A-EQ---Ea-------------
0012 2x0lA   ---qIEHW----kKI-VKTQEELK-ELLNKMVNLKEKIKELHQQYKEAS---efL---VKSKHRDLTALCKEYDELAETQGKLEEKL-Q-EL---E-aNP----PSDVyl
0013 8fbiA   ---aARIAktarlII-ELTLMVLK-ASLDLLRRILEELKEMLERLEKNP-----D----KDVIVKVLKVIVKAIEASVDNQRVSADN-Q-KM------------------
0014 5c21A   ---kYQKE----lNL-DKKRAERL-TILARINRYENLSRVEKSRLDDFR---khA---VLEQENKYVEAANELRVYKSQLEQIESEI-L-SA---KeeYQ----LVTQlf
0015 6msrA   ----gseY-----EI-RKALEELK-ASTAELKRATASLRASTEELKKNP---------SEDALVENNRLIVEHNAIIVENNRIIAAV-L-EL------------------
0016 5nikD   ---eqaEN-----QI-KEVEATLM-ELRAQRQQAEAELKLARVTYSRQQ---qqD---LDNAATEMAVKQAQIGTIDAQIKRNQASL-D-TA---K-tNL----DYTR--
0017 5i6rA   ---qseLV-----QRcQQLQSRLS-TLKIENEEVKKTMEATLQTIQDIV---raN---QQETEQFYFTKMKEYLEGRNLITKLQAKH-DlLQ---K--TLgesq------
0018 4b6xA   --------------------GAGA-ALRQEIEDKQLMVNNLTDELQDAI---paE---IANTSQQLRHARADLADLQRRFAVLRNEDrR-IN---Q--------------
0019 8ij9C   -----rKH-----LE-EVLEMKQE-ALLAAISEKDANIALLELSS---------S---KKKTQEEVAALKREKDRLVQQLKQQTQNR-M-KL------------------
0020 7w1mD   tstlaamK-----LM-TALVNVAL-NLSIHQDNTQRQYEAERNKM---------I---GKRANERLELLLQKRKELQENQDEIENMM-N-SI------------------
0021 8fihA   ---akkGE-----ASeEHAAALLA-EAAVLELKAVLLTLEARRLYKELG-----G---DERAREALEAAERAREAAREAREVAEKAY-D-AA---S--------------

0001 s001A   ----HHHL-----LL-LHHHHHHHLHHHHHHHHHHHHHHHHHHHHHLLL-----L---HHHHHHHHHHHHHHHHHHHHHHHHHHHHH-H-HL---L--LL----HHHL--
0002 6cnnA   ----LHHH-----HH-HHHHHHHH-HHHHHHHHHHHHHHHHHHHHHHHH---hhH---HHHHHHHHHHHHHHHHHHHHHHHHHHHHH-H-HH---L--------------
0003 5xg2A   ------LH-----HHhHHHHHHHH-HHHHHHHHHHHHHHHHHHHHHHHH---llH---HHHHHHHHHHHHHHHHHHHHHHHHHHHHH-H-HL------------------
0004 1lrzA   ----EE--llhleeeeeEHHHHHH-HHHHHHHHHHHHHHHHHHHHHHLL-----L---LHHHHHHHHHHHHHHHHHHHHHHHHHHHH-H-HH---L--------------
0005 2qupA   ----lLLH-----HH-HHHHHHHH-HHHHHHHHHHHHHHHHHHHHHH-H-----L---LHHHHHHHHHHHHHHHHHHHHHHHHHHLL-----------------------
0006 4l0rA   -------L-----HH-HHHHHHHH-HHHHHHHHHHHHHHHHHHHHHLL----hhH---HHHHHHHHHHHHHHHHHHHHHHHHHHHHH-H-HHhll---------------
0007 5j10A   ------LL-----HH-HHHHHHHH-HHHHHHHHHHHHHHHHHHHHHHLL---------LHHHHHHHHHHHHHHHHHHHHHHHHHHHH-H-HH---Hl-------------
0008 8ap9G   ------LL-----HH-HHHHHHHH-HHHHHHHHHHHHHHHHHHHHHHHLlhhhhH---HHHHHHHHHHHHHHHHHHHHHHHHHHHHH-H-HH---HhhHH----HHHHhh
0009 6njpG   ----------------LLHHHHHH-HHHHHHHHHHHHHHHHHHLLLLL------L---HHHHHHHHHHHHHHHHHHHHHHHLLLLLL-L-LL------------------
0010 5xbjA   --------------L-HHHHHHHH-HHHHHHHHHHHHHHHHHHHHHHLL-----LllhHHHHHHHHHHHHHHHHHHHHHHHHHHHHH-H-HL---L--L-----------
0011 5h69A   -------------LH-HHHHHHHH-HHHHHHHHHHHHHHHHHHHHHHHH---hhH---HHHHHHHHHHHHHHHHHHHHHHHHHHHHH-H-HH---Ll-------------
0012 2x0lA   ---hHHHH----hHH-HHHHHHHH-HHHHHHHHHHHHHHHHHHHHHHLL---hhH---HHHHHHHHHHHHHHHHHHHHHHHHHHHHH-H-HH---L-lLL----LLLLll
0013 8fbiA   ---hHHHHhhhhhHH-HHHHHHHH-HHHHHHHHHHHHHHHHHHHHHHLL-----L----HHHHHHHHHHHHHHHHHHHHHHHHHHHH-H-HH------------------
0014 5c21A   ---hHHHH----hHH-HHHHHHHH-HHHHHHHHHHHHHHHHHHHHHHHH---hhH---HHHHHHHHHHHHHHHHHHHHHHHHHHHHH-H-HH---HhhHH----HHHHhh
0015 6msrA   ----llhH-----HH-HHHHHHHH-HHHHHHHHHHHHHHHHHHHHHHLL---------LHHHHHHHHHHHHHHHHHHHHHHHHHHHH-H-HH------------------
0016 5nikD   ---hhhHH-----HH-HHHHHHHH-HHHHHHHHHHHHHHHHHHHHHHHH---lhH---HHHHHHHHHHHHHHHHHHHHHHHHHHHHH-H-HH---H-hHH----HHHE--
0017 5i6rA   ---hhhHH-----HHhHHHHHHHH-HHHHHHHHHHHHHHHHHHHHHHHH---hhH---HHHHHHHHHHHHHHHHHHHHHHHHHHHHH-HhHH---H--HHllll------
0018 4b6xA   --------------------LLLH-HHHHHHHHHHHHHHHHHHHHHHHH---hhH---HHHHHHHHHHHHHHHHHHHHHHHHHHHHHhH-HH---L--------------
0019 8ij9C   -----lHH-----HH-HHHHHHHH-HHHHHHHHHHHHHHHHHHLL---------L---LHHHHHHHHHHHHHHHHHHHHHHHHHHHH-H-HH------------------
0020 7w1mD   hhhhhhhH-----HH-HHHHHHHH-HHHHHHHHHHHHHHHHHHLL---------L---LHHHHHHHHHHHHHHHHHHHHHHHHHHHH-H-HH------------------
0021 8fihA   ---lllLH-----HHhHHHHHHHH-HHHHHHHHHHHHHHHHHHHHHHLL-----L---LHHHHHHHHHHHHHHHHHHHHHHHHHHHH-H-HH---L--------------
```

```
Job: Type-2 BREX; BrxX-MTase MBN9519353 (C-terminal CC-like Helical Extension)
Query: s001A
No: Chain Z rmsd lali nres id PDB Description
1: 8yad-C 5.0 3.6 83 1811 2 MOLECULE: SPATACSIN;
2: 9ivk-A 4.8 5.2 99 278 12 MOLECULE: HBC599 MEMBRANE PROTEIN BINDER;
3: 4k2p-A 4.8 7.1 90 238 7 MOLECULE: T-LYMPHOMA INVASION AND METASTASIS-INDUCING PROTE
4: 8eki-A 4.8 5.7 103 170 13 MOLECULE: PROTEIN TRANSPORT PROTEIN SEC20;
5: 6sny-A 4.7 3.4 88 107 10 MOLECULE: SYNTHETIC EPCR BINDING PROTEIN;
6: 8ap9-G 4.7 3.0 70 279 7 MOLECULE: ATP SYNTHASE GAMMA SUBUNIT;
7: 8w6e-B 4.7 4.6 94 161 14 MOLECULE: HBC599 IN COMPLEX WITH WFAP1.1;
8: 6dkm-D 4.7 2.9 68 79 13 MOLECULE: DHD131_A;
9: 6y1y-B 4.6 2.5 67 128 15 MOLECULE: CHEA;
10: 8bbg-B 4.6 7.3 82 1426 9 MOLECULE: WD REPEAT-CONTAINING PROTEIN 19;
11: 7mx2-B 4.6 4.9 83 648 7 MOLECULE: N-ALPHA-ACETYLTRANSFERASE 35, NATC AUXILIARY SUBU
12: 9clb-F 4.5 4.1 92 115 9 MOLECULE: BCL-2 HOMOLOGOUS ANTAGONIST/KILLER;
13: 3aai-A 4.5 3.3 74 78 5 MOLECULE: COPPER HOMEOSTASIS OPERON REGULATORY PROTEIN;
14: 6wc3-B 4.5 4.6 79 94 8 MOLECULE: PROTEIN TRANSPORT PROTEIN TIP20;
15: 4b6x-A 4.5 3.4 69 69 10 MOLECULE: AVIRULENCE PROTEIN;
16: 6dmp-B 4.5 3.3 70 82 17 MOLECULE: DESIGNED ORTHOGONAL PROTEIN DHD13_XAAA_A;
17: 5h69-A 4.4 2.7 68 252 9 MOLECULE: CHROMOSOME PARTITION PROTEIN SMC;
18: 2gd5-D 4.4 6.3 91 162 9 MOLECULE: CHARGED MULTIVESICULAR BODY PROTEIN 3;
19: 8ibh-A 4.4 2.7 65 75 15 MOLECULE: CENTROSOMAL PROTEIN OF 57 KDA;
20: 5n77-A 4.4 5.4 89 257 11 MOLECULE: MAGNESIUM TRANSPORT PROTEIN CORA;
21: 9c3i-S 4.4 3.1 84 231 7 MOLECULE: CCR4-NOT TRANSCRIPTION COMPLEX SUBUNIT 3;
22: 6dlm-B 4.4 2.6 65 72 12 MOLECULE: DHD127_A;
23: 6ezn-B 4.4 3.0 73 110 10 MOLECULE: DOLICHYL-DIPHOSPHOOLIGOSACCHARIDE--PROTEIN
24: 4hkr-A 4.3 5.1 83 165 8 MOLECULE: CALCIUM RELEASE-ACTIVATED CALCIUM CHANNEL PROTEIN
25: 8hr5-A 4.3 8.1 64 466 13 MOLECULE: TRANSPOSASE;
26: 8wt9-A 4.3 3.8 73 319 10 MOLECULE: IS621 TRANSPOSASE;
27: 7qlr-A 4.3 6.5 73 603 12 MOLECULE: CDHS1_22 PUTATIVE TAIL FIBER PROTEIN;
28: 7wo9-A 4.3 3.4 86 1581 10 MOLECULE: NUCLEOPORIN NUP188;
29: 3whj-A 4.3 4.2 73 111 11 MOLECULE: PROBABLE 26S PROTEASOME REGULATORY SUBUNIT P27;
30: 8ipr-C 4.3 8.2 87 515 5 MOLECULE: COMPONENT LINKED WITH THE ASSEMBLY OF CYTOCHROME'
31: 3fx7-B 4.3 2.9 68 87 9 MOLECULE: PUTATIVE UNCHARACTERIZED PROTEIN;
32: 6vbu-2 4.3 6.5 75 659 8 MOLECULE: BARDET-BIEDL SYNDROME 18 PROTEIN;
33: 2qyw-A 4.3 3.6 77 96 10 MOLECULE: VESICLE TRANSPORT THROUGH INTERACTION WITH T-SNAR
34: 9f0l-A 4.3 4.9 93 791 11 MOLECULE: DE NOVO DESIGNED PROTEIN K10;
35: 9o48-A 4.2 3.8 101 373 7 MOLECULE: INTERMEDIATE CONDUCTANCE CALCIUM-ACTIVATED POTASS
36: 2oer-B 4.2 4.8 88 189 16 MOLECULE: PROBABLE TRANSCRIPTIONAL REGULATOR;
37: 4j2c-A 4.2 3.8 80 108 8 MOLECULE: SYNTAXIN-6;
38: 8xyv-A 4.2 3.6 75 99 8 MOLECULE: DE NOVO DESIGNED PROTEIN 0705-5;
```

```
39: 5j9q-D 4.2 4.2 67 120 10 MOLECULE: HISTONE ACETYLTRANSFERASE ESA1;
40: 8cih-A 4.2 5.2 65 188 15 MOLECULE: CYCLIN-DEPENDENT KINASE 2-INTERACTING PROTEIN;

0001 s001A   ----HKPLSLSSRLDEIARQLGELA--ASRILERW------AKG-E-------------D--L-KKLIEEAEAKVVRFRGMMIALQEELDW-ECY-RHY---G--M-LA-----DDL--R----Y-PG-D-D-LPIGLIERP--EYK--RRWS-----T-ELWADQVR-RASE-NWLL-DRLE
0002 8yadC   npevDAGLLTDIMKQLLFSAKMMFV--K-----------------------------------------aGQSQDLALCDSYISKVDVLNIL-VAA------------A-YR-----H-V---P----S-LD--------YQLGVE-vsTK--TG----------------------------
0003 9ivkA   --deeRLKEILFFLLLIIIFVVFLL---iVDYKFL-----eEFK-E---knvtdkeefnE--V-IKIDEAVMLISAFLLAIAAALLKELEE-LIR-KR-------F-EE-----W--------------------RRELEKI--LGV--LLYV-----GaFLMVIAKF-L---eWYEFg----
0004 4k2pA   rllksEIKKLEQKIDMDEKLKKMGE--L-QLSSVT---------------------------D-SKAAATILDQIFVWEQNLEQFQMDLFR-FRC-YLA---S--LggE-----LPN--P----------krllA---------fhalvAART---------------------------
0005 8ekiA   -----mvVTFLQDLEVLQDALLNNLqkLSAISRRK----------E-------------E--E-EIEFEDLVNIIESKVSDFESVLKCSIV-EMT-YKY---P--E-L-----------------------------veeldfvlqfvDWFY-----CvKEILRQHH-KRDL-AWND-EKRD
0006 6snyA   nnweqQKKNIEDDLDRYKKRAEELRkeAEKARKE-----------K-------------E--W-EKRCKELEERARKLEDEAKDRVNDLFD-SNFfQVI---Y--S-----------------------------EEEWKK--ekdraeKEIE-----EfKRIKEKCE-EIK----------
0007 8ap9G   ---sgKLRLYKEKLEGYNRFYSIVKtiKMVTLAKY------RAaqS-------------E--Q-AARLVAVEGQLTNISSLQQRTSSLYNK-TRQ-FGI---T--A-ALieils---------------------------------------------------------------
0008 8w6eB   -mdeeRLKEILEELERIIEEVKRLL---eKDERLLfyrrdkeEF-R-------------R--V-IKLDEEVMKRSEELLKRAEELLRELEE-LIR--RI---P--F-----------------------------RRELE-E--ILRrlKELY--eeaK-RLMEKAKEeIVREe---------
0009 6dkmD   sdeddELERLLREYHRVLREYEKLLeeLRRLYEEY-----kRGE-V-----------seE--E-SDRILREIKEILDKSERLWDLSEEVWR-TLL-YQA---E--------------------------------------------------------------------------------
0010 6y1yB   anridYLLNLVSETVITKASLNQSTieFAELYDKFeyssllleVF-G-------------D--F-DSLMKAAVTKFKSSSQNLGRISGELQE-GVM-KIR---Mvp------------------------------------------------------------------------------
0011 8bbgB   qveideyQNYDKAHGALTEAYKCLA--KAKA-----------------------------------KSPLDQETRLAQLQSRMALVKRFIQA-RRT-Y-T---E--D-PK---esIKQ--C--elL-LE-E-PdLD--------------------------------------------------
0012 7mx2B   vtdlrVTGMLKDVEDDMQRRVKSTR--S-------------------------------------RDPEVELEHQQCLAVFSRVKFTRVL-LTV-LIA---F--TaVA-----EA-------qklmvqaadllS-------------------------------------------------
0013 9clbF   adpkkvLDQAKDQMENVVRTLKQEL---eELAKEA-----rKLD-L----------tqsE--K-IELKLRYIVAHLAAIGDIEEAIREAKE-EAD-KL-------K-RA-----G--------------------FDEFKR--rleelhKEAD--rkrA-DYAEEFRN-KLE----------
0014 3aaiA   ----hlDPKVREEARRRLLSAKGHL--E-GILRXL-----eDEK-V-----------------Y-CVDVLKQLKAVEGALDRVGEXVLRAHL-KDH---------------------------------------VEEIVEE-lXEA--LK----------------------------------
0015 6wc3B   ------sMPYATQLALLQDELLDML----EPRDGE---------------------------------glRTADIIDKTLRFRELLGCYRL-QVE-K--------S-TR-----Q--------------------QA--palaqlllweRFLA-----DrRRLDAAIV-HEHE-ATAA-R---
0016 4b6xA   ----GAGAALRQEIEDKQLMVNNLT--D-ELQDAI------D---E-------------A--N-PAEIANTSQQLRHARADLADLQRRFAV-LRN-EDR---R--I-NQ--------------------------------------------------------------------------
0017 6dmpB   krlleEAERAHREQKEIIKKAQELH---RRLEEIV-----sGSS-E-------------E--A-KKEAKKILEEIRELSKRSLELLREILY-LSQ-E----------qK-----GSL--V----Pr--------------------------------------------------------
0018 5h69A   aaqktELEQHEALLHQARQYRQQTKarQQWLEEMQggaakktAS-L-------------L-sR-NRELEMLSAKLQEMDETIARLERAVAA-KRH-ELA---E--Q-----------------------------------------------------------------------------
0019 2gd5D   lkirkeXRVVDRQIRDIQREEEKVK---RSVKDAA------K---K-------------G--Q-KDVCIVLAKEXIRSRKAVSKLYASKAH-XNS-VLX---G--XeVX-----KAXqsL----V-KI-P-E-------------------------------------------------
0020 8ibhA   eelseVLQTLQDEFGQMSFDHQQLA--K-LIQESP--------------------------T-VELKDKLECELEALVGRMEAKANQITK-VRK-YQA---Q--L-EKq-----------------------------------------------------------------------
0021 5n77A   dlfetKIEQLADEIENIYSDLEQLSrvIMEGH---------------------------Q-GDEYDEALSTLAELEDIGWKVRLCLMD-TQR-ALN---F--LqaR-----EIL-----------------------rdiesllphnESLF--qkvN-FLM-QAAM-GFIN-IEQN-RIIK
0022 9c3iS   qwltntIDTLNMQVDQFESEVESLS--V-QTRKKK-------------------------------GDKDKQDRIEGLKRHIEKHRYHVRM-LET-ILR---M--LsIL-----VDairkikddV-EY-YvD-SS-------------------------------------------------
0023 6dlmB   eyiikDILDSQEHLLRLIEELLETQkeLLEILKRR------P---D-------------S--V-E-RVRELVRRSKEIADEIRRQSDRNVR-LLE-EVS---K-----------------------------------------------------------------------------
0024 6eznB   iekyPKLKLIDTFCFFLVLLGVIQC---TFIILIR----------------------------D--N-FP-FNAFLAGFIICVGQFVLLMSLRLQ-LCN-S---------fPG-----ISKnrA----F-AEfI-V-AS-----------------------------------------------
0025 4hkrA   vqmliAFAICTTLLVAVHMLALMIStcILPN-----------------------------P--H-ERLHWYIETAWAFSTLLGLILFLLEIA-ILC-WVK---F--YaAW-----SATvvL----I-PVmI-I-FM------------------------------------lemLK-EQME
0026 8hr5A   mnesgLEIRTIKDLKDCEKDIDKNK---KEIEKL--------------------------------KISLVENKIEDYKLKIVELNKILEE-TQK---E---R--M-DI-----Q--------------------------------------------------------------------
0027 8wt9A   hpleRALRALVVRHQALTDMHTQEL--N-RTET------------------------aR-EVQRPSIDAHLLWLEAELKRLEKQIKD-LTD-D-D---P--D-M-------------------------RKLLESipgIGE--K----------------------------------------
0028 7qlrA   pnlleXKYNLQTSLNYINTIKSNIAsgKGQIX-AI------TY--P-------------I--L-KNAVKNYDGXETTRNISQRDIEEKIKI-SDA-ILE---S--S-IA--------------------------------------------------------------------------
0029 7wo9A   ndrfnVVGKLISIISSVIQNYDIIT--ASSLA-HD------YNN-D-------------qD--M-FTIVSLVQLKKFSDLKFILQILQILNL-MIL-NTKvpiL--G-LNtsiaqfdiqsplymdtetfdtvnsal--------------------------------------------------
0030 3whjA   qidsfKLSELMVLKTDIETQLEAYF--SVLEQQGI--gmdsaYP-R-------------SdvD-VLQVTMIRKNVNMLKNDLNHLLQRSHV-LLN-QH-------F-DN-----M--------------------SNQ------------------------------------------------
0031 8iprC   -----SYFQSNVVIAGCTIASAVVL---AHIVAGI-----tNPA-T-------------A-lggETDWAPGLVALAVLWSVRVVAQWFQGR-LSQ-RGA---T--AlRP-----YFTgylpavvlagiltpaalv-----------------------------------------vsvGL-RLVF
0032 3fx7B   ---qmDTEEVREFVGHLERFKELLR---EEVNSLS-----hFHN-LeswrdarrdkfseV--L-DNLKSTFNEFDEAAQEQIAWLKERIRV-LEE-DYL---E--H--------------------------------------------------------------------------------
0033 6vbu2   rkvlvKVDEYHSVHQKLSADMADNSnlIRSLLVQA-----rDMK-T-----------mkN--R-YKELYDLNKDLLNGYKIRCNNHTELLG-SLK-AVN---Q--A-IQ-----------------------------------------------------------------------------
0034 2qywA   ---xaASAASSEHFEKLHEIFRGLLedLQGVPERL-----gTEE-K------------kK--L-VRDFDEKQQEANETLAEXEEELRYAPL--------------------------------------------tfrnpxxSKLR-----NrKDLAKLHR-EVR----------
0035 9f0lA   rtpagLREFYDLRIAYLEKTVADLTarADRVLTD------sVPG-S-----------paE--R-EARAAGLRARAREAQLQLELTRLLRRLrVEN-A--------T-LS-----R--------------------------------------------------------------------
0036 9o48A   ralfEKRKRLSDYALIFGMFGIVVM--V-IETELS------WGA-Y-------------D--KaSLYSLALKCLISLSTIILLGLIIVYHA-REI-QL-------F-MV-----D-------------------MTYER-I--FFIclEILVcaihpitttadVDIIkltvR-ACER-YHDQ
0037 2oerB   qyfpNKAAILFRLQSDEWRRTTRLL--G-EILEDT------T---R-------------P--P-LERLRRLVLAFVRSECEEAAIRVALSD-AAP-------------------------------------------------lyRD--ADEA--revK-AEG-ARVF-QAFL-REAL-PEVA
0038 4j2cA   gameDPFFVVKGEVQKAVNTAQGLFqrWTELLQDP------S---T-------------A--T-REEIDWTTNELRNNLRSIEWDLEDLDE-TIS-IV-------E-AN-----P-------------------------lsirkafiT--STRQvvrdmK-DQMST----------------
0039 8xyvA   --mspEIEDLLKKILELLEKAFALW---aEAKK-----------E-------------G--D-LEKAISTLKELIATIEEVIVLTKKALE-LAE-KEG---N--P----------------------------------eiveqakklldLAE-----AakAELARAL-SSl----------
0040 5j9qD   rylleEIGSNDLKLIEEKKKYEQKE--S-QIHKFI-----qGSI-P-------------K--H-P-QEDGLDKEIKESLLKCQSLQREKCV-LAN-TAL---F--L---------------------------------------------------------------------------
0041 8cihA   etlndAGFTTANNIANLKISLLNK----------------------S-------------S--S-PASKENEEKVCLEYNEELEKLCEELQA-TLD-GLT---K--I-QV-------------------------LLL--------------------------------------------

0001 s001A   ----LLLHHHHHHHHHHHHHHHHHHLL--HHHHHHHL------LLL-L-------------L--H-HHHHHHHHHHHHHHHHHHHHHHHHHHHH-HHH-HHL---L--L-LL-----LLL--L----L-LL-L-L-LLHHHHLLL--LLL--LLLL-----L-LLHHHHHH-HHHH-HHHH-HHHH
0002 8yadC   llllLHHHHHHHHHHHHHHHHHHHH--L-----------------------------------------lLLHHHHHHHHHHHHHHHHHHHHH-HLL----------L-LL-----L-L--L----L-HH--------HHHHHH-hhHH--HL----------------------------
0003 9ivkA   --lhhHHHHHHHHHHHHHHHHHHHH---hHHHHHH-----hHHH-H---llllllhhhhhH--H-LHHHHHHHHHHHHHHHHHHHHHHHHHHH-HHH-HH-------H-HH-----H--------------------HHHHHHH--HHH--HHHH-----HhHHHHHHHH-H---hHHHHl----
0004 4k2pA   hhhhhHHHHHHHHHHHHHHHHHHHH--H-HLLLLL---------------------------L-HHHHHHHHHHHHHHHHHHHHHHHHHHHH-HHH-HHH---H--HllL-----LLL--H----------hhhhL---------hhhhhHLLL---------------------------
0005 8ekiA   -----llLLHHHHHHHHHHHHHHHHHhhHHHHHHHH----------H-------------H--H-HHHHHHHHHHHHHHHHHHHHHHHHHHHH-HHH-HHL---L--L-L-----------------------------hhhhhhhhhhhHHHH-----HhHHHHHHHH-HHHH-HHHH-HHHH
0006 6snyA   lhhhhHHHHHHHHHHHHHHHHHHHHhhHHHLLLL-----------L-------------L--L-LHHHHHHHHHHHHHHHHHHHHHHHHHHH-HLHhHHH---L--L-----------------------------HHHHHH--hhhhhhHHHH-----HhHHHHHHHH-HHL----------
0007 8ap9G   ---llHHHHHHHHHHHHHHHHHHHHhhHHHHHHHH------HHhlH-------------H--H-HHHHHHHHHHHHHHHHHHHHHHHHHHHH-HHH-HHH---H--H-HHhhhhl---------------------------------------------------------------
0008 8w6eB   -lhhhHHHHHHHHHHHHHHHHHHHH---hHHHHHHhhhllhhHH-H-------------H--H-HHHHHHHHHHHHHHHHHHHHHHHHHHHH-HHH--HL---L--L-----------------------------HHHHH-H--HHHhhHHHH--hhhH-HHHHHHHHhHHHHl---------
0009 6dkmD   hhhhhHHHHHHHHHHHHHHHHHHHHhhHHHHHHHH-----lLLL-L-----------lhH--H-HHHHHHHHHHHHHHHHHHHHHHHHHHHH-HHH-HHH---L--------------------------------------------------------------------------------
0010 6y1yB   hhhhhHHHHHHHHHHHHHHHHHHHHhhHHHHHHHHhhhhhhlLL-L-------------L--H-HHHHHHHHHHHHHHHHHHHHHHHHHHHH-HHH-HHL---Lll------------------------------------------------------------------------------
0011 8bbgB   hhhhhllLLHHHHHHHHHHHHHHHHH--HLLL-----------------------------------LLHHHHHHHHHHHHHHHHHHHHHHHHH-HHH-L-L---L--L-HH---hhHHH--H--hhH-HL-L-LlHH--------------------------------------------------
0012 7mx2B   llhhhHHHHHHHHHHHHHHHHHHLL--L-------------------------------------LLHHHHHHHHHHHHHHHHHHHHHHHHH-HHH-HHH---H--HhHH-----LH-------hhhhhhhhhhhH-------------------------------------------------
0013 9clbF   lhhhhhHHHHHHHHHHHHHHHHHHH---hHHHHHH-----lLLL-L----------lhhH--H-HHHHHHHHHHHHHHHHHHHHHHHHHHHH-HHH-HH-------H-HH-----L--------------------HHHHHH--hhhhhhHHHH--hhhH-HHHHHHHHH-HHL----------
0014 3aaiA   ----llLHHHHHHHHHHHHHHHHHHH--H-HHHHHH-----lLLE-E-----------------L-HHHHHHHHHHHHHHHHHHHHHHHHHHHH-HHL---------------------------------------HHHHHHH-hHHH--HL----------------------------------
0015 6wc3B   ------lLLHHHHHHHHHHHHHHHHHL----LLLLLL--------------------------------llLHHHHHHHHHHHHHHHHHHHHHH-HHH-L--------L-LL-----L--------------------LH--hhhhhhhhhhHHHH-----HhHHHHHHHH-HHHH-HLLL-L---
0016 4b6xA   ----LLLHHHHHHHHHHHHHHHHHHH--H-HHHHHH------H---L-------------L--L-HHHHHHHHHHHHHHHHHHHHHHHHHHHH-HHH-HHH---H--H-HL--------------------------------------------------------------------------
0017 6dmpB   lhhhhHHHHHHHHHHHHHHHHHHHH---HHHHHHH-----hLLL-H-------------H--H-HHHHHHHHHHHHHHHHHHHHHHHHHHHH-HHL-L----------lL-----LLL--L----Ll--------------------------------------------------------
0018 5h69A   hhhhhHHHHHHHHHHHHHHHHHHHHhhHHHHHHHHelllllllLL-H-------------H-hH-HHHHHHHHHHHHHHHHHHHHHHHHHHHH-HHH-HHH---H--H-----------------------------------------------------------------------------
0019 2gd5D   hhhhhhHHHHHHHHHHHHHHHHHHH---HHHHHHH------H---L-------------L--L-HHHHHHHHHHHHHHHHHHHHHHHHHHHH-HHH-HHH---H--HhHH-----HHHhhL----L-LL-L-L-------------------------------------------------
0020 8ibhA   hhhhhHHHHHHHHHHHHHHHHHHHH--H-HHHHLL----------------------------L-HHHHHHHHHHHHHHHHHHHHHHHHHHHH-HHH-HHH---H--H-LLl-----------------------------------------------------------------------
0021 5n77A   hhhhhHHHHHHHHHHHHHHHHHHHHhhHHHLL-----------------------------L-LHHHHHHHHHHHHHHHHHHHHHHHHHHH-HHH-HHH---H--HhH-----HHH----------------------hhhhhhhhhhHHHH--hhhH-HHH-HHHH-HHHH-HHHH-HHHL
0022 9c3iS   hhhhhhHHHHHHHHHHHHHHHHHHH--H-HHHHHL-------------------------------LLHHHHHHHHHHHHHHHHHHHHHHHHH-HHH-HHH---H--HlLL-----HHhhhhhhhhH-HH-HhH-LL-------------------------------------------------
0023 6dlmB   llhhhHHHHHHHHHHHHHHHHHHHHhhHHHHHLL------L---L-------------L--L-H-HHHHHHHHHHHHHHHHHHHHHHHHHHH-HHH-HHH---L-----------------------------------------------------------------------------
0024 6eznB   hhhlHHHHHHHHHHHHHHHHHHHHH---HHHHHLL----------------------------L--L-LL-LHHHHHHHHHHHHHHHHHHHHHHHHH-HHL-L---------lLL-----LLLlhH----H-HHhH-H-HH-----------------------------------------------
0025 4hkrA   hllhhHHHHHHHHHHHHHHHHHHHHhhLLLL-----------------------------L--H-HHHHHHHHHHHHHHHHHHHHHHHHHHHH-HHH-HHH---H--LhHH-----HHHhhL----H-HHhH-H-HH------------------------------------hhhHH-HHHH
0026 8hr5A   hhlhhHHHHHHHHHHHHHHHHHHHH---HHHHHL---------------------------------LHHHHHHHHHHHHHHHHHHHHHHHHH-HLL---L---L--L-LH-----H--------------------------------------------------------------------
0027 8wt9A   lhhhHHHHHHHHHHHHHHHHHHHHH--H-HHHH--------------------------lL-LLLHHHHHHHHHHHHHHHHHHHHHHHHH-HHH-H-L---H--H-H-------------------------HHHHLLlllLLH--H----------------------------------------
0028 7qlrA   hhhhhHHHHHHHHHHHHHHHHHHHHhhHHHHH-LL------EE--H-------------H--H-HHHHHHHHHHHHHHHHHHHHHHHHHHHH-HHH-HHL---L--H-HH--------------------------------------------------------------------------
0029 7wo9A   llhhhHHHHHHHHHHHHHLLLLLH--HHHHH-LL------LLL-L-----------hH--H-HHHHHHHHHHHHHHHHHHHHHHHHHHHH-HLL-LLLllhL--L-EEllllleellllhhhhlllhhhhhhhhh--------------------------------------------------
0030 3whjA   lllllLHHHHHHHHHHHHHHHHHHH--HHHHHLLL--lllllLL-L-------------LhhH-HHHHHHHHHHHHHHHHHHHHHHHHHHHH-HHH-HH-------H-HH-----L--------------------LLL------------------------------------------------
0031 8iprC   -----LHHHHHHHHHHHHHHHHHHH---HHHHHHH-----hLLL-L-------------L-lllLLLLHHHHHHHHHHHHHHHHHHHHHHHH-HHH-HHH---H--HhHH-----HHLllhhhhhhhhhhhhhhhhh-----------------------------------------hhhHH-HHHL
0032 3fx7B   ---llLHHHHHHHHHHHHHHHHHHH---HHHHHHH-----hHHH-LllllllhhhhhhhhH--H-HHHHHHHHHHHHHHHHHHHHHHHHHHHH-HHH-HHH---H--L--------------------------------------------------------------------------------
0033 6vbu2   hhhhhHHHHHHHHHHHHHHHHHHHHhhHHHHHHHH-----lLHH-H-----------hhH--H-HHHHHHHHHHHHHHHHHHHHHHHHHHHH-HHH-HHH---H--H-HH-----------------------------------------------------------------------------
0034 2qywA   ---lhHHHHHHHHHHHHHHHHHHHHhhHHHHHHHH-----lLHH-H-------------hH--H-HHHHHHHHHHHHHHHHHHHHHHHHLLLH--------------------------------------------hhhhhhhHHHH-----HhHHHHHHHH-HHL----------
0035 9f0lA   llhhhHHHHHHHHHHHHHHHHHHHHhhHHHHHHL------lLLL-L-----------hhH--H-HHHHHHHHHHHHHHHHHHHHHHHHHHHHhLLL-L---------L-LL-----H--------------------------------------------------------------------
0036 9o48A   hhhhHHHHHHHHHHHHHHHHHHHHH--H-HHHHHH------HHL-L-------------L--LlLHHHHHHHHHHHHHHHHHHHHHHHHHHH-HHH-HH-------H-HH-----H-------------------LLHHH-H--HHHhhHHHHhlllllleeeeeLLHHllhhH-HHLL-LLLL
0037 2oerB   hhllLHHHHHHHHHHHHHHHHHHHH--H-HHHLLL------L---L-------------L--H-HHHHHHHHHHHHHHHHHHHHHHHHHHHH-HLL-------------------------------------------------llLL--LHHH--hhhH-HHH-HHHH-HHHH-HHHL-LLLL
0038 4j2cA   llllLLHHHHHHHHHHHHHHHHHHHHhhHHHHHHLL------L---L-------------L--L-HHHHHHHHHHHHHHHHHHHHHHHHHHHH-HHH-HH-------H-HL-----H-------------------------hhhhhhhhH--HHHHhhhhhH-HHHHL----------------
0039 8xyvA   --llhHHHHHHHHHHHHHHHHHHHH---hHHHH-----------L-------------L--L-HHHHHHHHHHHHHHHHHHHHHHHHHHHH-HHH-HHL---L--H----------------------------------hhhhhhhhhhHHHH-----HhhHHHHHHHH-HHl----------
0040 5j9qD   hhhhhHHHHHHHHHHHHHHHHHHHH--H-HHHHHH-----hLLL-L-------------L--L-L-LHHHHHHHHHHHHHHHHHHHHHHHHH-HHH-HHH---H--H---------------------------------------------------------------------------
```

```
0041 8cihA   hhhhhHHHHHHHHHHHHHHHHHLL---------------------L-------------L--L-HHHHHHHHHHHHHHHHHHHHHHHHHHH-HHH-HHH---H--H-HH--------------------------HHH----------------------------------------------

Job: Type-1 BREX; BrxX-MTase MBZ5596028 wHTH (Type-1 BREX Specific)
Query: s001A
No: Chain Z rmsd lali nres id PDB Description
1: 8c45-A 6.8 1.9 109 1219 33 MOLECULE: SITE-SPECIFIC DNA-METHYLTRANSFERASE (ADENINE-SPEC
2: 3s93-B 5.6 3.4 75 81 9 MOLECULE: TUDOR DOMAIN-CONTAINING PROTEIN 5;
3: 2od5-A 5.4 2.7 67 91 12 MOLECULE: HYPOTHETICAL PROTEIN;
4: 8e4y-A 5.0 4.0 83 651 6 MOLECULE: GLYCEROL-3-PHOSPHATE ACYLTRANSFERASE 1, MITOCHOND
5: 8ffz-B 4.9 4.2 78 905 10 MOLECULE: TRANSCRIPTION FACTOR IIIA;
6: 9gm7-C 4.8 3.4 69 440 14 MOLECULE: CHROMOSOME PARTITION PROTEIN MUKF;
7: 1ldd-A 4.7 3.0 70 74 4 MOLECULE: ANAPHASE PROMOTING COMPLEX;
8: 5yad-A 4.7 2.7 62 73 5 MOLECULE: MEIOSIS REGULATOR AND MRNA STABILITY FACTOR 1;
9: 7asv-A 4.6 3.1 71 155 10 MOLECULE: DNA-DIRECTED RNA POLYMERASE III SUBUNIT RPC5;
10: 7vwv-B 4.6 2.6 64 74 3 MOLECULE: I73R;
11: 2lh9-A 4.6 3.0 69 78 14 MOLECULE: TUDOR DOMAIN-CONTAINING PROTEIN 7;
12: 6sj9-A 4.5 3.4 69 642 9 MOLECULE: PROTEASOME ACCESSORY FACTOR B/C (PAFBC);
13: 7z8b-C 4.5 4.8 83 1224 13 MOLECULE: CULLIN-7;
14: 2zme-B 4.5 3.6 77 215 12 MOLECULE: VACUOLAR-SORTING PROTEIN SNF8;
15: 6r7n-B 4.5 3.0 68 443 4 MOLECULE: COP9 SIGNALOSOME COMPLEX SUBUNIT 1;
16: 6aht-A 4.5 4.4 77 111 4 MOLECULE: CONSERVED HYPOTHETICAL PLASMID PROTEIN;
17: 7abi-7 4.2 3.4 74 236 5 MOLECULE: U5 SMALL NUCLEAR RIBONUCLEOPROTEIN 40 KDA PROTEIN
18: 3zco-A 4.2 3.9 80 127 10 MOLECULE: REGULATORY PROTEIN SIR3;
19: 6wge-C 4.2 2.5 61 174 8 MOLECULE: STRUCTURAL MAINTENANCE OF CHROMOSOMES PROTEIN 1A;
20: 7cv0-A 4.2 2.5 57 173 9 MOLECULE: TRANSCRIPTIONAL REGULATOR NIAR;
21: 7nyw-E 4.2 3.6 79 212 8 MOLECULE: CHROMOSOME PARTITION PROTEIN MUKB;
22: 8uuc-A 4.2 3.3 72 274 6 MOLECULE: ADENINE DNA GLYCOSYLASE;
23: 7qen-C 4.1 3.0 72 291 7 MOLECULE: DNA (35-MER);
24: 2iu5-A 4.1 3.2 72 179 6 MOLECULE: HTH-TYPE DHAKLM OPERON TRANSCRIPTIONAL ACTIVATOR
25: 2obp-A 4.1 2.7 62 81 10 MOLECULE: PUTATIVE DNA-BINDING PROTEIN;
26: 3qkx-A 4.0 3.1 72 183 7 MOLECULE: UNCHARACTERIZED HTH-TYPE TRANSCRIPTIONAL REGULATO
27: 7yw2-A 4.0 3.5 66 216 9 MOLECULE: TRNA 2'-PHOSPHOTRANSFERASE 1;
28: 1w5s-B 4.0 3.8 76 396 7 MOLECULE: ORIGIN RECOGNITION COMPLEX SUBUNIT 2 ORC2;
29: 2lnb-A 4.0 3.6 62 80 8 MOLECULE: Z-DNA-BINDING PROTEIN 1;
30: 3elk-A 3.9 2.9 64 104 8 MOLECULE: PUTATIVE TRANSCRIPTIONAL REGULATOR TA0346;
31: 6qpq-D 3.9 2.8 66 82 12 MOLECULE: STRUCTURAL MAINTENANCE OF CHROMOSOMES PROTEIN,STR
32: 7jgr-D 3.8 4.3 77 441 9 MOLECULE: ORIGIN RECOGNITION COMPLEX SUBUNIT 2;
33: 5d1w-D 3.8 4.2 81 196 4 MOLECULE: RV3249C TRANSCRIPTIONAL REGULATOR;
34: 4nb5-B 3.8 3.8 65 149 5 MOLECULE: DNA BINDING PROTEIN;
35: 7wze-A 3.8 4.1 64 161 6 MOLECULE: UNCHARACTERIZED HTH-TYPE TRANSCRIPTIONAL REGULATO
36: 8r7k-B 3.8 3.1 72 366 4 MOLECULE: GERMINAL-CENTER ASSOCIATED NUCLEAR PROTEIN;
37: 1yyv-A 3.8 3.6 69 114 7 MOLECULE: PUTATIVE TRANSCRIPTIONAL REGULATOR;
38: 8hih-N 3.8 2.7 61 217 2 MOLECULE: DNA-DIRECTED RNA POLYMERASE SUBUNIT ALPHA;
39: 5g5p-B 3.8 3.4 79 455 6 MOLECULE: NUCLEAR MRNA EXPORT PROTEIN SAC3;
40: 7z1n-O 3.8 2.7 69 570 9 MOLECULE: DNA-DIRECTED RNA POLYMERASE III SUBUNIT RPC1;
41: 4y66-C 3.8 3.0 62 197 2 MOLECULE: MND1;
42: 5xfo-A 3.8 3.9 80 315 10 MOLECULE: PHD FINGER PROTEIN 1;
43: 8fo9-F 3.8 3.5 72 2289 11 MOLECULE: LEUCINE-RICH REPEAT SERINE/THREONINE-PROTEIN KINA
44: 4kyw-A 3.8 3.4 68 254 7 MOLECULE: TYPE-2 RESTRICTION ENZYME DPNI;
45: 4i99-C 3.8 2.6 62 70 5 MOLECULE: CHROMOSOME PARTITION PROTEIN SMC;
46: 6pco-B 3.7 4.1 64 135 16 MOLECULE: MARR-FAMILY TRANSCRIPTIONAL REGULATOR;
47: 6wg3-C 3.7 23.2 65 248 9 MOLECULE: STRUCTURAL MAINTENANCE OF CHROMOSOMES PROTEIN 1A;
48: 5z7b-B 3.7 3.0 61 197 15 MOLECULE: PADR FAMILY TRANSCRIPTIONAL REGULATOR;
49: 9baq-A 3.7 3.9 83 1002 4 MOLECULE: DNA (CYTOSINE-5-)-METHYLTRANSFERASE;
50: 5uuj-A 3.7 3.5 70 371 13 MOLECULE: ALKZ;

0001 s001A   --VEEL--D-E--SS-AA-AV--V--ASY-AI-GCCFGRWD------WDGILVDD--EGHARDVV-ARVR-EVLRVVWKD-R---ADEIE-DAVCKTLGV-----KDLR-SYFRRNFFEDHIKRYS---KSRRRAP-VYWL-L-Q--SP-RK--S-Y--GVW-LY-----------
0002 8c45A   -tLLCN-kY-Y--TD-IT-ID--I--LSY-II-GCMMGRYSlda--dNDGILPLMddEWFDDDVT-SRVK-EFVRTVWGEeH---LQENL-EFIAESLClyasalDTIR-RYLSTQFWKDHMKMYK-------KRP-IYWL-F-S--SG-EK--A-F--ECL-VYlhryndatlar
0003 3s93B   ----GM--S-E--QE-RI-QE--C--LRK-EI-RSLLIS--------tKDGLSPQ-------ELE-KEYL-LMVG----------------hlPLRILGY-----R-----STME-LVLDMP-------------D-VVRV-C-P--GA-G---G-T--VIL-KAip---------
0004 2od5A   --------E-T--ES-XK-TV--R--IRE-KI-KKFLG----------DRPRNT-------aEIL-EHIN-ST-------------XRHG----------------tTS-QQLG-NVLSKD---------------K-DIVKvG-Y--IK-RsgyD-I--CEW-ATrnwvaehcpew
0005 8e4yA   -fnSFF--W-N-nyG-CV-RvdiM--STH-IV-ACLLLY-------rhRQGIDLS-------TLV-EDFF-VMKEEVLAR-D---FD----LGFS----------GNSE-DVVM-HAIQLLG--------------N-CVTI-T-H--TS-R--nD-E--FFI-TPsttvpsvfeln
0006 8ffzB   -lFRIY--D---eRS-LR-SL--Q--RQR-AI-LKVMNT---------iGGVAYLR--E----QFY-ESVS-KYM-----------GSTT------------------tLDK-KTV-rGDVDLMVES-------------E-KLGA-R-T--EP-VS--G-R--KII-FLptvgedaiqry
0007 9gm7C   ------------iSL-PVeRL--A--FLM-AI-AVLNS-----e--rLDGEMSEG-------ELI-DAFR-EVCK-----------GFEQ---------------svaVRAN-NAI-NDMVRQK----------------lLNR-F-T--SE-L--dG-N--AIY-RLtplgisisdyy
0008 1lddA   ------------kYE-LT-LQ--R--SLP-FI-EGMLTN---------lGAMKL-------hKIH-SFLK-ITV-----------PKDW---GYNR-----------ITL-QQLEGYLNTLADE---------------G-RLKY-I-A--N-----------GSY-EIv---------
0009 5yadA   --------G-A--AN-KS-LS--L--LST-ET-XSILQ-----d--aPACCLPLF-------KFI-DIYE-KKYG------------------------------hklNV-SDL-YKLTD------------------tIAI-R-E--QG-----N-G--RLV-CLlpsnq------
0010 7asvA   -------------aptPV-AR--E--LKA-FV-EATFQR---------qFVLTLS-------ELK-RLFN-LHLAS-LPP-G-----HTL-FS-------------gISD-RXLQ-DTVLAA------------------GCKQ-I-L--VP-F----eQ--KVF-ALwesgdxsdqhr
0011 7vwvB   -------------EF-ME-TQ--K--LIS-MV-KEALE--------kYQYPLTA-------kNIK-VVIQ-KEHN------------------------------vvLPT-GSIN-SILYSNS--------------E-LFEK-I-D--KT------yP--PLW-IRkn---------
0012 2lh9A   -------------ghmLE-AD--L--VSK-ML-RAVLQS--------hKNGIVLP-------RLQ-GEYR-SLTG----------------wiPFKQLGY-----P-----TLEAYLRSVP--------------A-VVRI-E-A--SR-S---G-E--IVC-YAva---------
0013 6sj9A   --------------G-AS-RT--E--RLL-NL-LLALLN--------tKVGLPRA-------VLR-EKVY-----------------hDS-AD----------ndVAFG-RXF-ERDKVDLKQFG----------F-EIET-L-X--D---P--A-S--ARY-RIgkdsnrlpdvs
0014 7z8bC   -qAEGE-gQ-N--LE-KR-RN--L--LNC-LI-VRILKA-------hGDEGLHID-------QLV-CLVL-EAWQKGPC-----pPRGLV-sslgKGSA-------cSS-TDV-lSCILHLLG----------KG-TLRR-H-D--D----------pQVL-SYav---------
0015 2zmeB   -eDETI------sGT-QY-HM--Q--LAK-QLaGILQV-----e--eRGGIMSLT-------EVY-CLVN-RA-------------RGMEL-----------------LSP-EDLV-NACKMLEALK-----------L-PLRL-R-V--FD-SG----V--MVI-ELqshkeeemvas
0016 6r7nB   -rEGEF-eH-I--EE-LL-RN--I--RTQ-VL-IKLIK----------PYTRIHIP-------FIS-KELN-----------------------------------iDV-ADVE-SLLVQCILDN----------tI-HGRI-D-Q--V--------N--QLL-ELdhqkrggaryt
0017 6ahtA   -xSSSE-iD-V--LC-EN-LN--D---GI-WA-LRVLYA---------eGAXNKE-------KLW-DYIN-QYHKDYQIEnekdyeGKKI------------lpsRYAL-DIX-tARLEGAG----------------lISF-K-A--IG-----R-V--RIY-DVtdlgnvlikel
0018 7abi7   -------qQ-F--MD-YF-SE--E--FRNdFL-ELLRR--------rfGTKRVH---------NN-IVYN-EYISHRE--------hiHM-NAT----QW-----ET-L-TDFTKWLGR-----------------E-GLCK-V-D--ET-P--kG-W--YIQ-YIetirrqlelek
0019 3zcoA   ------------iskkiigmS--L--LMR-TF-LYTLAQ---g--tNRHTLALE-------TVL-IKMV-KMLRD-NPG-Yk-aSKEIK-KVI--------itiEKLKqFSWI-SVVNDLVGE-----------K-LVVV-V-L--EE-PS--A-S--IMV-ELklpleinyafs
0020 6wgeC   -------------reimKR-TQ--Q--MLH-GL-QRALA----k--tGAESISLL-------ELC-R------------------------------ntnRKQA-AAKF-YSFLVLKKQ-----------Q-AIEL-T-Q--EE-P--yS-D--IIA-TPgprfhi-----
0021 7cv0A   ---------------IL-GE--E--RRS-LL-IKWLKA-------sDTPLTGA-------ELA-KRTN------------------------------------vSR-QVIV-QDVSLLKAKN----------H-PILA-T-A--------------qGY-IYmkeantvqaqr
0022 7nywE   -iPRSV-------lS-EL-DM--M--VGK-IL-CYLYLS----l--aNQGIFTSQ-------ELY-EELI-SLA-------D---EGKLM-KF-VNQR----ldkQKLQ-EKVR-TTLNRLRRL-----------G-MVYF-L---------------nNKF-TIteavfrfgadv
0023 8uucA   -hHTRQ--S-T--F-egS-RR--Q--KRA-EL-VRLVLA---------ePGIGID-------ELA-ERLD-AFERD---A-G---RK------------------gVDA-ATFTSIVADLVAE-----------G-FFRR-E-G-------------DAF-FA---------
0024 7qenC   ---------ewivskkvD-VR--R--LKK-NV-WRSINN----srknreKELKFS-------DII-QGIS-KMY-----------SDDTL-KD-------------IST-SFC-FICLLHLANE-----------H-GLQI-T-H--TE-NY--N-D--LIV-NYedla-------
0025 2iu5A   ----mE--K-S--I--IT-QK--I--IAK-AF-KDLMQ----------sNAYH--------QISV-SDIM-QTAK---------iRRQTF-YNYF-----------QNQ-EELLSWIFENDFAELInqnELLLL--yqkifvI-D--KNf------------------------
0026 2obpA   ----------G--ID-PA-IV--E--VLL-VL-REAGI-----e--nGATPWSL-------pKIA-KRAQ------------------------------------lPX-SVLR-RVLTQLQAA-----------G-LADV-S-V--EA-DG----R--GHAs-----------
0027 3qkxA   -----R--Q-A--KT-DL-AE--Q--IFS-AT-DRLXA------------REGL---------NQLSxLKLA-KEAN---------vAAGTI-YLYF----------KNKD-ELL-EQFAHRVFSXFX--aTLEKdfdnlkQY-E-Sl-----------------------------
0028 7yw2A   -------------EQ-DR-NV--Q--LSK-AL-SYALRHG---m--rADGFVPLQ-------ALL-Q--L-PQF------------------------------hsfSI-EDV-QLVVNTNE------------KQ-RFTL-Q-P--GE-P--sT-G--LLI-RAnqghslqvpel
0029 1w5sB   -qsMLS--StH--EL-EA-LS--I--HEL-II-LRLIAE----l--gGMEWINAG-------LLR-QRYE-DASLTMY-------NVKPR-------------gyTQYH-IYL-KHLTSLG----------------lVDA-K-P--S--------T--TLF-RLaphlpadrlie
```

```
0030 2lnbA   hhhshM--A-D--P--GR-EG--H--LEQ-RI-LQVLT--------eAGSPVKLA-------QLV-KECQ------------------------------------aPK-RELN-QVLYRMKKE-----------L-KVSL-T-S--P-----------ATW-CLggtdpe-----
0031 3elkA   ----------R--ER-IL-HG--L--ITL-YI-LKELV---------kRPXHG-------yELQ-KSXF-ETTG--------------------------------qalpqGSIY-ILLKTXKER-----------G-FVIS-E-S--SV-N--qQ-L--TVY-HItdagkkflxdh
0032 6qpqD   ----------------KA-IV--Q--MAK-IL-RKELS---------EEKEVIFT-------DVL-KSQA-N------------------------------nitKREA-SRGF-FDILSLATE-----------G-CIGL-S-Q--TE-AF--G-N--IKI-DAkpalferfi--
0033 7jgrD   -fEGDD---lC--GL-SVlEL--C--LII-AI-KHHSQI-------yDRDSFNFE-------IIY-ARFS-KFAKV--ST-T---MQAV-------------erSIVL-KAF-EHLRIAE-----------------lIMP-L-------VQ--K-E-fEMH-KLaltysqihhcm
0034 5dlwD   --IPYA-eA-S--RA-LL-RD--S--VLD-AM-RDLLL----------tRDWS-------AITL-SDVA-RAAG---------iSRQTI-YNEF-----------GSRQ-GLA-QGYALRLADRLV--dNVHAsldaDPLVislI--TT-DS--An-------------------
0035 4nb5B   ---------eS--RS-L---T--R--LAG-RL-LGWLLV-------cDPERQSSE-------ELA-TALA------------------------------------aSS-GGIS-TNARMLIQF-----------G-FIER-L-A--VA-GD--R-R--TYF-RLrpnafaagere
0036 7wzeA   -------eH-Y--FAgRG-LS--E--GKF-KI-LXLLFD-------aKDHRLSP-------tELA-KRSN------------------------------------vTK-ATIT-GLLDGLARD-----------G-FVSR-R-H--H------R-K--ISI-ELttegkarleqf
0037 8r7kB   cgifLI--L-E--K--LK-II--T--YRN-LF-KKVYL-----l--lKTHQLSLD-------AFL-VALK-F------------MQVE----------------dVDI-DEVQCILANLIYMGH-----------V-KGYI-S-H--Q--------H--QKL-VVskqnpfppl--
0038 1yyvA   -aEQCP--S-R--EV-LK-HV--TsrWGV-LI-LVALR----------dGTHRFS-------DLR-RXXG------------------------------------gVSE-XXLA-QSLQALEQD-----------G-FLNR-V-S--YP-V--pP-H--VEY-SLtplgeqvsdxv
0039 8hihN   ------------dlT-YK-EF--E--LLK-YL-AQHA-----------gRVFTRA-------QLL-HEVW-G--------------------------------ydffggT-RTV-DVHVRRLRAKLG------PEHEaLIGT-V-R------------N--VGY-KAvrpa-------
0040 5g5pB   -lNSQVhvL-L--EK-LP-MV--T--YRN-LI-KTVIKS----e--wGQNKLPYS-------LIE-RVLQ-LS------------iGPTFedNGIH------------SP-KNVE-NVLVTLINLG---------lL-RANC-F-P--Q---------L--QLC-VVkkttmiqeivp
0041 7z1nO   ---------kktrvG-SV-TA--Q--VYK-IA-LRLTEqkspkeektpGLTFNA-------iDLA-RHLP-----------------AEL-D--LRGSL---srkPHSA-SLIN-SHLKILASS---------NFP-FLNE-T-K--------------pGVY-YVpysklmpvlks
0042 4y66C   --------T-S--LD-EK-KE--R--LLE-EM-LKRG-----------EIYSNK-------TIE-T-LS-KPT------------------------------issMVIK-NVL-QALVNEDL-----------------VDT-D-K--IG-----A-S--TYY-WCfaskrsqaart
0043 5xfoA   ---------revrlqlRW-VD--V--AHL-VL-YHLSVC-------cKKKYFDFD--R----EIL-PFTS-ENWDSLL-------LGEL--SDTP---------kGERS-SRLL-SALNSHK-------------D-RFIS-G-R--EI-KK--R-K--CLF-GLharmpppve--
0044 8fo9F   qqrlKK--A-VplIP-DC-YV--E--LEK-II-LSERKN----p--iEFPVIDRK-------RLL-QLVR-ENQ------------------------------lqLDE-NEL-PHAVHFLNES-----------G-VLLH-F-Q--DP-A----lS--DLY-FVepkwlckimaq
0045 4kywA   -ksSLS--S-R-----GW-TI--E--ILN-CI-DKIE-----------GSEFTL-------EDM-YRFE-------------sdlKNIF-VK-------------nNHIK-EKIR-QQLQILRD-----------KE-IIEF-K-G------------R--GKY-RKl---------
0046 4i99C   -----------------D-IE--K--YVE-EL-YKVVKK-----e--kTGTPIKFW-------DLV-P----------------------------------dvePKII-ARTF-LYLLFLENM-----------G-RVEI-I-Q--EE-PF--G-E--ILV-VPm---------
0047 6pcoB   aifqQT--I-P--DS-K--LT--A--AQF-VV-LCALRD---------qGACSLV-------DVV-KATA-------------------------------------iDQ-ATVR-GVIERLKAR-----------K-LLAV-S-H--RR-------K--VLV-TLtpdgralveem
0048 6wg3C   -------ferC--LT-KR-TQ--Q--MLH-GL-QRALA-----k--tGAESISLL-------ELC-R----------------------------ntnR-----KQA--AAKF-YSFLVLKKQ-----------Q-AIEL-T-Q--EE-PY--S-D--IIA-TPgprfhi-----
0049 5z7bB   -------------SA-KD-LM--T--LRS-AL-LALLS----------sGPLTG-------yDAS-QRFGaSVGFV--------------------------wsgSD-SQIY-PELRKMEAE-----------E-LLVG-S-D--V----------ktEY-ALsekgwealrka
0050 9baqA   feyqRY--Y-Q--PF-LW-IA--D--LGK-HV-VDYCTR----v--eRKREVTLG-------CFK-SDFI-QWASKAHGK-Sk-aFQNWR-AQ-HPSD--------dFR-TSVA-ANIGYIWKE------------gdqLF-R-EliHT-YM--A-Ntx----------------
0051 5uujA   ------------vTP-RQ-VD--E--LTE-EV-AGILD----------GVVLTRD-------ELV-TRLV-A-----------------dKRF-VS-----------xeERLR-SGW-GSVLKPLAWR-----------G-VLCH-GpN--RG-N------K--ITF-TLpasqfgadwgk

0001 s001A   --LLLL--L-H--HH-HH-HH--H--HHH-HH-HHHLLLLL------LLLEEELL--LLLLLLHH-HHHH-HHHHHHHHH-H---HHHHH-HHHHHHHLL-----LLHH-HHHHHLHHHHHHHHLE---ELLEELL-LLEE-E-E--LL-LL--L-L--EEE-EL----------
0002 8c45A   -lLLLL-hH-H--HH-HH-HH--H--HHH-HH-HHHHLLLLlll--lLLLEEELLllLLLLLLHH-HHHH-HHHHHHHLLlL---HHHHH-HHHHHHHLllllhhHHHH-HHHHHLHHHHHHHHLL-------LLL-LEEE-E-E--LL-LL--L-L--EEE-EElllllllhhhh
0003 3s93B   ----LH--H-H--HH-HH-HH--H--HHH-HH-HHHHHL---------lLLLLLHH-------HHH-HHHH-HHHL----------------llLLHHHLL-----L-----LHHH-HHHLLL------------L-LEEE-E-E--LH-H---H-L--EEE-EEll---------
0004 2od5A   --------L-L--HH-HH-HH--H--HHH-HH-HHHLL-----------LLLEEH-------hHHH-HHHH-LL-------------LLLL-----------------lLH-HHHH-HHHHHL-------------L-LEEEeE-E--EE-EeeeE-E--EEE-EEhhhhhhhllll
0005 8e4yA   -hhHHL--L-L-llL-LE-EeelL--HHH-HH-HHHHHH-------llLLLEEHH-------HHH-HHHH-HHHHHHHHHL-L---LL----LLLL-----------LLHH-HHHH-HHHHHLL------------L-LEEE-E-E--LL-L---lL-L--EEE-EElllhhhhhhhh
0006 8ffzB   -eEEEE--E---lLL-HH-HH--H--HHH-HH-HHHHHL---------lLLEEELL--H----HHH-HHHH-HHH------------LLLL-----------------lLLH-HHH-hHHHHHHHHL-------------L-LLEE-E-E--LL-LL--L-L--EEE-ELllllhhhhhhhh
0007 9gm7C   ------------eEE-LHhHH--H--HHH-HH-HHHHH-----l--lLLLLEEHH-------HHH-HHHH-HHHH-----------HLLL--------------lhhHHHH-HHH-HHHHHLL-----------------lEEE-E-E--LH-H--lL-L--EEE-EElhhhhhhhhhh
0008 1lddA   ------------lLH-HH-HH--H--HHH-HH-HHHHHH----------hLLEEH-------hHHH-HHHH-HHL-----------LHHH---LLLL----------LLH-HHHHHHHHHHHHL-------------L-LEEL-L-L--L-----------LEE-EEl---------
0009 5yadA   --------L-H--HH-HH-HH--H--HHH-HH-HHHHH-----l--lHHHEEEHH-------HHH-HHHH-HHHL-----------------------------lllLH-HHH-LLLLL--------------------lEEE-E-L--LL------L-L--LEE-EElhhhl------
0010 7asvA   ------------lllLH-HH--H--HHH-HH-HHHHHH----------hLEEEHH-------HHH-HHHH-HHHHL-LLL-L-----LHH-HL-------------lLLH-HHHH-HHHHHL-------------------LLEE-E-L--LL-L-----hH--LEE-ELllllllhhhhh
0011 7vwvB   ------------LL-LH-HH--H--HHH-HH-HHHHH---------hLLLLLLH-------hHHH-HHHH-HHHL-----------------------------llLLH-HHHH-HHHHHLL---------------L-LEEE-L-L--LL------lL--LLE-EEll---------
0012 2lh9A   ------------lllLH-HH--H--HHH-HH-HHHHHH---------lLLLEEHH-------HHH-HHHH-HHHL-----------------llLHHHHLL-----L-----LHHHHHHLLL--------------L-LEEE-E-E--LL-L---L-L--EEE-EEll---------
0013 6sj9A   --------------L-HH-HH--H--HHH-HH-HHHHHH---------lLLLEEHH-------HHH-HHHL------------------lLL-LL-----------lhHHHH-HHH-HHHHHHHHHLL----------L-LLEE-E-L--L---L--L-L--LEE-ELlllllllleee
0014 7z8bC   -lLLLH-hH-H--HH-HH-HH--H--HHH-HH-HHHHHL--------lLLLLEEHH-------HHH-HHHH-HHHHLLLL-----lLHHHH-hhllLLLL-------lLH-HHH-hHHHHHHHH------------HL-LEEE-L-L--L----------lLEE-EEll---------
0015 2zmeB   -lLHHH------lLL-HH-HH--H--HHH-HHhHHHHH-----h--lLLLEEEHH-------HHH-HHHH-HH-------------LLLLL-----------------LLH-HHHH-HHHHLLHHHL-------------L-LEEE-E-E--LL-LL----L--EEE-EEllllhhhhhhhh
0016 6r7nB   -hHLLH-hH-H--HH-HH-HH--H--HHH-HH-HHHHL----------LLLEEEHH-------HHH-HHHL-------------------------------------lLH-HHHH-HHHHHHHHHL---------lL-LEEE-E-L--L--------L--LEE-EEllllllllhhh
0017 6ahtA   -lLHHH-hH-H--HH-HH-HH--H---HH-HH-HHHHHH----------hLLEEHH-------HHH-HHHH-HHHHHHHHHHhlllllLLLL------------lllHHHH-HHH-hHHHHHLL----------------lEEE-E-E--EL-----L-E--EEE-EElhhhhhhhhhh
0018 7abi7   -------hH-H--HH-HH-HH--H--HHHhHH-HHHHH---------hhLLLEEE----------HH-HHHH-HHHLLLL--------llLH-HHL----LL------LL-H-HHHHHHHHH-----------------H-LLEE-E-E--EL-L--lL-E--EEE-ELhhhhhhhhhhh
0019 3zcoA   ------------hhhhhhllL--H--HHH-HH-HHHHHH-----l--lLLLEEEHH-------HHH-HHHH-HHHHH-LLL-Lh-hHHHHH-HHH-----------llhHHHLlLLHH-HHHHHHHHL-----------L-LEEE-E-E--LL-LL--L-L--EEE-EEllhhhhhhhhh
0020 6wgeC   ------------llllLH-HH--H--HHH-HH-HHHHL-----l--lLLLLEEHH-------HHL-L-------------------------------------lllHHHH-HHHH-HHHHHHHHL-----------L-LEEE-E-L--LL-L--lL-L--LEE-EEllllll-----
0021 7cv0A   -----------------LH-HH--H--HHH-HH-HHHHHH---------lLLLLLHH-------HHH-HHHL----------------------------------------lLH-HHHH-HHHHHHHHLL----------L-LEEE-E-L----------------lEE-EElllllllleee
0022 7nywE   -lLEEE-------lL-HH-HH--H--HHH-HH-HHHHHL----h--hHLLEEEHH-------HHH-HHHH-HHL-------L---HHHHH-HH-HLLL----hhhHHHH-HHHH-HHHHHHHHL-----------L-LEEE-L---------------lLEE-EElhhhhhhhlll
0023 8uucA   -lLLLL--L-L--L-llL-HH--H--HHH-HH-HHHHHH---------lLLEEHH-------HHH-HHHH-HHHHH---L-L---LL-------------------lLLH-HHHHHHHHHHHHH-----------L-LLEE-E-L-----------------LEE-EL----------
0024 7qenC   ---------hhhllllllL-HH--H--HHH-HH-HHHHHH----hhhhhlLEEELH-------HHH-HHHL-LLL-----------LLLLL-LL--------------LLH-HHH-HHHHHHHHHHH-----------L-LLEE-E-E--LL-LL--L-L--EEE-ELllll-------
0025 2iu5A   ----lL--L-L--L--HH-HH-HH--H--HHH-HH-HHHHH----------hLLHH--------HLLH-HHHH-HHHL---------lLHHHH-HHLL--------------LLH-HHHHHHHHHHHHHHHlhhHHHHH--hhhhhhH-L--LLh-----------------------
0026 2obpA   ----------L--LL-HH-HH--H--HHH-HH-HHHLL-----l--lLLLLLLH-------hHHH-HHHL-------------------------------------lLH-HHHH-HHHHHHHHL-----------L-LEEE-E-E--LL-LL----L--EEEe------------
0027 3qkxA   -----L--L-L--HH-HH-HH--H--HHH-HH-HHHHH------------HLLL---------LLLLhHHHH-HHHL---------lLHHHH-HHHL----------LLHH-HHH-HHHHHHHHHHHH--hHHHLlllhhhHH-L-Ll------------------------------
0028 7yw2A   -------------LH-HH-HH--H--HHH-HH-HHHHHLL---l--lLLLLEEHH-------HHH-L--L-HHH-----------------------------lllLH-HHH-HHHHHHLL-----------LL-LEEE-E-E--LL-L--lL-L--EEE-EEllllllllll
0029 1w5sB   -hhHHL--LlH--HH-HH-LL--H--HHH-HH-HHHHHH----l--lLLLLEEHH-------HHH-HHHH-HHHLLLL-------LLLLL-------------lhHHHH-HHH-HHHHHLL------------------lEEE-E-L--L---------L--LEE-EElllllhhhhhh
0030 2lnbA   lllllL--L-L--L--LH-HH--H--HHH-HH-HHHHH----------hHLLLEEHH-------HHH-HHHL---------------------------------------lLH-HHHH-HHHHHHHHL-----------L-LEEE-E-E--L-----------LEE-EEllllll-----
0031 3elkA   ----------L--LH-HH-HH--H--HHH-HH-HHHHH-----------hLLEEH-------hHHH-HHHH-HHHL-----------------------------lllllLHHH-HHHHHHHHH-----------L-LEEE-E-E--EE-L--lE-E--EEE-EElhhhhhhhhhl
0032 6qpqD   -----------------LH-HH--H--HHH-HH-HHHHL----------LLLLEEHH-------HHH-HHHH-L----------------------------hllHHHH-HHHH-HHHHHHHHL-----------L-LEEE-E-L--LL-LL--L-L--LEE-EElhhhhllll--
0033 7jgrD   -hHLLL---hL--LL-LHhHH--H--HHH-HH-HHHHHH--------hLLLLLLHH-------HHH-HHHH-HHHLL--LL-L---LLLL---------------lhHHHH-HHH-HHHHHLL------------------lEEE-L-------LL--L-L-lLLE-EElllhhhhhhhh
0034 5dlwD   --LLHH-hH-H--HH-HH-HH--H--HHH-HH-HHHLL-----------lLLHH--------HLLH-HHHH-HHHL---------lLHHHH-HHHL----------LLHH-HHH-HHHHHHHHHHHH--hLHHHhhllLHHHhhhH--LL-LL--Hl-------------------
0035 4nb5B   ---------hL--LL-L---L--H--HHH-HH-HHHHHH--------lLLLLEEHH-------HHH-HHLL-------------------------------------lLH-HHHH-HHHHHHHHH-----------L-LEEE-E-L--LL-LL--L-L--LEE-EElllhhhhhhhh
0036 7wzeA   -------hH-H--HHlLL-LL--H--HHH-HH-HHHHHH--------lHHHEELH-------hHHH-HHLL-------------------------------------lLH-HHHH-HHHHHHHHH-----------L-LEEE-E-E--L------L-L--EEE-EElhhhhhhhhhh
0037 8r7kB   lllhHH--H-L--L--LH-HH--H--HHH-HH-HHHHH-----h--hLLLEEEHH-------HHH-HHHH-H-------------HLLL-----------------lLLH-HHHHHHHHHHHHHLL-----------L-LLEE-E-L--L----------L--LEE-ELllllllll--
0038 1yyvA   -lLLLL--H-H--HH-HH-HH--HlhHHH-HH-HHHHH-----------hLLEEHH-------HHH-HHLL-------------------------------------lLLH-HHHH-HHHHHHHHHH-----------L-LEEE-E-E--EL-L--lL-E--EEE-EElhhhhhhhhhh
0039 8hihN   ------------llL-HH-HH--H--HHH-HH-HLLL------------lLLEEHH-------HHH-HHHL-L--------------------------lllllllL-LHH-HHHHHHHHLLLL------HHHHlLEEE-E-L-------------L--LEE-EEllll------
0040 5g5pB   -hLLLHhhH-H--HH-HH-HH--H--HHH-HH-HHHHHH----l--lLLLEEEHH-------HHH-HHHH-HH-----------hLLLLllLLLL-------------LL-LLHH-HHHHHHHHLL---------lL-LEEE-E-L--L----------L--LEE-EElllllhhhhll
0041 7z1nO   ---------leehlL-LH-HH--H--HHH-HH-HHHLLlllllhhhllLLLLLH-------hHHH-LLLL-----------------LLL-L--LLLLE---lllLLLH-HHHH-HHHHHHHLL---------LLL-LLEE-E-E----------------lLEE-ELlhhhlhhhhhh
0042 4y66C   --------L-H--HH-HH-HH--H--HHH-HH-HHHL-------------LLLLLL-------HHH-H-HH-HHH---------------------------lllLLHH-HHH-HHHHHLLL----------------LEE-E-E--LL-----L-L--EEE-ELlllhhhhhhhhh
0043 5xfoA   ---------llleelllLH-HH--H--HHH-HH-HHHHHH---------hLLLLEELL--L----LHH-HHHH-HLHHHHL-------LHHH--LLLL----------lHHHH-HHHH-HHHHHLL-------------L-LEEE-E-E--EH-HH--L-E--EEE-EEllllllll--
0044 8fo9F   hhhhHL--E-EeeEE-HH-HH--H--HHH-HH-HHHLLL----l--lLLLEEEHH-------HHH-HHHH-HLL----------------------------llLLL-LLH-HHHHHHHHHL-----------L-LEEL-L-L--LL-L----lL--LEE-EElhhhhhhhhll
0045 4kywA   -llLLL--L-L-----LH-HH--H--HHH-HH-HHLL------------LLEEEH-------HHH-HHHH--------------hhhHHHL-LL-----------lLLHH-HHHH-HHHHHHHH----------LL-LEEE-L-L-------------L--LEE-EEl---------
0046 4i99C   -----------------L-HH-HH--H--HHH-HH-HHHHHH----h--hHLLLEEHH-------HHL-L----------------------------------lllHHHH-HHHH-HHHHHHHHL-----------L-LEEE-E-L--LL-LL--L-L--LEE-EEl---------
0047 6pcoB   hhhhHH--L-H--HH-L--LL--H--HHH-HH-HHHHHH-----------hLLLLHH-------HHH-HHHL-------------------------------------lLH-HHHH-HHHHHHHHL-----------L-LEEE-E-L--LL---------L--LEE-EElhhhhhhhhh
0048 6wg3C   -------hhhL--LL-LH-HH--H--HHH-HH-HHHHL-----l--lLLLLLLHH-------HHL-L---------------------------lllH-----HHH--HHHH-HHHHHHHHL-----------L-LEEE-E-L--LL-LL--L-L--LEE-EEllllll-----
0049 5z7bB   -------------LH-HH-HH--H--HHH-HH-HHHHL-----------lLLEEH-------hHHH-HHHHlLHHHL-----------------------------lllLH-HHHH-HHHHHHHHL-----------L-LEEE-E-E--E----------eeEE-EElhhhhhhhhhh
0050 9baqA   hhhhHH--H-H--HH-HH-HH--H--HHH-HH-HHHHHH----h--lLLLLLLLL-------HHH-LHHH-HHHHHHLLL-Lh-hHHHHH-HL-LLLL---------lLH-HHHH-HLHHHHHHH-----------hlhHH-H-HlhHH-HH--H-Hll----------------
0051 5uujA   ------------lLH-HH-HH--H--HHH-HH-HHHLL------------LLLEEHH-------HHH-HHHH-L--------------lLLL-HH-----------hhHHHH-HLH-HHHHHHHHHH-----------L-LLEE-LlL--LL-L------L--LEE-ELhhhhhhhhlll
```

```
##################################################################################################################################################################################################################################################################################################################################
##################################################################################################################################################################################################################################################################################################################################
##################################################################################################################################################################################################################################################################################################################################
```

Job: PglZ NPV81619 N-terminal Inactive P-loop Domain of Swi2/Snf2-Helicase
Query: s001A
No: Chain Z rmsd lali nres id PDB Description
14: 6l8o-A 5.7 3.3 89 838 9 MOLECULE: DNA REPAIR PROTEIN RAD5;
2: 6o16-A 7.0 3.2 91 798 4 MOLECULE: DEAH (ASP-GLU-ALA-HIS) BOX POLYPEPTIDE 37;
3: 6eud-A 6.9 2.8 90 808 14 MOLECULE: ATP-DEPENDENT RNA HELICASE HRPB;

4: 3k1j-A 6.8 3.2 90 566 10 MOLECULE: ATP-DEPENDENT PROTEASE LON;
5: 8umy-A 6.6 3.2 87 546 11 MOLECULE: CHROMOSOME TRANSMISSION FIDELITY PROTEIN 18 HOMOL
6: 6ut5-A 6.5 2.7 85 395 6 MOLECULE: GTPASE SUBUNIT OF RESTRICTION ENDONUCLEASE;
7: 8val-E 6.2 2.9 88 337 9 MOLECULE: DNA POLYMERASE III SUBUNIT DELTA;
8: 5lkl-A 6.2 2.9 77 704 10 MOLECULE: GENOME POLYPROTEIN;
9: 8k8v-A 5.9 3.1 90 719 13 MOLECULE: ENDOPEPTIDASE LA;
10: 8btg-B 5.8 2.8 85 335 7 MOLECULE: CHROMOSOMAL REPLICATION INITIATOR PROTEIN DNAA;
11: 8tw7-2 5.8 3.4 86 339 9 MOLECULE: REPLICATION FACTOR C SUBUNIT 5;
12: 5yww-A 5.8 2.8 81 485 6 MOLECULE: NUCLEOTIDE BINDING PROTEIN PINC;
13: 8fcv-R 5.8 3.5 89 343 7 MOLECULE: DNA (60-MER);
15: 6pxk-K 5.7 2.9 90 429 12 MOLECULE: ATP-DEPENDENT PROTEASE ATPASE SUBUNIT HSLU;
16: 4zo4-A 5.7 2.9 71 202 11 MOLECULE: DEPHOSPHO-COA KINASE;
17: 7st9-E 5.6 3.6 87 354 14 MOLECULE: CHECKPOINT PROTEIN RAD24;
18: 8y6p-Q 5.6 2.8 80 1228 9 MOLECULE: APAF-1 RELATED KILLER DARK;
19: 6l1q-A 5.6 3.1 81 267 16 MOLECULE: CBBQ PROTEIN;
20: 7w42-B 5.6 3.2 84 402 14 MOLECULE: UNCHARACTERIZED ATPASE YJOB;
21: 5x71-A 5.5 2.7 76 796 11 MOLECULE: MRNA CAPPING ENZYME P5;
22: 9emc-D 5.5 3.5 87 337 17 MOLECULE: RUVB-LIKE 1;
23: 7plh-A 5.5 2.9 84 260 11 MOLECULE: SHTNSC;
24: 9e22-A 5.4 3.6 90 2925 6 MOLECULE: CYTOPLASMIC DYNEIN 1 HEAVY CHAIN 1;
25: 8dgf-A 5.4 3.1 87 1541 14 MOLECULE: ATP-BINDING PROTEIN AVS4;
26: 3glf-G 5.4 3.3 87 378 13 MOLECULE: DNA POLYMERASE III SUBUNIT DELTA;
27: 3bos-B 5.4 3.0 88 231 9 MOLECULE: PUTATIVE DNA REPLICATION FACTOR;
28: 8s35-M 5.4 3.0 81 624 11 MOLECULE: CRISPR TYPE AFERR-ASSOCIATED PROTEIN CSF2;
29: 7qo4-J 5.3 2.9 87 405 14 MOLECULE: 26S PROTEASOME REGULATORY SUBUNIT RPN1;
30: 5zr1-A 5.3 3.2 85 494 7 MOLECULE: ORIGIN RECOGNITION COMPLEX SUBUNIT 1;
31: 8thc-A 5.3 3.0 86 479 10 MOLECULE: ELG1 ISOFORM 1;
32: 7rzy-1 5.3 3.3 87 311 11 MOLECULE: TN6677 VIBRIO CHOLERAE TRANSPOSON TNSC (VCHTNSC);
33: 2p0e-A 5.2 2.6 70 190 7 MOLECULE: NICOTINAMIDE RIBOSIDE KINASE 1;
34: 6z1f-3 5.1 3.3 85 284 7 MOLECULE: RIBULOSE BISPHOSPHATE CARBOXYLASE/OXYGENASE ACTIV
35: 9ri9-B 5.1 3.1 83 868 8 MOLECULE: SLNRC3;
36: 6yvh-K 5.1 3.4 78 164 9 MOLECULE: PRE-MRNA-SPLICING FACTOR CWC22 HOMOLOG;
37: 8jqb-E 5.1 3.6 86 499 14 MOLECULE: ENDONUCLEASE GAJA;
38: 8bob-C 5.1 3.4 86 412 17 MOLECULE: PROTEIN MALY;
39: 5v8f-D 5.1 3.2 82 456 6 MOLECULE: DNA REPLICATION LICENSING FACTOR MCM2;
40: 8xks-B 5.1 24.8 90 603 10 MOLECULE: CTAP1;
41: 8dr5-A 5.1 3.4 85 646 6 MOLECULE: REPLICATION FACTOR C SUBUNIT 1;
42: 8dgc-A 5.0 3.2 87 2028 11 MOLECULE: SEAVS3;
43: 3pvs-D 5.0 2.9 80 424 10 MOLECULE: REPLICATION-ASSOCIATED RECOMBINATION PROTEIN A;
44: 6nak-D 5.0 3.3 86 165 8 MOLECULE: TRNA THREONYLCARBAMOYLADENOSINE BIOSYNTHESIS PROT
45: 9jix-A 5.0 2.7 75 154 9 MOLECULE: DE NOVO DESIGNED ATPASE, PL2X4_2;
46: 7mi1-A 5.0 3.2 87 2628 14 MOLECULE: CHIMERA PROTEIN OF DYNEIN AND ENDOLYSIN;
47: 1ixs-B 5.0 3.3 87 315 9 MOLECULE: HOLLIDAY JUNCTION DNA HELICASE RUVA;
48: 2is6-A 5.0 3.3 86 654 9 MOLECULE: 5'-D(*CP*GP*AP*GP*CP*AP*CP*TP*GP*CP*AP*GP*TP*GP*C
49: 2zpa-B 5.0 3.3 88 662 7 MOLECULE: UNCHARACTERIZED PROTEIN YPFI;
50: 7mi8-A 4.9 3.2 88 868 16 MOLECULE: FUSION PROTEIN OF DYNEIN AND ENDOLYSIN;

```
0001 s001A   ---MASW-R--DH-IL-K-EFTPRV-S----R----LTLVADP-D-G-LLLEILEGIRE-R---G--FELI--P--F---E-DHI--AF-RYAYESRFR-S-R-W-D-AALVVVLRS----GL-E-A--LDVLPYDLLQAG-RKLSF-----------------
0015 6l8oA   --sHFQStI--KA-LL-R-HLIQET---spgE----QIIVFSQ-F-SsFLDILRSHLPR-D---Q--VIIY--K--F---DkERT--RI-LEQFHDKD------L-S-CIKLLLLSL----K--T-G--GVGLN---LTCAsRAFMMdpwwspgmedqaidrih
0003 6o16A   --qEERL-lpiLA-EEqA-IME-AE-H----P----IVIVCGE-TgS-GKTTVPQFLYE-A--yK--VVII--D-eA---H-ERV--YT-DILLGL-LS-R-I-VlP-LKLLIMSAT----L----R--VEDFTQNFTTPP-PVIKVesrfpvtvhfnddysge
0004 6eudA   --aMSSL--pvAA-VL-P-ELLTDC-A----P----QVLLSAP-TgA-GKSTLPLQLLA-H-----gLVIL--D-eF---H-ERL--QA-DLALAL-LL-D-V-QdD-LKLLIMSAT----L----D--NDRLQQML-PEA-PVVISegrsfpverrylplpah
0005 3k1jA   lidqvIG-Q--EH-AV-E-VIKNQK-R---------HVLLIGE-PgT-GKSMLGQAMAE-L---L-gVLFI--D--E-iaT-LSL--KM-QQSLLTAMQ-E-KkC-D-FVLVAAGNL-------D-T--VDKMHPALRSRIgYEVYMrttmpdtienrrklvqf
0006 8umyA   --ytELL-S-dDF-TN-R-CLWLKLwQ----K----VALLCGP-PgL-GKTTLAHVIAR----pN--CLVI--D--EidgA-PVA--AI-NVLLS-ILN-RgL-L-M-RPIICICN----------D--QFA-LRQLKQQA-FLLHFpptlpsrlvqrlqevsl
0007 6ut5A   ----GHN-I--KE-DY-F-RVD-NK-K----G----QVILYGP-PgT-GKTWaRKYVVE-E--kF--YLII--DeiN---R-GN----I-SKIFGelitlA-V-P-PnLYIIGTMNT----A-dR-S--IALLDVALRRRF-AFIEVeprpeflekenlkkire
0008 8valE   hmrwyPW-L--RP-DF-EkLVASRG-H----H----ALLIQALpG-M-GDDALIYALSR-Y---L-kVVWV-tD--A---A-LLTdaAA-NALLK-TLE-E-P-PaE-TWFFLATR----------E--PERLLATLRSRC-RLHYLapppeqyavtwlsrevt
0009 5lklA   lmsgaTD-L--TE-MV-K-KTTMN-----rgE----FRQITLA-TgA-GKTTLPRSVIE----yS--FIFL--D-eY---H-CAT-pEQ-LAIMGKI-H-R-F-SeN-LRVVAMTA----------T--PAGvNYDWS--------------------------
0010 8k8vA   --lPPPP-P--FF--R-E-ALEIRG-G----F----HAYLVGP-PsL-GKHELLAYLstqsvegG--YLIL--D--A---LlKRE--GT-WEAFKRALR-N-G-I-Q-MQVILVGTP-------E-A--FEGLEPAFSELFrIRAEFsptlpaspenctalggw
0011 8btgB   pkytfDT-F--VIgsH-A-ASLAKA-Y----N----PLFIYGG-VgL-GKTHLMHAIGH-Y---V-dVLLI--D-dI---Q-FLA-gkeqQEEFFHTFN-T-L-HeS-KQIVISSDR---------P--PKE-RSRFE-WG-LITDItppdletriailrkkak
0012 8tw72   nldevTA-Q--DH-AV-T-VLK-AN-L----P----HMLFYGP-PgT-GKTSTILALTK----yK--IIIL--D-eA---D-SMD--AQ-SA-LRRTME-T-Y-SgV-TRFCLICN----------Y--VTRIIDPLASRC-SKFRFkaldasnaidrlrfise
0013 5ywwA   vrkrlED-Y--NL-------lLEER-A----E----GIIIAGA-PgM-GKTTFAQALAE-Y---Y-dYTVY-dE--M---R-ND---ED-FKLYVDLRL-A-G-V----GMVGVVHA----TSpI-D--AIHRFVN--diD-TIIFInsgnvskvytlemtvkv
0014 8fcvR   --tVAHP-L--KE-VY-E-ILMIAE-P-agaS----FIFVYGA-SgV-GKTTLRLRVEQ----pD--VFFV--D-eA---Q-HFklqDQ-LDCLKSLAN-M-T-G---ILHCLLGTY---------E--LLTFRNQLSRRS-VDIHFrrycadspedvqafksv
0016 6pxkK   ldkhiIG-Q--DN-AK-R-SVALRN-P----K----NILXIGP-TgV-GKTEIARRLAK----hG--IVFIdeI--D---K-IEG--VQ-RDLLP-LVE-GkT-D-H-ILFIASGAF----Q-iA-K--PSDLIPELQGRLpIRVELqalttsdferiltepna
0017 4zo4A   ----------------------------axkN----AFFVTAS-I-AcGKSTFIEIANS-L---G--FKSI--S--A---DkIRA--KI-LEQXQ-ILD-K-E-N---KAFFVEIPL---------F--FESGA--YENLG-KVIVIytpkelslkrixqrdkl
0018 7st9E   alshnEE-L--TN-FL-K-SLSPRD-L----P----HLLLYGP-NgT-GKKTRCMALLE-S-----kCVII--N-eA---N-SLD--AQ-AA-LRRTME-K-Y-SkN-IRLIMVCDS------------MSPIIAPIKSRC-LLIRCpapsdseistilsdvvt
0019 8y6pQ   fakynVS-R--LQ-PY-L-KLRLLE-L-rpaK----NVLIDGV-LgS-GKTWVALDVCL-S-----cLLVL--L-nV---Q-NAK--AW-NAFNL-----------sCKILLTTRF----K--Q-V--TDFL---SAATT-THISLdhhsmtltpdevkslll
0020 6l1qA   gkepfyQ-P--QHdEV-A-LFEYRK-R----L----PVMVKGP-TgC-GKSRFVEFMAW----gA--ICYL--D-eI---V-EAR---qdTVVIH-PLT-D----pD-FQLVISYNP----G-----slmkDLKQSTKQRF-TGFEFdypnaeleagilvqetg
0021 7w42B   revqrSQ-V--IM--K-K-EISIDQ-dipykR----GILLYGP-P-GnGKTTLVKSIAG----pA--VLVI--E-dI---D-SMPedVR-SFFLNT-LD-G-A--eG-LFLIGTTN----------Y--PEEIDPGLMNRD-RAYEIglpdeelrleymkmrgf
0022 5x71A   --------------------------S----P----LVVFCLS-H-NeLAKKYAQDVSMsS---G--THVH--I--I---DeITV--SL-YRTFR-TIA-T-Q-LgR-MQIVVFVTV----DksvvS--TQVMKSIAWARG-SFVELrnqsvdsstlvsklenl
0023 9emcD   asqgmVG-Q--LA-AR-R-AVLEM--gkiagR----AVLIAGQ-PgT-GKTAIAMGMAQ----pG--VLFI--D-eV---H-MLD-iES-FSFLNRALE-S-D-M-A-PVLIMATNR----GitrirqsPHGIPIDLLDRL-LIVSTtpysekdtkqilrirce
0024 7plhA   --iVPLQ-V--KT-LH-D-WLDKAR-K---------SCRVVGE-SrT-GKTVYRY--------vE--MLII--D-eA---D-RLK-----PETFADVRD--eD-L-G-IAVVLVGTD----------rlDAVIKEQVLERFrAHLRFgklsgedfkntvemweq
0025 9e22A   plvlfNE-V--LD-HV-L-RIDRQP-Q----G----HLLLIGV-S-GaGKTTSRFVAWM-N-----iAFIM--D--E---SnVLD--S-gLERMNTLLA-N-G-ErN-LHVVFTM-N----PS-S-EkdrAATSPALFNRC-VLNWFgdwstealyqvgkefts
0026 8dgfA   --sIEID-R--RH-CI-E-LLHLVQ-K----K----IVIVSGE-GgV-GKTAVIKKIYE----rK--VIVV--D-sA---E-KLltnidpKEFLTV-LI-K-D-K---WQVVFTTRN-------N-Y--LADLNYAFIITP-GNLVIknlergelielsdnngf
0027 3glfG   tfadvVG-Q--EH-VL-T-ALA-RI-H----H----AYLFSGT-RgV-GKTSIARLLAK-G---L-kVYLI--D-eV---H-MLSrhSF-NALLK-TLE-E-P-PeH-VKFLLATT----------D--PQKLPVTILSRC-LQFHLkaldveqirhqlehiln
0028 3bosB   ddetfTS-Y--YPaaI-G-ALKSDG-V----Q----AIYLWGP-VkS-GRTHLIHAACA-R---A-dLICIddV--D---A-VHP--LW-EEAIFDLYN-R-V-AkR-GSLIVSASA---------S--PXE-LVSRXHWG-LTYQLqpxxddeklaalqrraa
0029 8s35M   --fsGQW-L--KA-VV-E-QLSC-I-Q----G----PALVLTA-S-HyETRMIANQLGE-V---S--QPVY--I--QkagQ-ALS--EI-IKQYQEK-----------pGILISAG--------aS--VGVSPRGgEQIFqDLIITripflppdrmkaeslyg
0030 7qo4J   tydmvGG-L--TK-QI-K-EIKEVI-P----K----GVILYGP-PgT-GKTLLARAVAH----pS--IIFM--D--E---I-DGG--DS-EVQRT-MLE-L-L-NkN-IKIIMATNR------------LDILDPALLRID-RKIEFpppsvaaraeilrihsr
0031 5zr1A   ssyylPA-R--EN-EF-A-SIAYSA-esdsaT----TIYVAGT-PgV-GKTLTVREVVK-E-----iVVLL-dE--L---D-AQD--IM-YNFF-nWTT-Y-E-A-K-LIVIAVANT-----M-D-L--PER-TSRI--GF-TRIMFtgytheelkniidlrlk
0032 8thcA   --tlKQV-LiePK-LK-L-RIIETS-ptefvP----LMILHGN-S-I-GKKTLIQTIMR----yG--LVLF--N-dV---D-VLhdrGY-WAMISK-LC-E-F-S-R-RPLVLTCK----------D--LSLVPSELIALAnSLFHTkkiststvyafltkylk
0033 7rzy1   --aFVSTpV--RK-IL-S-YMCRDL-lesepT----CMMVYGA-SgV-GKTTVIKKYLN----vK--LIII--D-eF---Q-HLnrvltqGNWLKMILN-K-T-K---CPIVIFGMP-------Y-S--KVVLNSQLHGRFsIQVELrpfsynggrgvfktfle
0034 2p0eA   --------------------------rgsktF----IIGISGV-TnS-GKTTaKNLQKH-L---P-nCSVI--S--Q---D-DXE--KX-XSAISC-WX-E-S-AeI-PILIIEGFL---------L--FNY-LDTIW--N-RSYFLtipyeeckrrrstrvyq
0035 6z1f3   ---SYYI-A--PR-FL-D-KLHITK-P----L----ILGIHGR-KgE-GKTFCELA-------mC--VLMI--NdlD---A-GAntqlV-NATLMN-IA-D-N-PrR-VPIIVTGN----------D--FSTLYAPLIRRM-EKFYWepnrddkvgivggifae
0036 9ri9B   --dDVVG-F--DD-EA-K-TVILIG-GsdyvE----VVPVVGM-PgL-GKTTLAYKIYK-D-----yLIVL--D-dV---W-TME--AW-DRIK-IAFP-N-N--kR-NRVLMTTR------------QSNV--RCND---KPHDLkfltkdeswellekkvf
0037 6yvhK   --vEREE----WK-FD-T-LCYDTL-I----T----QAVIFCN-T-KrKVDWLTEKMRE-A---N--FTVS--S--M----kERE--SI-MKEFRSGA-----------sRVLISTD------------vWGLD---VPQVsLIINYdlpnnrelyihrigrsg
0038 8jqbE   ----MIE-D--EM-SR-EqIIKDG-------G----NILVTAG-A-GsGKTTILVSKIE-A---DlfKIFI--D-eY---Q-DSK--DM-HNLFMYLKD-Q-L-K-I--KLFIVGDP--kqsiyiwrenFNGLIEN-STDF-NKYHLtsnfrccqdiqnysnlf
0039 8bobC   rldhtVV-R--ER-LL-A-KLSGAN-F----R----LALITSP-AgY-GKTTLISQWAA----pL--YLVI--D-dY---H-LInpvIH-ESMRF-FIR-H-Q-PeN-LTLVVLSRN------------LPQLGANLRVQL-LEIGSqqlafthqeaneffdcr
```

```
0040 5v8fD   --iiFTY-L--QD-CQ-Q-EIIKQS-iqkesH----SVILVGP-RqS-YKTYLLDYELS-------vVFIF--D-eI---D-TFA--GpvQTLLYNLFD------vP-VCIFGCTTK----L----N--ILE-KSRF--SQ-RVIYMpqiqnlddmvdavrnll
0041 8xksB   --eLPEG-N--KQ-AK-V-EVVDFA-P----K----GVLLVGP-PgN-GKTLMARAVAG----pC--IIFI--D-eL---D-AVD--NT-VNQLLTEMD-A-E-Q-QgIVVMGATN----------R--KDVLDAALTRPD-RSIEVrrpdfqgrleaslaslm
0042 8dr5A   nlqqvCG-N--KG-SV-M-KLLANW-F----R----AAMLYGP-PgI-GKTTAAHLVAQ----hF--VIIM--D-eV---D-GMG--GV-GQLA-QFCR---K-T-S-TPLILICN----------E--RNL-MRPFDRVC-LDIQFrrpdansiksrlmtiai
0043 8dgcA   --nSVSR----TQ-EA-T-LVQVEA-F---gA----PVIIHAD-AgV-GKSIFSTHIEE-H-----lCIII--D-aA---D-NAS--FI-KDLIRE--K-L-P-D-G-VCLVALCRP-------Y-R--RELLD--PPPEA-LTLSLqtfnrdetaahlhqkfp
0044 3pvsD   rpenlAQ-Y--IG-Qqhllapa GHL-H---------SMILWGP-PgT-GKTTLAEVIAR-Y-----tILFV--D-eV---H-RFN-----KSQQDA-FL-P-H-G-T-ITFIGATTE---------N--PSFLNSALLSRA-RVYLLkslstedieqvltqame
0045 6nakD   --eNLTE-E--QL-KR-L-ALTENL---kggE----VVILSGN-LgA-GKTTFVKGMIR-A---I-tIYHL--D--L---Y-RTD--FL-SLDVED-IL-E-D-E-D-GIMVVEW-------------gDLFD-GFWPEDS-IKVKIeiadeshrnveilipee
0046 9jixA   ----------------------------mgaI----VILVVGP-PgS-GKSQLIKAIEK-L--qP--VVTT--S--V---T-SED--EA-KKVLEELLK-K-D-P-N-AIVVIEIKN----P--R-I--AERVAKRV-dVL-VVVVSspevarelrenlpnviv
0047 7mi1A   iperlIY-T--PL-LL-I-GFA-TD-S----LhqkyGGCFFGP-AgT-GKTETVKAFGQ----gA--WGCFdqF--N---R-LDE-kVL-SAVSANI-Q-Q-I-QpH-TAVFITLNP----gynG-R--SE-LPENLKKSF-REFSMkspqsgtiaemilqimg
0048 1ixsB   tldeyIG-Q--ER-LK-Q-KLRVYL-L----E----HLLLFGP-PgL-GKTTLAHVIAH-E--gD--ILFI--D-eI---H-RLSrqAE-EH-LYPAME-D-F--pR-FTLIGATTR---------P--GLITAP-LLSRFgIVEHLeyytpeelaqgvmrdar
0049 2is6A   vsyllDS-L--ND-KQ-R-EVAAP--R----S----NLLVLAG-AgS-GKTRVLVHRIA-W---LmtnILV--DefQ---D-TNI--QY-AWIRL-LAG-D-T-G----KVMIVGDD----DQ-S-igaqVENIQRFLNGA-ETIRLeqnyrstsnilsaanal
0050 2zpaB   --tGAPQpQ--QQ-LLkQ-LXTX-P-P----G----VAAVTAA-RgR-GKSALAGQLIS----aD--WLVV--D-eA---A-AIP-----APLLHQLVS-----R-F-PRTLLTTTVqgyeGT-G-RgfLLKFCARF-PHL-HRFELqqpirwaqgcplekxvs
0051 7mi8A   iperlIY-T--PL-LL-I-GFA-TD-S----LhqkyGGCFFGP-AgT-GKTETVKAFGQ----gA--WGCF--D-qF---N-RLDekVL-SAVSAN-IQ-Q-I-QpH-TAVFITLNP----GynG-R--SE-LPENLKKSF-REFSMkspeeysiigkhsvkif

0001 s001A   ---LLLH-H--HH-HH-L-LLLLLL-L----L----EEEEELL-L-L-HHHLHHHHHHH-L---L--EEEE--E--L---L-LHH--HH-HHHHHHHLL-L-L-L-L-LEEEEEELL----LH-H-H--HHHLLHHHHHHL-EEEEL----------------
0015 6l8oA   --lLLLLhH--HH-HH-H-HHHHHH---lllL----EEEEEEL-L-HhHHHHHHHHLLL-L---L--EEEE--E--L---LhHHH--HH-HHHHLLLL------L-L-LEEEEEEEL----L--L-L--LLLLL---LLLEeEEEELlllllhhhhhhhhhlll
0002 9nv3C   ----LHH-H--HH-HH-H-HHHHLL-L----L----EEEEELL-L-L-LLLLLLL-lL-L---L--LEEE--E--L---L-LL---LH-HHHHH-HHH-LlL-L-L-LLEEEEEL----------L--LLL-LHHHHHHL-EELLLllhhhhhhhlllllll
0003 6o16A   --hHHHH-lhhHH-HHhH-HHH-HH-L----L----EEEEEEL-LlL-LHHHHHHHHHH-L--eE--EEEE--L-lH---H-HLH--HH-HHHHHH-HH-H-H-HlL-LEEEEEEELL----L----L--LHHHHLLLLLLL-LEEEElllllleeeeelllhhhh
0004 6eudA   --lLLLL--llHH-HH-H-HHHHLL-L----L----LEEEELL-HhH-LLLLHHHHHHL-L-----eEEEE--L-lH---H-HLH--HH-HHHHHH-HH-H-H-LlL-LEEEEEELL----L----L--LLLHHHHL-LLL-LEEELlllllleeeeellllll
0005 3k1jA   hhhhlLL-L--HH-HH-H-HHHHLL-L---------LEEEELL-LlL-LHHHHHHHHHH-L---L-lEEEE--L---L-hhH-LLH--HH-HHHHHHHHH-H-LeL-L-LEEEEEELH-------H-H--HHHLLHHHHHHHlEEEELlleeellhhhhhhhhhh
0006 8umyA   --llLLL-L-lHH-HH-H-HHHHHHhL----L----EEEEELL-LlL-LHHHHHHHHHH----lE--EEEE--E--LhhhL-LHH--HH-HHHHH-HHL-LlL-L-L-LLEEEEEL----------L--LLL-HHHHHLLL-EEEELllllhhhhhhhhhhhh
0007 6ut5A   ----LLH-H--HH-HH-H-HHH-HH-H----L----EEEEEEL-LlL-LHHHhHHHHHH-H--lE--EEEE--EllL---L-LL----H-HHHLHhhhhhL-L-L-LlEEEEEEEEL----L-lL-L--LLLLLHHHHHHL-EEEELlllhhhhlhhhhhhhhl
0008 8valE   lllllHH-H--HH-HH-HhHHHHLL-L----L----EEEEELLlL-L-LHHHHHHHHHH-H---H-eEEEE-lL--H---H-HLLhhHH-HHHHH-HHH-L-L-LlL-EEEEEEEL----------L--HHHLLLLLHHHL-EEEELllllhhhhhhhhhhhll
0009 5lklA   eellHH-H--HH-HH-H-HHLLL-----llL----EEEEELL-HhH-LLLLHHHHHHH----lL--EEEE--E-lL---L-LLL-hHH-HHHHHHH-H-L-L-HhH-LLEEEEEL----------L--LLLlHHHHL------------------------
0010 8k8vA   --lLLLL-L--LL--H-H-HHHHHL-L----L----EEEEELL-LlL-LHHHHHHHHlllllllL--EEEE--E--H---HhHHL--LL-HHHHHHHHH-H-L-L-L-LEEEEEELH-------H-H--HHLLLLLHHHHEeEEEELlleeellhhhhhhhhhh
0011 8btgB   lllllLL-L--LLllH-H-HHHHLL-L----L----EEEEEEL-LlL-LHHHHHHHHHH-H---H-lEEEE--E-lH---H-HHL-llhhHHHHHHHHH-H-H-HlL-LEEEEEELL----------L--LLL-HHHHL-LL-EEEEElllhhhhhhhhhhhhh
0012 8tw72   lllllLL-L--HH-HH-H-HHH-LL-L----L----LEEEELL-LlL-LHHHHHHHHHH----lE--EEEE--L-lH---H-HLH--HH-HH-HHHHHH-H-L-LlL-EEEEEEEL----------L--HHHLLHHHHHHL-EEEELllllhhhhhhhhhhhh
0013 5ywwA   llllhHH-H--LL-------hHHHL-L----L----EEEEELL-LlL-LHHHHHHHHHH-H---H-lEEEE-lL--L---L-LH---HH-HHHHHHHHH-L-L-L----EEEEELLL----LLhH-H--HHLLLLL--lhL-EEEEEelleeeeeeeeeeeel
0014 8fcvR   --lLLLH-H--HH-HH-H-HHHHHL-L-lllL----EEEEEEL-LlL-LHHHHHHHHHH----lL--EEEE--E-lH---H-HHhhhHH-HHHHHHHHH-H-H-L---LEEEEEELH---------H--HHHHLLHHHHLE-EEEEElllllllhhhhhhhhhh
0016 6pxkK   hllllLL-L--HH-HH-H-HHHHHH-L----L----LEEEELL-LlL-LHHHHHHHHHH----hL--EEEEelH--H---H-HHH--HH-HHHHH-HHL-LeL-L-LEEEEEELL----L-lL-L--HHHLLHHHHLLLlEEEELllllhhhhhhhhhllll
0017 4zo4A   ----------------------------lllL----EEEEEEL-L-HhHHHHHHHHHHH-L---L--LEEE--E--H---HhHHH--HH-HHHHH-HHH-H-L-L---LLEEEELLL---------L--LLLLL--HHHLL-EEEEEllllllhhhhhhhhhll
0018 7st9E   hllllHH-H--HH-HH-H-HHLHHH-L----L----LEEEELL-LlL-LHHHHHHHHHH-H-----eEEEE--L-lH---H-HLH--HH-HH-HHHHHH-H-L-LlL-EEEEEEELL------------LLLLLHHHHHLL-EEEELllllhhhhhhhhhhhh
0019 8y6pQ   hhhllLL-L--HH-HH-H-HHHHHH-L-lllL----EEEEELL-LlL-LHHHHHHHHHH-L-----lEEEE--E-eE---L-LHH--HH-HHHLL-----------lLEEEEEELL----H--H-H--HHHL---LLLLE-EEEELlllllllhhhhhhhhhh
0020 6l1qA   llllllL-L--LLlHH-H-HHHHHL-L----L----LEEEELL-LlL-LHHHHHHHHHH----lL--EEEE--E-lH---H-HLL---hhHHHHH-HHL-L----lL-LEEEEEELL----L-----llllLLLHHHHLLL-EEEELllllhhhhhhhhhhhl
0021 7w42B   llllhHH-L--LL--H-H-HHHHHH-lllllE----EEEEELL-L-LlLHHHHHHHHHH----lE--EEEE--L-lH---H-HLLhhHH-HHHHHH-HH-L-L--lL-EEEEEEEL----------L--HHHLLHHHLLLL-EEEELllllhhhhhhhhhhlh
0022 5x71A   ---------------------------L----L----EEEEEEL-L-HhHHHHHHHHHHhH---L--LEEE--E--E---LlHHH--HH-HHHHH-HHL-L-L-LlL-LLEEEEELL----LllllL--HHHHHHHHHHLL-EEEEEllllllhhhhhhhhhh
0023 9emcD   eelleEL-L--HH-HH-H-HHHHH--lllllL----EEEEEEL-LlL-LHHHHHHHHHH----eL--EEEE--E-lH---H-HLL-hHH-HHHHHHHLL-L-L-L-L-LEEEEEELL----LleelleeHHHLLHHHHLLL-EEEEEllllhhhhhhhhhhhh
0024 7plhA   --lLLLH-H--HH-HH-H-HHHHLL-L---------LEEEELL-LlL-LHHHHHH--------lL--EEEE--L-lL---L-LLL-----HHHHHHHHH--hH-H-L-LEEEEEELL---------hhHHHHHLHHHHHLlEELLLllllhhhhhhhhhhhh
0025 9e22A   lllllHH-H--HH-HH-H-HHHHLL-L----L----EEEEELL-L-LlLLLLHHHHHHH-L-----eEEEE--E--H---HhLLL--L-lHHHHHHHHH-H-L-LhL-EEEEEEE-L----LL-L-LlllLLLLHHHHHE-EEEELllllhhhhhhhhhhhl
0026 8dgfA   --eELLL-L--HH-HH-H-HHHHHH-L----L----EEEEELL-LlL-LHHHHHHHHHL----lL--EEEE--E-lH---H-HHllllhhHHHHHH-HH-H-L-L---LEEEEEELH-------H-H--HHHHHHHHHLLL-EEEELllllhhhhhhhhhhll
0027 3glfG   lllllLL-L--HH-HH-H-HHH-LL-L----L----EEEEELL-LlL-LHHHHHHHHHH-H---L-eEEEE--E-lH---H-HLLhhHH-HHHHH-HHH-L-L-LlL-EEEEEEELL---------L--HHHLLHHHHLLL-EEEELllllhhhhhhhhhhhh
0028 3bosB   lllllLL-L--LLllH-H-HHHHLL-L----L----EEEEELL-LlL-LHHHHHHHHHH-H---H-lEEEEelH--H---H-HLH--HH-HHHHHHHHH-H-H-HlL-LEEEEEELL---------L--LLL-HHHHHHHL-EEEELllllhhhhhhhhhhhh
0029 8s35M   --elHHH-H--HH-HH-H-HHHL-L-L----L----LEEEELL-L-HhHHHHHHHHLLL-L---L--LLEE--E--LlllL-LHH--HH-HHHHHHL-----------lLEEEELL--------lL--LLLLLLlLLLLlEEEELllllllllhhhhhhhhh
0030 7qo4J   lllllLL-L--HH-HH-H-HHHHHL-L----L----LEEEELL-LlL-LHHHHHHHHHH----lE--EEEE--L--L---L-LLH--HH-HHHHH-HHH-H-H-HlL-LEEEEEELL------------LLLLLLLLLLLL-LEEELllllhhhhhhhhhhhl
0031 5zr1A   lllllLL-L--LL-LH-H-HHHHHH-hllllE----EEEEEEL-LlL-LHHHHHHHHHH-H-----eEEEE-eL--L---L-LLL--LH-HHHH-hHHH-L-L-L-L-EEEEEELLL-----L-L-H--HHH-HHHH--LL-EEEEEllllhhhhhhhlhhhl
0032 8thcA   --llLLL-LllHH-HH-H-HHHHHH-lllllL----LLLLEEL-L-L-LLLHHHHHHHH----eE--EEEE--L-lL---L-LLlllLH-HHHHHH-HH-H-L-L-L-LLEEELLL---------L--HHHLLHHHHHHHlLLLEEllllhhhhhhhhhhhh
0033 7rzy1   --lLLLLhH--HH-HH-H-HHHHHH-lllllL----LLEELLL-LlL-LHHHHHHHHHH-----lL--EEEE--L-lL---H-HHlllllhHHHHHHHHH-H-L-L---LLLLLLLLH-------H-H--HHHHLHHHHHHLlLEELLllllllllllhhhhhhh
0034 2p0eA   ---------------------------lllllE----EEEEEEL-LlL-LHHHhHHHHLL-L---L-lEEEE--E--H---H-HHH--HH-HHHHHH-HH-H-H-HlL-LEEEEELLL---------L--LLL-HLLLL--L-EEEEEellhhhhhhhhhhhllll
0035 6z1f3   ---LLLL-L--HH-HH-H-HHHHHH-L----L----EEEEELL-LlL-LHHHHHHH-------lE--EEEE--LllL---L-LLhhhhH-HHHHHH-HH-H-L-LlL-LLEEEEEL---------L--LLLLLHHHLLLE-EEEELlllhhhhhhhhhhhlll
0036 9ri9B   --lLLLL-L--HH-HH-H-HHHHHL-LllllE----EEEEELL-LlL-LHHHHHHHHHL-L-----eEEEE--E-lL---L-LHH--HH-HHHH-HHLL-L-L--lL-LEEEEEEL------------LHHH--HHLL---LLEELllllhhhhhhhhhhhh
0037 6yvhK   --eELHH----HH-HH-H-HHHHHL-L----L----EEEEELL-L-HhHHHHHHHHHHH-L---L--LLEE--E--E----lHHH--HH-HHHHHLLL-----------lLEEEELL------------lLLLL---LLLEeEEEELlllllhhhhhhhhhllll
0038 8jqbE   ----LHH-H--HH-HH-HhHHLLL-------L----LEEEELL-L-LlLHHHHHHHHHH-H---HheEEEE--L-lL---L-LLH--HH-HHHHHHHHH-L-L-L-L--EEEEEELL--lllllllllHHHLLLL-LLLL-EEELLlllllllllhhhhhhhll
0039 8bobC   lllllLL-L--HH-HH-H-HHHLHH-L----L----EEEEELL-LlL-LHHHHHHHHHL----lE--EEEE--E-lH---H-HLlhhHH-HHHHH-HHH-H-L-LlL-EEEEEEELL------------LLLLLHHHHHLE-EEELHhhhlllhhhhhhhhhhhh
0040 5v8fD   --llLHH-H--HH-HH-H-HHHHHH-lllllL----EEEEELL-LlL-LHHHHHHHHHH-------eEEEE--E-lH---H-HLL--LllLHHHHHHHH------lL-EEEEELLLL----L----L--HHH-HHHL--LL-LEEELlllllhhhhhhhhhhhh
0041 8xksB   --lLLLL-L--LL-HH-H-HHHHHL-L----L----EEEEELL-LlL-LHHHHHHHHHH----lL--EEEE--E-lH---H-HLH--HH-HHHHHHHHL-L-L-L-LlLEEEEEEL---------L--LLLLLLLLLLL-LLLLLllllhhhhhhhllhhhhl
0042 8dr5A   lllllLL-L--HH-HH-H-HHHHLH-L----L----EEEEELL-LlL-LHHHHHHHHHH----lE--EEEE--L-lH---H-HLL--HH-HHHH-HHHH---L-L-L-LLEEEEEL---------L--LLL-HHHHLLLL-EEEELllllllllhhhhhhhhh
0043 8dgcA   --lLLLL----LH-HH-H-HHHHLL-L---lL----LEEEELL-LlL-LHHHHHHHHHH-H-----eEEEE--E-lH---H-HHL--HH-HHHLLL--L-L-L-L-L-EEEEEEELL-------L-L--HHHLL--LLLLL-EEEELllllhhhhhhhhlllll
0044 3pvsD   lllllLL-L--LL-Lhhhhlhh LLL-L---------LEEEELL-LlL-LHHHHHHHHHH-H-----eEEEE--E-lH---H-HLL-----HHHHHL-LH-H-H-L-L-LEEEEEELL---------L--HHHLLHHHHLLL-EEEELllllhhhhhhhhhhhh
0045 6nakD   --lLLLH-H--HH-HH-H-HHHHLL---lllL----EEEEELL-LlL-LHHHHHHHHLL-L---L-lEEEE--E--L---L-LLH--HH-HLLLHH-HH-L-L-L-L-LEEEEEL------------lLLLL-LLLLLLL-EEEEElllllllllleeellllll
0046 9jixA   ----------------------------lleE----EEEEELL-LlL-LHHHHHHHHHH-H--lL--EEEE--E--E---L-LHH--HH-HHHHHHHLL-L-L-L-L-LEEEEEELL----H--H-H--HHHHHHHH-lEE-EEEELlhhhhhhhhhhllleee
0047 7mi1A   lllllLL-L--HH-HH-H-HHH-HH-H----HhlllEEEEELL-LlL-LHHHHHHHHHH----lL--EEEEelL--L---L-LLH-hHH-HHHHHHH-H-H-H-HlL-LEEEEEELL----lllL-L--LL-LLLLLLLE-EEEELllllhhhhhhhhhhhhl
0048 1ixsB   lhhhlLL-L--HH-HH-H-HHHHHH-L----L----LEEEELL-LlL-LHHHHHHHHHH-H--lL--EEEE--E-lH---H-HLLhhHH-HH-HHHHHH-H-L--lL-LEEEEEELL---------L--LLLLLH-HHHHLlEEEELllllhhhhhhhhhhhh
0049 2is6A   hhhhhLL-L--LH-HH-H-HHLLL--L----L----LEEEELL-LlL-LHHHHHHHHHH-H---HhleEEE--LlhH---H-LLH--HH-HHHHH-HHL-L-L-L----EEEEEELH----HH-L-llllLHHHHHHHHLL-EEEELlllllllhhhhhhhhhh
0050 2zpaB   --lLLLLhH--HH-HLlL-LLLL-L-L----L----EEEEEEL-LlL-LHHHHHHHLLL----lL--LEEE--L-lH---H-HLL-----HHHHHHHHH-----H-L-LLEEEEEEELllllLL-L-HhhHHHHHLLL-LLE-EEEELllllllllllhhhhhhh
0051 7mi8A   lllllLL-L--HH-HH-H-HHH-HH-H----HhlllEEEEELL-LlL-LHHHHHHHHHH----lL--EEEE--L-lL---L-LLLhhHH-HHHHHH-HH-H-H-HlL-LEEEEEELL----LllL-L--LL-LLHHHLLLE-EEEELllllhhhhhhhhhhhh
```

Job: Structure-based Sequence Alignment of "BREX PglZ N-terminal Inactive P-loop Swi2/Snf2-Helicase" with various inactive P-loop domain from Swi2/Snf2-Helicases;
Query: s001A
No: Chain Z rmsd lali nres id PDB
1: s001-A 22.6 0.0 95 95 100 Type1.PglZ.NTD
2: s002-A 9.0 2.1 82 97 11 Type3.PglZ.NTD
3: s022-B 7.2 3.0 90 164 6 6nmi
4: s003-A 6.8 2.7 88 139 11 6l80
5: s007-A 6.5 2.8 86 155 8 7vdv
6: s015-A 6.4 2.9 86 149 10 9ii8
7: s021-A 6.4 2.5 83 150 11 8zns
8: s016-A 6.3 3.1 81 143 7 6acx
9: s010-K 6.3 3.1 88 156 8 9n6k
10: s009-A 5.9 3.0 85 149 8 6bog
11: s012-G 5.9 2.7 83 142 10 9c57
12: s013-A 5.8 3.2 84 160 8 3i5x
13: s011-A 5.7 2.7 78 148 8 2d7d
14: s008-E 5.7 2.7 85 158 12 7zke
15: s018-A 5.5 2.3 74 136 7 4buj
16: s005-A 5.3 2.9 83 140 6 8t5s
17: s019-A 5.2 2.8 79 144 9 5lst

```
18: s020-A 5.1 2.5 77 124 10 2fzl
19: s014-C 5.1 3.1 83 161 7 7smo
20: s006-A 4.3 2.8 67 123 3 9el5
21: s004-A 4.1 2.9 69 114 6 8qy7
22: s017-A 3.5 3.3 70 158 9 7w0b

s001A_Type1.PglZ.NTD  MASSRITEALLNA-F-NKYRIVFWDDDKE-KL-IDEFNSL---ELP-D-VTKIKL-D-N------NE-FSVKY-RILVEETDK--KFLIYRPA-G--EPDVKDNWLLDVQL-AS-YKFVAE----------------
s002A_Type3.PglZ.NTD  SWRDHILKEFT---P-RVSRLTLVAD--P-DG-L--LLEEileGIR-EgFELIPF-E-D----HIAF-RYAYEsRFRS-RWDA--ALVVVLRSgA--LDV----lPYDLLQ-AG-RKLSF-----------------
s003A_6l80            TKIKALLRHLKQI-QeTSPGQIIVFSQFS-SFlDILEIES---HLPdQ-VIIYKF-D-G-RLDMKER-TRILE-QFH--DKDLscIKLLLLSL-T--GGV--GLNL-TCAS----RAFMMDpwwspgmedqaidrih
s005A_8t5s            PMISKTVQYIVEQ-N-LADSRTIIFVRTR-YEaTILNKVLsneELL-MgIKSEWM-SglISASKQKQ-MEKLK-MFAD-----geiRILVSTS-V--AEE--GLDVP---E-CS-LVIKYNyatneiahvqrrgrgr
s007A_7vdv            GKFELLDRILPKL-R-ATNHKVLLFCQMT-SL-MTIMEDY---FyR-G-FKYLRL-D--GTTKAEDR-GMLLK-TFNE-PGSE--YFIFLLST--raGGLG---LNLQSA---D-TVIIFDsdwnphqdlqaqdrah
s008E_7zke            PKLGALRDLLVDC-GigKPHRALIFCQMK-EMlDMVQNTVlkqMLP-S-VSYLRL-D--GSVEANKR-QDIVN-KFNS--DPS--YDVLLLTT--svGGLG---LNLTGA---D-TVIFVEhdwnpqkdlqamdrah
s009A_6bog            wNFDPRVEWLXGY-L-TSHQKVLVICAKA-AT-ALQLEQV-lrERE-G-IRAAVF-H--EGXSIIER-DRAAA-WFA--EEDT-gaQVLLCSE----IGSE-GRNF-QFAS----HXVXFDlpfnpdlleqrigrld
s010K_9n6k            GKMVLLDQLLTRL-K-KDGHRVLIFSQMV-RM-LDILGDY---LiK-G-INFQRL-D--GTVPSAQR-RISID-HFNSPDS-N--DFVFLLS--T--RAGGLGIN-LMTA---D-TVVIFDsdwnpqadlqamarah
s011A_2d7d            GQIDDLIGEIQAR-I-ERNERVLVTTLTK-KMsEDLTDYK---EIG---IKVNYL-H--SEIKTLER-IEIIR-DL-----rlgkyDVLVG-I-N--LL---rEGLD--IP-EVsLVAILDadkegflrsersliqt
s012G_9c57            GKLEALAILLQKL-KsEGRR-VLILSQMI-LM-LDILEMN---FHY---LTYVRI-D--ENASSEQR-QELMR-SFN--RDRR--IFCAILST-H--SR---tTGINLV--eaD-TVVFYDndlnpvmdakaqewcd
s013A_3i5x            NSIFAAVEHIKKQ-I-KSNYKAIIFAPTV-KFtSFLCSIL-knEFK-KdLPILEF-H--GKITQNKR-TSLVK-RFKK---dE--SGILVCTD-G--ARG----MDFPNV---H-EVLQIGvpselanyihrigrta
s014C_7smo            RNESEKWRHIANL-C-RAEAQSIVFTFSR-KRtHELAAY-----SK-G-LKAKPY-H-S-GLPYKQR-KLTEM-EFLAQR-----lDVVVTT--A--ALGA-GVDF----P-AS-QVIFESlamgnkwlsvrefhqm
s015A_9ii8            EYVASIVSYIIEY-T-TTSSKCLVLFTSY-KMmHMVQDML---NFE-D-YVVLTQ-QqN------QN-Y-KIV-QQF-NNFDK--AILLGTST-F--FEG-----FDFQangIK-CVMIAKlpfmntstfkeyvlpd
s016A_6acx            -DDKQVAAALRRE-L-LRDGQAFYIHNRVrTI-DEAAARV-rqLVP-E-ARVVVA-H-G-QMNEETL-EKTVE-GFWN-----reyDILVC-T-T--IV---eTGLD--IS-NAnTLIVERadtfglsqlhqlrgrv
s018A_4buj            -SKKTWPEIVNYL-R-KELLPMVVFVFSK-KRcEEYADWL---EGI-N---IAVHvK-E------LIeILFSK-GF---------iKVLFA-T-E--TF--aMGLNL----ptR-TVIFS-----------------
s019A_5lst            DTDQALLTLLQGkrF-QNLDSIIIYCNRR-EDtERIAALL-rtCLH-AktTAEAY-H--AGMCSRER-RRVQR-AFMQ-----gqlRVVVA-T-V--AF---gMGLDR-pD-VR-AVLHLGlppsfesyvqavgrag
s020A_2fzl            NKIRKLREILER--H-RKD-KIIIFTRHN-ELvYRISKVF----------lIPAI-T--HRTSREER-EEILE-GFR---TGR--FRAIVSSQ----VLDE-GIDV---pD-AN-VGVIMSgsgsareyiqrlgril
s021A_8zns            -SWQKIADEIAAR------PCVLAVVNTR-KH-AQKLFAA---LPS-N-GIKLHLhC-S------EViALVRR-YLALySLHK--PLWLVSTQ-L--IEA--GVDL----D-FP-CVYRAMagldsiaqaagrcnre
s022B_6nmi            AVIRNYGNLLLEM-S-AVVPGIVAFFTSYqYM-ESTVASWileNIQ-RnKLLFIE-T-Q---DGAET-SVALE-KYQE-ENGR--GAILLSVA-RgkVSEG--IDFVHHY--gR-AVIMFGvpirendfltfdamrh
s004A_8qy7            ------------------KDKVIIFTELR-DLqRELQHAQ---NFG---frPVII-N-GKSQSQNSR-QRLID-DFQ--AQPG--FGVIIL---S--TVAVGFGVN---VQ-KAnHVIHFTrcwnpakedqatdray
s006A_9el5            ------------------ETRVMIFSSFR-DSvQEIAEMS---QHQpI-IRVMTF-V-GKGFTQKEQ-LEVVK-QFRD-----ggyNTLVSTC---vGEEG--LDIG---E-VD-LIICFDsqkspirlvqrmgrtg
s017A_7w0b            -------------dkdPKDICCLVFVERR-YTcKCIYGlN---YIQnV-LTPQFM-V-gSVLERKWQ-KSAIQ-QFRDGN-----aNLMICSS-V--LEE--GIDV---QA-CN-HVFILDpvktfnmyvqskgrar

s001A_Type1.PglZ.NTD  LHHHHHHHHHHHH-H-HHLLEEEEELHHH-LL-HHHHHHL---LLL-L-EEEEEL-L-L------LH-HHHHH-HHHLLLLLL--EEEEEELL-L--LLLLLLLLLHHHHH-LL-EEELLL----------------
s002A_Type3.PglZ.NTD  LHHHHHHLLLL---L-LLLLEEEEEL--L-LL-H--HHLHhhhHHH-HlEEEEEL-L-L----HHHH-HHHHHhHLLL-LLLL--EEEEEELLlH--HHH----lLHHHHH-HL-EEEEL-----------------
s003A_6l80            HHHHHHHHHHHHH-HhHLLLEEEEEELLH-HHhHHHHHHHH---HLLlL-EEEEEL-L-L-LLLHHHH-HHHHH-HHL--LLLLllEEEEEEEL-L--LLL--LLLL-LLEE----EEEELLllllhhhhhhhhhlll
s005A_8t5s            HHHHHHHHHHHHH-H-HLLLLEEEELLLH-HHhHHHHHHHllhHHH-HlLLEELL-LllLLLLHHHH-HHHHH-HHHH-----lllLEEEELH-H--HHL--LLLLL---L-LL-EEEEELllllhhhhhhhhhlll
s007A_7vdv            HHHHHHHHHHHHH-H-HHLLLEEEEELLH-HH-HHHHHHH---HhL-L-LLEEEE-L--LLLLHHHH-HHHHH-HHHL-LLLL--LLEEEEEH--hhHLLL---LLLLLL---L-EEEELLllllhhhhhhhhllll
s008E_7zke            HHHHHHHHHHHHL-LllLLLLEEEEELLH-HHhHHHHHHHHhhlLLL-L-LLEEEE-L--LLLLHHHH-HHHHH-HHHH--LLL--LLEEEEEH--hhHLLL---LLLLLL---L-EEEELLllllhhhhhhhhhlll
s009A_6bog            lLLLHHHHHHHHH-H-HHLLLEEEEELLH-HH-HHHHHHH-hhHHH-L-LLEEEE-L--LLLLHHHH-HHHHH-HHH--LLLL-llLEEEEEL----LLLL-LLLL-LLLL----EEEELLllllhhhhhhhhhlll
s010K_9n6k            LLHHHHHHHHHHH-H-HLLLLEEEEELLH-HH-HHHHHHH---HhH-L-LLEEEE-L--LLLLHHHH-HHHHH-HHLLLLL-L--LLEEEEE--L--LLLLLLLL-LLLL---L-EEEELLllllhhhhhhhhllll
s011A_2d7d            LHHHHHHHHHHHH-H-LLLLEEEEELLLH-HHhHHHHHHHH---HLL---LLEEEE-L--LLLLHHHH-HHHHH-HH-----hhlllLEEEE-L-L--LL---lLLLL--LL-LEeEEEELLlllllllllhhhhhhh
s012G_9c57            HHHHHHHHHHHHH-HhHLLL-EEEEELLH-HH-HHHHHHH---HLL---LLEEEE-L--LLLLHHHH-HHHHH-HHH--HLLL--LLEEEEEL-L--LL---lLLLLLL--llL-EEEELLllllhhhhhhhhhhhh
s013A_3i5x            HHHHHHHHHHHHH-H-HLLLEEEEELLLH-HHhHHHHHHH-hhHHL-LlLLEEEE-L--LLLLHHHH-HHHHH-HHHH---lL--LEEEEELH-H--LLL----LLLLLL---L-EEEEELllllllhhhhhhllll
s014C_7smo            LLHHHHHHHHHHH-H-HHHHLEEEELLLH-HHhHHHHHH-----HL-L-LLEEEL-L-L-LLLHHHH-HHHHH-HHHLLL-----lLEEEEL--L--LLLL-LLLL----L-LL-EEEELLllllllllllhhhhhh
s015A_9ii8            HHHHHHHHHHHHH-H-HLLLLEEEELLLH-HHhHHHHHHHH---HHL-L-LEEEEL-LlL------LL-L-HHH-HLL-LLLLL--LEEEEELL-L--LLL-----LLLLlllLE-EEEELLlllllllhhhhlhhhh
s016A_6acx            -LHHHHHHHHHHH-H-HLLLEEEEELLLHhHH-HHHHHHH-hhHLL-L-LLEEEE-L-L-LLLHHHH-HHHHH-HHHL-----lllLEEEE-L-H--HH---hLLLL--LL-LEeEEEELLhhhllhhhhhhhhhll
s018A_4buj            -LLLHHHHHHHHH-H-HLLLLEEEELLLH-HHhHHHHHLL---LLL-L---LEEEhH-H------HHhHHHHL-LL---------lLEEEE-L-H--HH--hHLLLL----llL-EEEEL-----------------
s019A_5lst            LHHHHHHHHHHLllL-LLLLLEEEEELLH-HHhHHHHHHH-hhHLL-LleEEEEE-L--LLLLHHHH-HHHHH-HHHL-----lleEEEEE-E-L--LL---lLLLLL-lL-LL-EEEEELllllhhhhhhhhllll
s020A_2fzl            HHHHHHHHHHHH--L-LLL-LEEEELLLH-HHhHHHHHHL----------lLLEE-L--LLLLLHHH-HHHHH-HHL---LLL--LLEEEELL----LLLL-LLLL---lL-LL-EEEEELllllhhhhhhhhhhhl
s021A_8zns            -LHHHHHHHHHLL------LEEEEEELLH-HH-HHHHHHH---LLL-L-LEEEEEhH-H------HHhHHHHH-HHHHhLLLL--LEEEEEEH-H--HLL--LLLL----L-LL-EEEEELllhhhhhhhhllllll
s022B_6nmi            HHHHHHHHHHHHH-H-HHLLLEEEEELLHhHH-HHHHHLLlhhHHH-HhLEEEEL-L-L---LLLHH-HHHHH-HHHH-LLLL--LEEEEEEL-LlhHHLL--LLLLLLL--lL-EEEEELllllhhhhhhhhhhhh
s004A_8qy7            ------------------LLEEEEELLLH-HHhHHHHHHHH---HHL---llLEEE-L-LLLHHHHHH-HHHHH-HHH--HLLL--LEEEEE---L--LLLLLLLLL---LL-LEeEEEELLllllhhhhhhhhllll
s006A_9el5            ------------------LLLEEEELLLH-HHhHHHHHHHH---LLLlL-LLEEEE-L-LLLLLHHHH-HHHHH-HHHL-----lllLEEEELH---hHLLL--LLLL---L-LL-EEEELLlllllhhhhhhhllll
s017A_7w0b            -------------lllHHHLLEEEELLLH-HHhHHHHHhH---HHHlL-LLLLLL-L-lLLLHHHHH-HHHHH-HHHLLL-----lLLLEELL-L--HHH--LLLL---LL-LL-EEEELLllllhhhhhhhhhlll

Job: Type1 PglZ WP_219938192 (Third Tri-Helical HTH-like Element)
Query: s001A
No: Chain Z rmsd lali nres id PDB Description
1: 8b3d-c 4.6 3.6 49 264 10 MOLECULE: DNA-DIRECTED RNA POLYMERASE SUBUNIT;
2: 6zce-q 4.5 2.9 52 636 6 MOLECULE: EUKARYOTIC TRANSLATION INITIATION FACTOR 3 SUBUNI
3: 6w4x-C 4.3 3.6 55 375 7 MOLECULE: RIBONUCLEOSIDE-DIPHOSPHATE REDUCTASE 1 SUBUNIT AL
4: 8bfi-A 4.3 3.6 45 601 9 MOLECULE: CCR4-NOT TRANSCRIPTION COMPLEX SUBUNIT 1;
6: 7st9-E 4.2 4.0 53 354 8 MOLECULE: CHECKPOINT PROTEIN RAD24;
7: 8dgc-A 4.2 3.6 52 2028 8 MOLECULE: SEAVS3;
9: 6q82-A 4.1 3.3 51 1027 14 MOLECULE: IMPORTIN BETA-LIKE PROTEIN KAP122;
10: 8cmk-A 4.1 3.4 52 913 15 MOLECULE: TRANSPORTIN-3;
11: 6vk5-B 4.1 3.2 56 392 5 MOLECULE: METHANE MONOOXYGENASE COMPONENT A ALPHA CHAIN;
12: 8zqc-A 4.1 4.1 51 930 8 MOLECULE: CAS12X2;
13: 4cej-B 4.1 3.6 51 1156 2 MOLECULE: ATP-DEPENDENT HELICASE/NUCLEASE SUBUNIT A;
14: 4owt-A 4.1 3.2 54 466 9 MOLECULE: INTEGRATOR COMPLEX SUBUNIT 3;
15: 7uic-e 4.1 3.4 47 729 6 MOLECULE: MEDIATOR OF RNA POLYMERASE II TRANSCRIPTION SUBUN
16: 3t92-A 4.0 2.7 50 112 6 MOLECULE: HISTONE ACETYLTRANSFERASE P300 TAZ2-CCAAT/ENHANCE
17: 9c57-G 4.0 3.2 48 878 13 MOLECULE: RUVB-LIKE 1;
18: 5kqj-A 4.0 3.6 50 185 6 MOLECULE: 2''-AMINOGLYCOSIDE NUCLEOTIDYLTRANSFERASE;
19: 7vu7-A 3.9 4.2 52 89 13 MOLECULE: FLAGELLIFORM FIBROIN;
20: 4in3-D 3.9 4.5 53 632 4 MOLECULE: CHITIN BIOSYNTHESIS PROTEIN CHS5;
21: 8hpp-A 3.9 3.0 49 395 8 MOLECULE: INTEGRATOR COMPLEX SUBUNIT 3;
22: 2iqc-A 3.9 4.1 57 173 4 MOLECULE: FANCONI ANEMIA GROUP F PROTEIN;
23: 7dhg-C 3.9 2.7 48 470 6 MOLECULE: MITOCHONDRIAL IMPORT RECEPTOR SUBUNIT TOM70;
24: 8j2x-A 3.8 3.9 50 337 8 MOLECULE: COBALAMIN-BINDING PROTEIN;
25: 6vg5-A 3.8 2.7 42 81 2 MOLECULE: CAPSID PREMEMBRANE PROTEIN;
26: 3jc6-5 3.8 3.0 50 254 10 MOLECULE: DNA REPLICATION LICENSING FACTOR MCM2;
27: 4d0l-E 3.8 3.5 50 479 6 MOLECULE: PHOSPHATIDYLINOSITOL 4-KINASE BETA;
28: 7xsq-A 3.8 3.8 51 1289 8 MOLECULE: RAMP SUPERFAMILY PROTEIN;
29: 8j1p-A 3.7 3.5 55 1234 7 MOLECULE: UBIQUITIN FUSION DEGRADATION PROTEIN 4;
30: 7t5p-A 3.7 3.8 52 403 8 MOLECULE: SUMO-INTERACTING MOTIF-CONTAINING PROTEIN 1;
31: 6tdw-C 3.7 3.8 50 157 8 MOLECULE: ATPTB1;
32: 8t1l-S 3.7 3.6 53 913 2 MOLECULE: MEDIATOR OF RNA POLYMERASE II TRANSCRIPTION SUBUN
```

```
33: 7ozs-F 3.7 3.0 51 571 8 MOLECULE: PUTATIVE RIBOSOMAL PROTEIN;
34: 1z2c-B 3.7 3.0 51 346 12 MOLECULE: RHO-RELATED GTP-BINDING PROTEIN RHOC;
35: 8a1h-A 3.7 2.9 49 516 8 MOLECULE: 6-4 PHOTOLYASE (FES-BCP, CRYPRO);
36: 3chl-A 3.7 3.2 49 315 4 MOLECULE: ALPHA-14 GIARDIN;
37: 7kpx-C 3.7 3.7 49 1352 10 MOLECULE: MEIOTIC MRNA STABILITY PROTEIN KINASE SSN3;
38: 6hb3-B 3.6 2.4 44 360 7 MOLECULE: PROTEIN HGH1;
39: 7zke-E 3.6 3.3 48 1623 8 MOLECULE: DNA (36-MER);
40: 8q6o-2 3.6 2.8 49 267 12 MOLECULE: DNA REPLICATION LICENSING FACTOR MCM2;
41: 7ywq-A 3.6 2.6 50 79 4 MOLECULE: GENOME POLYPROTEIN;
42: 5jp6-A 3.6 4.4 54 339 9 MOLECULE: PUTATIVE POLYSACCHARIDE DEACETYLASE;
43: 7wo9-A 3.6 3.3 53 1581 6 MOLECULE: NUCLEOPORIN NUP188;
44: 5tk6-A 3.5 3.9 46 191 9 MOLECULE: OXSA PROTEIN;
45: 7zol-B 3.5 4.0 51 1702 12 MOLECULE: CAS7-11;
46: 6n7p-X 3.5 3.9 49 826 4 MOLECULE: U1 SMALL NUCLEAR RIBONUCLEOPROTEIN 70 KDA HOMOLOG
47: 5ufl-A 3.5 4.1 50 523 4 MOLECULE: PROTEIN CIP2A;
48: 7wa4-A 3.5 3.1 49 529 6 MOLECULE: PROTEIN GIGANTEA;
49: 8d07-A 3.5 3.9 50 65 8 MOLECULE: HALC3_109;
50: 7v99-A 3.5 3.7 51 991 14 MOLECULE: TELOMERASE REVERSE TRANSCRIPTASE;
51: 8tp8-D 3.5 3.2 51 326 2 MOLECULE: DEOR-FAMILY TRANSCRIPTIONAL REGULATOR;
52: 8fib-A 3.5 3.9 57 418 5 MOLECULE: CYTOCHROME P450;
53: 4y5j-A 3.5 3.2 47 227 13 MOLECULE: MINI SPINDLES TOG3;
54: 7jtk-X 3.5 3.3 50 379 6 MOLECULE: FLAGELLAR RADIAL SPOKE PROTEIN 1;
55: 6v8o-N 3.5 3.4 50 412 12 MOLECULE: HIGH TEMPERATURE LETHAL PROTEIN 1;
56: 7wtw-w 3.5 3.9 51 332 6 MOLECULE: 18S RRNA;
57: 6bq1-E 3.5 3.9 54 1510 7 MOLECULE: PHOSPHATIDYLINOSITOL 4-KINASE III ALPHA (PI4KA);
58: 5tmc-F 3.4 2.6 51 351 8 MOLECULE: DNA-DIRECTED RNA POLYMERASE SUBUNIT ALPHA;
59: 8e5t-6 3.4 3.8 52 514 17 MOLECULE: RIBOSOME BIOGENESIS PROTEIN MAK21;
60: 9esh-N 3.4 3.5 56 1284 7 MOLECULE: PRE-MRNA;
61: 8d00-A 3.4 3.9 47 299 6 MOLECULE: MICROTUBULE-ASSOCIATED PROTEIN TORTIFOLIA1;
62: 3dad-A 3.4 2.8 54 324 11 MOLECULE: FH1/FH2 DOMAIN-CONTAINING PROTEIN 1;
63: 5cos-D 3.4 3.5 48 61 6 MOLECULE: SIDEROPHORE-INTERACTING PROTEIN;
64: 5t9f-B 3.4 2.7 51 254 4 MOLECULE: PREPHENATE DEHYDROGENASE 1;
65: 7e34-B 3.4 3.8 46 142 11 MOLECULE: CYCLIN-DEPENDENT KINASE 2;
66: 9jwg-A 3.4 3.0 52 1221 0 MOLECULE: E3 UBIQUITIN-PROTEIN LIGASE IPAH1.4;
67: 3few-X 3.4 4.0 52 431 2 MOLECULE: COLICIN S4;
68: 5jhf-D 3.4 2.6 42 82 0 MOLECULE: KLTH0D11660P;
69: 5l7s-A 3.3 3.7 48 267 4 MOLECULE: SECRETED RXLR EFFECTOR PEPTIDE PROTEIN;
70: 8gaq-A 3.3 4.6 44 147 7 MOLECULE: C5HR2_4R;
71: 7te2-A 3.3 3.4 47 205 11 MOLECULE: AERR;
72: 6ayi-A 3.3 3.2 45 183 7 MOLECULE: HTH-TYPE TRANSCRIPTIONAL REGULATOR UIDR;
73: 8boq-C 3.3 3.4 45 119 9 MOLECULE: NITROGENASE PROTEIN ALPHA CHAIN;
74: 2ii2-A 3.3 2.5 43 304 12 MOLECULE: ALPHA-11 GIARDIN;
75: 8vwk-A 3.3 3.4 55 430 11 MOLECULE: CYTOCHROME P450;
76: 8qat-C 3.3 4.5 54 606 6 MOLECULE: PROTEIN HOOK HOMOLOG 3;
77: 2a3v-B 3.3 2.8 42 320 5 MOLECULE: DNA (31-MER);
78: 1rjg-A 3.3 3.1 48 292 6 MOLECULE: CARBOXY METHYL TRANSFERASE FOR PROTEIN
79: 8gju-F 3.2 3.3 44 332 11 MOLECULE: METHYLMALONIC ACIDURIA TYPE A PROTEIN, MITOCHONDR
80: 1cf7-B 3.2 3.0 46 82 11 MOLECULE: DNA (5'-

0001 s001A   --------GLSL------H---DFA--LKIFND-SLKKQ-LGEP---SE--L---------S-N-EV-AMFLG-NWQ-D-S----K---N-----Y-G-ENFN-E-W-T-EK-A-A-T-E-L-N---------------
0002 8b3dc   -----------M------D---QKL--SKLVEE-LTTS--GEPR----------------lN-P-EK-MKELK-KIC-K-S----------------S-EEQL-S-R-A-YR-L-L-I-A-Q-Ltqehaeirlsafqiv
0003 6zceq   hssdylirlrDE------Q---SIY--NLILRT-QLYFE-ATL----------ehdleraL-T-RP-FVKRL-DHI-Y-Y--------K-----S-E-NLIK-I-M-E-TA-A-W-N-I-I-Paqfkskftskdqlds
0004 6w4xC   gktvtvslreLK------K---KLY--LCLMSV-NALEA-IRFY--egnalH---------L-T-GT-QHMLN-LLR-S-G-addP---E-----M-A-EIAE-E-C-K-QE-C-Y-D-L-F-Vqaaqqekdwadylfr
0005 8bfiA   ssssdlrgfaaqfikqelistgTPF--AFVIDL-AALAS-RR-----------------eyL-KL-DKWLT-DKI-R-E---------------H-G-EPFI-Q-A-C-MT-F-L-K-R-Rl---------------
0006 8i21A   ksveslrdFQNK------K---G-Q--LLPYVK-VKDHK-EKL----------emrdtpeF-Q-SW-MREIR-SY--D-Q----D---T-----K-E-KLNK-V-A-E-KY-E-E-E-G-N-Fnlsfvqdvldklese
0007 7st9E   lalksssp IIKP----dwI---IVI--HKLTRK-IVKER-S----------------vnsL-I-EC-RAVLY-DLL-A-H----C---I-----P-A-NIIL-K-E-L-TF-S-L-L-D-V-Etlnttnkssiieyss
0008 8dgcA   ilaelcpsSALAiisrveD---SLA--GYIWAR-LGS---PEAE---M--------------rW-QA-AHAVL-ALC-R-M---------------S-R-TCVI-Q-G-I-FQ-H-A-I-N-A-Ttlpfcdrnlpfytlh
0009 8q7eJ   aqawsrdiAEDH----egV---SAL--GWLLDQ-YLEQR-ETSR------sF---------A-S-RV-RRLCH-LLV-H-V----EppsS-----S-L-RNIT-Q-C-W-LS-V-V-Q-E-Q-Vsrflaaawrapdfvp
0010 6q82A   khwtsfilPQFL---------gDLV--YQIXYG-LFHT-----Q---RS--D---------L-N-SY-TDLLR-ALV-A-K---------------F-P-IEAR-E-W-L-VA-V-L-P-Q-Ia---------------
0011 8cmkA   keligqvmnqLG------Q---QLV--SQLLHT-CCFC----LP---PY--T---------L-P-DV-AEVLW-EIM-Q-V---------------D-R-PTFC-R-W-L-EN-S-L-K-G-L-Pvtvthkqltdfhkqv
0012 6vk5B   lfnahssvGRDC---------qDWN--EILWAGhAVYDA-TFGQ--tltptT---------R-G-AI-DDLFVyCLA-N-D----S---E-----F-G-AHNR-T-F-L-NA-W-T-E-H-Y-Lassvaalkdfvglya
0013 8zqcA   edtlhllecvKE------Y---NEL--SKAYMD-YMFKL-KEDH----------lnlcqkgL-L-PI--PEFA-KNV-E-K--------------lN-K-NVLY-G-I-I-SQ-A-G-S-R-M-Ksflkcddltqqnhln
0014 4cejB   sdeeqlmyvvNK------S---VAQ--SFTASQ-LRLWT-REYD---I-------------S-D-VW-WSTYN-VLM-S-E----Q----------D-R-LQSK-K-L-F-S--S-L-F-Fr-----------------
0015 4owtA   wllttctsnVAA------S---NAK--LALFYD-WLFF--SPDK----------------dS-I-XN-IEPAI-LVX-H-H----S---XkphpaI-T-ATLL-D-F-X-CR-I-I-P-N-F-Yppleghvrqgvfssl
0016 7uice   -------dmkLL------R---YYK--NIWLNN-KIINW------------------eisN-P-DF-LSKYS-A----qN----L---D-----QlL-TDLI-E-T-S-FT-C-F-A-Q-F-Vsnkqyhqansnltll
0017 3t92A   ------dsrrLS------I---QRA--IQSLVH-AAQCR------------------nancS-L-PS-CQKMK-RVV-Q-H-nggC---P-----I-C-KQLI-A-L-A-AY-H-A-K-H-C-Qenkcpvpfclnikqk
0018 9c57G   iaastareieCF------W---SNI--EQVVEI-KLRVE-LEEKrksvkfdyqklkngtkdL-E-EL-ADFME-QL---------------------T-PIEK-Y-A-L-NY-L-E-L-F-H-Tsieqekernsedavm
0019 5kqjA   pawwadeaYEIA------E---ApqgiWDYFYY-ADEVP-------pvD--W---------P-T-KH-IESYR-LAC-T-S--------------lG-A-EKVE-V-L-R-AA-F-R-S-R-Y-Aalehhhhhh------
0020 7vu7A   --------GSSR------L---PSL--INGIMS-SM-QG-GG-----------------fnY-Q-NF-GNVLS-QFA-T---gtcN---S-----N-D-LNLL-M-D-A-LL-S-A-L-H-T-Lsyqgmgtvpsypsps
0021 4in3D   ssqilssindID------S---SII--DLVVKI-CCWNH-RWYI---E---F---------S-I-IL-IDALS-VAV-Q-D--------------mG-I-TKVH-N-E-I-AS-R-F-S-D-Pv---------------
0022 8hppA   qlqkgsdtEAQC----svG---KPL--YLIFRN-LCQMQ------------------ednssF-S-LL-LDLLS-ELY-Q-K----------------Q-PKIG-Y-H-L-LY-Y-L-R-A-S-Kaaagkmnlyesfaqa
0023 2iqcA   ltlvtsrhpALS------P---VYL--GLLTDW-GQRLHgWVGT--sqD--V---------P-WeEL-HNRFQ-SLC-Q-A----P---P----pL-K-DKVL-T-A-L-ET-C-K-A-Q-D-Gdfevpglsiwtdlll
0024 7dhgC   rrakahekLDNK------K---ECL--EDVTAV-CILEG------------------fqN-Q-QS-MLLAD-KvlllsqpsemE---M-----A-H-LYSL-C-D-A-AH-A-Q-T-E-V-Akk------------
0025 8j2xA   ---------VDI------L---AAG--REELMA-ALAEG---------------------D-E-HAaVDLAM-RLL-D-G----G---------vP-A-DVVL-LeL-V-AD-A-Q-V-E-I-Gvlwqanrwsvaqeha
0026 6vg5A   ---------------------rgPL--KLFMAL-VAFLR-FLTI---P------------pT-A-GI-LKRWG-TI------------------------KK-S-K-A-IN-V-L-R-G-F-Rkeigrmlnilnrrrr
0027 3jc65   ---------DDD------N---TEI--IKSFKN-FILEF-RL-----------sltvnmehlI-GY-NEDIY-KKL-S-D---------------E-P-SDII-P-L-F-ET-A-I-T-Q-V-Akrisilsrnslptfq
0028 4d0lE   gnrlfcfrneDV------D---FYL--PQLLNM-YIHM-----D---ED--V---------G-D-AI-KPYIV-HRC-R-Q----------------S-INFS-L-Q-C-AL-L-L-G-A-Y-Ssrgtklrklilsdrl
0029 7xsqA   lcrieviksеDH------N---DEL--RKQAEV-IVEAF-KQNdklgkeevQ---------G-T-A--LRTKL-KEL-W-Q---------snkdiG-W-RKFT-E-M-L-GS-N-L-Y-L-I-Ykketggvstrfrilg
0030 8j1pA   dslvqcliPILV----inD---QLI--KLIGSI-LA-QK-ETA---eAG--T---------LlV-GG-LSLLD-LIC-K-K---------------F-S-ELFF-P-S-I-KR-E-G-S-G-F-Eftstglassitkrit
0031 7t5pA   sianmvlscdkQ------P---HNV--RDVIKW-LVKAV-TE------dglI---------V-C-QL-QRMLS-IAV-E-V---dR---T-----P-T-CSSN-K-I-A-EM-M-F-G-F-V-Ldipersqremffttm
0032 6tdwC   lacgpgkvDALT------E---KHL--LETGKA-RLEEL-TAGL--------------gnkdegvnafrKEVE-QEG-K-Y---aN---W-----P-A-EKSK-A-L-A-DK-V-I-A-A-S----------------
0033 8t1lS   pdhpcfrpDSTK---------acLS--ISAAIL-EILNA-WENG---V------------lA-F-ES-IQKIT-DNI---K----G---K-----V---CSLA-V-C-A-VA-W-L-V-A-H-Vrmlgldereks lqmi
0034 7ozsF   ldvltaieHAAK----tkQ---TIL--RLLFRL-ISAD-----I---AP--Q-------diY-E-EA-ISCLT-TLS-E-D-----------------N-LKVG-Q-A-I-TD---D-Q-E-T-Hvydvllklatgtdpr
0035 1z2cB   nnkfgiktmleT------E---EGI--LLLVRA-M--DP-AVPN---M---------------mI-DA-AKLLS-ALC-I-L----PqpeD-----M-N-ERVL-E-A-M-TE-R-A-E-M-D-Everfqplldglksgt
0036 8a1hA   dycascayQVKL----acP---LNS--LYWRFM-LKHRD-RL--------------annprI-G-ML-YKTWD-KM--------------------T-S-DSQQ-A-I-L-ST-A-D-A-Y-L-Sqiesl----------
```

```
0037 3chlA   navatgdriprD------T---HEL--EEDLVL-VRKAI-ETKG------kD---------E-V-ST-WIRIF-ATY--------------------T-RADF-R-Q-L-HK-M-Y-S-A-K-Yngdslragvedefqg
0038 7kpxC   kimennepaMAM------E---DLK--NFIFQI-IEITN-SN----------------------dlcsQIFDQ-LKDqT-I----E---M--itqI-V-EKDF-C-T-S-CL-Q-N-N-N-Q-Kiddnyivvvieiits
0039 6hb3B   dklvrniilsdD------K---KFL--KFLVWK-IVD-----LT---NP--N---------A-D-IM-CILLS-NLA-K------------------D-DGIL---A-V-LN-I-K-Rn--------------------
0040 7zkeE   lipmakefvmMR------R---SAL--DSLINI-VWESL-SN------------gddlsasT-G-KI-MDLLA-TLC-S------------------F-PEVL-E-A-M-KV-S-A-S-Q-D-Eersftllvprlypfl
0041 8q6o2   -tvrewvsmAAT------R---LEI--YHRFKN-FLRTH-VD------------eslpvnyedlaAR-EHVLA-YFL-P-E--------------A-P-AEML-K-I-F-DE-A-A-K-E-V-Vlvmypkydriareih
0042 7ywqA   tktrqprvqmPN------G---LVL--MRMMGI-LWHAV--AGT---A---R---------N-P-VL-KAFWN-SVP--------------------L-KQAT-A-A-L-RK-I-K-R-T-V-Salmvglqkrgkrr--
0043 5jp6A   ------------------M---SQI--GSSVRQ-AVSDN-QSA---nSE--A---------NpE-AL-FANWR-HEF-MvD----S---S-----K-R-ESMK-T-E-L-CK-E-L-Q-A-L-Paqdltlfeneirden
0044 7wo9A   alidtefanFEL------KdifNGW--SLVGRI-LQNLL-HSY---lQH--E---------LmI-SI-IKLVT-NVV-DkT----S---I-----EkS-SEIL-S-Y-L-SN-S-Ld----------------------
0045 5tk6A   tilnnndleIQE------I---VEH--ADHVDA-YCKSC-IEVH----------gnkdfisiqR-SL-GDKLD-NLT-K-E----------------Y-PYLK-E-F-Q-NL-Fl------------------------
0046 7zolB   dasvleisrppY------W---NNM--LNLAGT-ISEAF-ERE-----------wdieinkK-V-KL-RNWLF-WIF-N-E-frdtyayF-----D-W-RTFC-E-A-L-GQ-A-L-Y-L-E-Akkqvpnqfsserpvg
0047 6n7pX   imneygheDYFN------N---ALL--STLNAV-VVEQP-Q--------------------kqA-AI-ALLTM-VVN-S-K----------------N-NVAG-K-S-I-IN-Y-F-F-E-E-Lqkwckqtyndefkst
0048 5uflA   ssltlneeVGEK----fhA---RNI--HQTFQL-IFNIL-INGD--------------gtltrK-YS-VDLLM-DLL-K------------------N-PKIA-D-Y-L-TR-Y---E-H-F-Ssclhqvlgllngkdp
0049 7wa4A   vvltvcvshgPV------A---AFD--SYVLAA-VCALA-CEVQ----------ldsaishT-R-RI-LAILE-ALF-S-L--aaA---X-----V-A-AHIS-E-L-F-RR-Sk------------------------
0050 8d07A   ---reeieeAVK------E---AEL--KVLAIV-LVALR-SVSH-----------yeplsrlyE-SF-LDALK-KAL------------------S-E-EELK-E-V-E-KE-A-E-R-I-E-Kk--------------
0051 7v99A   gakgaagplpSE------A---VQW--LCHQAF-LLKLT-RHR-------pL---------L-G-SL-RTAQT-QLS-R-K-------------lP-G-TTLT-A-L-E-AA---A-N-P-A-Lpsdfktild------
0052 8tp8D   ripsgldfqtpT------A---EEL--AALRVA-ADSLR-ASGA---------------daraS-SL-YALEA-KLL-S-A----L---R---gsA-R-RRVA-P-D-V-EA-L-V-Q-A-Et----------------
0053 8fibA   ifaelfgmtQAD------I---FEL--AASIRT-ILEGL-NPHA--eQL--A---------AsD-AA-SLEVE-RYF-E---lsdT---E-----L-IsMLWG-I-L-L-GG-F-A-T-T-A-Amidhaildmlafpeq
0054 4y5jA   vaenypltttTV------D---LVI--NEIIEK-L-ADA-K--------------------ngA-AA-ADVLS-AFA-E-A-------------tK-L-EYVV-G-K-V-LS-F-A-F-E-Q-Kspkvqseafnwvnrs
0055 7jtkX   dstrraleaCGS------G----AV--LPRLME-LALLE-AQGG------gR---------A-Q-QG-LVLLF-TCT-Q-A----R---H-----N---AGIL-S-Q-LvDV-A-Qa----------------------
0056 6v8oN   kaneesaadNLD------H---KTL--SLIVSF-LLL-----EC---DS--E---------LiI-AS-LDFLY-QYI-L-P----G---S----qR-I-TELF-K-S---KE-C-S-Li--------------------
0057 7wtww   ipalsnweQILY---------rfyN--LVLLPR-VRDDV-AEYK---R------------lN-F-HL-YMALK-KAL-F----------------K-P-GAWF-K-GiL-IP-L-C-E-S-G-Tctlreaiivgsiitk
0058 6bq1E   aktkeneeELER----wsG---TVL--KTMLDI-LQTLS-LSLsadiVK--D---------FaA-RC-GMILQ-EAM-K-W--------------A-P-TVTK-S-H-L-QE-Y-L-N-Kh------------------
0059 5tmcF   aikklseitglD------P---DLI--REVVRA-KILGS---AR---VR-lD---------P-K-TV-EEIDQ-KLK-S-L--------------P-K-EHKR-Y---L-HI-A-R-E-G-E-Aarqhlieanlrlvvs
0060 8e5t6   lmpyllsyrrIL------K---ELI--KSIVGV-WSTT----RE---LE--T---------Q-I-AS-FAFLI-NTT-K-E--------------F-K-KSML-E-T-T-LK-T-T-Y-S-T-Fikscrktnmrsmpli
0061 9eshN   sivekfplHNLL------S---RWI--HSLLIK-SISYA-QTE-------tP---------L-L-AI-INMSL-VLL-S-A----F---P-----T-R-RFAH-P-V-I-ED-S-C-L-R-T-Sfpekyaikvdfeflk
0062 8d00A   --gshmltsFQA------M---VEL--KQKILT-SISKL-AD--------------rdtyqiaV-ED-LEKTI-QSL--------------------T-PETL-P-M-F-LN-C-L-Y-D-S-Csdpkpavkkeclhll
0063 3dadA   lvpefvhsEGLS----ahS---DTI--QWLYTL-CAS-----LS---RL--V----------vK-TA-LKLLL-VFV-EyS----E---N-----N-A-PLFI-R-A-V-NS-V-A-S-T-T-Gappwanlvsileekn
0064 5cosD   ---gatsipgEV------A---EQA--MHWHLE-LQEPA------------V---------S-A-AT-LAACM-SWR-Q-A----------------H-PLHE-H-A-W-QR-T-Q-V-F-A-Qrlremr---------
0065 5t9fB   ----------------aaK---SQF--ITHTIG-RTLGE-MDI---kET--M---------M-R-DS-FDLYS-GLF-V-Y----------------N-RFAR-Q-E-L-EN-L-E-H-A-L-Hkvketlmiqrty---
0066 7e34B   krakftisEHTR------I---NFF--IALYLA-NTVEE-------------------------dEE-ETKYE------lG----K---N-----W-R-KLFP-N-F-L-KL-R-D-Q-L-W-Dridyraivsrrccee
0067 9jwgA   asveylqevARI------R---LCL--DRAADF-LSE---PEGG-----------pemakeK-Q-CY-LQQVK-QFC-I-R----V---E-----N---DWHR-V-Y-L-VR-K-L-S-S-Q-Rgmefvqglskpgrph
0068 3fewX   lgniskafkfAD------V---VMK--VEKVRE-KSIEG-YET------------------gnW-GP-LMLEV-ESW-V-L----S---G----iA-S-AVAL-G-V-F-SA-T-L-G-A-Y-Alslgapaiavgivgi
0069 5jhfD   vdplkfywdlER------D---RSL--WSSVSK-LDNTK-K---------------------------tiDWK-RLS-R------------efkaP-E-HFIR-K-R-S-YA-L-F-A-K-H-Lklle-----------
0070 5l7sA   qmltlfqkTMTE-----eE---DII--KLVHML-R-NNP-G--------------------mkS-HA-DKLER-YLL-L-T----------------S-ESSH-K-T-M-AD-V-W-L-K-A-Retpeevfrilrlaek
0071 8gaqA   ------------------------V--EELKRK-LRQAK-EDGD------eL---------L-E-RV-KNEML-LLA-V-V----------------D-PRVL-V-E-V-LN-T-A-Ke--------------------
0072 7te2A   ----------SL------Q---TVL--VNRMVE-LAT-G-PEL-----------------gamD-LL-FDEFR-AAH-----------------vP-V-EEMAtH-Y-I-PE-A-A-R-Q-I-Gaawdsdrigfaqvti
0073 6ayiA   spgtlyhhfISK------E---ALI--QAIILQ-DQERA-LAR-----------ltheafgQ-R-AL-VVEIM-AEG-M-R----------------N-PQVA-A-M-L-KNk--------------------------
0074 8boqC   -----veapvhP------M---DAR--IDELTD-YIMKN-CL------------vcladdyR-E-HY--PWIN-SM-------------------S-K-EEIG-S-L-M-QG-L-K-D-R-M-Dyltitgslneelsdk
0075 2ii2A   ------------------Y---GDA--IPEVKA-ILEAK-----------------------nE-EE-LVTFT-SRW------------------S-A-EERK-E-L-R-TQ-F-Q-D-T-T-Glefiaflkkcikngp
0076 8vwkA   frwagipwsRQE------M---DRQ--ARRMSR-LLDTF-GRPA---T-----------hpisW-FE-RFRLD-RTF-TeK----T---A-----A-V-ELQN-L-T-R-PN-V-A-V-A-R-Faafagaalvehpewa
0077 8qatC   aaprlllfSRLV---------sYFC--PVLATG-LSALY-SSL---whclaL---------F-M-SS-LEFCN-AVI-Q-V----A---H---plV-Q-KQLV-D-Y-I-HN-G-L-V-P-Vm----------------
0078 2a3vB   refmqtryyAKK------T---IEA--YLHWIT-RYIHF-HNKK----------pslmgdkeveefltylavQ-GKV--------------------A-TKTQ-S-L-A-LN-S-L-S-F-L-Ykeilktplsleirfq
0079 1rjgA   ----------------------TDY--DALSCK-LAAIS-VGYL---PsskY---------T-E-WH-RSYLI-TLK-K-F--------------S-R-RAFG-K-V-D-KA-M-R-Ss--------------------
0080 8gjuF   ---------------------qrFV--DKLYTG-LIQG------------------------qR-AC-LAEAI-TLV-E-S----T---H---srK-K-ELAQ-V-L-L-QK-V-L-L-Y-H-Reqeqsnkgkplafrv
0081 1cf7B   ---------gkG------L---RHF--SMKVCE-KVQRK-GT--------------------tsynevaDELV-SEF-TdS----A---Y-----D-Q-KNIR-R-R-V-YD-A-L-N-V-L-Mamniiskekkeikwi

0001 s001A   --------LLLH------H---HHH--HHHHHH-HHHHH-LLLL---LL--L---------L-L-HH-HHHHH-HHH-L-L----L---L-----L-H-HHHH-H-H-H-HH-H-H-H-H-L-L---------------
0002 8b3dc   -----------L------H---HHH--HHHHHH-HHHL--LLLL----------------lL-H-HH-HHHHH-HHH-H-H----------------L-HHHH-H-H-H-HH-H-H-H-H-H-Hhlllhhhhhhhhhhh
0003 6zceq   llhhhhhhhhHH------H---HHH--HHHHHH-HHHHH-HHL-----------hhhhhhhH-H-HH-HHHHH-HLL-L-L--------L-----L-H-HHHH-H-H-H-HH-H-H-H-H-L-Llllllllllllllll
0004 6w4xC   leeeeelhhhHH------H---HHH--HHHHHH-HHHHH-LHHH--hhhhhH---------H-H-HH-HHHHH-HHH-H-L-lllH---H-----H-H-HHHH-H-H-H-HH-H-H-H-H-H-Hhhhhhhhhhhhhhll
0005 8bfiA   lllhhhhhhhhhhhhhhhhhhlLLH--HHHHHH-HHHHH-HL-------------------llL-LH-HHHHH-HHH-H-H----------------H-HHHH-H-H-H-HH-H-H-H-H-Ll----------------
0006 8i21A   hhhhhhhhHHHH------H---L-L--HHHHHH-HHHHH-HHH-----------hllllllL-H-HH-HHHHL-LL--L-L----L---L-----H-H-HHHH-H-H-H-HH-H-H-H-L-L-Llhhhhhhhhhhhhhl
0007 7st9E   leelllllLLLL----hhH---HHH--HHHHHH-HHHLL-L-----------------hhhH-H-HH-HHHHH-HHH-H-L----L---L-----L-H-HHHH-H-H-H-HH-H-H-H-L-L-Llllhhhhhhhhhhhh
0008 8dgcA   hhhhhlhhHHHHhhhhhhH---HHH--HHHHHH-HLL---LLHH---H--------------hH-HH-HHHHH-HHH-L-L--------------L-L-HHHH-H-H-H-HH-H-H-H-L-L-Llhhhllllllllhhh
0009 8q7eJ   hhhhhhhhHHLH----llL---LHH--HHHHHH-HHHHL-LLLL------hH---------H-H-HH-HHHHH-HHH-L-L----LlllL-----H-H-HHHH-H-H-H-HH-H-H-H-H-H-Hhhhhhhllllllhhh
0010 6q82A   llllllhhHHHH---------hHHH--HHHHHH-HHHL-----L---LL--L---------L-H-HH-HHHHH-HHH-H-H--------------L-H-HHHH-H-H-H-HH-H-L-L-L-Ll----------------
0011 8cmkA   hhhhhhhhhhHH------H---HHH--HHHHHH-HHLL----LL---HH--H---------H-H-HH-HHHHH-HHH-H-H--------------H-H-HHHH-H-H-H-HH-H-H-H-H-L-Llllhhhhhhhhhhhh
0012 6vk5B   hhhhhhhhHHHL---------lLHH--HHHHHHhHLHHH-HHHH--llhhhH---------H-H-HH-HHHHHhHHH-L-L----L---L-----L-H-HHHH-H-H-H-HH-H-H-H-H-H-Hhhhhhhhhhhhhhhh
0013 8zqcA   hhhhhhhhhhHH------H---HHH--HHHHHH-HHHHH-HHHL----------hhhhhhlL-L-LL--HHHH-HHL-L-L-------------lL-H-HHHH-H-H-H-HH-H-H-H-H-H-Hhhhhhhhhhhhhhhh
0014 4cejB   lhhhhhhhllLH------H---HHH--HHHHHH-HHHHH-LLLL---L-------------L-H-HH-HHHHH-HHH-H-L----L---------L-H-HHHH-H-H-H-H--H-H-H-Ll------------------
0015 4owtA   hhhhllllhHHH------H---HHH--HHHLLL-LLLL--LLLL----------------lL-H-HH-HHHHH-HHH-H-H----H---LlllhhH-H-HHHH-H-H-H-HH-H-H-H-H-L-Lhhhhhhhhhhhhhhh
0016 7uice   -------lhhHH------H---HHH--HHHHHH-HHHLL-------------------lllL-L-LH-HHHHH-H----lL----H---H-----HhH-HHHH-H-H-H-HH-H-H-H-H-H-Hhllllllllllllhh
0017 3t92A   ------lhhhHH------H---HHH--HHHHHH-HHHLL------------------llllL-L-HH-HHHHH-HHH-H-H-lllL---H-----H-H-HHHH-H-H-H-HH-H-H-L-L-L-Lllllllllhhhhhhh
0018 9c57G   hhhhhhhhhhHH------H---HHH--HHHHHH-HHHHH-HHHHhhllllhhhhlllllllL-H-HH-HHHHL-LL---------------------L-HHHH-H-H-H-HH-H-H-H-H-L-Llllllllhhhhhhhh
0019 5kqjA   eeeeelleEEEL------L---LlllhHHHHHH-HHHLL-------llL--L---------L-H-HH-HHHHH-HHH-H-H-------------hL-H-HHHH-H-H-H-HH-H-H-H-H-H-Hhhhhhhhhl------
0020 7vu7A   --------LLLH------H---HHH--HHHHHH-HH-LL-LL-----------------llH-H-HH-HHHHH-HHL-L---lllL---H-----H-H-HHHH-H-H-H-HH-H-H-H-H-H-Hhhhllllllllllhh
0021 4in3D   hhhhhhhhhhHH------H---HHH--HHHHHH-HHHHH-HLLL---L---L---------L-H-HH-HHHHH-HHH-H-H-------------lL-H-HHHH-H-H-H-HH-H-L-L-H-Hh----------------
0022 8hppA   hhhhlllhHHHH----hlL---LHH--HHHHHH-HHLLL-----------------lllllH-H-HH-HHHHH-HHL-L-L----------------L-LLHH-H-H-H-HH-H-H-H-H-L-Lllllllhhhhhhhll
0023 2iqcA   hhhhhhhlhHHH------H---HHH--HHHHHH-HHLLElEELL--llL--L---------L-HhHH-HHHHH-HHH-L-L----L---H----hH-H-HHHH-H-H-H-HH-H-H-H-H-L-Lllllllllhhhhhhh
0024 7dhgC   hhhhhhhhLLLH------H---HHH--HHHHHH-HHHLL--------------------llL-H-HH-HHHHH-HhhhhllllhhH---H-----H-H-HHHH-H-H-H-HH-H-H-H-H-H-Hll-------------
0025 8j2xA   ---------LLH------H---HHH--HHHHHH-HHHHL----------------------L-H-HHhHHHHH-HHH-H-L----L--------lL-H-HHHH-HhL-H-HH-H-H-H-H-H-Hhhhhlllllhhhhhh
0026 6vg5A   ---------------------llLH--HHHHHH-HHHHH-HLLL---L------------lL-H-HH-HHHHL-LL-------------------------LH-H-H-H-HH-H-H-H-H-H-Hhhhhhhhhhhhhhll
0027 3jc65   ---------LHH------H---HHH--HHHHHH-HHHLL-LL------------eeeelhhhhH-HH-LHHHH-HHH-L-L--------------L-H-HHHH-H-H-H-HH-H-H-H-H-H-Hhhhllllllllllle
0028 4d0lE   hhhhhhllhhHH------H---LLH--HHHHHH-HHHL-----L---HH--H---------H-H-HH-HHHHH-HHH-H-H----------------L-HHHH-H-H-H-HH-H-H-H-H-H-Lllhhhhhhhhhhlll
0029 7xsqA   eeeeeeellhHH------H---HHH--HHHHHH-HHHHH-HHLllllllllL---------L-L-L--HHHHH-HHH-H-H-----------llhhhL-H-HHHH-H-H-H-HH-H-H-H-H-H-Hhhhhlllllllllll
0030 8j1pA   hhhhhhhhHHHH----hhH---HHH--HHHHHH-LL-LH-HHH---hHH--H---------HhH-HH-HHHHH-HHH-H-H--------------L-H-HHHH-H-H-H-HH-H-L-L-H-H-Hhhhllhhhhhhhhhh
0031 7t5pA   lhhhhhhllllL------H---HHH--HHHHHH-HHHHH-HL------llhH---------H-H-HH-HHHHH-HHH-H-H---lL---L-----H-H-HHHH-H-H-H-HH-H-H-H-H-H-Hllllhhhhhhhhhll
0032 6tdwC   llllhhhhHHHH------H---HHH--HHHHHH-HHHHH-HHHH--------------llhhhhhhhhhHHHH-HHH-H-H---lL---L-----L-H-HHHH-H-H-H-HH-H-H-H-H-L-----------------
0033 8t11S   hhllllllLLLL---------llLL--HHHHHH-HHHHH-LLLL---L------------lL-L-HH-HHHHH-HHL---L----L---L-----L---LHHH-H-H-H-HH-H-H-H-H-L-Llllllllhhhhhhhh
0034 7ozsF   llhhhhhhHHHH----hlH---HHH--HHHHHH-HHHL-----L---LL--H-------hhH-H-HH-HHHHH-HHL-L-L----------------L-HHHH-H-H-H-HL---L-L-L-L-Lhhhhhhhhhhlllll
0035 1z2cB   llhhhhhhhhlL------L---LHH--HHHHHL-L--LL-LLHH---H--------------hH-HH-HHHHH-HHH-L-L----LlllL-----H-H-HHHH-H-H-H-HH-H-H-H-H-L-Lllllhhhhhhhllll
0036 8a1hA   lhhhhlllLLLL----llH---HHH--HHHHHH-HHLHH-HH--------------lllhhH-H-LH-HHHHH-LL---------------------L-HHHH-H-H-H-HH-H-H-H-H-H-Hlhhhl----------
0037 3chlA   hhhhlllllllL------H---HHH--HHHHHH-HHHHH-HLLL------hH---------H-H-HH-HHHHH-HHL--------------------L-HHHH-H-H-H-HH-H-H-H-H-L-Lllllhhhhhhhhllh
0038 7kpxC   hhhhllllhHHH------H---HHH--HHHHHH-HHHHH-LL----------------------lhhhHHHHH-HHHhH-H----H---H--lhhH-H-HHHH-H-H-H-HH-H-H-H-H-H-Hhhhhhhlllhhhhhh
0039 6hb3B   lhhhhhhhhhlL------L---HHH--HHHHHH-HLL-----LL---LL--L---------H-H-HH-HHHHH-HHL-L------------------L-HHHH---H-H-HH-L-L-Ll--------------------
```

```
0040 7zkeE   hhhhhhhhllLL------H---HHH--HHHHHH-HHHHH-HL-----------lllllllH-H-HH-HHHHH-HHL-L-----------------L-HHHH-H-H-H-HH-H-H-L-L-L-Hhhlhhhhhhhhhhhl
0041 8q6o2   -lhhhhhllHHH------H---HHH--HHHHHH-HHHLL-LL-----------leeeeehhhhHH-LHHHH-HHL-L-L--------------L-H-HHHH-H-H-H-HH-H-H-H-H-H-Hhhhlllllllllle
0042 7ywqA   llllllllllLH------H---HHH--HHHHHH-HHHHH--LLL---L---L---------L-H-HH-HHHHH-LLL------------------L-HHHH-H-H-H-HH-H-H-H-H-H-Hhhhhhhllllll--
0043 5jp6A   -----------------L---HHH--HHHHHH-HHHHH-HHL---lLL--L---------LhH-HH-HHHHH-HHH-HhL----H---H-----H-H-HHHH-H-H-H-HH-H-H-H-L-L-Lhhhhhhhhhhhhlhh
0044 7wo9A   llllhhhhhHHH------HleeLHH--HHHHHH-HHHHH-HHH---hHH--H---------HhH-HH-HHHHH-HHL-LlL----L---H-----HhH-HHHH-H-H-H-LL-L-Ll---------------------
0045 5tk6A   hhhllllhhHHH------H---HHH--HHHHHH-HHHHH-HHHH----------llhhhhhhH-H-HH-HHHHH-HHH-H-H----------------L-HHHH-H-H-H-HH-Hl----------------------
0046 7zolB   lllllllllllL------H---HHH--HHHHHH-HHHHH-HHL----------lllllllL-L-LH-HHHHH-HHH-H-H-hhhhlllL-----L-H-HHHH-H-H-H-HH-H-H-H-H-H-Hhhhllllllllllll
0047 6n7pX   hhhhllllLLLH------H---HHH--HHHHHH-LLLLH-H--------------------hhH-HH-HHHHH-HHH-H-H----------------L-HHHH-H-H-H-HH-H-H-H-H-H-Hhhhhhlllllhhhhl
0048 5uflA   hhhhllllLLHH----hlL---LLH--HHHHHH-HHHHH-HHLL--------------llhhhH-HH-HHHHH-HHH-L------------------L-HHHH-H-H-H-HL-L---L-L-H-Hhhhhhhhhhhhhlllh
0049 7wa4A   hhhhhhhhllHH------H---HHH--HHHHHH-HHHHH-HHHH----------llhhhhhH-H-HH-HHHHH-HHH-H-L--lhH---H-----H-H-HHHH-H-H-H-LL-Lh----------------------
0050 8d07A   ---lllhhhHHH------H---HHH--HHHHHH-HHHHH-HLLL-----------lllhhhhhH-HH-HHHHH-HHL-----------------L-H-HHHH-H-H-H-HH-H-H-H-H-H-Hl-------------
0051 7v99A   llllllllllHH------H---HHH--HHHHHH-HHHHL-LLL-------hH---------H-H-HH-HHHHH-HHH-H-H-------------lL-H-HHHH-H-H-H-HH---L-L-L-L-Llllllllll------
0052 8tp8D   ellllllllllL------H---HHH--HHHHHH-HHHHH-HHLL---------------hhhhH-HH-HHHHH-HHH-H-L----L---L---hhH-H-HHHH-H-H-H-HH-H-H-H-L-Le---------------
0053 8fibA   hhhhhhlllHHH------H---HHH--HHHHHH-HHHHH-LLLL--hHH--H---------HhH-HH-HHHHH-HHH-H---llhH---H-----H-HhHHHH-H-H-H-HL-L-L-H-H-H-Hhhhhhhhhhhhlhhh
0054 4y5jA   hhhhllllhhHH------H---HHH--HHHHHH-H-HLL-L--------------------lhH-HH-HHHHH-HHH-H-H-------------lL-H-HHHH-H-H-H-HH-H-H-H-H-L-Llhhhhhhhhhhhhhh
0055 7jtkX   hhhhhhhhlLLL------L----LH--HHHHHH-HHHHH-HLLL------hH---------H-H-HH-HHHHH-HHH-L-L----L---L-----L---HHHH-H-H-HhHH-H-Lh---------------------
0056 6v8oN   llllllhhhHLL------H---HHH--HHHHHH-HHL-----LL---LH--H---------HhH-HH-HHHHH-HHH-L-L----L---L----hH-H-HHHH-H-L---HH-H-H-Hh------------------
0057 7wtww   llllllhhHHHH---------hhhH--HLHHHH-HHHHH-HHHL---L------------lL-H-HH-HHHHH-HHL-L-----------------L-H-HHHH-H-HlH-HH-H-H-H-H-L-Lllhhhhhhhhhhhhh
0058 6bq1E   lllhhhhhHHHH----llH---HHH--HHHHHH-HHHHH-HHHhllhHH--H---------HhH-HH-HHHHH-HHH-H-L---------------L-H-HHHH-H-H-H-HH-H-L-L-Lh-----------------
0059 5tmcF   hhhhhhhhhllL------H---HHH--HHHHHH-HHLLL---LL---LL-lL---------L-L-HH-HHHHH-HHH-L-L---------------L-H-HHHH-H---H-HH-H-H-H-H-H-Hhhhhhhhhlhhhhhh
0060 8e5t6   lhhhhlllllLH------H---HHH--HHHHHH-HHHL----LL---HH--H---------H-H-HH-HHHHH-HHH-H-H---------------H-L-LLLH-H-H-H-HH-H-H-H-H-H-Hhllllllllllhhhh
0061 9eshN   hhhhhlllHHHH------H---HHH--HHHHHH-HHHHH-HHL-------hH---------H-H-HH-HHHHH-HHH-H-L----H---H-----H-H-HHHH-H-H-H-HH-H-L-L-L-L-Llllllllllhhhhh
0062 8d00A   --lhhhhhhHHH------H---HHH--HHHHHH-HHHHL-LL--------------llllhhhH-HH-HHHHH-HLL------------------L-LLLH-H-H-H-HH-H-H-H-H-H-Lllllhhhhhhhhhhh
0063 3dadA   hhhhhhhlLHHH----hlH---HHH--HHHHHH-HHL-----LL---HH--H----------hH-HH-HHHHH-HHH-HhL----H---H-----H-H-HHHH-H-H-H-HH-H-H-H-H-H-Lllllhhhhhhhllll
0064 5cosD   ---lllllllhHH------H---HHH--HHHHHH-HHLLE------------E---------L-H-HH-HHHHH-HHH-H-H----------------L-HHHH-H-H-H-HH-H-H-H-H-H-Hhhhlll---------
0065 5t9fB   ----------------hhH---LHH--HHHHHH-HHHHH-HLL---hHH--H---------L-L-LL-HHHHH-HHH-H-L---------------L-LLHH-H-H-H-HH-H-H-H-H-H-Hhhhhhhhhhhll---
0066 7e34B   hhhlllhhHLLH------H---HHH--HHHHHH-HHHHL-----------------------lLL-LHHHH------lH----H---H-----H-H-HHHH-H-H-H-HH-H-H-H-H-H-Hhlllllllllhhhhhh
0067 9jwgA   llhhhhhhhHHH------H---HHH--HHHHHH-HLL---LLLL-----------lllhhhH-H-HH-HHHHH-HHH-H-H----H---L-----L---LLHH-H-H-H-HH-H-H-H-H-H-Llhhhhhhllllllll
0068 3fewX   hllllhhhllLL------L---HHH--HHHHHH-HHHHH-HHH-----------------llL-HH-HHHHH-HHH-H-H----L---L----lL-H-HHHH-H-H-H-HH-H-H-H-H-H-Hhhllllllhhhhhhh
0069 5jhfD   lllllllllhHH------H---HHH--HHHHHH-HHLLL-L---------------------------llLHH-HHH-H-----------hlllL-H-HHHH-H-H-H-HH-H-H-H-H-H-Hhhhl-----------
0070 5l7sA   hhhhhhhlLLLL-----hH---HHH--HHHHHH-L-LLL-L--------------------lhH-HH-HHHHH-HHH-L-L---------------L-LHHH-H-H-H-HH-H-H-H-H-L-Lllhhhhhhhhhlllll
0071 8gaqA   ------------------------L--HHHHHH-HHHHH-HHLL------hH---------H-H-HH-HHHHH-HHH-H-H---------------L-HHHH-H-H-H-HH-H-H-Hh--------------------
0072 7te2A   ----------LH------H---HHH--HHHHHH-HHH-L-LLL-----------------lhhH-HH-HHHHH-HLL----------------lL-H-HHHHhL-H-H-HH-H-H-H-H-H-Hhhhhlllllhhhhhh
0073 6ayiA   lhhhhhhhlLLH------H---HHH--HHHHHH-HHHHH-HHH-----------hllllhhH-H-HH-HHHHH-HHH-L-L---------------L-HHHH-H-H-H-HHh-------------------------
0074 8boqC   -----lllllllL------L---HHH--HHHHHH-HHHHH-LL------------hhhhhhhH-H-HL--HHHH-HL-----------------L-H-HHHH-H-H-H-HH-H-H-H-H-H-Hhhhllllllllllll
0075 2ii2A   ------------------L---LLH--HHHHHH-HHHLL-----------------------lH-HH-HHHHH-LLL-----------------L-H-HHHH-H-H-H-HH-H-H-H-H-H-Lllhhhhhhhhllllh
0076 8vwkA   hhhhlllllHHH------H---HHH--HHHHHH-HHHHL-LLLL---L-----------hhhhH-HH-HHHHH-HHH-HhH----H---H-----H-H-HHHL-L-L-H-HH-H-H-H-H-H-Hhhhhhhhhhlhhhh
0077 8qatC   lllllhhhHLLH---------lLHH--HHHHHH-HHHHL-LLL---lllllhH---------H-H-HH-HHHHH-HHH-H-H----L---L---hhH-H-HHHH-H-H-H-HH-H-L-L-L-Lh---------------
0078 2a3vB   hhhhhhlllLHH------H---HHH--HHHHHH-HHHLL-LLLL----------hhhllhhhhhhhhhhhhlL-LLL------------------L-HHHH-H-H-H-HH-H-H-H-H-H-Hhllllllllllllll
0079 1rjgA   -----------------------LLH--HHHHHH-HHHHH-HLLL---LllhH---------H-H-HH-HHHHH-HHH-H-H---------------L-H-HHHH-H-H-H-HH-H-H-Ll--------------------
0080 8gjuF   ----------------------llLL--HHHHHH-HHLL-------------------------lH-HH-HHHHH-HHH-L-L----L---L---hhH-H-HHHH-H-H-H-HH-H-H-H-H-H-Hhhhhlllllllleee
0081 1cf7B   ---------llH------H---HHH--HHHHHH-HHHHH-LE-------------------elhhhhhHHHH-HHH-HhL----H---H-----H-H-HHHH-H-H-H-HH-H-H-H-H-H-Hhllllllllleell
```

Job: PglZ Core Enzymatic Phosphatase (NPP-Superfamily)
Query: s001A
No: Chain Z rmsd lali nres id PDB Description
1: 8tef-A 20.7 3.2 253 516 13 MOLECULE: RESPONSE REGULATOR RECEIVER PROTEIN;
2: 9nv3-C 16.4 3.0 270 866 36 MOLECULE: DUF1788 DOMAIN-CONTAINING PROTEIN;
3: 6c01-A 14.5 3.3 226 819 12 MOLECULE: ECTONUCLEOTIDE PYROPHOSPHATASE/PHOSPHODIESTERASE
4: 5xwk-A 14.2 3.5 233 530 14 MOLECULE: ALKALINE PHOSPHATASE PHOK;
5: 3szy-A 14.0 2.9 221 413 16 MOLECULE: PHOSPHONOACETATE HYDROLASE;
6: 6x1p-A 11.8 3.7 168 586 15 MOLECULE: LPS BINDING PROTEIN;
7: 5fgn-A 11.4 2.9 173 536 14 MOLECULE: LIPOOLIGOSACCHARIDE PHOSPHOETHANOLAMINE TRANSFERA
8: 6s21-A 11.0 3.0 174 486 11 MOLECULE: ENDO-4-O-SULFATASE;
9: 3igy-B 10.7 4.0 172 549 16 MOLECULE: COFACTOR-INDEPENDENT PHOSPHOGLYCERATE MUTASE;
10: 3b5q-B 10.6 3.1 173 467 12 MOLECULE: PUTATIVE SULFATASE YIDJ;
11: 7ebp-A 10.1 3.3 172 526 16 MOLECULE: SULFATASE;
12: 6uss-A 10.0 3.3 171 485 15 MOLECULE: SULFATASE;
13: 4n7t-A 10.0 3.3 163 402 15 MOLECULE: PHOSPHOPENTOMUTASE;
14: 4kjg-A 9.9 3.3 163 486 12 MOLECULE: INTESTINAL-TYPE ALKALINE PHOSPHATASE 1;
15: 5fql-A 9.9 3.1 170 507 12 MOLECULE: IDURONATE-2-SULFATASE;
16: 8eg3-A 9.9 3.6 177 554 10 MOLECULE: STERYL-SULFATASE;
17: 4upl-A 9.7 2.9 169 555 12 MOLECULE: SULFATASE FAMILY PROTEIN;
18: 5olt-A 9.6 3.5 169 383 17 MOLECULE: CELLULOSE BIOSYNTHESIS PROTEIN BCSG;
19: 4miv-D 9.6 3.2 168 480 15 MOLECULE: N-SULPHOGLUCOSAMINE SULPHOHYDROLASE;
20: 2zkt-B 9.5 3.3 161 381 12 MOLECULE: 2,3-BISPHOSPHOGLYCERATE-INDEPENDENT PHOSPHOGLYCER
21: 7qow-B 9.5 3.6 181 505 15 MOLECULE: ALKALINE PHOSPHATASE;
22: 4cys-A 9.0 3.6 166 534 10 MOLECULE: ARYLSULFATASE;
23: 2w5q-A 9.0 3.7 169 424 8 MOLECULE: PROCESSED GLYCEROL PHOSPHATE LIPOTEICHOIC ACID
24: 3lxq-A 8.9 3.6 166 409 9 MOLECULE: UNCHARACTERIZED PROTEIN VP1736;
25: 9fvt-A 8.7 3.7 171 519 11 MOLECULE: SULFATASE;
26: 9bba-A 8.5 3.7 164 602 12 MOLECULE: S1_8C SULFATASE;
27: 6dgm-B 8.3 4.1 167 382 11 MOLECULE: PHOSPHOGLYCEROL TRANSFERASE GACH;
28: 7kwd-A 7.8 3.7 170 473 12 MOLECULE: ALKALINE PHOSPHATASE;
29: 2d1g-A 7.5 3.5 159 481 8 MOLECULE: ACID PHOSPHATASE;
30: 8haw-A 7.3 3.7 160 479 9 MOLECULE: NON-SPECIFIC PHOSPHOLIPASE C4;
31: 8yk5-A 6.5 3.8 154 664 8 MOLECULE: PHOSPHOLIPASE C;

```
0001 s001A   ETVRTAVIISDAMRY-EVGR-ELFQKLSDVSISEMISTVPS-YTQLGMAALLPHKEL-EMTE-DY-KV-LADGLSTKI-LKNYN-P----SAVCVP--FDEIKNMSRDELRGVITGKSLLYIYHNQIDARGDKPATEDEVFRACEEAVDEIAALISNLTNAGNTVHYIVTADHGFLYRRDPVQEADKIDVSTDDVDVWVNRRFIVSDRPMKAPGVMTLPLDQVLGKKSGLTVSVPTGNTVFK-VQGGGANYVHGGPSPQETLIPVIECKTD
0002 8tefA   EIKPILFVVIDNLRY-DQWK-SFETVISNLKEVPYFSILPT-ATQYARNAIFSGLMP-L----------DMEKQFpEF-LSAQInI----KEDYFK--IYAGGKKlaENFKA-LKGNDLVTVVYNFVDMLSHKTEM--AYRSLTLSWFKPLLEIIQQAQL--LGFKLILTTDHGTIN------vkNPSKV---VGKN-LNLRYKTGRSLTYEKDVYVVKPKTIG-LPAISSFIFAKNDFFLAhYVSYYNTYQHGGISLEEMIIPFLVFNPK
0003 9nv3C   QIKRVFVIISDALRY-EVAE-ELGNQINTAELRSQLGVLPS-YTQLGMAALLPHDEI-CYQP-GD-IV-YADGLSTTI-LKKY-------KGMAVK--SDDLLKWKNQQGRDLIRDYEVVYIWHNTIDAMGDSASTEFEACNAVVELKDL----VTRVIN-rHGTRIIVTADHGFLFQQQPLSGQDKTTLQ--IKDNTNHKRFIIGHQLPADDFCWKGKVADTAGVSDNSEFLIPKGIQRFH-F-sgGARFVHGGAMLQEVCVPVLQVKA-
0004 6c01A   cLPPVILFSMDGFRA-EYLYmPNINKLKTGIHSYMRAMYPT-KTFPNHYTIVTGlSH-GI-K-NF-S--WWHGQ---P-MWLTA-MyqglKAATYFvpFEERIST-----lKWLDRPRFYTMYFEEPDSSGHAG-vSARVIKALQVVDHAFGMLMEGLKQRNLHVNIILLADHGMDQ---TYCN-KMEYMTDYF-YMYE-GPAPRIRahcrkQHFKPYLrlHYAKNVRIDVHLFVDQQWLAVksNTNCGGGNHGYNFRSM-EAIFLAHGP-
0005 5xwkA   PPKLIVAISVDQFSA-DLFS--gLKRLTSGAVFRGYQHAAT-ETCPGHSTILTGsRT-GI-wfnL-YC-A-sPLHLPT-LGGRM-KnpatRVVSVAamTLAFAAA--aiENMQL-QTDIISIGLSATDYVGHTF-eGTESCIQVDRLDTELGAFFDKLDKDGIDYVVVLTADHGGHDL-ERHRMEQRVDMAlKALWSDGP--SGDIYYdrAHPQVTVFTeaRASFYSRSGLLLLLKPRVMIP-EQMGSV-ATHGSPWDTDRRVPILFWRKG
0006 3szyA   WPVPAIAVCLDGCEP-AYLD-AlmPALKRGAVRLAHSVIPS-FTNPNNLSIATGsVH-GI-nyVM-MN-FLRAP---T-IFQAF-YdagaRVAVVTefVFAA---gvklLREFR--PDIMYLTTT--DYVQH-----KYAPPEANSFYEMFDRYLAELDG--LGAAIVVTADHGMKP--ADGS-pDVIYVQDLLdRVILLGSFATAYlpKAIQGDVVLACRRFELPEDRIIVLVSSENKTLGrhDLAAPLRSHGGLT--EQEVPFIVNR--
0007 6x1pA   TGQNVLLITVDGLNY-SRFEpALAGFAE-NISFTHMSSG-N-TTDNGIFGLFYG-pS-YMDG-IL------tRTPA-A-LITAL-NqqgyQLGLFSqtATQWINWLG-RYAQ--DNRWFSWVSFNGTN--IDDSQAFAKYSRAAGNVDDQINRVLNALRDSGKLTVVITAGRGIPLSE-----------------------------------------------------------------------------etDWSHGHLQVPLVIHWPG
```

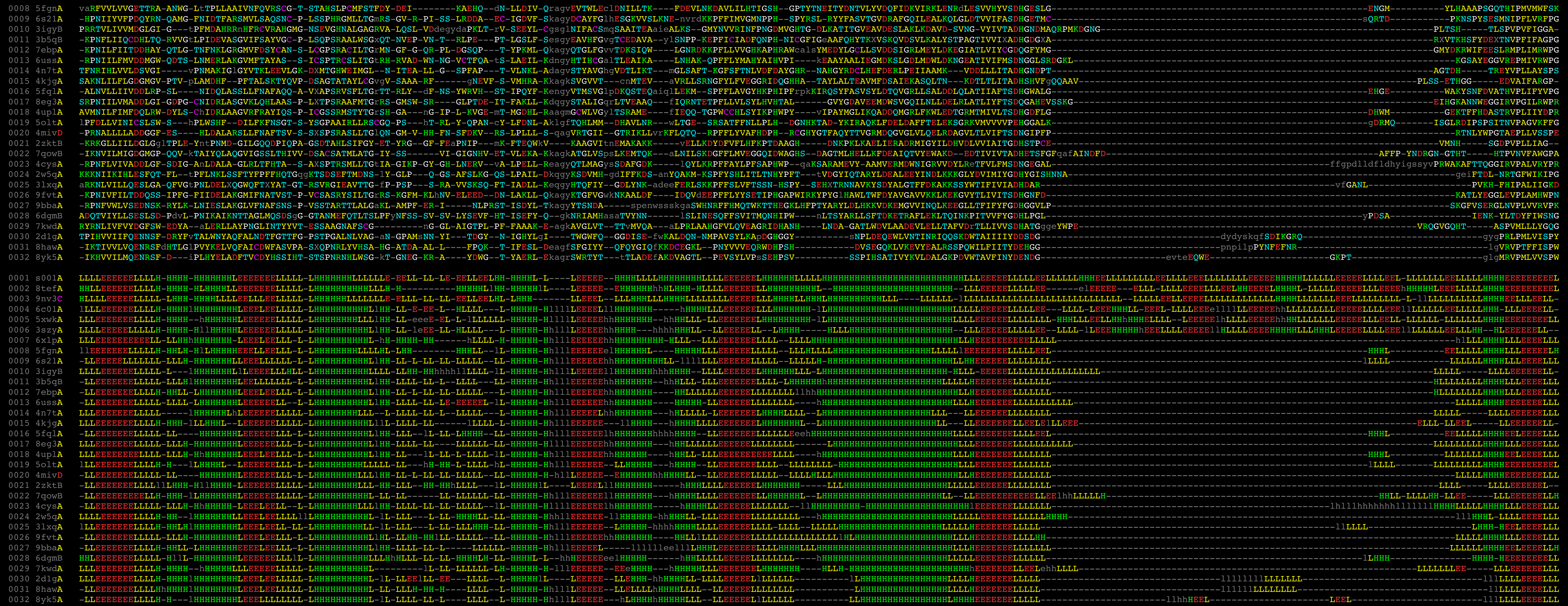

```
Job: Type-1 BREX; PglZ WP_219938192 CTD Beta-Sandwich (IG-Like)
Query: s001A
No: Chain Z rmsd lali nres id PDB Description
1: 8gul-A 9.5 2.5 92 464 14 MOLECULE: GLCNAC-BINDING PROTEIN A;
2: 6za2-B 9.1 3.1 101 1083 8 MOLECULE: POR SECRETION SYSTEM PROTEIN PORU;
3: 6fqa-A 8.8 2.3 90 229 7 MOLECULE: CSUC;
4: 5ghu-A 8.6 3.1 92 201 5 MOLECULE: PROBABLE FIMBRIAL CHAPERONE YADV;
5: 8u01-A 8.5 3.1 93 909 10 MOLECULE: GLYCOSYL HYDROLASE FAMILY 2, SUGAR BINDING DOMAIN
6: 8s3o-A 8.3 2.7 90 1324 8 MOLECULE: CD109 ANTIGEN;
7: 7rsk-A 8.2 3.1 95 775 6 MOLECULE: GLYCOSYL HYDROLASE FAMILY 2, SUGAR BINDING DOMAIN
8: 7a5p-q 8.1 2.8 93 1896 11 MOLECULE: U2 SNRNA;
9: 2co6-B 8.1 2.7 94 208 9 MOLECULE: PUTATIVE OUTER MEMBRANE PROTEIN;
10: 9ayr-B 8.0 3.2 99 424 10 MOLECULE: GUANINE NUCLEOTIDE EXCHANGE FACTOR SUBUNIT RIC1;
11: 6ej7-A 8.0 2.6 93 706 9 MOLECULE: XYLOSYLTRANSFERASE 1;
12: 7s63-A 7.9 2.6 90 1420 10 MOLECULE: ALPHA 2-MACROGLOBULIN;
13: 6cpd-B 7.9 3.0 89 127 16 MOLECULE: PMOD;
14: 8wae-A 7.9 2.9 94 107 14 MOLECULE: BETA-AGARASE;
15: 4be5-B 7.9 3.2 99 234 6 MOLECULE: RBMA;
16: 1cvr-A 7.8 2.3 76 433 14 MOLECULE: GINGIPAIN R;
17: 2j2z-A 7.8 3.4 92 216 9 MOLECULE: CHAPERONE PROTEIN PAPD;
18: 5axh-B 7.8 2.9 95 587 6 MOLECULE: DEXTRANASE;
19: 4e0s-A 7.8 2.9 91 1552 11 MOLECULE: COMPLEMENT C5;
20: 3q48-A 7.8 3.0 88 194 3 MOLECULE: CHAPERONE CUPB2;
21: 6ed1-A 7.7 3.1 99 860 8 MOLECULE: GLYCOSYL HYDROLASE FAMILY 2, SUGAR BINDING DOMAIN
22: 5cl2-A 7.6 3.1 95 246 11 MOLECULE: SPORULATION-CONTROL PROTEIN SPO0M;
23: 4b0m-M 7.6 2.6 91 212 10 MOLECULE: F1 CAPSULE-ANCHORING PROTEIN;
```

```
24: 5ag8-A 7.6 2.3 76 149 16 MOLECULE: GINGIPAIN R2;
25: 7ptb-A 7.5 2.5 92 317 5 MOLECULE: CENTROSOMAL PROTEIN OF 192 KDA;
26: 3vmn-A 7.5 3.2 90 633 4 MOLECULE: DEXTRANASE;
27: 8iua-A 7.5 2.5 82 555 7 MOLECULE: CANDIDATE DEXTRANASE GLYCOSIDE HYDROLASE FAMILY 6
28: 7lr3-D 7.5 3.1 94 110 9 MOLECULE: D3_2/6.14 FAB LIGHT CHAIN;
29: 2pn5-A 7.5 3.1 91 1283 5 MOLECULE: THIOESTER-CONTAINING PROTEIN I;
30: 8alz-B 7.5 2.5 94 1783 12 MOLECULE: ACTIVATING SIGNAL COINTEGRATOR 1;
31: 5vhv-A 7.4 3.1 93 361 14 MOLECULE: ALKYLPURINE DNA GLYCOSYLASE ALKC;
32: 3wnk-A 7.4 2.9 94 712 5 MOLECULE: CYCLOISOMALTOOLIGOSACCHARIDE GLUCANOTRANSFERASE;
33: 5dfk-A 7.4 2.4 82 185 10 MOLECULE: PROBABLE FIMBRIAL CHAPERONE ECPB;
34: 7xdi-E 7.4 3.1 88 185 6 MOLECULE: VP1;
35: 5uj6-B 7.4 3.1 98 840 8 MOLECULE: GLYCOSYL HYDROLASES FAMILY 2, SUGAR BINDING DOMAI
36: 8qb1-A 7.4 3.2 76 88 5 MOLECULE: MIROLASE;
37: 3fn9-C 7.4 2.9 96 673 9 MOLECULE: PUTATIVE BETA-GALACTOSIDASE;
38: 5t99-A 7.4 3.1 91 757 14 MOLECULE: GLYCOSIDE HYDROLASE;
39: 4ncd-A 7.3 2.8 90 211 7 MOLECULE: GRAM-NEGATIVE PILI ASSEMBLY CHAPERONE, N-TERMINAL
40: 9au7-C 7.3 2.9 98 285 4 MOLECULE: VPS35 ENDOSOMAL PROTEIN-SORTING FACTOR-LIKE;
41: 8cmt-A 7.3 2.7 92 722 10 MOLECULE: COAGULATION FACTOR XIII A CHAIN;
42: 6hpd-A 7.3 3.1 97 955 9 MOLECULE: BETA-GALACTOSIDASE (GH2);
43: 3eif-A 7.3 3.2 102 936 8 MOLECULE: C5A PEPTIDASE;
44: 7c7u-A 7.2 2.4 85 579 14 MOLECULE: BIOFILM-ASSOCIATED SURFACE PROTEIN;
45: 6pal-A 7.2 2.9 86 404 8 MOLECULE: UNCHARACTERIZED PROTEIN;
46: 5w0p-C 7.2 2.9 100 850 18 MOLECULE: ENDOLYSIN,RHODOPSIN,S-ARRESTIN;
47: 5n6u-A 7.1 3.0 91 811 19 MOLECULE: BETA-MANNOSIDASE;
48: 3tp4-B 7.1 3.2 84 457 11 MOLECULE: COMPUTATIONAL DESIGN OF ENZYME;
49: 8v2b-C 7.1 3.5 87 703 7 MOLECULE: ONCOSTATIN-M;
50: 7q5z-A 7.1 2.9 88 1344 10 MOLECULE: ALPHA-2-MACROGLOBULIN-LIKE PROTEIN 1;
51: 7usy-J 7.1 3.0 95 361 12 MOLECULE: TRANSMEMBRANE CHANNEL-LIKE PROTEIN 1;
52: 8wac-A 7.0 3.0 90 119 10 MOLECULE: AGARASE;
53: 6lvb-A 7.0 2.6 83 762 14 MOLECULE: N,N-DIMETHYLFORMAMIDASE LARGE SUBUNIT;
54: 1m1s-A 7.0 3.1 88 109 6 MOLECULE: WR4;
55: 8sgi-A 7.0 3.2 88 751 8 MOLECULE: SODIUM/CALCIUM EXCHANGER 1;

0001 s001A   ---DVSE--VKVK-VI---SR-ERIITS------G-QILITFYQ--EQPVEDKVK-GI-SI-KAGFFR-------D-G-VP-VSN---------I--ED-L-EFNKET-----TNVEEREQ-K-R-QFK-LS-T---G-ID--S-GS-VILKLTKRVE--N-----TDQY-AP----YDEIT-FSIR--Q----
0002 8gulA   -nsTAPE--YDLE-VY---GL-ESEYII--gdsaT-QLDLTLEA--T--------gDI-KT-EMTVYN------hH-H-ES-LS----------S--HS-A-ELSD-----------gQVE-A-A-TMT-LS-K-----SE--P-GH-HMLVVVVK--------dqQGKV-IE----QNTLD-FHLI--Eeqtp
0003 6za2B   svdGIAP--DVAD-VI---LF-PNPVRE--------SATFRIFH--NRPG----S-DL-NV-VVEIYD------fT-G-RL-VN----------S--LP-V-KTYSSS-----Y---GEPI-E-I-KWD-LTgV---K-IG--N-GF-YLYRCVVNSP--G--------GQ-TA----SMAKK-MIVV--Aq---
0004 6fqaA   ---------ATFL-IW---PI-YPKIEE------K-ATAVWLQN--TGKT------DA-MV-QIRVFK-dglkdnY-S-EQsEII---------PspPV-A-KIKA-----------gEKH-M-L-RLT-KS-V---N-L-gkE-QS-YRLIVDELPI------------fQM----RYSIP-LFAY--Gkgig
0005 5ghuA   ---------mDIV-IS---GT-RVIYKQ------K-SVNVRLEN--KGNN------PL-LV-QSWLDT-------G-D-TV-PFT---------AtpPV-S-RIDA-----------kRGQ-T-I-KLM-YT-A---S---drE-SV-FWFNVLEVPP--L-----LQLA-F-----RTRIK-LFYR--Pdglk
0006 8u01A   -gvxrprnsTVPL-LR---LP-KLIYTN-----qE-TVKGSLEV--AHF-GAAPLeVT-ST-VWTLKT------kE-G-KT-IA----------S--GT-L-AHVGIG--------nCIPLgQ-L-EIP-L--D---K-VD-vP-SC-LTLEATLGD------------------yANSWH-IWVY--Paava
0007 8s3oA   -psLPLNkrISVF-IQ---TD-KALYKP-----kQ-EVKFRIVT--LF----sdfyKT-SL-NILIKD-----pK-S-NL-I-Q---------Q--WLsQ-QSDL-----------GVI-S-K-TFQ-LS-S---H-PI--L-GD-WSIQVQVN---------------DQ----TYYQS-FQVS--Eyvlp
0008 7rskA   -lkitnP--LHFG-TF---V---tTPVA--tadkA-EVHVQSVL--AN-tekvTG-KV-IL-ETRIVD------kN-N-HT-VA----------R--KE-Q-LVTLDN-----KEK--TEV-G-H-ALE-VL-----A-PQ--LpYL-YQVVNRLLQD---------DKV-ID----EEYIS-IGIR--Niafs
0009 7a5pq   -kyvhLF--PKLE-LS---VH-L-qpiT------L-KVELTITP--DFQWDEKVHsSE-AF-WILVED-----vdS-E-VI-L-H---------H--EY-F-LLKAK---------yaQDEH-L-I-TFF-VP-V---FepL--P-PQ-YFIRVVSDRW-------------lSC----ETQLP-VSFRh------
0010 2co6B   -saTKLF---SVK-LG---AT-RVIYHA------gtGATLSVSN--PQNY------PI-LV-QSSVKA-----adK-S-SPaPFL---------VmpPL-F-RLEA-----------nQQS-Q-L-RIV-RT-G---G-DMdrE-TL-QWVCIKAVPP--E---------lSI----NACDK-LIFR--Pdavk
0011 9ayrB   -inrnNE--TMAK-VS---LS-APFYKT------D-DINLVIELdpITTP---LL-KVtSL-TVSLES-------FkG-NS-VY----------E--KH-F-ICFD-----------eCKSV-S-V-KLL-PP-R-tnQ-IT--G-QK-WMIGLKFVIIanL-----EGEE-F-----TCYVP-IPIL--Ctsed
0012 6ej7A   -iaspDF--GRLF-SE---VF-GGLLGM------D-EPVGMQKW--GKGP------NV-TV-TVIWVD------pV-N-VI-A-A---------T--YD-I-LIEST-----------AEFT-H-Y-KPP-LN-L---P-LR--P-GV-WTVKILHH-------------W-VP----VAETK-FLVA--Pltfs
0013 7s63A   ------K--VQYA-LT---I--PALLKS-----gE-TQRACVNL--IGYH----Q-PL-AL-SVVLEH-------Q-R-VN--IS---------I--FS-E-KVQP-----------pHYFK-C-N-KFM-VP-T---V-iT--N-AP-DFVTLSVSGG------------edI----KDRKA-VVIA--Plnti
0014 6cpdB   -------gyDMIH-IT---VY-CDEIPA-----tG-RTIMAFDI--ENPA-----rDL-PL-ELRIIR-------D-PlLT-EL----------H--LP-AkKYSK------------GTF-S-V-EHN-F--------AN--N-GH-YIGLVTLTRE--S-----G-QQ-ET-----AQFK-FMVG-------
0015 8waeA   --aVIGN--ESIT-IN---S-pSTNVES------DtKVNVTLAY--T-----aNA-TR-DI-VAEFWS-------S-T-GW-L-G---------Q--AV-K-TVSAG-----------nRTE-T-L-TIN-LN-N---A-PA--T-GSgYVVKASIRP---------vGTN-WtsniaTDQVNgLNVI--P----
0016 4be5B   -lpelNG--LTID-IK--nQF-GINSVG------G-FVPFTVDL--NN-gREGEA-NV-EF-WMTAVG-------PdG-LI-I-P---------V--NArE-KWVIAS-----G--dtYSK-V-R-GIN-FD-K---S-YP--A-GE-YTINAQVVIV--S-----G---eRV----EQSMT-VVKK-------
0017 1cvrA   -vrtLVP--TEMQ-VT---A--PANISA------Q-TFEVACD--------------ynGA-IATLSD-------D-G-DM-VG----------T--AI-V-----------------kDG-K-A-IIK-LN-E---S-IA-dE-TN-LTLTVVGYN------------------kvTVIKD-VKVEx------
0018 2j2zA   ----------AVS-LD---RT-RAVFDG----seK-SMTLDISN--DNKQ----L-PY-LA-QAWIEN----enqE-K-IIpVIA---------T-pPV-Q-RLEP-----------gAKS-M-V-RLS-TT-P---D-I-drE-SL-FYFNLREIpR--S-----EK--iAL----QTKIK-LFYR--Paaik
0019 5axhB   -valtkG--KLID-VY---TD-KARYYS------D-KVTVKIEL--NN-elQEDF-RG-TI-YIFYKH-------L-E-SI-VG----------K--AK-I-QVNIK-------sgqkKQL-N-I-FWE-AP-K---D--D--F-KG-YLVEVYAVKG---------NKA-I-----dNKNTA-VDVS--Sdwsk
0020 4e0sA   -kiFRVGdnGFLF-IH---TD-KPVYTP------DqSVKVRVYS--LNDD----laKR-ET-VLTFID------pE-G-SE-V-D---------M--VE-E-ID-------------hIGII-S-FpDFK-IP-S---N-PR--Y-GM-WTIKAKYKED----------------fstTGTAY-FEVK--Eyvlp
0021 3q48A   ------------I-AQ---GT-RVVFPE------R-EVTLRVSN--TSG-----T-PV-LA-QAWIDD-------G-R-QV-PFS---------VtpAV-T-RVEP-----------nGGA-V-L-RIA-YL-K---A---drE-SL-FWLNILEVPP--R--------FS-FR-----SRFK-LFFR--Psqlk
0022 6ed1A   -lqwteK--VHFP-VR---I---qTPEV--svseA-SMQIVAFL--KN-dFTDTK-HV-HV-NVFLCD------eM-N-RI-VK----------E--KQ-L-KLKLIP-----GRK--YPI-S-T-SVG-RI-E---N-PH--LpYL-YTVKVQVCDA--K-----NGEM-----yqEVISP-VGFR--Wfsvd
0023 5cl2A   ----iGA--AKVD-TI---LE-KDAYFP-----gE-EVQGTVHV--KGGKI--AQ-DIrYI-DLQLST-------RkY-AT-I-S---------F--RV-T-G-TIQP-----G--eeHQF-P-F-TFT-LP-L---D-TP--I-TE-VAVVTDLD--------------iDK----SDHDR-IFVE--Ahpwi
0024 4b0mM   -kfASKE--YGVT-IG---ES-RIIYPA------A-GVMVSVKN--TQDY------PV-LI-QSRIYD----enkE-K-EDpFVV---------T-pPL-F-RLDA-----------kQQN-S-L-RIA-QA-G---G---dkE-SL-KWLCVKGIPP--K---------vfvqfainNCIK-LLVR--Pnelk
0025 5ag8A   -----VP--TKMQ-VT---A--PANISA------Q-TFEVACD--------------ynGA-IATLSD-------D-G-DM-VG----------T--AI-V-----------------kDG-K-A-IIK-LN-E---S-IA-dE-TN-LTLTVVGYN------------------kvTVIKD-VKVE--Gkpyt
0026 7ptbA   -sgyggT--SNLI-LE---GV-KVTVNGlvpgkeS-KIVFSVRN--TGS-----R-AA-FV-KAVGFK---dsqkK-V-LLvLRI---------F-pDK-F-VLKE-----------rTQE-N-V-TLI-YN-P---S--dtaT-EL-STVYLFG-RQ--Q-----YRrasXC----KIILS-VIGE--Frdci
0027 3vmnA   -------ngnMIN-LT---TD-KAVYQA-----gE-AVHLNLTL--N-NTTSLAQ-NI-TA-TAEVYS-------L-E-NK-L-K---------T--LQ-Y-TKYLL-------pnesYTT-QkF-VIP------anslAN--N-RG-YLLKVNIS--------dsQNNI-----leQGNRA-IAVE--Ddwrt
0028 8iuaA   -------vtaKIT-LK---TD-KASYKG------E-TVNFTADK-----------vfN-SS-LIRYTH-------L-G-KV-I-K---------E--ET-F-S-----------------gT-S-W-SWL-PP-S---D--D--F-QG-YMVAIYQTNT--D-----G-TQ-TI----LGTVG-IDVS--Sdwak
0029 7lr3D   ---------ATITHVT---IP-N-dCAS------N-ECVLIIHV--WNnnKFVGS-QF-SC-SIACTN----ketD-Q-LAhINP---------I--AP-V-RAFIG-------pnknYAF-Y-F-IIK-FL-I---N--K--E-IT-TLCKAIVKDS---------NGKE-----xsIEEFE-LQSK--Es---
0030 2pn5A   -lfwediqpFYIV-EN---L--PYSIKR-----gE-AVVLQFTL--FN-nLGAEY-IA-DV-TLYNV------------AN-QTEfvgrpntdlS--YT-K-SVSVPP-----KVG--VPI-S-F-LIK-AR----------kL-GE-MAVRVKASLG--H-----E---------tdALEKV-IRVM--Peslv
0031 8alzB   -qcvhQI--PSVM-ME---A----SIQP-itrtvL-RVTLSIYA--DFTWNDQVHvGE-PW-WIWVED-------PnD-HI-Y-H---------S--EY-F-LALKKq----vISKE--AQ-L-L-VFT-IP-I---FepL--P-SQ-YYIRAVSDRW-----------------lgaEAVCI-INFQhs-----
0032 5vhvA   -agakaE--VKIH-HL---MvtPAVINL-----gE-RINLSFTL--ES-tAPAPQ-KL-VV-DYAIDY--vkstgH-G-AA-----------kvF--KL-K-AFSLGA-----G--aqQHI-R-R-EQH-IR-D-trK-HY--P-GR-HVVHVLVN-------------G-ER----LGSAE-FELR--A----
0033 3wnkA   -rgSHMSgsGGIErVF---TD-KARYNG------D-AVSIRVQA--KN-gtgSSW-SG-AA-RLEIFH-------L-E-NS-V-Y---------T--SS-Q-SLSLTN-----G--qsTTL-T-F-TWT-AP-S---T--D--F-RG-YFVRIDAGT------------------lgQGATA-IDVS--Sdftk
0034 5dfkA   ----------MLD-VG---DI-SSFMNS------S-TLSKTIQN--STDS------GR-LI-NIRLER-------L---iaeLLL---------T-pAS-L-LLPA-----------qASE-V-I-RFF-YK-G---P---ekE-RY-YRIVWFDQ---------------aA----RIGTI-LVVA--Prqan
0035 7xdiE   ---------gTIS-LL---STpPVNPPY------S-YFYIEFQF--LPTN---NT-PQ-PY-AIFVGP-------NpN-NL-T-E---------V--AE-G-YTLS-----------NGTG-Y-A-RVP-V--------IN--A-QT-EYVDIVWV-------------N-QN----YTMFE-IFPQ--Iqnat
0036 5uj6B   -ltavpN--QHFG-LF---I---STPQV--seeqA-TLSIRGEV--KN-dAPEKA-TL-EL-THTIYR-----pD-G-TL-L-Q---------T--LK-K-NIQLKA-----G--etYAF-S-N-EAT-PV-L---K-PE--LpRL-YRVETTLRNR--K-----TKTL-----ldQSNHY-TAFR--Wfrfd
0037 8qb1A   -----------GS-LT---L-sPNPA-T------D-VVTLQLTE--TDEVSglsverS-AY-EIQIWS-------G-X-RX-L-----------rS--FR-T-N----------------eP-T-F-QIP-XA-G-----LP--A-GL-YFVRVVKN---------------GQ----TYTQK-LIKK-------
0038 3fn9C   -livteQ--NNIG-VY---I---tQKDV--skksA-DITVKVKL--DN-aGLQPA-AV-TL-ENTIYT-----qE-G-QK-V-G---------T--HS-R-SFLSPQ-----G---tQTY-L-S-TFK-L--K---N-PH--LpYL-YKVVCRLMAD---------GKV-ID----EVVQP-LGVR--Kyeiv
0039 5t99A   -lrvdnK--VYIG-VY---V---tTPTV--tkskA-VVQIETTV--KN-aTFGNG-KF-NI-RHSIIN-------AqG-ET-V-A---------I--LN-D-NVEVA-------ageqGKT-L-A-YIN-ML-N-----PN--I-WM-YKLKTEIY---------dGKDL-----tdTYFTD-FGIR--Kicft
0040 4ncdA   ----------NFM-IY---P-iSKDLKN------GnSELVRVYS--KSKE------IQ-YI-KIYTKK-gtteeyE-V-DIgLVV---------T-pQK-V-ILPA-----------gASK-S-I-RLT-Q--F---K-IPkkE-EV-YRVYFEAVKP--D-----S---vNI----IYAAL-IRSL--Pseqn
0041 9au7C   -raLLPK--FLLR-GH---LN-STNCVT------Q-PLTGELVV--ES----sEA-AIrSV-ELQLVR-------VdA-TE-I-Q---------N--IQiA-DGDVC-----------rGLSV-P-I-YMV-FP-R---LfTC--P-TE-FEVNIVVLLH--P-----D---hLI----TENFP-LKLC--Ri---
0042 8cmtA   -mksRSN--VDMD-FE---V---ENAVG------K-DFKLSITF--RN-nSHNRY-TI-TA-YLSANI-tfytgvP-K-AE-F-K---------K--ET-F-DVTLE-------plsfKKE-A-V-LIQgEY-M---G-Q-leQ-AS-LHFFVTARIN-------------etrdvlAKQKS-TVLT--Ipeii
0043 6hpdA   -lhtmdP--LHIG-TY---V---YAENI--seksA-DITVETEI--QN-eHAENK-NI-TL-VTQIVD------nD-G-AV-VA----------H--SN-K-NVAIPS-----G--qkMKV-T-T-VTN-IQ-N-----PQ--L-WM-YKVVSAIKES---------NKV-ID----TYESP-LGIR--Nfdfn
0044 3eifA   -kasAAT--MYVT-DKsskVH-LNNV-S------D-KFEVTVTV--HN-KSDKpQ-EL-YY-QATVQT-------DaP-KA-LYE---------T--SW-Q-KITIPA-----N--ssKQV-T-V-PID-AS-R---F-SkknG-YF-LEGFVRFKQD-------pKKEE-LM----SIPYI-GFRG--Dfgnl
0045 7c7uA   -erQLSLtsPLVG-KP---Q---ADITD------T-VVSGSVPL--KE------G-AA-TQ-TAKVIN------aE-G-TT-VG----------T--AT-V-----------------nsDG-T-F-SVS-IP-N-----SP--E-GT-YTIAIDSPN-------------Y-DN----DEVNT-FEIV--Dnskl
0046 6palA   -elDETA--PIVG-MT---ID-NVRIKG------T-LFKAEVSV--TDK-------SL-AY-VWEVLK-ggsyepR-P-ER-MGD---------V--AV-S-D----------------kN-V-Y-ETM-I---------KV--P-GE-YRLYVYVLDN--T-----G-------fvsTANIP-FQVI--D----
0047 5w0pC   -fmsDKP--LNLS-VS---LS-KEIYFH------GePIPVTVTV--TNNTD----vVK-KI-KVSVEQ-------IyV-KP-VA----------S--EE-TqEKVQPN---------stLTK-T-L-VLV-PL-LadtN-LA--S-SS-YHIKVKLTVS--GflgelTSSE-V-----ATEVP-FRLM--Hpqp-
0048 5n6uA   -lkgwNK--ARLD-VW---V---pvrsN------A-QINIELDI--ELQES---I-PV-DV-AFRISH-------K---KP-VL----------E--QR-L-RFTLPE-----G---rVFL-K-I-PLT-IK-----N-PK--LqNL-YTLQLVLLDE---------KGEV-----ldKVEER-FGIR--Kvelf
0049 3tp4B   -aaAPAQ--PVVH-IA---SadGARVAS------A-PTTVRVRV-GGTD------VQ-SV-TVEVAQ-------G-G-TV-V-D---------T--LD-L-A-----------ydGALWW-T-A-PWS-PT-S----------nST-YTVTATAT----------------taagtlDVTNE-VAAA--L----
0050 8v2bC   -pknvscpkapQD-VT---LK-I--iGA------T-KANMTWKV---HSHG-nNY-TL-LC-QVKLQY-------G-E-VI---H---------E--HN-V-SVH------------MSA-N-Y-LFS-DL-------DP--D-TK-YKAFVRCAaN--H-----FWKW-SD----WTQKE-FSTP--Etaps
0051 7q5zA   -liQRQG--NGTF-VQ---TD-KPLYTP-----gQ-QVYFRIVT--MD----snfvND-KYsMVELQD------pN-S-NR-I-A---------Q--WLeV-VPE------------QGIV-D-L-SFQ-LA-P---E-AM--L-GT-YTVAVA-----------------EG----KTFGT-FSVE--Eyvlp
0052 7usyJ   --gmDYI--SSFD-IR---LN-KDVYYA-----gE-TISGSVLL--ENTEN----ikirGI-RVLLRG-------KqY-VL-D-E---------K--QL-L-WgLARG---------vHQF-S-F-NFD-LP-Q---SsLP--C-SR-YYFKVIIDIP--Y-----A---sSP----QGIKY-FTII--Gphid
```

```
0053 8wacA   ---------QSIT-WN---NK-QTDIQP-----gE-TIPLNITY--D--agvGNT-VY-YV-SVVLQE-mnaswqT-Q-NN-Y-N---------T--TY-P-V---sg----snqpnaSTI-D-F-NYT-ID-S---N-IP--L-SF-YLLKIFISVN--------tDGA-FA----NDNTQ-ITLL--Nnle-
0054 6lvbA   -------kdiAIR-GY---CD-RPSVAT-----gE-TIRFYVSA--NE-------tRG-TF-DAELVRlagpgykE-E-AI-KSL---------E--GQ-Y-PAvadpdaedclddcrwEK-D-F-EFT-VP-E---G-LK--S-DF-YAVKIRYE---------------DT----EDYIP-FFVL--Pprgt
0055 1m1sA   --------hsXIN-VD---P---GNYPG------G-NSTHNITS--ESDS------RL-AF-KVKSSN-----------NE-HYR---------vrpVY-G-FVDA-----------kGKS-K-L-DIN-RL-P-----GP--P-KE-DKIVIQYAPF--K-----AGAQ-QG----EIIVK-LIAA-------
0056 8sgiA   -fddDHA--GIFT-FE---EP-VTHVSI------G-IMEVKVLR--TSGA---RG-NV-IV-PYKTIE-------G---arfEDT---------C--GE-L-EFQND-----------eIVK-T-I-SVK-VI-D-deeyeK--N-KT-FFLEIGEPR--emsekkalllngrpilgehTKLE-VIIE--Esyef

0001 s001A   ---LLEE--LLEE-EE---LL-LLEELL------L-EEEEEEEE--LLLLLLLEE-LE-EE-EEEEEE-------L-L-EE-LLL---------L--EE-E-EELLLL-----LLLHHHEE-E-E-EEE-LL-L---L-LL--E-EE-EEEEEEEELL--L-----LLLE-EE----EEEEE-EEEE--L----
0002 8gulA   -llLLLL--LEEE-EE---LL-LLEEEL--llllL-EEEEEEEE--E--------lLE-EE-EEEEEL------lL-L-LE-EE---------E--EE-E-LLLL-----------lLEE-E-E-EEE-LL-L-----LL--L-EE-EEEEEEEE--------elLLLE-EE----EEEEE-EEEE--Lllll
0003 6za2B   lllLLLL--LLEE-EE---EE-LLLLLL--------LEEEEEEE--LLLL----L-LL-EE-EEEEEE------lL-L-LE-EE---------E--EE-E-ELLLLL-----L---LLLE-E-E-EEL-LLlL---L-LL--L-EE-EEEEEEEEEL--L-------EE-EE----LLLEE-EEEL--Ll---
0004 6fqaA   ---------LLEE-EE---LL-LLEELL------L-EEEEEEEE--LLLL------LE-EE-EEEEEE-hhhlleE-E-LLlLEE---------EelLE-E-EELL-----------lLEE-E-E-EEE-EL-L---L-L-llE-EE-EEEEEEEELL------------eEE----EEEEE-EEEE--Lllll
0005 5ghuA   ---------lLEE-EL---LL-EEEEEL------L-LEEEEEEE--LLLL------LE-EE-EEEEEL-------L-L-LL-LEE---------EelLE-E-EELL-----------lLEE-E-E-EEE-EL-L---L---llL-EE-EEEEEEEELL--L-----LLLL-E-----EEEEE-EEEE--Lllll
0006 8u01A   -lleelhllEEEE-EE---LL-LLEEEL-----lL-EEEEEEEE--EEL-LLLLLlLL-EE-EEEEEL------lL-L-LE-EE---------E--EE-E-ELLLLE--------eEEEEeE-E-EEE-L--L---L-LL-lL-EE-EEEEEEELL------------------eEEEEE-EEEE--Lllll
0007 8s3oA   -llLLLLllLEEE-EE---EL-LLEELL-----lL-EEEEEEEE--EL----llllLL-EE-EEEEEL------lL-L-LE-E-E---------E--EEeE-ELLL-------------LEE-E-E-EEE-LL-L---L-LL--L-EE-LEEEEEEL---------------LE----EEEEL-LEEL--Lllll
0008 7rskA   -eeeelL--EEEL-LE---E---eEEEE--llleE-EEEEEEEE--EE-llllLE-EE-EE-EEEEEL------lL-L-LE-EE---------E--EE-E-EEEELL-----LLE--EEE-E-E-EEE-EE-----L-LL--LlLL-EEEEEEEEEL---------LEE-EE----EEEEE-ELLL--Leeee
0009 7a5pq   -hhhhHL--LLEE-EE---EE-E-eelE------E-EEEEEEEE--LLLLLLLLlEE-EE-EEEEEL-----llL-L-LE-E-E---------E--EE-E-EEELL---------llLLLE-E-E-EEE-EE-L---LllL--L-LE-EEEEEEELLL------------lLL----EEEEE-EELLl------
0010 2co6B   -llLEEE---EEE-EL---LL-EEEEEL------llLEEEEEEL--LLLL------LE-EE-EEEEEE-----llL-L-LLlLEE---------EelLE-E-EELL-----------lLEE-E-E-EEE-EL-L---L-LLllL-EE-EEEEEEEELL--L---------eEE----EEEEE-EEEE--Lllll
0011 9ayrB   -eeelLE--EEEE-EE---EL-LLLEEL------L-LEEEEEELllLLLL---LE-EEeEE-EEEEEE-------ElL-EE-EE---------E--EE-E-ELLL-----------lLLEE-E-E-EEL-LL-E-leE-LL--L-LE-EEEEEEEEEEeeE-----EEEL-L-----EEEEE-LLEE--Lllll
0012 6ej7A   -elllLL--LLEE-EE---EL-LLLLLL------L-LLEEEEEE--LLLL------LE-EE-EEEEEL------lL-L-LE-E-E---------E--EE-E-EELLL-----------LLEE-E-E-LLL-LL-L---L-LL--L-EE-EEEEEEEL-------------L-EE----EEEEE-EEEL--Lllee
0013 7s63A   ------L--LLLL-EE---E--ELEEEL-----lL-EEEEEEEE--LLLL----L-LE-EL-EEEEEL-------L-L-LL--LL---------L--LL-L-EELL-----------lLEEE-E-E-EEE-LL-L---L-lL--L-EE-EEEEEELLLL-------------llL----EEEEE-EEEE--Lllle
0014 6cpdB   -------llEEEE-EE---EE-LLEELL-----lE-EEEEEEEE--LLHH-----hHL-LE-EEEEEE-------L-LlLE-EE---------E--EE-EeLLLL-------------LEE-E-E-EEE-E---------LL--L-EE-EEEEEEEELL--L-----L-LE-EE-----EEEE-EEEL-------
0015 8waeA   --lLLLL--LEEE-EE---E-lLLEEEL------LlEEEEEEEE--E-----eLL-LE-EE-EEEEEE-------L-L-EE-E-E---------E--EE-E-EELLE-----------eEEE-E-E-EEE-LL-L---L-LL--L-EEeEEEEEEEEE---------lLLL-HhhlleEEEEEeEEEE--L----
0016 4be5B   -lleeLL--EEEE-EE--eLL-LLLEEL------E-EEEEEEEE--EE-lLLLLE-EE-EE-EEEEEL-------HhH-LE-E-E---------E--EEeE-EEEELL-----L--leEEE-E-E-EEE-EL-L---L-LL--L-EE-EEEEEEEELL--L-----L---lEE----EEEEE-EEEL-------
0017 1cvrA   -lllLLL--LLLL-EE---L--LLEELL------L-EEEEEEL--------------llLL-EEEEEE-------L-L-EE-EE---------E--EE-L-----------------lLL-E-E-EEE-LL-L---L-LL-lL-LE-EEEEEELLL-----------------llLEEEE-EEELl------
0018 2j2zA   ----------LEE-EL---LL-EEEEEL----llL-EEEEEEEE--LLLL----L-LE-EE-EEEEEL----lllL-E-ELlEEE---------E-lLE-E-EELL-----------lLEE-E-E-EEE-EL-H---H-H-llL-EE-EEEEEEEElL--L-----LL--eEE----EEEEE-EEEE--Lhhhl
0019 5axhB   -hhhhlL--LLEE-EE---EL-LLLLLL------L-EEEEEEEE--EL-llLLLE-EE-EE-EEEEEE-------L-L-EE-EE---------E--EE-E-EEEEL-------llleEEE-E-E-EEE-LL-L---L--L--L-EE-EEEEEEEEEL---------LEE-E----eEEEEE-EEEL--Lllll
0020 4e0sA   -llEELLllLEEE-EE---EL-LLEELL------LlEEEEEEEE--ELLL----llLL-LE-EEEEEL------lL-L-LE-E-E---------E--EE-E-LL-----------lLLEE-E-EeEEE-LL-L---L-LL--L-EE-EEEEEEELLL-----------------lllEEEEE-EEEL--Lllll
0021 3q48A   ------------L-LL---LL-EEEEEL------L-EEEEEEEE--LLL-----L-LE-EE-EEEEEL-------L-L-LL-LEE---------EelLE-E-EELL-----------lEEE-E-E-EEE-EL-L---L---llL-EE-EEEEEEEELL--L-------LL-EE-----EEEE-EEEE--Lllll
0022 6ed1A   -eeeelL--EEEL-LE---E---eLLLL--llllL-LEEEEEEE--EE-lLLLLE-EE-EE-EEEEEL------lL-L-LE-EE---------E--EE-E-EEEELL-----LLL--EEE-E-E-EEL-LL-L---L-LL--LlLL-EEEEEEEEEL--L-----LLLE-----eeEEEEE-ELLL--Leeee
0023 5cl2A   ----lLL--LEEE-EE---EL-LLEELL-----lL-EEEEEEEE--ELLLL--LE-EEeEE-EEEEEE-------EeE-EE-E-E---------E--EE-E-L-EELL-----L--leEEE-E-E-EEE-LL-L---L-LL--L-LL-EEEEEEEE---------------lLE----EEEEE-EEEL--Llhhh
0024 4b0mM   -llLEEE--EEEE-EL---LL-EEEEEL------L-LEEEEEEE--LLLL------LE-EE-EEEEEL----lllL-L-LLlEEE---------E-lLE-E-EELL-----------lLEE-E-E-EEE-EL-L---L---llL-EE-EEEEEEEELL--L---------eeeeeeeeEEEE-EEEE--Lllll
0025 5ag8A   -----LL--LLLL-EE---L--LLEEEL------L-EEEEEEL--------------llLL-EEEEEE-------L-L-EE-EE---------E--EE-L-----------------lLL-E-E-EEE-LL-L---L-LL-lL-LE-EEEEEELLL-----------------llLEEEE-EEEE--Lllle
0026 7ptbA   -eeeelL--LLEE-EE---LL-EEEEEEelllllE-EEEEEEEE--LLL-----L-LE-EE-EEEEEE---elllL-E-ELlEEE---------E-lLE-E-EELL-----------lEEE-E-E-EEE-EL-L---L--lllL-EE-EEEEEEE-HH--H-----HHhhhLE----EEEEE-EEEE--Ehhhh
0027 3vmnA   -------lllLLL-LE---EL-LLLLLL-----lL-LEEEELEE--E-LLLLLLE-EE-EE-EEEEEE-------L-L-EE-E-E---------E--EE-E-EEEEL-------llleEEL-LlE-EEL------llllLL--L-EE-EEEEEEEE---------llLLLE-----eeEEEEE-EEEL--Lllll
0028 8iuaA   -------hhlLLL-EE---EL-LLLLLL------L-LEEEEELL------------llL-LE-EEEEEE-------L-L-EE-E-E---------E--EE-L-L------------------lL-E-E-EEL-LL-L---L--L--L-EE-EEEEEEEELL--L-----L-LE-EE----EEEEE-EEEL--Lllll
0029 7lr3D   ---------LEEEeEE---LL-L-lLLL------L-EEEEEEEE--ELllLLLLE-EE-EE-EELLLL----lllL-L-LLlLLL---------L--LL-E-EEEEL-------llleEEE-E-E-EEE-LL-L---L--L--L-LL-EEEEEEEEEL---------LLLE-----eeEEEEE-ELLL--Ll---
0030 2pn5A   -lhhhhhllEEEE-EE---L--LLEEEL-----lL-EEEEEEEE--EE-lLLLLE-EE-EE-EEELH-----------HH-LEEellllllllE--EE-E-EEEELL-----LLL--EEE-E-E-EEE-EL----------lL-EE-EEEEEEEELL--L-----L---------eeEEEEE-EEEE--Lllll
0031 8alzB   -hhhlLL--LLEE-EE---E----ELLL-lllllL-LEEEEEEE--LLLLLHHHHlLE-EE-EEEEEL-------LlL-LL-E-E---------E--EE-E-EELHHh----hHHLL--LE-E-E-EEL-LL-L---LllL--L-LE-EEEEEEELLL-----------------lllEEEEE-EELLll-----
0032 5vhvA   -lllllL--EEEE-EE---EeeLLEELL-----lL-EEEEEEEE--EE-lLLLLE-EE-EE-EEEEEL--lllllL-L-LL-----------eeE--EE-E-EEEELL-----L--leEEE-E-E-EEE-LL-L-llL-LL--L-EE-EEEEEEEL-------------L-EE----EEEEE-EEEL--L----
0033 3wnkA   -llLLLLllLEEEeEE---EL-LLLLLL------L-EEEEEEEE--EL-lllLLE-EE-EE-EEEEEE-------L-L-EE-E-E---------E--EE-E-EEEELL-----L--leEEE-E-E-EEE-LL-L---L--L--L-EE-EEEEEELHH-----------------hlEEEEE-EEEL--Lllll
0034 5dfkA   -----------LLL-LL---LL-EEEELL------L-EEEEEEEE--LLLL------LE-EE-EEEEEE-------E---ellEEE---------E-lLE-E-EELL-----------lEEE-E-E-EEE-EL-L---L---llL-EE-EEEEEELL-----------------lL----EEEEE-EEEL--Lllll
0035 7xdiE   ---------lLEE-EL---LLlLLLLLL------L-EEEEEEEE--LLLL---LL-LL-EE-EEEEEL-------LlL-LL-E-E---------E--EE-E-ELLL-----------LLEE-E-E-EEE-L--------LL--L-LL-EEEEEELL-------------L-LL----EEEEE-ELLL--Lllll
0036 5uj6B   -eeeelL--EEEL-EE---E---ELLLL--llleE-LLEEEEEE--EE-lLLLLE-EE-EE-EEEEEL------lL-L-LE-E-E---------E--EE-E-EEEELL-----L--leEEE-E-E-ELL-LE-E---L-LL--LlLL-EEEEEEEEEL--L-----LLLE-----eeEEEEE-ELLL--Leeee
0037 8qb1A   ------------LE-EE---E-eLLLL-L------L-EEEEEEEE--ELLLLlleellL-LE-EEEEEE-------L-L-EE-E----------eE--EE-E-L-----------------lL-E-E-EEE-LL-L-----LL--L-EE-EEEEEEEL---------------LE----EEEEE-EEEL-------
0038 3fn9C   -eeeelL--EEEL-EE---E---eEEEE--elleE-EEEEEEEE--EE-lLLLLE-EE-EE-EEEEEL------lL-L-LE-E-E---------E--EE-E-EEELLL-----L---lEEE-E-E-EEE-E--E---L-LL--LeLL-EEEEEEEEEL---------LEE-EE----EEEEE-ELLL--Leeee
0039 5t99A   -eeeelL--EELL-EE---E---eEEEE--llleE-EEEEEEEE--EE-lLLLLE-EE-EE-EEEEEL-------LlL-LE-E-E---------E--EE-E-EEEEL-------llleEEE-E-E-EEE-EE-L-----LL--L-LL-EEEEEEEE---------eLLEE-----eeEEEEE-ELLL--Leeee
0040 4ncdA   -----------LEE-EE---L-lEEEELL------LlLEEEEEEE--LLLL------LE-EE-EEEEEE-lllleE-E-EElEEE---------E-lLE-E-EELL-----------lLEE-E-E-EEE-E--L---L-LLllL-EE-EEEEEEEELL--L-----L---lLL----EEEEE-EEEL--Lllll
0041 9au7C   -hhHLLL--EEEE-EE---EL-LLLLLL------L-LEEEEEEE--EE----eLL-LEeEE-EEEEEE-------EeE-EE-E-E---------E--EEeE-ELLLL---------lLLEE-E-E-EEL-LL-L---LlLL--L-LE-EEEEEEEEEL--L-----L---lLL----LEEEE-ELLL--Ll---
0042 8cmtA   -lllLLL--EEEE-EE---E---LLLLL------L-LEEEEEEE--EE-lLLLLE-EE-EE-EEEEEE-ellllE-E-EE-E-E---------E--EE-E-EEEEL-------llleEEE-E-E-EELhHH-L---L-L-lhH-HE-EEEEEEEEEL-------------lllleeEEEEE-EELL--Lllle
0043 6hpdA   -eeeelL--EEEL-EE---E---EEELL--llleE-EEEEEEEE--EE-lLLLLE-EE-EE-EEEEEL------lL-L-LE-EE---------E--EE-E-EEEELL-----L--leEEE-E-E-EEE-EE-L-----LL--L-LL-EEEEEEEELL---------LLE-EE----EEEEE-ELLL--Leeee
0044 3eifA   -hhhHLL--EEEE-ELlllEE-EEEE-L------L-EEEEEEEE--EE-LLLLlE-EE-EE-EEEEEE-------EeE-EE-EEE---------L--LL-E-EEEELL-----L--eeEEE-E-E-EEE-LH-H---H-HhllL-EE-EEEEEEEELL-------lLLLL-LE----EEEEE-EEEL--Lhhhl
0045 7c7uA   -hhHLLLllLLLL-LL---E---LLEEL------L-EEEEELLL--LL------L-LL-LL-EEEEEL------lL-L-LE-EE---------E--EE-E-L----------------llLL-E-E-EEE-LL-L-----LL--L-EE-EEEEEELLL-------------L-LL----LEEEE-EEEE--Lllll
0046 6palA   -llLLLL--LLEE-EE---EH-LLEEEL------L-LEEEEEEE--LLL-------LL-EE-EEEEEE-llllllL-L-LE-EEE---------E--EE-E-L----------------lL-E-E-EEL-L--------LL--L-EE-EEEEEEEELL--L-----L--------leeEEEEE-EEEE--L----
0047 5w0pC   -lllLLL--EEEE-EE---EL-LLEEEL------LlLEEEEEEE--EELLL----lEE-EE-EEEEEE-------EeE-EE-EE---------E--EE-E-ElLLLLLL---------eeEEE-E-E-EEL-LL-HhllL-LL--L-LE-EEEEEEEEEE--LlllllLEEE-E-----EEEEE-EEEE--Llll-
0048 5n6uA   -eeeeLL--EEEE-EE---L---leeeL------E-EELLEEEE--EELLL---L-EE-EE-EEEEEL-------L---LL-LE---------E--EE-E-EEELLL-----E---eEEE-E-L-LEE-EL-----L-LL--LlLL-EEEEEEEELL--------LLLE-----eeEEEEE-ELLL--Leeee
0049 3tp4B   -eeLLLL--LEEE-EE---EllLLEELL------L-LEEEEEEE--ELLL------EE-EE-EEEEEL-------L-L-LE-E-E---------E--EE-L-E-----------elLLLLE-E-E-EEL-LL-L----------lLE-EEEEEEEE---------------elleeeEEEEE-EEEL--L----
0050 8v2bC   -llllllllllEE-EE---EE-E--lLL------L-LEEEEEEL---LLLL-lLL-LE-EE-EEEEEE-------L-L-EE---E---------E--LL-E-EEL-------------LLL-L-L-LLL-LL-------LL--L-LE-EEEEEEEElL--L-----LLLL-LL----LEEEE-EELL--Lllll
0051 7q5zA   -eeELLL--LEEE-EE---EL-LLEELL-----lL-EEEEEEEE--EL----llllLL-LEeEEEEEL------lL-L-LE-E-E---------E--EEeE-LLL-------------LLEE-E-E-EEE-LL-L---L-LL--L-EE-EEEEEL-----------------LL----LEEEE-EEEL--Lllll
0052 7usyJ   --llLLE--EEEE-EE---LL-LLEELL-----lL-EEEEEEEE--EELLL----llllEE-EEEEEE-------EeE-EE-E-E---------E--EE-E-EeLLLE----------eEEE-E-E-EEE-LL-L---LlLL--L-LE-EEEEEEEELL--L-----L---lLL----EEEEE-EEEL--Lllll
0053 8wacA   ---------LEEE-EL---LL-LLEELL-----lL-EEEEEEEE--E--lllLLL-EE-EE-EEEEEE-ellllE-E-EE-E-E---------E--EE-E-E---ll----llllleEEE-E-E-EEE-LL-L---L-LL--L-HE-EEEEEEEEEL--------lLLE-EE----ELLEE-EEEL--Llll-
0054 6lvbA   -------lllLEE-EE---EL-LLEELL-----lL-EEEEEEEE--LL-------lLL-EE-EEEEEEellllleE-E-EE-LLL---------L--EE-E-ELellllllllllllllLL-L-E-EEE-LL-L---L-LL--L-LE-EEEEEELL-----------------LL----EEEEE-EEEL--Lllll
0055 1m1sA   --------llLEE-EE---L---EEELL------E-EEEEEEEE--LLLL------EE-EE-EEEELL-----------LL-LEE---------eelLE-E-EELL-----------lLEE-E-E-EEE-EL-L-----LL--L-LE-EEEEEEEEHH--H-----LLLL-LE----EEEEE-EEEL-------
0056 8sgiA   -ellLLL--LEEE-EL---LL-EEEEEL------L-EEEEEEEE--LLLL---LL-EE-EE-EEEEEE-------L---lllLLL---------E--EE-E-EELLL----------lLEE-E-E-EEE-LL-L-lllllL--E-EE-EEEEELLLE--eeehhhhhhhllllleelllLEEE-EEEE--Llhhh
```

Job: Type-2 BREX; PglZ QBI18515 CTD wHTH (Type-2 BREX-Specific)
Query: s001A
No: Chain Z rmsd lali nres id PDB Description
1: 7wze-A 8.6 3.1 74 161 15 MOLECULE: UNCHARACTERIZED HTH-TYPE TRANSCRIPTIONAL REGULATO
2: 5a31-N 8.6 2.9 74 703 15 MOLECULE: ANAPHASE-PROMOTING COMPLEX SUBUNIT 1;
3: 3w6k-C 8.4 4.5 70 87 11 MOLECULE: SCPA;
4: 8xt8-B 8.4 2.5 69 145 16 MOLECULE: MARR FAMILY TRANSCRIPTIONAL REGULATOR;
5: 5xpq-A 8.3 3.1 74 100 20 MOLECULE: UNCHARACTERIZED HTH-TYPE TRANSCRIPTIONAL REGULATO
6: 7el3-A 8.2 2.7 67 138 9 MOLECULE: HOMOPROTOCATECHUATE DEGRADATION OPERON REGULATOR
7: 8ylg-A 8.2 2.5 67 161 19 MOLECULE: MARR FAMILY TRANSCRIPTIONAL REGULATOR;
8: 8b9x-A 8.2 1.9 60 256 12 MOLECULE: DDRGK DOMAIN-CONTAINING PROTEIN 1,E3 UFM1-PROTEIN
9: 2z99-A 8.2 4.2 70 160 21 MOLECULE: PUTATIVE UNCHARACTERIZED PROTEIN;
10: 8jri-V 8.1 3.9 68 508 6 MOLECULE: 26S PROTEASE REGULATORY SUBUNIT 7;
11: 3bdd-B 8.1 3.2 72 133 13 MOLECULE: REGULATORY PROTEIN MARR;
12: 1lva-A 8.1 2.5 61 258 10 MOLECULE: SELENOCYSTEINE-SPECIFIC ELONGATION FACTOR;
13: 6pco-B 8.0 2.6 68 135 13 MOLECULE: MARR-FAMILY TRANSCRIPTIONAL REGULATOR;
14: 2xrn-B 8.0 2.2 68 241 16 MOLECULE: HTH-TYPE TRANSCRIPTIONAL REGULATOR TTGV;
15: 5j6x-A 8.0 1.8 59 66 15 MOLECULE: Z-DNA BINDING PROTEIN KINASE;
16: 6uvu-B 8.0 2.7 69 114 20 MOLECULE: ARSR FAMILY TRANSCRIPTIONAL REGULATOR;
17: 4i98-B 8.0 4.5 68 167 18 MOLECULE: SEGREGATION AND CONDENSATION PROTEIN A;
18: 6zwt-C 7.9 2.9 79 101 14 MOLECULE: TWO-COMPONENT RESPONSE REGULATOR;
19: 7rhe-A 7.9 2.9 67 368 18 MOLECULE: ROK FAMILY PROTEIN;
20: 2nyx-B 7.9 2.6 69 147 12 MOLECULE: PROBABLE TRANSCRIPTIONAL REGULATORY PROTEIN, RV14

```
21: 8cli-A 7.9 2.8 65 542 15 MOLECULE: GENERAL TRANSCRIPTION FACTOR 3C POLYPEPTIDE 1;
22: 6juv-A 7.8 4.3 72 192 17 MOLECULE: SEGREGATION AND CONDENSATION PROTEIN B;
23: 6j0e-B 7.8 3.1 79 125 14 MOLECULE: ARSENIC RESPONSIVE REPRESSOR ARSR;
24: 8iue-P 7.8 3.0 66 303 12 MOLECULE: DNA-DIRECTED RNA POLYMERASE III SUBUNIT RPC1;
25: 2qlz-B 7.7 2.9 76 220 22 MOLECULE: TRANSCRIPTION FACTOR PF0095;
26: 2wte-A 7.7 3.0 70 212 17 MOLECULE: CSA3;
27: 7l1i-A 7.7 2.9 67 169 13 MOLECULE: MARR FAMILY MULTIDRUG RESISTANCE PUMP TRANSCRIPTI
28: 4ija-A 7.7 2.6 70 363 14 MOLECULE: XYLR PROTEIN;
29: 8qfc-B 7.7 1.8 60 631 12 MOLECULE: 60S RIBOSOMAL PROTEIN L10A;
30: 7qcd-D 7.7 3.3 70 326 13 MOLECULE: STRUCTURAL MAINTENANCE OF CHROMOSOMES PROTEIN 5;
31: 2rdp-A 7.7 2.5 64 140 16 MOLECULE: PUTATIVE TRANSCRIPTIONAL REGULATOR MARR;
32: 8a5y-T 7.6 3.2 68 650 13 MOLECULE: ANAPHASE-PROMOTING COMPLEX SUBUNIT CDC27;
33: 3s2w-A 7.6 3.0 72 146 18 MOLECULE: TRANSCRIPTIONAL REGULATOR, MARR FAMILY;
34: 5f7p-A 7.6 2.1 64 359 16 MOLECULE: LMO0178 PROTEIN;
35: 4fht-B 7.6 2.7 64 142 14 MOLECULE: PCAV TRANSCRIPTIONAL REGULATOR;
36: 1fx7-A 7.6 3.0 64 230 16 MOLECULE: IRON-DEPENDENT REPRESSOR IDER;
37: 7z1n-O 7.6 2.2 75 570 12 MOLECULE: DNA-DIRECTED RNA POLYMERASE III SUBUNIT RPC1;
38: 5a5t-M 7.6 3.0 70 365 11 MOLECULE: EUKARYOTIC TRANSLATION INITIATION FACTOR 3 SUBUNI
39: 1c0w-C 7.5 3.3 65 175 14 MOLECULE: DNA (5'-
40: 6c2j-A 7.5 2.9 85 265 12 MOLECULE: RNAP1;
41: 2lnb-A 7.4 2.0 64 80 20 MOLECULE: Z-DNA-BINDING PROTEIN 1;
42: 5y6i-B 7.4 2.3 68 234 18 MOLECULE: TRANSCRIPTIONAL REGULATOR KDGR;
43: 1sfx-A 7.4 2.7 72 109 13 MOLECULE: CONSERVED HYPOTHETICAL PROTEIN AF2008;
44: 7cv0-A 7.4 2.1 59 173 10 MOLECULE: TRANSCRIPTIONAL REGULATOR NIAR;
45: 2zkz-C 7.4 3.4 71 87 10 MOLECULE: TRANSCRIPTIONAL REPRESSOR PAGR;
46: 3cuq-A 7.4 2.8 71 219 15 MOLECULE: VACUOLAR-SORTING PROTEIN SNF8;
47: 4mtd-D 7.4 2.4 80 151 13 MOLECULE: ZINC UPTAKE REGULATION PROTEIN;
48: 7dvr-A 7.3 2.5 66 147 6 MOLECULE: HTH MARR-TYPE DOMAIN-CONTAINING PROTEIN;
49: 7c0j-B 7.3 2.0 56 62 14 MOLECULE: HISTONE H5,DOUBLE-STRANDED RNA-SPECIFIC ADENOSINE
50: 6r7n-B 7.3 4.1 65 443 12 MOLECULE: COP9 SIGNALOSOME COMPLEX SUBUNIT 1;
51: 6j05-A 7.3 3.8 81 101 15 MOLECULE: TRANSCRIPTIONAL REGULATOR ARSR;
52: 5ju7-A 7.3 2.5 78 107 17 MOLECULE: TRANSCRIPTIONAL ACTIVATOR CADC;
53: 6ndl-A 7.3 2.7 67 323 7 MOLECULE: BIOTIN PROTEIN LIGASE;
54: 4hw0-A 7.3 1.5 57 98 11 MOLECULE: DNA-BINDING PROTEIN SSO10A-2;
55: 8jxk-D 7.3 1.9 59 166 12 MOLECULE: CONSERVED PROTEIN;
56: 3r4k-A 7.3 2.4 66 255 12 MOLECULE: TRANSCRIPTIONAL REGULATOR, ICLR FAMILY;
57: 2eth-A 7.3 2.9 72 141 10 MOLECULE: TRANSCRIPTIONAL REGULATOR, PUTATIVE, MAR FAMILY;
58: 2qww-A 7.3 2.7 65 146 17 MOLECULE: TRANSCRIPTIONAL REGULATOR, MARR FAMILY;
59: 2mlg-A 7.2 2.3 59 77 19 MOLECULE: SULFOLOBUS TRANSCRIPTION FACTOR 76 AMINOACID PROT
60: 6qfd-B 7.2 3.5 67 116 18 MOLECULE: DNA-BINDING PROTEIN;
61: 3k69-A 7.2 2.6 65 151 14 MOLECULE: PUTATIVE TRANSCRIPTION REGULATOR;
62: 8qfc-D 7.1 4.0 68 185 15 MOLECULE: 60S RIBOSOMAL PROTEIN L10A;
63: 3g3z-A 7.1 2.7 65 142 12 MOLECULE: TRANSCRIPTIONAL REGULATOR, MARR FAMILY;
64: 7bzh-A 7.1 2.2 59 59 10 MOLECULE: SUL7S;
65: 2obp-A 7.1 2.2 61 81 21 MOLECULE: PUTATIVE DNA-BINDING PROTEIN;
66: 3oop-A 7.1 2.8 67 139 9 MOLECULE: LIN2960 PROTEIN;
67: 8f8u-A 7.1 3.6 63 297 16 MOLECULE: BIFUNCTIONAL LIGASE/REPRESSOR BIRA;
68: 1yyv-A 7.0 2.5 68 114 18 MOLECULE: PUTATIVE TRANSCRIPTIONAL REGULATOR;
69: 4hbl-D 7.0 3.4 72 147 14 MOLECULE: TRANSCRIPTIONAL REGULATOR, MARR FAMILY;
70: 4yif-B 7.0 2.3 63 141 17 MOLECULE: MARR FAMILY PROTEIN RV0880;
71: 2d1h-A 7.0 2.4 78 102 12 MOLECULE: 109AA LONG HYPOTHETICAL TRANSCRIPTIONAL REGULATOR
72: 2l4m-A 7.0 2.1 62 69 13 MOLECULE: UNCHARACTERIZED PROTEIN;
73: 3mq0-B 7.0 2.8 69 248 16 MOLECULE: TRANSCRIPTIONAL REPRESSOR OF THE BLCABC OPERON;
74: 9c5g-B 7.0 2.5 64 290 13 MOLECULE: CAPW;
75: 8r3g-A 7.0 2.4 61 335 20 MOLECULE: CENTRAL GLYCOLYTIC GENES REGULATOR;
76: 3bpx-A 7.0 2.6 70 147 14 MOLECULE: TRANSCRIPTIONAL REGULATOR;
77: 3f72-B 6.9 3.5 76 108 16 MOLECULE: CADMIUM EFFLUX SYSTEM ACCESSORY PROTEIN;
78: 2pg4-A 6.9 2.2 66 92 18 MOLECULE: UNCHARACTERIZED PROTEIN;
79: 3gfi-A 6.9 2.7 63 143 11 MOLECULE: 146AA LONG HYPOTHETICAL TRANSCRIPTIONAL REGULATOR
80: 4nb5-B 6.9 2.7 71 149 8 MOLECULE: DNA BINDING PROTEIN;
```

```
0001 s001A   GQPA-WV-TELLASPLLAEQRARAGRAALSD-DDLAAFLAVLARHG--DV-ATAGVLQRE-TG-V--K-G-VRLR-SKLQALRRTLS-V-AYD-V-VRVD-T--DGTVRLNR-Q-L-L-AEQFQ--LE--QA
0002 7wzeA   agsllslehYFAGR--------------gLS-EGKFKILXLLFDAK-dHR-LSPTELAKR-SN-V--T-K-ATIT-GLLDGLARDG--------F-VSRR-HhrKISIELTK-A-R-L-E---Q--FL--PG
0003 5a31N   vTMD-V-tLSVA-----------------VT-PVQAVILLYFQDQ---AS-WTLEELSKA-VK-M--P-V-ALLR-RRMSVWLQQG--------V-LREE-P--PGTFSVIE-E-E-----RPQ--DRd---
0004 3w6kC   ----------------------------aLK-PAKAIVEALLFAAG---G-LSLSQIAAV-LE-V--S-E-LEAK-AVIEELQQDCR-R-ERgiQ-LVEL-G--G-VFLLA----T-K-KE--H--AP--Y-
0005 8xt8B   mkdfyeakddmFKQNGYD-----------iT-TDHYALLRFLWEQ---DG-ISQIDLCEK-SC-K--D-K-SNTT-RILDVMKNKG--------L-IVRK-V--DFQIFLTR-E-L-E-E------------
0006 5xpqA   --------------yklKAEF----fkTLAH-PARIRILELLVER----D-RSVGELLSSdVG-L--E-S-SNLS-QQLGVLRRAG--------V-VAAR-RgnAMIYSIAA-P-D-I-AEL---aVA--RK
0007 7el3A   -pirtlafrPALNE--------------gLT-EQQWRIIRILYQY---EE-LESNQLAEL-AC-I--L-K-PSLT-GILNRMVEQK--------L-IQKR-KqrISLISLTG-L-E-C--------------
0008 8ylgA   ssmirlqnPLFARY--------------glQ-PGEFDVLATLRRSGapYA-LTPTALYDA-AM-I--S-S-GSMT-NRIDRLEKAG--------W-VERR-A--NTLVALGR-A-Li---------------
0009 8b9xA   rtqdriqiddfiyilaavanfirqrgrvtPA-QISKEMRDELHVRG--GR-VNIVDLQQV-IN-V--D-L-IHIE-NRIGDIIKSEK-------H-VQLV----LGQLIDENy-------------------
0010 2z99A   ----------------------------dAD-ELKRVLEALLLVID---P-VTADALAAA-TE-Q--P-V-YRVA-AKLQLMADELT-G-DSgiD-LRHT-S--E-GWRMY----T-R-AR--F--AP--Y-
0011 8jriV   alrkhtaivvlGEIP-----drlqfrqprHN-VIKTGVRMISLSY---SR-ISLADIAQK-LQ-L-dS-P-EDAE-FIVAKAIRDG------viE-ASIN-H-eKGYVQSKE-M------------ID--IY
0012 3bddB   -----qedetlFEKQLG------------iS-LTRYSILQTLLKD---AP-LHQLALQER-LQ-I--D-R-AAVT-RHLKLLEESG--------Y-IIRK----EVLVWPTE---Q-A-REAtN--PS--A-
0013 1lvaA   ------------------------------G-SPEKILAQIIQEHR--EG-LDWQEAATR-AS-L--S-L-EETR-KLLQSXAAAG--------Q-VTLL-R--VLYAISRY-Q-Aw----------------
0014 6pcoB   --gpseqhvaqtIPDSK------------LT-AAQFVVLCALRDQ---GA-CSLVDVVKA-TA-I--D-Q-ATVR-GVIERLKAR-------K-L-LAVS-HrrKVLVTLTP-D-G-R-A------------
0015 2xrnB   ----------------------------qVI-ARAASIMRALGSHP---G-LSLAAIAQL-VG-L--P-R-STVQ-RIINALEEEF--------L-VEALgP--AGGFRLG---pA-L-GQ-LI--NQ--AQ
0016 5j6xA   ----------------------------aEN-EIEMRICDYLRRH---GR-STVQDIFKE-LK-L--E-K-STVN-RHLYSLQASK--------Q-VFKT-VnkRPVWDLVE--------------------
0017 6uvuB   -------dlieagEVVVLFK--------laN-DTRLRLLHALARSG---G-LCVTDLAAA-VG-M--K-P-QAVS-NQLQRLADR-------R-I-LRAA-RgnNIHYRIVD-P-C-V-L------------
0018 4i98B   --------------------------------STLAKIEALLFVAG---G-IRVRQLAEL-LS-L--P-P-TGIQ-QSLGKLAQKYEkD-DSslA-LIET----SGAYRLV----T-K-PQ--F--AE--I-
0019 6zwtC   EVVSwSL-DLTLTRE-------------tLT-TGEFAVLKALVQHP--REpLTRDKLMNL-AR----grE-RSID-VQVSRLRRLIE-DpARA-RyIQTV-W--GVGYVFVP-D-Ga---------------
0020 7rheA   ----------------------------vRR-YNERLLLKTLRRA---GS-ASKADLARL-AN-M--T-G-TAVG-SIIASLADAK--------L-IEFA----ASLIRLDP---R-G-AFG---aGD--VL
0021 2nyxB   esvddalaHSIAQV--------------nIT-IPQFRTLVILSNH---GP-INLATLATL-LG-V--Q-P-SATG-RXVDRLVGAE--------L-IDRL-PrrELLAALTK---R-G-RD------------
0022 8cliA   rlgrqgevdahyhrknglitmQSHVirlKYD-ILMEKLSVMLSTRN--HI-ETLGKLREE-LG-L--C-E-RTFK-RLYQYMLNAG--------L-AKVV-S--LRCLKLLKt-------------------
0023 6juvA   ----------------------------gLL-EDKALVEAALFVAG--RP-LSLKELSKA-LG-I--KsL-EYLE-KLIELIASEYE-E-KSaiE-VVKV-L--GDKWVXQ----L-K-QE----sQK--VI
0024 6j0eB   ----lhtlatssdESEHYADLF----kVLGD-PVRLRILSQLAAGG--CP-VSVNELTDL-MG-L--S-Q-PTIS-HHLKKMTEAG--------F-LDRV-PgrVVLHRVRP---E-L-FAELR--T-----
```

```
0025 8iueP   ---------------------------dPV-EIENRIIELCHQFP--HG-ITDQVIQNE-MPhI--E-A-QQRA-VAINRLLSMG--------Q-LDLL-RntGLLYRIDS-Q-N-----DNQ--E-----
0026 2qlzB   yrsS-LX-RKIKEA-ylKEIG---------D-XTQLAILHYLLLN---GR-ATVEELSDR-LN-L--K-E-REVR-EKISEXARFV--------P-VKII-N--DNTVVLDE-D-Q-I-LR----------
0027 2wteA   llgirkrsrvndnTIRALXR--------dYS-REEXKLLNVLYET---KG-TGITELAKX-LD-K--S-E-KTLI-NKIAELKKFG--------I-LTQK-G-kDRKVELNE-L-G-L-N-----------
0028 7l1iA   nfpfsqviKKLG----------------ID-NTRRKIILSTNAL---GE-ASITDIANL-ST-L--K-L-TTAT-KAVYRLVEDG--------I-VEVY-S--SSMVKLTA-----K-GVE---------
0029 4ijaA   ----------------------------pMN-DNEKRVLREIYNHH---N-ISRTQISKN-LE-I--N-K-ATIS-SILNKLKYKS--------L-VNEV-GrkPILLKVNH---L-Y-GYYNG--LL--GV
0030 8qfcB   ----------------------------tPA-QISKEMRDELHVRG--GR-VNIVDLQQV-IN-V--D-L-IHIE-NRIGDIIKSE------K-H-VQLV----LGQLIDENy------------------
0031 7qcdD   ----------------------------tGD-ATAKYLLQYILSAR--GI-CHENALILA-LMtlnqW-V-DKLN-DYINAINVKLN-L-lGY-K-IIRI-N--HRFFVYV----N-L-AS----eTK--LA
0032 2rdpA   axneaelkqrILTN--------------pIT-PPQFVALQWLLEE---GD-LTVGELSNK-XY-L--A-C-STTT-DLVDRXERNG--------L-VARV-RehVVRIRLLE-K-----------------
0033 8a5yT   ysdilkpkVEIQlaRKLV----------dVS-LEQCSVINQFDSpP--IC-LSLEQLSES-LN-I--A-P-PRLT-HLLDFWIQKG--------V-LLKE-N---GTYSVIE-H-Se--------------
0034 3s2wA   ishacdkrygIGKK--IEPYG-------IGS-G-QFPFLXRLYRE---DG-INQESLSDY-LK-I--D-K-GTTA-RAIQKLVDEG--------Y-VFRQ-R--DYRVFLTK-K-L-E-P-----------
0035 5f7pA   -----------------------------D-INRYTVLNLIREK---GE-ITRTEIAKK-CD-F--G-M-STLT-YILDDLQQEG--------I-ILEG-----KLVRFNK---D-Y-GF-HL--LGvl--
0036 4fhtB   hmaaathhyltmVSEE------------TT-SPQYAVLNALVAE---PG-LDQRTVGER-VG-L--D-R-STIA-EVVSRLGRRG--------L-LDKV-RgrRSLLRLTD-E-----------------
0037 1fx7A   ----------------------------elV-DTTEMYLRTIYDLE--eT-PLRARIAER-LD-Q--S-G-PTVS-QTVSRMERDG--------L-LRVA-G--DRHLELGR-A-L---------pIP--GL
0038 7z1nO   pdLF-LYkELVKAH--------------lG-ERAASVIGMLVALG---R-LSVRELVEK-IDgM--D-V-DSVK-TTLVSLTQLR--------C-VKYL-QkkTTYYYYNE---E-G-IHILLym------
0039 5a5tM   sytesqalkdFLFDHL----------ltnMA-KMRLLTFMGMAVEN--KE-ISFDTMQQE-LQ-I--G-A-DDVE-AFVIDAVRTK------mvY-CKID-Q--TQRKVVVS------------rTF--GK
0040 1c0wC   ----------------------------dLV-DTTEMYLRTIYELE---vtPLRARIAER-LE-Q--S-G-PTVS-QTVARMERDG--------L-VVVA-S--DRSLQMGR-T-L-----tiP--GL--DE
0041 6c2jA   LLPR-MR-QEFEE-IDFKAFFAHI---GIDY-KFGIDAMVQMALH---KR-ADLPTLVGT-LR---kS-A-QEVA-DNLFKMASE-------D-C-FNFD-P-tIDKFIVIY-------------iSD--DV
0042 2lnbA   ----------------------------rEG-HLEQRILQVLTEAG--SP-VKLAQLVKE-CQ-A--P-K-RELN-QVLYRMKKEL--------K-VSLT-S--PATWCLGG---T-D-PE----------
0043 5y6iB   ----------------------------rSA-EVGTDILKALAELS--PA-TSLSRLAEH-VG-M--P-A-SKVH-RYLQALIASG--------F-AVQDaS--TNHYSL-A-L-R-V-GL--A--AL--D-
0044 1sfxA   -----------snplkalEKLS--------FK-PSDVRIYSLLLER---GG-XRVSEIARE-LD-L--S-A-RFVR-DRLKVLLKRG--------F-VRRE-IwvGYIYSAEK-E-K-V-LKEFK--S-----
0045 7cv0A   ----------------------------lGE-ERRSLLIKWLKASD---P-LTGAELAKR-TN-V--S-R-QVIV-QDVSLLKAKN-------hP-ILAT----AQGYIYMK-Ef----------------
0046 2zkzC   -----------eyxSLEDDAELL----kTXAH-PXRLKIVNELYKH---KA-LNVTQIIQI-LK-L--P-Q-STVS-QHLCKXR--------gK-V-LKRN-RglEIYYSINN-P-K-V-------------
0047 3cuqA   PVGG----TYLI----qSVPA--------eLN-MDHTVVLQLAEK-N--GY-VTVSEIKAS-LK-W--E-T-ERAR-QVLEHLLKE-------G-L-AWLD-LpgEAHYWLPAl-----------------
0048 4mtdD   ttQE-LL-AQAEKI--CAQRN--------rLT-PQRLEVLRLMSLQD--GA-ISAYDLLDL-LRqA--K-P-PTVY-RALDFLLEQG--------F-VHKV-E-sTNSYVLCH---L-F-DQP---------
0049 7dvrA   ----menlekryAKEYD------------HLA-GPQGHLVMHLYKHP---D-MSIKDAEEI-LH-I--S-K-SVAS-NLVKRMEKNG--------F-IAIV-P--SKYLYLTH-L----------------
0050 7c0jB   -----------------------------P-TYSEMIAAAIRAEG---G-SSRQSIQAY-IK---vN-K-KEIN-RVLYSLLAAG--------V-LKQT-G--VGSWALA-----------------
0051 6r7nB   feafdeslvllmKSGInpfdsqeakpyklRN-IRTQVLIKLIKP-Y--TR-IHIPFISKE-LN-I--D-V-ADVE-SLLVQCILDN------tiH-GRIDqV--NQLLELDH-Q-Kr---------------
0052 6j05A   -------------QDPAQIVARL----eALAS-PVRLEIFRLLVEQE---G-LVSGDIAEH-LG-Q--P-H-NGIS-FHLKNLQHAG--------L-VTVQ-R--EQRYRAAM-P-V-V-RALVA--YLt---
0053 5ju7A   pvvrwLV-TPQISRRQLT-----------LE-PRLIDLLVFFAQHS--GEvLSRDELIDN-VW---vT-N-HVVT-QSISELRKSLK-D-SPV-Y-IATV-P--KRGYKLMV-P-----------------
0054 6ndlA   -----------------------------S-KYSQDVLQLLYKNK---Y-ISGQSIAES-LN-I--S-R-TAVK-KVIDQLKLEG-------cK-IDSV-N--HKGHLLQQ-----L-PDIW--lDR--YQ
0055 4hw0A   ----------------------------gTX-EIXFDILRNCE-----PK-CGITRVIYG-AG-I--N-Y-VVAQ-KYLDQLVKVG--------A-LNIK-TndRKIYEITEk---------------
0056 8jxkD   -----------------------------EF-MLELAILGLLIES----P-MHGYELRKR-LTaF--S-Y-GSLY-PALRRMQADG--------L-IAEN-A--ARVYQLTD-K-----------------
0057 3r4kA   ----------------------------gTV-SKALTLLTYFN--R--LE-IGLSDLTRL-SG-X--N-K-ATVY-RLXSELQEAG--------F-VEQVeG--ARSYRLG---pQ-V-LR-LA--AL--R-
0058 2ethA   daletlfsNEEIS---------------dXK-TTELYAFLYVALF---GP-KKXKEIAEF-LS-T--T-K-SNVT-NVVDSLEKRG--------L-VVRE-XrrTYRVVLTK-E-I-F-G-----iLS--NF
0059 2qwwA   tenilktadQNAAS--------------gLT-IQQLAXINVIYST---PG-ISVADLTKR-LI-I--T-G-SSAA-ANVDGLISLG--------L-VVKL-NsxDLTLKLSK-K-----------------
0060 2mlgA   ----------------------------akL-SLTQLILIRLSNR---GC-QTLEELEEF-TQ-A--K-R-EVLL-VTLTRLHQRG--------V-IYRK-WrkYREYCLKH-R-----------------
0061 6qfdB   --------------pDARSD--------dLT-AFQKNILTVLGEE----A-RYGLAIKRE-LEeV--N-H-GRLY-PNLDDLVNKG--------L-VEKS-E--LNEYALTN-------------lEW--TL
0062 3k69A   ----------------------------lDF-SVAVHSILYLDAHR---K-VASRELAQS-LH-L--N-P-VXIR-NILSVLHKHG--------Y-LTGT-V--GGGYQLDL-----A-LADXNl-------
0063 8qfcD   ----lrkeaereersqreaewkkeeerlqSQ-SFLTEFINYIKQS---KV-VLLEDLASQ-VG-L--R-T-QDTI-NRIQDLLAEGT------iT-GVID-D--RGKFIYIT-P-E-E-LAAVAn-------
0064 3g3zA   xnqlgtriGQQD----------------lN-YNLFAVLYTLATE---GS-RTQKHIGEK-WS-L--P-K-QTVS-GVCKTLAGQ-------G-L-IEWQ-ErrKRLLSLTK-A-Y-A-------------
0065 7bzhA   -----------------------------ME-DVKQSVEKIIKDR---EW-VTFNDLLKY-IP-Y--P-A-PEVY-DALSQLIKEN--------K-VGRR----GRYFYYIK-R-----------------
0066 2obpA   ----------------------------gID-PAIVEVLLVLREAGienP-WSLPKIAKR-AQ-L--P-X-SVLR-RVLTQLQAAG--------L-ADVS-VdgRGHASLGA-A-La---------------
0067 3oopA   gyydfdvxrSIASY--------------dVT-PEQWSVLEGIEAN---EP-ISQKEIALW-TK-K--D-T-PTVN-RIVDVLLRKE--------L-IVRE-IrrISLLSLTR-K-E-T-------------
0068 8f8uA   ----------------------------kdH-TIPLTLISILA-DG--EF-HSGEQLGEQ-LG-M--S-R-AAIN-KHIQTLRDWGV-------D-VFTV-P--GKGYSLPE-------------dEK--KI
0069 1yyvA   -----------lregeqcPSRE----vlKHVTsRWGVLILVALR--D--GT-HRFSDLRRX-XGgV--S-E-XXLA-QSLQALEQDG--------F-LNRV-SppHVEYSLTP-L-G-E-Q-----------
0070 4hblD   rlanfsaekKLKQF--------------giT-YSQYLVXLTLWEE---NP-QTLNSIGRH-LD-L--S-S-NTLT-PXLKRLEQSG--------W-VKRE-R--QLIITLGQ-Q-Q-Q-EA-fE--AI--S-
0071 4yifB   ----arlsrqRNPS--------------pVS-LSQLSALTTLANE---GA-MTPGALAIR-ER-V--R-P-PSMT-RVIASLADMG--------F-VDRA-P--HVLVSVSEs-----------------
0072 2d1hA   klES-KK-DEIRCC--------------kIT-DTDVAVLLKXVEIE--KP-ITSEELADI-FK-L--S-K-TTVE-NSLKKLIELG--------L-VVRT-KtpKYYYSISI-L-E-K-IRNDLl-------
0073 2l4mA   -------mASPQ----------------fSQ-QREEDIYRFLKDN---GP-QRALVIAQA-LG-M--R-TaKDVN-RDLYRMKSRH---------lLDMD-E-qSKAWTIYR-------------------
0074 3mq0B   ----------------------------pAL-RRAVRILDLVAGSP--RD-LTAAELTRF-LD-L--P-K-SSAH-GLLAVMTELD--------L-LARS-A--DGTLRIGP---H-S-LR--W--AN--GF
0075 9c5gB   ----------------------------mQN-RRFEFIEWKLFW-E--GA-LNRSDLEET-FE-I--S-T-PQTS-IDLRRYRELAG-----D-N-IEYD-A-tDKTFKPTK-G-M-K-------------
0076 8r3gA   ----------------------------lVX-QKRFEILQYIRLT---EP-IGRRSLSAS-LG-I--S-E-RVLR-GEVQFLKEQN--------L-VDIK-T--N-GXTLTY-E-L-Lr-------------
0077 3bpxA   ----mdrlrsfIGRE-LGHLN--------lT-DAQVACLLRIHRE---PG-IKQDELATF-FH-V--D-K-GTIA-RTLRRLEES-------G-F-IERE-Q--DYILEVTG-E-E-I-------------
0078 3f72B   -------ekvqgdlISGVSQI----lkAIAD-ENRAKITYALCQDE---E-LCVCDIANI-LG-V--T-I-ANAS-HHLRTLYKQG---------vVNFR-KgkLALYSLGG-E-A-I-RQ----mIA--LA
0079 2pg4A   -----------------XDDE----tlRLQF-GHLIRILPTLLEFE--kE-PSLAEIVKA-SG-V--S-E-KTFFxGLKDRLIRAG--------L-VKEE-T--LKTLKLTE-K-----------------
0080 3gfiA   enristinrRLGE---------------nLS-YLDFLVLRATS--D--GP-KTMAYLANR-YF-V--T-Q-SAIT-ASVDKLEEMG--------L-VVRV-RrrKILIEITE-K-----------------
0081 4nb5B   EFVE------------------------sLT-RLAGRLLGWLLVCD-pER-QSSEELATA-LA-A--S-S-GGIS-TNARMLIQFG--------F-IERL-AdrRTYFRLRP---N-A-FAAGEr-------

0001 s001A   LLLH-HH-HHHHLLHHHHHHHHHHHHHHLLLH-HHHHHHHHHHHHHLL--LE-EEHHHHHHH-HL-L--L-L-HHHH-HHHHHHHHHHL-L-LLL-L-EEEL-L--LLEEEELH-H-H-H-HHHHL--LL--LL
0002 7wzeA   hhhhhhhhhHHHLL--------------lLL-HHHHHHHHHHHHHLH-hHE-ELHHHHHHH-LL-L--L-H-HHHH-HHHHHHHHHL--------L-EEEE-EllLEEEEELH-H-H-H-H---H--HH--HH
0003 5a31N   eEEE-E-lEELL----------------LL-HHHHHHHHHHHHLL---LL-LLHHHHHHH-HL-L--L-H-HHHH-HHHHHHHHHL--------L-LLLL-L--LLLLLLLL-L-L-----LLL--LLl---
0004 3w6kC   ----------------------------lLH-HHHHHHHHHHHHHHH---L-LLHHHHHHH-HL-L--L-H-HHHH-HHHHHHHHHHL-L-LLleE-EEEE-L--L-EEEEE----E-L-HH--H--HH--H-
0005 8xt8B   lllhhhhhhhHHHLLLL-----------lL-HHHHHHHHHHHHHL---LL-LLHHHHHHH-HL-L--L-L-HHHH-HHHHHHHLLL--------L-EEEE-E--LEEEEELH-H-L-H-H-----------
0006 5xpqA   --------------hhhHHHH----hhHLLL-HHHHHHHHHHHHHL----L-EEHHHHHLLlLL-L--L-H-HHHH-HHHHHHHHLL--------L-EEEE-EllLEEEEELL-L-H-H-HHH---hHH--HH
0007 7el3A   -lllhhhlhHHHHH--------------lLL-HHHHHHHHHHHHHH---LE-EEHHHHHHH-HL-L--L-H-HHHH-HHHHHHHHLL--------L-EEEE-EeeEEEEEELH-H-H-H-------------
0008 8ylgA   lhhhhhhhHHHHHL--------------llL-HHHHHHHHHHHHHLLllLE-ELHHHHHHH-HL-L--L-H-HHHH-HHHHHHHHHL--------L-EEEE-E--LEEEEEHH-H-Hh---------------
0009 8b9xA   lhhhhhhelleeelhhhhhhhhhhleelHH-HHHHHHHHHHHHHLL--LE-EEHHHHHHH-HL-L--L-L-HHHH-LLHHHHHHHLL-------L-EEEE----LLEEEEHHh-----------------
0010 2z99A   ----------------------------lHH-HHHHHHHHHHHHHLL---L-LLHHHHHHH-HL-L--L-H-HHHH-HHHHHHHHHHH-H-LLleE-EEEE-L--L-EEEEE----E-L-HH--H--HH--H-
0011 8jriV   hhhhlllhhhhLLLL-----llllllllhHH-HHHHHHHHHHHHHL---LE-ELHHHHHHH-HL-L-lL-H-HHHH-HHHHHHHHLL------llL-LEEL-L-lLLLEELLL-L------------LL--HH
0012 3bddB   -----lhhhhhHHHHHL-----------lL-HHHHHHHHHHHHHL---LL-EEHHHHHHH-HL-L--L-H-HHHH-HHHHHHHHLL--------L-EEEL----LLEEEELH---H-H-HHHlL--LL--H-
0013 1lvaA   -----------------------------L-LHHHHHHHHHHHLLL--LL-EEHHHHHHH-HL-L--L-H-HHHH-HHHHHHHHLL--------L-EEEE-E--EEEEEEHH-H-Hh---------------
0014 6pcoB   --llhhlhhhhhLHHHL-----------LL-HHHHHHHHHHHHHH---LL-LLHHHHHHH-HL-L--L-H-HHHH-HHHHHHHHL-------L-L-EEEE-LllLLEEEELH-H-H-H-H-----------
0015 2xrnB   ----------------------------hHH-HHHHHHHHHHHHLLL---L-EEHHHHHHH-LL-L--L-H-HHHH-HHHHHHHLLL--------L-EEELhH--HLEEEEL---lH-H-HH-HH--HH--HH
0016 5j6xA   ----------------------------hHH-HHHHHHHHHHHHHH---LL-LLHHHHHHH-LL-L--L-H-HHHH-HHHHHHHHLL--------L-EEEE-LllLLEEEELL-------------------
0017 6uvuB   -------lllhhhHHHHHHHH--------hlL-HHHHHHHHHHHHHHL---L-LLHHHHHHH-LL-L--L-H-HHHH-HHHHHHHHL-------L-L-EEEE-EllEEEEEELL-H-H-H-H-----------
0018 4i98B   ------------------------------LHHHHHHHHHHHHLL---L-EEHHHHHHH-HL-L--L-H-HHHH-HHHHHHHHHHHhL-LLleE-EEEE----LLEEEEE----E-L-HH--H--HH--H-
0019 6zwtC   LEEEeEE-ELEEEEL------------lLL-HHHHHHHHHHHLLL--LLlEEHHHHHHH-HH----llL-LHHH-HHHHHHHHHL-LhHHL-LlEEEE-L--LLEEEELL-L-Ll-------------
0020 7rheA   ----------------------------hHH-HHHHHHHHHHHHHH---LL-EEHHHHHHH-HH-L--L-H-HHHH-HHHHHHHHLL--------L-EEEL----LLEEEELL---L-L-LEE---lLL--EE
0021 2nyxB   llllhhhhHHHHHH--------------lLL-HHHHHHHHHHHHHH---LL-EEHHHHHHH-HL-L--L-H-HHHH-HHHHHHHHLL--------L-EEEE-LllLLEEEELH---H-H-HH-----------
0022 8cliA   hhhlllllllhhhhhhhlleeeEEEEeelHHH-HHHHHHHHHHHHHLL--LE-EEHHHHHHL-LL-L--L-H-HHHH-HHHHHHHHLL--------L-EEEE-E--EEEEEELLl-----------------
0023 6juvA   ----------------------------lHH-HHHHHHHHHHHHHHL--LL-EEHHHHHHH-HL-L--LlH-HHHH-HHHHHHHHHHH-H-LLleE-EEEE-L--LLEEEEE----E-L-HH----hLL--LH
0024 6j0eB   ----eeellhhllLHHHHHHHH----hHHLL-HHHHHHHHHHHHHLL--LL-EEHHHHHHH-HL-L--L-H-HHHH-HHHHHHHHLL--------L-EEEE-EllEEEEEELH---H-H-HHHHH--H-----
0025 8iueP   ----------------------------lHH-HHHHHHHHHHHHHLL--LL-LLHHHHHHH-LLlL--L-H-HHHH-HHHHHHHHHL--------L-EEEE-EllEEEEEEHH-H-L-----LHH--H-----
0026 2qlzB   hhhH-HH-HHHHHH-hhHHLL---------L-HHHHHHHHHHHHHH---LE-EEHHHHHHH-HL-L--L-H-HHHH-HHHHHHLLLL--------L-EEEE-L--LLEEEELH-H-H-H-HL-----------
0027 2wteA   hhhhlllllleehhHHHHHHL--------lLL-HHHHHHHHHHHHHH---LL-LLHHHHHHH-HL-L--L-H-HHHH-HHHHHHHHLL--------L-EEEE-L-lLLEEEELH-H-H-H-H-----------
```

```
0028 7l1iA   hlhhhhhhLLLL-----------------LL-HHHHHHHHHHHHH---LL-EEHHHHHHHH-LL-L--L-H-HHHH-HHHHHHHHLL--------L-EEEE-E--LEEEEELH-----H-HHH----------
0029 4ijaA   ----------------------------lLH-HHHHHHHHHHHHHLL---L-LLHHHHHHH-HL-L--L-H-HHHH-HHHHHHHHLL--------L-EEEL-LllLLLEEELH---H-H-LELLL--EE--EE
0030 8qfcB   ----------------------------lHH-HHHHHHHHHHHHHLL--LE-EEHHHHHHH-HL-L--L-H-HHHH-HHHHHHHHHL------L-L-EEEL----LLEEEEHHh-------------------
0031 7qcdD   ----------------------------hHH-HHHHHHHHHHHHHLL--LE-EEHHHHHHH-HHlllhH-H-HHHH-HHHHHHHHHHH-H-lLE-E-EEEE-E--LEEEEEE----E-L-LL----hHH--LL
0032 2rdpA   lllhhhhhhhHHLL--------------lLL-HHHHHHHHHHHHH---LL-LLHHHHHHHH-HL-L--L-H-HHHH-HHHHHHHHLL--------L-EEEE-EllLEEEEELH-H------------------
0033 8a5yT   hhhhhlleEEEEeeLEEE----------eEE-HHHHHHHHLLLLlL--LL-EEHHHHHHHH-HL-L--L-H-HHHH-HHHHHHHHLL--------L-EEEE-L---LEEEELL-L-Ll---------------
0034 3s2wA   hhhhlllhhhHHHH--HHHHL-------LLL-L-LHHHHHHHHHL---LL-EEHHHHHHHH-HL-L--L-H-HHHH-HHHHHHHHLL--------L-EEEE-E--LEEEEELH-H-H-H-H------------
0035 5f7pA   ------------------------------L-HHHHHHHHHHHHH---LE-EEHHHHHHHL-LL-L--L-H-HHHH-HHHHHHHHHL--------L-EEEL-----LEEEELL---L-L-LE-LE--EEel--
0036 4fhtB   llllhhlhhhhhLLLL-------------LL-HHHHHHHHHHHHL---LL-LLHHHHHHHH-HL-L--L-H-HHHH-HHHHHHHHHL--------L-EEEE-EllLEEEEELH-H------------------
0037 1fx7A   ----------------------------llL-LHHHHHHHHHHHHH--hL-LLHHHHHHHH-HL-L--L-H-HHHH-HHHHHHHHLL--------L-EEEL-L--LLLEEEHH-H-H---------lLL--LH
0038 7z1nO   hhHH-HHhHHHHHH---------------lL-HHHHHHHHHHHHHL---L-EEHHHHHHHH-LLlL--L-H-HHHH-HHHHHHHHLL--------L-LLEE-EleEEEELLLH---H-H-HHHHHll------
0039 5a5tM   hlllhhhhhlLLLHHH----------hhhHH-HHHHHHHHHHHHHL--LL-LLHHHHHHHH-HL-L--L-H-HHHH-HHHHHHHHHL------llL-LLLL-L--LLLLLLL--------------lLH--HH
0040 1c0wC   ----------------------------lLL-LHHHHHHHHHHHHH---llLLHHHHHHHH-LL-L--L-H-HHHH-HHHHHHHHLL--------L-EEEL-L--LLLEEEHH-H-H-----hlL--LL--LL
0041 6c2jA   HHHH-HH-HHHLL-LLHHHHHHHL---LLLH-HHHHHHHHHHHHH---LE-ELHHHHHHHH-HH---lL-H-HHHH-HHHHHHHHHL------L-L-LEEE-L-lLLEEELLL-------------lLH--HH
0042 2lnbA   ----------------------------hHH-HHHHHHHHHHHHHL--LL-EEHHHHHHHH-HL-L--L-H-HHHH-HHHHHHHHLL--------L-EEEE-E--LLEEEELL---L-L-LL-----------
0043 5y6iB   ----------------------------lHH-HHHHHHHHHHHHHLL--LL-EEHHHHHHH-HL-L--L-H-HHHH-HHHHHHHHHL--------L-EEELlL--LLEEEE-H-H-H-H-HH--H--HH--H-
0044 1sfxA   ----------llhhhhhHHLL--------LL-HHHHHHHHHHHHH---LL-LLHHHHHHHH-HL-L--L-H-HHHH-HHHHHHHHLL--------L-EEEE-EleEEEEEELL-H-H-H-HHHHH--H-----
0045 7cv0A   ----------------------------hHH-HHHHHHHHHHHHHLL---L-LLHHHHHHH-HL-L--L-H-HHHH-HHHHHHHHLL-------lL-EEEE----LLEEEELL-Ll-----------------
0046 2zkzC   ----------lhhHHHHHHHHH----hHHLL-HHHHHHHHHHHHH---LL-EEHHHHHHHH-HL-L--L-H-HHHH-HHHHHHL--------lL-L-EEEE-EllEEEEEELL-H-H-H--------------
0047 3cuqA   EELL----EEEE----eLLLL-------lLL-HHHHHHHHHHLL-L--LE-ELHHHHHHHH-HL-L--L-H-HHHH-HHHHHHHHH-------L-L-LEEE-LllLLEEELLLl-------------------
0048 4mtdD   lhHH-HH-HHHHHH--HHHLL-------lLL-HHHHHHHHHHHLLL--LL-EEHHHHHHHH-HHlL--L-H-HHHH-HHHHHHHHLL--------L-EEEE-L-lLLEEEELL---L-L-LLL----------
0049 7dvrA   ----lllhhhhhhHHHLL----------LLL-LHHHHHHHHHHHHLL---L-EEHHHHHHH-HL-L--L-H-HHHH-HHHHHHHHLL--------L-EEEE-E--LEEEEELH-H------------------
0050 7c0jB   ------------------------------L-LHHHHHHHHHHHHLL---L-EEHHHHHHH-HH---lL-H-HHHH-HHHHHHHHLL--------L-EEEL-L--LLEEEEL---------------------
0051 6r7nB   hhhhhhhhhhhhLLLLlllllllhhhhlhHH-HHHHHHHHHHLL-L--LE-EEHHHHHHHH-HL-L--L-H-HHHH-HHHHHHHHHL------llL-EEEElL--LLEEEELL-L-Ll---------------
0052 6j05A   ------------LLHHHHHHHH----hHHLL-HHHHHHHHHHHHHLL---L-EEHHHHHHH-HL-L--L-H-HHHH-HHHHHHHHLL--------L-EEEE-E--EEEEEELH-H-H-H-HHHHH--HHl---
0053 5ju7A   lleeeEE-EHEEELLEEE-----------LL-HHHHHHHHHHHHHLL--LLeELHHHHHHH-HL---lL-H-HHHH-HHHHHHHHHHH-L-LLL-L-EEEE-L--LLEEEELL-L------------------
0054 6ndlA   ------------------------------L-LLHHHHHHHHHHHL---L-LLHHHHHHHH-HL-L--L-H-HHHH-HHHHHHHHLL-------lE-EEEE-L--LLEEEEEE-----L-LLLL--lLH--HH
0055 4hw0A   ----------------------------lHH-HHHHHHHLLLL-----LE-ELHHHHHHHH-HL-L--L-H-HHHH-HHHHHHHHLL--------L-EEEE-EllEEEEEELHh-------------------
0056 8jxkD   -----------------------------LH-HHHHHHHHHHHHL----L-EEHHHHHHHH-HHlL--L-L-LLHH-HHHHHHHHLL--------L-EEEL-L--LLEEEELH-H------------------
0057 3r4kA   ----------------------------lHH-HHHHHHHLLLL--L--LE-EEHHHHHHHH-HL-L--L-H-HHHH-HHHHHHHHLL--------L-EEELlL--LLEEEEL---lL-H-HH-HH--HH--H-
0058 2ethA   lhhhhhhlLHHHH---------------hLL-HHHHHHHHHHHHH---LL-LLHHHHHHHH-LL-L--L-H-HHHH-HHHHHHHHLL--------L-EEEE-EllLEEEEELH-H-H-H-H-----hHH--HH
0059 2qwwA   hhhhhhhhhHHHHH--------------lLL-HHHHHHHHHHHHL---LL-EEHHHHHHHH-HL-L--L-H-HHHH-HHHHHHHHLL--------L-EEEL-LllLLEEEELH-H------------------
0060 2mlgA   ----------------------------llL-LHHHHHHHHHHHL---LE-EEHHHHHHHH-HL-L--L-H-HHHH-HHHHHHHHLL--------L-EEEE-EeeEEEEEELL-L------------------
0061 6qfdB   --------------lLLHHH--------lLL-HHHHHHHHHHHHL----L-EEHHHHHHHH-HHlL--L-H-HHHH-HHHHHHHHLL--------L-EEEE-E--EEEEEELH-------------hHH--HH
0062 3k69A   ----------------------------hHH-HHHHHHHHHHHLLL---L-LLHHHHHHHH-HL-L--L-H-HHLH-HHHHHHHHLL--------L-LEEE-L--LLEEELLL-----L-HHHLLh-------
0063 8qfcD   ----hhhhhhhhhhhhhhhhhhhhhhhhhHH-HHHHHHHHHHHHH---LE-ELLLHHHHHH-HL-L--L-H-HHHH-HHHHHHHHHLL------lL-EEEE-L--LLEEEELL-H-H-H-HHHHHh-------
0064 3g3zA   llhhhhhhHLLL-----------------lL-HHHHHHHHHHHHH---LL-LLHHHHHHHH-HL-L--L-H-HHHH-HHHHHHHHHL------L-L-EEEL-LhhHLLEEELH-H-H-H--------------
0065 7bzhA   -----------------------------LL-LHHHHHHHHHHHH---LE-EEHHHHHHHH-LL-L--L-H-HHHH-HHHHHHHHLL--------L-EEEE----LLEEEELL-L------------------
0066 2obpA   ----------------------------lLL-HHHHHHHHHHHHHLlllL-LLHHHHHHHH-HL-L--L-H-HHHH-HHHHHHHHLL--------L-EEEE-EllLEEEEEHH-H-Hh---------------
0067 3oopA   hhhhhhhhhHLLLL--------------lLL-HHHHHHHHHHHHH---LL-EEHHHHHHHH-HL-L--L-H-HHHH-HHHHHHHHLL--------L-EEEE-LllLLEEEELH-H-H-H--------------
0068 8f8uA   ----------------------------llL-HHHHHHHHHHL-LL--LL-LLHHHHHHHH-HL-L--L-H-HHHH-HHHHHHHHLLL-------L-LEEE-L--LLEEELLL-------------lHH--HH
0069 1yyvA   ----------lllllllLHHH----hhHHHHlHHHHHHHHHHHH--H-LL-EEHHHHHHHH-LLlL--L-H-HHHH-HHHHHHHHHL--------L-EEEE-EllEEEEEELH-H-H-H-H------------
0070 4hblD   hhhhhhhhhHHLLL--------------llL-HHHHHHHHHHHHL---LL-EEHHHHHHHH-HL-L--L-H-HHHH-HHHHHHHHLL--------L-EEEE-L--LLEEEEHH-H-H-H-HH-hH--HH--H-
0071 4yifB   ----hhhhhhLLLL--------------lLL-HHHHHHHHHHHHH---LL-LLHHHHHHHH-HL-L--L-H-HHHH-HHHHHHHHLL--------L-EEEE-E--LEEEEELHh-------------------
0072 2d1hA   hhHH-HH-HHHHHH--------------lLL-HHHHHHHHHHHHHL--LL-EEHHHHHHHH-HL-L--L-H-HHHH-HHHHHHHHLL--------L-EEEE-EllLEEEEELH-H-H-HHHHHh---------
0073 2l4mA   -------lLLLL----------------lLL-HHHHHHHHHHHHH---LL-EEHHHHHHHH-HL-L--L-LhHHHH-HHHHHHHLLL---------lEEEL-L-lLLEEEELL--------------------
0074 3mq0B   ----------------------------hHH-HHHHHHHHHHHHHLL--LL-EEHHHHHLL-LL-L--L-H-HHHH-HHHHHHHHLL--------L-EEEL-L--LLEEEELL---L-H-HH--H--HH--HH
0075 9c5gB   ----------------------------hHH-HHHHHHHHHHHHH-L--LL-EEHHHHHHH-HL-L--L-H-HHHH-HHHHHHHHHHL-----L-L-LEEE-L-lLLEEELLL-L-L-L--------------
0076 8r3gA   ----------------------------hHH-HHHHHHHHHHHHH---LL-LLHHHHHHHH-HL-L--L-H-HHHH-HHHHHHHHLL--------L-EEEE-L--L-EEEELH-H-H-Hl-------------
0077 3bpxA   ----lllhhhhHHHH-HHHHL--------lL-HHHHHHHHHHHLL---LL-LLHHHHHHHH-HL-L--L-H-HHHH-HHHHHHHHL-------L-L-EEEE-E--EEEEEELH-H-H-H--------------
0078 3f72B   -------hhhhhhhHHHHHHH----hhHHHL-HHHHHHHHHLLLLL---L-EEHHHHHHHH-HL-L--L-H-HHHH-HHHHHHHHLL---------lEEEE-EllLEEEEELL-H-H-H-HH----hHH--HH
0079 2pg4A   -----------------LLLH----hhHLLH-HHHHHHHHHHHHHH--hL-LLHHHHHHHH-HL-L--L-H-HHHHlLHHHHHHHLL--------L-EEEE-E--EEEEEELH-H------------------
0080 3gfiA   hhhhhhhhhHHHH---------------lLL-HHHHHHHHHHL--L--LL-EEHHHHHHHH-LL-L--L-H-HHHH-HHHHHHHHLL--------L-EEEE-EeeEEEEEELH-H------------------
0081 4nb5B   HHHH------------------------lLL-HHHHHHHHHHHHLL-lLL-EEHHHHHHHH-LL-L--L-H-HHHH-HHHHHHHHHL--------L-EEEE-LllLLEEEELL---L-H-HHHHHh-------
```

```
Job: Type-4 BREX; PglZ MBQ0159480 N-terminal iSTAND-NTPase (Type-4 BREX-specific Component)
Query: s001A
No: Chain Z rmsd lali nres id PDB Description
1: 6mdm-D 10.1 3.0 133 713 5 MOLECULE: VESICLE-FUSING ATPASE;
2: 8fcv-R 9.7 3.3 128 343 5 MOLECULE: DNA (60-MER);
3: 3te6-A 9.3 3.1 125 304 10 MOLECULE: REGULATORY PROTEIN SIR3;
4: 7tjj-I 9.1 3.2 117 380 7 MOLECULE: ORIGIN RECOGNITION COMPLEX SUBUNIT 1;
5: 7jgr-G 9.1 3.3 127 372 4 MOLECULE: ORIGIN RECOGNITION COMPLEX SUBUNIT 2;
6: 5zr1-A 8.9 3.4 126 494 2 MOLECULE: ORIGIN RECOGNITION COMPLEX SUBUNIT 1;
7: 8c0v-A 8.4 3.6 132 823 11 MOLECULE: PEROXISOMAL ATPASE PEX1;
8: 7jgs-C 8.4 3.7 121 614 9 MOLECULE: ORIGIN RECOGNITION COMPLEX SUBUNIT 2;
9: 7z6h-K 8.3 3.0 125 439 8 MOLECULE: CELL CYCLE CHECKPOINT CONTROL PROTEIN RAD9A;
10: 7st9-A 8.3 3.3 131 522 7 MOLECULE: CHECKPOINT PROTEIN RAD24;
11: 8tp1-A 8.3 3.0 121 419 12 MOLECULE: MITOCHONDRIAL CHAPERONE BCS1;
12: 5zr1-E 8.3 3.1 123 460 11 MOLECULE: ORIGIN RECOGNITION COMPLEX SUBUNIT 1;
13: 8umy-A 8.0 3.4 122 546 9 MOLECULE: CHROMOSOME TRANSMISSION FIDELITY PROTEIN 18 HOMOL
14: 7ykk-A 7.9 3.0 120 735 12 MOLECULE: ATPASE FAMILY GENE 2 PROTEIN;
15: 2qby-B 7.8 3.5 127 368 5 MOLECULE: CELL DIVISION CONTROL PROTEIN 6 HOMOLOG 1;
16: 6z1f-3 7.6 3.3 125 284 4 MOLECULE: RIBULOSE BISPHOSPHATE CARBOXYLASE/OXYGENASE ACTIV
17: 7w42-B 7.6 3.3 124 402 10 MOLECULE: UNCHARACTERIZED ATPASE YJOB;
18: 8dr5-A 7.6 3.6 124 646 5 MOLECULE: REPLICATION FACTOR C SUBUNIT 1;
19: 9lgo-B 7.6 3.7 128 701 9 MOLECULE: ATPASE FAMILY GENE 2 PROTEIN HOMOLOG A;
20: 8dyu-A 7.6 3.0 111 2562 5 MOLECULE: CYTOPLASMIC DYNEIN 1 HEAVY CHAIN 1;
21: 4ai6-A 7.6 3.0 119 2650 8 MOLECULE: GLUTATHIONE S-TRANSFERASE CLASS-MU 26 KDA ISOZYME
22: 7mi8-A 7.5 3.7 115 868 8 MOLECULE: FUSION PROTEIN OF DYNEIN AND ENDOLYSIN;
23: 8xku-B 7.5 3.2 125 845 9 MOLECULE: PROBABLE INACTIVE ATP-DEPENDENT ZINC METALLOPROTE
24: 7lcc-A 7.5 3.6 114 1369 10 MOLECULE: HELRAISER K1068Q;
25: 5e7p-A 7.4 3.7 130 719 5 MOLECULE: CELL DIVISION CONTROL PROTEIN CDC48;
26: 8xks-B 7.4 3.3 122 603 8 MOLECULE: CTAP1;
27: 6vvo-A 7.4 3.6 124 448 6 MOLECULE: REPLICATION FACTOR C SUBUNIT 1;
```

```
28: 4xgc-E 7.4 3.9 123 402 8 MOLECULE: ORIGIN RECOGNITION COMPLEX SUBUNIT 2;
29: 7jgr-D 7.4 4.0 126 441 6 MOLECULE: ORIGIN RECOGNITION COMPLEX SUBUNIT 2;
30: 8dgc-A 7.3 4.0 121 2028 7 MOLECULE: SEAVS3;
31: 8rwv-E 7.3 3.8 120 431 8 MOLECULE: DNA REPLICATION LICENSING FACTOR MCM2;
32: 7vcs-A 7.2 3.3 126 766 9 MOLECULE: TRANSITIONAL ENDOPLASMIC RETICULUM ATPASE;
33: 5g4f-A 7.2 3.7 130 726 8 MOLECULE: VCP-LIKE ATPASE;
34: 3bos-B 7.2 3.6 116 231 4 MOLECULE: PUTATIVE DNA REPLICATION FACTOR;
35: 8xku-F 7.2 3.2 123 478 10 MOLECULE: PROBABLE INACTIVE ATP-DEPENDENT ZINC METALLOPROTE
36: 7uqj-A 7.1 3.5 131 585 6 MOLECULE: ATPASE HISTONE CHAPERONE YTA7;
37: 8xku-A 7.1 3.3 124 730 8 MOLECULE: PROBABLE INACTIVE ATP-DEPENDENT ZINC METALLOPROTE
38: 7rzy-1 7.1 3.5 123 311 11 MOLECULE: TN6677 VIBRIO CHOLERAE TRANSPOSON TNSC (VCHTNSC);
39: 8r64-B 7.1 3.2 118 303 9 MOLECULE: FIDGETIN-LIKE PROTEIN 1;
40: 2gno-A 7.1 3.4 114 296 8 MOLECULE: DNA POLYMERASE III, GAMMA SUBUNIT-RELATED PROTEIN
41: 8zr0-A 7.0 3.5 117 480 2 MOLECULE: CASEINOLYTIC PEPTIDASE B PROTEIN HOMOLOG;
42: 9fnn-U 7.0 2.8 113 511 9 MOLECULE: CELLULOSE SYNTHASE CATALYTIC SUBUNIT [UDP-FORMING
43: 8k8v-A 7.0 3.4 114 719 6 MOLECULE: ENDOPEPTIDASE LA;
44: 8bob-C 7.0 3.7 109 412 7 MOLECULE: PROTEIN MALY;
45: 8xku-D 7.0 4.0 122 805 8 MOLECULE: PROBABLE INACTIVE ATP-DEPENDENT ZINC METALLOPROTE
46: 5j1s-A 7.0 3.6 124 277 9 MOLECULE: TORSIN-1A;
47: 8fnu-A 7.0 3.5 122 796 6 MOLECULE: KAP NTPASE DOMAIN-CONTAINING PROTEIN;
48: 1xwi-A 7.0 3.8 126 322 6 MOLECULE: SKD1 PROTEIN;
49: 5v8f-D 7.0 3.6 118 456 11 MOLECULE: DNA REPLICATION LICENSING FACTOR MCM2;
50: 6p8v-C 6.9 3.5 124 303 8 MOLECULE: ATPASE, AAA FAMILY;
51: 7qo4-M 6.9 3.2 122 421 7 MOLECULE: 26S PROTEASOME REGULATORY SUBUNIT RPN1;
52: 6nyy-C 6.9 3.6 124 491 10 MOLECULE: AFG3-LIKE PROTEIN 2;
53: 6pdw-D 6.9 3.5 125 299 9 MOLECULE: MEMBRANE-SPANNING ATPASE-LIKE PROTEIN;
54: 7qo4-K 6.9 3.2 119 394 7 MOLECULE: 26S PROTEASOME REGULATORY SUBUNIT RPN1;
55: 8xks-D 6.8 3.3 121 971 8 MOLECULE: CTAP1;
56: 8xku-C 6.8 3.5 130 921 9 MOLECULE: PROBABLE INACTIVE ATP-DEPENDENT ZINC METALLOPROTE
57: 6rla-A 6.8 3.5 116 2750 7 MOLECULE: O6-ALKYLGUANINE-DNA ALKYLTRANSFERASE MUTANT,DYNC2
58: 8ui7-C 6.8 3.4 113 339 9 MOLECULE: ATPASE FAMILY AAA DOMAIN-CONTAINING PROTEIN 5;
59: 7mi1-A 6.7 3.0 120 2628 5 MOLECULE: CHIMERA PROTEIN OF DYNEIN AND ENDOLYSIN;
60: 5nug-A 6.7 3.1 112 2920 7 MOLECULE: CYTOPLASMIC DYNEIN 1 HEAVY CHAIN 1;

0001 s001A   MLFW---SFSQLTQE-I-ER-D-K-DS---RD-ML---AQRYAVRFIML-N-NF-NE-F-KEL-AKFMTN-I---G-------V-DML-D-LENL--IDEDDTW--ITKDTLKDAIK-ACT-----K-STFVTPF-S--E-VAR--F-YN--DDDFRGFFNEI-M-L-L-E--D--IH--NP-N--KRIYIPLIG-LQ-NRFTDFLNHFARI-KES--A-P-VWRYD-------------------------
0002 6mdmD   kwgD---PVTRVLDD-G-EL-L-V-QQ-tkNS-DR---TP-LVSVLLEG-P-PH-SG-KtALA-AKIAEE-S---N--------fIKI-C-SDKM--I---GFS--EKCQAMKKIFD-DAY-ksqlS-CVVVDDI-E--R-LLD--Y--F--SNLVLQALLVL-L-K-K-A--P-pQG--RK----LLIIGTTS--RK-DVLQEME-----mlnaF--S-T-TIHVPniatgeqllealellgnfkdkertti
0003 8fcvR   vahP---RLKEVYEI-L-MR-T-I-AE-----------PAGASFIFVYG-A-SG-VG-K-TTL-RLRVEQ-K---LsdrarvpV-VGI-E-AI------aPESR-yFNWKEYYTR-----P-----D-VFFVDEA-Q----HFG--K-VS--GYKLQDQLDCL-K-SlA-N--M--T--------gILHCLLGTY-EL-LTFRNL---SGQL-SRR--S-V-DIHFRrycadspedvqafksvlltfqqhlpl
0004 3te6A   sqvE---DFTRIFLP-I-YD-S-L-XS---S----------qNKLFYITNaD-DS-TK-F-QLV-NDVXDE-L---IkelpifdY-IHI-D-ALEL-------------XDALYEKIW-FAIakkrkT-LILIQNP-E--N-LL-----------SEKILQYF-E-K-WiS--S--KN--SK----LSIICVGGH-NV-T-IREQINIXPSL-KAH--F-T-EIKLNkvdknelqqxiitrlksllkpfhvkv
0005 7tjjI   ------pARTAEYEQ-V-MN-F-L-AI---SE-H------RSDSLYITG-P-PG-TG-K-TAQ-LDMIIR-Q---KlswfelpdvTSI-N-CISL--G---------EPSSIFQKI----T-----F-VVVLDEM-D----RLL--H-AS--ETQSVRTILEL-F-L-LaK--L--PT--VS----FVLIGMANS-----------------GLL--P-Q-TIVFQpytaeqmyeiviqkmsslptiifqpm
0006 7jgrG   ------pGRESQLQE-L-RE-F-F-SN-hlES-QT------SGSLYVSG-Q-PG-TG-K-TAC-LSLLLR-D----dfskrlqR-VYI-N-CTSI-----------ASVGAVYKKL----M-----L-LLVLDEI-D--Q-LC---------TSRQ-EVLYTI-F-E-WpA--L--PG--SR----ILLVGIAN--SL-DLTDRALMRLNAR-CEL-kP-R-LMHFPpyskqqiveifksrlaeaevldvfpp
0007 5zr1A   pvirk-pARENEFAS-I-YL-S-A-YI---ES-D------saTTIYVAG-T-PG-VG-K-TLT-VREVVK-E---L--lsssaqyVEI-N-GLKM--------V--KPT-DCYETLW-N-T-----I-VVLLDEL-D--A-MVT--K-S-------QDIMYNF-F-N-WtT--Y--EN--AK----LIVIAVAN--TM-DLPERQL-GNKIT-SRI-gF-T-RIMFTgytheelkniidlrlkglndsffyvd
0008 8c0vA   ggsll-iTVNSIKKE-M-VN-Y-L-TS---PI-------IATPAIILDG-K-QG-IG-K-TRL-LKELIN-E---VekdhhifV-KYA-D-CET-------lHE--TSTQKLIMEWC-SFCywygpS-LIVLDNV-E--A-LFG--K-Pq-qWDNASKLLNFF-I-N-QkI--F--NK--DN-K-rIRVLFSGKQ--K-TQINPLL--FDKH----fvS-E-TWSLRapdkharaklleyffsknqimklnrd
0009 7jgsC   ------rSYARTLEQ-L-VD-F-V-VQ---AE-R-----eVLPTAALLT-G-NQ-PD-H-LSQ-FTALTQ-R---LhaqraamV-CVL-Q-SRD-----------cATLKAAVETL-----serrqL-VVILPDF-E--C-FN-------------asVLQDL-I-L-IaH--C--GS--LP----FVLVLGVAT-AM-TAVHGTL-PYHVS-SKI--RlR-VFQTQaaptglnevldkvllspkyafhlsgk
0010 7z6hK   vhkK---KIEEVETW-L-KA-Q-V-LE---RQ-PK-----QGGSILLIT-G-PC-GK-T-TTL-KILSKE-H---G--------I-QVQ-E-WI-N--PVLP------QIAVFKEFLL-RATdlrtdK-KIIL--V-E--D-LpnqfY-RD--SHTLHEVLRKY-V-R-I-G--R-------------CPLIFIISsgQ-RLL--FPKEQEEC-------sI-SNISFnpvaptimmkflnrivtieankngk
0011 7st9A   ------aIHKRKLKD-V-QE-A-L-DF---LP-N-----AKHRILLLSG---PS-GC----SK-STVIKE-L---SqnsngtsfkVTE-F-RGDC--IVN-dLP--QM-ESFSEFLK-GARvmsnlS-LILIEDL------PNV--F-HI---DTRRRFQQLI-L-Q-WyS--S--EP--LL----PPLVICITEfSA-ETI--MNKEI-lM-HPR--L-K-RIKFNpinstllkkhlkficvqnmkmlkekn
0012 8tp1A   lqqG---LADRIVKD-I-RE-F-I-DN--pKW-YI--rgIPYRRGYLLY-G-GC-GK-S-SFI-TALAGE-L---E--------H-SIC-L-LS------ltdSS--LSDDRLNHLLS-VAP-----Q-QSLVLLE-D--V--dA--A-FLlgrlTFSGLLNAL-D-A-S-T--E------------ARIVFMTTN-YI-DR-LDPA---------lirpvD-LKEYVgycshwqltqmfqrfypgqapslaen
0013 5zr1E   ------aFREYQTNC-L-AS-Y-I-SA---DP-DI---T--pSNLILQG-Y-SG-TG-K-TYT-LKKYFN-A-nlH--------A-VWL-E-PVEL-----------VSWKPLLQAIA-RTVqektcL-FLILDGF-D--S--LQ--D-L------DAALFNKY-I-K-L-N-lpkdSK--IN----IKFIYTMLE--T-SFLQRYST-------hC--I-P-TVMFPrynvdevstilvmsrcgelmedsclr
0014 8umyA   ------lSDDFTNRC-L-LK-W-L-KLweaGL-DP--qRPKQKVALLCG-P-PG-LG-K-TTLaHVIARH-A-gyS--------V-VEM-N-ASDD-----------RSPEVFRTRIE-AATaggkpN-CLVIDEI-D--G-A-------------PVAAINVL-L-S-I-L--nrkGL--LM----RPIICICN--DQ-FAP--RQLK------qQ--A-F-LLHFPptlpsrlvqrlqevslrqgmradpgv
0015 7ykkA   gldK---EIESLKSA-I-EI-PlH-QP---TF-GV---SP---pRGILL-HgKT-ML-L-RVV-ANTS-------N--------A-HVL-T-IN--------gPS--IVEAALRDIFN-EAY-----Q-PSII-FI-D--E--ID--S-IE--SRVVATLLTLM-D-G-M-G--A---------A--GKVVVIAAT--P-NSVDPAL------rrpG--R-F-DQEVEigipdvdarfdiltkqfsrmssdrhv
0016 2qbyB   ------pFREDILRD-A-AI-A-I-RY-fvKN-EV---K---FSNLFLG-L-TG-TG-K-TFV-SKYIFN-E---IeeykdvkQ-AYV-NcREVG-----------GTPQAVLSSLA-GKLtrnirA-IIYLDEV-D--T-LVK--R-R-----GGDIVLYQL-L-R-S-D--A-----------nISVIMISN--DI-NVRDYME-PRVL--SSL--G-P-SVIFKpydaeqlkfilskyaeyglikgtydd
0017 6z1f3   ------yiAPRFLDK-L-AV-H-I-TK---nG-VR---V--PLILGIHG-R-KG-EGkT-FQC-ELAFEK-M--gI--------E-VTL-I-S----------ggelesPARLIRLRYR-ETAvrgkmC-VLMINDL-D--A-GAG--R--Y--TVNTQLVNATL-M-A-D-N--P--TP--IR-R--VPIIVTGN--DF-ST--LYAP--liR-DGR--M-E-KFYWEpnrddkvgivggifaedglsqreieq
0018 7w42B   svyay-iMNPLLKKE-I-YR-S-I-DQ-ffHS-DK--dipYKRGILLYG-P-PG-NG-K-TTL-VKSIAG-S---I---------pVAY-W-Q---------itEF--TSSETIEEVFQ-AAR-rlapA-VLVIEDI-D--S--MP--E-D-----VRSFFLNTL---D-G-A--T--SK--EG----LFLIGTTN--YP-EEIDPG----lmN-RAGrfD-R-AYEIGlpdeelrleymkmrgfgiflsegeik
0019 8dr5A   qqvC---GNKGSVMK-L-KN-W-L-AN--wEN-SKk-gSGVFRAAMLYG-P-PG-IG-K-TTA-AHLVAQ-E-gyD--------I-LEQ-N-ASDV-----------RSKTLLNAGVK-NALlngkhF-VIIMDEV-D--G--MS--G-G---drGGVGQLAQF-C-R-K-T--S------------TPLILICN--ER-NL-PKMR-----pfdRV--C-L-DIQFRrpdansiksrlmtiairekfkldpnv
0020 9lgoB   phllt-gGLESIKLK-L-EQ-A-VeWP---LKhIQ---PP---kGVLLY-G-PG-CS-K-TMI-AKALANeS---G--------L-NFL-A-I---kgpelMNKYvgESERAVRETFR-KAV-----A-PSIIFFD-Q--L--dA--L-AV-vADRVLAQLLTE-M-D-G-I--E--Q----L-K--DVTILAATN-RP-DR-IDKA---lmR-PGR--I-D-RIIYVplpdaatrreifklqfhsmpvsnevd
0021 8dyuA   vqtP---LTDRCYLT-M-TQ-A-L-EA----------------rlGGSPF-G-PA-GT----GK-TESVKA-L---G--hqlgrF-VLV-F-N---------cdeT--FDFQAMGRIFV-GLG-----A-WGCFD-E-F--N-R-L--E-ER----MLSAVSQQV-Q-C-I-Q-nkqvKV--SP-D--MAIFITMNP---rsNLPDN---------lkK--L-F-RSLAMtkpdrqliaqvmlysqgfrtaevlan
0022 4ai6A   ADRLv-vDHILRIDR-A-LK-Q-V-------------------qGHMML-I-GT-GK-T-ILT-RFVAWL-N---G--------L-KIV-Q-PK------ihRHS--NDFDMILKKAI-SDE-----S-RTCLI-I-D--E-SNI--L-ET----AFLERMNTLlA-N-A-D-elydWF--VG-EiaKNLHVVFTIptS-SAMISSP-------------anR-CIINWmgdwdtktmsqvannmvdvipmeftd
0023 7mi8A   iytP---LLLIGFAT-L-TD-S-L-HQ---------------kyGGCFFG---PA-GT----GK-TETVKA-F---G-qnlgrvV-VVF-N-CDD----------S--FDYQVLSRLLV-GIG-----A-WGCFDQF-N--R---L--D-EK----VLSAVSANI-Q-Q-InG--L--QVglSP-H--TAVFITLNP-GR-SEL--------penlkKS--F-R-EFSMKspeeysiigkhsvkifsmlekfgqfh
0024 8xkuB   vlgG---DVWDLLDE-L-MI-Y-M-GN-yyEK-DV---A---fvRGVLL-S-GT-GK-T-LFA-RTLAKE-S---G--------L-PFV-F-A---------sgAE--FTAAKINEMFS-IAN-----A-PAFVFVD-E------ID--A-IR--RRATFEALIAQ-L-D-G-E-gI--DR--FS-L-rQAVIFICAT--P-DELDLEF-------vrsG--R-I-DRRLYiglpdakqrvqifgvhsagknlaedi
0025 7lccA   slnD---EQLAAFQT-I-TS-A-I-ED---QT-VH------PKCFFLDG-P-GG-SG-Q-TYL-YKVLTH-Y---I-rgrggtV-LPT-A-ST--------------afHSQYKlpipIKK-----AqLLIIDEC-T--M-----------------aSSHAINAI-D--rleI--M--NL-afG-G--KVLLLGGD--FR-QClsiSLKYCNVW-G-C--F-R-KLSLKtnmrsedsaysewlvklgdgkldssf
0026 5e7pA   llrgv-gDMVETKRA-L-TEaV-L-WP----qhID---PP--RGVLLYG-P-PG-CG-K-TFV-VRALAS-S---G---------sVHA-V-KSEL--M-dKWVG--SSEKAVRELFA-RAR-dsapS-LVFLDEI-D--A-LAP-----V--TDKVVASLLTE-L-DgI-E--P--L----R-D--VVVLGATN--RP-DL-IDPA---llR-PGR-lE-R-LVFVEppdaaarrdilrtagksipladdvdl
0027 8xksB   egnK---QAKVELME-V-VD-F-FrTP---EK-GA---RA--pKGVLLV-G-PPgNG-K-TLM-ARAVAG-E--sG--------V-AFI-S-S---------saaefieGAARVRDLFN-TAV-----A-PCIIFID-E------LD--A-VE--RDNTVNQLLTE-M-DeA-E--Q--Q---------GIVVMGATN-RK-DV-LDAA---ltR-PGR---fD-RSIEVrrpdfqgrleaslaslmggmsgaqia
0028 6vvoA   ktiI---GQQSCANK-L-LR-W-L-RN--wQK-SS---dgSSFKAALLSG-P-PG-VG-K-TTT-ASLVCQ-E-gyS--------Y-VEL-N-ASDT-----------RSKSSLKAIVA-ESLsvstkH-ALIMDEV-D--G-M-A--G-NE--drGGIQELIGL-I-K-H-T--K------------IPIICMCND-RN-HPKIRS-------lvhY--C-F-DLRFQrprveqikgammsiafkeglkipppa
0029 4xgcE   ------pCREAAIET-L-GE-L-IgDS---SE--------TYPSAIYLFG-H-SG-TG-KtALT-RAFLKE-C---G-krqnvrT-AHL-N-AIEC-----------YTTKIMLEILL-D--vedqgF-LIAVDNA-E----RLR--D-M------DANVLPVL-L-R-L-Q--E-lTN--LN----LCVILLSQL-PF-EKF--YNKTG----lsE--I-V-CLHLAqynkaetqrilgsdfqqvrnqlleqk
0030 7jgrD   ------rGYAKERSN-V-RL-L-L-QA---EM-G-------ESNSLLLLG-P-RG-SG-K-TTL-INSVLA-D---LsfgentlI-VHL-D-GNLH-----------TDDRVALKSIT-V--khsksV-IFILEEF-D--L-FCA---------hHNQTLLYNL-F-DvS-Q--S--AQ--AP----ICVLGVT-C-RL-D-VIELL--EKRVkSRF--ShR-QVFLFpslrrfedyvdlcrdllslptgnsll
0031 8dgcA   hiegq-vSRTQ-EAT-L-VQ-R-V-VE-----------AFGA-PVIIHA-D-AG-VG-K-SIF-STHIEE-H---L--ptgsvS-ILY-D-CFGlgqYRNASSY--RHHRTALVQ-----aeplavL-CIIIDAA-D--N-AQM--A-AE-gETRS--FIKDL-I-R-E-K--L------PD-G--VCLVALCR---P-YRRE-----ldpP-PEA----L-TLSLQtfnrdetaahlhqkfpdasesdvdef
0032 8rwvE   ------lCRESQVSI-L-QS-L-F-GE---RH-HF---S--FPSIFIYG-H-TA-SG-K-TYV-TQTLLK-T---L----elpH-VFV-N-CVEC-----------FTLRLLLEQ------lkdqtV-YIVLDKA-E----YLR--D-M-----EANL-LPGF-L-R-L-Q---elAD--RN----VTVLFLSEI-VW-EKF--RPNTG----cfE--P-F-VLYFPdysignlqkilshdhppeysadfyaa
0033 7vcsA   qvane-gGLEDVKRE-L-QE-L-V-QY---PehMT---PS--KGVLFYG-P-PG-CG-K-TLL-AKAIAN-E-cqA--------N-FIS-I-K------gpelLT--MWEANVREIFD-KAR--qaaP-CVLFFDE-L-----DS--I-Aa-aADRVINQILTE-M-D-G-M--S--TK--K--N--VFIIGATN--RP-DI-IDPA---ilR-PGR-lD-Q-LIYIPlpdeksrvailkanlrkspvakdvdl
0034 5g4fA   eemad-gGLEDVKRE-I-KE-T-V-EL---PlkIR---PS--KGFLLYG-P-PG-VG-K-TLL-AKAVAT-E---S---------nFIS-I-KEVL--S-kwVGE--SE-KAIREIFK-KAK-qvapA-IVFLDEI-D----SIA--P--V--TERIVNQLLTS-L-DgI-E--V--MN--G-----VVVIGATN--RP-DIMDPAL----lR-AGR-fD-K-LIYIPppdkearlsilkvhtknmplapdvdl
0035 3bosB   ypaA---GNDELIGA-L-KS-A-A-SG-------------dgvQAIYLWG-P-VK-SG-R-THL-IHAACA-R---A-nelerrS-FYI-P-LGIH--A-----S--IST-ALLE----LEQ-----FdLICIDDV-D--A--VA--G-H---PLWE-EAIFDL-Y-NrV-A--E--Q----K-R--GSLIVSASA-SP-XEAG----ldlvsrxhW--G-L-TYQLQpxxddeklaalqrraaxrglqlpedv
0036 8xkuF   aglG---KIRLELEE-I-VK-F-FtHG---EM-GV---KI--pGGILLC-G-PG-VG-K-TLL-AKAVAG-E--aG--------V-NFF-S-I---------sasqfveGASRVRALYQ-EAN-----A-PSVVFID-E------LD--A-VE--RDATLNQLLVS-L-D--------gfEG--RG----EVITIASTN-RP-DILDPAL----vR-PGR---fD-RKIFIpkpgligrmeilqvharkkpmaedld
0037 7uqjA   ddiG---GLDNYIDQ-L-KE-MvA-LP---L-lNI---TP-pRGVLFHG-P-PG-TG-K-TLM-ARALAA-S---C-ssderkI-TFF-M-R-------kgaDIL--SAERQLRLLFE-EAH-----Q-PSIIFFD-E--IdGLA--P--I--HASIVSTLLAL-M-D-G-M--D--NR--G-----QVIVIGATN-RP-DAVDPAL----rR-PGR-fD-R-EFYFPlpdvkarfkilqiqtrkwssplstnf
0038 8xkuA   agqE---YIKRELQE-I-VR-I-LkND---EE-GI---YC--pKGVLLH-G-GT-GK-T-LLA-KAIAGE-A---G--------L-PFF-A-A---------ngtdfveAASRVKDLFA-SSY-----A-PSIIFID-E------ID--A-IR--EQGLLQILTEM-D-G-F-K--V--TT--S-----QVLVIGATN-RL-DI-LDPA-----llRKG--RfD-KIIRVglpskdgrlailkvharnkffrsede
0039 7rzy1   vstP---SVRKILSY-M-DR-C-R-DL---SD-ES---EP--tCMMVYG-A-SG-VG-K-TTV-IKKYLN-Q---NaggdiipV-LHI-E-LP------------dnAKPVDAARELL-V-V-----K-LIIIDEF-Q----HLV--E-E-rsnrvLTQVGNWL-K-MiL-N--K--T--------kCPIVIFGMP-YS-KVVLQA---NSQL-hGR--F-SiQVELRpfsynggrgvfktfleyldkalpfek
0040 8r64B   agvE---FAKATIKE-IvVW-P-M-LF---TG-LR---G---ppKGILL-F-GT-GK-T-LIG-KCIASQ-S---G--------A-TFF-S-IS--------aSS--LTEKMVRALFA-VAQ-----Q-PAVIFIdqI--D-SLL-----S--SRRIKTEFLVQ-LdT-T-S--S---------E--DRILVVGAT--P-QEIDEAA--------------rrlVKRLYiplpeasarkqivinlmskeqcclse
0041 2gnoA   ----------DQLET-L-KR-I-I-EK-------------SEGISILING-E-DL-SY-P-REV-SLELPE-Y---V--------EdVLE-I-DPE-----------geNIGIDDIRTIK-DFLelytrK-YVIVHDC-E----RXT--Q--------QAANAFLKA-L-E-E-P--P-------E-Y--AVIVLNTR--RW-HYLLPT--------iksR--V-F-RVVVNvpkefrdlvkekigdlweelpllerd
0042 8zr0A   gcsal-iGQEGAINT-V-AS-A-I-RR-ngWY-DE----eHPLVFLFLG---SS-GI----GK-TELAKQ-V---AhkdikkgF-IRM-D-M--------------seEVAKFILT-----S-----A-VVLFDEV-E--K-A-------------HPDVLTVM-L-QlF-D--E--GE--CK-D--AIFIMTSNA-AirPILKAHF-RRDEF-lGR--I-NeIVYFLpfchsellqlvsrelhywakkakqrh
0043 9fnnU   -----pvfsislwDE-L-RH-M-P------------------agGVWWFNV-D-RH-ED-A-ISL-ANQTIA-S---Q--aetahV-AVI-S-MDSdpkiklfsmL--NHEKGLYYL-T-RDLidphnY-LFILVC---anN-AWQ--N-I------PAERLRSW-L-M-N-K--W-sRL--NH----CSLLVINPG--N-DKQFSLLLE---E-YRS-lF-G-LASLRfqgdqhlldiafwcnekgvsarqqls
0044 8k8vA   ppfF---GQERAREA-L-EL-A-I-RG---------------gfHAYLVG-P-PS-LG-KhEAL-LAYLST-QsgtP--------P-PIV-Y-E-----------------TAPRLFGAVH-RAQ-----G-GYLIL-D-A--L-SLK--R-EG----TWEAFKRAL-R-N-G-Q------vevepfpiqMQVILVGTPeAF-EGLE--EDPA---fsEL--F-RiRAEFSptlpaspenctalggwllaqgfqltq
0045 8bobC   ------vvRERLLAK-L-SG-A-N-----------------nFRLALITS-P-AG-YG-K-TTL-ISQWAA-G---K-----ndI-GWY-S-LD-----------egdNQQERFASYLI-AAVaewhsplYLVIDDY-H--L-I----T-NP--V-IHESMRFFI-----------------qPE-N--LTLVVLSRN-L--------GIAN--lR-VRD-qL-L-EIGSQqlafthqeaneffdcrlsspieaaes
0046 8xkuD   npvkpqmieQPGTIY-L-RY-L-V-DI-kkYL-MNytitypSRSILVIG-S-IG-TG-R-SYL-VKYLAT-N---S----yvpF-ITV-F-LNKF--LDNK-----dDRFYITLQFE-LAP-----C-IIWIPNI-H--D-L----------sylalGLLVNS-L-S-R-D--CerCS--TR-N--ILVIASTH--IP-QK-VDPA---liA-PNK-lN-T-CIKIRrllipqqrkhfftlsytrgfhlekkm
0047 5j1sA   gqhL---AKKIILNA-V-FG-F-I-NN---P--------KPKK-PLTLSL-H-GT-GK-N-FVS-KIIAEN-I---Y---------dYVH-L-F-vatlhfpHASN---YKDQLQLWIR-GNC-----A-RSIFI-F-D--Q-MdK--M-HA---GLIDAIK-PF-L-D-Y-Y--D--L---vdgvsyqKAMFIFLS-NAnnknSGFWH-SSLI-DRN--L-I-DYFVPflpleykhlkmcirvemqsrgyeide
0048 8fnuA   ------yIFSSQLDK-V-NL-I-L-EH-mdTV-GG----iHSRNIAITG-D-TG-KT---SFI-ETLKLV-L---E--kqnyyV-FDIvS-PTV----------lsSHLN-ILEIVisiqD-----L-VLIIDDL-D--L---V--E-NN----LVYDLLRDI-Q-L-D-S--Q------------LIVIFAYK---E-GQLEQSMFIERFL-TKL--VsN-RIPLFkqdellnktigeflasldpsygvgen
0049 1xwiA   ------aGLEGAKEA-L-KE-AvI-LP---I-kRT---PW--RGILLFG-P-PG-TG-K-SYL-AKAVAT-E-anN--------S-TFF-S-IS--ssdlVSKWL---SEKLVKNLFQ-LAR--enkP-SIIFIDE-I--D-SLC--G--A--ARRIKTEFLVQ-M-Q-G--------D--ND-G--ILVLGATNI--P-WVLDSAI--------rrrfE-K-RIYIPlpepharaamfklhlgttqnsltead
0050 5v8fD   ------tYLQDCQQE-I-DR-I-I-KI---IQ-K------esHSVILVG---PR-QS----YK-TYLLDY-E---L----sllqiTIR-L-N---------gfI--HSEQTAINGI----T-----V-VFIFDEI-D----TFA--G-----PVRQ-TLLYNL-F-DmV-E--H--SR--VP----VCIFGCTTK-LN---ILEYLEKR-vK-SRF--SqR-VIYMPqiqnlddmvdavrnlltvrseispwv
0051 6p8vC   arlV---GLDEHKDR-L-SK-I-L-GL-hpdD-TV---LRRPPLVVLAG-D-VG-SG-K-TELaETIGDA-V-idI-------T-LYP---LSLA--------trgqXTQLVSAAFD-YTIkkargavLLLIDQA-D--A-LAQ-----E--DRAGVNAFIRG-I-D-RiA--N--QK--L--P--AAVLXCTN--RL-KALDPA--------vqrraA-E-ILTFSrpndeqrhyllhskltglglnstave
```

```
0052 7qo4M   gglD---KQIEELVE-AiVL-P-MkRA---DK-GI---RA--pKGALMY-G-GT-GK-T-LLA-RACAAQ-T---N-------A-TFL-K-L--------aaPQ--LVAKLVRDAFA-LAK---ekA-PTIIFID-E-----LD--A-I-revqRTMLELLNQ-L-D-G-F--S-------sdD--RVKVLAATN-RV-DV-LDPA-----llRSG--RlD-RKIEFplpsedsraqilqihsrkmttdddin
0053 6nyyC   ------aGCEEAKLE-I-ME-F-V-NF----knAK---IP--KGAILTG-P-PG-TG-K-TLL-AKATAG-E---A----nvpF-ITV-S-GSEF--LEM---fvgVGPARVRDLFA-LAR-knapC-ILFIDQI-D--A-VGR------seqeNTLNQLLVE-M-DnT-T--T--N---------VVILAGTN--RP-DI-LDPA---llR-PGR-fD-R-QIFIGppdikgrasifkvhlrplkldstlek
0054 6pdwD   ------gGLDDIIEE-L-KEtI-I-YP-mphG-AL---LA-apSGVLLY-G-PG-CG-K-TML-AKAVAHeS---G-------A-SFI-NlHIST---------lteSNKIVRAVFS-LAL-----Q-PSIIFIDeI--D-AVL--G--A--SGMVKAEFMTL-W-D-GtS--T--NA-gVP-N--RIVVLGATN-RI-NDIDEAI-----------lmP-KQFPVplpgleqrrrilelvlrgtkrdpdfd
0055 7qo4K   gglD---MQKQEIREaV-EL-PlV-QA---DG-ID---P----pRGVLL-Y-GT-GK-T-MLV-KAVANS-T---K-------A-AFI-R-V--------ngSE--FVPRMVRDVFR-LAN-----A-PSIIFID-E-----VD--S-IE--VQRILIELLTQ-M-D-G----------fdQ-S--TNVKVIMAT---aDTLDPAL------lrpG--R-L-DRKIEfpslrdrrerrlifgtiaskmslape
0056 8xksD   vqlld-aGMEFLVTE-M-RE-I-V-RM-gdeG-AK---C---pKGIIFQ-G-PG-TG-K-TYL-ARAIAG-E---A--------pFFS-S-V---------gsefveAAARVNSLFY-NAA-----P-AIIFIDE-I--D-AIG--R--E--RESALLAMLVQ-M-D-GiA--N--KT--E-----QVLTIGATN-LA-QE-LDAA-----llRPG--RfE-VVYEVpqpgpsarmailryhakgkplegdgq
0057 8xkuC   knfA---SIESMREE-I-NE-V-V-AF-npkG-AR---A--pRGVLIVG-E-RG-TG-K-TSL-ALAIAA-E--aR-------V-PVV-N-VE--------aQE--LEAANVRELFQ-TAL-----A-PVIIFVE-DfdL-FAGvrgqQD--HESFINQLLVE-L-D-G-F--E--K----Q-D--GVVLMATTR-NH-KQI---DEAL-rR-PGR-mD-R-VFHLQsptemererilhnaaeetmdrelvdl
0058 6rlaA   vytP---LTDKCYLT-L-TQ-A-M-KM--------------glGGNPY-G-PG-TG-KtESV-KALGGL-L--gR-------Q-VLV-F-N--------cdEG--IDVKSMGRIFV-GLC-----G-AWGCFDE-F--N---R--L-EE---SVLSAVSMQI-Q-T-IdA--L--KNhvNS-N--SGIFITMNP---rqKLPDN--------lkQ--L-F-RPVAMshpdneliaevilysegfkdakvlsr
0059 8ui7C   ------iSHQDILST-I-QK-F-I-NE---D---------rlpHLLLYG-P-PG-TG-K-TST-ILACAK-Q---LdkefgsmV-L-EL-N-ASDD-----------RGIDIIRGPIL-SFAfkkgfK-LVILDEA-D--A-M------------TQDAQNAL-R-R-VkF--T--EN--------TRFCLICN--YL-SKI--IPAL-----qsR--C-T-RFRFGpltpelmvprlehvveeekvdisedg
0060 7mi1A   ADRLv-vDHILRIDR-A-LK-Q-V-----------------qgHMMLI-G-AT-GK-T-ILT-RFVAWL-N---G-------L-KIV-Q-PK------ihRHS--NDFDMILKKAI-SDC-slkeS-RTCLIID-E--S-N-I--L-ET----AFLERMNTL-L-A-N-AdwF--VG--EiaK--NLHVVFTICptS-SAMISSP---------alfN-R-CIINWmgdwdtktmsqvannmvdvipmeftd
0061 5nugA   RGMLhmeqpLDLTHI-V-GT-E-----------------vkpNTPVLMC-S-VY-DA-S-GHV-EDLAAE-Q---N-------T-QIT-S-I-------aigsA--EGFNQADKAIN-TAS-----G-RW-VMLK-N--V--hL--A-PG----WLMQLEKKL-H-S-L-Q--P-------H-A--CFRLFLTME-IN-PKVPVN----------llrA-G-RIFVFepppgvkanmlrtfssipvsricksp

0001 s001A   LEEL---LHHHHHHHH-H-HH-H-H-HL---LL-HH---HHHLLEEEEEEE-L-LH-HH-H-HHH-HHHHHH-L---L-------L-EEE-E-HHHH--LLLLLLL--LLHHHHHHHHH-HLL-----L-LEEEELL-H--H-HHL--L-LL--HHHHHHHHHHH-L-L-L-L--L--LL--LL-L--LLEEEEEEEL-LH-HHHHHHHHHLLLL-LLL--L-L-LEEEL------------------------
0002 6mdmD   lllH---HHHHHHHH-H-HH-H-H-HH-hhHL-LL---LL-EEEEEEEL-L-LL-LL-HhHHH-HHHHHH-H---L---------lEEE-E-LLLL--L---LLL--HHHHHHHHHHH-HHH-llleE-EEEELLH-H--H-HLL--L--L--LHHHHHHHHHH-H-L-L-L--L-lLL--LE----EEEEEEEL--LL-LLHHHLL-----llllL--L-E-EEELLllllhhhhhhhhhhhllllhhhhhhh
0003 8fcvR   lllH---HHHHHHHH-H-HH-H-H-HL-----------LLLLLEEEEEE-L-LL-LL-H-HHH-HHHHHH-H---HhlllllllE-EEE-E-LL------lLLLL-lLLHHHHHHH-----L-----L-EEEEELH-H----HHH--L-LL--LLHHHHHHHHH-H-HhH-H--H--H--------lLEEEEEELH-HH-HHHLLL---LHHH-HHL--E-E-EEEELllllllhhhhhhhhhhhhhhhllll
0004 3te6A   hhhH---HHHHHHHH-H-HH-H-H-HL---L----------lLLEEEEELlL-LH-HH-H-HHH-HHHHHH-H---HllllleeE-EEE-E-LLLL-------------LHHHHHHHH-HHHhhlleE-EEEEELL-L--L-LL------------LLHHHHHH-H-H-HhH--L--LL--LL----EEEEEELLL-LL-L-LHHHHHLLHHH-HLL--E-E-EEELLlllhhhhhhhhhhhhhhhllleeeee
0005 7tjjI   ------lLLHHHHHHH-H-HH-H-H-HH---LL-L------LLLEEEEEE-L-LL-LL-H-HHH-HHHHHH-H---HeeeelllleEEE-E-LLLL--L----------LHHHHHHHH----E-----E-EEEEELH-H----HHL--L-LL--HHHHLLLHHHH-H-H-HlL--L--LL--LL----EEEEEEEEL-----------------LLL--L-E-EEELLlllhhhhhhhhhhhlllllllllhh
0006 7jgrG   ------lLLLHHHHH-H-HH-H-H-HH-hhHL-LL------LLLEEEEE-L-LL-LL-H-HHH-HHHHLL-L----hhhhheeE-EEE-E-LLLL-----------LLHHHHHHHH----E-----E-EEEEELH-H--H-HL---------LLLL-HHHHHH-H-L-LlL--L--LL--LL----EEEEEEEL--LL-LHHHHHLHHHHHL-LLL-lL-E-EEEELlllhhhhhhhhhhhhlllhhhllh
0007 5zr1A   lllll-lLLLLLHHH-H-HH-H-H-HH---HL-L------llEEEEEEE-L-LL-LL-H-HHH-HHHHHH-H---H--hhhhhleEEE-E-HHHL--------L--LLL-HHHHHHH-H-E-----E-EEEEELL-L--L-LLL--L-L--------LLLHHHH-H-H-HhH--L--LL--LL----EEEEEELL--LL-LHHHHHL-LHHHH-HHH-lL-E-EEEELlllhhhhhhhhhlhhhlllllleeeeee
0008 8c0vA   lllee-lLLLLHHHH-H-HH-H-H-HL---LL-------LLLLEEEELL-L-LL-LL-H-HHH-HHHHHH-H---HhhhhlleE-LLL-L-LLL-------lLL--LLHHHHHHHHH-HHHhhlllE-EEEELLL-H--H-HHL--L-Ll-hLLHHHHHHHHH-H-H-HhH--H--LL--LL-L-lLLEEEELLL--L-LLLLLHH--HHLL----leE-E-EELLLlllhhhhhhhhhhhhhhhllllllll
0009 7jgsC   ------hHHHHHHHH-H-HH-H-H-HH---HH-L-----lLLLEELEEL-L-LH-HH-L-HHH-HHHHHH-H---HhhllleeE-EEL-L-HHH-----------lLLHHHHHHHH-----lllleE-EEELLLL-L--L-LL-------------hhHHHHH-H-H-HhL--L--LL--LL----EEEELLLLL-LH-HHHHHHL-LHHHH-LLL--LlL-LEELLlhhhhhhhhhhhllllllllleelhh
0010 7z6hK   llhH---HHHHHHHH-H-HH-H-H-HL---LL-LL-----LLLLEEEEEE-L-LL-LH-H-HHH-HHHHHH-L---L-------L-EEE-L-LL-L--LLLL------HHHHHHHHHH-HHHlllllL-EEEE--E-L--L-LlhhhH-HH--LLHHHHHHHHH-H-H-H-L--L-------------LLEEEELLllH-HHH--LLLLHHHH-------lL-LLEELlllhhhhhhhhhhhhhhhhhlllll
0011 7st9A   ------lLLHHHHHHH-H-HH-H-H-HL---LL-L-----LLLLEEEEEEE---LL-LL----LH-HHHHHH-H---HllllllllllEEE-L-LLLL--LLL-lLL--LL-HHHHHHHH-HHLlhhhlL-EEEELLL------LLL--L-LH---HHHHHHHHHHH-H-H-HhL--L--LL--LL----LLEEEEEEELeLH-HHH--LLHHH-hL-LLL--E-E-EEEELLlllhhhhhhhhhhhhlhhhhhhhl
0012 8tp1A   lllL---HHHHHHHH-H-HH-H-H-HL--lHH-HH--llLLLEEEEEEE-L-LL-LH-H-HHH-HHHHHH-L---L-------L-EEE-E-EL------lllLL--LLHHHHHHHHH-LLL-----L-LEEEEEE-L--H--hH--L-LLllllLHHHHHHHHH-L-L-L-L--L------------LEEEEEEEL-LH-HH-LLLL----------lllllL-EEEELlllhhhhhhhhhhhllllhhhhhh
0013 5zr1E   ------lLLHHHHHHH-H-HH-H-L-LL---LH-HH---L--lLEEEEEE-L-LL-LL-L-LHH-HHHHHH-L-llE-------E-EEE-L-LLLL-----------LLHHHHHHHH-HHHllleeE-EEEEELL-L--L--LL--L-L------LLLLHHHH-H-H-H-L-llllLL--EE----EEEEEEELL--L-LLHHHHHH-------hL--L-L-EEEELlllhhhhhhhhhhhllhhhhlhhhh
0014 8umyA   ------lLLHHHHHHH-H-HH-H-H-HHhllLL-LL--lLLLLLEEEEEL-L-LL-LL-H-HHHhHHHHHH-L-llE-------E-EEE-L-LLLL-----------LLHHHHHHHHH-HHHlllllE-EEEEELH-H--H-L-------------LHHHHHHH-H-H-H-H--lllLL--LL----LLEEEEEL--LL-LLH--HHHH------lL--L-E-EEELLlllhhhhhhhhhhhhhhlllllllhhh
0015 7ykkA   llhH---HHHHHHHH-H-HH-HhH-LH---HH-LL---LL---lLLEEE-ElLH-HH-H-HHH-HHHL-------L-------L-LEE-L-LL--------lHH--HLHHHHHHHHH-HHL-----L-LEEE-EE-E--L--HH--H-HL--HHHHHHHHHHH-H-L-L-L--L---------L--LLEEEEEEEE--L-LLLLHHH------llhH--H-H-LEEEElllllhhhhhhhhhhhhhhhllllll
0016 2qbyB   ------lLLHHHHHHH-H-HH-H-H-HH-hhLL-LL---L---LEEEEEEE-L-LL-LL-H-HHH-HHHHHH-H---HllllllleE-EEE-EhHHHL-----------LLHHHHHHHHH-HHHhllllE-EEEEELL-H--H-HHH--L-L-----LLHHHHHHHH-H-L-L-L--L-----------lEEEEEELL--LL-LLLLLLL-HHHH--HLL--L-L-EEEELllllhhhhhhhhhhhhhhllllllll
0017 6z1f3   ------llLHHHHHHH-H-HH-H-H-HH---hL-LL---L--LLEEEEEL-L-LL-LLhH-HHH-HHHHHH-H--lL-------E-EEE-E-L---------hhhlllHHHHHHHHHH-HHHhhlllE-EEEELLL-L--L-LLL--L--L--LHHHHHHHHHH-H-H-H-L--L--LL--LL-L--LLEEEEEL--LL-LL--LLHH--hlL-LLL--E-E-EEELLllhhhhhhhhhhlllllllhhhhhh
0018 7w42B   eeeee-lLLHHHHHHH-H-HH-H-H-HH-hhHL-LL--lllLLEEEEEEL-L-LL-LL-H-HHH-HHHHHH-H---L---------lEEE-E-E---------llLL--LLHHHHHHHH-HHH-hhllE-EEEELLH-H--H--LL--H-H-----HHHHHHHHH---H-L-L--L--LL--LL----EEEEEEEL--LH-HHLLHH----hlL-LLLllL-E-EEELLllhhhhhhhhhhhlhhhllhhhhh
0019 8dr5A   lllL---LLHHHHHH-H-HH-H-H-HL--hHH-HHh-lLLLLLEEEEEL-L-LL-LL-H-HHH-HHHHHH-H-llE-------E-EEE-L-LLLL-----------LLHHHHLLLHH-HHLhhhhlE-EEEELLH-H--H--LL--L-L---llLHHHHHHHH-H-H-L-L--L------------LLEEEEEL--LL-LL-HHHH-----hhlLL--L-E-EEELLlllllllhhhhhhhhhhhhleelhhh
0020 9lgoB   lllll-lLLLHHHHH-H-HH-H-HlHH---HHlLL---LL---lEEEEE-L-LL-LH-H-HHH-HHHHHHhL---L-------L-EEE-E-E---ehhhhLLLLhhHHHHHHHHHHH-HHL-----L-LEEEEEE-L--H--hH--L-LL-hHHHHHHHHHHH-H-H-H-L--L--L----L-L--LEEEEEEEL-LH-HH-LLLL---llL-LLL--E-E-EEEELllllhhhhhhhhhhhhlllllllll
0021 8dyuA   lllH---HHHHHHHH-H-HH-H-H-HL---------------llEEEEE-L-LL-LL----LH-HHHHHH-H---H--hhhllL-EEE-E-L--------lllL--LLHHHHHHHHH-HHL-----L-EEEEL-L-L--L-L-L--L-HH----HHHHHHHHH-H-H-H-H-lleeLL--LL-L--LEEEELLLL---llLLLHH---------hhL--L-E-EEEELlllhhhhhhhhhhhhllllhhhhhh
0022 4ai6A   LLLLl-hHHHHHHHH-H-HH-L-L-----------------lEEEEE-E-LL-LH-H-HHH-HHHHHH-L---L-------L-EEE-L-LL------llLLL--LHHHHHHHHH-HHL-----L-LEEEE-E-E--L-LLL--L-LH----HHHHHHHHHhH-L-L-L-hhhhHH--HH-HhhHHLEEEEEEEllH-HHHHHLH------------hhH-LEEEElllllhhhhhhhhhhhhllllllllll
0023 7mi8A   lllH---HHHHHHHH-H-HH-H-H-HL--------------llEEEEEL---LL-LL----LH-HHHHHH-H---H-hhllllL-EEE-L-LLL---------L--LLHHHHHHHHH-HHL-----L-EEEELLL-L--L---L--L-HH----HHHHHHHHH-H-H-HhH--H--HHllLL-L--LEEEEEELL-LL-LLL--------lhhhlLL--E-E-EEELLllllhhhhhhhhhhhhhhhhhhhhl
0024 8xkuB   hllL---LLHHHHHH-H-HH-H-H-LL-hhLL-LL---L---llLEEEE-E-LL-LH-H-HHH-HHHHHH-H---L-------L-LEE-E-E--------elLL--LLLHHHHHHHH-HHL-----L-LEEEEEE-L-----HH--H-LH--HHHHHHHHHHH-H-L-L-L-lL--LL--LL-H-hHLEEEEEEL--H-HHLLLLL------lllL--L-L-LEEEElllllhhhhhhhhhhlllllllllll
0025 7lccA   hllH---HHHHHHHH-H-HH-H-H-HL---LL-LL------LLEEEELL-L-LL-HH-H-HHH-HHHHHH-H---H-hhllllE-EEE-L-LL-------------hhHHHLLllllHHH-----LlEEEELLL-L--L--------------lLHHHHHHH-H--hhhH--L--LL-hhH-H--LEEELLLL--LL-LLlllLHHHLHHH-H-H--L-E-EELLLlllllllhhhhhhhhhhhlllllll
0026 5e7pA   hllll-lLLHHHHHHH-H-HHhL-H-HH----hlLL---LL--LEEEEEL-L-LL-LL-L-LHH-HHHHLL-L---L-------eEEE-E-EHHH--H-hLLLL--LHHHHHHHHHH-HHH-hlllE-EEEEELH-H--H-HLL-----H--HHHHHHHHHHH-H-HhH-L--L--L----L-L--EEEEEEEL--LH-HH-LLHH---hhL-LLL-eE-E-EEELLlllhhhhhhhhhhhlllllllllllh
0027 8xksB   lllL---LHHHHHHHH-H-HH-H-HhLL---LH-LL---LL--lLEEEEE-L-LLlLL-H-HHH-HHHHHH-H--hL-------L-LEE-L-L--------llllllHHHHHHHHHH-HHH-----L-LLEEEEE-L-----HH--H-LH--HHHHHHHHHHH-H-LlL-L--L--L---------LLEEEEEEL-LL-LL-LLLL---llL-LLL---lL-LLLLLlllhhhhhhllhhhhlllllhhhhh
0028 6vvoA   lllL---LLLLHHHH-H-HH-H-H-HL--hHH-LL--llLLLLEEEEEEL-L-LL-LL-H-HHH-HHHHHH-H-llE-------E-EEE-L-LLLL-----------LLHHHHHHHLH-HHHlllllE-EEEELLH-H--H-L-L--L-LL--llLHHHHHHHH-H-H-L-L--L------------LLEEEELLL-LL-LHHHHL-------lhhH--L-E-EEELLlllhhhhhhhhhhhhhllllllhhh
0029 4xgcE   ------lLLHHHHHHH-H-HH-H-HlLL---LL-------LLLLEEEEEL-L-LL-LL-HhHHH-HHHHHH-H---H-hhlleeE-EEE-E-LLLL-----------LLHHHHHHHHH-H--lllleE-EEEEELH-H----HHH--H-L------LLLHHHHH-L-L-H-H--H-hHL--LL----EEEEEEELL-LH-HHL--LLLLL----llL--L-E-EEELLlllhhhhhhhhhhlhhhhhhhhhhll
0030 7jgrD   ------lLLLHHHHHH-H-HH-H-H-HH---HH-L------LLEEEEEEL-L-LL-LL-L-HHH-HHHHHH-H---HllllleeE-EEE-E-LLLL-----------LLHHHHHHHHH-H--lllleE-EEEEELH-H--H-HLL---------lHHHHHHHH-H-HhH-L--L--LL--LL----LEEEEEL-L-LL-L-LLLLL--LHHHhHHL--LlL-LEELLllllhhhhhhhhhhhhllllhhhhh
0031 8dgcA   eeell-lLLLH-HHH-H-HH-H-H-HL-----------LLLL-LEEEEL-L-LL-LL-H-HHH-HHHHHH-H---L--lllleE-EEE-E-LLLlllLLLHHHL--LLHHHHHHH-----hlllllE-EEEEELH-H--H-HHH--H-HH-lLLLL--HHHHH-L-L-L-L--L------LL-L--EEEEEEEL---L-LLHH-----lllL-LLL----E-EEELLlllhhhhhhhhhlllllllllhhhhhhh
0032 8rwvE   ------lLLLLLHHH-H-HH-H-H-LL---LL-LL---L--LLEEEEEEL-L-LL-LH-H-HHH-HHHHLL-L---L----lllE-EEE-E-LLLL-----------LLHHHHHHH------lllllE-EEEEELL-L----LLL--L-L-----LLLL-HHHH-H-L-H-H---hhLL--LL----EEEEEEELL-LL-LLL--LLLLL----llL--L-E-EEELLlllhhhhhhhhlllllllllllhhhhh
0033 7vcsA   hhhhh-lLLHHHHHHH-H-HH-H-H-HH---HhlLL---LL--LEEEEEEL-L-LL-LL-H-HHH-HHHHHH-H-llL-------E-EEE-E-L------hhhhHH--HHLHHHHHHHH-HHH--hllL-EEEEELL-L-----LH--H-Hh-lLLHHHHHHHHH-H-H-L-L--L--LL--L--L--EEEEEEEL--LH-HH-LLLL---llL-LLL-eE-E-EEELLlllhhhhhhhhhhhllllllllllllh
0034 5g4fA   hhhhl-lLLHHHHHHH-H-HH-H-L-HH---HhlLL---LL--LEEEEEL-L-LL-LL-H-HHH-HHHHHH-H---L--------eEEE-E-LHHH--H-hhLLL--LH-HHHHHHHHH-HHH-hlllE-EEEEELH-H----HHL--L--L--LLHHHHHHHHH-H-HhL-L--L--LL--L-----LLEEEELL--LL-LLLLHHH----hL-LLL-lL-L-EEELLlllhhhhhhhhhhhhhhllllllllh
0035 3bosB   lllL---LLHHHHHHH-H-HH-H-H-HL-----------lllLEEEEEL-L-LL-LL-H-HHH-HHHHHH-H---H-hhllllE-EEE-E-HHHH--H-----H--LLH-HHHL----HHH-----LlEEEEELH-H--H--HL--L-L---HHHH-HHHHHH-H-HhH-H--L--L----L-L--LEEEEEELL-LL-LLLL----lhhhhhhhH--L-E-EEELLlllhhhhhhhhhhhhhhhllllllhhh
0036 8xkuF   lllL---HHHHHHHH-H-HH-H-HhLH---HH-LL---LL--lLEEEEE-L-LL-LL-H-HHH-HHHHHH-H--hL-------L-EEE-E-E--------ehhhlllHHHHHHHHHH-HHL-----L-LEEEEEE-L-----HH--H-LH--HHHHHHHHHHH-H-L--------llLL--LL----LEEEEEEEL-LH-HHLLLLL----lL-LLL---lL-EEEELllllhhhhhhhhhhhlllllllllll
0037 7uqjA   hhlL---LLHHHHHHH-H-HL-LlH-HH---H-hLL---LL-lLEEEEEL-L-LL-LL-H-HHH-HHHHHL-L---L-llllllL-EEE-E-E------lllLLL--LHHHHHHHHHH-HHL-----L-LEEEEEL-L--HhHHL--L--L--LHHHHHHHHHH-L-L-L-L--L--LL--L-----LEEEEEEEL-LL-LLLLLLL----lL-LLL-lL-E-EEELLlllhhhhhhhhhhhllllllllllhhh
0038 8xkuA   lllH---HHHHHHHH-H-HH-H-HhLH---HH-LL---LL--lLEEEEE-L-LL-LH-H-HHH-HHHHHH-H---L-------L-LEE-E-E--------ehhhlllHHHHHHHHHH-HHL-----L-LEEEEEE-L-----LL--L-LH--HHHHHHHHHHH-H-L-L-L--L--LL--L-----LEEEEEELL-LL-LL-LLLL-----llLLL--LlL-EEEELllllhhhhhhhhhhhlllllllllhhh
0039 7rzy1   lllH---HHHHHHHH-H-HH-H-H-HH---LL-LL---LL--lLLEELL-L-LL-LL-H-HHH-HHHHHH-H---HhhlllllE-EEE-L-LL-----------llLLHHHHHHHHH-H-L-----L-EEEELLL-H----HHL--L-L-lllllLLHHHHHH-H-HhH-H--H--L--------lLLLLLLLH-HH-HHHHHH---LHHH-hHH--L-LlEELLLlllllllllhhhhhhhhhhhhhlllll
0040 8r64B   lllH---HHHHHHHH-HhHH-H-H-HL---LH-HH---L---llLLEEE-E-LL-LH-H-HHH-HHHHHH-H---L-------L-EEE-E-EL--------hHH--HLHHHHHHHHH-HHL-----L-LEEEEEelL--L-LLL-----H--HHHHHHHHHHH-HlL-L-L--L---------L--LLEEEEEEEE--H-HHLLHHH------------hhlLEEEElllllhhhhhhhhhhhhhlllllllh
0041 2gnoA   ----------LHHHH-H-HH-H-H-HL-----------LLLEEEEEEEL-L-LL-LH-H-HHH-HHHHHH-H---H-------HlEEE-E-LLL----------llLLLHHHHHHHHH-HHHlllllE-EEEELLH-H----HLL--H-------HHHHHLHHH-H-H-L-L--L-------L-L--EEEEEEEL--LH-HHLLHH-------hhlL--L-E-EEELLllhhhhhhhhhhllhhhhlhhhhhll
0042 8zr0A   lllhh-lLLHHHHHHH-H-HH-H-H-HH-hlLL-LL----lLLEEEEEEL---LL-LL----LH-HHHHHH-H---HhlllllllE-EEE-E-H-------------hhHHHHHLLH-----L-----L-EEEEELL-L--L-L-------------LHHHHHHH-H-HhH-H--H--LL--LL-L--LEEEEEEELL-LhhHHHHHHH-LLHHH-hHL--L-LeEEELLlllhhhhhhhhhhhhhhhhhhhhhhhl
0043 9fnnU   -----lleellllHH-H-HE-E-L----------------llLEEEEEE-L-LH-HH-H-HHH-HHHHHH-H---L--lllleE-EEE-E-ELLlhleeeeeeL--LLHHHHHHH-H-HHHlllllE-EEEEEL---llL-LLL--L-L------LHHHHHHH-H-H-H-H--H-hHH--HL----EEEEEEEEL--L-HHHHHHHHH---L-LLL-lL-E-EEEEEellllleeeeeeeeelleeeeeeee
0044 8k8vA   lllL---LLHHHHHH-H-HH-H-H-HL--------------llEEEEEL-L-LL-LL-HhHHH-HHHHLL-LlllL-------L-LEE-E-L---------------LHHHHHLHHH-HLL-----L-LEEEE-E-H--H-HHH--H-LL----LHHHHHHHH-H-H-L-E------elelleellLEEEEEELHhHH-HLLL--LLLL---hhHH--E-EeEEELLleeellhhhhhhhhhhhhhllleelh
0045 8bobC   ------llLHHHHHH-H-HL-H-H----------------hLLEEEEEL-L-LL-LL-H-HHH-HHHHHL-L---L-----llE-EEE-E-LL----------hhhHLHHHHHHHHH-HHHhhllllEEEEEELH-H--H-L----L-LH--H-HHHHHHHHH---------------lLL-L--EEEEEEELL-L-------LHHH--hH-HLL-lE-E-EELHHhhlllhhhhhhhhhhhhlllllllhhhh
0046 8xkuD   lllhhllllLLHHHH-H-HH-H-H-HH-hlLL-LLlhhhllLLLEEEEL-L-LL-LL-L-HHH-HHHHHL-L---L----lllE-EEL-L-HHHH--LLLL-----lHHHHHHHHHH-HHL-----L-EEEEELH-H--H-L----------lhhhhHHHHHH-H-H-H-L--LllLL--LL-L--LLLLEEEL--LL-LL-LLHH---hlL-LLL-lE-E-EELLLlllhhhhhhhhhhhhhllllleellll
0047 5j1sA   llhH---HHHHHHHH-H-HH-H-H-LL---L-------LLLL-LEEEEE-E-EL-LH-H-HHH-HHHHHH-H---L--------lLEE-E-E-ehhhhllLHHH---HHHHHHHHHH-HHL-----L-LLEEE-E-E--L-HhH--L-LH---HHHHHHH-HH-H-L-L-L--L--L---lllllllLLEEEEEEE-LLlhhhHHHLL-LLLL-LLL--L-L-LEEEEellllhhhhhhhhhhhhhhlllllh
0048 8fnuA   ------lLLHHHHHHH-H-HH-H-H-HH-hhHL-LL----lLLLEEEEEE-L-LL-LL---LHH-HHHHHH-H---H--hllleE-LLLlL-HHH---------hlLLLL-HHHHHhhhhL-----E-EEELLLH-H--H---L--L-LH----HHHHHHHHH-H-L-L-L--L------------LEEELLLL---L-LHHHHHHHHHHHH-HHH--LlL-EEEELlhhhhhhllhhhhhhhhhllleelll
0049 1xwiA   ------lLLHHHHHHH-H-HH-HhH-HH---H-hLL---LL--LEEEEEL-L-LL-LL-H-HHH-HHHHHH-H-llL-------L-EEE-E-EE--llllLLLLL---LHHHHHHHHHH-HHH--hllL-EEEEEEL-L--L-HHH--L--H--HHHHHHHHHHH-H-H-L--------L--LL-L--EEEEEEELL--L-LLLLHHH--------hhllL-E-EEELLlllhhhhhhhhhhhhlllllllllhhh
0050 5v8fD   ------hHHHHHHHH-H-HH-H-H-HH---LL-L------llLEEEEEL---LL-LL----LH-HHHHHH-H---H----hhhheEEE-E-E---------llL--LLLLHHHHHH----E-----E-EEEEELH-H----HLL--L-----LLLL-HHHHHH-H-HhH-H--H--LL--LL----EEEEELLLL-LL---HHHHLLHH-hH-HHL--LlL-EEELLlllhhhhhhhhhhhlllllllllh
0051 6p8vC   hhlL---LLHHHHHH-H-HH-H-H-HH-lllH-HH---HHLLLEEEEEE-L-LL-LL-H-HHHhHHHHHH-H-leE-------E-EEE---LLHH--------hhllHHHHHHHHHH-HHHllleeeEEEELLH-H--H-HLL-----H--HHHHHHHHHHH-H-H-HhH--H--LL--L--L--EEEEEEEL--LH-HHLLHH--------hhhllL-E-EEEELlllhhhhhhhhhhhllllllhhhhh
0052 7qo4M   lllH---HHHHHHHH-HhHH-H-HhLH---HH-LL---LL--lLLEEEE-L-LL-LH-H-HHH-HHHHHH-H---L-------L-EEE-E-E--------ehHH--HLHHHHHHHHH-HHH---hlL-LEEEEEE-L-----LL--L-L-hhhhHHHHHHHHHL-L-L-L-L--L-------llL--LEEEEEEELL-LL-LL-LLHH-----hhLLL--LlL-EEEELllllhhhhhhhhhhhhhhllllllll
0053 6nyyC   ------lLLHHHHHHH-H-HH-H-H-HH----hlLL---LL--LEEEEEL-L-LL-LL-L-LHH-HHHHHH-H---L----lllE-EEE-E-LLLL--LLL---lllLLHHHHHHHHH-HHH-hlllL-EEEEELH-H--H-LLL------hhhhHHHHHHHHH-H-LlL-L--L--L---------LEEEEELL--LL-LL-LLLL---llL-LLL-lL-E-EEELLlllhhhhhhhhhhlllllllllllh
0054 6pdwD   ------lLLLHHHHH-H-HHhL-H-HH-lhhL-HH---HL-llLEEEEE-L-LL-LL-H-HHH-HHHHHHhH---L-------L-LEE-EeLHHH---------hllHHHHHHHHHH-HHL-----L-LEEEEEElH--H-HLL--L--H--HHHHHHHHHHH-H-L-LlL--L--LL-lLL-L--LEEEEEEEL-LH-HHLLHHH------------hlL-EEEELlllhhhhhhhhhhhhllllllllll
0055 7qo4K   lllH---HHHHHHHHhL-HH-HhH-LL---HL-LL---L----lLLEEE-L-LL-LH-H-HHH-HHHHHH-H---L-------L-EEE-E-E--------llLL--LLHHHHHHHHH-HHL-----L-LEEEEEE-E-----LL--L-LH--HHHHHHHHHHH-H-H-H----------hlL-L--LLEEEEEEL---lLLLLLLL------lllL--L-L-LEEELlllllhhhhhhhhhhhhhhlllllll
0056 8xksD   eeell-lLLHHHHHHH-H-HH-H-H-HH-lllL-LL---L---lLEEEEE-L-LL-LL-H-HHH-HHHHHH-H---H--------lEEE-E-E---------hhhhllHHHHHHHHHH-HHL-----L-LEEEEEL-L--H-HHH--L--H--HHHHHHHHHHH-H-H-HhH--H--LL--L-----LLEEEEEEL-LH-HH-LLLL-----llLLL--LlL-EEEELllllhhhhhhhhhhhllllllllllhh
0057 8xkuC   lllL---LLLLLHHH-H-HH-H-H-HH-lhhL-LL---L--lLEEEEEE-L-LL-LL-H-HHH-HHHHHH-H--hL-------L-LEE-E-EL--------hHH--HLHHHHHHHHH-HHL-----L-LLEEEEL-LllL-LLLllllHH--HHHHHHHHHHH-H-L-L-L--L--L----L-L--LLEEEEEEL-LL-LLL---LHHH-lL-LLL-lL-E-EEEELlllhhhhhhhhhhhhhhhllhhhlll
0058 6rlaA   lllL---HHHHHHHH-H-HH-H-H-HL--------------llLLEEE-L-LL-LL-HhHHH-HHHHHH-H--lL-------L-EEE-L-L---------llLL--LLHHHHHHHHH-HHH-----L-LEEEEEL-L--L---L--L-LH---HHHHHHHHHH-H-H-HhH--H--HLllLL-L--LLEEEELLL---llLLLHH--------hhL--L-L-LEEELllllhhhhhhhhhhhhllllhhhhhh
0059 8ui7C   ------lLLHHHHHHH-H-HH-H-H-HL---L----------lllLEEEEL-L-LL-LL-H-HHH-HHHHHH-H---HlllhhhhE-EEE-L-LLLL-----------LLLLLLLLHHH-HHHlllllE-EEEELLH-H--H-L-------------LHHHHHHH-H-H-HlL--L--LL---------EEEEEEEL--LL-LLL--LHHH------hhL--L-E-EEELLlllhhhhhhhhhhhhhhhllllhhh
0060 7mi1A   LLLLl-hHHHHHHHH-H-HH-L-L------------------llEEEEE-L-LL-LH-H-HHH-HHHHHH-L---L-------L-EEE-L-LL------llLLL--LHHHHHHHHH-HHH-hlllL-LEEEEEE-L--L-L-L--L-LH----HHHHHHHHH-H-L-L-LlhH--HH--HhhH--HEEEEEEELllH-HHHHHLH----------hhhH-H-LEEEElllllhhhhhhhhhhlllllllll
0061 5nugA   HLLLhllllLLHHHH-H-HH-L-----------------lllLLLEEEL-L-LL-LL-H-HHH-HHHHHH-H---L-------L-LEE-E-E-------elllH--HHHHHHHHHHH-HHL-----L-LE-EEEE-L--H--hH--L-HH----HHHHHHHHH-H-H-L-L--L-------L-L--LLLEEEELL-LL-LLLLHH----------hhhH-L-EEELLlllllhhhhhhhhhhhllhhhhllll

####################################################################################################################################################################################
####################################################################################################################################################################################
####################################################################################################################################################################################

Job: Type-1 BREX; BrxL MCM-AAA ATPase
Query: s001A
No: Chain Z rmsd lali nres id PDB Description
1: 8emh-B 47.5 0.8 319 676 71 MOLECULE: PROTEASE LON-RELATED BREX SYSTEM PROTEIN BRXL;
2: 5udb-7 16.8 3.4 256 726 14 MOLECULE: DNA REPLICATION LICENSING FACTOR MCM2;
3: 3f8t-A 16.4 3.4 246 459 13 MOLECULE: PREDICTED ATPASE INVOLVED IN REPLICATION CONTROL,
4: 5v8f-6 16.3 3.2 241 692 15 MOLECULE: DNA REPLICATION LICENSING FACTOR MCM2;
5: 3nbx-X 14.8 3.8 229 481 14 MOLECULE: ATPASE RAVA;
```

```
6: 8q6p-2 14.8 3.8 248 431 9 MOLECULE: DNA REPLICATION LICENSING FACTOR MCM2;
7: 2r44-A 13.9 4.0 236 330 11 MOLECULE: UNCHARACTERIZED PROTEIN;
8: 5udb-4 13.4 3.4 253 751 13 MOLECULE: DNA REPLICATION LICENSING FACTOR MCM2;
9: 8osg-B 12.1 3.7 223 323 15 MOLECULE: MAGNESIUM-CHELATASE SUBUNIT CHLI;
10: 8rxd-A 11.1 4.2 218 486 13 MOLECULE: COMPETENCE RELATED PROTEIN COMM;
11: 5dgk-A 11.1 3.7 209 519 11 MOLECULE: ACTIVE HELICASE;
12: 6iy8-A 8.7 5.4 163 469 10 MOLECULE: POSITIVE REGULATOR CAPR;
13: 3bos-B 8.6 3.0 132 231 13 MOLECULE: PUTATIVE DNA REPLICATION FACTOR;
14: 9e22-A 8.2 4.5 154 2925 14 MOLECULE: CYTOPLASMIC DYNEIN 1 HEAVY CHAIN 1;
15: 7ola-A 7.7 4.3 182 441 9 MOLECULE: DNA PRIMASE;
16: 7v2b-D 7.6 4.4 158 376 10 MOLECULE: VPSR;
17: 8iqi-C 7.6 4.2 182 916 12 MOLECULE: PUTATIVE PRIMASE C962R;
18: 7mi8-A 7.6 8.8 198 868 11 MOLECULE: FUSION PROTEIN OF DYNEIN AND ENDOLYSIN;
19: 8umy-A 7.5 7.8 134 546 12 MOLECULE: CHROMOSOME TRANSMISSION FIDELITY PROTEIN 18 HOMOL
20: 1ixs-B 7.4 3.4 138 315 14 MOLECULE: HOLLIDAY JUNCTION DNA HELICASE RUVA;
21: 7mi1-A 7.4 4.3 151 2628 13 MOLECULE: CHIMERA PROTEIN OF DYNEIN AND ENDOLYSIN;
22: 6hyp-A 7.3 4.6 164 2272 12 MOLECULE: MIDASIN,MIDASIN;
23: 6l1q-A 7.2 3.8 147 267 12 MOLECULE: CBBQ PROTEIN;
24: 8k8v-A 7.2 5.8 150 719 13 MOLECULE: ENDOPEPTIDASE LA;
25: 5v8f-D 7.2 3.7 136 456 10 MOLECULE: DNA REPLICATION LICENSING FACTOR MCM2;
26: 5zr1-A 7.2 3.9 135 494 11 MOLECULE: ORIGIN RECOGNITION COMPLEX SUBUNIT 1;
27: 9emc-D 7.1 3.5 135 337 14 MOLECULE: RUVB-LIKE 1;
28: 8btg-B 7.1 3.4 132 335 13 MOLECULE: CHROMOSOMAL REPLICATION INITIATOR PROTEIN DNAA;
29: 8fcv-R 7.0 3.8 127 343 14 MOLECULE: DNA (60-MER);
30: 6k9c-A 7.0 4.1 173 416 12 MOLECULE: PRIMASE;

0001 s001A   [illegible]
0002 8emhB   [illegible]
0003 5udb7   [illegible]
0004 3f8tA   [illegible]
0005 5v8f6   [illegible]
0006 3nbxX   [illegible]
0007 8q6p2   [illegible]
0008 2r44A   [illegible]
0009 5udb4   [illegible]
0010 8osgB   [illegible]
0011 8rxdA   [illegible]
0012 5dgkA   [illegible]
0013 6iy8A   [illegible]
0014 3bosB   [illegible]
0015 9e22A   [illegible]
0016 7olaA   [illegible]
0017 7v2bD   [illegible]
0018 8iqiC   [illegible]
0019 7mi8A   [illegible]
0020 8umyA   [illegible]
0021 1ixsB   [illegible]
0022 7mi1A   [illegible]
0023 6hypA   [illegible]
0024 6l1qA   [illegible]
0025 8k8vA   [illegible]
0026 5v8fD   [illegible]
0027 5zr1A   [illegible]
0028 9emcD   [illegible]
0029 8btgB   [illegible]
0030 8fcvR   [illegible]
0031 6k9cA   [illegible]

0001 s001A   [illegible]
0002 8emhB   [illegible]
0003 5udb7   [illegible]
0004 3f8tA   [illegible]
0005 5v8f6   [illegible]
0006 3nbxX   [illegible]
0007 8q6p2   [illegible]
0008 2r44A   [illegible]
0009 5udb4   [illegible]
0010 8osgB   [illegible]
0011 8rxdA   [illegible]
0012 5dgkA   [illegible]
0013 6iy8A   [illegible]
0014 3bosB   [illegible]
0015 9e22A   [illegible]
0016 7olaA   [illegible]
0017 7v2bD   [illegible]
0018 8iqiC   [illegible]
0019 7mi8A   [illegible]
0020 8umyA   [illegible]
0021 1ixsB   [illegible]
0022 7mi1A   [illegible]
0023 6hypA   [illegible]
0024 6l1qA   [illegible]
0025 8k8vA   [illegible]
0026 5v8fD   [illegible]
0027 5zr1A   [illegible]
```

```
0028 9emcD   ---------------lLLLLllleELLEE---------lLHHHHH-HHHHHHHHL-L-LEEEEEELLLLLHHH-HHHHHHL--LEEEEE-LL-LLHHHH-H----------HHHHL-LeLEEEEELHHH-LL-----hHH-HHH-HHHHLL-L----------LLL-L-EEEEEELLL-----leelllllleE-HHH-------LLHH--HHLLL-EEEEE--L-LLLHHHH-HH--HH------------------------------------------------------------------------------------------------------------
0029 8btgB   -----------------llllllllLLLL-----LL-LLLHHHHH-HHHHHHHLL-L-LEEEEEELLLLLHHH-HHHHHHH--HEEEEEHHH-HHHHHH-----------LLLL-----EEEEELHHH-HlllhhhHHH-HHH-HHHHHH-LL------------L-EEEEEELL--------------LL-LLL--L--L--LLHH-HHHHLLEEEEE--L-LLLHHHH-HH--HH------------------------------------------------------------------------------------------------------------
0030 8fcvR   --------------------------lhhhhlLLL-HHHHHHHH-HHHHHHHLL---lEEEEEELLLLLHHH-HHHHHHHllLEEEEELlH-HHHHHH-HH----------hHL-L-LEEEEELHHH-HhllllhhHH-HHH-HHHHHH-H----------HL-L-EEEEEELHH-------------hH-HHL--------LLLHHHHHLE-EEEEELLLlHHHHHH---------------------------------------------------------------------------------------------------------------hlLLL-
0031 6k9cA   LLLL-----------lllllHHHHH-------hhhhllLLHHHHH-HHHHhHHHH---LEEEEEELLLLLHHH-HHHHHHHhhHEEEEELHH-HLLLLL------------HHHLL-L-LEEEEELLLL------lhhhhlHHH-HHHHHH-LLEEEELLEEEELL-L-EEEEEELL---------------L-LLL--------LLLL-lLLLE-EEEEE--L-LLHLLLLLLL----hHLHHHHHH-HH------hLLLLL----------------------LLLL------------------------LLLL-HHHHHHHHHHLLHh------
```

Job: Type-1 BREX; BrxL HAM28421 NTD1 SIGMA-HTH-like
Query: s001A
No: Chain Z rmsd lali nres id PDB Description
1: 8emh-B 7.2 1.5 64 676 52 MOLECULE: PROTEASE LON-RELATED BREX SYSTEM PROTEIN BRXL;
2: 3frh-A 5.0 2.3 49 242 16 MOLECULE: 16S RRNA METHYLASE;
3: 8val-E 4.9 5.6 44 337 16 MOLECULE: DNA POLYMERASE III SUBUNIT DELTA;
4: 6h3a-B 4.7 3.0 51 57 14 MOLECULE: SWI/SNF-RELATED MATRIX-ASSOCIATED ACTIN-DEPENDENT
5: 2ejs-A 4.6 1.6 44 58 14 MOLECULE: AUTOCRINE MOTILITY FACTOR RECEPTOR, ISOFORM 2;
6: 4oci-A 4.6 1.7 46 139 4 MOLECULE: CALMODULIN, PUTATIVE;
7: 7qog-C 4.6 1.9 50 225 12 MOLECULE: PORTAL PROTEIN GP20;
8: 2qho-D 4.5 1.1 39 49 10 MOLECULE: UBIQUITIN;
9: 1otr-A 4.5 1.5 40 49 10 MOLECULE: PROTEIN CUE2;
10: 3bq3-A 4.5 1.4 40 250 10 MOLECULE: DEFECTIVE IN CULLIN NEDDYLATION PROTEIN 1;
11: 1mx9-D 4.4 2.1 50 533 6 MOLECULE: LIVER CARBOXYLESTERASE I;
12: 1xb2-B 4.4 1.2 40 276 18 MOLECULE: ELONGATION FACTOR TU, MITOCHONDRIAL;
13: 6c95-D 4.3 3.6 43 95 14 MOLECULE: N-ALPHA-ACETYLTRANSFERASE 15, NATA AUXILIARY SUBU
14: 7qoj-D 4.3 2.0 50 226 8 MOLECULE: PORTAL PROTEIN GP20;
15: 2n5j-A 4.2 2.9 45 49 11 MOLECULE: RIBONUCLEASE ZC3H12A;
16: 4eei-B 4.2 4.9 53 423 8 MOLECULE: ADENYLOSUCCINATE LYASE;
17: 8hkc-E 4.2 3.4 58 262 5 MOLECULE: DNA-DIRECTED RNA POLYMERASE SUBUNIT ALPHA;
18: 9cc7-C 4.2 2.1 49 160 10 MOLECULE: PHITE ADAPTOR PROTEIN;
19: 2cp8-A 4.2 2.8 44 54 9 MOLECULE: NEXT TO BRCA1 GENE 1 PROTEIN;
20: 9fvr-C 4.2 3.5 54 153 17 MOLECULE: TRANSCRIPTIONAL REPRESSOR NRDR;
21: 1nxh-A 4.2 2.9 56 124 13 MOLECULE: MTH396 PROTEIN;
22: 6q00-B 4.1 1.4 39 45 15 MOLECULE: UBIQUITIN;
23: 1aip-C 4.1 1.4 40 195 10 MOLECULE: ELONGATION FACTOR TU;
24: 8x6g-H 4.1 4.5 57 244 4 MOLECULE: DNA-DIRECTED RNA POLYMERASE SUBUNIT ALPHA;
25: 3e46-A 4.1 2.7 44 202 9 MOLECULE: UBIQUITIN-CONJUGATING ENZYME E2-25 KDA;
26: 6vvo-A 4.1 5.0 54 448 6 MOLECULE: REPLICATION FACTOR C SUBUNIT 1;
27: 5he9-E 4.0 2.2 44 56 7 MOLECULE: HELICASE LOADER;
28: 3b74-A 4.0 3.0 47 307 9 MOLECULE: UNCHARACTERIZED PROTEIN YKL091C;
29: 2cp9-A 3.9 1.9 45 64 16 MOLECULE: ELONGATION FACTOR TS, MITOCHONDRIAL;
30: 1k17-A 3.9 2.3 49 509 10 MOLECULE: THREONINE SYNTHASE;
31: 7p37-A 3.9 3.5 52 147 15 MOLECULE: TRANSCRIPTIONAL REPRESSOR NRDR;
32: 7lt2-A 3.8 3.3 52 387 13 MOLECULE: MAB-21 DOMAIN-CONTAINING PROTEIN;
33: 2ekf-A 3.8 4.1 43 61 14 MOLECULE: ANCIENT UBIQUITOUS PROTEIN 1;
34: 6jcx-F 3.8 4.5 58 186 7 MOLECULE: DNA-DIRECTED RNA POLYMERASE SUBUNIT ALPHA;
35: 2o7g-A 3.8 3.8 55 88 7 MOLECULE: PROBABLE RNA POLYMERASE SIGMA-C FACTOR;
36: 6ajf-A 3.8 3.6 52 901 6 MOLECULE: DRUG EXPORTERS OF THE RND SUPERFAMILY-LIKE PROTEI
37: 6jbq-F 3.8 3.9 60 186 7 MOLECULE: DNA-DIRECTED RNA POLYMERASE SUBUNIT ALPHA;
38: 5wuq-A 3.8 4.6 57 158 4 MOLECULE: ECF RNA POLYMERASE SIGMA FACTOR SIGW;
39: 2cos-A 3.8 2.7 45 54 18 MOLECULE: SERINE/THREONINE PROTEIN KINASE LATS2;
40: 2dna-A 3.7 2.3 48 67 8 MOLECULE: UNNAMED PROTEIN PRODUCT;
41: 8vjl-B 3.7 4.2 57 116 7 MOLECULE: STAGE IV SPORULATION PROTEIN FB;
42: 7mvv-A 3.7 4.0 51 1542 4 MOLECULE: NUCLEOPORIN NUP192;
43: 6e6y-A 3.7 5.7 54 268 6 MOLECULE: DIECKMANN CYCLASE, NCMC;
44: 1wgl-A 3.7 1.3 40 59 18 MOLECULE: TOLL-INTERACTING PROTEIN;
45: 2qsf-X 3.7 2.9 46 104 2 MOLECULE: DNA REPAIR PROTEIN RAD4;
46: 4fli-A 3.7 1.5 39 328 15 MOLECULE: 5'-TYROSYL-DNA PHOSPHODIESTERASE;
47: 9kgb-A 3.7 3.7 52 336 12 MOLECULE: TERPENE SYNTHASE;
48: 8yhu-B 3.7 2.6 46 56 20 MOLECULE: TOLL-LIKE RECEPTOR 3;
49: 6tdv-D 3.7 2.4 46 186 15 MOLECULE: ATPTB1;
50: 6toa-D 3.7 5.5 54 195 13 MOLECULE: ADAPTOR PROTEIN RCC01688;

```
0001 s001A   --MDVLDIKLNEAFPG-----------RKDLLHEI-K-RA-VN-V--PS-FVLEFLLSRYC---A--S--------E----D---P--EEIEEGKKAVLQTIEK-C--Y-------
0002 8emhB   -nDKELDQLLNEHFAG--------rvvRKDLTKLI-K-EG-AN-V--PV-YVLEYLLGMYC---A--S--------D----D---P--EIIEQGLRNVKTVLAE-N--Yvrpdeae
0003 3frhA   -----------------------ypmnINDALTSI-L-ASkRA-L--CP-DTVRRILTEEW---G-rH--------K----S---P--KQTVEAARTRLHGICG-Ay---------
0004 8valE   -rPDFE--yqagrghhallihylapppEQYAVTWL-S-RE-VT-M--SQ-DALLAALRLSA-------------------------------gSPGAALALFQG-D--Nwqaretl
0005 6h3aB   --sELEDLSEL-------------edlKDAKLQTL-K-EL-FQ-R--SD-NDLLKLIESTS---T-----------------------MDGAIAAALLMFGDAg------------
0006 2ejsA   -gsSGAS-----------------nsqLNAMAHQI-Q-EM-FQ-V--PY-HLVLQDLQLTR--------------------------------SVEITTDNILE-G--Riqvpf--
0007 4ociA   --------------------dgdgyltLNEFESLV-R-VL-VV-X--ET-SAIASTYNSN----S--K--------V----R------gxSYELFTSCFSQLKT-K--Sfnkdeik
0008 7qogC   -------------------------mtYNELIYMV-L-DE-LKyY--TP-DHVIFLLVKYR---SffP--------I----E---D--ALVPPLIELVVKELRG-P--Eyspkded
0009 2qhoD   --------------------sipasviPEELISQA-Q-VV-LQgK--SR-SVIIRELQRTN-------------------------------lDVNLAVNNLLS-R----------
0010 1otrA   ------------------------nddHESKLSIL-M-DM-FA-I--SK-SKLQVHLLENN-------------------------------nDLDLTIGLLLK-E--Nddks---
0011 3bq3A   -------------------------spEQEAIESF-T-SL-TK-C--DP-KVSRKYLQRNH-------------------------------wNINYALNDYYD-K--Eigvahpp
0012 1mx9D   --------ggesvsvlvlsplsegqldQKTAMSLL-W-KS-YC-I--AK-ELIPEATEKYL---G-gT--------D----D---T--VKKKDLFLDLIADVMF-G--Vpsvivar
0013 1xb2B   ------------------------sasSKELLMKL-R-RK-TG-Y--SF-INCKKALETCG-------------------------------gDLKQAESWLHK-Q--Aqkegwsk
0014 6c95D   --------qkaKQERE----elakvtiKKEDLELI-M-TE-ME-I--SR-AAAERSLREHM-------------------------------gNVVEALIALTN------------
0015 7qojD   -------------------------mtNKEFSDGF-S-TL-LT-L--DE-YEKSTFLTNAQeqlC--E--------L----N---P--VVHRAILERAVQLAII-S--Ktqlt---
0016 2n5jA   ---GPHM-----------------tseLQMKVDFF-R-KL--G-Y--SS-SEIHSVLQKLG---V--Q------------------------aDTNTVLGELVK-H--G-------
0017 4eeiB   rshgMFAEedrVRST--------saylSSFYLHFL-V-AN-TP-F--MR-EDCYKIVQQVA---F--D-------------------lESFSKKLQKVMHDEHNi-----------
0018 8hkcE   -aNAWPMLleaaKTLI-----lshlrfVVHIARNY-A--G-YG-L--PQ-ADLIQEGNIGL---M-rrfnpevgvrL----V---S--FAVHWIKAEIHEYVLR-N--Wrivkvat
0019 9cc7C   -------------------------mtNEQVIELV-R-VL-L-eI--SD-QTIIFFWTKWK---Y--D--------L----D-nrP--EKIPAALYNTVVDCVR-W--Livqevss
0020 2cp8A   ------gSSGS-------------sgqTAALMAHL-F-EM-GF-C--DR-QLNLRLLKKHN-------------------------------yNILQVVTELLQ-L--Sgpssg--
0021 9fvrC   --------fevAELVX----dvrepfnEEKLRSGX-L-RA-LEpV--SS-DDVEXAINHIK---S--QlratgereV----P---S--KXIGNLVXEQLKKLDK-Va---------
0022 1nxhA   -lhPRFESAR-------------prciREKLHSDLqL-CW-LI-I--SV-DDAEALKDEIT---E--L----vlagR----E---Y--SEALSEGRRRLHEILR-S----------
0023 6q00B   ------------------------snaRRLLXVEF-A-SV-AS-C--DA-AVAQCFLAEND-------------------------------wEMERALNSYFE-Pp---------
0024 1aipC   --------------------------sQMELIKKL-R-EA-TG-A--GM-MDVKRALEDAG-------------------------------wDEEKAVQLLRE-R--Gamkaakk
0025 8x6gH   ehqeNKNTDAQDK---------lvkhyQKLIESLA-Y-KY-SK-gqsHH-EDLVQVGMVGL---I--G-ainrfdmsferkF---E--AFLVPTVIGEIKRYLR-D--Ktwsvhvp
0026 3e46A   --------TPYEGG------apvsspeYTKKIENL-C-AA--G-F--DR-NAVIVALSSKS-------------------------------wDVETATELLLS-N----------
```

```
0027 6vvoA   -qQGDQSCcfdLRFQ---------rprVEQIKGAM-M-SI-AK-I--PP-PAMNEIILGAN---Q-------------------------DIRQVLHNLSMWCA-R--Skalmgpf
0028 5he9E   ------------------------snaMMVTKEFL-K-TK-LE-C--SD-MYAQKLID-EA---Q--G----------------------DENRLYDLFIQKLA-E--Rhtrpaiv
0029 3b74A   --------iCSPNALP----pgnltkeQEEALLQF-R-SI-LLrL--DD-STLLRFLRARK-------------------------------fDINASVEMFVE-T--Erwreeyg
0030 2cp9A   -gSSGSS------------------gsSKELLMKL-R-RK-TG-Y--SF-VNCKKALETCG-------------------------------gDLKQAEIWLHK-E--Aqkegwsk
0031 1kl7A   --------------------dwsklsfQDLAFAIX-R-LY-IE-I--PD-ADLKDLIKRSY---S--T--------FrsdevtyaF--KDVALQFVGNLFEYFL-Q--Rtnanlpe
0032 7p37A   fttvETCS------------gvtepfsRTKVINGV-R-KA-CQpV--TE-DALAQLGQRVE---E--avratgsaeL----T---T--HDVGLAILGPLQELDL-Va---------
0033 7lt2A   --------slsfQEQE----lidnknrLKPAVKLI-K-KL-KN-I--AS-YYIKTVFLHI------fW--------N----K---SlrEVFMTTLREYNEFIAD-Q--Sipyywcr
0034 2ekfA   -sgSSGS----------------pdvqLATLAQRV-K-EV-LH-V--PL-GVIQRDLAKTG--------------------------------CVDLTITNLLE-Ga---------
0035 6jcxF   -tdEELTARFER----------daiplLDQLYGGA-L-RM-TR-NpaDA-EDLLQETMVKA---Y-agfrsfrhgtN----L---K--AWLYRILTNTYINSYR-K--Kqrqpaey
0036 2o7gA   alalsaaKGNGRALE------afikatQQDVWRFV-A-YL-SDvG--SA-DDLTQETFLRA---I-gaiprfsarsS----A---R--TWLLAIARHVVADHIR------------
0037 6ajfA   fisiplqgIPLGQlslggisfdklplmALILIVTT-T-VL-MFvL--PI-KAALMSALTL------nytpqplmapM----I---G--LIIAVIWGLSTDYEVF-L--Vsrmvear
0038 6jbqF   -tDQVLVEkgdQKAFN-----llvvryQHKVASLV-S-RY-VP-S-gDV-PDVVQEAFIKA---Y-raldsfrgdsA----F---Y--TWLYRIAVNTAKNYLV-A--Qgrrppss
0039 5wuqA   -vKKGD-QDAFA---------divdiyKDKIYQLC-Y-RM-L--G--NV-HEAEDIAQEAF---I--RayvnidsfdinrkF---S--TWLYRIATNLTIDRIR-K--Kkpdyyld
0040 2cosA   --GSSGS-----------------sgvNRQMLQEL-V-NA-G--C--DQ-EMAGRALKQTG------------------------------sRSIEAALEYISK-M--Sgpssg--
0041 2dnaA   -sSGPS-HSLQ-----------apevrFSKEMECL-Q-AM-GF-V--NY-NANLQALIATD-------------------------------gDTNAAIYKLKS-S--Qgfsgpss
0042 8vjlB   lmakGDEHARNM-----------liehNLRLVAHI-V-KK-FEgE--DA-EDLISIGTIGL---I--K-giesysagkgtkL---A--TYAARCIENEILMHLR-A--Lkktk---
0043 7mvvA   --------lkigEEEY------slnedFVNDCLKL-A-DE-LD-L--NE-KESARILIDCD---A------------egdvE---T--QS-RPLWECGVIRFHQ-E--Rkylldcm
0044 6e6yA   -cAGSA--------------spttstlYYQFRKVV-E-SL-AGeQ--AA-REALAEGTAAA---D-rI--------G----D---V--EGLGAELVRVFTAAGR-A--Acaaadld
0045 1wglA   --------------------ssgssgcSEEDLKAI-Q-DM-FN-M--DQ-EVIRSVLEAQR-------------------------------gNKDAAINSLLQ-M--Geepsgps
0046 2qsfX   ----igLTVEDLLS------qvdytpeDDQAISRL-C-E--LG-F--ER-DLVIQVYFACD-------------------------------kNEEAAANILFS-D----------
0047 4f1iA   --------------------qgklsxsDEQKLHEF-A-II-TA-T--DE-AFAQSILQDVD-------------------------------wDLKKALDVFYG-Sea--------
0048 9kgbA   --------sgiPAVDR-----ahvlqtALSIYPEV-E-NW-VAiL--PQ-R-MGGMCLGMV---A-aP--------Y----A---Q----PSVLVEASIMALIA-F--Aiddited
0049 8yhuB   ----------------------sleeeAERVVEEL-V-KE-FN-L--SR-TQEIALRRYA----A--A--------R----A-asE--EVIEELLRDVAERLS-------------
0050 6tdvD   ----------------------thaelHLFDLDEF-MqTY-KR-L--QtqESVFGEMAREL---V--R--------G-----------kTADEATTSVMARVEK-E--Ilgvh---
0051 6toaD   ----MMLN---EVTAV---------pgtALPVAEF-R-DH-LRdlgaED-AALLSYLRAAI---A-eidftagfgaswsalP---V--DLAQAVFLLAAQYYEL-Rh---------

0001 s001A   --LLHHHHHHHHHLLL-----------LHHHHHHH-H-LL-LL-L--LH-HHHHHHHHHHL---L--L--------L----L---H--HHHHHHHHHHHHHHHH-H--L-------
0002 8emhB   -hHHHHHHHHHHHLLL--------leeEHHHHHHH-L-LL-LL-L--LH-HHHHHHHHHLL---L--L--------L----L---H--HHHHHHHHHHHHHHHH-H--Llllllhh
0003 3frhA   -----------------------llllHHHHHHHH-H-HLlLL-L--LH-HHHHHHHHHHH---L-lL--------L----L---H--HHHHHHHHHHHHHHHL-Ll---------
0004 8valE   -hHHHH--hhlllllleeeeeelllllHHHHHHHH-H-HH-LL-L--LH-HHHHHHHHHLL-------------------------------lLHHHHHHHHHL-L--Hhhhhhhh
0005 6h3aB   --lHHHHHHHH-------------hhhHHHHHHHH-H-HL-LL-L--LH-HHHHHHHHHLL---L------------------------LLLHHHHHHHHHLLLLl----------
0006 2ejsA   -llLLLL-----------------llhHHHHHHHH-H-HH-LL-L--LH-HHHHHHHHHHL--------------------------------LHHHHHHHHHH-L--Llllll--
0007 4ociA   --------------------lllllllHHHHHHHH-H-HL-LL-L--LH-HHHHHHHHHL----L--L--------L----L------llLHHHHHHHHHHHHH-L--Lllhhhhh
0008 7qogC   -------------------------llHHHHHHHH-H-HH-HLlL--LH-HHHHHHHHHHH---HhlL--------L----L---L--LLHHHHHHHHHHHHHH-H--Hlllllll
0009 2qhoD   --------------------lllhhhlLHHHHHHH-H-HH-LLlL--LH-HHHHHHHHHLL-------------------------------lLHHHHHHHHHH-L----------
0010 1otrA   ------------------------lllHHHHHHHH-H-HH-LL-L--LH-HHHHHHHHHLL-------------------------------lLLHHHHHHHHH-H--Lllll---
0011 3bq3A   -------------------------lhHHHHHHHH-H-HH-HL-L--LH-HHHHHHHHLLL-------------------------------lLHHHHHHHHHH-H--Hlllllll
0012 1mx9D   --------hhhhhhhllllllllllllHHHHHHHH-H-HL-HL-L--LL-LLHHHHHHHHH---L-lL--------L----L---H--HHHHHHHHHHHHHHHL-H--Hhhhhhhh
0013 1xb2B   ------------------------lhhHHHHHHHH-H-HH-HL-L--LH-HHHHHHHHHLL-------------------------------lLHHHHHHHHHH-H--Hhhhhhhh
0014 6c95D   --------hhhHHHHH----hhhllllLHHHHHHH-H-HH-LL-L--LH-HHHHHHHHHLL-------------------------------lLHHHHHHHHHL------------
0015 7qojD   -------------------------llHHHHHHHH-H-HH-HL-L--LH-HHHHHHHHHHHhhhL--L--------L----L---H--HHHHHHHHHHHHHHHH-H--Hllll---
0016 2n5jA   ---LLLL-----------------llhHHHHHHHH-H-HH--L-L--LH-HHHHHHHHHHL---L--L------------------------lLHHHHHHHHHH-H--L-------
0017 4eeiB   eellEEEEhhhHHLL--------lllhHHHHHHHH-H-HH-LL-L--LH-HHHHHHHHHHH---H--H-------------------lLLHHHHHHHHHHHHHLl-----------
0018 8hkcE   -hHHLLLLhhhhHHHH-----hhlhhhHHHHHHHH-H--H-HL-L--LH-HHHHHHHHHHH---H-hlllhhhlllH----H---H--HHHHHHHHHHHHHHHH-H--Llllllll
0019 9cc7C   -------------------------llHHHHHHHH-H-HH-L-lL--LH-HHHHHHHHHHH---H--L--------L----L-llH--HHHHHHHHHHHHHHHH-H--Hhhhhlll
0020 2cp8A   ------lLLLL-------------lllHHHHHHHH-H-HH-LL-L--LH-HHHHHHHLLLL-------------------------------lLHHHHHHHHHH-H--Llllll--
0021 9fvrC   --------eeeELLLL----lleelllHHHHHHHH-H-HH-LLlL--LH-HHHHHHHHHHH---H--HhhhlllleE----E---H--HHHHHHHHHHHHHHLH-Hh---------
0022 1nxhA   -hhHHHHLLH-------------hhhhHHHHHHHLlH-HH-HL-L--LH-HHHHHHHHHHH---H--H----hhllL----L---H--HHHHHHHHHHHHHHHL-L----------
0023 6q00B   ------------------------lhhHHHHHHHH-H-HH-HL-L--LH-HHHHHHHHHLL-------------------------------lLHHHHHHHHHL-Ll---------
0024 1aipC   --------------------------lHHHHHHHH-H-HH-HL-L--LH-HHHHHHHHHLL-------------------------------lLHHHHHHHHHH-H--Hhhhhhhh
0025 8x6gH   hhhhHLLHHHHHH---------hhhhhHHHHHHHH-H-HH-HL-lllLH-HHHHHHHHHHH---H--H-hhhhllllllllL---H--HHHHHHHHHHHHHHHH-H--Llllllll
0026 3e46A   --------LLLLLL------lllllhhHHHHHHHH-H-HL--L-L--LH-HHHHHHHHHLL-------------------------------lLHHHHHHHHHH-L----------
0027 6vvoA   -lLLLLLHleeEELL---------lllHHHHHHHH-H-HH-HL-L--LH-HHHHHHHHHLL---L-------------------------LHHHHHHHHHHHHH-H--Hhllllhh
0028 5he9E   ------------------------llhHHHHHHHH-H-HH-HL-L--LH-HHHHHHHH-HH---L--L----------------------LHHHHHHHHHHHHH-H--Hlllllee
0029 3b74A   --------lLLLLLLL----lllllhhHHHHHHHH-H-HH-HHlL--LH-HHHHHHHHHLL-------------------------------lLHHHHHHHHHH-H--Hhhhhhhl
0030 2cp9A   -lLLLLL------------------llLLHHHHHH-H-HH-HL-L--LH-HHHHHHHHHHL-------------------------------lLHHHHHHHHHH-H--Hhhhllll
0031 1kl7A   --------------------llllllhHHHHHHHH-H-LL-LL-L--LH-HHHHHHHHHHL---L--L--------LllllllllL--HHHHHHHHHHHHHHHH-H--Hhhlllll
0032 7p37A   eeeeEELL------------lleeellHHHHHHHH-H-HH-LLlL--LH-HHHHHHHHHHH---H--hhhhlllleE----E---H--HHHHHHHHHHHHHHLH-Hh---------
0033 7lt2A   --------eeelHHHH----hllllllHHHHHHHH-H-HH-HL-L--LH-HHHHHHHHHH------hH--------H----L---LhhHHHHHHHHHHHHHHHH-L--Llllllll
0034 2ekfA   -llLLLL----------------llllHHHHHHHH-H-HH-LL-L--LH-HHHHHHHHLLL--------------------------------LHHHHHHHHHL-Ll---------
0035 6jcxF   -llHHHHHHHHH----------hhhhhHHHHHHHH-H-HH-HL-LhhHH-HHHHHHHHHHH---H-hlllllllllL----H---H--HHHHHHHHHHHHHHHH-H--Hlllllee
0036 2o7gA   hhhhhhhLLLHHHHH------hhhhhhHHHHHHHH-H-HH-LLhH--HH-HHHHHHHHHHH---H-hhhhhlllllL----H---H--HHHHHHHHHHHHHHLL------------
0037 6ajfA   eeelllllHHHHHlleelllhhhhhhhHHHHHHHH-H-HH-HHhH--HH-HHHHHHHHHH------lllllleehhH----H---H--HHHHHHHHHHHHHHHH-H--Hhhhhhhh
0038 6jbqF   -lLHHHHHhllLLLHH-----hhhhhhHHHHHHHH-L-LL-LL-L-lLH-HHHHHHHHHHH---H-llhhhlllllL----H---H--HHHHHHHHHHHHHHHH-H--Hhhlllll
0039 5wuqA   -hHLLL-LLLHH---------hhhhhhHHHHHHHH-H-HH-H--L--LH-HHHHHHHHHHH---H--HhhhlhhhllllllH---H--HHHHHHHHHHHHHHHH-H--Hlllllll
0040 2cosA   --LLLLL-----------------lllLHHHHHHH-H-HH-H--L--LH-HHHHHHHHHHL------------------------------lLLHHHHHHHHHH-H--Llllll--
0041 2dnaA   -lLLLL-LLLL-----------lhhhhLHHHHHHH-H-HH-LL-L--LH-HHHHHHHHHLL-------------------------------lLHHHHHHHHHH-L--Llllllll
0042 8vjlB   hhhhLLHHHHHHH----------hhhlLHHHHHHH-H-HH-LLlL--LH-HHHHHHHHHHH---H--H-hhhlllllllllH---H--HHHHHHHHHHHHHHHH-H--Hhhhl---
0043 7mvvA   --------eeelLEEE------ellhhHHHHHHHH-H-HH-HL-L--LH-HHHHHHHHHHH---H------------hllhH---H--HL-LLHHHHHHHHHHH-H--Hhhhhhhh
0044 6e6yA   -lLHHH--------------lllhhhhHHHHHHHH-H-HL-HHhH--HH-HHHHHHHHHHH---H-hL--------L----L---H--HHHHHHHHHHHHHHHH-H--Hhhlllll
0045 1wglA   --------------------lllllllLHHHHHHH-H-HH-LL-L--LH-HHHHHHHLLLL-------------------------------lLHHHHHHHHHH-L--Llllllll
0046 2qsfX   ----llLLHHHHHH------lllllhhHHHHHHHH-H-L--LL-L--LH-HHHHHHHHHLL-------------------------------lLHHHHHHHHLL-L----------
0047 4f1iA   --------------------lllllllHHHHHHHH-H-HH-HL-L--LH-HHHHHHHLLLL-------------------------------lLHHHHHHHHHH-Lhl--------
0048 9kgbA   --------hhlLLLLH-----hhhhhhHHHHHHHH-H-HH-HLlL--LL-L-LHHHHHHHH---H-lL--------L----L---L----HHHHHHHHHHHHHH-H--Hhhhhhll
0049 8yhuB   ----------------------lhhhhHHHHHHHH-H-HH-LL-L--LH-HHHHHHHHHH----H--H--------H----H-llL--LLHHHHHHHHHLLL--------------
0050 6tdvD   ----------------------llhhhHHHHHHHH-HhHL-HH-H--LllLLHHHHHHHHH---H--H--------L-----------lLHHHHHHHHHHHHHH-H--Lllll---
0051 6toaD   ----LLLE---ELLLL---------lllLLLHHHH-H-HH-LLllllLH-HHHHHHHHHHH---H-eeeeeelllllhhhlL---H--HHHHHHHHHHHHHHHL-Ll---------

Job: Type-1 BREX; BrxL HAM28421 NTD2 OB-Fold
Query: s001A
No: Chain Z rmsd lali nres id PDB Description
1: 8emh-B 16.2 1.4 98 676 36 MOLECULE: PROTEASE LON-RELATED BREX SYSTEM PROTEIN BRXL;
2: 7xut-B 6.3 3.2 82 140 12 MOLECULE: FUSION PROTEIN OF REPLICATION PROTEIN A 70 KDA DN
3: 1u5k-A 6.1 3.4 73 242 4 MOLECULE: HYPOTHETICAL PROTEIN;
4: 7yny-A 6.1 3.0 69 332 9 MOLECULE: LEF3;
```

```
5: 4gnx-C 6.0 3.0 84 433 5 MOLECULE: PUTATIVE UNCHARACTERIZED PROTEIN;
6: 4gs3-A 5.8 3.7 74 90 11 MOLECULE: SINGLE-STRANDED DNA-BINDING PROTEIN;
7: 6d6v-E 5.5 2.9 77 149 5 MOLECULE: TELOMERASE REVERSE TRANSCRIPTASE;
8: 1m4v-B 5.5 2.9 69 197 3 MOLECULE: SET3, SUPERANTIGEN-LIKE PROTEIN;
9: 8aas-C 5.5 3.4 72 113 17 MOLECULE: REPLICATION FACTOR A;
10: 3u58-A 5.5 3.6 77 213 6 MOLECULE: TETRAHYMENA TEB1 AB;
11: 7lma-D 5.4 2.8 79 187 10 MOLECULE: TELOMERASE LA-RELATED PROTEIN P65;
12: 7ymo-A 5.4 3.9 69 206 14 MOLECULE: RECOMBINATION PROTEIN RECO;
13: 4gn5-A 5.4 3.1 75 113 4 MOLECULE: OBODY AM3L15;
14: 3jc6-5 5.2 3.7 79 254 6 MOLECULE: DNA REPLICATION LICENSING FACTOR MCM2;
15: 6pqh-B 5.2 3.5 78 479 4 MOLECULE: ASPARAGINE--TRNA LIGASE;
16: 1gm5-A 5.2 4.0 80 729 11 MOLECULE: RECG;
17: 8ez6-A 5.2 2.6 61 65 13 MOLECULE: CELL CYCLE AND APOPTOSIS REGULATOR PROTEIN 2;
18: 3tdq-A 5.2 3.3 68 86 6 MOLECULE: PILY2 PROTEIN;
19: 8soj-B 5.1 3.8 76 428 8 MOLECULE: CST COMPLEX SUBUNIT CTC1;
20: 5m1x-D 5.1 3.3 82 133 10 MOLECULE: REPLICATION FACTOR A PROTEIN 1;
21: 1nnx-A 5.1 3.1 74 93 7 MOLECULE: PROTEIN YGIW;
22: 8y30-A 5.1 4.2 78 756 10 MOLECULE: SINGLE-STRANDED-DNA-SPECIFIC EXONUCLEASE RECJ;
23: 3f2b-A 5.1 3.9 78 994 13 MOLECULE: DNA-DIRECTED DNA POLYMERASE III ALPHA CHAIN;
24: 5z7d-B 5.1 3.7 79 397 5 MOLECULE: INTERFERON-ACTIVABLE PROTEIN 204;
25: 6lbt-B 5.1 3.2 79 303 15 MOLECULE: KLLA0F20922P,KLLA0F20922P;
26: 6lbr-A 5.1 3.5 85 519 15 MOLECULE: KLLA0F20922P;
27: 4o2d-B 5.1 3.4 76 516 7 MOLECULE: ASPARTATE--TRNA LIGASE;
28: 6sw9-N 5.1 3.2 75 146 7 MOLECULE: 16S RIBOSOMAL RNA;
29: 7dpd-B 5.0 3.6 73 273 5 MOLECULE: DNA HELICASE MCM9;
30: 4hce-B 5.0 3.3 81 130 6 MOLECULE: CELL DIVISION CONTROL PROTEIN 13;
31: 3klw-A 5.0 3.4 70 98 4 MOLECULE: PRIMOSOMAL REPLICATION PROTEIN N;
32: 1ue1-B 5.0 4.2 76 119 8 MOLECULE: SINGLE-STRAND BINDING PROTEIN;
33: 8peu-A 5.0 3.3 85 419 8 MOLECULE: TRANSCRIPTION TERMINATION FACTOR RHO;
34: 3rd4-B 5.0 2.3 64 82 8 MOLECULE: UNCHARACTERIZED PROTEIN;
35: 8ehb-A 4.9 3.1 75 102 13 MOLECULE: PUTATIVE LIPOPROTEIN;
36: 8txr-C 4.9 3.5 71 431 14 MOLECULE: EXODEOXYRIBONUCLEASE 7 LARGE SUBUNIT;
37: 8d0b-C 4.9 3.5 77 121 6 MOLECULE: CST COMPLEX SUBUNIT CTC1;
38: 1miu-A 4.9 3.1 78 671 13 MOLECULE: DELETED IN SPLIT HAND/SPLIT FOOT PROTEIN 1;
39: 3k8a-B 4.9 3.3 70 103 9 MOLECULE: PUTATIVE PRIMOSOMAL REPLICATION PROTEIN;
40: 3e0e-A 4.9 3.5 74 91 8 MOLECULE: REPLICATION PROTEIN A;
41: 4dap-A 4.9 3.3 69 232 7 MOLECULE: SUGAR FERMENTATION STIMULATION PROTEIN A;
42: 1eqr-A 4.9 3.6 75 590 8 MOLECULE: ASPARTYL-TRNA SYNTHETASE;
43: 3en2-A 4.8 2.9 65 91 12 MOLECULE: PROBABLE PRIMOSOMAL REPLICATION PROTEIN N;
44: 3kf8-D 4.8 2.9 72 123 8 MOLECULE: PROTEIN STN1;
45: 7uy7-B 4.8 4.1 79 291 9 MOLECULE: TELOMERASE-ASSOCIATED PROTEIN OF 75 KDA;
46: 7w7p-D 4.8 3.9 79 284 6 MOLECULE: DNA HELICASE MCM9;
47: 2z4h-B 4.8 2.7 71 194 6 MOLECULE: COPPER HOMEOSTASIS PROTEIN CUTF;
48: 6cqm-D 4.8 4.3 72 99 8 MOLECULE: SINGLE-STRANDED DNA-BINDING PROTEIN RIM1, MITOCHO
49: 2i0q-A 4.7 4.1 78 461 18 MOLECULE: 5'-D(*GP*GP*GP*TP*TP*TP*TP*GP*GP*GP*G)-3';
50: 6lbu-B 4.7 2.8 73 135 15 MOLECULE: KLLA0C11825P;

0001 s001A   -VRPDE--SNKAQ--AIVE-Q-KKR-HKF-I-DKI-HVKYVER-EK-R-Y-WAEME-N-F---ASKRIAI-NS-N-FY-Q--Q-N--EKL----LESG--IWAEV-T-LAYN-E-V--EED--D-YA-F-FIE--D-L-RPIQ-I--A-H-FD-
0002 8emhB   -VRPDE--AEKVK--SLVR-E-RGS-YKV-I-DRV-TVKLNER-KD-K-Y-EASFS-N-L---GIKDAEI-SA-G-IV-K--E-Y--EKL----LVGG--IWVIA-T-LSYY-F-E--EQT--S-SP-F-GVS--L-L-KPIQ-M--P-N-MNm
0003 7xutB   ------qlseGAI--AAIMqK-GDTnIKP-I-LQViNIRPITTspP-R-Y-RLLMS-D--glnTLSSFMLqLN-P-LV-E--E-E--QLS------SN--CVCQIhR-FIVN-T-L--KDG--R-RV-V-ILM--E-L-EVLKs----------
0004 1u5kA   --------------------R-SRT-ANR-S-GIViRRRVTP--AG-D-I-IVTLL-T-G---KLK-AIA-RG-P-LS---------SSL----N-LF--HHVGV-Q-VYQG-P-H---------DL-A-SVK--Q-A-VLEG-A--L-P-T--
0005 7ynyA   --------------------K-LRT-LNV-K-GQLlTKTTMSInED-Y-Y-LFKFL-V-K---SID-YYG-TQ-T-QF-F--S-L--IN--------N--KTYEL-V-LQYS-R-K----------K-L-LIK--S-Y-EQCEd----------
0006 4gnxC   gaerrT--IVQVK-dENLG-MkpDY-FNV-R-ATV-VYIKQEN-LY-taL-STNVA-D-G---QMW-LSG-FN-E-DA-T--Q-L--Igm---lHRAA-nRMYMF-N-CRAK-M-D--TFdtaR-VR-Y-TIS--R-A-APVD-Fa--------
0007 4gs3A   --------------------E-NNT-VTL-V-GKV-fLEFSHE----F-F-NFILE-V-D---YLP-ITI-SN-R-LF-E--G-M--NL------EVG--TRVKI-E-GQLR-S-Y--NKL--I-LT-V-FAR--D-I-SVVP-E---------
0008 6d6vE   ------lnCFMIN--QIakenaFEN-AII-T-AKFeFLQSIND-SP-K-I-MGKLS-D--stgCIEVVIqpEF-V-QA-Y--E-I--E--lqnngNRH--KYVRA-M-LKMR-K-N---------AQ-I-QLL--Y-F-SIVN-Da--------
0009 1m4vB   ------akyenvtkdifdyyS-GAS-KEL-KnVTG-YRYSK---GG-K-H-YLIFD-K-K---FTR-IQI-FG-K-DI-E--R-L--KT------RKN--PGLDI-F-VVKE-A-----------vF-S-YG---G-V-TKKN-Q--Gr-----
0010 8aasC   ------rkISEI-----rE-E-DTR-VSL-I-GRViKVDKM------D-Y-MFWLD-D---gtGVAIIES-ES-D-LP-----------------kVG--QVVRV-I-GRII-R-N---EE--G-IH-I-YAE--V-I-QDFS-D--A------
0011 3u58A   ------llISEVL--KT---S-KQY-LSV-L-AQVvDIQSSD---K-N-I-RLKIC-D-Q---ELK-VVI-FC-Y-EW-R--D-K--FS-------IN--KWYYF-NeFVRQ-I-Y--N------vQ-L-KNS--S-I-KESD-D---------
0012 7lmaD   ------ssIEEII--QQAQ-AqkKE-FYV-Y-GNL-VSIQM-K-NK-LyL-RAFVQ-D--stgTIPV-MI-FD-Q-QS-S--Q-L--InqqlfskLDF--ARFIF-E-IQFE-N-K--EF-----iA-Y-KVL--K-I-EKEN-Ik--------
0013 7ymoA   --------------------------DPM-Q-GFIlHTQKVKD------L-IVYIL-S-M---LIK-AYR-FY----G-L--R-H--SSI----L-SG--YKIDF-A-LEEN-P-S---------FL-P-RLK--D-V-LHLG-F-wI-M-QRd
0014 4gn5A   ------thwtAEI--T-nL-H-GSE-VVV-A-GWVaHLGDYG---R-V-K-IVKVS-D-reggAAVPVYLhLF-K-VF----A-E--LS-------RE--DVVVI-K-GIVE-A-G--WPV----vE-I-FPS--E-I-WILNk---------
0015 3jc65   lanqiP--LRDLD--S-eH-V-SKI-VRL-S-GIIiSTSVLSS-R--htQ-FLKLQ-E-R---NLT-MTC-DR-Y-LT-N----K--VIP-------G--TRVTI-V-GIYS-I-Y--NS-----tpyI-KIL--G-I-QSDV-E--T-S----
0016 6pqhB   -mhkqT--IKEVL--E-kF-L-HHD-ITV-Y-GWVrAFRS---------R-FIALN-D-gstiNNLQIVV-DF-EnFD-E--N-L-iKNI----N-TA--SSLKI-V-GEVV-E-S--Q------vE-I-IAK--K-I-IVLG-D--N-Ft---
0017 1gm5A   -----frkifKLN--D-LL-P-GEK-VTT-Q-GKI-VVETKKFnMN-I-L-TAVLS-D-V---HVP-LKW-FD-Y-LQ-T--YlK--QLT-------G--KEVFV-T-GTVK-S-N-aYTG--Q-yE-I-HNA--E-V-TPKEg----------
0018 8ez6A   ----------------------EKQ-RVF-T-GIV-TSLHD--------F-GVVD----------EEVFF-QL-S-V---kgR-L--PQL-------G--EKVLV-K-AAYN-P-G----Q--A-VP-W-NAV--K-V-QTLS----------
0019 3tdqA   -------------------eD-PQT-FEG-A-GVVfEVQVE------K-N-LVDI----D---HRL-YRL-PNsP-SL-F--Q------V----KPG---SVVSY-S-GTVS-Q-----------PW-S-TIT--D-I-YIHK-Q--X-S-EQe
0020 8sojB   vfaklY--IRDIL--DM--kepIKQ-VDV-L-GTViGVRER---DA-F-Y-SYGVD-D-G---VIN-CIC-WK-KlntsveqK-T--KIE------IG--DTIRV-R-GSIR-T-Y--RE-----rE-I-HAT--T-Y-YKVD-Dp--------
0021 5m1xD   -qlSRG--DfHSI--FtRY-D-N-P-TGG-V-YQV-YNTR----KS-D-GaLIXIS-D---giYHXKALL-RA-S-KF-Q--S-X--ELQ------RG--DIIRViI-AEPA-I-V--RRK--K-YV-L-LVD--D-F-ELVQs----------
0022 1nnxA   ------ttVESAK--SL-R-D-DTW-VTL-R-GNIvERIS-------D-D-LYVFK-D---asGTINVDI-DH-K-RW-N--G-V--TV--------tpkDTVEI-Q-GEVD-K-D--W-N--S-VE-I-DVK--Q-I-RKV-----------
0023 8y30A   vaaflaltENDI-----iknisrPI-FEM-DdLSV-SVKAIGQ----H-L-KLTLG-E-S---NI-AALF-WQ-N---gH--L-E--PEL-----QDE--QPINI-L-GSVQ-I-N--EW----nPQ-I-IIQ--D-I-AMNE-Q-fS-Q-LYi
0024 3f2bA   ---vrR--LET-I--VEEE----RR-VVV-Q-GYVfDAEVSEL------L-TMKIT-D-N---SIL-VKM-FS-RdkeaE--L-M--SGV-----KKG--MWVKV-R-GSVQ--nD--TFV--R-DLvI-IAN--D-L-NEIAa----------
0025 5z7dB   rnatpK--ISYLF--SQ-A-R-GTF-VSG-E-YLVnKKTER---NK-F-I-YYGIG-D-G---KME-VVV-YG-R-LT-N----V--RCE------PG--SKLRL-VcFELT-S-T--E------wQ-L-RSS--Y-M-QVIN-A---------
0026 6lbtB   ---------DLKP--DS-I-S-KAL-YHIsD-GKIlGFIPnSL-IE-F-L-LIYVY-T-vpeyNCYEIAI-TA-K-FF-S--K-I--G-----eLKEL--IRYDI-T-IYKK-E-F--TI-----sK-W-ALK--D-V-VLRSg----------
0027 6lbrA   ------dpfDLKP--DS-I-S-KAL-YHIsD-GKIlGFIPNSL-IE-F-L-LIYVY-TlN---CYE-IAI-TA-K-FF-S--K-I--G-----lKELIdnNRYDI-T-IYKK-E-F--TI-----sK-W-ALK--D-V-VLRS-A-qA-F-YDd
0028 4o2dB   ------thaaGSL--R-aD-A-GQT-VTL-A-GWVaRRRDH---GG-V-I-FIDLR-D-G---VSQ-VVF-RL-A-AA----H-R--LR-------AE--FCVAV-T-GVVE-VrP--EGN--EniE-V-NAT--E-L-TVLGe----------
0029 6sw9N   ------keksDPL--E----G-APQ-ARG---IVL-EKIAVEAsGM-R-K-AVRVQnG-K---VV-TAFC-PG-G-AI-K--F-------------iD--EHDEV-I-IEGI-G-GpkGGS--MgIR-Y-KVV--K-V-N-rK-P--R-R----
0030 7dpdB   ------lvreHIP--K-kD-V-GHF-LSV-T-GTViRTSLVKV-L--yyQ-EIKIQ-E-R---SMK-VIL-ED-D-LV------D--SCK------SG--DDLTI-Y-GIVM-Q-R--WK--pfqeivL-KAN--Y-I-QVNN-----------
0031 4hceB   ----IS--YEQLS--LA-S-V-GSV-ERL-E-GKIvGMNPpFK-YC-T-L-KLYFT-QpN---CIE-IVI-P--E-LF-G------------kISDI--LRISV-E-VERI-L-W--DPG--M-AV-W-SLK--N-I-STDT-----------
0032 3klwA   ----------------------XNT-LEL-S-ARVlECGAXR-------L-ELLLV-HeL---TIS-AVA-LG-D-LA-L--L-----------aDTPL-gTEXQV-Q-GFLA-PaR--KDS-vK-VK-L-HLQ--Q-A-RRIA-G---------
0033 1ue1B   -------------------aG-DTT-ITI-V-GNL-TPELRFT----V-A-NFTVA-SdL---FLR-CNI-WE-N-VA----E-S--LT--------RG--ARVIV-S-GRLK-Q-R--SFT--V-IE-V-EVD--E-I-GPSL-T--A-K-V--
0034 8peuA   -LTELKrkQDIIF--AILK-Q-HAD-IFG-D-G-V-LEILQ----D-G-F-GFLRS-A-sylagpdDIYV-SP-S-QI-R--R-F--NLR----T--G--DTISG-K-IRPP-K-E----G--E-RY-F-ALL--K-VnEVNF-N--A-R-N--
0035 3rd4B   ----------------------aPV-KLY-M-VEViDKKEIAAiTH-Y-Y-QVTFR-L-K---DLV-LNI-DK-S-SY-Q--N------I----EPE---MKGRL-F-MQG-----------------S-RFV--Q-F-ETDV-P--------
0036 8ehbA   -----------eshasccveE-KIPiVTL-KnENA-HFRYXKR-RN-D-F-ALEIE-N-KelvRGLYLIPrGC-D-I-pK--K-Y--K-----eDGLP--VIISG-E-VFDC-S-E--YKR--D-PV-Y-FIK--L-S-TIK-----------
0037 8txrC   ------iftvsrlnqtvlehE-MGQ-VWI-S-GEI-SFTQPA--SG-H-W-YFTLK-D-A---QVR-CAM-FR-N-snrV--T-F--RP------QHG--QQVLV-R-ANIT-L-Y--ERG--D-YQ-I-IVE--S-M-QP-------------
0038 8d0bC   lgtyyL--PWEVS--AGqV-P-DGT-LRT-F-GRL-CLYDM----I-Q-S-RVTLM-A-gsdqHQVLVCTlVE-P-F-------H--AQV-------G--SLYIV-L-GELQ-H-Q--QDR----sV-V-KAR--V-L-TCVE-Gm--------
```

```
0039 1miuA  -srrSA--LKKILerDDTA-----A-KTL-V-LCI-SDIV---------D-TIELT-D-Y---AVR-AQL-DP-P-LM-A--L-VksGKL----T-VG--QKIIT-QgAELV-G-S--P-apdS-LR-L-KISanS-T-RPAR-Whe-------
0040 3k8aB  -----------------hmgF-TNL-VSL-A-ALIeKAFP---------L-DIILK-HwL---EIP-ARI-LG-R-QA-E--E-W--QYR------QG--DCATV-E-GFLA-QkS--RRS--L-MP-MlRIQ--N-I-KEYKg----------
0041 3e0eA  ------ykISEL------X-P-NLS-GTI-N-AEVvTAYPKKE----L-K-SLFLK-D-G---SIR-GTL-WN-E-LA-D----F--EVK------KG--DIAEV-S-GYVK-Q-----------lE-I-SVD--N-I-GIIE-K--S------
0042 4dapA  -----------------mefS-PPL-QRA---TLI-QRYK---------F-LADVIdG-R---EL-TLHC-PN-T----G--A-M--TG------caT--PGDTV-W-YSTS-D-N--TKR--K-YP-H-TWE--L-T-QSQS---tL-A-ENe
0043 1eqrA  ------teycgQL--RLsH-V-GQQ-VTL-C-GWVnRRRDLG---S-L-I-FIDMRrE-G---IV-QVFF-DL-K-LA----S-E--LR-------NE--FCIQV-T-GTVR-arD--EKN--IniE-V-LAS--S-L-TIINr----------
0044 3en2A  --------------------A-INR-LQL-V-ATL-VEREV--------paCLLSY-S-rqveFSIEALGkXA-S-VL-D----R--IAP-------G--TVLEC-V-GFLA-R-K--H----A-LV-F-HIS--G-L-EHH------------
0045 3kf8D  ------lipSNIP--QE----npER-LRI-L-AQVkDFIPH------E-S-TIVID-K-eqstYINI-CI-FN---LL-E--AcS--SRV----LVPG--TLVNI-D-AFYD-G-E----------S-I-NPV--D-I-YEVN-Ga--------
0046 7uy7B  -felvF--LKELP--SL-P-D-FSK-VCF-T-GLIlSFSNFPS-S----K-IAIIQ-D-G---EAE-LFL-DM----Y-K--F-C--Q-----eISVF--KAITG-I-GVLKkK-N--IG-----cK-I-IVE--R-F-RIIH-Sa--------
0047 7w7pD  ------tqLKNVR--ANCY---GKY-IAL-R-GTVvRVSNIKP-LctW-Q-SVKVQ-EmR---TIE-CEL-VQ-D-LV-D-------------scVPG--DVVTI-T-GVVK-V-S--STEcvF-LL-YiEAN--S-V-SNSK-----------
0048 2z4hB  ------iesqfnytleasL-P-MTP-MTL---RGM-YFYMA------D-A-ATFTD-C-G---KRFMVAN-NA-E-LE-R--S-Y--LA-arghsEKP--VLLSV-E-GHFT-L----eAP--T-KV-L-APD--TaG-KFYPn----------
0049 6cqmD  ---------------------dFSK-MSI-V-GRI-GFTEHTSanR-Y-L-KYSIA-S-rgqtNWYNITVqIN-F-LT-E--Y-V--RK--------G--ALVYV-E-ADAA-N-Y--VFT--T-LS-L-VQK--D-I-NLLKn----------
0050 2i0qA  vdmytA--LNKAQ--AQ-----KGD-FDV-V-AKIlQVHELD--EY-T-N-ELKLK-D-Q---VFY-TLS-LL-K-FP------H--VRT-------G--EVVRI-RsATYDeT-S--TQ-----kV-L-ILS--N-I-IT-F-I--Q-Ss---
0051 6lbuB  -kiitD--LDTIA--GK----tlLR-LRI-F-AQF-QDI----rtD-G-I-YLHFS-N-aeerSYYF-LI-DE-T-IY-D--E-A--F--sgerpHKG--DILDX-R-CCYR-K-Y-------D-KV-V-EIX--H-L-KVIS-Ia--------

0001 s001A  -LLHHH--HHHHH--HHHH-H-HLE-EEE-E-EEE-EEEEELL-LL-E-E-EEEEL-L-L---LEEEEEL-LH-H-HH-H--H-L--HHH----HLLL--EEEEE-E-EEEL-L-L--LLL--L-LL-E-EEE--E-E-EELL-L--L-L-LL-
0002 8emhB  -LLLLL--HHHHH--HHHH-H-HLE-EEE-E-EEE-EEEEELL-LL-E-E-EEEEL-L-L---LLLLEEE-LH-H-HH-H--H-L--HHH----HLLL--EEEEE-E-EEEL-L-L--LLL--L-LL-E-EEE--E-E-EELL-L--L-L-LLh
0003 7xutB  ------hlllLHH--HHHHhH-LLLlLLL-E-EEEeEEEEELLllL-E-E-EEEEE-L--lleEEEEEEEhHH-H-HH-H--L-L--LLL------LL--LEEEEeE-EEEE-E-L--LLL--L-EE-E-EEE--E-E-EEEEl----------
0004 1u5kA  --------------------L-LLE-EEE-E-EEEeEEEELL--LL-L-E-EEEEE-E-E---EEE-EEE-LL-L-LL---------LLL----L-LL--LEEEE-E-EELL-L-L---------LL-E-EEE--E-E-EEEE-L--L-H-H--
0005 7ynyA  --------------------L-LLE-EEE-E-EEEeEEEEEEElEE-E-E-EEEEE-L-L---EEE-EEE-LH-H-HH-H--H-L--LL--------L--LLEEE-E-EEEE-L-L----------E-E-EEE--E-E-LLLLl----------
0006 4gnxC  lllllL--HHHHH-hLLLL-LllEE-EEE-E-EEE-EEELLLL-LE-eeE-EEEEE-E-E---EEE-EEE-EH-H-HH-H--H-H--Hll---hHHHL-lEEEEE-E-EEEE-E-E--EEleeE-EE-E-EEE--E-E-EELL-Hh--------
0007 4gs3A  --------------------L-LLL-EEE-E-EEE-lLEEEEE----E-E-EEEEE-E-E---EEE-EEE-EH-H-HL-L--L-L--LL------LLL--LEEEE-E-EEEE-E-E--ELE--E-EE-E-EEE--E-E-EEEL-L---------
0008 6d6vE  ------llHHHHH--HHhhhllLLE-EEE-E-EEEeEEELLLL-LL-E-E-EEEEE-E--lleEEEEEElhHH-H-HH-H--H-H--H--lllllLLL--EEEEE-E-EELL-L-L---------LL-L-EEE--E-E-EELL-Lh--------
0009 1m4vB  ------lllllllhhhhhhhL-LLL-EEE-EeEEE-EEEEE---LL-E-E-EEEEE-E-E---EEE-EEE-LL-H-HH-H--H-L--LL------LEE--EEEEE-E-ELLL-E-----------eE-E-EL---L-E-EELL-L--Ll-----
0010 8aasC  ------llHHHL-----lL-L-LLE-EEE-E-EEEeEEELL------L-L-EEEEE-L---llLEEEEEL-LL-L-LL-----------------lLL--LEEEE-E-EEEE-E-E---LL--E-EE-E-EEE--E-E-EELL-L--L------
0011 3u58A  ------llHHHHL--LL---L-LLE-EEE-E-EEEeEEEELL---L-L-E-EEEEE-L-L---LEE-EEE-LL-H-HH-L--L-L--LL-------LL--LEEEE-EeEEEL-L-L--L------lE-E-ELL--E-E-EELL-L---------
0012 7lmaD  ------llHHHHH--HHHL-LleEE-EEE-L-LEE-ELLLL-L-LL-LlE-EEEEE-L--lllEEEE-EE-LH-H-HH-H--H-H--HhhhhhhlLLL--EELEE-E-EEEE-L-L--EE-----lE-E-EEE--E-E-ELLL-Hh--------
0013 7ymoA  --------------------------LLE-E-EEEeEEEEELL------E-EEEEE-L-L---EEE-EEE-LL----H-H--H-L--LLL----L-LL--LEEEE-E-EEEE-L-L---------EE-E-EEE--E-E-EEEL-L-lL-L-LHh
0014 4gn5A  ------lllhHHL--L-hH-L-LLE-EEE-E-EEEeEEEELL---L-E-E-EEEEE-L-llllLLEEEEEhHH-H-HH----L-L--LL-------LL--LEEEE-E-EEEE-L-L--LLH----lE-E-EEE--E-E-EEEEl----------
0015 3jc65  ellllL--HHHLL--L-lL-L-LLL-LLE-E-EEEeEELLLLL-L--llL-LEEEE-E-L---EEE-EEL-LH-H-HL-L----L--LLL-------L--LEEEE-L-LLEE-E-E--LL-----eeeE-ELL--L-L-EELL-L--L-L----
0016 6pqhB  -llllL--HHHHH--H-hH-L-LLE-EEE-E-EEEeEEEL---------L-EEEEE-L-llllLLEEEEL-LH-HhLL-H--H-H-hLLL----L-LL--LEEEE-E-EEEE-E-L--L------eE-E-EEE--E-E-EELL-L--L-Lh---
0017 1gm5A  -----lllllLLL--L-LL-L-LLL-LEE-E-ELL-LLEEEELlLE-E-E-EEEEL-L-L---LEE-EEE-LL-L-LH-H--HhH--LLL-------L--LLEEE-E-EEEL-L-L-lLLL--L-lL-E-EEE--E-E-ELLLl----------
0018 8ez6A  ----------------------LLE-EEE-E-EEE-EEELL--------E-EEEL----------LLEEE-EH-H-H---llL-L--LLL-------L--LEEEE-E-EEEL-L-L----L--L-LL-E-EEE--E-E-EELL-----------
0019 3tdqA  -------------------lL-LLE-EEE-E-EEEeEEEHH------H-L-EEEE----L---LEE-EEL-LLlE-HH-H--L------L----LLL---LEEEE-E-EELL-L-----------LL-L-EEE--E-E-EELL-L--L-L-EEh
0020 8sojB  heellL--HHHHH--HL--eeeELE-EEE-E-EEEeEEEEL---LL-E-E-EEEEE-L-L---EEE-EEE-EL-LlllllhhH-H--LLL------LL--LEEEE-E-EEEE-E-E--LL-----eE-E-EEE--E-E-EEEL-Ll--------
0021 5m1xD  -llLLL--HhHHH--HhHH-H-L-L-LLL-E-EEE-EELL----HH-H-LlEEEEE-L---llEEEEEEE-EH-H-HH-H--H-L--LLL------LL--LEEEEeE-EEEE-E-E--LLL--E-EE-E-EEE--E-E-EEEEl----------
0022 1nnxA  ------llHHHHL--LL-L-L-LEE-EEE-E-EEEeEEEE-------L-L-EEEEE-E---llEEEEEEL-LH-H-HL-L--L-L--LL--------lllLLEEE-E-EEEE-E-E--L-L--E-EE-E-EEE--E-E-EEL------------
0023 8y30A  lhhhhhllHHHL-----hhhhllLL-EEE-EeEEE-EEEEELL----E-E-EEEEL-L-L---LE-EEEE-LL-L---lL--L-H--HHH-----LLL--LEEEE-E-EEEE-E-E--EE----lEE-E-EEE--E-E-EELL-L-lL-E-EEe
0024 3f2bA  ---llL--HHH-L--LLLE----EE-EEE-E-EEEeEEEEEEL------E-EEEEE-L-L---EEE-EEE-EL-LlhhhH--H-H--HLL-----LLL--LEEEE-E-EEEE--eE--LLL--L-EEeE-EEE--E-E-EEELl----------
0025 5z7dB  hllllL--HHHHH--LL-L-L-LLL-EEE-E-EEEeEEEEE---LL-E-E-EEEEE-E-E---ELE-EEE-EH-H-HH-H----L--LLL------LL--LEEEE-EeEEEE-E-E--L------eE-E-ELL--E-E-EEEL-L---------
0026 6lbtB  ---------LLLL--LL-H-H-HLE-EEEeE-EEEeEEELlLL-LL-L-L-EEEEE-E-llllLLEEEEL-LH-H-HH-H--H-H--L-----hHHHH--HEEEE-E-EEEE-E-E--EE-----eE-E-EEE--E-E-EEELl----------
0027 6lbrA  ------lllLLLL--LL-H-H-HLL-EEEeE-EEEeEEELLLL-LL-L-E-EEEEE-LlL---LEE-EEE-LH-H-HH-H--H-H--L-----hHHHHhhLEEEE-E-EEEE-E-E--EE-----eE-E-EEE--E-E-EEEE-L-lL-L-LLl
0028 4o2dB  ------lllhHHL--L-hH-L-LLE-EEE-E-EEEeEEEEE---LL-E-E-EEEEE-E-E---EEE-EEE-LH-H-HH----L-L--LL-------LL--LEEEE-E-EEEE-ElL--LLL--LleE-E-EEE--E-E-EEEEl----------
0029 6sw9N  ------llllLLL--L----L-LLL-EEE---EEL-LLEEEELlLE-E-E-EEEEElL-L---EE-EEEL-LL-L-HH-H--L-------------lL--LLLLE-E-EELL-L-LhhHLL--LlLL-L-EEE--E-E-L-lL-L--L-L----
0030 7dpdB  ------leelLLL--L-hH-L-LLE-EEE-E-EEEeEELLLEE-E--llE-EEEEE-E-L---EEE-EEE-EH-H-HL------L--LLL------LL--LEEEE-E-EEEE-E-E--EE--llleeeE-EEE--E-E-EELL-----------
0031 4hceB  ----LH--HHHHL--LL-L-L-LLE-EEE-E-EEEeEEELlHL-LL-L-E-EEEEE-LlL---LEE-EEE-L--H-HH-L-------------hHHHH--LLEEE-E-EEEE-E-E--LLL--E-EE-E-EEE--E-E-ELLL-----------
0032 3klwA  ----------------------LEE-EEE-E-EEEeEELLLE-------E-EEEEE-EeE---EEE-EEE-EL-H-HH-H--H----------lLLLL-lLEEEE-E-EEEE-ElL--LLL-lL-EE-E-EEE--E-E-EEEL-L---------
0033 1ue1B  -------------------lL-LLE-EEE-E-EEE-LLEEEEL----E-E-EEEEE-ElE---EEE-EEE-EH-H-HH----H-H--LL-------LL--LEEEE-E-EEEE-E-E--ELE--E-EE-E-EEE--E-E-EELL-L--L-E-E--
0034 8peuA  -HHHHHlhHHHHH--HHHH-H-HHL-LLL-E-E-E-EEELL----L-L-L-EEEEL-L-lllllllLEEE-LH-H-HH-H--H-L--LLL----L--L--LEEEL-L-EELL-L-L----L--L-LL-L-EEL--L-LlEELL-L--L-L-L--
0035 3rd4B  ----------------------lLE-EEE-E-EEEeEEEEEEEeEE-E-E-EEEEE-E-E---EEE-EEE-LH-H-HH-L--L------L----LLL---LEEEE-E-EEL-----------------L-EEE--E-E-EELL-L---------
0036 8ehbA  -----------leellllllL-EEEeEEE-EeEEE-EEEEELL-LL-E-E-EEEEL-L-HhhlLLLLEELlLL-L-L-lH--H-H--L-----lLLLE--EEEEE-E-EEEE-H-H--HLL--L-LL-E-EEE--E-E-EEE------------
0037 8txrC  ------eeehhhhhhhhhhhH-LLE-EEE-E-EEE-EEELLL--LL-L-E-EEEEE-L-L---EEE-EEE-LH-H-hhlL--L-L--LL------LLL--LEEEE-E-EEEE-E-E--LLL--E-EE-E-EEE--E-E-EL-------------
0038 8d0bC  lleelL--HHHHL--LLlL-L-LLE-EEE-E-EEE-EEEEL----L-L-L-EEEEE-E-lleeEEEEEELlLL-L-L-------L--LLL-------L--LEEEE-E-EEEE-E-L--LLL----eE-E-EEE--E-E-EELL-Ll--------
0039 1miuA  -lllLH--HHHHHllLLLL-----L-LLE-E-LEE-LLLL---------L-LLLEE-L-L---EEL-LLL-LH-H-HH-H--H-HhlLLL----L-LL--LLLLL-LlLLEE-L-L--L-lllL-LE-E-LLLhhH-L-LLLL-Lll-------
0040 3k8aB  -----------------lllL-LEE-EEE-E-EEEeEELL---------E-EEEEE-EeE---EEE-EEE-EH-H-HH-H--H-L--LLL------LL--LEEEE-E-EEEE-ElL--LLL--L-LE-EeEEE--E-E-EELLl----------
0041 3e0eA  ------elHHHL------L-L-LEE-EEE-E-EEEeEELLLEE----E-E-EEEEE-E-E---EEE-EEE-EH-H-HH-H----L--LLL------LL--LEEEE-E-EEEE-E-----------eE-E-EEE--E-E-EEEE-L--L------
0042 4dapA  -----------------lllL-LLL-EEE---EEE-EEEL---------L-EEEEElL-L---EE-EEEL-LL-L----L--L-L--LL------llL--LLLEE-E-EEEL-L-L--LLL--L-LL-E-EEE--E-E-ELLL---lE-E-ELl
0043 1eqrA  ------lllllLL--LHhH-L-LLE-EEE-E-EEEeEEEEEL---L-E-E-EEEEElL-E---EE-EEEE-LH-H-HH----L-L--LL-------LL--LEEEE-E-EEEE-elL--HHH--LleE-E-EEE--E-E-EEEEl----------
0044 3en2A  --------------------L-LLL-EEE-E-EEE-EEELL--------llEEEEE-E-eeeeEEEEEEEhHH-H-HH-L----L--LLL-------L--LEEEE-E-EEEE-E-L--L----L-EE-E-EEE--E-E-EEL------------
0045 3kf8D  ------llhHHHH--HH----llEE-EEE-E-EEEeEEELL------L-L-EEEEE-E-llllLEEE-EL-LL---LL-L--LlL--HHH----HLLL--LEEEE-E-EEEL-L-L----------L-E-EEE--E-E-EELL-Hh--------
0046 7uy7B  -llllL--HHHLL--LL-L-L-LLE-EEE-E-EEElLLLLLLL-L----L-EEEEE-L-L---EEE-EEL-LL----L-L--L-L--L-----lLLLL--LLEEE-E-EEEEeE-E--LL-----eE-E-EEE--E-E-EELL-Lh--------
0047 7w7pD  ------llLLLLL--LLLL---LLL-LLL-L-EEEeEELLLEE-EeeE-E-EEEEE-ElL---EEE-EEE-EH-H-HL-L-------------llLLL--LEEEE-E-EEEE-E-L--LLLllL-LE-EeEEE--E-E-ELLL-----------
0048 2z4hB  ------leeeeeleeeelL-L-LLL-EEE---EEE-EEELL------L-L-EEEEE-L-L---LEEEELL-LH-H-HH-H--H-H--HH-hllllLLL--EEEEE-E-EEEE-E----eLL--E-EE-E-EEL--LlL-EEELl----------
0049 6cqmD  ---------------------lEEE-EEE-E-EEE-LLEEEELllE-E-E-EEEEE-E-llllEEEEEEEhHH-H-HH-H--H-L--LL--------L--LEEEE-E-EEEE-E-E--EEE--E-EE-E-EEE--E-E-EEEEl----------
0050 2i0qA  lhhllL--HHHHH--HL-----LLL-EEE-E-EEEeEEEELL--LL-E-E-EEEEE-L-L---EEE-EEE-EL-L-LL------L--LLL-------L--LEEEE-EeEEELlL-L--LL-----lE-E-EEE--E-E-EE-E-L--L-Ll---
0051 6lbuB  -eeelL--LLHHH--HH----llEE-EEE-E-EEE-EEL----llL-L-E-EEEEE-L-llllLEEE-EE-LH-H-HH-H--H-H--H--lllllLLL--LEEEE-E-EEEE-L-L-------L-LE-E-EEE--E-E-EELL-Hh--------
```

Job: Type-1 BREX; BrxL_HAM28421.1 C-Terminal HKD-EndoDNase
Query: s001A
No: Chain Z rmsd lali nres id PDB Description
1: 2ymb-D 18.7 1.5 152 231 34 MOLECULE: MIT DOMAIN-CONTAINING PROTEIN 1;
2: 7clg-A 8.9 4.1 130 838 8 MOLECULE: PUTATIVE HELICASE;
3: 4rct-A 8.9 3.1 124 347 15 MOLECULE: RESTRICTION ENDONUCLEASE R.NGOVII;
4: 3hsi-B 8.1 3.2 134 451 9 MOLECULE: PHOSPHATIDYLSERINE SYNTHASE;
5: 6ehi-D 8.0 2.7 112 160 5 MOLECULE: NUCLEASE NUCT;
6: 7v53-A 8.0 3.9 132 930 8 MOLECULE: PHOSPHOLIPASE D;
7: 5nwa-A 7.9 3.0 117 441 8 MOLECULE: TYROSYL-DNA PHOSPHODIESTERASE 1;
8: 1byr-A 7.8 3.3 116 152 8 MOLECULE: PROTEIN (ENDONUCLEASE);
9: 7e0m-A 7.6 3.4 127 388 8 MOLECULE: PHOSPHOLIPASE;
10: 2c1l-A 7.5 3.1 118 358 10 MOLECULE: RESTRICTION ENDONUCLEASE;
11: 4gel-B 7.4 3.6 120 198 7 MOLECULE: MITOCHONDRIAL CARDIOLIPIN HYDROLASE;
12: 7wdk-A 7.3 3.9 135 867 7 MOLECULE: PHOSPHOLIPASE D;
13: 4urj-D 6.9 3.4 116 174 6 MOLECULE: PROTEIN FAM83A;
14: 7wul-E 6.7 3.4 144 545 13 MOLECULE: PHOSPHOLIPASE D;
15: 3qph-A 6.5 4.5 112 335 13 MOLECULE: TRMB, A GLOBAL TRANSCRIPTION REGULATOR;
16: 5box-A 6.5 5.9 120 263 8 MOLECULE: PUTATIVE HTH-TYPE TRANSCRIPTIONAL REGULATOR TRMBL

```
17: 6kz8-A 6.3 4.3 137 784 7 MOLECULE: PHOSPHOLIPASE D ALPHA 1;
18: 3igy-B 5.4 3.0 107 549 13 MOLECULE: COFACTOR-INDEPENDENT PHOSPHOGLYCERATE MUTASE;
19: 1xdo-A 5.2 3.3 104 687 13 MOLECULE: POLYPHOSPHATE KINASE;
20: 5b3g-B 5.0 3.4 117 406 8 MOLECULE: PROTEIN SCARECROW;
21: 6s6v-A 4.8 4.5 102 386 7 MOLECULE: NUCLEASE SBCCD SUBUNIT D;

0001 s001A   --VEDG--SL---E--KTVDVQYGDGYG-YDDL-FAEY-LK-GA-SIVMLEEPYLT-QVANL-VRFV-EILVKI-G---D----CKVFRLVT-K-P---GE---T-P--EESTL-ISDSLQRL--KNTLDD--M---DDVSMAFEY-EFD----E-N-SHDRYIRTS-N-----NWDITLGRGL---H---FYQNLKTRFTFM-K---RN--
0002 2ymbD   -------------H--KQIKIEENAGFS-YESL-FREY-LNeTV-TEVWIEDPYIR-QLYNF-LRFC-EMLIK--C---K----VKTIHLLT-S-L---DE---------EQVQ-QSRGLQEI--EESLRS--------HGVLLEV-QYS----S-S-IHDREIRFN-N-----GWMIKIGRGL---D---YFKKHETTVDIF-H---KK--
0003 7clgA   --SHKG--NY---P--KLLVN--nKEKV-L-ST-IIDE-LQ-KC-ETFYFSVAFI--TESGL-ASLK-AQLLDL-S------nkgVKGKILT-S-N---YL---G--------F-NSPKXYGE--LLKL-------------KNVEV-RLT----D-I-FHAKGYIFE-H--kdySSXVIGSSNLtsnA---LKV-NYEHNVLL-S---TX--
0004 4rctA   -------------M--NTVFSNIA-tekslnAV-WMDL-FK-SA-DEVLMATGYV--SNDAV-VELH-KILELN-D---H----IQKIDLLVgM-H---YL--------eGFSHlQYDSLXKL--NDFLRH--E---------RGAV-YVS--pfV-K-FHGKMYSFK-N--yqkINGLIGSANL---T-------TYETMLHL-N---GKpa
0005 3hsiB   tkraeQ--NL-lsV--SPLFGLG-AGN-elnQV-IEDL-FL-QV-KKLVICTPYF---NFPR--TLQ-HKIATLlE---N----GKRVEIIV-G-DkvaND---F-YigALPYL-YESNLRRF--CEKFET------qieSGQLVV-RLW----RnT-YHLKGVWVD-D-----RYILLTGNNL-npR---AWR-DAENGLLI-Y---DQ--
0006 6ehiD   -------------N--SLFVLPY--RDA-L-NS-LISG-IS-SA-ESVKIAIYS-----fTH-RDIA-RAIKSV-A------srgIKVQIIY-D-Y---E---------snHNN-KqstIGYL--DKYP-------------NTKV-CLL----K-G-MNQKVAIID-D-----KIVFLGSANWsknA---FEN-NYEVLLKT-D---DT--
0007 7v53A   -------------V--RFFTT----rDY-FS-D-LASA-LD-SA-SSIFITGWQ---NYDV--rSLW-QCLRQA-L----erspaLKVYVMP-W-L---SP---S-G--SL-gtyDFETMLAV--FQLNAG--L---EGGA-RAFC-TPA----I-Q-HHQKSVVID-N-----RIGYVGGIDL---A-ygRRDDWQDVHCRI-E---GP--
0008 5nwaA   -----------kgnpfQFYLTRVS-ALH-IKDI-LSP-lfG-TL-VSSAQFNYCF-----DV-DWLV-KQY-PP-E---F---rKKPILLVH-G-D---------------KRE-AKAHLHAQ--AKPYE------------NISL-CQA----KgT-HHTKMMLLL-Y--eegLRVVIHTSNLihaD---WHQ-KTQGIWLSpL---YP--
0009 1byrA   --------EP---V--QVGYSPE--sarvlvlS-AIDS-A---K-TSIRMMAYSF--TAPDI-MKAL-VAAK-K-R---G----V-DVKIVI-D-E---R---------gnTGRA-SIAAMNYI--ANSG-------------IPL-RTD----SpI-QHDKVIIVD-N-----VTVETGSFNFtkaA---ETK-NSENAVVI-W---NM--
0010 7e0mA   yqptAA--TP--gN--YLVASPRAYIDS-Q-VE-LPRL-LA-SA-QRVRVQVMDY--APLSygAVID-NALRSAaA---R----GVQIELMV-A-N---W---------ntKKP-DIAWLKSL--ALVP-------------NVQI-KVV----TrV-IHSKLMTID-G-----ETAWVGTSNWtggY---LDN-SRNLELVL-H---SP--
0011 2c1lA   -------------N--FFSLHPNVYrpkgliGM-LENV-WV----GTLYLISGFS--NYNGG-VRFY-ETFTEH-I------nqgGRVIAIL-G-G---S----T-S--QR---lSSRQVVEE--LLNR-------------GVEV-HII----NrI-LHAKLYGTSlG-----ESLVVSSGNFtgpG---MSQ-NIEASLLL-DnntTQ--
0012 4gelB   -rSKREkaSR---H--EVIIFNELGLRN-V-AK-IVEQ-ID-RA-YSIDLAIYT-----fTS-LFLA-DSIKRAlQ---R----GVIIRIIS-D-G---E---------mvYSK-G-sqISML--AQLG-------------VPV-RVP--ttN-L-MHNKFCIID-G--pcySIVISGSVNW---T-------NWENCIIT-A---DD--
0013 7wdkA   -------------V--RFFTT----gRD-YFSD-LASA-LD-SA-SSIFITGWQV--NYDV--rSLW-QCLRQA-L----erspaLKVYVMP-W-L---SP---S-G--SL-gtyDFETMLAV--FQLNAG--L---EGGA-RAFC-TPA----IfS-HHQKSVVID-N-----RIGYVGGIDL---A---YGAYWQDVHCRI-E---GPsv
0014 4urjD   -------------A--TVYFQ---TNNI-R-DL-VRRC-IT-RT-QVLVILMDV-----fTD-VEIF-CDILEA-AnkrG----VFVCVLLD-Q-G-----------------gvKLFQEMcdkVQIS---D---SHLKNISIR-SVE----GgQ-IREKFIISD-W-----RFVLSGSYSFtwlC---GHV-HRNILSKF-T---GQav
0015 7wu1E   vtfskpstNY---KpgWILDTHFTfPADkILDA-IYDN-IV-SA-RSVDIVTLQPM-SGAFT-ATIK-NALSQL-AkntqysdhhITVRLLQ-G-S---FT---PeE--EEIRQ-LSLTQTNY--LSEIASvlP---EVNNLDITV-GSV----RaW-NHGKIINVD-N-----QSVITGGHNL---WgadYLQRVNDLSINI-L---GY--
0016 3qphA   -mKKLE-------I--WRSRS-----fdeaiEM-FRES-LY-SA-NEVIVVTPSE--FFETI-REDL-IKTLER-G---------VTVSLYI-D-K-----------------IPDL--SE--FKGK------------GNFFV-RQF----Y-K-LNHLIGMTD-G-----KEVVTIQN-------------IGPPSFKS-T---YP--
0017 5boxA   --TEVP--KY---V--WVVRG----iKN-STLK-TKEM-LE-EA-NEILLADDG------FI-AVLE-DDIIKAvD---R----GVKTKILL-T-K---N---------------LLPRLKAS-kIIDYAK--E---------KLEL-RAL----D-K-FDLPMLICD--------EEVFFALE-------------NYETQVWI-K---DH--
0018 6kz8A   iksaKF--PGv--V--QLFRSIDG-DRS-iqDA-YIHA-IR-RA-DFIYVENQYF--LGSSfaedIP-KELSLK-IvskiekgekFRVYVVV-P-M---W----pvQ--AILDW-QRRTMEMM--YKDVIQ--A---LRaqgYLTF-FCLgnfmI-Y-VHTKMMIVD-D-----EYIIIGSANInqrS---MDG-DSEIAMGG-Y---QA--
0019 3igyB   -----------vghnalgagrVALQSGE-IYTGeGYRYlHG-AF-STLHLIG-LLS-RDNQI-YSII-EHAVKD-G--------AKRIRVHA-L-Y---DGrdvP-D--GSSFR-FTDELEAV--LAKVRQ---------NGCDAAIaSGG----GmF-VTMdeaitttdqyvpkVRYAGMM-------------dlgIPNN---------
0020 1xdoA   -----------pvtfdYLMVS----QNS-R-RL-LYEM-VD-REiSGITLKLNN-----lVD-KGLV-DRLYAA-S------ssgVPVNLLV-R---------------------gmcslI--PNL---------EGISDNIRA-ISI----VyL-EHDRVYIFE-N--ggdKKVYLSSADWmtrN---IDY-RIEVATPL-L----D--
0021 5b3gB   -----------kwadsvlleaarafaTF-GHVA-ANGA-IL-E--IHIVDIS-------sTF-CTQW-PTLLEA-LatrsddtphLRLTTVV-V-A---NK---F-V--NDQTA-SHRMMKEI--GNRMEK--FarlMGVPFKFNI-IHH---vG-D-LSefsprdarP-----RIVTVVEE-----------vgPVVWASAW-R---PT--
0022 6s6vA   -----------------------------------M-RILHTSDWHLGqEHQAF-LDWLlETAQTH-----Q----VDAIIVAG-DvF---DT---G-S---PPSY-ARTLYNRF--VVNLQQ--------TGCHLVV-LAG----NasaHIHRYVHLV-T--lesVENLN------vpV---TQPM----------------

0001 s001A   --LLLL--LL---L--EEEEELLLLLLL-HHHH-HHHH-HL-LL-LEEEEELLLLL-HHHHH-HHHH-HHHHHH-L---L----LLEEEEEE-L-L---LL---L-H--HHHHH-HHHHHHHH--HHHHHL--L---LLLLLEEEE-EEL----L-L-LLLLEEEEL-L-----LEEEEELLLL---L---LLLLLLEEEEEE-E---LL--
0002 2ymbD   -------------L--EEEEELLLLLLL-HHHH-LLLL-LLlLL-LEEEEELLLLL-HHHHH-HHHH-HHLLL--L---L----LLEEEEEE-L-L---LL---------LLHH-HHHHHHHH--HHHHHL--------LLLEEEE-EEL----L-L-LLLLEEEEL-L-----LLEEEELLLL---L---LLLLLLEEEEEE-L---LL--
0003 7clgA   --LLLL--LL---L--EEELL--lLLLH-H-HH-HHHH-HH-LL-LEEEEEELLL--LHHHH-HHHH-HHHHHH-H------hllLLEEEEE-L-L---LL---L--------L-LLHHHHHH--HHHL------------LLEEE-EEL----L-L-LLLEEEEEE-L--lleEEEEEELLLLlhhH---HHL-LLLLEEEE-E---EE--
0004 4rctA   -------------E--EEEELLLL-llllhhHH-HHHH-HH-HL-LEEEEEELLL--LHHHH-HHHH-HHHHHL-L---L----LLEEEEEElL-H---HH--------hLLLHhHHHHHHHH--HHHHHH--L---------LEEE-EEL--llL-L-LLLLEEEEE-E--lleEEEEEELLLL---L-------LLLEEEEE-E---HHhh
0005 3hsiB   hhhhhH--HH-leE--EEEEEEL-LLL-hhhHH-HHHH-HH-LL-LEEEEELLLL---LLLH--HHH-HHHHHHhH---L----LLEEEEEE-E-LhhhLL---L-LlhHHHHH-HHHHHHHH--HHHLHH------hhhHLLEEE-EEE----LlE-ELLLEEEEL-L-----LEEEEELLLL-lhH---HHH-LEEEEEEE-E---LL--
0006 6ehiD   -------------E--EEEEELL--HHH-H-HH-HHHH-HH-LL-LEEEEEELL-----lLL-HHHH-HHHHHH-H------hllLEEEEEE-E-H---H---------hhLLL-LlllHHHH--HLLL-------------LEEE-EEE----L-L-LLLEEEEEL-L-----LEEEEELLLLlhhH---HHL-LLEEEEEE-L---LH--
0007 7v53A   -------------E--EEELL----hHH-HH-H-HHHH-HH-HL-LEEEEEELL---LLLL--lLHH-HHHHHH-H----hhlllLEEEEEE-L-L---LL---L-H--HH-lllHHHHHHHH--HHLLLL--L---LLLL-LEEE-LLL----L-L-LLLLEEEEL-L-----LEEEEELLLL---L-llLLLLEELLEEEE-E---LL--
0008 5nwaA   -----------lllllLEELLLLL-LLL-HHHH-LLH-hhL-LE-EEEEEELLLL-----LH-HHHH-HHL-LL-L---L---lLLLEEEEE-L-L---------------LLH-HHHHHHHH--LLLLL------------LEEE-EEL----LlL-LLLLEEEEE-E--lllEEEEEELLLLlhhH---LLL-LLLLEEELlL---LL--
0009 1byrA   --------LL---E--EEEEELL--hhhhhhhH-HHHH-L---L-LEEEEEELLL--LLHHH-HHHH-HHHH-H-L---L----L-EEEEEE-E-L---L---------llLLHH-HHHHHHHH--HHLL-------------LLE-EEE----LlL-LLLLEEEEL-L-----LEEEEELLLLlhhH---HHL-LLEEEEEE-E---LL--
0010 7e0mA   llllLL--LL--lE--EEEEELHHHEEH-H-HH-HHHH-HH-HL-LEEEEELLLL--LLLLllLHHH-HHHHHHhH---L----LLEEEEEE-E-H---H---------hhLLL-HHHHHHHH--HLLL-------------LEEE-EEE----LlE-ELLLEEEEL-L-----LEEEEELLLLlllH---HHH-LLEEEEEE-L---LH--
0011 2c1lA   -------------L--EEEEELLLElllllhhHH-HHHH-HL----LEEEEEELLL--LHHHL-LLLH-HHHHHH-H------hllLEEEEEE-E-L---L----L-L--LL---lLLHHHHHH--HHHL-------------LLEE-EEE----ElL-LLLEEEEEElE-----EEEEEELLLLlllL---LLL-LLEEEEEE-LhhhHH--
0012 4gelB   -lHHHHhhLL---E--EEEEELLLLHHH-H-HH-HHHH-HH-LL-LEEEEEELL-----lLL-HHHH-HHHHHHhH---L----LLEEEEEE-L-L---L---------hhHLL-L-llHHHH--HHLL--------------LLE-ELL--llL-L-LLLEEEEEL-L--lllLEEEEELLLL---L-------LLEEEEEE-L---LH--
0013 7wdkA   -------------L--EEELL----hHH-HHHH-HHHH-HH-HL-LEEEEEELLL--LLLL--lLHH-HHHHHH-H----hhlllLEEEEEE-L-L---LL---L-L--LL-lllLLLHHHHH--HHHLLL--L---LLLL-LEEE-LLL----LlL-EELLEEEEL-L-----LEEEEELLLL---L---LLLLEELLEEEE-E---LLhh
0014 4urjD   -------------E--EEEEL---LLHH-H-HH-HHHH-HH-LL-LEEEEEELL-----lLL-HHHH-HHHHHH-HhhlL----LEEEEEEE-L-L-----------------lhHHHHHHhhhLLLL---L---LLLLLEEEE-EEL----LlL-LLLLEEEEL-L-----LEEEEELLLLllhH---HLL-LLLEEEEE-E---HHhh
0015 7wu1E   llllllleeEE---LllLEEEELLLlHHHhHHHH-HHHH-HH-LL-LEEEEEEELLL-LLHHH-HHHH-HHHHHH-HhhlllllllEEEEEEE-E-L---LL---LhH--HHHHH-HHHHHHHH--HHHHHLllL---LLLLEEEEE-EEE----EeE-LLLLEEEEL-L-----LEEEEELLLL---LhhhHLLLLLEEEEEE-E---LL--
0016 3qphA   -hHHHH-------E--EEELL-----hhhhhHH-HHHH-HH-HL-LEEEEEELHH--HHHHH-HHHH-HHHHHL-L---------LEEEEEE-L-L-----------------LLLL--HH--HLLL------------LEEEE-EEL----L-L-LLLEEEEEL-L-----LEEEEELL-------------LLLLEEEE-L---LH--
0017 5boxA   --LLLL--LL---E--EEELL----hHH-HHHH-HHHH-HH-LL-LEEEEEELL------LL-LLHH-HHHHHHhH---L----LLEEEEEE-E-H---H---------------HHHHHLLL-hHHHHHL--L---------LLEE-EEE----L-L-LLLLEEEEL-------LEEEEELL-------------LLLLEEEE-E---LH--
0018 6kz8A   llllLL--LLl--E--EEEEEEEL-EEH-hhHH-HHHH-HH-LL-LEEEEEELLL--LLLHhhhhHH-HHHHHH-HhhhhhhlllLEEEEEL-L-L---L----lhH--HHHHH-HHHHHHHH--HHHHHH--H---HHhllLEEE-EEEeeeeL-E-ELLEEEEEL-L-----LEEEEELLLLlhhH---HLL-LLEEEEEE-E---LL--
0019 3igyB   -----------hhhhhhhllLLLLHLH-HHLLhHHHHhHH-HH-LLEEEEE-ELL-LHHHH-HHHH-HHHHHL-L--------LLEEEEEE-E-E---LLlllL-L--LLHHH-HHHHHHHH--HHHHHL---------LLLEEEEeEEE----ElL-LLLlhhhhhlllllllLEEEEEE-------------lllLLLL---------
0020 1xdoA   -----------lllllLLEEE----LLL-H-HH-HHHH-HH-HHhLLEEEEEELL-----lLL-HHHH-HHHHHH-H------hllLLEEEEE-L-------------------lllllL--LLL---------LLLLLLEEE-EEE----LlE-ELLEEEEEL-L--lllLEEEEELLLLlhhH---HHL-LLEEEEEL-L----L--
0021 5b3gB   -----------lhhhhhhhhhhhhhhLH-HHHH-HHHH-HH-H--EEEEEEL-------lLL-LLLL-HHHHHH-HhhhllllleEEEEEEE-E-E---LL---L-L--LLHHH-HHHHHHHH--HHHHHH--HhhhHLLLEEEEE-EEE---eL-L-HHhllhhhhlL-----LEEEEEEE-----------llEEEEEEEE-E---EL--
0022 6s6vA   -----------------------------------L-EEEEELLLLLLlHHHHH-HHHHhHHHHHL-----L----LLEEEEELL-LlL---LL---L-L---LLHH-HHHHHHHH--HHHHLL--------LLLLEEE-ELL----LlllLLLLEEEEE-E--eeeEEEEE------llL---LLLL----------------

############################################################################################################################################################################################################################################################################################################
############################################################################################################################################################################################################################################################################################################
############################################################################################################################################################################################################################################################################################################

Job: Type-1 BREX; BrxA BAY35696 N-Terminal HTH1 (Found in a SubSet of Type-1 BrxA, and in All Type-3 BrxA)
Query: s001A
No: Chain Z rmsd lali nres id PDB Description
1: 7zge-A 5.8 3.0 69 199 14 MOLECULE: BRXA, A BREX PHAGE DEFENCE PROTEIN;
2: 5ue0-A 5.2 3.0 67 280 9 MOLECULE: CT622 PROTEIN;
3: 2qww-A 5.2 2.5 63 146 14 MOLECULE: TRANSCRIPTIONAL REGULATOR, MARR FAMILY;
4: 2p8t-A 5.1 2.3 58 184 9 MOLECULE: HYPOTHETICAL PROTEIN PH0730;
5: 5z7b-B 5.1 2.8 59 197 7 MOLECULE: PADR FAMILY TRANSCRIPTIONAL REGULATOR;
6: 5f6f-A 5.1 2.7 63 141 13 MOLECULE: MARR FAMILY REGULATORY PROTEIN;
7: 1stz-A 5.0 2.7 64 323 6 MOLECULE: HEAT-INDUCIBLE TRANSCRIPTION REPRESSOR HRCA HOMOL
8: 8ioi-A 5.0 2.7 61 190 10 MOLECULE: PADR FAMILY TRANSCRIPTIONAL REGULATOR;
9: 2vla-A 4.8 3.1 67 280 9 MOLECULE: RESTRICTION ENDONUCLEASE R.BPUJI;
10: 8r3g-A 4.7 2.7 58 335 9 MOLECULE: CENTRAL GLYCOLYTIC GENES REGULATOR;
11: 4rul-A 4.7 3.2 66 821 9 MOLECULE: DNA TOPOISOMERASE 1;
12: 9qe1-J 4.7 2.7 65 108 3 MOLECULE: JETC;
13: 6j5u-A 4.6 3.7 71 663 10 MOLECULE: DISEASE RESISTANCE RPP13-LIKE PROTEIN 4;
14: 3cdh-B 4.6 2.7 60 136 12 MOLECULE: TRANSCRIPTIONAL REGULATOR, MARR FAMILY;
15: 2fa5-B 4.6 2.7 62 142 8 MOLECULE: TRANSCRIPTIONAL REGULATOR MARR/EMRR FAMILY;
16: 4asn-A 4.6 2.4 59 90 10 MOLECULE: TUBR;
17: 3elk-A 4.5 2.9 60 104 7 MOLECULE: PUTATIVE TRANSCRIPTIONAL REGULATOR TA0346;
18: 8jxk-D 4.5 2.7 59 166 8 MOLECULE: CONSERVED PROTEIN;
19: 6qfd-B 4.5 2.5 59 116 3 MOLECULE: DNA-BINDING PROTEIN;
20: 5hso-A 4.5 2.7 58 122 12 MOLECULE: UNCHARACTERIZED HTH-TYPE TRANSCRIPTIONAL REGULATO
21: 5x11-E 4.5 2.8 63 181 5 MOLECULE: DNA (28-MER);
22: 7dvr-A 4.5 2.5 61 147 15 MOLECULE: HTH MARR-TYPE DOMAIN-CONTAINING PROTEIN;
23: 3df8-A 4.4 2.8 63 109 3 MOLECULE: POSSIBLE HXLR FAMILY TRANSCRIPTIONAL FACTOR;
24: 2h09-A 4.4 2.5 55 127 7 MOLECULE: TRANSCRIPTIONAL REGULATOR MNTR;
25: 2wte-A 4.4 3.2 56 212 11 MOLECULE: CSA3;
26: 3m8e-A 4.4 2.4 61 101 3 MOLECULE: PUTATIVE DNA-BINDING PROTEIN;
```

```
27: 2nyx-B 4.4 2.3 59 147 10 MOLECULE: PROBABLE TRANSCRIPTIONAL REGULATORY PROTEIN, RV14
28: 4fht-B 4.3 2.6 58 142 5 MOLECULE: PCAV TRANSCRIPTIONAL REGULATOR;
29: 2co5-B 4.3 2.7 60 94 7 MOLECULE: VIRAL PROTEIN F93;
30: 7l1i-A 4.3 2.9 60 169 10 MOLECULE: MARR FAMILY MULTIDRUG RESISTANCE PUMP TRANSCRIPTI
31: 1p4x-A 4.2 2.9 64 250 9 MOLECULE: STAPHYLOCOCCAL ACCESSORY REGULATOR A HOMOLOGUE;
32: 3l9f-A 4.2 2.7 60 170 3 MOLECULE: PUTATIVE UNCHARACTERIZED PROTEIN SMU.1604C;
33: 5y7q-A 4.2 2.6 64 546 6 MOLECULE: FANCONI-ASSOCIATED NUCLEASE 1 HOMOLOG;
34: 8ylg-A 4.2 2.5 58 161 7 MOLECULE: MARR FAMILY TRANSCRIPTIONAL REGULATOR;
35: 5zqh-A 4.2 2.6 58 101 2 MOLECULE: PADR FAMILY TRANSCRIPTIONAL REGULATOR;
36: 9ri9-B 4.1 3.6 62 868 10 MOLECULE: SLNRC3;
37: 2fxa-B 4.1 2.3 56 180 5 MOLECULE: PROTEASE PRODUCTION REGULATORY PROTEIN HPR;
38: 5e20-A 4.1 2.8 63 169 5 MOLECULE: TRANSCRIPTIONAL REGULATOR, MARR FAMILY;
39: 2id3-A 4.0 2.8 53 191 6 MOLECULE: PUTATIVE TRANSCRIPTIONAL REGULATOR;
40: 1fok-A 4.0 3.1 67 568 9 MOLECULE: DNA (5'-
41: 4u03-A 4.0 3.3 59 390 3 MOLECULE: CYCLIC AMP-GMP SYNTHASE;
42: 8rkd-B 4.0 4.2 56 411 5 MOLECULE: PILUS ASSEMBLY ATPASE CPAF;
43: 3oop-A 4.0 2.3 54 139 7 MOLECULE: LIN2960 PROTEIN;
44: 6jbx-A 4.0 2.5 56 146 13 MOLECULE: FATTY ACID BIOSYNTHESIS TRANSCRIPTIONAL REGULATOR
45: 1ub9-A 4.0 2.6 59 100 10 MOLECULE: HYPOTHETICAL PROTEIN PH1061;
46: 1yyv-A 4.0 2.5 59 114 10 MOLECULE: PUTATIVE TRANSCRIPTIONAL REGULATOR;
47: 4hw0-A 3.9 3.0 56 98 13 MOLECULE: DNA-BINDING PROTEIN SSO10A-2;
48: 5j9q-E 3.9 3.2 59 293 7 MOLECULE: HISTONE ACETYLTRANSFERASE ESA1;
49: 4hqe-B 3.9 2.8 60 108 7 MOLECULE: TRANSCRIPTIONAL REGULATOR QSRR;
50: 3mq0-B 3.9 2.6 52 248 8 MOLECULE: TRANSCRIPTIONAL REPRESSOR OF THE BLCABC OPERON;

0001 s001A   --GTAMVN--ETRHLIQQWHP--TE-NVD--TFVKRVQAE-DL-LG-RQTAQRAKDIAR-VFA-A--R-FLLP--------------NNKP-ARL-LK-QIV-EAG-L-
0002 7zgeA   -gSSLLIT--ESRIIAESLLK--KL-PED--EWKSLIVEQ-NV-LQ-KKSGQTAIRYAR-TIR-W--R-IE----------------GLGD--EF-MT-DLL-AAS-Er
0003 5ue0A   -aAETILQ--VLDSLMSTINP--QA-NQE--EIMQXLT---AS-IS-KqSVDSLQKFAA-QLE-R--E-FVDG--------E----RSLAE-SQE-NAfRKQ-PA----
0004 2qwwA   aaslGLTI--QQLAXINVIYS--TP-G----ISVADLT---KR-LI--ITGSSAAANVD-GLI-S-lG-LVV-klnkpnDSXDLTLKLSKK-GED-LS-KRS-TAN-Af
0005 2p8tA   ---eyTVE--DVLAVIFLLK---------epLGRKQIS---ER-LE--LGEGSVRTLLR-KLS-H-lD-IIR----------SKGHFLTLK-GKE-IR-DKL-LSM-Fs
0006 5z7bB   -sakDLMT--LRSALLALLS--------sgpLTGYDASQR-F---gwSGSDSQIYPELR-KME-A-eE-LLV-vpwgskGATKTEYALSEK-GWE-AL-RKA-WYE-Pv
0007 5f6fA   leqfDITN--EQRHTLGYLYQ--QD-G----LTQNDIA---KA-LQ--RTGPTVSNLLR-NLE-R-kK-LIY-ryvdaqDTRRKNIGLTTS-GIK-LV-EAF-TSI-Fd
0008 1stzA   ----KLND--RQRKVLYCIV--nKK-PV----SSQRVLEV-SN-IE--FSSATIRNDMK-KLE-Y-lG-YIY------qPHTSAGRIPTDK-GLR-FY-YEE-MLK-Is
0009 8ioiA   ---PEFMA--LPHAILVSLS--------eqaSSGYELARR-F-dfW-TATHQQIYRTLR-VME-N-nN-WVR-tvlqhgRPDKKVYAISDS-GRA-EL-ARW-IAE-Pl
0010 2vlaA   -irGKAKN--LLPAYANIIDC--PC-DKA--SFVKDFN---NR-LIeETTKKTLDNHRTeIAG-KlfG-MFYE--------DDEVIFPSGR-TNK-YI-ED--SDQ-Pa
0011 8r3gA   pdllLVXQ--KRFEILQYIR-------ltepIGRRSLS---AS-LG--ISERVLRGEVQ-FLK-E-qN-LVD--------IKTNGXTLTEE-GYE-LL-SVL-EDTx--
0012 4rulA   arglSAGR--VQSVAVRLVVE--RF-SEA--SLVKELEKR-GI-G----RPSTYASIIS-TIQ-D--RgYVRV--------ENRRFYAEKM-GEI-VT-DRL-EEN-Fr
0013 9qe1J   -ltADSAA--HYRTILRYFY--mRD-FIA----PEELL---EH-MRaDFQEDQLHQQLA-QLVkW--N-NLIAktieeyKKKRFRYQCTPY-TVE-IE-RMI-VQL-Ek
0014 6j5uA   -tDRWSPS--HLKSCILTLSL--YI-PKQ--QLVHGWIGE-GF-VM-WSATESGEDCFS-GLT-N--RcLIEVktysgtIIT----CKIHD-vRD-LV-IDI-AKK-Ds
0015 3cdhB   iraqGLRV--PEWRVLACLVD--ND-A-------xXITR--KL-SL--XEQSRXTRIVD-QXD-A-rG-LVT-----rvADARVRVRLTDD-GRA-LA-ESL-VAS-Ar
0016 2fa5B   gdryGXAI--PEWRVITILAL--YP-G----SSASEVSDR-T------XDKVAVSRAVA-RLL-E-rG-FIR---------RESXLALSPA-GRQ-VY-ETV-APL-Vn
0017 4asnA   evxrKLTI--EDVSILGWLAN--AV---fkaikKSSIA---DE-LE--YSTANFRKTLN-KLE-A-iH-FIG----tvtGGKEHKLYLTEY-GQQ-AV-QQA-IHH---
0018 3elkA   reriLHGL--ITLYILKELV--------krpXHGYELQ---KS-XFqALPQGSIYILLK-TXK-E-rG-FVI-essvnkGQQLTVYHITDA-GKK-FL-XDH-SQA-Lq
0019 8jxkD   -----EFM--LELAILGLLI--------espMHGYELR---KR-LTrAFSYGSLYPALR-RMQ-A-dG-LIA---enaaPAGRRVYQLTDK-GRR-RF-GEL-VAD-Tg
0020 6qfdB   sdarDLTA--FQKNILTVLG--------eeaRYGLAIKRE-L----eEVNHGRLYPNLD-DLV-N-kG-LVE---kselDKRTNEYALTNE-GFD-AV-VDD-LEW-Tl
0021 5hsoA   lsplGLTL--PEFVCLRMLS----------qspgLSSAEL-RH-AS--VTPQAMNTVLR-KLE-D-aG-AVA------------PATLTAR-GRA-LA-KRA-EAV-Vr
0022 5x11E   ---dPMRV--LKYAILGLLRK--GE------LSGYDITSY-F-kfW-SAKHSQIYPELK-KLT-D-eG-FIT-ttiqgtKLEKKMYTLTDS-GKQ-EL-HDW-LIR-Hq
0023 7dvrA   ------AG--PQGHLVMHLYP--DK-D----MSIKDAE---EI-LH--ISKSVASNLVK-RME-K-nG-FIA-ivpsktDKRVKYLYLTHL-GKQ-KA-TQF-EIF-Le
0024 3df8A   esvlHLLGkkYTXLIISVLGG--ST-R----QNFNDIR---SS-IP-GISSTILSRRIK-DLI-D-sG-LVE-----rrSGQITTYALTEK-GXN-VR-NSL-XPLl--
0025 2h09A   vreaHRRE--LIDDYVELIS-------dlirevgeaRQVD-M--rL-GVSQPTVAKMLK-RLA-T-mG-LIE-------MIPWRGVFLTAE-GEK-LA-QES-RER-Hq
0026 2wteA   alxrDYSR--EEXKLLNVLYE--TK---------gTGITE-L--xL-DKSEKTLINKIA-ELK-K-fG-ILT------qKGKDRKVELNEL-GLN-VI-KLN-K-----
0027 3m8eA   ------DD--CAYQVLMAFIE--AQ-M----LNKTAVA---EM-IQ--LSKPTVFATVN-SFY-C--AgYIDE----trVGRSKIYTLSDL-GVE-IV-EC--FKQ-Ka
0028 2nyxB   qvdeNITI--PQFRTLVILS-------nhgpINLATLA---TL-LG--VQPSATGRXVD-RLV-G-aE-LID-rlphptSRRELLAALTKR-GRD-VV-RQV-TEH-Rr
0029 4fhtB   mvseETTS--PQYAVLNALV-------aepgLDQRTVG---ER-VG--lDRSTIAEVVS-RLG-R-rG-LLD-kvrdpqDGRRSLLRLTDE-GLR-VH-RRL-GVR-Ia
0030 2co5B   ------RI--NYYIILKVLV--nGS-RLE----KKRLRSE----dI-DISDGVLYPLID-SLI-D-dK-ILR----eeeAPDGKVLFLTEK-GMK-EF-EEL-HEF-Fk
0031 7l1iA   ikklGIDN--TRRKIILSTNA--LG-E-------aSITD--NL-ST--LKLTTATKAVY-RLV-E-dG-IVE-vyssttDERISMVKLTAK-GVE-LV-EQI-NQI-Sv
0032 1p4xA   kpevDMTI--KEFILLTYLF--qQE-NTL--P-FKKIVSD-L-----CYKQSDLVQHIK-VLV-K-hS-YISkrntyisISE----EQREK-IAE-RV-TLF-DQI-Ik
0033 3l9fA   ------MQ--GKDIILGILSK--KE------RSGYEINDI-LqnyD-GTY-GMIYPTLR-KLE-K-dG-KIT-vviqdgRPNKNIYAITES-GKK-EL-ASY-LQS-Dv
0034 5y7qA   -vHYVENG--LINSLFGLLCL--GRsYRD--AIRCRYRDK-FglYW-EL---lGEELLE-QAL-D--C-L-----------------PAAH-LRA-WF-ERL-LED-Ip
0035 8ylgA   farygLQP--GEFDVLATLSG--AP-------yalTPTA--DA-AM--ISSGSMTNRID-RLE-K-aG-WVE-rranpaDGRGTLVALTSA-GRA-LI-DDA-VVA-Hv
0036 5zqhA   etqllKGV--LEGCVLDMIG--------qkeRYGYELVQT-L--fD-TIV-GTIYPLLQ-KLE-K-nQ-WIR-mrpspdGPDRKYFSLMKE-GEE-RV-SVF-WQQ-Wd
0037 9ri9B   msydRLPY--DLKACFLYC---sQI-PAK--KLIRLWIAE-GF-IQ-YQGpLALEDVAE-DHL-N--D-LVN----------------rNL-VMvmLH-EFC-RHEa--
0038 2fxaB   lkpyDLNI--NEHHILWIAY----------qlngaSISE--KF-GV--XHVSTAFNFSK-KLE-E-rG-YLR-fskrlnDKRNTYVQLTEE-GTE-VF-WSL-LEE-Fd
0039 5e20A   lkslNLST--EKFAILHELML--GG-E----STPHTLARR--I-VF-E--PHSVSAIVS-RME-K-dG-LII-ktkdldKKHMVRIKLSEK-AID-TF-YQA-LEI-Sn
0040 2id3A   --gGRTAR--IREAVLLAAG--dAL-DLG--EIARRAG-----------------vGKT-TVY-R--R-WG----------------TPGG-LAA-DL-LAD-XAE-Qs
0041 1fokA   -qNPGKFE--NLKRVVQVFDRnhDL-IYT--Y--KELVGtgAT-IAgYIDNWSSDGFLR-WAH-A--LgFIEY------INKSDSFVITDV-GLA-YS-KS--ADG-Sa
0042 4u03A   ------RD--VFEEAKGIAKK--KS-ALTfeIIQEKVS-T-TQ-IK-HLSDSEQREVAK-LIY-E--M-------------------DDDA-RDE-FL-GLTx------
0043 8rkdB   ---DYYHA--TKTTIFNALTI--DLqAGE--EIRDIVAEL-V--nV-SMSVAEQEHLVQ-DII-N----DVLG-----------------------------fRDN-Lq
0044 3oopA   ------TP--EQWSVLEGIE----------anepiSQKE--LW-TK--KDTPTVNRIVD-VLL-R-kE-LIV-----reIRRISLLSLTDK-GRK-ET-TEL-RDI-Ve
0045 6jbxA   srfkDISI--KEMHTIDVIG----------kapdvTPSQ--KE-LM--VTLGTVTTSLN-NLE-R-kG-YIE-rvrseqDRRVVHLHLTKK-GRL-IH-RLH-KRF-Hk
0046 1ub9A   ------NP--VRLGIMIFLLP--RR-K----APFSQIQKV-L------lTPGNLDSHIR-VLE-R-nG-LVK-tykviaDRPRTVVEITDF-GME-EA-KRF-LSS-Lk
0047 1yyvA   revlKHVTsrWGVLILVALR--------dgtHRFSDLR---RX-XG-GVSEXXLAQSLQ-ALE-Q-dG-FLN-rvsypvVPPHVEYSLTPL-GEQ-VS-DXV-AALa--
0048 4hw0A   --krGTXE--IXFDILRNCEP------------kcGITR--YG-AG--INYVVAQKYLD-QLV-K-vG-ALN----iktENDRKIYEITEK-GKL-LR-THI-EEFi--
0049 5j9qE   -llSYRAY--WSDTLITLLEH--QK-E----ITIDEIS-S-MT-S-------mTTTDIL-HTA-K--TlNILR---yykGQH----IIFLN--eD-IL-DRY-NRL-Ka
0050 4hqeB   eetfKILGrsWNGLIINYLS-----rcndcsAHFSDMKRD-L---K-TITPRALSLKLS-ELA-Q-wE-LVE-kqiistSPVQIIYVLTEK-GKA-LA-EAL-HPI-Ea
0051 3mq0B   sdtvPALR--RAVRILDLVAS--PR-D----LTAAELT---RF-LD--LPKSSAHGLLA-VMT-E-lD-LLA--rsadgTLR----IGPHSl-----------------

0001 s001A   --LLLLHH--HHHHHHHHLLL--LL-LHH--HHHHHHHHL-LL-LL-LLLHHHHHHHHH-HHH-H--H-HLLL--------------LLHH-HHH-HH-HHH-HLL-L-
0002 7zgeA   -lLLLLHH--HHHHHHHHHLL--LL-LLL--HHHHHHLLL-LL-LL-LLLHHHHHHHHH-HHH-H--H-HL----------------LLLH--HH-HH-HHH-HLL-Hh
0003 5ue0A   -hHHHHHH--HHHHHHHHHLL--LL-LHH--HHHHHHH---HH-HH-LlLHHHHHHHHH-HHH-H--H-HLLH--------H----HHHHH-HHH-HHhHHH-HH----
0004 2qwwA   hhhhLLLH--HHHHHHHHHHH--LL-L----EEHHHHH---HH-HL--LLHHHHHHHHH-HHH-H-lL-LEE-elllllLLLLLEEEELHH-HHH-HH-HHH-HLL-Hh
0005 2p8tA   ---llLHH--HHHHHHHHLL---------llLLHHHHH---HH-HL--LLHHHHHHHHH-HHH-H-lL-LEE----------ELLEEELHH-HHH-HH-HHH-HLL-Ll
0006 5z7bB   -lhhHHHH--HHHHHHHHHL--------lllEEHHHHHHH-H---hlLLLHHHHHHHHH-HHH-H-lL-LEE-eeelllLEEEEEEEELHH-HHH-HH-HHH-HHL-Ll
0007 5f6fA   hlllLLLH--HHHHHHHHHHH--HH-L----LLHHHHH---HH-HL--LLHHHHHHHHH-HHH-H-lL-LEE-eeelllLLLLEEEEELHH-HHH-HH-HHH-HHH-Hh
0008 1stzA   ----LLLH--HHHHHHHHHH--hLL-LL----LHHHHHHH-LL-LL--LLHHHHHHHHH-HHH-H-lL-LEE------lLLLLLLLEELHH-HHH-HH-HHH-HHH-Hl
```

```
0009 8ioiA   ---LLLLL--HHHHHHHHHH--------hllLLHHHHHHH-H-hlL-LLLHHHHHHHHH-HHH-H-lL-LEE-eelllllLLLEEEEEELHH-HHH-HH-HHH-HHL-Ll
0010 2vlaA   -llLLLLH--HHHHHHHHHHL--LE-EHH--HHHHHHH---HH-HHlLLLHHHHHHHHHhHHH-HhlL-LEEE--------ELLEEEELHH-HHH-HH-HH--LLH-Hh
0011 8r3gA   llhhHHHH--HHHHHHHHHH-------hhllLLHHHHH---HH-HL--LLHHHHHHHHH-HHH-H-lL-LEE--------EELLEEEELHH-HHH-HH-HHH-HHHh--
0012 4rulA   llllLLLL--LHHHHHHHHHH--HL-LHH--HHHHHHHHL-LL-L----LLLLHHHHHH-HLL-L--LlLEEE--------ELLEEEELHH-HHH-HH-HHH-HHH-Ll
0013 9qe1J   -hhLLLHH--HHHHHHHHHH--lLL-LLL----HHHHH---HH-HHlLLLHHHHHHHHH-HHHhL--L-LEEEllhhhhHLLLEEEEELHH-HHH-HH-HHH-HHH-Hl
0014 6j5uA   -lLLLLLH--HHHHHHHHHHH--LE-EHH--HHHHHHHHH-LL-LL-LLLHHHHHHHHH-HHH-H--LlLLEEllllllLLE----EELLH-hHH-HH-HHH-HHL-Ll
0015 3cdhB   hhhlLLLH--HHHHHHHHHHH--LL-L-------eEHHH--HH-HL--LLHHHHHHHHH-HHH-H-hL-LEE-----elLLLLLEEEELHH-HHH-HH-HHH-HHH-Hh
0016 2fa5B   hhhhLLLH--HHHHHHHHHHH--LL-L----LLHHHHHHH-H------LLHHHHHHHHH-HHH-H-hL-LEE---------ELLLEEELHH-HHH-HH-HHH-HHH-Hh
0017 4asnA   hhhhLLLH--HHHHHHHHHLL--LH---hhleeHHHHH---LL-LL--LLLHHHHHHHH-HHH-H-lL-LEE----eelLLLLLEEEELHH-HHH-HH-HHH-LLL---
0018 3elkA   llhhHHHH--HHHHHHHHHH--------hllEEHHHHH---HH-HHlLLLLLHHHHHHH-HHH-H-hL-LEE-eeeellLLEEEEEEELHH-HHH-HH-HHL-LLL-Hh
0019 8jxkD   -----LHH--HHHHHHHHHH--------hllEEHHHHH---HH-HHlLLLLLLHHHHHH-HHH-H-lL-LEE---elllLLLLLEEEELHH-HHH-HH-HHH-HHL-Ll
0020 6qfdB   hhhlLLLH--HHHHHHHHHH--------hllEEHHHHHHH-H----lLLLHHHHHHHHH-HHH-H-lL-LEE---eeeeELLEEEEEELHH-HHH-HH-HHH-HHH-Hh
0021 5hsoA   hhhhLLLH--HHHHHHHHHH----------hlllLLHHHH-HH-LL--LLHHHHHHHHH-HHH-H-lL-LLL------------LLLLLHH-HHH-HH-HHH-HHH-Hh
0022 5x11E   ---lLLLH--HHHHHHHHLLL--LL------LLHHHHHHH-H-hlL-LLLHHHHHHHHH-HHH-H-lL-LEE-eeelllLLEEEEEEELHH-HHH-HH-HHH-HHL-Ll
0023 7dvrA   ------LL--HHHHHHHHHHL--LL-L----EEHHHHH---HH-HL--LLHHHHHHHHH-HHH-H-lL-LEE-eeelllLLLLEEEEELHH-HHH-HH-HHH-HHH-Hh
0024 3df8A   lllhHHHHllLHHHHHHHHLL--LL-L----LLHHHHH---HL-LL-LLLHHHHHHHHH-HHH-H-lL-LEE-----eeELLLEEEEELHH-HHH-HH-HHH-HHHh--
0025 2h09A   hhhhHHHH--HHHHHHHHHH-------hhhhhhlllLHHH-H--hH-LLLHHHHHHHHH-HHH-H-lL-LEE-------EELLLEEEELHH-HHH-HH-HHH-HHH-Hh
0026 2wteA   hhhlLLLH--HHHHHHHHHHH--HL---------lLLHHH-H--hH-LLLHHHHHHHHH-HHH-H-lL-LEE------eELLLLEEEELHH-HHH-HH-HHL-L-----
0027 3m8eA   ------LH--HHHHHHHHLLL--LL-L----LLHHHHH---HL-LL--LLHHHHHHHHH-HHH-H--LlLEEE----eeELLEEEEEELHH-HHH-HH-HL--LLL-Ll
0028 2nyxB   hhllLLLH--HHHHHHHHHH-------hhllEEHHHHH---HH-HL--LLHHHHHHHHH-HHH-H-lL-LEE-eellllLLLLLEEEELHH-HHH-HH-HHH-HHH-Hh
0029 4fhtB   hlllLLLH--HHHHHHHHHH-------hlllLLHHHHH---HH-HL--lLHHHHHHHHH-HHH-H-hL-LEE-eeelllLLLLEEEEELHH-HHH-HH-HHH-HHH-Hh
0030 2co5B   ------LH--HHHHHHHHHH--lLL-EEE----HHHHHHH----lL-LLLHHHHHHHHH-HHH-H-lL-LEE----eelLLLLLEEEELHH-HHH-HH-HHH-HHH-Hh
0031 7l1iA   hlllLLLH--HHHHHHHHHHH--HL-L-------eEHHH--HH-LL--LLHHHHHHHHH-HHH-H-lL-LEE-eeelllLLLLEEEEELHH-HHH-HH-HHH-HHH-Hh
0032 1p4xA   llllLLLH--HHHHHHHHHH--lLL-LEE--E-HHHHHHH-L-----LLLHHHLHHHHH-HHH-H-lL-LLEelleeeeLLH----HHHHH-HHH-HH-HHH-HHH-Hh
0033 3l9fA   ------LL--HHHHHHHHLLL--LL------EEHHHHHHH-HhheE-LLL-LLHHHHHH-HHH-H-lL-LEE-eellllLLLEEEEEELHH-HHH-HH-HHH-HHL-Ll
0034 5y7qA   -eEELLLH--HHHHHHHHHHH--HLlHHH--HHHHHHHHH-LllLL-LL---lLHHHHH-HHH-H--H-L-----------------LHHH-HHH-HH-HHH-HHL-Hh
0035 8ylgA   hhhllLLH--HHHHHHHHHLL--LL-------leeLHHH--HH-HL--LLHHHHHHHHH-HHH-H-hL-LEE-eeelllLLLLEEEEELHH-HHH-HH-HHH-HHH-Hh
0036 5zqhA   llhhhHHH--HHHHHHHHHH--------hllEEHHHHHHH-H--lL-LLL-LLLHHHHH-HHH-H-lL-LEE-eeelllLLEEEEEEELHH-HHH-HH-HHH-HHH-Hh
0037 9ri9B   hhhhLLLH--HHHHHHHHL---lLE-EHH--HHHHHHHHL-LL-LL-LLLlLLHHHHHH-HHH-H--H-HHH----------------lLL-LEehHH-HHH-HHHh--
0038 2fxaB   hhhhLLLH--HHHHHHHHHH----------hhlleEHHH--HH-HL--LLHHHHHHHHH-HHH-H-hL-LEE-eellllLLLLLEEEELHH-HHH-HH-HHH-HHH-Ll
0039 5e20A   lhhhLLLH--HHHHHHHHHHL--LL-E----ELHHHHHHH--L-LL-L--HHHHHHHHH-HHH-H-lL-LEE-eeelllLLLLEEEEELHH-HHH-HH-HHH-HHH-Hh
0040 2id3A   --lHHHHH--HHHHHHHHHH--hHL-LHH--HHHHHHL----------------lLHH-HHH-H--H-HL----------------LHHH-HHH-HH-HHH-HHH-Hl
0041 1fokA   -lLLLLHH--HHHHHHHLLLLllLL-LEE--H--HHHHLllHH-LLlLLLHHHHHHHHH-HHH-H--LlLEEE------ELLLLEEEELHH-HHH-HH-HL--LLL-Lh
0042 4u03A   ------HH--HHHHHHHHHHH--HL-LLLhhHHHHHHH-H-LH-HH-HLLHHHHHHHHH-HHH-H--L-------------------LHHH-HHH-HH-LLLl------
0043 8rkdB   ---LLHHH--HHHHHHHHHHL--LHhHHH--HHHHHHHHH-H--lL-LLLHHHHHHHHH-HHH-H----HHLL-----------------------------lLLH-Hh
0044 3oopA   ------LH--HHHHHHHHHH----------hhlleEHHH--HH-HL--LLHHHHHHHHH-HHH-H-lL-LEE-----eeLLLLLEEEELHH-HHH-HH-HHH-HHH-Hh
0045 6jbxA   llllLLLH--HHHHHHHHHH----------hllllLHHH--HH-HL--LLHHHHHHHHH-HHH-H-hL-LEE-eeelllLLLLEEEEELHH-HHH-HH-HHH-HHH-Hh
0046 1ub9A   ------LH--HHHHHHHHHHH--HL-E----EEHHHHHHH-L------lLHHHHHHHHH-HHH-H-lL-LEE-eeeellLLLEEEEEELHH-HHH-HH-HHH-HHH-Hh
0047 1yyvA   hhhhHHHHlhHHHHHHHHHH--------hllEEHHHHH---HH-LL-LLLHHHHHHHHH-HHH-H-hL-LEE-eeeellLLLEEEEEELHH-HHH-HH-HHH-HHHh--
0048 4hw0A   --llLHHH--HHHHHHLLLLL------------eeLHHH--HH-HL--LLHHHHHHHHH-HHH-H-lL-LEE----eeeELLEEEEEELHH-HHH-HH-HHH-HHHh--
0049 5j9qE   -hhHHHHH--HHHHHHHHHHH--LL-L----LLHHHHH-H-HH-L-------lLHHHHH-HHH-H--HlLLEE---ellLLE----EELLL--hH-HH-HHH-HHH-Hh
0050 4hqeB   hhhhHHHLllLHHHHHHHHL-----llhhheELHHHHHHH-H---L-LLLHHHHHHHHH-HHH-H-lL-LEE-eeeellLLLEEEEEELHH-HHH-HH-HLL-HHH-Hh
0051 3mq0B   llllHHHH--HHHHHHHHHL--LL-L----EEHHHHH---LL-LL--LLHHHHHHHHH-HHH-H-lL-LEE--elllllEEE----ELLLHh----------------
```

Job: Type-1 BREX; BrxA BAY35696 HTH2
Query: s001A
No: Chain Z rmsd lali nres id PDB Description
1: 7zge-A 10.3 1.4 80 199 18 MOLECULE: BRXA, A BREX PHAGE DEFENCE PROTEIN;
2: 4kyw-A 7.1 2.3 67 254 10 MOLECULE: TYPE-2 RESTRICTION ENZYME DPNI;
3: 1cf7-B 6.1 2.7 70 82 10 MOLECULE: DNA (5'-
4: 7qcd-D 5.7 3.5 73 326 10 MOLECULE: STRUCTURAL MAINTENANCE OF CHROMOSOMES PROTEIN 5;
5: 8c5s-A 5.5 3.8 75 924 12 MOLECULE: MITOCHONDRIAL TRANSCRIPTION FACTOR 1;
6: 4ets-B 5.5 3.0 61 149 7 MOLECULE: FERRIC UPTAKE REGULATION PROTEIN;
7: 3cuq-A 5.5 2.0 54 219 11 MOLECULE: VACUOLAR-SORTING PROTEIN SNF8;
8: 1sfu-A 5.3 2.0 58 70 3 MOLECULE: 5'-D(*T*CP*GP*CP*GP*CP*G)-3';
9: 8cli-A 5.2 2.4 65 542 8 MOLECULE: GENERAL TRANSCRIPTION FACTOR 3C POLYPEPTIDE 1;
10: 5nl9-A 5.2 2.6 60 146 8 MOLECULE: TRANSCRIPTIONAL REGULATOR (FUR FAMILY);
11: 7nyw-E 5.2 3.2 72 212 14 MOLECULE: CHROMOSOME PARTITION PROTEIN MUKB;
12: 5v8f-6 5.2 2.5 62 692 8 MOLECULE: DNA REPLICATION LICENSING FACTOR MCM2;
13: 4hga-A 5.1 2.9 65 207 14 MOLECULE: DEATH DOMAIN-ASSOCIATED PROTEIN 6;
14: 9qe1-D 5.1 2.7 70 384 9 MOLECULE: JETC;
15: 5dca-A 5.0 2.1 67 1948 9 MOLECULE: PRE-MRNA-SPLICING HELICASE BRR2;
16: 6qfd-B 5.0 2.4 57 116 9 MOLECULE: DNA-BINDING PROTEIN;
17: 6zvh-y 5.0 2.7 64 72 11 MOLECULE: 18S RRNA;
18: 9mj5-B 5.0 2.3 59 208 12 MOLECULE: REPLICATION PROTEIN A 14 KDA SUBUNIT;
19: 1lva-A 4.9 2.2 54 258 13 MOLECULE: SELENOCYSTEINE-SPECIFIC ELONGATION FACTOR;
20: 8fef-I 4.9 2.7 65 253 2 MOLECULE: VIRULENCE FACTOR MCE FAMILY PROTEIN;
21: 2qby-B 4.9 2.7 59 368 5 MOLECULE: CELL DIVISION CONTROL PROTEIN 6 HOMOLOG 1;
22: 6j05-A 4.8 2.3 60 101 7 MOLECULE: TRANSCRIPTIONAL REGULATOR ARSR;
23: 7uy7-B 4.8 2.6 61 291 13 MOLECULE: TELOMERASE-ASSOCIATED PROTEIN OF 75 KDA;
24: 2vwa-A 4.8 2.9 68 99 9 MOLECULE: PUTATIVE UNCHARACTERIZED PROTEIN PF13_0012;
25: 5l0p-A 4.8 2.4 56 311 11 MOLECULE: TRANSCRIPTION FACTOR ETV6, TRANSCRIPTION FACTOR E
26: 9jbn-C 4.8 2.4 61 423 20 MOLECULE: PROTEIN EDS1;
27: 7rhe-A 4.8 2.5 57 368 12 MOLECULE: ROK FAMILY PROTEIN;
28: 4yo2-A 4.7 3.5 71 186 11 MOLECULE: TRANSCRIPTION FACTOR E2F8;
29: 3k69-A 4.7 2.7 61 151 13 MOLECULE: PUTATIVE TRANSCRIPTION REGULATOR;
30: 7xx7-U 4.7 2.6 68 213 4 MOLECULE: HISTONE H3.1;
31: 3elk-A 4.6 2.7 59 104 12 MOLECULE: PUTATIVE TRANSCRIPTIONAL REGULATOR TA0346;
32: 1ldd-A 4.6 3.0 63 74 17 MOLECULE: ANAPHASE PROMOTING COMPLEX;
33: 8jxk-D 4.6 2.8 59 166 5 MOLECULE: CONSERVED PROTEIN;
34: 2ns0-A 4.6 3.1 63 85 16 MOLECULE: HYPOTHETICAL PROTEIN;
35: 4lmy-B 4.5 2.2 62 157 10 MOLECULE: PEROXIDE STRESS REGULATOR PERR, FUR FAMILY;
36: 7b0c-A 4.5 2.9 62 144 10 MOLECULE: HTH-TYPE TRANSCRIPTIONAL REPRESSOR NSRR;
37: 8rkd-B 4.5 3.8 64 411 8 MOLECULE: PILUS ASSEMBLY ATPASE CPAF;
38: 3bpx-A 4.4 2.3 55 147 18 MOLECULE: TRANSCRIPTIONAL REGULATOR;

```
39: 9dlr-B 4.3 2.9 64 395 14 MOLECULE: LEUKOCYTE RECEPTOR CLUSTER MEMBER 8;
40: 3lwf-A 4.2 2.7 60 144 12 MOLECULE: PUTATIVE TRANSCRIPTIONAL REGULATOR;

0001 s001A   --------DNLLYDFTLNIYWAAVAQ---R---R-KK--I--FT-Q-D-VLDFFESAAVD-GRI--L-SP--W---S-EGTQVRTARGLL-ATLRD-V-G-FLRDVS-R-EIIP-Y--YI
0002 7zgeA   lmmsllihSPVVADFMRHTLAEARRT---Y---K-PA--L--TA-D-A-WSEFYDTRVRA-Y-A--E-LG-gF---S-DSTIKKMGNNAI-KALVD-S-G-YLSDSR-K-KIQP-V--YL
0003 4kywA   lrksslssRGWTIEILN-CIDKI------E---G-SE--F--TL-E-D-MYRFESDLK---NIF--V-KN--N-------HIKEKIRQQL-QILRD-K-E-IIEFKG-Y-RKL-------
0004 1cf7B   ----gkglRHFSMKVCE-KVQRKG------------T--T--SY-N-E-VADELVSEFTN-SnnhlaaDS--A---YdQKNIRRRVYDAL-NVLMA-M-N-IISKEK-I-KWIG-L--P-
0005 7qcdD   ----pvtgDATAKYLLQ-YILSAR-----------gI--C--HE-N-A-LILALMRLETDaSTL--N-TE--W---S-IQQWVDKLNDYI-NAINV-K-L-KIIRINfF-VYV--N--LA
0006 8c5sA   pyltlidpGKMCVITIL-ELLKLNSTggvI---E-GM-rtarAV-I-S-VGKAIEMEFRS-EqvlkK-IL--W---P-QSIRARIGSVLI-SMLIQ-V-AkAFAHhn-V-LKIH-L--IR
0007 4etsB   vllerfkyTKQREVLL-KTLYHSD-----------tH--Y--TPeS-L-YMEIKQAEPD--------------------lnvGIATVYRTL-NLLEE-A-E-MVTSgK-Y-ELA--N--KP
0008 3cuqA   iqsvpaelNMDHTVVLQ-LAEKNG------------Y--V--TV-S-E-IKASLK-------------------------wETERARQVL-EHLLK-E-G-LAWLeA-Y-WLPAl-----
0009 1sfuA   ---ctvndAEIFSLVKK-EVLSL--N---T---N-DY--T--TA-I-S-LSNRLK-------------------------iNKKKINQQL-YKLQK-E-D-TVKMpP-W-FKNY-Nc---
0010 8cliA   evretyrlLKRRNLIIE-AVTNLR-----------lI--E--SL-F-T-IQKMIMDQEKQ-E---gV-ST--K-------cCKKSIVRLV-RNLSE-E-G-LL-RLYlV-VHPS-Mdq--
0011 5nl9A   ledaginvTVQRLQMAN-LLLSK----------P-QH--L--TAdQ-V-FQLINEHMP----------------------naSRATIFNNL-KLFAE-K-G-IVNLiT-Y-DSNV-I--H-
0012 7nywE   prsvlseldMMVGKILCYLYLSpeRL---A---NqGI--F--TS-Q-E-LYEELISLA----DE--G-KL--MkdlD-KQKLQEKVRTTL-NRLRR-L-G-MVYFNN-F-TITE-Avf--
0013 5v8f6   ttvtydkyVSMMNMIVR-KIAEVDRE---G---A-EE--L--TA-V-D-IVDWYLLQKEN-D-lgslaEY--W---EeRRLAFKVIKRLVkDRIL---------MEI------------
0014 4hgaA   ipyrgtryPEVNRRIE-RLINK--PG---P---D-TF--P--DY-G-D-VLRAVEKAAA---RH--S-LG--L---P-RQQLQLMAQDAF-RDVG----I-RLQ-------eRR-H--L-
0015 9qe1D   qdfqepmdYAIFCCALA-FLEGKA-----V---D-EQ--F--LL-S-E-LCQEIQADY----PG--D-FP----dwT-LYTHRKSLIRAV-KVLME-F-Q-LIRTIDlY-EAST-Ysr--
0016 5dcaA   lpiesqfvSKLVDNLNA-EVVAG--------------niK--CR-N-D-AVNWLAYT----YLY--V-RMdgQ---L-KKFRESLVHSAL-CILKE-Q-E-LVLYDA-I-EATD-Lgn--
0017 6qfdB   arsdardlTAFQKNILT-VLGEE---------------aR--YG-L-A-IKRELEEYYGE--------------------evNHGRLYPNL-DDLVN-K-G-LV-EKS---ALTN-Egf--
0018 6zvhy   -------kfNWKGTIKA-ILKQA--P---D---N-E---I--TI-K-K-LRKKVLAQYYT--VT--D-EH-hR---S-EEELLVIFNKKI-SKN---P-T-FK-LLK-Kv----------
0019 9mj5B   sfmpanglTVAQNQVLN-LIKAC--P---R---P-EG--L--NF-Q-D-LKNQLKH------------------------mSVSSIKQAV-DFLSN-E-G-HIYSdD-H-FKST-Da---
0020 1lvaA   --------GSPEKILAQ-IIQEH----------R-EG--L--DW-Q-EaATRAS--------------------------lSLEETRKLL-QSXAA-A-G-QV-TLLyA-ISTEr-----
0021 8fefI   lptlavsiPLTVLIIFT-LNILLAEF---G---AaDV--S--G--A-G-AALGAVTQ--lgpLV--T-VlfmL---N-GAVITIGLVGGF-FFGVY-I-Q-NVS-AG-Yv----------
0022 2qbyB   lieavkalPFHYKLAL-RSLIES---------------eD--VM-S-A-HKMYTDLCNKF-KQK---------------plSYRRFSDII-SELDM-F-G-IV-KIR---LVEDk-----
0023 6j05A   varlealaSPVRLEIFR-LLVEQE---------P-TG--L--VS-G-D-IAEHLG-------------------------qPHNGISFHL-KNLQH-A-G-LVTVQRrY-RAAM-V--RA
0024 7uy7B   yqvsigffQNILDIAT-KTVKDRG---------A-LG--C--NY-K-Y-LKDKLTKALNL-Q--qiS-YP--L-------iSESYISYLV-HLFQD-F-N-II-EIE------------
0025 2vwaA   dvsikrynNFVDIARL-SIQKHFEHL---SndqK-DS--HvnNX-E-Y-XQKFVQGLQEN-RNI-----S--L---S-KYQENKAVXDLK-YHLQ----K-VYA--------NY-L--SQ
0026 5l0pA   lkkaglkvTLPRLKILE-VLQEPD---------nhH--V--SA-E-D-LYKRLIDMG-----------------------eeiGLATVYRVL-NQFDD-A-G-IV-TRHvF-ELT-------
0027 9jbnC   ieatlenlDPKTRDCFLV-LGAF--P---E---D-KK--I--PL-DvL-INVLVELHD----------------------lEDATAFAVI-VDLAN-R-N-LL-TLVfV-TQHD-Vlr--
0028 7rheA   -----nvrRYNERLLLK-TLRRAG------------S--A--SK-A-D-LARLAN-------------------------mTGTAVGSII-ASLAD-A-K-LIE-aS-I-RLDP-G--AF
0029 4yo2A   nsrkdkslRVMSQKFV-MLFL---VS---T---P--Q--I--VS-L-E-VAAKILIGEDH---vedL-DK--S---K-FKTKIRRLYDIA-NVLSSlD-L-IKKV-H-T-EERG-R--KP
0030 3k69A   ----xkldFSVAVHSIL-YLDAHR---------D-SK--V--AS-R-E-LAQSLH-------------------------lNPVXIRNIL-SVLHK-H-G-YLTGTV-G-YQLD-L--AL
0031 7xx7U   nskkknqpGKYSQLVVE-TIRRLG-----E---R-NG--S--SL-A-K-IYTEAKKV--P-WFD--------------qQNGRTYLKYSI-KALVQ-NdT-LLQVKG-S-FKlN-R--KK
0032 3elkA   --rerilhGLITLYILK-ELVKR-------------P--X--HG-Y-E-LQKSXFETTGQ------------------alPQGS-IYILL-KTXKE-R-G-FV-ISEvY-HITD-Agk--
0033 1lddA   --kyeltlQRSLPFIEG-MLTNLG------------A--M--KL-H-K-IHSFLKITVPK-DWG--Y------------nrITLQQLEGYL-NTLAD-E-G-RLKYnG-Y-EIV-------
0034 8jxkD   -------eFMLELAILG-LLIES---------------pM--HG-Y-E-LRKRLTGLLG-------A-FR--A-------fSYGSLYPAL-RRMQA-D-G-LI-AEN---qLTD-Kgr--
0035 2ns0A   ----xtvsdRELEECIRALLDAR--A---D---S-AS--I--CP-S-D-VARAVAPDD---------------------wrPLXEPVREAA-GRLAD-A-G-EV-EVTrI-RWTR-T--D-
0036 4lmyB   lrekhiriTETRKAIIS-YMIQS----------T-EH--P--SA-D-K-IYRDLQPNFP---------------------nmSLATVYNNL-KVLVD-E-G-FVSEtT-Y-DFM--G--HQ
0037 7b0cA   --mrltkfTDLALRSLM-RLAVV-RD---G---D-EP--L--AT-R-EvAEVVG--------------------------vPYTHAAKAI-TRLQH-L-G-VVEARR-L-TLTD-R--RV
0038 8rkdB   -dyyhatkTTIFNALLNTIDLSQL-----A---Q-LDlkQ--AG-E-E-IRDIVAELVAI-K----N-VS--M---S-VAEQEHLVQDII-NDVLG-Y------------------R--IV
0039 3bpxA   relghlnlTDAQVACLL-RIHREP------------G--I--KQ-D-E-LATFFH-------------------------vDKGTIARTL-RRLEE-S-G-FIEREQiL-EVTR-Rge--
0040 9dlrB   lileklkiITYRNLFK-KVYLLLK---------T-HQ--L--SL-D-A-FLVALKFMQ---------------------vEDVDIDEVQCIL-ANLIY-M-G-VKGYISkL-VVSK-Q--N-
0041 3lwfA   qgxkittkGRYGLTITL-ELAKRI-----G---D-GP--I--SL-R-S-IAQDKN-------------------------lSEHYLEQLI-GPLRN-A-G-IVKSIRgY-VLNG-D--PE

0001 s001A   --------LHHHHHHHHHLHHHHHHL---L---L-LL--L--LH-H-H-HHHHHHHHHHL-LLL--L-LL--L---L-HHHHHHHHHHHH-HHHHH-L-L-LLLLLL-L-LLLL-L--LL
0002 7zgeA   hhhhhhhhLHHHHHHHHHLHHHHHHL---L---L-LL--L--LL-L-H-HHHHHHHHHHH-L-H--H-HH-lL---L-HHHHHHHHHHHH-HHHHH-L-L-LLLLLL-L-LLLL-L--LL
0003 4kywA   llllllllLLHHHHHHH-HHHHL------L---L-LE--E--EH-H-H-HHHHHHHHH---HHL--L-LL--L-------LHHHHHHHHH-HHHHH-L-L-LEEELL-E-EEL-------
0004 1cf7B   ----llhhHHHHHHHHH-HHHHHL------------E--E--LH-H-H-HHHHHHHHHHL-LllllhhHL--H---HhHHHHHHHHHHH-HHHHH-L-L-LLLLLL-E-ELLL-L--L-
0005 7qcdD   ----lhhhHHHHHHHHH-HHHHLL-----------lE--E--EH-H-H-HHHHHHHHHHHhLLL--L-LL--L---L-HHHHHHHHHHHH-HHHHH-H-H-EEEEEEeE-EEE--E--LL
0006 8c5sA   hhhllllhHHHHHHHHH-HHLLLLLLlllL---L-EE-ehhhHH-H-H-HHHHHHHHHHH-HhhllL-LL--L---L-HHHHHHHHHHHH-HHHHH-H-LlLEEEll-E-EEEL-H--HH
0007 4etsB   hhhhhhllLHHHHHHH-HHHHHLL-----------lL--L--LHhH-H-HHHHHHHLHH--------------------hllLHHHHHHHH-HHLLL-L-L-LEELeE-L-EEL--L--LL
0008 3cuqA   eellllllLHHHHHHHH-HHLLLL------------E--E--LH-H-H-HHHHHL-------------------------lLHHHHHHHH-HHHHH-H-L-LLEElL-E-ELLLl-----
0009 1sfuA   ---lllllHHHHHHHHH-HHHLL--L---L---L-LE--E--LH-H-H-HHHHLL-------------------------lLHHHHHHHH-HHHHH-L-L-LEEElL-E-EELL-Ll---
0010 8cliA   elllhhhhHHHHHHHHH-HHHHLL-----------eE--L--LH-H-H-HHHHHHHHHHH-H---lL-LL--L-------lLHHHHHHHH-HHHHH-L-L-LL-EEEeE-ELLL-Lll--
0011 5nl9A   hhhlllllLHHHHHHHH-HHHLL----------L-LE--E--LHhH-H-HHHHHHHLL----------------------llLHHHHHHHH-HHHHH-L-L-LLEElE-E-ELLL-L--L-
0012 7nywE   leeellhhhHHHHHHHHHHHHLlhHH---H---HlLE--E--EH-H-H-HHHHHHHHL----LH--H-HH--HhhhH-HHHHHHHHHHHH-HHHHH-L-L-LEEELL-E-EELH-Hhh--
0013 5v8f6   llllhhhhHHHHHHHHH-HHHHHHHL---L---L-LL--L--LH-H-H-HHHHHHHHLLL-L-lllhhHH--H---HhHHHHHHHHHHHhLLLL---------LLL------------
0014 4hgaA   llllllllHHHHHHHH-HHHLL--LL---L---L-LL--L--LH-H-H-HHHHHHHHHH---HL--L-LL--L---L-HHHHHHHHHHHH-HHHH----H-HHH-------hHH-H--H-
0015 9qe1D   lllllhhhHHHHHHHHH-HHLLLL-----L---L-LE--E--EH-H-H-HHHHHHHHL----LL--L-LL----llL-LHHHHHHHHHHH-HHHHH-L-L-LEEEELeE-EELH-Hhh--
0016 5dcaA   llllllhhHHHHHHHHH-HHHLL--------------llL--LH-H-H-HHHHHLLL----HHH--H-HHhlL---L-HHHHHHHHHHHH-HHHHH-L-L-LEEEEL-E-EELH-Hhh--
0017 6qfdB   lhhhhlllLHHHHHHHH-HHHHL---------------lE--EH-H-H-HHHHHHHHHLL--------------------llLHHHHHHHH-HHHHH-L-L-LE-EEE---EELH-Hhh--
0018 6zvhy   -------llLHHHHHHH-HHHLL--H---H---H-E---E--EH-H-H-HHHHHHHHHHH--HL--L-LL-lL---L-HHHHHHHHHHHH-HLL---L-L-EE-ELL-Ee----------
0019 9mj5B   llllllllLHHHHHHHH-HHHHL--L---L---L-LL--L--LH-H-H-HHHHLLL-------------------------lLHHHHHHHH-HHHHH-H-L-LEELlL-L-LEEL-Ll---
0020 1lvaA   --------LLHHHHHHH-HHHLL----------L-LL--E--EH-H-HhHHHHL--------------------------lLHHHHHHHH-HHHHH-L-L-LE-EEEeE-EEHHh-----
0021 8fefI   hhhhhhhhHHHHHHHHH-HHHHHHHH---L---LlLL--H--H--H-H-HHHHHHHL--hhhHH--H-HhhhH---H-HHHHHHHHHHHH-HHHHH-L-L-LLL-HH-Hh----------
0022 2qbyB   hhhhhhllLHHHHHHH-HHHHLL---------------lL--HH-H-H-HHHHHHHHHHL-LLL----------------llLHHHHHHHH-HHHHH-L-L-LE-EEE---EELLl-----
0023 6j05A   hhhhhhhlLHHHHHHHH-HHHHLL---------L-LL--E--EH-H-H-HHHHHL-------------------------lLHHHHHHHH-HHHHH-L-L-LEEEEEeE-EELH-H--HH
0024 7uy7B   eeelhhhhHHHHHHHH-HHHHHHL---------L-LL--L--LH-H-H-HHHHHHHHHLL-L--llL-LL--L-------lLHHHHHHHH-HHHHH-H-L-LL-LLL------------
0025 2vwaA   lllhhhhhHHHHHHHHH-HHHHHHHHL---LhhhH-HH--HllLH-H-H-HHHHHHHHHHH-LLL-----L--L---L-HHHHHHHHHHHH-HHHH----H-HHH--------HH-H--HH
0026 5l0pA   hhhlllllLHHHHHHHH-HHLLLL----------llE--E--EH-H-H-HHHHHHHLL---------------------lllLHHHHHHHH-HHHHH-L-L-LE-EEEeE-EEL-------
0027 9jbnC   hhhhhlllLHHHHHHHHH-HHHL--L---L---L-LL--E--EH-HhH-HHHHHHHHL----------------------lLHHHHHHHH-HHHHH-L-L-LL-EELeE-ELLH-Hhh--
0028 7rheA   -----lhhHHHHHHHHH-HHHHHL------------L--E--EH-H-H-HHHHHH-------------------------lLHHHHHHHH-HHHHH-L-L-LEE-lL-E-EELL-L--LE
0029 4yo2A   lllllllhHHHHHHHH-HHLL---LL---L---L--L--L--LH-H-H-HHHHHHHHLLL---lllL-LL--L---L-HHHHHHHHHHHH-HHLLLlL-L-LLLL-L-L-LLLL-L--LL
0030 3k69A   ----llhhHHHHHHHHH-HHHLLL---------L-LL--L--LH-H-H-HHHHHL-------------------------lLHHHLHHHH-HHHHH-L-L-LLEEEL-E-EELL-L--LH
0031 7xx7U   llllllllLHHHHHHHH-HHHHLL-----L---L-LL--L--LH-H-H-HHHHHHLL--L-LLL---------------lLLHHHHHHHH-HHHHH-LlL-EEEELL-E-EEeL-L--LL
0032 3elkA   --llhhhhHHHHHHHHH-HHHHL-------------L--E--EH-H-H-HHHHHHHHHLL-------------------llLLLH-HHHHH-HHHHH-H-L-LE-EEEeE-EELH-Hhh--
0033 1lddA   --llhhhhHHHHHHHHH-HHHHHL------------L--E--EH-H-H-HHHHHHHHLLH-HHL--L------------llLLHHHHHHHH-HHHHH-L-L-LEELlL-E-EEL-------
0034 8jxkD   -------lHHHHHHHHH-HHHHL---------------lE--EH-H-H-HHHHHHHHHLL-------L-LL--L-------lLLLLHHHHH-HHHHH-L-L-LE-EEL---eELH-Hhh--
0035 2ns0A   ----llllhHHHHHHHHHHHHHL--L---L---L-LL--L--LH-H-H-HHHHHLLLL--------------------lhHHHHHHHHHH-HHHHH-L-L-LE-EEEeE-EELL-L--L-
0036 4lmyB   hhhlllllLHHHHHHHH-HHHHL----------L-LL--L--LH-H-H-HHHHHLLLLL--------------------llLHHHHHHHH-HHHHH-L-L-LEEElL-E-EEL--L--LE
0037 7b0cA   --llllhhHHHHHHHHH-HHHLL-LL---L---L-LL--L--LH-H-HhHHHHL--------------------------lLHHHHHHHH-HHHHH-H-L-LEEELL-E-EELH-H--HL
0038 8rkdB   -llhhhhhHHHHHHHHHHLLHHHL-----L---L-LLhhH--HH-H-H-HHHHHHHHHHH-L----L-LL--L---L-HHHHHHHHHHHH-HHHLL-L------------------H--HH
0039 3bpxA   hhhhhhllLHHHHHHHH-HHHLLL------------L--L--LH-H-H-HHHHHL-------------------------lLHHHHHHHH-HHHHH-L-L-LEEEEEeE-EELH-Hhh--
0040 9dlrB   hhhllhhhHHHHHHHH-HHHHHHL---------L-LE--E--EH-H-H-HHHHHHHLL---------------------lLLLLHHHHHHHH-HHHHH-H-L-LLEEEEeE-EELL-L--L-
```

```
0041 3lwfA   llllllhhHHHHHHHHH-HHHHLL-----L---L-LL--L--LH-H-H-HHHHHL-------------------------lLHHHHHHHH-HHHHH-L-L-LEEEELeE-EELL-L--LL

Job: Type-1 BREX; BrxA BAY35696 wHTH
Query: s001A
No: Chain Z rmsd lali nres id PDB Description
1: 1jhf-A 6.1 2.6 63 197 13 MOLECULE: LEXA REPRESSOR;
2: 6lb9-A 6.0 2.4 66 345 11 MOLECULE: DUF4007 DOMAIN-CONTAINING PROTEIN;
3: 9fhl-c 5.8 2.6 64 109 8 MOLECULE: RRNA 16S SSO;
4: 1ylf-C 5.6 2.5 62 119 6 MOLECULE: RRF2 FAMILY PROTEIN;
5: 4o5v-A 5.6 3.0 62 213 11 MOLECULE: IRON-DEPENDENT TRANSCRIPTION REPRESSOR RELATED PR
6: 1fx7-A 5.4 3.0 62 230 11 MOLECULE: IRON-DEPENDENT REPRESSOR IDER;
7: 2jt1-A 5.4 3.1 61 71 15 MOLECULE: PEFI PROTEIN;
8: 3dv8-A 5.3 3.4 63 216 14 MOLECULE: TRANSCRIPTIONAL REGULATOR, CRP/FNR FAMILY;
9: 2ns0-A 5.2 3.2 62 85 16 MOLECULE: HYPOTHETICAL PROTEIN;
10: 5yad-A 5.2 3.4 62 73 8 MOLECULE: MEIOSIS REGULATOR AND MRNA STABILITY FACTOR 1;
11: 3ov8-A 5.2 3.8 66 91 11 MOLECULE: PROTEIN AF_1382;
12: 6qpq-D 5.2 2.8 63 82 8 MOLECULE: STRUCTURAL MAINTENANCE OF CHROMOSOMES PROTEIN,STR
13: 6wge-C 5.2 3.1 62 174 5 MOLECULE: STRUCTURAL MAINTENANCE OF CHROMOSOMES PROTEIN 1A;
14: 4i99-C 5.2 2.9 59 70 8 MOLECULE: CHROMOSOME PARTITION PROTEIN SMC;
15: 4kyw-A 5.2 3.2 62 254 6 MOLECULE: TYPE-2 RESTRICTION ENZYME DPNI;
16: 7y43-A 5.1 3.4 62 79 11 MOLECULE: HISTONE ACETYLTRANSFERASE KAT6A;
17: 2od5-A 5.0 3.1 67 91 7 MOLECULE: HYPOTHETICAL PROTEIN;
18: 3lmm-D 5.0 3.0 63 556 8 MOLECULE: UNCHARACTERIZED PROTEIN;
19: 5trd-A 5.0 3.0 60 219 3 MOLECULE: RIBOFLAVIN KINASE;
20: 5xpq-A 4.9 3.2 63 100 10 MOLECULE: UNCHARACTERIZED HTH-TYPE TRANSCRIPTIONAL REGULATO
21: 3h0d-A 4.9 3.1 64 155 16 MOLECULE: CTSR;
22: 8xt8-B 4.9 2.9 58 145 10 MOLECULE: MARR FAMILY TRANSCRIPTIONAL REGULATOR;
23: 5j6x-A 4.9 3.1 60 66 7 MOLECULE: Z-DNA BINDING PROTEIN KINASE;
24: 9gm7-C 4.8 3.6 67 440 3 MOLECULE: CHROMOSOME PARTITION PROTEIN MUKF;
25: 6uvu-B 4.8 2.9 60 114 12 MOLECULE: ARSR FAMILY TRANSCRIPTIONAL REGULATOR;
26: 8as8-C 4.8 2.7 65 195 14 MOLECULE: JETC;
27: 8qto-A 4.7 3.8 65 234 17 MOLECULE: FNR TYPE REGULATOR;
28: 2mh2-A 4.7 3.2 61 64 13 MOLECULE: HOMOLOGOUS-PAIRING PROTEIN 2 HOMOLOG;
29: 7yw2-A 4.7 3.1 65 216 9 MOLECULE: TRNA 2'-PHOSPHOTRANSFERASE 1;
30: 2h09-A 4.7 3.5 62 127 8 MOLECULE: TRANSCRIPTIONAL REGULATOR MNTR;
31: 1ucr-B 4.7 3.2 61 75 15 MOLECULE: PROTEIN DSVD;
32: 2obp-A 4.6 3.4 65 81 9 MOLECULE: PUTATIVE DNA-BINDING PROTEIN;
33: 1yyv-A 4.6 3.2 65 114 11 MOLECULE: PUTATIVE TRANSCRIPTIONAL REGULATOR;
34: 3bpx-A 4.6 3.0 60 147 7 MOLECULE: TRANSCRIPTIONAL REGULATOR;
35: 5v9x-A 4.6 3.3 66 772 14 MOLECULE: ATP-DEPENDENT DNA HELICASE;
36: 9dlr-B 4.6 2.6 64 395 8 MOLECULE: LEUKOCYTE RECEPTOR CLUSTER MEMBER 8;
37: 8wxs-A 4.6 2.8 59 76 14 MOLECULE: FI02030P;
38: 8ylg-A 4.6 2.9 60 161 10 MOLECULE: MARR FAMILY TRANSCRIPTIONAL REGULATOR;
39: 2qby-B 4.6 3.0 66 368 11 MOLECULE: CELL DIVISION CONTROL PROTEIN 6 HOMOLOG 1;
40: 7yk3-B 4.6 2.6 62 188 8 MOLECULE: DNA ADP-RIBOSYL TRANSFERASE;
41: 1xsd-A 4.6 3.4 64 125 3 MOLECULE: 5'-
42: 2g9w-A 4.6 2.8 60 119 10 MOLECULE: CONSERVED HYPOTHETICAL PROTEIN;
43: 8yz7-A 4.6 3.4 65 191 15 MOLECULE: TRANSCRIPTIONAL REGULATOR, FNR/CRP FAMILY;
44: 6qfd-B 4.6 3.4 66 116 8 MOLECULE: DNA-BINDING PROTEIN;
45: 2zkz-C 4.6 3.4 60 87 12 MOLECULE: TRANSCRIPTIONAL REPRESSOR PAGR;
46: 5a31-N 4.5 2.9 61 703 5 MOLECULE: ANAPHASE-PROMOTING COMPLEX SUBUNIT 1;
47: 7dvr-A 4.5 3.1 62 147 6 MOLECULE: HTH MARR-TYPE DOMAIN-CONTAINING PROTEIN;
48: 7qen-C 4.5 3.0 69 291 10 MOLECULE: DNA (35-MER);
49: 1lva-A 4.5 2.8 56 258 5 MOLECULE: SELENOCYSTEINE-SPECIFIC ELONGATION FACTOR;
50: 3elk-A 4.5 3.1 64 104 6 MOLECULE: PUTATIVE TRANSCRIPTIONAL REGULATOR TA0346;
```

```
0001 s001A   -----PYYI--S--DEG--VACLARYL-HESG-IS-N-S-A-LCE-----H--QDWN-I---F--GL---NREQVCDRLDQL-GEHR-G-LII-QKA---G--SVVSITW---KV-N-
0002 1jhfA   ----kaltA--R--QQE--VFDLIRDH-ISQT-GM-P----pTRA-----E---iAQ-R---L--GF--rSPNAAEEHLKAL-ARKG-V-IEI-VSG------ASRGIRL---LQ-Ee
0003 6lb9A   ftsgsrPSL--P--ARI--IAYACLDY-AART----L-A-R-LANepgapG--RAFR---------I---READIAAALEKV-AASHqE-LQL-VEA---V--GQRSLTF---TS-G-
0004 9fhlc   keiisraVT--I--DEE--TKKKVLDE-IKKE-SI-I-TpY-ALA--------TKSG---------I---SISVARKILKEL-ENQN-V-VKLySKN---R--RLEIYIA---AS---
0005 1ylfC   ------ISS--R---FS--IAVHILSI-LKNN----ssL-C-TSD-----YxaESVN---------T---NPVVIRKIXSYL-KQAG-F-VYV-NRG-------PGGAGL---LK-D-
0006 4o5vA   -----IRSV--T--EED--YLKIIQEL-VLYK-GY-A-----TLA-----DisRSLN---------V---KRQSVRDEINHL-ISLS-X-AEK-I---------eRGKYRL---TP-Sg
0007 1fx7A   -----MNELvdT--TEM--YLRTIYDL-EEEG-VT-P-----LRA-----RiaERLD---------Q---SGPTVSQTVSRM-ERDG-L-LRV-AG--------DRHLEL---TEk--
0008 2jt1A   -----mSES--I--VTK--IISIVQER-QNMD-----dgapvKTR-----DiaDAAG---------L---SIYQVRLYLEQL-HDVG-V-LEK-VNA-gkG--VPGLWRL---LE---
0009 3dv8A   ----iXWKS--L--DKR--VASFLLEE-TSIE----L-K-I-THE-----TiaNHLG---------S---HREVITRXLRYF-QVEG-L-VKL-SR---------GKITI---LD-S-
0010 2ns0A   ------XTV--S--DRE--LEECIRAL-LDAR----adsasiCPS-----DvaRAVA-P-------dwrpLXEPVREAAGRL-ADAG-E-VEV-TQA---R--GPIRIRW---TR-Td
0011 5yadA   -----GAAN-kS--LSL--LSTETXSI-LQDA----L-F-K-FID-----IyekKYG-H-----------KLNV--SDLYKL-T--D-T-IAI-REQ---G--NGRLVCL---LP-S-
0012 3ov8A   ----lLFKE--K--ALE--ILMTIYYE-SLGG-ND-V-----YIQ-----YiaSKVN---------S---PHSYVWLIIKKF-EEAK-M-VEC-ELE---G--RTKIIRL---TD-Kg
0013 6qpqD   --------K--A--IVQ--MAKILRKE-LSEEkeV-I-F-T-DVL-----K---SQA-N------titkrEASRGFFDILSL-ATEG-C-IGL-SQT-eaF--GNIKIDA---KP-Al
0014 6wgeC   gmddreimK--R--TQQ--MLHGLQRA-LAKT----S-L-L-ELC-----R--N----------tnRK---QAAAKFYSFLVL-KKQQ-A-IEL-TQE-epY--SDIIATP-gpRF-H-
0015 4i99C   ------DIE--K-yVEE--LYKVVKKI-YEKT----K-F-W-DLV-----P--D-------------vepkIIARTFLYLLFL-ENMG-R-VEI-IQE-epF--GEILVVP---M----
0016 4kywA   ----lSSRG--W--TIE--ILNCIDK--IEGS-eF-T-L-E-DMY-----R---FES-D---L--KknnhIKEKIRQQLQIL-RDKE-I-IEF-KG--------RGKYRK---L----
0017 7y43A   -----------klaNPL--YTEWILEA-IKKVqrP-S-E-ErICN-----AvsSSHG---------L---DRKTVLEQLELS-VKDG-T-ILK-VSN---K--GLNSYKD---PD-Np
0018 2od5A   -----ETES-xK--TVR--IREKIKKF-LGDR-pR-N-T-A-EIL-----E--HINS-T---XrhGT---TSQQLGNVLSKD-K----D-IVKvGYI---KrsDICEWAT---RN-Wv
0019 3lmmD   fspvrylST--D--QAE--LTNAAXLW-LSEV-GD-L-----ATS-----DlxAXCG---------V---SRGTAKACVDGL-VDEE-R-VVA-VGG---G--RSRRYRL---VE---
0020 5trdA   --------D--Q--YYR--AIKKIKEA-AEAS-nR-A-Y-L------tssklaDMLG---------I---SQQSASRIIIDL-EKNG-Y-ITR-TVT---K--RGQILNI---TE-Kg
0021 5xpqA   eplyklkaeffktlaHP--ARIRILEL-LVER-DR-S-V-G-ELL-----S--SDVG---------L---ESSNLSQQLGVL-RRAG-V-VAA-RRD---G--NAMIYSI---AA-P-
0022 3h0dA   ------pNI--S--D-I--IEQYLKQV-LNXSdiV-E-I-K--RS-----E--I-AN-K---F--RC---VPSQINYVINTRfTLER-G-YIV-ESK---R--GYIRIXK---VKt--
0023 8xt8B   kddlkkmfkqngydITT--DHYALLRF-LWEQ----Q-I-D-LCE-----K----------------K---DKSNTTRILDVM-KNKG-L-IVR-KVDvkdR--RKFQIFLtdlGR-E-
0024 5j6xA   -------mS--A--ENE--IEMRICDY-LRRHgrS-T-V-QdIFK-----E--LKL---------------EKSTVNRHLYSL-QASK-Q-VFK-TVE------KRPVWDL---VE---
0025 9gm7C   ndfsislPV--E--RLA--FLMAIAVL-NSERgeM-S-E-G-ELI-----DafREVC-K---G--FtaesVAVRANNAINDM-VRQK-L-LNR-FTS---E--GNAIYRL---TP-Lg
0026 6uvuB   saeagevvvlfkvlaND--TRLRLLHA-LARSggL-C-V-TdLAA-----A--VGM---------------KPQAVSNQLQRL-ADRR-I-LRA-ARC---G--NNIHYRI---VD-P-
0027 8as8C   lvrrqrlnL--E--QSL--LVAILRQH-FVAWaqI-A-I-D-DL------L--PQLQ-I---Y--LseskERTRLLTLLDQL-KG-H-G-LVT-SPD---A--HERIVIR--pII-A-
0028 8qtoA   ----lSKKN--A--EER--LAAFLYNL-STRFreF-R-L-T-mtR-----G--DIGN-Y---L--GL---TVETISRLLGRF-QKTE-M-LTV-KG---------KYITI---ND-H-
0029 2mh2A   --------G--A--PGI--ILRYLQEQ-NR------S-A-Q-DVF-----G---NLQ-K--eH--GL---GKAAVVKALDQL-AQEG-K-IKE-KTY---G--KQKIYFA---D----
```

```
0030 7yw2A   ------eQD--R--NVQ--LSKALSYAlRHGAlglpL-Q-A-LLQ-----L--PQFH----------S---FSIEDVQLVVNT-NEKQ-R-FTL-QPG---E--TGLLIRA---NQ-G-
0031 2h09A   ----rEAHRreL--IDD--YVELISDL-IREV-GE-A-----RQV-----DmaARLG----------V---SQPTVAKMLKRL-ATMG-L-IEM-IP--------WRGVFL---TAe--
0032 1ucrB   --------------MEE--AKQKVVDF-LNS-skF-Y-F-N-DFT--------DLFP---------DM---KQREVKKILTAL-VNDE-V-LEY-WSS---G--STTMYGL---KG-Ag
0033 2obpA   -----GIDP--A--IVE--VLLVLREA-GIE-tpW-S-L-P-KIA--------KRAQ----------L---PXSVLRRVLTQL-QAAG-L-ADV-SVE--aD--GRGHASL---TQ-Eg
0034 1yyvA   faeqcpsrevlK--HVTsrWGVLILVA-LRDG-TH-R-F-S-DLR--------RXXG---------GV---SEXXLAQSLQAL-EQDG-F-LNR-VSY---P--PHVEYSLtplGE-Q-
0035 3bpxA   shrvfigrelghlnLTD--AQVACLLR-IHRE----Q-D-E-LAT-----F--FH------------V---DKGTIARTLRRL-EESG-F-IER-EQDpenR--RRYILEVtrrGE-E-
0036 5v9xA   ----rVPAN--P--LDV--LAQHTVAV-AAlepVD-A-D-A-W-F-----D--AVRR-SapfA--TL---PRSAFEATLDLL-SlRP-R-LVY-DRD-------TGTLTA---RP-G-
0037 9dlrB   rcgiflileklK--IIT--YRNLFKKV-YLLLhqL-S-L-D-AFL-----V---ALK-F---M--QV---DIDEVQCILANL-IMGH-VkGYI-SHQ-------HQKLVV---SK-Q-
0038 8wxsA   --------dggE--LSL--VKKVVHSL-VVSS----V-E-Q-LMR-----DyrSAAG----------C---TLPYSESFLRSI-P--D-T-VTV-TGH---G--QMAWITA---VA-Ta
0039 8ylgA   iardrlnplfarygLQP--GEFDVLAT-LRRS----P-T-A-LYD-----A--AMI---------------SSGSMTNRIDRL-EKAG-W-VER-RANpadG--RGTLVALtsaGR-A-
0040 2qbyB   vdyeqerlieavkaLPF--HYKLALRS-LI-E-SE-DvM-S-AHK-----MytDLCN-Q---K--PL---SYRRFSDIISEL-DMFG-I-VKI-RII---N--VKKYALV---ED-K-
0041 7yk3B   --------------MTW--GRAVILEA-MRRYamepH-L-E-IQK-----LmyFANE-AdpdL--AL---DSERVRHLLQGM-EG-A-F-TVG-L-V---L--ANQPISL---TT-Kg
0042 1xsdA   -------tnkqveiSMA--E-WDVMNI-IWDKksV-S-A-NeIVV-----E---IQK-Y---K--EV---SDKTIRTLITRL-YKKE-I-IKR-YKS---E--NIYFYSS---NI-K-
0043 2g9wA   ----------kltrlGD--LERAVXDH-LWSR----V-R-Q-VHE-----A---LSA-R---R--DL---AYTTVXAVLQRL-AKKN-L-VLQ-IR--------AHRYAP---VH-G-
0044 8yz7A   ----lVGKR--L--RAR--IAGELLEL-KDTA-LAtI-Y-A-THD-----ElaAAVG----------S---VRETVTKVVGEL-SREG-V-ISA-GY---------GKITL---KD-E-
0045 6qfdB   -----pdarsdardLTA--FQKNILTV-LGEE-AR-G-L-A-IKR-----E-lEEYY-G---E--EV---NHGRLYPNLDDL-VNKG-L-VEK-S-D---K--RTNEYALtneGF-D-
0046 2zkzC   yxsleddaellktxaHP--XRLKIVNE-LYKHkaL-N-V-T-QII--------QILK----------L---PQSTVSQHLCKX-RG-K-V-LKR-NRQ---G--LEIYYSI---NN-P-
0047 5a31N   nmvlikEEE--L---LL--FWTYIQAM-LTNLslS-L-D-R-IYN-----MlrMFVVtL---A--EI---DLQELQGYLQKK-VRDQ-Q-LVY-S---------aGVYRL---P----
0048 7dvrA   ldryakeydveH--LAG--PQGHLVMH-LYKHdkD-M-S-I-kDA-----E--EILH----------I---SKSVASNLVKRM-EKNG-F-IAI-VPSktdK--RVKYLYL---TH-Lg
0049 7qenC   ----kKVDV--RrlKKN--VWRSINNL-IQEHkeL-K-F-S-DII-----Q--gISK-M---YsddI---STSFCFICLLHL-ANEH-G-LQI-THT-enY--NDLIVNY---ED-L-
0050 1lvaA   ---------------gS--PEKILAQI-IQEHegL-D-W-QeAAT-----R----------------L---SLEETRKLLQSX-AAAG-Q-VTL-LRV---E--NDLYAIS---TE-Ry
0051 3elkA   -------reriL--HGL--ITLYILKE-LV-K-RP-X-----HGY-----ElqKSXF-EttgQ--AL---PQGSIYILLKTX-KERG-F-VIS-ESS---V--QLTVYHI---TD-Ag

0001 s001A   -----LLLL--L--HHH--HHHHHHHH-HHLL-LL-H-H-H-HHL-----L--HHHH-H---H--LL---LHHHHHHHHHHH-LHHH-L-EEE-EEE---L--LEEEEEE---LL-L-
0002 1jhfA   ----llllH--H--HHH--HHHHHHHH-HHHH-LL-L----lLHH-----H---hHH-H---L--LL--lLHHHHHHHHHHH-HHLL-L-EEE-LLL------LLLLEEE---LL-Ll
0003 6lb9A   elllllLLL--L--HHH--HHHHHHHH-HHLL----H-H-H-HHHllllhH--HHHL---------L---LHHHHHHHHHHH-HLLLlL-EEE-EEL---L--LLEEEEE---LL-L-
0004 9fhlc   lllllllLL--L--LHH--HHHHHHHH-HHHL-LL-L-LhH-HHH--------HHHL---------L---LHHHHHHHHHHH-HHLL-L-EEEeEEL---L--LLEEEEE---LL---
0005 1ylfC   ------LLH--H---HH--HHHHHHHH-HHHL----llL-L-LHH-----HhhHHLL---------L---LHHHHHHHHHHH-HHHL-L-EEE-LLL-------LLLEEE---LL-L-
0006 4o5vA   -----LLLL--L--HHH--HHHHHHHH-HHHH-LL-L-----LHH-----HhhHHHL---------L---LHHHHHHHHHHH-HHLL-L-EEE-E--------eLLEEEE---LH-Hh
0007 1fx7A   -----LLLLllH--HHH--HHHHHHHH-HHHL-LL-L-----LHH-----HhhHHHL---------L---LHHHHHHHHHHH-HHLL-L-EEE-LL--------LLLEEE---LHh--
0008 2jt1A   -----lLLL--H--HHH--HHHHHHHH-HHHH-----llllleEHH-----HhhHHHL---------L---LHHHHHHHHHHH-HHLL-L-EEE-ELL-llL--LLLEEEE---LL---
0009 3dv8A   ----hHHLL--H--HHH--HHHHHHHH-HHHH----E-L-L-LHH-----HhhHHHL---------L---LHHHHHHHHHHH-HHLL-L-EEE-EL---------LEEEE---LL-H-
0010 2ns0A   ------LLL--L--HHH--HHHHHHHH-HHHL----llllllLHH-----HhhHHHL-L-------llhhHHHHHHHHHHHH-HHLL-L-EEE-EEL---L--LLLEEEE---LL-Ll
0011 5yadA   -----LHHH-hH--HHH--HHHHHHHH-HHLL----H-H-H-HHH-----HhhhHHL-L-----------LLLH--HHHLLL-L--L-L-EEE-ELL---L--LLLEEEE---LH-H-
0012 3ov8A   ----hHLLH--H--HHH--HHHHHHHH-HHLL-LL-E-----EHH-----HhhHHLL---------L---LHHHHHHHHHHH-HHLL-L-EEE-EEE---L--LEEEEEE---LH-Hh
0013 6qpqD   --------L--H--HHH--HHHHHHHH-HLLLllE-E-H-H-HHH-----H---HHH-L------lllhhHHHHHHHHHHHH-HHLL-L-EEE-ELL-llL--LLLEEEE---LH-Hh
0014 6wgeC   llllllllL--H--HHH--HHHHHHHH-HLLL----E-H-H-HHL-----L--L---------llHH---HHHHHHHHHHHH-HHLL-L-EEE-ELL-llL--LLLEEEE-llLL-L-
0015 4i99C   ------LHH--H-hHHH--HHHHHHHH-HHHH----E-H-H-HHL-----L--L------------llhhHHHHHHHHHHHH-HHLL-L-EEE-ELL-llL--LLLEEEE---L----
0016 4kywA   ----lLLLL--H--HHH--HHHHHHH--LLLL-eE-E-H-H-HHH-----H---HHH-H---H--HllllHHHHHHHHHHHH-HHLL-L-EEE-LL--------LLEEEE---L----
0017 7y43A   -----------lllLHH--HHHHHHHH-HHHHllL-L-H-HhHHH-----HhhHHHL---------L---LHHHHHHHHHHH-HHHL-L-LEE-EEE---L--LEEEEEL---LL-Ll
0018 2od5A   -----LLHH-hH--HHH--HHHHHHHH-LLLL-lE-E-H-H-HHH-----H--HHHL-L---LllLL---LHHHHHHHHHHL-L----L-EEEeEEE---EeeEEEEEEE---HH-Hh
0019 3lmmD   lllllllLL--L--HHH--HHHHHHHH-HHHL-LL-E-----EHH-----HhhHHLL---------L---LHHHHHHHHHHH-HHLL-L-EEE-ELL---L--LLLEEEE---LL---
0020 5trdA   --------L--H--HHH--HHHHHHHH-HHHH-lE-E-E-E------lhhhhhHHHL---------L---LHHHHHHHHHHH-HHLL-L-EEE-EEE---L--LEEEEEE---LH-Hh
0021 5xpqA   lhhhhhhhhhhhhllLH--HHHHHHHH-HHHL-LE-E-H-H-HHH-----L--LLLL---------L---LHHHHHHHHHHH-HHLL-L-EEE-EEL---L--LLEEEEE---LL-L-
0022 3h0dA   ------lLH--H--H-H--HHHHHHHH-HLLLleE-E-E-L--HH-----H--H-HH-H---L--LL---LLHHHHHHHHHlLHHH-L-EEE-EEE---L--LEEEEEE---EEe--
0023 8xt8B   hhhhhhhhhhllllLLH--HHHHHHHH-HHHL----H-H-H-HHH-----H---------------L---LLHHHHHHHHHH-HLLL-L-EEE-EELlllL--LLEEEEElhhHH-H-
0024 5j6xA   -------lL--H--HHH--HHHHHHHH-HHHHllL-L-H-HhHHH-----H--LLL---------------LHHHHHHHHHHH-HHLL-L-EEE-ELL------LLLEEEE---LL---
0025 9gm7C   hllleeeLH--H--HHH--HHHHHHHH-HHLLllE-E-H-H-HHH-----HhhHHHH-H---H--LllllHHHHHHHHHHHH-HHLL-L-EEE-EEL---H--LLEEEEE---LH-Hh
0026 6uvuB   hhhhhhhhhhhhhhlLH--HHHHHHHH-HHHHllL-L-H-HhHHH-----H--LLL---------------LHHHHHHHHHHH-HHLL-L-EEE-EEE---L--LEEEEEE---LL-H-
0027 8as8C   llllllllH--H--HHH--HHHHHHHH-HHHHleE-E-H-H-HH------H--HHHH-H---H--HlhhhHHHHHHHHHHHH-HH-L-L-LLL-LLL---L--LLEEELL--lLH-H-
0028 8qtoA   ----hHHLL--H--HHH--HHHHHHHH-HHHHleE-E-L-L-llH-----H--HHHH-H---H--LL---LHHHHHHHHHHH-HHLL-L-EEE-EL---------LEEEE---LL-H-
0029 2mh2A   --------L--H--HHH--HHHHHHHH-LL------E-H-H-HHH-----H---HHH-L--lL--LL---LHHHHHHHHHHH-HHHL-L-EEE-EEE---L--LEEEEEE---L----
0030 7yw2A   ------lHH--H--HHH--HHHHHHHHhHLLLllllH-H-H-HHL-----L--HHHL---------L---LLHHHHHHHHHH-LLLL-L-EEE-EEL---L--LLEEEEE---LL-L-
0031 2h09A   ----hHHHHhhH--HHH--HHHHHHHH-HHHH-LL-L-----LHH-----HhhHHHL---------L---LHHHHHHHHHHH-HHLL-L-EEE-EL--------LLEEEE---LHh--
0032 1ucrB   --------------LLH--HHHHHHHH-HLL-llE-E-H-H-HHH--------HHLL--------LL---LHHHHHHHHHHH-HHLL-L-EEE-EEE---L--LEEEEEE---LL-Hh
0033 2obpA   -----LLLH--H--HHH--HHHHHHHH-LLL-llL-L-H-H-HHH--------HHHL---------L---LHHHHHHHHHHH-HHLL-L-EEE-EEL--lL--LLEEEEE---LH-Hh
0034 1yyvA   llllllhhhhhH--HHHlhHHHHHHHH-HHHL-LE-E-H-H-HHH--------HHLL--------LL---LHHHHHHHHHHH-HHHL-L-EEE-EEE---L--LEEEEEElhhHH-H-
0035 3bpxA   hhhhhhhhhhhhhlLLH--HHHHHHHH-HHLL----H-H-H-HHH-----H--HL-----------L---LHHHHHHHHHHH-HHLL-L-EEE-EEElleE--EEEEEEElhhHH-H-
0036 5v9xA   ----lLLLL--L--HHH--HHHHHHHH-LLlllEE-H-H-H-H-H-----H--HHHH-LlllL--LL---LHHHHHHHHHHH-HlLL-L-EEE-ELL-------LLEEEE---LL-L-
0037 9dlrB   hhllhhhhllhH--HHH--HHHHHHHH-HHHHleE-E-H-H-HHH-----H---HHH-H---L--LL---LHHHHHHHHHHH-HHLL-LlEEE-ELL-------LLEEEE---LL-L-
0038 8wxsA   --------lllH--HHH--HHHHHHHH-HHLL----H-H-H-HHH-----HhhHHHL---------L---LLLLlHHHHHLL-L--L-L-EEE-ELL---L--LLLEEEE---LL-Ll
0039 8ylgA   hhhhlhhhhhhhllLLH--HHHHHHHH-HHHL----H-H-H-HHH-----H--HLL---------------LHHHHHHHHHHH-HHHL-L-EEE-EELlllL--LLEEEEElhhHH-H-
0040 2qbyB   hhhhhhhhhhhhhlLLH--HHHHHHHH-HH-L-LL-LhH-H-HHH-----HhhHHHH-L---L--LL---LHHHHHHHHHHH-HHLL-L-EEE-EEE---L--LEEEEEE---LL-L-
0041 7yk3B   --------------LLH--HHHHHHHH-HHHHhhllH-H-H-HHH-----HhhHHHH-HlhhH--LL---LLHHHHHHHHHL-LL-L-L-EEL-L-L---L--LLLEEEE---LH-Hh
0042 1xsdA   -------lllllllLLL--L-HHHHHH-HHHHllE-E-H-HhHHH-----H---HHL-L---L--LL---LHHHHHHHHHHH-HHLL-L-EEE-EEL---L--LLEEEEE---LL-L-
0043 2g9wA   ----------lhhhlLH--HHHHHHHH-HHLL----H-H-H-HHH-----H---HLL-L---L--LL---LHHHHHHHHHHH-HHLL-L-EEE-EL--------LLEEEE---LL-L-
0044 8yz7A   ----hHHLL--H--HHH--HHHHHHHH-LLLL-LEeE-E-L-LHH-----HhhHHHL---------L---LHHHHHHHHHHH-HHLL-L-EEE-LL---------LEEEE---LL-H-
0045 6qfdB   -----lllhhhhllLLH--HHHHHHHH-HHHL-LE-H-H-H-HHH-----H-hHHHH-L---L--LL---LHHHHHHHHHHH-HHLL-L-EEE-E-E---L--LEEEEEElhhHH-H-
0046 2zkzC   hhhhhhhhhhhhhhlLH--HHHHHHHH-HHHHllE-E-H-H-HHH--------HHHL---------L---LHHHHHHHHHHH-LL-L-L-EEE-EEE---L--LEEEEEE---LL-H-
0047 5a31N   llllllHHH--H---HH--HHHHHHHH-HHHHllL-H-H-H-HHH-----HhhHHLLlL---L--LL---LHHHHHHHHHHH-HHLL-L-LEE-E---------lLEEEL---L----
0048 7dvrA   hhhhhhhllllL--LLL--HHHHHHHH-HHHLllL-E-E-H-hHH-----H--HHHL---------L---LHHHHHHHHHHH-HHLL-L-EEE-EELlllL--LLEEEEE---LH-Hh
0049 7qenC   ----lLLLH--HhhHHH--HHHHHHHH-HHHHleE-E-L-H-HHH-----H--hHLL-L---LlllL---LHHHHHHHHHHH-HHHL-L-LEE-EEL-llL--LLEEEEL---LL-L-
0050 1lvaA   ---------------lL--HHHHHHHH-HHLLllE-E-H-HhHHH-----H---------------L---LHHHHHHHHHHH-HHLL-L-EEE-EEE---L--LEEEEEE---HH-Hh
0051 3elkA   -------llhhH--HHH--HHHHHHHH-HH-H-LL-E-----EHH-----HhhHHHH-HhhlL--LL---LLLHHHHHHHHH-HHHL-L-EEE-EEE---E--EEEEEEE---LH-Hh

#######################################################################################################################################################################################################################################################################
#######################################################################################################################################################################################################################################################################
#######################################################################################################################################################################################################################################################################

Job: Type-1 BREX; BrxB Inactive STAND-NTPase WP_102077887
Query: s001A
No: Chain Z rmsd lali nres id PDB Description
1: 9nv3-A 12.0 5.1 166 199 14 MOLECULE: DUF1788 DOMAIN-CONTAINING PROTEIN;
2: 8dr5-A 8.6 3.2 130 646 12 MOLECULE: REPLICATION FACTOR C SUBUNIT 1;
3: 8fnu-A 8.4 3.1 125 796 10 MOLECULE: KAP NTPASE DOMAIN-CONTAINING PROTEIN;
```

```
4: 8umy-A 8.2 3.3 128 546 10 MOLECULE: CHROMOSOME TRANSMISSION FIDELITY PROTEIN 18 HOMOL
6: 7z6h-K 7.8 3.3 128 439 10 MOLECULE: CELL CYCLE CHECKPOINT CONTROL PROTEIN RAD9A;
7: 8fcv-R 7.6 3.5 127 343 11 MOLECULE: DNA (60-MER);
8: 7st9-A 7.6 3.5 130 522 8 MOLECULE: CHECKPOINT PROTEIN RAD24;
9: 5j1s-A 7.5 3.7 134 277 9 MOLECULE: TORSIN-1A;
10: 3bos-B 7.5 3.4 120 231 11 MOLECULE: PUTATIVE DNA REPLICATION FACTOR;
11: 7rzy-1 7.4 3.3 122 311 8 MOLECULE: TN6677 VIBRIO CHOLERAE TRANSPOSON TNSC (VCHTNSC);
14: 6pxk-K 7.2 3.3 128 429 8 MOLECULE: ATP-DEPENDENT PROTEASE ATPASE SUBUNIT HSLU;
15: 8thc-A 7.1 3.6 126 479 12 MOLECULE: ELG1 ISOFORM 1;
16: 8glw-A 7.0 3.8 124 483 11 MOLECULE: TRANSPOSON TN7 TRANSPOSITION PROTEIN TNSC;
17: 7ykk-A 7.0 3.7 128 735 14 MOLECULE: ATPASE FAMILY GENE 2 PROTEIN;
18: 7uqj-A 6.9 3.7 130 585 8 MOLECULE: ATPASE HISTONE CHAPERONE YTA7;
19: 5e7p-A 6.8 3.6 127 719 11 MOLECULE: CELL DIVISION CONTROL PROTEIN CDC48;
20: 6n9x-B 6.8 3.4 115 500 8 MOLECULE: DNA PRIMASE/HELICASE;
21: 7qo4-M 6.8 3.6 131 421 9 MOLECULE: 26S PROTEASOME REGULATORY SUBUNIT RPN1;
22: 2qby-B 6.8 3.4 118 368 7 MOLECULE: CELL DIVISION CONTROL PROTEIN 6 HOMOLOG 1;
23: 7mca-C 6.7 3.9 122 584 5 MOLECULE: ORIGIN RECOGNITION COMPLEX SUBUNIT 1;
24: 9klv-D 6.7 3.6 132 338 5 MOLECULE: PROTEIN SUPPRESSOR OF MAX2 1;
25: 9emc-D 6.7 3.4 124 337 10 MOLECULE: RUVB-LIKE 1;
26: 8hra-C 6.6 3.5 126 852 8 MOLECULE: ARCHAEAL ATPASE;
28: 5zr1-E 6.6 3.2 117 460 8 MOLECULE: ORIGIN RECOGNITION COMPLEX SUBUNIT 1;
29: 6f0x-B 6.6 3.6 135 398 6 MOLECULE: PACHYTENE CHECKPOINT PROTEIN 2 HOMOLOG;
30: 6p8v-C 6.6 3.7 145 303 6 MOLECULE: ATPASE, AAA FAMILY;
31: 8xku-A 6.5 3.6 130 730 11 MOLECULE: PROBABLE INACTIVE ATP-DEPENDENT ZINC METALLOPROTE
35: 8xku-D 6.4 3.5 123 805 6 MOLECULE: PROBABLE INACTIVE ATP-DEPENDENT ZINC METALLOPROTE
36: 8tp1-A 6.4 3.5 126 419 12 MOLECULE: MITOCHONDRIAL CHAPERONE BCS1;
38: 8btg-B 6.4 3.9 120 335 10 MOLECULE: CHROMOSOMAL REPLICATION INITIATOR PROTEIN DNAA;
40: 8dgf-A 6.3 3.6 113 1541 9 MOLECULE: ATP-BINDING PROTEIN AVS4;
41: 5zr1-A 6.3 3.7 127 494 5 MOLECULE: ORIGIN RECOGNITION COMPLEX SUBUNIT 1;
44: 7plh-A 6.2 3.4 116 260 9 MOLECULE: SHTNSC;
45: 8c0v-B 6.2 3.3 135 1030 6 MOLECULE: PEROXISOMAL ATPASE PEX1;
46: 8bob-C 6.2 3.4 103 412 11 MOLECULE: PROTEIN MALY;
47: 8k8v-A 6.2 3.4 116 719 13 MOLECULE: ENDOPEPTIDASE LA;
48: 8dgc-A 6.2 3.9 123 2028 10 MOLECULE: SEAVS3;
49: 7jgr-D 6.2 3.9 121 441 7 MOLECULE: ORIGIN RECOGNITION COMPLEX SUBUNIT 2;
50: 8c0v-A 6.1 3.2 123 823 8 MOLECULE: PEROXISOMAL ATPASE PEX1;
51: 1w5s-B 6.1 3.6 124 396 4 MOLECULE: ORIGIN RECOGNITION COMPLEX SUBUNIT 2 ORC2;
53: 5j1s-B 6.1 3.8 134 235 7 MOLECULE: TORSIN-1A;
55: 7w42-B 6.0 3.5 125 402 11 MOLECULE: UNCHARACTERIZED ATPASE YJOB;
57: 8tw7-2 6.0 3.5 116 339 9 MOLECULE: REPLICATION FACTOR C SUBUNIT 5;
58: 8xku-B 6.0 3.6 129 845 12 MOLECULE: PROBABLE INACTIVE ATP-DEPENDENT ZINC METALLOPROTE
59: 9bf5-C 5.9 3.1 103 1190 4 MOLECULE: SSDNA1;
60: 6ut5-A 5.9 3.5 112 395 11 MOLECULE: GTPASE SUBUNIT OF RESTRICTION ENDONUCLEASE;
61: 6l1q-A 5.9 3.1 110 267 8 MOLECULE: CBBQ PROTEIN;
62: 2gno-A 5.9 3.5 119 296 8 MOLECULE: DNA POLYMERASE III, GAMMA SUBUNIT-RELATED PROTEIN
63: 9fnn-U 5.9 3.0 114 511 14 MOLECULE: CELLULOSE SYNTHASE CATALYTIC SUBUNIT [UDP-FORMING
64: 3syk-A 5.9 4.2 140 290 8 MOLECULE: PROTEIN CBBX;
65: 6bbm-D 5.8 3.2 114 455 9 MOLECULE: REPLICATIVE DNA HELICASE;
68: 8xvb-A 5.8 3.4 112 246 5 MOLECULE: DNA (5'-
69: 8ouy-C 5.8 3.4 117 308 9 MOLECULE: DNA REPAIR PROTEIN RAD51 HOMOLOG 2;

0001 s001A   -MLNT-DFNEL--MERVR-AGR-EFGH------AS---F--E-P-IFYLIFDP----QKI--LKIK-RQLPAWAAK-LRNE-------GW--DVH-IFS-MA-K-AVQEVF---D-E-MPVFNKSLAEALTKKN-ALQNKL-E-A--K-L-F-G-RP--N-S-I-LLVSD--I-E--A-L-HPY--L--R---I-GSMESQ-L--Q-GK-F-----H----VPTIFFYP-GM--RTGKQLKFLGFY--PE--D-GNYRS-V-HVG-G------
0002 9nv3A   pVLEY-RLSQV--QSRIS-EFL-KNNS------GN---E--I-G-FWIFDYPA----QNE--LQVR-EHLKYLLRN-LE-K-------DH--KFA-HLN-IF-Q-IIVDML---T-ErGLFDkqlvgLLNQ--K-KIADYI-A-KkvD-L-Q-N-----Q-E-F-VILTG--M-G--N-A-WPL--V--R---G-HELMSA-L--Q-DV-M-----G---fTPLLMFYP-GT--YSGHDLSPLAGI--DS--R-NYYRA-FrLVP-Esgpaat
0003 8dr5A   cGNKG-SVMKL--KNWLA-NWE-NSKK------NS---FkhF-R-AAMLYGP-------P--GIG---KTTAAHLV-AQEL-------GY--DIL-EQN--A-S-DV-----------------------rskT-LLNAGV-K-N--A-L-D-N-MK--H-F-V-IIMDE--V-D--G-M-SGG--D-rG---G-VGQLAQ-F--C-RK-T-----S----TPLILICN-ER--N----------L--PK--M-RPFVC-L-DIQi-------
0004 8fnuA   yIFSS-QLDKV--NLILE-HMD-TVGG------I----H--S-R-NIAITG-------DR--GTgktSFIETLKLV-LEKQ-------NY--YVFdIVS-P--T-VLSSH-----------------------LNILEI-V-I----------K--D-L-V-LIIDD--L-D--L-V-EN---N--L---V-YDLLRD-I--Q-HY-L---dsQ----LIVIFAYK-E---------GQLEQ------lnhLTKLVsN-RIP-Lfkqdel
0005 8umyA   lSDDF-TNRCL--LKWLK-LWD-LGLD------PSqrpK--Q-K-VALLCGP-------P--GLG---KTTLAHVI-ARHA-------GY--SVV-EMN--A-S-DD-----------------------rspE-VFRTRI-E-A--A-T-Q-M-EK--P-N-C-LVIDE--I-D--G-A-P----V--A---A-INVLLS-I--L-NL-L-----M----RPIICICN-D--------QFAP--------sL-RQLQA-F-LLH-Fpptlps
0006 7z6hK   avhkK-KIEEV--ETWLK-AQV-LERQ------PK---Q--GgS-ILLITG-------PP--GCG---KTTTLKIL-SKEH-------GI--QVQ-EW-----I-NPV---------------lpyQSQI--A-VFKEFL-L-R--A-T-K-ddTD--K-K-I-ILVED--L---pnqF-YRD--S-hT---L-HEVLRK-Y--V-RI-G-----R----CPLIFIIS-D-----slsgdnNQRL-lfP--K-EIQSI-S-NIS-Fnpvapt
0007 8fcvR   pRLKE-VYEIL--MRTIA-EPA---------------G--A-S-FIFVYGAS----GV----GKT-TLRLRVEQK-LTEL-------AarvPVV-GIE-A----IAPE-----------------SRYFN--W-KEYYTR-A-LitleepL-I-HR--H-PdV-FFVDE--A-Q--H-F-GKV----------lqDQLDC-L--K-SL-A--nmtG----ILHCLLGT-Y----------ELLT-frnL--S-GQLRS-V-DIH-Frrycad
0008 7st9A   kRKLK-DVQEA--LDAMF---L-PNA-------------K--H-R-ILLLSGPS----GC----SKS-TVIKELSKI-LVPK-------YRqnsKV-TEF------RGDC-I---V-N-D---------LPQM--E-SFSEFL-K-G--A-R-Y-L-VM-nL-S-L-ILIED--L----P-N-VFH---idtrrrF-QQLILQ-W--L-YS---seplL----PPLVICIT-------------ECEIpendnmN-KEILL-K-RIK-Fnpinst
0009 3bosB   aagND-ELIGA--LKSAG-DGV---------------------Q-AIYLWGPV----KS----GRT-HLIHAACAR-ANEL-------ER--RSF-YIP-LG-I--HASI-------------------------STALL-E----G-L-E-Q------fD-L-ICIDD--V-D--A-V-AGH--P--L---W-EEAIFD-L--Y-NR-V-----AeqkrGSLIVSAS-AS------pXEAG--------dL-VSRWG-L-TYQ-------
0010 6pxkK   iigQD-NAKRS--VAIAL-RNR-WRRR------HE---V--T-PkNILXIG-------PT--GVG---KTEIARRL-AKLA-------NA--PFI-KVE-AT---KFTEG-----------------------KEVDSI-I-R--DltdpsV-EQ--H-G-I-VFIDE--I-D--K-I-CVS--R--E--gV-QRDLLP-L--V-EGcD-----H----ILFIASGA-F------------QIAK-----lI-PELRL-P-IRV-Elqaltt
0011 8thcA   pKLKL-RIKNW--IETSF-HTL-EKPT-------E---F--V-P-LMILHGN-------S--IGKK-TLIQTIMRE-IAGD-----dnSY--QIY-EVN--S-N-MN-----------------------rsK-KDLLDilL-D--F-T-T-T-HD--Y-G-L-VLFND--V-D--V-L-FKE-----hdrgY-WAMISK-L--C-EF-S-----R----RPLVLTCK-D---------LSLV---------P-SEL-snS-LFH-Tkkists
0012 8glwA   lGTHL-LLSER--ISVMI-RGG-YVGA------RS---T--A-Q-SLLLIGCS----GS----GKT-TSLHRILAT--YPQ-------VeqvVYL-KID-CS---EICL-------------yerryglkrHGI-E----------------N-A-HA--L-G-L-LVIDE--I-Q--H-L-SRS--rsggsqeM-LNFFVT-M--V-NI-I-----G----VPVMLIGT-P----------KARE--------fE-ADLGF-G-AIF-Wdpiqqt
0013 7ykkA   dKEIE-SLKSA--IEIPL-HQP-TLFS---sfgVS---P--P-R-GILLHGPP----GT----GKT-MLLRVVANT-S-----------NA--HVL-TIN-GP-S-IV-----------------------SKYLG-ETEAAL-R-D--I-F-R-K-YQ--P-S-I-IFIDE--I-D--S-I-AP-----------esRVVAT-L--L-TL-M--dgmgaagkVVVIAATN-R---------PNSV------------PALRD-Q-EVE-Igipdvd
0014 7uqjA   pLLAD-QLNNL--KNKLD-YML-NaES------QI---C--K-P-RLLINGPK----GN----GQQ-YVGAAILNY-LE----------EF--NVQ-NLD--L-A-SLVSES---S---------------------rTIEAAV-V-Q--S-F-K-K-RQ--P-S-V-VFIPN--LdI--W-I-NTI--P--E----vILVLSGlF--R-SL---qsneK----ILLLCLAEnLD--IS--------------------SDFAFkN-IFQ-Lhkpske
0015 5e7pA   kvtHP-QAVKL--DEWLR-LSL-DEPEllktlgAT---P--H-L-GVLVSG-------PA--GVG---kATMVRAV-CA----------SR--RVV-ELD-GP-E-VGALQ-----------------------vD-ERLRSV-T-S--A-V-A-T-ES--G-G-V-LFIAD--V-D--AlL-PAGnemrppepvA-TLILAE-L--R-KA-V-----A-tpgVAFIATSA-V---------PENV---------dA-RLRAD-R-ELG-Lslpdat
0016 7qo4M   dKQIE-ELVEA--IVLPM-KRA-DKFK----dmGI---R--A-P-KGALMYGP----PGT---GKT-LLARACAAQ-TN--------------ATF-LKL-AA-P-QLV-----------------QMYI-GEGA-KLVRDA-F-A-lA-K-E-K------aP-T-IIFID-eL-D--A-I--------------gtmLELLN-QldG-FS-S----dD----RVKVLAAT-N-------------RVDV--LD--P-LRSGRlD-RKI-Efplpse
0017 2qbyB   pFRED-ILRDA--AIAIR-YFV-KNE---------------vkF-SNLFLGLT----GT----GKT-FVSKYIFNE-IEEV-------KV--KQA-YVN-CR-E-VG-----------------------gTPQAV-L-S--S-L-A----I--R-A-I-IYLDE--V-D--T-L---------vkrrgG-DIVLYQ-L--L-RS-----daN----ISVIMISN-DI--N-------------vrD--Y-MESLG-P-SVI-Fkpydae
0018 7mcaC   dLKAE-ISDLL--YSE----TT-QK-------------R--R-C-FNTIFLL----GSD--S--T-TKI--ELKD-E--S-------SR--YNV-LIE-LTpK-ESP-----------------------nvR-MMLRRS-M-Y--K---V-E-NF-kD-L-A-MVFNF--K-DvdS-I-NFN--T--L-----DNFIIL-L--K-SA-F------kydhVKISLIFN-I----------ntNLSNieKN--L-RIRLLrN-YHK-Ldvssnk
0019 9klvD   nDAAA-AVAAT--VSQCK-LGN----V------LS---K--G-D-VWLLFSG-----PDR--VGKR-KMVSALSSL-V-----------Y--GTN-PIM-IQ---agDGNS---S-F-R--------GKTA-lD-KIAETV-K-R--S-P--------------F-SVILL--E-D-ideA-DML--V--R-gsI-KQAMDR-G--RihS-L-----G----NVIFVMTA-SWhkLSLQ-----cvP--FA--F-HDMRV-D-DAV-Afra---
0020 8hraC   lIQKS-LRDQL--VESIR-NSI-A--------------G--R-N-VFFVDGTR----GA----GKT-TFINSVVKS-LNSD-----qdDV--KVNiKCL-PT----IDPT-----------------------K-LPRHEP-I-L--------eiCK--A-I-L-ITFDD--I-D--T-Q-F----D--A---G-WDVLES-I--R-KF-F-----N-srkLVVVATGD----------LRLYsQL--IRgsvrLLKLFqK-RIQ-Lktmlql
0021 7crcB   vGMRA-HMDML--EQLLR---L-VLD-------------E--V-R-MIGIWGPP----GI----GKT-TIARFLFNQ-VS-D-------RF--QSA-IMVnIK-G-CYP---------------PCFDEYSAQ--L-QLQNQM-L-S--Q---------K--K-V-F-LVLDE--V-D--Q-L------G--Q---L-DaLAKE----T-RW-F-----G--pgSRIIITTE-D-----------------------KAHGInH-VYK-Vgypsnd
0022 5zr1E   eYQTN-CLASY--IS--A-DPD-I----------------tpS-NLILQGYS----GT----GKT-YTLKKYFNA-N--P-------NL--HAV-WLE-PV-E-LV-----------------------swK-PLLQAI-A-R--T-V-Q----T--C-L-F-LILDG--F-D--S-L--QD--L--D-----aALFNK-Y--I-KL-NellpkdskinIKFIYTML-------------ETSF-lqR--Y-STHCI-P-TVM-Fprynvd
0023 6p8vC   vGLDE-HKDRL--SKILG-LnP-YGIV------DT---V--R-R-PPLVVLAG----DV---SGKT-ELAETIGDA-VARQ------eDI--DIT-LYP-LS----LATRG-----------RVGEXTQLV--S-AAFDYT-I-E--A-A-D-L-KK--ArG-A-VLLLIdqA-D--A-L-AaG--V--N---AfIRGIDR-I--A-NQ-K-----L----P-AAVLXC-T-----------nRLKA--lD--P-AVRRA-A-EIL-Tfsrpnd
0024 8xkuA   eYIKR-ELQEI--VRILK-N-D-EEFQ-------nkgiY--C-P-KGVLLHGP----PG---TGKT-LLAKAIAGE-AG--------------LPF-FAA-NG-T-DF--VE---M-F-----------VGVA--A-SRVKDL-F-A-sS-R-S-Y------aP-S-IIFID-eI-D--A-I-GS-----------glLQILT-E-mD-GF-K--vttS----QVLVIGAT-N-------------RLDI--LD--P-ARKGRfD-KII-Rvglpsk
0025 8xkuD   qPGTI-YLRYL--VDIHKkYLM-NYEF----ntsclaerritypsRSILVIGS----I----TGRS-YLVKYLATN-SY-----------V--PFI-TVF-LN---KFLDNK-----------------dmmLEID-RfITLQ-F-E--L-A-K-A-MS--P-C-I-IWIPN--I-H--D-L---------------aLGLLVN-S-lS-RD-C----ercstrnILVIAST-H-------------IPQK-----vD-PALKL-N-TCI-Kirrlli
0026 8btgB   sGNRF-AHAAS--LAV-A-PAK-------------------ayN-PLFIYGGV----GL----GKT-HLMHAIGHY-VIDH-------NA--KVV-YLS-SE-K-FTNEFI---NsI-RDN-----------------kavdfrnR--Y-R-N-V--------D-V-LLIDD--I-Q--F-L-AGK--E--------tqEEFFH-T----NT-L-----H-eesKQIVISSD-RP--------PKEI---------lR-SRFWG-L-ITD-Itppdle
0027 4hutA   --------yqqrqqkvkvdaaqaQEER---------------gI-IIVFT-GN----GK----GKT-TAAFGTAAR-AVGH-------GK--NVG-VVQ-FI-KgVMATGF-----------R-EADTAA--cM-AVWQHG-K-R--MlA-D-P-L----lD-M-VVLDE-lT-Y--M-VaYDY--L--P-----LEEVIS-A--L-NA-R-----P--ghQTVIITGR-G----------------------cH-RDILA-D-TVS-Elrpvkh
0028 8dgfA   iDRRH-CIELL--HENLV-QK--------------------K-IVIVSGEG----GV----GKT-AVIKKIYEA-EKQ-------YT--PFY-VFK-A--S-EFK-----------------------KDSI-NELF----------------ahEL--R-K-V-IVVDS--A-E--K-L-LEL-----------idpFKEF-L--T-VL-I---kdK----WQVVFTTR-N---------NYLA-----------YAFIP-G-NLV-Iknlerg
0029 5zr1A   eNEFA-SIYLS--AYSAI-ESD-------------------sA-TTIYVAGT----PG---VGKT-LTVREVVKE-LLSS-------SD--FLY-VEI-NG-L--KMVK-----------------------ptDCYETL-W-N--K-V---vpKK--K-T-I-VVLLD-eL-D--A-M-VTKsqD--I---M-YNFFN-------YE-N-----A----KLIVIAVA-NT--M-----DLPER--qlG--N-KITIG-F-TRI-Mftgyth
0030 8e2wA   eVLRP-LLKQA--AEKTA-AG----------------------kIVFAEAAT----GT----GKG-RMIASLAAA-AAIK-------GD--TVV-VSA-PL-AvltlslgrpnF-I-SPqrLLLAEDLpadslllthFML-A-A------L-P-HF--I-D-T-LIVDE--A---------------hL---L-EQAfaA-L--L-GL-W-----N--atGGATLVSA-TF--TTGDNG-----------------SLTRA-A-FLP-Pvhpawt
0031 8rwvC   nLFDN-LIEFL--QKSHS-GFQ-KNSG------QI---K--L-reIPTAALVL----GvnvtLTFG-SLTEALQNN-V---------------PY-VVS-LQ----AKDC-----------------------PDMK--HFLQKL-I-S------W-Y-MTvqS-P-P-VVVI---L-K--D-M-ESF--AtkV---L-QDFIII-S--S-QH-L----hE----FPLILIFG-I-----------atSPIIihRL--L-PSSLL-C-IEL-Fqslsck
0032 7plhA   qQVKT-LHDWL--DGKRK-AR--------------------K-SCRVVGES----RT----GKT-VACDAYRYR-HKPqqeagrppTV--PVV-YIR-PH-Q-KC-----------------------GPKDL-F-K--K---L-K-GC--G-VeM-LIIDE--A-D--R-L---------------kPETFAD-V--R-DI-A--edlG----IAVVLVGT-D----------RLDA--vikR--D-EQVRF-R-AHL-Rfgklsg
0033 8c0vB   lhiFP-YVRQL--VNILE-TSF-NCSG------IT---L--N-A-SVLLHST-------TN--NVG---KATMVRFA-SKYL-------GI--HLL-EI--DC-L-S--LTS---N---------SRQLDST--S-KIIGYI-RaK--C-E-N-VlAS--P-A-V-IFLAH--LdS--I-L-LviklqksI---N-FEMSKL-L--D-DF-T-----fkfpgTTFVGSVN-N---------IDNV----------P-SSFHM-R-FEI-Lvpvpse
0034 8bobC   vvrER-LLAKL--SG-AN-NF---------------------R-LALITSPA----GY----GKT-TLISQWAAG-KN-------------DIG-WYS-LD---gDNQQE-----------cetmaqkrqYAS-L-----------------W-HS--P-L-Y-LVIDD--Y-H--L-I-TNP----------ihESMRF-F-iR-HQ-P-----E---nLTLVVLSR-N---------------------ANLRL-L-EIG-Sqqlaft
```

```
0035 7jgrD   rGYAK-ERSNV--RLLLQ-RTA-EMG---------------S-N-SLLLLGPR----GS----GKT-TLINSVLAD-LLPN--------T--LIV-HLD-GN-L-HT----------------------------DDRVA-L-K--------ggSK--S-V-I-FILEE--F-D--LfC-AHH--N--Q------TLLYN-L--F-DV-SqsaqaP----ICVLGVTC-RL--------DVIE----------KSRFShR-QVF-Lfpslrr
0036 8c0vA   nSIKK-EMVNY--L-tSP-II------------------------aTPAIILDGkqgiGKT--RLLK-ELINEVEKD-HH---------------IF-VKY-AD-C---ETLH--------------ETSN-LDKTqKLIMEW-C-S-fC-Y-W-Y-------G-P-SLIVL--D-N----V-EAL--F--GkpqalLNFFIN-Q-vT-KI-F------nkdnKRIRVLFS-G-----------KQKT--Q--iN-PLLHF-V-SET-Wslrapd
0037 1w5sB   rVRRG-EAEAL--ARIYL-NRL-LSGA---------------S-D-VNMIYGS---igRVG--IGKT-TLAKFTVKR-VSEA-aakeglTV--KQA-YVN-AF-N-AP-----------------------------NLYTI-L-S--L-----lyNH--Y-L-L-VILDE--F-Q--S-MlSSP--R--------aaEDLYT-L--L-RV-srdgvnR----IGFLLVAS-DV---------RALS--------kI-PQVQI-G-FKL-Hlpayks
0038 6fhsC   cAARE-ACGVV--VDLIK-AHK-------------------A-G-RGVLLAGG----PG---TGKT-ALALAISQE-L-GT--------KI--PFC-PIT-GS-E-I-----------------------YSTEV-KKTEVL-M-E--N-F-R-RalL--I-P-GvLFIDE--A-H--M-L---------------D-IECFTY-L--N-KA-L--espI----APIVVLAS-N------------RGIA--T--iP-PDFQR-L-LII-Pthpyep
0039 5j1sB   gQSSF-LWQRG--RKFLQ-KHL-NASN------PT---E--P-A-TIIFTAAR----EGR--ETLK-CLSHhvadA-YTSS--------QV--SPI-QID-GA---gRTWQ------------------DSDT----VKLLV-D-L--E-L-F-E-NG--Q-K-A-AVVHH--F-E--S-F--AG--S--T-------LIFYK-Y--CdAA-F------K---dVALVLTVL-LT--EEKvRDLL----------kftlWSRIsH-LVL-Pvqpvss
0040 3zh9B   -MVFD-VWKSlkkGEVH---------------------------P-VYCLYGK------ET--YLLQ-ETVSRIRQTvVDQE--------TKdfNLS-VFD-LE-E-D---------------------------PLDQA-I-A--D-A-E-T-FP-eR-R-L-VIVKN--P-Y---fltgKI--E--H----nVSALES-Y-iQ-SP-A------P---yTVFVLLAP-YK--LDE-------------R--K-KLTHA-F-MME--------
0041 7w42B   vIMNPlLKKEI--YRSID-QFFhSDKSfyqtydIP---Y--K-R-GILL-YGP----PG---NGKT-TLVKSIAGS-I-----------DA--PVA-YWQ------ITEF---------------------tsS-ETIEEV-F-Q-aA-R-R-L-A---P-A-V-LVIED--I-D--S-M-PED--V--R------sFFLNT-L--D-GA---tskeG----LFLIGTTN-YP---------EEID------------MNRAfD-RAY-Eiglpde

0001 s001A   -LHHH-HHHHH--HHHHH-HHH-HLLL------LL---L--L-L-EEEEEELH----HHH--HHHH-HHHHHHHHH-HHHL--------LL--EEE-EEE-HH-H-HHHHHH---H-L-LHHHHHHHHHHHHHHH-HHHHHH-H-L--L-L-L-L-LL--L-E-E-EEEEL--H-H--H-H-LLL--L--L---H-HHHHHH-L--L-LL-L------L----LLEEEEEEE-EE--EELLEEEELLLL--LE--E-LLLLL-E-EEE-L------
0002 9nv3A   hHHHH-HHHHH--HHHHH-LHH-HLLL------LL---L--L-L-LEEEEELL----LLH--HHHH-HHHHHHHHH-HH-H--------LL--LEE-EEE-HH-H-HHHLLL---L-LlLHHHhhhhlLLLH--H-HHHHHH-H-HhlL-L-L-L------L-L-E-EEEEL--L-L--L-L-LLL--L--L---H-HHHHHH-H--H-HL-L------L---lLLLEEEEEE-EE--ELLLEEEELLLL--LL--E-ELLLL-EeLLL-Llllhhhl
0003 8dr5A   lLLHH-HHHHH--HHHHH-LHH-HHHH------LL---LllL-L-EEEEELL--------L--LLL---HHHHHHHH-HHHL--------LL--EEE-EEL--L-L-LL----------------------------llhH-HHLLLH-H-H--H-L-L-L-LH--L-E-E-EEELL--H-H--H-L-LLL--L-lL---H-HHHHHH-H--H-HL-L------L----LLEEEEEEL-LL--L------------L--HH--H-HHHLL-E-EEEl-------
0004 8fnuA   lLLHH-HHHHH--HHHHH-HHH-HLLL------L----L--L-L-EEEEEE--------LL--LLlllLHHHHHHHH-HHHL--------LL--EELlLLL-H--H-HHLLL-----------------------------LLHHHH-H-H------------L--L-E-E-EELLL--H-H--H-L-LL---H--H---H-HHHHHH-H--H-HL-L---llL----LEEELLLL-L----------LHHHH------hhlHHHHLlL-EEE-Ellhhhh
0005 8umyA   lLLHH-HHHHH--HHHHH-HHL-LLLL------LLlllL--L-L-EEEEELL--------L--LLL---HHHHHHHH-HHHL--------LL--EEE-EEL--L-L-LL----------------------------llhH-HHHHHH-H-H--H-H-L-L-LL--L-E-E-EEEEL--H-H--H-L-L----H--H---H-HHHHHH-H--H-LL-L------L----LLEEEEEEL-L---------LLLH---------hH-HHHLL-E-EEE-Llllllhh
0006 7z6hK   lllhH-HHHHH--HHHHH-HHH-HLLL------LL---L--LlL-EEEEEL--------LL--LLL---HHHHHHHH-HHHL--------LL--EEE-LL------L-LLL-------------------lllLLHH--H-HHHHHH-H-H--H-H-H-llLL--L-E-E-EEELL--L---lhhH-HHH--L-lH---H-HHHHHH-H--H-HH-L------L----LLEEEEELL-L------lllllhHHHH-hlL--L-LHHLL-L-LEE-Lllllhh
0007 8fcvR   hHHHH-HHHHH--HHHHH-LLL------------------L--L-L-EEEEEELL----LL----LHH-HHHHHHHHH-HHHH--------HlllLEE-EEE-L----LLLL-------------------LLLLL--H-HHHHHH-H-HhhhlllH-H-HH--L-LlE-EEEEL--H-H--H-H-HLL-----------hhHHHHHH-H--H-HH-H--hhhL----LEEEEEEL-H-----------HHHH-hllL--L-HHHLE-E-EEE-Elllllll
0008 7st9A   hHHHH-HHHHH--HHHHL---L-LLL-------------L--L-L-EEEEEELL----LL----LHH-HHHHHHHHH-HHHH--------HLlllLE-EEL-------LLLL-L---L-L-L---------LLLL--H-HHHHHH-H-H--H-L-L-L-LH-hL-L-E-EEELL--L-----L-L-LLL---hhhhhhH-HHHHHH-H--H-HL---llllL----LLEEEEEE--------------LEELllllllL-HHHHE-E-EEE-Llllllhh
0009 3bosB   lllLH-HHHHH--HHHHL-LLL-------------------L-EEEEELLL----LL----LHH-HHHHHHHHH-HHHL--------LL--LEE-EEE-HH-H--HHHL----------------------------LHHHH-L----L-H-H-H-------lL-E-EEEEL--H-H--H-H-LLL--H--H---H-HHHHHH-H--H-HH-H------HllllLEEEEEEL-LL-------lLLLL----------hH-HHHHL-E-EEE--------
0010 6pxkK   lllLH-HHHHH--HHHHH-HHH-HHHH------HH---L--L-LlLEEEEL--------LL--LLL---HHHHHHHH-HHHL--------LL--LEE-EEE-HH---HHLLL----------------------------LLLLHH-H-H--HhhhllH-HH--H-L-E-EEEEL--H-H--H-H-LHH--H--H--hH-HHHHHH-H--H-LLlL------L----LEEEEEEL-L------------LLLL------lL-HHHLL-L-EEE-Elllllh
0011 8thcA   hHHHH-HHHHH--HHHHH-HHH-HLLL-------L---L--L-L-LLLLEEL--------L--LLLL-HHHHHHHHH-HHLL------llLE--EEE-EEL--L-L-LL----------------------------llH-HHHHHhhH-H--H-H-H-L-LL--E-E-E-EEELL--L-L--L-L-LLL------llllH-HHHHHH-H--H-HL-L------L----LLEEELLL-L----------HHHL----------L-HHH-hlL-LLE-Elllllhh
0012 8glwA   lHHHH-HHHHH--HHHHH-HHL-LLLL------LL---L--L-L-LLEEEELL----LL----LHH-HHHHHHLLL--LLL--------EleeLEE-EEE-LL---HHHH--------------lhhhhllllLLH-H----------------H-H-HL--L-L-E-EEEEL--H-H--H-L-LLL--llllhhhH-HHHHHH-H--H-HH-H------L----LLEEEEEL-L----------LLHH---------hH-HHHLL-L-EEE-Elllllle
0013 7ykkA   hHHHH-HHHHH--HHHHH-HLH-HHHH---hhlLL---L--L-L-LEEEELLL----LL----LLH-HHHHHHHHH-L-----------LL--LEE-LLL-LH-H-HL-------------------------LLLHH-HHHHHH-H-H--H-H-H-H-LL--L-E-E-EEEEL--H-H--H-H-LL------------lhHHHHH-H--H-HH-H--hllllllllEEEEEEEL-L----------LLLL--------------HHHLL-E-EEE-Lllllhh
0014 7uqjA   hHHHH-HHHHH--HHHHH-HHH-LhHL------LL---L--L-L-EEEEEELL----LL----LHH-HHHHHHHHH-LL----------LL--LEE-ELL--H-H-HHLLLL---L-------------------lLHHHHH-H-H--H-H-H-H-LL--L-E-E-EEEEL--HhH--H-H-HHL--L--H-----hHHHHHHhH--H-LL---llllL-----EEEEEEEElLL--HH---------------------LLLLLlL-EEE-Lllllhh
0015 5e7pA   hllLL-HHHHH--HHHHH-HHH-LLHHhhhhhlLL---L--L-L-EEEEEL--------LL--LLL---hHHHHHHH-LL----------LL--LEE-EEE-HH-H-HHHLL-------------------------hH-HHHHHH-H-H--H-H-H-H-HH--L-E-E-EEEEL--L-L--LlL-LLLllllllllhH-HHHHHH-H--H-HH-H------L-lllEEEEEELL-L----------HHHL----------lL-LLLLL-E-EEE-Lllllhh
0016 7qo4M   hHHHH-HHHHH--HHHHH-HLH-HHHH----hhLL---L--L-L-LLEEEELL----LLL---LHH-HHHHHHHHH-HL--------------LEE-EEE-EH-H-HHL-------------------LLLL-LLLH-HHHHHH-H-H-hH-H-H-L-------lL-E-EEEEE-lL-L--L-L----------------lhhHHHHH-LllL-LL-L-----lL----LEEEEEEL-L-------------LLLL--LL--H-HLLLLlL-EEE-Elllllh
0017 2qbyB   lLLHH-HHHHH--HHHHH-HHH-LLL----------------llL-EEEEEELL----LL----LHH-HHHHHHHHH-HHHH--------HL--EEE-EEE-HH-H-HL-----------------------------lLHHHH-H-H--H-H-H-H-----L--L-E-E-EEEEL--L-H--H-H---------hhlllL-HHHHHH-H--H-LL------llL----EEEEEEELL-LL--L--------------llL--L-LLLLL-L-EEE-Ellllhh
0018 7mcaC   hHHHH-HHHHL--LLL-----LL-LL-------------L--L-L-LEEEEEE-----LLL--L--L-LLL--LLLL-L--L--------LL--LLE-EEE-ELlL-LLL------------------------lhH-HHHHHH-H-H--H---H-H-LH-lL-L-L-EEEEE--L-LllL-L-LHH--H--H------HHHHHH-H--H-HH-H-------hhlllLLLEEEEE-E-----------llLHHHllLL--L-LHHHHlL-EEE-Eelllll
0019 9klvD   hHHHH-HHHHH--HHHHH-HLL----L------LL---L--L-L-EEEEEEL------LLH--HHHH-HHHHHHHHH-H-------------L--LLL-LEE-EE---llLLLH---H-H-L---------LLLH-hH-HHHHHH-H-H--L-L--------------L-LEEEE--E-L-hhhL-LHH--H--H-hhH-HHHHHH-L--EelE-L------L----LLEEEEEL-LLllLLHH------hlL--HH--H-HHHHL-L-EEE-Elll---
0020 8hraC   lLLHH-HHHHH--HHHHH-HHH-L-------------------L--L-L-EEEEELLL----LL----LHH-HHHHHHHHH-HLLL------llLL--LLLlLLL-LL----LLLL-----------------------L-LLLHHH-H-H---------hhLL--L-E-E-EEELL--L-L--L-L-H-----H--H---H-HHHHHH-H--H-HH-L------L-lllEEEEEEEL-----------HHHHhHH--HHhllhHHHHLhH-LEE-Lllhhhh
0021 7crcB   lLLHH-HHHHH--HHHHL---L-LLL-------------L--L-L-EEEEELLL----LL----LHH-HHHHHHHHH-HH-H--------HL--LEE-EELlHH-H-HLL----------------LLLLHHHHH--H-HHHHHH-H-H--H---------L--L-E-E-EEEEL--L-L--L-H------H--H---H-HhHLLL-----H-HH-L------L--llLEEEEEEL-L--------------------------HHLLLlL-EEE-Lllllhh
0022 5zr1E   hHHHH-HHHHH--LL--L-LHH-H------------------llL-EEEEEELL----LL----LLL-HHHHHHHHL-L--L--------LL--EEE-EEL-LL-L-LL----------------------------lhH-HHHHHH-H-H--H-H-H----E--E-E-E-EEEEL--L-L--L-L--LL--L--L------lLLHHH-H--H-HH-LllllllllleeEEEEEEL----------------LLLL-hhH--H-HHHLL-L-EEE-Ellllhh
0023 6p8vC   lLLHH-HHHHH--HHHHH-HlL-HHHH------HH---H--H-L-LLEEEEEE----LL---LLHH-HHHHHHHHH-HHHH------lLL--EEE-EEE-LL----HHHHL-------------LLLHHHHHHH--H-HHHHHH-H-H--H-H-H-H-LL--LlE-E-EEEEEllH-H--H-H-LlH--H--H---HhHHHHHH-H--H-HL-L------L----L-EEEEEE-E------------lLHHH--lL--H-HHHLL-L-EEE-Eellllh
0024 8xkuA   hHHHH-HHHHH--HHHHH-L-H-HHHH--------llllL--L-L-LEEEEELL----LL---LLHH-HHHHHHHHH-HL--------------LLE-EEE-EH-H-HL--LL---L-L-----------LLHH--H-HHHHHH-H-H-hH-H-H-L-------lL-E-EEEEE-lL-L--L-L-LL-------------hhHHHHHH-H-hH-LL-L--lllL----LEEEEEEL-L-------------LLLL--LL--L-LLLLLlL-EEE-Elllllh
0025 8xkuD   lLHHH-HHHHH--HHHHHlLLL-LLLL----llllllhhhhhlllLLEEEELL----L-----LLLH-HHHHHHHLL-LL-----------L--LEE-ELL-HH---HHLLLL------------------lhhHHHH-HhHHHH-H-H--H-H-H-H-LL--L-L-E-EEEEL--H-H--H-L----------------hHHHHHH-H-hH-HL-L-----lllllllLLLLEEE-L-------------LLLL------lL-HHHLL-E-EEE-Lllllh
0026 8btgB   lLLHH-HHHHH--HHH-L-LLL-------------------llL-EEEEEELL----LL----LHH-HHHHHHHHH-HHHH--------LL--LEE-EEE-HH-H-HHHHHH---HhH-HHL-------------------lhhhhhhH--H-L-L-L---------L-E-EEEEL--H-H--H-H-LLL--H--------hhHHHHH-H-----HH-H------H-hllLEEEEEEL-LL--------LLLL----------hH-HHHLL-E-EEE-Ellllhh
0027 4hutA   --------lhhhhhhhhhhhhlLLLL-----------------lL-EEEEE-LL----LL----LHH-HHHHHHHHH-HHLL--------LL--LEE-EEE-LL-LlELLLLL------------H-HHHHHH--hH-HHHHHH-H-H--HlL-L-L-L-----lL-E-EEEEL-hH-H--H-HhLLL--L--L------HHHHHH-H--H-HL-L------L--llLEEEEEELL-L-----------------------lL-HHHHL-L-EEE-Eeeeeel
0028 8dgfA   lLLHH-HHHHH--HHHHH-HL----------------------L-EEEEELLL----LL----LHH-HHHHHHHLL-LLL--------LL--LEE-EEE-H--H-HHL--------------------------LLLH-HHHH---------------llLL--L-L-E-EEEEL--H-H--H-H-HHL------------lhhHHHH-H--H-HH-H---hlL----LEEEEEEL-H----------HHHH------------HHHHL-E-EEE-Lllllhh
0029 5zr1A   lLLHH-HHHHH--HHHHH-HLL--------------------lL-EEEEEEEL----LL---LLHH-HHHHHHHHH-HHHH--------HL--EEE-EEE-EH-H--HLLL--------------------------llHHHHHH-H-H--H-H---llLL--L-E-E-EEEEE-lL-L--L-L-LLLllL--L---H-HHHHH--------LL-L------L----LEEEEEEL-LL--L------LHHHH--hlL--H-HHHHL-L-EEE-Eellllh
0030 8e2wA   hHHHH-HHHHH--HHHHH-LL------------------------lEEEEELLL----LL----LHH-HHHHHHHHH-HHHL--------LL--LEE-EEL-LH-HheeellllhhhL-L-LHhhHHHLLLLlhhhhlllhHHH-H-H-------L-L-LL--E-E-E-EEEEL--H----------------hH---H-HHHhhH-H--H-HH-H------H--leEEEEEEEL-LL--LLLLLL----------------HHHHE-E-ELL-Llllhhh
0031 8rwvC   hHHHH-HHLHH--HHLLH-HHH-HHLL------LL---L--L-llEEEEEELL----LllllHHHH-HHHHHHHHH-L---------------LL-LLL-LL----LLLL---------------------LLLH--HHHHHH-H-H------H-H-HHhlL-L-L-EEEE---E-L--L-L-LLL--LllL---L-LLLLLL-L--L-LL-H-----hH----LLEEEEEL-L-----------llLHHHhhHH--L-LHHHE-E-EEE-Ellllll
0032 7plhA   hHHHH-HHHHH--HHHHH-LL------------------------L-LEEEELLL----LL----LHH-HHHHHHHHH-LLLlllllllllLL--LEE-EEL-LL-L-LL-----------------------------LHHHH-H-H--H---H-H-HL--L-LlE-EEELL--L-L--L-L----------------lHHHHHH-H--H-HH-H--hhhL-----LEEEEEEL-L----------HHHH--hhhL--L-LHHHL-L-EEL-Llllll
0033 8c0vB   lllLH-HHHHH--HHHHH-HHH-HLLL------LL---L--L-L-LEEEELL-------LL--LLL---HHHHHHHH-HHHH--------LL--EEE-EE--LL-L-L--LLL---L-----------LLLLLHH--H-HHHHHH-HhH--H-H-L-LhLL--L-E-E-EEELL--HhH--L-L-LlhhhhhhhH---H-HHHHHH-H--H-HH-H------hhlllEEEEEEEL-L---------HHHL-----------L-HHHLL-L-LEE-Elllllh
0034 8bobC   lllHH-HHHHH--HL-HH-HL----------------------L-EEEEELLL----LL----LHH-HHHHHHLL-LL---------------LEE-EEE-LL---hHHLHH-----------hhhhhhhllLLL-H-------------------L-LL--L-E-E-EEEEL--H-H--H-L-LLH-----------hhHHHHH-H-hH-HL-L------L---lEEEEEEEL-L---------------------------HHHHE-E-EEL-Hhhhlll
0035 7jgrD   lLLLH-HHHHH--HHHHH-HHH-HHL---------------L-E-EEEEELLL----LL----LLH-HHHHHHHHH-HHHH---------E--EEE-EEE-LL-L-LL----------------------------LHHHH-H-H---------llLL--E-E-E-EEEEL--H-H--HhL-LLH--H--H-------HHHHH-H--H-HH-HlllllL----LEEEEEELL-LL---------LLLL------------HHHLLlL-LEE-Llllllll
0036 8c0vA   lLHHH-HHHHH--H-hLL-LL-------------------------lLLEEEELLllllLHH--HHHH-HHHHHHHHH-HL---------------LE-ELL-LL-L---LLLL----------------LLLL-HHHHhHHHHHH-H-H-hH-H-H-L--------L-L-EEEEE--L-L----L-HHH--H--LllllhHHHHHH-H-hH-HH-H-------llllLLLLEEEE-L-------------LLLL--L--lL-LHHLL-E-EEE-Elllllll
0037 1w5sB   lLLLH-HHHHH--HHHHH-HHH-HHLL---------------L-L-EEEEEEL---llLLL--LLHH-HHHHHHHHH-HHHH-hhhlllLE--EEE-EEE-HH-H-LL-----------------------------LHHHH-H-H--H------hhLL--E-E-E-EEEEL--L-H--H-HhLLL--L---------lhHHHHH-H--H-LH-lllllleE----EEEEEEEE-EL---------HHHH---------hL-HHHLL-L-EEE-Elllllh
0038 6fhsC   hHHHH-HHHHH--HHHHH-LLL--------------------L-L-LEEEELLL----LL---LLHH-HHHHHHHHH-H-LL--------LL--LLL-LLL-HH-H-L-------------------------LLLLL-LLLLHH-H-H--H-H-H-HheE--E-E-LeEEEEL--L-L--L-L---------------L-HHHHHH-H--H-HH-H--lllL----LLEEEEEL-L-------------LLEE--E--lL-LLLHH-L-EEE-Lllllll
0039 5j1sB   lLLHH-HHHHH--HHHHH-HHH-LLLL------LL---L--L-E-EEEEEELH----HHH--HHHH-HHHHhhhhH-HLLL--------LL--LLE-EEE-LL---lLLLL-------------------LHHH----HHHHH-H-H--H-H-H-H-LL--L-L-E-EEEEL--H-H--H-L--HH--H--H-------HHHHH-H--HlLL-L------L---lLEEEEEEE-LH--HHHhHHHH----------hhlhHHHHlL-LEE-Ellllhh
0040 3zh9B   -LHHH-HHHHhhlLLLL----------------------------L-EEEEELL-------LH--HHHH-HHHHHHHHHhLLHH--------HHhhHEE-EEE-LL-L-L--------------------------------LHHHH-H-H--H-H-L-L-LL-lL-E-E-EEEEL--L-H---hhllLL--L--L-----lHHHHHH-H-hH-LL-L------L---lEEEEEEEL-LL--LLL--------------L--L-HHHHL-E-EEE--------
0041 7w42B   lLLLHhHHHHH--HHHHH-HHHhLLLHhhhhhllLL---L--L-E-EEEE-ELL----LL---LLHH-HHHHHHHHH-L-----------LL--LEE-EEE------LLLL-----------------------llH-HHHHHH-H-H-hH-H-H-L----L-E-E-EEELL--H-H--H-L-LHH--H--H------hHHHHHH-H--H-LL---llllL----EEEEEEEL-LH---------HHLL------------LLLLlL-EEE-Elllllh
```

Job: Type-3 BREX; BrxF Inactive STAND (BrxB-Homolog)
Query: s001A
No: Chain Z rmsd lali nres id PDB Description
1: 8fcv-R 9.6 3.3 127 343 13 MOLECULE: DNA (60-MER);
2: 2qby-B 9.4 2.9 124 368 12 MOLECULE: CELL DIVISION CONTROL PROTEIN 6 HOMOLOG 1;
3: 5e7p-A 9.4 3.3 130 719 15 MOLECULE: CELL DIVISION CONTROL PROTEIN CDC48;
4: 8umy-A 9.3 3.3 120 546 17 MOLECULE: CHROMOSOME TRANSMISSION FIDELITY PROTEIN 18 HOMOL
5: 8dr5-A 9.1 3.0 126 646 10 MOLECULE: REPLICATION FACTOR C SUBUNIT 1;
6: 3bos-B 8.9 2.8 113 231 9 MOLECULE: PUTATIVE DNA REPLICATION FACTOR;
7: 9nv3-A 8.7 3.1 136 199 14 MOLECULE: DUF1788 DOMAIN-CONTAINING PROTEIN;
8: 7st9-A 8.5 3.4 139 522 11 MOLECULE: CHECKPOINT PROTEIN RAD24;
9: 7crc-B 8.5 3.8 125 1112 14 MOLECULE: NAD+ HYDROLASE (NADASE);
10: 6z1f-3 8.5 3.1 128 284 14 MOLECULE: RIBULOSE BISPHOSPHATE CARBOXYLASE/OXYGENASE ACTIV
11: 8btg-B 8.4 3.7 126 335 13 MOLECULE: CHROMOSOMAL REPLICATION INITIATOR PROTEIN DNAA;
12: 7mi8-A 8.3 3.4 122 868 11 MOLECULE: FUSION PROTEIN OF DYNEIN AND ENDOLYSIN;
13: 7plh-A 8.3 3.4 120 260 17 MOLECULE: SHTNSC;
14: 9cru-C 8.3 3.3 133 741 17 MOLECULE: VESICULAR-FUSION PROTEIN SEC18;
15: 7jgr-D 8.2 3.4 123 441 16 MOLECULE: ORIGIN RECOGNITION COMPLEX SUBUNIT 2;
16: 6bbm-D 8.2 2.7 116 455 18 MOLECULE: REPLICATIVE DNA HELICASE;
17: 8ipr-A 8.2 3.4 110 504 23 MOLECULE: COMPONENT LINKED WITH THE ASSEMBLY OF CYTOCHROME'
18: 8dgc-A 8.1 3.5 121 2028 11 MOLECULE: SEAVS3;
19: 8c0v-A 8.1 3.3 134 823 13 MOLECULE: PEROXISOMAL ATPASE PEX1;
20: 5zr1-E 8.1 3.3 124 460 15 MOLECULE: ORIGIN RECOGNITION COMPLEX SUBUNIT 1;
21: 8xvb-A 8.0 3.0 116 246 16 MOLECULE: DNA (5'-
22: 5zr1-A 8.0 3.3 127 494 12 MOLECULE: ORIGIN RECOGNITION COMPLEX SUBUNIT 1;
23: 5yww-A 8.0 3.0 106 485 18 MOLECULE: NUCLEOTIDE BINDING PROTEIN PINC;
24: 7vcs-A 8.0 3.4 133 766 12 MOLECULE: TRANSITIONAL ENDOPLASMIC RETICULUM ATPASE;
25: 9bv1-A 7.9 3.2 124 405 16 MOLECULE: 26S PROTEASOME NON-ATPASE REGULATORY SUBUNIT 1;
26: 6n9x-B 7.9 3.2 117 500 11 MOLECULE: DNA PRIMASE/HELICASE;
27: 5j1s-A 7.9 3.4 129 277 9 MOLECULE: TORSIN-1A;
28: 4rh7-A 7.8 3.2 121 3005 13 MOLECULE: GREEN FLUORESCENT PROTEIN/CYTOPLASMIC DYNEIN 2 HE
29: 9emc-D 7.8 3.1 128 337 17 MOLECULE: RUVB-LIKE 1;
30: 1nlf-A 7.8 2.8 108 254 13 MOLECULE: REGULATORY PROTEIN REPA;
31: 9e22-A 7.8 3.3 116 2925 11 MOLECULE: CYTOPLASMIC DYNEIN 1 HEAVY CHAIN 1;
32: 4m9s-B 7.7 2.9 111 511 10 MOLECULE: CELL DEATH PROTEIN 4;

33: 6f0x-B 7.7 3.2 138 398 15 MOLECULE: PACHYTENE CHECKPOINT PROTEIN 2 HOMOLOG;
34: 7uqj-A 7.7 3.4 124 585 8 MOLECULE: ATPASE HISTONE CHAPERONE YTA7;
35: 6p8v-C 7.6 3.3 138 303 9 MOLECULE: ATPASE, AAA FAMILY;
36: 6dgd-A 7.6 3.6 115 704 11 MOLECULE: PRIMOSOMAL PROTEIN N';
37: 8zr0-A 7.6 3.3 124 480 11 MOLECULE: CASEINOLYTIC PEPTIDASE B PROTEIN HOMOLOG;
38: 8y6p-Q 7.5 3.0 112 1228 11 MOLECULE: APAF-1 RELATED KILLER DARK;
39: 7t8b-A 7.5 2.5 104 518 13 MOLECULE: TWINKLE MTDNA HELICASE;
40: 5juy-B 7.5 3.5 106 1234 16 MOLECULE: APOPTOTIC PROTEASE-ACTIVATING FACTOR 1;
41: 8gja-F 7.5 3.3 126 342 13 MOLECULE: RAD51C;
42: 8ui7-A 7.4 3.0 122 643 14 MOLECULE: ATPASE FAMILY AAA DOMAIN-CONTAINING PROTEIN 5;
44: 7qo4-M 7.4 3.3 130 421 15 MOLECULE: 26S PROTEASOME REGULATORY SUBUNIT RPN1;
45: 6qem-G 7.4 4.1 135 239 12 MOLECULE: REPLICATIVE DNA HELICASE;
46: 9ri9-B 7.4 3.1 109 868 11 MOLECULE: SLNRC3;
47: 8jx6-B 7.4 3.5 122 451 11 MOLECULE: DEEP-SEA HELICASE 9;
48: 4wia-A 7.4 3.2 115 229 17 MOLECULE: PUTATIVE FLAGELLA-RELATED PROTEIN H;
49: 8tp1-A 7.3 3.3 122 419 15 MOLECULE: MITOCHONDRIAL CHAPERONE BCS1;
50: 3j3r-A 7.3 3.7 133 798 17 MOLECULE: ADAPTER PROTEIN MECA 1;
51: 8xks-B 7.3 19.1 127 603 13 MOLECULE: CTAP1;
52: 7yqh-B 7.3 3.0 123 1066 15 MOLECULE: STRUCTURAL MAINTENANCE OF CHROMOSOMES PROTEIN 5;
54: 7qo4-J 7.3 3.3 128 405 12 MOLECULE: 26S PROTEASOME REGULATORY SUBUNIT RPN1;
55: 8bob-C 7.2 3.3 112 412 14 MOLECULE: PROTEIN MALY;
56: 7rzy-1 7.2 3.3 121 311 12 MOLECULE: TN6677 VIBRIO CHOLERAE TRANSPOSON TNSC (VCHTNSC);
57: 8xku-A 7.2 3.3 133 730 8 MOLECULE: PROBABLE INACTIVE ATP-DEPENDENT ZINC METALLOPROTE
58: 7lcc-A 7.2 3.5 118 1369 6 MOLECULE: HELRAISER K1068Q;
59: 6l1q-A 7.2 3.0 115 267 14 MOLECULE: CBBQ PROTEIN;
60: 8hqs-B 7.1 3.0 123 408 15 MOLECULE: STRUCTURAL MAINTENANCE OF CHROMOSOMES PROTEIN 5;

0001 s001A ----MAAS--LSSEIHDVLLK--TQ---DT-YY--K--LILAVGP-ARTGKTAALTELAAK--H----K------W-PRINVN--LCL-SEKLL-EL-THRQRAVR-VA-G-ILN-D-TVRDET-S-NV-VLLDN-I-E-LLFA-PE--L-A--QD--P-LKLLQSLSRN-RTIVAAWPGN-F--DGSSLTYSE-A---RRY---LT-P-Q-AVIVK-A----SETN------
0002 8fcvR ---aHPRLkeVYEILMRTIAE--P-----A-GA--S--FIFVYGA-SGVGKTTLRLRVEQK--L----TdrarvpV-VGIEA----IA-PE---------------fNW-K-EYY-T-RALITL-P-DV-FFVDE-A-Q-HFGK-VA-gY-K--LQ-dQ-L-DCLKSLANG-ILHCLLGTY-------elLTFR------nLS---GQ-L-R-SVDIH-F----RRYCadsped
0003 2qbyB keipfrED--ILRDAAIAIRY--FN---EV-K---F--SNLFLGL-TGTGKTFVSKYIFNE--I----EeykdvkQ-AYVNCR--EVG------------------gTP-Q-AVL-S-SLAGKL---AI-IYLDE-V-D-TLVK-----R-R--GG--D--IVLYQLLRN-ISVIMISNDI--------NVRD--Y---MEP-----S-S-L-GPSVI-F----KPYDaeqlkf
0004 5e7pA ---kVTHP--QAVKLDEWLRL--SL-lgAT-PH--L--GVLVSGP-AGVGKATMVRAVCAS--R----R------V-VELDGP--EVG-AL-----------QVDE-RL-R-SVT-S-AVAAVS-G-GV-LFIAD-VdA-LLP--PE--P-V--AT--liLAELRKAVATgVAFIATSAV---------PENV-------------R-A-P-ERELG-L----SLPDatarrs
0005 8umyA tellsdDF--TNRCLLKWLKL--WD-sqRP-KQ--K--VALLCGP-PGLGKTTLAHVIARHagY----S------V-VEMN-A--SDD------------------rSP-E-VFR-T-RIEAA--P-NC-LVIDE-I-D-GAP-----------VA--A-INVLLSILLM-RPIICICND---------QFAP------SLR---Q-kQ-Q-AFLLH-F----PPTLpsrlvq
0006 8dr5A qqvcgnKG--SVMKLKNWLAN--WE---NG-VF--R--AAMLYGP-PGIGKTTAAHLVAQE--L----G----ydI-LEQN-A--SDV--------------rskT-LL-N-GVK-N-ALDNMS-H-FV-IIMDE-V-D-GM-S-GG--D-R--GG--V--GQLAQFCTS-TPLILICNE---------RNLP--K---MRP----D-R-V-CLDIQ-F----RRPDansiks
0007 3bosB ypaagnDE--LIGALKSaGDG--V-----------Q--AIYLWGP-VKSGRTHLIHAACAR--A----N-elerrS-FYIPLG--I-H-ASI--------------------STA-L--LEGLQ-F-DL-ICIDD-V-D-AVAG-----H-P--LW--E--EAIFDLYNR-VSLIVSASAS-------pXEAG-------------lxH-W-GLTYQ-L----QPXXddekla
0008 9nv3A -idpvlEY--RLSQVQSRISEerFL---KN-EI--G--FWIFDYP-AQNQVREHLKYLLRN--L----E--kdhkF-AHLNIF--QII-VDMLT-ER-GLFdrvcqkIA-D-YIA-K-KVD--N-Q-EF-VILTG-M-G-NAWP-L---------E--L-MSALQDVMGF-TPLLMFYPGT-Y--SGHDLSPLA-I---DSR---NY-Y-R-AFRLVr-------------
0009 7st9A qvaihKRK--L-KDVQEALDA--MF-lpNA-KH--R--ILLLSGP-SGCSKSTVIKELSKI--L----V-------hKVTEF----RG-DC-IV-ND----LPQME-SF-S-EFL-K-GARYLN-L-SL-ILI-E-D-L-P-NV-FH--I-D--TR--F-QQLILQWLYL-PPLVICITE-----------CEIeT---IMN---KE-I-LrLKRIK-F----NPINstllkk
0010 7crcB ---vGMRA--HMDMLEQLLR---LV---LD-EV--R--MIGIWGP-PGIGKTTIARFLFNQ--V----S-drfqlS-AIMVIK--GCY----PR-PC----FDEYS-AQ-L-QLQ-N-QMLSQM---VF-LVLDE-V-D-QL-----------GQ--L-DaLAKE-TRW-FRIIITTED---------LGVL-------------A-H-G-IHVYK-V----GYPSndeafq
0011 6z1f3 -syyiAPR--FLDKLAVHITK--NF---LR-VP--L--ILGIHGR-KGEGKTFQCELAFEKmgI----E------V-TLIS-G--GEL-E----------sPDAGD-PA-R-LIR-L-RYRETA---CV-LMIND-LdA-GAG--YT--V-N--TQ--lvNATLMNIADNrVPIIVTGND---------FSTL-------yA---PL-I-R-MEKFY------WEPNrddkvg
0012 8btgB vigsgnRF--AHAASLAVAEA--------K-AY--N--PLFIYGG-VGLGKTHLMHAIGHY--V----IhnpsakV-VYLSSE--KFT-NEFINsIR-DN-------------dfR-N-RYRN---V-DV-LLIDD-I-Q-FLAG-K---E-Q--TQ-eefFHTFNTLHES-KQIVISSDRP---------PKEIP-L---ED------fE-W-GLITD-I----TPPDletria
0013 7mi8A ---yTPLLliGFATLTDSLHQ--K-----------Y--GGCFFGP-AGTGKTETVKAFGQN--L---gR------V-VVVFNC--D---DSFD------------yQ-VL-S-RLL-V-GITQIG-A--W-GCFDQ-F-N-R-LD-EK--V---lSA--V-SANIQQIQNGhTAVFITLNP-----------GYN---rselP---EN-L-KsFREFS-M----KSP-------
0014 7plhA ---vPLQQ---VKTLHDWLDG--KR---KA----rK--SCRVVGE-SRTGKTVACDAYRYR--H----KrpptvpV-VYIRPH--Q-------------------cGP-K-DLF-K-KITEYG-V-EM-LIIDE-A-D-RLK------------E--T-FADVRDIAEG-IAVVLVGTDR--------lDAVI------KRD---EL-E-R-FAHLR-F----GKLSgedfkn
0015 9cruC ---ySERVnsILKNGARYVRQ--VR-sdKS-RL--V--SLLIHGP-AGSGKTALAAEIALK--S----G---fpfI-RLI-SP--NE--LSGM---------SESA-KI-A-YID-N-TFRDAY---NI-LVIDS-L-E-TLVD-FS--N-N--IL-----QMLKVALKRrLLIMTTTSAY---------SVLQ--Q---MDI-----lS-C-FDNEI-A----VPNMtnldel
0016 7jgrD ttlrgyAK--ERSNVRLLLQR--TA--eMG-ES--N--SLLLLGP-RGSGKTTLINSVLAD--L----LfgentlI-VHLDGN--LHT-------------------DD-R-VAL-K-SITVQM---VI-FILEE-F-D-LFCA----hH-N--QT---lLYNLFD-vAP-ICVLGVTCRL---------DVIE------LLE---KS-R-F-SRQVF-L----FPSLrrfedy
0017 6bbmD qqphdgvtgvntgyddlnTAG--LQ---------psD--LIIVAAR-PSMGKTTFAMNLVEN--A----AmlqdkpV-LIFSLEmdDSS-G-----------------lTP-T-EVR-S-RARRIA-I-GL-IMIDY-L-Q-LMRV-PA--L-SdnrE--I-SRSLKALAKN-VPVVALSQL--P--VNSDL----rE---SGS----E-Q-D-ALIMF-I----YRDEvyhens
0018 8iprA tplrsrpD--VAT--VDLA---------------pgD--RLAVVGP-SGSGKTTMLMAIADR--L----NgetpqrA-AVFAED--A----HLFD-T-dtelvaalD-RV-GllLI-A-RALISA-F-PV-VLLDQ-P-T-E-NL-DA--G-D--AQ--M-LEGLLALFAAdRTVVVATHH---------------------lP---PG-F-D-CPIVR-C----T---------
0019 8dgcA ---vSRTQ---EATLVQRVVE--AF-------G--A--PVIIHAD-AGVGKSIFSTHIEEH--LptgsV------S-ILYDCF--G--lGQYRN-AS----------hHH-R-TAL-V-QMANEM-asLC-IIIDA-A-D-NAQM-aE--T-R--SF--I-KDLIRE----gVCLVALCRP---------YRRE-------------P-P-E-ALTLS-L----QTFNrdetaa
0020 8c0vA ---iTVNS--IKKEMVNYLTS---P---II-AT--P--AIILDGK-QGIGKTRLLKELINE--V----E-kdhhiF-VKYADC--E---TLHE-------tSNLDK-TQ-K-LIM-E-WCSFCG-P-SL-IVLDN-V-EaLFGK-P--wD-N--ASklL-NFFINQVTKIrIRVLFSGKQK-T------------qI---NPL-lfDK-H-F-VSETW-S----LRAPdkhara
0021 5zr1E pevafrEY--QTNCLASYISA--DP---DI-tP--S--NLILQGY-SGTGKTYTLKKYFNA--N----P---nlhA-VWLEPV--ELV-S-----------------wK-PL-L-QAI-A-RTVQYK-LkLF-LILDG-F-D-SLQD------lD--AA--L-FNKYIKL-lN-IKFIYTMLET---------SFLQ--------RY---ST-H-C-IPTVM-F----PRYNvdevst
0022 8xvbA pprfveTQ--TVKQIWTSMRF--AS---LT--E--S--IAVVCGN-PGVGKTEAAREYRRT--N----N-----nV-WMITIT--PSC-------------------aSV-L-ECL-T-ELAFEL---GL-VIIDE-A-D-HLG-------------E--V-LEELRLLQER-IGLVLMGNH------------------rvySNM---TF-S-R-IKRTA-I----NKTKkadvka
0023 5zr1A yylparEN--EFASIYLSAYS--AE---SD-S---A--TTIYVAGtPGVGKTLTVREVVKE--L----LreipdfL-YVEING--LKM-VK------------------pT-DCY-E-TLWNKV---TI-VVLLDeL-D-AMVT-K-----S--QD--I-MYNFFNWTTA-KLIVIAVANT-M-------DLPE--R---QLG---NS-R-I-GFTRI-M----FTGYtheelk
0024 5ywwA ---eDYNL---derlIKRLEE--R-----------aE--GIIIAGA-PGMGKTTFAQALAEY--Y----M-rlgkiV-KTIES-------------------------G-ELH-D-ILLLSR-P-DY-TVYDE-M-R-N--------------DE--D-FKLYVDLRLG-VGMVGVVHAT-S-----PIDA------iHRF---V-gtiL-DTIIF-I----NSG-------
0025 7vcsA ---gGCRK--QLAQIKEMVEL--PL-igVK-PP-----RGILLYGpPGTGKTLIARAVANE--T----G-----aF-FFLING--PEI-MSK-----------LAGE-SE-S-NLR-K-AFEEAN-A-PA-IIFID-E-LdAIAP-kE--R-R--IVsqL-LTLMDGL--A-HVIVMAATN----------RPNS-I---DPA----R-F-G-RDREV-D----IGIPdatgrl
0026 9bv1A sdvggcKE--QIEKLREVVET--PL-lgIE-PP--K--GVLLFGP-PGTGKTLCARAVANR--T--------dacF-IRVIG---SEL-V--QK-YV-------GE-GA-R-MVR-E-LFEMAK-A-CL-IFFDE-I-D-AIG-gDN--E-V--QrtmlelINQLD--GN-IKVLMATNRP-------------dT---LDP----R-P-G-RRKIE-F----SLPDlegrth
0027 6n9xB ---gCTGI----------ntLG--AR---------ggE--VIMVTSG-SGMGKSTFVRQQALQ--W----GtamgkkV-GLAMLQ--E------------sdslkreiidsT-D-RLL-A-KLAYMG-C-DV-IILDH-I-S-IVVS-SD-eR-K--MIdnL-MTKLKGFAKG-VVLVVICHL----pVSITDLR---G---SGA----R-Q-L-STIIA-L----ERNQqgdmpn
0028 5j1sA dnlfgqHL--AKKIILNAVFG--FI-pkPK-KP--L--TLSLHGW-TGTGKNFVSKIIAEN--I----YglnsdyV-HLFVAT--LHF-PH---------aSNITL-YK-D-QLQ-L-WIRGNV-R-SI-FIFDQ-MdK-MHA-----------GL--I-D-AIKPFLD-kAMFIFLSNAG-wraLSVSV------fnnKNS---GwrN-L-IDFVP-F----LPLEykhlkm
0029 4rh7A sklvyTPL--T-DKCYLTLTQ--AM---KM-GL-----GGNPYGP-AGTGKTESVKALGGL--L--grQ------V-LVFNCD-------------------------iDV-K-SMG-R-IFVGLC-G-AW-GCFDE-F-N-RLE----------eSV--L-SAVSMQIQTInSGIFITMNP------------AGK-R---QKL---PDlK-Q-LFPVA-M----SHPDneliae
0030 9emcD qgmvgqLA--ARRAAGVVLEM---G---KI-A--gR--AVLIAGQ-PGTGKTAIAMGMAQA--L----G--pdtpF-TAIA-G--SEI---FSL-E---------mS-KT-E-ALT-Q-AFRRSIgP-GV-LFIDE-V-H-MLD--------I--ES--F--SFLNRALEA-PVLIMATNR------------GIT-SphgIPI---D-lD-R-LLIVS-T----TPYSekdtkq
0031 1nlfA ileafaaappldyvLPNMV-------------agT--VGALVSP-GGAGKSMLALQLAAQ--I----AelptgpV-IYLPAE-rqLI-GSL-------pnimAPE-WF-D-GLK-R-AAE--G-R-RL-MVLDT-L-R-RFHI-E-----eenaQ--V-IGRMEAIAAG-CSIVFLHHA---------------------VL---VD-N-I-RWQSY-L----SSMTsaeaee
0032 9e22A ---qTPLTdrCYLTMTQALEA--R-----------L--GGSPFGP-AGTGKTESVKALGHQ--L----G------RfVLVFNC--D-E-TFD------------------F-Q-AMG-R-IFVGQV-G-A--WGCFDeF-N-RL----E--E-R--MA--V-SQQVQCIQEA-LAIFITMNP---------------------gsnlP---DN-L-KlFRSLA-M----TKPDrqliaq
0033 4m9sB xtcyirEY--HVDRVIKKLDE--XC---DL-DS--F--FLFLHGR-AGSGKSVIASQALSK--S-----ginydsI-VWLKDS-------------------gtAPK-ST-F-DLF-T-DILLXL---TL-FVFDD-V-V-QE-------------E--T-IRWAQE--LR-LRCLVTTRDV-E-----------------------S-Q-T-CEFIE-V----TSLEidecyd
0034 6f0xB ---yDVEV---KSHLLDYVMT--TL---LFsTW--N--RVVLLHGpPGTGKTSLCKALAQK--L----TssryryG-QLIEIN--S---HSLFS-K--WFSES-GK-LV-T-KMF-Q-KIQDLI-A-LV-FVLIDqV-E-SLTnaAI--R-V--VN-aV-LTQIDQIKRHsNVVILTTSN----------ITEK-I---DVA-----vD-R-ADIKQ-Y----IGPPsaaaif
0035 7uqjA ---kPLLA-dQLNNLKNKLDY--MLnesQI-CK--P--RLLINGP-KGNGQQYVGAAILNY--L----E-------nVQNLDL--ASL-VS-ES-SR--------T-IE-A-AVV-Q-SFMEAQ-P-SV-VFIPN-LdI-WINT--P--E-N--VI-----------rEK-ILLLCLAENL-D--I--------------sEV---KNaF-D-KNIFQ-L----HKPSkenitr
0036 6p8vC arlvglDE--HKDRLSKILGL--LV-dtVL-RR--P--PLVVLAGdVGSGKTELAETIGDA--V----ArqedidI-TLYPL----SL-AT-RG-QG-RV-GEXTQ-LV-S-AAF-D-YTIEAA-G-AV-LLLIDqA-D-ALAQsDR--A-G--VN-aF-IRGIDRIANQlPAAVLXCTN----------RLKA-L---DPA-----qR-R-AAEIL-T----FSRPndeqrh
0037 6dgdA erlrlNTE--Q-ATAVGAIHS--AA---DR--F--S--AWLLAGI-TGSGKTEVYLSVLEN--V----L-aqgrqA-LVM-VP--EIG-L-------fnapvevlhivigt-SSL-F---TPFK-L-GV-IVIDE-EhD-SSYK-QQ-egwR--YH--A-RDLAVWRAHQ-IPIILGSATP---------------------aLET---LH-NvK-YRQLT-L----S---------
0038 8zr0A ehiigqEG--AINTVASAIRR--KE-ngWY-DEehP--LVFLFLGsSGIGKTELAKQVARY--M----H-------kGFIRMD------------------fqEKHEVAK-FI----LT-K-QLKQSP-S----AVVLF-D-EvekAH-PD--V---lTV--M-LQLFDEG--K-DAIFIMTSNAadvvIRPI-----aH---FRR---DL-G-R-INEIV-Y----FLPFchsell
0039 8y6pQ kynvsrLQ--PYLKLRQALLE--LR------PA--K--NVLIDGV-LGSGKTWVALDVCLS--Y-----ckmdfkI-FWLNLK--N---------------cnSPE-TV-L-EML-Q-KLLYQI---CL-LVLLN-V-Q-NA---------------K--A-WNA--FNLS--CKILLTTRFK------------------qvtDFL---SA-A-T-TTHIS-LdhhsMTLTpdevks
0040 7t8bA snveqaaglrwsrfpdlnlKG--HR---------kgE--LTVFTGP-TGSGKTTFISEYALD--L----C-sqgvnT-LWGSFE--ItqtFHGQ--------------qSI-R-TVI-D-TMQHAD-I-CH-VIIDN-L-Q-FMM----------tdrY--I-IGVFRKFATN-CHVTLVIHPG----------------------S---AK-A-A-DNVLI-L----QDRGkrylqv
0041 5juyB vvfvtRKK--LVNAIQQKLSK--L-----KG-EP--G--WVTIHGM-AGCGKSVLAAEAVRD---------gcfpggV-HWVSVG--K------------------qDKSG-LL-M-KLQ-N-L--qsP-R-SL-LILDD-V-W-DS---------------W--V-LKAFD---SQ-CQILLTTRDK-S-------------------------tdsP-KYVVP-V----ESSLgkekgl
0042 8gjaF ---cQQLD---sflrgGILT---------------rT--LTEIAGE-SASGKTQLCMQLCLT--V----QgglgggA-VYICTE-eHAA-E---------------lD-DL-H-YCVsK-KVPVLL-V-KL-IIIDS-IaA-LFRC-EL--Q-E--RL--M-QLIASKLLQL-APAICVNQVS---------dVVEQ-P---TLG---IA-N-H-VVRLM-L----MRTNyklpvq
0043 8ui7A selignEL--AIKKLHSWLKD--WK-eeSR-LC--N--TVLITGP-TGVGKTAAVYACAQE--L---gF-------K-IFEVNA--SSQ------------------rSG-R-QIL-S-QLKEA--T-SL-ILFEE-V-D-V-IF-DE--D-A--GF--L--NAIKTFMTK-RPVILTTSDP-T----------------fsLMF---D--G-C-FEEIK-F----STPSllnvas
0045 7qo4M ---gGLDK--QIEELVEAIVL--PM-mgIR-AP--K--GALMYGP-PGTGKTLLARACAAQ--T----N----atF-LKLAA---PQL-V-----------qMYIGE-GA-K-LVR-D-AFALAK-A-PTiIFIDE-L-D-AIgD-RE--VqR--TM--LelLNQLD-DDR-VKVLAATNRV------------dV---LDP-alLR-S-GlDRKIE-F----PLPSedsraq
0046 6qemG ---eCEGQmnALSKARQYVEE--FD---GN--I--A--SFIFSGK-PGTGKNHLAAAICNE--L----L-lrgksV-LIITVA--DIM-SAMKD-tF-RNS-----sEE-Q-LLN-D-LSN----V-DL-LVIDE-I-G--vQT-ES--K-YekVI--I-NQIVDRRSSK-RPTGMLTNSN-M-------eEMTK-L---LGE----M-D-R-MSLWV-I----FNWDsyrsr-
0047 9ri9B ddvvgfDD--EAKTVIDRLIG---G---SD-YV--E--VVPVVGM-PGLGKTTLAYKIYKD--P-----eyefftR-VWVYVS--QTF-------------------KR-R-EIF-L-NIISK----YL-IVLDD-V-W-T-----------------E--A-WDRIKIAFPNrNRVLMTTRQS-N----------------------------aK-R-CNPHD-L----KFLTkdeswe
0048 8jx6B sateeqRY--IFTDFIKQAQL--AL---KS-DY--E--VFAIQGF-AGSGKTWLSALIIDE--L----L-elgmkV-AVTS-P--THK-A-----------------pgtiH-HFL-NlnvtLEY-V-DV-LIVDE-A-S-MVS------------GE--L-YDHALKTLGDcKIILFIGDSY-----------QLL-E---DS-sifLK-D-D-IFHYK-L----TKVVrqaedn
0049 4wiaA ---elaridlsrddldkrgGI--PH-------G--S--LIIIEGE-ESTGKSVLCQRLAYG--F----L-qnrysV-TYVST--iPVY-PL------iadnkkkDG-FL-K-KVX-E-T-RAFY-K-DV-IIFDS-IsA-LIAN--A--S-E--VN--V-DDLXAFFKRIkKIIICTVNPK----------ELPE-S---VLT-----rT-S-ATLIR-T----ELFTfggdlk
0050 8tp1A svvlqQGL--A-DRIVKDIRE--FI---DN-PY--R--RGYLLYGpPGCGKSSFITALAGE--L----E------H-SICLLS-----tDSSL----------sdD-RL-N-HLL-S-VA-----P-QQ-SLVLL-E-D-vdAA-FLsrL-T--FS-gL-LNALDG--TE-ARIVFMTTN----------YIDR-L---DPA----R-P-G-RDLKE-Y----VGYCshwqlt
0051 3j3rA srvigqDE--AVVAVAKAVRR--AK---DP-KR--PigSFIFLGP-TGVGKTELARALAES--IfgdeE------S-MIRIDM--SEYstsrlV-GS-PP-GYVGY-DEgG-QLT-E-KVRRK--P-YS-VVLLD-AiE-KAH-----------PD--V-FNILLQVLEDrNTILIMTSNVgM--GELK-----rA---FRP-----iN-R-IDIIV-F----HSLEkkhlte
0052 8xksB ---lPEGNkqAKVELMEVVDF-----sgAR-AP--K--GVLLVGP-PGNGKTLMARAVAGE--S---gV------A-FISSSA--AEF-I----------eMYMGL-GA-A-RVR-D-LFNTAA-P-CI-IFIDE-L-D-AVG--ND--E-R--D---ntvNQLLTEMD-gIVVMGATNRK-D-------------V---LDA-----R-P-G-RRSIE-V----RRPDfqgrle
0053 7yqhB ---hEHFE-------LELG-------------S--R--LNFIVGN-NGSGKSAILTAITIG--L----GsslkdlimCFLSAR--SFL---TAS-TslSGGEKSFS-QM-A-LLL-A-TWKPMR-S-RI-IALDE-F-D-VFMD-Q---V-N--RK-iG-TTLIVKLKDIrTQTIIITPQD-----------------igKI---AD-I-G-VSIHR-M----RDP-------
0054 5v8fD ---fTYLQ-dCQQEIDRIIKQ--SI---I--eS--H--SVILVGP-RQSYKTYLLDYELSL--L----Q-------iTIRLNG--FIH-S---------------eQ-TA-I-NGI-A-TQL----I-TV-VFIFDeI-D-TFAG-PV----R--QT--L-LYNLFDMVEV-PVCIFGCTTK-L------NILE--L---EK------rF-S-QRVIY-M----PQIQnlddmv
0055 7qo4J ---gGLTK--QIKEIKEVIEL--P--lgIA-QP--K--GVILYGP-PGTGKTLLARAVAHH--T----D------C-KFIRVS---gA-ELVQK-Y---------igE-GS-R-MVR-E-LFVMAH-A-PS-IIFMD-E-I---DS-IG-sggG--DS--EvQRTMLELLN-nIKIIM-ATNR-L------------dI---LDPallRP-G-R-IRKIE-F----PPPSvaarae
0056 8bobC dhtvvRER--LLAK-LSGA-----------NF--R--LALITSP-AGYGKTTLISQWAAG--K----N-----dI-GWYSLD-----eGDNQ-----------qE-RF-A-SYL-I-AAVQQA---LY-LVIDD-Y-H-LITN-----P-V--IHesM-RFF-----EN-LTLVVLSRNL------------------pqlGI---AN-LrQ-LLEIG-S--qqLAFThqeane
0057 7rzy1 rafvsTPS--V-RKILSYMDR-cRD---LS-EP--T--CMMVYGA-SGVGKTTVIKKYLNQ--N----RggdiipV-LHIELP-------------------------aKP-V-DAA-R-ELLVEM-V-KL-IIIDE-F-Q-HLVE-NR--V-L--TQ--V-GNWLKMILTK-CPIVIFGMP----------------skvvlQAN---SH-G-R-FIQVE-L----RPFSynggrg
0058 8xkuA ---aGQEY--IKRELQEIVRI--LK-kgIY-CP-----KGVLLHGpPGTGKTLLAKAIAGE--A----G-----lP-FFAANG--TDF-V----------EMFV-GV-AA-S-RVK-D-LFASSY-A-PS-IIFIDeI-D-AIGSkGA--E-R--E---qglLQILTEMDS-QVLVIGATN----------RLDI-L---DPA----R-K-G-RDKII-R----VGLPskdgrl
0059 7lccA linslNDE--Q-LAAFQTITS--AI---ED-HP--K--CFFLDGP-GGSGQTYLYKVLTHY--I----R-grggtV-LPTAST--GTF-HS-------qyklPIPL-----------nakTIKK-A-QL-LIIDE-C-T-MAS-----------SH--A-INAIDreFGG-KVLLLGGDF---------rqcls-qtslKYC---N-wG-C-FRKLS-L----KTNMrsedsa

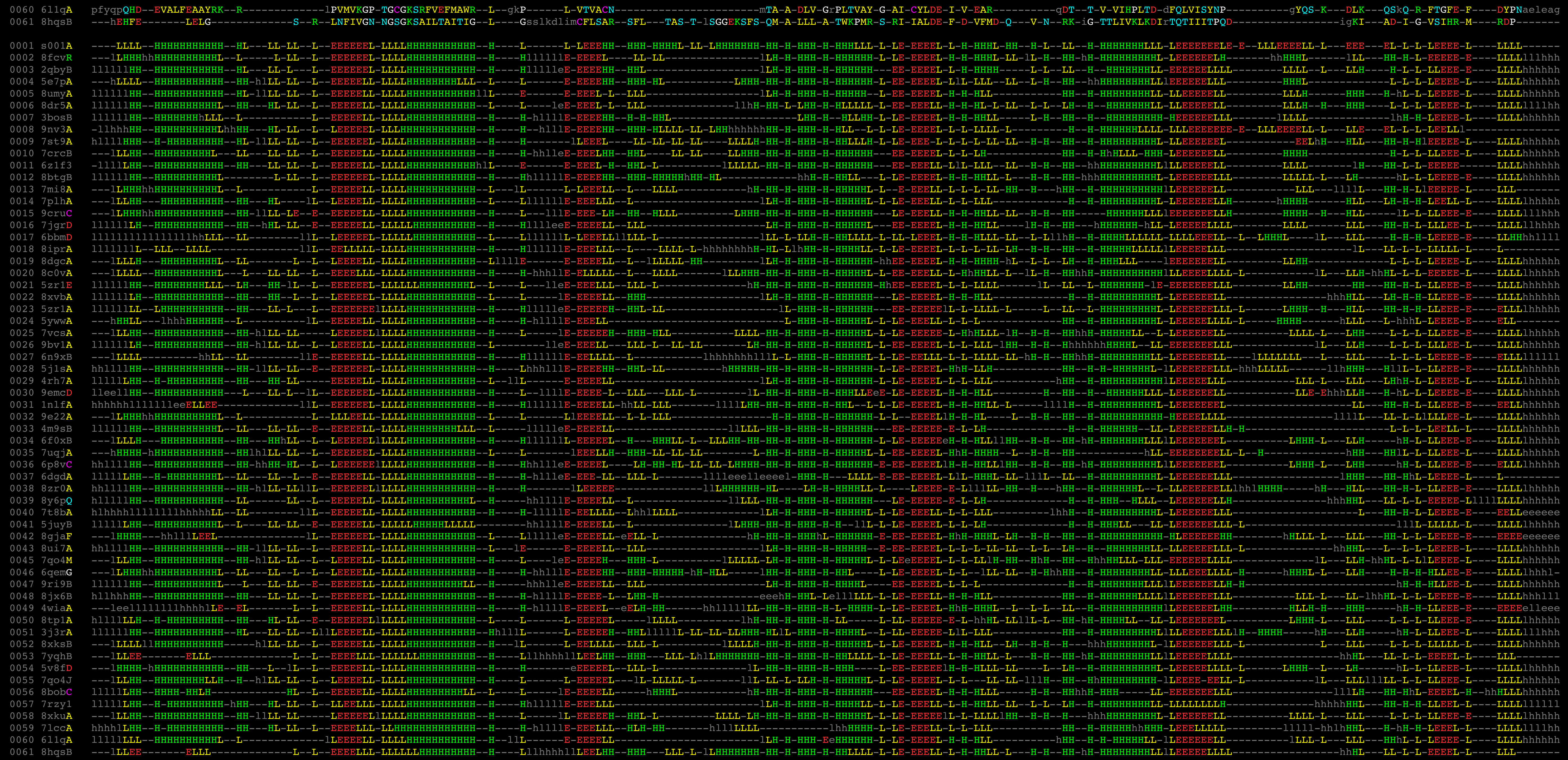
0060 6l1qA
0061 8hqsB
0001 s001A
0002 8fcvR
0003 2qbyB
0004 5e7pA
0005 8umyA
0006 8dr5A
0007 3bosB
0008 9nv3A
0009 7st9A
0010 7crcB
0011 6z1f3
0012 8btgB
0013 7mi8A
0014 7plhA
0015 9cruC
0016 7jgrD
0017 6bbmD
0018 8iprA
0019 8dgcA
0020 8c0vA
0021 5zr1E
0022 8xvbA
0023 5zr1A
0024 5ywwA
0025 7vcsA
0026 9bv1A
0027 6n9xB
0028 5j1sA
0029 4rh7A
0030 9emcD
0031 1nlfA
0032 9e22A
0033 4m9sB
0034 6f0xB
0035 7uqjA
0036 6p8vC
0037 6dgdA
0038 8zr0A
0039 8y6pQ
0040 7t8bA
0041 5juyB
0042 8gjaF
0043 8ui7A
0045 7qo4M
0046 6qemG
0047 9ri9B
0048 8jx6B
0049 4wiaA
0050 8tp1A
0051 3j3rA
0052 8xksB
0053 7yqhB
0054 5v8fD
0055 7qo4J
0056 8bobC
0057 7rzy1
0058 8xkuA
0059 7lccA
0060 6l1qA
0061 8hqsB

```
Job: Type-2 BREX; PglW_WP_185095299 1 wHTH1
Query: s001A
No: Chain Z rmsd lali nres id PDB Description
1: 5j6x-A 8.9 2.2 64 66 6 MOLECULE: Z-DNA BINDING PROTEIN KINASE;
2: 7qcd-D 8.9 2.7 80 326 9 MOLECULE: STRUCTURAL MAINTENANCE OF CHROMOSOMES PROTEIN 5;
3: 2mh2-A 8.3 1.8 63 64 16 MOLECULE: HOMOLOGOUS-PAIRING PROTEIN 2 HOMOLOG;
4: 6qfd-B 8.2 2.5 68 116 10 MOLECULE: DNA-BINDING PROTEIN;
5: 5yad-A 8.1 1.9 65 73 17 MOLECULE: MEIOSIS REGULATOR AND MRNA STABILITY FACTOR 1;
6: 7rhe-A 8.0 1.6 59 368 15 MOLECULE: ROK FAMILY PROTEIN;
7: 7vwv-B 8.0 2.4 69 74 9 MOLECULE: I73R;
8: 2l02-A 7.9 1.7 64 82 14 MOLECULE: UNCHARACTERIZED PROTEIN;
9: 1qgp-A 7.8 1.6 63 76 11 MOLECULE: PROTEIN (DOUBLE STRANDED RNA ADENOSINE
10: 1cf7-B 7.8 2.8 73 82 11 MOLECULE: DNA (5'-
11: 2xrn-B 7.7 1.9 61 241 10 MOLECULE: HTH-TYPE TRANSCRIPTIONAL REGULATOR TTGV;
12: 3cuq-A 7.7 2.4 74 219 14 MOLECULE: VACUOLAR-SORTING PROTEIN SNF8;
13: 1ku9-A 7.7 2.3 64 151 9 MOLECULE: HYPOTHETICAL PROTEIN MJ223;
14: 8e4y-A 7.7 2.2 67 651 7 MOLECULE: GLYCEROL-3-PHOSPHATE ACYLTRANSFERASE 1, MITOCHOND
15: 4ad9-A 7.7 2.2 74 288 5 MOLECULE: BETA-LACTAMASE-LIKE PROTEIN 2;
16: 3elk-A 7.6 2.3 70 104 10 MOLECULE: PUTATIVE TRANSCRIPTIONAL REGULATOR TA0346;
17: 7kbf-K 7.6 2.9 67 78 15 MOLECULE: HISTONE H3.2;
18: 2od5-A 7.6 1.8 66 91 14 MOLECULE: HYPOTHETICAL PROTEIN;
19: 8jxk-D 7.5 2.3 68 166 15 MOLECULE: CONSERVED PROTEIN;
20: 8iue-P 7.5 2.3 63 303 6 MOLECULE: DNA-DIRECTED RNA POLYMERASE III SUBUNIT RPC1;
21: 4ija-A 7.5 1.8 60 363 7 MOLECULE: XYLR PROTEIN;
22: 6qpq-D 7.5 2.0 66 82 5 MOLECULE: STRUCTURAL MAINTENANCE OF CHROMOSOMES PROTEIN,STR
23: 5y6i-B 7.4 2.2 63 234 11 MOLECULE: TRANSCRIPTIONAL REGULATOR KDGR;
24: 8xt8-B 7.4 1.6 61 145 10 MOLECULE: MARR FAMILY TRANSCRIPTIONAL REGULATOR;
25: 7cv0-A 7.4 2.2 61 173 8 MOLECULE: TRANSCRIPTIONAL REGULATOR NIAR;
26: 7y43-A 7.3 2.3 64 79 9 MOLECULE: HISTONE ACETYLTRANSFERASE KAT6A;
27: 2zme-B 7.3 2.1 64 215 9 MOLECULE: VACUOLAR-SORTING PROTEIN SNF8;
28: 3f72-B 7.2 1.7 61 108 18 MOLECULE: CADMIUM EFFLUX SYSTEM ACCESSORY PROTEIN;
29: 6sj9-A 7.2 2.8 73 642 5 MOLECULE: PROTEASOME ACCESSORY FACTOR B/C (PAFBC);
30: 4mtd-D 7.2 2.4 68 151 12 MOLECULE: ZINC UPTAKE REGULATION PROTEIN;
31: 3bpx-A 7.2 1.7 61 147 3 MOLECULE: TRANSCRIPTIONAL REGULATOR;
32: 4y66-C 7.2 2.4 67 197 7 MOLECULE: MND1;
33: 4i99-C 7.2 1.7 62 70 8 MOLECULE: CHROMOSOME PARTITION PROTEIN SMC;
34: 2cwe-A 7.1 1.7 59 191 12 MOLECULE: HYPOTHETICAL TRANSCRIPTION REGULATOR PROTEIN, PH1
35: 2vqc-A 7.1 2.6 66 70 9 MOLECULE: HYPOTHETICAL 13.2 KDA PROTEIN;
36: 3gfi-A 7.1 3.0 63 143 13 MOLECULE: 146AA LONG HYPOTHETICAL TRANSCRIPTIONAL REGULATOR
37: 7z8b-C 7.1 2.6 68 1224 12 MOLECULE: CULLIN-7;
38: 1xmk-A 7.1 2.2 65 79 8 MOLECULE: DOUBLE-STRANDED RNA-SPECIFIC ADENOSINE DEAMINASE;
39: 3k69-A 7.1 2.3 64 151 13 MOLECULE: PUTATIVE TRANSCRIPTION REGULATOR;
40: 3u2r-A 7.0 2.6 63 135 13 MOLECULE: REGULATORY PROTEIN MARR;
41: 6zvh-y 7.0 3.4 66 72 8 MOLECULE: 18S RRNA;
42: 5jbr-A 7.0 2.7 64 149 14 MOLECULE: UNCHARACTERIZED PROTEIN BCAV_2135;
43: 1xsd-A 7.0 2.2 65 125 6 MOLECULE: 5'-
44: 1yyv-A 6.9 2.5 65 114 6 MOLECULE: PUTATIVE TRANSCRIPTIONAL REGULATOR;
45: 6aht-A 6.9 3.5 70 111 9 MOLECULE: CONSERVED HYPOTHETICAL PLASMID PROTEIN;
46: 4fht-B 6.9 2.9 64 142 13 MOLECULE: PCAV TRANSCRIPTIONAL REGULATOR;
47: 7b0c-A 6.9 2.3 63 144 16 MOLECULE: HTH-TYPE TRANSCRIPTIONAL REPRESSOR NSRR;
48: 2g9w-A 6.8 1.9 62 119 19 MOLECULE: CONSERVED HYPOTHETICAL PROTEIN;
49: 3bdd-B 6.8 1.5 57 133 14 MOLECULE: REGULATORY PROTEIN MARR;
50: 1sfu-A 6.8 2.1 62 70 5 MOLECULE: 5'-D(*T*CP*GP*CP*GP*CP*G)-3';
51: 5n35-A 6.8 1.7 58 60 12 MOLECULE: POLB1 BINDING PROTEIN 2 (PBP2);
52: 5eri-A 6.8 1.7 61 153 8 MOLECULE: MARR FAMILY TRANSCRIPTIONAL REGULATOR;
53: 3f8f-A 6.8 2.7 69 114 9 MOLECULE: TRANSCRIPTIONAL REGULATOR, PADR-LIKE FAMILY;
54: 3r4k-A 6.8 2.1 60 255 5 MOLECULE: TRANSCRIPTIONAL REGULATOR, ICLR FAMILY;
55: 7c0j-B 6.8 1.9 59 62 7 MOLECULE: HISTONE H5,DOUBLE-STRANDED RNA-SPECIFIC ADENOSINE
56: 3w6k-C 6.8 2.1 61 87 16 MOLECULE: SCPA;
57: 2eth-A 6.8 2.1 60 141 12 MOLECULE: TRANSCRIPTIONAL REGULATOR, PUTATIVE, MAR FAMILY;
58: 1fok-A 6.8 1.9 65 568 9 MOLECULE: DNA (5'-
59: 5udb-4 6.8 2.6 69 751 9 MOLECULE: DNA REPLICATION LICENSING FACTOR MCM2;
60: 8as8-C 6.8 2.0 68 195 10 MOLECULE: JETC;
61: 2fxa-B 6.7 2.6 63 180 16 MOLECULE: PROTEASE PRODUCTION REGULATORY PROTEIN HPR;
62: 2lnb-A 6.7 2.1 62 80 10 MOLECULE: Z-DNA-BINDING PROTEIN 1;
63: 4hqe-B 6.6 2.6 65 108 8 MOLECULE: TRANSCRIPTIONAL REGULATOR QSRR;
64: 3bj6-A 6.6 1.7 60 152 15 MOLECULE: TRANSCRIPTIONAL REGULATOR, MARR FAMILY;
65: 4hw0-A 6.6 2.1 61 98 3 MOLECULE: DNA-BINDING PROTEIN SSO10A-2;
66: 2zkz-C 6.6 2.1 59 87 14 MOLECULE: TRANSCRIPTIONAL REPRESSOR PAGR;
67: 8uuc-A 6.6 2.6 63 274 16 MOLECULE: ADENINE DNA GLYCOSYLASE;
68: 2dql-A 6.6 2.7 67 115 9 MOLECULE: PEX PROTEIN;
69: 7xjg-J 6.6 2.1 64 306 9 MOLECULE: RNA-DIRECTED DNA POLYMERASE FROM RETRON EC86;
70: 3s93-B 6.6 2.3 63 81 14 MOLECULE: TUDOR DOMAIN-CONTAINING PROTEIN 5;
71: 2esh-A 6.5 2.8 66 114 9 MOLECULE: CONSERVED HYPOTHETICAL PROTEIN TM0937;
72: 9gm7-C 6.5 2.9 74 440 12 MOLECULE: CHROMOSOME PARTITION PROTEIN MUKF;
73: 5hs5-A 6.5 2.5 68 116 10 MOLECULE: HTH-TYPE TRANSCRIPTIONAL REGULATOR SARX;
74: 8soj-B 6.5 2.2 64 428 11 MOLECULE: CST COMPLEX SUBUNIT CTC1;
75: 8f8u-A 6.5 2.3 61 297 20 MOLECULE: BIFUNCTIONAL LIGASE/REPRESSOR BIRA;
76: 2xco-A 6.5 2.3 67 636 7 MOLECULE: DNA GYRASE SUBUNIT B, DNA GYRASE SUBUNIT A;
77: 2p4w-B 6.5 2.7 63 198 21 MOLECULE: TRANSCRIPTIONAL REGULATORY PROTEIN ARSR FAMILY;
```

```
78: 1ldd-A 6.5 3.2 69 74 9 MOLECULE: ANAPHASE PROMOTING COMPLEX;
79: 1w7p-D 6.5 2.8 72 171 13 MOLECULE: VPS22, YPL002C;
80: 9bz0-f 6.5 2.6 64 232 9 MOLECULE: DNA-DIRECTED RNA POLYMERASE SUBUNIT;
81: 5u8o-B 6.4 2.7 75 360 13 MOLECULE: ZN-DEPENDENT HYDROLASE;
82: 3l9f-A 6.4 2.2 61 170 11 MOLECULE: PUTATIVE UNCHARACTERIZED PROTEIN SMU.1604C;
83: 2ns0-A 6.4 2.4 67 85 7 MOLECULE: HYPOTHETICAL PROTEIN;
84: 6juv-A 6.4 2.7 61 192 11 MOLECULE: SEGREGATION AND CONDENSATION PROTEIN B;
85: 2qlz-B 6.4 2.5 61 220 15 MOLECULE: TRANSCRIPTION FACTOR PF0095;
86: 5l0p-A 6.4 2.5 68 311 18 MOLECULE: TRANSCRIPTION FACTOR ETV6, TRANSCRIPTION FACTOR E
87: 9qel-J 6.4 2.3 71 108 7 MOLECULE: JETC;
88: 5zqh-A 6.4 2.6 66 101 12 MOLECULE: PADR FAMILY TRANSCRIPTIONAL REGULATOR;
89: 3lmm-D 6.4 2.2 63 556 14 MOLECULE: UNCHARACTERIZED PROTEIN;
90: 7bzh-A 6.4 1.9 57 59 9 MOLECULE: SUL7S;
91: 5e20-A 6.4 1.8 62 169 10 MOLECULE: TRANSCRIPTIONAL REGULATOR, MARR FAMILY;
92: 1ylf-C 6.4 2.2 61 119 10 MOLECULE: RRF2 FAMILY PROTEIN;
93: 3lwf-A 6.3 2.3 63 144 10 MOLECULE: PUTATIVE TRANSCRIPTIONAL REGULATOR;
94: 7xx7-U 6.3 3.2 70 213 13 MOLECULE: HISTONE H3.1;
95: 4ru7-B 6.3 2.0 59 99 12 MOLECULE: ASPA;
96: 2co5-B 6.3 3.1 67 94 10 MOLECULE: VIRAL PROTEIN F93;
97: 5xfo-A 6.3 2.8 68 315 19 MOLECULE: PHD FINGER PROTEIN 1;
98: 8ylg-A 6.3 3.2 66 161 15 MOLECULE: MARR FAMILY TRANSCRIPTIONAL REGULATOR;
99: 4b8x-A 6.3 1.9 62 140 16 MOLECULE: POSSIBLE MARR-TRANSCRIPTIONAL REGULATOR;
100: 9f27-A 6.3 2.1 63 94 13 MOLECULE: RPA32 SUBUNIT OF THE HETERO-OLIGOMERIC COMPLEX IN

0001 s001A   NPVLLA--LRDEAVEIL-A-TL-G-RVASVA-ELGEALI-G--RRG----V--TQRDPQIRRALGFA-AARAAYEVDW--TR---SPRRMRS-RR---H--G-D-L-MLFALEV--------
0002 5j6xA   MSAENE--IEMRICDYL-R-RH---GRSTVQ-DIFKELK------------------------lEKS-TVNRHLYSLQ--AS-----KQVFK-TV---E--D-K-R-PVWDLVE--------
0003 7qcdD   PVTGDA--TAKYLLQYI-L-SA-R-GICHEN-ALILALM-R--LETdastL--NTE---WSIQQWVD-KLNDYINAIN--VKlnlLGYKIIR-IN---H--GiS-N-RFFVYVNlasteetk
0004 2mh2A   -----G--APGIILRYL-Q-EQ-N-RPYSAQ-DVFGNLQ-K--EHG-----------------lGKA-AVVKALDQLA--QE-----GKIKE-KT---Y--G-K-Q-KIYFAD---------
0005 6qfdB   aRDLTA--FQKNILTVL-G-EE----ARYGL-AIKRELE-E--YYG----E-----------eVNHG-RLYPNLDDLV--NK-----GLVEK-SE--lD--K-R-T-NEYALTNegfdavvd
0006 5yadA   NKSLSL--LSTETXSIL-Q-DA-PaCCLPLF-KFIDIYE-K--KYG----H--------------kL-NVSD-LYKLT---------DTIAI-RE---Q--G-N-G-RLVCLLPsnq-----
0007 7rheA   -NVRRY--NERLLLKTL-R-RA---GSASKA-DLARLAN------------------------mTGT-AVGSIIASLA--DA-----KLIEF-A-----------A-SLIRLDPrgafgigv
0008 7vwvB   FMETQK--LISMVKEAL-E-KY-Q-YPLTAK-NIKVVIQ-K--EHN----V-----------vLPTG-SINSILYSNS---------ELFEK-ID-ktN--T-I-YpPLWIRKN--------
0009 2l02A   KKIVGA--NAGKVWHAL-N-E--A-DGISIP-ELARKVN------------------------lSVE-STALAVGWLA--RE-----NKVVI-ER---K--N-G-L-IEIYNEGhfdfsfgl
0010 1qgpA   LSIYQD--QEQRILKFL-E-EL-G--ATTAH-DLSGKLG------------------------tPKK-EINRVLYSLA--KK-----GKLQK-EA---G----T-P-PLWKIAVsd------
0011 1cf7B   gkGLRH--FSMKVCEKV-Q-RK---GTTSYN-EVADELV-S--EFT----nshLAADSAYDQKNIRR-RVYDALNVLM--AM-----NIISK-EK------------KEIKWIGlp------
0012 2xrnB   sIQVIA--RAASIMRAL-G-SH-P--GLSLA-AIAQLVG------------------------lPRS-TVQRIINALE--EE-----FLVEA-LG---P------A-GGFRLGPalgqlinq
0013 3cuqA   GDFYYE--LGVQIIEVC-L-AL-KgGLITLE-ELHQQVL-K--GRG----K-------faQDVS-QD-DLIRAIKKLK--AL----GTGFGI-IP---V--G-G-T-YLIQSVPaelnmdht
0014 1ku9A   ihGLNK--SVGAVYAIL-Y-LS-D-KPLTIS-DIXEELK------------------------ISKG-NVSXSLKKLE--EL-----GFVRK-VW---I-gE-R-K-NYYEAVDgfssikdi
0015 8e4yA   SYDRiM--STHIVACLL-LyRH-R-QGIDLS-TLVEDFF-V--MKE------lardfdlgfsgNSED-VVMHAIQLLG---------NCVTI-TH---T-rN-D-E-FFITPSTtvpsvfel
0016 4ad9A   ISHRNI--REQQILTLF-R-EN-FeKSFTVM-ELVKIIY-K----N----T--PE----NLHEMAKH-NLLLHLKKLE--KE-----GKIFS-NT---D--P---D-KKWKAHL--------
0017 3elkA   RILHGL--ITLYILKEL-V-KR----PXHGY-ELQKSXF-E--TTG----Q---------ALPQ--G-SIYILLKTXK--ER-----GFVIS-ES---S--V-Q-L-TVYHITDagkkflxd
0018 7kbfK   ----pP--TLSMVVEVL-K-KN-T--GTSVQ-AIRTRIL-S--AHP----T-------vDPLRL-KF-LLRTALNKGL--EK-----GILIRpLN---S--S-gaT-GRFKLAK--------
0019 2od5A   SXKTVR--IREKIKKFL-G----D-RPRNTA-EILEHIN-S--TXR----H-----------gTTSQ-QLGNVLSKD----------KDIVKvGY---I--K-D-I-CEWATRNwvaehcpe
0020 8jxkD   ---EFM--LELAILGLL-I-ES----PMHGY-ELRKRLT-G--LLG----A--F------RAFS-YG-SLYPALRRMQ--AD-----GLIAE-NA---A--P-agR-RVYQLTDkgrrrfge
0021 8iueP   --DPVE--IENRIIELC-H-QF-P--GITDQ-VIQNE-M-P--HI-------------------eAQ-QRAVAINRLL--SM-----GQLDL-LR---S--N-T-G-LLYRIKDsqnagkmk
0022 4ijaA   --PMND--NEKRVLREI-Y-NH-H--NISRT-QISKNLE------------------------iNKA-TISSILNKLK--YK-----SLVNE-V------gR-K-P-ILLKVNHlygyfisl
0023 6qpqD   -----K--AIVQMAKIL-R-KE-L--EVIFT-DVLKSQA-N--------------tepENITKREAS-RGFFDILSLA--TE-----GCIGL-SQ---T--E-G-N-IKIDAKPalferfi-
0024 5y6iB   VRSA-E--VGTDILKAL-A-EL-S-PATSLS-RLAEHVG------------------------MPAS-KVHRYLQALI--AS-----GFAVQ-DA---S------T-NHYSLGRealrvgla
0025 8xt8B   ngydTT--DHYALLRFL-W-EQ---DGISQI-DLCEKSC------------------------KDKS-NTTRILDVMK--NK-----GLIVR-KV---D-dR-R-K-FQIFLTDlgreleep
0026 7cv0A   -ILGEE--RRSLLIKWL-K-AS-D--PLTGA-ELAKRTN------------------------VSRQ-VIVQDVSLLK--AK----NHPILA-TA------------QGYIYMKeantvqaq
0027 7y43A   klaNPL--YTEWILEAI-K-KV-K--rPSEE-RICNAVS-S--SHG-----------------lDRK-TVLEQLELSV--KD-----GTILK-VS---N--K-G-L-NSYKDPDnpgria--
0028 2zmeB   sHKEEE--MVASALETV-S-EK-G--SLTSE-EFAKLVG------------------------MSVL-LAKERLLLAE--KM-----GHLCR-DD---S--V-E-G-LRFYPNLfmtqs---
0029 3f72B   lkaiDE--NRAKITYAL-C-QD-E--ELCVC-DIANILG------------------------VTIA-NASHHLRTLY--KQ-----GVVNF-RK---E--G-K-L-ALYSLGGeairqimm
0030 6sj9A   GASRTE--RLLNLLLAL-L-NT-K--GLPRA-VLREKVY----HDS----A--------DNDVAFGR-XFERDKVDLK--QF---G-FEIET-L----X--D-P-AsARYRIGKdsnrlpdv
0031 4mtdD   nVRLTP--QRLEVLRLM-S-LQ-D-GAISAY-DLLDLLR-E--AEP----Q------------AKPP-TVYRALDFLL--EQ-----GFVHK-VE---S------T-NSYVLCHlfdqptht
0032 3bpxA   ghlnTD--AQVACLLRI-H-RE---PGIKQD-ELATFFH------------------------VDKG-TIARTLRRLE--ES-----GFIER-EQdpeN--R-R-R-YILEVTRrgeeiipl
0033 4y66C   -TSLDE--KKERLLEEM-L-KR-G-EIYSNK-TIETLSK-P--TG-----------------iSSMV-I-KNVLQALV--NE-----DLVDT-DK---I--G-A-S-TYYWCFAskrsqaar
0034 4i99C   --DIEK--YVEELYKVV-K-KI-Y--PIKFW-DLVP------------------------dVEPKIIA-RTFLYLLFLE--NM-----GRVEI-IQ---E--E-G-E-ILVVPM---------
0035 2cweA   ikvmED--TRRKILKLL-R----N-KEMTIS-QLSEILG------------------------kTPQ-TIYHHIEKLK--EA-----GLVEVkRT---E--M-V-E-KYYGRTAdvfyinly
0036 2vqcA   tlNSYK--XAEIXYKIL-E-KK---GELT-----LEDIL-A--QFE-------------iSVPSAYN-IQRALKAICE--RH----pDECEV-QY---K--N-R-K-TTFKWIK--------
0037 3gfiA   ELNLSY--LDFLVLRAT-S----D-GPKTMA-YLANRYF------------------------vTQS-AITASVDKLE--EM-----GLVVR-VR---D-dR-R-K-ILIEITEkgletfnk
0038 7z8bC   EKRRNL--LNCLIVRIL-K-AH-G--GLHID-QLVCLVL-E--AWQ----KgglvsslgkgsaCSST-DVLSCILHLL--GK-----GTLRR-HD---D-----rP-QVLSYAV--------
0039 1xmkA   SLDMAE--IKEKICDYL-F-NV---SDSSAL-NLAKNIG-----------------------LTKAR-DINAVLIDME--RQ-----GDVYR-QG---T----T-P-PIWHLTDkkrermqi
0040 3k69A   XKLDFS--VAVHSILYL-D-AH-R--KVASR-ELAQSLH------------------------LNPV-XIRNILSVLH--KH-----GYLTG-TV---G----K-N-GGYQLDLaladxnlg
0041 3u2rA   QFELSA--QQYNTLRLL-R-SV-HpEGXATL-QIADRL---------------------------AP-DITRLIDRLD--DR-----GLVLR-TR---K-nR-R-V-VEVALTDaglkllkd
0042 6zvhy   ----fN--WKGTIKAIL-K-QA-PdNEITIK-KLRKKVL-A--QYY--tvT--DEHH-rSEEEL-LV-IFNKKISKN----------PTFKL-LK------------DKVKLVK--------
0043 5jbrA   TWQLPR--TTGRTYGYL-L-LQ-S--ATSFQ-EIGADLG------------------------lSPG-AVSTSVRELV--AW-----GLART-IP---Q-gS-R-R-LLVEAAGgfeqllaa
0044 1xsdA   kqveSM--AEWDVMNII-W-DK---KSVSAN-EIVVEIQ-K--YKE-----------------VSDK-TIRTLITRLY--KK-----EIIKR-YK---S--E-N-I-YFYSSNIkeddikmk
0045 1yyvA   VLKHVTsrWGVLILVAL-R----D-GTHRFS-DLRRXXG-G----------------------VSEX-XLAQSLQALE--QD-----GFLNR-VS---Y--P-P-H-VEYSLTPlgeqvsdx
0046 6ahtA   CENLND---GIWALRVL-Y-AE---GAXNKE-KLWDYIN-Q--YHKdyqiendYEGK---kilpsRY-ALDIXTARLE--GA-----GLISF-KA---I--G-R-V-RIYDVTDlgnvlike
0047 4fhtB   SEETTS--PQYAVLNAL-V-AE---PGLDQR-TVGERVG------------------------lDRS-TIAEVVSRLG--RR-----GLLDK-VR---D-dG-R-R-SLLRLTDeglrvhrr
0048 7b0cA   LTKFTD--LALRSLMRL-A-VV-R--PLATR-EVAEVVG------------------------vPYT-HAAKAITRLQ--HL-----GVVEA-RR---G--R---G-GGLTLTDlgrrvsvg
0049 2g9wA   kltrGD--LERAVXDHL-W-SR-T--PQTVR-QVHEALS-A--RRD-----------------LAYT-TVXAVLQRLA--KK-----NLVLQ-IR----------A-HRYAPVHgrdelvag
0050 3bddB   kqlgSL--TRYSILQTL-L-KD---APLHQL-ALQERLQ------------------------IDRA-AVTRHLKLLE--ES-----GYIIR-K-----------E-VLVWPTEqarealit
0051 1sfuA   tVNDAE--IFSLVKKEV-L-SL-N--YTTAI-SLSNRLK------------------------iNKK-KINQQLYKLQ--KE-----DTVKM-VP---S----N-P-PKWFKNYnc------
0052 5n35A   --VNQK--EIEIAIEYF-K-NY---ISVGEI-VATMDLK-A--RG---------------------isNPQAVISKLI--EM-----GIIEK-GE------------GCYNLVR--------
0053 5eriA   kelkQK--GQFTFLTRI-C-EN---PGINLV-ELSNMLK------------------------VDKA-TTTKAIQKLI--KA-----GYVDK-KQdkfD--K-R-G-YNLTPTDkslevyel
0054 3f8fA   EMLRAQ--TNVILLNVL-K-QG----DNYVY-GIIKQVK-E--ASN----G---------emeLNEA-TLYTIFKRLE--KD-----GIISS-YW---G--D-R-R-KYYRLTEighenmrl
0055 3r4kA   -XGTVS--KALTLLTYF-N--hgR-LEIGLS-DLTRLSG------------------------xNKA-TVYRLXSELQ--EA-----GFVEQ-VE---G------A-RSYRLGPqvlrlaal
0056 7c0jB   ----pT--YSEMIAAAI-R-AE-G--GSSRQ-SIQAYIK-S--HYK-----------------vnKK-EINRVLYSLL--AA-----GVLKQ-TG---V------P-GSWALA---------
0057 3w6kC   mGALKP--AKAIVEALL-F-AA-G--GLSLS-QIAAVLE------------------------VSEL-EAKAVIEELQ--QD---CRrQLVE-LG------------GVFLLATkkehapyl
```

```
0058 2ethA   eisdKT--TELYAFLYV-A-LF---GPKKXK-EIAEFLS------------------------tTKS-NVTNVVDSLE--KR-----GLVVR-EXdpvD--R-R-T-YRVVLTEkgkeifge
0059 1fokA   KEYVRT--RRALILEIL-I-KA---GSLKIE-QIQDNLK-K--LG----------------fdEVIE-TIENDIKGLI--NT----GIFIEI-KG------------RFYQLKDhilqfvip
0060 5udb4   SVIQRK--LQEDLSREI-M-NV-LsDSMSFN-ELIKQIN-E---HS----Q--D------RVES--S-DIQEALSRLQ--QE-----DKVIV-LG----------E-GVRRSVR--------
0061 8as8C   rrqrNL--EQSLLVAIL-R-QH-FgtQIAID-DLLPQLQ-I--YLG----D-------pGSESKERT-RLLTLLDQLK--GH-----GLVTS-PD---A------H-ERIVIRPiiahladp
0062 2fxaB   pYDLNI--NEHHILWIA-Y-QL-N--GASIS-EIAKFGV------------------------xHVS-TAFNFSKKLE--ER-----GYLRF-SK---R-dK-R-N-TYVQLTEegtevfws
0063 2lnbA   PGREGH--LEQRILQVL-T-EA-G-SPVKLA-QLVKECQ------------------------aPKR-ELNQVLYRMK--KE-----LKVSL-TS----------P-ATWCLGGtdpe----
0064 4hqeB   LGRS----WNGLIINYL-S-RC-NdCSAHFS-DMKRDLK-T----------------------ITPR-ALSLKLSELA--QW-----ELVEK-QI---I--S-V-Q-IIYVLTEkgkalaea
0065 3bj6A   lregTV--GQRAILEGL-S-LT---PGATAP-QLGAALQ------------------------xKRQ-YISRILQEVQ--RA-----GLIER-RT---N-hA-R-S-HRYWLTPrgeaiita
0066 4hw0A   kRGTXE--IXFDILRNC-E------PKCGIT-RVIYGAG------------------------iNYV-VAQKYLDQLV--KV-----GALNI-KT---E--N-D-R-KIYEITEkgkllrth
0067 2zkzC   lktxHP--XRLKIVNEL-Y-KH---KALNVT-QIIQILK------------------------LPQS-TVSQHLCKXR--------gKVLKR-NR---Q--G-L-E-IYYSINNpkvegiik
0068 8uucA   egSRRQ--KRAELVRLV-L-AE---PGIGID-ELAERLD-A--FER----D-----agrkGVDA--A-TFTSIVADLV--AE-----GFFRR-EG------------DAFFA----------
0069 2dqlA   ylCQEV--AICYILYVL-L-Q----GESYGT-ELIQQLE-T--EHP----T-----------yRLSDtVLYSAIKFLE--DN-----RAITG-YW---K--K-P-R-RMYQVSPewqhqaed
0070 7xjgJ   gsftlY--AERFLLPCI-Y-LL-D--SVNYR-TLCELAF-K--AIK----Q---------DDVLSKI-IVRSVVSRLI--NE-----RKILQ-MT------------DGYQVTAlgasyvrs
0071 3s93B   ERIQEC--LRKEIRSLL-I-ST-K--GLSPQ-ELEKEYL-L--MVG----N-------hlplrilgyrsTMELVLDMP---------DVVRV-CP---G--A-G-T-VILKAIP--------
0072 2eshA   GFRGWW--LASTILLLV-A-EK----PSHGY-ELAERLA-E--FGI----E--------ipgighmG-NIYRVLADLE--ES-----GFLST-EW---D--T-P-R-KIYRITPqgklylre
0073 9gm7C   PVERLA--FLMAIAVLN-S-ERlD-GEMSEG-ELIDAFR-E--VCK-gfeQ--TAE---SVAVRANN-AINDMVRQKL-----------LNR-FT---S-aD-G-N-AIYRLTPlgisisdy
0074 5hs5A   SLNDLA--VLDLTXKHC-K----D-EKVLXQ-SFLKTAX-D--ELD-----------------LSRT-KLLVSIRRLI--EK-----ERLSK-VR---S-dE-R-K-IYIYLNNddiskfna
0075 8sojB   REDK-D--LHRKIHRIIqQ-DC-QkpGCHFL-HILACAR-L-sIRP----G------------LSEA-VLQQVLELLE--DQ-----SDIVS-TM----------E-HYYTA----------
0076 8f8uA   KDHT----IPLTLISIL-A-D--G-EFHSGE-QLGEQLG------------------------MSRA-AINKHIQTLR--DW----GVDVFT-VP----------G-KGYSLPEpihlldek
0077 2xcoA   vrdgKP--VHRRILYGL-N-EQ-G-MYKKSA-RIVGDVM-G-kYHP----H------------GDSS-IYEAMVRMAQdfSY---RYPLVDG-QG---N----mrY-TEARMTKitlellrd
0078 2p4wB   LDVLNE--TRRRILFLL-T-K----RPYFVS-ELSRELG------------------------vGQK-AVLEHLRILE--EA-----GLIES-RV---E--K-P-R-KYYMIKKglrleill
0079 1lddA   ELTLQR--SLPFIEGML-T-NL---GAMKLH-KIHSFLK-I--TVP----K--D---wgyNRIT-LQ-QLEGYLNTLA--DE-----GRLKY-IA---N--------GSYEIV---------
0080 1w7pD   ELFLDE--IAREIYEFT-L-SE-FyMIITLV-DLYAMYN-K-sMRI----G---------tglISPM-EMREACERFE--HL---GLNELKL-VK---V--N-R-I-LCVTSE---------
0081 9bz0f   IKYFVK--GRQAVLSMV-R-KA-KyRELLLS-ELLGR-R-A--PV-------------------vvrlGLTYHVHDLI--GA-----QLVDC-IS---T--T-S-G-TLLRLPEt-------
0082 5u8oB   RAHHDA--RLDEVRVAC-A----E-QPMSAA-DIVPIMF-R----R----R--EL--DIHQMTFALG-EAIAHLNLLW--LA-----GELVR-EQ---G-dD-G-V-LRFRIAR--------
0083 3l9fA   ----mQ--GKDIILGIL-S-KK----ERSGY-EINDILQnQ--LSY----F----------ydgtyG-MIYPTLRKLE--KD-----GKITK-EV---V--I-N-K-NIYAITEsgkkelas
0084 2ns0A   tvSDRE--LEECIRALL-D-AR-A--SICPS-DVARAVA-P------------------dDWRPLXE-PVREAAGRLA--DA-----GEVEV-TQ---KgaR-G-P-IRIRWTRtd------
0085 6juvA   kPELRA--GELKTLALI-A-YL---QPVEQS-KIIKLRG---------------------------S-QAYEHIKKLL--EX-----GLIYA-EP---Y--E-R-T-KLLGTTQkfaelygf
0086 2qlzB   EIGD-X--TQLAILHYL-L-L--N-GRATVE-ELSDRLN------------------------LKER-EVREKISEXA--RF-----VPVKI-IN----------D-NTVVLDEdqilr---
0087 5l0pA   KVTL----PRLKILEVL-Q-EP-DnHHVSAE-DLYKRLI-D--MGE----E------------IGLA-TVYRVLNQFD--DA-----GIVTR-HN---F--E-GgK-SVFELT---------
0088 9qe1J   TADSAA--HYRTILRYF-Y-HQ-HrDFIAPE-ELLEHMR-SipAFA----D------------FQED-QLHQQLAQLV--KW-----NNLIA-RQ---D--M-K-R-FRYQCTPytveierm
0089 5zqhA   QLLKGV--LEGCVLDMI-G-QK----ERYGY-ELVQTLR-E-aGFD----T--------------ivG-TIYPLLQKLE--KN-----QWIRG-DM---R--P-D-R-KYFSLMKegeervsv
0090 3lmmD   stDQAE--LTNAAXLWL-S-EV---GDLATS-DLXAXCG------------------------VSRG-TAKACVDGLV--DE-----ERVVA-VG---G--G-R-S-RRYRLVE--------
0091 7bzhA   ----ED--VKQSVEKII-K-DR---EWVTFN-DLLKYIP------------------------YPAP-EVYDALSQLI--KE-----NKVGR-RG------------RYFYYIKr-------
0092 5e20A   kslnST--EKFAILHEL-M-CL-G-GESTPH-TLARRIV------------------------FEPH-SVSAIVSRME--KD-----GLIIK-TK---D-kK-H-M-VRIKLSEkaidtfyq
0093 1ylfC   ISSRFS--IAVHILSIL-K-NN-P--lCTSD-YXAESVN------------------------tNPV-VIRKIXSYLK--QA----GFVYVN-RG----------P-GGAGLLKdlheitll
0094 3lwfA   ITTKGR--YGLTITLEL-A-KR-I--PISLR-SIAQDKN------------------------lSEH-YLEQLIGPLR--NA-----GIVKS-IR---G----A-H-GGYVLNGdpekitag
0095 7xx7U   NQPG-K--YSQLVVETI-R-RL-G--GSSLA-KIYTEAK----KVP----W-------fDQQNG-RT-YLKYSIKALV--QN-----DTLLQ-VK---G----A-N-GSFKLNRkkleggge
0096 4ru7B   ----lT--PRAYIIVHL-L-KV---GKAKAS-EISENTQ------------------------iPYQ-TVIQNIRWLL--AE-----GYVVK-EQ---K--G-E-E-IYYKLTDkgkqlata
0097 2co5B   KYMR-I--NYYIILKVL-V-IN-G-SRLEKK-RLRSEIL-K--RFD-------------iDISD---gvlYPLIDSLI--DD-----KILRE-EE---A--P-D-G-KVLFLTEkgmkefee
0098 5xfoA   vrrlLR--WVDVAHLVL-Y-HL-SkKYFDFDrEILPFTS-E--NWD------llgelsdTPKGERSS-RLLSALNSHK---------DRFIS-GR---E--I-R-K-CLFGLHArmpppve-
0099 8ylgA   RYGLQP--GEFDVLATL-R-RS-GpYALTPT-ALYDAAM-I-------------------SSGSMTN-RIDRLEKA-----------GWVER-RAnpaD--G-R-G-TLVALTSagralidd
0100 4b8xA   pygLTF--ARYEALVLL-T-FS-KsGELPMS-KIGERLM------------------------VHPT-SVTNTVDRLV--RS-----GLVAK-RP---N--P---G-TLATITDkgrevvea
0101 9f27A   NELLEK--AKEDILNIL-R-QK-R-TAISRK-YILKKLG-D--KY-------------------dEE-TIDDAITELL--AQ-----GEIYE-PE----------T-GYYKLL---------

0001 s001A   LHHHHH--HHHHHHHHH-H-HL-L-LEEEHH-HHHHHHH-H--HHL----L--LLLLHHHHHHHHHH-HHHHHHHHHH--LL---LLLLEEE-EE---E--L-L-E-EEEEELL--------
0002 5j6xA   LLHHHH--HHHHHHHHH-H-HH---LLLLHH-HHHHHLL------------------------lLHH-HHHHHHHHHH--HL-----LLEEE-EL---L--L-L-L-LEEEELL--------
0003 7qcdD   LHHHHH--HHHHHHHHH-H-HL-L-LEEEHH-HHHHHHH-H--HHHhhllL--LLL---LLHHHHHH-HHHHHHHHHH--HHhhhLLEEEEE-EE---L--LlL-L-EEEEEEEllllhhhh
0004 2mh2A   -----L--HHHHHHHHH-H-HH-L-LLEEHH-HHHHHHH-L--LLL-----------------lLHH-HHHHHHHHHH--HH-----LLEEE-EE---E--L-L-E-EEEEEL---------
0005 6qfdB   hLLLLH--HHHHHHHHH-H-HL----LEEHH-HHHHHHH-H--HHL----L-----------lLLHH-HHHHHHHHHH--HL-----LLEEE-EE--eE--L-L-E-EEEEELHhhhhhhhh
0006 5yadA   HHHHHH--HHHHHHHHH-H-LL-HhHEEEHH-HHHHHHH-H--HHL----L--------------lL-LHHH-HLLLL---------LLEEE-EL---L--L-L-L-LEEEELHhhl-----
0007 7rheA   -LHHHH--HHHHHHHHH-H-HH---LLEEHH-HHHHHHH------------------------lLHH-HHHHHHHHHH--HL-----LLEEE-L-----------L-LEEEELLllleeeee
0008 7vwvB   LLHHHH--HHHHHHHHH-H-HL-L-LLLLHH-HHHHHHH-H--HHL----L-----------lLLHH-HHHHHHHHLL---------LLEEE-LL-llL--L-L-LlLLEEELL--------
0009 2l02A   HHHHHH--HHHHHHHHH-H-H--L-LLLLHH-HHHHHHL------------------------lLHH-HHHHHHHHHH--LL-----LLEEE-EE---E--L-L-E-EEEEEHHhlllllll
0010 1qgpA   LHHHHH--HHHHHHHHH-H-HH-L--LEEHH-HHHHHHL------------------------lLHH-HHHHHHHHHH--HH-----LLEEE-EL---L----L-L-LEEEELLll------
0011 1cf7B   llHHHH--HHHHHHHHH-H-HH---LEELHH-HHHHHHH-H--HHH----lllLHHHLHHHHHHHHH-HHHHHHHHHH--HL-----LLLLL-LL------------LLEELLLll------
0012 2xrnB   lLHHHH--HHHHHHHHH-H-LL-L--LEEHH-HHHHHLL------------------------lLHH-HHHHHHHHHH--LL-----LLEEE-LH---H------H-LEEEELLhhhhhhhh
0013 3cuqA   HHHHHH--HHHHHHHHH-H-HH-HlLEEEHH-HHHHHHH-H--LLL----L-------llLLLL-HH-HHHHHHHHHH--HH----LLLLEE-EE---E--L-L-E-EEEELLLllllhhhh
0014 1ku9A   hlLLLH--HHHHHHHHH-H-HL-L-LLEEHH-HHHHHHL------------------------LLHH-HHHHHHHHHH--HL-----LLEEE-EL---L-lL-L-L-LEEEELLhhhhhhhh
0015 8e4yA   EELLlL--HHHHHHHHH-HhLL-L-LLEEHH-HHHHHHH-H--HHH------hhlllllllllLHHH-HHHHHHHHLL---------LLEEE-EE---L-lL-L-L-EEEEELLlhhhhhhh
0016 4ad9A   HHHHHH--HHHHHHHHH-H-HL-LlLLLLHH-HHHHHHL-L----L----L--LH----HHHHHHHH-HHHHHHHHHH--HL-----LLEEE-LL---L--L---L-LLEEELL--------
0017 3elkA   HHHHHH--HHHHHHHHH-H-HL----LEEHH-HHHHHHH-H--HHL----L---------LLLL--L-HHHHHHHHHH--HH-----LLEEE-EE---E--E-E-E-EEEEELHhhhhhhhh
0018 7kbfK   ----lL--HHHHHHHHH-H-HL-L--LEEHH-HHHHHHH-L--LLL----L-------lLLLLH-HH-HHHHHHHHHH--HL-----LLEEEeEE---L--L-llL-EEEEELL--------
0019 2od5A   HHHHHH--HHHHHHHHL-L----L-LLEEHH-HHHHHHH-L--LLL----L-----------lLLHH-HHHHHHHHL----------LLEEEeEE---E--E-E-E-EEEEEHHhhhhhlll
0020 8jxkD   ---LHH--HHHHHHHHH-H-HL----LEEHH-HHHHHHH-H--HLL----L--L------LLLL-LL-LHHHHHHHHH--HL-----LLEEE-LL---L--L-llL-LEEEELHhhhhhhhh
0021 8iueP   --LHHH--HHHHHHHHH-H-HL-L--LLLHH-HHHHH-L-L--LL-------------------lHH-HHHHHHHHHH--HH-----LLEEE-EE---E--L-L-E-EEEEELHhhllllll
0022 4ijaA   --LLHH--HHHHHHHHH-H-HL-L--LLLHH-HHHHHHL------------------------lLHH-HHHHHHHHHH--HL-----LLEEE-L------lL-L-L-LLEEELHhhleeeee
0023 6qpqD   -----L--HHHHHHHHH-H-HH-H--LEEHH-HHHHHHH-L--------------llhHHLLHHHHH-HHHHHHHHHH--HL-----LLEEE-EL---L--L-L-L-LEEEELHhhhllll-
0024 5y6iB   LLHH-H--HHHHHHHHH-H-HL-L-LLEEHH-HHHHHHL------------------------LLHH-HHHHHHHHHH--HH-----LLEEE-LL---L------L-LEEEELHhhhhhhhh
0025 8xt8B   llllLH--HHHHHHHHH-H-HL---LLLLHH-HHHHHHL------------------------LLLH-HHHHHHHHHH--LL-----LLEEE-EE---L-lL-L-L-EEEEELHhhhhlhhh
0026 7cv0A   -LHHHH--HHHHHHHHH-H-HL-L--LLLHH-HHHHHHL------------------------LLHH-HHHHHHHHHH--HL----LLLEEE-EL------------LEEEELLllllllee
0027 7y43A   lllLHH--HHHHHHHHH-H-HH-H--lLLHH-HHHHHHH-H--HHL-----------------lLHH-HHHHHHHHHH--HH-----LLLEE-EE---E--L-L-E-EEEELLLllllll--
0028 2zmeB   lLLHHH--HHHHHHHHH-H-HH-L--LLLHH-HHHHHHL------------------------LLHH-HHHHHHHHHH--HH-----LLEEE-EE---E--L-L-E-EEEEELHhhlll---
0029 3f72B   hhhhLH--HHHHHHHHL-L-LL-L--LEEHH-HHHHHHL------------------------LLHH-HHHHHHHHHH--HL-----LLEEE-EE---L--L-L-L-EEEEELLhhhhhhhh
0030 6sj9A   LHHHHH--HHHHHHHHH-H-HL-L--LEEHH-HHHHHHL----LLL----L--------LLHHHHHH-HHHHHHHHHH--HL---L-LLLEE-E----L--L-L-LlLEEELLLllllllee
0031 4mtdD   lLLLLH--HHHHHHHHH-H-LL-L-LLEEHH-HHHHHHH-H--HLL----L------------LLHH-HHHHHHHHHH--HL-----LLEEE-EL---L------L-LEEEELLllllllll
0032 3bpxA   hhhlLH--HHHHHHHHH-H-LL---LLLLHH-HHHHHHL------------------------LLHH-HHHHHHHHHH--HL-----LLEEE-EEellE--E-E-E-EEEEELHhhhhhhhh
0033 4y66C   -LHHHH--HHHHHHHHH-H-HH-L-LLLLLL-HHHHHHH-H--HL-----------------lLLLL-H-HHHHHHHH--HL-----LLLEE-EE---L--L-L-L-EEEELLLlhhhhhhh
0034 4i99C   --LHHH--HHHHHHHHH-H-HH-H--LEEHH-HHLL-----------------------lLLHHHHH-HHHHHHHHHH--HL-----LLEEE-EL---L--L-L-L-LEEEEL---------
0035 2cweA   hhhhLH--HHHHHHHHH-L----L-LLLLHH-HHHHHHL------------------------lLHH-HHHHHHHHHH--HL-----LLEEEeEE---E--E-E-E-EEEEELLleeeelll
0036 2vqcA   llLHHH--HHHHHHHHH-H-HH---LEEL-----HHHHH-H--HHL-------------lLHHHHHH-HHHHHHHHHH--HL----lLLEEE-EE---L--L-L-L-EEEEELL--------
```

```
0037 3gfiA   HHLLLH--HHHHHHHHH-L----L-LLEEHH-HHHHHLL-------------------------lLHH-HHHHHHHHHH--HL-----LLEEE-EE---E-eE-E-E-EEEEELHhhhhhhhh
0038 7z8bC   HHHHHH--HHHHHHHHH-H-LL-L--LEEHH-HHHHHHH-H--HHH----LlhhhhhlllllllLLHH-HHHHHHHHHH--HH-----LLEEE-LL---L-----lL-LEEEELL--------
0039 1xmkA   LHHHHH--HHHHHHHHH-H-HL---LLEEHH-HHHHHHL------------------------HHHHH-HHHHHHHHHH--HL-----LLEEE-EL---L----L-L-LEEEELHhhhlllll
0040 3k69A   LLHHHH--HHHHHHHHH-H-LL-L--LLLHH-HHHHHHL-------------------------LLHH-HLHHHHHHHH--HL-----LLLEE-EL---L----L-L-LEEELLLlhhhllhh
0041 3u2rA   LLLLLH--HHHHHHHHH-H-HH-LlLLEEHH-HHHHHL----------------------------LL-HHHHHHHHHH--HL-----LLEEE-EE---E-eE-E-E-EEEEELHhhhhhhhh
0042 6zvhy   ----lL--HHHHHHHHH-H-LL-HhHEEEHH-HHHHHHH-H--HHH--hhL--LLLL-lLHHHH-HH-HHHHHHHLL-----------LLEEE-LL------------LEEEELL--------
0043 5jbrA   HHLLLH--HHHHHHHHH-H-LL-L--LEEHH-HHHHHHL-------------------------lLHH-HHHHHHHHHH--HL-----LLEEE-EE---E-lE-E-E-EEEEELLllhhhhhh
0044 1xsdA   llllLL--LLHHHHHHH-H-HH---LLEEHH-HHHHHHH-L--LLL------------------LLHH-HHHHHHHHHH--HL-----LLEEE-EE---L--L-L-L-EEEEELLlhhhhhhh
0045 1yyvA   HHHHHHlhHHHHHHHHH-H----H-LLEEHH-HHHHHLL-L-----------------------LLHH-HHHHHHHHHH--HH-----LLEEE-EE---E--L-L-E-EEEEELHhhhhhhhh
0046 6ahtA   HHHHHH---HHHHHHHH-H-HH---LLEEHH-HHHHHHH-H--HHHhhhhhhlLLLL----lllllHH-HHHHHHHHHH--HL-----LLEEE-EE---E--L-L-E-EEEEELHhhhhhhhh
0047 4fhtB   LLLLLH--HHHHHHHHH-H-HL---LLLLHH-HHHHHHL-------------------------lLHH-HHHHHHHHHH--HH-----LLEEE-EE---L-lL-L-L-EEEEELHhhhhhhhh
0048 7b0cA   LLHHHH--HHHHHHHHH-H-LL-L--LLLHH-HHHHHHL-------------------------lLHH-HHHHHHHHHH--HH-----LLEEE-LL---L--L---L-LLEEELHhhhhllhh
0049 2g9wA   lhhhLH--HHHHHHHHH-H-LL-L--LEEHH-HHHHHHL-L--LLL------------------LLHH-HHHHHHHHHH--HL-----LLEEE-EL----------L-LEEEELLlhhhhhhh
0050 3bddB   hhhlLH--HHHHHHHHH-H-HL---LLEEHH-HHHHHHL-------------------------LLHH-HHHHHHHHHH--HL-----LLEEE-L-----------L-LEEEELHhhhhhhll
0051 1sfuA   lLLLHH--HHHHHHHHH-H-LL-L--EELHH-HHHHHLL-------------------------lLHH-HHHHHHHHHH--HL-----LLEEE-EL---L----L-L-LEEEELLll------
0052 5n35A   --LLHH--HHHHHHHHH-H-HL---LEEEHH-HHHHHHH-H--LL---------------------llLHHHHHHHHHH--HL-----LLEEE-LL------------LEEEELL--------
0053 5eriA   llllLL--LHHHHHHHH-H-HL---LLEEHH-HHHHHHL-------------------------LLHH-HHHHHHHHHH--HL-----LLEEE-EElllL--L-L-L-EEEEELHhhhhhhhh
0054 3f8fA   HHHHHH--HHHHHHHHH-H-HL----LLLHH-HHHHHHH-H--HLL----L----------lllLLHH-HHHHHHHHHH--HL-----LLEEE-EE---L--L-L-L-EEEEELHhhhhhhhh
0055 3r4kA   -LLHHH--HHHHHHLLL-L--llL-LEEEHH-HHHHHHL-------------------------lLHH-HHHHHHHHHH--HL-----LLEEE-LL---L------L-LEEEELLlhhhhhhh
0056 7c0jB   ----lL--HHHHHHHHH-H-HL-L--LEEHH-HHHHHHH-H--HLL------------------llHH-HHHHHHHHHH--HL-----LLEEE-LL---L------L-LEEEEL---------
0057 3w6kC   lLLLHH--HHHHHHHHH-H-HH-H--LLLHH-HHHHHHL-------------------------LLHH-HHHHHHHHHH--HH---HLlEEEE-EL------------LEEEEEElhhhhhhh
0058 2ethA   hhhhLH--HHHHHHHHH-H-HH---LLLLHH-HHHHHLL-------------------------lLHH-HHHHHHHHHH--HL-----LLEEE-EElllL--L-L-L-EEEEELHhhhhhhhh
0059 1fokA   HHHHHH--HHHHHHHHH-H-HL---LLEEHH-HHHHHHH-H--HL-----------------llLLHH-HHHHHHHHHH--HL----LLLEEE-EL------------LEEEELLllllllll
0060 5udb4   LLLHHH--HHHHHHHHH-H-HH-HlLLLLHH-HHHHHLL-L---LL----L--L-------LLLL--H-HHHHHHHHHH--HL-----LLEEL-LL----------L-LLLEELL--------
0061 8as8C   llllLH--HHHHHHHHH-H-HH-HllEEEHH-HHHHHHH-H--HHL----L--------lLLHHHHHH-HHHHHHHHHH--HL-----LLLLL-LL---L------L-LEEELLLlhhhhllh
0062 2fxaB   hHLLLH--HHHHHHHHH-H-HH-L--LEEHH-HHHHHHL-------------------------lLHH-HHHHHHHHHH--HH-----LLEEE-EL---L-lL-L-L-LEEEELHhhhhhhhh
0063 2lnbA   LLHHHH--HHHHHHHHH-H-HH-L-LLEEHH-HHHHHHL-------------------------lLHH-HHHHHHHHHH--HL-----LLEEE-EE----------L-LEEEELLllll----
0064 4hqeB   HLLL----LHHHHHHHH-L-LL-HhHEELHH-HHHHHHL-L-----------------------LLHH-HHHHHHHHHH--HL-----LLEEE-EE---E--L-L-E-EEEEELHhhhhhhhl
0065 3bj6A   hhllLH--HHHHHHHHH-H-HL---LLEEHH-HHHHHHL-------------------------lLHH-HHHHHHHHHH--HL-----LLEEE-EL---L-lL-L-L-LEEEELHhhhhhhhh
0066 4hw0A   lLLHHH--HHHHHHLLL-L------LEELHH-HHHHHHL-------------------------lLHH-HHHHHHHHHH--HL-----LLEEE-EE---E--L-L-E-EEEEELHhhhhhhhh
0067 2zkzC   hhhhLH--HHHHHHHHH-H-HH---LLEEHH-HHHHHHL-------------------------LLHH-HHHHHHHHHL--------lLLEEE-EE---E--L-L-E-EEEEELLhhhhhhhh
0068 8uucA   llLHHH--HHHHHHHHH-H-HL---LLEEHH-HHHHHHH-H--HHH----H------llllLLLH--H-HHHHHHHHHH--HH-----LLLEE-EL------------LEEEL----------
0069 2dqlA   elLHHH--HHHHHHHHH-L-L----LLLLHH-HHHHHHH-H--HLL----L------------eELLHhHHHHHHHHHH--HL-----LLEEE-EE---E--L-L-E-EEEEELHhhhhhhhh
0070 7xjgJ   hhhhlL--LHHHHHHHH-H-HL-L--LLLHH-HHHHHHH-H--HLL----L----------LLHHHHH-HHHHHHHHHH--HL-----LLEEE-EL------------LEEEELHhhhhhhhh
0071 3s93B   HHHHHH--HHHHHHHHH-H-LL-L--LLLHH-HHHHHHH-H--HHL----L--------llllhhhllllHHHHHHLLL---------LLEEE-EE---L--H-H-L-EEEEELL--------
0072 2eshA   HHHHHH--HHHHHHHHH-H-HL----LLLHH-HHHHHHH-L--LLL----L---------lllllllL-LHHHHHHHHH--HL-----LLEEE-EE---E--L-L-E-EEEEELHhhhhhhhh
0073 9gm7C   LHHHHH--HHHHHHHHH-H-LLlL-LLEEHH-HHHHHHH-H--HHH-hllL--LLL----LHHHHHHH-HHHHHHHLLL-----------EEE-EE---L-hL-L-L-EEEEELHhhhhhhhh
0074 5hs5A   LHHHHH--HHHHHHHHH-L----L-LLEEHH-HHHHHHH-H--HHL------------------LLHH-HHHHHHHHHH--HL-----LLLEE-EL---L-lL-L-L-LEEELLHhhhhhhhh
0075 8sojB   HHLH-H--HHHHHHHHHlL-LL-LllLEEHH-HHHHHHH-H-hLLL----L-------------LLHH-HHHHHHHHHH--HH-----LLLEE-EE----------L-LEEEL----------
0076 8f8uA   LLLH----HHHHHHHHH-L-L--L-LLLLHH-HHHHHHL-------------------------LLHH-HHHHHHHHHH--HL----LLLLEE-EL----------L-LEEELLLllllllhh
0077 2xcoA   llllLH--HHHHHHHHH-H-HL-L-LLEELH-HHHHHHH-H-hLLL----L-------------LHHH-HHHHHHHHLLllLL---LLLLEEE-EL---L----llL-LEEEELHhhhlllll
0078 2p4wB   HHHLLH--HHHHHHHHH-L-L----LLEEHH-HHHHHHL-------------------------lLHH-HHHHHHHHHH--HL-----LLEEE-EE---L--L-L-L-EEEEELLleeeeeee
0079 1lddA   HHHHHH--HHHHHHHHH-H-HH---LLEEHH-HHHHHHH-H--HLL-----H--H---hllLLLL-HH-HHHHHHHHHH--HL-----LLEEL-LL---L--------LEEEEL---------
0080 1w7pD   HHHHHH--HHHHHHHHH-H-HL-LlLEEEHH-HHHHHHH-H-hLLL----L----------lllLLHH-HHHHHHLLHH--HH---LLLLLEE-EE---L--L-L-L-EEEELL---------
0081 9bz0f   HHHHHH--HHHHHHHHH-H-HL-HhHEEEHH-HHHHL-L-L--LL-------------------llllLHHHHHHHHHH--HL-----LLLEE-EL---L--L-L-L-LEEELLLl-------
0082 5u8oB   HHHHHH--HHHHHHHHH-H----H-LLLLHH-HHHHHHL-L----L----L---LL--LHHHHHHHHH-HHHHHHHHHH--HL-----LLEEE-EE---L-lL-L-L-EEEEELL--------
0083 3l9fA   ----lL--HHHHHHHHL-L-LL----LEEHH-HHHHHHHhL--LLL----L-----------eelllL-LHHHHHHHHH--HL-----LLEEE-EE---E--L-L-E-EEEEELHhhhhhhhh
0084 2ns0A   llLHHH--HHHHHHHHH-H-HL-L--LLLHH-HHHHHHL-L-------------------lLLHHHHH-HHHHHHHHHH--HL-----LLEEE-EE---LllL-L-L-LEEEELLll------
0085 6juvA   lLLLLH--HHHHHHHHH-H-HH---LLEEHH-HHHHLLH----------------------------H-HHHHHHHHHH--HL-----LLEEE-EE---E--L-L-E-EEEEELHhhhhhhll
0086 2qlzB   HLLL-H--HHHHHHHHH-H-H--H-LEEEHH-HHHHHHL-------------------------LLHH-HHHHHHHHHL--LL-----LLEEE-EL----------L-LEEEELHhhhhl---
0087 5l0pA   LLLH----HHHHHHHHH-L-LL-LlLEEEHH-HHHHHHH-H--LLL----L-------------LLHH-HHHHHHHHHH--HL-----LLEEE-EL---L--L-LlL-LEEEEL---------
0088 9qe1J   HLLLHH--HHHHHHHHH-H-HH-HlLLLLHH-HHHHHHH-HllLLL----L-------------LLHH-HHHHHHHHHH--HL-----LLEEE-EE---L--L-L-L-EEEEELHhhhhhhhh
0089 5zqhA   HHHHHH--HHHHHHHHH-H-HL----LEEHH-HHHHHHH-H-hLLL----L--------------llL-LLHHHHHHHH--HL-----LLEEE-EE---E--E-E-E-EEEEELHhhhhhhhh
0090 3lmmD   llLHHH--HHHHHHHHH-H-HL---LLEEHH-HHHHHLL-------------------------LLHH-HHHHHHHHHH--HL-----LLEEE-EL---L--L-L-L-LEEEELL--------
0091 7bzhA   ----LL--HHHHHHHHH-H-HH---LEEEHH-HHHHHLL-------------------------LLHH-HHHHHHHHHH--HL-----LLEEE-EL------------LEEEELLl-------
0092 5e20A   hhhlLH--HHHHHHHHH-H-HL-L-LEELHH-HHHHHLL-------------------------LLHH-HHHHHHHHHH--HL-----LLEEE-EE---L-lL-L-L-EEEEELHhhhhhhhh
0093 1ylfC   LLHHHH--HHHHHHHHH-H-HL-L--lLLHH-HHHHHLL-------------------------lLHH-HHHHHHHHHH--HH----LLEEEL-LL----------L-LLEEELLlhhhllhh
0094 3lwfA   LLHHHH--HHHHHHHHH-H-HL-L--LLLHH-HHHHHHL-------------------------lLHH-HHHHHHHHHH--HL-----LLEEE-EL---L----L-L-LEEEELLllllllhh
0095 7xx7U   LLLL-H--HHHHHHHHH-H-HL-L--LLLHH-HHHHHHH----LLL----L--------lLLLLH-HH-HHHHHHHHHH--HL-----LLEEE-EL---L----L-L-LEEEELLllllllll
0096 4ru7B   ----lL--HHHHHHHHH-H-LL---LEELHH-HHHHHHL-------------------------lLHH-HHHHHHHHHH--HH-----LLEEL-LL---L--L-L-L-LLEEELHhhhhhhhh
0097 2co5B   LLLL-H--HHHHHHHHH-H-HL-L-LEEEHH-HHHHHHH-H--HHL--------------lLLLH---hhhHHHHHHHH--HL-----LLEEE-EL---L--L-L-L-LEEEELHhhhhhhhh
0098 5xfoA   eeelLL--HHHHHHHHH-H-HH-HlLLEELLlLHHHHHH-H--LHH-------llhhhllLLLHHHHH-HHHHHHHHLL---------LLEEE-EE---E--H-L-E-EEEEELLlllllll-
0099 8ylgA   HLLLLH--HHHHHHHHH-H-HL-LlLEELHH-HHHHHHL-L--------------------LHHHHHH-HHHHHHHH-----------LLEEE-EElllL--L-L-L-EEEEELHhhhhhhhh
0100 4b8xA   hhlLLH--HHHHHHHHH-H-LL-HhHEEEHH-HHHHHHL-------------------------LLHH-HHHHHHHHHH--HL-----LLEEE-EE---L--L---L-EEEEELHhhhhhhhh
0101 9f27A   LHHHHH--HHHHHHHHH-H-HH-L-LLEEHH-HHHHHHH-H--LL--------------------lHH-HHHHHHHHHH--HH-----LLEEE-EE----------L-LEEEEL---------
```

Job: Type-2 BREX; PglW_WP_185095299 wHTH2
Query: s001A
No: Chain Z rmsd lali nres id PDB Description
1: 2od5-A 4.1 2.6 60 91 10 MOLECULE: HYPOTHETICAL PROTEIN;
5: 3pvl-A 3.9 3.3 57 597 12 MOLECULE: MYOSIN VIIA ISOFORM 1;
6: 5z2i-B 3.9 2.4 59 94 2 MOLECULE: DICTYOSTELIUM DISCOIDEUM MITOCHONDRIAL CALCIUM UN
7: 5j9q-E 3.8 2.7 60 293 10 MOLECULE: HISTONE ACETYLTRANSFERASE ESA1;
8: 8d2z-A 3.8 2.7 59 306 10 MOLECULE: METALLO-BETA-LACTAMASE SUPERFAMILY PROTEIN;
9: 7asv-A 3.8 3.4 60 155 7 MOLECULE: DNA-DIRECTED RNA POLYMERASE III SUBUNIT RPC5;
10: 4txa-A 3.7 2.4 53 367 15 MOLECULE: ROQUIN-1;
12: 9ko8-A 3.7 3.1 53 61 8 MOLECULE: KINESIN-LIKE PROTEIN KIF1C;
13: 4k51-A 3.6 3.3 71 213 8 MOLECULE: EUKARYOTIC TRANSLATION INITIATION FACTOR 3 SUBUNI
14: 7mex-A 3.6 2.8 58 1737 7 MOLECULE: UBIQUITIN;
15: 2lnb-A 3.6 3.8 60 80 7 MOLECULE: Z-DNA-BINDING PROTEIN 1;
17: 8uqu-C 3.5 3.4 62 253 6 MOLECULE: FIDO DOMAIN-CONTAINING PROTEIN;

```
18: 6i9b-A 3.5 3.9 64 177 9 MOLECULE: LA-RELATED PROTEIN 4;
20: 7slp-B 3.5 2.6 63 321 10 MOLECULE: 7SK SNRNA METHYLPHOSPHATE CAPPING ENZYME;
22: 3vw4-A 3.4 3.1 59 122 7 MOLECULE: REP;
23: 7yw2-A 3.4 2.6 58 216 16 MOLECULE: TRNA 2'-PHOSPHOTRANSFERASE 1;
24: 5chh-A 3.4 5.2 60 315 13 MOLECULE: ARAC FAMILY TRANSCRIPTIONAL REGULATOR;
25: 5zuz-A 3.4 3.1 64 103 9 MOLECULE: ROK;
26: 8zna-A 3.4 2.5 56 93 7 MOLECULE: ROMX;
27: 7ymd-C 3.3 3.3 67 285 0 MOLECULE: NON-STRUCTURAL MAINTENANCE OF CHROMOSOME ELEMENT
28: 7pb9-A 3.3 3.1 64 173 3 MOLECULE: TANDEM WH DOMAINS OF VPS25;
29: 4ps2-A 3.3 3.9 58 79 12 MOLECULE: PUTATIVE TYPE VI SECRETION PROTEIN;
30: 6taz-B 3.2 2.6 52 141 10 MOLECULE: PROTEIN TIMELESS HOMOLOG;
31: 7aah-A 3.2 2.7 62 147 6 MOLECULE: MARGINAL ZONE B- AND B1-CELL-SPECIFIC PROTEIN;
32: 6zfw-A 3.2 3.7 56 70 9 MOLECULE: PEROXIN-14;
33: 8qfc-D 3.2 3.2 59 185 8 MOLECULE: 60S RIBOSOMAL PROTEIN L10A;
34: 9k49-B 3.2 3.5 56 217 13 MOLECULE: TOL-PAL SYSTEM PROTEIN TOLQ;
35: 9jm7-B 3.2 3.0 50 65 4 MOLECULE: LRD-2A;
36: 5edl-A 3.2 2.9 62 197 5 MOLECULE: PUTATIVE HMP/THIAMINE PERMEASE PROTEIN YKOE;
37: 4cej-B 3.1 3.5 61 1156 5 MOLECULE: ATP-DEPENDENT HELICASE/NUCLEASE SUBUNIT A;
38: 2k85-A 3.1 2.8 56 65 7 MOLECULE: GLUCOCORTICOID RECEPTOR DNA-BINDING FACTOR 1;
39: 1ylf-C 3.1 2.8 54 119 4 MOLECULE: RRF2 FAMILY PROTEIN;
40: 3cuq-A 3.1 3.1 70 219 10 MOLECULE: VACUOLAR-SORTING PROTEIN SNF8;
41: 5dwa-A 3.1 2.7 56 278 9 MOLECULE: TYPE-2 RESTRICTION ENZYME AGEI;
42: 7c4o-A 3.1 3.4 45 48 2 MOLECULE: TRANSCRIPTION FACTOR HES-1;
43: 8slm-A 3.1 3.0 58 248 17 MOLECULE: ZN DEPENDENT HYDROLASE FUSED TO HTH DOMAIN, IRRE
44: 8f2a-E 3.1 2.8 55 117 9 MOLECULE: RECEPTOR ACTIVITY-MODIFYING PROTEIN 3;
45: 7m5w-A 3.1 3.1 53 138 8 MOLECULE: PROTEIN CAPICUA HOMOLOG;
46: 8j56-C 3.1 2.0 43 160 16 MOLECULE: FLAGELLAR TRANSCRIPTIONAL REGULATOR FLHD;
47: 2e62-A 3.1 2.6 45 61 9 MOLECULE: PROTEIN AT5G25060;
48: 8iue-P 3.0 3.7 70 303 13 MOLECULE: DNA-DIRECTED RNA POLYMERASE III SUBUNIT RPC1;
49: 5fhp-C 3.0 3.2 57 209 7 MOLECULE: NICR;
50: 2o7t-A 3.0 3.1 57 188 11 MOLECULE: TRANSCRIPTIONAL REGULATOR;
51: 8dh3-E 3.0 4.0 70 825 10 MOLECULE: T7 RNA POLYMERASE;
52: 2vwa-A 3.0 3.0 61 99 8 MOLECULE: PUTATIVE UNCHARACTERIZED PROTEIN PF13_0012;
53: 6i7g-B 3.0 3.4 62 340 10 MOLECULE: ADENOSINE MONOPHOSPHATE-PROTEIN TRANSFERASE FICD;
54: 2c5q-E 3.0 3.6 44 233 7 MOLECULE: RRAA-LIKE PROTEIN YER010C;
55: 9kor-A 3.0 3.5 49 1229 8 MOLECULE: CRISPR-ASSOCIATED ENDONUCLEASE CAS9;
56: 1sfu-A 3.0 3.0 57 70 4 MOLECULE: 5'-D(*T*CP*GP*CP*GP*CP*G)-3';
57: 5y6i-B 2.9 3.0 55 234 15 MOLECULE: TRANSCRIPTIONAL REGULATOR KDGR;
58: 5a31-N 2.9 3.1 58 703 3 MOLECULE: ANAPHASE-PROMOTING COMPLEX SUBUNIT 1;
59: 6hrz-B 2.9 4.1 53 202 4 MOLECULE: PROBABLE TRANSCRIPTIONAL REGULATORY PROTEIN;
60: 2lh9-A 2.9 3.5 62 78 6 MOLECULE: TUDOR DOMAIN-CONTAINING PROTEIN 7;
61: 1szi-A 2.9 4.1 52 194 6 MOLECULE: MANNOSE-6-PHOSPHATE RECEPTOR BINDING PROTEIN 1;
62: 2zop-A 2.9 3.3 59 113 5 MOLECULE: PUTATIVE UNCHARACTERIZED PROTEIN TTHB164;
63: 2pn2-A 2.9 2.4 49 137 6 MOLECULE: UNCHARACTERIZED PROTEIN;
64: 7amv-W 2.8 3.5 66 637 5 MOLECULE: DNA-DIRECTED RNA POLYMERASE 147 KDA POLYPEPTIDE;
65: 9iac-B 2.8 2.7 53 269 8 MOLECULE: SIMILAR TO TR|Q3MCC8|Q3MCC8_ANAVT HYPOTHETICAL PR
66: 6j5u-A 2.8 3.2 67 663 3 MOLECULE: DISEASE RESISTANCE RPP13-LIKE PROTEIN 4;
67: 5g5p-B 2.8 2.8 67 455 6 MOLECULE: NUCLEAR MRNA EXPORT PROTEIN SAC3;
68: 2h09-A 2.8 2.8 56 127 9 MOLECULE: TRANSCRIPTIONAL REGULATOR MNTR;
69: 3mn2-A 2.8 5.1 57 108 18 MOLECULE: PROBABLE ARAC FAMILY TRANSCRIPTIONAL REGULATOR;
70: 3s63-B 2.8 3.4 58 90 9 MOLECULE: SAPOSIN-LIKE PROTEIN;
71: 7r3w-D 2.8 4.3 60 287 8 MOLECULE: PUTATIVE BACTERIAL REGULATORY HELIX-TURN-HELIX PR
72: 2mp8-A 2.8 3.1 48 64 17 MOLECULE: NKR-5-3B;
73: 7pc1-A 2.8 2.8 51 72 2 MOLECULE: STBA;
74: 6xwi-A 2.8 3.2 50 66 12 MOLECULE: S0_2.126;
75: 7e1n-A 2.8 2.9 55 210 4 MOLECULE: DUF1956 DOMAIN-CONTAINING PROTEIN;

0001 s001A   PDTPSAPALLNYADRLGK-AAD-L--LA-A---------R------D--VLASPTTVLE--QIV-A--A---D----A-PGTR-I-S---L-D--EG--R-MVTLAAAAS----GTAAANA--R-LEIYSRS--------
0002 2od5A   ------ETESXKTVRIRE-KIK-K--FL-G---------D---------RPRNTAEILE--HIN-S--T---X----R----------hgT-T--SQ--Q-LGNV-lskD----KDIVK---dI-CEWATRNwvaehcpe
0003 2qkmB   ----TNATFSQVLDDLSArFIL-N-----L---------P------A--EEERLCFQIE--QAH-W--FyedF----I-RAQL-P-S---L-G--LR--V-FSAKLFAHC----PLLwhEE--A-F--------------
0004 3s93B   ----GMSEQERIQECLRK-EIR-S--LL-I--------sT------K--DGLSPQELEK--EyN-H--L---P---------I-L-G---YrS--TM--E-LVLD----------DVVRV--tV-ILKAIP---------
0005 1qgpA   -LSSHFQE-LSIYQDQEQ-RIL-K--FL-E---------E---lgeG--KATTAHDLSG--KLG-T-----------------------------PK--K-EINRVLYSL-akkGKLQ-------LWKIAVsd-------
0006 3pvlA   ffrkRCEKEDDLAELASQ-QYF-V--DY-G---------S----------EMILERLLS--LVP-T--Y---I----P---DR-E-ItplK-N--LE--K-WAQLAIAAH----K-------------------------
0007 5z2iB   ----GELKTIXGQAKVSK-LQE-K--XK-L---------D------P--RKITFNDFKG--IAK-E--V---G--------------ieeK-E--IN--S-VSNA------------SIIY--L--VFTKPAhiyqsleh
0008 5j9qE   qkPLSDLGLLSYRAYWSD-TLI-T--LL-V--------eH------Q--KEITIDEISS--MTS-M-----------------------------TT--T-DILHTAKTL----N-ILRY-------iIFLNedildryn
0009 8d2zA   qleqGLDYTKDFVTTLLA-QGR-K--AV-E---------R----------NLDLKAAMA--LTR-E--AmdpK----F-GHVFiY-E---H-C--LP--F-DVSRAF--------eASGI--------------------
0010 7asvA   aTFQRESGD--XSDQHRQ-VLL-E--IF-S---------K------N--YRVRRNXIQS--RLT-Q--E---X------------eD---L-S--K---Q-EVDKVLK--------CCVS------wYLKGtvqs-----
0011 4txaA   -----nflGPARQEEALK-LVL-L--AL-E---------D------G--SALSRKVLVL--FVV-Q--R---L----E-PRFP-Q---asktS--IG--H-VVQL----------------------mQLKEefrtyeal
0012 7udkA   ------DREEAAFLAASI-LIQ-H--AH-E---------Q----------GKDDRELEK--ILE-I--A-irI----L-EK-D-R-E---E------aaF-LAASILI------qhdkIL--E-I---------------
0013 9ko8A   -----ATMADLKMQAVKE-ICY-E--VA-L---------A------D--FRHGRAEIEA--LAA-L--K-mrE----L-CRT---yG---D-A--WR--A-VARDV----------------------------------
0014 4k51A   KTYFSQYIAPLRDVIMRR-VFV-A--AS-Q---------K------F--TTVSQSELYK--LAT----L---P----A-P--------ldL-S--AW--D-IEKSLLQAA-vedYVSITID--H--VTFAK---------
0015 7mexA   kVSEKSSLKDRRMDQIKN-SII-Y--NL-Y---------M---------KPLSYSKLLR--SVP-D-----------------------yltE--DT--T-EFDEALE------eVSVF--------fKLKAslyakvdp
0016 2lnbA   hhsHMADP--GREGHLEQ-RIL-Q--VL-T--------eA------G--SPVKLAQLVK--ECQ-A-----------------------------PK--R-ELNQVLYRM-kkeLKVS----sP-ATWCLGGtdpe----
0017 4wziB   eDHYHYHNSLHAADVAQS-THV-L--LS-T-------paL------D--AVFTDLEILA--AIF-A--A--aI----H-DVDV-G-F---K-----------------------tkvyTD--R-I---------------
0018 8uquC   mpKNRSEQEIAGYRDALA-LIH-E--SA-T---------H-----------PFSEGVVL--QLH-T--L--lY----R-YMPQ--fP---D-G--N---G-RMSRLL-------tLLLL---------YHFDyavgryis
0019 6i9bA   --NSAVS-TEDLKECLKK-QLE-F--CF-S------renL------S--KD-PIWTVAN--M-I-K--K---L-----------------ttD--PD--L-ILEV-LRSS----PMVQVDG--E-KV-RPSHkrcivilr
0020 5mmcA   EVEHTHSEREKRVSNAVE-FLL-DsrVR-R---------T------------PTSSKVH--FLK-S--K---G---------------------lSA--E-EICEAFTKV----G--QPKTl-----------------
0021 7slpB   ------SRVKQVLADIAK-QVD-F--WF-GdanlhkdrfL------R--EQVDISLLVS--F-M-K--K---L--------------------ttDG--K-LIARALRSS----AVVELDE--G-TRIRRKKplgerpkd
0022 8twjA   SSKSNRAYCQSMYNSIRS-AGD-E--IS-R---------G-----------TSFEELWG--RAT-E--W---R---lS-KLQ---dqagsA-A--KA--E-FAARAIA------sAHGI---------------------
0023 3vw4A   ----HHHRNYHLFEKVRK-WAY-R--AI-R---------Q---------GWPVFSQWLD--AVI-Q--R-veM----Y-NALS-P-A---EcraigK--S-IAK------------THRK--------FSPEgfsavqaa
0024 7yw2A   --------EQDRNVQLSK-ALS-Y--AL-R----hgalklglpmraD--GFVPLQALLQlpqFH-S--F--------------------------SI--E-DVQLVVNT--nekQRFTLQP-tG-LLIRANQghslqvpe
```

```
0025 5chhA  GGYR-TNYREVLDLVRHD-RAR-Q--LL-R---------D---------ERLRIEEVAE--RLG-Y-------------------------xD--TS--S-FRHAFRRWT-------CAND-------YRQA--------
0026 5zuzA  rrgTARP--GSKAAKLRE-AAI-K--TL-K--------rH------N--AAIKSSELQK--EIEkE--S---G----L----------eiP-N--MT--T-FMQSLIKmY----PEVK--R--G-QY-ILEGeiesaesa
0027 8znaA  ---XTDEEKVKAXRLARA-IAS-D--IS-L-------ynE------Q--KI-LKEELEE--GRE-LykS---R----V-S--E-I-F---T------kxN-FFERAI--------nDIVL--------------------
0028 7ymdC  dfkiLSTDRDLVYKGVLS-VIL-C--IV-F--------fS------K--NNILHQELIK--FLE-T--F---G--------AI-L-N---I-T--IE--D-LIKSLEK-------eYIVRL--E--SYRIGRrtqaelgl
0029 7pb9A  slpSKTEDFEDWLNQWSD-FTL-Q--WFkI---------N------K--LQISLVELMGekPFS-Y-----------------------lQ-N--KS--K-ALTVIVENL--iaRNFC--Y--K-SIrVFWRgyrdwsev
0030 4ps2A  EQVARQIPRLKAMLAMRS-LLR-D--LK-A---------N-----------LDNVTFRK--ELE-K--I--lR----D-PA-S-Q-T---L------rdE-LRAL-------------VPE--K-AW-------------
0031 6tazB  ------twSPEEEAHLRE-LYL-A--NK-D---------V------E--GQ----DVVE--AILaH--L---N----T------V-P---R-T--RK--Q-IIHHL---------gLADSV--K-DF-------------
0032 7aahA  SAHMRCDACRAVAYQMWQ-NLA-K--AEtK---------L------H--TELSELVYTD--VLD-R--S---CsrnwqrLSRT--gE---F-G--ED--Q-IYEAHQQGRvsat--------------------------
0033 6zfwA  --GAMAWPEESEKRKRVS-SAV-Q--FL-H---------D------S--RVTPAANKIQ--FLK-S--K------------------------glTT--E-EVCEAF--------KAGQTI--Pl---------------
0034 8qfcD  GETMTEEQSQSFLTEFIN-YIK-Q--SK----------------------VVLLEDLAS--QVG-L-----------------------rtqD--TI--N-RIQ------laegTITGVID-rG-KFIYITPeelaavan
0035 9k49B  -----nTGSEQIFYSGFK-EFV-R--LH-R---------A---------NSHAPEAVVE--GAS-R--A-mrI----S-MNNY-D-NfmeE-F--TA--I--LHRQ-----------AFTV-------------------
0036 9jm7B  ---GVEAAKKEIKKLKEE-VLK-K--YK-K---------G----------EINEEEAIK--EFV-E--K---A--lkL-VKAdkF-A---I-E----eaK-ALV------------------------------------
0037 5edlA  --SWKVKEIVIMSVISIV-FAV-V--YL-L--------fT------H--FG-IYGIWF---IVS-V--I---A--ayM-IRKP-G-----------aalV-SEIIAAL---vecLLGNVIV--I-G--------------
0038 4cejB  FFKLEAPDIGQLFHSSLK-LIS-D--RL-R--------dE------K--LD-QCELFSY--DAE-R--L---A--pkL-QKEI-LyV---K-E--K---L-QKIV------------SGILse-----------------
0039 2k85A  -----SQQIATAKDKYEW-LVS-R--IV-K---------N----------NENWLSVSR--KMQ-A--S---P----E-----vY-L---E-G--TQ--K-AKKL----------lQHIH--------RLKHe-------
0040 1ylfC  ---------ISSRFSIAV-HIL-S--IL-K---------N----npS--SLCTSDYXAE--SVN-T-----------------------------NP--V-VIRKIXSYL----K-----G--P-GGAGLLKdlheitll
0041 3cuqA  KHKQEMLGVGDFYYELGV-QII-E--VC-L---------A-lkhrnG--GLITLEELHQ--QVL-K-------------------gkfaqD-V--SQ--D-DLIRAIKKL----KGFGIIP-gT-YLIQSVPaelnmdht
0042 5dwaA  -----lnVSEEQYQQISD-YFV-P--LV-N---------K------P--KLKSR-DAIG--QAF-V--M-atE----V-CP-D-A-N---P-SdlWH--H-VLYRIY----------gsWV--R-T--------------
0043 7c4oA  DPSVLGKYRAGFSECMNE-VTR-F--LS-T---------C------E--GVNT-EVRTR--LLG-H--L-anC----M-TQ-----------------------------------------------------------
0044 8slmA  -----TGALAPAKARMRE-LAT-A--YA-R--------rL------P--GLlahEISHA--LLL-G--D---DdllsD-LHDArL-E---Q-V--IE--T-LCNVGAA--------------------ILMP--------
0045 8f2aE  --CNETGMLERL-PLCGK-AFA-D--MM-G---------K------V--DV-EFIVYYE--SFT-N--C-teM----E-ANCY-W-P---N-----------plFITG---------QFFSn------------------
0046 7m5wA  nPYSSLRRTLDQRRALVM-QLF-Q--DH-G--------------------FPSAQATAA--FQA-R--Y-----------------adifP-S--KV--C-LQLKIREVR----Q-------------------------
0047 8j56C  ---KSVLQDANQTQLAIE-LIG-L--G----------------------------aRLQ--VLE-A--E---T----T-------------lS--RD--R-LIRL------------LRGV--Sp---------------
0048 2e62A  ----QRQKRRRIEVALIE-YRE-T--LE-E---------Q---------GMKNPEEIER--KVE-I--N-rkR----L-EVYG-L-S-----------------------------------------------------
0049 8iueP  VTGGQDFE-SEFVEVLNQ-QCF-K--FL-Q---------S------K--AFASSHEVWK--YICeL--G---I----S-----kV-E---L-S--ME--D-IETI-LNTL-iydGKVE--M--K-LY-RAVNpiipptgl
0050 5fhpC  fxeVYRRGLSDIDKKRQN-YLD-E--LK-S---------R------P--EGYNTYDIVR--TYL-R--P---Q-----hFARL--lY---D-H--TL--K-AFI--------------EIX--Ec---------------
0051 2o7tA  ----XRADALKRREHIIT-TTC-N--LY-R---------T----------HHDS-LTXE--NIAeQ--A---G----V----gyR-N---F-P--DR-fT-LDXA-CAQY----L------------gSLVPalapesld
0052 8dh3E  gevaQEIKPEAVAYITIK-TTL-A--CL-T---------S------A--DNTTVQAVAS--AIG-R--A---I--edE-ARFG--hkedsihV--GV--R-CIEMLIEST----GM-VSLH--E-TI-ELAPeyaeaiat
0053 2vwaA  nVDVSIKRYNNFVDIARL-SIQ-K--HF-E---------H------L--S--NXEYXQK--FVQ-G--LqenR----N-IS--lS-K---Y-Q--EN--K-AVXDLKY-------------------kVYANylsqee--
0054 6i7gB  GKSLEEQNEVIGMHAAMK-YINtT--LV-S---------R------I--GSVT-ISDVL--EIH-R--R-vlG----Y-VD----iD---G-N--G---R-TSRL------------NLIL-------MQAGyppitirk
0055 2c5qE  VQETDISKLVTYIEKSIE-VDL-L--VS-E-------diK------N--GIPAKQAQND--RRS-V--L---K----K--------------------------------------------------------------
0056 9korA  QTRHRIRSQQRFVLARRL-TYI-L--IE-G--------kL------K--RKLSPREKEA--ISS-L--L---RrrgyssltekdkkR---K-D--SN--Gk---------------------------------------
0057 1sfuA  -----TVNDAEIFSLVKK-EVL-S-----L---------N------T--NDY---tTAI--SLSnR--L---K----I----------nkK-K--IN--Q-QLYK-----lqkeDTVKMVP--S-PKWFKNYnc------
0058 5y6iB  ------VRSAEVGTDILK-ALA-E--LS-P--------------------ATSLSRLAE--HVG-M-----------------------pasK--VH--R-YLQA-----liasGFAVQDS--T-NHYSLGRealrvgla
0059 5a31N  lFINEeliKEEELLLFWT-YIQ-A--ML-T---------N------L--ESLSLDRIYN--MLRmF--V---V----T-------palaeI-D--LQ--E-LQGY----------qkLVYS-----VYRLP---------
0060 6hrzB  HMEIKRRTQEERSAATRE-ALI-T--GA-R---------K----------lVGTPEIAT--EAG------------------------------vTR--G-AMYHQ------------FAD--K---rDVVEvveqdvma
0061 2lh9A  ----HMLEADLVSKMLRA-VLQ-S--HK-N--------------------GIVLPRLQG--EYR-S--L---T---------Q-L-G---YpT--LE--A-YLRSVPAVV-------RIEA-gE-IVCY-----------
0062 1sziA  slqqARQKAQETLQQLTS-VLG-L--ME-S---------V------K--Q--VEARALS--MFR-D--I-tqQ----L-QSMC--vN---D-L--QA--T---------------sfVLAQt------------------
0063 2zopA  ----XRTRSQVWAQKAYE-KVR-E--AA-K---------G------E--GRdAHKALGN--DLA-Q--V---L----G----R-L-T---R-E--VL--A-AAEWFKRFA----QAL-----------------------
0064 2pn2A  tyqgTDLLATSLASCXLT-IIG-I--KA-R---------D------X--EIvTQKIFYN--TAL-T--C---P----VkSIHPdI-F---Q-K--VIih-----------------------------------------
0065 7amvW  sLPPHPIVKVLLGELVRQ-FFY-N--NS----------------------RIKYND--T--KLL-K--M---V---tS-VIKN--kedarN-Y--ID--D-IVN--hFFV----SNkLYKY-eN-DIITVPFrlsyepfv
0066 9iacB  lkpaQPQKIGKLAQRGVF-EFH-Q--EL-----------------------SGSH-GVE--QVAeI--L---Q----LnQESPiQ-A---R-V--LV--I-LNNY----------------------iLLNKeiinlsrg
0067 6j5uA  -----NGTDPSHLKSCIL-T------LS-L---------Y------PedCVIPKQQLVH--GWI-GegF---V----M-WRNG-R-S-atE-S--GE--D-CFSGLTNR------cLIEVI--I-TC-KIHDmvrdlvid
0068 5g5pB  idnwIVLLEKLPMVTYRN-LIK-T--VI-K---------SwttewgQ--NKLPYSLIER--VLQ-L--S---I-----tiYNG-I-H--spkN--VE--N-VLVTLIN---------RANC--F-LCVVKKTtmiqeivp
0069 2h09A  --EGFRQVREAHRRELID-DYV-E--LI-S---------D--lireV--GEARQVDMAA--R------------------------lgvsqpT--VA--K-MLKR-----latmGLIEMI------GVFLTAegeklaqe
0070 3mn2A  RSRGYSPXAFAKRVRLQH-AHN-L--LS-D---------G------A--TPT---TVTA--AAL-S--C---G-----------------fsN--LG--H-FARDYRDXF---------------SETL-----------
0071 3s63B  ----PKETCDLCQIALRT-VFG-H--FG-G---------N----------IPSRRKLVH--QLK-H--E--cK----R-HFNY-R-RrclL-L--MK--V--------------------D--L-IFMTDGSfkpmevcl

0001 s001A  LLLLLHHHHHHHHHHHHH-HHH-H--HH-L---------L------L--LLLLHHHHHH--HHH-L--L---L----L-LLLL-L-L---L-L--HH--H-HHHHHHHLL----LLEEELL--L-LLEEELL--------
0002 2od5A  ------LLHHHHHHHHHH-HHH-H--HL-L---------L---------LLEEHHHHHH--HHH-L--L---L----L----------llL-L--HH--H-HHHH-hhhL----LLEEE---eE-EEEEEHHhhhhhlll
0003 2qkmB  ----HLLLHHHHHHHHHHhHLL-L-----L---------L------H--HHHHHHHHHH--HHH-H--HhhhL----L-HHLL-L-L---L-L--HH--H-HHHHHHLLL----HHHlhHH--H-H--------------
0004 3s93B  ----LHHHHHHHHHHHHH-HHH-H--HH-H--------lL------L--LLLLHHHHHH--HhL-L--L---L---------H-H-L---LlL--HH--H-HHHL----------LLEEE--lE-EEEELL---------
0005 1qgpA  -LHHHHLL-LHHHHHHHH-HHH-H--HH-H---------H---hllL--LLEEHHHHHH--HHL-L-----------------------------LH--H-HHHHHHHHH-hhhLLEE--------EEEELLll------
0006 3pvlA  eeeeLLLLHHHHHHHHHH-HHH-H--HH-L---------L----------LLLHHHHHH--HHH-H--H---L----L---HH-H-LlllL-L--HH--H-HHHHHHHHH----H-------------------------
0007 5z2iB  ----LHHHHHHHHHHHHH-HHH-H--HH-H---------L------L--LEEEHHHHHH--HHH-H--L---L--------------llhH-H--HH--H-HHHH------------LLEE--L--EELLLHhhhhhhhh
0008 5j9qE  llLLLHHHHHHHHHHHHH-HHH-H--HH-H--------hH------L--LLLLHHHHHH--HHL-L-----------------------------LH--H-HHHHHHHHL----L-LEEE-------eELLLhhhhhhhh
0009 8d2zA  llllHHHHHHHHHHHHHH-HHH-H--HH-H---------L----------LLLHHHHHH--HHH-H--HhhhH----H-LLLLlH-H---H-H--HH--H-HHHHHH--------hHLLL--------------------
0010 7asvA  hHHHHLLLL--LHHHHHH-HHH-H--HH-H---------H------L--LEELHHHHHH--HHH-H--H---H------------lL---L-L--H---H-HHHHHHH---------HEEE------eEELLllll----
0011 4txaA  -----hllLHHHHHHHHH-HHH-H--HH-L---------L------L--LLLLHHHHHH--HHH-H--H---L----L-LLLL-L---llhhH--HH--H-HHHH----------------------eEELHhhllhhhh
0012 7udkA  ------LHHHHHHHHHHH-HHH-H--HH-H---------H----------LLLHHHHHH--HHH-H--H-hhH----H-HH-L-H-H---H------hhH-HHHHHHH-------hhlhHH--H-H--------------
0013 9ko8A  -----LLHHHHHHHHHHH-HHH-H--HH-L---------L------L--LLLLHHHHHH--HHH-H--H-hhH----H-HLL---lL---L-H--HH--H-HHHHH----------------------------------
0014 4k51A  LLLLHHHHHHHHHHHHHH-HHH-H--HH-H---------H------L--LEEEHHHHHH--HHL----L---L----H-H--------hlL-L--HH--H-HHHHHHHHH-hllLLLLLLL--L--EELLL---------
0015 7mexA  hHHHHLLHHHHHHHHHHH-HHH-H--HH-L---------L---------LLEEHHHHHH--HLL-H-----------------------hhlL--LH--H-HHHHHHH-------hHEEE-------eEELLhhhhhlll
0016 2lnbA  lllLLLLL--LHHHHHHH-HHH-H--HH-H--------hH------L--LLEEHHHHHH--HHL-L-----------------------------LH--H-HHHHHHHHH-hhlLLEE----eL-LEEEELLllll----
0017 4wziB  hHLLLLLLHHHHHHHHHH-HHH-H--HL-L-------hhH------L--LLLLHHHHHH--HHH-H--H--hH----L-LLLH-H-H---H------------------------lllhHH--H-H--------------
0018 8uquC  llLLHHHHHHHHHHHHHH-HHH-H--HH-H---------H-----------LLLHHHHH--HHH-H--H--hL----L-LLLL--lL---L-L--H---H-HHHHHH--------hHHHH--------HHLLlhhhhlll
0019 6i9bA  --LLLLL-HHHHHHHHHH-HHH-H--HL-L------hhhH------L--LL-LHHHHHL--L-H-H--L---L-----------------llL--HH--H-HHHH-HLLL----LLEEELL--L-LE-EELLllleeeel
0020 5mmcA  LLLLLHHHHHHHHHHHHH-HHL-LhhHH-H---------L------------LHHHHHH--HHH-H--L---L---------------------lLH--H-HHHHHHHHL----L--LLLLh------------------
0021 7slpB  ------LHHHHHHHHHHH-HHH-H--HL-LhhhhhhllhH------H--HHEEHHHHHL--L-H-H--L---L--------------------llLH--H-HHHHHLLLL----LLLEELL--L-LEEELLLllllllll
0022 8twjA  LLHHHHHHHHHHHHHHHH-HHH-H--HH-L---------L-----------LLHHHHHH--HHH-H--H---H---hL-LLL---llllhH-H--HH--H-HHHHHHH-------hHLLL--------------------
0023 3vw4A  ----LHHHHHHHHHHHHH-HHH-H--HL-L---------L---------LLLLHHHHHH--HHH-H--H-hhH----H-HHLL-H-H---HhhhhhH--H-HHH-------------HHHH-------LLHHhhhhhhhh
0024 7yw2A  --------LHHHHHHHHH-HHH-H--HH-H----llllllllllllL--LLEEHHHHHLlhhHL-L--L--------------------------LH--H-HHHHHHHH--lllLLEEEEE-lL-EEEEELLllllllll
0025 5chhA  LLHH-LLHHHHHHHHHHH-HHH-H--HL-L---------L---------LLLLHHHHHH--HLL-L-------------------------lL--HH--H-HHHHHHHHH-------LHHH-------HHHL--------
0026 5zuzA  hhhLLLL--LLHHHHHHH-HHH-H--HH-H--------hL------L--LLEEHHHHHH--HHHhH--H---L----L----------llL-L--HH--H-HHHHHHHhL----LLEE--L--L-EE-EELLllllllll
0027 8znaA  ---LLHHHHHHHHHHHHH-HHH-H--HH-H-------hlH------H--HH-LHHHHHH--HHH-HhhH---H----L-L--H-H-H---H------hlL-HHHHHH--------hHHHH--------------------
0028 7ymdC  hhhhLLLLLHHHHHHHHH-HHH-H--HH-H--------hL------L--LLEEHHHHHH--HHH-H--H---L--------LL-L-L---L-L--HH--H-HHHHHHH-------lLEEEE--E--EEEELHhhhhhllh
0029 7pb9A  lllLLHHHHHHHHHHHHH-HHH-H--HHhH---------L------L--LEEEHHHHLLllLHH-H-----------------------lL-L--HH--H-HHHHHHHHH--hhLLLE--L--L-EEeELLLlhhhhhhh
0030 4ps2A  HHHHHHHHHHHHHHHHHH-HHH-H--HH-L---------L-----------LLHHHHHH--HHH-H--H--hL----L-LL-L-H-H---H------hhH-HHLL-------------LLH--H-HL-------------
0031 6tazB  ------llLHHHHHHHHH-HHH-H--HL-L---------L------L--LL----LHHH--HHHhH--L---L----L------L-L---L-L--HH--H-HHHHH---------lLLLLH--H-HH-------------
0032 7aahA  LLLLHHHHHHHHHHHHHH-HHH-H--HHhH---------L------L--LLLLHHHHHH--HHH-H--H---LlllllhHHHH--hH---H-L--HH--H-HHHHHHHLHllll--------------------------
0033 6zfwA  --LLLLHHHHHHHHHHHH-HHH-H--HH-H---------L------H--HHLLHHHHHH--HHH-H--L------------------------llLH--H-HHHHHH--------HLLLLL--Lh---------------
```

```
0034 8qfcD   LLLLLHHHHHHHHHHHHH-HHH-H--HL----------------------EELLLHHHH--HHL-L-----------------------lhhH--HH--H-HHH------hhhlLLLEEEE-lL-EEEELLHhhhhhhhh
0035 9k49B   -----lLLHHHHHHHHHH-HHH-H--HH-H---------L---------LLLLHHHHHH--HHH-H--H-hhH----H-HHHH-H-HhhhH-H--HH--H--HHHL-----------LLLL-------------------
0036 9jm7B   ---LHHHHHHHHHHHHHH-HHH-H--HH-H---------L----------LLLHHHHHH--HHH-H--H---H--hhH-HHHlhH-H---H-H----hhH-HHH------------------------------------
0037 5edlA   --LLLHHHHHHHHHHHHH-HHH-H--HH-H--------hH------H--HH-HHHHHH---HHH-H--H---H--hhH-HLLL-L-----------hhhH-HHHHHHH---hhhHLLLHHH--H-H--------------
0038 4cejB   LLLLLHHHHHHHHHHHHH-HHH-H--HH-H--------hL------L--LL-HHHHHHH--HHH-H--H---H--hhL-LLLH-HhH---H-H--H---H-HHHH------------HHHHhl-----------------
0039 2k85A   -----LLLLLHHHHHHHH-HHH-H--HL-L---------L----------LLLHHHHHH--HHL-L--L---H----H-----hH-H---L-L--HH--H-HHHH----------hHHHH--------HHHHl-------
0040 1ylfC   ---------LLHHHHHHH-HHH-H--HH-H---------H----llL--LLLLHHHHHH--HLL-L-----------------------------LH--H-HHHHHHHHH----H-----L--L-LLEEELLlhhhllhh
0041 3cuqA   HHHHHHHLHHHHHHHHHH-HHH-H--HH-H---------H-hhhhhL--LEEEHHHHHH--HHH-H-------------------lllllL-L--LH--H-HHHHHHHHH----HLLEEEE-lE-EEEELLLllllhhhh
0042 5dwaA   -----llLLHHHHHHHHH-HHH-H--HH-L---------L------L--LLLHH-HHHH--HHH-H--H-hhH----H-LL-L-L-L---H-HhhHH--H-LHHHHH----------lhHH--H-H--------------
0043 7c4oA   LHHHHHHHHHHHHHHHHH-HHH-H--HL-L---------L------L--LLLH-HHHHH--HHH-H--H-hhH----H-HH-----------------------------------------------------------
0044 8slmA   -----LLLHHHHHHHHHH-HHH-H--HH-H--------hL------L--LLlhhHHHHH--HHH-H--L---HhhhhH-HHLLhH-H---H-H--HH--H-HHHHHHH-------------------HHLL---------
0045 8f2aE   --LLHHHHHHHL-HHHHH-HHH-H--HL-L---------L------L--LL-HHHLHHH--HHH-H--H-hhH----L-LLLL-L-L---L-----------llHHHH---------HHLLl------------------
0046 7m5wA   lLLHHHHHHHHHHHHHHH-HHH-H--HH-L--------------------LLLHHHHHH--HHH-H--L-----------------llllL-L--HH--H-HHHHHHHHH----H-------------------------
0047 8j56C   ---LLHHHHHHHHHHHHH-HHH-L--L----------------------------lLHH--HHH-H--H---L----L-------------lL--HH--H-HHHH------------HLLL--Ll---------------
0048 2e62A   ----HHHHHHHHHHHHHH-HHH-H--HH-H---------H---------LLLLHHHHHH--HHH-H--H-hhH----H-HHLL-L-L-----------------------------------------------------
0049 8iueP   LLLLLEEL-HHHHHHHHH-HHH-H--HH-H---------H------H--HLEEHHHHHH--HHHhH--L---L----L-----lL-L---L-L--HH--H-HHHH-HHHH-hhlLLEE--E--E-EE-EELLlllllllh
0050 5fhpC   hhhHHHHHHHHHHHHHHH-HHH-H--HH-L---------L------L--LLLLHHHHHH--HHH-H--H---H-----hHHHH--hL---H-H--HH--H-HHH--------------HHH--Hl---------------
0051 2o7tA   ----LLHHHHHHHHHHHH-HHH-H--HH-H---------H----------LHHH-LLHH--HHHhH--H---L----L----lhH-H---L-L--LH-hH-HHHH-HHHH----H------------hHHHHhhllllhh
0052 8dh3E   llllHHLLHHHHHHHHHH-HHH-H--HH-H---------H------H--LLLLHHHHHH--HHH-H--H---H--hhH-HHHH--lhhhhhhH--HH--H-HHHHHHHHL----LL-EEEL--L-LE-EELHhhhhhhhh
0053 2vwaA   lLLLLHHHHHHHHHHHHH-HHH-H--HH-H---------H------L--L--LHHHHHH--HHH-H--HhhhL----L-LL--lL-H---H-H--HH--H-HHHHHHH-------------------hHHHHhhhhhl--
0054 6i7gB   LLLHHHHHHHHHHHHHHH-HHHhH--LL-L---------L------L--LLLL-HHHHH--HHH-H--H-hlL----L-LL----lL---L-H--H---H-HHHH------------HHHH-------HHHLllllllh
0055 2c5qE   HHHLLHHHHHHHHHHHHH-HHH-H--HH-H-------hhH------L--LLLHHHHHHH--HHH-H--H---H----H--------------------------------------------------------------
0056 9korA   HHHHHHHHHHHHHHHHHH-HHH-H--HH-H--------hH------L--LLLLHHHHHH--HHH-H--H---LllllllhhhhhlheE---E-L--LL--Ll---------------------------------------
0057 1sfuA   -----LLLLHHHHHHHHH-HHH-L-----L---------L------L--LLE---eLHH--HHHhH--L---L----L----------lhH-H--HH--H-HHHH-----hhhlLLEEEEL--L-LEEEELLll------
0058 5y6iB   ------LLHHHHHHHHHH-HHH-H--LL-L--------------------LEEHHHHHH--HHL-L-----------------------lhhH--HH--H-HHHH-----hhhhLLEEELL--L-LEEEELHhhhhhhhh
0059 5a31N   hHHHHlllLHHHHHHHHH-HHH-H--HH-H---------H------H--LLLLHHHHHH--HHHhH--L---L----L-------lllllL-L--HH--H-HHHH----------hhLEEE-----EEELL---------
0060 6hrzB   LLHHHHHHHHHHHHHHHH-HHH-H--HH-H---------H----------hLLHHHHHH--HHL------------------------------lLH--H-HHHHH------------LLL--H---hHHHHhhhhhhhh
0061 2lh9A   ----LLLHHHHHHHHHHH-HHH-H--LL-L--------------------LEEHHHHHH--HHH-H--H---H---------H-H-L---LlL--HH--H-HHHLLLLLE-------EEEE-lL-EEEE-----------
0062 1sziA   hhhhHHHHHHHHHHHHHH-HHH-H--HH-H---------H------H--L--HHHHHHH--HHH-H--H-hhH----H-HHHH--hH---H-H--HH--H---------------lhHHHHh------------------
0063 2zopA   ----LLLHHHHHHHHHHH-HHH-H--HL-L---------L------L--LHhHHHHHHH--HHH-H--H---L----L----H-H-H---H-H--HH--H-HHHHHHHHH----HHH-----------------------
0064 2pn2A   eeeeHHHHHHHHHHHHHH-HHH-H--HH-H---------H------L--LLeHHHHHHH--HHH-L--L---L----LlLLLLlL-E---E-E--EEel-----------------------------------------
0065 7amvW   lLLLLLLHHHHHHHHHHH-HHH-H--LL----------------------LEELLL--H--HHH-H--H---H---hL-LLLL--hhhhhH-H--HH--H-HHH--lLEE----LLlEEEL-lL-EEEEEELllllllle
0066 9iacB   hlllHHHHHHHHHHHHHH-HHH-H--LL-----------------------HLLL-HHH--HHHhH--L---L----HhHLLHhH-H---H-H--HH--H-HHHH----------------------lLLLLeeeeelll
0067 6j5uA   -----LLLLLHHHHHHHH-H------HH-H---------L------LllLLEEHHHHHH--HHH-HhlL---L----L-LLLL-L-L-lhH-H--HH--H-HHHHHHHL------lLLEEL--L-EE-ELLHhhhhhhhh
0068 5g5pB   hhhhHHHHHHHHHHHHHH-HHH-H--HH-H---------HhhllllL--LEEEHHHHHH--HHH-H--H---H-----llLLL-L-L--lllL--HH--H-HHHHHHH---------LEEE--E-EEEELLLllhhhhll
0069 2h09A   --LLLHHHHHHHHHHHHH-HHH-H--HH-H---------H--hhhhH--LLLLHHHHHH--H------------------------hlllhhH--HH--H-HHHH-----hhhlLLEEEE------EEEELHhhhhhhhh
0070 3mn2A   HHLLLLHHHHHHHHHHHH-HHH-H--HH-L---------L------L--LLL---LHHH--HHH-H--L---L-----------------llL--HH--H-HHHHHHHHH---------------HHHH-----------
0071 3s63B   ----HHHHHHHHHHHHHH-HHH-H--LL-L---------L----------LLLHHHHHH--HHH-H--H--hL----L-LLLL-H-HhhhH-H--HH--H--------------------H--H-HHHHLLLllhhhhhh
```

Job: Type-2 BREX; PglW_WP_185095299 wHTH3
Query: s001A
No: Chain Z rmsd lali nres id PDB Description
1: 8zjr-M 4.5 2.8 61 82 10 MOLECULE: HISTONE H3.2;
2: 7ymd-C 3.9 2.9 65 285 9 MOLECULE: NON-STRUCTURAL MAINTENANCE OF CHROMOSOME ELEMENT
3: 5n19-A 3.9 3.1 60 146 13 MOLECULE: TRANSCRIPTIONAL REGULATOR (FUR FAMILY);
4: 7asv-A 3.8 2.8 61 155 13 MOLECULE: DNA-DIRECTED RNA POLYMERASE III SUBUNIT RPC5;
5: 7kbf-K 3.8 2.9 58 78 14 MOLECULE: HISTONE H3.2;
6: 7db7-B 3.6 3.4 48 836 17 MOLECULE: PHENYLALANINE--TRNA LIGASE ALPHA SUBUNIT;
7: 5a48-B 3.6 2.9 63 96 11 MOLECULE: MATERNAL EFFECT PROTEIN OSKAR;
8: 4ets-B 3.6 3.5 61 149 15 MOLECULE: FERRIC UPTAKE REGULATION PROTEIN;
9: 5okc-B 3.6 3.4 60 377 10 MOLECULE: SISTER CHROMATID COHESION PROTEIN DCC1;
10: 7z8b-C 3.4 3.0 59 1224 12 MOLECULE: CULLIN-7;
11: 1ka8-A 3.3 2.9 63 100 10 MOLECULE: PUTATIVE P4-SPECIFIC DNA PRIMASE;
12: 8e4y-A 3.3 2.9 57 651 9 MOLECULE: GLYCEROL-3-PHOSPHATE ACYLTRANSFERASE 1, MITOCHOND
13: 8e4v-A 3.2 2.9 53 84 11 MOLECULE: ISOFORM 3 OF HISTONE ACETYLTRANSFERASE KAT6B;
14: 2qby-B 3.2 2.9 57 368 11 MOLECULE: CELL DIVISION CONTROL PROTEIN 6 HOMOLOG 1;
15: 2lh9-A 3.0 2.5 50 78 12 MOLECULE: TUDOR DOMAIN-CONTAINING PROTEIN 7;
16: 2ly1-A 3.0 2.8 52 180 12 MOLECULE: TUDOR DOMAIN-CONTAINING PROTEIN 7;
17: 7nyw-E 3.0 3.3 58 212 7 MOLECULE: CHROMOSOME PARTITION PROTEIN MUKB;
18: 5l0p-A 2.9 2.7 52 311 12 MOLECULE: TRANSCRIPTION FACTOR ETV6, TRANSCRIPTION FACTOR E
19: 9c5g-B 2.9 3.0 53 290 8 MOLECULE: CAPW;
20: 4txa-A 2.9 3.2 55 367 15 MOLECULE: ROQUIN-1;
21: 6qfd-B 2.9 3.0 53 116 9 MOLECULE: DNA-BINDING PROTEIN;
22: 6l0o-A 2.9 3.0 51 71 10 MOLECULE: DNA HELICASE MCM8;
23: 7sol-A 2.9 5.2 57 1011 9 MOLECULE: UBIQUITIN-LIKE MODIFIER-ACTIVATING ENZYME 6;
24: 1zel-B 2.9 2.8 55 295 18 MOLECULE: HYPOTHETICAL PROTEIN RV2827C;
25: 7bzh-A 2.8 2.8 50 59 8 MOLECULE: SUL7S;
26: 8ioi-A 2.8 3.2 54 190 7 MOLECULE: PADR FAMILY TRANSCRIPTIONAL REGULATOR;
27: 6zwt-C 2.8 3.8 57 101 11 MOLECULE: TWO-COMPONENT RESPONSE REGULATOR;
28: 5uuj-A 2.8 3.8 65 371 15 MOLECULE: ALKZ;
29: 7pd7-B 2.8 2.8 48 251 15 MOLECULE: METHYLTRANSFERASE;
30: 1tbx-A 2.7 3.0 57 94 19 MOLECULE: HYPOTHETICAL 11.0 KDA PROTEIN;
31: 4k51-A 2.7 3.2 58 213 5 MOLECULE: EUKARYOTIC TRANSLATION INITIATION FACTOR 3 SUBUNI
32: 5yad-A 2.7 2.5 50 73 8 MOLECULE: MEIOSIS REGULATOR AND MRNA STABILITY FACTOR 1;
33: 7abi-7 2.7 4.3 60 236 3 MOLECULE: U5 SMALL NUCLEAR RIBONUCLEOPROTEIN 40 KDA PROTEIN
34: 8wxs-A 2.7 2.5 51 76 12 MOLECULE: FI02030P;
35: 2zme-B 2.7 4.1 62 215 11 MOLECULE: VACUOLAR-SORTING PROTEIN SNF8;
36: 7qen-C 2.7 3.2 57 291 7 MOLECULE: DNA (35-MER);
37: 1w7p-D 2.7 3.8 61 171 8 MOLECULE: VPS22, YPL002C;
38: 6zca-D 2.6 3.3 57 140 5 MOLECULE: PROBABLE DNA-DIRECTED RNA POLYMERASE SUBUNIT DELT
39: 7c0j-B 2.6 2.9 49 62 10 MOLECULE: HISTONE H5,DOUBLE-STRANDED RNA-SPECIFIC ADENOSINE

```
40: 3s93-B 2.6 2.5 50 81 10 MOLECULE: TUDOR DOMAIN-CONTAINING PROTEIN 5;
41: 8iue-P 2.6 3.2 60 303 5 MOLECULE: DNA-DIRECTED RNA POLYMERASE III SUBUNIT RPC1;
42: 8fo9-F 2.6 3.9 58 2289 9 MOLECULE: LEUCINE-RICH REPEAT SERINE/THREONINE-PROTEIN KINA
43: 2wb6-A 2.6 2.9 62 114 2 MOLECULE: AFV1-102;
44: 2esh-A 2.6 3.1 52 114 12 MOLECULE: CONSERVED HYPOTHETICAL PROTEIN TM0937;
45: 8ffz-B 2.6 2.9 53 905 11 MOLECULE: TRANSCRIPTION FACTOR IIIA;
46: 8jri-V 2.6 2.9 50 508 8 MOLECULE: 26S PROTEASE REGULATORY SUBUNIT 7;
47: 3cuq-A 2.5 3.8 63 219 16 MOLECULE: VACUOLAR-SORTING PROTEIN SNF8;
48: 3l9f-A 2.5 2.9 52 170 8 MOLECULE: PUTATIVE UNCHARACTERIZED PROTEIN SMU.1604C;
49: 8gcc-A 2.5 3.1 55 707 9 MOLECULE: DNA TOPOISOMERASE 2;
50: 8k5l-A 2.5 3.2 45 201 18 MOLECULE: TRNA (CMO5U34)-METHYLTRANSFERASE;

0001 s001A   -LQPVRALRLT-QA---GL-FL----PD-GLTIETIHQRVHNR--F-P-D------LS-T-R--LP-DQ--PAPLSRLLS--QA-----G------FAL-KW-RIRG-R---TVFLPE-H
0002 8zjrM   yMYAYRWIRNH-L----------ehtDT-CLPKQSVYDAYRKY--C-E-S--------lR-P--LS-TA----NFGKIIR--EI-----F------PDI-KA-RRLS-K---YCYSG---
0003 7ymdC   ivkenPVARKMvRY---IL---sesqNS-IITRNKLQSVIHEA--A-ReN------IA-K-P--SF-S-----KMFMDIN--AI-----L-----ynEL-QG-LPSK---raQKFILL-N
0004 5nl9A   vTVQRLQMANL-L-----------lskpqHLTADQVFQLINEH--M-P-N------ASrA-T--IF------nnLKLFAE--K--------------GIV-NL-LELG-I---TLYDNV-I
0005 7asvA   arELKAFVE---AT---FQ-------rqfVLTLSELKRLFNLH--L-A-SlppghtLF-S-G--IS-DR----XLQDTVL--A-------------AGC-KQ-ILVPfQ---KVFALW-E
0006 7kbfK   hPPTLSMVV---EV---LKnTE----RK-GTSVQAIRTRILSA--H-P-T------VD--fL--LR-T-----aLNKG------------------IL-IR-PLNS---atGRFKLA-K
0007 7db7B   -----------------------------mRLPYSWLREVVAVG--A-S------------gW--DV-T---PGELEQTLL--RI-----G------HEVeEV-IPgvttgddVVFHLA-I
0008 5a48B   yPDIDSEVR---AI---LLsHA----QN-GITISSIKSEYRKL--T-G-N----------pF--PL-HD----NVTDFLL--TI-----P------NVT-AE-CSES-G---KRIFN---
0009 4etsB   yTKQREVLLKT-L----------yhsdT-HYTPESLYMEIKQA--E-P-D------L--N-V--GI-A--tvyRTLNLLE-----------------MV-TSiSFGS---agKKYELA-N
0010 5okcB   lfKALHVLLXS-AxaesLD-LQ-------HLNVEDTHHAVGK---D-I-E------DF-N-P--YT-RE----iIETVLN-----------------KFA-VQ-EQE--N---NTWRLR-I
0011 7z8bC   ekRRNLLNCLIvRI---LKaHG----DE-GLHIDQLVCLVLEA--W-Q-KgpcpprgA-C-S--ST-------dvlsCIL--H--------------lTL-RR-HDDR-P---QVLSYA-V
0012 1ka8A   aDPTFDFIGY---------lpQ----TS-GMYMGYLYHAYLAY--M-EnG------YR-N-V--LS-LKmfGLGLPVMLK--EY-----G------LNY-EK-RHTK-G---IQTN----
0013 8e4yA   aIMSTHIVACL-L----------lyrhrqGIDLSTLVEDFFVM--K-E-E------VS-G-N--SE------dvVMHAIQ--LL-----G-------VT-IT-HTSR-N--dEFFIT---
0014 8e4vA   rNVDW--nkllrRA---IElEE----PN-GSSLKNIEKYLRSQsdL-T-S------TP-A-F--QQ---rlrlGAKR-------------------aRL-LK-DG-------PQYRVN-Y
0015 2qbyB   lpFHYKLAL---RS---LI----------esEDVMSAHKMYTDL--C-N-K------FQ-K-P--LS-YR----RFSDIIS--EL-dmfgI------VKI--R-IING-V---KKY-----
0016 2lh9A   aDLVSKMLRAV-L-----------qshkngIVLPRLQGEYRSL--T-G-------------dwipfpTL--EAYLRS--V--PA-----V------V-R-IE-ASRS-G---EIV-----
0017 2ly1A   mpDVKEKVAEL-L-----------gkyssgLWASALPKAFEDM--YkV-K------FPeD-A--LK--------NLASLS--D------V------CTI--N-YISG-Q---KAI-----
0018 7nywE   lsELDMMVGKI-L------laN----QG-IFTSQELYEELISL--A-DgK------LM-KkL--QE-K------VRTTLN--RL---------------MV-YF-LPN----nnNKFTITv-
0019 5l0pA   vtLPRLKIL---EV---LQ-----epdnhHVSAEDLYKRLIDM--G------------eE-I--GL-A-tvYRVLNQ--------------------fIV-TR-HNFE-K---SVFELT--
0020 9c5gB   -pwmQNRRF---EF---IEkLF----WEgALNRSDLEETFE--------------------tP--QT-S-----IDLRRYR--E--------------NI-EY-DAT--D---KTFKPT-K
0021 4txaA   qEEALKLVL---LA---LE-------dgsALSRKVLVLFVVQR--L-E-P-----------tS--IG-H--vvQLLYR--------------------CF-KV-TKRD-S---SLMQLK-E
0022 6qfdB   ltAFQKNILTV-L-------------geeaRYGLAIKRELEEY--Y-G-E----------eV--NH-G-rlYPNLDDLVN--K-----gL------VEK--S-ELDK-R---TNE-----
0023 6l0oA   -rSTAKRFISA-L---------vrtyNN-IFQFHQLRQIAKEL--N----------iQV-A-D--FE------nfIGSLND--Q-------------GYL-LK-KG------pKVYQ----
0024 7solA   pRNWSQCVE---LA---RLlqlkpspIK-FDHLSFLQNAAKLY--A-TyC------IP-F-A-eeD-LS--ADALLNILS---------E------VKI------------------Q-E
0025 1zelB   ipTWASRVVSG-L---arD-RP----V--VVTKEDLTQRLTEA--G---------------gR--DP-DS-aiRELRR---------------------WL-VQ-LPV--K---GTWA-A-I
0026 7bzhA   meDVKQSV---eKI---IK-DR------eWVTFNDLLKYIP--------------------Y--PA-P--evyDALSQLI--K--------------KV-GR-RG-------RYFYYI-K
0027 8ioiA   fmALPHAILVS-L-------------seqaSSGYELARRFDRS--I-GyF------WT-A-T--HQ-------qiyRTLR--VM-----E--nnnwVRA-TT-VLQR-P---DKKVY---
0028 6zwtC   lTTGEFAVL---KA---LVqHP----RE-PLTRDKLMNLARGR-----------------------------eRSIDVQVS--RL-----RrliednpYI-QT-VW---G---VGYVF-aE
0029 5uujA   vdELTEEVA---GI---LD--------gvVLTRDELVTRLV-A--D-K-R-----fVS-X-EerLR-SG--WGSVLKPLA--WR-----G------VLC-HG-PNRG-K---ITF--Q-F
0030 7pd7B   raGLLDVLKRI-H---------rrpggrgIIQIA----haahapkrtlvmlfrfrpaeyvlL--SM-TD--AHAFAACAR--EA-----G------FQV-TV-YSSK-R-pnPLVCVE--
0031 1tbxA   fFYPEAIVL---AY---LY-DN------eGIATYDLYKKVNAE--F-P----------stA-T--FY-------DAKKFLI--Q-----eG------FVK-ER-QER--G---EKRLY---
0032 4k51A   dVIMRRVFVAA-S-------------qkftTVSQSELYKLAT-------lP------AP-L-D--LS-AWdiEKSLLQAAV--E-----dY------VSI-TI-DHE--S---AKVTFA--
0033 5yadA   lsLLSTETXSI-L----------qdapaccLPLFKFIDIYEKK--Y-G-H-----------K--LN-VS----DLYKL-T--DT-----I--------A-IR-EQG--N---GRL-----
0034 7abi7   sEEFRNDFLEL-L----------rrfgtkRVHNNIVYNEYI----S-H-R------E---hI--HM-NT--LTDFTKWLG--R-----eG------LCK-VD-ETP--K---GWYIQY-I
0035 8wxsA   lsLVKKVVHSL-V-------------vsspLTVEQLMRDYRSA--A-G-----------cT-L--PYkDA--ESFLRSI-P--DT-----V-------TV-TG-H-------gQMAW----
0036 2zmeB   hmQLAKQLAGI-L------eeR----GG-IMSLTEVYCLVNRA----R-G------ME---L--LS-PE----DLVNACK--ML-----E-alklpLRL-R--VFDS-G---VMV--K-E
0037 7qenC   vrRLKKNVWRS-I---------snreKE-LK-FSDIIQGIS-K--M-Y-S---ddtLK-D-I--ST-S-----FCFICLL--HL-----A------nQI-TH-TEN--Y---NDLIV---
0038 1w7pD   ldEIAREIYEF-T---------dsdtnymIITLVDLYAMYNKS--MrI-G------TG---L--IS-PM----EMREACE--RF-----EhlglneLKL-V--KVNK-R---ILC-EK-F
0039 6zcaD   eMALVEIA---hEL---FE-----ehKK-PVPFQELLNEIASL--L-G-V-------lgD-R--IA-------qfyTDLN--------iD------GRF-LA-LS---D---QTWGLRwY
0040 7c0jB   -PTYSEMIA---AA---IR-------aegGSSRQSIQAYIKSH--Y-K-----------------vNK-KE-iNRVLYSL-------laagV------LKQ-T-------V---PGSW----
0041 3s93B   qeCLRKEIRSL-L-----------istkdgLSPQELEKEYLLM--V-G-N----------ilgyrS-TM--E-LVLDM-P--DV-----V--------R-VC-PGAG-G---TVI-----
0042 8iueP   veVLNQQCFKF-LqskaetqrN----SS-FASSHEVWKYICE---L-G-I------SK-V-E--LS-ME----dIETILN--TL-----I--ydgkVEM-TI-IAAK-G---HMKL----
0043 8fo9F   iPDCYVELEKI-I---------kpieFP-VIDRKRLLQLVREN--Q----------------lQ--LD-EN----ELPHAVH--FL-----N--esgvLLH-F-----Q-L---SDLYF---
0044 2wb6A   -LESTSLYKKA-G---seN-LY----FQgivFLDSMFLVI-EK--L-GeR------VK-LlgamcskLP--LSSFIEIVT---R-----K------AKV-MY-NE-------KEVTLV-A
0045 2eshA   gWWLASTILLL-V-------------aekpSHGYELAERLA-E--F-G-I-------G-H-M--GN--------iyRVLA--DL-----E--esgfLST-EW-DTVS-P---PRKI----
0046 8ffzB   slQRQRAILKV-M----------ntiggvaYLREQFYESVSKY--M-G-S-----------lD--KK-T---vrGDVDLMV--E----seK------LGA--R-TEPS-G---RKIIF---
0047 8jriV   hNVIKTGVRMI-S-------------lsysRISLADIAQKLQ--------------------ldspE-DA--E-FIVAKAIrdGV-----I------E-A-SI-NHE--K---GYVQS---
0048 3cuqA   yyELGVQIIEV-C------hrN----GG-LITLEELHQQVLKG--R-GkF------AQ-D-V--SQ-D-----DLIRAIK--KL-----K-algtgFGI-IP-VG---G---TYL---aE
0049 3l9fA   -MQGKDIILGI-L-------------skkeRSGYEINDILQNQ--L-SyF------YD-G-T----------ygmIYPTLR--KL-ekdgK------ITK--E-VVIQ-N---KNI-----
0050 8gccA   lkPGQRKIIFS-S----------krrltrSIKVVQLAGYVSEH--A-A--------YH-H-G--EQ---slvQTIVGLAQnfvgsnnvpL------LQQ-DG-Q--trR---YIF--tya
0051 8k51A   iEEPQSFLEKI-Y---------ndsngllIISIF----------------------ywkktfnnqiLS--PEYVEGLLK--ES-----G------FKI-ER-ICEV---stDMWVVR--

0001 s001A   -LLHHHHHHHL-LL---LL-LL----LL-LLLHHHHHHHHHHH--L-L-L------LL-L-L--LL-LL--HHHHHHHHH--HH-----L------LLE-EE-EELL-L---EEEEEL-L
0002 8zjrM   hHHHHHHHHHH-E----------eeeEE-EEEHHHHHHHHHHH--H-L-L--------lL-L--LL-HH----HHHHHHH--HH-----L------LLL-EE-EEEL-E---EEEEE---
0003 7ymdC   hhhhlHHHHHHhHH---HH---hhhlLL-LEEHHHHHHHHHHH--H-HhL------LL-L-L--LH-H-----HHHHHHH--HH-----H-----hhEE-EE-ELLL---llLEEEEE-E
0004 5nl9A   lLHHHHHHHHH-H-----------hllllEELHHHHHHHHHHH--L-L-L------LLhH-H--HH------hhHHHHHH--L--------------LLL-EE-EELL-L---EEEELL-L
0005 7asvA   hhHHHHHHH---HH---HH-------hhlEEEHHHHHHHHHHH--H-H-LllllhHH-L-L--LL-HH----HHHHHHH--H--------------LLL-EE-ELLLlH---LEEELL-L
0006 7kbfK   lLLHHHHHH---HH---HHlLL----LL-LEEHHHHHHHHHLL--L-L-L------LL--hH--HH-H-----hHHHH------------------LE-EE-EEEL---llEEEEEL-L
0007 7db7B   -----------------------------lEEEHHHHHHHHHLL--L-L------------lL--LL-L---HHHHHHHHH--HH-----L------LEEeEE-EEleellllEEEEEE-L
0008 5a48B   lLLHHHHHH---HH---HHhLL----LL-LLLHHHHHHHHHHH--H-L-L----------lL--LL-LL----LHHHHHL--LL-----L------LEE-EE-ELLL-L---LEEEE---
0009 4etsB   lLHHHHHHHHH-H----------hhllL-LLLHHHHHHHHHHH--L-H-H------H--L-L--LH-H--hhhHHHHHLL-----------------LE-ELlLEEL---leELLEEL-L
0010 5okcB   hhHHHHHHHHH-HhhllLL-LL-------LLLHHHHHHHHHLL---L-L-L------HH-L-L--LL-HH----hHHHHHH-----------------HHE-EE-LLL--L---LLEEEL-H
0011 7z8bC   hhHHHHHHHHHhHH---HHlLL----LL-LEEHHHHHHHHHHH--H-H-LllllhhL-L-L--HH-------hhhhHHH--H--------------hLE-EE-LLLL-L---LEEEEL-L
0012 1ka8A   lLHHHHHHHH---------llL----LL-LLLLLLHHHHHHHH--H-HlL------LL-L-L--LL-HHhhHHHHHHHHH--HL-----L------LLL-LE-EEEL-E---EEEL----
0013 8e4yA   lLLHHHHHHHH-H----------hhllllLEEHHHHHHHHHHH--H-H-H------HL-L-L--HH------hhHHHHHH--HL-----L-------EE-EE-ELLL-L--lLEEEE---
0014 8e4vA   lLLLH--hhhhhHH---HHhLL----LL-LLLHHHHHHHHHLllL-H-H------HL-H-H--HH---hhhhHHHH-------------------hLE-EE-EL-------LEEEEL-L
0015 2qbyB   llHHHHHHH---HH---HH----------llLLHHHHHHHHHHH--H-H-H------LL-L-L--LL-HH----HHHHHHH--HH-hhllL------EEE--E-EELL-L---EEE-----
0016 2lh9A   hHHHHHHHHHH-H-----------hhllllEEHHHHHHHHHHH--H-L-------------llllhlLH--HHHHHL--L--LL-----L------E-E-EE-ELLL-L---LEE-----
0017 2ly1A   llHHHHHHHHH-H-----------hllllllEELLHHHHHHHHH--HlL-L------LLhH-H--HL--------LHHHHH--H------H------EEE--E-EELL-L---EEE-----
```

```
0018 7nywE   llHHHHHHHHH-H------hhH----LL-EEEHHHHHHHHHHH--L-LhH------HH-HhH--HH-H------HHHHHH--HH-------------LE-EE-LLL----llLEEEELl-
0019 5l0pA   llHHHHHHH---HH---HL-----lllllEEEHHHHHHHHHHL--L------------lL-L--LH-H-hhHHHHHH-------------------hLE-EE-ELLL-L---LEEEEL--
0020 9c5gB   -lhhHHHHH---HH---HHhHH----HLlLEEHHHHHHHHL-------------------hH--HH-H-----HHHHHHH--H--------------LL-EE-ELL--L---LEEELL-L
0021 4txaA   hHHHHHHHH---HH---HL-------lllLLLHHHHHHHHHHH--L-L-L----------hH--HH-H--hhHHHHH--------------------LE-EE-EELL-L---EEEEEL-H
0022 6qfdB   llHHHHHHHHH-H-------------hhllEEHHHHHHHHHHH--H-L-L----------lL--LH-H-hhHHHHHHHHH--L-----lL------EEE--E-EEEL-L---EEE-----
0023 6l0oA   -lHHHHHHHHH-H---------hhhlLL-EEEHHHHHHHHHHL--L---------lLL-L-L--HH------hhHHHHHH--H-------------LLE-EE-LL------lLEEE----
0024 7solA   lLLHHHHHH---HH---HHhhhllllLL-LLHHHHHHHHHHHH--H-HlL------LL-L-L-hhH-HL--HHHHHHHHH---------L------LLL------------------L-L
0025 1zelB   llHHHHHHHHH-H---hhH-LL----L--LEEHHHHHHHHHHL--L--------------lL--LH-HH-hhHHHHH--------------------LE-EE-LLL--L---LEEE-E-E
0026 7bzhA   llLHHHHH---hHH---HH-HH------lEEEHHHHHHHLL--------------------L--LH-H--hhhHHHHHHH--H--------------LE-EE-EL-------LEEEEL-L
0027 8ioiA   llLHHHHHHHH-H-------------hhllLLHHHHHHHHHHHL--H-HhL------LL-L-L--HH-------hhhHHHH--HH-----H--hlllEEE-EE-ELLL-L---LEEEE---
0028 6zwtC   lLHHHHHHH---HH---HHlLL----LL-LEEHHHHHHHHHLL----------------------------lLHHHHHHH--HH-----HhhhlllhLE-EE-EL---L---LEEEE-lL
0029 5uujA   hhHHHHHHH---HH---LL--------llLEEHHHHHHHHH-L--L-L-L-----lHH-H-HhhHH-HL--HHHHHHHHH--HH-----L------LLE-EL-LLLL-L---LEE--H-H
0030 7pd7B   hhHHHHHHHHH-H---------heeeeeeEEELL----lhhhllllleeeeeeeeeleeeeE--EL-LL--HHHHHHHHH--HL-----L------LEE-EE-ELLL-L-llLEEEEEE--
0031 1tbxA   lLLHHHHHH---HH---HL-LL------lLLLHHHHHHHHHLL--L-L----------lhH-H--HH-------HHHHHHH--H-----lL------LEE-EE-EEL--L---EEEEE---
0032 4k51A   hHHHHHHHHHH-H------------hhllEEEHHHHHHHHL-------lL------HH-H-L--LL-HHhhHHHHHHHHH--L-----lL------LLL-LL-LLL--L---LEEELL--
0033 5yadA   hhHHHHHHHHH-H----------hllhhheEEHHHHHHHHHHH--H-L-L-----------L--LL-HH----HHLLL-L--LL-----E--------E-EE-LLL--L---LLE-----
0034 7abi7   hHHHHHHHHHH-H----------hhhlllEEEHHHHHHHHH----L-L-L------L---lL--LH-HL--HHHHHHHHH--H-----hL------LEE-EE-ELL--L---LEEEEE-L
0035 8wxsA   hhHHHHHHHHH-H-------------hlllLLHHHHHHHHHHH--H-L---------lL-L--LLlLH--HHHHHLL-L--LL-----E-------EE-EL-L-------lLLLE----
0036 2zmeB   hhHHHHHHHHH-H------hlL----LL-EEEHHHHHHHHHHH----L-L------LL---L-LL-HH----HHHHHHH--LL-----H-hhlllEEE-E--ELLL-L---LEE--L-H
0037 7qenC   hhHHHHHHHHH-H---------hhhlLE-EE-LHHHHHHHL-L--L-L-L---lllLL-L-L--LH-H-----HHHHHHH--HH-----H------hEE-EE-LLL--L---LLEEE---
0038 1w7pD   hhHHHHHHHHH-H---------lllllllEEEHHHHHHHHHHH--LlL-L------LL---L-LL-HH----HHHHHHL--LH-----HhhllllLEE-E--ELLL-L---LEE-LL-H
0039 6zcaD   hLLHHHHH---hHH---HH-----hhLL-LLLHHHHHHHHHHH--L-L-L-------lhH-H--HH-------hhhHHHH--------hL------LLE-EL-LL---L---LLLEEHhL
0040 7c0jB   -LLHHHHHH---HH---HH-------hllLEEHHHHHHHHHHH--L-L---------------lLH-HH-hHHHHHHH-------hhllL------EEE-L-------L---LLEE----
0041 3s93B   hhHHHHHHHHH-H-----------hlllllLLHHHHHHHHHHH--H-L-L----------hhlllL-HH--H-HHHLL-L--LL-----E--------E-EE-ELHH-H---LEE-----
0042 8iueP   hhHHHHHHHHH-HhhhhhhhhH----HH-LEEHHHHHHHHHH---H-L-L------LL-L-L--LL-HH----hHHHHHH--HH-----H--hlllEEE-EE-ELLL-L---LEEE----
0043 8fo9F   eEHHHHHHHHH-H---------llllLL-EEEHHHHHHHHHHL--L--------------lL--LL-LL----LHHHHHH--HH-----H--hlllEEL-L-----L-L---LLEEE---
0044 2wb6A   -LLHHHHHLLL-L---lhH-HH----HHleeHHHHHHHHH-HL--L-LlL------LL-LllleeeeEE--HHHHHHHHL---H-----H------HHE-EE-LL-------LEEEEE-E
0045 2eshA   hHHHHHHHHHH-H-------------hhllLLHHHHHHHHH-L--L-L-L-------L-L-L--LL--------hhHHHH--HH-----H--hlllEEE-EE-ELLL-L---LEEE----
0046 8ffzB   hhHHHHHHHHH-H----------hllllleeELLHHHHHHHHHH--H-L-L----------lL--HH-H---hhHHHHHHH--H----llL------LEE--E-ELLL-L---LEEEE---
0047 8jriV   hHHHHHHHHHH-H------------hhllEELHHHHHHHHL--------------------lllhH-HH--H-HHHHHHHhlLL-----L------L-L-EE-LLL--L---LLEEL---
0048 3cuqA   hhHHHHHHHHH-H------hhH----LL-EEEHHHHHHHHHHL--L-LlL------LL-L-L--LH-H-----HHHHHHH--HH-----H-hhlllLEE-EE-EL---L---EEE---lL
0049 3l9fA   -LLHHHHHHHH-L-------------llllEEHHHHHHHHHHL--L-LlL------EE-L-L---------lllHHHHHH--HH-hhllL------EEE--E-EELL-L---EEE-----
0050 8gccA   llHHHHHHHHH-H----------hhllllLEEHHHHHHHHHHH--L-L--------LL-L-L--HH---hhhHHHHHHHHLllllllllL------EEE-EL-L--llL---LLE--lhh
0051 8k51A   lLLHHHHHHHH-H---------heeeeeeEEEEE-----------------------hhhhhhhlhlLL--HHHHHHHHH--HH-----L------LEE-EE-EEEE---leEEEEEE--
```

Job: Type-2 BREX; PglW_WP_185095299 1 iSTAND
Query: s001A
No: Chain Z rmsd lali nres id PDB Description
1: 8umy-A 9.5 11.6 130 546 8 MOLECULE: CHROMOSOME TRANSMISSION FIDELITY PROTEIN 18 HOMOL
2: 7jgr-D 8.9 5.4 139 441 9 MOLECULE: ORIGIN RECOGNITION COMPLEX SUBUNIT 2;
3: 3bos-B 8.9 3.9 128 231 10 MOLECULE: PUTATIVE DNA REPLICATION FACTOR;
4: 8dr5-A 8.5 3.6 125 646 10 MOLECULE: REPLICATION FACTOR C SUBUNIT 1;
5: 8thc-A 8.5 4.9 132 479 11 MOLECULE: ELG1 ISOFORM 1;
7: 7mi1-A 8.4 9.7 123 2628 10 MOLECULE: CHIMERA PROTEIN OF DYNEIN AND ENDOLYSIN;
8: 5e7p-A 8.2 5.5 139 719 10 MOLECULE: CELL DIVISION CONTROL PROTEIN CDC48;
9: 5zr1-A 8.2 6.0 137 494 11 MOLECULE: ORIGIN RECOGNITION COMPLEX SUBUNIT 1;
10: 3n70-A 8.1 3.3 121 142 8 MOLECULE: TRANSPORT ACTIVATOR;
11: 8k8v-A 8.1 4.4 130 719 13 MOLECULE: ENDOPEPTIDASE LA;
12: 8btg-B 8.1 4.2 139 335 8 MOLECULE: CHROMOSOMAL REPLICATION INITIATOR PROTEIN DNAA;
14: 4rh7-A 8.0 4.1 140 3005 4 MOLECULE: GREEN FLUORESCENT PROTEIN/CYTOPLASMIC DYNEIN 2 HE
15: 2qby-B 8.0 5.0 133 368 11 MOLECULE: CELL DIVISION CONTROL PROTEIN 6 HOMOLOG 1;
16: 6hyp-A 7.9 4.6 126 2272 7 MOLECULE: MIDASIN,MIDASIN;
17: 3j3r-A 7.9 8.5 143 798 11 MOLECULE: ADAPTER PROTEIN MECA 1;
18: 6n9x-B 7.8 3.6 125 500 14 MOLECULE: DNA PRIMASE/HELICASE;
19: 8ui7-A 7.8 3.3 129 643 10 MOLECULE: ATPASE FAMILY AAA DOMAIN-CONTAINING PROTEIN 5;
20: 7vcs-A 7.7 3.3 132 766 6 MOLECULE: TRANSITIONAL ENDOPLASMIC RETICULUM ATPASE;
21: 7mi8-A 7.6 9.6 124 868 6 MOLECULE: FUSION PROTEIN OF DYNEIN AND ENDOLYSIN;
22: 5j1s-B 7.5 7.5 135 235 14 MOLECULE: TORSIN-1A;
23: 6iy8-A 7.5 5.9 138 469 6 MOLECULE: POSITIVE REGULATOR CAPR;
24: 2gno-A 7.4 4.5 117 296 7 MOLECULE: DNA POLYMERASE III, GAMMA SUBUNIT-RELATED PROTEIN
25: 9e22-A 7.4 10.1 129 2925 4 MOLECULE: CYTOPLASMIC DYNEIN 1 HEAVY CHAIN 1;
26: 7uqj-A 7.4 3.7 129 585 9 MOLECULE: ATPASE HISTONE CHAPERONE YTA7;
27: 8xks-B 7.4 23.4 130 603 9 MOLECULE: CTAP1;
28: 8fwj-A 7.3 3.6 134 552 11 MOLECULE: CIRCADIAN CLOCK PROTEIN KAIC;
29: 7ykk-A 7.3 6.1 137 735 12 MOLECULE: ATPASE FAMILY GENE 2 PROTEIN;
30: 7plh-A 7.3 6.4 129 260 8 MOLECULE: SHTNSC;
31: 6z1f-3 7.3 4.0 130 284 8 MOLECULE: RIBULOSE BISPHOSPHATE CARBOXYLASE/OXYGENASE ACTIV
32: 8xvb-A 7.2 4.7 129 246 12 MOLECULE: DNA (5'-
33: 5zr1-B 7.2 9.1 124 374 10 MOLECULE: ORIGIN RECOGNITION COMPLEX SUBUNIT 1;
34: 1nlf-A 7.2 3.9 119 254 12 MOLECULE: REGULATORY PROTEIN REPA;
35: 9bv1-A 7.2 3.3 124 405 10 MOLECULE: 26S PROTEASOME NON-ATPASE REGULATORY SUBUNIT 1;
36: 8bob-C 7.2 4.4 122 412 11 MOLECULE: PROTEIN MALY;
37: 7st9-A 7.2 4.9 133 522 9 MOLECULE: CHECKPOINT PROTEIN RAD24;
38: 8fcv-R 7.2 6.0 139 343 12 MOLECULE: DNA (60-MER);
39: 8tpl-A 7.1 9.2 123 419 12 MOLECULE: MITOCHONDRIAL CHAPERONE BCS1;
40: 6qem-G 7.1 8.4 136 239 7 MOLECULE: REPLICATIVE DNA HELICASE;
41: 3pvs-D 7.1 3.5 113 424 12 MOLECULE: REPLICATION-ASSOCIATED RECOMBINATION PROTEIN A;
42: 8tw7-2 7.1 5.3 121 339 12 MOLECULE: REPLICATION FACTOR C SUBUNIT 5;
43: 9cru-C 7.0 8.6 136 741 6 MOLECULE: VESICULAR-FUSION PROTEIN SEC18;
44: 5zr1-E 7.0 4.0 120 460 10 MOLECULE: ORIGIN RECOGNITION COMPLEX SUBUNIT 1;
45: 9klv-D 7.0 3.8 125 338 8 MOLECULE: PROTEIN SUPPRESSOR OF MAX2 1;

```
46: 3co5-A 6.9 3.2 107 134 10 MOLECULE: PUTATIVE TWO-COMPONENT SYSTEM TRANSCRIPTIONAL RES
47: 8xku-A 6.9 3.5 132 730 11 MOLECULE: PROBABLE INACTIVE ATP-DEPENDENT ZINC METALLOPROTE
48: 6l1q-A 6.9 3.7 113 267 5 MOLECULE: CBBQ PROTEIN;
49: 8dgc-A 6.9 5.6 130 2028 8 MOLECULE: SEAVS3;
50: 7st9-E 6.9 3.4 120 354 10 MOLECULE: CHECKPOINT PROTEIN RAD24;

0001 s001A   NADRTSAVT-QRR----E-QKLRYRANAQLLES-A-AR-P--GFRLLTVRYD-D-QRL-ALLT-LTEEWR---AEPVDVS--I-LFLTSLRRLILEADNAVPGSRDAMKFGEYTAAAWGEVE-KELASN-SRPLLLHD-A-S--VM-ARYG-G-T-G-ILQRLADHAR-R--G--GRGLWLLSPLNAVP--RLEDWTVALED-REEWIRLN-H-SVND-PEDP
0002 8umyA   lvLRAS---clap-----ellsDDFTNRCLLKW-L-KL-WdlKVALLCGPPG-L---G-KTTL-A-HVIAgysVVEMN-A--S-DDR-----------------------------SPEVFR-TRIEAAkPNCLVIDE-IdG--AP-V--A-A-I-N-VLLSILNR--gL--L--MRPIICICND---Q--FAPsLRQLKQQ---FLLHFP-P-TsrL-VQRV
0003 7jgrD   ADRESIRRK-ERLqrdyttlrGYAKERSNVRLL-L-QReS--NSLLLLGPRG-S---G-KTTL-I-NSVL----VHLDGN--L-HT------------------------------DDRVAL-KSITVQ-MVIFILEE-F-D--LF-CAHH-N-Q-T-LLYNLFDVSQ-SaqA--PICVLGVTCR-LDV--I-ELLEvRFSH---RQVFLF-P-SedY-VDLL
0004 3bosB   -------------tsyyP-AAGNDELIGALKSA-A-SG----QAIYLWGPVK-S---G-RTHL-IH-AAC---AFYIPLG--I--HASI-------------------------------ST-ALLEGLqFDLICIDD-V-D--AV-AGHP-W-E-E-AIFDLYNRVA-E--Q-kRGSLIVSASA-spX--EAGFVVSRXHW---LTYQLQ-P-XdeK-LAAR
0005 8dr5A   arrkenTKE-A--ptnlqqvcGNKGSVMKLKNW-L-AN-W--RAAMLYGPPG-I---G-KTTA-A-HLVAgydILEQN-A--S-DV-----------------------------rsKTLLN-AGVKNAkHFVIIMDE-V-D--GM-SGRG-G-V-G-QLAQFCRKT--------STPLILICN---ER--NL-PKMRFDRV---LDIQFR-R-PanS-IKSL
0006 8thcA   VLLGTNIK-qLFK-vtlkqvlIEPKLKLRIKNW-I-ET-S--PLMILHGN-----sIG-KKTL-I-QTIMsyqIYEVN-S--N-MN-----------------------------rSKKDLL-DILLDF-TGLVLFND-V-D--VL---RG-Y-W-A-MISKLCEFS--------RRPLVLTCKD---L--SLV-PSELASE-QNSLFHTK-K-IstV-YAFT
0007 8emhB   ----sdldD-LTK----L-KEDVQWHLLARMVP-F-VE-N--YNVCELGPRG-T---G-KSHI-YKECSP--nSILVSGG--Q-TT-------------------------------VANLF-YGLVGL-WDVVAFDQ-V-A--G--ISFK-G-V-Q-IMKDYMAS--greqmeaSASMVFVGNINLLA--PFPEAMdFFDR-fHAYIPGW-E--KMR-PEFT
0008 7mi1A   SSDGV------------------WNIADVVANS----E-N--NYFTMASERD-V-D---GTFK-L-IELAkesLKIIPL------GSIE---------------------------NLNYAQ-EEISKSeGGWILLQN-I-Q--MS-L--S-W-V-KtYLHKHVEE--tK--A--AEKMFMTCHLT-------DKLPLLQR----DRVVYE-D-I-PG-ILD-
0009 5e7pA   RGVVRLHpadHTPsvtlddvgdMVETKRALTEA-V-LWpP--RGVLLYGPPG-C---G-KTFV-V-RALArlsVHAVKGS--E-LMDKWV-------------------------GSSEKAV-RELFAR-ASLVFLDE-IdA--LA-PTDK-V-V-A-SLLTELDGIE-P--L--RVVVLGATNR--------LIDPLLRP---gRLVFVE-P-PrdI-LRTG
0010 5zr1A   IVETIFVKK-Q--lnssyylpaRENEFASIYLS-A-YSsA--TTIYVAGTPG-V---G-KTLT-VREVVK----VEINGL--K-MV------------------------------KPTDCY-ETLWNK-VIVVLLDE-L-D--AM-VTKS---Q-D-IMYNFFNWTT-YenA--KLIVIAVANT--MD--LPERQLgITSR---TRIMFT-G-YeEL-KNIR
0011 3n70A   -----------------WiNQYRRRLQQL-SET-D-------IAVWLYGAPG-T---G-RXTG-A-RYLHqgeFVYRELT-pD-NAP---------------------------------QL-NDFIALqGGTLVLSH-P-E--HL------T-R-E-QQYHLVQLQS-QehR--PFRLIGIGDT-lVE--LASNHIILYYCfAXTQIAC-----LT------
0012 8btgB   -------------dtfvI-GSGNRFAHAASLAVaE-AP-A--NPLFIYGGVG-L---G-KTHL-MH-AIG---HVYLSSE--K-FTNEFINSI-----------------------kaVDFR-NRYRNV--DVLLIDD-I-Q--FL-AGKE-T-Q-E-EFFHTFNTLH-E--E--SKQIVISSDR-pPK--EIPTLElRFEW-G-LITDIT-P-PetR-IAIK
0013 5v8fD   -DPGFLQRRlqLP--ekiiFTYLQDCQQEIDRI-I-KE-S--HSVILVGPRQ-S---Y-KTYL-L-DYELqfiTIRLNGF--I-HS-------------------------------EQTAI-NGIATQ-LVVFIFDE-I-D--TFaGPVR---Q-T-LLYNLFDMVE-H--SrvPVCIFGCTTK-LNI--L-EYLEvRFSQ---RVIYMP-Q-IvdA-VRNL
0014 4rh7A   dTSSE-dpL-ILDasklvyTPLTDKCYLTLTQA-M-KM-G--LGGNPYGPAG-T---G-KTES-VKALGGgrqVLVFNCD--E-GI------------------------------DVKSMG-RIFVGLcGAWGCFDE-FnR--LE-E--S-V-L-S-AVSMQIQTIQ-D--A--LSGIFITMNPA-kgyggRQKLPLKQL----RPVAMS-H-P-DN-ELIE
0015 2qbyB   -----KNPK-VSV-----eipFREDILRDAAIA-I-RYvK--FSNLFLGLTG-T---G-KTFV-S-KYIF----AYVNCR--E-VG-----------------------------gTPQAVL-SSLAGK-LAIIYLDE-VdT--LV-KRRG-G-D-I-VLYQLLRS---D--A--NISVIMISND--iN--VRDYMEVLSS----PSVIFK-P-YeqL-KFIY
0016 6hypA   alepqgltikptlqahyiiTPFVEKNMMNLVRA-T-SG-K--FPVLIQGPTS-S---G-KTSM-I-KYLAhkfVRINNHE--H-TD--------------------------------LQEY-LGVLVEkGYWIVLDE-LnL--AP-T--D-V-L-E-ALNRLLDdnrpH--P--DFLLFATQNPP------iKILSFRNR----LELHFD-D-IpqD-ELEL
0017 3j3rA   QENSEVT--ssetnilhsrvigQDEAVVAVAKA-V-RRpI--GSFIFLG----P-TGVgKTEL-A-RALAeesMIRIDMS--E-YM-kHSTSV---------------------pGYVGYDEgGQLTEK-VSVVLLDA-I-E--KA-HP-D-V-F-N-ILLQVLEDG-tvdfR--NTILIMTSNVGGEL--K-AFRPFINR----EIIVFH-S-LKHL-T---
0018 6n9xB   -------------ssvgllfsgCTGINKTLG---A-RG-G--EVIMVTSGGM-G-KST-FVRQ-QALQWGgkkVGLAMLQ--EsveeedligriengkfdqwfdelfgndtfhlydsTDRLL-AKLAYMgCDVIILDH-I-S--IV-VS-M-I-D-N-LMTKLKGFAK-S--T--GVVLVVICHL-TDL--R-GSGALRQL--sDTIIALE-R-pLEP-S-SS
0019 8ui7A   TEEVLLLEF-PLLpqtaseliGNELAIKKLHSW-L-KD-W--NTVLITGPTG-V---G-KTAA-V-YACAgfkIFEVN-A--S-SQ-----------------------------rSGRQIL-SQLKEA-TSLILFEE-V-D--VI--DAG-F-L-N-AIKTFMATT--------KRPVILTTSD-PTF--S----MFDGC----EEIKFS-T-PlnV-ASY-
0020 7vcsA   -------------diggC-RKQLAQIKEMVEL-----P-P--RGILLYGPPG-T---G-KTLI-A-RAVAgafFFLINGP--E-IMSKLA-------------------------GESESNL-RKAFEE-AAIIFIDE-LdA--IA-PERR-I-V-S-QLLTLMDG-L-K--Q--RVIVMAATNR--PN--SI--DPALRRFrfDREVDIG-I-PatG-RLE-
0021 7mi8A   SGYL-----nlvf---------eWNIADVVAN----SE-N--NYFTMASERD-V-D---GTFK-L-IELAkesLKIIPL------GSIE---------------------------NLNYAQ-EEISKSeGGWILLQN-I-Q--MS-L--S-W-V-KtYLHKHVE---eH--E--KFKMFMTCHLT-------DKLPLLQR----DRVVYE-D--IPG-IL--
0022 5j1sB   YSSPAQ---knflqledkfpgQSSFLWQRGRKF-L-QKeP--ATIIFTAARE-G-RET-LKCL-SH--HV---ADQIDGA--G-------------------------rtwqDSDTVKLLVD-LELSYGgQKAAVVHH-FeS--FP-A--G-S-T-L-IFYKYCD---aF--K--DVALVLTVLLEEKVrdLLWAKdLWSR----HLVLPV-Q--VSSi----
0023 6iy8A   YELQLVSL---------Q-TPAYQTVRNMMDKA-A-QG-K--VSVLLLGETG-V---G-KEVI-A-RSVHaepFVAVNCA--A---IPPD--------------------------LIESEL-FGRFERdKGTIFLDE-V-I--EL-S-PR-A-Q-A-SLLRVLQE--dnrtrkiDVRVIAATH--EDL--AeGRFRLYYR-lNVFPVAIP-A-LreD-IPLF
0024 2gnoA   -----------------------DQLETLKRII-E-KSeG--ISILINGE---D-LSY-PREV-S-LELP----LEIDpE--N-IG--------------------------------iDDI-RTIKDF-LKYVIVHD-C-E--RX-T-QQ-A-A-N-AFLKALEEP--P--E--YAVIVLNTRR---W--HYL-LPTIKS----FRVVVN-V-PkeF-RDLK
0025 9e22A   GEAVD----gvem------GGMWVEKVLQLYQI-T-QI-N--HGLMMVGPSG-S---G-KSMA-W-RVLLegvAHIIDPK--A-IS-------------------------------KDHLY-GLFTHV-LQWIVFDG-D-V--D-----E-W-V-E-NLN---SVLD-D------VRIMFEVQD------LKYATLTVSR----GMVWFS-E-D-LS-TDM-
0026 7uqjA   -------------nvnfddiggLDNYIDQLKEM-V-AP-P--RGVLFHGPPG-T---G-KTLM-A-RALAkitFFMRKGA--DiLSKW--------------------------VGEAERQL-RLLFEE-ASIIFFDE-IdG--LA-PIHA-SiV-S-TLLALM-DGM-D--N--RVIVIGATNR-pDA--VD---PALRRP--gREFYFP-L-PkAR-FKIQ
0027 8xksB   -----------------G-nkQAKVELMEVVDF-F--A-P--KGVLLVGPPG-N---G-KTLM-A-RAVAgvaFISSSAA--E-FI---EMYM----------------------GLGAARV-RDLFNT-ACIIFIDE-L-D-aVG-RQDN-T-V-N-QLLTEMD---eQ--Q--GIVVMGATNR--KD--VLDAALTRPG----RSIEVR-R-PfqG-RLE-
0028 8fwjA   -------------ksptgiqGFDELTLG--GLP-T----G--RPSLVCGSAGcG-KTL-FAST-FLINGVgepGVFVTFEieH-IAV----------------dpsEVAE--igdYDLEGLF-LRLELA-IKRVVLDT-I-E--SL-FSAF-L-R-A-EIRRLFDWLK-E--R--GLTTVITAERGALT--RQ---GLEE--vSDCVILLD---nFSV-LPVL
0029 7ykkA   VTLK---------kvywsdigGQEELKTKMKEM-I-QL----KGVLLYGPPG-CsKTL-TAKA-LATES-ginFLAVKGP--E-IF---NKYV----------------------GESERAI-REIFRK-ASIIFFDE-I-D--AL-SPNH-V-L-T-SLLNEID---eL--K--GVVIVAATNR---P--DEIDAALLRPGrlDRHIYVG-P-PnAR-LEIC
0030 7plhA   dEWLQAaRL-KG--ksivpLQQVKTLHDWLDGK-R-KA-R--KSCRVVGESR-TgKTV-ACDA-YRYRH-tvpVVYIRPH--Q--------------------------------kcGPKDL-FKKIT-gVEMLIIDE-A-D--RL------K-P-E-TFADVRDIAE-D--L--GIAVVLVGTD-rlD--AVI---VLER----AHLRFG-K-LedF-KNTW
0031 6z1f3   ------------------syyiAPRFLDKLAVH-I-TK-N--LILGIHGRKG-E---G-KTFQ-C-ELAFgieVTLISG---G-ELE--SPDA---------------------GDPARLIR-LRYRET-ACVLMIND-LdA--GA-GTQL-V-N-A-TLMNIADN----------VPIIVTGND---F--STLYAPLIRDG-RMEKFYWE-P-NvgI-VGGA
0032 8xvbA   ------LPE-PPR----vetqTVKQIWTSMRFA-S-LT-E--SIAVVCGNPG-V---G-KTEA-A-REYRnnnVWMITIT--P-SC-----------------------------aSVLECL-TELAFE-LGLVIIDE-A-D--HL------G-A-E-VLEELRLLQE-S--T--RIGLVLMGNH-rvY--SNMT--LFSR--iAKRTAIN-K-TkaD-VKAA
0033 5zr1B   mSMAP------------F-EIQKK-MFPQYWFE-L-TQ-G--FSLLFYGVG--S-KRN-FLEEfAIDYLSsipCLILNGN--P-SC------------------------------NYRDVF-KEITDLiKLILVVHN-L-DgpSI-RKNT-F-Q-T-MLSFLSVI-------R--QIAIVASTDHI-----yAPLLWKAQN----VFHDIS-Nf--------
0034 1nlfA   -------------thkpinileafpplyvLPNM---VA-G--TVGALVSPGGaG-KSM-LALQ-LAAQIAtgpVIYLPAEiqP-LIGSL------------------------pnimAPEWF-DGLKRAgRRLMVLDT-L-R--RF-HIE--pmA-Q-VIGRMEAIAA-D--T--GCSIVFLHHA-----------VLVDN--iRWQSYLSfV-RLKP-AV-E
0035 9bv1A   -------------vtysdvggCKEQ-IEKLREV-V-E-----KGVLLFGPPG-TgKTL-CARA-VANRT-dacFIRVIG---S-ELV--QKYV----------------------GEGARMV-RELFEM-ACLIFFDE-I-D--AI-GGQR-T-M-L-ELINQLD---pR--G--NIKVLMATNR------PDTLDPMRPG----RKIEFS-L-PthI-FKI-
0036 8bobC   -------------pvrldhtvvRERLLAKLSGA---NN-F--RLALITSPAG-Y---G-KTTL-ISQWAA----GWYSLD--E-GDN-----------------------------QQERFA-SYLIAA-VlYLVIDD-Y-H--L--ITNP-I-H-E-SMRFFIRH-Q-P--E--NLTLVVLSRN-----------QLGILR-VRLEIGSQ----AFT-HQEF
0037 7st9A   ---------eKFK--cleqvaiHKRKLKDVQEA-L-DA-M--RILLLSGPSG-C---S-KSTV-I-KELS----VTEFRG--D-C----IVND---------------------lPQMESFS-EFLKGAnLSLILIED-L----PN-VFHR-F-Q-Q-LILQWLYS---sepL--LPPLVICITECFSA--E--TIMNILMH---KRIKFN-P-ItLL-KKHI
0038 8fcvR   lLTRPERLAfENY----vaHPRLKEVYEILMRT-I-AE-P--SFIFVYGASG-V---G-KTTL-R-LRVErvpVVGIEA-----IAPE-----------------------SRYF-NWKEYY-TRAlithPDVFFVDE-A-Q--HF-GKVK-L-Q-D-QLDCLKSLAN-M--T--GILHCLLGTY--------llTFRNLSR-R-VDIHFR-R-YqaF-KSVF
0039 8tp1A   RTQH---------svvlQ-QGLADRIVKDIREF-I-DN----YRRGYLLYgC-G-KSS-FITA-LAGELE---HSICLLS--L---TDSS-------------------------lSDDRLN-HLLSVA-PQQSLVLL---------eaFS-G-LlN-ALDGvaSTEA----------RIVFMTTN---idrLDPALIRPG----DLKEYV-G--ycS-HWQM
0040 6qemG   wQKEQ----rpsf---vE-CEGQMNALSKARQY-V-EE-F--ASFIFSGKPG-T---G-KNHL-AA-AIC---NLIITVA--D-IMSAMKDTF----------------------tSEEQLL-NDLSNV--DLLVIDE-I--------EKV-IiN-Q-IVDRRSSS---------KRPTGMLTNS-EEM--T-LLGEmMRLG--NSLWVIF-N-W--------
0041 3pvsD   -------------laqyigqQHLLPLPRAIGHL-H--------SMILWGPPG-T---G-KTTL-A-EVIAnadVERISAV--T-SG--------------------------------VKEI-REAIER-ATILFVDE-V-H--RF------N-K-S-QQDAFLPHIE-D--G--TITFIGATTE--nP--SFElNSALLS----RVYLLK-S-LteD-IEQ-
0042 8tw72   ----lelAQ-QP-pknldevtAQDHAVTVLKKtlksAN-L--PHMLFYGPPG-T---G-KTST-I-LALTksrILELN-A--S-DE------------------------------rGISIVrEKVKNF-AKIIILDE-A-D--SM------T-A-D-AQSALRRTME-T--Y--STRFCLICNY-vTR--II----LASR----SKFRFK-A-LsNA-IDRI
0043 9cruC   MSDDVN-------ggmmlySERVNSILKNGARY-V-RQrL--VSLLIHGPAG-S---G-KTAL-AAEIAL----RLIS-P--N-ELSGMS------------------------ESAKIAYI-DNTFRD-ANILVIDS-L-E--TL-VDgN-I-L-Q-MLKVALKR---kppqdrRLLIMTTTSA-YSV--L-qQMDILCFD-N--EIAVP-N-M-tN-LDEV
0044 5zr1E   -------------nvttpevafREYQTNCLASY-I-ST-P--SNLILQGYSG-T---G-KTYT-LKKYF-nlhAVWLEPV--E-LV------------------------------SWKPLL-QAIART-VLFLILDG-F-D--SL--QDL-D-A-A-LFNKYIKL--lpkdskiNIKFIYTMLE-TSF--L----QRYSTH-CIPTVMFP-R-YdEV-STI-
0045 9klvD   ---------kalnekvwwqNDAAAAVAATVSQC-K-LG-D--VWLLFSGP---D-RVG-KRKM-V-SALSgtnPIMIQLG--SrqdaGNSSFR------------------------gKTAL-DKIAET-VSVILLED-IdE--AD-M--L-V-R-G-SIKQAMD---sL--G--NVIFVMTASWHSL-----PFAFMVSR--vDDAVAFR-A----------
0046 3co5A   ----------------gN-SAAIQEXNREVEAA-A-KR-T--SPVFLTGEAG-S---P-FETV-A--RYF---H-vSPAR--V-EY-------------------------------liDXP-XELLQKeGGVLYVGD-IaQ--YS-R--N-I-Q-T-GITFIIGKAE-R----cRVRVIASCSYASCE--EK---AGLFSEs--------------------
0047 8xkuA   -------------dfagQ-EYIKRELQEIVRIL-K-ND----pKGVLLHGGT-G-KTL-LAKA-IAGEAG---LFFAANG--T-DFV-----------------------EMFVGVAASRVK-DLFASSyAPSIIFIDeI-D--AI-GSEQ-G-L-L-QILTEMDGFK-V-tT--SQVLVIGATNR-------LDPALLRKG---DKIIRV-G--kdG-RLA-
0048 6l1qA   -------------vgkepfyqPQHDEVALFEAA-Y-RK-R--LPVMVKGPTG-C---G-KSRF-V-EFMAgkpLVTVACN----------------------------------edmTAADL-VGLTVAyGAICYLDE-I-V--EA------R-Q-D-TTVVI-HPLT-D--H---FQLVISYNPG-------MKDLTKQR----TGFEFD-Y-PelE-AGIV
0049 8dgcA   -------------iensvsRTQEATLVQRVVEA----F-G--APVIIHADAGvG-KSI-FSTH-IEEHL-gsvSILYDCF--G---------------------lgqyrNASSYrhHHRTAL-VQMANE-MLCIIIDA-A-D--NA-QMAA-etR-S-FIKDLIRE-K-L--P--DVCLVALCRP-YRR--E----LLDPPP-EALTLSLQ-T---fN-RDEH
0050 7st9E   -------------alshN-EELTNFLKSL-SDQ-P-RD-L--PHLLLYGPNG-T---G-KKTR-CM-ALL---ELEITPS--D--MGNN---------------------------dRIVIQ-ELLKEV--KCVIINE-AnS--LT-K--D-A-Q-A-ALRRTMEKYS----K--NIRLIMVCD----S--MSPiIAPIKS----LLIRCP-A-PseI-STI-
0051 7z6hK   ---------wvETelavH-KKKIEEVETWLKAQ-V-L-----SILLITGPPG-C---G-KTTT-LKILSI---QVQEW------INPV-----------------------lpyqSQIAVFK-EFLLRA-TKIILVED-L--pnQF-YRDH-T-L-HeVLRKYVRIG--------RCPLIFIISDS-sgdnNQRLLFpIQEE---SNISFN-P-V--A-PTI-
0052 6pxkK   -----------seseldkhiiGQDNAKRSVAIA-L-RNtP--KNILXIGPTG-V---G-KTEI-A-RRLAnapFIKVEAT----KFTEG------------------------------KEV-DSIIRD-LGIVFIDE-I-D--KI-CREG-V-Q-R-DLLPLVEG--xvktD--HILFIASGAFQ---iaKPSDLILQGR--lPIRVELQ-A-LtsD-FER-
0053 8ouyC   -------------ailstgiGSLDKLLD--AGL-Y--T-G--EVTEIVGGPGsG-KTQ-VCLC-MAANVAqqnVLYVDSNvvH-AFD------------------------------IFQML-DVLQELtVKVVVVDS-V-T--AV-VSPLlM-M-Q-LARELKTLAR-D--L--GMAVVVTNHITLKP--ALGRSWSFV-----TRILLD----DIG-T---
0054 6p8vC   -------------qerlarlvGLDEHKDRLSKI-L-GL----PLVVLAGDVG-S---G-KTEL-AETIGDditLYPLS-L--A-TR--GQGRV---------------------GEXTQLVS-AAFDYT-IVLLLIDQ-AdA--LA-Q-RA-G-V-N-AFIRGIDRIA-N--Q---AAVLXCTNR---L--KAL-DPAVQR-raAEILTFS-R-PeQR-HYLK
0055 3f8tA   ipevDPAE-eeDKaraiapLPGAEEVGKMLALQ-L-FSeR--LHVLLAG----Y-PVV-CSEI-L-HHVL----VYVDLR--R-TE--------------------------------LTDL-TGAAVLdGGILAVDH-LeG--AP-E--P-H-R-W-ALMEAMDK--dgialnaRCAVLAAINPGARI--DL-DQDFLSH--fDLIAFLG-Vp---------
0056 1g4aE   -----------seseldkhiiGQDNAKRSVAIA-L-RNtP--KNILMIGPTG-V---G-KTEI-A-RRLAnapFIKVEAT--K-FTE--VGYV-------------------------gKEV-DSIIRD-LGIVFIDE-I-D---kicREG-V-Q-R-DLLPLVEG--mvktD--HILFIASGAFQaKP--SDLiPELQG--rlPIRVELQ-A-LtsD-FER-

0001 s001A   LLLLLLLLL-LLL----H-HHHHHHHHHHHHHH-H-HL-L--LEEEEEEEHH-H-HHH-HHHH-HHHLLL---LEEEEHH--H-HHHHHHHHHHHHHHLLLLLLHHHHHHHHHHHHHHHHHH-HHHHHL-LLLEEEEL-L-H--HH-HHLL-L-H-H-HHHHHHHHHH-L--L--LLLEEEEEEELLLL--EELLEEELLLL-HHHEEELL-H-HHLL-HHHL
0002 8umyA   eeLLLL---llll-----llllLHHHHHHHHHH-H-HH-HllLEEEEELLLL-L---L-HHHH-H-HHHHlleEEEEL-L--L-LLL-----------------------------LHHHHH-HHHHHHlLEEEEEEL-HhH--LL-H--H-H-H-H-HHHHHHLL--lL--L--LLLEEEEELL---L--LLHhHHHHHLL---EEEELL-L-LhhH-HHHH
0003 7jgrD   HHHHHHHHH-HHHhlllllllLLLHHHHHHHHH-H-HHlL--EEEEEELLLL-L---L-LHHH-H-HHHH----EEEELL--L-LL------------------------------LHHHHH-HHHHHH-LEEEEEEL-H-H--HH-LLLH-H-H-H-HHHHHHHHHL-LllL--LLEEEEELLL-LLL--L-LLLLhHLLL---LLEELL-L-LhhH-HHHH
0004 3bosB   -------------llllL-LLLLHHHHHHHHHH-H-HL----LEEEEELLLL-L---L-HHHH-HH-HHH---HEEEEHH--H--HHHL-------------------------------LH-HHHLLHhLLEEEEEL-H-H--HH-LLLH-H-H-H-HHHHHHHHHH-L--L-lLLEEEEEELL-llL--LLLLLHHHHHH---EEEELL-L-LhhH-HHHH
0005 8dr5A   hhhhhhHHH-H--llllllllLLHHHHHHHHHH-H-HL-H--LEEEEELLLL-L---L-HHHH-H-HHHHlleEEEEL-L--L-LL-----------------------------llHHHHL-LLHHHHhLEEEEELL-H-H--HL-LLLL-H-H-H-HHHHHHHLL--------LLLEEEEEL---LL--LL-HHHHHLLL---EEEELL-L-LllL-LHHH
0006 8thcA   EELLLLLL-hHLL-lllllllLLHHHHHHHHHH-H-HH-H--LLLLLEEL-----lLL-LLHH-H-HHHHleeEEEEL-L--L-LL-----------------------------lLHHHHH-HHHHHH-HEEEEELL-L-L--LL---LL-H-H-H-HHHHHHHLL--------LLLEEELLLL---H--HHL-LHHHHHH-LLLLLEEL-L-LhhH-HHHH
0007 8emhB   ----hhhhH-HHH----L-LHHHHHHHHHHHHH-H-LL-L--LLEEEEELLL-L---L-LLHH-HHLLLL--lEEEELLL--L-LL-------------------------------HHHHL-ELHHHL-LLEEEELL-L-L--L--LLLL-H-H-H-HHHHHHHH--lleeeelLLEEEEEEELLLLL--LLLHHHlHHLL-lLLEEEHH-H--LLL-LLLL
0008 7mi1A   LLLLL------------------LLHHHHHHHL----L-L--LEEEEELLLL-L-L---LHHH-H-HHHHlllEEEEEL------LLHH---------------------------HHHHHH-HHHHHHhLLEEEEEL-H-H--HH-H--H-H-H-LlLHHHHHHH--lL--L--LLLEEEEELLL-------LLLLHHHH----EEEELL-L-L-LL-HHH-
0009 5e7pA   LLEEEELhehLLLlllhhhlllLHHHHHHHHHH-L-HHlL--LEEEEELLLL-L---L-LLHH-H-HHHLlleEEEEEHH--H-HHHLLL-------------------------LLHHHHH-HHHHHH-HEEEEEEL-HhH--HL-LHHH-H-H-H-HHHHHHHHHL-L--L--LEEEEEEELL--------HLLHHHLL---lEEEELL-L-LhhH-HHHL
0010 5zr1A   LLLLHHHHH-H--lllllllllLLLLHHHHHHH-H-HHlL--EEEEEEELLL-L---L-HHHH-HHHHHH----EEEEHH--H-LL------------------------------LLLHHH-HHHHHH-HEEEEEEL-L-L--LL-LLLL---L-L-LHHHHHHHHH-LllL--LEEEEEELLL--LL--HHHHHLlHHHH---EEEEEL-L-LhHH-HHHH
0011 3n70A   -----------------HhHHHHHHHHHH-LLL-L-------LLEEEELLLL-L---L-HHHH-H-HHHHlllLEEEELL-lL-LLL---------------------------------LH-HHHHHHlLLLEEEEL-H-H--HL------L-H-H-HHHHHHHHHH-LllL--LLLEEEEELL-hHH--HHHLLLLHHHHhHHHEEEL-----LL------
0012 8btgB   -------------llllL-LLLLHHHHHHHHHHhH-LL-L--LEEEEEELLL-L---L-HHHH-HH-HHH---HEEEEHH--H-HHHHHHHHH-----------------------lhHHHH-HHHLLL--LEEEEEL-H-H--HH-LLLH-H-H-H-HHHHHHHHHH-H--L--LLEEEEEELL-lLL--LLLLLLhHHLL-L-EEEEEL-L-LhhH-HHHH
0013 5v8fD   -LHHHHHHHhhLL--hlllLHHHHHHHHHHHHH-H-HL-L--LEEEEELLLL-L---L-HHHH-H-HHHHleeEEEEELL--L-LL-------------------------------LLHHH-HHHHHH-HEEEEEEL-H-H--HLlLLLL---L-H-HHHHHHHHHH-H--LllLEEEEELLLL-LLH--H-HHLLhHLLL---LEEELL-L-LhhH-HHHH
0014 4rh7A   hHHLL-llH-HHHllllllLHHHHHHHHHHHHH-H-HH-L--LEEEEELLLL-L---L-HHHH-HHHHHHlllEEEEELL--L-LL------------------------------LHHHHH-HHHHHHhLLEEEEEL-LlL--LL-H--H-H-H-H-HHHHHHHHHH-H--H--HLEEEEELLLL-lllllLLLLLHHLL----EEEELL-L-L-LL-HHHH
0015 2qbyB   -----LLLL-LHH-----lllLLHHHHHHHHHH-H-HHlL--LEEEEEELLL-L---L-HHHH-H-HHHH----EEEEHH--H-HL-----------------------------lLHHHHH-HHHHHH-HEEEEEEL-LhH--HH-HLLL-L-H-H-HHHHHHLL---L--L--LEEEEEELLL--lL--LLLLLLHHHL----LEEEEL-L-LhhH-HHHH
0016 6hypA   lllhlllllllllllllllLHHHHHHHHHHHHH-L-LL-L--LLLEEELLLL-L---L-HHHH-H-HHHHlllLEEELLL--L-LL--------------------------------LLLL-LLHHHHlLLEEEELL-LlL--LL-L--L-L-L-L-LLLLLLLllllL--L--LLLLEEEELLL------lLLLLLLLL----LEEELL-L-LllH-HHHH
0017 3j3rA   HHLLLLL--hhhhhhhllllllLHHHHHHHHHH-H-HHlL--LLEEEEL----L-LLLlHHHH-H-HHHHlllEEEEEHH--H-LL-lLLLLL---------------------lLLHHHHLlLHHHHH-HLEEEEEL-L-L--LL-LH-H-H-H-H-HHHHHHHLL-eeelL--LLEEEEELLLLHHH--H-HLLHHHHL----EEEELL-L-LLLH-H---
0018 6n9xB   -------------lllllllllLLLLHHHLL---L-LL-L--EEEEEELLLL-L-HHH-HHHH-HHHHHHlllEEELLLL--LlhhhhhhhhlllllhhhhhhhhlllllleeeellLLHHH-HHHHHHlLLEEELLL-L-L--LL-LL-H-H-H-H-HHHHHHHHHL-L--L--LLEEEEEELL-LLL--L-LLLLLLLL--lLEEEEEE-L-lLEE-L-LL
```

```
0019 8ui7A   LHHHHHHHH-HHHlllhhhllLLHHHHHHHHHH-H-HH-H--LEEEEELLLL-L---L-HHHH-H-HHHHleeEEEEL-L--L-LL-------------------------------lLHHHHH-HHHHHH-HEEEEEEL-L-L--LL--LLL-H-H-H-HHHHHHHHHL--------LLLEEEEELL-LLH--H-----HLLLL-----EEEELL-L-LhhH-HHH-
0020 7vcsA   -------------llllL-HHHHHHHHHHHHH-----L-L--LEEEEELLLL-L---L-HHHH-H-HHHHlleEEEEEHH--H-HHLLLL---------------------------LLHHHHH-HHHHHH-HEEEEEEL-HhH--LL-LHHH-H-H-H-HHHHHHHHL-L-L--L--LEEEEEELLL--LL--LL--LHHHHLLllLEEEELL-L-LhhH-HHH-
0021 7mi8A   LLEE-----hlee---------lLLHHHHHHH----LL-L--LEEEEEELLL-L-L---LHHH-H-HHHHlllLEEEEL------LLHH-----------------------------HHHHHH-HHHHHHhLLLEEEEL-L-L--LL-H--H-H-H-HhLHHHHHHH---hH--H--LLLEEEEEEELL-------LLLLHHHH-----EEELLL-L--LLL-HH--
0022 5j1sB   LLLHHH---llhhhhhhhlllLLHHHHHHHHHH-H-HHlL--EEEEEEELHH-H-HHH-HHHH-HH--HH---HHEEELL--L---------------------------llllLHHHHHHHHH-HHHHHHlLLEEEEEL-HhH--LL-H--H-H-H-H-HHHHHHHL---lL--L--LLEEEEEEEELLHHHhhHHHHHlHHHH-----LLEEEL-L--LHHh----
0023 6iy8A   HHHHHHHH---------L-LHHHHHHHHHHHHH-H-HL-L--LLEEEELLLL-L---L-HHHH-H-HHHHlllEEEEELL--L---LLLL----------------------------HHHHHH-HLHHHHlLLEEEEEL-H-H--HL-L-HH-H-H-H-HHHHHHHHH--llllleelLLEEEEEEEL--LLL--LlHLLLHHHH-hLLLEEELL-L-HhhH-HHHH
0024 2gnoA   -----------------------LHHHHHHHHH-H-LLlL--EEEEEELL---L-LLH-HHHH-H-HHHH----EEELlL--L-LL----------------------------------hHHH-HHHHHH-HEEEEELL-H-H--HL-L-HH-H-H-H-HLHHHHHHLL--L--L--LEEEEEEELL---H--HHL-LHHHHHL-----EEEELL-L-LhhH-HHHH
0025 9e22A   LLLLL----llll------LHHHHHHHHHHHHH-H-HH-L--LLEEELLLLL-L---L-HHHH-H-HHHHleeEEEELLL--L-LL---------------------------------HHHHH-LHHHHH-HEEEEEEL-L-L--L-----L-L-L-L-LLH---HHHL-L-------EEEEEELLL-------LLLLLHHHHL-----EEELLL-L-L-LL-HHH-
0026 7uqjA   -------------lllhhhlllLHHHHHHHHLL-L-HL-L--LEEEEELLLL-L---L-HHHH-H-HHHHlleEEEELLL--LlLLLL----------------------------LLHHHHHH-HHHHHH-HEEEEELL-HhH--HL-LLLH-HhH-H-HHHHHL-LLL-L--L--LEEEEEEEELL-lLL--LL---LLLLLL--lEEEELL-L-LhHH-HHHL
0027 8xksB   -----------------L-llLHHHHHHHHHHH-H--L-L--LEEEEELLLL-L---L-HHHH-H-HHHHlllEELLLLL--L-LL---LLLL------------------------LHHHHHH-HHHHHH-HLEEEEEL-H-H-hLL-LLHH-H-H-H-HHHHHHHL---lL--L--LLEEEEEEELL--LL--LLLLLLLLLL-----LLLLLL-L-LhhH-HHH-
0028 8fwjA   -------------eellllhHHHHHHLL--LEE-L----L--LEEEEELLLLlL-HHH-HHHH-HHHHHHlllEEEEELLeeE-LLL------------------lllLEEE--ellLLLHHHH-HHHHHH-HLEEEEEL-L-H--HH-HHHL-H-H-H-HHHHHHHHHHH-H--H--LLEEEEEEELLLLLL--LL---LLLL--lLLEEEEEEE---lEEL-LLLL
0029 7ykkA   LLLH---------llllllllLLHHHHHHHHHH-H-HH----LEEEEELLLL-LlLLH-HHHH-HHHHL-lllEEEELHH--H-HL---LLLL------------------------LHHHHHH-HHHHHH-HLEEEELL-L-L--LL-LLHH-H-H-H-HHHHHHHL---lL--L--LLEEEEEEELL---H--HHLLLLLLLLLLllLEEELLL-L-LhHH-HHHH
0030 7plhA   hHHHHHhHH-HL--lllllLHHHHHHHHHHHHH-H-HL-L--LLEEEELLLL-LlHHH-HHHH-HHHHL-lllEEEELLL--L----------------------------------llLHHHH-HHHHH-lLLEEEELL-L-L--LL------L-H-H-HHHHHHHHHHH-H--H--LLEEEEEEELL-hhH--HHH---HHHH-----EELLLL-L-LhhH-HHHH
0031 6z1f3   ------------------llllLHHHHHHHHHH-H-HH-H--LEEEEELLLL-L---L-HHHH-H-HHHHlleEEEELH---H-HLL--LLLL-----------------------LLHHHHHH-HHHHHH-HEEEEELL-LlL--LL-LHHH-H-H-H-HHHHHHHHL----------LLEEEEEELL----L--LLLLHHHLLLL-LEEEEELL-L-LhhH-HHHL
0032 8xvbA   ------LLL-LLL----lllhHHHHHHHHHHHH-H-HH-L--LLEEEEELLL-L---L-HHHH-H-HHHHlllEEEEELL--H-HH-------------------------------lLHHHHH-HHHHHH-HLEEEEEL-H-H--HL------L-H-H-HHHHHHHHHHH-H--H--LLEEEEEEELL-hhH--HLLL--HHHH--lLLEEEEL-L-LhhH-HHHH
0033 5zr1B   lLLLL------------H-HHLLL-LHHHHHHH-H-LL-L--LEEEEELLL--L-LHH-HHHHhHHLLHHlllEEEEELL--L-LL--------------------------------LHHHHH-HHHHHHlLEEEEEEL-L-LllLL-LLHH-H-H-H-HHHHHHLL-------L--LEEEEEELLLL------lHHHHLHHHH-----EEEELL-Ll---------
0034 1nlfA   -------------llllllhhhhhllleeELLE---EL-L--LEEEEEELLLlL-HHH-HHHH-HHHHHHlllEEEEELLelL-LLLLL--------------------------llllLHHHH-HHHHHHlLLEEEEEL-H-H--HH-LLL--hhH-H-HHHHHHHHHHH-H--H--LLEEEEEEEEL------------LLHHH--lLLEEEEEeE-EEEE-LL-L
0035 9bv1A   -------------llllllllLLHH-HHHHHHH-H-H-----LLEEEELLLL-LlHHH-HHHH-HHHHL-lleEEEELL---L-LLL--LLLL------------------------LHHHHHH-HHHHHH-HEEEEEEL-L-L--LL-LLHH-H-H-H-HHHHHHHL---lL--L--LEEEEEEEELL------LLLLLHHLLL-----LLEELL-L-LhhH-HHH-
0036 8bobC   -------------lllllllllLHHHHHHHHLH---HH-L--LEEEEELLLL-L---L-HHHH-HHHHHL----EEEELL--H-HHH-------------------------------LHHHHH-HHHHHH-HeEEEEEL-H-H--H--LLLH-H-H-H-HHHHHHHHH-L-L--L--LEEEEEEEELL-----------LLLHHH-HLEEELHH----LLL-HHHH
0037 7st9A   ---------hHLL--lhhhlllLHHHHHHHHHH-H-HH-H--LEEEEEELLL-L---L-HHHH-H-HHHH----EEELLL--L-L----LLLL-----------------------lLLLHHHH-HHHHHHhLLEEEELL-L----LL-LLLH-H-H-H-HHHHHHHHL---lllL--LLLEEEEEEELEELH--H--HHLLHHLL----EEEELL-L-LhHH-HHHH
0038 8fcvR   hHLLLHHHHhHHL----llLHHHHHHHHHHHHH-H-HL-L--LEEEEEELLL-L---L-HHHH-H-HHHHlllEEEEEL-----LLLL-------------------------LLLL-LHHHHH-HHHhhhlLLEEEEEL-H-H--HH-HLLH-H-H-H-HHHHHHHHHHH-H--H--LLEEEEEEELH---------hhHHLLHHH-L-EEEEEL-L-LhhH-HHHH
0039 8tp1A   LLLE---------hlllL-LLHHHHHHHHHHHH-H-HL----LEEEEEEElL-L-HHH-HHHH-HHHHLL---LEEEEEL--L---LLLL---------------------------lLHHHHH-HHHHLL-LLLEEEEE---------ehHH-H-HhH-HHLLllLLLL-----------EEEEEEEL---hhhLLLLLLLL----LEEEEL-L--llL-HHHH
0040 6qemG   hHHHH----lhll---lL-LHHHHHHHHHHHHH-H-HL-L--LLEEEELLLL-L---L-HHHH-HH-HHH---HEEEEHH--H-HHHHHHHHH------------------------lLHHHHH-HHHHLL--LEEEEEL-L--------HHH-HhH-H-HHHHHHHLL---------LLLLEEELLL-HHH--H-LLLHhHLLL--LLLEEEL-L-L----------
0041 3pvsD   -------------lllllllHHHHHHHHHHHLLL-L-------LEEEELLLL-L---L-HHHH-H-HHHHlleEEEEELL--L-LL----------------------------------HHHH-HHHHHH-HEEEEEEL-H-H--HL------L-H-H-HHHLLHHHHH-L--L--LLEEEEEEELL--lH--HHHlLHHHHL-----EEEELL-L-LhhH-HHH-
0042 8tw72   ----hhhHL-LL-llllllllLLHHHHHHHHHhhhlLL-L--LLEEEELLLL-L---L-HHHH-H-HHHHlllEEEEL-L--L-LL--------------------------------lLHHHHhHHHHHH-HEEEEELL-H-H--HL------L-H-H-HHHHHHHHHHH-H--L--LEEEEEEEELL-hHH--LL----HHHH-----EEEELL-L-LhHH-HHHH
0043 9cruC   LLLLLL-------llllllLHHHHHHHHHHHHH-H-HHlE--EEEEEELLLL-L---L-HHHH-HHHHHH----EEEL-H--H-HHLLLL--------------------------HHHHHHHH-HHHHHH-HLEEEELL-H-H--HH-LLlH-H-H-H-HHHHHLLL---llllllLEEEEEEEELL-HHH--H-hHHLLLLLL-E--EEELL-L-L-lL-LHHH
0044 5zr1E   -------------lllllllllLHHHHHHHHHH-L-LL-L--LEEEEEELLL-L---L-LLHH-HHHHH-lleEEEELLL--L-LL--------------------------------LHHHHH-HHHHHH-HEEEEEEL-L-L--LL--LLL-L-L-L-LHHHHHHHH--lllllleEEEEEEEEELL-LLL--H----HHHHHH-LLLEEEEL-L-LhHH-HHH-
0045 9klvD   ---------hhhlhhlhhhHHHHHHHHHHHHHH-H-HL-L--EEEEEELL---L-HHH-HHHH-H-HHHHlllLEEEELL--LllllLLHHHL--------------------------lLLHH-HHHHHH-HLEEEEEL-HhH--LL-H--H-H-H-H-HHHHHHHH---eL--L--LLEEEEELLLLH-----LHHHHHHH--lLEEEELL-L----------
0046 3co5A   ----------------lL-LHHHHHHHHHHHHH-H-LL-L--LLEEEEEELL-L---L-HHHH-H--HHH---L-eLLLL--L-LH---------------------------------hhHLH-HHHHHHlLLEEEEEE-LlL--LL-H--H-H-H-H-HHHHHHHHHL-L----lLLEEEEEEEELLHH--HH---HHHLLLl----------------------
0047 8xkuA   -------------llllL-HHHHHHHHHHHHHH-H-LH----lLEEEEELLL-L-HHH-HHHH-HHHHHL---LEEEEEH--H-HLL-------------------------LLLLLHHHHHHH-HHHHHHlLLEEEEEElL-L--LL-LLHH-H-H-H-HHHHHHHLLL-L-lL--LLEEEEEEELLL-------LLLLLLLLL---LEEEEL-L--hhH-HHH-
0048 6l1qA   -------------llllllllLLLLHHHHHHHH-H-HL-L--LLEEEELLLL-L---L-HHHH-H-HHHHlllEEEEELL------------------------------------lllLHHHH-HEHHHHhLLEEEEEL-H-H--HL------L-H-H-HHHHHH-HHHL-L--L---LEEEEEEELLL-------LLLLHHLL-----EEEELL-L-LhhH-HHHH
0049 8dgcA   -------------llllllLLHHHHHHHHHHLL----L-L--LLEEEELLLLlL-HHH-HHHH-HHHHL-lleEEEEELL--L-----------------------lllllLHHHLllLHHHHH-HHHHHH-HEEEEEEL-H-H--HH-HHHH-llL-L-HHHHHLLL-L-L--L--LEEEEEEEELL-LLH--H----HLLLLL-LLEEEELL-L---lL-HHHH
0050 7st9E   -------------hlllL-HHHHHHHHHH-LLL-H-HH-L--LLEEEELLLL-L---L-HHHH-HH-HHH---HEEELHH--H--HHHH-----------------------------hHHHHH-HHHHHH--EEEEELL-HhH--LL-H--H-H-H-H-HHHHHHHHHLL----L--LEEEEEEEL----L--LLLlLHHHHH-----EEEELL-L-LhhH-HHH-
0051 7z6hK   ---------hhLLhlllL-HHHHHHHHHHHHHH-H-H-----LEEEEELLLL-L---L-HHHH-HHHHHL---EEELL------LLLL-------------------------llllLHHHHHH-HHHHHH-HEEEEELL-L--lhHH-HHHL-H-H-HhHHHHHHHHHL---------LLLEEEELLLL-lllhHHHHHLlHHHH---LLEELL-L-L--L-HHH-
0052 6pxkK   -----------llhhhlllllLLHHHHHHHHHH-H-HHlL--LLEEEELLLL-L---L-HHHH-H-HHHHlllEEEEEHH----HHLLL--------------------------------LLL-LHHHHH-HLEEEEEL-H-H--HH-LHHH-H-H-H-HHHHHHHLL--eeelL--LLEEEEEELLL---llLHHHLLHHLL--lLEEEELL-L-LhhH-HHH-
0053 8ouyC   -------------leellllHHHHHHLL--LLE-E--L-L--LEEEEELLLLlL-HHH-HHHH-HHHHHHlllEEEEELLeeE-LLL--------------------------------HHHHH-HHHHHHlLLEEEEEL-L-L--LL-LHHHlH-H-H-HHHHHHHHHHH-H--H--LLEEEEEEELLEEEL--LLLHHHHLL-----EEEEEE----LLL-L---
0054 6p8vC   -------------hhhhhhllLLHHHHHHHHHH-H-HH----LEEEEEELLL-L---L-HHHH-HHHHHHeeeEEELL-H--H-HH--LLLLL-----------------------LHHHHHHH-HHHHHH-HEEEEELL-HhH--HL-L-HH-H-H-H-HHHHHHHHHHH-H--L---EEEEEEELL---H--HHL-LHHHHH-llLEEEEEL-L-LhHH-HHHH
0055 3f8tA   llllLHHH-hhHLhhhhllLLLLHHHHHHHHHH-H-LLhL--LLEEEEL----L-HHH-HHHH-H-HHHH----EEEEHH--H-LL----------------------------------HHHH-LLHHHHlLLEEEEEL-LlL--LL-H--H-H-H-H-HHHHHHHHH--lleeeelLLEEEEEEELLLHHL--LL-LHHHHLL--lLEEEELL-Ll----------
0056 1g4aE   -----------llhhhhhhllLLHHHHHHHHHH-H-HHlL--LLEEEELLLL-L---L-HHHH-H-HHHHlllEEEEELL--H-HHL--LLLL---------------------------lLLH-HHHHHH-HLEEEEEL-L-L---lllHHH-H-H-H-LLHHHHHHL--eeehH--HLEEEELLLLLlLH--HHLlHHHHH--llLEEEELL-L-LhhH-HHH-

############################################################################################################################################################################################################################################################################################################
############################################################################################################################################################################################################################################################################################################
############################################################################################################################################################################################################################################################################################################

Job: Type-2 BREX; BrxHI-Helicase MBK7581083 wHTH PDB50
Query: s001A
No: Chain Z rmsd lali nres id PDB Description
1: 3elk-A 8.5 2.6 71 104 4 MOLECULE: PUTATIVE TRANSCRIPTIONAL REGULATOR TA0346;
2: 7wjp-A 8.5 2.4 69 98 7 MOLECULE: PADR FAMILY TRANSCRIPTIONAL REGULATOR;
3: 5a31-N 8.4 1.8 65 703 14 MOLECULE: ANAPHASE-PROMOTING COMPLEX SUBUNIT 1;
4: 5zqh-A 8.4 2.3 71 101 11 MOLECULE: PADR FAMILY TRANSCRIPTIONAL REGULATOR;
5: 8iue-P 8.4 2.5 63 303 13 MOLECULE: DNA-DIRECTED RNA POLYMERASE III SUBUNIT RPC1;
6: 7wze-A 8.3 2.2 65 161 11 MOLECULE: UNCHARACTERIZED HTH-TYPE TRANSCRIPTIONAL REGULATO
7: 1ldd-A 8.2 1.9 64 74 8 MOLECULE: ANAPHASE PROMOTING COMPLEX;
8: 5v9x-A 8.2 2.7 73 772 19 MOLECULE: ATP-DEPENDENT DNA HELICASE;
9: 3nw0-A 8.2 2.1 72 236 10 MOLECULE: MELANOMA-ASSOCIATED ANTIGEN G1;
10: 8jxk-D 8.1 2.9 71 166 17 MOLECULE: CONSERVED PROTEIN;
11: 1yg2-A 8.1 2.4 68 169 10 MOLECULE: GENE ACTIVATOR APHA;
12: 1xma-B 8.1 2.4 71 103 7 MOLECULE: PREDICTED TRANSCRIPTIONAL REGULATOR;
13: 7xjg-J 8.0 2.4 72 306 8 MOLECULE: RNA-DIRECTED DNA POLYMERASE FROM RETRON EC86;
14: 6abq-A 8.0 2.5 72 111 10 MOLECULE: PADR FAMILY TRANSCRIPTIONAL REGULATOR;
15: 2o0y-C 8.0 1.8 65 255 17 MOLECULE: TRANSCRIPTIONAL REGULATOR;
16: 1xmk-A 7.9 2.0 65 79 6 MOLECULE: DOUBLE-STRANDED RNA-SPECIFIC ADENOSINE DEAMINASE;
17: 1xsx-A 7.9 2.1 64 95 14 MOLECULE: SSO10A;
18: 5dym-A 7.9 2.3 68 96 9 MOLECULE: PADR-FAMILY TRANSCRIPTIONAL REGULATOR;
19: 5h20-A 7.9 2.5 71 103 13 MOLECULE: PUTATIVE PADR-FAMILY TRANSCRIPTIONAL REGULATORY P
20: 3bdd-A 7.9 2.1 65 140 5 MOLECULE: REGULATORY PROTEIN MARR;
21: 2co5-B 7.8 2.5 70 94 11 MOLECULE: VIRAL PROTEIN F93;
22: 3u2r-A 7.8 2.2 62 135 10 MOLECULE: REGULATORY PROTEIN MARR;
23: 3l7w-A 7.8 2.3 70 106 10 MOLECULE: PUTATIVE UNCHARACTERIZED PROTEIN SMU.1704;
24: 2nyx-B 7.7 2.1 64 147 11 MOLECULE: PROBABLE TRANSCRIPTIONAL REGULATORY PROTEIN, RV14
25: 2dql-A 7.7 2.3 68 115 6 MOLECULE: PEX PROTEIN;
26: 3bpx-A 7.7 2.3 64 147 6 MOLECULE: TRANSCRIPTIONAL REGULATOR;
27: 2xrn-B 7.7 2.2 65 241 12 MOLECULE: HTH-TYPE TRANSCRIPTIONAL REGULATOR TTGV;
28: 2rdp-A 7.7 2.2 64 140 11 MOLECULE: PUTATIVE TRANSCRIPTIONAL REGULATOR MARR;
29: 3gfi-A 7.7 2.2 63 143 8 MOLECULE: 146AA LONG HYPOTHETICAL TRANSCRIPTIONAL REGULATOR
30: 8r3g-A 7.7 2.3 66 335 8 MOLECULE: CENTRAL GLYCOLYTIC GENES REGULATOR;
31: 2wte-A 7.6 2.1 64 212 13 MOLECULE: CSA3;
32: 7ymd-C 7.6 2.5 71 285 10 MOLECULE: NON-STRUCTURAL MAINTENANCE OF CHROMOSOME ELEMENT
33: 5x11-E 7.6 2.5 69 181 6 MOLECULE: DNA (28-MER);
34: 1yyv-A 7.6 2.3 65 114 11 MOLECULE: PUTATIVE TRANSCRIPTIONAL REGULATOR;
35: 5z7b-B 7.6 2.6 70 197 11 MOLECULE: PADR FAMILY TRANSCRIPTIONAL REGULATOR;
```

```
36: 2p8t-A 7.5 1.7 61 184 10 MOLECULE: HYPOTHETICAL PROTEIN PH0730;
37: 8uuc-A 7.5 2.1 62 274 11 MOLECULE: ADENINE DNA GLYCOSYLASE;
38: 6qfd-B 7.5 2.6 69 116 9 MOLECULE: DNA-BINDING PROTEIN;
39: 8ioi-A 7.5 2.4 68 190 7 MOLECULE: PADR FAMILY TRANSCRIPTIONAL REGULATOR;
40: 2qww-A 7.5 2.2 64 146 20 MOLECULE: TRANSCRIPTIONAL REGULATOR, MARR FAMILY;
41: 2esh-A 7.5 2.5 70 114 10 MOLECULE: CONSERVED HYPOTHETICAL PROTEIN TM0937;
42: 3g3z-A 7.5 2.1 64 142 6 MOLECULE: TRANSCRIPTIONAL REGULATOR, MARR FAMILY;
43: 8ffz-B 7.5 2.4 65 905 12 MOLECULE: TRANSCRIPTION FACTOR IIIA;
44: 1z7u-A 7.5 2.2 65 111 11 MOLECULE: HYPOTHETICAL PROTEIN EF0647;
45: 4fht-B 7.4 2.2 64 142 14 MOLECULE: PCAV TRANSCRIPTIONAL REGULATOR;
46: 2fa5-B 7.4 1.9 63 142 16 MOLECULE: TRANSCRIPTIONAL REGULATOR MARR/EMRR FAMILY;
47: 2zkz-C 7.4 1.9 61 87 7 MOLECULE: TRANSCRIPTIONAL REPRESSOR PAGR;
48: 8yli-B 7.4 2.3 64 162 9 MOLECULE: REGULATORY PROTEIN;
49: 5hs5-A 7.4 2.6 68 116 12 MOLECULE: HTH-TYPE TRANSCRIPTIONAL REGULATOR SARX;
50: 6aht-A 7.3 2.6 69 111 4 MOLECULE: CONSERVED HYPOTHETICAL PLASMID PROTEIN;
51: 7el3-A 7.3 2.2 64 138 11 MOLECULE: HOMOPROTOCATECHUATE DEGRADATION OPERON REGULATOR
52: 2qen-A 7.3 2.6 65 350 3 MOLECULE: WALKER-TYPE ATPASE;
53: 7xux-A 7.3 2.6 65 365 17 MOLECULE: FIDO DOMAIN-CONTAINING PROTEIN;
54: 5aip-A 7.3 2.0 64 141 13 MOLECULE: TRANSCRIPTIONAL REGULATOR, MARR FAMILY;
55: 2fna-A 7.2 2.8 65 352 9 MOLECULE: CONSERVED HYPOTHETICAL PROTEIN;
56: 4asn-A 7.2 2.2 65 90 12 MOLECULE: TUBR;
57: 3mq0-B 7.2 2.2 65 248 18 MOLECULE: TRANSCRIPTIONAL REPRESSOR OF THE BLCABC OPERON;
58: 3f8f-A 7.2 2.4 71 114 11 MOLECULE: TRANSCRIPTIONAL REGULATOR, PADR-LIKE FAMILY;
59: 8xt8-B 7.2 2.2 62 145 15 MOLECULE: MARR FAMILY TRANSCRIPTIONAL REGULATOR;
60: 5h3r-A 7.2 2.3 64 141 9 MOLECULE: DNA (5'-

0001 s001A   -------------ASHI-FAHQLMTLGIQ-L---D------GVLPQDV-DYWL-R----G--S------AAF------SRL---GSAERKAIVDHMLS----QEILADH---S----------GKLWLG--PVGERR----F--GR----------------------
0002 3elkA   ---------reriLHGL-ITLYILKELVK-R----------PXHGYEL-QKSXfE----T--T------G--------QAL---PQGSIYILLKTXKE----RGFVISE---S--svnkgqqlTVYHIT--DAGKKF----L--XDhsqalqlarkiiddllstvd--
0003 7wjpA   ----------pqfKKGV-LELCCLFLIQK-K----------DCYGYEL-ANQVsK----Y--I------E----------V---AEGAIYPVLRRLVK----EEYCSTY---L----vespsrKYYQLT--VKGEIY----L--NElisewnnftdsvakllteg---
0004 5a31N   pqdrdnmvlikeeELLL-FWTYIQAMLTN-L---E------SLSLDRI-YNML-RmfvvT--G------PAL------AEI---DLQELQGYLQKKVR----DQQLVYS---A----------GVYRLP---------------------------------------
0005 5zqhA   ---------etqlLKGV-LEGCVLDMIGQ-K----------ERYGYEL-VQTL-R----E--A------G-F------DTI---VG-TIYPLLQKLEK----NQWIRGD---M-rpspdgpdrKYFSLM--KEGEER----V--SVfwqqwddlsqkvegikn-----
0006 8iueP   -------------DPVE-IENRIIELCHQ-F--pH------GITDQVI-QNEM-P----H--------------------I---EAQQRAVAINRLLS----MGQLDLL---R------sntgLLYRIK-------D----S--QNagkmkgsdnqeklvyqiiedag
0007 7wzeA   kxnhvxehyfagrglSE-GKFKILXLLFD-A-kdH------RLSPTEL-AKRS-N-------------------------V---TKATITGLLDGLAR----DGFVSRR---H-------hrkISIELT--TEGKAR----L--EQflpghfskisavxenysdeekd
0008 1lddA   ---------kyelTLQR-SLPFIEGMLTN-L---G------AMKLHKI-HSFL-K----I----tvpkdWGY------NRI---TLQQLEGYLNTLAD----EGRLKYI---A---------nGSYEIV---------------------------------------
0009 5v9xA   qtgdietlrvpanPLDV-LAQHTVAVAAL-----E------PVDADAW-FDAV-R----R--S------APF------ATL---PRSAFEATLDLLSGkaelRPRLVYD-rdT----------GTLTAR--PGAQRL----A--VTsggaipdrgmftvylaseteel
0010 3nw0A   ttsiskmatdfaeNELD-LFRKALELIID-S-etG------FASSTNI-LN-L-V----D--Q------lKG------KKM---RKKEAEQVLQKFVQ----NKWLIEK---E----------GEFTLH--GRAILE----M--EQyiretypdavkicnichslliq
0011 8jxkD   --------------EFM-LELAILGLLIE-S----------PMHGYEL-RKRLtG----L--L------GA------fRAF---SYGSLYPALRRMQA----DGLIAEN---A-----apagrRVYQLT--DKGRRR----F--GElvadtgphnytddgfgvhlaff
0012 1yg2A   ----------------S-LPHVILTVLST-R----------DATGYDI-TKEF-S----AsiG------YFW------K-A---SHQQVYRELNKMGE----QGLVTCV---L----------eVYSIT--QAGRSA----L--GEwfdqptahptvrdefsaklmac
0013 1xmaB   -------vissdvIRGY-VDTIILSLLIE-G----------DSYGYEI-SKNI-R-iktD--E------LYV--------I---KETTLYSAFARLEK----NGYIKSY---Y----geetrrTYYRIT--PEGIKY----Y--KQkceeweltkkvinkfvk-----
0014 7xjgJ   kvrkenkvskdigNILY-AERFLLPCIYL-L---D------SVNYRTL-CELA-F----K--A--------I------KQDdvlSKIIVRSVVSRLIN----ERKILQM---T----------DGYQVT--ALGASY----V--RSvfdrktldrlrleimnfenrrk
0015 6abqA   gsakdpmkgltelLKGS-LEGMILERISR-----G------ETYGYEI-TKYL-N----D--L------G-F------DEI---VEGTVYTILVRLEK----KGLVEIE---K-kkselgpprKFYTLS--PAGEEE----L--AIfwkrwdfiqgkimqvkg-----
0016 2o0yC   dsaekpavadagvRSVT-RVIDLLELFDAaH---P------TRSLKEL-VEGT-K----------------------------lPKTTVVRLVATXCA----RSVLTSR--aD----------GSYSLG--PEXL-R----W--VRlagrtwappeevvdixrqlsad
0017 1xmkA   ------gshmaslDMAE-IKEKICDYLFN-V---S------DSSALNL-AKNI-G---------------------------ltKARDINAVLIDMER----QGDVYRQ---G-------ttpPIWHLT--DKKR-E----R--MQik--------------------
0018 1xsxA   ----------akkKSKLeIIQAILEACK------S------GSPKTRI-MYGA-N----------------------------lSYALTGRYIKMLMD----LEIIRQE---G----------KQYMLT--KKGEEL----L--EDirkfnemrknmdqlkekinsvl
0019 5dymA   ---------------GY-IDILIVSILEK-K----------DCYGYEI-AKQVrE----R--S------EF---------L---KEGTMYLALKRMES----KNLIKSY---YsneqssggrrKYYNLT--NEGKDF----L--EIkkqewrfikkvmnqflg-----
0020 5h20A   ------dnvksqmRKGM-LEYCIMLLLHK-E----------PAYASDI-IQKL-K----E--A------RLI--------V---VEGTLYPLLTRLKN----DDLLSYE---W-vestqgpprKYYKLT--GKGESF----L--GEleaswkelnetvnhia------
0021 3bddA   adetisnlfekqlgiSL-TRYSILQTLLK-D---A------PLHQLAL-QERL-Q-------------------------I---DRAAVTRHLKLLEE----SGYIIRK---R---npdnqreVLVWPT--EQAREA----L--ITnpsahhqaiktsxnqiltvees
0022 2co5B   --------mkirkymRI-NYYIILKVLVI-N---G-----sRLEKKRL-RSEI-L----K-rF------DID--------I---SDGVLYPLIDSLID----DKILREE---E------apdgKVLFLT--EKGMKE----F--EElheffkkivch-----------
0023 3u2rA   rxkaieeeifsqfelSA-QQYNTLRLLRS-V-hpE------GXATLQI-ADRL--------------------------------APDITRLIDRLDD----RGLVLRT---R---kpenrrvVEVALT--DAGLKL----L--KDleepvrqcherqlghlaadelh
0024 3l7wA   --------xyypvSALL-IEYLILAIVSK-----H------DSYGYDI-SQTI-K--------------LIA------S-I---KESTLYPILKKLEK----AGYLSTY---T--qehqgrrrKYYHLT--DSGEKH----L--VYltkewsvykxtidgivegrirh
0025 2nyxB   vaisahsiaqvdeniTI-PQFRTLVILSN-H---G------PINLATL-ATLL-G----------------------------vQPSATGRXVDRLVG----AELIDRL---P---hptsrreLLAALT--KRGRDV----V--RQvtehrrteiariveqxapaerh
0026 2dqlA   iyqffenppptylCQEV-AICYILYVLLQ-----G------ESYGTEL-IQQL-E--teH--P------TYR--------L---SDTVLYSAIKFLED----NRAITGY---WkklegrgrprRMYQVS--PE-WQH----Qae------------------------
0027 3bpxA   shrvfigrelghlnlTD-AQVACLLRIHR-E---P------GIKQDEL-ATFF-H----------------------------vDKGTIARTLRRLEE----SGFIERE---Q---dpenrrrYILEVT--RRGEEI----I--PLilkveerwedllfrdftederk
0028 2xrnB   -----------siQVIA-RAASIMRALGS-H---P-----hGLSLAAI-AQLV-G----------------------------lPRSTVQRIINALEE----EFLVEAL---G--------paGGFRLG--PA-LGQ----L--INqaqtdilslvkpylrslaeeld
0029 2rdpA   nlkqrgreiltnypiTP-PQFVALQWLLE-E---G------DLTVGEL-SNKX-Y----------------------------lACSTTTDLVDRXER----NGLVARV---R------dehvVRIRLL--EKGERI----I--EEviekrqrdlanvlesfsdeeiv
0030 3gfiA   amsrelnrrlgelnlSY-LDFLVLRATSD-----G------PKTMAYL-ANRY-F----------------------------vTQSAITASVDKLEE----MGLVVRV---R---dredrrkILIEIT--EKGLET----F--NKgieiykklanevtgdlsedevi
0031 8r3gA   liqaqkkllpdllLVXQ-KRFEILQYIRL-T---E------PIGRRSL-SASL-G----------------------------iSERVLRGEVQFLKE----QNLVDIK---T----------NGXTLT--EEGYEL----L--SVledtxkdvlgltllektlkerl
0032 2wteA   isfndntiralxrdySR-EEXKLLNVLYE-T---K------GTGITEL-AKXL-D----------------------------kSEKTLINKIAELKK----FGILTQK---G--------kdRKVELN--ELGLNV----I--KLnk--------------------
0033 7ymdC   ntlesklstdrdlVYKG-VLSVILCIVFF-S--kN------NILHQEL-IKFL-E----T--F------GipsdgskiAIL---N-ITIEDLIKSLEK----REYIVRL---E-eksdtdgevISYRIG--RRTQAE----L--GLesleklvqeimglekeqtkslh
0034 5x11E   ------------dpmRV-LKYAILGLLRK-----G------ELSGYDI-TSYF-K----EelG------QFW-------sA---KHSQIYPELKKLTD----EGFITFR---T-tiqgtklekKMYTLT--DSGKQE----L--HDwlirhqpipetvkdefmlkayf
0035 1yyvA   faeqcpsrevlkhvtSR-WGVLILVALRD-----G------THRFSDL-RRXX-G------------------------GV---SEXXLAQSLQALEQ----DGFLNRV---S---ypvvpphVEYSLT--PLGEQV----S--DXvaaladwielnlpqvlaqre--
0036 5z7bB   ----------sakDLMT-LRSALLALLSS-----G------PLTGYDAsQRFG-A----svgF------VWS--------G---SDSQIYPELRKMEA----EELLVGS---DvpwgskgatkTEYALS--EKGWEA----L--RKawyepvtygptrdparlkaayf
0037 2p8tA   ------------eyTVE-DVLAVIFLLK------E------PLGRKQI-SERL-E----------------------------lGEGSVRTLLRKLSH----LDIIRSK---------------GHFLT--LKGKEI----R--DKllsmfsepigvsvdgypgiaiv
0038 8uucA   rsahhtrqstfegSRRQ-KRAELVRLVLA-E---P------GIGIDEL-AERLdA----F--E------R----dagrKGV---DAATFTSIVADLVA----EGFFRRE---G----------DAFFA----------------------------------------
0039 6qfdB   -----pdarsdardlTA-FQKNILTVLGE-E----------ARYGLAI-KRELeE----Y--Y------G--------EEV---NHGRLYPNLDDLVN----KGLVEKS---E-----ldkrtNEYALT--NEGFDA----V--VDdlewtlskfvadadrrerveti
0040 8ioiA   ------------pefMA-LPHAILVSLSE-Q----------ASSGYELaRRFD-R----sigY------FWT--------A---THQQIYRTLRVMEN----NNWVRAT---T-vlqhgrpdkKVYAIS--DSGRAE----L--ARwiaeplsptrpgrgsaltdsst
0041 2qwwA   isagyadqnaaslglTI-QQLAXINVIYS-T---P------GISVADL-TKRL-I----------------------------iTGSSAAANVDGLIS----LGLVVKL---N---kpndsxdLTLKLS--KKGEDL----S--KRstanafxykaxxkvfenltene
0042 2eshA   -------rggrgfRGWW-LASTILLLVAE-K----------PSHGYEL-AERL-A----E--F------GI--eipgiGHM---G--NIYRVLADLEE----SGFLSTE---W--dttvspprKIYRIT--PQGKLY----L--REilrsledmkrrietleerikrv
0043 3g3zA   licnvfdkwigqqdlNY-NLFAVLYTLAT-E---G------SRTQKHI-GEKW-S----------------------------lPKQTVSGVCKTLAG----QGLIEWQ---E---geqdrrkRLLSLT--ETGKAY----A--APltesaqefsdkvfatfgdkrtt
0044 8ffzB   rlwvngsgsirpnIWKQ-VVTMVVNEIIF-H---P------GITLSRL-QSRC-R----E--V--------------------lSLHEISEICKWLLE----RQVLITT---D---------fDGYWVN--HNWYSIyest---------------------------
0045 1z7uA   tdkqtsinlalstinGK-WKLSLXDELFQ-----G------TKRNGEL-XRAL-D------------------------GI---TQRVLTDRLREXEK----DGLVHRE---S---fnelpprVEYTLT--PEGYAL----Y--DAlsslchwgetfaqkkarln---
0046 4fhtB   qahyllwntmvseetTS-PQYAVLNALVA-E---P------GLDQRTV-GERV-G----------------------------lDRSTIAEVVSRLGR----RGLLDKV---R---dpqdgrrSLLRLT--DEGLRV----H--RRlgvriarmnqvflaplaadeqa
0047 2fa5B   isgniakvygdrygxAI-PEWRVITILAL-Y---P------GSSASEV-SDRT-A----------------------------xDKVAVSRAVARLLE----RGFIRRE--------------SXLALS--PAGRQV----Y--ETvaplvnexeqrlxsvfsaeeqq
0048 2zkzC   yxsleddaellktxaHP-XRLKIVNELYK-H---K------ALNVTQI-IQIL-K----------------------------lPQSTVSQHLCKXR-----GKVLKRN---R------qgleIYYSIN--N--PKV----E--GIiklln-----------------
0049 8yliB   lmdqrlescfsrfdlSS-WEFDMLATLRR-AgapH------CLSPTEL-FSTL-M----------------------------vTSGTMTHRLKRLET----RGFIERV---Q---neldarsTLVQLT--SSGLEL----I--NRaveahienerqvlsvlpaevla
0050 5hs5A   lseyidkkyklslNDLA-VLDLTXKHCK-----dE------KVLXQSF-LKTA---------------xDEL------D-L---SRTKLLVSIRRLIE----KERLSKV---R---sskderkIYIYLN--NDDISK----F--NAlfedveqflnilehh-------
0051 6ahtA   isxssseiidvlcenLN-DGIWALRVLYA-E---G------AXNKEKLwDYIN-QyhkdyqienekdyeGKK------IlP---SRYALDIXTARLEG----AGLISFK---A------igrvRIYDVT--DLGNVL----I--KElekr------------------
0052 7el3A   aimshfrpalnevglTE-QQWRIIRILYQ-Y---E------ELESNQL-AELA-C----------------------------iLKPSLTGILNRMVE----QKLIQKR---K---dyddqriSLISLT--ESGLEC----F--KTqavkmeasyqkiqeqygeekmk
0053 2qenA   akglimgeleelrrrSP-RYVDILRAIAL-G----------yNRWSLI-RDYL-A----V--K------GTK--------I---PEPRLYALLENLKK----MNWIVEE---D----------NTYKIA-----DPV----V--ATvlri------------------
0054 7xuxA   vlypaleyskgrgviNE-TESKILKRTIS-Q---G------TVKTSDL-KEVL-P----G--------------------L---KPAQITYQIGKLVD----RGLLQPVevgS----------RIYTAGfsKSDL-M----R--GVihalrkegfipd----------
0055 5aipA   alxtqfrpilnqaniTD-QQWRIIRLLAE-N---G------TLDFQDL-ANQA-C----------------------------iLRPSLTGILTRLEK----AGLVVRL---K---psndqrrVFLKLT--AEGEKL----Y--EEigeevderydaieevlgrekxl
```

```
0056 2fnaA   ilkefenflhgreIARK-RYLNIXRTLS------K------CGKWSDV-KRALeL----E--E-----G--------IEI---SDSEIYNYLTQLTK----HSWIIKE---G----------EKYCPS-----EPL----Isl------------------------
0057 4asnA   ----sdyfeevxrklTI-EDVSILGWLFQ-N---Eanavfk AIKKSSI-ADEL-E------------------------Y---STANFRKTLNKLEA----IHFIGTV---T------ggkeHKLYLT--EYGQQA----V--QQaihh-----------------
0058 3mq0B   ---------sdtvPALR-RAVRILDLVAG-S--pR------DLTAAEL-TRFL-------------------------dL---PKSSAHGLLAVMTE----LDLLARS---A---------dGTLRIG--PHS-LR----W--ANgflshldivstfndhlaqrhdl
0059 3f8fA   -----maeipkemLRAQ-TNVILLNVLKQ-G----------DNYVYGI-IKQV-K-easN--G-----EME--------L---NEATLYTIFKRLEK----DGIISSY---W---gdeggrrKYYRLT--EIGHEN----M--RLafeswsrvdkiienleankkse
0060 8xt8B   kddlkkmfkqngydiTT-DHYALLRFLWE-Q---D------GISQIDL-CEKS-C---------------------------kdKSNTTRILDVMKN----KGLIVRK---V---dvkdrrkFQIFLT--DLGR-E----L--EEplneiasiyatetfksisdeel
0061 5h3rA   kkdrllneylspldiTA-AQFKVLCSIRC-A---A------CITPVEL-KKVL-S---------------------------vDLGALTRMLDRLVS----KGWVERL---P---npndkrgVLVKLT--TGGAAI----C--EQchqlvgqdlhqeltknltadev

0001 s001A   -------------LHHH-HHHHHHHHHHH-H---L------LLLHHHH-HHHH-L----L--L-----HHH------LLL---LHHHHHHHHHHHHH----LLLEEEE---L----------LEEEEL--HHHHHH----H--LL----------------------
0002 3elkA   ---------llhhHHHH-HHHHHHHHHHH-L----------LEEHHHH-HHHHhH----H--H-----L--------LLL---LLLHHHHHHHHHHH----HLLEEEE---E--eellllleeEEEEEL--HHHHHH----H--HHllllhhhhhhhhhhhhllll--
0003 7wjpA   ----------llhHHHH-HHHHHHHHHHH-L----------LEEHHHH-HHHHlL----L--L-----L----------L---LLLLHHHHHHHHHH----LLLEEEE---E----elllleEEEEEL--HHHHHH----H--HHhhhhhhhhhhhhhhhhhlll--
0004 5a31N   lllllllllllhhHHHH-HHHHHHHHHHH-H---L------LLLHHHH-HHHH-HhhllL--L-----LLL------LLL---LHHHHHHHHHHHHH----LLLLEEE---L----------LEEELL---------------------------------------
0005 5zqhA   ---------llhhHHHH-HHHHHHHHHHH-L----------LEEHHHH-HHHH-H----H--H-----L-L------LLL---LL-LLHHHHHHHHH----LLLEEEE---E-eelllllleeEEEEEL--HHHHHH----H--HHhhhhhhhhhhhhhhhhl-----
0006 8iueP   -------------LHHH-HHHHHHHHHHH-L--lL------LLLHHHH-HHHL-L----L-------------------L---LHHHHHHHHHHHHH----HLLEEEE---E------elleEEEEEL-------H----H--HLllllllllhhhhhhhhhhhhhh
0007 7wzeA   hhhhhhhhhhhllllLH-HHHHHHHHHHH-L-hhH------EELHHHH-HHHL-L------------------------L---LHHHHHHHHHHHHH----HLLEEEE---E-------lllEEEEEL--HHHHHH----H--HHhhhhhhhhhhhhhllllhhhhh
0008 1lddA   ---------llhhHHHH-HHHHHHHHHHH-H---L------LEEHHHH-HHHH-H----H---hllhhHLL------LLL---LHHHHHHHHHHHHH----LLLEELL---L---------lLEEEEL---------------------------------------
0009 5v9xA   hhlllllllllllLHHH-HHHHHHHHLLL-----L------LEEHHHH-HHHH-H----H--L-----LLL------LLL---LHHHHHHHHHHHHLllllLLLEEEE-llL----------LEEEEL--LLHHHH----H--HLllllllllllllllllllllll
0010 3nw0A   llllhhhhllllhHHHH-HHHHHHHHHHH-L-llL------LEEHHHH-HH-H-H----L--L-----lLL------LLL---LHHHHHHHHHHHHH----LLLEEEE---L----------LEEEEL--HHHHHH----H--HHhhhhhhllllllllllllllll
0011 8jxkD   --------------LHH-HHHHHHHHHHH-L----------LEEHHHH-HHHHhH----H--L-----LL------lLLL---LLLLHHHHHHHHHH----LLLEEEL---L-----lllllLEEEEL--HHHHHH----H--HHhhhlllhhhllhhhhhhhhhhh
0012 1yg2A   ----------------L-HHHHHHHHHHH-L----------LLLHHHH-HHHH-L----LhhH-----HLL------L-L---LHHHHHHHHHHHHH----LLLEEEL---L---------lLEEEL--HHHHHH----H--HHhhhlllllllllllhhhhhhhh
0013 1xmaB   -------lllhhhHHHH-HHHHHHHHHHH-L----------LEEHHHH-HHHH-H-hhlL--L-----LLL--------L---LHHHHHHHHHHHHH----LLLEEEE---E----llllllEEEEEL--HHHHHH----H--HHhhhhhhhhhhhhhhhll-----
0014 7xjgJ   hhhhhllllllllLLLL-LHHHHHHHHHH-L---L------LLLHHHH-HHHH-H----H--H-------L------LLLlhhHHHHHHHHHHHHHH----LLLEEEE---L----------LEEEEL--HHHHHH----H--HHhllhhhhhhhhhhhhhhhhlll
0015 6abqA   lllllllllllhhHLLH-HHHHHHHHHHL-----L------LLLHHHH-HHHH-H----H--L-----L-L------LLL---LHHHHHHHHHHHHH----LLLEEEE---E-eelllllleeEEEEEL--HHHHHH----H--HHhhhhhhhhhhhhhhhhl-----
0016 2o0yC   lllllllllllllHHHH-HHHHHHHLLLLlL---L------LLLHHHH-HHHH-L---------------------------lLHHHHHHHHHHHHH----LLLEEEL--lL----------LLEEEL--HHHH-H----H--HHhhhhhhlllhhhhhhhhhhhhh
0017 1xmkA   ------llllllhHHHH-HHHHHHHHHHH-L---L------LEEHHHH-HHHH-L--------------------------hhHHHHHHHHHHHHHH----LLLEEEE---L-------lllLEEEEL--HHHH-L----L--LLll--------------------
0018 1xsxA   ----------lllLLLLhHHHHHHHHHL------L------LLLHHHH-HLLL-L---------------------------lLHHHHHHHHHHHHH----LLLEEEE---L----------LEEEEL--HHHHHH----H--HHhhhhhhhhhhhhhhhhhhhhhh
0019 5dymA   ---------------LL-HHHHHHHHHLL-L----------LEEHHHH-HHHHhH----H--L-----LL---------L---LHHHHHHHHHHHHH----LLLEEEE---ElllllllllllEEEEEL--HHHHHH----H--HHhhhhhhhhhhhhhhhhl-----
0020 5h20A   ------lhhhhhhHHHH-HHHHHHHHHLL-L----------LLLHHHH-HHHH-H----H--L-----LLL--------L---LHHHHHHHHHHHHH----LLLEEEE---E-ellllllleEEEEEL--HHHHHH----H--HHhhhhhhhhlllhhhhhl-----
0021 3bddA   hhhhhhhhhhhhhllLH-HHHHHHHHHHH-H---L------LLLHHHH-HHHH-L------------------------L---LHHHHHHHHHHHHH----LLLEEEE---E---lllllllEEEEEL--HHHHHH----H--LLlllhhhhhhhhhhhllllhhhh
0022 2co5B   --------lllllllLH-HHHHHHHHHHH-L---L-----lEEEHHHH-HHHH-H----H-hH-----LLL--------L---LHHHHHHHHHHHHH----LLLEEEE---L------llllLEEEEL--HHHHHH----H--HHhhhhhhhhhhl-----------
0023 3u2rA   hhhhhhhhhhhhlllLH-HHHHHHHHHHH-H-llL------LEEHHHH-HHHL-------------------------------LLHHHHHHHHHHH----LLLEEEE---E---elleeeeEEEEEL--HHHHHH----H--HHhhhhhhhhhhhhhhhhhhhhhh
0024 3l7wA   --------lllllLHHH-HHHHHHHHHHH-----L------LEEHHHH-HHHH-L-------------LLL------L-L---LHHHHHHHHHHHHH----LLLEEEE---E--eeelleeeEEEEEL--HHHHHH----H--HHhhhhhhhhhhhhhhhhhlllll
0025 2nyxB   hhhhhhhhhhhhlllLH-HHHHHHHHHHH-H---L------LEEHHHH-HHHH-L---------------------------lLHHHHHHHHHHHHH----LLLEEEE---L---lllllllLEEEEL--HHHHHH----H--HHhhhhhhhhhhhhhllllhhhll
0026 2dqlA   hhhhhhlllleelLHHH-HHHHHHHHHLL-----L------LLLHHHH-HHHH-H--hhL--L-----LEE--------L---LHHHHHHHHHHHHH----LLLEEEE---EellllllllleEEEEEL--HH-HHH----Hhh------------------------
0027 3bpxA   hhhhhhhhhhhhhllLH-HHHHHHHHHHL-L---L------LLLHHHH-HHHH-L---------------------------lLHHHHHHHHHHHHH----LLLEEEE---E---elleeeEEEEEL--HHHHHH----H--HHhhhhhhhhhhhhhllllhhhhh
0028 2xrnB   -----------llHHHH-HHHHHHHHHHL-L---L-----lLEEHHHH-HHHL-L---------------------------lLHHHHHHHHHHHHL----LLLEEEL---H--------hhLEEEEL--LH-HHH----H--HHhhhhhhhhhhhhhhhhhhhhhl
0029 2rdpA   hhhhhhhhhhlllllLH-HHHHHHHHHHH-H---L------LLLHHHH-HHHH-L---------------------------lLHHHHHHHHHHHHH----LLLEEEE---E------llllEEEEEL--HHHHHH----H--HHhhhhhhhhhhhhhhhllhhhhh
0030 3gfiA   hhhhhhhhhhhhhllLH-HHHHHHHHHLL-----L------LEEHHHH-HHHL-L---------------------------lLHHHHHHHHHHHHH----LLLEEEE---E---elleeeEEEEEL--HHHHHH----H--HHhhhhhhhhhhhhhllllhhhhh
0031 8r3gA   hhhhhhhhlllhhHHHH-HHHHHHHHHHH-H---L------LLLHHHH-HHHH-L---------------------------lLHHHHHHHHHHHHH----LLLEEEE---L----------LEEEEL--HHHHHH----H--HHhhhhhhhlhhhhhhhhhhhhhl
0032 2wteA   eeeehhhhhhhhlllLH-HHHHHHHHHHH-H---L------LLLHHHH-HHHH-L---------------------------lLHHHHHHHHHHHHH----LLLEEEE---L--------llLEEEEL--HHHHHH----H--HHll--------------------
0033 7ymdC   lllllllllllhhHHHH-HHHHHHHHHHH-L--lL------LEEHHHH-HHHH-H----H--H-----Lllllllllll---L-LLHHHHHHHHHH----LLLEEEE---E-lllllllllEEEEEL--HHHHHH----L--LHhhhhhhhhhhhlllhhhhlllh
0034 5x11E   ------------lllLH-HHHHHHHHLLL-----L------LLLHHHH-HHHH-H----LlhH-----HLL-------lL---LHHHHHHHHHHHHH----LLLEEEE---E-eelllllleeEEEEEL--HHHHHH----H--HHhhhllllllllllllhhhhhhl
0035 1yyvA   llllllhhhhhhhhhLH-HHHHHHHHHHH-----L------LEEHHHH-HHHL-L-----------------------LL---LHHHHHHHHHHHHH----HLLEEEE---E---ellllleEEEEEL--HHHHHH----H--HHhhhhhhhhhhhhhhhhllll--
0036 5z7bB   ----------lhhHHHH-HHHHHHHHHLL-----L------LEEHHHHhHHHH-L----lhhH-----LLL--------L---LHHHHHHHHHHHHH----LLLEEEE---EeeelllleeeEEEEEL--HHHHHH----H--HHhhhlllllllllllhhhhhhlh
0037 2p8tA   ------------llLHH-HHHHHHHHLL------L------LLLHHHH-HHHH-L---------------------------lLHHHHHHHHHHHHH----LLLEEEL---------------LEEEL--HHHHHH----H--HHhhllllllleeeeelleeeeee
0038 8uucA   hllllllllllllLHHH-HHHHHHHHHHH-L---L------LEEHHHH-HHHHhH----H--H-----H----hlllLLL---LHHHHHHHHHHHHH----HLLLEEE---L----------LEEEL----------------------------------------
0039 6qfdB   -----lllhhhhlllLH-HHHHHHHHHHH-L----------LEEHHHH-HHHHhH----H--H-----L--------LLL---LHHHHHHHHHHHHH----LLLEEEE---E------elleEEEEEL--HHHHHH----H--HHhhhhhhhhhhhlllhhhhhhhh
0040 8ioiA   ------------lllLL-HHHHHHHHHHH-L----------LLLHHHHhHHHH-H----lhhH-----LLL--------L---LHHHHHHHHHHHHH----LLLEEEE---E-ellllllleEEEEEL--HHHHHH----H--HHhhhllllllllllllllllllhh
0041 2qwwA   hhhhhhhhhhhhhllLH-HHHHHHHHHHH-L---L------LEEHHHH-HHHH-L---------------------------lLHHHHHHHHHHHHH----LLLEEEL---L---lllllllLEEEEL--HHHHHH----H--HHhhllhhhhhhhhhhhllllhhh
0042 2eshA   -------llllhhHHHH-HHHHHHHHHHH-L----------LLLHHHH-HHHH-H----L--L-----LL--lllllLLL---L--LHHHHHHHHHH----LLLEEEE---E-ellllllleEEEEEL--HHHHHH----H--HHhhhhhhhhhhhhhhhhhhhhhh
0043 3g3zA   hhhhhhhhhhhhlllLH-HHHHHHHHHHH-H---L------LLLHHHH-HHHH-L---------------------------lLHHHHHHHHHHHHH----LLLEEEL---L---llllhhhLLEEEL--HHHHHH----H--HHhhhhhhhhhhhhhhhhlhhhhh
0044 8ffzB   lllllllllllhhHHHH-HHHHHHHHHHH-L---L------LEEHHHH-HHHL-L----L--L-------------------lLHHHHHHHHHHHHH----LLLEEEE---L---------lLEEEEL--LLLLLllll----------------------------
0045 1z7uA   llhhhhhhhhhhlllLL-LHHHHHHHHHH-----L------LLLHHHH-HHHL-L-----------------------LL---LHHHHHHHHHHHHH----HLLEEEE---E---ellllleEEEEEL--HHHHHH----H--HHhhhhhhhhhhhhhhhhhhl---
0046 4fhtB   hhhhhhhhhhlllllLH-HHHHHHHHHHH-L---L------LLLHHHH-HHHH-L---------------------------lLHHHHHHHHHHHHH----HLLEEEE---E---lllllllEEEEEL--HHHHHH----H--HHhhhhhhhhhhhhhllllhhhhh
0047 2fa5B   hhhhhhhhlhhhhllLH-HHHHHHHHHHH-L---L------LLLHHHH-HHHH-L---------------------------lLHHHHHHHHHHHHH----HLLEEEL--------------LLEEEL--HHHHHH----H--HHhhhhhhhhhhhhhllllhhhhh
0048 2zkzC   hhhhhhhhhhhhhhlLH-HHHHHHHHHHH-H---L------LEEHHHH-HHHH-L---------------------------lLHHHHHHHHHHHL-----LLLEEEE---E------elleEEEEEL--L--HHH----H--HHhhhhl-----------------
0049 8yliB   hhhhhhhhhhhhlllLH-HHHHHHHHHHH-HlllL------EELHHHH-HHHH-L---------------------------lLHHHHHHHHHHHHH----LLLEEEE---E---lllllllEEEEEL--HHHHHH----H--HHhhhhhhhhhhhhhllllhhhhh
0050 5hs5A   hhhhhhhhhlllhHHHH-HHHHHHHHHHL----lL------LEEHHHH-HHHH--------------hHHH------L-L---LHHHHHHHHHHHHH----LLLLEEE---L---lllllllLEEELL--HHHHHH----H--HHhhhhhhhhhhhhhhlll-----
0051 6ahtA   llllhhhhhhhhhhhHH-HHHHHHHHHHH-H---L------LEEHHHHhHHHH-HhhhhhhhhlllllLLL------LlL---LHHHHHHHHHHHHH----LLLEEEE---E------elleEEEEEL--HHHHHH----H--HHhhhl------------------
0052 7el3A   hhhlllhhhhhhhllLH-HHHHHHHHHHH-H---L------EEEHHHH-HHHH-L---------------------------lLHHHHHHHHHHHHH----LLLEEEE---E---elleeeeEEEEEL--HHHHHH----H--HHhhhhhhhhhhhhhhhhlhhhhh
0053 2qenA   hhhhhhhhhhhhhhhLH-HHHHHHHHHHL-L----------lLLHHHH-HHHH-H----H--L-----LLL--------L---LHHHHHHHHHHHHH----LLLEEEE---L----------LEEEEL-----LHH----H--HHhhll------------------
0054 7xuxA   lhhhhhhhhhhhlllLH-HHHHHHHHHHH-H---L------LLLHHHH-LLLL-L----L-------------------L---LHHHHHHHHHHHHH----LLLEEELlllL----------LLEEELllLLLH-H----H--HHhhhhhhllllll----------
0055 5aipA   hhhhhhhhhhhhlllLH-HHHHHHHHHHH-H---L------LEEHHHH-HHHH-L---------------------------lLHHHHHHHHHHHHH----LLLEEEE---L---lllllllLEEEEL--HHHHHH----H--HHhhhhhhhhhhhhhhhhlhhhhh
0056 2fnaA   hhhhhhhhhlllhHHHH-HHHHHHHHHL------L------LLLHHHH-HHHHhH----H--H-----L--------LLL---LHHHHHHHHHHHHH----LLLEEEL---L----------LLEEEL-----LHH----Hhh------------------------
0057 4asnA   ----lhhhhhhhhllLH-HHHHHHHHHHH-L---LlllhhhLEEHHHH-HLLL-L------------------------L---LLHHHHHHHHHHHH----LLLEEEE---L------llllLEEEEL--HHHHHH----H--HHhlll------------------
0058 3mq0B   ---------llllHHHH-HHHHHHHHHHH-L--lL------LEEHHHH-HLLL-------------------------lL---LHHHHHHHHHHHHH----LLLEEEL---L---------lLEEEEL--LLH-HH----H--HHhhhhlllhhhhhhhhhlllhhh
0059 3f8fA   -----lllllhhhHHHH-HHHHHHHHHHH-L----------LLLHHHH-HHHH-H-hhlL--L-----LLL--------L---LHHHHHHHHHHHHH----LLLEEEE---E---lllllllEEEEEL--HHHHHH----H--HHhhhhhhhhhhhhhhhhhhhhhh
0060 8xt8B   hhhhhhhhhhlllllLH-HHHHHHHHHHH-L---L------LLLHHHH-HHHH-L--------------------------llLHHHHHHHHHHHHL----LLLEEEE---E---lllllllEEEEEL--HHHH-H----L--HHhhhhhhhhhhhhlllllllhh
0061 5h3rA   hhhhhhhhhlhhhllLH-HHHHHHHHHHH-H---L------LLLHHHH-HHHH-L---------------------------lLHHHHHHHHHHHHH----LLLEEEE---E---lllllllEEEEEL--HHHHHH----H--HHhhhhllhhhhhhhlllllhhhh
```

Job: Type-2 BREX; BrxHI-Helicase MBK7581083 Small-Beta barrel pdb25
Query: s001A
No: Chain Z rmsd lali nres id PDB Description
1: 6znp-A 6.3 2.3 58 748 10 MOLECULE: UNCHARACTERIZED ATP-DEPENDENT HELICASE YPRA;
2: 7wrs-A 5.9 2.7 61 518 5 MOLECULE: GLUTAMYL-TRNA SYNTHETASE;
3: 5dm3-C 4.4 2.4 52 396 6 MOLECULE: L-GLUTAMINE SYNTHETASE;
4: 2det-A 4.4 2.6 54 365 15 MOLECULE: TRNA;
5: 7bgm-A 4.0 3.3 55 213 9 MOLECULE: PHOSPHORIBOSYL-AMP CYCLOHYDROLASE;
6: 9fhl-G 4.0 2.4 53 213 9 MOLECULE: RRNA 16S SSO;
7: 8fn2-X 3.8 3.1 55 181 5 MOLECULE: 23S RIBOSOMAL RNA;
8: 1zps-B 3.8 2.9 51 128 12 MOLECULE: PHOSPHORIBOSYL-AMP CYCLOHYDROLASE;
9: 6fan-C 3.5 2.3 42 62 5 MOLECULE: COOT;

```
10: 8s8k-B 3.4 3.4 49 229 8 MOLECULE: 18S RIBOSOMAL RNA;
11: 4hpp-A 3.3 2.4 51 427 4 MOLECULE: PROBABLE GLUTAMINE SYNTHETASE;
12: 7kpj-E 3.2 2.9 44 218 9 MOLECULE: 338E6 FAB LIGHT CHAIN KAPPA;
13: 2j9i-A 3.1 2.8 51 421 10 MOLECULE: GLUTAMATE-AMMONIA LIGASE DOMAIN-CONTAINING PROTEI
14: 7aoh-B 3.0 3.8 44 1134 9 MOLECULE: DNA-DIRECTED RNA POLYMERASE 147 KDA POLYPEPTIDE;
15: 2kco-A 3.0 3.3 52 133 10 MOLECULE: 30S RIBOSOMAL PROTEIN S8E;
16: 3wwl-A 2.9 2.5 42 54 12 MOLECULE: ALPHA-AMINOADIPATE CARRIER PROTEIN LYSW;
17: 9nvu-P 2.8 2.5 43 603 9 MOLECULE: NTS;
18: 6pew-A 2.8 3.6 55 521 2 MOLECULE: GLUTAMINE SYNTHETASE;
19: 6fai-G 2.8 2.5 49 232 6 MOLECULE: 40S RIBOSOMAL PROTEIN S27-A;
20: 8xlj-A 2.7 3.5 53 372 4 MOLECULE: GLUTAMINE SYNTHETASE;
21: 1o70-A 2.7 2.4 48 296 8 MOLECULE: FASCICLIN I;
22: 5o60-W 2.7 2.8 56 192 14 MOLECULE: 50S RIBOSOMAL PROTEIN BL37;
23: 2e28-A 2.6 3.0 50 587 14 MOLECULE: PYRUVATE KINASE;
24: 4m00-A 2.5 3.4 44 501 7 MOLECULE: SERINE-RICH ADHESIN FOR PLATELETS;
25: 1bml-C 2.5 2.8 47 318 11 MOLECULE: PLASMIN;

0001 s001A   --------------------------APRDITVRA------G-----P-DEV-GTV---E--A--RF-LAA--I--D---P---N-PGSF-ML-A-G-K-------S-WEII-T--VEW-----D-R------GVCVVRP--S----P-----------
0002 6znpA   lhrngeryhwasesfpasnislrs-aSQENVVIVD------QsdianV-RII-GEM---D--R--FS-AMT--L--L---H---D-EAIY-LH-E-G-V-------Q-YQVE-K--LDW-----D-H------KKAYVRK--V----D-----------
0003 7wrsA   mewdkiwsfnkkvidpvaprytal-lKDAVVPVNV------P-----EvGSK-VLI---E--G--AD-AET--L--T---E-----GEVV-TF-InW-G-------N-IIIT-K--LNR-nssgK-I------VSIDTKL--N----L-----------
0004 5dm3C   -------------------------geiDTVLVCI------V-dmqgR-LMG-KRL---H--A--RH-FVD--H-----------gwEET-HC-C-NlY-------I-MKPD-L-aTLR-----C-V--legtAMVLCDL--L----D-----------
0005 2detA   akkkdstgicfigerkfreflgry-lPAQPGKIIT-----vD-----G-DEI-GEH---Q--G-----LMY--H--T---L-----GQRKgLG-I-G-GtkegteeP-WYVV-D--KDV-----E-N------NILVVAQ--G----Hehprlmsvgli
0006 7bgmA   ---------snavdslldsvkwdn-kGLAVAIAQN------V---dtG-AIL-MQG---F--A--NreAVA--T--T---I---S-SRKatKG-E-TnN-------F-INVH-D--VFL-----DdS------IIYLGKP--D----Gptchtgaetcy
0007 9fhlG   ----------------------------PDFKIVI------SayrtkT-LQI-SVD---QnkA--TN-LVG--L--K---I-----GDVF-EA-NlP-V-------K-LKIT-G--GSD-----NpnensqeiVQINTII--V----R-----------
0008 8fn2X   --ensrvlscqyrssfgssnarri-rAKSEIPAVVygqgkdV-----S-HL---RI---K--S--SE-FNKkfA--K---F---TdNTVL-IL-DlE-R-------C-VFVK-D--AAEniaskL-I------YHIDFYE--V----Drnvelekyvpi
0009 1zpsB   -------skgdvnillnfrhning-eDLIIAVAQD------H---etG-EVL-MVA---Y--M--NreALR--R-----tL---E-TGTAhywstsG-H-------V-QRVK-D--VLV-----D----dgdaVVLKVEQ--E----Ggachtgyrscf
0010 6fanC   ----------------------------CEASAFI------V-ngdkE-ELF-LER---V--D--KL----------------------ifgqrK-V-I-------K-AKIK-R--LEL-----V-D------HRILLER--E----D-----------
0011 8s8kB   ----avgknkrlskglkkrvvdpf-tRKEWYDIKA------P-----SfRNV-GKT---L--V--N----------------dslkGRVV-EV-C-LkV-------K-LRVD-E--VQG--------------KNLLTNF--H----G-----------
0012 4hppA   --------------------mnrl-qPVRLVSFVT------T-dlagI-TRG-RSL---P--L--AT-LEE--Q-----------lASGC-GW--paspwgshgdlR-LLPD-PnsRVR-----V----apalDYLHGNL--V----E-----------
0013 7kpjE   -----------------------e-vQNVKINYYD------E---daE-KQV-AEV---P----idtscVN--M--A--------------------pE-------G-YALV-S--SDC-----I----rdgyVYVSVKK--D----V-----------
0014 2j9iA   -------------------------haaAFVRFEA------TdaagaS-RSK--SI---P--A--QF--FE--K--V--iH---A-RG---YL-E-DdI-------V-LMPE-L-stAA-----V-A--aaatAAVICDG--G----A-----------
0015 7aohB   lvsqlsvlssitniltseyldlekkyfETGFPITI------E-----N-ALV-ASL---N--P--NM-ICD--FvtD---F---R-R-----------rkrmgffgnlevgI-T--LVR----dH-M------NEIRINI--G----Agrlvrpflvvd
0016 2kcoA   ptfttlsaedirikdrtlggnfkv-rLKYTTTANV------LpatntA-KKV-KI---------aRR-GI---------iI---R-GAKI-RT-E-A-G-------L-AVVT-S--RPG-----Q-D------GVINAVL--L----K-----------
0017 3wwlA   ------------------------------MVGTC------P-------GAE-LRL---E--N----------------pE---L-GELV-VC-EcG-A-------E-LEVV-G--LD----------------PLRLEP--A----P-----------
0018 9nvuP   ----------------------------RYVYVLD-----vD-----G-KPL-MPT---C------R-FGKvrR--M---L---K-SG---------------------QAK-A--VDT-----L-P------FTIQLTY--Kprta------------
0019 6pewA   ----mksvsfsnnaelyeyikdkk-nDVEIVACII------T-nllgT-YFK-CFF---Y--V--KE--IT--L--N---K----lESGF-SF-D-AdF-------F-IKVD-H-stCY-----L-E--gkniLNIMCDI--K----R-----------
0020 6faiG   ------------------------------MKLNI------SpvngsQ-KTF-EIddehR--I--RV-FFD--K--R---I-----GQEV-DG----gY-------V-FKIS-G--GND-----KdL------AVLALVI--V----Kkgeqelegltd
0021 8xljA   --matsasshlnkgikqmymnlpq-gEKIQLMYIW------V------gLRC-KTR---T--L--DC-DP-------kcvE---E-LPEW-NF-DsD-M-------Y-LHPV-A--MFR-----D-P--rdpnKLVFCEV--F----Kynrkpaetnlr
0022 1o70A   engalrkfyevimdnggavlddin-sLTEVTILAP------S-----NmilnmHII---K----drlnVDK--I--RqknansdT-VITV-EG-G-G-V-------N-ATVI-Q--ADV-----A-Q---tngYVHIIDHvv-----------------
0023 5o60W   anipnkltanvrtrtgkgasrqar-rDGKVPAVLY------G--hgtD-PQH-LEL---N--A--RD-FAA--VlrshgtN---A-ILTL-DI-E-G-T-------E-QLAL-T-kALD-----ViQ------HADLLVV--Q----Rgekvtvevtvl
0024 2e28A   ------------------------------------------evlgtP-EKI-SVT---Y--P--SL-IDD--V--S---V-----GAKI-LL-DgL-I-------S-LEVN-A--VDK-----Q-A------GEIVTTV--L----N-----------
0025 4m00A   smtastggatnlqqvqfgtfeyte-sAVTQVRYVD----vtT-----G-KDIiPPK---T----tID-NQ-------------------------tA-K-------G-YNYT-S--VDS-----S-YasagqsVTYYFTD--V----Kaptvtvgnqti
0026 1bmlC   dlisekyyvlkkgekpydpfdrsh-lKLFTIKYVD----vnT-----N-ELL-KSE---QlltaaKL-LY------------------NN-LDaF-G-I-------MdYTLTgK--VED-----N----tnriITVYMGK--R----------------

0001 s001A   --------------------------LLLEEEEEE------L-----L-EEE-EEE---E--H--HH-HHL--L--L---L---L-LLEE-EE-L-L-E-------E-EEEE-E--EEL-----L-L------LEEEEEE--L----L-----------
0002 6znpA   eeelleeeellllllhhhllllll-lLLLEEEEEE------LllhhhL-EEE-EEE---E--H--HH-HHH--H--L---L---L-LLEE-EE-L-L-E-------E-EEEE-E--EEL-----L-L------LEEEEEE--L----L-----------
0003 7wrsA   llhhhhhhhhhhhhlllllleelee-eHHHLEEEEL------L-----LeLLE-EEE---L--H--HH-HLL--L--L---L-----LLEE-EE-LlL-E-------E-EEEE-E--EEE-llllL-E------EEEEEEE--L----L-----------
0004 5dm3C   -------------------------lllLEEEEEE------E-llllL-EEE-EEE---E--H--HH-HHH--L-----------llLLE-EL-L-LlL-------E-EEEL-H-hHLE-----E-L--llleEEEEEEE--E----L-----------
0005 2detA   llllllllllllllllhhhhhlll-lLLLLLLLLL-----lL-----L-LLL-LLL---L--L-----LLL--L--L---L-----LLLLlLL-L-L-LlllllllL-EEEE-E--LLL-----L-L------LLLEEEE--L----Llhhheeeeeee
0006 7bgmA   ---------llhhhhhhhllllll-lLLEEEEEEE------L---llL-LEE-EEE---E--E--LhhHHH--H--H---H---H-HLLleLL-L-LlL-------L-EEEE-E--EEE-----ElE------EEEEEEE--L----Llllllllllle
0007 9fhlG   ----------------------------LEEEEEE------EeeellE-EEE-EEE---LhhH--LL-LLL--L--L---L-----LLEE-EE-LeE-E-------E-EEEE-E--EEE-----LllllllleEEEEEEE--L----L-----------
0008 8fn2X   --lhhheeeeeelllllhhhhhhh-hHLLEEEEEEelllllL-----E-EE---EE---E--H--HH-HHHhhL--L---L---LlLLEE-EE-ElE-E-------E-EEEE-E--EEEellllE-E------EEEEEEE--L----Llllleeeeeee
0009 1zpsB   -------lhhhhhhhlllleeell-eEEEEEEEEE------L---llL-LEE-EEE---E--E--LhhHHH--H-----hH---H-HLLLeeeellL-L-------L-EEEE-E--EEE-----L----llleEEEEEEE--L----Lllllllllll
0010 6fanC   ----------------------------LLEEEEE------L-llllL-EEE-EEE---E--E--EE----------------------ellleE-E-E-------L-EEEE-E--EEL-----L-L------LEEEEEE--L----L-----------
0011 8s8kB   ----llllllllllllllllllll-lLEEEEEEEL------L-----LlLEE-EEE---E--E--E----------------hhhlLLEE-EE-E-HeE-------E-EEEE-E--ELL--------------LEEEEEE--E----E-----------
0012 4hppA   --------------------llll-lLLLEEEEEE------E-llllL-EEE-EEE---E--H--HH-HHH--H-----------hLLLE-EE--hhllllllllleE-EEEL-HhhLEE-----E----llleEEEEEEE--E----L-----------
0013 7kpjE   -----------------------l-lEEEEEEEEE------L---llL-EEE-EEE---E----lllllLL--H--H--------------------lL-------L-EEEE-E--LLL-----L----llleEEEEEEE--L----L-----------
0014 2j9iA   -------------------------lllLEEEEEE------ElllllE-EEE--EE---E--H--HH--LL--L--L--hH---H-HL---EE-E-LlE-------E-EEEL-L-llLE-----E-L--llleEEEEEEE--E----L-----------
0015 7aohB   lleelllllleellllhhhhhhhhhllLLLEEEEE------L-----L-EEE-EEE---L--H--HH-HHH--HhhH---H---H-H-----------hhhlllllllllleE-E--EEL----lL-L------LEEEEEL--L----Llleeeeeeeee
0016 2kcoA   llllllhhhhhhhhhlllllllll-lLLLLLEEEE------ElllleE-EEE-EE---------hLL-LL---------lL---L-LLEE-EE-L-L-L-------E-EEEE-E--EEL-----L-L------LLEEEEE--L----L-----------
0017 3wwlA   ------------------------------LEEEL------L-------LLE-EEL---L--L----------------lL---L-LLEE-EL-LlL-L-------E-EEEE-E--LL----------------LLEEEE--L----L-----------
0018 9nvuP   ----------------------------LLLEEEL-----lL-----L-LLL-LLL---L------L-HHHhhH--H---H---H-LL---------------------LEE-E--EEE-----L-L------EEEEELL--Lllll------------
0019 6pewA   ----lleelllhhhhhhhhhllll-lLEEEEEEEE------L-llllL-LEE-EEE---E--H--HH--LL--L--H---H----hHLLL-LL-L-LlL-------L-LEEE-E-eeEE-----L-L--leeeEEEEEEL--L----L-----------
0020 6faiG   ------------------------------LEEEE------ElllleE-EEE-ELllllL--L--LL-LLL--L--L---L-----LLEE-EH----lL-------E-EEEE-E--EEE-----LlE------EEEEEEE--E----Ellllllllll
0021 8xljA   --lllllhhhllhhhhhhhhllll-lLLEEEEEEE------E------lEEE-EEE---E--E--LL-LL-------llhH---H-LLLE-EE-ElE-E-------E-EEEE-E--EEE-----L-L--llllEEEEEEE--E----Llllllllllhh
0022 1o70A   lhhhhhhhhhhhhhlllhhhhhhh-lLLLEEEEEE------L-----HhhhhhLEE---L----llllHHH--H--HhhlllhhL-EEEE-EE-L-L-E-------E-EEEE-E--EEE-----E-L---lleEEEEELLll-----------------
0023 5o60W   lllleeeeeellllllhhhhhhhh-hLLEEEEEEE------L--lllL-LEE-EEE---E--H--HH-HHH--HhhhhllL---L-EEEE-EE-L-L-E-------E-EEEE-E-eEEE-----EeE------EEEEEEL--L----Llleeeeeeeee
0024 2e28A   ------------------------------------------lllllL-LEE-ELL---L--L--LL-LLL--L--L---L-----LLEE-EE-LlL-E-------E-EEEE-E--EEL-----L-L------LEEEEEL--L----L-----------
0025 4m00A   eeeeellllllleeeeeeeeeeee-eLEEEEEEEE----llL-----L-LLLlLLE---E----eLL-LL-------------------------hH-L-------L-EEEE-E--EEL-----L-LllllleeEEEEEEE--L----Llleeellleee
0026 1bmlC   eeeeeeeeeellllllllllllll-lEEEEEEEEE----llL-----L-LEE-EEE---EeeellLL-LL------------------LL-LHhH-L-L-------LlEEEEeE--EEE-----L----lleeEEEEEEE--L----------------

Job: Type-2 BREX; BrxHI-Helicase MBK7581083 CTD (Lhr-helicase CTD)
Query: s001A
No: Chain Z rmsd lali nres id PDB Description
1: 5v9x-A 10.3 2.7 162 772 13 MOLECULE: ATP-DEPENDENT DNA HELICASE;
2: 5jb3-1 8.1 2.3 69 83 13 MOLECULE: 16S RIBOSOMAL RNA;
3: 1d1r-A 7.1 2.6 72 83 11 MOLECULE: HYPOTHETICAL 11.4 KD PROTEIN YCIH IN PYRF-OSMB
4: 6vpq-A 7.0 3.9 67 87 9 MOLECULE: DENSITY-REGULATED PROTEIN;
5: 5oa3-0 6.8 8.8 82 479 7 MOLECULE: EUKARYOTIC TRANSLATION INITIATION FACTOR 2D;
6: 9iad-A 6.6 7.6 72 685 13 MOLECULE: PROTEIN ARGONAUTE;
7: 1xxa-C 6.5 1.7 56 74 14 MOLECULE: ARGININE REPRESSOR;
```

```
8: 7nad-U 6.4 2.0 63 102 11 MOLECULE: 25S RRNA;
9: 3mml-B 6.4 2.8 69 210 4 MOLECULE: ALLOPHANATE HYDROLASE SUBUNIT 2;
10: 5e68-B 6.3 3.2 111 172 5 MOLECULE: S-RIBOSYLHOMOCYSTEINE LYASE;
11: 4y2i-A 6.1 1.9 58 65 14 MOLECULE: PUTATIVE METAL-BINDING TRANSPORT PROTEIN;
12: 6znp-A 5.9 3.6 91 748 14 MOLECULE: UNCHARACTERIZED ATP-DEPENDENT HELICASE YPRA;
13: 2kwa-A 5.8 2.2 69 101 6 MOLECULE: KINASE A INHIBITOR;
14: 7bj2-a 5.8 9.9 66 306 9 MOLECULE: FLAGELLAR P-RING PROTEIN;
15: 7y8u-F 5.7 5.9 107 347 7 MOLECULE: ALBE HOMOLOG;
16: 5euf-B 5.4 5.6 110 416 9 MOLECULE: PROTEASE;
17: 2if1-A 5.4 4.8 82 126 11 MOLECULE: EIF1;
18: 2p5m-A 5.3 1.9 55 82 11 MOLECULE: ARGININE REPRESSOR;
19: 4u9r-A 5.3 2.0 55 147 13 MOLECULE: CZCP CATION EFFLUX P1-ATPASE;
20: 8b6j-b 5.3 6.8 120 460 10 MOLECULE: PEPTIDASE M16 INACTIVE DOMAIN PROTEIN;
21: 8q3b-C 5.2 7.9 83 359 10 MOLECULE: DNA-DIRECTED RNA POLYMERASE RPB1 HOMOLOG;
22: 7y8v-E 5.2 6.8 108 374 5 MOLECULE: ALBE HOMOLOG;
23: 7si6-A 5.1 4.0 65 873 5 MOLECULE: P-TYPE CU(+) TRANSPORTER;
24: 4v1a-l 5.1 6.2 67 133 10 MOLECULE: MITORIBOSOMAL PROTEIN ML37, MRPL37;
25: 8bep-B 5.0 5.9 113 487 16 MOLECULE: PROBABLE MITOCHONDRIAL-PROCESSING PEPTIDASE SUBUN

0001 s001A   HLRG-SESSLIE----AP------D--RITWWN--F-A-----GG-GANALLRGMLE--QEL-G---------E-KVVA-T--N-------A-AIKLE-------G-----D-------AS--GAAVREVLA-R--W---RP--T--AEDALRA--A--D----PSRTR--V-SKF---QVC-P-EALLAELVVEKV--LDVEGARRALS-A--P-PT-A-T-S--G-GQ--
0002 5v9xA   VVPSdTTFVVER----FR-delgdW--RVILHS--P-Y-----GL-RVHGPLALAVG--RRL-R---erygidE-KPTA-S--D-------D-GIIVR-------L-----P------dsP--GADL--FVF-D-aD---EI--E---PIVTAE--V--GgsalFASRF-----rE---CAA-I-VLEAVRECLQDV--YDVPALIELMH-K--IrIV-E-V-E--T-AT--
0003 5jb31   iakeeqKIKIYV----TK-rrfgkL--MTIIEG--F-D-----TsvIDLKELAKKLK--DIC-A---------C-GGTV-K--D-------N-TIELQ-------G-------------DH--RKKVAEELV-K--M---G--------------------------------------------------------------------fSR-D--S-IE-I-R----------
0004 1d1rA   --kgdgVVRIQR----QTsgrkgkG--VCLITG-vD-L-----DD-AELTKLAAELK--KKC-G---------C-GGAV-K--D-------G-VIEIQ-------G-----D--------K--RDLLKSLLE-A--K-----------------------------------------------------------------------G-M--K-VK-L-A-G--G-LE--
0005 6vpqA   ------------prakkK------Y--VTRVCG--L-A-----tfeiDLKEAQRFFA--QKF-S---------X-GASVtG--E-------D-EIIIQ-------G-------------DF--TDDIIDVIQ-E--K----------------------------------------------------------------------WP-E--VsIE-D-L-G--E-----
0006 5oa30   kgrI-CPIDITL----AQ-rasnkK--VTVVRN--L-E-----ayglDPYSVAAILQ--QRC-Q---------A-STTV-NpdS-------L-QVQIQ-------G-------------NQ--VHHLGWLLL-E--E---Y---------------------------------------------------------------------qkH--I-QG-L-E-Kag-----
0007 9iadA   ------------lditpK------N--FICFRLtpE-I-----ER-KTGNSFSWRFS--QKF-P--------dA-VVIW-H--N-------K-FFWVL-------AkpnrpM-------PS--QEQWREKLL-E--I-ceEL--K--K--------------------------------------------------------digdrT-Y--A-IQ-W-V-S--Ql----
0008 1xxaC   -plknlvLDIDY----ND------A--VVVIHT--S-P-----G---AAQLIARLLD--SLG-K-----aegiL-GTIA-G--D-------D-TIFTT-------P-----A-ngftvkdL--YEAILELFDr--------------------------------------------------------------------------------------------
0009 7nadU   ---------------iA------K--TFTVDV--S---sptengvfDPASYAKYLI--DHI-KgavgnlgnaV-TVTE-D--G-------T-VVTVV-------S---taK-------FS--GKYLKYLTK-K-yL---KK--N-----------------------------------------------------qlrdW-I--R-FV-S-T-K--T-----
0010 3mmlB   ----stlGTVHN---yGD------Q--ALLLEF----D-----ST-AEVLAWTETLR--EAE-L--------lgvvDIVP-A--A-------R-TVLVK-------L-----A-gpryqapT--RQRLGKLR----vR---PE--A--IThqppgdtP--G----XW-----V-QFRavg--------------------------------------------------
0011 5e68B   MQ----aPAVRT----AK------TitVFDLRF--CiM-----PE-KGIHTLEHLFA--GFM-RhlngngveiI-DISP-M-xR-------T-GFYMS-------L-----I------tPD--EQRVADAWK-AamA---DV--LkdQNQIPE---L--N----VYQCG--T----------------------yQM--HSLSEAQDIAReR--D-VR-V-N-S--N-KE--
0012 4y2iA   ---------------------------GMQFHI----T-----CG-GCASTVKKTIL--TLD-A--------nA-TVRT-D--P-----atR-LVDVE-------T-----S-------LS--AEQIAAALQ-K--A---G--------------------------------------------------------------------fP-PR-E-R----------
0013 6znpA   ktkekSRTSLHY----GD------VtsAAWLEI----digektLE-QLLLGISNVLQ--HIV-PvyimcdrndV-HVVS-Q--IkaahtglP-TIFLYdhypggiG-----L-------AEevFKRFSDINE-A--A---KqchD--GCP-SCI--G---------------------------------tEIEG--ikAKERILQLLD-Q--M-S-------------
0014 2kwaA   -gsmtvRYQIEQ---lGD------S--AMMIRF--G-eeineqVN-GIVHAAAAYIE--EQPgF---------I-ECIP-A--F-------T-SLTVF-------Y-----DmlpqgissP--FESVKRDVE-E--R-------------------------------------------------------------laeIAEDY-E--V-NR-------------
0015 7bj2a   -erirdlTSVQGtqfgaG------N--TINLQL--N-E-----DF-TMAQQITDAIN--RAR-G--------yG-SATAlD--A-------R-TVQVR-------V-----P-gnssqvrF--LADIQNM---E--V---NV--T--P------------------------vvIN---SRTg---------------------------------------------------
0016 7y8uF   ---------------vnK------D--MILLPV--E-HgdylkNR-MICHLIADMIK--HLA-Q----qleydV-SVGL-F--I-stnqhsC-YLKVK-------K-----S-------D---QKRFSSLIQ-Q--L---S-hfI--ETYIKDY--QwrF----MNELV-iN-FNQ-------geytvaelfgsLDS--VDKLDILAVRN-EliN-QL-T-V----------
0017 5eufB   KQDG--------gvhleW------V--ALGYKV--P--afkhkDQ-VALDALSKLLG--SWL-QvdkkrlasqA-FSHN-X--Q---lqdeS-VFLFI-------A-----G-pnikaeaL--QKEIVALLE-K--LkkgEI--T--QAELDKI--KinQ----KADFI-sN-LES-------qndlqgltdyQQQFldLKVSDLVRVAN-E-yF-KD-Tq----------
0018 2if1A   agTE-DYIHIRI----QQ-rngrkT--LTTVQG--I-A-----D-dYDKKKLVKAFK--KKF-A---------C-NGTV-I--E--hpeygE-VIQLQ-------G-------------dQ--RKNICQFLV-E--I---G--------------------------------------------------------------LAK-DdqL-KV-H-G-F--------
0019 2p5mA   ralmdafVKIDS----AS------H--MIVLKT--M-P-----G---NAQAIGALMD--NLD-W------demM-GTIC-G--D-------D-TILII-------C-----R-tpedtegV--KNRLLELL---------------------------------------------------------------------------------------------
0020 4u9rA   ------------gveaahiktanvdfgHVLWQLkgV-W-----HE-RRARTVASQLR--ALPgV---------I-EAEV-S--A-------SgIARVE-------F-----D----ndriS--AAGIEQALS-K-------------------------------------------------------------------------R--G-LA-------------
0021 8b6jb   G-----------eesnvT------L--ALLFQS--V--pwssaDI-VAFNVAAALLN--LRL-KlqkyayfdqA-EALN-F--H---ftdsG-LFGLR-------T-----S-sadrakdI--LNHSIAELK-A--I-asGV--N--ADELLTA--KaaL----KNSVLsaL-ERQ-------kiqhtDYVKQIDS---VTADQVAKAVA-K-vL-TS-NpvnT--Y-DAir
0022 8q3bC   QP------ELVL----FS------LktKAIITI--K-E-----TH-TIGNLLKTCIY--EMI-P-------diS-FVGY-Q--C-vphkqeM-VLTII-------H-----K-------AS--QEDLITLLE-KsiQ---NI--I--QTF------------------------qileknVDE-Ia-----------------------------------------------
0023 7y8vE   NE----------tdfssI------L--TCAYTF----hvtdleRE-ILIPMFDGYIG--SVL-F-ktlrlvdhI-SSHY-D--Q-----esN-LLFIT-------V-----L-sknqlstI--LTKIQHTIE-S--I---EF--D--SVSFSLS--KvmF----ENESRfrfeYKN---PE------sflKAIQNT----dIADFYRLKA-N--M-RWv-----------
0024 7si6A   ------------vedlgfgasvmeG--NVELIItgM-T-----CA-SCVHNIESRLM--RTP-G---------IlQASV-A--L-----atC-KAQVK-------F-----Dp-----iVG--PRDIIRIIE-G--I--------------------------------------------------------------------G-F--Q-AS-L-A-----hVG--
0025 4v1al   RSRM-HniPVYR----DI-thgnrQ--MTVIRK--V-E-----G---DIWALQKDVE--DFL-S---pllgktP-VTQV-N--E-----vtG-TLRVK-------G-------------YF--DQQLKAWLL-E--Kgf-------------------------------------------------------------------------------------
0026 8bepB   G-----------dlplaQ------F--AVAFEG--A--swtdpDS-VALMVMQTMLGswSDL-TvaineiaesI-MAFN-T--N---ykdtG-LFGVY-------A-----V-kadclddL--SYAIMYEVT-K--L-ayRV--S--DADVTRA--RnqL----KSSLL-------------riptaelFARIDA---VDASTVKRVAN-KyiY-DK-D-iqD--Y-NKfr

0001 s001A   HHLL-LLLEEEE----EL------L--EEEEEL--L-L-----LH-HHHHHHHHHHH--HHH-L---------L-EEEE-L--L-------L-EEEEE-------L-----L-------LL--HHHHHHHHH-H--L---LL--L--HHHHHHH--L--L----LLLLL--L-LLL---HHH-L-HHHHHHHHHHHH--LLHHHHHHHLL-L--L-LE-E-E-E--L-LL--
0002 5v9xA   LLLLlLEEEEEE----EE-lllllE--EEEEEL--L-L-----LH-HHHHHHHHHHH--HHH-H---hhllllL-LLEE-E--L-------L-EEEEE-------E-----L------llL--LHHH--LLL-L-hH---HH--H---HHHHHH--H--HhlhhHHHHH-----hH---HHH-H-HHHHHHHHHHLL--LLHHHHHHHHH-H--HeEE-E-E-E--L-LL--
0003 5jb31   llllllLEEEEE----LL-lllllL--EEEEEL--L-L-----LllLLHHHHHHHHH--HHH-L---------L-LEEE-E--L-------L-EEEEE-------L-------------LL--HHHHHHHHH-H--L---L------------------------------------------------------------------lLL-L--L-EE-E-L----------
0004 1d1rA   --llllEEEEEE----LLllllllL--LEEEEL-lL-L-----LH-HHHHHHHHHHL--LLL-L---------L-LLLL-L--L-------L-LEEEL-------L-----L--------L--HHHHHHHHH-H--H----------------------------------------------------------------------L-L--E-EL-L-L-L--L-LL--
0005 6vpqA   ------------ellllL------E--EEEEEL--H-H-----hhllLHHHHHHHHH--HHH-L---------L-LEEEeE--L-------L-EEEEE-------L-------------LL--LLLHHHHHH-H--H---------------------------------------------------------------------LL-L--LhEE-E-L-L--L------
0006 5oa30   ellL-LLEEEEE----EE-ellleE--EEEEEL--L-H-----hhllLHHHHHHHHH--HHH-L---------L-LEEE-EelL-------E-EEEEE-------L-------------LL--HHHHHHHHH-L--L---L--------------------------------------------------------------------lhH--E-EL-L-L-Lll------
0007 9iadA   ------------eelllL------L--EEEEEElllL-L-----LH-HHHHHHHHHHH--HHL-L--------lE-EEEE-E--L-------L-EEEEE-------ElllllL-------LL--HHHHHHHHH-H--H-hhLL--H--H--------------------------------------------------------hhlllL-L--L-EE-E-L-L--Ll----
0008 1xxaC   -llhhheEEEEE----LL------L--LEEEEE--L-L-----L---LHHHHHHHHL--LLL-H-----hhleE-EEEE-L--L-------L-EEEEE-------E-----L-llllhhhH--HHHHHHHHLl--------------------------------------------------------------------------------------------
0009 7nadU   ----------------lL------E--EEEEEL--H---hhhhllllLHHHHHHHHH--HLL-LlllllllllE-EEEE-L--L-------L-EEEEE-------E---llL-------LL--HHHHHHHHH-H-hH---HH--L----------------------------------------------------------llllL-E--E-EE-E-E-E--L-----
0010 3mmlB   ----lllLLEEE---lLL------L--EEEEEL----L-----LH-HHHHHHHHHHH--HHL-L-------lleeEEEE-L--L-------L-EEEEE-------E-----L-lhhhhhhH--HHHHLLLL----lL---LL--L--LLlllhhhlL--L----LE-----E-EEEell------------------------------------------------------
0011 5e68B   LL----lLEEEE----EE------EeeEEEEEL--LlL-----LH-HHHHHHHHHHH--HHH-HhhllllleeE-EEEE-L-lL-------L-EEEEE-------E-----E------lLL--HHHHHHHHH-HhhH---HH--HllHHHLLL---L--L----LLLLL--L----------------------lLL--LLHHHHHHHHHhH--L-LE-E-L-L--H-HH--
0012 4y2iA   ---------------------------LEEEEL----L-----LH-HHHHHHHHHHH--HHL-L--------lL-EEEE-E--L-----llL-EEEEE-------L-----L-------LL--HHHHHHHHH-H--L---L----------------------------------------------------------------------lL-LE-E-L----------
0013 6znpA   eeeeeLLEEEEE----EE------EeeEEEEEE----lllhhhHH-HHHHHHHHHHH--HHH-HhhlllllllE-EEEE-E--LlllllllL-EEEEEellllllL-----H----lllL-----H-------HHhhHHLHHHHHH-H--H---HhllL--LLL-LLL--L-----------------------------lLLLL--llHHHHHHHHHH-H--L-L-------------
0014 2kwaA   -lllllLLEEEE---lLL------L--EEEEEL--L-llllhhHH-HHHHHHHHHHH--HLLlE---------E-EEEE-L--L-------L-EEEEE-------E-----LhllllllllH--HHHHHHHHH-H--H---------------------------------------------------------hhhHLHHH-L--L-LL-------------
0015 7bj2a   -llhhhlEEELLllllllL------L--EEEEEE--L-L-----LH-HHHHHHHHHHH--HHL-L--------lL-LEEElL--L-------L-EEEEE-------L-----L-lhhhhhhH--HHHHHLL---L--L---LL--L--L-------------------------eeEL---LLLl---------------------------------------------------
0016 7y8uF   ---------------llL------L--EEEEEE--L-LllhhhHH-HHHHHHHHHHH--HHH-H----hllllL-LEEE-E--L-llllllE-EEEEE-------H-----H-------H---HHHHHHHHH-H--H---L-hhH--HHHHHHH--HhhH----HHHHH-hH-LLL-------llllhhhhhhhHHL--LLHHHHHHHHH-HhhH-HH-H-L-----------
0017 5eufB   LLLL--------lllllE------E--EEEEEE--L--lllllLH-HHHHHHHHHHL--LLH-HhllllleeeE-EEEE-L--L---llllE-EEEEE-------E-----E-llllhhhH--HHHHHHHHH-H--HhllLL--L--HHHHHHH--HhhH----HHHHH-hH-LLL-------lllhhhhhlhHHHHhlLLHHHHHHHHH-H-hL-LH-Hh-----------
0018 2if1A   llLL-LLEELLL----LL-lllllL--LLEELL--L-L-----L-lLLHHHHHLLHH--HHL-L---------L-LEEE-E--L--lllllL-EEEEL-------L-------------lL--HHHHHHHHH-H--H---L-------------------------------------------------------------LLL-LllE-EL-L-L-L--------
0019 2p5mA   hhhhhheEEEEE----EL------L--EEEEEE--L-L-----L---LHHHHHHHHH--LLL-L------lllL-EEEE-L--L-------L-EEEEE-------L-----L-lhhhhhhH--HHHHHLLL------------------------------------------------------------------------------------------
0020 4u9rA   ------------leeeeeeellllheeEEEEELllL-L-----LH-HHHHHHHHHHH--LLLlE---------E-EEEE-L--L-------LlEEEEE-------E-----E----llllL--HHHHHHHHH-L------------------------------------------------------------------------L--L-LL-------------
0021 8b6jb   L-----------llleeE------E--EEEELL--L--lllllLH-HHHHHHHHHHH--LHH-HllllllleeeE-EEEE-E--E---llllE-EEEEE-------E-----E-elllhhhH--HHHHHHHHH-H--H-hhLL--L--HHHHHHH--HhhH----HHHHHhhH-HLH-------lllhhHHHHHHHHH---LLHHHHHHHHH-H-hH-LL-LlllL--H-HHhh
0022 8q3bC   LL------LEEE----EE------LleEEEEEE--E-L-----LH-HHHHHHHHHHH--HHL-L--------llL-EEEE-E--E-elllleE-EEEEE-------E-----L-------LL--HHHHHHHHH-HhhH---HH--H--HHH------------------------hhhhhhHHH-Hl-----------------------------------------------
0023 7y8vE   LL----------lllllE------E--EEEEEE----lllhhhHH-LHHHHHHHHHH--LHH-H-hhhhllllE-EEEE-E--L-----llL-EEEEE-------E-----E-lhhhhhhH--HHHHHHHHH-L--L---LL--L--HHHHHHH--HhhH----HHHHHhhhhHHL---HH------hhhHHHHLL----lHHHHHHHHH-L--E-EEl-------------
0024 7si6A   ------------hhhllleeeellL--EEEEEElllL-L-----LH-HHHHHHHHHHH--HLL-L---------EeEEEE-L--L-----llL-EEEEE-------E-----Ll-----lLL--HHHHHHHHH-H--H-------------------------------------------------------------------L-L--L-EE-E-L-----eLH--
0025 4v1al   LLLL-LllLEEE----EE-ellleE--EEEELL--E-E-----E---LHHHHHHHHH--HHH-H---hhhlllL-LEEE-E--L-----llL-EEEEE-------L-------------LL--HHHHHHHHH-H--Lll-----------------------------------------------------------------------------------------
0026 8bepB   L-----------lllleE------E--EEEEEL--L--lllllHH-HHHHHHHHHHLeeLHH-HhhhhllllE-EEEE-E--E---llllE-EEEEE-------E-----E-lhhhhhhH--HHHHHHHHH-H--L-llLL--L--HHHHHHH--HhhH----HHHHH-------------lllhhhhHHHHHL---LLHHHHHHHHH-HhlL-LL-L-llL--H-HHhh

################################################################################################################################################################################################################################################################################################################################
################################################################################################################################################################################################################################################################################################################################
################################################################################################################################################################################################################################################################################################################################

Job: Type-2 BREX; BrxD-ATPase (Standalone ORC/CDC6 AAA+ ATPase)
Query: s001A
No: Chain Z rmsd lali nres id PDB Description
1: 1w5s-B 17.4 3.5 268 396 13 MOLECULE: ORIGIN RECOGNITION COMPLEX SUBUNIT 2 ORC2;
```

```
2: 2qby-B 17.0 3.2 251 368 16 MOLECULE: CELL DIVISION CONTROL PROTEIN 6 HOMOLOG 1;
3: 2fna-A 13.3 3.5 246 352 12 MOLECULE: CONSERVED HYPOTHETICAL PROTEIN;
4: 7rzy-1 13.3 3.4 252 311 15 MOLECULE: TN6677 VIBRIO CHOLERAE TRANSPOSON TNSC (VCHTNSC);
5: 8fcv-R 13.2 4.1 266 343 15 MOLECULE: DNA (60-MER);
6: 6fhs-C 13.1 5.1 267 459 13 MOLECULE: RUVB-LIKE HELICASE;
7: 5zr1-A 13.0 4.1 244 494 15 MOLECULE: ORIGIN RECOGNITION COMPLEX SUBUNIT 1;
8: 9emc-D 13.0 4.1 258 337 14 MOLECULE: RUVB-LIKE 1;
9: 8btg-B 12.9 4.1 223 335 13 MOLECULE: CHROMOSOMAL REPLICATION INITIATOR PROTEIN DNAA;
10: 7jgr-D 12.9 3.9 255 441 10 MOLECULE: ORIGIN RECOGNITION COMPLEX SUBUNIT 2;
11: 3bos-B 12.7 3.7 202 231 12 MOLECULE: PUTATIVE DNA REPLICATION FACTOR;
12: 7plh-A 12.3 3.3 230 260 10 MOLECULE: SHTNSC;
13: 6pxk-K 12.3 3.9 238 429 16 MOLECULE: ATP-DEPENDENT PROTEASE ATPASE SUBUNIT HSLU;
15: 1g4a-E 12.1 4.6 245 356 16 MOLECULE: ATP-DEPENDENT HSL PROTEASE ATP-BINDING SUBUNIT
16: 5zr1-E 12.1 4.2 239 460 10 MOLECULE: ORIGIN RECOGNITION COMPLEX SUBUNIT 1;
17: 6p8v-C 12.0 3.6 225 303 10 MOLECULE: ATPASE, AAA FAMILY;
18: 7uqj-A 11.9 4.4 229 585 11 MOLECULE: ATPASE HISTONE CHAPERONE YTA7;
19: 8xvb-A 11.8 3.6 227 246 11 MOLECULE: DNA (5'-
20: 7vcs-A 11.4 5.2 229 766 10 MOLECULE: TRANSITIONAL ENDOPLASMIC RETICULUM ATPASE;
21: 8tw7-2 11.4 3.6 227 339 15 MOLECULE: REPLICATION FACTOR C SUBUNIT 5;
22: 7st9-A 11.4 4.5 224 522 12 MOLECULE: CHECKPOINT PROTEIN RAD24;
24: 8dr5-A 11.3 4.2 233 646 14 MOLECULE: REPLICATION FACTOR C SUBUNIT 1;
25: 6l1q-A 11.1 3.3 208 267 15 MOLECULE: CBBQ PROTEIN;
26: 3glf-G 11.1 4.8 217 378 18 MOLECULE: DNA POLYMERASE III SUBUNIT DELTA;
27: 5e7p-A 11.0 4.1 221 719 16 MOLECULE: CELL DIVISION CONTROL PROTEIN CDC48;
28: 5kzf-K 10.9 3.8 231 498 8 MOLECULE: PROTEASOME-ASSOCIATED ATPASE;
29: 8umy-A 10.8 4.1 228 546 14 MOLECULE: CHROMOSOME TRANSMISSION FIDELITY PROTEIN 18 HOMOL
30: 7fd4-A 10.7 5.5 223 779 14 MOLECULE: LON PROTEASE;
31: 7st9-E 10.7 3.9 221 354 14 MOLECULE: CHECKPOINT PROTEIN RAD24;
32: 7pkq-x 10.7 4.3 228 368 18 MOLECULE: MS35;
33: 1ixs-B 10.6 4.0 223 315 11 MOLECULE: HOLLIDAY JUNCTION DNA HELICASE RUVA;
34: 3pvs-D 10.6 5.4 207 424 15 MOLECULE: REPLICATION-ASSOCIATED RECOMBINATION PROTEIN A;
35: 8csp-X 10.5 4.3 232 352 11 MOLECULE: 28S RIBOSOMAL PROTEIN S34, MITOCHONDRIAL;
36: 9cru-C 10.5 3.8 202 741 16 MOLECULE: VESICULAR-FUSION PROTEIN SEC18;
37: 8thc-A 10.5 3.8 211 479 12 MOLECULE: ELG1 ISOFORM 1;
38: 9bv1-A 10.4 5.0 213 405 12 MOLECULE: 26S PROTEASOME NON-ATPASE REGULATORY SUBUNIT 1;
39: 8zr0-A 10.3 4.1 209 480 13 MOLECULE: CASEINOLYTIC PEPTIDASE B PROTEIN HOMOLOG;
40: 7w42-B 10.3 3.1 197 402 14 MOLECULE: UNCHARACTERIZED ATPASE YJOB;
41: 7crc-B 10.2 4.7 232 1112 15 MOLECULE: NAD+ HYDROLASE (NADASE);
42: 5j1s-A 10.1 3.4 195 277 11 MOLECULE: TORSIN-1A;
43: 4rh7-A 10.1 3.6 252 3005 10 MOLECULE: GREEN FLUORESCENT PROTEIN/CYTOPLASMIC DYNEIN 2 HE
44: 3u5z-B 10.1 4.0 214 320 14 MOLECULE: DNA POLYMERASE ACCESSORY PROTEIN 44;
45: 2gno-A 10.0 3.7 189 296 12 MOLECULE: DNA POLYMERASE III, GAMMA SUBUNIT-RELATED PROTEIN
46: 8xku-A 10.0 4.2 219 730 13 MOLECULE: PROBABLE INACTIVE ATP-DEPENDENT ZINC METALLOPROTE
47: 8dgc-A 10.0 4.3 238 2028 15 MOLECULE: SEAVS3;
48: 7qo4-M 10.0 4.5 215 421 13 MOLECULE: 26S PROTEASOME REGULATORY SUBUNIT RPN1;
50: 6z1f-3 9.9 3.6 212 284 11 MOLECULE: RIBULOSE BISPHOSPHATE CARBOXYLASE/OXYGENASE ACTIV

0001 s001A   ETFAVGLERFERSVAEDLAAVERGAGTFKAIRGEYGSGKTFFVRWLQEQALRRGFLAAEVQISEAETPLHRLETVYRRMIERLHGALRSVIDRWLYLGGQPSVVKGDVDHFAALGFIQGLLVAARGLLIVLDEVETLQRVRR-DVREKALNALRQWIDEIDGGRFPGLFLVITGTPSFFDGVQVPLSQRLVQIRLRNFDLPSLAEVGRKVREIFVAVRRAVDDDYVVALATAVTGKLGKVGVPRMFLKKLVAEVLDRVELYPDFDPRRDYALTLNE
0002 1w5sB   PE-LRVRRGEEALARIYLNRLLGAGLSVNMIYGSVGIGKTTLAKFTVKRVSEATVKQAYVNA--FNAP--NLYTILSLIVRQ--TGYP--------IQVR------GAPALDILKALVDNLYVENYLLVILDEFQSLSSP------rIAAEDLYTLLRVHEEIPvNRIGFLLVADVRALSYMREQVESQIFKLHLPAYKSRELYTILEQRAELGL-RDTVWEPRHLELISDVYG---GGDG-ARRAIVALK-MACEMAEAMGRDSLSEDLrSENE-
0003 2qbyB   KE-IPFREDILRDAAIAIRYFVKEVKFSNLFLGLTGTGKTFVSKYIFNEIEEVKVKQAYVNCR-EVGGTPQ--AVLSSLAGKL--------------SVPKH-----GINLGEYIDKIKNGTRNI-RAIIYLDEVDTL------vkRRGGDIVLYQLLR---SDAN--ISVIMISDINVR-YME-RVLSSLPSVIFKPYDAEQLKFILSKYAEYGL-IKGTYDDEILSYIAAISA---KEHGDARKAVNLLF-RAAQLAS--GGGIIRKEDKAIYEQ
0004 2fnaA   RKDFFDREKEIEKLKGLR--------PITLVLGLRRTGKSSIIKIGINELN---LPYIYLDLR------kDFLLELQKEIN---KLVKRLPS-----IKFN--------RLSFANLLESFEQASKNVIIVLDEAQELVK------LRGV--NLLPALAYAYDN-LKRIKFIXSGEXGLLYYLRVPLFGR-STVELKPFSREEAIEFLRRGFQEAD---IDFK--DYEVVYEKIG--------PGWLTYFGFI-YLDNK-----------FAINTLE
0005 7rzy1   -FVSTPVRKILSYMDRCRDL-SDLEPTCMMVYGASGVGKTTVIKKYLNQNRRESIPVLHIELPDN--AKPV--DAARELLVEM--------------PLALY-----ETDLARLTKRLTELIPAVGVKLIIIDEFQHLVEESN-RVLTQVGNWLKMILNKTK------CPIVIFGMPYSK-VLQAQLHGRFIQVELRPFSYGVFKTFLEYLDKALP------kqAGLANLQKKLYAF--SQGNMRSLRNLIYQ-ASIEAIDNQ---ITEEDFSKLTS
0006 8fcvR   -TVAHPLKEVYEILMRTIAEP-----SFIFVYGASGVGKTTLRLRVEQKLTELAPVVGIEAIAPE---sFNWKEYYTRALITL--EEPL--IDHK-V-------eSKVVAPALRRALENALIHRHPDVFFVDEAQHFG-KVA-SG-YKLQDQLDCLKSLA--NMTG-ILHCLLGTYELLTFRNLQLSRRSVDIHFRRYDVQAFKSVLLTFQQHLP--LAETP--NLVDHWEYFYER--TLGCIGTLKDWLKR-VLSDALDRE-TTIT--KDLQLSV
0007 6fhsC   KAGFVGQCAAREACGVVVDLIKAHKMRGVLLAGGPGTGKTALALAISQELGT-KIPFCPITGSEIY----KTEVLMENFRR---------glRVREA---naRKPKMTEITDklrmenkvvknqgPGVLFIDEAHML--------DIECFTYLNKALE---SPIA--PIVVLASNRG--IGIPPDFLQRLLIIPTHPYEPDEIRRIVRIRAQTEG---vQLTDAAVDRVAEHGVRI------LRYCLQLL-APASILARVNGRT-DVQDIAEELFL
0008 5zr1A   --yLPARENEFASIYLSAYSAISDSATTIYVAGTPGVGKTLTVREVVKELLSSSFLYVEINGKMVK-PTDCYETLWNKVS------------------GERL---twaASMESLEFYFKrvpNKKKTIVVLLDELDAMVT-----KSQD---IMYNFFNWTTYENA-KLIVIAVANTMDL-PERQKITSRITRIMFTGYTHEELKNIIDLR-LKGLNvrLRMSADAIEIASRKVA---SVSGDARRALKVCK-RAAEIAEKHYMgVHIT-KALNTLN
0009 9emcD   LQGMVGQLAARRAAGVVLEMIEGKIARAVLIAGQPGTGKTAIAMGMAQALGP-DTPFTAIAGSEIF----KTEALTQAFR----------gvRIKE--GDTG-------eksevreqnakveeegPGVLFIDEVHML--------DIESFSFLNRALE---SDMA--PVLIMATNRG--IGIPIDLLDRLLIVSTTPYSEKDTKQILRIRCEEED---vEMSEDAYTVLTRIGLET------LRYAIQLIT-AASLVCRKRKGTEVQVDDiYSLFL
0010 8btgB   FDTFVInHAASLAVAEAPAK------NPLFIYGGVGLGKTHLMHAIGHYVIDHNAKVVYLSSE---KFTNEFIN----------------------------sirDNKAVDFRNRY--------vDVLLIDDIQFLA------GKEQTQEEFFHTFNTLHEE---SKQIVISSDR-PPK--TLRLRSRFLITDITPPDLETRIAILRKKAKAEG-----IPNEVMLYIANQID-------SIRELEGALI-RVVAYSSLINKDINADLALKDI--
0011 7jgrD   ---LRGYAKERSNVRLLLQRTAMGESNSLLLLGPRGSGKTTLINSVLADLLPNNTLIVHLDGN--LHTDDR--VALKSITVQ--------------AGKVF--gsfaENLAFLLQCLKAG-KHSKSVIFILEEFDLF------cAHHN-QTLLYNLFDVSQSAQA-PICVLGVTCLDVI-LLE-RVKSRFRQVFLFPSLFEDYVDLCRDLL--SLPWNKQIVLATaRSTLQALHD--FDIS-EAYLKNFLFRL-VAHL-RPQSPHTAEKAVGSEGD
0012 3bosB   FTSYYPNDELIGALKSASGDG-----QAIYLWGPVKSGRTHLIHAACARANELERRSFYIPLIHASI---STALLEG--------------------------------------fDLICIDDVDAVA------gHPLWEEAIFDLYNRVAEQ--KRGSLIVSASA-SPXGFV-DLVSRXLTYQLQPXXDDEKLAALQRRAAXRG-----LPEDVGRFLLNRXA--------LRTLFDVLDR-LDKASXVH-QRKLIPFVXLRL--
0013 7plhA   -IVPLQVKTLHDWLDGKRKA-----RKSCRVVGESRTGKTVACDAYRYR-HKPQVPVVYIRPHQK--CGPK--DLFKKITEYL--------------YRVT------KGTVSDFRDRTIEVLKGCGVEMLIIDEADRL---------KPETFADVRDIAEDLG------IAVVLVGTDRLDAVIKRQVLERFAHLRFGKLSGEDFKNTVEMWEQVLKLSN---LKSKMLRILTSAT----EGYI-GRLDEILRE-AAIRSLSRGL--DKAVLQKEYK-
0014 6pxkK   DKHIIGQDNAKRSVAIALRNRWRRTPKNILXIGPTGVGKTEIARRLAKLAN---APFIKVEAT----KFTE-KEVDS-IIRDLrvqaiekELKQ-----------------------------HGIVFIDEIDKICK-PD-VSREGVQRDLLPLVEG---vKTDHILFIASGAFAKPSLIP-ELQGRLIRVELQALTTSDFERILTSITVQYKAIE--FTDSGIKRIAEAAWQVNSENIGARRLHTVL-ERLXEEISYDA--qYVSKHDALVSR
0015 8rwvE   LNVVLCRESQVSILQSLFGE-RHHFSFSIFIYGHTASGKTYVTQTLLK---TLELPHVFVNCV--ECFT--LRLLLEQILNKL---NHLSSSE---I----------TCETNDFVRLFKQVTTNLKTVYIVLDKAEYLRD-----MEAN---LLPGFLRLQ-ELADRNVTVLFLSEI-VWE-FRPG-CFEPFVLYFPDYSIGNLQKILSHDH---PPSADF-YAAYINILLGVFYT--VCRD-LKELRHLAVLN-FPKY-CEPVvrnEPHLAMQTLR
0016 1g4aE   DKHIIGQDNAKRSVAIALRNRWRRTPKNILMIGPTGVGKTEIARRLAKLAN---APFIKVEAT---KFTE--KEVDS-IIRDLtDAAVKMVRVQA-----------------------eelkDAIDHGIVFIDEIDKICKGPD-VSREGVQRDLLPLVEG---vKTDHILFIASGAFAKPSLIP-ELQGRLIRVELQALTTSDFERILTSITVQYKAIE--FTDSGIKRIAEAAWQVNS---IARRLHTVLERL-MEEISYDA--qYVSKHDALVSR
0017 5zr1E   -peVAFREYTNCLASYISA--DPDI-SNLILQGYSGTGKTYTLKKYFNA--NPNLHAVWLEPV--ELVSW---PLLQAIARTVqYKLKTLYPNIP-DPL-------QVEEFLLVKTLHN--iqyeCLFLILDGFDSLQDL-----DAAL---FNKYIKLNELL-skNIKFIYTMLESFLQ----rYSTHCPTVMFPRYNVDEVSTILVMRCGELMEFQNVAA-NFIHLIVQAFHSY-TGND-IFALNDLIDFKW-PKYVSRIT-ikAIKLSTDDNL
0018 6p8vC   LARLVGLDEHKDRLSKILGLLVN---PLVVLAGDVGSGKTELAETIGDAVARIDITLYPLS-----LATRLVSAA--------------------------------FDYTIEAADKLKAVLLLIDQADAL--eXH-HEDRAGVNAFIRGIDRIAN-QKLPAAVLXCTNLKAL------DPAVQEILTFSRPNDEQRHYLLHSKLT-----GLGLNSTAVEELVRLTGPRdPGFT-FSDITQRLIPSI-ILAAYPYNAVSVHSALNKXT-
0019 7uqjA   FDDIGGLDNYIDQLKEMVALPLL---RGVLFHGPPGTGKTLMARALAASCSSRKITFFMRKGADILSK--AERQLRL--------------------------------LFEEAKKHQ----PSIIFFDEIDGL--sEQ-IH-ASIVSTLLALM-DGMDN-RGQVIVIGATNRPDA-VDPA-LRRP-REFYFPLPDVKARFKILQIQTRKWS-----LSTNFIDKLAFLT----KGYG-GADLRSLCTEA-ALISIQRSIKVKVS-LALKK--
0020 8xvbA   -FVETQVKQIWTsMRFASLTE-----SIAVVCGNPGVGKTEAAREYRRTNN----NVWMITIT-PSCA--SVLECLTELAFEL--------------DAP--------RRKGPLSRALRRRLE---QGLVIIDEADHL------------GAEVLEELRLLQ--ESTR-IGLVLMGNHRVYSNMTGRLFSRIKRTAINKTKKADVKAIADAWQ----------NGEKELELLQQIA----QKPGLRILNHSLR-LAAMTAHGKG-ERVN-DYLRRELD
0021 7vcsA   YDDIGGCRKQLAQIKEVELPLRHP--RGILLYGPPGTGKTLIARAVANETG---AFFFLINGIMSK----SESNLRKA--------------------------------FEEAEKNA----PAIIFIDELDAIA-THG-EVERRIVSQLLTLMDGL-KQRA-HVIVMAATN--RPNIDPA-LRRF-REVDIGIPDATGRLEILQIHTKNM--KLAD--dVDLEQVANET----HGHV-GADLAALCS-EAALQAIRnSLAVTMD-WALSQ--
0022 8tw72   -deVTAQDHAVTVLKKTLKSA-----PHMLFYGPPGTGKTSTILALTKELYGPDLRILELNAS--DERGIIVREKV--------------------------------KNFARLTV---SYKIIILDEADSM---------TADAQSALRRTMETY----SGVTRFCLICNYVTR-IIDP-LASRCSKFRFKALDASNAIDRLRFISEQEN---vKCDDGVLERILDI------SAGDLRRGITLLQ-SASKGAQYLGDKNIT--qELAGV-
0023 7st9A   LEQVAIHKRLKDVQEALDAMFLPNA-RILLLSGPSGCSKSTVIKELSKILVPKYtKVTEFR-GDCIvnpESFSEFL--------------------------------kGARY-LVMSN-lSLILIEDL-PNVF-HI-DTRRRFQQLILQWLYS--SEPLL-PPLVICITEFSAEIMN-EILMH-KRIKFNPINSTLLKKHLKFICVQNM-KWNKR-QEVIDYIAQE----------IRSAITTLQ-FWATSS----------SLPI----
0024 5v8fD   -KIIFYLQDCQQEIDRIIKQSIQKESHSVILVGPRQSYKTYLLDYELSLLQQSYFITIRLNGF--IHSEQTAINGIATQLE---QQLQKIH-----TISS-----GSLTFEKILLLLDS------TVVFIFDEIDTF------aGPVR-QTLLYNLFDMVEH-SRVPVCIFGCTTLNIL-YLE-RVKSRFRVIYMPQINLDDMVDAVRNLLT--VRWNETKELSdlNRHIRMNF---TFR--LPTLKNSI-IPLVAT-sKNFGSAIKS-SFLDNKN
0025 8dr5A   Q--VCGNKGSVMKLKNWLANWENSK-RAAMLYGPPGIGKTTAAHLVAQELG---YDILEQNAS---DVRSLLNAGV--------------------------------KNALDNMS-VVGHFVIIMDEVDGMS-----GGDRGGVGQLAQFCRKT------sTPLILICNE--RNMR---PFDRVLDIQFRRPDANSIKSRLMTIAIREK-----FKLD--PNVIDRLIQT--TRGDIRQVINLLSTI-STTTK------NHENIEISKA-
0026 6l1qA   F--YQPQHDEVALFEAAYRKR-----LPVMVKGPTGCGKSRFVEFMAWRLG---KPLVTVACNED--MTAA--DLVG-----------------------RWLLDgtRWQD--gpltvAARY-----GAICYLDEIVEA--------RQDTTVVIHPLTD--HRRTHPDFQLVISYNPG--sDL--STKQRFTGFEFDYPNAELEAGILVQETG---------APSIAAQLVTVAATARLKghITRLLVYAAML-MDDGV-----------AACRM--
0027 3glfG   AD-VVGQEHVLTALANGLSL------HAYLFSGTRGVGKTSIARLLAKGLNCEpqDLIEIDAAS--RTKVDTRDLL---------------------------------DNVQ-YAPAR-FKVYLIDEVHML---------SRHSFNALLKTLEEP----PEHVKFLLATDPQK--LPVT-ILSRCLQFHLKALDVEQIRHQLEHILNEEH---iAHEPRALQLLARAAE-------GLRDALSLTD-QAIASGDG-----STQAAMLGLDD
0028 5e7pA   LDDVGDMVETKRALTEALWPLQHPPPRGVLLYGPPGCGKTFVVRALASS---GRLSVHAVKGSMDKWvSEKA--VREL--------------------------------FARARDSA----PSLVFLDEIDALpQNFDsGVTDKVVASLLTELDGIE--PLRDVVVLGATN--RPD----iDPALLRLVFVEPPDAAARRDILRTAGKSI--PLAD--dVDLDSLADDL----DGYS-AADCVALLR-ESAMTAMRRSI-VTAA-KARET--
0029 5kzfK   YADIGLSRQIRDAVELPFLH------KGVLLYGPPGCGKTLIAKAVANSLAKKKSYFLNIKGLLNK----TERHIRLI--------------------------------FQRAREKASEG-PVIVFFDEMDSIFR-VE-TT---VVPQLLSEIDGVE--GLENVIVIGASREDM--IDPA-ILRP-VKIKIERPDAEAAQDIYSKYLT--EFRSA--IKAMIEKVVDRMYAeiDFNS-GAMIQNVV-DRAKKNaiKSVLGLRIQ-DSIVFAE
0030 8umyA   YTELLSDDFTNRCLLKWLKLWDLVVhKVALLCGPPGLGKTTLAHVIARHAG---YSVVEMNAS--DDRSPVFRTRI--------------------------------EAATQME---SVPNCLVIDEIDGA--------PVAAINVLLSILNR--KGLLM-RPIICICN--DQFSLR----QLKFLLHFPPTLPSRLVQRLQEVSLRQG-----MRAD--PGVLAALCEK--TDNDIRACINTLQFLY-SRGQ-----ELSV--DVQAT-
0031 7fd4A   D--HYGLKDVKERILEYLAVrlLDVRaILVLVGPPGVGKTSLGRSIARSMN---RKFHRISLGG----rAEIRGKLIHAMK--QVGV---------------------------------------NPVILLDEIDKM-SSDW-RG--DPASAMLEVLD-PEQNNLSKVFFITTAN--TLQIPRP-LLDRMEVIEIPGYTNMEKQAIARQLWPKQVRIE--VTDAAILRVISEYT----REAGVRGLERELG-KIARKGAKFWL-gDIPTYGIPR--
0032 7st9E   NALSHNENFLKSLSDQ-PRDL-----PHLLLYGPNGTGKKTRCMALLESIFG-fvYHLEITPSDMGnNDRIVQELL--------------------------------keVAQM---EQVD--YKCVIINEANSL--------TKDAQAALRRTMEKY----SKNIRLIMVCDSMS--IIA-PIKSRCLLIRCPAPSDSEISTILSDVVTNER---iQLETK---DILKRIAQA--SNGNLRVSLLMLE-SMALNNE------LKSSSPIIKPD
0033 7pkqx   -ASILYRPIMAALNGAVAAG------PRLLLTGPAGCGKSLALLGLVEWARQQGWLVVYVSCLA---LVRGyLTSAQQLLKGVmDAHGPLLQSLP-R-GLSS-dnaQLAVDSALQLIRQLQLL-gRVLFALDDYNYLYGPTV-LDAGELiRGLRllSELGTNP-vGGAVVVAATTARSLA------LEVPTVVEVPGFDEAETAAALAHYAATG-----aRAAS--AAEARHLFA--LTGGNGRELRAKAGA---------------------
0034 1ixsB   D-EYIGKQKLRVYLEAAKARKE----EHLLLFGPPGLGKTTLAHVIAHELG---VNLRVTSGP----AIEKPGDLAAI--------------------------------lANSLE-------GDILFIDEIHRL--------SRQAEEHLYPAMEDFvmELPR-FTLIGATT--RPGTAP--LLSRFIVEHLEYYTPEELAQGVMRDARLLG---vRITEEAALEIGRRSR-------GMRVAKRLFR-RVRDFAQVAG-eRALEAALGLEKR
```

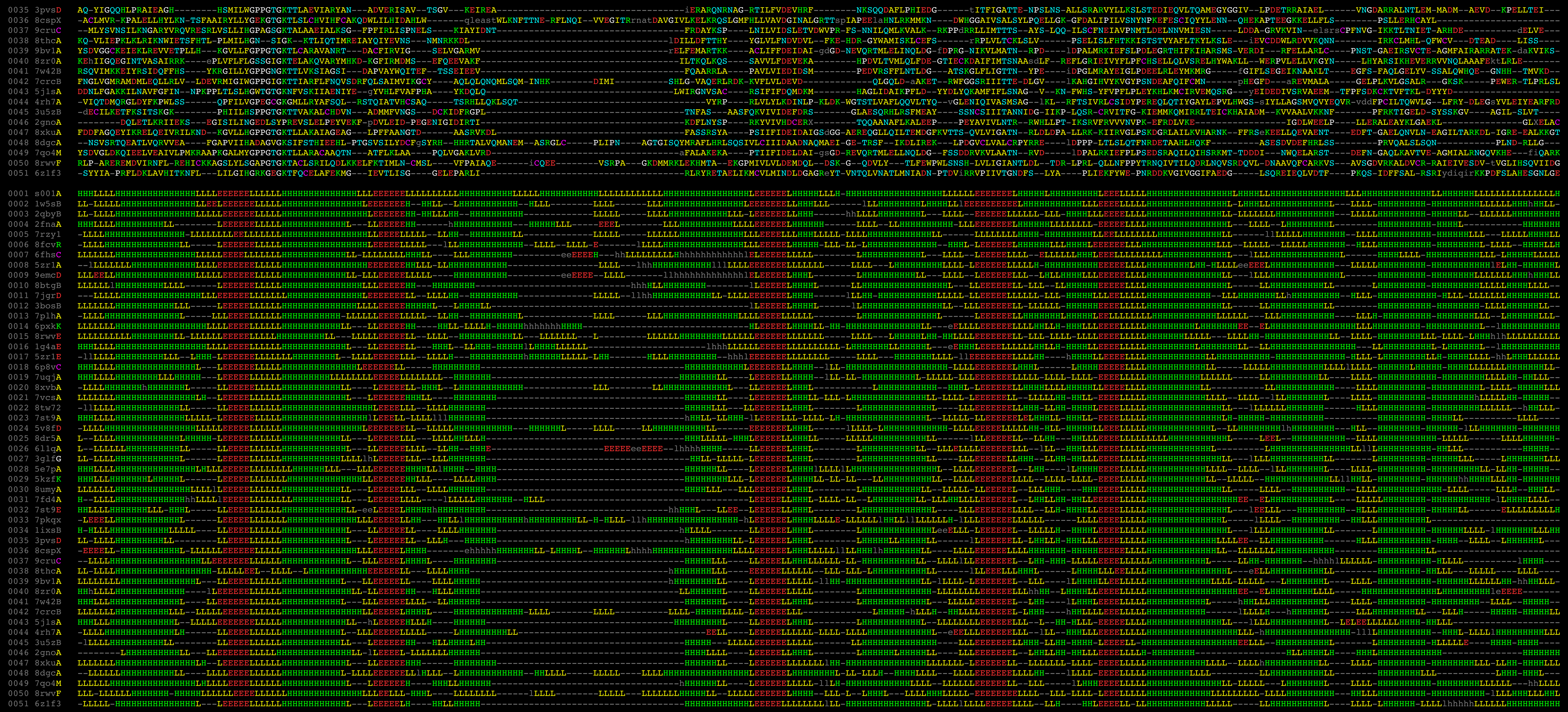

```
Job: Type-3 BREX; MTase MBW2068239 HTH1
Query: s001A
No: Chain Z rmsd lali nres id PDB Description
1: 5a31-N 6.3 2.0 54 703 11 MOLECULE: ANAPHASE-PROMOTING COMPLEX SUBUNIT 1;
2: 1ldd-A 5.7 2.3 53 74 9 MOLECULE: ANAPHASE PROMOTING COMPLEX;
3: 9bwl-M 5.7 2.7 56 779 13 MOLECULE: LE_POLYA;
4: 1hlv-A 5.4 2.2 53 131 9 MOLECULE: CENP-B BOX DNA;
5: 2nvu-A 5.4 3.0 63 530 5 MOLECULE: NEDD8-ACTIVATING ENZYME E1 REGULATORY SUBUNIT;
6: 9kbd-A 4.9 2.0 51 737 4 MOLECULE: S-PHASE KINASE-ASSOCIATED PROTEIN 1;
7: 3mq0-B 4.8 2.5 48 248 8 MOLECULE: TRANSCRIPTIONAL REPRESSOR OF THE BLCABC OPERON;
8: 3c1d-B 4.8 2.5 52 154 12 MOLECULE: REGULATORY PROTEIN RECX;
9: 3fhz-D 4.7 2.1 53 167 15 MOLECULE: ARGININE REPRESSOR;
10: 6lb9-A 4.6 2.8 62 345 11 MOLECULE: DUF4007 DOMAIN-CONTAINING PROTEIN;
11: 8j56-C 4.6 2.5 54 160 6 MOLECULE: FLAGELLAR TRANSCRIPTIONAL REGULATOR FLHD;
12: 8eki-D 4.6 3.2 60 684 7 MOLECULE: PROTEIN TRANSPORT PROTEIN SEC20;
13: 2od5-A 4.6 2.0 47 91 6 MOLECULE: HYPOTHETICAL PROTEIN;
14: 8cli-A 4.5 3.0 58 542 10 MOLECULE: GENERAL TRANSCRIPTION FACTOR 3C POLYPEPTIDE 1;
15: 5of3-C 4.4 3.0 57 107 5 MOLECULE: DNA PRIMASE SMALL SUBUNIT PRIS;
16: 7z0o-G 4.3 2.2 49 84 2 MOLECULE: HISTONE H3;
17: 4fcy-B 4.3 2.2 49 476 10 MOLECULE: TRANSPOSASE;
18: 8s0c-A 4.3 2.8 52 113 10 MOLECULE: ORIGIN RECOGNITION COMPLEX SUBUNIT 1;
19: 2dob-A 4.3 3.2 56 82 9 MOLECULE: PROACTIVATOR POLYPEPTIDE;
20: 2rkh-A 4.2 2.1 50 164 10 MOLECULE: PUTATIVE APHA-LIKE TRANSCRIPTION FACTOR;
21: 3v4g-A 4.2 2.0 48 145 6 MOLECULE: ARGININE REPRESSOR;
22: 6zvh-y 4.2 2.0 47 72 17 MOLECULE: 18S RRNA;
23: 6v9i-C 4.2 2.2 52 730 8 MOLECULE: IMMUNOGLOBULIN G-BINDING PROTEIN G,CULLIN-5;
24: 6qfd-B 4.1 2.1 45 116 11 MOLECULE: DNA-BINDING PROTEIN;
25: 6zca-D 4.1 2.7 58 140 10 MOLECULE: PROBABLE DNA-DIRECTED RNA POLYMERASE SUBUNIT DELT
26: 7z8b-C 4.1 2.3 52 1224 8 MOLECULE: CULLIN-7;
27: 7pik-C 4.0 2.6 52 557 12 MOLECULE: TRANSPOSON TN7 TRANSPOSITION PROTEIN TNSB;
28: 2qby-B 4.0 2.2 50 368 6 MOLECULE: CELL DIVISION CONTROL PROTEIN 6 HOMOLOG 1;
29: 1d3y-B 4.0 2.1 48 290 6 MOLECULE: DNA TOPOISOMERASE VI A SUBUNIT;
30: 1cf7-B 4.0 2.1 52 82 10 MOLECULE: DNA (5'-
31: 6zfb-d 4.0 2.6 58 139 10 MOLECULE: DNA-DIRECTED RNA POLYMERASE SUBUNIT DELTA;
32: 7oqe-K 4.0 2.0 46 406 4 MOLECULE: PROTEIN NAM8;
33: 3e3v-A 4.0 2.3 45 154 7 MOLECULE: REGULATORY PROTEIN RECX;
34: 7kbf-K 3.9 2.4 50 78 16 MOLECULE: HISTONE H3.2;
35: 3s63-B 3.9 3.0 56 90 5 MOLECULE: SAPOSIN-LIKE PROTEIN;
36: 8ea3-W 3.9 2.7 53 514 11 MOLECULE: TARGET_LE;
37: 5b7i-C 3.8 2.8 63 138 14 MOLECULE: CRISPR-ASSOCIATED NUCLEASE/HELICASE CAS3 SUBTYPE
38: 1fnn-A 3.8 2.9 54 379 9 MOLECULE: CELL DIVISION CONTROL PROTEIN 6;
39: 1cf7-A 3.8 2.8 48 67 6 MOLECULE: DNA (5'-
40: 7dh7-C 3.8 2.4 50 150 14 MOLECULE: TRANSCRIPTIONAL REGULATOR FUR FAMILY;
41: 5zi8-A 3.8 2.9 52 104 13 MOLECULE: TRANSCRIPTIONAL REGULATOR;
42: 8as8-E 3.8 2.9 51 498 6 MOLECULE: JETC;
43: 1dp7-P 3.8 2.0 46 76 11 MOLECULE: DNA (5'-D(*CP*GP*(BRU)P*TP*AP*CP*CP*AP*(BRU)
44: 2k85-A 3.8 2.0 46 65 11 MOLECULE: GLUCOCORTICOID RECEPTOR DNA-BINDING FACTOR 1;
45: 3al0-B 3.8 2.5 53 482 8 MOLECULE: GLUTAMYL-TRNA(GLN) AMIDOTRANSFERASE SUBUNIT A;
46: 6tda-L 3.8 3.9 52 596 8 MOLECULE: HISTONE H3.2;
47: 8yb6-K 3.8 2.9 52 165 10 MOLECULE: CRISPR SYSTEM CASCADE SUBUNIT CASD;
48: 8u8m-A 3.7 2.5 51 116 8 MOLECULE: DOUBLE-STRAND TELOMERIC DNA-BINDING PROTEINS 1;
49: 3elk-A 3.7 2.2 47 104 11 MOLECULE: PUTATIVE TRANSCRIPTIONAL REGULATOR TA0346;
50: 8yzt-C 3.7 3.2 59 121 5 MOLECULE: PROTEIN BANP;
51: 8jxk-D 3.7 2.1 46 166 17 MOLECULE: CONSERVED PROTEIN;
52: 3k69-A 3.7 2.4 45 151 9 MOLECULE: PUTATIVE TRANSCRIPTION REGULATOR;
53: 1j5y-A 3.7 3.0 46 172 9 MOLECULE: TRANSCRIPTIONAL REGULATOR, BIOTIN REPRESSOR FAMIL
54: 2hye-C 3.7 2.0 49 719 8 MOLECULE: DNA DAMAGE-BINDING PROTEIN 1;
55: 3o2p-E 3.7 2.2 51 86 6 MOLECULE: DEFECTIVE IN CULLIN NEDDYLATION PROTEIN 1;
56: 3h20-A 3.7 3.0 59 288 7 MOLECULE: REPLICATION PROTEIN B;
57: 6rfl-I 3.7 2.6 50 773 4 MOLECULE: DNA-DEPENDENT RNA POLYMERASE SUBUNIT RPO132;
58: 5dcm-B 3.7 3.0 52 98 8 MOLECULE: PHOB FAMILY TRANSCRIPTIONAL REGULATOR;
59: 8amy-A 3.6 2.7 49 85 6 MOLECULE: MU8.1 CONOTOXIN;
60: 5nl9-A 3.6 2.9 52 146 13 MOLECULE: TRANSCRIPTIONAL REGULATOR (FUR FAMILY);
61: 6jx3-B 3.6 3.8 51 85 4 MOLECULE: TFUB1;
62: 6vqx-A 3.6 2.6 61 97 10 MOLECULE: ACRF6;
63: 3sqn-A 3.6 2.7 54 468 7 MOLECULE: CONSERVED DOMAIN PROTEIN;
64: 7vwv-B 3.6 2.7 50 74 12 MOLECULE: I73R;
65: 5u30-A 3.6 2.9 50 1085 6 MOLECULE: CRISPR-ASSOCIATED ENDONUCLEASE C2C1;
66: 7qvg-T 3.6 3.2 51 267 6 MOLECULE: 26S PROTEASOME REGULATORY SUBUNIT 7;
67: 6dk4-A 3.6 2.2 47 135 17 MOLECULE: FERRIC UPTAKE REGULATION PROTEIN;
68: 8iue-P 3.6 2.4 52 303 10 MOLECULE: DNA-DIRECTED RNA POLYMERASE III SUBUNIT RPC1;
69: 1m12-A 3.6 2.5 53 84 8 MOLECULE: SAPOSIN C;
70: 8bpz-A 3.6 2.2 50 322 8 MOLECULE: SPBETA PROPHAGE-DERIVED UNCHARACTERIZED PROTEIN Y
71: 8xjg-A 3.6 2.8 50 148 8 MOLECULE: YQEY;
72: 2rre-A 3.6 2.2 51 74 6 MOLECULE: PUTATIVE UNCHARACTERIZED PROTEIN;
73: 5nwt-M 3.6 3.4 56 431 7 MOLECULE: DNA-DIRECTED RNA POLYMERASE SUBUNIT ALPHA;
74: 1xma-B 3.6 2.4 49 103 10 MOLECULE: PREDICTED TRANSCRIPTIONAL REGULATOR;
75: 3a1y-A 3.5 2.6 46 58 9 MOLECULE: 50S RIBOSOMAL PROTEIN P1 (L12P);
76: 7apd-B 3.5 2.3 49 289 8 MOLECULE: REPLICATION PROTEIN E1;
77: 2z99-A 3.5 2.3 46 160 7 MOLECULE: PUTATIVE UNCHARACTERIZED PROTEIN;
```

```
78: 7sol-A 3.5 2.7 62 1011 3 MOLECULE: UBIQUITIN-LIKE MODIFIER-ACTIVATING ENZYME 6;
79: 1q1v-A 3.5 2.6 50 70 6 MOLECULE: DEK PROTEIN;
80: 1z0x-A 3.5 2.6 48 216 8 MOLECULE: TRANSCRIPTIONAL REGULATOR, TETR FAMILY;
81: 6c2j-A 3.5 3.4 52 265 10 MOLECULE: RNAP1;
82: 5tjj-A 3.5 2.7 45 254 9 MOLECULE: TRANSCRIPTIONAL REGULATOR, ICLR FAMILY;
83: 2lnb-A 3.5 2.9 48 80 13 MOLECULE: Z-DNA-BINDING PROTEIN 1;
84: 8b9x-A 3.5 2.5 43 256 7 MOLECULE: DDRGK DOMAIN-CONTAINING PROTEIN 1,E3 UFM1-PROTEIN
85: 7o4i-R 3.5 2.0 44 268 11 MOLECULE: GENERAL TRANSCRIPTION AND DNA REPAIR FACTOR IIH H

0001 s001A   -GGLE-ERFK-L-T-A---G--T--EE-GV-W-DFVRTH-LK-QL-P----AER---QNYLLFDRMVAF-HVQ--R--G---VT-V--P--M--S-A-AEFYVG-LA-QRF-P---------------
0002 5a31N   eqppfgvyavilevpedieE--E--LL-LF-W-TYIQAM-LT-NL------eSL---SLDRIYNMLRMFvVTG--P--A---LA-E-----I--D-L-QELQGY-LQ-KKV-Rdqqlvysagvyrlp-
0003 1lddA   ---------------------lT--LQ-RS-L-PFIEGM-LT-NL------gAM---KLHKIHSFLKIT-VP---K--D---WG-Y-nR--I--T-L-QQLEGY-LN-TLA-Degrlkyiangsyeiv
0004 9bw1M   qkygcgyigllP-E-D---T-----LA-IM-E-KFILEDyET-LK-------QK---RMWEVHAALVRA-CEE--A--G---VI-A-----P--S-Y-KAFTKE-VQ-RRT-Gyeqtkkrqgrraayq
0005 1hlvA   ---------------------sP--YD-KL-E-GLLIAW-FQ-QI-R--aglpV---KGIILKEKALRI-AEE--L--G---MDdF--T--A--S---NGWLDR-FR-RRR-S---------------
0006 2nvuA   dDRCI-N-IT-K-Q-T------P--SF-WI-L-ARALKE-FV-AK----ekakK---DAAAVGNHVAKL-LQS--I--G---QA-P-eS--I--S-E-KELKLL-CS-NSA-Flrvvrcrslaeeygl
0007 9kbdA   vaqfvglelyklkdgedlvykdR--KL-LI-Q-AAIVRI-MK-MR------kVL---KHQQLLGEVLTQ-LS------S---RF-K-----P--R-V-PVIKKC-ID-ILI-Ekeylervdgekdtys
0008 3mq0B   -----------S-D-T---V--P--AL-RR-A-VRILDL-VA-GS-P----rDL---TAAELTRFLD------------------------L--P-K-SSAHGL-LA-VMT-Eldllarsadgtlrig
0009 3c1dB   ---------------------pA--YA-RL-L-DRAVRI-LA-VR-------dH---SEQELRRKLAA--PIM--G-gP---EE-I--D--A--T-A-EDYERV-IA-WCH-Ehgylddsrfvarfia
0010 3fhzD   apvaG-PEV-------A---A--N-RA-GR-Q-ARIVAI-LS-SA------QVR---SQNELAALLAAE-G------------------ieV--T-Q-ATLSRD-LE-ELG-Avklrgadggtgiyvv
0011 6lb9A   dsRLA-EAA----H-S---S--FaswA-PS-W-YVAFHL-A--PF-S----R-F---TLADLTQVIVRH-VNL-sF--P---EG-P--V-eA--S-I-AKDVDC-IT-KMY-Vpaqrlrggedllscp
0012 8j56C   ---------------------wL--PN-IH-S-SLFFSAyQF-MV-Q--tvgiR---AVVAAYRLYLEH-VSL--L--G---GE-I--V--L--S-F-TRAWTL-VR-FFE-Snmlqlsrctccggqf
0013 8ekiD   aFKPS--NIL-E-Y-R---D--------CP-L-DIISNL-LE-LN-P---rlyK---DLPTTKSLLFGI-YDS--L--S---IN---gqtgK--V-E-VDLMVL-HI-DYA-Lvnldfgtayelgkqv
0014 2od5A   ---------------------xK--TV-RI-R-EKIKKF-LG-DR-------PR---NTAEILEHINST-XR-----------------hgT--T-S-QQLGNV-LS-KDK-Divkvgyikrsgilsg
0015 8cliA   dlsrqyQREK-----------yR--LL-KR-R-NLIIEA-VT-NL-R----LIE---SLFTIQKMIMDQ-EKQ--E--G---VS-----tkC--C-K-KSIVRL-VR-NLS-Eegllrlyrttviqdg
0016 5of3C   ySWIE-KV---mglqD---S--R--KR-FI-LyVASRYLvNV-KG--------V---NEDEALQTLKEF-YYK--L--Q---------sgkV--Y-E-SWLKSV-IN-GVK-Kkgllpwslkrieerd
0017 7z0oG   ----------------------E--LN-DY-S-TMIDIL-LS-DM-D---letV---TTKKVRMALKEV-YA-------------I--D--V--E-G-KAINKL-IR-KHL-Dlvkerprfersledl
0018 4fcyB   ---------------------eF--DE-DA-W-QFLIAD-YL-RP-E-----kP---AFRKCYERLELA-ARE---------HG-W--S--I-------PSRAT-AF-RRI-Qqldeamvvacregeh
0019 8s0cA   ---------------------sV--LE-QS-F-LRAILAeFR-RS-G---leeA---TFQQIYSQHVAL-CRM--E--G---LP-Y-----P--T-M-SETMAV-CS-HLG-----------------
0020 2dobA   -------gsL-P-C-D------I--CK-DV-V-TAAGDX-LK-DN--------A---TEEEILVYLEKT-CDW--LpxS---AS-C-eI--V--D-S--YLPVI-LD-IIK-Gexsrpgevcsalnlc
0021 2rkhA   ---------------------tP--KE-AV-R-LCALGT-IA-SQ-------pX---RYSELAGSVRHF-TSRixG--P---SLeL--X--G--I-----SIEL-LR-GLV-Eavdaxlaisaagrre
0022 3v4gA   ----------------------K--QD-NL-V-RAFKAL-LK-EE------rFG---SQGEIVEALKQE-G-------------fE--N--I--N-Q-SKVSRM-LT-KFG-Avrtrnakmemvyclp
0023 6zvhy   --------------------------kfNW-K-GTIKAI-LK-QA-P---dneI---TIKKLRKKVLAQ-YYT--V--T--------rseeE--L-L-VIFNKK-IS-PTF-Kllkdkvklvk-----
0024 6v9iC   ykknhsgrklhnevgqydqL--R--IL-RT-Q-EAIIQI-MK-MR------kKI---SNAQLQTELVEI-LK------N---MF-L-----P--Q-K-KMIKEQ-IE-WLI-Ehkyirrdesdintfi
0025 6qfdB   ------------------------dlT-AF-Q-KNILTV-LG-EE-------AR---YGLAIKRELEEY-YGE--------------------vN-H-GRLYPN-LD-DLV-Nkglvekseldkrtne
0026 6zcaD   -MGIK--QYS-QeE-L---K--E--MA--L-V-EIAHEL-FEeHK-K----PV----PFQELLNEIASL-LGV-----------------kkedR-I-AQFYTD-LN-IDG-Rflalsdqtwglrswy
0027 7z8bC   ffdvflrylcqhraskltwpvkR--RN-LL-N-CLIVRI-LK-AH-G---deGL---HIDQLVCLVLEA-WQK--G--PcpprglvssA--C--S-S-TDVLSC-IL-HLL-Gkgtlrrhddrpqvls
0028 7pikC   inevvlfdndpmqisadkgT--K--VTpEI-E-RLFRLT-IE-KH----nqkgT---KTTVAYRRFVDL-FAQ--Y--F---P----dY--P--T-L-RQFRYF-YD-REY-Pkalgpgsryeidati
0029 2qbyB   ---------------------imialP-FH-Y-KLALRS-LI-E--------SE---DVMSAHKMYTDL-CNK--F--K---QK-P-----L--S-Y-RRFSDI-IS-ELD-Mfgivkiriinrgrag
0030 1d3yB   ---------------------aK--IF-AQ-T-TKMLEF-AK-QL-L---ddFS---TLREAYYVSKNW-GE----------------arfD--D-Q-QASNNV-IE-DLE-Aalgvlrehlgfipee
0031 1cf7B   ---------------G---K--G--LR-HF-S-MKVCEK-VQ-RK-G-----tT---SYNEVADELVSE-FTN--S--N-----nhlydqkN--I-R-RRVYDA-LN-VLM-Amniiskekkeikwig
0032 6zfbd   -MGIK-QYsQ-E-E-L---K--E---M-AL-V-EIAHEL-FEeHK-K----PV----PFQELLNEIASL-LGV-----------------kkedR-I-AQFYTD-LN-IDG-Rflalsdqtwglrswy
0033 7oqeK   ---------------------eA--EK-EF-I-TMLKEN-Q--------vdstW---SFSRIISELGTR-DP--------rywM-V--D--D--D-P-LWKKEM-FE-KYL-Snrkfkeafqkmlqnn
0034 3e3vA   ---------------------dD--IS-KG-Y-NAALNY-LS-YQ-------lR---TRKEVEDKLRSL-D--------------------I--H-E-DYISEI-IN-KLI-Dldlindknyaesyvr
0035 7kbfK   -------------------------hP-PT-L-SMVVEV-LK-KN-T---rKGT---SVQAIRTRILSA-H-----------PT-V--DplR--L-K-FLLRTA-LN-KGL-Ekgilirplnssatga
0036 3s63B   ----ltpkeT-C-D-L---------CQ-IA-L-RTVFGH-FG-GN-------iP---SRRKLVHQLKHE-CKR--H--F-nyRR-R--CllL--M-K-VNSDLI-FR-EMT-Dgsfkpmevclimrec
0037 8ea3W   ------------------eF--W--EN-FI-T-KTYKEG--N-KG-S----krM---TPKQVALRVEAK-ARE--L--K---DS---kP--P--N-Y-KTVLRV-LA-PIL-Ekqqkaksirspgwrg
0038 5b7iC   vARSV-IEAA-rfI-Q---S--WeqVL-AA-A-GFAARL-HE-GL-QatfadwG---WWYRIANVXLAT-ASQ--N-vG---VT-W-------rV-H-GRLXAI-FQ-DKF-Kqryeeq---------
0039 1fnnA   ---------------------pL--HE-KLfL-LAIVRS-LK-IS-H---tpYI---TFGDAEESYKIV-CEE--Y--G---ER-P-----R--V-H-SQLWSY-LN-DLR-Ekgivetrqnttlisi
0040 1cf7A   ---------S-R-H-E---K--S--LG-LL-T-TKFVSL-LQ-EA-K---dgVL---DLKLAADTLA-----------------------vR--Q-K-R-RIYD-IT-NVL-Egigliekksknsiqw
0041 7dh7C   ------------------------rlT-PI-R-ANVLRL-IA-DA-G----KPV---KAYELLDWVREG-KG----------VG-A--D-----A-P-PTVYRA-LD-FLM-Angfvhklesvnafva
0042 5zi8A   ---amdpefM-R-E-F------Q--RA-AV-R-LHILHH-AA-DN-------EV---HGAWLTQELSRH-G--------------Y--R--V--S-P-GTLYPT-LH-RLE-Adgllvseqrvvdgra
0043 8as8E   ---------------------rR--AP-LV-L-SCLKTL-FE-KS-H----dGI---PLEEAIQSLSSI-LIE--H--V---sqeqynpflQ--A-S-RELREW-IK-RLI-Verdgrifatdaleva
0044 1dp7P   -----------------------------T-V-QWLLDN-YE-------egvsL---PRSTLYNHYLLH-SQE--Q--K---LE-P-----V--N-A-ASFGKL-IR-SVF-Mglrtrrlgtrgnsky
0045 2k85A   ---------------------tA--KD-KY-E-WLVSRI-VK-N-------hnE---NWLSVSRKMQAS-P------------eyQ--D--Y--VyT-QKAKKL-FL-QHI-Hrlkhe----------
0046 3al0B   kiakeifpevfgltqI---N--D--EK-LI-E-ELVKKA-ME-QN-P----KAVqdkAAGFFVGYVMRE-TK-----------G-K--A--N--P---ELTNRI-IQ-KLL-Ege-------------
0047 6tdaL   wFDIS-KiheirfktP---K--A--YK-DT-R-NFIINT-YR-LSpY----EYL---TITAVRRNVA------------------------M--D-V-ASIVKI-HA-FLE-Kwglinyqidprtkps
0048 8yb6K   ---------------N---R--G--TV-DF-I-ASLEN--LK-EG----ilrklrglPGFDLFSALWWP-LRQ--K------NQ-R--A--P--K-R-EVAWLI-AK-LFA-Efrfeqregatlpilm
0049 8u8mA   ------------------------iSD-SF-E-KAMWNH-VS-QN-A---gKFL---MTEQFWAELLQS-QP------N---IP-I-------kS-A-HIVLHH-FN-EMM-Lenlwkqamdpdaklq
0050 3elkA   ------------------rI--L--HG-LI-T-LYILKE-LV-KR-------pX---HGYELQKSXFET-TG-----------------qaL--P-Q-GSIYIL-LK-TXK-Ergfvisessvnkgqq
0051 8yztC   iPSDM-LHIS-T-N-C---R--T-aEK-MA-L-TLLDYL-FHrevqavs-kKQL---D-PLTIYGIRCH-LFY--K--F-------------gsD-W-YRIKQS-ID-SKC-Rtawrrkqrg------
0052 8jxkD   -------------------------EF-ML-E-LAILGL-LI-ES-------PM---HGYELRKRLTGL-LGA--------------------fS-Y-GSLYPA-LR-RMQ-Adgliaenaapagrrv
0053 3k69A   ------------------xK--L--DF-SV-A-VHSILY-LD-AHrD----SKV---ASRELAQSLH--------------------------lN-P-VXIRNI-LS-VLH-Khgyltgtvgknggyq
0054 1j5yA   -------------X-K---T--V--RQ-ER-L-KSIVRI-LE-RS-K----ePV---SGAQLAEELS--------------------------vS-R-QVIVQD-IA-YLR-Slgynivatprgyvla
0055 2hyeC   klheavravqsavenlcsslldR--QY-QI-D-AAIVRI-MK-MR-K-----tL---GHNLLVSELYNQ-L------------K-F--P--V--K-P-GDLKKR-IE-SLI-Drdymerdkdnpnqyh
0056 3o2pE   ---------------------eR--QI-FL-E-ACIVRI-MK-AK------rNL---PHTTLVNECIAQ-SH------Q---RF-N-----A--K-V-SMVKRA-ID-SLI-Qkgylqrgddgesyay
0057 3h20A   rSEMA-GLVK-R-F-G--dD--L--SK-CD-F-IAAQKL-AS-RG--------R---SAEEIGKAMAEA--------------S-P--A--LahE-A-DYIERT-VS-KVM-Glpsvqlaraelarap
0058 6rflI   ------------------eT--I--LI-EI-I-PKIKAY-LL-DA-N---iSPK---SYDDFISRNK-N-IFV--------------nvstI--T-E-EDIRLL-YT-TIE-Qnidaddqtlvaifsy
0059 5dcmB   tLTRE-------------------ilS-PT-E-NKILSI-LL-MHpK----QVV---SKESLLEKLWEN-D--------------S--F--I--D-Q-NTLNVN-MT-RLR-Kkivpigfdyihtvrg
0060 8amyA   ------------------hC--R--LF-EF-R-LCLLEC-MS-LT-------------ldHCYARCTTV-ITQ--I-hG---SD-T--N----dC-T-IFKTCY-YR-CYV-Lgktedhcwkgtatsv
0061 5nl9A   kDSYE-------------------nvT-VQ-R-LQMANL-LL-SK-P----QHL---TADQVFQLINEH-M--------------P--N--A--S-R-ATIFNN-LK-LFA-Ekgivnllelksgitl
0062 6jx3B   sVAQTdYGMV--------------qlN-DT-A-ALIVQR-LL-DG--------H---SPADVAQFLTS----------------eY--E--V--E-R-TDAERD-IA-ALV-Tslkengmalp-----
0063 6vqxA   iLTIE-QIIE-A-I-N-----sA--PE-IAgY-YAWDAA-TD-AL-E--dleqL---TEDDFVAHLE-V-LEE--R--G---AK-I--D--R--D----aAIAV-AL-QFQ-Aaavndlhs-------
0064 3sqnA   --XYS-XLKR-V-I-T---E--K--DL-LR-Q-IRLLEQ-LL-NV-P----QLT---AKRL-AAQIQ--------------------------tT-E-RTVFSD-LQ-YIR-Sqlpadwsietdssgi
0065 7vwvB   ---------------------mE--TQ-KL-I-SMVKEA-LE-KY-Q----yPL---TAKNIKVVIQKE-HN-------------V--V--L--P-T-GSINSI-LYsELF-Ekidktntiypplwir
0066 5u30A   rwvrxreagewdrtadvllR--G--SD-KV-F-EKWGKL-A----------pdA---PFDLYDAEIKNV-QRR--N--T---RR-F--G--S--H---DLFAKL-AE-EYQ-Alwredasfltryavy
0067 7qvgT   fMEGAynrvlsM-D-L---L--A--KT-VR-D-EIAGCS-EK-AY-D-----TL---SVNDARQLLL--------------------------sS-D-KDLLEY-IK-EEH-Peweikdgcvvfqksk
0068 6dk4A   ---------------------lK--AT-PQ-R-LCVLKI-LK-RH------eHP---NIDELYIEIKKE-YP------------------sI--S-L-ATVYKN-LN-TLQ-Eqglvveinvlnqktc
0069 8iueP   ---------------------eF--VE-VL-N-QQCFKF-LQ-SK-Aet-ssFA---SSHEVWKYICEL-GI----------SK-V--E--L--S-M-EDIETI-LN-TLI-Ydgkvemtiiaakegt
0070 1m12A   ---------------------eV--CE-FL-V-KEVTKL-ID-NN--------K---TEKEILDAFDKM-CSK--L--PkslSE-E--CqeV--V-D-TYGSSI-LS-ILL-Eevspelvcsmlhlcs
0071 8bpzA   nyqskavlrL-L-F-E---G-iV--RS-GH-L-YRAYQL-YK-EK-------gV---IDNSVRYQIIDD-FN-----------------lrvkrA-V-YSMQDYiNE-EEV-Nkyyaeelglk-----
0072 8xjgA   yeaagrqdladP-Q-P---L--T--EE-EV-A-ALIEKA-IA-ES-----------qDMGKVMGVLKPQ-IQ-----------G-R--A--D--M---GKVSGL-VR-AKL-----------------
```

```
0073 2rreA  ------------------------vDR-KL-K-QRVIQY-LS-SN-----gKYV---DTGILASDLQRL-YS------V---DY-G-rkrnA--F-R-IQVEKV-FS-IIS-Sekelkn---------
0074 5nwtM  feQGE-E-YX-K-P----sA--S--ST-AI-R-ALVKKL-IA-AE-N---aKPL---SDSKLTSXLSEQ-G----------------------vA-R-RTVAKY-RE-SLS-Ippsnqr---------
0075 1xmaB  ------------------dV--I--RG-YV-D-TIILSL-LI-EG-------dS---YGYEISKNIRIK-TDE--L-------------yvI--K-E-TTLYSA-FA-RLE-Kngyiksyygeetrrt
0076 3a1yA  --------------------------M-EY-V-YAALLL-HS-VG-------------keiNEENLKAV-LQA--A--G---VE-P--E--E--A-RiKALVAA-LE-GVN-Ideviekaa-------
0077 7apdB  ------------------kF--D--FG-TM-V-QWAYDH-K-----------yA---EESKIAYEYALA--AG--S--DsnaRA-F--L----nS-Q-AKHVKD-CA-TMV-Rhylraetqalsmpay
0078 2z99A  --------------aE---L--D--AD-EL-K-RVLEAL-LL-VI-D----TPV---TADALAAATE--------------------------qP-V-YRVAAK-LQ-LMA-Deltgrdsgidlrhts
0079 7solA  esLER-QLKH-P------pL--E--IH-TA-M-LALDQF-QE-KY-S--vgcqQ---DSEELLKLATSI-SET--L-eE---KP-D--V--N----A-DIVHWL-SW-TAQ-Gflsplaaavggvasq
0080 1q1vA  -----------klkkP---P--T--DE-EL-K-ETIKKL-LA-SA-N---leeV---TMKQICKKVYEN-YP------T---YD-L-tE--R--K-D--FIKT----tELI-Sleh------------
0081 1z0xA  ---------------K---L--S--KD-TI-I-AAAFSL-LE-KS------pTL---EQ-LSXRKVAKQ-LG---------vqapA--I--Y--W--yfKNKQA-LL-QSX-Aeaieehfqepalcge
0082 6c2jA  pRMRQ-EFEE-----------sgiiDY-KF-G-IDAMVQ-MA-LH------kRA---DLPTLVGTLR------------------H--H--C--K-A-QEVADN-LF-KMA-Sedcfnfdptidkfiv
0083 5tjjA  ------------xdyQ---V--P--SV-AL-A-ARVLKL-LS-RH-K---yrQS---TLTEIAERLG--------------------------vN-K-TTCLRV-LR-TLE-Redfvsydpqsrrysl
0084 2lnbA  ghhhhhhshM-A-D-P---G--R--EG-HL-E-QRILQV-LT-EA-G----sPV---KLAQLVKECQ--------------------------aP-K-RELNQV-LY-RMK-Kelkvsltspatwclg
0085 8b9xA  ------------gmtE---E--Q--SQ-SF-L-TEFINY-IK-QS------kVV---LLEDLASQVG--------------------------lR-T-QDTINR-IQ-DLLf----------------
0086 7o4iR  gkirinkdgskpheydlerM--P--KK-EI-L-DYLFKL-FD-EY------dYW---SLKGLKERTR------------------------Q--P-E-AHLKEC-LD-KVA-Tlvkkgpyafkytlrp

0001 s001A  -LLHH-HHHH-H-H-L---L--L--HH-HH-H-HHHHHH-HH-HL-L----LLL---LHHHHHHHHHHH-HHH--L--L---LL-L--L--L--L-H-HHHHHH-HH-HHL-L---------------
0002 5a31N  lllllllllllllllhhhhH--H--HH-HH-H-HHHHHH-HH-HH------lLL---LHHHHHHHHHHHlLLL--L--L---LL-L-----L--L-H-HHHHHH-HH-HHH-Hlllleeelleeell-
0003 1lddA  ---------------------hH--HH-HH-H-HHHHHH-HH-HH------lLE---EHHHHHHHHHHH-LL---H--H---HL-L-lL--L--L-H-HHHHHH-HH-HHH-Hllleellllleeeel
0004 9bw1M  hhlllhhhhhlL-H-H---H-----HH-HH-H-HHHHHHlLL-LL-------LL---LHHHHHHHHHHH-HHH--H--L---LL-L-----L--L-H-HHHHHH-HL-LLL-Lhhhhhhhllhhhhhh
0005 1hlvA  ---------------------lL--LH-HH-H-HHHHHH-HH-HH-H--hlllL---LHHHHHHHHHHH-HHH--H--L---LLlL--L--L--L---HHHHHH-HH-HHL-L---------------
0006 2nvuA  lHHHH-L-LL-L-L-L------L--HH-HH-H-HHHHHH-HH-HL----hhhhH---HHHHHHLLLHHH-HHH--H--L---LL-L-lL--L--L-H-HHHHHH-HH-LHH-Hleeellllhhhhhll
0007 9kbdA  llllllhhhhhhlllllllhhhH--HH-HH-H-HHHHHH-HH-HH------lLE---EHHHHHHHHHHH-HL------L---LL-L-----L--L-H-HHHHHH-HH-HHH-Llllllllllllleel
0008 3mq0B  -----------L-L-L---L--H--HH-HH-H-HHHHHH-HH-HL-L----lLE---EHHHHHLLLL------------------------L--L-H-HHHHHH-HH-HHH-Hllleellllleeeel
0009 3c1dB  ---------------------hH--HH-HH-H-HHHHHH-HL-LL-------lL---LHHHHHHHHLL--LEE--E-eE---EL-L--L--L--L-H-HHHHHH-HH-HHH-Hlllllhhhhhhhhhh
0010 3fhzD  llllL-LEE------L---L--L--HH-HH-H-HHHHHH-HH-HL------LLL---LHHHHHHHHHLL-L------------------llL--L-H-HHHHHH-HH-HLL-Leellllllllleeel
0011 6lb9A  llHHH-HHL----L-L---L--LlllL-HH-H-HHHHHL-L--LL-L----E-E---LHHHHHHHHHHH-HHH-hL--L---LL-L--L-hH--H-H-HHHHHH-HH-HHH-Llhhhllllllllllh
0012 8j56C  ---------------------hH--HH-HH-H-HHHHHHhHH-HH-H--lllhH---HHHHHHHHHHHH-HHH--H--L---LL-L--L--L--L-H-HHHHHH-HH-HHH-Llleeeeellllllee
0013 8ekiD  lLLLL--HHH-H-H-H---H--------LH-H-HHHHHH-HH-HL-L---lhhH---LHHHHHHHHHHH-HHH--L--L---LL---llhhH--H-H-HHHHHH-HH-HHH-Hhlllhhhhhhhhhhh
0014 2od5A  ---------------------hH--HH-HH-H-HHHHHH-LL-LL-------LE---EHHHHHHHHHLL-LL-----------------llL--L-H-HHHHHH-HH-HLL-Leeeeeeeeeeellle
0015 8cliA  hhhhhhHHHH-----------hH--HH-HH-H-HHHHHH-HH-HL-L----EEL---LHHHHHHHHHHH-HHH--H--L---LL-----llL--L-H-HHHHHH-HH-HHH-Hlllleeeeleeeell
0016 5of3C  lHHHH-HH---hlllL---L--H--HH-HH-HhLHHHHHhHL-LL--------L---LHHHHHHHHHHH-HLL--L--L---------lllL--L-H-HHHHHH-HH-HHH-Hhlllllllhhhhhhl
0017 7z0oG  ----------------------L--HH-HH-H-HHHHHH-HL-LL-L---lllL---LHHHHHHHHHHH-HL-------------L--L--L--L-H-HHHHHH-HH-HHH-Hhllllllllllhhhh
0018 4fcyB  ---------------------lL--LH-HH-H-HHHHHH-HL-LL-L-----lL---LHHHHHHHHHHH-HHH---------HL-L--L--L-------LLHHH-HH-HHH-Hhhllhhhhhhhllll
0019 8s0cA  ---------------------lH--HH-HH-H-HHHHHHhHH-HH-L---lllL---LHHHHHHHHHHH-HHH--H--L---LL-L-----L--L-H-HHHHHH-HH-HHH-----------------
0020 2dobA  -------llH-H-H-H------H--HH-HH-H-HHHHHH-HH-LL--------L---LHHHHHHHHHHH-HHH--LlhH---HH-H-hH--H--H-H--HHHHH-HH-HLL-Lllllhhhhhhhllll
0021 2rkhA  ---------------------lH--HH-HH-H-HHHHHH-HH-HL-------lE---EHHHHHHHHHHH-HHHhhL--L---LhHH--L--L--L-----LLHH-HH-LLE-Elllleeeelhhhhhh
0022 3v4gA  ----------------------L--HH-HH-H-HHHHHH-HH-HL------lLL---LHHHHHHHHHHL-L-------------lL--L--L--L-H-HHHHHH-HH-HLL-Leeeellllleeeell
0023 6zvhy  --------------------------llLH-H-HHHHHH-HH-LL-H---hheE---EHHHHHHHHHHH-HHH--H--L--------llhhH--H-H-HHHHHH-HH-LLE-Eellleeeell-----
0024 6v9iC  hhhhllleeeeelleeeehH--H--HH-HH-H-HHHHHH-HH-HL------lEE---EHHHHHHHHHHH-HL------L---LL-L-----L--L-H-HHHHHH-HH-HHH-Hllleeeelleeeeee
0025 6qfdB  ------------------------llL-HH-H-HHHHHH-HH-HL-------LE---EHHHHHHHHHHH-HLL--------------------lL-H-HHHHHH-HH-HHH-Hllleeeeeeelleee
0026 6zcaD  -LLLL--LLL-HhH-H---H--H--LL--H-H-HHHHHH-HHhHL-L----LL----LHHHHHHHHHHH-LLL-----------------lllhH-H-HHHHHH-HH-HLL-Leellllllleehhhl
0027 7z8bC  lhhhhhhhhlhhhhhhhllllhH--HH-HH-H-HHHHHH-HH-LL-L---llLE---EHHHHHHHHHHH-HHL--L--LlllhhhhhlL--L--L-H-HHHHHH-HH-HHH-Hhlleeelllllleee
0028 7pikC  llleeeellleeellllllL--L--LLhHH-H-HHHHHH-HH-HH----llllL---LHHHHHHHHHHH-HHH--H--L---L----hL--L--L-H-HHHHHH-HH-HHL-Lllllllllleeeeee
0029 2qbyB  ---------------------eeellL-HH-H-HHHHHH-HH-L--------LL---LHHHHHHHHHHH-HHH--L--L---LL-L-----L--L-H-HHHHHH-HH-HHH-Hllleeeeeellllll
0030 1d3yB  ---------------------hH--HH-HH-H-HHHHHH-HH-HH-H---llLE---EHHHHHHHHHHL-HH----------------hllL--L-H-HHHHHH-HH-HHH-Hhhlllhhhhleelll
0031 1cf7B  ---------------L---L--H--HH-HH-H-HHHHHH-HH-HH-L-----eE---LHHHHHHHHHHH-HHL--L--L-----lllhhhhH--H-H-HHHHHH-HH-HHH-Hlllllllllleelll
0032 6zfbd  -LLLL-LLlH-H-H-H---H--H---L-LH-H-HHHHHH-HHhHL-L----LL----LHHHHHHHHHHL-LLL-----------------lllhH-H-HHHHHH-HH-HLL-Leellllllleehhhl
0033 7oqeK  ---------------------lH--HH-HH-H-HHHHHL-L--------llllL---LHHHHHHHHHHH-LL--------hhhH-L--L--L--L-H-HHHHHH-HH-HHH-Hlllhhhhhhhhhhhl
0034 3e3vA  ---------------------hH--HH-HH-H-HHHHHH-HH-LL-------lL---LHHHHHLLLHHH-L--------------------L--L-H-HHHHHH-HH-HHH-Hlllllhhhhhhhhhh
0035 7kbfK  -------------------------lL-LH-H-HHHHHH-HH-HL-L---lLLE---EHHHHHHHHHLL-L-----------LL-L--LllL--H-H-HHHHHH-HH-HHH-Hllleeeeeellllll
0036 3s63B  ----llhhhH-H-H-H---------HH-HH-H-HHHHHH-LL-LL-------lL---LHHHHHHHHHHH-HLL--L--L-llHH-H--HhhH--H-H-HLHHHH-HH-HHH-Lllllhhhhhhhllll
0037 8ea3W  ------------------hH--H--HH-HH-H-HHHHHH--L-LL-L----llL---LHHHHHHHHHHH-HHH--H--L---LL---lL--L--L-H-HHHHHH-LH-HHH-Hhhhhhllllllllll
0038 5b7iC  hLLLH-HHHH-hhH-H---H--HlhHH-HH-H-HHHHHH-HH-HH-HhhllllL---HHHHHHHHHHHH-HHH--H-hL---LL-L-------hH-H-HHHHHH-HH-HHH-Hhhllll---------
0039 1fnnA  ---------------------lH--HH-HHhH-HHHHHH-HH-HH-L---llLE---EHHHHHHHHHHH-HHH--L--L---LL-L-----L--L-H-HHHHHH-HH-HHH-Hlllleeeelleeeel
0040 1cf7A  ---------L-L-L-L---L--L--HH-HH-H-HHHHHH-HH-HL-L---llEE---EHHHHHHHLL-----------------------lL--L-L-H-HHHH-HH-HHH-Hhhlleeeeelleeee
0041 7dh7C  ------------------------llL-HH-H-HHHHHH-HH-HL-L----LLL---LHHHHHHHHHHH-HL----------LL-L--L-----L-H-HHHHHH-HH-HHH-Hllleeeehhhleeee
0042 5zi8A  ---lllhhhH-H-H-H------H--HH-HH-H-HHHHHH-HH-HL-------LL---LHHHHHHHHHHL-L--------------L--L--L--L-H-HHHHHH-HH-HHH-Hllleeeeeeeellee
0043 8as8E  ---------------------lL--HH-HH-H-HHHHHH-HH-HL-L----lLE---EHHHHHHHHHHH-HHH--L--L---llllllhhhH--H-H-HHHHHH-HH-LLE-Eeelleeeelhhhhhh
0044 1dp7P  -----------------------------L-H-HHHHHH-EE-------eeeeE---EHHHHHHHHHHH-HHH--L--L---LL-L-----L--L-H-HHHHHH-HH-HHL-Llleeeeellllllee
0045 2k85A  ---------------------hH--HH-HH-H-HHHHHH-LL-L-------llL---LHHHHHHHHLLL-H------------hhH--H--H--HhH-HHHHHH-HH-HHH-Hhhhhl----------
0046 3al0B  lhhhhlhhhhhllllL---L--L--LL-LH-H-HHHHHH-HH-LL-H----HHHhhlLLHHHHHHHHHL-LL-----------L-L--L--L--H---HHHHHH-HH-HHL-Lll-------------
0047 6tdaL  lLLLL-LllhhllllH---H--H--HH-HH-H-HHHHHH-HH-LLlL----LLL---LHHHHHHHLL------------------------L--L-H-HHHHHH-HH-HHH-Hlllllllllllllll
0048 8yb6K  ---------------L---L--H--HH-HH-H-HHHHL--LL-HH----hhhllllhHHHHHHHHHHHH-HHH--H------LL-L--L--L--L-H-HHHHHH-HH-HHH-Hllleelllllhhhhh
0049 8u8mA  ------------------------eLH-HH-H-HHHHHH-HH-HL-H---hHHH---LLHHHHHHHHHH-LL------L---LL-E-------eL-H-HHHHHH-HH-HHL-Hhhlllllllhhhhhh
0050 3elkA  ------------------hH--H--HH-HH-H-HHHHHH-HH-HL-------lE---EHHHHHHHHHHH-HL-----------------llL--L-L-LHHHHH-HH-HHH-Hhlleeeeeeelllle
0051 8yztC  lHHHH-HHHH-H-H-L---L--L-hHH-HH-H-HHHHHH-LLhhhhhle-lEEL---L-HHHHHHHHHH-HHH--H--H-------------lhH-H-HHHHHH-HH-HHH-Hhhhhhhlll------
0052 8jxkD  -------------------------LH-HH-H-HHHHHH-HH-HL-------LE---EHHHHHHHHHHH-LLL--------------------lL-L-LLHHHH-HH-HHH-Hllleellllllllle
0053 3k69A  ------------------lL--H--HH-HH-H-HHHHHH-HH-LLlL----LLL---LHHHHHHHHL--------------------------lL-H-HHLHHH-HH-HHH-Hlllleeellllleee
0054 1j5yA  -------------L-H---H--H--HH-HH-H-HHHHHH-HH-HL-L----lLL---LHHHHHHHHL--------------------------lL-H-HHHHHH-HH-HHH-Hhlllleeelleeell
0055 2hyeC  hhhhhhhllllhhhhhllhhhhH--HH-HH-H-HHHHHH-HH-HL-L-----eE---ELHHHHHHHHHH-L------------L-L--L--L--L-H-HHHHHH-HH-HHH-Hlllllllllllleee
0056 3o2pE  ---------------------hH--HH-HH-H-HHHHHH-HH-HH------lEE---EHHHHHHHHHHH-HL------L---LL-L-----L--L-H-HHHHHH-HH-HHH-Hllleelllllleeee
0057 3h20A  hHHHH-HHHH-H-L-L--lL--H--HH-HH-H-HHHHHH-HH-LL--------L---LHHHHHHHHHHH--------------L-H--H--HhlH-H-HHHHHH-HH-HHH-Llhhhhhhhhhhhlll
0058 6rflI  ------------------hH--H--HH-HH-H-HHHHHH-HH-LL-L---lLLL---LHHHHHHHLL-H-HHH--------------llllL--L-H-HHHHHH-HH-HHH-Hlllllhhhhhhhhhh
0059 5dcmB  eELLL-------------------elL-HH-H-HHHHHH-HH-LLlL----LEE---EHHHHHHHHHLL-L--------------L--L--L--L-H-HHHHHH-HH-HHH-Hhhhhhlllleeeell
0060 8amyA  ------------------hH--L--HH-HH-H-HHHHHH-HH-LL-------------llLHHHHHHHH-HHH--H-hL---HH-H--H----hH-H-HHHHHH-HH-HHH-Llllhhhhhhhhhhhl
0061 5nl9A  hHHHH-------------------llL-HH-H-HHHHHH-HH-LL-L----LEE---LHHHHHHHHHHH-L--------------L--L--L--L-H-HHHHHH-HH-HHH-Hlllleeeelllllee
0062 6jx3B  eEEELlLLEE--------------eeL-HH-H-HHHHHH-HH-LL--------L---LHHHHHHHHHH----------------hL--L--L--L-H-HHHHHH-HH-HHH-Hhhhhllllll-----
0063 6vqxA  lLLHH-HHHH-H-H-H-----lH--HH-HHhH-HHHHHH-HH-LL-L--lhhhL---LLHHHHHHHH-H-HHL--L--L---LL-L--L--H--H----hHHHH-HH-HHH-Hhhhhllll-------
0064 3sqnA  --LLH-HHHH-H-L-L---L--H--HH-HH-H-HHHHHH-HH-HL-L----LLL---LHHH-HHHHL--------------------------lL-H-HHHHHH-HH-HHH-Llllllleeeeellee
0065 7vwvB  ---------------------lH--HH-HH-H-HHHHHH-HH-HL-L----lLL---LHHHHHHHHHHH-HL-------------L--L--L--L-H-HHHHHH-HHhLLE-Eellllllllllleee
0066 5u30A  hhhhhhhlllllhhhhhhlL--L--HH-HH-H-HHHHLL-L----------llL---LHHHHHHHHHHH-HHH--L--L---LL-L--L--L--H---HHHHHH-LL-LLL-Hhhhllllhhhhhhhh
```

```
0067 7qvgT   hHHLLhhhhhhH-H-H---H--H--HH-HH-H-HHHHHH-HH-HL-L-----EE---EHHHHHHHHL--------------------------lL-H-HHHHHH-HH-HHL-Llleeelleeelllll
0068 6dk4A   ---------------------hH--HH-HH-H-HHHHHH-HL-LL------lLL---LHHHHHHHHHLL-LL------------------lL--L-H-HHHHHH-HH-HHH-Hllleeeellllllle
0069 8iueP   ---------------------hH--HH-HH-H-HHHHHH-HH-HH-Hhh-hhLE---EHHHHHHHHHHH-LL----------LL-L--L--L--L-H-HHHHHH-HH-HHH-Hllleeeeeellllll
0070 1m12A   ---------------------hH--HH-HH-H-HHHHHH-HH-LL--------L---LHHHHHHHLLLL-HHH--L--LlllHH-H--HhhH--H-H-HHHHHH-HH-HHL-Llllllhhhhhhlllll
0071 8bpzA   lhhhhhhhhH-H-H-H---L-hH--HH-HH-H-HHHHHH-HH-HH-------lL---LLHHHHHHHHHH-LL-----------------llllhH-H-HHHLLLlLH-HHH-Hhhllllllll-----
0072 8xjgA   hhhlllhhhhhL-L-L---L--L--HH-HH-H-HHHHHH-HH-HH-----------hHHHHHHHHHHHH-HL-----------L-L--L--L--H---HHHHHH-HH-HHL-----------------
0073 2rreA   ------------------------lLH-HH-H-HHHHHH-HH-HL-----lLLL---LHHHHHHHHHHH-LL------L---LL-L-llhhH--H-H-HHHHHH-HH-HHH-Hhlllll---------
0074 5nwtM   llLLL-L-LL-L-L----lL--H--HH-HH-H-HHHHHH-HH-LL-L---lLLL---LHHHHHHHHHHH-L----------------------lL-H-HHHHHH-HH-HHL-Lllllll---------
0075 1xmaB   ------------------hH--H--HH-HH-H-HHHHHH-HH-HL-------lE---EHHHHHHHHHHH-LLL--L-------------llL--L-H-HHHHHH-HH-HHH-Hllleeeeelllllle
0076 3a1yA   --------------------------L-HH-H-HHHHHH-HH-LL-------------lllLHHHHHHH-HHH--L--L---LL-L--L--H--H-HhHHHHHH-HL-LLL-Hhhhhhhhl-------
0077 7apdB   ------------------lL--L--HH-HH-H-HHHHHH-L-----------lL---LHHHHHHHHHLL--LL--L--LhhhHH-H--H----lL-H-HHHHHH-HH-HHH-Hhhhhhhhhhllhhhh
0078 2z99A   --------------lL---L--L--HH-HH-H-HHHHHH-HH-HL-L----LLL---LHHHHHHHHL--------------------------lL-H-HHHHHH-HH-HHH-Hhhhhlllleeeeeel
0079 7solA   llHHH-HHHL-L------hH--H--HH-HH-H-HHHHHH-HH-HH-L--lllhH---HHHHHHHHHHHH-HHH--L-lL---LL-L--L--L----H-HHHHHH-HH-HLL-Lllhhhhhhhhhhhhh
0080 1q1vA   -----------llllL---L--L--HH-HH-H-HHHHHH-HL-LL-L---hhhL---LHHHHHHHHHHH-LL------L---LL-L-lH--H--H-H--HHHH----hHHH-Hlll------------
0081 1z0xA   ---------------L---L--L--HH-HH-H-HHHHHH-HH-HL------lLH---HH-LLHHHHHHH-HL---------llhhH--H--H--L--llLLHHH-HH-HHH-Hhhhhhhlllllllll
0082 6c2jA   hHHHH-HHLL-----------llllLH-HH-H-HHHHHH-HH-HH------lEE---LHHHHHHHHH------------------H--H--L--L-H-HHHHHH-HH-HHH-Hllllleeellllleeel
0083 5tjjA   ------------lllL---L--H--HH-HH-H-HHHHHH-HL-LH-H---hlLE---EHHHHHHHHL--------------------------lL-H-HHHHHH-HH-HHH-Hllleeellllleeee
0084 2lnbA   lhhhlllllL-L-L-L---L--H--HH-HH-H-HHHHHH-HH-HH-L----lLE---EHHHHHHHHL--------------------------lL-H-HHHHHH-HH-HHH-Hllleeeeelleeeel
0085 8b9xA   ------------llhH---H--H--HH-HH-H-HHHHHH-HH-HL------lEE---EHHHHHHHLL--------------------------lL-H-HHHHHH-HH-HHHl----------------
0086 7o4iR   eeeeeellllllleeeeelL--L--HH-HH-H-HHHHHH-HH-HL------lLE---EHHHHHHHHL------------------------L--L-H-HHHHHH-HH-HHE-Eeellllllllleeeelh
```

Type-3 BREX; MTase MBW2068239 HTH2 FoldSeek Top-Hits

| | | | | | |
|---|---|---|---|---|---|
| job | A | AF-A0A7V7ZHA6-F1-model v4 HTH luxR-type domain-containing protein | 3.807E+00 | LEAVRAGFKKAWQERDYATIIAVARKIPENVLQEDSKLLMWYDQALTRMG | Chloroflexia bacterium |
| job | A | AF-A0A3A4PC77-F1-model v4 Uncharacterized protein | 3.130E+00 | EAVRAGFKKAWQERDYATIIAVARKIPENVLQEDSKLLMWYDQALTRMG | Actinomycetia bacterium |
| job | A | AF-A0A2U3AIG9-F1-model v4 Uncharacterized protein | 3.130E+00 | FRLEAVRAGFKKAWQERDYATIIAVARKIPENVLQEDSKLLMWYDQALTRMGGK | Kurthia sibirica |
| job | A | AF-A0A538U0Z4-F1-model v4 BTAD domain-containing protein | 3.807E+00 | VRAGFKKAWQERDYATIIAVARKIPENVLQEDSKLLMWYDQALTRMGG | Candidatus Eisenbacteria |
| job | A | AF-A0A2T5JU15-F1-model v4 Transcriptional regulator | 3.807E+00 | LEAVRAGFKKAWQERDYATIIAVARKIPENVLQEDSKLLMWYDQAL | Nitrosospira sp. Nsp2 |
| job | A | AF-A0A7X9H9P0-F1-model v4 Uncharacterized protein | 3.130E+00 | AVRAGFKKAWQERDYATIIAVARKIPENVLQEDSKLLMWYDQALTRMG | Bacteroidales bacterium |
| job | A | AF-A0A535SKQ3-F1-model v4 TPR MalT domain-containing protein | 3.341E+00 | VRAGFKKAWQERDYATIIAVARKIPENVLQEDSKLLMWYDQALTRMG | Chloroflexi bacterium |
| job | A | AF-A0A2N2BPY1-F1-model v4 HTH luxR-type domain-containing protein | 3.807E+00 | VRAGFKKAWQERDYATIIAVARKIPENVLQEDSKLLMWYDQALTRMG | Firmicutes bacterium HGW-4 |
| job | A | AF-X1E1S1-F1-model v4 Uncharacterized protein | 2.412E+00 | LEAVRAGFKKAWQERDYATIIAVARKIPENVLQEDSKLLMWYDQALT | marine sediment metagenome |
| job | A | AF-C0C6U7-F1-model v4 Tetratricopeptide repeat protein | 4.629E+00 | VRAGFKKAWQERDYATIIAVARKIPENVLQEDSKLLMWYDQALTRMGG | [Clostridium] hylemonae DSM |
| job | A | AF-A0A3B8VLG3-F1-model v4 TPR MalT domain-containing protein | 6.008E+00 | VRAGFKKAWQERDYATIIAVARKIPENVLQEDSKLLMWYDQALTRMG | Dehalococcoidia bacterium |
| job | A | AF-A0A345UKZ8-F1-model v4 MCP methyltransferase, CheR-type | 2.259E+00 | QRKLKVF---RLEAVRAGFKKAWQERDYATIIAVARKIPENVLQEDSKLLMWYDQALTRMG | Candidatus Cyclonatronum |
| job | A | AF-A0A6N6KE84-F1-model v4 AAA domain-containing protein | 3.566E+00 | LEAVRAGFKKAWQERDYATIIAVARKIPENVLQEDSKLLMWYDQALTRMG | Bacteroidetes bacterium |
| job | A | AF-A0A1B0CMM2-F1-model v4 Putative transcription factor six | 2.259E+00 | QRKLKVFRLEAVRAGFKKAWQERDYATIIAVARKIPE-NVLQEDSKLLMWYDQALTRMG | Lutzomyia longipalpis |
| job | A | AF-A0A2V7HP17-F1-model v4 Uncharacterized protein | 4.629E+00 | FRLEAVRAGFKKAWQERDYATIIAVARKIPENVLQEDSKLLMWYDQA | Candidatus Rokubacteria |
| job | A | AF-A0A183Q8K0-F1-model v4 Uncharacterized protein | 1.858E+00 | KVFRLEAVRAGFKKAWQERDYATIIAVARKIPENVLQE---DSKLLMWYDQALTRMG | Schistosoma mattheei |
| job | A | AF-A0A2V7RC08-F1-model v4 Uncharacterized protein | 5.629E+00 | QRKLKVFRL---EAVRAGFKKAWQERDYATIIAVARKIPENVLQEDSKLLMWYDQALTRMG | Gemmatimonadetes bacterium |
| job | A | AF-A0A2N5ZC89-F1-model v4 Uncharacterized protein | 3.130E+00 | EAVRAGFKKAWQERDYATIIAVARKIPENVLQEDSKLLMWYDQALTRMG | Marinilabiliales bacterium |
| job | A | AF-A0A352UHV2-F1-model v4 Uncharacterized protein | 6.008E+00 | VRAGFKKAWQERDYATIIAVARKIPENVLQEDSKLLMWYDQALTRMG | Dehalococcoidia bacterium |
| job | A | AF-A0A6I7X4I9-F1-model v4 Uncharacterized protein | 2.574E+00 | EAVRAGFKKAWQERDYATIIAVARKIPENVLQEDSKLLMWYDQAL | Methylococcaceae bacteriu |
| job | A | AF-A0A7C4J6Y3-F1-model v4 HTH luxR-type domain-containing protein | 5.274E+00 | LEAVRAGFKKAWQERDYATIIAVARKIPENVLQEDSKLLMWYDQALTRMG | Chloroflexi bacterium |
| job | A | AF-B8CZU7-F1-model v4 Regulatory protein LuxR | 6.008E+00 | VRAGFKKAWQERDYATIIAVARKIPENVLQEDSKLLMWYDQALTRMG | Halothermothrix orenii H 168 |
| job | A | AF-A0A2G8L558-F1-model v4 Putative homeobox protein SIX4 | 3.130E+00 | LKVFRLEAVRAGFKKAWQERDYATIIAVARKIP-ENVLQEDSKLLMWYDQALTRMG | Apostichopus japonicus |
| job | A | AF-A0A202E176-F1-model v4 Uncharacterized protein | 2.259E+00 | EAVRAGFKKAWQERDYATIIAVARKIPENVLQEDSKLLMWYDQALTRMG | bacterium K02(2017) |
| job | A | AF-A0A3B0UHN1-F1-model v4 Uncharacterized protein | 2.933E+00 | VRAGFKKAWQERDYATIIAVARKIPENVLQEDSKLLMWYDQAL | hydrothermal vent metagenome |
| job | A | AF-A0A3D1TRP5-F1-model v4 Uncharacterized protein | 4.941E+00 | LEAVRAGFKKAW-QERDYATIIAVARKIPENVLQEDSKLLMWYDQALTRMG | Chloroflexi bacterium |
| job | A | AF-A0A1I1QWZ7-F1-model v4 Soluble lytic murein transglycosylase | 2.748E+00 | KVFRLEAVRAGFKKAWQERDYATIIAVARKIPENVLQEDSKLLMWYDQALTRMG | Thiohalospira halophila DSM |
| job | A | AF-A0A2N2BK61-F1-model v4 HTH luxR-type domain-containing protein | 4.941E+00 | VRAGFKKAWQERDYATIIAVARKIPENVLQEDSKLLMWYDQALTRMG | Firmicutes bacterium HGW-3 |
| job | A | AF-A0A2K9LKH4-F1-model v4 HTH luxR-type domain-containing protein | 6.413E+00 | EAVRAGFKKAWQERDYATIIAVARKIPENVLQEDSKLLMWYDQAL | Ketobacter alkanivorans |
| job | A | AF-A0A2N2AXE2-F1-model v4 Uncharacterized protein | 5.274E+00 | VRAGFKKAWQERDYATIIAVARKIPENVLQEDSKLLMWYDQALTRMGG | Firmicutes bacterium HGW-7 |
| job | A | AF-A0A328CYM2-F1-model v4 Uncharacterized protein | 5.629E+00 | EAVRAGFKKAWQERDYATIIAVARKIPENVLQEDSKLLMWYDQALTRMG | Cuscuta australis |
| job | A | AF-A0A5E4R7C1-F1-model v4 SIX1 SD domain-containing protein | 3.566E+00 | RKLKVFRLEAVRAGFKKAWQERDYATIIAVARKIPENVLQE-DSKLLMWYDQALTRMG | Leptidea sinapis |
| job | A | AF-A0A1Y6CXP0-F1-model v4 Glycosyl transferase family 2 | 5.274E+00 | LEAVRAGFKKAWQERDYATIIAVARKIPENVLQEDSKLLMWYDQALTRMG | Methylomagnum ishizawai |
| job | A | AF-A0A7Z8JWP3-F1-model v4 Helix-turn-helix transcriptional regulator | 6.845E+00 | VRAGFKKAWQERDYATIIAVARKIPENVLQEDSKLLMWYDQALTRMG | Cellulomonas hominis |
| job | A | AF-A0A4Q3QPK3-F1-model v4 HTH luxR-type domain-containing protein | 4.941E+00 | LEAVRAGFKKAWQERDYATIIAVARKIPENVLQEDSKLLMWYDQALTRMG | Cytophagaceae bacterium |
| job | A | AF-A0A402A047-F1-model v4 LuxR family transcriptional regulator | 5.274E+00 | LEAVRAGFKKAWQERDYATIIAVARKIPENVLQEDSKLLMWYDQALTRMG | Tengunoibacter tsumagoiensis |
| job | A | AF-A0A6A7SGG8-F1-model v4 Serine/threonine-protein kinase PknK | 6.413E+00 | EAVRAGFKKAWQERDYATIIAVARKIPENVLQEDSKLLMWYDQAL | wastewater metagenome |
| job | A | AF-A0A4Q5J8M4-F1-model v4 Helix-turn-helix transcriptional regulator | 6.008E+00 | VRAGFKKAWQERDYATIIAVARKIPENVLQEDSKLLMWYDQALTRMG | Nocardioides iriomotensis |
| job | A | AF-A0A7Z8QAW1-F1-model v4 Uncharacterized protein | 1.858E+00 | LKVFRLEA----VRAGFKKAWQERDYATIIAVARKIPENVLQEDSKLLMWYDQA-LTRMGGK | Methylococcaceae bacterium |
| job | A | AF-A0A212F9Z5-F1-model v4 Homeobox domain-containing protein | 3.807E+00 | QRKLKVFRLEAVRAGFKKAWQERDYATIIAVARKIPENVL-QEDSKLLMWYDQALTRMG | Danaus plexippus plexippus |
| job | A | AF-A0A816A7M8-F1-model v4 Hypothetical protein | 6.008E+00 | LKVFRLEAVRAGFKKAWQERDYATIIAVARKIPENVLQEDSKLLMWYDQALTRMGG | Adineta steineri |
| job | A | AF-A0A1Z5HE73-F1-model v4 Uncharacterized protein | 3.130E+00 | VRAGFKKAWQERDYATIIAVARKIPENVLQEDSKLLMWYDQAL | Bathymodiolus platifrons |
| job | A | AF-A0A0R3Q074-F1-model v4 Dolichyl-phosphate-mannose--protein mannosyltransferase | 7.306E+00 | EAVRAGFKKAWQERDYATIIAVARKIPENVLQEDSKLLMWYDQALTRMG | Angiostrongylus costaricensis |
| job | A | AF-A0A2M9Q9I4-F1-model v4 SMI1 KNR4 domain-containing protein | 5.629E+00 | AVRAGFKKAWQERDYATIIAVARKIPENVLQEDSKLLMWYDQALTRMG | Lysinibacillus xylanilyticus |
| job | A | AF-A0A7C7GAQ4-F1-model v4 Tetratricopeptide repeat protein | 7.306E+00 | VRAGFKKAWQERDYATIIAVARKIPENVLQEDSKLLMWYDQALTRMG | Dehalococcoidia bacterium |
| job | A | AF-A0A1A7QMR6-F1-model v4 LuxR family maltose regulon positive regulatory protein | 4.629E+00 | LEAVRAGFKKAWQERDYATIIAVARKIPENVLQEDSKLLMWYDQALTRMG | Gelidibacter algens |
| job | A | AF-A0A6L3EVV1-F1-model v4 LuxR family transcriptional regulator | 6.845E+00 | VRAGFKKAWQERDYATIIAVARKIPENVLQEDSKLLMWYDQALTRMG | Chloroflexi bacterium |
| job | A | AF-A0A225MLE2-F1-model v4 HTH luxR-type domain-containing protein | 7.799E+00 | LEAVRAGFKKAWQERDYATIIAVARKIPENVLQEDSKLLMWYDQALT | Candidimonas nitroreducens |
| job | A | AF-U6NQR7-F1-model v4 Transcription factor Six1 | 2.933E+00 | KVFRLEAVRAGFKKAWQERDYATIIAVARKIPENVLQE---DSKLLMWYDQALTRMG | Haemonchus contortus |
| job | A | AF-Q0IEI2-F1-model v4 AAEL010327-PA | 3.341E+00 | QRKLKVFRLEAVRAGFKKAWQERDYATIIAVARKIPE-NVLQEDSKLLMWYDQALTRMG | Aedes aegypti |
| job | A | AF-A0A1B0EZS4-F1-model v4 SIX1 SD domain-containing protein | 3.566E+00 | QRKLKVFRLEAVRAGFKKAWQERDYATIIAVARKIPE-NVLQEDSKLLMWYDQALTRMG | Phlebotomus papatasi |
| job | A | AF-A0A1B6G3Q3-F1-model v4 Uncharacterized protein | 4.337E+00 | LKVFRLEAVRAGFKKAWQERDYATIIAVARKIPENVLQEDSKLLMWYDQALTRMGGK | Cuerna arida |
| job | A | AF-A0A496Z8W3-F1-model v4 Uncharacterized protein | 5.274E+00 | EAVRAGFKKAWQERDYATIIAVARKIPENVLQEDSKLLMWYDQALTRMG | Deltaproteobacteria bacterium |
| job | A | AF-X1RTA1-F1-model v4 Uncharacterized protein | 7.306E+00 | EAVRAGFKKAWQERDYATIIAVARKIPENVLQEDSKLLMWYDQALTRMG | marine sediment metagenome |
| job | A | AF-A0A3N5RDJ8-F1-model v4 Uncharacterized protein | 6.845E+00 | VRAGFKKAWQERDYATIIAVARKIPENVLQEDSKLLMWYDQALTRMG | Chloroflexi bacterium |
| job | A | AF-A0A1F9LJY2-F1-model v4 Uncharacterized protein | 5.629E+00 | VFRLEAVRAGFKKAWQERDYATIIAVARKIPENVLQE---DSKLLMWYDQALTRMG | Deltaproteobacteria bacterium |
| job | A | AF-A0A352SD74-F1-model v4 Serine/threonine protein kinase | 5.629E+00 | VRAGFKKAWQERDYATIIAVARKIPENVLQEDSKLLMWYDQALT | Cupriavidus sp. |
| job | A | AF-A0A829YIW5-F1-model v4 LuxR family transcriptional regulator | 7.799E+00 | RLEAVRAGFKKAWQERDYATIIAVARKIPENVLQEDSKLLMWYDQAL | Steroidobacter agaridevorans |

| | | | | | |
|---|---|---|---|---|---|
| job | A | AF-A0A1R1CYN4-F1-model v4 Uncharacterized protein | 7.306E+00 | RLEAVRAGFKKAWQERDYATIIAVARKIPENVLQEDSKLLMWYDQALTRMG | Paenibacillus sp. FSL H8-0259 |
| job | A | AF-A0A484KDR7-F1-model v4 Uncharacterized protein | 6.845E+00 | EAVRAGFKKAWQERDYATIIAVARKIPENVLQEDSKLLMWYDQALTRMG | Cuscuta campestris |
| job | A | AF-A0A1V3N6V0-F1-model v4 Uncharacterized protein | 6.008E+00 | LEAVRAGFKKAWQERDYATIIAVARKIPENVLQEDSKLLMWYDQALT | Thioalkalivibrio denitrificans |
| job | A | AF-A0A7I0ZTV4-F1-model v4 homeobox protein six1a-like isoform X2 | 4.337E+00 | QRKLKVFRLEAVRAGFKKAWQERDYATIIAVARKIPENVL-QEDSKLLMWYDQALTRMG | Danaus plexippus plexippus |
| job | A | AF-A0A536V4J1-F1-model v4 Protein-glutamate O-methyltransferase CheR | 2.748E+00 | QRKLKVFRLEAVRAGFKKAWQERDYATIIAVARKIPENVLQEDSKLLMWYDQALTRMG | Betaproteobacteria bacterium |
| job | A | AF-A0A7Z8VPL5-F1-model v4 Uncharacterized protein | 2.574E+00 | VRAGFKKAWQERDYATIIAVARKIPENVLQEDSKLLMWYDQA-LTRMGGK | Methylococcaceae bacterium |
| job | A | AF-A0A329ZWU5-F1-model v4 Uncharacterized protein | 3.341E+00 | VRAGFKKAWQERDYATIIAVARKIPENVLQEDSKLLMWYDQALT | Helicobacter sp. 15-1451 |
| job | A | AF-A0A7V4D6T6-F1-model v4 Tetratricopeptide repeat protein | 6.413E+00 | EAVRAGFKKAWQERDYATIIAVARKIPENVLQEDSKLLMWYDQALTRMG | Spirochaetes bacterium |
| job | A | AF-A0A6N7M497-F1-model v4 BTAD domain-containing protein | 6.008E+00 | EAVRAGFKKAWQERDYATIIAVARKIPENVLQEDSKLLMWYDQAL | Spirochaeta sp. |
| job | A | AF-A0A1G8HSG4-F1-model v4 Non-specific serine/threonine protein kinase | 4.941E+00 | VRAGFKKAWQERDYATIIAVARKIPENVLQEDSKLLMWYDQA--LTRMG | Rhodococcus triatomae |
| job | A | AF-A0A3L8BXX2-F1-model v4 HTH luxR-type domain-containing protein | 9.483E+00 | LEAVRAGFKKAWQERDYATIIAVARKIPENVLQEDSKLLMWYDQAL | Ketobacter sp. |
| job | A | AF-A0A1Z3HJV7-F1-model v4 HTH luxR-type domain-containing protein | 6.008E+00 | VRAGFKKAWQERDYATIIAVARKIPENVLQEDSKLLMWYDQALTRMG | Halomicronema hongdechloris |
| job | A | AF-A0A7V3RIN7-F1-model v4 Tetratricopeptide repeat protein | 5.274E+00 | EAVRAGFKKAWQERDYATIIAVARKIPENVLQEDSKLLMWYDQALT | candidate division WOR-3 |
| job | A | AF-A0A3B1BRW7-F1-model v4 Uncharacterized protein | 4.337E+00 | VRAGFKKAWQERDYATIIAVARKIPENVLQEDSKLLMWYDQALTRM | hydrothermal vent metagenome |
| job | A | AF-A0A0K9I8E1-F1-model v4 Uncharacterized protein | 6.413E+00 | LEAVRAGFKKAWQERDYATIIAVARKIPENVLQEDSKLLMWYDQAL | Desulfocarbo indianensis |
| job | A | AF-A0A6G0UZW5-F1-model v4 SIX1 SD domain-containing protein | 2.574E+00 | FRLEAVRAGFKKAWQERDYATIIAVARKIPENVLQ---EDSKLLMWYDQALTRMG | Halicephalobus sp. NKZ332 |
| job | A | AF-A0A1I8I735-F1-model v4 SIX1 SD domain-containing protein | 3.566E+00 | FRLEAVRAGFKKAWQERDYATIIAVARKIPE-NVLQEDSKLLMWYDQALTRMGGK | Macrostomum lignano |
| job | A | AF-A0A838VI86-F1-model v4 Rhomboid family intramembrane serine protease | 7.799E+00 | VRAGFKKAWQERDYATIIAVARKIPENVLQEDSKLLMWYDQALTRMG | Nostocaceae cyanobacterium |
| job | A | AF-A0A7R9CCC0-F1-model v4 Hypothetical protein | 4.337E+00 | KLKVFRLEAVRAGFKKAWQERDYATIIAVARKIPENVLQE-DSKLLMWYDQALTRMG | Timema cristinae |
| job | A | AF-A0A2Z4FRJ3-F1-model v4 Six4/5 | 4.629E+00 | KVFRLEAVRAGFKKAWQERDYATIIAVARKIPENVLQEDS-KLLMWYDQALTRMG | Branchiostoma lanceolatum |
| job | A | AF-A0A815KZC7-F1-model v4 Hypothetical protein | 8.885E+00 | LKVFRLEAVRAGFKKAWQERDYATIIAVARKIPENVLQEDSKLLMWYDQALTRMGG | Adineta steineri |
| job | A | AF-A0A661U499-F1-model v4 Uncharacterized protein | 7.306E+00 | LEAVRAGFKKAWQERDYATIIAVARKIPENVLQEDSKLLMWYDQALTRMG | Candidatus Coatesbacteria |
| job | A | AF-A0A022QS49-F1-model v4 AAA domain-containing protein | 4.063E+00 | QRKLKVFRLEAVRAGFKKAWQERDYATIIAVARKIPENVLQEDSKLLMWYDQALTRMG | Erythranthe guttata |
| job | A | AF-A0A2P5P8R0-F1-model v4 Tetratricopeptide repeat protein | 6.413E+00 | VRAGFKKAWQERDYATIIAVARKIPENVLQEDSKLLMWYDQALTRMG | Candidatus Dehalogenimonas |
| job | A | AF-A0A358PYK4-F1-model v4 HTH luxR-type domain-containing protein | 9.483E+00 | EAVRAGFKKAWQERDYATIIAVARKIPENVLQEDSKLLMWYDQALTRMG | Desulfosporosinus sp. |
| job | A | AF-A0A3R7XB52-F1-model v4 Helix-turn-helix transcriptional regulator | 6.008E+00 | EAVRAGFKKAWQERDYATIIAVARKIPENVLQEDSKLLMWYDQAL | Alteromonadaceae bacterium |
| job | A | AF-A0A328AZR3-F1-model v4 TIR domain-containing protein | 5.274E+00 | LKVFRLEAVRAGFKKAWQERDYATIIAVARKIPENVLQEDSKLLMWYDQAL | Phenylobacterium hankyongense |
| job | A | AF-A0A518GGT5-F1-model v4 ASPIC and UnbV | 5.274E+00 | KVFRLEAVRAGFKKAWQERDYATIIAVARKIPENVLQEDSKLLMWYDQALTRMG | Aureliella helgolandensis |
| job | A | AF-A0A7I8VY92-F1-model v4 DgyrCDS9858 | 4.337E+00 | QRKLKVFRLEAVRAGFKKAWQERDYATIIAVARKIPENVLQEDS-KLLMWYDQALTRMG | Dimorphilus gyrociliatus |
| job | A | AF-A0A661NSX6-F1-model v4 Uncharacterized protein | 7.306E+00 | EAVRAGFKKAWQERDYATIIAVARKIPENVLQEDSKLLMWYDQAL | Deltaproteobacteria bacterium |
| job | A | AF-A0A6J4I1X2-F1-model v4 Transcriptional activator of maltose regulon, MalT | 7.306E+00 | VRAGFKKAWQERDYATIIAVARKIPENVLQEDSKLLMWYDQALTRMG | uncultured Chloroflexia |
| job | A | AF-A0A3D1UWW4-F1-model v4 Uncharacterized protein | 6.008E+00 | VRAGFKKAWQERDYATIIAVARKIPENVLQEDSKLLMWYDQALTRMG | Lentisphaeria bacterium |
| job | A | AF-A0A1F8LSQ5-F1-model v4 Uncharacterized protein | 8.885E+00 | EAVRAGFKKAWQERDYATIIAVARKIPENVLQEDSKLLMWYDQALTRMG | Chloroflexi bacterium |
| job | A | AF-A0A7C3LEI3-F1-model v4 HTH luxR-type domain-containing protein | 5.629E+00 | VRAGFKKAWQERDYATIIAVARKIPENVLQEDSKLLMWYDQALT | Anaerolineae bacterium |
| job | A | AF-A0A4Y7B8F5-F1-model v4 HTH luxR-type domain-containing protein | 6.413E+00 | LEAVRAGFKKAWQERDYATIIAVARKIPENVLQEDSKLLMWYDQALT | Ramlibacter sp. WS9 |
| job | A | AF-A0A0V7ZLE3-F1-model v4 HTH luxR-type domain-containing protein | 8.324E+00 | LEAVRAGFKKAWQERDYATIIAVARKIPENVLQEDSKLLMWYDQALTRMGGK | Mastigocoleus testarum BC008 |
| job | A | AF-A0A1M5QGM0-F1-model v4 ATP-, maltotriose-and DNA-dependent transcriptional regulator MalT | 8.324E+00 | VRAGFKKAWQERDYATIIAVARKIPENVLQEDSKLLMWYDQALTRMGGK | Thermosyntropha lipolytica |
| job | A | AF-V2RJQ2-F1-model v4 Uncharacterized protein | 7.306E+00 | FRLEAVRAGFKKAWQERDYATIIAVARKIPENVLQEDSKLLMWYDQALT | Mucispirillum schaedleri |
| job | A | AF-A0A7R8Z9Z2-F1-model v4 Hypothetical protein | 5.629E+00 | KVFRLEAVRAGFKKAWQERDYATIIAVARKIPENVLQE-DSKLLMWYDQALTRMG | Timema douglasi |
| job | A | AF-A0A1J1IEB6-F1-model v4 CLUMA CG012018, isoform A | 4.941E+00 | VFRLEAVRAGFKKAWQERDYATIIAVARKIPENVLQ-EDSKLLMWYDQALTRMG | Clunio marinus |
| job | A | AF-A0A3D0CXQ8-F1-model v4 TPR MalT domain-containing protein | 6.845E+00 | VRAGFKKAWQERDYATIIAVARKIPENVLQEDSKLLMWYDQALTRMG | Chloroflexi bacterium |
| job | A | AF-A0A178MBV7-F1-model v4 Uncharacterized protein | 4.941E+00 | FRLEAVRAG-FKKAWQERDYATIIAVARKIPENVLQEDSKLLMWYDQALTRMGGK | Chloroflexus islandicus |
| job | A | AF-A0A354TB23-F1-model v4 Uncharacterized protein | 5.629E+00 | VFRLE--AVRAGFKKAWQERDYATIIAVARKIPENVLQEDSKLLMWYDQALTRMGGK | Verrucomicrobiales bacterium |
| job | A | AF-A0A497A7T7-F1-model v4 Uncharacterized protein | 8.324E+00 | LEAVRAGFKKAWQERDYATIIAVARKIPENVLQEDSKLLMWYDQALTRMG | Chloroflexi bacterium |
| job | A | AF-A0A0F7GBT7-F1-model v4 BTAD domain-containing protein | 7.799E+00 | VRAGFKKAWQERDYATIIAVARKIPENVLQEDSKLLMWYDQALTRMG | Dehalogenimonas sp. WBC-2 |
| job | A | AF-A0A3B0XSH3-F1-model v4 Uncharacterized protein | 6.413E+00 | EAVRAGFKKAWQERDYATIIAVARKIPENVLQEDSKLLMWYDQAL | hydrothermal vent metagenome |
| job | A | AF-A0A419DBD6-F1-model v4 BTAD domain-containing protein | 9.483E+00 | VRAGFKKAWQERDYATIIAVARKIPENVLQEDSKLLMWYDQALTRMG | Dehalococcoidia bacterium |
| job | A | AF-A0A850A478-F1-model v4 Tetratricopeptide repeat protein | 6.845E+00 | LEAVRAGFKKAWQERDYATIIAVARKIPENVLQEDSKLLMWYDQALTRMG | Caldilineales bacterium |
| job | A | AF-A0A7V1FY58-F1-model v4 Uncharacterized protein | 5.274E+00 | FRLEAVRAGFKKAWQERDYATIIAVARKIPENVLQEDSKLLMWYDQALTRM | Phycisphaerales bacterium |
| job | A | AF-A0A1A9V577-F1-model v4 Homeobox domain-containing protein | 4.941E+00 | LKVFRLEAVRAGFKKAWQERDYATIIAVARKIPE-NVLQEDSKLLMWYDQALTRMG | Glossina austeni |
| job | A | AF-A0A7Z9ZKJ6-F1-model v4 Tetratricopeptide repeat protein | 7.306E+00 | EAVRAGFKKAWQERDYATIIAVARKIPENVLQEDSKLLMWYDQALTRMG | Anaerolineae bacterium |
| job | A | AF-A0A6P8YI81-F1-model v4 homeobox protein SIX1-like isoform X1 | 6.413E+00 | VFRLEAVRAGFKKAWQERDYATIIAVARKIPENVLQEDSKLLMWYDQALTRMG | Thrips palmi |
| job | A | AF-A0A0N1IN86-F1-model v4 Homeobox protein SIX4 | 6.008E+00 | VFRLEAVRAGFKKAWQERDYATIIAVARKIPENVL-QEDSKLLMWYDQALTRMG | Papilio xuthus |
| job | A | AF-A0A3D0DB67-F1-model v4 TPR MalT domain-containing protein | 8.885E+00 | LEAVRAGFKKAWQERDYATIIAVARKIPENVLQEDSKLLMWYDQALTRMG | Chloroflexi bacterium |
| job | A | AF-A0A497C9T8-F1-model v4 Helix-turn-helix transcriptional regulator | 7.799E+00 | LEAVRAGFKKAWQERDYATIIAVARKIPENVLQEDSKLLMWYDQALTRMGGK | Chloroflexi bacterium |
| job | A | AF-A0A4P6K0U0-F1-model v4 Tetratricopeptide repeat protein | 8.885E+00 | LEAVRAGFKKAWQERDYATIIAVARKIPENVLQEDSKLLMWYDQALT | Ktedonosporobacter rubrisoli |
| job | A | AF-A0A354J8B5-F1-model v4 Uncharacterized protein | 7.799E+00 | EAVRAGFKKAWQERDYATIIAVARKIPENVLQEDSKLLMWYDQALTRMG | Lachnospiraceae bacterium |
| job | A | AF-A0A840SF85-F1-model v4 Tetratricopeptide (TPR) repeat protein | 4.063E+00 | VFRLEAVRAGFKKAWQERDYATIIAVARKIPENVLQE---DSKLLMWYDQALTRMG | Treponema rectale |
| job | A | AF-A0A6J4KIA3-F1-model v4 HTH luxR-type domain-containing protein | 7.306E+00 | VRAGFKKAWQERDYATIIAVARKIPENVLQEDSKLLMWYDQALT | uncultured Chloroflexia |
| job | A | AF-A0A1M7Y8L6-F1-model v4 Uncharacterized protein | 5.629E+00 | QRKLKVFRLEAVRAGFKKAWQERDYATIIAVARKIPENVLQE-DSKLLMWYDQALTRM | Desulfopila aestuarii |
| job | A | AF-A0A317CP45-F1-model v4 Uncharacterized protein | 4.941E+00 | EAVRAGFKKAWQERDYATIIAVARKIPENVLQEDSKLLMWYDQALTRMG | Leucothrix pacifica |
| job | A | AF-A0A662C7Z5-F1-model v4 HTH luxR-type domain-containing protein | 8.885E+00 | LEAVRAGFKKAWQERDYATIIAVARKIPENVLQEDSKLLMWYDQALTRMG | Bacteroidetes bacterium |
| job | A | AF-A0A3B9D0A3-F1-model v4 Uncharacterized protein | 4.063E+00 | LKVFRLEAVRAGFKKAWQERDYATIIAVARKIPENVLQEDSKLLMWYDQALTRMG | Planctomycetaceae bacterium |
| job | A | AF-A0A7W9RUA0-F1-model v4 LuxR family maltose regulon positive regulatory protein | 7.799E+00 | LEAVRAGFKKAWQERDYATIIAVARKIPENVLQEDSKLLMWYDQALT | Acidovorax konjaci |
| job | A | AF-A0A1M3K8W3-F1-model v4 HTH luxR-type domain-containing protein | 6.413E+00 | EAVRAGFKKAWQERDYATIIAVARKIPENVLQEDSKLLMWYDQALT | Chloroflexi bacterium 44-23 |
| job | A | AF-A0A6J4JZV2-F1-model v4 Transcriptional activator of maltose regulon, MalT | 8.324E+00 | VRAGFKKAWQERDYATIIAVARKIPENVLQEDSKLLMWYDQALT | uncultured Chloroflexia |
| job | A | AF-A0A2G9TDS3-F1-model v4 DUF4071 domain-containing protein | 6.413E+00 | VFRLEAVRAGFKKAWQERDYATIIAVARK---IPENVLQEDSKLLMWYDQALT | Teladorsagia circumcincta |
| job | A | AF-A0A369B873-F1-model v4 Tetratricopeptide repeat protein | 9.483E+00 | FRLEAVRAGFKKAWQERDYATIIAVARKIPENVLQEDSKLLMWYDQALTRMG | Fontibacillus phaseoli |
| job | A | AF-A0A3N5I8U9-F1-model v4 Uncharacterized protein | 8.324E+00 | VRAGFKKAWQERDYATIIAVARKIPENVLQEDSKLLMWYDQAL | Desulfobacteraceae bacterium |
| job | A | AF-A0A7V7XG44-F1-model v4 Tetratricopeptide repeat protein | 8.324E+00 | LEAVRAGFKKAWQERDYATIIAVARKIPENVLQEDSKLLMWYDQAL | Anaerolineae bacterium |
| job | A | AF-A0A1F8Q221-F1-model v4 HTH luxR-type domain-containing protein | 8.885E+00 | EAVRAGFKKAWQERDYATIIAVARKIPENVLQEDSKLLMWYDQALTRMG | Chloroflexi bacterium |
| job | A | AF-A0A0S8AW68-F1-model v4 HTH luxR-type domain-containing protein | 8.885E+00 | LEAVRAGFKKAWQERDYATIIAVARKIPENVLQEDSKLLMWYDQAL | Anaerolineae bacterium SG8 19 |
| job | A | AF-A0A5N8V799-F1-model v4 Tetratricopeptide repeat protein | 9.483E+00 | VRAGFKKAWQERDYATIIAVARKIPENVLQEDSKLLMWYDQALTRMGGK | Streptomyces adustus |
| job | A | AF-A0A517Z1Q5-F1-model v4 ASPIC and UnbV | 3.566E+00 | KVFRLEAVRAGFKKAWQERDYATIIAVARKIPENVLQEDS---KLLMWYDQALTRMG | Maioricimonas rarisocia |
| job | A | AF-A0A0S8GJC0-F1-model v4 BTAD domain-containing protein | 8.324E+00 | EAVRAGFKKAWQERDYATIIAVARKIPENVLQEDSKLLMWYDQALTRMG | candidate division WOR 3 |
| job | A | AF-A0A7T7BC48-F1-model v4 HTH-type transcriptional regulator MalT | 7.799E+00 | VRAGFKKAWQERDYATIIAVARKIPENVLQEDSKLLMWYDQAL | Ralstonia pickettii |
| job | A | AF-A0A4Z0WBJ0-F1-model v4 BTAD domain-containing protein | 7.799E+00 | LEAVRAGFKKAWQERDYATIIAVARKIPENVLQEDSKLLMWYDQAL | Natronospirillum operosum |
| job | A | AF-A0A0V8HL48-F1-model v4 Uncharacterized protein | 9.483E+00 | RLEAVRAGFKKAWQERDYATIIAVARKIPENVLQEDSKLLMWYDQALTRMGGK | Bacillus enclensis |
| job | A | AF-A0A4U3KWY6-F1-model v4 DUF4071 domain-containing protein | 7.799E+00 | LKVFRLEAVRAGFKKAWQERDYATIIAVARKIPENVLQEDSKLLMWYDQALTRMG | Ilyomonas limi |
| job | A | AF-A0A437AS29-F1-model v4 SIX1 SD domain-containing protein | 7.306E+00 | RKLKVFRLEAVRAGFKKAWQERDYATIIAVARKIPENVL-QEDSKLLMWYDQALTRMG | Chilo suppressalis |
| job | A | AF-A0A382D4E2-F1-model v4 Uncharacterized protein | 7.306E+00 | KVF--RLEAVRAGFKKAWQERDYATIIAVARKIPENVLQEDS----KLLMWYDQALTRMG | marine metagenome |

```
job A   AF-A0A5S3WVC1-F1-model v4 Uncharacterized protein                           4.629E+00   LEAVRAGFKKAWQERDYATIIAVARKIPENVLQE-DSKLLMWYDQALTRMGGK          Pseudoalteromonas rubra
job A   AF-A0A818RWE8-F1-model v4 Hypothetical protein                              6.413E+00   KVFRLEAVRAGFKKAWQERDYATIIAVARKIPE-NVLQEDSKLLMWYDQALTRMG        Adineta steineri
job A   AF-A0A7R9JQM7-F1-model v4 Hypothetical protein                              6.413E+00   KVFRLEAVRAGFKKAWQERDYATIIAVARKIPENVLQE-DSKLLMWYDQALTRMG        Timema genevievae
job A   AF-A0A3D0PK83-F1-model v4 Uncharacterized protein                           9.483E+00   EAVRAGFKKAWQERDYATIIAVARKIPENVLQEDSKLLMWYDQALTRMG              Lachnospiraceae bacterium
job A   AF-A0A0F9G5F1-F1-model v4 Uncharacterized protein                           7.799E+00   VRAGFKKAWQERDYATIIAVARKIPENVLQEDSKLLMWYDQALTRMG                marine sediment metagenome
job A   AF-A0A849HSF9-F1-model v4 Transglycosylase SLT domain-containing protein    7.799E+00   VFRLEAVRAGFKKAWQERDYATIIAVARKIPENVLQEDSKLLMWYDQALTRMG          Legionellales bacterium
job A   AF-A0A317XH78-F1-model v4 F-box domain-containing protein                   5.629E+00   LEAVRAGFKKAWQERDYATIIAVAR----KIPENVLQEDSKLLMWYDQALTRMG         Testicularia cyperi
job A   AF-A0A7Y7P3C5-F1-model v4 Tetratricopeptide repeat protein                  6.413E+00   VFRLEAVRAGFKKAWQERDYATIIAVARKIPENVLQE--DSKLLMWYDQALTRMG        Bacteroidales bacterium
job A   AF-A0A4Q5VWA5-F1-model v4 TIR domain-containing protein                     5.629E+00   VRAGFKKAWQERDYATIIAVARKIPENVLQEDS----KLLMWYDQALTRMGG           Chitinophagaceae bacterium
job A   AF-A0A7V5AL18-F1-model v4 Glycosyltransferase                               8.885E+00   FRLEAVRAGFKKAWQERDYATIIAVARKIPENVLQEDSKLLMWYDQALTRMG           Syntrophobacterales
job A   AF-A0A536M2I7-F1-model v4 Tetratricopeptide repeat protein                  5.274E+00   LEAVRAGFKKA-WQ---ERDYATIIAVARKIPENVLQEDSKLLMWYDQALTRMG         Chloroflexi bacterium

Job: Type-3 BREX; MTase MBW2068239 HTH2
Query: s001A
No: Chain Z rmsd lali nres id PDB Description
1: 8qgy-B 8.8 1.9 55 809 18 MOLECULE: MITOGEN-ACTIVATED PROTEIN KINASE KINASE KINASE 5;
2: 6z0f-A 8.1 2.1 54 384 9 MOLECULE: ESX SECRETION SYSTEM PROTEIN YUKC;
3: 8h38-H 6.8 2.4 56 177 9 MOLECULE: COP9 SIGNALOSOME COMPLEX SUBUNIT 1;
4: 4n5c-D 6.7 2.2 52 757 12 MOLECULE: CARGO-TRANSPORT PROTEIN YPP1;
5: 6xt9-D 6.5 2.4 54 396 13 MOLECULE: BARDET-BIEDL SYNDROME 1 PROTEIN;
6: 2yhe-A 6.3 2.2 57 639 14 MOLECULE: SEC-ALKYL SULFATASE;
7: 6vbu-8 6.2 2.4 49 475 12 MOLECULE: BARDET-BIEDL SYNDROME 18 PROTEIN;
8: 5vxv-A 6.1 2.3 51 217 14 MOLECULE: PEROXISOMAL MEMBRANE PROTEIN PEX15;
9: 5a63-D 6.1 2.2 54 100 9 MOLECULE: NICASTRIN;
10: 2uy1-A 6.0 1.8 50 438 12 MOLECULE: CLEAVAGE STIMULATION FACTOR 77;
11: 6z2w-E 6.0 2.4 51 2325 12 MOLECULE: DNA DAMAGE CHECKPOINT PROTEIN LCD1;
12: 2c2l-A 5.9 2.3 52 281 12 MOLECULE: CARBOXY TERMINUS OF HSP70-INTERACTING PROTEIN;
13: 8sxf-C 5.8 3.0 55 532 5 MOLECULE: PROBABLE CARBOXYL-TERMINAL PROTEASE;
14: 8s4g-J 5.7 2.2 50 512 6 MOLECULE: ANAPHASE-PROMOTING COMPLEX SUBUNIT 10;
15: 4rib-B 5.7 2.5 50 628 12 MOLECULE: FANCONI-ASSOCIATED NUCLEASE 1;
16: 6sli-A 5.7 2.7 51 482 14 MOLECULE: LIPOPROTEIN RAGB;
17: 2btp-A 5.7 2.4 58 248 10 MOLECULE: 14-3-3 PROTEIN TAU;
18: 9lth-A 5.6 2.8 52 285 4 MOLECULE: TRANSCRIPTIONAL REGULATOR;
19: 3l22-A 5.6 2.2 50 429 8 MOLECULE: SUSD SUPERFAMILY PROTEIN;
20: 4cgu-A 5.6 2.5 51 112 12 MOLECULE: TPR REPEAT-CONTAINING PROTEIN ASSOCIATED WITH HSP
21: 2grm-A 5.6 3.3 55 315 5 MOLECULE: PRGX;
22: 6hs5-A 5.6 3.1 53 249 19 MOLECULE: TSSA;
23: 2o8p-A 5.5 2.3 52 220 4 MOLECULE: 14-3-3 DOMAIN CONTAINING PROTEIN;
24: 2vkj-A 5.5 3.1 56 106 9 MOLECULE: TM1634;
25: 4m57-A 5.5 3.1 58 702 9 MOLECULE: CHLOROPLAST PENTATRICOPEPTIDE REPEAT PROTEIN 10;
26: 5vbg-A 5.5 2.9 52 543 8 MOLECULE: PENICILLIN-BINDING PROTEIN ACTIVATOR LPOA;
27: 8hmd-C 5.5 2.8 51 1334 12 MOLECULE: INTRAFLAGELLAR TRANSPORT PROTEIN 122 HOMOLOG;
28: 5xw7-C 5.4 2.5 51 245 6 MOLECULE: CELLULOSE SYNTHASE SUBUNIT C;
29: 8cbk-E 5.4 3.0 54 470 7 MOLECULE: 3-HYDROXYACYL-COA DEHYDROGENASE TYPE-2;
30: 2pzi-A 5.3 2.8 52 654 10 MOLECULE: PROBABLE SERINE/THREONINE-PROTEIN KINASE PKNG;
31: 8rg0-u 5.3 2.2 53 603 8 MOLECULE: EUKARYOTIC TRANSLATION INITIATION FACTOR 3 SUBUNI
32: 7y4i-A 5.3 2.3 49 822 6 MOLECULE: PROBABLE UDP-N-ACETYLGLUCOSAMINE--PEPTIDE N-
33: 7tj4-A 5.3 2.6 50 122 10 MOLECULE: ACTH;
34: 8q9t-E 5.3 2.2 52 1060 10 MOLECULE: ANTIVIRAL HELICASE SKI2;
35: 3qdn-A 5.3 2.2 53 285 13 MOLECULE: PUTATIVE THIOREDOXIN PROTEIN;
36: 8ara-C 5.3 2.5 54 112 11 MOLECULE: CHAPERONE PROTEIN YSCY;
37: 9dt9-A 5.3 2.9 52 112 15 MOLECULE: 231_C2_TRKA;
38: 4it4-B 5.2 2.4 53 210 4 MOLECULE: CG17282;
39: 7zty-A 5.2 3.1 50 345 6 MOLECULE: CNH DOMAIN-CONTAINING PROTEIN;
40: 5a1v-K 5.2 2.3 49 1125 10 MOLECULE: ADP-RIBOSYLATION FACTOR 1;
41: 8rbk-A 5.2 2.6 53 739 2 MOLECULE: MGC151858 PROTEIN;
42: 7dkh-I 5.2 2.2 51 914 14 MOLECULE: RNA POLYMERASE-ASSOCIATED PROTEIN CTR9;
43: 1y8m-A 5.2 2.2 53 144 6 MOLECULE: FIS1;
44: 7dhg-C 5.2 2.6 52 470 13 MOLECULE: MITOCHONDRIAL IMPORT RECEPTOR SUBUNIT TOM70;
45: 8qt5-A 5.2 2.1 53 248 11 MOLECULE: 14-3-3-LIKE PROTEIN G-BOX FACTOR 14 LAMBDA,PROTEI
46: 4ymr-A 5.1 2.0 47 134 6 MOLECULE: PROTEIN SNX21;
47: 8g0l-B 5.1 2.8 51 925 12 MOLECULE: N-ALPHA-ACETYLTRANSFERASE 20;
48: 6erp-A 5.1 2.6 52 1011 12 MOLECULE: TRANSCRIPTION FACTOR A, MITOCHONDRIAL;
49: 5an3-B 5.1 2.1 51 136 14 MOLECULE: SGT1;
50: 2mv3-A 5.1 2.9 51 88 20 MOLECULE: MITOCHONDRIAL INNER MEMBRANE I-AAA PROTEASE SUPER
51: 3jq1-B 5.1 2.6 51 464 18 MOLECULE: SUSD SUPERFAMILY PROTEIN;
52: 8vx7-A 5.1 2.2 53 161 17 MOLECULE: CID7;
53: 6au8-A 5.1 2.9 54 280 9 MOLECULE: GOLGI TO ER TRAFFIC PROTEIN 4 HOMOLOG;
54: 3u4t-A 5.1 2.3 48 258 10 MOLECULE: TPR REPEAT-CONTAINING PROTEIN;
55: 6f1t-f 5.1 3.0 56 929 11 MOLECULE: ARP1 ACTIN RELATED PROTEIN 1 HOMOLOG A;
56: 2pl2-A 5.0 2.5 51 194 14 MOLECULE: HYPOTHETICAL CONSERVED PROTEIN TTC0263;
57: 5wft-A 5.0 2.1 52 118 12 MOLECULE: PELB;
58: 8gaa-A 5.0 3.5 54 197 2 MOLECULE: C6HR1_4R;
59: 5y7q-A 5.0 2.8 51 546 14 MOLECULE: FANCONI-ASSOCIATED NUCLEASE 1 HOMOLOG;
60: 2lxl-A 5.0 2.5 55 163 9 MOLECULE: VACUOLAR PROTEIN SORTING-ASSOCIATED PROTEIN VTA1
61: 8ebf-A 5.0 2.2 54 667 17 MOLECULE: CYTOPLASMIC MEMBRANE PROTEIN;
62: 2bb6-A 5.0 2.8 54 414 6 MOLECULE: TRANSCOBALAMIN II;
63: 5cd6-A 5.0 2.7 50 575 8 MOLECULE: TPR-DOMAIN CONTAINING PROTEIN;
64: 4fym-D 5.0 2.7 55 227 7 MOLECULE: OROTATE PHOSPHORIBOSYLTRANSFERASE;
65: 2kat-A 5.0 2.6 53 115 11 MOLECULE: UNCHARACTERIZED PROTEIN;
66: 1pc2-A 5.0 2.6 55 152 9 MOLECULE: MITOCHONDRIA FISSION PROTEIN;
```

```
67: 5u1s-A 5.0 3.0 59 1488 10 MOLECULE: SEPARIN;
68: 2kck-A 5.0 2.5 53 112 13 MOLECULE: TPR REPEAT;
69: 6yj6-A 5.0 2.5 55 819 4 MOLECULE: TRANSCRIPTION FACTOR TAU 131 KDA SUBUNIT;
71: 2kc7-A 4.9 2.9 50 99 8 MOLECULE: BFR218_PROTEIN;
72: 5wd8-A 4.9 2.2 50 90 6 MOLECULE: UNCHARACTERIZED PROTEIN;
73: 8f5o-C 4.9 2.8 50 1179 12 MOLECULE: INTRAFLAGELLAR TRANSPORT PROTEIN 122B, PUTATIVE;
74: 5efr-A 4.9 2.2 51 403 10 MOLECULE: BAMA-BAMD FUSION PROTEIN;
75: 4eba-A 4.9 3.1 54 585 9 MOLECULE: MRNA 3'-END-PROCESSING PROTEIN RNA14;
76: 9l4f-A 4.9 3.3 53 2383 9 MOLECULE: SERINE/THREONINE-PROTEIN KINASE ATR;
77: 6tdy-J 4.9 2.8 51 170 10 MOLECULE: ATP SYNTHASE SUBUNIT ALPHA;
78: 4bt9-B 4.9 2.8 53 238 17 MOLECULE: PROLYL 4-HYDROXYLASE SUBUNIT ALPHA-1;
79: 4uzy-A 4.8 2.6 53 624 9 MOLECULE: FLAGELLAR ASSOCIATED PROTEIN;
80: 8f5p-D 4.8 2.4 48 612 17 MOLECULE: NET DOMAIN-CONTAINING PROTEIN;
81: 7t6d-A 4.8 3.0 53 389 8 MOLECULE: LIPOPOLYSACCHARIDE ASSEMBLY PROTEIN B;
82: 3as5-A 4.8 2.5 51 183 12 MOLECULE: MAMA;
83: 5w94-A 4.8 2.9 52 617 6 MOLECULE: MAU2 CHROMATID COHESION FACTOR HOMOLOG;
84: 8h7g-C 4.8 2.6 47 3261 9 MOLECULE: TRANSFORMATION/TRANSCRIPTION DOMAIN-ASSOCIATED PR
85: 8j0n-B 4.8 2.3 53 296 4 MOLECULE: ER MEMBRANE PROTEIN COMPLEX SUBUNIT 1;

0001 s001A   ----QRKL----K-----V--F---R---L--EAVRAG-FKKAW-QE---R------DYATIIA-VARK---I-----PE----NVL--QE----D-SKLLMWYDQALT-R-M--G--G--K--------------
0002 8qgyB   elarirqrvdniE-----V--L---T---A--DIVINL-LLSYR-DI---Q------DYDSIVK-LVET---L-----EK-lptfDL--AS----H-HHVKFHYAFALN-R-R--N--L--Pgdrakaldimipmv
0003 6z0fA   vpaliysmyalfF-----A--Q---P---K--HQAIVD-SNRAF-LN---K------QYSEVIS-TLSK---Y-----DA----ESL--------P-ESVQYQLATSYV-E-V--E--N--Lgsaktkniennlvt
0004 8h38H   dqcenqeleapgG-----I--A---T---P--PVYGQL-LALYL-LH---N------DMNNARY-LWKR---I-----PP----AIK--SA----N-SELGGIWSVGQR-I-W--Q--R--Dfpgiyttinahqws
0005 4n5cD   --------------------kA---L---R--ELQFKY-SYTLA-QQ---R------HIETAIK-TLES---L----iLS----KN---PN----Y-YKAWHLLALCRS---V--Q--E--Dkexsykivcsvlea
0006 6xt9D   ---------------tydP--T---N---Y--KAILAA-GSMMQ-TH---G------DFDVALT-KYRV---V-----AC----AV---PE----S-PPLWNNIGMCFF-G-K--K--K--Yvaaisclkranyla
0007 2yheA   pfppvEAG----K-----R-mG---G---A--DAVLKQ-MRAAI-DK---G------DYRWAVQ-LGNH---L----vFA----DP----A----N-KDARALQADAME-Q-L--G--Y--Qtenalwrnmymtga
0008 6vbu8   --------------------------------MEPLLL-AWSYF-RR---R------RFQLCAD-LCTQ---M----lEK----SP----C----D-QAAWILKARALT-E-M--V--Y--Vdeidvdeegiaemi
0009 5vxvA   --------------------------------SEVFQE-CVNLF-IK---R------DIKDCLE-KXSEvgfI-----DI----TVF--KS----N-PXILDLFVSACD-I-X--P--S--Ftklgltlqseilni
0010 5a63D   ------nL----E-----R--V---S---N--EEKLNL-CRKYY-LG---G--flpfLWLVNIF-WFFR---E---afLV----PA-------teQ-SQIKGYVWRSAV-G-F--L--F--Wvivltswitifqiy
0011 2uy1A   ---------------------S---S---P--SAIMEH-ARRLY-MS---K------DYRSLES-LFGR---C-----LK----KS----Y----N-LDLWMLYIEYVR-K-V--S--------------------
0012 6z2wE   hrifshfqppkltdggkfS--D---D---P--KTTTRM-LKSMY-DH---Q------LYSQIISnSSFH------ssdGK----IS-------pdV-KEWYSIGLEAAN-L-E--G--N--Vqtlknwveqieslr
0013 2c2lA   ------------------S--P---S---A--QELKEQ-GNRLF-VG---R------KYPEAAA-CYGR--aI-----TR----NP----L----V-AVYYTNRALCYL-K-M--Q--Qp-------------
0014 8sxfC   altlledN----S-----A--S--hE---V--APLLLR-SRLLQ-SM---K------RSDEALP-LLKA---G-----iK----EHP---D----D-KRVRLAYARLLV-E-Q--N--R--Lddakaefaglvqqf
0015 8s4gJ   ---------------yaeV--H---S---E--EALFLL-ATCYY-RS---G------KAYKAYR-LLKG---H------------SC--TT------PQCKYLLAKCCV-D-L--S--Kl-------------
0016 4ribB   --------------------yA---A---A--THMLSD-ISSAM-AN---G------NWEEAKE-LAQC-akR-----DW----N--------wiY-TRILSRFVEILQ-R-L--H--M--Yeeavreleslls qr
0017 6sliA   ---------------vlyV--S---R---D--YAYALR-ARIYL-AL---G------EYGKAAA-DAKM---V----vDK----YP--gaadafsV-AEVYLILVESAL-Q-T--G--Dt-------------
0018 2btpA   sgvdLGTEnlyfQ-----S--M---E---K--TELIQK-AKLAE-QA---E------RYDDMAT-CMKA-vtE-----QG----A----EL----S-NEERNLLSVAYK-N-V--V--G--Grrsawrvissieqk
0019 9lthA   --------------------lE---K---E--FTDMEK-IKIYF-EK---N------NIVGLQN-LLTS---Y----sSQ----IN--vKE----K-IIYALISSLLGR-L-T--N--Ts-------------
0020 3l22A   --------------------aT---Q---A--AANXLK-XRVYX-AX---N------EWDKAIT-AGEL---V-----TG----YS-------frY-AETLLDLAECYA-N-K--A--Gg-------------
0021 4cguA   --------------------xS---Q---F--EKQKEQ-GNSLF-KQ---G------LYREAVH-CYDQ--lI-----TA----QP----Q----N-PVGYSNKAXALI-K-L--G--Ey-------------
0022 2grmA   siikPMYP----I-----V-----tI---Q--TVLKNA-LTISI-MN---R------NLKEAQY-YINQ--fE-----HL----K--------cyD-LEINYLKQIYQF-L-T--D--K--Nidsylnavniinif
0023 6hs5A   ---------------psgD--D---L---L-fSNEFDA-IQDAR-RYdkeA------DWGFVVD-HAGE---L----lRT----RT----K----D-LRLAVWLTEALA-L-E--D--G--Itgltegyalleglc
0024 2o8pA   --------------------xD---E---R--LLQKYR-AQVFE-WG---G------CFDKXFE-ALKS--lI-----YL----SE--seF----D-DEERHLLTLCIK-H-K--I--S--Dyrtxtsqvlqeqtk
0025 2vkjA   elyifSDY----E-------eK---K---A--RSLIAE-GKDLF-ET---A------NYGEALV-FFEK-alN-----LS----DN--eEI----K-KIASFYLEECRK-K-L--A--Gd-------------
0026 4m57A   alldETPL----P--pgsR--L---D---V--RAYTTV-LHALS-RA---G------RYERALE-LFAE---L---rrQG----VAP--------T-LVTYNVVLDVYG-R-X--G--R--Swprivalldexraa
0027 5vbgA   ---------------lndA--Q---K---L--DRALIE-ARISA-AK---N------ANEVAQN-QLRA---L-----DL----NK-------sqK-SRYYETLAIVAE-N-R--K--D--Xieavkariexdknl
0028 8hmdC   --------------------mQ---T---I--LIAQSQ-VYYYI-RE---G------FWSTMQR-FCQE---Q----yKA----FG---------D-PFFIFWKAYGLY-Q-E--G--L--Pneaineltsiqhkk
0029 5xw7C   --------------------pT---A---Q--QQLLSQ-VRLGE-AT---K------REDLVRQ-SLYR--lE-----LI----DP----D----N-PDVIAARFRYLL-R-Q--G--Dn-------------
0030 8cbkE   vidvfeimkaryK-----T--L---E---P--RGYSLL-IRGLI-HS---D------RWREALL-LLED---I----kKV----ITP--------S-KKNYNDCIQGALlH-Q--D--V--Ntawnlyqellghdi
0031 2pziA   --------------------gW---R---W--RLVWYR-AVAEL-LT---G------DYDSATK-HFTE---V----lDT----FP----G----E-LAPKLALAATAE-L-A--G--N--Tdehkfyqtvwstnd
0032 8rg0u   -----MPA----Y-----F--Q---R---P--ENALKR-ANEFL-EV---G------KKQPALD-VLYD---V-----MK--------qkI----H-EPIMLKYLELCV-D-L--Rqk------------------
0033 7y4iA   -------------------------------sNDTLSY-ANILR-AR---N------KFADALA-LYEA---M----lEK----DS----K----N-VEAHIGKGICLQ-T-Q--N--K--Gnlafdcfseairld
0034 7tj4A   ------------------------kE---D--NIYNKL-IKDDM-TS---G------NYDNAQN-IAKQ---T----iNK----NYA--------D-DQTYYLSGMIMA-T-I--N--Sk-------------
0035 8q9tE   ---------------vkcK--N---N---I--LAHRIL-CQYYL-LT---K------EYEAALP-YIKN-giS-----LI----A---plT----K-REFSLDLATVYT-Y-V--D--P--Kdhnaalklydnils
0036 3qdnA   ---------------qlsS--Q---N---S--EIGLLL-AEAQI-AL---N------RSEEAEA-VLKT---I-----PL----QDQ--------D-TRYQGLVAQIEL-L-K--Q--A--Adtpeiqqlqqqvad
0037 8araC   ------------I-----T--L---TkrqQ--EFLLLN-GWLQL-QC---G------HAERACI-LLDA---L-----lT----LNP---E----H-LAGRRCRLVALL-NnN--Q--G--Eraekeaqwlishdp
0038 9dt9A   ---------------------S---G---K--EEIKEA-IKKAV-VR---A--gdpkYLEEAKA-LLEK--lK-----EL----DE----E----D-KDVEKFEKAIKQ-V-E--A--E--Ltlkeakevvkrlfe
0039 4it4B   ---------------qveK--L---S---A--AEIYEV-ALRLK-ES---G--fafdYFVRAAK-LLIT---Y-----K------PF--DK--inG-QAVEELFIQIQT-N-L--A--A--Cllqekryehviy--
0040 7ztyA   ipwqaapdgigyS-----Y--P---YilalqaTDYDSQ-VEELV-RG---G------KLDEAIS-VLTX---L-----ED----ALL----------KNKTETLREVKX-Q-K--A--------------------
0041 5a1vK   qplfvsggddykikvlqpV--H---M---I--LVLRTA-LNLFF-KL---K------NFKTAAT-FARR--lL-----EL----GP-------evA-QQTRKILSACEK-N-Pt----------------------
0042 8rbkA   dllgcyeqalelF-----P--D---D---E--VICNSM-GEHLF-RM---G------FRDEAAG-YFHK--------avK----LNP---D----F-NDAKENFYRVAN-W-L--V--E--Rwhfimlndtkrnti
0043 7dkhI   --------------------qH---N---P--VLLTLL-QTYYY-FK---G------DYQTVLD-IYHH---R----iLK----XS-------ivL-SESSFWCGRAHY-A-L--G--D--Yrksfixfqeslkkn
0044 1y8mA   ---------------eaeS--R---R---R--ECLYYL-TIGCY-KL---G------EYSMAKR-YVDT---L----fEH----ER----N----N-KQVGALKSMVED-K-I--Q--K--Etlkgvvvaggvhhh
0045 7dhgC   --------------------vD---L---S--TFYQNR-AAAFE-QL---Q------KWKEVAQ-DCTK--aV-----EL----NP----K----Y-VKALFRRAKAHE-K-L--D--N--Kkecledvtavcile
0046 8qt5A   ------------------T--L---G---R--DQYVYM-AKLAE-QA---E------RYEEMVQ-FMEQ-lvT-----GA----T---eEL----T-VEERNLLSVAYK-N-V--I--G--Slraawrivssieqk
0047 4ymrA   -----------------------------V--LPELRR-AQSLT-CT---G------LYREALA-LWAN-awQ-----LQ----T--------drP-LLTLAGLAVCHQ-E-L--E--Dp-------------
0048 8g0lB   ---------------------D---P---N--DRRLRP-IYDYL-DN---G------NNKMAIQ-QADK---L----lKK----HK----D----L-HCAKVLKAIGLQ-R-T--G--Kqi------------
0049 6erpA   --------------------sG---Q---Q--QRLLAF-FKCCL-LT---D------QLPLAHH-LLVV---HhgqrqKR----KLL--------T-LDMYNAVMLGWA-R-Q--G--A--Fkelvyvlfmvkdag
0050 5an3B   -------------------------P---V--EKDLKT-AYKALyDE---K------EPLKALH-LYDE---I----lKG----SP----T----N-LTALIFKAACLE-K-L--Y--F--Gfsdwhsdatmenak
0051 2mv3A   atreqeaN----K-----D--L--tS---P--DAQAAF-YKLLL-QS---N------YPQYVVS-RFET---P-----G--------------asS-PECMELYMEALQ-R-I--G--R--Hseadavrqnlehhh
0052 3jq1B   --------------------pT---Q---G--AAYAAL-GKIYV-YE---E------NWQEAIN-VLEP---L----tQN----PYtysifelirY-ADVLLXLAEAYX-N-K--G--A--Ldtsigyinqirrra
0053 8vx7A   keemeklL----E-----E--G---D---L--DALKKA-LQLAV-NV---A--gdkeLLAHAAE-VIKR---A----lDL----AL--lqS----A-KYLASLALWIAK-R-A--G--------------------
0054 6au8A   -----rvE----G-----K--L--eA---H--QMYRTL-FFRYM-SQ---S------KHTEARE-LMYS-gaL-----LF----F---qnS----A-ADLSMLVLESLE-K-A--E--V--Evadellenlakvfs
0055 3u4tA   ---------------------------------VEFRY-ADFLF-KN---N------NYAEAIE-VFNK--lE-----AK----KY----N----S-PYIYNRRAVCYY-E-L--A--K--Ydlaqkdietyfskv
0056 6f1tf   lgtaknaN----E-----M--FriqL---I--QRVKDD-IESLH-DK---FkvwakqIDRQLTA-YMKR---V-----EdvlgkGWE--NH--veG-QKLKQDGDSFRM-K-L--N--T--Qeifddwarkvqqrn
0057 2pl2A   -------------------------A---E--QNPLRL-GVQLY-AL---G------RYDAALT-LFER--aL-----KE----NP----Q----D-PEALYWLARTQL-K-L--G--L--Vnpalengktlvart
0058 5wftA   ---------------lagA--D---D---P--ALREHA-WRLSL-QX---F------DFDSAIE-LLAP---I----gAQ----RQX--------T-DEELDALVYSHE-T-R--G--Tp-------------
0059 8gaaA   lvcvylmkelgmP-----N--E---E--lI--KLLEEL-VEVLR-IL---A--vdkrVLDKAEV-CIEE---M----eEL----GM----P----E-EKIKELREELKF-V-R--E--I--Ldki-----------
0060 5y7qA   ---------------rqsS--H---P---G---ARLRA-IRSLE-RG---E------RFAEAHA-LARE---A-----SC----AP-------aeR-QGLARLLPRLQG-K-L--G--L--Prqaraaapeidrld
0061 2lxlA   ayycRLYA------------flskvG---C--AHLENY-ALKMF-LY---A--nmikSFYTASL-LIDV---I----tVF----GEL--------T-DENVKHRKYARW-K-A--T--Y--Ihnclkngetpq---
0062 8ebfA   ---------------lppE--I---Q---R--ELRYFK-GWVDY-MS---G------DFAASLN-EWSG---F-----ED----EEE--YN----H-STLLMGKANAFF-Y-T--G--Qy-------------
```

```
0063 2bb6A   slgilALC----V--hqkR--V---H---D--SVVGKL-LYAVE-H-------vsvdTMAMAGM-AFSC---L----eLS----NL-------kqR-NRINLALKRVQE-K-I--L--K--Aqtpegyfgnvystp
0064 5cd6A   ---------------inaS--S---D---E--WVVERK-VQLLI-DK---K------QYQEAKD-LLLS---T-----HF----Q--------vhqtYTRTGLWEQINE-G-L--G--L--Spqpvpeqlgedrla
0065 4fymD   ------------E-----F--L---C---D--EEIYKS-FVHLK-DK---I--syssYIKEMKK-LLKV---V---llKY----KAlkvLN----N-IVSSNIICFLLS-E-L--I--L--Knklsfdyllgasyk
0066 2katA   ---------------aqgT--D---N---M--LLRFTL-GKTYA-EH---E------QFDAALP-HLRA---A----lDF----DP----T----Y-SVAWKWLGKTLQ-G-Q--G--D--Ragarqawesglaaa
0067 1pc2A   ivlleellpkgsK-----E--E---Q---R--DYVFYL-AVGNY-RL---K------EYEKALK-YVRG---L-----LQ----TE---PQ----N-NQAKELERLIDK-A-M--K--K--Dglvgmaivggmalg
0068 5u1sA   klyiNKWL----Q-----K-----fE---M--DFVKML-LCYLN-FN---N------FDKLSIE-LSLC---I---ksKE----KYY--SS----IvPYADNYLLEAYL-S-L--Y--M--Iddalmmknqlqktm
0069 2kckA   ---------------qldP--E---E---S--KYWLMK-GKALY-NL---E------RYEEAVD-CYNY---V--invIE----DE----Y----N-KDVWAAKADALR-Y-I--E--G--Keveaeiaearakle
0070 6yj6A   alnhfqclydetF-----S--D---V---A--DLYFEA-ATALT-RA---E------KYKEAID-FFTP---L----lSL----EEW--R-----T-TDVFKPLARCYK-E-I--E--S--Yetakefyelaikse
0072 2kc7A   -----------------------------M--DQL-KT-IKELI-NQ---G------DIENALQ-ALEE--fL-----QT----EP---VG----K-DEAYYLMGNAYR-K-L--G--D--Wqkalnnyqsaieln
0073 5wd8A   --------------------vT---E---L--TRLKEY-XEDQI-AK---A--aqlkFLENAHT-EHFV--kX-----GS----L--------rlK-IEIRSLYEEXLE-L-K--D--K--Crdqiqqyet-----
0074 8f5oC   htsvrwsetadavkgpinV--G---K---G--AILYTL-SRIAN-RL---E------MVRTARA-VFEK--lQ-----GV----IL--vsM----M-EQVDIETLLVRS-K-Pv----------------------
0075 5efrA   ---------------pdtP--W---A---D--DALVGA-MRAYI-AY---A--rqpeRYRRAVE-LYER---L--lqiFP----DS-------plL-RTAEELYTRARQ-R-L--T--E-----------------
0076 4ebaA   ckvlelgL----K-----Y--F--tD---G--EYINKY-LDFLI-YV---N------EESQVKS-LFES---S-----ID----KI-------hlL-KMIFQKVIFFES-K-V--G--S--Lnsvrtlekrffekf
0077 9l4fA   vdlfcrnsqhecqlkqleP--D---Q---I--IHYHGV-VKSML-GL---G------QLSTVIT-QVNG--vH-----AN----RS---eW----T-DELNTYRVEAAW-K-L--S--Q--Wdlvenylaadgkst
0078 6tdyJ   ---------------sgiK--P---N---A--ESWGYV-LKELV-QA---G------DFRLGWV-CIAG--mK-----SL----GIT-----------PDQALVDANEA-N-A--A--K--Akaagtdfpaylkka
0079 4bt9B   iskgnlpgvkhkS-----F--L---T---A--EDCFEL-GKVAY-TE---A------DYYHTEL-WMEQ-alR-----QL----D---tiD----K-VSVLDYLSYAVY-Q-Q--G--D--Ldkallltkklleld
0080 4uzyA   ---------------qnvP--E---S---R--AALSLL-GYCYY-YT---G------QYDMASQ-MYEQ---L----vTL----YP----S----N-EDYKLYYAQSLY-KgG--M--Y--Peaskavvkveghqk
0081 8f5pD   --------------------------------ELSVRV-GRALV-AA---H------DYAKAIR-YYQD---A----lVT----DP----H----L-SIVRADLATLQW-R-L--G--Hi----------------
0082 7t6dA   ---------------kedT--G---T---V--EAHLTL-GNLFR-SR---G------EVDRAIR-IHQT---L---meSA----SL--yeQ----R-LLAIQQLGRDYM-A-A--G--L--Ydraedmfnqltdet
0083 3as5A   -----------------dD--I---R---Q--VYYRDK-GISHA-KA---G------RYSQAVM-LLEQ--vY-----DA----D----aF----D-VDVALHLGIAYV-K-T--G--Av----------------
0084 5w94A   --------------------eE---I---M--RCEFLLlHDLPL-MR---D---skfHYKIALR-NCNE---L----vQY----MV--qnW----A-SVFQYVGVMLCI-K-L--K--Q--Hrrvktsfhgllsqc
0085 8h7gC   takepynyflllraleeipqhA---S---A--SAIIQY-GKIAR-KQ---G------LVNVALD-ILSR---I-----HT----I--------vdC-FQKIRQQVKCYL-Q-Lnl---------------------
0086 8j0nB   ---------------mtnP--H---N---H--LYCQQY-AEVKY-TQ---G---gleNLELSRK-YFAQ---A----lKL----NN----R----N-MRALFGLYMSAS-H-I--A--S--Npkasaktkkdnmky

0001 s001A   ----LLLL----L-----L--L---L---H--HHHHHH-HHHHH-HL---L------LHHHHHH-HHLL---L-----LH----HHH--HH----L-HHHHHHHHHHHH-H-H--L--L--L--------------
0002 8qgyB   hhhhhhhhhlllL-----L--L---L---H--HHHHHH-HHHHH-HL---L------LLHHHHH-HHHH---H-----HH-llllLL--LL----L-LHHHHHHHHHHH-H-H--L--L--Llhhhhhhhhhhhhh
0003 6z0fA   hhhhhhhhhhhhL-----H--H---H---H--HHHHHH-HHHHH-HL---L------LHHHHHH-HLLL---L-----LH----HHL--------L-HHHHHHHHHHHH-H-H--H--L--Llhhhhhhhhhhlll
0004 8h38H   hhhhhhhhllllL-----L--L---L---H--HHHHHH-HHHHH-HL---L------LHHHHHH-HHHH---L-----LH----HHH--HH----L-HHHHHHHHHHHH-H-H--H--L--Lhhhhhhhhhhllll
0005 4n5cD   --------------------hH---H---H--HHHHHH-HHHHH-HL---L------LHHHHHH-HHHH---L----lLL----LL---LL----L-HHHHHHHHHHHH---L--L--L--Llhhhhhhhhhhhhh
0006 6xt9D   ---------------hhlL--L---L---H--HHHHHH-HHHHH-LL---L------LHHHHHH-HHHH---H-----LL----LL---LL----L-HHHHHHHHHHHH-H-L--L--L--Hhhhhhhhhhhhhhl
0007 2yheA   lllhhHHH----H-----H-lL---H---H--HHHHHH-HHHHH-HH---L------LHHHHHH-HHHH---H----hHH----LL----L----L-HHHHHHHHHHHH-H-H--H--H--Hlllhhhhhhhhhhh
0008 6vbu8   --------------------------------LLHHHH-HHHHH-HL---L------LLLHHHH-HHHH---H----hHH----LL----L----L-HHHHHHHHHHHH-H-H--L--L--Llllllllllhhhhh
0009 5vxvA   --------------------------------LHHHHH-HHHHH-HL---L------LHHHHHH-HHHHhllL-----LH----HHH--HH----L-HHHHHHHHHHHH-H-L--L--L--Lllllhhhhhhhhhh
0010 5a63D   ------lH----H-----H--L---L---H--HHHHHH-HHHHH-HH---L--llhhHHHHHHH-LLHH---H---hhHL----LL-------llH-HHHHHHHHHHHH-H-H--H--H--Hhhhhhhhhhhhhhh
0011 2uy1A   ---------------------L---L---H--HHHHHH-HHHHH-HL---L------LHHHHHH-HHHH---H-----LL----LL----L----L-HHHHHHHHHHHH-H-H--L--------------------
0012 6z2wE   hhhhhhllllllllllllL--L---L---L--HHHHHH-HHHHH-HL---L------LHHHHHLlLLLL------lllLL----LL-------llH-HHHHHHHHHHHH-H-L--L--L--Hhhhhlhhhhhllll
0013 2c2lA   ------------------L--L---L---H--HHHHHH-HHHHH-HL---L------LHHHHHH-HHHH--hH-----HH----LL----L----L-HHHHHHHHHHHH-H-L--L--Lh----------------
0014 8sxfC   hhhhhhhL----H-----H--H--hL---L--HHHHHH-HHHHH-HH---L------LHHHHHH-HHHH---H-----hH----HLL---L----L-HHHHHHHHHHHH-H-H--L--L--Lhhhhhhhhhhhhhl
0015 8s4gJ   ---------------hhhH--L---L---H--HHHHHH-HHHHH-HL---L------LHHHHHH-HHHH---L------------LL--LL------HHHHHHHHHHHH-H-L--L--Lh----------------
0016 4ribB   --------------------hH---H---H--HHHHHH-HHHHH-HL---L------LHHHHHH-HHHH-hhH-----HH----H--------hhH-HHHHHHHHHHHH-H-L--L--L--Hhhhhhhhhhhhhll
0017 6sliA   ---------------lllL--L---H---H--HHHHHH-HHHHH-HH---L------LHHHHHH-HHHH---H----hLL----LL--llllheeH-HHHHHHHHHHHH-H-H--L--Ll----------------
0018 2btpA   llllLLLLlhhhH-----L--L---L---H--HHHHHH-HHHHH-HL---L------LHHHHHH-HHHH-hhH-----LL----L----LL----L-HHHHHHHHHHHH-H-H--H--H--Hhhhhhhhhhhhhhh
0019 9lthA   --------------------lL---H---H--HHHHHH-HHHHH-HL---L------LHHHHHH-HHHH---L----lLL----LL--hHH----H-HHHHHHHHHHHH-H-L--L--Ll----------------
0020 3l22A   --------------------lL---H---H--HHHHHH-HHHHH-HL---L------LHHHHHH-HHHL---L-----LL----LL-------eeH-HHHHHHHHHHHH-L-L--L--Ll----------------
0021 4cguA   --------------------lL---H---H--HHHHHH-HHHHH-HH---L------LHHHHHH-HHHH--hH-----HH----LL----L----L-HHHHHHHHHHHH-H-L--L--Lh----------------
0022 2grmA   hhhhHHLL----L-----L-----hH---H--HHHHHH-HHHHH-HL---L------LHHHHHH-HHHH--hH-----HH----L--------hhH-HHHHHHHHHHHH-H-H--H--L--Lhhhhhhhhhhhhhh
0023 6hs5A   ---------------lllL--L---L---L-lLHHHHH-HHHHH-LLlllL------LHHHHHH-HHHH---H----hHH----LL----L----L-HHHHHHHHHHHH-H-H--H--L--Hhhhhhhhhhhhhhh
0024 2o8pA   --------------------lL---H---H--HHHHHH-HHHHH-HL---L------LHHHHHH-HHHH--hH-----HH----HH--llL----L-HHHHHHHHHHHH-H-H--H--H--Hhhhhhhhhhhhhhh
0025 2vkjA   hhhhhLLH----H-------hH---H---H--HHHHHH-HHHHH-HL---L------LHHHHHH-HHHH-hhH-----HL----LL--hHH----H-HHHHHHHHHHHH-H-H--H--Ll----------------
0026 4m57A   hhhhHLLL----L--lhhH--L---L---H--HHLHHH-HHHHH-HL---L------LHHHHHH-HHHH---H---hlLL----LLL--------L-HHHHHHHHHHHH-H-H--L--L--Lhhhhhhhhhhhhhl
0027 5vbgA   ---------------llhH--H---H---H--HHHHHH-HHHHH-HL---L------LHHHHHH-HHHL---L-----LH----HH-------hhH-HHHHHHHHHHHH-H-L--L--L--Hhhhhhhhhhhhhll
0028 8hmdC   --------------------hH---H---H--HHHHHH-HHHHH-HH---L------LHHHHHH-HHHH---H----hHH----HL---------L-HHHHHHHHHHHH-H-L--L--L--Hhhhhhhhhlllllh
0029 5xw7C   --------------------lL---H---H--HHHHHH-HHHHH-HL---L------LHHHHHH-HHHH--hH-----HH----LL----L----L-HHHHHHHHHHHH-H-L--L--Lh----------------
0030 8cbkE   hhhhhhhhhhhhL-----L--L---L---L--LHHHHH-HHHHL-LL---L------LHHHHHH-HHHH---H----hHH----LLL--------L-HHHHHHHHHHHHhL-L--L--H--Hhhhhhhhhhhhhll
0031 2pziA   --------------------lL---L---H--HHHHHH-HHHHH-HH---L------LHHHHHH-HHHH---H----hHH----LL----L----L-LHHHHHHHHHHH-H-H--L--L--Lllllhhhhhhhhll
0032 8rg0u   -----LLL----L-----L--L---L---H--HHHHHH-HHHHH-HH---L------LHHHHHH-HHHH---H-----HH--------llL----H-HHHHHHHHHHHH-H-L--Lhl------------------
0033 7y4iA   -------------------------------lLHHHHH-HHHHH-HL---L------LHHHHHH-HHHH---H----hHH----LL----L----L-HHHHHHHHHHHH-H-L--L--L--Hhhhhhhhhhhhhhl
0034 7tj4A   ------------------------lL---L--LHHHHH-HHHHH-HL---L------LHHHHHH-HHHH---H----hHL----LLL--------L-HHHHHHHHHHHH-H-H--L--Lh----------------
0035 8q9tE   ---------------hhlL--L---L---H--HHHHHH-HHHHH-HL---L------LLLLLHH-HHHH-hhH-----HH----H---hhH----H-HHHHHHHHHHHH-H-H--L--L--Llhhhhhhhhhhhhh
0036 3qdnA   ---------------hhlL--L---L---H--HHHHHH-HHHHH-HL---L------LHHHHHH-HHLL---L-----LH----HHL--------L-HHHHHHHHHHHH-H-H--H--H--Lllhhhhhhhhhhhl
0037 8araC   ------------L-----L--L---LhhhH--HHHHHH-HHHHH-HL---L------LHHHHHH-HHHH---H-----hH----HLL---L----L-HHHHHHHHHHHH-HlL--L--H--Hhhhhhhhhhhhhll
0038 9dt9A   ---------------------L---H---H--HHHHHH-HHHHH-HH---H--llhhHHHHHHH-HHHH--hH-----HH----LL----L----L-LLHHHHHHHHHH-H-H--H--H--Hhhhhhhhhhhhhhh
0039 4it4B   ---------------lhhH--L---L---H--HHHHHH-HHHHH-HH---H--hhhhHHHHHHH-HHHL---L-----L------LL--LL--llH-HHHHHHHHHHHH-H-H--H--H--Hhhhhhhllllll--
0040 7ztyA   lllllllleeeeE-----L--L---EeeeeeleLLHHHH-HHHHH-HL---L------LHHHHHH-HHHH---L-----LH----HHL----------LLHHHHHHHHHH-H-H--L--------------------
0041 5a1vK   lleeeeelllleeeellhH--H---H---H--HHHHHH-HHHHH-HH---L------LHHHHHH-HHHH--hH-----HH----LL-------hhH-HHHHHHHHHHHH-L-Ll----------------------
0042 8rbkA   hhhhhhhhhhhhL-----L--L---L---H--HHHHHH-HHHHH-HL---L------LHHHHHH-HHHH--------hhH----HLL---L----L-HHHHHHHHHHHH-H-H--L--L--Hhhhhhhllhhhhhh
0043 7dkhI   --------------------lL---L---H--HHHHHH-HHHHH-HL---L------LHHHHHH-HHHH---L----lHH----HH-------hhH-HHHHHHHHHHHH-H-L--L--L--Hhhhhhhhhhhhhhl
0044 1y8mA   ---------------hllL--L---H---H--HHHHHH-HHHHH-LL---L------LHHHHHH-HHHH---H----hHL----LL----L----L-HHHHHHHHHHHH-H-H--H--H--Lllllllllllllll
0045 7dhgC   --------------------hH---H---H--HHHHHH-HHHHH-HL---L------LHHHHHH-HHHH--hH-----HH----LL----L----L-HHHHHHHHHHHH-H-L--L--L--Hhhhhhhhhhhhhhl
0046 8qt5A   ------------------L--L---L---H--HHHHHH-HHHHH-HL---L------LHHHHHH-HHHH-hhH-----HH----L---lLL----L-HHHHHHHHHHHH-H-H--H--H--Hhhhhhhhhhhhhhh
0047 4ymrA   -----------------------------L--HHHHHH-HHHHH-HH---L------LHHHHHH-HHHH-hhH-----HH----H--------lhH-HHHHHHHHHHHH-H-L--L--Lh----------------
0048 8g0lB   ---------------------L---H---H--HHHHHH-HHHHH-HL---L------LHHHHHH-HHHH---H----hHH----LL----L----L-HHHHHHHHHHHH-H-L--L--Lll---------------
0049 6erpA   --------------------lH---H---H--HHHHHH-HHHHH-HH---L------LHHHHHH-HHHH---HhhlhhHH----LLL--------L-HHHHHHHHHHHH-H-H--L--L--Hhhhhhhhhhhhhll
0050 5an3B   -------------------------L---H--HHHHHH-HHHHHhLL---L------LHHHHHH-HHHH---H----hHH----LL----L----L-HHHHHHHHHHHH-H-H--H--L--Llhhhllhhhhhhhh
0051 2mv3A   hhhhhhhH----H-----L--L--lL---H--HHHHHH-HHHHH-HL---L------LHHHHHH-HHLL---L-----L--------------llL-HHHHHHHHHHHH-H-L--L--L--Hhhhhhhhhhhhlll
0052 3jq1B   --------------------lL---H---H--HHHHHH-HHHHH-HL---L------LHHHHHH-HHHH---H----hLL----LLlleeeeeeeH-HHHHHHHHHHHH-H-H--L--L--Hhhhhhhhhhhhhhl
0053 8vx7A   hhhhhhhH----H-----H--L---L---H--HHHHHH-HHHHH-HH---H--llhhHHHHHHH-HHHH---H----hHH----HH--hhH----H-HHHHHHHHHHHH-H-H--L--------------------
0054 6au8A   -----lhH----H-----H--H--hH---H--HHHHHH-HHHHH-HL---L------LHHHHHH-HHHH-hhH-----HH----H---hhH----H-HHHHHHHHHHHH-H-L--L--L--Llhhhhhhhhhhhhh
0055 3u4tA   ---------------------------------LHHHH-HHHHH-LL---L------LHHHHHH-HHHH--hH-----HL----LL----L----L-LLLHHHHHHHHH-H-L--L--L--Hhhhhhhhhhhhlll
0056 6f1tf   hhhlllhH----H-----H--HhhhH---H--HHHHHH-HHHHH-HH---HhlhhhhHHHHHHH-HHHH---H-----HhhhllLLL--LL--llH-HHHHHHHHHHHH-H-L--L--L--Hhhhhhhhhhhllll
0057 2pl2A   -------------------------L---L--HHHHHH-HHHHH-HL---L------LHHHHHH-HHHH--hH-----LL----LL----L----L-HHHHHHHHHHHH-H-L--L--L--Hhhhhhhhhhhhhhl
```

```
0058 5wftA   ---------------hhhL--L---L---H--HHHHHH-HHHHH-HL---L------LHHHHHH-HHLL---H----hHH----LLL--------L-HHHHHHHHHHHH-H-L--L--Lh----------------
0059 8gaaA   hhhhhhhhhhllL-----H--H---H--hH--HHHHHH-HHHHH-HH---H--lllhHHHHHHH-HHHH---H----hHH----LL----L----H-HHHHHHHHHHHH-H-H--H--H--Hlll-----------
0060 5y7qA   ---------------hllL--L---L---L---HHHHH-HHHHH-HL---L------LHHHHHH-HHHH---H-----HH----LL-------hhH-HHHHHHHHHHHH-H-L--L--L--Llllllllllleee
0061 2lxlA   hhhhHHHH------------hhhhhH---H--HHHHHH-HHHHH-HH---H--hhhhHHHHHHH-HHHH---H----hLL----LLL--------L-HHHHHHHHHHHH-H-H--H--H--Hhhhhhllllll---
0062 8ebfA   ---------------llhH--H---H---H--HHHHHH-HHHHH-HL---L------LHHHHHH-HHLL---L-----LH----HHH--LL----L-HHHHHHHHHHHH-H-L--L--Lh----------------
0063 2bb6A   hhhhhHHH----H--lllL--L---L---H--HHHHHH-HHHHH-L-------lhhhHHHHHHH-HHHH---H----hHH----LL-------hhH-HHHHHHHHHHHH-H-H--H--H--Llllllllllllhh
0064 5cd6A   ---------------lllL--L---L---H--HHHHHH-HHHHH-HL---L------LHHHHHH-HHHL---L-----LL----L-------llllLHHHHHHHHHHHH-H-L--L--L--Llllllhhhllllll
0065 4fymD   ------------L-----L--L---L---H--HHHHHH-HHHHH-HH---H--hhhhHHHHHHH-HHHH---H---hhHL----LLeehHL----L-HHHHHHHHHHHH-H-H--H--H--Hlllllleeeellll
0066 2katA   ---------------lllL--L---L---H--HHHHHH-HHHHH-HL---L------LHHHHHH-HHHH---H----hHH----LL----L----L-HHHHHHHHHHHH-H-H--L--L--Hhhhhhhhhhhhhhh
0067 1pc2A   hhhhhhhhhhllH-----H--H---H---H--HHHHHH-HHHHH-HL---L------LHHHHHH-HHHH---H-----HH----HL---LL----L-HHHHHHHHHHHH-H-H--H--H--Lllllllllllllll
0068 5u1sA   hhhhHHLH----H-----H-----hH---H--HHHHHH-HHHHH-HL---L------LHHHHHH-HHHH---H---hhLH----HHH--HH----HhHHHHHHHHHHH-H-L--L--L--Hhhhhhhhhhhhhhl
0069 2kckA   ---------------hhlL--L---L---H--HHHHHH-HHHHH-HL---L------LHHHHHH-HHHH---H--hhlLL----LL----L----L-HHHHHHHHHHHL-L-L--L--L--Llhhhhhhhhhhhhh
0070 6yj6A   hhhhhhhhhlllL-----L--L---L---H--HHHHHH-HHHHH-HL---L------LHHHHHH-HHHH---H----hHL----LLL--L-----L-HHHHHHHHHHHH-H-L--L--L--Hhhhhhhhhhhllll
0072 2kc7A   -----------------------------L--LLH-HH-HHHHH-HH---L------LHHHHHH-HHHH--hH-----HH----LL---LL----H-HHHHHHHHHHHH-H-H--L--L--Hhhhhhhhhhhhhhl
0073 5wd8A   --------------------hH---H---H--HHHHHH-HHHHH-HH---H--hhhhHHHHHHH-HHHH--hH-----HL----H--------hhH-HHHHHHHHHHHH-H-H--H--H--Hhhhhhhhhl-----
0074 8f5oC   lleeeeeeellllllllllL--L---H---H--HHHHHH-HHHHH-HL---L------LHHHHHH-HHHH--hL-----LL----LL--llL----H-HHHHHHHHHHHH-L-Ll---------------------
0075 5efrA   ---------------lllL--H---H---H--HHHHHH-HHHHH-HH---H--hhhhHHHHHHH-HHHH---H--hhhLL----LL-------llH-HHHHHHHHHHHH-H-H--H--L-----------------
0076 4ebaA   hhhhhhlH----H-----H--H--lL---H--HHHHHH-HHHHH-HH---L------LHHHHHH-HHLL---L-----LL----LL-------hhH-HHHHHHHHHHHH-H-H--L--L--Hhhhhhhhhhhhhhl
0077 9l4fA   lllllllllllllllhhlL--L---L---H--HHHHHH-HHHHH-HH---L------LHHHHHH-HHHH--hH-----HH----LL---lL----L-LLLHHHHHHHHH-H-H--L--L--Hhhhhhhhhllllll
0078 6tdyJ   ---------------lllL--L---L---H--HHHHHH-HHHHH-HH---L------LHHHHHH-HHHH--hL-----LL----LLL----------LLHHHHHHHHHH-H-H--H--H--Hhhllllllhhhhll
0079 4bt9B   hhhlllllllllL-----L--L---L---H--HHHHHH-HHHHH-HL---L------LHHHHHH-HHHH-hhH-----HH----H---llL----H-HHHHHHHHHHHH-H-L--L--L--Hhhhhhhhhhhhhhl
0080 4uzyA   ---------------hhlL--L---L---H--HHHHHH-HHHHH-HH---L------LHHHHHH-HHHH---H----hHH----LL----L----L-HHHHHHHHHHHH-HlL--L--H--Hhhhhhhlllllllhh
0081 8f5pD   --------------------------------LLHHHH-HHHHH-HL---L------LLHHHHH-HHHH---H----hHH----LL----L----L-HHHHHHHHHHHH-H-L--L--Lh----------------
0082 7t6dA   ---------------lllL--L---L---H--HHHHHH-HHHHH-HH---L------LHHHHHH-HHHH---H---hlLL----LL--hhH----H-HHHHHHHHHHHH-H-H--L--L--Hhhhhhhhhhhlllh
0083 3as5A   -----------------lL--H---H---H--HHHHHH-HHHHH-HH---L------LHHHHHH-HHLL--lL-----LL----L----lL----L-HHHHHHHHHHHH-H-L--L--Lh----------------
0084 5w94A   --------------------hH---H---H--HHHHHHhLHHHH-HH---L---lhhHHHHHHH-HHHH---H----hHH----HL--hhH----H-HHHHHHHHHHHH-H-L--L--L--Hhhhhhhhhhhhhhl
0085 8h7gC   eelllhhhhhhhhhhhhlllhH---H---H--HHHHHH-HHHHH-HL---L------LLHHHHH-HHHH---H-----HH----L--------hhH-HHHHHHHHHHHH-H-Lll---------------------
0086 8j0nB   ---------------hhlL--L---L---H--HHHHHH-HHHHH-HH---L---lhhHHHHHHH-HHHH---H----hHH----HL----L----L-LHHHHHHHHHHH-H-H--H--H--Llllllhhhhhhhhhh

Job: Type-3 BREX; MTase MBW2068239 wHTH
Query: s001A
No: Chain Z rmsd lali nres id PDB Description
1: 6qfd-B 6.6 3.1 55 116 5 MOLECULE: DNA-BINDING PROTEIN;
2: 6zvh-y 6.4 2.4 55 72 16 MOLECULE: 18S RRNA;
3: 8qkf-A 6.4 2.3 50 99 14 MOLECULE: ARSR FAMILY TRANSCRIPTIONAL REGULATOR;
4: 5dym-A 6.3 3.7 56 96 13 MOLECULE: PADR-FAMILY TRANSCRIPTIONAL REGULATOR;
5: 5j6x-A 6.2 2.7 51 66 12 MOLECULE: Z-DNA BINDING PROTEIN KINASE;
6: 7wjp-A 6.2 3.1 54 98 13 MOLECULE: PADR FAMILY TRANSCRIPTIONAL REGULATOR;
7: 8jxk-D 6.1 2.9 55 166 15 MOLECULE: CONSERVED PROTEIN;
8: 1ldd-A 6.1 2.8 57 74 9 MOLECULE: ANAPHASE PROMOTING COMPLEX;
9: 2od5-A 6.1 1.6 51 91 14 MOLECULE: HYPOTHETICAL PROTEIN;
10: 1sfx-A 6.0 3.5 51 109 8 MOLECULE: CONSERVED HYPOTHETICAL PROTEIN AF2008;
11: 2esh-A 6.0 4.0 60 114 8 MOLECULE: CONSERVED HYPOTHETICAL PROTEIN TM0937;
12: 5a31-N 6.0 1.7 51 703 10 MOLECULE: ANAPHASE-PROMOTING COMPLEX SUBUNIT 1;
13: 3elk-A 6.0 3.1 55 104 15 MOLECULE: PUTATIVE TRANSCRIPTIONAL REGULATOR TA0346;
14: 8soj-B 6.0 2.3 53 428 8 MOLECULE: CST COMPLEX SUBUNIT CTC1;
15: 5zqh-A 6.0 2.3 54 101 9 MOLECULE: PADR FAMILY TRANSCRIPTIONAL REGULATOR;
16: 5jls-A 6.0 2.8 51 134 6 MOLECULE: ADHESIN COMPETENCE REPRESSOR;
17: 2zkz-C 5.9 2.2 50 87 8 MOLECULE: TRANSCRIPTIONAL REPRESSOR PAGR;
18: 5xpq-A 5.9 2.3 49 100 12 MOLECULE: UNCHARACTERIZED HTH-TYPE TRANSCRIPTIONAL REGULATO
19: 4ejo-A 5.9 4.0 54 112 13 MOLECULE: TRANSCRIPTIONAL REGULATOR, PADR-LIKE FAMILY;
20: 7z8b-C 5.9 2.2 58 1224 9 MOLECULE: CULLIN-7;
21: 4omz-C 5.9 2.4 50 100 10 MOLECULE: NOLR;
22: 5hs7-A 5.9 3.2 53 102 17 MOLECULE: HTH-TYPE TRANSCRIPTIONAL REGULATOR YODB;
23: 8uuc-A 5.8 2.6 54 274 15 MOLECULE: ADENINE DNA GLYCOSYLASE;
24: 8iue-P 5.8 2.5 52 303 6 MOLECULE: DNA-DIRECTED RNA POLYMERASE III SUBUNIT RPC1;
25: 3mq0-B 5.8 1.7 48 248 13 MOLECULE: TRANSCRIPTIONAL REPRESSOR OF THE BLCABC OPERON;
26: 3bdd-A 5.8 2.8 52 140 13 MOLECULE: REGULATORY PROTEIN MARR;
27: 2l4m-A 5.7 1.7 49 69 10 MOLECULE: UNCHARACTERIZED PROTEIN;
28: 6uvu-B 5.7 1.9 48 114 8 MOLECULE: ARSR FAMILY TRANSCRIPTIONAL REGULATOR;
29: 2rdp-A 5.7 2.4 50 140 16 MOLECULE: PUTATIVE TRANSCRIPTIONAL REGULATOR MARR;
30: 2oqg-A 5.7 1.8 49 109 10 MOLECULE: POSSIBLE TRANSCRIPTIONAL REGULATOR, ARSR FAMILY P
31: 5zi8-A 5.7 3.5 57 104 9 MOLECULE: TRANSCRIPTIONAL REGULATOR;
32: 2fe3-B 5.7 3.2 56 143 7 MOLECULE: PEROXIDE OPERON REGULATOR;
33: 7mex-A 5.7 3.1 55 1737 20 MOLECULE: UBIQUITIN;
34: 7z1n-O 5.6 3.1 53 570 9 MOLECULE: DNA-DIRECTED RNA POLYMERASE III SUBUNIT RPC1;
35: 2xrn-B 5.6 1.4 48 241 2 MOLECULE: HTH-TYPE TRANSCRIPTIONAL REGULATOR TTGV;
36: 3s93-B 5.6 3.0 58 81 16 MOLECULE: TUDOR DOMAIN-CONTAINING PROTEIN 5;
37: 6j05-A 5.5 2.6 49 101 6 MOLECULE: TRANSCRIPTIONAL REGULATOR ARSR;
38: 3l9f-A 5.5 3.5 57 170 11 MOLECULE: PUTATIVE UNCHARACTERIZED PROTEIN SMU.1604C;
39: 2wte-A 5.5 1.6 48 212 13 MOLECULE: CSA3;
40: 3b73-B 5.5 2.0 49 89 6 MOLECULE: PHIH1 REPRESSOR-LIKE PROTEIN;
41: 2nyx-B 5.5 2.8 50 147 8 MOLECULE: PROBABLE TRANSCRIPTIONAL REGULATORY PROTEIN, RV14
42: 6jyi-A 5.5 3.6 56 174 13 MOLECULE: TRANSCRIPTIONAL REPRESSOR PADR;
43: 3f6v-A 5.5 2.2 49 96 18 MOLECULE: POSSIBLE TRANSCRIPTIONAL REGULATOR, ARSR FAMILY P
44: 5fmf-V 5.5 2.7 51 174 10 MOLECULE: DNA REPAIR HELICASE RAD25, SSL2;
45: 6lui-A 5.5 2.4 53 71 13 MOLECULE: ATHERIN;
46: 1xmk-A 5.4 2.4 51 79 12 MOLECULE: DOUBLE-STRANDED RNA-SPECIFIC ADENOSINE DEAMINASE;
47: 2eth-A 5.4 2.8 52 141 8 MOLECULE: TRANSCRIPTIONAL REGULATOR, PUTATIVE, MAR FAMILY;
48: 2p4w-B 5.4 2.7 50 198 18 MOLECULE: TRANSCRIPTIONAL REGULATORY PROTEIN ARSR FAMILY;
49: 3u21-A 5.4 2.6 58 109 12 MOLECULE: NUCLEAR FACTOR RELATED TO KAPPA-B-BINDING PROTEIN
```

```
50: 3l7w-A 5.4 2.4 54 106 7 MOLECULE: PUTATIVE UNCHARACTERIZED PROTEIN SMU.1704;
51: 7xjg-J 5.3 2.6 53 306 11 MOLECULE: RNA-DIRECTED DNA POLYMERASE FROM RETRON EC86;
52: 8ioi-A 5.3 4.0 56 190 13 MOLECULE: PADR FAMILY TRANSCRIPTIONAL REGULATOR;
53: 3gfi-A 5.3 3.6 53 143 9 MOLECULE: 146AA LONG HYPOTHETICAL TRANSCRIPTIONAL REGULATOR
54: 4ija-A 5.3 1.7 47 363 4 MOLECULE: XYLR PROTEIN;
55: 8xt8-B 5.3 2.9 51 145 12 MOLECULE: MARR FAMILY TRANSCRIPTIONAL REGULATOR;
56: 6j0e-B 5.3 2.3 49 125 18 MOLECULE: ARSENIC RESPONSIVE REPRESSOR ARSR;
57: 3k69-A 5.3 1.9 49 151 12 MOLECULE: PUTATIVE TRANSCRIPTION REGULATOR;
58: 7wze-A 5.3 1.7 48 161 15 MOLECULE: UNCHARACTERIZED HTH-TYPE TRANSCRIPTIONAL REGULATO
59: 3fhz-D 5.3 3.5 56 167 18 MOLECULE: ARGININE REPRESSOR;
60: 4xrf-A 5.3 2.3 50 142 10 MOLECULE: TRANSCRIPTIONAL REGULATOR, MARR FAMILY;
61: 6v9i-C 5.3 2.5 55 730 13 MOLECULE: IMMUNOGLOBULIN G-BINDING PROTEIN G,CULLIN-5;
62: 3f8f-A 5.3 3.5 54 114 13 MOLECULE: TRANSCRIPTIONAL REGULATOR, PADR-LIKE FAMILY;
63: 7el3-A 5.2 2.6 50 138 8 MOLECULE: HOMOPROTOCATECHUATE DEGRADATION OPERON REGULATOR
64: 2g9w-A 5.2 2.3 52 119 13 MOLECULE: CONSERVED HYPOTHETICAL PROTEIN;
65: 3f72-B 5.2 2.2 49 108 4 MOLECULE: CADMIUM EFFLUX SYSTEM ACCESSORY PROTEIN;
66: 2mh2-A 5.2 2.5 50 64 14 MOLECULE: HOMOLOGOUS-PAIRING PROTEIN 2 HOMOLOG;
67: 5okc-B 5.2 2.8 55 377 11 MOLECULE: SISTER CHROMATID COHESION PROTEIN DCC1;
68: 2cwe-A 5.2 2.6 49 191 10 MOLECULE: HYPOTHETICAL TRANSCRIPTION REGULATOR PROTEIN, PH1
69: 7vwv-B 5.2 3.3 55 74 24 MOLECULE: I73R;
70: 7kbf-K 5.2 3.1 54 78 15 MOLECULE: HISTONE H3.2;
71: 7asv-A 5.2 3.8 62 155 11 MOLECULE: DNA-DIRECTED RNA POLYMERASE III SUBUNIT RPC5;
72: 1yg2-A 5.2 2.4 50 169 10 MOLECULE: GENE ACTIVATOR APHA;
73: 6o8o-B 5.2 2.4 50 108 8 MOLECULE: TRANSCRIPTIONAL REGULATOR, ARSR FAMILY;
74: 3v4g-A 5.2 3.1 54 145 9 MOLECULE: ARGININE REPRESSOR;
75: 4hqe-B 5.2 3.2 53 108 9 MOLECULE: TRANSCRIPTIONAL REGULATOR QSRR;
76: 3u2r-A 5.1 2.5 49 135 8 MOLECULE: REGULATORY PROTEIN MARR;
77: 5n35-A 5.1 1.7 49 60 2 MOLECULE: POLB1 BINDING PROTEIN 2 (PBP2);
78: 4txa-A 5.1 3.0 59 367 7 MOLECULE: ROQUIN-1;
79: 6a6i-A 5.1 3.1 57 94 14 MOLECULE: EXCISION REPAIR CROSS-COMPLEMENTING RODENT REPAIR
80: 3bpx-A 5.1 3.0 51 147 16 MOLECULE: TRANSCRIPTIONAL REGULATOR;
81: 4yif-B 5.1 3.2 51 141 10 MOLECULE: MARR FAMILY PROTEIN RV0880;
82: 6yj6-B 5.1 2.7 55 508 7 MOLECULE: TRANSCRIPTION FACTOR TAU 131 KDA SUBUNIT;
83: 7bzd-A 5.1 3.3 54 110 17 MOLECULE: HTH-TYPE TRANSCRIPTIONAL ACTIVATOR HXLR;
84: 2gai-A 5.1 2.3 51 581 8 MOLECULE: DNA TOPOISOMERASE I;
85: 5z7b-B 5.1 3.1 55 197 15 MOLECULE: PADR FAMILY TRANSCRIPTIONAL REGULATOR;

0001 s001A   --EASAIQWLKQQLT-K-----K---P-Q-T-FQELQ-PQFL-R-EL----HK--AKH---EK--L--P---ELIELLEQ-----N---------F-----LR-Y-DGKGPIP-------KDRWYV------
0002 6qfdB   rdLTAFQKNILTVLG-E-----E---A-R-Y-GLAIK-RELE-E-YY----G-----------eevnhG---RLYPNLDD-----L----vnkglV-----EK-S-ELDK---------rTNEYALtnegfd
0003 6zvhy   --kFNWKGTIKAILK-Q-----A--nE-I-T-IKKLR-KKVL-A-QY----Y--dEHH---RSeeellV---IFNKKISK-----N-------ptF-----KL-L-K-------------DKVKLvk----
0004 8qkfA   alAAPLRIAIVLQLK-Q-----S---Q-R-C-VHELV-DALD----------------------vpqP---LVSQHLRI-----L----kqagvV-----SS-E-RAG----------rEVLYRLvdhhla
0005 5dymA   ---GYIDILIVSILE-K-----K---D-C-Y-GYEIA-KQVR-E-RS----EF-----------lkeG---TMYLALKR-----M----esknlI-----KS-Y-YSNEQssgg----rRKYYNLtnegkd
0006 5j6xA   saENEIEMRICDYLR-R----hG---R-S-T-VQDIF-KELK----------------------lekS---TVNRHLYS-----L----qaskqV-----FK-T-VEDN---------kRPVWDLve----
0007 7wjpA   fkKGVLELCCLFLIQ-K-----K---D-C-Y-GYELA-NQVS-K-YI----E---------------vaegAIYPVLRR-----L----vkeeyC-----ST-Y-LVES----p----sRKYYQLtvkgei
0008 8jxkD   --EFMLELAILGLLI-E-----S---P-M-H-GYELR-KRLT-G-LL----G---------afrafsyG---SLYPALRR-----M----qadglI-----AE-N-AAPAG---------rRVYQLtdkgrr
0009 1lddA   ltLQRSLPFIEGMLT-N----lG---A-M-K-LHKIH-SFLK-I-TV----PK--D--wgyNR--I--T-lqQLEGYLNT-----L----adegrL-----KY-I-A-------------NGSYEIv-----
0010 2od5A   xkTVRIREKIKKFLG-D-----R---P-R-N-TAEIL-EHIN-S-TX----RH-------------gttsqQLGNVLSK-----D-------kdI-----VK-V-GYikrsgilsggydicEWATrnwvae
0011 1sfxA   lsFKPSDVRIYSLLL-E-----R--gG-X-R-VSEIA-RELD----------------------lsarFVRDRLKV-----L----lkrgfV-----RR-E-IVEKG---w----vGYIYSAekpekv
0012 2eshA   frGWWLASTILLLVA-E-----K---P-S-H-GYELA-ERLA-E-FG----IE--IPG---I------ghmgNIYRVLAD-----L----eesgfL-----ST-E-WDTTV--sp----pRKIYRItpqgkl
0013 5a31N   eeLLLFWTYIQAMLT-N-----L--eS-L-S-LDRIY-NMLR-M-FV----V------tgpalaeidlQ---ELQGYLQK-----K----vrdqqL-----VY-S---------------AGVYRLp-----
0014 3elkA   ilHGLITLYILKELV-K-----R---P-X-H-GYELQ-KSXF-E-TT----GQ-------------alpqgSIYILLKT-----X----kergfV-----IS-E-SSVN--kgq----qLTVYHItdagkk
0015 8sojB   reDKDLHRKIHRIIQ-Q-----D--kG-C-H-FLHIL-ACAR-LsIR----PG------------lseA---VLQQVLEL-----L----edqsdI-----VS-T-M-------------EHYYTA------
0016 5zqhA   llKGVLEGCVLDMIG-Q-----K---E-R-Y-GYELV-QTLR-EaGF----DT-------------ivG---TIYPLLQK-----L----eknqwI-----RG-D-MR-pspdgp----dRKYFSLmkegee
0017 5jlsA   qkLTNTQEHILMLLS-Q-----Q---R-L-T-NTDLA-KALN----------------------isqA---AVTKAIKS-----L----vkqdmL-----AG-T-KDTV---da----rVTYFELtelakp
0018 2zkzC   txAHPXRLKIVNELY-K-----H--kA-L-N-VTQII-QILK----------------------lpqS---TVSQHLCK-----X-----rgkvL-----KR-N-RQG----------lEIYYSInnpkve
0019 5xpqA   tlaHPARIRILELLV-E-----R---D-R-S-VGELLsSDVG----------------------lesS---NLSQQLGV-----L----rragvV-----AA-R-RDG----------nAMIYSIaapdia
0020 4ejoA   lrRGTLVXLVLSQLR-------E---P-A-Y-GYALV-KSLA-D-HG----------------ipieaN---TLYPLXRR-----L----esqglL-----AS-E-WDNGG--sk----pRKYYRTtdeglr
0021 7z8bC   krRNLLNCLIVRILK-A-----H--eG-L-H-IDQLV-CLVL-E-AW----QkgsLGK---GS-acssT---DVLSCILH-----L----lgkgtL-----RR-H-DD-----------rPQVLSYav----
0022 4omzC   amANPKRLLILDSLV-K-----E---E-M-A-VGALA-NKVG----------------------lsqS---ALSQHLSK-----L----raqnlV-----ST-R-RDA----------qTIYYSSssdsvm
0023 5hs7A   llgKRWNGLIIHVLM-D-----G---P-K-R-FKEIT-ETIP-M-I-------------------sqK---MLAERLKE-----L----eqneiV-----ER-Q-VLPET---p----vKVIYTLtekgta
0024 8uucA   gsRRQKRAELVRLVL-A-----E---PgI-G-IDELA-ERLD-A-FE--rdAG--RK-------gvdaA---TFTSIVAD-----L----vaegfF-----RR-E---------------GDAFFA------
0025 8iueP   -dPVEIENRIIELCH-Q-----F--hG-I-T-DQVIQ-NEMP-H-I-------------------eaQ---QRAVAINR-----L----lsmgqL-----DL-L-RSN----------tGLLYRIkdsqna
0026 3mq0B   vpALRRAVRILDLVA-G-----S--rD-L-T-AAELT-RFLD----------------------lpkS---SAHGLLAV-----M----teldlL-----AR-S-A-------------DGTLRIgphslr
0027 3bddA   lgISLTRYSILQTLL-K-----D---ApL-H-QLALQ-ERLQ----------------------idrA---AVTRHLKL-----L----eesgyI-----IR-K-RNPDN---q----rEVLVWPteqare
0028 2l4mA   qfSQQREEDIYRFLK-D----nG---P-Q-R-ALVIA-QALG-M-------------------rtakDVNRDLYR-----M----ksrhlL-----DM-D-EQ------------SKAWTIyr----
0029 6uvuB   vlaNDTRLRLLHALA-R-----S--gG-L-C-VTDLA-AAVG----------------------mkpqAVSNQLQR-----L----adrriL-----RA-A-RCG----------nNIHYRIvdpcvl
0030 2rdpA   ypITPPQFVALQWLL-E----eG---D-L-T-VGELS-NKXY----------------------lacsTTTDLVDR-----X----ernglV-----AR-V-RDEH----------VVRIRLlekger
0031 2oqgA   alsDETRWEILTELG-R-----A---D-Q-S-ASSLA-TRLP----------------------vsrQ---AIAKHLNA-----L----qacglV-----ES-V-KVG----------rEIRYRAlgaeln
0032 5zi8A   fqRAAVRLHILHHAA-D-----N---E-V-H-GAWLT-QELS-R-HG----YR-------------vspG---TLYPTLHR-----L----eadglL-----VS-E-QRVVD--gr----aRRVYRAtpagra
0033 2fe3B   vrITPQRHAILEYLV-N-----S--aH-P-T-ADDIY-KALE-G-KF----PN-------------msvA---TVYNNLRV-----F----resglV-----KE-L-TYGD---------aSSRFDFvtsdhy
0034 7mexA   rrMDQIKNSIIYNLY-M-----K---P-L-S-YSKLL-RSVP-D-YL------------------tedtT---EFDEALEE-----V---------S-----VF-V-EPKGL---a----dNGVFKLkaslya
0035 7z1nO   ahLGERAASVIGMLV-A----lG---R-L-S-VRELV-EKID-G-M-------------------dvD---SVKTTLVS-----L----tqlrcV-----KY-L-QETA-isgk----kTTYYYYneegih
0036 2xrnB   iqVIARAASIMRALG-S-----H--hG-L-S-LAAIA-QLVG----------------------lprsTVQRIINA-----L----eeeflV-----EA-L-GP------------AGGFRLgpalgq
0037 3s93B   riQECLRKEIRSLLI-S-----T--dG-L-S-PQELE-KEYLlM-VG----N----hlplrIL--G--Y--rSTMELVLD-----M-------pdvV-----RV-C-PGAG---------gTVILKAip----
0038 6j05A   alASPVRLEIFRLLV-E-----Q--tG-L-V-SGDIA-EHLG-Q------------------------phnGISFHLKN-----L----qhaglV-----TV-Q-REG----------ryQRYRAampvvr
0039 3l9fA   ---MQGKDIILGILS-K-----K---E-R-S-GYEIN-DILQ-NqLS----YF------ydgT--Y--G---MIYPTLRK-----L----ekdgkI-----TK-E-VVIQ-dgrp----nKNIYAItesgkk
0040 2wteA   rdYSREEXKLLNVLY-E-----T--kG-T-G-ITELA-KXLD----------------------ksekTLINKIAE-----L----kkfgiL-----TQ-K-GK-------------DRKVELnelgln
0041 3b73B   swXTIWDDRILEIIH-E-----E--gN-G-S-PKELE-DR-D-E-IR--------------------isksSVSRRLKK-----L----adhdlL-----QP-L-A-------------NGVYVIteegea
0042 2nyxB   enITIPQFRTLVILS-N-----H--gP-I-N-LATLA-TLLG----------------------vqpsATGRXVDR-----L----vgaelI-----DR-L-PHPT---sr----rELLAALtkrgrd
0043 6jyiA   kdpXKGRDVVLGLLX-Q-----K---E-L-S-GYDIK-IVFE-DvFT----HF----------fdgsfG---XIYPTLRQ-----L----enegkI-----KK-E-VVXQK---p----nKKXYFItdegre
0044 3f6vA   vaAEPTRRRLVQLLT-S-----G---E-Q-T-VNNLA-AHFP----------------------asrsAISQHLRV-----L----teaglV-----TP-R-KDG----------rFRYYRLdpqgla
```

```
0045 5fmfV   mpKKEILDYLFKLFD-E-----Y--dY-W-S-LKGLK-ERTR-----------------------qpeA---HLKECLDK-----V---------A-----TL-V-KKGP---------yAFKYTLrpeykk
0046 6luiA   --SPHYQEWILDTID-S-----L--karP-D-LERIC-RXVR-R-RH----G-------------pepE---RTRAELEK-----L----iqqraV-----LR-V-SYK----------gSISYRNaar---
0047 1xmkA   ldMAEIKEKICDYLFnV-----S---D-S-S-ALNLA-KNIG-L-TK-----------------------arDINAVLID-----M----erqgdV-----YR-Q-GT-----------tPPIWHLtdkkre
0048 2ethA   sdXKTTELYAFLYVA-L----fG---P-K-K-XKEIA-EFLS-----------------------ttkS---NVTNVVDS-----L----ekrglV-----VR-E-XDPVD---r----rTYRVVLtekgke
0049 2p4wB   vlGNETRRRILFLLT-K-----R---P-Y-F-VSELS-RELG--------------------------vgqkAVLEHLRI-----L----eeaglI-----ES-R-VEKI-prgr----pRKYYMIkkglrl
0050 3u21A   eisSSFFSLLLEILL-L-----E--sQ-A-S-LPXLE-ERVL-D-WQsspaSS--LNswfsAA--P--NwaeLVLPALQY-----LavpssfspfV-----EF-X-EX------------TQQWXLlgdnex
0051 3l7wA   vsALLIEYLILAIVS-K-----H---D-S-Y-GYDIS-QTIK-L-IA----S-------------ikeS---TLYPILKK-----L----ekagyL-----ST-Y-TQE--hqgr----rRKYYHLtdsgek
0052 7xjgJ   gnILYAERFLLPCIY-L-----L--dS-V-N-YRTLC-ELAF-K-AI----KQ--D-------dvlskI---IVRSVVSR-----L----inerkI-----LQ-M---------------TDGYQVtalgas
0053 8ioiA   pefMALPHAILVSLS-E-----Q---A-S-S-GYELA-RRFD-RsIG----YF----------wtathQ---QIYRTLRV-----M----ennnwV-----RA-T-TVLQH-grp----dKKVYAIsdsgra
0054 3gfiA   lnLSYLDFLVLRATS-D-----G---P-K-T-MAYLA-NRYF-----------------------vtqS---AITASVDK-----L----eemglV-----VR-V-RDREdRR-------KILIEItekgle
0055 4ijaA   -pMNDNEKRVLREIY-N-----H--hN-I-S-RTQIS-KNLE-----------------------inkA---TISSILNK-----L----kykslV-----NE-V-G---------ggrkpILLKVnhlygy
0056 8xt8B   ydITTDHYALLRFLW-E-----Q---DgI-S-QIDLC-EKSC-----------------------kdkS---NTTRILDV-----M----knkglI-----VR-K-VDVK---dr----rKFQIFLtdlgre
0057 6j0eB   vlgDPVRLRILSQLA-A-----G--gP-V-S-VNELT-DLMG-----------------------lsqP---TISHHLKK-----M----teagfL-----DR-V-PEG----------rVVLHRVrpelfa
0058 3k69A   klDFSVAVHSILYLD-A-----H--sK-V-A-SRELA-QSLH-L-------------------------npvXIRNILSV-----L----hkhgyL-----TG-T-VG-----------kNGGYQLdlalad
0059 7wzeA   rgLSEGKFKILXLLF-D-----A--hR-L-S-PTELA-KRSN--------------------------vtkaTITGLLDG-----L----ardgfV-----SR-R-HH-----------rKISIELttegka
0060 3fhzD   anRAGRQARIVAILS-S-----A---Q-VrS-QNELA-ALLA-A-EG----------------ievtqA---TLSRDLEE-----L--------gA-----VK-L-RGADGG--------TGIYVVpedgsp
0061 4xrfA   tgLTPPQFYILKILD-H-----Y--gA-S-R-ATTLA-KKMY--------------------------vkpsAITVMIDR-----L----idqelV-----ER-Y-HDKD---dr----rVVIIELtkkgka
0062 6v9iC   lrILRTQEAIIQIMK-M-----R--kK-I-S-NAQLQ-TELV-E-IL----KN--M-------flpqkK---MIKEQIEW-----L----iehkyI-----RR-D-ES-----------dINTFIYma----
0063 3f8fA   mlRAQTNVILLNVLK-Q-----G---D-N-Y-VYGII-KQVK-E-AS-------------ngemelneA---TLYTIFKR-----L----ekdgiI-----SS-Y-WGDE---gg----rRKYYRLteighe
0064 7el3A   vgLTEQQWRIIRILY-Q-----Y--eE-L-E-SNQLA-ELAC--------------------------ilkpSLTGILNR-----M----veqklI-----QK-R-KDYD---dq----rISLISLtesgle
0065 2g9wA   trLGDLERAVXDHLW-S-----R--eP-Q-T-VRQVH-EALS-A-RR----D-------------layT---TVXAVLQR-----L----akknlV-----LQ-I-R-------------AHRYAPvhgrde
0066 3f72B   aiADENRAKITYALC-Q-----D--eE-L-C-VCDIA-NILG--------------------------vtiaNASHHLRT-----L----ykqgvV-----NF-R-KEG----------kLALYSLggeair
0067 2mh2A   ----GAPGIILRYLQ-E-----Q--rP-Y-S-AQDVF-GNLQ-K-EH-----------------glgkA---AVVKALDQ-----L----aqegkI-----KE-K-TYG----------kqKIYFAd-----
0068 5okcB   tlpxDPKERFKVLFR-L-----Q--sQ-W-D-LEDIK-PLIE---EL----NS---------R--G--X---KIDSFIXK-----Y---------A-----RR-K-RLGK----------KTVVTSr-----
0069 2cweA   vmLEDTRRKILKLLR-N-----K---E-M-T-ISQLS-EILG--------------------------ktpqTIYHHIEKlkeagL---------V-----EVkR-TEM--kgnl----vEKYYGRtadvfy
0070 7vwvB   meTQKLISMVKEALE-K-----Y--yP-L-T-AKNIK-VVIQ-K-EH----N--------------vvlptgSINSILYS-----N------selF-----EK-I-DKTNTI-------ypPLWIRkn----
0071 7kbfK   --hPPTLSMVVEVLK-K-----N--kG-T-S-VQAIR-TRIL-S-AH----PT--------vdplrlkF---LLRTALNK-----G----lekgiL-----IR-PlNSSA-------tgatGRFKLak----
0072 7asvA   pvARELKAFVEATFQ-R-----Q--fV-L-T-LSELK-RLFN-L-HL---aSL--PPG---HTlfS--G---ISDRXLQD-----T---------VlaagcKQ-I-LVPFPpqtaaspdeqKVFALwesgdx
0073 1yg2A   ----SLPHVILTVLS-T-----R---D-A-T-GYDIT-KEFS-AsIG----YF----------wkashQ---QVYRELNK-----M----geqglV-----TC-V-LE--------------VYSItqagrs
0074 6o8oB   alAHEGRLXIXCYLA-S-----G---E-K-S-VTELE-TRLS-----------------------trqA---AVSQQLAR-----L----reglV-----QS-R-REG----------kTIYYSLsdpraa
0075 3v4gA   -kQDNLVRAFKALLK-E-----E---R-F-GsQGEIV-EALK-QeGF----EN------------inqS---KVSRMLTK-----F--------gA-----VR-T---rnaKM-------EMVYCLptvsss
0076 4hqeB   ilgRSWNGLIINYLS-R-----C--cS-A-H-FSDMK-RDLK-T-I--------------------tpR---ALSLKLSE-----L----aqwelV-----EK-Q-IISTS---p----vQIIYVLtekgka
0077 3u2rA   feLSAQQYNTLRLLR-S-----V--eG-X-A-TLQIA-DRL-----------------------------apDITRLIDR-----L----ddrglV-----LR-T-RKPE---nr----rVVEVALtdaglk
0078 5n35A   -vNQKEIEIAIEYFK-N-----Y--iS-V-G-EIVAT-MDLK-A-RG-----------------------isNPQAVISK-----L----iemgiI-----EK-G---------------EGCYNLvr----
0079 4txaA   arQEEALKLVLLALE-D----gS---A-L-S-RKVLV-LFVV-Q-RL----EP--R-F---PQ--A---sktSIGHVVQL-----L---------YrascfKV-T-KRD-----------SSLMQLkeefrt
0080 6a6iA   teHDDLLVEMRNFIA-FqahtdG---Q-A-S-TREIL-QEFE-S-KL----SA--S----------qsC---VFRELLRN-----L---------C-----TF-H-RTSG---------gEGIWKLkpeyc-
0081 3bpxA   lnLTDAQVACLLRIH-R-----E---PgI-K-QDELA-TFFH-----------------------vdkG---TIARTLRR-----L----eesgfI-----ER-E-QDPE---nr----rRYILEVtrrgee
0082 4yifB   spVSLSQLSALTTLA-N----eG---A-M-T-PGALA-IRER--------------------------vrppSMTRVIAS-----L----admgfV-----DR-A-PHPID---g----rQVLVSVsesgae
0083 6yj6B   esLEECLKILRELFA-R-----R--pI-W-V-KRHLD-GIVP-K-KI--------------------hH---TMKIALAL-----I---------S-----YR-F-TMG--PW-------RNTYIKfgidpr
0084 7bzdA   viGGKWKMLILWHLG-K----eG---T-K-R-FNELK-TLIP-D-I--------------------tqK---ILVNQLRE-----L----eqdmiV-----HR-E-VYPVV---p----pKVEYSLtphges
0085 2gaiA   lsAGRVQSATLKLVC-D-----RerpR-Y-T-EGSLV-KEME-R-LG---------------igrP--S---TYASTIKL-----L----lnrgyI-----KK-I---------------RGYLYPtivgsv
0086 5z7bB   kdLMTLRSALLALLS-S-----G---P-L-T-GYDAS-QRFG-AsVG----FV----------wsgsdS---QIYPELRK-----M----eaeelL-----VG-S-DVPwgskga----tKTEYALsekgwe

0001 s001A   --LHHHHHHHHHHHH-H-----L---L-E-E-HHHHH-HHHH-H-HL----LL--LLL---LL--L--L---LHHHHHHH-----H---------L-----EE-L-LLLLLLL-------LLEEEL------
0002 6qfdB   llLLHHHHHHHHHHH-H-----L---L-E-E-HHHHH-HHHH-H-HH----L-----------llllhH---HHHHHHHH-----H----hhlllE-----EE-E-EEEL---------lEEEEEElhhhhh
0003 6zvhy   --lLLHHHHHHHHHH-L-----L--hE-E-E-HHHHH-HHHH-H-HH----H--lLLL---LLhhhhhH---HHHHHHHL-----L-------llE-----EE-L-L--------------LEEEEll----
0004 8qkfA   hhLLHHHHHHHHHHH-H-----L---L-L-L-HHHHH-HHHL-----------------------llhH---HHHHHHHH-----H----hhlllE-----EE-E-EEL----------lEEEEEEllllhh
0005 5dymA   ---LLHHHHHHHHHL-L-----L---L-E-E-HHHHH-HHHH-H-HL----LL------------llhH---HHHHHHHH-----H----hhlllE-----EE-E-ELLLLllll----lLEEEEElhhhhh
0006 5j6xA   lhHHHHHHHHHHHHH-H----hL---L-L-L-HHHHH-HHLL-----------------------llhH---HHHHHHHH-----H----hhlllE-----EE-E-LLLL---------lLLEEEEll----
0007 7wjpA   hhHHHHHHHHHHHHH-H-----L---L-E-E-HHHHH-HHHL-L-LL----L----------------llllLHHHHHHH-----H----hhlllE-----EE-E-EELL----l----lEEEEEElhhhhh
0008 8jxkD   --LHHHHHHHHHHHH-H-----L---L-E-E-HHHHH-HHHH-H-HL----L---------lllllllL---LHHHHHHH-----H----hhlllE-----EE-L-LLLLL---------lLEEEElhhhhh
0009 1lddA   hhHHHHHHHHHHHHH-H----hL---L-E-E-HHHHH-HHHH-H-HL----LH--H--hllLL--L--L-hhHHHHHHHH-----H----hhlllE-----EL-L-L-------------LLEEEEl-----
0010 2od5A   hhHHHHHHHHHHHLL-L-----L---L-E-E-HHHHH-HHHH-L-LL----LL--------------lllhhHHHHHHHH-----L-------llE-----EE-E-EEeeeeellleeeeeeEEEEhhhhhh
0011 1sfxA   llLLHHHHHHHHHHH-H-----H--lL-L-L-HHHHH-HHHL--------------------------llhhHHHHHHHH-----H----hhlllE-----EE-E-EEELL---l----eEEEEEEllhhhh
0012 2eshA   hhHHHHHHHHHHHHH-H-----L---L-L-L-HHHHH-HHHH-L-LL----LL--LLL---L------llllLHHHHHHH-----H----hhlllE-----EE-E-EELLL--ll----lEEEEEElhhhhh
0013 5a31N   hhHHHHHHHHHHHHH-H-----H--lL-L-L-HHHHH-HHHH-H-HL----L------lllllllllhH---HHHHHHHH-----H----hhlllL-----EE-E---------------LLEEELl-----
0014 3elkA   hhHHHHHHHHHHHHH-H-----L---L-E-E-HHHHH-HHHH-H-HH----LL--------------lllllHHHHHHHH-----H----hhhllE-----EE-E-EEEL--lll----eEEEEEElhhhhh
0015 8sojB   hhLHHHHHHHHHHHL-L-----L--lL-E-E-HHHHH-HHHH-HhLL----LL------------llhH---HHHHHHHH-----H----hhhllL-----EE-E-E-------------LLEEEL------
0016 5zqhA   hhHHHHHHHHHHHHH-H-----L---L-E-E-HHHHH-HHHH-HhLL----LL-------------llL---LLHHHHHH-----H----hhlllE-----EE-E-EE-elllll----eEEEEEElhhhhh
0017 5jlsA   llLLHHHHHHHHHHH-H-----L---L-L-L-HHHHH-HHHL-----------------------llhH---HHHHHHHH-----H----hhlllE-----EE-E-ELLL---ll----lLEEEEEllllhh
0018 2zkzC   hhLLHHHHHHHHHHH-H-----H--lL-E-E-HHHHH-HHHL-----------------------llhH---HHHHHHHH-----H-----lllE-----EE-E-EEL----------lEEEEEEllhhhh
0019 5xpqA   hllLHHHHHHHHHHH-H-----L---L-E-E-HHHHHlLLLL-----------------------llhH---HHHHHHHH-----H----hhlllE-----EE-E-ELL----------lLEEEEElllhhh
0020 4ejoA   hhHHHHHHHHHHHLL-------L---L-E-E-HHHHH-HHHH-H-LL----------------llllhH---HHHHHHHH-----H----hhlllE-----EE-E-EEELL--ll----eEEEEEElhhhhh
0021 7z8bC   hhHHHHHHHHHHHHH-L-----L--lL-E-E-HHHHH-HHHH-H-HH----HllhLLL---LL-lllhH---HHHHHHHH-----H----hhhllE-----EE-L-LL-----------lLLEEEEll----
0022 4omzC   hhLLHHHHHHHHHHH-H-----L---L-E-E-HHHHH-HHLL-----------------------llhH---HHHHHHHH-----H----hhlllL-----EE-E-EEL----------lEEEEELllhhhh
0023 5hs7A   hhhLLLHHHHHHHHL-L-----L---L-E-E-HHHHH-HHLL-L-L--------------------lhH---HHHHHHHH-----H----hhlllE-----EE-E-EELLL---l----lEEEEEElhhhhl
0024 8uucA   llHHHHHHHHHHHHH-H-----L---LlE-E-HHHHH-HHHH-H-HH--hhLL--LL-------lllhH---HHHHHHHH-----H----hhhllL-----EE-E---------------LLEEEL------
0025 8iueP   -lHHHHHHHHHHHHH-H-----L--lL-L-L-HHHHH-HHLL-L-L--------------------lhH---HHHHHHHH-----H----hhhllE-----EE-E-EEL----------lEEEEEElhhhll
0026 3mq0B   lhHHHHHHHHHHHHH-H-----L--lL-E-E-HHHHH-LLLL-----------------------llhH---HHHHHHHH-----H----hhlllE-----EE-L-L-------------LLEEEElllhhh
0027 3bddA   hlLLHHHHHHHHHHH-H-----H---LlL-L-HHHHH-HHHL-----------------------llhH---HHHHHHHH-----H----hhlllE-----EE-E-ELLLL---l----lLEEEEElhhhhh
0028 2l4mA   llLLHHHHHHHHHHH-H----hL---L-E-E-HHHHH-HHHL-L------------------------llhhHHHHHHHH-----H----hllllE-----EE-L-LL------------LLEEEEll----
0029 6uvuB   hhlLHHHHHHHHHHH-H-----H--lL-L-L-HHHHH-HHLL--------------------------llhhHHHHHHHH-----H----hhlllE-----EE-E-EEL----------lEEEEEEllhhhh
0030 2rdpA   llLLHHHHHHHHHHH-H----hL---L-L-L-HHHHH-HHHL--------------------------llhhHHHHHHHH-----H----hhlllE-----EE-E-ELLL----------LEEEEElhhhhh
0031 2oqgA   hllLHHHHHHHHHHH-H-----L---L-L-L-HHHHH-HHLL-----------------------llhH---HHHHHHHH-----H----hhlllE-----EE-E-EEL----------lEEEEEEllhhhh
0032 5zi8A   hhHHHHHHHHHHHHH-H-----L---L-L-L-HHHHH-HHHH-H-LL----LL------------llhH---HHHHHHHH-----H----hhlllE-----EE-E-EEEEL--le----eEEEEEElhhhhh
0033 2fe3B   llLLHHHHHHHHHHH-H-----L--lL-L-L-HHHHH-HHHL-L-LL----LL------------llhH---HHHHHHHH-----H----hhlllE-----EE-E-LLLL---------lLLEEEEllllee
0034 7mexA   hhHHHHHHHHHHHHL-L-----L---L-E-E-HHHHH-HHLL-H-HH-----------------lllhH---HHHHHHHH-----H---------E-----EE-E-LLLLL---l----lLLEEEEllhhhh
0035 7z1nO   hhLLHHHHHHHHHHH-H----hL---L-E-E-HHHHH-HHLL-L-L--------------------lhH---HHHHHHHH-----H----hhlllL-----LE-E-EEEL-llll----eEEEELLlhhhhh
0036 2xrnB   lhHHHHHHHHHHHHH-L-----L--lL-E-E-HHHHH-HHLL--------------------------llhhHHHHHHHH-----H----hllllE-----EE-L-HH-----------HLEEEEEllhhhh
0037 3s93B   hhHHHHHHHHHHHHH-L-----L--lL-L-L-HHHHH-HHHHhH-HL----L----llllhHH--L--L--lLHHHHHHL-----L-------llE-----EE-E-ELHH---------hLEEEEEll----
0038 6j05A   hhLLHHHHHHHHHHH-H-----L--lL-E-E-HHHHH-HHHL-L-------------------------lhhHHHHHHHH-----H----hhlllE-----EE-E-EEL----------leEEEEElhhhhh
```

```
0039 3l9fA   ---LLHHHHHHHHLL-L-----L---L-E-E-HHHHH-HHHH-HlLL----LL------eelL--L--L---LHHHHHHH-----H----hhlllE-----EE-E-EELL-llll----lEEEEEElhhhhh
0040 2wteA   llLLHHHHHHHHHHH-H-----H--lL-L-L-HHHHH-HHHL--------------------------llhhHHHHHHHH-----H----hhlllE-----EE-E-LL------------LLEEEElhhhhh
0041 3b73B   llLLHHHHHHHHHHH-H-----H--lE-E-L-HHHHH-LL-L-L-LL---------------------llhhHHHHHHHH-----H----hhlllE-----EE-E-H-------------HHEEEElhhhhh
0042 2nyxB   llLLHHHHHHHHHHH-H-----H--lL-E-E-HHHHH-HHHL--------------------------llhhHHHHHHHH-----H----hhlllE-----EE-E-LLLL---ll----lLLEEEElhhhhh
0043 6jyiA   lllLHHHHHHHHHHH-H-----L---L-L-L-HHHHH-HHHH-HlHH----HL----------llllhH---HHHHHHHH-----H----hhlllE-----EE-E-EELLL---l----lEEEEEElhhhhh
0044 3f6vA   hhLLHHHHHHHHHHH-H-----L---L-E-E-HHHHH-LLLL--------------------------llhhHHHHHHHH-----H----hhlllE-----EE-E-EEL----------lEEEEEElhhhhh
0045 5fmfV   hhHHHHHHHHHHHHH-H-----L--lL-L-L-HHHHH-HHLL-----------------------llhH---HHHHHHHH-----H---------L-----LL-L-LLLL---------lLLLLLLllllll
0046 6luiA   --LLLHHHHHHHHHH-H-----H--lllL-L-HHHHH-HHHH-H-HH----L-------------llhH---HHHHHHHH-----H----hhlllE-----EE-E-EEL----------lEEEEEElll---
0047 1xmkA   hhHHHHHHHHHHHHHhL-----L---L-E-E-HHHHH-HHHL-H-HH-----------------------hhHHHHHHHH-----H----hhlllE-----EE-E-LL-----------lLLEEEElhhhhl
0048 2ethA   hhLLHHHHHHHHHHH-H----hL---L-L-L-HHHHH-HHLL-----------------------llhH---HHHHHHHH-----H----hhlllE-----EE-E-ELLLL---l----lLEEEEElhhhhh
0049 2p4wB   hlLLHHHHHHHHHHL-L-----L---L-E-E-HHHHH-HHHL--------------------------llhhHHHHHHHH-----H----hhlllE-----EE-E-ELLL-llll----lLEEEEElleee
0050 3u21A   lllLLHHHHHHHHHH-L-----L--lE-E-E-HHHHH-HHHH-H-HHllhhHH--HLllhhHL--L--LlhhHHHHHHHH-----HlllllllllE-----EE-E-LL------------LLEEEEllllhh
0051 3l7wA   llHHHHHHHHHHHHH-H-----L---L-E-E-HHHHH-HHHL-L-LL----L-------------llhH---HHHHHHHH-----H----hhlllE-----EE-E-EEE--elle----eEEEEEElhhhhh
0052 7xjgJ   llLLLLHHHHHHHHH-H-----L--lL-L-L-HHHHH-HHHH-H-HL----LL--L-------lhhhhH---HHHHHHHH-----H----hhlllE-----EE-E---------------LLEEEElhhhhh
0053 8ioiA   lllLLHHHHHHHHHH-H-----L---L-L-L-HHHHH-HHHH-HlHH----HL----------llllhH---HHHHHHHH-----H----hhlllE-----EE-E-EELLL-lll----lEEEEEElhhhhh
0054 3gfiA   hlLLHHHHHHHHHHL-L-----L---L-E-E-HHHHH-HHLL-----------------------llhH---HHHHHHHH-----H----hhlllE-----EE-E-EELLeEE-------EEEEEElhhhhh
0055 4ijaA   -lLHHHHHHHHHHHH-H-----L--lL-L-L-HHHHH-HHHL-----------------------llhH---HHHHHHHH-----H----hhlllE-----EE-L-L---------lllllLLEEElhhhle
0056 8xt8B   llLLHHHHHHHHHHH-H-----L---LlL-L-HHHHH-HHHL-----------------------lllH---HHHHHHHH-----H----hllllE-----EE-E-ELLL---ll----lLEEEEElhhhhh
0057 6j0eB   hhlLHHHHHHHHHHH-H-----L--lL-E-E-HHHHH-HHHL-----------------------llhH---HHHHHHHH-----H----hhlllE-----EE-E-EEL----------lEEEEEElhhhhh
0058 3k69A   lhHHHHHHHHHHHHH-L-----L--lL-L-L-HHHHH-HHHL-L-------------------------lhhHLHHHHHH-----H----hhlllL-----EE-E-LL-----------lLLEEELlllhhh
0059 7wzeA   llLLHHHHHHHHHHH-H-----L--hE-E-L-HHHHH-HHLL--------------------------llhhHHHHHHHH-----H----hhhllE-----EE-E-EL-----------lLEEEEElhhhhh
0060 3fhzD   llHHHHHHHHHHHHH-H-----L---L-LlL-HHHHH-HHHH-L-LL----------------llllhH---HHHHHHHH-----L--------lL-----EE-E-LLLLLL--------LLEEELllllll
0061 4xrfA   hlLLHHHHHHHHHHH-H-----H--lL-E-E-HHHHH-HHHL--------------------------llhhHHHHHHHH-----H----hhlllE-----EE-E-ELLL---ll----lLEEEEElhhhhh
0062 6v9iC   hhHHHHHHHHHHHHH-H-----L--lE-E-E-HHHHH-HHHH-H-HH----LL--L-------llllhH---HHHHHHHH-----H----hhlllE-----EE-E-LL-----------eEEEEEEll----
0063 3f8fA   hhHHHHHHHHHHHHH-H-----L---L-L-L-HHHHH-HHHH-H-HL-------------lllllllhH---HHHHHHHH-----H----hhlllE-----EE-E-ELLL---ll----lLEEEEElhhhhh
0064 7el3A   hlLLHHHHHHHHHHH-H-----H--lE-E-E-HHHHH-HHHL--------------------------llhhHHHHHHHH-----H----hhlllE-----EE-E-EELL---ee----eEEEEEElhhhhh
0065 2g9wA   hhLLHHHHHHHHHHH-L-----L--lL-E-E-HHHHH-HHHL-L-LL----L-------------llhH---HHHHHHHH-----H----hhlllE-----EE-E-L-------------LLEEEElllhhh
0066 3f72B   hhHLHHHHHHHHHLL-L-----L--lL-E-E-HHHHH-HHHL--------------------------llhhHHHHHHHH-----H----hhlllE-----EE-E-ELL----------lLEEEEEllhhhh
0067 2mh2A   ----LHHHHHHHHHH-H-----H--lL-E-E-HHHHH-HHHH-L-LL-----------------lllhH---HHHHHHHH-----H----hhhllE-----EE-E-EEL----------leEEEEEl-----
0068 5okcB   hlllLHHHHHHHHHH-H-----L--lL-L-L-HHHHH-HHHH---HH----LL---------L--L--L---LHHHHHHH-----H---------E-----EE-E-ELLL----------LEEEEEl-----
0069 2cweA   hhLLHHHHHHHHHHL-L-----L---L-L-L-HHHHH-HHHL--------------------------llhhHHHHHHHHhhhllL---------E-----EEeE-EEE--elle----eEEEEEElleee
0070 7vwvB   lhHHHHHHHHHHHHH-H-----L--lL-L-L-HHHHH-HHHH-H-HH----L--------------llllhhHHHHHHHH-----L------lllE-----EE-L-LLLLLL-------llLLEEEll----
0071 7kbfK   --lLLHHHHHHHHHH-H-----L--lL-E-E-HHHHH-HHHH-L-LL----LL--------lllllhhH---HHHHHHHH-----H----hhlllE-----EE-EeELLL-------llllEEEEEll----
0072 7asvA   lhHHHHHHHHHHHHH-H-----H--lE-E-E-HHHHH-HHHH-H-HH---hLL--LLL---LHhhL--L---LLHHHHHH-----H---------HhhlllEE-E-LLLLLlllllllhhhLEEELllllll
0073 1yg2A   ----LHHHHHHHHHH-H-----L---L-L-L-HHHHH-HHHL-LhHH----HL----------llllhH---HHHHHHHH-----H----hhlllE-----EE-L-LL--------------LEEElhhhhh
0074 6o8oB   hhLLHHHHHHHHHHH-H-----L---L-E-E-HHHHH-HHLL-----------------------lllL---HHHHHHHH-----H----hhlllE-----EE-E-ELL----------lLEEEEEllhhhh
0075 3v4gA   -lHHHHHHHHHHHHH-H-----L---L-L-LlHHHHH-HHHH-HlLL----LL------------llhH---HHHHHHHH-----L--------lL-----EE-E---ellLL-------LEEEELllllll
0076 4hqeB   hhlLLLHHHHHHHHL-L-----L--hE-E-L-HHHHH-HHHL-L-L--------------------lhH---HHHHHHHH-----H----hhlllE-----EE-E-EELLL---l----lEEEEEElhhhhh
0077 3u2rA   llLLHHHHHHHHHHH-H-----H--lL-E-E-HHHHH-HHL-----------------------------llHHHHHHHH-----H----hhlllE-----EE-E-EELL---ee----eEEEEEElhhhhh
0078 5n35A   -lLHHHHHHHHHHHH-H-----L--lE-E-E-HHHHH-HHHH-H-LL-----------------------llLHHHHHHH-----H----hhlllE-----EE-L---------------LLEEEEll----
0079 4txaA   hhHHHHHHHHHHHHL-L----lL---L-L-L-HHHHH-HHHH-H-HL----LL--L-L---LL--L---lhhHHHHHHHH-----H---------HhllleEE-E-ELL-----------LEEEEElhhhll
0080 6a6iA   llLHHHHHHHHHHHH-HlllllL---E-E-E-HHHHH-HHHL-L-LL----LH--H----------hhH---HHHHHHHH-----H---------E-----EE-E-ELLL---------lLEEEEElhhhl-
0081 3bpxA   hlLLHHHHHHHHHHH-L-----L---LlL-L-HHHHH-HHHL-----------------------llhH---HHHHHHHH-----H----hhlllE-----EE-E-EELL---ee----eEEEEEElhhhhh
0082 4yifB   llLLHHHHHHHHHHH-H----hL---L-L-L-HHHHH-HHHL--------------------------llhhHHHHHHHH-----H----hhlllE-----EE-E-ELLLL---l----lLEEEEElhhhhh
0083 6yj6B   hhHHHHHHHHHHHHH-H-----L--lE-E-L-HHHHL-LLLL-L-LL--------------------lL---LHHHHLLL-----L---------E-----EE-E-LLL--LL-------LLLEEEllllhh
0084 7bzdA   hhLLLLHHHHHHHHH-H----hL---L-E-E-HHHHH-HHLL-L-L--------------------lhH---HHHHHHHH-----H----hhlllE-----EE-E-EELLL---l----lEEEEEElhhhhh
0085 2gaiA   llLHHHHHHHHHHHH-H-----HhhlL-L-L-HHHHH-HHHH-H-HL---------------lllL--L---LHHHHHHH-----H----hhlllE-----EE-E---------------LLEEEElhhhhh
0086 5z7bB   hhHHHHHHHHHHHHL-L-----L---L-E-E-HHHHH-HHHH-LlHH----HL----------llllhH---HHHHHHHH-----H----hhlllE-----EE-E-EEEelllle----eEEEEEElhhhhh

###########################################################################################################################################################################################################################################
###########################################################################################################################################################################################################################################
###########################################################################################################################################################################################################################################
```

Job: Type-3 BREX; BrxHII-Helicase WP_162523516 (N-Terminal TUDOR Domain)
Query: s001A
No: Chain Z rmsd lali nres id PDB Description
1: 6hq9-A 6.9 1.5 47 60 4 MOLECULE: DNA EXCISION REPAIR PROTEIN ERCC-6-LIKE 2;
2: 6if4-B 6.4 1.6 48 61 10 MOLECULE: HISTONE ACETYLTRANSFERASE;
3: 3fc3-A 6.3 1.3 44 189 9 MOLECULE: RESTRICTION ENDONUCLEASE HPY99I;
4: 7abi-7 6.2 1.6 45 236 16 MOLECULE: U5 SMALL NUCLEAR RIBONUCLEOPROTEIN 40 KDA PROTEIN
5: 7ef1-A 6.2 2.0 48 148 8 MOLECULE: HB TRANSCRIPTION FACTOR;
6: 1m1g-C 6.1 1.8 46 244 15 MOLECULE: TRANSCRIPTION ANTITERMINATION PROTEIN NUSG;
7: 1vie-A 6.1 1.9 48 60 10 MOLECULE: DIHYDROFOLATE REDUCTASE;
8: 5hh7-A 6.1 1.4 47 192 2 MOLECULE: ORIGIN OF REPLICATION COMPLEX SUBUNIT 1B;
9: 2lcc-A 6.1 2.0 51 76 12 MOLECULE: AT-RICH INTERACTIVE DOMAIN-CONTAINING PROTEIN 4A;
10: 8xv7-A 6.0 1.5 48 227 10 MOLECULE: E3 UBIQUITIN-PROTEIN LIGASE UHRF1;
11: 8jw0-e 5.9 1.9 48 74 10 MOLECULE: PHOTOSYSTEM I PSAA;
12: 6kcp-C 5.9 1.3 43 81 16 MOLECULE: LD23804P;
13: 7cce-A 5.8 1.5 48 159 13 MOLECULE: BROMO-ADJACENT HOMOLOGY (BAH) DOMAIN-CONTAINING P
14: 2dig-A 5.8 1.3 45 68 16 MOLECULE: LAMIN-B RECEPTOR;
15: 4m4z-A 5.8 1.3 41 152 22 MOLECULE: SRC-LIKE-ADAPTER 2;
16: 2lq8-A 5.7 1.7 46 177 11 MOLECULE: TRANSCRIPTION ANTITERMINATION PROTEIN NUSG;
17: 7cfd-F 5.7 1.4 44 182 7 MOLECULE: FI20010P1;
18: 5m9n-A 5.7 1.7 45 202 9 MOLECULE: TUDOR DOMAIN-CONTAINING PROTEIN 1;
19: 8jvz-A 5.5 1.8 42 50 14 MOLECULE: MKAL2_V1_SH3;
20: 6ted-Z 5.5 1.4 44 510 7 MOLECULE: DNA-DIRECTED RNA POLYMERASE SUBUNIT;
21: 6cbl-Z 5.5 2.0 48 135 15 MOLECULE: 35S PRE-RIBOSOMAL RNA MISCRNA;
22: 1vbv-A 5.5 1.6 47 77 9 MOLECULE: HYPOTHETICAL PROTEIN B0966;
23: 6bim-A 5.5 1.8 44 278 14 MOLECULE: CLAN CA, FAMILY C40, NLPC/P60 SUPERFAMILY CYSTEIN
24: 2jt4-A 5.4 2.2 48 71 10 MOLECULE: CYTOSKELETON ASSEMBLY CONTROL PROTEIN SLA1;

```
25: 2e70-A 5.4 1.8 46 71 9 MOLECULE: TRANSCRIPTION ELONGATION FACTOR SPT5;
26: 1sf9-A 5.4 1.6 44 118 9 MOLECULE: YFHH HYPOTHETICAL PROTEIN;
27: 5z8l-A 5.4 1.5 46 204 9 MOLECULE: CHROMATIN REMODELING PROTEIN EBS;
28: 8tqc-D 5.4 2.5 49 969 12 MOLECULE: MEDIATOR OF RNA POLYMERASE II TRANSCRIPTION SUBUN
29: 7k9b-A 5.4 2.0 45 98 7 MOLECULE: OLE-ASSOCIATED PROTEIN B;
30: 8rsw-A 5.4 2.5 48 67 15 MOLECULE: PROTEIN KINASE C AND CASEIN KINASE SUBSTRATE IN N
31: 8qsz-Y 5.3 1.3 44 72 16 MOLECULE: DNA-DIRECTED RNA POLYMERASE II SUBUNIT RPB1;
32: 8s7v-L 5.3 1.6 43 47 5 MOLECULE: METHYL-COENZYME M REDUCTASE SUBUNIT GAMMA;
33: 7aoa-A 5.3 1.5 46 343 15 MOLECULE: METHYL-CPG-BINDING DOMAIN PROTEIN 2;
34: 7csp-A 5.3 2.5 47 455 9 MOLECULE: RHO GUANINE NUCLEOTIDE EXCHANGE FACTOR 16;
35: 6vld-B 5.3 2.6 48 470 15 MOLECULE: ALPHA-(1,6)-FUCOSYLTRANSFERASE;
36: 4cos-A 5.3 1.7 47 313 11 MOLECULE: PROTEIN KINASE C-BINDING PROTEIN 1;
37: 3qwx-X 5.3 2.3 46 171 11 MOLECULE: CELL DEATH ABNORMALITY PROTEIN 2;
38: 3ob9-D 5.2 1.6 48 93 13 MOLECULE: MALE-SPECIFIC LETHAL 3-LIKE 1 (DROSOPHILA), ISOFO
39: 2n5u-A 5.2 1.8 47 79 9 MOLECULE: TSR0524 PROTEIN;
40: 5z3g-Q 5.2 2.1 45 137 18 MOLECULE: 25S RRNA;
41: 4anj-A 5.2 1.6 40 995 8 MOLECULE: UNCONVENTIONAL MYOSIN-VI, GREEN FLUORESCENT PROTE
42: 2bud-A 5.1 2.2 50 92 12 MOLECULE: MALES-ABSENT ON THE FIRST PROTEIN;
43: 2lcs-A 5.1 2.2 48 67 8 MOLECULE: NAP1-BINDING PROTEIN 2;
44: 1tg0-A 5.1 1.9 46 66 9 MOLECULE: MYOSIN TAIL REGION-INTERACTING PROTEIN MTI1;
45: 8hyj-W 5.1 1.2 38 44 11 MOLECULE: DNA-DIRECTED RNA POLYMERASE V SUBUNIT 1;
46: 6rwl-A 5.1 2.3 45 270 13 MOLECULE: POL PROTEIN;
47: 1wfw-A 5.0 2.3 47 74 17 MOLECULE: KALIRIN-9A;
48: 6bog-A 5.0 1.8 44 967 9 MOLECULE: RNA POLYMERASE-ASSOCIATED PROTEIN RAPA;
49: 7ud6-A 5.0 1.7 42 266 14 MOLECULE: TYROSINE-PROTEIN KINASE FYN,CATECHOL O-METHYLTRAN
50: 5zwx-A 5.0 1.4 45 148 13 MOLECULE: DUF724 DOMAIN-CONTAINING PROTEIN 6-LIKE;

0001 s001A   -------MGENVHWVFDRW-------N--------N--KPVKAVE-RY-EL---W---N-TT-NCLVYDP---T-S----G--R---IYTLPA--DGVGD-----
0002 6hq9A   ----mkdiwhPGERCLAPG-------K--------L--CEASIKS-IT-VD--eN---G-KS-FAVVLYA-----D----F--Q---ERKIPL--KQLQEvk---
0003 6if4B   -----mvmfeVRQKVYATL-----heT--------F--HAAIIQE-VA-HD-ahT---G-QL-LYYVHYV---E-Q----D--S-rmDRWLPG--SALRErr---
0004 3fc3A   -------mlkNDDFVIAKN-qlgnivP--------N--SVGVIRA-VN-G----------K-SAMVLFI---G-L----N--E---LKRVDF--SELEAidiyr
0005 7abi7   -tartdywlqPEIIVKIITkklgekyH--------K--KKAIVKE-VI-D----------KY-TAVVKMI---D-S----G--D---KLKLDQ--THLETvipap
0006 7ef1A   -eatecvavlPGDLILCFQ-egkdqaL--------Y--YDAHVLD-AQ-RR---R---HcRC-RFLVRYD---H-D----S--S---EEIVPL--RKVCRrpetd
0007 1m1gC   -vkpskvefeKGDQVRVIE----gpfM--------N--FTGTVEE-VH-PE---------KR-KLTVMIS---I-R----M--T---PVELDF--DQVEKi----
0008 1vieA   ---psnatfgMGDRVRKKS------gA--------A--WQGQIVG-WY-CT---N---LtPE-GYAVESE---A-H----P-gS---VQIYPV--AALERin---
0009 5hh7A   -vefdetefeIGDDVYVKRpeiedcqikartmrekL--WAARIDK-LW-KE-vdD---G-VY-WIRARWY---M-T----N--D---FADIEM--ECILRhcsvk
0010 2lccA   -----edMEPCLTKVKVKY-grgktqK--------I--YEASIKS-TE-ID---D---G-EV-LYLVHYY---G-W----N--V-ryDEWVKA--DRIIWpldkg
0011 8xv7A   --gplgslykVNEYVDARD---tnmgA--------W--FEAQVVR-VT-RK---A-leE-DV-IYHVKYD---D-Y----PenG---VVQMNS--RDVRArarti
0012 8jw0e   ----awvgpkKGSQVRILR--pesywF--------Q--QRGTVVN-VN-QK---T---N-IKyPVTVKFD---F-VnyanV--N---TNGFAL--WEVDElkege
0013 6kcpC   ------asysIGDLVFAKV----kgyP--------P--WPAKITK-SN------------K-KYNVYFY---G-T----G--E---TANIKL--EDLFPyasnk
0014 7cceA   -faydgntydLEVPVLLVP-------E--------P--YVAIIKD-IT-QT--kD---G-SM-MILGQWF---YrS----F--H---RDEVPA--ESVMHrcvvy
0015 2digA   -sgmpsrkfaDGEVVRGRW---pgssL--------Y--YEVEILS-HD-ST---------SQ-LYTVKYK----D----G--T---ELELKE--NDIKSgpssg
0016 4m4zA   -------------TAVALGsfplslrL--------G--EPLTIVS-ED-G----------D-WWTVLSE---V-S----G--R---EYNIPS--VHVAKvshgw
0017 2lq8A   -pvkvelgfkVGDMVKIIS----gpfE--------D--FAGVIKE-ID-PE---------RQ-ELKVNVT---I-R----E--T---PVVLHV--SEVEKie---
0018 7cfdF   -krtfkskprLLDIVLALY----sdgC--------F--YRAQIID-EF-P----------S-EYMIFYV---D-Y----G--N---TEFVPL--SCLAPcenvd
0019 5m9nA   -pprsdfypaIGDICCAQF---seddQ--------W--YRASVLA-YA-S----------EE-SVLVGYV---D-Y----G--N---FEILSL--MRLCPiipkl
0020 8jvzA   -------gvkVGDVVEVKK----------------K--VVARVVE-LL-HD---P-arN-AP-VARVRFE----D----G--E---ERLILV--P---------
0021 6tedZ   -aqelrkyfkMGDHVKVIA----grfE--------G--DTGLIVR-VE-E-----------N-FVILFSD---L-T----M--H---ELKVLP--RDLQLcseta
0022 6cb1Z   -----akflkAGKVAVVVR----gryA--------G--KKVVIVK-PHdEG---S---KpFG-HALVAGI---E-K----P--F---IKVVNY--NHLLPtrytl
0023 1vbvA   -----askfgIGQQVRHSL-------L--------G--YLGVVVD-ID-PV-------A-AP-WYHVVMEddnG-L----P--V---HTYLAE--AQLSSelqde
0024 6bimA   -wiskdsleEITIPTQSVT-------EpsrtgtivN--SQVTILD-FN-C----------DG-FYRINYR----------G--Y---IGYILE--DALQYkwkqi
0025 2jt4A   ------mASKSKKRGIVQY-------DfmaesqdeG--DKVYILD-DK-K---------sKD-WWMCQLV---D-S----G--K---SGLVPA--QFIEPvrdkk
0026 2e70A   -rgrgrrdNELIQTVRISQ----gpyK--------G--YIGVVKD-AT-E-----------S-TARVELH---S-T----C--Q---TISVDR--QRLTTvgsrr
0027 1sf9A   -yllnpedysPGETYRVEN-------T--------E--DEFTISY-LN-G-----------V-FAWGYRT---S-S----P-qQ---EEALPI--SVLQEke---
0028 5z8lA   -ikgtnkvvrAGDCVLMRP---------------sP--YVARVEK-IE-AD---A-----NV-KVHCRWY---Y-S----D--H---FDVQSA--HTIEGkcivh
0029 8tqcD   -hitlaqQSNSPFQVILCP-------F--------G--LNGTLTGqAF-KM---SdscS-LA-AVEVLVA----------G--V---RMIYPA--CFVLVpqsqt
0030 7k9bA   -----gpspeIGQIVKIVK----grdR--------D--QFSVIIK-RV-D----------DR-FVYIADG---D-K----R--KvdrAKRKNM--NHLKLidhis
0031 8rswA   ---gsrrASVGSVRVRALY-------DyegqeqdeG--DELTKME-NE-DE---------QG-WCKGRLD----N----G--Q---VGLYPA--NYVEPiq---
0032 8qszY   ------rdkaIGATVRIRR----gpmK--------G--LLGVIKD-TT-D-----------A-NARVELH---T-G----N--K---MVTIPK--ENLLYttktg
0033 8s7vL   --------ieIGSYVRYIN-------T--------G--THGTVKA-IE-PK---N---D-EE-WVLLEN------------------DIYYRP--ELLELve---
0034 7aoaA   -----------GDYVYFEN----------------Y--LIRRIEE-LN-KT--aN---G-NV-EAKVVCF---Y-S----R--Q---LESLPA--THIRGkcsvt
0035 7cspA   -qwqgitNKGELPQVEVTK-------AyfakqadeA--DIVLVLQ-EE-D-----------G-WLHGERL---R-D----G--E---TGWFPE--SFAHSitsrv
0036 6vldB   gddiyyfGGQNAHNQIAIY-------AhqprtadeG--DIIGVAG-NH-W----------DG-YSKGVNR---K-L----G--R---TGLYPS--YKVREkietv
0037 4cosA   -nwfcepCSNPHPLVWAKL----kgfP--------F--WPAKALR-DK-D-----------G-QVDARFF---G-Q----H--D---RAWVPI--NNCYLmskei
0038 3qwxX   -sllaaykKPIIEVVVGTF-------KftgeretdG--ERLEILS-KT-N----------QD-WWEARNA----L----G--T---TGLVPA--NYVQIq----
0039 3ob9D   -segmkfkfhSGEKVLCFE-------P------dpL--YDAKIVD-VI-VG---K-grK-IP-EYLIHFN---G-W----NrsW---DRWAAE--DHVLRdtden
0040 2n5uA   -fekiegrmdVGQKVRVCRaqdiiqkL--------G--QVGQITG-FK-MT---D---GsGV-GVIVTFD----D----R--S---STWFFE--DEVEVvg---
0041 5z3gQ   -kasnwrlveVGRVVLIKK----gqsA--------G--KLAAIVE-II-D----------QK-KVLIDGP---K-A----G-vP---RQAINL--GQVVLtpltf
0042 4anjA   -----------GKPVWAPH----ptdG--------F--QVGNIVD-IG-P-----------D-SLTIEPL-------------K---TFLALI--NQVFPaeeds
0043 2budA   -lmqkidiSENPKIYFIRR----edgT--------V--HRGQVLQ-SR-TTenaA---A-PD-EYYVHYV---G-L----NrrL---DGWVGR--HRISDnaddl
0044 2lcsA   -------MAIVNQRAVALY-------DfependneG--DIVFISY-KH-G----------QG-WLVAENE---S-G----S--K---TGLVPE--EFVSYiqpel
0045 1tg0A   -------epEVPFKVVAQF-------PyksdyeddD--QEIIVTS-VE-D----------AE-WYFGEYQ---D-D----V--I---EGIFPK--SFVAVqg---
0046 8hyjW   -------tysIGQKLRIRV----gplK--------G--YLCRVIA-----------------TVKLD---S-Q----H--K---IFTVKS--EHLAE-----
0047 6rwlA   -klqnqiSKIQNFRVYFRE--grdqqW--------K--GPATLIW-KG-E-----------G-AVVIQDG----------Q--D---LKVVPR--RKCKIik---
0048 1wfwA   -----gssGSSGSTMTVIK-------DyyalkeneG--EVVQVLA-VN-Q----------QN-MCLVYQP---A-P----A--A---EGWVPG--SILAPfsgps
0049 6bogA   -------pftLGQRWISDT-------E--------SelGLGTVVA-VD-A-----------R-TVTLLFP---S-T----G--E---NRLYARsdSPVTRvxfnp
0050 7ud6A   ------------MLFVALY--------yearteddG--EKFVILR-SS-E----------GD-WWLAVSL---T-T----G--E---QGYIPS--NYVAPvgggt
0051 5zwxA   -erekrrevaVGDDVDAFY-----sdG--------W--WEGTVTE-VX-G----------DG-RXSVYFR---A-S----K--E---QIRFRR--DELRFhrewv

0001 s001A   -------LLLLLEEEEELL-------L--------L--EEEEEEE-EE-EE---L---L-EE-EEEEELL---L-L----L--L---EEEEEH--HHEEL-----
0002 6hq9A   ----llllllLLLEEEEEL-------L--------E--EEEEEEE-EE-EE--lL---L-LE-EEEEELL-----L----L--L---EEEEEH--HHLLLll---
```

```
0003 6if4B   -----lllllLLLEEEEEEE-----llE--------E--EEEEEEE-EE-EL-llL---L-LE-EEEEEEL---L-L----L--H-hhLEEEEH--HHEEEll---
0004 3fc3A   -------lllLLLEEEELL-llllllL---------L--LEEEEEE-EE-L-----------L-EEEEEEL---L-L----L--E---EEEEEH--HHEEEllhhh
0005 7abi7   -lllllllllLLLEEEELLllllhhhL---------L--EEEEEEE-EE-L----------LL-EEEEEEL---L-L----L--L---EEEEEH--HHEEElllll
0006 7ef1A   -lhhhhhhllLLLEEEEEEE-elllleE--------E--EEEEEEE-EE-LL---L---LlLL-EEEEEEL---L-L----L--L---EEEEEH--HHEEElhhhh
0007 1m1gC   -lllllllllLLLEEEELL----lllL---------L--LEEEEEE-EE-LL---------LL-EEEEEEE---E-E----E--E---EEEEEH--HHEEEl----
0008 1vieA   ---lllllllLLLEEEELL------lL---------L--LEEEEEE-EE-LL---L---LlLL-EEEEEEL---L-L----L-lL---EEEEEH--HHEEEll---
0009 5hh7A   -eeelleeeeLLLEEEELLlllllllllllllhhhhL--EEEEEEE-EE-EE-llL---L-LE-EEEEEEE---E-E----E--E---EEEEEH--HHEEEeeeee
0010 2lccA   -----llLLLLLLEEEEEEE-eelleeE--------E--EEEEEEE-EE-EE---L---L-EE-EEEEEEL---L-L----L--L-llLEEEEH--HHEELlllll
0011 8xv7A   --llllllllLLLEEEEEL---llllL---------E--EEEEEEE-EE-EE---L-llL-EE-EEEEEEL---L-L----HhhL---EEEEEH--HHEEElllll
0012 8jw0e   ----llllllLLLEEEELL--lllllL---------L--LEEEEEE-EL-LL---L---L-LLlLEEEELL---L-LllllL--L---EEEELH--HHEEElllll
0013 6kcpC   ------llllLLLEEEELL----lllL---------L--EEEEEEE-EL-------------L-EEEEEEL---L-L----L--L---EEEEEH--HHEEEhhhhh
0014 7cceA   -eeelleeelLLLEEEELL-------L---------L--EEEEEEE-EE-EL--lL---L-LE-EEEEEEE---ElE----E--E---EEEEEH--HHEEEellee
0015 2digA   -lllllllllLLLEEEEEL---llllL---------E--EEEEEEE-EE-LL---------LL-EEEEELL-----L----L--L---EEEEEL--LLEELlllll
0016 4m4zA   -------------LEEELLlllllllL---------L--LEEEEEE-EE-L-----------L-EEEEEEL---L-L----L--L---EEEEEH--HHEEEeelll
0017 2lq8A   -lllllllllLLLEEEELL----lllL---------L--LEEEEEE-EE-LL---------LL-EEEEEEE---E-E----E--E---EEEEEH--HHEELll---
0018 7cfdF   -hhlllllllLLLEEEEEL----lllE---------E--EEEEEEE-EL-L-----------L-LEEEEEL---L-L----L--L---EEEELH--HHEEEllhhh
0019 5m9nA   -lllllllllLLLEEEEEL---llllL---------E--EEEEEEE-EE-E----------LL-EEEEEEL---L-L----L--L---EEEEEH--HHEELllhhh
0020 8jvzA   -------lllLLLEEEEEEE----------------E--EEEEEEE-EE-EE---L-llL-EE-EEEEEEL-----L----L--L---EEEEEL--L---------
0021 6tedZ   -hhhllllllLLLEEELLL----lllL---------L--LEEEEEE-EL-L-----------L-LEEEEEL---L-L----L--L---LLLLLH--HHLLLlllll
0022 6cb1Z   -----lllllLLEEEEELL----lllL---------L--EEEEEEE-ELlLL---L---LlLL-EEEEEEE---E-E----E--E---EEEEEL--LLEEEeeeel
0023 1vbvA   -----lllllLLLEEEELL-------L---------L--LEEEEEE-EE-LL-------L-LL-EEEEEEElllL-L----E--E---EEEEEH--HHEEElllll
0024 6bimA   -eeehhheeEELEEEELLL-------LllllllleeL--LEEEEEE-EE-E----------LL-EEEEEEL----------L--E---EEEEEH--HHLLLlllll
0025 2jt4A   ------lLLLLLEEEEELL-------LlllllllllL--LEEEEEE-LL-L---------lLL-EEEEEEL---L-L----L--L---EEEEEH--HHEEElllll
0026 2e70A   -lllllllLLLLLEEEELL----lllL---------L--LEEEEEE-EL-L-----------L-LEEEEEL---L-L----L--L---EEEELL--LLEEElllll
0027 1sf9A   -hhllhhhllLLLEEEELL-------L---------L--LEEEEEE-EE-L-----------L-EEEEEEL---L-E----E-eE---EEEEEH--HHEEEll---
0028 5z81A   -lllllleelLLLEEEELL----------------lL--EEEEEEE-EE-EL---L-----LE-EEEEEEE---E-E----E--E---EEEEEH--HHEEEeeeee
0029 8tqcD   -hhhhhhHLLLLEEEEELL-------L---------L--EEEEEEEeEE-EL---LlllL-EE-EEEEEEL----------L--E---EEEEEL--LLLEEellll
0030 7k9bA   -----lllllLLEEEEELL----lllL---------L--LEEEEEE-EL-L----------LL-EEEEELL---L-L----L--LhhhLEEEEH--HHEEEeeeel
0031 8rswA   ---llhhHLLLLEEEEELL-------LlllllllllL--LEEEELL-LL-LL---------LL-EEEEELL-----L----L--L---EEEEEH--HHEEEll---
0032 8qszY   ------llllLLLEEEELL----lllL---------L--LEEEEEE-EL-L-----------L-EEEEEEL---L-L----L--L---EEEEEH--HHEEEellll
0033 8s7vL   --------llLLLEEEELL-------L---------L--LEEELLE-EL-LL---L---L-LL-LEELLL------------------LLEELH--HHEEEll---
0034 7aoaA   -----------LLEEEELL-----------------L--EEEEEEE-EE-EL--lL---L-LE-EEEEEEE---E-E----E--E---EEEEEH--HHEEEeeeee
0035 7cspA   -hhhhhhLHHHLLEEEELL-------LlllllllllL--LEEEEEE-EE-L-----------L-EEEEEEL---L-L----L--L---EEEEEH--HHEEEellhh
0036 6vldB   lllllllLLLLLLEEEELL-------LlllllllllL--LEEEEEE-EL-L----------LL-EEEEEEL---L-L----L--E---EEEEEH--HHEEElllee
0037 4cosA   -lhhhllLLLLLLEEEELL----lllL---------L--EEEEEEE-EE-L-----------L-EEEEEEL---L-L----L--L---EEEEEH--HHEEElllll
0038 3qwxX   -llleellLLLLEEEEELL-------LlllllllllL--LEEEEEE-LL-L----------LL-EEEEELL-----L----L--L---EEEEEH--HHEEEl----
0039 3ob9D   -lllllllllLLLEEEEEL-------L-------llE--EEEEEEE-EE-EE---E-llE-EE-EEEEEEL---L-L----LhhH---LEEEEH--HHEEEllhhh
0040 2n5uA   -lllhhhlllLLLEEEELLlllhhhhL---------L--LEEEEEE-EE-EL---L---LlLE-EEEEEEL-----L----L--L---EEEEEL--LLEEEel---
0041 5z3gQ   -lllllleelLLLEEEELL----lllL---------L--EEEEEEE-EL-L----------LL-EEEEELL---L-L----L-lL---LEEEEH--HHEELlllll
0042 4anjA   -----------LLLEEEEE----lllE---------E--EEEEEEE-EL-L-----------L-EEEEEEL-------------L---LEEEEH--HHLEElllll
0043 2budA   -lllllllLLLLLLEEEEL----lllL---------E--EEEEEEE-EE-LLlllL---L-LL-EEEEELL---L-L----LllL---LEEEEL--LLEELlhhhh
0044 2lcsA   -------LLLLLEEEEELL-------LlllllllllL--LEEEEEE-EE-L----------LL-EEEEELL---L-L----L--L---EEEEEL--LLEEElllll
0045 1tg0A   -------llLLLEEEEELL-------LlllllllllL--LEEEEEE-EL-L----------LL-EEEEEEE---L-L----E--E---EEEEEH--HHEEEll---
0046 8hyjW   -------lllLLLLEEELL----lllL---------L--LEELLLL--------------------LEELL---L-L----L--L---EELLLH--HHEEL-----
0047 6rwlA   -hhhhhhHLLLLLEEEELL--lllllL---------L--LLEEEEE-EL-L-----------L-EEEEELL----------L--L---EEEEEH--HHEEEel---
0048 1wfwA   -----lllLLLLLEEELLL-------LlllllllllL--LEEEEEE-EL-L----------LL-EEEEEEL---L-L----L--E---EEEEEH--HHLLLlllll
0049 6bogA   -------lllLLLEEEELL-------L---------HhhLEEEEEE-EL-L-----------L-EEEEEEL---L-L----L--E---EEEEELllLLEEElllll
0050 7ud6A   -------------LEEEELL--------llllllllL--LEEEEEE-EL-L----------LL-LEEEEEL---L-L----L--L---EEEELH--HHEEElllll
0051 5zwxA   -hhhhhllllLLLEEEEEEE-----llE--------E--EEEEEEE-EE-L----------LL-EEEEEEL---L-L----L--E---EEEEEH--HHEEElleee
```

Job: Type-3 BREX; BrxHII-Helicase WP_162523516 C-Terminal Inactive PDDEXK-REase Fold
Query: s001A
No: Chain Z rmsd lali nres id PDB Description
1: 6bog-A 6.1 2.8 76 967 14 MOLECULE: RNA POLYMERASE-ASSOCIATED PROTEIN RAPA;
2: 5lth-A 4.6 2.8 63 334 10 MOLECULE: HEME DEPENDENT OXIDATIVE N-DEMETHYLASE;
3: 4qbn-A 4.4 3.2 59 93 10 MOLECULE: NUCLEASE;
4: 6hcz-A 3.9 3.7 55 65 7 MOLECULE: SJOEGREN SYNDROME/SCLERODERMA AUTOANTIGEN 1;
5: 6m1u-A 3.5 4.0 59 122 10 MOLECULE: RNA N6-ADENOSINE-METHYLTRANSFERASE METTL16,RNA N6
6: 5wi2-B 3.2 2.9 53 93 11 MOLECULE: CDNA FLJ56409, HIGHLY SIMILAR TO SERINE/THREONINE
7: 6rrv-A 3.0 3.4 53 127 2 MOLECULE: REGULATORY PROTEIN SIR4;
8: 2okf-A 3.0 3.5 55 129 5 MOLECULE: FDXN ELEMENT EXCISION CONTROLLING FACTOR PROTEIN;
9: 5ux0-D 3.0 3.7 56 639 14 MOLECULE: ARGONAUTE PROTEIN;
10: 1v5s-A 2.9 4.3 60 126 3 MOLECULE: MAP/MICROTUBULE AFFINITY-REGULATING KINASE 3;
11: 6rfl-I 2.9 5.6 58 773 14 MOLECULE: DNA-DEPENDENT RNA POLYMERASE SUBUNIT RPO132;
12: 3vn5-A 2.9 2.4 45 255 11 MOLECULE: RIBONUCLEASE HIII;
13: 2fug-7 2.8 3.9 59 127 12 MOLECULE: NADH-QUINONE OXIDOREDUCTASE CHAIN 1;
14: 8ka7-A 2.8 2.6 49 115 8 MOLECULE: DE NOVO DESIGN PROTEIN -NB7;
15: 9j8p-A 2.8 3.2 55 623 7 MOLECULE: SPECKLE TARGETED PIP5K1A-REGULATED POLY(A) POLYME
16: 6hpb-A 2.8 2.1 43 57 16 MOLECULE: MRNA INTERFERASE TOXIN HICA;
17: 3t4n-A 2.7 3.0 55 139 2 MOLECULE: CARBON CATABOLITE-DEREPRESSING PROTEIN KINASE;
18: 6l7x-A 2.7 5.0 53 203 17 MOLECULE: MRNA_TRIPASE DOMAIN-CONTAINING PROTEIN;
19: 9nl2-A 2.7 3.7 72 1107 8 MOLECULE: R2 RETROTRANSPOSON PROTEIN;
20: 2p09-A 2.7 2.8 47 69 4 MOLECULE: A NON-BIOLOGICAL ATP BINDING PROTEIN WITH TWO MUT

```
0001 s001A   ------ELSI-DILKNI---KYGEIYELLRTMFINYVLY-NG------GTV-KEGKN-------------------G-F-DITIDG-------V-------------RRYKGVT-FD--------------PSGN-GA--N-AIT-LNHE-Y-V-KK-M-I-QE--
0002 6bogA   eihsngGEKAqALAESI--eEQDDD-TNLIAFAXNLFDI-IG------INQ-DDRGD------------------nX-I-VLTPS-------sE-------------DGIT-IT-FD--------------REVR-ED--AqFIT-WEHP-L-I-RN-G-L-DLil
0003 5lthA   rarvleldpgrclvmpH---MAQAAWDTLAMLMEHLARD-YP----qhFRL-TRQGD-------------------A-W-HWQNLA------lG-------------IDQR-FT-FG--------------D----PA--S-LpP-LEY---iT-RQ-M-Q-GDfa
0004 4qbnA   ----------------------ATKEGRVQKYAKERFEA-LG------GLV-RKLSP-------------------D-L-LVILPR-------G-------------VIWF-VE-VK----------kdenTKPD---nvF-VVG-SFK---qV-DK-Li------
0005 6hczA   ----swEPPT-EAETKV--lQARRERQDRISRLMGDYLL-RG------YRM-LTCAD-----------------cgT-I-LLQDKQ-------R-------------KIY----------------------------vaC-QEL---------------------
0006 6m1uA   asvmkelslkasplRSE---T-AEGIVVVTTWIEKILTD-LK------VQH-KRVPC--------------gkeevS-L-FLTAI-------yL-------------FKCL-IN-VK--------------KE---------------CT-Y-I-RN-Q-I-FRlv
0007 5wi2B   ------------------rfftkLDADKSYQCLKETCEK-LG------YQW-KKSC------------------mnQ-V-TISTT-------nK-------------LIFK-VN-LL--------------EM---------------FL-K-I-KG-K-L-IDiv
```

```
0008 6rrvA   klisvslvdefpselSD---SDRQIINEKMQLLKDIFANnLK------SAI-SNN--------------------------------------------------freSDIIIL--K--------------GEIE-DyrfW-SFM---RF-V-SnFD-I-Q-----
0009 2okfA   -------------------------RDVFHEVVKTALKK-DG------WQItDDPLT----------isvggvnlkL-I-AAER-Q-------G-------------QKIA-VE-VKsflkqssaisefhtALGQ-F-vlY-LAV-PLktiiyD-VE-Qe------
0010 5ux0D   nvfqykvekeeiikeEE---DKKLFVKTLYKYIKKLFLD-ND------FYF-KK----------------------G-N-NFISNS-------EvfsldsnenvnahLTYK-IK-IH--------------NIsylsiyniksgfgilLG-F-L--N-K-I-TNln
0011 1v5sA   hlihnvhkeE-HAHahnkfqgssGSSGDMMREIRKVLGA-NN------CDY-EQRE------------------rfL-L-FCVHGD------nL-------------VQWE-ME-VC--------------KLplnGV--R-F-----SK-I-A-NE-L-Kl----
0012 6rflI   eeistsLSFN-D--KNT---TD-EMTYNLYDLFFNTL------------DM-YLRVK-------------------Y-V-YLEN-----------------------TGKIYKNsFS--------------ED-H-NN--S-------GK-V-I-IPl--------
0013 3vn5A   --------------------pslkISPSEAEKIQNYLVS-SG------FRK-INAPY-------------------TlW-ALEGN--------G-------------VKVY-------------------------------------LK-E-V-LN-L-Le----
0014 2fug7   -------------ASSE--rELYEAWVELLSWMREYAQA-KG------VRF-EKEADfpdfiyrmerpydlpttimT-A-SLSD-G-------L-------------GEPflLA-DV--------------SP-----lhahyekpLTKE-R-F-FA-La------
0015 8ka7A   aavaalraadpgaarrrv-gPDAARVQALADAVVAALER-EG------FKL-EKKEE---------ntdaagnagaK-Y-EGE------------------------GGLV-LN-VK--------------QG---------peT-LKITr---------------
0016 9j8pA   qrrssrgrdwgllpllqpiplplAPFTQLTAALVQVFREaLG------CHI-EQASA-------------------S-W-RCALWHrvwqgrpL-------------LSFV-AS-VS--------------PA---------------QV-F-L-PQ-A-I-RHlk
0017 6hpbA   ----------------------------KQSEFRRWLES-QG------VDV-ANGSN-------------------H-L-KLRF-H-------G-------------RRSV-XP-RH------------------------pcdK-EPLR-Ks-------------
0018 3t4nA   gspaaskisplvtkkskt-irSRSYPLDVMGEIYIALKN-LG------AEW-AKPS---------------eedlwT-I-KLRWK-------dL-------------MKMV-IQ-LF--------------QI---------------TT-K-L-IM-E-L-AVns
0019 6l7xA   emrlgqfkvE-EDA-----cvSTEDYERIKTYLMTEME---N------SSM-TRSVD-------------------R-L-SITD-G-------M-------------FRYD------------------mtqvtvlMH--E-VE----AM-R-L-ATl--------
0020 9nl2A   crnctaSYES-LSHILGq-gARIRRHNKLCSMLKREAKE-LK------WVV-YEERK-------------------P-DlIFVK-E-------E-------------MALV-VD-VT--vrfeykekvfedaaAE-KVeyF-GFPpEINE--pdykR-T-A-KRfs
0021 2p09A   --------------------DDDDKKTNWLKRIYRV-RP-CVkckvapRDW-KVKNK-------------------H-L-RIYNM-------yH-------------GHVD-WL-MY--------------ADS--------------------------------

0001 s001A   ------LLLL-HHHHLL---LHHHHHHHHHHHHHHHHHH-LL------LEE-EEELL-------------------E-E-EEEELL-------L-------------EEEEEEL-LL--------------LLLL-LL--L-LLL-LLLH-H-H-HH-H-H-HL--
0002 6bogA   hhhhhlHHHHhHHHHHH--hHHLLL-HHHHHHHHHHHHH-HL------LEE-EELLL------------------lE-E-EEELL-------lL-------------LLEE-EE-LL--------------HHHL-LL--LeELL-LLLH-H-H-HH-H-H-HHhh
0003 5lthA   hhhhhhhlhhheeelhH---HHHHHHHHHHHHHHHHHHH-LL----llEEE-EEELL-------------------E-E-EEEELL------lL-------------EEEE-EE-LL--------------L----HH--H-LlH-HHH---hH-LL-L-L-LEee
0004 4qbnA   ----------------------LLHHHHHHHHHHHHHHH-LL------LEE-EELLL-------------------L-E-EEEEHH-------H-------------EEEE-EE-EL----------llllLLLL---eeE-EEL-LHH---hH-HH-Hh------
0005 6hczA   ----llLLLL-HHHHHH--hHHHHHHHHHHHHHHHHHHH-LL------LEE-EELLL-----------------llL-E-EEELLL-------L-------------LEE----------------------------llL-LLL---------------------
0006 6m1uA   hhhhhhhhhhhlllLLL---L-LLHHHHHHHHHHHHHHH-LL------LEE-EEELL--------------llleeE-E-EEEEE-------eE-------------EEEE-EE-EE--------------EE---------------HH-H-H-HH-H-H-HHhh
0007 5wi2B   ------------------eeeelLLHHHHHHHHHHHHHH-LL------LEE-EEEE------------------llE-E-EEEEE-------lE-------------EEEE-EE-EE--------------EL---------------HH-H-H-HH-H-L-HHhe
0008 6rrvA   llleeeellllllllLH---HHHHHHHHHHHHHHHHHHHlLL------LEE-ELL--------------------------------------------------hhhLLEEEE--L--------------LLHH-HleeE-EHH---HH-H-HhLL-L-L-----
0009 2okfA   -------------------------LHHHHHHHHHHHHH-LL------LEEeELLLL----------leelleellL-E-EEEE-L-------L-------------EEEE-EE-ELlllllllhhhhhhhHHHH-H-eeE-EEE-EHhlhheE-LL-Ll------
0010 5ux0D   hhhhheeeeeeeeelLL---LHHHHHHHHHHHHHHHHHH-LL------EEE-EL----------------------L-L-EEEEEE-------EeeelllllleeeeEEEE-EE-EE--------------EEleeeeeellllehllLH-H-H--H-H-L-LLll
0011 1v5sA   lllllllllL-LLLlllllllllLLHHHHHHHHHHHHHH-LL------EEE-EEEE------------------llE-E-EEEELL-------lL-------------EEEE-EE-LL--------------LLlllLL--E-E-----HH-H-H-HH-L-Ll----
0012 6rflI   eeeehhHLLL-L--LLL---LL-LHHHHHHHHHHHL------------LE-EEELL-------------------E-E-EELL-----------------------LLLEEELlLL--------------LL-L-LL--H-------HH-H-H-HHh--------
0013 3vn5A   --------------------leeeLLHHHHHHHHHHHHH-LL------LEE-LLLLL-------------------LlE-EEEEL--------L-------------EEEE-------------------------------------HH-H-H-HL-L-Ll----
0014 2fug7   -------------LHHH--hHHHHHHHHHHHHHHHHHHH-HL------LEE-EEEEElhhhhllllllllllllleeE-E-EEEL-L-------L-------------LLEeeEE-EE--------------LL-----eeeeeellLLHH-H-H-HH-Hh------
0015 8ka7A   hhhhhhhhhlllhhhlle-eLLHHHHHHHHHHHHHHHHH-LL------LLL-EEEEE---------eellllleeeE-E-ELH------------------------HHLE-EE-EE--------------EL---------llE-EEEEl---------------
0016 9j8pA   hlllllllllllhhhhlllllllLLHHHHHHHHHHHHHLlLL------LEE-EELLE-------------------E-E-EEEELLlllllhlL-------------EEEE-EE-EL--------------LL---------------HH-H-H-HH-H-H-HHll
0017 6hpbA   ----------------------------LHHHHHHHHHH-LL------LEE-ELLLL-------------------L-E-EEEE-L-------L-------------EEEE-EL-LL------------------------lllL-HHHH-Hl-------------
0018 3t4nA   lllhhhhlllllllllll-eeELLLHHHHHHHHHHHHHH-HL------LEE-ELLL---------------hhhllE-E-EEEEL-------lE-------------EEEE-EE-EE--------------EE---------------HH-H-H-HH-H-H-HHll
0019 6l7xA   eeeeeeellL-LLL-----llLHHHHHHHHHHHHHHLL---L------LEE-EEEEE-------------------E-E-EEEE-L-------L-------------EEEE------------------eeeeeeeEE--E-EE----HH-H-H-HLl--------
0020 9nl2A   llllllLLLL-HHHHHHl-hHHHHHHHHHHHHHHHHHHH-LL------LEE-EELLL-------------------L-LeEEEL-L-------L-------------LEEE-EL-EE--eellllllhhhhhhHH-HHelL-LEElHHHH--lhhhH-H-H-HHhh
0021 2p09A   --------------------LHHHHHHHHHHHHHHH-LL-LLllllllLLE-EEELL-------------------E-E-EEELL-------lL-------------LEEE-EE-LL--------------LLL--------------------------------

Job: Structural Search Anchored on "C-terminal Inactive PDDEXK-REase Domain" Found in RapA-Helicase;
Query: s001A
No: Chain Z rmsd lali nres id PDB Description
1: 6bog-A 17.4 0.0 86 967 100 MOLECULE: RNA POLYMERASE-ASSOCIATED PROTEIN RAPA;
2: 4qbn-A 4.1 2.9 63 93 10 MOLECULE: NUCLEASE;
3: 2x3l-B 3.9 3.4 62 423 13 MOLECULE: ORN/LYS/ARG DECARBOXYLASE FAMILY PROTEIN;
4: 1y88-A 3.6 3.3 67 184 12 MOLECULE: HYPOTHETICAL PROTEIN AF1548;
5: 2xnk-A 3.3 2.9 58 291 3 MOLECULE: DNA TOPOISOMERASE 2-BINDING PROTEIN 1;
6: 2okf-A 3.3 2.8 60 129 8 MOLECULE: FDXN ELEMENT EXCISION CONTROLLING FACTOR PROTEIN;
7: 9esh-m 3.3 3.1 51 81 6 MOLECULE: PRE-MRNA;
8: 5y88-U 3.3 3.0 49 488 4 MOLECULE: PRE-MRNA-SPLICING FACTOR 8;
9: 7p0j-A 3.2 2.9 56 195 11 MOLECULE: DNA DAMAGE RESPONSE PROTEIN MDB1;
10: 5lth-A 3.1 3.2 58 334 12 MOLECULE: HEME DEPENDENT OXIDATIVE N-DEMETHYLASE;
11: 5wi2-B 3.1 3.1 54 93 11 MOLECULE: CDNA FLJ56409, HIGHLY SIMILAR TO SERINE/THREONINE
12: 9n82-F 3.1 2.9 55 699 11 MOLECULE: X-RAY REPAIR CROSS-COMPLEMENTING PROTEIN 6;
13: 2zyz-C 3.1 2.7 56 96 7 MOLECULE: PUTATIVE UNCHARACTERIZED PROTEIN PAE0789;
14: 5hy3-A 3.1 4.0 55 252 15 MOLECULE: MRNA ENDORIBONUCLEASE LSOA;
15: 9n83-F 3.0 2.9 55 866 11 MOLECULE: X-RAY REPAIR CROSS-COMPLEMENTING PROTEIN 6;
16: 8yax-A 2.9 3.7 55 1314 11 MOLECULE: PAPAIN-LIKE PROTEASE NSP3;
17: 9nl2-A 2.9 3.4 67 1107 6 MOLECULE: R2 RETROTRANSPOSON PROTEIN;
18: 3cxj-A 2.9 3.1 52 144 12 MOLECULE: UNCHARACTERIZED PROTEIN;
19: 5vzl-C 2.9 4.5 59 87 5 MOLECULE: SINGLE GUIDE RNA (116-MER);
20: 2czr-A 2.9 4.8 67 226 6 MOLECULE: TBP-INTERACTING PROTEIN;
21: 6m1u-A 2.8 4.4 60 122 12 MOLECULE: RNA N6-ADENOSINE-METHYLTRANSFERASE METTL16,RNA N6
22: 9u48-A 2.8 3.5 58 336 7 MOLECULE: U6 SMALL NUCLEAR RNA (ADENINE-(43)-N(6))-METHYLTR
23: 8v9u-A 2.8 3.6 51 81 4 MOLECULE: DNA (CYTOSINE-5)-METHYLTRANSFERASE 1;
24: 9bf5-C 2.8 3.4 62 1190 5 MOLECULE: SSDNA1;
25: 1fok-A 2.8 3.4 72 568 13 MOLECULE: DNA (5'-
26: 6c9j-A 2.8 3.3 54 386 6 MOLECULE: 5'-AMP-ACTIVATED PROTEIN KINASE CATALYTIC SUBUNIT
27: 7lyc-N 2.8 3.5 56 335 9 MOLECULE: HISTONE H3.1;
28: 4a0g-A 2.8 3.6 61 769 5 MOLECULE: ADENOSYLMETHIONINE-8-AMINO-7-OXONONANOATE
29: 2qsr-A 2.7 3.9 73 161 12 MOLECULE: TRANSCRIPTION-REPAIR COUPLING FACTOR;
30: 3r3p-B 2.7 3.5 57 98 11 MOLECULE: MOBILE INTRON PROTEIN;
31: 5u0p-Q 2.7 2.9 54 508 7 MOLECULE: MEDIATOR COMPLEX SUBUNIT 14;
32: 8a5w-B 2.7 3.6 61 367 10 MOLECULE: PHOSPHOSERINE AMINOTRANSFERASE;
33: 2hc5-A 2.7 3.3 61 116 10 MOLECULE: HYPOTHETICAL PROTEIN YVYC;
34: 2ost-A 2.7 3.3 60 148 7 MOLECULE: SYNTHETIC DNA 29 MER;
35: 1whz-A 2.7 2.9 52 70 10 MOLECULE: HYPOTHETICAL PROTEIN;
36: 9j8p-A 2.7 3.4 56 623 13 MOLECULE: SPECKLE TARGETED PIP5K1A-REGULATED POLY(A) POLYME
37: 8que-E 2.6 4.3 59 493 12 MOLECULE: PHIKZ055;
38: 6l30-A 2.6 4.0 55 518 4 MOLECULE: PROTEIN ECT2;
39: 5yrz-B 2.6 2.9 48 58 15 MOLECULE: HICB;
40: 6wm6-A 2.6 3.3 61 536 13 MOLECULE: EXTRACELLULAR SOLUTE-BINDING PROTEIN, FAMILY 5;
41: 6hpb-A 2.6 2.4 43 57 5 MOLECULE: MRNA INTERFERASE TOXIN HICA;
```

```
42: 7t8b-A 2.6 5.4 52 518 8 MOLECULE: TWINKLE MTDNA HELICASE;
43: 4wl5-A 2.6 3.4 72 594 11 MOLECULE: HETERODIMERIC RESTRICTION ENDONUCLEASE R.BSPD6I L
44: 8q3b-E 2.6 3.6 62 205 6 MOLECULE: DNA-DIRECTED RNA POLYMERASE RPB1 HOMOLOG;
45: 6ijb-B 2.5 3.1 58 538 9 MOLECULE: AMP-BINDING DOMAIN PROTEIN;
46: 9dev-A 2.5 2.8 50 97 6 MOLECULE: PROTEIN MONO-ADP-RIBOSYLTRANSFERASE PARP4;
47: 7myj-C 2.5 2.9 53 464 4 MOLECULE: 5'-AMP-ACTIVATED PROTEIN KINASE CATALYTIC SUBUNIT
48: 1ob8-B 2.5 3.9 64 127 13 MOLECULE: HOLLIDAY-JUNCTION RESOLVASE;
49: 5oe9-C 2.5 3.8 55 415 11 MOLECULE: LARGE SUBUNIT TERMINASE;
50: 7wae-A 2.5 3.5 48 877 10 MOLECULE: CYANOPHYCIN SYNTHASE;

0001 s001A   ----DDTN-LIAFAXNLFDI---IGINQDDRG---------DNXIVLTPSDHX----LVPD--F--PGL--SEDGITITFDR---------E-V--A----LARE-----D-AQFITWEH-----PLIRNGLDLIL
0002 6bogA   eeqdDDTN-LIAFAXNLFDI---IGINQDDRG---------DNXIVLTPSDHX----LVPD--F--PGL--SEDGITITFDR---------E-V--A----LARE-----D-AQFITWEH-----PLIRNGLDLIL
0003 4qbnA   --atKEGR-VQKYAKERFEA---LGGLVRKLS---yegrsgAPDLLVILPR---------------------GVIWFVEVnT---------K-P--Dph-qLREH------nVFVVSFKQ---vdKLIEH------
0004 2x3lB   ydstLFFA-KRAQLIECLEN---KGFEMLQVD---------DLKLLIKYEG-ftghdiqNW--F--M--laDDYQALAILPL-----------------------------------LFD-----SLLRKIEDMI-
0005 1y88A   ----NLYF-QGHMVARLLEE---HGFETKTNV-vqgncveqEIDVVAERD----------------------GERYMIECKfhnipvytglK-E--A----MYTY------qPWIFTNTwsypekEGIEVLLESKG
0006 2xnkA   sctslEKE-KREEVHKYVQX---XGGRVYRD---------------------------------------lnvSVTHLAGEV---------G-SkkY----LVAA--nlkK-PILLP--S-----WIKTLWEKSQE
0007 2okfA   -----RDV-FHEVVKTALKK---DGWQITDPL---------TKLIAAERQ----------------------GQKIAVEVAL---------G-Q--F----INYR------vLYLAVPLqldfpkeiIFQW-----
0008 9eshm   -------S-FRELVEEFCAE---NSLLFVPLR---------RALFRISTQA-------------------sKARGITVYLRN-----diiwkkspgase-dTPYD-------------------pIGFNE------
0009 5y88U   elgkvsSS-FKDVVEDYCLE---KGYLISKIPnygrdqdciVPLFEIRNG----------------------KKKMEVALK---------hdilwveds-sGTFK---------------I-----YLWA-------
0010 7p0jA   airdSMVGdSIHGLYSILET---SGAEIVGDI---------K---------------------------aqeKDTIILAQnD---------Q-E--G----RNMS-atglN-VYKI---E-----LVALSILRD--
0011 5lthA   vmphMAQA-AWDTLAMLMEH---LAFRLTRQG----------DAWHWQN-------------lA--LGI-----DQRFTGDP---------A------------------------PLEY-------TRQMQG---
0012 5wi2B   ftklDADK-SYQCLKETCEK---LGYQWKKSC---------MNQVTISTTD------------------rnnKLIFKVNLLE---------M---------------------------F-----LKIKGKLI---
0013 9n82F   vmsgtDSQ-PKPDLENRIAE---FGGYIVQN--------------------------------------pgpDTYCVIAGSE---------NiR--V----KNIIlsnkhD-VVKP---A-----WLLECFKTKS-
0014 2zyzC   -----MDV-LQEQVFKDLKS---RGFKIEQLD---------DKIFIAEK-----------------------KERYLFYVM---vegvevtiqT--L----LSVI------pVVLALVTV-----TY---------
0015 5hy3A   ipkfsDDD-RANLFEFLSE----EGITITEDN---------NHQYIMTT-----------------------SNGDRVRAKIS------------------------------------A-----SLINDFMCS--
0016 9n83F   vmsgtDSQ-PKPDLENRIAE---FGGYIVQN--------------------------------------pgpDTYCVIAGSE---------NiR--V----KNIIlsnkhD-VVKP---A-----WLLECFKTKS-
0017 8yaxA   ltsssKTP-EEHFIETISLA---GSYSGQSTQ---------L-GIEF-------------------lkR--GDKSVYYTSNP----------------------------------TFDN-----LKTLLSL--R
0018 9nl2A   arirRHNK-LCSMLKREAKE---LKWVVYEEP-httekelrKPDLIFVK----------------------EEMALVVDVaA---------E-K--V----RHYK------eYFGFPLIN-----EK-vGMPDQQK
0019 3cxjA   -------L-SQEXIKKWLDE---EGFLRXEVP---------DFHYVVNY---------------------peDHVIDIIQPA---------G------------------------FFDG-----LSKDRLIS---
0020 5vzlC   -------M-NINDLIREIKN---KDYTVKLSG----tdsnsITQLIIRV--------------------nnDGNEYVIS--------------E--S----EN--------sieKFISAF-----K-MQTITLKSE
0021 2czrA   hmyaelSP-GTKKVYTQVRY---LDYHWEIEG----------STITGIHKK--------------------sNVKVVIDVAD---------S-L--A----GKD-----ViHIVAIPDL-----TYRYLKA----
0022 6m1uA   etaeGIVV-VTTWIEKILTD---LKVQHKRVP---------CVSLFLTAIE--------------------lqyLFKCLINVKK---------E------------------------C-----TYIRNQIFRLV
0023 9u48A   ldpelCAQ-IDDILQKFLDD---NKIPWSKKG---------S-VLEISTKSIT----WSRK---------legQMKCELNVID--------------------------------------C-----SALARALRD--
0024 8v9uA   dlerdSLT-EKECVKEKLNL---LH-------------------------EF----LQTE--IknQLC--D-lETKL----------------------rkE-----E-LSEE---A-----KVKSLLNKDLS
0025 9bf5C   tniyKGAL-GEQAVEAVLTA---FDFTFEEVE---------RFDNRVIFA--------------------gIEQPIWLDS------kywkhegnesseg-ySSKI------kFIYVNALI-----PALI--TNRT-
0026 1fokA   rnstQDRI-LEMKVMEFFMK--vYGYRGKHLG------gsrKPDGAIYTVG------------------spiDYGVIVDTkaysggynlpiG-Q--A----DEMQryveenkFLFVSGHV-----LSVEELLIGGE
0027 6c9jA   rsqsRPND-IMAEVCRAIKQ---LDYEWKVVN---------PYYLRVRRKN------------------tstYSKMSLQLYQ---------V-------------------------yA-----NLIKILAQ---
0028 7lycN   igsglSSE-QQKMLSELAVI---LKAKKYTEF---------D------------------------------sTVTHVVsT---------L-K--C----MLGI---lnG-CWILK--F-----EWVKACLRRKV
0029 4a0gA   sqgktlrelwDEELVQQISS---HVQRVVV-I---------GTLFALELK------slyaK--S--LL-rpLGNVIYLMCGP---------C-T--S---------------piCRRL-T-----KLYKRLGEFNR
0030 2qsrA   pdvvAYLL-EIGLVKSYLDK---VFVRVERK---------DNKITIQFEKVTq--rLFLAqdY--FKAnkgLXELVFD-vQ---------N-K--K---------------yEILE-L-----IFGESLLEIKE
0031 3r3pB   -----sTT-PERRVKEILDE---MDIVYFTHH----vvegwNVAFYLG---------------------KKLAIEVN-----gvywaskqknvnk-dKRKL------rVLTIEDDElndidKVKQQIQK---
0032 5u0pQ   ifyyNLNQ-ESLEFQRWLKQ---RDISFKFMP---------NISWRIMV--------------------snfEPAIFIQHTP---------T------------------------SSLK-----NVCQYIE----
0033 8a5wB   amekLSSI-KSQTIYEIIDN---SFYVCPV------------MNIPFRIGNakgddaleKR--F--L--hrsvGGIRASLYN---------A------------------------DVQK------LAAFMKKFLE
0034 2hc5A   pvhqvSYT-NLAEMVGEMNK---LHLKFELHD---------KYYVKVIEDS------------------tnEVIREIPP----------------------------------KRWL--D-----FYAAMTEFLGL
0035 2ostA   stklKGDI-AQQAAIMRALK---MGWGVLKPL---------SYDLVFDVE--------------------GILLKVQVnyvvdnrrtrtnrrnivrs-pYRGN-----D-FFAVAYLF-----YVtEAWH----
0036 1whzA   ---xwxpP-RPEEVARKLRR---LGFVERXAK---------GGHRLYTHPD-------------------grIVVVPFHS--------------------------------LPKG---tfKRIL-LTEEE-
0037 9j8pA   lplaPFTQ-LTAALVQVFRE--aLGCHIEQA----------ASWRCAL---------wHR--V--WqgtePLLSFVASVSP---------A------------------------H-----FLQVFLPQA--
0038 8queE   dpntgvnM-TRRYIDSLFDI---SYEGNRACT-------plkYMIDLMFSY------------------gemLYPRPMLLPA---------FkrgnmvtingakyigspvlkqdhhLMSE------FNYAVSMFGY
0039 6l30A   ftgfRKKE-ELVRLVTLVHH---MGGVIRKDF------------------------------------nskvTHLVANCT---------Q-G----e-kFRVAvslgtP-IMKP--------ewIYKAWERR--
0040 5yrzB   -------pmTQKEMVKLLTA---HGWIKTRGK---------GSHIKMEKQG------------------ERPITILHG-------------------------------------eLNKYR-----GIRK-------
0041 6wm6A   vtsalGNN-AVVRTVTLPIVpsgGPYMVSFDP---------GQLVTLVPNPKW----YGEK--G--PYL--D--KLKFRIIT---------DstqqltalenGEV-----D-----afayqlptaliAYNT-----
0042 6hpbA   ---------KQSEFRRWLES---QGVDVANGS---------N-HLKLRF--------------------HGRRSVXPRH---------------------------------KEPL-----RK---------
0043 7t8bA   ------PV-TATEIRQYLRG---HGIPFQDGH----------SCLRAL-------------------sP--FSFSLFIDKT----------------------------------SWE-----DFQASV-EGEF
0044 4wl5A   fianKNTV-FEWLTWNGFII---LGaEYKNNFvpvthaagnQPAMEIIY--------------------EDFIVLGEVES---------E-P--V----TRHY------yCLFIAPLK-----QFNMLLMVQKK
0045 8q3bE   ---maMQK-LFTYIYEFIEY---RKMVLLE------------FRINAETL--------------------nHGIVSVFIF------gangkyvhhggd-mRTLL------eLILIVYPY-----HLF---NIPKV
0046 6ijbB   gtlaWNNR-RHLEIYYAASG---AGFVCHTIN---------P-----------------------------nhaQDRVLFFDAT-----------F--L----PLVA------hFVLMG--D-----ELI-ETGD--T
0047 9devA   kvkylPQQ-QKKKLQTDIKE---NGGKFSFSL--------------------------------------npQCTHIILDNA---------D-V-----------------hIANP---D-----FIWKSIREKR-
0048 7myjC   rsqsKPYD-IMAEVYRAMKQ---LDFEWKVVN---------AYHLRVRRKN------------------tgnYVKMSLQLYL---------V-------------------------dffemcASLITTLA---
0049 1ob8B   ---dIGKN-AERELVSILRG---EGFNAVRIP------tnpLPDIFATK--------------------GNTLLSIECstwenkvkvkeH-Q--V----RKLL-----DvPLIAIKFtidnsiPIEDLFKI---
0050 5oe9C   ktigALRRnVITPLKRMLKS---RGYRVKDHR--------aDNYLTITF--------------------KGKTNYFYLFG--------gkdessqdliqgITL-----A-----FFDE---------------
0051 7waeA   knqaslGP-STEAIVKEAEA---RGIPWTQLG--------aRFMIQFG-------------------Y--GVNQKKIQA---------tlsnQ--T----GI-------------kllarnvagAVMDM------

0001 s001A   ----LLHH-HHHHHHHHHHH---HLLEEEELL---------LLEEEEEELLLL----LLLL--L--LLL--LLLLEEEELLH---------H-H--H----HHLL-----L-LEELLLLL-----HHHHHHHHHHH
0002 6bogA   hhhlLLHH-HHHHHHHHHHH---HLLEEEELL---------LLEEEEEELLLL----LLLL--L--LLL--LLLLEEEELLH---------H-H--H----HHLL-----L-LEELLLLL-----HHHHHHHHHHH
0003 4qbnA   --llHHHH-HHHHHHHHHHH---LLLEEEELL---llllllLLLEEEEEHH---------------------HEEEEEEElL---------L-L--Lhh-hHHHH------eEEEELHHH---hhHHHHH------
0004 2x3lB   lllhHHHH-HHHHHHHHHHH---LLLEEEELL---------LLEEEEEELLL-llhhhhhHH--H--H--eeLLLLEEEELLL-----------------------------------LHH-----HHHHHHHHLL-
0005 1y88A   ----LHHH-HHHHHHHHHHL---LLLEEEEEE-eellllleeEEEEEEEEL----------------------LEEEEEEELlllllleelhH-H--H----HHHH------eEEEELLLlllllLLHHHHHHLLL
0006 2xnkA   eeellLHH-HHHHHHHHHHH---LLLEELLL--------------------------------------lllLLLEEELLL---------L-LhhH----HHHH--hllL-LEELL--H-----HHHHHHHHHHL
0007 2okfA   -----LHH-HHHHHHHHHHH---LLLEEELLL---------LLLEEEEEL--------------------LEEEEEEEHH---------H-H--H----HHHH------eEEEEEEHhlhhhhhEEEE-----
0008 9eshm   -------L-HHHHHHHHHHH---LLLEEEEEE---------EEEEEEELLL------------------lLLLEEEEEEEL-----leeeelllllll-lLLLE-------------------eLLHHH------
0009 5y88U   llllllLL-HHHHHHHHHHH---HLLEEELLLllllllleeeEELEEEELL--------------------LLLEEEELL---------llleeeell-lLLEE---------------E-----LLLL-------
0010 7p0jA   hhhhHLLLhHHHHHHHHHHH---LLLEEELLL---------L---------------------------hhlLLLEEELLlL---------H-H--H----HHHH-hhllL-LLLL---H-----HHHHHHHHL--
0011 5lthA   elhhHHHH-HHHHHHHHHHH---HHEEEEEEL----------LEEEEEE-------------lL--LLE-----EEEEELLH---------H------------------------HHHH-------HLLLLL---
0012 5wi2B   eellLHHH-HHHHHHHHHHH---LLLEEEEEE---------LLEEEEEEEL------------------lllEEEEEEEEEE---------L------------------------H-----HHHHHHLH---
0013 9n82F   ellllLLL-LHHHHHHHHHH---LLLEELLL--------------------------------------lllLLLEEELLLL---------LhH--H----HHHHhllllL-EELH---H-----HHHHHHHHLL-
0014 2zyzC   -----LLH-HHHHHHHHHHH---LLLEEEEEE---------LLEEEEEL----------------------LLEEEEEEE---elllllllhhH--H----HHHH------lEEEEEELE-----EE---------
0015 5hy3A   ellllLLH-HHHHHHHHHH----LLLEEEEEL---------LLEEEEEL---------------------LLLLLLLLLLL-------------------------------------H-----HHHHHHHHH--
0016 9n83F   eeellLLL-LHHHHHHHHHL---LLLEELLL--------------------------------------lllLLLEEEELLL---------LhH--H----HHHHhhlllL-EELH---H-----HHHHHHHHLL-
0017 8yaxA   hhlllLLH-HHHHHHHHHHH---LELLLLLLL---------L-LEEE-------------------eeE--LLEEEEEEELLL-----------------------------------LHHH------HHHHHHL--L
0018 9nl2A   hhhhHHHH-HHHHHHHHHHH---LLLEEEELL-llllllllLLLEEEEL----------------------LLLEEEELEhH---------H-H--H----LLLH------eLLLEEEHH-----HH-hLLLHHHH
0019 3cxjA   -------L-HHHHHHHHHHH---LLLEEEELL---------LEEEEEEL---------------------llLLEEEEEEEL---------L-------------------------EHHH-----LLHHHHHH---
```

```
0020 5vzlC   -------L-LHHHHHHHHHH---LLLLLLLLL----lllllLLLEELLL-------------------llLLLLLEEL-------------L--L----LL--------lhhHHHHHL-----L-HHHHHHLLL
0021 2czrA   hhhhllLH-HHHHHHHHHLL---LLEEEEEEL----------LEEEEEELL--------------------lLLEEEEEEHH---------H-H--L-----LLL-----LeEEEEELLE-----EHHHHHH----
0022 6m1uA   llllHHHH-HHHHHHHHHHH---LLLEEEEEL---------LEEEEEEEEE-----------------lleEEEEEEEEEE---------E-------------------------H-----HHHHHHHHHHH
0023 9u48A   llllhHHH-HHHHHHHHHHH---LLLLLLLLL---------L-LLLLLLLLL----LLHH--------lllLLEEEEELLL---------------------------------H-----HHHHHHHHH--
0024 8v9uA   hhhhhLLL-HHHHHHHHHHH---HH------------------------LL----LLHH--HhhHHH--H-hHHHH-----------------------hhL-----L-LLHH---H-----HHHHHHHHHHH
0025 9bf5C   hllhHHHH-HHHHHHHHHHH---LLLEEELLL---------LLLEEEELL--------------------lLLLLEEEEE------lllllllllllhh-hHHHH------eEEEELLLE-----LLLL--LLHH-
0026 1fokA   llhhHHHH-HHHHHHHHHHH--lLLLEEEELL------lllLLLEEEELLL------------------lllLEEEEEEEelllllllllhH-H--H----HHHHhhhhhleEEEEELLE-----EEHHHHHHHHH
0027 6c9jA   eellLHHH-HHHHHHHHHHH---LLLEEEEEE---------LLEEEEEEEL------------------lllEEEEEEEEEE---------L--------------------------eH-----HHHHHHHL---
0028 7lycN   eelllLHH-HHHHHHHHHHH---HLLEEELLL---------L-----------------------------lLLLEEElL---------H-H--H----HHHH---hlL-LEEEE--H-----HHHHHHHHHLL
0029 4a0gA   llllllllllLHHHHHHHHH---LEEEEEE-E---------LLEEEEEEL------lhhhH--H--HH-llLLLEEEEELLL---------L-L--L---------------hhHHHH-H-----HHHHHHLLLLL
0030 2qsrA   lhhhHHHH-HHHHHHHHHHH---LLEEEEEL----------LLEEEEEELLLHh--hHLLHhhH--HHHellEEEEEEE-lL---------L-L--L----------------hHHHH-H-----HHHHHHHHHHH
0031 3r3pB   -----lLL-HHHHHHHHHHH---LLLLLEEEE----eelleEEEEEEE-------------------------LLEEEEEE-----llllllllllhhh-hHHHH------eEEEEEHHHhhhhhHHHHHHHHH---
0032 5u0pQ   hhhhHHHH-HHHHHHHHHHL---LLLLLLEEL---------LLEELLLL--------------------lllLLLLEEELLL---------L------------------------LLHH-----HHHHHHH----
0033 8a5wB   hhhhHHHH-HHHHHHHHHHL---LLEELLL------------LEEEEEELLllllhhhhHH--H--H--llllLLEEEELLL---------L------------------------HHHH------HHHHHHHHHH
0034 2hc5A   lllllLHH-HHHHHHHHHHH---HLEEEEEEE---------EEEEEEEELL--------------------llEEEEEELH-------------------------------HHHH--H-----HHHHHHHHHHH
0035 2ostA   llhhHHHH-HHHHHHHHHHH---LLLEEEEEL---------LLLEEEEEL----------------------LEEEEEEEeeeeelleeellllllleee-lLLLL-----L-LEEEEEEE-----EElLLLH----
0036 1whzA   ---llllL-LHHHHHHHHHH---LLLEEEEEEE---------LLLEEEEELLL--------------------llEEEEELLL-----------------------------------LLHH---hhHHHH-LLHHH-
0037 9j8pA   llllLHHH-HHHHHHHHHHL--lLLLEEEEL------------EEEEEEE---------lLL--L--Llllll LLEEEEEEELL---------L--------------------------H-----HHHHHHHHH--
0038 8queE   lllllllH-HHHHHHHHHHH---HEEEEEELL-------hhhEEEEEEEEE------------------leeLLLEEEEELL---------LllllleeeelleeeeelleeeeeeeHLHH------HHHHHHHHHH
0039 6l30A   eellLLHH-HHHHHHHHHHH---HLLEEELLL--------------------------------------llllLEEEELLL---------L-L----h-hHHHHhhhllL-EELL--------hhHHHHHHHL--
0040 5yrzB   -------llLHHHHHHHHHH---LLLEELLLL---------LLEEEEELLL--------------------LLLEEEELL------------------------------------lLLHHH-----HHHH-------
0041 6wm6A   lllllLLL-HHHHHHHLLLLlllLLEEEEEEL---------LLEEEEEELLLL----LLLL--L--LLL--L--EEEEEELL---------LhhhhhhhhhlLLL-----L-----hlleeeelleeHHHH-----
0042 6hpbA   ---------LHHHHHHHHHH---LLLEEELLL---------L-LEEEEE----------------------LLEEEEELLL-----------------------------------LHHH-----HH---------
0043 7t8bA   ------LL-LHHHHHHHHHH---LLLLEELLL----------LLEEEL--------------------lL--LLLLEEELLL-----------------------------------LHH-----HHHHHL-LLLL
0044 4wl5A   llllHHHH-HHHHHHHHHHH---HLeEEEELLeeeelllllLLLEEEEE----------------------LLEEEEEEELL---------H-H--H----HHHH------eEEEEELHH-----HHHHHHHHHHH
0045 8q3bE   ---lhHHH-HHHHHHHHHHH---LLLEELL------------EEEEEELL---------------------lLLEEEEEEE------llllllllllhhh-hHHHH------eEEEELELL-----HHH---LHHHL
0046 6ijbB   eeelLLLH-HHHHHHHHHHH---LLLEEEELL---------L-----------------------------hhhLLLEEEELHH-----------H--H----HHHH------eEEELL--H-----HHH-HHLL--L
0047 9devA   ellllLHH-HHHHHHHHHHH---LLLEEELLL-------------------------------------llLLLEEEELLL---------L-L------------------eEELH---H-----HHHHHHHHLL-
0048 7myjC   eellLHHH-HHHHHHHHHHH---LLLEEEELL---------LLEEEEEEEL------------------lllEEEEEEEEEE---------L---------------------------hhhhhhHHHHHHHL---
0049 1ob8B   ---lLLHH-HHHHHHHHHHH---LLLEEEELL------lllLLLEEEEE----------------------LLEEEEEEEeellleeeelhH-H--H----HHHH-----HeEEEEEEEellllEHHHHHHH---
0050 5oe9C   llhhHHHHhLHHHHHHHHHH---LLLEEEEEL--------lLLEEEEEE----------------------LLEEEEEEEEE--------lllhhhhhhhhhLLL-----L-----EEEL---------------
0051 7waeA   hhhlllLH-HHHHHHHHHHH---LLLLEEELL--------lLLLEEEL--------------------L--HHHLEEELL---------llllL--L----LH-------------ehllllhhhHHHHH------
```

Job: Type-3 BREX; BrxHII-Helicase_WP_162523516 (C-Terminal Most Alpha+Beta Domain)
Query: s001A
No: Chain Z rmsd lali nres id PDB Description
1: 6bog-A 5.6 3.5 146 967 8 MOLECULE: RNA POLYMERASE-ASSOCIATED PROTEIN RAPA;
2: 2i1k-A 5.6 10.3 104 545 4 MOLECULE: MOESIN;
3: 6b2z-M 5.5 6.8 81 249 4 MOLECULE: ATP SYNTHASE SUBUNIT C, MITOCHONDRIAL;
4: 8ek4-B 5.5 4.4 90 135 8 MOLECULE: ICE-BINDING PROTEIN TIP-99A;
5: 4dci-B 5.4 3.1 84 148 13 MOLECULE: UNCHARACTERIZED PROTEIN;
6: 9gm9-A 5.4 9.2 94 999 10 MOLECULE: CHROMOSOME PARTITION PROTEIN MUKB;
7: 5ziy-A 5.4 5.5 79 195 4 MOLECULE: FLAGELLAR HOOK-ASSOCIATED PROTEIN 3;
8: 8rt9-F 5.3 5.7 86 272 7 MOLECULE: TRWJ PROTEIN;
9: 5y9o-A 5.2 10.2 109 401 8 MOLECULE: WIPA;
10: 6aay-A 5.2 5.4 75 1199 11 MOLECULE: BERGEYELLA ZOOHELCUM CAS13B (R1177A) MUTANT;
11: 7wdk-B 5.0 5.9 79 348 9 MOLECULE: PHOSPHOLIPASE D;
12: 2x0l-A 5.0 11.5 96 670 9 MOLECULE: LYSINE-SPECIFIC HISTONE DEMETHYLASE 1;
13: 6zbj-A 5.0 9.6 100 666 10 MOLECULE: PRECURSOR OF THE MAJOR MEROZOITE SURFACE ANTIGENS
14: 5iun-E 4.8 7.1 85 220 11 MOLECULE: SENSOR HISTIDINE KINASE DESK;
15: 8bsb-B 4.7 3.8 80 162 8 MOLECULE: METHYL-ACCEPTING CHEMOTAXIS PROTEIN;
16: 6rw8-B 4.6 10.6 109 2337 5 MOLECULE: A COMPONENT OF INSECTICIDAL TOXIN COMPLEX (TC);
17: 4dyl-A 4.6 9.1 97 376 4 MOLECULE: TYROSINE-PROTEIN KINASE FES/FPS;
18: 2xco-A 4.6 8.4 97 636 5 MOLECULE: DNA GYRASE SUBUNIT B, DNA GYRASE SUBUNIT A;
19: 4mt8-A 4.6 5.3 81 100 9 MOLECULE: ETHYLENE RESPONSE SENSOR 1;
20: 4zm1-B 4.6 4.5 113 238 8 MOLECULE: CHAIN LENGTH DETERMINANT PROTEIN;
21: 6th1-R 4.6 6.0 98 360 7 MOLECULE: IMMEDIATE EARLY PROTEIN 1;
22: 7f3t-A 4.6 8.6 88 330 7 MOLECULE: TRANSMEMBRANE PROTEIN 120A;
23: 2oev-A 4.6 4.8 92 697 8 MOLECULE: PROGRAMMED CELL DEATH 6-INTERACTING PROTEIN;
24: 7p5v-B 4.5 6.6 101 732 8 MOLECULE: VOLUME-REGULATED ANION CHANNEL SUBUNIT LRRC8A;
25: 6zh3-A 4.5 8.2 88 170 8 MOLECULE: VACUOLAR PROTEIN-SORTING-ASSOCIATED PROTEIN 24;
26: 7t7t-A 4.5 11.4 84 466 7 MOLECULE: PROTEIN TONSOKU;
27: 9io5-G 4.5 6.8 89 260 4 MOLECULE: G1-ATPASE SUBUNIT BETA;
28: 8ap7-a 4.5 11.4 106 231 4 MOLECULE: ATP SYNTHASE SUBUNIT A;
29: 8k89-A 4.3 6.8 75 78 13 MOLECULE: NUCLEAR FACTOR INTERLEUKIN-3-REGULATED PROTEIN;
30: 7ag9-B 4.3 10.0 97 384 6 MOLECULE: KAR9;

```
0001 s001A   PKIKIDGLAN-EEGYWSLWQITL---NDSPND--KIFPFFINNEGKM-RLPSAEAIWDRLVS-TDAHITYDGMK-EIE-D-GVYGELYKRVSEY-A-S-SMFYE-IRDAYIKNRDEERKRM-EYAFTLKKEMI-GRV-G-LETVKRHRLNELEKEKKAWEDKF-A-KS-RN-IIPSL--Y-P--L--C--IL-Y-ME-
0002 6bogA   TISLLKNkalgTLLVELIYVVEA---QaNRFL--TPVRXLLDKNGNN-LAAQ--VEFETF----nRQLNAVN----vQ-Q-DVHAIL-QLGEAQ-I-E-KSARA-LIDAARNEADEKLSAE-LSRLEALRAVN--------------PNIR-DDELTAIESNR-Q-QVxES-LDAGW--R-L--D--A--LR-L-IVv
0003 2i1kA   ----------------------------------------ksmnvrvtiQQTT-TGKQLF-------eVWFF-----eY-Q-DRLRQMQEEMERSqA-N-LLEAQ-DMILRLEEQLRQLQAA-KEELEQRQNEL-QAM--dEIRAKQEEVSRIQQEVELKDSET-R-RL-QE-E--raaaA-L--L--A-aTT-P-QHh
0004 6b2zM   feirtlfglqllvitslytltnnnnkiisqeawglftlfiislsiviykhGWVF---------------------fvpagTPLP-LVPLLVII-E-T-LSYFA-RAISLGLRLGSNILAG-HLLMVILAGLT-FNFmT-LVFG-FVPLAMILAIMMLEFAIG-I-IQ-GYv------------------------
0005 8ek4B   ----------eevreklkrmekkfddslIREIkeAEKKKKRN-----------------------------------------PYEAV-VTT-L-R-AILKA-VETKIRAIIKALKTEL-DALIKAMETIL-KAH-D----kNDELKKEVEDIIKKMRDKL-T-KL-IK-AKELK--K-Ka--------------
0006 4dciB   -------------------------------------------------------------------------------------raVVTPtW-K-EEAER-EISNGIANADQQLAQL-EQEGQTVVDQV-RRQ-SpLDPRVQEQVANIQQQVAGKRSEL-E-EQkRN-LL-xQ--G-Q--L----qSI-E-E--
0007 9gm9A   ----------frsltlvnwngffartfdtlsgipdfrnystldvinsrlqqvagrdrkvdikpfmirenritle-AIR-VtQSDRDLFKHLITE-A-T-SYVSA-DYMRHANERRTHLDEA-LALRGELFGSH-KQLahaEMENVQKKLRKLERDYYQIREQV-V-SA-KA-RPER-----L--R--E--RR-Q-DIi
0008 5ziyA   ----------------------------------------AASKSF-----------------------------------------hSLANM-E-Q-MQKDI-ADSKNVLTQTENTLQG-VLKSLTRADQL-TVQ-AkELQAIGVEIDQILKQVVYLANT----KE-QG---delseQ-N--V--Al---------
0009 8rt9F   ------------------------------------------------------------------------------------aF-ELFTPLFNKIDQT-T-AtYVTDI-SSRAIAAITPVVSVGL-TLGFITYGWLI-IRG------aVEMPVAEFLNRCLRIGIIV-S-IA-LG-GLYIA--N-A---------F-A-AVf
0010 5y9oA   ednlgSISL-------------------------------------naIKLIHFLFRHK----------------TE-I-INFHEAYQQFVTI-YeQ-YDDMV-QEYLEIRTLLQLIQIK-ITNAQQRILDI-EQK-LeFSQSLLQLKKPIEANLQMAEKSK-A-GL-ES-GLKlrfnK-F--M--T-qIE-I-NDi
0011 6aayA   kitvfgrlseweifpnpnydfpkenisviaGS--L-----------------------------------------kilkpyqdgDKEKLIKDL-K-Q-EQNIL-QKLKDEQTVREKEYND-FIAYQDKNREI-NKV-R-DRNHKQYLKDNLKRKYPEAP----------------------------kSE-N-K--
0012 7wdkB   -------------------------------------dasrvwqisevtavgdngsYDYDS------------------------mifkvDKNE-F-H-NASNY-IKGTAEIYQD-HLREN-IKLDKKAISTL-QKN-K----sEEKSIERIKKGIAEMEAKI---PL-AK-IYEHD--L-----------A-R-FRt
0013 2x0lA   vlvhrvhsyltgkviiigsgvsglaaarmdvtgnyamvqkcplyeankekDEMVEQEFNRL-----fnvlnNKP-VSL-GqALEVVIQLQEKHV-KdE-QIEHW-KKIVKTQEELKELLNK-MVNLKEKIKEL-HQQ--fLVKSKHRDLTALCKEYDELAETQ-G-KL-EEk------------------------
0014 6zbjA   gIVYP-----------------------------------------------------LPL-TDIfsssnnsv-YNV-QkLKKALSYLEDYSL-R-K-GISEK-DFNHYYTLKTGLEADI-KKLTEEIKSSE-NKI--ekEVSDIVKLQVQKVLLIKKIEDL-R-KI-EL-KNASI--H-V--P--Nii--------
```

```
0015 5iunE   -----------------------------------------------------------------------iikL-RKE-IeRLEEKLEDANERI-A-E-LVKLE-ERQRIARDLHDTLGQK-LSLIGLKSDLA-RKL-K-DPEQAARELKSVQQTARTSLNEV-R-KI-VS-S----------------------------
0016 8bsbB   ------------------------------------------------------------------------------------GPLGSSELDKI-Q-S-ELLNYtDDTLPAMENVDAIKDK-MSYWRRTQFAV-LPM-K-DEAQIRQTIERNNRVQAEINDSL-V-AY-GT-VWP--------------------------
0017 6rw8B   glSENEL--------------------------------settalqhdfhaVIMRCGSYATEIlTALElgaltaeqvRQ-D-AEALAEILQTQGS-E-L-ALQSI-KMQDKVMAEIDADKLA-LQESRHGAQSRfDSF-NeWEIQRNNAEAEIKQIDAQLATLA-V-RReAA-VL----------qlafL--QS-Kf---
0018 4dylA   --------------------smgfsseLCSP-------gvlqqmqeakwmaqrvksdreyagllhheglsrLLR-QhaedlnSGPLSKLSLLIR-E-R-QQLRK-TYSEQWQQLQQELTKThSQDIEKLKSQY-RAL--rSLWKLFAHHNRYVLGVRAAQLHH-QqLL-LP-RSLQmacI-L--K--E---I-S-SLv
0019 2xcoA   eamvrmaqdfaamrytearmtkitlelltidf--FPNlvlslsknpD-ISIAELMEDIEGpdfptaSIQMRSR---mialvngrPKLI-nlkEA-L-V-HYLEH-QKTVVRRRTQYNLRKA-KDRAHILEGLR-IAR-L-TG-LERDKIEAEYNELLNYISEL-E-TIlAD-EE---------------------------
0020 4mt8A   -----------------------------------------------------------------------ashA-AILeE-SMHARDQLMEQNF-A-L-DKARQ-EAEMAVHARNDFLAVM-NHEMRTPMHAIiSLS-SmIETILKSSNLVATLISDVLDL-S-R-LE---------------------------------
0021 4zm1B   ---------awtsTAIITqpdvgqiaGYNNam--LIGrdnqeePEKL-T---IEPS-VKNQ---------------tA-E-GAQMKLAQYIQQV-D-D-KVNQE-LEKDLKDNIALGRKNL-QDSLRTQEVVA-QEQ-KeaTRPL--VFSPNYYQTRQNLLDI-E--K-LK-FLDIH----Y--R--Y--VMk-----
0022 6th1R   skkaavqslvrttciqmanesyaradivASQG--YT-------------------------------nfmpvnaN-ESE-K-RAWMEMLGECTSH-G-N-KLCEM-ANAQVEQETRDIINIM-FKNIDDVVTQT-TRA-MrLCLSILTSAKERIDIIYHSARSQ-H-LA-CV-RMNvatfI-L--T--Na---------
0023 7f3tA   ----------------------------------------------------------------------GPLGdcDWE-DlQQDFQNIQETHRL-Y-R-LKLEE-LTKLQNNCTSSITRQK-KRLQELALALK-KCK-L-PAEAEGAAQELENQMKERQGLFF-D-ME-AY-L----------------------------
0024 2oevA   ------------------------------------------------------------------------------aA-I-EDvsQSILTKSRS-V-IeQGGIQ-TVDQLIKELPELLQRN-REILDESLRLL-DEE-EpSNELYKPLRAEGTNFRTVLDKAV-Q-AD-GQ-VefAY--D-Nfr---------------
0025 7p5vB   kdscndstgpnrlhwfakyfpylvllhtsNFWkfpLEHF--------------vsillkcfdspwtseTGVLDK-KegeqaKALFEKVKKFRTH-V-E-EG-DI-VYRLYMRQTIIKVIKF-ALIICYTVYYV-HNI-KpLATLFKILASFYISLVIFYGLIC-M-YT-LW--LKKYsfesF--A--V--F-------
0026 6zh3A   ------------------------------------------------------------------------------M-D-YIKKAIWKEQQRR-I-R-SVLRK-NGRNIEKSLRELT-VL-QNKTQQLIKKS-AKK------nDVRTVRLYAKELYQINKQY-D-RM-YT-AQLDI--D-E--AirM--NT-L-S--
0027 7t7tA   DahnnigxlqedddGRSR--------------------HHNLgnvyxeHIEQDIIICKKIE---------------elhyrvqkycyqlaqsxededA-LASQI-DQNIETVKKAIEVXDE-LKKEEQNLKKL-TRN--sQERKSLLQQNASLDCLIEKSSXIf--------------------------------------
0028 9io5G   ---------------------------------------------------------------------------M-KRK-D-VEEKKQNLDFYHN-Y-I-DISKI-KVLQKLNEEIASLNML-KLQKGESHYLL-NRI--rivpfFYEQFVKSVFKQELFEFKS-I-SViEE-LK-KK--K-R----------K-W-NRa
0029 8ap7a   dfllfCLFDL----------ylfvglclMLFN------yycitylnllYIAFLFLFCFLCD---------------RF-L-LCFLEcFSLLCRC-L-S-tflRL-FCN-LLSSHFLLLMFF-DFFYFIFVFFF-YGV-F-CY-WFILFIFVFCFCLLFYVFLY-L-LD-LF-ILQL-----------------D-F-LLf
0030 8k89A   ------------------------------------------------------------------------KDA-XEK-R-RKNNEAAKRSREK-R-R-LNDLV-LENKLIALGEENATLK-AELLSLKLKFG-L------------iSSTAYAQEXQKLSNST-A-VY-F-------------------------------
0031 7ag9B   SRDH------mdtldDLIN------------krhtdqlsrkflilkR-NIPPIEQSLTEILP-QR-----------ininlladfkrirfMMNE-I-K-DLKIE-LIDKRWNILFINLNNE-LEYIIEEVRLL-LKK-ItIKDRFNSQLAKKSKIITKTFNII-Y-RAlEF-SL---------------------------

0001 s001A   EEEEELLLLL-LLEEEEEEEEEEE---LLLLLL--EEEEEEEELLLLL-LHHHHHHHHHHHHL-LLLLEEEEEEEE-LLL-L-LHHHHHHHHHHHH-H-H-HHHHH-HHHHHHHHHHHHHHHHH-HHHHHHHHHHH-HLL-L-LHHHHHHHHHHHHHHHHHHHHHHH-H-HH-LL-LLEEE--E-E--E--E--EE-E-EL-
0002 6bogA   EEEEEELllllLEEEEEEEEEEEL---LlHHLL--EEEEEEELLLLLL-LLLL--LLHHHH----hHLLLLLL----lH-H-HHHHHH-HHHHHH-H-H-HHHHH-HHHHHHHHHHHHHHHHH-HHHHHHHHHLLL---------------LLLL-HHHHHHHHHHHH-H-HHhHH-HLLEE--E-E--E--E--EE-E-EEe
0003 2i1kA   ----------------------------------------lleeeeeeeLLLL-LHHHHH-------lHHHE-----hH-H-HHHHHHHHHHHHHhH-H-HHHHH-HHHHHHHHHHHHHHHHH-HHHHHHHHHHH-HHH--hHHHHHHHHHHHHHHHHHHHHHHHH-H-HH-HH-H--hhhhH-H--H--L-lLL-L-HHh
0004 6b2zM   hllllllllllhhhhhhlllllllllllhhhhlhhhhhhhhhhhhhhhhhhhhHHHH----------------------hllllLLHH-HHHHHHHHH-H-H-HHHHL-HHHHHHHHHHHHHHHHH-HHHHHHHHHHH-HHHlH-LLLL-HHHHHHHHHHHHHHHHHHH-H-HH-HHh---------------------------
0005 8ek4B   ----------lhhhhhhhhhhhhhhhhhhHHHHhhHHHHHHHL--------------------------------------------LHHHH-HHH-H-H-HHHHH-HHHHHHHHHHHHHHHHH-HHHHHHHHHHH-HHH-L----lLLLLHHHHHHHHHHHHHHHH-H-HH-HH-HHHHH--H-Hh---------------
0006 4dciB   -------------------------------------------------------------------------------------------eeELLHhH-H-HHHHH-HHHHHHHHHHHHHHHHH-HHHHHHHHHHH-HLL-LlLLHHHHHHHHHHHHHHHHHHHHHH-H-HHhHH-HH-lE--E-E--E----eEE-E-L--
0007 9gm9A   ----------eeeeeeeelleeeeeeeeeeellllllleeeeeeeelleeeeeelllllleeeeeeeeeehhhhhhhh-HHH-HhHHHHHHHHHHHHH-H-H-HHHHH-HHHHHHHHHHHHHHHHH-HHHHHHHHHHH-HHHhhhHHHHHHHHHHHHHHHHHHHHHHHH-H-HH-HH-LLHH-----H--H--H--LL-L-LLl
0008 5ziyA   ------------------------------------------LHHHHH------------------------------------------hHHHHH-H-H-HHHHH-HHHHHHHHHHHHHHHHH-HHHHHHHHHHH-HHH-HhHHHHHHHHHHHHHHHHHHHLL----EE-LL---lhhhhH-H--H--Hh---------
0009 8rt9F   ---------------------------------------------------------------------------------lL-LLHHHHHHHHHHH-H-HhHHLHH-HHHHHHHHHHHHHHHHH-HHHHHHHHHHH-HLL------lLLLLLLHHHHHHHHHHHHHH-H-HH-HL-LLHHH--H-H----------H-H-HHh
0010 5y9oA   lllllEEEE---------------------------------------lhHHHHHHHHHHLL----------------LL-L-LLHHHHHHHHHHHH-HhH-HHHHH-HHHHHHHHHHHHHHHHH-HHHHHHHHHHH-HHH-HhHHHHHHHHHHHHHHHHHHHHHH-H-HH-HH-HHHhhhhH-H--H--L-lEE-E-LLl
0011 6aayA   eeeeellhhhelllllleellllllllllllLL--L------------------------------------------hhhllllllLHHHHHHHH-H-H-HHHHH-HHHHHHHHHHHHHHHHH-HHHHHHHHHHH-HLL-L-LHHHHHHHHHHHHHHHLLLLL-------------------------------lLL-H-H--
0012 7wdkB   ---------------------------------------lhhhhhhlllllllllleeELLLL------------------------eeellLHHH-H-H-HHHHH-HHHHHHHHHHH-HHHHH-HHHHHHHHHHH-LLL-L----lLLLLHHHHHHHHHHHHHLL---HH-HL-LEEEL--L-----------H-H-EEl
0013 2x0lA   hhhhhhhhhhlleeeeelllhhhhhhhhhleeellelleleeeeeeellllllh-----lllllLLL-LLH-HhHHHHHHHHHHHH-HhH-HHHHH-HHHHHHHHHHHHHHHHH-HHHHHHHHHHH-HHH--hHHHHHHHHHHHHHHHHHHHHHHHH-H-HH-HHh---------------------------
0014 6zbjA   lLLLL--------------------------------------------------------LLH-HHHhhhhhhhh-HHH-HhHHHHHHHHHHHH-H-H-HHHLL-LHHHHHHHHHHHHHHHH-HHHHHHHHHHH-HHH--llLLHHHHHHHHHHHHHHHHHHHHH-H-HH-HH-HHHLL--L-L--L--Lll--------
0015 5iunE   ------------------------------------------------------------------------lhhH-HHH-HhHHHHHHHHHHHH-H-H-HHHHH-HHHHHHHHHHHHHHHHH-HHHHHHHHHHH-HHH-H-LHHHHHHHHHHHHHHHHHHHHHHH-H-HH-HH-L--------------------------
0016 8bsbB   --------------------------------------------------------------------------------------LLLLHHHHHHH-H-H-HHHHHhHLHHHHHHHHHHHHHHH-HHHHHHHHHHHL-LLL-L-LHHHHHHHHHHHHHHHHHHHHHHH-H-HH-HL-LLL------------------------
0017 6rw8B   llLLLLH--------------------------------lllllllllhhhHHHHHHHHHHHHhHHHHlllllllhHH-H-HHHHHHHHHHHHH-H-H-HHHHH-HHHHHHHHHHHHHHHHH-HHHHHHHHHHHhHHH-HhHHHHHHHHHHHHHHHHHHHHHHHH-H-HHhHH-HH---------hhhhH--HH-Ll---
0018 4dylA   --------------------lllhhhhLLLH-------hhhhhhhhhhhhhhhhhhhhhhhhhhhhhhhhhhHHH-HhhhhhhHLHHHHHHHHHH-H-H-HHHHH-HHHHHHHHHHHHHHHHHhLHHHHHHHHHH-HHH--hHHHHHHHHHHHHHHHHHHHHHHHH-HhLH-HH-HHHHhhhH-H--H--H---H-H-LLl
0019 2xcoA   hhhhhhllllllllllleeeelhhhhllllllle--LLHhhhhhhhllL-LLHHHHHHHLLLlllllllEEEEELE---leeeelleEEEL-lhhHH-H-H-HHHHH-HHHHHHHHHHHHHHHHH-HHHHHHHHHHH-HHH-H-LL-LHHHHHHHHHHHHHHHHHHHH-H-HHhHL-HH-------------------------
0020 4mt8A   ------------------------------------------------------------------------llhH-HHHhH-HHHHHHHHHHHHH-H-H-HHHHH-HHHHHHHHHHHHHHHHH-HHHHHHHHHHHhHHH-HhHHHHHHHHHHHHHHHHHHHHH-H-L-LL-------------------------------
0021 4zm1B   ---------leeeEEEEEllllhhhhHHHHhh--HHHhhllllLLLE-E---EEEL-LLLL---------------lH-H-HHHHHHHHHHHHH-H-H-HHHHH-HHHHHHHHHHHHHHHHH-HHHHHHHHHHH-HHH-HhhHLLL--LLLHHHHHHHHHHHHHH-L--L-LL-LLLLL----L--E--E--EEl-----
0022 6th1R   hhhhhhhhhhhhhhhhhhhhhhhhhhhhhHHHL--LL-------------------------------hhllllL-LLL-H-HHHHHHHHHHHHH-H-H-HHHHH-HHHHHHHHHHHHHHHHH-HHHHHHHHHHH-HHH-HhHHHHHHHHHHHHHHHHHHHHHHH-H-HH-HH-HHHhhhhH-H--H--Hh---------
0023 7f3tA   -----------------------------------------------------------------------LLHHhhHHH-HhHHHHHHHHHHHH-H-H-HHHHH-HHHHHHHHHHHHHHHHH-HHHHHHHHHHH-HHL-L-LHHHHHHHHHHHHHHHHHHHHHHHH-H-HH-LL-L--------------------------
0024 2oevA   ------------------------------------------------------------------------------lL-L-LLllHHHHHHHHH-H-HlLLLHH-HHHHHHHHHHHHHHHHH-HHHHHHHHHHH-HHH-HlHHHHLLHHHHHHHHHHHHHHHHHH-H-HH-HH-HhlHH--H-Hhl--------------
0025 7p5vB   llllllllllhhllhhhhhhhhhhhhhhHHHhlhHHHH--------------hhhhhhhhlllhhllLLLLLH-HhhhhhHHHHHHHHHHHHH-H-L-LL-LL-HHHHHHHHHHHHHHHHH-HHHHHHHHHHH-HHL-LlLHHHHHHHHHHHHHHHHHHHHHHHH-H-HH-HH--LLLLllhhH--H--H--H-------
0026 6zh3A   -------------------------------------------------------------------------------L-L-HHHHHHHHHHHHH-H-H-HHHHH-HHHHHHHHHHHHH-HH-HHHHHHHHHHH-HHH------lLHHHHHHHHHHHHHHHHHHH-H-HH-HH-HHHHH--H-H--HhhL--LL-H-H--
0027 7t7tA   HhhhhhhhhhllllhHHHH---------------------HHHHhhhhhhHHHHHHHHHHHHL---------------hhhhhlllhhhhhhllllllhH-HHHHH-HHHHHHHHHHHHHHHHH-HHHHHHHHHHH-HHH--lHHHHHHHHHHHHHHHHHHHHHHHLl------------------------------------
0028 9io5G   ---------------------------------------------------------------------------L-LHH-H-HHHHHHHHHHHHH-H-H-HHHHH-HHHHHHHHHHHHHHHHH-HHHHHHHHHHH-HHH--eeellLHHHHHHHHHHHHHHHHHHH-H-HHhHH-HH-HH--H-H----------H-H-HHh
0029 8ap7a   llleeLLLLL----------llhhhhhhHHHH------llllllllhhHHHHHHHHHHLLL---------------HH-H-HHHHHhHHHHHHH-H-H-hhhHH-HHH-HHHHHHHHHHHHH-HHHHHHHHHHH-HHH-H-LL-LHHHHHHHHHHHHHHHHHHHH-H-HH-HH-HHHH----------------L-L-LLl
0030 8k89A   -------------------------------------------------------------------------LLH-HHH-H-HHHHHHHHHHHHH-H-H-HHHHH-HHHHHHHHHHHHHHHHH-HHHHHHHHHLL-L------------lLHHHHHHHHHHHHHHHH-H-LL-L-----------------------------
0031 7ag9B   HHLL------hhhhhHHHH------------hlllhhhhhhhhhhhhH-HHHHHHHHHHHHHH-HH-----------llhhhhhhhhhhhhHHHH-H-H-HHHHH-HLHHHHHHHHHHHHHHH-HHHHHHHHHHH-HHH-HhHHHHHHHHHHHHHHHHHHHHHHH-H-LLhHH-HL-------------------------
```

```
Job: BR-systems; DUF499-ATPase CTD Ferredoxin-fold/RNA-Recognition-Motif (Representative_1)
Query: s001A
No: Chain Z rmsd lali nres id PDB Description
1: 4kyz-A 7.1 2.4 99 167 10 MOLECULE: DESIGNED PROTEIN OR327;
2: 5cw9-A 6.4 3.1 99 146 12 MOLECULE: DE NOVO DESIGNED FERREDOXIN-FERREDOXIN DOMAIN INS
3: 4ney-B 6.3 5.0 81 173 12 MOLECULE: ENGINEERED PROTEIN OR277;
4: 3n0v-A 6.2 2.8 78 280 13 MOLECULE: FORMYLTETRAHYDROFOLATE DEFORMYLASE;
5: 4v1a-k 6.0 4.4 89 131 4 MOLECULE: MITORIBOSOMAL PROTEIN ML37, MRPL37;
6: 1r3n-A 6.0 4.7 88 438 7 MOLECULE: BETA-ALANINE SYNTHASE;
7: 2nzc-D 5.9 3.3 78 82 12 MOLECULE: HYPOTHETICAL PROTEIN;
8: 3n5f-A 5.8 3.5 87 406 13 MOLECULE: N-CARBAMOYL-L-AMINO ACID HYDROLASE;
9: 1ysj-A 5.8 3.4 89 359 13 MOLECULE: PROTEIN YXEP;
10: 6wi5-A 5.8 3.0 81 90 12 MOLECULE: DE NOVO DESIGNED PROTEIN FOLDIT4;
11: 3fgv-A 5.7 2.6 80 94 6 MOLECULE: UNCHARACTERIZED PROTEIN WITH FERREDOXIN-LIKE FOLD
12: 3r2c-K 5.7 2.8 75 80 11 MOLECULE: N UTILIZATION SUBSTANCE PROTEIN B;
13: 1zpv-C 5.6 3.6 80 88 6 MOLECULE: ACT DOMAIN PROTEIN;
14: 2fb0-A 5.6 2.7 81 94 6 MOLECULE: CONSERVED HYPOTHETICAL PROTEIN;
15: 8p6p-I 5.6 3.7 81 104 6 MOLECULE: 23S RIBOSOMAL RNA;
16: 1u8s-B 5.6 3.2 79 172 10 MOLECULE: GLYCINE CLEAVAGE SYSTEM TRANSCRIPTIONAL
17: 1z2l-A 5.6 3.5 88 411 10 MOLECULE: ALLANTOATE AMIDOHYDROLASE;
18: 8d8k-J 5.5 4.7 87 144 3 MOLECULE: PROBABLE S-ADENOSYL-L-METHIONINE-DEPENDENT RNA
19: 7c50-A 5.5 5.2 93 192 8 MOLECULE: SIMPL DOMAIN-CONTAINING PROTEIN;
20: 5i4m-A 5.5 4.9 93 414 10 MOLECULE: AMIDASE, HYDANTOINASE/CARBAMOYLASE FAMILY;
21: 4uer-J 5.4 4.0 84 107 6 MOLECULE: EIF1A;
22: 7m6u-B 5.3 3.6 98 392 12 MOLECULE: CARBOXYPEPTIDASE G2 CIRCULAR PERMUATION PRO-DOMAI
23: 2bbe-A 5.3 3.3 85 103 8 MOLECULE: HYPOTHETICAL PROTEIN SO0527;
24: 7ljh-A 5.3 3.3 92 400 4 MOLECULE: POLY(ASPARTIC ACID) HYDROLASE;
25: 8tcf-C 5.3 2.4 71 75 11 MOLECULE: INTEGRIN ALPHA-V HEAVY CHAIN;
26: 2nyi-A 5.3 3.3 82 171 12 MOLECULE: UNKNOWN PROTEIN;
27: 3ce8-A 5.3 4.6 79 90 11 MOLECULE: PUTATIVE PII-LIKE NITROGEN REGULATORY PROTEIN;
28: 1y0h-A 5.3 3.2 83 101 8 MOLECULE: HYPOTHETICAL PROTEIN RV0793;
29: 4npo-B 5.2 2.7 80 105 10 MOLECULE: UNCHARACTERIZED PROTEIN;
30: 3gz7-A 5.2 3.9 82 99 9 MOLECULE: PUTATIVE ANTIBIOTIC BIOSYNTHESIS MONOOXYGENASE;
31: 4zos-A 5.1 3.6 81 105 5 MOLECULE: PROTEIN YE0340 FROM YERSINIA ENTEROCOLITICA SUBSP
32: 2bvf-A 5.1 3.9 85 453 13 MOLECULE: 6-HYDROXY-D-NICOTINE OXIDASE;
33: 1x7v-C 5.1 3.3 80 99 10 MOLECULE: PA3566 PROTEIN;
34: 6slf-A 5.0 4.0 93 398 11 MOLECULE: N-ALPHA-ACYL-GLUTAMINE AMINOACYLASE;
35: 2rb7-A 5.0 4.8 97 360 9 MOLECULE: PEPTIDASE, M20/M25/M40 FAMILY;
36: 6az1-M 5.0 4.1 77 102 10 MOLECULE: RIBOSOMAL PROTEIN S1E;
37: 2omo-A 5.0 4.0 85 105 8 MOLECULE: DUF176;
38: 5jlp-A 4.9 2.4 74 397 14 MOLECULE: PHOSPHOSERINE PHOSPHATASE;
39: 3obi-A 4.9 3.1 77 285 6 MOLECULE: FORMYLTETRAHYDROFOLATE DEFORMYLASE;
40: 2ril-A 4.9 3.2 77 95 5 MOLECULE: ANTIBIOTIC BIOSYNTHESIS MONOOXYGENASE;
41: 4q7a-A 4.9 2.8 80 361 15 MOLECULE: N-ACETYL-ORNITHINE/N-ACETYL-LYSINE DEACETYLASE;
42: 4pxc-A 4.9 4.6 93 424 11 MOLECULE: UREIDOGLYCOLATE HYDROLASE;
43: 8ecx-C 4.9 3.5 79 102 10 MOLECULE: ANTIBIOTIC BIOSYNTHESIS MONOOXYGENASE;
44: 4hl9-A 4.9 3.1 80 95 6 MOLECULE: ANTIBIOTIC BIOSYNTHESIS MONOOXYGENASE;
45: 4mmo-A 4.9 4.2 93 437 15 MOLECULE: SSO-CP2 METALLO-CARBOXYPETIDASE;
46: 4ewt-A 4.9 3.1 88 389 9 MOLECULE: PEPTIDASE, M20/M25/M40 FAMILY;
47: 2gx8-C 4.8 4.1 81 364 10 MOLECULE: NIF3-RELATED PROTEIN;
48: 3bb5-D 4.8 2.5 80 108 5 MOLECULE: STRESS RESPONSIVE ALPHA-BETA PROTEIN;
49: 1iuj-B 4.8 3.5 81 103 5 MOLECULE: HYPOTHETICAL PROTEIN TT1380;
50: 7qa5-A 4.8 3.8 80 93 8 MOLECULE: PROTEIN MGTC;
51: 2f7v-A 4.8 4.1 98 360 12 MOLECULE: AECTYLCITRULLINE DEACETYLASE;
52: 3kg0-B 4.8 3.4 79 98 4 MOLECULE: SNOAB;
53: 2qmw-A 4.8 4.1 83 264 12 MOLECULE: PREPHENATE DEHYDRATASE;
54: 3ced-A 4.8 3.6 78 98 10 MOLECULE: METHIONINE IMPORT ATP-BINDING PROTEIN METN 2;
55: 6c0d-A 4.8 3.7 87 404 14 MOLECULE: AMIDASE, HYDANTOINASE/CARBAMOYLASE FAMILY;
56: 6dgd-A 4.7 4.2 89 704 4 MOLECULE: PRIMOSOMAL PROTEIN N';
57: 5jb3-L 4.7 4.1 83 102 5 MOLECULE: 16S RIBOSOMAL RNA;
58: 3bm7-A 4.7 3.2 87 106 5 MOLECULE: PROTEIN OF UNKNOWN FUNCTION WITH FERREDOXIN-LIKE
59: 2od4-A 4.7 3.0 79 101 6 MOLECULE: HYPOTHETICAL PROTEIN;
60: 2gff-B 4.7 3.7 82 100 7 MOLECULE: LSRG PROTEIN;
61: 3oq2-B 4.7 2.8 72 101 14 MOLECULE: CRISPR-ASSOCIATED PROTEIN CAS2;
62: 5xvn-F 4.6 2.7 74 108 9 MOLECULE: CRISPR-ASSOCIATED ENDONUCLEASE CAS1;
63: 8dew-A 4.6 3.9 80 1058 10 MOLECULE: EFFLUX PUMP MEMBRANE TRANSPORTER;
64: 7bio-B 4.6 3.5 79 111 4 MOLECULE: MONOOXYGENASE/PUTATIVE ANTHRONOXYGENASE;
65: 2n51-A 4.6 4.4 82 133 9 MOLECULE: ELONGATION FACTOR 1-DELTA;
66: 2dcl-A 4.6 3.5 77 99 18 MOLECULE: HYPOTHETICAL UPF0166 PROTEIN PH1503;
67: 2peb-A 4.6 3.3 78 115 9 MOLECULE: PUTATIVE DIOXYGENASE;
68: 6nuk-A 4.6 2.5 73 101 11 MOLECULE: FERREDOG-DIESEL;
69: 1o51-A 4.6 3.7 73 89 5 MOLECULE: HYPOTHETICAL PROTEIN TM0021;
70: 4usj-C 4.5 4.0 92 143 11 MOLECULE: ACETYLGLUTAMATE KINASE, CHLOROPLASTIC;

0001 s001A   ---PPKEARMHDRN-VAA---LAVLQLAVDG-D----G----SE--GVREL-RSLGL-AVPQL-G------P----GQFRVEVSA-TG--EL--EGGSIRLEFRGPWERYKDLKDAVVSVLERASA----A-QV-RARLDWRADP---LT--PEGINQVRDVLAHL-I--PGRVRITAEPAT-G-E--V-D
0002 4kyzA   --kRVRISFLAILA-----dinRKXTVRFRG-D----------D--LEALE-KALKE-XIRQAkF------A----GTVTYTLDG------------------------------------------------N-DLEITITGVP---RQ----VLEELAKEAERL-A--KITITITVTVEG-Q-Lg----
0003 5cw9A   ----------------------MEMDIRFRG----------------ddpeykALRE-MIRQAkF------A----GTVTVTLII-RF--RgddLEALLEIRITGV--PPQVILELVKEAIRLAIT----V-TV-ELVIRITG-----vP--EQVRKELAKEAERL-A--KITVTYTIRL-----------
0004 4neyB   ---------------------xGEXXIIFEG-D----------D--LEALE-KALKE-XIRQAkF------A----GTVEYWLSG------------------------------------------------N-RLXIRITG-----VP--EQVRKELAKEAERL-K--AIQVEYQIRSGS-G-S-tG-V
0005 3n0vA   ----------------------DTWILTADC-P----S----XL----GTV-DVVTR-YLFEQ--------R----CYVEHHSFD-DR--Q----------------------------------S----G-RF-FIRVEFRQDD---F-----DEAGFRAGLAER-S--EXAFELTAPN-----------
0006 4v1ak   --------piNLGT-DYE---YGVLNIHLIA-Y----------D--MALAE-SYAQY-VHNLChL------A----IKVESYAMT-KT--ME--VL------------------------qlqmVL----T-TH-ERVVQISG-----LS--ATFAEIFLEIIHSN-L--PVKLSVRE-------------
```

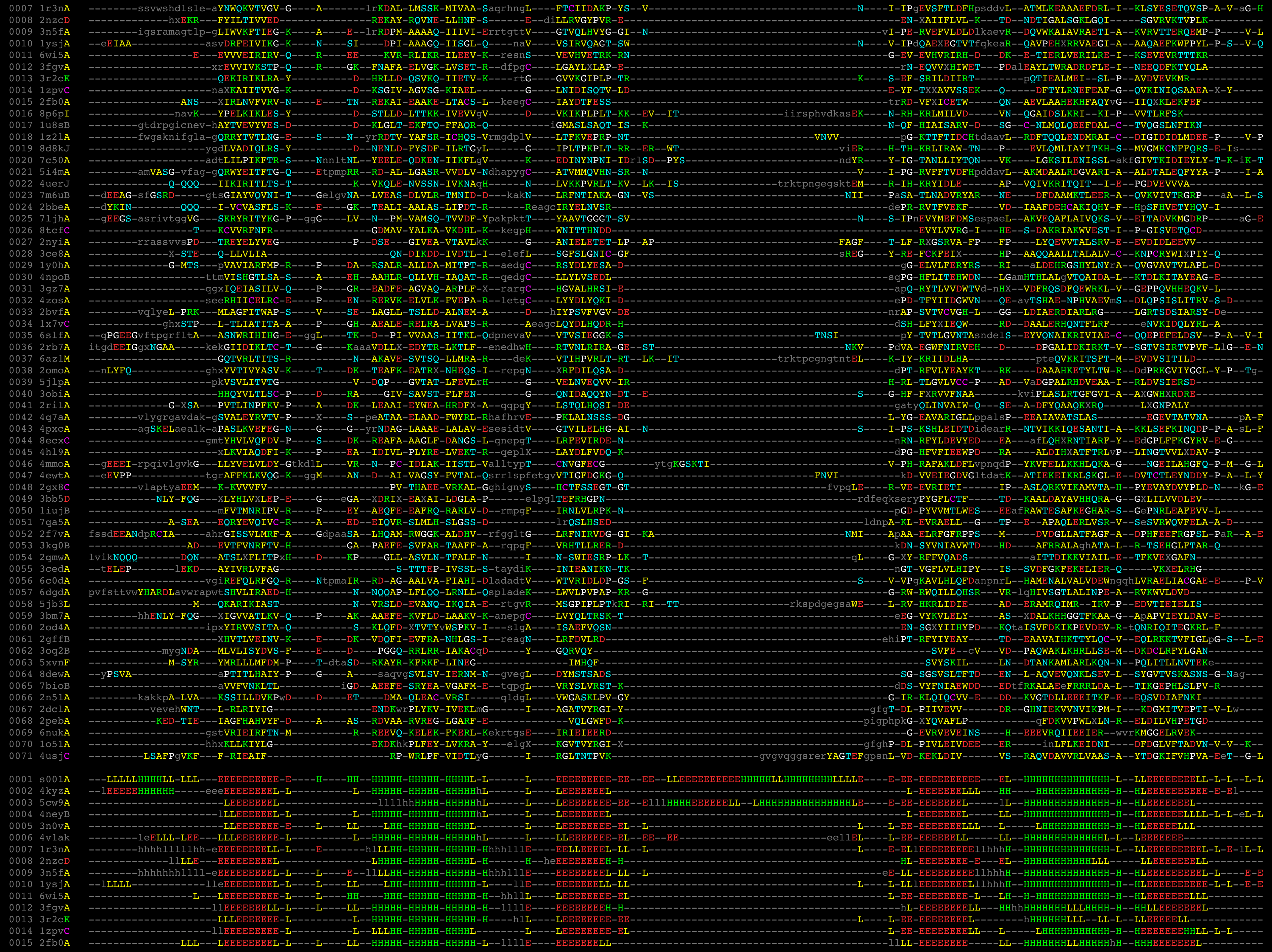

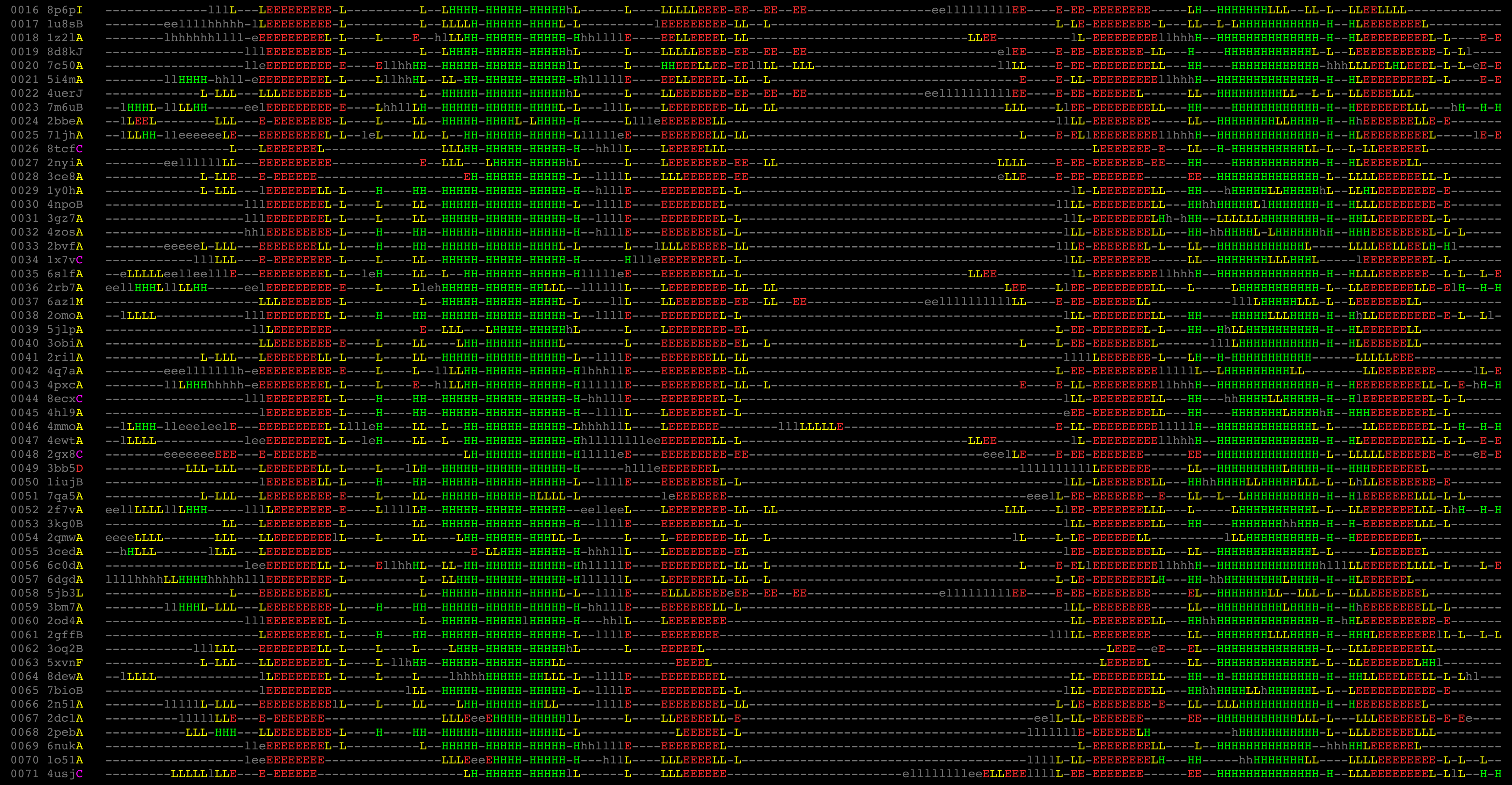


Job: BR-systems; DUF499-ATPase CTD Ferredoxin-fold/RNA-Recognition-Motif (Representative_2)
Query: s001A
No: Chain Z rmsd lali nres id PDB Description
1: 9bk5-A 6.8 3.1 73 78 11 MOLECULE: LNG BINDER;
2: 4kyz-A 6.1 3.3 84 167 4 MOLECULE: DESIGNED PROTEIN OR327;
3: 3nd1-B 6.0 2.7 70 250 13 MOLECULE: PRECORRIN-6A SYNTHASE/COBF PROTEIN;
4: 2npn-A 5.7 3.6 71 239 8 MOLECULE: PUTATIVE COBALAMIN SYNTHESIS RELATED PROTEIN;
5: 5cw9-A 5.5 3.2 75 146 12 MOLECULE: DE NOVO DESIGNED FERREDOXIN-FERREDOXIN DOMAIN INS
9: 2p6y-A 5.0 3.5 78 131 6 MOLECULE: HYPOTHETICAL PROTEIN VCA0587;
10: 3oha-A 4.9 4.5 83 516 10 MOLECULE: DNA POLYMERASE ETA;
12: 7dkk-A 4.8 4.2 75 89 12 MOLECULE: DE NOVO DESIGN PROTEIN XM2H;
13: 3qph-A 4.7 3.5 65 335 5 MOLECULE: TRMB, A GLOBAL TRANSCRIPTION REGULATOR;
14: 1ecg-B 4.5 4.5 67 501 4 MOLECULE: GLUTAMINE PHOSPHORIBOSYLPYROPHOSPHATE AMIDOTRANSF
15: 8k0k-C 4.5 3.6 76 304 4 MOLECULE: CSY1;
16: 3htn-A 4.5 3.2 71 143 6 MOLECULE: PUTATIVE DNA BINDING PROTEIN;
17: 8adb-A 4.5 3.8 73 209 10 MOLECULE: WC-VDT1;
18: 3h20-A 4.4 3.9 60 288 8 MOLECULE: REPLICATION PROTEIN B;
19: 7r8q-B 4.4 5.0 63 389 2 MOLECULE: ATP-GRASP DOMAIN-CONTAINING PROTEIN;
20: 6vqv-J 4.2 3.6 75 337 9 MOLECULE: ACRF9;
21: 6pij-A 4.2 4.0 78 351 9 MOLECULE: CAS7 TYPE I-F CRISPR-ASSOCIATED PROTEIN CSY3;
22: 6ahr-G 4.2 4.3 57 131 9 MOLECULE: H1 RNA;
23: 6x73-A 4.2 4.9 82 439 4 MOLECULE: DNA (5'-D(*GP*GP*GP*GP*TP*GP*TP*GP*GP*TP*AP*GP*C)
24: 6cst-A 4.1 4.8 77 446 8 MOLECULE: DNA POLYMERASE KAPPA;
25: 3fds-A 4.1 5.2 81 352 10 MOLECULE: DNA POLYMERASE IV;

```
26: 7a02-A 4.1 3.3 67 112 9 MOLECULE: DUF3992 DOMAIN-CONTAINING PROTEIN;
27: 2hx0-A 4.1 3.8 72 138 11 MOLECULE: PUTATIVE DNA-BINDING PROTEIN;
28: 2ip4-A 4.1 4.3 65 414 9 MOLECULE: PHOSPHORIBOSYLAMINE--GLYCINE LIGASE;
29: 5i47-B 4.1 4.6 63 264 14 MOLECULE: RIMK DOMAIN PROTEIN ATP-GRASP;
30: 1wyz-B 4.1 3.2 67 227 6 MOLECULE: PUTATIVE S-ADENOSYLMETHIONINE-DEPENDENT METHYLTRA
31: 2dfx-I 4.0 2.7 60 106 5 MOLECULE: COLICIN-E5;
32: 1vi7-A 4.0 4.5 68 206 4 MOLECULE: HYPOTHETICAL PROTEIN YIGZ;
33: 1ukf-A 4.0 4.4 74 188 7 MOLECULE: AVIRULENCE PROTEIN AVRPPH3;
34: 4ecw-A 4.0 4.4 74 432 5 MOLECULE: DNA POLYMERASE ETA;
35: 4a2b-A 4.0 4.6 61 386 5 MOLECULE: CELL DIVISION PROTEIN FTSA, PUTATIVE;
36: 3vpd-A 4.0 4.9 61 281 13 MOLECULE: RIBOSOMAL PROTEIN S6 MODIFICATION PROTEIN;
37: 2h6l-A 3.9 3.2 68 140 6 MOLECULE: HYPOTHETICAL PROTEIN;
38: 5mmj-j 3.9 4.2 58 99 3 MOLECULE: 50S RIBOSOMAL PROTEIN L31;
39: 7dko-A 3.9 4.0 61 90 13 MOLECULE: AM2M;
40: 3vpb-B 3.9 5.1 65 283 6 MOLECULE: PUTATIVE ACETYLORNITHINE DEACETYLASE;
41: 4ir1-A 3.9 4.9 75 342 12 MOLECULE: DNA POLYMERASE IV;
42: 3mjf-A 3.9 3.5 63 429 6 MOLECULE: PHOSPHORIBOSYLAMINE--GLYCINE LIGASE;
43: 2gjh-A 3.9 2.2 48 57 2 MOLECULE: DESIGNED PROTEIN;
44: 4uer-J 3.8 4.2 60 107 5 MOLECULE: EIF1A;
45: 3lp8-A 3.8 4.7 62 421 6 MOLECULE: PHOSPHORIBOSYLAMINE-GLYCINE LIGASE;
46: 5u4j-v 3.7 1.9 51 132 6 MOLECULE: 16S RRNA;
47: 2cve-A 3.7 3.8 59 190 5 MOLECULE: HYPOTHETICAL PROTEIN TTHA1053;
48: 2j6h-A 3.7 3.6 65 609 8 MOLECULE: GLUCOSAMINE-FRUCTOSE-6-PHOSPHATE AMINOTRANSFERASE
49: 7yll-A 3.6 5.3 67 329 1 MOLECULE: DNA POLYMERASE IV;
50: 5l9w-B 3.6 5.0 67 711 7 MOLECULE: ACETOPHENONE CARBOXYLASE DELTA SUBUNIT;
51: 2ihr-1 3.6 1.9 54 351 13 MOLECULE: PEPTIDE CHAIN RELEASE FACTOR 2;
52: 4aw7-A 3.6 5.2 70 565 3 MOLECULE: GH86A BETA-PORPHYRANASE;
53: 8uy1-A 3.5 4.3 59 598 2 MOLECULE: METHYLENETETRAHYDROFOLATE REDUCTASE-LIKE PROTEIN;
54: 1gsa-A 3.5 3.6 59 314 5 MOLECULE: GLUTATHIONE SYNTHETASE;
55: 2qbu-B 3.5 3.3 53 230 11 MOLECULE: PRECORRIN-2 METHYLTRANSFERASE;
56: 5m41-A 3.5 4.0 66 712 8 MOLECULE: NIGRITOXINE;
57: 6bjv-A 3.5 3.6 67 148 10 MOLECULE: RNA SILENCING SUPPRESSOR P19;
58: 4iwx-A 3.5 4.2 59 295 8 MOLECULE: RIBOSOMAL PROTEIN S6 MODIFICATION PROTEIN;
59: 9j46-F 3.5 3.5 70 124 6 MOLECULE: E146L;
60: 5k2m-D 3.5 5.0 60 270 7 MOLECULE: RIMK-RELATED LYSINE BIOSYNTHESIS PROTEIN;
61: 7lvo-A 3.5 4.6 62 435 6 MOLECULE: PHOSPHORIBOSYL-GLYCINAMIDE (GAR) SYNTHETASE;
62: 5yoy-A 3.5 3.0 70 154 11 MOLECULE: TUMOR NECROSIS FACTOR;
63: 7rjf-A 3.4 2.9 46 47 11 MOLECULE: [L47W]MOPD-1;
64: 3v68-A 3.4 2.8 64 248 3 MOLECULE: PUTATIVE UNCHARACTERIZED PROTEIN;
65: 5ta1-A 3.4 3.4 63 627 6 MOLECULE: GLYCOSIDE HYDROLASE;
66: 5wx8-A 3.4 3.9 68 165 4 MOLECULE: IMMEDIATE-EARLY PROTEIN 2;
67: 2dt4-A 3.4 3.3 66 143 8 MOLECULE: HYPOTHETICAL PROTEIN PH0802;
68: 3gqc-B 3.4 5.4 84 440 11 MOLECULE: DNA REPAIR PROTEIN REV1;
69: 6p5b-A 3.4 6.0 77 388 9 MOLECULE: MAVC;
70: 3wvq-A 3.4 5.0 69 433 9 MOLECULE: PGM1;
71: 5jb3-L 3.4 4.5 59 102 5 MOLECULE: 16S RIBOSOMAL RNA;
72: 2okq-A 3.3 2.9 52 127 8 MOLECULE: HYPOTHETICAL PROTEIN YBAA;
73: 7dov-A 3.3 2.8 65 148 5 MOLECULE: LYMPHOTOXIN-ALPHA;
74: 1aly-A 3.3 3.2 65 146 15 MOLECULE: CD40 LIGAND;
75: 8z9m-A 3.2 3.8 59 332 5 MOLECULE: PROTEIN FAM91A1;

0001 s001A   -AALAVLQLAVDGD-G-S-EGVRELRS-L-G-LA-VPQL-G-PG-QFRVEVSAT-G-ELE-G-G-SIRLEFRGP-W-E-RYK-D-L-K-DAVV-SV-L-E-R-A-S-A-AQVRARLDW-R-A-RD-
0002 9bk5A   --------GRIVVR-G-D---VAIAEA-V-V-RKvGEVA------GKEVILLIS-Y-RKN-G-E-WITYQRNLE--pE-DVE-R-T-I-AVIR-EI-Y-E-E-S-G-D-FILAIFSD---------
0003 4kyzA   kQGAKRVRISITAR------SSKEAYK-F-L-AIlAKVF-AlGY-NDNRKXTVR-F-R-L-E-K-ALEITITG------VPR-Q-V-L-EELA-KE-AeR-L-A-K-T-ITITVTVEG-Q-L-GSl
0004 3nd1B   gwpAGTETVVAML-------DGEC--S-F-Q-SL-PP-------dGLTIFWGAC----------mPEEVLIRGP-V-A-EVT-D-E-I-LQAR-AD-L-R-ArH-G-W-VMDIYLLRR-N-V----
0005 2npnA   rIGEA-RNCVVX-------LDGK--TA-W-Q-DV-AT-------eHTYXWWGAF----------tEQQVLRKGY-V-H-EIG-A-Q-V-AELK-QQ-L-R-TeH-G-W-IXDTYLLRE-L-D----
0006 5cw9A   ----MEMDIRFRG----------eayA-L-R-EM-IRQAkF-AG-TVTVTLIIR-F-RGD-DgN-DLEIRITG-----vPPQ-V-I-L-ELVK-EA-I-R-L-AnI-T-VTVELVIRI-T-G-VPe
0010 2p6yA   ----XIHLIALRLT-R-G---XDLKQQ-I-V-QL-VQQH-R-IH-AGSISCVGC-L-StV-S-A-PFEISLSGT-LhC-HLHgG-H-L-L--E-GN-L----------iNTAELXIHH-Y-P-QHh
0011 3ohaA   sSRPVVKSMMSNKN-LscvDCISWLEV-F-C-AElTSRI-QkIV-IPTVSISLK-T-KS------YEVYRKSGP-V-AhELL-K-V-G-IKFV-T-------sY-Y-P-LKLSMTINF-D-I-IDl
0013 7dkkA   ------MHSWSATV-D-seAVRAAARR-L-A-ER-LLAA-G--I-SGKIKIEVE-A-N-------GIKYEYEVE--tE-EVA-K-KiV-EYAV-AA-A-L-R-AgA-T-SVTITVGLE--------
0014 3qphA   eiignPKDIRFF-----------AMFH-A-V-DF-VKNHlK-NR-NIYAEITGK-N-LE----S-GRLETLTGR-V-V-G--eatengvkvGG-MF-A-V-I--------eTEIKFIM-G-V-DLq
0015 1ecgB   -------CGIVGIA-G--mpVNQSIYD-AlT-VL-QH-------gqDAAGIITI-D-AN------NCFRLRKAN-V-S-DV----F-E-ARHM-QR-L----------gNMGIGHVRY-P-T-AGs
0016 8k0kC   pHNKNTLLVKYNVK-F-VtlayRYVYN-I-AgRT-LWRR-V-GAeSIETVITVN-------------DQTFTFS-D-L----dV-D-V-AEIA-DM-V-A-G-VeG-F-VTLKVEHYM-L-L-G--
0017 3htnA   sykkiGNKYIVSIN-N-H-T--EIVKA-L-N-AF-CKEK-G-iL-SGSINGIGA-I-G----rE-QXEINLTGN-IyL-HLHgrgH-L-LSA---------------N-GAGEFVVED-Y-S-E--
0018 8adbA   lntfELKRLPYAG-------TLDSFAK-M-M-EK-ASDS-KlPK-PVALVMTKS-------------NMTITIV-IgK-YWLdE-L-I-KKIK-EI-F-P-K-T-SyF-NSFEAYAVR-R------
0019 3h20A   ------------------------rtA-I-G-RQ-LKAM-----gcERFDIGVR-D-AT----T-GQMMNREWS-A-A-EVL-Q-N-T-PWLK-RM-N-A-Q-G-----NDVYIRPAE-R-H-G--
0020 7r8qB   tfnnqqfPKRKVV-------KIEQFEN-E-I-KN-W---------eFPLVIKP-------------YGVMICY-H-D-ADL-Q-K-A-ITRI-KE-A-T-A-E----tNSLIIEQKI-E-E-KAn
0021 6vqvJ   pSDADTLKVRFTLR-V-LvndqgyAHN-L-AaRF-LWRR-V-GAeAVEVRINHI-------------ARAWRFD-A-L-A--dA-E-L-DALA-EL-I-A-S-GsG-H-VLLEVVAFA-R-I-G--
0022 6pijA   pYGASHIECSFSVS-F-SetkigYLMN-I-CgKW-LWKT-R-KAyCWNIVLTPW-P----------NGEKVGFE-D-I----nK-N-W-SAIV-EM-I-K-T-AdG-L-AIFEVRATL-H-L-PTn
0023 6ahrG   rgqnaCSEIYIHGL-G-L-AINRAINI-A-L-QL-QAGS-F-G--SLQVAANTS-T-VEL-V------------------------------------------rN-N-SAIHIRVFR-V-T-P--
0024 6x73A   eVLQR-KSLSIDIN-W-G-IrfkFIER-G-C-QYlLEKL-NiNK-TTQITLKLM-R-RCK-D-ArCDSFSRSSR----aTEM-K-SlY-RTL--GC-P-P-M-E----lRGLALQFKL-V-D-VGp
0025 6cstA   tRDGERKSMSVERT-F-SeeqySLCQE-L-C-SElAQDLeR-LK-GRTVTIKLK-N-VN------FEVKTRAST----fAIA-K-ElL-KTE--------hP-L-R-L-RLMGVRISS-F-P-N--
0026 3fdsA   irTRVRKSIGRIVT-MnseEIKPYLFR-A-I-EEsYYKL-D-KR-IPAIHVVAV-T-ED------LDIVSRGRT-K-E-TAY-S-E-S-VKLL---------eR-K-I-RRIGVRFSK-F-I-EAi
0027 7a02A   nGQKTIVQDKVCID-W-T-----------iY-AD-NI-------qDIYASGYLK-VdTGT-GaT-GSSASFTVR-R-----F-D-T-V---TI-LG--------aE-T-GEFCMTIRY-T-L-S--
0028 2hx0A   hnASTARFYALRLL-P-G-Q---vfSQ-L-H-AF-VQQN--qlR-AAWIGCTGS-L-T----tG-TFEVSLNGT-LgE-HLH--gvxlghXXP-GC-T---------R-TTLELVIGE-L-P-ALt
0029 2ip4A   erYGIPTARYRVFR------EPLEALA-Y-L-EE-V----------gPVVVKD-------------KGVTV------aFDL-H-Q-A-KQAV-AN-I-L-N-RaE-G-GEVVVEEYL-E-G-E--
0030 5i47B   lADIPVPETRVCF-------GEEAIFA-A-I-AE-I----------gPVVLKS-------------GFPVAL------vEDQ-D-A-A-EAIV-EH-R-I-X-L-G-G-ERVLVQQFI--------
0031 1wyzB   rvyAESQTQLFIET-P-Y-RNHKXIED-I-L-QN-CR-------pqTKLCIAAN----------cEGEFIQTRT-V-K-DWK-G-H-I------PE-L-S-------K-IPCIFLLYK-L------
0032 2dfxI   ---------nasLS-P-K-AAIEVCNE-A-A-KK-G-------------wLGID-G-G----dS-SASWTYDMY-K-S-KIP-E-N-N-RLAI-EN-I-K-D-DyT-A-FIITLKM---------
0033 1vi7A   eeiKKSRFITMLAH-TvE-AAKAFVES-V-R-AE-HPD-------aRHHCVAWV-A-GA-dD-S-QQLGFSDDG-E-P-A-------------------------gV-G-EITAVVVRY-Y-G-GIl
0034 1ukfA   daGFSLRPKTVHAS-G-G-S-aQLGQT-V-A-HD-VAQ--S-GR-KHLLSLRFA-------------VQGHAIAcK-L-FD-fqqL-I-KGLI-DH-Y-N-S-L----yDVACVNEFR-V-S-V--
```

```
0035 4ecwA   kPRQLPKTIGCSKN-FrewWLLQLAQE-L-E-ER-LTKD-RnDR-VAQLVVSIR-V-QG--------SLRRCCA-L-T----hK-M-S-HdaftikncntwS-P-P-L-TMLFLCATK-F-S-A--
0036 4a2bA   sVSFEREDTVIERD-F--sitlDILSE-M-Q-SEaLEKL-KtpL-HIFSRYLLD--------------ERIVF------------------nPL-DM-K----------aSKIAIEYTS-I-V-VPl
0037 3vpdA   gerpeaaPKTALAT------DREEALR-L-M-EA-F----------gPVVLKP--------------RLLAK------vTDR-A-A-A-EALL-EH-K-EvL-G-G-FhQLFYIQEYV-E-K-PGr
0038 2h6lA   fefEVGKGFLLRLD-Y-G-----lvRQ-I-E-EF-LEEK-G-iH-AAHISAIGA-V-R----xE-PLEISLSGNvF-C-HI-lggH-L-FSA----------------vFACEVFVLP-L-S-GEa
0039 5mmjj   ----QKIRIKLRSY-----WVPLIEDS-C-K-QI-MDAAtT-NA-KIMPVPLTK-K-RIY-C-------------------------------------------fE-I-RTHQRLIDI-L-Y-PTa
0040 7dkoA   --------ASAEAE-V-KeeirAAARR-L-A-EAlRKAG-----vsGPVTVTAE-A-G-------DVSFSYTAD-L-DeeGL-K-R-VeaivraIA-A-L-K--tK-P-VLLSAVL----------
0041 3vpbB   rEGIPIPDSIIAL-------SAEAALK-A-Y-EQ-R----------gPLIDKP--------------RLVSL------iRDV-F-E-G-KTII-EH-R-E-L-M-G-NaLKAHIVQE-lE-H-PNk
0042 4ir1A   nSERLRKSVGVERT-MwsaIIERLYPE-L-E-RR-LAKV-K-pdlIARQGVKLK-F-DD------FQQTTQEHV-W-PrlNK-A-D-L-IA-------------rG-V-RLVGLHVTL-L-D-P--
0043 3mjfA   arHNIPSAEYQNFT------DVEAALA-Y-V-RQ-K----------gPIVIKAD-------------KGVIV------aXTQ-E-E-A-ETAV-NDxL-A-G-NdA-G-HRIVVEEFLk-------
0044 2gjhA   ----ERVRISITAR-----TKKEAEKF-A-A-IL-IKVE-L-GYnDINVTWDG----------------------------------------------------------DTVTVEG-Q-L-EGg
0045 4uerJ   qqQIIKIRITLTST-----KVKQLENV-S-S-NI-VKNAqH-NL-VKKPVRLTK-V-LKI-S-------------------------------------------yE-M-RIHKRYIDL-E-A-PVq
0046 3lp8A   mrYGIPTAKYGYFV------DTNSAYK-F-I-DK-HK----------PLVVKAD-------------KGTVI------cHTH-E-E-A-YNAV-DAmL-V-H-H---ggCAIIIEEFL--------
0047 5u4jv   -----DCYLDIQAGgT-E-AQDWASML-E-R-MY-LRWAsR-GF-KTEIEESEG-E-------------------------------------------------------gKSVTIKI-S-G-DYa
0048 2cveA   eeiQKSRFIAKAAP-VeE-EALAFLAE-N-R------------eeaTHNGHAYK-I-G-------LLYRFSDDG-E-P-S-------------------------grpD-RVAVLVVRY-F-G-GVk
0049 2j6hA   -----CGIVGAIAQ-------RDVAEI-L-L-EG-LRRE-Y-RGdSAGLAVVDA------------EGHMTRLR--vQ-MLA-Q-A-A-EEHP-----------------hGTGIAHT-R-W-ATh
0050 7yllA   vETMREIKSIGKEK-TiQ-HLKEFSEI-V-S-EE-LIKE-R---lyCRTVTVKI-K-TAD---F-AVHTKSKT----------------------------------yV-R-LIGLSVSNL-S-P-V--
0051 5l9wB   gsSIMDIVHMYEQS-R-RehfnQTVDT-M-I-ERaRQEL-RgledDASFGLELD-M-LYG-G-QnLKRMSS-------------pL-L-HIrtadalkvyfsgV-F-L-DNFVLRVTV-P-T----
0052 2ihr1   phAEKNAILTIQP-gT-E-ACDWAEML-L-R-MY-TRFArQ-GF-QVEVDLTPG-P-E-----------------------------------------------------gDYAQILV-K-G-ENa
0053 4aw7A   aFTNSIIRNKYYEY-L-K---piFPFT-----GI-ES-------gSGRASLRMS-I-GRP-ViF-FGMIEVPFD-I-Q-LL----kngnnvDI-TF--------gG-H-VSMILQVEK-Y-T-VS-
0054 8uy1A   gyvyQKAYLEFFV-------SPELYPE-I-K-RR-IESH-P----DLTYHAVTK------------SGNLETNA-----qsdtiawkdayhlgmwarcydvL-L-E-EnTWWLVNIVN-N-D-FHq
0055 1gsaA   geqdlpftPETLVT-R-N---KAQLKA-F-W-EK-H------------DIILKP------------ASIFR----K-E-GD--P-N-L-GVIA-ET-L-T-E-HgT-R-YCMAQNYLP-Aq----
0056 2qbuB   veGDEIDACVIMK-------TSRHGRR-A-M-EV-VESD-P--R-GKDVVSVAN----------mDDEVVER-----------------------------------sgYLATTLVRF--------
0057 5m41A   llvastSGTQLLMS-AaV-SVIQGAAG-L-T-AD-AIEG-K-eipLDVISLSLA--------knaETGVDIVGS-S-R-DLI-E-G-FkGNFT-DI-I-N-GlV-S-V-A----------------
0058 6bjvA   raIQGNtKVVFKRY-L--rvlgSWTGS-V-N-YA-ASRFlG-AN-QVGCTYSIR-F-R-------GVSVTISGG-S-R-TLQ-H-L-C-EMAI-RS-K-Q-E-L-Lq------------------
0059 4iwxA   kgrklpaPVTGI--------AHSPddD-L-I-DM----------ggAPLVVKLV-E-G------tQGIGVVLAE-R-Q-AAE-S-V-I-DAFR-GL-N----------aHILVQEYIK-E-Ae---
0060 9j46F   ---MFTGWSPFKYS-K-G-N----tVT-F-K----TPDE-S-S-aYMRFRCVFT-F-TDP-KgS-LHSIDVTE--N-N-MA-gfssftgghcqaLN-A-F----nC-D-ATLTGLQRI-I-H-HHh
0061 5k2mD   ddllfplPEWKAAL------SEGGALR-V-P-DS-L----------gPLVSKP--------------rLLAK------vNDR-D-S-L-EAVL-EH-R-K-WkN-P-L-YIHYFQEFV-E-K-PGr
0062 7lvoA   rHNIPTAAFRS--------fTSTQYED-A-V-AY-IKSK--ftS-GRSVIKASG---------------GVLIP-D-E-EAF-A-A-L-KSVM-VD-K------------DEVVVEE---------
0063 5yoyA   rtPSDKPVAHVVANeG-Q-LQ--lnrnalnQ-LV-V--P-S-EG-LYLIYSQVL-F-KGQkP-W-YEPIYLGGV-F--------ekgdlsaEI-NR---P-D-Y-L-D-FQVYFGIIA-L-T-S--
0064 7rjfA   -----IQIREYKRC-G-Q-DEERVRRE-C-K-ERgERQN-------CHYVIHKE-G---------------------------------------------------------NCYVCGIIC-W-----
0065 3v68A   ftakhEEWIVGENVnI-A-MFLSRVSN-T-V-SS-KIPGgelsiieglHINFTA-R-YG----S-WIVVKRMID-I-A-RLL-A-S-I-NETA-VN-K-I-K-De--------------------
0066 5ta1A   yvdGKNVYFIINNL-D-F-KPVDL-------nLS-VN-gkD-AK-SIEVRHLYL-K-GGK---D-GVPILDVYD-A-K-------------------------------feTEATCVICY-N-F-DRk
0067 5wx8A   ----------------------KNLKE-A-V-YD-ICCN-G---lsNNAAIIXY-F-TRS-K-K-VkTXIIYAA-P-I-DFV-G-A-V-KTCN-KY-A-K-D-NpK-E-IVLRVCSI-eF-K-SKf
0068 2dt4A   gmfSLGRTYLFRVP-E-G-----llTY-I-K-NF-CKKE-G-iE-TAIINGIGT-----------lYELILIGN-VfV-HAHgngH-L-VEGE---------------vFVAEIFLQE-L-K-GEk
0069 3gqcB   tEKERKSVSAEIGI-R-FkEAEAFLLS-L-S-EE-IQRRaT-GM-KGRLTLKIM-V-RKP-G-AiCDNIARTVT----gkAM-L-NmF-HTM------kL-N-I-S-D-MRGVGIHVNqL-V-PTn
0070 6p5bA   kLEKTgtqpYQVL-------SGDG-SN-L-K-GS-LPN-----K-NLLVRVEMD-R-FNAiD-C-WTTHSFTIF-VtL-YPYekgY-I-EKLA-LL-G-T-T--fS-P-LRVKFKVTE-V-N-PEi
0071 3wvqA   eafagrdPAGAVCA------DRRRAHR-H-V-TRlLDEG-------SPVILKQD-------------dGNEILS-R-TrVLA-DaA-L-DAYL-DE-R-W-DeG-G-R-HRVVVERYH-P-G-SRa
0072 5jb3L   ---MQKARIKIAST-----NVRSLDEV-A-N-QI-KQIA-EtGV-RMSPIPLPTkR-IRI-T-------------------------------------------wE-L-RVHKRLIDI-E-A-DEr
0073 2okqA   namKYVDGFVVAVP-A-D-KKDAYREM-A-A-KA-APLF-KgAL-RIVECWA------------------------------------------------------vpdkeNEEVVFSWI-E-Y-PSk
0074 7dovA   ------AAIHLTGG-Y-NktLDWRddqafrE-II-I--P-D-DG-IYFVYSQVS-L-HISnL-W-YNTIYLGAA-F--------regdlgtDT-TT----aL-L-P-M-VKTFFGVFG-L------
0075 1alyA   gDQNPQIAAHVISE-A-S--sktekgtmskQ-LT-V--K-R-QG-LYYIYAQVT-F-CSN-RcG-QQSIHLGGV-F--------qpgavfvNV-TD---P-S-Q-V----FTSFGLLK-L------
0076 8z9mA   lrhnkdlPPSLLLS-K-G-T---------lP-DI-FQS-------yDRLLITSW------------GHDPGVVP-T-S--------N-V-LTML-ND-A-L-T-H-----SAVLIQGHGnA-S-SQl

0001 s001A   -LLLLEEEEEEEEE-H-H-HHHHHHHH-H-H-HH-HHHL-L-LE-EEEEEEEEE-E-EEL-L-E-EEEEEEEEE-H-H-HHH-L-L-H-HHHH-HH-H-L-L-L-L-E-EEEEEEEEE-E-E-LL-
0002 9bk5A   --------LEEEEE-L-L---HHHHHH-H-H-HHhHHLL------LLLEEEEEE-E-EEL-L-E-EEEEEEEEL--hH-HHH-H-H-H-HHHH-HH-H-H-H-H-L-L-EEEEEELL---------
0003 4kyzA   hHLLLEEEEEEEEL------LHHHHHH-H-H-HHhHHHH-HhLE-EEEEEEEEE-E-E-H-H-H-HEEEEEEL------LLH-H-H-H-HHHH-HH-HhH-H-H-H-E-EEEEEEEEE-E-E-LLh
0004 3nd1B   lllLLLLEEEEEL-------LLLL--H-H-H-HL-LL-------lLEEEEEEEL----------lLLLEEEEEE-H-H-HHH-H-H-H-HHHH-HH-H-H-HhH-L-L-LLEEEEEEEE-L-L----
0005 2npnA   lLLLL-HEEEEE-------LLLL--LL-H-H-HH-LL-------lLEEEEEEEL----------lLLLEEEEEE-H-H-HHH-H-H-H-HHHH-HH-H-H-HhH-L-L-LLEEEEEEEE-L-L----
0006 5cw9A   ----LEEEEEEEL----------lhhH-H-H-HH-HHHHhL-LL-EEEEEEEEE-E-ELL-LlL-EEEEEEEL-----lLHH-H-H-H-HHHH-HH-H-H-H-HlL-E-EEEEEEEEE-E-L-LLh
0010 2p6yA   ----LLEEEEEEEL-L-L---LLHHHH-H-H-HH-HHHL-L-LL-LEEEEEEEE-E-EeE-L-L-LEEEEEEEE-ElE-EEEeE-E-E-L--L-LL-E----------eLLEEEEEEE-L-L-LEe
0011 3ohaA   lLLLLLLLEEEEEE-LllhHHHHHHHH-H-H-HHhHHHH-HeEE-EEEEEEEEE-L-LL------LLEEEEEEE-L-LhHHH-H-H-H-HHHH-H-------lL-L-L-LEEEEEEEE-E-E-EEl
0013 7dkkA   ------LEEEEEEE-L-lhHHHHHHHH-H-H-HH-HHHH-L--L-LLLEEEEEE-E-L-------LEEEEEEEL--lH-HHH-H-HhH-HHHH-HH-H-H-H-HlL-L-LEEEEEEEL--------
0014 3qphA   eeellLLLEEEL-----------LHHH-H-H-HH-HHHHlL-LL-LEEEEEEEE-E-LL----L-LLEEEEEEEE-E-E-E--llelleeeeEL-LL-L-L-L--------eEEEEEEE-L-L-LLl
0015 1ecgB   -------LEEEEEE-L--llLHHHHHH-HhH-HH-HH-------llLEEEEEEE-L-LL------LLEEEEEEEE-H-H-HH----L-L-HHHH-HH-L----------lLEEEEEEEL-L-L-LLl
0016 8k0kC   lLLLLEEEEEEEEE-E-EhhhhHHHHH-H-HlHH-HHHH-L-LLlLEEEEEEEL-------------LEEEEEL-L-L----lH-H-H-HHHH-HH-H-H-H-HlL-E-EEEEEEEEE-E-L-L--
0017 3htnA   eeeeeLLEEEEEEL-L-L-L--LHHHH-H-H-HH-HHHH-L-lL-LEEEEEEEE-E-E----lL-LEEEEEEEE-EeE-EEEeleE-E-EEE---------------E-EEEEEEEEE-L-L-L--
0018 8adbA   hhhhLLEEELEEE-------EHHHHHH-H-H-HH-HLLL-LlLL-LEEEEEEEL-------------LEEEEEE-ElL-EEEhH-H-H-HHHH-HH-L-L-L-L-LlH-HEEEEEEEE-L------
0019 3h20A   ------------------------lhH-H-H-HH-HHHH-----llLEEEEEEE-E-LL----L-LLEEEEEEEE-H-H-HHH-H-L-H-HHHH-HH-H-H-L-L-----EEEEEEELL-L-L-L--
0020 7r8qB   eellhheLLEEEE-------ELLLHHH-H-H-HH-L---------lLLEEEEE--------------LLLEEEL-L-H-HHH-H-H-H-HHHH-HH-L-L-L-L----lLEEEEEEELL-L-L-LEe
0021 6vqvJ   lLLLLEEEEEEEEE-E-LhllllhHHH-H-HlLL-LLLL-L-LLlEEEEEEEEE-------------EEELLEE-L-L-L--lL-H-H-HHHH-HH-H-H-H-HlL-L-EEEEEEEEE-E-L-L--
0022 6pijA   lLLLLEEEEEEEEE-E-EhhhllHHHL-L-LlHH-HLLL-L-LLlEEEEEEEEE-L----------LLLLEEEL-L-L----lL-L-H-HHHH-HH-H-H-H-HlL-E-EEEEEEEEE-E-L-LLl
0023 6ahrG   lllllLLEEEEEEL-L-L-HHHHHHHH-H-H-HH-HHHH-L-L--LEEEEEEEE-E-EEE-E-------------------------------------------eE-E-EEEEEEEEE-L-L-L--
0024 6x73A   hHLLL-LLEEEEEL-L-L-LlllHHHH-H-H-HHhHHHH-HlLE-EEEEEEEEE-E-ELL-L-LlEEEEEEEEE----hHHH-H-HhH-HHH--LL-L-H-H-H----eEEEEEEEEE-E-E-LLl
0025 6cstA   lLLLLLLEEEEEEE-E-EhhhhHHHHH-H-H-HHhHHHHlL-LL-EEEEEEEEE-E-LL------LLEEEEEEE----hHHH-H-HhH-HHH--------lL-L-L-E-EEEEEEEEL-L-L-L--
0026 3fdsA   llLLLLLEEEEEEE-EeehHHHHHHHH-H-H-HHhHHHH-L-LL-EEEEEEEEE-E-LL------LLEEEEEEE-H-H-HHH-H-H-H-HHHH---------lL-L-E-EEEEEEEEE-E-E-LLl
0027 7a02A   lLLLLLEEEEEEEE-E-E-----------eE-EL-LL-------lLLEEEEEEE-EeELL-LlL-LLEEEEEEEE-L-----L-L-E-E---EE-EL--------lE-E-EEEEEEEEE-L-L-L--
0028 2hx0A   llLLLLEEEEEEEL-L-L-L---hhHH-H-H-HH-HHHH--llL-LEEEEEEEE-E-E----eE-EEEEEEEEE-EeE-EEE--lleeeeELL-LL-E---------E-EEEEEEEEE-L-L-LEe
0029 2ip4A   hhLLLLLLLEEEEL------LHHHHHH-H-H-HH-H----------lLEEEEL--------------LLLEE------eLLH-H-H-H-HHHH-HH-H-L-L-LlL-L-LLEEEEELL-L-L-L--
0030 5i47B   hLLLLLLLEEEEL-------LHHHHHH-H-H-HH-H----------lLEEEEE-------------LLLLEE------eLLH-H-H-H-HHHH-HH-H-H-H-L-L-L-LLEEEEEELL--------
0031 1wyzB   hhhHHLLEEEEEEL-H-H-HHHHHHHH-H-H-HH-LL-------llLEEEEELL----------lLLLLEEEEE-H-H-HHL-L-L-L------LL-L-L-------L-LLEEEEEEL-L------
0032 2dfxI   ---------lllLL-H-H-HHHHHHHH-H-H-HH-L-------------lEEEE-E-E----lH-HHLEELLLL-H-H-HHH-H-H-H-HHHH-HH-H-H-H-HlL-E-EEEEELL----------
0033 1vi7A   eeeLLEEEEEEEEE-LhH-HHHHHHHH-H-H-HH-LLL-------lLLLLEEEE-L-LL-lL-L-LLLEEELLL-L-L-L-------------------------lL-L-LEEEELLEE-L-L-LLl
0034 1ukfA   hlLLEELLEEEELL-L-L-H-hHHHHH-H-H-HH-HLL--L-LL-EEEEEEEEL-------------LEEEEEEeE-E-EE-eehH-H-HHHH-HH-H-H-H-L----lLEEEEEEEE-E-E-L--
0035 4ecwA   lLLLLLLLEEEEEE-LhhhHHHHHHHH-H-H-HH-HHHH-HhLE-EEEEEEEEE-E-LL--------LEEEEEE-L-L----hH-H-H-HhhhhhhhhlleE-E-E-E-EEEEEEEEE-E-E-E--
0036 4a2bA   lLLLEEEEEEEEEE-L--ellhHHHHH-H-H-HHhHHHH-HeeE-EEEEEEEEL-------------LEEEL-------------------lLL-LL-E----------eLEEEEEEEE-E-E-EEh
0037 3vpdA   llllhhhLLEEEEL------LHHHHHH-H-H-HH-H----------lLEEEEL--------------LLLEE------eLLH-H-H-H-HHHH-HH-H-HhH-L-L-HhHLEEEEELL-L-L-LLe
0038 2h6lA   eeeELLEEEEEELL-L-L-----hhHH-H-H-HH-HHHH-L-lL-LEEEEEEEE-E-E----lL-LEEEEEEEEeE-E-EE-eeeE-E-EEE---------------eEEEEEEEE-L-L-LLl
0039 5mmjj   ----LLEEEEEEEL-----LHHHHHHH-H-H-HH-HHHHhL-LL-LEEEEEEEE-E-EEE-E-------------------------------------------eE-E-EEEEEEEEE-L-L-LLh
0040 7dkoA   --------LEEEEE-E-LhhhhHHHHH-H-H-HHhHLLL-----llEEEEEEEE-E-L-------LEEEEEEEE-E-LhhHH-H-H-HhhhhhHH-H-H-H--lL-L-EEEEEEL----------
0041 3vpbB   hLLLLLLLEEEEL-------LHHHHHH-H-H-HH-H----------lLEEEEL--------------LLLEE------eLLH-H-H-H-HHHH-HH-H-L-L-L-L-LhHHLEEEEE-eE-E-LLl
0042 4ir1A   lLLLLLLEEEEEEE-EhhhHHHHHHHH-H-H-HH-HHHH-L-lllLLEEEEEEE-E-LL------LLEEEEEEE-L-LllLH-H-H-H-HH-------------lL-E-EEEEEEEEL-L-L-L--
```

```
0043 3mjfA   hhLLLLLLLEEEEL------LHHHHHH-H-H-HH-H----------lLEEEEEL-------------LLEEE------eLLH-H-H-H-HHHH-HHhH-L-L-HlL-L-LLEEEEEELLl-------
0044 2gjhA   ----LEEEEEEELL-----LHHHHHHH-H-H-HH-HHHH-L-LLlLLEEEELL-----------------------------------------------------------LLEEEEEE-E-L-LLl
0045 4uerJ   llLLLLEEEEEEEEL-----LHHHHHHH-H-H-HH-HHHHhL-LL-LEEEEEEEE-E-EEE-E-------------------------------------------eE-E-EEEEEEEEE-L-L-LHh
0046 3lp8A   hhHLLLLLLEEEEL------LHHHHHH-H-H-HH-LL----------LEEEEEL-------------LLEEE------eLLH-H-H-H-HHHH-HHhH-L-L-L---hhLLEEEEEELL-------
0047 5u4jv   -----LLEEEEEEEElL-H-HHHHHHHH-H-H-HH-HHHHhH-LL-LLEEEEEEL-L-------------------------------------------------------lEEEEEEL-L-L-LLh
0048 2cveA   eeeLLEEEEEEEEE-LhH-HHHHHHHH-H-L------------lllLEEEEEEEE-E-L-------LEEEEELLL-L-L-L------------------------hhhL-LEEEEEEEE-L-L-LLl
0049 2j6hA   -----LEEEEEEELL-------LLLHHH-H-H-HH-HHHH-H-HLlEEEEEEEELL------------LLLEEEEEE--hH-HHH-H-H-H-HHLL-----------------lLEEEEEE-E-L-LLl
0050 7yllA   lLLLLLLLEEEEEE-EhH-HHHHHHHH-H-H-HH-HHHH-L---llEEEEEEEE-E-ELL---L-LEEEEEEE-------------------------------lE-E-EEEEEEEEE-L-L-L--
0051 5l9wB   hhHHLLEEEEEEEEE-E-EhhhhHHHHH-H-H-HHhHHHH-HlllhHLEEEEEEEE-E-EEL-L-LlLEEEEL-------------lL-L-LLllhhhhhhhhhlE-E-E-EEEEEEEEE-L-L----
0052 2ihr1   llLLLEEEEEEEEE-lH-H-HHHHHHHH-H-H-HH-HHHHlL-LL-EEEEEEEEL-L-L----------------------------------------------------lEEEEEEE-E-L-LLh
0053 4aw7A   lLLEEEEEEEEEELL-L-E---elEEEE-----EE-LL-------lLEEEEEEEE-E-EEE-LeE-EEEEEEEEL-H-H-HL----lleeeeEE-EL--------lL-E-EEEEEEEEE-E-E-LL-
0054 8uy1A   leeeELEEEEEEEE-------LLLLHHH-H-H-HH-HHLL-L----LEEEEEEEL------------LLLEEELL-----llleehhhhhhhhhhhllllhH-H-H-HhHLEEEEEEEE-L-L-LLl
0055 1gsaA   eeeeeehlLLEEEE-L-L---HHHHHH-H-H-HH-H------------LEEEEL------------LLLEE----L-L-LL--L-L-H-HHHH-HH-H-L-L-LlL-L-LEEEEEELLH-Hl-----
0056 2qbuB   llLLLLLEEEELL-------HHHHHHH-H-H-HH-HHLL-L--L-LLEEEEEEL----------lLLLEEEE------------------------------------llLLEEEEEEEL-------
0057 5m41A   hhhhhlLLLLEEEE-LhH-HHHHHHHH-H-H-HH-HHHL-L-lllLLEEEEEEE--------hheLLEEEEEEEE-H-H-HHH-H-H-HhLLLH-HH-H-H-HhH-H-H-H----------------
0058 6bjvA   llLLHHlLEEEEEEE-E--hhhlLLLHH-H-H-HH-HLLLlL-LL-LLEEEEEEEE-E-L-------LEEEEEEEEE-H-H-HHH-H-H-H-HHHH-HH-H-H-H-H-Ll-------------------
0059 4iwxA   lleellhLLEEE--------ELLLllH-H-H-HL----------llLLEEEEEEL-L-L------lLLLEEEEEEL-H-H-HHH-H-H-H-HHHH-LH-H----------hLLEEEEEELL-L-Ll---
0060 9j46F   ---LLLLLEEEEEEE-L-L-L----lLE-E-E----LLLL-L-L-hHEEEEEEEEE-E-ELL-LlL-EEEEELHH--H-H-HH-llleeellllllLL-L-L----lL-L-EEEEEEEEE-L-L-LLl
0061 5k2mD   hhleelhLLEEEEL------LHHHHLL-H-H-HH-H----------lLEEEEL--------------lLLEE------eLLH-H-H-H-HHHH-HH-H-H-HlL-H-H-HLEEEEEELL-L-L-LLe
0062 7lvoA   hLLLLLLLEEE--------eEHHHHHH-H-H-HH-HHLL--llL-LLEEEEEELL---------------LEELL-H-H-HHH-H-H-H-HHHH-LL-L------------HEEEEEEE--------
0063 5yoyA   llLLLLLEEEEEELlL-L-LL--ellllelE-EE-L--L-L-LE-EEEEEEEEE-E-EEElE-E-EEEEEEEEE-E--------lllleeeEE-LL---H-H-H-L-L-LHLEEEEEEE-L-L-L--
0064 7rjfA   -----LEEEEEEEEE-L-L-LHHHHHHH-H-H-HHhHHHL-------LEEEEEEEE-L-----------------------------------------------------LEEEEEEEEE-L------
0065 3v68A   eeeeeLLEEEEEEELhH-H-HHHHHHHH-H-H-HH-HHHHllhllllllhEEEEEEE-E-EL----L-EEEEEEEEEH-H-H-HHH-H-H-H-HHHH-HH-H-H-H-Hl---------------------
0066 5ta1A   eeeLLEEEEEEEEEL-L-L-LLEEE-------eEE-EE-llL-LL-EEEEEEEEE-E-LLL---L-LEEEEEEEEE-E-L-----------------------------elLLLEEEEEEE-E-L-LLl
0067 5wx8A   ----------------------LHHHH-H-H-HH-HHHL-L---llLLEEEEEEE-E-LLL-L-L-HlEEEEEEEL-H-H-HHH-H-H-H-HHHH-HH-H-H-H-LlL-L-EEEEEEEEE-hH-H-HHh
0068 2dt4A   lleEEEEEEEEEEL-L-L-----hhHH-H-H-HH-HHHH-L-lL-LEEEEEEEEE-----------eEEEEEEEE-EeE-EEEeleE-E-EEEE---------------eEEEEEEEEE-E-E-ELl
0069 3gqcB   lLLLLLLEEEEELL-L-LhHHHHHHHH-H-H-HH-HHHHhH-LE-EEEEEEEEE-E-ELL-L-LlEEEEEEEEEE----hhHH-H-HhH-HLL------lL-L-H-H-H-EEEEEEEEEeE-E-ELl
0070 6p5bA   lLLLLhhllLEEL-------LLLL-HH-H-H-LL-LLL-----L-LEEEEEEEEE-E-ELLlL-L-EEEEEEEEE-ElL-EEEhhhH-H-HHHH-HH-H-H-L--lL-L-EEEEEEEEE-L-L-HHh
0071 3wvqA   hhhlllhLLEEEEL------LHHHHHH-H-H-HHhHHLL-------LLEEEEEL-------------hHEEEEE-L-LeELL-LhH-H-HHHH-HH-H-H-HlL-L-L-LLEEEEEELL-L-L-LEe
0072 5jb3L   ---LEEEEEEEEEL-----LHHHHHHH-H-H-HH-HHHL-LlLL-EELLEEEEEeE-EEE-E-------------------------------------------eE-E-EEEEEEEEE-E-E-LHh
0073 2okqA   hllLEEEEEEEEEEE-H-H-HHHHHHHH-H-H-HH-HHHH-HlLL-EEEEEEEE-----------------------------------------------------lllllLEEEEEEEE-E-E-LLh
0074 7dovA   ------LEEEEEEE-E-LllEEEElllllelL-EE-L--L-L-LE-EEEEEEEEE-E-EELlL-E-EEEEEEEEE-E--------lllleeeEE-LL----lL-H-H-H-LLLEEEEEEE-L------
0075 1alyA   lLLLLLLEEEEEEL-L-L--lllllleellE-EE-E--L-L-LE-EEEEEEEEE-E-EEL-LlE-EEEEEEEEE-E--------lllleeeEE-LL---H-H-H-L----LLEEEEEEE-L------
0076 8z9mA   llllllleLLEEEEL-L-L-L---------lL-HH-HLL-------lLEEEEEEL------------LLLLEEEE-L-L-------L-L-HHHH-HH-H-L-L-L-----LLEEEEEEEEeL-L-LLl
```

Job: BR-systems; WP_041948680 DUF499-ATPase CTD FN-III like Domain (Type-2 BR-system Specific)
Query: s001A
No: Chain Z rmsd lali nres id PDB Description
1: 6q63-A 8.9 2.4 73 751 12 MOLECULE: BETA-HEXOSAMINIDASE;
2: 8ayr-A 8.5 2.2 70 677 11 MOLECULE: COAGULATION FACTOR 5/8 TYPE DOMAIN PROTEIN;
3: 2w1n-A 6.7 2.8 73 228 7 MOLECULE: O-GLCNACASE NAGJ;
4: 6smc-C 6.6 2.7 69 303 12 MOLECULE: INTERLEUKIN-12 SUBUNIT BETA;
5: 2vkx-E 6.5 2.7 70 202 9 MOLECULE: NEURAL CELL ADHESION MOLECULE;
6: 6bt9-B 6.4 2.8 69 626 12 MOLECULE: CHITINASE;
7: 2rb8-A 6.2 2.7 68 93 12 MOLECULE: TENASCIN;
8: 6nmy-F 6.1 2.3 68 270 6 MOLECULE: INTERLEUKIN-3 RECEPTOR SUBUNIT ALPHA;
9: 2yn3-C 6.1 2.9 69 284 4 MOLECULE: PUTATIVE INNER MEMBRANE PROTEIN;
10: 2c26-A 6.1 2.7 70 250 10 MOLECULE: ENDOGLUCANASE;
11: 1tdq-A 6.1 3.0 69 271 4 MOLECULE: TENASCIN-R;
12: 1fnf-A 6.1 2.6 68 368 6 MOLECULE: FIBRONECTIN;
13: 3wcy-A 6.0 2.7 68 318 7 MOLECULE: INTERFERON ALPHA/BETA RECEPTOR 1;
14: 6q9m-B 6.0 3.1 71 269 7 MOLECULE: RIM-BINDING PROTEIN, ISOFORM F;
15: 7y8i-E 6.0 2.3 65 97 15 MOLECULE: DSCAM;
16: 2dkm-A 6.0 2.6 69 104 10 MOLECULE: COLLAGEN ALPHA-1(XX) CHAIN;
17: 4jmh-A 5.9 2.7 69 200 6 MOLECULE: CLAMP SHC1_PY239/240;
18: 5mzv-A 5.9 3.0 69 303 6 MOLECULE: INTERLEUKIN-12 SUBUNIT BETA;
19: 6m76-A 5.9 2.9 72 923 13 MOLECULE: LPXTG-MOTIF CELL WALL ANCHOR DOMAIN PROTEIN;
20: 1qr4-A 5.9 2.8 65 175 11 MOLECULE: PROTEIN (TENASCIN);
21: 6aem-A 5.9 2.3 66 84 11 MOLECULE: PKD DOMAIN;
22: 6xax-B 5.9 2.9 67 272 4 MOLECULE: FIBRONECTIN;
23: 3t1w-A 5.9 2.6 67 368 6 MOLECULE: FOUR-DOMAIN FIBRONECTIN FRAGMENT;
24: 7u08-A 5.8 2.8 66 193 17 MOLECULE: RECEPTOR-TYPE TYROSINE-PROTEIN PHOSPHATASE ETA;
25: 3bes-R 5.8 2.5 67 250 12 MOLECULE: INTERFERON GAMMA;
26: 6mfa-A 5.8 2.8 67 363 12 MOLECULE: FIBRONECTIN;
27: 3lb6-D 5.8 2.8 71 286 10 MOLECULE: INTERLEUKIN-13;
28: 3og6-B 5.8 2.5 68 200 7 MOLECULE: INTERLEUKIN-29;
29: 3r8q-A 5.8 2.9 67 271 6 MOLECULE: FIBRONECTIN;
30: 7uww-A 5.7 2.5 69 632 6 MOLECULE: STARCH ADHERENCE SYSTEM PROTEIN 6 (SAS6);
31: 3b83-C 5.7 2.8 67 99 12 MOLECULE: TEN-D3;
32: 7z0l-B 5.7 2.5 69 370 6 MOLECULE: INTERLEUKIN-6 RECEPTOR SUBUNIT BETA;
33: 4doh-E 5.7 2.7 69 204 10 MOLECULE: INTERLEUKIN-20;
34: 2w91-A 5.7 3.4 74 635 8 MOLECULE: ENDO-BETA-N-ACETYLGLUCOSAMINIDASE D;
35: 2v5y-A 5.7 3.1 68 564 9 MOLECULE: RECEPTOR-TYPE TYROSINE-PROTEIN PHOSPHATASE MU;
36: 6x3a-A 5.7 2.5 66 306 8 MOLECULE: RECEPTOR-TYPE TYROSINE-PROTEIN PHOSPHATASE DELTA;
37: 7y8h-B 5.7 2.5 64 93 11 MOLECULE: DOWN SYNDROME CELL ADHESION MOLECULES;
38: 1k85-A 5.7 3.2 71 88 11 MOLECULE: CHITINASE A1;
39: 2jll-A 5.6 2.9 67 381 12 MOLECULE: NEURAL CELL ADHESION MOLECULE 2;
40: 1ug9-A 5.6 2.7 67 1019 13 MOLECULE: GLUCODEXTRANASE;
41: 4yg8-A 5.6 2.7 70 276 7 MOLECULE: CHITIN BIOSYNTHESIS PROTEIN CHS5;
42: 3bpl-B 5.6 2.8 68 202 9 MOLECULE: INTERLEUKIN-4;
43: 5gzt-B 5.6 3.0 69 1134 7 MOLECULE: CHITINASE;

44: 1l0q-A 5.5 2.6 67 391 12 MOLECULE: SURFACE LAYER PROTEIN;
45: 4a2m-B 5.5 2.6 71 750 6 MOLECULE: TWO-COMPONENT SYSTEM SENSOR HISTIDINE KINASE/RESP
46: 6h41-A 5.5 3.5 67 309 6 MOLECULE: INTERLEUKIN-5 RECEPTOR SUBUNIT ALPHA;
47: 5e4e-C 5.5 2.5 66 303 8 MOLECULE: INTERLEUKIN-13;
48: 8bw0-C 5.5 2.7 68 177 7 MOLECULE: TUSAMITAMAB FAB HEAVY CHAIN;
49: 8v2b-C 5.5 3.1 69 703 10 MOLECULE: ONCOSTATIN-M;
50: 8din-C 5.5 2.7 63 262 8 MOLECULE: IG GAMMA-1 FC CHAIN;
51: 2qbw-A 5.5 3.3 66 189 5 MOLECULE: PDZ-FIBRONECTIN FUSION PROTEIN;
52: 7y8s-B 5.5 2.8 68 288 6 MOLECULE: DSCAM;
53: 7zlv-h 5.4 3.0 76 527 13 MOLECULE: PROBABLE CENTRAL STRAIGHT FIBER;
54: 7biz-A 5.4 2.5 64 477 6 MOLECULE: CELL SURFACE PROTEIN;
55: 4xed-A 5.4 2.9 67 97 13 MOLECULE: PEPTIDASE M14, CARBOXYPEPTIDASE A;
56: 4doh-B 5.4 2.4 65 193 6 MOLECULE: INTERLEUKIN-20;
57: 4eq2-A 5.4 2.6 67 206 4 MOLECULE: INTERFERON GAMMA RECEPTOR 1;
58: 6grt-B 5.3 2.9 72 594 8 MOLECULE: PAIRED IMMUNOGLOBULIN-LIKE RECEPTOR B;
59: 8wm1-A 5.3 2.2 64 464 11 MOLECULE: 3-DEHYDROSHIKIMATE DEHYDRATASE (DHS DEHYDRATASE);
60: 1hwh-B 5.3 3.3 71 196 17 MOLECULE: GROWTH HORMONE;

```
0001 s001A   ---------AAKTDVIINQVLRDE--------E----TGAVKLRVEPQH-----G------------DKVYVDYGADP-----TTASELIH--------HGNFETA--R-----MEVRFLCVDST-----GKH----PQGPVRIWKNT----------
0002 6q63A   ydaegynyakhvFDVKAEFTPNPA-------------DGTLDITLTTID-----N------------APIHYTLGTEP-----TSTSPVYD--------G-ALKIK--E-----NDFSAIAIR--------PT----GNSRVVSEKIDfskssmkpiv
0003 8ayrA   lcevslwkyPVILAPAVNYDRN------------------GRVTLASAE-----------------nVVIRYTTGTEP-----GPQSAMYR--------N-PFFLA--G-----GTVKAAAEYRG-----R-------KSSVTTQIIPvptrdwkvva
0004 2w1nA   niiegtspeIVVNPVRFKASEIN--------------KKNVTVTWTEPE-ttegL------------EGYILYKDGKK--------VAEID--------ETSYTFK-klnRHTIYNFKIAAKYSN-----G-------EVSSKESLTLRta-------
0005 6smcC   stsffirdiikpDPPKLQMKPLK--------------NSQVEVSWEYP--------dswstphsyfsLKFFVRIQ--rkGC-NQKGAFLVE--------KTSTEVQ--C---KGGNVCVQAQDRY-----YNS----SCSKWACVPCRvr--------
0006 2vkxE   aasefktqpVREPSPKLEGQRGED-------------GNSIKVNLIKQDdggspI------------RHYLVRYR---aLSSEWKPEIRLG--------SDHVMLK-sldWNAEYEVYVVAEN--------QQ----GKSKAAHFVFRtaa-------
0007 6bt9B   ggpinqkdtepPTNVKIVVTNKN--------------SNSVQLNWTAST-dnvgV------------TEYEITAGE---------EKWSTT--------TNSITIK-nlkPNTEYTFSIIAKDAA-----G------NKSQPTALTVKtdetatfsvt
0008 2rb8A   ---------MRLDAPSIEVKDVT--------------DTTALITWMPPP-----V------------DGFELTYG--ikDVPGDRTTIDLT------edENQYSIG-nlkPDTEYEVSLISRRGD-----------mSSNPAKETFTtgl-------
0009 6nmyF   dkfvvfsqiEILTPPQMTA-KCNK--------------THSFMHWKMRS-------------hfnrkFRYELQIQ----KRMQPVITEQVR-------dRTSFQLL----NPGTYTVQIRARERV-----Y-----eFLSAWSPQRFEcdq-------
0010 2yn3C   ----------PPNAPVVTYSDIV--------------NDLIIXQGTAEA-----------------kSQLIITDSE--------GNTYTL---tvpdngKWSXAIP--Y--PSEGKFTITSVDAI-----G------NRSDDVPLDIXkevpvislsp
0011 2c26A   -------peNQAPKAIFTFSP-PV--------T----DENVVFNANSID-edgtI------------AYYVWDFG--------DGYEGTST--------TPTITYK--ykNPGTYKVKLIVTDNQ-----G-------ASSSFTATIKvtsatgdnsk
0012 1tdqA   sipatmnarTELDSPRLMVTASS--------------ETSISLIWTKAS---gpI------------DHYRITFT----PSSGISSEVTVP------rdRTSYTLT-dlePGAEYIISITAERGR------------QQSLESTVDAF----------
0013 1fnfA   splligqqsTVSDVPRLEVVAAT--------------PTSLLISWDAPA---vtV------------RYYRITYG--etGGNSPVQEFTVP------gsKSTATIS-glkPGVDYTITVYAVTGR-------gdspaSSKPISINYRt----------
0014 3wcyA   vdpfipfytAHMSPPEVRLEAED---------------KAILVHISPP-------qdgnmwalekpsFSYTIRIW---qKSSSDKKTINST--------YYVEKIP-ellPETTYCLEVKAIHPS-----LKK-----HSNYSVQCISttvankmpvp
0015 6q9mB   ------lgsdNVPAPKLTLERQL--------------NKSVLIGWSPP---eplI------------DSYHVYVDGVL--------KVTVE--------RTRALIE-gvdSTRPHRISVRSVTQN-----R------QTSRDCTMIIGrdtahlgpsa
0016 7y8iE   ---------GASPPPSISISHVT--------------SSSVQLNWE-----------nsqavpastiKQYLLEFR----GDNKDWIKLHIP------nnRKSFVLN-gldSSRRYQLRLAAYN--------RY----GRGDFAVIGFTtahke-----
0017 2dkmA   ---gssgssGPLPPPRLTLAAVT--------------PRTVHLTWQPSA-----gA-----------THYLVRCSpaspKGEEEEREVQVG--------RPEVLLD-glePGRDYEVSVQSLRGP-----E-------GSEARGIRARtptsgpssg-
0018 4jmhA   flwvggsggsVSSVPKLEVVAAT--------------PTSLLISWDAIW-ypyyV------------SYYRITYG--etGGNSPVQEFTVP------gySSTATIS-glsPGVDYTITIYAKYHR-----AK-----ySSPISINYRt---------
0019 5mzvA   tssffirdiikPDPPNLQL-KPLK--------N----SRQVEVSWEYP--------dtwstphsyfsLTFCVQVQ----GKREKKDRVFTD--------KTSATVI--C--RKNASISVRAQDRY-----YSS----SWSEWASVPC----------
0020 6m76A   vdggnttvdDQLTIPAIAINKTTP--------------SSLTLQWDQVE-----A------------TSYEVERDGTV--------FGNIQ--------TNTATFD-gfsFLSEHTFRVRAVGKN-----G-------VSEWSPIKGKtq--------
0021 1qr4A   ------------DNPKLEVSDPT--------------ETTLSLRWRRPV---akF------------DRYRLTYV----SPSGKKNEMEIP------vdSTSFILR-gldAGTEYTISLVAEKGR-----------hKSKPTTIKGStvvgspkgis
0022 6aemA   ---------eNQAPVANFEL-KTDG-------------LSVSAFNYSHD-edgeL------------VSYAWDFG--------NGQMSSE----------MAPSWS--ytRAGQYTVSLTVTDDK-----G-------ATNTTTRTTQvevp------
0023 6xaxB   ----------EIDKPSMQVTDVQ--------------DNSISVKWLPSS---spV------------TGYRVTTT--pkNGPGPTKTKTAG------pdQTEMTIE-glqPTVEYVVSVYAQNPS-----------gESQPLVQTAVtnidrpkgla
0024 3t1wA   stplrgrqkTGLDSPTIDFSDIT--------------ANSFTVHWIAPR---atI------------TGYRIRHH--peHFSGRPREDRV------phsRNSITLT-nltPGTEYVVSIVALNG-----------rEESPLLIGQQStvsa------
0025 7u08A   -----------PSPVFIKAVSIS--------------PTNVILTWKSND-------------taaseYKYVVKHK-----XENEKTITVVH--------QPWCNIT-glrPATSYVFSITPGIGN-----E------tWGDPRVIKVItepipvsdlr
0026 3besR   ctglcievkIGPPTVTLTEYDD-------------------HINLYIEHpyatrgykrndmcdiyllYTANFTFGD-------SEEPVIYdydctstgcSIDFATT--E------KVCVMAQGATeglldKIT----PWSSEVCLTPKknvytcairs
0027 6mfaA   ----------TVPSPRLQFVEVT--------------DVKVTIMWTPPA-----V------------TGYRVDVIpvnlPGEHGQRLPISR--------NTFAEVT-glsPGVTYYFKVFAVSHG-----------rESKPLTAQQTtkldaptnlq
0028 3lb6D   --------eIKVNPPQFEIV-DPG--------Y----LGYLYLQWQPPL--------------sectVEYELKYR---nIGSETWXTIITK--------NLHYKDG--fdLNXGIEAKIHTLLPW-----QCtngseVQSSWAETTYWispqgipetk
0029 3og6B   yldylfevePAPPVLVLTQTE-------------------EILSANATY-----qlppcmppldlkYEVAFWKEG-------AGNKTLFP----------VQITLQaaS-----EHHCLSARTIYtfsvpKYS----KFSKPTCFLLEvp--------
0030 3r8qA   sspvvidasTAIDAPSLRFLATT--------------PNSLLVSWQPPR---arI------------TGYIIKYE--kpGSPPREVvPRPR------pgVTEATIT-glePGTEYTIYVIALKNN-----------qKSEPLIGRKKt---------
0031 7uwwA   kqwidygkpDDHAYGYTLT-anFS--------T----E-SLDVKLALKN-----A------------DKGYYSVDG--------SAKKEFA-------nGDSVKVGegkiGNSKVTLTLYATG--------AD----GVETEQTYTFKktftaskttf
0032 3b83C   ----------MLQPPFIKVTNIT--------------LTTAVVTWQPP---ilpI------------EGILVTFG--rkNDPSDETTVDLS--------ITSLTLT-nlePNTTYEIRIVARNGQ------------QYSPPVSTTFTtgslehhhhh
0033 7z0lB   ----------SQPRVQCHASRY-----------------PVAVDCSWTP-----------lqtrstsFIATYRLGV----A-TQQQSQPCL--------QCTIPVH--L-----FPYMLNVTAVH-----PGA-----SSSLLAFVAEriikpdppeg
0034 4dohE   esgrfypflETQIGPEVAL-TTDE--------------KSISVVLTAP-----ekdlpvsmqqiysnLKYNVSVL---nTKSNRTWSQCVT--------NHTLVLT-wlePNTLYCVHVESFVPG-----PPR----RAQPSEKQCARtlkd------
0035 2w91A   gqltisdnhQEPQSPTFSVVKQSL--------K-naqEAEAVVQFKGND-----A------------DFYEVYESWKL---------lTGSS--------STTIYLPsasaQGTTQELKVVAVGKN-----G------VRSEAATTTFDwgmtvkd---
0036 2v5yA   isnyaelvvKEPPVPaPQLASVG--------------ATYLWIQLNA-ingdgpI------------VAREVEYC---tASGSWNDRQPVD--------STSYKIG-hldPDTEYEISVLLTR-P-----GEG----GTGSPGALRTRtkcadpmrgp
0037 6x3aA   kpklvsttgavPGKPRLVI-NHTQ-------------MNTALIQWHPPVdtfgpL------------QGYRLKFG---rKDMEPLTTLEFK--------EDHFTAT-dihKGASYVFRLSARNKV-----------gFGEEMVKEISipe-------
0038 7y8hB   -------gasppSPPRIKVSEVT--------------TRTARLSWQSP------------------vVTYIVRYW-rdeESRSQLHQLTFQ--------VTSANLK-dlhPGTSYAVQILAENDV------------GASIPSLVQFRtiee------
0039 1k85A   --------hMAPTAPTLASTAQT--------------TSSITLSWTAS-tdnvgV------------TGYDVYNGTAL--------aTTVT--------GTTATIS-glaADTSYTFTVKAKDAA-----G------NVSAASAVSVKt---------
0040 2jllA   kieifqtlpVREPPPSIHGQPSS--------------GKSFKLSITKQDdggapI------------LEYIVKYR-------SQWLEKKVQ------gnKDHIILE-hlqWTMGYEVQITAAN-------------lGYSEPTVYEFSmppkpn----
0041 1ug9A   aaryaagtpLSSPELSVTAP----------ealstadSATAVVRGTTN------A------------AKVYVSVNG---------TATEAP--------VFSLDVA--L-TGAKNKVTVAAVAA--------D----GGTAVEDRTVLyygsrigals
0042 4yg8A   akilekygtHKPESPVLKIVNVT--------------QTSCVLAWDPLKlgsakL------------KSLILYRKGI--------RsMVIP----npfkVTTTKIS-glsVDTPYEFQLKLITT--------S----GTLWSEKVILRthkmtdmsgi
0043 3bplB   sfkpsehvkPRAPGLTVHTQVS------------------DTLLLTWSN------yppdnylynhltYAVNIWSEN-------DPADFRIY--qvtylepSLRIALK--S-----GSYRARVRAWAqcyntTWS----EWSPSTKWHNS----------
0044 5gztB   ggygggsgsSSNPVVKLISTASNN-------------DRQLVLKFNAYG-----W------------DNATIVLT----SPAGKQTTYTYE----knsaqVTFTSD-kglAAGTWLYSVKTAKGS-----------VTSDTVTGKAFvqgkivsyip
0045 1l0qA   qfigsipvqPVYPSADFKSNItIF--------L----SEPVQFTDLSKD-----A------------TEWKWDFG--------DGSSSKK----------QNPTHT--ysETGIYTVRLTVSN---------S----NGTDSQISTVNvvlkgsptps
0046 4a2mB   frpelllldnPYTPPVVITKLQLFnkvvrtlksW----QTAFSIEFVVSN-----------yisgqhNTFAYKLE-----G-YDKEWYYLD--------SRTVSYS--nlPQGTYQFLVKAANSD-------G----KWNIPTALEIIvlpi------
0047 6h41A   --------kISLLPPVFTIKVTGL--------------AQVLLQWKPN----------pdqeqrnvnLEYQVKIN------APKEDDYETR--------ITESKAV--tiLHKGFSASVRTI-LQ-----ND--hsllASSWASAELHappgspgtsi
0048 5e4eC   -----------QPPVNLSV-SVEN-------------LCTVIWTWNPPE-----------gassncsLWYFSHFGDK-------QDKKIAP--------ETRRSIE--vpLNERICLQVGSQCS--------tnesekPSILVEKCISppegdpesav
0049 8bw0C   rsdpvtldvLYGPTPIISP--pYL--------S----GANLNLSCHSAS---------------npsPQYSWRING---------IPQQHT----------QVLFIAnnN-----GTYACFVSN-------LAT----GRNNSIVKSITvsa-------
0050 8v2bC   ---------PLPLTPEIHKVSFQL--------K----LQEVNLEWTV----------paltheelnMIFQIEIS--rlNISNTIWVENY-sttvkreeAVRWNWTdiplECVKHFIRIRALVDD-tkslpQS----SWGNWSWKEVNakvsvepdks
0051 8dinC   ---------APKAVIKLQP------------pwvsvfQEESVTLHCEV----------phlpgsssTQWFLN-------------GTAIQ--------TYHITAS--E-----DSGEYRCQR-------GLS----GRSDPIQLEVHrgwlllqvss
0052 2qbwA   kqeirvrvekdsSVPTLEVVAAT--------------PTSLLISWDasG-----V------------SYYRITYG--etGGNSPVQEFTVS--------SSTATIS-glkPGVDYTITVYAYSDY-----YgS----HHYSPISINYRt---------
0053 7y8sB   qelivktldGVLPPPSVSLLSAS--------------DSTISVKWGHTD---epV------------TGYTLHYR----KKVGHWLHVPLD--------QTRYTLT-gldSDTTYNVYVTANNRY-----------gRGDPSILSVRtgd-------
0054 7zlvh   fevrynfvpPAAPIIVFKAARIAD--------G----KERYDLRVQWDW---------nrgaganvreFVLSYIDSAefvRT-GWTKAQKIN---------ATIIFP--W-----KEHKFKVSSIAwgpdaqDV----TDSAVQTFILNestpldnsfv
0055 7bizA   ----------TPPVISLAL-sqVV--------R----NTDYTFTPDIQH-----S--------dvegFKIEWVREG---------KIVSTE---------NTYTFN--ekELGVYTVTINASN--------------IGTTTKDVSVEvvetmpyvvk
0056 4xedA   --------aNDPPNPNVTG---GN--------I----GTTYNYTFVTTD-------------pngdnITYYIEWG--------DGFTEEWI--gpyasgeEVIFSHT--wdKKGTYVVRAKAKDIL-----N------YESSWGTLEVTxpk-------
0057 4dohB   hpfnrqstiLTRPGMEITKDG-------------------FHLVIELE------------dlgpqfeFLVAYWRRE-------PGAEEHVK--mvrsggiPVHLTME--P----GAAYCVKAQTFV-kaigRYS----AFSQTECVEV----------
0058 4eq2A   efvlqkhgkIGPPKLNLSRHG-------------------AEIIVDVYH----perpwmreiyselsYSVIFRNSE--------NESRKNFvadcemnecNLSIPVP--S---EGSTYCVSAKGHF-fddlIVG----ASSEESCIWVPi---------
0059 6grtB   ----------SLPKPILRVQP-VS--------R----WTKVTFFCEETI-----G------------AEYRLYKDGKL--------YKTVT--------kAEFSLN--A-----GQYECSYSTQY------------KSSGYSPLKLVvtghywtpsl
0060 8wm1A   eqgcnaqpnQGIKAPKLTANK-------------------GLVAVEVNG-------------lpnqrYQVEFFSNqnaaSKEAEQYLGAIvatdaqgiaKANWKPK--V-----ASITANITDRF-----G------ATSELSALQTK----------
0061 1hwhB   ekcfsvdeiVQPDPPILNWTLLNV--------SltgiHADIQVRWEAP--------rnadiqkgwmvLEYELQYK--evNETKWKMMDPIL--------TTSVPVY-slkVDKEYEVRVRSKQRN-----SG-----NYGEFSVLYVTlpqms-----

0001 s001A   ---------LLLLEEEEEEEEEELL--------L----LLLEEEEEEEEL-----L------------LEEEEELLLLL-----LLLLEELL--------LLEEEEL--L-----LEEEEEEEELLL-----LLL----LLLLLEEEELL----------
```

```
0002 6q63A  hhhllllllllhhHLLEEEEEEEELL-------------LLEEEEEEEELLL-----L------------LLEEEELLLLL------LLLLLLL---------L-LEEEL--L-----LEEEEEELL---------LL----LLLLLEEEEELlllllllllee
0003 8ayrA  eleeeeellLLLLLLEEEELLL--------------------LEEEEELLL------------------lLEEEEELLLLL------LLLLEELL---------L-LEELL--L-----EEEEEEEEELL------E-------ELLLEEEEELlllllllleeee
0004 2w1nA  eeellllllLLLLLLEEEEEEEEL---------------LLLEEEEEEELLL-llllE------------EEEEEEELLEE---------EEEEL---------LLEEEEL-lllLLLEEEEEEEEEELL------L------LLLLLEEEEEEll---------
0005 6smcC  eeeelhhhllllLLLEEEEEEELL---------------LLLEEEEEEELL-------llllllllllllEEEEEEEE--llLL-LLLLLEEEL----------LLEEEEL--L---LLLEEEEEEEEELL-----LLL----LLLLLEEEELLll---------
0006 2vkxE  lleeeellLLLLLLEEEEEEELLL--------------LLLEEEEEEELLLlllllE------------EEEEEEEE---eLLLLLLLLEEEL---------LLEEEEL-lllLLLEEEEEEEEEEE--------LL----EELLLEEEEEElll-------
0007 6bt9B  lllllllllllLLLLEEEEEEEEL---------------LLLEEEEEEELLL-llllL------------LEEEEEELL----------EEEEEL---------LLEEEEL-lllLLLEEEEEEEEEELL-----L------LLLLLEEEEEElllleeeeee
0008 2rb8A  ---------LLLLLLEEEEELLL---------------LLLEEEEEEELLL-----L------------LEEEEEEE--elLLLLLLEEEEEE------llLLEEEEL-lllLLLEEEEEEEEEELL------------eELLLEEEEEElll-------
0009 6nmyF  eeeeehhhlLLLLLLEEEE-EELL---------------LEEEEEEELLL-------------lllllEEEEEEEE----ELLLLLEEELLL-------lLLEEEEE----LLLLEEEEEEEEELL-----L-----lLLLLLLEEELlll-------
0010 2yn3C  ----------LLLLLEEEEEEEE---------------LLEEEEEEELLL-----------------lLEEEEEELL--------LLEEEE---ellllEEEEEEE--L--LHHHEEEEEEELLL-----L------LLLLLEEEELLlllleeeelh
0011 2c26A  -------llLLLLLLLLEEEL-LL--------L----LLLEEEELLLLL-llllE------------EEEEEELL--------LLLEEEEL--------LLEEEEL--llLLEEEEEEEEEEELL-----L-------LEEEEEEEEEellllllll
0012 1tdqA  llleeeeeLLLLLLEEEEEEEL---------------LLLEEEEEEELLL---llL------------LEEEEEEE----LLLLLLEEEEEE------llLLEEEEL-lllLLLLEEEEEEEEEELL-----------EELLLEEEEEL---------
0013 1fnfA  llleeeeeLLLLLLEEEEEEEE---------------LLEEEEEEEELLL---llL------------LEEEEEEE--elLLLLLLEEEEEE------llLLEEEEL-lllLLLLEEEEEEEEEELLL--------lllllLLLLEEEEELl---------
0014 3wcyA  llllllhhhhEELLLLEEEEEELL--------------LLEEEEEEELL-------llllllllllllLEEEEEEE---eLLLLLLEEEEEL--------LLEEEEL-lllLLLEEEEEEEEEELLL-----LLL-----LLLLLLEEEEllllllllll
0015 6q9mB  ------llllLLLLLEEEEEEEEE--------------LLEEEEEEEELL---lllE------------EEEEEEELLEE--------EEEEL--------LLEEEEE-lllLLLLEEEEEEEEEELL-----L------LLLLHLEEEELlllllllllee
0016 7y8iE  ---------LLLLLLLLEEELLL--------------LLLEEEELL-----------llllllllllLEEEEEEE----ELLLLLEEELLL------llLLEEEEL-lllLLLEEEEEEEEEEE---------LL----EELLLEEEEEElllll-----
0017 2dkmA  ---llllllLLLLLLLLEEEEEL--------------LLEEEEELLLLL----lL------------LEEEEEEEelllLLLLLLEEEEEL--------LLEEEEE-lllLLLEEEEEEEEEELLL-----L------LLLLEEEELLllllllllll-
0018 4jmhA  ellllllllLLLLLEEEEEEEEE--------------LLEEEEEEEELLL-lhhhE------------EEEEEEEE--elLLLLLLEEEEEE------llLLEEEEL-lllLLLEEEEEEEEEELL-----LL-----lLLLLEEEEEEl---------
0019 5mzvA  eeeelhhhlllLLLLEEEL-LLLL--------L----LLEEEEEEEELL--------llllllllllllEEEEEEEE----LLLLLLLLEEEEL--------LLEEEEE--L--LLLLEEEEEEEEELL-----LLL----LLLLLEEEEEL----------
0020 6m76A  eelllllllLLLLLLLLEELHLLL---------------LLEEEELLLLL-----L------------LEEEEEEELLEE--------EEEEL--------LLEEEEL-lllLLLEEEEEEEEEELL-----E-------ELLLLLEEEEll--------
0021 1qr4A  ------------LLLEEEEELLL--------------LLLEEEEEEELLL---llL------------LEEEEEEE----LLLLLEEEEEEL------llLLEEEEE-lllLLLEEEEEEEEEELLL-----------lLLLLEEEEEEllllllllee
0022 6aemA  --------lLLLLLLLEEE-EEEL--------------LEEEEEELLLL-llllE------------EEEEEELL--------LLLEELL----------LLLEEE--llLLEEEEEEEEEEELL-----L-------LEEEEEEEEEelll------
0023 6xaxB  ----------LLLLLEEEEEEEEL--------------LLLEEEEEEELLL---llL------------LEEEEEEE--elLLLLLLEEEEEL------llLLEEEEL-lllLLLEEEEEEEEEELLL-----------lLLLLEEEEEElllllleeee
0024 3t1wA  llleeeeeLLLLLLEEEEELLL--------------LLLEEEEEEELLL---llL------------LEEEEEEE--elLLLLLLEEEEE------ellLLEEEEL-lllLLLEEEEEEEEEEEL-----------lEELLLEEEEEElllll------
0025 7u08A  -----------LLLLEEEEEELL--------------LLLEEEEEEELLL-------------lllllLEEEEEEL-----LLLLLEEEEEL--------LLEEEEL-lllLLLEEEEEEEEEELLL-----L------lLLLLEEEEEElllllleeee
0026 3besR  ellhhhhllLLLLEEEEEEEELL-------------------EEEEEEELleeeellllhhhlllllleEEEEEEELL-------LLLLEEEhhhllllleeEEEELLL--L------LEEEEEEEELllllllLLL----LLLLLEEEELLLlllllllll
0027 6mfaA  ----------LLLLLEEEEEEEEL--------------LLLEEEEEEELLL-----L------------LEEEEEEEellllLLLLLEEEEEEL--------LLEEEEL-lllLLLEEEEEEEEEELL-----------eELLLEEEEEEllllllleeee
0028 3lb6D  --------lLLLLLLEEEEE-LLL--------L----LLEEEEEEEELLL--------------llllLEEEEEEE---eLLLLLLEEEEEL--------LLEEEEE--llLLLLEEEEEEEEEELH-----HHhllllEELLLEEEEEELllllllllll
0029 3og6B  lllhhhhllLLLLEEEEEEEEL--------------------LEEEEEEEL------lllllllllllleeEEEEEEELL-------LLLLEELL----------EEEELLllL-----LEEEEEEEEEEellleEEL----LLLLLEEEELLll--------
0030 3r8qA  llleeeeeLLLLLLEEEEEEEE--------------LLLEEEEEEELLL---llL------------LEEEEEEE--llLLLLEELlLLLL------llLLEEEEL-lllLLLEEEEEEEEEELL-----------eELLLEEEEEEl---------
0031 7uwwA  lleeelllllLLLLEEEELL-llEL--------L----L-LEEEEEEEEL-----L------------LLEEEEELL--------LLLEEEL-------lLLEEEELllllLLEEEEEEEEEEL---------LL----LLEEEEEEEEEellllllllee
0032 3b83C  ----------LLLLLEEEEELLL--------------LLLEEEEEELL---lllL------------LEEEEEEE--elLLLLLLEEEEEL--------EEEEEEL-lllLLLEEEEEEEEEELL-----------EELLLEEEEEEllllllllll
0033 7z01B  ----------LLLEEEEELLLL------------------LLEEEEEEEL-----------lllllleEEEEEEELL----L-LLLLLEELL--------LEEEELL--L-----LLEEEEEEEEL-----LLE-----EEEEEEELLLllllllllee
0034 4dohE  elllllhhhHLLLLLEEEE-EELL--------------LLEEEEEELL-----llllllllhhhhlllLEEEEEEE---eLLLLEEEEEEEL--------LLEEEEL-lllLLLEEEEEEEEEELLL-----LLL----LLLLLLLEEEElllll------
0035 2w91A  eeeeeelllLLLLLLEEEEEEEEE--------E-ellEEEEEEEEELLL-----E------------EEEEEEEEEEEE--------eEEEL--------LLEEEEElllLLLEEEEEEEEEEELL-----L------LLLLLEEEEEEElllllll---
0036 2v5yA  ellleeeeeELLLLLlLEEEEEL--------------LLLEEEELLL-llllllL------------LEEEEEEE---eLLLLLEEEEELL--------LLEEEEL-lllLLLEEEEEEEEEEEL-L-----LLL----LLLLLLLEEEEllllllllll
0037 6x3aA  lleeeellllLLLLEEEE-EEEL--------------LLEEEEEEEELLLllllllL------------LEEEEEEE---eLLLLLLEEEEEL--------LLEEEEE-lllLLLEEEEEEEEEELLL-----------lLLLLEEEEEElll-------
0038 7y8hB  -------lllllLLLEEEEELLL--------------LLLEEEEEEELL------------------lLEEEEEEE-ellLLLLLLEEEEEL--------LLEEEEL-lllLLLEEEEEEEEEELLL-----------LLLLLLLEEEElllll-----
0039 1k85A  --------lLLLLLLEEEEEEEEL--------------LLLEEEEEEELL-lllllE------------EEEEEEEELLLE--------eEEEL--------LLEEEEL-lllLLLEEEEEEEEEELL-----L------EELLLLLEEEEl---------
0040 2jllA  lleeeelllLLLLLLLEEEEEEL--------------LLEEEEEEELLLLllllllL------------LEEEEEEE-------LLLLLLLEEL------llLLEEEEL-lllLLLEEEEEEEEEEL-------------lLLLLLEEEEEEllllll----
0041 1ug9A  hhhhlllllLLLLLEEEEEL----------lleeellLLEEEEEEEEEL------L------------LEEEEEELL----------EEEEEL--------LEEEEEE--L-LLLEEEEEEEEEELL--------L----LLEEEEEEEEEElllleeeeeee
0042 4yg8A  hhhhhhhhlLLLLLLEEEEEEEL--------------LLLEEEEEELLLLllllllE------------EEEEEEELLLL--------LlLLLL----llllLLEEEEL-lllLLLEEEEEEEEEEEL--------L----EEEEEEEEEEEElllllllllll
0043 3bplB  eelhhhlllLLLLEEEEELLLL-----------------LEEEEEEEL------llllllllhhhleEEEEEEELL------LLLLEEEE--eellllllEEEEELL--L-----LEEEEEEEEELhhhllLLL----LLLLLEEEELL----------
0044 5gztB  llllllllllLLLLLEEEEEEEEELL--------------LLLEEEEEEELL-----L------------LLEEEEEE----LLLLLEEEEEHH----hhhhhEEEELL-lllLLEEEEEEEEEEEELL-----------EELLLEEEEEEelleeeeeee
0045 1l0qA  lleeellllLLLLLLLEEELLlEE--------L----LLLEEEEELLLL-----L------------LEEEEELL--------LLLEELL----------LLLEEL--llLLEEEEEEEEEEE----------L----LEEEEEEEEEEeellllllll
0046 4a2mB  ellllllllLLLLLEEEEEEEELleelleellL----LLLEEEEEEELL-----------llllllLEEEEEEE-----L-LLLLLEEEL--------LLEEEEL--llLLEEEEEEEEEEELLL--------L----LLLLLEEEEEEEelll------
0047 6h41A  --------lLLLLLLEEEEEEEEEL--------------LEEEEEEELL----------llllllllllLEEEEEEE------ELLLEEEEEL--------LLEEEEE--llLLLLEEEEEEEEE-EE-----LL--lleeELLLEEEEELllllllllll
0048 5e4eC  -----------LLLLEEEE-EEEL--------------LLEEEEEEEELLL-----------llllllllLEEEEEEELLL-------EEEEEEL--------LLEEEEE--llLLLLEEEEEEEEEELL--------llllllLLLEEEEEELllllllllll
0049 8bw0C  elllllllllLLLLLLEELL--lEL--------L----LLLEEEEEEELLL---------------lllLEEEEEELL--------EEEELL----------LEEEELllL-----EEEEEEEEL--------LLL----LLEEEEEEEEEEell-------
0050 8v2bC  ---------LLLLLLEEEEEEEEEL--------L----LLEEEEEEEL-----------llllllleeeEEEEEEEE--elLLLLEEEEEEEE-eeeeellllLEEEEEEllllLLLLEEEEEEEEEELL-lllllLL----LLLLLLLEEELlllllllllle
0051 8dinC  ---------LLLLEEEEEL-------------llleeeLLLLEEEEEEEL----------lllllllllEEEEEL-------------LEELL--------LEEELLL--H-----HLEEEEEEE--------LLL----LLLLLEEEEEElleeeeeell
0052 2qbwA  eeeeeeeeeellllLLLLLEEEEEE---------------LLEEEEEELLllL-----L------------LEEEEEEE--elLLLLLLEEEEEL--------LLEEEEL-lllLLLEEEEEEEEEELLL-----LlL----LLLLLEEEEEEl---------
0053 7y8sB  lleeeellllLLLLLLLEEEEEEL--------------LLLEEEEELLLL---llL------------LEEEEEEE----ELLLLLEEEEEL--------LLEEEEL-lllLLLEEEEEEEEEELL-----------eELLLLLEEEElll-------
0054 7zlvh  eelllllllLLLLEEEEEEELLLL--------L----LLLLEEEEEEEL--------lllllllllllEEEEEEEHHhhhhHL-LLLLLEEEE----------EEELLL--L-----LLEEEEEEEELllllllLE----EELLLEELLLLlllllllllll
0055 7bizA  ----------LLLEEELLL-llEE--------L----LEEEEELLEEEL-----L-----------llllLEEEEEELL--------EEEELL----------LLLEEL--llLLEEEEEEEEEEL--------------LLEEEEEEEEEeeellllleee
0056 4xedA  --------lLLLLLLEEEL---LL--------L----LLLEEEEEEELLL-------------lllllEEEEEELL-------LLLEEEEE--eeellllLEEEEL--llLLEEEEEEEEEEELL-----L------LLLLLEEEEEEEell-------
0057 4dohB  llllhhhllLLLLLEEEEEEEL--------------------LEEEEEEL-------------llllllleEEEEEEELL------LLLLLEEE--eellllLEEEEELL--L----LLLEEEEEEEEE-hhhlEEL----LLLLLEEELL----------
0058 4eq2A  leehhhhllLLLLEEEEEEEEL--------------------LEEEEEEEL----lllhhhhhhhlleeEEEEEEELL-------LLLEEEEeellhhhhleEEEEELL--L---LLEEEEEEEEEEE-llllEEL----LLLLLEEEEELl---------
0059 6grtB  ----------LLLLLEEEEEL-EE--------L----LLLEEEEEEELLL-----L------------LEEEEEEELLEE--------EEEEE---------lEEEEEH--L-----EEEEEEEEELL-----------EELLLLLEEEEeelllllllee
0060 8wm1A  llllllllhHHLLLLEEEEEEL--------------------LEEEEEEEEL-------------lllleEEEEEEEEllllLLLLLEEEEEEEeelllllleeEEEELLL--L-----LEEEEEEEELL-----L------LLLLLLEELL----------
0061 1hwhB  eeeeehhhhLLLLLLEEEEEEEEEL--------LllllEEEEEEEEELL--------lllllllllllEEEEEEEE--elLLLLLEELLLLL--------LLEEEEE-eeeLLLLEEEEEEEEEELL-----LL-----LLLLLLLEEELlllll-----
```

```
Job: DUF3780 (Type-2 BR-system Specific)
Query: s001A
No: Chain Z rmsd lali nres id PDB Description
1: 8aj8-B 4.3 3.7 85 558 6 MOLECULE: PHOSPHATIDYLINOSITOL 4,5-BISPHOSPHATE 3-KINASE CA
2: 2ipc-A 3.9 12.3 80 939 9 MOLECULE: PREPROTEIN TRANSLOCASE SECA SUBUNIT;
3: 5h1q-A 3.9 10.8 79 360 6 MOLECULE: INNEXIN-6;
4: 1yns-A 3.9 8.3 63 254 8 MOLECULE: E-1 ENZYME;
5: 5yet-A 3.8 12.6 70 397 3 MOLECULE: UNCHARACTERIZED PROTEIN R354;
6: 8ppq-a 3.6 13.9 59 147 2 MOLECULE: ENVELOPE PROTEIN E;
7: 5mlp-A 3.6 16.0 65 255 3 MOLECULE: UNCHARACTERIZED PROTEIN;
8: 3k7d-A 3.5 3.1 83 497 7 MOLECULE: GLUTAMATE-AMMONIA-LIGASE ADENYLYLTRANSFERASE;
9: 4gmq-A 3.5 2.6 58 92 5 MOLECULE: PUTATIVE RIBOSOME ASSOCIATED PROTEIN;
10: 6d9z-C 3.5 8.8 104 241 8 MOLECULE: SULFATE TRANSPORTER CYSZ;
11: 6xqj-A 3.5 2.9 49 173 10 MOLECULE: PROTEIN VPR,UV EXCISION REPAIR PROTEIN RAD23 HOMO
12: 4myy-B 3.5 4.4 57 84 14 MOLECULE: CURG, CURH FUSION PROTEIN;
13: 7x34-C 3.4 3.9 71 100 10 MOLECULE: ZUOTIN;
```

```
14: 8c81-B 3.3 2.8 48 539 6 MOLECULE: PROTEIN ORM1;
15: 8rxa-A 3.3 4.8 76 102 11 MOLECULE: AP2 DOMAIN TRANSCRIPTION FACTOR AP2-O5, PUTATIVE;
16: 6cgh-A 3.3 4.3 66 89 8 MOLECULE: DNAJ HOMOLOG SUBFAMILY C MEMBER 2;
17: 6x6o-B 3.3 3.3 63 76 5 MOLECULE: PROTEIN SPACKLE;
18: 9fk0-D 3.2 7.4 52 75 4 MOLECULE: ENVELOPE PROTEIN E;
19: 4v1a-h 3.2 5.4 78 289 6 MOLECULE: MITORIBOSOMAL PROTEIN ML37, MRPL37;
20: 5u4w-H 3.1 3.4 47 53 4 MOLECULE: E PROTEIN;
21: 9m31-A 3.1 12.6 76 945 8 MOLECULE: CASRX;
22: 9qx2-A 3.1 12.4 72 947 4 MOLECULE: UBR-TYPE DOMAIN-CONTAINING PROTEIN;
23: 6jdp-A 3.1 4.1 47 241 4 MOLECULE: IMM52 FAMILY PROTEIN;
24: 8rjj-A 3.1 17.4 62 183 5 MOLECULE: GENOME POLYPROTEIN;
25: 4p96-A 3.1 7.9 61 278 7 MOLECULE: FATTY ACID METABOLISM REGULATOR PROTEIN;
26: 6c6r-A 3.0 14.6 80 451 8 MOLECULE: SQUALENE MONOOXYGENASE;
27: 3cxj-A 3.0 5.4 50 144 14 MOLECULE: UNCHARACTERIZED PROTEIN;
28: 7e9d-A 3.0 7.4 55 443 5 MOLECULE: IRG1;
29: 8an4-A 3.0 13.1 78 195 10 MOLECULE: BACTERIAL TOXIN;
30: 9qx0-B 3.0 13.2 74 1090 3 MOLECULE: E3 UBIQUITIN-PROTEIN LIGASE KCMF1;
31: 5cuf-C 3.0 13.3 63 370 6 MOLECULE: SESTRIN-2;
32: 5tf3-A 2.9 2.3 62 131 3 MOLECULE: PUTATIVE MEMBRANE PROTEIN;
33: 6w4x-C 2.9 9.6 56 375 9 MOLECULE: RIBONUCLEOSIDE-DIPHOSPHATE REDUCTASE 1 SUBUNIT AL
34: 2ffl-A 2.9 19.4 90 732 8 MOLECULE: DICER;
35: 6s7t-A 2.9 6.2 88 706 3 MOLECULE: DOLICHYL-DIPHOSPHOOLIGOSACCHARIDE--PROTEIN
36: 9e41-B 2.9 6.5 52 75 4 MOLECULE: E GLYCOPROTEIN;
37: 5mlq-A 2.9 15.4 59 226 8 MOLECULE: CDPS;
38: 8ul9-A 2.9 6.8 95 387 8 MOLECULE: CHOLINEPHOSPHOTRANSFERASE 1;
39: 9jc2-A 2.9 3.8 49 166 8 MOLECULE: ATP SYNTHASE SUBUNIT A;
40: 6mca-A 2.9 14.8 92 460 12 MOLECULE: ANKYRIN REPEAT DOMAIN PROTEIN;
```

```
0001 s001A   [illegible]
0002 8aj8B   [illegible]
0003 2ipcA   [illegible]
0004 5h1qA   [illegible]
0005 1ynsA   [illegible]
0006 5yetA   [illegible]
0007 8ppqa   [illegible]
0008 5mlpA   [illegible]
0009 3k7dA   [illegible]
0010 4gmgA   [illegible]
0011 6d9zC   [illegible]
0012 6xqjA   [illegible]
0013 4myyB   [illegible]
0014 7x34C   [illegible]
0015 8c81B   [illegible]
0016 8rxaA   [illegible]
0017 6cghA   [illegible]
0018 6x6oB   [illegible]
0019 9fk0D   [illegible]
0020 4v1ah   [illegible]
0021 5u4wH   [illegible]
0022 9m31A   [illegible]
0023 9qx2A   [illegible]
0024 6jdpA   [illegible]
0025 8rjjA   [illegible]
0026 4p96A   [illegible]
0027 6c6rA   [illegible]
0028 3cxjA   [illegible]
0029 7e9dA   [illegible]
0030 8an4A   [illegible]
0031 9qx0B   [illegible]
0032 5cufC   [illegible]
0033 5tf3A   [illegible]
0034 6w4xC   [illegible]
0035 2fflA   [illegible]
0036 6s7tA   [illegible]
0037 9e41B   [illegible]
0038 5mlqA   [illegible]
0039 8ul9A   [illegible]
0040 9jc2A   [illegible]
0041 6mcaA   [illegible]

0001 s001A   [illegible]
0002 8aj8B   [illegible]
0003 2ipcA   [illegible]
0004 5h1qA   [illegible]
0005 1ynsA   [illegible]
0006 5yetA   [illegible]
0007 8ppqa   [illegible]
0008 5mlpA   [illegible]
0009 3k7dA   [illegible]
0010 4gmgA   [illegible]
0011 6d9zC   [illegible]
```

```
0012 6xqjA   ----------------------------------------------------------------------------------------------------------------------------------------------------------lhhhhH-HH--H----H-HHHHHHHHHL-------LHHHHHHHHHHH---H-HHH------L--L----------------lHHHHHHHHHHHH--H-H-H--Hhh
0013 4myyB   ----------------------------------------lHH-HH-------------------------------hhHHHH-------------------------------------------------hhllhHHH--HHHH-HHHHH---HHH-LL-L--hHHH-HH-HHHHHHHHHHL-------LHHHHHHHHHHH---H-HHHhhhhll-----------------------------------------
0014 7x34C   ------------------LHH--HhhHH-H-HHH--HH---------------------------------------------------------------------------------------------------HHH--HHHH-HHHHH---HHH-------lLHH-HHhHHHHHHHHHLL-------LHHHHHHHHHHH---H-HLL--------------------------LLLH-HHHHH------L-L--L--
0015 8c81B   -------------------------------------------------------------------------------------------------------------------------------------------------------------------hHHH-HH-HHHHHHHHHL-------LL-LHHHHHHHHH---H--HL------L--L------L-----HH---H-HHHHHHHHH-----H-H--L--
0016 8rxaA   --------------------------LL-L-HHH--H----------------------------------------------------------------------------------------------------HHH--HHHH-HHHHH---HHHhHH-LL-hHHH-HH-HHHHHHHHH--llllhhHLHHHHHHLHHH---HhLLL------L-------hhH-----LL---HHHHHHHHHHH---H-H-H--H--
0017 6cghA   ---------------------------------------------------------------------------------------------------------------------------------------llLLH--HHHH-HHHHH---HHH-HH-LL-lLHH-HH-HHHHHHHHHH---hhhlLHHHHHHHHHHH---L-LLL----------------------H---HHHHHHHHHHH-----H-H--H--
0018 6x6oB   --------------------------------------------------------------------------hHHLL-----------------------------------------------------------LL--HHHH-HHHHH---HHH-HH-HH-hLHH-HH-HHHHHHHHHH-------L---LHHHHHHHHH---H-LL--------------------------llLLHHHHHHHH-H-L-L-L-----
0019 9fk0D   -------------------------------------------------------lllL-LL-L---------------------------------------------------------------------------------------------------hHHH-HH-HHHHHHHLLL-------HH-HHHHHHHHH---H-HHL--------lL-HH---H--------hhHHHHHHHHLL--L-L-L-L-----
0020 4v1ah   hhhhhhhhhHL--L--LLLHH-hH--HL-L---------------------------------------------------------------------------------------------------------lllHH--HHHHhHHHHH---HHH-HH-HH--HhH-HH-HHHHHHLLHhhllhhhhHHHHHHHHHHHH---H-HLL------H--------------------HHHHHHHHHLHH-H-L-L--L--
0021 5u4wH   ----------------------------------------------------------------------------------------------------------------------------------------------------------------------LH-HH-HHHHHHHHHL-------LH-HHHHHHHHH---H-LL------lL--L-HH---H--------hhHHHHHHHHHL-LL-L-L-------
0022 9m31A   -hHHLE--EEE--EeellllhhlL--LL-LlHHH--HL-----hhHHLL----------------------------------------------------------------------------lhhhHH-HHhhhhhhhhhllhhhhhhllllhhhhhhhhhhHHH-HL-HHHHHHHHHH-llllllHHHHHHHHHHHH---L-LllllllhhhhlH-HH---H---------hHHHHHHHHHHHHH-H-H-H--H--
0023 9qx2A   -hhHLL--LLH--HhlLLLLL--H--HhhH-HHH--H-------hhhhHHLL-------------------------llllhhhhhhhhlllhhhhhlllhhHHHH------------------------------------------------------------hllLL-HHHHHHHHHL-------LHHHHHHHHHHH---H-HHH------H-hL-LL---H----hHH---HHHHHHHHHH-------------
0024 6jdpA   ------------------------------------------------------------lL-L-LE--EE--------------------------------------------------------------------------------------------------eeeeLHHHHHLL-------LHHHHHHHHHHH---H-HHHhhhlhhhlhhlHH---H-----HH---H-HHHHHL-LLEE-Ee--------
0025 8rjjA   -lLLLE--EL------------eeeellhhhhhhhhhhhhhhhhhhhHLLHH---hhhhhHH-H-H--------------------------------------------------------------------------------------------------hHHH-HH-HHHHHHHHHH----hhlLLHHHHHHHHHH---H-LLL----------------L-----HH---H-HHHHHHHH-HH-H-Hl------
0026 4p96A   ----------------------hhhhhhllleelllllllleelllllllhHH----HH-HH-H-H-----------------------------------hHHHH-----------------------------------------------------------hHHH-HH-HHHHHHHHHH----hhhLHHHHHHHHHHH---H-HHH-----------HH---H-----HH---H-HHHHHHHHhHH-H-Hh------
0027 6c6rA   -----L--LLL----leeelL--L--HH-H---------HLLLlLEEEL------lllL-EE-E-EeelHHH---LlhhhhhhhhhhhhhhhhhlhhhhhhhhllhhhhHHHHL------------------------------------------------------LH-HH-HHHHHHHHHH-----hhLLLHHHHHHHHHhllL-LLL-----------------------------HHHHHHHHHHHHH-H-H-H--H--
0028 3cxjA   -------------------------------------------------------lhhhhellL-LL--EE----eeeeelllLLEE------------------------------------------------------------------------------eeeeL-LLHHHHHHHHL-------LHHHHHHHHHHH---H-HHH-----------llhhhH-----HH---H-HHHHHHHHHL-----------
0029 7e9dA   -------------------------------------------------------hhlL-LH-H----------------------------------------------------------------------------------------------------hHHH-HH-HHHHHL-LLL-------H--HHHHHHHHH---H-HH-------L--L-LL---L-----EE-elHHHHHHHHHHHHH-hhL-L--L--
0030 8an4A   ----------------------hH--LL-L-EEE-------------------------------------------------------------------------eeHHHHHHHHlllllllleeeeeelllEEEEelHH--HLeehhhhHH---HH--hH-LL--LLL-HHhHHHHHHHHHL-------LHHHHHHHHHHH---H-HHH------H--L-LL---L-----LL---LLHHHHHHHHH-------------
0031 9qx0B   ----------------------------lL-LLLllLLLL---LLLLlhHHH----H----hH-HH--H----hhllllhhhhhhhhhllhH---HHHH------------------------------------------------------hhhhHH-HH-lHHH-HHhHHHHHHHHHL-------LHHHHHHHHHHH---H-LL-------------------------hhHHHHHHHHHHhhH-L-L-H--Hhh
0032 5cufC   ---------------llhhhH--L--LL-L-------------------------lllllLH-H-HH-------------------------------------------------------------------------------------------------lLEE-HH-HLLLHHHHHH----hhhLHHHHHHHHHHH---H-HHH-----------HH---H-----HhhhhHHHHHHHHHHHHHH-H-H-H--L--
0033 5tf3A   -------------------------------------------------------------------------------------------------------------------------------------------L--HHHH-HHHHH---HHH-HH-HH--LL--LL-HHHHHHHHHLL-------LHHHHHHHHHHH---H-HLL---------------------------HHHHHHHHHHH---H-L-L--Lll
0034 6w4xC   -------------------------------------------------------hhHH-HHhH-HL--L---------------------------------------------------------------------------------------llL-LL--L---LL-HHHHHHHHHL-------LHHHHHHHHHHH---H-HHH------H--H-HHhhhH-----HH---H-HHHHHLLL--------------
0035 2fflA   -lHHHH----------------lllllllllLLH--HH-----------hhHH----LHhHH-H-HH-lllllhhhhhhhhhHHHH-HL--LE---------------------------------------hhhhhhHH--HHHH-HHHHH---HHH-HH-HH--HHH-HH-HLLLLHHHHH-lllhhhHHHHHHHHHHHH---H-HHL------H--------------------HHHHHHHHHLHH-Hl--------
0036 6s7tA   hhhllL--LLL-hH--HHHHH--H--HH-------hhhHHHH--------------------------------hhhlllLLLLH-H-HH--HHlllLLLLHH------------------------------------------------------hhhhLL--LL--HH-HHHHHHHHHH-hhlllhHHHHHHHHHHHH---H-HHH------L-lL-LL---L-----HH---H--HHHHHHHHHH-H-H-H--H--
0037 9e41B   -------------------------------------------------------lllL-LL-L----------------------------------------------------------------------------------------------------hHHH-HH-HHHHHHHHHL-------LH-HHHHHHHHH---H-HHL---------lL-HH---H--------hhHHHHHHHHH-HH-L-L-------
0038 5mlqA   ----------------------------lllllleeeeELLL-H-----------eeeeeHH-H-H-------------------------------------------------------------------------------hhlllllleeeeehhhhH--LLLlHH-HHHHHHHHHHH-----hhLHHHHHHHHHHH---H-HHH---------lH-HH---H--------hhHHHHHHHHHH-HH-H-Lh------
0039 8ul9A   lllllllhHHL--L--LLL------------------------------------hhhllhH-H-LL-----------------------------------LLL-HHHHHHHHHHHHHHHHHHHhhhlllllllhhhHH--HHHH-HHHHHhhlHHH-L----------L-HHHHHHHHHH-lllllhHHHHHHHHHHHH---H-HHH-------------lllL-----LL---H-HHHHHHHHHHH---H-H--H--
0040 9jc2A   --------------------------------------------------------------------------------------------------------------------------------------------------------------------hHHH-HH-HHHHHHHHHH-llllllHHHHHHHHHHHH---H-HHH-----------lL---L-----HH---HHHHHHHHHH-HH-H-Hh------
0041 6mcaA   -lLLLL--LlllhL--LLLlL--L--LL-E-EE---------------eELLLllllLL-HH-H-----------------------------hhhhhlllhhhhhhhhHHHHHHHHHHHLLLLH--------------------hhhhhhhhhhHH-LL-lL--LHH-HH-HHHHHHHHHH---llllLLHHHHHHHHHH--lL-LLL--------------------------LHHHHHHHHHhH-L-L-Ll----
```

```
Job: Type-3 BR-systems Inactive STAND-NTPase
Query: s001A
Chain Z rmsd lali nres id PDB Description
4dt1-A 4.7 3.3 93 193 10 PDB MOLECULE: CHROMOSOME SEGREGATION IN MEIOSIS PROTEIN 2;
8c0v-B 4.4 3.7 93 1030 9 PDB MOLECULE: PEROXISOMAL ATPASE PEX1;
7vcs-A 4.3 3.3 91 766 9 PDB MOLECULE: TRANSITIONAL ENDOPLASMIC RETICULUM ATPASE;
7mi1-A 4.3 2.9 80 2628 11 PDB MOLECULE: CHIMERA PROTEIN OF DYNEIN AND ENDOLYSIN;
4rh7-A 4.1 3.1 90 3005 8 PDB MOLECULE: GREEN FLUORESCENT PROTEIN/CYTOPLASMIC DYNEIN 2 HE
9e22-A 4.0 3.4 88 2925 9 PDB MOLECULE: CYTOPLASMIC DYNEIN 1 HEAVY CHAIN 1;
7xp9-A 3.9 2.6 51 76 8 PDB MOLECULE: RXLR EFFECTOR PROTEIN AVR-VNT11;
8xks-B 3.8 3.4 87 603 8 PDB MOLECULE: CTAP1;
7ykk-A 3.8 3.3 83 735 6 PDB MOLECULE: ATPASE FAMILY GENE 2 PROTEIN;
3io5-B 3.8 3.2 84 272 8 PDB MOLECULE: RECOMBINATION AND REPAIR PROTEIN;
7mi8-A 3.7 3.5 93 868 14 PDB MOLECULE: FUSION PROTEIN OF DYNEIN AND ENDOLYSIN;
5fsz-A 3.7 3.2 85 245 5 PDB MOLECULE: MACRODOMAIN;
9fnn-U 3.7 3.3 84 511 13 PDB MOLECULE: CELLULOSE SYNTHASE CATALYTIC SUBUNIT [UDP-FORMING
8xku-E 3.7 3.6 89 498 12 PDB MOLECULE: PROBABLE INACTIVE ATP-DEPENDENT ZINC METALLOPROTE
6p8v-C 3.4 3.0 78 303 9 PDB MOLECULE: ATPASE, AAA FAMILY;
8c0v-A 3.3 3.2 82 823 9 PDB MOLECULE: PEROXISOMAL ATPASE PEX1;
5e7p-A 3.3 3.3 84 719 10 PDB MOLECULE: CELL DIVISION CONTROL PROTEIN CDC48;
6wc3-B 3.3 3.1 53 94 13 PDB MOLECULE: PROTEIN TRANSPORT PROTEIN TIP20;
8glw-A 3.3 3.4 81 483 10 PDB MOLECULE: TRANSPOSON TN7 TRANSPOSITION PROTEIN TNSC;
8btg-B 3.3 3.8 88 335 7 PDB MOLECULE: CHROMOSOMAL REPLICATION INITIATOR PROTEIN DNAA;
1g4a-E 3.2 4.0 88 356 11 PDB MOLECULE: ATP-DEPENDENT HSL PROTEASE ATP-BINDING SUBUNIT
7mca-C 3.2 3.3 81 584 6 PDB MOLECULE: ORIGIN RECOGNITION COMPLEX SUBUNIT 1;
6tdw-C 3.1 5.5 56 157 16 PDB MOLECULE: ATPTB1;
8rg0-5 3.1 3.4 63 520 6 PDB MOLECULE: EUKARYOTIC TRANSLATION INITIATION FACTOR 3 SUBUNI
7st9-A 3.0 3.2 85 522 6 PDB MOLECULE: CHECKPOINT PROTEIN RAD24;
7uqj-A 3.0 2.9 82 585 11 PDB MOLECULE: ATPASE HISTONE CHAPERONE YTA7;
```

```
0001 s001A   ------------MKGYEIFE------QKAPYE---NKIGF---LDFLRELKSGTQGIP--SSFMVVGI-D-DVLYL-AGRDERLALTIHKILQS-SA-KVLD-QK-II-EVQIVCKG-RLYKGESFWSEYRGEKLP-LDYIFG-TPN
0002 4dt1A   -------meyedLELITIWP------SP----tqlFFIDatssEELSAITVKLLQIL-lkIILYINGL-E-VMFRN-SQ-PQRSHELLRDTLLK-LR-VMGN-DA-SI-RTLLEFPKeQLLDKNGDS--------L-AEY-IW-KY-
0003 8c0vB   etsfncsqrgitLNASVLLH------SKATM-ihlLEID----gYIRAKCENVLPYAP--AVIFLAHL-D-SILLD-VNaQKSINFEMSKLLDD-FT-FKF---P-GT-TFVGSVNN---------------iDNV-PSSFRS-HMR
0004 7vcsA   ehpdkflkfgmtPSKGVLFYgppgcgKTLLAKanfISIKG---PEseANVREIFDKA-apCVLFFDEL-D-SIAKA-RGG-gGAADRVINQILTeMD-GMsT-KK-NV-FIIGATNR---------------pDII-DPA-IL-RP-
0005 7mi1A   dewniadvvansENNYFTMA-serdvDGTF-L---KIIPL---nYAQEEISK-KIEGG---WILLQNI-Q-MSL-------swvKTYLHKHVEE--T-KAAE-EHeKF-KMFMTCH-----------LTGD---KL-PAPLLQr---
0006 4rh7A   dkcyltltqamkMGLGGNPYgpagtgKTESVKrqvLVFNC-----mGRIFVG-VKCGA---WGCFDEF-N-RL-------EESVLSAVSMQIQT-IQ-DALK-NS-NS-GIFITMNP------------AGKGyKL-PDNLKQ-rgn
0007 9e22A   drcyltmtqaleARLGGSPF------GtESVKrfvLVFNC-----mGRIFVGLCQVGA---WGCFDEF-N-RL-------EERMLSAVSQQVQC-IQ-EALR-EP-DM-AIFITMNP-G---------------SN-LPDNLK-Klp
0008 7xp9A   -----------------------------keatrkYY---LDLFKRADFTLPKLA--KKG--daL-K-KLRKA-G--SEEKFAELKGAAAK-YA-DDWY-RI-Y--------------------------------------------
0009 8xksB   rtpekfkasgarAPKGVLLV------GKTLMAvafISSSA---A-vRDLFNT-RSVAP--CIIFIDEL-D-AVG---RQRQDNTVNQLLTEMDG-fE-AEQ---Q-GI-VVMGATNR-KD--------------VL-DAALTR-PG-
0010 7ykkA   easetfarlgisAPKGVLLY------G----F---LAVKG-----irEIFRK-RSAAP--SIIFFDEI-D-ALSP--DRDGNHVLTSLLNEIDG----EELK----GV-VIVAATNR---------------pDEI-DAALLR-PDR
0011 3io5B   nialsgeitggmQSGLLILAsksfksNFGL--avcLFYD----EQLRIDMVNQLDAI-ekVVVFIDSL-G-NLA-------kTRAKTMKSLFRI-VT-PYFS-TK-NI-PCIAINHT-Y--------------------TGPMY-EKS
0012 7mi8A   ligfatltdslhQKYGGCFFgpagtgKTETVKrvvVVFNC-----lSRLLVGITQIGA---WGCFDQF-N-RL-------DEKVLSAVSANIQQ-IQ-NGLQ-VP-HT-AVFITLNP------------GYNGRSE-LPENLK-K--
0013 5fszA   delkpvdsshdilRHIALYN------GDLQL---dAIVNaankgqYRSILSLAHRNRL--RSIGFCCV----STGVyGYPLIPATRIAVDETIE-YL-KQ-H-FS-AFdLCCFACFK-LE---------------nA-YTDCLR-A--
0014 9fnnU   isslwdelrhmpaGGVWWFN------VLANQTahvAVIS----kGLYYLT-RDLQC--hnYLFILVCanN-AWQN--------iPAERLRSWLDK-MN-KWSR-LN-HC-SLLVINPG---------------NNNDkQFSLLL-E-F
0015 8xkuE   knpdlfdkmgikPPHGVLLE------GTLVAKvpfYQMAG---SEgsARIRDLFKRA-kpSVIFIDEI-D-AL-------ATQERETTLNQLLI-EL-DGFDtGK-GV-IFLGATNR-RD--------------LL-DP-ALL-R--
```

```
0016 6p8vC   pdaraavdtvlrrpPLVVLA------gLAETIitlYPLS------vSAAFDYAADKkA--VLLLIDQA-D-ALA----qsDRAGVNAFIRGIDR-IA-NQ---KL-PA-AVLXCT-N-RLK-------------AL-DPAVQRr---
0017 8c0vA   kemvnyltspiiATPAIILD------G-----ifvKYAD----kLIMEWCSFCYWYGP--SLIVLDNV-E-ALF----anWDNASKLLNFFINQ-VT-KIFN-KD-NI-RVLFSGKQ-KT-------------QI-NPL-LF-DK-
0018 5e7pA   qhpdtfsrlgidpPRGVLLYgppgcgKTFVVRlsvhAVKG-----vRELFAR-RDSAP--SLVFLDEI-D-ALAPR-RGQTDKVVASLLTELDG-IE-PL----R-DV-VVLGATNR--------------pDLI-DPALLR-P--
0019 6wc3B   --------------------------------smpyatQL---ALLQDELLDMLEPLR--TADiieLL-G-CYRLQ-VEkALAQLLLWERFLAD-YR-RRLD-AA-I-----------------------------------------
0020 8glwA   eletfrfeearsTAQSLLLI------Gslhr Y---LKIDC---kEICLnLALMSQIAL--GLLVIDEI-Q-HL--S-RSRS-GGSQEMLNFFVT-MV-NIIG-----V-PVMLIGTP-----------------KA-REI-FE-ADL
0021 8btgB   aaslavaeapakAYNPLFIY------GLMHAIakvVYLSS---EKFTNE--FRNR-NV--DVLLIDDI-Q-FLA---------GKEQTQEEFFH-TF-NTLH-EE-SK-QIVISSDR-PPK-------------PT-LED-LR-SR-
0022 1g4aE   rmqlneelrhevTPKNILMI------GKTEIAapfIKVEA---TKgkEVDS--IRDLvqhGIVFIDEI-D-KICKR-GGPDVSRE-GVQRDLLP-LveVKTD----HI-LFIASGAF-Q---------------SD-LIPELQ-G--
0023 7mcaC   isdllysettqkRRCFNTIF------L----N---VLIEL-----mMLRRsYDLSLV-kdLAMVFNFK---DVDS--------iNFNTLDNFIIL-LK-S-AF-KYdHV-KISLIFNI------------NTNLSNI-EknlTI-RL-
0024 6tdwC   ----------------------------------davKF---QTLYNVLTGlqvNLT--ASWKAKWF-DsEYAPK-L--PAAERDSAKKSLDL-YL-KRVD-LT-R-----------------------------------------
0025 8rg05   kiwnvhsvlnvlhslvdksnrqlevytageyhsrvpecQV---TTYYYVGFAYLRRYQ---rvfaLYI-Q-RTKSM-FQ-YEMINKQNEQMHAL-LA-IALT-MY-PM-RID--------------------------------------
0026 7st9A   qealdamflpnaKHRILLLSgpsgcsKSTVIKehkVTEF------fSEFLKGARYLVL--SLILIEDL----PNVF----HIDTRRRFQQLILQ-WL-YSsE-PL-LP-PLVICIT------------ECEIpeeT-IMN-IL-MHP
0027 7uqjA   lypelyqnfnitPPRGVLFHgppgtgKTLMARitfFMRK------lRLLFEEAKKHQP--SIIFFDEI-D-GLApvrsQIHASIVSTLLALMD-gmD-NRG-----QV-IVIGAT-N-RPD-------------AV-DPA-LR-RP-

0001 s001A   ------------LLLEEEEL------LLLLLL---LEEEH---HHHHHHHHHLLLLLL--LEEEEELH-H-HHHHH-LLLLHHHHHHHHHHHHHH-HH-HHHH-HL-LL-EEEEELLL-EEEELLLEEEEELLEEEE-LHHHHL-LLE
0002 4dt1A   -------llhhhLLLEEEEE------LL----eeeEEEEllllHHHHHHHHHHHHHH-eeEEEEEELH-H-HHHHH-HH-HHHHHHHHHHHHHHH-HH-HHHH-LE-EE-EEEEEELHhHHHHLLLLL--------H-HHH-HH-HH-
0003 8c0vB   hhhhhlllllllLLLLEEEE------LHHHH-leeEEEL----hHHHHHHHLLHHHLL--EEEEELLH-H-HLLLL-LLlHHHHHHHHHHHHHH-HH-HHL---L-LE-EEEEEELL---------------hHHL-LHHHHL-LLL
0004 7vcsA   hlhhhhhhllllLLLEEEEEllllllHHHHHHleeEEELH---HHllHHHHHHHHHH-llEEEEELLL-L-HHHHH-HLL-lLLLLHHHHHHHHhHH-LLlL-LL-LE-EEEEEELL---------------hHHL-LLL-LL-LL-
0005 7mi1A   llllhhhhhhhlLLLEEEEE-lllllLLHH-E---EEEEL---hHHHHHHHH-HHHLL---EEEEELH-H-HHH-------hhhLLLHHHHHHH--L-LLLL-LLlLL-LEEEEEL-----------LLLL---LL-LHHHHHh---
0006 4rh7A   hhhhhhhhhhhhHLLEEEEEllllllHHHHHHlleEEEEL-----hHHHHHH-HHHLL---EEEEELL-L-LL-------LHHHHHHHHHHHHH-HH-HHHH-HL-LL-EEEEELLL------------LLLLlLL-LHHHHL-hll
0007 9e22A   hhhhhhhhhhhhLLLLLLEE------LhHHHHlleEEELL-----hHHHHHHHHHHLL---EEEELLH-H-HL-------LHHHHHHHHHHHHH-HH-HHHH-LL-LL-EEEELLLL-L---------------LL-LLLLLL-Lhl
0008 7xp9A   --------------------------------hhhhhhHH---HHHHHHHHLHHHHHH--LLL--hhH-H-HHHHH-L--LHHHHHHHHHHHHH-HH-HHHH-HH-H----------------------------------------
0009 8xksB   hlllhhhhhlllLLLEEEEE------LHHHHHlleELLLL---L-hHHHHHH-HHHLL--LEEEEELH-H-HLL---LLLLHHHHHHHHHHHHLL-lL-LLL---L-LL-EEEEEELL-LL-------------LL-LLLLLL-LL-
0010 7ykkA   hlhhhhhhhlllLLLEEEEE------L----E---EEELH-----hhHHHHH-HHLLL--LEEEELLL-L-LLLL--LLLLHHHHHHHHHHHHLL----LLLL----LL-EEEEEELL---------------hHHL-LLLLLL-LLE
0011 3io5B   hhhhhlllllllLLEEEEEEllllhhHHHH--leeEEEE----HHHHHHHHHHHHLL-llEEEEEELL-L-LLL-------lLHHHHHHHHHHH-HH-HHHH-HL-LL-EEEEEEEE-L-------------------LLHHH-EEL
0012 7mi8A   hhhhhhhhhhhhLLLEEEEEllllllHHHHHHlllEEELL-----hHHHHHHHHHHLL---EEEELLL-L-LL-------LHHHHHHHHHHHHH-HH-HHHH-HL-LL-EEEEEELL------------LLLLLLL-LLHHL-L--
0013 5fszA   hhlllllllllhHHEEEEL------LHEEL---lEEEEellllHHHHHHHHHHLLL--LEEEELLL----LLLLlLLLHHHHHHHHHHHHHH-HH-HH-L-LL-LLlEEEEELLL-HH-------------hH-HHHHHH-H--
0014 9fnnU   lllllhhhheellLLEEEEE------EHHHHHleeEEEE----hHHHHHH-HHHHH--llEEEEEELllL-LLLL-------lLHHHHHHHHHH-HH-HHHH-HH-LE-EEEEEEEL---------------LLLHhHHHHHH-H-E
0015 8xkuE   hlhhhhhhllllLLLEEEEE------LHHHHHlleEEEEH---HHhhHHHHHHHHHH-llEEEEEELH-H-HL-------HHHHHHHHHHHHHH-HH-HLLLhHH-LE-EEEEEELL-LL-------------LL-LL-LLL-L--
0016 6p8vC   lllhhhhhhhhllLEEEEE------eHHHHHeeeEELL------hHHHHHHHHHHlE--EEEEELLH-H-HHL----llHHHHHHHHHHHHHH-HH-HL---LL-LE-EEEEEE-L-LHH-------------HL-LHHHHHl---
0017 8c0vA   hhhhhhhhllllLLLEEEEL------L-----leeLLLL----hHHHHHHHHHHLLL--EEEEELLL-H-HHH----llLLHHHHHHHHHHHHH-HH-HHHL-LL-LL-LEEEELLL-LL-------------LL-LLH-HH-HL-
0018 5e7pA   hlhhhhhhlllllLLEEEEEllllllLLHHHHleeeEEEH-----hHHHHHH-HHLLL--EEEEEELH-H-HHLLL-LLLHHHHHHHHHHHHHH-HL-LL----L-LE-EEEEEELL---------------hHHL-LHHHHL-L--
0019 6wc3B   --------------------------------lllhhhHH---HHHHHHHHHLLLLL--HHHhhhHH-H-HHHHH-HHlHHHHHHHHHHHHHH-HH-HHHH-HH-H----------------------------------------
0020 8glwA   llllllllllllLLLLLEEE------EhhhhE---EEEEL---lHHHHhHHHHHHHHL--LEEEEELH-H-HL--L-LLLL-LLHHHHHHHHHH-HH-HHHL-----L-LEEEEELL-----------------LL-HHH-HH-HHH
0021 8btgB   hhhhhhhhllllLLLEEEEE------EHHHHHlleEEEEH---HHHHHH--HHHH-LL--LEEEEELH-H-HHL---------LLHHHHHHHHH-HH-HHHH-HL-LL-EEEEEELL-LLL-------------LL-LLH-HH-HH-
0022 1g4aE   hllllhhhhlllLLLLEEEE------LHHHHHlleEEEEL---LHllLHHH--HHHHhhlLEEEEELL-L-LLLLL-LLLLLHHH-HHHHLLHH-HhhEEHH----HL-EEEELLLL-L---------------HH-LLHHHH-H--
0023 7mcaC   hhhhlllllllllLLLLEEEE------E----L---EEEEE-----hHHHHhLLLHHH-llLLEEEEEL---LLLL-------lLHHHHHHHHHH-HH-H-HH-HHlLL-LLEEEEEE-----------LLLHHHL-LlllHH-HH-
0024 6tdwC   ----------------------------------lhhHH---HHHHHHHHLlllHHH--LLHHHHHH-HhLLLL-L--LHHHHHHHHHHHHH-HH-HHHH-HH-L----------------------------------------
0025 8rg05   llllhhhhhhhhhhhhhhhhlhhhhhhhllhhhhhlhhhHH---HHHHHHHHHHLLHH---hhhhHHH-H-HHHHH-HL-HHHHHHHHHHHHHH-HH-HHHH-HL-LL-LLL------------------------------------
0026 7st9A   hhhhhhhlllllLLLEEEEEelllllHHHHHHlllEEEL------hHHHHHHHLLLLL--LEEEELLL----LLLL----LHHHHHHHHHHHHH-HH-HLlL-LL-LL-LEEEEEE------------LEELllhH-HLL-HH-LLL
0027 7uqjA   hlhhhhlllllLLLEEEEEllllllHHHHHHleeEEEL------hHHHHHHHHHLLL--EEEEELLH-H-HHLllllLLLHHHHHHHHHHHLL-llL-LLL-----LE-EEEEEE-L-LLL-------------LL-LLL-LL-LL-

@
@
@

Job: BREX-PAPS.NLT83667 Alpha+Beta Domain
Query: s001A
No: Chain Z rmsd lali nres id PDB Description
1: 5iri-A 8.6 2.4 82 105 13 MOLECULE: SERINE/THREONINE-PROTEIN KINASE BRSK1;
2: 6ipv-A 6.8 2.7 78 221 4 MOLECULE: CQSB2;
3: 9j8p-A 6.5 2.7 75 623 8 MOLECULE: SPECKLE TARGETED PIP5K1A-REGULATED POLY(A) POLYME
4: 1q9u-A 6.5 2.8 77 128 12 MOLECULE: UNCHARACTERIZED PROTEIN APC35924;
5: 3c6k-D 6.5 2.3 69 348 9 MOLECULE: SPERMINE SYNTHASE;
6: 6pny-A 6.4 3.0 81 116 12 MOLECULE: FLPP3;
7: 6xrb-A 6.4 3.9 77 150 14 MOLECULE: SCIW;
8: 9hg5-C 6.3 2.9 83 318 5 MOLECULE: OUTER MEMBRANE PROTEIN ASSEMBLY FACTOR BAMA;
9: 3tvr-A 6.2 2.6 79 172 6 MOLECULE: POLYKETIDE CYCLASE;
10: 2iv9-A 6.0 5.0 84 236 10 MOLECULE: AP-2 COMPLEX SUBUNIT BETA-2;
11: 6e8a-A 5.9 3.8 76 148 9 MOLECULE: DUF1795 DOMAIN-CONTAINING PROTEIN;
12: 3w9k-A 5.9 2.3 74 135 7 MOLECULE: FATTY ACID-BINDING PROTEIN;
13: 2ehb-D 5.8 2.5 75 126 12 MOLECULE: CALCINEURIN B-LIKE PROTEIN 4;
14: 6m1u-A 5.8 2.7 77 122 14 MOLECULE: RNA N6-ADENOSINE-METHYLTRANSFERASE METTL16,RNA N6
15: 6ser-A 5.8 2.8 82 245 5 MOLECULE: START DOMAIN-CONTAINING PROTEIN 10;
16: 7tj1-D 5.7 2.5 77 133 6 MOLECULE: PUTATIVE FLUORIDE ION TRANSPORTER CRCB;
17: 1v8c-A 5.7 3.1 73 165 12 MOLECULE: MOAD RELATED PROTEIN;
18: 4e6f-B 5.7 2.7 79 173 11 MOLECULE: UNCHARACTERIZED PROTEIN;
19: 8fg6-B 5.6 2.2 61 151 10 MOLECULE: AMYLOIDOGENIC PEPTIDE;
20: 3mah-A 5.5 2.5 63 139 5 MOLECULE: ASPARTOKINASE;
21: 4low-A 5.5 2.3 65 84 3 MOLECULE: ACRAF;
22: 1zxf-A 5.5 2.6 70 155 10 MOLECULE: CALC;
23: 6y2p-A 5.4 2.8 75 354 7 MOLECULE: MRNA ENDORIBONUCLEASE TOXIN LS;
24: 2e3m-A 5.4 2.3 81 237 1 MOLECULE: LIPID-TRANSFER PROTEIN CERT;
25: 5cwa-A 5.4 2.6 77 505 5 MOLECULE: ANTHRANILATE SYNTHASE COMPONENT 1;
26: 2jmu-A 5.4 2.7 77 224 17 MOLECULE: THIAMINE-TRIPHOSPHATASE;
27: 6v04-A 5.3 2.3 70 268 9 MOLECULE: UNCHARACTERIZED SRPBCC DOMAIN-CONTAINING PROTEIN;
28: 7ns9-A 5.3 3.2 74 178 16 MOLECULE: TRIPHOSPHATE TUNNEL METALLOENZYME SACI_0718;
29: 6hlu-A 5.3 3.8 81 907 6 MOLECULE: RAB FAMILY PROTEIN;
30: 1vjh-A 5.2 2.9 77 120 9 MOLECULE: BET V I ALLERGEN FAMILY;
31: 3osm-A 5.2 2.7 76 119 9 MOLECULE: SERINE/THREONINE-PROTEIN KINASE KCC4;
32: 3put-B 5.2 2.3 70 155 6 MOLECULE: HYPOTHETICAL CONSERVED PROTEIN;
33: 2mj7-A 5.2 2.9 75 141 7 MOLECULE: AP-4 COMPLEX SUBUNIT BETA-1;
```

```
34: 3t4n-A 5.1 3.0 72 139 10 MOLECULE: CARBON CATABOLITE-DEREPRESSING PROTEIN KINASE;
35: 3bfm-A 5.1 3.3 85 230 9 MOLECULE: BIOTIN PROTEIN LIGASE-LIKE PROTEIN OF UNKNOWN FUN
36: 7ohi-A 5.1 4.4 79 250 9 MOLECULE: AP-2 COMPLEX SUBUNIT ALPHA-2;
37: 5a1u-E 5.1 5.2 75 822 8 MOLECULE: ADP-RIBOSYLATION FACTOR 1;
38: 8ba1-A 5.1 2.8 65 119 12 MOLECULE: CLEAVAGE AND POLYADENYLATION SPECIFICITY FACTOR S
39: 1x2g-C 5.1 3.1 86 337 5 MOLECULE: LIPOATE-PROTEIN LIGASE A;
40: 5n9j-Y 5.1 3.1 76 185 1 MOLECULE: MEDIATOR OF RNA POLYMERASE II TRANSCRIPTION SUBUN

0001 s001A   -DIIQKE-EG-QRFVLND---PG----D---R--FEE-W-IKVLG-------PVRVASDN-------GKLEGDLL-VS---N---------R-TLHFSVDKNT-A-------GRAIVDIDGI-KGDK-LLLGRL-NKVAY--KSTYC
0002 5iriA   -ISLDKE-EQ-IFLVLKD---KP----L--sS--IKA-D-IHAFL--sipslSHSVLSQ----------TSFRAE-YK-qkP---------V-RFQVDISSS----------GIYSVTFTL--SRRFkRVVETI-QAQLL--S----
0003 6ipvA   geifgqY-CS-LAEHIN-----------pydI--VFE-Y-AARSLpntdiyiRAEAQKGP-------EHGLVVYP-CA-elW---------M-RYYMTIIDSSdK-------PGTVVLWTN--gFHA-GHSIEM-GNLKR--ILEHR
0004 9j8pA   psspsSL-LSaTPIPLPL---A------ltaA--LVQ-V-FRLGC-------HIEQA-----------SASWRCA-LWhepL---------L-SFVASVSPAD-----------RMLTVTP--GLFP-DLHHFL-QVFLP--QAIRH
0005 1q9uA   -----aM-FH-YTVDVST----------tieR--LEE-S-LKEGF-------GVWQFSVT-gldfstPMVILEVX-NP---Q------vgyflPXKLVVYQEN-G--------TTKIGMPKP-TMAA-DIEKRL-AACLD--RCR--
0006 3c6kD   ---gsrH-ST-LDFML------------dgeT--ILK-G-LQSIF--qeqgmAESVHTWQ------dHGYLATYT-N--knG---------S-FANLRIYP------------HGLVLLDL---EID-SILNKV-EERMK--EL---
0007 6pnyA   eGRQYKD-GY-YITTLN-----------nfnT--VYN-A-TQAIQqtfdyksnpVNKNNG-------TDAEIVSA--S---T---------D-SLQVAMKKLP-N-------NATRISIKY--QGNS-IRSSAL-IGIIE--GNIRY
0008 6xrbA   -NIFILEtSP-SLNISRD----------yidR--QIA-L-MKNLG-------QHRVSRAP-ndalmgEQIAATHK-SG---K---------T-EVYQRQAGFI-A---tpgkVLVFTLTSPRp---------------------
0009 9hg5C   -RTQFTG-DT-ASLLVEN----------lwpQ--VVS-V-LQKNY-------TIQRDDA---------GQTLTTD-WV-qyR---------G-RYQISVKPQG-Y--------QQAVTVKL---MQR-YsTEMM-NVISA--GLDKS
0010 3tvrA   -VPRGHMaGH-TDNEITI-------aaplfseyasvevlgrdthW-------VSERVADP-----vtRTVRAQRveTG---P---------FqYMNIVWEYAE-T----aegTVMRWTQDFA--DDA-WMTDNI-NRNSR--TQMAL
0011 2iv9A   wkdipNE-NE-LQFQIKE---CH----L--nadtVSS-K-LQN--------nNVYTAKRN---vegqDMLYQSLK-LT--nG---------I-WILAELRIQPgN-------PNYTLSLKC--RAPE--VSQYI-YQVYD--SILKN
0012 6e8aA   nNMHVYSkAV-IVIVGDN---TD----EalpV--LAN-RlLEQQQ-------VVTNKSIE---lkghTLQLDSI-IS-gqT---------A-YSSIVLGK-V-D-------NQLLTIQVTLp---------------------
0013 3w9kA   --------MK-VEKEIKT----------mtifsdpaF-T-IPQ--lafsykvkGRVYKGV-------DEVRIIYD-SD---R---------G-NGILYIRKKD-N-------NTLQIILEHD-NKAF-LGKPYV-SSNLD--RLAEN
0014 2ehbD   rrqdfvK-RQ-TRFVSRR---E-------psE--IIA-N-IAVAN---smgfKSHTRN-----------FKTRLE-GG---Q---------L-AVVIEIYEVA-P-------SLFMVDVRK--TLEYhKFYKKL-CSKLE--NII--
0015 6m1uA   --------KP-ITFVVLA---SV----MgivV--VTT-WiEKILV-------QHKRVCGK-------EEVSLFLT-AI-qyL---------F-KCLINVKK-E-V-------DDALVEMHW--RDLMnQLCTYI-RNQIF--R----
0016 6serA   aGVSVWVkIK-CRMECCD---V------paeT--LYD-V-LHDeiknrdvitLRSWLPMG-------ADYIIMNYsvV---S---------I-QTGYLIQSTG-P-------KSCVITYLA--qSQF-LAPKAM-KKMYK--ACLKY
0017 7tj1D   ---IIDS-GL-VTVESR-----------svaE--TIE-R-VAKAK--sxgxnVFTRVDHG-aglglpPTELIIFG-NP---Q------igldlPIRALAWEDG-S-------GKVWLTVNDP-ArXV-TGTGTVtKYAAG--D----
0018 1v8cA   -------gGG-FERTFGA---F------ppwL--LER-Y-LEEWG-------GTREGE-----------GVYRLP------------------GAVVRFREVE-PkvgslsiPQLRVEVEGE------eaERWF-ERIAF--AASR-
0019 4e6fB   vpevdgK-VV-FSKEFQI----------txtK--WXD-E-RLKEN----kniDSRIFSDE-----akGTIAGVGE-EW-rtL---------V-NYQITVTC-K-P-------GNCLVELEKIrnKTV-DFADDX-FXDVA--VAFGA
0020 8fg6B   --------GS-VTFEFEN---LseeaaE---K--VKK-Y-VKEV----------------------------aEK-LG-----------------VEVKVEE-E-E-------GKLKIYVENL---KE-EDALDI-FKYAA--LAAEL
0021 3mahA   -AAKDGIkDX-VIICIVG----------feaR--IIN-A-LKGV-----------------------------------------------pvRXISYGGsN-------YNVSVLVKAE-------DKKKA-LIALS--NKLFN
0022 4lowA   hSWREQGpPM-LFKRFAF----------fldA--LAA-L-SEETG----------------------------------------------qhpQNINF-G-T-------TYVNITLD-A-ATLG-EAERAF-AARVD--ALAGS
0023 1zxfA   --nydpF-VR-HSVTVKA----------drkT--AFK-T-FL--wdeqgeehtfKVDE-----------pDTLVIG-WR-dnS---------S-EFTVTFVADG-Q-------KKTRVDVEH--hMDK-GWPTIL-QSFQD--KIDEE
0024 6y2pA   inpaeFE-QV-NMVLQG-------fveT---S--VLP-V-LELSI-------EFRESRNA-------HTVVWKII-SY---Q---------D-ELTVSLHITT-----------GKLQIQGRP-L----sCYRV--TFNLA--ALLDL
0025 2e3mA   gEMKVYRpLK-ATHAVKG---V------tghE--VCN-Y-FWNdesqrdvlyLSVIRKIP-ltendpETWIVCNF----raK---------I-NVAMICQTLV-SqeisrdnILCKITYVA--nAKR-EYPKFL-KRFTS--YVQEK
0026 5cwaA   adLAATTpVT-RKVLADS---E------tplS--AYR-K-LAA------nrpGTFLLESS-------RWSFIGAG-AP---TaltvrelllA-TDVAAVDHHE-----------GTITLIAN-ADAV-ARLDVM-TAALG--Q----
0027 2jmuA   saqgliE-VE-RKFAP----------gP---D--TEE-R-LQEL--------GATLHRVTfvasfitTRSSWKLA-LS-gqE---------P-QLTIDLDSAD-F-------GYAVGEVEAM-VEVP-AALEKI-ITVSS--MLG--
0028 6v04A   ------D-DW-VRVMVSV----------avtdprrvaqwfgsmtF-------DIEVHVEP------rDRLLFRWS-FL-gvG---------P-ECQVGWTLTG-G----aeaTTLTVDDSC---EVA-QLKAGW-LDFVG--RLARY
0029 7ns9A   --gsyiE-RE-IKLRVI----SP----S---LeeIEE-R-IR---------nNYTFNEEH-sfqvekIRKNYKYA------------------DFIISLDSIK-E-------LGEFIEIEG--KTEK-ELISFV-DEFVKkhQIQY-
0030 6hluA   ditegeP-LR-FIMKYDY----------lpsT--IIP-R-LMAMQ-------HqMQWR-----------YGMVLK-SQ-heG---------A-LAKVVAETKD-----------STITIAIQ-GEKR-EYLSII-WYEIK--KINAN
0031 1vjhA   -stlkgA-LS-VKFDVK-----------padK--FFSaF-VEDTT-------EIEVDLV---------KKTXTIQ-XS-fkT---------L-KGSIAVTPIG-V------gDGSHVVWTF--DPHS-IIDESV-KYFKK--LDEAI
0032 3osmA   -------iLE-LEIHAKI----------lyeG--LHK-L-LEGLK-------NLVFNIT---------NMIITGK-LV-lrS---------T-LFEIMVLPNG-D-------GRSLIKFNK---STK-TLTKLA-TEIQI--ILQKE
0033 3putB   -trsaeH-TT-FVIERRL-----tapvaachgewvpleyalrgtY-------DARYDIVP------dTRIIYAYE-XK-trI---------S-ASLVTVAFDV-E----psgTRXVFTEQV---DNG-ARLQGT-EIGLD--NLELF
0034 2mj7A   wlslkvA-HQ-QVLPWRG---E-------fhpdtlQM-A-LQNIQ-------TIAMSRAG------sRPWKAYLS-AQ-gcL---------F-LTELLLEPGN-----------SEMQISV---TLN-SFISVL-ETVIG--TIEEI
0035 3t4nA   -------trwhfGIRSRS---Y----pL---D--VMG-EiYIALA-------EWAKPSEE------dlWTIKLR-WK-pdL---------M-KMVIQLFQIE-T-------NNYLVDFKF--AYPFlHLTTKL-IMELA--V----
0036 3bfmA   -VYELGT-DV-LRAALVL----------xlpV--CGV-G-FNALGalappevAVHLDW----------NGALRIN------------------gARCGRLRIAAdW-------LVVGLDLPLW-PgAD-VAAPRL-LESWA--RHCLH
0037 7ohiA   kqlsnPQ-QE-VQNIFKA---KH-----pmdteitKA-K-IIGFG--------SALLENP-------ANFVGAGI-IH-ttQ---------I-GCLLRLEPNLqA-------QMYRLTLRTS-KDT--vsQRLC-ELLSE--QF---
0038 5a1uE   -------eKE-ETFTLST---I------tleE--AVG-N-IVKGM-------HPCDKVPK-------NTHTLLLA-GV-ghD---------I-LVRSRLLLLD-----------TVTMQVTAR--EEL-PVDIIL-ASV--------
0039 8ba1A   ykemciY-ER-QRIPY-----NS----T---AvlLKQ-V-LVGFE-------ELVEVE-----------SGFMVG------------------NVHIVLSG------------QEVVLEWK-SSDD-VLAISI-SKMIS--D----
0040 1x2gC   -FLWRNAlGN-TCFTFXA----------stsI--VLN-A-LNALV-------SAEASGR----------NDLVVK-------------tvegdRKVSGSAYREdR-------GFHHGTLLLN-ADkG-ITHEQV-CEAIT--EAFFA
0041 5n9jY   ------P-VH-GVIYYSSpsmAT----F---LspAQD-N-LVRTY-------FAQHKKWV-rqsftiEGSIYEVG------------------SFKVAIANVLvW-------KGILFHVTYD-GTRP-IIQEFF-LKCFL--Q----

0001 s001A   -EEEELL-LE-EEEEEEL---LL----L---L--HHH-H-HHHLL-------LEEEEEEL-------LEEEEEEE-EL---L---------E-EEEEEEELLL-L-------LEEEEEEELL-LLLH-HHHHHH-HHHHH--HHHHL
0002 5iriA   -EEELLL-LE-EEEEELL---LL----H--hH--HHH-H-HHHHH--hllleEEEEEEL----------LEEEEE-EH-llL---------E-EEEEEEEEL----------LLEEEEEEE--HHHHhHHHHHH-HHHHH--L----
0003 6ipvA   hhhhllE-EE-EEEEEL-----------lhhH--HHH-H-HHHHHllleeeeLLEEEELL-------LEEEEEEE-EE-llL---------E-EEEEEEEELHlL-------LLEEEEEEE--hHHH-HHHHHH-HHHHH--HHHHH
0004 9j8pA   lllllLL-LLlLLLLLLL---L------hhhH--HHH-H-HHLLL-------EEEEL-----------LEEEEEE-ELlllL---------E-EEEEEEELLLL-----------LLEEEEE--LLHH-HHHHHH-HHHHH--HHHHH
0005 1q9uA   -----lL-LE-EEEELLL----------hhhH--HHH-H-HHLLL-------EEEEEEHH-llllllLEEEEEEE-LH---H------hhhhlLEEEEEEEEL-L--------EEEEEEELH-HHHH-HHHHHH-HHHHH--LLL--
0006 3c6kD   ---leeE-EE-EEEEL------------lhhH--HHH-H-HHHHH--hllllEEEEEELL------lLLEEEEEE-L--llL---------L-EEEEEELL------------LLEEEEEE---HHH-HHHHHH-HHHHH--HL---
0007 6pnyA   lLLEEEL-LE-EEEEEL-----------lhhH--HHH-H-HHHHHlllllllllEEEELL-------LEEEEEEE--E---E---------E-EEEEEEEEEL-L-------LEEEEEEEE--LLLH-HHHHHH-HHHHH--HHHHL
0008 6xrbA   -EEEELLlLL-EEEEEEE----------hhhH--HHH-H-HHHLL-------LLEEEEEE-hhleeeEEEEEEEE-LL---L---------L-EEEEEEEEEL-L---llllEEEEEEEELLl---------------------
0009 9hg5C   -LEEEEL-LE-EEEELLL----------hhhH--HHH-H-HHLLL-------LEEEELL---------LLEEEEE-EE-leE---------E-EEEEEEEELL-L--------LEEEEEEE---HHH-HhHHHH-HHHHH--HHHHH
0010 3tvrA   -EELLLLlLE-EEEEEEE-------lllhllleeeeeeeeelelE-------EEEEEEEL-----llLEEEEEEllLL---L---------EeEEEEEEEEEE-E----lleEEEEEEEEEE--LHH-HHHHHH-HHHHH--HHHHH
0011 2iv9A   hhhllLL-LL-EEEEEEL---LL----L--lhhhHHH-H-HHL--------lLLEEEEEE---elleEEEEEEEE-LL--lL---------L-EEEEEEEELLlL-------LEEEEEEEL--LLHH--HHHHH-HHHHH--HHHHL
0012 6e8aA   eEEEEEElEE-EEEEEEL---LL----LlhhH--HHH-HhHHHHE-------EEEEEEEE---elleEEEEEEEE-EE-leE---------E-EEEEEEEE-L-L-------LEEEEEEEEEe---------------------
0013 3w9kA   --------LE-EEEEEEL----------hhhhllhhH-H-HHH--lleeeeeeEEEEEEL-------LEEEEEEE-EL---L---------L-LEEEEEEEEE-L-------LEEEEEEEEL-LHHH-HHHHHH-HHHHH--HHHHH
0014 2ehbD   llllllL-LL-LEEEELL---L-------hhH--HHH-H-HHHHH---hlleEEEELL-----------LEEEEE-EH---L---------L-EEEEEEEEEE-L-------LEEEEEEEE--HHHHhHHHHHH-HHHLL--LLL--
0015 6m1uA   --------LL-EEEEELH---HH----HhhhH--HHH-HhHHHHL-------EEEEELLL-------LEEEEEEE-EE-leE---------E-EEEEEEEE-E-L-------LEEEEEEEE--LHHHhHHHHHH-HHHHH--H----
0016 6serA   lLEEEEElEE-EEEEELL---L------lhhH--HHH-H-HHLheleeeeeeEEEEEEEL-------LEEEEEEEeeE---E---------E-EEEEEEEELL-L-------LLEEEEEEE--eHHH-HHHHHH-HHHHH--HHHHH
0017 7tj1D   ---LEEL-LE-EEEEEL-----------lhhH--HHH-H-HHHHH--hllleEEEEEEHH-llllllLEEEEEEE-LH---H------hhhllLEEEEEEEELL-L-------LLEEEEEELH-HhHH-HHHHHHhHHHHL--L----
0018 1v8cA   -------lEE-EEEEEEL---L------lhhH--HHH-H-HHHLL-------LEEEEL-----------LEEEEL------------------LEEEEEEEEE-EeelleeeEEEEEEEEEL------lhHHHH-HHHHH--HHLL-
0019 4e6fB   lleellE-EL-EEEEEEL----------hhhH--HHH-H-HHHLL----lllLLEEEEEL-----llLEEEEEEE-EE-eeE---------E-EEEEEEEE-E-L-------LEEEEEEEEEelHHH-HHHHHH-HHHHH--HHLLL
0020 8fg6B   --------LL-EEEEEEL---LlhhhhH---H--HHH-H-HHHH----------------------------hHH-LL-----------------LLEEEEE-E-L-------LEEEEEELLL---LH-HHHHHH-HHHHH--HHLLL
0021 3mahA   -EEEEEEeEE-EEEEEEL----------hhhH--HHH-L-LLLL-----------------------------------------------llLEEEELLlL-------LLEEEEEEHH-------HHHHH-HHHHH--HHHHL
0022 4lowA   lLLEEELlLE-EEEEEEL----------hhhH--HHH-H-HHHHL----------------------------------------------lllLEEEE-L-L-------LEEEEEEE-L-LLLL-HHHHHH-HHHHH--HHLLL
0023 1zxfA   --llllL-EE-EEEEELL----------hhhH--HHH-H-HL--llllllllllEEEL-----------lLEEEEE-LL-llL---------L-LEEEEEEEEL-L-------LEEEEEEEE--lHLL-LHHHHH-HHHHH--HHHHH
0024 6y2pA   lllllLL-LE-EEEEEL-------llhH---H--LHH-H-HHHHE-------EEEEEELL-------LEEEEEEE-EL---L---------L-EEEEEEELLL-----------LEEEEEELL-L----lLLLL--LHHHH--HHLLL
0025 2e3mA   lLEEEEElEE-EEEEEEL---L------lhhH--HHH-H-HHLhelleeeeeEEEEEEEL-llllllLEEEEEEE----elE---------E-EEEEEEEEEE-LlllhhhEEEEEEEEE--eHHH-HHHHHH-HHHHH--HHHHH
0026 5cwaA   llLLLLLeEE-EEEELLL---L------lhhH--HHH-H-HLL------lllLLEEEELL-------LEEEEELL-LL---LeeeeeleeeE-LEEEEEEELLL-----------LEEEEEEE-EHHH-HHHHHH-HHHHH--L----
0027 2jmuA   llllleE-EE-EEEEE----------lL---L--HHH-H-HHHH--------LLEEEEEEeeeeeeeEEEEEEEE-LL-llL---------L-EEEEEEEEEL-L-------LEEEEEEEEE-EHHH-HHHHHH-HHHHH--HHE--
0028 6v04A   ------L-LL-EEEEEEE----------hhhlhhhhlllllllleE-------EEEEEEEL------lLEEEEEEE-EL-llL---------L-LEEEEEEEEE-L----lllEEEEEEEEEL---HHH-HHHHHH-HHHHH--HHHHH
0029 7ns9A   --lleeE-EE-EEEEEE----EL----L---HhhHHH-H-HL---------lLLEEEEEE-eeeeeeEEEEEEEL------------------LEEEEEEEEL-L-------LEEEEEEEE--LLHH-HHHHHH-HHHHHhlLLEE-
0030 6hluA   llllllE-EE-EEEEELL----------lllL--HHH-H-HHHLH-------HhEEEL-----------LEEEEE-LL-llL---------L-EEEEEEEELLL-----------LEEEEEEE-LLHH-HHHHHH-HHHHH--HHHLL
0031 1vjhA   -llleeE-EE-EEEEEL-----------lhhH--HHHhH-HHHLE-------EEEEELL---------LLEEEEE-EE-eeE---------E-EEEEEEEELL-L------lLLEEEEEEE--LLHH-HHHHHH-HHHHH--HHHHH
```

```
0032 3osmA   -------eLL-LLEEELL----------hhhH--HHH-H-HHLLE-------EEEEEHH---------HLEEEEE-EE-llE---------E-EEEEEEEELL-L-------LLEEEEEEE---LHH-HHHHHH-HHHHH--HHHHH
0033 3putB   -llleeE-EE-EEEEEEE-----lllhhlllllleeeeeeelleE-------EEEEEEEL------lLEEEEEEE-EE-eeE---------E-EEEEEEEEEE-L----lllEEEEEEEEE---LLL-HHHHHH-HHHHH--HHHHH
0034 2mj7A   hhhlllL-EE-EEEELLL---L-------llhhhhHH-H-HHLLE-------EEEELLLL------lLLEEEEEE-EE-llE---------E-EEEEEELLLL-----------LEEEEEE---HHH-HHHHHH-HHHHH--HHHLL
0035 3t4nA   -------llleeLEEELL---L----hH---H--HHH-HhHHHHL-------EEELLLHH-------hlLEEEEE-EL-llE---------E-EEEEEEEEEE-L-------LEEEEEEEE--LHHHhHHHHHH-HHHHH--H----
0036 3bfmA   -EEEELL-LE-EEEEEEE----------hhhH--HHH-H-HHHHHhhlllllLEEELL----------LLEEEEL-----------------lEEEEEEEEEElL-------EEEEEEEELL-LlLL-LLHHHH-HHHHH--HHHHH
0037 7ohiA   hhlllHH-HE-EEEEEEL---LL-----lllhhhhHH-H-HHHHL--------LEELLLL-------LLEEEEEE-EE-llE---------E-EEEEEEEEELlL-------LEEEEEEEEL-LHH--hhHHHH-HHHLL--LL---
0038 5a1uE   -------lEE-EEEEELL---L------lhhH--HHH-H-HHHLL-------EELLLLLL-------LEEEEEEE-EE-leE---------E-EEEEEEEELL----------LEEEEEEEE--LHH-HHHHHH-HHL---------
0039 8ba1A   leeleeE-EE-EEEEL-----LL----L---HhhHHH-H-HHHLL-------LLEEEL-----------LEEEEL------------------LEEEEEEL-------------LEEEEEEL-LLHH-HHHHHH-HHHHH--L----
0040 1x2gC   -EEELLLlLE-EEEEEEE----------hhhH--HHH-H-HHHHL-------LEELLLL-----------LLEEEL-------------lllllEEEEEEEEEElE-------EEEEEEEELL-LLlL-LLHHHH-HHHHH--HHHHH
0041 5n9jY   ------L-EE-EEEELLLhhhHL----L---HhhHHH-H-HHHHH-------LLEEEEEE-lleeeeEEEEEEEL------------------LEEEEEEEEElL-------LLEEEEEEEL-LLHH-HHHHHH-HHHLL--L----

@
@
@

Job: BREX PAPS (4Fe-4S) iron-sulfur cluster-binding Fer4_7
Query: s001A
No: Chain Z rmsd lali nres id PDB Description
1: 9eri-B 4.9 2.1 43 333 26 MOLECULE: NA(+)-TRANSLOCATING FERREDOXIN:NAD(+) OXIDOREDUCT
2: 7np8-B 4.9 2.1 42 620 24 MOLECULE: COENZYME F420-DEPENDENT SULFITE REDUCTASE;
3: 9ilt-B 4.9 2.3 42 952 26 MOLECULE: CYTOCHROME C7-LIKE DOMAIN-CONTAINING PROTEIN;
4: 1dur-A 4.7 2.3 42 55 31 MOLECULE: 2[4FE-4S] FERREDOXIN;
5: 6x6u-B 4.6 2.1 43 167 40 MOLECULE: FORMALDEHYDE:FERREDOXIN OXIDOREDUCTASE WOR5;
6: 7bkd-A 4.5 2.2 43 664 37 MOLECULE: COB--COM HETERODISULFIDE REDUCTASE IRON-SULFUR SU
7: 9mqx-E 4.4 2.0 41 152 37 MOLECULE: NADH:UBIQUINONE OXIDOREDUCTASE CHAIN G-LIKE PROTE
8: 8zqd-A 4.3 1.5 38 626 29 MOLECULE: [FEFE]-HYDROGENASE;
9: 6u8y-n 4.1 2.2 42 176 31 MOLECULE: MONOVALENT CATION/H+ ANTIPORTER SUBUNIT E;
10: 8c0z-C 3.8 2.1 42 158 26 MOLECULE: ALDEHYDE:FERREDOXIN OXIDOREDUCTASE,TUNGSTEN-CONTA
11: 5c4i-E 3.7 2.2 42 312 24 MOLECULE: OXALATE OXIDOREDUCTASE SUBUNIT ALPHA;
12: 4hea-9 3.7 2.0 42 180 36 MOLECULE: NADH-QUINONE OXIDOREDUCTASE SUBUNIT 1;
13: 7bkb-k 3.6 2.6 41 386 29 MOLECULE: COB--COM HETERODISULFIDE REDUCTASE IRON-SULFUR SU
14: 2fgo-A 3.6 2.3 42 81 36 MOLECULE: FERREDOXIN;
15: 7z0s-F 3.5 2.1 42 164 31 MOLECULE: FORMATE HYDROGENLYASE SUBUNIT 3;
16: 7p63-I 3.5 2.0 43 180 35 MOLECULE: NADH-QUINONE OXIDOREDUCTASE SUBUNIT F;
17: 7arc-I 3.4 1.9 42 199 26 MOLECULE: PSST;
18: 3gyx-B 3.4 2.3 42 166 29 MOLECULE: ADENYLYLSULFATE REDUCTASE;
19: 6f0k-B 3.4 2.4 43 961 28 MOLECULE: CYTOCHROME C FAMILY PROTEIN;
20: 6tmf-d 3.4 2.1 41 582 22 MOLECULE: 16S RIBOSOMAL RNA;
21: 8rja-L 3.4 2.2 42 78 36 MOLECULE: FORMYLMETHANOFURAN DEHYDROGENASE SUBUNIT A;
22: 5t5m-F 3.4 1.9 42 342 31 MOLECULE: TUNGSTEN FORMYLMETHANOFURAN DEHYDROGENASE SUBUNIT
23: 7koe-D 3.4 2.4 43 92 19 MOLECULE: ELECTRON TRANSFER FLAVOPROTEIN, BETA SUBUNIT;
24: 8jjr-c 3.4 2.2 42 86 24 MOLECULE: PCPI-7;
25: 3bk7-A 3.2 2.4 43 593 21 MOLECULE: ABC TRANSPORTER ATP-BINDING PROTEIN;

0001 s001A   ---------------CAHC-----GVCEVECPTG---ALSVI-------PSVLV-NTSRCIHCYSCLDF----VEKGC----------------------
0002 9eriB   eamiasggSKGCRYGCLGY-----GTCKAVCPFD---AIVIG-----edGLPKV-DPEKCTSCGKCVEA----CPKSIMTLVPEAqevivkchnfdkgki
0003 7np8B   igiagvkyPKVNEEKCNGC-----GRCAEVCKVE---AIDIR-------GTSYT-NYNVCVGCGKCIKN----cPNEAREVKEEGylvyvggktgrevve
0004 9iltB   gedldnpsIYMMPVNCMQC---ekAPCEVVCPVA---ATVHD-----yeGLNNM-VYNRCVGTKYCSNN----cPYKVRRFNFLQysdtttetfklafnp
0005 1durA   --------AYVINDSCIAC-----GACKPECPVN---CIQEG-------SIYAI-DADSCIDCGSCASV----cPVGAPNPED-----------------
0006 6x6uB   irvfelfpGINVPHTCVQC---pdYPCVNACPTN---ALSVD----ektGAVVV-NEEKCITCGACVLA----CPGKVPRIPAGKgsvvicdlcggnpkc
0007 7bkdA   evelepyfAMCIDELCAGC-----GMCVNLCPYS---ALSLG--ekngrTVMVV-TEAKCKGCGTCGGF----CPGGAIKMQHFTtpqivaqidaffag-
0008 9mqxE   vtvyrfeaGANVPMTCQQC---ddAPCISVCKAG---ALAR-----dekNVVQV-DSSKCIGCRMCVMA----cPFGNMSYHWEQstaikcdqcngspyc
0009 8zqdA   tsqcnhlvKLTITKKCIGC-----GACKRACPVD---CINGE-----lkKKHEI-DYNRCTHCGACVSAcpvd---------------------------
0010 6u8yn   papryrgfHTLDWKKCIGC-----NMCGQICPAR---AIEMT-----wiPHPKI-DYGRCTFCQFCVDV----cPTGALGFIETYmltttwreeellyd
0011 8c0zC   kvfsfeheGRKVPYTCTQC---teAWCLHSCPVD---AIRL----dlttGAKMV-FEDTCVGCKVCTIA----CPFGTINYNQDTgkvqkcdlcegdpac
0012 5c4iE   tgnwriqrPIIDREACTEC-----YTCWIYCPDS---CITRT------eEGPVF-NMKYCKGCGLCTAV----cPSGALTNVPELdfkd-----------
0013 4hea9   hgrhvltrHPNGLEKCIGC-----SLCAAACPAY---AIYVE-------PVYEI-NMLRCIFCGLCEEA----cPTGAIVLGYDFemadyeysdlvygke
0014 7bkbk   nkifegeiTFHAEKCPGGC-----STCVDVCPAN---AIYLP-------TkIAV-NKDFCILCGACVNA----GEDI-----------------------
0015 2fgoA   --------SLKITDDCINC-----DVCEPECPNG---AISQG-------EIYVI-DPNLCTECPQCQQV----cPVDCIPLDDANveskdqlmekyrkit
0016 7z0sF   vdknfrgkPEQNPQQCIGC-----AACVNACPSN---ALTVEtdlatgeLAWEF-NLGHCIFCGRCEEV----cPTAAIKLSQEYelavwkkedflqqsr
0017 7p63I   rgrivltrDPDGEERCVAC-----NLCAVACPVG---CISLQkdgrwypEFFRI-NFSRCIFCGLCEEA----CPTTAIQLTPDFemgeykrqdlvyeke
0018 7arcI   rgehalrrYPTGEERCISC-----KLCEAICPAQ---AITIE-------ARYDI-DMTKCIYCGFCQEA----cPVDAIVEGPNFefstetreellydkq
0019 3gyxB   -------pTYVDPSKCDGCkggekTACMYICPND---LMILD-------PKAFNqEPEACWECYSCIKI----cPQGAITARPYAdfapmggtciplrgs
0020 6f0kB   eahaddpqIVVQPVPCMHC---enAPCESVCPVA---ATVHS-----pdGLNEM-VYNRCIGTRYCSNN----CPYKVRRFNWFNwvktlpiqvqmaqnp
0021 6tmfd   -----mrvAVINYDFCKPD--kcnLECINFCPVD-gkAIEL---seivkGKPVI-YEETCIGCGICVKK----cPYEAISIVNLPdelegevihrykvng
0022 8rjaL   ----msfkKVKVSEECVGC-----GVCETVCPVNnklAIKVT-------NEAAV-DEEVCLTCGTCTFN----cPSGAVYAEYEP---------------
0023 5t5mF   rtgeenrrLIFQDCLCAVC-----GLCGEICPVS---AIEVN-------PKIAI-DENKCVLCGMCSSI----cPFQALDLQIDGtsikelaeypkiiks
0024 7koeD   yrtdeenpHLKIKDESICAekcsdRPCVSCCPAD---VYEWT-------EGMEV-KFEGCLECGTCRIV----CPFGNIEWNYPRgnygvlykfg-----
0025 8jjrc   ------shAVKIYDTCIGC-----TLCVRACPTD---VLEMVatvnaakQVASSpRVEDCVGCKRCETA----tDFLSIRVYLQEneetqyslgldladw
0026 3bk7A   -mvrkmriAVIDYDKCNPD-kcghFLCERVCPVN-geAIIID----eenYKPII-QEASCTGCGICVHK----CPFNAISIVNLPeqldedcvhrygvna

0001 s001A   ---------------LLLL-----LHHHHLLLLL---LEELL-------LLLEE-LLLLLLLLLHHHHL----LLLLL----------------------
0002 9eriB   hhhhllllLLLLLLLLLLL-----LLHHHHLLLL---LEEEL-----llLLEEE-LLLLLLLLLHHHHH----LLLLLLEEEELLllleelllllllhhh
0003 7np8B   eeeeeeeeEEELLLLLLLL-----LHHHHHLLLL---LEEEE-------LEEEE-LLLLLLLLLHHHHL----lLLLLEEEEEEEeeeeellllllllll
0004 9iltB   llllllllLLLEEELLLLL---llLLLLLLLLLL---LEEEL-----llLLEEE-LHHHLLLLLHHHHL----lLLLLLEELLLLllllllhhhhhhlll
0005 1durA   --------LEEELLLLLLL-----LLLHHHLLLL---LEELL-------LLLEE-LLLLLLLLLHHHHH----lLLLLEEELL-----------------
0006 6x6uB   leeellllLLLEEELLLLL---llLHHHHHLLLL---LEEEL----lllLLEEE-LHHHLLLLLHHHHH----LLLLLLLLLLLLlllllllhhhhllhh
0007 7bkdA   eeelllllEEELLLLLLLL-----LHHHHHLLLL---LEEEE--eelleEEEEE-LLLLLLLLLHHHHH----LLLLLEEELLLLhhhhhhhhhhhlll-
0008 9mqxE   eeeeelhhHLEEEEELLLL---llLHHHHHLLLL---LEEE-----lllLLEEE-LHHHLLLLLHHHHH----lLLLLLEEELLLleeelllhhhhllhh
0009 8zqdA   llllllllLLEELLLLLLL-----LHHHHHLLLL---LEELL-----llLLLEE-LLLLLLLLLHHHHHllll---------------------------
0010 6u8yn   llllllleEEELLLLLLLL-----LLHHHLLLLL---LLLLL-----llLLLLL-LLLLLLLLLLLLLL----lLLLLEEEELLLlleeellhhhlhhhl
```

```
0011 8c0zC   eeeeelllLEEEEELLLLL---llLHHHHLLLLL---LEEL----llllLLLEE-LLLLLLLLLHHHH----LLLLLLEEELLLleeelllhhhhllll
0012 5c4iE   lhhhlleeEEELLLLLLLL-----LHHHHHLLLL---LEEEE------lLEEEE-LLLLLLLLHHHHH----lLLLLEEEEEHHhlll----------
0013 4hea9   llleeellLLLLLLLLLLL-----LHHHHLLLLL---LEEEE-------EEEEE-ELLLLLLLHHHHH----lLLLLEEELLLLllllllhhhheeehh
0014 7bkbk   llleeeeeEELHHHLLLLL-----LHHHHHLLLL---LLLLL-------LlLLL-LLLLLLLLLHHHHH----LLLL---------------------
0015 2fgoA   --------LLLLLLLLLLL-----LLLHHHLLLL---LEEEL-------LLEEE-LLLLLLLLLHHHHH----lLLLLLLLLLLLlllhhhhhhhhhhh
0016 7z0sF   llllllllEEELLLLLLLL-----LHHHHHLLLL---LEEEEeellllleEEEEE-ELLLLLLLHHHHH----lLLLLEEELLLLllllllhhhheeeee
0017 7p63I   llleeellLLLLLLLLLLL-----LHHHHHLLLL---LEEEEllllleeeEEEEE-ELLLLLLLHHHHL----LLLLLEEELLLLlllllllllleelhh
0018 7arcI   llleeellLLLLLLLLLLL-----LHHHHHLLLL---LEEEE-------EEEEE-ELLLLLLLHHHHH----lLLLLEEELLLLllllllhhhheelhh
0019 3gyxB   -------lEEELLLLLLLLlllllLHHHHHLLLL---LEEEE-------LEEEElLHHHLLLLHHHHH----lLLLLEEELLLLllllllleeeeeell
0020 6f0kB   llllllllLEELLLLLLLL---llLHHHHHLLLL---LEELL-----llLLLEE-LLLLLLLLLHHHHL----LLLLLLEELLLLllllllllhhhllll
0021 6tmfd   -----leeEEELLLLLLLL--lllLHHHHLLHHH-llLEEE---lllllLLEEE-LLLLLLLLLHHHHH----lLLLLEEEEELLllllllllleellll
0022 8rjaL   ----llllEEEELLLLLLL-----LHHHHHLHHHhllLEEEE-------LEEEE-LLLLLLLLLHHHHH----lLLLLEEEELLL--------------
0023 5t5mF   elllllleeEEELHHHLLLL-----LHHHHHLLLL---LEEEL-------LLEEE-LLLLLLLLLHHHHH----lLLLLEEEEELLeehhhllllllllle
0024 7koeD   eellllllLEELLLLLHHHhhlllLHHHHLLLLL---LEEEE-------LEEEE-LHHHLLLLLHHHHH----LLLLLLEELLLLllleellllll-----
0025 8jjrc   ------llEEEEELLLLLL-----LHHHHHLLLL---LLEEElllllllEEEELlLHHHLLLLLHHHHH----lLLLLEEEELLLllllhhhhlllllll
0026 3bk7A   -llllleeeEELLHHHLLLL-lllllLHHHHHLHHH-llLEEEE----lllLEEEE-LLLLLLLLLHHHHH----LLLLLLEEEEELllhhhleeeelllll

@@
@@
@@

Job: BREX-Related Capture Systems (BRC) HerA/FtsK
Query: s001A
No: Chain Z rmsd lali nres id PDB Description
1: 4d2i-A 30.1 3.3 323 464 23 MOLECULE: HERA;
2: 7es4-A 28.8 3.0 310 373 16 MOLECULE: DNA PHOSPHOROTHIOATION-DEPENDENT RESTRICTION PROT--DndH CTD HerA ATPase
3: 4ag6-B 27.1 3.0 310 376 18 MOLECULE: TYPE IV SECRETORY PATHWAY VIRB4 COMPONENTS-LIKE P
4: 7wrx-C 26.4 3.8 345 594 16 MOLECULE: HERA;
5: 8wet-A 22.6 3.6 311 584 19 MOLECULE: TDPB;
6: 1e9s-A 20.0 4.1 298 427 15 MOLECULE: CONJUGAL TRANSFER PROTEIN TRWB;
7: 4kfr-B 19.0 2.6 200 208 16 MOLECULE: GENOME PACKAGING NTPASE B204;
8: 8rtb-B 18.6 3.8 233 661 14 MOLECULE: TRWM PROTEIN;
9: 4lya-A 18.4 3.0 217 521 14 MOLECULE: UNCHARACTERIZED PROTEIN;
10: 4nh0-A 18.4 2.6 219 861 20 MOLECULE: CELL DIVISIONFTSK/SPOIIIE;
11: 2iut-A 18.3 2.8 215 408 17 MOLECULE: DNA TRANSLOCASE FTSK;
12: 8rtd-T 18.3 4.2 308 808 15 MOLECULE: TRWJ PROTEIN;
13: 6sz9-A 16.7 3.6 265 473 16 MOLECULE: ICMO (DOTL);
14: 8dol-A 15.6 3.6 299 540 10 MOLECULE: CAG PATHOGENICITY ISLAND PROTEIN (CAG5);
15: 6tv1-A 13.9 3.1 190 228 13 MOLECULE: ESSC PROTEIN;
16: 4wia-A 12.7 3.3 179 229 11 MOLECULE: PUTATIVE FLAGELLA-RELATED PROTEIN H;
17: 8faz-X 12.2 3.7 181 236 16 MOLECULE: DNA REPAIR PROTEIN RAD51 HOMOLOG 2;
18: 8ouy-C 11.9 3.2 182 308 14 MOLECULE: DNA REPAIR PROTEIN RAD51 HOMOLOG 2;
19: 2r2a-A 11.7 3.0 156 191 16 MOLECULE: UNCHARACTERIZED PROTEIN;
20: 3io5-B 11.5 2.9 166 272 10 MOLECULE: RECOMBINATION AND REPAIR PROTEIN;
21: 2f1h-A 11.5 3.4 187 313 11 MOLECULE: DNA REPAIR AND RECOMBINATION PROTEIN RADA;
22: 7t8b-A 11.4 3.4 173 518 8 MOLECULE: TWINKLE MTDNA HELICASE;
23: 3cmu-A 11.1 3.2 186 1937 11 MOLECULE: DNA (5'-
24: 8peu-A 11.0 3.2 177 419 11 MOLECULE: TRANSCRIPTION TERMINATION FACTOR RHO;
25: 8gja-F 10.9 3.4 180 342 10 MOLECULE: RAD51C;
26: 6bbm-D 10.5 3.4 177 455 12 MOLECULE: REPLICATIVE DNA HELICASE;
27: 4hut-A 10.3 4.0 156 191 13 MOLECULE: COB(I)YRINIC ACID A,C-DIAMIDE ADENOSYLTRANSFERASE
28: 7njn-C 10.3 5.2 188 536 11 MOLECULE: ATP SYNTHASE SUBUNIT ALPHA;
29: 8dtp-A 10.2 4.3 185 432 8 MOLECULE: DNAB-LIKE REPLICATIVE HELICASE;
30: 6tdz-C 10.0 4.6 185 530 18 MOLECULE: OLIGOMYCIN SENSITIVITY CONFERRING PROTEIN (OSCP);

0001 s001A   MLLQVGRDIETREQVFLEGDRARTVFICGKRGSGKSYTLGVLVEELFEKSGGVTVILVDPMGIFHTMVLPKGLPVRLLVPGRGVDVLGLRLNPSDLTPENWCSLFDLSI-NDLMGIALYRAITGLQERKPY-YLQDIERAIMRDTRAQDKTKEALTNRLSIAHRWDIFSDGPGANWGQLFRADSVNIVDVSVLDPGGLRSLVVDLITKQIFSVRISARRQMPRVWLALDEAHQFVPSGSSLCKESLTRWVKEGRQPGLGLIAATQQPSAMDSDLLSQCDLIIAHKITSLEDVQALN-RLSATYMT-GEMKSVLRNIGQRGEAVMVDDEKESVTTVAIRPRR
0002 4d2iA   gQIRIGSLIGKNVEVKLNINSARHLAILAATGSGKSNTVAVLSQRISELG--GSVLIFDYHGEYYDSD---IKNLNRIEP---------KLNPLYMTPREFSTLLEIREnAIIQYRILRRAFIKVTNGIRaqFYELMADAL----------KDEVLNKFEEFMDSNVID-LTSSDIIEKVKRGKVNVVSLTQLDE-DSMDAVVSHYLRRILDSRKDFKRGLFPIIAVIEEAHVFLSNENTLTKYWASRIAREGRKFGVGLTIVSQRPKGLDENILSQMTNKIILKIIEPTDKKYIL-ESSD--LS-EDLAEQLSSL-DVGEAIIIGKIVKLPAVVKIDMFE
0003 7es4A   LQILFGHDAVRQNPLYWEPTNNTNTGIIGTXGTGKTQFTKSLVTQLXRNQSKIGLLIFDYKSDYVAFLE--ATGAKRYQLS-------LPYNPLSTAXAFAETXYNLG---VKQRXKLVTLIXECYDLVAP-TIEDVWQQYLAQEKVDE---DSLYAALYNLAGFQIFETDKXTSLYDLVD--GVTVIELAGYPS-EIQNLVVALTLDLFYAQXQKRGKdrlTKXILVDEADNFXR--QDFA--SLRKILKEGREYGVGAILSTQEITHFKTNYASYILTWVIHRVSEIR-nSDIK-AVFNIdKS-EQLXGQIRQL-EKHFSLYIDG-NKKVRKXRDKAFW
0004 4ag6B   SGIVLGKDR-DGGLVLVDIWKNSNWTILAKPGAGKSFTAKXLLLREYXQG--SRVIIIDPEREYKEXCR--KLGGVWINCT------EGKINPLQVHIQTLRTFLRDL--TDTEKAALEDALVEVYKEAWP-TVKELYEYCVKK---NPETYGRLSVLLKRAADSYLWAGP-TAVE--ADSD--FIVFDVHDLAEDAQYFNVLSFAWNILERDR------RERTVLVVDEAWXLV-PQTPQAIAFLRDTSKRIRKYNGSLIVISQNVIDFGQALLDNPTYKLLLAQGE-KDLEAIT-TLXN--LS-EAEHDLLVNA-KRGEGLFVAG--TQRIHIKIEAYE
0005 7wrxC   AAFPGGLL-ADGQPLPLNFRFIGHINISGISGATKTSYALFLLHSIFRSGTAGRALIFNVKEDLLFLDKPEPFDVQLLAPP----VTPFVFTIARRM---LPYVFSLNL-----GFVIGNIEEKLFRLAlq-TFEQLISYLEYKllQSPGTLRAFTRRLRGVQKYPLIRGDLGYRP--DPLRIQLTVVDIHALSA-HAQMFVVGVLLREVFEYKERVG--QDTVFVVLDELNKYAPEGDSPIKDVLLDIAERGRSLGIILIGAQQTASEVERRIVSNAAIRVVGRLDLAEAEREYR-F-----lP-QSFRGRAGIL-QPGTMLVSQPDVPNPVLVNYPFPA
0006 8wetA   RRYAFGHLAEYPEVKEVDPAVGRRTANFGKTGFGKSNENKVILTLLAHAFPRVGMLILDQNAEYLLLAQALGIRIRFYT-AEYVEVLPLKVDF--LAVALAYQ-RRRLqgAEPP-QYLENAFYNLEDW-sesqrgdnkGGARE-------lYSRA-KVFS-FLRA-FHAPEANFTIKEDLgEGKVVILDLPSLG--EAADFFTLRLMDLLFDRAVELYG--ANFLVVLEEAHNFLE----dkaGIFYRVAKEGRKYGIGMLYSTQSPASIPMEILSQTENFLVKHLSSEEDVKVLK-RAKA--PF-AFVADFLLSEPIIGYSYVYFEPYQFVVPLRVKLLE
0007 1e9sA   KQVTVAG-------VPMPRDEPRHLLVNGATGTGKSVLLRELAYTGLLRG--DRMVIVDPNGDMLSKF----RDKDIILNP-----RTKGWSFFNEDWQRYAL-vtdeewasyGRLLLRETAKKLALIGTP-SMRELFHWTtiESLFASKALTSARFVLSDKLPETMPD--GDFSIRSWLPNGG-NLFITWR-MGPPLISAWVDVVCTSILS--LPEE--pRRLWLFIDELASL-----EKLA-SLADALTKGRKAGLRVVAGLQSTSQLAQTLRASFRSLVVLGGSDPKTNEDMS-LSLGEHrE-RVVPAEIANL-PDLTAYVGFAGNRPIAKVPLEIKQ
0008 4kfrB   ------------------MNPDDIVVLVGRKKSGKSYLIKYFIPVLKAHK--ISYIIDDHNSEYSKF-------GYNATS--------------------------------------------------------------------------------------------lSDIV-SKQY---VVVYDRAK----NDDF-FEKLWQASKLHSK----KYGTTVLIIDEAYYHFKKQKV--TPAIDEALHANRHAGLGLILSTQRVYDLMPIVYKQADLIIMFYTREPNELRWIS-KYIS-----AEAAEKVKTLKQ-YHFLIYDVNSQTIKIHKPILEH
0009 8rtbB   PAVTILKTV-SGTPLYFNF-LLGNTMLIGQSSSGKTVLLGFLLAQA-QKFK-PTIVAFDKDRGMEIS---rAMGGRYLPL----------------------------------------------------------------------------------------------LDLSTHQ---IYGFDITEFLDNAR-TPVMMYLLYRTESMID-----GRRFMYVFDEFWKPLQ--DEYFEDLAKNKQKTIRKQNGIFVFATQEPSDAAKTLIQQCATYIFLANPKAD-yEDYTqGFKL---T-DSEFELVRGLGFSRRFLIKQG--DQSALAEMNL--
0010 4lyaA   NAFPIGLKDESQSDYFYQWLEDGNIGIFGSAGYGKSTTXXTLLLSFAGAYNPLHYYIFDFNSALLPL-----RQLP-----------------------------------------------------------------------------------------------------------HTADYFRLDD------eKKIEKFIKFXKEEXEQRKQ-LPIIIVALDNFDVVK-EEXPDFETQLIQYARDGQSLGIFFIXTATRVSGIRPPLXNNLKTKIVHYFIDSSEKFSLI-----------GRTPY--DVDPPGRALIKK---DNAALTQIYLPA
0011 4nh0A   LRVPIGLDA-DGRPLELDIKEaPHGLCIGATGSGKSELLRTLVLALAMTHSPLNFVLVDKGGAFLGM-----EGLR----------------------------------------------------------------------------------------------------------HVSAIITNLEEE--LPLVDRMYDALHGEMVRRQEHLR-MPTLFIVLDEFSELLS-aKPDFAELFVMIGRLGRSLGVHLLLASQRLEEKLRGLDTHLSYRIGLRTFSAMESRVVL-G-----------VPDAYELPSPGNGYLKFA-TEPLVRFKAAYVS
0012 2iutA   VPLALGHDI-GGRPIITDLAKMPHLLVAGTTGSGKSVGVNAMLLSILFKSTPARLIMIDPKMELSI------YEGI----------------------------------------------------------------------------------------------------------PHLLVVTD---------MKEAANALRWSVAEMERRYRTLPTIVVVVDEFADMMMivGKKVEELIARIAQKARAAGIHLILATQRPSVITGLIKANIPTRIAFQVSSKIDSRTIL-D-----------QGGAEQLLGHGDMLYLPPGTGLPIRVHGAFVS
0013 8rtdT   PAVTILKTVSGTPLYFNFHaLLGNTMLIGQSSSGKTVLLGFLLAQ-AQKF--PTIVAFDKDRGMEIS---rAMGGRYLPL------pSGFNPFQLFLKQFVKKLAAaEV-THRDEEEIDQAITAMMSKSLR-RLSLLLQF--------------VHARLVKWCYGWLFDNPTDADLST----HQIYGFDITEFLDN-ARTPVMMYLLYRTESMID-----GRRFMYVFDEFWKPLQ--DEYFEDLAKNKQKTIRKQNGIFVFATQEPSDAAKTLIQQCATYIFLANPKAD-yEDYTqGFKL---T-DSEFELVRGLGFSRRFLIKQG--DQSALAEMNLfL
0014 6sz9A   GITFFGNDRKTGEELWFANDMRTHALIFGSTGSGKTETLVSLSYNALVQG--SGFIYVDGKNSLYAVFSMSMGRLLLINF-----SNTLNPF-SSML---------tvvslmMWKGRAIAFVEALRLLV--tIRNYFDL----plrgVLEQHGFITMQLVRSFSSLadIRTNLADFKDVLNRR-ILVVLLP-aKSPNLGKIIVSSLKAMMAAGLG---R-atPYMCILDEYGYY-------AVQGF---AVVPRSLGFSAIFAGQDLPAFAASIGANTNIKICMKL---------------------------------GEAHIFFK--SKIVRARMFYAN
0015 8dolA   REVIVGRR--gLGDFIAYA-GQAFIGLIAPTRSGKGVFIMPNMINYP-----QNIVVFDPKADTETCG--KIREVFIYEPF------THRFNPFAGLAKLVFP---enekDPFFSNQARNLFVINCNIkTP-TMFFIGSMASGareTYSSVQGVYTSAFAPYNNRNFT--SANDDFRR--iDEVSIGVIAN--PKEIV-GPILELFFNVMIYSNLILPICKRSCLMLMDEFTLC-----GYLE-TFVKAVGIMAEYNMRPAFVFQSKAQLAKTILDNLSLNMYYGINYYEHFE-----klsKVLGkLMTPDELMTMG--DELIILENTL-KPIKCHKALYY
0016 6tv1A   GALPIGLDYEGVTLQKIKL--TEPAMISSENPREIAHIAEIMMKE-IDILNEYAICIADSSGEFKA------YRHQ-----------------------------------------------------------------------------------------------------------VANFAE---------EREDIKAIHQLMIEDLKQREMFEKDSLYIINDFKTFI--dcTYIPEDVKKLITKGPELGLNILFVGILIDA-IDVARKMINQFSIGIRISD--QQFF---------------qrePVIK-ENEAYMVAN--QAYQKIRWFK--
0017 4wiaA   ARID-------lsDDLDGIPHGSLIIIEGEESTGKSVLCQRLAYGFLQNR--YSVTYVSTQLTFIKQXNSYSIN---------------------------------------------------------------------------------------------------------kLSGALLYIPVYPADNKDGFLKKVXET-RAFY----------eKDVIIFDSISALIAaSEVN-VDDLXAFFKRITALKKIIICTVNPKEL-LTIIRTSATXLIRTE-----------------------------lfgLKNLAKILYNXASYQKNIVFRV--
0018 8fazX   KEIE---------PNLFedVHGDILEFHGPEGTGKTEMLYHLTARCILGLEV-EVLFIDDYHFDLRLV--TILE-------EII------------------------------------------------------------------------------------------------kyGRFFLVYC-------SSSTHLLLTLYSLESMFCSH---PSLCLLILDSLSAFY-lQESTLRKCSQCLEKLVNDYRLVLFATTQT-PYLCKAWQQLVKHRMFFS------------------------------kSNQFSLVSRCLKSNSLKKHFFIIGE
0019 8ouyC   LSTGIGS---ldklDAGLY-TGEVTEIVGGPGSGKTQVCLCMAANVAHGLQQ-NVLYVDSNGGL-TASR-QLL--------------------------------------------------------------------------------------------------------qataeRRIQVVHAF-------DIFQMLDVLQELRGTVAQQ--SGTVKVVVVDSVTAVVSGLAL--MMQLARELKTLRDLGMAVVVTNHIKPALGRSWSFVPSTRILLD-----------------------------tISGGRMACLAKSSRQPTFQEMVDI--
0020 2r2aA   --------------------XAEICLITGTPGSGKTLKXVSXXANXFKP-GIRKVFTNI--KGLKI---------------------------------------------------------------------------------------------------------------------pHTYIET-----dKLPksSAHDXYEWIKKP------ENIGSIVIVDEAQDVWPRS--skiPENVQWLNTHRHQGIDIFVLTQGPKLLDQNLRTLVRKHYHIA--------------------------snKXGXR--TLLEWKI---SAFSSYTLDVY
0021 3io5B   VVRT--------kPMMNGMQS-GLLILAGPSKSFKSNFGLTMVSSYMRQYPDAVCLFYDSEFGITYLRS-mGVDPE-----------------------------------------------------------------------------------------------------------RVIHTPV-------QSLEQLRIDMVNQLDAIE-----GEKVVVFIDSLGNL--RAKT-mKSLFRIVTPYFSTKNIPCIAINHT----yTGPMYSADTVFIIG------------------------------KRQF-VLNVESRTVKEKSKFFI----
0022 2f1hA   WKLSSSSE-----ldGGLE-SQSVTEFAGVFGSGKTQIMHQSCVNLQNP-VAPKAVYIDTDGTFRPRIMQDGTVLD-----------------------------------------------------------------------------------------------------------NTFVARAY-------NSDMQMLFAEKIEDLIQE---GNNIKLVVIDSLTSTFRLAERKLGRHMATLNKLADLFNCVVLVTNQVQAIGGHIVGHAATFRFFVR------------------------------kGDKRVAKLYDSPHLPDAEAIFRI--
0023 7t8bA   LRWS--------rPDLNGHRKGELTVFTGPTGSGKTTFISEYALDLCSQG--VNTLWGSFEIRLARMLTQ-----------WA-------------------------------------------------------------------------------------------------rfLPLYFM-TFHG---QQSIRTVIDTMQHAVYV-------YDICHVIIDNLQFM--IAA-yiIGVFRKFATDNN---CHVTLVIHPGS---AKASQEADNVLILQ------------------------------DRGKRYLQVSNRFDGDVGVFPLEF--
0024 3cmuA   ETISGSLS-----diGGLP-MGRIVEIYGPESSGKTTLTLQVIAAAQREG--KTCAFIDEHALDPYARK-lGVDID-----------------------------------------------------------------------------------------------------------NLLCSQP-------DTGEQALEICDALARSG-------AVDVIVVDSVAAL--MGLAMMSQAMRKLAGNLKQSNTLLIFINQITTTGGNALKFYASVRLDIR------------------------rigavkengETRVKVVNKIAAPFKQAEFQI-Y
0025 8peuA   LRMETARV----lLASPIG-RGQRGLIVAPPKAGKTMLLQNIAQSIAYNHPDVLMVLLIDEREVTEMQ---RLVK------------------------------------------------------------------------------------------------------------GEVVASTFDEPA-SRHVQVAEMVIEKAKRLVEHK----KDVIILLDSITRLARANALhRPKRFFGA---ARNVSLTIIATALI-EVIYEEFKGTGNMELHLS--------------------------RKIARVFPAIDSGTRKEE------------
0026 8gjaF   HLTT------cQQLDGGIL-TRTLTEIAGESASGKTQLCMQLCLTVQLGLGG-GAVYICTDVFPNRLVQSkdCFTD-----------------------------------------------------------------------------------------------------------NIFIEHA-------AELDDLHYCVSKKVPV------QRHVKLIIIDSIAALFRLQERrLMQLIASKLLQLNQFNVPAICVNQVSDTLGISWANHVTVRLMLM---------------------lpqniegdvilQIRTMEVLFAPHLPNSLCRFVDQD
```

0027 6bbmD VNTG-------yDDLNAGLQPSDLIIVAARPSMGKTTFAMNLVENAAMLQD-KPVLIFSEMPSIMMRSLS[illegible]gqdedwaillRNIYIDDS-------SGLT-PTEVRSRARRIA-----HGGIGLIMIDYLQLMrvTLE--iaEISRSLKALAKELNVPVVALSQLLRESGS-IEQDADLIMFIY[illegible]RDEVAEIIIGQRNGPIGTVRLTFNG-
0028 4hutA -----yqqrVDAR----qeERGIIIVFTGNG-KGKTTAAFGTAARAVGHG--KNVGVVQIKGTwNGER---NLLEP[illegible]HGVEFQVMATGFTRDAACMAVWQHGKRMLADP[illegible]LLDMVVLDELTYMVA-YDYLPLEEVISALNARPG-HQTVIITGRG---CHRDILDLADTVSELRP[illegible]VKHA[illegible]
0029 7njnC lQTGI---------KAIDPIGRGQRQLIIGDRKTGKTAVCVDTILN-QREAWQVRCVYVAIIASVKALEE--GGAM[illegible]EYTTIVAAP-ASDAGFKWLAPYTGSAIGQHWMYNG----KHVLIVFDDLSKQADFPGD-vfYLHSRLLERCAKLSMTGLPIIET-AFIPTNVISITDGQCFLES[illegible]DLFNVRPAINSVSRVGGglelLKQPQYS
0030 8dtpA VPFKL---------RILNGAETGTLNVLMAGVNVGKSLGLCSLAADYLQLG--HNVLYISMEMVCAKIDAN[illegible]KM[illegible]ksGRLIVKQY-------PTGGADANTFRSLLNE------NFVPTIIIVDYLGIC-------KSCRiEELRALAVETETVLWTAAQVGIAESAGLPATADFMLAVI[illegible]etEELAEQQLIKQKSRYGDKWNKFLMGVQK
0031 6tdzC lLTGYKV-----iTLIPVG-RGQRELILGDRQTGKTSIALSTILN-QTKVNkVLSVYVSGQRCIHRLLTE-YDAM[illegible]KYCTIVAATADPA-GLQYLAPYAGTTLGEEFRNSG----RHILLVYDDLSKQAvPGDVyLHSRLLERS--AMMSSLTSLPIVET-AYIVTNVISITDGQIYLD[illegible]AKLFGQRPAVNSVSRVGS-------QKGP

0001 s001A [illegible]
0002 4d2iA [illegible]
0003 7es4A [illegible]
0004 4ag6B [illegible]
0005 7wrxC [illegible]
0006 8wetA [illegible]
0007 1e9sA [illegible]
0008 4kfrB [illegible]
0009 8rtbB [illegible]
0010 4lyaA [illegible]
0011 4nh0A [illegible]
0012 2iutA [illegible]
0013 8rtdT [illegible]
0014 6sz9A [illegible]
0015 8do1A [illegible]
0016 6tv1A [illegible]
0017 4wiaA [illegible]
0018 8fazX [illegible]
0019 8ouyC [illegible]
0020 2r2aA [illegible]
0021 3io5B [illegible]
0022 2f1hA [illegible]
0023 7t8bA [illegible]
0024 3cmuA [illegible]
0025 8peuA [illegible]
0026 8gjaF [illegible]
0027 6bbmD [illegible]
0028 4hutA [illegible]
0029 7njnC [illegible]
0030 8dtpA [illegible]
0031 6tdzC [illegible]
