# Supplementary material for: Expanding the landscape of BREX diversity: uncovering multi-layered functional frameworks and identification of novel BREX-related defense systems
Source: Nucleic Acids Res. 2026 Jan 27;54(3):gkag035. doi: 10.1093/nar/gkag035 (PMC12839542; doi:10.1093/nar/gkag035)

# Supplementary Data S3

*The PDF presents two distinct datasets organized as follows:*

**i. Representative MSAs of all analysed domains present in various protein components of BREX and their related systems.** Alignments are color-coded and annotated as in Supplementary Data S2. The corresponding topology denoting the boundaries of secondary structural elements are marked at the top of each alignment. Domain architecture and their boundaries are marked accordingly on top of each alignment. Consensus for each alignment at percent identity (70% to 100%) are marked below the alignments.

**ii. Shannon entropy plots for fast-evolving domains along with their corresponding alignment.** The bar plots represent positional entropy for each corresponding alignment: yellow-to-red indicates absolute entropy, while light-to-dark blue represents amino acid property-based entropy.


1. Type-1 BREX; BrxC-ATPase….4
2. Type-2 BREX; BrxC-ATPase….5
3. Type-3 BREX; BrxC-ATPase….6
4. Type-4 BREX; BrxC-ATPase (Version 1 and 2)….7-8
5. Type-1 BREX; BrxX/PglX-MTase….9
6. Type-2 BREX; BrxX/PglX-MTase….10
7. Type-3 BREX; PglXI/BrxXI-MTase….11
8. Type-1 BREX; PglZ (Representative 1, 2 and 3)….12
9. Type-2 BREX; PglZ (Representative 1 and 2)….13
10. Type-3 BREX; PglZ (Representative 1 and 2)….14
11. Type-4 BREX; PglZ (Representative 1, 2, 3 and 4)….15
12. BREX-PglZ and PorX PglZ core NPP phosphatase; comparison of residues for coordinating metal ions….16
13. BREX-PglZ, PorX PglZ, and Type-3 BR-system standalone PglZ; comparison of residues for coordinating metal ions….17
14. Type-1 BREX; BrxL (C-terminal inactive LonP version)….18
15. Type-1 BREX; BrxL (C-terminal active HKD-DNase version)….19
16. Type-4 BREX; BrxL (SIGMA HTH-like+OB-Fold+MCM)….20
17. Type-1 BREX; BrxA (Representative 1)….21
18. Type-1 BREX; BrxA (Representative 2)….21
19. Type-1 BREX; BrxA (Representative 3)….21
20. SSP systems BrxA homolog - SspB….22
21. Type-2 BREX; PglW BrxA-like Tripartite DNA-binding component….23
22. Type-3 BREX; BrxA….24
23. Type-4 BREX; DUF4007 (BrxA homolog; Representative 1)….25
24. Type-4 BREX; DUF4007 (BrxA homolog; Representative 2)….25
25. Type-1 BREX; BrxB-iSTAND….26
26. Type-2 BREX; PglW C-terminal iSTAND….27
27. Type-3 BREX; BrxF iSTAND (Representative 1 and 2)….28
28. Type-3 BREX; BrxF STAND (Representative 3, with Walker A & B intact)….28
29. Type-4 BREX; PglZ N-terminal iSTAND (Representative 1 and 2)….29

30. Type-1 BrxB-iSTAND and Type-2 BREX PglW C-terminal iSTAND (subset) alignment comparison….30
31. vWA-MoxR system; iSTAND….31
32. Type-2 BREX; PglW complete protein (Representative 1)….32
33. Type-2 BREX; PglW complete protein (Representative 2)….33
34. Type-2 BREX; BrxHI-Helicase….34
35. Type-2 BREX BrxHI-Helicase and Ski2-like Helicase alignment comparison….35
36. Type-2 BREX; BrxD AAA+ ATPase….36
37. Type-3 BREX; BrxHII-Helicase….37
38. Type-3 BREX BrxHII-Helicase and Swi2/Snf2-Helicase alignment comparison….38
39. **Representative alignments of core protein components associated with DUF499-centered Type-1 BREX-related systems**
   (i) DUF499-ATPase (Representative 1)….39
   (ii) DUF499-ATPase (Representative 2; HEPN fusion variant)….39
   (iii) DUF499-ATPase (Representative 3)….39
   (iv) Methyltransferase (PglXI-homolog)….40
   (v) Helicase-nuclease fusion component (BrxHII homolog)….41
40. **Representative alignments of core protein components associated with DUF499-centered Type-2 BREX-related systems**
   (i) DUF499-ATPase (Representative 1)….42
   (ii) DUF499-ATPase (Representative 2)….42
   (iii) DUF499-ATPase (Representative 3)….42
   (iv) Methyltransferase (PglXI-homolog)….43
   (v) Nuclease-Helicase fusion component (BrxHII homolog)….44
   (vi) DUF3780….45
   (vii) DUF3780+RAMA….45
41. **Representative alignments of core protein components associated with DUF499-centered Type-2 BREX-related systems**
   (i) DUF499-ATPase (Representative 1)….46
   (ii) Methyltransferase (PglXI-homolog; Representative 1 and 2)….47
   (iii) Standalone PglZ….48
   (iv) Inactive STAND (BrxB/BrxF-homolog; Representative 1 and 2)….49
   (v) Helicase without nuclease fusion (BrxHII homolog)….50
42. Type-4 BREX; Cysteine desulfurase….51
43. Type-4 BREX; BrxP (DndC homolog; PAPS-reduct)….51
44. Dnd-system PAPS-reductase; DndC….51
45. SSP-systems PAPS-reductase; SspD….51
46. OLD-ABC+TOPRIM (Previously classified OLD-ABCs associated with ABC-ATPase centric conflict systems)….52
47. BREX-systems OLD-ABCs (TOPRIM associated)….52
48. **Representative alignments of core protein components associated with the novel HerA/FtsK-PglZ anchored Capture systems**
   (i) HerA translocase….53

(ii) GNAT….54
(iii) tRNA-guanine transglycosylase (TGT)….55
(iv) DUF6079 (BrxC-counterpart)….56
(v) PglZ (BrxZ/PglZ counterpart)….57
(vi) DUF4007 (BrxA counterpart)….58
(vii) STAND-NTPase (BrxB counterpart….59
49. **Fast-evolving domain candidates with high entropy (Represented in the shannon entropy plot alongside their corresponding sequence alignment)**
(i) Type-1 BREX; BrxC (α+β-domain + wHTH)….61
(ii) Type-3 BREX; BrxC (α+β-domain + wHTH)….63
(iii) Type-4 BREX; BrxC (α+β-domain + wHTH)….65
(iv) BR-systems; DUF499-ATPase (α+β-domain + wHTH)….67
(v) Type-1 BREX; PglZ iSwi1/Snf2….69
(vi) Type-2 BREX; PglZ iSwi1/Snf2….71
(vii) Type-3 BREX; PglZ iSwi1/Snf2….73
(viii) Type-4 BREX; PglZ iSwi1/Snf2 (Representative 1)….75
(ix) Type-4 BREX; PglZ iSwi1/Snf2 (Representative 2)….77
(x) BRC-systems; PglZ iSwi1/Snf2….79
(xi) Type-1 BREX; PglZ C-terminal β-sandwich IG-like….81
(xii) Type-3 BREX; PglZ C-terminal β-sandwich IG-like (Representative 1)….83
(xiii) Type-3 BREX; PglZ C-terminal β-sandwich IG-like (Representative 2)….85
(xiv) Type-1 BREX; BrxB-iSTAND….87
(xv) Type-2 BREX; PglW C-terminal iSTAND….89
(xvi) Type-3 BREX; BrxF-iSTAND….91
(xvii) Type-4 BREX; PglZ N-terminal iSTAND….93
(xviii) Type-3 BR-systems; iSTAND….95
(xix) MoxR-vWA Ternary systems; iSTAND….97
(xx) Type-3 BREX; BrxHII-Helicase C-terminal iREase….99
(xxi) BR-systems; Helicase iREase (Representative 1)….101
(xxii) BR-systems; Helicase iREase (Representative 2)….103
(xxiii) BR-system; DUF499 C-terminal RRM/Ferredoxin (Representative 1)….105
(xxiii) BR-system; DUF499 C-terminal RRM/Ferredoxin (Representative 2)….107
(xxiv) BR-system; DUF499 C-terminal RRM/Ferredoxin (Representative 3)….109
(xxv) BR-system; DUF499 C-terminal RRM/Ferredoxin (Representative 4)….111
(xxvi) BR-system; DUF499 C-terminal RRM/Ferredoxin (Representative 5)….113
(xxvii) Type-2 BR-system; DUF499 C-terminal FnIII+FnIII….115

***Titles are internally hyperlinked. Click on title to access the material***

## ; Type1_BrxC-ATPase; All-Merged Reference Alignment (ORC-CDC6-AAA+ATPase + wHTH + Alpha+Beta-Domain + wHTH + Coiled-Coil)

[illegible]

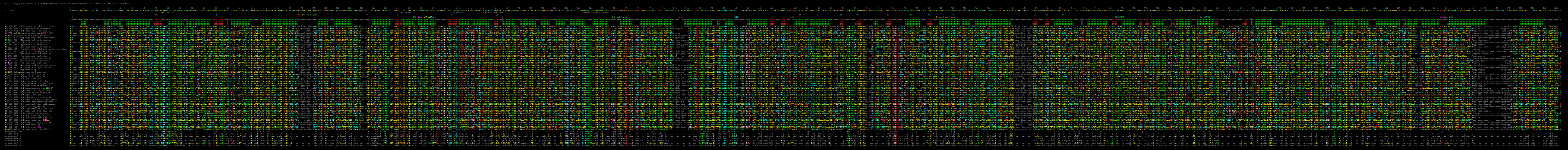
## ; Type2_BrxC-ATPase; ORC-Cdc6-AAA+ATPase + wHTH + Alpha+Beta-Domain + 3S-wHTH1 + 3SwHTH2 + Coiled-Coil
OrigSeq
--Walker-A--
WP_038566393.1_Dermacoccus_nishinomiyaensis
WP_015443641.1_Ilumatobacter_coccineus
WP_276096852.1_Streptomyces_silvisoli
EEP73860.1_Micromonospora_sp._ATCC_39149
HUP68595.1_Acidimicrobiales_bacterium
HUY49759.1_Streptosporangiaceae_bacterium
MBK6942983.1_Planctomycetota_bacterium
MBK9578953.1_Fibrobacterota_bacterium
MBL8678385.1_Myxococcales_bacterium
MBL8842560.1_Planctomycetota_bacterium
MBX3168148.1_Candidatus_Eremiobacteraeota_bacterium
MBX3732320.1_Verrucomicrobiae_bacterium
MCB9398403.1_Acidobacteriota_bacterium
MCB9544268.1_Myxococcales_bacterium
MCB9660515.1_Sandaracinaceae_bacterium
MCE9632143.1_Planctomycetia_bacterium
MCG5052490.1_Myxococcales_bacterium
MCP5560212.1_Verrucomicrobiaceae_bacterium
MDJ0841533.1_Acidobacteriota_bacterium
MDX2267520.1_Bryobacter_sp.
MSW49533.1_Actinomycetia_bacterium
WP_030506727.1_Microbispora_rosea
WP_052424042.1_Nonomuraea_candida
WP_061229212.1_Dietzia_cinnamea
WP_071086063.1_Parafrankia_colletiae
WP_073883613.1_Corynebacterium_variabile
WP_075083674.1_Mariniblastus_fucicola
WP_123558026.1_Micromonospora_sp._HM5-17
WP_123661993.1_Actinocorallia_herbida
WP_131153514.1_Egibacter_rhizosphaerae
WP_144981417.1_Gimesia_aquarii
WP_161103223.1_Actinomadura_rayongensis
WP_165632998.1_unclassified_Actinopolyspora
WP_184886687.1_Sphaerisporangium_siamense
WP_191733017.1_Corynebacterium_gallinarum
WP_199044191.1_Glycomyces_salinus
WP_200346143.1_Halochromatium_glycolicum
WP_222632854.1_unclassified_Rhodococcus
WP_245209875.1_Ralstonia_solanacearum
WP_247539401.1_Bradyrhizobium_sp._168
WP_256246191.1_Pseudarthrobacter_sp._R1
WP_267686051.1_Nannocystis_sp._SCPEA4
WP_270679261.1_Nocardiopsis_sp._LSu2-4
WP_296554869.1_Rhodococcus_sp.
WP_322753649.1_Frankia_sp._Cas3
WUA45283.1_Streptomyces_sp._NBC_01367
consensus/100%
consensus/95%
consensus/90%
consensus/85%
consensus/80%
consensus/75%
consensus/70%

## ; Type3_BrxC-ATPase; STAND/ORC-Cdc6-AAA+ATPase + wHTH + Alpha+Beta-Domain + 4S-wHTH + Coiled-Coil

```
OrigSeq [illegible]
WP_164569833.1_Rhizobium_leguminosarum [illegible]
SDB48716.1_Belnapia_rosea [illegible]
MBA4157055.1_Gemmatimonadota_bacterium [illegible]
WP_190509459.1_Oscillatoria_sp. [illegible]
RJQ83728.1_Desulfobacteraceae_bacterium [illegible]
WP_013219148.1_Nitrosococcus_watsonii [illegible]
GHU24474.1_Spirochaetia_bacterium [illegible]
WP_209691006.1_Methanomicrobium_sp. [illegible]
NMB01453.1_Bacillota_bacterium [illegible]
MCK9328444.1_Candidatus_Cloacimonadota_bacterium [illegible]
WP_047754172.1_Kosmotoga_pacifica [illegible]
MCB5228480.1_Candidatus_Cloacimonadota_bacterium [illegible]
OBW63497.1_Dehalococcoides_mccartyi [illegible]
MBX3742594.1_Akkermansiaceae_bacterium [illegible]
MBT3747344.1_Bacteroidota_bacterium [illegible]
WP_106343732.1_Planifilum_fimeticola [illegible]
WP_254843978.1_Virgibacillus_dokdonensis [illegible]
WP_235850150.1_Niallia_circulans [illegible]
WP_146342557.1_Dellaglioa_algida [illegible]
WP_229736080.1_Kroppenstedtia_guangzhouensis [illegible]
MCP4110753.1_Desulfobacteraceae_bacterium [illegible]
NUM38593.1_Armatimonadota_bacterium [illegible]
MCM1566961.1_Dehalobacter_sp. [illegible]
MBU0460852.1_Nanoarchaeota_archaeon [illegible]
KFI36375.1_Peptococcaceae_bacterium [illegible]
EMR73852.1_Thermoplasmatales_archaeon [illegible]
WP_114474457.1_Halanaerobium_sp. [illegible]
WP_098658108.1_Bacillus_toyonensis [illegible]
MCL4298469.1_Anaerolineae_bacterium [illegible]
MBP7946969.1_Verrucomicrobiota_bacterium [illegible]
MBT9159326.1_Dehalococcoidia_bacterium [illegible]
NLF03150.1_Anaerolineales_bacterium [illegible]
GFP32117.1_Candidatus_Hakubanella_thermoalkaliphilus [illegible]
HIH77326.1_Halobacteria_archaeon [illegible]
MBP6974014.1_Syntrophorhabdus_sp. [illegible]
HHW29753.1_Syntrophomonadaceae_bacterium [illegible]
MBT6646600.1_Nitrososphaerota_archaeon [illegible]
WP_053959405.1_Sulfobacillus_thermosulfidooxidans [illegible]
WP_020614466.1_Sediminispirochaeta_bajacaliforniensis [illegible]
WP_149544905.1_Calorimonas_adulescens [illegible]
NMA94940.1_Clostridiales_bacterium [illegible]
WP_209451817.1_Lactimicrobium_massiliense [illegible]
EGD52432.1_Thermoanaerobacter_ethanolicus [illegible]
NLV27395.1_Methanomicrobiales_archaeon [illegible]
consensus/100% [illegible]
consensus/95% [illegible]
consensus/90% [illegible]
consensus/85% [illegible]
consensus/80% [illegible]
consensus/75% [illegible]
consensus/70% [illegible]
```

## ; Type4_BrxC-ATPase (Version 1); ORC-Cdc6-AAA+ATPase + wHTH + Alpha+Beta-Domain + wHTH + Coiled-Coil

OrigSeq [illegible]

--Walker-A--

S1 S2 <---HEH-Insert-Region--- [illegible]

```
WP_165846692.1_Candidatus_Cryosericum_hinesii [illegible]
WP_169366114.1_Pseudanabaena_yagii [illegible]
HAR58250.1_Acholeplasmatales_bacterium [illegible]
HHW21153.1_Thermodesulfovibrio_thiophilus [illegible]
KUK66683.1_Parcubacteria_bacterium_34_609 [illegible]
MAU00653.1_Anaerolineaceae_bacterium [illegible]
MBD3337553.1_Candidatus_Lokiarchaeota_archaeon [illegible]
MBF0303406.1_Desulfamplus_sp. [illegible]
MBF2075132.1_Synechococcales_cyanobacterium [illegible]
MBK9943588.1_Kouleothrix_sp. [illegible]
MBN2678363.1_Anaerolineaceae_bacterium [illegible]
MBR3710350.1_Bacteroidales_bacterium [illegible]
MBU7048060.1_Theionarchaea_archaeon [illegible]
MBX7251231.1_Candidatus_Promineofilum_sp. [illegible]
MCI9127450.1_Eubacterium_sp. [illegible]
MCK4843012.1_Methylococcales_bacterium [illegible]
MCL6576920.1_Kyrpidia_sp. [illegible]
NJL05929.1_Chloroflexaceae_bacterium [illegible]
NLT83665.1_Coprothermobacter_proteolyticus [illegible]
OQC38485.1_Bacteroidetes_bacterium_ADurb.Bin041 [illegible]
OQC39308.1_Bacteroidetes_bacterium_ADurb.Bin037 [illegible]
PMQ01344.1_Dictyoglomus_sp._NZ13-RE01 [illegible]
PSB31008.1_Stenomitos_frigidus_ULC18 [illegible]
QEP42413.1_Ectothiorhodospiraceae_bacterium [illegible]
WP_048175326.1_Methanothermobacter_sp. [illegible]
WP_055301605.1_Roseburia_inulinivorans [illegible]
WP_062196491.1_Anaerolinea_thermolimosa [illegible]
WP_062417761.1_Levilinea_saccharolytica [illegible]
WP_139515175.1_Hymenobacter_jejuensis [illegible]
WP_140468254.1_Hymenobacter_nivis [illegible]
WP_143714756.1_Thermomicrobium_roseum [illegible]
WP_189070975.1_Deinococcus_radiotolerans [illegible]
WP_195558544.1_Bacteroides [illegible]
WP_201382002.1_Ktedonobacter_sp. [illegible]
WP_214462063.1_Bacteroidaceae [illegible]
WP_219156489.1_Hymenobacter_profundi [illegible]
WP_223980148.1_Rivularia_sp. [illegible]
WP_235137407.1_Dyadobacter_fanqingshengii [illegible]
consensus/100% [illegible]
consensus/95% [illegible]
consensus/90% [illegible]
consensus/85% [illegible]
consensus/80% [illegible]
consensus/75% [illegible]
consensus/70% [illegible]
```

## ; Type4_BrxC-ATPase (Version 2); ORC-Cdc6-AAA+ATPase + wHTH + Alpha+Beta-Domain + wHTH + Coiled-Coil

1---------11---------21 [illegible]

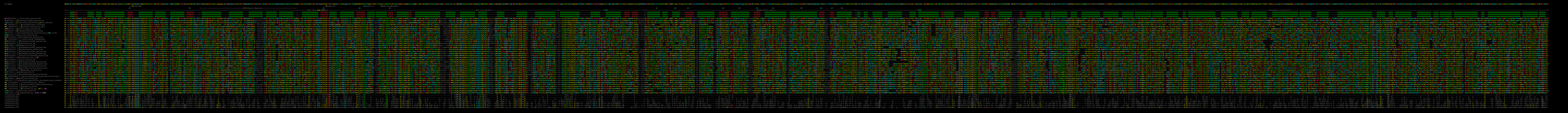

OrigSeq
--Walker-A--
S1
S2
HEH-Insert-Region
MCD8003208.1_Clostridia_bacterium
HCH97636.1_Lachnospiraceae_bacterium
OQB25825.1_Firmicutes_bacterium_ADurb.Bin182
WP_048095117.1_Archaeoglobus_fulgidus
WP_198469750.1_Acetomicrobium_sp._S15
NPV08139.1_Anaerolineae_bacterium
HED00229.1_Pseudomonadota_bacterium
SHE91973.1_Desulfofundulus_australicus_DSM_11792
NSW92344.1_Bacillota_bacterium
MBF0318625.1_Nitrospirota_bacterium
WP_195464807.1_Clostridium_jeddahense
MBR4835363.1_Thermoguttaceae_bacterium
MBR6984229.1_Ruminococcus_sp.
NLY01889.1_Rhodopirellula_sp.
MBR0237621.1_Thermoguttaceae_bacterium
MBP6982276.1_Thermotogota_bacterium
MBR2693833.1_Thermoguttaceae_bacterium
MBQ7111119.1_Thermoguttaceae_bacterium
MCL2016625.1_Defluviitaleaceae_bacterium
NBJ16968.1_Dehalobacter_sp._4CP
MBD5156842.1_Butyrivibrio_sp.
MBQ4143519.1_Thermoguttaceae_bacterium
MBR2520311.1_Selenomonadaceae_bacterium
MBQ9371615.1_Thermoguttaceae_bacterium
MBQ7256248.1_Abditibacteriota_bacterium
WP_215692602.1_Gemmiger_formicilis
NBK77201.1_bacterium_D16-76
MBR2215718.1_Selenomonadaceae_bacterium
WP_268058969.1_Caproiciproducens_galactitolivorans
MZQ76088.1_Peptoclostridium_sp.
HIS35237.1_Candidatus_Scatousia_excrementigallinarum
WP_103226893.1_Clostridium_sp._chh4-2
WP_031492453.1_Succinivibrio_dextrinosolvens
WP_123481433.1_Lepagella_muris
WP_118016040.1_Mitsuokella_sp._AF21-1AC
RKI68143.1_bacterium_1xD42-67
ONI45222.1_Epulopiscium_sp._SCG-C07WGA
consensus/100%
consensus/95%
consensus/90%
consensus/85%
consensus/80%
consensus/75%
consensus/70%

## ; Type 1 BREX MTase; N-Terminal-Domain + N6_METHYLASE + Target-Recognition-Domain(TRD) + Double_Helical_Spacer + wHTH + Coiled-Coil(CC)

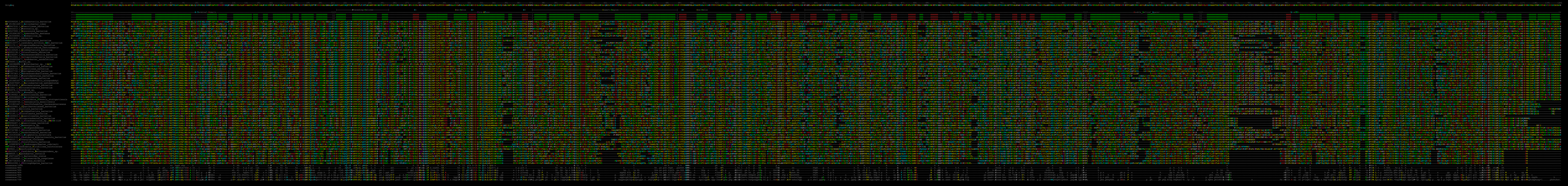

## ; Type 2 BREX MTase; N-Terminal-Domain + N6_METHYLASE + Target-Recognition-Domain(TRD) + Coiled-Coil(CC)

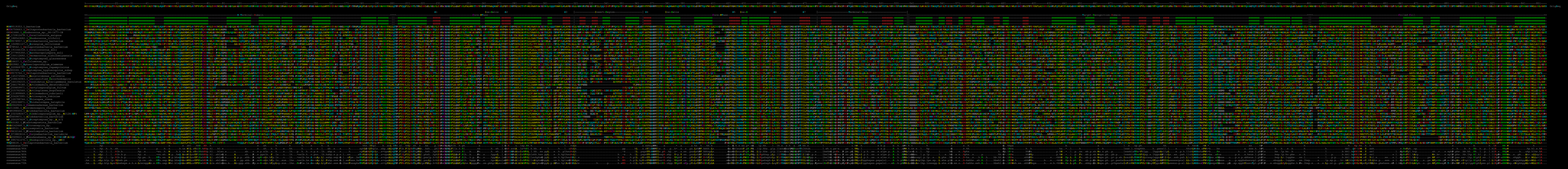

## ; Type 3 BREX MTase (DUF499 system like MTase); N-terminal-Preceding-Region + N6_METHYLASE(TRD-Insert) + HTH1 + wHTH + HTH2

OrigSeq

[illegible]

## ; Type_1_BREX PglZ Representative_1; iSwi2/SNF2-Helicase + 3-HTH + Helical-Connector + Alkaline-Phosphatase + All-Beta-Sandwich-IG-like

```
OrigSeq [illegible]
WP_238754868.1_Leptospira_bandrabouensis [illegible]
MBF0258418.1_Desulfamplus_sp. [illegible]
WP_009097832.1_Rhodopirellula_sp._SWK7 [illegible]
MBX3247846.1_Myxococcales_bacterium [illegible]
MBT9554533.1_Myxococcales_bacterium [illegible]
MCC6649976.1_Candidatus_Eisenbacteria_bacterium [illegible]
KPQ33026.1_Phormidesmis_priestleyi_Ana [illegible]
MBD2549130.1_Microcystis_elabens_FACHB-917 [illegible]
WP_096450969.1_Thauera_sp._K11 [illegible]
MBY3465673.1_Rhizobium_laguerreae [illegible]
MBY0113210.1_Phycisphaerales_bacterium [illegible]
WP_273378697.1_Symbiobacterium_thermophilum [illegible]
WP_209466794.1_Symbiobacterium_terraclitae [illegible]
MBX6362428.1_Gemmatimonadota_bacterium [illegible]
MBW2308572.1_Deltaproteobacteria_bacterium [illegible]
WP_083610988.1_Desulfatibacillum_alkenivorans [illegible]
NIM12894.1_Candidatus_Aminicenantes_bacterium [illegible]
MBN1165673.1_Methanospirillaceae_archaeon [illegible]
MCI0601819.1_bacterium [illegible]
WP_062190142.1_Anaerolinea_thermolimosa [illegible]
WP_150114001.1_Leptospirillum_ferriphilum [illegible]
WP_156746889.1_Mycobacterium_sp._E2733 [illegible]
MBN2413376.1_candidate_division_KSB1_bacterium [illegible]
WP_164995432.1_Candidatus_Kuenenia_stuttgartiensis [illegible]
MBV6422080.1_Ignavibacteriaceae_bacterium [illegible]
WP_083423498.1_Stigmatella_aurantiaca [illegible]
WP_218933729.1_Rubripirellula_lacrimiformis [illegible]
NLN06912.1_Bacillota_bacterium [illegible]
WP_055669757.1_Desnuesiella_massiliensis [illegible]
WP_242609850.1_Comamonas_thiooxydans [illegible]
WP_015282546.1_Thioflavicoccus_mobilis [illegible]
WP_243520949.1_Hymenobacter_monticola [illegible]
OQB98762.1_Spirochaetes_bacterium_ADurb.Bin110 [illegible]
WP_166386808.1_Polaribacter_sp._11A2H [illegible]
WP_235164441.1_Dyadobacter_chenhuakuii [illegible]
MCP5535438.1_Akkermansiaceae_bacterium [illegible]
consensus/100% [illegible]
consensus/95% [illegible]
consensus/90% [illegible]
consensus/85% [illegible]
consensus/80% [illegible]
consensus/75% [illegible]
consensus/70% [illegible]
```

## ; Type_1_BREX PglZ Representative_2; iSwi2/SNF2-Helicase + 3-HTH + Helical-Connector + Alkaline-Phosphatase + All-Beta-Sandwich-IG-Like

```
OrigSeq [illegible]
MCC6145260.1_Candidatus_Hydrogenedentes_bacterium [illegible]
MBN9589012.1_Alphaproteobacteria_bacterium [illegible]
SUA58557.1_Oligella_ureolytica [illegible]
WP_197678758.1_Janthinobacterium_lividum [illegible]
WP_002628220.1_Cystobacter_fuscus [illegible]
WP_100919682.1_Candidatus_Thiodictyon_syntrophicum [illegible]
MBW6486418.1_Syntrophobacterales_bacterium [illegible]
WP_004078483.1_Methanoplanus_limicola [illegible]
WP_011448105.1_Methanospirillum_hungatei [illegible]
WP_217922213.1_Miltoncostaea_oceani [illegible]
WP_104992436.1_Deinococcus_sp._NW-56 [illegible]
WP_030150652.1_Oerskovia_turbata [illegible]
WP_248598775.1_Agromyces_sp._C10 [illegible]
WP_222877737.1_Mycolicibacterium_austroafricanum [illegible]
MXZ45724.1_Chloroflexota_bacterium [illegible]
MBK9494689.1_Xanthomonadales_bacterium [illegible]
MBA4013264.1_Phenylobacterium_sp. [illegible]
WP_146522118.1_Stieleria_varia [illegible]
MXX75215.1_Holophagales_bacterium [illegible]
NBT25026.1_Actinomycetia_bacterium [illegible]
GDX95760.1_Planctomycetia_bacterium [illegible]
WP_246481782.1_Natronogracilivirga_saccharolytica [illegible]
MBQ6007748.1_Kiritimatiellia_bacterium [illegible]
WP_091825284.1_Butyrivibrio_sp._ob235 [illegible]
WP_215427522.1_Akkermansia_muciniphila [illegible]
MBP8650767.1_Deltaproteobacteria_bacterium [illegible]
consensus/100% [illegible]
consensus/95% [illegible]
consensus/90% [illegible]
consensus/85% [illegible]
consensus/80% [illegible]
consensus/75% [illegible]
consensus/70% [illegible]
```

## ; Type_1_BREX PglZ Representative_3; iSwi2/SNF2-Helicase + HTH + Helical-Connector + Alkaline-Phosphatase + All-Beta-Sandwich-IG-Like

```
OrigSeq [illegible]
WP_219938192.1_Succinivibrio_faecicola [illegible]
MCK6605714.1_Ignavibacteriaceae_bacterium [illegible]
MCH3907672.1_Sphaerochaeta_sp. [illegible]
WP_251178637.1_Adlercreutzia_agrestimuris [illegible]
MCC6107936.1_Atopobium_sp. [illegible]
MCI1665139.1_Atopobiaceae_bacterium [illegible]
MBQ6502306.1_Flexilinea_sp. [illegible]
WP_237187173.1_Rothia_nasimurium [illegible]
WP_149431761.1_Rhodococcus_cavernicola [illegible]
OFQ22229.1_Actinomyces_sp._HMSC062G12 [illegible]
MBX3116434.1_Cryobacterium_sp. [illegible]
HJG42481.1_Corynebacterium_phoceense [illegible]
WP_219100161.1_Bifidobacterium_pluvialisilvae [illegible]
MBI9030469.1_bacterium [illegible]
MBU6197218.1_Cyanobacteria_bacterium_REEB446 [illegible]
MBD1206695.1_Paracoccaceae_bacterium [illegible]
HBJ35655.1_Planctomycetaceae_bacterium [illegible]
QNI65417.1_Synechococcus_sp._A15-44 [illegible]
WP_147932466.1_Neolewinella_aurantiaca [illegible]
WP_013460554.1_Sulfuricurvum_kujiense [illegible]
WP_260916970.1_Aliarcobacter_butzleri [illegible]
MCF7790535.1_Victivallales_bacterium [illegible]
WP_153112503.1_Prevotella_copri [illegible]
MBK9103302.1_Saprospiraceae_bacterium [illegible]
WP_006745114.1_Bacteroides_coprosuis [illegible]
MAO64830.1_Balneola_sp. [illegible]
WP_053405041.1_Persicobacter_sp._CCB-QB2 [illegible]
NLN95591.1_Bacteroidales_bacterium [illegible]
MBW8002950.1_Planctomycetota_bacterium [illegible]
WP_262567766.1_Endozoicomonas_gorgoniicola [illegible]
MBQ2845006.1_Alphaproteobacteria_bacterium [illegible]
WP_156790427.1_Alcanivorax_sp._DG881 [illegible]
OYU71397.1_Alphaproteobacteria_bacterium_PA2 [illegible]
MBN1905583.1_Deltaproteobacteria_bacterium [illegible]
MCI7405010.1_Desulfovibrio_piger [illegible]
TXI44184.1_Methylophilus_sp. [illegible]
WP_006086471.1_Shewanella_baltica [illegible]
WP_216328025.1_Deinococcus_aestuarii [illegible]
WP_151792240.1_Acinetobacter_seifertii [illegible]
MCA6503069.1_Pseudanabaena_sp. [illegible]
WP_186816219.1_Deinococcus_cellulosilyticus [illegible]
MBP7846546.1_Burkholderiales_bacterium [illegible]
consensus/100% [illegible]
consensus/95% [illegible]
consensus/90% [illegible]
consensus/85% [illegible]
consensus/80% [illegible]
consensus/75% [illegible]
consensus/70% [illegible]
```

## ; Type_2_BREX PglZ Representative_1; iSwi2/SNF2-Helicase + 3-HTH + Helical-Connector + Alkaline-Phosphatase + 3s-wHTH

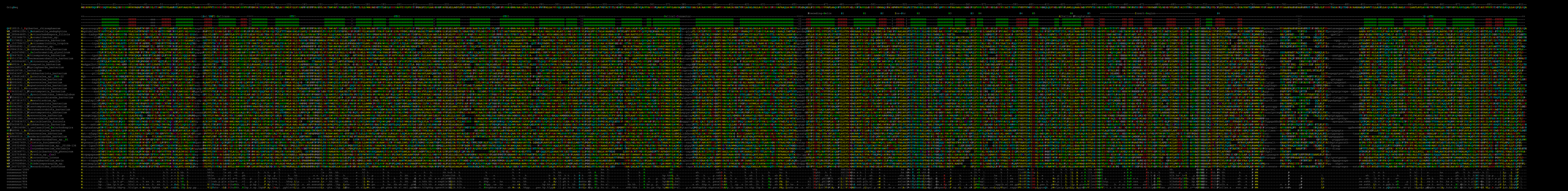

## ; Type_2_BREX PglZ Representative_2; iSwi2/SNF2-Helicase + 3-HTH + Helical-Connector + Alkaline-Phosphatase + 3s-wHTH

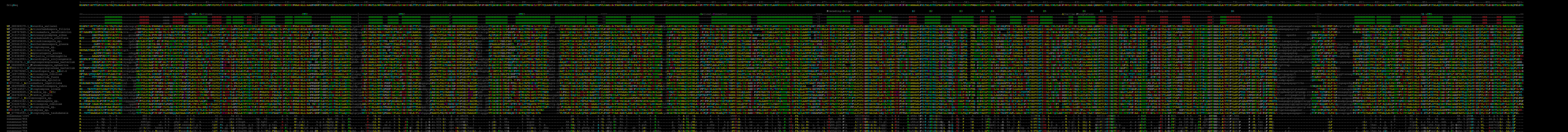

## ; Type_3_BREX PglZ Representative_1; iSwi2/SNF2-Helicase + 3-HTH + Helical-Connector + Alkaline-Phosphatase

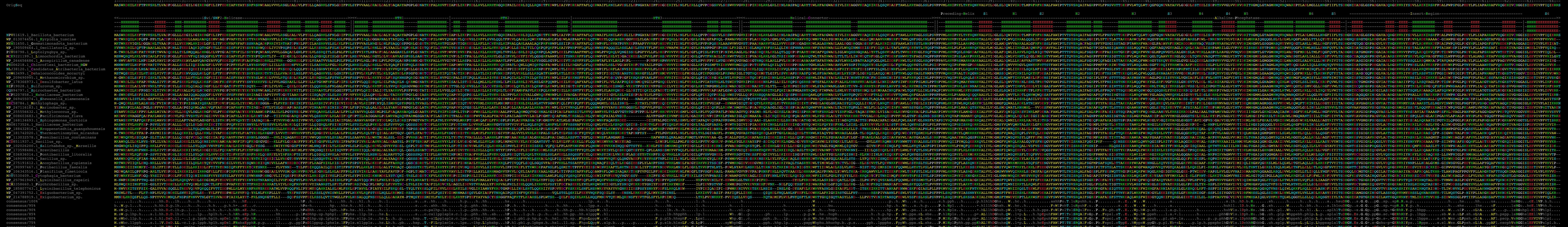

## ; Type_3_BREX PglZ Representative_2; iSwi2/SNF2-Helicase + HTH + Helical-Connector + Alkaline-Phosphatase

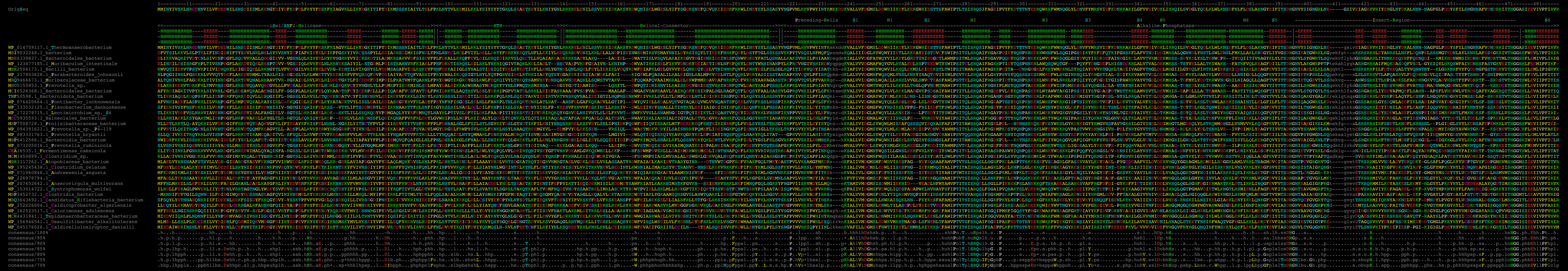

## ; Type_4_BREX PglZ Representative_1; iSTAND-NTPase + iSwi/SNF2-Helicase + 3-HTH + Helical-Connector + Alkaline-Phosphatase + All-Beta-Sandwich-IG-Like

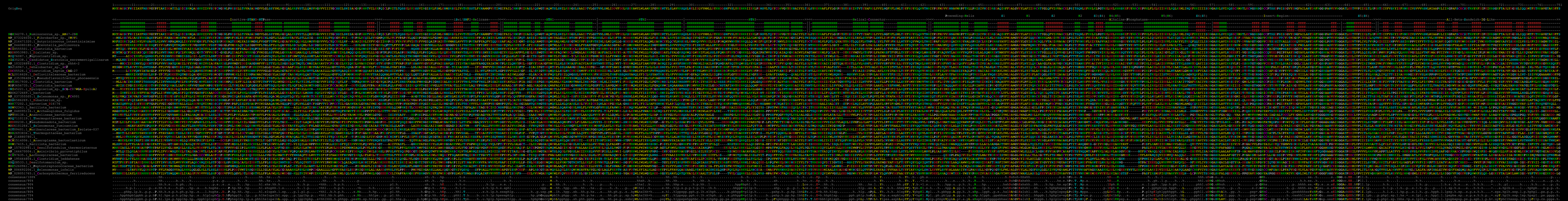

## ; Type_4_BREX PglZ Representative_2; iSTAND-NTPase + iSwi/SNF2-Helicase + 3-HTH + Helical-Connector + Alkaline-Phosphatase + All-Beta-Sandwich-IG-Like

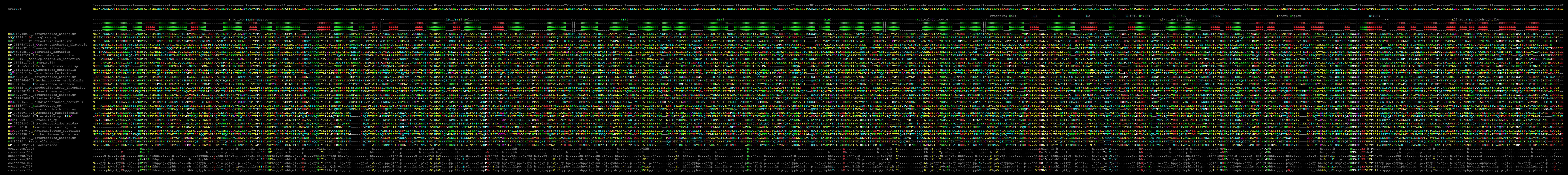

## ; Type_4_BREX PglZ Representative_3; iSwi/SNF2-Helicase + 3-HTH + Helical-Connector + Alkaline-Phosphatase + All-Beta-Sandwich-IG-Like

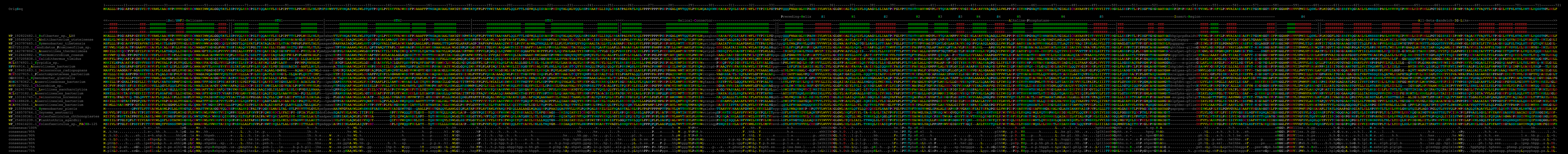

## ; Type_4_BREX PglZ Representative_4; iSwi/SNF2-Helicase + 2-HTH + Helical-Connector + Alkaline-Phosphatase + All-Beta-Sandwich-IG-Like

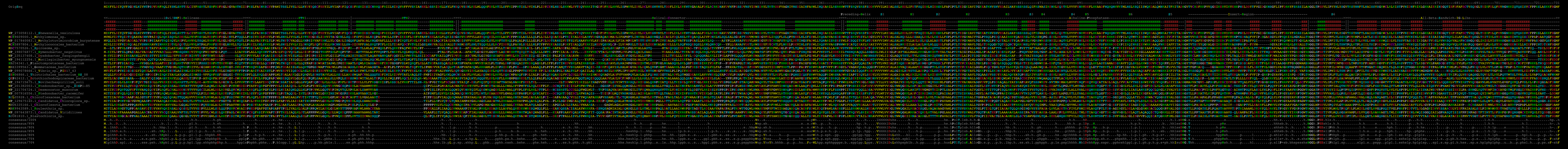

```
## ; BREX PglZ Phosphatase and PorX PglZ Phosphatase Comparison Alignment

                                                   Alkaline-Phosphatase [illegible]
                                                   [illegible]
                                                   [illegible]
                                                   [illegible]
WP_238754868.1_Leptospira_bandrabouensis           [illegible]
MBF0258418.1_Desulfamplus_sp.                      [illegible]
MBX3247846.1_Myxococcales_bacterium                [illegible]
MBD2549130.1_Microcystis_elabens_FACHB-917         [illegible]
MBY0113210.1_Phycisphaerales_bacterium             [illegible]
MBX6362428.1_Gemmatimonadota_bacterium             [illegible]
WP_083610988.1_Desulfatibacillum_alkenivorans      [illegible]
MBN1165673.1_Methanospirillaceae_archaeon          [illegible]
WP_156746889.1_Mycobacterium_sp._E2733             [illegible]
MBV6422080.1_Ignavibacteriaceae_bacterium          [illegible]
WP_083423498.1_Stigmatella_aurantiaca              [illegible]
NLN06912.1_Bacillota_bacterium                     [illegible]
WP_242609850.1_Comamonas_thiooxydans               [illegible]
WP_243520949.1_Hymenobacter_monticola              [illegible]
OQB98762.1_Spirochaetes_bacterium_ADurb.Bin110     [illegible]
WP_166386808.1_Polaribacter_sp._11A2H              [illegible]
WP_235164441.1_Dyadobacter_chenhuakuii             [illegible]
MCP5535438.1_Akkermansiaceae_bacterium             [illegible]
MCC6145260.1_Candidatus_Hydrogenedentes_bacterium  [illegible]
MBN9589012.1_Alphaproteobacteria_bacterium         [illegible]
WP_100919682.1_Candidatus_Thiodictyon_syntrophicum [illegible]
WP_011448105.1_Methanospirillum_hungatei           [illegible]
NBT25026.1_Actinomycetia_bacterium                 [illegible]
WP_246481782.1_Natronogracilivirga_saccharolytica  [illegible]
MBQ6007748.1_Kiritimatiellia_bacterium             [illegible]
WP_091825284.1_Butyrivibrio_sp._ob235              [illegible]
WP_219938192.1_Succinivibrio_faecicola             [illegible]
MCK6605714.1_Ignavibacteriaceae_bacterium          [illegible]
MCH3907672.1_Sphaerochaeta_sp.                     [illegible]
WP_251178637.1_Adlercreutzia_agrestimuris          [illegible]
MBQ6502306.1_Flexilinea_sp.                        [illegible]
WP_237187173.1_Rothia_nasimurium                   [illegible]
OFQ22229.1_Actinomyces_sp._HMSC062G12              [illegible]
QNI65417.1_Synechococcus_sp._A15-44                [illegible]
MCF7790535.1_Victivallales_bacterium               [illegible]
MBW8002950.1_Planctomycetota_bacterium             [illegible]
WP_262567766.1_Endozoicomonas_gorgoniicola         [illegible]
MBQ2845006.1_Alphaproteobacteria_bacterium         [illegible]
WP_216328025.1_Deinococcus_aestuarii               [illegible]
WP_151792240.1_Acinetobacter_seifertii             [illegible]
MBP7846546.1_Burkholderiales_bacterium             [illegible]
7PVK_1|Chains                                      [illegible]
7PVA_1|Chains                                      [illegible]
8TED_1|Chains                                      [illegible]
8TFF_1|Chains                                      [illegible]
8THP_1|Chains                                      [illegible]
consensus/100%                                     [illegible]
consensus/95%                                      [illegible]
consensus/90%                                      [illegible]
consensus/85%                                      [illegible]
consensus/80%                                      [illegible]
consensus/75%                                      [illegible]
consensus/70%                                      [illegible]
Metal-ion-Co-ordinating Residues: ^                [illegible]
```

```
## ; BREX_PglZ - PorX_PglZ - Type-3 BR system PglZ; Comparison Alignment

WP_238754868.1_Leptospira_bandrabouensis            [illegible]
MBF0258418.1_Desulfamplus_sp.                       [illegible]
MBX3247846.1_Myxococcales_bacterium                 [illegible]
MBD2549130.1_Microcystis_elabens_FACHB-917          [illegible]
MBY0113210.1_Phycisphaerales_bacterium              [illegible]
MBX6362428.1_Gemmatimonadota_bacterium              [illegible]
WP_083610988.1_Desulfatibacillum_alkenivorans       [illegible]
MBN1165673.1_Methanospirillaceae_archaeon           [illegible]
WP_156746889.1_Mycobacterium_sp._E2733              [illegible]
MBV6422080.1_Ignavibacteriaceae_bacterium           [illegible]
WP_083423498.1_Stigmatella_aurantiaca               [illegible]
NLN06912.1_Bacillota_bacterium                      [illegible]
WP_242609850.1_Comamonas_thiooxydans                [illegible]
WP_243520949.1_Hymenobacter_monticola               [illegible]
OQB98762.1_Spirochaetes_bacterium_ADurb.Bin110      [illegible]
WP_166386808.1_Polaribacter_sp._11A2H               [illegible]
WP_235164441.1_Dyadobacter_chenhuakuii              [illegible]
MCP5535438.1_Akkermansiaceae_bacterium              [illegible]
MCC6145260.1_Candidatus_Hydrogenedentes_bacterium   [illegible]
MBN9589012.1_Alphaproteobacteria_bacterium          [illegible]
WP_100919682.1_Candidatus_Thiodictyon_syntrophicum  [illegible]
WP_011448105.1_Methanospirillum_hungatei            [illegible]
NBT25026.1_Actinomycetia_bacterium                  [illegible]
WP_246481782.1_Natronogracilivirga_saccharolytica   [illegible]
MBQ6007748.1_Kiritimatiellia_bacterium              [illegible]
WP_091825284.1_Butyrivibrio_sp._ob235               [illegible]
WP_219938192.1_Succinivibrio_faecicola              [illegible]
MCK6605714.1_Ignavibacteriaceae_bacterium           [illegible]
MCH3907672.1_Sphaerochaeta_sp.                      [illegible]
WP_251178637.1_Adlercreutzia_agrestimuris           [illegible]
MBQ6502306.1_Flexilinea_sp.                         [illegible]
WP_237187173.1_Rothia_nasimurium                    [illegible]
OFQ22229.1_Actinomyces_sp._HMSC062G12               [illegible]
QNI65417.1_Synechococcus_sp._A15-44                 [illegible]
MCF7790535.1_Victivallales_bacterium                [illegible]
MBW8002950.1_Planctomycetota_bacterium              [illegible]
WP_262567766.1_Endozoicomonas_gorgoniicola          [illegible]
MBQ2845006.1_Alphaproteobacteria_bacterium          [illegible]
WP_216328025.1_Deinococcus_aestuarii                [illegible]
WP_151792240.1_Acinetobacter_seifertii              [illegible]
MBP7846546.1_Burkholderiales_bacterium              [illegible]
7PVK_1|Chains                                       [illegible]
7PVA_1|Chains                                       [illegible]
8TED_1|Chains                                       [illegible]
8TFF_1|Chains                                       [illegible]
8THP_1|Chains                                       [illegible]
HCF99179.1_Chloroflexota_bacterium                  [illegible]
CAD6492703.1_Candidatus_Argoarchaeum_ethanivorans   [illegible]
HDN59917.1_Candidatus_Marinimicrobia_bacterium      [illegible]
HDQ70819.1_Chloroflexota_bacterium                  [illegible]
HEU5078765.1_Opitutaceae_bacterium                  [illegible]
HIE29205.1_Candidatus_Poribacteria_bacterium        [illegible]
HLE25735.1_Thermodesulfobacteriota_bacterium        [illegible]
HNQ22500.1_Phycisphaerae_bacterium                  [illegible]
HZX48547.1_Nitrospirota_bacterium                   [illegible]
KXA95579.1_candidate_division_MSBL1_archaeon        [illegible]
MBA3936147.1_Planctomycetota_bacterium              [illegible]
MBC7332925.1_Actinomycetia_bacterium                [illegible]
MBC7429624.1_Bacteriovorax_sp.                      [illegible]
MBE7508222.1_Planctomycetia_bacterium               [illegible]
MBI4523255.1_Deltaproteobacteria_bacterium          [illegible]
MBK6914963.1_Ignavibacteriales_bacterium            [illegible]
MBK8636967.1_Chromatiaceae_bacterium                [illegible]
MBM4466104.1_Chloroflexota_bacterium                [illegible]
MBN2145087.1_Candidatus_Auribacterota_bacterium     [illegible]
MBP6702581.1_Vicinamibacteria_bacterium             [illegible]
MBQ8111586.1_Kiritimatiellia_bacterium              [illegible]
MCD6507459.1_Candidatus_Poribacteria_bacterium      [illegible]
MCK8825904.1_Fuchsiella_alkaliacetigena             [illegible]
MCK9588325.1_Terrimicrobiaceae_bacterium            [illegible]
MCL0058393.1_Dehalococcoidia_bacterium              [illegible]
MCL0097130.1_Dehalococcoidia_bacterium              [illegible]
MDB6140354.1_Verrucomicrobiaceae_bacterium          [illegible]
MDD3269498.1_Syntrophomonadaceae_bacterium          [illegible]
MDD4099138.1_Lentisphaeria_bacterium                [illegible]
MDT3473331.1_Stenotrophomonas_maltophilia           [illegible]
MDU2146555.1_Paeniclostridium_sordellii             [illegible]
MEA1926980.1_Candidatus_Auribacterota_bacterium     [illegible]
MEA3344887.1_Chloroflexota_bacterium                [illegible]
MEA3401059.1_Armatimonadota_bacterium               [illegible]
NLX99245.1_Rhodopirellula_sp.                       [illegible]
NMX21981.1_ANME-1_cluster_archaeon_GoMg4            [illegible]
RLF12261.1_Thermoprotei_archaeon                    [illegible]
RLF82337.1_Thermococci_archaeon                     [illegible]
TEU13000.1_Anaerolineales_bacterium                 [illegible]
WP_083335243.1_Planococcus_glaciei                  [illegible]
WP_084067307.1_Desulfocicer_vacuolatum              [illegible]
WP_121919201.1_Haloplanus_aerogenes                 [illegible]
WP_125672296.1_Candidatus_Methanodesulfokores       [illegible]
WP_149537233.1_Siccirubricoccus_phaeus              [illegible]
WP_218932320.1_Adhaeretor_mobilis                   [illegible]
WP_257627367.1_Haloplanus_salinarum                 [illegible]
WP_259101347.1_Candidatus_Fervidibacter_sacchari    [illegible]
WP_260594794.1_Salinirubellus_salinus               [illegible]
WP_283022679.1_Bradyrhizobium_sp.                   [illegible]
consensus/85%                                       [illegible]
consensus/80%                                       [illegible]
consensus/75%                                       [illegible]
consensus/70%                                       [illegible]
```

```
## ; Type_1 BREX BrxL Representative_1; (SIGMA-HTH + OB-Fold + MCM-family-AAA+ATPase + Inactive-LonP)

OrigSeq                                             [illegible]

                                                    [illegible]
                                                    [illegible]
                                                    [illegible]
                                                    [illegible]
WP_270096522.1_Methanococcoides_alaskense           [illegible]
WP_199585971.1_Collinsella_sp._AF11-11              [illegible]
MBI2765421.1_Chloroflexota_bacterium                [illegible]
MBN1450020.1_Anaerolineales_bacterium               [illegible]
KYF76802.1_Sorangium_cellulosum                     [illegible]
MCD6506894.1_Candidatus_Poribacteria_bacterium      [illegible]
MXY51051.1_Gammaproteobacteria_bacterium            [illegible]
MCI0407274.1_Acidobacteriota_bacterium              [illegible]
WP_005980888.1_Fusobacterium_ulcerans               [illegible]
WP_244056943.1_Aeromonas_caviae                     [illegible]
SFX61200.1_Ruminococcus_flavefaciens                [illegible]
HBO56052.1_Leuconostoc_mesenteroides                [illegible]
MBK7688412.1_Elusimicrobiota_bacterium              [illegible]
MBI5489026.1_Deltaproteobacteria_bacterium          [illegible]
MBI4023516.1_Verrucomicrobiota_bacterium            [illegible]
MCD6317877.1_Candidatus_Aerophobetes_bacterium      [illegible]
MBU1143922.1_Bacillota_bacterium                    [illegible]
CCU85339.1_Mesotoga_infera                          [illegible]
OQC41885.1_Acidobacteria_bacterium.                 [illegible]
MBR4647499.1_Bacteroidales_bacterium                [illegible]
WP_242979527.1_Subdoligranulum_sp.                  [illegible]
AKB55566.1_Methanosarcina_barkeri_MS                [illegible]
OGO91754.1_Clostridiales_bacterium_GWF2_38_85       [illegible]
MBC8432678.1_Desulfobacterales_bacterium            [illegible]
MBX9637029.1_Nitrosomonas_sp.                       [illegible]
WP_110300682.1_Halanaerobium_congolense             [illegible]
WP_254547351.1_Halomarina_sp._BND7                  [illegible]
WP_264798996.1_Brevibacterium_sp._JSBI002           [illegible]
consensus/100%                                      [illegible]
consensus/95%                                       [illegible]
consensus/90%                                       [illegible]
consensus/85%                                       [illegible]
consensus/80%                                       [illegible]
consensus/75%                                       [illegible]
consensus/70%                                       [illegible]
```

## ; Type_1 BREX BrxL Representative_2; (SIGMA-HTH + OB-Fold + MCM-family-AAA+ATPase + HKD-DNase)

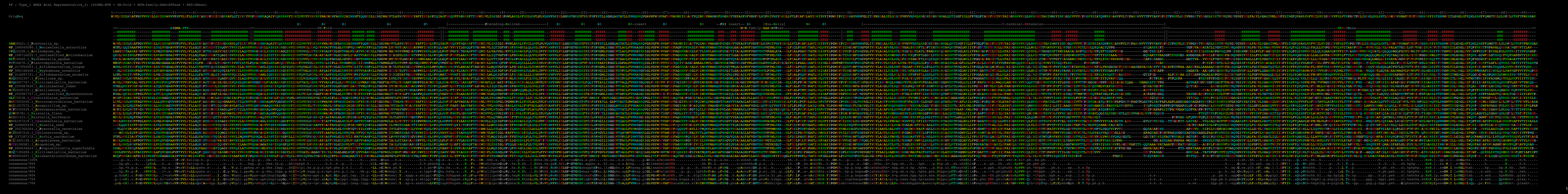

```
## ; Type_4 BREX BrxL Representative Alignment; (SIGMA-HTH + OB-Fold + MCM-family-AAA+ATPase)

OrigSeq                                        [illegible]

BDF48892.1_Lachnospiraceae_bacterium           [illegible]
AFZ48537.1_Cyanobacterium_stanieri_PCC_7202    [illegible]
HAR58248.1_Acholeplasmatales_bacterium         [illegible]
HCF89836.1_Bacillota_bacterium                 [illegible]
HED00231.1_Pseudomonadota_bacterium            [illegible]
KUK07752.1_Archaeoglobus_fulgidus              [illegible]
MAU00661.1_Anaerolineaceae_bacterium           [illegible]
MBE6363380.1_Lentisphaerota_bacterium          [illegible]
MBH8560404.1_Hymenobacter_negativus            [illegible]
MBI2377776.1_Deltaproteobacteria_bacterium     [illegible]
MBI9047159.1_Anaerolineaceae_bacterium         [illegible]
MBN2258341.1_Anaerolineaceae_bacterium         [illegible]
MBR5399200.1_Bacteroidales_bacterium           [illegible]
MBU6335165.1_Chloroflexota_bacterium           [illegible]
MCD4818677.1_Candidatus_Cloacimonadota_bacterium [illegible]
MCH4147879.1_Prevotella_sp.                    [illegible]
MCI7180755.1_Lachnospiraceae_bacterium         [illegible]
NBI83184.1_Clostridiaceae_bacterium            [illegible]
NLC55396.1_Armatimonadota_bacterium            [illegible]
NLG95480.1_Acetomicrobium_flavidum             [illegible]
NLI70094.1_Bacillota_bacterium                 [illegible]
RKY16067.1_Planctomycetota_bacterium           [illegible]
WP_007916555.1_Ktedonobacter_racemifer         [illegible]
WP_119090030.1_Candidatus_Cryosericum_hinesii  [illegible]
WP_219156485.1_Hymenobacter_profundi           [illegible]
WP_219722098.1_Deinococcus_planocerae          [illegible]
WP_251958558.1_Nostoc_commune                  [illegible]
WP_259125643.1_Salinibacter_ruber              [illegible]
consensus/100%                                 [illegible]
consensus/95%                                  [illegible]
consensus/90%                                  [illegible]
consensus/85%                                  [illegible]
consensus/80%                                  [illegible]
consensus/75%                                  [illegible]
consensus/70%                                  [illegible]
```

## ; Type1 BrxA DNA-Binding Component Representative_1; (HTH1 + HTH2)

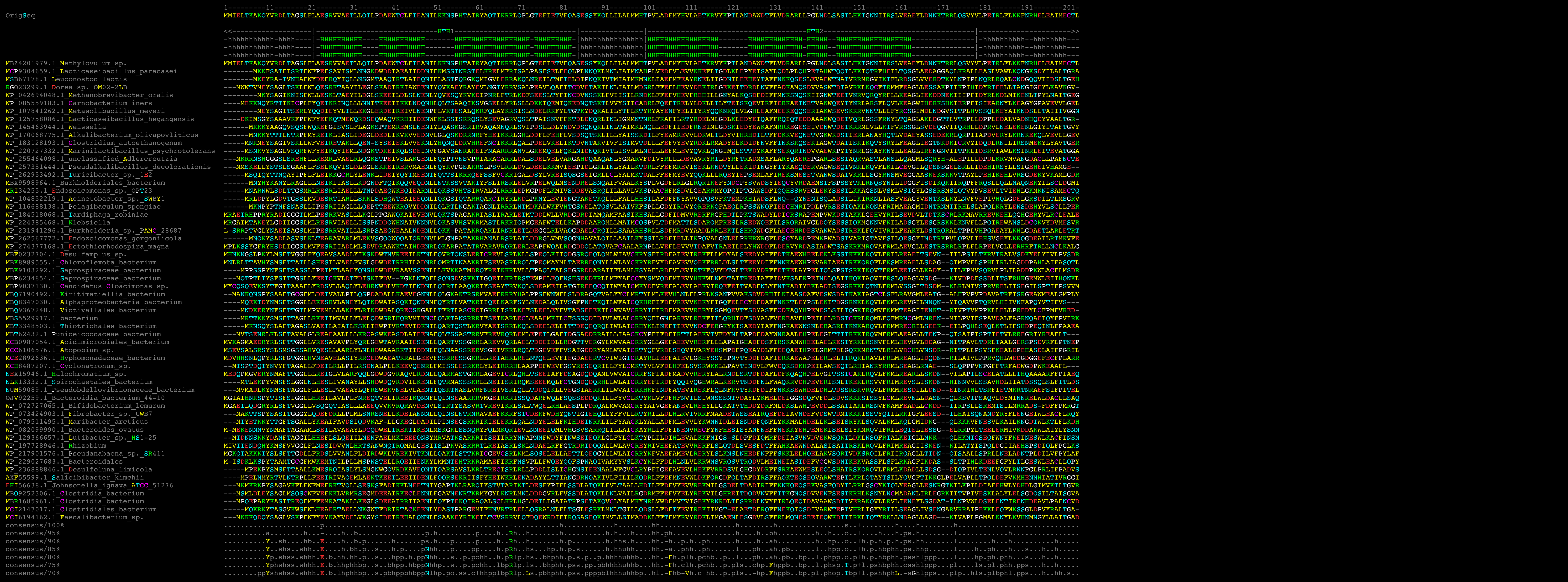

## ; Type1 BrxA DNA-Binding Component Representative_2; (HTH1 + HTH2 + wHTH1)

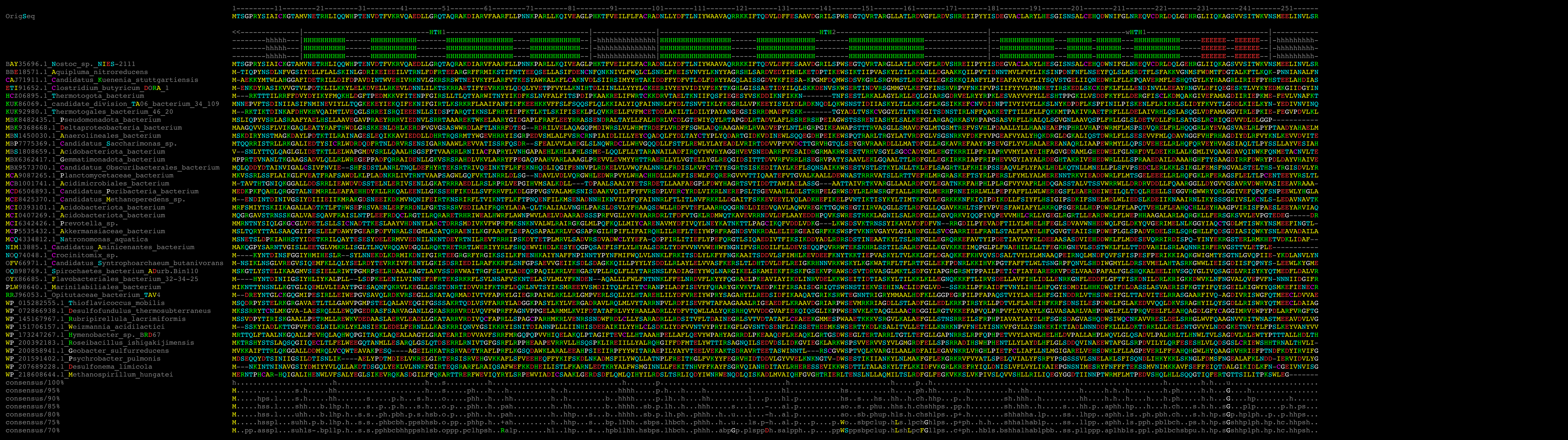

## ; Type1 BrxA DNA-Binding Component Representative_3; (HTH1 + HTH2 + wHTH1)

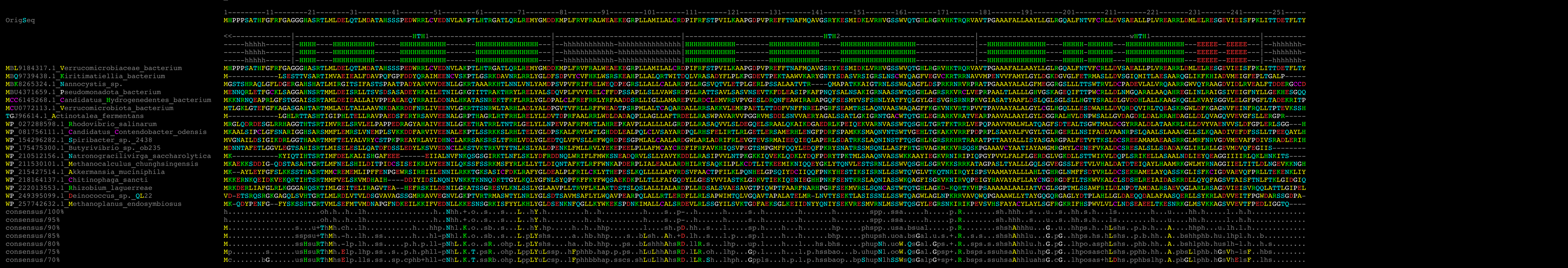

## ; SSP systems SspB component (DUF4007/BrxA Homolog; wHTH1 + wHTH2 + wHTH3)

```
OrigSeq         MDSIDGAFGRHETFTIRYGWLKRGFDHAVADPSLFYSDDAHHRLGVGKNMAKSIRFWLSAARLMEEVPAVDNMVPTYFGLALLNDEGFDPFLERLESWWLIHWMMLSPHSRLPVWWTAFNTFGAVSFTPEVLLDHVLAQIEATSPRPPSQSTVKKDVLALLRAYAGTLGSRKADKLDDTIDAPMVPLSLVRAAGSGFRFAVGRKPGMSPAVVAFACLDFLDRTGFTARSALISSLANEVGGPGKAFKLAERDLVEMLQEAAAEQPNLIAVSSTASSPTLVTVSELRTVKLLARHYGLF

                <<------------------------wHTH1------------------------||--------------||------------------------wHTH2------------------------>><<------------------------wHTH3------------------------>>
                [illegible]

ABF41279.1      MDSIDGAFGRHETFTIRYGWLKRGFDHAVADPSLFYSDDAHHRLGVGKNMAKSIRFWLSAARLMEEVpavdnMVPTYFGLALLndeGFDPFLERLESWWLIHWMMLSPHSRLPVWWTAFNTFGAVSFTPEVLLDHVLAQIEAtsPRPPSQSTVKKDVLALLRAYAGTlgsrkADKLDDTIDAPMVPLSLVRAAGSGFRFAVGRKPGMSPAVVAFACLdfldrtgfTARSALISSLANEVGGPGKAFKLAERDLVEMLQEAAAEQpnLIAVSSTASSPTLVTVSELRTVKLLARHYGLF
CVH75683.1      [illegible]
EQM88114.1      [illegible]
GIV87097.1      [illegible]
HAA81079.1      [illegible]
HAR69672.1      [illegible]
HBP89809.1      [illegible]
HCM67585.1      [illegible]
KQC86134.1      [illegible]
MBE5916694.1    [illegible]
MBI2830764.1    [illegible]
MBK7200303.1    [illegible]
MBL8604950.1    [illegible]
MBL92279.1      [illegible]
MBO5175219.1    [illegible]
MBP3324817.1    [illegible]
MBQ8068457.1    [illegible]
MBR0652113.1    [illegible]
MBR3316189.1    [illegible]
MBR4189802.1    [illegible]
MBU1223689.1    [illegible]
MBX3576086.1    [illegible]
MCG8529187.1    [illegible]
MCI8826050.1    [illegible]
MCI9434830.1    [illegible]
MCM1218777.1    [illegible]
MXZ30902.1      [illegible]
MYD94248.1      [illegible]
NFA97008.1      [illegible]
PSQ83801.1      [illegible]
QZE15890.1      [illegible]
RKU27179.1      [illegible]
RWH12898.1      [illegible]
TAE87575.1      [illegible]
TDK99179.1      [illegible]
TIL65601.1      [illegible]
TVQ60190.1      [illegible]
TXH96997.1      [illegible]
WP_004641460.1  [illegible]
WP_026835191.1  [illegible]
WP_081469353.1  [illegible]
WP_092098984.1  [illegible]
WP_091150728.1  [illegible]
WP_098996779.1  [illegible]
WP_144167758.1  [illegible]
WP_192703942.1  [illegible]
WP_217354883.1  [illegible]
WP_223131378.1  [illegible]
WP_230657396.1  [illegible]
WP_237123961.1  [illegible]
WP_248278535.1  [illegible]
WP_248960911.1  [illegible]
WP_252020420.1  [illegible]
consensus/100%  [illegible]
consensus/95%   [illegible]
consensus/90%   [illegible]
consensus/85%   [illegible]
```

## ; Type2 BREX PglW Tripartite-DNA-binding component; (HTH + wHTH1 + wHTH2)

```
<<------------HTH------------>><<------------wHTH1------------>><<------------wHTH2------------>>
[illegible]
WP_185095299.1_Streptomyces_sp._CEV_2-1          [illegible]
WP_268746228.1_Dermacoccus_nishinomiyaensis      [illegible]
GII83978.1_Sphaerisporangium_siamense            [illegible]
WP_030506725.1_Microbispora_rosea                [illegible]
RJQ78831.1_Pseudonocardiaceae_bacterium          [illegible]
MCB0954589.1_Ilumatobacter_sp.                   [illegible]
WP_208577501.1_Micromonospora_tulbaghiae         [illegible]
MCP9485019.1_Candidatus_Gaiellasilicea_maunaloa  [illegible]
WP_193120346.1_Nocardiopsis_coralli              [illegible]
RKN35100.1_Streptomyces_hoynatensis              [illegible]
WP_212908910.1_Streptomyces_sp._TS71-3           [illegible]
GES07632.1_Acrocarpospora_macrocephala           [illegible]
OAA24551.1_Frankia_sp._EI5c                      [illegible]
WP_213171153.1_Natronoglycomyces_albus           [illegible]
WP_144820010.1_Micrococcus_luteus                [illegible]
MBL8842562.1_Planctomycetota_bacterium           [illegible]
MBX7222607.1_Blastocatellia_bacterium            [illegible]
MBO9539711.1_bacterium                           [illegible]
MBN9688999.1_Verrucomicrobiota_bacterium         [illegible]
KAA0252897.1_Acidobacteriota_bacterium           [illegible]
MBK9387522.1_Planctomycetota_bacterium           [illegible]
WP_144981411.1_Gimesia_aquarii                   [illegible]
WP_090664774.1_Belnapia_rosea                    [illegible]
WP_136411732.1_Luteimonas_yindakuii              [illegible]
KRE61575.1_Tetrasphaera_sp._Soil756              [illegible]
WP_052424041.1_Nonomuraea_candida                [illegible]
WP_093484991.1_unclassified_Streptomyces         [illegible]
WP_109776035.1_Quadrisphaera_granulorum          [illegible]
WP_090069475.1_Lentzea_flaviverrucosa            [illegible]
WP_257478501.1_Acidipropionibacterium_jensenii   [illegible]
MBL9044566.1_Myxococcales_bacterium              [illegible]
MCP5560207.1_Verrucomicrobiaceae_bacterium       [illegible]
MBK6942988.1_Planctomycetota_bacterium           [illegible]
WP_217637676.1_Actinopolyspora_saharensis        [illegible]
TAF23825.1_Verrucomicrobiota_bacterium           [illegible]
MBZ5659870.1_Acidobacteriia_bacterium            [illegible]
MBO0801654.1_Nocardiopsaceae_bacterium           [illegible]
WP_200346137.1_Halochromatium_glycolicum         [illegible]
MBK9978482.1_Gemmatimonadota_bacterium           [illegible]
WP_130480151.1_Leptothrix_mobilis                [illegible]
MBI4602629.1_Planctomycetota_bacterium           [illegible]
MCO6436553.1_Phycisphaerae_bacterium             [illegible]
RLS37136.1_Planctomycetota_bacterium             [illegible]
WP_085387166.1_Cellulosimicrobium_sp._TH-20      [illegible]
consensus/100%                                   [illegible]
consensus/95%                                    [illegible]
consensus/90%                                    [illegible]
consensus/85%                                    [illegible]
consensus/80%                                    [illegible]
consensus/75%                                    [illegible]
consensus/70%                                    [illegible]
```

```
## ; Type3 BrxA DNA-Binding Component; (HTH + wHTH1 + wHTH2)

                                          1---------11--------21--------31--------41--------51--------61--------71--------81--------91--------101-------111-------121-------131-------141-------151-------161-------171-------181-------191-------201-------211-------221-------231-----
OrigSeq                                   MRASQIGFSQRIRLEWLEQTANLVLAGNDKTEINDSLQELLKNKVSGEANREKVISILMKTWLTVPRGLEALRDEGLQIHQGLPRKDRIAVHWGMALAAYPFWGAVAAHTGRLLRLQGTAAAAHVQRRVKEQYGERETASRAARRVLRSFIDWNVLNETDKGVYAQGIRYSIQDPRLISWMVEASLHARANGSAATKDLLDGPSIFPFRLAITAEHLASSPRLDLLRHGLDEDLMMLR

                                          <<--------|------------------------HTH-------------------------|--------------------------------|--------------------------------wHTH1-----------------------------|---------------------------------wHTH2----------------------------->>
                                          -hhh------|--HHHHHHHHHHHHH---HHHHHHHHHHHHHHH---HHHHHHHHHHHHHHHH--|--hhhhhhhhhhhhhh--hhhhhhhhhhhhhhhh|HHHHHHHHHHHHHHHHH----HHHHHHHHHHHH---HHHHHHHHHHHHHHHHHH-EEEE----EEEE---|----HHHHHHHHHHHHHH----EEHHHHHH-----------HHHHHHHH---EEEEEE---EEEEEE-
                                          -hhh------|--HHHHHHHHHHHHH---HHHHHHHHHHHHHHH---HHHHHHHHHHHHHHHH--|--hhhhhhhhhhhhhh--hhhhhhhhhhhhhhhh|HHHHHHHHHHHHHHHHH----HHHHHHHHHHHH---HHHHHHHHHHHHHHHHHH-EEEE----EEEE---|----HHHHHHHHHHHHHH----EEHHHHHH-----------HHHHHHHH---EEEEEE---EEEEEE-
                                          -hhh------|--HHHHHHHHHHHHH---HHHHHHHHHHHHHHH---HHHHHHHHHHHHHHHH--|--hhhhhhhhhhhhhh--hhhhhhhhhhhhhhhh|HHHHHHHHHHHHHHHHH----HHHHHHHHHHHH---HHHHHHHHHHHHHHHHHH-EEEE----EEEE---|----HHHHHHHHHHHHHH----EEHHHHHH-----------HHHHHHHH---EEEEEE---EEEEEE-
MBW1792999.1_Deltaproteobacteria_bacterium MRASQIGFSQRIRLEWLEQTANLVLAGNDKTEINDSLQELLKNKVSGEANREKVISILMKTWLTVPRGLEALRDEGLQIHQGLPRKDRIAVHWGMALAAYPFWGAVAAHTGRLLRLQGTAAAAHVQRRVKEQYGERETASRAARRVLRSFIDWNVLNETDKGVYAQGIRYSIQDPRLISWMVEASLHARANGSAATKDLLDGPSIFPFRLAITAEHLASSPRLDLLRHGLDEDLMMLR
WP_013627486.1_Rubinisphaera_brasiliensis -MTAVVGFDRKLKLEWLDDLADQVAKGEDVSTLRTFLHERLEEDHPGETARGKSITVLMRIWVFVPDEHHEIREQAFELLKRINAKDRIWLHWGMCILAYPLFRDTATAIGRLLKLQDEFTLAQLQRRLIDSWGERSTVVRAFQRVVRSMVEWHTLEDATPGHFRAPTKVTTKSKSLQIWFLRACHAATGKEMVEVDQLLSLPSSFPFKITVAKSDLRNSKDFSIHRQGLDMDMVTTT
MBT3379348.1_Lentisphaerota_bacterium      SRHEVIGIKQVVRREWYDFALDRLISGDSTAAIRAGLDEFISERLQGDQTYTKAVTQIMRSWVTPGNDLVPFRDAALDHARSADRFERVVLHWAVTIAAYPFWHRVAVVVGRLLNLQEAVSQSQIRQRCYEAFGERTTIERSARRVIRSFVTWGLLDDSTKGMYRRSAPVVVTNHDLAALLIEGVLLAIPEGRAPQSQVLSNPGLYPFQLPMNGGMIAASERIDVGRFSLDDELLRLR
WP_271916069.1_Polyangium_sp._rjm3         TERRTIGFDRPVQLEWLDALAYRLAAGASAKEAREVIWNLLDGVVAGETARGKTLTVVSRIWLTVPPGVSPMRDAALLLIRHASVEERQAIHWALMSAAYTFFVDLASSIGKLIALNGEISLSQLTRRLVEVWGDRSTLRPAAQRVVRSMIRWGVLREGRPGQYLPPTKRIVVSAEVSELLLEGLLLAAGRGGMPLSQLLMHPAAFPFELRFDPTRLRKSARLRLHRQGDQADYVERE
WP_189000175.1_Paenibacillus_nasutitermitis GLKKAVGFDQKLQLHQLDHIAREFSRVENRQQLYDLVDQNLMADIAGAKARLNARTILFKIWVLVDNENVELRDRGLQLFANASREERLLLHWGMKLLAYPFFRDVADQVGRLFQLHGEFSSLQLSRKIFALYGERRRVSVSMGAVLGTFKHLEVISEQ-KKLYVNAEKIPIHQLEFKLWLIEIMLKATEKSAIELKQIATEPCLFPFNLDITESELRN-SRLQVTRQGIDMVMVVLR
WP_156173096.1_Kosmotoga_pacifica          ISSKMIGFDRQLKKEWLDLVIEMLHDGETRDKITEALNKYIGNDLKGKDAIRKTRLVINRMWFNPNFQLEFLRRSALELFNEARENATSIIHWGMCIACYPYFRDIARICGRLINLQGYFKPSQVRQRTMEIWGERPVVRRSVDRVIQTIMYWGLIEQEASDKYVVSKFVRINNPKLVNWLIRAYLVSRESKTASIDDLRNDPVLFPFSININIDNLLLNKEIELFNQGLKSEILILR
MBX7228944.1_Burkholderiaceae_bacterium    MKQGKIGFDRALKLRWLNATAEWTAHGFSPNEIRVRLDQLLEGEVAGEDAKGKTKTVLLHIWTLVPDHLKPLRNEGLGLIGELPPNNQLAVHWGMCLATYPFFRVVVEATGRLIKLQREFTKAQIQRRVEEVYGTRETVTRATRRVLQSLVEWDALVEDERGLYQANSLLNIANAKLAIWMTEAILASSDFSSATLTAVTQSPALFPFILTLNPRDIEANERLELLRYGLDQEFVKLR
NMB01449.1_Bacillota_bacterium             MRHDKIAFKQIVRLEWMNETLSLVLAGYPESEIRVKLDQYLSTQRQGNPATYGMSLGLLSCWFRKEPELEDFRKSLLTQATRMYQEDWLPLHWAMLAASYPFFAWVCEHVGRLFSLQESITSAQVYERMKQMFGDKEMIARNTRYVVRTLVSWNLLEDERKGQYSQPSVFQIQDTQLTQLLLEGLLLSMPDERCELNALLRHRALYGFSIGTSPQELVRNGRIVATQVGLTTGMLEIN
OQB94798.1_Spirochaetes_bacterium_ADurb.Bin110 QRTGIIGIKQTIRLEWMEKTANLILAGLPAEKVRQELTRYLANRRGSEQDCKFAVAILMNCWVNPLPELEDLRTQGLLLLREG-VEDHTAIHFGILCAAYPYWFNIAVQTGRLFNLQDAVTASQIVTRLKEIYGDRQTIMRTSRYVLRSFIAWDLIRDSRKGNYIKSEPIHIHDMRNVAFLLLSAFYAMGNPQADFNVILHSPAFFAFNMHIAAETLHQSDNIEIAQIGADVLVMKK-
MSP14283.1_Chloroflexota_bacterium         -MDKGIGFNRNIFLPWLDAVAALRAGTADAAQIRAQLDPIVQQDIASAENRRKAIDILINIWVTGGVSPRLHQQALHFFQTTPPGDRLWLHYGMTLLYYPFFRETAAAIGQLSRYTEGITTALVKQRLIADRGELGSLNKAVERVVFSLRKWGIVQDARRNTYVPQRTLTANTQDLEAWLLACALWSHPAEELPFNDLVRLPELFPFCFSIGIDCIRQNSAFIVHRQGVSMDMVRLS
MCF7763175.1_Verrucomicrobiota_bacterium   MRFVKVGIQRELLVDWFDHAAGIHAMGADRIEARKKCYTYLDTAPGSPPTKAYIANAMVKTWINPDRELISIRNRALDLLTQD-RNDRTAIHWALLCAAYPFWFNLASITGRLLNLQDQATQSQIVLRLKEKYGDRPTVSRRARYVIRSFIAWKTVRDTNKGSYERANVIPIAQPEVTVLLVEAALQATPNGTCELAQLINCPAWFPFHVHPLRSDLIACSGIEVIGFGPDQDLLKLK
WP_209690993.1_Methanomicrobium_sp._W14    -RHDIIGIKQSVRLEWMQKTSDLLLEGKNEKEIRNELKEYLSERMGSETACNFSVSMLMRIWVVPDKDLLPFRDAGLKFLKNSSEDEQVAIHFCMLSAAYPFWYNVSKQIGMLLRLQENVTIQQIIQRTKEYYGDRQTVDRNTQFVIRSLYYWNLLEETLRGCYKKGINIEILSETLNSWIIEAQLHSISDGKAVLSSLQNDMALFSFNTKLNSTVISKNKWIEVFQSGMSDEIMMIK
MBC7752535.1_Candidatus_Saccharibacteria_bacterium SKQPLVGFERFVAFDWLDQTAKWVIEGKSPKEVHDLIDDYLASQISGDTSKRKTKNVLSGVWVKSDGVDPEYKEQAKNLYLMANRTEKIAINYGLFIASYPFFYSLGRILGRLFKLQDDVTNAEFYRRCVEANGDRESIKRAAARYLQSVVEWGLLDDAESAKVKPTKKIVIDNPSIVTWLMTSVLFSSDRERLSVDELLSDPIWFPFEISTRDFQIGESQLLEVVHQNVGSVLLALK
OGK77266.1_Candidatus_Rokubacteria_bacterium -KPGEIGFDRRLRREWLDFVADCAAAHVAPDVIRAKLHDLLGPVVAESGARSKTITVLLRLWVVFDPRTDGLRLDALRRLPTCAPGERLTLHWGLAMAVYPFFADVVSSIGRLLRLQPTVSLAEVVRRTKEKWGDRERVARSARHVLQSIRDWAVLAETSAGVYQAAPPQAALRGDLALWMIEAVLQGSGTSIGSLRQLERAPALFPFTLTVRSAEIERGSRLELVRQAGDEDTVGLR
WP_152691916.1_Aeromonas_hydrophila        -MVKTPGFCRHVEKEWLEQSLRWVANGAEYTQLNDNLDNFLSAYIVSKENRRKARNLLTSVWSQPVDQYKNFFDKGIVLHKEAE-SFSLPIHWGMLIARKPFFAEVARFIGRQSRLNNTFTYAQVFRRMSMLYGETEATSRALSAVLRTIMELGILKRNSPASYSIVNQRVSINSHLANWLLDALLISSSKESIALDDALNDQALFPFHLDINLSNLDM-SAFECLQQGNSIVIFKKG
MBL9185845.1_Opitutaceae_bacterium         -TNQRIGFGKKVRWDWLLLAVRLRAKAVSFDDAREELERLIEETNAGKVAITKTMSVLRQLVFLPTPGCRLFSEHGLELFRRRGAAVAMPVIWGLSISAYSFFASCAETVGRLLRLHGEFTAAEMLRRLTEREGDRAFVIRIARFNLSSMLDWGLLEFDGSKRYRRGKLTAAHDPEILGWLAEGILLASDKTSMPQSQLFASNLFFPFDTKIAATQLIGNPRLQVIRQSLNEEIVALS
PKO06219.1_Chloroflexi_bacterium_HGW       SRDGQIGYNRQVELEWLDFTASLIASGETPQSIKSSLDEYLKDRLSSDASRGKTISILLSIWVTVPKHLTKLRDDALLLMRHLSASEHLALHWGMTMTAYPFFGVVADTAGRMLGLQETVSTLTLKNRMREIYGQREQVIRSTRFVLYSFTEWGVLIETERGVYRLSETKNLKDHQLQEWLLKAFLISEGKTLAPVNTAIRSPKLFPFKFE-EPFRSENYSDLEFFRQGLDEEMVMIK
WP_044665230.1_Syntrophaceticus_schinkii   --MQRIGFDRMIKLDWIEYYAKISRHEEDTGKLKEQMHETLFEECPQMASRKKNITILQRIWTRVEAEHRLLRRGALNILDDVEMPERLALHWGMSLLAYPFFREIAACCGRFLKLQKNFSRQQIKEQITATWGARPTLERALPRVLESMVDWGALSHARKSLYRAQPEIQVTDEQLRLWLLYVCLE-IEGGCLSLERAASLPCLFPFSFNISLAEIQQANCFSVTRERNNSIVVTCQ
WP_257961950.1_Lysinibacillus_capsici      -MTKAVGFDQKIYLDHLDKTASLLRHHIADEKIMYNLDGHLIGTIKGDKSRKNAITMLMKIWNFVDDSVVDLRNLALEEYPYLTQIEKKFIHYCLACLAYPFFSEQVCYIGKQFKQADRIYSRTVLAHMKDLYGERRRVEVATGAVFSSIKEWEIVSMPKAGQYEVKKEISAHNLLFSSFLIEVLLKYYGAGSLTLETLNNSAIFFPFDYHVGIGDLDK-QRFTTFSTVRDTVIERNT
WP_106343515.1_Planifilum_fimeticola       -AKMIIGFDYRVTLEDMEFAAFQDPNR-SRGDLITLFREHVGRNRPGRTTQESTATIMLNIWHGRSGETSHLRMRARSLLTETEARQRRALHWGMALLVYPFFRHFAKQVGLALQLDREVLAERINRKIKRIYGERRKVEVASKAVISSMREWGALKMVRHGVYVPTERLAIDHPDLRLWLAEVLIYASEAEMVPLDVINRSPELFPFSVNLSIPDLRASERLEVDRQGLDLIMVGCR
KUK47748.1_Actinobacteria_bacterium_66_15  -MSQMIGFDRSLRLDWLDAAAGVSRETEDIEQIRNYLHAYLAESYPHYTARRKTITVLTRVWSRIPDSHRLLRERAFVLLPTLNQTDRIWLHWGMCLLAYPFFRDVVRTVGHSIRYYGSFTKQEIIQRMSETWGERATLPRAVQRVIESLNHWGVIAGCQKGRYRCAVPLSTSNKDVEIWLLKVALCANPNDSFPVDQVHTLSEAFAFSCSLSIPEILGSREFEVSPMGNNRFALRCS
NLI08746.1_Thermotogaceae_bacterium        MKTISVGADRFLALQWVNYAYELFISSLDQDSSRKALREYLENQMLGQVSTRKTTDQLFRLWLYSGDPYQTLREMTLSIAKPIDLAQMAVFHLGMAINVFPIYRETVRVIGTLERLTDQISTKAISTRVIEKFPTTTSIPRTVNRVLQTLDDWHFIELT-QGLVVVKE-ILLLDPKLVNWFILTTLRAIQQNEITLQELDTCPFKLGINFSHPRNVVNDSEDLFFGRNQMGLEVIRIK
MBX3742602.1_Akkermansiaceae_bacterium     SSARPIAFNRRLRASWLEEGLRLRSDGLDGPVWVERMEELIKVDISGKDSIAKSMRYLRHVWADSGED-EAMRSEAIAIFRSSPSETAIILSWGMVIATYPFLNDVAATVGRMIRVQPEVKLEQILRKLSETYGERETVRRSGRYALGLITDFGFVKRASPGCYVLGNPIKI-DPTLSGWFVRSWFKALGSTVIDRVGMAGAPSLVFFDSALISDAIER-RILVPERLSFSQEVLRLN
MBN1459904.1_Armatimonadota_bacterium      -DRSRIGIDRALHLDWLDAAAGLAANGATCDTARTALLEVLGRELSSGGAADKTASVLLRTWFKIPAEAAPLRKDATLAMRHADGSGRLAAHWSMLLAAYPFFIDCTSVAGRLLRLSGGFQRSEYSRRVAELWGEKPTANRASGRVLETLVALGVLRQ--EGAHCTQTGRFTVDGPAAAMLAEGLLLGEDRMAVPVESVSSHAAMFPFEMPPIATAVVCSSRLGIEVRGGDTEMLVRT
MBI3175734.1_Chloroflexota_bacterium       MSEYLVGFDRFIALDWANYTLGLLSYPGDEAAKMSELKSWLSLYISGKEASQKTANVLARLWLRATPEISIFKKYAVSLAPELRKDELMYLHWGMSLLVFPLFHEVCTQVGRLLFLQSSVSRREIQIRMAEKYSNQSTIPRSVNRILQTLVAWGLLQEISPRELSAKR-QAPSNILIKAWLIECAVFSAPSKKVSLHGLYKNPILFPFEFHEDLSWIVNSDKLFLERDGNNLEYVVWL
WP_075724812.1_Tissierella_creatinophila   -MSKQVGMSRNISLDYLNKTIEFMEIEDDFEKVRENLREYIGLYIDDNTNIRKTANILSNIWLEDDKENRKIKDYAREEVAKSSSNLRLVAHWCMILITYKVFEDIASVIGKLEYMQIEITSKLIREKMTDMWGERPTLIHAIPKNIRTMRDTSVLETVKQGVYKVKK-HRVDDERAIILIVATLIHLKDKLYLSFDELIDDSLMFPFDYSIDVGVLEESNMFSFDRFGGELAISLKE
WP_020614472.1_Sediminispirochaeta_bajacaliforniensis MREKTIGYDRLLKNDLLNKVAYFVGDGNNPEDVKSKLRASFEDQTS---ALRKTINLLMGIWSTVPEEIVPLRDQAIKLFLKAYDSEKRIIHYFMSMANYPFFFDVCLLTGRALRLSNSFTSRQILTKIQEKWGDRSTTIRAVQRTLKTIEQWGWLNSTKQRDYSLNDMKTIIPKEWHGFIANCVLFGSLRSSMLIEEITQNPSLFPFHIEFNIHEMKQWNGIRLEQRGRGDLLVSLK
KAF0196958.1_Bacillota_bacterium           -MSKQIGFDRFIKLQWAEDLAWRMRETKDEVSLKRIIEDILSKELPTQETRRKTSIVLLKAFYRIPPQHERLRNEALELLPQVGVNEAKALYWGLMLLAFPIFTDTARHIGQLFSLQSDASAALVQRRVIETWGQRGSLHSTLNKVVKTMAEWGCIDLTHKKVLVPKA-PTPLGKPASLWLTRVALTVSETPLVPVDQALRPSICFPFTMDIHAQDFSD-GEFLLQRMDGRTEYVGLR
WP_270641086.1_Limosilactobacillus_mucosae -MKKEVGLFRPLRFDQLDLAASLLSNY-ELNDAFEIMNEKLSNELKGNVGRRKVINNITRVWSGGKKQPSNFQKNVLSRYCNANHDSKIAYQYLMTAIAFPFFRDTVNIVGKYLRMTGKISNAFLLKEMENSYGSAESVYKGTNAVVRGLIDWNLVDGMEKGKLIEIDKLRISDEFLKQLIVKVLMEHYNTEYIQLDQLNEDAIFFPFNYHLSESYFDNLTNYEVIDDHSGKYVHMIG
WP_270691668.1_Bacillus_cereus_group_sp.   -MTKPVGFDQKVLLHHLDFAANHTKKY-SRKDMYEVLDGYLRNDITGAKSRKNAVTMLMKIWYLVDKDLIEIRDRVLEVLPVLTKEERVFIHWGMTLAAYPFFKDTIHELGKLFQLQDTVPSTVIGKRMKEEYGDRRRVEVATSAVLMSIKAWGIIIPTKNRSYQLPDKVFISNPLLQVFIVKIMLHVLEHTALQIDILKNHPMLFPFDHEFDLLELRQNEWLTLHHQGVDTLIVECK
WP_140397540.1_Flavonifractor_sp._An135    -MAKAVGLSRKITLKWLNKAHEFFSEGLTEEEFKTQINDYLSFEIDSPTVLRKTREILMHVWFYEDNYHTLIRRDADDLLKRFP-EYDIAIHWCMLLSVYPVFADICKLIGRISDFTDIVTLSQLKQKLYDEWGERSTLYHSTDKIIATMKELDAISSEKPGKYTIKK-HTIARSEVALFMAMIAMKVDGNSYYSFSELHDFELLFPFEYKTSKEELMTNERFTVSTFGGEVSVSLSV
MBT6646604.1_Nitrososphaerota_archaeon     -MLKNVGFLQYVTKDWLDATVAFYQPHQNIDEVKASLDTLIKFDVPKNEERRKTIDVLTRIWARIPPKFDSLRLDALSFYDDLDPNERIILHWGMALLAYPFFRDIVFSIGSLLKIQDDFTSTQLRNKLISNWGHRTTIIRAIDRNIQSLKNWNILIIDKS--LKQNSKMIIKNESLCLWFLECIFRAENLDSMLFEKLVSSQTMFPFELKIPVYKIKQAKNFEYQIHGMNIEMISLR
HEC58782.1_Methylophaga_sp.                -MKVSIGFNRHLETEWLTQTASWVAKDIEGDELKSQIDLMLAPVFTSQVAKDKTRNLLFGIWNKLPAHIKAFQDDAAKLIMEHS-ELNLVFHWGMMLAKYPFFYLVVGQIGRLTKLNDAFLYAQLEQRVVENFGDTSTIKRSMQFVVRTLINLDLLANPKQGLYQTQKPIKVDSSSVKAWLVEASLYSESQSSRSLTTISDAPVWFPFELHFQASDLSQNGRLEVHHQSSDVIVFINN
WP_243191721.1_Vibrio_parahaemolyticus     -MSKRVNFSRHLEIQWLDQTARWVASGLDKATLNENVDRMLEPHIKCKVNKGKTRNQLINLWFSVSRDMDSFQNKAIEFVKSSD-ELPLTLHWGQLVAKNVFFSDVARFVGRKLKHGDSFTYAQAQKHLVELYGDTETVKRALRSALKTLVEFRLVSRESNRSYSVNKLEMYVDTRYKSWLLLALMYNQGFHNRSLSNLLDDLVWFPFDFSVSFNEIDA-SLFELHQQGNELVLFRKE
WP_149544908.1_Calorimonas_adulescens      -MIKKVGFSRALKLEWLNSTVEIYENTKDLETTRKLVKEYLTYKIKGRVSLSKCTGMLMDTWVDVPDEYRSFINKGIELYDGSDSEQKIILHWGALMVAFPIFTDISSTIGRFLYLHDSFNASSVKKRIYELWGERSTVERVVRYSLVTMSDFGVISRDKAGEYKRGKCIVVDNPEHKLFLINSYMLATNRKTLQYDTIEEQYPLPFPFKMNLATHEINGSNVLTLNRFDNQTFVVMK-
WP_045174404.1_Caldicellulosiruptor_danielii --MKAIGFLKPVRVEWLNITVEELIKGKEKNEIRDVLKRIVSNYYKSEDTIRKILAILMSTWVNVSEKNMCLRDRGVDLFKIANPDEKIALHFGMLMVTYPIFNDVVFIIGKAFYLENKFKLEYLKKRIFEKWGERETLRFSIDRIIKTLKDWGLIVSKNSGVYEPGTKVTITSKEIKAFLIACYLKANERNYININEIENLYSFFPFNISVNLNELMNVDNLSVNKIDSIITVAVNR
WP_010252938.1_Treponema_primitia          -MLKSVGFLRAVSNAWLDKTVELYLTGLDPDKIREELTNHISFELKGKESIRKTIDVLMATWININTENQQARKTAITAYKNCA-ENHNIQHYCMLLLAYPVFNDFCNLIGKISKMQDTFTKSWLRDKLFEQWGDRVSIDNSLKYLIQTLLSLQILERIGVGKYQIKK-HEIMDNQNKILLLHALALLGGTKYCVLSSANFQPILFPYIVEFSPELILNNSILELCIFQGKAIISLKK
WP_140396494.1_Gordonibacter               -MARMVGFTRIIRLPWLNKTIELAGEGLEPTQMREELEEYLKFEISSDTNRRKTREILLLPWVAEDAALEALRPKALELALSHP-YELLAVHWGMLVVAFPMFGDLVRLIGKMCEYQDEFTAAQLKLKILDEWGERSTVVKGSEKMLASLNAIGVIQRVKQGRYVLAP-RVDADEELSLFLLHADMLAYQSGYRSYTELQALPELFPFVLHASKERLVEDARFSIGNFGREFTVSLNQ
WP_122014531.1_Biomaibacter_acetigenes     -MMQTVGFNRFIKREWLNFTAFLAGREKDEKIIRKNLNEYLEKETKAEITRQKTLVELLRIWVNVPASHEDLKEKALDLFEKVPEDEKMLFHWSIMILAFPIFIDVASSLGKIFSLQDRVSLKILEKRIFEKWGERSTVKYALPKIVGSMVEWGVITRAKVGEYTKAQTIEIKNKDCKLFFINCYMQALEKDYLRFIEANNLSAAFPFKLNLGLDDFNNSNTLSLSRMGSDVVIIKR-
WP_035270489.1_Desulfitibacter_alkalitolerans -MLKRVGFDRQIKREWLDKTVELLINKEDSKDIKNILDGIVKEDIASNDNRRKAISILMHSWINVETDHIAIRSKALKLWETGNSTERLAIHWCMLSLAYPLFFNISEIIGKLINLQDEFSLNMVRRRVYDVWGERSTLDYAINRVVWSMKDWGTIGQNKVGEYKKIERIVIEDKEVQLLLLEAYFIASQRSNIPCAELEMINALFPFKTDITINDLQSNHRFKINNMGNNLVVSL--
WP_028274685.1_Atopococcus_tabaci          -NKKTIGLFRVLTVEDLDRTARLLKKY-SPQDAYDILNEKLMEKAPGDVGRRKLVNNLMMIWGNGKKEPANYQKKAMEVYNELPKEEQLVLHYLLALIAFPFLTFEARLAGKYLGLMDTVSSKVFMDEVMNKYGNSGTITRSVSNGFGILREFGFLKKEKAGRYSLGNRIIVKSTLLKNYIVLAALLNTDSDTLSLEAVQHDKMFFGLDFILYSSDLDP-NMFEVTSDRVHTYIKKR-
WP_202065228.1_Enterococcus                -MKKMVGLFRVMTFEELDKAAEVMVKY-SPTDAYQILDETFAKTVKGDTFRRKALNNLMKVWGNGQKPLSDFQEKVLERYAISNYDEKKSLQVLMMLYSFSFFSDTINIIGKYLRMQDTFQSKTIMEEVRSTYGMTETVNKGVLTVLGSLVDWGFLFRSKPGVYELTKKILITDEFIKNVLIRSILAHTDAEYLPLEIINNQAGLFMFDYQVSRLEVHL-DDLNVLRERTDTFI----
MBP3452731.1_Clostridia_bacterium          -MAERVGLSRNISEEYLNVVADCKLADKPLLEARDILNALIGTHIHAETNIKNTRVILLNTWYRNE---LWFQNTCIEIAKEVTVPERLPLHWALLMVAYPVFFDLCTIIGTMLDYRDEFSATQVKERIFETWSARTTLIHALSKNLQTMKDMRILEPQKNGVYKPIS-HHVDNKKTVCVLAAAILKCSGLEYMTWEEIVHHPALFPFNIEVTQADLAACDRFLLERMGDNIVIRFRT
MBQ3643691.1_Candidatus_Riflebacteria_bacterium -MTKNIGLSQKLKFSWLEKTVEIVSSE-EDTFIKKKISEYLSCEIKSPTNIRKITKILMNIWVSNN---NDLQINALELIKKYP-EYSLVINWCLILTAYPVFTDICTIIGKIAKFQEVITNNQIKEKMFEEWGERSTLFYSVEKTLGTIIELGALKPIKKGSYSLCK-YAINNDEIIDFMLKSILLSNTKESKTINELQNLACFFPFKYKINENRIINNNSFKTVNLGGNLNISVA-
consensus/100%                             ......sh.b.h....h.................h...h................h...h.........h...................h........hh.......G........h...h.p....s...........h..h.....l...........................hh...................h......h.h.............h..............
consensus/95%                              .....luh.b.l....hp..h...............h...l........s.......l...W.........hb..s...h..........hpa.h....asha...s...Gp...h...h....h..c....bu.p..h..s....l.sh..h..l.......h.........p.....hhh...h............h.....hasa........h.....h..........h....
consensus/90%                              .....lGh.b.l..pbhp..h............h...hp..l........s..p....l...W.........hb..s...h..........hpash.h.sasha...s...Gph..hp..h....h.p+h.p.aGpp.pl..s....l.oh..h.hl.......h.........p..h..hhh.s.h..........hp.h.....hFsFp.......h.....h.h.........h....
consensus/85%                              .....lGh.b.l..pahp.sh.b.....s..phb..lp..l.....s..sb.ps...L.p.W.........hbp.uh.bh..........haash.hhsaPha.p.s..hGph..hp..hp...l.p+h.p.aGpp.pl..s....l.oh..h.hl.......a.........s..h..hhh.shh.s.....h.hp.h.p...hFsFphp.....l.....h.h.p.s.p..h....
consensus/80%                              .....lGhpb.lpbcalp.sh.h.....s..phbp.lppbl..ph.u..sb.ps.shL.p.W...s.....hbppuhphh.p.....pbhlHash.hhsaPhF.p.s..hG+hhphp.phs..bl.p+h.p.aGpp.sl..u.p.sl.oh.phshl...p.s.Y...p...h.s.ph..hhh.shh.s.....h.hp.l.p...hFPFphph....l...p.hph.p.s.p..h....
consensus/75%                              .b.p.lGhp+.lphcWLs.shphh.p..s.pphbp.lpphl..ph.up.sbpKs.shLhphWh.ss.p...h+ppulphh.p.s.ppphhlHauhhhhsYPhF.shsp.lG+hhphpspho..blbp+h.-.aG-Rpol.+u.pbsl.oh.pashlpp.p.u.Y...p.b.h.s.ph..allpshhbs..p..hshppl.p.s.hFPFphphs..pl..sp.hpl.pbu.s..hh..p
consensus/70%                              .b.p.lGasR.lph-WLsbshphh.ps.s.pph+p.lcphl..ph.upssbpKs.shLhphWhpss.p...h+ppulplh.p.s.ppphhlHWuhhhhsYPhF.clsp.lG+hhplpspho..blbp+hb-.aG-Rpol.+uhpbllboh.pashlppsp.G.Yp..p.b.l.s.pl..allpshlbu.spp.hshppl.p.s.hFPFphphs.ppl.pspphpl.pbu.s..hh.bp
```

```
## ; Type4 DUF4007 Representative_1 DNA-Binding Component; (HTH + wHTH1 + wHTH2)

                                          [illegible]
OrigSeq                                   [illegible]

                                          [illegible]
                                          [illegible]
                                          [illegible]
                                          [illegible]
CDE63278.1_Alistipes_putredinis_CAG:67    [illegible]
CVI65225.1_Alistipes_sp._CHKCI003         [illegible]
GFI28163.1_Lachnospiraceae_bacterium      [illegible]
GHU63481.1_Clostridia_bacterium           [illegible]
HAR58252.1_Acholeplasmatales_bacterium    [illegible]
HBZ34376.1_Bacteroidales_bacterium        [illegible]
HED00227.1_Pseudomonadota_bacterium       [illegible]
HHW21154.1_Thermodesulfovibrio_thiophilus [illegible]
HHY36030.1_Bacillota_bacterium            [illegible]
HIQ39048.1_Methanothermococcus_okinawensis [illegible]
KUK07756.1_Archaeoglobus_fulgidus         [illegible]
KUK66685.1_Parcubacteria_bacterium_34_609 [illegible]
MBB6067916.1_Methanococcus_maripaludis    [illegible]
MBD5142972.1_Ruminococcus_sp.             [illegible]
MBD8953985.1_Dialister_sp.                [illegible]
MBE5813906.1_Clostridiales_bacterium      [illegible]
MBE6363377.1_Lentisphaerota_bacterium     [illegible]
MBE6838774.1_Ruminococcus_sp.             [illegible]
MBF0554174.1_Nitrospirota_bacterium       [illegible]
MBK5260963.1_Peptostreptococcaceae_bacterium [illegible]
MBO6251941.1_Muribaculaceae_bacterium     [illegible]
MBQ6210587.1_Prevotella_sp.               [illegible]
MBQ7110121.1_Thermoguttaceae_bacterium    [illegible]
MBR2732786.1_Selenomonadaceae_bacterium   [illegible]
MBR4291499.1_Oscillospiraceae_bacterium   [illegible]
MBR5214694.1_Bacteroidales_bacterium      [illegible]
MBR6984230.1_Ruminococcus_sp.             [illegible]
MBR7025509.1_Selenomonadaceae_bacterium   [illegible]
MCC8021079.1_Akkermansia_sp.              [illegible]
MCI9323608.1_Lachnospiraceae_bacterium    [illegible]
MCI9677951.1_Oscillospiraceae_bacterium   [illegible]
MCL2016624.1_Defluviitaleaceae_bacterium  [illegible]
MCM1232923.1_Ruminococcus_flavefaciens    [illegible]
MZQ76089.1_Peptoclostridium_sp.           [illegible]
NBJ16969.1_Dehalobacter_sp._4CP           [illegible]
NLT83667.1_Coprothermobacter_proteolyticus [illegible]
NPV08141.1_Anaerolineae_bacterium         [illegible]
NSW92345.1_Bacillota_bacterium            [illegible]
ONI45223.1_Epulopiscium_sp._SCG-C07WGA-EpuloA2 [illegible]
OQC39309.1_Bacteroidetes_bacterium_ADurb.Bin037 [illegible]
WP_024333181.1_Desulfotignum_balticum     [illegible]
WP_062281146.1_Flexilinea_flocculi        [illegible]
WP_071148138.1_Bacteroides_ndongoniae     [illegible]
WP_126850816.1_Candidatus_Cryosericum_hinesii [illegible]
WP_138286703.1_Phocaeicola_vulgatus       [illegible]
WP_197538712.1_Methanothermobacter_sp._CaT2 [illegible]
WP_207678197.1_Desulfonema_magnum         [illegible]
WP_268058970.1_Caproiciproducens_galactitolivorans [illegible]
consensus/100%                            [illegible]
consensus/95%                             [illegible]
consensus/90%                             [illegible]
consensus/85%                             [illegible]
consensus/80%                             [illegible]
consensus/75%                             [illegible]
consensus/70%                             [illegible]

## ; Type4 BrxA DUF4007 Representative_2 DNA-Binding Component; (HTH + wHTH1 + wHTH2)

                                          [illegible]
OrigSeq                                   [illegible]

                                          [illegible]
                                          [illegible]
                                          [illegible]
                                          [illegible]
KEF34343.1_Deinococcus_sp._RL             [illegible]
EBT6907987.1_Salmonella_enterica          [illegible]
GIV81896.1_Anaerolineae_bacterium         [illegible]
MAU00652.1_Anaerolineaceae_bacterium      [illegible]
MBH8560400.1_Hymenobacter_negativus       [illegible]
MBI2377783.1_Deltaproteobacteria_bacterium [illegible]
MBI2842135.1_Armatimonadota_bacterium     [illegible]
MBM4284315.1_Deltaproteobacteria_bacterium [illegible]
MBU6335169.1_Chloroflexota_bacterium      [illegible]
MBU6335172.1_Chloroflexota_bacterium      [illegible]
MBV5327605.1_Chlorobium_sp.               [illegible]
MCK5872694.1_Methylococcales_bacterium    [illegible]
MCK9608994.1_Methylomonas_sp.             [illegible]
MCL4273185.1_Anaerolineales_bacterium     [illegible]
MCL4273188.1_Anaerolineales_bacterium     [illegible]
NJL05931.1_Chloroflexaceae_bacterium      [illegible]
NJL05934.1_Chloroflexaceae_bacterium      [illegible]
NPV84910.1_Anaerolineae_bacterium         [illegible]
OAB59991.1_Phormidium_willei_BDU_130791   [illegible]
PIQ01002.1_Shewanella_sp.                 [illegible]
PNY80835.1_Deinococcus_koreensis          [illegible]
PSB31010.1_Stenomitos_frigidus_ULC18      [illegible]
QEP42420.1_Ectothiorhodospiraceae_bacterium_BW-2 [illegible]
SEH06869.1_Thiotrichales_bacterium_HS_08  [illegible]
SEH06873.1_Thiotrichales_bacterium_HS_08  [illegible]
TPG64628.1_Hymenobacter_nivis             [illegible]
WP_027878022.1_Meiothermus_cerbereus      [illegible]
WP_062417763.1_Levilinea_saccharolytica   [illegible]
WP_083924616.1_Lamprocystis_purpurea      [illegible]
WP_086759844.1_Nostoc_sp._106C            [illegible]
WP_129675134.1_Candidatus_Chloroploca_sp._Khr17 [illegible]
WP_165774646.1_Candidatus_Viridilinea_mediisalina [illegible]
WP_169366112.1_Pseudanabaena_yagii        [illegible]
WP_192822488.1_Rufibacter_sp._LB8         [illegible]
WP_194112251.1_Mucilaginibacter_myungsuensis [illegible]
WP_194715673.1_Noviherbaspirillum_soli    [illegible]
WP_210653449.1_Gemmata_palustris          [illegible]
WP_219156497.1_Hymenobacter_profundi      [illegible]
WP_235137402.1_Dyadobacter_fanqingshengii [illegible]
WP_235137405.1_Dyadobacter_fanqingshengii [illegible]
WP_254031242.1_Planktothrix_agardhii      [illegible]
consensus/100%                            [illegible]
consensus/95%                             [illegible]
consensus/90%                             [illegible]
consensus/85%                             [illegible]
consensus/80%                             [illegible]
consensus/75%                             [illegible]
consensus/70%                             [illegible]
```

```
## ; Type1 BREX; BrxB iSTAND ATPase; Representative Alignment

                                                    1---------11--------21--------31--------41--------51--------61--------71--------81--------91--------101-------111-------121-------131-------141-------151-------161-------171-------181-------
OrigSeq                                             MLQQQFENHYRVITSAGFLQRQGLANEVPFFISTFSADQQVEAEGLVNSLFMRLQTQGVDVLKIDLFEFCLELLEQQGVLEDYLAMESQIKKADLKDALVGALSVQDKIAPAIARKLAEQNWKVLFLCGVGRAYPILRTHTVLSNLQSIVVSQPTVLFFPGRYTFVSLDLFGNLNEERYYRAFNLNNYQL

                                                                                S1                            S2   ------------HEH-Extension----------                            S3                              S4                          S5
                                                    -HHHHHHHHHHHHH-HHHHH---------EEEEEE----HHHHHHHHHHHHHHHHHHH---EEEEE|HHHHHHHHHHH-HHHHHHHHHHH--HHHHHHHHHH-|HHHHHHHHHHHHHHHHH----EEEEE-HHHH-----HHHHHHHHHHH-----EEEEE-|EEE---EEE---EEE--|--EEEE-----
                                                    -HHHHHHHHHHHHH-HHHHH---------EEEEEE----HHHHHHHHHHHHHHHHHHH---EEEEE|HHHHHHHHHHH-HHHHHHHHHHH--HHHHHHHHHH-|HHHHHHHHHHHHHHHHH----EEEEE-HHHH-----HHHHHHHHHHH-----EEEEE-|EEE---EEE---EEE--|--EEEE-----
                                                    -HHHHHHHHHHHHH-HHHHH---------EEEEEE----HHHHHHHHHHHHHHHHHHH---EEEEE|HHHHHHHHHHH-HHHHHHHHHHH--HHHHHHHHHH-|HHHHHHHHHHHHHHHHH----EEEEE-HHHH-----HHHHHHHHHHH-----EEEEE-|EEE---EEE---EEE--|--EEEE-----
WP_102077887.1_unclassified_Psychrobacter           MLNTDFNELMERVRAG---REFGHASFEPIFYLIFDPQKILKIKRQLPAWAAKLRNEGWDVHIFSMAKAVQEVFDEMPVFQDSAALENRDQWQKTNKSLAEALTKKNALQNKLEAKLFGRPNSILLVSDIEALHPYLRIGSMESQLQGKFH-VPTIFFYPGMRTGKQLKFLGFYPEDGNYRSVHVGG---
MAE16012.1_Deltaproteobacteria_bacterium            MLQQQFENHYRVITSAGFLQRQGLANEVPFFISTFSADQQVEAEGLVNSLFMRLQTQGVDVLKIDLFEFCLELLEQQGVLEDYLAMESQIKKADLKDALVGALSVQDKIAPAIARKLAEQNWKVLFLCGVGRAYPILRTHTVLSNLQSIVVSQPTVLFFPGRYTFVSLDLFGNLNEERYYRAFNLNNYQL
MCF2581773.1_Bacteroides_caecigallinarum            MMDTVFEKVYQKLSSPDF--GKNLGGELPLYIQPIPVSGQTELNTQVERLVSRLSKLGKNSIVVDLYRLALEIIDEEGILETLLEDEKNIDKDDLNATFESIFDTKEILIPRMRSMIEENKYDFVFITGVGRVYPFIRSHSIVNNMEGLADNANIVLFFPGEYNRLQISLFGKLPADNHYRAHNLNDIET
AUX12765.1_Latilactobacillus_sakei                  MTNERLNELQARISDNQFLTKQGLGNEVSFYIFDYNPADELLVRSRLPEIQRYTNKLDTPIKEFNLFDIATRFFEERQYMEKNYQMEQTKDSFTLYDRMQRALKTATEVIKYIREH--CEDNTITFITGVGSAYPLVRAHTVLNNLQPIIVQKPLILFYPGVYEGNKLSLFNQFKDDNYYRAFRIVE---
ETR72220.1_Candidatus_Magnetoglobus_multicellularis MIESRIKILKDDLLNPL-GPSISTNQNYPFAIFHYPPHEEFKMREKLIELLDTLRNKAWVILNVDLFATLIEYLSIQEDIDALVQEEKLQPLNVLKNSLDIVLKNTDFVFDKILNKIE-ISKTVIFLSRIGGLYPFYRTSTLLRMLDNGIQ-VPTIVLYPGERTEQYLRFMGEMDADRDYRPRIY-----
HIU24259.1_Candidatus_Coprovicinus_avistercoris     MLQERLDGIAKRISSQEFLENKGLGNEVGIHVFAYDPHYEWQVRAAVANLMHASGKLPCCIHERNLWEAVLEVCRQKRILDKIDALEHKRGSKALQKSLQPIVTPEA-LVASMNWQPHEHGRDVLFITGVGQAYPFVRAHQILENGQHVFDDIPVVLFYPGTYNDRELALFGRHDQSNYYRAFSLI----
KAB1640464.1_Ellagibacter_isourolithinifaciens      MFSHCCEALRAHLLDVNFLENKGIGNEIGFFTFCYDPALEMQARTFFAALERESGDKPYRIVSRNLYDVFLNICEKRRILKAIPVQEAKHGTASQLKQLSKICTPAA-FAEAIDYEPH-ERGDVLVLTGVGEVYPFLRIHTLLDNLHVRFSDIPVVIAYPGSYTGHSFSLFNSLSDGNYYRAFDLV----
KAF3303186.1_Carnobacterium_sp._PL17RED31           MINELLDKFEETIMEEKFIESRSIGDDNNYYVFDYPHEKEMEVRNRIESMIIKSGAYPSELILFDIYDFTIDLLEKRGFLEMAYQFEEKNGMAFVADTVQSLLSKDGIFIDHIERN--SNTDSVILVGGIGKIHPIIKAKTILSSLQSTSNQSSTILLFPGTFTGLQLNLFEKISEENYYRALKLLD---
MBD1206698.1_Paracoccaceae_bacterium                MKPGLKDHLRSIITGPRVLGGDTPGNDVPFFLCPYDPEGEAAMQRLIADLAAEIGAAGTRVLTLDLYRIAVDLLQRTGRWDRLLSREARDDKVRFLALLQSTLNEEEVLVPEIRDLVDRTPHDLIFLTGFAPAFPMIRSYTILSRLQTVLKVAPTVIFFPGRYTQDSLKMLGRLRERNYYRAHNILHMRG
MBD5357713.1_Bacteroides_sp.                        MSEIVVDKLYERLCQPDFQDTPHGDLFYNYFIVPYDPAKEYETRERIQWYHDNLPTNFVNALHIDIFEEFCNYLDNKPFLKYLIEKESKDSSAGVARSLTQQAQCDAYIRDRIVAHKEENKTPYVFISGFGNIYPYLRLSTFLNKYEEHTHAYKIFVFYPGEPDGNSYKLFDRFADQHVYRAALILDFRN
MBE7502026.1_Verrucomicrobiales_bacterium           MLKDNFETLRQRLGHG---REIDSTGTEPIYYLVFPVAEILEVKRQTKTWIARLANDGWNVVPLSLADTIAGIFQNHKLRKLWLASEQQLDPSEINKTLAKALSDNSELRVRLDAAVAAQPNGLLLLTDLEALHPYLRINTIEDYLRGRIR-HPVIVLYPGQREGKSLRFLEFYPPDPNYRSDHIG----
MBP6234855.1_Saprospiraceae_bacterium               M---QFDKILNVLARKDFQDPSTGNLFFPVYIYTYDTKDEALYKEQIPEMISHLPSNHLHTLQIDVWKEIMIFFEEKTFLELIKNKEN--TGEDVTQWVRHIFNSQNWLKNKIDAHYRLKFKPYIIVNGFTEAFPYMRPGEFLKNTESLPEHYKIILFYPGKVEGENFKLFGTLESESIYRANILNN---
MBP6733458.1_Chromatiaceae_bacterium                MLAARFEHLVNVIGSRRFLKMQGLNNDLPFYICEFRAAEAVEMQRLRRQLIHKLQGRGVRILDINLYDLSIALLQAREGWDDLLAIETEVDKDQLLELLQNVLGIEEYLIPAIGERLAQAEYDVIFLSGIGEVFPFIRSHNVLNNLQSRIKGKPLVMFFPGEYRHSSLELFGLLRDDKYYRAFNIYEIQA
MBQ6248037.1_Kiritimatiellia_bacterium              MTTEIFHQILPVLSSKPFLDGSLAAPGIPFYVCPYEAEREQETVAMIGNLVRQLAAKGVAVTVVDAFRLMVERMKENGDLDYDLEDEPGYTRAEFLEHLQGILDVPKEIVPAICARVEEEKPRLIFVTGVGAAWPVLRAHSLLMNLESAVK-IPLVLFFPGAYRQISLYLFDKLPGDGYYRAFNLLDIHP
MBQ6343446.1_Anaerolineaceae_bacterium              MLSKRIEYLYKVFTSDRFLSMQGIGRETSYYICPFSPSEANEFSKQRELLVKRLTTNGIGVCEIDLYDLSVEILKEEDAWDGIMEQETAIDKSDLLETIQATIDLGGTFLRKIQNHVSRGDHKIIFISGIGEVYPWIRTHNLLENMQTVINGEPVVLFFPGSYKQTSLELFDTFKNERYYRAYNIFNYGV
MBQ8347029.1_Alphaproteobacteria_bacterium          MIEEKYNHLKKIISSPDFLSGKVLCNDLPIFIADYDISEEKDFLIMEKNLIKTLEAMEIKVLSVDLYDLIIEILKKNQGWDWVMSPDR--NKEELLEELKGILGIEDTVIPAIANKMANGKYDVLFLNGCGNLFPFLRTHTILNNLHSHIQ-KPVIVFFPGINVSKAFNLFGRIDEGNDYRATNIFQIRG
MBR3160179.1_Atopobiaceae_bacterium                 MLSSKKNHILKVLASPQVIRGDRTMSRSTAIIVPYPPEDEVEWDDMAHTLVGDLGAQGIAASIANLYDIVLRILDDDGLYDEKVEIERTHGKARLIQELKGAADIELDIVPVIEDIIDDEGPQLLFLTGVGACHPFLRTSQLLEKLDDTID-VPVVVFYPGRYQRKPLEILNIPRGNAFYQGRNVFD---
MBR4606064.1_Lachnospiraceae_bacterium              MFYARLEQVPDRLLSKDLLHNIHIGNEIGFYIFDYPPDKELVVREWLPSLPEQVSEQKITLLSINIFQEVIRYIKNTNKYEKIIQVEKERGNDATLRGLGSLLNTTA-ITSIFKDVIN-TNADIILMHGVGAAWPLIRIHSFLNILQPIVERVPLVLFYPGTYNSSEIKLFNRLPSENYYRAFRLLP---
MBR6253007.1_Clostridia_bacterium                   MIENRLQEVYDKLKTKKLSETQSLGNGLKFFIFDYDPEDEEKVYNYFETYLN--VKDDFHLRIVDVYDCIINILKEKGYLEKVYKMEKEKGIDYTNGVITSTIGVGSLLLNEITKD--LKKDEILVLKGIGKCFGVVRAHTILSNLEKVVDENPVLLLYPGGYDENGLRLFNKLKPNDYYKAIQLVY---
MBU6197214.1_Cyanobacteria_bacterium_REEB446        MRKQRFDLLLRFTSNKDNLDYRKY-NDIPFFIVPYEISEQNKIKEDIPSLESHLIKNDIRHLSINLYELSLELLDNNLDLKYFLETETERDKKRFIEDLQGVLTSQK-VAEKIGDECEKFKPELLLISGVGEVFPYLRLSKLLENLSCKVKEIVVLIFFPGIYCQSYLRLFSCLDYDHHYRAFNIEDLNL
MBX6362418.1_Gemmatimonadota_bacterium              MLKQRLEQLEQKILAG---RPLGPSTDIPFGIFVYDPAEELEMRRQASLFATRLENGGKRVRSVDLGALLWACLDESPL-PRALEVEEAFELEAILHQAHTMVAGQHPLERRIIEQLADRVRDFALLSRAGELFPIYRTSALLERLIGHVR-IPTLLLYPGTMTGPELRFMGVCDPSPNYRPTIYA----
MCB1062256.1_Verrucomicrobiae_bacterium             MLDATFERLFSVMQSERFLTSTGIGNEVSLFVQPYEIEVQDPVEKRIKALASRLSAKGLGVLCINLLELVAEFTSREDRLKRLLEKEPTFTRDKMLSTMARWTDAKEHLVPAMKAKFDAEKWDITLVYGCGSVFPFLRTHTILENLQSDMDKRPIVFFFPGEYQHRDLRLFGKLSHKGYYRAFNLDHYHL
MCC6107939.1_Atopobium_sp.                          MVNEAELFLVSKLSSDRFLKSAGAFSNSSMFVAPYPPEQQEDAAKMVDALALDLRSRGVTASVVDLFSVFTSVLDDQGLWEPYCQIEGTVPTKDFIIALKDAADLETQICPRVVAQVKEEHAQLLIVTGMGACYPVIRTHRLVEVLDPQ---VPVLVMFPGRQTHKSLDILATPAGEGQYRARNIFE---
MCF0260591.1_Erysipelotrichaceae_bacterium          MVTKRLENLMEKISTSAFLNNKGISNEVGLHIFTYKPSEEMIIKDFVRRLKKDH---PCRIVEFDLYKSLLKALENDGILDEVGEVEKEISHEELLEQLDTNLDQ---LMEAAELPLK-LGEDVLLITGIGDVFPFIRAHYVLNSIQQVVS-VPVVVFYPGEYTGRDLKLFNRIDDKNYYRAFNIC----
MCH3907668.1_Sphaerochaeta_sp.                      MYNERYAFLLDLLDDDKFIRKKYVGAEIPNYICAFPPEDVSSYNDMLENLAKALTSKGRKVLKVNVYEIMIEMLKDSGDFEDYLE-EPSLTHKKIMEDFEGVLDNDTEFAPKVASIINESVPGIVLMNGIGEAYPFIRIHTLLEKLPTLLKRVPIVVFYPGNYDKVALQLFGKLEHKNYYRAFNIFT---
MCI6194161.1_Faecalibacterium_sp.                   MTDQKLIKLDDFLHTFSTETGDGLCGEAGYYVFSYDPKDEMKVRRWTECETQILQQSNRPVVEFDLYKIMTGLLEEKHPRQKYEDLELRTSMTRVVNAVNSVLHIGQLMLNYIVDRIP-KKDAIILLTGVGKCYPLLRSHKILNDLHDRLADGIVLMLFPGRYEDGYLYLFGDVKDGNNYRAKPI-----
MCI9596747.1_Bacillota_bacterium                    MLAVRLDKMEAAIRKPAFRQSSGKANEVNYWIFDYPPEAELRVRERIQHLKNKNGNDVFELVVFDLYDIIIDFLENRNFLDKCFDFEKRHGLDRITKAVNNSMKINDLIIQRIKEN--TPKNAIVFLTGIGKCYPILRSHKVLNNLHQAFVSVPVVLFFPGTYNEQELILFNEIKDDNYYRAFRLVK---
MCK6605710.1_Ignavibacteriaceae_bacterium           MISDKFDNLFNYILNSDLTAEAKTGGQLPFYISCYDIESEALIDQHINALYNRLTSSGKIITVIDLFEIVTGVLTMNNELQHLLHEESSYDYEERKEFFTEIHNPEKYVAPLIKNKADETNAQLILIKGISKIYPHLRTGHLLAVLERFISDMPVIVFFPGTYDGINLSLFGRFTD--SYRAFVLDALVN
MCK7578718.1_Chromatiales_bacterium                 MTSDPTEHLFGILSHPDFLAMKGLANEVPIFIHTYEPRDEDATRRRVANLAARLKTMGIVLAVVDLFDLVLEQLEEEGRLHRIIEKEAAMGKQKLLELLNNLSDPKTRLVPRLIQRIGGNEIQLTLLTGVGRVFPFLRTHTLLESLQPAMMRHPVVLFFPGQYTQEQLRLFGSLSNRPYYRAFNLAHYRL
NLA16136.1_Bacteroidales_bacterium                  MLSASINQAFEKMRNPKFLQKEGLGGEIPFFIFPYDIKYERQIDDTISGLKKRLNREGICVLELNLYTICIDILQSKGGIEKVLKLEERHDRPNFLKALQSALNIQEVFIPHIKNIVDTIEHHLIFITGVAPVFPYIRSHVILNNLHSVTDKVPTVMFYPGTYTGKTLNLFDRLKSNNYYRAYNLNEYQL
PZV04978.1_Cyanobium_sp.                            MLQQQLEPV---LTAADSAAGLSTYTDMPFGIFLYPPEDEWELRGELGDLVIRLENAGKTVQRLSMAVLLDQAIEAAGLMEEIAQGEADLGIAIVRDTIRDVLSGKAPLDQLVAEAVDPTSSHIVFLERVGALFGLHRPSALLDNLYRPVT-HPLVLFYPGQRDGPGLRFMDEAEADHNYRPKLFA----
RKX87881.1_Spirochaetota_bacterium                  -TARQFENLFSIMSHEKFLNMEGLGNEVPFFIHAYDIREQNEIYEHIHLITRRLTTAGVETKLIGLYDMVLDILGETGSLEDVFDLEQESSKKELKETFSTLFNQDE-IKSYFTSKLREGDQQIVMIYQVGEVFPFLRTHDILNQLQSIVTDTPLVVFFPGEYVMSHLNLFGKFSGP-YYRAFRLDDYFA
RRF95307.1_Coriobacteriaceae_bacterium              MINDARGFIVDKLSDDALLGRNGYMMRQALYIIDYDPAQQQYAADLVRAICEKDPRRGVTPVVVNLYDLVLGYLDEQDLWEPLVEAEPDTPRLDLIQMLQDTVGVKDVVAPRVNEAIASPEADIAFVTGVGETFPYVRTHTLLEEISSP---IPVVLVFPGRYEQHALNILGLTQASTYYRATRVFD---
TVQ28582.1_Spirochaetaceae_bacterium                MTAKRFDRVYQIISHPRFLARQGLGNEVPYFIETYEPANEFAVTTEVRTIHERLLHNGIPAVLLPMYDIVIECLESDGRLAQVFEKEPAMGRQRFFAMLDAFTRPDAPVHDAIVRRLDAPDHKLVLMHQLGTVFPFLRTHTLLTNLHSTIYQVPLVAFFPGTYVSSYLSLFGTFKGD-YYRAFQLSDYIE
UCC65223.1_Anaerolineae_bacterium                   MLEADFEKLRQRLGDP---DALNPAHSDPIFYFVYPPSQILTMRKLLPGWIARLRNEGLKVETLSLSEIMWELIDASGRWDDWLELEPEHDLDAVNEAIRDVLRAGNALVERVAERAASRENTVLFITDVELLHPYFRSRVIENYLNNKVL-IPTVFFYPGRRTGQGLHFLEFYPEDPGYRSTLIGGLE-
WP_004808450.1_Actinomycetaceae                     MLQETLDIALRVMTSKRFLNREGLGNEVPYYVLRYRIEWDRSFDQSLRQMLSHL-NESVPTLHIDVYQLAIQIWRDCGYWDQILAQEAGMDRQDFADGLAQILDAERVLAPAIAERIQAPDSRVVILSGVHHLFPIMRAHRLLNCLQPLTGDVPVVLTFPGSYRQSALVLFDQVSEDNYYRAFDLLDQQY
WP_012411903.1_Nostoc_punctiforme                   MINQRLDALLPKLQDARLLSNRGIGNEIGFYIFDYDAEYEPLVQRYVDRLKLQLSPIQLILLEIDLYKTILDILSERGVLKKAFELEATKGNAALAKTVAPLVRPDQ-VIAYIQKKLR-GDEQLVIMTGVGASWPLLRSHSILNNLHPVLDKIPLVMFFPGSYDGHELRLFDTFKDDNYYRAFPLIPHQQ
WP_013452129.1_Calditerrivibrio_nitroreducens       MLTSKLNCLINILKDENYFRLEHIANEVPFYIFDYDPEAELDVRKTIKIISDS-DQLKYRIEVVNIYDFIISYLQEKDFLEKIFEMEQKLSPEKFLTRLKSIIDPEL-IINKIVQLNDEKNPDVFILTGIGNSYPIIRIHQIINRLQEKVCNKVVIVMYPGTFDVNKLILFNSLESKAFYRAFKICG---
WP_019974717.1_Empedobacter_brevis                  MIKQDFDTIFKITSSEGFLKMEALNGEIPFWIAPYEVKLHNEYEKQIENLRKKLKTNSIQALVIDLFQLASEIIFEDFTVEQIEKLEKRKSKEQFKRALQATLNIHDIFIPKIKECLESNDYNLLILKNVGSLYPFMRSHTILNNLQSAIKTKPVLIFYPGAYTGQSLNLFGELIDDNYYRANNILKHKK
WP_029256830.1_Rhodococcus_erythropolis             MLTHEEKHVYDVLRSGRFLRMEGLSKEVPFFIYHFPPAWALDVDGMRDRVTKKLSDDGINVVEINLYNLAVELLNERGVWDRVLQAEPTMDKADFRDMLQGMLDPHDHLAPAIRARLDEPNTTVVFLTGIGEVFPFIRTHTVLENLQSVVVGRPMLAWFPGTYEFTQLRALNLGASDSYYRAKDILEQET
WP_048345182.1_Lactobacillaceae                     MLEERFQHLKDKIQSPSFQHNQGLSNEVGYYIFDYPVTETLKMRTLLKRFLDSPMAAQLNIRCFNVFDIMIKEIKSFDYLNELEKVEERNGMDYLIEQVGNIMEITKAIVKYIQNRLP-KNESIIFITGLGEVYPLLRTHKILNAMTQVIDNYPVIFFYPGKYDSFSLQAFGEVKDQNYYRAFRID----
WP_094203898.1_Finegoldia_magna                     MLNERLDNLKKIIQEPDFLEGKGLSNEVNINIFCYDPKEEMKIRYFVEKLKQDNSLKNCNILAFDLFELFIEVSNDRKLIHKYVEKEKKSGKEALLNSIKKVVSYDK-IIDKMDLSNKHMNNEVVIIYGVGRVHPFLKLHMILNEIQKV-TDLPIIALYPGKYDGLTVSLFGEFESSSYYRAFN------
WP_118234408.1_Fusobacterium_mortiferum             MINLRFRELVTKIKSDEFYNNRGLANEVPFYIFDYQPEDELSMRDNIKNLLLPADDVRLKAIEIDLFELLIESLNNDGIFESVFKMEERRGTKILYEKLKKSFNMEV-ICNYFKEK--SKDKNFVIITGVGKIFPLVRTHALLNNLQVIFNETKVLLFFPGEYTTTDLRVFG-FKDNHYYRAFRID----
WP_126029123.1_Bifidobacterium_callimiconis         MIEREFESLFAIMRRPSFRTGSDTAGEPANYIYAYPPVKELEVERRTDQLANRLAETAPGVLIIDLYETAIQVLRDSRIFERVLRKEPRLIPDMFTQSLIDKLSPDKSIAEQYRQAREHKQGDIVFITGVGKVYPYIRTHILMERIQLVFEQRPVVLFFPGTYAKTTMRLFDRLESSNYYRAFSLNAMED
WP_135754989.1_Leptospira_bouyouniensis             MLLKRLNDLKKDILHPE-GIQVTQSQNYPFSIFIYPPADEFTVRAKFVEMIQDIKKENIEILEINLAHECLELLKQRDGIDEIIQKEKEFTFALVNDVFSPILEDENGISKSILNIMEEGRKGIVFITRAGFLYPFYRTSSLLKFLTNTRG-LSVVFLYPGTRTSESLSYMGVMSPDSNYRPRMY-----
WP_151792237.1_Acinetobacter_seifertii              MVDDRLSLIFERLNNPKFLENQSLSNDMPYWVFDYNPEDELIVRDYLAHKVPRFEKEGLNFHTIDLYDVMLSILDSRNLLERTYNLELSKGFDHLKKTMDSVLEQKK-VAKHIVDHLK-DNPSFIILHGVGNCFPYIKAPALLNALHGATGCTPVLLFFPGNYDGNMFETLNVVEPDPYYRAFRLVPEKT
WP_163588065.1_Ligilactobacillus_murinus            MIKDSLSILDETIKSEDFLKNRGLGNEVGYYIFDYDPKDELLVREYVRELCEQNTKDGYTIKVFNLYKIMIELLEEKKYLDIISKWEKQHDIMFVATRINRLLKMEEYFTKYIMDR--VEENSVVILTGIGEIYPLVRAHSVLNKMHLVYTKRPVIMMYPGEYDNKRLTILNTNRDANYYRAFRIH----
WP_168675170.1_Hymenobacter_artigasi                MIPEKAERLFQTISGSRFLKRELLGGDIHFFISTHAAEQQTEMRQAIAALIKRLDNTGIQVLEINLFKLALSVLDSEIGLPALFEFEEQESAAEFREALHSAMDMKQVLLPAIERHVQSATPQVYFLTGIGEVYPFIRSHSILNNLHHLVERAPLVAFFPGTYSGEQLKLFGLLADDNYYRAFNLDTFTE
WP_187771726.1_Phascolarctobacterium_faecium        MLSERLGKIKNIITNSNFLNKTGNANEVSYFIFDYPPDKDIIVEDYIQRLATEIMEKDMQIKIFDIYGLIMDYLKDKKYIDKCILLEQKKGSAGLIKAIKDTLHLDSYIVNHIDENTI-QDKDIVFLVGVGRAFPIVRVRELLSVLNNLYTKCPIVLMLPGSYDGQHVTAFNKLKMENYYRAFKLVQ---
WP_200392182.1_Roseibacillus_ishigakijimensis       MFDQKIDELIRRLGAPE--GTTNARQGDQVFNVHYPPHLWAELRTRLPKLMARLKDQSYAPRHCSLLALMEKIFGDNGKIALLRRSEQQSHQA-FTDTLHSILANKKPIVAAIEEEIQSKPNGLLLITDIEMAHPLLRVSAFEQVLQGRFA-VPTVFFYPGERGNINPSYLGVYPSDGNYRSTHIY----
WP_217359511.1_Anabaena_sp._UHCC_0204               NLDTAIAALKNDLLDPG-SPKISTMRNYRFAILQYDPREEYKLRDRIRKLTDELKSQGWNVLSVSLKSLFLNRLKKEDTLDSIIRTEKRLGINHLKDKISLYIEGADGIAQDVINLINKADHTLILLERAGSLYPFFRSSALLKHIDGKTNNLPVVLLYPGERRDLALSFMGELPADRDYRPRIYS----
WP_222635590.1_unclassified_Rhodococcus             MLTARFERAYRVMRSPSFLEMKGQIGEVPYFVLAYKPSDEPRVGAEHKRLVTRLRTDGLEVCDIDLWALANELWSATGQWELILEQETALPKAVFSEGVGNVISPKDYLSPRIARIVERPRPQVVLLSNVGRLYPLVRAHTVLNTLQPLLADLPIVLVFPGTYRQSSLVLFDRLTDDDYYRAFDLLELEN
WP_224137561.1_Candidatus_Kaistella_beijingensis    MPNNINDKIFSLLSEEEFKDPDTGLLFFPVYIYTYNPEKEFQIREEIKELDRKLPSNNLNCLVLNIYNEFIDYLKHNIFLEQIFELEEIDALDFIAEKAQEKNDNDAHIGEKLKAYFDSPDRVYLLLYGFGSIFPYLRLSEFLKKTEEYVRNYKLIAFYPGELKNDHYSLFGIFGDENVYRANHLNQISQ
WP_235341611.1_Bifidobacterium_boum                 MIDEDFQHLFDVMKQSSFRHSKGIAGEQPFYIYDYPPECELEVDRHIPWLMQKLDDYAPVVRRVDLYDVVLGILKRRKILDRVLKIEPRYSQDKFLGMLESILGADSQLPNAVVEAMHTVKPGIIFITGVGAVYPYVRAHTLLNTLQGMIEDIPVVLFYPGTFQSSTMTLFNCLEPDNYYRVMRIRDLNV
WP_243520940.1_Hymenobacter_monticola               MLNEAFTQLLDALNTRD--RTINPTGAAQFYYLVFPPEQALEVKRQLHKWKKRLTDQRYKTREYSFAKEMPGLAEKHPLVSRSLQLEAAKTAAEKFAASPKLLHQLTKLAADITARFG-SEKTVLLLTDLEALHPAVRIGTIENQLTQTLK-GPIIVFYPGQRTAKALKFLGFYPEDGNYRSTHFEATTA
WP_245747814.1_Methanolobus_profundi                MLEERLELLKEELQKEDFLKARGLGNEVPFWIFDYPPEKELLVRDTITKVIPALEKKSIQVLEIDLYDVCLEILEKKISAEKIGSFEGNKGSDELLKKLKLMLKPDI-LKNAIQQKMNEDGFQLIFLTGVGKAWPMVRSHSVLNNLQPLLGNIPLVTFYPGKYSGFDLSLFGKFRDANYYRAFRLINTDN
WP_251490388.1_Otoolea_muris                        MIKERLDKVRALIQKPEFLEGRGLSNEVNIRIFCYEPENEMMVRHFVNQLETDP---DGHLKICNLYKTFLSVCGDMDITDAIPGMEEADGSAYLLEQLSFAIGNRE-FIDKIQYEPH-EPGDVLMLTGVGEVFPFMRIHTLLEALQPYFSDVPILVMYPGEFDGRHLKLFNRLKPNDYYRAFSVIE---
WP_258185035.1_unclassified_Bifidobacterium         MIEQDFDRLFRYMSSKRFRNGEDTGGEPANFIYAYPPEKELEVRRRIPMLVNRLDDVAPAVLVIDLYEMAIGIMKDNDVFDSAIEQEPEMQEDDFYQALAGMLGPDNALRKCYQQAAASSEADMVFLTGMGQVYPYIRTHALLNCIQDVVPSTPVVVFFPGTYEKTVMRLFDRLDADNYYRAFSLNTMEG
consensus/100%                                      ................................................................sh....................-.....................h...h..........h.hh..h...as..+...h.............h..hPG.........h........Yps........
consensus/95%                                       M.......h...h..................h..a..............h.............hsh.......h............E...........h.........h...h..........h.hh..h...aPhh+...hbp...........lhhaPG......h.hh........YRs..h.....
consensus/90%                                       M....b..h...h...............sh.h..a.......h......h.............hslh..h..hhp.p..hp.....E...........h...h.....h...h..........hhhl..h..haPhhR...hhp.hp......s.lhhaPG.bp...h.hhs.......YRs..h.....
consensus/85%                                       M....hp.l...h.p...........p.shalh.a.s.....hp..h..h..p.......h..hslhp.h..hhppp..hp.h...E...s...h.p.h...h.....h...h..........llhlpshu.haPhhR.p.llp.lp......s.lhhaPGpbp...hphhs.h..p..YRu..l.....
consensus/80%                                       Mhppphp.lbp.l.p....p..s.ssp.shalhsY.sp.p..hppbh..h..p....s..h..hslap.hhphhppp..hc.hhp.E...s...h.p.l...hp.p..l...l.........pllhlpslG.haPhlR.p.lLp.lp..h...PllhhaPGpbp...lphhs.h..p..YRu.pl.....
consensus/75%                                       Mhppphp.lbp.lpp.phbp.ps.ssphshalhsYssppp.bhcpbl..l..ph.p.sh.h..hslachhhphhppp..hc.hhp.E.p.s...h.p.lp.hhp.pp.l...l...h...p.pllhlsGlG.haPhlRspplLppLp..h...PllhhaPGphp.p.Lphhs.h.sps.YRAbpl.....
consensus/70%                                       Mlppphpplbp.lps.phbp.ps.ss-hshaIhsYssppp.bhcpbl.pl.pcl.p.shpl..lsLachhlphlcpp..h-phhpbE.p.s...h.p.lp.hls.pp.lhs.l.p.h...p.pllhlsGlGphaPhlRs+slLppLps.hp..PlllhaPGpas.ppLplFsphpsssaYRAbpl.....
```

```
## ; Type 2 BREX systems PglW C-terminal iSTAND-AAA+ATPase

---1211------1221------1231------1241------1251------1261------1271------1281------1291------1301------1311------1321------1331------1341------1351------1361------1371------1381------1391------1401--
SAVTQRRITASTPEQKLRYRANAQLLESAARPGFRLLTVRYDDQRLALLTLTEEWRAEPVDVSILFLTSLRRLVEARPRPWDTILEADNAVPGSRDAMKFGEYTAAAWGEVEKELASNSRPLLLHDASVMARYGGTGILQRLADHARRGGRGLWLLSPLNDATAVPRLEDWTVALEDREEWIRLNHSWVVNDPEDAPAA

                                   S1                    S2 -----------HEH-Extension--------                             S3                              S4                         S5
|------HHHHH-HHHHHHHHHHHHHHHHHH---EEEEEEE-HHHHHHHHHHHHHH--EEEE|HHHHHHHHHHHHHH----HHHHHHHHH---|-HHHHHHHHHHHHHHHHHHHHHHH----EEEE-HHHHHHH-HHHHHHHHHHHHHH----EEEEEEE-----------HHHHHHHHH-EEEE-HHHHH---------
|------HHHHH-HHHHHHHHHHHHHHHHHH---EEEEEEE-HHHHHHHHHHHHHH--EEEE|HHHHHHHHHHHHHH----HHHHHHHHH---|-HHHHHHHHHHHHHHHHHHHHHHH----EEEE-HHHHHHH-HHHHHHHHHHHHHH----EEEEEEE-----------HHHHHHHHH-EEEE-HHHHH---------
|------HHHHH-HHHHHHHHHHHHHHHHHH---EEEEEEE-HHHHHHHHHHHHHH--EEEE|HHHHHHHHHHHHHH----HHHHHHHHH---|-HHHHHHHHHHHHHHHHHHHHHHH----EEEE-HHHHHHH-HHHHHHHHHHHHHH----EEEEEEE-----------HHHHHHHHH-EEEE-HHHHH---------
WP_185095299.1_Streptomyces_sp. SAVTQRRITASTPEQKLRYRANAQLLESAARPGFRLLTVRYDDQRLALLTLTEEWRAEPVDVSILFLTSLRRLVEARPRPWDTILEADNAVPGSRDAMKFGEYTAAAWGEVEKELASNSRPLLLHDASVMARYGGTGILQRLADHARRGGRGLWLLSPLNDATAVPRLEDWTVALEDREEWIRLNHSWVVNDPEDAPAA
WP_268746228.1_Dermacoccus_nishinomiyaensis -----SRRPTHDVASSITGRFGVRLDDSVSSRSFLALGARSERLVEAVETLRRAYGAEVVDLSELLVDALKQQTVGGRLTWDHVRAADAAAPHTKAAQGLGQLVRRAWPQIENHLASAERPLLLTDAAPLARYGHMDMLARWSDLAR--SRPVWLLVPQVSGNHGAVLDGAPLPLAAPSQFVPLDSDWIDTHAAAAKEH
GII83978.1_Sphaerisporangium_siamense -----GQTSTLGGVGRNLAAFEQRLTRSIAEHAFVTLAVDWRFHARAAAALAERFGVTPVDVTADLLAAMRREAEEHQVDWSLVLRSDRPDAAPQDAANLRTLVAMAAASLESRLEAETRPLLIVEAAPLARYDRLAVLERLADQAR--PAARWLLLPVE-QDGVPSIDGRPAP--GALGALRLSTTWITRPRDEAVS-
KEZ35702.1_Frankia_sp._CeD VTSRGRWSTRVS---DEVLAADDRLTRSLVDGGWLVLSVPPRRLARAERCLAA---VTVVDAEQALLAGMREFCAQHRVQWSIVLAADAADRSSRDWANLSRVAQAGLAGVRASIEAAGPAVLITNAGVVARYDPAVVLDELRASVRMPVRTVWLLVPWADVDKQPLLDGAPVPQ-FGNQGLALSEEWIVRHESRGAA-
WP_030506725.1_Microbispora_rosea -----SASRTAAPRQVVDSAAMQRLTGAAGRGGFLALNVDLRRAVLAVRVIPESLDVMPIDVGGEFLRALTDLASERDRPWSDVLRADERFSDGILPRGLAGYVREAWQRLATRLSQPSGTVLLHNAGVFARYPGGDLLVEIQQAARFGPRGVWLLCPTGTPKQRPILDGLVVEVIGENEWLELTAELLRSLDEKAA--
RJQ78831.1_Pseudonocardiaceae_bacterium ----SISLTTVGGVLTAREVVASKLTSAVQRGGFLALTLRGGELPGTAAMLAARFPVQPVDVDQEFLRTFRALADERGADWQKVRTLDLRLSTGEPTPGLASFARKAWDGVRSRVGAGETVLLLHHLSLLQRYYQWEFLTELQNDARYSPHGLWLLCPAENEKHDPHLDDRIVVECTDSEWAVLSKKFLNALRGQGAE-
WP_212516928.1_Actinospica_acidithermotolerans -----ASGTGGAASGAVVDAAVKRLGEAVEDGGFRALTVSVRHLPGLSAAIERRFPLISVDGNAEFLACFRELAAEQGLEWERVLRVDARLQRGEAPRSYAEYVAEVASRLHAKWSSPRSTVLLHNAGLLARYWDIDLVVGLQQSARRAPRGLWLLCPAESARSSPNLDGRLVETVVAGEWIVLGSAAVEELKCQDGHE
WP_208577501.1_Micromonospora_tulbaghiae ------TATTHTPLLTAADEASARLAAAIERRSFLAVLAPWRRLGPARRALLARLPLTEVDVTAILLDRLRAL----GFPWEAIVAADNGNPQDADFRSLMDLVHQVVPAIGEALAAVDGPALLTEAAPLARYGQLGLLQELADPTR--AAARLLLVPARRPE-PAVLDGVQLPLTSPGQSLWLSGDWIEAAGVASADH
MCP9485019.1_Candidatus_Gaiellasilicea_maunaloa --RHVRRPTYEDPEVLDAAQFERRLETSTRRGGFLCLVGHDRSLDDIRDELARRFDAQHVSLDRLLIHHLRLAAGRRNVVWQTVVDADAATRDSRAWGNLQQLFVEAVERAEGELLAYEGTVLATDPGLLARYGQLEMIDRLRDRAS-SLSTLWLLVPDDNQSDRPAIDGQAIPIIGAGQWARVPSAWIENRHRGRAA-
WP_193120346.1_Nocardiopsis_coralli ------HTGHREPGRAVSQETWERLGRAVERGGYRALKTWLPDTERSAEQIGNMPGVVPFDVSAEFVQAMDEVRQGKGLTWRHLTQADSSRPRPRP---FQTMLELALQRVGERVAAGHGVVLLHRATPLARYEQGALLGRLAEEARESPHGLWLLCPMRGPQSPALLDGEPAGIIADAEQVLLPRGFSAPE-------
WP_103561562.1_Actinomadura_rubteroloni -ASGVRRGPHSDPALAAAARAEDQLVAAASRDGFRVLTVRMMLFSEALEELTGRFGGTEVSATRLFLDALHSLTAPGKRPWETILRADNAEPNEKSAHKFAEYATTALGRVEPHLASGNGPIILTDATVFAHYNAMDTLSRIADAAR-GKRGLWLVCPQSDPAREPRLGRRAVPYQPASEWVELNDHWARNRHRSERSA
WP_248824789.1_Frankia_sp._Ag45/Mut15 ATPSVRQPTYDSPEQAAASLTEQRLAAAAEAGGFRALTVRRSRYLAARTELALRFAAAPVDVAAVFLDALHAQVDPRPRPWATILRADTAEPGSRAAGNLAEYVRTAWERAAPLLGGGPAPLLLHDAGIFGRYAGRDVLFALAERAR-GGRPVWVLCPTTEPTRPPRLDGALVQVATESEWVALPDAWTTNWHRSGKAS
WP_213171153.1_Natronoglycomyces_albus -------TTGVGSGADEAARFTYRIDKAVESGGFRAIKTGVAETCAAAKWLAARPGVVALDVQAVFLRLLRQYVAERNKPWERVLAADTVPPPAP----LTNLLVEVWNRFETALTAEGRVLFLYNATPLGRYSGGKVLHELLSAARRRPHGVWLLCPTDALRHVAHLDGYNVGAIDSGEQIRFPAGVLKPRLDEDSGQ
WP_144820010.1_Micrococcus_luteus -----TREATITLPSSELGAIGQRLLDSAAQRGYLVLGVPAHRMNRSRELLAARFGAEHVDLADLFLTRLRETVDRSPVSWEMALSADSAPPGTKAGRALQAWEAAALPQVVEHVSQAGRPVFLTGVAVLTRLGPLQPVARWADLGR--SRAVWVGVPQAPGNIGPVLDGVPVPTSSRSQFVRLEPEWIDTAARTSPA-
MBN9688999.1_Verrucomicrobiota_bacterium --STATKGNGVTAEKLTANQFTDRLQRSIQTAGYLVLVSTTTNYLVVEQRLREGFPVAVCDIDTLLLNELKAQAESLKVKWEKILEADQAVETSTDWNKLNQLVGYVMPKLKAHLVAQDGPVLLTNIGLLARFNQIPILEQLREAIL-DPRSLWVLVPSDDQNRLPTLHGRPVPVATRGQWEQIPEVWLNLTTSATS--
KAA0252897.1_Acidobacteriota_bacterium -----APTTHVPTRDDPAAEFDRRLAQAAREGGYLVLLAPERELTRAREAIAARAGIEIRSLDRLFLDALKATAESKKVKWDAVLRADAAPADGPEGTKLRTLVGNALPRLEEALAASGDRLLLTEPGLLVRYRRLDVLDRLRDRVS--PGAAWVLVPADEQTDRPVLDGVAIPVLTPNQWARVPLAWIEAQATAGEVS
QQS02053.1_Austwickia_sp. -----TATPTAGGAASLRDPVAARLAESVATRSFLALGVGALDLARVQDVLRERYDAHLLDVTGLLIDAMRAAAARHGVPWDVVLASDAATPGTRDRANLEQLVGMCLPEVWEAIHAKDAPVVLTDVAPLARYRHLGELSRWSDVAR--ERAVWVLAPQTPTSQGPVIDGQPLPLSSPGQYVRLDLRRLYASDDQNARA
MBK9387522.1_Planctomycetota_bacterium SSLLPKAPLILSDEATDHRLFEKKLRHYAKDGGLLVLSTAPKSFVAAEQALVTFLALERCDLDALIVRAMHAALANGIDWQTVLLADAPDASASDRANLHQLFHLALPEIEAALLAPGPAILVRKPGLLARYGALRIFALLKERAR--RPSLWFLLPSSQQSSVPRLFEEPLPI-SVDRHLRIPESWPRQQLSPRSTT
WP_090664774.1_Belnapia_rosea -ATLIRHATEATPDVLAARAIEDKIVFAAEKGVFLALTVEPRRARDAETELLRRFPREVVSLERLMLHAMRTRAEERRIAWPKALTADAAARDSTDFKNLLRLAAEAAPRVAKEVLALRQPALLTRPGLIARYDLMTMLDEFSQASA--SPSLWLLIPQATPG-RPEIDGAILPVISAANWTRLTDPWLANAHRAGVPS
WP_136411732.1_Luteimonas_yindakuii TSTLRRMTTADSPEAREARAFDATVRRALDAGRVVLVTADLRRVEDAVPELSQQYDLAAISLDRLLIDALKAQTSQIGGDWRVVRQADAAVQSSADWRRLTALVERAMPAVVAQLRDDPRPLLLQHLGLLVRYGHTGVIQTLRDAAS--APARLLLLPGDSHQ-PPMLDGVVLPVITPADWTHLPRSWLENRHRAAPTA
WP_150933330.1_Microbispora_cellulosiformans ----ATRTTTGLPGLLGPTDAINRLVTVADRGGFLVVKARIKQAAALPELLSAELPVSCVDVTKAFIAALREVRDEWGVPWPIVLATDSPDAIPEDRVGLAEMTTEAFALVEDRVRAMEGVVLLHDATPLARYAGGELLARLADAARSSPHGLWLLAPMADPREPAQLDGQAIPVIPGNEELALSSDTVDALEDSSKAS
WP_090069475.1_Lentzea_flaviverrucosa ---PSTSTGTMSPPLTAASDVDARLVEAARHGGVRIITVRRKRWERARTAVEIALGMPVIDVSVAFVAAVRQVARQLNIPFDRVLLADAAEPGSNDQLNLHRVVAQACELLEAEWSR--HPVPLDALTPLGRYPAGALLERLASRARYGPDALVLISPAGDETKVPRIGDHVIRVNTPEEWVIARSPWPSGVQPRSQAG
WP_183357094.1_Garicola_koreensis -----KTHHTIPVGRKQPERLVRELRESVGARSFLAIGIPARQVERTTSDLLSTYGGVQVNVTDLLLGGLRSQAEEKSVPWDAVLAADAAESGTRAADGLAALVRQSISTIETAISEATQPVVVTDAAPLARYGHLRVIARLADVAR--SNAVWLIVPQG-PEPGAHLDGTPIPLSYSSQFLNLDNRVAVPAAAEETQ-
WP_257478501.1_Acidipropionibacterium_jensenii -----ARDATFGVDLVEDSATTRRLEESVRYRSYLVLGAEPTRLHRLVRALGSRFHAPELDLSKCFLDALHRLSDDRRVPWPVLVAADAQPEASRAHIGLSKVIELAMPEVRSTLSDSPAPLVLTNVAPLVRCGYVKILRELSDLTR--GRAVWVVVPQYGLHVGPEVDDVRL-VTSPNQFVEIDRTWIDAETDAETE-
MBL9044566.1_Myxococcales_bacterium TPGLARLTHPSGPEEEEALALSAKLDRASREGGLLILSVLPQYADAAKQKLAQRFALSVYSIEEAILRHLKAFAHSKQIQWPVILLADAADRKSADFRKLQTAMSAALAQMVDEIRHAPSPLLLIRPGLLARYDRLDVIESIRVSVEPPPPGVWLLLPSDELDLGPRLDGQTVPVLTPAYWRAISPHFLRAALPAVRSS
MCP5560207.1_Verrucomicrobiaceae_bacterium EKSSHRMRTVMQEDVATALLLEEKLSSCATRGSFLVLSVPPDRLARAESELLARFPVQRVDGDALFLRLLHAQAAEYEIPWDTILLADAAKEGTEDNQNLRRLVDMIKPQLRQALVSPAQTLLLVNPGLFARYGLMGMLAELRNDVR-APHGLWLLIPAVVQSALPKLNGQPVPVISEHQHISLPQAWLDNAHGKA---
MBK6942988.1_Planctomycetota_bacterium TRSTPRAATEISDEVAAARAFEERLARAQKDGVFLVLAITPSHALDAERELLRGFALERRSVDVLLLDAMRRHATANEVPWSVVLNADRARPDSVDGKNLRRLVRMAIADVEQQLATSTRTILLTQPGLLARFDCIDVLERLRDRVRVTLFGVWVLVPCDDQREPPVLNGVAVPVITRAQWARIPISWVKNRHRGSESR
WP_217637676.1_Actinopolyspora_saharensis -----SRVGTVPAPSSRGDYLGATLAESISARSFLALGVAVDEADRAAHVLALRHGGTVLDVTGMLVDEMKTVAGKKGLPWDLVRGADAAQEGSRDAQGLHALLNKAFPAVGERLRAGAGPLILTELSPLARYGQLKMVARWSELAR--SRAVWVVLPQLSWMRGALVDSKPVQLGSNGQFVELDADWLATQEAETATR
TAF23825.1_Verrucomicrobiota_bacterium -TIGSRRGTPVSEEVAEAQRLEDKLTRARAQGSYLVLTIDPKYLNDAANQL-ERIGAKRLNASELFLTAMKDKAADLGVPWEVVLEADAAEKGSADWTRLQELIRRSMPGVLEPLRNSNEDILLTDAGLFARYGRMNELDALRNDTT-QPHSLWLLVPGHGAATTPTLCGEAVPISNEAQFEQLTPQWARNHHRGGVA-
MBO0801654.1_Nocardiopsaceae_bacterium -----RRKPSTATLLDETARASRRLEQASRRGGFVAVKAPLRDAARIGDVIAGMDGVTGVNVTREFVRLLRDEASKRPKLWETVLSADSADAPPGARRGFGELLRAVWGALEARIAGDGGIVLLHDATPLARYTGGELLARLAVAARDKPFGLWLLCPMENPKGSPLLDKSTVGVIPGMEQLDLPEGFGSGAESRAS--
WP_238634082.1_unclassified_Halorhodospira -----TRDTTDDEQGERAAEVERKVRRVASEGRFLALRIGPRHMVKAQRRLASGYGLQAYNLEGELLRAMRDVADERRVQWSKVLEADAAGPKGPDWKKLLQVVGEAVRRMRPHVIEPPQPLLLLNPGLLVRYGHIALLNQVRDACE--QPGVLLLLPGSSATTMPVIDDQALPIVHASEWLHADRHWLDAPANA----
MBK9978482.1_Gemmatimonadota_bacterium --TQTHTPNILDDAMDEARMLEERIAAARSDGGFLVLSTEPARFRRAELALAERHALTPMSLERLFVESMRKTAEANRVHWPLVLQADAAPHHDRAWRNLRLLAEKARPMMEATLAALVRPTLLLHPGLLARYDLLAVFEPLQDRSR--GPGVVLLLPSDGMSAMPVIDLVPLPMVYPSDWARLPREWFRLARSA----
WP_130480151.1_Leptothrix_mobilis STYLGRHATARLARLADASALEQRLQHHLQQGGLLSLTVEPRLARHAEAELLRRFGLQRLSLDALLLRELRTQAYARRVDWATVLRADAAPAGSRDWTNLLRLVQLTLPALQQALLNSPAPLLVVHAGLLARYGLMRLVTELEDQSR--PPAAWLLLPGQRPG-VAEIDGTPVPLVHAQAVAALQPAWIGNEHRSAERP
MCO6436553.1_Phycisphaerae_bacterium SGTPSRFPTQIPPEIAEARQFEERLRYAEKDGAFLALTAKPNLYERARQELLNRFDVRPLDLERVFLDSLRQAAEEVGADWNVVVKADAAGPNSTDWRNLNHLISKVVPKVEEAIVQDGKTVVIYHLNWLGRYDQVVMLSRVQQAVQ--DHGTWLLIPGSPQTEMPLLDGKAVPVITNNQWAAVPKSWCQNLHRTDEKA
WP_181762535.1_Pseudarthrobacter_sp. -------TTHTKLSSTQILEIERQLAQSLTRKSAVTLAVRPRDFIRTSKALTQVFGLKLVDVSAEVVKAIRATAQSKNVDWDVVLRADAK-SSPSDHANLAQLVRMAVGPLWVELVDDPNPLLLTNIGPLLKFGFGAEVARLFDMSVPRPAARWVVAPKRGSANTPSSDGRVPPM-GPDGFVDISPGLASALSKRESTM
MBK9741405.1_Actinomycetia_bacterium -------HVLTPPEVPPYDEIAARLQGSLRAGGYLTLAVDPRRVDQAAVLARTYGLTRVDVTAELLAAAKEYAVEVGADWSTILDADKE-VGTRDRALLDSFVKAALDRRFPDILAATDPLLITDASILGRYGQQHWLADLADLAGNRPAARWLLVPHHASGGRPTLDAQPAPL-GADGYLVINAEFLAARQASEQSA
NMM16638.1_Cellulomonas_sp. -------TNLTRVATEPADVVDGYLRASLTRSSALTLCVHPREHDRAVATLTTLYGVTPVDVSERLLTAVRALADRDGVDWSLVLRSDAE-PSGGDWENLRGLVQDALTEPWQQILATRRPLLLLNAAPLARYGLTAWLSDLLDQSQPRPAARWVLVPRRGHQAVPTLDGRPVPL-GADRWIDLPYDLSSLRPSSGVSA
WP_136709615.1_Agromyces_sp._H66 -------LSRTLAKSLPASDLHARLLGSIGSNAALTLAVNPRRYTEARRYLTNRYDVTTIDLAAELVRRMHAVADERGAKWAVVTRADAS-ESSNDFKRVSQIARQAFEPYWRELSAEPNPLLLTNAAPIARYGLTEYLAEMFDLARPRPAARWLLVPHKAAKPTPDLDGRPIPL-GPDRWVSLPSDLAELFPSSSGVV
WP_210768105.1_Cellulomonas_humilata -------GSSVHRGLVPASQVAAQLVSSLRTKSAVTLCAHPDAYVSTTRMLGRVFEIRTVDVGAELVRAARADAKVVGASWEFVLGADVE-PTSPLGSDLRVFLEPTVTAVWDRLMADTSPLLLTNAGPLLRYGFTSALAEMLDLATDRPAARWLLVPRPRAVAIPTLDGHAIPL-GADRPVDVPLDLESAVSGA----
WP_067228577.1_Microterricola_viridarii -------RSTTLQGSLPNDTLAPTLAESVQHRSALVLTTTWGRYDDSATALAARFGVEVVDLAALAVEQLHATAASVGADWLVVVDADKP-DSTIDKRNLQALAKQAMSSAWAEINDRDTALLFTNAAVLAHLGLVDLIARTIDLSTKRAAARWFLLPKHGAAAVPDLDGTPMPI-GAGKWIELPTTLAAVLPIPSSIA
WP_129336322.1_Cellulomonas_endophytica -------LTSIATRTSTVPQVLAVLQRSVETRSGLLLAVTPERHEHASQQLAAAVGLVVLDVADALVREARRVADEDGADWSFVL--GADAE-PGSPLRSQLHEFLEAAVDAVWEQVVSTDAPLLLVHAGPLLRHGFEDRLDHLLDLATARRAARWVLAPRPSSSGVPLLDGHPVPV-GAGRWTDLPADPLPTLGAPS-
RKN35100.1_Streptomyces_hoynatensis LTSASWGASDEDTAVAAAVKAEQRLAHSARQDGFRVLTVPQRRAEEAVRLLTEPYGAQPLSLTGLFLAELRGVVGEAARPWETVLKADAAETGSRAHQQLRIRTDKAWGRLEPELTADDGPLLLTDTAVLARYDAFDLLGRLAEEAR-GGRALWLLVVQSDPARAPRLAGQSVPYQAGDEWIVVPDAWVARRHLVSRAI
WP_262103914.1_Arthrobacter_sp._Marseille -------STYTFIASGSVSELERRLRDSLDRKSALTLAAHPRDYLKAASTLAQRFGVKRIDIAAEVLRVIKSTARQADVSWELVLRSDSE-PSSGDFTNLTRLAQAAIRPMWGEMMQVPGPVLLTNIGPLIRYGFGPDVSKLLDLSHQRPAARWILAHRRGYDETPKVDGKPVPM-GPDGWIDMPRNLSDLLTTNDLNS
MBZ5659870.1_Acidobacteriia_bacterium --SLHRFQTELPREMEEAVEFERRLHAAYRSPSYLVLATEPKYLEMARQNLAKHFPMTIFHCEREFLAALQKEAESKHIRWDVILRADTSKPGARDWDNLRKLAANAAHRVAEQVQSRTQSMLLIYPGLLGRYGQLSILDELADSLT---YSLWVLAGSEHQAASPMMDGQAIPA-RPTQWAWIPAKWLDNEFRKSTA-
MCB0954589.1_Ilumatobacter_sp. GASGVRTGTAVAHEREEALAVHARLQRQEAEGGYLVMTVEPRWQRRAIE-LLRGYGPHTVDVDGWLVGAMRAYTDRRTIKWPAILGTDAAGPEGERWPRLLTVAKDAMAPLQDGLAQ--RHLLFVHPGLLGRYNLLGMLDQLRERTRPALRTVWVLVAADDPAATPTVAGRAVPVSTPAEHLVLPEPWLHNLHKTEGVA
MBL8842562.1_Planctomycetota_bacterium DATSSRHTTEVDPEVVNAAEVERRLTLSHRRGGFLALSVLPRHYLAARAELRARFALDGLSFDAALLARMKRIAQEKGVDWQLVRATDAAGEGAAGWRNLVTLASLATGELLAELSAAGRPLLLTELGLLKRYEQIERLAALRERARRPLHGLWLLLAQSSPGPRPQIDGRLVPVLANGEWLALDSAWVKNRHRSVPRP
MBO9539711.1_bacterium -STLHRLSTLPSDEILDARAFETRLERAVTDRRFLLLAIEPKYLLRAEAEITKRFEVHRVSFESLLVRKLRSAAQAVGAKWEVVVSADGTPRDSNAWRNLRGLVKRAMGEVEQELSTSSTPVLMVENALIARYDHMELLERLRDDCR-SAPGYLVLIASDDQSTAPKLERQAVPLITASEWVRIPHAWLENRHRSTPTP
WP_144981411.1_Gimesia_aquarii SSIGSRRDTSTSPETVEARQTEDRLQHAYRDGGFLVLTVKPSYLRPCEHNLVHRFPLERVSFDDLLFEQLRAKSKEYEFPWSDLYEADSEGTNTDDWANLLHLMSEVGPNIETELMNSERPLLLVHPGLIARYQMMSVLQTLRDRVH--DPTVWVLIATDSQNEMPYLDGVQIPLISKGQRAAVSEYWIDNLHRGRSGS
WP_093484991.1_unclassified_Streptomyces -LSGSGSGTPLDPE-STAARADAQLAGAARRDGLRVLTVRARNAPVVGRELRRQYGARAVSVTELFLKGLRGELTPGAMPWEVVVKADVL-PAGQRPTGFTNLVGAVWQAVQGVLAADPAPVLLTDAAVFARYDGMHVLDRLTALAR-GGRPLWVLVAQDDETAVPKLAGRPVPYLEAQEWIPIPKSWTRMHEPNAATT
WP_200346137.1_Halochromatium_glycolicum -YDSSRFGSAALAAEQRQQAFDDRVRTALDEHRFLALSVRPADAQRAAQRLQARFGLVAISFDALLRQLRRLCDARPPDWQVVLRADAAAPGSIDWSRLQGLVQRVLPELTAALGAAGAPVLLTDTGLIGRYGLVSWLAELRRRLE--GHALLLLIASDGTGPGARIEGVSVPDPGRREHAAIPSQWLQAAADG----
WP_252180113.1_Endozoicomonas_sp._4G ----SRTATVDTTTLEEVKRLEQEIATAIETSRFLALTVKPQLMVTARDKLCKDYPLKHISFDELLFHHLQELFNPKAPRWDVVLKADAAQKNSVDWGRLQMLVSKVLPAMANEIKSAGQPVLLTEPGLIARYDLVSWLNELRQHLNAEVHGMILLVAADAQRTAAVIDGTIIPRTGAAEFARIASVWLEASNDSQQA-
RLS37136.1_Planctomycetota_bacterium SSSVGRWSTTLAPGLETALHFEDRLSRSRRQPNFLTLLVEPRGYGKARAALVQRFQAEIIDLEGFFLAALRHTATALGVEWELVLRADSH-RLPQEWMNLMALVRRAAPDFEERLMATDKALLLVQPGLLARYGLMDQLDKLVDQAR--RPAVWLLLAGD----RALIDGQAVPLIHAGLQVRVPLEWLENRHRAAELS
WP_241482799.1_Kocuria_polaris -------VSTTQFAPTPAAEVARKLTESLERHSALTLTVAPKRYVAAARALASKFDIDVLDVAELVVSATREQIEKAGGIWSGLLGYDAL-DDSKQWKALEGVVQRSVEPVWGERLSAHRPLLLTNAGPLVRYGMTSLLATLLDTGTKRPAARWLLVAKAGDARAPLLEGRSVPL-SPSGWIDLPRDMAQLADDKTIQL
consensus/100% ........................l...........h.............l.........p....hh...p.........a..h...D...........h.......................h...s.h.+......h............s.hh..........s.................h...............
consensus/95% .......................pl........h..l.............l........hsh...hl..hp.........W..l..sD.....s.....h..h...s...h...h......hhh...s.h.+h.....l..h.........s.hllhs.......s.l....h..........h...............
consensus/90% ........s...........h..pl..s....shb.l.........s...l.....h..hsh...hl..h+.........W..l..sD.....s.....h..hh..sh..h...l.....slll.p.s.h.Ra.....l..h.p.sp....s.hllhs.......s.l.s..l..........ls..h...........
consensus/85% ........s...........h..pL..u..p.shbsl.s....h..s...L...hsh..hsh...hl..h+..s......W..lh.uDs....s.....L..hh..sh..h...l.....slll.p.u.lsRYs....l.ph.p.sp....u.hlLhs.......s.lss..lsh......h.ls..h...........
consensus/80% ........s........s..h..cL..uhpp.uhlsLss..p.h..s...L...hsh..lshs..hl..h+..s......Wp.ll.ADs...ss.s...L..hh..sh..h...l.s...slLl.psu.lsRYs...hl.pl.p.sp....u.WlLhs.......s.l-u.slsh.ssspbh.ls..hh..........
consensus/75% .......ss..ss....s..h..+L.puhpp.uhlsLss..pph..u.p.L...ashp.lsls.bhlp.h+..s....h.Wp.ll.ADs...ss.s...L..hh..shs.lb..l.sss.slLL.psu.luRYs...hL.cL.p.sp..s.uhWlLhP..s....P.l-G.slPh.ssspah.ls..hhps........
consensus/70% .......ss..ss....s..hp.+L.puhpp.uaLsLss.spph..u.p.L..pashp.lsls.hhlp.h+..s....hsWp.lL.ADus..ss.s..pL.plhp.uhs.lb.pl.sss.slLLppsu.LuRYs...hLscL.-.sp..s.uhWlLhP.ps....P.lDG.slPl.ssspah.ls..ahps..p.s...
```

## ; Type 3 BREX; BrxF iSTAND ATPase; Representative_1

```
                                          1---------11--------21--------31--------41--------51--------61--------71--------81--------91--------101-------111-------121-------131-------141-------151-
OrigSeq                                   MLSFDIDNIQKAIATAQSRYYKLVLAIPGTLLDGWIGMMAALGTTPVNLGLLLSQSLLEVEPRFRSLEFQDLFARIIRSAEGDLVAFDHAEILFQRDLQLDPLRMLLQHSRNKVLIVRWPGVFSGDKLTYGEPHHPEYRCYEQVDAVVCCMNQG

                                                               S1                        S2  -----------HEH-Extension--------    S3                             S4                                    S5
                                          -HHHHHHHHHHHHHHHHHH----EEEEE-------HHHHHHHH-EEEE|HHHHHHHHHH---HHHHHHHHHHHHHHHHHHH-|--EEEE--HHHHH-HHH---HHHHHHHH----EEEEE--|EEE--EEEE--------EEE--|--EEEE----
                                          -HHHHHHHHHHHHHHHHHH----EEEEE-------HHHHHHHH-EEEE|HHHHHHHHHH---HHHHHHHHHHHHHHHHHHH-|--EEEE--HHHHH-HHH---HHHHHHHH----EEEEE--|EEE--EEEE--------EEE--|--EEEE----
                                          -HHHHHHHHHHHHHHHHHH----EEEEE-------HHHHHHHH-EEEE|HHHHHHHHHH---HHHHHHHHHHHHHHHHHHH-|--EEEE--HHHHH-HHH---HHHHHHHH----EEEEE--|EEE--EEEE--------EEE--|--EEEE----
CEO89248.1_Syntrophaceticus_schinkii      MLSFDIDNIQKAIATAQSRYYKLVLAIPGTLLDGWIGMMAALGTTPVNLGLLLSQSLLEVEPRFRSLEFQDLFARIIRSAEGDLVAFDHAEILFQRDLQLDPLRMLLQHSRNKVLIVRWPGVFSGDKLTYGEPHHPEYRCYEQVDAVVCCMNQG
CEA16532.1_Fermentimonas_caenicola        -MSE----ISKQITHFAAPRYKFVVAPNERM---------LKDFSAFDLGYNLSLVLKENKNEELTFIVHEELTKLLNDNITDYICLSNIGILFEKELKINILQTFQRLSKNTLLILLWDGEVKGNTLFFLSENSKNKIDLREINHIIL-----
HHY32855.1_Bacillota_bacterium            -MR-----LHDLIKQMETNYYKMLVIVDNGHQPKIIGPLQAEGWTAVDVTDAVLKLIEGIPGEKVRVRIGGKLKEWFHSLP-DRLVLYNTSILYSPELRLNPVGAFKYKSRKEKEVVVLLEGRLACNRIQYSQYGRPDYAEMDVSEVIHADIDG-
KUO63975.1_Lutibacter_sp._BRH_c52         -MDKP---IEKHITTYSQSRNKVVLFKEN-----------IFDFKPINVGHILSQALYNLDKDKLPMLVSKELNTILNASITRMLAISNLGILLEPDLKQDINSLFEKYSNNNVLFVQWEGELDHNNLYFLTKEKGIIINIKNLSHIAI-----
MBI3973658.1_Chloroflexota_bacterium      MMASEVHTISAAVDQLDPGYYACLVLVHYDVTHQGSAVLATYRWPHLSVGQELSEALLQEQPSRYPTAASKWLTDRVSSLSPGPVQVTAIDLLFGPALDLDPLILFRQASRTTRLVILWPGTYLNGVLSYAVPEHGHYRTWPKPDIQITTLQ--
MBI9032244.1_bacterium                    -M------LKDRLKEIQKLRNKLCLIPVNGVTKIIYTFNNTYNINSINIGFELSKVLKNELGKNLPIIAVESFRKIIEGSTSETIMVYNLGILLEDDLNLNLESILLEISKNIVVILLWEGEISKQGLHFLSIKHGKLLELTEKHYLQLEI---
CCX79045.1_Parabacteroides_johnsonii      -MDIT------AINKYSAPRYRLVVLKDEMP---------PDDIPKFDIGKSISAYIISEHSNDLKMLVNAEFMRLFREIASNAIALCNFGIVFEEKLGLDMRTILKDLSRNYLMFIQWEGVVKDDRLCFLTSDSSNFIDLSEISHLTI-----
MBK9455368.1_Bacteroidota_bacterium       -MENN-RHIDTYLALANSLRFKLWAIVGEDLIKAVIQYLQKHGYLLVDVGAELGSLYKELTNEEPSHDIGQKIKDWFLSKP-EKLILTNASILYHNAFKISPVGAFKYNSRNKSTVLFLDERIISNRISYGQAGSEEYYDKDINDILITIDIEDN
MBL9119763.1_Phycisphaerae_bacterium      MLRGNLKDLCAAVLAAEERHTRLVLVVGTDEGALIAEFGLRLTTRVANLGLVVAERLINLPARHRPVTAADAAADFIRDAEPGPALVSNLQLLFLPELMLDPLKLLSDSARSRVVVASWPGERTGSCLRYAAPSHPEFREYHNPDCLVVEAPPP
MBO8173527.1_Bacillaceae_bacterium        -MYEEKQSVQFALEQLEGRRHRLLLLVSEDQRKVLNDVASKHQIPILNMNLSLSEELINVPVDRRPRKVGEIVEKILRHQKKDLVIISHIELLFDHSLHLNPIKLFEELSKTKTLIVGWNGKYENNVLSYADPGHSDYRSYYDCDAIIFSIK--
MBQ9466676.1_Muribaculaceae_bacterium     -MKPL---IDQRLTKFSAPRHKVVVLLNDSV---------GASLPAIDVGRELAAYLEAYTSHRAMDEAKQFLTKLIAQKTQPWVALRNLGILFERDLDISPANLIDNLSRNTLFIIQWPGLVNDGKLYFLDRGSRYFIDLSQISHITL-----
MBT3747343.1_Bacteroidota_bacterium       -MEIHISAISELIADMSDVREKYVIVCKNKHQPDFMHIAEQLNASYININLELSRRLLDIPKKKIPFKANRYLEDIINNSNKNIILLDRIEILFDPLISIDPLYFIESNSKPVTMIVNWPGEFSSKILSYAKPVHPEYKSYPNISGRVISIK--
MCA6513726.1_Chitinophagaceae_bacterium   -MQ--IQTMSTPIKDITTERYKLYTLMCNSNELDKIAFVKSQNINVINIGKELANYIDSLDFRYLNIDVYDHTKKLLDKHRSDVVAIYNLGILMEPALELNAVQLFKEFSKSTSLIIIWDNQSENDRLDWQTQKQNFFLDFSDIQLKKLQYAI-
MCF1431077.1_Shewanella_sp.               -MEPG---LRQAIEQAQNYHSQLVVVAGESASLLLEHTASTDGLAVVNLGLELSERLIEVPRQDRAKSAASLFADLLHERNTRVLLLNHIEILFDKTLSIEPLKLLQSSAKNLTLVVAWPGEKTASSLTYAVPSHPEYRYYKASDFGETFVDAG
MCM1450902.1_Clostridium_sp.              -MAETSESVIADLLRFNGPRHRLVFVKDTNR---------TNSNIGVDVGWEVAKYLHDNSLGNIKAESQNFIKKLIYDNISKYIYLRNIGVLFEPELGLNVDMFLTNISRNTLLLLDWDGQMKYPYMYFLHTDSKHRIKIDTLNYMTL-----
NLI12135.1_Peptococcaceae_bacterium       -MSGNIYNIKKELAHIGYRRHQLLIICGVEHAKIIQDITRELAIPCINLSLKLSEELLEVPVVQRGRKVNQLVDKIVSESTGEILCFEHIELLFHPQLQQDPMRILENISRNKIIVVSWSGRYANGRLTYAEPEHPEYRVYNELEASVITID--
OJX40547.1_Chloroflexi_bacterium_44-23    -MP--------KLEIIFHELFDCLILVNPSIQIETANKIKEENISVINISQELGGFLLNISAMDRSRSTQNWMVDKLASHPHDPILCVCPDLLFDPTLQIDPFALFRQIARIKHIVVMWPGNFSSGILSYAVPEHHHFRTWKITDTIINQFPTS
PAD23514.1_Niallia_circulans              -MNTHLEQIENAISAVSEQYFKQIFVYDYINGNSVRTFAESHSLPYINVNVEISLLLQKVPINRRAFLITEVFQGLIGKYSDGVICLDYYELLFDNSLAIDPMILIKNNSRNKTLIISWRGKIIGDALIHAEPGHPEYKKFIVQDAVIIK----
PKO06211.1_Chloroflexi_bacterium          -MQAFQDELLVRLAIAPQQYHRLIIIILNPDSRISKLDIAGKFGLRYINLGLILSQMLLDLTERQKALKMPQLVDQIIGCIEDKPAVLDHIEILFEPSLQQDPLRLLQGMSRNRTILAIWNGQVNNGYLTYAATEHPEYRKYSIRDLNILSLSDY
PZV08804.1_Leptolyngbya_sp.               -MGNYIAPLADLLAIAQGQYHQLVFATVEADSTYWRSVAVALDGHYLNLNLALSQRLLDIPQRQWPHKVMELIDAVVLSTDLRVTVLHCIEVLFDPQLRLDPLKSLQQIARRHNLLVVWPGTYDGRYLAYAEPWHPEYCRYPAKDLIVFPSA--
ROS80359.1_Muribaculaceae_bacterium       --------MRDKIAQYYDPRNKLIFAPSL-----------DQHLHSVDVGFELAKSIEGDSSPHLPMIAEDYLNAILRRSIKDYIALENWGILFEPALKLNLVSIFDSHSKSNTLILVNCGQADYENFHLVNKHFNTTFPIGNLHPYILQ----
TDP26837.1_Halanaerobium_congolense       -MT----HIDDLIKSNRGSHYKLLTLVGSEENHNIIDYLKNNGWNVYDIEEVILDLIEDIPENKIGLKIGDKIKEWLSDQE-NKIVLTNTSIIYSPELLIDPVETFRYAMRDKEAVIFIEGKMRDDKVIYSTPDKQDHKDIDISRIVSEEVEVN
TNF50602.1_Bacteroidota_bacterium         --------MKDKLLTYGSSRNKLVITSEK-----------IPGVHYLDVGETLSLSLANSPISWNSMKIKSEISKLFAESIESILSIENIGILLESEVQFDFKHFLEQYSRDMAIFLKWDGDHDESTLFFLSKDKGIKIPLTNLSYIKL-----
WP_061808754.1_Rossellomorea_vietnamensis -MNFS-EQLETLIEAIKGQRHQFLLLSFQANNKKIIDTLKEIRATTFNVNLLLANKLIELPISRRKRAVKDTLREALNEVENEVVYFERIEYLFDEELNQDPLKLFEYLSGNKVLLINWPGDVRCNGLSYATPDHPEYYQVDGYRDHLIVI---
WP_073205029.1_Prevotella_ruminicola      -M------MNSNLSSYKNPKYRLVFDLSS-----------SLEVESVDVGTYLADALQTKQKANLNMYASDAIRKMIRQHTHRYVALCNIGILFEPALSFNLETIVEQTAKDELLIIQAKGGIANERFYFLSQQNNYSFSLKNLTYTIVE----
WP_075981269.1_Bacillus_massilionigeriensis -MS--LQQIKSEIETLDSRWYKLLIICGLKPYQ-DKDMANQLDIPFININLKLSEKLKDISKAKYALKVPEILQSILEQYEESYYWLGDIEILFDKQLQQNPVRLLENLGKRYSIIVTWPGQYANGQLTYATPEHPEFFSCHETDGKILEL---
WP_086717961.1_Bacillus_cereus_group      -MD--LYILEKEMALMHNWWHKLIFICASNELGIGEKIATNEDIYTVNLNLIMSETLVNISEEKYPLYVEEVTRETFDN-PFKIYLLQHIDVLFDPVIKINPIRLLENISKKSKLIVIWPGEYKNNQLVYAQEGHPEYFLSGSFEGKVILI---
WP_106450119.1_Trichococcus_alkaliphilus  -LNYQLNQLDREVPYLKNKYHKLLYLYEYKKGDTLKYYSEANKVPYINVNLYLSENLADVKPNRRPYKVVDLM-NLLGDTEDDILCLDYFEILFEPSLMLQAFDIFKNLSKRKVVLVAWRDEIKDGYLIHAKPGHPEYTKELIADTTIIY----
WP_113032580.1_Paenibacillus_contaminans  -MSGKLERLRAEMAGVRSRRYKLLFLLPETASAAIAGEMEREGIAAINVSLLLSDKLRHVAPDKRPYEVAKHVKQAINEYKADVVCLYHIEYLFDLELKQNPIKLLEALSGNIVLLVAWPGGAEQGVLYYADPEHPEYDSNGEYGSCILPY---
WP_118194774.1_Pedobacter_indicus         -MID----CKDKIVSFLIPRNRLIISTDFFD---------TEKVNWMNIGKLLAHELGNTDKSKIVFRINQLLDDVIVKNKIETIAIENIGILLEPELKFNVEKFLSDYSQNNTLILKWEGEIDTNQLYFLTKNDNHKIDLNNISHIVI-----
WP_119865508.1_Clostridium_isatidis       -MEAEIYRMTDAVAAAKSKRNKLIIVNTKKVA--FEKAIAAIGLDCINLNLLLSEKLLEIPVSKRSRSVDSFIKDLIKSNNMNTLALSNYELLFLPELKQDPMRLFEDLSKERVILIVWEGRYENNSLYYAEPWHREYREYKDLDAIIVQGNI-
WP_155743288.1_Scytonema_sp._UIC_10036    -MGTSSSQISSYFDQVVERYRLIVVVVPATRSNFCQVAESTNSRYVNINLELSGRLLELRQLHRATQVQQLLAEIIGNTNNGAVFLDRLEILFDESLKLKPLERLQKLARDRTIVAVWNGNIENNSLIYAKSGHPEYCRCPTDDCIVINL---
MBR1791000.1_Bacteroidaceae_bacterium     -MS-----LSEQVISFSSSHHHIVFCREM-----------MPELTFVNVGLVLSEKILEEDSPMLDFATDDFIQEIMSRHESNYLALTNIGILFEPCLKYNIPELFKSHSRNKILFIHTQAVIKDDVLYFNDPSDNITADLSGVHYIIV-----
WP_169099405.1_Bacillus_sp._DNRA2         -MSADVKKIEQELEGISTRYYKMILVCELSKGTAVKKAAESFGIPLLNISMLLSEKLKEYPSKRMAGRVHSLLSDILRNAGSSTLCLEHIEVLFDPTLKQDVIQLLQPLSRNYTLVVSWKGSYDGRRFVYAVPGHPEYYECKEFDGIIVTE---
WP_173978270.1_Magnetospirillum_sp._UT-4  -MQD-IGVVLQEITDVAGTYHRLLLLVGRVPAECIEAVAAHLDRPVLNLNAELSSALLEVPHRQRQLKAHKVVGDIIDGGPADIVLFDHTDILFSPSLRLDALALLKSLSRNKTVVATWSGTAKDGKLRHAEEWHPEYQSHAIEDIKVITVDQG
WP_181742022.1_Thermoactinomyces_mirandus MINTQVEAILTSLKEITRHRHQLIFLAGDCGKYDFRTLAEKIDAIYLNLSLELSHMLMEIPVHKRPRKVDICFREILTGYSQSLFLLDHIDVLFDSSLQLNPGELLENMSRKVVFVIPVYGQVTDSELIYADPGHLEHVRIPINEAMIIQI---
WP_214689525.1_Exiguobacterium_sp._s163   -MNLSVEKIQSSLPQVTNQYYKLMYVNEYLHGNTISYYAKNFETGYLNLGIELSLALLDIPLKRRPFKVTDIMENLIQKQHNKIICIDRIEVLFEPSLKTNPFQMLKQLSKNHTLIIAWRGIVSDQTLMYAKPNHPEYFKYTATEEWIL-----
consensus/100%                            ...........h.........h.....................hsh...h...h.................h.........h....hlh...h..p.....h.........hh..........h.........................
consensus/95%                             ...........h..h.....phh....................hsls..l...l..................h.........h..h...hlh...l.bp...h....u....hhh......s.......h.a....p.........p.........
consensus/90%                             .h......h...l..h.....phhhh.................hsls..lu..l.p.........h.p.h.phh.......hhh.p..lLhp..l.bs...hp..upp..hhh.h.u......h.a....p.........p.........
consensus/85%                             .h......h...l..h...b.phlhh.................hsls..lu..l.p......pb...h.p.h.phh.p...p.hhl.ph.lLap..L.bs...hhp..u+p..hll.h.G.h......l.a....p..b.ph...p...h.....
consensus/80%                             .M......hp..l..h...b.+hlhh................h..lslsb.Lu..Lbp....pbs..h.p.h.phl.p...p.lhlpph.lLac.pLpbs...hhpp.u+p..lll.W.Gphpss.L.ahp..p.pbbph...p...h.....
consensus/75%                             .M......lpp.l..hp..ba+llhl.s..............sh..lslsbbLup.Lbp.s.ppbsbbh.p.hpchl.p...p.lhlpph.lLF-.pLphsshphhpp.S+sp.lll.W.Gphpss.L.ahp..cscabph...p..hl.....
consensus/70%                             .Mp.....lpp.l..hps.ba+Llhl.s............p.sh.hlNlshbLup.Lbphs.pcbsbbh.phhpcllpp...phlhlppl.lLF-spLphsshphhcphS+sp.lll.W.Gphpss.L.ahp..+scabph...c.bll.....
```

## ; Type 3 BREX; BrxF iSTAND+ATPase; Representative_2

```
                                               1---------11--------21--------31--------41--------51--------61--------71--------81--------91--------101-------111-------121-------131-------141----
OrigSeq                                        MKDELKRARASRAAEYCSHNLVLVVLEPEVRDSQIEDVELINVSKSLSQRIVCLSANERIKRLDVEFEEVAQECNENIWLTKLDLLFEPSLKANPILLLKWLSKSRPVVAIWPGQIYEKTLVYAEPGSEDYRTYSLQELKDIQVVNAC

                                                                  S1                  S2  -----------HEH-Extension--------    S3                              S4                                 S5
                                               -HHHHHHHHHHHHHHHHH---EEEEE--HHHH-------EEE|HHHHHHHHHH---HHHHHHHHHHHHHHHHHHH--|-EEEE-HHHHH-HHH---HHHHHHHHHHH--EEEEE-|EEE--EEEE--------EEEE|HHH--EEEE---
                                               -HHHHHHHHHHHHHHHHH---EEEEE--HHHH-------EEE|HHHHHHHHHH---HHHHHHHHHHHHHHHHHHH--|-EEEE-HHHHH-HHH---HHHHHHHHHHH--EEEEE-|EEE--EEEE--------EEEE|HHH--EEEE---
                                               -HHHHHHHHHHHHHHHHH---EEEEE--HHHH-------EEE|HHHHHHHHHH---HHHHHHHHHHHHHHHHHHH--|-EEEE-HHHHH-HHH---HHHHHHHHHHH--EEEEE-|EEE--EEEE--------EEEE|HHH--EEEE---
EGR2106511.1_Vibrio_cholerae                   MKDELKRARASRAAEYCSHNLVLVVLEPEVRDSQIEDVELINVSKSLSQRIVCLSANERIKRLDVEFEEVAQECNENIWLTKLDLLFEPSLKANPILLLKWLSKSRPVVAIWPGQIYEKTLVYAEPGSEDYRTYSLQELKDIQVVNAC
CCU85050.1_Mesotoga_infera                     MVKGDYSKNSLECLQAKEPGLVLLSGGNDPEVANEMNTEVLNLNFVLSKEMKTLSINERPLRASRFLSELVNSRE-SLLLDHIEILFEPQLKLLPIQILRRASRTTPIVANWPGSIENEFAIYAQRNHPEFMEVRLDSEIIFKIE---
HBR02122.1_Ruminiclostridium_sp.               MLGTLIRISEIIETIRKVTIPIIFCSLAPRLKKLINDYQELSLNKELAKALVEKDVIIRSRFVADEVMKIVSLIQGPILLTDYEMLFDPRYNIDVLKLFYELSRRAKMVIKWCGALDDNRLVYATPAYSDFHSYNIHDYDIICVI---
HCH5614386.1_Vibrio_parahaemolyticus           MTEQLLQQNACLEAEFRSFKLVLVVIEPEDSREILNNSIGFVNLSKELARKLISLSISERVRQLETEVKIIANECNSSVWLTKLDVLFEPALANDPMRLLKGIAKSKAIVAIWPGEITETSVVYSKPGKPDYKTYPLVELNDIQVIDAC
HHV38844.1_Tepidimicrobium_sp.                 -MGIDCNIKDLRIWNINNHYPVFSILQVDRIANEFKDFYYIALGEILSSSLLKYGKDDRKDYVIYEMDNIFHNMDSKLIIDHIDILFNPDYRIDILGYFIQLARNRRIIVIWPGEYVSGTLTYATPEYRNYKKYSIKDYNIICLI---
MBD9124683.1_Lachnospiraceae_bacterium         -MGRIIQYRLLSENQTGLMRPVVYCDEKYCES-----LHQVSLNEKIAALLIKIKPERRTMRIERCVQEVLTNIPEKSCIRDFDVLFNPAYKIDVLQLLTVANRSKSFSVLWPGTVIDGKLVYAEDGYADYKEYDVEQYDITCVV---
MBL0521045.1_Aeromonas_enteropelogenes         MAYELQANKALLRMSVQSSALVLVINSPTQIARQTEGLTYLELSEQLCERLLPLSCNDRSRHVSDIIAQMLNGIASVVWLDRIQVLFEPTLELDPLRQLQDLARLKPIVAIWPGQITERFLTFSVPGRDDYQSYSANDLANVPIITEQ
MBN2003265.1_Anaerolineae_bacterium            -DALALSQQRI-MAQIRSHALLVLVGMTDLVTALPDDSVYINVGLELAKHLRQ-NLPTGSLRAAQIMEELLESSGASLILDRLEILFALTLHLDVLRLLLDLNRRIPIAAIWPGEYRDARLTYAMPGHPEYREYEYPEAIFPITILAP
MBO2493598.1_Clostridia_bacterium              -GDNELSLRQVQGYTSNAGNIMVACVDKELVKAFFSNWKWINLGLVVSEQLCRLPKEQRKRMVADVVINAIKKTPSNIVVENIDLLFSPEYSLDVIRTLQLAGKNKRLLVIWAGDYQDGVLTYAQPDCPDFHRYEIKNYGVCCITR--
MBP3452736.1_Clostridia_bacterium              -MGAKIRRSDFRQYPAVYQGLFVFSDA----KRLPKDQPVINLGYVLSERLMGIPADRRCFEIDNCLRSIVETYT-AVSLSHIEILFTPSLKLDAIGVLLSLCRNRKICLSWPGRISGNTLTYAEPSSPEYYEADYTRFIDTYVITE-
MBP8682476.1_Trichococcus_sp.                  -MGSTIKQRDLAVIKQTRQGVFVLAPN----VTAPVNASVVNVGLLLGERMFRVKTEGSSTSITRELDDILDEEHRDILLTNIDILFAPEYELDVIKLLLQLGRNQRLYLPWPGELVGERLTYSEPIRFDFKEYIVKNYVDTYVV---
MBR6410258.1_Clostridia_bacterium              --MYLLKSSELPENLKQSGVPLVFCSESPRICKRVESYIELKINIILSERLCRIPRDERAKLVEFKLNNLLNYYHAPVLISDFEMLFDPRYKIDILRFFCEKARIINVAIKWPGQYADGKLTYANPADPDYHEFDCNAYQIRIVQ---
MBX3742593.1_Akkermansiaceae_bacterium         MIAEKIAQVDIARASGEHFRLVWLAGGTPSELAEAEDGVVVDVGKILSSSFIEVPVSLRSASVEDCFTACLGESPSLTCLDHLEILFEPSLRVNPVALIKGASRHAVLVASWPGTTSTGHLFFGAADHPSHMDMSEHELESVLHL---
MCC5856368.1_Idiomarina_sp.                    MEEQLPSNEQIDKLALQSHQMLIVVGANTEQLTLLDNIPRLAISELLCEKLLPLASQRRVDEVEALTADVIASQATSVLLERIHLLFEPSLQLDVLRCLKALSKQRTLIIDWPGVYNGAALSFSMPGKPDYFNYSETELSSVPVLLTG
MCD8293624.1_Clostridia_bacterium              -MGTIIPAKIFPTDLTGLLKPIIYCCSYDGL-----DARALPINVPLARELLKYESAARTMLMKECFTNVVEEFPDDCTIKNFDVMFNPAYKIDVLGIMVSVCKIKPFNLIWSGTLDSNKLIYAENGFKDHTVYEIDDYDITCIAKK-
MCP5271842.1_Burkholderiaceae_bacterium        ----MLDLRLVNDAGALQSRLVLILGGDDRRAAAHFGTQSLPLGIDFARRISAVARRQRPLQASSLLRDSVDAAFPPALLDRTEILFDRTLQFHALVELKRLAHSRTVVAAWPGQLRDQRLIYAPANHPEHCDHPAAGFLVHHIS---
MCR4442916.1_Peptococcaceae_bacterium          -MGKEILLREASEYGTGKGPVFVACTNREVIKQLFPDWPWFNLGLCISQKVLSYKKEKRPNYIVDIIQEMVSASESRVIIDNIDLLFAPYYQLDVLKLFIQLGRNKKLIIMWDGAFRDGLLSSSVPELPDYHLYDIKNYDVYCLTK--
NMM63085.1_Clostridium_muellerianum            MMGATFKFSSMKVEIEKLMKPLILCVRQDKITSYISEYNVLSVNGLLSKNLLRYEFDKRSIFITDEFNKIIDNINDPILIKDFEILFDPAFQLDVLKLFIMANRKKRVTVLWCGKYKNGRLIFAEPEYEDYKSYNISDYDISCIV---
NPE22819.1_Fusibacter_sp._A1                   -MGQIIKYYDLSYFITSTNSLIVICNNKKLKKDIGLQYHHFSLNQELAKKMNEIEVEKKALFVIDILNEMFE-INENIVVSDFEMLFNPSYQLDPLKYFINLSNKRKIILEWCGDFDGENLTYATPNYLDYIAYKVDKHNITCVI---
QQS04458.1_Fibrobacterota_bacterium            ----MHSAQILSELKERHFKLVLLVGINPQEWANDCLTSVTQLGRALSPRLAELSVRSRPLEVSGALEELAKEVTTLLFLDGLEVLFDDSLKIDPFDTLKRLSLRRLVFAVWPGDYLRGRLTYAKVGHPEFRDCAPEGVLLYPRSMQ-
RKL62728.1_Thermoanaerobacteraceae_bacterium_SP2 --MLKALQNAIKILPHKYYKLILVVGPKERQMSLEFGYDYINLNLKLSEKLIRIPFDERCFYVEDYVNEIVESNHGLLILDNIEILFEKHLKIDPLLLLKNLSRYKACIAHWNGKVEDGHLIYAVPQHPDFVKYRMDGHDYIVIDTAQ
RRF92483.1_Coriobacteriaceae_bacterium         -MGALISRRQFSHKQLNYARPLVFCCDINGLT-----EDSVPLNVPLARRLAGIPAPSRSRRLEECLVDCLESLPNGVAIRDFDVLFNPSYEVDIVTLLVSAYRRHSFSVIWPGTLDGDELVYAEEGCADYRRYDINRYDITCIF---
WP_013778924.1_Tepidanaerobacter_acetatoxydans --MGLTDTNALMIFSHKFYQLLIAGDDSHKIAESGNYAYVNLNLALSERLMMIPSHEHSLFVNQCIDEIINGISATVIFDHIEILFEKPLNTDPMVLLKNISRHRKLISDWPGIIKDDILIYAKPGHREYVKYPVE-VDYTIIDIDQ
WP_013787752.1_Thermoanaerobacterium_xylanolyticum --MLLCERDALAILSNEYHKLIIVLTQLDDKISNKYAYDVLNVNLRLSEKLISVPISERWIYVDEYFKKLLNDYKTVIVLNNIEILFNPHLKVDPLRLLKSVSRYRKIIAIWTGNYTDGVLTYAEPSHPEYRRYKNDDLDFIMVCSNC
WP_028243927.1_Pseudobutyrivibrio_ruminis      -MANVMEWYKFLDNISGLMKPIIFCTSSEIEEL----SEAYSMNIPVAKELQKSGSSMRTMKMESILSSIISKLGDNPIIKDIDVMFNPDYQLDVLRTLINVSKRKSFMVVWPGTYKDGKLIYAQEGYSDYKSFDLNDYDITVVI---
WP_052235486.1_Alteromonas_marina              MMDDIFTQQSLSSGEMQSSCLIFLILEDSELSTLFSDALFLNTSKLLAERLITMSRREREANVASALKSIVLAGDNMCYLQRIQLLFARELNINPVKLLQQLSRKKPVVAVWPGGLDCSNLYYAKPGHPEYVSYQLSEFQDVQVISTC
WP_071138484.1_Bacillota                       -MAEIYTKYNFLDLIKRNKLLGLLCMSADEFHIEVKDLSIINLGLDLSHKLKEYPMEYRNSKVLDELTNILAKAQTYIVVKNIDILFNPDYKLNILSYFINLSRSRLIFVEWPGRLKGRMLEYADINSPDYHKYNIDDYKIILIK---
WP_108775977.1_Lactimicrobium_massiliense      -MGVITRIDKLDDK---YMKPVIYTARNYASG-----IEQISMNRPLAERLIKLTTPQRGMRMGKVLLDCLKDQPDDVVITDIDVMFNPDYPIDVLQVLIDTCRVKAFQVLWPGSYESGALIYSVPDRPDYKKYLIENYDVTVII---
WP_164569834.1_Rhizobium_leguminosarum         MPAAAAAKQLLEATGVSGGKPVLLVGSEHDRLATLSNTSSINLNIHLVQALIDASDA-----RVDIISTISALEPSVLLLDRIQILMLPQLMVKATDVLCRVARRRSVCASWPGRLDRGRLLYAEPNHPEFLDEDASRALVIDLS---
WP_191750182.1_Clostridium_gallinarum          -MGDVIGIREVEMLIQQENKVIILCTNYKSIQSIFKEFKRISLGVILSERLLRYPIEIRNSILIQELDKLLLNTDDKLLIKDIDILFNPEYKIDILKYFYSLARGKKVAIEWNGSIKNNYLQYSEIGYLDYKRYSINKYDIICIK---
WP_243187470.1_Clostridium_celatum             -MEKVSKYKELSKINKLTNGLILITNSFDKAEIKIQDYKYISLGESLSKKLIKNDKVIRNRKVKLELDNIILNNDNSLVVDNIDILFNPEYKLNILKYFENVSKKKKIILVWPGNLDKNELVYSKLEFNDYKRYKIKDYNFILLK---
WP_271679096.1_Thermomonas_sp._2C3345          MWSVALEQSLMADTAQLQSKLILLVGGTPAELQRSQHATPLHLGVSMASALTELPQRERHLQAGNLLREIAQSHEPPLLIEGVELLFDASLKVNVLDLLKRQSQARPVVAIWPGEWRNGRLTYAVLGNPEYQDHAANGVIVFEI---
consensus/100%                                 ....................h....................s..hs..h............................hp..phhh...h.......h.......h...WsG.....h..u.....pa................
consensus/95%                                  ........h..........hhh.................h.hs..hs..h...........h....h...hh........lp.hphhF...h..p.h....h....sp...h.h.WsG.h....l.au.....pa................
consensus/90%                                  ........h..........hlh.................h.ls..lup.h.......+...h....h.phh......hhlpphclLF...hphs.h..h..hs+...h.h.WsG.h.....L.au......-a.ph..p.h.....l....
consensus/85%                                  ........p.h........hlhh................lsls..luppl..h....+...h....h.phh......hhlpph-lLF...hpls.l..h..hs+.p.h.h.WsG.h.....L.au......-a.ph.hp.h.....l....
consensus/80%                                  ........p.h.........llhhs....b.........lslsb.Luppl.phs...R..bh.p.hpphh....s.lhlpph-lLFpsphplssl.hhb.hs+.+.hhh.WsGphps..L.Yu..s...-a.pa.hpph.....l....
consensus/75%                                  .....hpbp.h.........llhhs....b.........lslsb.LuccLhphs.pbRs.bl.p.lppllp...s.lhlppl-lLFpPphplcslphhbphu+p+.lhh.WsGphpss.L.Yu.ss.s-a.paphpph.h..l....
consensus/70%                                  ....hhpbp.h.....p.....llhhs..p.b.....sh..lslsb.LuccLhphs.pbRsbbl.p.lppllpp..sslhlppl-lLFpPphplDslphhbplu+p+.lhh.WPGphpss.L.YApss.s-appYphpph.h..l....
```

## ; Type 3 BREX; BrxF iSTAND ATPase; Representative_3 (Walker A and Walker B intact)

```
                                               1---------11--------21--------31--------41--------51--------61--------71--------81--------91--------101-------111-------121-------131-------141-------151-----
OrigSeq                                        MAASLSSEIHDVLLKTQDTYYKLILAVGPARTGKTAALTELAAKHKWPRINVNLCLSEKLLELTHRQRAVRVAGILNDTVRDETSNVVLLDNIELLFAPELAQDPLKLLQSLSRNRTIVAAWPGNFDGSSLTYSEPGHPEARRYLTPQAVIVKASETN

                                                                        S1                          S2  -----------HEH-Extension--------    S3                                S4                                  S5
                                               ----HHHHHHHHHHHHHH-----EEEEEE-----HHHHHHHHHHHHH---EEE|HHHHHHHHH---HHHHHHHHHHHHHHHHHHHH-|--EEEEE--HHHH-HHH---HHHHHHHHHH--EEEEEEE|EEE--EEEE--------EEEE-|-EEEEE-----
                                               ----HHHHHHHHHHHHHH-----EEEEEE-----HHHHHHHHHHHHH---EEE|HHHHHHHHH---HHHHHHHHHHHHHHHHHHHH-|--EEEEE--HHHH-HHH---HHHHHHHHHH--EEEEEEE|EEE--EEEE--------EEEE-|-EEEEE-----
                                               ----HHHHHHHHHHHHHH-----EEEEEE-----HHHHHHHHHHHHH---EEE|HHHHHHHHH---HHHHHHHHHHHHHHHHHHHH-|--EEEEE--HHHH-HHH---HHHHHHHHHH--EEEEEEE|EEE--EEEE--------EEEE-|-EEEEE-----
WP_063830324.1_Bradyrhizobium_murdochi         MAASLSSEIHDVLLKTQDTYYKLILAVGPARTGKTAALTELAAKHKWPRINVNLCLSEKLLELTHRQRAVRVAGILNDTVRDETSNVVLLDNIELLFAPELAQDPLKLLQSLSRNRTIVAAWPGNFDGSSLTYSEPGHPEARRYLTPQAVIVKASETN
OQZ06365.1_Planctomycetes_bacterium_UTPLA1     MVQSLLNRLSQLVEDATSQYARLVLIVGQHGTGKSAVLREFAHRGGHPVFDLGADLPQGIFEVPARRRPVAASDRVGEQLR--NDDVAFVDNIEILFQPGLNLDPLKLLQDNARNRVIVATWPGTFDGRDLTFAEPGHPEFRLYPAAEVRILTL----
WP_211295643.1_Planifilum_fimeticola           MDRTAFQALMEAVRQMQTRRHRLILVVGPSGSGKTALLRAAAKILAAPLLNINLRVSARLHEVPRSRHPYEVGRIVRHMIADTASGTVFLDNLEMLFAPELQLDPLLLLENESRHRLLVASWAGRLEGEVLVYAEPDHPEYRRYTNFHAHLISVGGKV
WP_254950516.1_unclassified_Cyanobium          M-RDLARAIAEQAELAAKSFYRLVVVAGPPGSGKTIAFNELQVARGWSLLNLNKVLSERLLDLTSKQRRLRTADIVRDLIEEQNQTVTMLDNIGLLFHPALQQEPMLALQRASRNHTVIAAWQGEVVAGKLIYGSPDHPEYRRLDVKDVQIVTANGHC
NMD20860.1_Verrucomicrobiota_bacterium         -TDVSFPQLLDTIRATQGGYYKLTILAGSARAGKTDLLKQVAAQLDLPLINLSLHLSQRLLSQNRRQRVLNAEDVATEVIDENYKSGLCLDNTELLFDSTLQLNPLGFLQGVSRNRLIVATWNGILAGGKLRFGHTGHPDFFRQPVNGYPVVSVAEDK
MBL9185852.1_Opitutaceae_bacterium             -SSGFPEKIDETIRHARSGRHRLVLVIGDAGSGKSAFLQSIAAARAMQFLSLGEPLGIRLLETSPRTRPLVIEDTVRQFID--TRAGVCIDGTDILFNPALRCDPLRLACSISQNTFVAFTLTGRIEGKRFVHGYPDHPEFYSEEVPSVPIITLDRSE
MBA4157056.1_Gemmatimonadota_bacterium         -SPSAMDRLLPLLAAAPRQFYRLVLVVGASGAGKTATLRALGEHLGVGVVNLGLRLSERLLDVPVRYRPVEVLGLLDDILATSGDLPVLLDNTEILFELALEQQPLQVLQRLSRNRTVVATWSGVVQGGRLTYAPVGHPARREYAVDNLLISLPGE-
ACD82385.1_Methylacidiphilum_infernorum_V4     MAEPLVDQVLRKIREARELYHRLILVVCPAGSGKTGALWEVSASTSAPLVNVNLELSRRMLDLAKSQRARKLQRILGEIMGEATGELVLLDNIEILFDVHLEHDPLRLLQRLSRNKTIVAAWNGSIVDGYVNYAVPDHREYRRYPIRDFMVACPEMTT
SCX35221.1_Agrobacterium_rosae                 MPEPIQKIRDAIKQSEGLYHRLVLLVGENGSGKTTAIRDAAHALDADVINVNLALSTKLLETAKQRALRLAELLGEVVENS-APVALLDNTEILFDHNLQQDPLRLLQSMSRNRCVVVSWNGTIRDNKLTYAEAGHPEYRRYDLTETLVVEIRQSI
GGE39031.1_Primorskyibacter_flagellatus        ----MIDRLDRVVEDISTVHSKLVLLIGEPGAGKSKLLCQLAERRAAPILNVGAALGRELLTVPRARRHLHVAEIFRRLADDLSQRILTVDNIELLFDRTLKLSPLDLLRRQASRRIIAVWPGGMRDNRLTYAETGHPEHQDYAIDGLVPFIVH---
MBI1186275.1_Alphaproteobacteria_bacterium     ----MLDDLVRLVEDVAGLNSKLVLLIGPPRSGKSRLLEQLSMRRKVPVVRLGEALGRLLAPLPASQRHLMAPDLLSGLAGGAGRDLLLLDNIEILFDRTLRLSPLDLMRRHSHGRRVVAAWPGELRDGRLVYAVPGHPEHQNYGTEGWVPCSVC---
MBK6654764.1_Zoogloea_sp.                      ----MISRLETLTSEISALHSRLILLIGPPRSGKTALLNSFGERMGMVPLNIGSELGRQLMPVPHKQRHLQAGNLLRELADIHTQDLLLIDNIELLFDPTLQLSPLDLLKKQAHTKKVVAVWPGELRGSRLTYAEMGHPEQRDCATNGVVLFEIQQ--
MBQ0714976.1_Paraperlucidibaca_sp.             M---TIERALHLLTEVGNTNTQLILIVGSRGAGKSAFLRECSLKLGIQHRNVSYELSRQLGFASCSERSFLVGDMLRDLAEKNRNIPLLLDNLELLFEPSLKISPLDLLKRLSHSRPILAVWPGLLQGENLTYADMTHPEYRNYSLDGITILKIKNS-
MBS3911805.1_Hydrogenophaga_sp.                --MLMLTKLERYVDQLGGLHTKLIVLAGPRGSGKTKLLQELGAKLGVKPLNVNLELGRRLSATPHAARGFSAGQLLRDIADKQKEEVLLLDNIELLFERGLQINPLELIKRLAHSKRVVAVWPGELRGNRLYYAEMTHPEYRDYDADGVVALEI----
NMB01454.1_Bacillota_bacterium                 -TNLLIPALQRAVTSIRSNRVRLILLVASSGKGKTKILQKYAVENDFPLLSLGLLITKAIQEGILPEKLII---YLRQEFE--LGETVLLDNIEVLFQPSLRLQVLQLLKLLSRERTLIATFPGRMIDGSLVYAEASYPEYKQYEIKDFVILDMNGEL
QCB54648.1_Sphingopyxis_sp._PAMC25046          MERRMLEHLQQLVADAAQLNSKLVLVVANSGGGKTALLAEFAKTHDLQIINVGSELSNLLLPIPSSQRSFEVLGVLRGMLSHHDRGVVVLDNIEVMFDAELKINPFDTIKRLAHAMTVVVAWPGDFKDGRLLYAKVGHPEHCDVAVDGAIVLELN---
WP_198322149.1_Methylococcus                   ----MLSRLDSLVDETGALQSKLILLVGRPGCGKTRLLAMLANRRGAKVMNVGAALGKRLAALPLRQRALQANVVMRELAQSYSGDLLLLDNIELLFDQMLKLDPLDLLKRHAKALRVVAVWPGELRDGRLIYAEMGHPEYQDYGLDGLVPFEIDKFA
WP_202949433.1_Pseudomonas_sp._GM55            MRMEVVEELEKLLADVPALHNQLILLIGPPGSGKTAALLGLAKRLGVMLLNVGSALGTRLATVPGKARSLEATHLLREMADEHRNSVLLLDNIELLFDRHLRLDPLDLLKRQAQSRPVVAAWSGALHDGRLTYAHMGHPEYQDYAAIGLVHLQIDA--
WP_210246760.1_Bradyrhizobium_sp._UNPA324      MSEETLGRLERLVDEIESLNCKLVLVIGRSDAGKTELLAELAKRRQTSSLSVGAALGKALLSVPASRRHLQAPDLLREMTGNSANGLILLDDIEVLFDRTLRLNPLDLLRRNAQTRRVVASWPGELQNNRLSYAAMGHPEHQDYDAEGIVAFRM----
QVL49523.1_Thiocapsa_sp.                       ----MLTRMSALVDDVASLQNKLILLVGPPGCGKTALLDAYAEHRGVERRPLGSALGRRLAALPRTQRHLLAGSLLREIAETTTASPLLVDNIELLFDASLKLNPLALLKRLAQARVIVAVWPGELRHPRLTYAEPGHPEYRDDALDGVVTLDLQD--
WP_210879911.1_Roseovarius_autotrophicus       ----MIERLEKLVDDITSLNSKLVLLIGPPRSGKTALLVQLSQHRNVPVLNVGAALGQQLLMVPSTRRHLHAVDLMKDLTDETREGLLLLDNIELLFDRTLLLNPLDFLKRHAHARRVICVWPGEVRDGRLSYGRAGHPEYQDYGINGLVPFEISDGG
PSR33880.1_Sulfobacillus_benefaciens           MMRDSWETARHFVSQIQELPQPLVIIAGARKTGKTPLAKALATQGLGTYINLSLLLSRYLATDPDADDNLTASTMVRLLRDISPTGPMIVDNIEAVFQPELRLSPLRWFRQIARDMPLVVVWPGAVVEGEFRYSMPNRPDYFYSREPSLVVVNLETEF
HBM74931.1_Clostridiaceae_bacterium            --MPVVEDIERIVKLFPQQYYKLLLLIGPSGSGKTFVLKKCMQVLMCKYINLNLELSRKLKEVPVVERCYYVQDYIDEIVKNNDSDIIIFDDIEILFSKHLKIDPLSTLKNISKYKKFIAAWPGYIKGGYLVYAEEGHEDYIRYNLNELECSYLNLGR
KUJ90861.1_Thermoanaerobacter_thermocopriae    MDMSIYKDIQDAFILLPYKYYKLILLVGPIGSGKTQILKKISSDYNYDYINLNLKLSEKLINIPFEERCYYVDDFMEEILSQSKTDVTILDNIEILFAPHLKIDPLKMLKNMSKYKKIITSWGGSYINGILTYAEPGYPEYRRYKKDDLDIIVINVEG
PKO02178.1_Chloroflexi_bacterium_HGW-Chloroflexi-5 -KSAIKGDLITAIQTVQSDRHKLIILLGKFGSGKTAILKQTAVEVRGNYLNLNMELSEKLLGIPTSQYLTTVHAIIDQLCEDAHQEPLLVDNLEILFSPELKINPIDTFKRIARERPVVIALPAYRQGSSAIYSSSGHEDYVVMPLEEYIVIEIPEE-
WP_026486464.1_Caldanaerobius_polysaccharolyticus -ELIRKEDIDEALKKLKYRYYKLILVVGPMGSGKTDFIKSISLKYAFDYINLNLELSEKLINIPVDERCYYVEDFIDEILAGKRTEILAIDNIVILFGPHLKLEPLRLFKNMSKYRGMIVAWGGDLDDGYLTYARPGHLEFRKYSQNEIECMVIKLGG
WP_051534279.1_Desulfitibacter_alkalitolerans  --MATKDEILGCINKLDSHYYKLILLVGKTGSGKTRMLKALAAATDGAVADLNVELSKMLKNLPKMERCHYVQDFLDVVLRQSSGQPLFLDNTEILFSSHLKVDPIGMLKNASRYQPLVVTINGVIDNNKLTYAEPNHPDFIVYKVSELGCRYYSFRG
WP_053959406.1_Sulfobacillus_thermosulfidooxidans -MQESWDVISQQVKQLPTQLHPFYVIAGKPGTGKTAMARRIADEGLGVYVNLSFALAQYLLRQQDID-LVSVHDLLMCCEEIYSSGLFVFDNIELLMTLKLQVVPL--FQEFSRYHPAVVIWPGTVEDGIFQYSMPNLDDYYYFRDMSVLVTTLEHKS
WP_241751529.1_Bacillus_cereus_group           -DIVKEEKLLKAIKGASNDHFKLIVISGRFKTGKTSLMKRIANDFNYMYLNLNLIMSKRLLQIKEEAYVTQSQDIIKGIFDEIDDKILFVDNIELLFSKEVSLNPVEVFKNLARDKVIILSLPGRVKGDRIEYSTIDRNDYKLMDVFGLTVINMEE--
consensus/100%                                 ........h.............h.lh.s....GKo..h.............ls..hs..h..............h.............hDs..hhh...l...sh..hp..up...hh..h.u......h.au..s.....................
consensus/95%                                  ........h...h.........Lhllhu...sGKo.hh..h.........sls..ls..l..............h............hhDs..hlF...L..pPh..hp..up...hlhshsG.h....h.au..s..-.........h.....h....
consensus/90%                                  ........h...h.........bLlllhu...sGKo.hh..hu.......hsls..lup.l..............hh...........hhlDs.-lLF...Lp.pPl..hpp..up...hlhshsG.h.s..h.au..s..-.b......h.....h....
consensus/85%                                  ......ppl.p.l..h.....+LlllhG...sGKo.hl..hu.......hsls..LupbL...s..p+.h.h..hhp.hh.......hhlDN.ElLFp..LpbsPl.hhpp.up.b.llhsasG.h.ss.l.Yu..s+s-ab.h.....h..h.h....
consensus/80%                                  ......ppl.phlp.h.....+LlllhG...sGKT.hl..hu.......lsls..LupbL...s..p+.h.h..hlpphh.......hhlDNlElLFp..LphsPL.hhpp.u+.+.llhsWsG.h.ss.l.Yu..sHs-ab.h.....h..hph....
consensus/75%                                  .....hpcl.phlpph.....+LlLllG..ssGKT.hLpphu.p.sh.hlNlsh..LupbLh.ls.ppR.h.h.shlcphhp...p..lhlDNlElLFs.pLplsPLphlpp.u+.+.llhsWsG.lpssbL.Yup.sHP-abphs...h.hhplp...
consensus/70%                                  ....hhpcl.phlpph.s...+LlLllG.sGuGKT.hLpphu.p.sh.hlNlsh..LupbLh.ls.ppR.h.h.shlcphhpp.pp..lhlDNIElLFs.pLplsPLphlppbu+s+.llhsWsG.lpssbL.YupssHPEabphshpsh.hhplp...
```

## ; Type 4 BREX systems PglZ N-terminal iSTAND-AAA+ATPase; Representative_1

```
                                                         1---------11--------21--------31--------41--------51--------61--------71--------81--------91--------101-------111-------121------131------
OrigSeq                                                  MRYDAESCFNEIIAYFNSEVGYPFIANIDDATILQDICSKMQADSKKIIRVSDYCNGDNLPNESELLTHVASADNGVVLGLSSYYMLRGEQALKKSVSTLLQMSVHGHVVIIIYGCSSILSNISADGRPDHRTVILEK

                                                                                S1                      S2 -----HEH-Extension----   S3                                  S4                        S5
                                                         ----HHHHHHHHHHHHH------EEEEE--HHHHHHHHHHHHHHH--EEEEHHHH--------HHHHHHHHHH--EEEE-HHHHHHHHH-HHHHHHHHHHHHH-----EEEEEE--HHHHHHHH---------EEEE--
                                                         ----HHHHHHHHHHHHH------EEEEE--HHHHHHHHHHHHHHH--EEEEHHHH--------HHHHHHHHHH--EEEE-HHHHHHHHH-HHHHHHHHHHHHH-----EEEEEE--HHHHHHHH---------EEEE--
                                                         ----HHHHHHHHHHHHH------EEEEE--HHHHHHHHHHHHHHH--EEEEHHHH--------HHHHHHHHHH--EEEE-HHHHHHHHH-HHHHHHHHHHHHH-----EEEEEE--HHHHHHHH---------EEEE--
RGH34270.1_Ruminococcus_sp._AM47-2BH                     MRYDAESCFNEIIAYFNSEVGYPFIANIDDATILQDICSKMQADSKKIIRVSDYCNGDNLPNESELLTHVASADNGVVLGLSSYYMLRGEQALKKSVSTLLQMSVHGHVVIIIYGCSSILSNISADGRPDHRTVILEK
WP_073324030.1_Fibrobacter_sp._UWOV1                     --MNLESCVLEAKSYRKSAQSYPLWIDFANAEDMRSFMENFLA--DKKISVEKYCNEDSMPRFENLYSDFRMEDFAFICNLSGFLKLLGSRKTAEVLSTLLSIG--SKKIILTVQCGVDFRI--TDPRLKCQILSVEG
WP_216471320.1_Butyricicoccus_intestinisimiae            MRFSIEKCIQAIDRYLVRSASTLRLVNVDNPAQMNQLIERYRVKGNEFLTVAQYSKPDEKAQTEEMLYAISKMGNLFVSGFTTFWKLEGEAEVKKQLDRLLHMSVKGHVVVLCYQCEHLLNF--ADARIERLIYFVDG
WP_044080180.1_Prevotella_pectinovora                    --MKYEEVIRRIDKYKDSDKHQPIIVDLPNVQVYNQMVKHYDVGSNVFKDVSSFCNGEGLPVMDKLQYCLSIGGVVFLTGLSFYLKLQGEVSLRSCLRQMLDLSCSGKLVILTLNCSCVLNH---DSRLSGRISIVDG
MCI8555501.1_Clostridia_bacterium                        --MTIQE-VNYVKEYLFEGDNETCILNIESLDMYNNIVKAIKELNINIVRLSSFCNDNVSPNMENFYNALEINQPTAVIGISQFFKFIGKDRLKNEISVALSKTLKNKALFITFQCFDVLKELSQDIRLHNRITILGE
MBD8953983.1_Dialister_sp.                               MENTMDQCFDRIEAYCQKEIGHPLLVNTENAHDFQCLVKRMQLDGKTCVFVHDYC-GKGLPQIDALFRDVKKKGCFVMIGLSSYVMLLGKKRAEELMARLQELSVAGHVIVLLSHMKEIRLSMQQDQHLSYRVLLLEG
MZQ76087.1_Peptoclostridium_sp.                          MT-----AIDCIKKFLKQSNSKFLIVNINNVEDYMNIKSSLDIE-LSTIKLSNFCEYDDMPDIEKFVKCCRTDKSSLVLGVSQYLKLKNKESFEMWTQVLLSSMTLAKVIMIYFDCENKLKELDRDVRFQNQIILVKG
HIS35238.1_Candidatus_Scatousia_excrementigallinarum     --MQLNSCIEKIEKHIHRGSSKPIFINLNNSQDVKELIDHFNVGGNKIVNVASNDQIDELPTLDAILGLIHRDETIFITGISSFARLMGQSQFASILNQIINSQSVSKAIVLLYQCEDILHDISKDPRIDSHIFFIDG
WP_103226892.1_Clostridium_sp._chh4-2                    ----MNDCIERINNYLQDENTYPLFVNVENATQKMEILDYYRVGDNKIVHIADFAKKDSLPNVMGVIDDIKASGNMFVFELTTYLKLISGSELQKTMQTLCNMSIAKKAVIVCFHCGDILKKIQKDLRVAKRIVAVDS
NBJ16967.1_Dehalobacter_sp._4CP                          MYSGWDRTLNEVTKYLSNTSNAP-FFLVVGDGNYESIKTQLSEAGLKSVKVSELCNTDKRPNLDSLIGNVDPDKKIVILGLGEYLALCGEEAAFDRLSKIKDMKVNARVVILLRGVTSVVLRQNADPRFDGRRVLFTD
CDC05526.1_Clostridium_leptum_CAG:27                     M------LCKEVQNYIATAKGLP-FFYVVGDEDYATVLDELRQANVSIVRMSDFCKDDKFPSVDELVDYFRRNNKFVVVGLGEYLALRGTAIVDKELRRLKNTTLNARAILLLRGVSTQASQIRDDNKMEQQRAYISA
MCL2016626.1_Defluviitaleaceae_bacterium                 -------MNKKIKNYFSSELSKP-YFCFLNDEEYRQTMSELQADGVKIIRVSDMCGTDRPPNMDKLIDIIKNPAKMAIVGLGEYLAISGFDTARDNLNRLQATETRQKVVLLLRGVFSQIPKMRNDRRFDERVHYTSA
WP_235396406.1_Pseudoflavonifractor_phocaeensis          --MTIQEVIKKIDRYLKKDNVEPLIVDVQNKADLEAIMLHYQLPQNVFLCASDFCKPDEFPVIPNILERLAENTNFFVSEITSFLKLKGEKALVQELKELLSMSTEGHAVILTFQCVEYLHTIKNDRRLDSRICVLEG
MBR3817930.1_Clostridia_bacterium                        --MLLDACINRINNFITSSSSQPLIINVNNSSQFNELVSHFNVGDNRVISSKDYCKNDEMPQIDLVFDLISADGIVFITNFTTYLRMYGEDELRKTLKTIINLSTRGKVVFISYQCEQYLSF--TNPAQKNRVCLVDG
MBP3656735.1_Clostridia_bacterium                        --MKISDIYGRIERYLRSDDTRPRIVNFQDRASLIAFRDHFNVGTAAFKMPSDYSKVDEDILTENLYAEIQFQGNLFLIGFTSQLKLKGRDTLHQFIAQIAHMTRSGKLILVCFCCAEMLHF--DDTRIQGLVYPADD
ONI45221.1_Epulopiscium_sp._SCG-C07WGA-EpuloA2           M---VDEYIRSIDTYFKGEKNSPFIVNFENILDLQEAIAHFEVDGNTIVKTSTLANKDNLPSLDNVLSNCDITGQIFVTEYSSFLKLRGHNSLIETFRILLHHN--KKLVIFTYKCERQLRELNTDLRHRNNIYFHCG
MBO6272669.1_bacterium                                   M---IRNYIEKIDRYFVRADTQPLLIDFNNKQMLNEFIQHYNVGAYNFIKAAECCREDEILNIVDLFHKLKSSNPTFLVNISAYLHLLGEKEAKNILKDLMSNN-HQKLVIVGYQICSYLGE--TDPRLKNRMVLVND
WP_037355505.1_Selenomonas_sp._FC4001                    MRLDVMQCINDVASYCNNVSGFPLIIDTEDYDSFHDLIFRLRIDVKKFIYVSDFCKEGAIPNIEQAYSSAFADGVHVLVGLSQACMLQSQSTLQEYLDRAVNETCREHTIILLMHCREYLQKCRQDVRLERRIRFLGN
MBS6586269.1_Eubacterium_sp.                             MINDVSECFEQISAYFSGDCGFFLLVDTEDYDTFQKVLQRLQADGKKCVYVSEHCSRNGLPDVDSAVRAVCGDGDSVLVGVSQARMLQSGEALDRSLDDLLSRPVSGHCVALLDHCRQVLQKLHRDIRLKNRVVLVEE
NBK77202.1_bacterium_D16-76                              MKASIQNVKQEIDSYEEHFTGAPLVVGIDGGYNYTALLSLLKDDYKQVIRMSDSCSVDFPPDPAFQISLVSKAKPVIWIGAAQAKMLYGQQPTEQFLVNLLGTCFSGPVTVICPFCCTILEGARNYAKLGYNAIVMSD
NSW92343.1_Bacillota_bacterium                           MKYTIEELKQELLRDKAHRDRFPVFILIKGRDNWHKVVTLLEDMTDYTYKLSERCGEDTIPNLNDLTTKIESYSNMLVIPLAEYLRIFENRR--AILRELATIENKCRAYIPLFEVENIFFEMKGVSRFCAKYFSINT
WP_048095116.1_Archaeoglobus_fulgidus                    MAGSVSRLITKLKDEANIRERFPVFILIEGSNAWKELLDGLKSFTDKVVLLSSFCDNDTYPDVNKAISMAKTGKRVIVLPFSEILRVDSDAE--RLIYELLDFQASGRIYIPLYGVTSQFNKWRTYDKEHSPPYFVED
NPV08138.1_Anaerolineae_bacterium                        MTHDVETLLTEVSYEAVSYTRFPVFILVTGVNAWRELIPALRAQMDACIDLSSLCSEDVLPYLPKVLALAREVPRAVVLPVAEALRLHPASS--SLLSQLAQLEKHQRIYVPLLEVEDLFGEVARMDRYLHPGWRLGT
MBQ7110119.1_Thermoguttaceae_bacterium                   MN-----ANKALLAYLASKEATP-FFYVVGDGEYLEAKKSLLEMGMQVVKTSDFCDADRAPRFDALLDALRAGGTRVLLGLGEYLALKGEETARRQLDILRNLQIGGKAILLLRGVAGVVAARARDPRFDGRRYWASE
HED00230.1_Pseudomonadota_bacterium                      MIPDLRDLFHELSKDAAKRARFPIFILVERLDHWKEVIEHVKAPANKLIKLSHLCDEDRFPKIDELKEILEGAGSVVILPLSEYVRLTGEK---EILTDLAQWELKGRVYIPLYGFASEFIEFVHVARYIHPGWHLSG
MBE6101264.1_Selenomonas_ruminantium                     M------DMSAVEAYFVSPQHKP-FYLVVGDEDYKDALVTLSNRVTSVLRVSNYCTPDRIPNLDKMKDDLQEHNRVLVLGLGEYLALEGEEYAKSYLQSLKDFNLAGKAVLLLRGVAEQVKSIKGDRRLESGRIAIAS
ROT09401.1_Muribaculaceae_bacterium                      MQMTLEQVKIKIEKYLNSTDIMPRIVNVSKADDLVELKNYFEIGNIKFIDMGDFASVDSMPLFDKLLNSISNKDITFLYGISTFLKLLGSDIMNLMLRNILEIVPQNKVIIIILVACDQYIKL--QDPRIVDRGAYIMC
MBR2693834.1_Thermoguttaceae_bacterium                   --MNKTDLIEAVKRYAVSKSKYP-FFAVSANRDYSSLRENIHEYGFKKVFVSDFCGPDTIPDWDELIDCVKAGEKILVIGLSEYFAFQGEEFALRKLKELKDIRLGGRAVLLFRSIGAWIKSLKSDPRFDGARYCDAA
WP_216880780.1                                           --MNLDDTKKRVQKYLNSNKRYPLIVDVQTREDLANIAEYFKVGSNKFPAIEEFCNQDEAIKLDELYASVSNSGNTFITGLSGFLMLFGENVAKQALKTLITTSISGHVVIITYQCEKYLRF--TDPRISGRLVIVDG
MCM1514006.1_Anaeroplasma_bactoclasticum                 ---MHERLKNKINSFIDSNDNRPLIIDVSNVEERDNLLQDFFATPSIFELVKS---ECELPEISSLYEYMSKEKTVILTDLGTYFKLLGQDSLQQNIHALLEKSFATKFIILTLQCGQYLNE--KNPKNASKIVVSNQ
HHW17415.1_Bacillota_bacterium                           MLSSVDELVTYLRAESDKGERFATFILLEGTEAWDRLTARLQYEVDSTVHLSEFCEDDVFPDMSRLISYLKRCKKLLITPLSEWVRLNPERTD-VIIQILAEWPPVRRIYVPLLSIKDSFYQIERVVRYLPQEWYLSE
WP_157832981.1_Desulfofundulus_thermocisternus           MRKEIASLLDFIKNEAKVPTRYPAFILVRGLDAWQELLKSLRLEVDEVFTLSTLCNDDTFPYVEGIIPILKKTTKILVLPLAECLRFTGESR--LVLRELATWEDYKRVYIPLMELDDIFGYMNMIAQSLPEVLSFND
WP_166670004.1_Aminivibrio_pyruvatiphilus                --MTLQEILQRLADEKKVESRFPVLIFVDDWSQYKELETALENACDVTLELADFCAPDVLPNFSKIFKKIEREKHILLLSLGEYLRLTIAREVQPQKANIPSLWARTRVIIPLPCCRDLWDRIPYDERQRDCIWELSD
HCH97635.1_Lachnospiraceae_bacterium                     --MTITDILQQEDLERKIESRFPVVIFCESMEEYRKLVTRLRGACDCCWNLAEFCEPDKYPKFRRLIQKIEQDKHILLLSVGEYLRMATKFEVYNNSAQFYDLWVKTRIFIPIFAAREYFNRVGTIERQLDFLWELDT
WP_195464804.1_Clostridium_jeddahense                    MVNNFSELRTYLRKDRAESSLNPIFINVESMEMWVEVKKLLLSMSSGHLFLSEFCTEDTTPNIRRLIAKMKSTDSICVLPISEYLRINPEIAQ-PTILDIITKERLFRIYIPMYRMRSILYTSDNDPRRKGSILLLET
HBQ28533.1_Desulfotomaculum_sp.                          MIFTFKEFESFIAEDIKRKDLRIFFINVESLTLWVKVKNFLAQKCHNQIRLSTFCGDDLSPKINRLCAKLEIDDKTLLFPLSEHLRINNTKSD-EVLSQIVSTEISVHVYIPMYRMKDCLQAIAQNSRLNDNIIFLAE
MBK5260961.1_Peptostreptococcaceae_bacterium             MLKDYSSFTQYIDLDCGKADLNPVFINVETMKMWAQVKSYLTSKSQVLIKLSDFCNEDIAPNMNQLKKKIRVKDKTLVVPLSEYLRINRPLAT-KTLYDILHANTDLRIYIILYRMSEVLST----PREMNAVLIISE
WP_006692206.1_Selenomonas_infelix                       MV-----DLTAVERYLQDRERTP-FFLFAGGSVYAQLRHRLQGLGLHLLTLSSLSEEDRLPLPDDIVMTIKSGEKILLIGLAEYLALCGEAAARRLFSRLKSMPCDGAALLLLRGFFRYASELSEDPRIGERRLALDA
WP_028051765.1_Carboxydothermus_ferrireducens             MVSSREDLIAYLREERKKKERFATFILVTSWQQWRELIKALEFEVDVVVSLSHFCGDDVFPYLQGVLDTLEEGKTVLLFPLAEWLRLDWEDGS-KLLTKIVEWPEIKRLYVPLLAAREMLEQLRAFNRYLPPVWHLKG
consensus/100%                                           ........................hh.............h..........p...............h......hh..hs....h............h..........h....h.....................
consensus/95%                                            ..........h...........hh.......b..h...h...........p.s..p.......h...h......hh..hs....h............h.........hh....h...h.......p..........
consensus/90%                                            ..........l...........hh.h.s...h.ph...h...........p.s..s..s....h...hp.....hl.shs.hh.h.........h..h.p.....+hhh....h.p.h.......+..........
consensus/85%                                            .......h.p.l.......p..s.hh.h.s...h.ph...hp..s...h.huphs..-..P.hp.hh..hp.....hl.shuphh.l.s.......h..l.p.....+hhl....h.p.h.......+b....h.h..
consensus/80%                                            ......phbp.lp.b..p.p..P.hh.h.s.p.h.ph.p.hp..s...l.huphs..D..P.hp.hh..hp.....hl.slupah.l.sp....p.l.pl.p.....+hhl..b.h.p.h.....s.Rb....h.hp.
consensus/75%                                            ....hpphbpblppbh.p.p.bP.hl.hps.p.h.plbp.hp..s..hlpluphCp.D.hPphpplh..lp...p.hlhslupalbl.spp...p.lpplhp.p...+hhl.hb.h.phh.....s.Rb....hhlps
consensus/70%                                            ....hpphhpblppbh.p.p.bP.hl.lps.p.hpplbp.hp..spphlpluphCp.D.hPphsplhp.lp.s.phhlhslupalbL.upp...p.lpplhpbp..s+hhlhhh.h.phhpp..pssRh.sp.hhlss
```

## ; Type 4 BREX systems PglZ N-terminal iSTAND-AAA+ATPase; Representative_2

```
                                                    1---------11--------21--------31--------41--------51--------61--------71--------81--------91--------101-------111-------121-------131-------141-------151
OrigSeq                                             MLFWSFSQLTQEIERDKDSRDMLAQRYAVRFIMLNNFNEFKELAKFMTNIGVDMLDLENLIDEDDTWITKDTLKDAIKACTKSTFVTPFSEVARFYNDDDFRGFFNEIMLLEDIHNPNKRIYIPLIGLQNRFTDFLNHFARIKESAPVWRYDA

                                                                              S1                      S2 -----HEH-Extension----      S3                                       S4                          S5
                                                    -----HHHHHHHHHHHHH---HHHHH--EEEEE--HHHHHHHHHHHHHHHH--EEEEHHHH---------HHHHHHHHHH----EEEE--HHHHHH--HHHHHHHHHHHHH---------EEEEEEE--HHHHHHHHHH---HHH----EEE--
                                                    -----HHHHHHHHHHHHH---HHHHH--EEEEE--HHHHHHHHHHHHHHHH--EEEEHHHH---------HHHHHHHHHH----EEEE--HHHHHH--HHHHHHHHHHHHH---------EEEEEEE--HHHHHHHHHH---HHH----EEE--
                                                    -----HHHHHHHHHHHHH---HHHHH--EEEEE--HHHHHHHHHHHHHHHH--EEEEHHHH---------HHHHHHHHHH----EEEE--HHHHHH--HHHHHHHHHHHHH---------EEEEEEE--HHHHHHHHHH---HHH----EEE--
MBQ0159480.1_Bacteroidales_bacterium                MLFWSFSQLTQEIERDKDSRDMLAQRYAVRFIMLNNFNEFKELAKFMTNIGVDMLDLENLIDEDDTWITKDTLKDAIKACTKSTFVTPFSEVARFYNDDDFRGFFNEIMLLEDIHNPNKRIYIPLIGLQNRFTDFLNHFARIKESAPVWRYDA
PMQ01343.1_Dictyoglomus_sp._NZ13-RE01               MIFGNLDELIEYLNKDRVSRNTYSIRFPTRLIMLSNYKLLHELINKLN--KANVISIGDILLNEDRWLTQSNIYNLIKNLQNDFIILGISEFLRFFSDNELYSFINFLLEIEGSPERNRRIYIPLIGMRNRIEAFLNKYHRRFEWDPLWIIND
KUK66682.1_Parcubacteria_bacterium_34_609           MIPLEENKLISLIKDDQEKFGISYGRYPVRFILIDNFEDMKKIIETMAKNGVETLDLSNLENDSNGWVSTYELIDVIKKLEKDYIVFPISEILRFLDNDDFYSLLTSLMEIENSTNSKRRIYIPILGLFSRFRSFLNRYHRRSEFSFVWKIGN
WP_018963723.1_Coprothermobacter_platensis          MNVNSMESLIELIDEDKSDNTPSATRYPVRFIFTKSVWAVNDITMLLKLRELDILDASELFPFSDGFPSLSTILQAIREKKDDVVVVSLGNLVRFWPDEDFYSLMMGLMEIENSPKPDRRIYVILEGLIERFDVFWSRRTRQIGSDPRWTLES
MBR3117632.1_Oceanobacillus_sp.                     M--NDVSSIVKQIIEDKQKSG-SYDRYPIRFLFMDLNNDTQQDIVKIVGISIRIKDISEELSFNDAWITKSKMLSIVSDLNTDYFITGFSELARFYSRQDLEALLLSFTNIENTSGGKQRIYFVCFSLFDKIAQELRTNSRNESINPIINGNK
MBQ4585185.1_Clostridia_bacterium                   M--TEQQEIIKLILDDKKKSN-TYDRYPVRFLFMKLSQNSEDELITLVELDIQFINLHTLLSFEDGWITKSHLLNFIASLNKDYIITGFSELIRFYSRNDLEALIISFTNIESVSKNKQRIYFVCYSLFEKIAIELRSNSRNESIDPILKPST
WP_024292379.1_Lacrimispora_indolis                 M---DIDSMVDIIQSDLNRQG-SYDRYPIRFFSMKLSNGTSNYLMKLRKVCVEILDFQNFLLHDDGWITIDRFRKKVYALDKSYIIVGFSEYARFLSNAEFITLLLGLLELENSGYVKRRIYIPCFAIYSQVRIVKANNRRMDVYNPFLNEMD
HAR58249.1_Acholeplasmatales_bacterium              M--SYTDELLNLMKEDKNLSN-TYDRYPIRFLFTRLSQETYNDISELIKMKLEVIRLSDYLNFNDGWLTKNSVYNIIKCLDKDYLIFGFSELARFYSKADLQSLIISFTDIESNSKSKQRIYFVCFSLFEKLSEVLKINGRNENINPIIYHMD
WP_117621172.1_Hungatella                           M---DVRGMVELIKKDIGKTN-SYDRYPIRFFSVHYEEGVADKLIQLQQINAEICDVKDFLAREDGWFSPDAFCKKICGLPKNYIVVGFSEYVRFINEPKFVSMLLTLLEIENTANRKRRIYIPCFALYNQISTIRKMHRRMDVYNPLMNETD
WP_191216537.1_Methanothermobacter_sp._THM-1        MEFNSFNKLFLEMENDLQTDN----RYPVRFIFLNSFEELKKFMEYIGEI-SKTVSLDGML-DGSEWLSFDNILKNIKEADSRICVVPLSEFLRFSPKKDFYALLKGLSEIET--NDKTKIYIPLVGLYKRFSEFMEEFYRKDEWAPIWCLNG
WP_011944160.1_Thermotoga_petrophila                MNFYSLEKLYDRIKQDKNSKRISDRRFPIRLIFINSFEELQEIIRFLKDCGAESKEITELLSSKDQWLTPNEIVNWIKGIYNNAVVTSLSEFLRFQNKENFYITLKKLTEIET--QNNIRIYIPLVGLCERFKEFWTNFYRKEEWAPIWKLES
OQC39307.1_Bacteroidetes_bacterium_ADurb.Bin037     MKFESIEALISEIKYDKATNDILSRRYPVRLIFLQRFETFRVLIEKLSSIGIENYHLEKDLPHHDGWITKDTLISIVKKNSKDTAVVPFSEIVRFYSKEDFKNFFNQLLLIEN--ELSRRIYLPLIGVEERFEDFFQDFTRKNESAPYWKISR
MBQ4420212.1_Bacteroidales_bacterium                IDF-SFDELKAEIMQDASSKDMLPRRYPARFIMLDNFETFRELRRFLHNLGAKLLQFDSLLDDVDGWLTSDQLIEIISNTTEPTLITPFSELVRFYEENKFSGFMHDISLLEDLSHPNKRLYIPLIGLENRVEGFLKGFTRIAESAPIWRCRT
WP_183546997.1_Methanococcus_maripaludis            MEYRDIECLYDAISKDLNSKEYLANRFSLRFIFVPDFDHMAKLCNYLTSMNLKEINVSKMLPHDDGQVTPQRIVETIKNEDKNLLITSLSEISRFYGEDDFLSLFLDLAYIEK----KNKIYVPVVGIYERFREVMPVFSRSNESAPIWALSL
MBU7048061.1_Theionarchaea_archaeon                 MEFDSIDELVSHIQDDKKTPRLSFRRYPVRFIFLPNLWILKDLVHALVNIGIYRIELEDSLPGPDGWLTIDDIMNIFKKLKKDHVVISFSELTRFCSDDDFIGILNKLIEIENTENQERRLYIPMIGIFERFS---TTFSCRREWAPIWRISG
HHW21152.1_Thermodesulfovibrio_thiophilus           MVFENINYLIQKIKEDKKTKNVTEKRFPVRYIFLSNFDTLRDLIKETSKIGITTFEISQFLPKDDGWITKQEFIDKIKSLNKDYLLLPFSEIARFYNKQDFNNLFSQLTELENIANSNQRIYIPLMGIKVRFEEFYKNFNRKIEYSFVWEIYE
WP_207678199.1_Desulfonema_magnum                   MNFKFIEELYSEIDSEKKDKSPAVQRYPIRLIFLNSFNSLNLITDYFSKQAVDVLELNKLLPHNDGWLTSDDIINSIKLLEDDTVIVPFSEILRFKNTAEFNTILTGLFEIENSDKKIKRIYIPLVGMKERFEDFWKTFHRRGNWAPIWYLEE
MBR5399199.1_Bacteroidales_bacterium                MKYQSFEELVDAIKEDIKTRDFLSRRYGVRFIMLNSFDILRKLVLELQNLDVEMVTLDGLISEPDSWISTDTLKERVFSCTKPSLITPFSELVRFYPDEQFRGFFSEIILSER--SKTNRIYIPIIGLENRFIDFLSHFGRLSESAPIWQYNE
WP_022459608.1_Bacteroidales                        MVFYSLDDLKEAISVDIKSHDVLAQRYCVRFIMLNNFDAFRELTKFLVELGVEKFDLEKLALGSDKTIDIDTLSDAVRNLKVSSIVTPFSELARFFKEDEFKGFFNDIILSEDIAHPQKRIYIPIIGLHNRFTDFLKSFGRIEESAPIWQYYT
MCQ2340464.1_Paludibacteraceae_bacterium            M---------KEIQQDAASKETIAQRYPVRFIMLDNFDTFRDLSNELTSAGVETFPLESILEEDDAWITTDELKDAICQCTSSTMITPFSELARFYSDEEFVGFFNEIILHEDLAHPGKRIYIPLIGLKNRVEGFLSRFGRITESAPIWRLNS
MBE6838777.1_Ruminococcus_sp.                       M---NLEELKQKIADDKAKNTSSYRRYPVRFLFMELNQDTQNDIQDLVSSNGELLELSDFIMKDDGWLTKSRFLQVVKTAGKDIFIVGFSELIRFYSRKDIESTVLSLFDIENIMSSSRRIYFICFSMMDNVYVLQNSFARKDLLDPFINSDY
MBR3710351.1_Bacteroidales_bacterium                MRFDSYEALLDEIKRDMTDGDPLRCRYPVRFILLNNFDVFTKFAQDLAHHGVSPLNLEELIETPDRWITTDELCQAIKDRKVRTLVTPFSELVRFYSENDFRGFFNEIINTEDVDRPEKRIYIPLIGLQNRFSNFLNSFARIEESAPVWAYLS
WP_205109179.1_Marseilla_massiliensis               -SFNSLDALFAYIQEDKHWTGSHYNRYPVRFVLFDNFSDFNEFIVNRP--GIYKHSIDTMIDSPDDFLSYTELSREIRAFINDFVIYPFSEMARFYDNEEFDALITTIRASQAPEEEHVRIYIPIVGMQKKMGRFMN-----DNSTFVWEFKS
WP_172204608.1_Prevotella_sp._PTAC                  -DFEHIDSLIEEVKKDKAADGSILNRYPVRFVLFDNFADSKDFVSELILGVTKMQKIVDWMDHPDQILTHSCLANCIRQYDSDCIIVPFSELARFYDNHTFETLVSDIKGIQSATNNRQRVYIPMIGQYGKMSKFFS-----DSQSVIWHLVG
MCH4183501.1_Prevotella_sp.                         -EVTDFDGLKALVKKDKASAGSTRNRYPIRLLLLDNFRDCSDFVDFVRELKTMVYSVEKWLDYPDIMLTHTQLADNIRKQGKDCVIVLFSELARFYDNNEFEALLRTIKAIEATWVNHQRIYVLIVGLEGKMEAFSN-----DSQSFIWRLKR
WP_196042834.1_Parabacteroides_merdae               -IFTNIEALLWEVQADRHIEGSTANRFPVRFVLFDNFRDCCAFVDDCLLPNIAIQRIDDWMDYPDQFRSHKFIADKILNLTEYRIIMPFSELARFYNNEPFDALIATVKGFDTNRQYKQRIYIPIVGLEGKIQHFRD-----DSQSFIWYFHN
MCI6981218.1_Akkermansia_muciniphila                M-------------DKEAHYSGLNRYPVRFILFNSVEDYSKFIDVCAARGAYVQRISDWLLGNDKLLTYTELAEHIKEKGEDFVITPFSEIARFFGNSKFRSLMKTLGAWEASPRAHQRVYIPLMGISGMLEKLIT-----EPGTNVWDCCS
MCD7797870.1_Akkermansiaceae_bacterium              MPYQSLKDLEAAINDDKKRGG---NRFPCRFVLFDDYKNFDTFIQTRRKDGAFMCPLAEAL-SDDKQLTMKSLCDWIKGKKNDLVVFPISEIARFYNDNEFKCFITTIKGIES--DEQQRVYLPFIGQQSRMYNFVN-----DPAIVIWDNCN
MBO6251938.1_Muribaculaceae_bacterium               MKFSNIDELIQAVVVDKNAEGTVQNRYPIRFVLFDNFKDSYAFTIRLVELHITIEDIQQWIDYPDIIISHQKLATEIERCGESKIIAPFSELARFYDNHDFDTLIRTIKNIQTSAEKKQRIYIPIVGLEGKMSTFFG-----DTQATIWYMRS
MBR4838437.1_Bacteroidales_bacterium                -CFSHIDELMDEVRTDKWCNGTTLDRYPVRFVLFDNFRDSREFVATMQEMGCLVKNVNDWLDYDDRMMTHSKLAKQIEAFVKDYVIAPFSELARFYDNKDFDALVKTIKGKESRYQSRYRLYIPIVGLEGKMSQFHN-----DSQIFIWYLKS
RGX95620.1_Prevotella_copri                         MTIASFEELKALVEEDKNINGATANRYPIRFVLFDNFHDSFEFVMYMLECGVAVQSVDKWIDYPDLMITYQELSEHIADYGRDSVITPFSELARFYDNQSFDALIKTMKSIEASVENQQRIYIPIIGLEGKMSTFEN-----DTQINVWHLHD
WP_254009599.1_Bacteroides                          MYVENIAQLIELVKEDKLTQNYLRDRYPIRFVLFDNFNDSFEFVSQLMDCGCILKSVEDWVDYPDIMLTYNDLSRRIKNFSNDYVIAPFSELARFYDNNEFESLLRTIKGIESTSNNHQRIYIPIVGLEGKMQSILP-----DTQIIIWQLKS
consensus/100%                                      ..............-.........Ras.Rhh......................h.......s...s...h.p.h.......hl..huph.RF..p..h...h..h...p.......+lYh.h.u....h................h....
consensus/95%                                       ............h..D.........RashRhh......................h...h...s.b.o.p.h.p.l.......hl.shSEh.RF..p.ph.s.h..h...p.......+lYh.hhub..bh..............hh....
consensus/90%                                       ......p.h...l..Db.......pRaslRhlhhp..p....b...h........plpphh...D.hho.pph.p.l....ps.hl.shSEhhRFhsp.ph.shh..h...-s....ppRlYhshhuhb.bh..h............hh....
consensus/85%                                       ....p.p.l...l..Db.......pRaPlRhlhhp..p...pb...h....h...plpphh...D.hlo.pph.p.l....ps.hl.shSEhhRFhsppph.shh..l...Es..p.ppRlYlshhuhb.+h..hbp........s.lh....
consensus/80%                                       M...php.L...lppDb..ps...pRYPlRFlhhpphp..pphh..h...sh..bplpphl..sD.hlo.ppl.p.lp...ps.ll.shSElhRFhsppcF.shh.pl..hEs..p.ppRIYlPhhGlb.+hp.hbp.....pp.s.lW...p
consensus/75%                                       M...shp.LbpblppDb.pps...pRYPlRFlhhsshps.pphh..h.p.sh..bplpphl..sD.hlo.splhp.Ipph.ps.llssFSElhRFaspp-F.uhh.pl..lEs..p.ppRIYlPhhGlb.+hp.hhp.....pp.sslWphps
consensus/70%                                       M.h.shcpLhpblppDbppps...pRYPlRFlhhssaps.pchh..h.p.shb.bplpphls.sD.hlT.splhp.Icphpps.llssFSElhRFaspp-F.uhhpslb.lEs..p.ppRIYIPllGlbp+hpphhp.....pp.sslWphps
```

```
## ; Type 1 BrxB-iSTAND and Type 2 BREX/Pgl systems PglW C-terminal iSTAND(subset) alignment comparison

WP_102077887.1_unclassified_Psychrobacter          MLNTDFNELMERVRAGREFGH------ASFEPIFYLIFDPQKILKIKRQLPAWAAKLRNEGWDVHIFSMAKAVQEVFDEMPVFQDSAALENRDQWQKTNKSLAEALTKKNALQNKLEAKLFGRPNSILLVSDIEALHPYLRIGSMESQLQGKFH-VPT---IFFYPGMRTGKQ----------------LKFLGFYPEDGNYRSVHVGG
MCF2581773.1_Bacteroides_caecigallinarum           MMDTVFEKVYQKLSSPDF--GKNL---GGELPLYIQPIPVSGQTELNTQVERLVSRLSKLGKNSIVVDLYRLALEIIDEEGILETLLEDEKNIDKDDLNATFESIFDTKEILIPRMRSMIEENKYDFVFITGVGRVYPFIRSHSIVNNMEGLADNANI---VLFFPGEYNRLQ----------------ISLFGKLPADNHYRAHNLND
RRF95307.1_Coriobacteriaceae_bacterium             MINDARGFIVDKLSDDALLGRNGY---MMRQALYIIDYDPAQQQYAADLVRAICEKDPRRGVTPVVVNLYDLVLGYLDEQDLWEPLVEAEPDTPRLDLIQMLQDTVGVKDVVAPRVNEAIASPEADIAFVTGVGETFPYVRTHTLLEEISSP---IPV---VLVFPGRYEQHA----------------LNILGLTQASTYYRATRVFD
TVQ28582.1_Spirochaetaceae_bacterium               MTAKRFDRVYQIISHPRFLARQGL---GNEVPYFIETYEPANEFAVTTEVRTIHERLLHNGIPAVLLPMYDIVIECLESDGRLAQVFEKEPAMGRQRFFAMLDAFTRPDAPVHDAIVRRLDAPDHKLVLMHQLGTVFPFLRTHTLLTNLHSTIYQVPL---VAFFPGTYVSSY----------------LSLFGTFKGD-YYRAFQLSD
UCC65223.1_Anaerolineae_bacterium                  MLEADFEKLRQRLGDPDALNP------AHSDPIFYFVYPPSQILTMRKLLPGWIARLRNEGLKVETLSLSEIMWELIDASGRWDDWLELEPEHDLDAVNEAIRDVLRAGNALVERVAERAASRENTVLFITDVELLHPYFRSRVIENYLNNKVL-IPT---VFFYPGRRTGQG----------------LHFLEFYPEDPGYRSTLIGG
WP_004808450.1_Actinomycetaceae                    MLQETLDIALRVMTSKRFLNREGL---GNEVPYYVLRYRIEWDRSFDQSLRQMLSHL-NESVPTLHIDVYQLAIQIWRDCGYWDQILAQEAGMDRQDFADGLAQILDAERVLAPAIAERIQAPDSRVVILSGVHHLFPIMRAHRLLNCLQPLTGDVPV---VLTFPGSYRQSA----------------LVLFDQVSEDNYYRAFDLLD
WP_126029123.1_Bifidobacterium_callimiconis        MIEREFESLFAIMRRPSFRTGSDT---AGEPANYIYAYPPVKELEVERRTDQLANRLAETAPGVLIIDLYETAIQVLRDSRIFERVLRKEPRLIPDMFTQSLIDKLSPDKSIAEQYRQAREHKQGDIVFITGVGKVYPYIRTHILMERIQLVFEQRPV---VLFFPGTYAKTT----------------MRLFDRLESSNYYRAFSLNA
WP_135754989.1_Leptospira_bouyouniensis            MLLKRLNDLKKDILHPEGIQVTQ----SQNYPFSIFIYPPADEFTVRAKFVEMIQDIKKENIEILEINLAHECLELLKQRDGIDEIIQKEKEFTFALVNDVFSPILEDENGISKSILNIMEEGRKGIVFITRAGFLYPFYRTSSLLKFLTNTRG-LSV---VFLYPGTRTSES----------------LSYMGVMSPDSNYRPRMY--
WP_168675170.1_Hymenobacter_artigasi               MIPEKAERLFQTISGSRFLKRELL---GGDIHFFISTHAAEQQTEMRQAIAALIKRLDNTGIQVLEINLFKLALSVLDSEIGLPALFEFEEQESAAEFREALHSAMDMKQVLLPAIERHVQSATPQVYFLTGIGEVYPFIRSHSILNNLHHLVERAPL---VAFFPGTYSGEQ----------------LKLFGLLADDNYYRAFNLDT
WP_187771726.1_Phascolarctobacterium_faecium       MLSERLGKIKNIITNSNFLNKTGN---ANEVSYFIFDYPPDKDIIVEDYIQRLATEIMEKDMQIKIFDIYGLIMDYLKDKKYIDKCILLEQKKGSAGLIKAIKDTLHLDSYIVNHIDENTIQ-DKDIVFLVGVGRAFPIVRVRELLSVLNNLYTKCPI---VLMLPGSYDGQH----------------VTAFNKLKMENYYRAFKLVQ
WP_200392182.1_Roseibacillus_ishigakijimensis      MFDQKIDELIRRLGAPEGTTNA-----RQGDQVFNVHYPPHLWAELRTRLPKLMARLKDQSYAPRHCSLLALMEKIFGDNGKI-ALLRRSEQQSHQAFTDTLHSILANKKPIVAAIEEEIQSKPNGLLLITDIEMAHPLLRVSAFEQVLQGRFA-VPT---VFFYPGERGNIN----------------PSYLGVYPSDGNYRSTHIY-
WP_222635590.1_unclassified_Rhodococcus            MLTARFERAYRVMRSPSFLEMKGQ---IGEVPYFVLAYKPSDEPRVGAEHKRLVTRLRTDGLEVCDIDLWALANELWSATGQWELILEQETALPKAVFSEGVGNVISPKDYLSPRIARIVERPRPQVVLLSNVGRLYPLVRAHTVLNTLQPLLADLPI---VLVFPGTYRQSS----------------LVLFDRLTDDDYYRAFDLLE
WP_224137561.1_Candidatus_Kaistella_beijingensis   MPNNINDKIFSLLSEEEFKDPDTG---LLFFPVYIYTYNPEKEFQIREEIKELDRKLPSNNLNCLVLNIYNEFIDYLKHNIFLEQIFELEEIDALDFIAEKAQEKNDNDAHIGEKLKAYFDSPDRVYLLLYGFGSIFPYLRLSEFLKKTEEYVRNYKL---IAFYPGELKNDH----------------YSLFGIFGDENVYRANHLNQ
WP_235341611.1_Bifidobacterium_boum                MIDEDFQHLFDVMKQSSFRHSKGI---AGEQPFYIYDYPPECELEVDRHIPWLMQKLDDYAPVVRRVDLYDVVLGILKRRKILDRVLKIEPRYSQDKFLGMLESILGADSQLPNAVVEAMHTVKPGIIFITGVGAVYPYVRAHTLLNTLQGMIEDIPV---VLFYPGTFQSST----------------MTLFNCLEPDNYYRVMRIRD
G8417454.1_                                        --RDPVAKALGAERSPHEAVRDMLLDAAGSRGFRILVTPPEHHADIGPSVAR--------ALGGTWVSFADAFFRDHGAN--------IAALERAERFVAQRDALTEAAEDTMFRLLD--EHGRPDHTAVVGDTGLLGVCDALDLPRRLYDETLSGSRGFWVLVVPGVIYKSQPHFNEGPSMWQLAGATFSLLTALPD-----------
MBK8541194.1_Ardenticatenia_bacterium              --PDPLPTFLPALTSPGDRAAAWLRSSRGMSSWRLVVTPPERHAELGRQVAA--------ALDARYVSFEAAFLKRIEDR--------FADFETAERMSALRGRLKKEAEGVLEALLA--EHGRPGTATVLGDTALLGLCEAKHLVTRVYERALGGALGFWVVVVPGVVHQQQPLLNEVDNLGSWPGMVLPLAEDVGV-----------
MDO9017995.1_Myxococcales_bacterium                --RDTLPAEFIAHRSPDEIVVGLLRAARKSRGFRMIVAPPERHAEVARSVAQ--------ALDARFVSFEHGLLAGMEPE--------FAVYERAERFKAQRSRLTRAAEGLFEALLA--EHGRPGTCTVLGDMGILGLCDAAHMIRRLYDETQTGARGFWVVVVPGVIFQRQPLFNEKQPLFHVDGTVLPVDGELGR-----------
WP_075011136.1_Stigmatella_aurantiaca              --QDPLPSDLSTQRSYEEVVADRLREASRGRGYRLVVAPPEKHVEVAHSVRR--------ALSAELVSFEARLLQRMEPD--------FPAFERAERFLAQKPKLKKEAVALLDALLE--QHGRPGNVVVLADTGILGVCGVKDVPRRLYDRVTERDLGFWVLVIPGVVSERQPLFNEQEPVLNLQ--VLPVPEPLDV-----------
MCA9600002.1_Myxococcales_bacterium                --RDPVPDDLAPDRTPEQVLVARLRDAAERREWRLLVTPPEHHADYGCSVAA--------AINAEYISFEQRLLERMEPS--------FADFESALRMKARRRKLTRAAEALLEELLR--ERGRPDCRTVLGDTALLQMCDAAHLVRALYDETQGGQRGFWAFVIPGVIQERQPLLNEKDPVFHLPGATLPLSGPLPP-----------
TVQ88636.1_Deltaproteobacteria_bacterium           --PDEIPELLEERRTPEEVARDVLRDAVKRSSYRLIVADPGQVGPIAKSVIR--------ALDATPIDLADAWFQRHAAT--------VKVDAHAERFPAMKAVLAQKAHKLFEELVA--EHGERGRAIVVYNTGLLDAIGGVVQVRNLYERVQGGQHGFWVLVVPGVISGRTPLFNERTQLWHQPGLTLPLRKPLPD-----------
KYF91864.1_Sorangium_cellulosum                    --PDELPPEL--VKDPAEAARDILRSVAKTRGYRLVVAPPDDHVEIGRSVAR--------ALEAAFVSFEHEFFRRVDAQ--------IEVFDRAERFAAQRPRLRKEAEALLDALVQ--EHGKPGSTVVLGDTALFGTCDALHLVRRLYDLTATGGKGFWALVIPGVLHKRQPLFNEKATVFSIDGAVLPLAREIPE-----------
MBI4699810.1_Deltaproteobacteria_bacterium         --SDPVPPELVAAKSPEEVAADLLRGAASGDGFRLVVSPAELAPEVGRSLAR--------ALEVRFVSFEQELLGRMDGA--------FDGFVRAERFKAQRGKLTREADGLLADLLA--REGRSGATVVLGDTAIFAVCEALHLVRKLYDETSAGGRGFWIMVVPGVVYQKQPLFLERAPIFHPE-STLPILRAIEG-----------
consensus/100%                                     .............................................h..............s.....hsh...h..........................................h.............h.........h......p..............hh.hPG........................h.................
consensus/95%                                      .........b......................hbh....s.....h..............s.....hsh...hb.......................h.............l...h.............l.....h.shh....h.p.h............hhhhPG......................h.h...h.............
consensus/90%                                      ...p.h...b......................hbh....s.....h...h..........s..s..hsh...hb..h..p.................h...b.........l...l.......p.....l.....h.shhcs..h.p.l............lhhhPG....p.................h.h...h.............
consensus/85%                                      ...p.h...h...ppsp..............shbhh..ss.....h.p.l..........uh.s..lsh...hh..hp.p........b........h...b....p..p.l...l.....p.p....hl.s...lhshhcs..h.p.l.......s....lhhhPG.b..p.................h.h...h.............
consensus/80%                                      ...p.h...h...ppspb.............shblhs.sP.p..ph.ppl..........uh.s..lsh...hhp.hc.p........b........h...b.ph.p..p.l...l....pp.c.s.hhl.s...lhshhcs.ph.p.l..b....s....lhhhPG.h..pp................hshh..h.............
consensus/75%                                      ...p.h.phh..hpsspb.............shblhshsPppp.pl.ppl..........uh.s..lshbp.hhp.hc.p........bp.......h...b.ph.p..p.l...l....ppsc.s.hhl.sss.lhshhcs.plhppL.sb....s....VhhhPG.h.ppp................lslh..h.............
consensus/70%                                      ..pc.hsphh..hpsspbhs.p.....s.p.shblhshsPppp.pl.ppl..........ulps..lshbp.hhpbhcsp........bcsb.p.p.h...b.ph.p..pslh..l.p..ppscsshhhl.sss.lashhcs.pllppLbsb....s....VhhhPGshpppp................lslhs.hss...........
```

```
## ; vWA-MoxR subsystem 2: the iSTAND ternary systems

                                               1---------11--------21--------31-------41--------51--------61--------71--------81--------91--------101-------111-------121-------131-------141-------151-------161-------171-------181-------191-------201-------211-------221-------231-------241-------251-------261-------271-----
OrigSeq                                        TFLEICQRLVKEIADFAVHDSDDLDIQVCVNRLDSNQLHKALLCLNYTKQENLFREFV----RHQIGACLIHGEPEHGQRWLLNRLLGL-VPNGRTESVIPLNL-LVATRSSSIDALWRELGKAVGAARL-ATCDEIAQKVGNLWKSQTVILIFHNLDQLPEDYFNEFICKFCSSQAN-VRNYRLLAFLMDLSGCVD-SWGITWAETIDLNWEPHTPLKLPKITKLSHNELSYWLEKSPHPLPAEIDKIALDILNSDDGKP----EAVLDYICELCD

                                                                                                                                  S1                              S2     -------------HEH-Extension---------------     S3                                 S4                             S5     -------------Degenerated-Helical-Bundle-------------
                                               -------------------------------------HHHHHHHH--HHHHHHHHHHH-------EEEEEEE------HHHHHHHHHHH------HHHEEEEE-|HHHHHH--HHHHHHHHHHHH--------HHHHHHHHHHHHHH|--EEEEEE-HHH--HHHHHHHHHHHHHH-HHHHH--EEEEEEEE-------HHH------------EEEEEE---|---HHHHHHHHHH------HHHH--HHHHHHHHHHH-----HHHHHHHHHHHH
                                               -------------------------------------HHHHHHHH--HHHHHHHHHHH-------EEEEEEE------HHHHHHHHHHH------HHHEEEEE-|HHHHHH--HHHHHHHHHHHH--------HHHHHHHHHHHHHH|--EEEEEE-HHH--HHHHHHHHHHHHHH-HHHHH--EEEEEEEE-------HHH------------EEEEEE---|---HHHHHHHHHH------HHHH--HHHHHHHHHHH-----HHHHHHHHHHHH
                                               -------------------------------------HHHHHHHH--HHHHHHHHHHH-------EEEEEEE------HHHHHHHHHHH------HHHEEEEE-|HHHHHH--HHHHHHHHHHHH--------HHHHHHHHHHHHHH|--EEEEEE-HHH--HHHHHHHHHHHHHH-HHHHH--EEEEEEEE-------HHH------------EEEEEE---|---HHHHHHHHHH------HHHH--HHHHHHHHHHH-----HHHHHHHHHHHH
OCQ97673.1_Nostoc_sp._MBR_210                  TFLEICQRLVKEIADFAVHDSDDLDIQVCVNRLDSNQLHKALLCLNYTKQENLFREFV----RHQIGACLIHGEPEHGQRWLLNRLLGL-VPNGRTESVIPLNL-LVATRSSSIDALWRELGKAVGAARL-ATCDEIAQKVGNLWKSQTVILIFHNLDQLPEDYFNEFICKFCSSQAN-VRNYRLLAFLMDLSGCVD-SWGITWAETIDLNWEPHTPLKLPKITKLSHNELSYWLEKSPHPLPAEIDKIALDILNSDDGKP----EAVLDYICELCD
BAI89278.1_Arthrospira_platensis_NIES-39       MMETGFEKLETNINQNLQTIQESLNELIQTLNLSEQSIKDKLLHLDYREQVRSLEDFL----QDRVGAFWIYGSNQSEPHWLVTSLIENKIETSNTSSRVSLSF-VGNTQSHCLDNIIDSISKAFQIENPTEKLTYIIEQICIRWQTKSVVILAEIKKGVDPEIVEEFINKLRQKHYN-IYNYSLMLFLVDVHGCTN-NWEINFTEDILSEQIPEIPIRLTEIQPIPREQLRQWFRTHL---PDQLIEKMLDLIYHNN--Q----EKVFYQICKLFE
BAY43400.1_Scytonema_sp._HK-05                 WVSLIFVKL-----------------IADDSQIKNQQLYHVLLKLDYTDQEQLFSRFI----TNQIAAFLIHGSQDYGQHWLTNRLLKK-IRRVSDKPVL-VDL-DCLVHNRNSQTMWRELGRHFGGIK--DSPDLIAGKVLKSWTTKNIYIVVDNVQSMSEQLLQELIQKFQSIAS--QTNFKLLMFLIDNVSKVESSWNVSFAENFEPTWRCHQPVILPKNNVFSDIALRDWINHESVYLPENIDGVIQTILNSEAGIP----IPAFREICNLCE
KPA13794.1_Candidatus_Magnetomorum_sp._HK-1    FRPNGIQRL------------------------PEKLRENFPYSVNRTEQVQGIKDIG----EKKPLVWLIHGDRNQSHERFLECVQWFHWENIKKGSKIEPRF---YYSNNFENDCMTEIWKNFSQDGQKDNWRDLSPLIDNNCNK--PVILYTMIRCSQQRLFYVLLDIQKKLAGKRKYP-LFLCILIIIYTHKHKR-----YQRKITRQLKKYNKYTLPLLSNIESEDVDEWQNMSF----KNERKIVKDIFDDIEQIPMTELAKIFKHCLYHQD
KPA12842.1_Candidatus_Magnetomorum_sp._HK-1    TKKAIFSDITKEINDTTKDTTDDKIV-------THHCGKFVHLMCDRANQVVEFRKTFSNENHKKPQFYVIHGKERNGHLSLVERLKIQVIQPFVNEQAVSLLV-EKGDLDMKMDILLDALFAEIFNQNDSIQYSLKALIQSDPIKADHAVIIAHRIPANHLDLANRYISDFADLSIVDTIPLFIVFFNIEYPRLVKRMKELE--YKIRSMVKELTNQLIKELQTINTHHVIDWFNSHQKNM-PEKFKAVDSIFDGQDEC-MFEIEDLLKKY---VP
ETR67363.1_Candidatus_Magnetoglobus_multicellularis LENLLMKQLDRVIPETIPDIPPTDQQ---SQAPIATIGDDINLYCNRITHVGEFLFTYRDDCGNMPQFYFVYGCKDECPESLFLRLRDIELRDHFKPQVPPIDERSNSSFEARKDVLLKSLFKTLTGDYQSNKFKLTDLYQSNAIKDNNVIVVPHFLSFDQQKLMEWYIKDY-------DAPHFMLFFIITCIPKKNFPKHVQ--KKLKKFSSQLSAYLFDPLHKIDKSEVAPLFKQFK----HEINKHIDDLFKDQELMNMSDIEKYLLRLKQTIK
OQX97257.1_Bacteroidetes_bacterium             LQDTSCRDV-----------------NSNNKENISLDRNYYRYSCNCYKQNNYFENVFKKNKNEKIQFYVIHAKAEQCPEILPQRFFNEHINDNDNNSIF-----QVDNIDSLKIKTLYQIFSFFNEE---KEACLESLFNSKTLQKKKYITINFIVESRQHEFIEFFINDFCKNQIKKTKLRFIFFWSIIYTSEIKDRETKK--DKIQKCIKNLEFDVLPEIQIINLDDIRTWIKSIE----SKINISLFNIFTNKKEFTMEEVLEMFDFLSEKYN
WP_020569053.1_Neolewinella_persica            EQANINGSLLYVLGESSADLKQDGAK--------EFIPPFHAFGVDRAPQEGQFFDEQFEAFDQKVFFYYLHGDDRQQASSLAERLGFKLVGLQLTADDLDIEALNGMREEHCRALLVHAITQQFMGGAVGLNLTILDLLNRPKLKRHPIVCINVTVGHSYPGMLRKFYETFCKVELPEDAPTFYFFFGLEHCDNKPT-----VRKEVAEAIAGRQNSALPELEPVLISDIVNWFTHHG---PYDAEQLVAENFTEGDQLDMLVVEADLKRLIDKFN
RMG26468.1_Bacteroidota_bacterium              WEAQQEEELPWQLYGRHTDWRLALPG-------KKRSGRLLHLMCDRDPQAESFLDRFLSREAGDPQYYFIHGPRGERHKSLVRRFREIEIRHHLGRMPALVDF-TTGDLDMRKRNLKRSIARSRELPGIVGSKWESPALMALLAQRGNLVVFQHQLAAEKFRLLEWYIHDF-------ASFHFLLFVNIVYPEKWGSRQDIW--EDMQALKRKTPETILAELQPITPEDVAAWVDTYF---PDNMQHLPGTIFDKQKRLPMKVVETQLKLAIERLE
KPA17452.1_Candidatus_Magnetomorum_sp._HK-1    EMNTVCKNIALEAKNKSIIINSYDEK--------KFDGRIIPTLCNRDIHVEKFYKFFEKKHKKLPLFFIIHGFENQGHRSLIKRLRFTLIRAYLQKQKIELPF-DKGKLSDRKQILARQLFNAFQKMDDIKTYDLKRLNQLKAIETNNIVIISHNIEASLKDLLSWYIENYVDNFKYDDIPQFIIFLNIQYPVAIKKHSTIKVKKELTVFTQNFSKKLIDELNSITQQHVKDWICKYKSNM-YEIEEEIESIFENSEHMSMIEIEKKLKNILLK-S
WP_028158837.1_Bradyrhizobium_japonicum        NLDELVAELHRFVNDGAAP-------DQLGKARSKKFARLLPYLCDRRMQRRHFRDAVMERDVVRPQIFFVLGADSECADKFVEQLFFVDMPELLQRNGLEDRLADVSDIGDYLGELQYEFADKLG-----LKPRSSVTEIERRLQRHTALFLYFDVDIRKRRLLREWIGWWNSLVVMGRRYPIIVVGRFCSANQGIWSPTMRLVDDLTALSTTAGKTVLPQLGRVSFEDIEAWINENADFAPPDLRQRLRRFFIGERKVSMIQAATLLKTIMNEMK
RKZ89871.1_Candidatus_Parabeggiatoa_sp.        APSESAKQLIELIEQYPQGLTHLSEVVAPQKKFKLNVDDLRPHLPNRRPQESKLGRAITHHHKQRPLLCLIHSDECQCSDRFVDRLVQYYLPKVVPGDGIFVHC-HFSQGIDLHQEISEQLASRLGI----NPFAQPSEIVEAIMRERSPIIFHTDMCTKNMKAVHNFIEFWAKLDIPSTHNHLLICLYFNYKDIKPSKKSIN--DQIRAEFRQLENIVLPELMDIEKEEVYAWARDHLSSF-DSVQPKIDDLFSPDETIPMCDLARSLREILEEFP
OCR02031.1_Oscillatoriales_cyanobacterium_USR001 AVKQAIKKLAKTLASWNVPQVIVQNTNLPITQQSHDKVHQALLRLNYTHQVRLFRAFF----HHPIGAYLIHGEEESGQKWLLNRLMKF-ITDSRTAKVIPVKL-SRKSCGTYTEALWRQLGGHVGLMNQ-PSQTQIIKNVSELCKNQIVVLIFDELNAMPEECMSQFIGQFRDLT---QSQSRLLMFLVDNIGCVE-QWNVAFAETLDPNWEPHIPIKLPKINPIEQSDLVSWVRNAVDDLPNQMEEIVQNFFNGETILP----EAAMIKICDECD
RMG84230.1_Bacteroidota_bacterium              KIAQVKKKLKATLAPEPQP---AGSE-------RVVFHEYHRYTCNRQPQNVQFHSSFVSKDKQKTHFFYMYGLNVQSPEGLFYRFSYDRLLDYLNPDLESVRAQLSPDETYNKQEVLKNLFAALSVPYAHKKRLEDVCLSSPTIRKEDYVCIFLGVSSFDPALAHWFITRFCACTLPEDAPTFLFFLAIIYEEDDPQ-----IRAEVKETVENSDLIGLPELGDVSLQDVKRWFFNYQTIFPKTRQQLIEERFGDAQTFEMEEVEIRLERIIDEYN
WP_028091549.1_Dolichospermum_circinale        EMNSLYEEL-EKIDQETKGLKSPIKK-------LSAQRSFLSYLANRSSQEFELTEAIKNRDKSRLLVCIIHGDELQSHYKFLERMRNDFLPDVLDLDQNKIKKSELKNLNELPKYLLMNLAKNLV-----NKGNTSLEEINEILNKSSPMIVHTDLISDDLHILNKLLEFWQDWHNQNTLNKVIICICVKYIRIEWTYQQIN--KKMRKHIQEFSLIILSELEAVGRADVENWACEHTKNF-QQLKDKIGDMFKSSSKIPMRYLADNLLEILEHFH
WP_018400346.1_filamentous_cyanobacterium_ESFC-1 SLSQLFEALDRELINIENIALEDQDHHQTTTASLSQDLSELLFLLNCIQQENAFR---ARATANPAGTFLVRAKDLSLQKWLVNRLVRL-VPGY-ENAYCPFNV-YSPQIRLNFNLLWENLAANLNTT---ATIENVAEALIERYCHQTVILVLYGVHKLQDHQIAEFIQ-FQERYTR-ARRSRLILFLVEDTEEH--NYSFDCVESIDYQCL-EKPLRLSPLGQISKAEALTWFDEVYNQL-EQIDLLLQDI-NDPN--P-NEVSISLDEICMTCG
KPA18269.1_Candidatus_Magnetomorum_sp._HK-1    RIDFDIERLINAIEKNKERSDNKSTDNKTTGFKQPNNPKKLHLLCNRKKQALDFTLYFKNLKKNTIQLIFFYGPENECPYSFIERLAIKEIKDYWENQYDKINKEKKNDVNDQKNTLIQELYNSFLLIA--NDYKLSAFYQHPTFDKHNVIILYHEISIDKLKLLEWYINEHSQFFCHNNVKPLLLFFYIKVDGFKEKCQKML--DKLKKKIDKKFKLLLEPLQPVDKDDVREWFSNLE---PSIVEPEIEQIFGKENQMPMRKIITKLDIFITNID
WP_066426097.1_Anabaena_sp._4-3                TLESFFDKLVTKCQEKTSP-------KQSVENNSSENIINALKKLNYNEQEKDFRDYIKIVENKYAATFLIHGQYGYGQRWLVNRLCEQ-VDDGLTTAFCPINL---TKKHRDIQRIWDDIAAKVGSSS--RNPQTIVDVIYQHWQTKPILLCFHNVDVVGCQELNKFIEPFKHPNT--VNTQPLLLFLIDNYGQQK-FSNLF--NQISKEYDIENNLEIKELDKFSDAEIKIWTKCYQKIF-SNLSEITQEIIYNNT--P----IQAIEYICQCFD
KPQ40445.1_Phormidium_sp._OSCR                 SYESDVSRHTEAVLRAMDELRGTGQESFPVNSSNKNNFIKILGRFNYVQQKRRFQVFL-EDANPRIGAFWLRGERDSAQKWLLSQLWLQNVPNSTKAIKKTLTM----TTRQNTERILEKLATWLQVE---ATPEALIKRILENCKTETVALVFYQVEYLDKGTLEELINTFQKTYLRHSLDNPLLLFMVDLGKKGDH-----CPINHSSDYNAEHPEQFVELTQRFQDRDFRWLTDNQTELPVSLEAIKKDMIDDNH--P----EEVFTELCDYFG
WP_071187406.1_Trichormus_sp._NMC-1            KLSRKAERLLKEIQEKLSELDKNSSN-----------LEKSFQKIDFSKSREIAESLKKQYETEGAILLFLQKSKKQLGKYCIEEFIDVIISNQIETCPRTINF-ESNTYKSNKYGFINAISGYLGIEISSSIINEIHEKIVSSLSLDTIFFEIKSINRLEEEFIDWFIKIFKSLFN--QKRNRLIFALIAESPFPINSSNFC--QEVTLNHDK---IEIPLL-DWTVEDISNWLISHHKLFPDEIHKEAKNI-EESEGTP----ESVCSKLQEYFY
SEH05005.1_Candidatus_Venteria_ishoeyi         VFNGVAQRLLQWVPALTDVLYHARKSINSTPNISKIIPNFAPYLVNYTKQQTCFEDIVQPTLGNHPLIFLLHGQESNTTHGLAARLKQNLEENCLHHGETTVRL-CPFDCDERPGFLLTTIEKNLKDK---FAVENLEILHESAFEA--TTLFYSEVEAKHKKLLQDFLNFWNDDGWPEQNHLFIVCLFIDYRPGPLRKMQLT--QRLKAKNIDICNMLLPELTPIPREDFTPWYNLMKTYYSDELEIRVTELFDENNKLSMEYLGPKLSKLLADLC
WP_017306429.1_Spirulina_subsalsa              TLRDIYKRIKDKCNNREQQFQAWLKGTTLDQELLEYLLNDTLKKLNYDHQKRPVKDSF-PEINQRINAFIVQGQSHHAPRWLTNQLLYY-VPHHDNAKKHSIVL----KTHFRFNRLCEGIASGMDCG---AEPQNIVSALAQNLETQSVVLALRDIHQAKNGILSSFINEIQTNESLSTGQHYLILFLVDGDSSSSK-----WSTELNPFIQTDSPCSLPPLENFTQGTITLWRGRKEKILPDQYPQAIDEFIDTEE--P----KGFLDFIYDSCL
OCR02924.1_Oscillatoriales_cyanobacterium      DFETAYSNLTEVLLKGINP-------AVQNSATVENKLYNALLSLDYIRQESLFQEFV----NNQIGACLIHGKQEYGQRWLLHRLLRA-IPNNNTASLTKISF-LRQVQNGSLDALWGEIKRKLKLKCQ-PLPDELAKAVCNLWQTQTVVLIFDDADQIEEAHLEKFINDFNSKPPK-SRNSRLLVFLIDFDGCAP-KWSIPFADALDTTWKPHIPLKLPPIKPLREMELAYWLEKAFNDLPDSIEATVQSILNSDNGLDLHEFELAMAHICELCD
KIF28928.1_Hassallia_byssoidea_VB512170        EMDELAQQI-----------------DRLERASPVERLYRALLKLNYREQTKKFRRII----DNSVAAFLIHGLPDYGQGWLLNRLVQQHVPNSITAKVVQFNL-NRLARKSDVPSLWRELSRGVGLGGQ-GLPPDIADRVYQWWQTQNVLLIFRDVDFLSEIYLRELLENFTSANAK-VSQFQLIMFLIDEKGCVS-KWNVPFAEEPNSNWQPDSPVKLPEISGFSQEELAKWLDYESDDLPEQLEQTVQTILNSDNGVP----ELAMEQICYLSG
CUR12819.1_Planktothrix_paucivesiculata_PCC    PDDMLWTQL-----------------CQQQVPQSEHKLFKSLLRIDFDEQEEAVMEVL-SPSYNKTAAFLVHGEEKFGQETLVTRLSQL--SKLRNGRRIKIKV----GGMEDISNLWNEVGKHLLDKNQTLPETKIIDQIFELLQTQHLIFIFSDAHRTYISFLPELIQEF-------QRETYLAMFLVDNKGYIC-KSGLSLAWQVNHPDLPRIPLSLPPASRFPVRKLNEWVAVAAEVVP------VETLLESQGGVP----DLVYQRICYYCG
OQW38836.1_Proteobacteria_bacterium_SG         DEEEAETRLLSKIQDRPRPVFKKSES----PQYLSNQAEQKLGQINRKKHRDHLAIQIWQNENRKTHGFVISGEQQECPEDIRFKLSYLLQEDLINHNPKGLRTKSYRIGKSAEQFLWELLAKRLGSC---NPEKAAIEMRLNQVDR--SYLFFRELPSDEQQFLVDLLVAWSNLSLASTSHSHFLLLIHAAENSGNSKPSWR--DQIESLLKQHEVALLPEFHPPSKKEIRDWKGLHI----SVLRDGIEELFLKHESIPLGKFKAALPLLKKHYS
KPQ40050.1_Phormidium_sp._OSCR                 RLDSGFYKLESEITEIWKKLDEDTKEQLILEDSLPKRVHQCLLNFDYQKQEYEFDEFI----STQLGICIVNGLQKCGQPWFLYRMFQT--KYYFDDTSTPFNK-ISCFSRTQKLSLKEEICHFFRLPVN-ESIERIYQEIANHLEHKSLVFIVKNIHLTTQEYLDYFVKQFQQQPPN-NPENRLLFLLVDLEGVSH-HWEMSSEQELLSHYRSHTIYRLKQIKPLSWAERQDCLKQLAWDLP---DEISQKVILTEKFVP----ETVLEYICNCCN
ETR71372.1_Candidatus_Magnetoglobus            VLTSFADEIFQLLQSQPLE------------KKSTSFGSASVLMCNRIPQLRSFLTNY--RVHKMPQFYFIHGREQDQHDSLVTRYNQTIIHKRIQEIYDPYMCEYGKKSDDQQNTLLYDIGHELTGESTHDITSFEKLVNATIFEQQNAIIISHKIEEDFKDLLTWYIQEFLKKYKHERIPQFILFFNIIYE-----KKKMI--RWINEITPKNSTVLIPELEPVIRPDVQSWFNTYTSDLPSKRNECIKQLFPKPKSRPMAVVEEKLVGF-----
WP_020531248.1_Flexithrix_dorotheae            YHKNFVKKIELALKKKVNSIFNTS---------KNLIPEKFYLGCNRTIQFQDLQYIL-EISTKKPCMCILYGNREDCHRSLIERFYYYLLNK-------LINI-GKWDVRNRKERIIKSSFEAFNLTDIKAVPDNAQMIIDSYNQKCDVLLIEIEIDCNEKSTLKWYINEF-----------LKVLHLIKLVSKPSNERRIE--KDLRDLYSSEIRKVLPKLSPIKKYHVKELL----KGL-ASYVDKIDKLFLKKSEITMLNFEMFVT--KEFLN
GAK59821.1_Candidatus_Vecturithrix_granuli     LDNPDAQRLLWAITGQRELIADASAS-------QPFLGDIVYKMCNRGPQENDFLRFFLRENPRQPHFYFLHGSDDECHESLIERLARTRIKKYVEQTCGDVQS-IRGDLETRQEDLALNFMKTFDSDDLMPNELISGLCHLAVLRKHPCVILRHNLDAARERFIRWYIKAWLQPEWTADLPQFLIFLNIIYPRRHHRQKTIS--KQLCRIHTSSGEQLLPELKMVTPFDVSDWFSYYR---PWQRKEKIALIFGNQERCSMSDVEKHLKMIIDECT
KIJ79200.1_Tolypothrix_campylonemoides         GYCVLFEDLVRKVQERVSSWVTSVGGPLRTAASNEQPTEAEIIQLGHKSLSRQFEDSLLSSEQAAVVCFLIHGASGYGHKEMVVRLRQI-LEQQRSVQARKAAF-SPLWREKSLAKLLELIGDQIEQGWIPDSPEVLAQRLHKMLETSDVILEITEIQRFNGSLLS-FIKDF-------PTQHRLVVLLTLEQTILPEQSPLA--MDDDSEIDSMRPIKLAELKEFTEKELTSWLSKWR---PNDALVLAKTLMDETKGNP----QLLYNKLRDDST
RAM51670.1_Hapalosiphonaceae_cyanobacterium    SLDRLFSSLNLDLERESS--------IDQKPQKPTKEFMIALYDLDYLHQRTSFKTAI----DVKAATFLIHGKPHYGQRWLLNQLKSQ-VPYHTNAWQKSIYI---KPHRKDIEVIWQDLAQELKTS---ASPKDVAKELYQHWLKSTVILAIHDVDLIAGSPLKQFMDEFKKAGQL-QSSYRMLLFLVDNINSKSKEKSLSFLENIDIN-EPHIPFVLKELEPFGKNLIEAWVGVQEQSF-ASIKEVMCEIVRDSQ--P----ISVFTDICQCFD
WP_088278871.1_Ideonella_sp._A_288             AWSSVSRDLFPLIETSTITAPSRSTQ-----QPLTTVDLPLPYLCNQEPLTRAFEDAAQRGGAKHAMLVLLAGGHNDAPEHFARRLQRHHLPRRLKTIGVAVHTPAAGTTLRHSMPLLRALFEALDDDLVPTEFDQAAQVLCEHLRARTAAVLMAWLPDRDLPALLRFIDAA-----------RLVLICVGEARGSVW-------RAVRKSSQA---AALEPMAEIDVAALTKWHKEHG----RVDLDRCETLFRAGR---AREFARHFQTFCEGLR
OCQ93508.1_Nostoc_sp._MBR_210                  TQNLRYHQLADELERYITSIFTAEKDDQPLSLLFNSQLFDCLLSIDFKDQEACVTQAIWQKKRQRTVAFLINGDDKYGQSAFIERLFRK-IPEFKHEKEIKIKL----ESGWETSNILQEIAKFFGLDYISDIQDKIINKACECLQFKNLIFVFYIPQRFFVGLLADLINDF-------HKDTYLTMFLVESNHKEKGLQELS--NKLAPREDDSSYLYLPLLSKFSRDHLEKWLENGVEDE-TQQKNFVDTLLETEWGIP----ERVYKKICNHFS
KPV52199.1_Kouleothrix_aurantiaca              QIKQTLRAVDDEPNDDVRAGGAALGQETQRQNSGDGKLYQALQGLNYSAQKSAFVLSV----ARTVAAYLIDGQRNHGQRWLLNLLLKR-YEHALQA---SIDM-RRLGRGKSIADLCQEIGRKAGVAA--STPEQLAPVLCRWWETQHTFLIFHSVDLLGEAGLREILEHFGDPQHH-NAANKLLLFLVDNQGVAA-KWNLPLAQNPA---NAGGPERLPTIARFSEDELDRWMMASLLDLPERTD--VQTILNTDDGIP----ESVLEEICVICD
PKO80078.1_Betaproteobacteria_bacterium        ---------LQTVKEWAADLGKALSSLAGRRQASGDIPLAELGRIGRDTQRMALVDALRSVKGERAAAFLVHGPENHGQREFAEFLNVW-DNEWEDAQPI--------ADTDTADALIRWTCGQLGTPMIDAAIPELAQTLAARLRRGPVIIIQRSIGHRQ-ERLTRFIGGF-----------RLYWFLIDHCPLPQPEGVIA--PEAGADTADYDDLALPALGDISAGQVRKWLKELRKSAP-------DEILTTPDGNP----SDVYNRLWASAS
RKZ83713.1_Candidatus_Parabeggiatoa_sp.        ADNRLDNSITDSLVKAWPKLDP----DRTPKPKRKKYKRTLLPYLPNRRDQELELGELIAHDDKHRPLLCVVHGDEYQCSDKFLDRIREESLPNIIPEQMHGVDL-FVCDTFQSVNELHRKMLANLGELFINNMYATCDEVAQAIAQRQCPVILHTDMSTTDINIIHGFIQFWADWPVLSAQNHLLVCLCFNYRDIEKMRPSVN--QAIRNTFKDLASRVLPELPSIERTHVEQWAKIYLSEF-YELIPYIRELFPPIEKIAMEPLAQQLKQILKECC
RMG30280.1_Bacteroidota_bacterium              YKEEAAEVLNWRLPQQATQ--------------KGNLSMLDKYTCNRAENSRFKLNFKSRGNKKFQYYIIHGEEQESPYGLFQRFVLEHISTAYQPYFY----EEATSLEEAKINFLTALFQALELNPNRDQLTLANLLKAPAAQDMQCIALKLRVYSSERDMIQWVLQEFRESELSPNAPDLLMFISIIYDLQQASSKNPY--EKLIKTLAEFPEGLLPVLPPVKISDVNKWFDRIT---PEEKKQQIATYFDAQPEYHMVTVEKKLEQIIEVYN
PZN77012.1_Candidatus_Methylumidiphilus_alinenensis SARDINGPV-----------------YTTQNPRIPPVLSLLPLMFGRETQEEQLQDYLCFHKGQQIIAVVLPGPHRESPAHFVERYAKLSLRDAYQRHWREYVWEKHSPLQQRLDFLLRDLCRNIDGVSF-GEEGHVRDRLVSQFADQSHQILYYFLTADRANLIGEWIGFWQKLKLDTAKNMVSIIFCLHSEPSILPEWSSRCVKALRTLCPNAPDLMLEKLPPIKEQDSYNWAKEHESTFPAILYQRAKNAFRKRR--PLLNLMTKLEEAVAKAA
WP_072720293.1_Planktothrix_tepida             TTLNIYKRIVGEINKDKQEFSEWLQTGSNQEDEQAEQVYQSLLRLDFRKSSGIFADWE----QNKAACLLVYGKKKHGQEWIMRRLMTK--PKYSKRMFYTCSF---SSYISDSKKLWKWLSEKIGIQFNRENQLRIIKALKEQLQYNNIVLIFTDLQANSLEYPQEFIDQFFSQQAQPNSCNKLLVFLLADEPEIK-NWCQNWVSNINENWSITEPIQLPEPPICPEDDLIPWLKQETDYLPVNPRKTAQEILKTNQGVP----EAVLMYICQYDQ
WP_020536784.1_Lewinella_cohaerens             GILYLLEECKKSVGTSTTDAGG------------AALHEYHAYTCDRVAHSDTFRQLF-GEQKQPVQFYFLYGGDLQSHEGLFRRISYDRLQDYLNPELKSLQLEFSRQLEHYKQNILRSFFSMLGLPPNEEKDLTYALERSPRLQNTDYVCVFLHISQYDPAAARWFIKEFCQEGKVNTSVAILFFLAIEYDEEDAD-----LKSEVVAALEMAERQPLPELDMVAQRDIGRWLEHYKQVSPRTRKELMKTVFGNSREHYMEDVELELKKIIDQYN
ETR70369.1_Candidatus_Magnetoglobus_multicellularis RLKEIATELSQYMQNQPAALPDRTAS-------MLSRNLLPYMPDRSELIQEMSLALKKNQAGKPLVWIIHGNDNQCHYDLMDCIEGCYWERFLYPTSKLLRY-YQYETNQWRDLVAMELKHKFDIQK--EAPEDVFDAINRRCDKASPVMLYDVVDTDQQTVCQRFIDFWNQWPGMCCQNPLIVCLLVRYKSFPVSKKDPN--LEFKETILTLKNCVFPELPDISKKDTEDWYHNYKNYIPKNVSQCIEDIFDANQKFPMQSLAREIEQ------
PZN72517.1_Candidatus_Methylumidiphilus_alinenensis SEDGIEPNFESSPNANPTAIPSTRDNLSSQSNGLENTPAFKAGLVDKDEQATHVIDHLKEETHNNPLVYLLYGASTQWPDALLNILYYQQLPRFNKNSSPTLKYRILRGNIDPVEYLYELLDEELTCGLNR-------KAIERRLSKEKPLIFYRILTAAEPALVQGMLVAWQKLELSINSPRHILIFYYEYIETPKPEKTLI--NHLKASLPTEMSIFLPRLKSPNKKLVHDWISIHIQKL-EEAKKYVEDKYNAKKAYAKDDYHHDLKNILNEFC
consensus/90%                                  .......l.............................hsb..p...h.............hhhl.G........hh.ph..........................h...h.........h...h...h.................hp....hhh...h.......h..hl..a...........hhhh.h................h...........l..h..h...ph..W.................phh...p..........h..h.....
consensus/85%                                  .......pl............................b.hsb..p...h...h.....p...hhhl.G.p......hhpph....h.p..p.....h.............lh..l.........lh..l...h.................hpp...hhh...l.......h..hlp.a...........hhhh.h...............ph...........l..l..h...pl..Wh..............h.phh...p..s.......h..h.....
consensus/80%                                  ..p...ppl...h........................b.hsb..p...h...h.....p...hhhl.G.ppps.p.hhpph....h.p..p.....h.........p.....lhp.l.....p....lhp.l.p.h.................hpppp.hhh...lp.....hhp.hlp.a..........blhhh.l..............pphp..........Ls.l..h...cl..Whp........p....hpphh..pp..s......hp.h...h.
consensus/75%                                  .bp..hppl...h........................b.hsb..Q...h.p.h.....pp..hhhl.G.ppps.c.hhpcl.b..l..p..p.....hph......pp.....lhp.l.....pp....lhp.l.p.h.................hpppshlhl...lp..p..hlpbhlp.a.p........bllhhhl..............pplp..........Lsbl..hp..cl..Whp........p.p..hpplh.ppp..s......hpblh..h.
consensus/70%                                  .bpp.hppl...l.p.....................b..h.hsbppQpp.h.p.h....ppps.hallaG.ppps.c.hhpRl.b..l..p..p.....lph......ppp.p.lhppl.....ppp.p.lhppl.p.h.........p.......p.hpppshlllbpplp..p.phlpbaIp.a.p.....p...bllhhhlp.......p.ph...pplp...p....b.Lsbl..hsbpcl.pWhpp.......pbp..hpplhpspp..s....b..hpblhp.hs
```

## ; Type 2 BREX/Pgl system PglW Representative_1; NERD-REase + iPKinase + PKinase + HTH + wHTH1 + wHTH2 + wHTH3 + CTD-iSTAND-NTPase

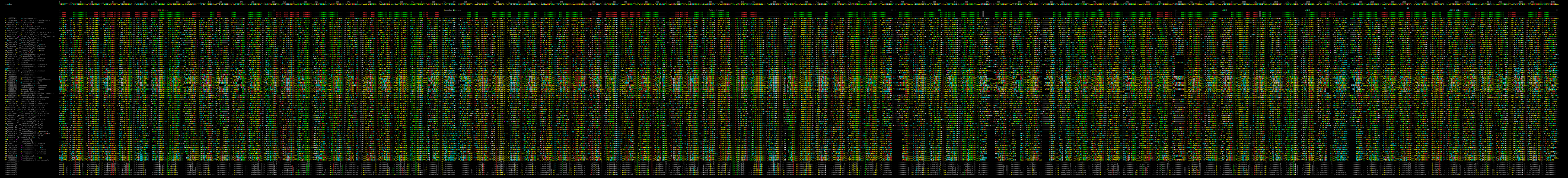

```
## ; Type 2 BREX/Pgl system PglW Representative_2; NERD-REase + iPKinase + PKinase + HTH1 + HTH2 + wHTH1 + wHTH2 + wHTH3 + CTD-iSTAND-NTPase(Type1-BREX-like-iSTAND)

                                      1---------11--------21--------31--------41--------51--------61--------71--------81--------91--------101-------111-------121-------131-------141-------151-------161-------171-------181-------191-------201- [illegible]
OrigSeq                               MAAHFISMGEPAHDAERQAIRFLVEGLPTDYTVFGNAWLVERSGVVYELDAVVAAPHAIYVVEIKSYRGRIEGTDHTWYIPEPVPSPIKRNRKTAQVLKEQLKRRSYQAGQVWVQGLVFLSATTNVGVKGPASRDRIHTRKTILAALQDPGLVERLSGRQSLVPTESAQSDLLYVLTGTRSGPRPLRRVREYDIVDTLDHHDTYTE [illegible]

                                      <<------------------------------------------------------------------------------------NERD-REase---------------------------------------------------------------------------------->><<- [illegible]
                                      ---EEEE------HHHHHHHHHHHHH----EEEEE-EEEEE--EEEEEE-EEEEE--EEEEEEEE----------EEE---EEE-HHHHHHHHHHHHHHHHHHH-HHHHH--EEEEEEE------EEE-------EEE--HHHHHHH-HHHHHHHH-------HHHHHHHHHHHHHHHH-----||----EEEEEEEEEE--EEE [illegible]
                                      ---EEEE------HHHHHHHHHHHHH----EEEEE-EEEEE--EEEEEE-EEEEE--EEEEEEEE----------EEE---EEE-HHHHHHHHHHHHHHHHHHH-HHHHH--EEEEEEE------EEE-------EEE--HHHHHHH-HHHHHHHH-------HHHHHHHHHHHHHHHH-----||----EEEEEEEEEE--EEE [illegible]
                                      ---EEEE------HHHHHHHHHHHHH----EEEEE-EEEEE--EEEEEE-EEEEE--EEEEEEEE----------EEE---EEE-HHHHHHHHHHHHHHHHHHH-HHHHH--EEEEEEE------EEE-------EEE--HHHHHHH-HHHHHHHH-------HHHHHHHHHHHHHHHH-----||----EEEEEEEEEE--EEE [illegible]
MBL0196963.1_Myxococcales_bacterium   MTAKFVPIGEPAHDAERQAIRFLVEGLPDTYTVYGNPWLVERSGVIYELDAVVAPQAVFVVEIKSYRGIIQGTDSDWTPEKRPSPLRLNRKTAQVLKSHLRDTTYQAGQVWTEGFVFLSATTDSRVRGLASNDRVHTRLTLLAALQSPELIERLSGGRARTPSSAAERELLELFTGAQQGPKPVRRVREYEIVETLDASDTFTE [illegible]
MBK8541194.1_Ardenticatenia_bacterium MTARYQAVGKPAHRQEIDALKFLIRGLPESHMLYANSWLAERDGALYELDAVVVAPHAVFVIEIKGYTGAIRGTDHDWYTPSPMRSPLRNNRLTAQMLKSALERENAALRGLVWVEGYVFLSHTDDVAISGIGSEQRVHTRGSILKALTDPQFLTEHITRGPAPVGQEAAEAVHRILTGGDRRHPPPRRVREYELGDIVERGEDYLE [illegible]
MDO9017995.1_Myxococcales_bacterium   MAAKHIPLGDPAHDAERIAILHLVGNLPGSYTIWTNAWLTDRNGTIHELDAVVAAPHAVYIVEIKSYRGHVEGNENDWYLDDPIRSPLRLNRLTAQKLKDALRRDDFNAAQVWVEGLVFLSHATSFSPLGTAAIGRVFLRNGICAELQNPDSVQQRNGRRTGTVEPHVLAALTRVFQSERGRRPPQRKIREYRLETAIDRTDRYVE [illegible]
MBL8718633.1_Myxococcales_bacterium   MGAKFIHIGDPAHDAERQALRFLVEGLPESFTVYGNAWLVERSGVLYELDAVVVAPHAVFVVEIKSYRGRIEGTDNDWWLPEKIRSPLKLNRITAQVLKAHLKNTNYQAGQLWTEGLVFLSATTDVGVRGPASNDRVHTRKTILAALQDEALLKRVSGGRAATPSSAAERELLELFTGAQNGPRPVRRVREYEVIETLDHHETFTE [illegible]
MCB9654435.1_Deltaproteobacteria_bacterium MAAKFIPIGDPAHDAERQALRFLVEGLPADYTVYGNPWLVERNGSVYELDAVVVAPHALFVVEIKSYRGRIDGTDNDWYIPHPIKSPIRLNRRTAQVLKSQLKRESYQAGQVWVEGFVFLSATTNVGIKGPASDDRIHTRKSILAAIQDQALIKRLSQRHSLTPTDDAESDLLRLLTGAKNGPRPTRRVREYVIDRVDLQEHFVE [illegible]
WP_075011136.1_Stigmatella_aurantiaca MSARHHSLGEPAHEAERQAIRLIVDGLPEDYTVYSNAWLVERTGVVHELDVVVVAPHGFYVVELKAYRGVVRGTDHDWDVPQPIRSPLKKNRQTAQVLASELKRLSAEAARRWVEHFVCLSHASEHKVEGPASRGRVHTRHTILEALQDARGFHDRCPRGCPEVDEHTQRTVHTLLTQQDPALRPPERIGGWRLEYTVEATERWDE [illegible]
MCA9600002.1_Myxococcales_bacterium   MPAKFIKVGEPAHDAERQAIKFLVDGLDERCTVYGNPWIVERSGGIYELDAVVVAPHALFVVEIKAYRGIRGTDFDWYVPAPVRSPLRLNRKTAQVLASELRRRSFDAGRPWVDFLVFLSHAAGVELQGPLSRLHVHTKRSVLDAISDPHSVYQRARSGSPPVDDHCARTLHELLTGVDKTLAPPRRIREYELENVLDTTDDYTE [illegible]
MBK7192658.1_Myxococcales_bacterium   MAARFIKLGEPAHDSERQALRFLVDSLPATFTVYGNAWLVERSGVVHELDAVVVGPHAVFVVEIKGYRGTIEGTDYDWYLPEPVKSPIEKNRKTAQILHGLMKRVSYEAGQVWTQGLVFLSATTRCNVRGPASKDRIHTRTIIAALTDPAFVDRIANRAATPVTARTLADVDKILTSSAASPRTRRVREYEIQAALESHDTERVE [illegible]
TVQ88636.1_Deltaproteobacteria_bacterium MAARIIKVGEPAHANEQAAIEHLASELPSSVTVFANPWLIEPNGAVYELDAVVVMPHAIYVVEIKGWRGTIITGDHDWYLPAPQRSPLHLARKTAQILNNLLRRKDATAGAVWVQELVYLAAASRVELHGDAAKRRVATRQTIHAILHDEQRIRSLSRRMLTMVDADVESALVKVLDSKPP-RPPQRTILGFEVIDTVDTNERYRE [illegible]
MCA9674838.1_Myxococcales_bacterium   MPAKFIPLGQPAHSAEVQGLKFLVDGLPSTYTVYGNPWLVERSGVVYETDAIVVAPHAVFVVELKAYRGVIDGTDYDWYTPHPVKSPIGLNRKTAQVLNSLMKRDSYEAGQIWIQGLVFLSAASGCQVRGPASRDRIHTRATIIAALTQPELVTRLASRATAHNTPSTQSALHRILTSEDTRARPARRVREYEIERALETHDNCKE [illegible]
WP_153825183.1_Polyangium_spumosum    MPAKHIQLGETAHEVEKAAIRYLVDGLPASFTVYSNAFIAERTGAVYELDAVVVAPHAVYVVEIKGYRGEIFGNDNDWYPEPIRSPLRLNRKTAQVLQSCLRRASEDAGRVWVEGLVFLYQARSVQIIGPASESRVHARETILSELQNPAALQKRSSRGTPPLDKHAAEELDRLLRGADARQRPTKRIREYEIEATLEQTERFVE [illegible]
MBI2893937.1_Deltaproteobacteria_bacterium MSAKHIPLGEPAHDAERQAQRFLVEGLPETFTVYTNAWLVERSGVVHELDAVVAAPHAVFVVEIKSNRGAVEGTDYDWYVPEPIRSPVPENRKTAQALHSLLKRESHDVGQIWVQGLVFLSASASCGVRGPGSRDRIHTRKSILAALQDQSFIERIAQRPIAAVSQSGAEALHRVLTSAPKSARPTRRIREYEILGTNETHETSAEE [illegible]
NUN14841.1_Myxococcales_bacterium     M-ATFFQIGQPAHDAERQAIHFLVEGLPDSYIVVGNPWIVERTGVIYEIDALVIAPHAIYVIEIKSYRGRIEGTDTNWYLPQPIASPLRKIRVCAQVLNSRLRHENYEAGRVWVQELVYLSATTDVGIKGPASDGRIFNRSNILAALQDESLVLRLSRRSQLVPTGEAMHELNRIFAANASGPTPARQIREYDIIDTLSFSEQFSE [illegible]
KYF91864.1_Sorangium_cellulosum       M-ATFIPIGEPASDRERQALRYLVDGLPEDYAVYGNPWLVERSGAVYEIDALVVAPHALYVVEIKSHSGVVSGNDNDWYVPAPIRSPLKLNRKTAQIFADQLKRHSEFAGRPYVEGFVFLSHADGCRVTGPASAERLHTRRTILQALLDRAAFVRRHGE-REPVDEHVRRVVDELLTGVDRAHPPPRRIREWEIEATLERTERYVE [illegible]
MBX7096841.1_Myxococcaceae_bacterium  MPAKFIQIAESRHDHERRALRLLVDGLPSTFTVYGNPWLVERTGVIHELDAVVVSAPHGIFVVEIKSYRGRIEGTDTNWYIPETITSPIKLNRITAQVLKNQLKDHDSAAGRSWVEGFIFLSETLNCGVRGPASNDRIYTKTDKILLALQDTELLKRLSRGMSTPVNPATDRALLAIFQANQNPLRPVRSVREYDVVRKVSVEETFSE [illegible]
MBI4699810.1_Deltaproteobacteria_bacterium MPAKHIPIGEPAHDAERQGIRFLVDGLDERFTVYSNAWLAERSGVIHELDAVVVSPHGFYVVELKSWRGDIKALDHDWYVPHAIRSPLKTTRVLAQKLKDEIKRRSADAARPWVEFFVLLSHTGQVSVFGPLNTGRVHTRKTIIEALGNAKTFYDRAGGQMPKVDAHAAKVLDDLLRGVDKRFAPPRRIREYELGDVLERSNRYVE [illegible]
consensus/100%                        M.Ap...hups.p..E..ub..lh..Ls.p..lhsNsal.-.sGslaE.DhlV..PpuhallElKu.pG.l.s.-.sW.s.....SPl...R..AQ.h...hcp.s..h...a.p.hlhL..s.......G..s..+l..+..l...l.p...h.p...........s....l..lh.......P.cpl..acl....p..pp..- [illegible]
consensus/95%                         M.Ap...hups.p..E..ub..lh..Ls.p..lhsNsal.-.sGslaE.DhlV..PpuhallElKu.pG.l.s.-.sW.s.....SPl...R..AQ.h...hcp.s..h...a.p.hlhL..s.......G..s..+l..+..l...l.p...h.p...........s....l..lh.......P.cpl..acl....p..pp..- [illegible]
consensus/90%                         M.Apa..lGpsAHp.Eb.ulc.LlpsLs.ph.lasNsWlhERsGslaElDAlVhuPHuhalVElKuapG.l.usD.sWa.P....SPl..sR.sAQ.L.s.h+c.s..hu..Wsp.hVhLu.s....l.G..u..Rlas+.slh..l.s...h.p........ss..s...l..lhpu.....P.+plb.acl...lp.p-ph.E [illegible]
consensus/85%                         MsA+ah.lGcPAHc.Eb.Al+aLVcuLP.sasVauNsWlhERsGslaElDAlVhuPHulaVVElKuaRG.lpGsD.sWa.P.sh.SPlcbsRbTAQhL.s.l+c.s..Aup.Wsp.hVaLS.ss.h.l.Gsuup.RlasR.sIl.hLps...h.pbs......ss..s.p.l.plhpu.....P.RplREa-l...l-.p-ph.E [illegible]
```

## ; Type 2 BREX/Pgl systems Helicase (BrxHI-Helicase); SF1-SF2-Helicase(Ski2-like-Helicase) + wHTH + Small-B-Barrel(HAS) + Lhr-Helicase-CTD

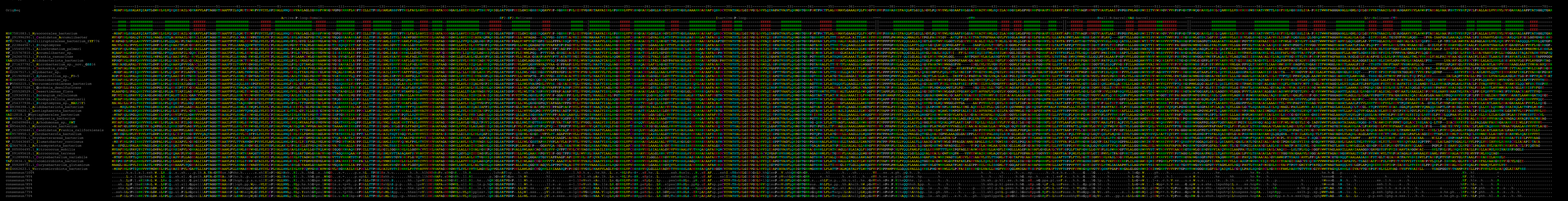

## ; Type 2 BREX helicase and Ski2-Like-RNA-Helicase; Core Components Comparison Alignment;

```
MBK7581083.1_Myxococcales_bacterium             --MSAFDRLSSA[illegible]
WP_291996290.1_Candidatus_Accumulibacter        M-VEGFERLHGA[illegible]
MTV27845.1_Nitriliruptoraceae_bacterium         M-DAPAGELHPL[illegible]
WP_223844567.1_Streptomyces                     M-ADVLDRLDPV[illegible]
WP_155450775.1_Allochromatium_palmeri           M-GFSFDQLHPA[illegible]
WP_015767810.1_Accumulibacter_sp.               M-QAAFDRLHPA[illegible]
WP_242667013.1_Frankia_casuarinae               M-APGFAALDPA[illegible]
WP_271637792.1_Microbacterium_sp._nov.          M-MSEASGLAPA[illegible]
WP_133290010.1_Dankookia_rubra                  --MTAFEALHPA[illegible]
MDX2267517.1_Bryobacter_sp.                     --MSGFDALHPS[illegible]
MDR3405892.1_Chthoniobacter_sp.                 M-VSAFDRLDPL[illegible]
MCP5560215.1_Verrucomicrobiaceae_bacterium      M-ESSFELLHPL[illegible]
WP_205528529.1_Desertimonas_flava               M-LAAFGRLDPA[illegible]
WP_129345931.1_Sorangium_cellulosum             M-PDVIDRLSPA[illegible]
WP_250928728.1_Rhodopirellula_aestuarii         --MKAFDRLHPA[illegible]
MCB9398398.1_Acidobacteriota_bacterium          -----MSQLHPA[illegible]
HAI12818.1_Phycisphaerales_bacterium            --MTAFDQLHPG[illegible]
MSW49536.1_Actinomycetia_bacterium              M-SEAFGRLHPA[illegible]
MCB9544265.1_Myxococcales_bacterium             --MDAFDGLSPM[illegible]
MBK9578950.1_Fibrobacterota_bacterium           --MSESGRLHPL[illegible]
WP_015443645.1_Ilumatobacter_coccineus          --MSDFDRLHPS[illegible]
MDQ3647618.1_Actinomycetota_bacterium           M---SFGLLHPA[illegible]
WP_005004495.1_Nitrococcus_mobilis              M-VSAFERLHPA[illegible]
WP_091695618.1_Micrococcus_terreus              M-VEGFDVLAPG[illegible]
WP_059289751.1_Corynebacterium_glutamicum       M-AAGLDALHPV[illegible]
Q8IY21-DDX60_HUMAN                              LQYMGHYLIRD-[illegible]
Q8N3C0-HELC1_HUMAN                              LQPLPITAL-G-[illegible]
A2PYH4-HFM1_HUMAN                               GSLKAVTEI-P-[illegible]
Q8TDG4-HL308_HUMAN                              NDLGPFYSL-P-[illegible]
P42285-SK2L2_HUMAN                              PAEEDYLPLKP-[illegible]
Q15477-SKIV2_HUMAN                              SPVGDFYRLIP-[illegible]
O75643-U5200_HUMAN                              EQLLPVEKL-P-[illegible]
P32639-BRR2_YEAST                               YELKEITSL-P-[illegible]
P51979-HFM1_YEAST                               KKCLSTTIL-P-[illegible]
P47047-MTR4_YEAST                               PPNYDYTPIAE-[illegible]
P35207-SKI2_YEAST                               HKIENFDELIP-[illegible]
P53327-SLH1_YEAST                               LRPLPTSAL-Q-[illegible]
Q6XGE6-Q6XGE6_ECOLX                             HERLPLVPVGG-[illegible]
consensus/90%                                   ........l.s.[illegible]
consensus/85%                                   ....sh..L.s.[illegible]
consensus/80%                                   ....sh..L.P.[illegible]
consensus/75%                                   ....shs.L.P.[illegible]
consensus/70%                                   ....shs.L.P.[illegible]
```

## ; Type 2 BREX/Pgl systems BrxD ORC/Cdc6-AAA+ATPase (Type 2 BREX systems)

```
OrigSeq                                          [illegible]

                                                 |---Preceding-Helices---| S1  H1  S2  H2 |---Insert-Region---| S3  H3  S4  H4  S5 |---Helical-Bundle---|

MBK8233499.1_Candidatus_Eisenbacteria_bacterium  [illegible]
WP_264762807.1_Frankia_gtarii                    [illegible]
MCB9654431.1_Deltaproteobacteria_bacterium       [illegible]
WP_175348386.1_Cellulomonas_humilata             [illegible]
WP_242518426.1_Halochromatium_roseum             [illegible]
WP_274519969.1_unclassified_Halorhodospira       [illegible]
WP_170320004.1_Polyangium_spumosum               [illegible]
WP_091695616.1_Micrococcus_terreus               [illegible]
MBK7077332.1_Myxococcales_bacterium              [illegible]
TVQ88632.1_Deltaproteobacteria_bacterium         [illegible]
MBK7069431.1_Deltaproteobacteria_bacterium       [illegible]
HBQ10062.1_Myxococcales_bacterium                [illegible]
RZO66311.1_Sandaracinaceae_bacterium             [illegible]
WP_012240294.1_Sorangium_cellulosum              [illegible]
MBK8541202.1_Ardenticatenia_bacterium            [illegible]
WP_014010194.1_Corynebacterium_variabile         [illegible]
MBI4699820.1_Deltaproteobacteria_bacterium       [illegible]
MCE9575758.1_Deltaproteobacteria_bacterium       [illegible]
WP_095346964.1_Micrococcus_luteus                [illegible]
MCG5052492.1_Myxococcales_bacterium              [illegible]
MBO9539707.1_bacterium                           [illegible]
WP_165633000.1_unclassified_Actinopolyspora      [illegible]
WP_179642186.1_Spinactinospora_alkalitolerans    [illegible]
WP_201724034.1_Rhodopirellula_sp._SM50           [illegible]
MBL8678387.1_Myxococcales_bacterium              [illegible]
MSW90815.1_Actinomycetia_bacterium               [illegible]
WP_006368783.1_Gordonia_polyisoprenivorans       [illegible]
CCD90938.1_Bradyrhizobium_sp._ORS_375            [illegible]
MCP9485024.1_Candidatus_Gaiellasilicea_maunaloa  [illegible]
WED64128.1_Opitutaceae_bacterium_LMO-M01         [illegible]
WP_015443644.1_Ilumatobacter_coccineus           [illegible]
MCP5560214.1_Verrucomicrobiaceae_bacterium       [illegible]
TAF23833.1_Verrucomicrobiota_bacterium           [illegible]
MBX3732322.1_Verrucomicrobiae_bacterium          [illegible]
MCM2371083.1_Rhodopirellula_aestuarii            [illegible]
WP_134500748.1_Microvirga_pakistanensis          [illegible]
MCB1051860.1_Acidobacteriota_bacterium           [illegible]
WP_253862669.1_Prauserella_halophila             [illegible]
MBK9578951.1_Fibrobacterota_bacterium            [illegible]
MCB9544266.1_Myxococcales_bacterium              [illegible]
WP_007626462.1_Dietzia_cinnamea                  [illegible]
WP_206824863.1_Microbacterium_esteraromaticum    [illegible]
WP_256246189.1_Pseudarthrobacter_sp._R1          [illegible]
WP_188543685.1_Rhodococcus_trifolii              [illegible]
consensus/100%                                   [illegible]
consensus/95%                                    [illegible]
consensus/90%                                    [illegible]
consensus/85%                                    [illegible]
consensus/80%                                    [illegible]
consensus/75%                                    [illegible]
consensus/70%                                    [illegible]
```

```
## ; Type 3 BREX; BrxHII-Helicase Representative Alignment; TUDOR + Swi2/SNF2-Helicase + Inactive-REase + CTD(Alpha+Beta)

                                                   1---------11--------21--------31--------41--------51--------61--------71--------81--------91--------101-------111-[illegible]
OrigSeq                                            MGENVHWVFDRWNNKPVKAVERYELWNTTNCLVYDPTSGRIYTLPADGVGDINEGYVYDEHRIRYILAGAKLKELIARQSILSPIGSNIIPLPHQIYALVRAISSNRIRFLLAD[illegible]
                                                   <<-------------------TUDOR-------------------------->><<[illegible]
                                                                     ---------------Preceding-Helices--------------    S1 [illegible]
                                                   -----EEEEE---EEEEEEEEEEEE--EEEEEEEE---EEEEEEE-HHH-----||---HHHHHHHHHHHHHHHHHHHH----HHHH------HHHHHHHHHHHH----EEEEE-[illegible]
                                                   -----EEEEE---EEEEEEEEEEEE--EEEEEEEE---EEEEEEE-HHH-----||---HHHHHHHHHHHHHHHHHHHH----HHHH------HHHHHHHHHHHH----EEEEE-[illegible]
                                                   -----EEEEE---EEEEEEEEEEEE--EEEEEEEE---EEEEEEE-HHH-----||---HHHHHHHHHHHHHHHHHHHH----HHHH------HHHHHHHHHHHH----EEEEE-[illegible]
WP_162523516.1_Calorimonas_adulescens              MGENVHWVFDRWNNKPVKAVERYELWNTTNCLVYDPTSGRIYTLPADGVGDINEGYVYDEHRIRYILAGAKLKELIARQSILSPIGSNIIPLPHQIYALVRAISSNRIRFLLAD[illegible]
MCL4298460.1_Anaerolineae_bacterium                MLKIGDFA----TGQTGRVIGLATLFGQTYADIFIQPNGPVQRIPLSDLRPLADPLQTIASLFVARLAALQLRAMLTQSGVLSAANFRVTPLPHQVLAVDFVLNQLRPRALLAD[illegible]
WP_207425053.1_Pedobacter_sp._SYSU_D00535          ----------VFNGDDAEVIELSKVFGVEKAQIKLLRSGNIIEVKVDQLEEQLA--IFSNVKIRFLAIAAKIKAEIANQAMIAPLESNIIPLPHQILALEKVMNGQSLRFLIAD[illegible]
WP_218808307.1_Methylococcus_sp._Mc7               -WSVGAWCWHARQASPCR[illegible]
NPV81618.1_Bacillota_bacterium                     M--GGVWAFSPAHSEPCR[illegible]
ELY62516.1_Natronococcus_jeotgali_DSM_18795        MLQSGQQIHID--GQPAE[illegible]
WP_053959402.1_Sulfobacillus_thermosulfidooxidans  M----NWRYLPSYRQMVR[illegible]
MCD8293627.1_Clostridia_bacterium                  -MNTGDYVYDKVAKTRVR[illegible]
WP_162251488.1_unclassified_Nocardioides           WLIPGILV--------VE[illegible]
NMB01451.1_Bacillota_bacterium                     MYQIGQWLMYQPDFSECQ[illegible]
WP_009146898.1_Thiorhodovibrio_sp._970             MSVGQEWRYSTVHNSACL[illegible]
HIP19029.1_Sulfurovum_sp.                          M--NSNWQYSTTYNSPCK[illegible]
MBT5530410.1_Cytophagia_bacterium                  ------------------[illegible]
WP_200891843.1_Caldicellulosiruptor_danielii       M--KDHFVYYVKSLKKYAK[illegible]
NLE00371.1_Fibrobacter_sp.                         MMQPGDLVTCLLHEDKGK[illegible]
EMR73855.1_Thermoplasmatales_archaeon              M--IGQKVISDLYAEQGI[illegible]
WP_073324029.1_Marisediminitalea_aggregata         MWQAGCWLWSKSQECYVQ[illegible]
MBN1459902.1_Armatimonadota_bacterium              MYSSGEWVSIELTGQAAR[illegible]
WP_068347600.1_Kosmotoga_arenicorallina            M----EWYWLEEKKQPCV[illegible]
WP_202636300.1_Rugosibacter_aromaticivorans        MLGVGDWCWFTRHASPCR[illegible]
WP_020614470.1_Sediminispirochaeta_bajacaliforniensis MYSIGELVWSRQHKKEAK[illegible]
MBT6646602.1_Nitrososphaerota_archaeon             MYSEEQWVWSKVHQEPLK[illegible]
MBV6489839.1_Fimbriimonadaceae_bacterium           MLVVGQRLWSPTLRAWVQ[illegible]
WP_073205019.1_Prevotella_ruminicola               M----------RGKSIE[illegible]
NSW90672.1_Bacillota_bacterium                     MFDIGQWAYDTVKGVRVQ[illegible]
WP_148854793.1_Moorella_thermoacetica              M--PGILVRSTIYPEKGL[illegible]
MBU0460857.1_Nanoarchaeota_archaeon                M--IGDLVRSPDYKDLGR[illegible]
KAF0196956.1_Bacillota_bacterium                   MYNTGDWVCYR-GKNTAK[illegible]
consensus/100%                                     ..................[illegible]
consensus/95%                                      ..................[illegible]
consensus/90%                                      ..................b[illegible]
consensus/85%                                      h.....b.......p.sb[illegible]
consensus/80%                                      M.....b.......p.sb[illegible]
consensus/75%                                      M...sphh......p.sb[illegible]
consensus/70%                                      M...sphh......p.sb[illegible]
```

## ; Type3 BREX BrxHII-Helicase and Eukaryotic Swi2/SNF2-Helicase Alignment Comparison

```
WP_162523516.1_Calorimonas_adulescens            [illegible]
MCL4298460.1_Anaerolineae_bacterium              [illegible]
WP_207425053.1_Pedobacter_sp._SYSU_D00535        [illegible]
NPV81618.1_Bacillota_bacterium                   [illegible]
ELY62516.1_Natronococcus_jeotgali_DSM_18795      [illegible]
WP_053959402.1_Sulfobacillus_thermosulfidooxidans [illegible]
MCD8293627.1_Clostridia_bacterium                [illegible]
WP_162251488.1_unclassified_Nocardioides         [illegible]
NLE00371.1_Fibrobacter_sp.                       [illegible]
EMR73855.1_Thermoplasmatales_archaeon            [illegible]
MBT6646602.1_Nitrososphaerota_archaeon           [illegible]
MBV6489839.1_Fimbriimonadaceae_bacterium         [illegible]
P46100-ATRX_HUMAN                                [illegible]
O14981-BTAF1_HUMAN                               [illegible]
Q12873-CHD3_HUMAN                                [illegible]
Q96L91-EP400_HUMAN                               [illegible]
Q03468-ERCC6_HUMAN                               [illegible]
Q9NRZ9-HELLS_HUMAN                               [illegible]
Q14527-HLTF_HUMAN                                [illegible]
Q9Y620-RAD54B_HUMAN                              [illegible]
Q149N8-SHPRH_HUMAN                               [illegible]
P51531-SMCA2_HUMAN                               [illegible]
Q9H4L7-SMRCD_HUMAN                               [illegible]
Q6ZRS2-SRCAP_HUMAN                               [illegible]
Q9UNY4-TTF2_HUMAN                                [illegible]
Q5FWF4-ZRAB3_HUMAN                               [illegible]
P53115-INO80_YEAST                               [illegible]
P38144-ISW1_YEAST                                [illegible]
P31244-RAD16_YEAST                               [illegible]
P38086-RDH54_YEAST                               [illegible]
Q08562-RIS1_YEAST                                [illegible]
P32849-RAD5_YEAST                                [illegible]
Q05471-SWR1_YEAST                                [illegible]
P31380-YAB9_YEAST                                [illegible]
P43610-YFK8_YEAST                                [illegible]
Q06554-YL247_YEAST                               [illegible]
P60240-RAPA_ECOLI                                [illegible]
consensus/90%                                    [illegible]
consensus/85%                                    [illegible]
consensus/80%                                    [illegible]
consensus/75%                                    [illegible]
consensus/70%                                    [illegible]
```

Representative Alignments of Core Protien Components in DUF499 Associated Type-1 BREX-related System;
1. DUF499 ATPase
2. METHYLASE
3. HELICASE

## ; DUF499 AAA-ATPase Representative_1; ORC-CDC6-AAA+ATPase + wHTH + Alpha+Beta-Domain + 3S-wHTH + Degenerated-RRM(Ferredoxin-Fold)

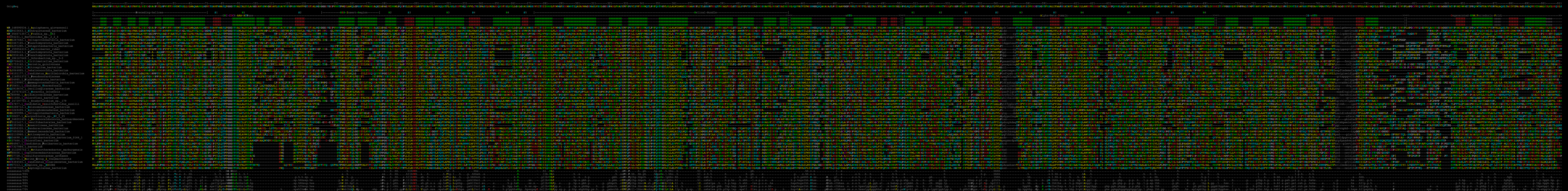

## ; DUF499 AAA-ATPase Representative_2 (HEPN fusion Version); HEPN-EndoRNase + ORC-CDC6-AAA+ATPase + Alpha+Beta-Domain + 3S-wHTH + Degenerated-RRM(Ferredoxin-Fold)

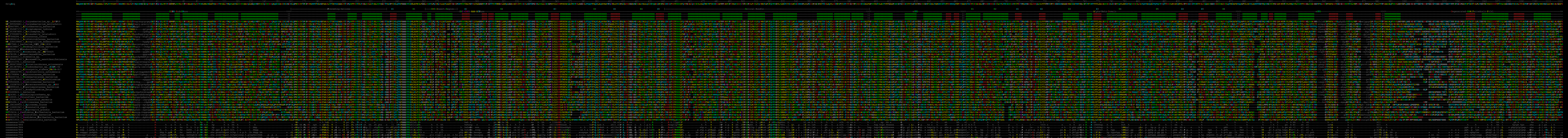

## ; DUF499 AAA-ATPase Representative_3; ORC-CDC6-AAA+ATPase + wHTH + Alpha+Beta-Domain + 3-wHTH + Degenerated-RRM(Ferredoxin-Fold)

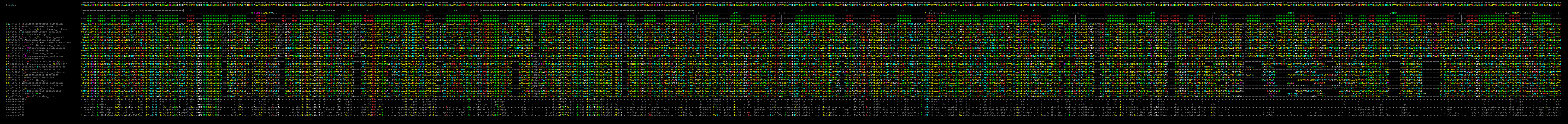

## ; Type 1 BR systems; METHYLASE; NTD(DUF1156) + N6_METHYLASE(TRD-Insert) + HTH + wHTH + HTH

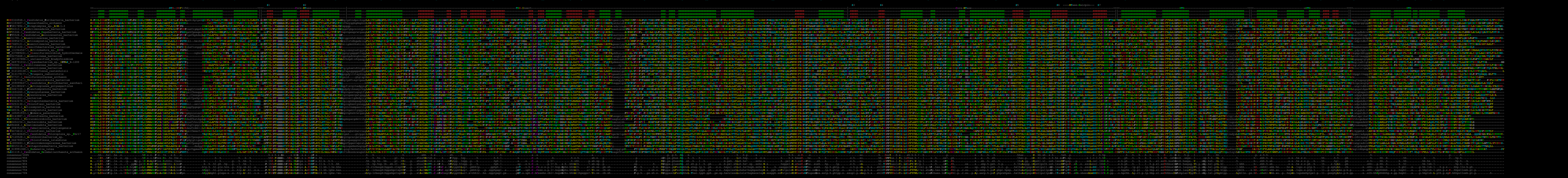

##; Type 1 BR systems; HELICASE (BrxHII-Helicase Counterpart); TUDOR + Swi2/SNF2-Helicase + Inactive-REase + CTD(Alpha+Beta) + Active-PDDEXK-REase

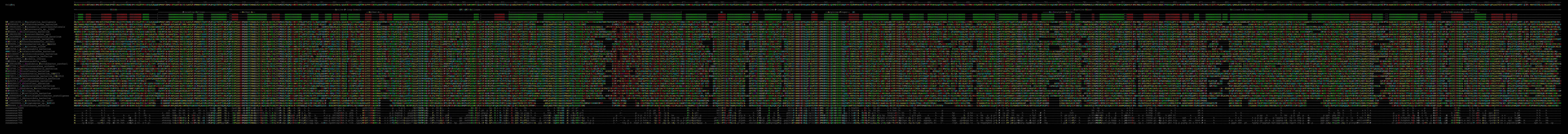

Representative Alignments of Core Protien Components in DUF499 Centered Type-2 Brex-Related System;
1. DUF499 ATPase
2. METHYLASE
3. HELICASE
4. DUF3780

## ; AAA-ATPase Representative_1; ORC-CDC6-AAA+ATPase + wHTH + Alpha+Beta-Domain + 4S-wHTH + RRM/Ferredoxin-Fold

OrigSeq MSLPTIFDKCTPRQDVLIGELAEDQFAASLADVAHSDAPEVYANPQLFFEKTFPTEGLQELLGRLATRFVSYHDQSNGVLRLDTSFGGGKTHNQIAAYHLAERPDAVPDLTDFLNDEQTALGLDVNTAVFVGTHVDGTSARCDDPNA[illegible]

WP_015408759.1_Natronomonas_moolapensis MSLPTIFDKCTPRQDVLIGELAEDQFAASLADVAHSDAPEVYANPQLFFEKTFPTEGLQELLGRLATRFVSYHDQSNGVLRLDTSFGGGKTHNQIAAYHLAERPDAVPDLTDFLNDEQTALGLDVNTAVFVGTHVDGTSARCDDPNA[illegible]

[illegible]

WP_285255572.1_Halobacterium_salinarum MSIKTLFEACEPRSDVLEGSLAEEEFAADLGDVVYGDGDSIYDDPGDFFEKTYPTDGLEKLLGNLMGQVIDGSG--SSVIDLDTTFGGGKTHNLIAAYHFLTAPATIDQPERFI-DIEKRDDKSVSVAAFVGTDVDALDASMSSEG[illegible]

[illegible]

## ; AAA-ATPase Representative_2; ORC-CDC6-AAA+ATPase + wHTH + Alpha+Beta-Domain + 2-wHTH + RRM/Ferredoxin-Fold

OrigSeq MTMSILDTCTPRKDLLAGTFNPEIFTANLMQVIGHyrgetgVVENIYTDPLAFFGEGTYPTEGMRLMLRNIFGRLKGDASFPAIQRSETAFGGGKTHTLIAATHLAYRGTEIADAARELIDADLLLAPGAVTVVGIAGDRVAIHETK[illegible]

ESQ16532.1_Thiohalocapsa_sp._PB-PSB1 MTMSILDTCTPRKDLLAGTFNPEIFTANLMQVIGHyrgetgVVENIYTDPLAFFGEGTYPTEGMRLMLRNIFGRLKGDASFPAIQRSETAFGGGKTHTLIAATHLAYRGTEIADAARELIDADLLLAPGAVTVVGIAGDRVAIHETK[illegible]
KAE8440079.1_Halomonas_piezotolerans MMRDVLSTCQPRPEILAGTFNPEIFTASLSRVLGDyrkgeaGASSLYSDPVAFFRDATHPTQGLCTILDNALARLTNDLSRPAMQRLDTAFGGGKTHTLIALAHAAMQGQPLAEHMTGIVEPERLPEPNLVQVVGIIGDTVDTLRET[illegible]

[illegible]

WP_284505300.1_Streptomyces_cellostaticus -MVALRDVCVPREDVLAGRLTDKHFAAQLDKIVRDp-----EHYAVYGDADKFFE-LTYPTSGIQSMLSRTFGRLTQDEGKHAILRASTSFGGGKTHSLIAVYHVAKGAR--PGNLSEFVDPALLPD-GSVQITAVVGDVLPVNGLE[illegible]

[illegible]

## ; AAA-ATPase Representative_3; ORC-CDC6-AAA+ATPase + wHTH + Alpha+Beta-Domain + wHTH + Fn-III+Fn-III + RRM/Ferredoxin-Fold

OrigSeq MLKTVQQLCSPRKSLFETRFKDYALNLTDLAQGKIDAEEFFTENFVTDGMKTLLDTAFERFEGKSNISVVKLTQAMGGGKTHNMIALALLAMNPDLRKKIGLK---TNVKTAIRVASFSGRESDSEYGIWGSIAQQIGKLDDFADY[illegible]

AFM14204.1_Turneriella_parva_DSM_21527 MLKTVQQLCSPRKSLFETRFKDYALNLTDLAQGKIDAEEFFTENFVTDGMKTLLDTAFERFEGKSNISVVKLTQAMGGGKTHNMIALALLAMNPDLRKKIGLK---TNVKTAIRVASFSGRESDSEYGIWGSIAQQIGKLDDFADY[illegible]
MBX3108928.1_Fimbriimonadaceae_bacterium -MPSLKQACTPRASVFDSNKRDTVHDILDLVKGNIDPNEFFTENYVTQGMRTFLTESFKRLEGNSAQAIFRLSQSMGGGKTHNLIAFGLLCQNPELRDSVMGDFHKVGNLGKVRVVAFSGRQTDAQHGIWGEIAKQLGKQEQFSHF[illegible]
AGC68793.1_Thermoclostridium_stercorarium -LKTIFELCKPREETLKGGSIEDAQELNLFLDKKIDPNDFFSKTYVTKNMTLLFRKAFARFIGQSGSGLIVLKQSMGGGKTHNMIALGLLAQYPSMRKQILGADFPYLYEGKIEIVGFTGRNTT--ELPWIEIARRLNRLNLLVPS[illegible]

[illegible]

## ; Type-2 Brex-Related System;; METHYLASE; NTD(DUF1156) + N6_METHYLASE(TRD-Insert) + HTH + wHTH + HTH

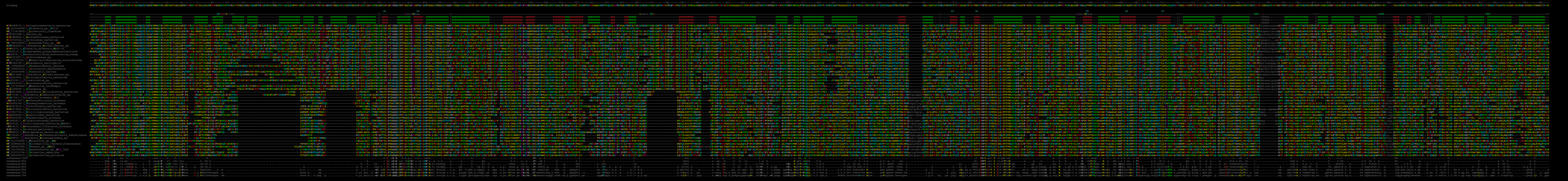

## ; Type-2 Brex-Related System;; HELICASE (BrxHII-Helicase Counterpart); HKD-EndoDNase + Swi2/SNF2-Helciase + Inactive-REase + CTD(Alpha+Beta-domain)

```
                                              1---------11--------21--------31--------41--------51--------61--------71--------81--------91--------101-------111-------121-------131-------141-------151-------161---- [illegible]
OrigSeq                                       MPSLRSHPFRFSYGQ-RDDRLHEFYIPALERSVRYDRSAGFFSSAALAIAAAGIVRLIANGGRMRLLCGAQLTKEDVEAIRRGDDGRVGAAMQGCLGDPKDGSLRARLEALAWMVANDRLVIRVVLPRGK-DGHPLAAADAHDYYHPKEGVFADDQGNRLGFSGSSN [illegible]
                                                                                                                                                                      -----HKD-Catalytic-Motif----- [illegible]
                                              <<--------------------------------------------------------------------------------------------------------------------HKD-EndoDNase--------------------------- [illegible]
                                              ---HHH-------------HHHHHHHHHHHH-EEEEEEE----HHHHHHHH--HHHHHHH--EEEEEEE----HHHHHHHHH--HHHHHHHHHHH------HHHHHHHHHHHHHHHHH--EEEEEEEE--------------------EEEEEE--EEEEEEEE--- [illegible]
                                              ---HHH-------------HHHHHHHHHHHH-EEEEEEE----HHHHHHHH--HHHHHHH--EEEEEEE----HHHHHHHHH--HHHHHHHHHHH------HHHHHHHHHHHHHHHHH--EEEEEEEE--------------------EEEEEE--EEEEEEEE--- [illegible]
                                              ---HHH-------------HHHHHHHHHHHH-EEEEEEE----HHHHHHHH--HHHHHHH--EEEEEEE----HHHHHHHHH--HHHHHHHHHHH------HHHHHHHHHHHHHHHHH--EEEEEEEE--------------------EEEEEE--EEEEEEEE--- [illegible]
MCK6447153.1_Planctomycetota_bacterium        [illegible]
AKC83094.1_Verrucomicrobia_bacterium          [illegible]
MCB9841664.1_Phycisphaeraceae_bacterium       [illegible]
WP_090215779.1_Pseudomonas_guangdongensis     [illegible]
WP_196479964.1_Burkholderia_pseudomultivorans [illegible]
HCG7747318.1_Vibrio_parahaemolyticus          [illegible]
MCE9614803.1_Lentisphaerota_bacterium         [illegible]
MTE20051.1_Streptomyces_taklimakanensis       [illegible]
WP_206882508.1_Alicyclobacillus_fructus       [illegible]
WP_084460301.1_Aminiphilus_circumscriptus     [illegible]
MBI3972513.1_Chloroflexota_bacterium          [illegible]
KPC75468.1_Thermoactinomyces_vulgaris         [illegible]
MBX6754325.1_Thermorudis_peleae               [illegible]
MCF8137375.1_Desulfotignum_sp.                [illegible]
GIW50774.1_Gemmatimonadales_bacterium         [illegible]
MCI7613155.1_Selenomonadaceae_bacterium       [illegible]
MYD03943.1_Acidimicrobiia_bacterium           [illegible]
WP_073234049.1_Desulforamulus_putei           [illegible]
WP_036720040.1_Paracoccus_sphaerophysae       [illegible]
WP_161844016.1_Pseudoflavonifractor_sp.       [illegible]
WP_100917763.1_Candidatus_Thiodictyon_syntrophicum [illegible]
AXH34461.1_Humibacter_sp._BT305               [illegible]
ELZ44607.1_Halorubrum_distributum_JCM_9100    [illegible]
MBD5553416.1_Desulfovibrio_sp.                [illegible]
MBF6555076.1_Acidimicrobiales_bacterium       [illegible]
MBP7066719.1_Methanothrix_sp.                 [illegible]
MBV9397056.1_Bryobacterales_bacterium         [illegible]
MCD8487848.1_Desertifilum_sp.                 [illegible]
MCK6511424.1_Myxococcota_bacterium            [illegible]
MCY4068113.1_Acidimicrobiaceae_bacterium      [illegible]
MCY4434136.1_bacterium                        [illegible]
MDE0094453.1_Gammaproteobacteria_bacterium    [illegible]
MXW96682.1_Acidimicrobiaceae_bacterium        [illegible]
NLA99646.1_Methanomicrobiales_archaeon        [illegible]
NLJ36707.1_candidate_division_WS1_bacterium   [illegible]
OYY89820.1_Sphingomonas_sp.                   [illegible]
PZV04180.1_Cyanobium_sp.                      [illegible]
SNR42896.1_Halorubrum_vacuolatum              [illegible]
WP_054466705.1_Planktothricoides_sp.          [illegible]
WP_103287095.1_Candidatus_Nitrosocaldus       [illegible]
WP_128478225.1_Halorussus_pelagicus           [illegible]
WP_164002139.1_Pyxidicoccus_caerfyrddinensis  [illegible]
WP_176737426.1_Micromonospora_citrea          [illegible]
WP_179052052.1_Nostoc_sp._TCL26-01            [illegible]
WP_285255575.1_Halobacterium_salinarum        [illegible]
consensus/100%                                [illegible]
consensus/95%                                 [illegible]
consensus/90%                                 [illegible]
consensus/85%                                 [illegible]
consensus/80%                                 [illegible]
consensus/75%                                 [illegible]
consensus/70%                                 [illegible]
```

## ; Type-2 Brex-Related System; DUF3780 Component

```
                                                  1---------11--------21--------31--------41--------51--------61--------71--------81--------91--------101-------111-------121-------131-------141-------151-------161-
OrigSeq                                           MPnl---------AKSFNMQEFQLRVPTDNNNAIELYQCAYKKAEKkrpaaKRIGRLKSSTLIQVRE----MVYNCLKANKYDPKTLRQAHYILNEETGVNLALLFQTIQPLKIEKINNIAEGIQAMSYEESHYWFAKISNGk-----RSQ-ALKAIRVLLGD-

                                                                      S1         S2                S3           H1                         S4        H2               H3              H4                     H5
                                                  ---------------------EEEE--------EEEEE-------------EEEEEE-HHHHHHHHHHHHHHHHHHHHHH-----------EEEE-HHHHHHHHHHHHHH---HHHHHHHHHHHHH--HHHHHHHHHHHH--------HHHHHHHHHHHHH---
                                                  ---------------------EEEE--------EEEEE-------------EEEEEE-HHHHHHHHHHHHHHHHHHHHHH-----------EEEE-HHHHHHHHHHHHHH---HHHHHHHHHHHHH--HHHHHHHHHHHH--------HHHHHHHHHHHHH---
                                                  ---------------------EEEE--------EEEEE-------------EEEEEE-HHHHHHHHHHHHHHHHHHHHHH-----------EEEE-HHHHHHHHHHHHHH---HHHHHHHHHHHHH--HHHHHHHHHHHH--------HHHHHHHHHHHHH---
EFA73813.1_Raphidiopsis_brookii_D9                MPnl---------AKSFNMQEFQLRVPTDNNNAIELYQCAYKKAEKkrpaaKRIGRLKSSTLIQVRE----MVYNCLKANKYDPKTLRQAHYILNEETGVNLALLFQTIQPLKIEKINNIAEGIQAMSYEESHYWFAKISNGk-----RSQ-ALKAIRVLLGD-
MAO03350.1_Citromicrobium_sp.                     MAtrakssvgfgvPTDEDPQRFIVRIAPSSREPVTIIEDFGISGPQtnlqaVVRCKLDPKRWRAIAQstkqVFNERLKGRKIPVGKWEAHDNPVHRLLGRELCVLAWAIESCDSALIPAAIDAWVGLRPEERWWLYAMAVHStghaddADKGWRKAIRIGLTEG
RZB36986.1_Desulfobacteraceae_bacterium_Eth-SRB2  MNrekkkqnvlgfNPDLTDHYFLVTIPRAKSAEVMISEHFEWQKPEkgkslHIKVILKRFMWDDIAEetkaEFNRRLRTYGIGPGKWKKGQIHLERTLGKELVLLAWAIEECDPVLISTAVRNWLGLAPEERWWLYTMTNAStghainRNKGWRKAIRFALTEN
HJI24375.1_Akkermansia_muciniphila                MEwidfga[illegible]
KJE19504.1_Frankia_torreyi                        MT[illegible]
MBA2278683.1_Chloroflexia_bacterium               MSggrnap[illegible]
MBF6555078.1_Acidimicrobiales_bacterium           MS[illegible]
MBL9158411.1_Verrucomicrobiales_bacterium         MP[illegible]
MBM63539.1_Acidobacteriota_bacterium              MAgkpith[illegible]
MBO6043630.1_Acetobacter_sp.                      MSnvspna[illegible]
MBP6739049.1_Leptospiraceae_bacterium             MR[illegible]
MBR2123972.1_Acetobacter_sp.                      MRnplist[illegible]
MBS0652530.1_Verrucomicrobiota_bacterium          MKnreafe[illegible]
MCA3288088.1_Roseomonas_sp.                       MNelslkp[illegible]
MCB0857326.1_Solirubrobacterales_bacterium        MSla[illegible]
MCC5947762.1_Nitriliruptoraceae_bacterium         MPas[illegible]
MCE2689651.1_Rubrivivax_sp.                       MTvatalds[illegible]
MCI6483251.1_Selenomonadaceae_bacterium           MKrkktig[illegible]
MCK6511428.1_Myxococcota_bacterium                MLkkhprk[illegible]
MCM8748013.1_Thermomicrobiaceae_bacterium_CFH_74404 MT[illegible]
MCY3677711.1_Gemmatimonadetes_bacterium           MPst[illegible]
MCZ2109948.1_Dehalococcoidia_bacterium            MAavaf[illegible]
MCZ8140258.1_Acetobacteraceae_bacterium           MAsl[illegible]
MDA7889827.1_Akkermansiaceae_bacterium            MSdsdrln[illegible]
MDA8285698.1_Actinomycetota_bacterium             MTll[illegible]
MDD2765363.1_Opitutaceae_bacterium                MAterrgv[illegible]
MDD3461081.1_Mesotoga_sp.                         MI[illegible]
MDE0094451.1_Gammaproteobacteria_bacterium        MNfsi[illegible]
MDE0603885.1_bacterium                            MT[illegible]
MXZ29853.1_Acidimicrobiia_bacterium               MA[illegible]
NBC12559.1_Gammaproteobacteria_bacterium          [illegible]
NBT25740.1_Actinomycetia_bacterium                MT[illegible]
NJN57313.1_Leptolyngbyaceae_cyanobacterium_SL_5_9 MKlkkpve[illegible]
NLJ36705.1_candidate_division_WS1_bacterium       MK[illegible]
NLT83946.1_Coprothermobacter_proteolyticus        MSpakitt[illegible]
OQB28031.1_Chloroflexi_bacterium_ADurb.Bin180     MV[illegible]
PKO21513.1_Chloroflexi_bacterium_HGW-Chloroflexi-1 MKtlspvs[illegible]
RLC95458.1_Chloroflexota_bacterium                MTva[illegible]
RWG75420.1_Mesorhizobium_sp.                      MSqreslr[illegible]
TVQ96847.1_Desulfovibrionales_bacterium           MArtakas[illegible]
WP_013403050.1_Caldicellulosiruptor_hydrothermalis MSkskkda[illegible]
WP_017869319.1_Deinococcus_wulumuqiensis          MTkqstta[illegible]
WP_044150173.1_Epulopiscium_sp._'N.t._morphotype_B' MGfgf[illegible]
WP_079233153.1_Halolamina_sp._CBA1230             MSkglaeg[illegible]
WP_090140690.1_Limnohabitans_sp._DM1              MNtptapt[illegible]
WP_141612029.1_Litorilinea_aerophila              MSgstpwt[illegible]
WP_161844018.1_Pseudoflavonifractor_sp._524-17    MG[illegible]
WP_169718982.1_Haloplanus_natans                  MTshpgpe[illegible]
WP_171833828.1_Lichenicola_cladoniae              MSgsgagf[illegible]
WP_217438619.1_Fertoeibacter_niger                MTwrwimaa[illegible]
WP_228579681.1_Acidiferrobacter_thiooxydans       MTvrfaqv[illegible]
WP_252129088.1_Thermomicrobium_sp._CFH_73360      MT[illegible]
WP_262057849.1_Streptomyces_sp._STR69             MT[illegible]
consensus/90%                                     M[illegible]
consensus/85%                                     M[illegible]
consensus/80%                                     M[illegible]
consensus/75%                                     Ms[illegible]
consensus/70%                                     Ms[illegible]
```

## ; Type-2 Brex-Related System; DUF3780 + RAMA

```
AFT74738.1_Alteromonas_macleodii_str              MPKTTPKS[illegible]
ARA42376.1_Klebsiella_pneumoniae                  MAKAQSNI[illegible]
EFA6413128.1_Escherichia_coli                     MAKAPSKT[illegible]
EUM08186.1_Enterobacter_sp._BIDMC_29              MAKAQSNK[illegible]
HAT2492741.1_Aeromonas_hydrophila                 MAKTPISR[illegible]
HBC5231501.1_Klebsiella_oxytoca                   MAKAQSNK[illegible]
HCT3226644.1_Enterobacter_hormaechei              MAKALPKA[illegible]
MBY6226421.1_Ferrimonas_balearica                 MAKAT--T[illegible]
QYK01737.1_Shewanella_sp._FJAT-53749              MAKVKTST[illegible]
WP_017062797.1_Vibrio_crassostreae                MSKVT--A[illegible]
WP_036795922.1_Photobacterium_kishitanii          MSKVT--T[illegible]
WP_038193802.1_Xenorhabdus_bovienii               MAKAQSNI[illegible]
WP_126492064.1_Shewanella_baltica                 MVKASSNT[illegible]
WP_227830709.1_unclassified_Clostridioides        M-------[illegible]
WP_256684214.1_Aeromonas_salmonicida              MAKTPISR[illegible]
WP_285145129.1_unclassified_Lelliottia            MAKAQLNI[illegible]
consensus/100%                                    M[illegible]
consensus/95%                                     M[illegible]
consensus/90%                                     MsKs[illegible]
```

Representative Alignments of Core Protien Components in DUF499 Centered Type-3 Brex-Related System;

1. DUF499 ATPase
2. METHYLASE
3. PglZ-Phosphatase (Standalone-Domain)
4. Inactive STAND-NTPase
5. HELICASE

## ; DUF499 AAA-ATPase; ORC-CDC6-AAA+ATPase + wHTH + Alpha+Beta-Domain + wHTH + RRM/Ferredoxin-Fold

| Sequence | Alignment |
|---|---|
| OrigSeq | [illegible] |
| CAD6492704.1_Candidatus_Argoarchaeum_ethanivorans | [illegible] |
| HCF99178.1_Chloroflexi_bacterium | [illegible] |
| HIE29204.1_Candidatus_Poribacteria_bacterium | [illegible] |
| HPN33675.1_bacterium | [illegible] |
| HUT59382.1_Phycisphaerae_bacterium | [illegible] |
| HVA45320.1_Pirellulales_bacterium | [illegible] |
| HXK27027.1_Candidatus_Binatia_bacterium | [illegible] |
| HZM03188.1_Candidatus_Saccharimonadales_bacterium | [illegible] |
| KOX91592.1_Haloarcula_rubripromontorii | [illegible] |
| KXA95606.1_candidate_division_MSBL1_archaeon | [illegible] |
| MBA3936148.1_Planctomycetota_bacterium | [illegible] |
| MBC7429623.1_Bacteriovorax_sp. | [illegible] |
| MBK6914962.1_Ignavibacteriales_bacterium | [illegible] |
| MBM4461756.1_Chloroflexi_bacterium | [illegible] |
| MBM4466103.1_Chloroflexi_bacterium | [illegible] |
| MBN2391962.1_Anaerolineae_bacterium | [illegible] |
| MBQ8111585.1_Kiritimatiellae_bacterium | [illegible] |
| MBU4312937.1_Candidatus_Omnitrophica_bacterium | [illegible] |
| MBX3129295.1_Polyangiaceae_bacterium | [illegible] |
| MBY8216331.1_Vibrio_fluvialis | [illegible] |
| MCE0498498.1_Methylacidiphilales_bacterium | [illegible] |
| MCG2864876.1_Vulcanisaeta_sp. | [illegible] |
| MCK8825903.1_Fuchsiella_alkaliacetigena | [illegible] |
| MCL0058394.1_Dehalococcoidia_bacterium | [illegible] |
| MDD3269499.1_Syntrophomonadaceae_bacterium | [illegible] |
| MDH4162607.1_Nitrospirota_bacterium | [illegible] |
| MDM7274691.1_Thermoprotei_archaeon | [illegible] |
| MDR0374944.1_Treponema_sp. | [illegible] |
| MDR0392109.1_Planctomycetaceae_bacterium | [illegible] |
| MDR2561287.1_Holophagales_bacterium | [illegible] |
| MDU2146556.1_Paeniclostridium_sordellii | [illegible] |
| MEA3344886.1_Chloroflexota_bacterium | [illegible] |
| MEA3401058.1_Armatimonadota_bacterium | [illegible] |
| MEA3428074.1_Thermodesulfobacteriota_bacterium | [illegible] |
| MEB3759607.1_Desulfurococcales_archaeon | [illegible] |
| NLX24424.1_Lentisphaerae_bacterium | [illegible] |
| NMX21982.1_ANME-1_cluster_archaeon_GoMg4 | [illegible] |
| OCQ93485.1_Nostoc_sp._MBR_210 | [illegible] |
| RLF82338.1_Thermococci_archaeon | [illegible] |
| SFC23913.1_Halobiforma_haloterrestris | [illegible] |
| TEU12999.1_Anaerolineales_bacterium | [illegible] |
| WP_011944180.1_Thermotoga_petrophila | [illegible] |
| WP_012608229.1_Desulfurococcus_amylolyticus | [illegible] |
| WP_125670094.1_Candidatus_Methanodesulfokores_washburnensis | [illegible] |
| WP_125672294.1_Candidatus_Methanodesulfokores_washburnensis | [illegible] |
| WP_259101344.1_Candidatus_Fervidibacter_sacchari | [illegible] |
| WP_260594793.1_Salinirubellus_salinus | [illegible] |
| WP_283022678.1_Bradyrhizobium_sp._CB1717 | [illegible] |
| WP_286167649.1_Ochrobactrum_sp._3-3 | [illegible] |
| consensus/100% | [illegible] |
| consensus/95% | [illegible] |
| consensus/90% | [illegible] |
| consensus/85% | [illegible] |
| consensus/80% | [illegible] |
| consensus/75% | [illegible] |
| consensus/70% | [illegible] |

## ; Type-3 Brex-Related System; METHYLASE; NTD(DUF1156) + N6_METHYLASE(TRD-Insert) + HTH + wHTH + HTH

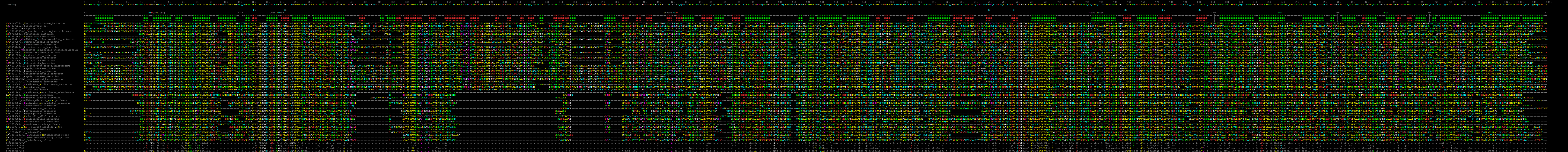

## ; Type-3 Brex-Related System; METHYLASE; NTD(DUF1156) + N6_METHYLASE(TRD-Insert) + HTH + wHTH + HTH

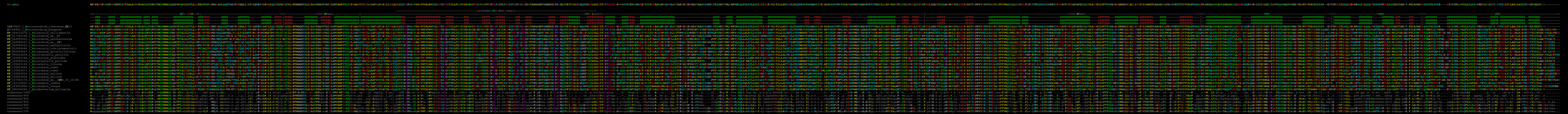

```
## ; Type-3 Brex-Related System; PglZ-Phosphatase; (Standalone-Domain)

         1---------11--------21--------31--------41--------51--------61--------71--------81--------91--------101-------111-------121-------131-------141-------151-------161-------171-------181-------191-------201-------211-------221-------231-------241-------251-------261-------271-------281-------291-------301-------311
OrigSeq  MYSPSDL-ELIFEGFDSVFGILRR-IWDPEN-KSISKYYRNGEIDTNRLEETLLELYPALYDELLERCMAENVAEITRKKKRYCLVIMDSLSLREAMLLENDLKDK-YSV-NLSYSFSSLPSETESFKQKVFGRT-------PDFKYLHDLD---GISVLPESDTLTIWTQAPDDKLLHHTRSGHSEPWSMDDVYADTQQLVHEILRTCR-HDDVIITSDHGYVDLTAGCTFQISKEWKGVLGNQF-KNRWAKENNWELEQLYEKGFIRYAGEYYVVAGRYSWTSRGSVNARKHGGLSIMECMTPVLNVKVN

                                                                                         S1                   S2                                S3                   S4                                          S5                  ----------------------Insert-Region----------------                S6
         -----HHHHHH---HHHHHHHHHHHHHHHH---------HHHHHHHHHHHHHHHHHHH-HHHHHHHHHHH---HHHHHHH-----EEEEEEE---HHHHHHHHHHHHHHH-----EEEEEE------HHHHHHHH----HHHH------EEEE---------------EEEEEE--HHHHHHHH---HHHHHHHHHHHHHH-HHHHHHHHHHHHH---EEEEE-------HHH--------HHHHHHHHHHH---EEE---------EEEE-------EEEEE--------------------HHHH-EEEEEE---
         -----HHHHHH---HHHHHHHHHHHHHHHH---------HHHHHHHHHHHHHHHHHHH-HHHHHHHHHHH---HHHHHHH-----EEEEEEE---HHHHHHHHHHHHHHH-----EEEEEE------HHHHHHHH----HHHH------EEEE---------------EEEEEE--HHHHHHHH---HHHHHHHHHHHHHH-HHHHHHHHHHHHH---EEEEE-------HHH--------HHHHHHHHHHH---EEE---------EEEE-------EEEEE--------------------HHHH-EEEEEE---
         -----HHHHHH---HHHHHHHHHHHHHHHH---------HHHHHHHHHHHHHHHHHHH-HHHHHHHHHHH---HHHHHHH-----EEEEEEE---HHHHHHHHHHHHHHH-----EEEEEE------HHHHHHHH----HHHH------EEEE---------------EEEEEE--HHHHHHHH---HHHHHHHHHHHHHH-HHHHHHHHHHHHH---EEEEE-------HHH--------HHHHHHHHHHH---EEE---------EEEE-------EEEEE--------------------HHHH-EEEEEE---

HCF99179.1_Chloroflexota_bacterium  MTAVDWASLVLTGGTAERWQTLFDGVADLAMPG-------EFEQALDSGDRLIADAAFDLWTEFPSAAPSSVAELQSWGGSGRAVLVLDALSVREMHIILRVAAERGVTVTSSRVCGSAVPTDTQAFASALGLSGRAQLKASGRNFVFASSKPWTGFDHVPPAPNVFLWDPWPDKLLHDSANRSSGPKHFAATVQQRGDGFWKLVERLRQGRRVAITADHGYAVSHRFVDLPP--GLGEKMRLQFGASRSGAVPSALLH-PLYM----SVNGRAAVVGPWKWKSPGGYPHLNGGLTLGEVCVPVITFEAI
CAD6492703.1_Candidatus_Argoarchaeum_ethanivorans  MYSPSDL-ELIFEGFDSVFGILRR-IWDPEN-KSISKYYRNGEIDTNRLEETLLELYPALYDELLERCMAENVAEITRKKKRYCLVIMDSLSLREAMLLENDLKDK-YSV-NLSYSFSSLPSETESFKQKVFGRT-------PDFKYLHDLD---GISVLPESDTLTIWTQAPDDKLLHHTRSGHSEPWSMDDVYADTQQLVHEILRTCR-HDDVIITSDHGYVDLTAGCTFQISKEWKGVLGNQF-KNRWAKENNWELEQLYEKGFIRYAGEYYVVAGRYSWTSRGSVNARKHGGLSIMECMTPVLNVKVN
HDN59917.1_Candidatus_Marinimicrobia_bacterium  MVKINNINPLIRGDVDGIFISLNE-IWNYAW-SEDKKYYPLGEKLTSKFEEQVSKLYPEIYDSIMQNAERSNTISEIMSKDRFCLIIMDGMSLREVVPLLKDLKKYG-DV-RYRFSYSAIPSETEFFTKRYFDALRPNEK--YYYVHLQREDAIEDIP--LGKNKLLIWSLYPDDMFRYFRSGFE-TQDLQEVFIKTKNILFKLLEHLSSYNEIFITSDHGYFVD----TF-----SWKGLNDFPSGERY----------ESLKKYCKQFNDYWVLIGRYNTIKRGKYVHVRHGGLSFLETIVPLIEIKRN
HDQ70819.1_Chloroflexota_bacterium  M-DLSLIKQLIHIDLQTLYEVLNGEIWTPERHGDLHGYLREGEHAASRLEQAVYAIYRDVYALLEADDVRRDRVDVFRQREAYTLLLIDGLSLRELPLIQEVLNDRGLDC-EVDFALAPLPTETADFARRHYNASRNDHRYPFAFRHVANEDWRLDFS--PSERQRFIWYAFPDDYFGLKE-----TEYGRHVVQPIEAILAAVLGDPDLVRPLAITGDHSYLWQGNQCAWPVADRERAVLAEHFKLGRCTRTATEALA-RTGKAW--VAGSLGAARGRFAWGGRGPTSLFKHGGVSLMECLVPWVRI---
HEU5078765.1_Opitutaceae_bacterium  MLPHVTTQRLLTEDASAAWATIRSELQSQFFQGNVVEELIRREKAIEPLELLLGGAAWTLWEEFATAVPRGSARLLEWGHGGKAVLILDGLSAREMPVLMAEAAKRGFFVNLPELCGSELPAETNAYARALGFSHRGALDNNGGAHKVTG--AYTQLPSLSHESRLFVWHEWPDVRVHDLAGHGKGIRELLPEVVQRSDDFWTLVAKLATGREVLITSDHGYADAGSFRDAET--DEKNYLRDNFGANRYRQSELTT---PLALTLDCPSGRWSLALGRTKWAVAGGNKTLSHGGLTLLETFVPFLTLSKI
HIE29205.1_Candidatus_Poribacteria_bacterium  M-SVELLHRLIETEVHLIWEILTQTIWTYEQEHGADGYFAVCEQKTSEIERNINAVYFEIYERIFEQSLKEQKLKQLLNQDTYCLIIFDGLSLREAPVIANTLAEAGYRV-NQSYALAQIPSETEIFTQYVFGFDKSNLD--FQYYFIPDDSNT-EMS--RNTDKQVFWVRFPDVLLHGKNKGKTQILSYQEILQRTLTILKSLLK----AKEIVITSDHGYINLAPGCFWDLPGSEQKLIGKKFGWGRSTSTGLSELESLPNRV--KVIPGYASILGRYAWPTKGQASALNHGGLSFMEVIVPYLKARKT
HLE25735.1_Thermodesulfobacteriota_bacterium  MLILNNFASLIRPGIEWLEKWLLGEVWTTVRSLSPIEYLNDGESKVNELEEIIAQAAYRLYDEFLEEPRQGRNILGFINDESSALVIFDGLSLREIPVLLNLAQKSNFIVQEVGVSYSALPTETISYIEQRLKLGRREIKEKISAYYYDSQSQRHLLD--PDARKLFLWSAFPDNTYSDSGARF--AQHFEQIHTLLETAWLNTVQQIPRGRRILITSDHGYVYLGAGLTFARSNSELRPLSEYLGGERHRDLTEEGNQPEHPDLAVYPNHKVAVLRGRIQTHPPGATRLYKHGGLSIMEMLVPWIVLEAK
HNQ22500.1_Phycisphaerae_bacterium  MLTPTIFEKLIQPGLPWLADWLLGEVWSQHNQMPPGQYLQSGEQSVNEVEEMLCAAAARVYDEVLLPMPDARHLRQYLSGPPCAAVVFDGVSLREIPLLKRLAEQSGLRILEEGWSVAATPSETVDFIAARLGVGCAALRADITCHYLGQVNERCSLD--AAARAVLVWSSFPDYRYTERDAKF--PELFENLHNMMMTAWQNSVQAVVQGRRILVTSDHGYVYFGSGCSFGWDNATVAPLTGYFGGDRFARLAEKPNPPDHRGVHVLADRGVAMIRGRVQTHPRGARKLYKHGGLSIMEMLVPWLVLEAA
HZX48547.1_Nitrospirota_bacterium  MLTHELLDNLIRPGLPWIKKWLIEKVWTLPLSMSHIEYLKAGEDKVNRFEELASFTADRIYRELLSPPDPDKRLLNVL--EDTAVVVFDGLSLREIPIILKLSERSGFNVKEVDCSIAAIPSETMDFVEREFQCGRSELKGRITAIYTNNITQGINAA--DGNSPLLVWSAFPDNTYTDSGSKF--ENHFENIHIQFETSWMHTVQQIKGRKTIIITSDHGYIFFGTGMDRTSSDREIRELNRYFGNNRNISFLDSPHPPNSDDIIIDESKGIAMIKGRIKTRSTGATKLYKHGGLSLMEMLTPWVVLEIG
KXA95579.1_candidate_division_MSBL1_archaeon  MIENKIVQELMESDFPILFEYLLEEIWTPK--PGIKEYYAQ-EQEMNKFERFLSNVYGEIYSELLPRYCMDKDKN---LDMEHSIILLDSLSLREAILLKKDLQEEGFDV-NLSFSYSSLPSDTKFYKEKISYEERK-----NKTKKIQDPD---SIS--LEGDEDIIWSDFPDAWLENISAGRTKLNKIEDMFNNCKNLLFNILDQMENTPAVTITSDHGYVRSEAAYSFKTNESDQKELRQVMGGSRYKPIENANGEECVKAGYLLNFNGYYLAKERHTWTIGGKYEIFQHGGVSLLECLIPRLEVEG-
MBA3936147.1_Planctomycetota_bacterium  MPSTAWRERIFGAGPSAIWSAIFGDV-----ACAKLGDGQRLETHCLDGDQLLADAGWDLWRSYADDADRTSDRLIAWPGPPPAVLVIDALSMRELPLLLQAGARRGVVPHTVDAAGAEVPTDTNAFAKALGLSQRAAIKNNPAAFRLQA--AFTRIPAIQHDPKIFVWHDLIDAHLSDIRS----QAQLDDLLRPDGDGFWRFVDHLRHGRRLVITGDHGYATKHHARPIDG--NLGAQVRDIFGGQRLESAAKALPNGPWVV---MTTGSHHVIVGNRHWRVPGSPKEAYHGGLSLLETIVPWIAFPAK
MBC7332925.1_Actinomycetia_bacterium  MLDTDFIVSLIKSEIYSVWESLLN-IWLNNT-SDLKEYYYHGEEDAFLFEDFLLKTYDEFFTSHLPKACLNEPSLFQKETHNVCFIVMDGMSIREGVLIFNALQKQGIAP-KMSYFLSGIPSDTQSFREKIKVDL------SDKFVEINNPK---KIR--ISEGECYIWSYFPDVLLDKIQVGHTVISDLENMYKTSEKIILEVLERIN-IKKIIIILSDHGYIRSEPGFSFSVSDPAKKKLKEAFGSSRYIPMDKADLSDLVQAGLVVDFAGYWLVKSRYIWPLPGRYNIYLHGGISLMKSIRPDAEIRKL
MBC7429624.1_Bacteriovorax_sp.  MLSTEWLNKVLSKSPDEAWVDIFAHLKGLVPAQEAKTSYRKLEDDVAYIDHVLSASAWDLWRDFSS-APRASTYVQNFTVGPKAVLILDSLSIREINPICTEISKLGLKIKNIELAGSEIPSETDIYASAIGLSGRASLAKGPKGFLISTENTFVNTPKITSEKNIFIWHGWPDDSLHVYGKHDDAFSEFIEHVTDTSEGFGSCIQKLAQGRELLITSDHGYCNTSSFTSAVG--EHHQELKLL-GHTRAKKSADLVKGAPATMEMTSTNELYRIAVGRRR-PSDKGFPALTHGGLSLMECAVPLIHIASN
MBE7508222.1_Planctomycetia_bacterium  M--RPWEDIILGNLARDAWTELFASLCEIARPL-------ELHNKTIPPDRLLAEAGWDLWQAYPGAAPRSSEALREWIASGRAVLILDAFSLREMFVLLAAAEQRQIVPTQIRVTGSEVPSDTDNFAAMGAQSRARLATGPAGFALASDDLAVNHPAIRPRRDLFVWHTWLDDILHVHERS---PDQVNSVARQQGPGFWRFIDALRQGRRLIITSDHGYALARSFATNETDEEVVTALRDTFSASRCKPASAAWN--PVVF----THGGHHVVMGQRKWKVQGGYPYLCHGGMSLLEVAVPFVELPAL
MBI4523255.1_Deltaproteobacteria_bacterium  MLDRALLDELTQPGLPRLINWLMTEVWTMDRGQKPAEYLLQGETLVHHRETLLTLAGESLYDELAAAPPPNRSIRAFLQYPGASVVILDGCSIRELPRLAELAAASGRPILEQSYGFAAVPSETEIFIGQRMGLGRRELREQIQAHWIRQPTDQIRIP--EGTETVLLWMRFPDMRFMDTLAAT--AALFDSIWDMLQTVWIRTVQKLPQARPVLVTSDHGYIFLGPGLSEPRLNGVDRPLE----GRRYREFSSAEELPKRPGLWIDPARRLAVIAGRVHNRPQASNSIYRHGGLSLMEMLTPWLVLETQ
MBK6914963.1_Ignavibacteriales_bacterium  MDFNTVFDLLTTPGIPLIHRWMKENVWSESASMDIKTYFESGEKQIQKLEELILTSSNGIYDELSALAEKNSSLLEEL-QSKTALIIFDGTSIRELPLLESLAVNTGFQIIESNYQASALPSDTVSFVEQRLVGKRKELSSRIKSYYYDTPIRHFEIK--SSELNLYLLWSSFPDGTYTNFEARN--SLHFETFIKQFDLVWKNIVMAIPRGYRIIITSDHGYVYLNNGFESSKGDMALRFLN----QERFRYYSEDEEIPNNSELKIISNRRLGILKGRIKNRPKGANKVFRHGGLSMLEVLTPWLIIQRS
MBK8636967.1_Chromatiaceae_bacterium  M-DKALHADVFTLPADQAWPRIFDWAWSVCAAASVPSILGERERGLAPLDNLLAGSAWDLWDGFEAHAPRTSAALATFTPGGKAILILDALSLREVPWLLEQAQARGFTIHAARATASELPAETTPFARAIGFAQRSALAHNGKSPIFPG--ALTDLPLVPPHPGIVFWHTWPDERMHKLAEDSGGSRTLAKEAAQAGDDFWALIATLATGRRLVVTSDHGYANVGLFPDVEGADTQGQALKGVFKSGRSATGDDATAAGPLTRRLSTAHGDWLLVLGRKKWKSQGGYPTLAHGGLTLLEVAVPYIELSA-
MBM4466104.1_Chloroflexota_bacterium  MDESTLARDLITADLAVLFAALNQ-VWQADD-DDVNAFFTKRERETNQLERFVLETFFDVYDLLLAQVRQGRGRSRF-EGTTGPIIVLDGCSIREANLLMGQLQEAGYQVADYGYALSEVPSGTVDFNRNVFGTARQWHQ--YQVVSIESG----QLPLFPAETEVLVWISYPDELLHKVRGE---AVTPQEAFDKTAKVLLGTLDGLE-ADEFLVTSDHGYLYVENATLFKASGGDEKVLRQLFGGRRAVPAGDVGPQ-PKSRTYALFDDEGCYVRGRYHWSVQGPAPDIAHGGISLMECLVPVMRIRRG
MBN2145087.1_Candidatus_Auribacterota_bacterium  MVPEELIKKLLQPGLGWLKEWLSREVWSSENELSPLDYLNRGEKIICDFEELLVSAAEQVYDELISSAEKQFSIPDAL-DKDTAVVIFDGLSVREMPMLKVLAEKSGMKIKTADYSLSSVPSETLDFIEQKIQVGRKEFKEKISAFYVSGHNETVTL---SGNNSMLLWSSFPDNTYKDSGARF--DSHFSNIRDVFETAWINCVQAIRNKKRIIITSDHGYIFLGSGLDFPRNTKDLRVLNDIFGNERCVSLDKLKGVETLEDVYLDKVRNLALLKGRVKTVGTGASRLYKHGGLSLMEMFTPWLELEIK
MBP6702581.1_Vicinamibacteria_bacterium  MVTAESLGRLLGGEPKEAWAEVFSLLWKGLSAPDVAAEMIRRDKSFESLDILLSGAAWDLWLSFEESVESTADALAAWASGGRAVLILDALSLRETPWILGEAAKRGYTVKSARPTASALPSDTTSFARALGLPQRSSLSAGVPSLRLAG--AATEQPTLPNAPDVLAWHDWPDSLMHDLSGAGPGLRQLAEDARKVGDDFWHFVDRMATGRRLVITSDHGYAASGLFADVPD--DQKTYFQQTFGAERHSATPGAAP--PVDLHLQTARGPHRFALGRRGWRVQSGRKNLSHGGLSVLETAVPFIELTKT
MBQ8111586.1_Kiritimatiellia_bacterium  MINAEWTDLLLAAHPAVTWHGVAQALAQLALPE-------ELHRMTVLPDRILAESAFDLWKAFPTAAPRTSARLAQWCTGSKAVLIMDALSLREMKVIVEEAKRRELAV-SAEVTFSEAPSDTTAFAKALGTPTRASLANNSGSFKLFGGRCSTSVPPVPPVPSVVIWHSFVDDLIHAGNKT---AAEVFKLTRQQDDGFWKLVDALRTGRQLVITSDHGYADAAKFSTEIKEPSAVDFLKANFHGKRFAENVSPQ---PLFL----VSNGFCMVTGQRKWPVQGGFPKICHGGLSLLEVASPWIELPAK
MCD6507459.1_Candidatus_Poribacteria_bacterium  MAIDDFLKRLIEEDLIVIWQQMLE-LYQVDDPSDLIRFYNERETRLSQLEEALYHGYWEIYDLCFRRSQAQSGDYVFELREGYVVIIADSLSLREVGIMGKLLNDLGWYIEEKTFAVASFPTLTESLSRILLGTTKDVPA--FRYRYVAGPG---EVPNLPVGEPTLIWLRLPDKKLEQVTVAQ--VTTIADAFNATMETLIQLLEQIT-TRPVFITSDHGYLYATDISHYKMPGSIEKEVRKLFRESRAQPSDKEGMERQGNQRYFVRSDTHVGILGRYWWASAKGDRCTAHGGLSLIETLVPAIKVRKN
MCK8825904.1_Fuchsiella_alkaliacetigena  M-RLKLVRELLASEVEVVWKQLQE-IYITEKTDNILEYYSRTEKELLELEKFLFKSYFEFYQEDLKELANREDLTLVRDNPNLTYLLMDALSLRELGLFYDSLEQKGYQL-KIDYTFSALPSQTRFYKEKMRFDIRN-----IKFREIRDYQ---DIK--VRGNEQLIWSRYPDTLIEDIRSGQSLKSDLEETYKKTKQVLFTVLDNLD-KEKIIICSDHGYTRTEAGYNIKINNTVKKWLKELFGGSRFAAKEEANKELLLDRNLIVEVEDHYLPISHYSWPVSGKYSTFSHGGLSLLEVITPRIILTK-
MCK9588325.1_Terrimicrobiaceae_bacterium  MDIHFHHQGLTGEGLQAITGWLTETVWSRERGLSSEDYLRKGELAVCDLEALIAAGAPHVWDELESASAGATTLADWLLPSPHAAVVFDGLSLREMPMLLERAAASGFRVKSARAVATCLPTETRSFVEQRLLGKRREFAEKAQAFYFGQPNSRDNFP---VGRSLLVWSHYPDGLFFDDNARS--EQLFSTFASYIETIWKCTVQAIPAGVPIVVTSDHGYVFFGAGGETGALQADAGALL---EQSRWREFPPEDRFPWHPDLQLVPSRRLALLRGRIRTRKPGSNRLYQHGGFSLMETLVPWIELERK
MCL0058393.1_Dehalococcoidia_bacterium  MSTEVALRRLIGRELSVVWDTLVE-IWRVDSPKDLDGFYRDKEIESCRLEETLEQTYFEAYQELLNGTAVHKTSPWA-GEEERLAIIVDAMSVREACLLSPILEGQGYRVESLSFSLSALPSHTEAFARKHFNMGRQGI-------FVSPPGH--PVPQFPPYDLCFVWLRYPDRGLHAT------ILSPAEVFGKTSQYILEILRNSG-RNTFLLTSDHGYIYADTADHAPIAQSVQKEMRKVFGGSRYVQGPKEALATVYEKTL--SVGDTLLVRGRYLWRQ--RDKRYFHGGLSFVECLIPVIKAKTK
MCL0097130.1_Dehalococcoidia_bacterium  MNLSSLAAQLLTQAIGLLFQAINE-VWATSNANDIDAFYARQERETNHLEDFIKDTYFEIYELLVEWIKSHDRAAEF-ASYDSSIVILDGLSLREANWLMPRLRENGFEIRQYCYAFSRMPSTTQSFFNDVFGVARKWQG--FNFRSIKSG----DVPMFLDGPKSLIWLSFPDELMHPMRGK---GVTPSFALQRTEEFLLKILNLLE-TEDVVLTSDHGYMYAPSASSFHPAVSSRKVLQKLFGAKRGVLFDGKKVEEPKDQTYVMFDDKGCYVRGRYYWSAPGKQSDIAHGGVSLMECLVPMHLRRK
MDB6140354.1_Verrucomicrobiaceae_bacterium  MLSSHTLQSLLTASEAEAWSSIRDLMAETWLAGAELSEVRRREAGLASLDLLLSHSIWPLWQNFPAGVPRAATQLVKFQLGGRAVLLLDSLSLRELPLLIMAAQSRGFSVHDSAVCASELPPETTTFAKALGFSSRGALDNNAASIAFPG--AFTNLPAVPPAPNIFCWHHWPDERLHQLDRADGGLEKLFPEMLRTGDDFWQFVQRLATGRRLVITSDHGYAATGAFPNVPA--EQKDYLRETFAAQRFRSGAIDA---PLTTTVTTASGLVTFVLGRQKWQVPGGFPALSHGGLTLMETFVPWIELSLS
MDD3269498.1_Syntrophomonadaceae_bacterium  MKQQALIIQLLDQRSKACWQSIIEYLGSMVYPA-------SLHRATVNLDRFLAESAWNLWQDYPASVTTTARTLQAWAPGGCAVLVLDALSLRELPLILQYGKEQGIEPLQAVVTGSEIPTETDAFAHALGASSRASLKNNGSSFILGGAAAYSSAPEIPPLKNLVVWHTWLDRQIHEHRRE---PDELIRLVHKSSPGFWQLLQRLRLRQGRKLIVTSDHGYANCRLFASSETNGPAKEIMARIFGQTRSCPAQLDFP--PLAL----TMNNHHVVLGQRRWTQVAGGYPHMTHGGLSLLEALVPFIEYAEV
MDD4099138.1_Lentisphaeria_bacterium  M-SQDWNSLILDSRPAEAWAGIFSALLSVARPV-------ALHEATVEPDRLLAEAAWELWHAFPGSAAHTAAELIRWAHHAQAILIIDALSLRELPLLLSAASNRNILPLRVAVTGAECPSTTDHFAKAIGLPSRSAMANDPASFALFSEACYTSMPAVPSVPNVVLWHSWLDDLIHQQKL----PDQIASITATKGDGFWSFIDRLRKGRKLVITSDHGYAVSKLFSSEVADPDAIDILRSTFGASRNTAAATPWQ--PIVM----THNKQHVVMGQRKWKVQGGFPHVCHGGMSLLETVVPWIELPHR
MDT3473331.1_Stenotrophomonas_maltophilia  MLDAQILQRLTQDSPKEAWRIITDRVLEIASESNAPSEVVKRDREIAELDLFLASSGWDLWRVFGQGVERTSDRLARWHSVARAVLVILDALSLRELPWLLQGAKERGFTLHTVGATASELPSETNPFARALGLTSRSQLQANGLKPLQ--AQTDLPLVSGAKNWVFWHHWPDAKLHDYGA-GQGLELLTRDAAQSSDDFWSFIERLATGRRLVITSDHGYAATGYFPDADG--EVASHLKQTFSSGRSKAGNGDA---PVALQIDSPHGAHLLALGRKKWRSQGGYPTLTHGGLSLLETVLSPGFRRAAI
MDU2146555.1_Paeniclostridium_sordellii  MISLASLDNILSKRPEESWKYISDCVWNIFSETCIENEASLRDSLTNDIESLLATSCSDLWDEFDRCISKTSDEVKSFESSDGKAVLILDGLSMRELPFLLQGLESRNYTIDKACITGAELPSHTNSFAKAIGISSRSSLTNNSKKSIFNN--AYTGLSIISSQPNLFIWHEYPDKKVHELDSYGCTIQNLSKNMYSSDEKFWEFIKKISTGRDVIITSDHGYAHTGMFMDVTDN-DVKDYLKKQYKSARYRKTQNNE---AIEKSINSNHGDYTFVLNKIKWKVPGGYPTLSHGGLSLLEVMVPFIQFRQE
MEA1926980.1_Candidatus_Auribacterota_bacterium  MLTTDILTKLVDPSLSWITDWLLDDVWSTERSTTPEKYLTAGENQVHALEELLAASADRFYNELAGAASQTKKLTERV--PDTAIVVFDGASIREIPLFLEKAHQSGYRVIERSYSFAALPSETTFDVEQRLLGKRKELKERIKAFYFHDAISTNHIT--AGTEQLLLWSSFPDVTYQDSESRF--ARHFADMQQLYDTVWKNTVMQIPHDRRILITSDHGYIFFGTGVDSIRPNIACELLN----QNRFRIFDQDENLPEIKDLQIFPERRLAMLRGRLRNRPRGTNRVYRHGGLSLMEMLVPWIILEKE
MEA3344887.1_Chloroflexota_bacterium  MQLAELLSQLIQADMPVIWTYLQS-LWRPEE-ESGETYFKQGEKRTMYLEKVLGAAYPELYREILPERCARCNLDV--TRVEVPIIWMDALSLREALLLCSDLDADC----ELSFAYSRLPSETTFYKETIFYPW-------AKTVEIKDVN---WLS--LDGDERGVWCPIPDKELEDIKGRVK-VRTQTDIYEKSRQVLLNICEMLY-GNEFLVTSDHGYISTETHF-LTLPKRLKGAMQNLFDASRYVEVETKADA-LIEDGYIVEHDGYYLVSNRYAWPTRGKYKVMLHGGISLLECLVPVLRI---
MEA3401059.1_Armatimonadota_bacterium  M-KLDLLDRLLTEELVPPFEELRG-LWSVESAGDLQAYLSDNEHRASAFEREIWDVYADVYQVLEGEHPRKQFVSEVLQQDEFTLVVFDGLSLREVPCLLQVMEERDIEA-EVDFALAPVPTETSVFAQEHFGASQSGAE--LAFEHVKQTTWEPSFG--REQRRRLIWAPHPDNIFTLNSDT---VEYGGHVVEPVQAILAAILDSDP-VLPLYITSDHGYLWQGGACAWALDDDEEALLAREFKAGRSADDPTAELT-SGEKVWSDAERQVAAARGRFAWGGTGPPRNFKHGGVSLVECLVPALRLSGE
NLX99245.1_Rhodopirellula_sp.  MPAEEWINLILNPAPTDTWTAIFARLRDFALSPIGKGWAESLHRETIVPDRLLAESAWDLWQDFARCAPTVVDELKKFTVSGTAVLILDGLSLRELPLIVQAGQSRGLPPVRVEVRGSEVPSETDRFAAALGLPGRSKLYNNPGTFAFAGPDVHTDAPSIPTSPRLFIWHKWPDELIHLHDDKKDGPAVVAAQTKAVSDGFWALVDRIRQGRRLVITGDHGYATSSDFSNEEKNEDTIKLLRNHFGASRCVQENPQSPWPPLVV----RIDGWLVVMGQRKWAVQGGFPHLCHRGLSLLEASVPVIEYPAK
NMX21981.1_ANME-1_cluster_archaeon_GoMg4  MLSNEFVEDLICEDLDRIWEYLVEDVWVHD--KSVSAYYNQGEKKTGSFERFFKDAYFEIYDLMAKESLGKRKKLRDIERSEYCLIVSDGLSIREANLLLRKLEENGYNIKKYTYEISNLPSDTIAFSRDFLNISRTSGEWRGKSVYIEDDKT--NYP-VPYSDQVLIYSIFPDADFDTVTAKI--GDSLEEIYLKASNIILKQIENTN-HDQIVITSDHGYCTKHPGSNWSIPVSEKEIFQEVFGSARFKEESKIFGDMLDKTDRLKKVGDNVIVNGRYIWPVPGSKKQIYHGGISFMENFVPLIEVEK-
RLF12261.1_Thermoprotei_archaeon  M-LPSHVKLLVESKLCEVWRILTE-IWRPLD-DLKEHFSKDREKIASRFEYQLNRVFYEIYQHLHTSIHEST------KRFKSPLLIVDGLSVREGGLLARDLELKGYKVLRYWYSFSALPSNTSSFRKVLK----------GRFREVKSG----RVP--YIDFSVPVWISYPDEILHHAGR----IISPYEAYERTRSVILKVLDRIVESCVVTITSDHGYVVRDVQP-WPISKAVRTFLRSIFGGSRFVKVEDIDKNKPEEDSYVFIDDEYCYVKGRYFWPIQGYRSNVDHGGLSLTECIVPLIEVEV-
RLF82337.1_Thermococci_archaeon  MIPEALLEKIITTGTKEIGNFIVKDFSKA--ASPDFQYYSEFEQKVSLFEQWLYENWTEVYEVLLIPENKEEISIITAFSEINANVLIADGFSIRELIFLINEF---GKDRVTYEFSYAYPPTITSKAATIVFGSPKEIIITGWKTRLIETPM---DPPRIGHETGFAFYSYYPDAPLHNAQIHGTTLQNIGEVIKK----LINMVKELSRYTNLVITGDHGYIFLGNNPNL--------YLWKPIKSPRFGENLKKPP--------YLIVENEAVAIGRFHALK--RDSFITHGGISIMESLVPVVIIRRE
TEU13000.1_Anaerolineales_bacterium  MIDEQFITELIKDQLAAVWRQMER-VYTQTSCGDLRGLMDDRESALNDLERGVYRGFFEAYELLLERAGAQDENYVLSFDECPFVIITDSLSVREAVLLEKFWEGEAWSVERKGFAVAPFPPMTESLSNLLLGVPRDRPQ--FTYRYVAGPE---HIPAMPPSGSLLVWMRLPDTALEEVTVAQ--THTVADAFHSTVRTLEAVLETSG-RDAALATSDHGYLYARSPTQYPMPGGVEEIARSAFRASRVRPLTDERARGRVEQRFFAFGEAHAAMRGRHWWGSASNDRCTAHGGLSFVECLVPVLKIWRR
WP_083335243.1_Planococcus_glaciei  MIDKELLNSMLLGTDQRAWEAIEETLFNVFTEQSPVQEILKREGKISGLEALIAGAGWDLWHSFETNVSKTSELLIKFNHDGKAVFIIDGLSLREMPFFLTESQKRGYTIHQSKITASALPSDTTYFAKKLGFSQRSSLENNGLNHFFTG--AKTDMPLIGNDPNLIFWHTAFDDRIHEYKVPGKGLRELSLKSSETSEGFWELIEKASLGRRVIITSDHGYASTGEFTNISG--EQGDYLKETYKSQRYTKNKERRP--PIEIQMENSQGLFSYVLGRRRWKNSGGYPTLAHGGLSLMETMVPFIEITK-
WP_084067307.1_Desulfocicer_vacuolatum  MSSLEFVRKIQNESSKDAWHSILSYAWNFCSRTNPASEIIKRDRAVGNIDHYLATAGWDLWSTYEQSVPQTSNALINWHDTGRGVLILDAFSLREVPWLLQQAKERGFTIHKAGPVCAELPADTTPFAKALGFNQRSSLANNGSAHHLPG--AVTDMELIGSEPDWVFWHHFPDHRLHHHDAAGKGISSLVDEIKFTGDSFWSLIHRLTQGRRVIITSDHGYAASGLFPDANK--QQSDYLKKQFKSGRWHNNEMDTG--PIDLEIESRHGAYGYVNGRRKWKSAGGYPTLTHGGLTVLEIAVPYIEISRS
WP_121919201.1_Haloplanus_aerogenes  MLTSEWPKSLVEADVDVVWDALWD-IWWPPS-GPSDHYDHDREWASVELERTLVDIYWEFYSEVLPWRCVNEASIE--IPDDGAFLVMDAMSVREASLFAAALESDGYDV-KVSYSYATVPSETTPYKKRVGYADRE-----YPSATVRDL----DPS--LSGDERLVWSRYPDALLENIQEGKTELSSVEDAYDDSLRVFRSILDQLD-TDRIIVGADHGYVREESGYSFAISEHEKNRLRDTFGGQRFVGVDQADADDLVEDRLAVEADGYYMPVGRYTWPVRGKYSVYQHGGLSLMECLTPRLEVQR-
WP_125672296.1_Candidatus_Methanodesulfokores  M-TENFVLSLIREKTSVIFSKMIE-LAESKLLSDLDKSYEDIEDELLRLEKWLFDAWVDLYRYHLAREKNKLAISKAFSEKGANVLISDGYSFREHVVVKNSIKAE----VDEDFGLSATPSITSEASKIFFDVPRRFIEGSWEMCIVDKIK---NPPKTGKKKGIAFLTYYPDAPLHEAKKHGIILQSITAVVGD----LVSLIDELSSNVPLVVTGDHGYIFTKGNPGL--------FLWKNWPKNRYGESYGEFQ---------LKIDETTVAIGRKHAPK--TESMITHGGVSLAESLVPITIHKK
WP_149537233.1_Siccirubricoccus_phaeus  MLPLPLLRQLQEADPAAAWAALAQHALDVCDGKASDTAARLRDEALQAVDAWLAGAGWDLWAAFGDAMPRTADRLVDWHGAGAAVLILDGLSLRELPLLTQGALGHGFAVVRAEATAAELPGDTNSFAQALGVPARNTLANNPAGFRLLG--ATTDLPLVKAEPRWLFWHEWPDARFHA----GSSFQALARNVADSDAAFWGFLRRLATGRRLLVTSDHGYAHIGGFHDTYE--DQKAWLRGVFAGGRTAADGET----PLALSLQGRQERVRLPLGRRKWPVPGGNANLGHGGLTLLEALSPFIELRLQ
WP_218932320.1_Adhaeretor_mobilis  MLSRGLLDTITSGSPQQAWDAIGQSLWETFSKQTATSEIVNRDANINELENVVSGSCWDLWQHFGDCVPRTSQSLIEFVGGGKAVLILDGLSLRESPWLLGALKERGYTIHKAGYRASELPCETTPFARSLGFQQRADLENNSKTHSLTG--AATGLPSVKSQEHFVYWHHWPDSRIHELAKPGEGLRKLAAEAQSSEDAFWEFVERLAHGRRLVITADHGFAACGNFPDLGK--EQSNYMKALFKSGRTAVVPPSTEPTPIDLELETEHGKHRFALGRRKWKVSGKNRLLAHGGLSLLEVFVPFFELST-
WP_257627367.1_Haloplanus_salinarum  MYGSEQLSELVRGELDGAFECLRR-IWSRPD-ALDGQTLTDSEYRTRLLEHHLAAQEPKLYDELNASMGDHPIT----RLDSGCGVIMDALSLREGFQLLSDLDEHGWDVTLEWSGVERLPSETTFICREWFDAHR------DDYRFVGDL----DVPQLPASDPAFVWTRHPDRRLEESFKGNYSSEAMTDIYSDTKGLLEEIVAESV-HDEFLVTSDHGYVNVQDTNPYVLSDSNEDALSSKF-SGRHREVADDYALDLHDAGVIERAGGHYVVKGHYHPTKPGATKHIRHGGLSLPECMTPVLRITT-
WP_259101347.1_Candidatus_Fervidibacter_sacchari  MLNESLVRELVEAELPVIWKAMVR-IWKPNGPETLKEFYDEREEQMFRLERFLSDTVSDLYNFLLQRAINQDGSYVLKLQGNNVVIIADSLSVREAALLTHWFPQINFAK-DQPFAVAPFPTLTESLAQKLLGVIRDTEK--FTYRYIAGPG---ALESFPEDRPLLIWLRLPDAELEQVTEAQ--TTKIADVLRVTVEALGKLFQRLC-GRKVVITSDHGYFYGASSKHF----S-FRGLEQLPRERRARRGEASSLS-PLEQDAFIGHGEWIALKGRYWRSGSGNARHTSHGGLSLTEVLVPILEI---
WP_260594794.1_Salinirubellus_salinus  MYGIDEVRHLVNDDFDALFECLRR-IWDADD-AFEAKQVADREYRTRLLEREISRQYPDLYDDLVDRVGDHPLM----ELTDGCGIIMDGMSLREGFRLAADLEERDWTVEYDWAACECGLPTETKFIAQPWFNANKH-----DHVAYIGEP----EVPPLPGTSPEYVWSRFPDKRLHNSQEGQYTLTELVEVYDEAKELVIDIIEESV-HDRFLVSSDHGYVNFSGNNPYRLAEEDEAILKEKF-SGRYREVEHSGLLRLERDGIIHRAGGHYLIRGHHSWTKRGATSRIDHGGLTLVETMTPLLTVDTT
WP_283022679.1_Bradyrhizobium_sp._CB1717  MLDRDVLATILTQTPSVAWSAIVERVHSICRDKDLIRSVREREREVGELDDLISTSAWDLWDTFQDGVPRTSALLQTFPAHGKAILILDGLSLRELPHLISGAEAHGLKLISETVTASEFPPETDPFAAALGFASRSSLRNAPTSKFFPG--AKTDMDAVMPDPNIILWHQMPDDRIHSLENDGDALGRLATELASQGVDFWGLVKKLTTGRSLIITSDHGYAAGGEFYELPD--GPTGFLRDNFKARRSLAGDGEL---PLVMRFDTPRGPYRLALGRRKWKVSGGFPKLTHGGLTLFEVLSPFVEITQ-
consensus/100%  M.......h..........h..h..............c.....-..h.....ha......................hh.Du.ShRE...h................Ps.T..h...................................hh......D..h.......................h...h.......h.h.uDHua..................h.......R.......................................H.Ghoh.c...P.......
consensus/95%  M.......l..........h..h..............c.....-..l.....ha.....................shll.DuhShRE...h................s..Ps.T..h................h..................ha..h.D..h.......................h...l.........hhlsuDHGYh..............h.......R.......................h..sp...........HGGhoh.E.h.P.h.h...
consensus/90%  M.......l..........h..h..............c.....-..l.....phap.h..................shllhDuhSlRE...h.......s....p..h..u.hPs.T..h.......p.........h..............hhW..h.D..hp.......................h...l.........hhlTuDHGYh..............h.......R....................hh.up............h.HGGlohhE.hsP.h.h...
consensus/85%  M.......lh.....hh..l...lhp..........p.-.....h-..l..s..clap.h.................shllhDuhSlREh.hl...h..psh...p..hs.u.hPspT..a.p......+.........h.s.....hs.......hhW..hPD..hp.........h.........hb..lp.....p.lllTuDHGYh..............h...h...R.....................hh.G+b......u....h.HGGlolhEshsPhl.h...
consensus/80%  M.....hpplhp.....ha..l.p.lhp..........h.p.-p....h-p.l..sh.clap.h.......p..........s.slllhDuhSlREhshl...h..psh...p..hshu.hPopTp.a.p.b....+.........h.s.....phs.......hhW..aPD..hp..........ph.p.h......hbphlp.h...pplllTSDHGYh..............lp..h...R.....................hh.G+b.....G....h.HGGlSlhEshsPhl.l...
consensus/75%  M...phhppllp.....hap.l.p.lhp.....s...bh.p.Ep....h-phl..sh.clap.h...s.ppp.....b...s.slllhDuhSlREhshlhp.hpppGh.l.p..hshu.lPocTp.asp.h..s.R..........l.s.....phs...s..phhlWppaPD.bhcp.........pph.p.h....p.hhphlppl..scplllTSDHGYh......sh.......p.Lpp.a.s.R.....................hhbGRb.h.s.Gs...h.HGGlSLhEshsPhlplp..
consensus/70%  M.s.phlppLlp.....hap.l.p.lap.....s...bh.p.Ep....h-phls.sh.-lap.h...s.ppp.....b...s.slllhDuLSlREhshlhp.hpppGhpl.p.phshu.lPo-TpsFsp.h..s.R..........bl.s.....phs...s..phhlWppaPD.bhcp.p.......pph.p.hpp..p.hhphlppl..scplllTSDHGYh...s..sh.......p.Lpp.F.upR...................s...hhbGRbpW.s.Gs...hpHGGLSLhEshlPhlplp..
```

```
## ; Type-3 Brex-Related System; Inactive-STAND-NTPase Representative_1;

                                                  1---------11--------21--------31--------41--------51--------61--------71--------81--------91--------101-------111-------121-------131-------141-------151-------161-----
OrigSeq                                           MSPINDKSFVRKGMSLIWDPELLAEIIAYHKAVTIRQFCQMKGKWPESLPSKDEHALVVVGLEGCMDVLKPSDAEKWLEQDIKPLILDFQDEYQGQCALIFWIPSGLKKIKHVSSTDNYVWQYSTPHSGKVEIGRILWAGAQEVRPIITGSADVADSNWKGLHHPRIS

                                                               S1               S2                       S3                                      S4      -------Insert-Region---------           S5           Terminal-Insert-Str
                                                  -------HHHH--EEEE--HHHHHHHHHHH-EEE-HHHHHH--------------EEEEE-HHHHHHH--HHHHHHHHHHHHHHHHHHHHHHHHH----EEEEEE--|---EEEE---EEEEE--------EEE-HHH|-------EEE--------HHH-EEEEE----
                                                  -------HHHH--EEEE--HHHHHHHHHHH-EEE-HHHHHH--------------EEEEE-HHHHHHH--HHHHHHHHHHHHHHHHHHHHHHHHH----EEEEEE--|---EEEE---EEEEE--------EEE-HHH|-------EEE--------HHH-EEEEE----
                                                  -------HHHH--EEEE--HHHHHHHHHHH-EEE-HHHHHH--------------EEEEE-HHHHHHH--HHHHHHHHHHHHHHHHHHHHHHHHH----EEEEEE--|---EEEE---EEEEE--------EEE-HHH|-------EEE--------HHH-EEEEE----
MDO8527158.1_Deltaproteobacteria_bacterium        MSPINDKSFVRKGMSLIWDPELLAEIIAYHKAVTIRQFCQMKGKWPESLPSKDEHALVVVGLEGCMDVLKPSDAEKWLEQDIKPLILDFQDEYQGQCALIFWIPSGLKKIKHVSSTDNYVWqystphsgKVEIGRILWAGAQEVRPIITGSADvadsnwKGLHHPRIS
EFN9589256.1_Escherichia_coli                     MSDVTNDKWKRRGVSVLWCGKTLAELNAASQVISLRQFIGYYEAWPDDMPLLNDDGLYVAGLDVAVDALSPEDALEWLESEIYEMIYDFQNH--ADAALIFWMPDQG-RWKEDLTTSTYHWclagkydaMFPLGQCIWNGAQDVRRIESTSGGkt-newLGLYLERIS
MBJ2281507.1_Pseudomonas_sp._MF6767               MRQVLDTVWQRRGTSWLWDEEARNQVCKAEEVWSLRQFLLSAQAWPDDLPSNNSRTLIVAGLEGSLDLLAPDQGEVWLGDTIKRAILSFQDEYAGEAALIFWLPQKHGRLKVQASTDAVSWlceapnrgQLDFGRLLWGEANYPQEILLREGAqt----AGLFHLRIT
WP_283022681.1_Bradyrhizobium_sp._CB1717          MRQIEDEVWRRRGFTVLWSPTALGLLCSTTEIISMRELFRVCTNWPDTLPTSNGNALVVTGLEGCLDRISPPDAEAWLDEVVQPLMLRFQRQYSDDAALIFWMPTGEKRVELVNLDDTFSWefappfkkPFSISRALFGGAHDAGKIVASEQDdrgqawIGLHLSRIA
MDU2146553.1_Paeniclostridium_sordellii           MRGRKSEIWKRSGISVLWELPPVIECIRNSEVITLNQLFRFSVEWPENLPSFSDSAIIVSGLEYILDLLSPSEAKEWLDQRLYPLLLEFQKEYEGYAALIFVIPQGDERIKYITVKDTFYWicncnd--EIPFSSSLWNGAEDAKYIDIDKYIk-----CGLYHPRIS
KYH32453.1_Moorella_mulderi_DSM_14980             MRE-FSSAWLRRGSSVVFDKYVLGHLIESASLVSLRQALAWLKAWPVEPP-AGRNTVLVSGLETLLEVLDPVEAEAFLRKRVKPLIMEFQYRW-DQCGLVFGFAAPERSFEVTVADEEVLFlr---rdgRVHLSFALWDGSTDVTRLVRDEPQtgr---VGYHVARIS
OPX92064.1_Pelotomaculum_sp._PtaB.Bin013          MRE-RGNGWLRSGSSLIWDAQLLSPLLENGVQIPLHKALNWIGGLPSTIPSGDGKTIFVVGLQTVLETLDSEAAFTFLRNRVQRLIVRVQDFY-NNVGIVFGCNCNWRQWRIDS-NEHAYLrl---rggELNVTFALWNGVAEAQIIMVEDKNvhreigGGFYVRRYS
MBE2220371.1_Anaerolineae_bacterium               MRE-SGTRWQRRGSSIVFDPETLSPLLIGQSLISLRAFLGWREDWPDDPP-GDGKTVLVGGLEPCLELYSPEEAESLLRYRIRPVVEEFQTRW-DQRGLVFGFGKPARAFQLDPTSDEMTYir---qdlVVRLAYSLWNGGADLTQIIRSTAKgse---IGYYVPRLS
MBP6702579.1_Vicinamibacteria_bacterium           MRAPEIDSWKRQGHTILWDAASLASLCHAEKAVTLRHFIRMHAAWPDDATLVKGRALVVAGLDAALDALDPDDALAWLEKTVYPAVISFEKNVASQAALVFWFADGR-RFKHSMADLVAWWdcslafgqQIPLTRGVWNGSSSMQEIRVKNSEgk-vvhVGYHVQKIS
HEU5078767.1_Opitutaceae_bacterium                MRADNDSVWTKRGVSVVWDAPELAKRCTLAKALSLRQWFRWQAEWPDYYTGKDGRTIIVAGLDAAIDSMTPETATEWMQAKLLAAIREFQAEVAQEAALIFWMVNRR-RFEHRRADDSVIWksapgyrgTIPISHCIWNGAQDIARIVPPGCAdl-ehgIGLFLQRVS
MBW4553277.1_Aphanocapsa_sp._GSE-SYN-MK-11-07L    MQPVFGTGWTRKGFSLLWDVTALSKLVESDQIISMRRFFNLVGQWQDDLPAANGDALVVAGLDASLDALTADDASQWLEEKLRSALLSFQDYYQGDAALIFWLPGGRSRIEVQRATNTYLWlcgstsgrRLPLGQALWAGAEDARHIMAVNDPqtdkdwIGLHLPRIS
MBP7052832.1_Phycisphaerae_bacterium              MQE-TASKWKRTGSSIIWHPDLLADLIRDIEPTPLRTLIGWLKAFPEDPP-GGKQTVLVGGLQTAVETMTPDAVADWLRNHALAAVRAWKSHW-PDVGLVFVMDGPGSLFEFNEGDEIVYFgrgr-drsKVMLSLAIWNGAAAGAYQILVEGTgev---GGYFVRWLS
WP_149537235.1_Siccirubricoccus_phaeus            MP--LADAWARRGLTLLWGADAFRELASPDQVMPLRALFGERLNWPVDLVSNGGRALVATGLEASLDCLAPADAETWIATDLRDLVREFQDAYQGERALVLWLPSGAQRVTYDAPNDAWLWatadn---RLPLGRLLFSGAEDVERVSPAGWRgpapglIGLRQARLS
MCX6876430.1_Verrucomicrobia_bacterium            MNPPTDDVWKRKGISVLWDVAGLASLGSLQTAISLREFFLWNADWREDSTSGGARRIVVAGLEAALDCMEPEAAEEWMAQQLLPVINRCAEILFTGGALLFWMVKHD-RFRVKLENGEILWtcdgehrdKILFSHGLWNGAYDVQRIAPLRTEt--epgLGFYLQRIS
MDR4485162.1_Nitrospirales_bacterium              MNPDHDNTWLYCGTSLLWEAEALNHICSAEAVRSLREFLLLHQAWPDTLRLSNNRTLVVAGLEAAMDTLHPAKAIEWIEQKVYPAVLDFQESVAREAALILWLADHK-RIFHLPSENTYHWhcsaahrkSIPLGRCIWNGAESARRIVTKDTAkk-eiwAGLFQARIS
MCP3685978.1_bacterium                            MMVLRDDAWIRRGFTLLWGLKTLGKVVRPPQVVSMRKFFSMANAWPEELPASNGNALVVSGFEGCLDVLTGQDAERWIEKDIKDVILSFQDEYEGQAGLIFWVPSGHNRISMVGSSEEYHWkhraskssGLHIGRLLWSGAEEVERLIDDQRStadkawVGLHHPRIS
MBI5511448.1_Deltaproteobacteria_bacterium        MLPPADRGWVRRGLSLVWNAEALGTVAGPDAAWTMRQLLTRSRAWPAALPSSNGEAVVVAGFDSCLDAFGPDAAEVWIQRELVPVMLSFQEEYDGQAALIFWLPEALRRLTHSSADGSYSWrwagrtsvPLALGRCLWAGAEDLRRIVVPAKGkvdpgwIGLHLARLS
MBQ8111589.1_Kiritimatiellia_bacterium            MKTTNYTLMLRGGSSVLFGKKELSELLTQIEPISLREFLLLGDDLPDDPPPEFEDGILVSGLETVLDAMPVGEAEEFLAGRLRGVIQQIQNHW-TRTGIVFGCSLSEKAFEKQGVSESVVFtt---kddELALSDALWGRSQDIMRLEHKEPGere---VGYHVPRIS
MBA3936145.1_Planctomycetota_bacterium            MKR-GGTSWMRQGSTVITHLETASMLLAQAGLEPLRTALRWAAEWPAE-LAN-VSTLVVNGLSAVLDRCTPEDSAEFLRTKAKLIIRRSQEQW-TRTGILFLFPTHPSTLSVDL-DDRVIYrl---pggSVALSHELWNGAADLREI-----Hadv---IGYHVLRLS
MBN8780446.1_Thiobacillus_sp.                     MKILQGDVWKRRGVSLLWGGEALSVLAQPQQVVSIRQFFAMVGQWPDDLPCNDGNTLVVAGLEGCVDLMDPIEGEAWMKSDLLPAVLAFQDEYSLEAALVFWLPTGKNRVKMNRATEAYSWvcsaphsnHLDLGRILWAGAEDVGRIIDPDCTnsdpgwIGLHHPRLS
MBR2124994.1_Acetobacter_sp.                      MKELRDTVWQRRGISWLWDNTALDHVCKAHEVGSLRQFLQLYGEYPKSLERSFGRAFVVGGLDTSLDLLTPEEAKTWLREDIKKAIKAFQQRWAEGAALIFWLPRGDSRFDI-SSTDVLTWrcrppyg-KIDFGRILWGQCD-PQHIMQGDKK------IGFFYPRLS
MBK8636965.1_Chromatiaceae_bacterium              MKASEDDVWTRRGINVLWDGAALARIGPVAAAISLRRFFTLDALWPDETPLVDDQALVVAGLDVALDALDPADIEDWIEQVLYPRIQEFQSVFAGHRALIFWMPDKG-RWLENSGEGAFEWrlgsgdqgRLPLARLLWDGAYDVQRIEEPGDKgv-nrwIGLYLARIS
OCQ93482.1_Nostoc_sp._MBR_210                     MHK-SSSAWQRRGSSIVFHRDLLVPLIESGAMVSLREALSWANNWPLEPP-NGSNTVLIVGLETCLEVMVPSEAEIFLRRTIRPFLLESRNHW-DRCGLIFGFGCSEKRFCIDPQESILFVyl---dgtHIRLSEGLWNGAAELFYLTTPEQTknh---GGYHV-RLS
MBC7429626.1_Bacteriovorax_sp.                    MEEIEAGVWFKRGCSIIWDNRCLTRLVKDNSITPISTALRMSKAWPDKLPASSGRAMVVVGLDGCLESLEDDQREKWLREHVKPLVLSFYRKFQNEASLIFFFERGTASF-IDHIDEAFVWqptn-kqgPLPLVKFLYGGRFEIKRLVSEHDPkke---WGLYVANPS
MBF0302144.1_Desulfamplus_sp.                     MDT-SNTVWQRKGSSVIFDQDSLAPFVKDEAVVSLREALSWMKKLPTTPP-VKGKTIVVCGLETMIETLPEDEIDDFLTQRIRPLIIELQNRW-TGNGLVFGFSSHPNTFEESAMQEEVLFkr---rgnKVRLSEGLWDGSANMKRIITSEREsgq---IGYYVARIS
WP_145058250.1_Adhaeretor_mobilis                 MDKPNDDLWKRRGISILWDADSLNSLCRSDEVISLRHFRQLFNDWSDDASMVDEETLVVAGLESCIDALPPEEATKWLQDVFYKAMVSYQGEVATQAALILWFVDSR-RLKYRTSDDGWSMhcagefkgQIPLGRCLFNGAADLKEIQDADGNs-----LGLYHPRIS
HCF99181.1_Chloroflexota_bacterium                MDE-AGDMWRRTGTSLVWDGDALHTAATEGVTVSLRTALMWAEVMPEDPP-DGKRVLVVTGLRTAIDVLTEAERDAVLEK-VARLVRAQSRYW-PEAAIVFALPETTRLL-VAPPTGTVVLdlgg---dSLDIGNRIWGGAGGANQLIESRLDskgrqaIGYWLRRVS
MBX3394309.1_Phycisphaerae_bacterium              MDEFDSKMWLTRGSSVIFDKVMLGPLLTEGALIPLRQALSWLSSWPSATP-TGGKTVLVGGLETVLEMLAVSDAEDFVRRYIRPLIAEFQSCW-PEHGLVFGLNTEPGRLIEDPVSEMVNLkr---rddTICLSTELWNGSADMRRLVRTDRAsgq---GGYYAPR--
WP_270720981.1_unclassified_Bacillus_cereus_group MASSLGDTWTRRGISLLWDSNSLSLITEIKDVLSVREFLRYYQTWPNELHSANGYGMVVSGLETILDYTESQSLIDWMEKDFYPALLSFQSMYEGQCSLIFWLPDGQKRL-EQKTDGLIYWnctgayygQIPLSQCLWNGAADAKLIIKNTNVlsmkncLGIYHPRIS
consensus/100%                                    M.......h...G.shl................shp.h......h..........hhhsGhp..h-.........hh...h...h...........ullh.h......h.....p..h.h.........h.hs..las.....................Gh......
consensus/95%                                     M.....p.a.ppG.ohla....h..h.......shp.hh.....h.........pshhlsGhps.h-.h.......hh...h..hh......h....ullh.hs.....h.....p..h.h.........h.hs..las.s.....l.............Gha..p.s
consensus/90%                                     M....sp.W.+pG.olla....h..h.......sh+phh.b...hs........pshlVsGLps.l-.h.s.....ah...hb.hh...p..h....ullh.hs.....h.....p..h.h.........l.hs..las.s.....l.............Gha..+ho
consensus/85%                                     Mp...sp.W.RpG.ollap...Ls.lh....h.slRphh.h...aP.p.s....psllVsGL-shl-.h.s.p...alpp.lb.hlbphpp.h.spsuLlF.hsp...ph.....p..h.h.........l.lup.lWsGu.ph.bl.............Gha..RlS
consensus/80%                                     Mp...ss.W.RpG.ollas...Ls.lh...hhslRphh.h...aPpp.s.ss.psllVsGL-shl-.hss.ph..alppplb.hlbphpp.h.spsuLlF.hsp...ph.....s..h.a.........l.lup.lWsGu.ph.bl...p.......hGha..RlS
consensus/75%                                     Mp...ss.WbRpG.SllWc.psLs.lhp..hholRphh.h...WPpp.s.ssspsllVsGL-shl-.hss.pup.alppplbshlbpaQppa.spsuLlFhhsp...php.p.ssp.h.a.......pl.lup.LWsGu.-hpbl..p.......hGha..RlS
consensus/70%                                     Mp..psssWbR+G.SllWc.psLs.lhps..hloLRphh.h...WPss.s.ssspsllVsGL-shlDhhsspcuppalcpplbshlbpaQppa.spsuLlFhhsp...php.p.ssp.h.a......splslup.LWsGu.-hpcl..sp.......hGha..RlS

## ; Type-3 Brex-Related System; Inactive-STAND-NTPase Representative_2;

                                                  1---------11--------21--------31--------41--------51--------61--------71--------81--------91--------101-------111-------121-------131
OrigSeq                                           ---MKGYEIFEQKAPYENKIGFLDFLRELKSGTQGIPSSFMVVGIDDVLYLAGRDERLALTIHKILQSSAKVLDQKIIEVQIVCKGRLYKGESFWSEYRGEKLPLDYIFGTPNKRRECPVYSTGFNLSS---

                                                        S1          S2                    S3                                        S4   ----Insert-Region-----      S5  Terminal-Insert-Str
                                                  ------EEEE--------EEE-HHHHHHHHH-------EEEEE-HHHHHHHH---HHHHHHHHHHHHHHHHHHHHHH--EEEEE-|-EEEE---EEEEE--EEEE-|HHH--EEE-----EEEE---------
                                                  ------EEEE--------EEE-HHHHHHHHH-------EEEEE-HHHHHHHH---HHHHHHHHHHHHHHHHHHHHHH--EEEEE-|-EEEE---EEEEE--EEEE-|HHH--EEE-----EEEE---------
                                                  ------EEEE--------EEE-HHHHHHHHH-------EEEEE-HHHHHHHH---HHHHHHHHHHHHHHHHHHHHHH--EEEEE-|-EEEE---EEEEE--EEEE-|HHH--EEE-----EEEE---------
CBX30016.1_uncultured_Desulfobacterium_sp.        ---MKGYEIFEQKAPYENKIGFLDFLRELKSGTQGIPSSFMVVGIDDVLYLAGRDERLALTIHKILQSSAKVLDQKIIEVQIVCKGRLYKGESFWSEYRGEKLPLDYIFGTPNKRRECPVYSTGFNLSS---
HIE51614.1_Armatimonadota_bacterium               --MRPGFYLITRRQRPAKTMSLIALLRALHRGE-ALPSAVCVVGLVGLLESASDLRQAARLLAQRFQRHRSALMRQSPVIVFPVEHLSPTPRGVQVGARGSNFIAEWIFP-RLEPKVPRAYYSPF-------
KXA95577.1_candidate_division_MSBL1_archaeon      --MEEGFHIIEKSLPIFKKMSIVNWIKNIRNEGFNQFESYTVVGLEDLLEKISNPQDFSEKLRKILIDQSNYLVSTGNIFQFLIDNSIVWEKNPTVKTASGVIDLSAIFG-ELERKDVNWFYQQLNIQS---
MBM4461752.1_Chloroflexota_bacterium              EGLKAGYNLVERGEDDSEVMSIVQFLQRLQARD-TLPRRVKVAGLDRIMATMSDLKP----LHDILYEAGERLLRRSPVVLLPVD-KLTMDVEPKMYWRNQEIRLRPLFGNRLEPQGVGHFYSPFNIP----
MBN2391965.1_Anaerolineae_bacterium               MS-SSGYVLIEQRRSPRLQRGLLDFLRDLAEDELPYPSLVTVTGLDEVLYAAQPDEELAHEIHGRLNQAASRLESRLILIEMRLKGQIIRGDPFRLRHRGIDLPLHIIFDTPGKAADGNYYLVRCHLS----
MBP1716767.1_Deltaproteobacteria_bacterium        ---MGNYLLIEERLPNPKRMGFLDFLRELGQEVFPFSNRLCVVGLEEVLLQAGSGENVSLEIRKILTARANELEANLGQVQIVFRRPLKRSDDFWFDPGGNRMSLRPIFGSPIKQNGNEYYMTGFNLT----
MBU7037416.1_Theionarchaea_archaeon               MDLKRGFHVIEGQFTLIHKTSIINLLRLIETNSHQ--KLTIVEGLDRFLKYSTDE--AIEQARKVLNTGITDMLAIDASLIFVVECDIRIPDNPSIH----DRPLAMILPRPHDIMEPGYLYYPIM------
MBW1935302.1_Deltaproteobacteria_bacterium        ---MPGYQLLELREPYENKMGLLDFLREITQDPFPFPYRVQILGLEDVLFAARPQEELALKIQRILRSAAQNLEKKLVEIQIVFRGRLVRGESLWVEYRNAKLPIGHIFGSPIRQSRNQFYKKSFSLTNGVT
MCF8267322.1_Ignavibacteriales_bacterium          ---MPAYLLVEKKPANTQKIGFLDFLRDLEKDEFPYNSRLMIVGIEETLLAASPDIHAAIQIRNLLQKAANRLSNNCSDIQIVFRGTLQRGDQLVVRHVPQDIPIYLIFGTPIQEYGEKVYRPSFNRSSN--
MCK8825906.1_Fuchsiella_alkaliacetigena           --MQQGYNLMTQNDDFVERMSIMKFLKMVQEENKYIDYSLEIYGLEEFLCFLGKED-ANDLLTEILGKGVNYLTDNFATIKVITENKIIWNNKAVLKYSNEEFPLGNIFGSSLRQEDTEYWVANLNIDS---
NLY06148.1_Candidatus_Atribacteria_bacterium      VEINIGFNIIHRSDIFRERISIIQFLGDLNRGS-KFPPDFAVFGLDSLLYYVEDREKMAKYIRHTLQDNANYLTSGNYIIQIVIEGDIESDEKPRVVYKDEEFPLYSVIG-RLKRVDLKHFYAPLNLQS---
NMX21979.1_ANME-1_cluster_archaeon_GoMg4          MNPTQGYNVLLKEPEGFAKMSIIKFLREIRRGQ-ELPKKVAIYGLESLLLTNKDARAGTKFIRNMFIENNRLFREKGYTFLFIPRKELYVNRDVYLKAEDKKANVSSLFGLRLQIKEVGIYYADFEF-----
WP_089768781.1_Halobellus_clavatus                MSLNAGYNLVTDREDTEEELPALAFFRRLANNG-TLPPRLTVTRLEDLLYETDEEERAVRELRDVLRESGSF--RGPKAIQFVFDGDLVDDDVFSVRIERGYLPVGNLFVEEPRVVEAGHAVA------R-K
WP_103424089.1_Salinigranum_rubrum                SDLDIGYHVIDEDGMFDEEMSIVEFLRRLKRRE-EIPLDMTVRGLDDYLLGVDDADVACDYIHRLLRDRVNYLSLRNPRVQFVVD-DVENWSGPVIPTGDEPIKLNRIFHGSMEQSGPGWYSSNLNVQS---
AKH97513.1_Halanaeroarchaeum_sulfurireducens      MPLRPGYNIVSEPAETNSSLAALEFFRQVANNE-SIETPVTVTGLEDLLYNSAENERVLGRLRQALRDTGSI--GSMDAVQLHVDGKLVDDVEFRVRIEKAYLDIGQLFVEAPQLISPTHAVA------R-K
CAD6492699.1_Candidatus_Argoarchaeum_ethanivorans MRLKRGFNIVLNEDHFEDTMTLIEFLGDIRRNK-QIPSRVTVKGLDTLLLNSCDQEEMGLFIGELLRDGQSKLIRTSTVVQFIVDGKITKDIHTKIKVRNEYINLENLFYGKVSRLAPDWVHA------V-R
consensus/100%                                    .....sa.lh.........shh.hh..l...........l..l...h...........h...h....p.......h.h..c.................h..lh....p.....h..........
consensus/95%                                     .....sa.lh.........shh.hh..l...........l..l...h...........h...h....p.......h.h..c.................h..lh....p.....h..........
consensus/90%                                     .....ua.lh........pbshl.hh..l.p....b...h.l.Gl-phL............l.p.h.p..p........hbh..c..l........h......h.l..lh....p......h..........
consensus/85%                                     .....Ga.llppp....pphuhlpalp.l.p....h...h.l.GL-phLb.....p.h..blpp.L.p..p.h........lbh.hc.pl..s.....h......h.l..lFs...p...s..h..........
consensus/80%                                     .....Ga.llppp....pphuhlpFL+pl.p....hs..h.l.GL-phLh.s.p.c.hs.blpphL.p.sp.h..p.....lbh.hc.pl..sp....l....s..hsl..lFs...p....s..h.s.h.......
consensus/75%                                     ...p.Gapllppp....pphuhlpFL+plpppp..hs.ph.V.GL-clLh.sss.c.hs.bl+phLppsssbL.pp...lbhlhc.pl..spp..lc..s..hsl..lFs...cb..s.aa.s.h.......
consensus/70%                                     ...p.Gapllppp....pphuhlpFL+plpppp..hs.ph.V.GL-clLh.sss.c.hs.bl+phLppsssbL.pp...lbhlhc.pl..spp..lc..s..hsl..lFs...cb..s.aa.s.h.......
```

## ; Type-3 Brex-Related System; HELICASE (BrxHII-Helicase Counterpart); TUDOR + Swi2/SNF2-Helicase + Inactive-REase + CTD(Alpha+Beta);

```
OrigSeq                                          [illegible]
MBC7429627.1_Bacteriovorax_sp.                   [illegible]
MBE7508219.1_Planctomycetia_bacterium            [illegible]
MBI2192005.1_Planctomycetota_bacterium           [illegible]
MBI5511449.1_Deltaproteobacteria_bacterium       [illegible]
MBI5901621.1_Rhodocyclales_bacterium             [illegible]
MBA3936144.1_Planctomycetota_bacterium           [illegible]
MBM4322262.1_Deltaproteobacteria_bacterium       [illegible]
MBN2145092.1_Candidatus_Auribacterota_bacterium  [illegible]
MBN2391966.1_Anaerolineae_bacterium              [illegible]
MBP6702578.1_Vicinamibacteria_bacterium          [illegible]
MBQ8111590.1_Kiritimatiellia_bacterium           [illegible]
MBU4312941.1_Candidatus_Omnitrophota_bacterium   [illegible]
MBW1935303.1_Deltaproteobacteria_bacterium       [illegible]
MBW4553276.1_Aphanocapsa_sp._GSE-SYN-MK-11-07L   [illegible]
MBX3410397.1_Phycisphaeraceae_bacterium          [illegible]
MCC7337860.1_Pirellulaceae_bacterium             [illegible]
MCX6876429.1_Verrucomicrobia_bacterium           [illegible]
MDR1607843.1_Deltaproteobacteria_bacterium       [illegible]
MDR2179037.1_Synergistaceae_bacterium            [illegible]
NLG01394.1_Lentisphaerota_bacterium              [illegible]
NPV69259.1_Bacillota_bacterium                   [illegible]
UJS21265.1_Candidatus_Brocadia_sp.               [illegible]
WP_026299088.1_Deinococcus_aquatilis             [illegible]
WP_058554674.1_Thiohalocapsa_sp._ML1             [illegible]
WP_277443845.1_Pelotomaculum_isophthalicicum     [illegible]
WP_282552852.1_Providencia_stuartii              [illegible]
WP_283022682.1_Bradyrhizobium_sp._CB1717         [illegible]
WP_308999799.1_Paenibacillus_sp._LHD-38          [illegible]
WP_309896344.1_Archangium_sp.                    [illegible]
consensus/100%                                   [illegible]
consensus/95%                                    [illegible]
consensus/90%                                    [illegible]
consensus/85%                                    [illegible]
consensus/80%                                    [illegible]
consensus/75%                                    [illegible]
consensus/70%                                    [illegible]
```

## ; Type4 BREX Systems; Cysteine Desulfurases

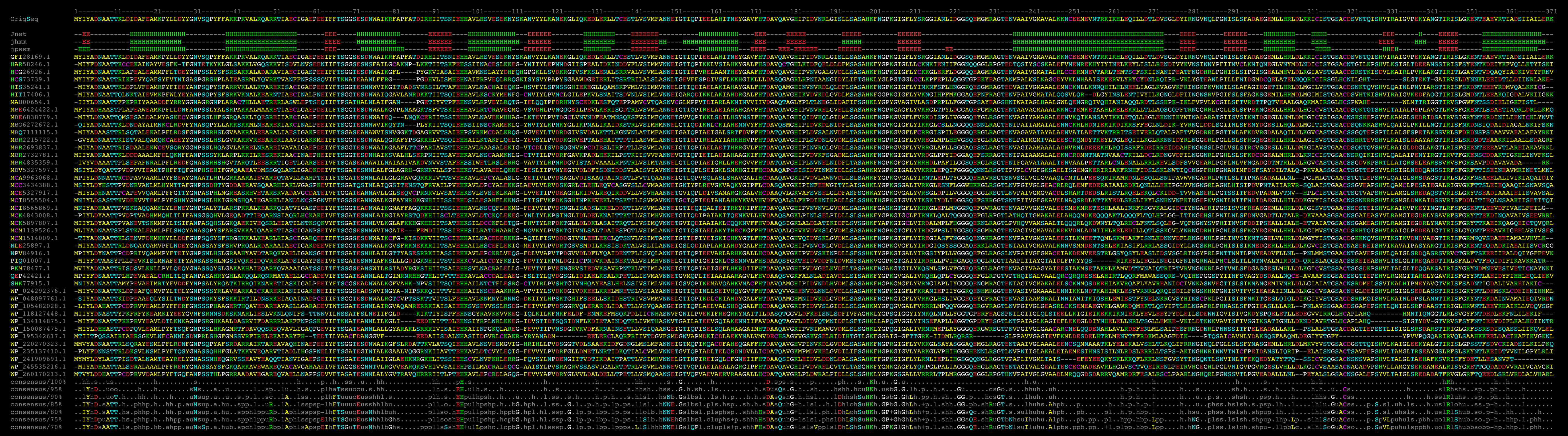

## ; Type-4 BREX Systems; (BrxP Component) PAPS-Reduct (DndC Homolog); (PAPS-reduct + A+B-Domain + Fer4_9 + DUF4007)

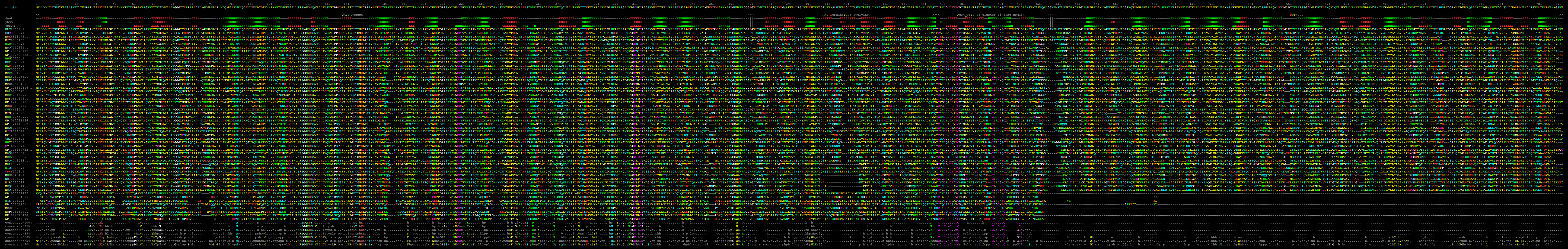

## ; Dnd-System PAPS-reduct DndC;

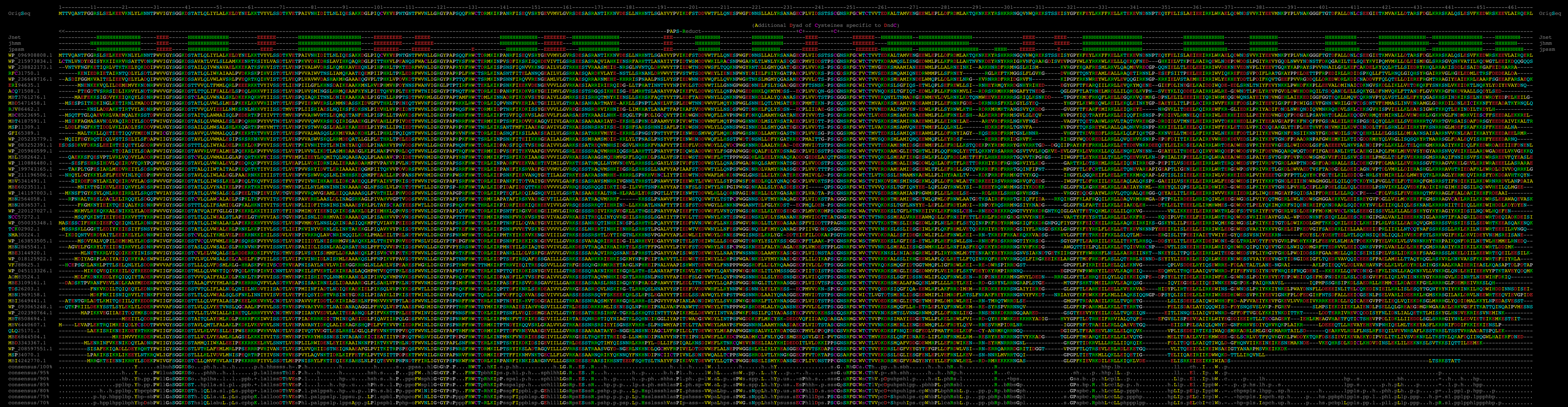

## ; SSP-systems PAPS-Reduct (SspD); (PAPS-reduct + A+B-Domain + Fer4_9)

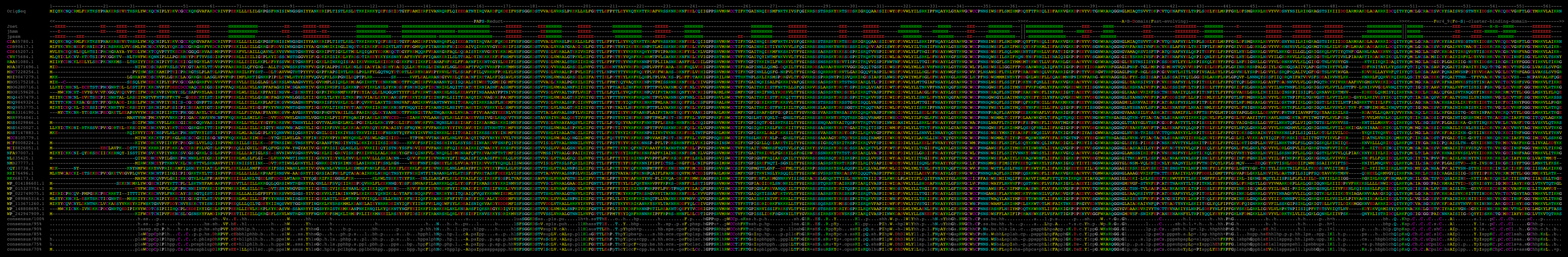

# ; OLD+TOPRIM (Previously Classified OLD-ABCs, associated with ABC-ATPase centric Conflict Systems)

[illegible]

# ; BREX-systems OLD-ABC (TOPRIM-linked); Representative_1;

[illegible]

# ; BREX-systems OLD-ABC (TOPRIM-linked); Representative_2;

[illegible]

# ; BREX-systems OLD-ABC (TOPRIM-linked); Representative_3;

[illegible]

# ; BREX-related (DUF499 centered) systems OLD-ABC (Associated with TOPRIM/REase/HNH)

[illegible]

##Representative Alignments of Core Protien Components in Novel HerA/FtsK Anchored Conflict Systems; (BREX-Related Capture Systems)
##1. HerA Translocase
##2. GNAT; (Partially Conserved in System)
##3. TGT; (Partially Conserved in System)
##4. DUF6079 ATPase (BrxC Counterpart)
##5. PglZ (BrxZ/PglZ Counterpart)
##6. DUF4007 (BrxA Counterpart)
##7. STAND-NTPase (BrxB Counterpart)

## ; BREX-Related Capture Systems; HerA/FtsK Transloacase Representative Alignment;

```
                                                        1---------11--------21--------31--------41--------51--------61--------71--------81--------91--------101-------11 [illegible]
OrigSeq                                                 MLLQVGRDIETREQVFLEGDRARTVFICGKRGSGKSYTLGVLVEELFEKSGGDVTVILVDPMGIFHTMVLPNKDQSRELLEWGLVEKGLPVRLLVPGDPVRRFGDSEIVSTL[illegible]

                                                        |-Preceding-Hairpin-|  [illegible] -Alpha-Helical-Insert(2-insert-Strands)- [illegible] -R-Finger- -Sensor-1- [illegible] -C-Terminal-Extension-
                                                        S1  H1  S2  [illegible]  H2  S3  H3  S4  H3  S5
                                                        -EEEEEEE---EEEEEEE----EEEEEE-------HHHHHHHHHHHHHHH---EEEEEEE---HHHHHH------HHHHHHH--------EEEEEE--HHHHH--HHHHHHH[illegible]
                                                        -EEEEEEE---EEEEEEE----EEEEEE-------HHHHHHHHHHHHHHH---EEEEEEE---HHHHHH------HHHHHHH--------EEEEEE--HHHHH--HHHHHHH[illegible]
                                                        -EEEEEEE---EEEEEEE----EEEEEE-------HHHHHHHHHHHHHHH---EEEEEEE---HHHHHH------HHHHHHH--------EEEEEE--HHHHH--HHHHHHH[illegible]
HHW27503.1_Candidatus_Fermentithermobacillaceae_bacterium   MLLQVGRDIETREQVFLEGDRARTVFICGKRGSGKSYTLGVLVEELFEKSGGDVTVILVDPMGIFHTMVLPNKDQSRELLEWGLVEKGLPVRLLVPGDPVRRFGDSEIVSTL[illegible]
HQD80870.1_Bacillota_bacterium                          -FLNIGKDSETGKKLYIEADRSRAIFICGKRGSGKSYTLGVLIEELFENAKGEALIVVCDPMGIYHTMVLPNERQIQELYSWGLAKRGFPVRLLIPRDPVAQFGSTEIVERI[illegible]
GIK42764.1_Chloroflexota_bacterium                      -KLLLGKDAHNEEQVFLDARGSRAILICGKRGSGKSYTLGVLIEELVSVGSTEVIPIVVDPMGVYHTMSQTNIIQQDELFKWGLSARSYPVRLLVPGDPLKLY-DRDILKIF[illegible]
RLC84906.1_Chloroflexota_bacterium                      -KLLLGRD[illegible]
RKU21514.1_Candidatus_Poribacteria_bacterium            -NLRVGTD[illegible]
MFZ1536133.1_Chromatiaceae_bacterium                    -QLMLGRD[illegible]
MFN8469221.1_Caldilineaceae_bacterium                   -LLNLGTK[illegible]
MDQ2805419.1_Chloroflexota_bacterium                    -KLFLGKD[illegible]
MBW7959470.1_Candidatus_Promineofilum_sp.               -KLFLGQE[illegible]
MFQ6042699.1_Candidatus_Poribacteria_bacterium          -MLTIGKE[illegible]
HHW40998.1_Syntrophomonadaceae_bacterium                -MLRIGKD[illegible]
WP_052723405.1_Methanosarcina_barkeri                   -NLFIGKD[illegible]
MFA0740147.1_Candidatus_Fervidibacter_sacchari          MMISLGRD[illegible]
TEU15944.1_Anaerolineales_bacterium                     -SILLGKE[illegible]
WP_089881367.1_Halogeometricum_limi                     -ELFIGKE[illegible]
RJP56335.1_Deltaproteobacteria_bacterium                -QLLIGKD[illegible]
NBT26026.1_Actinomycetota_bacterium                     -MLYVGRT[illegible]
MCK4444003.1_Thermoplasmata_archaeon                    -MIRIGKS[illegible]
WP_423222230.1_Candidatus_Amarolinea_aalborgensis       -MLSIGRD[illegible]
WP_338740222.1_Haloplanus_salilacus                     -RLFIGEE[illegible]
WP_089309286.1_Halorubrum_ezzemoulense                  -EIFIGEE[illegible]
MEI6180765.1_Chloroflexales_bacterium                   -MINLGKD[illegible]
WP_348254530.1_Leptolyngbya_sp._ST-U4                   -MIFLGRD[illegible]
MBN1891047.1_Thermoflexales_bacterium                   -MLFVGRN[illegible]
NPV62498.1_Methanotrichaceae_archaeon                   -MINIGNE[illegible]
consensus/100%                                          ..h.lGpp[illegible]
consensus/95%                                           ..l.lGpp[illegible]
consensus/90%                                           ..l.lGpp[illegible]
consensus/85%                                           ..l.lGpp[illegible]
```

## ; BREX-Related Capture Systems; GCN5-related N-acetyltransferases (GNAT; Partially Conserved in System) Representative Alignment;

```
                                                       1---------11--------21--------31--------41--------51--------61--------71--------81--------91--------101-------111-------121-------131-------141-------151
OrigSeq                                                MVFGAETLVIRKATPADLDAIKAIADAHRHELGFVLRPALAESIGRGEVLVAENHQGLIGFAEYHHRRDAQTTLYHIAVIPQCRQQGVGRALVNALCAEASALGKLTVFLKCPADLSARGFYACLGFEVLGEEPGNGRSLIVWTLSLTENTQ

                                                       --------EEEE--HHHHHHHHHHHHHHHHHHH----HHHHHHHHHH--EEEEE-----EEEEEEEE----EEEEEEEEE---------HHHHHHHHHHHHHHH---EEEEEEE---HHHHHHHHH--EEEEEEE----EEEEEEEEE-----
                                                       --------EEEE--HHHHHHHHHHHHHHHHHHH----HHHHHHHHHH--EEEEE-----EEEEEEEE----EEEEEEEEE---------HHHHHHHHHHHHHHH---EEEEEEE---HHHHHHHHH--EEEEEEE----EEEEEEEEE-----
                                                       --------EEEE--HHHHHHHHHHHHHHHHHHH----HHHHHHHHHH--EEEEE-----EEEEEEEE----EEEEEEEEE---------HHHHHHHHHHHHHHH---EEEEEEE---HHHHHHHHH--EEEEEEE----EEEEEEEEE-----
RLC84907.1_Chloroflexota_bacterium                     MVFGAETLVIRKATPADLDAIKAIADAHRHELGFVLRPALAESIGRGEVLVAENHQGLIGFAEYHHRRDAQTTLYHIAVIPQCRQQGVGRALVNALCAEASALGKLTVFLKCPADLSARGFYACLGFEVLGEEPGNGRSLIVWTLSLTENTQ
WP_048108637.1_Methanosarcina_barkeri                  MNNFNSDVEVTKARLEEVPQIKSIADKNKNLIGFILRGSLVESIEKENLVVLKYKKIIIGFINYHHRKDSQTTLYEICVDENNRNKGYGKILINYLLQEAKSQKKKTLLLNCPIDGIAHSFYLRCGFKIKSTRTHTKKKLVTWVYKLDDNNT
GAB4153040.1_Candidatus_Promineifilaceae_bacterium     MTSG-NEVLIRAGRSEDIDGIKQLADAHKRELGFLRRPALLEAIQRGELLVAQNGFTIVGFIEYRHRRDHQTTLYNVVVHPDYRRLGIGRQLVLALEKEAIQREKSQVVLKCPEDLPANDFYEQTGYERIHVEPGKLRRLNVWRKFLMNGCR
MBW7959469.1_Candidatus_Promineofilum_sp.              MPSG-SDLIIRKAVFTDLETVKRIADAHRHELGFVVLSALRERIERNEMLVAVRDNELIGFVDYHKRRDGQLTLYRIAVDEEDKGQRVGQALIDNLVAIARAQGCSQIILKCPVDLRANQFYEQHGFTPAGTVEGRRRSLNVWAMSLDHTTQ
HFD39725.1_Anaerolineae_bacterium                      M----SDPEIRQATMFDLNAIKELADAHRHELGFVRRPALARSIERGELFVAQNGQGVIGFVEYHHRRDGQTTLYHLAVQADYRRQGLGRRLVQALVHDAGQHSQEFIQLKCPVDLEANRFYQQIGFAQIDLQRGKRRDLAIWRLSLAPGCD
MBC8263372.1_Anaerolineales_bacterium                  M----TSLMIRKAIPADLDIVKGLADAHKNELGFVLRPALASSIDRGEVIVAENSRGIVGFVEYHHRQDEQTTLYHIAVAPQHRRQGIGQRLVNALVYDAGEHNQRFVLLKCPADLEANKFYERLGFSHVDTQPGKQRELSIWRLPLSSAIR
NLE46404.1_Chloroflexota_bacterium                     MTSGNQVSIVRKAVLDDLDTIKAIADAHRDELGFVIRRALQESIERGELSVAIDGDRLAGFVEYRHRRDQQTTLYHIAVVEQWRQRGIGRLLVEAVRRDALANGKNVIRLKCPESLPANEFYTRLGWQKIAVESGKRRRLVVWELPLVLEAK
MBV6438331.1_Anaerolineae_bacterium                    MISG-GNFVIRRARLDDIDNAKALVDRHKNELGFVIRSALVTSVSRGELLVALANDDIVGLVHFRHRRDKQSTLYSIVVSENVRSQSIGRALLAELVDECQRLGQERIVLKCPVELPANSIYPKFGFTLTGTENGKHRRLNIWQLELPGSN-
MBN2393031.1_Anaerolineae_bacterium                    MISGNAEITLRKATEEDLDNIKIMADAHRRELGFVRRPALLEAIHRKEIIVAQNSRHLAGFVHYRHRRDEQTTLYDIVVAAEYRLIGIGKALVQTLVAEIQALGKQTLILKCPAELPANTFYTHLGFERWKEEPGKHRPLIVWRLSIPSQQ-
MBI5668050.1_Chloroflexota_bacterium                   MISGNNNVTVRKATIEDLEAIKRLSDQHRAELGFVLKPALEKAILDGEVFVAV-APQIVGFVHYHHRRDSQTTLYHIVVNMEERRQGIGRALLSRLKVEATERGQSSILLKCPTELPANKFYEQCGYSHIDVEPGKHRRLNVWQISL-----
MFZ4828883.1_Phototrophicaceae_bacterium               MIFGSDDFVLRKASEIDLTSIKEIADENKKELGFINRGTIIESIKRYEVAIVEDGYGIVGFIHYRHRLDSQTTLYNLAVKHPFRNCGMGRRLIEFLKQDALASQKLTIRLKCPEELASNQFYERYGFKLMSVENGKHRRLNIWVLNL-----
GIK42763.1_Chloroflexota_bacterium                     MTSGVSEIIITKAEFADIGGIKQIADAHRNELGFVRRPALIEAIDRSEILIAKQNGNIVGFVEYRHRRDEQTTLYNIAVMPENRHTGIGRKLFQALVKEAEKRDKSHILLKCPEELAANKFYRALGLHLTELEPGKQRRLNIWQWEL-----
GAB4528674.1_Anaerolineae_bacterium                    MISGNSRFTLRKATLDDIDAIKALADAHRRELGFVRRPALIESINRQEIIVMQNSRDVIGFVEYHHRKDDQTTLYHICVEPAYRRRGVGRLLIEALQEEARRYGKHMIRIRCPANLPANQFYRRLGCFLRGKELGKKRCLAIWDIFV-----
MBP8947513.1_Promineifilum_sp.                         MTSG-NSLLVRPAQLADLTPIKALADAHRHELGFVLLPSLREQMERGQMLVAEQDGTLLGFVDYHRRRDGQITLYHIVVKPGVRGQGTGRALLAALEACGRDANCRRIVLKCPIDLASNHFYERYGFILDGTIEGRKRPLNVWMLNL-----
MDQ2805418.1_Chloroflexota_bacterium                   M----ESVIIRTATSADLDAVKALADRHKRDVGFVRRGALLESINRAHVLLALAGEDPVGFVQFHHRRDGQTTIHLIMVASEHRRRGIAALLLNQLRTACQGLGQQRIGLKCPVDLSANSFYQQAGFTLIAQEPGKIRALNLWQLPL-----
HET59441.1_Chloroflexota_bacterium                     M------IKIRKAKPVDLDAIKSLVDSHKHELGFVLRPALMKSIDRSEVFVANGGKTIVGVLDYHHRLDQQTTIYHFVVAPQLRRQGVGRLLVEALKEEAKEYGKSHILLKCPEDLESNDFYGRVGFQLTAIENGRQRRLNVWELVLIS---
NPV62500.1_Methanotrichaceae_archaeon                  -------MLIKKATIDDLEGIKLLADKNRNELGFVLNSALVRSINRGELIIAL-DETIVGFLEYHHRKDCQTTIYHFVIEENYRLRGISKILLNFLIRECESQGMRYVFLKCPADLNANNIYPKLGFKNVGLEGGKKRKLNLWNLNIYLS--
MFQ5758745.1_Candidatus_Bathyarchaeia_archaeon         M------VVVRKATLDDLDVIKALADSHRYELGFVRCAALQRAIEQNEVFVAQNRQYITGFVDYHHRRDGQTTLYHIGVSPALRGQGIGRSLVMALYEEAQQRGSRTIVLKCPADLPANDFYAALSFHLIGEERSKNRPLRIWSLSL-----
HHW27508.1_Candidatus_Fermentithermobacillaceae         MP------IVRLAEQADLRDVKSLADCHRKEIGFLPRAAFEESALHQCLIVACDDKGVVGFTRFRHRRDGVTVIYEIC--SQLSGSGAGGQMIDWLKADCREHGQRSIILKCPSDLPSNRFYESKGFRKVGVEAGKAQPLNIWSLEV-----
WP_310922608.1_Halogeometricum_sp._S1BR25-6            MTDQQSDFLVKTVESDRLGQLNDFFNSPKSELHFTHRDTLERAAVRNDLYYYR-FDEILGWCESRVLEDHQAQIRLVAVHPAYREYGIGRYLVKSAIKFAKVWEKSTMIADVAADASAVGFWKACGFHQVDQYQTKGREMYQMQRRI-----
WP_086215089.1_Halorubrum_sp._SD683                    MEKSGADFTVKAVNLSLVTAVNEFFNSTKSELHFTHRDTLERASERNDLLYIR-RETVIAWCKSRVLEDDQAQIRLVAVDPAYRNYGLGAYLVSAAVDFAQFFGKKEMIADVAAEAPAVTFWEHCDFAKKSEYSTEGRLMYRMWKPI-----
WP_232571137.1_Halobacterium_litoreum                  MSRGKKLMKVSSASPDDVERINAFFNSTKTDLHFTYRDTLDRAFEREDLLYVDDDGEIIGWCQSRVLDPGEAQIRLVAVSPDNRDEGLGRLLCECAEAFARRQSQDRMIADVVASAPAVEFWKSIGYDPLEEWETRGRSMLTVQKPL-----
WP_089673069.1_Halohasta_litchfieldiae                 MKSKLTVLPIQAASINEVEAINDFFNSSKNDLHFTYRDTLKRAFERGDLYYVETSGTLIGWCESRILDKHEAQIRLVAVSRVGRGAGIGRYLCEEAEEFARSHGKEQMIADVVKGSSAVEFWKSIGYEVQSGWETKKTEMLTVEKWL-----
WP_089881364.1_Halogeometricum_limi                    MTSN-ERSAIKTARPEDIDRIQRLYNHNKKELHYTYRDGLERAVSNEGILFTDETEEVIGWCESRVLKPGQAQVRLIAVHPDFRSTGLGADLIEASENYAKEAGKTVLIAETAASSDAKQFWLTMDFQVTSTRTTDGREMALMKKEI-----
WP_256533862.1_Halovivax_cerinus                       MKKP-EPITIKHAEEEDVTRINEFFNSKKQELHFTYRTTLSRAFSRDDLFYVE-NESLVGWCESRVLDDDEAQIRLVAVSPDRREEGIAARLCENAEEFATYHGQERVSADVACDSPAVNFWTSIEYDIEYEWQTDGREMYRMMKAL-----
WP_338740224.1_Haloplanus_salilacus                    MTQP-KSIAVEQVDEDDVGRVNDFFNLRKRDLHFTYRDTLDRAFCRDDLFYIE-DATIMAWCESRVLDDDEAQIRLVAVKPEHRGQGFGRALCERAEQFAFEFGEQKMSADVMAESQAVEFWQSLGYTIEEEWTTDNRSMYRVSKPI-----
WP_117592509.1_Haloprofundus_halophilus                MS---DEFPVLSAEPETIEFLLNFFNSPKKELHFTHRATVERAFERDDLFYTVGDNTIIGWCESRVLEPHEAQIRLVAVDLDYRDCGIGKQLCDAAEGFAKECEKKAMIADVAEKSPAVSFWHACGYKTQKKWSTKNRPMLRVEKSL-----
consensus/100%                                         ........l..s....l..h..h.s..+..l.a....sh.p...p..h.h........uh.p.+.b......l..h......p....u..hh.............h.hps.....u..ha....h........p.p.h..h.b.l.....
consensus/95%                                          M........lp.s...pl..lp.hhs..+.-l.F...ssl.p.h.p.phhh......lhuhhp.+.b.s.b.plb.hhl....+..uhu..Lh..h...h....p..h.hcss.p..u..ha...sh.....b.sp.p.h..h.b.l.....
consensus/90%                                          M........lp.s...cl..lp.hhs.p+p-l.F..bssl.cuh.c.plhhh.....lhGahp.+.bps.bsplb.lhV....R..Ghu..Lh..h...h....pp.h.hcss.p..u.pFa...sa.....b.sc.+.h..h.b.l.....
consensus/85%                                          M......h.lp.Ap..-lp.lp.hhs.p+p-L.Fs.+ssL.cuhp+.clhhh.....llGahc.+.bcD.bsplb.lhV....R..GhG..Lhp.h...h....pp.h.hcss.p..AspFa.phGap....b.sc.R.h..h.b.l.....
```

## ; BREX-Related Capture Systems; tRNA-guanine transglycosylase Representative Alignment; (TGT; Partially Conserved in System)

```
                                                 1---------11--------21--------31--------41--------51--------61--------71--------81--------91--------101-------111-------121-------131-------141-------151-------161-------171-------181-------191-------201-------211-------221-------231-------241-------251-------261-------271-------281-------291-------301-------311-------321-------331------
OrigSeq                                          MILNFFTALTLCPSDIRKIRRLLDMHRIDMNPLRHILITPLFASPESLKLVRNLKEHNGSTVMFDSGGYYVQVGKLTYQELYYPLLEFYKANPWADIYTLPDHVPTSQDSEQVVWQKIRDTADYTALFYQEMPHHLQSRALAVVQGHTYEQVDLCLKTYLQLGVQRIGFGSFGTIGKNNGINIATNSAVDLARYVVDIAHKNSVKVHFFGLGAPALIAMIYGAGADSFDSSAWIKSAGFGQVYLPFTRGYNISHRSELQQGIQFKEFETLKILTGHSCPFCASIDELQAHKMHRALHNLIAIQQSVNMINSDAGDTIERIYRNGSPRYREEYQKWLNGI

                                                 ---EEEEE----HHHHHHHHHHHHHH--------EEEE------HHHHHHHHHHHHHH--EEEE---HHHHHH----HHHHHHHHHHHHHH-----EEEE----------HHHHHHHHHHHHHHHHHHHHH--HHHHHH-EEEE----HHHHHHHHHHHHHH---EEEE---EEE----EEEE--HHHHHHHHHHHHHHHHH--EEEE-----HHHHHHHHHH---EEEE-HHHHHHHH------------------------HHHHHHHHHHH---------HHHHHHHHHHHHHHHHHHHHHHHHHHH---HHHHHHHHHH--HHHHHHHHHHH---
                                                 ---EEEEE----HHHHHHHHHHHHHH--------EEEE------HHHHHHHHHHHHHH--EEEE---HHHHHH----HHHHHHHHHHHHHH-----EEEE----------HHHHHHHHHHHHHHHHHHHHH--HHHHHH-EEEE----HHHHHHHHHHHHHH---EEEE---EEE----EEEE--HHHHHHHHHHHHHHHHH--EEEE-----HHHHHHHHHH---EEEE-HHHHHHHH------------------------HHHHHHHHHHH---------HHHHHHHHHHHHHHHHHHHHHHHHHHH---HHHHHHHHHH--HHHHHHHHHHH---
                                                 ---EEEEE----HHHHHHHHHHHHHH--------EEEE------HHHHHHHHHHHHHH--EEEE---HHHHHH----HHHHHHHHHHHHHH-----EEEE----------HHHHHHHHHHHHHHHHHHHHH--HHHHHH-EEEE----HHHHHHHHHHHHHH---EEEE---EEE----EEEE--HHHHHHHHHHHHHHHHH--EEEE-----HHHHHHHHHH---EEEE-HHHHHHHH------------------------HHHHHHHHHHH---------HHHHHHHHHHHHHHHHHHHHHHHHHHH---HHHHHHHHHH--HHHHHHHHHHH---
MBN2393039.1_Anaerolineae_bacterium              MILNFFTALTLCPSDIRKIRRLLDMHRIDMNPLRHILITPLFASPESLKLVRNLKEHNGSTVMFDSGGYYVQVGKLTYQELYYPLLEFYKANPWADIYTLPDHVPTSQDSEQVVWQKIRDTADYTALFYQEMPHHLQSRALAVVQGHTYEQVDLCLKTYLQLGVQRIGFGSFGTIGKNNGINIATNSAVDLARYVVDIAHKNSVKVHFFGLGAPALIAMIYGAGADSFDSSAWIKSAGFGQVYLPFTRGYNISHRSELQQGIQFKEFETLKILTGHSCPFCASIDELQAHKMHRALHNLIAIQQSVNMINSDAGDTIERIYRNGSPRYREEYQKWLNGI
MBV6438325.1_Anaerolineae_bacterium              M-LRFVASMTVCPSDIRKIRNHVGAAGTHLHPLQDILITPMFVQKQSLALARELAEEN-SNVTFDSGGYYVQLGRTKYEELYMPLLRAYRENRWASVYTLPDHVPTSQDTTEIVDRKVQDTIRFSTLFFQEMPDELKPRAMPVVQGHTYKQVEACLQAYIQLGVRQIGFGSFGTQGQNSEINVATKSAIEIARHVIAVAHQHDMRVHLFGLGTPSLVAMIKGIGADSFDSATWLKSAGFGQVFLPFMRAYNISYGSELQKGITFSQFQEWKALTGHRCRLCDDLDRLNSQKMYRAAHNLVVMAETVEMVNSGNPQMIKAIYKAGSPKYRDEFEKWLQRN
WP_086215083.1_Halorubrum_sp._SD683              M-IEFVASVTINKSQFNSLIDRLEEHGLVEDVLGNLMFCPLTAGESTSEIIERLATDYDIHVACDSGGYESQINKYSMRDIYQFDRKYYLETDWPTEYVMPDQVPLETDDEMTVEHKVQDTITLSRMLYRELPPEKQKRAVPVVQGHKREQIVNCLDAYRRLNVQKIGFGSFSTGGVNGGVNYLNEDNIELLQFVVKEAKKYGLDVHAFGIGGPTSIPILYECGVDTFDSTGWIRSAGYGNVFFPFKSRLNITHLDRSGPAVFREELSNLKTETNHSCPFCESFEDLQENREDRVLHNLVVMSEMADAIGEYSTDELLDIMNPRS-QYTKYLKKLTAI-
SDG37465.1_Halorientalis_regularis               M-LTFYAALSVSPSSMRKTFARLEDGYGSLEPLENIMFTPISGGEKAHQTIRELNDHVGINIMFDSGGYEVQVGNKTFDELQAYLHDFYGENHWGHRYVLPDNVPVSQDSSEVVQRKVEQTADISALFYRQMPDPLKPRALAVVQGHTQDQLQHCLNKYASLGLQHVGFGSFGTSGVSNGVNMLTTEAYHNLEWAVNQAHEAGLSVHAFGVGGPTSIPLLYHAGVDSFDTTSWMRSSGYGNVFFPFKSRYNASHRKRGGNLLTKDELPHIKAETEHECPFCQEMSQLRSSRWNRIMHNLIVIHEMTNRVEDMTREEILKAMDEES-KYRRRLKKVTSSN
MBI5668044.1_Chloroflexota_bacterium             M-LQFVASVTICPSDIQKVRKHVEIAKTATDPLHSVLITPLFANPRSLMMVRDLAESG-KQVYFDSGGYYVQIGRLRYEELYMPLLQAYRANPWASIFTLPDHVPTTQDDPDTVECKVHDTITYSTLFFQELPDNLKPRAMPVVQGHTVRQIDACLQAYIRLGAKQIGFGSFGTSGKKSEINVATNGSVGLAKYVIQVAHSHGMKVHVFGLGAPALVAMLKGIKADSFDSSSWLKAAGFGQVFLPFMRAYNISHNSELQRGITFNQFDEWKTLTGHHCKLCEELDNLQKHKMYRAAHNLIVLAETIAMVNQGDFSRIKQIYVKGSGKYRQEFDRWLQPN
WP_114450123.1_Haloplanus_salinus                M-LTFYAGMNISPSSMRKLFARLQNEHPTLAPLENIIFTPIAHGNGTHSEIESLSKELDLNLMFDSGGYEVQVGNKTFDELYEYLLEYYDENRWGQRYVLPDNVPMSDDDVQTVQEKVDETISATRMCFRRLPEDVREDSLAVIQGHTREQISRCIDEYKELGLTKVGFGSFATGGVNGGVNMITSEAFKNLRWATELAHEHGLSVHAFGVGGPTSIPLLYEAGVDSFDTTSWMRTSGYGNVYFPFKGRLNASHRHRSGNVLTRQELPHLKAETDHSCAFCEDISRLSNNRWDRILHNLIVTHEVVERIDDMTQQEVIDLMDQSS-RYRKRLENITPRK
MCZ7554452.1_Anaerolineales_bacterium            --MKFVASLTMDASLLKKLLALWDASGDSRNPFESVLVTPLFASASTLKMIRELKEKRGAQVYFDSGGYYVQQGKIGFEQLYQDVRAWYSENQWADWYVLPDNVPTSGDCGEVVEQKVNETVAAAKMFFAEAPAAVLERCMAVVHGHELWQVNRCIESYLGLGVKYLGFGSFGTGGGDNSINQLDKHSAEIVAHLASGLCSCDVLLHTFGISTPPAVYAFKKLGFSSFDSLGWQRSAGYGKVYMPFTRAYNVSHRSIHNSALTRDEFVRLCLLTGHECPFCVDFRQLSEDRLYRSLHNLVVLMETGNGTGESRQEKIARLIEDWSPRYYRLFQQVYNG-
GAB4528646.1_Anaerolineae_bacterium              M-LQFSTTLTLCPSDIRKICQLLDEHDIAATPLKHILVTPLFVQKESLQRIKAIKEQQGSTIMFDSGGYYVQIGKLTYEELYYPLLQFYRENPWADVYTLPDHVPTSQDTENDVWRKVRETVRYSILFHQELPADIQTRAMPVVQGHTLEQVDHCLEAYLSLSIRYIGFGSFGTIGKNSEVNVATNSAVNLAKYVVKVVQARGVKVHFFGLGAPALVAMIYGAGASSFDSSSWIKSAGFGQIYLPFMRGYNISHRSELQRGITVEEFQDLRLLTGHHCPFCSSISELQRRKLYRALHNLLSIQETVDMINSGDHRTIKTIYEQGSPRYKQEYEKWLAPA
MBC8263378.1_Anaerolineales_bacterium            --MQLVTSLTVDASLLRKLLRLWDESGDPRNPFATVLVTPLFASPSTLRLIRELKERRGATVYFDSGGYYVQQRKIAFEELCCTLRDYYQDNQWADWYVLPDHVPTSTDSPAIVEEKINDTVTAVRMFYAEMPSIVQERSMPVIQGHTFKQVNRCIEAYRELGLKYIGFGSFGTSGTNNSINVADSRSAATVAHIVRELDNDGIRLHTFGVSTPPVIYAFQRLGIYSFDSLAWQRSAGYGKVYMPFMRAYNVSHRSTRNSALSQVEFELIKERTGHHCPFCENFSHLSKRRLYRSLHNLVAVMDTVKVDQRQDSQSIASLIAWKSQRYYKMFKEIYLGQ
GAB4153075.1_Candidatus_Promineifilaceae_bacterium  --MQFVASITRNASDIRKISRLWQGSGDTRNPLKNILITPLFTPPSTLKLIREMRERGEEQVYFDSGGYFVQMGRLSYEDMYWYLLQFYRANHWADWYVLPDYVPLSSDKEDEVWYKVRRTADGSKLFFLEMPSSLQDKALPVAQGSESKQIEYCLETYVRLGVQQIGFGSFGTSGKSSSVNAVTSNSLKMLGELSEILTQERLNLHAFGVGNPPVIYLLNQAGVDSFDSVGWMKTAGFGKIFMPLTRAYNITYRDPMARGLKEDAFYTLKELSNHDCYFCQSFQKLRESRDYRVMHNLTVVLDTLEMIDKGLSNLVERILQTYSPAYAKLGRRDRDA-
MBW7959475.1_Candidatus_Promineofilum_sp.        --MQFVASLTRGAAEIKRLIRHWNDSEDPRNPFANILVTPIFTPPSTLKLVRELKEEGVKQVFFDSGGFFVQMGRISYEDMYWQLLHFYRSNPWGDWYTLPDYVPLSSDDEETCWHKVRQTADGSRLFYQELPYEIKAKALAVIQGRTIAQVDYCLETYLRTGPERWGFGSFGTNGKQSNANIVTEEAFILLNYVSEIAVSNNRHLHAFGVSTPPAIYLLNESRVDSFDSIGWMKTAGYGKVYLPFVRAYNITYRDKGARGMPPQVFEKLKIRSGHECSFCTDFRMLETSRTHRILHNLAVIQDMVDMIRERTGDDLLQIMEEYASTYARLRSIAES--
MBP7045033.1_Chloroflexota_bacterium             --MEFIASITLDVSYVHKIRRLWKMSGDPRNPLKNVLVSPLFTHPKTLAKFRELRENNEEQIYFDSGAYSVQKGRSSYEEMYWSLLNYYRENDWADWYVLPDYVPLSSDTPEDVWYKVQTTANMGSLFFAEMPEHLRLRALPVVQGHSIDQIEFCLESFLKMGIQRIGFGSFPTNGKDSSVNTLSPSALTFLSHLTKILDSKGIGLHAFGVGTPPVIFLLHQAGVKSFDSVGWMKTAGFGKIFMPFVRAYNITYRDRTARGLTKETFLELKRMTNHDCFFCQSFDTLAQSRDFRFMHNLIVVMDTIDQLGNLQSVDVGKILDIYSPYYAKMNIGANT--
HHW27507.1_Candidatus_Fermentithermobacillaceae   M-VRFVTALGIRPGDLAQIALAWGNVKDRNTPLANVLISPLFADPASIQMIRRLKEQGANTVYFDSGGYQVQKGRIDYNTLYNRLLQYYEANDWADYYVMPDFPPVSGDSDSIVQEKVRITVDGTKRFFYDLPSTLRSRALAVVQGRRVEQVKFCLVNYHDLGISYVGFGSFPTNGAQSSINHLDAGAAEMLACIRRQYPS--VRIHAFGVGNPPVAYILNHMGIHSFDSSGWIKAAAYGNIYFPFTRAYNITYREPGRGALDAESFERIKTLTGHSCPYCSDFYELSRNRTSRFLHNLICMMETVEDISAPL---ASEVISHWAPAYKRFLTGRAL--
WP_117592495.1_Haloprofundus_halophilus          --MDFYAALSVNRSSIRKIAKRLEEEYSSFDPLQNIIFTPITQGETSHQEIRRVAEKYDVNLMFDSGGYEVQTGHFEFDDLYEYLIDYYSDNQWASRYVLPDNVPVSGDSPEVVEQKVTETLSASRSCFKRLPEFVQENAVGVIQGHTREQISRCVNEYSEFNLKHLGFGSFATSGGQNSVNMLTSESLQNLRWAVKLAKEHGFTIHAFGIGGPTSIPLLEEAGVDSFDTSSWMKSSGYGNVFFPFKSRYNASHRLRSGKVLMADNLEDLKEETNHDCPFCRDVAKLREDRLTRVMHNLVVTHELSNRVSGMARDQMAGQMDTNS-MYRRRLETM----
WP_089881382.1_Halogeometricum_limi              --MKLYASMTVSPSTVRKFKEHLLREYGTLEALDPLIYTPISTGDGLTEEMKQLSKE-GVNVMFDSGGYHVQTNNGDFDELYNYIMPFYDKNRWAHQYVLPDNVPLSDDDDETVERKVKETRTNAKLSYHFLPEELKSRAVPVIQGSKYPHITDCIDTYSELQIQTVGFGSFETFGKNNGINFISERVAETLRYAVNLAHERGLKIHAFGVGGPTAIPVLGKLGVDSFDCSSWLRAAGYGDIYFPFRTPRNVTHKDRTGPKVFRSDLEELKKETGHKCPFCASYDKLRDSRIIRILHNLIVLRDMAESINSYSTEEMLDLMNDKS-RYTTILRSIS---
MDW8029827.1_Armatimonadota_bacterium            L-PTFFVTLTHEATEIRKILRLWDESGDRRNPFAHLIFTPLFSKPATIATIRQLKDQRGSTVMFDSGGYQVQTGKATYEELFERLLRFYRENDWADFYVLPDHVPRSSDSDREVDFKVRETVDFARLFLKMMPSDFAAKAVGVVHGRTFEQVRYCVQAYVDMGISYIGFGSFGTSGPNGKVNMLSQKSLRLLKLVQTLACEFGLRLHIFGIGSPSHLIRLQQGAIDSFDSAGWWKAGGFGNIFFPAGRQLHITAINAEATLL---GIERQKRHSGHQCPFCDNSKLLRLSRVKRILHNLAAMLDTLKQVYGGQ--------------------------
WP_048108638.1_Methanosarcina_barkeri            --------MSAHPGLIKKMRDSLKDFEKIKWPLKNVLYTPVFSTKKTSEAIKDLKDNDGSKIMFDSGGFFVQQGKITTQELFSKLTDFYSQNEWGDYYVLPDSPPVNGDSDLIVEYKVRDTITNAKIFHDQLKENIQKKCIPVVQGHNKEQIFRCVREYSDLNVSRIGFGSFGTCGSNNGINFLTSKSMENVEYMMNLLKDYSLEVHAFGVGGTSSLSKLCEAGFTSFDSAGWKRAAAYGDVLFPFHSTHNITHREKSCSILSRNNFEKVKTESNHACYFCEDFNKLQSQVYYRYLHNMIAIFDTVEFLKKYPISSHNDI-------------------
consensus/100%                                   ........hs...s.h.p....h........sh..lhhsPh.........hc.h.pp...pl.hDSGua.sQ..p...pph......hY..s.Wsp.ashPD.sP.p.Dp...h..Klp.T...s......h......pshsVhpGpp..pl..Cl..a.....p.hGFGSF.T.G.ps.hN.hs..sh...h..h...h......lH.FGlusss.h..h....h.oFDs.sWb+suuaGplhhPh....pho........h....h...p..o.H.C.hC.p...L..p...R.hHNh.s..p...................................
consensus/95%                                    ........hs...s.h.p....h........sh..lhhsPh.........hc.h.pp...pl.hDSGua.sQ..p...pph......hY..s.Wsp.ashPD.sP.p.Dp...h..Klp.T...s......h......pshsVhpGpp..pl..Cl..a.....p.hGFGSF.T.G.ps.hN.hs..sh...h..h...h......lH.FGlusss.h..h....h.oFDs.sWb+suuaGplhhPh....pho........h....h...p..o.H.C.hC.p...L..p...R.hHNh.s..p...................................
consensus/90%                                    ...ph.sshs.p.u.hpph...h........sh.plhhoPl.....s...hcpl.cp...plhFDSGGa.VQ.sp..hpphh..l..hY..N.Wup.YshPD.sP.s.Ds...V..KlppThp.s.bha.bhP..hb.cshsVlpGpp..Ql..Clp.Y.phshp.lGFGSFsT.G.ps.lN.hs..sh...h..h.p.h.p..h.lH.FGlusPs.l..h....hpSFDo.uWb+suuaGplahPh.p.bNhoa.p.....l....h.ph+.bosHpC.hC.ph..Lp.p+..R.hHNLhs..-..p................................
consensus/85%                                    ..hphhssho.psu.hp+h...h.......pPh.pllhoPl.s...o...h+pl.cp...plhFDSGGYbVQ.sp.pap-ha..lbpaYp.N.WupbYsLPD.VP.o.Ds...Vb.KVppThs.s.hha.bhP..lb.+uhsVlQG+p..Qlp.Clp.Y.phslp.lGFGSFsT.G.ps.lN.hsppuh...h..h.p.hpp.shplHhFGlusPs.l.hl..huhpSFDo.uWb+suGaGplahPF.p.hNhoabp.....l..p.h.phK.bosHpC.aC.shppLppp+..RhhHNLhsh.-..p.l......h..hh...u..Y.pb.........
```

## ; BREX-Related Capture Systems; DUF6079 ATPase Representative Alignment (BrxC ATPase Homolog); ORC/CDC6-AAA-ATPase + wHTH + Alpha+Beta-Domain + 2-wHTH + Extended-Coiled-Coil

```
OrigSeq                                      [illegible]
                                             |------Preceding-Helices------  S1  S2  |------HEH-Insert-Region------|  [illegible]
                                             <<------------------------------ORC/CDC6-AAA-ATPase [illegible]
BAL53915.1_uncultured_prokaryote             [illegible]
MDT7893011.1_Armatimonadota_bacterium        [illegible]
MCS7186064.1_Armatimonadota_bacterium        [illegible]
HEY9694182.1_Oculatellaceae_cyanobacterium   [illegible]
HIE11924.1_Desulfotomaculum_sp.              [illegible]
MDX2239253.1_Leptolyngbyaceae_cyanobacterium [illegible]
HEY9694177.1_Oculatellaceae_cyanobacterium   [illegible]
WP_310922611.1_Halogeometricum_sp._S1BR25-6  [illegible]
WP_338740219.1_Haloplanus_salilacus          [illegible]
WP_147270938.1_Haloplanus_salinus            [illegible]
MYC73120.1_Gemmatimonadota_bacterium         [illegible]
MFN8469223.1_Caldilineaceae_bacterium        [illegible]
RKU21510.1_Candidatus_Poribacteria_bacterium [illegible]
MYB94331.1_Candidatus_Poribacteria_bacterium [illegible]
HET59438.1_Chloroflexota_bacterium           [illegible]
HFD39728.1_Anaerolineae_bacterium            [illegible]
MCZ7554455.1_Anaerolineales_bacterium        [illegible]
MBI5668047.1_Chloroflexota_bacterium         [illegible]
GAB4528661.1_Anaerolineae_bacterium          [illegible]
MBN2393034.1_Anaerolineae_bacterium          [illegible]
MBV6438328.1_Anaerolineae_bacterium          [illegible]
MBP7045036.1_Chloroflexota_bacterium         [illegible]
MBW7959472.1_Candidatus_Promineofilum_sp.    [illegible]
consensus/100%                               [illegible]
consensus/95%                                [illegible]
consensus/90%                                [illegible]
consensus/85%                                [illegible]
```

```
## ; BREX-Related Capture Systems; PglZ Representative Alignment; iSwi2/SNF2-Helicase + PglZ-Phosphatase

OrigSeq                                             [illegible]

                                                    [illegible]

MDS0297782.1_Halogeometricum_sp._S1BR25-6           [illegible]
WP_271955572.1_Halorubrum_ezzemoulense              [illegible]
WP_139229723.1_Halogeometricum_limi                 [illegible]
ATW88247.1_Halohasta_litchfieldiae                  [illegible]
WP_338740220.1_Haloplanus_salilacus                 [illegible]
WP_143414662.1_Halorubrum_sp._SD683                 [illegible]
WP_048108644.1_Methanosarcina_barkeri               [illegible]
NPV62497.1_Methanotrichaceae_archaeon               [illegible]
MCK4444004.1_Thermoplasmata_archaeon                [illegible]
MBI2842128.1_Armatimonadota_bacterium               [illegible]
HHW27504.1_Candidatus_Fermentithermobacillaceae     [illegible]
HIE11925.1_Desulfotomaculum_sp.                     [illegible]
HHW40997.1_Syntrophomonadaceae_bacterium            [illegible]
HQD80871.1_Bacillota_bacterium                      [illegible]
MBE2199248.1_Anaerolinea_sp.                        [illegible]
MBC8263374.1_Anaerolineales_bacterium               [illegible]
MCZ7554456.1_Anaerolineales_bacterium               [illegible]
HID64303.1_Anaerolineae_bacterium                   [illegible]
MBN2393033.1_Anaerolineae_bacterium                 [illegible]
MBV6438329.1_Anaerolineae_bacterium                 [illegible]
MBW7959471.1_Candidatus_Promineofilum_sp.           [illegible]
MFN8469222.1_Caldilineaceae_bacterium               [illegible]
MBI5668048.1_Chloroflexota_bacterium                [illegible]
NLE46402.1_Chloroflexota_bacterium                  [illegible]
RLC84905.1_Chloroflexota_bacterium                  [illegible]
MDX2239252.1_Leptolyngbyaceae_cyanobacterium_bins.302 [illegible]
MDE0397489.1_Candidatus_Poribacteria_bacterium      [illegible]
MYC73121.1_Gemmatimonadota_bacterium                [illegible]
RJP56334.1_Deltaproteobacteria_bacterium            [illegible]
MFZ4828881.1_Phototrophicaceae_bacterium            [illegible]
consensus/100%                                      [illegible]
consensus/95%                                       [illegible]
consensus/90%                                       [illegible]
consensus/85%                                       [illegible]
```

## ; BREX-Related Capture Systems; DUF4007 (BrxA Tripartite DNA binding Homolog); DNA-Binding Component Representative Alignment; (HTH + HTH + wHTH)

```
                                          1---------11--------21--------31--------41--------51--------61--------71--------81--------91--------101-------111-------121-------131-------141-------151-------161-------171-------181-------191-------201-------211-------221-------231-------241-------251-------261
OrigSeq                                   MSTKLHIEGTGRPDAIKSYLDLIERREYNSASAVMEDAYVEFNLTREVDTKGMLTTLTNLGLVEENNQQLTELGEEFIDVLIYNEDAFFDLFHLLYSTAYYRNSNTGISWSYFQISDAYRRRAPTNFRQEVIEEVMNRADPIFNDPGPLSKRSLNSYKRFVEKLVPPVLKDGTFDLRSFAKNELVIAAVDSLYRSDILRYGDRLELSNESREFLSTVLLVYEDDLPELLEHTASMDGRLSIESDYSIRIRLTEEVNIDDLA

                                          <<-------------------------------HTH--------------------------------|----------------------------------|-----------------------------HTH------------------------------>><<-----------------------------------------------wHTH-------------------------------------->>
                                          ------------HHHHHHHHHHHHHH----HHHHHHHHHHHH------HHHHHHHHHHHHH-------|-hhhhhhhhhhhh-hhhhhhhhhhhhhhhhhhh-|-HHHHHHHHHHHHHHHHH-----HHHHHHHHHHHHHHH--------HHHHHHHHHHHHH-----||-----------HHHHHHHHHHHHH-------------HHHHHHHHHHH---HHHHHHHHHHHHHH---EEEE-----EEEEE---------
                                          ------------HHHHHHHHHHHHHH----HHHHHHHHHHHH------HHHHHHHHHHHHH-------|-hhhhhhhhhhhh-hhhhhhhhhhhhhhhhhhh-|-HHHHHHHHHHHHHHHHH-----HHHHHHHHHHHHHHH--------HHHHHHHHHHHHH-----||-----------HHHHHHHHHHHHH-------------HHHHHHHHHHH---HHHHHHHHHHHHHH---EEEE-----EEEEE---------
                                          ------------HHHHHHHHHHHHHH----HHHHHHHHHHHH------HHHHHHHHHHHHH-------|-hhhhhhhhhhhh-hhhhhhhhhhhhhhhhhhh-|-HHHHHHHHHHHHHHHHH-----HHHHHHHHHHHHHHH--------HHHHHHHHHHHHH-----||-----------HHHHHHHHHHHHH-------------HHHHHHHHHHH---HHHHHHHHHHHHHH---EEEE-----EEEEE---------
WP_162551688.1_Halohasta_litchfieldiae    MSTKLHIEGTGRPDAIKSYLDLIERREYNSASAVMEDAYVEFNLTREVDTKGMLTTLTNLGLVEENNQQLTELGEEFIDVLIYNEDAFFDLFHLLYSTAYYRNSNTGISWSYFQISDAYRRRAPTNFRQEVIEEVMNRADPIFNDPGPLSKRSLNSYKRFVEKLVPPVLKDGTFDLRSFAKNELVIAAVDSLYRSDILRYGDRLELSNESREFLSTVLLVYEDDLPELLEHTASMDGRLSIESDYSIRIRLTEEVNIDDLA
WP_310922613.1_Halogeometricum_sp._S1BR25-6 MSNKLHIVSAGRPEAIKRYLDLLDRRDYDTVESVMSDAYHEFGLSQHINTKDMTNTLARLGLVQEDNLALTEFGQELVEVLIHNEQRFYDLLHFVYASAYYRNPKRLISWSYYQITREYCERAPVDFKQEIVEAVLQTAETGFDNYGTLSKKSINNYRRFIDELEPPVLQEGQFVLREFVPSELVVASIDLLYRTDFQDYGGLMSLSGAPAEALQTLCLVQEDSLSDVVEQAVKMDSRLSLKADYTMHVRLSEPVNLNDLA
WP_117592497.1_Haloprofundus_halophilus   MSNKIHIEGDRRPVAMRDYLDLIEQYEYESEEDVITDAYQQ-GYEGEIPTKNMVSTLSSLGIVAESDQELTDFGRDLVDVLLYDEQLFYEMLHFRFSTVYGINPNNMMSWAYYEVSNILYNTAPIESKQDLADDIIYRSDPGFDEPGAFSRKSINGYQKFIEVLEPPVIEGGVIDLRSYAREELIISAIDYIYRSEVLSYGDLLELSNEIEEVIGTVCLLAPQSIDEMIEQTAAGYDSCSVESDHRVRVRLREEVNLSDLT
WP_338740217.1_Haloplanus_salilacus       M-AKLHIEAKHRPEAIKHYLDFIDQRDYDSVEGVMDDVYRETGLD-VVNTKNMQTTLAKLGLVDDSDQQLTDYGRELVEVLLYNDQLFYELLHFTYATAYYRDSDRAISWAYYQICDEFRRRAPVEFKQEIVESVDLTAEEGLSDSGPLSTTSLNNYARFIKELDPDVYDDSEVTLRSFAPTELVLATVDHLYRTDIRNYGDPVELSGDTTEIICNILLLQEEQLPDVIEHVASMDARLSIKSDYQLRVRLSDPVEINDLA
MDW8029828.1_Armatimonadota_bacterium     MRLPFHIQQNLTAERLWVIL-----CCGFDGTVDAAHIAEKCDLAVSVLEKVVLPFVRQLGLIRNTDFSLTDLGEQFYQLGKHSLILLPEAMHHLLYTLHHNDKGKHFSWAYAKVVDALWLSGERNLMAQLVGMVVDEAVQTFNEQIAFSRDSIRGVLNWLRALDPPVVKANKFRRRYFCPIPAFFWGLDFLYRINKIAYGVRMFLTSECIEQLCKVCVLDPSGLENVLMMAKRISDYYGTEGGFGRWLLLASPCPLPKLP
HIE11922.1_Desulfotomaculum_sp.           MRVVLHIQHNIDPPKLAKIL------SGVATGGNILQIARELGIKEDSLPKRMFPFIRQLGMLDSRGCRLTNLGLRCHCLAQH--PLFPEAIHHLLYTGHLLDNTKAFSWAYATVADTLWATGSSVLKAQLVGHVVQEAQQTLGDRIAFSINSVRGALNWLGALSPQVVRANSFSRRFYCEPATFLWAVDFLYRFEGKPYGVRLFLTPERVERLCKLCILDPSGLHNVLLYTKNRSDYFGTEGGFGQWILLTRPCEVPSLP
WP_348254523.1_Leptolyngbya_sp._ST-U4     MKLTLHLQQNTTFERVVSIV-----EFCNQEETDLDELGVTCGLGTSVLQKVIFPFLRSISILSKKNPTLTNLGWVAARIHQENPAILSEFFHSLIYNLHYQEPDKRFSWVYSTVVQQLWQRKEVVLKNSLVSQVIEAATEKFDDEIAFSSNSITGVVNWLRALEPSVVKAEHFNLRYFCAAPAIVKAVDNLYQQGDRTYGVKIFLRESVQDTLCQMLLLDPSGLPGVLDNAKATYDYWGYEGGYGQWVMLNKPVDWLELI
MFA0782867.1_Candidatus_Fervidibacter_sacchari MQLPFHIQHNTTLTVIVKTL-----RFPTNEQITPETLSGALGCAKGTVEKVIAPALRQLGVLDG--WRLSEFGERLKVLGQSHPDLLAEALHVHLYTLHWRQPDAYFSFAYATICDWLWERGTWVLKAELVGVVVNAAAEKFGNQIAFSQNSVHGAINWLKALNPSVIKSGRFSLRDACPVQTLLWSIDALYHHPKRDYGVRIILDDERLTQLCKICLVDLDGLEKMLDFAVRTCSRIGTEGGFGRWLSLTEPIPVGMLW
MBV6438326.1_Anaerolineae_bacterium       MKLSIHVPPDANPDNVSTTISILSENPSFSSSKEMLEYLTSGGVA---TRQELPYIAHAMGILERSKVTISENG---RALSDIREEARGDILHFLMYAGWDAEMTFPQSWGYRNCCDRYWAAHEVQLLDRQVEETIDEAYRTFEDEVAFSRKSLTGAHKWMLALDPPVLKDRVFRRRDFCQPELAVLALAWALRNESTVTGVDILLTTEKREAISKVCLLSPESLDQVLDWAIPTFPEAGTSAGYGRFVRLKRLPSLQDLA
MBI5668045.1_Chloroflexota_bacterium      MKLAIHIPPDANPVNITEMVVAIARDTVFESVRDLLEYTESQGIG---SRTEMQSTAVYLGLLEKSSLGLTRTG---FALAKIREDVRSDLLHYLMYTGWEESVNFLPSWSYRICCDRYWEGQEVTLLNRQVEETINQAREAFAAEVSFSRKSLEGAHKWLGALQPPVIENKVFTRRSFCPPELLLLAIGYVMKDETDATDIDILLTREKRDRICKVCLLDHNALDRALDWMLPIFPAPGTSAGYGRFVRLHKLPTLEDVV
GAB4528652.1_Anaerolineae_bacterium       MRLTFHVPQEGEPDKLIPLLEAMYAGMRFPTVKALLEFAREQGLG---GRTEMQILASACGLLDTDGDGWIIMSETAKVIVQLKPEVRPDLIHYLLYTGWQPESANTILWSYREITDSLWEKPSANVANVVAEEVRNRTQQVFIGGVSFSPKSIRGVRKWLEAVTPPVIEDDLFARRHFCPPELALLATGWVAQAMGGEIGIDFLLTPECREAICRVCLLELSALDRVLDWMLPLYVEPGTSAGYGRFLHFLKWPQVSDLA
HET59436.1_Chloroflexota_bacterium        MDFVFHVR-NAQPGDMIAILATLEDYPGLSTIAEIVDQADTLGFTRDRARLEALMTARELDFVEMDKNLLTDKGKALVRLEMNKPDLFPDIVHGYQYTLWDKKSIHCFSWTYREICKHLWQLGSYHMKKDLTSEIEGLARTHFESDIALSAKSVGGAFLWIEELVPGVIPSELFARRTFCPPELFILGVNFVYRGNEMDYGTNLLLGEEPRNTICQICLLEPDGFDRVLDYATAQFD-KGIGGGWGQYLTLYRAPVVEDFV
MCZ7554453.1_Anaerolineales_bacterium     MASSFHVR-NAQPDDLLAALTVLQAYPGLSSVADIAVRAETMGLSRDRLRLEALMTARELGLVHGDSNELTLDGEKALELEGQKPELFGDIVHGLQYMLWVPFWEHCFSWTYQAVCRSLWRQVRVEV---LASELEVEARTQFANDIALSGKSVRGVLMWLRELRPRVIEDDNYLSRHFCPPELFLLALDYVYRSEVADYGTNLLLNEERRDAICQMCLLDTEAFDRVLQYAVGQFP-RGIGGGWGHYMSLERRVTLGVLG
RKU21509.1_Candidatus_Poribacteria_bacterium M-ARIHIL--RGFSDLDGALWVLKYHPELRTRSEIVATILENGLISDRTRFEILQLLQEWGIAESNGYQLTLKGKHFYSLWEMKPEIAIDVLHGLQYNLWTKHDQNQASWAYQRVCDYLWEYQILPEENDLTAYVYEMREENLGIANAFSSKSVKGAYDWLLPLDPPVLRRASFTRRTHCASALFLMGLSWVSRESEIGFGELVELTDERRIDVCRFCLIEESSFDIMLNQALTRFD-MQRIGR--LFVIIQREPQIADF-
MYD63886.1_Gemmatimonadota_bacterium      M-ARSHIL--KGFSDLESALWIHKYHPGLTIRRQMVKVALENGYSTDSARYEAVQLLSEWGLADGKSGSLTPEGQLFYSLWETKRSTAIDFLHGRQYGLWTQNDQNIASWAYKNICDYLWTRQTLPDKGDLVTYINDLRAEVVIMGNAFSNKSINDAYDWLRPLDPPVLNGATFAQRAFCSTLLFLMGLGYIVRETNTVFGDLVKIDDTRKQAVCCFCLISEIGFDLMLDEALRQVS-VQRGWD-GIYMELNQKPEVRDFI
MYB94330.1_Candidatus_Poribacteria_bacterium M-SRIHIL--KGFSDVDSALWVLKYHPGTRSRADLVQIVRDNGYTANKD-YEIILLLTAWELVQRKPQGLTQKGMAFYHLWEVKRDVAIDVLHGLQYGLWTEHTQNTESWSYREVCNYLWDSDALPEKDDIISHIYSSLSLEIDEKAAFDGKSVNHAYDWLLPLSPPVLRNATFTRRSHCSTALFLMGLDWVARESGIIYGDLWMIGEEQRRQVCRFCLIDETRFDFMFNETLRRFSVVRRSGG--LYIVINNEPQISDF-
TEU15948.1_Anaerolineales_bacterium       MKEDVHFLDNAPPAEVEYVLACLELDIAYSTEDLRKRLLSEWGYSAQRNLTFSTRRLLDLGLADKANHVITQLGEKVRGILSIDRELYTEIMHFLHYTSYDGSSSRKLLWSYRTCCEIVWARKQVPPTPELVAEVQSRIAELFPVGGNFNAGGVSGWKPWLAQLQPSPFEGRALVPRHSHRLELAALALDHVYRSRGYRYGDPVILDEPLLDEIARVFFLDPICCRELIDLAARLTKASDTFTG--TSATLLAPYDVERL-
RJP56331.1_Deltaproteobacteria_bacterium  MFEDTHFYDNASPVEVTNVLRVLSPSHEFTPKEISDTLRDSYGFSMQKDYTLSPRRLFDLGLTGQNKYTLTELGSKVQNILGIDPALAMDLLHYLHYSGYTGKTQRKYLWSYRRCCEIVWGNMRALRSTELASTIQSEMREKFDTGARFNDKAAVQVYSWLRSLEPSPFSSSTVTPRKIDRYEIALLALDEAYQTSDYTYGDPVIMDNEFIKLVSGVFMLDPECCKGLLGIGARINQSSETLSG--ASINLMRPFKIDSI-
MBI2842131.1_Armatimonadota_bacterium     MEEDTHFYDSAPPDEVTRVLRVLAADVCLSPDEIADTLDSQYGEPMQRDHTYTPRRLFDLGLAEQMRYCLTSRGLRVQTVQASDAAFARDLLHYLHFSGYGGSTDRKYLWSYRRCCELMWANGKMIPAVELAALIQAEMVSAFPAGGRFNHKAASCVSSWLGCLTPPAFRGRHVQPRIVDRYELAALALDDVYRSRRYRYGDAVVMDDSLLNQVAGVFFLDRGCCANLLRLAASITPSSDTLAG--PSVNLTRGFTIEDL-
consensus/100%                            M....Hh........h...l......................s.............h..h.h.......h...u.....l.........-hhH......a.........asY..hsp.h.......................h.....hs..uh..h..ah..l.P.sh....h..R.......hh.s.s.h.p......s..h.h.......l..hhhl....h..hh...................h.h.....h..h.
consensus/95%                             M....Hh........h...l......................s.............h..h.h.......h...u.....l.........-hhH......a.........asY..hsp.h.......................h.....hs..uh..h..ah..l.P.sh....h..R.......hh.s.s.h.p......s..h.h.......l..hhhl....h..hh...................h.h.....h..h.
consensus/90%                             M....Hh........h...l....................p.sh............h..hshh......lo..G.....l...p.....-hhH....s.a..p......WsY..hsp.hb......................h.....hs.puh..h..al..L.P.shp...h..R..s....hh.uls.hhp......G..h.hs....p.ls.hhhl....h..hl.............ss....h.l.p...l..h.
consensus/85%                             M....Hh........l..hl.........p......p...p.Gh............h..hulhp.....lo..G..h..l...p..h..-hhH...hs.a..p......WsY.phsp.hh.........plh..l.......h....shs.pulpsh..al..LpPsshps..h..R..s...hhhhuls.lhp.....hG..hbls.p..p.lsphhhlp..sh..hl...s....s....us....h.l.p...l.ph.
```

```
## ; BREX-Related Capture Systems; STAND-NTPase Representative Alignment; (Enzymatically Active)

                                                1---------11--------21--------31--------41--------51--------61--------71--------81--------91--------101-------111-------121-------131-------141-------151-------161-------171
                                                MSRLVNFQNRIRRFFSTQWLTTNQKQTYDRLRQLLSFQDSVNLYGQSGVGKTFLIWVLSKEVSTDFYSNWEQLEEKEVDSDSIAVVDPHPWRRDAVRQTLSTLHRLGYRKIIIVSDEPVADQIPSCQLTLAQSDLGKIYQNWSEVSIPVDELPESFNGVNLHQALCSIAL

                                                                                        S1                      S2                 S3                         S4             S5
                                                --HHHHHHHHHHHH--HHH--HHHHHHHHHHHHHHHH--EEEEE------HHHHHHHHHHH--EEEE---------------EEEEE----HHHHHHHHHHHHHHH---EEEEEEE---------EEE----HHHHHHHHHHHHHHH----------HHHHHHHHHHH---
                                                --HHHHHHHHHHHH--HHH--HHHHHHHHHHHHHHHH--EEEEE------HHHHHHHHHHH--EEEE---------------EEEEE----HHHHHHHHHHHHHHH---EEEEEEE---------EEE----HHHHHHHHHHHHHHH----------HHHHHHHHHHH---
                                                --HHHHHHHHHHHH--HHH--HHHHHHHHHHHHHHHH--EEEEE------HHHHHHHHHHH--EEEE---------------EEEEE----HHHHHHHHHHHHHHH---EEEEEEE---------EEE----HHHHHHHHHHHHHHH----------HHHHHHHHHHH---
HEY9694178.1_Oculatellaceae_cyanobacterium      MSRLVNFQNRIRRFFSTQWLTTNQKQTYDRLRQLLSFQDSVNLYGQSGVGKTFLIWVLSKEVSTDFYSNWEQLEEKEVDSDSIAVVDPHPWRRDAVRQTLSTLHRLGYRKIIIVSDEPVADQIPSCQLTLAQSDLGKIYQNWSEVSIPVDELPESFNGVNLHQALCSIAL
HQD80873.1_Bacillota_bacterium                  VGKWFEIHNLIKARCSSSWLTPSQQKALRTIRELTSTFGRCNLHGNVGVGKTFVAWVLSKEHGLIYFPTPNLLLDVVGTHGKFAIIDNYSERTLSYRKLAPEISIRGFLGFILISREPIADHIHKVELTLTEADYVWVNRTLENLGITVTYVGGLWRNISP---------
MFA0782864.1_Candidatus_Fervidibacter_sacchari  MNRFVALLNRIRKEADWGWLTESQREASEQLRDFLGVSDVVNLFGFHGVGKTFLAWVWQKEGRIAYFPSVRLVMPV--ELHRLAIVDNLPSDRTSVRDALRKCRFCGFQRVILITTYSADDQIPKVRLNLTEQDAKQVSEQLRQLGYPPDEPRNLWELVVPFDFV-----
RKU21511.1_Candidatus_Poribacteria_bacterium    MNWLTNHKRLSRLAETDETLTPSQLSARDVLLDTIHEETRINLWGGPGTGKTFLAHYLHHRADVIYFSYQHHYDRR-VSQHSVVAIDNAPYIRQEARGLYDSIRWGDGPKVILITRKPIADAVRRIELTLTDTDIVHIENIIQQFGESDRQPSGLWKNL-----------
WP_348254525.1_Leptolyngbya_sp._ST-U4           MSRLVEFRNRIYHRFRREWLSPSQEQAYTRLKNLLVFQDAINLCGSSGSGKTFMTWVLAKEGPVCFLTQPKQIVEQSVAENTVAIADPHSPERSAIRATLTQCRQFGCRKVIFVSDEPVADQIPICQLFLTKRDLEKIRQNWESIAIPVQELQEATENLNLHMALREIVL
MYD63885.1_Gemmatimonadota_bacterium            MHWQSYHRKLLALAETGETLTTSQQAVCEFLHQAIEYQQRINLWGSPGVGKTFLAHYLHYCAEGLYFFSPENCPSTEISPNSVVIVDNAPSGRSLARSTFGNVLWAGASSVILVTRQPIDDAVWRIELALTDRDRAQLEGVMKLFAHLPHYRSGIWKVL-----------
MBP7045035.1_Chloroflexota_bacterium            MSQLLTLLNRLKAEARLERLTVSQQTAWHNIQQQLRFPERINLYGAAGSGKTFLAWALANEQNAAYFVSPEVLFQSNFMNESLIIVDNGVSDTGELRRLLAELQMRNGRSTLIITRHANQIGLPLVHLPLMSQDITITYRNLSLLEKYALTEGNMWQIIMSTLTA-----
MBW7959473.1_Candidatus_Promineofilum_sp.       MSRLLAAHNHLKAQADIDRLASHQRQAYESIINQWRFPTWLNLHGSAGAGKTYLGWVVAESQGAHHLSTPEQLGKATIRPGQALVIDNANSDSYVLRQLLATLDLYNIRRVLIITCVSNTNILPTVNLVTTAEDIELVRRNLKDAGYYSTQHTNLWRIIHSTLV------
BAL53914.1_uncultured_prokaryote                MSRFVDTLNRIRQSMKPEWMTPGQRAAYDLLRERLRFLDEVNLWGGPGVGKTFLGWVLHVQGLAIYMPLLARVEEELPLPRTTVIVDNLGWRREEVRQALHLCRSKGYEKVVLITSEPAQEQMAIVELRLTEEDIEKVKANLLGISVVPDTPRNLWDLVSPMPLWE----
HET59437.1_Chloroflexota_bacterium              MSEFIRLLNQIKTHRNRAWLTPSQVNAMRALQKTLRIPGTVNLFGSVGVGKTFLGWSLAEEMRYVYLAHIDCLADLQDQEIIGLIVDNCAPDRESHRNILKHLSFRKIRYAVLITQQMIGDYTSYVELELEAQDWDRVVENLKSIKVFRSSASNLWQLLNPYLSQG----
MCZ7554454.1_Anaerolineales_bacterium           MSGFVRNLNRIKTLCNQDWLSPSQKAALDTLTQGILYPGTTNLWGPAGVGKTFLAWILQQETGAEYVPHISAVATSREMRGCTMIVDNCSPDRISHRNTLNQLRLANVSSAILVSRELIQDYTSHVELVCSEADLNHIRSVLVPLGIFTEDISSLWYVVNPYFALD----
HFD39729.1_Anaerolineae_bacterium               MSKFVRTLNSIKTHRCAEWLTPSQSRALAALRRELHAPGTVNLYGPAGVGKTFLAWILADELDLTYFPHLEYLAQAERVTASGVIIDNGQSSRQAHRNTLKVLQLEGVVRAVLITRELVHDYTRYVELSLLPEDKEKASLNLATIGHPAAQVPNLWHLVNPYLGEV----
MBN2393037.1_Anaerolineae_bacterium             MSRLLVLLNRLKAEARHDWLTESQAATLSEIERLWRFPERINLYGPPGAGKTFLAWSVARALNAVYYPSARVYHIASVRDQARVVIDNAPDDVVALRRLLAALQLNKARTALMITAQPGRLGLPAVGLPVTPQDVDTIYRNLSLLDYYAAYDANLWVVVMQVL-------
MBN1891044.1_Thermoflexales_bacterium           MISRTEVENQIKAQSDASWFFPSQVVAYQELLPFLGLHRVVNLYGAQGVGKTFLAHTLCKENRIDYVSSPDLIRSA----SHPLAIDNAPFDRTAVRGMRNQARKFDLRQVILITRYRVEDTIPSFALSLTPEDIRCFRATLRSLDLRLCAALNLWKLI-----------
GAB4528656.1_Anaerolineae_bacterium             MSRMLCLLNRIKAEARRDLLTGNQLIAFEEIKRLWQFPERVNLWGPPGSGKTMVGWVVGRSLHATIYSSPDTFRERSQYGEARVIVDNVPYDQHALRTVLAEMQLKNIRTALLITIQPNRLGLPTVALRPTTEDIDVLYRNLSLMEHYALREGNFWDIIYSVL-------
MBC8263376.1_Anaerolineales_bacterium           MSDLIRALNLIKTRRDPAWLTVSQQKALAALQEALRVPCTVNLFGATGVGKTFLAWTLADELGYTYFPHLDHFERIEDLHTTGVVLDNCRPDRRTHRHTLKTLSFQNVRYAVLITRQMIQDYTHYVELALTPADRTKAWDNLATLGIFRKELPNLWHLVNPHL-------
WP_147270937.1_Haloplanus_salinus               EADLLTRMNTLKQVDIKQHLTSTQKGALEEIRQHREGEPFINLHGPRNSGKTFLCWALRE-HGWKYQQAHFQRVTE---DVTAVVYDHGKPDRSTTRQLRNNVDLSGLSNVVYVTKSPAKEVYPRVHLSVDEPHYQSVAANWDTLGLDTANLPSVDATVNTNTNTN----
WP_338740218.1_Haloplanus_salilacus             EAPLLRQTNVLKEAADESLLTDAQKDARDAIRQHREGARFINLHGPRHAGKTFLCWVLHLSEGWDYYQTGSKSVDT------TVLYDHGEPERRETRKLRNQASINGLATIVYVTDRPADELYPRVELWPEDEHYQQIANTWDDLGLDTENAPTPIQQ-----------
WP_232571133.1_Halobacterium_litoreum           NALLLQRMNALKDAASVERLSAAQQEAMDQIRKHRDDARFINLYGPEEAGKTFLCWALREAEDWEYHPQMPESADE------VVIYDHGEADRMATRNLRNHASINGLATIVYVTHRPAEEVFPRVELAPGEDHYQTVRANWEALGLSVEHAPTM-------------
consensus/100%                                  ...b....p..........h...Q..s...l..........NL.G...sGKThh.a.h.........................hhhD.........R.....h........lhlo..........h.L.....c.......h..h..........................
consensus/95%                                   ...b....p..........h...Q..s...l..........NL.G...sGKThh.a.h.........................hhhD.........R.....h........lhlo..........h.L.....c.......h..h..........................
consensus/90%                                   ...b....p.lb...p...hs.sQ..s...l.p.b.....hNLaG...sGKTalsa.l.......a.................hhhD.....p...R..b..hpb.....hlhlo..........h.L.....c...h...h..h..........................
consensus/85%                                   bs.h....N.l+...p...LossQ..sbp.lbp.b.....lNLaGs.ssGKTFlsa.l.....h.ah.......p........hlhDp...pp...R..b..hpb.sh..hlhlop.........h.L.....cb..h..sh..h.......sh....l............
```

# ; Alpha+Beta-Domain + wHTH (BrxC-ATPase Type-1 BREX)

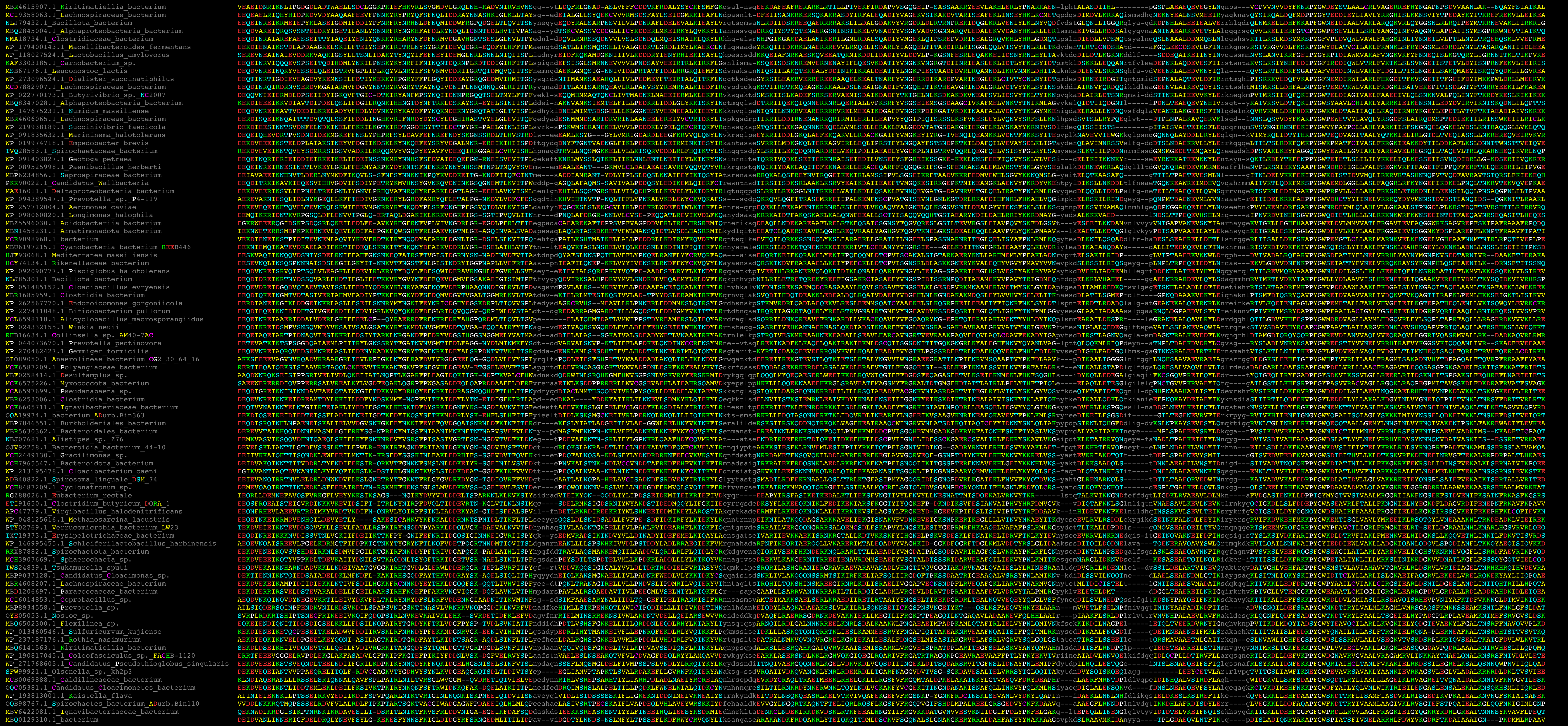

## Type-1 BREX BrxC Alpha+Beta-Domain + wHTH Entropy Plot

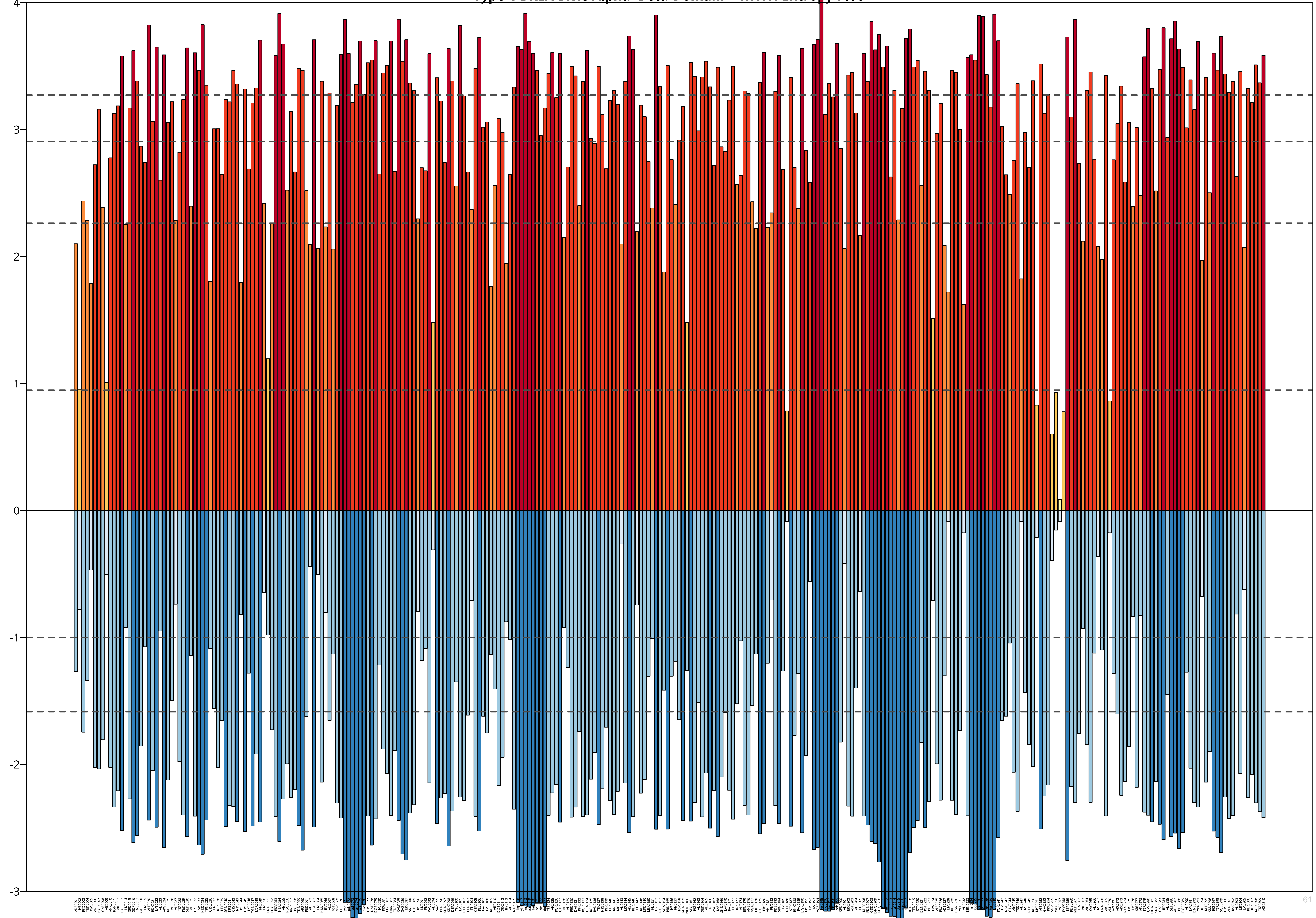

```
# ; Alpha+Beta-Domain + wHTH (BrxC-ATPase Type-3 BREX)

WP_164569833.1_Rhizobium_leguminosarum                DVDYDAEI[illegible]
SDB48716.1_Belnapia_rosea                             DVDYDAQI[illegible]
MBA4157055.1_Gemmatimonadota_bacterium                TVDYDAQI[illegible]
WP_190509459.1_Oscillatoria_sp.                       TTDYDAQI[illegible]
RJQ83728.1_Desulfobacteraceae_bacterium               TDDFDALI[illegible]
WP_013219148.1_Nitrosococcus_watsonii                 TDDFDALI[illegible]
GHU24474.1_Spirochaetia_bacterium                     TEDFDTLI[illegible]
WP_209691006.1_Methanomicrobium_sp.                   TEDYDAII[illegible]
NMB01453.1_Bacillota_bacterium                        TVDFDQII[illegible]
MCK9328444.1_Candidatus_Cloacimonadota_bacterium      TFDFEQTI[illegible]
WP_047754172.1_Kosmotoga_pacifica                     TIDFVALI[illegible]
MCB5228480.1_Candidatus_Cloacimonadota_bacterium      VDDFDAII[illegible]
OBW63497.1_Dehalococcoides_mccartyi                   VEDYEARI[illegible]
MBX3742594.1_Akkermansiaceae_bacterium                TDDYDALV[illegible]
MBT3747344.1_Bacteroidota_bacterium                   DIDYDEKI[illegible]
WP_106343732.1_Planifilum_fimeticola                  GVDVERLI[illegible]
WP_254843978.1_Virgibacillus_dokdonensis              SIPVDELI[illegible]
WP_235850150.1_Niallia_circulans                      DIDVDSLI[illegible]
WP_146342557.1_Dellaglioa_algida                      DIDIESLI[illegible]
WP_229736080.1_Kroppenstedtia_guangzhouensis          DIAVDDLI[illegible]
MCP4110753.1_Desulfobacteraceae_bacterium             GVDPEQKI[illegible]
NUM38593.1_Armatimonadota_bacterium                   DRDYEQEV[illegible]
MCM1566961.1_Dehalobacter_sp.                         DKDYEQDI[illegible]
MBU0460852.1_Nanoarchaeota_archaeon                   TQDYDVII[illegible]
KFI36375.1_Peptococcaceae_bacterium                   TVDYDQVI[illegible]
EMR73852.1_Thermoplasmatales_archaeon                 SIDYDEVV[illegible]
WP_114474457.1_Halanaerobium_sp.                      SVDYDVVI[illegible]
WP_098658108.1_Bacillus_toyonensis                    VQDFDVLI[illegible]
MCL4298469.1_Anaerolineae_bacterium                   IDDYDAII[illegible]
MBP7946969.1_Verrucomicrobiota_bacterium              QVNPVKFF[illegible]
MBT9159326.1_Dehalococcoidia_bacterium                GLSAPQRF[illegible]
NLF03150.1_Anaerolineales_bacterium                   PLAVLDLF[illegible]
GFP32117.1_Candidatus_Hakubanella_thermoalkaliphilus  EAKPAQIF[illegible]
HIH77326.1_Halobacteria_archaeon                      NSEVIKTF[illegible]
MBP6974014.1_Syntrophorhabdus_sp.                     DVDYDKKI[illegible]
HHW29753.1_Syntrophomonadaceae_bacterium              GEDPDVLI[illegible]
MBT6646600.1_Nitrososphaerota_archaeon                NIDYNAKI[illegible]
WP_053959405.1_Sulfobacillus_thermosulfidooxidans     MVDYDGML[illegible]
WP_020614466.1_Sediminispirochaeta_bajacaliforniensis GKDPNQEI[illegible]
WP_149544905.1_Calorimonas_adulescens                 DIDYDAKI[illegible]
NMA94940.1_Clostridiales_bacterium                    DIDYDVEI[illegible]
WP_209451817.1_Lactimicrobium_massiliense             VVDYDEKI[illegible]
EGD52432.1_Thermoanaerobacter_ethanolicus             GINYDAKI[illegible]
NLV27395.1_Methanomicrobiales_archaeon                SVDIDLLI[illegible]
```

## Type-3 BREX BrxC Alpha+Beta-Domain + wHTH Entropy Plot

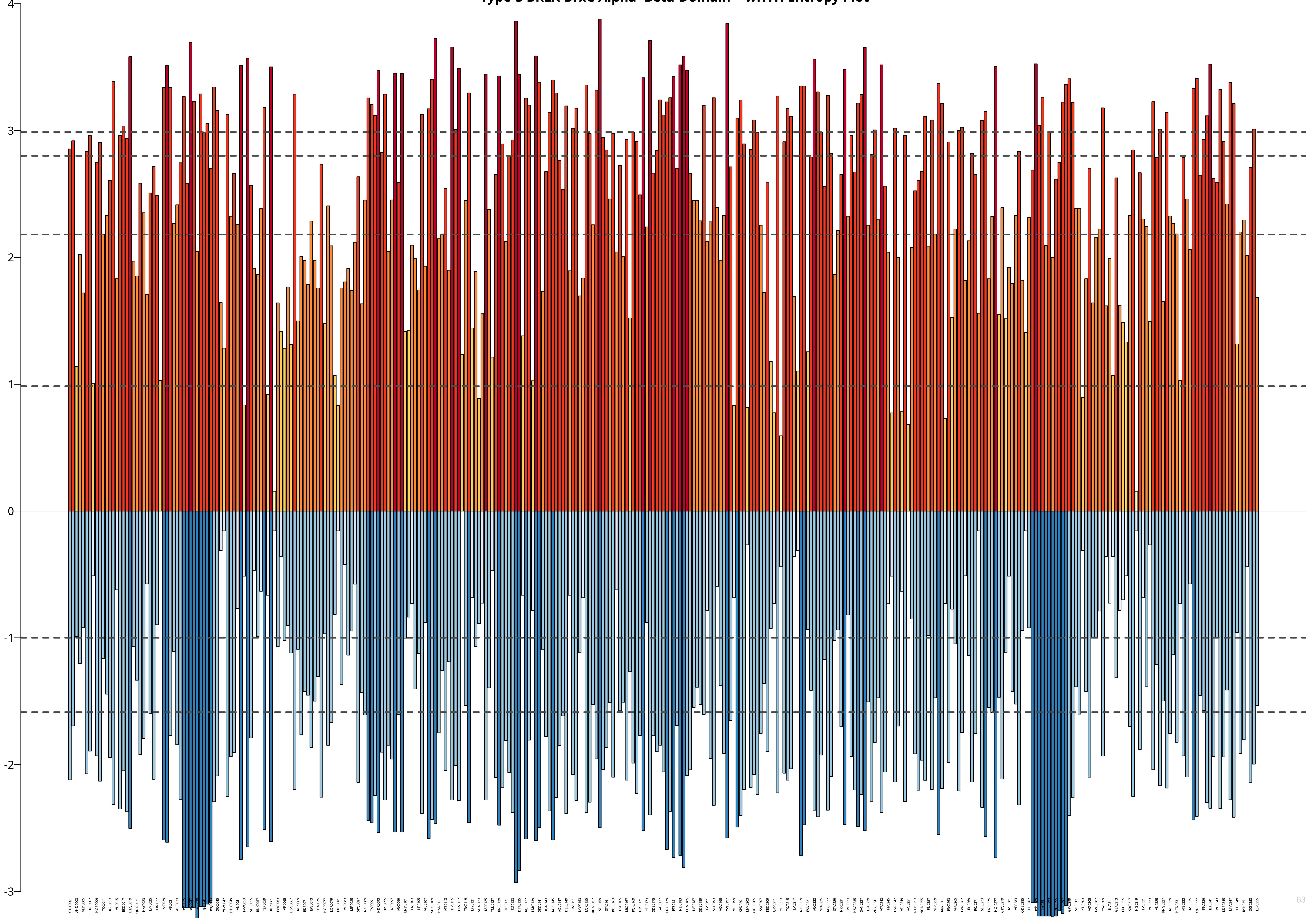

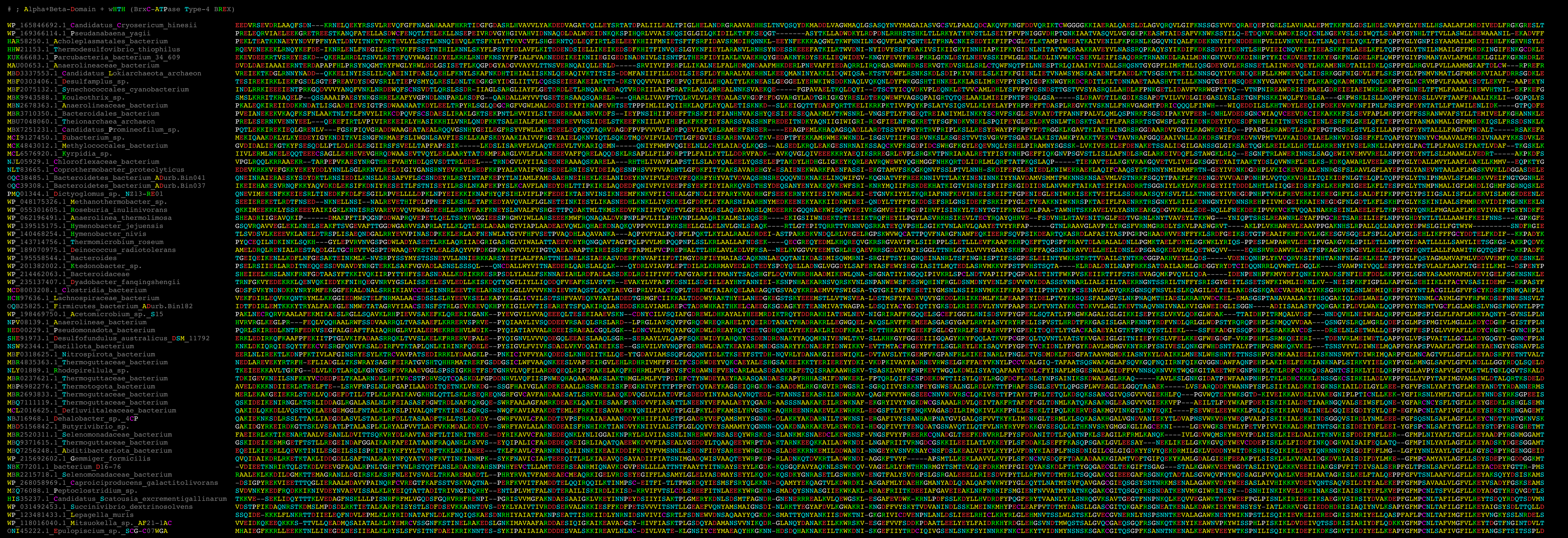

# ; Alpha+Beta-Domain + wHTH (BrxC-ATPase Type-4 BREX)
WP_165846692.1_Candidatus_Cryosericum_hinesii
WP_169366114.1_Pseudanabaena_yagii
HAR58250.1_Acholeplasmatales_bacterium
HHW21153.1_Thermodesulfovibrio_thiophilus
KUK66683.1_Parcubacteria_bacterium_34_609
MAU00653.1_Anaerolineaceae_bacterium
MBD3337553.1_Candidatus_Lokiarchaeota_archaeon
MBF0303406.1_Desulfamplus_sp.
MBF2075132.1_Synechococcales_cyanobacterium
MBK9943588.1_Kouleothrix_sp.
MBN2678363.1_Anaerolineaceae_bacterium
MBR3710350.1_Bacteroidales_bacterium
MBU7048060.1_Theionarchaea_archaeon
MBX7251231.1_Candidatus_Promineofilum_sp.
MCI9127450.1_Eubacterium_sp.
MCK4843012.1_Methylococcales_bacterium
MCL6576920.1_Kyrpidia_sp.
NJL05929.1_Chloroflexaceae_bacterium
NLT83665.1_Coprothermobacter_proteolyticus
OQC38485.1_Bacteroidetes_bacterium_ADurb.Bin041
OQC39308.1_Bacteroidetes_bacterium_ADurb.Bin037
PMQ01344.1_Dictyoglomus_sp._NZ13-RE01
WP_048175326.1_Methanothermobacter_sp.
WP_055301605.1_Roseburia_inulinivorans
WP_062196491.1_Anaerolinea_thermolimosa
WP_139515175.1_Hymenobacter_jejuensis
WP_140468254.1_Hymenobacter_nivis
WP_143714756.1_Thermomicrobium_roseum
WP_189070975.1_Deinococcus_radiotolerans
WP_195558544.1_Bacteroides
WP_201382002.1_Ktedonobacter_sp.
WP_214462063.1_Bacteroidaceae
WP_235137407.1_Dyadobacter_fanqingshengii
MCD8003208.1_Clostridia_bacterium
HCH97636.1_Lachnospiraceae_bacterium
OQB25825.1_Firmicutes_bacterium_ADurb.Bin182
WP_198469750.1_Acetomicrobium_sp._S15
NPV08139.1_Anaerolineae_bacterium
HED00229.1_Pseudomonadota_bacterium
SHE91973.1_Desulfofundulus_australicus_DSM_11792
NSW92344.1_Bacillota_bacterium
MBF0318625.1_Nitrospirota_bacterium
MBR4835363.1_Thermoguttaceae_bacterium
NLY01889.1_Rhodopirellula_sp.
MBR0237621.1_Thermoguttaceae_bacterium
MBP6982276.1_Thermotogota_bacterium
MBR2693833.1_Thermoguttaceae_bacterium
MBQ7111119.1_Thermoguttaceae_bacterium
MCL2016625.1_Defluviitaleaceae_bacterium
NBJ16968.1_Dehalobacter_sp._4CP
MBD5156842.1_Butyrivibrio_sp.
MBR2520311.1_Selenomonadaceae_bacterium
MBQ9371615.1_Thermoguttaceae_bacterium
MBQ7256248.1_Abditibacteriota_bacterium
WP_215692602.1_Gemmiger_formicilis
NBK77201.1_bacterium_D16-76
MBR2215718.1_Selenomonadaceae_bacterium
WP_268058969.1_Caproiciproducens_galactitolivorans
MZQ76088.1_Peptoclostridium_sp.
HIS35237.1_Candidatus_Scatousia_excrementigallinarum
WP_031492453.1_Succinivibrio_dextrinosolvens
WP_123481433.1_Lepagella_muris
WP_118016040.1_Mitsuokella_sp._AF21-1AC
ONI45222.1_Epulopiscium_sp._SCG-C07WGA

Type-4 BREX BrxC Alpha+Beta-Domain + wHTH Entropy Plot

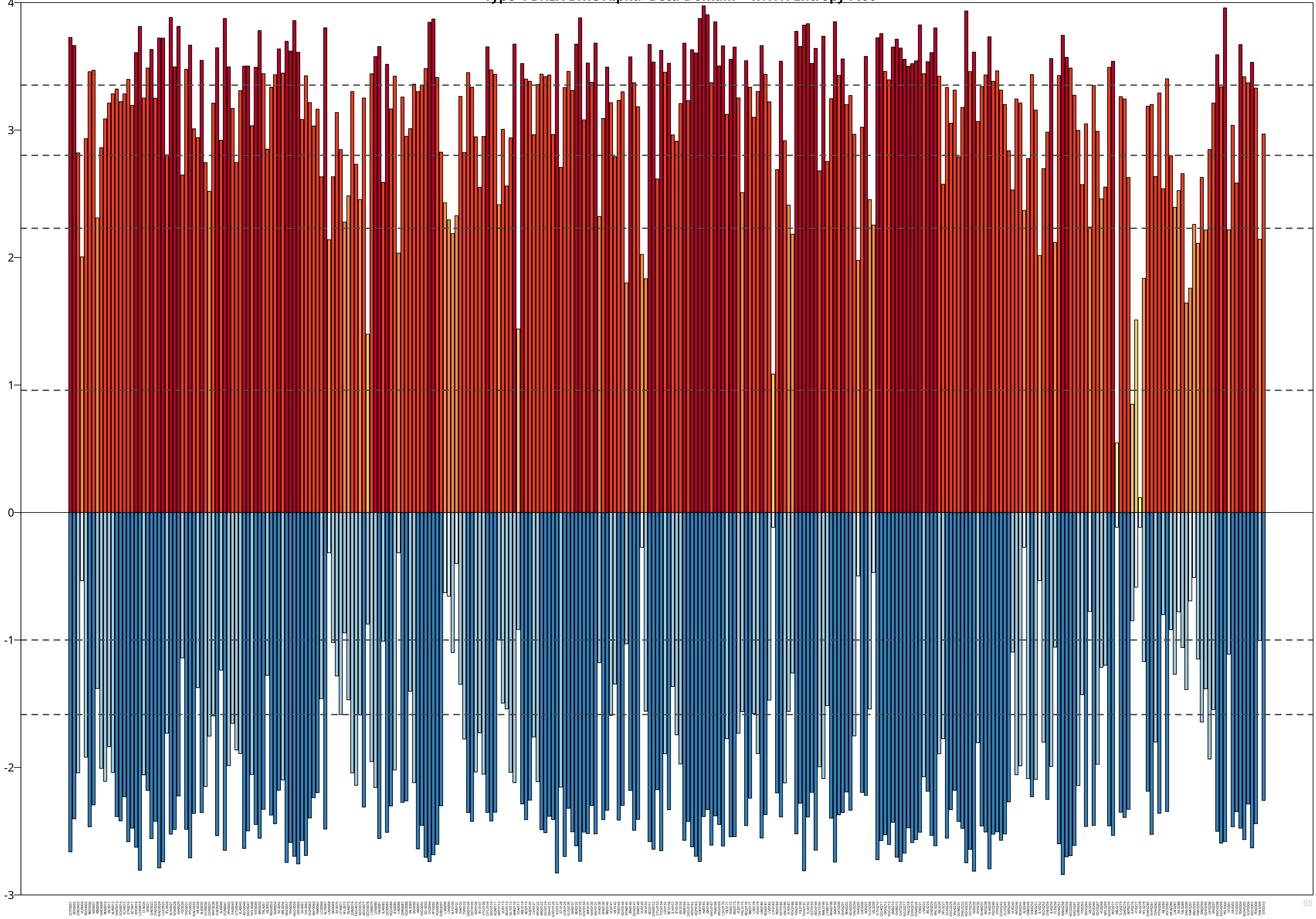

# ; Alpha+Beta-Domain + wHTH (DUF499-ATPase BR-systems)

WP_148590534.1_Aquisphaera_giovannonii
MBQ9433643.1_Synergistaceae_bacterium
WP_054571357.1_Frankia_sp._R43
EEO54231.1_Bacteroides_sp._2_2_4
MBM4287682.1_Deltaproteobacteria_bacterium
MBI3467145.1_Planctomycetota_bacterium
WP_259054315.1_Salinibacter_ruber
MBQ9726423.1_Kiritimatiellae_bacterium
CAI1753142.1_Serratia_quinivorans
MCL0064885.1_Dehalococcoidia_bacterium
MCD6101177.1_Candidatus_Marinimicrobia_bacterium
WP_116051372.1_Pseudonocardiaceae
RME04985.1_Planctomycetota_bacterium
MDE0129995.1_Gammaproteobacteria_bacterium
PJF35651.1_Candidatus_Thermofonsia
MBQ3918676.1_Oscillospiraceae_bacterium
WP_157574125.1_Nocardia_jejuensis
MYJ80998.1_Acidimicrobiaceae_bacterium
TME11450.1_Chloroflexi_bacterium
WP_247297741.1_Bradyrhizobium_sp._179
HEC67877.1_Candidatus_Desulfofervidus_auxilii
MBR0288712.1_Selenomonadaceae_bacterium
MBM4285998.1_Deltaproteobacteria_bacterium
NJK30457.1_Acaryochloris_sp._SU_5_25
WP_048201423.1_Methanocaldococcus_bathoardescens
MBF0121417.1_Desulfobacterales_bacterium
MCS6856320.1_Sandaracinaceae_bacterium
MBT7692838.1_Gemmatimonadales_bacterium
MBU2110960.1_Actinomycetia_bacterium
UGS34698.1_Solirubrobacterales_bacterium_0166_1
RIK37466.1_Chloroflexi_bacterium
MXV84967.1_Candidatus_Poribacteria_bacterium
MBC7327984.1_bacterium
WP_013295752.1_Methanothermobacter_marburgensis
NPV88406.1_Coprothermobacteraceae_bacterium
PWV37728.1_Desulfurococcaceae_archaeon
KEQ56705.1_Marine_Group_I_thaumarchaeote
MBC8384565.1_Candidatus_Cloacimonetes_bacterium
NET07313.1_Symploca_sp._SIO2B6
MCX7997938.1_Leptospiraceae_bacterium
WP_244804062.1_Corynebacterium_sp._LD5P10
WP_005520661.1_Corynebacterium_matruchotii
MCL5447091.1_Actinobacteria_bacterium
WP_052687173.1_Streptomyces_lydicus
WP_053587850.1_Actinomyces_sp.
WP_270528574.1_Mitsuokella_jalaludinii
MCV7506294.1_Micrococcus_luteus
MCY3894210.1_Acidimicrobiaceae_bacterium
WP_238518868.1_Azospirillum_doebereinerae
HBM97560.1_bacterium_UBP9_UBA11836
MBC6439887.1_Rhodospirillales_bacterium
OZG52261.1_Pseudoscardovia_radai
MCF6470725.1_Nonomuraea_sp._MG754425
MXX78159.1_Gemmatimonadales_bacterium
MCI1646461.1_Olsenella_sp.
WP_094451589.1_Parenemella_sanctibonifatiensis
MCG3151370.1_bacterium
WP_092673506.1_Siccationidurans_arizonensis
WP_201387653.1_Ktedonobacter_sp._SOSP1-52
MBD2550060.1_Microcystis_elabens_FACHB-917
WP_188816795.1_Calditerricola_satsumensis
MCL1790896.1_Peptococcaceae_bacterium
MCY4445744.1_Rhodobacteraceae_bacterium
WP_127367955.1_unclassified_Mesorhizobium
MCZ0940408.1_Caldilineaceae_bacterium
WP_132287513.1_Paracraurococcus_sp._NE82
CAG0999165.1_Planctomycetaceae_bacterium
WP_151650193.1_Bradyrhizobium_betae
MDE2480824.1_bacterium
HDQ14361.1_Sediminispirochaeta_sp.
MYD10935.1_Chloroflexi_bacterium
WP_075830034.1_Deinococcus_marmoris
MYA05496.1_Caldilineaceae_bacterium
WP_106140933.1_Spirosoma_oryzae
WP_118201547.1_Prevotella_copri
WP_051018640.1_Saprospira_grandis
RKU15614.1_Candidatus_Poribacteria_bacterium
MCY4474618.1_Chloroflexi_bacterium
MDE0020779.1_Candidatus_Poribacteria_bacterium
MCA9831553.1_Dehalococcoidia_bacterium

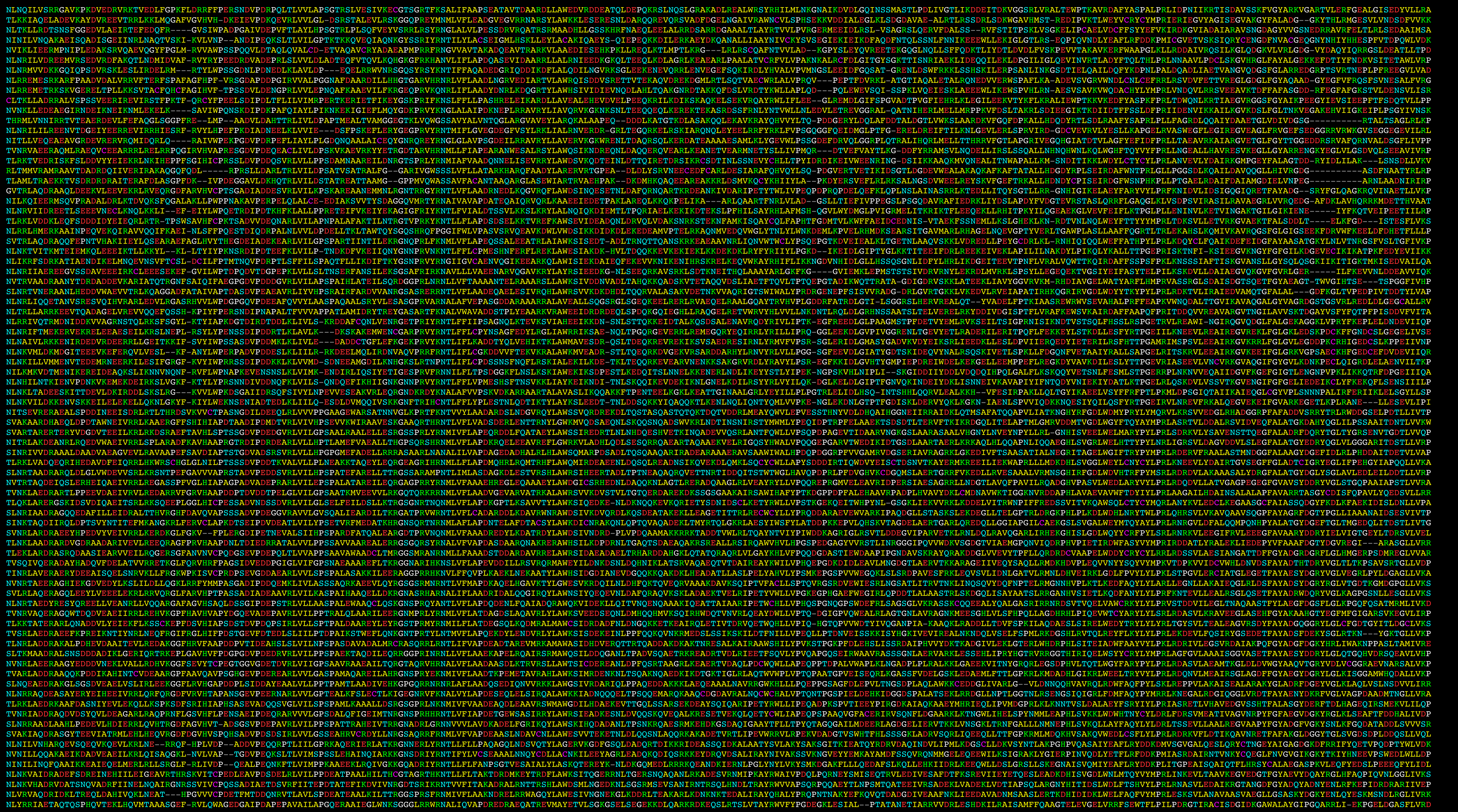

**BR-system DUF499-ATPase Alpha+Beta-Domain + wHTH Entropy Plot**

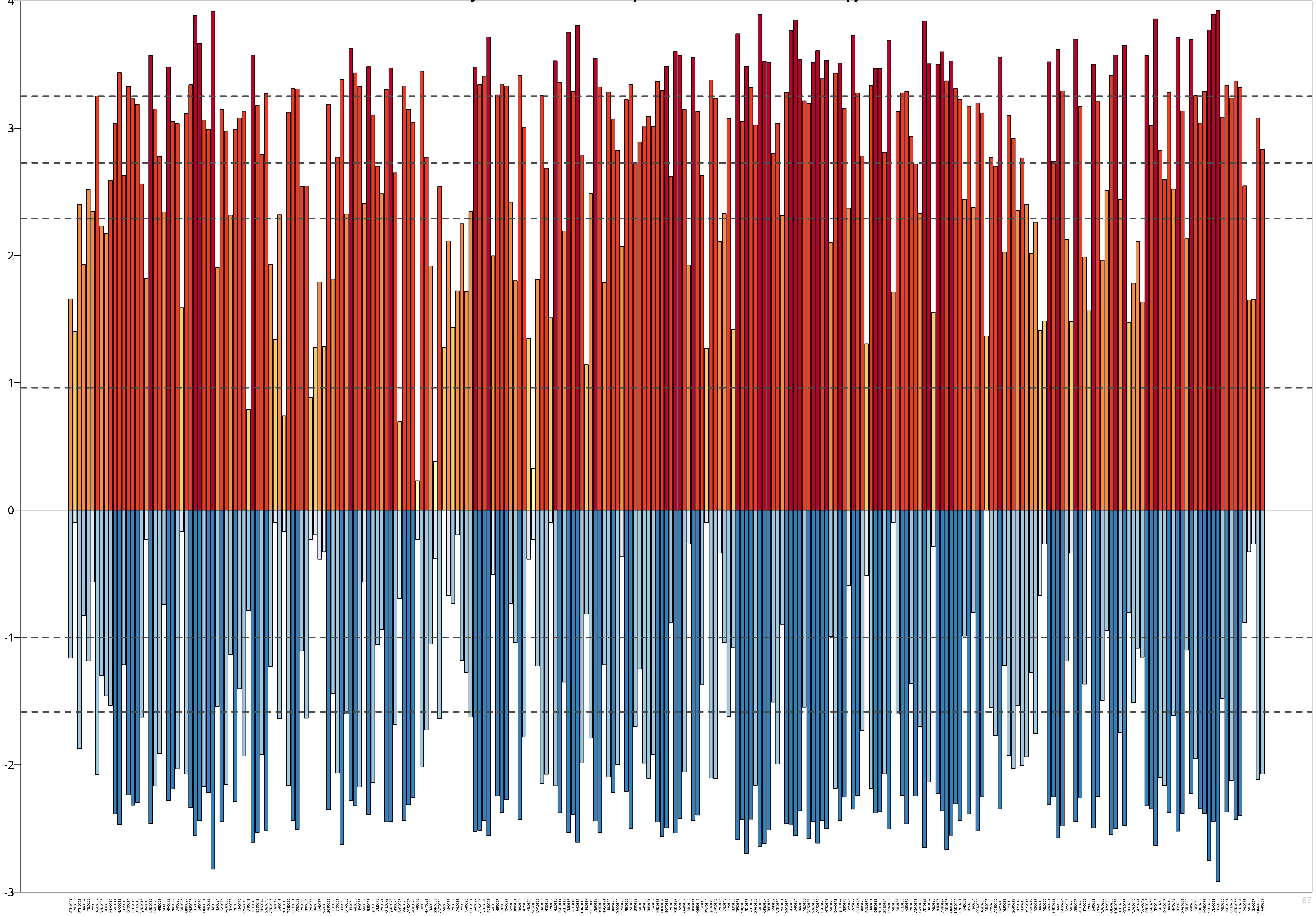

```
# ; Type_1 BREX PglZ NTD iSwi2/SNF2-Helicase

WP_238754868.1_Leptospira_bandrabouensis          MLYNAITSYIDLKINRECLVSWIDPTGEFSGYIDQLEKKKYHLVRFKGSFLESILALHQDVKKPDCLVYIPFISEKTPFLEVLKSSEQVPS
MBF0258418.1_Desulfamplus_sp.                     MIAEHIKRKLEQDLRTKGLLVWLDKENQFTPLVDTWILFHYDIFAFRGSFLELMVKSQSGRDIPKCVIHMPGFNKETPVLEAYKAGQRWRV
WP_009097832.1_Rhodopirellula_sp._SWK7            MVSKALESELRALIGQHDIVIWLDAGSIYTDFVDSLAKLPYAVHAFRGSHLQLLFDLRSSPSRHPMVIHMPGFNKQTPILELYLAGRSFEK
MCC6649976.1_Candidatus_Eisenbacteria_bacterium   MASRALAAEVAKKVRERGLVVWVDAERKFDAFVDAIRGFSYPVVAYRGSYLELMLALKNGLYPEHVLIHLPGINRETPVFELFKAGTVFEK
MBD2549130.1_Microcystis_elabens_FACHB-917        MLQARFCQELSRQLSLHRVVLLFDPAEQLRSLLDALAELQAQWVVAGTSLFALRHQLECADLPDPLLVYLPQRQGRATLLELIRAGTVFEA
WP_096450969.1_Thauera_sp._K11                    MLHQQIASTLDRLLRERRIVVFYDLREEFQPFLDELEDTLACLARFDGSFFALKAAVEAAPLPEPLLVYVPGYAVWNVLFELDCAGKSYEP
MBY3465673.1_Rhizobium_laguerreae                 MLHDYIAARIGDRVKDRRVVVIYDERKEYTAFFDEILSRKASVVRFEGSFLELRYAVEGGDNIEDVVIYLPGVREGSLLLEIEKLGTHYAP
MBY0113210.1_Phycisphaerales_bacterium            MFHDYLASRLAEKVAKRQVAVWYDPREEFRGFIAELRGRDVRLICKNGSLFETKLVAEDGETIEPLLIYLPGERTLNALLELEKAGTPVEW
WP_273378697.1_Symbiobacterium_thermophilum       MVSQYVAQLVRRKLDQHLVLVWYDPTRVFAELVDHLDPPGTRLARFAGSYFELRAEAEARMTAAPLLVYVPAAPQIDPLLGLAAAGGVLKE
WP_209466794.1_Symbiobacterium_terraclitae        MVSQWVVDHVSRVLKEESLVVWFDPERYYETLLPYLEEPNRVVIPYRGSYYEQRRAAENNLSGKQLLLYVPAKQDASPLLEFVLAGRALEV
MBW2308572.1_Deltaproteobacteria_bacterium        MILDELRRILEKQLDNAGIVVWYDPEGIYETVAHRLPIEGATFITFDGSYFKVRREIEISVNRARVLVYVKSPRKLPPLIELEAAGVVCET
WP_083610988.1_Desulfatibacillum_alkenivorans     MLSDRIRSMIARQVKDRGIVVWYDPEKAYAGLIQNLDLPETAVLQYADSFFRLRHELEEFVNAPNVVVYVPMERTSFALVEAETAGVVVES
MBN1165673.1_Methanospirillaceae_archaeon         MATEYLKTQITSSLKNHHLVVLFDPEKHYSDIIKNLDLNGIPIHTYTGSFIELRKEIATGEKPPDLLLYIPMAESNNALIGFTKPGVTLQT
MCI0601819.1_bacterium                            MIADHLQEMLARQVAEQGIVLWFDPDQTYSSLIETLKIPSCKIARYNTSYYDLRAQIERGPHRPHMLVYVPLHRTKQALAELAALGAVAQT
WP_062190142.1_Anaerolinea_thermolimosa           MVSQYLIQTLRQQIQEHHIVVWFDPEKQYQALVRAENFPNVQLFSYQKGFMALRRELEGTFEPPQLLIYVPTSESNHALVEYTTAGVTVQS
WP_156746889.1_Mycobacterium_sp._E2733            MLRDQLSAELAKAVRQAGLVVWQDEHREYASSARSMCPESVQVVAYDGSWYGLRWEVEAGDRAPELVVYVPSAAPEDPLAEIRSAGKEFKR
MBN2413376.1_candidate_division_KSB1_bacterium    MVNEFISAYLLDHIREYGIVLWYDPEQLYTEMLNDLTLPNVPVFIYEGSFLKLRYNIEAGEKKPNMLIYINLARTEYAMIEAESAGCYLMT
MBV6422080.1_Ignavibacteriaceae_bacterium         MLKTYLQDLISTQLDEKRIVLWYDEKGFFKNYIGDFVDSD--IIHFNESYFELRDLLKENPEESKILIYLITKRKNLPLNEIESIGFIFEL
WP_083423498.1_Stigmatella_aurantiaca            MVRDYVVQQVMERLRDRRILVWYDPEGDFYDLFREFPMPRLIRADARHSVLEARRAADRKTFPDPVLIYVPKVRRTEPFEAFALAGAAFGE
WP_218933729.1_Rubripirellula_lacrimiformis       MVSTFIHRRLVDLLEEQRVVVWYDAEQSFTELVAKLTVPNCIVVDTSLSTLQSRRESDAGINHKHLLVYCPRGRYEDPFEVFAVIGAAFGD
WP_055669757.1_Desnuesiella_massiliensis         MITEFLNNSLLEKLDKYRVVVWYDGKHEFKDFIDKFTAPNCVVINATKSIIIARREAEKKMNQKNMIIYLPFFPLDNQFELFSRVGTVFGE
WP_242609850.1_Comamonas_thiooxydans              MIAEFIRESLRPRLKQSGCLVVYDADRRYREQCFDLAADKVRVVDASESSIESREAALREVGLEGVLIYVPAKRQTDPFALYAECGAVFPD
WP_243520949.1_Hymenobacter_monticola             MIRAFIQNQIQQKLTKRRCVVVYDPQHLYAELVAELAAPQAVVVAALPGPTQALLSAWASLGGPPLVVYVATAARADPFWALAVAGCAFGE
WP_235164441.1_Dyadobacter_chenhuakuii            MINQYIADYLRQKLVDSPTLLVYDPNGRYHDLVQTLASGLVHVVDASKSFIKAREEAQTELPQDRLVLYLPLKSIQDPYFMFGLGGAIFPD
MCP5535438.1_Akkermansiaceae_bacterium            MFLKFIANHWQQRLSSNRSIVIYDGSGALRGALALLDLKELHVVDTSESSMRSRLEAWLAVGQTRLLVYVPQNLLADPYQPFRLAGCHFPD
MCC6145260.1_Candidatus_Hydrogenedentes_bacterium MVLDALVSRLRALEAAPVAILWMDEKREWEGVLPRLKEAMPELFTLTGPGVWLRMAADGFAPGETAILYLPGVGRTDPIAELQFRGCFWRQ
MBN9589012.1_Alphaproteobacteria_bacterium        MLIHGLARALIAGEGWPAALLWPDPERQWHFAFADLRRRLASLYALIGPAIWLRCLIEAPDDAKHSVILLPGISWRDMLVDLQYRGDVFRQ
MBW6486418.1_Syntrophobacterales_bacterium        MILESILKYLRHDLAPPRVILWPDEEGLWKRCIEPLRANYPALWSLTGPAVWLRYQLEVHEGEDIPVIYLPGIGRYAHLFALQFQGQFWTQ
WP_004078483.1_Methanoplanus_limicola             MFIEEVKKAILERLSAPVALIWTDHNREWEPVVEKLVKDM-PIATFKGPAIYIRCLISHNLKEGTPVIYLPGYSKDVQLFELQYRGIVWSQ
WP_011448105.1_Methanospirillum_hungatei          MLDEVICASLQKDVVAPLSVFWFDKTGEWQPVIDRLKKRF-TIYTYTGPAIYIRTSVMHHESRNIPILYLPGYSREEPLMGLYHRSVSWTS
WP_030150652.1_Oerskovia_turbata                  MVRAVVAESLRDAQERPVAVLWPDPDRTWEPAIRSLQEAV-PILVLQGPALWIRAVLAAPETANPWVLHLPGIHRHEPLADIALRSTWWGP
WP_248598775.1_Agromyces_sp._C10                  MFAESVHQSILADEIPPAAILWPDPIQAWKPVVSHLRRAGAAILTLRGPAIWIRSQLAAASDDMPRIVYLPGKDRATPIAEVQFRSNWWAE
MBA4013264.1_Phenylobacterium_sp.                 MPLDALIEALEDVQARPEAVVWCDPDASFGPVMPMLRARLLRLHSLTAPAFALRLVTERAEGEPPPILWLPNVGREAPLAWLAVAGAFFGH
NBT25026.1_Actinomycetia_bacterium                MLIQAMVAAWEDGVVPPVAVLWTDHAGEWNKLWPTVLDAFPAL-RLTGPAIWIRAVLAGALPGQCPVVVLPGIERNQPLVELLYRGTGWWH
WP_246481782.1_Natronogracilivirga_saccharolytica MLKQTLINSIRGNLTAPVVVLWPDPEKQWSSVIAILQEDMPELMVLTGPAIWLKCMVASTLASLVPVIYLPGISKNDPLMEYQYTGTLWTH
WP_091825284.1_Butyrivibrio_sp._ob235             MIIDILIRKIRSAEVKPAVILWPDAERRFEYAIKVLQTEMNELFVLTGPAIWLRCAMTGTVKDRVPVIYMPGVGREKPLIPLQYLGRFFSQ
WP_219938192.1_Succinivibrio_faecicola            MASSRITEALLNAFNKYRIVFWDDDKEKLIDEFNSLELPDVTKIKLDNNEFSVKYRILVEETDKKFLIYRPAGEPDVWLLDVQLASYKFVA
MCK6605714.1_Ignavibacteriaceae_bacterium         M---DLRPVLAEYFQKTDVVFWYDKEGEYNIEINTLLPEGVTLLRLDNNHFGIKYRIYNSAAGSRFLIYSCNEKPLPPLLGLLLTGREFLP
WP_251178637.1_Adlercreutzia_agrestimuris         MTAIEIAGRLAAYFGKHRLVFWEDEDGGYSEILDNLSVDGATIVDVTGRELSSKRRILRDERDGKFVIYRQGALPAPFLYDLKLAALPFNV
WP_149431761.1_Rhodococcus_cavernicola            MSAERIAELLADRFATTPILTWRDVEGEHADALDAVAAAELTTHRVSGNEFGIKHQLYAQHEWARHLLYRTGEPPQDWLLDIEAGYGLFTA
MBX3116434.1_Cryobacterium_sp.                    MGLGVLKKSLTDRFGDRHIAFWRDQEGEYEEDLDQLELGDVKVIRVDGDEFAVKHRLLKEEPEQAFVVYRKGDVPEGWLWDLELAYGLFKA
HJG42481.1_Corynebacterium_phoceense              MSLESVLPHLEEKFAAQRIVFWHDDEAEFSAEIDSLAIPGVTVVKVANNELGIKDKLITSADDEKFLVYRAGTVPKGWLLDQELAYGNFSA
MBU6197218.1_Cyanobacteria_bacterium_REEB446      MILRQIKQSLGKLLDLYDVFFWYDTKAEFRREYEKLNLENLIKYELNNNEFNLRYEIHAEKTEAKFLIYSNSARLADCLLDLLLSNHEFIA
QNI65417.1_Synechococcus_sp._A15-44               MPTSRIHESLDAALARKRVVLWYDPNGEWASEFDDYQSAAAEKLRVEGNEFSVKVAISRAPLEQRFLLYLPCAKPPEWLLDLLLAGHEFTA
WP_147932466.1_Neolewinella_aurantiaca            M--STLNQRLHKLFDDHRIVLWYNDSSEMTAEFEAMELAGVTKLNVQNQALKTKFSVQVTDDKDKFLLFSPGKVPSDWLLDLQLAYPLFST
WP_153112503.1_Prevotella_copri                   MTNEKIKQVLTSMVEQNRYVFWYDEGGQMKDIATSLDVPGISVLEMEDNAFSIKFQMQKKQPETGFVIYSAKPMPKDWLLDYQEEGALFSA
MBK9103302.1_Saprospiraceae_bacterium             MSSDKVKLTLEKIFQQHRLIFWYDEQAEMKDLFDDLSFENIGKLVIDKNEFGLKYHVLIEEPTKSFLIYKAGEKPEDWLLDLELSNYEFHT
MAO64830.1_Balneola_sp.                           MTQNKLEKNLKGLFEKHRIIFWYDSEQEFSEAFSSLNLDAVEKVNCDGNPFFVKHLVSKKQPEQKFLLYFPYEQPKDWLLDLQLAHKVYHS
WP_262567766.1_Endozoicomonas_gorgoniicola        MDTGKVLEDIQRRFSSHRLVFWHDTDRVYADELDELMLPDVALIRLDNEMLGVKTRIEIDEPDQPFLLFSEKAKPEPWLLDVRLYSDEVAV
MBQ2845006.1_Alphaproteobacteria_bacterium        MNETQIKQALQKYFEDNRIVLWYDDNAEFSELVAEI---DVKVINLKEIALQARVMFDVDNPTEKFLLYAPFPKPEYWFLDVSKYAPIFKT
WP_156790427.1_Alcanivorax_sp._DG881              MSIERIEKGLAKLLAGNRIVWWSDPDGEFADAVADLSLEEADVVSLGDTALAVKRQIELESPASPFVLYEGQEAPDPWLLDIRLYAEPFAA
TXI44184.1_Methylophilus_sp.                      MLNQKIIDSIQSQLTKERFLFWYDTEKEYLECISSLALEGVRLVMTDNSALAIKLEIAQSDASTRFLFYSNNPEPERWLLSYRLKGKSFSA
WP_006086471.1_Shewanella_baltica                 MQISELAQGVAAKLEQSRIVFWYDPEQSFTEELEHLAPNDLTILNMDNEVLAVKKRIEVDEREAKFLIYFPSAEPEPWLLDVRLYSEQFFA
WP_151792240.1_Acinetobacter_seifertii            LDIDQLLNNILLLSQDSRILFWYDAGKQFEQALEQLSLESIELINLSGRTFEVKKILERDKPQQNFLIYAPFAEPEYPLLDIQKYSKQFHA
MBP7846546.1_Burkholderiales_bacterium            MDIDKIIKQLQELCKNNRIIFWQD-DGYFEKILAEIELSDVTIINTSLVDLEIKVRVELEQINDKFIIYRTTE-PLVWLLNIKCYAKAFSA
consensus/100%                                    h....h...h.........h.h.s.......h..................b...........lh.........h........h..
consensus/95%                                     M....l...l.........hhh.D....h...h..h.............h..b...........lla.........h..h......h..
consensus/90%                                     M....l...l........hlhh.D....h...h..h.......h.h....h..+..h.........lla.s.......hh.h...s..h..
consensus/85%                                     M..p.l...l...h....hlhW.D.p..a...h..l......hh.hp.s.h.h+..h.........llY.s.......hh.h...u..h..
consensus/80%                                     M..p.l.p.l...h....hlhW.D.p..a..hh.pl......lh.hpss.h.h+..h........hllY.s.......lh.h...u..a..
consensus/75%                                     M..p.l.p.l.p.h.p..hlhW.D.p.bap.hh.pl....h.lh.hpss.h.l+..h........hllY.s.......lh.h...u..a..
consensus/70%                                     M..p.l.p.l.pbh.p..llhWbDsp.bap.hlppL....h.lhphpss.h.l+..lb.......hllYhP....ps.Lh-lbb.u..a..
```

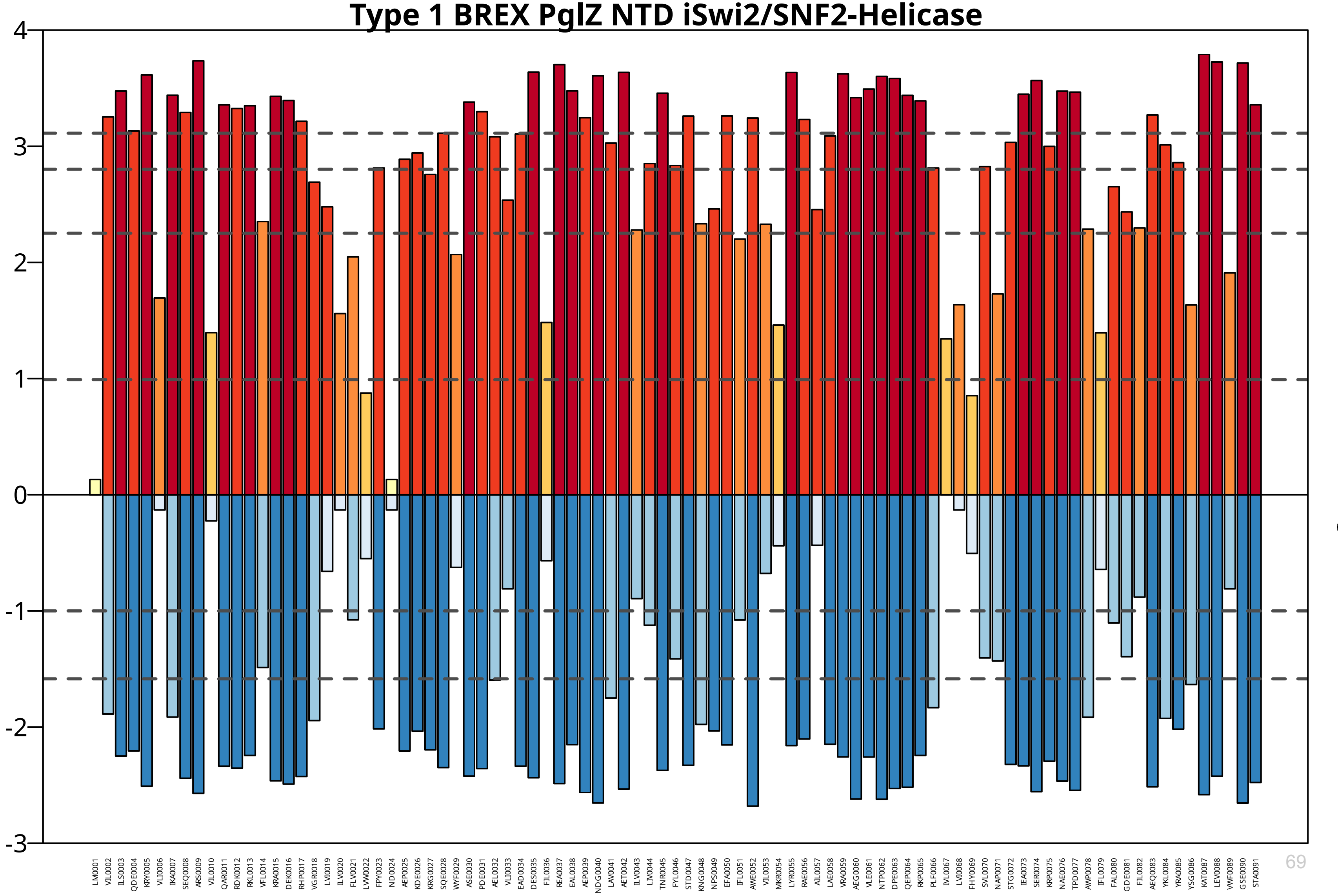

Type 1 BREX PglZ NTD iSwi2/SNF2-Helicase
4
3
2
1
0
-1
-2
-3
LM0001
VIL0002
ILS0003
QDE0004
KRY0005
VLI0006
IKA0007
SEQ0008
ARS0009
VIL0010
QAR0011
RDK0012
RKL0013
VFL0014
KRA0015
DEK0016
RHP0017
VGR0018
LVI0019
ILV0020
FLV0021
LVW0022
FPY0023
ND0024
AEP0025
KDE0026
KRG0027
SQE0028
WYF0029
ASE0030
PDE0031
AEL0032
VLI0033
EAD0034
DES0035
FIL0036
REA0037
EAL0038
AEP0039
NDG0040
LAV0041
AET0042
ILV0043
LIV0044
TNR0045
FYL0046
STD0047
KNG0048
NPS0049
EFA0050
IFL0051
AWE0052
VIL0053
MKR0054
LYR0055
RAE0056
AIL0057
LAE0058
VRA0059
AEG0060
VLE0061
NTP0062
DPE0063
QEP0064
RKP0065
PLF0066
IVL0067
LVI0068
FHY0069
SVL0070
NAP0071
STG0072
IEA0073
SER0074
KRP0075
NAE0076
TPD0077
AWP0078
IFL0079
FAL0080
GDE0081
FIL0082
AEQ0083
YKL0084
YRA0085
YSG0086
KAT0087
LEV0088
VWF0089
GSE0090
STA0091

# ; Type_2 BREX PglZ NTD iSwi2/SNF2-Helicase

```
QBI18515.1_Egibacter_rhizosphaerae              MGEAVVDESQVRAIAAQIGERRRMVAVAGAPTWEGPERLETDAGTVRVLAAPSTLAVRAALVAHRAAELLVVVTDRDERELGEEVVAELWDHRVVRP
WP_188941324.1_Nakamurella_endophytica          MSPTTVDRSTVIAQIRLMRRKRRVLAILATPVWSGTDVLEADGSTYRVWPCPSRLAVRDALRHRHETPAPVILTDLEAAELGDGICDHLVDHRPLSP
WP_332348679.1_Accumulibacter_sp.               MNVLLPSVRQIVAQVDAVLRHDRVIAIRAQHDESWPSTLAVGEREFSVAWCPSPIAVREQLLALADGPGLVVLTPLPDHVLGADVLARFSRGKVFSV
MBT3378822.1_Lentisphaerota_bacterium           MAG--IRTSQVKALIKSVRNKRDFIGISSPFLWLEDESVEVDGETYDIVQCCSELHIREALAAATRKRSTVVITSLDDPDLGEDLRLRFHKRSLRKL
MCB0954592.1_Ilumatobacter_sp.                  MPA--ADAQQLLALVNSVRRQEDAIAVRVDPVWAGPDTLD-GPVPIRIVPCPSPLAVREALAEHERTQVVMLLTGCGDSELGPDVVARLAKGRVVSL
MDJ0841534.1_Acidobacteriota_bacterium          MN---TRARAVKVLVRDIAKKSPPLAVAWDGVW--PEAERDDGEVLLFRRAVGPLAVRRVLAEPREAEMSVILTPTAEKDLGADVITRLWGRRLFQV
WP_005004490.1_Nitrococcus_mobilis              MS---LTPTQLATHAAKALQQATTLAYHWPQPWNGGDSIVVDGQTLPLRHCVSPLEIREALAEASD-VSRVLLVSQQETGLGQDVLARVFRHRLLHV
MSW49534.1_Actinomycetia_bacterium              MADGPVSTSHLKRHLERWLDGPPRFLIHGEWPAERPTELVAFGHTVDVVSCPSELSVREELVRDRPDRPLVMVTPID--RVGSDVIARLTKRRMISL
MBO9539708.1_bacterium                          MSA--LSGPQLSALIASLRAKRAVIGVHAPDGWQGASELRVGNETWTVAVCETPLAVSVALSERPEASPMAVLTRLTEADLTQDILVRFPRRELVRL
MCX6543497.1_Acidobacteriota_bacterium          MTPTLLSPAHVRAEALDLRKRVTVVGIRYPGQWSGPSAFETEHGTVSVLCSQSPLEIRERLVDHERTVPLVILTSLSEQDLGDDVTARLAGRRLRQP
MCG5052491.1_Myxococcales_bacterium             MKA--LTRQDLAVELQRFHKPPENTAFYGFGERDRVSVIGADSHDFEIVPVKCELELRKELGRIEREAQAAFLVDFTE-RLPLDLGGRLASGDLQWV
MBF0261196.1_Magnetococcales_bacterium          ME---LTESQIVAQVEAILKKDPAIGIRSMGPWRGGETIVAGDRQVRVRHCATPLAFRERLVAVKESEVSVLLTPLDTNSLGQDVLARIWRQQLIAI
TAF23832.1_Verrucomicrobiota_bacterium          MTETMLIPEQLRAQIDRWRDRRGVFAVRTDFDYAGPAQITCDGLAVTVADCRSDLEARDLLLGLNSQHGLLMLMRTDEDRIGEDVRARFAGKTVHTL
MCE9632142.1_Planctomycetia_bacterium           MAAA-LSFNQIKAEVTALGRKKQPIGIRAPSRWIGEAVHTDGEAGYLIRQCDSPLAMRLALREAPPPVVKVIVTNLPDAEISRDIFARLHRQRLFSI
WP_330928032.1_Synoicihabitans_lomoniglobus     MTT--VTAGQIRAQVAGLRKKRNAFALRVNGTWTGPDHMEIDGTAHLIRSCASDLQAREALHDATEKSPIILLCSIASDRLGEDVIARFARNRVLAP
MCP5560213.1_Verrucomicrobiaceae_bacterium      M----LTPPILSAQVKAARAKTRPLAFRSYDTWSGDETLAFEDKHLTVKECRSDLETREAMGLGAP-DELVLLVRASDGLLAEDTLSRCAKGRLLDL
WP_287381673.1_Mesorhizobium_sp.                MEAPGPELADIERALDSLLLKDEILAMRSPIRRAWPEAVERHGRRFCIAWCPSELEVRERLDEVERNEGLVVVTPLDAATLGDDIVARFPRARLDQT
MCI0393077.1_Acidobacteriota_bacterium          MSQSLSLPPQIRAHVELLHKKHGVFGIRMSVPAPVSERSEVNGRECFFNWCDSVLQIRELLFEREKDALQVVLTNLSEADLGQDLLARFAKKRFFTD
MBK6942982.1_Planctomycetota_bacterium          MTSVVVGEAELKAHLQQFLRKTEVIALRTASTWNGPSSIEVDGVTFDVVACSSTLAVRESMAKFEDTHRLVVLTDVADRELGDDVLAHVAKGRGVPP
MBI4662621.1_Verrucomicrobiota_bacterium        M----LTLKQVRAQVEAIRRRSPAIGLVSAVRWTGENELKVDGERYDLRAAETELEMRTVLVEAKRKSRVVVLTNLDPSAIGKDLLARFARRRFFSI
MBL8842559.1_Planctomycetota_bacterium          MSELALTTATLRIAVNDVLDRRRVVAVQSSTPWRGGSEIDLGESRARVICCASPLEVREALTESEAALPLVVLTPADERQLGQDVLARLARAQLHPL
MBK6462401.1_Myxococcales_bacterium             MEAAGLSQADVRLEVERLFRRDHLLALFGRGQ---PGRFELDGLKWDIIPTACELSLRSRLPAPGEPTGRVYLVDWAEDALPLDVSCRLAGGRIYHV
MCZ2341280.1_Bacteroidales_bacterium            MSIGTLSSQQLRSLLEDKWQRDAVIGLHVAQPWKTPSVVDFEAGKAQVVRADTVFQVREVLGDAERNERIILLTKLRQGDLGNDVVGRLARCRLFAI
WP_075083673.1_Mariniblastus_fucicola           MSD--LKRSHLIAGVSSL---KKVVGVRSSISWSGDIESEVNGIRYCIRQCDSPLAVREFIRSAVDGAINIAVTNMDEETIGMDAMLRMASTRLLTI
MBZ5659873.1_Acidobacteriia_bacterium           MANSEMKESVLLSQIESIRSKREAIGVHSQSRWQGRDKILVAQETWRVAQCDSVLELRQRLSEQID-TPLVLITPLPTTEIGDDVRARLFKQQLLPV
WP_238634086.1_unclassified_Halorhodospira      MTNPTATPRTIEARVTGLVSRGEVVAIGMSGHWSAGETVEINSTTWPVRQCDSVLSMGLALTELETQGRGVVLTSLSESDLPEDMLARLQGRRLHRL
MBL8678386.1_Myxococcales_bacterium             MATNFVSRDALVEELKALHKRWHDTLLYARLERELPSIVDTNEGPFVVRTAECELELRAALVDGVREDPVVVLVDFDLPRVPADLLGRVATGKVLRI
MCB9660516.1_Sandaracinaceae_bacterium          MSAAAVSRQDLAEDLKRLYESPRLFAYYGTGS---EDAVDLNAARVTVVPVRSELELRMKLPLLDDDARVAFLVPFK-GEVPLDLQARFALGRVRRI
WP_154827459.1_Dietzia_kunjamensis              MTPVSATPADLIPLLTSYRDKRHLLVVHARPEWDG-TVLRVDDLPVPVHCCRTVLEIRDVLRHRNDPPWRVVLTDLHDDQIPQGVKEHFRPGRSLEL
WP_105324909.1_Corynebacterium_sp._J010B-136    MTSLQVTPRDIEQHVEKLRNKNYTVAIRTSADFVGRDQWVSNGQNIEVQRVNTPLLLRAALRRSDEAPWRFFLTTLPDSEMPLDLQEKLYPGRVFTP
WP_312777491.1_Corynebacterium_variabile        MSARLIRQRDIDDQIDYLTGKNYVLVLHTDQGWDGPASYTRSGDLVAVTTVRTPLALRHALVQAEQQPWNVFLTPLGDADLPADVRDRLRPHDVQVV
WP_322753648.1_Frankia_sp._Cas3                 MTVPAATVAIVRAMIDKALEKRYVIGVRARPVWSGPETFTHRDRPARVVVCRSALAVREALRERSAGRWLVVITDRDDADLGAGILAHLVFQRLRTP
WP_144825789.1_Micrococcus_luteus               MTRTRITGAAVRSVIADAQRQYAVVGIYADPAGVAELGVVVDGAAVTVLPAASTLAALDQVHHAPRGSWSALVTNRPEEDLGVGVLSALVGGVLRTP
WP_259849919.1_Brachybacterium_muris            MTVTTVPRAAVLATLDAVRRRESVVGIRASRGLDDTTPSDAHHEPITVRWCPSVLSLWDALTGWNEQGWLVLLTDRTEEEIGSGLLARMVQQRLHRP
WP_243731729.1_Nocardioides_ochotonae           MTWTLAEPGYVRQRAAAVRNHDPVLILHAVPQWSGEPIFTVGDAVVHVRACESQLAVLDAYAQLPEGDYLVVLTDRTAQDLGDAVLLRAWRRRVEMP
WP_280369379.1_Nocardia_wallacei                MSAPALKTADVAQYLSAARGERTVVLLRAEPAWAGSAELAIGERRARVVPAPSALAVHELVLRHLPPKVLVVLTDREHEELDPAILARTHRKRISL-
WP_285664605.1_Actinorhabdospora_filicis        MTVTAVQPHLILGEARGLRKRPPVMLLRASADWTGDELLKIDAHTVRVRPARSVLAVLDAIDSRSEHELLIVLSPFREEELGPNVLARVVEQRVFS-
WP_148767669.1_Actinomadura_decatromicini       MTEAAATSGILIAEVRRARRRGGTLFLRAAAEWRGGDVLDLDGATVQVAPCTTLLGVLDAVSAKRT-DFLVVLSAFERRELGESLLAHAIGHDIVA-
WP_158645688.1_Stackebrandtia_albiflava         MSVGTARQTMVHAKVVELPGESAVLLLQAAPVWNGPETFPVPGAEVRVVPADSPLAVMEALSGASKDEVTVIVSPYGD-ELGANVLARVYKRRAQS-
WP_169440218.1_Streptomyces_sp.                 MGVTVLDQRVVAGLLTAHLAKRRLVLVQGKYAAGSPEEFTLSRRSVRVVHSTSVLGITDAWQAHLRRDVLVVTTDVPDDRLGLDLVGEMMRSHVIK-
WP_326854225.1_Actinocrinis_sp.                 MAVAAVSRGMLESLVRRERKASRLLLVYAQYAAQGPARFRVQRYVVTVTACSSVLAVADAWESFQASRVLVITSTLAAQEIGLSLRAFAARMGTIA-
WP_018383907.1_Wenjunlia_vitaminophila          MTAAAVGRRTIEALLDTHAGRRRLMLVHGHYPPTAAPEFTVRTRRVHVVDRSSILGILQAWHDHQSRDLLVVTTAVDDAQLGWDIRGHAVRRRTLT-
WP_051042993.1_Nocardiopsis_xinjiangensis       MNSPAVNAEAVAGALERARSGATALALSANPRWDGSARLTVHPRSVRVVGADSVISLVYALSGHDDTDHVVVLTPLESADLGEALLGRFLGEEVVR-
MBV9853173.1_Streptosporangiaceae_bacterium     MSTPGANRTVVTAELRRAAGPSRVLLLRAAPEWLDEPRLTVSTVTANVAGCPTVLAVLDALATRPEDGYLVILTPCDDDELGDSVLARVIANQVHT-
WP_189653453.1_Nonomuraea_spiralis              MSD--LNDLVVREFVRTLMRRQDIILLYGRYEPGAPTELKVEERQVRVADCPSVLSVLEAEVGHPG-GLLVIVTPRSDDELGNTLRSRAVKQRALR-
WP_066069029.1_Frankia_sp._EI5c                 MSVAAVTTAMLDGRVARLGERARILLVRAQPEWPGPGELLVSGRTVRVVACVSPLGVLEEVARWGPDGWLVLLTDADEETLGDTVLAQVHRQRVDV-
QGN48447.1_Micromonospora_sp.                   MRQTQISPIALRQKVTQQRHRYPVLVVRAEPAWPHDPAMQLPGRRVRVVPCVSPLAIWEHLVAERGDEVLVLLTDLAESSLGAGVRGRIFRQQVIM-
WP_034267102.1_Actinospica_robiniae             MSITSAARPLHLAALRQLDDEPRVYLVRGAPSWNGPATLPGPGHDVRIATAVSPLEVWELIVRHADESLLAIITDAEEHDLGPNVLAHVFRYRVLI-
consensus/100%                                  M.............h...........h....................h....s.h...............hh........hs...............
consensus/95%                                   M.........l...h.........h.h..................h.l..s.s.l.h...h.........hh.s......ls.sh..ph...p....
consensus/90%                                   M....h....l...h.........h.h..................h.l..s.o.L.h...h.........hhlss.....ls.sl..ch...p....
consensus/85%                                   Ms...hp...l...l..h..p...h.l.s.........h......h.l..s.o.L.hb..l.........lhlss....pls.sl.s+h...ph...
consensus/80%                                   Ms...hp...l...lp.h..p..hh.l.s...h..s..h......hpl..s.o.L.lbp.l.........lllos..p.pls.slbs+h...ch...
consensus/75%                                   Ms...hs...l.s.lp.h.pc..hl.lpu...h.ss..h.hss..hpl..s.osL.lbc.l....p....VllTshsp.pLG.slbu+hh..cl...
consensus/70%                                   Ms...ls...l.sblp.hbp+p.hlhlpu...a.uss.hphss.phpV..ssSsL.lbchL....p....VllTshsc.cLG.slhuRhhp.+lb..
```

# Type 2 BREX PglZ NTD iSwi2/SNF2-Helicase

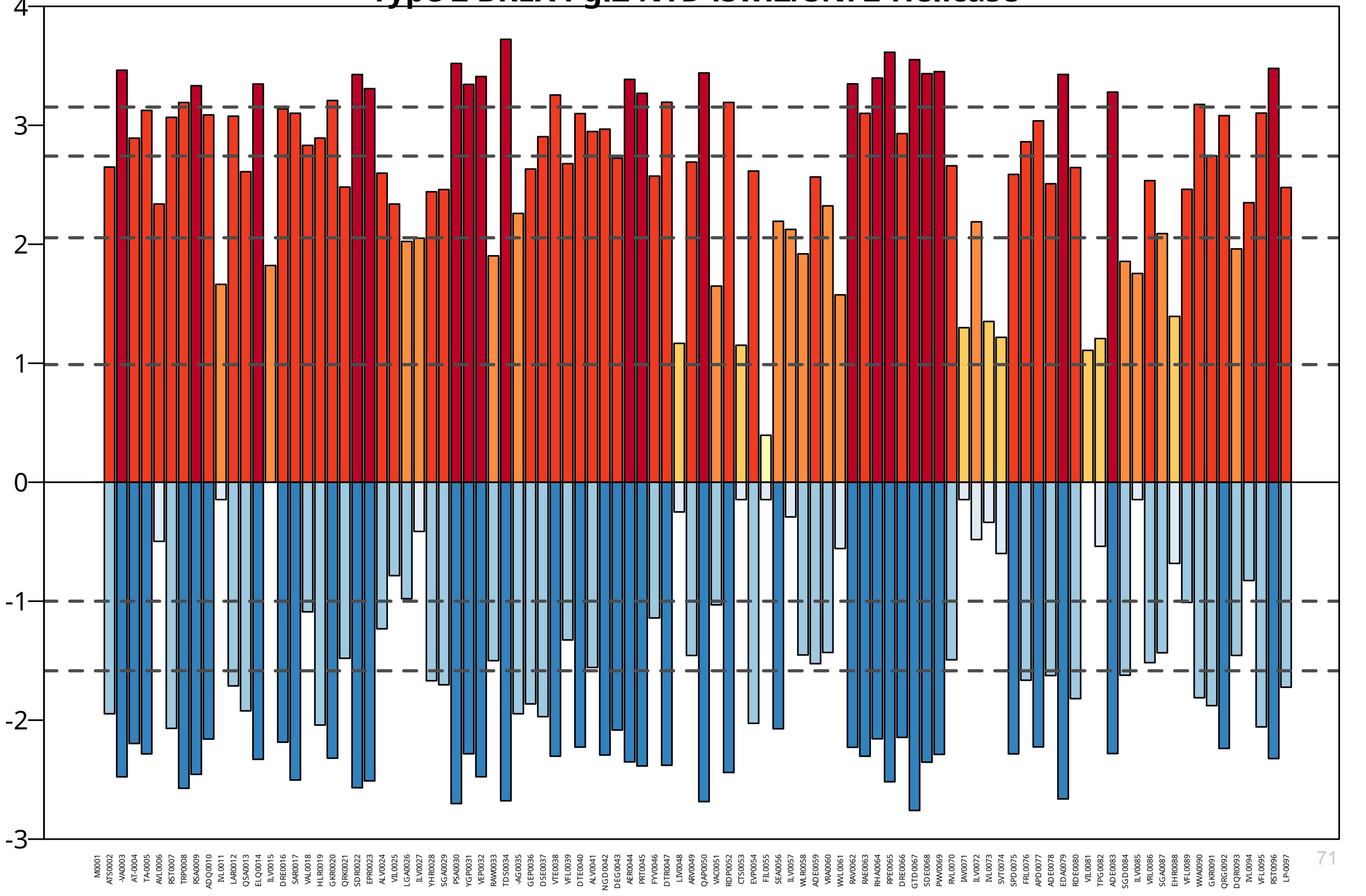

# ; Type_3 BREX PglZ NTD iSwi2/SNF2-Helicase

```
NPV81619.1_Bacillota_bacterium                 MWRDHILKEFTPRVSRLTLVADPDGLLLEEGILEGIRERGFELIPFEDHIAFRYAYESRFRSRDAALVVVLRSGDVLPYDLLQAGRKLSFNLGDIFP
WP_264656686.1_Azospirillum_canadense          MWVDAVTKDLNPEIAPLVAVIDPDGIFREEAVLAAVQARGYAVVQFEDEIQFRVEFEAKFRGRDRHLLIVHHGGEDLPYDLVSAATVVSASLPSLFP
WP_069455074.1_Shewanella_xiamenensis          MWVKELISTLNLDGAFVYLLDDPDGLCYEPQIASFFQDKRAVLIDEPDPLAIRLTYEEWLRENNQVSLIIRISDHSIPFDIALHAKRRSFNLAEICP
HEC58784.1_Methylophaga_sp.                    MWQDRLLNKLALNESLCFVINDPDRICYEPHISNAIKQSKAIIFDDVDPIALHLIYEEWLEKESGGAFLFRYSDINIPYELLRKSKIVDFHISEVIE
WP_247116183.1_Marinobacter_sp.                YWRKPILRALEPGLSDFFVVEDTDGLLAEPGIQKMLQARDVQFYAYDDPIAFRHFYETRIRGDEVTLVISLGEDRSLPFDVINRARVESINISDCFP
WP_008603683.1_Pacificimonas_flava             MWVDHVAGGFQAEFAKLWAVSDPDDVLFGDTVSRTLVERGIDVVEYAEPIAFRLLYERDLRENEAPTIIHVRGDQELPWDVLQSSRHVDLRLAEIFD
WP_181742020.1_Thermoactinomyces_mirandus      MWRDEVLKYFADP-PAVMIISDPDRLLQEKKILDQLLEKNLEIVAFEDSIRFRYLFEATYRDRGNFCLVVHTEEHHVPYFLYQHGEIVELRLADLFP
WP_113032578.1_Paenibacillus_contaminans       MWRQKVLESIQAP-SLITIAHDPDLLLQDEGIVCELQERGIEIADFQDRAVFRYWYESRNRLSGGRHVLIRQAGEDIPYDVWVQGHHVSLSKSALFP
MCD8511937.1_Bacillus_sp.                      MWRQLILDKLNVS-YPLILVADRDDLLSDEELILYLQENSIHVHEAMDRAEMRFIFEQFKEGKVGEKLLFKLGDEFFPYDFLTEGHFVSLSVSEIFP
WP_102026200.1_Salirhabdus_sp._Marseille       MWRQKLISQIPFQ-HKLLFVYDRDDILSDEELLSYLQQNEVNVFSFKDRAELRIHYEGQLEYSSGKWIYKVGEGFTFPFDMLNNQEPIELTLHIAFP
WP_036836014.1_Pontibacillus_litoralis         MWREEVLKQFERP-SSVYIVADPDHLISDEKILSYLQKEKLHVVDIKDTIDFRYIFESKFREKSKEYLVIRFSRTSIPYDFLQIGHQINVSLADVFP
WP_169099399.1_Bacillus_sp.                    MWRDQVLKQFIAN-NALYLVLDNDQLLLDELILGKLKEDNIQVVNFNESIIFRYEYERDYRTKEEICLLVREGTNVFPYDLIQSGQRTRLDVSIIFP
WP_190997467.1_Peribacillus_faecalis           MWRDIIINKMKYC-ESLIFFIDPDYLLSDEILLRKIEDLEFTITRYENSIQFRFFYEKYCRYSNKKFL-VYNNETELPFDIKQQSAILQIDISTIFP
MBT5530409.1_Cytophagia_bacterium              MWRKYILNHFDGQ-YRSIIVEDPDDLFFDETIQDNIRRNSFKVYSYSTSLAFYYFYESVWRNNNGDVLIIVQSEEFAPYELHRDIQKIEVSLPILFP
MCM3358660.1_Psychrobacillus_sp.               MWQERIINHFTSK-GSLYIVTDEHELLREKTVQEMLRIQNCTLHFYDDPVAFRYEYETTFRHIHRATLVIVLDHNHVPYDVYINANRSTLSFETLFP
WP_214788919.1_Exiguobacterium_sp.             MDLHRI-QSFLLQSNPYYVVRDMNQLFNSPSFRNVVTNLGYTIHVADDPIAIRLAYETFRLSNKGTFLLISQDFSSVPFDILKSSLQVTITWGLLFP
WP_014759117.1_Thermoanaerobacterium           MMINYIVSKLNRCDNNVILVFDEDNILSNSDIISMLKENGYDIYDFKDPDLFRYYFESKFKIAGVKLLIRYDGKITIPYDIKMSSSNIAITLTELFP
MBI9032248.1_bacterium                         DFVTSILKKVLSLPTELIFINDIDNIFTYSEVLNRLNELSYKVSNYICPIAFRIEYELEIKPFGSKYIVRCSSSPYLLEDIAEHSDIMKISFNDLYP
MBE6339837.1_Bacteroidales_bacterium           DLIKKVAQRITVDYCNLSIVENPDGFLSQDVVRALLQDCGIEVV-VGSNLQLRIHYELHYKVSGRRYVYICKSKDTILADMKRSAHVVDFSISDVFP
WP_123477185.1_Muribaculum_intestinale         ELINEILDSISRGFHPLTIVSNPDGFLAHEDVIQELRESPTDVV-VGSDIQLRVHYELNLRRNEKSAIYILSTG-DMLPDISTKANNVRFKIAELFP
MBR2506173.1_Bacilli_bacterium                 RWVQTIIEKVTPSTVKPIIVIDHQRYLEIGEIQELLRKNGYTLVFVDCGILVRIKYELEVKGK-ENTILVIKGNYSLVDDMSASAFVIDLKSKEVFN
WP_217803628.1_Parabacteroides_johnsonii       NLPQGIINRLFGSEKRLVVVQTYDEFLAREDVRMMLTYASLRIS-EGDSLTLRLWWETTVKEENIRFVFVQREQFDVPDDIAIYADTTQFVARHIFP
MBQ9466673.1_Muribaculaceae_bacterium          ALTQNIVNRLFAGDKRLTIVINRDGFLSRTDVQNALRHAAVTVR-HGKAIDLRVLVELHIDDDTGKTLFICDDEFDIMPDILPRATVMTFQARNLFP
MBO5155853.1_Prevotella_sp.                    ELAKRIEEKVTDRHFHLTMVRNSDGFLSRSDVQQAVQECGVLLLPVCSALELRVRYELNDRLSEQKVCYIVNGNLTLLPDMKPYIKDKMINLSDLMP
TNF50598.1_Bacteroidota_bacterium              TLIEHIAQDLKDQPSKFLLIENPDRFFQRKDIQVLLESQGIIFL-SGSSLYLRVSLETQDEKNDATVCYFISNENHVLEDIREKVFKKKLSINDYLK
WP_076420664.1_Pontibacter_lucknowensis        AFVNHIAEEFLASPKRLILVRNPDGFLRMPEVQKALKAYSIRLC-DKQGIDLRIDFELRYKATADKTVYLISSLEAILEDIASEGEYRVFHLADYFP
WP_133532125.1_Flavobacterium_dankookense      SFIEHIVSYFNSFDKKIILVENPDKFLLREDIIKIFEDNNISIV-NGTGIEQRIKFEL--KSSVTTLIFTSDDNENYLEDIKRNAAKTTFHLSNFIK
WP_172278715.1_Lentimicrobium_sp._S6           TLIKKIVDDFVSLNRRVIAVENNDGFLFREDVIEALFANGIELV-SGTSLDHRIAYELRDK--SKTLILLSKQKQDYVPDMGAQLYYYEFEMNQYLN
MCC5935593.1_Balneolales_bacterium             ELLRNIAEKLKSYGARTMLIENPDGFLNRPDVAKELLKYGLTLH-RGTQLEQRIQYEL--RDPEERLVLLNKDNSSFLQDIKQNAFPVKFRLDDYLG
MBP7508728.1_Prolixibacteraceae_bacterium      NLLETLRKTLQAY-QKHILVFDKDGIFFHSEVQTEAQEVGFKLIFIEDAYKFRISFELNHNLN-GRILYITNCTFPVLPDIIREVKTITVSFRDFFP
WP_083331763.1_Prevotella_bryantii             SLLQCIVKEITRQYRRLSVISNPDGFLRRKDTIRAMEQADITVL-SFSQLELRVWFETVFKEDANRFVVLMDDTTKLVADIYNQAFVTVFKTRDLLL
MBP6412566.1_Bacteroidia_bacterium             NWIDKFKKVLISSHKEHIVVADIDDLFEYSELKQAFETDGYKIETCKSSLAVRLFFELNVRNSKDKFLIIAPAYYFPLPDIEMQVQFQAIGLLNLFP
WP_073205016.1_Prevotella_ruminicola           SLVSNIVHNIIQENVSISIIRYADSLLMREDIYSRLEESGVKFV-TGSNIELRFHYELHKNDDGQHYCYLLGTGQMLMPDIMRKCYVTDFKLTDLFS
MCM1450899.1_Clostridium_sp.                   RLLADIVNRIVGGDKRLFVVDNRDGFIMKYRQALETYGNRCRIF-GGTSLDLRIVRDYEMMLDESRFIFVPSEDFTVLDDVAADSERVTINVQRFFA
MBR3317262.1_Atopobiaceae_bacterium            MLAELVYRNSNAAFSTSVLLVDH-GIEARCGYAEVFERNGFVVIHWVDDLRFRIEWEDQLKRG-ERLAVIAPDGAYVPYDLAQLMQRYDVSLRNLFP
WP_195245438.1_Clostridium_celatum             MFDEYLEDRLALKYEKYIMVLDEDDILSRYNIIEKIKENKFNVEQYNDVEKFRYLFETKIKKNTEKYFVIVNSDIYIPYDIRNFFYQVNISFQNLFP
WP_071060846.1_Andreesenia_angusta             MFKDHMIKMLSIDYESRILVFDYDDMESKYGYNEILVENGFRIIKYEDPTEFRFIYESEIKHSDEKFAVIVKTEGYVPYDIIKSFYSVELRLEDIFP
WP_226978794.1_                                MFADLLYEKSSAAYEKHVLLIEEDALEDKTEYCAYFAGKGFRIIRYENDLVYRVKHEEAVRAGDSKILMLASSGVYIPYDVQKCFRVFKVSLAKLFP
WP_153014722.1_Syntrophomonas_wolfei           ILRDLLFEKAGLPWVERLIIYDTDNLVSRTAGTNGLYWDEYSVFEVKCSEELRFIYERDCRQTESTIFIVPSLDIKIPYDIYKQFTIVTLGLDTVFP
MBP3452732.1_Clostridia_bacterium              M-KNRIFEVTGILSQNNCILFDIDGIWQEYDCSSNASEAGFQVINIHNEMELRYEHEKKLMHNEDRILLMHQLDMYIPVDIRRLFTITMLTYDRLFQ
MBQ3643692.1_Candidatus_Riflebacteria_bacterium SFSQYLYETNNAEQNKRIIFIDEENLEEKFSISDYFKKQGYDVY-YSSKTPFVFVFVGELQESDKQILLIVRSDGDIPNEIRDICVVKRINIEEIFP
WP_252226004.1_Caldicoprobacter_algeriensis    LLCQYILEKMAVDYENQILLFDYDDLCERSGALRFAHNKGFQIIEYADPEEFRYFYELKLRDNDVKVIIVMNEKIYVPYDIRKRYYNVFVDYASVFP
WP_162523517.1_Calorimonas_adulescens          MFYEELLNGISSRKGQIIIIHDPDGFFSEVTV-SELFDGSIDVHYFNDPDLFRYEYERDYRQRDKVFMVICSNNRGIPYDVLSKGSLVDMELSRIFP
MCR4431941.1_Tepidanaerobacteraceae_bacterium  MIEDVIIQRLPLNQNNFILLYNPDDMVAGEKIVSRISSCGYTLINYEDPDMFRFYFEQILNKGGDKIILRLTRNTYVPYDIQSIFQEIRITIKDIFP
WP_045176026.1_Caldicellulosiruptor_danielii   MIEDAIWEKINSNLYDFLLVYDYDNLLCEFNVITKIFDEGYEVFYYKDIEEYRIFYETEIRYTDKKVILIVTENVYIPWDVKCDYHEVLIRVKDLFF
consensus/100%                                 .....h...........hh.....h...............h....p..................h...........h........h....hh.
consensus/95%                                  .h.p.l.p.........hh.s.p.h.........h....h.h....p....+h..E..........hh.......h..-h.........h....hh.
consensus/90%                                  .h.p.l.p.h........hl.s.-.hh....h...h....h.l....s...hRh.aE...p......hh.......h..Dh.........hph..hh.
consensus/85%                                  .h.p.lhp.h........hl.s.D.hh.p..h...h....hpl....s...hRh.aE...p......hh.......h..Dh.........hph.phhs
consensus/80%                                  .h.p.lhpph.....p.hll.s.D.hh.c..l.p.hbp.shpl....s.h.hRh.aE...c.p..phlhh.......l..Dl........phph.plFs
consensus/75%                                  .h.p.lhpph.....p.hll.s.D.hh.c.pl.p.lbppshplh...ssh.hRh.aE.phc.p..phlhl.psp..l..Dlbpp....phph.plFs
consensus/70%                                  bh.p.lhpph.....phhll.s.Dshh.c.pl.p.lbppshplh.h.ssh.hRhbaE.ph+.s..phlhl.psp..ls.Dlbppsp..phplpplFP
```

## Type 3 BREX PglZ NTD iSwi2/SNF2-Helicase

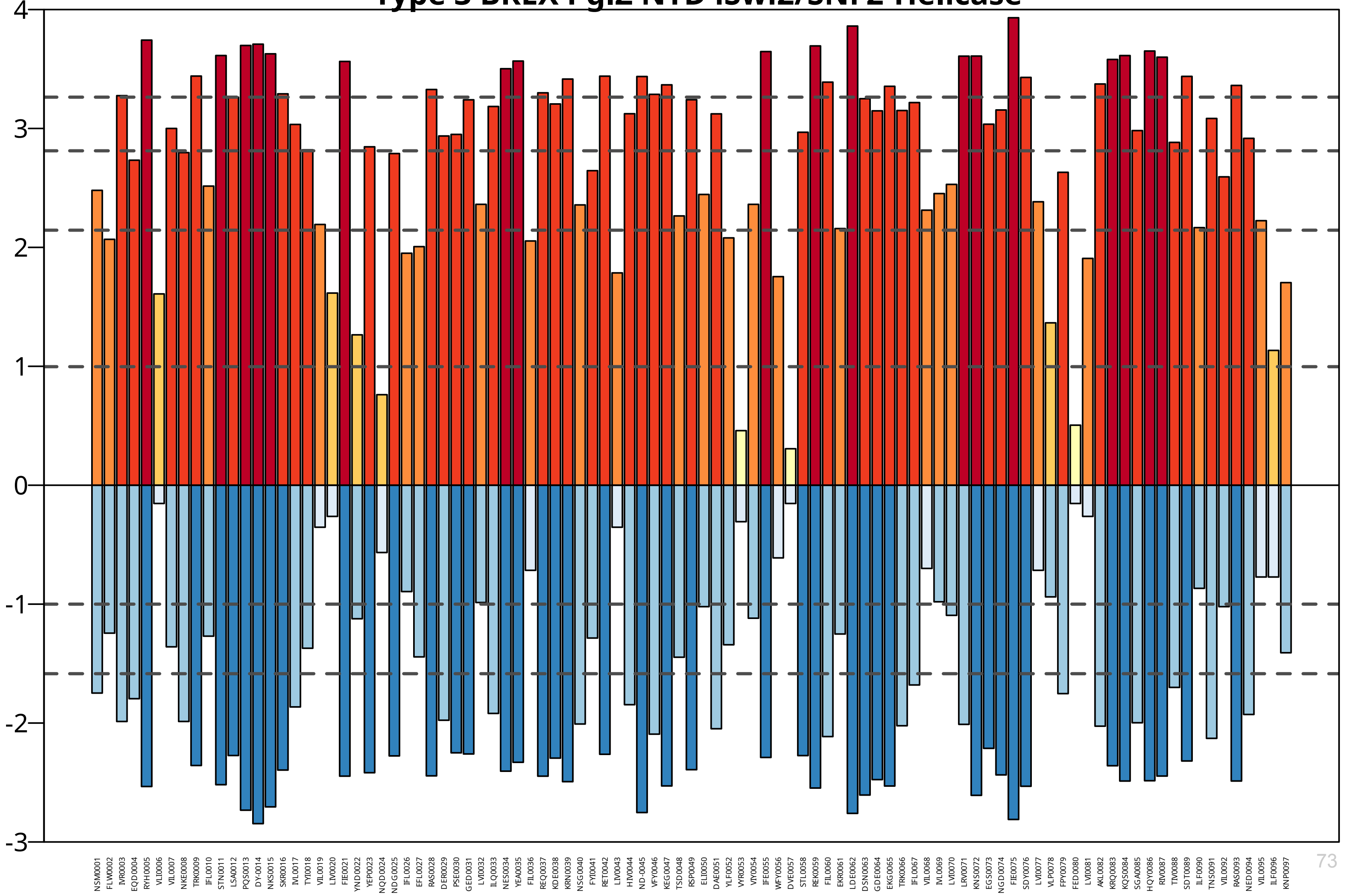

```
# ; Type_4 BREX PglZ NTD iSwi2/SNF2-Helicase Rep_1

RGH34270.1_Ruminococcus_sp._AM47-2BH                      PQICLPKITLTQSSELEKDSSVIKGISKLFSMLENMSKSELIVHTHYSSTLFKNAMMPVDTING
WP_073324030.1_Fibrobacter_sp._UWOV1                      MPQKYLDVCFVPAPEYLQGIRPYGGVHLFGSMFEQQDDAVAYILTSKKKRDFPDAKINIRELNN
WP_216471320.1_Butyricicoccus_intestinisimiae             EIIRRPEICFYREVDFVIETEFVVGIQAIPEAIETRVMMSLAVVTTKPKTAFPYAQFVIQQEES
WP_044080180.1_Prevotella_pectinovora                     EKLQPTTLFFVKPCLADSAQACIKGVNNLLKLLECGNGGVISIITSKTKRDYPYSMYDIEEYSS
MCI8555501.1_Clostridia_bacterium                         KSIDNFCFDFIKVDN---QSNEIRGFKNLLAKMEDNLSKDYRCFTTIDDRIFKEHCYAINIINN
MBD8953983.1_Dialister_sp.                                DPTPLPSIYLASPEENSFGQLVLPNIQALLEKLEQYDETSFAVRTSLHKEVFSESMYSIRSSKG
MZQ76087.1_Peptoclostridium_sp.                           DRLENATLNLVNSQITNKNGNVINGIKNYLDQCEEECIVKLPVSTRHYNYDYSEAGVNIRKISD
HIS35238.1_Candidatus_Scatousia_excrementigallinarum      DKQTLPKIIFVKENVAKLAQPIIIGMAALIKEVEYTNKKVLYVKSHFKKSNFTESIYYIEELNS
WP_103226892.1_Clostridium_sp._chh4-2                     TVDSVPHITFVAPEFSFDSANVVEGVEFLPVAIESAVQSEIYIRTAKKAAVFANGQYAIATMDT
CDC05526.1_Clostridium_leptum_CAG:27                      SPLTNISIINVPNDM---GVVTKSGVKYLLHNLEDGVFGNVYASTS---LILDNTLFPSSTLSG
MCL2016626.1_Defluviitaleaceae_bacterium                  IQQNALNVTVLASDLQDLQGTILPNFKALLAELEKAAEDKILAITNL--SNVANSTFEINKINN
WP_235396406.1_Pseudoflavonifractor_phocaeensis           IPSARPKLIFTAEGIQPDASALVKGLRGMAKAVESAAAEILYIETAKSKDQYPFSLYTISDLKN
MBR3817930.1_Clostridia_bacterium                         EYQAIPQLIFISNSKFANASFMNHGLPSIGNSIEALSRDKIFIVTNHLTTNFTNSLIPISAIKS
MBP3656735.1_Clostridia_bacterium                         ATSNLPQLRFVGDDISIKDQKVTKGIDKVGLLVETVYADTLLIRTKKLAQDYPNSMLHIEELVD
ONI45221.1_Epulopiscium_sp._SCG-C07WGA-EpuloA2            KYELEKTIVFVTNNLRIDDVKILPGINYIAPEIEKSTEEKIYIYTAKSKDIYRNSLLEIVELNN
MBO6272669.1_bacterium                                    RTDKLPEIYFCEDSDYFENKNVINGINLLGAELENSSLLTVYVKTKKNKSDFPYSCLKIQNLKN
WP_037355505.1_Selenomonas_sp._FC4001                     KNQPLPQIIIERNPA---RGKTYNSIGELLSALEQMTSLMISLVTKYSPTLFNKAVYSVSQGPE
MBS6586269.1_Eubacterium_sp.                              NSSPLPKIRLVKSAELCVGAEPLNGIPGLLGYLEKMSQPVLNVLCGLNPGLFSRAAYYVSAADG
NBK77202.1_bacterium_D16-76                               GERNIPAIHVVPENVAYPGVDSVQGIKELLRILENGKGTDIYLTTSCKVVYLERSMYPVSEGMN
NSW92343.1_Bacillota_bacterium                            GEDEKVSLQIMPRNIETLCEDVIRGIKQYMQIWEKTSRQNSFLITGFAQAKETAGDYCIRICNS
WP_048095116.1_Archaeoglobus_fulgidus                     LEKTTVDVWIITDRLTKRNVVAVDGFKEYLKLWENQVIHDILVYSILLHSTPIAGNVKISPITT
MBQ7110119.1_Thermoguttaceae_bacterium                    RAECDIRVDLYDETTLGGLKHCIDGIKNALETLEAGRARTVAVRTR---EDFSSSLCSVRRVAS
HED00230.1_Pseudomonadota_bacterium                       -RPEEINISVFQRQIEGSRFFWINGIKNYMGLWENAKVSSICLSTRFADRGAQVSVIRIDVFKS
ROT09401.1_Muribaculaceae_bacterium_Isolate-037           DDCKPIKLTLINKSLAKFYETNTYGINQISKLIELIGLNEILIETKHKRHEFPDSIFEIHERNS
MBR2693834.1_Thermoguttaceae_bacterium                    DPKDSFSVIRVPLGS---NASFCDGFKAFLHEAEKGEPREYWVSSA---LTFEEAKFSVKTVSS
MCM1514006.1_Anaeroplasma_bactoclasticum                  NYSSTPAIVFVNNEYQ--YVRAENGLNVALRNVERNTGVKIYVLTSYKKKDFKSPLFNIEECKD
WP_166670004.1_Aminivibrio_pyruvatiphilus                 HSLEPFSLNVYSQEFGECGTDIMQGVQTWFYKWSGMIRKSCRLVTNLWRNQISSSLINIKIISD
HCH97635.1_Lachnospiraceae_bacterium                      DDEKTYSLTVYSEKFKNPLENIAHNLKEWLEKWQDCFFSQAMIMTGQIENEKTYGKVTIDIVEY
WP_195464804.1_Clostridium_jeddahense                     GEETDYSLTIIQKGLDVLSGNEIYGFKKYLQYWEQNPDKPLILHTSNAVYENNVFFDDVKVIVT
WP_006692206.1_Selenomonas_infelix                        AEDSVVSLSVLPPEP---MEVGIHGARSLLAALEQGRDGTHIVQS---RQSFPHALLRVHHVRD
WP_028051765.1_Carboxydothermus_ferrireducens             GEEEKCELIVAPFARKGKEFVSRNGVKDYFSFWEKDGVPSLWLVTSWASHPSQAINFKVKVYTD
MBQ0159480.1_Bacteroidales_bacterium                      -ETQSVEVFFSKYKNES-VQCQLNSLREWLMFWKKQAPQERIVCTSAPKYSKPDNIFNFTRIAN
KUK66682.1_Parcubacteria_bacterium                        -GSIKYNLVFCKFASIE-GFTIVHTTKDFLELWKKDDISSSILISSELNRAIDDELFTLTKIKD
WP_018963723.1_Coprothermobacter_platensis                -PRDKVQVYLIKFDYID-DSNALPDVKSLMKLWEKSKSDE-FICFSKTSCFCSDDVVSVNNVLT
MBR3117632.1_Oceanobacillus_sp.                           -YKDKICVYYTNSSLEV-LGNKISSSKEWLNIYKNGNYDKGIVCVSDAEQAKPDNFVDIEKLDN
WP_024292379.1_Lacrimispora_indolis                       -FEDLPRIFFIDDNLSA-YENEVTNSSEWFGMWRKSDVTKPIICTSKVEKASPDNVYNIKRIST
WP_191216537.1_Methanothermobacter_sp                     -TSDQLTIYHADFDFLE-EFEIIRNTHDWFNLWKKTYYNDRFLSFLPVEEWLPDEIFNLRIIKN
OQC39307.1_Bacteroidetes_bacterium                        -ETNSIKVYLTSQTLID-NYETIANTEEWLKFWKRKSPCD-VICYSKPKNTLPDTIFTIEQVDN
MBQ4420212.1_Bacteroidales_bacterium                      -EKQATRVYLMSETPNS-HITVLKNAKEWLQFWKTQAPQDVVICTSRPNYSQPDNIFSFLPIND
WP_183546997.1_Methanococcus_maripaludis                  -QDTPNQIFIPKFEYND-QYHVVKNVRDWINLWNNQSITKNIVCTSRFENVFPDYFFKFNILDN
MBU7048061.1_Theionarchaea_archaeon                       -KNDKIEVFLTDFLVSV-HFSVIENTRDWLSLWKGREKRK-IICHSRSKNAIFDKAFEIEKVED
HHW21152.1_Thermodesulfovibrio_thiophilus                 -SIQRTNIFLYKDLFIN-TIQKVKGSKEWLTLWKKSLSTP-ILVLSKTDNAKPDEFFDLKKINN
WP_207678199.1_Desulfonema_magnum                         -PFKKIKIYQISFEALC-NFKTINNTLGWLDLWKQNDLEV-VICKSGTKDFLPDMMFFPEEIST
MBE6838777.1_Ruminococcus_sp.                             -ELEYRQICFVSDEYNI-KTNKITTSVEWIGLWRSADFTKPIWCCSASQEASPDNAFQIDSISN
WP_205109179.1_Marseilla_massiliensis                     -EIGTYNLVVTNGTISD-KYTYVKTLYEWLKLWEKGKIKQTIISSSPNHFAQPDNAFTYTECKN
MCH4183501.1_Prevotella_sp.                               -PNFSYRLILTDGETET-HYTIVNNMHEWLSLWRDEQENPNIVCTSHSGYAQPDHAFSYIICPD
MCI6981218.1_Akkermansia_muciniphila                      -PADKYRIILTNGTTKD-SCVICHNTREWVKSWEDLDGRRTFICSSKTQNISPNDCFEYVRCET
MCD7797870.1_Akkermansiaceae_bacterium                    -QANCYSLILTDDVKSN-KYSICSSLKDWVNLWQPNKIKHKIILSSKTSLPTSDNAIDVKFYPD
consensus/100%                                            .......h................s...h...hp.......h.....................
consensus/95%                                             ......pl................s...h...hp.......h.............h.h....s
consensus/90%                                             ......pl.h..............s...h...hc.......l.............h.h....s
consensus/85%                                             ......pl.h...........h.s.p.hh..hcp.....hl.s............h.hp.h.s
consensus/80%                                             ...p..pl.h...........h.s.p.hh..hcp.....hl.o............h.lp.hps
consensus/75%                                             ...p..pl.hhp.p......p.hpshp.hh.bhcp...pp.hl.os..p..bspshhplp.hps
consensus/70%                                             ..pp..pl.hhp.p......p.lpshpphhpbhcp...pp.hl.os..pp.bssshhplp.hps
```

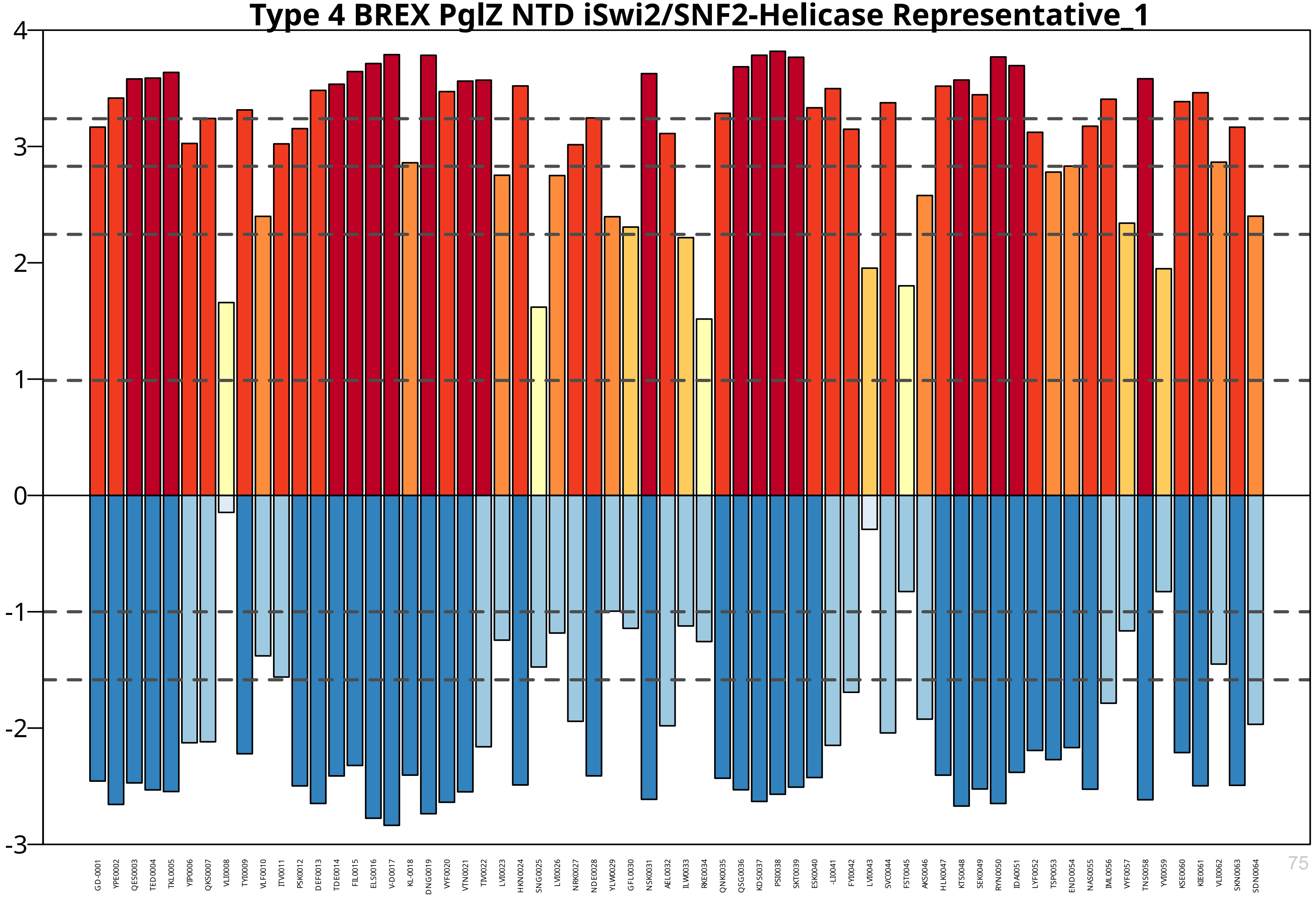

Type 4 BREX PglZ NTD iSwi2/SNF2-Helicase Representative_1
4
3
2
1
0
-1
-2
-3
GD-0001
YPE0002
QES0003
TED0004
TKL0005
YIP0006
QKS0007
VLI0008
TYI0009
VLF0010
ITV0011
PSK0012
DEF0013
TDE0014
FIL0015
ELS0016
V-D0017
KL-0018
DNG0019
VYF0020
VTN0021
TIV0022
LVI0023
HKN0024
SNG0025
LVI0026
NRK0027
NDE0028
YLW0029
GFL0030
NSK0031
AEL0032
ILW0033
RKE0034
QNK0035
QSG0036
KDS0037
PSI0038
SKT0039
ESK0040
-LI0041
FYI0042
LVI0043
SVC0044
FST0045
AKS0046
HLK0047
KTS0048
SEK0049
RYN0050
IDA0051
LYF0052
TSP0053
END0054
NAS0055
IML0056
VYF0057
TNS0058
YVI0059
KSE0060
KIE0061
VLI0062
SKN0063
SDN0064

```
# ; Type_4 BREX PglZ NTD iSwi2/SNF2-Helicase Rep_2

WP_192822482.1_Rufibacter_sp._LB8                      MKALLLDPSGDAPAPEGYSVIDTDVAWLKAADNVPKVVVRGVERCNWAYIWWQALGGQTATLIS
WP_105482027.1_Abditibacterium_utsteinense             MSSLRLDWKGDAEIPEGARLVTNEVEWLRVAPDAQDVWVRGATVCKWASQWWQAAGGKCEEVRG
WP_243797665.1_Hymenobacter_tibetensis                 MSFLLLDPHGDARVPVGYAVVATELEWLRETLSAPPIVVRGLELTRWVRKWWEARGGECQELVS
MBX7251230.1_Candidatus_Promineofilum_sp.              MTDVWLDRLGDADTPEGAAVVECEADFLRCALDEPRLLVRGERLCEWAGAFYRGRGLPVMETHS
WP_162787804.1_Anaerolinea_thermolimosa                MMEILLDHEQDCPVPDGYILVENEVDFLRLATSGQSLLIRGRELCAWAEGFYSLRNLPVRVVES
WP_012642882.1_Thermomicrobium_roseum                  MREVWFDQDGLCPPRPGTVSISDDVSFLEEIDTDRPLLIRGRQRCEWARALADARNWRVHEVQP
MCL6576921.1_Kyrpidia_sp.                              MRKIYLNPNADAYKPEGTRVVSSETEFWIYALRPEPLVVQGLRLCEWAREFAEGRGLEVEELPS
WP_139401683.1_Deinococcus_radiopugnans                MARFYLDAAGGAEV-AGAHSVNSEAEFLRLALGDGPLLVHGEALCRWARRFGAGRGIPTQEVVA
MCE5327915.1_Planctomycetaceae_bacterium               MRELLLDIHGEANPVPGCRRIETELDFLQALSEGKPVLVCGEHLCEWAGAIWQARGWRVQRLTS
MBV5327602.1_Chlorobium_sp.                            MICFVFDPSGTESFESEYRLVEDDVSLLQGVLYQQSLFVRGERLCRWASLIADARGYDQVWRKA
MCC6188628.1_Anaerolineales_bacterium                  MSEILLDREGTARIPPGFQVAETEVEFLRVAPEAEALVVRGARLCDWAATFFRGRRATVRTLVP
WP_168570764.1_Oxynema_aestuarii                       MSDVVLDPTELYDIPAEYTPISTEVEWLEHVITSNPLWIRGEFLYKWTREWLRVWNRTVEIKQP
WP_006100361.1_Coleofasciculus_chthonoplastes          MIEIVLDPSKTYAIPPGYHLIASELDWMRDFINGSPYWVQGESLCNWTQTWLRCWNRSDQIKRP
WP_273058112.1_Shewanella_vesiculosa                   MIKFYLDIYQVFSGDSDVDRFRQLIYSNLSRTTDLCIYFHSKTLYSRFRDFEGLDGVAHTNIID
MCK9609000.1_Methylomonas_sp.                          MVMLVIDTYQIDTEQPAIEQLRQKISQHLKDEHLLRVRFKSKALYERFKDFEGLDNVLPTQQLL
WP_017840083.1_Methylotuvimicrobium_buryatense         MVQLILDYFGVDADDPSIAAVTRQIHQYLREPVLHRVRLTSQALYHRFEHLEGLDGVLKTQTLI
MCK5897804.1_Methylococcales_bacterium                 MIRLIIDYFNIDKQDKAVNINRKKIDTHLEQDTLLRVRLNTPALFRHFSDYEGLNRVLLTQKLL
MBC7570526.1_Spirosoma_sp.                             M--LTLDPFRLSPPDTAYVEARRQILAYRLGSKAPTFVVYETPYLFWFNDLPDEERTD----VT
WP_198076877.1_Hymenobacter_negativus                  M-KIYLDPFSLQPASASYVVARCLLLDFMRSQSNRDVIVRQKPWIEWFGDLPQTLIAR----VS
WP_235137408.1_Dyadobacter_fanqingshengii              M-KVIIDPYKIYKSASTYVISRTSLLDYFVSEDFENIHVTEHPFTKWYDDLGEHLQV-----IK
WP_194112254.1_Mucilaginibacter_myungsuensis           M-KVIIDRHHLYKTTQTYQKARGLLLEFLLSAGYDDIHVFKEPFVDWFDDIPELVAP-----VT
HBJ34341.1_Planctomycetaceae_bacterium                 MITLIFDPFSIAEKQKAELLAFEEINDSLRRTVVPKVYIIPSSSIEHFRTYREVKGVNIQT-VT
MBX3399563.1_Gemmataceae_bacterium                     MIELVFDPYRIYTGESQEVAAFHTVGEMLRQTRRPKIYVLDGIAWEHFRSLRSVRGVRVRE-FT
WP_099078666.1_Vibrio_sp._PID17_43                     MVEFLVDPYNIDSDSASVREARLRINENLASIRNLRIRIVHESLLRRFSDYLGINGVKVTFTPS
SEH06866.1_Thiotrichales_bacterium_HS_08               MIQLLVDDFDLEDGEDDIPHIRQKIYAHLAYQGALSIRM-SSVFFQRFKSFEGGQGITVKR-IS
QEP42412.1_Ectothiorhodospiraceae_bacterium            MVILEADSWGISANADQISGARSKIRHYLLQAEELTIPV-KPAYRQRFADFIGFDGVE-WREVR
WP_194715675.1_Noviherbaspirillum_soli                 MVEILVDSLKLSRKPSDLLHVRERIRDHLRRSTTLRVRL-DEARYRKVADLDGINDLLVVH-VT
WP_201382003.1_Ktedonobacter_sp._SOSP1-85              MITIYCDFYSILQVEQDYIPLRQRIRSALDKNTAYTVYVQNPILAQWLKDLRGYDPHVRWEDID
NJL05928.1_Chloroflexaceae_bacterium                   MITIIADVHSCEAAPTEYVPLRQQIQRALTTQQALVVVIRHATLVHWLADLRQYPSSIVWRTVD
WP_129675140.1_Candidatus_Chloroploca_sp.              MITIIADPHNYKGDVRDYVAARQAIRRALDLDNSLTLHVTSHLLLHWLEDFNGYTGVD-WKQIA
MBU6335166.1_Chloroflexota_bacterium                   MITILHDRLGVLPPPEDYVAVRRAVRVALDLGQALTVVVHDPQVSRALADLRTYPAAVVWRDAD
WP_097643873.1_Candidatus_Viridilinea                  MITIIADRFAAAAAPADYVEVRCQIKAALVNFTSLTVYVTDPVVLCWLSDLQRYPASLCWHEVD
consensus/100%                                         M..h..s.....................b.......h.h...........h.............
consensus/95%                                          M..h.hD.................h...b.......h.h...........h.............
consensus/90%                                          M..hhhD.............h.p.l...b.......l.l......p.h..h.............
consensus/85%                                          M..lhhD..........h..hpp.l...b.......l.l....hhpbh..h.............
consensus/80%                                          M.plhhD.........sh..hpppl...b...p...l.l....hhpbh.ph....s.......s
consensus/75%                                          M.plhhD.......s.sh..hppplp.hb...p...l.lps..hhpah.sh..h.s.......s
consensus/70%                                          M.plhhD.......s.sh..hppplp.hb...ps.sl.lps..hhcahpsh..h.s....p..s
```

# Type 4 BREX PglZ NTD iSwi2/SNF2-Helicase Representative_2

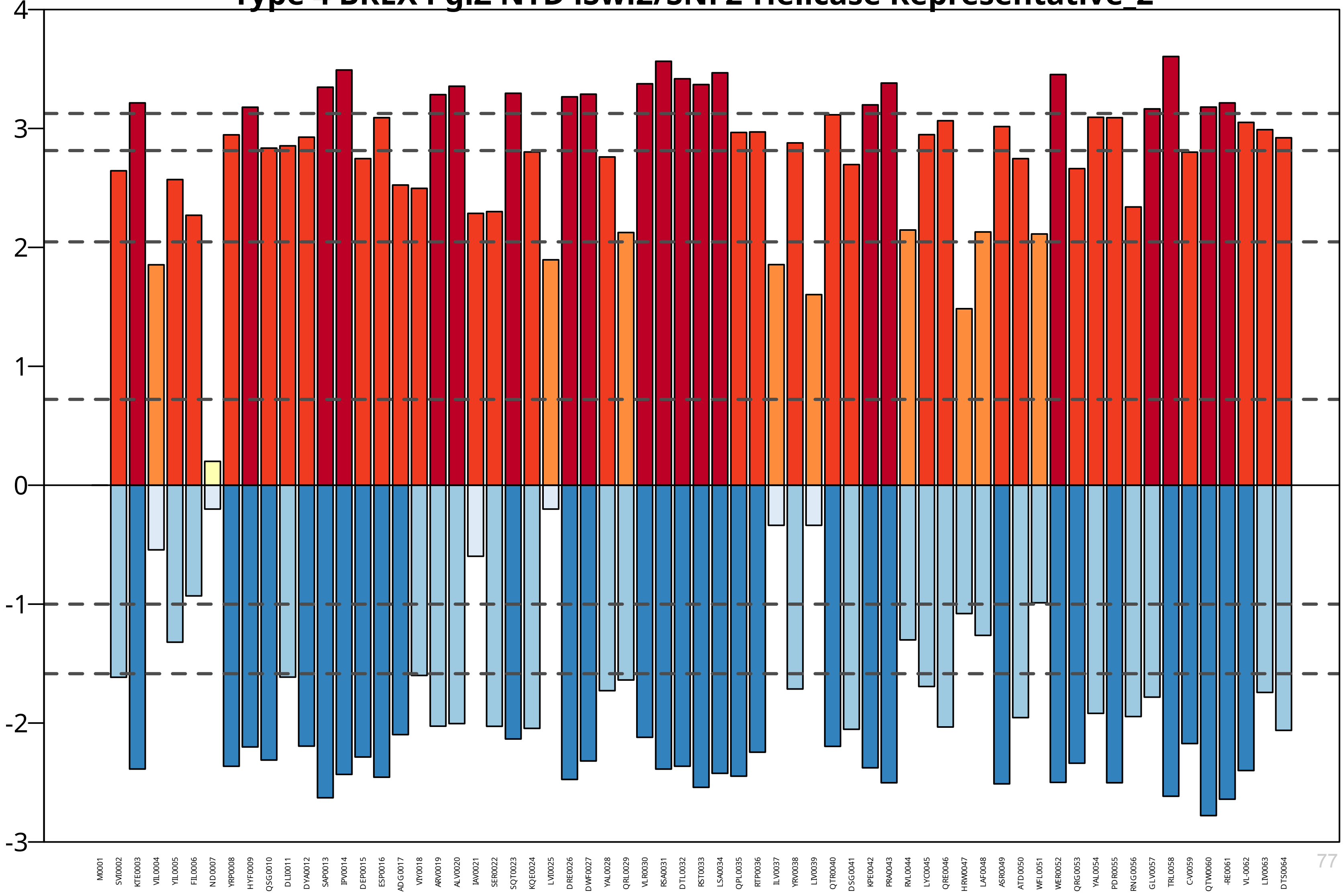

```
# ;BRC-system PglZ NTD iSwi2/SNF2-Helicase

MDS0297782.1_Halogeometricum_sp._S1BR25-6             MPTFTEIAEHAKVSPVVCSLPKLASFYLDDLAVYTSLDNFHHDLYDPDYRFEEDVVIDTDEHHPDYVRLSCDEEPV
WP_271955572.1_Halorubrum_ezzemoulense                IESLSALAEATRDGPVVANIGGLESRVLQNIDTVESLEEFYRLYESPTNLIETDVIIRDQGPKTLYPSVEYTGTVY
WP_139229723.1_Halogeometricum_limi                   MAKLEEVVDEAKSHPVVVDVHPLSYLYLQELELFESEKQFEDELDNPSKSFERNVAIKEPRVKTTYPSMSIDHDPL
ATW88247.1_Halohasta_litchfieldiae                    MAYFNEIKERAHQQPVIVPLPKLAQFYLEDFNIYSSIDDFQTDFYHPDFLFENDVIIDADEHHPDFDRLSCDEETV
WP_338740220.1_Haloplanus_salilacus                   MASFETLADRASEAPVISPLPALATFYLDGLSIYRSVDTFHRDLYDPDYQFEQDVVIDDDSRYHEYPYINCTTDPI
WP_143414662.1_Halorubrum_sp._SD683                   MEDIKSIADRAHEEPIRADLPALAERILQQIDVISTTTEFYQRFEDPDHRFSADVLINDTTPKTTYPTIPYTGDPY
WP_048108644.1_Methanosarcina_barkeri                 -MKLSELDNLLSKTPVYLSLDSLVSLCYLDITIFHNRRDFWRKIDQLEPLKLCRYVILDDPD-YRLPYVRLKHNPF
NPV62497.1_Methanotrichaceae_archaeon                 RVTLEELDALLNKAPIAADMNPVSRCYLPN--RFANLDDVVKELDSPRPVDPSRTLIIGPRKDEKFLNVRVVHEPF
MCK4444004.1_Thermoplasmata_archaeon                  -MRVESFLELLRRTPVVLKGNELPLLYVDRTRIIRDIEELNQQIDSPNPINDAEIVIVDDDKGWSFPVAEVSEDLM
MBI2842128.1_Armatimonadota_bacterium                 DALVSGLSATLNSYPPAFRGNPLNLLFLDDARPIREASAFLNLLDHPALCDERREYLLDDPAN-PFPAYNISRRPD
HHW27504.1_Candidatus_Fermentithermobacillaceae       -MDVPRLIRMARETPVGIPNDLFLRLRLPDCNVFPNAQSFREALNGHRPMPNSPTLIVCESGDEQMPFEDTHYDFM
HIE11925.1_Desulfotomaculum_sp.                       KVDLEWLREMLRQTPVMAEIHPLLLALLPKVVVFGDVWELIARLDSPELQPTRPMLCTQVKSEHRLPAIFCDHDPL
HHW40997.1_Syntrophomonadaceae_bacterium              QLSLDEFIALLQKTPVITAWNPWLKVKLGKVVVFPSLSEFRIAVNRFHPLADMPTVIFAGQNEHAFPKVQCTYDPK
HQD80871.1_Bacillota_bacterium                        MIRREHVPSIIDQYPVCVRFDPLLYMRLYGCEVLSSPEEFREHLDDPSYFCGPTLVMCDNLGESIYPLQTLDVV-L
MBE2199248.1_Anaerolinea_sp.                          --MIELLLKQLNIAPVMGQFHPLWSLCFPPVVIAHSAKQVAEQLDGRERHEEKPILITEYNPADRFLSHHLPDCPP
MBC8263374.1_Anaerolineales_bacterium                 MMSLDDLLTLLDCTPVIAEIPPVSICYLPEVTVLASAAEAAWQLDAPEPMLPGRLLITASDIQDRFPHVRCDHDPF
MCZ7554456.1_Anaerolineales_bacterium                 VLEWDDLLAALDASPMIVDMHPVLYAFLPDVRVVHDSAEASWLMDSPDPTLLSKMIIQREAALGRFPHVKCSRDPF
HID64303.1_Anaerolineae_bacterium                     SATMSQLDDTLDRTPVVYEGEPLLLAELDNLVVERDVWRYVQRVDNPAPVAVERTVLLGPVTSDRWPVVRMDARPD
MBN2393033.1_Anaerolineae_bacterium                   ETGFMRLLQALQRGPVMTEISPLALLCLPPIDIASDAETLVERLDILEPHSERPILIPCIDTRHRFQVESLTYTP-
MBV6438329.1_Anaerolineae_bacterium                   ALSVDELEAMLSRTPVIAKVHPLVPTALSEVRIVSDANAFQIELDEHAPAPELPTLLLSSNAAARFPRVTSLGLPP
MBW7959471.1_Candidatus_Promineofilum_sp.             IEQIDTLRKQLSLAPVMGHFHPLLPLCLPAVMIVTEARRVALAMDARGPVPESQILITRIDPTDRFLSLSLTETPP
MFN8469222.1_Caldilineaceae_bacterium                 ---MDW--SELNVIPHIVQLSCLWRAVLLQILVPITLHQWKGILDA-APRATAPTVFVDLSPNYGFPQVQMPLLPD
MBI5668048.1_Chloroflexota_bacterium                  ATTFNELAQLLAKIPVIGHVHPLIPLCLPPTQVVNDIQGFGYEIDSPELHAARPTLLIRHDKAARFPLVNVLNHPP
NLE46402.1_Chloroflexota_bacterium                    VESINYLLDLLARAPVMGSFHPLLPLALPTVTIADSAQKVAEALDALEQHEPCPILINEYDVRDRFLVERFTELPP
RLC84905.1_Chloroflexota_bacterium                    MTDPAELARLLMQAPVMGPLHPLQALCLPPTEVLADAAALAEELDDLAHHAVRPIWLTDVQPRDRFLQADASTPPP
MDX2239252.1_Leptolyngbyaceae_cyanobacterium_bins.302 EVSLDKLPDYLEAGPIMAQMHPLSLAFLPILKVFDSAAELVAALDNPANECSDAMLALSYDANDRLLSLKINHQPL
MDE0397489.1_Candidatus_Poribacteria_bacterium        ------MAQQLCTIPCITEIRALWELCLTDVWCPVSLEEWRERIDSPREIDADSTLFVNLSPSFGFPAVKFPALPS
MYC73121.1_Gemmatimonadota_bacterium                  ------MIDILEKIPCLTTLQGLWDICLSDIWCPNSLAEWRDVVDAPQDDSTKATLFVNLPETFGFSTVDLPTFPP
RJP56334.1_Deltaproteobacteria_bacterium              GADVTKLSTAIDAYPAAFRGNRLNLLFLDDAHPIDSSREFLRKVDSLQLQEERREYLISDKEG-HFPTISIESRPD
MFZ4828881.1_Phototrophicaceae_bacterium              GKNPEILGGVLQRSPAIVKAHPLFPLALPKVKIVTDGLDILEKIDNREHLVNHQTVILDQHPLDRFPTLEFPTHPS
consensus/100%                                        ..............P.......h....h.......p...h...h...........hh........h..........
consensus/95%                                         ......h...h...P.......h....h.......p...h...h...........hh........h..........
consensus/90%                                         ......h...hp..Phh.....l..h.L.......s...h...hp..........hh........h...ph...s.
consensus/85%                                         ......h...hp..Phh.....l..hhL..h.hh.s..ph...hp..........lh........a...ph...s.
```

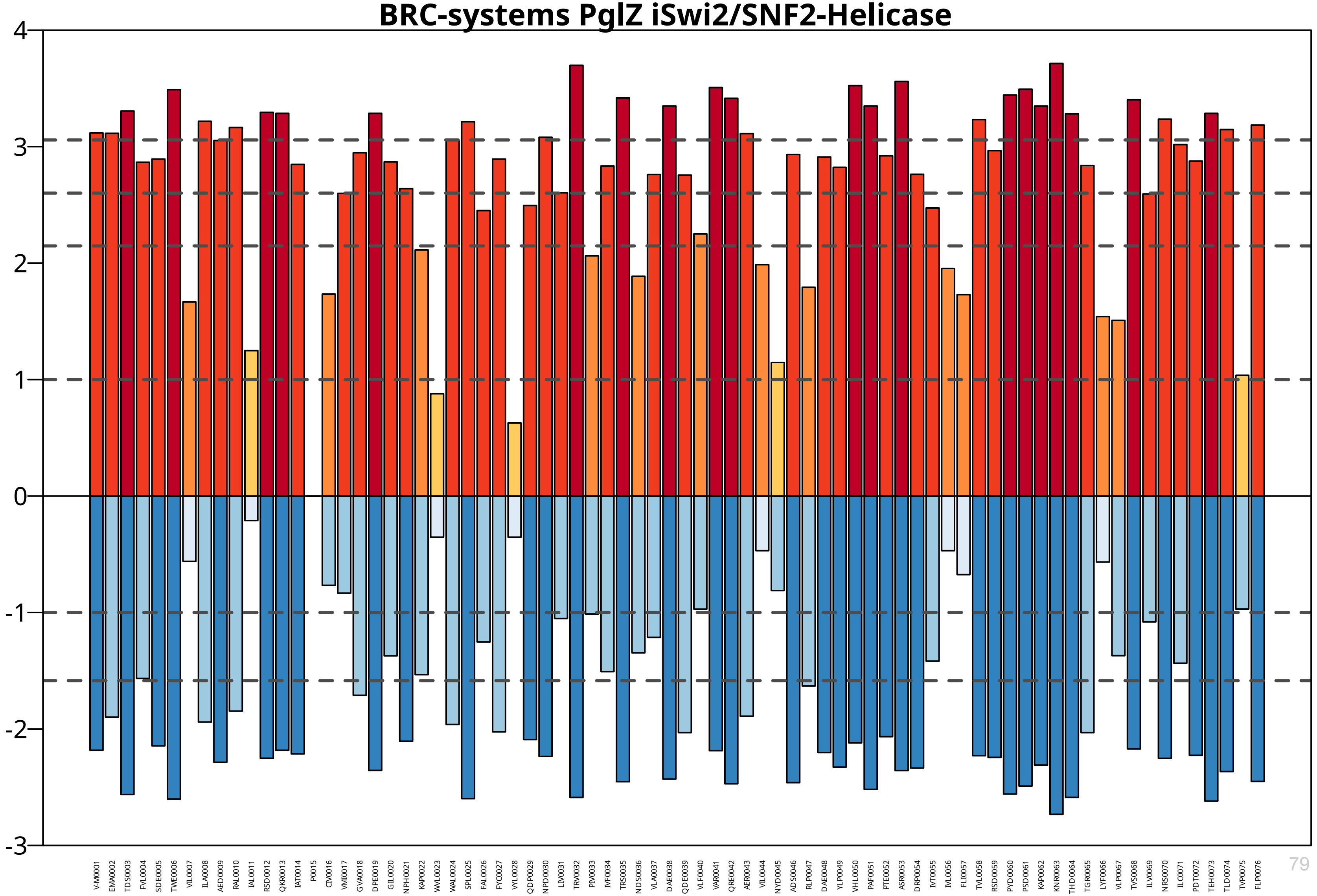
BRC-systems PglZ iSwi2/SNF2-Helicase
4
3
2
1
0
-1
-2
-3
V-M0001
EMA0002
TDS0003
FVL0004
SDE0005
TWE0006
VIL0007
ILA0008
AED0009
RAL0010
IAL0011
RSD0012
QKR0013
IAT0014
P0015
CIV0016
VMI0017
GVA0018
DPE0019
GIL0020
NPH0021
KAP0022
WL0023
WAL0024
SPL0025
FAL0026
FYC0027
VYL0028
QDP0029
NPD0030
LIV0031
TRV0032
PIV0033
IVF0034
TRS0035
NDS0036
VLA0037
DAE0038
QDE0039
VLF0040
VAR0041
QRE0042
AER0043
VIL0044
NYD0045
ADS0046
RLP0047
DAE0048
YLP0049
VHL0050
PAF0051
PTE0052
ASR0053
DRP0054
IVT0055
IVL0056
FLI0057
TVL0058
RSD0059
PYD0060
PSD0061
KAP0062
KNR0063
THD0064
TGR0065
LYF0066
VLP0067
TVS0068
ILV0069
NRS0070
ILC0071
PDT0072
TEH0073
TLD0074
TVP0075
FLP0076

```
# ; Type_1 BREX PglZ CTD IG-like

WP_238754868.1_Leptospira_bandrabouensis       NDISYEILAELFIGKGKHRIELSLENVLFKTLIVELDSVDGARLKIISSNSYDNNGIVVEPNTKVNLEFEITTDYQEKASVKIWSPTLNSSFVKVPL
MBF0258418.1_Desulfamplus_sp.                  GTFELTIQKPDALGFHRIGVLPLSTPGFFSIPHVEVQMFSDQEIGDIVGAQRKGNILTLPLDRESEIYFKLSKGACSKAQIYFKATQQRVLLTNSEY
MBX3247846.1_Myxococcales_bacterium            LRYVVRGSREPLAGLQCVRAVVEVAQRSLEFLELALRATDDAELVDVRGGRTDAGTVLARVGQELEIFFRLATSAVERAQIELSHPSKMALYFEVMP
MBT9554533.1_Myxococcales_bacterium            AKYRTEVLPADVLGLRRVRVRVLPDLSALEFVDVELVATGRPTLKDATGGVVKGGRLAVRVGEWTEVFFGLEGPREEMVALEVVGPGPVAAFFSVSG
MCC6649976.1_Candidatus_Eisenbacteria          SKYEVAARAPAHLGRQRLRVAVRLETASLGFISLALRVPGRPSLLDAGPPTLSDGRVLVPPNREALIEFELEALADEKVRVEIYHPDAREVFFDAGR
KPQ33026.1_Phormidesmis_priestleyi_Ana         VPYVIEAEASAIAGFDRVQVKVKPAQGVLTFLNLSLRVPNRPNIAEATGAQLKNQQVQLAIDQWVEILFKLKGPKDERIKLEVYHSDGAEIYFEVAG
MBD2549130.1_Microcystis_elabens_FACHB-917     TDPAWPGALPEKITNRILMLPIELPVTLLNQRQVVLGAYDSKTPIQAIGADTGTNRLTLTPGQAATIGLMLPAESARKLYLELRDAATDLVLHKSPE
WP_096450969.1_Thauera_sp._K11                 ASSKVALEGPSVLTNRTFGMRVLVAAELFSQVPVRLILLADGRCGMALDADRASGVLMLQPGKPANVAMMLTRDEVNKIRIVAQDPATDAVLAQSAD
MBY3465673.1_Rhizobium_laguerreae              EKNAVSVKLFDAVTNRIFSVEISLSGGMFAERRVRPVAICDGSAKMTSNGAVEDGVVVVEPGKAINVAFLLSDDQIKTLKIQMLDADTDAVLYASPD
MBY0113210.1_Phycisphaerales_bacterium         ATLTATVGDPAKITNRTFRVTLTLADGLFTDASVRVSLVHKGSVGHAIDADAVSKTVKLEIGKAATILMLLQRDDAKQARLVIEDATSRAVLYQSDT
WP_273378697.1_Symbiobacterium_thermophilum    AGDVFKIRLPPRVTNRFFMVTVLYVRSLLSPRRVRLELVANGHAKASQYGDPSSEEITLESGKDNTVTLFFKADASGDLVIQMKDAESGAVLAEEGP
WP_209466794.1_Symbiobacterium_terraclitae     RGSDVRLAPSDPVTNRAFKVDLLYA-SVIEPRTVRLTVLLGDEVMVAEG-NDQTKEVTLVSQKPNTVFLKLSSDGQGEFTVQVVDSRTGLVLAQRN-
MBX6362428.1_Gemmatimonadota_bacterium         KKPAVTLELGRKVTNAIFQVTLSFALDLFDERRFRVTVVDPSKTALATDGREAGAEVELDTGRTSTLVLQLTDPGSGDLELHVADVDAGETILRKK-
MBW2308572.1_Deltaproteobacteria_bacterium     VDIGYELEITPRVTTRMFTVRVVYPAGLFVPVKVAFRVLKGRTAATSMYGQEGTREVILEKERENYVTVLLEGELTGKVSVRMLDADTDAELQRIDD
WP_083610988.1_Desulfatibacillum_alkenivorans  TSVTLKLEFKSAITNRFFSMTATLTHGLFESVRVRAAVLSNKFCAMAAYGEEGSREITMRVNEPNALTFMLSEDGIEKVTIQIVDCHTQLELATMAD
NIM12894.1_Candidatus_Aminicenantes_bacterium  ESIEIKLSISKKITSRHFSVTVIYLKSLFGPIRIKAQVKSGNNAVMSAYGEGGTKEILLKKDKENHITFMLTEEDIQHVFVQILDTKSQIELKRSDK
MBN1165673.1_Methanospirillaceae_archaeon      SSISWSLAIGDKISSRMVSVKINGITSITCDPKVRVEIRSGDNPLTAHYDREATGDISMRINEENTAIMQVHTIPKGQATIHLLDSETDVELTKIGP
MCI0601819.1_bacterium                         VKISWKLALTPKITSRFFSVTVTGESRVLEMPRIKVEARVGNIPVSGSYGNQTTADFALRESEPNTIALMLTEKASGSVTIHLIDSLTGMELVKPNP
WP_062190142.1_Anaerolinea_thermolimosa        KEFHWEVILSKQISTRFFSVTINGATELLATPKIRVELKSGNMPVAATYGDETTKDVKMKIEIPNTVTLFIDDIGTVTVDLDLINSETGLALTQVQN
WP_156746889.1_Mycobacterium_sp._E2733         QKLDIKLTIGGRITTGVFAATFEFLGDLFTDVTFRVVVRGETRIVSGDGYDSESGSVTIAQGKPSVLTFQVISNKDAHIGVQVLDARTGRRLAESVA
MBN2413376.1_candidate_division_KSB1           GDAMFRVSMKKEITNRFFTLQIRYTADLFLPRRVHLDIEYDKQIVTASYGDPRSGDILLEKNKDNSIAVMLNEPIPPTLDLILSDTETLAVLSKTTK
WP_164995432.1_Candidatus_Kuenenia             TAPEIKIILKAKITNRLFSVQLVWQRKLLGTKRVRVAVKSGKEAVHAVYGEDGTKDIILEQNKPNLVTIMLTGEAQNVLYLVLVDAETLVELARLEN
MBV6422080.1_Ignavibacteriaceae_bacterium      PAEDFSIKLKDQITNRIFMIDVLFKLDFVAEEKVKLTIHSQESVVAAKSGNESNRSLELKNNELNTITIMLSDVGISKFDITMYEYESDVLLASKKD
WP_083423498.1_Stigmatella_aurantiaca          QSVRVDLTFSGQFTNRILLITATAFGDMFTDVPVRITVQHASILGDLDERDDATGLVSLKPRQPVKLPIRIADDFRGDIVIRALHATPDVEYGRLV-
NLN06912.1_Bacillota_bacterium                 GKPVIEIRYSDRFITQVIGLKIYLQENLFKQEQVLVEAYDGKEAADCDARDERTRLVTLQAGQEIFVPVLIYPDFNGPVQIRVIDPQTRVVWAKKD-
WP_055669757.1_Desnuesiella_massiliensis       KNENVTISYRNFFTNRVFSINICF-DSLIPSLRTKVDVYDGTTVADCPSRDETTHEVILNSGCPVHIPILIDSDFNGDIEIRVIDSTSKVIWTRLK-
WP_242609850.1_Comamonas_thiooxydans           RKVVVELSYAKRITTRLPVIEVILADDMFSQVEILLEAQDSKEPRPGGDVNPATRTVTLMPGQRKQIALRMDDEFRGKFSVKALNPTTLAAYSNLA-
WP_243520949.1_Hymenobacter_monticola          KRTSLSLTYKPVVTTRQPILTAEAGGSLLDFVEFQLVVTAADKLGLHTAVDVATQLVRLSVQEKARLPVRLDEEFEGAFTVRALHPDTQVELASIT-
OQB98762.1_Spirochaetes_bacterium              HEVGVSLSYTTKITTMRPSFDLSVHTDPFKGIAIAIAARAGGRAQASAGYDPGSGCFRLKPGKSIKITLAMSEEYRGSFTVSAFDPVTHELYAKLD-
WP_166386808.1_Polaribacter_sp._11A2H          QDYQINLTYSGYVTTRRPSIEISCGDSFFDEIDIMLEAIGDNGPAASENVNSTSGFAEIKPGQSLKITMSLHDDFEGKFTIYAKAPATGVILSEIN-
WP_235164441.1_Dyadobacter_chenhuakuii         SKADVTLSHKGFITGLRPSIQVAASESLFAELVVRIEAVAGGTLADQTGVNLLAGTVELTPGTTSSLTLEMEEDFEGNFDVVAINPATGKQYHFLS-
MCP5535438.1_Akkermansiaceae_bacterium         FSFEVNLNYSKKIMTRRPSIEIGIQEGLFAGVRFQLEAVHKKRPAPCQYVDPSTNYVQMPPQSFAKLTLIMDEEFNGEFELRAIDPDTKMPLGKQAK
WP_219938192.1_Succinivibrio_faecicola         DDVSEVISRERIITSGQILITFYQEQPVVKGISIKAGFFRDGVPVSNIEDLEFNKETTNVEEREQKRQFKLSTGIDSGVILKLTKRVENTPYDEITF
MCK6605714.1_Ignavibacteriaceae_bacterium      ENISKVISRSTTLSSNRPVFELVQKSPITLPRELSVGIYSDGLLISDTVRVMFDSKSEDLRERTKKITLRMQSGIENKVILKLSEISEKVPYHDFTL
MCH3907672.1_Sphaerochaeta_sp.                 DDITDVLTNLKTITTGSVAVTFFQEDYVVQGYEATFGFYQDGTAISDIEKRTIASEAIDPKKREFQIVFYLNKESERRVFLRIQKILPSGMIAEKEC
WP_251178637.1_Adlercreutzia_agrestimuris      KKTYETFATGLSITGSTVAVDVYQVQPCILPVTVKVGLYPDGKLLSATKTLVLESENKASDARKSRVTLDVTNDIDRTAMVRISKQVGQTSVWEQEY
MCC6107936.1_Atopobium_sp.                     RSAAPVLTGGKVITGSFVRFEIFQTEPVVLPVSLQVGLYADGQLRSETKPIEAASSSRSAEERRIPVTLAVAQDVAGALTLRVNQRYGTTTVAQAEY
MCI1665139.1_Atopobiaceae_bacterium            RKAETVLTGGTLITGNAVALDLYQTDPVASKIHVRVFASAGEAAISPVEELDLASESEDTAARISHIRLPLASTVRGQVTVKVQRRVGETDLASQDY
MBQ6502306.1_Flexilinea_sp.                    HINQKVANNTRIITTSVKTVVFFQQDPVIQPLTVTAGFYPEGEAVSDEPVITFNLTEPEDRLRQVQQNFTFGNNAVGKVELRLTEQLSATTQTLGVY
WP_237187173.1_Rothia_nasimurium               NTAQTVAEVPQRMTTNLLTIKLLQQEPILKPLKLRAGIYAD-KLISDQPEATFHFTSEMPSDRQIKLTLELTRAADAGADIRLESYNTTTHYDKTSI
WP_149431761.1_Rhodococcus_cavernicola         KDTSKVNTSSPTITSSAVLVTLTQREPVRRGRSLSIGVFDDGTLLSNQRAVVVDSRADDIRERHIAVELVLGDDAMGQVQIRADEVSHDTGYKSTSV
OFQ22229.1_Actinomyces_sp._HMSC062G12          TDVRDVHYDDTSLKVNRFSPRVIQQEPVRRPLTVTIGLEATGERLSSTRVITLDADQRSEVSLKQSSELILNDGVLGQARIVAYKTVNGQAVAVHEL
MBX3116434.1_Cryobacterium_sp.                 SEVKPVKPKSDVLTTAIVRIGLLQAEPIAQARELRASLYGD-KLISDQVVVVFNATGDE-RDRIQEVKLQIRPDAGGAVEFRLEQQSTISTPYKHLF
HJG42481.1_Corynebacterium_phoceense           STNSVVFQNSTVITTQQLIIKMTQEKRVIQPAQVRLSLYKRQVLISDQPKITLGSTSHESRDWEVEVKLSLNSDADGTVTAVIEIPLPNTTLTTQTY
WP_219100161.1_Bifidobacterium_pluvialisilvae  ADVRPVLQRTDRITTGQLTVEFQQTEPVVAQRTVFAGLYVGGEPISNEVPLTFRSESPEARDRHVAATFLLTSEADDTVELRLSERIAGSLEQKADY
MBI9030469.1_bacterium                         DDVKEVISQAKNITSGQISVTLYQSEAVIKACYISVGLYQDDILLSDKHEVSFDSESELSRDREVKVTLRLNEKAEEQILLKLKKKMSGTDYKSDNY
MBD1206695.1_Paracoccaceae_bacterium           DDIEEVIGAGRIITTGQLTIRLYQAKPSRKSVRLRIGLEPDRTKISNEEDRIFDFTSEDPRQRETSVRLLLSKAAEGGVRLVMRRLDHKGVYRSETY
QNI65417.1_Synechococcus_sp._A15-44            DESREVLRVPEKITTARLKFSLFQDEPCFLPITLRISVVNDKELLCEPATLRLDSTAVEPREREQLIDLKLSNAAGNQLELRIDQMLEGVRLETKEV
WP_147932466.1_Neolewinella_aurantiaca         ADALPVIAGNGHITNNEYSVRFFQKQAVRGERRLRIGFYADEKLISDEKNIVFNSEAVEERNRETRLRFRFGADAGQRVKLRLRD-----IVFEHPF
WP_013460554.1_Sulfuricurvum_kujiense          DDVENVVKSFSRISSNQITLTLWQKTPAMLPRTLYAGFYGD-ELVSNEEQLVFGSTDEDSRNREVKVKFHFVQTISNQISLRLSELEPGTVYKQEQF
WP_260916970.1_Aliarcobacter_butzleri          NMINEVMPIS-TITTNNVNVSVYQEEPIIKPITISCGFYEDGIELSDKHKITLNSSDDENRNRENKLKFTFKEISNGKIKFVMKKVIENSIYKTYDV
MCF7790535.1_Victivallales_bacterium           DKVNQVLGKPERITSGQLSLKFYQLEPIRLSRDLRIGFYPDSKLISNREDVSFESVDPVGDNRTKRLTFIFSKDIDNKIELRLEEQIKNSTYKKYKF
WP_153112503.1_Prevotella_copri                QGVSEVLNKKSKITTNTQTISFYQNEPVVKEVTLRFGFYQADNLLSDSITLTFGSMSEDSTQREQKHRFMFKRQLSGQIYLRKEQQVAGSKLEDIAY
MBK9103302.1_Saprospiraceae_bacterium          SDIEQVISRSPNITSNTFGVIFYQTRPVLLPRKLRTGFFSSGQKISDLVTILFNSTDSDSAAREKRQTYIFSNEANAQVYLRMEELIEGTEYKKITY
WP_006745114.1_Bacteroides_coprosuis           DTLREVINQANRITTNQHRVQFYQKEPIVVARDIKAYFASEGKVISDIFTYTLDKKESRSQDREIDYLFTISSHSSE-VFLYIECRIRNSSLKKFRY
WP_053405041.1_Persicobacter_sp._CCB-QB2       NTVKAVIRSVERITTGALAVSFMQSEPVCLAQKIRAGIYQDGVLLSDVFTYNFDSEAAMERQREQTHHFKLNYEASNQVALRLETPVEGTKYKEFFY
MBW8002950.1_Planctomycetota_bacterium         DDVREVMSKSEAITTNQIAVSLFQEEPVIQSRKITLRFETEGQVLSNQRDIVFDSNDPNAQNRTLLASFVFSKEIETQIYLCFYQDRYGQIIDPVPY
WP_262567766.1_Endozoicomonas_gorgoniicola     QRVSDVLGDNHRITTPTHYFTMLQASVVVKAVSLRAEIRDSSKAVSDQQILTFDSASASMDERKKRVRFTLKALQFDRAQYYLVLEKADTEVMSQPV
MBQ2845006.1_Alphaproteobacteria_bacterium     ARVQDVMAAPARITNKIQSFRLIQTDAVNQPAEIKVAVYEDENPITNVVNMTFDSESANMDERIQVATLTLISKPYDKKDYFLRITNTNDILSEYNV
WP_156790427.1_Alcanivorax_sp._DG881           ARVSKVLGQQHRITTNFHVFTLLQTEPVVLATRITIELVNQHEGISSREILNLDSDATELGERQSKVSLSLAAQAVKPNDHFLVLSDAESELQRIPV
OYU71397.1_Alphaproteobacteria_bacterium_PA2   TSIRKVLFHSTRVTTARHRFTLTQTEAVVQPATARLAIYDGDQQISNTAVVTFDSAVADMNLWRKEVWLTLANLPFDAKQYRLIIRDNDDDLPPFPI
MBN1905583.1_Deltaproteobacteria_bacterium     TKVKPVLGTIHKITTSRHKFDIIQMESVVKPVTLKIAIYDAEEPVTNIESVKFESVSDKIDDRKKTVMLVLQNRKYDKRQYRLILRDAETEQESRNI
MCI7405010.1_Desulfovibrio_piger               DILKQVLGQPSRIVNNIQKFEFIQTEKVCLPRKLLVSLSDGKESISDEKKLIFDSVSEDMENRKKSVKLALKNRTFDSATYWLTLRDEETEYLRVGM
TXI44184.1_Methylophilus_sp.                   AKTKSVLGQVLKVVTNMQRFDLMQTESVVLPRTLKLSIQDGHQPISDEQTITFDKSGSMLDDLKVSTLITLKAGDYDRKMYYFVAKDIVSEVIRQPI
WP_006086471.1_Shewanella_baltica              HEKQPVATQPIKLVNNIDKVRFIQTDPVFVARLLDIYIVDVKNEVSSREMINFDSVSKVMDERTREARLKLIGSQFDRAQYTLVLENAETRYSQYAV
WP_216328025.1_Deinococcus_aestuarii           QASRKVVARSRRVTNTLFSVPFVQAEAVVRPRTVTVRLVDAAREVTDVKQLTFESASPHPSEREQVARLSVTLQGPDVATYFLSVTDTEDELLREPW
WP_151792240.1_Acinetobacter_seifertii         KEVKPVLDRNIRFMNPVSFVRTMQTSIVTKSSRVEFYIKDDNQIRSSRIIENYDATKAKKFEKHVRINLVGRDFDSGQYFLVATNLETGQ-VIEYPV
WP_186816219.1_Deinococcus_cellulosilyticus    GVSRKVTSTDRTVRNNTFTVMVVQEEPVIRPRRVRIGLYEKEIAVTNEVLLDLASESSYATEREFPVKLIIGSRKTSSTPYLLEVRDAEDVVTSEEW
MBP7846546.1_Burkholderiales_bacterium         NKISLVLSANFKVTSYNFNVDVFQCEAVILADEVSIEVFDEHISVSEVKRISLASISHEISQRTFKVSLALKQSIYARNKYRLTMRGKAQTKDNQFI
consensus/100%                                 ...........h.......................................................h..........................
consensus/95%                                  ...........h.....h.h..........h.h.h..............................h.h........h.h.............
consensus/90%                                  ......h....hss....h.h....h.....h.h.h..............s.......b....h.h........h.h........h.p...
consensus/85%                                  ......h....lss....hph....shh...pl.h.h.......s.....s.......b...h.h.h......h.h.h........h.p...
consensus/80%                                  .p....h....lss....hph.b.pshh...plph.h.....hss.....s.......b...h.h.h.....ph.h.h........h.p...
consensus/75%                                  .p.p.sl..s..los....lph.b.pslh...plph.h.ss.p.hss.....s........c..pl.h.lpp...sph.lbh.p......h.p...
consensus/70%                                  pphp.sl..s..lTsp.h.lph.b.pslh.s.plpl.hbss.p.hss.....ssps....pcp.pl.h.lppp..sphplbh.p..s.s.h.p.s.
```

# Type 1 BREX PglZ all-Beta IG-like

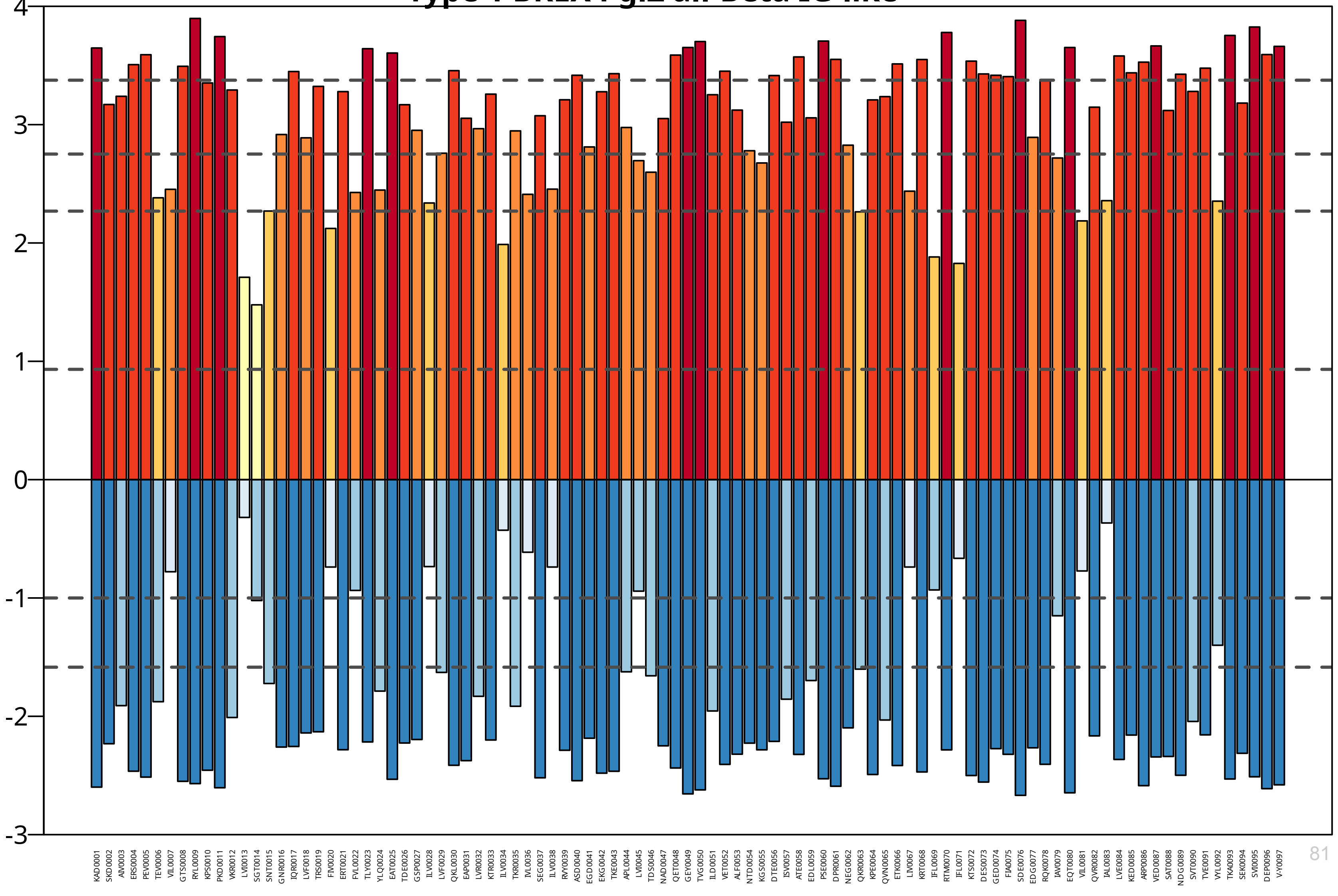

# ; Type_3 BREX PglZ CTD IG-Like Rep_1

```
RGH34270.1_Ruminococcus_sp._AM47-2BH          KTDIYFVSKYIEFHIVSVVVHSNLMIAAPKLIIKGPAFSCECDTKHYKFDIPEKRSGKFTADLYDGDSLIQMTFETKKAVGTKDFF
WP_073324030.1_Fibrobacter_sp._UWOV1          NVEIWIETPEVTVGNAKIKIASKTVLKNVFMEINGKKIPAIESGQKFSFDVPKIKAGMYDVRVFENGNLLKLKCEIKKKMAQRNLL
WP_216471320.1_Butyricicoccus_intestinisimiae EIEIIIENPVVESSPPVIYFYTKTKLENIAVDIQGTKYSVEWDEHSFCIVMDKTRKGMYKASVISDEHILVLEFKIEKAGMKKDIL
WP_044080180.1_Prevotella_pectinovora         NITCRLTDKPIMVSKARLKLFVDIDSDNLAVSVNGKFYALAKDISYYIAEMPEKTVGIYKFNVYEDESLIAMEFEVKKEGASRKFF
MCI8555501.1_Clostridia_bacterium             QINVKLINTLIKVSKAILGIETSIKLQNPYVIINGKKYNAENNEKLAEFILDDKKPGDYIAKLYDGGNQIGLNFIIESKVSSNTLF
MBD8953983.1_Dialister_sp.                    DPMVEFQPALVQLRVGELLLVTNFDMADPYIIVNEKIYRGKLHRRRAIIPLPDKRTHDYMADIYDGDTLLKVAFHVKKEVAKNLGF
MZQ76087.1_Peptoclostridium_sp.               IIRANLVEDVITVSKAVLKIFVTKKFEKIKVKLNNNEYESTEDGRYYTVPIYDIKLGEYEGEVYANEQYIDIKFNVIKPTSNKDYG
HIS35238.1_Candidatus_Scatousia               DINIEVLTKIIQTGKPILKFVSNHLLNNVNVLIGDKRYTAVTDGKHFEVELKGNREKEYSFIVLSNNNHIVLKFKIKTAGMSNDLL
WP_103226892.1_Clostridium_sp._chh4-2         VWEFKVMNRKVIFSQPVLVIWSKNELNNLSVKVNGKMYAGKQDKKTFRFELDKEKACDCSADIYVSSNCVKVKFKFEREGVKSQKF
NBJ16967.1_Dehalobacter_sp._4CP               DTSVEVVDDRLIASPLEFMLFSKTELQRVSVVIKGTPFVAKKDKNHYRIRTEIKRPGKYLADVFDSDNLIGINLDVLSETQKSGDF
CDC05526.1_Clostridium_leptum_CAG:27          TGVIVVIHNDITADGVTLNLYISDTSENIRLVIEDKIYQGISDESHYTFELKDKRAKTYTADVFDGADLIGVSFRVKGKTATKADF
MCL2016626.1_Defluviitaleaceae_bacterium      QLIVQITTEIIKADGIEFEVSLNKPPNPVILVLNNEKYTATPINNKYNVKMPNKKAGTYQAEVHIGNDLINISIKVQGAAAMDDFF
WP_235396406.1_Pseudoflavonifractor_phocaeensis AVEVKIMPPEIFVSKAAIKVFSTEKLVDVSVVIDGHTYEAKPADNFVVEEMTERRAKTYSVDVFAGGMPVALPLRVKKESGSKNIL
MBR3817930.1_Clostridia_bacterium             DNEFIVTTPIVTASEPVLKLFIKYPMDKISIVAEGTSYEGESGKGNYEFVLSDKKVGDYSFDVYYCNGIIALNFKLKKEGQNRKFF
MBP3656735.1_Clostridia_bacterium             EVEVQVETKIVEYSQPVLKLFSKTILTNASVLINGKWYEADADGQNFSFMLAGLKKGQYTAALYANRSEVALTFELRSKAGSTGKL
WP_037355505.1_Selenomonas_sp._FC4001         RLTYAFENDSIERKPTKLVLFCNKEMTNPQLRVKNKFYSGKLDNKHAEFILDDKRKSIYLAEIYDDNKATGLQFKIVKKTHEDLGF
MBS6586269.1_Eubacterium_sp.                  NVEFCFTEQVITLVVPQLTLYANVPMARPRLLIDGEFIDGELDSRHAKFLLPKKRRGEYLAEVYDGDVSRGLAFTAQKNTKEDLFF
NBK77202.1_bacterium_D16-76                   EQQVFFVETSVLCSGSRIRMFANPSLKKPRLVVNGQSYDGKFDKHNVIFDMPDRRKGHYEAEIYNGGIKVALTFETKRQTGTKQLL
NSW92343.1_Bacillota_bacterium                ENRFSLHSSTIRLNEAVLNLTLSDRVEKLSLVIRSVRFEARYKDNKWEITLRNLKPGQYRGTLWADNKNIGVTFELTKGITINDMG
WP_048095116.1_Archaeoglobus_fulgidus         IPLIKVLESKLPAYKPVLHVVVSPPVENVGLRILSELVSGKRDETLWEFDLKSLKPGIYTATVEVGSFKKEIQLEIESGIEEEELL
NPV08138.1_Anaerolineae_bacterium             ELVFKVLSPTVRVSEGHLETVTSLPLEELRLVVAGHWLKGSRGRDRWAFDVSRLRAGHYKGRLESPGQSPGVEFEVRRGIAEDDMG
MBQ7110119.1_Thermoguttaceae_bacterium        EGLVELKTTEVAVKAPVVELFALQRLNGVEIEVDGKRYTASSDGQHFSAELSDKKKGDYSICFGIGAEVLEFDIRVVQGGVKNAMF
HED00230.1_Pseudomonadota_bacterium           KKEFRLWPEKISLDRAVIEIRFDPDLDELHVLLSSPLIKAEKGVGVFRCEIQGLRSGRQWVDLISHGKNIGASFEVVVGMEEEDLG
MBE6101264.1_Selenomonas_ruminantium          SFSVKITNDNITADGIKFNLSLSKSNENLALGYDNQRYLAQRDDYNYEVEISAIKAGTYSLDAYLGDNLVELSIVVSGKSAMNDDF
ROT09401.1_Muribaculaceae_bacterium_Isolate-037 KIECNILDETIFISKAEIKLFISTLSEQYRILINGKFYSAKLDVKYYDFVIPDKKSGNYLFDLYDGDNIVVLSFIVKKEGSNRNLF
MBR2693834.1_Thermoguttaceae_bacterium        KDKIKLVNPEILLRTPELLLSASWSYKEITVEINGRRFTVSADGRQYRVHLGGGKKGVYTANVFSGEEFLAISFCIASKGMQKKDF
MCM1514006.1_Anaeroplasma_bactoclasticum      NVEIRVITEVIKVSIAVLKFYSSSKLSNVTVHVNGASYSAESDGFNFLSELIDKRSGNYSFEVWANNKLVSNNFKIEKESAKNNLW
HHW17415.1_Bacillota_bacterium                ELQFELVDNVVRLNEGVLTMRCNRVVHDLRLLLRGQIISGKSNGREWVFLLRMLKSSQHTAEVYCGTKPVGINFRTVKGITQSDLG
WP_157832981.1_Desulfofundulus_thermocisternus DGAIAIFTSVVKLNEGCLEVELTAPVEKVILQVAGQVYPGILGVQKWVIGIRNLKAGRYRGRLEYEGGFLGIEFEVTRGLMEEDLG
WP_166670004.1_Aminivibrio_pyruvatiphilus     LAPPTLLATFVYRESVSLRVKFSKKISVLSAFSGEKRADCSPSGREWQIHFSEMKQGKHLLRFEADGKMLECSFEVGTRGIADDVL
HCH97635.1_Lachnospiraceae_bacterium          PGRYKINNKKI-LSKATLRVEFNSPVQSLKIITNNGECECIQNVEEWTAHFSNLHEGEAELEIIADNKMIKKIMQVGSRGIQNSML
WP_195464804.1_Clostridium_jeddahense         KVKKKIVNKEFKISVVTIEFRLEVVMEAVSVFLRGKRIVCEKCDSDYTFKYKPDEETEATATIFAGSDEICTKFSIRRPLTTNKKF
HBQ28533.1_Desulfotomaculum_sp.               EKKITLFEAVIRLKLPKVKFKIDKPFTELYATVDAKKYFCHR-EDYWYFEPEVGKKERYVAKISSKGNIGEFEYRIIKGRTDSDKF
MBK5260961.1_Peptostreptococcaceae_bacterium  LSEITVISQLIKIPDIEVQFSVDMSLNKIGVIVDNQKFDCEYNNGIYSFIQHLSKKTDYVAKVVSDGHIIGFKYRVVRPMEKRQEF
WP_006692206.1_Selenomonas_infelix            QTVVSLADKNVFVDGLCLALFADAPINALGIVWEGKFYAGQMDAQHFVVQIPETHAGKYSLAVYEGDTPLNLVVEAQSKGMKNDGF
WP_028051765.1_Carboxydothermus_ferrireducens AVRINVLNERVKLDEGWLEIEVDKPLASLELRIGGKVFSGEESPQRWKFSLQKLGEKEYTAELWELAWKIGVSFTVERKGIKDDLG
MBQ0159480.1_Bacteroidales_bacterium          NHQVKITSEDIMISNPVVSLTIIPEPKGAILSCEGRSYRMNRCGTTWTVVLEGITEGRHEVEVKPGANSINKPINVVGGVNSNDMF
PMQ01343.1_Dictyoglomus_sp.                   KFEVHILKTDISIRNPILEVKIKPKPKVKKIEYSGRLYEMNKESDIYSIKLENIKVGNLSIKIKIGEFEKPFEINIIGGMKERDIL
KUK66682.1_Parcubacteria_bacterium            EYKIELLTKEINIRTPILELKIKPDPEKVSFLVKNRDLNADRDKEIYKINLSGYKAGEYTLTVKIGLWTKDLKFKIRGGLREKDIL
WP_018963723.1_Coprothermobacter_platensis    KYEVKLESATISVRRRVFRFRIKPEPKSVHVTVGNTRYEVSKDGDWFEIKLSNIKSGNYTAKITLPGFEQEVEFAVKGGIEER---
MBR3117632.1_Oceanobacillus_sp.               KHSVELKSGLLSGLNRSVTIEILPPTEKAVIVEDGSKHIMKVSNNIWVSPINS--IKSQVLTVKVGNSDYKVSVSSSLGASQDDGF
WP_024292379.1_Lacrimispora_indolis           TYKVNPKDLKVSGMKKELEFKISPKPDSALMAKDGTNINMDQEKKIWIGKLKR--GIEQTIEVCIENQYFCFTIPPTKMG--DDLF
HAR58249.1_Acholeplasmatales_bacterium        NYNINPIKTTVNGLDKIIKIEITPKPKNQLYIDFGNQGVFNEGNNVWSVKLQV--ITNQNIKIAIGSFSDKLNVCSSSGITKDDGF
WP_191216537.1_Methanothermobacter_sp         IYRLKESEIEVEISEPKVPFEIYPTPTETKVLLNERELNITIGKGCYEIDVSDLKAGEYNVKINLKKQFLDLKINLKAGFRETDLF
OQC39307.1_Bacteroidetes_bacterium            DFSITIEKTEIPKKESLVSINITPKPSIANIDIKGKITKLNEMTQKWEVNLDKSLSGKIPIKVKIGKCEKEFTINIISGIIEEDLF
MBQ4420212.1_Bacteroidales_bacterium          NYTIRIKKNKIPLSNPLVEIFVSPKPTEVKMKIGSVYIDMHETGDMWVASLPDAKEGNIRIAIQPGGAAQNFDIEVFGGFGNGNMF
WP_183546997.1_Methanococcus_maripaludis      EYEIRLQNDIINTK---ISLEITPWPKSVLKDQDDNEYILYSDESFWNLQNPEKKASVVTYAVVIGNIIKNIEINTKGFIE-KDLG
MBU7048061.1_Theionarchaea_archaeon           SYKVAPQEQDISIRNPTIHLSIEPFPDIEPLAYKDRTFDFDSTEDKWSAILEGLKTGKYELEIRIGDWTGKISIEIKGGMKERDLL
HHW21152.1_Thermodesulfovibrio_thiophilus     TYNIKLITDEISIKNPVVIFEISPVPNKKFLAYKKKEIELKNEGGQYTNKININKAGQYKIKVKIGDFQQEFNINIKSGFKEEDLF
WP_207678199.1_Desulfonema_magnum             IYEIKLLTKELSTKNPIMDILIKPQPSTPVLTFKGSFIKLKQKDEKWRASLKGFKPGNYQFKLNVQEQKFEMEVSIEGGMKEKELF
MBR5399199.1_Bacteroidales_bacterium          DFHIIPVNDKVAVSEALVEVTIIPQPTAAFISFNGVEYEMKRKNTQWTAKIDGVSEGINNFIIIPAGEPQNISIEFFGGAGSDDEF
WP_022459608.1_Bacteroidales                  KYTIVPVEAKVPISDRKVAFKIIPEPLSSKAIFNGQEIALIRHGMQWEGIIPNATEGKHHITVIPRGKPVQLEIEFFGGFNSLDLD
MCQ2340464.1_Paludibacteraceae_bacterium      PYVIQVIQNRLPLSKPVVSMQIIPKPDSCKLMVDGVEYDMNTTKDIWETTINNPEERTYKCIVKPKGTNHPVDVEFYGGFGSNDIN
MBE6838777.1_Ruminococcus_sp.                 TYKVFDENLQVNGIDRVVRFSIIPDVEAAVVESSGEKHSLVKVKGMYQAELSS--GKEQNISVLIEDKVFGFTVNTAKKNMEDDGF
MBR3710351.1_Bacteroidales_bacterium          RFKYELVSKELEVSEPVVSVVIMPQPSKVKLTIDNQEYNMTRSAGKWSVRVEGLEEGEYKVDISIGGTSHSETIKVIGGFGGNDFL
WP_205109179.1_Marseilla_massiliensis         TYSVTIDNDEIDGRQPNLKFTIKGLASVDTLEYNGVIYHLNLGGDKFESERLNLVDTATRAVVRINDIFGTFNIKISTGAVEDDLF
WP_172204608.1_Prevotella_sp._PTAC            EYSISLIDDAVNGNNPILMFRIKGVTNLEKLIYNNTGYNLNQGHFRFESDRLELTQEVDEVEIRIGSYSQRLYALVP-----NHVF
MCH4183501.1_Prevotella_sp.                   NWTAELLTTEISGANPTFQFVINELPSTEYIVYDGTRYELNTEDDIYESDPVIINEKACEVQLVIGSKLYIKNVTVLTGAKEEDLF
WP_196042834.1_Parabacteroides_merdae         TWRAVFKSFEVEEANPVVVYSIAGLDNNQMVEYDGKFYSMIT-GNTYTSERLPLNKDENKVILHIGTWQKEPIFTIKMAVIEDDPF
MCI6981218.1_Akkermansia_muciniphila          ACSVKLITNAPYARLSRLEFEIRGLKKSDCVVYNSKKYLLRDKGVSFMTENIPICTQCKGIRVMIGVQSWDFSLEIRGGAVEDDFF
MCD7797870.1_Akkermansiaceae_bacterium        RINAKILTKKISPIRSNIQYQIKGITSSAEIEYNCKKYTLKVDESTFECPKVNIQQTETKVILRIGASFEQVTLIVKTGAEETDLF
MBO6251938.1_Muribaculaceae_bacterium         TWEARLVEVEVSATHPVVQIHITGLSAMDLLIYKGNSYKLNKGNNIYESERMPLEENECTLDLHIGDYSEILKITVNTGGHMDDMF
MBR4838437.1_Bacteroidales_bacterium          DWTATLQTKTLSESDPIVRYVFKGDTTNLRIVYNGKQYALRINDTLFESDRLTLDKDATTIKLVVGDIEQKDSIVVNLGATEDDLF
RGX95620.1_Prevotella_copri                   HWSVVVLSQELTTVNPQVRLRIKGLSASDYVVYNNKSYPLKLGMDEFMTAPLEANENDSELSVLVGDKVIDRSILWKKAAQEEDLF
WP_254009599.1_Bacteroides                    NWKANIISSNVSANKPVVSIEIKNAPIDAYLIYDGKRYEMEKQNEIFESEELILNADVKRIALYVGPDKKDFHIDFSKAADVDNMF
consensus/100%                                .................h............................h...............h..........h............
consensus/95%                                 ...h......l......h.h..........h...............h...............l..........h........p...
consensus/90%                                 ...h...p..l......l.h..........h...s..h........a.............h.l..........h........p...
consensus/85%                                 ...h.h.p..l......l.h......p...l.hpsp.h........a.......p.....h.l........hphph......pp..
consensus/80%                                 p.ph.l.p..l..p.s.l.h.hp...p...l.hpsp.h..p...p.a...b...p.sp.phpl..ss....hphph.....ppp.h
consensus/75%                                 p.phpl.sp.l..p.s.lph.hp...pp..l.hpspbhphp.s.p.aph.h...p.sp.phplb.ss....hphpl.....ppsbh
consensus/70%                                 phphpl.sp.lphpps.lph.hp...pp..l.hsspbaphp.s.p.aphph..bc.sphphplb.ss..b.hphpl.....ppchh
```

# Type 3 BREX PglZ all-Beta IG-like Representative_1

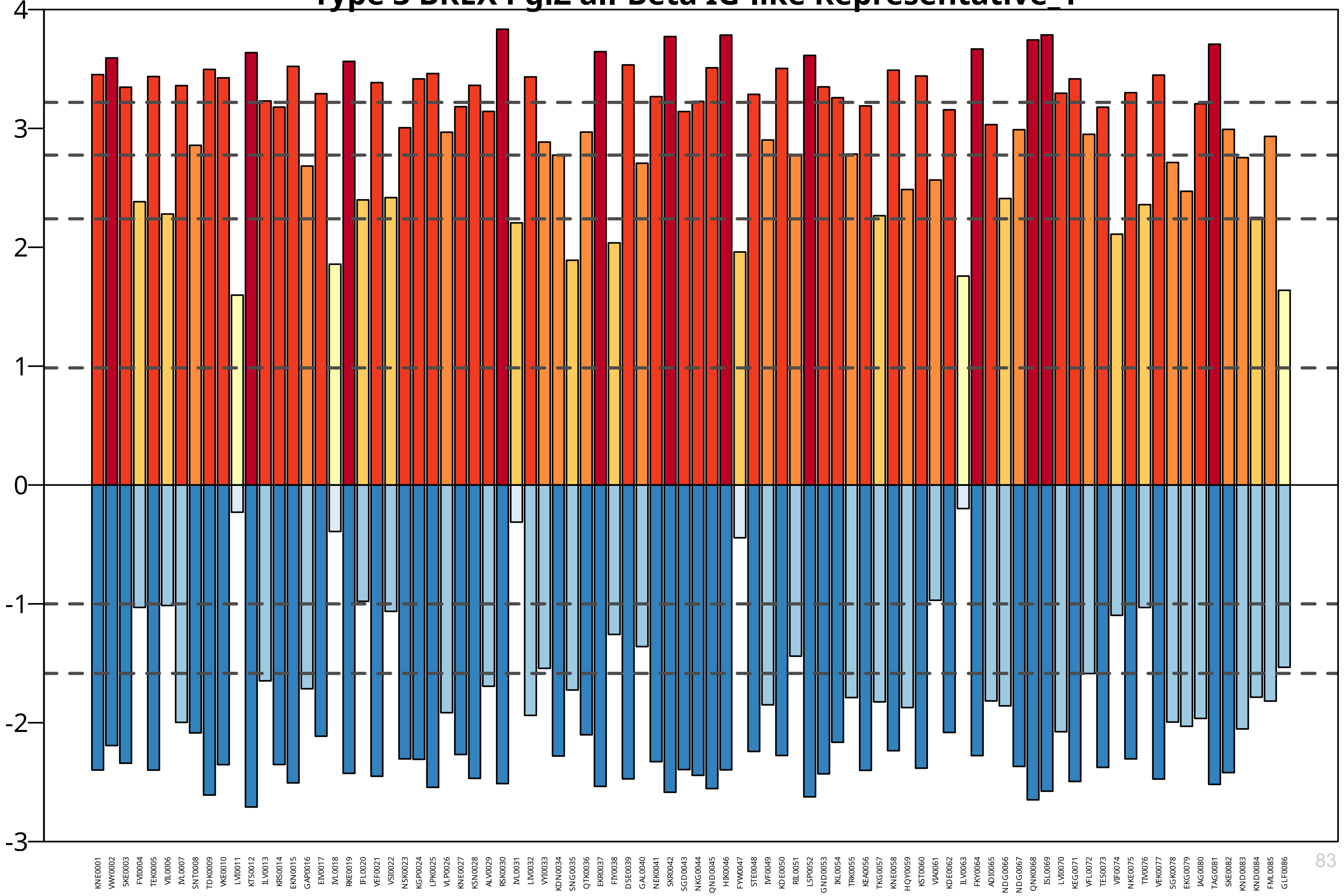

```
# ; Type_3 BREX PglZ CTD IG-Like Rep_2

WP_192822482.1_Rufibacter_sp._LB8          LMIGGFLSGESRSYRQGKAELHLNNSRPARLRKVKVQIPRVELLPGTIGISKSIEIPRWPDTAQAEKTVAQVTLETDGELHTFNLNTNLRNTDLRQSSTPLDDLF
WP_105482027.1_Abditibacterium             IRVEAVLSGKGRAGRLGKAALRLINFRALLMENIELRYGGKVTVAAFGHAEIEVQLDAWPETAQARDLTMLVLLRADGRLVQSVAEIHLQVEALQTRENILGDLI
WP_243797665.1_Hymenobacter_tibetensis     IKITGSATGRARAGSTGTITLQLFNERPVRLRRLQISWTGTTTLSGQQSTSVSVPLSNWPDGLRIGTATLFITVELDGREQQFSLTNQLSTDEFQTRQVLLGDL-
MBX7251230.1_Candidatus_Promineofilum_sp.  PEIGVTIRGRSVAGRPGQATVQVLNPVSVTLTWIDLRRPKGLTIAPRSQAQAMLDIDPWPTPAEVDELTAEAVFHLSGKRFDVSVSLELDSEEMYGRDNILEDLV
WP_162787804.1_Anaerolinea_thermolimosa    PELDISISGEGEANMSSKVFISILNSMSLECQEIEISGENYKEIPPLDKVIFQEILAPWPSKIIERNLTAKVTFTLNGKIYTQNVTPDLKVQILYQQSDLLKDLL
WP_012642882.1_Thermomicrobium_roseum      PSPVVAIKGDGRAYGSGTLVVMVTNEIPLQLLKLELEFRQRETIGPLSQVTITRQIESWPTPQDCREARVRLTYRLSGAHPTIQEKIELTSRTLYDQTNILEDLQ
WP_157205838.1_Calidithermus_timidus       KDIEAELLGKGVVERAGQIVLQVRNPISVVVLELSFGAKQQREVSAMGGLEHALEIPSWPSKRDVQSLRAQVSFLRDQRPAYVEAKVRLEVEEMYQQGDPLEDLL
MCL6576921.1_Kyrpidia_sp.                  LPTKAYLVGTGVAGRINNAVLVVENPAPVELVQLELILGGTSMANPMSRTEWSIDLNPWPEKKVLKNGQARLIYRLGGKQDAAPVELRLTSLEMYEQNDILGDLL
NPV84914.1_Anaerolineae_bacterium          PKISIEITGHGEANKTGTLLVKITNQVTLECLEISLSAVINCQISPLQETTFKFNTISWPTKSALPSLKAKLLFRMNGKTFEQEIMPKLEADELYSQPT-LKDLG
WP_139401683.1_Deinococcus_radiopugnans    RLPTLTAEGDGQAEEPGTFILTLTNPVPLVVERLGGPLGAQWTVAPQSQQRLTVALGSWPTSAALSALTLRATVRARSAAQDVAVTLKLKSVELYTSTAILGDLL
MCE5327915.1_Planctomycetaceae_bacterium   PNAVAKITGKAREGAEGRLTVAVTNSISLQATWLEIAFGTAHEIAPLTKREYVISVADWPAKPNAISARAWIGIRPAGPEIRIETEVVLDSEGFYVQDAILGDLT
OGX83061.1_Hymenobacter_glacialis          VKVAASLTGAGRAGFPGVAQLRLRNDHHLTVVHVSIRFGAAQPSVPLKTIDVSIDLPSWPHSSEVQSGQLVVVVETDGKQASFPVDANLTNTGLQDRGVIGAEFG
MBN2678362.1_Anaerolineaceae_bacterium     PNIEFAFSGAGRATLPGKIIVSTINPIDLDLTKAEINFGGDKNVPGLKKTEFEIDITTWPSSEQVSLGKANFTLRLSGEEFGVSSLVGISVTELYTRDKILEGLD
MBV5327602.1_Chlorobium_sp.                IILEATLTGKGVAKSTGVYLLEILNTVRIQVLEIELIQIGYFEVAAMDKKLINIPVTYWPTTLESNSLEASILYSLTGERKVLVIKPSLETEEMYQQEDILSDL-
WP_062417760.1_Levilinea_saccharolytica    PDATITISGNGRARRDGTFAVTVLNKIDLFLETVTLTLRTGQKIPARTQIPAEIPYSPWPSAGELETVHATVRLRLNKMVYEYSAETSVQSEDIYTRPEILEDLD
MCC6188628.1_Anaerolineales_bacterium      PQVEVTLTGTGRARRTGILRLTVMNMVPVELRSLTLDVGRSLKIGQRAKAEWEGDLSPWPSPSDCRAASAIAHCVPNGVSFDVTVSASLQSEDLYIAPDILEDLT
MCA9963066.1_Anaerolineales_bacterium      PAVTVTLRGSGQAGQSGQFILQLRNPISVTAVALSLLIAGQQAITPYSEQTFTLSWSSWLKRAEVSQTTVTLSLQLNGLSFTVPANPEIESIEMYRRDNNLGDLD
WP_215594968.1_Hymenobacter_piscis         PLITATLSGAAPAGRSGIATLELLNPRTLTLRELHVRLGNKGSCPGLQTLSVSVQLPDWPDSAQLQTAQAKVLLQTDLQEHTCSVGLNLRNTGLQDRTDILADLF
WP_006100361.1_Coleofasciculus             APIIIKCSGEGRPGENSPLRVDIYNPVAIEDLRLRVNQLVSVTVQPHQRQLIEISISTWPELPPTHELTGKLEFQYNAERGWADPESSIEVHQIFSSGEGLDDFL
WP_254031239.1_Planktothrix_agardhii       YPVIVTCRGEGKAKQPGELTITIHNHVSLTNLCLYIDQLNDITLKPNDKVTHKISISEYPELAPDKNLSGQLTFSFNTDSSQSNSQSYIIIKQIFSSGQGLDDFL
WP_273058112.1_Shewanella_vesiculosa       SYNNFTLNAISSVSGKWCLRLKISTPVNIDNFRIKLGKHAIERINEGQTIEFDFAFECTVSQEGLTDIKFDLQFEVNGNKKIQTQSISVCFPPKSLASKKFEGMF
MCK9609000.1_Methylomonas_sp.              VKMPLELATLSDSDKSWQISLELSAGINVKNINLKLGEESISAISAGTSQKLILAFSALTEQSGLTEITIHLSYDREGAHEINTKQISCIFPAPDAATQGFEDMF
WP_017840083.1_Methylotuvimicrobium        LETHLEVSLENNDDKHWQIDLVLTSSVTASDICIKIGAETVDSLRVNKSQKVALRFNCSNGQEGLIRIELVISYHHSGANEQLIKFIDVNFPASDGGVQSFEGMF
MBC7570526.1_Spirosoma_sp.                 VPLVLTYYGSSDGKTIKDFGIQVPNPFAIELLTLSIVEDLPMRIEPTASATLTGSIAQKKPQQGQLSLAVSITFRANAEESAQSATLTVPIQTSELDD--FDFDF
WP_198076877.1_Hymenobacter_negativus      VELAISYFGKKEGKTLKDVQFRIPNDFIVELRGLSLAEDLPAQLAPNSSVALKTKLPSSRPKEKSVSLTASLEYVVQGDPYTTQASVSVPIQQANELD-DFDFDI
WP_235137408.1_Dyadobacter_fanqingshengii  VKLSIRPIGFNEGKSERNFKFRISNINSIQISKVTVLQELPLDIPANAEADLTGTLHPRHSKDGKLNLELAVKYTLLGHHYEDVINLDILTERD-----EFENDF
WP_194112254.1_Mucilaginibacter            QQLQIRYTGIRDGKTEREFKLKVVGTTAVELQEFTILEDMPVTIGPGADKPLHSALPQKNAKGGKLDLNLEVTCLILGVRVVQSLKCTVTTEKD-----DFEDDF
HBJ34341.1_Planctomycetaceae_bacterium     TWPSVSFPEAGYDRGVFRFSVTLSRSVPTTSCTLRIGEVKLPAMSCGDSTKLDIVLPLRTSDCDTVAIVVTMRAKETGELIDKCNDFIVPRKKLTNDQDDFENMF
MBX3399563.1_Gemmataceae_bacterium         AFPDVTVPNGATDRGQWILTLQLSHPIQTNGGRIRVGEESYPALRPNATHMLPLEVPGQLPNDQELVLEVTLSPRGDTPAPDLTRHVPVARVRQTKDQAAFEDMF
WP_099078666.1_Vibrio_sp._PID17_43         HEQPFILTSDEVGEKRWNITLSAIKNASEEHVTFKMGNGNW----DSANEQLHLSLHSSVNHEGLVEVAFTVGFKG---KHVTTVTLEIDFPKSDKASSAFDDML
SEH06866.1_Thiotrichales_bacterium_HS_08   AKIPIKVNVLKPLEKGWHLELSILPRIRLNKLQLQFANTNYSDLQAHSETKIPLQLVSEQAQSGLTQVDLTLRYLPQTTWTSLTVNLSLDLVAPTQEADNFNKMF
QEP42412.1_Ectothiorhodospiraceae          PLPPLQLQPKRYAEQQWLVQLVIQLHETVERVKIRAGQVRFNQID--EALNLDLLVVANQAQRGVISLPLTLETERNHEVMVLEESVEVEFFASDESNQLFEGMF
WP_194715675.1_Noviherbaspirillum_soli     KTPDVQISSEQDSGEGWTVTFKVASATHLRRVQIVCGHCTVENILEQQSKLVTFNIRSTVHQEGAVNLMLNVAYVLGVGFQHVQLPVSLKLRPHEQGSADFDNMF
WP_201382003.1_Ktedonobacter_sp._SOSP1-85  FEPVINISGFLFSSKVNALQVTLMNPFPITVLQLLVADSIQSYVEPNAMITGEVILPAVVYRESARLVTWSLTCEGGGIRQVFRGDVSIPIRRLQTSD--VDEMF
WP_221704724.1_Desmonostoc_muscorum        ISPVVKIEGNFKPNQVSTLRVLVLNPVPLRKVRLEISNAIWSDVPAFSKSSIEEILPA-SHNGLKQTVEWFINCEAVGKTWQFSGQIEVAIRRFQVNS--VDELF
WP_007916556.1_Ktedonobacter_racemifer     LAPILQIEGSLRTGTISKLETIIPNNFPLKIIQFSIDNVKPTRIGANSTILIPIQVQT-TSNQSDQYIKWFLSYERGGQHTSIEGSVNIQVRRLYANT--LDDMF
NJL05928.1_Chloroflexaceae_bacterium       WALDVALHGQLRANQAGTLELEIPNPFPVQVQQLTLDTDAPFAVAALAKDTFPVAVAPPTTRADQLHVQWHITTTSPAGQQTQHGAADVPIRRLQTTDPDFDAMF
WP_129675140.1_Candidatus_Chloroploca_sp.  VNLQLTLDGQLQPQREGVLSLLLPNPAPIDGLVVRVADLTIERIAAQDRYETTIRFPARPINGTKLSLQWTLDGQVLGVAYHQQGEASIKVRRLQSGN-SIDDLF
MBU6335166.1_Chloroflexota_bacterium       HPPVLRLSGVPSAIDDATLVVELFNAGALEAVTLVLDGVPLPSLPGGGHARHAIAVPRCHSAAA--TIDWWLEYRCLGDAYRTPGPAPLAVRRLHHDDGGFDAMF
MBK9943589.1_Kouleothrix_sp.               LPLMIELSGTLRVGQPSQITVGLPNRFPIDTVTLDLGAGHIDRVESSARCDLVIEFPAIASALSELPIKWRMRYDTVAGTQTQDGRLLLPIRRLQAEDMGFDDFF
WP_097643873.1_Candidatus_Viridilinea      MPLELVVQGELRARRAGTLTMLIHNPFPIENLHWQISGGQIAILGPNASQTISISFPPINPAGDTFTINYTLDFSHFGKPEQNSGMCKIRVQHLYIQDEGLEDMF
NJO81810.1_Blastochloris_sp.               IPLDILLQGTLRAGQSGILTLIIINTFPVEQIVVTVTASEVARIEGLAKATAEIHIPMVTVSGTELPVTYEVQYYAFGNSYQTMGKTTIAVRRLQVEDTSFDDMF
consensus/100%                             ..................h..............h.....................................h................h................
consensus/95%                              ....h.h.s...s.....h.h.l..........h.h.......h...........h.............h.h................l...........h....
consensus/90%                              ....h.h.s...s.....h.h.l.s...hp...h.h.......h.s..p......h.............h.h.h..............l...........h..h.
consensus/85%                              ....h.h.u...s.....h.l.l.s..slp..plpl.......l.s..p..h...h...........hph.l.h...s..........l...........h.sh.
consensus/80%                              ....h.hpu...s.p...h.l.l.s..slp..plpl.......l.s..p..h.h.hss.s.......hph.l.hp..s.......p.pl........s..hpsh.
consensus/75%                              ....hplpG..ps.p..ph.l.l.N.hslp..plpl.......l.s..p.ph.h.lss.s.p....plphplphp..u.....p.phpl..p.b..ps..hcshh
consensus/70%                              ....lplpGp.pu.p..ph.lpl.Nshslp..plpls.....plss.sp.phpl.lssbssp....plphplphp.su...p.p.phpl..pphb.ps..h--hh
```

# Type 3 BREX PglZ all-Beta IG-like Representative_2

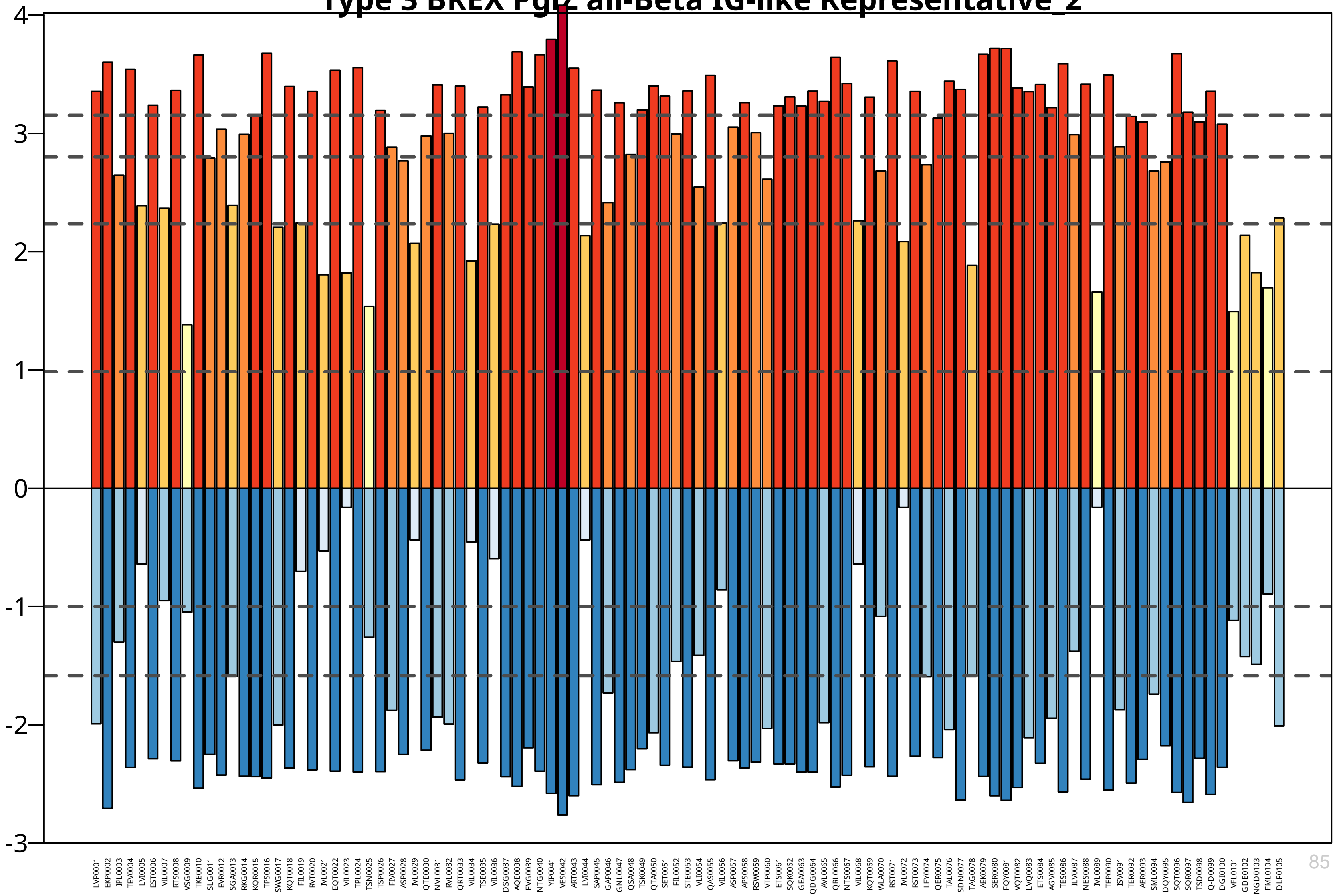

```
# ; Type 1 BREX BrxB-iSTAND NTPase; (For Supplementary ENTROPY)

WP_258176317.1_Halomonas_ventosae  [illegible]
WP_262567771.1_Endozoicomonas_gorgoniicola  [illegible]
WP_094203898.1_Finegoldia_magna  [illegible]
MCK7578718.1_Chromatiales_bacterium  [illegible]
MAE16012.1_Deltaproteobacteria_bacterium  [illegible]
WP_165775784.1_Bifidobacterium  [illegible]
HIU24259.1_Candidatus_Coprovicinus  [illegible]
WP_168040576.1_Neolewinella_antarctica  [illegible]
WP_151792237.1_Acinetobacter_seifertii  [illegible]
MAO64837.1_Balneola_sp.  [illegible]
WP_245747814.1_Methanolobus_profundi  [illegible]
WP_167658113.1_Pseudoalteromonas_sp._HF66  [illegible]
KAB1640464.1_Ellagibacter  [illegible]
MBU6197214.1_Cyanobacteria_bacterium_REEB446  [illegible]
WP_235341611.1_Bifidobacterium_boum  [illegible]
WP_012411903.1_Nostoc_punctiforme  [illegible]
MBR4606064.1_Lachnospiraceae_bacterium  [illegible]
MCB1062256.1_Verrucomicrobiae_bacterium  [illegible]
MBD1206698.1_Paracoccaceae_bacterium  [illegible]
WP_159471216.1_Dyadobacter_sp._3J3  [illegible]
WP_168675170.1_Hymenobacter_artigasi  [illegible]
MBP6733458.1_Chitinophagaceae_bacterium  [illegible]
WP_231782752.1_Rhodococcus_qingshengii  [illegible]
MCH3907668.1_Sphaerochaeta_sp.  [illegible]
WP_013460545.1_Sulfuricurvum_kujiense  [illegible]
MBQ6141564.1_Kiritimatiellia_bacterium  [illegible]
WP_048345182.1_Lactobacillaceae  [illegible]
MBQ6502299.1_Flexilinea_sp.  [illegible]
MBW7869162.1_Brumimicrobium_sp.  [illegible]
WP_019974717.1_Empedobacter_brevis  [illegible]
WP_222635590.1_unclassified_Rhodococcus  [illegible]
TVQ28582.1_Spirochaetaceae_bacterium  [illegible]
RKX87881.1_Spirochaetota_bacterium  [illegible]
WP_217359511.1_Anabaena_sp._UHCC_0204  [illegible]
WP_238754849.1_Leptospira_bandrabouensis  [illegible]
MBY0113215.1_Phycisphaerales_bacterium  [illegible]
PZV04978.1_Cyanobium_sp.  [illegible]
WP_024332154.1_Winkia_neuii  [illegible]
WP_251490388.1_Otoolea_muris  [illegible]
WP_153861779.1_Fundicoccus_ignavus  [illegible]
WP_196032769.1_Collinsella_aerofaciens  [illegible]
KAF3303186.1_Carnobacterium_sp._PL17RED31  [illegible]
HDN19843.1_Candidatus_Acetothermia  [illegible]
NBI63882.1_Clostridiales_bacterium  [illegible]
MBQ7190493.1_Kiritimatiellia_bacterium  [illegible]
ETR72220.1_Candidatus_Magnetoglobus  [illegible]
WP_224137561.1_Candidatus_Kaistella  [illegible]
WP_098060819.1_Longimonas_halophila  [illegible]
MCF0260591.1_Erysipelotrichaceae_bacterium  [illegible]
MBX6362418.1_Gemmatimonadota_bacterium  [illegible]
WP_270462425.1_Gemmiger_formicilis  [illegible]
WP_215020743.1_Bacillus_sp._ISL-40  [illegible]
MBR6253007.1_Clostridia_bacterium  [illegible]
WP_273738558.1_Natrinema_soli  [illegible]
MBK7566197.1_Bacteroidota_bacterium  [illegible]
WP_200392182.1_Roseibacillus_ishigakijimensis  [illegible]
WP_242820840.1_Deferrivibrio_essentukiensis  [illegible]
WP_235327429.1_Bacteroides_caecigallinarum  [illegible]
NMA18736.1_Clostridiaceae_bacterium  [illegible]
WP_163588065.1_Ligilactobacillus_murinus  [illegible]
AUX12765.1_Latilactobacillus_sakei  [illegible]
WP_162921703.1_Listeria_costaricensis  [illegible]
MBD5357713.1_Bacteroides_sp.  [illegible]
MBP6234855.1_Saprospiraceae_bacterium  [illegible]
WP_215708905.1_Bacteroides_uniformis  [illegible]
MBQ0129311.1_bacterium  [illegible]
WP_028665034.1_Runella_zeae  [illegible]
MCI5856702.1_Agathobacter_sp.  [illegible]
WP_061570111.1_Caldibacillus_debilis  [illegible]
WP_271398743.1_Salinicoccus_roseus  [illegible]
WP_091403824.1_Geotoga_petraea  [illegible]
MCK6587210.1_Polyangiaceae_bacterium  [illegible]
MBN1450029.1_Anaerolineales_bacterium  [illegible]
MBE7502026.1_Verrucomicrobiales_bacterium  [illegible]
WP_261427665.1_Serratia_proteamaculans  [illegible]
MBQ8347029.1_Alphaproteobacteria_bacterium  [illegible]
MBQ6248037.1_Kiritimatiellia_bacterium  [illegible]
OIO89049.1_Anaerolineae_bacterium_CG2_30_64_16  [illegible]
MBX9598965.1_Burkholderiales_bacterium  [illegible]
WP_191006190.1_Hymenobacter_montanus  [illegible]
MBR3160179.1_Atopobiaceae_bacterium  [illegible]
MCH8487208.1_Cyclonatronum_sp.  [illegible]
MBP2454131.1_Mycolicibacterium_lutetiense  [illegible]
MCK6605710.1_Ignavibacteriaceae_bacterium  [illegible]
GDY21839.1_Verrucomicrobiota_bacterium  [illegible]
WP_025641434.1_Schnuerera_ultunensis  [illegible]
WP_201561161.1_Psychrobacter_glacincola  [illegible]
RKY59329.1_Candidatus_Latescibacteria_bacterium  [illegible]
UCC65223.1_Anaerolineae_bacterium  [illegible]
WP_154435942.1_Olsenella_porci  [illegible]
WP_075010320.1_Stigmatella_aurantiaca  [illegible]
WP_187771726.1_Phascolarctobacterium_faecium  [illegible]
WP_252697946.1_Natribaculum_sp._CGA3  [illegible]
MCC6107939.1_Atopobium_sp.  [illegible]
WP_255679049.1_Methanosarcina_sp._DH1  [illegible]
MCL4835317.1_Caldilineaceae_bacterium  [illegible]
WP_012508723.1_Pelodictyon_phaeoclathratiforme  [illegible]
MBL9184318.1_Verrucomicrobiaceae_bacterium  [illegible]
WP_157203231.1_Methanofollis_liminatans  [illegible]
MCC6785238.1_Planctomycetota_bacterium  [illegible]
WP_050592628.1_Mesorhizobium_sp._L2C084A000  [illegible]
WP_100919676.1_Candidatus_Thiodictyon  [illegible]
EMR73246.1_Thaumarchaeota_archaeon_SCGC  [illegible]
MCC6224162.1_Thermoleophilia_bacterium  [illegible]
WP_247863886.1_Gordonia_sp._PP30  [illegible]
WP_131908314.1_Flavobacterium_caseinilyticum  [illegible]
WP_262301579.1_Prevotella_hominis  [illegible]
WP_011448111.1_Methanospirillum_hungatei  [illegible]
RDE50959.1_Candidatus_Accumulibacter_meliphilus  [illegible]
MCC5847926.1_Verrucomicrobiota_bacterium  [illegible]
MBA2375852.1_Rubrobacter_sp.  [illegible]
WP_019935797.1_Oceanimonas_smirnovii  [illegible]
WP_229574328.1_Rhizobium_sp.  [illegible]
WP_210512158.1_Natronogracilivirga  [illegible]
consensus/100%  [illegible]
consensus/95%  [illegible]
consensus/90%  [illegible]
consensus/85%  [illegible]
consensus/80%  [illegible]
consensus/75%  [illegible]
consensus/70%  [illegible]
```

## Type 1 BREX; BrxB Inactive STAND-NTPase Entropy

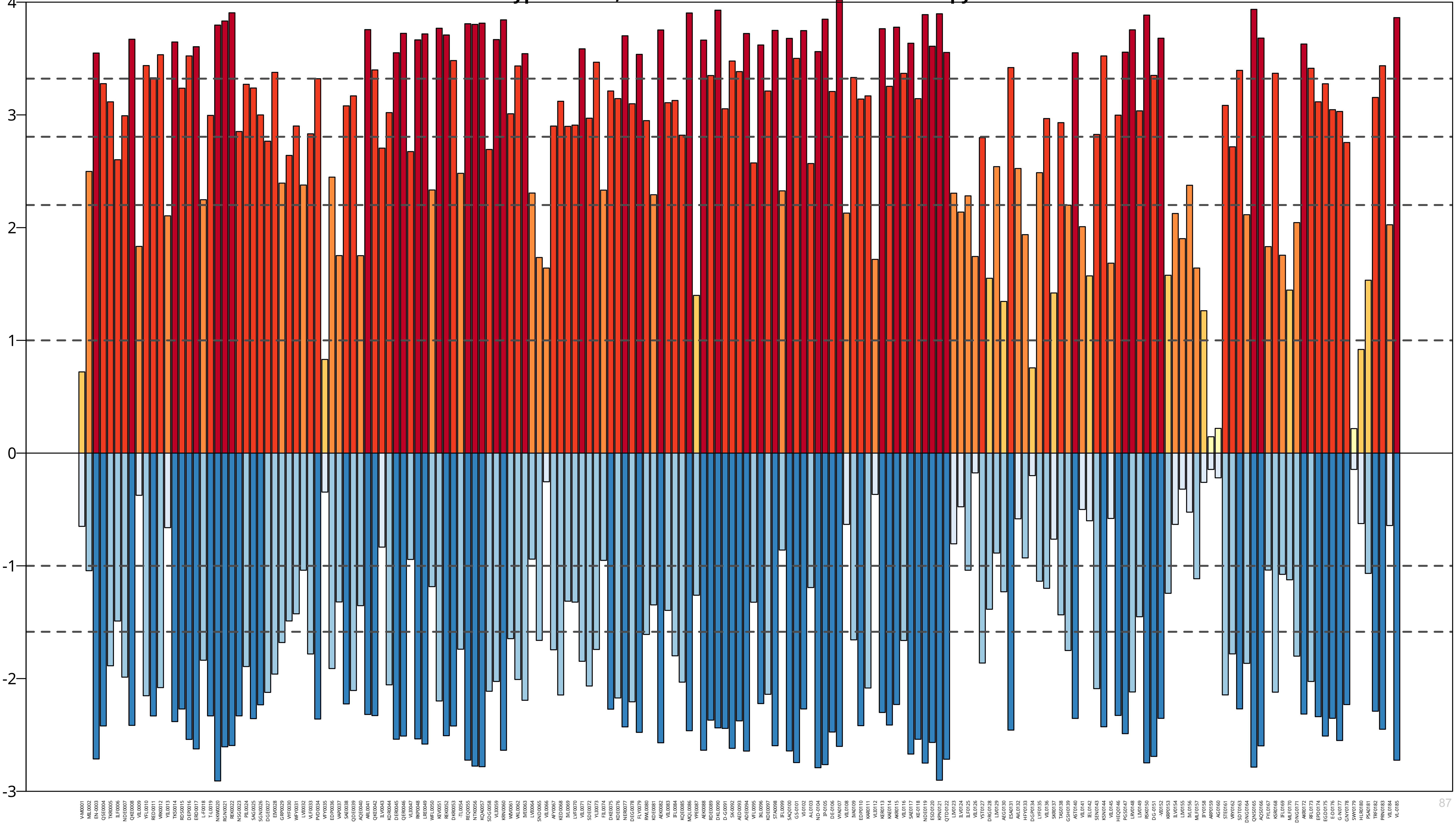

# ; Type 2 BREX PglW C-terminal iSTAND NTPase; (For Supplementary ENTROPY)

| | |
|---|---|
| WP_185095299.1_Streptomyces_sp. | ITASTPEQKLRYRANAQLLESAARPGFRLLTVRYDDQRLALLTLTEEWRAEPVDVSILFLTSLRRLVEARPRPWDTILEADNAVPGSRDAMKFGEYTAAAWGEVEKELASNSRPLLLHDASVMARYGGTGILQRLADHARGRGLWLLSPLNDATAVPRLEDWTVALEDREEWIRLNHSWVVNDPEDAPA |
| WP_268746228.1_Dermacoccus_nishinomiyaensis | RPTHDVASSITGRFGVRLDDSVSSRSFLALGARSERLVEAVETLRRAYGAEVVDLSELLVDALKQQTVGGRLTWDHVRAADAAAPHTKAAQGLGQLVRRAWPQIENHLASAERPLLLTDAAPLARYGHMDMLARWSDLARSRPVWLLVPQVSGNHGAVLDGAPLPLAAPSQFVPLDSDWIDTHAAAAKE |
| GII83978.1_Sphaerisporangium_siamense | TSTLGGVGRNLAAFEQRLTRSIAEHAFVTLAVDWRFHARAAAALAERFGVTPVDVTADLLAAMRREAEEHQVDWSLVLRSDRPDAAPQDAANLRTLVAMAAASLESRLEAETRPLLIVEAAPLARYDRLAVLERLADQARPAARWLLLPVE-QDGVPSIDGRPAP--GALGALRLSTTWITRPRDEAVS |
| KEZ35702.1_Frankia_sp._CeD | STRVS---DEVLAADDRLTRSLVDGGWLVLSVPPRRLARAERCLAA---VTVVDAEQALLAGMREFCAQHRVQWSIVLAADAADRSSRDWANLSRVAQAGLAGVRASIEAAGPAVLITNAGVVARYDPAVVLDELRASVRVRTVWLLVPWADVDKQPLLDGAPVPQ-FGNQGLALSEEWIVRHESRGAA |
| WP_030506725.1_Microbispora_rosea | SRTAAPRQVVDSAAMQRLTGAAGRGGFLALNVDLRRAVLAVRVIPESLDVMPIDVGGEFLRALTDLASERDRPWSDVLRADERFSDGILPRGLAGYVREAWQRLATRLSQPSGTVLLHNAGVFARYPGGDLLVEIQQAARPRGVWLLCPTGTPKQRPILDGLVVEVIGENEWLELTAELLRSLDEKAA- |
| RJQ78831.1_Pseudonocardiaceae_bacterium | LTTVGGVLTAREVVASKLTSAVQRGGFLALTLRGGELPGTAAMLAARFPVQPVDVDQEFLRTFRALADERGADWQKVRTLDLRLSTGEPTPGLASFARKAWDGVRSRVGAGETVLLLHHLSLLQRYYQWEFLTELQNDARPHGLWLLCPAENEKHDPHLDDRIVVECTDSEWAVLSKKFLNALRGQGAE |
| WP_212516928.1_Actinospica_acidithermotolerans | GTGGAASGAVVDAAVKRLGEAVEDGGFRALTVSVRHLPGLSAAIERRFPLISVDGNAEFLACFRELAAEQGLEWERVLRVDARLQRGEAPRSYAEYVAEVASRLHAKWSSPRSTVLLHNAGLLARYWDIDLVVGLQQSARPRGLWLLCPAESARSSPNLDGRLVETVVAGEWIVLGSAAVEELKCQDGH |
| WP_208577501.1_Micromonospora_tulbaghiae | ATTHTPLLTAADEASARLAAAIERRSFLAVLAPWRRLGPARRALLARLPLTEVDVTAILLDRLRAL----GFPWEAIVAADNGNPQDADFRSLMDLVHQVVPAIGEALAAVDGPALLTEAAPLARYGQLGLLQELADPTRAAARLLLVPARRPE-PAVLDGVQLPLTSPGQSLWLSGDWIEAAGVASAD |
| MCP9485019.1_Candidatus_Gaiellasilicea_maunaloa | PTYEDPEVLDAAQFERRLETSTRRGGFLCLVGHDRSLDDIRDELARRFDAQHVSLDRLLIHHLRLAAGRRNVVWQTVVDADAATRDSRAWGNLQQLFVEAVERAEGELLAYEGTVLATDPGLLARYGQLEMIDRLRDRASLSTLWLLVPDDNQSDRPAIDGQAIPIIGAGQWARVPSAWIENRHRGRAA |
| WP_193120346.1_Nocardiopsis_coralli | TGHREPGRAVSQETWERLGRAVERGGYRALKTWLPDTERSAEQIGNMPGVVPFDVSAEFVQAMDEVRQGKGLTWRHLTQADSSRPRPRP---FQTMLELALQRVGERVAAGHGVVLLHRATPLARYEQGALLGRLAEEARPHGLWLLCPMRGPQSPALLDGEPAGIIADAEQVLLPRGFSAPE------ |
| WP_103561562.1_Actinomadura_rubteroloni | GPHSDPALAAAARAEDQLVAAASRDGFRVLTVRMMLFSEALEELTGRFGGTEVSATRLFLDALHSLTAPGKRPWETILRADNAEPNEKSAHKFAEYATTALGRVEPHLASGNGPIILTDATVFAHYNAMDTLSRIADAARKRGLWLVCPQSDPAREPRLGRRAVPYQPASEWVELNDHWARNRHRSERS |
| WP_248824789.1_Frankia_sp._Ag45/Mut15 | PTYDSPEQAAASLTEQRLAAAAEAGGFRALTVRRSRYLAARTELALRFAAAPVDVAAVFLDALHAQVDPRPRPWATILRADTAEPGSRAAGNLAEYVRTAWERAAPLLGGGPAPLLLHDAGIFGRYAGRDVLFALAERARGRPVWVLCPTTEPTRPPRLDGALVQVATESEWVALPDAWTTNWHRSGKA |
| WP_213171153.1_Natronoglycomyces_albus | TTGVGSGADEAARFTYRIDKAVESGGFRAIKTGVAETCAAAKWLAARPGVVALDVQAVFLRLLRQYVAERNKPWERVLAADTVPPPAP----LTNLLVEVWNRFETALTAEGRVLFLYNATPLGRYSGGKVLHELLSAARPHGVWLLCPTDALRHVAHLDGYNVGAIDSGEQIRFPAGVLKPRLDEDSG |
| WP_144820010.1_Micrococcus_luteus | EATITLPSSELGAIGQRLLDSAAQRGYLVLGVPAHRMNRSRELLAARFGAEHVDLADLFLTRLRETVDRSPVSWEMALSADSAPPGTKAGRALQAWEAAALPQVVEHVSQAGRPVFLTGVAVLTRLGPLQPVARWADLGRSRAVWVGVPQAPGNIGPVLDGVPVPTSSRSQFVRLEPEWIDTAARTSPA |
| MBN9688999.1_Verrucomicrobiota_bacterium | GNGVTAEKLTANQFTDRLQRSIQTAGYLVLVSTTTNYLVVEQRLREGFPVAVCDIDTLLLNELKAQAESLKVKWEKILEADQAVETSTDWNKLNQLVGYVMPKLKAHLVAQDGPVLLTNIGLLARFNQIPILEQLREAILPRSLWVLVPSDDQNRLPTLHGRPVPVATRGQWEQIPEVWLNLTTSATS- |
| KAA0252897.1_Acidobacteriota_bacterium | TTHVPTRDDPAAEFDRRLAQAAREGGYLVLLAPERELTRAREAIAARAGIEIRSLDRLFLDALKATAESKKVKWDAVLRADAAPADGPEGTKLRTLVGNALPRLEEALAASGDRLLLTEPGLLVRYRRLDVLDRLRDRVSPGAAWVLVPADEQTDRPVLDGVAIPVLTPNQWARVPLAWIEAQATAGEV |
| QQS02053.1_Austwickia_sp. | TPTAGGAASLRDPVAARLAESVATRSFLALGVGALDLARVQDVLRERYDAHLLDVTGLLIDAMRAAAARHGVPWDVVLASDAATPGTRDRANLEQLVGMCLPEVWEAIHAKDAPVVLTDVAPLARYRHLGELSRWSDVARERAVWVLAPQTPTSQGPVIDGQPLPLSSPGQYVRLDLRRLYASDDQNAR |
| MBK9387522.1_Planctomycetota_bacterium | PLILSDEATDHRLFEKKLRHYAKDGGLLVLSTAPKSFVAAEQALVTFLALERCDLDALIVRAMHAAALANGIDWQTVLLADAPDASASDRANLHQLFHLALPEIEAALLAPGPAILVRKPGLLARYGALRIFALLKERARRPSLWFLLPSSQQSSVPRLFEEPLPI-SVDRHLRIPESWPRQQLSPRST |
| WP_090664774.1_Belnapia_rosea | ATEATPDVLAARAIEDKIVFAAEKGVFLALTVEPRRARDAETELLRRFPREVVSLERLMLHAMRTRAEERRIAWPKALTADAAARDSTDFKNLLRLAAEAAPRVAKEVLALRQPALLTRPGLIARYDLMTMLDEFSQASASPSLWLLIPQATPG-RPEIDGAILPVISAANWTRLTDPWLANAHRAGVP |
| WP_136411732.1_Luteimonas_yindakuii | TTADSPEAREARAFDATVRRALDAGRVVLVTADLRRVEDAVPELSQQYDLAAISLDRLLIDALKAQTSQIGGDWRVVRQADAAVQSSADWRRLTALVERAMPAVVAQLRDDPRPLLLQHLGLLVRYGHTGVIQTLRDAASAPARLLLLPGDSHQ-PPMLDGVVLPVITPADWTHLPRSWLENRHRAAPT |
| WP_150933330.1_Microbispora_cellulosiformans | TTTGLPGLLGPTDAINRLVTVADRGGFLVVKARIKQAAALPELLSAELPVSCVDVTKAFIAALREVRDEWGVPWPIVLATDSPDAIPEDRVGLAEMTTEAFALVEDRVRAMEGVVLLHDATPLARYAGGELLARLADAARPHGLWLLAPMADPREPAQLDGQAIPVIPGNEELALSSDTVDALEDSSKA |
| WP_090069475.1_Lentzea_flaviverrucosa | TGTMSPPLTAASDVDARLVEAARHGGVRIITVRRKRWERARTAVEIALGMPVIDVSVAFVAAVRQVARQLNIPFDRVLLADAAEPGSNDQLNLHRVVAQACELLEAEWSR--HPVPLDALTPLGRYPAGALLERLASRARPDALVLISPAGDETKVPRIGDHVIRVNTPEEWVIARSPWPSGVQPRSQA |
| WP_183357094.1_Garicola_koreensis | HHTIPVGRKQPERLVRELRESVGARSFLAIGIPARQVERTTSDLLSTYGGVQVNVTDLLLGGLRSQAEEKSVPWDAVLAADAAESGTRAADGLAALVRQSISTIETAISEATQPVVVTDAAPLARYGHLRVIARLADVARSNAVWLIVPQG-PEPGAHLDGTPIPLSYSSQFLNLDNRVAVPAAAEETQ |
| WP_257478501.1_Acidipropionibacterium_jensenii | DATFGVDLVEDSATTRRLEESVRYRSYLVLGAEPTRLHRLVRALGSRFHAPELDLSKCFLDALHRLSDDRRVPWPVLVAADAQPEASRAHIGLSKVIELAMPEVRSTLSDSPAPLVLTNVAPLVRCGYVKILRELSDLTRGRAVWVVVPQYGLHVGPEVDDVRL-VTSPNQFVEIDRTWIDAETDAETE |
| MBL9044566.1_Myxococcales_bacterium | THPSGPEEEEALALSAKLDRASREGGLLILSVLPQYADAAKQKLAQRFALSVYSIEEAILRHLKAFAHSKQIQWPVILLADAADRKSADFRKLQTAMSAALAQMVDEIRHAPSPLLLIRPGLLARYDRLDVIESIRVSVEPPGVWLLLPSDELDLGPRLDGQTVPVLTPAYWRAISPHFLRAALPAVRS |
| MCP5560207.1_Verrucomicrobiaceae_bacterium | RTVMQEDVATALLLEEKLSSCATRGSFLVLSVPPDRLARAESELLARFPVQRVDGDALFLRLLHAQAAEYEIPWDTILLADAAKEGTEDNQNLRRLVDMIKPQLRQALVSPAQTLLLVNPGLFARYGLMGMLAELRNDVRPHGLWLLIPAVVQSALPKLNGQPVPVISEHQHISLPQAWLDNAHGKA-- |
| MBK6942988.1_Planctomycetota_bacterium | ATEISDEVAAARAFEERLARAQKDGVFLVLAITPSHALDAERELLRGFALERRSVDVLLLDAMRRHATANEVPWSVVLNADRARPDSVDGKNLRRLVRMAIADVEQQLATSTRTILLTQPGLLARFDCIDVLERLRDRVRLFGVWVLVPCDDQREPPVLNGVAVPVITRAQWARIPISWVKNRHRGSES |
| WP_217637676.1_Actinopolyspora_saharensis | VGTVPAPSSRGDYLGATLAESISARSFLALGVAVDEADRAAHVLALRHGGTVLDVTGMLVDEMKTVAGKKGLPWDLVRGADAAQEGSRDAQGLHALLNKAFPAVGERLRAGAGPLILTELSPLARYGQLKMVARWSELARSRAVWVVLPQLSWMRGALVDSKPVQLGSNGQFVELDADWLATQEAETAT |
| TAF23825.1_Verrucomicrobiota_bacterium | GTPVSEEVAEAQRLEDKLTRARAQGSYLVLTIDPKYLNDAANQL-ERIGAKRLNASELFLTAMKDKAADLGVPWEVVLEADAAEKGSADWTRLQELIRRSMPGVLEPLRNSNEDILLTDAGLFARYGRMNELDALRNDTTPHSLWLLVPGHGAATTPTLCGEAVPISNEAQFEQLTPQWARNHHRGGVA |
| MBO801654.1_Nocardiopsaceae_bacterium | KPSTATLLDETARASRRLEQASRRGGFVAVKAPLRDAARIGDVIAGMDGVTGVNVTREFVRLLRDEASKRPKLWETVLSADSADAPPGARRGFGELLRAVWGALEARIAGDGGIVLLHDATPLARYTGGELLARLAVAARPFGLWLLCPMENPKGSPLLDKSTVGVIPGMEQLDLPEGFGSGAESRAS- |
| WP_238634082.1_unclassified_Halorhodospira | DTTDDEQGERAAEVERKVRRVASEGRFLALRIGPRHMVKAQRRLASGYGLQAYNLEGELLRAMRDVADERRVQWSKVLEADAAGPKGPDWKKLLQVVGEAVRRMRPHVIEPPQPLLLLNPGLLVRYGHIALLNQVRDACEQPGVLLLLPGSSATTMPVIDDQALPIVHASEWLHADRHWLDAPANA--- |
| MBK9978482.1_Gemmatimonadota_bacterium | PNILDDAMDEARMLEERIAAARSDGGFLVLSTEPARFRRAELALAERHALTPMSLERLFVESMRKTAEANRVHWPLVLQADAAPHHDRAWRNLRLLAEKARPMMEATLAALVRPTLLLHPGLLARYDLLAVFEPLQDRSRGPGVVLLLPSDGMSAMPVIDLVPLPMVYPSDWARLPREWFRLARSA--- |
| WP_130480151.1_Leptothrix_mobilis | ATARLARLADASALEQRLQHHLQQGGLLSLTVEPRLARHAEAELLRRFGLQRLSLDALLLRELRTQAYARRVDWATVLRADAAPAGSRDWTNLLRLVQLTLPALQQALLNSPAPLLVVHAGLLARYGLMRLVTELEDQSRPPAAWLLLPGQRPG-VAEIDGTPVPLVHAQAVAALQPAWIGNEHRSAER |
| MCO6436553.1_Phycisphaerae_bacterium | PTQIPPEIAEARQFEERLRYAEKDGAFLALTAKPNLYERARQELLNRFDVRPLDLERVFLDSLRQAAEEVGADWNVVVKADAAGPNSTDWRNLNHLISKVVPKVEEAIVQDGKTVVIYHLNWLGRYDQVVMLSRVQQAVQDHGTWLLIPGSPQTEMPLLDGKAVPVITNNQWAAVPKSWCQNLHRTDEK |
| WP_181762535.1_Pseudarthrobacter_sp. | TTHTKLSSTQILEIERQLAQSLTRKSAVTLAVRPRDFIRTSKALTQVFGLKLVDVSAEVVKAIRATAQSKNVDWDVVLRADAK-SSPSDHANLAQLVRMAVGPLWVELVDDPNPLLLTNIGPLLKFGFGAEVARLFDMSVPAARWVVAPKRGSANTPSSDGRVPPM-GPDGFVDISPGLASALSKREST |
| MBK9741405.1_Actinomycetia_bacterium | HVLTPPEVPPYDEIAARLQGSLRAGGYLTLAVDPRRVDQAAAVLARTYGLTRVDVTAELLAAAKEYAVEVGADWSTILDADKE-VGTRDRALLDSFVKAALDRRFPDILAATDPLLITDASILGRYGQQHWLADLADLAGPAARWLLVPHHASGGRPTLDAQPAPL-GADGYLVINAEFLAARQASEQS |
| NMM16638.1_Cellulomonas_sp. | TNLTRVATEPADVVDGYLRASLTRSSALTLCVHPREHDRAVATLTTLYGVTPVDVSERLLTAVRALADRDGVDWSLVLRSDAE-PSGGDWENLRGLVQDALTEPWQQILATRRPLLLLNAAPLARYGLTAWLSDLLDQSQPAARWVLVPRRGHQAVPTLDGRPVPL-GADRWIDLPYDLSSLRPSSGVS |
| WP_136709615.1_Agromyces_sp._H66 | LSRTLAKSLPASDLHARLLGSIGSNAALTLAVNPRRYTEARRYLTNRYDVTTIDLAAELVRRMHAVADERGAKWAVVTRADAS-ESSNDFKRVSQIARQAFEPYWRELSAEPNPLLLTNAAPIARYGLTEYLAEMFDLARPAARWLLVPHKAAKPTPDLDGRPIPL-GPDRWVSLPSDLAELFPSSSGV |
| WP_210768105.1_Cellulomonas_humilata | GSSVHRGLVPASQVAAQLVSSLRTKSAVTLCAHPDAYVSTTRMLGRVFEIRTVDVGAELVRAARADAKVVGASWEFVLGADVE-PTSPLGSDLRVFLEPTVTAVWDRLMADTSPLLLTNAGPLLRYGFTSALAEMLDLATPAARWLLVPRPRAVAIPTLDGHAIPL-GADRPVDVPLDLESAVSGA--- |
| WP_067228577.1_Microterricola_viridarii | RSTTLQGSLPNDTLAPTLAESVQHRSALVLTTTWGRYDDSATALAARFGVEVVDLAALAVEQLHATAASVGADWLVVVDADKP-DSTIDKRNLQALAKQAMSSAWAEINDRDTALLFTNAAVLAHLGLVDLIARTIDLSTAAARWFLLPKHGAAAVPDLDGTPMPI-GAGKWIELPTTLAAVLPIPSSI |
| WP_129336322.1_Cellulomonas_endophytica | LTSIATRTSTVPQVLAVLQRSVETRSGLLLAVTPERHEHASQQLAAAVGLVVLDVADALVREARRVADEDGADWSFVLGADAE-PGSPLRSQLHEFLEAAVDAVWEQVVSTDAPLLLVHAGPLLRHGFEDRLDHLLDLATRAARWVLAPRPSSSGVPLLDGHPVPV-GAGRWTDLPADPLPTLGAPS-- |
| RKN35100.1_Streptomyces_hoynatensis | ASDEDTAVAAAVKAEQRLAHSARQDGFRVLTVPQRRAEEAVRLLTEPYGAQPLSLTGLFLAELRGVVGEAARPWETVLKADAAETGSRAHQQLRIRTDKAWGRLEPELTADDGPLLLTDTAVLARYDAFDLLGRLAEEARGRALWLLVVQSDPARAPRLAGQSVPYQAGDEWIVVPDAWVARRHLVSRA |
| WP_262103914.1_Arthrobacter_sp._Marseille | STYTFIASGSVSELERRLRDSLDRKSALTLAAHPRDYLKAASTLAQRFGVKRIDIAAEVLRVIKSTARQADVSWELVLRSDSE-PSSGDFTNLTRLAQAAIRPMWGEMMQVPGPVLLTNIGPLIRYGFGPDVSKLLDLSHPAARWILAHRRGYDETPKVDGKPVPM-GPDGWIDMPRNLSDLLTTNDLN |
| MBZ5659870.1_Acidobacteriia_bacterium | QTELPREMEEAVEFERRLHAAYRSPSYLVLATEPKYLEMARQNLAKHFPMTIFHCEREFLAALQKEAESKHIRWDVILRADTSKPGARDWDNLRKLAANAAHRVAEQVQSRTQSMLLIYPGLLGRYGQLSILDELADSLT-YSLWVLAGSEHQAASPMMDGQAIPA-RPTQWAWIPAKWLDNEFRKSTA |
| MCB0954589.1_Ilumatobacter_sp. | GTAVAHEREEALAVHARLQRQEAEGGYLVMTVEPRWQRRAIE-LLRGYGPHTVDVDGWLVGAMRAYTDRRTIKWPAILGTDAAGPEGERWPRLLTVAKDAMAPLQDGLAQ--RHLLFVHPGLLGRYNLLGMLDQLRERTRLRTVWVLVAADDPAATPTVAGRAVPVSTPAEHLVLPEPWLHNLHKTEGV |
| MBL8842562.1_Planctomycetota_bacterium | TTEVDPEVVNAAEVERRLTLSHRRGGFLALSVLPRHYLAARAELRARFALDGLSFDAALLARMKRIAQEKGVDWQLVRATDAAGEGAAGWRNLVTLASLATGELLAELSAAGRPLLLTELGLLKRYEQIERLAALRERARLHGLWLLLAQSSPGPRPQIDGRLVPVLANGEWLALDSAWVKNRHRSVPR |
| MBO9539711.1_bacterium | STLPSDEILDARAFETRLERAVTDRRFLLLAIEPKYLLRAEAEITKRFEVHRVSFESLLVRKLRSAAQAVGAKWEVVVSADGTPRDSNAWRNLRGLVKRAMGEVEQELSTSSTPVLMVENALIARYDHMELLERLRDDCRAPGYLVLIASDDQSTAPKLERQAVPLITASEWVRIPHAWLENRHRSTPT |
| WP_144981411.1_Gimesia_aquarii | DTSTSPETVEARQTEDRLQHAYRDGGFLVLTVKPSYLRPCEHNLVHRFPLERVSFDDLLFEQLRAKSKEYEFPWSDLYEADSEGTNTDDWANLLHLMSEVGPNIETELMNSERPLLLVHPGLIARYQMMSVLQTLRDRVHDPTVWVLIATDSQNEMPYLDGVQIPLISKGQRAAVSEYWIDNLHRGRSG |
| WP_093484991.1_unclassified_Streptomyces | GTPLDPE-STAARADAQLAGAARRDGLRVLTVRARNAPVVGRELRRQYGARAVSVTELFLKGLRGELTPGAMPWEVVVKADVL-PAGQRPTGFTNLVGAVWQAVQGVLAADPAPVLLTDAAVFARYDGMHVLDRLTALARGRPLWVLVAQDDETAVPKLAGRPVPYLEAQEWIPIPKSWTRMHEPNAAT |
| WP_200436137.1_Halochromatium_glycolicum | GSAALAAEQRQQAFDDRVRTALDEHRFLALSVRPADAQRAAQRLQARFGLVAISFDALLLRQLRRLCDARPPDWQVVLRADAAAPGSIDWSRLQGLVQRVLPELTAALGAAGAPVLLTDTGLIGRYGLVSWLAELRRRLEGHALLLLIASDGTGPGARIEGVSVPDPGRREHAAIPSQWLQAAADG--- |
| WP_252180113.1_Endozoicomonas_sp._4G | ATVDTTTLEEVKRLEQEIATAIETSRFLALTVKPQLMVTARDKLCKDYPLKHISFDELLFHHLQELFNPKAPRWDVVLKADAAQKNSVDWGRLQMLVSKVLPAMANEIKSAGQPVLLTEPGLIARYDLVSWLNELRQHLNVHGMILLVAADAQRTAAVIDGTIIPRTGAAEFARIASVWLEASNDSQQA |
| RLS37136.1_Planctomycetota_bacterium | STTLAPGLETALHFEDRLSRSRRQPNFLTLLVEPRGYGKARAALVQRFQAEIIDLEGFFLAALRHTATALGVEWELVLRADSH-RLPQEWMNLMALVRRAAPDFEERLMATDKALLLVQPGLLARYGLMDQLDKLVDQARRPAVWLLLAGD----RALIDGQAVPLIHAGLQVRVPLEWLENRHRAAEL |
| WP_241482799.1_Kocuria_polaris | VSTTQFAPTPAAEVARKLTESLERHSALTLTVAPKRYVAAARALASKFDIDVLDVAELVVSATREQIEKAGGIWSGLLGYDAL-DDSKQWKALEGVVQRSVEPVWGERLSAHRPLLLTNAGPLVRYGMTSLLATLLDTGTPAARWLLVAKAGDARAPLLEGRSVPL-SPSGWIDLPRDMAQLADDKTIQ |
| G8417454.1_ | AKALGAERSPHEAVRDMLLDAAGSRGFRILVTPPEHHADIGPSVARALGGTWVSFADAFFRDHGANIAAL----------ERAERFVAQRDALTEAAEDTMFRLLDEHGRPDHTAVVGDTGLLGVCDALDLPRRLYDETLSRGFWVLVPGVIYKSQPHFNGATFSLLTALPD----------------- |
| MBK8541194.1_Ardenticatenia_bacterium | PTFLPALTSPGDRAAAWLRSSRGMSSWRLVVTPPERHAELGRQVAAALDARYVSFEAAFLKRIEDRFADF----------ETAERMSALRGRLKKEAEGVLEALLAEHGRPGTATVLGDTALLGLCEAKHLVTRVYERALALGFWVVVPGVVHQQQPLLNGMVLPLAEDVGV----------------- |
| MDO9017995.1_Myxococcales_bacterium | PAEFIAHRSPDEIVVGLLRAARKSRGFRMIVAPPERHAEVARSVAQALDARFVSFEHGLLAGMEPEFAVY----------ERAERFKAQRSRLTRAAEGLFEALLAEHGRPGTCTVLGDMGILGLCDAAHMIRRLYDETQARGFWVVVPGVIFQRQPLFNGTVLPVDGELGR----------------- |
| WP_075011136.1_Stigmatella_aurantiaca | PSDLSTQRSYEEVVADRLREASRGRGYRLVVAPPEKHVEVAHSVRRALSAELVSFEARLLQRMEPDFPAF----------ERAERFLAQKPKLKKEAVALLDALLEQHGRPGNVVVLADTGILGVCGVKDVPRRLYDRVTDLGFWVLVPGVVSERQPLFN-QVLPVPEPLDV----------------- |
| MCA9600002.1_Myxococcales_bacterium | PDDLAPDRTPEQVLVARLRDAAERREWRLLVTPPEHHADYGCSVAAAINAEYISFEQRLLERMEPSFADF----------ESALRMKARRRKLTRAAEALLEELLRERGRPDCRTVLGDTALLQMCDAAHLVRALYDETQQRGFWAFVPGVIQERQPLLNGATLPLSGPLPP----------------- |
| TVQ88636.1_Deltaproteobacteria_bacterium | PELLEERRTPEEVARDVLRDAVKRSSYRLIVADPGQVGPIAKSVIRALDATPIDLADAWFQRHAATVKVD----------AHAERFPAMKAVLAQKAHKLFEELVAEHGERGRAIVVYNTGLLDAIGGVVQVRNLYERVQQHGFWVLVPGVISGRTPLFNGLTLPLRKPLPD----------------- |
| KYF91864.1_Sorangium_cellulosum | PPELV--KDPAEAARDILRSVAKTRGYRLVVAPPDDHVEIGRSVARALEAAFVSFEHEFFRRVDAQIEVF----------DRAERFAAQRPRLRKEAEALLDALVQEHGKPGSTVVLGDTALFGTCDALHLVRRLYDLTAGKGFWALVPGVLHKRQPLFNGAVLPLAREIPE----------------- |
| MBI4699810.1_Deltaproteobacteria_bacterium | PPELVAAKSPEEVAADLLRGAASGDGFRLVVSPAELAPEVGRSLARALEVRFVSFEQELLGRMDGAFDGF----------VRAERFKAQRGKLTREADGLLADLLAREGRSGATVVLGDTAIFAVCEALHLVRKLYDETSGRGFWIMVPGVVYQKQPLFLESTLPILRAIEG----------------- |
| consensus/100% | .................l...........h.............l..........p...hh................................h.......................h...s.h...................s.hh..........s................................ |
| consensus/95% | .................l..s.....h..l.............l.........hsh..hh..hp................-...........h..........h...........lh.p.s.h..h.....h..h.......s.hhlhs.......s.h....h......................... |
| consensus/90% | .s...........h..bl..s....shb.l.s...........l...h.h...hsh..hl..hc................-.....s.....l..hh...h..h.........shll.p.s.h..h.....l..h.p.s...s.hllhs.......s.h....ls........................ |
| consensus/85% | .s...........h..bL..u..p.uhb.lss..p....h...l...hsh..lsh...hl..hc..h......W..h...D.....s.....L..hh..sh..h...h.....slll.p.u.ls+hs....l.pl.p.sp..u.Wllhs.......s.lss.slsh.........h............. |
| consensus/80% | ss...........h..bL..u.pp.uhbhlss..p....h...l...hsh..lshp..hl..h+..h......W..l..sD.....s.....L..hh..sh..h...l...s.slll.psu.lsRas...hl.pl.p.sp..u.WlLhP.......P.lsu.slPh..s.....ls..h.......... |
| consensus/75% | ss..ss....s..h..bL.puhpp.uabhLss.spph..h.p.L...hshp.lshp.bhlp.h+..h......W..lh.uDss...s.....L.phhp.sh..lb.pl..ss.sllL.psu.luRYs...hl.cL.-.sps.uhWlLhP.......P.lsG.slPl.ssspbh.ls..h.......... |
| consensus/70% | ss..ss....s..h..+L.puhpp.ualhLss.scph.ph.p.L...hshp.lsls.bhlp.h+..h......Wp.ll.ADss..ss.s..pL.phhp.shs.lb.pl.sss.slLLspsu.LuRYs..phl.cL.-.sps.uhWlLlP.ss.p..P.l-G.slPl.ssspbh.ls..hh......... |

**Type 2 BREX; PglW C-terminal Inactive STAND-NTPase Entropy**

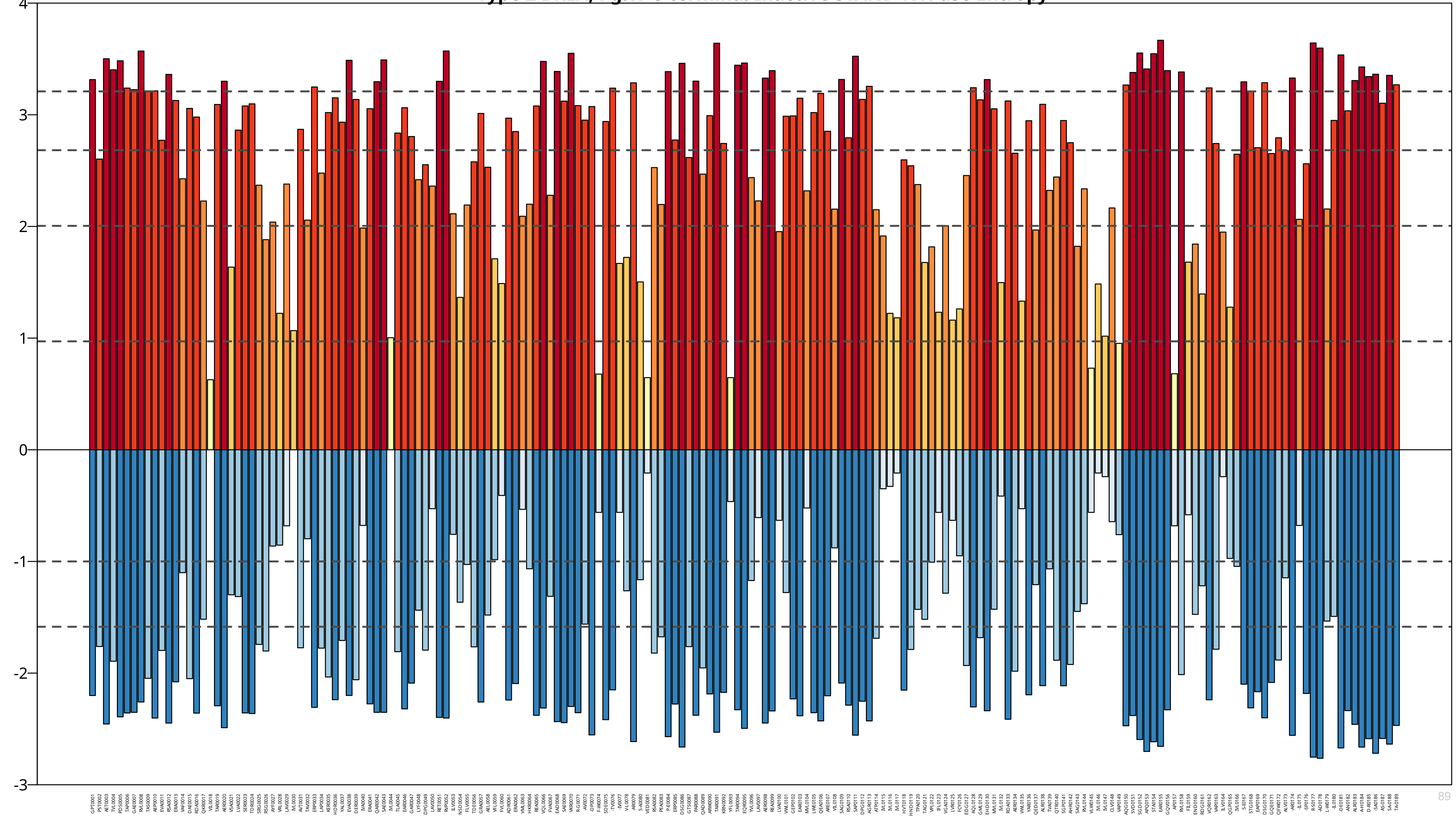

```
# ; Type 3 BREX BrxF-iSTAND NTPase; (For Supplementary ENTROPY)

MBI3973658.1_Chloroflexota_bacterium              MAAAVDQLDPGYYACLVLVHYLATYRWPHLSVGQELSEALLQEQPSRYPTAASKWLTDRVSSLSPGPVQVTAIDLLFGPALDLDPLILFRQASRTTRLVILWPGTYLNGVLSYAVPEHGHYRTWPKPDIQITT
MBO8173527.1_Bacillaceae_bacterium                MYFALEQLEGRRHRLLLLVSEASKHQIPILNMNLSLSEELINVPVDRRPRKVGEIVEKILRHQKKDLVIISHIELLFDHSLHLNPIKLFEELSKTKTLIVGWNGKYENNVLSYADPGHSDYRSYYDCDAIIFS
CEO89248.1_Syntrophaceticus_schinkii              LSKAIATAQSRYYKLVLAIPGMAALGTTPVNLGLLLSQSLLEVEPRFRSLEFQDLFARIIRSAEGDLVAFDHAEILFQRDLQLDPLRMLLQHSRNKVLIVRWPGVFSGDKLTYGEPHHPEYRCYEQVDAVVCC
CEA16532.1_Fermentimonas_caenicola                MSKQITHFAAPRYKFVVAPNERMLKDFSAFDLGYNLSLVLKENKNEELTFIVHEELTKLLNDNITDYICLSNIGILFEKELKINILQTFQRLSKNTLLILLWDGEVKGNTLFFLSENSKNKIDLREINHIIL-
HHY32855.1_Bacillota_bacterium                    MRDLIKQMETNYYKMLVIVDNLQAEGWTAVDVTDAVLKLIEGIPGEKVRVRIGGKLKEWFHS-LPDRLVLYNTSILYSPELRLNPVGAFKYKSREKEVVVLLEGRLACNRIQYSQYGRPDYAEMDVSEVIHAD
KUO63975.1_Lutibacter_sp._BRH_c52                 MDKHITTYSQSRNKVVLFK--ENIFDFKPINVGHILSQALYNLDKDKLPMLVSKELNTILNASITRMLAISNLGILLEPDLKQDINSLFEKYSNNNVLFVQWEGELDHNNLYFLTKEKGIIININIKNLSHIAI-
MBI9032244.1_bacterium                            M-DRLKEIQKLRNKLCLIPVNNNTYNINSINIGFELSKVLKNELGKNLPIIAVESFRKIIEGSTSETIMVYNLGILLEDDLNLNLESILLEISKNIVVILLWEGEISKQGLHFLSIKHGKLLELTEKHYLQLE
CCX79045.1_Parabacteroides_johnsonii              MDTAINKYSAPRYRLVVLKDEMPPDDIPKFDIGKSISAYIISEHSNDLKMLVNAEFMRLFREIASNAIALCNFGIVFEEKLGLDMRTILKDLSRNYLMFIQWEGVVKDDRLCFLTSDSSNFIDLSEISHLTI-
MBK9455368.1_Bacteroidota_bacterium               METYLALANSLRFKLWAIVGELQKHGYLLVDVGAELGSLYKELTNEEPSHDIGQKIKDWFLS-KPEKLILTNASILYHNAFKISPVGAFKYNSRNKSTVLFLDERIISNRISYGQAGSEEYYDKDINDILITD
MBL9119763.1_Phycisphaerae_bacterium              LRAAVLAAEERHTRLVLVVGTGLRLTTRVANLGLVVAERLINLPARHRPVTAADAAADFIRDAEPGPALVSNLQLLFLPELMLDPLKLLSDSARSRVVVASWPGERTGSCLRYAAPSHPEFREYHNPDCLVVE
MBQ9466676.1_Muribaculaceae_bacterium             MKQRLTKFSAPRHKVVVLLNDSVGASLPAIDVGRELAAYLEAYTSHRAMDEAKQFLTKLIAQKTQPWVALRNLGILFERDLDISPANLIDNLSRNTLFIIQWPGLVNDGKLYFLDRGSRYFIDLSQISHITL-
MBT3747343.1_Bacteroidota_bacterium               MEELIADMSDVREKYVIVCKNAEQLNASYININLELSRRLLDIPKKKIPFKANRYLEDIINNSNKNIILLDRIEILFDPLISIDPLYFIESNSKPVTMIVNWPGEFSSKILSYAKPVHPEYKSYPNISGRVIS
MCA6513726.1_Chitinophagaceae_bacterium           MQTPIKDITTERYKLYTLMCNVKSQNINVINIGKELANYIDSLDFRYLNIDVYDHTKKLLDKHRSDVVAIYNLGILMEPALELNAVQLFKEFSKSTSLIIIWDNQSENDRLDWQTQKQNFFLDFSDIQLKKLQ
MCF1431077.1_Shewanella_sp.                       MEQAIEQAQNYHSQLVVVAGEASTDGLAVVNLGLELSERLIEVPRQDRAKSAASLFADLLHERNTRVLLLNHIEILFDKTLSIEPLKLLQSSAKNLTLVVAWPGEKTASSLTYAVPSHPEYRYYKASDFGETF
MCM1450902.1_Clostridium_sp.                      MAADLLRFNGPRHRLVFVKDTNRTNSNIGVDVGWEVAKYLHDNSLGNIKAESQNFIKKLIYDNISKYIYLRNIGVLFEPELGLNVDMFLTNISRNTLLLLDWDGQMKYPYMYFLHTDSKHRIKIDTLNYMTL-
NLI12135.1_Peptococcaceae_bacterium               MSKELAHIGYRRHQLLIICGVTRELAIPCINLSLKLSEELLEVPVVQRGRKVNQLVDKIVSESTGEILCFEHIELLFHPQLQQDPMRILENISRNKIIVVSWSGRYANGRLTYAEPEHPEYRVYNELEASVIT
OJX40547.1_Chloroflexi_bacterium_44-23            M-PKLEIIFHELFDCLILVNPIKEENISVINISQELGGFLLNISAMDRSRSTQNWMVDKLASHPHDPILCVCPDLLFDPTLQIDPFALFRQIARIKHIVVMWPGNFSSGILSYAVPEHHHFRTWKITDTIINQ
PAD23514.1_Niallia_circulans                      MNNAISAVSEQYFKQIFVYDYAESHSLPYINVNVEISLLLQKVPINRRAFLITEVFQGLIGKYSDGVICLDYYELLFDNSLAIDPMILIKNNSRNKTLIISWRGKIIGDALIHAEPGHPEYKKFIVQDAVIIK
PKO06211.1_Chloroflexi_bacterium                  MQVRLAIAPQQYHRLIIILNPAGKFGLRYINLGLILSQMLLDLTERQKALKMPQLVDQIIGCIEDKPAVLDHIEILFEPSLQQDPLRLLQGMSRNRTILAIWNGQVNNGYLTYAATEHPEYRKYSIRDLNILS
PZV08804.1_Leptolyngbya_sp.                       MGDLLAIAQGQYHQLVFATVEAVALDGHYLNLNLALSQRLLDIPQRQWPHKVMELIDAVVLSTDLRVTVLHCIEVLFDPQLRLDPLKSLQQIARRHNLLVVWPGTYDGRYLAYAEPWHPEYCRYPAKDLIVFP
ROS80359.1_Muribaculaceae_bacterium               --DKIAQYYDPRNKLIFAP--SLDQHLHSVDVGFELAKSIEGDSSPHLPMIAEDYLNAILRRSIKDYIALENWGILFEPALKLNLVSIFDSHSKSNTLILVNCGQADYENFHLVNKHFNTTFPIGNLHPYILQ
TDP26837.1_Halanaerobium_congolense               MTDLIKSNRGSHYKLLTLVGSLKNNGWNVYDIEEVILDLIEDIPENKIGLKIGDKIKEWLSD-QENKIVLTNTSIIYSPELLIDPVETFRYAMRDKEAVIFIEGKMRDDKVIYSTPDKQDHKDIDISRIVSEE
TNF50602.1_Bacteroidota_bacterium                 MKDKLLTYGSSRNKLVITS--EKIPGVHYLDVGETLSLSLANSPISWNSMKIKSEISKLFAESIESILSIENIGILLESEVQFDFKHFLEQYSRDMAIFLKWDGDHDESTLFFLSKDKGIKIPLTNLSYIKL-
WP_061808754.1_Rossellomorea_vietnamensis         MNTLIEAIKGQRHQFLLLSFQLKEIRATTFNVNLLLANKLIELPISRRKRAVKDTLREALNEVENEVVYFERIEYLFDEELNQDPLKLFEYLSGNKVLLINWPGDVRCNGLSYATPDHPEYYQVDGYRDHLIV
WP_073205029.1_Prevotella_ruminicola              M-SNLSSYKNPKYRLVFDL--SSSLEVESVDVGTYLADALQTKQKANLNMYASDAIRKMIRQHTHRYVALCNIGILFEPALSFNLETIVEQTAKDELLIIQAKGGIANERFYFLSQQNNYSFSLKNLTYTIVE
WP_075981269.1_Bacillus_massilionigeriensis       MSSEIETLDSRWYKLLIICGLANQLDIPFININLKLSEKLKDISKAKYALKVPEILQSILEQYEESYYWLGDIEILFDKQLQQNPVRLLENLGKRYSIIVTWPGQYANGQLTYATPEHPEFFSCHETDGKILE
WP_086717961.1_Bacillus_cereus_group              MDKEMALMHNWWHKLIFICASATNEDIYTVNLNLIMSETLVNISEEKYPLYVEEVTRETFDN-PFKIYLLQHIDVLFDPVIKINPIRLLENISKKSKLIVIWPGEYKNNQLVYAQEGHPEYFLSGSFEGKVIL
WP_106450119.1_Trichococcus_alkaliphilus          LNREVPYLKNKYHKLLYLYEYSEANKVPYINVNLYLSENLADVKPNRRPYKVVDLM-NLLGDTEDDILCLDYFEILFEPSLMLQAFDIFKNLSKRKVVLVAWRDEIKDGYLIHAKPGHPEYTKELIADTTIIY
WP_113032580.1_Paenibacillus_contaminans          MSAEMAGVRSRRYKLLFLLPEMEREGIAAINVSLLLSDKLRHVAPDKRPYEVAKHVKQAINEYKADVVCLYHIEYLFDLELKQNPIKLLEALSGNIVLLVAWPGGAEQGVLYYADPEHPEYDSNGEYGSCILP
WP_118194774.1_Pedobacter_indicus                 MIDKIVSFLIPRNRLIISTDFFDTEKVNWMNIGKLLAHELGNTDKSKIVFRINQLLDDVIVKNKIETIAIENIGLLLEPELKFNVEKFLSDYSQNNTLILKWEGEIDTNQLYFLTKNDNHKIDLNNISHIVI-
WP_119865508.1_Clostridium_isatidis               MEDAVAAAKSKRNKLIIV--NIAAIGLDCINLNLLLSEKLLEIPVSKRSRSVDSFIKDLIKSNNMNTLALSNYELLFLPELKQDPMRLFEDLSKERVILIVWEGRYENNSLYYAEPWHREYREYKDLDAIIVQ
WP_155743288.1_Scytonema_sp._UIC_10036            MGSYFDQVVERYYRLIVVVVPAESTNSRYVNINLELSGRLLELRQLHRATQVQQLLAEIIGNTNNGAVFLDRLEILFDESLKLKPLERLQKLARDRTIVAVWNGNIENNSLIYAKSGHPEYCRCPTDDCIVIN
MBR1791000.1_Bacteroidaceae_bacterium             MSEQVISFSSSHHHIVFCR--EMMPELTFVNVGLVLSEKILEEDSPMLDFATDDFIQEIMSRHESNYLALTNIGILFEPCLKYNIPELFKSHSRNKILFIHTQAVIKDDVLYFNDPSDNITADLSGVHYIIV-
WP_169099405.1_Bacillus_sp._DNRA2                 MSQELEGISTRYYKMILVCELAESFGIPLLNISMLLSEKLKEYPSKRMAGRVHSLLSDILRNAGSSTLCLEHIEVLFDPTLKQDVIQLLQPLSRNYTLVVSWKGSYDGRRFVYAVPGHPEYYECKEFDGIIVT
WP_173978270.1_Magnetospirillum_sp._UT-4          MQQEITDVAGTYHRLLLLVGRAAHLDRPVLNLNAELSSALLEVPHRQRQLKAHKVVGDIIDGGPADIVLFDHTDILFSPSLRLDALALLKSLSRNKTVVATWSGTAKDGKLRHAEEWHPEYQSHAIEDIKVIT
WP_181742022.1_Thermoactinomyces_mirandus         INTSLKEITRHRHQLIFLAGDAEKIDAIYLNLSLELSHMLMEIPVHKRPRKVDICFREILTGYSQSLFLLDHIDVLFDSSLQLNPGELLENMSRKVVFVIPWYGQVTDSELIYADPGHLEHVRIPINEAMIIQ
WP_214689525.1_Exiguobacterium_sp._s163           MNSSLPQVTNQYYKLMYVNEYAKNFETGYLNLGIELSLALLDIPLKRRPFKVTDIMENLIQKQHNKIICIDRIEVLFEPSLKTNPFQMLKQLSKNHTLIIAWRGIVSDQTLMYAKPNHPEYFKYTATEEWIL-
EGR2106511.1_Vibrio_cholerae                      MKRASRAAEYCSHNLVLVVLESQIEDVELINVSKSLSQRIVCLSANERIKRLDVEFEEVAQE-CNENIWLTKLDLLFEPSLKANPILLLKWLSKSRPVVAIWPGQIYEKTLVYAEPGSEDYRTYSLQELKDIQ
CCU85050.1_Mesotoga_infera                        VKNSLECLQAKEPGLVLLSGGANEMNTEVLNLNFVLSKEMKTLSINERPLRASRFLSELVNS--RESLLLDHIEILFEPQLKLLPIQILRRASRTTPIVANWPGSIENEFAIYAQRNHPEFMEVRLDSEIIFK
HBR02122.1_Ruminiclostridium_sp.                  LGSEIIETIRKVTIPIIFCSLKLINDYQELSLNKELAKALVEKDVIIRSRFVADEVMKIVSL-IQGPILLTDYEMLFDPRYNIDVLKLFYELSRRAKMVIKWCGALDDNRLVYATPAYSDFHSYNIHDYDIIC
HHV38844.1_Tepidimicrobium_sp.                    MGKDLRIWNINNHYPVFSILQNEFKDFYYIALGEILSSSLLKYGKDDRKDYVIYEMDNIFHN-MDSKLIIDHIDILFNPDYRIDILGYFIQLARNRRIIVIWPGEYVSGTLTYATPEYRNYKKYSIKDYNIIC
MBD9124683.1_Lachnospiraceae_bacterium            MGRLLSENQTGLMRPVVYCDE--CESLHQVSLNEKIAALLIKIKPERRTMRIERCVQEVLTN-IPEKSCIRDFDVLFNPAYKIDVLQLLTVANRSKSFSVLWPGTVIDGKLVYAEDGYADYKEYDVEQYDITC
MBL0521045.1_Aeromonas_enteropelogenes            MAKALLRMSVQSSALVLVINSRQTEGLTYLELSEQLCERLLPLSCNDRSRHVSDIIAQMLNG-IASVVWLDRIQVLFEPTLELDPLRQLQDLARLKPIVAIWPGQITERFLTFSVPGRDDYQSYSANDLANVP
MBN2003265.1_Anaerolineae_bacterium               --QQRIMAQIRSHALLVLVGMALPDDSVYINVGLELAKHLRQ-NLPTGSLRAAQIMEELLES-SGASLILDRLEILFALTLHLDVLRLLLDLNRRIPIAAIWPGEYRDARLTYAMPGHPEYREYEYPEAFPIT
MBO2493598.1_Clostridia_bacterium                 GDRQVQGYTSNAGNIMVACVDAFFSNWKWINLGLVVSEQLCRLPKEQRKRMVADVVINAIKK-TPSNIVVENIDLLFSPEYSLDVIRTLQLAGKNKRLLVIWAGDYQDGVLTYAQPDCPDFHRYEIKNYGVCC
MBP3452736.1_Clostridia_bacterium                 MGSDFRQYPAVYQGLFVFS--RLPKDQPVINLGYVLSERLMGIPADRRCFEIDNCLRSIVET--YTAVSLSHIEILFTPSLKLDAIGVLLSLCRNRKICLSWPGRISGNTLTYAEPSSPEYYEADYTRFIDTY
MBP8682476.1_Trichococcus_sp.                     MGRDLAVIKQTRQGVFVLA--TAPVNASVVNVGLLLGERMFRVKTEGSSTSITRELDDILDE-EHRDILLTNIDILFAPEYELDVIKLLLQLGRNQRLYLPWPGELVGERLTYSEPIRFDFKEYIVKNYVDTY
MBR6410258.1_Clostridia_bacterium                 MYSELPENLKQSGVPLVFCSEKRVESYIELKINIILSERLCRIPRDERAKLVEFKLNNLLNY-YHAPVLISDFEMLFDPRYKIDILRFFCEKARIINVAIKWPGQYADGKLTYANPADPDYHEFDCNAYQIRI
MBX3742593.1_Akkermansiaceae_bacterium            IAVDIARASGEHFRLVWLAGGAEAEDGVVVDVGKILSSSFIEVPVSLRSASVEDCFTACLGE-SPSLTCLDHLEILFEPSLRVNPVALIKGASRHAVLVASWPGTTSTGHLFFGAADHPSHMDMSEHELSVLH
MCC5856368.1_Idiomarina_sp.                       MEEQIDKLALQSHQMLIVVGATLLDNIPRLAISELLCEKLLPLASQRRVDEVEALTADVIAS-QATSVLLERIHLLFEPSLQLDVLRCLKALSKQRTLIIDWPGVYNGAALSFSMPGKPDYFNYSETELSSVP
MCD8293624.1_Clostridia_bacterium                 MGKIFPTDLTGLLKPIIYCCS--GLDARALPINVPLARELLKYESAARTMLMKECFTNVVEE-FPDDCTIKNFDVMFNPAYKIDVLGIMVSVCKIKPFNLIWSGTLDSNKLIYAENGFKDHTVYEIDDYDITC
MCP5271842.1_Burkholderiaceae_bacterium           MLRLVNDAGALQSRLVLILGGAAHFGTQSLPLGIDFARRISAVARRQRPLQASSLLRDSVDA-AFPPALLDRTEILFDRTLQFHALVELKRLAHSRTVVAAWPGQLRDQRLIYAPANHPEHCDHPAAGFLVHH
MCR4442916.1_Peptococcaceae_bacterium             MGREASEYGTGKGPVFVACTNQLFPDWPWFNLGLCISQKVLSYKKEKRPNYIVDIIQEMVSA-SESRVIIDNIDLLFAPYYQLDVLKLFIQLGRNKKLIIMWDGAFRDGLLSSSVPELPDYHLYDIKNYDVYC
NPE22819.1_Fusibacter_sp._A1                      MGYDLSYFITSTNSLIVICNNDIGLQYHHFSLNQELAKKMNEIEVEKKALFVIDILNEMFE--INENIVVSDFEMLFNPSYQLDPLKYFINLSNKRKIILEWCGDFDGENLTYATPNYLDYIAYKVDKHNITC
QQS04458.1_Fibrobacterota_bacterium               MHQILSELKERHFKLVLLVGIANDCLTSVTQLGRALSPRLAELSVRSRPLEVSGALEELAKE-VTTLLFLDGLEVLFDDSLKIDPFDTLKRLSLRRLVFAVWPGDYLRGRLTYAKVGHPEFRDCAPEGVLLYP
RKL62728.1_Thermoanaerobacteraceae_bacterium_SP2  M-NAIKILPHKYYKLILVVGPSLEFGYDYINLNLKLSEKLIRIPFDERCFYVEDYVNEIVES-NHGLLILDNIEILFEKHLKIDPLLLLKNLSRYKACIAHWNGKVEDGHLIYAVPQHPDFVKYRMDGHDYIV
WP_028243927.1_Pseudobutyrivibrio_ruminis         MAYKFLDNISGLMKPIIFCTS-IEELSEAYSMNIPVAKELQKSGSSMRTMKMESILSSIISK-LGDNPIIKDIDVMFNPDYQLDVLRTLINVSKRKSFMVVWPGTYKDGKLIYAQEGYSDYKSFDLNDYDITV
WP_052235486.1_Alteromonas_marina                 MMQSLSSGEMQSSCLIFLILETLFSDALFLNTSKLLAERLITMSRREREANVASALKSIVLA-GDNMCYLQRIQLLFARELNINPVKLLQQLSRKKPVVAVWPGGLDCSNLYYAKPGHPEYVSYQLSEFQDVQ
WP_071138484.1_Bacillota                          MAYNFLDLIKRNKLLGLLCMSIEVKDLSIINLGLDLSHKLKEYPMEYRNSKVLDELTNILAK-AQTYIVVKNIDILFNPDYKLNILSYFINLSRSRLIFVEWPGRLKGRMLEYADINSPDYHKYNIDDYKIIL
WP_164569834.1_Rhizobium_leguminosarum            MPQLLEATGVSGGKPVLLVGSATLSNTSSINLNIHLVQALIDASDAR-----VDIISTISAL-EPSVLLLDRIQILMLPQLMVKATDVLCRVARRRSVCASWPGRLDRGRLLYAEPNHPEFLDEDASRALVID
WP_191750182.1_Clostridium_gallinarum             MGREVEMLIQQENKVIILCTNSIFKEFKRISLGVILSERLLRYPIEIRNSILIQELDKLLLN-TDDKLLIKDIDILFNPEYKIDILKYFYSLARGKKVAIEWNGSIKNNYLQYSEIGYLDYKRYSINKYDIIC
WP_271679096.1_Thermomonas_sp._2C3345             MWSLMADTAQLQSKLILLVGGQRSQHATPLHLGVSMASALTELPQRERHLQAGNLLREIAQS-HEPPLLIEGVELLFDASLKVNVLDLLKRQSQARPVVAIWPGEWRNGRLTYAVLGNPEYQDHAANGVIVFE
consensus/100%                                    ................................h..h..........................h....hhh...h.......h..........h..........h.....................
consensus/95%                                     h...h.........hh............h.ls..ls..h...............h...h.........h....hlh...h..p....h...sp...hhh.h.u......h.a....................
consensus/90%                                     h...h.........hhh...........hsls..lu..l..........h...h.phh.......hhlpph.lLa...h.bs....h...up...hhh.W.G.h....l.a......p.......p.....
consensus/85%                                     h...h..h......hlhh...........hslsb.lu..l.p.....b...h.p.h.phh.......hhlpph.lLFp..hphs...hhb..u+p..hhh.W.G.h....L.a....p.pa.ph...p.....
consensus/80%                                     M.p.l..h.....phlhh...........lslsb.lup.l.p...ppbs..h.p.hpphl.p...p.lhlpphplLFp.phphssh.hhbphu+p..lhh.W.Gph.s..L.au...p.ca.ph...p.....
consensus/75%                                     M.p.l..h.....phlhhh......sh..lslsb.Lup.Lbphs.ppbs.bh.p.hpphl.p...p.lhlpphplLFpsplplsshphhbphu+pp.lll.WsGphpss.L.aup..psca.ph...p...h.
consensus/70%                                     M.p.l..h...b.pllllhs.....sh..lslsb.LupbLbphs.ppbsbbh.phlppllpp...s.lhlpplplLFpspLplsslphhbphu+pp.lll.WsGphpss.L.aups.ps-abphp..ph.lh.
```

Type 3 BREX; BrxF Inactive STAND-NTPase Entropy

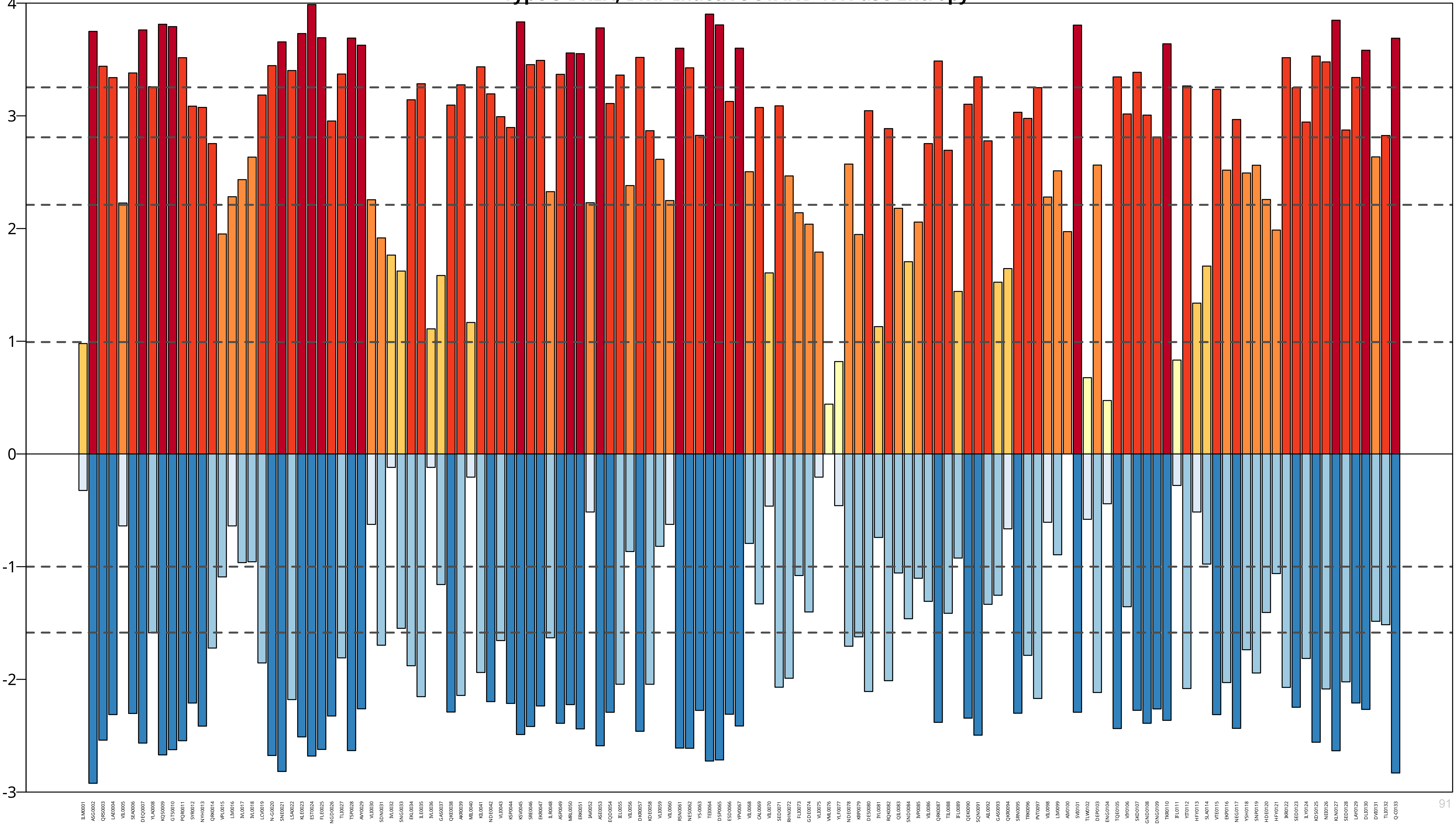

# ; Type 4 BREX PglZ N-terminal iSTAND NTPase; (For Supplementary ENTROPY)

```
RGH34270.1_Ruminococcus_sp._AM47-2BH                   MDAESCFNEIIAYFNSEVGYPFIANIDDATILQDICSKMQADSKKIIRVSDYCNGDNLPNESELLTHVASADNGVVLGLSSYYMLRGEQALKKSVSTLLQMSVHGHVVIIIYGCSSILSNISADGRPDHRTVILEK
MCI8555501.1_Clostridia_bacterium                      MTIQE-VNYVKEYLFEGDNETCILNIESLDMYNNIVKAIKELNINIVRLSSFCNDNVSPNMENFYNALEINQPTAVIGISQFFKFIGKDRLKNEISVALSKTLKNKALFITFQCFDVLKELSQDIRLHNRITILGE
MBD8953983.1_Dialister_sp.                             MTMDQCFDRIEAYCQKEIGHPLLVNTENAHDFQCLVKRMQLDGKTCVFVHDYCGK-GLPQIDALFRDVKKKGCFVMIGLSSYVMLLGKKRAEELMARLQELSVAGHVIVLLSHMKEIRLSMQQDQHLSYRVLLLEG
WP_073324030.1_Fibrobacter_sp._UWOV1                   MNLESCVLEAKSYRKSAQSYPLWIDFANAEDMRSFMENFLA--DKKISVEKYCNEDSMPRFENLYSDFRMEDFAFICNLSGFLKLLGSRKTAEVLSTLLSI--GSKKIILTVQCGVDFR--ITDPRLKCQILSVEG
WP_216471320.1_Butyricicoccus_intestinisimiae          MSIEKCIQAIDRYLVRSASTLRLVNVDNPAQMNQLIERYRVKGNEFLTVAQYSKPDEKAQTEEMLYAISKMGNLFVSGFTTFWKLEGEAEVKKQLDRLLHMSVKGHVVVLCYQCEHLLN--FADARIERLIYFVDG
WP_044080180.1_Prevotella_pectinovora                  MKYEEVIRRIDKYKDSDKHQPIIVDLPNVQVYNQMVKHYDVGSNVFKDVSSFCNGEGLPVMDKLQYCLSIGGVVFLTGLSFYLKLQGEVSLRSCLRQMLDLSCSGKLVILTLNCSCVLN---HDSRLSGRISIVDG
MZQ76087.1_Peptoclostridium_sp.                        M---TAIDCIKKFLKQSNSKFLIVNINNVEDYMNIKSSLDIE-LSTIKLSNFCEYDDMPDIEKFVKCCRTDKSSLVLGVSQYLKLKNKESFEMWTQVLLSSMTLAKVIMIYFDCENKLKELDRDVRFQNQIILVKG
HIS35238.1_Candidatus_Scatousia_excrementigallinarum   MQLNSCIEKIEKHIHRGSSKPIFINLNNSQDVKELIDHFNVGGNKIVNVASNDQIDELPTLDAILGLIHRDETIFITGISSFARLMGQSQFASILNQIINSQSVSKAIVLLYQCEDILHDISKDPRIDSHIFFIDG
WP_103226892.1_Clostridium_sp._chh4-2                  M--NDCIERINNYLQDENTYPLFVNVENATQKMEILDYYRVGDNKIVHIADFAKKDSLPNVMGVIDDIKASGNMFVFELTTYLKLISGSELQKTMQTLCNMSIAKKAVIVCFHCGDILKKIQKDLRVAKRIVAVDS
NBJ16967.1_Dehalobacter_sp._4CP                        MGWDRTLNEVTKYLSNTSNAPFFLVVGD-GNYESIKTQLSEAGLKSVKVSELCNTDKRPNLDSLIGNVDPDKKIVILGLGEYLALCGEEAAFDRLSKIKDMKVNARVVILLRGVTSVVLRQNADPRFDGRRVLFTD
CDC05526.1_Clostridium_leptum_CAG:27                   M----LCKEVQNYIATAKGLPFFYVVGD-EDYATVLDELRQANVSIVRMSDFCKDDKFPSVDELVDYFRRNNKFVVVGLGEYLALRGTAIVDKELRRLKNTTLNARAILLLRGVSTQASQIRDDNKMEQQRAYISA
MCL2016626.1_Defluviitaleaceae_bacterium               M------KKIKNYFSSELSKPYFCFLND-EEYRQTMSELQADGVKIIRVSDMCGTDRPPNMDKLIDIIKNPAKMAIVGLGEYLAISGFDTARDNLNRLQATETRQKVVLLLRGVFSQIPKMRNDRRFDERVHYTSA
WP_235396406.1_Pseudoflavonifractor_phocaeensis        MTIQEVIKKIDRYLKKDNVEPLIVDVQNKADLEAIMLHYQLPQNVFLCASDFCKPDEFPVIPNILERLAENTNFFVSEITSFLKLKGEKALVQELKELLSMSTEGHAVILTFQCVEYLHTIKNDRRLDSRICVLEG
MBR3817930.1_Clostridia_bacterium                      MLLDACINRINNFITSSSSQPLIINVNNSSQFNELVSHFNVGDNRVISSKDYCKNDEMPQIDLVFDLISADGIVFITNFTTYLRMYGEDELRKTLKTIINLSTRGKVVFISYQCEQYLS--FTNPAQKNRVCLVDG
MBP3656735.1_Clostridia_bacterium                      MKISDIYGRIERYLRSDDTRPRIVNFQDRASLIAFRDHFNVGTAAFKMPSDYSKVDEDILTENLYAEIQFQGNLFLIGFTSQLKLKGRDTLHQFIAQIAHMTRSGKLILVCFCCAEMLH--FDDTRIQGLVYPADD
ONI45221.1_Epulopiscium_sp._SCG-C07WGA-EpuloA2         M-VDEYIRSIDTYFKGEKNSPFIVNFENILDLQEAIAHFEVDGNTIVKTSTLANKDNLPSLDNVLSNCDITGQIFVTEYSSFLKLRGHNSLIETFRILLHH--NKKLVIFTYKCERQLRELNTDLRHRNNIYFHCG
MBO6272669.1_bacterium                                 M-IRNYIEKIDRYFVRADTQPLLIDFNNKQMLNEFIQHYNVGAYNFIKAAECCREDEILNIVDLFHKLKSSNPTFLVNISAYLHLLGEKEAKNILKDLMSNN-HQKLVIVGYQICSYLG--ETDPRLKNRMVLVND
WP_037355505.1_Selenomonas_sp._FC4001                  MDVMQCINDVASYCNNVSGFPLIIDTEDYDSFHDLIFRLRIDVKKFIYVSDFCKEGAIPNIEQAYSSAFADGVHVLVGLSQACMLQSQSTLQEYLDRAVNETCREHTIILLMHCREYLQKCRQDVRLERRIRFLGN
MBS6586269.1_Eubacterium_sp.                           MDVSECFEQISAYFSGDCGFFLLVDTEDYDTFQKVLQRLQADGKKCVYVSEHCSRNGLPDVDSAVRAVCGDGDSVLVGVSQARMLQSGEALDRSLDDLLSRPVSGHCVALLDHCRQVLQKLHRDIRLKNRVVLVEE
NBK77202.1_bacterium_D16-76                            MSIQNVKQEIDSYEEHFTGAPLVVGIDGGYNYTALLSLLKDDYKQVIRMSDSCSVDFPPDPAFQISLVSKAKPVIWIGAAQAKMLYGQQPTEQFLVNLLGTCFSGPVTVICPFCCTILEGARNYAKLGYNAIVMSD
NSW92343.1_Bacillota_bacterium                         MTIEELKQELLRDKAHRDRFPVFILIKGRDNWHKVVTLLEDMTDYTYKLSERCGEDTIPNLNDLTTKIESYSNMLVIPLAEYLRIFEN--RRAILRELATIENKCRAYIPLFEVENIFFEMKGVSRFCAKYFSINT
WP_048095116.1_Archaeoglobus_fulgidus                  MSVSRLITKLKDEANIRERFPVFILIEGSNAWKELLDGLKSFTDKVVLLSSFCDNDTYPDVNKAISMAKTGKRVIVLPFSEILRVDSD--AERLIYELLDFQASGRIYIPLYGVTSQFNKWRTYDKEHSPPYFVED
NPV08138.1_Anaerolineae_bacterium                      MDVETLLTEVSYEAVSYTRFPVFILVTGVNAWRELIPALRAQMDACIDLSSLCSEDVLPYLPKVLALAREVPRAVVLPVAEALRLHPA--SSSLLSQLAQLEKHQRIYVPLLEVEDLFGEVARMDRYLHPGWRLGT
HED00230.1_Pseudomonadota_bacterium                    MDLRDLFHELSKDAAKRARFPIFILVERLDHWKEVIEHVKAPANKLIKLSHLCDEDRFPKIDELKEILEGAGSVVILPLSEYVRLTGE---KEILTDLAQWELKGRVYIPLYGFASEFIEFVHVARYIHPGWHLSG
MBE6101264.1_Selenomonas_ruminantium                   M----DMSAVEAYFVSPQHKPFYLVVGD-EDYKDALVTLSNRVTSVLRVSNYCTPDRIPNLDKMKDDLQEHNRVLVLGLGEYLALEGEEYAKSYLQSLKDFNLAGKAVLLLRGVAEQVKSIKGDRRLESGRIAIAS
ROT09401.1_Muribaculaceae_bacterium                    MTLEQVKIKIEKYLNSTDIMPRIVNVSKADDLVELKNYFEIGNIKFIDMGDFASVDSMPLFDKLLNSISNKDITFLYGISTFLKLLGSDIMNLMLRNILEIVPQNKVIIILVACDQYIK--LQDPRIVDRGAYIMC
MBR2693834.1_Thermoguttaceae_bacterium                 MNKTDLIEAVKRYAVSKSKYPFFAVSAN-RDYSSLRENIHEYGFKKVFVSDFCGPDTIPDWDELIDCVKAGEKILVIGLSEYFAFQGEEFALRKLKELKDIRLGGRAVLLFRSIGAWIKSLKSDPRFDGARYCDAA
WP_216880780.1                                         MNLDDTKKRVQKYLNSNKRYPLIVDVQTREDLANIAEYFKVGSNKFPAIEEFCNQDEAIKLDELYASVSNSGNTFITGLSGFLMLFGENVAKQALKTLITTSISGHVVIITYQCEKYLR--FTDPRISGRLVIVDG
MCM1514006.1_Anaeroplasma_bactoclasticum               M-HERLKNKINSFIDSNDNRPLIIDVSNVEERDNLLQDFFATPSIFELVKSEC---ELPEISSLYEYMSKEKTVILTDLGTYFKLLGQDSLQQNIHALLEKSFATKFIILTLQCGQYLN--EKNPKNASKIVVSNQ
HHW17415.1_Bacillota_bacterium                         MSVDELVTYLRAESDKGERFATFILLEGTEAWDRLTARLQYEVDSTVHLSEFCEDDVFPDMSRLISYLKRCKKLLITPLSEWVRLNPE-RTDVIIQILAEWPPVRRIYVPLLSIKDSFYQIERVVRYLPQEWYLSE
WP_157832981.1_Desulfofundulus_thermocisternus         MEIASLLDFIKNEAKVPTRYPAFILVRGLDAWQELLKSLRLEVDEVFTLSTLCNDDTFPYVEGIIPILKKTTKILVLPLAECLRFTGE--SRLVLRELATWEDYKRVYIPLMELDDIFGYMNMIAQSLPEVLSFND
WP_166670004.1_Aminivibrio_pyruvatiphilus              MTLQEILQRLADEKKVESRFPVLIFVDDWSQYKELETALENACDVTLELADFCAPDVLPNFSKIFKKIEREKHILLLSLGEYLRLTIAREVQPQKANIPSLWARTRVIIPLPCCRDLWDRIPYDERQRDCIWELSD
WP_195464804.1_Clostridium_jeddahense                  MNFSELRTYLRKDRAESSLNPIFINVESMEMWVEVKKLLLSMSSGHLFLSEFCTEDTTPNIRRLIAKMKSTDSICVLPISEYLRINPE-IAQPTILDIITKERLFRIYIPMYRMRSILYTSDNDPRRKGSILLLET
HBQ28533.1_Desulfotomaculum_sp.                        MTFKEFESFIAEDIKRKDLRIFFINVESLTLWVKVKNFLAQKCHNQIRLSTFCGDDLSPKINRLCAKLEIDDKTLLFPLSEHLRINNT-KSDEVLSQIVSTEISVHVYIPMYRMKDCLQAIAQNSRLNDNIIFLAE
WP_006692206.1_Selenomonas_infelix                     M----DLTAVERYLQDRERTPFFLFAGG-SVYAQLRHRLQGLGLHLLTLSSLSEEDRLPLPDDIVMTIKSGEKILLIGLAEYLALCGEAAARRLFSRLKSMPCDGAALLLLRGFFRYASELSEDPRIGERRLALDA
MBQ0159480.1_Bacteroidales_bacterium                   MSFSQLTQEIERDKDSAQRYAVFIMLNNFNEFKELAKFMTNIGVDMLDLENIDEDDTWITKDTLKDAIKATKSTFVTPFSEVARFYNDDDFRGFFNEIMLLEDNKRIYIPLIGLQNRFTDFLNHFARIKEVWRYDA
PMQ01343.1_Dictyoglomus_sp._NZ13-RE01                  MNLDELIEYLNKDRVSSIRFPTLIMLSNYKLLHELINKLNK--ANVISIGDLLNEDRWLTQSNIYNLIKNQNDFIILGISEFLRFFSDNELYSFINFLLEIEGNRRIYIPLIGMRNRIEAFLNKYHRRFELWIIND
KUK66682.1_Parcubacteria_bacterium_34_609              MEENKLISLIKDDQEKYGRYPVFILIDNFEDMKKIIETMAKNGVETLDLSNENDSNGWVSTYELIDVIKKEKDYIVFPISEILRFLDNDDFYSLLTSLMEIENKRRIYIPILGLFSRFRSFLNRYHRRSEVWKIGN
WP_018963723.1_Coprothermobacter_platensis             MSMESLIELIDEDKSDATRYPVFIFTKSVWAVNDITMLLKLRELDILDASEFPFSDGFPSLSTILQAIREKDDVVVVSLGNLVRFWPDEDFYSLMMGLMEIENDRRIYVILEGLIERFDVFWSRRTRQIGRWTLES
MBR3117632.1_Oceanobacillus_sp.                        MDVSSIVKQIIEDKQKYDRYPIFLFMDLNNDTQQDIVKIVGISIRIKDISELSFNDAWITKSKMLSIVSDNTDYFITGFSELARFYSRQDLEALLLSFTNIENKQRIYFVCFSLFDKIAQELRTNSRNESIINGNK
WP_024292379.1_Lacrimispora_indolis                    MDIDSMVDIIQSDLNRYDRYPIFFSMKLSNGTSNYLMKLRKVCVEILDFQNLLHDDGWITIDRFRKKVYADKSYIIVGFSEYARFLSNAEFITLLLGLLELENKRRIYIPCFAIYSQVRIVKANNRRMDVFLNEMD
WP_183546997.1_Methanococcus_maripaludis               MDIECLYDAISKDLNSANRFSLFIFVPDFDHMAKLCNYLTSMNLKEINVSKLPHDDGQVTPQRIVETIKNDKNLLITSLSEISRFYGEDDFLSLFLDLAYIEKKNKIYVPVVGIYERFREVMPVFSRSNEIWALSL
HHW21152.1_Thermodesulfovibrio_thiophilus              MNINYLIQKIKEDKKTEKRFPVYIFLSNFDTLRDLIKETSKIGITTFEISQLPKDDGWITKQEFIDKIKSNKDYLLLPFSEIARFYNKQDFNNLFSQLTELENNQRIYIPLMGIKVRFEEFYKNFNRKIEVWEIYE
WP_207678199.1_Desulfonema_magnum                      MFIEELYSEIDSEKKDVQRYPILIFLNSFNSLNLITDYFSKQAVDVLELNKLPHNDGWLTSDDIINSIKLEDDTVIVPFSEILRFKNTAEFNTILTGLFEIENIKRIYIPLVGMKERFEDFWKTFHRRGNIWYLEE
MCI6981218.1_Akkermansia_muciniphila                   M----------DKEALNRYPVFILFNSVEDYSKFIDVCAARGAYVQRISDLLGNDKLLTYTELAEHIKEGEDFVITPFSEIARFFGNSKFRSLMKTLGAWEAHQRVYIPLMGISGMLEKLITEPGTN--VWDCCS
MCD7797870.1_Akkermansiaceae_bacterium                 MSLKDLEAAINDDKKRGNRFPCFVLFDDYKNFDTFIQTRRKDGAFMCPLAEALSDDKQLTMKSLCDWIKGKNDLVVFPISEIARFYNDNEFKCFITTIKGIESQQRVYLPFIGQQSRMYNFVNDPAIV--IWDNCN
MBO6251938.1_Muribaculaceae_bacterium                  MNIDELIQAVVVDKNAQNRYPIFVLFDNFKDSYAFTIRLVELHITIEDIQQIDYPDIIISHQKLATEIERGESKIIAPFSELARFYDNHDFDTLIRTIKNIQTKQRIYIPIVGLEGKMSTFFGDTQAT--IWYMRS
consensus/100%                                         M....................hh.........................p...............h......hh..hs....h............h..........h...........................
consensus/95%                                          M........l...........hh..........h...h..........p............h...h......hl..hs.h..h.s.......h..h.......+hhh....h...h.................
consensus/90%                                          M........l..........s.hh...s......h...h.........h.p.s..s.......h...hp.....hl.shsphh.h.s.......h..l.......+hhh....h...h.......p.....h....
consensus/85%                                          M...ph.p.l.....p.p.bshhl.h.s.p.h.ph...h.......h.h.phs..D.b.p.pphh..hp.....hl.shuphhbh.sp......h..l.p.....+hhl....h.pbh.......pb....h.h..
consensus/80%                                          M..pphbp.lpp.b.p.p.bPhhl.hps.p.h.ph...hp..s...h.huphs..D.b.p.pphh..lp...p.hl.shuphhbh.sp..h.p.h.pl.pbp...+hhl.hb.h.pbh.....s.pb....h.h..
consensus/75%                                          M.hpphhpblpp.bpp.ppbPhhl.hps.p.hpph.p.hp..s.p.lpluphsp.D.h.p.pplhp.lp...p.hl.shuphhbhbsp..hpp.lppl.pbp...+hhl.hh.h.pbh....ps.+b....h.hps
consensus/70%                                          MphpphhpblPP.bpp.ppbPhhl.hps.pphpplhp.hp..s.phlpluphsppD.hsshsplhp.lp.spphhlhslophh+hbupp.hppblpplhphp.p.+lhl.hh.h.pbhp.h.ps.+bp.phh.lss
```

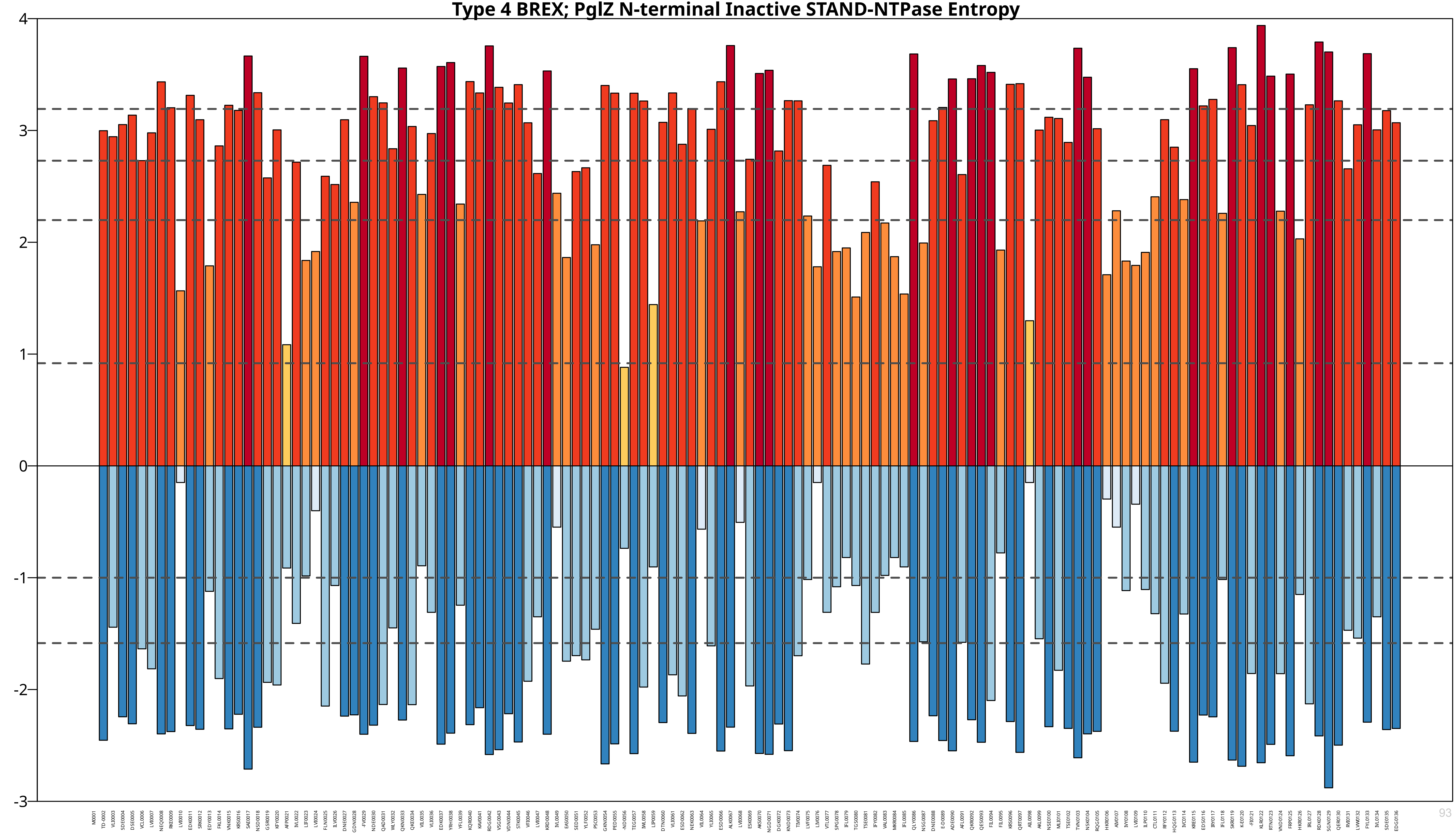
Type 4 BREX; PglZ N-terminal Inactive STAND-NTPase Entropy
4
3
2
1
0
-1
-2
-3

```
# ; Type-3 BRSystems iSTAND NTPase

MBP8199113.1_Nitrospira_sp.                     RPPLEIDTWKRRGHSILWDAASLASLCSSDQAVTLRQFLLHAPAAQTDVRGRALVVAGLDAALDDPDESLIWLEKFLYPAVISFENVADGALIFWFADVMSEICAHWHCSLAFIPLSRGLWNGSSVSMQEILETRTDGSMHVGYYVQKIS
MCX6876430.1_Verrucomicrobia_bacterium          NPPLTDDVWKRKGISVLWDVAGLASLGSLQTAISLREFFWNAREDSTHAGARRIVVAGLEAALDEPEAAEEWMAQQLLPVINRCAILFEGALLFWMVKHLENGEILWTCDGEHILFSHGLWNGAYRDVQRIAPLRTETEPGLGFYLQRIS
MCP3685978.1_bacterium                          VLTRRDDAWIRRGFTLLWGLKTLGKVVRPPQVVSMRKFFSMAPEELPASNGNALVVSGFEGCLDTGQDAERWIEKDIKDVILSFQDEYEGGLIFWVPSGGSSEEYHWKHRASKLHIGRLLWSGAEKEVERLIDDQRSTKAWVGLHHPRIS
WP_270720981.1_unclassified_Bacillus            SSVLLGDTWTRRGISLLWDSNSLSLITEIKDVLSVREFLYYQPNELHSANGYGMVVSGLETILDESQSLIDWMEKDFYPALLSFQSMYEGSLIFWLPDG-TDGLIYWNCTGAYIPLSQCLWNGAARDAKLIIKNTNVLKNCLGIYHPRIS
MSU35393.1_Pedosphaera_sp.                      NHDLLHNTWMRCGTSLLWDAQALNQVCSPEAVRSLREFLLHQPEGTLKIQNRTFVVAGLETAMDHPDEAVEWLEKTVYPVVLDFQEIVAGALIFWLADRASENTYHWHCTGEHIPLGRCIWNGAESDVRRIIAPDTQKTHHIGLFLQRIS
MBI5511448.1_Deltaproteobacteria_bacterium      LPPMADRGWVRRGLSLVWNAEALGTVAGPDAAWTMRQLLTRSPAALPSSNGEAVVVAGFDSCLDGPDAAEVWIQRELVPVMLSFQEEYDGALIFWLPEASADGSYSWRWAGRTLALGRCLWAGAEADLRRIVVPAKGKPGWIGLHLARLS
WP_263354237.1_Acidicapsa_acidisoli             MKVLHGDVWTRRGMNLLWDGKALSALAEPQSVVSIRQFFAMIPADLPCNEGKALVVAGLEGCLDKPGDAERWLDQHLGPAIDAFGRKYELALVFWMPTGAASEAYSWLCSAPHIEIGRILWAGAEADVRRIIDPKCANPAWIGLNLTRLS
WP_190375081.1_Leptolyngbya_sp._FACHB-17        MQPIFGTGWTRKGFSLLWDVTALSKIVESNQVISMRRFFGLVQDDLPAANGDALVVAGLDASLDTSDDASQWLEEKLRSAMLSFQDYYQGALIFWLPGGRATNTYLWLCGPTSLPLGQALWAGAEADAGHIIASSDFQKDWIGLHLPRIS
WP_125861692.1_Stutzerimonas_xanthomarina       MRQVLDTVWQRRGTCWLWDEEARNQVCVAGEVWSLRQFLAAQPDDLPSNNGRTLVVAGLEGSLDAPDQGEIWLGDTIKRAILSFQDEYAGALIFWLPQGASTDAVSWLCEAPHLDFGRLLWGEANEYPQEILLREGAK---AGLFHLRIT
OPX92064.1_Pelotomaculum_sp._PtaB.Bin013        MREIRGNGWLRSGSSLIWDAQLLSPLLENGVQIPLHKALWIGPSTIPSGDGKTIFVVGLQTVLEDSEAAFTFLRNRVQRLIVRVQDFYGNGIVFGCNCN-SNEHAYLRLRGGHLNVTFALWNGVAREAQIIMVEDKNVEIGGGFYVRRYS
WP_145058250.1_Adhaeretor_mobilis               DKPLNDDLWKRRGISILWDADSLNSLCRSDEVISLRHFRLFNSDVDASVDEETLVVAGLESCIDPPEEATKWLQDVFYKAMVSYQEVADGALILWFVDSTSDDGWSMHCAGEFIPLGRCLFNGAAKDLKEIQDADGNS---LGLYHPRIS
MBV5311590.1_Chromatium_okenii                  TAPLTDNVWTRRGINVLWDGAALARIGPVSGVISLRRFFLDQPEEATPTGGEALVVAGLDAAIDDAETVTHWIEQELYPRIQEFQSVFDGALIFWMADKTGKGCFEWRLGCGNLPLASLLWNGAHKDVRRIEASAEKGNRWIGLYLARIS
MBP7052832.1_Phycisphaerae_bacterium            MQEMTASKWKRTGSSIIWHPDLLADLIRDIEPTPLRTLIWLKPEDPPG-GKQTVLVGGLQTAVETPDAVADWLRNHALAAVRAWKSHWPDGLVFVMDGP-EGDEIVYFGRGRDVMLSLAIWNGAASGAGAYQILVEGTGEVGGYFVRWLS
MBQ8111589.1_Kiritimatiellia_bacterium          KTTTNYTLMLRGGSSVLFGKKELSELLTQIEPISLREFLLGDPDDPPPEFEDGILVSGLETVLDPVGEAEEFLAGRLRGVIQQIQNHWTRGIVFGCSLSGVSESVVFTTKDDTLALSDALWGRSQSGDIMRLEHKEPGSRRVGYHVPRIS
HCF99181.1_Chloroflexota_bacterium              MDEYAGDMWRRTGTSLVWDGDALHTAATEGVTVSLRTALWAEPEDPPD-GKRVLVVTGLRTAIDTEAERDAVL-EKVARLVRAQSRYWPEAIVFALPETPPTGTVVLDLGG--LDIGNRIWGGAGTGANQLIESRLDSRQAIGYWLRRVS
WP_149537235.1_Siccirubricoccus_phaeus          ----LADAWARRGLTLLWGADAFRELASPDQVMPLRALFGERPVDLVSNGGRALVATGLEASLDAPADAETWIATDLRDLVREFQDAYQGALVLWLPSGAPNDAWLWATAD--LPLGRLLFSGAERDVERVSPAGWRGPGLIGLRQARLS
MBF0302144.1_Desulfamplus_sp.                   MDTISNTVWQRKGSSVIFDQDSLAPFVKDEAVVSLREALWMKPTTPPV-KGKTIVVCGLETMIEPEDEIDDFLTQRIRPLIIELQNRWTGGLVFGFSSHAMQEEVLFKRRGNRVRLSEGLWDGSAAVNMKRIITSEREETIIGYYVARIS
MBX3394309.1_Phycisphaerae_bacterium            DEFVDSKMWLTRGSSVIFDKVMLGPLLTEGALIPLRQALWLSPSATPT-GGKTVLVGGLETVLEAVSDAEDFVRRYIRPLIAEFQSCWPEGLVFGLNTEPVSEMVNLKRRDDTICLSTELWNGSAVTDMRRLVRTDRAKVRGGYYAPRS-
MBN2415250.1_bacterium                          NPVLLDDVWLRKGISVIWDNSMLTKLVRNNRAVSLREFFYYKPEENMHINEEMLLVAGLDAALDDAQSAEEWVTSEVYERIYDFQNWAEGALVFWMSQKIESSRYTWLCDGKDIELGRGIWNGAQLSVSRIESES----RWIGLFLARIS
MBA3936145.1_Planctomycetota_bacterium          MKRIGGTSWMRQGSTVITHLETASMLLAQAGLEPLRTALWAAPAE---ANVSTLVVNGLSAVLDTPEDSAEFLRTKAKLIIRRSQEQWTRGILFLFPTH-LDDRVIYRLPG--VALSHELWNGAAKDLREIHADV---DTIIGYHVLRLS
OCQ93482.1_Nostoc_sp._MBR_210                   MHKMSSSAWQRRGSSIVFHRDLLVPLIESGAMVSLREALWANPLEPPN-GSNTVLIVGLETCLEVPSEAEIFLRRTIRPFLLESRNHWDRGLIFGFGCSPQESILFVYLDGTTIRLSEGLWNGAARNELFYLTTPEQTDVVGGYHV-RLS
MBE2220371.1_Anaerolineae_bacterium             MREESGTRWQRRGSSIVFDPETLSPLLIGQSLISLRAFLWREPDDPPG-DGKTVLVGGLEPCLESPEEAESLLRYRIRPVVEEFQTRWDQGLVFGFGKPPTSDEMTYIRQDLKVRLAYSLWNGGAAMDLTQIIRSTAK--VIGYYVPRLS
MBE7511465.1_Anaerolineales_bacterium           MRDMSASSWLRQGSSIVFDKTKLGPLVTTGCLISLREALWVDPAAPKN-GADTVLVGGLETCLDPAPDAETWIRTHVRRFIVKFQNEWDRGLVFGFSTP-SDDEVLFQSNN--IRLSHALWNGTATVDVARLVTDDRE---VGYHVRRIS
RKY62549.1_Candidatus_Latescibacteria           -------MILTYGLYAFEKQI-LTKLAQ---HRSILAFLLRH-RE---PLADEVLVTGLDRMLYAEDKAKD-AVKIFGSELYRHRADLAR-VLFPLEYVYWKAGIRYRPTGEPFRLEWFF-PRCEVTEIAG----------EPACYSMF-
OIO18554.1_Ignavibacteria_bacterium_CG1         ----------MAGYMLVEQRSFAVPQTPNRGKIGIADFLLEQ-EEFPFDENSSLMVTGIEEYLLSRPDMEV-TAREIRMKLQKAAGFFNDNVQIVFRQPKRGEHLIVDHVTQSIPIYLIFNTPIQTDIGG----------QTVFISQFN
MBW1935302.1_Deltaproteobacteria_bacterium      ----------MPGYQLLELRH-LRPNMV-EGKMGLLDFLITQ-DPFPFPKFYRVQILGLEDVLFARPQEEE-LALKIQRILRSAAQNLEK-IQIVFRGRVRGESLWVEYRNAKLPIGHIFGSPIRQTDASR----------NQFYKKSFS
MBU1206361.1_Pseudomonadota_bacterium           ----------MGNYLLIEERM-LQPYRP-KGRMGFLDFLLRQ-EVFPFSRGNKLCVVGLEEVLLGSGENFMKVSLGIRNILTSRANELEA-VQIVFRRSKRADDFWFDPGGRRVSLRPIFGSPIKQSDQNG----------NEYFMTGFN
CBX30016.1_uncultured_Desulfobacterium_sp.      ----------MKGYEIFEQKY-LKDYFGEEGKIGFLDFLLKS-GTQGIPSSSSFMVVGIDDVLYGRDERKM-LALTIHKILQSSAKVLDQ-VQIVCKGRYKGESFWSEYRGEKLPLDYIFGTPNKRDIRE----------CPVYSTGFN
MBN2391965.1_Anaerolineae_bacterium             --------MSSSGYVLIEQRE-PTPTILRDDQRGLLDFLLAE-DELPYPKWSLVTVTGLDEVLYAQPDEEA-LAHEIHGRLNQAASRLES-IEMRLKGQIRGDPFRLRHRGIDLPLHIIFDTPGKAADGN-------------YYLVRCH
WP_248664448.1_Fuchsiella_alkaliacetigena       ---------MQQGYNLMTQNY-NQQYYQ---RMSIMKFLVQE-EN---KIDYSLEIYGLEEFL-GKEDA----NDLLTEILGKGVNYLTD-IKVITENKIWNNKAVLKYSNEEFPLGNIFGSSLRQEDTE-------------YWVANLN
WP_103424089.1_Salinigranum_rubrum              -----MASDLDIGYHVIDEDA-LDD------EMSIVEFLLKR-RE---EIPLDMTVRGLDDYL-GVDDADV-ACDYIHRLLRDRVNYLSL-VQFVVDD-ENWSGPVIPTGDEPIKLNRIFHGSMEQSGPG-------------WYSSNLN
KXA95577.1_candidate_division_MSBL1_archaeon    ---------MEEGFHIIEKSS-VKERDL---KMSIVNWIIRN-EG---NQFESYTVVGLEDLL-KISNPQD-FSEKLRKILIDQSNYLVS-FQFLIDNSVWEKNPTVKTASEVIDLSAIFG-ELERKDVN-------------WFYQQLN
NLY06148.1_Candidatus_Atribacteria_bacterium    ---M---VEINIGFNIIHRSN----------RISIIQFLLNR-GS---KFPPDFAVFGLDSLL-YVEDREK-MAKYIRHTLQDNANYLTS-IQIVIEGDESDEKPRVVYKDEEFPLYSVIG-RLKRVDLK-------------HFYAPLN
HIE51614.1_Armatimonadota_bacterium             ---------MRPGFYLITRRQ-LGRAIQ---TMSLIALLLHR-GE---ALPSAVCVVGLVGLL-SASDLRQ-AARLLAQRFQRHRSALMR-IVFPVEHLPTPRGVQVGARGSQNFIAEWIFPRLEPLEKV-----------PRAYYSPF-
WP_285254567.1_Halobacterium_salinarum          ---MSDIDAVAGMYNIIVSSE----------RVPIEEFVLET-RD----LPDEICVAGLEEVL--TEDEDL--RDQLVSALRQEMDYLNSAIQFVVDGDGAGDSFDVEIDDEFHSLEPIFGRQIKKRDS-------------GWLVAPFR
MCR8454635.1_Thermoproteota_archaeon            ---MKSLKTMEVGYNIVEKSL-VGKEFE---KISIIEFLLYR-KQ---PVNRKIAVIGLEEAL-AGEET----ARYIRRILVDSTYMLRTIIQFQINGEILDKEPKIRYRSKEINLIPIFGNRIKPKTVG-------------YFHSPPN
WP_135823665.1_Halorussus_ruber                 ---MPEAGRLRGVYNIVTAGD----------QIPVDEFLLED-DD----VPEEVTVVGLEDAY---DDESF--IDELSRAMDRKADDLENMVQFEIDADRGQVAFDIFHEGEIYPLHEVFGRQIKPKDQT-----------GSWYTTPF-
WP_153952804.1_Halosegnis_longus                ---MPTDGQLRGVYNIVEHGT----------QLPIEAFELAE-GD----VPNQVTVVNLGEAF---TDQEF--INSLAKTMADHGNALATIIQFVVQGEHRGEPFDLFIDGEIHSLRPLFGRQMQALTDD-----------HSWLKAPF-
NMX21979.1_ANME-1_cluster_archaeon_GoMg4        -------MNPTQGYNVLLKE----DEPP---KMSIIKFLIRR-GQ---ELPKKVAIYGLESLL-TNKDARA-GTKFIRNMFIENNRLFRE-FLFIPRKEYVNRDVYLKAEDKKANVSSLFGLRLQIKEVG-------------IYYADFE
MCL4459198.1_Chloroflexota_bacterium            ---M---EGLKAGYNLVERAL-IGQRYE---VMSIVQFLLES-GE---NLPRRFKVTGLDRLM-AIDD-----SGTIRSILYEAGEKLLR-VLFPVDK-TMDAEPKMYWRNQEIRLRPLFGNRLEPQGVG-------------YFYSPFN
consensus/100%                                  ..............hh.................sh..h...............h.h.sh...h.............h...h.......h...h.h.....................h...h............................
consensus/95%                                   ............s..lh................sh..hh..............h.l.Glp..h.............h...h.......h...l.h.h..........h...s.....l...h......................h....h.
consensus/90%                                   ...........G..ll.................sh..hhh.............h.l.GLp.hl....p........h...h.p..p.h...l.h.h..........h...s..h.l...h......................h....h.
consensus/85%                                   .........p.G.pll.p.............hslbphhh...........p.h.VsGL-.hl....p.......p.l...h.p..p.h...l.h.h..........h.h...s..h.l...h.s..................ha...hp
consensus/80%                                   .........p.G.sllbp...h.........hslbphhh.p.........sp.lhVsGL-.hl....p......h.p.l..hl.p..p.h...l.h.h..........h.h...s..l.l...h.s.h................ha...hs
consensus/75%                                   .........p.G.sllbp...h.........hulbphlh.p..p......sp.lhVsGL-shL.s.pc.p..h.p.l..hl.p..p.h...l.F.h.....sp.h.h...s..l.Ls.hh.s.h....s..............ha...hs
consensus/70%                                   .......b.c.G.sllbc.p.ls.hh.....hulbpFLh.p..p......spslhVsGL-shL.ssp-.p..h.pplb.hlbp..s.hp..lbF.hsp...sp.h.h..ps..l.Ls.hh.sshp.ps.............shahsphs
```

Type-3 BR-systems; Inactive STAND-NTPase entropy

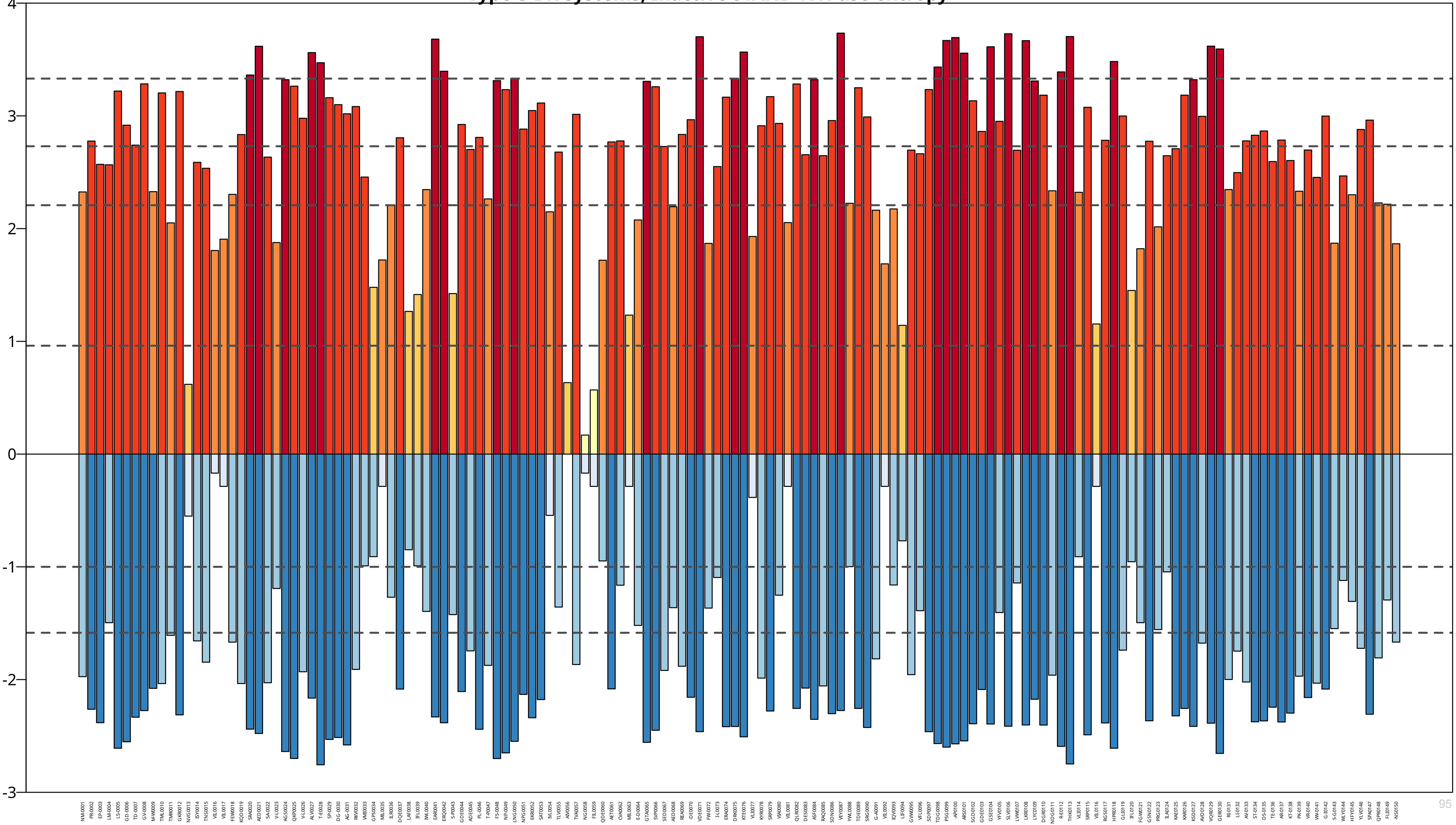

```
# ; vWA-MoxR subsystem 2: the iSTAND ternary systems; (For Supplementary ENTROPY)

OCQ97673.1_Nostoc_sp._MBR_210                      LHKALLCLNYTKQENLFREFVRHQIGACLIHGEPEHGQRWLLNRLLGLVPNGRTESVIPLNLTRSSSIDALWRELGKAVGAAATCDEIAQKVGNLWKSQTVILIFHNLDQLPEDYFNEFICKFCVRNYRLLAFLMDLSGCVDETIDLNWEPHTPLKLPKITKLSHNELSYWLEKSPAEIDKIALDILNSDDEAVLDYICELCD
BAI89278.1_Arthrospira_platensis_NIES-39           IKDKLLHLDYREQVRSLEDFLQDRVGAFWIYGSNQSEPHWLVTSLIENIETSNTSSRVSLSFTQSHCLDNIIDSISKAFQIEEKLTYIIEQICIRWQTKSVVILAEIKKGVDPEIVEEFINKLRIYNYSLMLFLVDVHGCTNEDILSEQIPEIPIRLTEIQPIPREQLRQWFRTHLDQLIEKMLDLIYHNNEKVFYQICKLFE
BAY43400.1_Scytonema_sp._HK-05                     LYHVLLKLDYTDQEQLFSRFITNQIAAFLIHGSQDYGQHWLTNRLLKKIRRVSDKPVL-VDLVHNRNSQTMWRELGRHFGGIDSPDLIAGKVLKSWTTKNIYIVVDNVQSMSEQLLQELIQKFQQTNFKLLMFLIDNVSKVEENFEPTWRCHQPVILPKNNVFSDIALRDWINHESENIDGVIQTILNSEAIPAFREICNLCE
KPA13794.1_Candidatus_Magnetomorum_sp._HK-1        RENFPYSVNRTEQVQGIKDIGEKKPLVWLIHGDRNQSHERFLECVQWFWENIKKGSKIEPRFYSNNFENDCMTEIWKNFSQDDNWRDLSPLIDNNCNK--PVILYTMIRCSQQRLFYVLLDIQKKYP-LFLCILIIYTHKHKRKITRQLKKYNKYTLPLLSNIESEDVDEWQNMSFKNERKIVKDIFDDIEAKIFKHCLYHQD
KPA12842.1_Candidatus_Magnetomorum_sp._HK-1        GKFVHLMCDRANQVVEFRKTFHKKPQFYVIHGKERNGHLSLVERLKIQIQPFVNEQAVSLLVDLDMKMDILLDALFAEIFNQIQYSLKALIQSDPIKADHAVIIAHRIPANHLDLANRYISDFATIPLFIVFFNIEYPRLVKYKIRSMVKELTNQLIKELQTINTHHVIDWFNSHQPEKFKAVDSIFDGQDEDLLKKY---VP
ETR67363.1_Candidatus_Magnetoglobus_multicellularis GDDINLYCNRITHVGEFLFTYGNMPQFYFVYGCKDECPESLFLRLRDILRDHFKPQVPPIDESFEARKDVLLKSLFKTLTGDNKFKLTDLYQSNAIKDNNVIVVPHFLSFDQQKLMEWYIKDY-DAPHFMLFFIITCIPKKNKKLKKFSSQLSAYLFDPLHKIDKSEVAPLFKQFKHEINKHIDDLFKDQEEKYLLRLKQTIK
OQX97257.1_Bacteroidetes_bacterium                 RNYYRYSCNCYKQNNYFENVFNEKIQFYVIHAKAEQCPEILPQRFFNEINDNDNNSIF----NIDSLKIKTLYQIFSFFNEEKEACLESLFNSKTLQKKKYITINFIVESRQHEFIEFFINDFCTKLRFIFFWSIIYTSEIKDKIQKCIKNLEFDVLPEIQIINLDDIRTWIKSIESKINISLFNIFTNKKLEMFDFLSEKYN
WP_020569053.1_Neolewinella_persica                PPFHAFGVDRAPQEGQFFDEQDQKVFFYYLHGDDRQQASSLAERLGFKVGLQLTADDLDIEAREEHCRALLVHAITQQFMGGLNLTILDLLNRPKLKRHPIVCINVTVGHSYPGMLRKFYETFCDAPTFYFFFGLEHCDNKPKEVAEAIAGRQNSALPELEPVLISDIVNWFTHHGYDAEQLVAENFTEGDEADLKRLIDKFN
RMG26468.1_Bacteroidota_bacterium                  GRLLHLMCDRDPQAESFLDRFAGDPQYYFIHGPRGERHKSLVRRFREIIRHHLGRMPALVDFDLDMRKRNLKRSIARSRELPGSKWESPALMALLAQRGNLVVFQHQLAAEKFRLLEWYIHDF-ASFHFLLFVNIVYPEKWGEDMQALKRKTPETILAELQPITPEDVAAWVDTYFDNMQHLPGTIFDKQKETQLKLAIERLE
KPA17452.1_Candidatus_Magnetomorum_sp._HK-1        GRIIPTLCNRDIHVEKFYKFFKKLPLFFIIHGFENQGHRSLIKRLRFTIRAYLQKQKIELPFKLSDRKQILARQLFNAFQKMKTYDLKRLNQLKAIETNNIVIISHNIEASLKDLLSWYIENYVDIPQFIIFLNIQYPVAIKKELTVFTQNFSKKLIDELNSITQQHVKDWICKYKYEIEEEIESIFENSEEKKLKNILLK-S
WP_028158837.1_Bradyrhizobium_japonicum            ARLLPYLCDRRMQRRHFRDAVVVRPQIFFVLGADSECADKFVEQLFFVMPELLQRNGLEDRLDIGDYLGELQYEFADKLG--LKPRSSVTEIERRLQRHTALFLYFDVDIRKRRLLREWIGWWNRRYPIIVVGRFCSANQGIDDLTALSTTAGKTVLPQLGRVSFEDIEAWINENAPDLRQRLRRFFIGERATLLKTIMNEMK
RKZ89871.1_Candidatus_Parabeggiatoa_sp.            DDLRPHLPNRRPQESKLGRAIKQRPLLCLIHSDECQCSDRFVDRLVQYLPKVVPGDGIFVHCQGIDLHQEISEQLASRLGI-NPFAQPSEIVEAIMRERSPIIFHTDMCTKNMKAVHNFIEFWATHNHLLICLYFNYKDIKPDQIRAEFRQLENIVLPELMDIEKEEVYAWARDHLDSVQPKIDDLFSPDEARSLREILEEFP
OCR02031.1_Oscillatoriales_cyanobacterium_USR001   VHQALLRLNYTHQVRLFRAFFHHPIGAYLIHGEEESGQKWLLNRLMKFITDSRTAKVIPVKLSCGTYTEALWRQLGGHVGLMPSQTQIIKNVSELCKNQIVVLIFDELNAMPEECMSQFIGQFRQSQSRLLMFLVDNIGCVEETLDPNWEPHIPIKLPKINPIEQSDLVSWVRNAVNQMEEIVQNFFNGETEAAMIKICDECD
RMG84230.1_Bacteroidota_bacterium                  HEYHRYTCNRQPQNVQFHSSFKQKTHFFYMYGLNVQSPEGLFYRFSYDLLDYLNPDLESVRADETYNKQEVLKNLFAALSVPKKRLEDVCLSSPTIRKEDYVCIFLGVSSFDPALAHWFITRFCDAPTFLFFLAIIYEEDDPAEVKETVENSDLIGLPELGDVSLQDVKRWFFNYQKTRQQLIEERFGDAQEIRLERIIDEYN
WP_028091549.1_Dolichospermum_circinale            RSFLSYLANRSSQEFELTEAIKSRLLVCIIHGDELQSHYKFLERMRNDLPDVLDLDQNKIKKNLNELPKYLLMNLAKNLV--NKGNTSLEEINEILNKSSPMIVHTDLISDDLHILNKLLEFWQTLNKVIICICVKYIRIEWKKMRKHIQEFSLIILSELEAVGRADVENWACEHTQQLKDKIGDMFKSSSADNLLEILEHFH
WP_018400346.1_filamentous_cyanobacterium_ESFC-1   LSELLFLLNCIQQENAFR---ANPAGTFLVRAKDLSLQKWLVNRLVRLVPGY-ENAYCPFNVQIRLNFNLLWENLAANLNTTATIENVAEALIERYCHQTVILVLYGVHKLQDHQIAEFIQ-FQARRSRLILFLVEDTEEH-ESIDYQCL-EKPLRLSPLGQISKAEALTWFDEVYEQIDLLLQDI-NDPNSISLDEICMTCG
KPA18269.1_Candidatus_Magnetomorum_sp._HK-1        PKKLHLLCNRKKQALDFTLYFKNTIQLIFFYGPENECPYSFIERLAIKIKDYWENQYDKINKDVNDQKNTLIQELYNSFLLINDYKLSAFYQHPTFDKHNVIILYHEISIDKLKLLEWYINEHSNVKPLLLFFYIKVDGFKEDKLKKKIDKKFKLLLEPLQPVDKDDVREWFSNLESIVEPEIEQIFGKENITKLDIFITNID
WP_066426097.1_Anabaena_sp._4-3                    IINALKKLNYNEQEKDFRDYINKYAATFLIHGQYGYGQRWLVNRLCEQVDDGLTTAFCPINLKKHRDIQRIWDDIAAKVGSSRNPQTIVDVIYQHWQTKPILLCFHNVDVVGCQELNKFIEPFKVNTQPLLLFLIDNYGQQKNQISKEYDIENNLEIKELDKFSDAEIKIWTKCYQSNLSEITQEIIYNNTIQAIEYICQCFD
KPQ40445.1_Phormidium_sp._OSCR                     FIKILGRFNYVQQKRRFQVFLNPRIGAFWLRGERDSAQKWLLSQLWLQVPNSTKAIKKTLTMTTRQNTERILEKLATWLQVEATPEALIKRILENCKTETVALVFYQVEYLDKGTLEELINTFQSLDNPLLLFMVDLGKKGDINHSSDYNAEHPEQFVELTQRFQDRDFRWLTDNQVSLEAIKKDMIDDNHEEVFTELCDYFG
WP_071187406.1_Trichormus_sp._NMC-1                LEKSFQKIDFSKSREIAESLKTEGAILLFLQKSKKQLGKYCIEEFIDVISNQIETCPRTINFTYKSNKYGFINAISGYLGIESIINEIHEKIVSSLSLDTIFFEIKSINRLEEEFIDWFIKIFKQKRNRLIFALIAESPFPIQEVTLNHDK---IEIPLL-DWTVEDISNWLISHHDEIHKEAKNI-EESEESVCSKLQEYFY
SEH05005.1_Candidatus_Venteria_ishoeyi             PNFAPYLVNYTKQQTCFEDIVGNHPLIFLLHGQESNTTHGLAARLKQNEENCLHHGETTVRLDCDERPGFLLTTIEKNLKDKFAVENLEILHESAFEA--TTLFYSEVEAKHKKLLQDFLNFWNQNHLFIVCLFIDYRPGPLQRLKAKNIDICNMLLPELTPIPREDFTPWYNLMKDELEIRVTELFDENNGPKLSKLLADLC
WP_017306429.1_Spirulina_subsalsa                  LNDTLKKLNYDHQKRPVKDSFNQRINAFIVQGQSHHAPRWLTNQLLYYVPHHDNAKKHSIVLKTHFRFNRLCEGIASGMDCGAEPQNIVSALAQNLETQSVVLALRDIHQAKNGILSSFINEIQTGQHYLILFLVDGDSSSSTELNPFIQTDSPCSLPPLENFTQGTITLWRGRKEDQYPQAIDEFIDTEEKGFLDFIYDSCL
OCR02924.1_Oscillatoriales_cyanobacterium          LYNALLSLDYIRQESLFQEFVNNQIGACLIHGKQEYGQRWLLHRLLRAIPNNNTASLTKISFVQNGSLDALWGEIKRKLKLKPLPDELAKAVCNLWQTQTVVLIFDDADQIEEAHLEKFINDFNSRNSRLLVFLIDFDGCAPDALDTTWKPHIPLKLPPIKPLREMELAYWLEKAFDSIEATVQSILNSDNELAMAHICELCD
KIF28928.1_Hassallia_byssoidea_VB512170            LYRALLKLNYREQTKKFRRIIDNSVAAFLIHGLPDYGQGWLLNRLVQQVPNSITAKVVQFNLARKSDVPSLWRELSRGVGLGGLPPDIADRVYQWWQTQNVLLIFRDVDFLSEIYLRELLENFTVSQFQLIMFLIDEKGCVSEEPNSNWQPDSPVKLPEISGFSQEELAKWLDYESEQLEQTVQTILNSDNELAMEQICYLSG
CUR12819.1_Planktothrix_paucivesiculata_PCC        LFKSLLRIDFDEQEEAVMEVLYNKTAAFLVHGEEKFGQETLVTRLSQL-SKLRNGRRIKIKVGGMEDISNLWNEVGKHLLDKLPETKIIDQIFELLQTQHLIFIFSDAHRTYISFLPELIQEF-QRETYLAMFLVDNKGYICWQVNHPDLPRIPLSLPPASRFPVRKLNEWVAVAA------VETLLESQGDLVYQRICYYCG
OQW38836.1_Proteobacteria_bacterium_SG             AEQKLGQINRKKHRDHLAIQINRKTHGFVISGEQQECPEDIRFKLSYLQEDLINHNPKGLRTIGKSAEQFLWELLAKRLGSCNPEKAAIEMRLNQVDR--SYLFFRELPSDEQQFLVDLLVAWSTSHSHFLLLIHAAENSGNDQIESLLKQHEVALLPEFHPPSKKEIRDWKGLHISVLRDGIEELFLKHEKAALPLLKKHYS
KPQ40050.1_Phormidium_sp._OSCR                     VHQCLLNFDYQKQEYEFDEFISTQLGICIVNGLQKCGQPWFLYRMFQT-KYYFDDTSTPFNKFSRTQKLSLKEEICHFFRLPESIERIYQEIANHLEHKSLVFIVKNIHLTTQEYLDYFVKQFQNPENRLLFLLVDLEGVSHQELLSHYRSHTIYRLKQIKPLSWAERQDCLKQLA---DEISQKVILTEKETVLEYICNCCN
ETR71372.1_Candidatus_Magnetoglobus                GSASVLMCNRIPQLRSFLTNYHKMPQFYFIHGREQDQHDSLVTRYNQTIHKRIQEIYDPYMCKSDDQQNTLLYDIGHELTGEDITSFEKLVNATIFEQQNAIIISHKIEEDFKDLLTWYIQEFLRIPQFILFFNIIYE----RWINEITPKNSTVLIPELEPVIRPDVQSWFNTYTSKRNECIKQLFPKPKEEKLVGF-----
WP_020531248.1_Flexithrix_dorotheae                PEKFYLGCNRTIQFQDLQYILTKKPCMCILYGNREDCHRSLIERFYYYLNK-------LINIDVRNRKERIIKSSFEAFNLTAVPDNAQMIIDSYNQKCDVLLIEIEIDCNEKSTLKWYINEF-----LKVLHLIKLVSKPSKDLRDLYSSEIRKVLPKLSPIKKYHVKELL----ASYVDKIDKLFLKKSEMFVT--KEFLN
GAK59821.1_Candidatus_Vecturithrix_granuli         GDIVYKMCNRGPQENDFLRFFPRQPHFYFLHGSDDECHESLIERLARTIKKYVEQTCGDVQSDLETRQEDLALNFMKTFDSDPNELISGLCHLAVLRKHPCVILRHNLDAARERFIRWYIKAWLDLPQFLIFLNIIYPRRHHKQLCRIHTSSGEQLLPELKMVTPFDVSDWFSYYRWQRKEKIALIFGNQEEKHLKMIIDECT
KIJ79200.1_Tolypothrix_campylonemoides             TEAEIIQLGHKSLSRQFEDSLQAAVVCFLIHGASGYGHKEMVVRLRQILEQQRSVQARKAAFWREKSLAKLLELIGDQIEQGDSPEVLAQRLHKMLETSDVILEITEIQRFNGSLLS-FIKDF-PTQHRLVVLLTLEQTILPMDDDSEIDSMRPIKLAELKEFTEKELTSWLSKWRNDALVLAKTLMDETKQLLYNKLRDDST
RAM51670.1_Hapalosiphonaceae_cyanobacterium        FMIALYDLDYLHQRTSFKTAIDVKAATFLIHGKPHYGQRWLLNQLKSQVPYHTNAWQKSIYIPHRKDIEVIWQDLAQELKTSASPKDVAKELYQHWLKSTVILAIHDVDLIAGSPLKQFMDEFKQSSYRMLLFLVDNINSKSENIDIN-EPHIPFVLKELEPFGKNLIEAWVGVQEASIKEVMCEIVRDSQISVFTDICQCFD
WP_088278871.1_Ideonella_sp._A_288                 DLPLPYLCNQEPLTRAFEDAAAKHAMLVLLAGGHNDAPEHFARRLQRHLPRRLKTIGVAVHTTTLRHSMPLLRALFEALDDDTEFDQAAQVLCEHLRARTAAVLMAWLPDRDLPALLRFIDAA-----RLVLICVGEARGSVRAVRKSSQA---AALEPMAEIDVAALTKWHKEHGRVDLDRCETLFRAGRARHFQTFCEGLR
OCQ93508.1_Nostoc_sp._MBR_210                      LFDCLLSIDFKDQEACVTQAIRQRTVAFLINGDDKYGQSAFIERLFRKIPEFKHEKEIKIKLESGWETSNILQEIAKFFGLDDIQDKIINKACECLQFKNLIFVFYIPQRFFVGLLADLINDF-HKDTYLTMFLVESNHKEKNKLAPREDDSSYLYLPLLSKFSRDHLEKWLENGVTQQKNFVDTLLETEWERVYKKICNHFS
KPV52199.1_Kouleothrix_aurantiaca                  LYQALQGLNYSAQKSAFVLSVARTVAAYLIDGQRNHGQRWLLNLLLKRYEHALQA---SIDMGRGKSIADLCQEIGRKAGVASTPEQLAPVLCRWWETQHTFLIFHSVDLLGEAGLREILEHFGNAANKLLLFLVDNQGVAAQNPA---NAGGPERLPTIARFSEDELDRWMMASLERTD--VQTILNTDDESVLEEICVICD
PKO80078.1_Betaproteobacteria_bacterium            PLAELGRIGRDTQRMALVDALGERAAAFLVHGPENHGQREFAEFLNVWDNEWEDAQPI----ADTDTADALIRWTCGQLGTPAAIPELAQTLAARLRRGPVIIIQRSIGHRQ-ERLTRFIGGF-----RLYWFLIDHCPLPQPEAGADTADYDDLALPALGDISAGQVRKWLKELR-------DEILTTPDSDVYNRLWASAS
RKZ83713.1_Candidatus_Parabeggiatoa_sp.            RTLLPYLPNRRDQELELGELIKHRPLLCVVHGDEYQCSDKFLDRIREELPNIIPEQMHGVDLDTFQSVNELHRKMLANLGELNMYATCDEVAQAIAQRQCPVILHTDMSTTDINIIHGFIQFWAAQNHLLVCLCFNYRDIEKQAIRNTFKDLASRVLPELPSIERTHVEQWAKIYLYELIPYIRELFPPIEAQQLKQILKECC
RMG30280.1_Bacteroidota_bacterium                  SMLDKYTCNRAEQNSRFKLNFNKKFQYYIIHGEEQESPYGLFQRFVLEISTAYQPYFY----SLEEAKINFLTALFQALELNDQLTLANLLKAPAAQDMQCIALKLRVYSSERDMIQWVLQEFRNAPDLLMFISIIYDLQQAEKLIKTLAEFPEGLLPVLPPVKISDVNKWFDRITEEKKQQIATYFDAQPEKKLEQIIEVYN
PZN77012.1_Candidatus_Methylumidiphilus_alinenensis LSLLPLMFGRETQEEQLQDYLGQQIIAVVLPGPHRESPAHFVERYAKLLRDAYQRHWREYVWPLQQRLDFLLRDLCRNIDGVGEEGHVRDRLVSQFADQSHQILYYFLTADRANLIGEWIGFWQAKNMVSIIFCLHSEPSILKALRTLCPNAPDLMLEKLPPIKEQDSYNWAKEHEAILYQRAKNAFRKRRMTKLEEAVAKAA
WP_072720293.1_Planktothrix_tepida                 VYQSLLRLDFRKSSGIFADWEQNKAACLLVYGKKKHGQEWIMRRLMTK-PKYSKRMFYTCSFSYISDSKKLWKWLSEKIGIQENQLRIIKALKEQLQYNNIVLIFTDLQANSLEYPQEFIDQFFNSCNKLLVFLLADEPEIKSNINENWSITEPIQLPEPPICPEDDLIPWLKQETVNPRKTAQEILKTNQEAVLMYICQYDQ
WP_020536784.1_Lewinella_cohaerens                 HEYHAYTCDRVAHSDTFRQLFKQPVQFYFLYGGDLQSHEGLFRRISYDLQDYLNPELKSLQLQLEHYKQNILRSFFSMLGLPEKDLTYALERSPRLQNTDYVCVFLHISQYDPAAARWFIKEFCTSVAILFFLAIEYDEEDASEVVAALEMAERQPLPELDMVAQRDIGRWLEHYKRTRKELMKTVFGNSRELELKKIIDQYN
ETR70369.1_Candidatus_Magnetoglobus_multicellularis RNLLPYMPDRSELIQEMSLALAGKPLVWIIHGNDNQCHYDLMDCIEGCWERFLYPTSKLLRYETNQWRDLVAMELKHKFDIQEAPEDVFDAINRRCDKASPVMLYDVVDTDQQTVCQRFIDFWNCQNPLIVCLLVRYKSFPVLEFKETILTLKNCVFPELPDISKKDTEDWYHNYKKNVSQCIEDIFDANQAREIEQ------
PZN72517.1_Candidatus_Methylumidiphilus_alinenensis PAFKAGLVDKDEQATHVIDHLHNNPLVYLLYGASTQWPDALLNILYYQLPRFNKNSSPTLKYGNIDPVEYLYELLDEELTCG-------KAIERRLSKEKPLIFYRILTAAEPALVQGMLVAWQNSPRHILIFYYEYIETPKNHLKASLPTEMSIFLPRLKSPNKKLVHDWISIHIEEAKKYVEDKYNAKKHHDLKNILNEFC
consensus/90%                                      ......hsb..p...h.........hhhl.G.......hh.ph.........................h...h...h...............hp....hhh...h.......h..hl..a......hhhh.h.........h...........l..h..h...ph..W............phh...p...h..h.....
consensus/85%                                      .....b.hsb..p...h...h.p...hhhl.G.p......hhpph...h.p..p.....h..........lh..l...h...............hpp...hhh...l.......h..hlp.a......hhhh.h........ph...........l..l..h...pl..Wh..........h.phh...p...h..h.....
consensus/80%                                      .....b.hsb..p...h...h.p...hhhl.G.ppps.p.hhpph...h.p..p.....h.....p....lhp.l.p.h...............hpppp.hhh...lp.....hhp.hlp.a.....blhhh.l.......pphp..........Ls.l..h...cl..Whp....p....hpphh..pp...hp.h...h.
consensus/75%                                      .....b.hsb..Q...h.p.h.pp..hhhl.G.ppps.c.hhpcl.b.l.p..p.....hph..pp....lhp.l.p.h...............hpppshlhl...lp..p..hlpbhlp.a.....bllhhhl.......pplp..........Lsbl..hp..cl..Whp....p.p..hpplh.ppp...hpblh..h.
consensus/70%                                      ..b..h.hsbppQpp.h.p.hppps.hallaG.ppps.c.hhpRl.b.l.p..p.....lph..ppp.p.lhppl.p.h......p.......p.hpppshlllbpplp..p.phlpbaIp.a.p...bllhhhlp......pplp...p....b.Lsbl..hsbpcl.pWhpp...pbp..hpplhpsppb..hpblhp.hs
```

**MoxR vWA Ternery systems; Inactive STAND-NTPase Entropy**

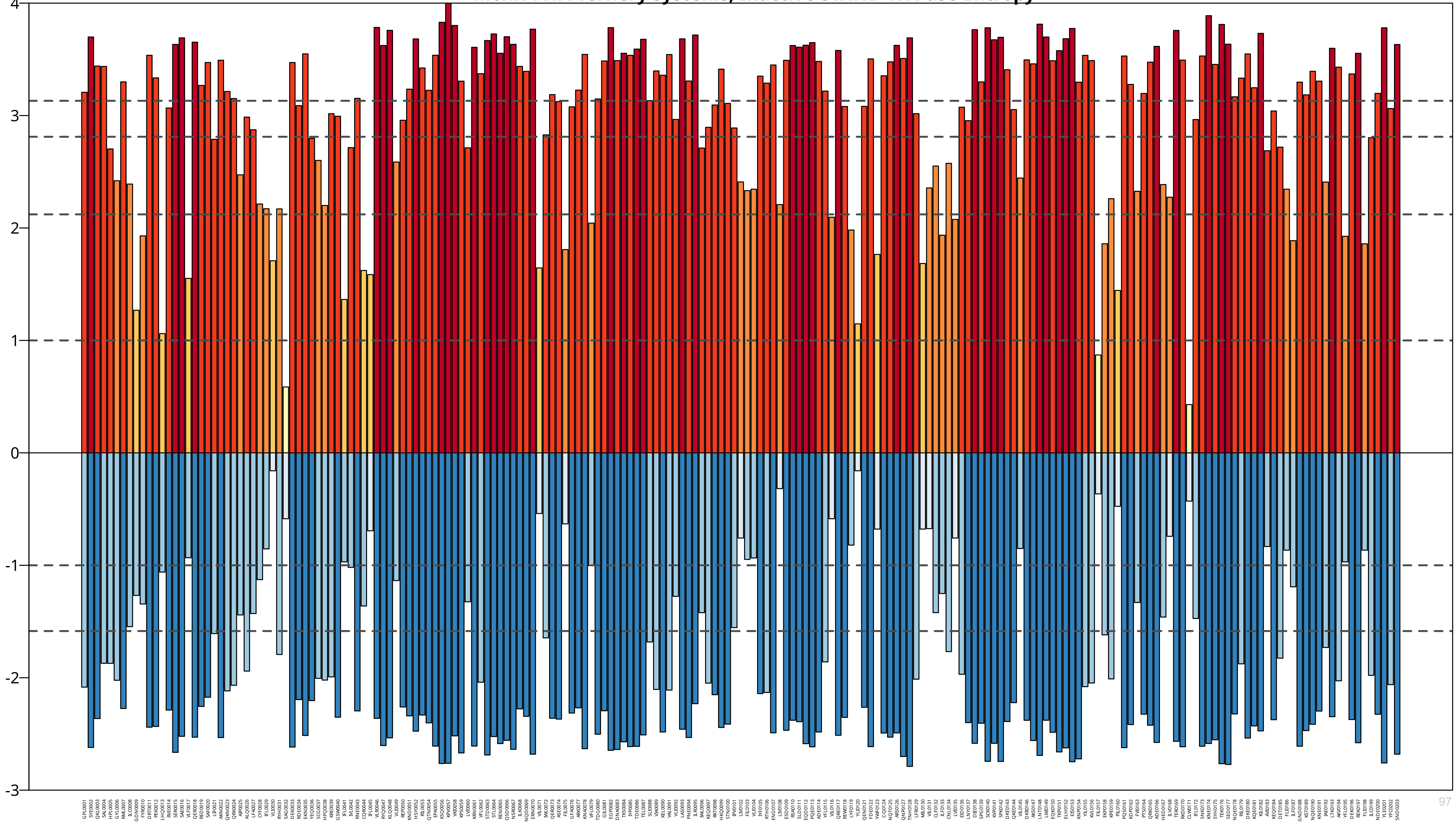

```
# ; Type_3 BREX Helicase CTD Inactive REase

WP_162523516.1_Calorimonas_adulescens                    DEKELSIDILKNIKYGEIYELLRTMFINYVLYNGGTVKEGKNGFDITIDGVRRYKGVTFDPSGNGANAITLNHEYVKKMIQELPVYPE
MCL4298460.1_Anaerolineae_bacterium                      PQTQAEVEQYRQRLIEISQAQVKAMLGGYLAAHGESLNEYSRRPGVYYFDKTRYSNVVFDRADDGLTYLHLNHPAVQTILTHLADD-R
WP_207425053.1_Pedobacter_sp._SYSU_D00535                TLAEIKADKVEAIKHSPLPKWLEGLTKSYLQIKGIGYKYLNDGINFKFP-GHQESIYTFNINNPIPEPISLQHEIIQTILKDAIHYTS
WP_218808307.1_Methylococcus_sp._Mc7                     EDHALEADDARKWRDHPAQYWLERAITAGLPARGGMAVREGNCWRIRWPDATESPRVCFDAQNPEMEWITLEDPRARAVISELPRCVA
NPV81618.1_Bacillota_bacterium                           IAQELDPGETQRLLSYPIPYWVERMTVSYLRAHGGKAEQNGHLWDLTWPDGKTQKGIVFSGEFPSGRHLTLEDPKIRGLIMRLPCFVP
ELY62516.1_Natronococcus_jeotgali_DSM_18795              STERIHEVMDESEDVFGSEAAIRSFVERSLDIFGGNLDRAGNLYRIEAPNSETRGPVTFSRNNEGIEHLSPDDPLIQRLRERILEDDR
WP_053959402.1_Sulfobacillus_thermosulfidooxidans        AL-PPDLEKVSKALGHPFPEWVEAMTEQYVMARGGMVERRLFGIDIHFPDGEVISQAVFRKRAAQSHYVGIHHPRIASLIDKVPRVTP
MCD8293627.1_Clostridia_bacterium                        ENKDLTQLVGTK-PNFDIDSALRSMLSHYESWQGR---------STQWID-----------------KISIADNEIVQHLRSKILQDE
WP_162251488.1_unclassified_Nocardioides                 EEAVLRQEMELARAASFQPDVVKDFTLTALARFRGDIAQAGSTWQVRHVPLPRYDRLIFGRSFVTPEMMSPGHPLLAALVAAVDT---
NMB01451.1_Bacillota_bacterium                           SIMKPTYDEIRKLQESLVPFWTENAVLGYLRYMGGSAVKSSKGWSLNWPDGTNQKFVGFQG---PKQLLSTASPKVLQLIQQAPDFHI
WP_009146898.1_Thiorhodovibrio_sp._970                   LSEEPDVQAAERLRSHPLPHWVERMTVAYINSHGGAATRKRSWWDLNWPDGEEHRKAVFSARLTDATLLNPENSRVRGLAMNLPQVAP
HIP19029.1_Sulfurovum_sp.                                ISDEHDPSAVKKLRTHPLPHWVERMTTSYIEAYGGQVTRKQHWWELKWPDDTLHKKVVFNIHYNNTNFLNLENDKLRGLTKNLPQVTD
MBT5530410.1_Cytophagia_bacterium                        DSDENMLELSKSYLDNQLPFWVEQMTTSFLDWSGGKVKKDLFGYSLLWPDGEHFKKIVFGWQSTGYRHLTLESPKVQELLGKDNRFIP
WP_200891843.1_Caldicellulosiruptor_danielii             DEKVLSVNLLAELPSDKVDEYIRKMYLYKEISLGN--------------------------LNISEETLSLENDKIKELLN-IPEYPI
NLE00371.1_Fibrobacter_sp.                               PF----STDAKELINTGINDIVACLVTSFLRYREIRINRYKENADMCYFSDPIIRNATFYNVSEKAELINADHHIVKNIRAFFASNPS
EMR73855.1_Thermoplasmatales_archaeon                    PFDQENISNIQISEIQNISKKIQKFTELFLQNRNLIITEYKDKTGVYYFKEKLYSKIIFDQDDEDAELLSFTHPYIKESINHSKQNGR
WP_073324029.1_Marisediminitalea_aggregata               GDIPLQLDEYKNALNHPMPYWVEQMVSSFLKALGKSINRNAKFLDITWPDGSTSNNVTFSGQTTNVNLLTLEEPKVSELLQKLAPWVP
MBN1459902.1_Armatimonadota_bacterium                    SA-DVQADTAARLLEGQIPIAVRTMTLAHLAERGREVGDSEQVYRLEWPDGSIVERAVFDNQFDDVTLVGLDDARVRGLLDRVVPVVD
WP_068347600.1_Kosmotoga_arenicorallina                  AYEITNPKEAQKLVQQPVNYWLERAVISYIKAYGGKATQIGDNWKIRYLETGREELITFDRKNPDVKKIAFNDQLVNDAIKHLPELSE
WP_202636300.1_Rugosibacter_aromaticivorans              DGHALEADDARKWRDHPAQFWLERAITSGLPARGGSAVKVDQAWRVKWVDGSESAQVCFDAQNPELEWITLEDPRARALISELPRCVA
WP_020614470.1_Sediminispirochaeta_bajacaliforniensis    STGSMDPGIAKEIEQHPIGFWIQQMVINYLNQNNARIEEGKTGYNIYWPDGDYSEEVTFDRDTYSLDLLSLESSIIQELLEKSTPLPE
MBT6646602.1_Nitrososphaerota_archaeon                   QDKNLDVEIAKTVNEHPLPLWIESMVLNYLKSNNGSIIEKNRGFDIVWPDGETSLSISFEKENKNLKFLSLEDEHVRKISKYISTFVK
MBV6489839.1_Fimbriimonadaceae_bacterium                 GQ----LADGDKIPTVPLRAWLDTLRGAHQ-------------------------RTLMDEE--------LSELVVERINSLRPYFCD
WP_073205019.1_Prevotella_ruminicola                     SIKEITKTAASEVKYSPLPIWLEDLMDLHCINVKGFYDKMLNLTLYHLPDGNL--GAIFDSDNPAAIHITLQHPIIQHIIRDIDGGI-
NSW90672.1_Bacillota_bacterium                           DDKTFDVALISNIKNNNVIDLLRRMYVNYKEW----------------------------KENIDQLMIDLNHEEIKTLVNQVLHWDK
WP_148854793.1_Moorella_thermoacetica                    PILAPEYVLASQRDLEKRAKRVQRLVEQYLQVYGASLHPYKLREGVYYFQGRRLHNVIFQQANEGAELLSFQHPYMVELLAHLEDALR
MBU0460857.1_Nanoarchaeota_archaeon                      PFDKKDIKEIEKSTVENESNIIKNLVETYAKFNNFDFKEYSKKKNIFYTDETKIKNAVFNKEDESLDLINITHPLVKEITNELIK--R
KAF0196956.1_Bacillota_bacterium                         ASEPLSPALAQQVAESPIPYWVEQMTKSFLTAEGAHIRYVGESYEVIWPGETTVEKVSFRETTEASGVASLEHPRIRALLDRLSPVVP
consensus/100%                                           ........................h................................................p..h............
consensus/95%                                            .....................lp.h...........................................h...p..h............
consensus/90%                                            .....................lp.h...........................................hs..p..h..h.........
consensus/85%                                            .....p...pp.........hlcph....h......h........h.h........h.F.........lshpp.bl..l.........
consensus/80%                                            .....p...pp......h..hlcph...ah...s..h........h.h........h.F.......p.lshpp.blp.l..ph.....
consensus/75%                                            s....p...hpph..p.hs.hlcph..sal...s..h.b..p..pl.a.s.p....h.Fp.p..s.p.lshpcsblp.llpphs..s.
consensus/70%                                            s....p.p.hpph..pshs.hlcph..sal...s..h.b..p.bpl.a.s.p....hsFp.ps.s.p.lslpcsblppllpplsphs
```

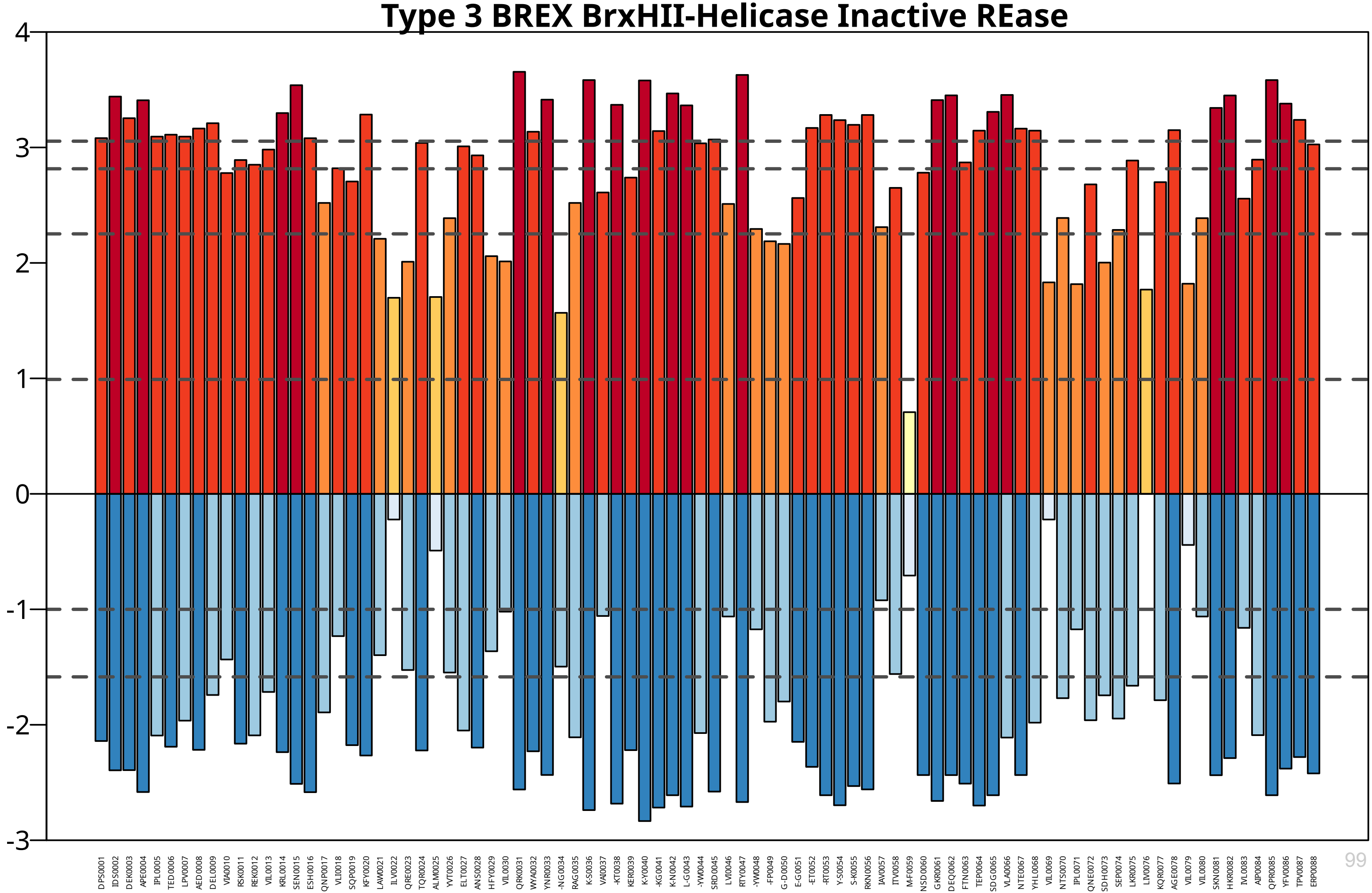
Type 3 BREX BrxHII-Helicase Inactive REase
4
3
2
1
0
-1
-2
-3

BR-system Helicase CTD Inactive REase Rep_1

```
WP_169116183.1_Tepidiphilus_baoligensis            QPFFIRSFFTQAFRRFQGAMHPREPGRYEITHPVLRRYERICFEKRHVRGAPMASLIHPGHPLMQALTDLVLEEHRPK
MCY3585835.1_Acidimicrobiaceae_bacterium           NPVIVEHFLERVASASLIGRAPLGDGFYYLSALVVTRHERSSAIAQGVDRAADAHMLGPSDTAFRALVEALRTRLETD
WP_094451591.1_Parenemella_sanctibonifatiensis     QPHHVEAFFHKAFKDLGGNLRMRESHRFEIKSPIVNTYHRVAFDRANVKGNHVATLLHPGHPLMAATIGCITDRHHDA
WP_042699659.1_Methanocorpusculum_bavaricum        QPYFVRSYFLNAFSKVGGVLNQRETGRYEISHPVVHKYDRVCFDKEDIEKNKQAVMLHPGHPLMRAVTSIILEKYGGL
SCM57150.1_Methanothermobacter_wolfeii             APEYVEEFFRTVFRRMGWKYTER-KGLYTLRTALRREYRNLTFNP----GTDEHEFISFGHPLLEATIRYTGKRFGDA
AXV39096.1_Methanobacterium_sp._BAmetb5            VPEYLEEFFKKAFKKVGGKFTTRKNGTLNIASRLNKSYPRLTFDKNFASENSDYDFLSFGHPLLEAVLKWVELNYSNT
WP_106140929.1_Spirosoma_oryzae                    QPVYIRQFFEKAFTYMGGKLQELQPQLFRITTLINPAELYLCFDKRLFLNLTKTIYLNPGNALFDALLSLIQQQCRPD
GIV15258.1_Armatimonadota_bacterium                VPWDVERFTRLAVQTVGGQFSERRPNVYRLSVDAFARGLRVAFQRAVAR--ENAEFFAPGHPLLEALIDHFLQPNLPV
NNN07583.1_Acidimicrobiaceae_bacterium             QPGFVEAFFTAALTDLGGRITPRERGRFEITRPLQSAYERVTFDKDRITDRVRAELLAPGNPLVKAVIETVLERYGAT
MDE0136609.1_Acidimicrobiaceae_bacterium           QPHYVQAFFEAALSDLGGTMREVEPGRFQIARAVLERYTRITFDKDLTEGAPDADLLAPGHRLLDVVVDLTLERHRDQ
OZG52264.1_Pseudoscardovia_radai                   QPGYIEAFFVEAILDLGGSISRRETGRWEILRPLANRYERVTFDPAHVDNQTKALLVTPGTPILNAVIAVVLRKYAGE
WP_259102394.1_Candidatus_Fervidibacter_sacchari   IPEYIERFFLRACESLKVTVKPREKGVFQVESEVKMAYRSLTFRKELAK--DTVEFVAPGHPLLEAIVDAVMAEGKEE
PWV37726.1_Desulfurococcaceae_archaeon             NPSHVEEFFRRAFEKAGGRIKILGDGFISIGSPVKDKYPKITFYKDLALKDPEAELVTLGHPLFEATLEWVRRKFFLS
RLC12746.1_Deltaproteobacteria_bacterium           MPRYVEAQFVAAARQVGLRVEPRADGLWRIEHKADSSYRKITFHKHHLEAHLDAVLMGPGHPLYAAVDEELNDRLALL
PZR94482.1_Chloroflexota_bacterium                 VPYFIASFFLEAFRNLGGEAVEREAGRYEIKFHIVTKYARICFDKADIAGHLKAEFIAPGHSLLDTVVELLLEREGSL
NJL06253.1_Chloroflexaceae_bacterium               VPEYIQDFVVQAFNTLAADLQRRNDAVWRIESTLDSSYPRLVFKRDLLEQYPNAEFVAPGHPLFVALLNLVRDRYGSL
MCY4618413.1_Chloroflexi_bacterium                 TPEFVERFFVDGLRYLGGRIARGDDRDWRLDHEFGEDGRIITFNKDRLRREPPAEFAAPGHPLFDTVLNRILDQGRPA
MCS6856322.1_Sandaracinaceae_bacterium             RQEEIHRFLEEAFAHLGVATEVIKKG--SILRTGKKEWVEVALDRASAI--EGVELVGPGHPLLVEVTKRVLREAKPH
GAK56805.1_Candidatus_Vecturithrix_granuli         MPAFIRYYIEELAPYLGLTLAGDIRGLFRFEDPVLLTYPRLTFEKTLALSILQAIYLHPGEIVFDRISSRFLAKYSRD
MCP9446721.1_Nitrospira_sp.                        VPETIARFMQEAAEFVPLTLKPIPPHTFEPGRALATKYPRCSTDRETAE--HNLEWVTPGHPLFEAIRRHTYDKARDA
MBN1909331.1_Pirellulales_bacterium                MPEYIERFFVEAYRSFGGTITPTKKGVWSIGRKIGNSYPQITFDKELLVGYSDMEFIGPGHPLFEGIVERVLTQYGPS
WP_059053655.1_Tractidigestivibacter_scatoligenes  QPHYIQSFFAWAVEDLGGHLASNRDGTWRIRKPLADSYSSLAFDKEDVADPGRTDLVAVGHPLLDAAVEETLAKHGGA
MCL5028417.1_Bacteroidota_bacterium                QPLYIERFFTKAFSYAGGFIKQHEQPVIYLGQPIRDYYNPFVFNKSLVSSPDGTKLLGPGHPLFDCLLNYLKKQAAEV
WP_090584037.1_Arthrobacter_sp._ov407              NPAIIEQYLHRLNLAGVLSVTAAGDGILRIQGLIATSGKALSDAQRGGALLSNVFTLGPGEPSFNELVDYAGAELEPD
WP_159000922.1_Streptomyces_sp._SBT349             NPVIVDAFLDQLARAQGWALAP---GLRLLTSLVAADGARLAINDGATD--DDVIVLGPTEEPFGELVELALRSGEMD
MBC7429627.1_Bacteriovorax_sp.                     DLKRLFSAFQAGLSLFDGSYSNLDNKTFEVSYPKGSNKTLVTFDRDLANGDSKLTLLGFDHPIVANMINTIESRKNL-
MBE7508219.1_Planctomycetia_bacterium              GMRRLVAFVQRAAALFGGQFVSKSGTRYVLIDP-DKAETPFTTDRDEALREENVELLGLEHPAVSRLIERCRQLEAE-
MBI2192005.1_Planctomycetota_bacterium             GLQRLAAFIKRAARQQDATLREREDGLWELLLR-GREALLCTLDRERALQSEGVDLLGLDHPIVKQWLEQCRALPAR-
MBI5511449.1_Deltaproteobacteria_bacterium         SVERMLRYFGAATADTGKTLATTGPGRFEVRDGSGAALLRFTTDRDLAREDDALDLFGLDHPLMQEALRRWREAAPE-
MBI5901621.1_Rhodocyclales_bacterium               TLAELGAWVRGMILQLGGAALP-DGQFWSFLLPEAPRYEQVCFDRDLALRARQSELGGIGHPLVDALLHEAKSPGFP-
MBA3936144.1_Planctomycetota_bacterium             SMMALLDFARRAAVAEGGRFDSAADGTHDFT-PVGQPPLRFSTDRAVALANGGIQLLGIDHPEVQKWLEKFASLPPE-
MBN2145092.1_Candidatus_Auribacterota_bacterium    TLSDLRLFVEKAVLRLGGGFIP-SGEIVEILVPESAVYKNSTFNRKMAVRKKGVELLGIGHPLINALLDYFKSDLGA-
MBP6702578.1_Vicinamibacteria_bacterium            GLGALDRFVAEAAREDGGRLEPMGCGLSRLVDPDGSTAAVFCHDRHRAMADEGLALMGLDHPAVDAWLRSSRDTPPE-
MBQ8111590.1_Kiritimatiellia_bacterium             NMARLVKFLREASSFAGYAYREEEGGDFTLTS--ANDTWSFTCEREKALKDDSLQLAGLEHPVVTAIIDACRRALAE-
MBW4553276.1_Aphanocapsa_sp._GSE-SYN-MK-11-07L     SMDRLIRFLSAALEEKKQTIVRVSNGIYELHNELGEKVLRFTTNRELAREQDGLDLMGLDHPIMQKAIEKWQNVVPE-
MBX3410397.1_Phycisphaeraceae_bacterium            GMQRLLAFTRECARAAGGAMRDAGGSIHEVSLP-GGRIERFTTDRAKAKDDDALALLGLEHPLVREFMDEYRALPAE-
MCC7337860.1_Pirellulaceae_bacterium               GMSRIVRFAQLATEETGGTWNTVGDGTFEFRGKDGRWPAVFTDSRERSLEDEHLQLLGLDHALIQGYLEDYRNVPAN-
MCX6876429.1_Verrucomicrobia_bacterium             DRKDIIGFAAQATTASGGNWSTISPDRFSCAVP-GETELVFTTDREQANASVSLELLGLDHPFVANQLRKWQSLPPE-
MDR1607843.1_Deltaproteobacteria_bacterium         GMRRLLFFAQKAAHLNGEEFLPIGGDIFELSRP-NSETVRFTTDRDQALEAEYLSLLGLEHPIVKQWLDSSASLQPR-
MDR2179037.1_Synergistaceae_bacterium              AMDRLVAFFSSALAAHGQKLLKTADGLYQLSSPDGFEKIIFTLDRDEATSREDIELLGLDHPLVKKELEAWRDLPPE-
NPV69259.1_Bacillota_bacterium                     GMERLVSFMSRVERLTERQMKQRDATLWELWQ--SDTPLLFTSDRERALQNEDLHLMGLEHPVVRERFKTCLGLPEA-
WP_026299088.1_Deinococcus_aquatilis               SFERMVQFLCLAIAEDDRHIVKIDQHIYAITASDGQQELMFTTDRDLAIRETQLELIGIDHPIMQQEIKRWQSTPPE-
WP_058554674.1_Thiohalocapsa_sp._ML1               SKHRLLAFLATAAEAAGGGLRRLDGQRFELHAGAGAEPMTCTLDRELAQAEEGLTLIGIDHPLVARFCAALQGMDPA-
WP_277443845.1_Pelotomaculum_isophthalicicum       GMLRLIHFATIALQYKNGKLIEVEKGKTYIADIGNGQSVRFTTQRDIAIEDENLSLLGLEHPLIQQLLEAYTNLPPE-
WP_282552852.1_Providencia_stuartii                SIDDLIRFMQKALPYEGEAIEIRGQKQWALVKD-GLEQMLFTTVREDATNSEKLHLIGLDHPVIAKQLDKYRNLDPE-
WP_283022682.1_Bradyrhizobium_sp._CB1717           GFRRLIDFLAASLPDQGRRLVRKEEDLYEVVDNAGKRTSFLTTDRAKAADREDLELVGLDHPLVEAELAKWRDLNPA-
WP_308999799.1_Paenibacillus_sp._LHD-38            GLEQIEMFIREAVTIEGGEYRKKSDDLIEISTNSGLSSIIFTTDRESYIEDDKVELLGIDHPLIANYIAQYQGLDPD-
WP_309896344.1_Archangium_sp.                      GLERVLEFLRTSLAADGRRVEAQDDGTYVVKDAEQQTVCRFTLDREAARARADLDLLGLDHPLVQEAIKRWQTTSPE-
consensus/100%                                     ....h....................................s..................p..h..............
consensus/95%                                      ....l..ah..........h.........h..........hs..............hh...p..h..............
consensus/90%                                      ....l..ah..s.......h.........h..........hs.pc..........hhs..cshh..............
consensus/85%                                      ....l..Fh..sh...s..h.......h.l..........hs.p+..h.....h.hhs.sHPlh...hp.hb......
consensus/80%                                      ...bl..Fh..uh...s..h.......hpl......p...hs.p+..h.....hphlu.sHPlh..hlp.hb......
consensus/75%                                      ...bl..Fh..Ah...G.ph....ss.apl....s.p..bhs.s+p.h.....hphlu.sHPlhp.hlc.hbpb....
consensus/70%                                      s..blb.FhppAhp..G.ph....ss.apl.p..spp..bho.c+c.h...pphpllu.sHPlhp.hlc.hbpb....
```

# BR-systems Helicase inactive REase Representative_1

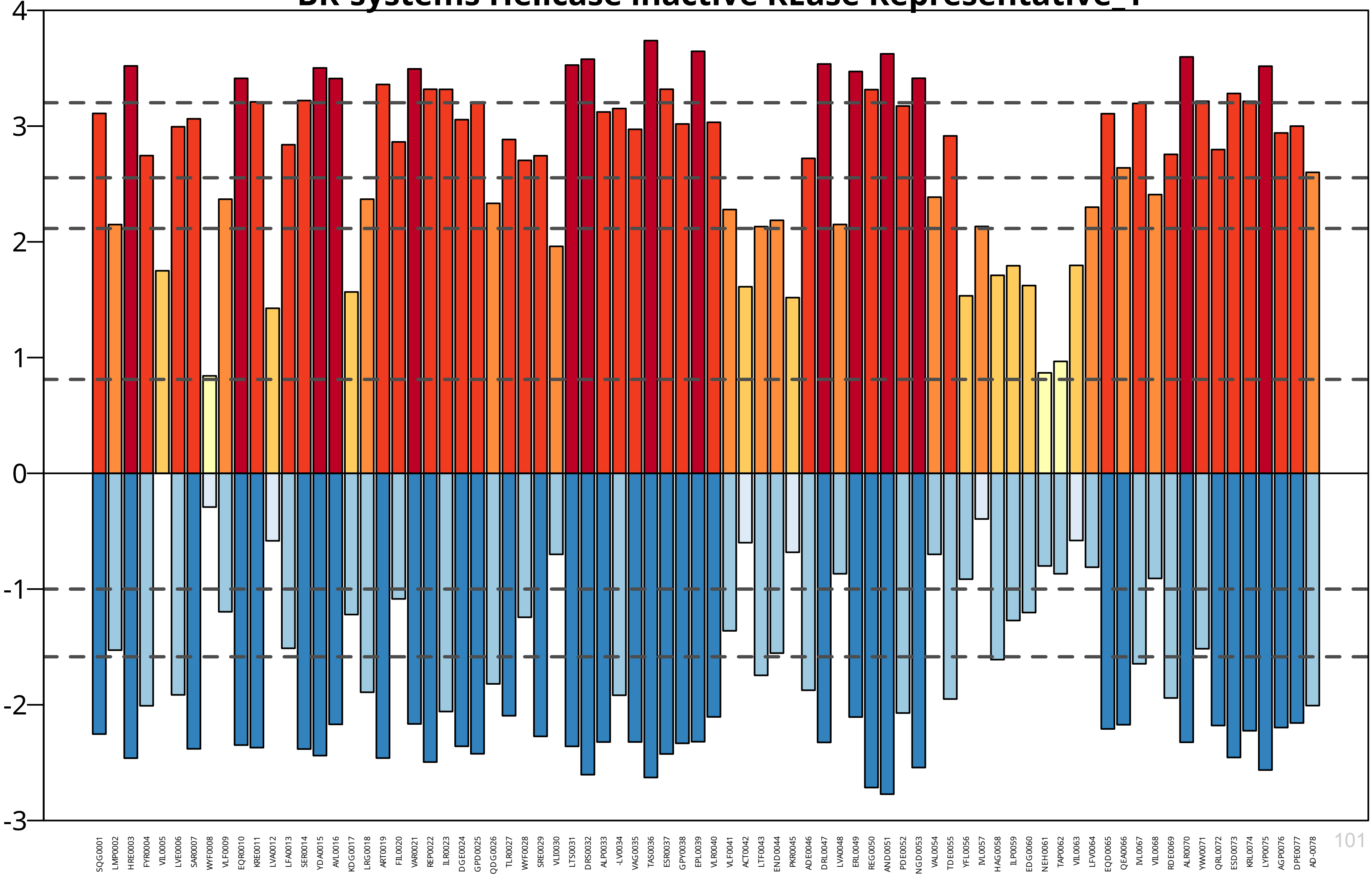

BR-systems Helicase CTD Inactive REase Rep_2

```
MCK6447153.1_Planctomycetota_bacterium          PPVTLQQLERAVVGSKTLGSKFTPHNQIAGAHALAWG--ASETVTFSPEVFDVHPNVKLLS
AKC83094.1_Verrucomicrobia_bacterium            PLATRDSVRCALYRLA----GTHATVSDSNRVGVS-GLGDAFSVGLTQEALEGDSDALPPT
MCB9841664.1_Phycisphaeraceae_bacterium         RVITLDDIVHALSESEYLRAGSKVRETGQPWVEIRGGLYTRFALTAKQDLYERGLALRFAS
WP_090215779.1_Pseudomonas_guangdongensis       LPVTLAQIWRSLIESEYLKSGCQFSPC-ARYITLNGPLIDGSNLTVDRELMDVGLPLHFAS
WP_196479964.1_Burkholderia_pseudomultivorans   LPITLEQIYSTLAASMTLKSGSEVIPSYGEALLVRGGS-ESFMLTVSRALYERGIALHFAT
HCG7747318.1_Vibrio_parahaemolyticus            IPVRLNDIWDAIVNSAFLKSGAKLESFNGEAVEINGGIPGRTLFTVSRELFEEGIDLVFAT
MCE9614803.1_Lentisphaerota_bacterium           PAITRADVIAALVTLQP---GLQTVGHAKGTVTIK-GLTKPIRLAFTDPALDADTAARPLT
MTE20051.1_Streptomyces_taklimakanensis         SPVTLEDLREVLVERFAAVL-------------------EGRPVTFDPSRASRDGETALAT
WP_206882508.1_Alicyclobacillus_fructus         GPVQLDDIWRALVESPFLRHGCHTEETHGPVFVVRGGVEDGTRLTVSPELYERGLPVHFAS
WP_084460301.1_Aminiphilus_circumscriptus       MPVTLESIWGLLANCRYLCDGCELVPGNEKVLRLAGGIPRGCCLTGDRAVLETGIPLHLAT
MBI3972513.1_Chloroflexota_bacterium            PPVEKEQLKAALARLVEADVGVSAEALDDGAWQIRT---NATTVGLDSAVLEAREDLRELP
KPC75468.1_Thermoactinomyces_vulgaris           APIDLQSIWSTLTESRYLQEGCKIFDENNLILEINHGVPDGSVLTVSRELYESGLPVHFAS
MBX6754325.1_Thermorudis_peleae                 SPVSLEDLARVVPSLPGLAERFSPHPTIPNAWLVSW---QLHTVTFDREVFDMHSQVRLLT
MCF8137375.1_Desulfotignum_sp.                  SPVGLDAFWQAVAGSRYLKDGCQTLDAADRMVCLSGKIIDGTFMTFSRKVYEEHPRLAFGT
GIW50774.1_Gemmatimonadales_bacterium           PPVSLSDLERVLLTSS-LRERFVAHPELAGAYRLDLG--RLWEVTFDPAVADAHPGVRLLT
MCI7613155.1_Selenomonadaceae_bacterium         LPATVDDIWQAFADSVALQAGLQVDGD---------GKMLEVAFTKDRGEVD----VHYLT
MYD03943.1_Acidimicrobiia_bacterium             PPATPSELEAFYTRSHSFGRLFRPDADLDGAYLL-----EGRSVTFRPELADQHPDLHFLT
WP_073234049.1_Desulforamulus_putei             PPVTPLELERIFTAT-LLPEGVSLQPGEKGVYLLSF---QSYRVTFNPRVYDRRPDVKLLA
WP_036720040.1_Paracoccus_sphaerophysae         APVSLADIDVALTSLAKVAPLLSVT--HPGIFALRYG--RVSHITASRELLDRPPPLEFAS
WP_161844016.1_Pseudoflavonifractor_sp.         PSACLEDLWAALTQSPYLQSGAFCSGP---VWRLPE---PALEGTIQRDAIT----VSFLT
WP_100917763.1_Candidatus_Thiodictyon_syntrophicum LPATLDGLWGVLTGSAYLKAGCACAVNGREPLLLRGGVLDGTALTTDRRLYEEGIELRFAT
AXH34461.1_Humibacter_sp._BT305                 GATTPEVLERLLTTNGLSRAAFEVDNQRPKVYRLKPGVRETLPVTFDRATWDESDDLVFLT
ELZ44607.1_Halorubrum_distributum_JCM_9100      APYQKSSLEAVLVENETLADGVTFTPVEETTYRLNWGMEDSVAVTFSSDCADEFPSIHHFA
MBD5553416.1_Desulfovibrio_sp.                  PAWDLSLLRRVLAEPRLMPPGYAASQLGSHDWQLIAGAAQPWRVSVDAEFYANHVDVAFWT
MBF6555076.1_Acidimicrobiales_bacterium         PVVTLEDVQRILLDSPATREQLSEHPMIDGAWLVEDG--NKIAVTFDRIVLDENSPVRLLS
MBP7066719.1_Methanothrix_sp.                   DDVLVERLRGFVVSAA----GLSTHELNDKLFV---GVPNGAALTSEK--------ANALT
MBV9397056.1_Bryobacterales_bacterium           PAYDLPVLDQAMILDASRPMDLEWRPLDARTYAARLGLPEPIRVTTSADVFEESSDHEFFS
MCD8487848.1_Desertifilum_sp.                   APMTPETIEQLFTTSTILRSGALFECQAHRTWQLTYG--QNYNVTFYPEVFDERPSLRLMT
MCK6511424.1_Myxococcota_bacterium              PPYDLRDLQRILAQPDLMPNGVTIRTLGPEEVGYRSQHVEEVRVTTSPTYFDQHTEVELWS
MCY4068113.1_Acidimicrobiaceae_bacterium        PAMTLEGLRERLLAVPAAARLLKPDPQRCGVWLLDLGADAPVPVAFDRSVCEAHDDVTLLT
MCY4434136.1_bacterium                          PAMTLNDLQDALLSIPWCRNRFTPHPDFSDAWLLSLGT-STQPVTFNPTRYQDTPHITLLT
MDE0094453.1_Gammaproteobacteria_bacterium      PAIDFNYLERALEDETLLPSGVNAIRKGVKEFHIKVGTHDTVRVTTNPSYYEAHSEVELWS
MXW96682.1_Acidimicrobiaceae_bacterium          PAMTLDGLCDRLLSLPAVATRLVADPQRVGVWLLSLGAEAGVPVTFDRSICAGYDDVALLT
NLA99646.1_Methanomicrobiales_archaeon          DDAVILRTAAFIWTAA----GFTVEERDARLYIVGSGIPNGASLAAGR--------ANALT
NLJ36707.1_candidate_division_WS1_bacterium     PLCQPEDIRDLILTSQAITANWTLEEHVAGVYTLTEKTDKTTTVTFRQDILEARPEHHCLA
OYY89820.1_Sphingomonas_sp.                     PALSLTDLARIVADHRLLPPGYAMNSLGEQDFAVEQESRRRVRATISREFYASHFDTDFWT
PZV04180.1_Cyanobium_sp.                        PPLTLADLEAILKQPALLPPGCSARSIGAYDFAWSQGLDKEMRVTCNASYYEDNSDCELWV
SNR42896.1_Halorubrum_vacuolatum                PLLNPEVVKHLFTQSQILRNGWTFEMLYQKLYRLEIASPDEVLVSFDPEVLEWYPSVIIPL
WP_054466705.1_Planktothricoides_sp.            SPITPAEIEQLFTQSEIMAEGIVFTPIGDKIWQLTYN--QEISVTFYPEVFDEHPSLRLMT
WP_103287095.1_Candidatus_Nitrosocaldus         PTFTEVEVSRVLERFVRLCCGMRLVKRGDLLFVEPL---ERLPVTV-RSFVENGLSPDALV
WP_128478225.1_Halorussus_pelagicus             PVFTVKAIKGVFIESETLNEGVHFDSLAESIYCLQHGDAEGIGVTFDSETADEYPSLRFLA
WP_164002139.1_Pyxidicoccus_caerfyrddinensis    PPATQAELAYWCLQHPA---GMQIRTGEPGTDRLATGTNEEVLATFSGEVADRHPPVRLLT
WP_176737426.1_Micromonospora_citrea            PSDTLADLAAAIVGNKAVAPWLTADPTEEGAWELALGA-PPQLVSFDRAVVDASKRVALFT
WP_179052052.1_Nostoc_sp._TCL26-01              PLYDLKTLDQILQSPSLLPPGIDVQLMQDGEYKFSMGMKETLRVTTKPDYFDEHPDTELWS
WP_285255575.1_Halobacterium_salinarum          PPYDPPIAASLLLDSQFLAIDVECEPLGDSEYRLQW---QVAVVTTNPE--QTRMETRLLT
consensus/100%                                  ...........h............................s................
consensus/95%                                   ..h....h...h...........................hs................
consensus/90%                                   ..hp...l...h...................h.h.........hs.p...h.......hhs
consensus/85%                                   .shs...l..hh..........h.........h.h.....p...lo.p...hp....h.hhs
consensus/80%                                   sshs...l..hh.p...h...hp.p......h.l.....p...lT.s..hhp....h.hhs
consensus/75%                                   sshsbp.l..hl.ps..h..shphps.....h.l..s..p...lThs.phh-....lphho
consensus/70%                                   sshsbpplb.hl.ps..h..shphps.....a.lp.u..ps..lThs.phh-...slphho
```

# BR-systems Helicase inactive REase Representative_2

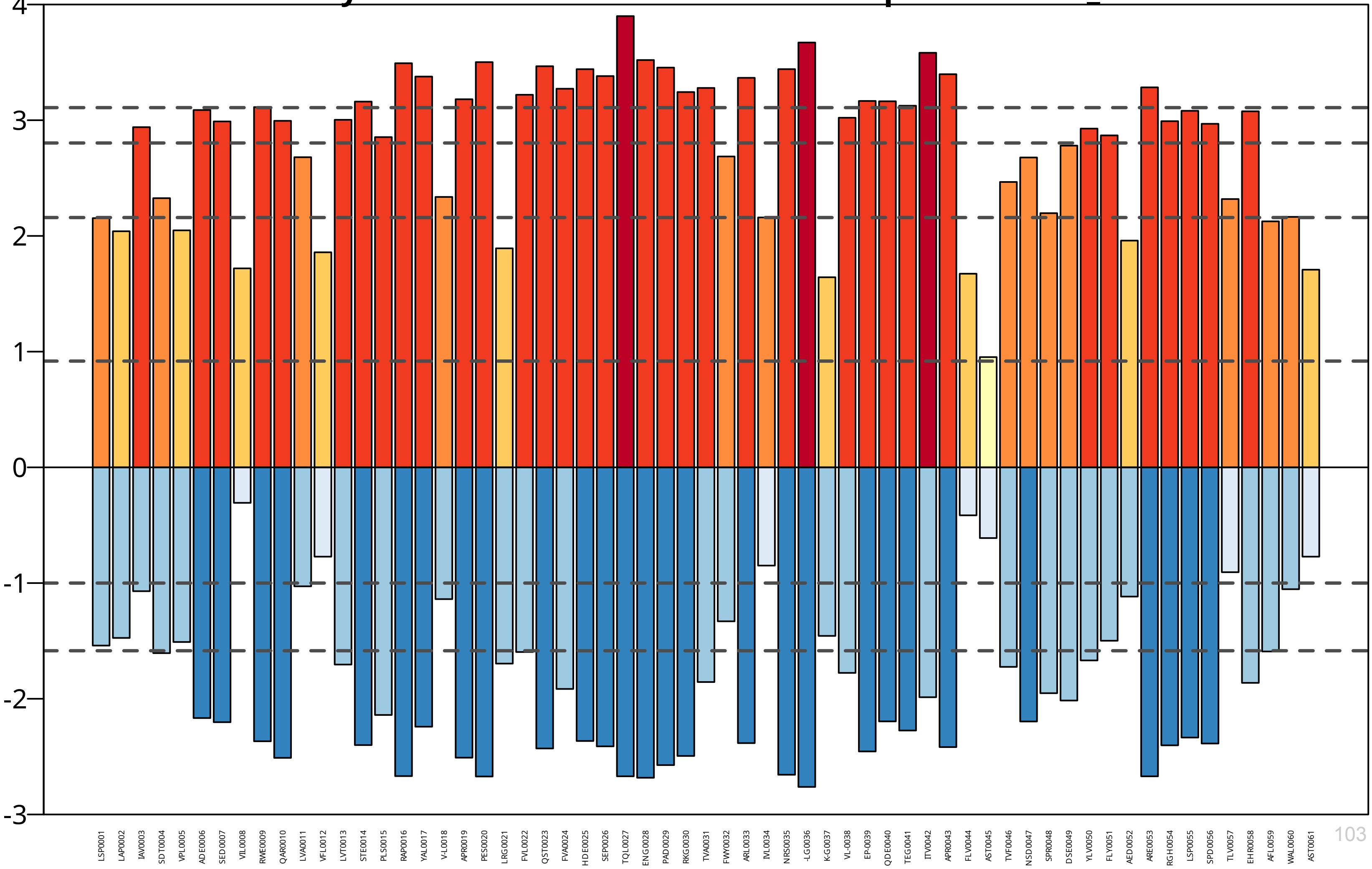

## Type-2 BR-systems

# ; DUF499_2 C-Terminal RRM Rep_1 (MAIN_FIG)

| | |
|---|---|
| WP_015408759.1_Natronomonas_moolapensis | ASTSSPYHVPRALKEMRADHPDEIITAVEGVWIRIEGADGWKGAWFTANKLGDSHTLVNFD--YTASESMVDITYRGDPSAFADFNMEPEDFSTPDGERTAEADFSIEFEGDPMLYGETFEALDDLLAVDNAFTVTMETEVEIRARD |
| NHV98109.1_Thaumarchaeota_archaeon | LIVLAESSV-ETLNDIEKRAIRDRFTKVNSVILKISSNLEIKNLLTRLG--PERNCNIRLSGSITRRSPSYTISFEINKEDLQKGKSMLDLLWRLKGIDSLQVRLELKWGEPVKPEDAA-----GVLRSLGDIVAGLEANVGA---- |
| MBD5553419.1_Desulfovibrio_sp. | LEAGANGAVAEAFAELEEKMKAAGMREIASLTAEAFLPRDGFGLTRLAAGVP-GRKQVALSASWATAGANFSLELEGSCQEAAEAGQFVQGQMRGAAEQSFSLALTVSFEPPLS----DLAPVRTQFCKLADAALFLVCRGRQ---- |
| MCK6511429.1_Myxococcota_bacterium | FTLDHENNLPEILRFLEQSLQQRKARSIRSFSCQLFHLPDFTQLTQSSNVFKDASPHLEFRATFAKNNSEGILSFEGHPDDIRSVLPFLQSQSQRRAEVEINATFSFDFSTPLPLPDPQ--AFSARIRPLEPNKCKISLTVSTKDT- |
| MBY0489937.1_Gemmatimonadaceae_bacterium | -------------SDLAAAIAAAVRPLERLVLTARTTADAKLLTQFAQPFGATSLGLTVSVSGDCGGGTMALMVDGVKPTHPTPLDMASAIANSTSATSFEASLTMDFEPGLLLAGDQLQQL-------AASSVTVRCRFGPANGS |
| OYR54791.1_Halorubrum_halodurans | DTATSQMAASRAFNEVRTHAPSDGTPGVSQVTVEVMGEDRLSKGSFIAQ---RDAFAVSVRMSYDVTGTSYSASYQGGLDDFSRVTNQPDPF--------GSINVTLKFRVDLDETDDEDDVLAELQAGLGQTNIDVKVQARGPVEV |
| GBD14658.1_bacterium_HR25 | ALLEATGDPGLALADLAARAKDARWTAIGQARVFWQGDPRLSALRTVLGHLAHARARVEVDLAVRYDGDEWESHFRGAAARYQSLAPTLEAQAGQAQDAHVNIALALEFPSGLPPEGPDYGDIRDVLDLAGLGHIRIVAWPLQGGDS |
| MBQ7457436.1_Desulfovibrio_sp. | TEFSKEGILKEAFVRLWEEIRHAKVAVISEFHIEAVEMADALRLMPLVQAIRNADKTLTITGILETHGAKLTLEYSGSIEEAQALRDYLSIQLRQSTSHSLEVAFGCLFPQGLDMNSNMPEAILDRLSKAAASTAHIRAKAKV---- |
| MBA3046419.1_Euryarchaeota_archaeon | IPPITEKTLQGIIGELLPILEDKNIEVIQSIGLKAKGRDDLARLLSAIPQFLGAKAYYIIEATLNEKGGFMQLDYRGDDAGFKVVKPVLLNYQPKEQFSSCDLVLTFSWDGGIS-RDEFVGILRDKVANFTGDSIYTAKVTPKEAEK |
| MYC64339.1_Caldilineaceae_bacterium_SB0661_bin_34 | SEFQSTRAPAEAIEDVVAQAQDASATRMEELILEWDAEGCVAKALHTLAQHPNVDPSLSVD--LDTEGKPFTLKMTCSAPQAQMMGDSLKAILRQGEGTACTTRIVLAFPASTSVEGNEKRTIQDAVRGTMSRSLTILKTA------ |
| MCA3396953.1_Roseomonas_sp. | ELLHAEGVLKEALNRIFEQAAARKVSAISRLTVRVFEPADGFRLLGVVSAVRTAEVRASIKGEFETAGSTVMIGFEGVPQDALPLRDFLDPQLKLAAEKDVSFVLSLGYKDGLPTSGEAPTKLIEQLTRFVTGAAYVEATAEPKP-- |
| MBR2123973.1_Acetobacter_sp. | NPISGEGVFKQILRSLLEETKRRSIEEITQIDIKITEPTDGFRFLGTIGNISLVQKELQMAVDYETTKGCFKVGYKGQAAEAIIFKEFLEPQLRTAEETNFTMVFSLKFEPSFSLRDEENRKLSDQLTRFIAAQVFVTIFPSS---- |
| QES21410.1_Streptomyces_venezuelae | TVFTAAGAAGSAFAQARSAAADTQRESLVELKIEVDHLPELLRLHSIIPPTLGADLTYDVDVVVTLDGQTAKISYVGTPRDYAPLREALKQLLPR--ESVLKASVTAAFADPLPLSGDVVERFAQAARDTGPTKCRISMRTESDG-- |
| NLV45647.1_Candidatus_Hydrogenedentes_bacterium | TLLSAEGPLRQALVELFDKARSQKVEYLKSISMRFYEAKGAWNAHQAVATYRDSETICQLSVGIEGGIESFEVQFSGTLNKANSVKSFLEPQLRSATNATFEANYTLTFATPFATVKEKTNDFIAAMTKYGASEAYVEAEADRAEGR |
| MCY4162822.1_bacterium | IIVRRSGTVGKAFRKIEDALGDRGGGPVAGLTVIAAADRDLRALGYCVSQLPRFDCRVTANIVVEYLDGSLSADLAGAATGWRQVEDALLGLADRGSAVGGTLTLEFTPAVPIPYGGPDWDQFRSVVTNNDPGAIEITVTLADGDSR |
| MYE76343.1_Acidimicrobiaceae_bacterium | RVLEAHGSAAAALQKLDDAMRDAGAQHLSQVAVRVSAEQPFRVLGMCVPQLARFDVALKLDATAEFPSGAASIRFEGSAAACTPVFEAFWGALSGASDVAGSLTLTASQADAIAYDGPDFRQFAEVLRRAAPGHVTLSAKVAETAAA |
| MCY3677712.1_Gemmatimonadetes_bacterium | LVVTVDAAPGRAFQRIADGFQDAKREFIYTLKITCEDLSDMRALGMAVPQFPKGEYRVDLQLVAEFADGSVEVRYRGLWDRYKRLRSTLEAQAGEATKVDVKIVLTASYPEGLAPGSRAFQTLRDVMVQLDMGRIEVRAEERPASDP |
| GBD21619.1_bacterium_HR28 | ELISLDGTLGQVSKGLLDVVRDKQLERLRDVTLRLETASEVRAFGLWVAQLGQLQPELEASLTATLQGGDLRLTFTGPPEQYVRYRSLFETPLRDARDLDARFLLRVT--NLVAKDVQQLVLLGEDELAAELGKVRLTAQGYREHA- |
| HHV54946.1_Firmicutes_bacterium | EALEASGTAQQALQGILDRMHDRNVAALAVLQLAVDGDRELRSLGLAVPQLGPGQFRVEVSATGELEGGSIRLEFRGPWERYKDLKDAVVSVLERASAAQVRARLDWRARDPLTPEG--INQVRDVLAHLIPGRVRITAEPATGEVD |
| OQA44952.1_Chloroflexi_bacterium_ADurb.Bin325 | TRFQGQGAAAQAFGQLVDLCAEHKVAHLRRLTITVDALADVRGLGLAIPQFGKGRYVVEQDVKAEFAAESFTLNFRGGWDRYKRFRSLVEAFGSEADELKATMRVAGEFEDGLEVAGAQFAAIRDVLTTLELGRISVEGIPLP---- |
| WP_014555848.1_Haloquadratum_walsbyi | GKTDSPAVVTEAVDTITAESTHEIKQSLDGLTLKLQGDDAWKKGEWIAKQMPEQNVAVELE--YEADDSHVETHFDGTGKMFADLKHTNKP--------SGDQYIEILIKADTSLELNRLDILEDIIPGATTIEMSVRTALELTEDE |
| WP_073234041.1_Desulforamulus_putei | KPLVAAGVPNKAFADLRDLCEKEKVEALASLTIKLKDIKSVQAVALMLPQYQKQNPVIEQDFTVELPDGSLELKFSGGWQNYREVKNFTERLAKSARETYVETRLTFEYPEPVSPHGEELAKMNESLNTFKVEKIEIEAKETKDE-- |
| SBW09990.1_uncultured_Desulfovibrio_sp. | AAVSQEGVLKEALAILWEKARQKHFLAIATLDLKIYDAQDAFTLLAAVASLSTYDKRVQLEVNFETDHSTISISLNGSTSSAQQLKDFLQPQLRAALDKEFSAIFTIYFANGLSLAETAPEDLAEKLTRVGGVTAYVTATAQRQGA- |
| NLJ36704.1_candidate_division_WS1_bacterium | QQFSSDISPQRAFAEMQDWVTDHKIERLEWLQIEVRDRQAWTGMTYFV----ELSIDVTFEGSIELQGGRIALDFAGSGQNYQRVYDFFHALAREQEGVQFVAIFRANLPAPLAPAAGEISDLRRRFADGDVQQIKLRAGAPREGAS |
| MDA8372907.1_Actinomycetota_bacterium | RFSNASMSPSAAADQVAASL---DASARKRLRVKLQENPGSTAWTVLISQSAKVNVLVVVE--LQGSGARLRVNLQGTAEEFKRASALIVEFAKRDVKTEGRVEV------GFVVTGAELVGFMESVKLLELQTIGLDLEEIQ---- |
| MBP6739048.1_Leptospiraceae_bacterium | KIFISEGPLVLALESLWTKLREAKIKSISKVRIKTYDSNSVSKIQQGAINYNLATKTISYNGKISTSIPDFSVNYSGDVGKFSSIRNFFESQIRSGGESSVEGEFQFEFNEPMKLSDSNPENFAKELSSYGGAEVYIEAEVKE---- |
| PSB37932.1_Aphanothece_cf._minutissima | GVIERTDNVKTALTQLAQQVSSSG-KAIKNLTWRMNSGDGFLPM-ALLKAEPGAMVRVEMEGGWSSDGSEWSFDFNGTPEEAAHLRGFIEGQWRRGGEKSLDITYQLRYEPPLRWEDGG-EGWITRLTRAGTATIRLELTAAAEGQA |
| MBY0527363.1_Gemmataceae_bacterium | VRLHAEGTPSQTFQTVTDQCHDQKVKALKRMFIRVEGLRDARSLGLAIPQIGKVTIALDQKMVLEFDGEKFTVEFAGSWERYKRIKALTDALGQEASNASVKLVLRADFDGGLDVSGDQYQTIRDVLGNLNIGRIAVDGEPMAEGQG |
| MCW5609142.1_Rubrivivax_sp. | GVVEAEDVLKAALNRVFELARHNGALTFGTMAIRPFDKGDAMKLLALVKSVPNATKRIELEAQFETSQSEATITFKGDLEDATPLKDYLEPQFRAASDSDASVTYRLHFEPPLSVNEKAQEALVQRLTRLVSPVAHVLAVVAEKRS- |
| SPE38928.1_Candidatus_Sulfopaludibacter_sp. | TELHAEGPLLEALREIWEKARKAKHARIQKVIIRLYDALAIFKVHGAMATLKDATVNCELEAELQFGVKEFLVRFHGPIDKANAVKTFLEPQARSASDRRVEAVYTLEFAVGLPLAGDGPESLTTSLTRYGGGEAYVEAHAAAPEPK |
| WP_091105663.1_Micromonospora_citrea | RIEAKTGQAGVALSNFRDELMDKG-KPLVEMRLTVRAEQDIDLLGMAVAQMPKQPAKVTIDVAASMLEGVIEIRLEGDKAKYQQASSKLGPLLKAAEEIDGTLTLTLDFGEGVTVTDNAFSTVDSVIRGLGPSELTVEGTLA----- |
| WP_142649215.1_Microbispora_sp._SCL1-1 | KTFTATGPGGVAFQHIVDQVTDFTIQTISRLTLKVTADHDIDLAVAALGMLQKQSITVRSTIRAEYLNGGLQFMGTADRMDFQSTYNHVKKALVSATKVAGDLTLNITFQPAVAVNDAVISQIHTVIKNLQMQQTSMTAEVTK---- |
| WP_164002142.1_Pyxidicoccus_caerfyrddinensis | QPVVQVGTPREALTAAWDALATARNVRIAELGLSVEDRESFDNLRVAWADRPKVAVHATLN------GQAVNVTYEG---RFETLKDLLAPLWRSQGELQVTITVALKFAEPPAVDDAALGTFRTALMNAGQGRVEVRLVPVRPRKT |
| WP_168134876.1_Sphingomonas_corticis | -VFVHEGVLKEALTVVFQKARSAKAVSLASVTISPFDYADAFKLLPVAEGVPDATKHVEMEGDLATAGGELSIRAEGTLTEMKGLREFVEPQLRAASEKRLEVVIRLDFANGLALDGDAPERIVDRLTRFGAAAAFVEASAKVNS-- |
| WP_199338599.1_Nostoc_sp._FACHB-145 | KIFKAEAPLREALSKIWEDARKAQIKKISRLSLRVFDVSDGFKLLGVINTVSGADKQVKLSAEYETNSSSFSIEFEGQVSDAQPIKDFLQPQLRAASETHLEITFTLTYANGLELNTDEPEKITEKLARFATGAAFVEAYAEA---- |
| WP_233194430.1_Haloferax_marisrubri | SHTSNTASAKRAFSEVREEAGNNATPTVESVSIEVSGDEVLKHAFFLA----QTTLHTTVSMIYRAGDDEFKANFNGSLASFNTLNQRLEGFADKSGQRNVDLTFTLSLPSSTEDETDVLAVLRDKLSG---TDVTLQVEGSGDIDT |
| WP_239638853.1_Halorubrum_californiense | VSTDSPSAITGAFSDVNSASSSDIVVEVDTIEIELEGDQVWERAWFISNKLKDLATSIVFEYVAQGSSSVFEAGFEGSAAEFADTNHIPESFISAGGDSEGEAVLIIDTESQDNTREATLDVLQEALEGLGNVELVVSGVVKEASEQ |
| WP_242016564.1_Pseudanabaena_sp._FACHB-1277 | EIIDLSGTVNATFTGFSDRCADYKVKAIHSIEIMIDQVTDYRKLMTAFPLLSKFVFEIDQKATIQMDKQFVRFDYQGGLRGFQSFSNPINALLAEGVRANVSLKLTFTFDPAIAPNGNELQTLQQALMRNPVDRLSLTTRVSY---- |
| WP_257121428.1_Dolichospermum_heterosporum | ETFNAEGTHNSVFTQLADFIKDNQAEGIELLEISVAEVMDYRKLLTAFPLLMKLPLQIDQTASITLGEQFIRLEYQGPYKGFNSFQNTLNTLLSKDVKADVFLKLEFKFLSPILPEGTEISNIKSTLNRNPVDRLNLMAKLTY---- |
| WP_285255572.1_Halobacterium_salinarum | RANDKPMGVLRALTELRDQTFENGAPVLDDLDITFTGNNAWDDAYDFV----DYNPFADVEGTDEFRGDEVECTASGDVEDMPSLS-------QNGGEQQISLSTTVE-PDAIVGDTSLLDTLIGSLEDGSRGTVIVEAEVHNVPEG |
| consensus/100% | ..............h..............h.h................................................................................................................... |
| consensus/95% | ...........h..h...........h..h.h.........h...h.........h..p.........h.h.h.u....h...................p..h.h....s..........h..........h.h............. |
| consensus/90% | .......s...hh..h.....p.....l..h.lph........h...h.........hp.p..hp.....h.h.hpG....h..h.................ph.h.h....s.........h...h........h.l......... |
| consensus/85% | .......s...hh..l...h.p.....l.pl.lph....sh..h..hh.........hphp..hp.....hphphpGs...h..h......b.........ph.h.h.h..sh...s.....h.p.h.......h.lp......... |
| consensus/80% | .....pss...hh.pl.p.h.p.....l.pl.lph....sh..h..hh......p..lphp..hp.....hphphpGs..ph..hpp.hpsbhp.s...phph.hphph.ssh...s.....h.p.l.....sph.lps.s...... |
| consensus/75% | ..hp.pss...Ahppl.p.h.p..h..l.plplph.s..sh..h..hh..h.p.p..lphp..hp..ssphplpapGs..ph..hps.hpsbhp.s.p.phph.hphpa.ssls..s.....h.p.l..hs.sph.lpsps...... |
| consensus/70% | ..hpspush.pAhppl.p.h.p..h..lpplplph.s..shb.h..hhs.h.p.s.plphp..hp.sssphplpapGs.pphpphpshlpsbhp.usp.phph.hphpa.ssls.pss..p.l.p.l.phsssph.lpsps...... |

# Type-2 BR-system DUF499-ATPase C-terminal RRM/Ferredoxin Fold Representative_1

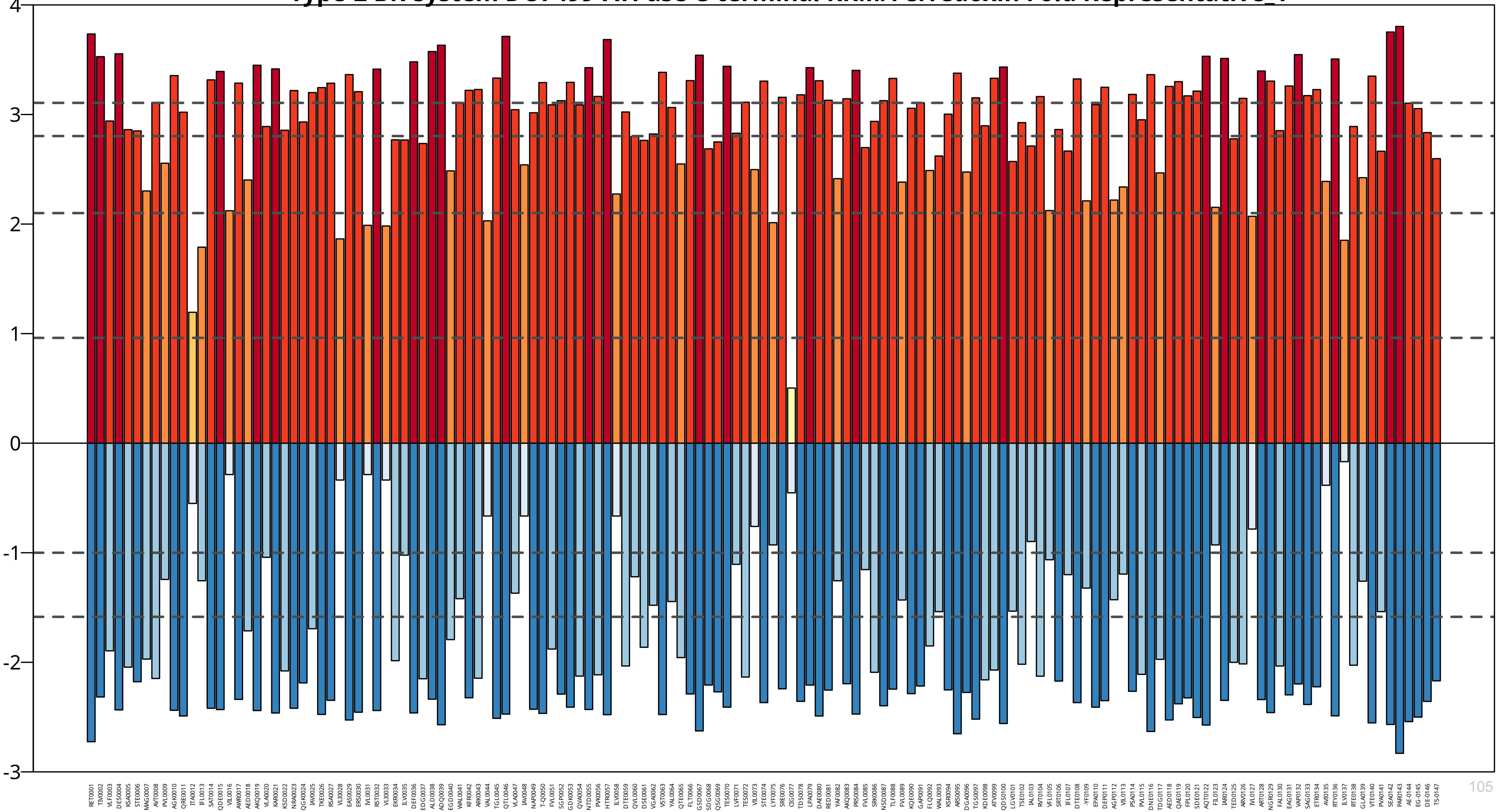

## Type-2 BR-systems

# ; DUF499_2 C-Terminal RRM Rep_2 (MAIN_FIG)

```
AFM14204.1_Turneriella_parva_DSM_21527              ESKAAIVIRRHKFDGTDKCYQLIESMRRYQISVSQVEVAIEAADTFVSYSTGGQIIRDATKLSELIDNLRKPFIEEYRIRLMIGEMHYRQGGDIQHFAGEMNLPGEPHEVRQ
MBX3108928.1_Fimbriimonadaceae_bacterium            PTKAYDWDRKFDKDSTMEAYEFLSLCRKHDVGMRGVQANADISHRFAEFLFDAKFPYMSDNVQSVLDLLQS-LLPESKLSLRVSGLNFANGQAFLDMLAELKVQLPPHEVKE
AGC68793.1_Thermoclostridium_stercorarium           PAKPVKYQPRVRFSDKVAYYYEIQKLKSSNAKPVKIYLDIRGSKREMTVILSEGEGIDIDLLQNLVENLSTEIIRDAQVSGRFETMRFETGKDFLEWLKLQNKKIESGEVEQ
EAZ58601.1_Pseudomonas_aeruginosa_2192              DAKPAKLNKKVSLDSTEKVFGVLNSFKSSPATFKGVRIQIGEGENTVSINFVER-EVTASVIEAAINGLRKALGNEETVTVQIRSAAFENGFAAKEFAKLSGVELRPGDVIQ
MBK7550235.1_Syntrophaceae_bacterium                RSKAAVWRCNLKKDSTGETYRLLETAKKHGAELGGVRLIIAREGRWIELNSPDDAYHAVQHFEHGADLLKE-FIPEGAISIDVGTLKFDSGQLLLEMVADLKTELKEGDVRQ
MBK8067117.1_Rhodanobacteraceae_bacterium           LNKPVAFQRNAQITSREKVFTALAKATERNIEFTDVVLRIQEGSAFGQFGLQGQ-SAKAERVQQALTMLAVGFSPSTPVTMQFRA-QFPSGQDLIDFAGTLELSY-AGDWKE
MBR2257284.1_Blautia_sp.                            DEKGLEYKSMKKCPDTASAYSEFGRLQKLAGTYIRHTVVITEADNYMEITTARV-PYDASNLQATVDIIRESAFANVVVEFEYKSILFTKGAIFKQWVDMNKLDMNTNEIIQ
MBS0652531.1_Verrucomicrobia_bacterium              PLRPAWLNQNNGTFADAQAWKLLAEARTLGATLYVRSVDLKDGSKTLALRALDAWETDPAHWEEAIQLHARHFGDKPILTLQISEIRFQTPQDLELLAERLQLSYKNAEVKQ
MBX3024939.1_bacterium                              PAAPARLAKLVSAPSTAEAFRVLAQARRDGIRLSEVSLEVGSGTRTASLRFGRDGVLTAEQLEQVIAALRAALGEDAEVRLSARRLEFTRGHDLSEFVKAWGLSVDANEVEQ
MCA3288087.1_Roseomonas_sp.                         AGKASSLLKRVERTDTQGAFELINQIKSARAQAFGAILTVGLGDASAMLRIGRDAGAEGAAIERAATLLRELVSDEAPVTLRLDRLDFPTGADLLALVDALHEPVEPAMVKQ
MCF7997122.1_Chromatiaceae_bacterium                TTQPATWASGFSFTTTRTAYGFVERLRRRNGKAAVTRIAVL-NNCWVDLNLADDVLLDPDQIQTTIEHLRS-LLEEGEVSIEAPSLWFPTGQDLLDYWAQR----EAAAVEN
MCK9217630.1_Firmicutes_bacterium                   KDKALKITEPVNYSNATDLYRELELLEKFNVSLSGLTLVVSDNKGYVQLMIGGGFVVSSQKIINELNHILNNLFEGFKANLTINEIEFKKGEDFERWISAKKEKIEKGKIRQ
MCL6686250.1_Akkermansia_muciniphila                PDAPARLDAAGYKSPGVSAAKALDVAEECGVTFREAQLLFGSGEQSVQLKLGRL-EIPAGRLKGLAAAILAPGDGGAGMELRFKTCLAPAARELERFLKNAAIAYQPEELRQ
MCP4350268.1_Desulfobacterales_bacterium            KDKALTLARRIKTNDTSETYGELNQVKKHSALLSQVFVTFYKGKGWIELSFDGTTKVDPKKLEIEMDNIRSNFMNEIDISLEYTGICFRTGQNFLDWVAEKKLALNEEEIIQ
MCX6251131.1_Bacteroidetes_bacterium                KMIPLTWKVRLKRDSTSESYKLVDALKKTGAKVSGIDINSH-AEDWVSLQTSPEMTFEASLVEEYLKFIQDHIYGKGMLGLQIDACHFETGQKFLDLIRELKTDYKENEIEQ
MCZ8170923.1_Brevundimonas_sp.                      NERPAKAKRDFQFASKGEVLRVVRAAKGKSVRFQGVSVTVGEGERSLRVRSGGDVSLSGADIETMIEGLRAALGKPAEVQLRFREVDFPDGYTLKDFATQVGIDIPVEDVEQ
MDA7889828.1_Akkermansiaceae_bacterium              PNIPATWKHTLTAATTAEVYNLLDHLQSCNAKISDIQITAMKGADCAELMITGPTKRAILEAKDTVKFLQD-LIPQAEVNIDIKASYFELGSELKKVLELLQVAAKAEEIKQ
MDD2765364.1_Opitutaceae_bacterium                  PDKPICWKPKEKFDASTG--RLVALLGQRNARLKKVGMGLAVGRSFGTIEFGDA-EVSAGFVDRLASMVRE-ETGATVTSLRFGEARFEKGEDLVALVKEMNLDLPPEEWLQ
MDD5268641.1_Methylococcales_bacterium              PSKPLTWNTHQGTLSAKAAFDFIERLIKFEGTVQGVIIDVFAESASLSYSAEGSTQYTGEEIKTLLEKLQG-VMKGSQVSISVEDVKFQRGQLLIDWLAEVGRQLQPGEVRQ
NCB71009.1_Clostridia_bacterium                     KDKPLVLSSVIQTGSTKQTYELMSNLIKVEASINGLSTNFSIGSEWFEFNSGGR-DINASEARNVIDSIVSLFGREVSITTQIESLKFPSGERFESWIAAEKKEITTMNIVQ
ORC33988.1_Marispirochaeta_aestuarii                PDKPLDWKTEYYNIPQTDAYSVFEKCTKYNARLANVFMTIIHSGETLSYTLPEGIGKTAHELSSLMAALQE-LIGEASVQVAIGKISFETGQAFLDWAQEEKFTPDPQEVEQ
PYG00798.1_Thioalkalivibrio_sp._ALE21               PHQPVRLQNSVRLDTTQSVFQVLVDFKDRNVSFTQVRLQVGEGEDAIEVNFNSR-AVTPAIIESTIESLRKATNDSAEVQMFIRNGDFATGEDLKRFAEIAGLQLKHDNVSQ
RDE52523.1_Candidatus_Accumulibacter_meliphilus     PDIPASVNGTAHLVTRSATYHLLGSLPADRLQMVQVKSTVAATDTTVTMTWDSKTQLAPQAVMEAFEFLDK-QVEGGEWSLRFKHLHFATGKSLLQWQVDTSSKIELSQVSQ
RQH12683.1_Bradyrhizobium_sp._RP6                   PDRPVVIKKRLKIASTKDVFVVINALKAAHGKVRGSSATVGQGDVNATTRFGPKTELSAEILEGFLSAGRAALANEAEVEVGFSEVQFSTGREMEDFIAAVGWEVQPNEVEQ
TPG55477.1_Roseomonas_nepalensis                    EAKPATLVLDIRQSSTEDFFSLVTALKAASAEAVVPQVTVGESTAALSMRLGGDLRLKGEGLSAVVEAVRASMGRPATVTSAVKSVSFPTGRDLIAFVDALKVDVDPRCVTQ
WP_005488051.1_Halanaerobium_saccharolyticum        RDKPVKLTEPQTPKGTKQTYEELEFLNETNATFKQAQFIITGGDFSITFMIDKVEIDDMNMLEEQLKNIKDNFVGEHDLTANITGIKFKSGRDFLRWLEKNEFNLEKNRFSQ
WP_012692622.1_Deinococcus_deserti                  PVTPARWARTHKTTATADSVKLMDRLEKHGAKAAGVKVMVDGGDQYGTATFSEGAAFTADALRELSEYVRKLVGPETNLHLDVKEIRFERGQNLSDWTAETGDKLNMSEVAQ
WP_018962942.1_Coprothermobacter_platensis          QAKPLLFSPKITAPSLRDLRTLVDTLARYNASIAEAYITADTQNATIYLRTKEGIYQKPDQLFDIVEKMSDLLKTDVNISAQIRTIYFEHGRDFSSWVNGQNFKFEDSEWEQ
WP_045169275.1_Caldicellulosiruptor_morganii        YEKPVRLKIAIQLYNMEEVNREIDLLRSFGGKPVKYSIDIYKDDNYIVITNNIPKGIEADEFLRLIEKVKSEFYNNQNVAAVITELYFEDARNFEEWLKQKGKTLQKEAITQ
WP_085411740.1_Fibrella_sp._ES10-3-2-2              KQKPARWNRTIQFDTTQKTYDFLTKMDKAQVNVVLAGAAIT-GIEYVELMLSEKLSFKPAQIQAAIDALRTNLYADGQVTLDVRALLFPDGQALEEYAQELRIDLKSHEIEQ
WP_090663037.1_Belnapia_rosea                       PARPAQWSSRFMFSERRKAFTALECLKRLGGQLGGARLNVQVSTRFMELTHGPAVFRKPDEIEALATDLETKLGAPPNLQLFAARLSFPTGRALTEAAKELGIALDTGEITQ
WP_119720856.1_Methanococcus_maripaludis            RDYPLTITKNEKLNSSQETYRCIDLFEKYCVKTSGNTIYIKGNEKWGQIELGTDVELNMQDLKKLIDVIRDQISNEINVSLRYTKAKFEKGSDFIRWTKDAEKDIEEGHTKQ
WP_171833827.1_Lichenicola_cladoniae                PNRPAKWKARLKFRTTEDTYNAVKAAKDAQATIMGLEINVTSSEAFASLVMGEGLGLPASDMEGVLADLQA-RVPGGEVTLAAPVVSFETGTDLNGFSNAIGQAYDVKDVVQ
WP_190610329.1_Synechococcus_sp._FACHB-909          PTRPARWEKGGKLDDSGAVWDLITRLEQNPAVLAYDHLTAQSGEQIVEYTGSLANGYCGAELKTVAGKLQN-IVQDGTLRMEIGELGFPTGQALIDWLNVVKEPFNLSYVSQ
WP_231170937.1_Bradyrhizobium                       DGLPVFVNYVVESKSTPQAYEIIEAIKAATGSLVKGRIEVMGEHDFLAATFGDTIALDSETIETLVRTLAGKAGTLPTVTLQINSIRFGTGRDFKNFADKFAIDFEKANWSQ
WP_274435612.1_Alicyclobacillus_sp._ALC3            RESPAIWHSPKRLDNSSKTYEGLKMAKEKSIEFEQVMLMVGSAPRAIHLSLGEI-KIDAEFIETELGHLQSLVGSEAPVIMTFKKAYMPTGYDLEQFAKQLGIEIGNGEVEQ
WP_277247933.1_Phascolarctobacterium_succinatutens  NAKSLKLKINKRYGNNADVYQYLSDMKKIGFTATLDSINVADDDCYFDITCSGQ-NLTAEQIENMLDTIRQVVMKDTKVELGIAEMHFTSGQSFRDWIAASKDEIKKGCIEQ
consensus/100%                                      ...s..h..............h..h................................h..................h....h..s..h..h..............p
consensus/95%                                       ...sh.h.......s.......h..h............h........h...........h...h...........h....h....h..u..h..h...........h.p
consensus/90%                                       ...sh.h.......s....h..h..h.....ph....h.h........h............h...h..l.........hph.h..h.F.pG..h..h.......h....h.Q
consensus/85%                                       ..bsh.h....p..sp..shp.l..hpp...ph....h.h..sp..hph..s......s..hb..h..l....h.....lph.h..h.F.pG..h.phh......hp..ph.Q
consensus/80%                                       ..bsh.hp...p.ssp.psaphlp.hpp..sph....h.h..sp..hph..s.....ps..lb..hp.lp..h....s.lph.h..h.F.pGp.hbphh......hp..plpQ
consensus/75%                                       .sbPhphp...p.sss.psaphlp.hpp.ssph..h.l.l..sp..hphp.s.....hssp.lbphhp.lpp.h...splphphpph.F.pGpshbpah.....php..plpQ
consensus/70%                                       .s+Phphppp.p.sss.psaphlp.hcp.ssph..hplsl..upp.hphphs....hssp.lbphlp.lpp.h..psplplphpphpF.sGpshbcah....bphp..clpQ
```

# Type-2 BR-system DUF499-ATPase C-terminal RRM/FerredoxinFold Representative_2

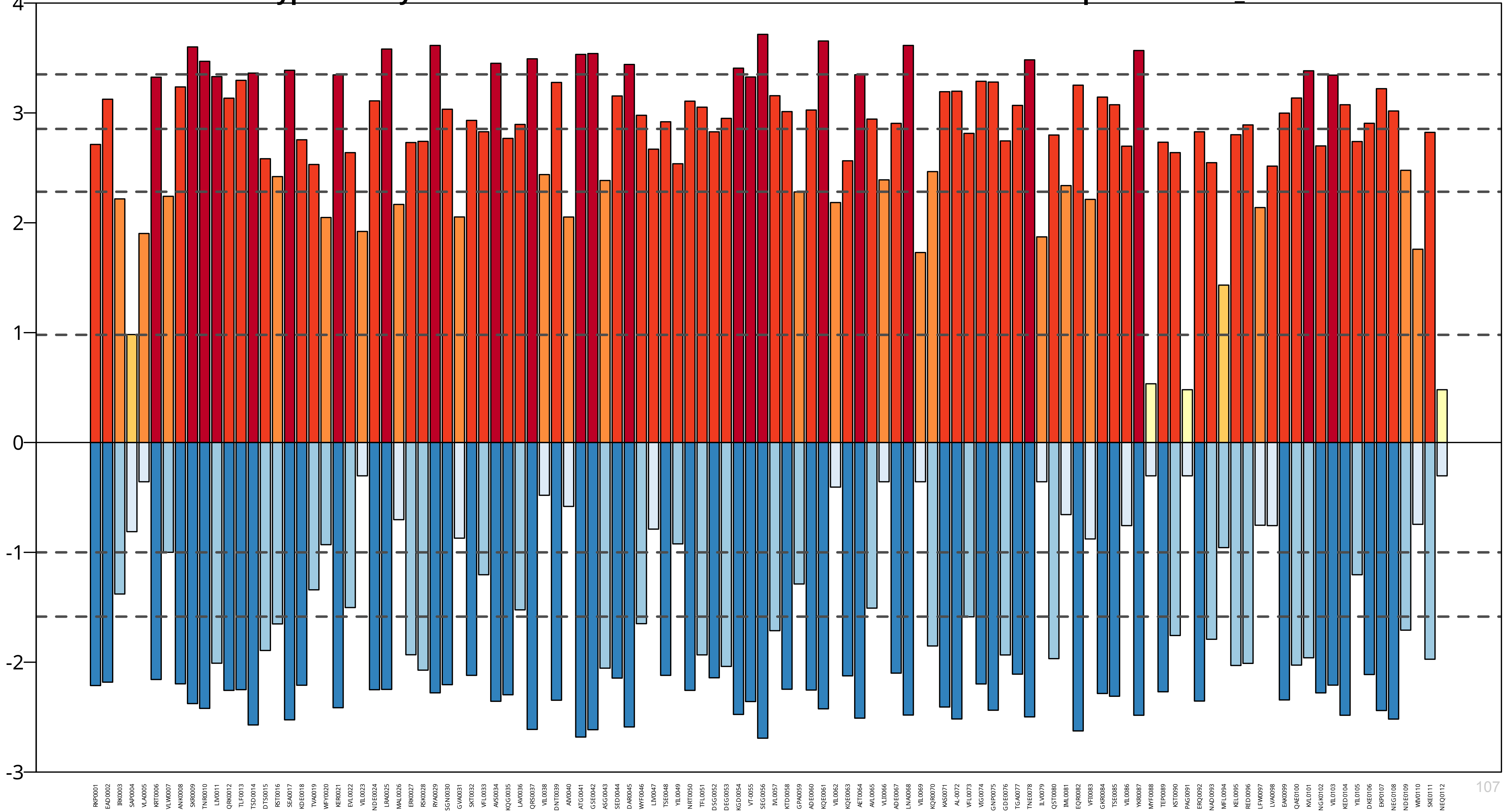

# Type-1 BR-systems

```
# ; DUF499_1 C-Terminal Degenerated RRM Rep_3

WP_148590534.1_Aquisphaera_giovannonii               AGGGTATKP---KMIRWRGTIPPQKWTNFYAKVVSPFALAQSLTAEIVIPADAGLSSAQVEKIRAALKDLKLDEQDLS
MBQ9433643.1_Synergistaceae_bacterium                ------TPAKLNRKCKVDAGKFQRDTRQIHDNIILCLQESPSIRVDIYMETPESISAAVESAIRDNIKSMDIDYC---
WP_054571357.1_Frankia_sp._R43                       VDQGPALPHDTVRTAQISAHFDRSGLFRLVRALSK-LRDVDRVEVDVKLRVEGELDRAEFRSVIEPIDEDGVDIQQLT
EEO54231.1_Bacteroides_sp._2_2_4                     -KSEHTAPSRKFKSIKVSGKVPVERWTDLFSSFVVPLKNNGEISFKAKTTSLNPLDESAYKVVKESAMQLGLNLEEE-
MBM4287682.1_Deltaproteobacteria_bacterium           --GGGAPPPAAARPLQLQLRVPNPVTANKLLALAQPLT----LDVTVTGPLKDGGTMNFAGEVKPTLQAIELSLNEAR
MBI3467145.1_Planctomycetota_bacterium               GTGGGSTVARRVSKVRVEMSLPWENWHDVYNEVIQPLANEGFCQVVVIAKGDAAIRENTVEGIRESLSQRDIEAD---
MBI4291280.1_Betaproteobacteria_bacterium            PLPQPEPPSAQPRSIG--------AKGVNLLGELEKWLP---HASLTFNGVSKE---------LRDLCMRL-LQLTEED
WP_259054315.1_Salinibacter_ruber                    GQEGEEEPLV--HRFSLRADIPTRKLTDLNRGVLMPITRDATFTIEFEVASTDGISERKIETVMETLRQLGATVEKDT
MCX5685413.1_Planctomycetota_bacterium               GVGGGTTPVVKTQRAD-----EPQAINTIVTAGDTWVNTILEAWTAITATSAGGAGRAVIDSLRELLSEVGLDYKEEK
MBA3936148.1_Planctomycetota_bacterium               PPGGPQTPPTESRR-------SPPAKTNLLSWIEQQIE----ASLRLTGVSIE---------LRKLLQSL-VEVQQGG
WP_286167649.1_Ochrobactrum_sp._3-3                  PGDSGETPSQQLTTLS-----NPATPLNLIGKLEGWIG---EVMLKVPAATAQ---------LKELLKKL-LSLD---
MBQ9726423.1_Kiritimatiellae_bacterium               ------QPPQAFRHYALDPQLAVARVAQIQDELLRLFNAKPSVKLDIEASSATPFDPNLVRAVRENASPSNLNLSTE-
CAI1753142.1_Serratia_quinivorans                    SGTGSVSPQSQKTRFRLDAIRAKMDFANIMDEVVQNFASQYEISIEINARSEKGFDANLQRAIKENCSMLKFGNAEE-
MCC7203047.1_Nitrospirae_bacterium                   TPRCGQRPCSLAFRLEYDG-------AEITKATYKIFQKDGRGNLEISITKNGRFVKN---QIEQQVESLGLIVENK-
MCL0064885.1_Dehalococcoidia_bacterium               KEEKQIEPKIEIVGIRWKGEVPYKQLMNFSTRVLSPFA--KSLQIDFEVYPPEGVSPEKVQEVKQALRELGLDETEMV
MCD6101177.1_Candidatus_Marinimicrobia_bacterium     PEEETEKPEGKITQITYTLKVNRQQLYKCFNALGNLVE--KTLSLKIEAQTKEGIDTNWLRAVEEPIEEAGVEIKEEK
WP_116051372.1_Pseudonocardiaceae                    ATSGAATGSKQAASFLANSG-NSDAFAALFKTLYMALDERQQGTLQLVVDADD---------LQQNMDELGISGTDI-
RME04985.1_Planctomycetota_bacterium                 ELEGERRVERVRRDLELVLGVEVGQMSTVSRVVRY-LKERF--------------CEAEYEKVLEALYQEGIRVEG--
MDE0129995.1_Gammaproteobacteria_bacterium           DSGAGGRPGSGSREITFSFVADQRSLYQAWNAMANLAD--LAVAINAKATAPEGYDESKVEGVLEPLRELGLLDEES-
PJF35651.1_Candidatus_Thermofonsia                   SEVHTAQPLIVKRRYKLNPLHANAEMSSVVEEILKHFLNFSELELEISARRAEGFDESLMRDINENSRSLKFTSYEED
MBQ3918676.1_Oscillospiraceae_bacterium              DDHGGETPPQKKTRFTLDNTRINRDVQNLVDEVISHISA-QKITLDVEAECSEGYTQPTIRAVNENCKTLHVKTFED-
WP_157574125.1_Nocardia_jejuensis                    VASNSGKPAQILATLANDPS-STDLLAQVFRRLFDLIDREAQGTMQLHLPSDD---------LATLIRELGIAVNDK-
MYJ80998.1_Acidimicrobiaceae_bacterium               VGDSTGQPDRHWQTLAVNPDDPDADLVHLLRELSNRLEEGNNQTTQITVTGQE--------AIEDLAKEAGTAVNQL-
TME11450.1_Chloroflexi_bacterium                     IADIQELPVPKYHRFIIDERRMGTQAGNIMEEVVKHLTSLYKVTLEIQAELQNGISEATVQTVMENCRTLKFESFEEE
WP_247297741.1_Bradyrhizobium_sp._179                --DLPLPPPADKRRLQLRISCVSAGDAQKVLGATQPLQ----IEAELTGDLKDGGRLSFVAEAKISLQTLGVVLEAR-
HEC67877.1_Candidatus_Desulfofervidus_auxilii        KTEDGKTPTTLPTTLQFKAGIPWDKLHTIIAGIITPLKDKGEITLEIKAHTSTGLDRTTLDKIKETLLQIGAKIEEE-
MBR0288712.1_Selenomonadaceae_bacterium              -GDAGTDPSLLPKHFELDKTRLNKSFNACIDEVASYLMHLPSIRLAINISAPEGIPEDLKEVISENCHTLKVRNFEN-
MDD3269499.1_Syntrophomonadaceae_bacterium           PMPGADRPEPVVKR-------SEEKILNLGGEFDVWME---QARLEFNGLTQD---------IKQLLRRL-LEIKE--
MBM4285998.1_Deltaproteobacteria_bacterium           PVGTGPTPPKQPVDIEWRGTMPKEKWNLFSHRVLARLSTAEQIEVTVKAKVPD---PTVKQQLNMALQDLDLEGE---
NJK30457.1_Acaryochloris_sp._SU_5_25                 ETSSTSYPQRQINRFSLDAYRVGKDAGRIAEEIIQHLSEVAEVKLDIQVQFSNQPTDQVIRTITENCKTLKFDDLESD
WP_048201423.1_Methanocaldococcus_bathoardescens     TIQKEDKIKPTINELTLEVKIPRGKLSEFYRYVMRNLENVFEITIKIKATNGN-LKKSDYEKIKETLNQINAEVLEDI
MBF0121417.1_Desulfobacterales_bacterium             FVRKELKVQIRKKSIRWSGEVPNSKWMVFYNKVLAKQVSKSKVRVSFEFETEESEQEASIESIKSELNELGFNTEEI-
MBN2391962.1_Anaerolineae_bacterium                  PPEASVSPSQLAFQFDAGD-------VRVLRVRFSIVEQSRRGSLEIVLTFDGEYAKS---AVEQMAEQLGLTLQQNS
MCS6856320.1_Sandaracinaceae_bacterium               EVSQESKSQGRLKKYELSAVIPAGMLSHFAKGVLMPLQMAEELEIHLRASSEVGIPLYVIKQVEETIRQIGGKIHEGQ
MBT7692838.1_Gemmatimonadales_bacterium              EGDGGGKPGTAKHQFDLDPVRSIKQLG----EILEHVGAKLEISLEVRAKNAEGFDDRTRRTVSENANNLGANEAE--
MBU2110960.1_Actinomycetia_bacterium                 -SNGPAAPAVERARYEVDASDADARLREVIDEVVRHIVSAPSIVLEVSAENSDGFSESVGRTVRENSRVLGFDKSEDV
UGS34698.1_Solirubrobacterales_bacterium_0166_1      PQNDPPAPQGDATGLALSITATEDDLFTLSQALGKLLLGEGGLQIEVRSATGEAIDRLKARTVVEPLEENGVTLSQTR
RIK37466.1_Chloroflexi_bacterium                     ASSSRTTPR---QSLEWTGELPHQKWMNFYMKVLTRFAGQHHIRLNVEISAPSGVSTQKIAEMQAALRELGLDDKE--
MXV84967.1_Candidatus_Poribacteria_bacterium         RQSGPETPPLPRKSIGIRFTLPQGKVSNVAQHFNQ-LQTNF-MQIELKASDGE-ISQDAYEELKENLRALGIEIEEI-
MBC7327984.1_bacterium                               KMKEEISAPPPLKEIYLKLQVPFGKLSDVIKMVNL-IKSKYEMFMEVKAKQGEGMTNTEYENLKEGLRQAGVDIEEK-
WP_013295752.1_Methanothermobacter_marburgensis      RVDGKDMPTGHIKWIELEFTTALENMSNIIRIKRT-ISEDFVAEIRIVARDGE-MDDTKYERILETFEQLGIDLKKVR
NPV88406.1_Coprothermobacteraceae_bacterium          DGGGEGEGEAVVKELELDLDVPVGSFSAIAQIVPF-MKLKFEIHVRIVAHGGS-LTNKDYEKVIEALKLANIKIVEEK
PWV37728.1_Desulfurococcaceae_archaeon               PSERRDKPELTYKELHLELKAKPGMISEIMRLVSSILKPKFTITVKIQAGEGE-ITREDYEEVREMLRQLGVEIKEEP
KEQ56705.1_Marine_Group_I_thaumarchaeote             -GEGGSMPDDSIENLDFGFTIPEGQINNVSTMLLK-IASHY-LKLKVEASDGK-MTKHDLDLIKETLSQIGSDL----
MBC8384565.1_Candidatus_Cloacimonetes_bacterium      KLEKPKQEETKKDHLNLSFEIPKGKVAGILGILNF-LQLKF-VKVDITSTNGE-ITESDYEKIKEALMQLGINFK---
NET07313.1_Symploca_sp._SIO2B6                       SEYKSETSSKKTARFKLNSTVNKAQVFQLFEVLQT-LSDKATIQIEIRAYTNQEFDPNWIRAIEEPLDELDIKASE--
MCX7997938.1_Leptospiraceae_bacterium                PTPQPFTPTSTKKEVKLEFSVPKGKVYEIAKMLHS-LHEKF-ITLTIHSIDGS-ITTQDYEQILETLKQLGIQYREE-
consensus/100%                                       ..............................................................h...............
consensus/95%                                        ..............h.....................h.........h...............l...h...........
consensus/90%                                        .......s......h.............h...h...h.......hph...............l...h....h......
consensus/85%                                        .......s.....ph.h..........ph...h...h.....hphph...............l.p.hpph.hp.....
consensus/80%                                        .......s....pph.hp.......h.ph...l...h.....hplplp....p.........lbp.hpph.hp..p..
consensus/75%                                        ......pP....pphphp...s..ph.phhp.l...l.....hplplph.s.p.........lbpshppl.hp.pp..
consensus/70%                                        ......pP....pphphp...s..ph.plhp.l.p.lp...phplclphpssp.hs....p.lb-shpplslp.pp..
```

## Type-1 BR-system DUF499-ATPase C-terminal RRM/Ferredoxin Fold Representative_3

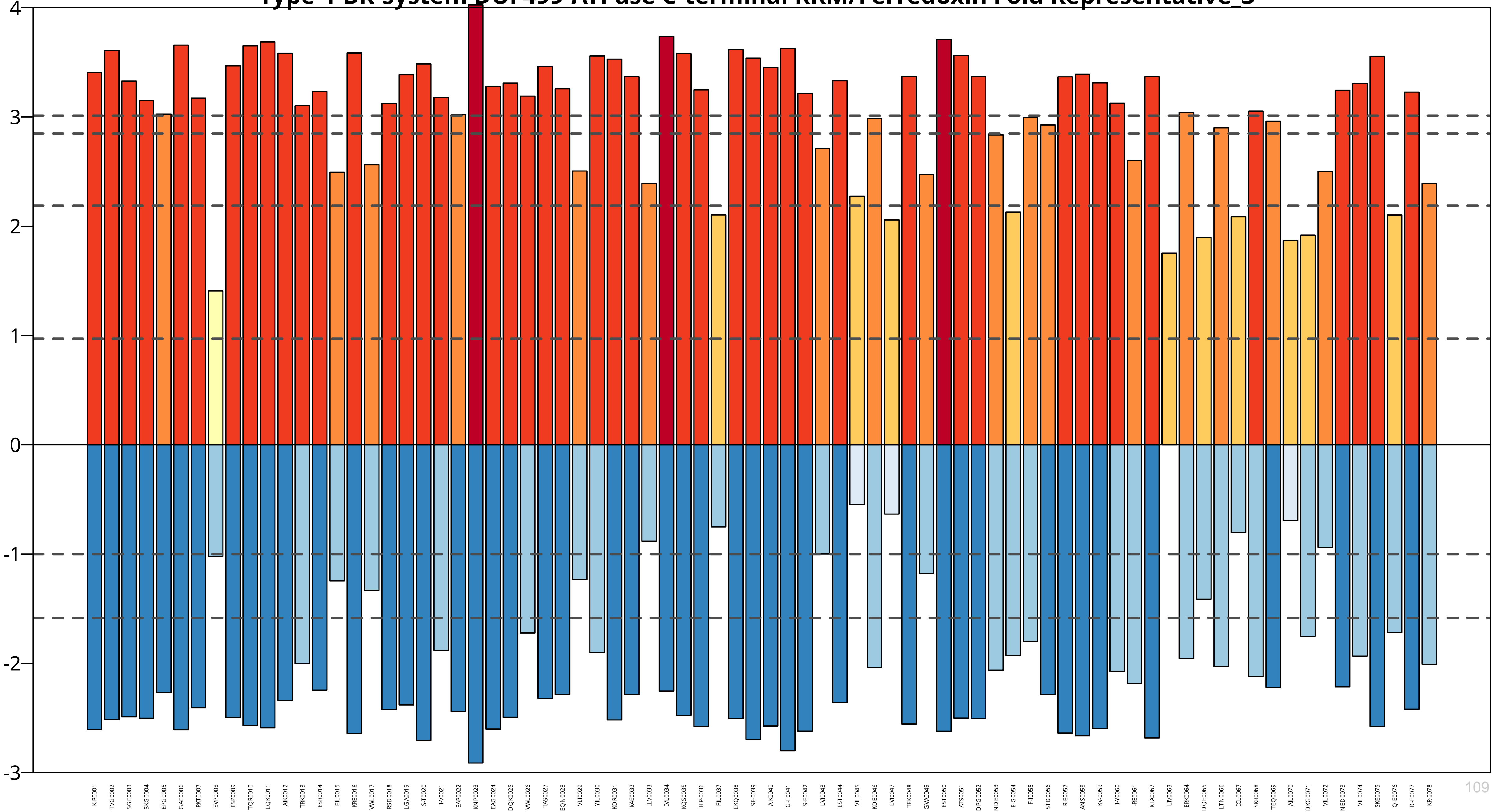

## Type-1 BR-systems

```
# ; DUF499_1 C-Terminal Degenerated RRM Rep_4

WP_244804062.1_Corynebacterium_sp._LD5P10                TTPPRPSGEPGATRFTATFDLPLTGGVGRYAIIDDEVFTHLKAGSGGSVSISLDVEATNPEGFSPAVQKMINGSLAEMSDADGAFE
WP_005520661.1_Corynebacterium_matruchotii               LSGGPTVVELPDTHYTGSVVINSDRYVRMVNNIIEEVIDRLA-GSGADLEITMNIHATKPKGFTETEKRIISENSQTLKFGYYGFE
MCL5447091.1_Actinobacteria_bacterium                    PIGTPGSGSRRPRRFHGSIVLHSDRLNRDFGQVVQEIVQQLQNANSADVEVTVEISATSNDGFDDAVMRTVTENARTLRFRDQGFE
WP_052687173.1_Streptomyces_lydicus                      GISVQPILPRTLNRFYATARLTPEQYSKNATDYVFEILQHLA-LSGTDVEVTVEIQASRPEGFPEDKVRILRENSNTLNLQAE-FE
WP_053587850.1_Actinomyces_sp.                           GGADPEAARPKNTRFTGSVELDPGDIAAQLASLAEEIIVHLRRGGADGLEINVNIDATRYAGFDQATVRTVGENARVLGLRPGRFE
WP_270528574.1_Mitsuokella_jalaludinii                   NGAASAHPGPQYSHFSMTQDLDSLRATKQVNDCLQEVVNHLSDLKGSQVTIKLEVQADLPANLPKDVVRTITENCQALGIKFT---
MCV7506294.1_Micrococcus_luteus                          PETEKSAARVPNARYSGRVEVPGADLPSTFQTLIDEVLSHLQNADPDTLEILVEVHAEKSSGFSTATSRTVGENARQLGFIKTHFL
MCY3894210.1_Acidimicrobiaceae_bacterium                 PRDDGDAVQRGLRVFRGSVKLDVSRPTQAFTRLSDGVLTRVLGHPDAVVDIRVEIEVHNPAGFDSDVIRNVNENTRELDFEEGGFT
WP_238518868.1_Azospirillum_doebereinerae                SPQPIAAAEKPKTRFFATLDLDPVRMMAKLPQVAQGVVSELARPEGARIRISLEIHVDAPDGFPNDVMDIVRDNLKDLGFPAGRFE
HBM97560.1_bacterium_UBP9_UBA11836                       KPLETEKSESLKTSLYASVKIDPNCCNQVVKKILENIVYDLVNLEDCELNLTFSVEANAKQGIPAEIVSAIEENSKSLNLDDFDLH
MBC6439887.1_Rhodospirillales_bacterium                  -NTDSPDPELPPTRFTGTVSISAERPARDMNRIVDEIVDHLKTQPSARISLRLEIDADLPDGMDPAKVRTLRENATTLGFIDQTIE
OZG52261.1_Pseudoscardovia_radai                         PSVPGPAPEPKQTRFYGSVVLDPDQYNRQIAKINEMIIDQLR-FSHAKMELRLDLQATQPDGFDPSIVQRISSSAETLGFTASGFD
MCF6470725.1_Nonomuraea_sp._MG754425                     PGPNPPAPPVRNTRFWGSLKVNPERYSRDLNSLAKDLLALLTAPDDVELEVEVQITARRTEGFSDDTVMKVLENLGHLKVEGK-FE
MXX78159.1_Gemmatimonadales_bacterium                    GETEEEAVEKPPARFFASIPVEPDRAGFVVARIMDALLVELTRSPGSNVRVTLDLEGNAGNGYPKDVVDIVKANARDLKIDKNGFE
MCI1646461.1_Olsenella_sp.                               GGGDRPAPEPRKHDVELEATLDQWSAGSDVGAIVEEILQRLYDLRGSSAHLTLSAEVSVPGGIDDTTLRIVTENAKALGVTLR---
WP_094451589.1_Parenemella_sanctibonifatiensis           GTGASSAAPAVPTRFHATKDLNTTRVVRDASQIYEEVISHFV-ASGINVTVTLDVESDQLDKLTGDQRTAIRENLKTLGFADHDWS
MCG3151370.1_bacterium                                   PTMNEPVAPALPTRFHGSVLLDPTRVGREAGKIADEVITHLEKLIGAKVEVRLEIQATLPEGYPEDVQRTVSENSRTLKFTGFGFE
WP_092673506.1_Siccationidurans_arizonensis              DDNTAKDSGTKRTRFYADANLDVTRFFRDAEALEKEIIKHLP----KGATVTMHIEARNVDGFTLDLQRVLQENMRTLGLGSAEFE
WP_201387653.1_Ktedonobacter_sp._SOSP1-52                AVPVTVKNETIKRRFYGSVKLNPRMMAGDAGKIMDEIVTHLTTLSKADVSITLEIQANIPGGVPAETIRTVMENSNTLKFESAEFE
MBD2550060.1_Microcystis_elabens_FACHB-917               PAPLPPPPAPQRTTFTGSLKLDPVRAGLQMGQFLEEVMSHLQALPGAEVNLSVEVHVKAPNGIDDQTARIVLQNAADLKLDNP---
WP_188816795.1_Calditerricola_satsumensis                VDPPIVPPPPKVRRLNLFANVPPERLIGKSSEIFQEVLTHLLSLDGAEVEVNLDITVRIPNGLPGHVRRVINENARVLGVQVVEYE
MCL1790896.1_Peptococcaceae_bacterium                    SVQPDIPPAGKNTHFYGSVRIDASKLGSTAGTINTEVLQHLNRLTGARTRITLDIQVSVPDGVPDHVAHTIRENCKFLKFDNAEFG
MCY4445744.1_Rhodobacteraceae_bacterium                  DNGIKPIKEKKYRKYKATTTLNPESPNPKFNDIANYILDPLL-RDVANVQVKVEIVAIMPNGFSDETIRTLNENSPLIDLDGEFLK
WP_127367955.1_unclassified_Mesorhizobium                PGGTRPSAPGKPRRFYGSVELDMVRPVKSFDAILNAVVMELQRTHSAKVKLTLEIEAEASDGFADSEVSVVRDNARQLKFKSEGFE
MCZ0940408.1_Caldilineaceae_bacterium                    VDVGSGREEPSLTRFYGQVELDSVRAIRDLESILKEVVSHLR-RAGKKVTISVEVNAEA-DGFDTHTVRVVSENATQLGFTSHEFE
WP_132287513.1_Paracraurococcus_sp._NE82                 SPGPAPNSPKPLTRYFGTAEIGADRAMRDFGTISDAVLAHLVTQRGAKVKLRIEIEAELPSGFTTDLVRVVTENGNTLKFTQQGFE
CAG0999165.1_Planctomycetaceae_bacterium                 TQSGKPVAPKLPTQFFGTVELSPDRVGSQAGKVAMEVLLHLMNQPDSKVKVTLDIEATLPKGADEAIQRTVLENCAALKFVNREFT
WP_151650193.1_Bradyrhizobium_betae                      PLPTGGTRAARPRRFYAKIVLDPNRPTPQVSNIAQSILSELDRVRGIRMTLTLDIDAEAADGFPEDVESVVRDNAASLRITDFGFE
MDE2480824.1_bacterium                                   VAVEQPQASGPPKHFFLRVKADDTKLPRVATQLANDIVAHLASIGGASVEVNIEVKADISNGINAQLEDRLRKNAAAHQFPDPEFN
HDQ14361.1_Sediminispirochaeta_sp.                       QRPVKPAEAAAKKRFYGTVDLNPHAPSGKMGTIAEEVLAHLA-ADGARVKVHLEIEAESAEGFSEETVNTVLENTRTLGFEQKGFE
MYD10935.1_Chloroflexi_bacterium                         PDALLPDPPKPKTRYFGSVRVDPQRAMRDFNQIADEIILRLASLPGADVSVTVEIKCERSDGFDDVTVRAVSENSNTLNFSAHGFE
WP_075830034.1_Deinococcus_marmoris                      PEPKPVVPPPLPQQVFAEGKLDDARMVRKFQTIYEEIIQQVI-DAGGDVSVELVIRGDVRGGLNTTQQRNLSENARNLGLKLQ---
MYA05496.1_Caldilineaceae_bacterium                      IIDPPPPPPPSSQRFFGAVKVDPERLGLEAARIADEVLTHLTTQPGVQVEVSLEINAVLPQGLTEDELRDIRENCSTLRFASFDFE
WP_106140933.1_Spirosoma_oryzae                          GSTTSPNSVPTGPRPIHTVTVHGKVDIANYSQVFQTFIMPLA-QQEVEIEIRIKGKSTTAKPLTETSPEVVIEGARQLGLRVE--E
WP_118201547.1_Prevotella_copri                          TNPLNSDSSENEVKTYSKVTISGSVPMENWVQLFPSFITTLK-KNNLEIEVKFTAKSTEQNPLTENSPTSIKESASQLGLDFE-ME
WP_051018640.1_Saprospira_grandis                        LSVQKVDEELKYTAFQTTG----QVGPKEYTKLFDYFIRPFL-REGDEISIDLSFNIKTGTSFDEKDERRAKEAAKQLGFDIRP--
RKU15614.1_Candidatus_Poribacteria_bacterium             ASVKKDKAKLGSKRIVARKIVEGELSLDDIGDLCQEIIGPLS-TDGGDVTIEIIITAYKEDGFSQNIERSIKENSIQLNIEVQSTN
MCY4474618.1_Chloroflexi_bacterium                       GEGMQPPPQTGPSRITATKQLQGNISFDEVSGIREEIIRNLT-DDGGDVTVTITVSASKEGGFSQNTIRAVRENGDALQLVLQQTE
MDE0020779.1_Candidatus_Poribacteria_bacterium           RRRKELDPPKLPKLFTMTAEAKPDTLKQAVEQLENEIMPSLR---NGEVSMRIIVTAKHARGFDENDARAVRENCKELGIELDEES
MCA9831553.1_Dehalococcoidia_bacterium                   VQTPVPQPATRHSRV----RIRVDMPVAKSTNLQPYLWKVLQ-GVDIDTKLSITVEVESAAGVPEDVLNRIVEGLEQLGIQVE---
consensus/100%                                           ..............................h...hh..h........ph.h.........hs......h..s...........
consensus/95%                                            ..............h.....h........h..h...hh..l.......hplph.hps....shs....p.l.ps...l.h......
consensus/90%                                            ............pph..p..l.s......h..l.p.ll..L....s.phplplplpsp...uhs....p.l.cs...L.h......
consensus/85%                                            ..s..s.s....pph..p..lps.....phspl.p.ll.pL....ssplplplplpsp...Ghs....p.l.-Ns..L.h......
consensus/80%                                            s.s..s.s....p+h.uphplss.....phspl.p.ll.pL....ssplplplplpsp.spGhs.p..p.l.ENsp.L.h.....p
consensus/75%                                            s.s..sss....p+a.ushplsspp...phsplhppll.pL....ssplplplplpup.spGhsps..c.lpENsppL.h....hp
consensus/70%                                            s.s..sss....p+a.uohplsspp.s.phsplhppll.cL...sssclplplclpup.ssGhsps..cslpENsppL.hp...hp
```

# Type-1 BR-system DUF499-ATPase C-terminal RRM/Ferredoxin Fold Representative_4

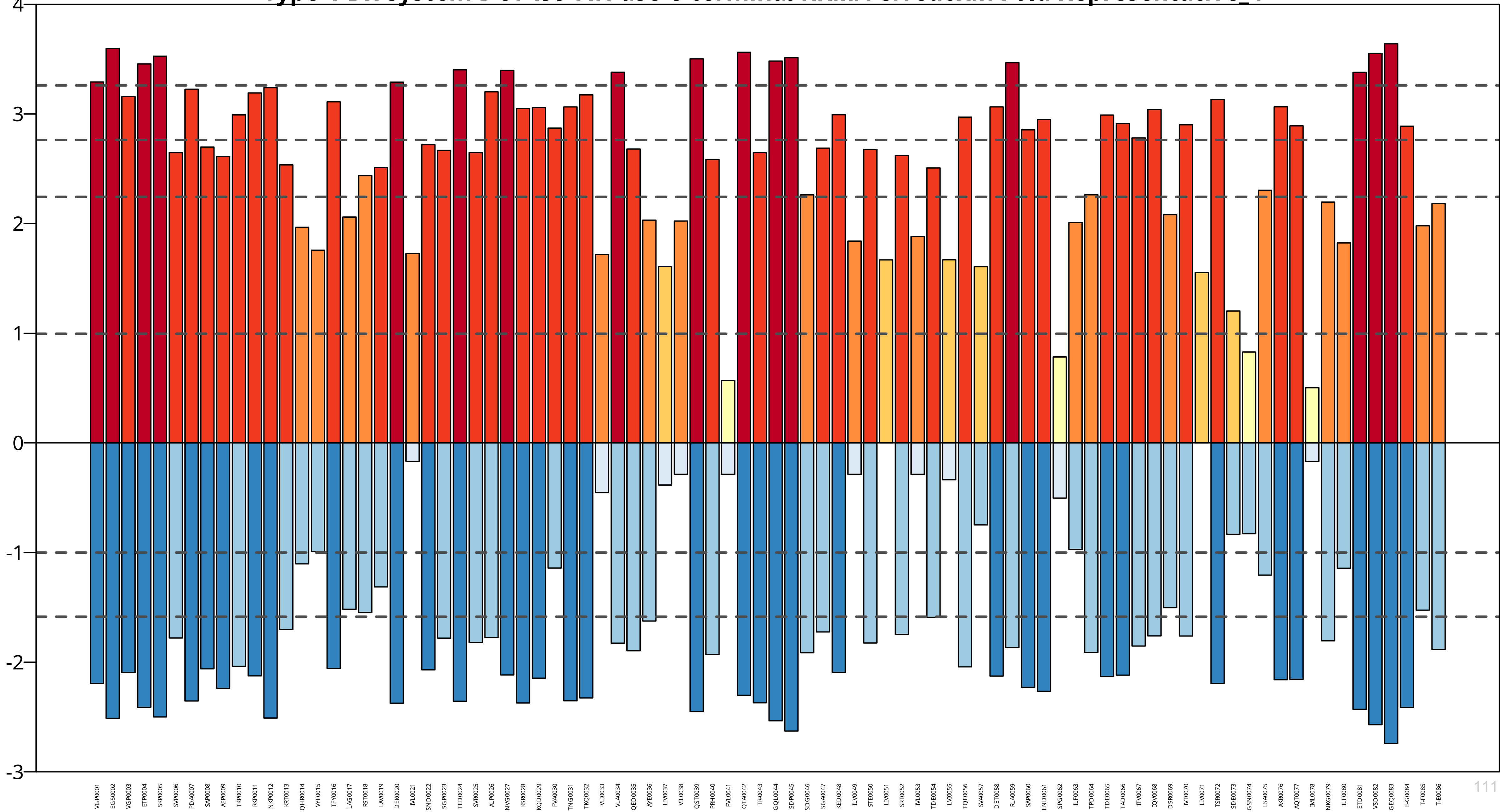

## Type-3 BR-systems

```
# ; DUF499_3 C-Terminal RRM Rep_5 (For MAIN_FIG)

CAD6492704.1_Candidatus_Argoarchaeum_ethanivorans             IGDEVAKTPPSKTVTVPPITAKPELIDKIDREINSGDILKEIDLSISGVDKNGFISFLRELKTPKIATFEVQMKVVRG
HCF99178.1_Chloroflexi_bacterium                              TPPTGGTSPAAKTLKSDDARIGLNLLGDVAKWGVQDREILSARLALEGLSYQELKELLQRIPPKLKASLEIDVEDQTP
HIE29204.1_Candidatus_Poribacteria_bacterium                  VKEGGSDEPTTETVQTAIFSNIGGLIDQLEKTVATTGTIRGVTIKMNTYTKNELLQNLNKLPNIPSGEIQAIVKVERN
HPN33675.1_bacterium                                          -LKTTSEVPQQSKIQTPFTNSLGQLRQEVAGRLLDADQITDLEFRIFAFSKAQVEQLVEALPVYQGATYKAEMSVLDQ
HUT59382.1_Phycisphaerae_bacterium                            SPSVATVSPAGETISIPFKSSKGLLRQEVASRLAQEGQVTSVRFQIFQLSKAQVEQFCEQLPDFPNAQYMARLEITGA
HVA45320.1_Pirellulales_bacterium                             APLAGGVQPAGVIHRSLGAKSGINLLGDLERWALPGQRVIQASLTISGATVKELRELCSRIPAKIVAELQITLPPEGD
HZM03188.1_Candidatus_Saccharimonadales_bacterium              TPAPGGGQTSGVTRRTDSESNTISLIGQLELWGVTDKMLTSAKVEFSNLTAQQLRRILQQIPSAHRANLEVSYQQQQT
KOX91592.1_Haloarcula_rubripromontorii                        GGSSEEDGGSSKQVT----PSARVLRSHAESRVNETDTVTHVDLSYDVLSKEELIELVEQLPGANHIEATVVIEREVQ
MBA3936148.1_Planctomycetota_bacterium                        QPAGGPQTPPSTESRRSPPASKTNLLSWIEQQRDIAGAPLEASLRLTGVSVIELRKLLQSLPVSVQPSVEVQWTADGQ
MBC7429623.1_Bacteriovorax_sp.                                TPIGAEASPSQKLMRLGYGRAPLQILDQLERWGVSETKLHDVSLVFTALSGEQLRKIIQTLDSDIELNLKLIQEEDK-
MBK6914962.1_Ignavibacteriales_bacterium                      TDNGISGIPGAINISTANFKSIGELRQEIASRLNEENVIENIRFTIFLFSKSQIEQLSEQLPSYQDAVYKATLKGYVK
MBM4461756.1_Chloroflexi_bacterium                            -TTEYERTPEGVPLASQEHTTPFKMVTELEGRLSSGDKVASVTISLRGMAKDELMKLLGKLPVPSEGIIKATLEVEKH
MBM4466103.1_Chloroflexi_bacterium                            KPPEEAEEPKAVVVRTEAHATPFSLQTDVEGKLRSGDRVTGLEVTIEGQQKQEILKLLDRLPIPVDGQVRATLEVESS
MBN2391962.1_Anaerolineae_bacterium                           EPPEASVSPSVRELAIRYQPTPGQLRQEIAFQFQDGDRVLRVRFSILVYAKSAVEQMAEQLPAIPGGEYTATLTLQAS
MBQ8111585.1_Kiritimatiellae_bacterium                        NPPTVLVAPKK----TAAPANGSTLQGKFEEWGLAGKKLKSATLSVEGISVSDLKTFLMRLPSKYKASMEVEEEA---
MBU4312937.1_Candidatus_Omnitrophica_bacterium                EPQIGTVTPAQQTISVPSQPGIGALRQAVAVRLQESEKVIKAVFTAYLFTKGQIESHIESLPNVKNAEYSARLDILIS
MBY8216331.1_Vibrio_fluvialis                                 EPTGNGSAASGKTVASAQNTSTLSLLGKLEQWGINGSKVKDVKLSTSQLTGAQLEHLLKNLPDGVTYSLDLDKEQ---
MCE0498498.1_Methylacidiphilales_bacterium                    SPNPGNYQPLPETDSTGFLKSRQEVRQEVARRLDAENKIVHVRIGLTYYTKAQVEQLMERLPDFTPGACRVTIQLQLR
MCK8825903.1_Fuchsiella_alkaliacetigena                       --ETAEQSNASVEIH----NSNRLLISQVETQLRAADQINKVVITINFFSKEDLKELLLKLPDKEG-SYSLEVRGERE
MCL0058394.1_Dehalococcoidia_bacterium                        EPPPTPLTPVR----TESRATPPGLRAEVYNKLEEKAKVIQFSIDITALSKANIIDLINKLPMPVADTITLNLEVEIE
MDD3269499.1_Syntrophomonadaceae_bacterium                    ENQVGIVMPGDTVVKRSEEKTILNLGGEFDVWGMETRKIAQARLEFNGLTVQDIKQLLRRLPPTTRAVLEIKLPGGAE
MDH4162607.1_Nitrospirota_bacterium                           QPAESDVEPGEEERSTPSCRSLGELRQQTAARLTDDDIVRTVRFTIFAMTKAGLEQHCEKLPNLSGASYMARFSVELQ
MDM7274691.1_Thermoprotei_archaeon                            STIAPPPPPPS--------RTLNEFRQRLDLLYHTGQLVNYMAVKADGHEVSHILNFLKHLSEKYKATLQLEIGFAEP
MDR0374944.1_Treponema_sp.                                    VPVLGTMSPPPVRTKSSAPRSSINLIGQFESWGLSDKTINKATLEFDGLSVFQIKQILQRIPIAYQAGLSISYDDIEN
MDR0392109.1_Planctomycetaceae_bacterium                      PP-----PPPTKHYATNDSTSGLNLLGRIEQWGISTTSVSNVKLTITQMSGKNLDLLLHVLPSGVKYELELDTEE---
MDR2561287.1_Holophagales_bacterium                           ASAVTTTGPATTVHKECPATSPLNLLGNMETWGVMNSITEKITLSISCATGAQLKEIIKKLPEDSSCELKLYIRAKD-
MEA3344886.1_Chloroflexota_bacterium                          EGEAVEGVPVSFTIS----LTPLGL-----QELGPQDTVREVAIVVDELDKATLEKLIAGLPEGTAIRLKLKVVRRG-
MEA3401058.1_Armatimonadota_bacterium                         ARAEQQQLPQPKTERNWVHLPKGAMVTAVVQEINKTDLLRNVNITLQKSDRDGLVALLNALPDDESMQVSLSLERMER
MEA3428074.1_Thermodesulfobacteriota_bacterium                TPPICGKDPCELTIRIPPQNSIGSLRQETAFKLQDEDIITRVTCKIFLFSKSQIEQHIESLPVISGADFSVDMEVEVI
MEB3759607.1_Desulfurococcales_archaeon                       LPPQKPFTPATISLE----PSGDDVQQKLTVFIEHKATVNELVFQLETVDVDTLRNLISRLSSKETGTVKVSITLGEP
NLX24424.1_Lentisphaerae_bacterium                            QTGETEKSPVPVTVETPFVHGMGALRQEVAIKLSDADIVKEMTFRIFYLGKSDVERVVESLPQFPGGEYKAELKILRP
NMX21982.1_ANME-1_cluster_archaeon_GoMg4                      -EEDEVVTPPSFKIE----STPWSLQGRLGNTLDESVDVGEVSVTMKRLKGKDVKGIVDNILEEHEDNLRMKLDSKVR
OCQ93485.1_Nostoc_sp._MBR_210                                 TPVGNGSIPPVKSQRTHEQSTGINLSGQFEKWGLAATPLSTAKLEFTDMTVQQLKQVLQKLPPVLKAHLEVSYLEDEQ
RLF82338.1_Thermococci_archaeon                               EVKKKEKKPKIKTVD----DGNINAPIIVESEEIGESGFGHLEISISEIERWKLIKIIRKLSLKELGFEKITLRIERE
SFC23913.1_Halobiforma_haloterrestris                         GAGQIFTCPQDTTTRFPVMDDKPSLIDTLERELGIEWTIHTVSLSYTGLSRDALLDVLLRLDVPDDASFEVWMEVEKG
TEU12999.1_Anaerolineales_bacterium                           PSGQRFPAPPDPSLIHDVSDTVDQFLRDLRARLREKARIQSARFQVSPVSKTAFLNLIGQLEQIEGVLVSVEVSGDVP
WP_011944180.1_Thermotoga_petrophila                          ---TGDDKNENDELS----NSPRVILSQIEARTSEKDEFEEILVEYRFLSKQEIMKFVKQLPFQEDCEIKAKIILWRG
WP_012608229.1_Desulfurococcus_amylolyticus                   -----------VEIQ----DAKSGLLEVLIKYLNRRNSITSCRLVVQLYEKDEALQMVKSMGLSPGGVRLIESREQ--
WP_125670094.1_Candidatus_Methanodesulfokores_washburnensis   KVPEIRRTPAQWTIETSEYQTPFRLSEEMERRIPESVKIQQIKIGFSKMDKKEAIKLIEKLPASLGGIIKASIEVKKV
WP_125672294.1_Candidatus_Methanodesulfokores_washburnensis   ELGKKEEIPPEIDIK----INGEKYRTPVQLKFERGEKVIVDSLEFDGRSVSVVANKIKNIPPEEISISTFKLEMSAR
WP_259101344.1_Candidatus_Fervidibacter_sacchari              TNGERYPEPPDQVLEISVTNAKDLWLGELENKLDEDAQVERVSCEIQCAGKEELLRWIESLPEGMSFVVSTKIEGKRP
WP_260594793.1_Salinirubellus_salinus                         TGTEVTDGPADSTVTFPAMDNKFSLVDKLERRMGADWEVHHLTLKIEALSKRRVLDIVEDVSVPRVATLSIRLEVMKH
WP_283022678.1_Bradyrhizobium_sp._CB1717                      TP-----APASRPRAVESPTSSLNLVGQLETWQVKATRVKNVTVTISDATGAQVEALLKKLPDGMVYGLQLDAEDDN-
WP_286167649.1_Ochrobactrum_sp._3-3                           TPMDSGETPSGRTTLSNPATSPLNLIGKLEGWGIGATPVKEVMLKVPAATGAQLKELLKKLPDGMTFELSLDKEDG--
consensus/100%                                                ........................h..................h.........h......h.................
consensus/95%                                                 ........s...........s...h......b......h..h.h.h.......h...h..l.........h.......
consensus/90%                                                 ........s...........s...h...h..b......l.ph.h.h..hp...lbp.h.pls......hph.......
consensus/85%                                                 ........P...........s...h...h..b......l.ph.hph..hs...lbphlppls......hphphp....
consensus/80%                                                 .s......P...........s...l..ph..b......l.phphph..hs..plbphlppLs......hphphp....
consensus/75%                                                 .s......Pss.p.p....ps...Lb.pl..b....s.l.phplphp.hs..plbphlppLP....s.hphphp....
consensus/70%                                                 .s......Pss.p.p....ps..pLb.pl..b..pssplpphplphp.hs..plbpllppLPs...sphphphp....
```

Type-3 BR-system DUF499-ATPase C-terminal RRM/Ferredoxin Fold Representative_5

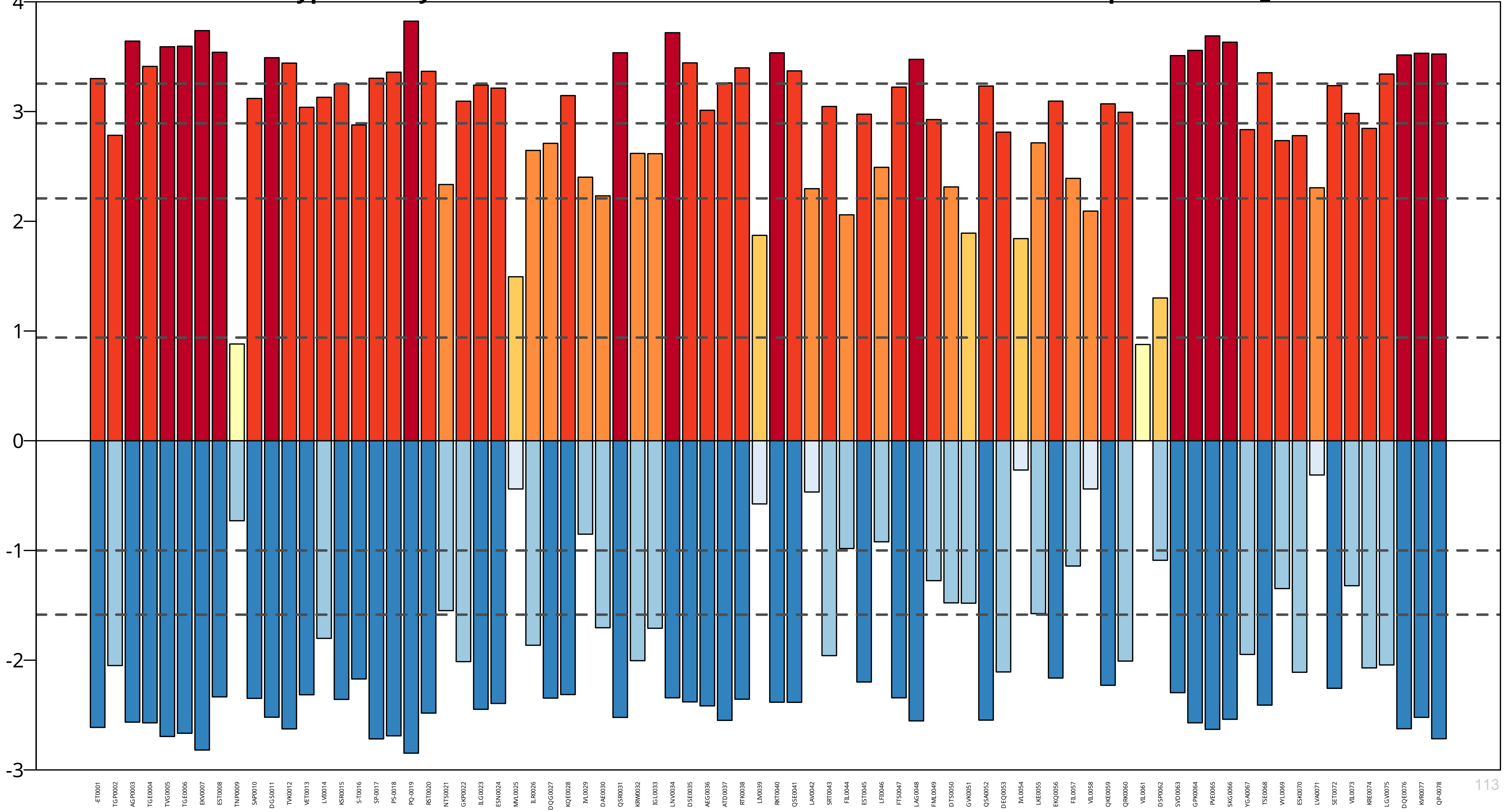

# Type-2 BR-systems

```
# ; DUF499_2 C-Terminal Fn-III + Fn-III

AFM14204.1_Turneriella_parva_DSM_21527            AAKTDVIINQVLRDEETGAVKLRVEPQHGDKVYVDYGADPTTASELIHHGNFETARMEVRFLCVDSTGKHPQGPVRIWKNTIALKYDTTPRDGKVFVTLRAIPDADIRYTTDGSAPRELGGEYTGVFEVKKKCT-VMAIATKEGIESQILSFEADPTASKGARI
MBX3108928.1_Fimbriimonadaceae_bacterium          QPQTEVRIQQVYRDSETGEATLKVTALHGDQVFVEVGGPATPGSRKLDSNDYKTKEMEVSFLAVDTTKVHEAGPAQTWKNTVTLKHRFFQDGEVMRCEVKAAPGGQIKFTTDGSNPTQNGGTYTEPFIVPPGSKFIQAVGTNGSVQSEVLRVSVPA-EPEKLVI
AGC68793.1_Thermoclostridium_stercorarium         KAAVTVD-SDVEYDFENDEIVLKLNVKNADTVYYNPGTTIDVTAKNVDWDEFRLKPGKFTFVAMDSSGANIDGDPYVFTCPIKLRKAEEADQGLKHIKVKSLPEADIYYTTDGSSPVTSDTLLEGEIIVPAQVQLLLIVAQNDKGYSNVLEINTKM----GLVI
EAZ58601.1_Pseudomonas_aeruginosa_2192            AEKTAVNVTVHAANEATGETTLALIPRNAPVVHYAPTAKVSTSDPKVELDNFTTDAATLYFLAVDSEGKHETGEPQRWVAELRVRHQVQAIADKRMVTLSCMPAADMRYTLDGSNPKD-GELYGDPFEVGPQAARLMVYACSGEATRTA-DFQIPAANDKQVQI
MBK7550235.1_Syntrophaceae_bacterium              KPATSVRVDVLSRDDQTGQATLRIRPLNGDTVYMETKGVATVSSKKIEEYDIKTTELKLSFLCVDSKGEHATGEAVMWTNSLSLKHRFFQKGAKRMCELKAVPAGRIRYTTDGSGVETKGIAYEKPFEIPADCRVILVVAEETGIPPQKASIPAPQGVDVAPTI
MBK8067117.1_Rhodanobacteraceae_bacterium         -KQAGVQVTPVGKMQDDGSTVLQIGIINATSVHYAEDGEVTEASPKLNDDKLTTKALRVAFLAVDHSGRTQKGAPYIWPNKLMVQWEIAPGQGKRVVTLKVLPRDVLRYTLNGAEPRN-GIGYTAPFTVGPEAAILLVFAEASGLETKA-DFRIPQAGGAEPEP
MBR2257284.1_Blautia_sp.                          KEPTEVSVEQLSYDEGTGEFTLKVRGVYGNTVYYDVGAEPTEASAIVP-TPFITRETDLWFLCVDQSDPHPTGDAKHFMCKVPLRAEQREAAVGTVMQLATHPGFEVRYTTDGSNPKESGGKYSGEFVIPKGCKYVRTAVYNHGELVEEKDIPITEQSSKKAQI
MBS0652531.1_Verrucomicrobia_bacterium            -KKPTVRITQVAPLRDDGTYELEVVAVHAPEIYYSEHPNVPKQRTQLNGQRFSTQKLRLYFLAVDPTGEAGEGAPVEHKGSIKITAMPPKVQGKRLVELKALPYGLLKYTLDGSDPKLRGSSVDGALDIGSEAVTLLVYAEADGLESHWKEM-YPSLGNGETVL
MBX3024939.1_bacterium                            KPKTSVILDERAYDEATGEATLEVTVRDAPRIYWDTTPNVTARSHRLTDSKLVTDATCVWVVAEDPAGGHERGDPVLWRNRLTITHQPREGVGSRSVELHVVPRGTIRYTLNGANPAE-GTVYDGPIEIGDGETTIWCHAEHEGVTERR-TFTIPPAGNRGIRI
MCA3288087.1_Roseomonas_sp.                       KARTTVSVSRIAYDSNSGEARIEVTARDAPVIRWSDTGPALPSSPELKELSFATSKLRLWFLAEDSTGDHPTGDAVLWRNEITILHEPRETSGGRVVELKAVPAGSLRWTLGGTSPRDMGAAYVGAVPIPLSGGLLRVLAEDGDISAEN-SFDFAPKGVEEKYV
MCF7997122.1_Chromatiaceae_bacterium              KPATQVKVQELSRDNDSGRVKLRATPINGDTIYVEVGAAATPASHKLSGRDYETDELEVSFLAVDSTSEHDTGDPLAWKNRITLQSRVYDAGGDKMIELRAAPPVPIRYTTDGSDPKVSGGTYDDAFVVPEGTRLVLMVAEDKGVASEVLQVPVNWDPNVEEPI
MCK9217630.1_Firmicutes_bacterium                 APETSLTVITVYEDEDIGEVTLKLIPENGDTIYYEIGQTATKASLKVETNNFKTKELKLSFLCVDSRGEHEEGPAVEWSKNVKVKYKPYDKEGKQYIELKATADVKILYSTDGSNPRESGGIYEDDFIVPVGAKFVQAIAEKLEVFSEPLQIEI---KEREFEI
MCL6686250.1_Akkermansia_muciniphila              -RRASLRWTREYGPDDSGLVRLAVDPVDGPEIHYEEDGAVTEESPCLTSDRLETRALRVEFLVKDPTGKFETGDPQCWRNELRLRVNRRPGTGAELLLAPARPDIPIRYTLDGSNPRD-GLLYEGPLEVGAGEVRIEAFAEHEGLETRE-SFTLPAESDPMAVL
MCP4350268.1_Desulfobacterales_bacterium          KEKTDVLIRELHRNTETGEVILMITPRYGDRVLYETGSAATPDSPPAELNKFRTSELKLSFLCIDTKGEHPAGEPKEWCNKIAIKYRVYDKEDDKVLELKSVPDIPIRYTVDGSNPKDYGVPYDGELKVPSDVTHVQAVAEEQDVYSEVLTVRIDRTKGATVGI
MCX6251131.1_Bacteroidetes_bacterium              KPPTKVTVQQIERNPDTGKVKLRIKPVHGDKILYQYGKGVTEQCPEWDQNIMETDAMDVEFLCIDSKNEHEKGKPVLWTNEITLKYGWIPQGDETMMEINVAPKAQVKYTTDGSEPLLNGGIYLSPFLVP--MTLVSIIGVTDSFHSEIKYVKVPA-KPTKFEI
MCZ8170923.1_Brevundimonas_sp.                    -PQPLIRVTRIGGEEATGESELELAVSNAPEVLVAPTRDGLDAGEVITVGTYKTTEVELWFQARNPE-TKEVSDPYRWSGSITITHERRDNAGMWQVTLAARPEAELRWNTTGINPKD-GTVYEGPIEIGRQKTTLYVYAVKGGVSAQR-SFTFDA-VGARRTI
MDA7889828.1_Akkermansiaceae_bacterium            KDKTSIRWTE-NRDPETQEVILNLVPEFGDTIHYEEGGVATESSPKIDKRTHKTTAMEISFLVVDSKGEHKMGDPMPWTNKLVIKHRVLENGAKRKCELKAYPDAVISYTTDNMDPAKSGSTYSAPFELPDTCKIVQAIATLGSHKTATPEINIAE-KGKKFEI
MDD2765364.1_Opitutaceae_bacterium                TPQAQVRNAIVSADEKRGLCRVSVQPVNGDSVVWEREALPGDSSPVWKGDAAAHDWLWAGFLGRDAANPAAPKGEVSWNPNVSIKHDFKKQPNTVLVLEAAPPRIRIFYTFDGSAPTNAQAAYDGPAEVPADCRAILVVGEFDGPEGVL-RTEVQKFPGNPLPP
MDD5268641.1_Methylococcales_bacterium            KPETQVNVQLLSRNPDTGEVNLKITPVNGNKVLYELGHSPSSASMDVAYSRFSTSDMRVTFACIDSRGEHDQGSVYVWENTLQLKHEVIQQGDKWRVELKAVPGAEIRYTTDGSDPSSLGASYDSVFEVPASSRFVQAIATKDGIQSRLEKIDMDQYRDKKVVI
NCB71009.1_Clostridia_bacterium                   QPQTDVGVHEVARTDD--TVILRISPLNGDTVYYEVGQPATTASMKINLNGFRTNELELHFLCIDSIGEHETGTEKIWRNITNCRYNTYDNHGDKMLELKSEAPLTIRYTLDGSDPRSSAATYVAAVPLPKGKVFVQAITSKYDFWGNALGVWIVNKPDTEIVI
ORC33988.1_Marispirochaeta_aestuarii              KDPTGVKVIVKNRDEHTGETILQITPEGGRVVYYEIGKKPSSASLQVTYQNFRTRELSLTFLCVDDSDPRPTGEPLYWKNQVTIKGQFYQQGDEIMFKALSIPPIPLKYTTDGSNPLSHGVPYNGDFLVPNGAQLIQVLAQKDDIQAKE-TFTVK--SATGPVV
PYG00798.1_Thioalkalivibrio_sp._ALE21             KEQTSVSVTPKSTDEATGELILQLVPKHSPRVHYCTDEGVSEQDPVADLEEFRTTEPTLYFLAVDPDGNHETGDATLWKAPIKIRHEVHTKPDHRVVELQSTPKAALYYTTDGSGPRE-GFEYTGPFPIEDGRVLVQVYAKAGASEQTE-KFTIQPSNDGRPEI
RDE52523.1_Candidatus_Accumulibacter              -EITGVSI-EYARDKDSGVITTTINLVHADKLFVREDAAEYRVHP---DQALVSGAMLIEFKAVDSSGKNKEGKPYRIENSIDLAHDFVPPAGHQVVKVKVVPPSTLLYSLDGSNPANNGKPYAKPGIEAPEGSTVRLHAAKGGVVRDI-SITVPK-GGTGPAI
RQH12683.1_Bradyrhizobium_sp._RP6                 AAKTSVKVLTRNYDDQTGVATIELTATDAGKIHFAATSDVSAKSPIVPDLITDRDETVLWFVAVDPDGKHETGEPVKWTNTLTLTYEPKEVMGKRSVALTVKPRGNIRWNTDGTNARE-GKPYTGPIPIGNDEVKIYAYAEDAGVETQK-IFTIRPVKGGEVQI
TPG55477.1_Roseomonas_nepalensis                  VDKTRVSVTPESRSLD-GEVRLSVVALDAPRIHFAKGTTVSTSDPEMTELSFSTTDLRIAFLAVDPKGVHPTGEPYVWRNEITIQHTVSPSADGKRVELFARPSATIRYTLNGTLARE-GTVYDGSFLVTGGSVLLRAIAIDGDLEGEE-TFHIPMTEGTDMFV
WP_005488051.1_Halanaerobium_saccharolyticum      KKETNVNITKMSTVSENGISTLRITPVNADEVYYEIGQEPTKASKKVEHNSFEATDLVYYFLAVDPDGVNETGDPVRWENDINLQYKELTIKGKDALKLQATPSCEIRYTTDGASPKENGGVYQEPIIIPKNAKYIQAVAEEHDITSDVLQYEV---KGKEVTV
WP_012692622.1_Deinococcus_deserti                LEPTSVTVRHDILDAAADRYRLTVTPKYGTQVHYAEGSTVDIAAPVMTDASMEVTGQTFTFLAVDPSKGHPTGQSFTWQAPTFLKGVWESG----EVRFEASPDVEIRVTFDGSAPE-KGQIYEPPITPPPGTHVVQAVATRGGVNSTPITL-----KPDVIPL
WP_018962942.1_Coprothermobacter_platensis        KIPEILSYDIAEYDFENETVTIRLYTRDADAIYWTFGNRLEISDANRELTELTINPENNTQITIIPAFGETLGDPTTINIPIKVKQTEKTDNGNIHVQLKVLPKDKLVYEYGSSTETFTASEATSELVVPTEASQVQVKAPQGEEYLPLTTVAVDK------HV
WP_045169275.1_Caldicellulosiruptor_morganii      KDKTTVRVQFVSADFESGEVVLKLIPMFGDVIHYEEGDEVTENSPVVQINEFRTKSAKLKFLCIDSTGYHPKGEIVKYECPIMLDDNIKYDEGKVHIKVRTAPQATVKYTTDGTNPRYNGKAENGDIVIPDDAKVIIVVAEKDNVFSELKHIPVKKDVVKTVEL
WP_085411740.1_Fibrella_sp._ES10-3-2-2            LPQTDLRFQQVARDTDTGEVILKLQPVHGDEVYYEIGAVATTASARVALKNFVTTELEISFLCHDSTGEHETGPAVTWNNQITLKYDLYDKGGQKMLELKAAPNALIRYTTDGSNPLTTGGTYTAPLAVPPATRLVLAIAEKKGIQSNALNIDIDWKAPNTVTL
WP_090663037.1_Belnapia_rosea                     PEPTEVLVTERAPQDDRGRVVLSVTAKRGDSVHWEVGSPPTAASAKVENGELATDEMRVTFIAVDSTGQVPTGNHFTWENRLRVKSETPYRDGTFRLRLKAVPESKLRYTFDGSDPKLGGGAYDGETPVPEGTRVILAVAENSGIYSRVERIDLPDAGDDGPAV
WP_119720856.1_Methanococcus_maripaludis          EPKTAVSLN-VKSELNNGEVILRVVPQYGEKVYYDIDSDPTTASSEVEINSFKTDELEVRFLCVDPTGKHETGDINIWRNIPKIKYDLISKGNKKQLELFALPKLEIKYTTDGSNPKEHGAVYVEPVLLPENCDTVCAIAKKYNLTSEKLEIKIPKNSGPKVKI
WP_171833827.1_Lichenicola_cladoniae              PDRTGVICRTRTRDETTGKTSLEVKAVHGDRVHWAPTSSVSAASPIVTGEVLDSDKVSLWFLCVDTKGHHETGDPVHWTGKIEVKIDPQSGATTRRMALSSIPPAAIRFTMDDSEPASSGTPYTGPIEIGDGGCTFAAIAEREGVRSEKVTYRFPPRGAEAPEI
WP_190610329.1_Synechococcus_sp._FACHB-909        LPTPSIDIRELSRDDDDGRTYLQLVPLHCDSIVFESGEEPTTASSPVPTAKFEAKGLQYKFLAFDSDNPERQSEVKTWTATLRLKKQLHDRGDHYEVELLAFPRVSIHYTTNGSSPITNAAVYDGPIRVPDSCRKVCAVAPAYSLTSQGIVQDIPKRGEEARTI
WP_231170937.1_Bradyrhizobium                     VTSVTARVEPAGDAHVRGTYILSLSSEDADTIYVSETGVPDARSEKVSGRQYETAAATVWFMAVDSTGKAATGPAYPWYAPIDVKPTLNSSSSGMKLSVSVMPRAVVLATFDGS-PAREGAEITTETKVPPGTEMVRVIGNVDGRYGQEISIVVQRPVPAKPEL
WP_274435612.1_Alicyclobacillus_sp._ALC3          -KTTEVIIGEDSSPDDSGTVRLKIDVANAPRIHYAEDSEVSESSPVLSDNTLVTKALRVQFLAVDPTGKNLTGAPKTWKNRLTLRNRFDEV--SRTVELFVAPKGSIKYTLDGSEARN-GTDYAGPIQLGDDETTVYVFAECDGLEEKR-NFTFAKSGSKEVPI
WP_277247933.1_Phascolarctobacterium              VPKTGLRIVETSRNGE--DVTLKLVPENGDVIRWEIEQDPTSASPIVKLGAFRTNEMSLSFICEDSTGKHEVGEVKTWTNKVEVQYNFFDDNGIKRCELEATNSVKIMYSTNGSNPR-NGAVYNGSFVVPKDASILLAVSEPLDLYGAELRAAIPAFPEPEIVI
consensus/100%                                    .....h.................h....s..l.h............................s....................h............h...s.s...h.hp.ss......................h...s........................
consensus/95%                                     ...s.l...............l.l....u..l.h........................h.h.s.s.......u....h.....l............h.h.s.s...l.hs.ss..s...s..h............l.h.s........................
consensus/90%                                     ...s.l.h.............lpl.s..us.l.h.....s...s..h....h..p...h.Fhs.Ds......G....a.s.l.lp.......s...h.h.s.P...lbao.sGs.s...u..h...h.ls.....l.hhu...........h.h.........l
consensus/85%                                     ...s.l.h.....s..ps...Lpl.s..us.l.h..s..ss..s..h....h.sp..ph.Fls.Dsps.p..G.s..aps.l.lp.p.....s...hpl.s.P...lbao.sGo.P...G..Y.s.h.ls.....lbhhu...s...p..ph.h....s....l
consensus/80%                                     ...s.lplp....s.psGph.Lpl.s.puspl.a..s..ss.su..hp...h.op.hpl.FlshDsps.p.pG.s..Wps.lplp.p.....s.b.hpl.s.P.s.lbYT.sGosP...G..Ypssh.ls.....lbhhAp..sh.sp..ph.h....s....l
consensus/75%                                     ...TpVplp....s.psGph.Lpl.s.pusplaap.s..so.su..lp..phpTp.hpl.FlslDspu.p.pGps..Wpsplplp.p.....s.b.lplps.P.s.l+YT.DGSsP.p.Gs.Ysssh.ls.ssp.lbhhApp.sh.sp..ph.h....s....l
consensus/70%                                     ..pTsVplp..s.sppsGph.Lplss.pusplaap.sssso.uS..lp.pphpTs.hpl.FLslDssG.c.pGps..Wpsplplc.p.bp.sspb.lcLpu.P.spl+YT.DGSsPbp.GssYsush.lssssp.lbhhApp.sl.sp..phpls...s....l
```

# Type-2 BR-system Type 2 DUF499 systems; DUF499-ATPase C-terminal FnIII+FnIII

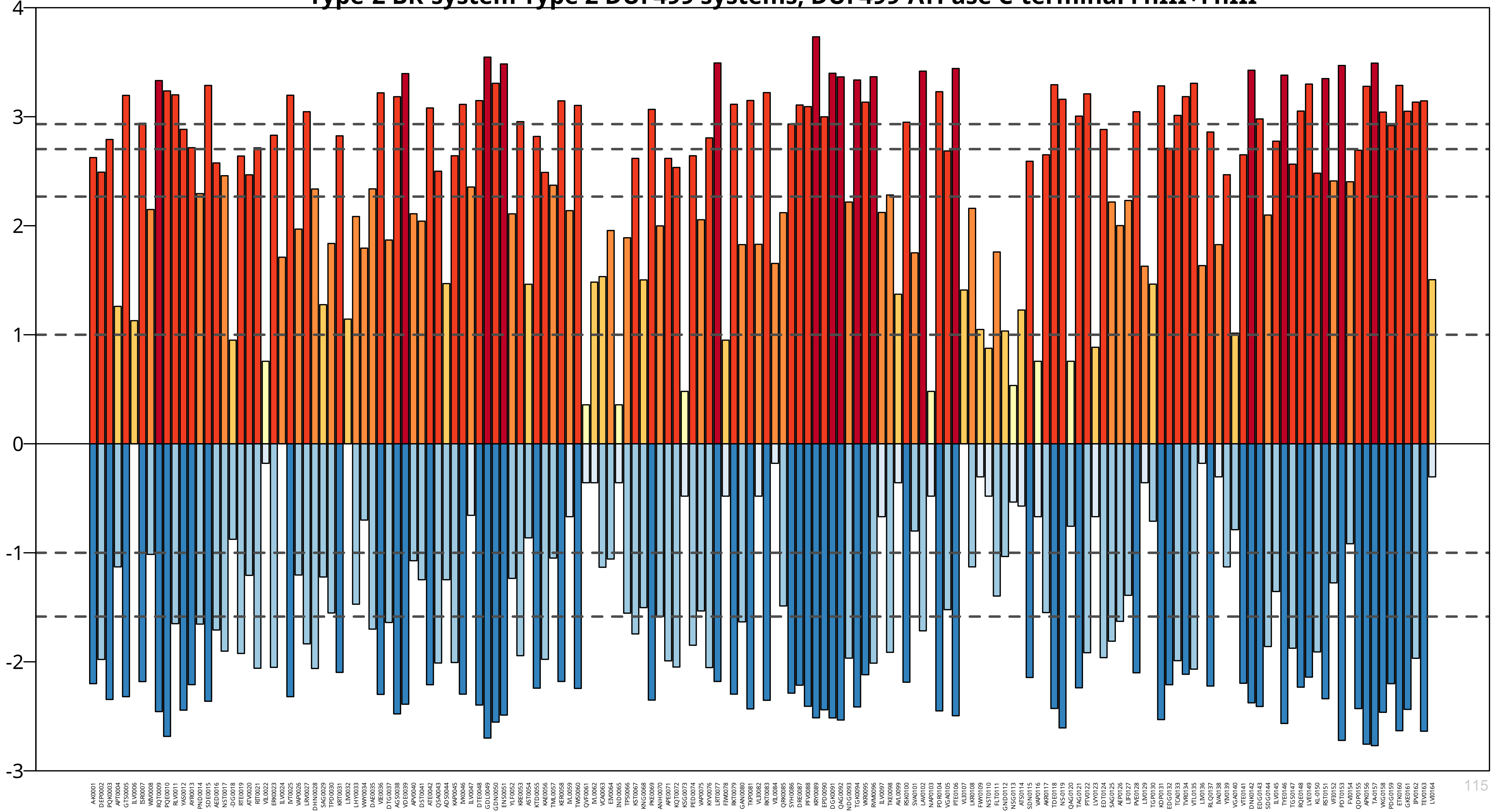

Supplement: gkag035_Supplemental_Files [file gkag035_supplemental_files.zip › Supplementary_Data_S3.pdf]
