# Supplementary material for: Expanding the landscape of BREX diversity: uncovering multi-layered functional frameworks and identification of novel BREX-related defense systems
Source: Nucleic Acids Res. 2026 Jan 27;54(3):gkag035. doi: 10.1093/nar/gkag035 (PMC12839542; doi:10.1093/nar/gkag035)

Supplementary Figures S1-S11 Index

Supplementary Figure S1: **(A and B)** Comparison of structure and domain architecture of DUF6079 (BrxC ATPase) with DUF499 ATPase; **(C and D)** Comparison of MSAs of C-terminal region from DUF6079 (BrxC ATPase) and DUF499 ATPase……………………………………………………………………………………………………...2

Supplementary Figure S2-S5: Mirror trees depicting maximum-likelihood phylogenies of BrxC ATPases (including homologs across multiple BREX subtypes) alongside their corresponding PglZ proteins, with both sets of sequences derived from the same genomic neighborhoods. Each figure from S2 to S5 represents phylogenies constructed using distinct combinations of BrxC (or homologous ATPases) and PglZ sequences sampled across different BREX systems. Across all sequence combinations analyzed, Type-5 and Type-6 BREX components consistently cluster with canonical Type-1 BREX systems. In addition, Supplementary Figure S2 includes homologous sequences from Type-3 BR systems (DUF499 ATPases and Type-3 BR–specific PglZ) as well as from BRC systems (BRC-associated DUF6079 ATPases and PglZ)………………… …………………………..3-6

Supplementary Figure S6: **(A)** Structural superimposition of BrxX MTase NTD with NTD localized at C-terminal of multiple Adenine Methyltransferases from Type-I RM. **(B)** DALI hitlist for BrxX MTase N-terminal domain. **(C)** Multiple sequence alignment highlighting conserved residues across various N-terminal domains from Type-1 RM systems……..7

Supplementary Figure S7: **(A)** Structure based alignment of PglZ N-terminal inactive P-loop domain, with multiple inactive P-loop from Swi2/Snf2 Helicase. **(B)** Structural superimposition of PglZ N-terminal inactive P-loop with inactive P-loop domain of Swi2/Snf2-Helicases……………………………………………………………………..8

Supplementary Figure S8: **(A and B)** 3D structure and domain architecture of Type-1 BREX PglZ, **(C)** Type-2 BREX PglZ, **(D)** Type-3 BREX PglZ, **(E)** Type-4 BREX PglZ, **(E)** Type-3 BR-system standalone PglZ and **(G)** BREX-related Capture systems PglZ ………..9

Supplementary Figure S9: **(A-F)** 3D structure and domain architecture of DNA binding HTHs (BrxA/DUF4007) from **(A)** Type-1 BREX (bipartite version 1), **(B)** Type-1 BREX (tripartite version 2), **(C)** Type-3 BREX, **(D)** Type-2 BREX, **(E)** Type-4 BREX, and **(F)** BRC system. **(G)** Maximum likelihood phylogeny of individual HTH/wHTH modules across BrxA/DUF4007 protein components……………………………………………………………………..10

Supplementary Figure S10: **(A)** Maximum likelihood phylogeny of BrxHI-Helicase core unit with previously classified members of SF1 and SF2 helicase superfamilies. **(B)** MSA of BrxHI helicase core unit with Ski2-like SF2-helicase superfamily……………………………………………………………………………………………………...11

Supplementary Figure S11: Heatmap illustrating the comparative phyletic distribution of all BREX subtypes and BREX-related systems across prokaryotes. Color intensity reflects the number of unique NCBI species-level TaxIDs, following the scheme used in Figure 1B. Type-1, 5 and 6 BREX systems are grouped together and highlighted with red borders, whereas all BR system subtypes are grouped and indicated with blue borders. Type-2 BREX, Type-3 BREX, Type-4 BREX, and BRC systems are displayed separately and are individually color-coded………12

# Titles are internally hyperlinked. Click on title to access material.

Supplementary Figure S1

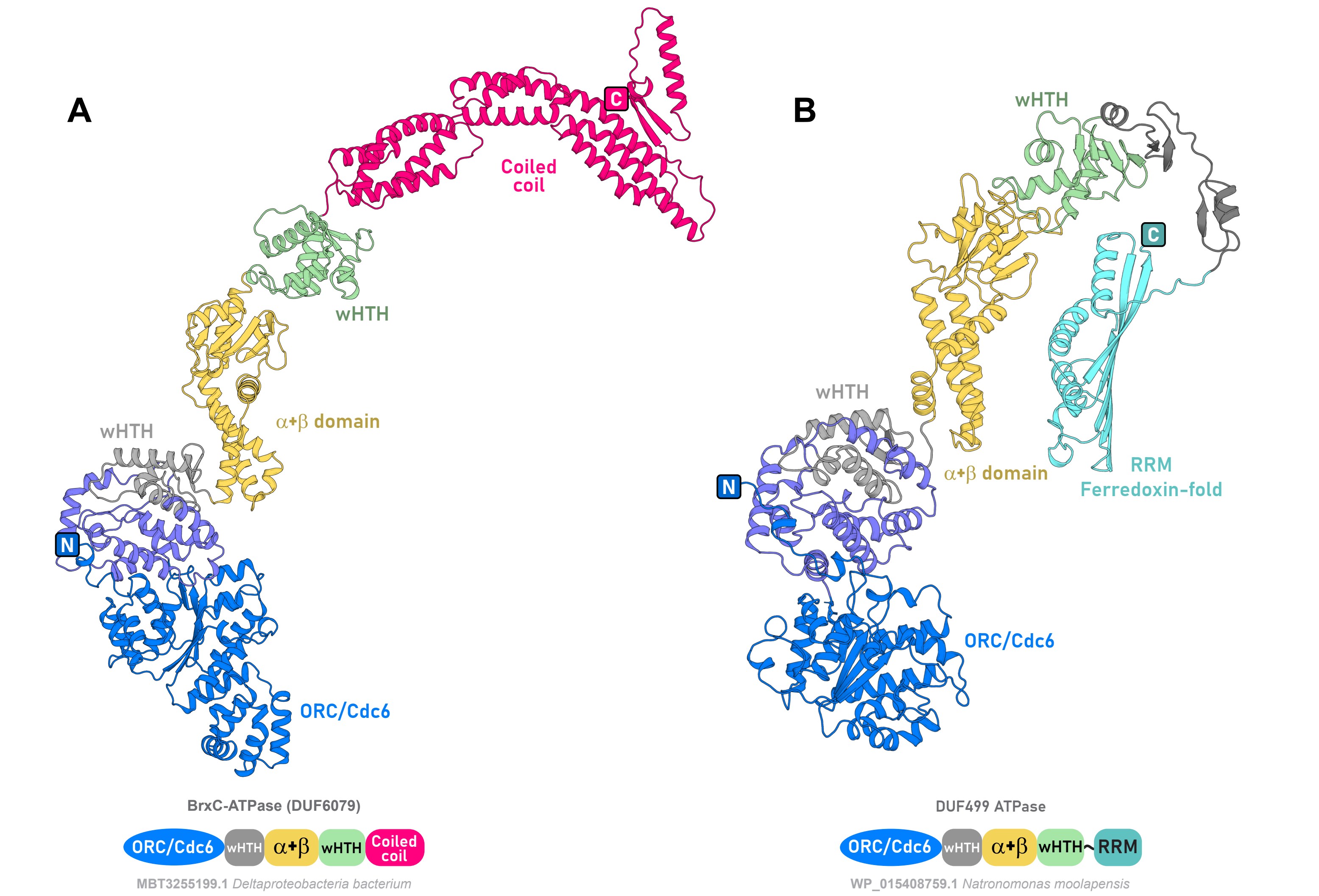

BREX system; BrxC-ATPase (DUF6079) C-Terminal Coiled-coil like Extension

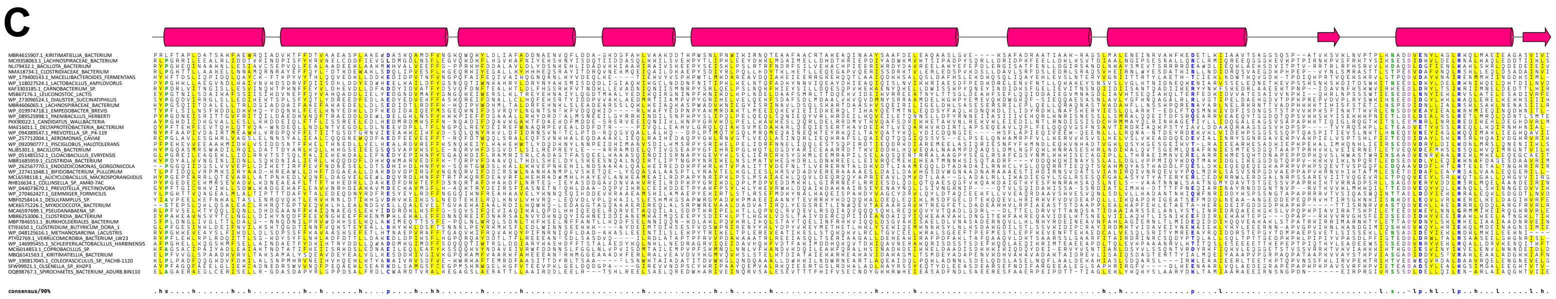

BR system; DUF499-ATPase C-Terminal RRM/Ferredoxin-like Domain

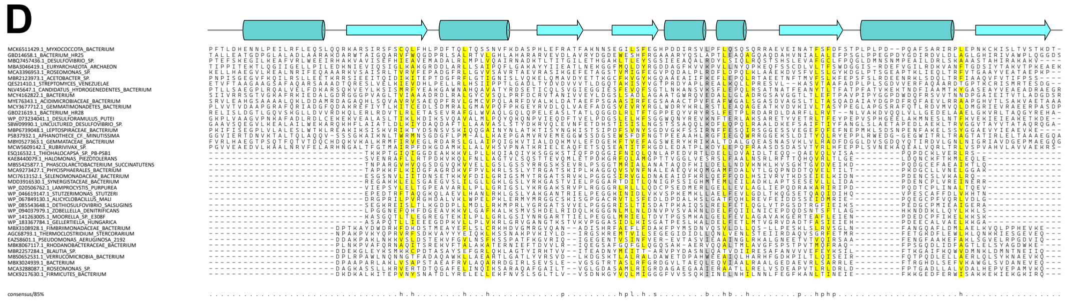

Supplementary Figure S2

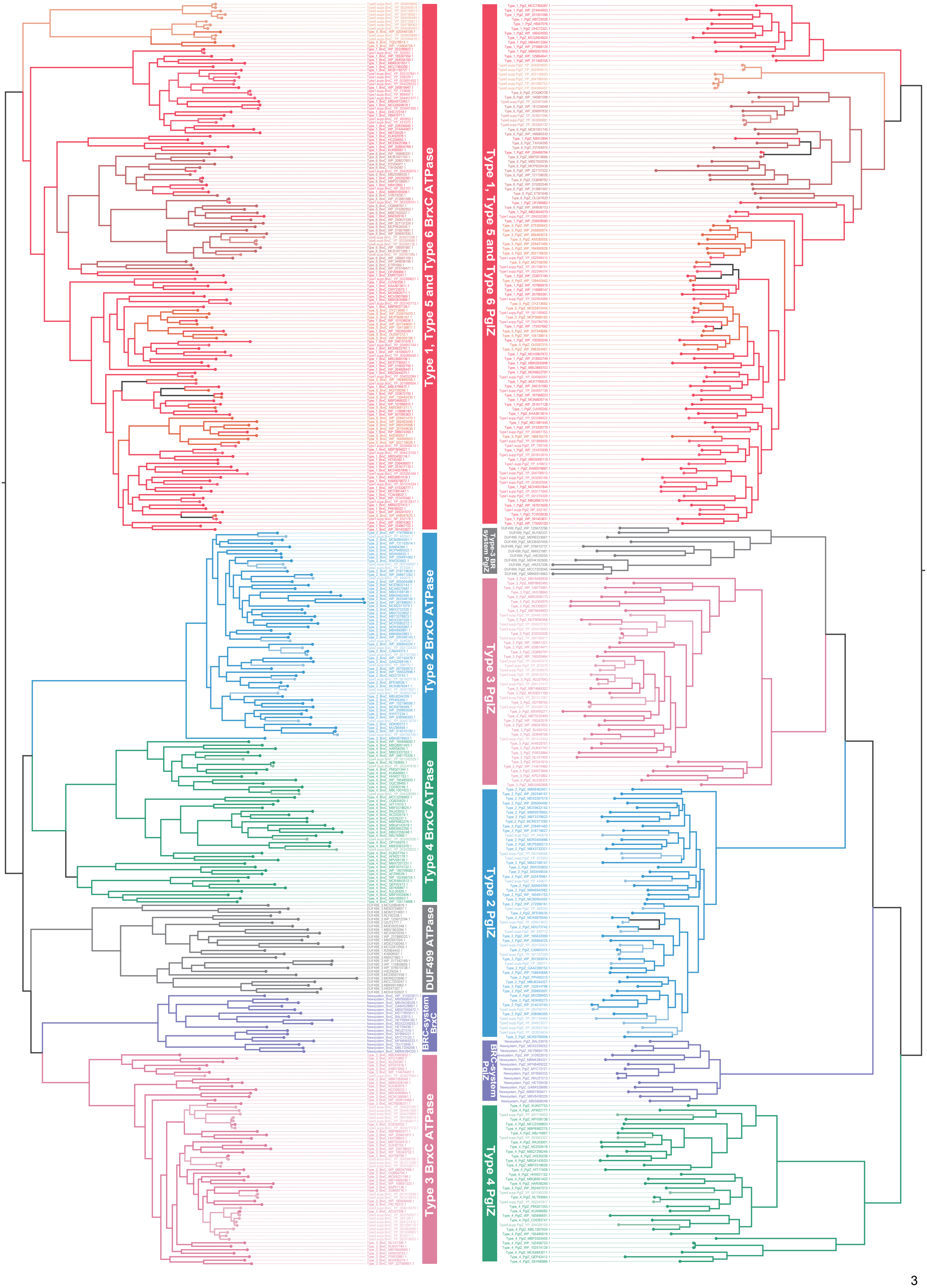

Supplementary Figure S3

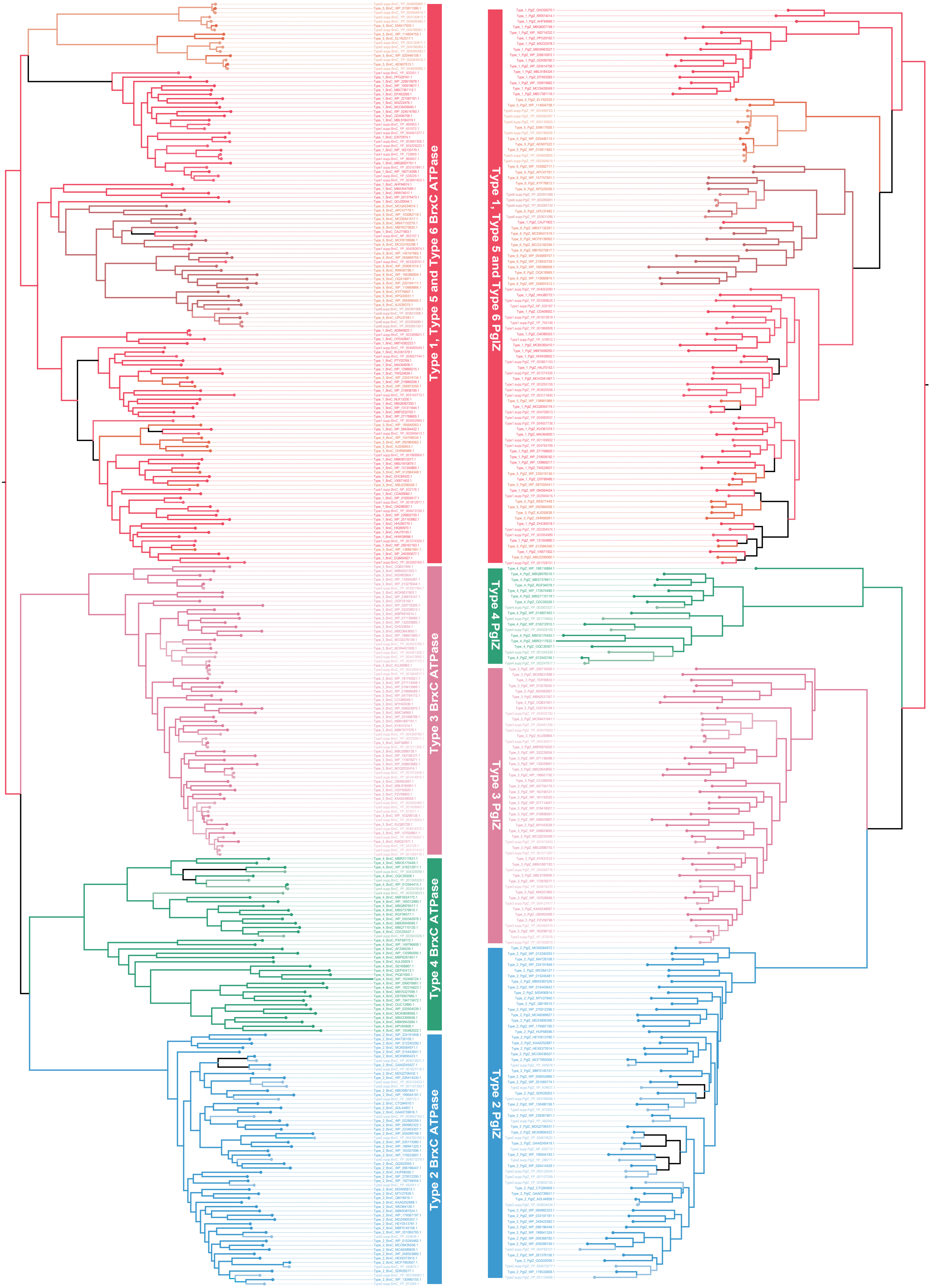

Supplementary Figure S4

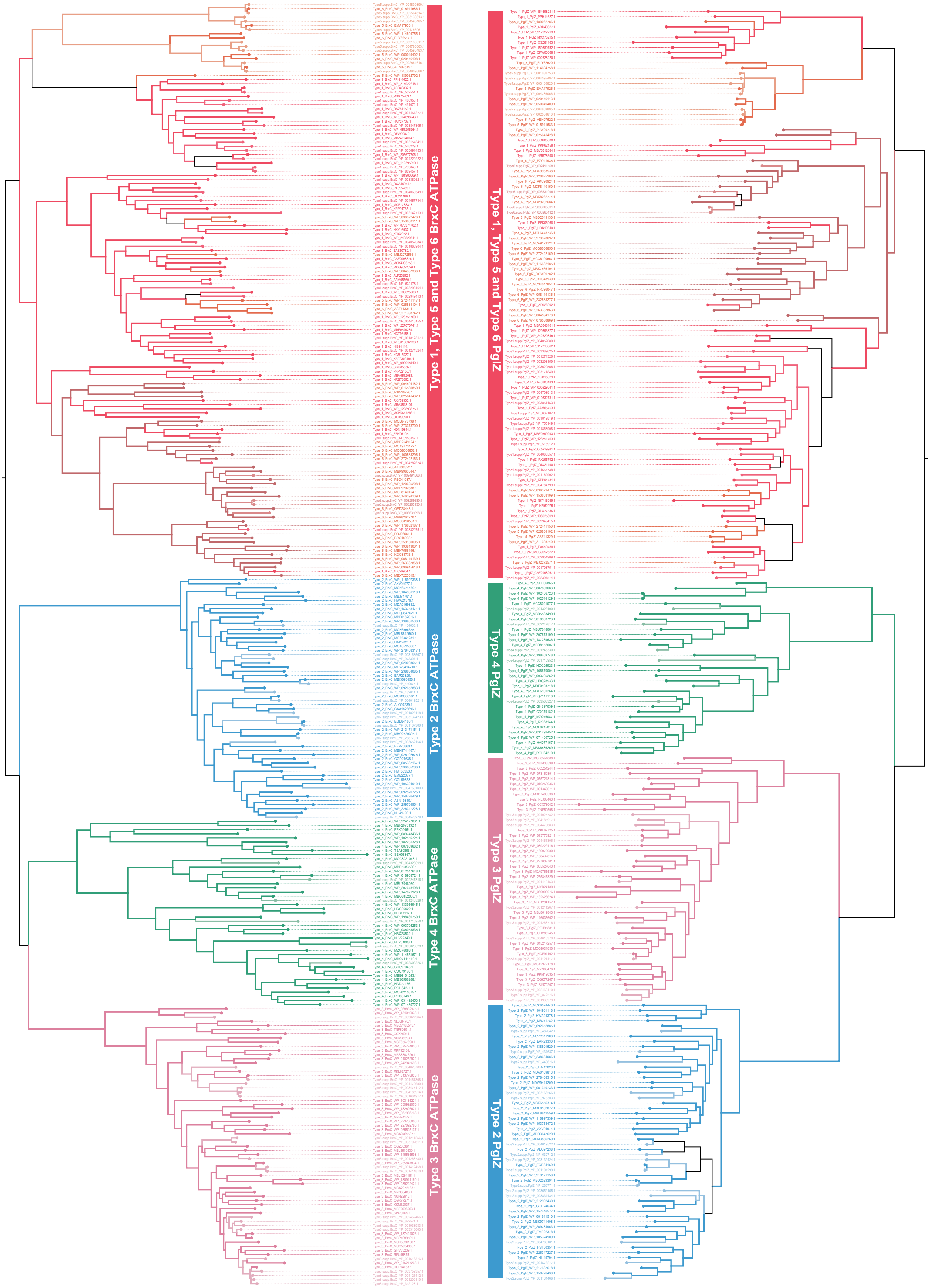

Supplementary Figure S5

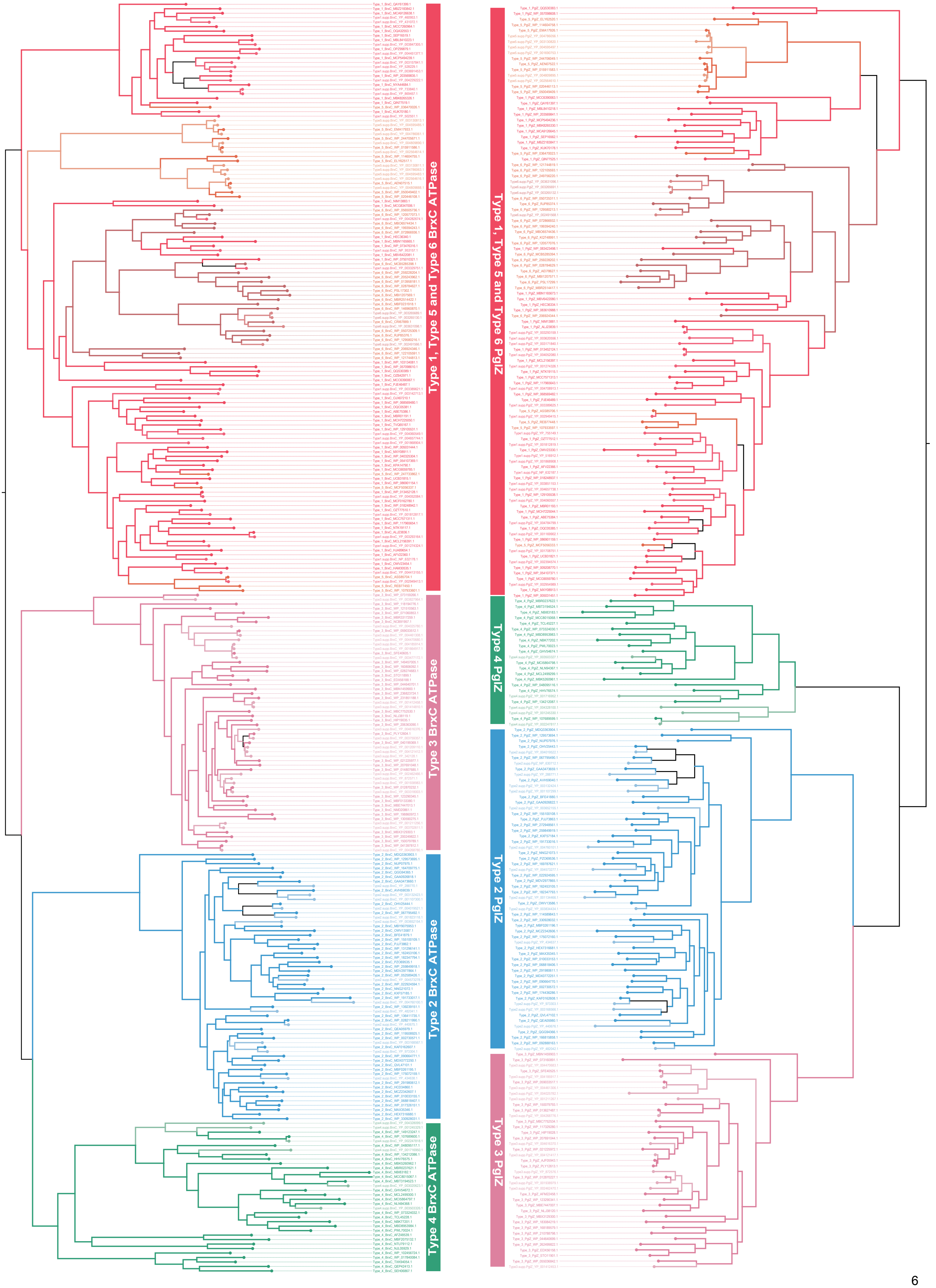

Supplementary Figure S6

Structural superimposition of BrxX MTase NTD with N-terminal domains localized at the C-terminus of adenine methyltransferases from Type-I RM

A

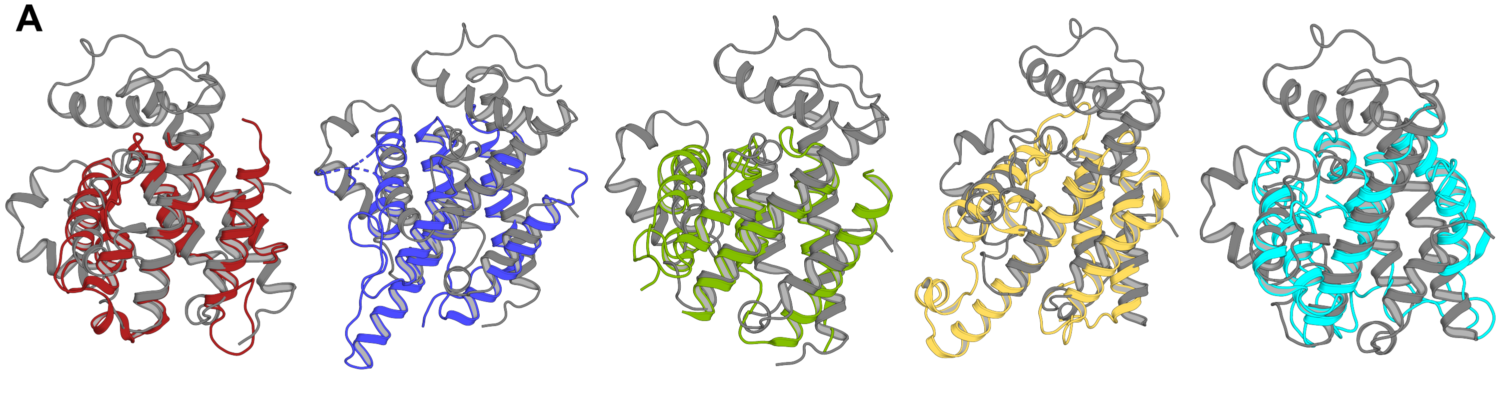

2Y7H-B; (RMSD: 5.33) Type I RM enzyme EcoKI (M2S1)

3UFB-A; (RMSD: 4.78) Type I RM enzyme Vibrio vulnificus YJ016

5YBB-A; (RMSD: 5.72) Type I RM system *Caldanaerobacter subterraneus* synthetic construct

7VRU-A; (RMSD: 5.89) Type I RM enzyme PacII_M1M2S-DNA-SAH complex

8W0P-A; (RMSD: 5.63) BsaXI Type IIB R-M system

B

DALI hitlist for BrxX-MTase N-terminal domain (NTD)

Job: Type-1_BREX; BrxX-MTase_MBZ5596028_NTD_domain
Query: s001A

No:	Chain	Z	rmsd	lali	nres	%id	PDB	Description
1:	8c45-A	17.8	2.4	192	1219	23	MOLECULE:	SITE-SPECIFIC_DNA-METHYLTRANSFERASE_(ADENINE-SPEC
2:	3khk-A	7.7	4.0	122	488	12	MOLECULE:	TYPE_I_RESTRICTION-MODIFICATION_SYSTEM
3:	2y7h-B	7.4	3.5	123	530	11	MOLECULE:	TYPE-1_RESTRICTION_ENZYME_ECOKI_SPECIFICITY_PROTE
4:	5ybb-A	6.8	4.0	127	488	15	MOLECULE:	TYPE_I_RESTRICTION-MODIFICATION_SYSTEM_METHYLTRAN
5:	3ufb-A	6.7	3.8	123	484	10	MOLECULE:	TYPE_I_RESTRICTION-MODIFICATION_SYSTEM_METHYLTRAN
6:	2okc-B	6.6	3.5	121	427	15	MOLECULE:	TYPE_I_RESTRICTION_ENZYME_STYSJI_M_PROTEIN;
9:	7vru-A	5.9	3.8	120	498	11	MOLECULE:	SITE-SPECIFIC_DNA-METHYLTRANSFERASE_(ADENINE-SPEC
10:	3lkd-B	5.9	3.6	118	472	10	MOLECULE:	TYPE_I_RESTRICTION-MODIFICATION_SYSTEM
11:	5hr4-J	5.6	3.5	126	752	13	MOLECULE:	MMEI;
12:	7eew-A	4.3	4.0	111	611	12	MOLECULE:	TYPE_I_RESTRICTION-MODIFICATION_SYSTEM_METHYLTRAN
13:	8w0p-A	3.9	4.4	118	917	9	MOLECULE:	RM.BSAXI;
16:	2f8l-A	3.6	3.5	84	324	8	MOLECULE:	HYPOTHETICAL_PROTEIN_LMO1582;
18:	3s1s-A	3.4	4.0	109	871	8	MOLECULE:	RESTRICTION_ENDONUCLEASE_BPUSI;

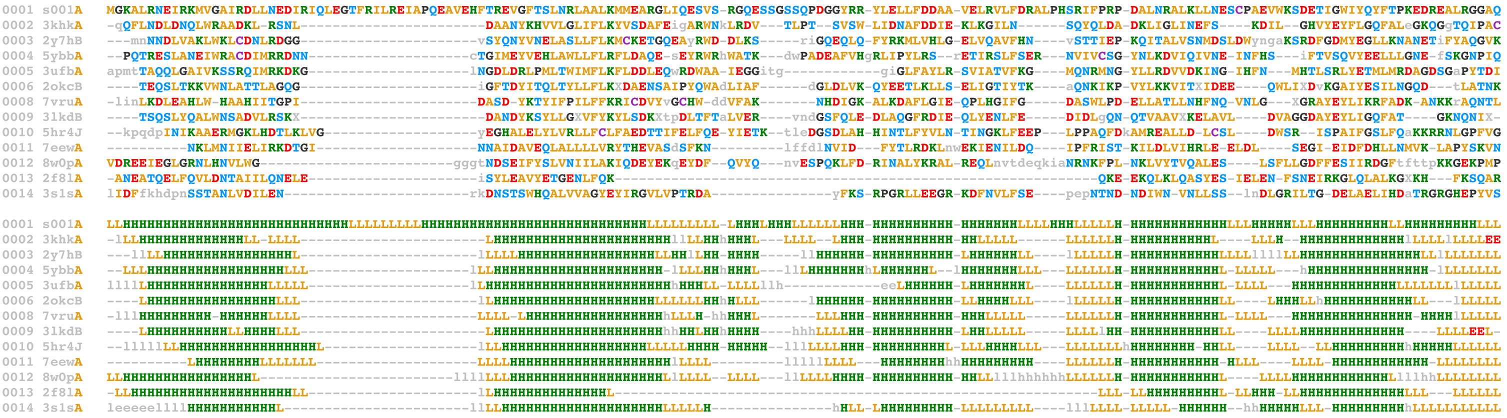

C

Multiple sequence alignment highlighting conserved residues across various N-terminal domains from MTases of Type-1 RM systems

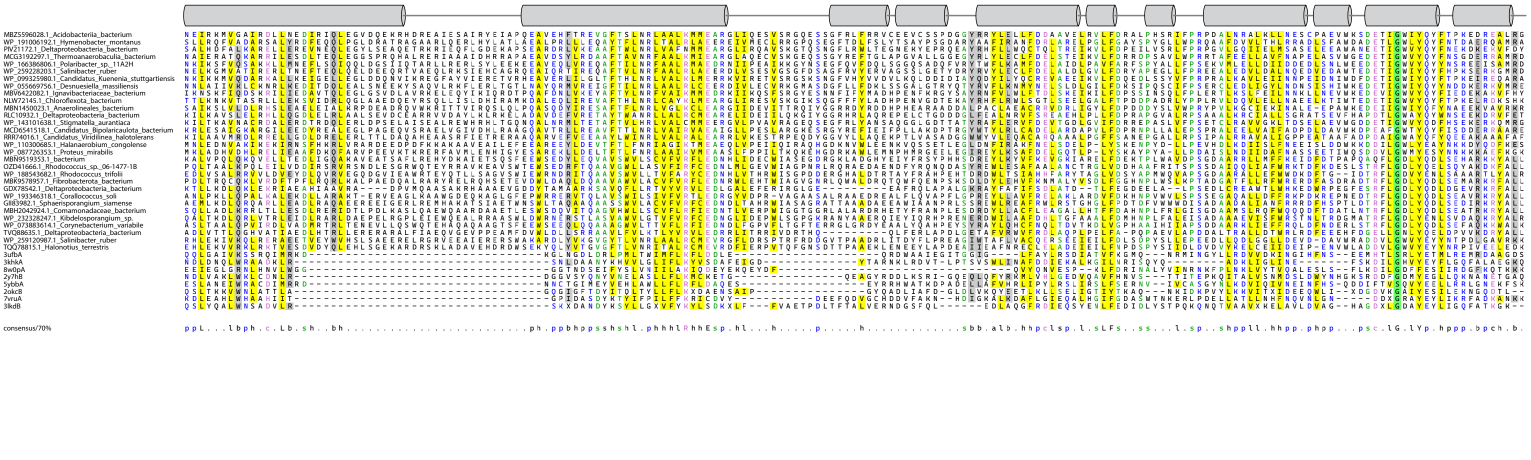

Supplementary Figure S7

Structure based DALI alignment of PglZ N-terminal inactive P-loop domain with inactive P-loop from multiple Swi2/Snf2 helicases

```
Job:Structure_based_Alignment_of_BREX_PglZ_N-terminal
Inactive_P-loop_Swi2/Snf2-Helicase
with_various_inactive_P-loop_domain
from_Swi2/Snf2-Helicases;
Query:        s001A
No:   Chain   Z     rmsd  lali  nres  %id  PDB
1:    s001-A  22.6  0.0   95    95    100  Type1.PglZ.NTD
2:    s002-A  9.0   2.1   82    97    11   Type3.PglZ.NTD
3:    s022-B  7.2   3.0   90    164   6    6nmi
4:    s003-A  6.8   2.7   88    139   11   6l80
5:    s007-A  6.5   2.8   86    155   8    7vdv
6:    s015-A  6.4   2.9   86    149   10   9ii8
7:    s021-A  6.4   2.5   83    150   11   8zns
8:    s016-A  6.3   3.1   81    143   7    6acx
9:    s010-K  6.3   3.1   88    156   8    9n6k
10:   s009-A  5.9   3.0   85    149   8    6bog
11:   s012-G  5.9   2.7   83    142   10   9c57
12:   s013-A  5.8   3.2   84    160   8    3i5x
13:   s011-A  5.7   2.7   78    148   8    2d7d
14:   s008-E  5.7   2.7   85    158   12   7zke
15:   s018-A  5.5   2.3   74    136   7    4buj
16:   s005-A  5.3   2.9   83    140   6    8t5s
17:   s019-A  5.2   2.8   79    144   9    51st
18:   s020-A  5.1   2.5   77    124   10   2fzl
19:   s014-C  5.1   3.1   83    161   7    7smo
20:   s006-A  4.3   2.8   67    123   3    9el5
21:   s004-A  4.1   2.9   69    114   6    8qy7
22:   s017-A  3.5   3.3   70    158   9    7w0b
```

```
s001A_Type1.PglZ.NTD  MASSRITEALLNA-F-NKYRIVFWDDDKE-KL-IDEFNSL---ELP-D-VTKIKL-D-N------NE-FSVKY-RILVEETDK--KFLIYRPA-G--EPDVKDNWLLDVQL-AS-YKFVAE----------------
s002A_Type3.PglZ.NTD  SWRDHILKEFT---P-RVSRLTLVAD--P-DG-L--LLEEileGIR-EgFELIPF-E-D----HIAF-RYAYEsRFRS-RWDA--ALVVVLRSgA--LDV----lPYDLLQ-AG-RKLSF----------------
s003A_6l80            TKIKALLRHLKQI-QeTSPGQIIVFSQFS-SFlDILEIES---HLPdQ-VIIYKF-D-G-RLDMKER-TRILE-QFH--DKDLscIKLLLLSL-T--GGV--GLNL-TCAS----RAFMMDpwwspgmedqaidrih
s005A_8t5s            PMISKTVQYIVEQ-N-LADSRTIIFVRTR-YEaTILNKVLsneELL-MgIKSEWM-SglISASKQKQ-MEKLK-MFAD-----geiRILVSTS-V--AEE--GLDVP---E-CS-LVIKYNyatneiahvqrrgrgr
s007A_7vdv            GKFELLDRILPKL-R-ATNHKVLLFCQMT-SL-MTIMEDY---FyR-G-FKYLRL-D--GTTKAEDR-GMLLK-TFNE-PGSE--YFIFLLST--raGGLG---LNLQSA---D-TVIIFDsdwnphqdlqaqdrah
s008E_7zke            PKLGALRDLLVDC-GigKPHRALIFCQMK-EMlDMVQNTVlkqMLP-S-VSYLRL-D--GSVEANKR-QDIVN-KFNS--DPS--YDVLLLTT--svGGLG---LNLTGA---D-TVIFVEhdwnpqkdlqamdrah
s009A_6bog            wNFDPRVEWLXGY-L-TSHQKVLVICAKA-AT-ALQLEQV-lrERE-G-IRAAVF-H--EGXSIIER-DRAAA-WFA--EEDT-gaQVLLCSE----IGSE-GRNF-QFAS----HXVXFDlpfnpdlleqrigrld
s010K_9n6k            GKMVLLDQLLTRL-K-KDGHRVLIFSQMV-RM-LDILGDY---LiK-G-INFQRL-D--GTVPSAQR-RISID-HFNSPDS-N--DFVFLLS---T--RAGGLGIN-LMTA---D-TVVIFDsdwnpqadlqamarah
s011A_2d7d            GQIDDLIGEIQAR-I-ERNERVLVTTLTK-KMsEDLTDYK---EIG---IKVNYL-H--SEIKTLER-IEIIR-DL-----rlgkyDVLVG-I-N--LL---rEGLD--IP-EVsLVAILDadkegflrsersliqt
s012G_9c57            GKLEALAILLQKL-KsEGRR-VLILSQMI-LM-LDILEMN---FHY---LTYVRI-D--ENASSEQR-QELMR-SFN--RDRR--IFCAILST-H--SR---tTGINLV--eaD-TVVFYDndlnpvmdakaqewcd
s013A_3i5x            NSIFAAVEHIKKQ-I-KSNYKAIIFAPTV-KFtSFLCSIL-knEFK-KdLPILEF-H--GKITQNKR-TSLVK-RFKK---dE--SGILVCTD-G--ARG----MDFPNV---H-EVLQIGvpselanyihrigrta
s014C_7smo            RNESEKWRHIANL-C-RAEAQSIVFTFSR-KRtHELAAY-----SK-G-LKAKPY-H-S-GLPYKQR-KLTEM-EFLAQR-----lDVVVTT--A--ALGA-GVDF----P-AS-QVIFESlamgnkwlsvrefhqm
s015A_9ii8            EYVASIVSYIIEY-T-TTSSKCLVLFTSY-KMmHMVQDML---NFE-D-YVVLTQ-QqN------QN-Y-KIV-QQF-NNFDK--AILLGTST-F--FEG-----FDFQangIK-CVMIAKlpfmntstfkeyvlpd
s016A_6acx            -DDKQVAAALRRE-L-LRDGQAFYIHNRVrTI-DEAAARV-rqLVP-E-ARVVVA-H-G-QMNEETL-EKTVE-GFWN-----reyDILVC-T-T--IV---eTGLD--IS-NAnTLIVERadtfglsqlhqlrgrv
s018A_4buj            -SKKTWPEIVNYL-R-KELLPMVVFVFSK-KRcEEYADWL---EGI-N---IAVHvK-E------LIeILFSK-GF---------iKVLFA-T-E--TF--aMGLNL----ptR-TVIFS----------------
s019A_51st            DTDQALLTLLQGkrF-QNLDSIIIYCNRR-EDtERIAALL-rtCLH-AktTAEAY-H--AGMCSRER-RRVQR-AFMQ-----gqlRVVVA-T-V--AF---gMGLDR-pD-VR-AVLHLGlppsfesyvqavgrag
s020A_2fzl            NKIRKLREILER--H-RKD-KIIIFTRHN-ELvYRISKVF-----------lIPAI-T--HRTSREER-EEILE-GFR---TGR--FRAIVSSQ----VLDE-GIDV---pD-AN-VGVIMSgsgsareyiqrlgril
s021A_8zns            -SWQKIADEIAAR------PCVLAVVNTR-KH-AQKLFAA---LPS-N-GIKLHLhC-S------EViALVRR-YLALySLHK--PLWLVSTQ-L--IEA--GVDL----D-FP-CVYRAMagldsiaqaagrcnre
s022B_6nmi            AVIRNYGNLLLEM-S-AVVPGIVAFFTSYqYM-ESTVASWileNIQ-RnKLLFIE-T-Q---DGAET-SVALE-KYQE-ENGR--GAILLSVA-RgkVSEG--IDFVHHY--gR-AVIMFGvpirendfltfdamrh
s004A_8qy7            ------------------KDKVIIFTELR-DLqRELQHAQ---NFG---frPVII-N-GKSQSQNSR-QRLID-DFQ--AQPG--FGVIIL---S--TVAVGFGVN---VQ-KAnHVIHFTrcwnpakedqatdray
s006A_9el5            ------------------ETRVMIFSSFR-DSvQEIAEMS---QHQpI-IRVMTF-V-GKGFTQKEQ-LEVVK-QFRD-----ggyNTLVSTC---vGEEG--LDIG---E-VD-LIICFDsqkspirlvqrmgrtg
s017A_7w0b            -------------dkdPKDICCLVFVERR-YTcKCIYGlN---YIQnV-LTPQFM-V-gSVLERKWQ-KSAIQ-QFRDGN-----aNLMICSS-V--LEE--GIDV---QA-CN-HVFILDpvktfnmyvqskgrar
```

```
s001A_Type1.PglZ.NTD  LHHHHHHHHHHHH-H-HHLLEEEEELHHH-LL-HHHHHHL---LLL-L-EEEEEL-L-L------LH-HHHHH-HHHLLLLLL--EEEEEELL-L--LLLLLLLLHHHHH-LL-EEELLL----------------
s002A_Type3.PglZ.NTD  LHHHHHHLLLL---L-LLLLEEEEEL--L-LL-H--HHLHhhhHHH-HlEEEEEL-L-L----HHHH-HHHHHhHLLL-LLLL--EEEEEELLlH--HHH----lLHHHHH-HL-EEEEL----------------
s003A_6l80            HHHHHHHHHHHHH-HhHLLLEEEEEELLH-HHhHHHHHHH---HLLlL-EEEEEL-L-L-LLLHHHH-HHHHH-HHL--LLLLllEEEEEEEL-L--LLL--LLLL-LLEE----EEEELLllllhhhhhhhhhlll
s005A_8t5s            HHHHHHHHHHHHH-H-HLLLLEEEELLLH-HHhHHHHHHHllhHHH-HlLLEELL-LllLLLLHHHH-HHHHH-HHHH-----lllLEEEELH-H--HHL--LLLLL---L-LL-EEEEELllllhhhhhhhhhlll
s007A_7vdv            HHHHHHHHHHHHH-H-HHLLLEEEEELLH-HH-HHHHHHH---HhL-L-LLEEEE-L--LLLLHHHH-HHHHH-HHHL-LLLL--LLEEEEEH--hhHLLL---LLLLLL---L-EEEELLllllhhhhhhhhllll
s008E_7zke            HHHHHHHHHHHHL-LllLLLLEEEEELLH-HHhHHHHHHHhhlLLL-L-LLEEEE-L--LLLLHHHH-HHHHH-HHHH--LLL--LLEEEEEH--hhHLLL---LLLLLL---L-EEEELLllllhhhhhhhhllll
s009A_6bog            lLLLHHHHHHHHH-H-HHLLLEEEEELLH-HH-HHHHHHH-hhHHH-L-LLEEEE-L--LLLLHHHH-HHHHH-HHH--LLLL-llLEEEEEL----LLLL-LLLL-LLLL----EEEELLllllhhhhhhhhllll
s010K_9n6k            LLHHHHHHHHHHH-H-HLLLLEEEEELLH-HH-HHHHHHH---HhH-L-LLEEEE-L--LLLLHHHH-HHHHH-HHLLLLL-L--LLEEEEE--L--LLLLLLLL-LLLL---L-EEEELLllllhhhhhhhhllll
s011A_2d7d            LHHHHHHHHHHHH-H-LLLLEEEEELLLH-HHhHHHHHHH---HLL---LLEEEE-L--LLLLHHHH-HHHHH-HH-----hhlllLEEEE-L-L--LL---lLLLL--LL-LEeEEEELLlllllllllhhhhhhh
s012G_9c57            HHHHHHHHHHHHH-HhHLLL-EEEEELLH-HH-HHHHHHH---HLL---LLEEEE-L--LLLLHHHH-HHHHH-HHH--HLLL--LLEEEEEL-L--LL---lLLLLLL--llL-EEEELLllllhhhhhhhhhhhh
s013A_3i5x            HHHHHHHHHHHHH-H-HLLLEEEEELLLH-HHhHHHHHHH-hhHHL-LlLLEEEE-L--LLLLHHHH-HHHHH-HHHH---lL--LEEEEELH-H--LLL----LLLLLL---L-EEEEELlllllhhhhhhhllll
s014C_7smo            LLHHHHHHHHHHH-H-HHHHLEEEELLLH-HHhHHHHHH-----HL-L-LLEEEL-L-L-LLLHHHH-HHHHH-HHHLLL-----lLEEEEL--L--LLLL-LLLL----L-LL-EEEELLlllllllllhhhhhhh
s015A_9ii8            HHHHHHHHHHHHH-H-HLLLLEEEELLLH-HHhHHHHHHH---HHL-L-LEEEEL-LlL------LL-L-HHH-HLL-LLLLL--LEEEEELL-L--LLL-----LLLLlllLE-EEEELLlllllllhhhhlhhhh
s016A_6acx            -LHHHHHHHHHHH-H-HLLLEEEEEELLLHhHH-HHHHHHH-hhHLL-L-LLEEEE-L-L-LLLHHHH-HHHHH-HHHL-----lllLEEEE-L-H--HH---hLLLL--LL-LEeEEEELLhhhllhhhhhhhhhll
s018A_4buj            -LLLHHHHHHHHH-H-HLLLLEEEELLLH-HHhHHHHHLL---LLL-L---LEEEhH-H------HHhHHHHL-LL----------lLEEEE-L-H--HH--hHLLLL----llL-EEEEL----------------
s019A_51st            LHHHHHHHHHHLllL-LLLLLEEEEELLH-HHhHHHHHHH-hhHLL-LleEEEEE-L--LLLLHHHH-HHHHH-HHHL-----lleEEEEE-E-L--LL---lLLLLL-lL-LL-EEEEELllllhhhhhhhhhlll
s020A_2fzl            HHHHHHHHHHHH--L-LLL-LEEEELLLH-HHhHHHHHHL----------lLLEE-L--LLLLLHHH-HHHHH-HHL---LLL--LLEEEELL----LLLL-LLLL---lL-LL-EEEEELlllllhhhhhhhhhhl
s021A_8zns            -LHHHHHHHHHLL------LEEEEEELLH-HH-HHHHHHH---LLL-L-LEEEEEhH-H------HHhHHHHH-HHHHhLLLL--LEEEEEEH-H--HLL--LLLL----L-LL-EEEEELllhhhhhhhhllllll
s022B_6nmi            HHHHHHHHHHHHH-H-HHLLLEEEEELLHhHH-HHHHHLLlhhHHH-HhLEEEEL-L-L---LLLHH-HHHHH-HHHH-LLLL--LEEEEEEL-LlhHHLL--LLLLLLL--lL-EEEEELllllhhhhhhhhhhhh
s004A_8qy7            ------------------LLEEEEELLLH-HHhHHHHHHH---HHL---llLEEE-L-LLLHHHHHH-HHHHH-HHH--HLLL--LEEEEE---L--LLLLLLLL---LL-LEeEEEELLllllhhhhhhhhllll
s006A_9el5            ------------------LLLEEEELLLH-HHhHHHHHHH---LLLlL-LLEEEE-L-LLLLLHHHH-HHHHH-HHHL-----lllLEEEELH---hHLLL--LLLL---L-LL-EEEELLlllllhhhhhhhllll
s017A_7w0b            -------------lllHHHLLEEEELLLH-HHhHHHHHhH---HHHlL-LLLLLL-L-lLLLHHHHH-HHHHH-HHHLLL-----lLLLEELL-L--HHH--LLLL---LL-LL-EEEELLllllhhhhhhhhllll
```

Structural supermposition of PglZ N-terminal inactive P-loop with inactive P-loop domain from multiple Swi2/Snf2 helicases

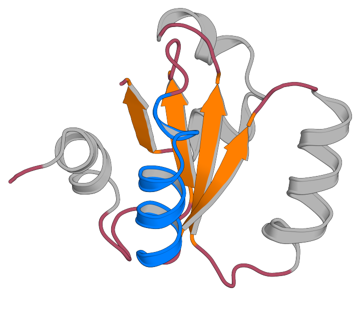

PglZ N-terminal inactive P-loop

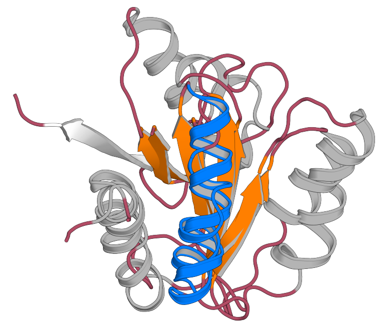

6L80; DNA repair protein RAD5 (RMSD: 3.48)

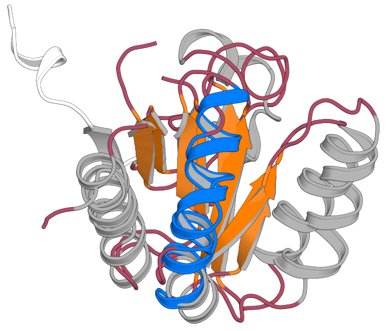

6NMI; DNA repair factor IIH helicase subunit XPB (RMSD: 3.64)

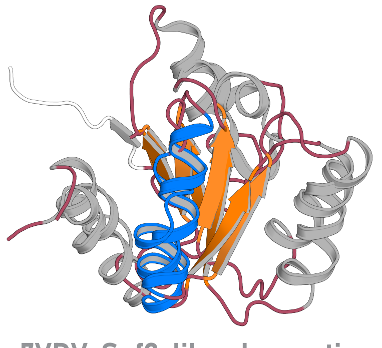

7VDV; Snf2-like chromatin remodellers (RMSD: 3.67)

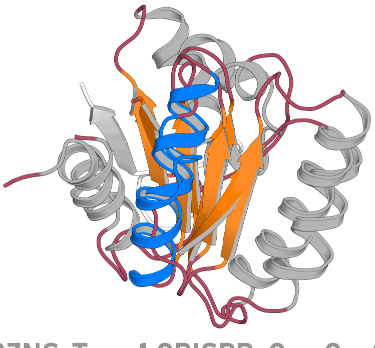

8ZNS; Type 1 CRISPR-Cas Cas3 Helicase-nuclease (RMSD: 3.52)

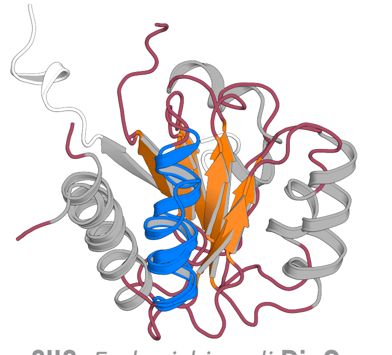

9II8; *Escherichia coli* DinG Helicase (RMSD: 4.11)

Supplementary Figure S8

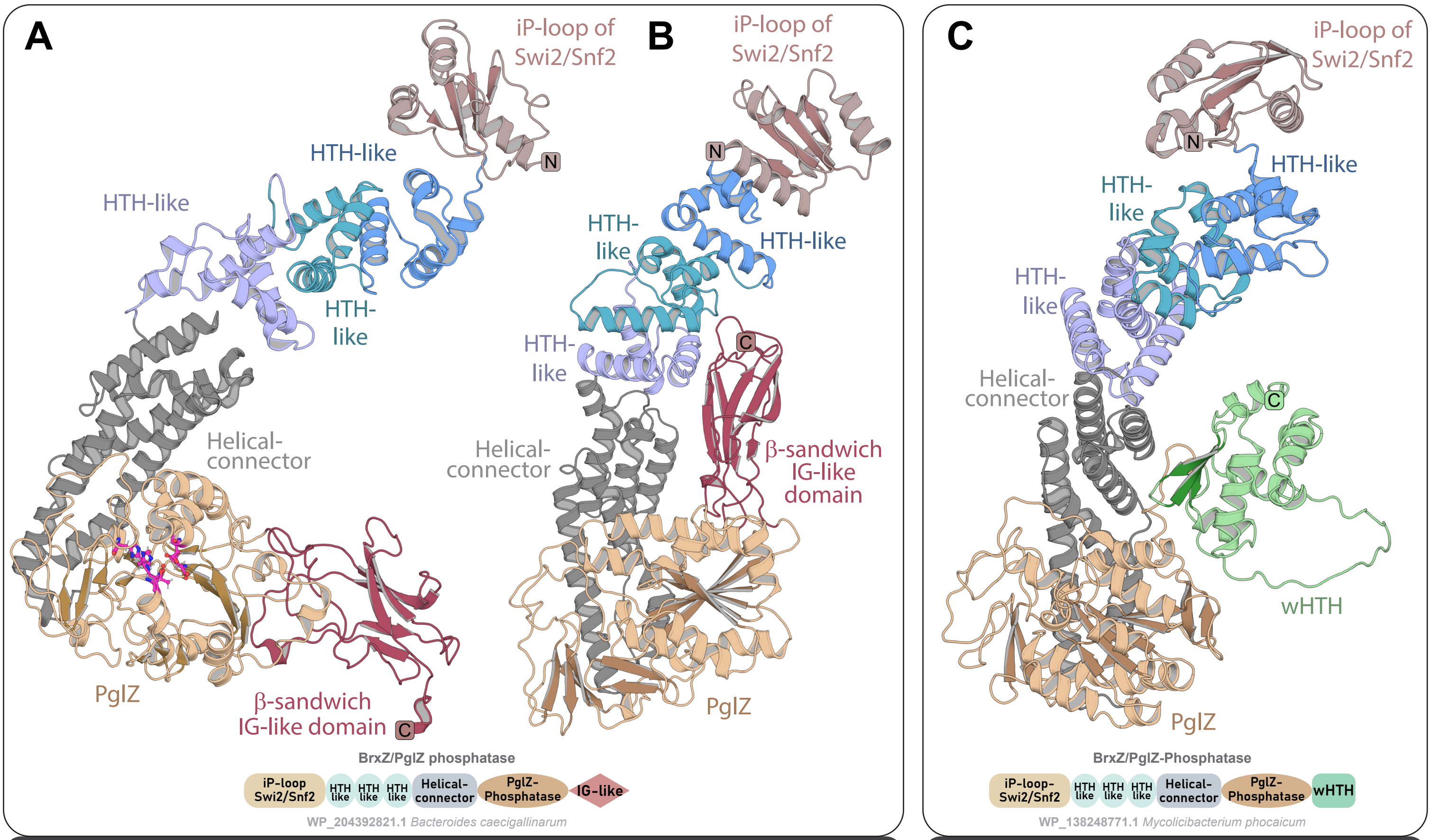

3D structure of Type 1 BREX PglZ:
(A) PDB ID: 6I8O and (B) WP_204392821.1 AF3 predicted structure

3D structure of Type 2 BREX PglZ

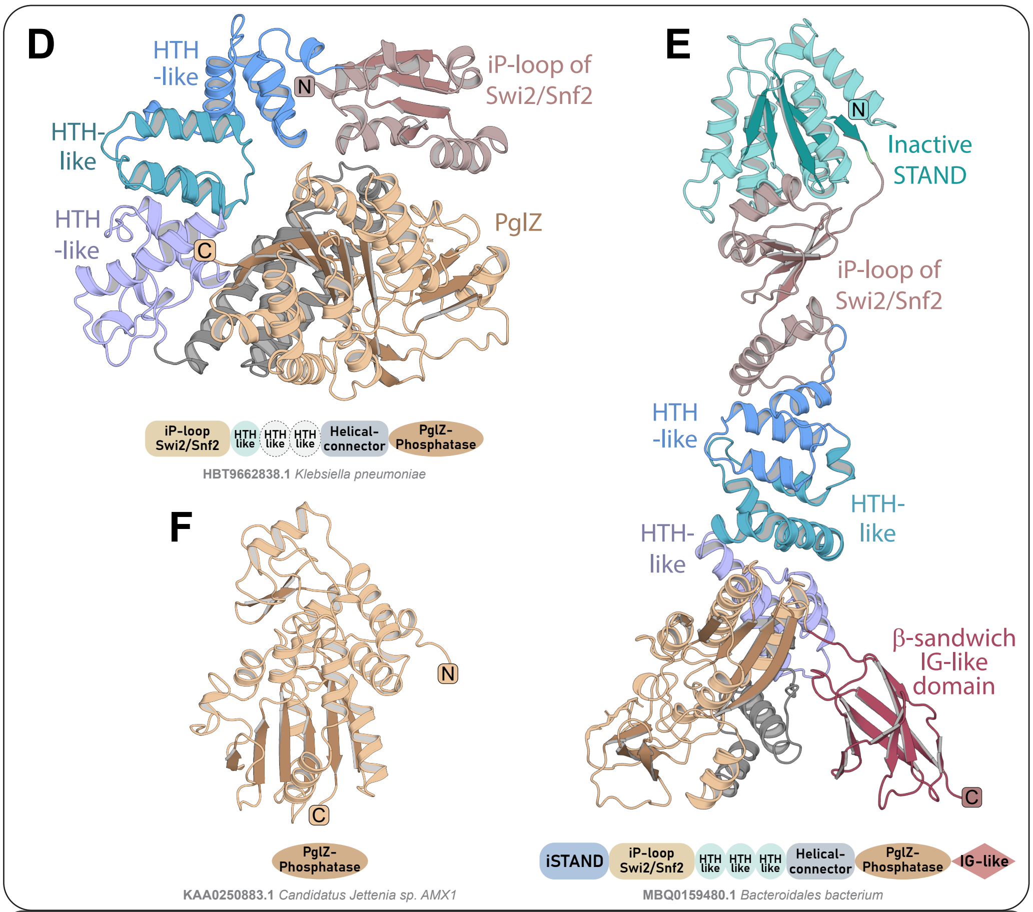

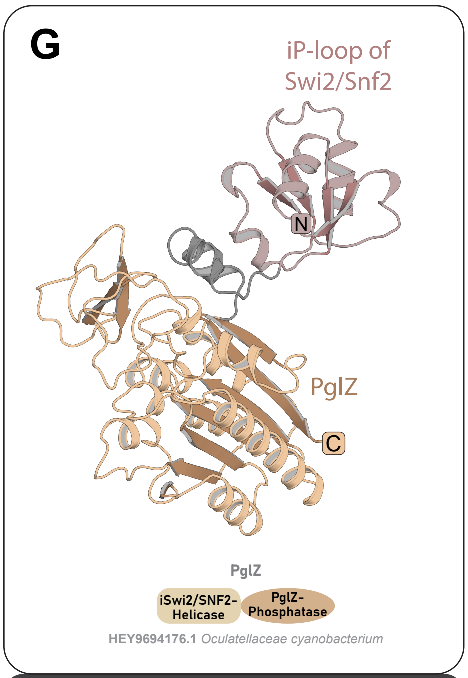

3D structure of BRC system PglZ

3D structure of (D) Type 3 BREX PglZ; (E) Type 4 BREX PglZ;
and (F) Type 3 BR system PglZ

Supplementary Figure S9

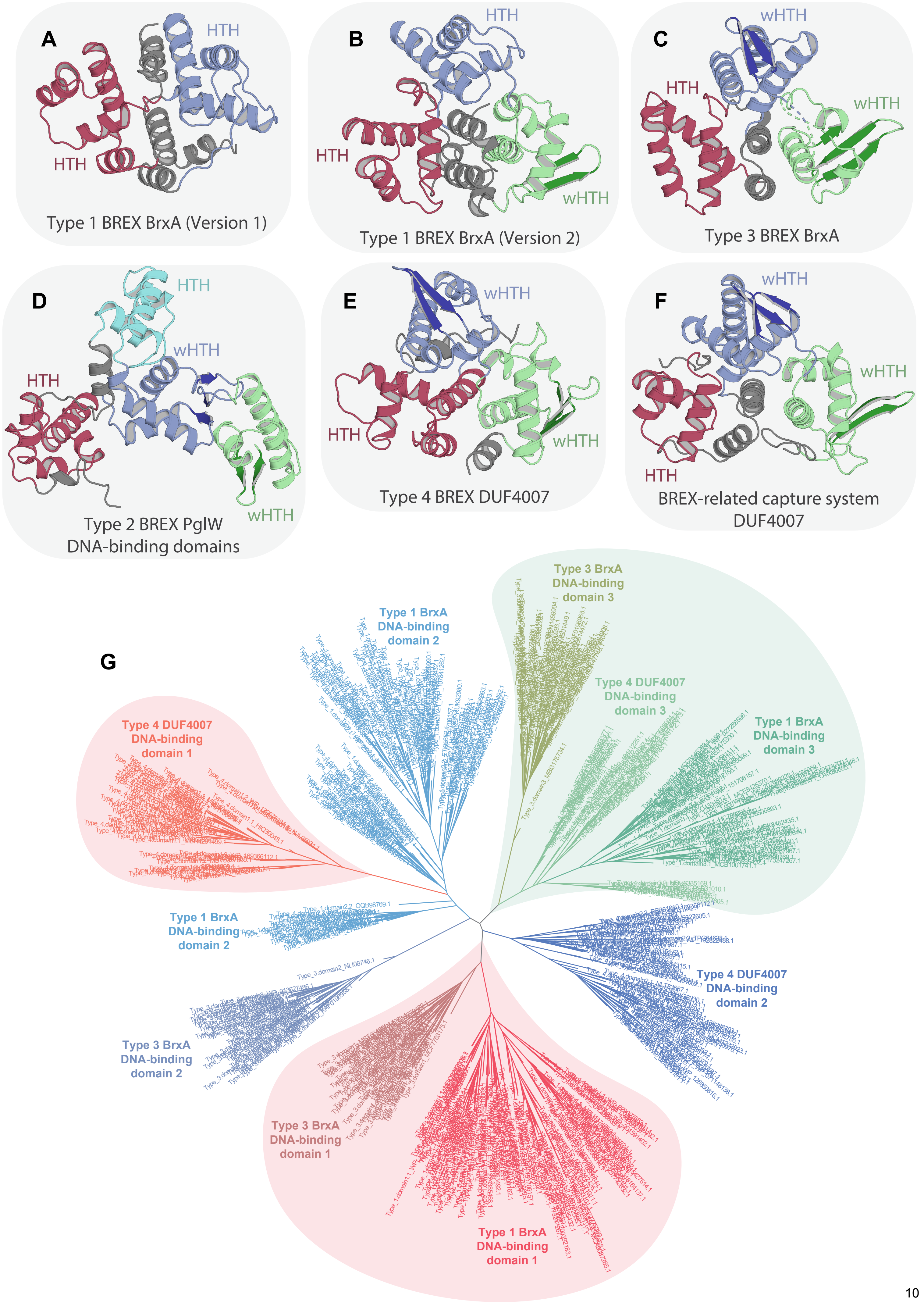

Supplementary Figure S10

Phylogenetic clustering and multiple sequence alignment comparison of BrxHI core helicase unit with previously classified members of SF1 and SF2 helicase superfamilies

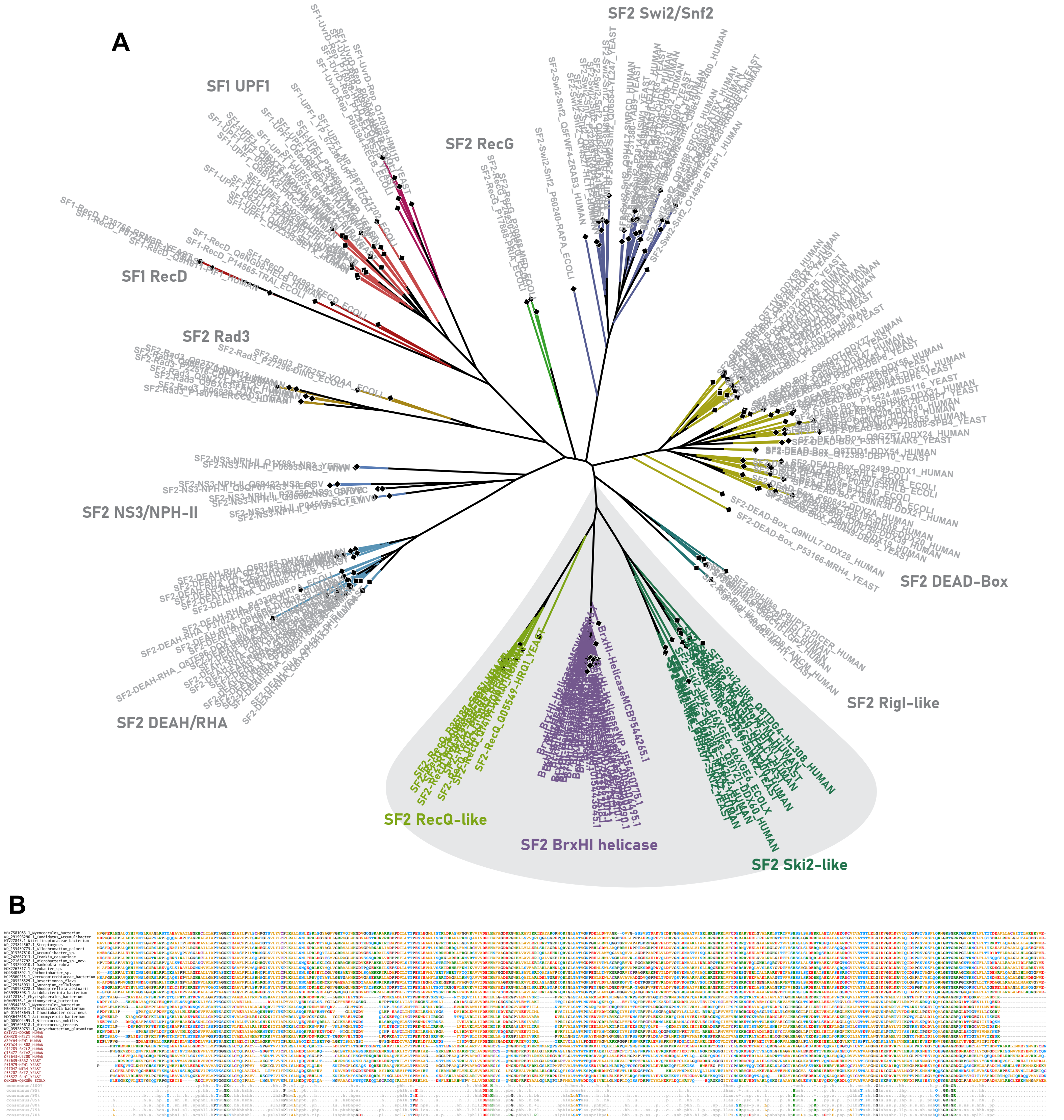

Supplementary Figure S11

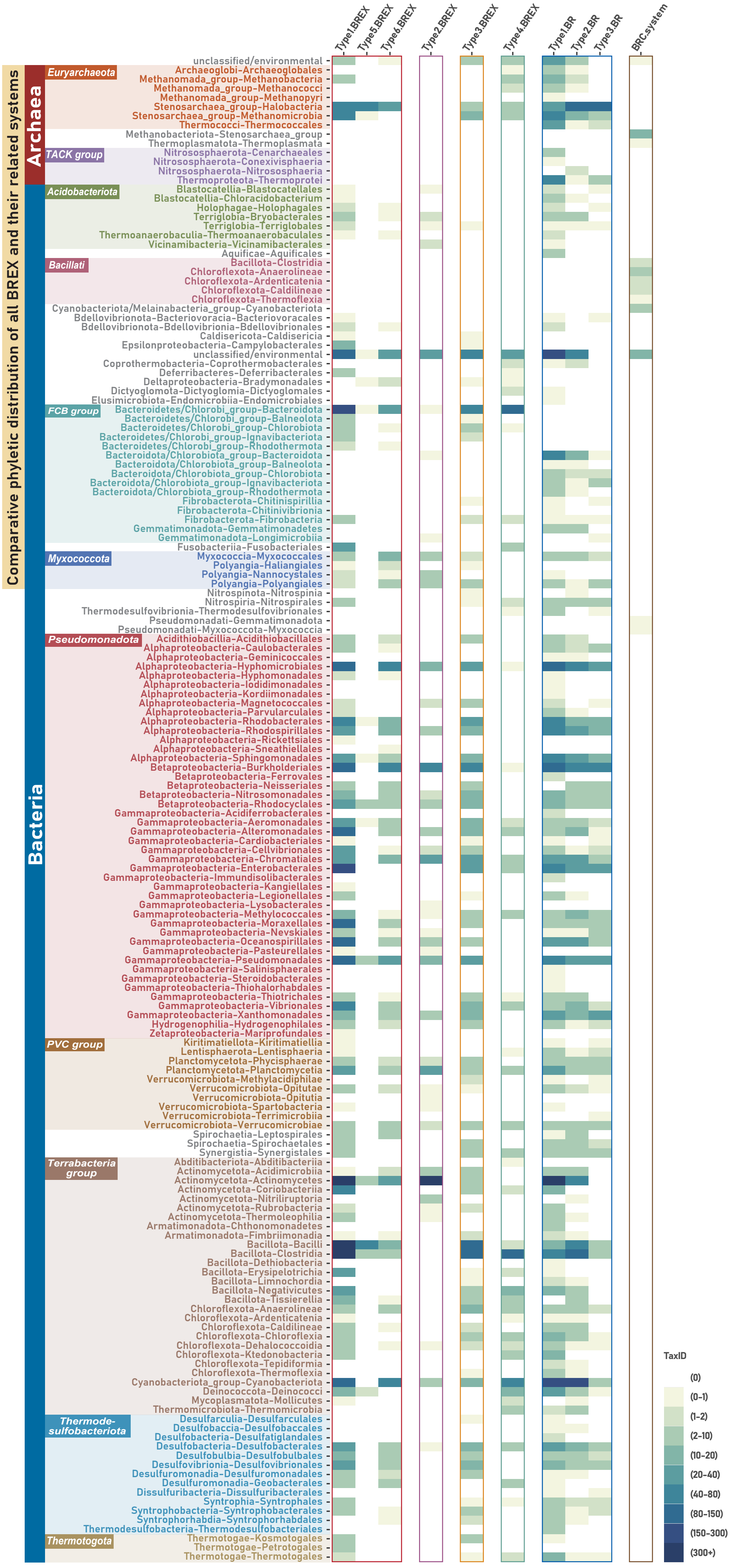

Supplement: gkag035_Supplemental_Files [file gkag035_supplemental_files.zip › Supplementary_Figures_S1-S11.pdf]
