# Supplementary material for: Expanding the landscape of BREX diversity: uncovering multi-layered functional frameworks and identification of novel BREX-related defense systems
Source: Nucleic Acids Res. 2026 Jan 27;54(3):gkag035. doi: 10.1093/nar/gkag035 (PMC12839542; doi:10.1093/nar/gkag035)
Supplement: gkag035_Supplemental_Files [file gkag035_supplemental_files.zip › Supplementary_Table_S1-S2.pdf]

Supplementary Table S1-S2 index

Supplementary Table S1: NCBI protein accession IDs and their Predicted template modeling (pTM) scores for all AlphaFold3 structures used in the main and supplementary figures……….2

Supplementary Table S2: Comprehensive synapomorphy table summarizing conserved sequence features, structural characteristics, and domain-level signatures of major protein components across all BREX systems and their related defense systems analyzed in this study………………………………………………………………………………………………….….3

Supplementary Table S1

Predicted template modeling (pTM) scores for all AlphaFold3 structures used in the figures.

The predicted template modeling (pTM) score measures the accuracy of the entire structure (Zhang and Skolnick, 2004; Xu and Zhang, 2010).
A pTM score above 0.5 means the overall predicted fold for the complex might be similar to the true structure.

System Subtype	Protein Name	Protein Accession ID	AF3 pTM score	Figures or Panels
**Main Figures in Manuscript**				
Type 1 BREX	BrxX/PglX MTase (N6 Adenine MTase)	MBE6254617.1	**0.91**	Figure 2D, (Now replaced with PDB ID: 9EWZ)
Type 1 BREX	BrxC ATPase (DUF6079)	MBT3255199.1	**0.63**	Figure 2C
Type 1 BREX	BrxZ/PglZ Phosphatase	WP_204392821.1	**0.83**	Figure 2E
Type 1 BREX	BrxL (C-terminal HKD-DNase)	WP_205503389.1	**0.84**	Figure 2F
Type 1 BREX	BrxA (DNA binding)	MBL9184317.1	**0.92**	Figure 2G
Type 1 BREX	BrxB (iSTAND NTPase)	OZT77509.1	**0.91**	Figure 2H
Type 2 BREX	PglW (Type 2 BREX Specific)	WP_091597006.1	**0.62**	Figure 2C
Type 2 BREX	BrxHI Helicase (Ski2-like SF2 Helicase)	HEU5155678.1	**0.91**	Figure 4E
Type 2 BREX	BrxD ATPase	WP_141928906.1	**0.88**	Figure 4G
Type 3 BREX	BrxF iSTAND NTPase	WP_189000167.1	**0.92**	Figure 5C
Type 3 BREX	PglXI MTase (N6 MTase with a TRD insert)	WP_264656684.1	**0.84**	Figure 5D
Type 3 BREX	BrxHII Helicase (Swi2/Snf2 like SF2-Helicase)	WP_249385182.1	**0.83**	Figure 5E
Type 2 BR systems	DUF499 ATPase (BrxC/DUF6079 homolog)	WP_213612192.1	**0.65**	Figure 6D
Type 1 BR systems	Helicase (TUDOR+ Swi2/Snf2+iREase+CTD+ Active-REase)	MCF7789459.1	**0.88**	Figure 6G
Type 2 BR systems	Helicase (HKD-DNase +Swi2/Snf2+iREase+CTD)	WP_036795919.1	**0.87**	Figure 6G
Type 2 BR systems	DUF3780	NJI20145.1	**0.5**	Figure 6H
Type 2 BR systems	DUF3780 + RAMA	AFT74738.1	**0.52**	Figure 6H
Type 3 BR systems	PglZ Phosphatase (standalone version)	KAA0250883.1	**0.93**	Figure 7C
Type 3 BR systems	Inactive STAND NTPase	EKO9556875.1	**0.91**	Figure 7D
Type 4 BREX	BrxP (DUF4007 + PAPS Reductase)	NLT83667.1	**0.74**	Figure 8C
Type 4 BREX	Cysteine Desulfurase	NLE35793.1	**0.96**	Figure 8D
BRC systems	PglZ (iSwi2/Snf2 + PglZ)	HEY9694176.1	**0.93**	Figure 10C
BRC systems	DUF4007 (BrxA DNA binding homolog)	MDW8029828.1	**0.94**	Figure 10D
BRC systems	Inactive STAND NTPase	HEY9694178.1	**0.77**	Figure 10E
BRC systems	HerA Translocase	HHW27504.1	**0.92**	Figure 10G
**Additional Figures in Supplementary Datasets**				
Type 1 BREX	BrxC ATPase (DUF6079)	MBT3255199.1	**0.63**	Supplementary Figure S1A
Type 2 BR system	DUF499 ATPase (BrxC homolog)	WP_015408759.1	**0.64**	Supplementary Figure S1B
Type 1 BREX	BrxZ/PglZ Phosphatase	WP_204392821.1	**0.83**	Supplementary Figure S8A
Type 2 BREX	BrxZ/PglZ Phosphatase	QBI18515.1	**0.72**	Supplementary Figure S8B
Type 3 BREX	BrxZ/PglZ Phosphatase	NPV81619.1	**0.83**	Supplementary Figure S8C
Type 4 BREX	BrxZ/PglZ Phosphatase	MBQ0159480.1	**0.78**	Supplementary Figure S8D
BRC systems	PglZ (iSwi2/Snf2 + PglZ)	HEY9694176.1	**0.93**	Supplementary Figure S8E
Type 3 BR systems	PglZ Phosphatase (standalone version)	KAA0250883.1	**0.93**	Supplementary Figure S8F
Type 1 BREX	BrxA (Tripartite DNA binding architecture)	MBL9184317.1	**0.92**	Supplementary Figure S9A
Type 3 BREX	BrxA (Tripartite DNA binding architecture)	MBW1792999.1	**0.87**	Supplementary Figure S9C
Type 2 BREX	PglW DNA (Tripartite DNA binding architecture)	WP_091597006.1	**0.62**	Supplementary Figure S9D
Type 4 BREX	DUF4007 (Tripartite DNA binding architecture)	CDE63278.1	**0.79**	Supplementary Figure S9E
BRC system	DUF4007 (Tripartite DNA binding architecture)	MDW8029828.1	**0.94**	Supplementary Figure S9F

Supplementary Table S2

SYSTEMS-->>	Type 1 BREX	Type 2 BREX/Pgl-systems	Type 3 BREX	Type 4 BREX	Type-1 BR-system	Type-2 BR-system	Type-3 BR-system
**Protein Components**							
**Core-ATPase**	✔	✔	✔	✔	✔	✔	✔
Designated-name	**BrxC-ATPase (DUF6079)**	**BrxC/PglY-ATPase (DUF6079)**	**BrxC-ATPase (DUF6079)**	**BrxC-ATPase (DUF6079)**	**DUF499 ATPase (DUF499)**	---	---
Domain architecture	**STAND/ORC-Cdc6-AAA+ATPase + wHTH + α+β-Domain + wHTH + Coiled-Coil**	**STAND/ORC-Cdc6-AAA+ATPase + wHTH + α+β-Domain + wHTH + Coiled-Coil**	**STAND/ORC-Cdc6-AAA+ATPase + wHTH + α+β-Domain + wHTH + Coiled-Coil**	**STAND/ORC-Cdc6-AAA+ATPase + wHTH + α+β-Domain + wHTH + Coiled-Coil**	**STAND/ORC-Cdc6-AAA+ATPase + wHTH + α+β-Domain + wHTH + RRM<br>or<br>STAND/ORC-Cdc6-AAA+ATPase + wHTH + α+β-Domain + 3wHTH + RRM<br>or<br>HEPN + STAND/ORC-Cdc6-AAA+ATPase + wHTH + α+β-Domain + wHTH + RRM**	**STAND/ORC-Cdc6-AAA+ATPase + wHTH + α+β-Domain + wHTH + RRM<br>or<br>STAND/ORC-Cdc6-AAA+ATPase + wHTH + α+β-Domain + 2wHTH + RRM<br>or<br>STAND/ORC-Cdc6-AAA+ATPase + wHTH + α+β-Domain + wHTH + Fn-III+Fn-III + RRM**	**STAND/ORC-Cdc6-AAA+ATPase + wHTH + α+β-Domain + wHTH + RRM**
Type-specific Synapomorphy	**AAA+ ATPase:**<br>Canonical features - Walker A and Walker B residues, Sensor-1 atop strand-4, Arginine-Finger at the base of strand-5, Sensor-2-Arginine at distal end of helical-bundle, elaborated HEH-insert region, C-terminal helical bundle.<br>**Conserved canonical wHTH:**<br>**α+β-Domain:**<br>Average length 185 residues;<br>Topology - (αH1-αH2 bi-helical connector)-βS1-βS2-βS3-αH3-βS4-(αH4-αH5 long helices forming bundled extension with bi-helical connector)-βS5-βS6-αH6.<br>Structurally distinguished into an α-helical region (formed by the bi-helical connector and αH4-αH5 helix extension), and an α/β/α globular region comprising of a six stranded central β-sheet;<br>Central β-sheet is flanked by two β-hairpins on either end, βS1-βS2 (N-terminal hairpin) and βS5-βS6 (C-terminal hairpin).<br>**wHTH:**<br>Average length 118 residues; Elaborated helical bundle and three stranded wHTH.<br>**Coiled-coil**:<br>Extended coiled-coil helices with an average length of 297 residues.	**AAA+ ATPase:**<br>Canonical features same as Type-1 BREX.<br>**Conserved canonical wHTH:**<br>**α+β-Domain:**<br>Average length 237 residues; Structural and topological features are same as Type-1 BREX;<br>Sequence profile is markedly distinct and relatively conserved within this subtype (primarily due to lesser phyletic distribution of Type-2 BREX);<br>However, like the α+β domain for every other BREX subtype, the sequence profile of α+β domain in Type-2 BREX systems is also unique, with no similarities identified across other variants of α+β domain;<br>**wHTH:**<br>*Elaborated into two closely packed three-stranded wHTH;*<br>wHTH1 average length is 57 residues, wHTH2 average length is 79 residues.<br>**Coiled-coil:**<br>Extended coiled-coil helices with an average length of 241 residues.	**AAA+ ATPase:**<br>Canonical features same as Type-1 BREX, HEH-insert region consists of usual 2-helix "helix-extension-helix" element.<br>**Conserved canonical wHTH:**<br>**α+β-Domain:**<br>Average length 209 residues; Structural and topological features are same as Type-1 BREX; H3 after core S4 is either degenerate or not predicted in AF3 models;<br>**wHTH:**<br>Single wHTH domain, elaborated into a four-stranded wHTH domain; Average length is 137 residues.<br>**Coiled-coil:**<br>Extended coiled-coil helices with an average length of 395 residues.<br>*Features a Type-3 specific 3C-H type of Zn-finger motif highlighted in the reference alignments and AF3 models.*	**AAA+ ATPase:**<br>Canonical features same as Type-1 BREX.<br>**Conserved canonical wHTH:**<br>**α+β-Domain:**<br>Average length 196 residues: Structural and topological features are same as Type-1 BREX; Absence of first βS1 hairpin;<br>**wHTH:**<br>Elaborated solitary three-stranded wHTH with an extended helical section, averaging over 150 residues in length.<br>**Coiled-coil:**<br>Extended coiled-coil helices.<br>Two distinct versions of coiled-coil observed within Type-4 BREX systems;<br>One is averaging 279 residues, other is markedly extended, averaging 497 residues in length.	**AAA+ ATPase:**<br>Canonical features same as Type-1 BREX. HEH-insert region consists of usual 2-helix "helix-insert-helix" element. N-terminal;<br>HEPN fusion occuring in a subset.<br>**Conserved canonical wHTH:**<br>**α+β-Domain:**<br>Average length 184 residues; Structural and topological features are same as Type-1 BREX;<br>Absence of first βS1 hairpin;<br>A conserved motif consisting of a glycine, followed by a highly conserved stretch of [Rxx+N] (+ denotes positively charged residues).<br>*The Rxx+N motif (particularly the conserved N) is a synapomorphy specifically found in DUF499 associated α+β-Domains;* Conserved alanine in H4.<br>**wHTH:**<br>Single three-stranded canonical wHTH, typified by a usual three-helical triangular core, with an average length of 103 residues;<br>Expanded into three distinct three-stranded wHTH, with a total average length of 268 residues, in a subset of Type-3 DUF499-ATPase.<br>**RRM:**<br>Degenerated RRM domain with hypervariable sequence profile; Average length of 103 residues;<br>*AF3 structure prediction is inconsistent due to divergent and degenerate sequence features.*	**AAA+ ATPase:**<br>Canonical features same as Type-1 BREX. HEH-insert region consists of usual 2-helix "helix-insert-helix" element.<br>**Conserved canonical wHTH:**<br>**α+β-Domain:** Average length of 197 residues;<br>Structural and topological features are same as Type-1 BREX;<br>Absence of first βS1 hairpin;<br>Similar sequence profile to the Type-1 DUF499 α+β-Domain.<br>**wHTH:**<br>Four-stranded wHTH with an average length of 92 residues;<br>In a subset, the 4S-wHTH is followed by another 3S-wHTH, separated by a short loop region;<br>*Features an additional downstream β-sandwich (Fn-III associated repeat) in a subset of Type-2 DUF499-ATPase.*<br>**RRM:**<br>*Structurally complete RRM domain with hypervariable sequence profile, maintaning the characteristic β-α-β-β-α-β core topology;* Average length of 143 residues.	**AAA+ ATPase:**<br>Canonical features same as Type-1 BREX;<br>HEH-insert region consists of usual 2-helix "helix-insert-helix" element.<br>**Conserved canonical wHTH:**<br>**α+β-Domain:**<br>Average length 170 residues;<br>Structural and topological features are same as Type-1 BREX;<br>Absence of first βS1 hairpin;<br>Sequence profile is significantly more divergent relative to the Type-1 and Type-2 BR systems;<br>The Rxxx+N motif is divergent, characterized by the loss of first Arginine, and the terminal "+N" is usually represented by a conserved [RN];<br>**wHTH:**<br>Single three-stranded canonical wHTH with an average length of 107 residues, typified by a usual three-helical triangular core.<br>**RRM:**<br>Structurally complete RRM domain with hypervariable sequence profile;<br>Relatively smaller with average length of 96 residues;<br>Maintains the characteristic β-α-β-β-α-β core topology.
**Methylase**	✔	✔	✔	---	✔	✔	✔
Designated-name	**BrxX/PglX-MTase**	**BrxX/PglX-MTase**	**PglXI-MTase**	---	**MTase**	---	---
Domain architecture	**NTD + N6-MTase + TRD + wHTH + CC-like helical ext**	**NTD + N6-MTase + TRD + CC-like helical ext**	**N-terminal ext + N6-MTase(TRD-Insert) + HTH1 + wHTH + HTH2**	---	**DUF1156 + N6-MTase(TRD-Insert) + HTH1 + wHTH + HTH2**	**DUF1156 + N6-MTase(TRD-Insert) + HTH1 + wHTH + HTH2**	**DUF1156 + N6-MTase(TRD-Insert) + HTH1 + wHTH + HTH2**
Type-specific Synapomorphy	**NTD**:<br>Average length 206 residues;<br>Conserved stretch in αH3 represented by [ExhhT] and [NRhh] in the middle of the helix, followed by a conserved [EsR] in the terminal end of the helix;<br>A conserved proline situated in the interior region of the core structure;<br>Terminal helices consists a stretch of conserved motifs represented by a dyad of negatively charged residues [DE], followed by a distinct motif [GWIYQ], which is shared with the Type-2 BREX-MTase NTD.<br>**N6-MTase:**<br>Central β-sheet comprise of nine β-strands;<br>Two additional anti-parallel β-strands are situated C-terminally to the βS6-βS7 MTase hairpin;<br>Large insert region after core βS3, forming 5 packed helices protruding outside from the core;<br>Core catalytic motifs for SAM and substrate binding includes:<br>(i) glycine rich loop after βS1, represented by conserved [DP] on top of βS1, followed by [CGxGHF] in the loop region;<br>(ii) Conserved [DID] on top of βS2 and<br>(iii) [NPP(Y/F)] motif on top of βS4.<br>**TRD:**<br>*Separate domain following the N6-MTase*, with an average length of 273 amino acid residues;<br>Conserved residues includes a glycine on top of βS1, followed by a threonine, following which the βS2 includes a conserved stretch of residues represented by [RFIRhhWE], extending throughout the β-strand. Top of βS3 includes a glycine rich loop represented by [KGG];<br>**C-terminal end:**<br>Three distinct region at the C-terminal end, following the TRD-domain;<br>(i) Two extended helical region with an average length of 91 residues, termed as double-helical spacer, suggested to mimic the coiled-coil spacers found in the specificity subunit (HsdS) of M.EcoKI;<br>(ii) *A three-stranded wHTH with an average length of 155 residues, restricted to only Type-1 BREX MTases;*<br>(iii) A C-terminal CC-like helical extension with an average length of 134 residues, and a sequence profile with multiple conserved residues specific to Type-1 BREX MTases.	**NTD:**<br>Average length 197 residues; Conserved stretch of amino acid residues in αH3, containing a glutamine, followed by a stretch of hydrophobic residues, ends with a conserved motif of [RhhEDN];<br>Terminal helices consists of a conserved stretch containing the motif [TRFL], immediatedly followed by a conserved [GDLYQ] comparable to that of Type-1 BREX-MTase NTD.<br>**N6-MTase:**<br>Sequence and structural profile is similar to the Type-1 BREX core N6-MTase;<br>Central β-sheet comprising nine β-strands with two additional anti-parallel β-strands, situated C-terminally to the βS6-βS7 MTase hairpin;<br>Relatively smaller insert region after core βS3, consisting of a β-hairpin and two small α-helices, leading into the core βS4; Core catalytic motifs for SAM and substrate binding are similar to those found in Type-1 BREX N6-MTases.<br>**TRD:**<br>*Separate domain following N6-MTase*, with an average length of 263 residues;<br>Structurally similar to the TRD of Type-1 BREX MTase with markedly distinct sequence profile;<br>Begins N-terminally with a glycine rich loop; Conserved glycine at the top of βS1;<br>Conserved dyad of negatively charged residues [D/E] at the base of βS2;<br>Conserved glycine followed by a downstream conserved motif [RDW] in the loop connecting βS3 and βS4;<br>Conserved motif [HNH] at the base of βS6;<br>Conserved stretch of polar residues [NSS] buried in the structural core, followed by a motif [FWhKQ] and [KG] in the downstream α-helix.<br>**C-terminal end:**<br>*Absence of the double helical spacer and the downstream three-stranded wHTH found in Type-1 BREX MTases;*<br>Features a C-terminal CC-like helical extension with an average length of 150 residues.	**N-terminal preceding extension:**<br>Average length 111 resdiues;<br>Structurally consists of a small incosistently occuring β-hairpin in its N-termini, followed by two fairly conserved helices and a β-strand lodged in the rightmost end of the central β-sheet of MTase rossmann fold;<br>Overall sequence profile is fairly conserved with multiple conserved stretches of amino acid residues, which are not shared with any other MTase NTD across all BREX subtypes.<br>**N6-MTase:**<br>Distinct from Type-1 and Type-2 BREX MTases;<br>Central β-sheet comprises eight β-strands, with one additional β-strands occuring from the N-terminal preceding extension, lodged in the rightmost end of the central β-sheet;<br>*Two distinct insert region, one small inconstently retained insert region occuring immediately downstream of the glycine rich loop, whereas the second insert region occurs after core βS2, forming a large TRD insert;*<br>Core catalytic motifs for SAM binding and substrate binding are retained in their usual structural positions, including the<br>(i) Conserved [DG] on top of βS1, followed by glycine rich loop [CGxGMTG] after βS1;<br>(ii) Conserved D on top of βS2 and<br>(iii) [DPPF] motif on top of βS4.<br>**Insert TRD:**<br>Large insert region after core βS2 with an average length of 254 residues;<br>Structurally composed of α-helices at the N-terminal and C-terminal end, whereas the β-sheet elements in the middle of the insert-TRD is protruding outside, potentially forming the specific DNA-binding interfaces;<br>4C Zinc finger at the apex of the insert-TRD;<br>A conserved motif R[N/T/Q] positioned atop the 4th α-helix; and a conserved negatively charged aspartate in the terminal β-sheet of the insert TRD.<br>**C-terminal end:**<br>*Consist of three distinct region, each encoding a DNA binding HTH-like domain,* sequentially occuring as HTH1, a canonical wHTH and the C-terminal most HTH2;<br>Structurally homologous domains also occur in the C-terminal end of DUF499 associated MTases;<br>However, the seqeunce profile in Type-3 BREX MTase, and in each subtype of DUF499 associated MTases are unique with no significant conservation of residues retained across these multiple subtypes.	---	**N-terminal DUF1156:**<br>Average length 101 residues; *Distinct from the N-terminal extension of Type-3 BREX MTase, the N-terminal all α-helical bundle in DUF499 associated MTases, is positioned in the anterior end of the core MTase, organised in four tightly packed α-helices;*<br>Conserved residues include two aspartate in the N-termini, followed by a motif [HxWWARR], where H, W, and R are positioned internally towards the interface of the core MTase and insert-TRD domain;<br>These conserved residues are retained across all DUF499 associated MTases in structurally superimposable regions.<br>**N6-MTase:**<br>Similar to the Type-3 BREX MTase;<br>Central β-sheet comprises seven β-strands;<br>The small insert region found in Type-3 BREX MTases, are absent in DUF499 associated MTases;<br>The core βS2 and the downstream helix leads to the large insert-TRD;<br>Core catalytic motifs for SAM binding and substrate binding are retained in their usual structural positions, including the<br>(i) Conserved [DP] on top of βS1, followed by glycine rich loop [sGGG] after βS1;<br>(ii) Conserved D on top of βS2 and<br>(iii) [DPPY] motif on top of βS4.<br>**Insert TRD:**<br>*Large insert region after core βS2 with an average length of 318 residues;*<br>*Structurally homologous to the insert-TRD of Type-3 BREX MTase;*<br>Also retains the 4C Zinc finger at the apex of the TRD, the conserved motif R[N/T/Q] atop the 4th α-helix, and a conserved negatively charged aspartate in the terminal β-sheet of the insert TRD.<br>**C-terminal end:**<br>Consist of three distinct globular region, each encoding a DNA binding HTH-like domain, sequentially occuring as HTH1, a canonical wHTH and the C-terminal most HTH2.	**N-terminal DUF1156:**<br>Average length 150 residues; extended α-helical bundle in a subset of representatives;<br>Sequence and structural profile same as Type-1 DUF499 associated MTases.<br>**N6-MTase:**<br>Similar to Type-1 DUF499 associated MTase, sharing all major sequence and structural synapomorphies.<br>**Insert TRD:**<br>Large insert region after core βS2; Structurally similar to the insert-TRD found in other DUF499 associated MTases and Type-3 BREX MTase;<br>Average length of 324 residues;<br>The 4C Zinc finger is inconsistely retained;<br>The conserved motif R[N/T/Q] atop the 4th α-helix is retained, however the aspartic acid in the terminal β-sheet is inconsistently retained.<br>**C-terminal end:**<br>Structurally and topologically similar to Type-3 BREX and Type-1 DUF499 associated MTases, with a distinctive sequence profile.	**N-terminal DUF1156:**<br>Average length 85 residues; Sequence and structural profile same as Type-1 DUF499 associated MTases.<br>**N6-MTase:**<br>Similar to Type-1 DUF499 associated MTase, sharing all major sequence and structural synapomorphies.<br>**Insert TRD:**<br>Large insert region after core βS2; Structurally similar to the insert-TRD found in other DUF499 associated MTases and Type-3 BREX MTase;<br>*Relatively more extended β-sheet elements, increasing the average length of the insert-TRD to 433 residues;*<br>*Often features two or three 4C Zinc finger compared to the single 4C zinc finger found in other subtypes;*<br>The conserved motif R[N/T/Q] atop the 4th α-helix; and a conserved negatively charged aspartate in the terminal β-sheet of the insert TRD, are both consistently retained.<br>**C-terminal end:**<br>Structurally and topologically similar to Type-3 BREX, Type-1 DUF499, and Type-2 DUF499 associated MTases, with a distinctive sequence profile.
**PglZ component**	✔	✔	✔	✔	---	---	✔
Designated-name	**BrxZ/PglZ-Phosphatase**	**PglZ-Phosphatase**	**PglZ-Phosphatase**	**PglZ-Phosphatase**	---	---	---
Domain architecture	**iSwi2/Snf2-Helicase + 3HTH + helical-connector + PglZ-Phosphatase + IG-like**	**iSwi2/Snf2-Helicase + 3HTH + helical-connector + PglZ-Phosphatase + 3S-wHTH**	**iSwi2/Snf2-Helicase + 3HTH + helical-connector + PglZ-Phosphatase<br>or<br>iSwi2/Snf2-Helicase + HTH + helical-connector + PglZ-Phosphatase**	**iSTAND + iSwi2/Snf2-Helicase + 3HTH + helical-connector + PglZ-Phosphatase + IG-like<br>or<br>iSwi2/Snf2-Helicase + 3HTH + helical-connector + PglZ-Phosphatase + IG-like**	---	---	**PglZ-Phosphatase**
Type-specific Synapomorphy	**iSwi2/Snf2-Helicase:**<br>*Small truncated inactive P-loop domain of the Swi2/Snf2-Helicases;*<br>Average length of 101 residues, typically represented by 4 short core-strands;<br>Highly variable at the sequence level with very few conserved residues, including a conserved stretch of hydrophobic residues in βS1, ending with the conserved D on top of βS1;<br>Conserved hydrophobic stretch in βS3, across all variants of Type-1 BREX PglZ.<br>**3HTH-like:**<br>Three structurally conserved HTH-like units arranged tandemly;<br>Highly divergent sequence profile with no conservation of residues;<br>Combined average length of 167 residues.<br>**Helical-connector:**<br>Five to six extended α-helix forming the connector region; Average length of 159 residues;<br>Conserved residues include an ariginine and tryptophan in αH2; tyrosine, aspartate and arginine in αH4;<br>Most conserved residues are pointing inwards towards the structural core, potentially forming stabilizing interactions.<br>**Phosphatase:**<br>Core topology in comparison with other nucleotide pyrophosphatase superfamily of enzymes - [Preceding(αH)-βS1-αH1-βS2-αH2-(β-hairpin *insert-1*)-αH3-βS3-αH4-βS4-αH5-αH6-βS5-(*Insert-Region-2*)-βS6].<br>Structurally includes a six-stranded central β-sheet of the core-domain and two distinct insert regions, insert region-1 forming a β-hairpin and insert region-2 with an elaborated cap-like subdomain;<br>Insert-region-2 forms a large elaborate cap-like subdomain consisting of six β-strands, βS1 to βS6, where βS2-βS3-βS4-βS5 forms a four stranded anti-parallel β-sheet, and βS1 and βS6 are arranged anti-parallely and often predicted as a loop region in AF3 predicted models;<br>Conservation of residues involved in coordination of Mg2+ and Zn+ ions includes;<br>(i) Aspartate on top of βS1, (ii) threonine at the base of αH2, (iii) aspartate in αH5,<br>(iv) Conserved motif [DHG] on top of βS5, and (v) conserved histidine in the loop preceding βS6.	**iSwi2/Snf2-Helicase:**<br>Average length of 102 residues;<br>Structurally and topologically similar to Type-1 BREX PglZ iSwi2/Snf2-helicases, with an additional small insert-strand placed in-between βS1 and βS2;<br>Highly divergent sequence profile distinguished from those of Type-1 BREX PglZ iSwi2/Snf2-helicases;<br>Conserved motifs unique to Type-2 occurs in the beginning of αH1, represented by [SxLhhR] and conserved strech in βS3 represented by [VIIT] (I-aliphatic amino acid).<br>**3HTH-like:**<br>Three structurally conserved HTH-like units arranged tandemly;<br>Highly divergent sequence profile with no significant conservation of sequences other than few sporadically conserved hydrophobic residues;<br>Combined average length of 222 residues.<br>**Helical-connector:**<br>Five extended α-helix forming the connector region; Average length of 135 residues;<br>Conserved residues include an arginine in αH1, histidine in αH2, alanine and arginine in [AhRL] motif in αH3, aspartate and alanine in [DbA] motif in αH4, arginine followed by [FA] in αH5;<br>Like Type-1 BREX PglZ, most conserved residues are pointing inwards towards the structural core.<br>**Phosphatase:**<br>Similar to Type-1 BREX PglZ phosphatase domains with minimal variation in secondary structural elements; Core topology - [Preceding region(βS-αH)-βS1-αH1-βS2-(insert βS)-αH2-αH3-βS3-αH4-βS4-αH5-αH6-βS5-(Insert-Region-2)-βS6-terminal strand];<br>Structurally retains the the six-stranded central β-sheet with a small additional terminal β-strand lodged beside the left-most core βS2;<br>Insert β-hairpin found after αH2 in Type-1 variants are absent;<br>Cap-like sub-domain is retained;<br>Catalytic residues involved in metal ion coordination are conserved.<br>**3S-wHTH:** Canonical wHTH, with an average length of 105 residues;<br>*Only in Type-2 BREX system's PglZ component, the C-terminal regions harbors a wHTH domain, instead of the IG-like domains found in other BREX subtypes;*	**iSwi2/Snf2-Helicase:** Average length of 100 residues;<br>Structurally and topologically similar to Type-1 BREX PglZ iSwi2/Snf2-helicases;<br>Divergent sequence profile distinguished from those of Type-1 and Type-2 PglZ iSwi2/Snf2-helicases;<br>Conserved motifs includes (i) Aspartate on top of βS1, similar to Type-1 PglZ;<br>(ii) Conserved motf [RhxaE] (a-aromatic amino acid) in αH2 and<br>(iii) conserved aspartate in αH3.<br>**3HTH-like:**<br>Three structurally conserved HTH-like units arranged tandemly;<br>Highly divergent sequence profile with no significant conservation of sequences apart from few sporadically conserved hydrophobic residues;<br>Combined average length of 154 residues;<br>*In a subset of Type-3 PglZ, second and third HTH-like elements are lost, retaining only the N-terminal HTH1-like unit, before leading to the helical connector.*<br>**Helical-connector:**<br>*Relatively shorter version of the helical connector made up of three helices.*<br>Average length of 78 residues;<br>Conserved residues include a tryptophan in αH2, and [FppW] motif in αH3, all of which are pointing inwards towards the structural core, potentially forming stabilizing interactions.<br>**Phosphatase:**<br>Similar to Type-1 BREX PglZ phosphatase domains;<br>Core topology: Same as Type-1 and Type-2 BREX PglZ Phosphatase;<br>Structurally retains the six-stranded central β-sheet βS2;<br>Insert β-hairpin found after αH2 in Type-1 variants are absent;<br>Cap-like sub-domain is retained;<br>Catalytic residues involved in metal ion coordination are conserved;<br><br>*Type-3 BREX PglZ is the only version across all BREX subtypes which does not feature any additional domain fusion towards the C-terminal end of the core phosphatase domain, making it the smallest version among all BREX subtypes.*	**iSwi2/Snf2-Helicase:**<br>Three distinct sub-variants of iSwi2/SNF2-Helicase are observed within Type-4 BREX PglZ N-termini, with extreme diversity in their sequence profile and no conservation of residues across the sub-variants;<br>Average length varies from 78 residues to 116 residues across these variants;<br>Most variants exhibit further reduction in its core structural elements typified by the loss of αH1 and αH2 usually found in other subtypes;<br>**iSTAND:**<br>*A subset of Type-4 PglZ, contains an iSTAND domain fused N-terminally to the iSwi2/Snf2-helicase;*<br>Features the typical iSTAND topology with the loss of walker A, walker B and C-terminal helical bundle.<br>**3HTH-like:**<br>Three HTH-like units arranged tandemly;<br>Divergent sequence profile with no significant conservation of sequences other than few sporadically conserved hydrophobic residues;<br>Combined average length of 164 residues;<br>*Occasionally, third HTH-like element is lost.*<br>**Helical-connector:**<br>Highly divergent helical connector, ranging from three to four helix extension;<br>No significant conservation of residues, except few stretches of tyrosine;<br>Average length of 61 residues;<br>In a subset of these, the region is highly elaborated to form eight helices.<br>**Phosphatase:**<br>Two distinct version of phosphatase domains are observed in Type-4 BREX PglZ;<br>*In the canonical versions of domain architecture with no N-terminal iSTAND, the structural and topological features are similar to other variants* found in Type-1, Type-2 and Type-3 BREX systems;<br>*The other version with an iSTAND fusion in its N-termini, is typified by the loss of βS3-αH4, occuring in the rightmost end of the core structure, thereby reducing the central β-sheet to a five-stranded sheet instead of the usual six-stranded β-sheet;*<br>Cap-like subdomain is retained;<br>Catalytic residues involved in metal ion coordination are conserved.	---	---	**Phosphatase:**<br>*Standalone PglZ Phosphatase domain with no additional domain fusions in its N-termini or C-termini;*<br>Core topology is similar to those of BREX systems;<br>Structurally retains the the six-stranded central β-sheet with an additional four-helix bundle in the N-terminal of the core domain;<br>Cap-like sub-domain is retained;<br>Catalytic residues involved in metal ion coordination are conserved.
**Inactive STAND**	✔	---	✔	---	---	---	✔
Designated-name	**BrxB**	---	**BrxF**	---	---	---	---
Domain architecture	**Inactive STAND-NTPase**	---	**Inactive STAND-NTPase**	---	---	---	**Inactive STAND-NTPase**
Type-specific Synapomorphy	Average length 186 residues; Absence of walker A, walker B and C-terminal helical bundle;<br>Central β-sheet consists of the usual five core-strands found in AAA+ ATPases;<br>Retention of STAND/ORC-Cdc6 specific HEH (Helix-extension-helix) insert region after core βS2;<br>The top of strand-4 consistently extends into a three-stranded antiparallel β-sheet, protruding outward to form a cap-like subdomain;<br>Conserved sequence elements includes a hydrophobic stretch ending with a tyrosine in βS1;<br>Conserved aspartate in the HEH insert region, conserved motif [GxG] on top of βS3;<br>Conserved motif [PhhR] in loop connecting βS3 and αH4;<br>*A stretch of conserved hydrophobic residues in βS4 which leads to a highly conserved proline-glycine dyad [PG] on top of βS4, leading into the cap-like subdomain;*<br>The conserved motifs of βS4 are also retained across Type-2 and Type-3 iSTAND components;	---	Average length 147 residues; Absence of Walker A, Walker B and C-terminal helical bundle;<br>Structurally similar to Type-1 BREX iSTAND(BrxB);<br>HEH insert region is smaller consisting of 'one-helix insert';<br>Strand-4 leads to the three-stranded antiparallel β-sheet insert region, protruding outward to form a cap-like subdomain;<br>Sequence profile is highly divergent, with multiple versions observed (see supplementary alignments);<br>Conserved motifs include a stretch of polar residues (represented by [NhN] motif) in loop connecting βS2 and HEH insert region;<br>Hydrophobic residues in αH3;<br>*Conserved tryptophan followed by proline-glycine dyad [WPG] on top of βS4 is somewhat retained, similar to the Type-1 BrxB component;*<br>*In a very limited subset of BrxF(iSTAND) specific to Type-3 BREX systems, the walker A and walker B residues are intact* (represented in supplementary alignment).	---	---	---	Average length 163 residues; Absence of Walker A, Walker B and C-terminal helical bundle;<br>Structurally similar to Type-1 BREX iSTAND(BrxB); Includes an additional terminal β-strand, inserted antiparallelly into the central five-stranded β-sheet, making the central sheet a six-stranded β-sheet;<br>Strand-4 leads to the three-stranded antiparallel β-sheet insert region, protruding outward to form a cap-like subdomain;<br>Overall sequence profile is markedly distinct from those of Type-2 and Type-3 BREX MTase, however, similar to Type-3 BREX iSTAND, the [WxG] motif on top of βS4 is retained;
**DNA-binding HTH**	✔	---	✔	✔	---	---	---
Designated-name	**BrxA**	---	**BrxA**	**DUF4007**	---	---	---
Domain architecture	**HTH + wHTH1 (Version 1)<br>or<br>HTH + wHTH1 + wHTH2 (Version 2)**	---	**HTH + wHTH1 + wHTH2**	**PAPS_Reduct + HTH + wHTH1 + wHTH2 (Version 1; DUF4007)<br>or<br>HTH + wHTH1 + wHTH2 (Version 2; DUF4007)**	---	---	---
Type-specific Synapomorphy	Two distinct versions of domain architecture found in BrxA component within Type-1 BREX systems;<br>One version has a single wHTH domain (wHTH1), whereas the second version has two wHTH domains towards the C-termini (wHTH1 and wHTH2);<br>***Version 1:*** Average length of 199 residues; Topologically contains a preceding helix region, followed by HTH, a small helical spacer, a wHTH and a C-terminal helix region;<br>***Version 2:*** Average sequence length is 254 residues;<br>Topologically retains the preceding helix region, the small helical connector between HTH and wHTH1, and the C-terminal most helical extension;	---	Average length 235 residues; Topologically contains a small preceding helix, followed by a three-helical HTH domain, a two-helical spacer region, wHTH1, and C-terminal wHTH2;<br>Absence of the terminal helical extension observed in Type-1 and Type-4 BrxA components;<br>The N-terminal HTH domain is clustered with HTH domain of Type-1 BrxA;	The BrxA component (denoted by the architechture of HTH+wHTH1+wHTH2, is often described as DUF4007 in the literature, because of the DUF4007 (UPF0758 entry) top hit, recovered from HHpred searches;<br>BrxA component found in Type-1 and Type-3 BREX systems, and DUF4007 component found in Type-4 BREX systems, both recover the BrxA PDB structure (7RZB) and DUF4007 profile as their top hits, suggesting the occurence of shared domains among these components;<br>Our analysis of domain architecture and sequence-structure features suggests that BrxA and DUF4007 represents homologous counterparts in various BREX subtypes;<br>Two distinct versions of domain architecture found in DUF4007 component within Type-4 BREX;<br>Version 1 is fused to an N-terminal PAPS reductase; Average length 248 residues;<br>Topology same as Type-1 BrxA;<br>Version 2 is standalone (HTH + wHTH1 + wHTH2) BrxA;	---	---	---
**AAA+ Helicase**	✔	---	---	✔	---	---	---
Designated-name	**BrxL**	---	---	**BrxL**	---	---	---
Domain architecture	**SIGMA-HTH like + OB-Fold + MCM-family-AAA+ATPase + iLon-Protease<br>or<br>SIGMA-HTH like + OB-Fold + MCM-family-AAA+ATPase + HKD-DNase**	---	---	**SIGMA-HTH like + OB-Fold + MCM-family-AAA+ATPase**	---	---	---
Type-specific Synapomorphy	**SIGMA-HTH:**<br>Average length 67 residues;<br>Begins N-terminally with a small α-helix, followed by a tri-helical SIGMA-HTH like domain;<br>Topologically the SIGMA-HTH includes [Preceding αH-Insert β-strand-αH1-αH2-αH3]; The small insert β-strand together with a small β-strand present in the C-terminal helical bundle of the downstream MCM-AAA+ATPase, forms a small β-bridge element;<br>The sequence profile of SIGMA-HTH is highly conserved, including (i) a stretch of highly conserved positively charged residues [lysine and arginine], in the insert β-strand and following loop region;<br>(ii) Hydrophobic stretch of residues and a highly conserved negatively charged residues [D/E] in the middle of αH1;<br>**OB-Fold:**<br>[αH1-βS1-βS2-βS3-αH2-βS4-βS5]; Average length 93 residues;<br>Typical topology associated with OB-fold from small β-barrel assemblage of proteins;<br>αH1 is followed by βS1-βS2-βS3, together forming the β-meander sheet-A;<br>βS3 is followed by a helix and βS4-βS5, which forms the three stranded β-sheet B, along with the N-terminal half of βS1.<br>Overall sequence profile within Type-1 BrxL is highly conserved with multiple conserved, residues but is relatively distinctive from the OB-fold of Type-4 BrxL component.<br>**MCM-family-AAA+ATPase:**<br>Average length 329 residues; Structural and sequence features are typical to those of MCM-clade of AAA+ ATPases, including the clade-specific H2-insert in core αH2, and the PSI (Pre-sensor 1) insert region after core αH3;<br>C-terminal helical bundle terminates with a three-stranded wHTH-like configuration; The small β-strand occuring after core βS5 towardds the C-terminal end, pairs along with the insert βS from the N-terminal SIGMA-HTH to form a β-bridge.<br>**iLon-Protease:**<br>Average length 191 residues;<br>Structurally and topologically similar to canonical Lon-Proteases from E.coli;<br>*Absence of catalytic serine-lysine dyad residues, suggesting that it is enzymatically inactive;*<br>**HKD-DNase:**<br>In a subset of Type-1 BrxL, the C-terminal iLonP is replaced with an HKD-DNase component; Average length of BrxL HKD-DNase is 171 residues;<br>Structurally and topologically similar to the canonical versions of HKD-DNases belonging to PLD superfamily of endonucleases in RM systems;<br>*Retains a catalytic triad represented by [HRD], instead of the usual [HKD], found inType-I/Type-III-like RM system's endonucleases;*	---	---	The sequence and structural profile of all individual domains including the N-terminal SIGMA-HTH, the following OB-fold and the MCM-family AAA+ATPases, are similar to those found in Type-1 BrxL component;<br>*The key distinction lies in the C-terminal end, where unlike the Type-1 BrxL component, no additional domain fusions (inactive LonP or HKD-endoDNase) are retained, thus reducing the overall domain architecture to a three-domain containing BrxL component.*	---	---	---
**PglW (NERD + Kinase)**	---	✔	---	---	---	---	---
Designated-name	---	**PglW**	---	---	---	---	---
Domain architecture	---	**NERD + iPkinase + Pkinase + HTH + wHTH1 + wHTH2 + wHTH3 + iSTAND(BrxB)**	---	---	---	---	---
Type-specific Synapomorphy	---	**NERD:**<br>Average length 198 residues; Structurally and topologically similar to canonical PDDEXK superfamily of nuclease and related domain (NERD);<br>Retention of PDDEXK catalytic residues.<br>**iPkinase:**<br>Average length 289 residues; Belongs to Serine/Threonine/Tyrosine (S/T/Y) class of protein kinases;<br>Retains the typical structural and topological features of S/T/Y kinases;<br>Absence of the glycine rich loop between S1-S2, used for ATP localization;<br>Degeneration of the HRD motif of the catalytic loop and DFG motif of the Mg+ binding loop in βS6 and βS9 respectively;<br>No conserved D in Mg+ binding loop;<br>Lack of all catalytic motifs and loop features makes this S/T/Y kinase enzymatically inactive.<br>**Pkinase:**<br>Average length 278 residues; Belongs to the same S/T/Y class of protein kinases as the previous domain;<br>Retains the typical structural, topological and sequence features of S/T/Y kinases;<br>Glycine rich loop is conserved;<br>Catalytic loop with HRD motif and Mg+ binding loop with DFx motif are both conserved, followed by the activation loop;<br>This is a functional copy of S/T/Y kinase domain which is preceded by a enzymatically inactive copy of S/T/Y kinase.<br>**HTH + wHTH1 + wHTH2 + wHTH3:**<br>*The first HTH domain resembles the CTD of the RNA polymerase α-subunit, which is followed by three set of wHTH domains, together forming a large DNA-binding module similar to BrxA architecture, found in Type-1 and Type-4 BREX systems;*<br>**iSTAND:**<br>The C-terminal most region encodes an iSTAND, characterized by the absence of Walker A, Walker B, and the C-terminal helical-bundle; Average length of 192 residues;<br>Central β-sheet consists of the usual five core-strands found in AAA+ ATPases;<br>Retention of STAND/ORC-Cdc6 specific HEH (Helix-extension-helix) insert region after core βS2;<br>Conserved residues includes a Tryptophan and Aspartate in HEH insert region, a conserved arginine (not arginine finger) and tyrosine in αH3, a conserved stretch of hydrophobic residues in βS4.<br>*Although the sequence profile of majority of PglW C-terminal iSTAND is distinct from the iSTAND BrxB or BrxF components, found in other BREX systems, one subset of iSTAND from Type-2 PglW shares its sequence and structural features with those of BrxB/BrxF;*<br>*This includes stretch of hydrophobic residues and a highly conserved [PG] residues in βS4, which is followed by the three-stranded cap-like subdomain insert region.*	---	---	---	---	---
**Helicase-I**	---	✔	---	---	---	---	---
Designated-name	---	**BrxHI-Helicase**	---	---	---	---	---
Domain architecture	---	**Ski2-like-RNA-Helicase + wHTH + Small-β-Barrel (HAS-barrel) + Lhr-Helicase-CTD**	---	---	---	---	---
Type-specific Synapomorphy	---	**Ski2-like-RNA-Helicase:**<br>SF2-helicase with all typical sequence and structural features, including two tandem P-loop domains (an active P-loop, followed by an inactive P-loop);<br>*Belongs to the Ski2-like helicase family, within SF2-Helicase superfamily;*<br>**wHTH:**<br>Canonical wHTH domain with an average length of 89 residues;<br>**HAS-barrel:**<br>Small All-β protein domain, belonging to the small β-barrel assemblage of proteins, with structural and topological features identifying it as a homolog of HAS-barrel domains;<br>**Lhr-helicase-CTD:**<br>A C-terminal α+β domain with an average length of 169 residues;<br>No structural homologs detected, apart from the C-terminal domain found in Lhr-helicases;<br>five-stranded central β-sheet flanked by α-helices and a distinct three-helical bundle extending outward;<br>Conserved residues includes:<br>(i) an arginine at the base of αH2;<br>(ii) a conserved motif [WWTaAG] (a-aromatic) in the loop connecting βS2-αH3;<br>(iii) a conserved asparagine in the beginning of αH3;<br>(iv) Conserved dyad [KF] in the beginning of αH6;<br>(v) A conserved arginine or lysing at the end of αH6.<br>Only the conserved arginine in αH2(i), conserved dyad [KF](iv) and the conserved lysine or arginine(v) are pointing outwards from the structure, rest all are buried within the central β-sheet and surrounding helices of the core structure;<br>A conserved dyad of lysine/arginine residues [KR] in the CTD of Lhr-helicase has been shown to be involved in DNA-binding.	---	---	---	---	---
**Standalone-ATPase**	---	✔	---	---	---	---	---
Designated-name	---	**BrxD-ATPase**	---	---	---	---	---
Domain architecture	---	**STAND/ORC-Cdc6-AAA+ATPase**	---	---	---	---	---
Type-specific Synapomorphy	---	**STAND/ORC-Cdc6-AAA+ATPase:**<br>Standalone canonical STAND NTPase with an average length of 435 residues;<br>Canonical features - Walker A and Walker B residues, Sensor-1 atop strand-4, Arginine-Finger at the base of strand-5, Sensor-2-Arginine at distal end of helical-bundle, C-terminal helical bundle, are all retained;<br>Similar to the BrxC-STAND/ORC-Cdc6, the HEH insert region after core βS2 is elaborated forming four closely pacled α-helices.	---	---	---	---	---
**Helicase-II**	---	---	✔	---	✔	✔	✔
Designated-name	---	---	**BrxHII-Helicase**	---	---	---	---
Domain architecture	---	---	**TUDOR + Swi2/Snf2-Helicase + iREase + CTD**	---	**TUDOR + Swi2/Snf2-Helicase + iREase + CTD + REase(PDDEXK)**	**HKD-DNase + Swi2/Snf2-Helicase + iREase + CTD**	**TUDOR + Swi2/Snf2-Helicase + iREase + CTD**
Type-specific Synapomorphy	---	---	**TUDOR:**<br>A Small-β-Barrel domain with homology to TUDOR domains;<br>**Swi2/SNF2-Helicase:**<br>Average length of 601 residues;<br>SF2-Helicase belonging to the Swi2/SNF2-helicase superfamily, with retention of all canonical features associated with these helicases, including two tandem P-loop domains (an active P-loop, followed by an inactive P-loop);<br>A large all-α helical insert region (average length 115 residues) occuring after the core βS2 of the 2nd inactive P-loop domain;<br>**iREase:**<br>Small degenerated α+β domain with an average length of 82 residues;<br>Topologically represented by [αH1-βS1-βS2-βS3-(small-degenerate βS)-αH2];<br>The central β-sheet consists of three-stranded β-sheet, and both αH1 and αH2 are located behind the central β-sheet;<br>The domain is structurally and sequentially homologous to the first small α+β domain of RapA;<br>Our comparative structural analysis reveals that both the first small α+β domain of RapA and BrxHII-Helicase adopts a small REase like fold;<br>*Structurally and topologically it is similar to various endonucleases related to the virus-type replication-repair (VRR) nuclease and holliday junction resolvases (HJC), which belongs to the PD-(D/E)-XK superfamily of nucleases;*<br>*Despite the retention of the PDDEXK-fold, the core β-sheet (particularly βS4) exhibits partial deterioration;*<br>βS4 in RapA is represented as a small degenerated β-strand, but in BrxHII-iREase, it takes the form of a loop section which leads directly to αH4 behind the central β-sheet;<br>The catalytic PD-(D/E)-XK motif is entirely degenerate, with no conservation of residues across all known representatives, and a fast-evolving sequence profile, suggesting that this REase module is potentially inactive.<br>**CTD α+β:**<br>An α+β domain with an average length of 166 residues;<br>Topologically represented by [βS1-βS2-βS3-αH1-βS4-αH2-αH3-βS4];<br>The five β-strands are arranged anti-parallely to form a five-stranded β-sheet;<br>Structural searches did not identify any true homologs beyond the C-terminal most α+β domain of RapA Swi2/Snf2-helicase, suggesting that this is unique to Type-2 helicases.	---	Overall features of all individual domains are same as those found in Type-3 BrxHII-Helicase;<br>The sequence profile of the C-terminal α+β domain is distinct from other variants found in Type-3 BREX and Type-2, Type-3 BR system's helicase components;<br>*The key distinction lies in the occurence of a canonical REase domain in the C-terminal end of the helicase component, belonging to the PDDEXK superfamily;*<br>The REase features the typical sequence and structural features associated with PDDEXK REases, and also features the catalytic residues, suggesting that unlike the degenerated version of an iREase found in the preceding region, this REase is enzymatically active and can potentially faciliate DNA degradation restriction activity.	**HKD-DNase:**<br>N-terminal TUDOR domain is absent;<br>*Instead a enzymatically active restriction endonuclease from the HKD-superfamily (also known as Phospholipase-D), is found in the N-terminal region;*<br>Catalytic residues [HKD] intact, resembling the canonical versions found in Type-I/Type-III RM-like systems;<br>**Swi2/SNF2-Helicase:**<br>Overall sequence and structural features are similar to those found in Type-3 BREX BrxHII-Helicase and the helicase component of Type-1 DUF499 associated systems;<br>**CTD α+β:**<br>The C-terminal α+β domain is slightly shortened;<br>Sequence profile is highly divergent and distinctive from other versions of C-terminal α+β domains, found across all variants of this helicase component.	*Structurally and architecturally similar to the Type-3 BREX system's helicase BrxHII;*<br>Begins with a standalone N-terminal TUDOR domain, followed by a Swi2/SNF2-Helicase, a degenerated inactive REase, and a C-terminal α+β domain;<br>*Compared to the helicase component of Type-1 and Type-2 BR systems, this lacks an active endonuclease domain;*<br>The C-terminal α+β domain of Type-3 associated DUF499 systems are relatively smaller with an average length of 108 residues, primarily due to presence of a four antiparallel strands in the central b-sheet, and reduction in size of the coiled-coi
