# Supplementary material for: Expanding the landscape of BREX diversity: uncovering multi-layered functional frameworks and identification of novel BREX-related defense systems
Source: Nucleic Acids Res. 2026 Jan 27;54(3):gkag035. doi: 10.1093/nar/gkag035 (PMC12839542; doi:10.1093/nar/gkag035)
Supplement: gkag035_Supplemental_Files [file Supplementary_Data_S4.pdf › Supplementary_Data_S4.pdf]

Supplementary Data S4

The PDF presents three distinct datasets organized as follows:

(i) Gene neighbourhoods of BREX and related systems containing auxiliary effectors from diverse superfamilies. The file follows the format of Supplementary Data S1, with column-specific information marked at the top of each page. Within each neighbourhood, core components of a given BREX subtype are highlighted in light blue, while nearby auxiliary nuclease effectors are shown in distinct colors for clarity. Nucleases occurring as fusion components or in consistent genomic association with specific core proteins (as discussed in the manuscript) are also color-coded for visualization.

(ii) Domain architectures and corresponding NCBI node information for all identified nuclease superfamilies associated with BREX and related systems.

(iii) Representative MSAs and domain boundary characterizations for all auxiliary nuclease superfamilies linked to BREX and related systems. Alignments are color-coded and annotated as in Supplementary Data S2 and S3.

1. **Backup effector nucleases associated with Type-1/5/6 BREX**
   - (i) HEPN-endoRNase………4-8
   - (ii) HKD-DNase(BrxL-Fusion)………9-24
   - (iii) HKD-DNase+OGG-like………24-28
   - (iv) ParB+HNH(GmrSD)………29-39
   - (v) OLD-ABC associated HNH………39-40
   - (vi) wHTH(SBD)+HNH………40-41
   - (vii) HNH (Standalone)………41-42
   - (viii) PIN-EndoRNase………43-44
   - (ix) BrxHII-Helicase+REase (Type-5 BREX)………45
   - (x) CR-ATPase+REase………45-46
   - (xi) (w)HTH+REase/HARE-HTH+REase………46-49
   - (xii) SF2-Helicase+REase………49-51
   - (xiii) Standalone REase; OLD-ABC associated REase………51-57
   - (xiv) Schlafen+HATPase+wHTH………58-67
   - (xv) OLD-ABC associated TOPRIM………68-79
2. **Backup effector nucleases associated with Type-2 BREX**
   - (i) HEPN-endoRNase………80-81
   - (ii) SF2-Helicase+HKD-DNase………82
   - (iii) ParB+HNH (GmrSD)………83-84
   - (iv) OLD-ABC associated HNH and SAD(SRA)+HNH………84
   - (v) PglW component………85-97
   - (vi) PIN-EndoRNase………98
   - (vii) REase; SF2-Helicase+REase; REase+SF2-Helicase………99-100
   - (viii) Schlafen+HATPase+wHTH………101
   - (ix) OLD-ABC associated TOPRIM………102
3. **Backup effector nucleases associated with Type-3 BREX**
   - (i) HEPN-endoRNase………103-104
   - (ii) HKD-DNase………105
   - (iii) ParB+HNH(GmrSD)………106
   - (iv) HNH(standalone) and wHTH(SBD)+HNH………106-107
   - (v) PIN-EndoRNase………108
   - (vi) BrxHII-Helicase+REase………109
   - (vii) (w)HTH+REase………109-110
   - (viii) Standalone REase; OLD-ABC associated REase; SF1-SF2-Helicase+REase………110-111
   - (ix) Schlafen+HATPase+wHTH………112-115
   - (x) OLD-ABC associated TOPRIM………116-117
4. **Backup effector nucleases associated with Type-4 BREX**

(i) HEPN-endoRNase……118
(ii) ParB+HNH(GmrSD)……119
(iii) wHTH(SBD)+HNH……119
(iv) HNH (standalone)……119-120
(v) PIN-EndoRNase……121
(vi) Standalone REase; HTH+REase……122
(vii) CR-ATPase+REase……122
(viii) Schlafen+HATPase+wHTH……123
(ix) OLD-ABC associated TOPRIM……124

5. Backup effector nucleases associated with Type-1 BREX-Related systems
(i) HEPN+DUF499……125-146
(ii) Standalone HEPN; DNA-PolB associated HEPN……146-148
(iii) HKD-DNase……149
(iv) ParB+HNH(GmrSD)……150-152
(v) wHTH(SBD)+HNH……153
(vi) HNH standalone; OLD-ABC associated HNH……153-155
(vii) PIN-EndoRNase; OLD-ABC associated PIN……156-161
(viii) Additional PDDEXK-REase alongside primary REase effector; OLD-ABC associated REase……162-167
(ix) REase, SF2-Helicase+REase……167-170
(x) Schlafen+HATPase+wHTH……171-174
(xi) OLD-ABC associated TOPRIM……175-178

6. Backup effector nucleases associated with Type-2 BREX-Related systems
(i) HEPN-EndoRNase……179
(ii) ParB+HNH(GmrSD)……180
(iii) HNH; Standalone versions, OLD-ABC associated and wHTH(SBD)+HNH……181
(iv) PIN-EndoRNase……182-183
(v) (w)HTH+REase……184-186
(vi) REase……186-187
(vii) Schlafen+HATPase+wHTH……188-189
(viii) OLD-ABC associated TOPRIM……190

7. Backup effector nucleases associated with Type-2 BREX-Related systems
(i) HEPN-EndoRNase……191
(ii) HKD-DNase……192
(iii) ParB+HNH(GmrSD)……193
(iv) HNH……193
(v) PIN-EndoRNase……194
(vi) REase……195
(vii) Schlafen+HATPase+wHTH……196
(viii) OLD-ABC associated TOPRIM……197

8. OLD-ABC associated nucleases other than TOPRIM (OLD ABC associated with HNH/REase/PIN)....198-207

9. Representative domain architectures and corresponding NCBI node information
(i) HKD-DNase+OGG-like……208-210
(ii) HNH-standalone……211-216
(iii) ParB+HNH……216-231
(iv) wHTH(SBD)+HNH……232
(v) SAD(SRA)+wHTH(SBD)+HNH……233

(vi) PIN-EndoRNase……234-242
(vii) CR-ATPase+REase……243
(viii) (w)HTH+REase……244-248
(ix) McrC-NTD+REase……248
(x) REase+SF2-Helicase……249-250
(xi) REase-standalone……250-260
(xii) SF2-Helicase+REase……261-263
(xiii) TUDOR+SF1-SF2-Helicase+iREase+CTD+REase……263-264
(xiv) Schlafen+HATPase+wHTH……265-278
(xv) OLD-ABC……279-291
(xvi) OLD-ABC+TOPRIM……291-294
(xv) TOPRIM……294-306
10. Representative alignments with topology and domain architectures of backup nuclease effectors
(i) HEPN-EndoRNase……307-309
(ii) CBS+HEPN……309
(iii) HKD-DNase+OGG-like……310
(iv) SIGMA-HTH+OB-Fold+MCM-AAA+HKD-DNase……310
(v) ParB+HNH……311-312
(vi) wHTH(SBD)+HNH……312
(vii) SAD(SRA)+wHTH(SBD)+HNH……312-313
(viii) Standalone HNH EndoDNase……313
(ix) PIN-EndoRNase……314-316
(x) Type-5 BREX and Type-3 BREX BrxHII-Helicase with C-terminal Active PDDEXK REase; (TUDOR+SF1-SF2-Helicase+iREase+CTD+REase)……317
(xi) REase+SF2-Helicase……317
(xii) SF2-Helicase+REase……318
(xiii) CR-ATPase+REase……318-319
(xiv) (w)HTH+REase……319-320
(xv) McrC-NTD+REase……320
(xvi) Standalone PDDEXK-REase……321-323
(xvii) Schlafen+HisKin+wHTH……324
(xviii) OLD-ABC+TOPRIM (Previously classified OLD-ABCs in association with ABC-ATPase centric conflict systems)……325
(xix) OLD-ABC (TOPRIM-linked)……325-327
(xx) BR-systems OLD-ABC……327
(xxi) OLD-ABC+TOPRIM……327
(xxii) TOPRIM-nuclease……328

#Titles are internally hyperlinked.
Click on the titles to access the material.

Protein-ID	System	Gene-Neighbourhoods	Length	Taxonomy	Assembly-ID	Strain	Locus-Tag	Genomic-coordinates	TaxID	Fasta-defline
--										
-- HEPN -EndoRNase										
--										
WP_015789649.1	Type1.BREX	<-REase<-?<-?||DOC+zhth-><-BirA-HTH<-?||?->BrxA-DNA-binding->DUF6079*->BrxB-BrxF-iSTAND->DUF6079-><-?<-?||?-><-OLD-ABC+TOPRIM||N6-METHYLASE->TBP+TBP->PglZ->	1236	Archaea>Euryarchaeota>Stenosarchaea_group>Halobacteria>Halobacteriales>Haloarculaceae	GCF_000023945.1	Halorhabdus_utahensis	HUTA_RS09330	1919731..1923441	146826	hypothetical-protein-[Halorhabdus-utahensis]
WP_015789651.1	Type1.BREX	DOC+zhth-><-BirA-HTH<-?||?->BrxA-DNA-binding->DUF6079->BrxB-BrxF-iSTAND->DUF6079*-><-?<-?||?-><-OLD-ABC+TOPRIM||N6-METHYLASE->TBP+TBP->PglZ->HEPN->TUDOR+SF1-SF2-Helicase+iREase+CTD->	1280	Archaea>Euryarchaeota>Stenosarchaea_group>Halobacteria>Halobacteriales>Haloarculaceae	GCF_000023945.1	Halorhabdus_utahensis	HUTA_RS09340	1924016..1927858	146826	hypothetical-protein-[Halorhabdus-utahensis]
WP_205252856.1	Type1.BREX	<-SF1-SF2-Helicase+REase<-TUDOR+SF1-SF2-Helicase+iREase+CTD<-HEPN<-PglZ<-?<-N6-METHYLASE||?->?-><-REase<-DUF6079*<-BrxB-BrxF-iSTAND<-DUF6079<-BrxA-DNA-binding||ORC-CDC6->ORC-CDC6+HTH-><-?||MarR-HTH->	1280	Archaea>Euryarchaeota>Stenosarchaea_group>Halobacteria>Halobacteriales>Halobacteriaceae	GCF_016917835.2	Halobacterium_sp._BOL4-2	JRZ79_RS24035	52137..55979	2810537	hypothetical-protein-[Halobacterium-sp.-BOL4-2]
WP_205252782.1	Type1.BREX	<-HEPN<-PglZ<-?<-N6-METHYLASE||?->?-><-REase<-DUF6079<-BrxB-BrxF-iSTAND<-DUF6079*<-BrxA-DNA-binding||ORC-CDC6->ORC-CDC6+HTH-><-?||MarR-HTH-><-?<-?||HTH_28->NTP_transf_5->	1227	Archaea>Euryarchaeota>Stenosarchaea_group>Halobacteria>Halobacteriales>Halobacteriaceae	GCF_016917835.2	Halobacterium_sp._BOL4-2	JRZ79_RS24045	56554..60237	2810537	hypothetical-protein-[Halobacterium-sp.-BOL4-2]
WP_004047662.1	Type5.BREX	Peptidase_M29-><-?<-BirA-HTH||?-><-?<-ParA-Soj-PloopNTPase<-?||?->BrxA-DNA-binding->DUF6079*->BrxB-BrxF-iSTAND->DUF6079->?->HEPN->N6-METHYLASE->PglZ->N6-METHYLASE->TUDOR+SF1-SF2-Helicase+iREase+CTD->	1249	Archaea>Euryarchaeota>Stenosarchaea_group>Halobacteria>Haloferacales>Halorubraceae	GCF_000337915.1	Halorubrum_saccharovorum	C471_RS07440	145399..149148	2248	hypothetical-protein-[Halorubrum-saccharovorum]
WP_004047666.1	Type5.BREX	<-BirA-HTH||?-><-?<-ParA-Soj-PloopNTPase<-?||?->BrxA-DNA-binding->DUF6079->BrxB-BrxF-iSTAND->DUF6079*->?->HEPN->N6-METHYLASE->PglZ->N6-METHYLASE->TUDOR+SF1-SF2-Helicase+iREase+CTD->?->?-><-DDE_Tnp_IS240	1276	Archaea>Euryarchaeota>Stenosarchaea_group>Halobacteria>Haloferacales>Halorubraceae	GCF_000337915.1	Halorubrum_saccharovorum	C471_RS07450	149762..153592	2248	hypothetical-protein-[Halorubrum-saccharovorum]
WP_137705565.1	Type1.BREX	<-ParA-Soj-PloopNTPase||?-><-PADR-HTH<-?<-?<-?<-?||BrxA-DNA-binding->DUF6079*->BrxB-BrxF-iSTAND->DUF6079->REase-><-?<-?||N6-METHYLASE->?->PglZ->HEPN->	1227	Archaea>Euryarchaeota>Stenosarchaea_group>Halobacteria>Haloferacales>Halorubraceae	GCF_005435115.1	Halorubrum_sp._ARQ200	EXE50_RS16210	7684..11367	1855872	hypothetical-protein-[Halorubrum-sp.-ARQ200]
WP_137705567.1	Type1.BREX	<-PADR-HTH<-?<-?<-?<-?||BrxA-DNA-binding->DUF6079->BrxB-BrxF-iSTAND->DUF6079*->REase-><-?<-?||N6-METHYLASE->?->PglZ->HEPN->TUDOR+SF1-SF2-Helicase+iREase+CTD-><-BirA-HTH	1280	Archaea>Euryarchaeota>Stenosarchaea_group>Halobacteria>Haloferacales>Halorubraceae	GCF_005435115.1	Halorubrum_sp._ARQ200	EXE50_RS16220	11942..15784	1855872	hypothetical-protein-[Halorubrum-sp.-ARQ200]
WP_138006592.1	Type5.BREX	<-TUDOR+SF1-SF2-Helicase+iREase+CTD<-PglZ<-N6-METHYLASE<-?<-HEPN<-Peptidase_M1<-DUF6079*<-BrxB-BrxF-iSTAND<-DUF6079<-BrxA-DNA-binding	1272	Archaea>Euryarchaeota>Stenosarchaea_group>Halobacteria>Haloferacales>Halorubraceae	GCF_005543295.1	Halalkalirubrum_salinum	E5K03_RS09380	11807..15625	2563889	hypothetical-protein-[Halalkalirubrum-salinum]
WP_138006594.1	Type5.BREX	<-TUDOR+SF1-SF2-Helicase+iREase+CTD<-PglZ<-N6-METHYLASE<-?<-HEPN<-Peptidase_M1<-DUF6079<-BrxB-BrxF-iSTAND<-DUF6079*<-BrxA-DNA-binding	1258	Archaea>Euryarchaeota>Stenosarchaea_group>Halobacteria>Haloferacales>Halorubraceae	GCF_005543295.1	Halalkalirubrum_salinum	E5K03_RS09390	16209..19985	2563889	hypothetical-protein-[Halalkalirubrum-salinum]
WP_121744813.1	Type6.BREX	Thermonuclease->?->?->wHTH+REase->SF1-SF2-Helicase->BrxE->BrxA-DNA-binding->BrxB-BrxF-iSTAND->DUF6079*-><-?||N6-METHYLASE-><-HAD<-HTH+PADR-HTH||?-><-?||PglZ->SIGMA+OB-Fold+AAA-ATPase+Lon-Protease-BrxL->HEPN->	1206	Archaea>Euryarchaeota>Stenosarchaea_group>Halobacteria>Natrialbales>Natrialbaceae	GCF_003670115.1	Natronorubrum_halophilum	DWB23_RS21360	42652..46272	1702106	BREX-system-P-loop-protein-BrxC-[Natronorubrum-halophilum]
MBU4220824.1	Type1.BREX	PAIREDC-HTH->?->?->?->?-><-?||BrxB-BrxF-iSTAND->DUF6079*->N6-METHYLASE->TIR->Schlafen-p->HEPN->Schlafen+HATPase+wHTH->?->?->PglZ-p->	1180	Archaea>Euryarchaeota>unclassified_Euryarchaeota	GCA_018898145.1	Euryarchaeota_archaeon	KKA10_04250	6252..9794	2026739	BREX-system-P-loop-protein-BrxC-[Euryarchaeota-archaeon]
MBD0371455.1	Type1.BREX	VirB11->PSBP->?-><-SIGMA+OB-Fold+AAA-ATPase+Lon-Protease-BrxL<-PglZ<-ParB+HNH<-NERD<-N6-METHYLASE<-HEPN<-DUF6079*<-BrxB-BrxF-iSTAND<-BrxA-DNA-binding	1218	Bacteria>Acidobacteriota>Blastocatellia>Blastocatellales>Pyrinomonadaceae>unclassified_Pyrinomonadaceae	GCA_014534425.1	Pyrinomonadaceae_bacterium	ICV60_11505	29495..33151	2283092	BREX-system-P-loop-protein-BrxC-[Pyrinomonadaceae-bacterium]
MCI0490100.1	Type1.BREX	BrxA-DNA-binding->BrxB-BrxF-iSTAND->DUF6079*->HEPN->Four-helical-protein-><-?<-?||N6-METHYLASE->Schlafen+HATPase+wHTH-p->	1218	Bacteria>Acidobacteriota>Blastocatellia>unclassified_Blastocatellia	GCA_022615215.1	Blastocatellia_bacterium	L0229_26200	2085..5741	2052146	BREX-system-P-loop-protein-BrxC-[Blastocatellia-bacterium]
MBK9165938.1	Type1.BREX	XIS-HTH-><-?<-SIGMA+OB-Fold+AAA-ATPase+Lon-Protease-BrxL<-PglZ<-PglZ<-PglZ<-?<-N6-METHYLASE<-HEPN<-DUF6079*<-BrxB-BrxF-iSTAND<-BrxA-DNA-binding<-CopG/RHH||?-><-ParD_antitoxin<-HTH_37||?->?->Tox-RelE->	1234	Bacteria>Acidobacteriota>Terriglobia>Bryobacterales>unclassified_Bryobacterales	GCA_016716385.1	Bryobacterales_bacterium	IPM24_00570	108049..111753	2026791	BREX-system-P-loop-protein-BrxC-[Bryobacterales-bacterium]
OFW17305.1	Type1.BREX	<-SF1-SF2-Helicase<-SIGMA+OB-Fold+AAA-ATPase+Lon-Protease-BrxL<-PglZ<-HATPase+wHTH<-HATPase_c_4<-Schlafen-p<-N6-METHYLASE<-HEPN<-PolB<-DUF6079*<-BrxB-BrxF-iSTAND<-BrxA-DNA-binding||wHTH+AbiEi_2->AbiEii-><-?<-?<-PIN	1156	Bacteria>Acidobacteriota>unclassified_Acidobacteriota	GCA_001767195.1	Acidobacteria_bacterium_RIFCSPLOWO2_02_FULL_67_21	A3H29_10520	11544..15014	1797183	hypothetical-protein-A3H29_10520-[Acidobacteria-bacterium-RIFCSPLOWO2_02_FULL_67_21]
OFW39743.1	Type1.BREX	PIN->?->?-><-AbiEii<-wHTH+AbiEi_2||BrxA-DNA-binding->BrxB-BrxF-iSTAND->DUF6079*->PolB->HEPN->N6-METHYLASE->Schlafen+HATPase+wHTH->HATPase+wHTH->PglZ->SIGMA+OB-Fold+AAA-ATPase+Lon-Protease-BrxL->SF1-SF2-Helicase->DDE_Tnp_1_4->	1156	Bacteria>Acidobacteriota>unclassified_Acidobacteriota	GCA_001767515.1	Acidobacteria_bacterium_RIFCSPLOWO2_12_FULL_67_14	A3F70_01760	7438..10908	1797192	hypothetical-protein-A3F70_01760-[Acidobacteria-bacterium-RIFCSPLOWO2_12_FULL_67_14]
MBS1828808.1	Type1.BREX	D5N+D5ATPase-><-?||ParB-><-?<-Phage_integrase+Phage_integrase<-?||CopG/RHH->BrxA-DNA-binding->BrxB-BrxF-iSTAND->DUF6079*->HEPN->N6-METHYLASE->PglZ->SIGMA+OB-Fold+AAA-ATPase+Lon-Protease-BrxL->REase->HEPN->?->Phage_integrase->	1226	Bacteria>Acidobacteriota>unclassified_Acidobacteriota	GCA_018268935.1	Acidobacteriota_bacterium	JST93_26130	24024..27704	1978231	BREX-system-P-loop-protein-BrxC-[Acidobacteriota-bacterium]
MCI0393103.1	Type1.BREX	BrxA-DNA-binding->BrxB-BrxF-iSTAND->DUF6079*-><-?||HEPN->N6-METHYLASE->Schlafen+HATPase+wHTH+HTH->PglZ->SIGMA+OB-Fold+AAA-ATPase+Lon-Protease-BrxL-><-MPTase<-HTH_3	1220	Bacteria>Acidobacteriota>unclassified_Acidobacteriota	GCA_022614935.1	Acidobacteriota_bacterium	MOB07_30595	1910..5572	1978231	BREX-system-P-loop-protein-BrxC-[Acidobacteriota-bacterium]
MCI0698477.1	Type1.BREX	<-Schlafen+HATPase+wHTH<-HEPN<-DUF6079*<-BrxB-BrxF-iSTAND<-BrxA-DNA-binding<-AbiEii+ZnR<-AbiEi_4+AbiEi_1	1215	Bacteria>Bacteria_incertae_sedis>Bacteria_candidate_phyla>candidate_division_KSB1>unclassified_candidate_division_KSB1	GCA_022616415.1	candidate_division_KSB1_bacterium	L0337_41575	2047..5694	2172550	BREX-system-P-loop-protein-BrxC-[candidate-division-KSB1-bacterium]
MBI2566519.1	Type1.BREX	NtrC-AAA+FIS-HTH->?->?-><-?<-?||BrxA-DNA-binding->BrxB-BrxF-iSTAND->DUF6079*->?->N6-METHYLASE->HEPN->ParA-Soj-PloopNTPase->?->PglZ->HNH-><-?<-NtrC-AAA+FIS-HTH	1109	Bacteria>Bacteria_incertae_sedis>Bacteria_candidate_phyla>Candidatus_Schekmanbacteria	GCA_016188575.1	Candidatus_Schekmanbacteria_bacterium	HYV63_05760	102186..105515	2419984	BREX-system-P-loop-protein-BrxC-[Candidatus-Schekmanbacteria-bacterium]
PKK90022.1	Type1.BREX	<-Amidohydro_1<-?<-?||BrxA-DNA-binding->BrxB-BrxF-iSTAND->DUF6079*->HEPN->N6-METHYLASE-p->?->HEPN->	1176	Bacteria>Bacteria_incertae_sedis>Bacteria_candidate_phyla>Candidatus_Wallbacteria	GCA_002838975.1	Candidatus_Wallbacteria_bacterium_HGW-Wallbacteria-1	CVV64_11925	128140..131670	2013854	BREX-system-P-loop-protein-BrxC-[Candidatus-Wallbacteria-bacterium-HGW-Wallbacteria-1]
WP_046997142.1	Type1.BREX	wHTH+WYL+WCX->BrxA-DNA-binding->BrxB-BrxF-iSTAND->DUF6079*->N6-METHYLASE->?->HEPN->Schlafen+HATPase+wHTH->PglZ->SIGMA+OB-Fold+AAA-ATPase+Lon-Protease-BrxL->	1202	Bacteria>Campylobacterota>Epsilonproteobacteria>Campylobacterales>Arcobacteraceae>Aliarcobacter	GCF_001010585.1	Aliarcobacter_butzleri	AA20_RS09495	10373..13981	28197	BREX-system-P-loop-protein-BrxC-[Aliarcobacter-butzleri]
WP_260968564.1	Type1.BREX	ABC-ATPase+SbcC->?->LuxR-HTH-><-?||Phage_integrase->?->wHTH+WYL+WCX->BrxA-DNA-binding->BrxB-BrxF-iSTAND->DUF6079*->N6-METHYLASE-p->HEPN+RES->N6-METHYLASE-p->Schlafen+HATPase+wHTH->Schlafen+HATPase+wHTH->PglZ->SIGMA+OB-Fold+AAA-ATPase+Lon-Protease-BrxL->Calcineurin-><-SIGMA-HTH	1162	Bacteria>Campylobacterota>Epsilonproteobacteria>Campylobacterales>Arcobacteraceae>Aliarcobacter	GCF_025335945.1	Aliarcobacter_cryaerophilus	N5S77_RS10385	37284..40772	28198	BREX-system-P-loop-protein-BrxC-[Aliarcobacter-cryaerophilus]
OZA03558.1	Type1.BREX	<-SAM-METHYLASE||?-><-TPR||?->BrxB-BrxF-iSTAND->ACET->XIS-HTH->BrxA-DNA-binding->BrxB-BrxF-iSTAND->DUF6079*->HEPN->N6-METHYLASE->?->ZnR+HEPN->?->PglZ->SIGMA+OB-Fold+AAA-ATPase+Lon-Protease-BrxL->	1158	Bacteria>Campylobacterota>Epsilonproteobacteria>Campylobacterales>Thiovulaceae>Sulfuricurvum	GCA_002282015.1	Sulfuricurvum_sp._17-40-25	B7X89_02520	35980..39456	1970439	MAG:-hypothetical-protein-B7X89_02520-[Sulfuricurvum-sp.-17-40-25]
WP_013460546.1	Type1.BREX	Phage_integrase-><-XIS-HTH<-HTH_36<-?||HTH_3-><-XIS-HTH||BrxA-DNA-binding->BrxB-BrxF-iSTAND->DUF6079*-><-?||N6-METHYLASE-p->?->?->HEPN->BrxD->N6-METHYLASE-p->CR-ATPase5+REase->PglZ->	1163	Bacteria>Campylobacterota>Epsilonproteobacteria>Campylobacterales>Thiovulaceae>Sulfuricurvum	GCF_000183725.1	Sulfuricurvum_kujiense	SULKU_RS08475	1692937..1696428	148813	BREX-system-P-loop-protein-BrxC-[Sulfuricurvum-kujiense]
QFR43750.1	Type1.BREX	Glycos_transf_4->?->?->tRNA->tRNA->XIS-HTH->BrxA-DNA-binding->BrxB-BrxF-iSTAND->DUF6079*->HEPN->N6-METHYLASE-p->?->DUF3644->?->N6-METHYLASE-p->?->PglZ->SIGMA+OB-Fold+AAA-ATPase+Lon-Protease-BrxL->	1163	Bacteria>Campylobacterota>Epsilonproteobacteria>Campylobacterales>Thiovulaceae>Sulfurimonas	GCA_009258045.1	Sulfurimonas_xiamenensis	FJR47_07440	1472966..1476457	2590021	BREX-system-P-loop-protein-BrxC-[Sulfurimonas-xiamenensis]
WP_188093712.1	Type1.BREX	Glycos_transf_4->?->?->tRNA->tRNA->XIS-HTH->BrxA-DNA-binding->BrxB-BrxF-iSTAND->DUF6079*->HEPN->N6-METHYLASE-p->?->DUF3644->?->N6-METHYLASE-p->?->PglZ->SIGMA+OB-Fold+AAA-ATPase+Lon-Protease-BrxL->	1160	Bacteria>Campylobacterota>Epsilonproteobacteria>Campylobacterales>Thiovulaceae>Sulfurimonas	GCF_009258045.1	Sulfurimonas_xiamenensis	FJR47_RS07440	1472975..1476457	2590021	BREX-system-P-loop-protein-BrxC-[Sulfurimonas-xiamenensis]
WP_193150356.1	Type1.BREX	<-Phage_integrase<-SIGMA+OB-Fold+AAA-ATPase+Lon-Protease-BrxL<-PglZ<-?<-SKI<-N6-METHYLASE-p<-RVT<-N6-METHYLASE-p<-HEPN<-DUF6079*<-BrxB-BrxF-iSTAND<-BrxA-DNA-binding<-DDE_Tnp<-?<-DDE_Tnp	1160	Bacteria>Campylobacterota>Epsilonproteobacteria>Campylobacterales>Thiovulaceae>Sulfurimonas	GCF_014905115.1	Sulfurimonas_sediminis	FJR45_RS09735	1812721..1816203	2590020	BREX-system-P-loop-protein-BrxC-[Sulfurimonas-sediminis]
OYZ34277.1	Type1.BREX	<-SIGMA+OB-Fold+AAA-ATPase+Lon-Protease-BrxL<-PglZ<-?<-ZnR+HEPN<-?<-N6-METHYLASE<-HEPN<-DUF6079*<-DUF6079-p<-BrxB-BrxF-iSTAND<-BrxA-DNA-binding<-XIS-HTH<-ACET<-BrxB-BrxF-iSTAND<-?||TPR->	1013	Bacteria>Campylobacterota>Epsilonproteobacteria>Campylobacterales>unclassified_Campylobacterales	GCA_002281685.1	Campylobacterales_bacterium_16-40-21	B7Y30_07055	21520..24561	1970501	hypothetical-protein-B7Y30_07055-[Campylobacterales-bacterium-16-40-21]
WP_013452128.1	Type1.BREX	CR-ATPase8+REase-><-?<-SIGMA+OB-Fold+AAA-ATPase+Lon-Protease-BrxL<-PglZ<-N6-METHYLASE<-HEPN<-N6-METHYLASE-p<-DUF6079*<-BrxB-BrxF-iSTAND<-BrxA-DNA-binding||CR-ATPase8+REase->	1161	Bacteria>Deferribacterota>Deferribacteres>Deferribacterales>Calditerrivibrionaceae>Calditerrivibrio	GCF_000183405.1	Calditerrivibrio_nitroreducens	CALNI_RS10260	2104217..2107702	477976	BREX-system-P-loop-protein-BrxC-[Calditerrivibrio-nitroreducens]
MBZ4644075.1	Type1.BREX	<-TPR<-ARG<-?||McrB-AAA->wHTH+REase->BrxA-DNA-binding->BrxB-BrxF-iSTAND->DUF6079*->N6-METHYLASE-p->HEPN->N6-METHYLASE->PglZ->SIGMA+OB-Fold+AAA-ATPase+Lon-Protease-BrxL->?->SF1-SF2-Helicase->	1168	Bacteria>Deferribacterota>Deferribacteres>Deferribacterales>Deferribacteraceae>unclassified_Deferribacteraceae	GCA_020054865.1	Deferribacteraceae_bacterium	JG767_1684	7812..11318	2053543	hypothetical-protein-[Deferribacteraceae-bacterium]
WP_242820841.1	Type1.BREX	NUDIX-><-TPR+S1<-ARG<-?<-?||McrB-AAA->wHTH+REase->BrxA-DNA-binding->BrxB-BrxF-iSTAND->DUF6079*->N6-METHYLASE-p->HEPN->N6-METHYLASE->PglZ->SIGMA+OB-Fold+AAA-ATPase+Lon-Protease-BrxL->?->SF1-SF2-Helicase->	1168	Bacteria>Deferribacterota>Deferribacteres>Deferribacterales>Deferrivibrionaceae>Deferrivibrio	GCF_020480685.1	Deferrivibrio_essentukiensis	LF845_RS09815	29861..33367	2880922	BREX-system-P-loop-protein-BrxC-[Deferrivibrio-essentukiensis]
EFK06105.1	Type1.BREX	<-Acyltransferase<-?<-?<-SIGMA+OB-Fold+AAA-ATPase+Lon-Protease-BrxL<-PglZ<-TOPRIM<-OLD-ABC<-N6-METHYLASE<-HEPN<-DUF6079*<-BrxB-BrxF-iSTAND-p||?-><-BrxA-DNA-binding||?->?->?-><-DnaJ	1215	Bacteria>delta/epsilon_subdivisions>Deltaproteobacteria>unclassified_Deltaproteobacteria	GCA_000179315.1	delta_proteobacterium_NaphS2	NPH_2628	46790..50437	88274	conserved-hypothetical-protein-[delta-proteobacterium-NaphS2]
MBI5543786.1	Type6.BREX	XIS-HTH->Classical-AAA->Peptidase_S8-><-SF1-SF2-Helicase+wHTH+Small-B-barrel+DUF1998<-BrxD-AAA<-PglZ<-HEPN+RES<-N6-METHYLASE<-DUF6079*<-BrxB-BrxF-iSTAND<-BrxA-DNA-binding<-BrxE	1206	Bacteria>delta/epsilon_subdivisions>Deltaproteobacteria>unclassified_Deltaproteobacteria	GCA_016218885.1	Deltaproteobacteria_bacterium	HY901_07870	25804..29424	2026735	BREX-system-P-loop-protein-BrxC-[Deltaproteobacteria-bacterium]
MCS4056789.1	Type1.BREX	<-Calcineurin-SbcD<-SIGMA+OB-Fold+AAA-ATPase+HKD-DNase-BrxL<-PglZ<-HEPN<-PolB||?-><-?<-N6-METHYLASE<-DOC+HTH<-DUF6079*<-BrxB-BrxF-iSTAND<-BrxA-DNA-binding<-?<-viral-protein<-?<-?||?-><-RHH	1165	Bacteria>FCB_group>Bacteroidetes/Chlorobi_group>Bacteroidota>Bacteroidetes_incertae_sedis>Rhodothermaceae	GCA_024809315.1	Salinibacter_ruber	GGP75_002131	31231..34728	146919	hypothetical-protein-[Salinibacter-ruber]
WP_259115052.1	Type1.BREX	<-SbcC+SbcC<-Calcineurin-SbcD<-SIGMA+OB-Fold+AAA-ATPase+HKD-DNase-BrxL<-PglZ<-HEPN<-PolB<-?<-N6-METHYLASE<-DOC+HTH<-DUF6079*<-BrxB-BrxF-iSTAND<-BrxA-DNA-binding<-?<-viral-protein<-?<-?||?-><-RHH||HTH_3+Peptidase_S24->	1167	Bacteria>FCB_group>Bacteroidetes/Chlorobi_group>Bacteroidota>Bacteroidetes_incertae_sedis>Rhodothermaceae	GCF_024809315.1	Salinibacter_ruber	GGP75_RS10390	31231..34734	146919	BREX-system-P-loop-protein-BrxC-[Salinibacter-ruber]
CDE09669.1	Type1.BREX	ArdA->HTH_3->BrxA-DNA-binding->BrxB-BrxF-iSTAND->DUF6079*->Pkinase->N6-METHYLASE->HEPN->?->?->PglZ->SIGMA+OB-Fold+AAA-ATPase+Lon-Protease-BrxL->	1221	Bacteria>FCB_group>Bacteroidetes/Chlorobi_group>Bacteroidota>Bacteroidia>Bacteroidales	GCA_000437495.1	Prevotella_sp._CAG:485	BN677_00784	6933..10598	1262927	putative-uncharacterized-protein-[Prevotella-sp.-CAG:485]
WP_191006191.1	Type6.BREX	HEPN->Phage_integrase+Phage_integrase->?-><-HEPN<-TIR||?->BrxE->BrxA-DNA-binding->BrxB-BrxF-iSTAND->DUF6079*->N6-METHYLASE->HEPN->PglZ->SIGMA+OB-Fold+AAA-ATPase+HKD-DNase-BrxL->N6_N4_METHYLASE->MORC+HISKIN->?-><-DOC+HTH	1204	Bacteria>FCB_group>Bacteroidetes/Chlorobi_group>Bacteroidota>Cytophagia>Cytophagales	GCF_014699115.1	Hymenobacter_montanus	IC235_RS15945	31815..35429	2771359	BREX-system-P-loop-protein-BrxC-[Hymenobacter-montanus]
WP_243520943.1	Type6.BREX	Glyoxalase-><-?<-HEPN<-?||?->?->BrxE->BrxA-DNA-binding->BrxB-BrxF-iSTAND->DUF6079*->N6-METHYLASE->HEPN->PglZ->SIGMA+OB-Fold+AAA-ATPase+HKD-DNase-BrxL->?->HKD-DNase->HTH+REase-><-CopG_antitoxin	1204	Bacteria>FCB_group>Bacteroidetes/Chlorobi_group>Bacteroidota>Cytophagia>Cytophagales	GCF_022811645.1	Hymenobacter_monticola	MTP16_RS25545	68480..72094	1705399	BREX-system-P-loop-protein-BrxC-[Hymenobacter-monticola]
WP_059014619.1	Type1.BREX	<-HEPN<-ParB+HNH<-N6-METHYLASE<-DUF6079*<-BrxB-BrxF-iSTAND<-BrxA-DNA-binding<-HTH_3<-Classical-AAA<-HTH_36<-HTH_36<-XIS-HTH<-Arm-DNA-bind_5+Phage_int_SAM_5+Phage_integrase	1169	Bacteria>FCB_group>Bacteroidetes/Chlorobi_group>Bacteroidota>Flavobacteriia>Flavobacteriales	GCF_001485515.1	Flavobacterium_psychrophilum	FPK15_RS07995	6803..10312	96345	BREX-system-P-loop-protein-BrxC-[Flavobacterium-psychrophilum]
WP_133546801.1	Type1.BREX	<-XIS-HTH<-?<-?<-?<-AraC-HTH<-Arm-DNA-bind_5+Phage_int_SAM_5+Phage_integrase||?->BrxA-DNA-binding->BrxB-BrxF-iSTAND->DUF6079*->N6-METHYLASE->REase+DUF5655->NERD+SF1-SF2-Helicase->TPR+S1->PglZ->SIGMA+OB-Fold+AAA-ATPase+HKD-DNase-BrxL->HEPN->?-><-HTH_36	1167	Bacteria>FCB_group>Bacteroidetes/Chlorobi_group>Bacteroidota>Flavobacteriia>Flavobacteriales	GCF_004362395.1	Salegentibacter_sp._24	DET49_RS03950	90042..93545	2183986	BREX-system-P-loop-protein-BrxC-[Salegentibacter-sp.-24]
WP_165150638.1	Type1.BREX	<-ABhydrolase<-REase+RNase<-?<-wHTH+WYL+WCX||?->?->?->BrxA-DNA-binding->BrxB-BrxF-iSTAND->DUF6079*->N6-METHYLASE->ParB+HNH->PglZ->SIGMA+OB-Fold+AAA-ATPase+Lon-Protease-BrxL->HEPN->?->?-><-FGase<-tRNA||SKI->	1198	Bacteria>FCB_group>Bacteroidetes/Chlorobi_group>Bacteroidota>Flavobacteriia>Flavobacteriales	GCF_011065475.1	Aequorivita_sp._KMM_9714	G3A46_RS13570	21223..24819	2707173	BREX-system-P-loop-protein-BrxC-[Aequorivita-sp.-KMM-9714]
WP_163432971.1	Type1.BREX	<-LexA-protease<-?||?->XIS-HTH->PIN->GDResolvase+HIN-HTH->HTH_3->BrxA-DNA-binding->BrxB-BrxF-iSTAND->DUF6079*->N6-METHYLASE->PglZ->HEPN-><-?||?->Relaxase->VirD4-TraG+VirD4-FtsK->	1189	Bacteria>FCB_group>Bacteroidetes/Chlorobi_group>Bacteroidota>Flavobacteriia>Flavobacteriales	GCF_014050215.1	unclassified_Flavobacterium	G7B00_RS14255	32052..35621	196869	MULTISPECIES:-BREX-system-P-loop-protein-BrxC-[unclassified-Flavobacterium]
WP_095606906.1	Type1.BREX	<-REase||?->?-><-SIGMA+OB-Fold+AAA-ATPase+Lon-Protease-BrxL<-PglZ<-N6-METHYLASE<-DUF6079*<-BrxB-BrxF-iSTAND<-BrxA-DNA-binding<-HEPN||?-><-XIS-HTH<-?<-?<-Phage_int_SAM_5+Phage_integrase	1213	Bacteria>FCB_group>Bacteroidetes/Chlorobi_group>Balneolota>Balneolia>Balneolales	GCF_002287075.1	Aliifodinibius_sp._WN023	CK503_RS11215	230173..233814	2032627	BREX-system-P-loop-protein-BrxC-[Aliifodinibius-sp.-WN023]
WP_265763895.1	Type1.BREX	<-HEPN<-SIGMA+OB-Fold+AAA-ATPase+HKD-DNase-BrxL<-PglZ<-?<-Schlafen+HATPase+wHTH<-REase<-?<-N6-METHYLASE<-DUF6079*<-BrxB-BrxF-iSTAND<-BrxA-DNA-binding<-tRNA||?->?->?-><-?<-?<-ABC-ATPase+SbcC	1165	Bacteria>FCB_group>Bacteroidetes/Chlorobi_group>Balneolota>Balneolia>Balneolales	GCF_026229185.1	Aliifodinibius_sp._1BSP15-2V2	J6I44_RS00130	34405..37902	2820877	BREX-system-P-loop-protein-BrxC-[Aliifodinibius-sp.-1BSP15-2V2]
OQC05381.1	Type1.BREX	BrxA-DNA-binding->BrxB-BrxF-iSTAND->DUF6079*->OLD-ABC->REase->N6-METHYLASE->PglZ->SIGMA+OB-Fold+AAA-ATPase+HKD-DNase-BrxL->HEPN->	1162	Bacteria>FCB_group>Candidatus_Cloacimonetes>unclassified_Candidatus_Cloacimonetes	GCA_002070875.1	Candidatus_Cloacimonetes_bacterium_ADurb.Bin089	BWX76_00750	1812..5300	1852836	MAG:-hypothetical-protein-BWX76_00750-[Candidatus-Cloacimonetes-bacterium-ADurb.Bin089]
NLV46851.1	Type1.BREX	Mrr_N->Mrr_N->Mrr_N->DUF4007->LON-AAA->N6-METHYLASE->DUF6079*->PglZ->SIGMA+OB-Fold+AAA-ATPase-BrxL->HEPN->?->Radical-SAM->MPTase->?->?->REase->	1339	Bacteria>FCB_group>Candidatus_Hydrogenedentes>unclassified_Candidatus_Hydrogenedentes	GCA_012730105.1	Candidatus_Hydrogenedentes_bacterium	GXY07_20415	9749..13768	2030809	hypothetical-protein-[Candidatus-Hydrogenedentes-bacterium]
NUM53512.1	Type1.BREX	<-SF1-SF2-Helicase<-OLD-ABC+TOPRIM<-?<-SIGMA+OB-Fold+AAA-ATPase+Lon-Protease-BrxL<-PglZ<-ArdCN+MPTase<-?<-N6-METHYLASE<-HEPN<-DUF6079*<-BrxB-BrxF-iSTAND<-BrxA-DNA-binding||?->HTH_3->MPTase->	1149	Bacteria>FCB_group>Candidatus_Hydrogenedentes>unclassified_Candidatus_Hydrogenedentes	GCA_013359265.1	Candidatus_Hydrogenedentes_bacterium	HUU46_07705	154693..158142	2030809	BREX-system-P-loop-protein-BrxC-[Candidatus-Hydrogenedentes-bacterium]
MCC6156024.1	Type1.BREX	<-SIGMA+OB-Fold+AAA-ATPase+Lon-Protease-BrxL<-PglZ<-ArdCN+MPTase<-N6-METHYLASE<-HEPN<-DUF6079*	1142	Bacteria>FCB_group>Candidatus_Hydrogenedentes>unclassified_Candidatus_Hydrogenedentes	GCA_020846215.1	Candidatus_Hydrogenedentes_bacterium	IT367_19820	10018..13447	2030809	BREX-system-P-loop-protein-BrxC-[Candidatus-Hydrogenedentes-bacterium]
ODT04869.1	Type6.BREX	<-SIGMA+OB-Fold+AAA-ATPase+Lon-Protease-BrxL<-PglZ<-N6-METHYLASE-p<-ORC-CDC6<-ParA-Soj-PloopNTPase<-HEPN<-N6-METHYLASE<-DUF6079*<-BrxB-BrxF-iSTAND<-BrxA-DNA-binding<-BrxE||McrB-AAA->wHTH+REase->REase->?->EVE+HNH->	1181	Bacteria>FCB_group>Gemmatimonadota>unclassified_Gemmatimonadota	GCA_001724275.1	Gemmatimonadetes_bacterium_SCN_70-22	ABS52_01570	17993..21538	1660163	hypothetical-protein-ABS52_01570-[Gemmatimonadetes-bacterium-SCN-70-22]
WP_098702328.1	Type1.BREX	SKI->JAB+sGTP->wHTH+WYL+WCX->?->?->ABC-ATPase+SbcC->?-><-?||DUF6079*->OLD-ABC->TOPRIM->N6-METHYLASE->HEPN->CR-ATPase8+REase->Classical-AAA->Peptidase_S8->PglZ->SIGMA+OB-Fold+AAA-ATPase+Lon-Protease-BrxL->	1250	Bacteria>Fusobacteriota>Fusobacteriia>Fusobacteriales>Fusobacteriaceae>Fusobacterium	GCF_002573625.1	Fusobacterium_nucleatum	RN96_RS03470	669523..673275	851	BREX-system-P-loop-protein-BrxC-[Fusobacterium-polymorphum]
WP_099959237.1	Type1.BREX	<-SIGMA+OB-Fold+AAA-ATPase+Lon-Protease-BrxL<-PglZ<-Peptidase_S8<-Classical-AAA<-CR-ATPase8+REase<-HEPN<-N6-METHYLASE<-TOPRIM<-OLD-ABC<-DUF6079*||?-><-?<-?<-Peptidase_M3_N+Peptidase_M3||RNaseH->?->?->?->Peptidase_M20->	1250	Bacteria>Fusobacteriota>Fusobacteriia>Fusobacteriales>Fusobacteriaceae>Fusobacterium	GCF_002761955.1	Fusobacterium_pseudoperiodonticum	CTM71_RS09955	1967192..1970944	2663009	BREX-system-P-loop-protein-BrxC-[Fusobacterium-pseudoperiodonticum]
WP_100024172.1	Type1.BREX	<-Peptidase_M20<-?<-?<-?<-RNaseH||Peptidase_M3_N+Peptidase_M3->?->?-><-?||DUF6079*->OLD-ABC->TOPRIM->N6-METHYLASE->HEPN->CR-ATPase8+REase->PglZ->SIGMA+OB-Fold+AAA-ATPase+Lon-Protease-BrxL->BrxA-DNA-binding->BrxB-BrxF-iSTAND->	1250	Bacteria>Fusobacteriota>Fusobacteriia>Fusobacteriales>Fusobacteriaceae>Fusobacterium	GCF_002763625.1	Fusobacterium_pseudoperiodonticum	CTM72_RS01085	199877..203629	2663009	BREX-system-P-loop-protein-BrxC-[Fusobacterium-pseudoperiodonticum]
WP_238959505.1	Type1.BREX	<-BrxB-BrxF-iSTAND<-BrxA-DNA-binding<-SIGMA+OB-Fold+AAA-ATPase+Lon-Protease-BrxL<-PglZ<-CR-ATPase8+REase<-HEPN<-MORC<-N6-METHYLASE<-Schlafen+HATPase+wHTH<-DUF6079*||?-><-?<-?<-ABC-ATPase+SbcC<-?<-?<-wHTH+WYL+WCX<-JAB+sGTP<-SKI	1250	Bacteria>Fusobacteriota>Fusobacteriia>Fusobacteriales>Fusobacteriaceae>Fusobacterium	GCF_022340045.1	Fusobacterium_nucleatum	K5I20_00280	65514..69266	851	BREX-system-P-loop-protein-BrxC-[Fusobacterium-nucleatum]
WP_238972693.1	Type1.BREX	SKI->JAB+sGTP->wHTH+WYL+WCX->?->ABC-ATPase+SbcC->?-><-?||DUF6079*->OLD-ABC->TOPRIM->Schlafen+HATPase+wHTH->N6-METHYLASE->MORC->HEPN->CR-ATPase8+REase->PglZ->SIGMA+OB-Fold+AAA-ATPase+Lon-Protease-BrxL->	1250	Bacteria>Fusobacteriota>Fusobacteriia>Fusobacteriales>Fusobacteriaceae>Fusobacterium	GCF_022340165.1	Fusobacterium_nucleatum	K5I19_RS02505	127932..131684	851	BREX-system-P-loop-protein-BrxC-[Fusobacterium-nucleatum]
WP_060798221.1	Type1.BREX	<-BrxA-DNA-binding<-SIGMA+OB-Fold+AAA-ATPase+Lon-Protease-BrxL<-PglZ<-CR-ATPase8+REase<-HEPN<-MORC<-N6-METHYLASE<-TOPRIM<-OLD-ABC<-DUF6079*<-?<-SWIM+DUF3670+SF1-SF2-Helicase<-?<-?<-?<-SF1-SF2-Helicase	1250	Bacteria>Fusobacteriota>Fusobacteriia>Fusobacteriales>Fusobacteriaceae>Fusobacterium	GCF_023383525.1	Fusobacterium_nucleatum	YWH7054_RS02310	21716..25468	851	BREX-system-P-loop-protein-BrxC-[Fusobacterium-nucleatum]
WP_249524183.1	Type1.BREX	<-BrxA-DNA-binding<-SIGMA+OB-Fold+AAA-ATPase+Lon-Protease-BrxL<-PglZ<-CR-ATPase8+REase<-HEPN<-MORC<-N6-METHYLASE<-TOPRIM<-OLD-ABC<-DUF6079*<-?<-SWIM+DUF3670+SF1-SF2-Helicase<-?<-?<-?<-SF1-SF2-Helicase	1250	Bacteria>Fusobacteriota>Fusobacteriia>Fusobacteriales>Fusobacteriaceae>Fusobacterium	GCF_023383605.1	Fusobacterium_nucleatum	YWH7056_RS07045	28639..32391	851	BREX-system-P-loop-protein-BrxC-[Fusobacterium-nucleatum]
WP_249526950.1	Type1.BREX	<-BrxB-BrxF-iSTAND<-BrxA-DNA-binding<-SIGMA+OB-Fold+AAA-ATPase+Lon-Protease-BrxL<-PglZ<-HEPN<-MORC<-N6-METHYLASE<-TOPRIM<-OLD-ABC<-DUF6079*<-?<-SWIM+DUF3670+SF1-SF2-Helicase<-?<-?<-?<-SF1-SF2-Helicase	1250	Bacteria>Fusobacteriota>Fusobacteriia>Fusobacteriales>Fusobacteriaceae>Fusobacterium	GCF_023383645.1	Fusobacterium_nucleatum	YWH7199_RS06535	22791..26543	851	BREX-system-P-loop-protein-BrxC-[Fusobacterium-nucleatum]
WP_008793539.1	Type1.BREX	<-RNaseH||Peptidase_M3_N+Peptidase_M3->?->?-><-?<-HTH_OrfB_IS605+OrfB_IS605+OrfB_Zn_ribbon||DUF6079*->OLD-ABC->RNase+PHP->Schlafen+HATPase+wHTH->N6-METHYLASE->HEPN->CR-ATPase8+REase->PglZ->SIGMA+OB-Fold+AAA-ATPase+Lon-Protease-BrxL->BrxA-DNA-binding->	1250	Bacteria>Fusobacteriota>Fusobacteriia>Fusobacteriales>Fusobacteriaceae>Fusobacterium	GCF_902373915.1	Fusobacterium_periodonticum	FY302_RS02975	615184..618936	860	BREX-system-P-loop-protein-BrxC-[Fusobacterium-periodonticum]
WP_026745399.1	Type1.BREX	Peptidase_M24->?->?->Peptidase_M18+Peptidase_M42->?->BrxA-DNA-binding->BrxB-BrxF-iSTAND->DUF6079*->REase->N6-METHYLASE->HEPN->?->HicA_toxin->HicB_lk_antitox->?->PglZ->SIGMA+OB-Fold+AAA-ATPase+Lon-Protease-BrxL->	1193	Bacteria>Fusobacteriota>Fusobacteriia>Fusobacteriales>Leptotrichiaceae>Leptotrichia	GCF_007990525.1	Leptotrichia_hofstadii	FVE77_RS00755	171012..174593	157688	BREX-system-P-loop-protein-BrxC-[Leptotrichia-hofstadii]
WP_151554984.1	Type1.BREX	<-REC+NtrC-AAA+FIS-HTH<-HAMP+PAS+HISKIN<-ACET<-?<-SIGMA+OB-Fold+AAA-ATPase+Lon-Protease-BrxL<-PglZ<-HEPN<-N6-METHYLASE<-DUF6079*<-BrxB-BrxF-iSTAND<-BrxA-DNA-binding||TRPR-HTH->Couple_hipA+HipA_C->SF1-SF2-Helicase->?-><-wHTH+REase<-McrB-AAA<-TraI-VirD2-RCRE	1205	Bacteria>Pseudomonadota>Alphaproteobacteria>Hyphomicrobiales>Brucellaceae>Brucella/Ochrobactrum_group	GCF_008932275.1	Brucella_tritici	F9K85_RS05045	164979..168596	94626	BREX-system-P-loop-protein-BrxC-[Brucella-tritici]
WP_080921652.1	Type1.BREX	HEPN->HEPN->?-><-DEDD_Tnp_IS110+Transposase_20||<-?<-HEPN<-SIGMA+OB-Fold+AAA-ATPase+HKD-DNase-BrxL<-PglZ<-N6-METHYLASE<-DUF6079*<-BrxB-BrxF-iSTAND<-BrxA-DNA-binding<-wHTH+WYL+WCX||Calcineurin-SbcD->SbcC+CCDC158->?->HTH_3->Peptidase+SF1-SF2-Helicase+REase->	1179	Bacteria>Pseudomonadota>Alphaproteobacteria>Hyphomicrobiales>Phyllobacteriaceae>Pseudaminobacter	GCF_002075885.1	Pseudaminobacter_manganicus	BFN67_RS22220	17195..20734	1873176	BREX-system-P-loop-protein-BrxC-[Pseudaminobacter-manganicus]
WP_088677288.1	Type1.BREX	<-SPOUT||tRNA->tRNA->?->?->?->tRNA->?->Classical-AAA+S2P->HEPN->BrxA-DNA-binding->BrxB-BrxF-iSTAND->DUF6079*->N6-METHYLASE->PglZ->SIGMA+OB-Fold+AAA-ATPase+HKD-DNase-BrxL-><-?||?->?->?->MORC+REase->RNase->	1194	Bacteria>Pseudomonadota>Alphaproteobacteria>Hyphomicrobiales>Rhizobiaceae>Rhizobium/Agrobacterium_group	GCF_002211395.1	Rhizobium_sp._R339	ATY76_RS24585	353019..356603	1764273	BREX-system-P-loop-protein-BrxC-[Rhizobium-sp.-R339]
WP_222013551.1	Type1.BREX	<-SIGMA+OB-Fold+AAA-ATPase+Lon-Protease-BrxL<-PglZ<-N6-METHYLASE<-DUF6079*<-BrxB-BrxF-iSTAND<-BrxA-DNA-binding<-HEPN<-?<-PIWI<-SIR2<-HARE-HTH||Phage_integrase->Phage_Integr_2+Phage_integrase->	1137	Bacteria>Pseudomonadota>Alphaproteobacteria>Hyphomicrobiales>Rhizobiaceae>Rhizobium/Agrobacterium_group	GCF_019779115.1	Rhizobium_laguerreae	HFO05_RS34495	7944..11357	1076926	BREX-system-P-loop-protein-BrxC-[Rhizobium-laguerreae]
PSL17302.1	Type6.BREX	<-HAMP+HISKIN||?->tRNA-><-SIR2<-SIGMA+OB-Fold+AAA-ATPase+HKD-DNase-BrxL<-PglZ<-N6-METHYLASE<-N6-METHYLASE<-DUF6079*<-BrxB-BrxF-iSTAND<-BrxA-DNA-binding<-BrxE<-XIS-HTH<-HEPN<-Polbeta<-wHTH+AbiEi_2||REase->	1216	Bacteria>Pseudomonadota>Alphaproteobacteria>Rhodobacterales>Roseobacteraceae>Shimia	GCA_003014475.1	Shimia_abyssi	CLV88_11975	80664..84314	1662395	hypothetical-protein-CLV88_11975-[Shimia-abyssi]
WP_207797113.1	Type6.BREX	<-HAMP+HISKIN||?->tRNA-><-SIR2<-SIGMA+OB-Fold+AAA-ATPase+HKD-DNase-BrxL<-PglZ<-N6-METHYLASE<-N6-METHYLASE<-DUF6079*<-BrxB-BrxF-iSTAND<-BrxA-DNA-binding<-BrxE<-XIS-HTH<-HEPN<-Polbeta<-wHTH+AbiEi_2||REase->	1207	Bacteria>Pseudomonadota>Alphaproteobacteria>Rhodobacterales>Roseobacteraceae>Shimia	GCF_003014475.1	Shimia_abyssi	CLV88_RS20200	80664..84287	1662395	BREX-system-P-loop-protein-BrxC-[Shimia-abyssi]
QKS29579.1	Type6.BREX	<-SIGMA+OB-Fold+AAA-ATPase+Lon-Protease-BrxL<-PglZ<-HNH<-OLD-ABC<-N6-METHYLASE<-ORC-CDC6<-ParA-Soj-PloopNTPase<-HEPN<-N6-METHYLASE<-DUF6079*<-BrxB-BrxF-iSTAND<-BrxA-DNA-binding<-BrxE<-XIS-HTH	1180	Bacteria>Pseudomonadota>Betaproteobacteria>Betaproteobacteria_incertae_sedis>Candidatus_Accumulibacter	GCA_013347225.1	Candidatus_Accumulibacter_similis	HT579_12075	2666587..2670129	2954388	MAG:-BREX-system-P-loop-protein-BrxC-[Candidatus-Accumulibacter-similis]
HCN68765.1	Type1.BREX	HTH_3-><-?<-?||?->wHTH+AbiEi_2->Polbeta->REase->BrxA-DNA-binding->BrxB-BrxF-iSTAND->DUF6079*->HEPN->N6-METHYLASE->OLD-ABC+TOPRIM->SF1-SF2-Helicase->N6-METHYLASE->PglZ->SIGMA+OB-Fold+AAA-ATPase+Lon-Protease-BrxL->	1154	Bacteria>Pseudomonadota>Betaproteobacteria>Betaproteobacteria_incertae_sedis>Candidatus_Accumulibacter>unclassified_Candidatus_Accumulibacter	GCA_003535635.1	Candidatus_Accumulibacter_sp.	DIT03_10970	31798..35262	2053492	BREX-system-P-loop-protein-BrxC-[Candidatus-Accumulibacter-sp.]
WP_043683814.1	Type1.BREX	DDE_Tnp_1_4->?->VirB5-><-DUF772+DDE_Tnp_1+DDE_Tnp_1_6<-?||wHTH+WYL+WCX->BrxA-DNA-binding->BrxB-BrxF-iSTAND->DUF6079*->HEPN->N6-METHYLASE->OLD-ABC->HNH->DUF772+DDE_Tnp_1+DDE_Tnp_1_6->HNH->PglZ->SIGMA+OB-Fold+AAA-ATPase+Lon-Protease-BrxL-><-YdaS_antitoxin	1153	Bacteria>Pseudomonadota>Betaproteobacteria>Burkholderiales>Alcaligenaceae>Castellaniella	GCF_000612685.1	Castellaniella_defragrans	BN940_RS13240	2967883..2971344	75697	BREX-system-P-loop-protein-BrxC-[Castellaniella-defragrans]
WP_183964658.1	Type6.BREX	<-SWIM<-?||NUDIX+NAAR1+NAAR2->?->?->?->HEPN->BrxE->BrxB-BrxF-iSTAND->DUF6079*->N6-METHYLASE->N6-METHYLASE-p->PglZ->SIGMA+OB-Fold+AAA-ATPase+HKD-DNase-BrxL-><-RVT<-?<-SAM-METHYLASE+wHTH+HNH<-BirA-HTH	1210	Bacteria>Pseudomonadota>Betaproteobacteria>Burkholderiales>Burkholderiaceae>Quisquiliibacterium	GCF_014202215.1	Quisquiliibacterium_transsilvanicum	HNQ70_RS04390	209103..212735	1549638	BREX-system-P-loop-protein-BrxC-[Quisquiliibacterium-transsilvanicum]
WP_086927320.1	Type1.BREX	<-SIGMA+OB-Fold+AAA-ATPase+Lon-Protease-BrxL<-PglZ<-ParB+HNH<-SF1-SF2-Helicase<-OLD-ABC+TOPRIM<-N6-METHYLASE<-HEPN<-DUF6079*<-BrxB-BrxF-iSTAND<-BrxA-DNA-binding<-RHH_5<-?<-XIS-HTH<-tRNA<-tRNA<-35exo<-?<-?<-DnaJ	1153	Bacteria>Pseudomonadota>Betaproteobacteria>Burkholderiales>Comamonadaceae>Acidovorax	GCF_002157125.1	Acidovorax_carolinensis	CBP36_RS10055	2162910..2166371	553814	BREX-system-P-loop-protein-BrxC-[Acidovorax-carolinensis]
WP_094097616.1	Type1.BREX	<-Peptidase_C39_2<-?||?-><-?||XIS-HTH->Phage_X->BrxA-DNA-binding->BrxB-BrxF-iSTAND->DUF6079*->HNH->HEPN->ParD_antitoxin->RelE-ParE->N6-METHYLASE->OLD-ABC->TOPRIM->ZnR+TOPRIM->PglZ->	1214	Bacteria>Pseudomonadota>Betaproteobacteria>Burkholderiales>Comamonadaceae>Acidovorax	GCF_002157145.1	Acidovorax_carolinensis	CBP34_RS06480	1379414..1383058	553814	BREX-system-P-loop-protein-BrxC-[Acidovorax-carolinensis]
WP_204247869.1	Type1.BREX	DnaJ->?->?->tRNA->tRNA->XIS-HTH->?->RHH_5->BrxA-DNA-binding->BrxB-BrxF-iSTAND->DUF6079-p*->DUF6079-p->HEPN->N6-METHYLASE->OLD-ABC+TOPRIM->SF1-SF2-Helicase->ParB+HNH->PglZ->SIGMA+OB-Fold+AAA-ATPase+Lon-Protease-BrxL->	590	Bacteria>Pseudomonadota>Betaproteobacteria>Burkholderiales>Comamonadaceae>Acidovorax	GCF_002157185.1	Acidovorax_carolinensis	CBP35_RS21920	1914645..1916417	553814	BREX-system-P-loop-protein-BrxC-[Acidovorax-carolinensis]
GAO25248.1	Type1.BREX	HTH_3-><-SIGMA+OB-Fold+AAA-ATPase+Lon-Protease-BrxL<-PglZ<-ZnR+TOPRIM<-TOPRIM<-OLD-ABC<-N6-METHYLASE<-HEPN<-HNH<-DUF6079*<-BrxB-BrxF-iSTAND<-BrxA-DNA-binding<-HAD+CHTH<-CHTH||?->AAA-ATPase-p->?-><-AAA-ATPase	1205	Bacteria>Pseudomonadota>Betaproteobacteria>Burkholderiales>Comamonadaceae>Alicycliphilus	GCA_000974645.1	Alicycliphilus_sp._B1	ALISP_5068	1538304..1541921	1603291	ATPase-like-protein-[Alicycliphilus-sp.-B1]
WP_103018746.1	Type1.BREX	APATPase->?-><-?||?->?->?->RHH_5->BrxA-DNA-binding->BrxB-BrxF-iSTAND->DUF6079*->HEPN-><-?||N6-METHYLASE->SF1-SF2-Helicase+PRTase->SLOG+BirA-HTH->?->TPR+REase->?->ParB+HNH->	1153	Bacteria>Pseudomonadota>Betaproteobacteria>Burkholderiales>Comamonadaceae>Alicycliphilus	GCF_002894355.1	Alicycliphilus_denitrificans	CUB93_RS20845	10909..14370	179636	BREX-system-P-loop-protein-BrxC-[Alicycliphilus-denitrificans]
WP_169107995.1	Type1.BREX	SIGMA+OB-Fold+AAA-ATPase+Lon-Protease-BrxL->tRNA->tRNA->Phage_integrase->?-><-XIS-HTH||?->RHH_5->BrxA-DNA-binding->BrxB-BrxF-iSTAND->DUF6079*->HEPN->N6-METHYLASE->PHP+SbcC->MarR-HTH->Polbeta->PglZ->SIGMA+OB-Fold+AAA-ATPase+Lon-Protease-BrxL->SF1-SF2-Helicase->PIN->	1153	Bacteria>Pseudomonadota>Betaproteobacteria>Burkholderiales>Comamonadaceae>Comamonas	GCF_012844455.2	Comamonas_suwonensis	HF327_RS09545	49446..52907	2606214	BREX-system-P-loop-protein-BrxC-[Comamonas-suwonensis]
WP_172207178.1	Type1.BREX	<-OLD-ABC||<-N6-METHYLASE-p<-RelE-ParE<-ParD_antitoxin<-PIN<-MazE_antitoxin||?-><-HEPN<-HNH<-DUF6079*<-BrxB-BrxF-iSTAND<-BrxA-DNA-binding<-Phage_X<-XIS-HTH||?-><-?||?->Peptidase_C39_2->	1213	Bacteria>Pseudomonadota>Betaproteobacteria>Burkholderiales>Comamonadaceae>Diaphorobacter	GCF_013154915.1	Diaphorobacter_sp._JS3050	HND92_RS11875	2478331..2481972	2735554	BREX-system-P-loop-protein-BrxC-[Diaphorobacter-sp.-JS3050]
WP_226462338.1	Type1.BREX	<-SIGMA+OB-Fold+AAA-ATPase+Lon-Protease-BrxL<-PglZ<-ZnR+TOPRIM<-?<-HEPN<-N6-METHYLASE<-N6-METHYLASE<-Couple_hipA+HipA_C<-HTH_3<-DUF6079*<-BrxB-BrxF-iSTAND<-BrxA-DNA-binding<-SbcC+CCDC158<-Calcineurin-SbcD<-?<-MoxR-AAA<-HEPN	1204	Bacteria>Pseudomonadota>Betaproteobacteria>Burkholderiales>Comamonadaceae>Hydrogenophaga	GCF_020510545.1	Hydrogenophaga_taeniospiralis	KI616_RS15915	3311609..3315223	65656	BREX-system-P-loop-protein-BrxC-[Hydrogenophaga-taeniospiralis]
WP_101103324.1	Type1.BREX	HTH_Tnp_1+HTH_21+rve+rve_3->RHH_5->BrxA-DNA-binding->BrxB-BrxF-iSTAND->DUF6079*->HEPN->N6-METHYLASE->OLD-ABC+TOPRIM->SF1-SF2-Helicase->OLD-ABC->HNH->ParB+HNH->PglZ->SIGMA+OB-Fold+AAA-ATPase+Lon-Protease-BrxL->	1153	Bacteria>Pseudomonadota>Betaproteobacteria>Burkholderiales>Comamonadaceae>Macromonas	GCF_002837135.1	Macromonas_bipunctata	CON76_RS10585	3906..7367	183670	BREX-system-P-loop-protein-BrxC-[Macromonas-bipunctata]
OOG58024.1	Type1.BREX	<-HTH_3<-?||?-><-SIGMA+OB-Fold+AAA-ATPase+Lon-Protease-BrxL<-PglZ<-HEPN<-N6-METHYLASE<-DUF6079*<-BrxB-BrxF-iSTAND<-BrxA-DNA-binding||?->HD+PspB->	1207	Bacteria>Pseudomonadota>Betaproteobacteria>Burkholderiales>Comamonadaceae>Polaromonas	GCA_002002705.1	Polaromonas_sp._C04	BOE49_04110	64714..68337	1945857	ATPase-[Polaromonas-sp.-C04]
WP_011485879.1	Type1.BREX	N6-METHYLASE-><-?<-?<-?<-REC<-HISKIN||HTH_3->BrxA-DNA-binding->BrxB-BrxF-iSTAND->DUF6079*->CHTH->Tox-RES->N6-METHYLASE->HEPN->NERD+Pkinase+Pkinase+SF1-SF2-Helicase->?->PglZ->SIGMA+OB-Fold+AAA-ATPase+Lon-Protease-BrxL-><-PHP+SbcC	1202	Bacteria>Pseudomonadota>Betaproteobacteria>Burkholderiales>Comamonadaceae>Polaromonas	GCF_000013865.1	Polaromonas_sp._JS666	BPRO_RS25145	121747..125355	296591	BREX-system-P-loop-protein-BrxC-[Polaromonas-sp.-JS666]
APW46516.1	Type1.BREX	Glyco_transf_9-><-?||?->?->TPR+TPR+TPR->?->tRNA-><-?||DUF6079-p*->MarR-HTH+Polbeta->HEPN->HEPN->N6-METHYLASE-p->N6-METHYLASE->?->?->?-><-DDE_Tnp_1+DDE_Tnp_1	269	Bacteria>Pseudomonadota>Betaproteobacteria>Burkholderiales>Comamonadaceae>Rhodoferax	GCA_001955735.1	Rhodoferax_antarcticus	RA876_09190	1894413..1895222	81479	hypothetical-protein-RA876_09190-[Rhodoferax-antarcticus]
MBP9058688.1	Type1.BREX	<-PglZ<-SLOG+BirA-HTH<-SF1-SF2-Helicase+PRTase<-?<-TPR+REase<-?<-N6-METHYLASE<-HEPN<-MarR-HTH+Polbeta<-DUF6079*<-BrxB-BrxF-iSTAND<-BrxA-DNA-binding<-RHH_5||N6-METHYLASE->ORC-CDC6+ZnR->	1153	Bacteria>Pseudomonadota>Betaproteobacteria>Burkholderiales>Comamonadaceae>Rhodoferax	GCA_018057375.1	Rhodoferax_sp.	KBF98_00065	15882..19343	50421	BREX-system-P-loop-protein-BrxC-[Rhodoferax-sp.]
MCF8211220.1	Type1.BREX	BrxA-DNA-binding->BrxB-BrxF-iSTAND->DUF6079*->MarR-HTH+Polbeta->OLD-ABC->REase->HEPN->N6-METHYLASE-p->	1153	Bacteria>Pseudomonadota>Betaproteobacteria>Burkholderiales>Comamonadaceae>Rhodoferax	GCA_021738345.1	Rhodoferax_sp.	K9K38_17735	1925..5386	50421	BREX-system-P-loop-protein-BrxC-[Rhodoferax-sp.]
WP_139313405.1	Type1.BREX	ABC-ATPase+SbcC->?->Glyco_transf_9-><-?||?->?->TPR+TPR+TPR->?->tRNA->||DUF6079-p*->MarR-HTH+Polbeta->HEPN->?->HEPN->N6-METHYLASE-p->N6-METHYLASE->?->?->RAMA->	323	Bacteria>Pseudomonadota>Betaproteobacteria>Burkholderiales>Comamonadaceae>Rhodoferax	GCF_001955735.1	Rhodoferax_antarcticus	RA876_RS09190	1894251..1895222	81479	hypothetical-protein-[Rhodoferax-antarcticus]
WP_264569449.1	Type1.BREX	<-Glyco_transf_9||?->?->?->TPR+TPR+TPR->?->tRNA->||DUF6079-p*->MarR-HTH+Polbeta->HEPN->?->HEPN->N6-METHYLASE-p->N6-METHYLASE-p->	323	Bacteria>Pseudomonadota>Betaproteobacteria>Burkholderiales>Comamonadaceae>Rhodoferax	GCF_025961535.1	Rhodoferax_antarcticus	M2244_RS19480	9793..10764	81479	hypothetical-protein-[Rhodoferax-antarcticus]
MBH2043138.1	Type1.BREX	RHH_5->BrxA-DNA-binding->BrxB-BrxF-iSTAND->DUF6079*->MarR-HTH+Polbeta->HEPN->OLD-ABC->HNH->PglZ->SIGMA+OB-Fold+AAA-ATPase+Lon-Protease-BrxL->	1153	Bacteria>Pseudomonadota>Betaproteobacteria>Burkholderiales>Comamonadaceae>unclassified_Comamonadaceae	GCA_016000285.1	Comamonadaceae_bacterium	I8H87_06315	1878..5339	1871071	BREX-system-P-loop-protein-BrxC-[Comamonadaceae-bacterium]
WP_096234088.1	Type1.BREX	HTH_3->?->?->?->BrxA-DNA-binding->BrxB-BrxF-iSTAND->DUF6079*->N6-METHYLASE->OLD-ABC->TOPRIM->PglZ->SIGMA+OB-Fold+AAA-ATPase+Lon-Protease-BrxL->HEPN-><-TPR	1204	Bacteria>Pseudomonadota>Betaproteobacteria>Burkholderiales>Oxalobacteraceae>Janthinobacterium	GCF_002327145.1	Janthinobacterium_svalbardensis	CNX70_RS06880	1564323..1567937	368607	BREX-system-P-loop-protein-BrxC-[Janthinobacterium-svalbardensis]
WP_203569835.1	Type1.BREX	<-HAD+HAD||?->HTH_Tnp_1+HTH_21+rve+rve_3->wHTH+WYL+WCX->BrxA-DNA-binding->BrxB-BrxF-iSTAND->DUF6079*->HEPN->N6-METHYLASE->?->?->?->PglZ->SIGMA+OB-Fold+AAA-ATPase+Lon-Protease-BrxL->	1156	Bacteria>Pseudomonadota>Betaproteobacteria>Neisseriales>Chromobacteriaceae	GCF_014042245.2	Deefgea	H1229_RS02985	646302..649772	400947	MULTISPECIES:-BREX-system-P-loop-protein-BrxC-[Deefgea]
MBK7491769.1	Type1.BREX	HIN-HTH->?-><-SF1-SF2-Helicase<-?||?->DUF772+DDE_Tnp_1->PIN+OST-HTH->BrxA-DNA-binding->BrxB-BrxF-iSTAND->DUF6079*->N6-METHYLASE->HEPN->HTH+REase->PglZ->SIGMA+OB-Fold+AAA-ATPase+Lon-Protease-BrxL->HIN-HTH->	1203	Bacteria>Pseudomonadota>Betaproteobacteria>Nitrosomonadales>Nitrosomonadaceae>Nitrosomonas	GCA_016707165.1	Nitrosomonas_sp.	IPI17_07005	52164..55775	42353	BREX-system-P-loop-protein-BrxC-[Nitrosomonas-sp.]
MBK7235806.1	Type6.BREX	<-PhdYeFM_antitox||?-><-tRNA<-SIGMA+OB-Fold+AAA-ATPase+Lon-Protease-BrxL<-PglZ<-N6-METHYLASE<-ORC-CDC6<-ParA-Soj-PloopNTPase<-HEPN<-N6-METHYLASE<-DUF6079*<-BrxB-BrxF-iSTAND<-BrxA-DNA-binding<-BrxE<-XIS-HTH<-NUDIX||?->?->Trypsin+PDZ->	1180	Bacteria>Pseudomonadota>Betaproteobacteria>Nitrosomonadales>Sterolibacteriaceae>unclassified_Sterolibacteriaceae	GCA_016707455.1	Sterolibacteriaceae_bacterium	IPI02_09355	160184..163726	2682146	BREX-system-P-loop-protein-BrxC-[Sterolibacteriaceae-bacterium]
MBK6972675.1	Type6.BREX	<-Trypsin+PDZ<-?<-?||NUDIX->XIS-HTH->BrxE->BrxA-DNA-binding->BrxB-BrxF-iSTAND->DUF6079*->N6-METHYLASE->HEPN->ParA-Soj-PloopNTPase->ORC-CDC6->N6-METHYLASE->PglZ->SIGMA+OB-Fold+AAA-ATPase+Lon-Protease-BrxL->tRNA-><-?||PhdYeFM_antitox->	1180	Bacteria>Pseudomonadota>Betaproteobacteria>Nitrosomonadales>Sterolibacteriaceae>unclassified_Sterolibacteriaceae	GCA_016708905.1	Sterolibacteriaceae_bacterium	IPH26_06870	229623..233165	2682146	BREX-system-P-loop-protein-BrxC-[Sterolibacteriaceae-bacterium]
MBL8465274.1	Type6.BREX	ABC-ATPase+SbcC->BrxE->BrxA-DNA-binding->BrxB-BrxF-iSTAND->DUF6079*->MNS+MarR-HTH->N6-METHYLASE->N6-METHYLASE->HEPN->ParA-Soj-PloopNTPase->ORC-CDC6->PglZ->SIGMA+OB-Fold+AAA-ATPase+HKD-DNase-BrxL->	1209	Bacteria>Pseudomonadota>Betaproteobacteria>Rhodocyclales>Zoogloeaceae>Thauera	GCA_016790365.1	Thauera_sp.	JNL37_10520	3981..7610	1905334	BREX-system-P-loop-protein-BrxC-[Thauera-sp.]
WP_096450985.1	Type6.BREX	<-SIGMA+OB-Fold+AAA-ATPase+Lon-Protease-BrxL<-PglZ<-HNH<-OLD-ABC<-N6-METHYLASE<-ORC-CDC6<-ParA-Soj-PloopNTPase<-HEPN<-N6-METHYLASE<-DUF6079*<-BrxB-BrxF-iSTAND<-BrxA-DNA-binding<-BrxE<-XIS-HTH<-HTH_3||?-><-ParB<-?||HTH_3->	1180	Bacteria>Pseudomonadota>Betaproteobacteria>Rhodocyclales>Zoogloeaceae>Thauera	GCF_002354895.1	Thauera_sp._K11	CCZ27_RS19540	4450815..4454357	2005884	BREX-system-P-loop-protein-BrxC-[Thauera-sp.-K11]
WP_247725420.1	Type6.BREX	<-wHTH<-ORC-CDC6<-ParA-Soj-PloopNTPase<-HEPN<-N6-METHYLASE-p<-PIN<-?<-N6-METHYLASE<-MNS+MarR-HTH<-DUF6079*<-BrxB-BrxF-iSTAND<-BrxA-DNA-binding<-BrxE<-?||ABC-ATPase+SbcC+ABC-ATPase+SbcC->HAD->	1209	Bacteria>Pseudomonadota>Betaproteobacteria>Rhodocyclales>Zoogloeaceae>Thauera	GCF_023093595.1	Thauera_aromatica	MXC99_RS10610	105350..108979	59405	BREX-system-P-loop-protein-BrxC-[Thauera-aromatica]
BDD08311.1	Type1.BREX	ARG->RHS->HTH_23+HTH_32+HTH_33->DDE_3->RHS->BrxA-DNA-binding->BrxB-BrxF-iSTAND->DUF6079-p-><-DDE_Tnp_1||DUF6079*->ParB+HNH->N6-METHYLASE->HEPN->PAIREDC-HTH+HIN-HTH->N6-METHYLASE-p->DUF3944->?->PglZ->SIGMA+OB-Fold+AAA-ATPase+Lon-Protease-BrxL->	1089	Bacteria>Pseudomonadota>Gammaproteobacteria>Aeromonadales>Aeromonadaceae>Aeromonas	GCA_024347775.1	Aeromonas_caviae	KAM643c_18840	2028863..2032132	648	hypothetical-protein-KAM643c_18840-[Aeromonas-caviae]
WP_059169685.1	Type1.BREX	<-PglZ<-N6-METHYLASE-p<-HEPN<-RVT<-N6-METHYLASE<-?<-HNH<-TOPRIM<-OLD-ABC<-DUF6079*<-BrxB-BrxF-iSTAND<-BrxA-DNA-binding<-RHS<-ARG<-RHS||LexA-protease->?-><-HDRnase<-Arm-DNA-bind_2+Phage_int_SAM_3+Phage_integrase	1240	Bacteria>Pseudomonadota>Gammaproteobacteria>Aeromonadales>Aeromonadaceae>Aeromonas	GCF_001481535.2	Aeromonas_salmonicida	VO70_RS19205	18975..22697	645	BREX-system-P-loop-protein-BrxC-[Aeromonas-salmonicida]
WP_101617899.1	Type1.BREX	<-TetR-HTH<-Phage_integrase+Phage_integrase<-Phage_integrase+Phage_integrase<-Phage_integrase<-LexA-protease||?->RHS->BrxA-DNA-binding->BrxB-BrxF-iSTAND->DUF6079*->OLD-ABC->TOPRIM->N6-METHYLASE->N6-METHYLASE-p->HEPN->N6-METHYLASE-p-><-AAA-ATPase<-HTH+rve||DUF3944->	1231	Bacteria>Pseudomonadota>Gammaproteobacteria>Aeromonadales>Aeromonadaceae>Aeromonas	GCF_002850695.3	Aeromonas_hydrophila	C1A23_RS07645	1215070..1218765	644	BREX-system-P-loop-protein-BrxC-[Aeromonas-hydrophila]
WP_160837717.1	Type1.BREX	<-N6-METHYLASE-p<-TIR<-HEPN<-N6-METHYLASE<-TOPRIM<-OLD-ABC<-DUF6079*<-BrxB-BrxF-iSTAND<-BrxA-DNA-binding<-RHS<-ZnR<-ARG<-RHS||LexA-protease->?-><-HDRnase	1231	Bacteria>Pseudomonadota>Gammaproteobacteria>Aeromonadales>Aeromonadaceae>Aeromonas	GCF_009831085.1	Aeromonas_caviae	GR302_RS08560	7464..11159	648	BREX-system-P-loop-protein-BrxC-[Aeromonas-caviae]
WP_171280306.1	Type1.BREX	AAA-ToxA+Classical-AAA->Classical-AAA->ARG->?->ZnR->RHS->BrxA-DNA-binding->BrxB-BrxF-iSTAND->DUF6079*->N6-METHYLASE->ParB+HNH->PglZ->HEPN+Sel1+SEC-C-><-SF1-SF2-Helicase+REase<-SF1-SF2-Helicase+REase||HIN-HTH->	1157	Bacteria>Pseudomonadota>Gammaproteobacteria>Aeromonadales>Aeromonadaceae>Aeromonas	GCF_013085505.1	Aeromonas_sp._2692-1	E5E97_RS12155	2531425..2534898	2560029	BREX-system-P-loop-protein-BrxC-[Aeromonas-sp.-2692-1]
WP_210557479.1	Type1.BREX	DEDD_Tnp_IS110+Transposase_20->||BrxA-DNA-binding->BrxB-BrxF-iSTAND->DUF6079*->HNH->?->N6-METHYLASE->N6-METHYLASE-p->HEPN->N6-METHYLASE-p->	1218	Bacteria>Pseudomonadota>Gammaproteobacteria>Aeromonadales>Aeromonadaceae>Aeromonas	GCF_017939245.1	Aeromonas_sp._PrichA-15	J9889_RS17255	4362..8018	2823360	BREX-system-P-loop-protein-BrxC-[Aeromonas-sp.-PrichA-15]
WP_213387487.1	Type1.BREX	<-LexA-protease||?-><-wHTH+WYL+WCX||ARG->?->ZnR->RHS->BrxA-DNA-binding->BrxB-BrxF-iSTAND->DUF6079*->N6-METHYLASE->OLD-ABC->HNH->PglZ->HEPN+Sel1+SEC-C->HIN-HTH-><-SF1-SF2-Helicase+REase<-SF1-SF2-Helicase+REase	1157	Bacteria>Pseudomonadota>Gammaproteobacteria>Aeromonadales>Aeromonadaceae>Aeromonas	GCF_018359765.1	Aeromonas_veronii	J4G64_RS11995	290568..294041	654	BREX-system-P-loop-protein-BrxC-[Aeromonas-veronii]
WP_234578020.1	Type1.BREX	<-SIGMA+OB-Fold+AAA-ATPase+Lon-Protease-BrxL<-PglZ<-?<-DUF3944<-N6-METHYLASE-p<-HEPN<-N6-METHYLASE<-TOPRIM<-OLD-ABC<-DUF6079*<-BrxB-BrxF-iSTAND<-BrxA-DNA-binding<-RHS<-ZnR+ZnR||wHTH+WYL+WCX-><-?||LexA-protease->?-><-SF1-SF2-Helicase	1231	Bacteria>Pseudomonadota>Gammaproteobacteria>Aeromonadales>Aeromonadaceae>Aeromonas	GCF_021440865.1	Aeromonas_salmonicida	LZ632_RS15270	23400..27095	645	BREX-system-P-loop-protein-BrxC-[Aeromonas-salmonicida]
WP_139392567.1	Type1.BREX	<-Phage_integrase+Phage_integrase<-Phage_integrase<-LexA-protease||?-><-wHTH+WYL+WCX||?->RHS->BrxA-DNA-binding->BrxB-BrxF-iSTAND->DUF6079*->OLD-ABC->TOPRIM->N6-METHYLASE->RVT->HEPN->N6-METHYLASE-p->DUF3944->?->PglZ->	1225	Bacteria>Pseudomonadota>Gammaproteobacteria>Aeromonadales>Aeromonadaceae>Aeromonas	GCF_021654355.1	Aeromonas_hydrophila	L6424_RS03515	743150..746827	644	BREX-system-P-loop-protein-BrxC-[Aeromonas-hydrophila]
WP_261925329.1	Type1.BREX	<-LexA-protease||ARG->ZnR->RHS->RHS->BrxA-DNA-binding->BrxB-BrxF-iSTAND->DUF6079-p-><-DDE_Tnp_1||DUF6079*->ParB+HNH->N6-METHYLASE->HEPN->N6-METHYLASE-p->PAIREDC-HTH+HIN-HTH->N6-METHYLASE-p->DUF3944->?->PglZ->	1089	Bacteria>Pseudomonadota>Gammaproteobacteria>Aeromonadales>Aeromonadaceae>Aeromonas	GCF_024347775.1	Aeromonas_caviae	OCX50_RS09760	2028863..2032132	648	BREX-system-P-loop-protein-BrxC-[Aeromonas-caviae]
WP_257712004.1	Type1.BREX	<-PglZ<-TOPRIM<-OLD-ABC<-N6-METHYLASE-p<-HEPN<-RVT<-N6-METHYLASE<-TOPRIM<-OLD-ABC<-DUF6079*<-BrxB-BrxF-iSTAND<-BrxA-DNA-binding<-RHS<-?||wHTH+WYL+WCX->	1281	Bacteria>Pseudomonadota>Gammaproteobacteria>Aeromonadales>Aeromonadaceae>Aeromonas	GCF_024649525.1	Aeromonas_caviae	NUK31_RS14230	17564..21409	648	BREX-system-P-loop-protein-BrxC-[Aeromonas-caviae]
WP_265783524.1	Type1.BREX	<-TOPRIM<-OLD-ABC<-N6-METHYLASE-p<-TIR<-HEPN<-N6-METHYLASE<-HEPN<-TOPRIM<-OLD-ABC<-DUF6079*<-BrxB-BrxF-iSTAND<-BrxA-DNA-binding<-RHS<-?||LexA-protease->?->PBPB+PBPB+PAS+PAS+HISKIN+REC+HPT->REC+EAL->	1235	Bacteria>Pseudomonadota>Gammaproteobacteria>Aeromonadales>Aeromonadaceae>Aeromonas	GCF_026230585.1	Aeromonas_veronii	OD810_RS03320	107717..111424	654	BREX-system-P-loop-protein-BrxC-[Aeromonas-veronii]
WP_270698108.1	Type1.BREX	<-LexA-protease||?-><-wHTH+WYL+WCX||?->RHS->BrxA-DNA-binding->BrxB-BrxF-iSTAND->DUF6079*->OLD-ABC->TOPRIM->N6-METHYLASE->RVT->HEPN->N6-METHYLASE->OLD-ABC->TOPRIM->PglZ->	1280	Bacteria>Pseudomonadota>Gammaproteobacteria>Aeromonadales>Aeromonadaceae>Aeromonas	GCF_027705185.1	Aeromonas_sp._QDB05	OKC32_RS09690	4766..8608	2990478	BREX-system-P-loop-protein-BrxC-[Aeromonas-sp.-QDB05]
WP_201934227.1	Type1.BREX	RHS->BrxA-DNA-binding->BrxB-BrxF-iSTAND->DUF6079*->ParB+HNH->N6-METHYLASE->HEPN->N6-METHYLASE-p->DUF3944->?->PglZ->SIGMA+OB-Fold+AAA-ATPase+Lon-Protease-BrxL->	1231	Bacteria>Pseudomonadota>Gammaproteobacteria>Aeromonadales>Aeromonadaceae	GCF_019972675.1	Aeromonas	MWG22_RS17445	1818..5513	642	MULTISPECIES:-BREX-system-P-loop-protein-BrxC-[Aeromonas]
WP_171273737.1	Type1.BREX	Arm-DNA-bind_2+Phage_int_SAM_3+Phage_integrase->HDRnase-><-?||RHS->ARG->ZnR->RHS->BrxA-DNA-binding->BrxB-BrxF-iSTAND->DUF6079*->OLD-ABC->TOPRIM->N6-METHYLASE->HEPN->TIR->N6-METHYLASE-p->DUF3944->?->||PglZ->	1231	Bacteria>Pseudomonadota>Gammaproteobacteria>Aeromonadales>Aeromonadaceae	GCF_021654375.1	Aeromonas	E4185_RS15845	3355159..3358854	642	MULTISPECIES:-BREX-system-P-loop-protein-BrxC-[Aeromonas]
GGW57088.1	Type1.BREX	<-wHTH+WYL+WCX-p||SIR2->?-><-?||HEPN-p->JAB->BrxA-DNA-binding->BrxB-BrxF-iSTAND->DUF6079*->N6-METHYLASE->OLD-ABC->HNH->PglZ->HEPN+Sel1+SEC-C->Immank+ANK+ANK->REase-><-?<-SF1-SF2-Helicase+REase	1114	Bacteria>Pseudomonadota>Gammaproteobacteria>Alteromonadales>Alteromonadaceae>Alishewanella	GCA_014652375.1	Alishewanella_tabrizica	GCM10008111_11430	80462..83806	671278	hypothetical-protein-GCM10008111_11430-[Alishewanella-tabrizica]
WP_229796961.1	Type1.BREX	<-wHTH+WYL+WCX-p||SIR2->?-><-?||HEPN-p->JAB->BrxA-DNA-binding->BrxB-BrxF-iSTAND->DUF6079*->N6-METHYLASE->OLD-ABC->HNH->PglZ->HEPN+Sel1+SEC-C->Immank+ANK+ANK->REase-><-?<-SF1-SF2-Helicase+REase	1125	Bacteria>Pseudomonadota>Gammaproteobacteria>Alteromonadales>Alteromonadaceae>Alishewanella	GCF_014652375.1	Alishewanella_tabrizica	IE070_RS05825	80429..83806	671278	BREX-system-P-loop-protein-BrxC-[Alishewanella-tabrizica]
WP_240919947.1	Type1.BREX	BrxA-DNA-binding->BrxB-BrxF-iSTAND->DUF6079*->N6-METHYLASE->ParB+HNH->OLD-ABC->Tox-RelE->PglZ->SIGMA+OB-Fold+AAA-ATPase+Lon-Protease-BrxL->HEPN->	1180	Bacteria>Pseudomonadota>Gammaproteobacteria>Alteromonadales>Alteromonadaceae>Paraglaciecola	GCF_011378905.1	Paraglaciecola_sp._20A4	GQS54_RS13935	91436..94978	2687288	BREX-system-P-loop-protein-BrxC-[Paraglaciecola-sp.-20A4]
WP_058373560.1	Type1.BREX	<-SIGMA+OB-Fold+AAA-ATPase+Lon-Protease-BrxL<-PglZ<-SF1-SF2-Helicase<-?<-N6-METHYLASE<-HEPN<-N6-METHYLASE<-DUF6079*<-BrxB-BrxF-iSTAND<-BrxA-DNA-binding<-wHTH+WYL+WCX<-HTH_26||Phage_AlpA->Arm-DNA-bind_2+Phage_int_SAM_3+Phage_integrase->HDRnase-><-Phage_integrase<-Phage_integrase+Phage_integrase	1229	Bacteria>Pseudomonadota>Gammaproteobacteria>Alteromonadales>Pseudoalteromonadaceae>Pseudoalteromonas	GCF_001465295.1	Pseudoalteromonas_translucida	PTRA_a2157	1987847..1991536	166935	BREX-system-P-loop-protein-BrxC-[Pseudoalteromonas-translucida]
WP_233998422.1	Type1.BREX	<-PglZ<-?<-?<-SF1-SF2-Helicase<-HEPN<-N6-METHYLASE<-N6-METHYLASE-p<-HATPase+wHTH<-Schlafen+HATPase+wHTH<-DUF6079*<-BrxB-BrxF-iSTAND<-BrxA-DNA-binding<-wHTH+WYL+WCX<-?<-?||?-><-?||?-><-SIR2	1252	Bacteria>Pseudomonadota>Gammaproteobacteria>Alteromonadales>Pseudoalteromonadaceae>Pseudoalteromonas	GCF_002964785.1	Pseudoalteromonas_sp._T11g48	C2M29_RS12220	655771..659529	2077100	BREX-system-P-loop-protein-BrxC-[Pseudoalteromonas-sp.-T11g48]

Protein-ID	System	Gene-Neighbourhoods	Length	Taxonomy	Assembly-ID	Strain	Locus-Tag	Genomic-coordinates	TaxID	Fasta-defline
WP_182739649.1	Type1.BREX	<-Phage_integrase<-DUF2787<-?||XIS-HTH-> HEPN ->?-> wHTH+WYL+WCX -> BrxA-DNA-binding -> BrxB-BrxF-iSTAND -> DUF6079 *-> N6-METHYLASE -p->	1235	Bacteria>Pseudomonadota>Gammaproteobacteria>Alteromonadales>Pseudoalteromonadaceae>Pseudoalteromonas	GCF_014163545.1	Pseudoalteromonas_sp._SR45-6	H5158_RS05620	135410..139117	2760927	BREX-system-P-loop-protein-BrxC-[Pseudoalteromonas-sp.-SR45-6]
WP_193038371.1	Type1.BREX	<-ZetaToxin<-?<- PglZ <-ParB+HNH<- N6-METHYLASE <- DUF6079 *<- BrxB-BrxF-iSTAND <- BrxA-DNA-binding <- wHTH+WYL+WCX <-?<- HEPN <-XIS-HTH||?->DUF2787->Phage_integrase->	1159	Bacteria>Pseudomonadota>Gammaproteobacteria>Alteromonadales>Pseudoalteromonadaceae>Pseudoalteromonas	GCF_014897925.1	Pseudoalteromonas_nigrifaciens	INT11_RS00030	7982..11461	28109	BREX-system-P-loop-protein-BrxC-[Pseudoalteromonas-nigrifaciens]
WP_248533819.1	Type1.BREX	<-MarR-HTH||?-><-?<-?|| BrxA-DNA-binding -> BrxB-BrxF-iSTAND -> DUF6079 *->SSB->GP+REase->SF1-SF2-Helicase+PRTase->SLOG+BirA-HTH-> N6-METHYLASE -> HEPN -> N6-METHYLASE ->DnaB+McrB-AAA->wHTH+REase+ZnR->	1229	Bacteria>Pseudomonadota>Gammaproteobacteria>Alteromonadales>Pseudoalteromonadaceae>Pseudoalteromonas	GCF_023161935.1	Pseudoalteromonas_sp._2CM28B	MTN96_RS29910	10677..14366	2929851	BREX-system-P-loop-protein-BrxC-[Pseudoalteromonas-sp.-2CM28B]
WP_037429580.1	Type1.BREX	<- PglZ <-ParB+HNH<- N6-METHYLASE <- DUF6079 *<- BrxB-BrxF-iSTAND <- BrxA-DNA-binding <-?<- HEPN <-?<-MoxR-AAA<-NtrC-AAA+FIS-HTH<-XIS-HTH	1157	Bacteria>Pseudomonadota>Gammaproteobacteria>Alteromonadales>Shewanellaceae	GCF_000282755.1	Shewanella	SHEWPOL2_RS15310	7136..10609	22	MULTISPECIES:-BREX-system-P-loop-protein-BrxC-[Shewanella]
HAY94623.1	Type1.BREX	<-DUF2787<-CbeA_antitoxin<-?<-?||XIS-HTH-> wHTH+WYL+WCX -p-> wHTH+WYL+WCX -> BrxA-DNA-binding -> BrxB-BrxF-iSTAND -> DUF6079 *->HNH->?->?->ParB+HNH-> N6-METHYLASE -> HEPN -> N6-METHYLASE -p->	1224	Bacteria>Pseudomonadota>Gammaproteobacteria>Alteromonadales>Shewanellaceae>Shewanella	GCA_003486485.1	Shewanella_sp.	DCY70_12125	4933..8607	50422	BREX-system-P-loop-protein-BrxC-[Shewanella-sp.]
MCD8561045.1	Type1.BREX	<- N6-METHYLASE -p<- HEPN <- N6-METHYLASE -p<- N6-METHYLASE -p<- N6-METHYLASE <-?<-HNH<- DUF6079 *<- BrxB-BrxF-iSTAND <- BrxA-DNA-binding <- wHTH+WYL+WCX <-XIS-HTH	1231	Bacteria>Pseudomonadota>Gammaproteobacteria>Alteromonadales>Shewanellaceae>Shewanella	GCA_021209305.1	Shewanella_xiamenensis	LRY75_19995	6155..9850	332186	BREX-system-P-loop-protein-BrxC-[Shewanella-xiamenensis]
WP_011622483.1	Type1.BREX	XIS-HTH->NtrC-AAA+FIS-HTH->MoxR-AAA->?-> HEPN ->?-> BrxA-DNA-binding -> BrxB-BrxF-iSTAND -> DUF6079 *-> N6-METHYLASE ->ParB+HNH-> PglZ ->ZetaToxin->?-><-SF1-SF2-Helicase+REase<-SF1-SF2-Helicase+REase||HerA/FtsK->	1157	Bacteria>Pseudomonadota>Gammaproteobacteria>Alteromonadales>Shewanellaceae>Shewanella	GCF_000014685.1	Shewanella_sp._MR-4	SHEWMR4_RS09030	2019364..2022837	60480	BREX-system-P-loop-protein-BrxC-[Shewanella-sp.-MR-4]
WP_011716828.1	Type1.BREX	XIS-HTH->NtrC-AAA+FIS-HTH->MoxR-AAA->?-> HEPN ->?-> BrxA-DNA-binding -> BrxB-BrxF-iSTAND -> DUF6079 *-> N6-METHYLASE ->ParB+HNH-> PglZ ->ZetaToxin->?-><-SF1-SF2-Helicase+REase<-SF1-SF2-Helicase+REase||HerA/FtsK->	1157	Bacteria>Pseudomonadota>Gammaproteobacteria>Alteromonadales>Shewanellaceae>Shewanella	GCF_000203935.1	Shewanella_sp._ANA-3	SHEWANA3_RS09590	2144277..2147750	94122	BREX-system-P-loop-protein-BrxC-[Shewanella-sp.-ANA-3]
WP_028764576.1	Type1.BREX	BrxA-DNA-binding -> BrxB-BrxF-iSTAND -> DUF6079 *-> N6-METHYLASE ->ParB+HNH-> PglZ -> HEPN +Sel1+SEC-C-><-SF1-SF2-Helicase+REase<-SF1-SF2-Helicase+REase<-?<-MerR-HTH	1157	Bacteria>Pseudomonadota>Gammaproteobacteria>Alteromonadales>Shewanellaceae>Shewanella	GCF_000518705.1	Shewanella_colwelliana	L876_RS0115155	4861..8334	23	BREX-system-P-loop-protein-BrxC-[Shewanella-colwelliana]
WP_055648628.1	Type1.BREX	<-PVC-AAA<-?||SF1-SF2-Helicase+REase->SF1-SF2-Helicase+REase-><-?<-ZetaToxin<- PglZ <-ParB+HNH<- N6-METHYLASE <- DUF6079 *<- BrxB-BrxF-iSTAND <- BrxA-DNA-binding <-?<- HEPN	1157	Bacteria>Pseudomonadota>Gammaproteobacteria>Alteromonadales>Shewanellaceae>Shewanella	GCF_001308045.1	Shewanella_sp._Sh95	AEA42_RS19845	36695..40168	1689868	BREX-system-P-loop-protein-BrxC-[Shewanella-sp.-Sh95]
WP_244159705.1	Type1.BREX	<-HDRnase<-DDE_Tnp_1<-Arm-DNA-bind_2+Phage_int_SAM_3+Phage_integrase<-Phage_AlpA||HTH_26-> wHTH+WYL+WCX -> BrxA-DNA-binding -> BrxB-BrxF-iSTAND -> DUF6079 *->HNH->?-> N6-METHYLASE -> N6-METHYLASE -p-> HEPN -> N6-METHYLASE -p->MULE-TRANSPOSASE-> N6-METHYLASE -p->	1254	Bacteria>Pseudomonadota>Gammaproteobacteria>Alteromonadales>Shewanellaceae>Shewanella	GCF_001723195.1	Shewanella_xiamenensis	ABT47_RS18205	4085889..4089653	332186	BREX-system-P-loop-protein-BrxC-[Shewanella-xiamenensis]
WP_069454895.1	Type1.BREX	<-HDRnase<-Arm-DNA-bind_2+Phage_int_SAM_3+Phage_integrase<-Phage_AlpA||HTH_26-> wHTH+WYL+WCX -> BrxA-DNA-binding -> BrxB-BrxF-iSTAND -> DUF6079 *->HNH->?-> N6-METHYLASE -> N6-METHYLASE -p-> HEPN -> N6-METHYLASE -p->MULE-TRANSPOSASE-> N6-METHYLASE -p->	1240	Bacteria>Pseudomonadota>Gammaproteobacteria>Alteromonadales>Shewanellaceae>Shewanella	GCF_002074855.1	Shewanella_xiamenensis	VO00_RS12940	4702..8424	332186	BREX-system-P-loop-protein-BrxC-[Shewanella-xiamenensis]
WP_080916542.1	Type1.BREX	<-Pkinase+STYKIN||?->?->?-> wHTH+WYL+WCX -> BrxA-DNA-binding -> BrxB-BrxF-iSTAND -> DUF6079 -p-> DUF6079 *->ParB+HNH->ParB+HNH-p-> HEPN -> PglZ -> SIGMA+OB-Fold+AAA-ATPase+Lon-Protease-BrxL -><-MarR-HTH||?->?->Phage_int_SAM_4->	1234	Bacteria>Pseudomonadota>Gammaproteobacteria>Alteromonadales>Shewanellaceae>Shewanella	GCF_002075795.1	Shewanella_japonica	SJ2017_RS16905	3936240..3939944	93973	BREX-system-P-loop-protein-BrxC-[Shewanella-japonica]
WP_088585687.1	Type1.BREX	ZnR-><- HEPN -p<- HEPN -p<- SIGMA+OB-Fold+AAA-ATPase+Lon-Protease-BrxL <- PglZ <- N6-METHYLASE -p||DDE_Tnp_1->?-><- N6-METHYLASE <- DUF6079 *<- BrxB-BrxF-iSTAND <- BrxA-DNA-binding <-DDE_Tnp_1<-Prok-JAB<- wHTH+WYL+WCX <-?<-XIS-HTH	1229	Bacteria>Pseudomonadota>Gammaproteobacteria>Alteromonadales>Shewanellaceae>Shewanella	GCF_002209245.2	Shewanella_sp._FDAARGOS_354	CEQ32_RS05910	1298859..1302548	1930557	BREX-system-P-loop-protein-BrxC-[Shewanella-sp.-FDAARGOS_354]
WP_208124435.1	Type1.BREX	XIS-HTH->NtrC-AAA+FIS-HTH->MoxR-AAA->?-> HEPN -> BrxA-DNA-binding -> BrxB-BrxF-iSTAND -> DUF6079 *-> N6-METHYLASE ->ParB+HNH-> PglZ ->ZetaToxin-><-SF1-SF2-Helicase+REase<-SF1-SF2-Helicase+REase||REase->HIN-HTH-><-GDResolvase+HIN-HTH	1157	Bacteria>Pseudomonadota>Gammaproteobacteria>Alteromonadales>Shewanellaceae>Shewanella	GCF_017570045.1	Shewanella_algae	JKK45_RS07515	1709365..1712838	38313	BREX-system-P-loop-protein-BrxC-[Shewanella-algae]
WP_144191359.1	Type1.BREX	XIS-HTH->NtrC-AAA+FIS-HTH->MoxR-AAA->?-> HEPN -> BrxA-DNA-binding -> BrxB-BrxF-iSTAND -> DUF6079 *-> N6-METHYLASE ->ParB+HNH-> PglZ ->ZetaToxin->?-><-SF1-SF2-Helicase+REase<-SF1-SF2-Helicase+REase	1157	Bacteria>Pseudomonadota>Gammaproteobacteria>Alteromonadales>Shewanellaceae>Shewanella	GCF_017580415.1	Shewanella_algae	I6M45_RS07395	495996..499469	38313	BREX-system-P-loop-protein-BrxC-[Shewanella-algae]
WP_224020606.1	Type1.BREX	<-HerA/FtsK||SF1-SF2-Helicase+REase->SF1-SF2-Helicase+REase-><-?<-ZetaToxin<- PglZ <-ParB+HNH<- N6-METHYLASE <- DUF6079 *<- BrxB-BrxF-iSTAND <- BrxA-DNA-binding <-?<- HEPN <-?<-MoxR-AAA<-NtrC-AAA+FIS-HTH<-XIS-HTH	1157	Bacteria>Pseudomonadota>Gammaproteobacteria>Alteromonadales>Shewanellaceae>Shewanella	GCF_019973655.1	Shewanella_xiamenensis	LDO18_RS12250	2746465..2749938	332186	BREX-system-P-loop-protein-BrxC-[Shewanella-xiamenensis]
WP_248084385.1	Type1.BREX	SF1-SF2-Helicase-><-DUF2787<-CbeA_antitoxin<-?<-?||XIS-HTH-> wHTH+WYL+WCX -> BrxA-DNA-binding -> BrxB-BrxF-iSTAND -> DUF6079 *->HNH->?->ParB+HNH-> N6-METHYLASE -> N6-METHYLASE -p-> HEPN -> N6-METHYLASE -p->	1231	Bacteria>Pseudomonadota>Gammaproteobacteria>Alteromonadales>Shewanellaceae>Shewanella	GCF_023159095.1	unclassified_Shewanella	MOD77_RS02670	216752..220447	196818	MULTISPECIES:-BREX-system-P-loop-protein-BrxC-[unclassified-Shewanella]
WP_249553736.1	Type1.BREX	<- SIGMA+OB-Fold+AAA-ATPase+Lon-Protease-BrxL <- PglZ <-?<- N6-METHYLASE <- HEPN <-RVT<- N6-METHYLASE -p<- N6-METHYLASE <- DUF6079 *<- BrxB-BrxF-iSTAND <- BrxA-DNA-binding <- wHTH+WYL+WCX ||?->?->CbeA_antitoxin->DUF2787->DUF2787->Phage_integrase->	1268	Bacteria>Pseudomonadota>Gammaproteobacteria>Alteromonadales>Shewanellaceae>Shewanella	GCF_023475945.1	Shewanella_sp._8A	M2897_RS12020	2756867..2760673	2943323	BREX-system-P-loop-protein-BrxC-[Shewanella-sp.-8A]
WP_259573703.1	Type1.BREX	<-CHTH<-REase<- SIGMA+OB-Fold+AAA-ATPase+Lon-Protease-BrxL <- PglZ <- N6-METHYLASE -p<- HEPN <-RVT<- N6-METHYLASE -p<- N6-METHYLASE <- DUF6079 *<- BrxB-BrxF-iSTAND <- BrxA-DNA-binding <- wHTH+WYL+WCX <-HTH_26||Phage_AlpA->Arm-DNA-bind_2+Phage_int_SAM_3+Phage_integrase->HDRnase->?-><-SF1-SF2-Helicase	1240	Bacteria>Pseudomonadota>Gammaproteobacteria>Alteromonadales>Shewanellaceae>Shewanella	GCF_024999165.1	Shewanella_baltica	G7A68_RS06675	25637..29359	62322	BREX-system-P-loop-protein-BrxC-[Shewanella-baltica]
WP_263197229.1	Type1.BREX	<- SIGMA+OB-Fold+AAA-ATPase+Lon-Protease-BrxL <- PglZ <- N6-METHYLASE -p<- HEPN <- N6-METHYLASE -p<- N6-METHYLASE <-?<-HNH<- DUF6079 *<- BrxB-BrxF-iSTAND <- BrxA-DNA-binding <- wHTH+WYL+WCX <-HTH_26||Phage_AlpA->Arm-DNA-bind_2+Phage_int_SAM_3+Phage_integrase->HDRnase->	1246	Bacteria>Pseudomonadota>Gammaproteobacteria>Alteromonadales>Shewanellaceae>Shewanella	GCF_025654545.1	Shewanella_sp._SM20	L5M18_RS13095	42119..45859	2912792	BREX-system-P-loop-protein-BrxC-[Shewanella-sp.-SM20]
WP_101520955.1	Type1.BREX	HEPN ->HNH+ Polbeta ->?->?->?->Peptidase_M29+DUF2971->XIS-HTH-> BrxA-DNA-binding -> BrxB-BrxF-iSTAND -> DUF6079 *-> N6-METHYLASE ->MarR-HTH-> Polbeta -> PglZ -> SIGMA+OB-Fold+AAA-ATPase+Lon-Protease-BrxL -> HEPN +SF1-SF2-Helicase+RNase+RNase->TPR-><-?<-Arm-DNA-bind_3+Phage_integrase	1151	Bacteria>Pseudomonadota>Gammaproteobacteria>Cellvibrionales>Halieaceae>Kineobactrum	GCF_002868715.1	Kineobactrum_sediminis	CWI75_RS08140	627029..630484	1905677	BREX-system-P-loop-protein-BrxC-[Kineobactrum-sediminis]
MBN49711.1	Type1.BREX	<- SIGMA+OB-Fold+AAA-ATPase+Lon-Protease-BrxL <- PglZ <-DOC+HTH<-?<-ZNR+RmuC<- HEPN <-?<- N6-METHYLASE <- DUF6079 *<- BrxB-BrxF-iSTAND <- BrxA-DNA-binding || wHTH+WYL+WCX ->?-><-?<-SF1-SF2-Helicase||?->?->Phage_integrase->	1193	Bacteria>Pseudomonadota>Gammaproteobacteria>Cellvibrionales>Spongiibacteraceae>unclassified_Spongiibacteraceae	GCA_002723235.1	Spongiibacteraceae_bacterium	CMN85_09225	177592..181173	2026793	hypothetical-protein-[Spongiibacteraceae-bacterium]
QVL47244.1	Type6.BREX	<-PIN<-RHH<- N6-METHYLASE <-TOPRIM<-OLD-ABC<- N6-METHYLASE -p<-ORC-CDC6<-ParA-Soj-PloopNTPase<- HEPN <- DUF6079 *<-?<- BrxB-BrxF-iSTAND <- BrxA-DNA-binding <- BrxE <-?<-NTP_transf_3+Hexapep+Hexapep	1179	Bacteria>Pseudomonadota>Gammaproteobacteria>Chromatiales>Chromatiaceae>Thiocapsa	GCA_018399035.1	Thiocapsa_sp.	KFB96_16145	3553103..3556642	2024551	MAG:-BREX-system-P-loop-protein-BrxC-[Thiocapsa-sp.]
MBK8639641.1	Type6.BREX	REC-><-?|| BrxE -> BrxA-DNA-binding -> BrxB-BrxF-iSTAND -> DUF6079 *->?-> N6-METHYLASE ->RHH->PIN-> PglZ -> PolB -> HEPN -> SIGMA+OB-Fold+AAA-ATPase+Lon-Protease-BrxL ->	1178	Bacteria>Pseudomonadota>Gammaproteobacteria>Chromatiales>Chromatiaceae>unclassified_Chromatiaceae	GCA_016714645.1	Chromatiaceae_bacterium	IPN92_15725	846355..849891	2049432	BREX-system-P-loop-protein-BrxC-[Chromatiaceae-bacterium]
MCF7977306.1	Type6.BREX	<-ParA-Soj-PloopNTPase<- HEPN <- DUF6079 *<- BrxB-BrxF-iSTAND <- BrxA-DNA-binding <- BrxE ||?->MlaE->ABC-ATPase+SbcC->	1182	Bacteria>Pseudomonadota>Gammaproteobacteria>Chromatiales>Chromatiaceae>unclassified_Chromatiaceae	GCA_021736685.1	Chromatiaceae_bacterium	K9L82_04715	1611..5159	2049432	BREX-system-P-loop-protein-BrxC-[Chromatiaceae-bacterium]
RJT19525.1	Type1.BREX	<- PglZ <-RVT<- HEPN <- N6-METHYLASE <-ParB+HNH<- DUF6079 *<- BrxB-BrxF-iSTAND <- BrxA-DNA-binding <-?<-XIS-HTH||?->?->DUF932->?->wHTH+WYL->	1233	Bacteria>Pseudomonadota>Gammaproteobacteria>Enterobacterales>Enterobacteriaceae>Buttiauxella	GCA_003601925.1	Buttiauxella_izardii	D6029_18525	11062..14763	82991	BREX-system-P-loop-protein-BrxC-[Buttiauxella-izardii]
WP_244212427.1	Type1.BREX	<- PglZ <- N6-METHYLASE -p<- N6-METHYLASE -p<-RVT<- HEPN <- N6-METHYLASE <-ParB+HNH<- DUF6079 *<- BrxB-BrxF-iSTAND <- BrxA-DNA-binding <-?<-XIS-HTH||?->?->DUF932->?->wHTH+WYL->	1237	Bacteria>Pseudomonadota>Gammaproteobacteria>Enterobacterales>Enterobacteriaceae>Buttiauxella	GCF_003601925.1	Buttiauxella_izardii	D6029_RS18475	11062..14775	82991	BREX-system-P-loop-protein-BrxC-[Buttiauxella-izardii]
WP_181630678.1	Type1.BREX	<-Sulfatase<-?<-?<-?<-Schlafen+HATPase+wHTH<-PHP+SbcC<-?|| BrxA-DNA-binding -> BrxB-BrxF-iSTAND -> DUF6079 *->ParB+HNH-> N6-METHYLASE -> HEPN -><-HIN-HTH|| N6-METHYLASE -p-> N6-METHYLASE -p-> PglZ -> SIGMA+OB-Fold+AAA-ATPase+Lon-Protease-BrxL -><-HTH_23+HTH_32+HTH_33+DDE_3	1233	Bacteria>Pseudomonadota>Gammaproteobacteria>Enterobacterales>Enterobacteriaceae>Citrobacter	-	Citrobacter_sp._RHBSTW-01013	HV350_21470	4470372..4474073	2742677	BREX-system-P-loop-protein-BrxC-[Citrobacter-sp.-RHBSTW-01013]
QLR26611.1	Type1.BREX	<-Sulfatase<-?<-?<-?<-Schlafen+HATPase+wHTH<-PHP+SbcC<-?|| BrxA-DNA-binding -> BrxB-BrxF-iSTAND -> DUF6079 *->ParB+HNH-> N6-METHYLASE -> HEPN -><-HIN-HTH|| N6-METHYLASE -p-> N6-METHYLASE -p-> PglZ -> SIGMA+OB-Fold+AAA-ATPase+Lon-Protease-BrxL -><-HTH_23+HTH_32+HTH_33+DDE_3	1233	Bacteria>Pseudomonadota>Gammaproteobacteria>Enterobacterales>Enterobacteriaceae>Citrobacter	GCA_013781985.1	Citrobacter_sp._RHBSTW-01013	HV350_21470	4470372..4474073	2742677	BREX-system-P-loop-protein-BrxC-[Citrobacter-sp.-RHBSTW-01013]
MBM9568541.1	Type1.BREX	<-wHTH+WYL<-DUF932<-nSTAND1+GTPase-AIG+sGTP<-CopG/RHH||XIS-HTH->?->?-> BrxA-DNA-binding -> BrxB-BrxF-iSTAND -> DUF6079 *->ParB+HNH-> N6-METHYLASE -> HEPN ->RVT-> N6-METHYLASE -> PglZ -> SIGMA+OB-Fold+AAA-ATPase+Lon-Protease-BrxL -><-Arm-DNA-bind_3+Phage_integrase	1233	Bacteria>Pseudomonadota>Gammaproteobacteria>Enterobacterales>Enterobacteriaceae>Citrobacter	GCA_014931625.2	Citrobacter_sedlakii	INP82_14060	158441..162142	67826	BREX-system-P-loop-protein-BrxC-[Citrobacter-sedlakii]
WP_249418019.1	Type1.BREX	<-Sulfatase<-?<-?<-?<-Schlafen+HATPase+wHTH<-PHP+SbcC<-?|| BrxA-DNA-binding -> BrxB-BrxF-iSTAND -> DUF6079 *->ParB+HNH-> N6-METHYLASE -> HEPN ->RVT-><-HIN-HTH||RVT-> N6-METHYLASE -p-> N6-METHYLASE -p-> PglZ ->	1240	Bacteria>Pseudomonadota>Gammaproteobacteria>Enterobacterales>Enterobacteriaceae>Citrobacter	GCF_013781985.1	Citrobacter_sp._RHBSTW-01013	HV350_RS21465	4470351..4474073	2742677	BREX-system-P-loop-protein-BrxC-[Citrobacter-sp.-RHBSTW-01013]
WP_275379687.1	Type1.BREX	Arm-DNA-bind_3+Phage_integrase-><- SIGMA+OB-Fold+AAA-ATPase+Lon-Protease-BrxL <- PglZ <- N6-METHYLASE -p<-RVT<- HEPN <- N6-METHYLASE <-ParB+HNH<- DUF6079 *<- BrxB-BrxF-iSTAND <- BrxA-DNA-binding <-?<-?<-XIS-HTH<-?||?->nSTAND1+GTPase-AIG+sGTP->DUF932->	1240	Bacteria>Pseudomonadota>Gammaproteobacteria>Enterobacterales>Enterobacteriaceae>Citrobacter	GCF_029104295.1	Citrobacter_freundii	L2W90_RS09965	166217..169939	546	BREX-system-P-loop-protein-BrxC-[Citrobacter-freundii]
CAF2998376.1	Type1.BREX	Arm-DNA-bind_3+Phage_integrase-><- SIGMA+OB-Fold+AAA-ATPase+Lon-Protease-BrxL <- PglZ <- N6-METHYLASE -p<-RVT<- HEPN <- N6-METHYLASE <-ParB+HNH<- DUF6079 *<- BrxB-BrxF-iSTAND <- BrxA-DNA-binding <-Glyoxalase<-ZnR<-SAM-METHYLASE<-YjhX_toxin<-Glyoxalase<-?<-Glycoside_Hydrolase	1240	Bacteria>Pseudomonadota>Gammaproteobacteria>Enterobacterales>Enterobacteriaceae>Enterobacter	-	Enterobacter_cloacae	AI2972V1_0508	557695..561417	550	hypothetical-protein-AI2972V1_0508-[Enterobacter-cloacae]
WP_039268698.1	Type1.BREX	Arm-DNA-bind_3+Phage_integrase-><- SIGMA+OB-Fold+AAA-ATPase+Lon-Protease-BrxL <- PglZ <- N6-METHYLASE -p<-RVT<- HEPN <-ParB+HNH<- DUF6079 *<- BrxB-BrxF-iSTAND <- BrxA-DNA-binding <-Glyoxalase<-ZnR<-SAM-METHYLASE<-YjhX_toxin<-Glyoxalase<-?<-Glycoside_Hydrolase	1233	Bacteria>Pseudomonadota>Gammaproteobacteria>Enterobacterales>Enterobacteriaceae>Enterobacter	-	Enterobacter_hormaechei	LI66_02640	557969..561670	158836	MULTISPECIES:-BREX-system-P-loop-protein-BrxC-[Enterobacter]
AFP68425.1	Type1.BREX	Arm-DNA-bind_3+Phage_integrase-><- SIGMA+OB-Fold+AAA-ATPase+Lon-Protease-BrxL <- PglZ <- N6-METHYLASE -p<-RVT<- HEPN <- N6-METHYLASE <-ParB+HNH<- DUF6079 *<- BrxB-BrxF-iSTAND <- BrxA-DNA-binding ||?->?-><-?<-?<-Glyoxalase<-ZnR<-METHYLASE	1233	Bacteria>Pseudomonadota>Gammaproteobacteria>Enterobacterales>Enterobacteriaceae>Enterobacter	GCA_000286275.1	Enterobacter_kobei	ECENHK_02660	581253..584954	208224	hypothetical-protein-ECENHK_02660-[Enterobacter-kobei]
AJB80307.1	Type1.BREX	Arm-DNA-bind_3+Phage_integrase-><- SIGMA+OB-Fold+AAA-ATPase+Lon-Protease-BrxL <- PglZ <- N6-METHYLASE -p<-RVT<- HEPN <-ParB+HNH<- DUF6079 *<- BrxB-BrxF-iSTAND <- BrxA-DNA-binding <-Glyoxalase<-ZnR<-SAM-METHYLASE<-YjhX_toxin<-Glyoxalase<-?<-Glycoside_Hydrolase	1233	Bacteria>Pseudomonadota>Gammaproteobacteria>Enterobacterales>Enterobacteriaceae>Enterobacter	GCA_000814225.1	Enterobacter_hormaechei_subsp._xiangfangensis	LI66_02640	557969..561670	1296536	DNA-repair-protein-[Enterobacter-hormaechei-subsp.-xiangfangensis]
MBG0523800.1	Type1.BREX	Arm-DNA-bind_3+Phage_integrase-><- SIGMA+OB-Fold+AAA-ATPase+Lon-Protease-BrxL <- PglZ <- N6-METHYLASE -p<-RVT<- HEPN <- N6-METHYLASE <-ParB+HNH<- DUF6079 *<- BrxB-BrxF-iSTAND <- BrxA-DNA-binding <-HIN-HTH<-REase<-wHTH+SAD+HNH||SymE_toxin->	1233	Bacteria>Pseudomonadota>Gammaproteobacteria>Enterobacterales>Enterobacteriaceae>Enterobacter	GCA_015704905.1	Enterobacter_cloacae	IOR24_17915	3774454..3778155	550	BREX-system-P-loop-protein-BrxC-[Enterobacter-cloacae]
MBK3145473.1	Type1.BREX	Glyoxalase->ACET->YjhX_toxin->SAM-METHYLASE->ZnR->Glyoxalase-> BrxA-DNA-binding -> BrxB-BrxF-iSTAND -> DUF6079 *->ParB+HNH-> N6-METHYLASE -> HEPN ->RVT-> N6-METHYLASE -p-> PglZ -> SIGMA+OB-Fold+AAA-ATPase+Lon-Protease-BrxL -><-Arm-DNA-bind_3+Phage_integrase	1233	Bacteria>Pseudomonadota>Gammaproteobacteria>Enterobacterales>Enterobacteriaceae>Enterobacter	GCA_016616795.1	Enterobacter_hormaechei	FKK11_12155	273388..277089	158836	BREX-system-P-loop-protein-BrxC-[Enterobacter-hormaechei]
WP_228371847.1	Type1.BREX	Arm-DNA-bind_3+Phage_integrase-><- SIGMA+OB-Fold+AAA-ATPase+Lon-Protease-BrxL <- PglZ <- N6-METHYLASE -p<-RVT<- HEPN <- N6-METHYLASE <-ParB+HNH<- DUF6079 *<- BrxB-BrxF-iSTAND <- BrxA-DNA-binding ||?-><-?<-Glyoxalase<-ZnR<-METHYLASE<-YjhX_toxin	1240	Bacteria>Pseudomonadota>Gammaproteobacteria>Enterobacterales>Enterobacteriaceae>Enterobacter	GCF_000286275.1	Enterobacter_kobei	ECENHK_RS02760	581253..584975	208224	BREX-system-P-loop-protein-BrxC-[Enterobacter-kobei]
WP_234704204.1	Type1.BREX	Arm-DNA-bind_3+Phage_integrase-><- SIGMA+OB-Fold+AAA-ATPase+Lon-Protease-BrxL <- PglZ <- N6-METHYLASE -p<-RVT<- HEPN <- N6-METHYLASE <-ParB+HNH<- DUF6079 *<- BrxB-BrxF-iSTAND <- BrxA-DNA-binding <-Glyoxalase<-ZnR<-SAM-METHYLASE<-YjhX_toxin<-ACET<-Glyoxalase	1240	Bacteria>Pseudomonadota>Gammaproteobacteria>Enterobacterales>Enterobacteriaceae>Enterobacter	GCF_000814225.1	Enterobacter_cloacae_complex	LI66_RS02640	557969..561691	354276	MULTISPECIES:-BREX-system-P-loop-protein-BrxC-[Enterobacter-cloacae-complex]
WP_230863208.1	Type1.BREX	Arm-DNA-bind_3+Phage_integrase-><- SIGMA+OB-Fold+AAA-ATPase+Lon-Protease-BrxL <- PglZ <- N6-METHYLASE -p<-RVT<- HEPN <- N6-METHYLASE <-ParB+HNH<- DUF6079 *<- BrxB-BrxF-iSTAND <- BrxA-DNA-binding <-REase<-wHTH+SAD+HNH||SymE_toxin->	1240	Bacteria>Pseudomonadota>Gammaproteobacteria>Enterobacterales>Enterobacteriaceae>Enterobacter	GCF_015704905.1	Enterobacter_cloacae	IOR24_RS17915	3774454..3778176	550	BREX-system-P-loop-protein-BrxC-[Enterobacter-cloacae]
WP_234450174.1	Type1.BREX	Glycoside_Hydrolase->?->Glyoxalase->YjhX_toxin->SAM-METHYLASE->ZnR->Glyoxalase-> BrxA-DNA-binding -> BrxB-BrxF-iSTAND -> DUF6079 *->ParB+HNH-> N6-METHYLASE -> HEPN ->RVT-> N6-METHYLASE -p-> PglZ -> SIGMA+OB-Fold+AAA-ATPase+Lon-Protease-BrxL -><-Arm-DNA-bind_3+Phage_integrase	1240	Bacteria>Pseudomonadota>Gammaproteobacteria>Enterobacterales>Enterobacteriaceae>Enterobacter	GCF_016616795.1	Enterobacter_hormaechei	FKK11_RS12010	273367..277089	158836	BREX-system-P-loop-protein-BrxC-[Enterobacter-hormaechei]
WP_174580485.1	Type1.BREX	DDE_Tnp_IS1->?->ZnR-> BrxA-DNA-binding -> BrxB-BrxF-iSTAND -> DUF6079 *->ParB+HNH-> N6-METHYLASE -> HEPN ->RVT-> N6-METHYLASE -p-> PglZ -> SIGMA+OB-Fold+AAA-ATPase+Lon-Protease-BrxL -><-Arm-DNA-bind_3+Phage_integrase||Phage_integrase->	1233	Bacteria>Pseudomonadota>Gammaproteobacteria>Enterobacterales>Enterobacteriaceae>Escherichia	-	Escherichia_coli	HUC40_15465	2945..6646	562	BREX-system-P-loop-protein-BrxC-[Escherichia-coli]
EEZ5714529.1	Type1.BREX	<-wHTH+WYL<-nSTAND1+sGTP<-?||XIS-HTH->?->?->ZnR-> BrxA-DNA-binding -> BrxB-BrxF-iSTAND -> DUF6079 *->ParB+HNH-> N6-METHYLASE -> HEPN ->RVT-> N6-METHYLASE -p-> PglZ -> SIGMA+OB-Fold+AAA-ATPase+Lon-Protease-BrxL -><-Arm-DNA-bind_3+Phage_integrase	1233	Bacteria>Pseudomonadota>Gammaproteobacteria>Enterobacterales>Enterobacteriaceae>Escherichia	GCA_012215525.1	Escherichia_coli_O25	DBU27_000026	20065..23766	1095709	BREX-system-P-loop-protein-BrxC-[Escherichia-coli-O25]
EFD0312067.1	Type1.BREX	GDResolvase+HIN-HTH-><- SIGMA+OB-Fold+AAA-ATPase+Lon-Protease-BrxL <- PglZ <- N6-METHYLASE -p<-RVT<- HEPN <- N6-METHYLASE <-ParB+HNH<- DUF6079 *<- BrxB-BrxF-iSTAND <- BrxA-DNA-binding <-?<-?<-XIS-HTH<-?||?->nSTAND1+GTPase-AIG+sGTP->DUF932->	1233	Bacteria>Pseudomonadota>Gammaproteobacteria>Enterobacterales>Enterobacteriaceae>Escherichia	GCA_012380475.1	Escherichia_coli	E6D44_20535	19474..23175	562	BREX-system-P-loop-protein-BrxC-[Escherichia-coli]
EFN5198232.1	Type1.BREX	<-TIR|| BrxA-DNA-binding -> BrxB-BrxF-iSTAND -> DUF6079 *->ParB+HNH-> N6-METHYLASE -> HEPN ->RVT-> N6-METHYLASE -p-> PglZ -> SIGMA+OB-Fold+AAA-ATPase+Lon-Protease-BrxL -> wHTH+WYL+WCX ->	1233	Bacteria>Pseudomonadota>Gammaproteobacteria>Enterobacterales>Enterobacteriaceae>Escherichia	GCA_012993025.1	Escherichia_coli	FQG10_00765	2767..6468	562	BREX-system-P-loop-protein-BrxC-[Escherichia-coli]
NUC65042.1	Type1.BREX	DDE_Tnp_IS1->?->ZnR-> BrxA-DNA-binding -> BrxB-BrxF-iSTAND -> DUF6079 *->ParB+HNH-> N6-METHYLASE -> HEPN ->RVT-> N6-METHYLASE -p-> PglZ -> SIGMA+OB-Fold+AAA-ATPase+Lon-Protease-BrxL -><-Arm-DNA-bind_3+Phage_integrase||Phage_integrase->	1233	Bacteria>Pseudomonadota>Gammaproteobacteria>Enterobacterales>Enterobacteriaceae>Escherichia	GCA_013342565.1	Escherichia_coli	HUC40_15465	2945..6646	562	BREX-system-P-loop-protein-BrxC-[Escherichia-coli]
WP_250907975.1	Type1.BREX	DDE_Tnp_IS1->?->ZnR-> BrxA-DNA-binding -> BrxB-BrxF-iSTAND -> DUF6079 *->ParB+HNH-> N6-METHYLASE -> HEPN ->RVT-> N6-METHYLASE -p-> PglZ -> SIGMA+OB-Fold+AAA-ATPase+Lon-Protease-BrxL -><-Arm-DNA-bind_3+Phage_integrase||Phage_integrase->	1240	Bacteria>Pseudomonadota>Gammaproteobacteria>Enterobacterales>Enterobacteriaceae>Escherichia	GCA_030820635.1	Escherichia_coli	HUC40_RS15465	2924..6646	562	BREX-system-P-loop-protein-BrxC-[Escherichia-coli]
WP_001019654.1	Type1.BREX	LexA-protease->||Arm-DNA-bind_3+Phage_integrase-> N6-METHYLASE ->ORC-CDC6+ZnR-> HEPN -><-Zn_Tnp_IS1+HTH_Tnp_IS1+DDE_Tnp_IS1<-REase|| BrxA-DNA-binding -> BrxB-BrxF-iSTAND -> DUF6079 *-> N6-METHYLASE -> PglZ -> SIGMA+OB-Fold+AAA-ATPase+Lon-Protease-BrxL -><-ZnR<-ZnR<-?||HTH_Tnp_1+HTH_21+rve+rve_3-><-LuxR-HTH	1213	Bacteria>Pseudomonadota>Gammaproteobacteria>Enterobacterales>Enterobacteriaceae>Escherichia	GCF_000017765.1	Escherichia_coli	ECHS_RS01710	342111..345752	562	BREX-system-P-loop-protein-BrxC-[Escherichia-coli]
WP_233470897.1	Type1.BREX	<-DUF932<-nSTAND1+GTPase-AIG+sGTP<-CopG/RHH||?->XIS-HTH->?->?-> BrxA-DNA-binding -> BrxB-BrxF-iSTAND -> DUF6079 *->ParB+HNH-> N6-METHYLASE -> HEPN ->RVT-> N6-METHYLASE -> PglZ -> SIGMA+OB-Fold+AAA-ATPase+Lon-Protease-BrxL -><-Arm-DNA-bind_3+Phage_integrase	1240	Bacteria>Pseudomonadota>Gammaproteobacteria>Enterobacterales>Enterobacteriaceae	GCF_014931625.2	Citrobacter	INP82_RS14360	158420..162142	544	MULTISPECIES:-BREX-system-P-loop-protein-BrxC-[Citrobacter]
WP_225990544.1	Type1.BREX	Glyoxalase->ACET->YjhX_toxin->SAM-METHYLASE->ZnR->Glyoxalase-> BrxA-DNA-binding -> BrxB-BrxF-iSTAND -> DUF6079 *->ParB+HNH-> N6-METHYLASE -> HEPN ->RVT-> N6-METHYLASE -p-> PglZ -> SIGMA+OB-Fold+AAA-ATPase+Lon-Protease-BrxL -><-Arm-DNA-bind_3+Phage_integrase	1240	Bacteria>Pseudomonadota>Gammaproteobacteria>Enterobacterales>Enterobacteriaceae	GCF_954736865.1	Enterobacter	CU081_RS00925	173954..177676	547	MULTISPECIES:-BREX-system-P-loop-protein-BrxC-[Enterobacter]
AUN75366.1	Type1.BREX	Arm-DNA-bind_3+Phage_integrase-><- SIGMA+OB-Fold+AAA-ATPase+Lon-Protease-BrxL <- PglZ <- N6-METHYLASE -p<-RVT<- HEPN <- N6-METHYLASE <-ParB+HNH<- DUF6079 *<- BrxB-BrxF-iSTAND <- BrxA-DNA-binding <-?<-?<-XIS-HTH||?->nSTAND1+sGTP->wHTH+WYL->	1233	Bacteria>Pseudomonadota>Gammaproteobacteria>Enterobacterales>Enterobacteriaceae>Klebsiella/Raoultella_group	GCA_002870985.1	Klebsiella_pneumoniae	C0077_01580	326913..330614	573	BREX-system-P-loop-protein-BrxC-[Klebsiella-pneumoniae]
QHX00137.1	Type1.BREX	YjhX_toxin->ACET->METHYLASE->ZnR->Glyoxalase-> BrxA-DNA-binding -> BrxB-BrxF-iSTAND -> DUF6079 *->ParB+HNH-> N6-METHYLASE -> HEPN ->RVT-> N6-METHYLASE -p-> PglZ -> SIGMA+OB-Fold+AAA-ATPase+Lon-Protease-BrxL -><-Arm-DNA-bind_3+Phage_integrase	1233	Bacteria>Pseudomonadota>Gammaproteobacteria>Enterobacterales>Enterobacteriaceae>Klebsiella/Raoultella_group	GCA_010365565.1	Klebsiella_variicola	GZS05_24295	4949707..4953408	244366	BREX-system-P-loop-protein-BrxC-[Klebsiella-variicola]
QJM45197.1	Type1.BREX	<-wHTH+WYL<-sGTP<-?||XIS-HTH->?->?-> BrxA-DNA-binding -> BrxB-BrxF-iSTAND -> DUF6079 *->ParB+HNH-> N6-METHYLASE -> HEPN ->RVT-> N6-METHYLASE -p-> PglZ -> SIGMA+OB-Fold+AAA-ATPase+Lon-Protease-BrxL -><-Arm-DNA-bind_3+Phage_integrase	1233	Bacteria>Pseudomonadota>Gammaproteobacteria>Enterobacterales>Enterobacteriaceae>Klebsiella/Raoultella_group	GCA_012970665.1	Klebsiella_pneumoniae	HJW71_23035	4620464..4624165	573	BREX-system-P-loop-protein-BrxC-[Klebsiella-pneumoniae]
EJG2380286.1	Type1.BREX	<-DUF905<-DUF932<-CopG/RHH||?->XIS-HTH-><-TIR<-TIR|| BrxA-DNA-binding -> BrxB-BrxF-iSTAND -> DUF6079 *->ParB+HNH-> N6-METHYLASE -> HEPN ->RVT-> N6-METHYLASE -p-> PglZ -> SIGMA+OB-Fold+AAA-ATPase+Lon-Protease-BrxL -> wHTH+WYL+WCX ->	1233	Bacteria>Pseudomonadota>Gammaproteobacteria>Enterobacterales>Enterobacteriaceae>Klebsiella/Raoultella_group	GCA_016088515.2	Raoultella_ornithinolytica	AM381_RS08835	19475..23176	54291	BREX-system-P-loop-protein-BrxC-[Raoultella-ornithinolytica]
MBN7733855.1	Type1.BREX	<-wHTH+WYL<-sGTP<-?||XIS-HTH->?->?-> BrxA-DNA-binding -> BrxB-BrxF-iSTAND -> DUF6079 *->ParB+HNH-> N6-METHYLASE -> HEPN ->RVT-> N6-METHYLASE -p-> PglZ -> SIGMA+OB-Fold+AAA-ATPase+Lon-Protease-BrxL -><-Arm-DNA-bind_3+Phage_integrase	1233	Bacteria>Pseudomonadota>Gammaproteobacteria>Enterobacterales>Enterobacteriaceae>Klebsiella/Raoultella_group	GCA_017255495.1	Klebsiella_pneumoniae	JX616_18220	31914..35615	573	BREX-system-P-loop-protein-BrxC-[Klebsiella-pneumoniae]
EIV3098057.1	Type1.BREX	<-wHTH+WYL<-nSTAND1+sGTP<-?||XIS-HTH->?->?-> BrxA-DNA-binding -> BrxB-BrxF-iSTAND -> DUF6079 *->ParB+HNH-> N6-METHYLASE -> HEPN ->RVT-> N6-METHYLASE -p-> PglZ -> SIGMA+OB-Fold+AAA-ATPase+Lon-Protease-BrxL -><-Arm-DNA-bind_3+Phage_integrase	1233	Bacteria>Pseudomonadota>Gammaproteobacteria>Enterobacterales>Enterobacteriaceae>Klebsiella/Raoultella_group	GCA_021854435.1	Klebsiella_pneumoniae	L7W77_004838	45573..49274	573	BREX-system-P-loop-protein-BrxC-[Klebsiella-pneumoniae]
HBR1784209.1	Type1.BREX	<-wHTH+WYL<-sGTP<-?||XIS-HTH->?->?-> BrxA-DNA-binding -> BrxB-BrxF-iSTAND -> DUF6079 *->ParB+HNH-> N6-METHYLASE -> HEPN ->RVT-> N6-METHYLASE -p-> PglZ -> SIGMA+OB-Fold+AAA-ATPase+Lon-Protease-BrxL -><-Arm-DNA-bind_3+Phage_integrase	1233	Bacteria>Pseudomonadota>Gammaproteobacteria>Enterobacterales>Enterobacteriaceae>Klebsiella/Raoultella_group	GCA_021911385.1	Klebsiella_quasipneumoniae_subsp._quasipneumoniae	L9M28_003408	20306..24007	1667327	BREX-system-P-loop-protein-BrxC-[Klebsiella-quasipneumoniae-subsp.-quasipneumoniae]
HCD1392381.1	Type1.BREX	<- wHTH+WYL+WCX <- SIGMA+OB-Fold+AAA-ATPase+Lon-Protease-BrxL <- PglZ <- N6-METHYLASE -p<-RVT<- HEPN <- N6-METHYLASE <-ParB+HNH<- DUF6079 *<- BrxB-BrxF-iSTAND <- BrxA-DNA-binding ||TIR->TIR-><-XIS-HTH<-?||CopG/RHH->DUF932->DUF905->	1233	Bacteria>Pseudomonadota>Gammaproteobacteria>Enterobacterales>Enterobacteriaceae>Klebsiella/Raoultella_group	GCA_023530865.1	Klebsiella_pneumoniae_subsp._pneumoniae	M9022_004645	23383..27084	72407	BREX-system-P-loop-protein-BrxC-[Klebsiella-pneumoniae-subsp.-pneumoniae]
MCS5967892.1	Type1.BREX	<-nSTAND1+sGTP<-?<-?||?-> BrxA-DNA-binding -> BrxB-BrxF-iSTAND -> DUF6079 -p*-> DUF6079 -p->?-> DUF6079 -p->ParB+HNH->ParB+HNH-> N6-METHYLASE -> HEPN ->?->?->RVT->	389	Bacteria>Pseudomonadota>Gammaproteobacteria>Enterobacterales>Enterobacteriaceae>Klebsiella/Raoultella_group	GCA_024990005.1	Klebsiella_variicola_subsp._variicola	LNP26_07865	1516930..1518099	2590157	BREX-system-P-loop-protein-BrxC-[Klebsiella-variicola-subsp.-variicola]
WEJ89011.1	Type1.BREX	<-wHTH+HNH||tRNA->Arm-DNA-bind_3+Phage_integrase-><- SIGMA+OB-Fold+AAA-ATPase+Lon-Protease-BrxL <- PglZ <- N6-METHYLASE -p<-RVT<- HEPN <- N6-METHYLASE <-ParB+HNH<- DUF6079 *<- BrxB-BrxF-iSTAND <- BrxA-DNA-binding <-ZnR<-XIS-HTH<-?||HIN-HTH->?->LZ_Tnp_IS66+zf-IS66+DDE_Tnp_IS66+DDE_Tnp_IS66_C->	1240	Bacteria>Pseudomonadota>Gammaproteobacteria>Enterobacterales>Enterobacteriaceae>Klebsiella/Raoultella_group	GCA_029202365.1	Klebsiella_huaxiensis	POY63_27795	5833350..5837072	2153354	MAG:-BREX-system-P-loop-protein-BrxC-[Klebsiella-huaxiensis]
WP_256991583.1	Type1.BREX	<-DUF905<-DUF932<-CopG/RHH||?->XIS-HTH->ZnR-><-TIR|| BrxA-DNA-binding -> BrxB-BrxF-iSTAND -> DUF6079 *->ParB+HNH-> N6-METHYLASE -> HEPN ->RVT-> N6-METHYLASE -p-> PglZ -> SIGMA+OB-Fold+AAA-ATPase+Lon-Protease-BrxL -> wHTH+WYL+WCX ->	1240	Bacteria>Pseudomonadota>Gammaproteobacteria>Enterobacterales>Enterobacteriaceae>Klebsiella/Raoultella_group	GCF_002264145.1	Raoultella_planticola	CIG23_RS11860	59081..62803	575	BREX-system-P-loop-protein-BrxC-[Raoultella-planticola]
WP_264757344.1	Type1.BREX	<-wHTH+WYL<-nSTAND1+sGTP<-?||XIS-HTH->?->?-> BrxA-DNA-binding -> BrxB-BrxF-iSTAND -><-DDE_Tnp_1_5+DDE_Tnp_1|| DUF6079 -p*->ParB+HNH-> N6-METHYLASE -> HEPN ->RVT-> N6-METHYLASE -p-> PglZ -> SIGMA+OB-Fold+AAA-ATPase+Lon-Protease-BrxL -><-Arm-DNA-bind_3+Phage_integrase	995	Bacteria>Pseudomonadota>Gammaproteobacteria>Enterobacterales>Enterobacteriaceae>Klebsiella/Raoultella_group	GCF_003660185.1	Klebsiella_pneumoniae	D9K63_RS23205	4667642..4670629	573	BREX-system-P-loop-protein-BrxC-[Klebsiella-pneumoniae]
WP_223814682.1	Type1.BREX	<-wHTH+WYL<-nSTAND1+sGTP<-?||XIS-HTH->?->?-> BrxA-DNA-binding -> BrxB-BrxF-iSTAND -> DUF6079 *->ParB+HNH-> N6-METHYLASE -> HEPN ->RVT-> N6-METHYLASE -p-> PglZ -> SIGMA+OB-Fold+AAA-ATPase+Lon-Protease-BrxL -><-Arm-DNA-bind_3+Phage_integrase	1240	Bacteria>Pseudomonadota>Gammaproteobacteria>Enterobacterales>Enterobacteriaceae>Klebsiella/Raoultella_group	GCF_004313365.1	Klebsiella	E0018_RS24630	24494..28216	570	MULTISPECIES:-BREX-system-P-loop-protein-BrxC-[Klebsiella]
WP_235410418.1	Type1.BREX	<-wHTH+WYL<-sGTP<-?||XIS-HTH->?->?-> BrxA-DNA-binding -> BrxB-BrxF-iSTAND -> DUF6079 *->ParB+HNH-> N6-METHYLASE -> HEPN ->RVT-> N6-METHYLASE -p-> PglZ -> SIGMA+OB-Fold+AAA-ATPase+Lon-Protease-BrxL -><-Arm-DNA-bind_3+Phage_integrase	1240	Bacteria>Pseudomonadota>Gammaproteobacteria>Enterobacterales>Enterobacteriaceae>Klebsiella/Raoultella_group	GCF_021532695.1	Klebsiella_pneumoniae	LZ602_RS12600	31915..35637	573	BREX-system-P-loop-protein-BrxC-[Klebsiella-pneumoniae]
WP_223339928.1	Type1.BREX	<-wHTH+WYL<-sGTP<-?||XIS-HTH->?->?-> BrxA-DNA-binding -> BrxB-BrxF-iSTAND -> DUF6079 *->ParB+HNH-> N6-METHYLASE -> HEPN ->RVT-> N6-METHYLASE -p-> PglZ -> SIGMA+OB-Fold+AAA-ATPase+Lon-Protease-BrxL -><-Arm-DNA-bind_3+Phage_integrase	1240	Bacteria>Pseudomonadota>Gammaproteobacteria>Enterobacterales>Enterobacteriaceae>Klebsiella/Raoultella_group	GCF_022353805.1	Klebsiella_pneumoniae	JX616_RS18220	31893..35615	573	BREX-system-P-loop-protein-BrxC-[Klebsiella-pneumoniae]
WP_227643422.1	Type1.BREX	Arm-DNA-bind_3+Phage_integrase-><- SIGMA+OB-Fold+AAA-ATPase+Lon-Protease-BrxL <- PglZ <- N6-METHYLASE -p<-RVT<- HEPN <- N6-METHYLASE <-ParB+HNH<- DUF6079 *<- BrxB-BrxF-iSTAND <- BrxA-DNA-binding <-?<-?<-XIS-HTH<-?||CopG/RHH->nSTAND1+GTPase-AIG+sGTP->DUF932->	1240	Bacteria>Pseudomonadota>Gammaproteobacteria>Enterobacterales>Enterobacteriaceae>Klebsiella/Raoultella_group	GCF_900173735.1	Klebsiella_pneumoniae	B7E22_RS15515	109572..113294	573	BREX-system-P-loop-protein-BrxC-[Klebsiella-pneumoniae]
WP_227635281.1	Type1.BREX	XIS-HTH-><-TIR<-TIR|| BrxA-DNA-binding -> BrxB-BrxF-iSTAND -> DUF6079 *->ParB+HNH-> N6-METHYLASE -> HEPN ->RVT-> N6-METHYLASE -p-> PglZ -> SIGMA+OB-Fold+AAA-ATPase+Lon-Protease-BrxL -> wHTH+WYL+WCX ->	1240	Bacteria>Pseudomonadota>Gammaproteobacteria>Enterobacterales>Enterobacteriaceae>Klebsiella/Raoultella_group	GCF_902159715.1	Klebsiella	PIK69_RS17535	2737..6459	570	MULTISPECIES:-BREX-system-P-loop-protein-BrxC-[Klebsiella]
WP_227627429.1	Type1.BREX	<- wHTH+WYL+WCX <-?<- SIGMA+OB-Fold+AAA-ATPase+Lon-Protease-BrxL <- PglZ <- N6-METHYLASE -p<-RVT<- HEPN <- N6-METHYLASE <-ParB+HNH<- DUF6079 *<- BrxB-BrxF-iSTAND <- BrxA-DNA-binding ||TIR-><-ZnR<-XIS-HTH<-?||?->DUF932->DUF905->	1240	Bacteria>Pseudomonadota>Gammaproteobacteria>Enterobacterales>Enterobacteriaceae>Klebsiella/Raoultella_group	-	Klebsiella_michiganensis	G4403_RS10440	98242..101964	1134687	BREX-system-P-loop-protein-BrxC-[Klebsiella-michiganensis]
WP_117042583.1	Type1.BREX	<-DUF905<-DUF932<-CopG/RHH||?->XIS-HTH-><-TIR<-TIR|| BrxA-DNA-binding -> BrxB-BrxF-iSTAND -> DUF6079 *->ParB+HNH-> N6-METHYLASE -> HEPN ->RVT-> N6-METHYLASE -p-> PglZ -> SIGMA+OB-Fold+AAA-ATPase+Lon-Protease-BrxL -> wHTH+WYL+WCX ->	1233	Bacteria>Pseudomonadota>Gammaproteobacteria>Enterobacterales>Enterobacteriaceae>Klebsiella/Raoultella_group	-	Klebsiella_pneumoniae	AM381_RS08835	19475..23176	573	MULTISPECIES:-BREX-system-P-loop-protein-BrxC-[Klebsiella]
WP_114263049.1	Type1.BREX	<-DUF932<-GspA-ATPase+GTPase<-CopG/RHH||?->XIS-HTH->?->?-> BrxA-DNA-binding -> BrxB-BrxF-iSTAND -> DUF6079 *->ParB+HNH-> N6-METHYLASE -> HEPN ->RVT-> N6-METHYLASE -p-> PglZ -> SIGMA+OB-Fold+AAA-ATPase+Lon-Protease-BrxL -><-Arm-DNA-bind_3+Phage_integrase	1233	Bacteria>Pseudomonadota>Gammaproteobacteria>Enterobacterales>Enterobacteriaceae>Klebsiella/Raoultella_group	-	Klebsiella_pneumoniae	AN2353V1_3685	3967252..3970953	573	MULTISPECIES:-BREX-system-P-loop-protein-BrxC-[Enterobacteriaceae]
WP_062729754.1	Type1.BREX	Arm-DNA-bind_3+Phage_integrase-><- SIGMA+OB-Fold+AAA-ATPase+Lon-Protease-BrxL <- PglZ <- N6-METHYLASE -p<-RVT<- HEPN <- N6-METHYLASE <-ParB+HNH<- DUF6079 *<- BrxB-BrxF-iSTAND <- BrxA-DNA-binding <-?<-?<-XIS-HTH||?->nSTAND1+sGTP->wHTH+WYL->	1233	Bacteria>Pseudomonadota>Gammaproteobacteria>Enterobacterales>Enterobacteriaceae>Klebsiella/Raoultella_group	-	Klebsiella_pneumoniae	C0077_01580	326913..330614	573	MULTISPECIES:-BREX-system-P-loop-protein-BrxC-[Enterobacteriaceae]
WP_073511310.1	Type1.BREX	<-wHTH+WYL<-sGTP<-?||XIS-HTH->?->?-> BrxA-DNA-binding -> BrxB-BrxF-iSTAND -> DUF6079 *->ParB+HNH-> N6-METHYLASE -> HEPN ->RVT-> N6-METHYLASE -p-> PglZ -> SIGMA+OB-Fold+AAA-ATPase+Lon-Protease-BrxL -><-Arm-DNA-bind_3+Phage_integrase	1233	Bacteria>Pseudomonadota>Gammaproteobacteria>Enterobacterales>Enterobacteriaceae>Klebsiella/Raoultella_group	-	Klebsiella_pneumoniae	L7V03_004584	32186..35887	573	BREX-system-P-loop-protein-BrxC-[Klebsiella-pneumoniae]
WP_117247358.1	Type1.BREX	<-wHTH+WYL<-nSTAND1+sGTP<-?||XIS-HTH->?->?-> BrxA-DNA-binding -> BrxB-BrxF-iSTAND -> DUF6079 *->ParB+HNH-> N6-METHYLASE -> HEPN ->RVT-> N6-METHYLASE -p-> PglZ -> SIGMA+OB-Fold+AAA-ATPase+Lon-Protease-BrxL -><-Arm-DNA-bind_3+Phage_integrase	1233	Bacteria>Pseudomonadota>Gammaproteobacteria>Enterobacterales>Enterobacteriaceae>Klebsiella/Raoultella_group	-	Klebsiella_pneumoniae	L7W77_004838	45573..49274	573	MULTISPECIES:-BREX-system-P-loop-protein-BrxC-[Klebsiella]
WP_094898295.1	Type1.BREX	<-TIR|| BrxA-DNA-binding -> BrxB-BrxF-iSTAND -> DUF6079 *->ParB+HNH-> N6-METHYLASE -> HEPN ->RVT-> N6-METHYLASE -p-> PglZ -> SIGMA+OB-Fold+AAA-ATPase+Lon-Protease-BrxL -> wHTH+WYL+WCX ->	1233	Bacteria>Pseudomonadota>Gammaproteobacteria>Enterobacterales>Enterobacteriaceae>Klebsiella/Raoultella_group	-	Raoultella_planticola	FQG10_00765	2767..6468	575	BREX-system-P-loop-protein-BrxC-[Raoultella-planticola]
QXB23949.1	Type1.BREX	XIS-HTH->?->ZnR->ZnR->?->|| BrxA-DNA-binding -> BrxB-BrxF-iSTAND -> DUF6079 *->ParB+HNH-> N6-METHYLASE -> HEPN ->RVT-> N6-METHYLASE -><-HIN-HTH|| PglZ -> SIGMA+OB-Fold+AAA-ATPase+Lon-Protease-BrxL ->	1233	Bacteria>Pseudomonadota>Gammaproteobacteria>Enterobacterales>Enterobacteriaceae>Lelliottia	GCA_019048185.1	Lelliottia_amnigena	I6L76_15755	3242307..3246008	61646	BREX-system-P-loop-protein-BrxC-[Lelliottia-amnigena]
WP_254208058.1	Type1.BREX	XIS-HTH->?->ZnR->ZnR->?->|| BrxA-DNA-binding -> BrxB-BrxF-iSTAND -> DUF6079 *->ParB+HNH-> N6-METHYLASE -> HEPN ->RVT-> N6-METHYLASE -><-HIN-HTH|| PglZ -> SIGMA+OB-Fold+AAA-ATPase+Lon-Protease-BrxL ->	1237	Bacteria>Pseudomonadota>Gammaproteobacteria>Enterobacterales>Enterobacteriaceae>Lelliottia	GCF_019048185.1	Lelliottia_amnigena	I6L76_RS15755	3242295..3246008	61646	BREX-system-P-loop-protein-BrxC-[Lelliottia-amnigena]
WP_216981896.1	Type1.BREX	XIS-HTH->?->ZnR->ZnR->?->|| BrxA-DNA-binding -> BrxB-BrxF-iSTAND -> DUF6079 *->ParB+HNH-> N6-METHYLASE -> HEPN ->RVT-> N6-METHYLASE -><-HIN-HTH|| PglZ -> SIGMA+OB-Fold+AAA-ATPase+Lon-Protease-BrxL ->	1233	Bacteria>Pseudomonadota>Gammaproteobacteria>Enterobacterales>Enterobacteriaceae>Lelliottia	-	Lelliottia_amnigena	I6L76_15755	3242307..3246008	61646	BREX-system-P-loop-protein-BrxC-[Lelliottia-amnigena]
EAA4435938.1	Type1.BREX	XIS-HTH->?->?-><-?<-ZnR|| BrxA-DNA-binding -> BrxB-BrxF-iSTAND -> DUF6079 *->ParB+HNH-> N6-METHYLASE -> HEPN ->RVT-> N6-METHYLASE -p-> PglZ -> SIGMA+OB-Fold+AAA-ATPase+Lon-Protease-BrxL -><-tRNA||?-><-SKI	1233	Bacteria>Pseudomonadota>Gammaproteobacteria>Enterobacterales>Enterobacteriaceae>Salmonella	GCA_004179155.1	Salmonella_enterica_subsp._salamae	DPE44_05505	71371..75072	59202	BREX-system-P-loop-protein-BrxC-[Salmonella-enterica-subsp.-salamae]
WP_079805580.1	Type1.BREX	SLOG->HAD-><- SIGMA+OB-Fold+AAA-ATPase+Lon-Protease-BrxL <- PglZ <- N6-METHYLASE -p<-RVT<- HEPN <- N6-METHYLASE <-ParB+HNH<- DUF6079 *<- BrxB-BrxF-iSTAND <- BrxA-DNA-binding ||Schlafen+HATPase+wHTH-><-?<-?<-XIS-HTH	1240	Bacteria>Pseudomonadota>Gammaproteobacteria>Enterobacterales>Enterobacteriaceae>Salmonella	GCF_016903075.1	Salmonella_enterica	AJH70_RS01330	291078..294800	28901	BREX-system-P-loop-protein-BrxC-[Salmonella-enterica]
UCB31815.1	Type1.BREX	<-wHTH+WYL<-?||XIS-HTH->?->?-> BrxA-DNA-binding -> BrxB-BrxF-iSTAND -> DUF6079 *->ParB+HNH-> N6-METHYLASE -> HEPN ->RVT-> N6-METHYLASE -p-> PglZ -> SIGMA+OB-Fold+AAA-ATPase+Lon-Protease-BrxL -><-Arm-DNA-bind_3+Phage_integrase	1240	Bacteria>Pseudomonadota>Gammaproteobacteria>Enterobacterales>Erwiniaceae>Duffyella	GCA_020342335.1	Duffyella_gerundensis	J9874_02366	2569295..2573017	1619313	hypothetical-protein-J9874_02366-[Duffyella-gerundensis]
WP_225700910.1	Type1.BREX	<-wHTH+WYL<-?||XIS-HTH->?->?-> BrxA-DNA-binding -> BrxB-BrxF-iSTAND -> DUF6079 *->ParB+HNH-> N6-METHYLASE -> HEPN ->RVT-> N6-METHYLASE -p-> PglZ -> SIGMA+OB-Fold+AAA-ATPase+Lon-Protease-BrxL -><-Arm-DNA-bind_3+Phage_integrase	1237	Bacteria>Pseudomonadota>Gammaproteobacteria>Enterobacterales>Erwiniaceae>Duffyella	GCF_020342335.1	Duffyella_gerundensis	J9874_RS11820	2569304..2573017	1619313	BREX-system-P-loop-protein-BrxC-[Duffyella-gerundensis]
WP_223177211.1	Type1.BREX	<-wHTH+WYL<-nSTAND1+sGTP<-?||XIS-HTH->?->?-> BrxA-DNA-binding -> BrxB-BrxF-iSTAND -> DUF6079 *->ParB+HNH-> N6-METHYLASE -> HEPN ->RVT-> N6-METHYLASE -p-> PglZ -> SIGMA+OB-Fold+AAA-ATPase+Lon-Protease-BrxL -><-Arm-DNA-bind_3+Phage_integrase	1240	Bacteria>Pseudomonadota>Gammaproteobacteria>Enterobacterales	GCF_004312085.1	Enterobacteriaceae	E0M53_RS14900	53286..57008	543	MULTISPECIES:-BREX-system-P-loop-protein-BrxC-[Enterobacteriaceae]
WP_175320436.1	Type1.BREX	<-DUF932<-nSTAND1+GTPase-AIG+sGTP<-?||?->XIS-HTH->?->?-> BrxA-DNA-binding -> BrxB-BrxF-iSTAND -> DUF6079 *->ParB+HNH-> N6-METHYLASE -> HEPN ->RVT-> N6-METHYLASE -p-> PglZ -> SIGMA+OB-Fold+AAA-ATPase+Lon-Protease-BrxL -><-Arm-DNA-bind_3+Phage_integrase	1233	Bacteria>Pseudomonadota>Gammaproteobacteria>Enterobacterales>Hafniaceae>Hafnia	GCF_013360205.1	Hafnia_paralvei	G4F99_RS14350	55379..59080	546367	BREX-system-P-loop-protein-BrxC-[Hafnia-paralvei]
WP_234619586.1	Type1.BREX	<-nSTAND1+GTPase-AIG+sGTP<-CopG/RHH||XIS-HTH->?->?->ZnR-> BrxA-DNA-binding -> BrxB-BrxF-iSTAND -> DUF6079 *->ParB+HNH-> N6-METHYLASE -> HEPN ->RVT-> N6-METHYLASE -p-> PglZ -> SIGMA+OB-Fold+AAA-ATPase+Lon-Protease-BrxL -><-HTH+MPTase	1237	Bacteria>Pseudomonadota>Gammaproteobacteria>Enterobacterales>Hafniaceae>Hafnia	GCF_021440285.1	Hafnia_alvei	LZ667_RS17495	58169..61882	569	BREX-system-P-loop-protein-BrxC-[Hafnia-alvei]
AUR32853.1	Type1.BREX	HTH->Pertactin+Autotransporter->?->wHTH+SAD+HNH->?->ZnR-> BrxA-DNA-binding -> BrxB-BrxF-iSTAND -> DUF6079 *->ParB+HNH-> N6-METHYLASE -> HEPN ->RVT-> N6-METHYLASE -p-> PglZ -> SIGMA+OB-Fold+AAA-ATPase+Lon-Protease-BrxL -><-Arm-DNA-bind_3+Phage_integrase	1233	Bacteria>Pseudomonadota>Gammaproteobacteria>Enterobacterales>Morganellaceae>Morganella	GCA_002891475.1	Morganella_morganii	C1070_15730	3342273..3345974	582	BREX-system-P-loop-protein-BrxC-[Morganella-morganii]
WP_225973345.1	Type1.BREX	HTH->Pertactin+Autotransporter->wHTH+SAD+HNH->?->ZnR-> BrxA-DNA-binding -> BrxB-BrxF-iSTAND -> DUF6079 *->ParB+HNH-> N6-METHYLASE -> HEPN ->RVT-> N6-METHYLASE -p-> PglZ -> SIGMA+OB-Fold+AAA-ATPase+Lon-Protease-BrxL -><-Arm-DNA-bind_3+Phage_integrase	1240	Bacteria>Pseudomonadota>Gammaproteobacteria>Enterobacterales>Morganellaceae>Morganella	GCF_002891475.1	Morganella_morganii	C1070_RS15730	3342252..3345974	582	BREX-system-P-loop-protein-BrxC-[Morganella-morganii]
EGS9996488.1	Type1.BREX	<-nSTAND1+GTPase-AIG+sGTP<-CopG/RHH||XIS-HTH->?-> BrxA-DNA-binding -> BrxB-BrxF-iSTAND -> DUF6079 *->ParB+HNH-> N6-METHYLASE -> HEPN ->RVT-> N6-METHYLASE -p-> PglZ -> SIGMA+OB-Fold+AAA-ATPase+Lon-Protease-BrxL -><-Arm-DNA-bind_3+Phage_integrase	1233	Bacteria>Pseudomonadota>Gammaproteobacteria>Enterobacterales>Yersiniaceae>Serratia	GCA_015929585.1	Serratia_marcescens	I9Y15_003567	178923..182624	615	BREX-system-P-loop-protein-BrxC-[Serratia-marcescens]
WP_260965787.1	Type1.BREX	Arm-DNA-bind_3+Phage_integrase-><- SIGMA+OB-Fold+AAA-ATPase+Lon-Protease-BrxL <- PglZ <- N6-METHYLASE -p<-RVT<- HEPN <- N6-METHYLASE <-ParB+HNH<- DUF6079 *<- BrxB-BrxF-iSTAND <- BrxA-DNA-binding <-?<-XIS-HTH<-?||?->GspA-ATPase+GTPase->	1240	Bacteria>Pseudomonadota>Gammaproteobacteria>Enterobacterales>Yersiniaceae>Serratia	GCF_025336405.1	Serratia_marcescens	N7751_RS05105	1087639..1091361	615	BREX-system-P-loop-protein-BrxC-[Serratia-marcescens]
WP_262254721.1	Type1.BREX	<-GTPase-AIG<-CopG/RHH||?->XIS-HTH-><-?<-?|| BrxA-DNA-binding -> BrxB-BrxF-iSTAND -> DUF6079 *->ParB+HNH-> N6-METHYLASE -> HEPN ->RVT-> N6-METHYLASE -p-> PglZ -> SIGMA+OB-Fold+AAA-ATPase+Lon-Protease-BrxL -><-Arm-DNA-bind_3+Phage_integrase	1240	Bacteria>Pseudomonadota>Gammaproteobacteria>Enterobacterales>Yersiniaceae>Serratia	GCF_946228755.1	Serratia_proteamaculans	OC575_RS10145	2133622..2137344	28151	BREX-system-P-loop-protein-BrxC-[Serratia-proteamaculans]
WP_132542053.1	Type1.BREX	<- SIGMA+OB-Fold+AAA-ATPase+Lon-Protease-BrxL <- PglZ <-HNH<-OLD-ABC<- N6-METHYLASE <- HEPN <- PolB <- DUF6079 *<- BrxB-BrxF-iSTAND <- BrxA-DNA-binding ||ABhydrolase-><-Acyl_transf_3<-?<-?<-?||Peptidase_M3-><-ABhydrolase	1153	Bacteria>Pseudomonadota>Gammaproteobacteria>Gammaproteobacteria_incertae_sedis>Candidatus_Competibacteraceae>Plasticicumulans	GCF_004341245.1	Plasticicumulans_lactativorans	EV699_RS09620	184945..188406	1133106	BREX-system-P-loop-protein-BrxC-[Plasticicumulans-lactativorans]
WP_223374968.1	Type1.BREX	Arm-DNA-bind_3+Phage_integrase-><- SIGMA+OB-Fold+AAA-ATPase+Lon-Protease-BrxL <- PglZ <- N6-METHYLASE -p<-RVT<- HEPN <- N6-METHYLASE <-ParB+HNH<- DUF6079 *<- BrxB-BrxF-iSTAND <- BrxA-DNA-binding ||?->PHP+SbcC->Schlafen+HATPase+wHTH->?->?->?->Sulfatase->	1240	Bacteria>Pseudomonadota>Gammaproteobacteria	GCF_003051945.2	Enterobacterales	N5D94_RS08195	82368..86090	91347	MULTISPECIES:-BREX-system-P-loop-protein-BrxC-[Enterobacterales]
WP_099433763.1	Type1.BREX	<-Schlafen+HATPase+wHTH<-PHP+SbcC<-?|| BrxA-DNA-binding -> BrxB-BrxF-iSTAND ->DUF772+DDE_Tnp_1-> DUF6079 *->ParB+HNH-> N6-METHYLASE -> HEPN ->RVT-> N6-METHYLASE -p-> PglZ -> SIGMA+OB-Fold+AAA-ATPase+Lon-Protease-BrxL -><-Arm-DNA-bind_3+Phage_integrase	1233	Bacteria>Pseudomonadota>Gammaproteobacteria	GCF_014168815.1	Enterobacterales	H7R56_RS21645	4484852..4488553	91347	MULTISPECIES:-BREX-system-P-loop-protein-BrxC-[Enterobacterales]
WP_253145790.1	Type1.BREX	DDE_Tnp_IS66+DDE_Tnp_IS66_C-><- HEPN <-?<- SIGMA+OB-Fold+AAA-ATPase+Lon-Protease-BrxL <- PglZ <-?<- N6-METHYLASE <- DUF6079 *<- BrxB-BrxF-iSTAND <- BrxA-DNA-binding <- wHTH+WYL+WCX ||DUF4236-><-?<-ParB+HNH-p<-CR-ATPase5+REase||HicA->HicB->	1222	Bacteria>Pseudomonadota>Gammaproteobacteria>Moraxellales>Moraxellaceae>Acinetobacter	GCF_024129855.1	Acinetobacter_lwoffii	NI465_RS14465	11210..14878	28090	BREX-system-P-loop-protein-BrxC-[Acinetobacter-lwoffii]
WP_161384457.1	Type1.BREX	<- wHTH+WYL+WCX ||?-><-RelE-ParE<-RHH_1||?-> HEPN ->?-> BrxA-DNA-binding -> BrxB-BrxF-iSTAND -> DUF6079 *->ParB+HNH-> N6-METHYLASE ->?-> HEPN ->ZNR+RmuC-> PglZ -> SIGMA+OB-Fold+AAA-ATPase+Lon-Protease-BrxL -><-?<-HATPase_c	1192	Bacteria>Pseudomonadota>Gammaproteobacteria>Oceanospirillales>Alcanivoracaceae>Alcanivorax	-	Alcanivorax_sp._ZXX171	GN155_RS10555	118232..121810	2695858	BREX-system-P-loop-protein-BrxC-[Alcanivorax-sp.-ZXX171]
WP_153499070.1	Type6.BREX	<-PHP<- wHTH+WYL+WCX ||?->?-> HEPN -p-> BrxE -> BrxA-DNA-binding -> BrxB-BrxF-iSTAND -> DUF6079 *-> N6-METHYLASE -> N6-METHYLASE ->?->ParB+HNH-> PglZ -> SIGMA+OB-Fold+AAA-ATPase+HKD-DNase-BrxL -><-?<-HTH_3	1214	Bacteria>Pseudomonadota>Gammaproteobacteria>Oceanospirillales>Alcanivoracaceae>Alcanivorax	GCF_009601165.1	Alcanivorax_sediminis	GFN93_RS03795	861896..865540	2663008	BREX-system-P-loop-protein-BrxC-[Alcanivorax-sediminis]
WP_145953601.1	Type1.BREX	<-RelE-ParE<-RelE-ParE<-RelB<- SIGMA+OB-Fold+AAA-ATPase+Lon-Protease-BrxL <- PglZ <-ZNR+RmuC<- HEPN <-?<- N6-METHYLASE <- DUF6079 *<- BrxB-BrxF-iSTAND <- BrxA-DNA-binding <-?<- HEPN ||?-> wHTH+WYL+WCX ->	1192	Bacteria>Pseudomonadota>Gammaproteobacteria>Oceanospirillales>Alcanivoracaceae>Alloalcanivorax	GCF_002072815.1	Alloalcanivorax_xenomutans	P40_RS14805	3223957..3227535	1094342	MULTISPECIES:-BREX-system-P-loop-protein-BrxC-[Gammaproteobacteria]
MBR2514422.1	Type6.BREX	Arm-DNA-bind_3+Phage_integrase-><-?<- SIGMA+OB-Fold+AAA-ATPase+HKD-DNase-BrxL <- PglZ <-ORC-CDC6<-ParA-Soj-PloopNTPase<- HEPN <- N6-METHYLASE <- DUF6079 *<- BrxB-BrxF-iSTAND <- BrxA-DNA-binding <- BrxE <-SIGMA-HTH<-?<-?<-?<-?<-XIS-HTH	1224	Bacteria>Pseudomonadota>Gammaproteobacteria>Oceanospirillales>Halomonadaceae>Halomonas	GCA_017515565.1	Halomonas_sp.	IKE45_10465	23089..26763	1486246	BREX-system-P-loop-protein-BrxC-[Halomonas-sp.]
WP_062374360.1	Type1.BREX	<-ABC-ATPase+SbcC<-ABC-ATPase+SbcC<-?<-?<-?<-TetR-HTH||?->tRNA->ncRNA-> BrxA-DNA-binding -> BrxB-BrxF-iSTAND -> DUF6079 *->Phd-YefM->PIN->Schlafen+HATPase+wHTH-> N6-METHYLASE -> N6-METHYLASE -p-> HEPN ->OLD-ABC->TOPRIM->	1227	Bacteria>Pseudomonadota>Gammaproteobacteria>Oceanospirillales>Halomonadaceae>Halomonas	GCF_001596805.1	Halomonas_sp._KX33721	A3220_RS08340	63501..67184	1819251	BREX-system-P-loop-protein-BrxC-[Halomonas-sp.-KX33721]
WP_101147206.1	Type1.BREX	<-DiS_P_DiS+Peptidase_A24<-?<-VirB11||?->?->?->tRNA-> BrxA-DNA-binding -> BrxB-BrxF-iSTAND -> DUF6079 *->Phd-YefM->PIN->Schlafen+HATPase+wHTH-> N6-METHYLASE -> HEPN -><-?||OLD-ABC->TOPRIM->	1230	Bacteria>Pseudomonadota>Gammaproteobacteria>Oceanospirillales>Halomonadaceae>Halomonas	GCF_002836035.1	Halomonas_sp._MES3-P3E	CXF87_RS15340	100201..103893	2058321	BREX-system-P-loop-protein-BrxC-[Halomonas-sp.-MES3-P3E]
WBU42642.1	Type1.BREX	<-Mrr-N||?->?->?->?->SWIM+DUF3670+SF1-SF2-Helicase->HTH_3-> BrxA-DNA-binding -> BrxB-BrxF-iSTAND -> DUF6079 *->DOC+HTH-> N6-METHYLASE ->?-> N6-METHYLASE -p-> HEPN ->?->TIR->?->TIR->	1221	Bacteria>Pseudomonadota>Gammaproteobacteria>Pseudomonadales>Marinobacteraceae>Marinobacter	GCA_027912435.1	Marinobacter_alkaliphilus	PBN92_07055	1547680..1551345	254719	BREX-system-P-loop-protein-BrxC-[Marinobacter-alkaliphilus]
WP_058091394.1	Type1.BREX	wHTH+WYL+WCX ->?-><-?<-Mrr-N||HTH_3-> BrxA-DNA-binding -> BrxB-BrxF-iSTAND -> DUF6079 *->DOC+HTH->SF1-SF2-Helicase-> N6-METHYLASE -> HEPN ->?->TIR->OLD-ABC->TOPRIM->	1221	Bacteria>Pseudomonadota>Gammaproteobacteria>Pseudomonadales>Marinobacteraceae>Marinobacter	GCF_001447155.2	Marinobacter_sp._LQ44	ASQ50_RS18570	4052781..4056446	1749259	BREX-system-P-loop-protein-BrxC-[Marinobacter-sp.-LQ44]
WP_273917578.1	Type1.BREX	<- SIGMA+OB-Fold+AAA-ATPase+Lon-Protease-BrxL <- PglZ <- HEPN <-TPR<-NERD+GspA-ATPase<- N6-METHYLASE <- N6-METHYLASE <-TOPRIM<-OLD-ABC<- DUF6079 *<- BrxB-BrxF-iSTAND <- BrxA-DNA-binding <- wHTH+WYL+WCX <- wHTH+WYL+WCX ||?->ARG->	1223	Bacteria>Pseudomonadota>Gammaproteobacteria>Pseudomonadales>Pseudomonadaceae>Pseudomonas	GCF_028656895.1	Pseudomonas_sp._TNT2022_ID1044	M5G20_RS01570	347595..351266	2942636	BREX-system-P-loop-protein-BrxC-[Pseudomonas-sp.-TNT2022-ID1044]
WP_053105412.1	Type1.BREX	METHYLASE->?->?-><-?<-?<-?|| wHTH+WYL+WCX -> BrxA-DNA-binding -> BrxB-BrxF-iSTAND -> DUF6079 *-> N6-METHYLASE ->ParB+HNH->OLD-ABC->TOPRIM-> HEPN -> PglZ -> SIGMA+OB-Fold+AAA-ATPase+Lon-Protease-BrxL ->Arm-DNA-bind_3+Phage_integrase-><- SIGMA+OB-Fold+AAA-ATPase+Lon-Protease-BrxL	1155	Bacteria>Pseudomonadota>Gammaproteobacteria>Pseudomonadales>Pseudomonadaceae>Thiopseudomonas	GCF_001267275.1	Thiopseudomonas_alkaliphila	AKN90_RS00615	123321..126788	1697053	BREX-system-P-loop-protein-BrxC-[Thiopseudomonas-alkaliphila]
WP_101840169.1	Type1.BREX	ZnR+TOPRIM+TOPRIM->SIGMA-HTH+SIGMA-HTH+SIGMA-HTH-><-?<- wHTH+WYL+WCX ||?-> HEPN -> BrxA-DNA-binding -> BrxB-BrxF-iSTAND -> DUF6079 *-> N6-METHYLASE ->	1210	Bacteria>Pseudomonadota>Gammaproteobacteria>Thiotrichales>Piscirickettsiaceae>Hydrogenovibrio	GCF_002848135.1	Hydrogenovibrio_sp._SC-1	CYQ88_RS04565	151062..154694	2065820	BREX-system-P-loop-protein-BrxC-[Hydrogenovibrio-sp.-SC-1]
MBK85426.1	Type1.BREX	HHH_5+SF1-SF2-Helicase-> BrxA-DNA-binding -> BrxB-BrxF-iSTAND -> DUF6079 *-> N6-METHYLASE -> HEPN ->Schlafen+HATPase+wHTH->TIR-> PglZ -> SIGMA+OB-Fold+AAA-ATPase+Lon-Protease-BrxL ->?->RNase-><-SWIM+DUF3670+SF1-SF2-Helicase	1217	Bacteria>Pseudomonadota>Gammaproteobacteria>unclassified_Gammaproteobacteria	GCA_002721025.1	Gammaproteobacteria_bacterium	CMD81_16515	7659..11312	1913989	ATPase-[Gammaproteobacteria-bacterium]
NLY14765.1	Type1.BREX	<- SIGMA+OB-Fold+AAA-ATPase+Lon-Protease-BrxL <- PglZ <- HEPN <-TOPRIM<-OLD-ABC<-ParB+HNH<- N6-METHYLASE <- DUF6079 -p*	438	Bacteria>Pseudomonadota>Gammaproteobacteria>unclassified_Gammaproteobacteria	GCA_012728415.1	Gammaproteobacteria_bacterium	GXZ05_00075	12795..14111	1913989	hypothetical-protein-[Gammaproteobacteria-bacterium]
MBK7728002.1	Type1.BREX	Peptidase-><-DDE_Tnp<- SIGMA+OB-Fold+AAA-ATPase+Lon-Protease-BrxL <- PglZ <- HEPN <-?||?-><- DUF6079 *<- BrxB-BrxF-iSTAND <- BrxA-DNA-binding ||URI-DNase->?-><-tRNA<-MerR-HTH	1048	Bacteria>Pseudomonadota>Gammaproteobacteria>unclassified_Gammaproteobacteria	GCA_016709565.1	Gammaproteobacteria_bacterium	IPJ33_05745	303360..306506	1913989	BREX-system-P-loop-protein-BrxC-[Gammaproteobacteria-bacterium]
MBT5223355.1	Type1.BREX	BrxA-DNA-binding -> BrxB-BrxF-iSTAND -> DUF6079 *->MazE_antitoxin->PIN-> N6-METHYLASE -> N6-METHYLASE -> HEPN ->GrpE->?->?->RelE-ParE->	1192	Bacteria>Pseudomonadota>Gammaproteobacteria>unclassified_Gammaproteobacteria	GCA_018661125.1	Gammaproteobacteria_bacterium	HOM15_11165	1377..4955	1913989	BREX-system-P-loop-protein-BrxC-[Gammaproteobacteria-bacterium]
MCG3145056.1	Type1.BREX	HTH_23+HTH_32+HTH_33->DDE_3-><- SIGMA+OB-Fold+AAA-ATPase+Lon-Protease-BrxL <- PglZ <-Schlafen+HATPase+wHTH+HTH<- N6-METHYLASE <- HEPN <- DUF6079 *<- BrxB-BrxF-iSTAND <- BrxA-DNA-binding <-?||?-><-ParB<-ParA-Soj-PloopNTPase||Glyoxalase-><-?||AbiEi_4+AbiEi_1->	1220	Bacteria>Pseudomonadota>Gammaproteobacteria>unclassified_Gammaproteobacteria	GCA_022072255.1	Gammaproteobacteria_bacterium	HONDAALG_02597	52539..56201	1913989	hypothetical-protein-[Gammaproteobacteria-bacterium]
WP_190395653.1	Type1.BREX	<-Couple_hipA+HipA_C<-HTH_3||?-><-RNase<-PALP|| wHTH+WYL+WCX -> BrxA-DNA-binding -> BrxB-BrxF-iSTAND -> DUF6079 *-> N6-METHYLASE ->OLD-ABC->TOPRIM-> PglZ -> HEPN +SF1-SF2-Helicase+RNase+RNase->TPR-><-SF1-SF2-Helicase+REase<-SF1-SF2-Helicase+REase<-Peptidase_M32	1157	Bacteria>Pseudomonadota>Gammaproteobacteria>Vibrionales>Vibrionaceae>Aliivibrio	GCF_014694495.1	Aliivibrio_sp._S10_S31	HC728_RS13680	57939..61412	2720224	BREX-system-P-loop-protein-BrxC-[Aliivibrio-sp.-S10_S31]
WP_159406353.1	Type1.BREX	<-GspA-ATPase<-?||CR-ATPase4->?-><-?<-LexA-protease|| wHTH+WYL+WCX -> BrxA-DNA-binding -> BrxB-BrxF-iSTAND -> DUF6079 *-> N6-METHYLASE ->ParB+HNH-> PglZ -> HEPN +SF1-SF2-Helicase+RNase+RNase->TPR-><-SF1-SF2-Helicase+REase<-SF1-SF2-Helicase+REase<-viral-protein<-AbiEi	1157	Bacteria>Pseudomonadota>Gammaproteobacteria>Vibrionales>Vibrionaceae	GCF_009883895.1	Vibrio	O8X55_RS08175	512098..515571	662	MULTISPECIES:-BREX-system-P-loop-protein-BrxC-[Vibrio]
WP_086713494.1	Type1.BREX	<-GrpE<-ANK+ANK+ANK||?->SF1-SF2-Helicase->?->?-> wHTH+WYL+WCX -> BrxA-DNA-binding -> BrxB-BrxF-iSTAND -> DUF6079 *-> N6-METHYLASE ->ParB+HNH-> PglZ -> HEPN +SF1-SF2-Helicase+RNase+RNase->TPR-><-SF1-SF2-Helicase+REase<-SF1-SF2-Helicase+REase<-?||AbiEi_4->	1157	Bacteria>Pseudomonadota>Gammaproteobacteria>Vibrionales>Vibrionaceae	GCF_024347515.1	Vibrio	EDB41_RS15380	67742..71215	662	MULTISPECIES:-BREX-system-P-loop-protein-BrxC-[Vibrio]
WP_232606822.1	Type1.BREX	ROK-HTH-><-?<-sGTP+sGTP||?->ANK+ANK-> wHTH+WYL+WCX -p-> BrxA-DNA-binding -> BrxB-BrxF-iSTAND -> DUF6079 *-> N6-METHYLASE -> N6-METHYLASE -> PglZ -> HEPN +SF1-SF2-Helicase+RNase+RNase->TPR-><-SF1-SF2-Helicase+REase<-SF1-SF2-Helicase+REase||McrB-AAA->wHTH+REase->	1157	Bacteria>Pseudomonadota>Gammaproteobacteria>Vibrionales>Vibrionaceae>Photobacterium	GCF_021237045.1	Photobacterium_phosphoreum	CJF26_RS18915	30954..34427	659	BREX-system-P-loop-protein-BrxC-[Photobacterium-phosphoreum]
MBE4310404.1	Type1.BREX	<-PIN+OST-HTH||CR-ATPase4->?-><-DUF4113||wHTH-><-HTH_Tnp_1+HTH_21+rve+rve_3|| wHTH+WYL+WCX -> BrxA-DNA-binding -> BrxB-BrxF-iSTAND -> DUF6079 *->ParB+HNH-> PglZ -> HEPN +SF1-SF2-Helicase+RNase+RNase->TPR-><-SF1-SF2-Helicase+REase<-SF1-SF2-Helicase+REase<-viral-protein<-AbiEi	1157	Bacteria>Pseudomonadota>Gammaproteobacteria>Vibrionales>Vibrionaceae>Vibrio	GCA_014921165.1	Vibrio_parahaemolyticus	HJ064_04290	963533..967006	670	BREX-system-P-loop-protein-BrxC-[Vibrio-parahaemolyticus]
EGR0719595.1	Type1.BREX	HTH_20-><- HEPN <-SbcC||CR-ATPase4->?-><-DUF4113|| wHTH+WYL+WCX -> BrxA-DNA-binding -> BrxB-BrxF-iSTAND -> DUF6079 *-> N6-METHYLASE ->ParB+HNH-> PglZ -> HEPN +SF1-SF2-Helicase+RNase+RNase->TPR-><-SF1-SF2-Helicase+REase<-SF1-SF2-Helicase+REase<-viral-protein<-AbiEi	1157	Bacteria>Pseudomonadota>Gammaproteobacteria>Vibrionales>Vibrionaceae>Vibrio	GCA_015789455.1	Vibrio_alginolyticus	EX464_05795	8586..12059	663	BREX-system-P-loop-protein-BrxC-[Vibrio-alginolyticus]
EGR4188113.1	Type1.BREX	AbiEi->viral-protein->SF1-SF2-Helicase+REase->SF1-SF2-Helicase+REase-><-TPR<- HEPN +SF1-SF2-Helicase+RNase+RNase<- PglZ <-ParB+HNH<- N6-METHYLASE <- DUF6079 *<- BrxB-BrxF-iSTAND <- BrxA-DNA-binding <- wHTH+WYL+WCX ||DDE_Tnp_1_5+DDE_Tnp_1->PIN+OST-HTH-><-?<-HTH_20	1157	Bacteria>Pseudomonadota>Gammaproteobacteria>Vibrionales>Vibrionaceae>Vibrio	GCA_015809855.1	Vibrio_cholerae	DDM99_15775	19819..23292	666	BREX-system-P-loop-protein-BrxC-[Vibrio-cholerae]
EGR4671255.1	Type1.BREX	AbiEi->viral-protein->SF1-SF2-Helicase+REase->SF1-SF2-Helicase+REase-><-TPR<- HEPN +SF1-SF2-Helicase+RNase+RNase<- PglZ <-ParB+HNH<- N6-METHYLASE <- DUF6079 *<- BrxB-BrxF-iSTAND <- BrxA-DNA-binding <- wHTH+WYL+WCX ||DUF4113-><-?<-CR-ATPase4||SbcC->	1157	Bacteria>Pseudomonadota>Gammaproteobacteria>Vibrionales>Vibrionaceae>Vibrio	GCA_015814545.1	Vibrio_parahaemolyticus	C4G45_02530	555237..558710	670	BREX-system-P-loop-protein-BrxC-[Vibrio-parahaemolyticus]

Protein-ID	System	Gene-Neighbourhoods	Length	Taxonomy	Assembly-ID	Strain	Locus-Tag	Genomic-coordinates	TaxID	Fasta-define
MBM4838763.1	Type1.BREX	AbiEi->viral-protein->SF1-SF2-Helicase+REase->SF1-SF2-Helicase+REase-><-TPR<- HEPN +SF1-SF2-Helicase+RNase+RNase<- PglZ <-ParB+HNH<- N6-METHYLASE <- DUF6079 *<- BrxB-BrxF-iSTAND <- BrxA-DNA-binding <- wHTH+WYL+WCX ||HTH_Tnp_1+HTH_21+rve+rve_3-><-wHTH||DUF4113-><-?<-CR-ATPase4||PIN+OST-HTH->	1157	Bacteria>Pseudomonadota>Gammaproteobacteria>Vibrionales>Vibrionaceae>Vibrio	GCA_016876235.1	Vibrio_parahaemolyticus	HY055_06530	138658..142131	670	BREX-system-P-loop-protein-BrxC-[Vibrio-parahaemolyticus]
MBM4875012.1	Type1.BREX	<-AAA-ATPase-p<- HEPN ||CR-ATPase4->?->|| wHTH+WYL+WCX -> BrxA-DNA-binding -> BrxB-BrxF-iSTAND -> DUF6079 *-> N6-METHYLASE ->ParB+HNH-> PglZ -> HEPN +SF1-SF2-Helicase+RNase+RNase->TPR-><-SF1-SF2-Helicase+REase<-SF1-SF2-Helicase+REase<-viral-protein<-AbiEi	1157	Bacteria>Pseudomonadota>Gammaproteobacteria>Vibrionales>Vibrionaceae>Vibrio	GCA_016876415.1	Vibrio_parahaemolyticus	HY045_05440	517620..521093	670	BREX-system-P-loop-protein-BrxC-[Vibrio-parahaemolyticus]
HBC3445072.1	Type1.BREX	<-ZnR<-?||AbiEi->viral-protein-> wHTH+WYL+WCX -> BrxA-DNA-binding -> BrxB-BrxF-iSTAND -> DUF6079 *-> N6-METHYLASE ->?->?-> PglZ -> SIGMA+OB-Fold+AAA-ATPase+Lon-Protease-BrxL -><-TPR<- HEPN +SF1-SF2-Helicase+RNase+RNase||<-?<-CR-ATPase4	1225	Bacteria>Pseudomonadota>Gammaproteobacteria>Vibrionales>Vibrionaceae>Vibrio	GCA_018119025.1	Vibrio_parahaemolyticus	KDY48_003788	6147..9824	670	BREX-system-P-loop-protein-BrxC-[Vibrio-parahaemolyticus]
WP_021709035.1	Type1.BREX	<-Thioredoxin<-?<-Phage_integrase||?->?-><-?|| wHTH+WYL+WCX -> BrxA-DNA-binding -> BrxB-BrxF-iSTAND -> DUF6079 *-> N6-METHYLASE -> HEPN -> PglZ -> SIGMA+OB-Fold+AAA-ATPase+Lon-Protease-BrxL ->GNAT-><-Peptidase	1229	Bacteria>Pseudomonadota>Gammaproteobacteria>Vibrionales>Vibrionaceae>Vibrio	GCF_000400265.1	Vibrio_azureus	VAN_RS15510	14465..18154	512649	BREX-system-P-loop-protein-BrxC-[Vibrio-azureus]
WP_053027640.1	Type1.BREX	DUF6079 -p*-> N6-METHYLASE -> PglZ -> SIGMA+OB-Fold+AAA-ATPase+Lon-Protease-BrxL -> HEPN ->HDRnase->VirD4-TraG+VirB4-FtsK->?->?-><-Glyoxalase_8	848	Bacteria>Pseudomonadota>Gammaproteobacteria>Vibrionales>Vibrionaceae>Vibrio	GCF_001259875.1	Vibrio_cholerae	ALV71_RS07765	696..3240	666	BREX-system-P-loop-protein-BrxC,-partial-[Vibrio-cholerae]
WP_096070868.1	Type1.BREX	HTH_20->CR-ATPase4->?-><-?<-LexA-protease|| wHTH+WYL+WCX -> BrxA-DNA-binding -> BrxB-BrxF-iSTAND -> DUF6079 *-> N6-METHYLASE ->ParB+HNH-> PglZ -> HEPN +SF1-SF2-Helicase+RNase+RNase->TPR-><-LZ_Tnp_IS66+zf-IS66+DDE_Tnp_IS66+DDE_Tnp_IS66_C<-?<-HTH_Tnp_1<-AAA-ATPase	1157	Bacteria>Pseudomonadota>Gammaproteobacteria>Vibrionales>Vibrionaceae>Vibrio	GCF_002313025.1	Vibrio_cholerae	FORC55_RS15555	276972..280445	666	BREX-system-P-loop-protein-BrxC-[Vibrio-cholerae]
WP_148513618.1	Type1.BREX	AbiEi->viral-protein->SF1-SF2-Helicase+REase->SF1-SF2-Helicase+REase-><-TPR<- HEPN +SF1-SF2-Helicase+RNase+RNase<- PglZ <-ParB+HNH<- N6-METHYLASE <- DUF6079 *<- BrxB-BrxF-iSTAND <- BrxA-DNA-binding <- wHTH+WYL+WCX ||DDE_Tnp_1_5+DDE_Tnp_1->	1157	Bacteria>Pseudomonadota>Gammaproteobacteria>Vibrionales>Vibrionaceae>Vibrio	GCF_008083315.1	Vibrio_cholerae	FXF10_RS00340	29414..32887	666	BREX-system-P-loop-protein-BrxC-[Vibrio-cholerae]
WP_148490856.1	Type1.BREX	<-DDE_Tnp_1_5+DDE_Tnp_1||HTH_Tnp_1+HTH_21+rve+rve_3-><-SbcC||CR-ATPase4->?-><-DUF4113|| wHTH+WYL+WCX -> BrxA-DNA-binding -> BrxB-BrxF-iSTAND -> DUF6079 *-> N6-METHYLASE ->ParB+HNH-> PglZ -> HEPN +SF1-SF2-Helicase+RNase+RNase->TPR-><-SF1-SF2-Helicase+REase<-SF1-SF2-Helicase+REase<-viral-protein<-AbiEi	1157	Bacteria>Pseudomonadota>Gammaproteobacteria>Vibrionales>Vibrionaceae>Vibrio	GCF_008084645.1	Vibrio_cholerae	FXE54_RS16715	26974..30447	666	BREX-system-P-loop-protein-BrxC-[Vibrio-cholerae]
WP_213899843.1	Type1.BREX	AbiEi->viral-protein->SF1-SF2-Helicase+REase->SF1-SF2-Helicase+REase-><-TPR<- HEPN +SF1-SF2-Helicase+RNase+RNase<- PglZ <-ParB+HNH<- N6-METHYLASE <- DUF6079 *<- BrxB-BrxF-iSTAND <- BrxA-DNA-binding <- wHTH+WYL+WCX ||<-?<-CR-ATPase4||SbcC-> HEPN -><-HTH_20	1157	Bacteria>Pseudomonadota>Gammaproteobacteria>Vibrionales>Vibrionaceae>Vibrio	GCF_018458525.1	Vibrio_alginolyticus	J4H25_RS19085	22843..26316	663	BREX-system-P-loop-protein-BrxC-[Vibrio-alginolyticus]
WP_248354663.1	Type1.BREX	AbiEi->viral-protein->SF1-SF2-Helicase+REase->SF1-SF2-Helicase+REase-><-TPR<- HEPN +SF1-SF2-Helicase+RNase+RNase<- PglZ <-ParB+HNH<- N6-METHYLASE <- DUF6079 *<- BrxB-BrxF-iSTAND <- BrxA-DNA-binding <- wHTH+WYL+WCX ||DDE_Tnp_1_5+DDE_Tnp_1->PIN+OST-HTH-><-?<-HTH_20	1157	Bacteria>Pseudomonadota>Gammaproteobacteria>Vibrionales>Vibrionaceae>Vibrio	GCF_024745175.1	Vibrio_parahaemolyticus	DDM99_15775	19819..23292	670	BREX-system-P-loop-protein-BrxC-[Vibrio-parahaemolyticus]
WP_272236739.1	Type1.BREX	TraJ-RHH->TraI-VirD2-RCRE->REase-><-AbiEi_4<-?||?-> wHTH+WYL+WCX -> BrxA-DNA-binding -> BrxB-BrxF-iSTAND -> DUF6079 *-> N6-METHYLASE -> HEPN -> PglZ -> SIGMA+OB-Fold+AAA-ATPase+Lon-Protease-BrxL -><-?<-?<-SF1-SF2-Helicase<-?<-Arm-DNA-bind_3	1229	Bacteria>Pseudomonadota>Gammaproteobacteria>Vibrionales>Vibrionaceae>Vibrio	GCF_028446685.1	Vibrio_europaeus	OPW18_RS21520	351695..355384	300876	BREX-system-P-loop-protein-BrxC-[Vibrio-europaeus]
WP_229611425.1	Type1.BREX	REase+SPF-cons-><-PIN+OST-HTH||CR-ATPase4->?->|| wHTH+WYL+WCX -> BrxA-DNA-binding -> BrxB-BrxF-iSTAND -> DUF6079 *-> N6-METHYLASE ->ParB+HNH-> PglZ -> HEPN +SF1-SF2-Helicase+RNase+RNase->TPR-><-SF1-SF2-Helicase+REase<-SF1-SF2-Helicase+REase<-viral-protein<-AbiEi	1157	Bacteria>Pseudomonadota>Gammaproteobacteria>Vibrionales>Vibrionaceae>Vibrio	GCF_905331955.1	Vibrio_parahaemolyticus	LPR99_RS16740	18511..21984	670	BREX-system-P-loop-protein-BrxC-[Vibrio-parahaemolyticus]
WP_107394298.1	Type1.BREX	<-HisKin<-SF1-SF2-Helicase+REase<-35Exo+REase|| wHTH+WYL+WCX -> BrxA-DNA-binding -> BrxB-BrxF-iSTAND -> DUF6079 *-> N6-METHYLASE -> HEPN -> PglZ -> SIGMA+OB-Fold+AAA-ATPase+Lon-Protease-BrxL -><-AbiEii	1140	Bacteria>Pseudomonadota	GCF_003034225.1	Alphaproteobacteria	CV103_RS05945	27177..30599	28211	MULTISPECIES:-BREX-system-P-loop-protein-BrxC-[Alphaproteobacteria]
WP_053322564.1	Type1.BREX	HNH->?->?->LON-AAA->Calcineurin->?-> BrxA-DNA-binding -> BrxB-BrxF-iSTAND -> DUF6079 *-> N6-METHYLASE -p->Schlafen+HATPase+wHTH-> HEPN ->REase-> N6-METHYLASE -p->HerA/FtsK-> PglZ -> SIGMA+OB-Fold+AAA-ATPase+HKD-DNase-BrxL ->	1181	Bacteria>Pseudomonadota>Zetaproteobacteria>Mariprofundales>Mariprofundaceae>Mariprofundus	GCF_001273615.1	Mariprofundus_ferrooxydans	AL013_RS02410	88551..92096	314344	BREX-system-P-loop-protein-BrxC-[Mariprofundus-ferrooxydans]
MCF7790541.1	Type1.BREX	<- HEPN <- SIGMA+OB-Fold+AAA-ATPase+HKD-DNase-BrxL <- PglZ <-TOPRIM<-OLD-ABC<-?<-?<-?<- DUF6079 *<- BrxB-BrxF-iSTAND <- BrxA-DNA-binding <- wHTH+WYL+WCX <-?||?-><-?<-?||S2P+PDZ->	1176	Bacteria>PVC_group>Lentisphaerota>Lentisphaeria>Victivallales>unclassified_Victivallales	GCA_021733985.1	Victivallales_bacterium	K9M56_00960	14578..18108	2212475	BREX-system-P-loop-protein-BrxC-[Victivallales-bacterium]
CAJ71903.1	Type1.BREX	Unstab_antitox->RelE-ParE->RelE-ParE-> HEPN ->ABhydrolase-><- HEPN <- Polbeta <- SIGMA+OB-Fold+AAA-ATPase+Lon-Protease-BrxL <- PglZ <- DUF6079 *<-?<-?<- N6-METHYLASE -p||?-><-DDE_Tnp_1_4<- N6-METHYLASE <- BrxB-BrxF-iSTAND <- BrxA-DNA-binding	1220	Bacteria>PVC_group>Planctomycetota>Candidatus_Brocadiia>Candidatus_Brocadiales>Candidatus_Brocadiaceae	-	Candidatus_Kuenenia_stuttgartiensis	kustc1158	789101..792763	174633	hypothetical-protein-kustc1158-[Candidatus-Kuenenia-stuttgartiensis]
MBQ9812620.1	Type1.BREX	[illegible]	596	Bacteria>PVC_group>Planctomycetota>Planctomycetia>Pirellulales>Thermoguttaceae	GCA_017441335.1	Thermoguttaceae_bacterium	IJM54_04830	4203..5993	2691551	BREX-system-P-loop-protein-BrxC-[Thermoguttaceae-bacterium]
WP_145192991.1	Type1.BREX	[illegible]	1220	Bacteria>PVC_group>Planctomycetota>Planctomycetia>Planctomycetales>Planctomycetaceae	GCF_007744075.1	Gimesia_chilikensis	HG66A1_RS30060	7903021..7906683	2605989	BREX-system-P-loop-protein-BrxC-[Gimesia-chilikensis]
TET34868.1	Type1.BREX	[illegible]	1347	Bacteria>PVC_group>Planctomycetota>unclassified_Planctomycetota	GCA_004376375.1	Planctomycetota_bacterium	E3J72_12085	41890..45933	2026780	MAG:-hypothetical-protein-E3J72_12085-[Planctomycetota-bacterium]
MBI3468946.1	Type1.BREX	[illegible]	1222	Bacteria>PVC_group>Planctomycetota>unclassified_Planctomycetota	GCA_016196255.1	Planctomycetota_bacterium	HY000_38570	1768..5436	2026780	BREX-system-P-loop-protein-BrxC-[Planctomycetota-bacterium]
MBL1217602.1	Type1.BREX	[illegible]	1165	Bacteria>PVC_group>Planctomycetota>unclassified_Planctomycetota	GCA_016743455.1	Planctomycetota_bacterium	D8M59_08910	1591..5088	2026780	BREX-system-P-loop-protein-BrxC-[Planctomycetota-bacterium]
MBW8002946.1	Type1.BREX	[illegible]	1164	Bacteria>PVC_group>Planctomycetota>unclassified_Planctomycetota	GCA_019456255.1	Planctomycetota_bacterium	FVQ80_13155	4626..8120	2026780	BREX-system-P-loop-protein-BrxC-[Planctomycetota-bacterium]
MCC6785237.1	Type1.BREX	[illegible]	1150	Bacteria>PVC_group>Planctomycetota>unclassified_Planctomycetota	GCA_020848735.1	Planctomycetota_bacterium	IT457_20475	22318..25770	2026780	BREX-system-P-loop-protein-BrxC-[Planctomycetota-bacterium]
MBI4023965.1	Type1.BREX	[illegible]	1148	Bacteria>PVC_group>Verrucomicrobiota>unclassified_Verrucomicrobiota	GCA_016198745.1	Verrucomicrobiota_bacterium	HY360_03230	10391..13837	2026799	BREX-system-P-loop-protein-BrxC-[Verrucomicrobiota-bacterium]
NLV85368.1	Type1.BREX	[illegible]	1160	Bacteria>Spirochaetota>Spirochaetia>Spirochaetales>unclassified_Spirochaetales	GCA_012729295.1	Spirochaetales_bacterium	GXY60_12460	48..3530	2026792	BREX-system-P-loop-protein-BrxC-[Spirochaetales-bacterium]
RKX87882.1	Type1.BREX	[illegible]	1168	Bacteria>Spirochaetota>unclassified_Spirochaetota	GCA_003644105.1	Spirochaetota_bacterium	DRP70_07570	3107..6613	2202144	BREX-system-P-loop-protein-BrxC-[Spirochaetota-bacterium]
WP_009648402.1	Type1.BREX	[illegible]	1169	Bacteria>Terrabacteria_group>Actinomycetota>Actinomycetes>Actinomycetales>Actinomycetaceae	GCF_000278725.1	Actinomyces_sp._ICM47	HMPREF1136_RS05845	15295..18804	936548	BREX-system-P-loop-protein-BrxC-[Actinomyces-sp.-ICM47]
KFI62072.1	Type1.BREX	[illegible]	1219	Bacteria>Terrabacteria_group>Actinomycetota>Actinomycetes>Bifidobacteriales>Bifidobacteriaceae	GCA_000741575.1	Bifidobacterium_cuniculi	BCUN_1388	51093..54752	1688	putative-ATP-binding-protein-[Bifidobacterium-cuniculi]
GDY95791.1	Type1.BREX	[illegible]	1223	Bacteria>Terrabacteria_group>Actinomycetota>Actinomycetes>Bifidobacteriales>Bifidobacteriaceae	GCA_018326565.1	Bifidobacteriaceae_bacterium_MCC01943	MCC01943_10780	5409..9080	2569488	hypothetical-protein-MCC01943_10780-[Bifidobacteriaceae-bacterium-MCC01943]
WP_032738770.1	Type1.BREX	[illegible]	1218	Bacteria>Terrabacteria_group>Actinomycetota>Actinomycetes>Bifidobacteriales>Bifidobacteriaceae	GCF_000730055.1	Bifidobacterium_longum	BL71B_RS01495	365977..369633	216816	BREX-system-P-loop-protein-BrxC-[Bifidobacterium-longum]
WP_051922710.1	Type1.BREX	[illegible]	1249	Bacteria>Terrabacteria_group>Actinomycetota>Actinomycetes>Bifidobacteriales>Bifidobacteriaceae	GCF_000741785.1	Bifidobacterium_stellenboschense	BSTEL_1465	61247..64996	762211	BREX-system-P-loop-protein-BrxC-[Bifidobacterium-stellenboschense]
WP_160257632.1	Type1.BREX	[illegible]	1215	Bacteria>Terrabacteria_group>Actinomycetota>Actinomycetes>Bifidobacteriales>Bifidobacteriaceae	GCF_000771465.1	Bifidobacterium_cuniculi	BCUN_RS07325	51105..54752	1688	BREX-system-P-loop-protein-BrxC-[Bifidobacterium-cuniculi]
WP_100495666.1	Type1.BREX	[illegible]	1240	Bacteria>Terrabacteria_group>Actinomycetota>Actinomycetes>Bifidobacteriales>Bifidobacteriaceae	GCF_002802865.1	Bifidobacterium_scaligerum	CUU80_RS01790	434556..438278	2052656	BREX-system-P-loop-protein-BrxC-[Bifidobacterium-scaligerum]
WP_180326342.1	Type1.BREX	[illegible]	865	Bacteria>Terrabacteria_group>Actinomycetota>Actinomycetes>Bifidobacteriales>Bifidobacteriaceae	GCF_002846775.1	Bifidobacterium_pseudolongum	CQR50_RS05955	514..3111	1694	BREX-system-P-loop-protein-BrxC-[Bifidobacterium-pseudolongum]
WP_109056521.1	Type1.BREX	[illegible]	1246	Bacteria>Terrabacteria_group>Actinomycetota>Actinomycetes>Bifidobacteriales>Bifidobacteriaceae	GCF_003129905.1	Bifidobacterium_callitrichidarum	DF196_RS03600	108404..112144	2052941	BREX-system-P-loop-protein-BrxC-[Bifidobacterium-callitrichidarum]
WP_115955260.1	Type1.BREX	[illegible]	1217	Bacteria>Terrabacteria_group>Actinomycetota>Actinomycetes>Bifidobacteriales>Bifidobacteriaceae	GCF_003390735.1	Bifidobacterium_bifidum	CJD48_RS06210	1417204..1420857	1681	BREX-system-P-loop-protein-BrxC-[Bifidobacterium-bifidum]
WP_117759621.1	Type1.BREX	[illegible]	1223	Bacteria>Terrabacteria_group>Actinomycetota>Actinomycetes>Bifidobacteriales>Bifidobacteriaceae	GCF_003458805.1	Bifidobacterium_adolescentis	DWX79_RS04615	1022537..1026208	1680	BREX-system-P-loop-protein-BrxC-[Bifidobacterium-adolescentis]
WP_126029122.1	Type1.BREX	[illegible]	1235	Bacteria>Terrabacteria_group>Actinomycetota>Actinomycetes>Bifidobacteriales>Bifidobacteriaceae	GCF_003952005.1	Bifidobacterium_callimiconis	D2E23_RS00850	182499..186206	2306973	BREX-system-P-loop-protein-BrxC-[Bifidobacterium-callimiconis]
WP_150379196.1	Type1.BREX	[illegible]	1267	Bacteria>Terrabacteria_group>Actinomycetota>Actinomycetes>Bifidobacteriales>Bifidobacteriaceae	GCF_008698155.1	Bifidobacterium_myosotis	EMO91_RS06000	83624..87427	1630166	BREX-system-P-loop-protein-BrxC-[Bifidobacterium-myosotis]
WP_152233646.1	Type1.BREX	[illegible]	1235	Bacteria>Terrabacteria_group>Actinomycetota>Actinomycetes>Bifidobacteriales>Bifidobacteriaceae	GCF_009193355.1	Bifidobacterium_leontopitheci	F7D09_RS01345	123122..126829	2650774	BREX-system-P-loop-protein-BrxC-[Bifidobacterium-leontopitheci]
WP_117762529.1	Type1.BREX	[illegible]	1223	Bacteria>Terrabacteria_group>Actinomycetota>Actinomycetes>Bifidobacteriales>Bifidobacteriaceae	GCF_009832825.1	Bifidobacterium_adolescentis	DWZ00_RS05085	1174565..1178236	1680	BREX-system-P-loop-protein-BrxC-[Bifidobacterium-adolescentis]
WP_196336889.1	Type1.BREX	[illegible]	1217	Bacteria>Terrabacteria_group>Actinomycetota>Actinomycetes>Bifidobacteriales>Bifidobacteriaceae	GCF_015703835.1	Bifidobacterium_dentium	IHQ82_RS06050	179848..183501	1689	BREX-system-P-loop-protein-BrxC-[Bifidobacterium-dentium]
WP_214374600.1	Type1.BREX	[illegible]	1249	Bacteria>Terrabacteria_group>Actinomycetota>Actinomycetes>Bifidobacteriales>Bifidobacteriaceae	GCF_018555335.1	Bifidobacterium	JS541_RS11260	52363..56112	1678	MULTISPECIES:-BREX-system-P-loop-protein-BrxC-[Bifidobacterium]
WP_217054570.1	Type1.BREX	[illegible]	1229	Bacteria>Terrabacteria_group>Actinomycetota>Actinomycetes>Bifidobacteriales>Bifidobacteriaceae	GCF_019041665.1	Bifidobacterium_longum	KTF47_RS04030	15252..18941	216816	BREX-system-P-loop-protein-BrxC-[Bifidobacterium-longum]
WP_270561184.1	Type1.BREX	[illegible]	1217	Bacteria>Terrabacteria_group>Actinomycetota>Actinomycetes>Bifidobacteriales>Bifidobacteriaceae	GCF_027698005.1	Bifidobacterium_dentium	PG823_RS09640	38589..42242	1689	BREX-system-P-loop-protein-BrxC-[Bifidobacterium-dentium]
WP_029253807.1	Type1.BREX	[illegible]	1160	Bacteria>Terrabacteria_group>Actinomycetota>Actinomycetes>Micrococcales>Cellulomonadaceae	GCF_000701465.1	Paraoerskovia_marina	Q343_RS0111945	114651..118133	545619	BREX-system-P-loop-protein-BrxC-[Paraoerskovia-marina]
WP_156999272.1	Type1.BREX	[illegible]	1173	Bacteria>Terrabacteria_group>Actinomycetota>Actinomycetes>Micrococcales>Microbacteriaceae	GCF_024172065.1	Agromyces_terreus	BJ978_RS07845	1788329..1791850	424795	BREX-system-P-loop-protein-BrxC-[Agromyces-terreus]
WP_085152401.1	Type1.BREX	[illegible]	1156	Bacteria>Terrabacteria_group>Actinomycetota>Actinomycetes>Mycobacteriales>Mycobacteriaceae	GCF_010729895.1	Mycolicibacterium_confluentis	H7K00_RS24370	129281..132751	28047	BREX-system-P-loop-protein-BrxC-[Mycolicibacterium-confluentis]
WP_094721084.1	Type1.BREX	[illegible]	1174	Bacteria>Terrabacteria_group>Actinomycetota>Actinomycetes>Mycobacteriales>Nocardiaceae	GCF_002259415.1	Rhodococcus_sp._14-2470-1b	CH293_RS18620	24333..27857	2023149	BREX-system-P-loop-protein-BrxC-[Rhodococcus-sp.-14-2470-1b]
MBW3578771.1	Type1.BREX	[illegible]	1145	Bacteria>Terrabacteria_group>Actinomycetota>Actinomycetes>unclassified_Actinomycetes	GCA_019347675.1	Actinomycetia_bacterium	KY462_13720	21398..24835	1883427	BREX-system-P-loop-protein-BrxC-[Actinomycetia-bacterium]
MBR3314870.1	Type1.BREX	[illegible]	1189	Bacteria>Terrabacteria_group>Actinomycetota>Coriobacteriia>Coriobacteriales>Atopobiaceae	GCA_017510745.1	Atopobiaceae_bacterium	IKG18_12110	33041..36610	2485926	BREX-system-P-loop-protein-BrxC-[Atopobiaceae-bacterium]
HIZ18751.1	Type1.BREX	[illegible]	1177	Bacteria>Terrabacteria_group>Actinomycetota>Coriobacteriia>Coriobacteriales>Atopobiaceae	GCA_019116265.1	Candidatus_Olsenella_stercoravium	IAA22_06560	20591..24124	2838713	BREX-system-P-loop-protein-BrxC-[Candidatus-Olsenella-stercoravium]
WP_204537521.1	Type1.BREX	[illegible]	1171	Bacteria>Terrabacteria_group>Actinomycetota>Coriobacteriia>Coriobacteriales>Coriobacteriaceae	-	Collinsella_tanakaei	H6A33_RS00145	33492..37007	626935	BREX-system-P-loop-protein-BrxC-[Collinsella-tanakaei]
MBD9244247.1	Type1.BREX	[illegible]	1191	Bacteria>Terrabacteria_group>Actinomycetota>Coriobacteriia>Coriobacteriales>Coriobacteriaceae	GCA_014847015.1	Coriobacteriaceae_bacterium	EGQ66_00120	16164..19739	2011094	BREX-system-P-loop-protein-BrxC-[Coriobacteriaceae-bacterium]
MBN2945151.1	Type1.BREX	[illegible]	1220	Bacteria>Terrabacteria_group>Actinomycetota>Coriobacteriia>Coriobacteriales>Coriobacteriaceae	GCA_016939135.1	Collinsella_sp.	JTJ24_07700	2990..6652	1965294	BREX-system-P-loop-protein-BrxC-[Collinsella-sp.]
MBN2939679.1	Type1.BREX	[illegible]	1196	Bacteria>Terrabacteria_group>Actinomycetota>Coriobacteriia>Coriobacteriales>Coriobacteriaceae	GCA_016939175.1	Collinsella_sp.	JTJ11_06140	12590..16180	1965294	BREX-system-P-loop-protein-BrxC-[Collinsella-sp.]
MBS5295771.1	Type1.BREX	[illegible]	1237	Bacteria>Terrabacteria_group>Actinomycetota>Coriobacteriia>Coriobacteriales>Coriobacteriaceae	GCA_018367145.1	Collinsella_sp.	KHY16_04805	17070..20783	1965294	BREX-system-P-loop-protein-BrxC-[Collinsella-sp.]
MBS6611921.1	Type1.BREX	[illegible]	1187	Bacteria>Terrabacteria_group>Actinomycetota>Coriobacteriia>Coriobacteriales>Coriobacteriaceae	GCA_018377935.1	Collinsella_intestinalis	KH279_01485	57380..60943	147207	BREX-system-P-loop-protein-BrxC-[Collinsella-intestinalis]
MBS6608517.1	Type1.BREX	[illegible]	1220	Bacteria>Terrabacteria_group>Actinomycetota>Coriobacteriia>Coriobacteriales>Coriobacteriaceae	GCA_018377985.1	Collinsella_sp.	KH291_01665	76425..80087	1965294	BREX-system-P-loop-protein-BrxC-[Collinsella-sp.]
MBS6695654.1	Type1.BREX	[illegible]	1220	Bacteria>Terrabacteria_group>Actinomycetota>Coriobacteriia>Coriobacteriales>Coriobacteriaceae	GCA_018378775.1	Collinsella_sp.	KH246_00985	77204..80866	1965294	BREX-system-P-loop-protein-BrxC-[Collinsella-sp.]
MBS6858450.1	Type1.BREX	[illegible]	1220	Bacteria>Terrabacteria_group>Actinomycetota>Coriobacteriia>Coriobacteriales>Coriobacteriaceae	GCA_018380015.1	Collinsella_sp.	KH187_04800	313170..316832	1965294	BREX-system-P-loop-protein-BrxC-[Collinsella-sp.]
MBS7081308.1	Type1.BREX	[illegible]	1220	Bacteria>Terrabacteria_group>Actinomycetota>Coriobacteriia>Coriobacteriales>Coriobacteriaceae	GCA_018382125.1	Collinsella_sp.	KH077_07095	14975..18637	1965294	BREX-system-P-loop-protein-BrxC-[Collinsella-sp.]
WP_006235452.1	Type1.BREX	[illegible]	1220	Bacteria>Terrabacteria_group>Actinomycetota>Coriobacteriia>Coriobacteriales>Coriobacteriaceae	GCF_000169035.1	Collinsella_aerofaciens	COLAER_RS06810	43874..47536	74426	BREX-system-P-loop-protein-BrxC-[Collinsella-aerofaciens]
WP_087204139.1	Type1.BREX	[illegible]	1199	Bacteria>Terrabacteria_group>Actinomycetota>Coriobacteriia>Coriobacteriales>Coriobacteriaceae	GCF_002159765.1	Collinsella_sp._An268	B5F70_RS03250	39703..43302	1965612	BREX-system-P-loop-protein-BrxC-[Collinsella-sp.-An268]
WP_099431905.1	Type1.BREX	[illegible]	1219	Bacteria>Terrabacteria_group>Actinomycetota>Coriobacteriia>Coriobacteriales>Coriobacteriaceae	GCF_002736145.1	Collinsella_aerofaciens	FX111_RS04100	928827..932486	74426	BREX-system-P-loop-protein-BrxC-[Collinsella-aerofaciens]
WP_117639687.1	Type1.BREX	[illegible]	1220	Bacteria>Terrabacteria_group>Actinomycetota>Coriobacteriia>Coriobacteriales>Coriobacteriaceae	GCF_003436395.1	Collinsella_sp._TM06-3	DXD56_RS01895	14278..17940	2292342	BREX-system-P-loop-protein-BrxC-[Collinsella-sp.-TM06-3]
WP_117735584.1	Type1.BREX	[illegible]	1196	Bacteria>Terrabacteria_group>Actinomycetota>Coriobacteriia>Coriobacteriales>Coriobacteriaceae	GCF_003436895.1	Collinsella_sp._TF09-1AT	DXC90_RS08415	29935..33525	2292334	BREX-system-P-loop-protein-BrxC-[Collinsella-sp.-TF09-1AT]
WP_117716812.1	Type1.BREX	[illegible]	1220	Bacteria>Terrabacteria_group>Actinomycetota>Coriobacteriia>Coriobacteriales>Coriobacteriaceae	GCF_003438115.1	Collinsella_sp._OM08-14AT	DXC18_RS07380	14092..17754	2292329	BREX-system-P-loop-protein-BrxC-[Collinsella-sp.-OM08-14AT]
WP_117746709.1	Type1.BREX	[illegible]	1220	Bacteria>Terrabacteria_group>Actinomycetota>Coriobacteriia>Coriobacteriales>Coriobacteriaceae	GCF_003438495.1	Collinsella_sp._TF05-9AC	DXC52_RS07155	15430..19092	2292330	BREX-system-P-loop-protein-BrxC-[Collinsella-sp.-TF05-9AC]
WP_117763119.1	Type1.BREX	[illegible]	1220	Bacteria>Terrabacteria_group>Actinomycetota>Coriobacteriia>Coriobacteriales>Coriobacteriaceae	GCF_003466125.1	Collinsella_sp._TM10-22	DXD94_RS02350	80369..84031	2292344	BREX-system-P-loop-protein-BrxC-[Collinsella-sp.-TM10-22]
WP_117786732.1	Type1.BREX	[illegible]	1224	Bacteria>Terrabacteria_group>Actinomycetota>Coriobacteriia>Coriobacteriales>Coriobacteriaceae	GCF_003467795.1	Collinsella_sp._AM36-4AA	DW850_RS07585	35525..39199	2292317	BREX-system-P-loop-protein-BrxC-[Collinsella-sp.-AM36-4AA]
WP_118226697.1	Type1.BREX	[illegible]	1224	Bacteria>Terrabacteria_group>Actinomycetota>Coriobacteriia>Coriobacteriales>Coriobacteriaceae	GCF_003470945.1	Collinsella_sp._AM23-17	DW645_RS05360	15377..19051	2292030	BREX-system-P-loop-protein-BrxC-[Collinsella-sp.-AM23-17]
WP_117583764.1	Type1.BREX	[illegible]	1220	Bacteria>Terrabacteria_group>Actinomycetota>Coriobacteriia>Coriobacteriales>Coriobacteriaceae	GCF_003472325.1	unclassified_Collinsella	DW136_RS06205	18574..22236	2637548	MULTISPECIES:-BREX-system-P-loop-protein-BrxC-[unclassified-Collinsella]
WP_118273143.1	Type1.BREX	[illegible]	1220	Bacteria>Terrabacteria_group>Actinomycetota>Coriobacteriia>Coriobacteriales>Coriobacteriaceae	GCF_003472465.1	unclassified_Collinsella	DW129_RS04200	27076..30738	2637548	MULTISPECIES:-BREX-system-P-loop-protein-BrxC-[unclassified-Collinsella]
WP_117629077.1	Type1.BREX	[illegible]	1220	Bacteria>Terrabacteria_group>Actinomycetota>Coriobacteriia>Coriobacteriales>Coriobacteriaceae	GCF_003474585.1	unclassified_Collinsella	DW037_RS01545	67055..70717	2637548	MULTISPECIES:-BREX-system-P-loop-protein-BrxC-[unclassified-Collinsella]
WP_118384652.1	Type1.BREX	[illegible]	1220	Bacteria>Terrabacteria_group>Actinomycetota>Coriobacteriia>Coriobacteriales>Coriobacteriaceae	GCF_003475325.1	Collinsella_sp._AF33-16	DWZ52_RS00745	185923..189585	2292012	BREX-system-P-loop-protein-BrxC-[Collinsella-sp.-AF33-16]
WP_124786083.1	Type1.BREX	[illegible]	1220	Bacteria>Terrabacteria_group>Actinomycetota>Coriobacteriia>Coriobacteriales>Coriobacteriaceae	GCF_003856815.1	Collinsella_aerofaciens	CV096_RS03705	854606..858268	74426	BREX-system-P-loop-protein-BrxC-[Collinsella-aerofaciens]
WP_129423162.1	Type1.BREX	[illegible]	1205	Bacteria>Terrabacteria_group>Actinomycetota>Coriobacteriia>Coriobacteriales>Coriobacteriaceae	GCF_004135645.1	Senegalimassilia_faecalis	ET524_RS02380	534170..537787	2509433	BREX-system-P-loop-protein-BrxC-[Senegalimassilia-faecalis]
WP_154515691.1	Type1.BREX	[illegible]	1219	Bacteria>Terrabacteria_group>Actinomycetota>Coriobacteriia>Coriobacteriales>Coriobacteriaceae	GCF_009696325.1	unclassified_Collinsella	FYJ40_RS02790	33433..37092	2637548	MULTISPECIES:-BREX-system-P-loop-protein-BrxC-[unclassified-Collinsella]
WP_204559883.1	Type1.BREX	[illegible]	1171	Bacteria>Terrabacteria_group>Actinomycetota>Coriobacteriia>Coriobacteriales>Coriobacteriaceae	GCF_016902615.1	Collinsella_tanakaei	H6A21_RS01790	55502..59017	626935	BREX-system-P-loop-protein-BrxC-[Collinsella-tanakaei]
WP_215694813.1	Type1.BREX	[illegible]	1220	Bacteria>Terrabacteria_group>Actinomycetota>Coriobacteriia>Coriobacteriales>Coriobacteriaceae	GCF_018785265.1	Collinsella_aerofaciens	GPK42_RS08810	321305..324967	74426	BREX-system-P-loop-protein-BrxC-[Collinsella-aerofaciens]
WP_270471419.1	Type1.BREX	[illegible]	1184	Bacteria>Terrabacteria_group>Actinomycetota>Coriobacteriia>Coriobacteriales>Coriobacteriaceae	GCF_027683425.1	Collinsella_intestinalis	PGX73_RS02685	38070..41624	147207	BREX-system-P-loop-protein-BrxC-[Collinsella-intestinalis]
WP_271743294.1	Type1.BREX	[illegible]	1220	Bacteria>Terrabacteria_group>Actinomycetota>Coriobacteriia>Coriobacteriales>Coriobacteriaceae	GCF_028206795.1	Collinsella_aerofaciens	PMX70_RS07965	7863..11525	74426	BREX-system-P-loop-protein-BrxC-[Collinsella-aerofaciens]
WP_271729196.1	Type1.BREX	[illegible]	1220	Bacteria>Terrabacteria_group>Actinomycetota>Coriobacteriia>Coriobacteriales>Coriobacteriaceae	GCF_028206955.1	Collinsella_aerofaciens	PMX73_RS03570	36978..40640	74426	BREX-system-P-loop-protein-BrxC-[Collinsella-aerofaciens]
WP_196032768.1	Type1.BREX	[illegible]	1196	Bacteria>Terrabacteria_group>Actinomycetota>Coriobacteriia>Coriobacteriales>Coriobacteriaceae	GCF_028209425.1	Collinsella_aerofaciens	PMX18_RS06205	35659..39249	74426	BREX-system-P-loop-protein-BrxC-[Collinsella-aerofaciens]
WP_261981442.1	Type1.BREX	[illegible]	1191	Bacteria>Terrabacteria_group>Actinomycetota>Coriobacteriia>Coriobacteriales>Coriobacteriaceae	GCF_901212495.1	Collinsella_aerofaciens	SFLMC1_RS10875	26184..29759	74426	BREX-system-P-loop-protein-BrxC-[Collinsella-aerofaciens]
WP_118094641.1	Type1.BREX	[illegible]	1220	Bacteria>Terrabacteria_group>Actinomycetota>Coriobacteriia>Coriobacteriales>Coriobacteriaceae	GCF_902364885.1	Collinsella	FY305_RS06610	68794..72456	102106	MULTISPECIES:-BREX-system-P-loop-protein-BrxC-[Collinsella]
WP_226814226.1	Type1.BREX	[illegible]	1191	Bacteria>Terrabacteria_group>Actinomycetota>Coriobacteriia>Coriobacteriales>Coriobacteriaceae	GCF_902501535.1	Collinsella_aerofaciens	GDK10_RS03455	524536..528111	74426	BREX-system-P-loop-protein-BrxC-[Collinsella-aerofaciens]
WP_136846141.1	Type1.BREX	[illegible]	1192	Bacteria>Terrabacteria_group>Actinomycetota>Coriobacteriia>Coriobacteriales>Coriobacteriaceae	GCF_948466405.1	Parvibacter_caecicola	E5982_RS08750	41748..45326	747645	BREX-system-P-loop-protein-BrxC-[Parvibacter-caecicola]
HIT45392.1	Type1.BREX	[illegible]	1225	Bacteria>Terrabacteria_group>Actinomycetota>Coriobacteriia>Coriobacteriia_incertae_sedis>Candidatus_Aphodovivens	GCA_018715935.1	Candidatus_Aphodovivens_excrementavium	IAC28_04775	5578..9255	2840682	BREX-system-P-loop-protein-BrxC-[Candidatus-Aphodovivens-excrementavium]
CDB32945.1	Type1.BREX	[illegible]	819	Bacteria>Terrabacteria_group>Actinomycetota>Coriobacteriia>Eggerthellales>Eggerthellaceae	GCA_000436075.1	Eggerthella_sp._CAG:209	BN534_00740	18377..20836	1262875	uncharacterized-protein-BN534_00740-[Eggerthella-sp.-CAG:209]
MCI9261625.1	Type1.BREX	[illegible]	1223	Bacteria>Terrabacteria_group>Actinomycetota>Coriobacteriia>Eggerthellales>Eggerthellaceae	GCA_022798975.1	Eggerthellaceae_bacterium	HFJ71_04015	1625..5296	1972561	BREX-system-P-loop-protein-BrxC-[Eggerthellaceae-bacterium]
WP_160709085.1	Type1.BREX	[illegible]	1222	Bacteria>Terrabacteria_group>Actinomycetota>Coriobacteriia>Eggerthellales>Eggerthellaceae	GCF_009911695.1	Enterorhabdus_sp._P55	D1643_RS04550	2737..6405	2304571	BREX-system-P-loop-protein-BrxC-[Enterorhabdus-sp.-P55]
WP_165048948.1	Type1.BREX	[illegible]	1188	Bacteria>Terrabacteria_group>Actinomycetota>Coriobacteriia>Eggerthellales>Eggerthellaceae	GCF_011038975.1	unclassified_Adlercreutzia	G7Y50_RS02135	57415..60981	2636013	MULTISPECIES:-BREX-system-P-loop-protein-BrxC-[unclassified-Adlercreutzia]
WP_195347157.1	Type1.BREX	[illegible]	1201	Bacteria>Terrabacteria_group>Actinomycetota>Coriobacteriia>Eggerthellales>Eggerthellaceae	GCF_028209285.1	Eggerthella_lenta	PMV52_RS07270	251002..254607	84112	unnamed-protein-product-[Eggerthella-lenta]
WP_251231669.1	Type1.BREX	[illegible]	1213	Bacteria>Terrabacteria_group>Actinomycetota>Coriobacteriia>Eggerthellales>Eggerthellaceae	GCF_943193145.1	Adlercreutzia_aquisgranensis	NFR41_RS09570	19774..23415	2941323	BREX-system-P-loop-protein-BrxC-[Adlercreutzia-aquisgranensis]
WP_227070741.1	Type1.BREX	[illegible]	1199	Bacteria>Terrabacteria_group>Actinomycetota>Coriobacteriia>Eggerthellales	GCF_015551405.1	Eggerthellaceae	I2D50_RS06370	44195..47794	1643826	MULTISPECIES:-BREX-system-P-loop-protein-BrxC-[Eggerthellaceae]
MBA3474041.1	Type1.BREX	[illegible]	1219	Bacteria>Terrabacteria_group>Actinomycetota>Rubrobacteria>Rubrobacterales>Rubrobacteraceae	GCA_013813465.1	Rubrobacter_sp.	HOT57_12585	3865..7524	2036010	BREX-system-P-loop-protein-BrxC-[Rubrobacter-sp.]
MCL6447596.1	Type1.BREX	[illegible]	1222	Bacteria>Terrabacteria_group>Armatimonadota>unclassified_Armatimonadota	GCA_023511535.1	Armatimonadota_bacterium	K6U04_05490	13164..16832	2033014	BREX-system-P-loop-protein-BrxC-[Armatimonadota-bacterium]
MCI1589808.1	Type1.BREX	[illegible]	1190	Bacteria>Terrabacteria_group>Bacillota>Bacilli>Bacillales>Bacillaceae	GCA_022646685.1	Heyndrickxia_oleronia	LKH79_04555	33937..37509	38875	BREX-system-P-loop-protein-BrxC-[Heyndrickxia-oleronia]
WP_031537547.1	Type1.BREX	[illegible]	1187	Bacteria>Terrabacteria_group>Bacillota>Bacilli>Bacillales>Bacillaceae	GCF_000701605.1	Bacillus_sp._MB2021	T349_RS0112475	16440..20003	1408303	BREX-system-P-loop-protein-BrxC-[Bacillus-sp.-MB2021]
WP_048824801.1	Type1.BREX	[illegible]	1190	Bacteria>Terrabacteria_group>Bacillota>Bacilli>Bacillales>Bacillaceae	GCF_000821085.1	Bacillus_sp._B-jedd	BN1002_01993	1962591..1966163	1476857	BREX-system-P-loop-protein-BrxC-[Bacillus-sp.-B-jedd]
WP_062353349.1	Type1.BREX	[illegible]	1190	Bacteria>Terrabacteria_group>Bacillota>Bacilli>Bacillales>Bacillaceae	GCF_001375515.1	Bacillus_kwashiorkori	BN1242_RS07000	23447..27019	1522318	BREX-system-P-loop-protein-BrxC-[Bacillus-kwashiorkori]
WP_108713788.1	Type1.BREX	[illegible]	1191	Bacteria>Terrabacteria_group>Bacillota>Bacilli>Bacillales>Bacillaceae	GCF_003073375.1	unclassified_Lysinibacillus	DCE79_RS14920	3050129..3053704	2636778	MULTISPECIES:-BREX-system-P-loop-protein-BrxC-[unclassified-Lysinibacillus]
WP_114371647.1	Type1.BREX	[illegible]	1205	Bacteria>Terrabacteria_group>Bacillota>Bacilli>Bacillales>Bacillaceae	GCF_003336365.1	Salicibibacter_kimchii	DT065_05890	1163447..1167064	2099786	BREX-system-P-loop-protein-BrxC-[Salicibibacter-kimchii]
WP_126293910.1	Type1.BREX	[illegible]	1190	Bacteria>Terrabacteria_group>Bacillota>Bacilli>Bacillales>Bacillaceae	GCF_003966145.1	Lysinibacillus_telephonicus	EKG35_RS07955	47066..50638	1714840	BREX-system-P-loop-protein-BrxC-[Lysinibacillus-telephonicus]
WP_144538762.1	Type1.BREX	[illegible]	1191	Bacteria>Terrabacteria_group>Bacillota>Bacilli>Bacillales>Bacillaceae	GCF_007677425.1	Bacillus_sp._FJAT-22090	FS503_RS11600	136183..139758	1581038	BREX-system-P-loop-protein-BrxC-[Bacillus-sp.-FJAT-22090]
WP_216486703.1	Type1.BREX	[illegible]	1194	Bacteria>Terrabacteria_group>Bacillota>Bacilli>Bacillales>Bacillaceae	GCF_018918075.1	Virgibacillus_sp._MSJ-26	KQI49_RS03110	240564..244148	2841522	BREX-system-P-loop-protein-BrxC-[Virgibacillus-sp.-MSJ-26]
WP_220158113.1	Type1.BREX	[illegible]	1190	Bacteria>Terrabacteria_group>Bacillota>Bacilli>Bacillales>Bacillaceae	GCF_019459225.1	Bacillus_sp._IITD106	KOH71_20660	11529..15101	2864214	BREX-system-P-loop-protein-BrxC-[Bacillus-sp.-IITD106]
WP_259548277.1	Type1.BREX	[illegible]	1187	Bacteria>Terrabacteria_group>Bacillota>Bacilli>Bacillales>Bacillaceae	GCF_024971915.1	Heyndrickxia_oleronia	KXF32_RS03880	741080..744643	38875	BREX-system-P-loop-protein-BrxC-[Heyndrickxia-oleronia]
WP_131470342.1	Type1.BREX	[illegible]	1188	Bacteria>Terrabacteria_group>Bacillota>Bacilli>Bacillales>Bacillales_incertae_sedis	GCF_004337105.1	Exiguobacterium_sp._SH3S1	EVJ26_RS09655	119198..122764	2510955	BREX-system-P-loop-protein-BrxC-[Exiguobacterium-sp.-SH3S1]
WP_214711985.1	Type1.BREX	[illegible]	1188	Bacteria>Terrabacteria_group>Bacillota>Bacilli>Bacillales>Bacillales_incertae_sedis	GCF_018617985.1	Exiguobacterium_sp._s55	KNP09_RS10355	38338..41904	2751245	BREX-system-P-loop-protein-BrxC-[Exiguobacterium-sp.-s55]
WP_148456431.1	Type1.BREX	[illegible]	1206	Bacteria>Terrabacteria_group>Bacillota>Bacilli>Bacillales>Paenibacillaceae	GCF_008084145.1	Paenibacillus_faecis	FRY98_RS22745	231357..234977	862114	BREX-system-P-loop-protein-BrxC-[Paenibacillus-faecis]
WP_212938911.1	Type1.BREX	[illegible]	1206	Bacteria>Terrabacteria_group>Bacillota>Bacilli>Bacillales>Paenibacillaceae	-	Paenibacillus_antibioticophila	KI368_RS07085	201985..205605	1274374	BREX-system-P-loop-protein-BrxC-[Paenibacillus-antibioticophila]
PIC94624.1	Type1.BREX	[illegible]	1186	Bacteria>Terrabacteria_group>Bacillota>Bacilli>Bacillales>Planococcaceae	GCA_002743245.1	Sporosarcina_sp._P26b	CSV69_15785	3496..7056	2048253	hypothetical-protein-CSV69_15785-[Sporosarcina-sp.-P26b]
WP_233192413.1	Type1.BREX	[illegible]	1188	Bacteria>Terrabacteria_group>Bacillota>Bacilli>Bacillales>Planococcaceae	GCF_002743245.1	Sporosarcina_sp._P26b	CSV69_RS15805	3490..7056	2048253	BREX-system-P-loop-protein-BrxC-[Sporosarcina-sp.-P26b]
WP_010024517.1	Type1.BREX	[illegible]	1189	Bacteria>Terrabacteria_group>Bacillota>Bacilli>Bacillales>Sporolactobacillaceae	GCF_000222445.2	Sporolactobacillus_inulinus	SINU_RS07430	4602..8171	2078	BREX-system-P-loop-protein-BrxC-[Sporolactobacillus-inulinus]
HJF20832.1	Type1.BREX	[illegible]	1239	Bacteria>Terrabacteria_group>Bacillota>Bacilli>Bacillales>Staphylococcaceae	GCA_020742605.1	Mammaliicoccus_lentus	K8U93_01665	12906..16625	42858	BREX-system-P-loop-protein-BrxC-[Mammaliicoccus-lentus]

Protein-ID	System	Gene-Neighbourhoods	Length	Taxonomy	Assembly-ID	Strain	Locus-Tag	Genomic-coordinates	TaxID	Fasta-defline
MBK0347250.1	Type1.BREX	HTH_36->?->HTH_26->BrxA-DNA-binding->BrxB-BrxF-iSTAND->DUF6079*->Schlafen+HATPase+wHTH->N6-METHYLASE->HEPN->PglZ->wHTH+WYL+WCX->?->ParA-Soj-PloopNTPase-><-DEOR-HTH	1198	Bacteria>Terrabacteria_group>Bacillota>Bacilli>Lactobacillales>Aerococcaceae	GCA_016522115.1	Aerococcaceae_bacterium_zg-1578	JDW15_01195	55878..59474	2796350	BREX-system-P-loop-protein-BrxC-[Aerococcaceae-bacterium-zg-1578]
WP_274731953.1	Type1.BREX	LACI-HTH+PBPI->?->HEPN->HTH_26->BrxA-DNA-binding->BrxB-BrxF-iSTAND->DUF6079*->N6-METHYLASE-p->	1193	Bacteria>Terrabacteria_group>Bacillota>Bacilli>Lactobacillales>Carnobacteriaceae	GCF_028863945.1	Jeotgalibaca_sp._YN-L-12	PVE40_RS10850	8874..12455	3028623	BREX-system-P-loop-protein-BrxC-[Jeotgalibaca-sp.-YN-L-12]
TXJ87453.1	Type1.BREX	HDRnase->CHC2+TOPRIM-DNAG->SIGMA-HTH+SIGMA-HTH+SIGMA-HTH->SIGMA-HTH+SIGMA-HTH->||HAD+HAD->||?->BrxA-DNA-binding->BrxB-BrxF-iSTAND->||DUF6079-p*->N6-METHYLASE->SIGMA+OB-Fold+AAA-ATPase+Lon-Protease-BrxL->HEPN->	917	Bacteria>Terrabacteria_group>Bacillota>Bacilli>Lactobacillales>Lactobacillaceae	GCA_008016355.1	Lactobacillus_delbrueckii	FU655_07470	24596..27349	1584	BREX-system-P-loop-protein-BrxC-[Lactobacillus-delbrueckii]
MBT8803912.1	Type1.BREX	ParB+HNH->BrxA-DNA-binding->BrxB-BrxF-iSTAND->DUF6079*->N6-METHYLASE->PglZ->SIGMA+OB-Fold+AAA-ATPase+Lon-Protease-BrxL->HEPN->?->O6MGT-HTH-><-?<-NUDIX||METHYLASE->	1178	Bacteria>Terrabacteria_group>Bacillota>Bacilli>Lactobacillales>Lactobacillaceae	GCA_018719835.1	Lactobacillus_delbrueckii_subsp._bulgaricus	BTJ20_08000	3864..7400	1585	ATPase-[Lactobacillus-delbrueckii-subsp.-bulgaricus]
MBT8901771.1	Type1.BREX	<-METHYLASE||NUDIX->?->||<-AbiGi<-HEPN<-SIGMA+OB-Fold+AAA-ATPase+Lon-Protease-BrxL<-PglZ<-N6-METHYLASE<-DUF6079*<-BrxB-BrxF-iSTAND<-BrxA-DNA-binding<-ParB+HNH<-?||<-HAD+HAD<-SIGMA-HTH+SIGMA-HTH<-SIGMA-HTH+SIGMA-HTH	1178	Bacteria>Terrabacteria_group>Bacillota>Bacilli>Lactobacillales>Lactobacillaceae	GCA_018720695.1	Lactobacillus_delbrueckii_subsp._bulgaricus	BTI52_06840	21471..25007	1585	ATPase-[Lactobacillus-delbrueckii-subsp.-bulgaricus]
MBT8914213.1	Type1.BREX	ParB+HNH->BrxA-DNA-binding->BrxB-BrxF-iSTAND->DUF6079*->N6-METHYLASE->PglZ->SIGMA+OB-Fold+AAA-ATPase+Lon-Protease-BrxL->HEPN->	1178	Bacteria>Terrabacteria_group>Bacillota>Bacilli>Lactobacillales>Lactobacillaceae	GCA_018720815.1	Lactobacillus_delbrueckii_subsp._bulgaricus	BTI61_07605	4087..7623	1585	ATPase-[Lactobacillus-delbrueckii-subsp.-bulgaricus]
MCT7861392.1	Type1.BREX	<-PglZ<-HEPN<-?<-?<-N6-METHYLASE<-?<-DUF6079*<-BrxB-BrxF-iSTAND<-BrxA-DNA-binding	1165	Bacteria>Terrabacteria_group>Bacillota>Bacilli>Lactobacillales>Lactobacillaceae	GCA_025347225.1	Lactobacillus_crispatus	N4Q24_00945	8507..12000	47770	BREX-system-P-loop-protein-BrxC-[Lactobacillus-crispatus]
WP_025283578.1	Type1.BREX	ASCH->Glyoxalase->Glyoxalase->ParB+HNH->BrxA-DNA-binding->BrxB-BrxF-iSTAND->DUF6079*->N6-METHYLASE->HEPN->HEPN->PglZ-><-?||DDE_Tnp_1-><-Calcineurin<-Peptidase_M13_N+Peptidase_M13	1243	Bacteria>Terrabacteria_group>Bacillota>Bacilli>Lactobacillales>Lactobacillaceae	GCF_000525715.1	Lactobacillus_helveticus	LBH_0939	971853..975584	1587	BREX-system-P-loop-protein-BrxC-[Lactobacillus-helveticus]
WP_060598044.1	Type1.BREX	<-METHYLASE||NUDIX->?-><-AraC-HTH+O6MGT-HTH<-?<-HEPN<-SIGMA+OB-Fold+AAA-ATPase+Lon-Protease-BrxL<-PglZ<-N6-METHYLASE<-DUF6079*<-BrxB-BrxF-iSTAND<-BrxA-DNA-binding<-ParB+HNH<-?<-?||<-HAD+HAD||<-SIGMA-HTH+SIGMA-HTH<-SIGMA-HTH+SIGMA-HTH+SIGMA-HTH<-SIGMA-HTH+SIGMA-HTH+SIGMA-HTH	1178	Bacteria>Terrabacteria_group>Bacillota>Bacilli>Lactobacillales>Lactobacillaceae	GCF_001469775.1	Lactobacillus_delbrueckii	AT236_RS05345	1068532..1072068	1584	BREX-system-P-loop-protein-BrxC-[Lactobacillus-delbrueckii]
WP_086355469.1	Type1.BREX	LZ_Tnp_IS66+zf-IS66+DDE_Tnp_IS66+DDE_Tnp_IS66_C-><-AbiGi<-?<-HEPN<-SIGMA+OB-Fold+AAA-ATPase+Lon-Protease-BrxL<-PglZ<-N6-METHYLASE<-DUF6079*<-BrxB-BrxF-iSTAND<-BrxA-DNA-binding<-ParB+HNH||<-ParB+HNH<-?<-?<-REase+N6_METHYLASE<-ABhydrolase+ABhydrolase<-ABhydrolase	1183	Bacteria>Terrabacteria_group>Bacillota>Bacilli>Lactobacillales>Lactobacillaceae	GCF_002142575.1	Lactobacillus_delbrueckii	B9N98_RS00590	112113..115664	1584	BREX-system-P-loop-protein-BrxC-[Lactobacillus-delbrueckii]
WP_087363710.1	Type1.BREX	Peptidase_M13_N+Peptidase_M13->Acetate_kinase-><-TerBN+TPR<-PglZ<-HEPN<-HEPN<-N6-METHYLASE<-DUF6079*<-BrxB-BrxF-iSTAND<-BrxA-DNA-binding<-ParB+HNH<-?<-OrfB_Zn_ribbon	1244	Bacteria>Terrabacteria_group>Bacillota>Bacilli>Lactobacillales>Lactobacillaceae	GCF_002160975.1	Lactobacillus_gallinarum	B5E63_RS08425	13791..17525	52242	BREX-system-P-loop-protein-BrxC-[Lactobacillus-gallinarum]
WP_113532397.1	Type1.BREX	YTCD-HTH-><-?<-?<-TetR-HTH<-TetR-HTH<-PglZ<-HEPN+REase<-nSTAND3<-N6-METHYLASE<-DUF6079*<-BrxB-BrxF-iSTAND<-BrxA-DNA-binding<-?<-?<-?<-NUDIX<-HAD+HAD	1239	Bacteria>Terrabacteria_group>Bacillota>Bacilli>Lactobacillales>Lactobacillaceae	GCF_003584685.1	Lactobacillus_paragasseri	LpgJCM5343_RS05125	1058950..1062669	2107999	BREX-system-P-loop-protein-BrxC-[Lactobacillus-paragasseri]
WP_172987666.1	Type1.BREX	ParB+HNH->BrxA-DNA-binding->BrxB-BrxF-iSTAND->DUF6079*->N6-METHYLASE->HEPN->HEPN->PglZ->TerBN+TPR->ParB->ParB+HNH->	1243	Bacteria>Terrabacteria_group>Bacillota>Bacilli>Lactobacillales>Lactobacillaceae	GCF_013280955.1	Lactobacillus_helveticus	IMAU50022_RS01295	3445..7176	1587	BREX-system-P-loop-protein-BrxC-[Lactobacillus-helveticus]
WP_173006741.1	Type1.BREX	Peptidase_M13_N+Peptidase_M13->?-><-TerBN+TPR<-PglZ<-HEPN<-HEPN<-N6-METHYLASE<-DUF6079*<-BrxB-BrxF-iSTAND<-BrxA-DNA-binding<-ParB+HNH||MODE-HTH+PBPII<-?<-?<-wHTH+WYL	1241	Bacteria>Terrabacteria_group>Bacillota>Bacilli>Lactobacillales>Lactobacillaceae	GCF_013281475.1	Lactobacillus_helveticus	IMAU30014_RS01855	25052..28777	1587	BREX-system-P-loop-protein-BrxC-[Lactobacillus-helveticus]
WP_220735234.1	Type1.BREX	Calcineurin-><-?||?->?->?-><-?||BrxA-DNA-binding->BrxB-BrxF-iSTAND->DUF6079*->N6-METHYLASE->HEPN->HEPN-><-Phage_int_SAM_5+Phage_integrase<-N6-METHYLASE||PglZ->SIGMA+OB-Fold+AAA-ATPase+Lon-Protease-BrxL->	1212	Bacteria>Terrabacteria_group>Bacillota>Bacilli>Lactobacillales>Lactobacillaceae	GCF_019656015.1	Leuconostoc_inhae	K5Q14_RS07795	32108..35746	178001	BREX-system-P-loop-protein-BrxC-[Leuconostoc-inhae]
WP_223660616.1	Type1.BREX	BrxA-DNA-binding->BrxB-BrxF-iSTAND->DUF6079*->N6-METHYLASE->nSTAND3->HEPN-p->PglZ->TetR-HTH->?-><-?||MORC+DNA_gyrase+TOPRIM->	1239	Bacteria>Terrabacteria_group>Bacillota>Bacilli>Lactobacillales>Lactobacillaceae	GCF_020026535.1	Lactobacillus_johnsonii	K7D69_RS01700	3964..7683	33959	BREX-system-P-loop-protein-BrxC-[Lactobacillus-johnsonii]
WP_231523637.1	Type1.BREX	GGDEF+PAS-><-ABhydrolase||ABhydrolase->ABhydrolase+ABhydrolase->ParB+HNH->||ParB+HNH->ParB+HNH->BrxA-DNA-binding->BrxB-BrxF-iSTAND->DUF6079*->N6-METHYLASE->PglZ->SIGMA+OB-Fold+AAA-ATPase+Lon-Protease-BrxL->HEPN->AbiGi-><-AraC-HTH+AraC-HTH||?->AraC-HTH+O6MGT-HTH->	1183	Bacteria>Terrabacteria_group>Bacillota>Bacilli>Lactobacillales>Lactobacillaceae	GCF_021134645.1	Lactobacillus_delbrueckii	LOB39_RS07020	33020..36571	1584	BREX-system-P-loop-protein-BrxC-[Lactobacillus-delbrueckii]
WP_236153484.1	Type1.BREX	ABhydrolase->ABhydrolase+ABhydrolase->?->OrfB_Zn_ribbon->HNH->ParB+HNH->ParB+HNH->BrxA-DNA-binding->BrxB-BrxF-iSTAND->DUF6079*->N6-METHYLASE->PglZ->SIGMA+OB-Fold+AAA-ATPase+Lon-Protease-BrxL->HEPN->AbiGi-><-AraC-HTH+AraC-HTH	1183	Bacteria>Terrabacteria_group>Bacillota>Bacilli>Lactobacillales>Lactobacillaceae	GCF_021600265.1	Lactobacillus_delbrueckii	ME802_14840	21460..25011	1584	BREX-system-P-loop-protein-BrxC-[Lactobacillus-delbrueckii]
WP_236149793.1	Type1.BREX	ABhydrolase->ABhydrolase+ABhydrolase->REase+N6_METHYLASE->?->?->ParB+HNH-p->ParB+HNH->BrxA-DNA-binding->BrxB-BrxF-iSTAND->DUF6079*->N6-METHYLASE->PglZ->SIGMA+OB-Fold+AAA-ATPase+Lon-Protease-BrxL->HEPN->AbiGi-><-AraC-HTH+AraC-HTH	1188	Bacteria>Terrabacteria_group>Bacillota>Bacilli>Lactobacillales>Lactobacillaceae	GCF_021600925.1	Lactobacillus_delbrueckii	L5981_RS02870	31328..34894	1584	BREX-system-P-loop-protein-BrxC-[Lactobacillus-delbrueckii]
WP_050952512.1	Type1.BREX	<-AraC-HTH+O6MGT-HTH<-?||AraC-HTH+AraC-HTH-><-AbiGi<-HEPN<-SIGMA+OB-Fold+AAA-ATPase+Lon-Protease-BrxL<-PglZ<-N6-METHYLASE<-DUF6079*<-BrxB-BrxF-iSTAND<-BrxA-DNA-binding<-ParB+HNH<-ParB+HNH-p<-?<-?<-REase+N6_METHYLASE<-ABhydrolase+ABhydrolase<-ABhydrolase	1188	Bacteria>Terrabacteria_group>Bacillota>Bacilli>Lactobacillales>Lactobacillaceae	GCF_021601025.1	Lactobacillus_delbrueckii	L5988_RS00790	158168..161734	1584	BREX-system-P-loop-protein-BrxC-[Lactobacillus-delbrueckii]
WP_268886389.1	Type1.BREX	<-AraC-HTH+O6MGT-HTH<-?||AraC-HTH+AraC-HTH-><-AbiGi<-HEPN<-SIGMA+OB-Fold+AAA-ATPase+Lon-Protease-BrxL<-PglZ<-N6-METHYLASE<-DUF6079*<-BrxB-BrxF-iSTAND<-BrxA-DNA-binding<-ParB+HNH<-ParB+HNH||<-ParB+HNH<-ABhydrolase+ABhydrolase<-ABhydrolase||ABhydrolase-><-GGDEF+PAS	1183	Bacteria>Terrabacteria_group>Bacillota>Bacilli>Lactobacillales>Lactobacillaceae	GCF_026891475.1	Lactobacillus_delbrueckii	O0549_RS05810	14866..18417	1584	BREX-system-P-loop-protein-BrxC-[Lactobacillus-delbrueckii]
WP_270447891.1	Type1.BREX	ABhydrolase+ABhydrolase-><-?||?->?->?->HTH+REase->BrxA-DNA-binding->BrxB-BrxF-iSTAND->DUF6079*->N6-METHYLASE->PglZ->SIGMA+OB-Fold+AAA-ATPase+Lon-Protease-BrxL->HEPN->HEPN->SF1-SF2-Helicase->?-><-AraC-HTH	1188	Bacteria>Terrabacteria_group>Bacillota>Bacilli>Lactobacillales>Lactobacillaceae	GCF_027681365.1	Lactobacillus_delbrueckii	PGX26_RS03415	69740..73306	1584	BREX-system-P-loop-protein-BrxC-[Lactobacillus-delbrueckii]
WP_271018243.1	Type1.BREX	BrxA-DNA-binding->BrxB-BrxF-iSTAND->DUF6079*->N6-METHYLASE->PglZ->SIGMA+OB-Fold+AAA-ATPase+Lon-Protease-BrxL->HEPN->HEPN->AbiGi-><-AraC-HTH+AraC-HTH||?->AraC-HTH+O6MGT-HTH->	1183	Bacteria>Terrabacteria_group>Bacillota>Bacilli>Lactobacillales>Lactobacillaceae	GCF_027858035.1	Lactobacillus_delbrueckii	PF599_RS07175	1642..5193	1584	BREX-system-P-loop-protein-BrxC-[Lactobacillus-delbrueckii]
WP_271873858.1	Type1.BREX	<-TerBN+TPR<-PglZ<-HEPN<-HEPN<-N6-METHYLASE<-DUF6079*<-BrxB-BrxF-iSTAND<-BrxA-DNA-binding<-ParB+HNH<-?<-?<-AAA_33	1244	Bacteria>Terrabacteria_group>Bacillota>Bacilli>Lactobacillales>Lactobacillaceae	GCF_028308865.1	Lactobacillus_amylovorus	ODV08_RS03455	54390..58124	1604	BREX-system-P-loop-protein-BrxC-[Lactobacillus-amylovorus]
WP_271897403.1	Type1.BREX	<-HIN-HTH+PAIREDC-HTH||?->CR-ATPase5+REase-><-TerBN+TPR<-PglZ<-HEPN<-HEPN<-N6-METHYLASE<-DUF6079*<-BrxB-BrxF-iSTAND<-BrxA-DNA-binding<-ParB+HNH<-?<-?<-AAA_33	1233	Bacteria>Terrabacteria_group>Bacillota>Bacilli>Lactobacillales>Lactobacillaceae	GCF_028308945.1	Lactobacillus_amylovorus	ODV12_RS07245	16097..19798	1604	BREX-system-P-loop-protein-BrxC-[Lactobacillus-amylovorus]
WP_064081740.1	Type1.BREX	ParB+HNH->BrxA-DNA-binding->BrxB-BrxF-iSTAND->DUF6079*->N6-METHYLASE->PglZ->SIGMA+OB-Fold+AAA-ATPase+Lon-Protease-BrxL->HEPN->?->?->O6MGT-HTH-><-?<-NUDIX	1178	Bacteria>Terrabacteria_group>Bacillota>Bacilli>Lactobacillales>Lactobacillaceae	GCF_925300675.1	Lactobacillus_delbrueckii	NGL01_RS07850	3981..7517	1584	BREX-system-P-loop-protein-BrxC-[Lactobacillus-delbrueckii]
WP_118669593.1	Type1.BREX	<-SIGMA+OB-Fold+AAA-ATPase+Lon-Protease-BrxL<-OLD-ABC+TOPRIM<-PglZ<-Schlafen+HATPase+wHTH<-HEPN<-TIR<-N6-METHYLASE<-DUF6079*<-BrxB-BrxF-iSTAND<-BrxA-DNA-binding<-HTH_26	1196	Bacteria>Terrabacteria_group>Bacillota>Clostridia>Eubacteriales>Clostridiaceae	-	Clostridium_sp._TF08-15	DXC82_15795	32814..36404	2293052	BREX-system-P-loop-protein-BrxC-[Clostridium-sp.-TF08-15]
CCZ63532.1	Type1.BREX	HTH_26->BrxA-DNA-binding->BrxB-BrxF-iSTAND->DUF6079*->N6-METHYLASE->PglZ->SIGMA+OB-Fold+AAA-ATPase+Lon-Protease-BrxL->HEPN->?->?->NUDIX->?->HTH+HisKin->	1215	Bacteria>Terrabacteria_group>Bacillota>Clostridia>Eubacteriales>Clostridiaceae	GCA_000433395.1	Clostridium_hathewayi_CAG:224	BN544_01435	55563..59210	1263067	putative-uncharacterized-protein-[Clostridium-hathewayi-CAG:224]
CDD75188.1	Type1.BREX	MACRODOMAIN->?->PIN->ZnR+ZnR+ZnR->HTH_26->BrxA-DNA-binding->BrxB-BrxF-iSTAND->DUF6079*->OLD-ABC->PIN->N6-METHYLASE->OLD-ABC->NERD->HEPN->Schlafen+HATPase+wHTH->PglZ->DUF4365+REase->	1200	Bacteria>Terrabacteria_group>Bacillota>Clostridia>Eubacteriales>Clostridiaceae	GCA_000436175.1	Clostridium_sp._CAG:62	BN737_01375	69547..73149	1262828	uncharacterized-protein-BN737_01375-[Clostridium-sp.-CAG:62]
RGF28780.1	Type1.BREX	<-OLD-ABC+TOPRIM<-PglZ<-Schlafen+HATPase+wHTH<-HEPN<-N6-METHYLASE<-DUF6079*<-BrxB-BrxF-iSTAND<-BrxA-DNA-binding<-HTH_26	1196	Bacteria>Terrabacteria_group>Bacillota>Clostridia>Eubacteriales>Clostridiaceae	GCA_003435925.1	Clostridium_sp._AF46-9NS	DW081_14990	34449..38039	2293020	BREX-system-P-loop-protein-BrxC-[Clostridium-sp.-AF46-9NS]
RHU61449.1	Type1.BREX	<-SIGMA+OB-Fold+AAA-ATPase+Lon-Protease-BrxL<-OLD-ABC+TOPRIM<-PglZ<-Schlafen+HATPase+wHTH<-HEPN<-TIR<-N6-METHYLASE<-DUF6079*<-BrxB-BrxF-iSTAND<-BrxA-DNA-binding<-HTH_26	1196	Bacteria>Terrabacteria_group>Bacillota>Clostridia>Eubacteriales>Clostridiaceae	GCA_003481275.1	Clostridium_sp._TF08-15	DXC82_15795	32814..36404	2293052	BREX-system-P-loop-protein-BrxC-[Clostridium-sp.-TF08-15]
MBS5781623.1	Type1.BREX	<-SMODS<-?<-SAVED<-SIGMA+OB-Fold+AAA-ATPase+Lon-Protease-BrxL<-PglZ<-HEPN<-N6-METHYLASE-p<-N6-METHYLASE-p<-N6-METHYLASE<-DUF6079*<-BrxB-BrxF-iSTAND<-BrxA-DNA-binding||?->?->?-><-?||XIS-HTH->ZnR+TOPRIM+AAA-helicase+wHTH->	1171	Bacteria>Terrabacteria_group>Bacillota>Clostridia>Eubacteriales>Clostridiaceae	GCA_018371215.1	Clostridium_sp.	KID04_01935	432250..435765	1506	BREX-system-P-loop-protein-BrxC-[Clostridium-sp.]
MCI9636406.1	Type1.BREX	<-SF1-SF2-Helicase+REase<-ParB<-?<-wHTH+WYL+WCX<-SIGMA+OB-Fold+AAA-ATPase+Lon-Protease-BrxL<-PglZ<-N6-METHYLASE-p<-HEPN<-N6-METHYLASE<-DUF6079*<-BrxB-BrxF-iSTAND<-BrxA-DNA-binding<-REase<-Sulfatase<-REC+AraC-HTH+AraC-HTH<-dCache_1+HAMP+HisKin+HATPase_c<-Sulfatase+DUF4976<-ABC-ATPase+SbcC	1182	Bacteria>Terrabacteria_group>Bacillota>Clostridia>Eubacteriales>Clostridiaceae	GCA_022802035.1	Hungatella_sp.	HFG77_08430	22407..25955	2613924	BREX-system-P-loop-protein-BrxC-[Hungatella-sp.]
WP_003411890.1	Type1.BREX	<-HEPN<-N6-METHYLASE<-DUF6079*<-BrxB-BrxF-iSTAND<-BrxA-DNA-binding<-?<-MORC||HTH_3-><-?<-Phage_integrase<-?<-Phage_integrase	1215	Bacteria>Terrabacteria_group>Bacillota>Clostridia>Eubacteriales>Clostridiaceae	GCF_000171115.1	Clostridium_butyricum	CBY_RS17320	4352..7999	1492	BREX-system-P-loop-protein-BrxC-[Clostridium-butyricum]
WP_002598576.1	Type1.BREX	<-CRISPR_Cas2<-Cas_Cas1<-?<-Cas9+HNH+RuvC<-N6-METHYLASE<-Ntox45<-HEPN<-DUF6079*<-BrxB-BrxF-iSTAND<-BrxA-DNA-binding<-?||TetR-HTH->?-><-DnaB+TnsC-ATPase<-?||HAD->	1208	Bacteria>Terrabacteria_group>Bacillota>Clostridia>Eubacteriales>Clostridiaceae	GCF_000371465.1	Clostridium_thermobutyricum	HMPREF1092_RS10115	2173864..2177490	29372	BREX-system-P-loop-protein-BrxC-[Clostridium-thermobutyricum]
WP_065077880.1	Type1.BREX	<-GNTR-HTH+UTRA||?->?->tRNA->HEPN->BrxA-DNA-binding->BrxB-BrxF-iSTAND->DUF6079*->N6-METHYLASE->	1188	Bacteria>Terrabacteria_group>Bacillota>Clostridia>Eubacteriales>Clostridiaceae	GCF_001675165.1	Clostridium_ragsdalei	CLRAG_RS07695	80363..83929	217158	BREX-system-P-loop-protein-BrxC-[Clostridium-ragsdalei]
WP_118731364.1	Type1.BREX	PALP->?->RVT->HTH_26->BrxA-DNA-binding->BrxB-BrxF-iSTAND->DUF6079*->N6-METHYLASE->HEPN->Schlafen+HATPase+wHTH->PglZ->OLD-ABC+TOPRIM->SIGMA+OB-Fold+AAA-ATPase+Lon-Protease-BrxL->	1196	Bacteria>Terrabacteria_group>Bacillota>Clostridia>Eubacteriales>Clostridiaceae	GCF_003480225.1	Clostridium_sp._AM34-9AC	DW835_RS14805	20266..23856	2293030	BREX-system-P-loop-protein-BrxC-[Clostridium-sp.-AM34-9AC]
WP_118742502.1	Type1.BREX	35exo+T7DNApol->?->?->?->HTH_26->BrxA-DNA-binding->BrxB-BrxF-iSTAND->DUF6079*->N6-METHYLASE->HEPN->Schlafen+HATPase+wHTH->PglZ->SIGMA+OB-Fold+AAA-ATPase+Lon-Protease-BrxL-><-MODE-HTH+PBPII	1182	Bacteria>Terrabacteria_group>Bacillota>Clostridia>Eubacteriales>Clostridiaceae	GCF_003480665.1	Clostridium_sp._AM28-20LB	DW739_RS01385	291245..294793	2293027	BREX-system-P-loop-protein-BrxC-[Clostridium-sp.-AM28-20LB]
WP_118744885.1	Type1.BREX	ACET->REase->?->?->?->?->HTH_26->BrxA-DNA-binding->BrxB-BrxF-iSTAND->DUF6079*->OLD-ABC->PIN->N6-METHYLASE->HEPN->?->OLD-ABC->HNH->?->TIR->	1203	Bacteria>Terrabacteria_group>Bacillota>Clostridia>Eubacteriales>Clostridiaceae	GCF_003480745.1	Clostridium_sp._AM27-31LB	DW721_RS03885	61324..64935	2293026	BREX-system-P-loop-protein-BrxC-[Clostridium-sp.-AM27-31LB]
WP_164950923.1	Type5.BREX	<-GDResolvase<-?<-?<-TUDOR+SF1-SF2-Helicase+iREase+CTD+REase<-PglZ<-TOPRIM<-OLD-ABC<-HEPN<-N6-METHYLASE<-DUF6079*<-BrxB-BrxF-iSTAND<-BrxA-DNA-binding<-?<-Calcineurin<-PARG+MACRODOMAIN<-Calcineurin<-HAD<-LRR+LRR	1191	Bacteria>Terrabacteria_group>Bacillota>Clostridia>Eubacteriales>Clostridiaceae	GCF_011065955.2	Clostridium_sp._FP2	G9F71_RS16320	3398773..3402348	2724481	BREX-system-P-loop-protein-BrxC-[Clostridium-sp.-FP2]
WP_164949146.1	Type1.BREX	<-HEPN<-SIGMA+OB-Fold+AAA-ATPase+Lon-Protease-BrxL<-PglZ<-HNH<-OLD-ABC<-N6-METHYLASE<-DUF6079*<-BrxB-BrxF-iSTAND<-BrxA-DNA-binding<-?<-CR-ATPase8+REase||?->?-><-?<-?||METHYLASE->	1215	Bacteria>Terrabacteria_group>Bacillota>Clostridia>Eubacteriales>Clostridiaceae	GCF_011065955.2	Clostridium_sp._FP2	G9F71_RS17965	3736090..3739737	2724481	BREX-system-P-loop-protein-BrxC-[Clostridium-sp.-FP2]
WP_077854163.1	Type1.BREX	<-HAD+HAD<-?<-Peptidase_M42<-HEPN<-SIGMA+OB-Fold+AAA-ATPase+Lon-Protease-BrxL<-?<-PglZ<-N6-METHYLASE<-DUF6079*<-BrxB-BrxF-iSTAND<-BrxA-DNA-binding<-MORC||HTH_3-><-?<-Phage_integrase<-?<-Phage_integrase<-Aminotran_1_2	1216	Bacteria>Terrabacteria_group>Bacillota>Clostridia>Eubacteriales>Clostridiaceae	GCF_013297045.1	Clostridium_beijerinckii	BCD95_RS19475	4461966..4465616	1520	BREX-system-P-loop-protein-BrxC-[Clostridium-beijerinckii]
WP_195540766.1	Type1.BREX	<-HNH<-ClpABC-AAA<-?<-SIGMA+OB-Fold+AAA-ATPase+Lon-Protease-BrxL<-PglZ<-Schlafen+HATPase+wHTH<-HEPN<-N6-METHYLASE<-?<-DUF6079*<-BrxB-BrxF-iSTAND<-BrxA-DNA-binding<-HTH_26<-?<-?<-?<-wHTH+WYL+WCX||?-><-NUDIX	1182	Bacteria>Terrabacteria_group>Bacillota>Clostridia>Eubacteriales>Clostridiaceae	GCF_015558125.1	Clostridium_sp._D43t1_170807_D5	I2G39_RS02195	465793..469341	2787150	BREX-system-P-loop-protein-BrxC-[Clostridium-sp.-D43t1_170807_D5]
WP_216574650.1	Type1.BREX	<-ABhydrolase||IG+IG->?->MazE_antitoxin+SpoVT_C->HTH_26->BrxA-DNA-binding->BrxB-BrxF-iSTAND->DUF6079*->N6-METHYLASE->HEPN->Schlafen+HATPase+wHTH->PglZ->SIGMA+OB-Fold+AAA-ATPase+Lon-Protease-BrxL->TIR->?->MazE_antitoxin+SpoVT_C->	1182	Bacteria>Terrabacteria_group>Bacillota>Clostridia>Eubacteriales>Clostridiaceae	GCF_018918265.1	Falcatimonas_sp._MSJ-15	KQI85_RS03175	188313..191861	2841515	BREX-system-P-loop-protein-BrxC-[Falcatimonas-sp.-MSJ-15]
WP_270807575.1	Type1.BREX	<-LRR_5+LRR_8<-SIGMA+OB-Fold+AAA-ATPase+Lon-Protease-BrxL<-?<-PIN<-SbcC<-PglZ<-HEPN<-N6-METHYLASE<-DUF6079*<-BrxB-BrxF-iSTAND<-BrxA-DNA-binding<-HTH_26<-PIN<-?<-MACRODOMAIN	1201	Bacteria>Terrabacteria_group>Bacillota>Clostridia>Eubacteriales>Clostridiaceae	GCF_027723425.1	Clostridium_fessum	PG965_RS01510	334129..337734	2126740	BREX-system-P-loop-protein-BrxC-[Clostridium-fessum]
WP_072525015.1	Type1.BREX	TRAM+METHYLASE->SymE_toxin->ParA-Soj-PloopNTPase->ParB->?->HTH_26->BrxA-DNA-binding->BrxB-BrxF-iSTAND->DUF6079*->N6-METHYLASE->PglZ->SIGMA+OB-Fold+AAA-ATPase+Lon-Protease-BrxL->HEPN->HEPN-><-XIS-HTH<-PemK_toxin	1199	Bacteria>Terrabacteria_group>Bacillota>Clostridia>Eubacteriales>Clostridiaceae	GCF_900120155.1	Clostridium_sp._Marseille-P3244	BQ7367_RS08330	1368431..1372030	1871020	BREX-system-P-loop-protein-BrxC-[Clostridium-sp.-Marseille-P3244]
WP_073007111.1	Type1.BREX	PAS+GGDEF->?->?->?->?->BrxA-DNA-binding->BrxB-BrxF-iSTAND->DUF6079*->N6-METHYLASE->HEPN->HipA_C+STYKinase->Couple_hipA+HIRAN->Cas9+HNH+RuvC->Cas_Cas1->CRISPR_Cas2->PglZ->SIGMA+OB-Fold+AAA-ATPase+Lon-Protease-BrxL->	1214	Bacteria>Terrabacteria_group>Bacillota>Clostridia>Eubacteriales>Clostridiaceae	GCF_900142075.1	Clostridium_amylolyticum	BUA82_RS09870	579511..583155	1121298	BREX-system-P-loop-protein-BrxC-[Clostridium-amylolyticum]
WP_117466987.1	Type1.BREX	<-OLD-ABC+TOPRIM<-PglZ<-Schlafen+HATPase+wHTH<-HEPN<-N6-METHYLASE<-DUF6079*<-BrxB-BrxF-iSTAND<-BrxA-DNA-binding<-HTH_26	1196	Bacteria>Terrabacteria_group>Bacillota>Clostridia>Eubacteriales>Clostridiaceae	-	unclassified_Clostridium	DW081_14990	34449..38039	2614128	MULTISPECIES:-BREX-system-P-loop-protein-BrxC-[unclassified-Clostridium]
WP_014901355.1	Type1.BREX	ANK+ANK->?->?->SMODS->SLATT->?->BrxA-DNA-binding->BrxB-BrxF-iSTAND->DUF6079*->N6-METHYLASE->HEPN->PglZ->SIGMA+OB-Fold+AAA-ATPase+Lon-Protease-BrxL->?->HEPN+PAS+DUF4011+SF1-SF2-Helicase+REase->?->TniQ->TniQ->	1203	Bacteria>Terrabacteria_group>Bacillota>Clostridia>Eubacteriales>Desulfitobacteriaceae	GCF_000231385.2	Desulfosporosinus_meridiei	Desmer_0377	362087..365698	79209	BREX-system-P-loop-protein-BrxC-[Desulfosporosinus-meridiei]
MCH3955225.1	Type1.BREX	<-RVT+HEPN||HIN-HTH+PAIREDC-HTH-><-RVT+HEPN<-SIGMA+OB-Fold+AAA-ATPase+Lon-Protease-BrxL<-PglZ<-N6-METHYLASE<-DUF6079*<-BrxB-BrxF-iSTAND<-BrxA-DNA-binding<-?<-?<-HNH+3C-ABC||SIGMA-HTH-><-Recombinase<-Resolvase+Recombinase+ZnR	1171	Bacteria>Terrabacteria_group>Bacillota>Clostridia>Eubacteriales>Eubacteriaceae	GCA_022482865.1	Eubacterium_sp.	LKE44_08530	1944805..1948320	142586	BREX-system-P-loop-protein-BrxC-[Eubacterium-sp.]
MCH4073094.1	Type1.BREX	<-ClpABC-AAA<-?<-HEPN<-HEPN<-SIGMA+OB-Fold+AAA-ATPase+Lon-Protease-BrxL<-PglZ<-Schlafen+HATPase+wHTH<-Schlafen+HATPase+wHTH<-N6-METHYLASE<-DUF6079*<-BrxB-BrxF-iSTAND<-BrxA-DNA-binding<-?<-TRAM+METHYLASE<-DTXR-HTH<-RADICAL-SAM<-?<-?<-HAD+HAD	1171	Bacteria>Terrabacteria_group>Bacillota>Clostridia>Eubacteriales>Eubacteriaceae	GCA_022483875.1	Pseudoramibacter_sp.	LKF02_09135	1854333..1857848	2034862	BREX-system-P-loop-protein-BrxC-[Pseudoramibacter-sp.]
MCI5510134.1	Type1.BREX	CopG/RHH->?->TraJ-RHH->TraI-VirD2-RCRE->?->HTH_26->BrxA-DNA-binding->BrxB-BrxF-iSTAND->DUF6079*->?->N6-METHYLASE->HEPN->Schlafen+HATPase+wHTH->PglZ->SIGMA+OB-Fold+AAA-ATPase+Lon-Protease-BrxL->OLD-ABC+TOPRIM->SF1-SF2-Helicase-><-HTH	1182	Bacteria>Terrabacteria_group>Bacillota>Clostridia>Eubacteriales>Eubacteriaceae	GCA_022764165.1	Eubacterium_sp.	MR436_06790	9461..13009	142586	BREX-system-P-loop-protein-BrxC-[Eubacterium-sp.]
CVI69463.1	Type1.BREX	<-Schlafen+wHTH<-HEPN<-N6-METHYLASE<-TIR<-DUF6079*<-BrxB-BrxF-iSTAND<-BrxA-DNA-binding<-HTH_26<-ParB+HNH	1179	Bacteria>Terrabacteria_group>Bacillota>Clostridia>Eubacteriales>Eubacteriaceae	GCA_900016875.1	Eubacteriaceae_bacterium_CHKCI004	BN3660_01565	6017..9556	1780380	hypothetical-protein-BN3660_01565-[Eubacteriaceae-bacterium-CHKCI004]
SEG33478.1	Type1.BREX	<-SbcC<-Calcineurin<-?<-?<-SIGMA+OB-Fold+AAA-ATPase+Lon-Protease-BrxL<-PglZ<-N6-METHYLASE<-N6-METHYLASE<-HEPN<-DUF6079*<-BrxB-BrxF-iSTAND<-BrxA-DNA-binding<-?<-wHTH+WYL+WCX<-35exo<-HTH_3<-Tox-RelA<-PTSEIIA1	1157	Bacteria>Terrabacteria_group>Bacillota>Clostridia>Eubacteriales>Eubacteriaceae	GCA_900108205.1	Eubacterium_ruminantium	SAMN04487934_11616	23559..27032	42322	hypothetical-protein-SAMN04487934_11616-[Eubacterium-ruminantium]
WP_055289382.1	Type1.BREX	HTH-><-?<-PHP+SbcC<-SIGMA+OB-Fold+AAA-ATPase+Lon-Protease-BrxL<-PglZ<-Schlafen+HATPase+wHTH<-HEPN<-N6-METHYLASE<-?<-DUF6079*<-BrxB-BrxF-iSTAND<-BrxA-DNA-binding<-HTH_26<-TraI-VirD2-RCRE<-TraJ-RHH<-?<-CopG/RHH<-?<-HTH_36	1183	Bacteria>Terrabacteria_group>Bacillota>Clostridia>Eubacteriales>Eubacteriaceae	GCF_001406295.1	Eubacterium_ramulus	ARA08_RS03325	204677..208228	39490	BREX-system-P-loop-protein-BrxC-[Eubacterium-ramulus]
WP_078766837.1	Type1.BREX	AbiEi_4->AbiEii->HTH_3->?->REase->wHTH+HNH->HTH_26->BrxA-DNA-binding->BrxB-BrxF-iSTAND->DUF6079*->N6-METHYLASE->HEPN->PglZ->SLATT->?->SIGMA+OB-Fold+AAA-ATPase+Lon-Protease-BrxL->RVT->SbcC->	1203	Bacteria>Terrabacteria_group>Bacillota>Clostridia>Eubacteriales>Eubacteriaceae	GCF_900167115.1	Eubacterium_uniforme	B5D16_RS09945	14532..18143	39495	BREX-system-P-loop-protein-BrxC-[Eubacterium-uniforme]
MCI6150868.1	Type1.BREX	<-wHTH+WYL<-?<-HTH+MPTase<-ZnR<-SIGMA+OB-Fold+AAA-ATPase+Lon-Protease-BrxL<-PglZ<-HEPN<-HKD-DNase<-N6-METHYLASE<-DUF6079*<-BrxB-BrxF-iSTAND<-BrxA-DNA-binding<-HMG+NLPC+NLPC<-VirB4-FtsK<-?||?-><-TrwI<-TraM<-VirD4-TraG+VirD4-FtsK	1179	Bacteria>Terrabacteria_group>Bacillota>Clostridia>Eubacteriales>Eubacteriales_incertae_sedis	GCA_022772165.1	Flintibacter_sp.	MR705_10640	43131..46670	1918624	BREX-system-P-loop-protein-BrxC-[Flintibacter-sp.]
WP_118637679.1	Type1.BREX	<-LexA-protease<-MNS+REase<-wHTH+WYL+WCX<-N6-METHYLASE<-HEPN<-ParB+HNH<-SIGMA+OB-Fold+AAA-ATPase+Lon-Protease-BrxL<-PglZ<-N6-METHYLASE<-DUF6079*<-BrxB-BrxF-iSTAND<-BrxA-DNA-binding<-HTH_26||TraJ-RHH->TraI-VirD2-RCRE->	1215	Bacteria>Terrabacteria_group>Bacillota>Clostridia>Eubacteriales	GCF_027924705.1	Oscillospiraceae	H6A32_RS13625	30472..34119	216572	MULTISPECIES:-BREX-system-P-loop-protein-BrxC-[Oscillospiraceae]
WP_256171760.1	Type1.BREX	HTH_36->?->CopG/RHH->?->TraJ-RHH->TraI-VirD2-RCRE->HTH_26->BrxA-DNA-binding->BrxB-BrxF-iSTAND->DUF6079*->?->N6-METHYLASE->HEPN->Schlafen+HATPase+wHTH->PglZ->SIGMA+OB-Fold+AAA-ATPase+Lon-Protease-BrxL->OLD-ABC+TOPRIM->SF1-SF2-Helicase-><-HTH	1182	Bacteria>Terrabacteria_group>Bacillota>Clostridia>Eubacteriales>Lachnospiraceae	-	Dorea_longicatena	NE607_07100	21358..24906	88431	BREX-system-P-loop-protein-BrxC-[Dorea-longicatena]
OOOOO080.1	Type1.BREX	OLD-ABC->?->RADICAL-SAM->?->?->?->BrxA-DNA-binding->BrxB-BrxF-iSTAND->DUF6079*-><-HEPN	1209	Bacteria>Terrabacteria_group>Bacillota>Clostridia>Eubacteriales>Lachnospiraceae	GCA_002007315.1	Epulopiscium_sp._Nele67-Bin004	ATN35_09340	17804..21433	1764962	MAG:-ATPase-[Epulopiscium-sp.-Nele67-Bin004]
RGI65884.1	Type1.BREX	HTH_26->BrxA-DNA-binding->BrxB-BrxF-iSTAND->DUF6079*->OLD-ABC->PIN->N6-METHYLASE->TIR->HEPN->Schlafen+HATPase+wHTH->PglZ->Tox-ART-HYD3->SIGMA+OB-Fold+AAA-ATPase+Lon-Protease-BrxL->	1196	Bacteria>Terrabacteria_group>Bacillota>Clostridia>Eubacteriales>Lachnospiraceae	GCA_003436035.1	[Eubacterium]_rectale	DXD95_13285	1619..5209	39491	BREX-system-P-loop-protein-BrxC-[[Eubacterium]
HBA06127.1	Type1.BREX	MNS+REase->TOPRIM+Topoisom_bac+TOPRIM_Crpt+TOPRIM_Crpt+TOPRIM_Crpt+TOPRIM_Crpt->?->?->?->HTH_26->BrxA-DNA-binding->BrxB-BrxF-iSTAND->DUF6079*->?->N6-METHYLASE->HEPN->Schlafen+HATPase+wHTH->PglZ->SIGMA+OB-Fold+AAA-ATPase+Lon-Protease-BrxL->?->Tox-ART-HYD3->AT-HTH-HYD2->	1182	Bacteria>Terrabacteria_group>Bacillota>Clostridia>Eubacteriales>Lachnospiraceae	GCA_003491515.1	Roseburia_sp.	DC452_03320	12130..15678	2049040	BREX-system-P-loop-protein-BrxC-[Roseburia-sp.]
TGX97927.1	Type1.BREX	<-OLD-ABC+TOPRIM-p<-OLD-ABC+TOPRIM-p<-PglZ<-HNH<-OLD-ABC<-N6-METHYLASE<-HEPN<-ParB+HNH<-N6-METHYLASE<-DUF6079*<-BrxB-BrxF-iSTAND<-BrxA-DNA-binding<-HTH_26<-ZnR+ZnR+ZnR<-35exo+ADA-DBD<-?<-MACRODOMAIN	1201	Bacteria>Terrabacteria_group>Bacillota>Clostridia>Eubacteriales>Lachnospiraceae	GCA_004793585.1	Lachnospiraceae_bacterium	E5357_10825	35909..39514	1898203	BREX-system-P-loop-protein-BrxC-[Lachnospiraceae-bacterium]
TYL56933.1	Type1.BREX	HTH_26->BrxA-DNA-binding->BrxB-BrxF-iSTAND->DUF6079*->N6-METHYLASE->REase->Schlafen+HATPase+wHTH->PglZ->?->HEPN->SIGMA+OB-Fold+AAA-ATPase+Lon-Protease-BrxL->?->LRR_5+LRR_8->	1196	Bacteria>Terrabacteria_group>Bacillota>Clostridia>Eubacteriales>Lachnospiraceae	GCA_008123415.1	[Eubacterium]_rectale	FYL31_13935	1744..5334	39491	BREX-system-P-loop-protein-BrxC-[[Eubacterium]
QNM03383.1	Type1.BREX	GNTR-HTH+Aminotran_1_2->?->HTH_26->BrxA-DNA-binding->BrxB-BrxF-iSTAND->DUF6079*->Tox-ART-HYD2->AT-HTH-HYD2-1->N6-METHYLASE->HEPN->Schlafen+HATPase+wHTH->PglZ->SIGMA+OB-Fold+AAA-ATPase+Lon-Protease-BrxL->?->RNase->	1215	Bacteria>Terrabacteria_group>Bacillota>Clostridia>Eubacteriales>Lachnospiraceae	GCA_014337175.1	Simiaoa_sunii	H9Q77_04470	956158..959805	2763672	BREX-system-P-loop-protein-BrxC-[Simiaoa-sunii]
MBR3244379.1	Type1.BREX	TraJ-RHH->TraI-VirD2-RCRE->XIS-HTH->BrxA-DNA-binding->BrxB-BrxF-iSTAND->DUF6079*->N6-METHYLASE->?->PglZ->HEPN+SH3-like+SF1-SF2-Helicase->	1207	Bacteria>Terrabacteria_group>Bacillota>Clostridia>Eubacteriales>Lachnospiraceae	GCA_017511245.1	Parasporobacterium_sp.	IKF90_17090	4939..8562	2740533	BREX-system-P-loop-protein-BrxC-[Parasporobacterium-sp.]
MBR4345194.1	Type1.BREX	RelB->PIN->BrxA-DNA-binding->BrxB-BrxF-iSTAND->DUF6079*->VbhA+DOC+HTH->N6-METHYLASE->N6-METHYLASE-p->HEPN->SbcC+SbcC->Schlafen+HATPase+wHTH->PglZ->SIGMA+OB-Fold+AAA-ATPase+Lon-Protease-BrxL->TIR->	1173	Bacteria>Terrabacteria_group>Bacillota>Clostridia>Eubacteriales>Lachnospiraceae	GCA_017542245.1	Lachnospiraceae_bacterium	IKP88_21230	56947..60468	1898203	BREX-system-P-loop-protein-BrxC-[Lachnospiraceae-bacterium]
MBP3802955.1	Type1.BREX	RelB-><-?||HTH_26->BrxA-DNA-binding->BrxB-BrxF-iSTAND->DUF6079*->N6-METHYLASE->?->Schlafen+BirA-HTH->PglZ->SIGMA+OB-Fold+AAA-ATPase+Lon-Protease-BrxL->HEPN->	1204	Bacteria>Terrabacteria_group>Bacillota>Clostridia>Eubacteriales>Lachnospiraceae	GCA_017940955.1	Oribacterium_sp.	J6I76_03520	9410..13024	1969407	BREX-system-P-loop-protein-BrxC-[Oribacterium-sp.]
MCB7506159.1	Type1.BREX	<-ClpABN-AAA+ClpABC-AAA||HTH-><-MULE-TRANSPOSASE<-PHP+SbcC<-SIGMA+OB-Fold+AAA-ATPase+Lon-Protease-BrxL<-PglZ<-Schlafen+HATPase+wHTH<-HEPN<-N6-METHYLASE<-DUF6079*<-BrxB-BrxF-iSTAND<-BrxA-DNA-binding<-HTH_26<-HTH_36	1182	Bacteria>Terrabacteria_group>Bacillota>Clostridia>Eubacteriales>Lachnospiraceae	GCA_020553125.1	Blautia_sp._MSK20_18	LI091_01875	117354..120902	2883186	BREX-system-P-loop-protein-BrxC-[Blautia-sp.-MSK20_18]
MCC2229908.1	Type1.BREX	HTH_36->?->CopG/RHH->TraJ-RHH->TraI-VirD2-RCRE->HTH_26->BrxA-DNA-binding->BrxB-BrxF-iSTAND->DUF6079*->?->N6-METHYLASE->HEPN->Schlafen+HATPase+wHTH->PglZ->SIGMA+OB-Fold+AAA-ATPase+Lon-Protease-BrxL->OLD-ABC+TOPRIM->SF1-SF2-Helicase-><-HTH	1182	Bacteria>Terrabacteria_group>Bacillota>Clostridia>Eubacteriales>Lachnospiraceae	GCA_020687485.1	Lachnospiraceae_bacterium_CLA-AA-H215	LKD81_02670	26793..30341	2885348	BREX-system-P-loop-protein-BrxC-[Lachnospiraceae-bacterium-CLA-AA-H215]
MCI8732218.1	Type1.BREX	<-STYKinase-like||OLD-ABC->MerR-HTH-><-?<-?||BrxA-DNA-binding->BrxB-BrxF-iSTAND->DUF6079*->N6-METHYLASE->HEPN->	1210	Bacteria>Terrabacteria_group>Bacillota>Clostridia>Eubacteriales>Lachnospiraceae	GCA_022794815.1	Lachnospiraceae_bacterium	HFH57_14060	7970..11602	1898203	BREX-system-P-loop-protein-BrxC-[Lachnospiraceae-bacterium]
MCI9358063.1	Type1.BREX	<-STYKinase-like||Schlafen+HATPase+wHTH-p->BirA-HTH-><-Peptidase_M50<-?||BrxA-DNA-binding->BrxB-BrxF-iSTAND->DUF6079*->N6-METHYLASE->PolB->HEPN->OLD-ABC->PglZ->?->nSTAND3->?->Calcineurin->	1213	Bacteria>Terrabacteria_group>Bacillota>Clostridia>Eubacteriales>Lachnospiraceae	GCA_022799775.1	Lachnospiraceae_bacterium	HFH59_11085	6660..10301	1898203	BREX-system-P-loop-protein-BrxC-[Lachnospiraceae-bacterium]
MCM1125306.1	Type1.BREX	MoxR-AAA->LRR_5->Glyco_hydro_3_C+Fn3-like+Glyco_hydro_3->Peptidase_C26->tRNA->tRNA-><-?<-MerR-HTH||HTH_26->BrxA-DNA-binding->BrxB-BrxF-iSTAND->DUF6079*->ParB+HNH->HEPN->N6-METHYLASE-p->?-><-tRNA||?->HAD+HAD->	1205	Bacteria>Terrabacteria_group>Bacillota>Clostridia>Eubacteriales>Lachnospiraceae	GCA_023663945.1	Lachnospiraceae_bacterium	NC429_02425	28301..31918	1898203	BREX-system-P-loop-protein-BrxC-[Lachnospiraceae-bacterium]
MCQ4892848.1	Type1.BREX	HTH_36->?->CopG/RHH->?->TraJ-RHH->TraI-VirD2-RCRE->HTH_26->BrxA-DNA-binding->BrxB-BrxF-iSTAND->DUF6079*->?->N6-METHYLASE->HEPN->Schlafen+HATPase+wHTH->PglZ->SIGMA+OB-Fold+AAA-ATPase+Lon-Protease-BrxL->OLD-ABC+TOPRIM->SF1-SF2-Helicase-><-HTH	1182	Bacteria>Terrabacteria_group>Bacillota>Clostridia>Eubacteriales>Lachnospiraceae	GCA_024461355.1	Dorea_longicatena	NE607_07100	21358..24906	88431	BREX-system-P-loop-protein-BrxC-[Dorea-longicatena]
MCR5727513.1	Type1.BREX	BrxA-DNA-binding->BrxB-BrxF-iSTAND->DUF6079*->VbhA+DOC+HTH->N6-METHYLASE->HEPN->SbcC+SbcC->?->PglZ->SIGMA+OB-Fold+AAA-ATPase+Lon-Protease-BrxL->?->SF1-SF2-Helicase+REase->	1173	Bacteria>Terrabacteria_group>Bacillota>Clostridia>Eubacteriales>Lachnospiraceae	GCA_024699435.1	Lachnospiraceae_bacterium	K6G24_08610	2669..6190	1898203	BREX-system-P-loop-protein-BrxC-[Lachnospiraceae-bacterium]
SCW50280.1	Type1.BREX	Arm-DNA-bind_4+Phage_int_SAM_3+Phage_integrase->TraJ-RHH->ParA-Soj-PloopNTPase->ParB->?->HTH_26->BrxA-DNA-binding->BrxB-BrxF-iSTAND->DUF6079*->N6-METHYLASE->?->PglZ->HEPN->SIGMA+OB-Fold+AAA-ATPase+Lon-Protease-BrxL->Peptidase_C14->	1181	Bacteria>Terrabacteria_group>Bacillota>Clostridia>Eubacteriales>Lachnospiraceae	GCA_900100095.1	Lachnospiraceae_bacterium_C10	SAMN02910400_01008	22627..26172	1520822	hypothetical-protein-SAMN02910400_01008-[Lachnospiraceae-bacterium-C10]
SNU04610.1	Type1.BREX	HTH_3->?->?->35exo->SbcC->?->wHTH+WYL+WCX->BrxA-DNA-binding->BrxB-BrxF-iSTAND->DUF6079*->KAP-NTPase->N6-METHYLASE->HEPN->PglZ->HEPN+SH3-like+SF1-SF2-Helicase+wHTH->MORC+DUF3684+HNH->MORC+DUF3684+HNH->SF1-SF2-Helicase->SF1-SF2-Helicase+REase->	1206	Bacteria>Terrabacteria_group>Bacillota>Clostridia>Eubacteriales>Lachnospiraceae	GCA_900199595.1	Lachnospiraceae_bacterium	SAMN06297422_101152	246659..250279	1898203	hypothetical-protein-SAMN06297422_101152-[Lachnospiraceae-bacterium]
WP_009536434.1	Type1.BREX	<-SF1-SF2-Helicase<-ParB<-SbcC<-SIGMA+OB-Fold+AAA-ATPase+Lon-Protease-BrxL<-PglZ<-Schlafen+HATPase+wHTH<-N6-METHYLASE-p<-HEPN<-N6-METHYLASE<-DUF6079*<-BrxB-BrxF-iSTAND<-BrxA-DNA-binding<-HTH_26<-HTH+rve<-Sigma-HTH<-TIR<-Peptidase_C14<-TIR||DEDD_Tnp_IS110->	1217	Bacteria>Terrabacteria_group>Bacillota>Clostridia>Eubacteriales>Lachnospiraceae	GCF_000238075.1	Oribacterium_asaccharolyticum	HMPREF9624_00544	316049..319702	1501332	BREX-system-P-loop-protein-BrxC-[Oribacterium-asaccharolyticum]
WP_002578404.1	Type1.BREX	HTH-><-SF1-SF2-Helicase<-OLD-ABC+TOPRIM<-SIGMA+OB-Fold+AAA-ATPase+Lon-Protease-BrxL<-PglZ<-Schlafen+HATPase+wHTH<-HEPN<-N6-METHYLASE<-?<-DUF6079*<-BrxB-BrxF-iSTAND<-BrxA-DNA-binding<-HTH_26<-HTH_36<-TraI-VirD2-RCRE<-TraJ-RHH<-CopG/RHH<-?<-HTH_36	1182	Bacteria>Terrabacteria_group>Bacillota>Clostridia>Eubacteriales>Lachnospiraceae	GCF_000371725.1	Enterocloster_bolteae	HMPREF1095_RS19895	1377442..1380990	208479	BREX-system-P-loop-protein-BrxC-[Enterocloster-bolteae]
WP_016216871.1	Type1.BREX	<-HTH||||HTH->||HTH_26->BrxA-DNA-binding->BrxB-BrxF-iSTAND->DUF6079*-><-DEDD_Tnp_IS110+Transposase_20||HEPN->N6-METHYLASE->HTH+REase->Schlafen+HATPase+wHTH->PglZ->	1182	Bacteria>Terrabacteria_group>Bacillota>Clostridia>Eubacteriales>Lachnospiraceae	GCF_000403455.2	Dorea_sp._5-2	C817_RS00440	102069..105617	1235798	BREX-system-P-loop-protein-BrxC-[Dorea-sp.-5-2]
WP_026519395.1	Type1.BREX	<-LRR_5<-BrxL<-SIGMA+OB-Fold+AAA-ATPase+Lon-Protease-BrxL<-PHP+SbcC<-PglZ<-HEPN<-N6-METHYLASE<-DUF6079*<-BrxB-BrxF-iSTAND<-BrxA-DNA-binding<-HTH_26<-?<-?<-?<-TraJ-RHH<-LPD2<-ZnR+TOPRIM	1194	Bacteria>Terrabacteria_group>Bacillota>Clostridia>Eubacteriales>Lachnospiraceae	GCF_000424525.1	Butyrivibrio_sp._FCS006	G633_RS0106250	43812..47396	1280684	BREX-system-P-loop-protein-BrxC-[Butyrivibrio-sp.-FCS006]
WP_029067206.1	Type1.BREX	HTH_26->BrxA-DNA-binding->BrxB-BrxF-iSTAND->DUF6079*->N6-METHYLASE->PglZ->ParB+HNH->SIGMA+OB-Fold+AAA-ATPase+Lon-Protease-BrxL->RVT+HEPN->HTH_38->HTH_26->BrxA-DNA-binding->BrxB-BrxF-iSTAND->	1182	Bacteria>Terrabacteria_group>Bacillota>Clostridia>Eubacteriales>Lachnospiraceae	GCF_000687515.1	Lachnobacterium_bovis	CC91_RS0108435	378442..381990	140626	BREX-system-P-loop-protein-BrxC-[Lachnobacterium-bovis]
WP_055216182.1	Type1.BREX	<-PIN<-ClpABC-AAA<-?<-SIGMA+OB-Fold+AAA-ATPase+Lon-Protease-BrxL<-PglZ<-HEPN<-N6-METHYLASE<-DUF6079*<-BrxB-BrxF-iSTAND<-BrxA-DNA-binding<-HTH_26<-?<-?<-?<-ArdCN+LPD32+LPD36+N6_Mtase+SF1-SF2-Helicase	1186	Bacteria>Terrabacteria_group>Bacillota>Clostridia>Eubacteriales>Lachnospiraceae	GCF_001404435.1	Lachnospira_eligens	ERS852490_02366	200381..203941	39485	BREX-system-P-loop-protein-BrxC-[Lachnospira-eligens]
WP_118348289.1	Type1.BREX	<-SIGMA+OB-Fold+AAA-ATPase+Lon-Protease-BrxL<-PglZ<-Schlafen+HATPase+wHTH<-HEPN<-N6-METHYLASE<-?<-DUF6079*<-BrxB-BrxF-iSTAND<-BrxA-DNA-binding<-HTH_26<-?<-TraI-VirD2-RCRE<-TraJ-RHH<-?<-CopG/RHH<-HTH_36	1182	Bacteria>Terrabacteria_group>Bacillota>Clostridia>Eubacteriales>Lachnospiraceae	GCF_003458885.1	Coprococcus_sp._AF21-14LB	DWX73_RS07815	11153..14701	2292231	BREX-system-P-loop-protein-BrxC-[Coprococcus-sp.-AF21-14LB]
WP_118051545.1	Type1.BREX	<-Phage_int_SAM_5+Phage_integrase||TraJ-RHH->?->?-><-?||HTH_26->BrxA-DNA-binding->BrxB-BrxF-iSTAND->DUF6079*->?->N6-METHYLASE->HEPN->Schlafen+HATPase+wHTH->PglZ->PHP+SbcC->	1183	Bacteria>Terrabacteria_group>Bacillota>Clostridia>Eubacteriales>Lachnospiraceae	GCF_003464645.1	Blautia_obeum	DWW07_RS08215	33645..37196	40520	BREX-system-P-loop-protein-BrxC-[Blautia-obeum]
WP_118592376.1	Type1.BREX	<-Schlafen+HATPase+wHTH-p<-ClpABC-AAA<-?<-SIGMA+OB-Fold+AAA-ATPase+Lon-Protease-BrxL<-PglZ<-HEPN<-N6-METHYLASE<-DUF6079*<-BrxB-BrxF-iSTAND<-BrxA-DNA-binding<-HTH_26<-?<-?<-?<-?<-SF1-SF2-Helicase<-N6_N4_METHYLASE	1186	Bacteria>Terrabacteria_group>Bacillota>Clostridia>Eubacteriales>Lachnospiraceae	GCF_003467035.1	Roseburia_intestinalis	DW927_RS18160	14462..18022	166486	BREX-system-P-loop-protein-BrxC-[Roseburia-intestinalis]
WP_118198114.1	Type1.BREX	<-SIGMA+OB-Fold+AAA-ATPase+Lon-Protease-BrxL<-?<-PHP<-PglZ<-Schlafen+HATPase+wHTH<-HEPN<-N6-METHYLASE<-?<-DUF6079*<-BrxB-BrxF-iSTAND<-BrxA-DNA-binding<-HTH_26||?-><-?<-TraJ-RHH||Phage_int_SAM_5+Phage_integrase->	1183	Bacteria>Terrabacteria_group>Bacillota>Clostridia>Eubacteriales>Lachnospiraceae	GCF_003471125.1	Blautia_obeum	DW272_RS15845	24197..27748	40520	BREX-system-P-loop-protein-BrxC-[Blautia-obeum]
WP_118742111.1	Type1.BREX	ZnR->?->HTH_26->BrxA-DNA-binding->BrxB-BrxF-iSTAND->DUF6079*->N6-METHYLASE->RAMA->HEPN->Schlafen+HATPase+wHTH->PglZ->SIGMA+OB-Fold+AAA-ATPase+Lon-Protease-BrxL->OLD-ABC+TOPRIM->SF1-SF2-Helicase->	1182	Bacteria>Terrabacteria_group>Bacillota>Clostridia>Eubacteriales>Lachnospiraceae	GCF_003480245.1	Blautia_sp._AM42-2	DW915_10705	4262..7810	2292976	BREX-system-P-loop-protein-BrxC-[Blautia-sp.-AM42-2]
WP_154780312.1	Type1.BREX	Peptidase->?->?->MACRODOMAIN->?->ZnR+ZnR+ZnR->HTH_26->BrxA-DNA-binding->BrxB-BrxF-iSTAND->DUF6079*->N6-METHYLASE->RVT->?->OLD-ABC->HNH->?->PglZ->HEPN->SIGMA+OB-Fold+AAA-ATPase+Lon-Protease-BrxL->	1201	Bacteria>Terrabacteria_group>Bacillota>Clostridia>Eubacteriales>Lachnospiraceae	GCF_009707925.1	Blautia_luti	GKZ57_RS08760	74022..77627	89014	BREX-system-P-loop-protein-BrxC-[Blautia-luti]
WP_249326641.1	Type1.BREX	GNTR-HTH+Aminotran_1_2->?->HTH_26->BrxA-DNA-binding->BrxB-BrxF-iSTAND->DUF6079*->Tox-ART-HYD2->AT-HTH-HYD2-1->N6-METHYLASE->HEPN->Schlafen+HATPase+wHTH->PglZ->SIGMA+OB-Fold+AAA-ATPase+Lon-Protease-BrxL->?->RNase->	1215	Bacteria>Terrabacteria_group>Bacillota>Clostridia>Eubacteriales>Lachnospiraceae	GCF_014337175.1	Simiaoa_sunii	H9Q77_RS04465	956158..959805	2763672	BREX-system-P-loop-protein-BrxC-[Simiaoa-sunii]
WP_128132381.1	Type1.BREX	MerR-HTH-><-?<-REase<-?<-SIGMA+OB-Fold+AAA-ATPase+Lon-Protease-BrxL<-PglZ<-HEPN<-N6-METHYLASE<-DUF6079*<-BrxB-BrxF-iSTAND<-BrxA-DNA-binding<-HTH_26<-HTH||?-><-?<-TraJ-RHH<-SF1-SF2-Helicase	1182	Bacteria>Terrabacteria_group>Bacillota>Clostridia>Eubacteriales>Lachnospiraceae	GCF_015547745.1	Enterocloster_bolteae	I2D45_RS23920	73790..77338	208479	BREX-system-P-loop-protein-BrxC-[Enterocloster-bolteae]
WP_195251421.1	Type1.BREX	MACRODOMAIN->?->35exo+ADA-DBD->HTH_26->BrxA-DNA-binding->BrxB-BrxF-iSTAND->DUF6079*->N6-METHYLASE->ParB+HNH->HEPN->	1201	Bacteria>Terrabacteria_group>Bacillota>Clostridia>Eubacteriales>Lachnospiraceae	GCF_015550485.1	Coprococcus_comes	I2A60_RS13430	4839..8444	410072	BREX-system-P-loop-protein-BrxC-[Coprococcus-comes]
WP_215726767.1	Type1.BREX	ParB->?-><-?||HTH_3->HTH_26->BrxA-DNA-binding->BrxB-BrxF-iSTAND->DUF6079*->?->N6-METHYLASE->HEPN+ZNR->HEPN->PglZ->?->SIGMA+OB-Fold+AAA-ATPase+Lon-Protease-BrxL->wHTH+WYL+WCX->HTH+REase->	1205	Bacteria>Terrabacteria_group>Bacillota>Clostridia>Eubacteriales>Lachnospiraceae	GCF_018784865.1	Dorea_longicatena	GPK48_RS02600	18956..22573	88431	BREX-system-P-loop-protein-BrxC-[Dorea-longicatena]
WP_252210442.1	Type1.BREX	<-TIR<-SIGMA+OB-Fold+AAA-ATPase+Lon-Protease-BrxL<-PglZ<-Schlafen+HATPase+wHTH<-HEPN<-AT-HTH-HYD2<-Tox-ART-HYD2<-AT-HTH-HYD2<-SIR2<-DUF6079*<-BrxB-BrxF-iSTAND<-BrxA-DNA-binding<-HTH_26<-HTH_36<-ABhydrolase<-?<-SAM-METHYLASE	1099	Bacteria>Terrabacteria_group>Bacillota>Clostridia>Eubacteriales>Lachnospiraceae	GCF_018785625.1	Blautia_faecis	GPK86_RS03045	13256..16555	871665	BREX-system-P-loop-protein-BrxC-[Blautia-faecis]
WP_216429335.1	Type1.BREX	HTH_26->BrxA-DNA-binding->BrxB-BrxF-iSTAND->DUF6079*->N6-METHYLASE->RAMA->HEPN->PglZ->SIGMA+OB-Fold+AAA-ATPase+Lon-Protease-BrxL->?->REase->?-><-HTH_3	1182	Bacteria>Terrabacteria_group>Bacillota>Clostridia>Eubacteriales>Lachnospiraceae	GCF_018918145.1	Anaerotignum_sp._MSJ-24	KQI66_RS10650	65878..69426	2841521	BREX-system-P-loop-protein-BrxC-[Anaerotignum-sp.-MSJ-24]
WP_265190723.1	Type1.BREX	<-ClpABN-AAA+ClpABC-AAA||HTH-><-MULE-TRANSPOSASE<-PHP+SbcC<-SIGMA+OB-Fold+AAA-ATPase+Lon-Protease-BrxL<-PglZ<-Schlafen+HATPase+wHTH<-HEPN<-N6-METHYLASE<-DUF6079*<-BrxB-BrxF-iSTAND<-BrxA-DNA-binding<-HTH_26<-HTH_36	1182	Bacteria>Terrabacteria_group>Bacillota>Clostridia>Eubacteriales>Lachnospiraceae	GCF_020553125.1	Blautia_sp._MSK20_18	LI091_RS01865	117354..120902	2883186	BREX-system-P-loop-protein-BrxC-[Blautia-sp.-MSK20_18]
WP_227165162.1	Type1.BREX	MACRODOMAIN->?->35exo+ADA-DBD->ZnR+ZnR+ZnR->HTH_26->BrxA-DNA-binding->BrxB-BrxF-iSTAND->DUF6079*->N6-METHYLASE->HEPN->PglZ->OLD-ABC+TOPRIM->SIGMA+OB-Fold+AAA-ATPase+Lon-Protease-BrxL->LRR_5+LRR_8->?->STYKIN->	1201	Bacteria>Terrabacteria_group>Bacillota>Clostridia>Eubacteriales>Lachnospiraceae	GCF_020560115.1	Mediterraneibacter_sp._210702-DFI.5.30	LI126_RS08100	75769..79374	2883232	BREX-system-P-loop-protein-BrxC-[Mediterraneibacter-sp.-210702-DFI.5.30]
WP_158358547.1	Type1.BREX	<-SIGMA+OB-Fold+AAA-ATPase+Lon-Protease-BrxL<-PglZ<-Schlafen+HATPase+wHTH<-HEPN+HEPN<-ParB+HNH<-AT-HTH-HYD2<-Tox-ART-HYD2<-AT-HTH-HYD2<-N6-METHYLASE<-DUF6079*<-BrxB-BrxF-iSTAND<-BrxA-DNA-binding<-HTH_26<-?<-?<-?<-DOC+HTH<-HTH_3<-PDZ_6+Peptidase_S55	1182	Bacteria>Terrabacteria_group>Bacillota>Clostridia>Eubacteriales>Lachnospiraceae	GCF_025567195.1	Alitiscatomonas_aceti	OCV69_RS08300	70137..73685	2981724	BREX-system-P-loop-protein-BrxC-[Alitiscatomonas-aceti]
WP_270422341.1	Type1.BREX	CopG/RHH->?->TraJ-RHH->TraI-VirD2-RCRE->?->HTH_26->BrxA-DNA-binding->BrxB-BrxF-iSTAND->DUF6079*->?->N6-METHYLASE->HEPN->Schlafen+HATPase+wHTH->PglZ->SIGMA+OB-Fold+AAA-ATPase+Lon-Protease-BrxL->OLD-ABC+TOPRIM->SF1-SF2-Helicase-><-HTH	1182	Bacteria>Terrabacteria_group>Bacillota>Clostridia>Eubacteriales>Lachnospiraceae	GCF_027661365.1	Fusicatenibacter_saccharivorans	PGQ16_RS03035	254369..257917	1150298	BREX-system-P-loop-protein-BrxC-[Fusicatenibacter-saccharivorans]
WP_119247667.1	Type1.BREX	HTH_36->?->CopG/RHH->TraJ-RHH->TraI-VirD2-RCRE->HTH_26->BrxA-DNA-binding->BrxB-BrxF-iSTAND->DUF6079*->?->N6-METHYLASE->HEPN->Schlafen+HATPase+wHTH->PglZ->SIGMA+OB-Fold+AAA-ATPase+Lon-Protease-BrxL->OLD-ABC+TOPRIM->SF1-SF2-Helicase-><-HTH	1182	Bacteria>Terrabacteria_group>Bacillota>Clostridia>Eubacteriales>Lachnospiraceae	GCF_027674485.1	Dorea_longicatena	PGV47_RS07575	41898..45446	88431	BREX-system-P-loop-protein-BrxC-[Dorea-longicatena]
WP_099385222.1	Type1.BREX	<-Tox-RES<-?<-URI-DNase<-SIGMA+OB-Fold+AAA-ATPase+Lon-Protease-BrxL<-?<-PglZ<-HEPN<-N6-METHYLASE<-N6-METHYLASE<-DUF6079*<-BrxB-BrxF-iSTAND<-BrxA-DNA-binding<-HTH_26<-ACET	1207	Bacteria>Terrabacteria_group>Bacillota>Clostridia>Eubacteriales>Lachnospiraceae	GCF_028539255.1	Agathobacter_ruminis	CSX02_RS00500	90797..94420	1712665	BREX-system-P-loop-protein-BrxC-[Agathobacter-ruminis]
WP_090167800.1	Type1.BREX	<-HKD-DNase<-?<-SIGMA+OB-Fold+AAA-ATPase+Lon-Protease-BrxL<-BrxD-AAA<-?<-PglZ<-HEPN<-N6-METHYLASE<-N6-METHYLASE<-DUF6079*<-BrxB-BrxF-iSTAND<-BrxA-DNA-binding<-HTH_26<-?<-?<-wHTH+WYL+WCX<-HNH<-?<-ARG	1201	Bacteria>Terrabacteria_group>Bacillota>Clostridia>Eubacteriales>Lachnospiraceae	GCF_900100545.1	Pseudobutyrivibrio_sp._49	BLP54_RS06100	185622..189227	1855344	BREX-system-P-loop-protein-BrxC-[Pseudobutyrivibrio-sp.-49]
WP_090393802.1	Type1.BREX	wHTH+WYL+WCX->?->?->HKD-DNase->HTH_26->BrxA-DNA-binding->BrxB-BrxF-iSTAND->DUF6079*->N6-METHYLASE->?->PglZ->SIGMA+OB-Fold+AAA-ATPase+Lon-Protease-BrxL->HEPN->?->HKD-DNase->SF1-SF2-Helicase->REase->	1182	Bacteria>Terrabacteria_group>Bacillota>Clostridia>Eubacteriales>Lachnospiraceae	GCF_900111635.1	Pseudobutyrivibrio_sp._C4	BMY66_RS00580	126012..129560	1520803	BREX-system-P-loop-protein-BrxC-[Pseudobutyrivibrio-sp.-C4]
WP_090545210.1	Type1.BREX	<-HKD-DNase<-?<-SIGMA+OB-Fold+AAA-ATPase+Lon-Protease-BrxL<-BrxD-AAA<-?<-PglZ<-HEPN<-N6-METHYLASE<-N6-METHYLASE<-DUF6079*<-BrxB-BrxF-iSTAND<-BrxA-DNA-binding<-HTH_26<-?<-?<-wHTH+WYL+WCX<-HNH<-?<-ARG	1201	Bacteria>Terrabacteria_group>Bacillota>Clostridia>Eubacteriales>Lachnospiraceae	GCF_900113655.1	Pseudobutyrivibrio_sp._OR37	BM097_RS01535	134498..138103	1798186	BREX-system-P-loop-protein-BrxC-[Pseudobutyrivibrio-sp.-OR37]
WP_072699981.1	Type1.BREX	<-HEPN<-?<-SIGMA+OB-Fold+AAA-ATPase+Lon-Protease-BrxL<-TIR<-PglZ<-RAMA<-N6-METHYLASE<-DUF6079*<-BrxB-BrxF-iSTAND<-BrxA-DNA-binding<-HTH_26<-?<-TraJ-RHH<-LPD2<-TOPRIM+Topoisom_bac<-?<-ArdCN+MPTase-inact+LPD19	1185	Bacteria>Terrabacteria_group>Bacillota>Clostridia>Eubacteriales>Lachnospiraceae	GCF_900143205.1	Butyrivibrio_hungatei	BUA02_RS00135	23142..26699	185008	BREX-system-P-loop-protein-BrxC-[Butyrivibrio-hungatei]
WP_102289727.1	Type1.BREX	<-TnpB_IS66||?-><-SIGMA+OB-Fold+AAA-ATPase+Lon-Protease-BrxL<-Schlafen+HATPase+wHTH<-PglZ<-HEPN<-N6-METHYLASE<-DUF6079*<-BrxB-BrxF-iSTAND<-BrxA-DNA-binding<-HTH_26<-?<-?<-?<-?<-?||HAMP+HisKin+HATPase_c->	1215	Bacteria>Terrabacteria_group>Bacillota>Clostridia>Eubacteriales>Lachnospiraceae	GCF_900243045.1	Eisenbergiella_massiliensis	C1733_RS26410	2142071..2145718	1720294	BREX-system-P-loop-protein-BrxC-[Eisenbergiella-massiliensis]
WP_055192172.1	Type1.BREX	<-MORC+DUF3684+HNH<-HEPN+SH3-like+SF1-SF2-Helicase+wHTH<-PglZ<-Schlafen+HATPase+wHTH<-HEPN<-?<-?<-N6-METHYLASE<-DUF6079*<-BrxB-BrxF-iSTAND<-BrxA-DNA-binding<-?<-35exo+wHTH+SF1-SF2-Helicase+HTH<-?<-35exo	1204	Bacteria>Terrabacteria_group>Bacillota>Clostridia>Eubacteriales>Oscillospiraceae	-	Faecalibacterium_prausnitzii	FXW87_RS13010	15894..19508	853	BREX-system-P-loop-protein-BrxC-[Faecalibacterium-prausnitzii]
MCL2124490.1	Type1.BREX	<-sGTP<-?||?->?->?->BrxA-DNA-binding->BrxB-BrxF-iSTAND->DUF6079*->N6-METHYLASE->NERD+SF1-SF2-Helicase->OLD-ABC->HNH->PolB->HEPN->PglZ->SIGMA+OB-Fold+AAA-ATPase+Lon-Protease-BrxL->	1207	Bacteria>Terrabacteria_group>Bacillota>Clostridia>Eubacteriales>Oscillospiraceae	GCA_009776905.1	Oscillospiraceae_bacterium	FWH33_00685	36555..40178	2485925	BREX-system-P-loop-protein-BrxC-[Oscillospiraceae-bacterium]
MBS7028836.1	Type1.BREX	<-RVT<-SIGMA+OB-Fold+AAA-ATPase+Lon-Protease-BrxL<-PglZ<-HEPN<-N6-METHYLASE<-?<-?<-DUF6079*<-BrxB-BrxF-iSTAND<-BrxA-DNA-binding<-DOC+HTH||DUF932->?->?->CR-ATPase5+REase->?->CR-ATPase8+REase->	1182	Bacteria>Terrabacteria_group>Bacillota>Clostridia>Eubacteriales>Oscillospiraceae	GCA_018381555.1	Faecalibacterium_prausnitzii	KH132_10095	50879..54427	853	BREX-system-P-loop-protein-BrxC-[Faecalibacterium-prausnitzii]
MCI6921905.1	Type1.BREX	ABC-ATPase+SbcC+ABC-ATPase+SbcC->?->?->?->LRR_5->HEPN->BrxA-DNA-binding->BrxB-BrxF-iSTAND->DUF6079*->N6-METHYLASE-p->	1161	Bacteria>Terrabacteria_group>Bacillota>Clostridia>Eubacteriales>Oscillospiraceae	GCA_022778185.1	Oscillospiraceae_bacterium	MR756_00805	25286..28771	2485925	BREX-system-P-loop-protein-BrxC-[Oscillospiraceae-bacterium]
UYJ36126.1	Type1.BREX	TraJ-RHH-><-CR-ATPase5+REase||TraI-VirD2-RCRE->?->?->?->BrxA-DNA-binding->BrxB-BrxF-iSTAND->DUF6079*->N6-METHYLASE->PglZ->HEPN+SH3-like+SF1-SF2-Helicase+wHTH->MORC+DUF3684+HNH->?->Tox-RelA->HKD-DNase+HKD-DNase->HKD-DNase+HKD-DNase->REC+OmpR-HTH->	1205	Bacteria>Terrabacteria_group>Bacillota>Clostridia>Eubacteriales>Oscillospiraceae	GCA_025757985.1	Oscillospiraceae_bacterium	OGM67_07425	1479517..1483134	2485925	MAG:-BREX-system-P-loop-protein-BrxC-[Oscillospiraceae-bacterium]
UYJ36609.1	Type1.BREX	HTH-><-TIR<-SIGMA+OB-Fold+AAA-ATPase+Lon-Protease-BrxL<-PglZ<-Schlafen+HATPase+wHTH<-HEPN<-N6-METHYLASE<-?<-DUF6079*<-BrxB-BrxF-iSTAND<-BrxA-DNA-binding<-HTH_26<-TraI-VirD2-RCRE<-TraJ-RHH<-?<-CopG/RHH<-?<-HTH_36	1182	Bacteria>Terrabacteria_group>Bacillota>Clostridia>Eubacteriales>Oscillospiraceae	GCA_025758005.1	Oscillospiraceae_bacterium	OGM10_10415	2214157..2217705	2485925	MAG:-BREX-system-P-loop-protein-BrxC-[Oscillospiraceae-bacterium]
WP_044960386.1	Type1.BREX	<-CR-ATPase8+REase<-?<-?<-DUF932||DOC+HTH->BrxA-DNA-binding->BrxB-BrxF-iSTAND->DUF6079*->?->N6-METHYLASE->HEPN->PglZ->SIGMA+OB-Fold+AAA-ATPase+Lon-Protease-BrxL->RVT->RVT->	1182	Bacteria>Terrabacteria_group>Bacillota>Clostridia>Eubacteriales>Oscillospiraceae	GCF_000209855.1	Faecalibacterium_prausnitzii	FPR_RS00845	148257..151805	853	BREX-system-P-loop-protein-BrxC-[Faecalibacterium-prausnitzii]
WP_087369154.1	Type1.BREX	<-GspA-ATPase<-SIR2<-?<-?<-?||BrxA-DNA-binding->BrxB-BrxF-iSTAND->DUF6079*->N6-METHYLASE->?->HEPN->PglZ->SIGMA+OB-Fold+AAA-ATPase+Lon-Protease-BrxL-><-Lysozyme+NLPC<-VirB4-FtsK	1178	Bacteria>Terrabacteria_group>Bacillota>Clostridia>Eubacteriales>Oscillospiraceae	GCF_002161675.1	Pseudoflavonifractor_sp._An44	B5F98_RS00420	121430..124966	1965635	BREX-system-P-loop-protein-BrxC-[Pseudoflavonifractor-sp.-An44]
WP_113992229.1	Type1.BREX	<-CR-ATPase5+REase<-?<-?<-DUF932<-NUDIX<-HKD-DNase+SF1-SF2-Helicase+HTH+HTH+SRA||XIS-HTH->BrxA-DNA-binding->BrxB-BrxF-iSTAND->DUF6079*->wHTH+ZnR->Four-helical-protein->N6-METHYLASE->HEPN->PglZ->SIGMA+OB-Fold+AAA-ATPase+Lon-Protease-BrxL->ParB+HNH->HKD-DNase+SF1-SF2-Helicase+HTH+HTH+SRA->nSTAND->	1218	Bacteria>Terrabacteria_group>Bacillota>Clostridia>Eubacteriales>Oscillospiraceae	GCF_003324185.1	Faecalibacterium_prausnitzii	C7J97_RS04695	32898..36554	853	BREX-system-P-loop-protein-BrxC-[Faecalibacterium-prausnitzii]
WP_158398728.1	Type1.BREX	<-ParB+HNH<-SIGMA+OB-Fold+AAA-ATPase+Lon-Protease-BrxL<-PglZ<-HEPN<-TIR<-?<-DUF6079*<-BrxB-BrxF-iSTAND<-BrxA-DNA-binding<-HTH_3<-?<-RepA_N+HTH||<-Peptidase_S51||SIGMA-HTH+SIGMA-HTH->ncRNA->RFC-AAA+RuvB-AAA->	1182	Bacteria>Terrabacteria_group>Bacillota>Clostridia>Eubacteriales>Oscillospiraceae	GCF_902167845.1	Faecalibacterium_prausnitzii	FQ260_RS07280	12413..15961	853	BREX-system-P-loop-protein-BrxC-[Faecalibacterium-prausnitzii]
WP_118142807.1	Type1.BREX	wHTH+WYL+WCX->?->?->?->HTH_26->BrxA-DNA-binding->BrxB-BrxF-iSTAND->DUF6079*->N6-METHYLASE->DUF4365+REase->PglZ->HEPN+SH3-like+SF1-SF2-Helicase+wHTH->MORC+DUF3684+HNH->SF1-SF2-Helicase->PIN->?->CHTH->	1203	Bacteria>Terrabacteria_group>Bacillota>Clostridia>Eubacteriales>Oscillospiraceae	-	Ruminococcus_sp._OM07-7	DXC06_07755	161101..164712	2293234	BREX-system-P-loop-protein-BrxC-[Ruminococcus-sp.-OM07-7]
WP_066638545.1	Type1.BREX	<-Glyoxalase<-?<-SIGMA+OB-Fold+AAA-ATPase+Lon-Protease-BrxL<-PglZ<-HEPN<-PolB<-N6-METHYLASE<-DUF6079*<-BrxB-BrxF-iSTAND<-BrxA-DNA-binding<-?<-SLATT<-MazG<-NUDIX<-35exo<-35exo	1184	Bacteria>Terrabacteria_group>Bacillota>Clostridia>Eubacteriales>Peptococcaceae	-	Desulfolucianica_intricata	DIN01_RS10945	45737..49291	1285191	BREX-system-P-loop-protein-BrxC-[Desulfolucianica-intricata]
WP_057552934.1	Type1.BREX	TetR-HTH->?-><-?||BrxA-DNA-binding->BrxB-BrxF-iSTAND->DUF6079*->N6-METHYLASE->Preader17->HEPN->?->?->OLD-ABC->HEPN->PglZ->SIGMA+OB-Fold+AAA-ATPase+Lon-Protease-BrxL->	1185	Bacteria>Terrabacteria_group>Bacillota>Clostridia>Eubacteriales>Peptostreptococcaceae	GCF_001372915.1	Paeniclostridium_sordellii	SSCC32135_RS03600	682084..685641	1505	BREX-system-P-loop-protein-BrxC-[Paeniclostridium-sordellii]

Protein-ID	System	Gene-Neighbourhoods	Length	Taxonomy	Assembly-ID	Strain	Locus-Tag	Genomic-coordinates	TaxID	Fasta-defline
WP_153971752.1	Type1.BREX	<-HTH_3||MORC->MORC-><-RHH||MarR-HTH->?->?-> BrxA-DNA-binding -> BrxB-BrxF-iSTAND -> DUF6079 *-> N6-METHYLASE ->GTPase-AIG+IIGP-C-> HEPN -> PglZ -> SIGMA+OB-Fold+AAA-ATPase+Lon-Protease-BrxL ->MazF->PspA->	1187	Bacteria>Terrabacteria_group>Bacillota>Clostridia>Eubacteriales>Peptostreptococcaceae	-	Romboutsia_ilealis	EAI30_RS08825	173166..176729	1115758	BREX-system-P-loop-protein-BrxC-[Romboutsia-ilealis]
MBY6275820.1	Type6.BREX	<- HEPN <- SIGMA+OB-Fold+AAA-ATPase+Lon-Protease-BrxL <- PglZ <- N6-METHYLASE <- N6-METHYLASE <- DUF6079 *<- BrxB-BrxF-iSTAND <- BrxA-DNA-binding <- BrxE ||wHTH+REase->AAA-ATPase->	1196	Bacteria>Terrabacteria_group>Bacillota>Clostridia>Eubacteriales>Symbiobacteriaceae	GCA_019806665.1	Symbiobacterium_thermophilum	CWE10_06285	9595..13185	2734	BREX-system-P-loop-protein-BrxC-[Symbiobacterium-thermophilum]
WP_273378700.1	Type6.BREX	<- HEPN <-?<- SIGMA+OB-Fold+AAA-ATPase+Lon-Protease-BrxL <- PglZ <- N6-METHYLASE <- N6-METHYLASE <- DUF6079 *<- BrxB-BrxF-iSTAND <- BrxA-DNA-binding <- BrxE ||wHTH+REase->	1196	Bacteria>Terrabacteria_group>Bacillota>Clostridia>Eubacteriales>Symbiobacteriaceae	GCF_019806665.1	Symbiobacterium_thermophilum	CWE10_RS06335	9595..13185	2734	BREX-system-P-loop-protein-BrxC-[Symbiobacterium-thermophilum]
OAD88367.1	Type1.BREX	DEOR-HTH+ISOCOT->SF1-SF2-Helicase->HAD->?->?->HTH_26-> BrxA-DNA-binding -> BrxB-BrxF-iSTAND -> DUF6079 *->OLD-ABC->?-> N6-METHYLASE -> HEPN ->AT-HTH-HYD2->AT-HTH-HYD2->SIR2->Schlafen+HATPase+wHTH-> PglZ ->	1209	Bacteria>Terrabacteria_group>Bacillota>Clostridia>Eubacteriales>unclassified_Eubacteriales	GCA_001641065.1	Clostridiales_bacterium_KLE1615	HMPREF2738_01633	60233..63862	1715004	hypothetical-protein-HMPREF2738_01633-[Clostridiales-bacterium-KLE1615]
OKZ97591.1	Type1.BREX	<-MORC+DUF3684+HNH<- HEPN +SH3-like+SF1-SF2-Helicase+wHTH<- PglZ <- N6-METHYLASE -p<-Schlafen+HATPase+wHTH<- HEPN <-?<-?<- N6-METHYLASE <- DUF6079 *<- BrxB-BrxF-iSTAND <- BrxA-DNA-binding <-?<-35exo+wHTH+SF1-SF2-Helicase+HTH<-?<-35exo<-?<-?||HTH->	1204	Bacteria>Terrabacteria_group>Bacillota>Clostridia>Eubacteriales>unclassified_Eubacteriales	GCA_001915795.1	Clostridiales_bacterium_42_27	BHV90_19800	17930..21544	1897045	hypothetical-protein-BHV90_19800-[Clostridiales-bacterium-42_27]
PWM66029.1	Type1.BREX	PAPS-reduct->ParB->?-><-?<-?<- SIGMA+OB-Fold+AAA-ATPase+Lon-Protease-BrxL <- PglZ <- HEPN <- N6-METHYLASE <- DUF6079 *<- BrxB-BrxF-iSTAND <- BrxA-DNA-binding <-HTH_26<-?<-?<-wHTH+REase<-McrB-AAA<-Phage_int_SAM_3+Phage_integrase	1180	Bacteria>Terrabacteria_group>Bacillota>Clostridia>Eubacteriales>unclassified_Eubacteriales	GCA_003343885.1	Clostridiales_bacterium	DBX61_08855	48363..51905	1898207	BREX-system-P-loop-protein-BrxC-[Clostridiales-bacterium]
NLC74531.1	Type1.BREX	TrwI->?->?->VirB4-FtsK->?->Lysozyme+NLPC->HTH_26-> BrxA-DNA-binding -> BrxB-BrxF-iSTAND -> DUF6079 *-> HEPN ->?-> N6-METHYLASE -p->	1161	Bacteria>Terrabacteria_group>Bacillota>Clostridia>Eubacteriales>unclassified_Eubacteriales	GCA_012512995.1	Clostridiales_bacterium	GX685_04750	28468..31953	1898207	BREX-system-P-loop-protein-BrxC-[Clostridiales-bacterium]
MBE6030170.1	Type1.BREX	<-ABC-ATPase+SbcC<-?<-HNH+ADA-DBD<-?<-?<- SIGMA+OB-Fold+AAA-ATPase+Lon-Protease-BrxL <- PglZ <- HEPN <- N6-METHYLASE <- DUF6079 *<- BrxB-BrxF-iSTAND <- BrxA-DNA-binding <-GDResolvase+HIN-HTH||?->HTH_3-><-RNasePH+RNasePH+KH+S1	1212	Bacteria>Terrabacteria_group>Bacillota>Clostridia>Eubacteriales>unclassified_Eubacteriales	GCA_015058155.1	Clostridiales_bacterium	E7225_01045	104723..108361	1898207	BREX-system-P-loop-protein-BrxC-[Clostridiales-bacterium]
MCD7920553.1	Type1.BREX	<-HTH_3<-?<-wHTH+TPR<-HTH_3<-?<- SIGMA+OB-Fold+AAA-ATPase+Lon-Protease-BrxL <- PglZ <- HEPN <- N6-METHYLASE <- DUF6079 *<- BrxB-BrxF-iSTAND <- BrxA-DNA-binding <-HTH_26||?->?->?-><-HTH_3	1211	Bacteria>Terrabacteria_group>Bacillota>Clostridia>Eubacteriales>unclassified_Eubacteriales	GCA_021201475.1	Clostridiales_bacterium	LUG45_10925	23806..27441	1898207	BREX-system-P-loop-protein-BrxC-[Clostridiales-bacterium]
WP_173717967.1	Type1.BREX	<- PglZ <-Schlafen+HATPase+wHTH<- HEPN <-AT-HTH-HYD2<-Tox-ART-HYD2<-AT-HTH-HYD2<-SIR2<- N6-METHYLASE <-TIR<- DUF6079 *<- BrxB-BrxF-iSTAND <- BrxA-DNA-binding <-HTH_26<-?<-ABhydrolase<-?<-SAM-METHYLASE	1179	Bacteria>Terrabacteria_group>Bacillota>Clostridia	GCF_013302415.1	Eubacteriales	G5A19_RS02670	21026..24565	186802	MULTISPECIES:-BREX-system-P-loop-protein-BrxC-[Eubacteriales]
WP_018248942.1	Type1.BREX	<-SF1-SF2-Helicase+REase<-SF1-SF2-Helicase+REase<- PglZ <-?<- N6-METHYLASE <-Ntox45<- HEPN <- DUF6079 *<- BrxB-BrxF-iSTAND <- BrxA-DNA-binding <-?<-?<-Peptidase_S78<- HEPN	1197	Bacteria>Terrabacteria_group>Bacillota>Clostridia>Halanaerobiales>Halobacteroidaceae	GCF_000379025.1	Orenia_marismortui	OREMA_RS0108995	158007..161600	46469	BREX-system-P-loop-protein-BrxC-[Orenia-marismortui]
MBN2931490.1	Type1.BREX	HEPN ->?->MqsA_antitoxin+DUF4065->?->?-> HEPN -> BrxA-DNA-binding -> BrxB-BrxF-iSTAND -> DUF6079 *-> N6-METHYLASE ->TIR-> PglZ ->SbcC-> SIGMA+OB-Fold+AAA-ATPase+Lon-Protease-BrxL ->?->MazG-><-TetR-HTH	1177	Bacteria>Terrabacteria_group>Bacillota>Erysipelotrichia>Erysipelotrichales>Coprobacillaceae	GCA_016939095.1	Catenibacterium_mitsuokai	JTJ30_05795	72892..76425	100886	BREX-system-P-loop-protein-BrxC-[Catenibacterium-mitsuokai]
WP_254151261.1	Type1.BREX	HEPN -> BrxA-DNA-binding -> BrxB-BrxF-iSTAND -> DUF6079 -p-> DUF6079 *-> N6-METHYLASE -p-> N6-METHYLASE -p->?-> PglZ ->SbcC-> SIGMA+OB-Fold+AAA-ATPase+Lon-Protease-BrxL ->?->MazG-><-TetR-HTH	1084	Bacteria>Terrabacteria_group>Bacillota>Erysipelotrichia>Erysipelotrichales>Coprobacillaceae	GCF_019041565.1	Catenibacterium_mitsuokai	KTF58_RS01735	17255..20509	100886	BREX-system-P-loop-protein-BrxC-[Catenibacterium-mitsuokai]
WP_226793210.1	Type1.BREX	TetR-HTH-><-MazG<-?<- SIGMA+OB-Fold+AAA-ATPase+Lon-Protease-BrxL <-SbcC<- PglZ <-?<- N6-METHYLASE <- DUF6079 *<- DUF6079 -p<- BrxB-BrxF-iSTAND <- BrxA-DNA-binding <- HEPN	1084	Bacteria>Terrabacteria_group>Bacillota>Erysipelotrichia>Erysipelotrichales>Coprobacillaceae	GCF_020537355.1	Catenibacterium_mitsuokai	LIP38_RS01520	50676..53930	100886	BREX-system-P-loop-protein-BrxC-[Catenibacterium-mitsuokai]
WP_270396150.1	Type1.BREX	BrxA-DNA-binding -> BrxB-BrxF-iSTAND -> DUF6079 *-> N6-METHYLASE ->ParB+HNH-> HEPN + HEPN -> PglZ ->SbcC-> SIGMA+OB-Fold+AAA-ATPase+Lon-Protease-BrxL ->REase->	1177	Bacteria>Terrabacteria_group>Bacillota>Erysipelotrichia>Erysipelotrichales>Coprobacillaceae	GCF_027659655.1	Catenibacterium_mitsuokai	PGD85_RS08450	1355..4888	100886	BREX-system-P-loop-protein-BrxC-[Catenibacterium-mitsuokai]
MBD8958802.1	Type1.BREX	<- HEPN <- SIGMA+OB-Fold+AAA-ATPase+Lon-Protease-BrxL <-REase+DUF4268<- PglZ <-SbcC<-?<-?<- N6-METHYLASE <-?<- DUF6079 *<- BrxB-BrxF-iSTAND <- BrxA-DNA-binding <-HTH_26	1182	Bacteria>Terrabacteria_group>Bacillota>Erysipelotrichia>Erysipelotrichales>Erysipelotrichaceae	GCA_014847315.1	Holdemanella_biformis	EGG83_10165	17990..21538	1735	BREX-system-P-loop-protein-BrxC-[Holdemanella-biformis]
WP_107029377.1	Type1.BREX	<- wHTH+WYL+WCX ||PIN->?->?->HTH_26-> BrxA-DNA-binding -> BrxB-BrxF-iSTAND -> DUF6079 *-> N6-METHYLASE -> HEPN -> PglZ -> SIGMA+OB-Fold+AAA-ATPase+Lon-Protease-BrxL ->	1178	Bacteria>Terrabacteria_group>Bacillota>Erysipelotrichia>Erysipelotrichales>Erysipelotrichaceae	GCF_027669645.1	Faecalibacillus_intestinalis	C7U54_RS03870	158603..162139	1982626	BREX-system-P-loop-protein-BrxC-[Faecalibacillus-intestinalis]
NBK98923.1	Type1.BREX	wHTH+WYL+WCX ->?->SPOUT-> PolB -> HEPN ->?-> BrxA-DNA-binding -> BrxB-BrxF-iSTAND -> DUF6079 -p*->	377	Bacteria>Terrabacteria_group>Bacillota>Erysipelotrichia>unclassified_Erysipelotrichia	GCA_009916335.1	Erysipelotrichia_bacterium	EOM50_13050	4855..5984	2184014	BREX-system-P-loop-protein-BrxC-[Erysipelotrichia-bacterium]
HAA34479.1	Type1.BREX	DOC-><-HicB_lk_antitox||?-> BrxA-DNA-binding -> BrxB-BrxF-iSTAND -> BrxB-BrxF-iSTAND -p-> DUF6079 *-> N6-METHYLASE -> PolB -> HEPN -> PglZ -> SIGMA+OB-Fold+AAA-ATPase+Lon-Protease-BrxL -p->	1003	Bacteria>Terrabacteria_group>Bacillota	GCA_003444595.1	Bacillota_bacterium	DCD97_04120	2822..5830	1879010	BREX-system-P-loop-protein-BrxC-[Bacillota-bacterium]
MTI67135.1	Type1.BREX	BrxA-DNA-binding -> BrxB-BrxF-iSTAND -> DUF6079 *-> N6-METHYLASE -> PglZ -> SIGMA+OB-Fold+AAA-ATPase+Lon-Protease-BrxL -> HEPN ->?-><-Phage_int_SAM_4+Phage_integrase	1184	Bacteria>Terrabacteria_group>Bacillota	GCA_009711765.1	Bacillota_bacterium	FH753_11140	12161..15715	1879010	BREX-system-P-loop-protein-BrxC-[Bacillota-bacterium]
NLX91917.1	Type1.BREX	SF1-SF2-Helicase+REase->SF1-SF2-Helicase+REase->TetR-HTH->?->?->?-> BrxA-DNA-binding -> BrxB-BrxF-iSTAND -> DUF6079 *-> PolB -> HEPN -> PglZ -> SIGMA+OB-Fold+AAA-ATPase+Lon-Protease-BrxL ->EVE+McrB-AAA->wHTH+REase->ARG-> HEPN ->RelE-ParE->	1176	Bacteria>Terrabacteria_group>Bacillota	GCA_012728375.1	Bacillota_bacterium	GXZ07_10120	36614..40144	1879010	BREX-system-P-loop-protein-BrxC-[Bacillota-bacterium]
OLA37697.1	Type1.BREX	<- HEPN <- SIGMA+OB-Fold+AAA-ATPase+Lon-Protease-BrxL <- PglZ <-RADICAL-SAM<-?<-REase<-OLD-ABC<- DUF6079 *<- BrxB-BrxF-iSTAND <- BrxA-DNA-binding	1207	Bacteria>Terrabacteria_group>Bacillota>Negativicutes>Acidaminococcales>Acidaminococcaceae	GCA_001916865.1	Phascolarctobacterium_succinatutens	BHW43_05680	71469..75092	626940	hypothetical-protein-BHW43_05680-[Phascolarctobacterium-succinatutens]
UYJ17329.1	Type1.BREX	<-Glyoxalase<-?<- HEPN <- SIGMA+OB-Fold+AAA-ATPase+Lon-Protease-BrxL <- PglZ <-?<-?<- N6-METHYLASE <- DUF6079 *<- BrxB-BrxF-iSTAND <- BrxA-DNA-binding <-HTH_26<-?<-HNH-p<-HNH+3C-ABC||SIGMA-HTH->MazG->D5N+D5ATPase->	1171	Bacteria>Terrabacteria_group>Bacillota>Negativicutes>Veillonellales>Veillonellaceae	GCA_025757885.1	Veillonellaceae_bacterium	OGM58_02475	522598..526113	2049049	MAG:-BREX-system-P-loop-protein-BrxC-[Veillonellaceae-bacterium]
WP_108850313.1	Type1.BREX	<-Glyoxalase<-?<- HEPN <- SIGMA+OB-Fold+AAA-ATPase+Lon-Protease-BrxL <- PglZ <-?<-?<- N6-METHYLASE <- DUF6079 *<- BrxB-BrxF-iSTAND <- BrxA-DNA-binding <-HTH_26<-?<-HNH+3C-ABC||D5N+D5ATPase->Vrr-REase->SF1-SF2-Helicase->SIGMA-HTH->	1171	Bacteria>Terrabacteria_group>Bacillota>Negativicutes>Veillonellales>Veillonellaceae	GCF_900343095.1	Dialister_massiliensis	DHT67_RS06445	1390627..1394142	2161821	BREX-system-P-loop-protein-BrxC-[Dialister-massiliensis]
WP_073050525.1	Type1.BREX	<-RNase<-NERD<-OLD-ABC<-REase<- SIGMA+OB-Fold+AAA-ATPase+Lon-Protease-BrxL <- PglZ <- HEPN <- N6-METHYLASE <-?<- DUF6079 *<- BrxB-BrxF-iSTAND <- BrxA-DNA-binding <-HTH_26	1214	Bacteria>Terrabacteria_group>Bacillota>Tissierellia>Tissierellia_incertae_sedis>Dethiosulfatibacter	GCF_900142005.1	Dethiosulfatibacter_aminovorans	BUB05_RS15460	41174..44818	332095	BREX-system-P-loop-protein-BrxC-[Dethiosulfatibacter-aminovorans]
RHP99067.1	Type1.BREX	GNTR-HTH+Aminotran_1_2->?->HTH_26-> BrxA-DNA-binding -> BrxB-BrxF-iSTAND -> DUF6079 *->Tox-ART-HYD2-> N6-METHYLASE -> HEPN ->Schlafen+HATPase+wHTH-> PglZ -> SIGMA+OB-Fold+AAA-ATPase+Lon-Protease-BrxL ->?->RNase->SKI->	1215	Bacteria>Terrabacteria_group>Bacillota>unclassified_Bacillota	GCA_003478205.1	Firmicutes_bacterium_AM59-13	DXA19_01120	43130..46777	2292897	BREX-system-P-loop-protein-BrxC-[Firmicutes-bacterium-AM59-13]
RHT37458.1	Type1.BREX	<-ClpABC-AAA<-?<- SIGMA+OB-Fold+AAA-ATPase+Lon-Protease-BrxL <- PglZ <-Schlafen+HATPase+wHTH<- HEPN <- N6-METHYLASE <-?<- DUF6079 *<- BrxB-BrxF-iSTAND <- BrxA-DNA-binding <-HTH_26<-HTH_36<-?<-?||DSBH-><-ABhydrolase	1182	Bacteria>Terrabacteria_group>Bacillota>unclassified_Bacillota	GCA_003480425.1	Firmicutes_bacterium_AM31-12AC	DW790_07855	25743..29291	2292892	BREX-system-P-loop-protein-BrxC-[Firmicutes-bacterium-AM31-12AC]
MAT43664.1	Type1.BREX	<-AAA-ATPase<-TPR+TPR+TPR+TPR<-?<-?<-?<-?<- SIGMA+OB-Fold+AAA-ATPase+HKD-DNase-BrxL <- PglZ <- N6-METHYLASE <- DUF6079 *<- BrxB-BrxF-iSTAND <- BrxA-DNA-binding <- CBS+ HEPN ||?->SF1-SF2-Helicase+DUF1998->ParB-><-?<-rve	1169	Bacteria>Terrabacteria_group>Chloroflexota>Anaerolineae>Anaerolineales>Anaerolineaceae	GCA_002702705.1	Anaerolineaceae_bacterium	CL609_15105	30474..33983	2024896	hypothetical-protein-[Anaerolineaceae-bacterium]
MBP9040834.1	Type1.BREX	SF1-SF2-Helicase-><- SIGMA+OB-Fold+AAA-ATPase+Lon-Protease-BrxL <- PglZ <- N6-METHYLASE <- HEPN <- DUF6079 *	1023	Bacteria>Terrabacteria_group>Chloroflexota>Anaerolineae>Anaerolineales>Anaerolineaceae	GCA_018057145.1	Anaerolineaceae_bacterium	KBF64_03565	8301..11374	2024896	BREX-system-P-loop-protein-BrxC-[Anaerolineaceae-bacterium]
WP_062190148.1	Type1.BREX	<-sGTP<-DDE_Tnp<- SIGMA+OB-Fold+AAA-ATPase+Lon-Protease-BrxL <- PglZ <- N6-METHYLASE <- HEPN <- DUF6079 *<-?<- N6-METHYLASE <-?<-?<- N6-METHYLASE <- BrxB-BrxF-iSTAND <- BrxA-DNA-binding <-HTH_26<-CHTH	1245	Bacteria>Terrabacteria_group>Chloroflexota>Anaerolineae>Anaerolineales>Anaerolineaceae	GCF_001050195.2	Anaerolinea_thermolimosa	ATHL_RS04285	935293..939030	229919	BREX-system-P-loop-protein-BrxC-[Anaerolinea-thermolimosa]
NLF02163.1	Type1.BREX	N6-METHYLASE -p-> DUF6079 *-> HEPN -> PglZ -> SIGMA+OB-Fold+AAA-ATPase+Lon-Protease-BrxL ->HIN-HTH->	1237	Bacteria>Terrabacteria_group>Chloroflexota>Anaerolineae>Anaerolineales>unclassified_Anaerolineales	GCA_012514695.1	Anaerolineales_bacterium	GX601_14420	1151..4864	2073117	BREX-system-P-loop-protein-BrxC-[Anaerolineales-bacterium]
MCA9933330.1	Type1.BREX	HTH_28+rve->XIS-HTH-><-?|| BrxA-DNA-binding -> BrxB-BrxF-iSTAND -> DUF6079 *-> N6-METHYLASE -> HEPN -> PglZ -> SIGMA+OB-Fold+AAA-ATPase+HKD-DNase-BrxL -><-CopG_antitoxin<-BrnT_toxin<-GDResolvase+HIN-HTH||?->TIR->	1174	Bacteria>Terrabacteria_group>Chloroflexota>Anaerolineae>Anaerolineales>unclassified_Anaerolineales	GCA_020432585.1	Anaerolineales_bacterium	KC415_05370	4959..8483	2073117	BREX-system-P-loop-protein-BrxC-[Anaerolineales-bacterium]
MCB0100628.1	Type1.BREX	PTSEIIA1->MarR-HTH->?-> BrxA-DNA-binding -> BrxB-BrxF-iSTAND -> DUF6079 *-> HEPN -> N6-METHYLASE -> HEPN -> PglZ -> SIGMA+OB-Fold+AAA-ATPase+Lon-Protease-BrxL ->ParB+HNH->?->HTH_3->	1152	Bacteria>Terrabacteria_group>Chloroflexota>Anaerolineae>Anaerolineales>unclassified_Anaerolineales	GCA_020433725.1	Anaerolineales_bacterium	KDD74_01185	15066..18524	2073117	BREX-system-P-loop-protein-BrxC-[Anaerolineales-bacterium]
HEC36340.1	Type1.BREX	<- SIGMA+OB-Fold+AAA-ATPase+Lon-Protease-BrxL <- PglZ <- N6-METHYLASE <-TPR<-HicB<-HicA_toxin<- N6-METHYLASE <- DUF6079 *<- HEPN <- N6-METHYLASE <-?<-MarR-HTH<-Schlafen+HATPase+wHTH+HTH||RNase->	1243	Bacteria>Terrabacteria_group>Chloroflexota>Anaerolineae>unclassified_Anaerolineae	GCA_011052725.1	Anaerolineae_bacterium	ENI39_07385	20646..24377	2052143	BREX-system-P-loop-protein-BrxC-[Anaerolineae-bacterium]
MBK9096490.1	Type1.BREX	<- SIGMA+OB-Fold+AAA-ATPase+Lon-Protease-BrxL <- PglZ <-ParB+HNH<- HEPN +REase<-?<- N6-METHYLASE <-TOPRIM<-OLD-ABC<- DUF6079 -p*<- DUF6079 -p<- BrxB-BrxF-iSTAND <- BrxA-DNA-binding <-?||?-> N6-METHYLASE -p->RVT->	900	Bacteria>Terrabacteria_group>Chloroflexota>Anaerolineae>unclassified_Anaerolineae	GCA_016719785.1	Anaerolineae_bacterium	IPM84_27780	244922..247624	2052143	hypothetical-protein-[Anaerolineae-bacterium]
KPV54581.1	Type1.BREX	<- N6-METHYLASE <-REase<- DUF6079 -p*<- DUF6079 -p<- HEPN <- BrxB-BrxF-iSTAND <- BrxA-DNA-binding <-XIS-HTH	802	Bacteria>Terrabacteria_group>Chloroflexota>Chloroflexia>Chloroflexales>Roseiflexineae	GCA_001399705.1	Kouleothrix_aurantiaca	SE17_02870	3869..6277	186479	hypothetical-protein-SE17_02870-[Kouleothrix-aurantiaca]
NLW72147.1	Type1.BREX	<-HTH_3<-?<- SIGMA+OB-Fold+AAA-ATPase+Lon-Protease-BrxL <- PglZ <- HEPN <- N6-METHYLASE <-?<- DUF6079 *<- BrxB-BrxF-iSTAND <- BrxA-DNA-binding <-HTH_26||Calcineurin->	1224	Bacteria>Terrabacteria_group>Chloroflexota>unclassified_Chloroflexota	GCA_012729755.1	Chloroflexota_bacterium	GXY37_04595	11724..15398	2026724	BREX-system-P-loop-protein-BrxC-[Chloroflexota-bacterium]
MBI2765415.1	Type1.BREX	SF1-SF2-Helicase-><-?<-?<-?<-VirB6|| BrxA-DNA-binding -> BrxB-BrxF-iSTAND -> DUF6079 -p-> DUF6079 *-> HEPN -><-?|| N6-METHYLASE -> N6-METHYLASE -p-> PglZ -> SIGMA+OB-Fold+AAA-ATPase+Lon-Protease-BrxL -><-LysM+wHTH+TPR	1100	Bacteria>Terrabacteria_group>Chloroflexota>unclassified_Chloroflexota	GCA_016191435.1	Chloroflexota_bacterium	HYX53_05820	46909..50211	2026724	BREX-system-P-loop-protein-BrxC-[Chloroflexota-bacterium]
MBI3732787.1	Type6.BREX	<-McrB-AAA<-?<- SIGMA+OB-Fold+AAA-ATPase+Lon-Protease-BrxL <- PglZ ||<- N6-METHYLASE <- HEPN <- DUF6079 *<- N6-METHYLASE <- BrxB-BrxF-iSTAND <- BrxA-DNA-binding <- BrxE <-?<-?<-?<-HerA/FtsK	1243	Bacteria>Terrabacteria_group>Chloroflexota>unclassified_Chloroflexota	GCA_016200765.1	Chloroflexota_bacterium	HY259_04935	12602..16333	2026724	BREX-system-P-loop-protein-BrxC-[Chloroflexota-bacterium]
MBK8030403.1	Type6.BREX	<-Phage_holin<-Relaxase<-MetJarc-HTH<-?|| BrxE -> BrxA-DNA-binding -> BrxB-BrxF-iSTAND -> N6-METHYLASE -> DUF6079 *-> HEPN -> N6-METHYLASE -> N6-METHYLASE -p-> PglZ -> PglZ -> SIGMA+OB-Fold+AAA-ATPase+Lon-Protease-BrxL ->	1258	Bacteria>Terrabacteria_group>Chloroflexota>unclassified_Chloroflexota	GCA_016713355.1	Chloroflexota_bacterium	IPK17_13050	320822..324598	2026724	BREX-system-P-loop-protein-BrxC-[Chloroflexota-bacterium]
MCC6629583.1	Type1.BREX	HIN-HTH-> BrxA-DNA-binding -> BrxB-BrxF-iSTAND -> DUF6079 *->?->PIN-> N6-METHYLASE -> PolB -> HEPN ->?-> PglZ -> SIGMA+OB-Fold+AAA-ATPase+Lon-Protease-BrxL ->	1204	Bacteria>Terrabacteria_group>Chloroflexota>unclassified_Chloroflexota	GCA_020849725.1	Chloroflexota_bacterium	IT340_19565	31214..34828	2026724	BREX-system-P-loop-protein-BrxC-[Chloroflexota-bacterium]
MCL4487040.1	Type1.BREX	BrxA-DNA-binding -> BrxB-BrxF-iSTAND ->Peptidase_M91-> N6-METHYLASE ->HKD-DNase-> DUF6079 *-> HEPN ->PhdYeFM_antitox->PIN-> PglZ ->?->?->?->MerR-HTH+TipAS->	1221	Bacteria>Terrabacteria_group>Chloroflexota>unclassified_Chloroflexota	GCA_023381515.1	Chloroflexota_bacterium	M1570_02770	10289..13954	2026724	BREX-system-P-loop-protein-BrxC-[Chloroflexota-bacterium]
WP_071453316.1	Type1.BREX	<- N6-METHYLASE <-RelE-ParE<-?<-REase+HTH||?-><-REase+HTH||?->PIN-><- HEPN <- DUF6079 *<- BrxB-BrxF-iSTAND <- BrxA-DNA-binding <-?||?->?->?->tRNA-><-?||MPTase->	1210	Bacteria>Terrabacteria_group>Cyanobacteriota/Melainabacteria_group>Cyanobacteriota>Cyanophyceae>Gloeomargaritales	GCF_001870225.1	Gloeomargarita_lithophora	GlitD10_RS01485	315738..319370	1188228	BREX-system-P-loop-protein-BrxC-[Gloeomargarita-lithophora]
WP_190963244.1	Type6.BREX	HTH_26->?->?->?->?-> HEPN -> BrxE -> BrxA-DNA-binding -> BrxB-BrxF-iSTAND -> DUF6079 *-> N6-METHYLASE ->MORC+DUF3684->MORC+DUF3684+REase-> PglZ -> BrxD-AAA -> SF1-SF2-Helicase+wHTH+Small-B-barrel+DUF1998 ->	1207	Bacteria>Terrabacteria_group>Cyanobacteriota/Melainabacteria_group>Cyanobacteriota>Cyanophyceae>Nostocales	-	Desmonostoc_muscorum	H6G91_22885	8366..11989	1179	BREX-system-P-loop-protein-BrxC-[Desmonostoc-muscorum]
WP_114083280.1	Type1.BREX	NACHT->?-> BrxA-DNA-binding -><- SIGMA+OB-Fold+AAA-ATPase+Lon-Protease-BrxL <- PglZ +Phosphodiest <-SbcC+ABC-ATPase<- N6-METHYLASE <- HEPN <- DUF6079 *<- BrxB-BrxF-iSTAND ||?->?->REase->Transposase->	1180	Bacteria>Terrabacteria_group>Cyanobacteriota/Melainabacteria_group>Cyanobacteriota>Cyanophyceae>Nostocales	GCF_009873495.1	Nostoc_sp._ATCC_53789	GJB62_RS33695	77191..80733	76335	BREX-system-P-loop-protein-BrxC-[Nostoc-sp.-ATCC-53789]
BBD57221.1	Type1.BREX	<-MerR-HTH||RHH-><-?<-?||?->TPR+TPR+TPR-><-Phage_integrase<-?|| BrxB-BrxF-iSTAND -> DUF6079 *->?-><-?<-REase||?->PIN-> N6-METHYLASE -> HEPN -> PglZ +Phosphodiest ->HKD-DNase-> SIGMA+OB-Fold+AAA-ATPase+Lon-Protease-BrxL ->	1176	Bacteria>Terrabacteria_group>Cyanobacteriota/Melainabacteria_group>Cyanobacteriota>Cyanophyceae>Oscillatoriophycideae	GCA_003609755.1	Planktothrix_agardhii_NIES-204	NIES204_45570	14633..18163	282423	hypothetical-protein-NIES204_45570-[Planktothrix-agardhii-NIES-204]
WP_027255660.1	Type1.BREX	<-MerR-HTH||?->PAS-><-?<-?<-?||?-> BrxB-BrxF-iSTAND -> DUF6079 *->?->PIN-> N6-METHYLASE -p-> N6-METHYLASE -> HEPN -> PglZ +Phosphodiest ->HTH_Tnp_IS630+DDE_3-><-DDE_Tnp_1||HKD-DNase->	1176	Bacteria>Terrabacteria_group>Cyanobacteriota/Melainabacteria_group>Cyanobacteriota>Cyanophyceae>Oscillatoriophycideae	GCF_000464825.1	Planktothrix_agardhii	N316_RS0124710	8429..11959	1160	BREX-system-P-loop-protein-BrxC-[Planktothrix-agardhii]
WP_190817045.1	Type1.BREX	HTH_28+HTH_32+rve+Mu-transpos_C->TnsC-ATPase-> BrxA-DNA-binding -> BrxB-BrxF-iSTAND -> DUF6079 *->DOC+HTH-> N6-METHYLASE ->ParB+HNH-> HEPN -> PglZ -> SIGMA+OB-Fold+AAA-ATPase+Lon-Protease-BrxL ->?-><-TPR+TPR+TPR<-AAA-ATPase	1234	Bacteria>Terrabacteria_group>Cyanobacteriota/Melainabacteria_group>Cyanobacteriota>Cyanophyceae>Oscillatoriophycideae	GCF_014698845.1	Coleofasciculus_sp._FACHB-1120	H6H02_RS09830	171769..175473	2692783	BREX-system-P-loop-protein-BrxC-[Coleofasciculus-sp.-FACHB-1120]
MBC6953093.1	Type1.BREX	<-SIR2<-?||Phage_integrase-><- SIGMA+OB-Fold+AAA-ATPase+Lon-Protease-BrxL <- PglZ <- HEPN <-?<-?<- N6-METHYLASE <- DUF6079 *<- BrxB-BrxF-iSTAND <- BrxA-DNA-binding ||?->?->XIS-HTH->Primpol+PriCT->Phage_integrase->	1150	Bacteria>Terrabacteria_group>Cyanobacteriota/Melainabacteria_group>Cyanobacteriota>Cyanophyceae>Pseudanabaenales	GCA_014338235.1	Leptolyngbya_sp.	DWB46_02725	400090..403542	47254	BREX-system-P-loop-protein-BrxC-[Leptolyngbya-sp.]
MCA6523516.1	Type1.BREX	PolB -> HEPN ->?->RelE-ParE-><-FGS<-?||?->?-> BrxB-BrxF-iSTAND -> DUF6079 *-> N6-METHYLASE -> PolB -> HEPN ->	1202	Bacteria>Terrabacteria_group>Cyanobacteriota/Melainabacteria_group>Cyanobacteriota>Cyanophyceae>Pseudanabaenales	GCA_020403865.1	Pseudanabaena_sp._M051S1SP2A07QC	IM596_17155	23967..27575	2771253	BREX-system-P-loop-protein-BrxC-[Pseudanabaena-sp.-M051S1SP2A07QC]
MCL1489692.1	Type1.BREX	<-SF1-SF2-Helicase+REase|| BrxA-DNA-binding -><- SIGMA+OB-Fold+AAA-ATPase+Lon-Protease-BrxL <- PglZ +Phosphodiest ||?->?-><- HEPN <- PolB <- N6-METHYLASE <- DUF6079 *<- BrxB-BrxF-iSTAND <-?||?->?-><-?<-ParB+HTH<-ParA-Soj-PloopNTPase<-pPIWI||HTH_Tnp_4+DDE_Tnp_4->	1200	Bacteria>Terrabacteria_group>Cyanobacteriota/Melainabacteria_group>Cyanobacteriota>Cyanophyceae>Pseudanabaenales	GCA_023336205.1	Pseudanabaena_sp._Salubria-1	M1G31_02900	157927..161529	2938918	BREX-system-P-loop-protein-BrxC-[Pseudanabaena-sp.-Salubria-1]
WP_015123655.1	Type1.BREX	<-REase+N6_Mtase||HD-><-?<-NACHT||TOPRIM+Primase->?-><-?|| BrxA-DNA-binding -> BrxB-BrxF-iSTAND -> DUF6079 *-> HEPN -> N6-METHYLASE ->?->?-> PglZ -> SIGMA+OB-Fold+AAA-ATPase+Lon-Protease-BrxL -><-?||HHH_5+SF1-SF2-Helicase->	1221	Bacteria>Terrabacteria_group>Cyanobacteriota/Melainabacteria_group>Cyanobacteriota>Cyanophyceae>Synechococcales	GCF_000316685.1	Synechococcus_sp._PCC_6312	SYN6312_RS04385	886176..889841	195253	BREX-system-P-loop-protein-BrxC-[Synechococcus-sp.-PCC-6312]
WP_193814304.1	Type1.BREX	<- SIGMA+OB-Fold+AAA-ATPase+Lon-Protease-BrxL <- PglZ <- N6-METHYLASE <-ORC-CDC6<-ParA-Soj-PloopNTPase<- HEPN <- N6-METHYLASE <- DUF6079 *<- BrxB-BrxF-iSTAND <- BrxA-DNA-binding <- BrxE	1182	Bacteria>Terrabacteria_group>Cyanobacteriota/Melainabacteria_group>Cyanobacteriota>Cyanophyceae>Synechococcales	GCF_015207535.1	Cyanobium_sp._LEGE_06143	IQ216_RS03215	12673..16221	945727	BREX-system-P-loop-protein-BrxC-[Cyanobium-sp.-LEGE-06143]
WP_255021816.1	Type6.BREX	<-SIGMA-HTH<- HEPN <- SIGMA+OB-Fold+AAA-ATPase+Lon-Protease-BrxL <-?<-Schlafen+HATPase+wHTH<- PglZ <- N6-METHYLASE <- DUF6079 *<- BrxB-BrxF-iSTAND <- BrxA-DNA-binding <- BrxE <-?||Sak4+WYL-><-?<-Thermonuclease||?->DUF4236+TPR->	1178	Bacteria>Terrabacteria_group>Cyanobacteriota/Melainabacteria_group>Cyanobacteriota>Cyanophyceae>Synechococcales	GCF_024345755.1	Cyanobium_sp._L1E-Cus	KBZ22_RS09750	55554..59090	2823714	BREX-system-P-loop-protein-BrxC-[Cyanobium-sp.-L1E-Cus]
WP_210391379.1	Type1.BREX	<-Peptidase_S66+Peptidase_S66C||Aminotran_1_2->?->?-><-?<-?||?-><-Zn_Tnp_IS1+HTH_Tnp_IS1|| BrxB-BrxF-iSTAND -> DUF6079 *-> Polbeta -> HEPN -> N6-METHYLASE -p-> N6-METHYLASE -p-><-DUF4096+DDE_Tnp_1+DDE_Tnp_1_2||MMR_HSR1+sGTP+sGTP->?-><-ZnR+SLATT	1155	Bacteria>Terrabacteria_group>Cyanobacteriota/Melainabacteria_group>Cyanobacteriota>Cyanophyceae>Synechococcales	-	Synechococcus_sp._PCC_7502	SYN7502_RS06685	1387178..1390645	1173263	BREX-system-P-loop-protein-BrxC-[Synechococcus-sp.-PCC-7502]
WP_073476316.1	Type1.BREX	<-MoxR-AAA||XIS-HTH-> BrxA-DNA-binding -> BrxB-BrxF-iSTAND -> DUF6079 *-> HEPN -> N6-METHYLASE ->OLD-ABC->TOPRIM-> PglZ -> SIGMA+OB-Fold+AAA-ATPase+Lon-Protease-BrxL ->?->Cap4_nuclease+SAVED->SMODS->	1215	Bacteria>Thermodesulfobacteriota>Desulfobacteria>Desulfobacterales>Desulfatibacillaceae>Desulfatibacillum	GCF_900142135.1	Desulfatibacillum_alkenivorans	BUE18_RS12840	56211..59858	259354	BREX-system-P-loop-protein-BrxC-[Desulfatibacillum-alkenivorans]
WP_207689226.1	Type1.BREX	<- PglZ <-?<-?<-?<- N6-METHYLASE -p<- N6-METHYLASE || PolB -> HEPN -><- DUF6079 *<- BrxB-BrxF-iSTAND <- BrxA-DNA-binding ||?->?->REase-><-?<-?<-PAS+NtrC-AAA+FIS-HTH	1199	Bacteria>Thermodesulfobacteriota>Desulfobacteria>Desulfobacterales>Desulfococcaceae>Desulfonema	GCF_017377355.1	Desulfonema_limicola	dnl_RS26385	6146847..6150446	45656	BREX-system-P-loop-protein-BrxC-[Desulfonema-limicola]
WP_181552438.1	Type1.BREX	<- N6-METHYLASE <- HEPN <- DUF6079 *<- BrxB-BrxF-iSTAND <- BrxA-DNA-binding <-?||HTH->Phage_int_SAM_3+Phage_integrase->	1209	Bacteria>Thermodesulfobacteriota>Desulfobacteria>Desulfobacterales>Desulfosalsimonadaceae>Desulfosalsimonas	GCF_013761005.1	Desulfosalsimonas_propionicica	HNR65_RS15495	3160..6789	332175	BREX-system-P-loop-protein-BrxC-[Desulfosalsimonas-propionicica]
MBF0120841.1	Type1.BREX	<- SIGMA+OB-Fold+AAA-ATPase+Lon-Protease-BrxL <- PglZ <- N6-METHYLASE <-CR-ATPase8+REase<- DUF6079 *<- BrxB-BrxF-iSTAND <- BrxA-DNA-binding <- HEPN	1201	Bacteria>Thermodesulfobacteriota>Desulfobacteria>Desulfobacterales>unclassified_Desulfobacterales	GCA_015228685.1	Desulfobacterales_bacterium	HQK79_18595	27077..30682	2044940	BREX-system-P-loop-protein-BrxC-[Desulfobacterales-bacterium]
MCG2778948.1	Type1.BREX	DUF6079 -p*-> HEPN -> N6-METHYLASE ->OLD-ABC->TOPRIM-> PglZ -> SIGMA+OB-Fold+AAA-ATPase+Lon-Protease-BrxL ->	757	Bacteria>Thermodesulfobacteriota>Desulfobacteria>Desulfobacterales>unclassified_Desulfobacterales	GCA_022013615.1	Desulfobacterales_bacterium	L6406_25005	1..2276	2044940	hypothetical-protein-[Desulfobacterales-bacterium]
WP_192624162.1	Type1.BREX	AbrB-HTH-> BrxA-DNA-binding -> BrxB-BrxF-iSTAND -> DUF6079 *-> HEPN -> N6-METHYLASE ->PHP+SbcC->Schlafen+HATPase+wHTH-> PglZ -> SIGMA+OB-Fold+AAA-ATPase+Lon-Protease-BrxL -> HEPN ->	1142	Bacteria>Thermodesulfobacteriota>Desulfovibrionia>Desulfovibrionales>Desulfomicrobiaceae>Desulfomicrobium	GCF_014873765.1	Desulfomicrobium_macestii	H4684_RS13755	68182..71610	90731	BREX-system-P-loop-protein-BrxC-[Desulfomicrobium-macestii]
MBG0773462.1	Type1.BREX	<- SIGMA+OB-Fold+AAA-ATPase+Lon-Protease-BrxL <- PglZ <- HEPN <- N6-METHYLASE <- DUF6079 *<- BrxB-BrxF-iSTAND <- BrxA-DNA-binding <-Phage_integrase<-tRNA||?->SIGMA-HTH->ncRNA->?->?->?->REC+PP2C->	1190	Bacteria>Thermodesulfobacteriota>Desulfovibrionia>Desulfovibrionales>Desulfovibrionaceae>Oleidesulfovibrio	GCA_015709285.1	Oleidesulfovibrio_alaskensis	HOS83_07675	9061..12633	58180	BREX-system-P-loop-protein-BrxC-[Oleidesulfovibrio-alaskensis]
WP_273238273.1	Type1.BREX	<- SIGMA+OB-Fold+AAA-ATPase+Lon-Protease-BrxL <- PglZ <- HEPN <- N6-METHYLASE <- DUF6079 *<- BrxB-BrxF-iSTAND <- BrxA-DNA-binding <-Phage_integrase<-tRNA||?->SIGMA-HTH->ncRNA->?->?->?->REC+PP2C->	1191	Bacteria>Thermodesulfobacteriota>Desulfovibrionia>Desulfovibrionales>Desulfovibrionaceae>Oleidesulfovibrio	GCF_015709285.1	Oleidesulfovibrio_alaskensis	HOS83_RS07680	9061..12636	58180	BREX-system-P-loop-protein-BrxC-[Oleidesulfovibrio-alaskensis]
SOB57200.1	Type1.BREX	<- SIGMA+OB-Fold+AAA-ATPase+Lon-Protease-BrxL <- PglZ <- HEPN <- N6-METHYLASE -p||HTH_Tnp_1->HTH_Tnp_1+HTH_21+rve+rve_3-><- N6-METHYLASE -p<- N6-METHYLASE -p<- DUF6079 *<- BrxB-BrxF-iSTAND <- BrxA-DNA-binding <-Phage_integrase<-tRNA<-?<-?<-?||?->?->HAD+HAD->	1190	Bacteria>Thermodesulfobacteriota>Desulfovibrionia>Desulfovibrionales>Desulfovibrionaceae>Pseudodesulfovibrio	GCA_900217235.1	Pseudodesulfovibrio_profundus	DPRO_0321	331811..335383	57320	conserved-protein-of-unknown-function-[Pseudodesulfovibrio-profundus]
WP_232005669.1	Type1.BREX	<- SIGMA+OB-Fold+AAA-ATPase+Lon-Protease-BrxL <- PglZ <- HEPN <- N6-METHYLASE -p||HTH_Tnp_1+HTH_21+rve+rve_3-><- N6-METHYLASE -p<- N6-METHYLASE -p<- DUF6079 *<- BrxB-BrxF-iSTAND <- BrxA-DNA-binding <-Phage_integrase<-tRNA<-?<-?<-?||?->?->HAD+HAD->	1191	Bacteria>Thermodesulfobacteriota>Desulfovibrionia>Desulfovibrionales>Desulfovibrionaceae>Pseudodesulfovibrio	GCF_900217235.1	Pseudodesulfovibrio_profundus	DPRO_RS01600	331811..335386	57320	BREX-system-P-loop-protein-BrxC-[Pseudodesulfovibrio-profundus]
WP_015862248.1	Type1.BREX	<-Phage_integrase<-?<-?||ParB+N6_N4_METHYLASE->PHP+SbcC-><- SIGMA+OB-Fold+AAA-ATPase+Lon-Protease-BrxL <- PglZ <- HEPN <- N6-METHYLASE <- DUF6079 *<- BrxB-BrxF-iSTAND <- BrxA-DNA-binding <-HKD-DNase<-?<-SbcC<-HAD<-?||?->Radical-SAM->	1188	Bacteria>Thermodesulfobacteriota>Desulfovibrionia>Desulfovibrionales>Desulfovibrionaceae>Solidesulfovibrio	GCF_000010665.1	Solidesulfovibrio_magneticus	DMR_RS17110	4099313..4102879	184917	BREX-system-P-loop-protein-BrxC-[Solidesulfovibrio-magneticus]
WP_010942753.1	Type1.BREX	<-Ferredoxin-RRM+Radical_SAM<-?<- SIGMA+OB-Fold+AAA-ATPase+Lon-Protease-BrxL <- PglZ <- N6-METHYLASE <- HEPN <- DUF6079 *<- BrxB-BrxF-iSTAND <- BrxA-DNA-binding <-?<-?||CHTH-><-?<-?||Phage_integrase->Arm-DNA-bind_2+Phage_int_SAM_3+Phage_integrase->	1214	Bacteria>Thermodesulfobacteriota>Desulfuromonadia>Geobacterales>Geobacteraceae>Geobacter	GCF_000007985.2	Geobacter_sulfurreducens	GS_RS10585	2325892..2329536	35554	BREX-system-P-loop-protein-BrxC-[Geobacter-sulfurreducens]
RKJ48602.1	Type1.BREX	HTH-><-PHP+SbcC<- SIGMA+OB-Fold+AAA-ATPase+Lon-Protease-BrxL <- PglZ <-Schlafen+HATPase+wHTH<- HEPN <- N6-METHYLASE <-TIR<-?<- DUF6079 *<- BrxB-BrxF-iSTAND <- BrxA-DNA-binding <-HTH_26<-?<-TraI-VirD2-RCRE<-TraJ-RHH<-?<-HTH_36	1182	Bacteria>unclassified_Bacteria	GCA_003612585.1	bacterium_1XD42-54	D7Y05_12400	29825..33373	2320110	BREX-system-P-loop-protein-BrxC-[bacterium-1XD42-54]
MCI0601822.1	Type1.BREX	<-MORC<-HTH+MPTase<-?<- SIGMA+OB-Fold+AAA-ATPase+Lon-Protease-BrxL <- PglZ <- N6-METHYLASE <- HEPN <- DUF6079 *<- BrxB-BrxF-iSTAND <- BrxA-DNA-binding	1220	Bacteria>unclassified_Bacteria	GCA_022616075.1	bacterium	L0156_02305	63267..66929	1869227	BREX-system-P-loop-protein-BrxC-[bacterium]
MCP4549289.1	Type6.BREX	<-XIS-HTH<-?|| BrxE -> BrxA-DNA-binding -> BrxB-BrxF-iSTAND -> N6-METHYLASE ->?->REase-> DUF6079 *-> HEPN -> N6-METHYLASE -><-?||?-> N6-METHYLASE ->AbiEi_4+AbiEi_1->AbiEii+ZnR->	1242	Bacteria>unclassified_Bacteria	GCA_024231955.1	bacterium	GY835_22785	11465..15193	1869227	BREX-system-P-loop-protein-BrxC-[bacterium]

Protein-ID	System	Gene-Neighbourhoods	Length	Taxonomy	Assembly-ID	Strain	Locus-Tag	Genomic-coordinates	TaxID	Fasta-defline
--										
-- HKD-DNase (BrxL-Fusion)										
--										
BDC48932.1	Type6.BREX	<-HKD-DNase+HKD-Rease<-?<-SIGMA+OB-Fold+AAA-ATPase+HKD-DNase-BrxL<-PglZ<-N6-METHYLASE<-DUF6079*<-BrxB-BrxF-iSTAND<-BrxA-DNA-binding<-BrxE<-NACHT+GTPase-AIG+sGTP<-NTP_transf_8<-REase+HTH	1202	Bacteria>Acidobacteriota>Terriglobia>Bryobacterales>unclassified_Bryobacterales	GCA_016865505.2	Bryobacterales_bacterium_F-183	F183_A12480	1423409..1427017	2802972	hypothetical-protein-F183_A12480-[Bryobacterales-bacterium-F-183]
NLV30311.1	Type1.BREX	DNA-glycosylase-><-?<-SH3||wHTH+WYL+WCX->SF1-SF2-Helicase->BrxA-DNA-binding->BrxB-BrxF-iSTAND->DUF6079*->Schlafen+HATPase+wHTH+HTH->N6-METHYLASE->PglZ->SIGMA+OB-Fold+AAA-ATPase+HKD-DNase-BrxL->HigB_toxin->HTH+MPTase->SF1-SF2-Helicase->?-><-DSBA+Thioredoxin	1169	Bacteria>Acidobacteriota>unclassified_Acidobacteriota	GCA_012729555.1	Acidobacteriota_bacterium	GXY47_04070	27594..31103	1978231	BREX-system-P-loop-protein-BrxC-[Acidobacteriota-bacterium]
MBS1808657.1	Type6.BREX	<-SF1-SF2-Helicase+DUF1998<-?<-35exo||NtrC-AAA+FIS-HTH-><-SIGMA+OB-Fold+AAA-ATPase+HKD-DNase-BrxL<-PglZ<-N6-METHYLASE<-DUF6079*<-BrxB-BrxF-iSTAND<-BrxA-DNA-binding<-BrxE||?->Cysteine-Desulfurase-><-Peptidase	1212	Bacteria>Acidobacteriota>unclassified_Acidobacteriota	GCA_018268995.1	Acidobacteriota_bacterium	JST84_10740	129628..133266	1978231	BREX-system-P-loop-protein-BrxC-[Acidobacteriota-bacterium]
MCK9474444.1	Type1.BREX	BrxA-DNA-binding->BrxB-BrxF-iSTAND->DUF6079*->OLD-ABC->NERD->N6-METHYLASE->PglZ->SIGMA+OB-Fold+AAA-ATPase+HKD-DNase-BrxL->?->ZnR->?->PadR-HTH->	1170	Bacteria>Bacteria_incertae_sedis>Bacteria_candidate_phyla>Candidatus_Muirbacteria>Candidatus_Muirbacterium	GCA_023228995.1	Candidatus_Muirbacterium_halophilum	MOR46_00860	28955..32467	2053465	BREX-system-P-loop-protein-BrxC-[Candidatus-Muirbacterium-halophilum]
PKL50472.1	Type1.BREX	<-MPTase<-HTH_19<-?||BrxA-DNA-binding->BrxB-BrxF-iSTAND->DUF6079*->Schlafen+HATPase+wHTH+HTH->OLD-ABC->REase->N6-METHYLASE->PglZ->SIGMA+OB-Fold+AAA-ATPase+HKD-DNase-BrxL->	1185	Bacteria>Bacteria_incertae_sedis>Bacteria_candidate_phyla>Candidatus_Riflebacteria>unclassified_Candidatus_Riflebacteria	GCA_002839435.1	Candidatus_Riflebacteria_bacterium_HGW-Riflebacteria-2	CVV42_02960	62937..66494	2013830	MAG:-BREX-system-P-loop-protein-BrxC-[Candidatus-Riflebacteria-bacterium-HGW-Riflebacteria-2]
NUM59087.1	Type1.BREX	Phage_integrase->Phage_integrase->Phage_int_SAM_1+Phage_integrase->?-><-?<-SbcC+SbcC<-SIGMA+OB-Fold+AAA-ATPase+HKD-DNase-BrxL<-PglZ<-N6-METHYLASE<-DUF6079*<-BrxB-BrxF-iSTAND<-BrxA-DNA-binding<-XIS-HTH<-?<-?<-ABhydrolase<-REC+NtrC-AAA+FIS-HTH<-HISKIN+MORC	1157	Bacteria>Bdellovibrionota>Bdellovibrionia>Bdellovibrionales>Pseudobdellovibrionaceae>unclassified_Pseudobdellovibrionaceae	GCA_013359135.1	Pseudobdellovibrionaceae_bacterium	HUU56_10670	15517..18990	2026715	BREX-system-P-loop-protein-BrxC-[Pseudobdellovibrionaceae-bacterium]
PIT99446.1	Type1.BREX	<-ABhydrolase<-REC+NtrC-AAA+FIS-HTH<-HISKIN+MORC<-?<-?||XIS-HTH->BrxA-DNA-binding->BrxB-BrxF-iSTAND->DUF6079*->N6-METHYLASE->PglZ->SIGMA+OB-Fold+AAA-ATPase+HKD-DNase-BrxL->SIR2-><-MuB-ATPase<-HTH_28+HTH_32+rve+rve_3	1164	Bacteria>Bdellovibrionota>Bdellovibrionia>Bdellovibrionales>unclassified_Bdellovibrionales	GCA_002778785.1	Bdellovibrionales_bacterium_CG10_big_fil_rev_8_21_14_0_10_45_34	COT74_10625	394529..398023	1973927	BREX-system-P-loop-protein-BrxC-[Bdellovibrionales-bacterium-CG10_big_fil_rev_8_21_14_0_10_45_34]
KAA3613611.1	Type1.BREX	<-PIN||?->?->BrxA-DNA-binding->BrxB-BrxF-iSTAND->DUF6079*->N6-METHYLASE->?->PglZ->SIGMA+OB-Fold+AAA-ATPase+HKD-DNase-BrxL->REase->CARF+NtrC-AAA+FIS-HTH->REase->	1159	Bacteria>Calditrichota>unclassified_Calditrichota	GCA_008501795.1	Calditrichota_bacterium	DWQ03_12295	106728..110207	2212469	BREX-system-P-loop-protein-BrxC-[Calditrichota-bacterium]
WP_187475233.1	Type1.BREX	METHYLASE->Peptidase_M3->?->?-><-DDE_Tnp_1_4||?->XIS-HTH->BrxA-DNA-binding->BrxB-BrxF-iSTAND->DUF6079*->OLD-ABC->REase->N6-METHYLASE->DrHyd->Schlafen+HATPase+wHTH->Trypsin->?->PglZ->SIGMA+OB-Fold+AAA-ATPase+HKD-DNase-BrxL->	1166	Bacteria>Campylobacterota>Epsilonproteobacteria>Campylobacterales>Arcobacteraceae>Aliarcobacter	GCF_014352915.1	Aliarcobacter_cryaerophilus	HOO34_RS05640	1060903..1064403	28198	BREX-system-P-loop-protein-BrxC-[Aliarcobacter-cryaerophilus]
WP_260896239.1	Type1.BREX	Peptidase->Phage_integrase->?->XIS-HTH->BrxA-DNA-binding->BrxB-BrxF-iSTAND->DUF6079*->?->N6-METHYLASE->DrHyd->Schlafen+HATPase+wHTH->REase->ACET->PglZ->SIGMA+OB-Fold+AAA-ATPase+HKD-DNase-BrxL->	1166	Bacteria>Campylobacterota>Epsilonproteobacteria>Campylobacterales>Arcobacteraceae>Aliarcobacter	GCF_025335025.1	Aliarcobacter_cryaerophilus	N5T57_RS09580	13633..17133	28198	BREX-system-P-loop-protein-BrxC-[Aliarcobacter-cryaerophilus]
TDB40153.1	Type1.BREX	Phage_integrase->SIR2-><-Thermonuclease<-?<-LexA-protease||?->BrxA-DNA-binding->BrxB-BrxF-iSTAND->DUF6079*->Schlafen+HATPase+wHTH+HTH->N6-METHYLASE->REase->PglZ->SIGMA+OB-Fold+AAA-ATPase+HKD-DNase-BrxL-><-?<-?<-35exo<-Calcineurin	1180	Bacteria>delta/epsilon_subdivisions>Deltaproteobacteria>unclassified_Deltaproteobacteria	GCA_004348915.1	Deltaproteobacteria_bacterium	D9V46_00535	112112..115654	2026735	BREX-system-P-loop-protein-BrxC-[Deltaproteobacteria-bacterium]
MBC8466717.1	Type1.BREX	<-RAMA<-SIGMA+OB-Fold+AAA-ATPase+HKD-DNase-BrxL<-PglZ<-N6-METHYLASE<-REase<-OLD-ABC<-DUF6079*<-BrxB-BrxF-iSTAND<-BrxA-DNA-binding	1172	Bacteria>delta/epsilon_subdivisions>Deltaproteobacteria>unclassified_Deltaproteobacteria	GCA_014384065.1	Deltaproteobacteria_bacterium	H8D55_02650	10326..13844	2026735	BREX-system-P-loop-protein-BrxC-[Deltaproteobacteria-bacterium]
MCC6503799.1	Type1.BREX	<-SbcC<-Calcineurin+Calcineurin-SbcD<-SbcC+APATPase+SbcC<-SIGMA+OB-Fold+AAA-ATPase+HKD-DNase-BrxL<-PglZ<-N6-METHYLASE<-REase<-OLD-ABC<-DUF6079*<-BrxB-BrxF-iSTAND<-BrxA-DNA-binding<-XIS-HTH	1181	Bacteria>delta/epsilon_subdivisions>Deltaproteobacteria>unclassified_Deltaproteobacteria	GCA_020846435.1	Deltaproteobacteria_bacterium	IT362_11790	16699..20244	2026735	BREX-system-P-loop-protein-BrxC-[Deltaproteobacteria-bacterium]
MCP4158880.1	Type1.BREX	<-CARF<-HKD-DNase+HKD-DNase<-?<-S1+SF1-SF2-Helicase+S1<-SIGMA+OB-Fold+AAA-ATPase+HKD-DNase-BrxL<-PglZ<-Couple_hipA+HipA_C<-HTH_3<-N6-METHYLASE<-DUF6079*	1006	Bacteria>delta/epsilon_subdivisions>Deltaproteobacteria>unclassified_Deltaproteobacteria	GCA_024224625.1	Deltaproteobacteria_bacterium	GY760_02295	25697..28718	2026735	BREX-system-P-loop-protein-BrxC-[Deltaproteobacteria-bacterium]
BAH89618.1	Type6.BREX	CHTH-><-?||?->ZnR+TOPRIM+AAA-helicase+wHTH-><-?||BrxE->BrxA-DNA-binding->BrxB-BrxF-iSTAND->DUF6079*->GreA_GreB+SF1-SF2-Helicase->N6-METHYLASE->N6-METHYLASE->DOC+HTH->PglZ->SIGMA+OB-Fold+AAA-ATPase+HKD-DNase-BrxL->?->?->RADICAL-SAM->	1211	Bacteria>environmental_samples	-	uncultured_bacterium	.	12703..16338	77133	ATPase-[uncultured-bacterium]
MCS4056789.1	Type1.BREX	<-Calcineurin-SbcD<-SIGMA+OB-Fold+AAA-ATPase+HKD-DNase-BrxL<-PglZ<-HEPN<-PolB||?-><-?<-N6-METHYLASE<-DOC+HTH<-DUF6079*<-BrxB-BrxF-iSTAND<-BrxA-DNA-binding<-?<-viral-protein<-?<-?||?-><-RHH	1165	Bacteria>FCB_group>Bacteroidetes/Chlorobi_group>Bacteroidota>Bacteroidetes_incertae_sedis>Rhodothermaceae	GCA_024809315.1	Salinibacter_ruber	GGP75_002131	31231..34728	146919	hypothetical-protein-[Salinibacter-ruber]
WP_119843039.1	Type1.BREX	<-HD<-SbcC+SbcC<-Calcineurin-SbcD<-HORMA<-SIGMA+OB-Fold+AAA-ATPase+HKD-DNase-BrxL<-PglZ<-N6-METHYLASE<-DUF6079*<-BrxB-BrxF-iSTAND<-BrxA-DNA-binding<-REase+RNase<-viral-protein||?-><-Phage_holin_2_4<-?<-?<-PAS+PAS+PAS+GAF+HISKIN	1201	Bacteria>FCB_group>Bacteroidetes/Chlorobi_group>Bacteroidota>Bacteroidetes_incertae_sedis>Rhodothermaceae	GCF_002290345.1	Salinibacter_ruber	B7W84_RS15115	76596..80201	146919	BREX-system-P-loop-protein-BrxC-[Salinibacter-ruber]
WP_259091014.1	Type6.BREX	SF1-SF2-Helicase+REase-><-HORMA||BrxE->BrxA-DNA-binding->BrxB-BrxF-iSTAND->DUF6079*->N6-METHYLASE->PglZ->SIGMA+OB-Fold+AAA-ATPase+HKD-DNase-BrxL->MULE-TRANSPOSASE->HORMA->Calcineurin-SbcD->SbcC+SbcC->HD->HNH->	1192	Bacteria>FCB_group>Bacteroidetes/Chlorobi_group>Bacteroidota>Bacteroidetes_incertae_sedis>Rhodothermaceae	GCF_024807395.1	Salinibacter_ruber	GGP54_RS13575	8828..12406	146919	BREX-system-P-loop-protein-BrxC-[Salinibacter-ruber]
WP_259130005.1	Type6.BREX	<-SbcC+SbcC<-Calcineurin-SbcD<-HORMA<-SIGMA+OB-Fold+AAA-ATPase+HKD-DNase-BrxL<-PglZ<-N6-METHYLASE<-DUF6079*<-BrxB-BrxF-iSTAND<-BrxA-DNA-binding<-BrxE<-REase+RNase<-viral-protein<-?<-?||?-><-HKD-DNase+MED12	1199	Bacteria>FCB_group>Bacteroidetes/Chlorobi_group>Bacteroidota>Bacteroidetes_incertae_sedis>Rhodothermaceae	GCF_024807455.1	Salinibacter_ruber	GGQ04_003012	22826..26425	146919	BREX-system-P-loop-protein-BrxC-[Salinibacter-ruber]
WP_259115052.1	Type1.BREX	<-SbcC+SbcC<-Calcineurin-SbcD<-SIGMA+OB-Fold+AAA-ATPase+HKD-DNase-BrxL<-PglZ<-HEPN<-PolB<-?<-N6-METHYLASE<-DOC+HTH<-DUF6079*<-BrxB-BrxF-iSTAND<-BrxA-DNA-binding<-?<-viral-protein<-?<-?||?-><-RHH||HTH_3+Peptidase_S24->	1167	Bacteria>FCB_group>Bacteroidetes/Chlorobi_group>Bacteroidota>Bacteroidetes_incertae_sedis>Rhodothermaceae	GCF_024809315.1	Salinibacter_ruber	GGP75_RS10390	31231..34734	146919	BREX-system-P-loop-protein-BrxC-[Salinibacter-ruber]
WP_259076695.1	Type1.BREX	TraJ-RHH->RHH->Phage_holin_2_4->||viral-protein->?->BrxA-DNA-binding->BrxB-BrxF-iSTAND->DUF6079*->DOC+HTH->N6-METHYLASE->?->?->PglZ->SIGMA+OB-Fold+AAA-ATPase+HKD-DNase-BrxL->Calcineurin-SbcD->SbcC+SbcC->	1167	Bacteria>FCB_group>Bacteroidetes/Chlorobi_group>Bacteroidota>Bacteroidetes_incertae_sedis>Rhodothermaceae	GCF_024809595.1	Salinibacter_ruber	GGP73_RS15155	19341..22844	146919	BREX-system-P-loop-protein-BrxC-[Salinibacter-ruber]
WP_259087839.1	Type1.BREX	URI-DNase-><-SIGMA+OB-Fold+AAA-ATPase+HKD-DNase-BrxL<-PglZ<-PIN<-XIS-HTH<-?<-?<-N6-METHYLASE<-DUF6079*<-BrxB-BrxF-iSTAND<-BrxA-DNA-binding<-?||Phage_integrase-><-TOPRIM<-?||XIS-HTH->Phage_int_SAM_4+Phage_integrase-><-tRNA||BetaPropeller+ABhydrolase->	1168	Bacteria>FCB_group>Bacteroidetes/Chlorobi_group>Bacteroidota>Bacteroidetes_incertae_sedis>Rhodothermaceae	GCF_024809725.1	Salinibacter_ruber	GGQ07_RS07865	19343..22849	146919	BREX-system-P-loop-protein-BrxC-[Salinibacter-ruber]
WP_259228204.1	Type6.BREX	<-REC+OmpR-HTH<-PAS+PAS+PAS+GAF+HISKIN<-Phage_integrase<-?<-?<-?<-SIGMA+OB-Fold+AAA-ATPase+HKD-DNase-BrxL<-PglZ<-N6-METHYLASE<-DUF6079*<-BrxB-BrxF-iSTAND<-BrxA-DNA-binding<-BrxE||?->?->?-><-viral-protein	1201	Bacteria>FCB_group>Bacteroidetes/Chlorobi_group>Bacteroidota>Bacteroidetes_incertae_sedis>Rhodothermaceae	GCF_024809755.1	Salinibacter_ruber	GGP93_RS14280	71185..74790	146919	BREX-system-P-loop-protein-BrxC-[Salinibacter-ruber]
WP_263820582.1	Type1.BREX	<-SbcC+SbcC<-Calcineurin-SbcD<-SIGMA+OB-Fold+AAA-ATPase+HKD-DNase-BrxL<-PglZ<-?<-N6-METHYLASE<-DOC+HTH<-DUF6079*<-BrxB-BrxF-iSTAND<-BrxA-DNA-binding<-DUF4258<-REase+RNase<-viral-protein<-?||CHTH-><-Phage_holin_2_4<-RHH	1167	Bacteria>FCB_group>Bacteroidetes/Chlorobi_group>Bacteroidota>Bacteroidetes_incertae_sedis>Rhodothermaceae	GCF_947077735.1	Salinibacter_sp.	OJA43_RS08805	45420..48923	2065818	BREX-system-P-loop-protein-BrxC-[Salinibacter-sp.]
WP_259248797.1	Type1.BREX	<-SbcC+SbcC<-Calcineurin-SbcD<-SIGMA+OB-Fold+AAA-ATPase+HKD-DNase-BrxL<-PglZ<-CHTH<-RVT<-N6-METHYLASE<-DOC+HTH<-DUF6079*<-BrxB-BrxF-iSTAND<-BrxA-DNA-binding<-DUF4258<-REase+RNase<-HKD-DNase+SF1-SF2-Helicase<-RHH||HTH_23+HTH_32+HTH_33->DDE_3-><-TraJ-RHH	1167	Bacteria>FCB_group>Bacteroidetes/Chlorobi_group>Bacteroidota>Bacteroidetes_incertae_sedis>Rhodothermaceae	-	Salinibacter_ruber	GGP55_RS14300	49306..52809	146919	BREX-system-P-loop-protein-BrxC-[Salinibacter-ruber]
WP_117964426.1	Type6.BREX	<-TRAM+METHYLASE||?-><-UDG<-?<-ARG||RHH+MobC->?-><-SIGMA+OB-Fold+AAA-ATPase+HKD-DNase-BrxL<-PglZ<-DUF6079-p*<-DUF6079-p<-BrxB-BrxF-iSTAND<-BrxA-DNA-binding<-BrxE<-HTH_3<-?||<-XIS-HTH<-AbiEii||HTH_3->	938	Bacteria>FCB_group>Bacteroidetes/Chlorobi_group>Bacteroidota>Bacteroidia>Bacteroidales	-	Bacteroides_uniformis	IYV59_08305	23505..26321	820	MULTISPECIES:-BREX-system-P-loop-protein-BrxC-[Bacteroides]
WP_118696414.1	Type1.BREX	<-Arm-DNA-bind_5+Phage_int_SAM_5+Phage_integrase||DUF4007->BrxB-BrxF-iSTAND->DUF6079-p->DUF6079-p*->N6-METHYLASE->PglZ->SIGMA+OB-Fold+AAA-ATPase+HKD-DNase-BrxL-><-?<-RHH+MobC||ARG->?->UDG->	938	Bacteria>FCB_group>Bacteroidetes/Chlorobi_group>Bacteroidota>Bacteroidia>Bacteroidales	-	Bacteroides_uniformis	KSX92_08935	3061..5877	820	BREX-system-P-loop-protein-BrxC-[Bacteroides-uniformis]
EFG14417.1	Type6.BREX	PRTase->?->?->tRNA->tRNA-><-?<-SIGMA+OB-Fold+AAA-ATPase+HKD-DNase-BrxL<-PglZ<-HNH<-OLD-ABC<-N6-METHYLASE<-DUF6079-p*<-DUF6079-p<-BrxB-BrxF-iSTAND<-BrxA-DNA-binding<-BrxE<-HTH_3<-?<-?<-TraJ-RHH<-ZnR+TOPRIM<-CR-ATPase4	762	Bacteria>FCB_group>Bacteroidetes/Chlorobi_group>Bacteroidota>Bacteroidia>Bacteroidales	GCA_000178215.1	Bacteroides_xylanisolvens_SD_CC_1b	CW3_0497	100900..103188	702447	hypothetical-protein-CW3_0497-[Bacteroides-xylanisolvens-SD-CC-1b]
EFF57551.1	Type6.BREX	CR-ATPase4->ZnR+TOPRIM->TraJ-RHH->?->HTH_3->BrxE->BrxA-DNA-binding->BrxB-BrxF-iSTAND->DUF6079-p->DUF6079-p*->N6-METHYLASE->OLD-ABC->HNH->PglZ->SIGMA+OB-Fold+AAA-ATPase+HKD-DNase-BrxL->	706	Bacteria>FCB_group>Bacteroidetes/Chlorobi_group>Bacteroidota>Bacteroidia>Bacteroidales	GCA_000178295.1	Bacteroides_xylanisolvens_SD_CC_2a	CW1_2295	25339..27459	702444	hypothetical-protein-CW1_2295-[Bacteroides-xylanisolvens-SD-CC-2a]
CDD81792.1	Type1.BREX	<-HIRAN<-RADICAL-SAM<-NADAR<-HTH_3<-SIGMA+OB-Fold+AAA-ATPase+HKD-DNase-BrxL<-PglZ<-Schlafen+HATPase+wHTH<-N6-METHYLASE<-Four-helical-protein<-DUF6079*<-BrxB-BrxF-iSTAND<-BrxA-DNA-binding<-HTH_3||?->?->?->?->?-><-XIS-HTH	1217	Bacteria>FCB_group>Bacteroidetes/Chlorobi_group>Bacteroidota>Bacteroidia>Bacteroidales	GCA_000435095.1	Bacteroides_sp._CAG:462	BN666_01223	46564..50217	1262740	putative-uncharacterized-protein-[Bacteroides-sp.-CAG:462]
CDM00531.1	Type6.BREX	PRTase->?->?->tRNA->tRNA-><-?<-SIGMA+OB-Fold+AAA-ATPase+HKD-DNase-BrxL<-PglZ<-HNH<-OLD-ABC<-N6-METHYLASE<-DUF6079-p*<-DUF6079-p<-BrxB-BrxF-iSTAND<-BrxA-DNA-binding<-BrxE<-HTH_3<-?<-?<-TraJ-RHH<-ZnR+TOPRIM	689	Bacteria>FCB_group>Bacteroidetes/Chlorobi_group>Bacteroidota>Bacteroidia>Bacteroidales	GCA_000577955.1	Bacteroides_xylanisolvens_SD_CC_2a	BN891_34560	156968..159037	702444	hypothetical-protein-BN891_34560-[Bacteroides-xylanisolvens-SD-CC-2a]
OCW94638.1	Type1.BREX	MarR-HTH->Polbeta->Calcineurin-><-DOC+HTH||?->HTH_3->BrxA-DNA-binding->BrxB-BrxF-iSTAND->DUF6079*->N6-METHYLASE->PglZ->SIGMA+OB-Fold+AAA-ATPase+HKD-DNase-BrxL->?->?->XIS-HTH->TOPRIM->XIS-HTH-><-AbiEii	1202	Bacteria>FCB_group>Bacteroidetes/Chlorobi_group>Bacteroidota>Bacteroidia>Bacteroidales	GCA_001704385.2	Macellibacteroides_sp._HH-ZS	A9168_06455	108865..112473	1857568	hypothetical-protein-A9168_06455-[Macellibacteroides-sp.-HH-ZS]
OKZ04598.1	Type1.BREX	<-SIGMA-HTH<-?||35exo+SF1-SF2-Helicase-><-SIGMA+OB-Fold+AAA-ATPase+HKD-DNase-BrxL<-PglZ<-Schlafen+HATPase+wHTH<-SIR2+SIR2<-N6-METHYLASE<-DUF6079*<-BrxB-BrxF-iSTAND<-BrxA-DNA-binding<-?||<-REase||HTH_3->DNA_METHYLASE->McrC-NTD+REase->SbcC->REase->	1202	Bacteria>FCB_group>Bacteroidetes/Chlorobi_group>Bacteroidota>Bacteroidia>Bacteroidales	GCA_001915435.1	Bacteroides_sp._41_26	BHV71_10470	85707..89315	1896973	MAG:-hypothetical-protein-BHV71_10470-[Bacteroides-sp.-41_26]
OKZ20119.1	Type1.BREX	<-REase<-?<-?<-?<-?<-SIGMA+OB-Fold+AAA-ATPase+HKD-DNase-BrxL<-PglZ<-Schlafen+HATPase+wHTH<-N6-METHYLASE<-DUF6079*<-BrxB-BrxF-iSTAND<-BrxA-DNA-binding<-HTH_3<-?<-?<-?||?->?->SIGMA-HTH->	1185	Bacteria>FCB_group>Bacteroidetes/Chlorobi_group>Bacteroidota>Bacteroidia>Bacteroidales	GCA_001915545.1	Bacteroides_sp._43_108	BHV77_10980	38384..41941	1896974	MAG:-hypothetical-protein-BHV77_10980-[Bacteroides-sp.-43_108]
HAH91106.1	Type1.BREX	<-HIRAN<-RADICAL-SAM<-ARG<-?<-?||HTH_3->wHTH+WYL+WCX->BrxA-DNA-binding->BrxB-BrxF-iSTAND->DUF6079*->OLD-ABC->TOPRIM->?->SF1-SF2-Helicase->PglZ->SIGMA+OB-Fold+AAA-ATPase+HKD-DNase-BrxL->McrB-AAA->McrC-NTD+REase->	1202	Bacteria>FCB_group>Bacteroidetes/Chlorobi_group>Bacteroidota>Bacteroidia>Bacteroidales	GCA_003450425.1	Prevotella_sp.	DCL96_05230	7787..11395	59823	BREX-system-P-loop-protein-BrxC-[Prevotella-sp.]
HAM28416.1	Type6.BREX	ChlI+Lon_C+AAA-><-RNase<-AraC-HTH||?-><-?||HTH_3->BrxE->BrxA-DNA-binding->BrxB-BrxF-iSTAND->DUF6079*->N6-METHYLASE->OLD-ABC->HNH->PglZ->SIGMA+OB-Fold+AAA-ATPase+HKD-DNase-BrxL->?->?->35exo->	1218	Bacteria>FCB_group>Bacteroidetes/Chlorobi_group>Bacteroidota>Bacteroidia>Bacteroidales	GCA_003453655.1	Prevotella_sp.	DCP79_00675	66224..69880	59823	BREX-system-P-loop-protein-BrxC-[Prevotella-sp.]
RHC75801.1	Type1.BREX	sGTP->Phage_int_SAM_5+Phage_integrase->?->XIS-HTH->SMS-KaiC->HTH_3->BrxA-DNA-binding->BrxB-BrxF-iSTAND->DUF6079-p*->DUF6079-p->OLD-ABC->REase->N6-METHYLASE->PglZ->SIGMA+OB-Fold+AAA-ATPase+HKD-DNase-BrxL-><-REC+AraC-HTH+AraC-HTH	691	Bacteria>FCB_group>Bacteroidetes/Chlorobi_group>Bacteroidota>Bacteroidia>Bacteroidales	GCA_003468465.1	Bacteroides_uniformis	DW831_02855	19890..21965	820	BREX-system-P-loop-protein-BrxC-[Bacteroides-uniformis]
RHK71295.1	Type6.BREX	<-ARG||RHH+MobC->?-><-?<-SIGMA+OB-Fold+AAA-ATPase+HKD-DNase-BrxL<-PglZ<-N6-METHYLASE<-DUF6079-p<-?<-DUF6079-p*<-BrxB-BrxF-iSTAND<-BrxA-DNA-binding<-BrxE<-HTH_3||<-XIS-HTH<-AbiEii<-?||HTH_3->HISKIN->	145	Bacteria>FCB_group>Bacteroidetes/Chlorobi_group>Bacteroidota>Bacteroidia>Bacteroidales	GCA_003474125.1	Bacteroides_uniformis	DW051_06915	97313..97750	820	hypothetical-protein-DW051_06915-[Bacteroides-uniformis]
RHR54760.1	Type1.BREX	ACET-><-?<-Cysteine-Desulfurase||tRNA-><-?<-?<-?<-?||BrxA-DNA-binding->BrxB-BrxF-iSTAND->DUF6079*->N6-METHYLASE->Schlafen+HATPase+wHTH->PglZ->SIGMA+OB-Fold+AAA-ATPase+HKD-DNase-BrxL-><-?||?->PolB->HEPN->	1216	Bacteria>FCB_group>Bacteroidetes/Chlorobi_group>Bacteroidota>Bacteroidia>Bacteroidales	GCA_003479205.1	Parabacteroides_sp._AF17-28	DWW90_15085	22918..26568	2292241	BREX-system-P-loop-protein-BrxC-[Parabacteroides-sp.-AF17-28]
HBQ57053.1	Type1.BREX	<-SF1-SF2-Helicase||tRNA-><-?<-?<-SIGMA+OB-Fold+AAA-ATPase+HKD-DNase-BrxL<-PglZ<-N6-METHYLASE<-DUF6079*<-BrxB-BrxF-iSTAND<-BrxA-DNA-binding<-?<-REase	1176	Bacteria>FCB_group>Bacteroidetes/Chlorobi_group>Bacteroidota>Bacteroidia>Bacteroidales	GCA_003511405.1	Porphyromonadaceae_bacterium	DD786_07890	28635..32165	2049046	BREX-system-P-loop-protein-BrxC-[Porphyromonadaceae-bacterium]
ROS89081.1	Type1.BREX	<-SF1-SF2-Helicase<-SIGMA+OB-Fold+AAA-ATPase+HKD-DNase-BrxL<-PglZ||?-><-Schlafen+HATPase+wHTH<-N6-METHYLASE<-DUF6079*<-BrxB-BrxF-iSTAND<-BrxA-DNA-binding<-HTH_3	1215	Bacteria>FCB_group>Bacteroidetes/Chlorobi_group>Bacteroidota>Bacteroidia>Bacteroidales	GCA_003762635.1	Muribaculaceae_bacterium_Isolate-043_(Harlan)	EEL36_13385	16553..20200	2486463	BREX-system-P-loop-protein-BrxC-[Muribaculaceae-bacterium-Isolate-043-(Harlan)]
ROT16603.1	Type1.BREX	<-SIGMA+OB-Fold+AAA-ATPase+HKD-DNase-BrxL<-PglZ<-MORC+DUF3684+SF1-SF2-Helicase<-ACET+TPR<-AT-HTH-HYD2<-Tox-ART-HYD2<-AT-HTH-HYD2<-N6-METHYLASE<-DUF6079*<-BrxB-BrxF-iSTAND<-BrxA-DNA-binding<-HTH_3||SAVED->SMODS->?->?-><-Phage_lysozyme	1195	Bacteria>FCB_group>Bacteroidetes/Chlorobi_group>Bacteroidota>Bacteroidia>Bacteroidales	GCA_003762745.1	Muribaculaceae_bacterium_Isolate-105_(HZI)	EEL50_01930	18926..22513	2486472	BREX-system-P-loop-protein-BrxC-[Muribaculaceae-bacterium-Isolate-105-(HZI)]
ROT18783.1	Type1.BREX	<-SIGMA+OB-Fold+AAA-ATPase+HKD-DNase-BrxL<-PglZ<-Schlafen+HATPase+wHTH<-REase<-N6-METHYLASE<-?<-Tox-ART-HYD2<-?<-DUF6079*<-Four-helical-protein<-BrxB-BrxF-iSTAND<-BrxA-DNA-binding<-HTH_3<-?<-AbiEii<-AbiEii	1223	Bacteria>FCB_group>Bacteroidetes/Chlorobi_group>Bacteroidota>Bacteroidia>Bacteroidales	GCA_003833095.1	Muribaculaceae_bacterium_Isolate-114_(HZI)	EEL53_11930	25441..29112	2486475	BREX-system-P-loop-protein-BrxC-[Muribaculaceae-bacterium-Isolate-114-(HZI)]
KAA3824840.1	Type1.BREX	DUF6079*->N6-METHYLASE->SIR2+SIR2->Schlafen+HATPase+wHTH->PglZ->SIGMA+OB-Fold+AAA-ATPase+HKD-DNase-BrxL->||BACON+Peptidase_M64->	1065	Bacteria>FCB_group>Bacteroidetes/Chlorobi_group>Bacteroidota>Bacteroidia>Bacteroidales	GCA_008565655.1	Bacteroides_ovatus	F3F58_10380	1..3200	28116	BREX-system-P-loop-protein-BrxC,-partial-[Bacteroides-ovatus]
KAA4088264.1	Type1.BREX	<-SIGMA+OB-Fold+AAA-ATPase+HKD-DNase-BrxL<-PglZ<-SWIM+DUF3670+SF1-SF2-Helicase<-?<-?<-?<-Peptidase_C39_2<-ACET+TPR<-N6-METHYLASE<-DUF6079*	1069	Bacteria>FCB_group>Bacteroidetes/Chlorobi_group>Bacteroidota>Bacteroidia>Bacteroidales	GCA_008566385.1	Bacteroides_ovatus	F3F49_00290	70210..73420	28116	BREX-system-P-loop-protein-BrxC,-partial-[Bacteroides-ovatus]
KAA4007861.1	Type1.BREX	<-NUDIX+NUDIX<-?<-?<-?||HTH_3->wHTH+WYL+WCX->BrxA-DNA-binding->BrxB-BrxF-iSTAND->DUF6079*->N6-METHYLASE->ACET+TPR->Peptidase_C39_2->?->?->?->SWIM+DUF3670+SF1-SF2-Helicase->PglZ->SIGMA+OB-Fold+AAA-ATPase+HKD-DNase-BrxL->	1208	Bacteria>FCB_group>Bacteroidetes/Chlorobi_group>Bacteroidota>Bacteroidia>Bacteroidales	GCA_008566415.1	Bacteroides_ovatus	F3F37_14290	84100..87726	28116	BREX-system-P-loop-protein-BrxC-[Bacteroides-ovatus]
KAA4743085.1	Type1.BREX	AbiEi_4-><-Phage_int_SAM_5+Phage_integrase||STYKinase-like->?->?->||HTH_3->BrxA-DNA-binding->BrxB-BrxF-iSTAND->DUF6079*->DUF6079-p->N6-METHYLASE->Schlafen+HATPase+wHTH->PglZ->SIGMA+OB-Fold+AAA-ATPase+HKD-DNase-BrxL->?->URI-DNase+RAMA->DOC+HTH->	1056	Bacteria>FCB_group>Bacteroidetes/Chlorobi_group>Bacteroidota>Bacteroidia>Bacteroidales	GCA_008569225.1	Bacteroides_fragilis	F3B36_09960	278156..281326	817	BREX-system-P-loop-protein-BrxC-[Bacteroides-fragilis]
KAB5317657.1	Type1.BREX	<-Phage_int_SAM_5<-Phage_integrase<-Phage_int_SAM_5+Phage_integrase<-SIGMA+OB-Fold+AAA-ATPase+HKD-DNase-BrxL<-PglZ<-N6-METHYLASE<-REase<-OLD-ABC<-DUF6079*<-BrxB-BrxF-iSTAND<-BrxA-DNA-binding<-HTH_3<-SMS-KaiC<-XIS-HTH	1196	Bacteria>FCB_group>Bacteroidetes/Chlorobi_group>Bacteroidota>Bacteroidia>Bacteroidales	GCA_009025145.1	Bacteroides_stercoris	F9949_11590	61674..65264	46506	BREX-system-P-loop-protein-BrxC-[Bacteroides-stercoris]
NBJ07681.1	Type1.BREX	LRR_5->HTH_3->PSBP->?->?->?->HTH_3->BrxA-DNA-binding->BrxB-BrxF-iSTAND->DUF6079*->N6-METHYLASE->REase->Schlafen+HATPase+wHTH->PglZ->SIGMA+OB-Fold+AAA-ATPase+HKD-DNase-BrxL->SF1-SF2-Helicase->?->?->MPTase->	1223	Bacteria>FCB_group>Bacteroidetes/Chlorobi_group>Bacteroidota>Bacteroidia>Bacteroidales	GCA_009911575.1	Alistipes_sp._Z76	D1647_16085	15947..19618	2304565	BREX-system-P-loop-protein-BrxC-[Alistipes-sp.-Z76]
NLA16135.1	Type1.BREX	<-HTH_3||HTH+REase-><-SIGMA+OB-Fold+AAA-ATPase+HKD-DNase-BrxL<-PglZ<-N6-METHYLASE<-DUF6079*<-BrxB-BrxF-iSTAND<-BrxA-DNA-binding<-HTH_3<-?||?->tRNA-><-?<-?||?->LuxR-HTH->	1174	Bacteria>FCB_group>Bacteroidetes/Chlorobi_group>Bacteroidota>Bacteroidia>Bacteroidales	GCA_012510445.1	Bacteroidales_bacterium	GX877_06335	9723..13247	2030927	BREX-system-P-loop-protein-BrxC-[Bacteroidales-bacterium]
NLN95595.1	Type1.BREX	PolB->HEPN->?-><-?<-SIGMA+OB-Fold+AAA-ATPase+HKD-DNase-BrxL<-PglZ<-?<-N6-METHYLASE<-?<-DUF6079*<-BrxB-BrxF-iSTAND<-BrxA-DNA-binding<-HTH_3||?->TRD+TRD->	1176	Bacteria>FCB_group>Bacteroidetes/Chlorobi_group>Bacteroidota>Bacteroidia>Bacteroidales	GCA_012522795.1	Bacteroidales_bacterium	GX128_05430	111716..115246	2030927	BREX-system-P-loop-protein-BrxC-[Bacteroidales-bacterium]
MBD5181931.1	Type1.BREX	<-Acyltransferase<-?<-?||HISKIN+MORC->REC+OmpR-HTH->BrxA-DNA-binding->BrxB-BrxF-iSTAND->DUF6079*->N6-METHYLASE->OLD-ABC->TOPRIM->PglZ->SIGMA+OB-Fold+AAA-ATPase+HKD-DNase-BrxL->	1193	Bacteria>FCB_group>Bacteroidetes/Chlorobi_group>Bacteroidota>Bacteroidia>Bacteroidales	GCA_014800235.1	Bacteroidales_bacterium	HDS98_05210	20716..24297	2030927	BREX-system-P-loop-protein-BrxC-[Bacteroidales-bacterium]
MBD5263514.1	Type1.BREX	<-Calcineurin<-?<-?<-Aminotran_1_2||?-><-?||BrxA-DNA-binding->BrxB-BrxF-iSTAND->DUF6079*->N6-METHYLASE->Schlafen+HATPase+wHTH->SF1-SF2-Helicase->BirA-HTH->PglZ->SIGMA+OB-Fold+AAA-ATPase+HKD-DNase-BrxL->	1221	Bacteria>FCB_group>Bacteroidetes/Chlorobi_group>Bacteroidota>Bacteroidia>Bacteroidales	GCA_014801365.1	Bacteroides_sp.	HDS39_05520	20086..23751	29523	BREX-system-P-loop-protein-BrxC-[Bacteroides-sp.]
MBD5349669.1	Type1.BREX	<-ABhydrolase<-JAB<-?<-?<-SIGMA+OB-Fold+AAA-ATPase+HKD-DNase-BrxL<-Schlafen+HATPase+wHTH<-?<-N6-METHYLASE<-Four-helical-protein<-DUF6079*<-BrxB-BrxF-iSTAND<-BrxA-DNA-binding<-HTH_3<-TraJ-RHH||Zn_Tnp_IS1595+DDE_Tnp_IS1595->?-><-?<-HTH_58<-METHYLASE	1219	Bacteria>FCB_group>Bacteroidetes/Chlorobi_group>Bacteroidota>Bacteroidia>Bacteroidales	GCA_014802205.1	Bacteroides_sp.	HDR89_02130	36441..40100	29523	BREX-system-P-loop-protein-BrxC-[Bacteroides-sp.]
MBD5357714.1	Type1.BREX	AAA+->?->XIS-HTH-><-?<-?<-?<-?||BrxA-DNA-binding->BrxB-BrxF-iSTAND->DUF6079*->N6-METHYLASE->HU-HIG+REase->PglZ->SIGMA+OB-Fold+AAA-ATPase+HKD-DNase-BrxL->REase->?->CASPASE->	1203	Bacteria>FCB_group>Bacteroidetes/Chlorobi_group>Bacteroidota>Bacteroidia>Bacteroidales	GCA_014802265.1	Bacteroides_sp.	HDR88_12035	22916..26527	29523	BREX-system-P-loop-protein-BrxC-[Bacteroides-sp.]
MBD5419378.1	Type1.BREX	BrxB-BrxF-iSTAND->DUF6079*->N6-METHYLASE->Schlafen+HATPase+wHTH->PglZ->SIGMA+OB-Fold+AAA-ATPase+HKD-DNase-BrxL->?->Calcineurin->Glyco_hydro_3->	1201	Bacteria>FCB_group>Bacteroidetes/Chlorobi_group>Bacteroidota>Bacteroidia>Bacteroidales	GCA_014803765.1	Bacteroides_sp.	HDR48_05020	1391..4996	29523	BREX-system-P-loop-protein-BrxC-[Bacteroides-sp.]
MBE6254618.1	Type1.BREX	<-SF1-SF2-Helicase<-?<-SIGMA+OB-Fold+AAA-ATPase+HKD-DNase-BrxL<-PglZ<-SIR2<-N6-METHYLASE<-DUF6079*<-BrxB-BrxF-iSTAND<-BrxA-DNA-binding<-wHTH+WYL+WCX	1211	Bacteria>FCB_group>Bacteroidetes/Chlorobi_group>Bacteroidota>Bacteroidia>Bacteroidales	GCA_015060555.1	Prevotella_sp.	E7105_03785	25107..28742	59823	BREX-system-P-loop-protein-BrxC-[Prevotella-sp.]
MBF7062545.1	Type6.BREX	<-TRAM+METHYLASE||?-><-UDG<-?<-ARG||RHH+MobC->?-><-SIGMA+OB-Fold+AAA-ATPase+HKD-DNase-BrxL<-PglZ<-DUF6079-p*<-DUF6079-p<-BrxB-BrxF-iSTAND<-BrxA-DNA-binding<-BrxE<-HTH_3<-?||<-XIS-HTH<-AbiEii||HTH_3->	938	Bacteria>FCB_group>Bacteroidetes/Chlorobi_group>Bacteroidota>Bacteroidia>Bacteroidales	GCA_015594615.1	Bacteroides_sp._HF-5613	ITJ87_11475	2819894..2822710	2785794	BREX-system-P-loop-protein-BrxC-[Bacteroides-sp.-HF-5613]
MBM6671973.1	Type6.BREX	<-SIGMA+OB-Fold+AAA-ATPase+HKD-DNase-BrxL<-PglZ<-N6-METHYLASE<-DUF6079*<-BrxB-BrxF-iSTAND<-BrxA-DNA-binding<-BrxE<-GNAT	1224	Bacteria>FCB_group>Bacteroidetes/Chlorobi_group>Bacteroidota>Bacteroidia>Bacteroidales	GCA_016900015.1	Phocaeicola_coprophilus	H6B14_14560	8845..12519	387090	BREX-system-P-loop-protein-BrxC-[Phocaeicola-coprophilus]
MBN2915288.1	Type1.BREX	<-McrC-NTD+REase<-McrB-AAA<-SIGMA+OB-Fold+AAA-ATPase+HKD-DNase-BrxL<-PglZ<-SIR2+SIR2<-N6-METHYLASE<-TOPRIM<-OLD-ABC<-DUF6079-p*	971	Bacteria>FCB_group>Bacteroidetes/Chlorobi_group>Bacteroidota>Bacteroidia>Bacteroidales	GCA_016939035.1	Prevotella_sp.	JTJ26_04215	25012..27927	59823	BREX-system-P-loop-protein-BrxC-[Prevotella-sp.]
MBQ6178640.1	Type1.BREX	ArdCN+MPTase->?->?->?-><-wHTH+WYL+WCX<-?||HD->BrxA-DNA-binding->BrxB-BrxF-iSTAND->DUF6079*->N6-METHYLASE->REase->PglZ->SIGMA+OB-Fold+AAA-ATPase+HKD-DNase-BrxL->	1202	Bacteria>FCB_group>Bacteroidetes/Chlorobi_group>Bacteroidota>Bacteroidia>Bacteroidales	GCA_017429185.1	Bacteroidales_bacterium	IJK32_03880	18867..22475	2030927	BREX-system-P-loop-protein-BrxC-[Bacteroidales-bacterium]
MBQ6872144.1	Type1.BREX	BrxA-DNA-binding->BrxB-BrxF-iSTAND->DUF6079*->N6-METHYLASE->PglZ->SIGMA+OB-Fold+AAA-ATPase+HKD-DNase-BrxL->	1190	Bacteria>FCB_group>Bacteroidetes/Chlorobi_group>Bacteroidota>Bacteroidia>Bacteroidales	GCA_017435685.1	Bacteroidales_bacterium	IJO23_07295	3964..7536	2030927	BREX-system-P-loop-protein-BrxC-[Bacteroidales-bacterium]
MBQ9588226.1	Type1.BREX	Peptidase->?-><-HD||BrxA-DNA-binding->BrxB-BrxF-iSTAND->DUF6079*->N6-METHYLASE->HTH+REase->N6-METHYLASE-p->PglZ->SIGMA+OB-Fold+AAA-ATPase+HKD-DNase-BrxL->	1183	Bacteria>FCB_group>Bacteroidetes/Chlorobi_group>Bacteroidota>Bacteroidia>Bacteroidales	GCA_017446745.1	Bacteroidales_bacterium	IJR26_10275	19779..23330	2030927	BREX-system-P-loop-protein-BrxC-[Bacteroidales-bacterium]
MBQ7672328.1	Type1.BREX	REase+35exo->?->?->LRR_5->LRR_5->BrxA-DNA-binding->BrxB-BrxF-iSTAND->DUF6079*->N6-METHYLASE->PglZ->SIGMA+OB-Fold+AAA-ATPase+HKD-DNase-BrxL->?->DOC+HTH->	1193	Bacteria>FCB_group>Bacteroidetes/Chlorobi_group>Bacteroidota>Bacteroidia>Bacteroidales	GCA_017450385.1	Paludibacteraceae_bacterium	IJS49_04535	76772..80353	2748031	BREX-system-P-loop-protein-BrxC-[Paludibacteraceae-bacterium]
MBR6863890.1	Type1.BREX	<-ParB+HNH<-wHTH+WYL+WCX<-?||?->Couple_hipA+HIRAN->EVE->HD->BrxA-DNA-binding->BrxB-BrxF-iSTAND->DUF6079*->N6-METHYLASE->PglZ->SIGMA+OB-Fold+AAA-ATPase+HKD-DNase-BrxL->?->SF1-SF2-Helicase->	1202	Bacteria>FCB_group>Bacteroidetes/Chlorobi_group>Bacteroidota>Bacteroidia>Bacteroidales	GCA_017531205.1	Bacteroidales_bacterium	IKM75_03420	106682..110290	2030927	BREX-system-P-loop-protein-BrxC-[Bacteroidales-bacterium]
MBR5893775.1	Type1.BREX	<-SIGMA+OB-Fold+AAA-ATPase+HKD-DNase-BrxL<-PglZ<-DOC+HTH<-N6-METHYLASE<-?<-DUF6079*<-BrxB-BrxF-iSTAND<-BrxA-DNA-binding<-?<-HAD+HAD	1185	Bacteria>FCB_group>Bacteroidetes/Chlorobi_group>Bacteroidota>Bacteroidia>Bacteroidales	GCA_017554605.1	Bacteroidaceae_bacterium	IKZ37_09095	11422..14979	2212467	BREX-system-P-loop-protein-BrxC-[Bacteroidaceae-bacterium]
MBR5630362.1	Type1.BREX	<-CopG/RHH||BrxA-DNA-binding->BrxB-BrxF-iSTAND->DUF6079*->N6-METHYLASE->PglZ->SIGMA+OB-Fold+AAA-ATPase+HKD-DNase-BrxL->	1171	Bacteria>FCB_group>Bacteroidetes/Chlorobi_group>Bacteroidota>Bacteroidia>Bacteroidales	GCA_017557685.1	Bacteroidales_bacterium	IKW82_01820	70807..74322	2030927	BREX-system-P-loop-protein-BrxC-[Bacteroidales-bacterium]
MBR5576788.1	Type1.BREX	<-LRR_5+LRR_5+LRR_5+LRR_5<-?<-?<-?<-?<-?<-?||BrxA-DNA-binding->BrxB-BrxF-iSTAND->DUF6079*->N6-METHYLASE->SbcC+SbcC->?->ACET+TPR->?->SF1-SF2-Helicase->PglZ->SIGMA+OB-Fold+AAA-ATPase+HKD-DNase-BrxL->	1209	Bacteria>FCB_group>Bacteroidetes/Chlorobi_group>Bacteroidota>Bacteroidia>Bacteroidales	GCA_017558255.1	Bacteroidaceae_bacterium	IKW22_05890	14217..17846	2212467	BREX-system-P-loop-protein-BrxC-[Bacteroidaceae-bacterium]
MBO7470104.1	Type1.BREX	BrxA-DNA-binding->BrxB-BrxF-iSTAND->DUF6079*->N6-METHYLASE->?->?->PglZ->SIGMA+OB-Fold+AAA-ATPase+HKD-DNase-BrxL->	1169	Bacteria>FCB_group>Bacteroidetes/Chlorobi_group>Bacteroidota>Bacteroidia>Bacteroidales	GCA_017649945.1	Bacteroidales_bacterium	J6T81_08275	1593..5102	2030927	BREX-system-P-loop-protein-BrxC-[Bacteroidales-bacterium]
MBS6238336.1	Type1.BREX	<-PAS+HISKIN||?-><-AraC-HTH+AraC-HTH<-SIGMA+OB-Fold+AAA-ATPase+HKD-DNase-BrxL<-PglZ<-Schlafen+HATPase+wHTH<-ParB+HNH<-ParB+HNH<-N6-METHYLASE<-DUF6079*<-BrxB-BrxF-iSTAND<-BrxA-DNA-binding<-HTH_3<-?<-?<-?<-?||SIGMA-HTH->	1205	Bacteria>FCB_group>Bacteroidetes/Chlorobi_group>Bacteroidota>Bacteroidia>Bacteroidales	GCA_018374845.1	Bacteroides_sp.	KH897_08200	204452..208069	29523	BREX-system-P-loop-protein-BrxC-[Bacteroides-sp.]
MBV3827782.1	Type6.BREX	<-UDG<-?<-ARG||RHH+MobC->?-><-?<-SIGMA+OB-Fold+AAA-ATPase+HKD-DNase-BrxL<-PglZ<-N6-METHYLASE<-DUF6079-p*<-DUF6079-p<-BrxB-BrxF-iSTAND<-BrxA-DNA-binding<-BrxE<-HTH_3||<-XIS-HTH<-AbiEii	938	Bacteria>FCB_group>Bacteroidetes/Chlorobi_group>Bacteroidota>Bacteroidia>Bacteroidales	GCA_019129685.1	Bacteroides_uniformis	DW051_06905	94105..96921	820	BREX-system-P-loop-protein-BrxC-[Bacteroides-uniformis]
MCB9017748.1	Type1.BREX	<-ACET||?-><-SIGMA+OB-Fold+AAA-ATPase+HKD-DNase-BrxL<-PglZ<-N6-METHYLASE-p<-HTH+REase<-?<-Schlafen+HATPase+wHTH<-DUF6079*<-BrxB-BrxF-iSTAND<-BrxA-DNA-binding<-?<-PALP	1207	Bacteria>FCB_group>Bacteroidetes/Chlorobi_group>Bacteroidota>Bacteroidia>Bacteroidales	GCA_020636675.1	Prevotellaceae_bacterium	H6544_03995	62697..66320	2049047	BREX-system-P-loop-protein-BrxC-[Prevotellaceae-bacterium]
HJF82219.1	Type1.BREX	<-SF1-SF2-Helicase-p<-HTH_3<-?<-?||BrxA-DNA-binding->BrxB-BrxF-iSTAND->DUF6079*->OLD-ABC->TOPRIM->N6-METHYLASE->SIR2+SIR2->PglZ->SIGMA+OB-Fold+AAA-ATPase+HKD-DNase-BrxL->	1195	Bacteria>FCB_group>Bacteroidetes/Chlorobi_group>Bacteroidota>Bacteroidia>Bacteroidales	GCA_020742805.1	Phocaeicola_plebeius	K8V40_11345	4107..7694	310297	BREX-system-P-loop-protein-BrxC-[Phocaeicola-plebeius]
MCC8037087.1	Type1.BREX	<-HAD+PRTase+PRTase+REase+SF1-SF2-Helicase<-?<-?<-?<-STYKIN<-?<-SIGMA+OB-Fold+AAA-ATPase+HKD-DNase-BrxL<-PglZ<-DUF6079*<-BrxB-BrxF-iSTAND<-BrxA-DNA-binding<-?<-?<-?<-?<-Acetate_kinase	1219	Bacteria>FCB_group>Bacteroidetes/Chlorobi_group>Bacteroidota>Bacteroidia>Bacteroidales	GCA_020859755.1	Bacteroidales_bacterium	LIP02_02980	18317..21976	2030927	BREX-system-P-loop-protein-BrxC-[Bacteroidales-bacterium]
MCC8115076.1	Type1.BREX	PglZ->SIGMA+OB-Fold+AAA-ATPase+HKD-DNase-BrxL->BrxA-DNA-binding->BrxB-BrxF-iSTAND->DUF6079-p*->DUF6079->N6-METHYLASE->	159	Bacteria>FCB_group>Bacteroidetes/Chlorobi_group>Bacteroidota>Bacteroidia>Bacteroidales	GCA_020860445.1	Bacteroidales_bacterium	LIP03_14010	6329..6808	2030927	hypothetical-protein-[Bacteroidales-bacterium]
MCC8115077.1	Type1.BREX	PglZ->SIGMA+OB-Fold+AAA-ATPase+HKD-DNase-BrxL->BrxA-DNA-binding->BrxB-BrxF-iSTAND->DUF6079-p->DUF6079*->N6-METHYLASE->	1040	Bacteria>FCB_group>Bacteroidetes/Chlorobi_group>Bacteroidota>Bacteroidia>Bacteroidales	GCA_020860445.1	Bacteroidales_bacterium	LIP03_14015	6789..9911	2030927	BREX-system-P-loop-protein-BrxC-[Bacteroidales-bacterium]
MCH4018978.1	Type1.BREX	XIS-HTH->?->AAA+-><-?||wHTH+WYL+WCX->HTH_3->BrxA-DNA-binding->BrxB-BrxF-iSTAND->DUF6079*->N6-METHYLASE->Schlafen+HATPase+wHTH->SIR2->PglZ->SIGMA+OB-Fold+AAA-ATPase+HKD-DNase-BrxL->SIR2+SIR2->ZnR->DDE_Tnp_ISL3->Tox-RelE->	1198	Bacteria>FCB_group>Bacteroidetes/Chlorobi_group>Bacteroidota>Bacteroidia>Bacteroidales	GCA_022483425.1	Prevotella_sp.	LKE74_11130	2793807..2797403	59823	BREX-system-P-loop-protein-BrxC-[Prevotella-sp.]
MCI1742488.1	Type1.BREX	<-HTH_31||tRNA-><-?<-SIR2+SIR2<-SIGMA+OB-Fold+AAA-ATPase+HKD-DNase-BrxL<-PglZ<-HTH+REase<-Schlafen+HATPase+wHTH<-N6-METHYLASE<-DUF6079*<-BrxB-BrxF-iSTAND<-BrxA-DNA-binding<-HTH_3<-wHTH+WYL+WCX||?-><-AAA+<-?<-XIS-HTH	1206	Bacteria>FCB_group>Bacteroidetes/Chlorobi_group>Bacteroidota>Bacteroidia>Bacteroidales	GCA_022647785.1	Prevotella_sp.	LKI18_09155	39395..43015	59823	BREX-system-P-loop-protein-BrxC-[Prevotella-sp.]
MCI2087046.1	Type1.BREX	<-SIGMA+OB-Fold+AAA-ATPase+HKD-DNase-BrxL<-PglZ<-HTH+REase<-Schlafen+HATPase+wHTH<-N6-METHYLASE<-DUF6079*<-BrxB-BrxF-iSTAND<-BrxA-DNA-binding<-HTH_3<-wHTH+WYL+WCX||?-><-AAA+<-?<-XIS-HTH	1198	Bacteria>FCB_group>Bacteroidetes/Chlorobi_group>Bacteroidota>Bacteroidia>Bacteroidales	GCA_022653375.1	Prevotella_sp.	LKK10_02020	11419..15015	59823	BREX-system-P-loop-protein-BrxC-[Prevotella-sp.]
MCI7017346.1	Type1.BREX	<-SIGMA+OB-Fold+AAA-ATPase+HKD-DNase-BrxL<-PglZ<-?<-Schlafen+HATPase+wHTH<-N6-METHYLASE<-AT-HTH-HYD2<-Tox-ART-HYD2<-AT-HTH-HYD2<-DUF6079*<-BrxB-BrxF-iSTAND<-BrxA-DNA-binding<-wHTH+WYL+WCX	1189	Bacteria>FCB_group>Bacteroidetes/Chlorobi_group>Bacteroidota>Bacteroidia>Bacteroidales	GCA_022775925.1	Prevotella_sp.	MR923_09655	21723..25292	59823	BREX-system-P-loop-protein-BrxC-[Prevotella-sp.]
MCI6342424.1	Type6.BREX	DnaB+TnsC-ATPase->?-><-SIGMA+OB-Fold+AAA-ATPase+HKD-DNase-BrxL<-PglZ<-HNH<-OLD-ABC<-N6-METHYLASE<-DUF6079*<-BrxB-BrxF-iSTAND<-BrxA-DNA-binding<-BrxE<-HTH_3	1217	Bacteria>FCB_group>Bacteroidetes/Chlorobi_group>Bacteroidota>Bacteroidia>Bacteroidales	GCA_022777695.1	Prevotella_sp.	MR788_07720	61686..65339	59823	BREX-system-P-loop-protein-BrxC-[Prevotella-sp.]
MCI7387531.1	Type6.BREX	Arm-DNA-bind_5-><-UDG<-?<-ARG||?-><-SIGMA+OB-Fold+AAA-ATPase+HKD-DNase-BrxL<-PglZ<-N6-METHYLASE<-DUF6079*<-BrxB-BrxF-iSTAND<-BrxA-DNA-binding<-BrxE<-HTH_3<-?||?-><-XIS-HTH<-AbiEii	1215	Bacteria>FCB_group>Bacteroidetes/Chlorobi_group>Bacteroidota>Bacteroidia>Bacteroidales	GCA_022784345.1	Bacteroides_uniformis	MSH64_12855	232214..235861	820	BREX-system-P-loop-protein-BrxC-[Bacteroides-uniformis]
MCK9398992.1	Type1.BREX	PIN-><-?||DDE_Tnp_1-><-?<-?||HTH_3->BrxA-DNA-binding->BrxB-BrxF-iSTAND->DUF6079*->N6-METHYLASE->PglZ->SIGMA+OB-Fold+AAA-ATPase+HKD-DNase-BrxL->?->?->?->?-><-tRNA||RADICAL-SAM->	1163	Bacteria>FCB_group>Bacteroidetes/Chlorobi_group>Bacteroidota>Bacteroidia>Bacteroidales	GCA_023229505.1	Bacteroidales_bacterium	MOQ51_03210	70739..74230	2030927	BREX-system-P-loop-protein-BrxC-[Bacteroidales-bacterium]
MCQ2071598.1	Type1.BREX	<-TOPRIM<-?<-?||Peptidase_M23->BrxA-DNA-binding->BrxB-BrxF-iSTAND->DUF6079*->N6-METHYLASE->Schlafen+HATPase+wHTH->PglZ->SIGMA+OB-Fold+AAA-ATPase+HKD-DNase-BrxL->XIS-HTH->wHTH+WYL+WCX->?->HHH_5+SF1-SF2-Helicase->	1190	Bacteria>FCB_group>Bacteroidetes/Chlorobi_group>Bacteroidota>Bacteroidia>Bacteroidales	GCA_024398655.1	Bacteroidaceae_bacterium	MJY96_00535	107887..111459	2212467	BREX-system-P-loop-protein-BrxC-[Bacteroidaceae-bacterium]
WP_156315612.1	Type1.BREX	XIS-HTH-><-?<-?||?->?->?->HTH_3->BrxA-DNA-binding->BrxB-BrxF-iSTAND->DUF6079-p*->DUF6079-p->N6-METHYLASE-p->N6-METHYLASE->PglZ-p->PglZ->SIGMA+OB-Fold+AAA-ATPase+HKD-DNase-BrxL->KAP-NTPase->	925	Bacteria>FCB_group>Bacteroidetes/Chlorobi_group>Bacteroidota>Bacteroidia>Bacteroidales	GCA_030820635.1	Phocaeicola_coprophilus	JCM13816_RS17810	17247..20024	387090	BREX-system-P-loop-protein-BrxC-[Phocaeicola-coprophilus]
SHK68061.1	Type1.BREX	<-Classical-AAA<-?||tRNA-><-?<-?<-HTH+DOC||?->HTH_3->BrxA-DNA-binding->BrxB-BrxF-iSTAND->DUF6079*->N6-METHYLASE->SbcC+SbcC->?->SIR2+SIR2->PglZ->SIGMA+OB-Fold+AAA-ATPase+HKD-DNase-BrxL->?->ParB+HNH->ParB+HNH->	1206	Bacteria>FCB_group>Bacteroidetes/Chlorobi_group>Bacteroidota>Bacteroidia>Bacteroidales	GCA_900142525.1	Prevotella_ruminicola	SAMN05216463_10942	46265..49885	839	hypothetical-protein-SAMN05216463_10942-[Prevotella-ruminicola]
WP_008760280.1	Type1.BREX	D5ATPase+DUF3874-><-?||ADH_N+ADH_zinc_N+METHYLASE->SPOUT-><-SIGMA+OB-Fold+AAA-ATPase+HKD-DNase-BrxL<-PglZ<-HTH+REase<-N6-METHYLASE-p<-N6-METHYLASE<-DUF6079*<-BrxB-BrxF-iSTAND<-BrxA-DNA-binding<-HTH_3<-?<-?<-?<-?<-TIR<-?<-TIR	1193	Bacteria>FCB_group>Bacteroidetes/Chlorobi_group>Bacteroidota>Bacteroidia>Bacteroidales	GCF_000162515.1	Bacteroides_thetaiotaomicron	HMPREF9007_RS20215	181866..185447	818	MULTISPECIES:-BREX-system-P-loop-protein-BrxC-[Bacteroides]
WP_232506819.1	Type6.BREX	PRTase->?->?->tRNA->tRNA-><-?<-SIGMA+OB-Fold+AAA-ATPase+HKD-DNase-BrxL<-PglZ<-HNH<-OLD-ABC<-N6-METHYLASE<-DUF6079-p*<-DUF6079-p<-BrxB-BrxF-iSTAND<-BrxA-DNA-binding<-BrxE<-HTH_3<-?<-?<-TraJ-RHH<-ZnR+TOPRIM	794	Bacteria>FCB_group>Bacteroidetes/Chlorobi_group>Bacteroidota>Bacteroidia>Bacteroidales	GCF_000178215.1	Bacteroides_xylanisolvens	CW3_RS0123000	100900..103284	371601	hypothetical-protein-[Bacteroides-xylanisolvens]
WP_008016782.1	Type6.BREX	<-SIGMA+OB-Fold+AAA-ATPase+HKD-DNase-BrxL<-PglZ<-HNH<-OLD-ABC<-N6-METHYLASE<-DUF6079-p<-DUF6079-p*<-BrxB-BrxF-iSTAND<-BrxA-DNA-binding<-BrxE<-HTH_3<-?<-?<-TraJ-RHH<-ZnR+TOPRIM<-CR-ATPase4	425	Bacteria>FCB_group>Bacteroidetes/Chlorobi_group>Bacteroidota>Bacteroidia>Bacteroidales	GCF_000178215.1	Bacteroides_xylanisolvens	CW3_RS26225	103281..104558	371601	DUF6079-family-protein-[Bacteroides-xylanisolvens]
WP_004312187.1	Type1.BREX	<-SIGMA+OB-Fold+AAA-ATPase+HKD-DNase-BrxL<-PglZ<-SWIM+DUF3670+SF1-SF2-Helicase<-?<-?<-?<-Peptidase_C39_2<-ACET+TPR<-N6-METHYLASE<-DUF6079*<-BrxB-BrxF-iSTAND<-BrxA-DNA-binding<-wHTH+WYL+WCX<-HTH_3||?->?->?->NUDIX+NUDIX->	1208	Bacteria>FCB_group>Bacteroidetes/Chlorobi_group>Bacteroidota>Bacteroidia>Bacteroidales	GCF_000178275.1	Bacteroidaceae	KOG46_RS13725	52737..56363	815	MULTISPECIES:-BREX-system-P-loop-protein-BrxC-[Bacteroidaceae]
WP_004314649.1	Type6.BREX	XIS-HTH->CR-ATPase4->ZnR+TOPRIM->TraJ-RHH->?->?->BrxE->BrxA-DNA-binding->BrxB-BrxF-iSTAND->DUF6079-p*->DUF6079-p->N6-METHYLASE->OLD-ABC->HNH->PglZ->SIGMA+OB-Fold+AAA-ATPase+HKD-DNase-BrxL->	515	Bacteria>FCB_group>Bacteroidetes/Chlorobi_group>Bacteroidota>Bacteroidia>Bacteroidales	GCF_000178295.1	Bacteroides_xylanisolvens	CW1_RS26610	23799..25346	371601	BREX-system-P-loop-protein-BrxC-[Bacteroides-xylanisolvens]
WP_232505074.1	Type6.BREX	CR-ATPase4->ZnR+TOPRIM->TraJ-RHH->?->?->BrxE->BrxA-DNA-binding->BrxB-BrxF-iSTAND->DUF6079-p->DUF6079-p*->N6-METHYLASE->OLD-ABC->HNH->PglZ->SIGMA+OB-Fold+AAA-ATPase+HKD-DNase-BrxL->	711	Bacteria>FCB_group>Bacteroidetes/Chlorobi_group>Bacteroidota>Bacteroidia>Bacteroidales	GCF_000178295.1	Bacteroides_xylanisolvens	CW1_RS26615	25324..27459	371601	hypothetical-protein-[Bacteroides-xylanisolvens]
WP_006745116.1	Type1.BREX	<-MODE-HTH+PBPII||?-><-?<-NUDIX||ABhydrolase->DNA-glycosylase-><-SIGMA+OB-Fold+AAA-ATPase+HKD-DNase-BrxL<-PglZ<-N6-METHYLASE<-DUF6079*<-BrxB-BrxF-iSTAND<-BrxA-DNA-binding<-?||?->REase-><-?<-?<-?<-SIGMA-HTH	1192	Bacteria>FCB_group>Bacteroidetes/Chlorobi_group>Bacteroidota>Bacteroidia>Bacteroidales	GCF_000212915.1	Bacteroides_coprosuis	BCOP_RS09060	2132867..2136445	151276	BREX-system-P-loop-protein-BrxC-[Bacteroides-coprosuis]
WP_018109295.1	Type1.BREX	<-FURR-HTH<-?<-Peptidase_M49<-AraC-HTH||SF1-SF2-Helicase+PRTase->tRNA->HTH_3->BrxA-DNA-binding->BrxB-BrxF-iSTAND->DUF6079*->N6-METHYLASE->PglZ->SIGMA+OB-Fold+AAA-ATPase+HKD-DNase-BrxL->?->?->XIS-HTH->HTH_3->MerR-HTH->TOPRIM->	1202	Bacteria>FCB_group>Bacteroidetes/Chlorobi_group>Bacteroidota>Bacteroidia>Bacteroidales	GCF_000375405.1	Bacteroides_propionicifaciens	B091_RS0106980	23999..27607	392838	BREX-system-P-loop-protein-BrxC-[Bacteroides-propionicifaciens]
WP_002561973.1	Type1.BREX	35exo+SF1-SF2-Helicase-><-DDE_Tnp||<-SIGMA+OB-Fold+AAA-ATPase+HKD-DNase-BrxL<-PglZ<-Schlafen+HATPase+wHTH<-SF1-SF2-Helicase<-OLD-ABC+TOPRIM<-N6-METHYLASE<-DUF6079*<-BrxB-BrxF-iSTAND<-BrxA-DNA-binding	1202	Bacteria>FCB_group>Bacteroidetes/Chlorobi_group>Bacteroidota>Bacteroidia>Bacteroidales	GCF_000382465.1	Bacteroides_sp._HPS0048	HMPREF1214_RS19625	483033..486641	1078089	BREX-system-P-loop-protein-BrxC-[Bacteroides-sp.-HPS0048]
WP_155941928.1	Type6.BREX	Glyoxalase-><-?<-?||HD->wHTH+WYL+WCX->HTH_3->BrxE->BrxA-DNA-binding->BrxB-BrxF-iSTAND->DUF6079*->N6-METHYLASE->PglZ->SIGMA+OB-Fold+AAA-ATPase+HKD-DNase-BrxL->KAP-NTPase->?->?->?->SWIM+DUF3670+SF1-SF2-Helicase->ACET->	1198	Bacteria>FCB_group>Bacteroidetes/Chlorobi_group>Bacteroidota>Bacteroidia>Bacteroidales	GCF_000424185.1	Prevotella_sp._AGR2160	G604_RS15270	9168..12764	1280674	BREX-system-P-loop-protein-BrxC-[Prevotella-sp.-AGR2160]
WP_024993540.1	Type1.BREX	<-wHTH+WYL+WCX<-wHTH+WYL+WCX<-SIGMA+OB-Fold+AAA-ATPase+HKD-DNase-BrxL<-PglZ<-Schlafen+HATPase+wHTH<-N6-METHYLASE<-DUF6079*<-BrxB-BrxF-iSTAND<-BrxA-DNA-binding<-HTH_3	1210	Bacteria>FCB_group>Bacteroidetes/Chlorobi_group>Bacteroidota>Bacteroidia>Bacteroidales	GCF_000613805.1	Phocaeicola_paurosaccharolyticus	JCM15092_RS04015	53285..56917	732242	BREX-system-P-loop-protein-BrxC-[Phocaeicola-paurosaccharolyticus]
WP_042520323.1	Type1.BREX	DDE_Tnp_ISL3-><-PARG+MACRODOMAIN<-REase<-SIGMA+OB-Fold+AAA-ATPase+HKD-DNase-BrxL<-PglZ<-N6-METHYLASE<-DUF6079*<-BrxB-BrxF-iSTAND<-BrxA-DNA-binding<-HTH_3	1204	Bacteria>FCB_group>Bacteroidetes/Chlorobi_group>Bacteroidota>Bacteroidia>Bacteroidales	GCF_000833975.1	Prevotella_pectinovora	ST44_RS13175	10747..14361	1602169	BREX-system-P-loop-protein-BrxC-[Prevotella-pectinovora]
WP_044064719.1	Type6.BREX	<-REase<-?<-SIGMA+OB-Fold+AAA-ATPase+HKD-DNase-BrxL<-PglZ<-HNH<-OLD-ABC<-N6-METHYLASE<-DUF6079*<-BrxB-BrxF-iSTAND<-BrxA-DNA-binding<-BrxE<-HTH_3<-AAA+<-?<-XIS-HTH<-?<-Phage_int_SAM_5+Phage_integrase	1217	Bacteria>FCB_group>Bacteroidetes/Chlorobi_group>Bacteroidota>Bacteroidia>Bacteroidales	GCF_000833995.1	Prevotella_pectinovora	ST45_RS02505	69518..73171	1602169	BREX-system-P-loop-protein-BrxC-[Prevotella-pectinovora]
WP_044073670.1	Type1.BREX	HTH_3->BrxA-DNA-binding->BrxB-BrxF-iSTAND->DUF6079*->N6-METHYLASE->PglZ->SIGMA+OB-Fold+AAA-ATPase+HKD-DNase-BrxL->REase->PARG+MACRODOMAIN-><-DDE_Tnp_ISL3	1206	Bacteria>FCB_group>Bacteroidetes/Chlorobi_group>Bacteroidota>Bacteroidia>Bacteroidales	GCF_000834015.1	Prevotella_pectinovora	ST42_RS12125	1627..5247	1602169	BREX-system-P-loop-protein-BrxC-[Prevotella-pectinovora]
WP_008139697.1	Type6.BREX	HTH_3->BrxE->BrxA-DNA-binding-p->BrxB-BrxF-iSTAND->DUF6079*->N6-METHYLASE-p->N6-METHYLASE-p->N6-METHYLASE-p->PglZ->PglZ->SIGMA+OB-Fold+AAA-ATPase+HKD-DNase-BrxL->KAP-NTPase->	1215	Bacteria>FCB_group>Bacteroidetes/Chlorobi_group>Bacteroidota>Bacteroidia>Bacteroidales	GCF_001315785.1	Phocaeicola_coprophilus	JCM13818_RS17975	17242..20889	387090	BREX-system-P-loop-protein-BrxC-[Phocaeicola-coprophilus]
WP_140392864.1	Type6.BREX	<-TatD_DNase<-?<-?<-KAP-NTPase<-SIGMA+OB-Fold+AAA-ATPase+HKD-DNase-BrxL<-PglZ<-N6-METHYLASE<-DUF6079*<-BrxB-BrxF-iSTAND<-BrxA-DNA-binding<-BrxE<-HTH_3	1211	Bacteria>FCB_group>Bacteroidetes/Chlorobi_group>Bacteroidota>Bacteroidia>Bacteroidales	GCF_002159645.1	Parabacteroides_sp._An277	B5F77_RS15595	54457..58092	1965619	BREX-system-P-loop-protein-BrxC-[Parabacteroides-sp.-An277]
WP_094389547.1	Type1.BREX	LRR_5->?->?->?-><-?||?->HTH_3->BrxA-DNA-binding->BrxB-BrxF-iSTAND->DUF6079*->N6-METHYLASE->PglZ->SIGMA+OB-Fold+AAA-ATPase+HKD-DNase-BrxL->REase->	1228	Bacteria>FCB_group>Bacteroidetes/Chlorobi_group>Bacteroidota>Bacteroidia>Bacteroidales	GCF_002251345.1	Prevotella_sp._P4-119	CIK89_RS00860	20388..24074	2024218	BREX-system-P-loop-protein-BrxC-[Prevotella-sp.-P4-119]
WP_118441918.1	Type1.BREX	HTH_Tnp_1+HTH_21+rve+rve_3-><-?<-?||BrxA-DNA-binding->BrxB-BrxF-iSTAND->DUF6079*->OLD-ABC->TOPRIM->N6-METHYLASE->?->PglZ->SIGMA+OB-Fold+AAA-ATPase+HKD-DNase-BrxL->?->?->Peptidase_M28->	1195	Bacteria>FCB_group>Bacteroidetes/Chlorobi_group>Bacteroidota>Bacteroidia>Bacteroidales	GCF_003474535.1	Phocaeicola_plebeius	DW041_RS13375	4564..8151	310297	BREX-system-P-loop-protein-BrxC-[Phocaeicola-plebeius]

Protein-ID	System	Gene-Neighbourhoods	Length	Taxonomy	Assembly-ID	Strain	Locus-Tag	Genomic-coordinates	TaxID	Fasta-defline
WP_135993587.1	Type1.BREX	<-SF1-SF2-Helicase<- SIGMA+OB-Fold+AAA-ATPase+ HKD-DNase -BrxL <- PglZ <-Schlafen+HATPase+wHTH<-SIR2<- N6-METHYLASE <- DUF6079 *<- BrxB-BrxF-iSTAND <- BrxA-DNA-binding <-HTH_3<-?||Phage_int_SAM_1+Phage_integrase->Phage_integrase->Phage_int_SAM_1+Phage_integrase-><-Peptidase_M23	1221	Bacteria>FCB_group>Bacteroidetes/Chlorobi_group>Bacteroidota>Bacteroidia>Bacteroidales	GCF_004793655.1	Muribaculum_intestinale	E5330_RS10970	18051..21716	1796646	BREX-system-P-loop-protein-BrxC-[Muribaculum-intestinale]
WP_172774764.1	Type1.BREX	OLD-ABC->TOPRIM->XIS-HTH-><-?<-?<-?||HTH_3-> BrxA-DNA-binding -> BrxB-BrxF-iSTAND -> DUF6079 *-> N6-METHYLASE ->OLD-ABC+TOPRIM->SF1-SF2-Helicase->Schlafen+HATPase+wHTH-> PglZ -> SIGMA+OB-Fold+AAA-ATPase+ HKD-DNase -BrxL ->NADAR->RADICAL-SAM->HIRAN->	1217	Bacteria>FCB_group>Bacteroidetes/Chlorobi_group>Bacteroidota>Bacteroidia>Bacteroidales	GCF_012976275.1	Phocaeicola_vulgatus	HKQ53_RS16515	79396..83049	821	BREX-system-P-loop-protein-BrxC-[Phocaeicola-vulgatus]
WP_175324259.1	Type1.BREX	<- SIGMA+OB-Fold+AAA-ATPase+ HKD-DNase -BrxL <- PglZ <-SWIM+DUF3670+SF1-SF2-Helicase<-?<-?<-?<-Peptidase_C39_2<-ACET+TPR<- N6-METHYLASE <- DUF6079 *<- BrxB-BrxF-iSTAND <- BrxA-DNA-binding <- wHTH+WYL+WCX <-HTH_3||?->?->?->NUDIX+NUDIX->	1208	Bacteria>FCB_group>Bacteroidetes/Chlorobi_group>Bacteroidota>Bacteroidia>Bacteroidales	GCF_013361175.1	Phocaeicola_vulgatus	HUV02_RS04070	75655..79281	821	BREX-system-P-loop-protein-BrxC-[Phocaeicola-vulgatus]
WP_181757796.1	Type6.BREX	<-DUF772+DDE_Tnp_1+DDE_Tnp_1_6||Arm-DNA-bind_5-><-UDG<-?<-ARG||?-><- SIGMA+OB-Fold+AAA-ATPase+ HKD-DNase -BrxL <- PglZ <- N6-METHYLASE <- DUF6079 -p*<- DUF6079 -p<- BrxB-BrxF-iSTAND <- BrxA-DNA-binding <- BrxE <-HTH_3<-?||?->?-><-XIS-HTH	722	Bacteria>FCB_group>Bacteroidetes/Chlorobi_group>Bacteroidota>Bacteroidia>Bacteroidales	GCF_013874375.1	Bacteroides_sp._CACC_737	H1A11_RS04980	1170653..1172821	2755405	hypothetical-protein-[Bacteroides-sp.-CACC-737]
WP_181757797.1	Type6.BREX	Arm-DNA-bind_5-><-UDG<-?<-ARG||?-><- SIGMA+OB-Fold+AAA-ATPase+ HKD-DNase -BrxL <- PglZ <- N6-METHYLASE <- DUF6079 -p<- DUF6079 -p*<- BrxB-BrxF-iSTAND <- BrxA-DNA-binding <- BrxE <-HTH_3<-?||?->?-><-XIS-HTH<-AbiEii	496	Bacteria>FCB_group>Bacteroidetes/Chlorobi_group>Bacteroidota>Bacteroidia>Bacteroidales	GCF_013874375.1	Bacteroides_sp._CACC_737	H1A11_RS04985	1172743..1174233	2755405	DUF6079-family-protein-[Bacteroides-sp.-CACC-737]
WP_186905779.1	Type6.BREX	<-TRAM+METHYLASE||?->Arm-DNA-bind_5-><-UDG<-?<-ARG||<- SIGMA+OB-Fold+AAA-ATPase+ HKD-DNase -BrxL <- PglZ <- N6-METHYLASE <- DUF6079 *<- BrxB-BrxF-iSTAND <- BrxA-DNA-binding <- BrxE <-HTH_3<-?||?->?-><-XIS-HTH	1213	Bacteria>FCB_group>Bacteroidetes/Chlorobi_group>Bacteroidota>Bacteroidia>Bacteroidales	GCF_014287575.1	Bacteroides_sp._NSJ-21	H8S53_RS11040	2778988..2782629	2763025	BREX-system-P-loop-protein-BrxC-[Bacteroides-sp.-NSJ-21]
WP_115498725.1	Type1.BREX	<- SIGMA+OB-Fold+AAA-ATPase+ HKD-DNase -BrxL <- PglZ <-Schlafen+HATPase+wHTH<- N6-METHYLASE <- DUF6079 *<- BrxB-BrxF-iSTAND <- BrxA-DNA-binding ||?->?->?->?-><-tRNA||Cysteine-Desulfurase->?-><-ACET	1211	Bacteria>FCB_group>Bacteroidetes/Chlorobi_group>Bacteroidota>Bacteroidia>Bacteroidales	GCF_014385205.1	Parabacteroides_acidifaciens	H8784_RS05825	88295..91930	2290935	BREX-system-P-loop-protein-BrxC-[Parabacteroides-acidifaciens]
WP_004319896.1	Type1.BREX	BrxA-DNA-binding -> BrxB-BrxF-iSTAND -> DUF6079 *-> N6-METHYLASE ->SIR2+SIR2->Schlafen+HATPase+wHTH-> PglZ -> SIGMA+OB-Fold+AAA-ATPase+ HKD-DNase -BrxL -><-35exo+SF1-SF2-Helicase||BACON+Peptidase_M64->	1202	Bacteria>FCB_group>Bacteroidetes/Chlorobi_group>Bacteroidota>Bacteroidia>Bacteroidales	GCF_015548155.1	Bacteroides	PO193_RS10880	44218..47826	816	MULTISPECIES:-BREX-system-P-loop-protein-BrxC-[Bacteroides]
WP_195290998.1	Type1.BREX	Calcineurin-><-?<- SIGMA+OB-Fold+AAA-ATPase+ HKD-DNase -BrxL <- PglZ <-Cas4+SF1-SF2-Helicase<-ACET+TPR<- N6-METHYLASE <- DUF6079 *<- BrxB-BrxF-iSTAND <- BrxA-DNA-binding <-HTH_3<-Arm-DNA-bind_3+Phage_integrase	1188	Bacteria>FCB_group>Bacteroidetes/Chlorobi_group>Bacteroidota>Bacteroidia>Bacteroidales	GCF_015548275.1	Alistipes_finegoldii	I2C47_RS09460	177258..180824	214856	BREX-system-P-loop-protein-BrxC-[Alistipes-finegoldii]
WP_195366148.1	Type6.BREX	<-TatD_DNase<-?<-KAP-glycosylase<-KAP-NTPase<- SIGMA+OB-Fold+AAA-ATPase+ HKD-DNase -BrxL <- PglZ <- N6-METHYLASE <- DUF6079 -p<- DUF6079 -p*<- BrxB-BrxF-iSTAND <- BrxA-DNA-binding <- BrxE <-HTH_3<-?<-?||?->?-><-XIS-HTH	921	Bacteria>FCB_group>Bacteroidetes/Chlorobi_group>Bacteroidota>Bacteroidia>Bacteroidales	GCF_015550085.1	Bacteroides_ovatus	PO368_RS20360	46610..49375	28116	BREX-system-P-loop-protein-BrxC-[Bacteroides-ovatus]
WP_118403962.1	Type1.BREX	<-Phage_int_SAM_5+Phage_integrase||STYKinase-like->?->?-><-HEPN||HTH_3-> BrxA-DNA-binding -> BrxB-BrxF-iSTAND -> DUF6079 *-> N6-METHYLASE ->Schlafen+HATPase+wHTH-> PglZ -> SIGMA+OB-Fold+AAA-ATPase+ HKD-DNase -BrxL ->?->URI-DNase+RAMA->	1211	Bacteria>FCB_group>Bacteroidetes/Chlorobi_group>Bacteroidota>Bacteroidia>Bacteroidales	GCF_015553065.1	Bacteroides_cellulosilyticus	I2F01_RS18130	73980..77615	246787	BREX-system-P-loop-protein-BrxC-[Bacteroides-cellulosilyticus]
WP_195571580.1	Type1.BREX	BrxA-DNA-binding -> BrxB-BrxF-iSTAND -> DUF6079 *-> N6-METHYLASE ->SIR2+SIR2->Schlafen+HATPase+wHTH-> PglZ -> SIGMA+OB-Fold+AAA-ATPase+ HKD-DNase -BrxL -><-35exo+SF1-SF2-Helicase||?->SIGMA-HTH->	1202	Bacteria>FCB_group>Bacteroidetes/Chlorobi_group>Bacteroidota>Bacteroidia>Bacteroidales	GCF_015557365.1	Bacteroides_nordii	I2G99_RS18035	12742..16350	291645	BREX-system-P-loop-protein-BrxC-[Bacteroides-nordii]
WP_195628684.1	Type1.BREX	<-MORC+REase<-?<-REase+nSTAND3||?->?-><- SIGMA+OB-Fold+AAA-ATPase+ HKD-DNase -BrxL <- PglZ <- N6-METHYLASE <- DUF6079 -p*||?->?->?->?->?->?-><-?<-ACET	769	Bacteria>FCB_group>Bacteroidetes/Chlorobi_group>Bacteroidota>Bacteroidia>Bacteroidales	GCF_015558155.1	Bacteroides_finegoldii	I2H47_RS02655	98961..101270	338188	hypothetical-protein-[Bacteroides-finegoldii]
WP_195663259.1	Type1.BREX	<- SIGMA+OB-Fold+AAA-ATPase+ HKD-DNase -BrxL <- PglZ <-SWIM+DUF3670+SF1-SF2-Helicase<-?<-?<-?<-?<-DOC+HTH<- N6-METHYLASE <- DUF6079 *<- BrxB-BrxF-iSTAND <- BrxA-DNA-binding <-HTH_3||?->?-><-?<-?<-RADICAL-SAM	1206	Bacteria>FCB_group>Bacteroidetes/Chlorobi_group>Bacteroidota>Bacteroidia>Bacteroidales	GCF_015559525.1	Bacteroides_congonensis	I2I22_RS15360	96982..100602	1871006	BREX-system-P-loop-protein-BrxC-[Bacteroides-congonensis]
WP_117964425.1	Type6.BREX	Arm-DNA-bind_5-><-UDG<-?<-ARG||RHH+MobC->?-><- SIGMA+OB-Fold+AAA-ATPase+ HKD-DNase -BrxL <- PglZ <- N6-METHYLASE <- DUF6079 -p*<- BrxB-BrxF-iSTAND <- BrxA-DNA-binding <- BrxE <-HTH_3<-?||?-><-XIS-HTH<-AbiEii	265	Bacteria>FCB_group>Bacteroidetes/Chlorobi_group>Bacteroidota>Bacteroidia>Bacteroidales	GCF_015679285.1	Bacteroides	ITJ87_RS11480	2822744..2823541	816	MULTISPECIES:-DUF6079-family-protein-[Bacteroides]
WP_203041477.1	Type1.BREX	Phage_lysozyme->PRTase-><-?<-?<-?||HTH_3-> wHTH+WYL+WCX -> BrxA-DNA-binding -> BrxB-BrxF-iSTAND -> DUF6079 *-> N6-METHYLASE ->SIR2+SIR2-> PglZ -> SIGMA+OB-Fold+AAA-ATPase+ HKD-DNase -BrxL ->	1207	Bacteria>FCB_group>Bacteroidetes/Chlorobi_group>Bacteroidota>Bacteroidia>Bacteroidales	GCF_016803185.1	Prevotella_copri	F8Q96_RS13415	22671..26294	165179	BREX-system-P-loop-protein-BrxC-[Prevotella-copri]
WP_203049164.1	Type6.BREX	<-TOPRIM<-NUDIX<-?<-DUF4369+Thioredoxin_8||RNasePH+RNasePH+KH+S1->HTH_3-> BrxE -> BrxA-DNA-binding -> BrxB-BrxF-iSTAND -> DUF6079 *-> N6-METHYLASE ->Schlafen+HATPase+wHTH-> PglZ -> SIGMA+OB-Fold+AAA-ATPase+ HKD-DNase -BrxL ->SLATT_4->	1216	Bacteria>FCB_group>Bacteroidetes/Chlorobi_group>Bacteroidota>Bacteroidia>Bacteroidales	GCF_016803255.1	Prevotella_copri	F8Q89_RS01960	71962..75612	165179	BREX-system-P-loop-protein-BrxC-[Prevotella-copri]
WP_204430319.1	Type1.BREX	<-NADAR<- SIGMA+OB-Fold+AAA-ATPase+ HKD-DNase -BrxL <- PglZ <-Schlafen+HATPase+wHTH<-SIR2<- N6-METHYLASE <- DUF6079 *<- BrxB-BrxF-iSTAND <- BrxA-DNA-binding <-HTH_3||?->?->?->?-><-XIS-HTH<-TOPRIM	1217	Bacteria>FCB_group>Bacteroidetes/Chlorobi_group>Bacteroidota>Bacteroidia>Bacteroidales	GCF_016899805.1	Bacteroides_gallinaceum	H6B13_RS12495	13542..17195	1462571	BREX-system-P-loop-protein-BrxC-[Bacteroides-gallinaceum]
WP_204392825.1	Type1.BREX	<-SbcC<- HKD-DNase <- SIGMA+OB-Fold+AAA-ATPase+ HKD-DNase -BrxL <- PglZ <- N6-METHYLASE <- DUF6079 *<- BrxB-BrxF-iSTAND <- BrxA-DNA-binding ||?-><-?<-Sulfatase+Nab6_mRNP_bdg+Phosphodiest<-Glyco_hydro_88||?->?-><-LexA-protease	1185	Bacteria>FCB_group>Bacteroidetes/Chlorobi_group>Bacteroidota>Bacteroidia>Bacteroidales	GCF_016901995.1	Bacteroides_caecigallinarum	H6A61_RS08410	10706..14263	1411144	BREX-system-P-loop-protein-BrxC-[Bacteroides-caecigallinarum]
WP_204506195.1	Type1.BREX	HTH_3-> BrxA-DNA-binding -> BrxB-BrxF-iSTAND -> DUF6079 *-> N6-METHYLASE ->Schlafen+HATPase+wHTH-> PglZ -> SIGMA+OB-Fold+AAA-ATPase+ HKD-DNase -BrxL ->?->?->?->?->REase->	1187	Bacteria>FCB_group>Bacteroidetes/Chlorobi_group>Bacteroidota>Bacteroidia>Bacteroidales	GCF_016902095.1	Phocaeicola_plebeius	H6A37_RS09755	27157..30720	310297	BREX-system-P-loop-protein-BrxC-[Phocaeicola-plebeius]
WP_211451926.1	Type1.BREX	Peptidase_M23-><-?<-?<-Calcineurin<- SIGMA+OB-Fold+AAA-ATPase+ HKD-DNase -BrxL <- PglZ <-SIR2<- N6-METHYLASE <- DUF6079 *<- BrxB-BrxF-iSTAND <- BrxA-DNA-binding <- wHTH+WYL+WCX <-HTH_3||?->?->?->NUDIX+NUDIX->	1208	Bacteria>FCB_group>Bacteroidetes/Chlorobi_group>Bacteroidota>Bacteroidia>Bacteroidales	GCF_018289355.1	Phocaeicola_vulgatus	INE85_RS07560	1903040..1906666	821	BREX-system-P-loop-protein-BrxC-[Phocaeicola-vulgatus]
WP_215652320.1	Type1.BREX	Phage_lysozyme->PRTase-><-?<-?<-?||HTH_3-> wHTH+WYL+WCX -> BrxA-DNA-binding -> BrxB-BrxF-iSTAND -> DUF6079 *-> N6-METHYLASE ->SIR2->Peptidase_C39_2-> PglZ -> SIGMA+OB-Fold+AAA-ATPase+ HKD-DNase -BrxL ->McrB-AAA->McrC-NTD+REase->	1206	Bacteria>FCB_group>Bacteroidetes/Chlorobi_group>Bacteroidota>Bacteroidia>Bacteroidales	GCF_018784085.1	Prevotella_copri	GPK65_RS08775	33741..37361	165179	BREX-system-P-loop-protein-BrxC-[Prevotella-copri]
WP_215708904.1	Type1.BREX	Arm-DNA-bind_5-><-UDG<-?<-ARG||<- SIGMA+OB-Fold+AAA-ATPase+ HKD-DNase -BrxL <- PglZ <- N6-METHYLASE <- DUF6079 *<- BrxB-BrxF-iSTAND <-DUF4007||Arm-DNA-bind_5+Phage_int_SAM_5+Phage_integrase->Virulence_RhuM-><-?||Mob_Pre-><-TPR	1215	Bacteria>FCB_group>Bacteroidetes/Chlorobi_group>Bacteroidota>Bacteroidia>Bacteroidales	GCF_018785865.1	Bacteroides_uniformis	GRD73_RS11230	15032..18679	820	BREX-system-P-loop-protein-BrxC-[Bacteroides-uniformis]
WP_215758559.1	Type6.BREX	Phage_int_SAM_5+Phage_integrase->?->XIS-HTH->?->AAA+->HTH_3-> BrxE -> BrxA-DNA-binding -> BrxB-BrxF-iSTAND -> DUF6079 *-> N6-METHYLASE ->OLD-ABC->HNH-> PglZ -> SIGMA+OB-Fold+AAA-ATPase+ HKD-DNase -BrxL -><-?||ParA-Soj-PloopNTPase->	1217	Bacteria>FCB_group>Bacteroidetes/Chlorobi_group>Bacteroidota>Bacteroidia>Bacteroidales	GCF_018789675.1	Hallella_faecis	KM934_00340	78197..81850	2841596	BREX-system-P-loop-protein-BrxC-[Hallella-faecis]
WP_216951216.1	Type1.BREX	<-HTH_Tnp<-?<-?||HTH_3-><-?|| wHTH+WYL+WCX -> BrxA-DNA-binding -> BrxB-BrxF-iSTAND -> DUF6079 *-> N6-METHYLASE ->ACET+TPR->Peptidase_C39_2->?->?->?->SWIM+DUF3670+SF1-SF2-Helicase-> PglZ -> SIGMA+OB-Fold+AAA-ATPase+ HKD-DNase -BrxL ->	1208	Bacteria>FCB_group>Bacteroidetes/Chlorobi_group>Bacteroidota>Bacteroidia>Bacteroidales	GCF_019042015.1	Phocaeicola_vulgatus	KTG13_RS11225	6191..9817	821	BREX-system-P-loop-protein-BrxC-[Phocaeicola-vulgatus]
WP_217745553.1	Type1.BREX	<-HIRAN<-RADICAL-SAM<-ARG<-?<-?||HTH_3-> wHTH+WYL+WCX -> BrxA-DNA-binding -> BrxB-BrxF-iSTAND -> DUF6079 *->OLD-ABC->TOPRIM-> N6-METHYLASE ->SIR2+SIR2-> PglZ -> SIGMA+OB-Fold+AAA-ATPase+ HKD-DNase -BrxL ->McrB-AAA->McrC-NTD+REase->	1206	Bacteria>FCB_group>Bacteroidetes/Chlorobi_group>Bacteroidota>Bacteroidia>Bacteroidales	GCF_019127365.1	Prevotella_copri	KSW85_RS06175	41108..44728	165179	BREX-system-P-loop-protein-BrxC-[Prevotella-copri]
WP_217718390.1	Type1.BREX	TOPRIM->XIS-HTH-><-?<-?<-?<-?||HTH_3-> BrxA-DNA-binding -> BrxB-BrxF-iSTAND -> DUF6079 *-> N6-METHYLASE ->OLD-ABC+TOPRIM->SF1-SF2-Helicase->Schlafen+HATPase+wHTH-> PglZ -> SIGMA+OB-Fold+AAA-ATPase+ HKD-DNase -BrxL ->NADAR->RADICAL-SAM->HIRAN->	1217	Bacteria>FCB_group>Bacteroidetes/Chlorobi_group>Bacteroidota>Bacteroidia>Bacteroidales	GCF_019127825.1	Bacteroides_uniformis	KSX11_RS12350	118043..121696	820	BREX-system-P-loop-protein-BrxC-[Bacteroides-uniformis]
WP_217725669.1	Type1.BREX	<-BACON+Peptidase_M64||35exo+SF1-SF2-Helicase-><- SIGMA+OB-Fold+AAA-ATPase+ HKD-DNase -BrxL <- PglZ <-Schlafen+HATPase+wHTH<-SIR2+SIR2<- N6-METHYLASE <- DUF6079 *<- BrxB-BrxF-iSTAND <- BrxA-DNA-binding	1202	Bacteria>FCB_group>Bacteroidetes/Chlorobi_group>Bacteroidota>Bacteroidia>Bacteroidales	GCF_019128915.1	unclassified_Bacteroides	KSX87_RS10005	229853..233461	2646097	MULTISPECIES:-BREX-system-P-loop-protein-BrxC-[unclassified-Bacteroides]
WP_217719995.1	Type1.BREX	DUF4007-> BrxB-BrxF-iSTAND -> DUF6079 -p*-> N6-METHYLASE -> PglZ -> SIGMA+OB-Fold+AAA-ATPase+ HKD-DNase -BrxL -><-RHH+MobC||ARG->?->UDG-><-Arm-DNA-bind_5	265	Bacteria>FCB_group>Bacteroidetes/Chlorobi_group>Bacteroidota>Bacteroidia>Bacteroidales	GCF_019412375.1	Bacteroides_uniformis	KSX92_RS08930	2230..3027	820	DUF6079-family-protein-[Bacteroides-uniformis]
WP_138333738.1	Type6.BREX	STYKinase-like->Pkinase->SIR2+SIR2->?->?-><-REase<- SIGMA+OB-Fold+AAA-ATPase+ HKD-DNase -BrxL <- PglZ <- N6-METHYLASE <- DUF6079 *<- BrxB-BrxF-iSTAND <- BrxA-DNA-binding <- BrxE <-HTH_3<-GNAT<-?<-TraJ-RHH<-ZnR+TOPRIM<-CR-ATPase4	1224	Bacteria>FCB_group>Bacteroidetes/Chlorobi_group>Bacteroidota>Bacteroidia>Bacteroidales	GCF_019412725.1	Bacteroides	GPL18_RS15350	126249..129923	816	MULTISPECIES:-BREX-system-P-loop-protein-BrxC-[Bacteroides]
WP_115473019.1	Type1.BREX	<-OLD-ABC||?-><-Phage_int_SAM_5+Phage_integrase||?->?-><-HEPN||HTH_3-> BrxA-DNA-binding -> BrxB-BrxF-iSTAND -> DUF6079 *-> N6-METHYLASE ->Schlafen+HATPase+wHTH-> PglZ -> SIGMA+OB-Fold+AAA-ATPase+ HKD-DNase -BrxL ->?-><-MULE-TRANSPOSASE||DOC+HTH->	1211	Bacteria>FCB_group>Bacteroidetes/Chlorobi_group>Bacteroidota>Bacteroidia>Bacteroidales	GCF_019733435.1	Bacteroides_fragilis	G7060_RS07670	242068..245703	817	BREX-system-P-loop-protein-BrxC-[Bacteroides-fragilis]
WP_120489887.1	Type1.BREX	BrxA-DNA-binding -> BrxB-BrxF-iSTAND -> DUF6079 *-> N6-METHYLASE ->SIR2->Schlafen+HATPase+wHTH-> PglZ -> SIGMA+OB-Fold+AAA-ATPase+ HKD-DNase -BrxL -><-35exo+SF1-SF2-Helicase||BACON+Peptidase_M64->	1202	Bacteria>FCB_group>Bacteroidetes/Chlorobi_group>Bacteroidota>Bacteroidia>Bacteroidales	GCF_019733535.1	Bacteroides_ovatus	G7086_RS03160	85646..89254	28116	BREX-system-P-loop-protein-BrxC-[Bacteroides-ovatus]
WP_130041244.1	Type1.BREX	Polbeta+HEPN->D5ATPase+DUF3874-><-?||ADH_N+ADH_zinc_N+METHYLASE->SPOUT-><- SIGMA+OB-Fold+AAA-ATPase+ HKD-DNase -BrxL <- PglZ <-HTH+REase<- N6-METHYLASE <- DUF6079 *<- BrxB-BrxF-iSTAND <- BrxA-DNA-binding <-HTH_3<-sGTP||?->Response_reg->NAD_binding_10->	1175	Bacteria>FCB_group>Bacteroidetes/Chlorobi_group>Bacteroidota>Bacteroidia>Bacteroidales	GCF_019733625.1	Bacteroides_thetaiotaomicron	EAJ10_RS04945	124743..128270	818	BREX-system-P-loop-protein-BrxC-[Bacteroides-thetaiotaomicron]
WP_221729077.1	Type1.BREX	<-DUF772+DDE_Tnp_1+DDE_Tnp_1_6<-?||HTH_3-> BrxA-DNA-binding -> BrxB-BrxF-iSTAND -> DUF6079 *-> N6-METHYLASE ->DOC+HTH-> N6-METHYLASE -p->SIR2-> PglZ -> SIGMA+OB-Fold+AAA-ATPase+ HKD-DNase -BrxL -><-TnpB_IS66	1206	Bacteria>FCB_group>Bacteroidetes/Chlorobi_group>Bacteroidota>Bacteroidia>Bacteroidales	GCF_019733655.1	Parabacteroides_merdae	HCH02_RS20805	4560..8180	46503	BREX-system-P-loop-protein-BrxC-[Parabacteroides-merdae]
WP_225659484.1	Type1.BREX	Polbeta+HEPN->D5ATPase+DUF3874-><-?||ADH_N+ADH_zinc_N+METHYLASE->SPOUT-><- SIGMA+OB-Fold+AAA-ATPase+ HKD-DNase -BrxL <- PglZ <-HTH+REase<- N6-METHYLASE <- DUF6079 *<- BrxB-BrxF-iSTAND <- BrxA-DNA-binding <-HTH_3<-sGTP||?->Response_reg->NAD_binding_10->	1175	Bacteria>FCB_group>Bacteroidetes/Chlorobi_group>Bacteroidota>Bacteroidia>Bacteroidales	GCF_020313445.1	Bacteroides_thetaiotaomicron	KFX61_RS09580	34477..38004	818	BREX-system-P-loop-protein-BrxC-[Bacteroides-thetaiotaomicron]
WP_217720628.1	Type1.BREX	<-NUDIX+NUDIX<-?<-?<-?||HTH_3-> wHTH+WYL+WCX -> BrxA-DNA-binding -> BrxB-BrxF-iSTAND -> DUF6079 *-> N6-METHYLASE ->ACET+TPR->Peptidase_C39_2->?->?->?->SWIM+DUF3670+SF1-SF2-Helicase-> PglZ -> SIGMA+OB-Fold+AAA-ATPase+ HKD-DNase -BrxL ->	1208	Bacteria>FCB_group>Bacteroidetes/Chlorobi_group>Bacteroidota>Bacteroidia>Bacteroidales	GCF_020827675.1	Bacteroidaceae	HZY58_RS04175	119839..123465	815	MULTISPECIES:-BREX-system-P-loop-protein-BrxC-[Bacteroidaceae]
WP_234117060.1	Type1.BREX	<-BACON+Peptidase_M64||35exo+SF1-SF2-Helicase-><- SIGMA+OB-Fold+AAA-ATPase+ HKD-DNase -BrxL <- PglZ <-Schlafen+HATPase+wHTH<-SIR2+SIR2<- N6-METHYLASE <- DUF6079 *<- BrxB-BrxF-iSTAND <- BrxA-DNA-binding	1202	Bacteria>FCB_group>Bacteroidetes/Chlorobi_group>Bacteroidota>Bacteroidia>Bacteroidales	GCF_021410745.1	Bacteroides_ovatus	KOG75_RS22190	19952..23560	28116	BREX-system-P-loop-protein-BrxC-[Bacteroides-ovatus]
WP_237798701.1	Type6.BREX	DSBH+AraC-HTH+AraC-HTH->||?->?-><-?<-SF1-SF2-Helicase||HTH_3-> BrxE -> BrxA-DNA-binding -> BrxB-BrxF-iSTAND -> DUF6079 *-> N6-METHYLASE -> PglZ -> SIGMA+OB-Fold+AAA-ATPase+ HKD-DNase -BrxL ->?-> wHTH+WYL+WCX ->EVE-><-PRTase	1216	Bacteria>FCB_group>Bacteroidetes/Chlorobi_group>Bacteroidota>Bacteroidia>Bacteroidales	GCF_022024195.1	Prevotella_bryantii	L6467_12860	2115705..2119355	77095	BREX-system-P-loop-protein-BrxC-[Prevotella-bryantii]
WP_249093422.1	Type1.BREX	TOPRIM->XIS-HTH-><-?<-?<-?<-?||HTH_3-> BrxA-DNA-binding -> BrxB-BrxF-iSTAND -> DUF6079 *-> N6-METHYLASE ->SIR2->Schlafen+HATPase+wHTH-> PglZ -> SIGMA+OB-Fold+AAA-ATPase+ HKD-DNase -BrxL ->NADAR->RADICAL-SAM->HIRAN->HHH_5+SF1-SF2-Helicase->	1217	Bacteria>FCB_group>Bacteroidetes/Chlorobi_group>Bacteroidota>Bacteroidia>Bacteroidales	GCF_023337025.1	Mediterranea_sp._ET5	M3087_RS02205	173446..177099	2939419	BREX-system-P-loop-protein-BrxC-[Mediterranea-sp.-ET5]
WP_250803006.1	Type1.BREX	<- SIGMA+OB-Fold+AAA-ATPase+ HKD-DNase -BrxL <- PglZ <-SWIM+DUF3670+SF1-SF2-Helicase<-?<-?<-?<-Peptidase_C39_2<-ACET+TPR<- N6-METHYLASE <- DUF6079 *<- BrxB-BrxF-iSTAND <- BrxA-DNA-binding <- wHTH+WYL+WCX <-HTH_3||?->?->?->NUDIX+NUDIX->DUF4365+REase->	1208	Bacteria>FCB_group>Bacteroidetes/Chlorobi_group>Bacteroidota>Bacteroidia>Bacteroidales	GCF_023668475.1	Phocaeicola_massiliensis	NCY86_RS00900	193015..196641	204516	BREX-system-P-loop-protein-BrxC-[Phocaeicola-massiliensis]
WP_252760249.1	Type1.BREX	<-TIR<-?<- SIGMA+OB-Fold+AAA-ATPase+ HKD-DNase -BrxL <- PglZ <- N6-METHYLASE -p<-?<- N6-METHYLASE <- DUF6079 *<- BrxB-BrxF-iSTAND <- BrxA-DNA-binding <-HTH_3<- wHTH+WYL+WCX ||?->?->?-><-?||ACET->	1205	Bacteria>FCB_group>Bacteroidetes/Chlorobi_group>Bacteroidota>Bacteroidia>Bacteroidales	GCF_024054555.1	Prevotella_cerevisiae	NG821_RS03330	12038..15655	2053716	BREX-system-P-loop-protein-BrxC-[Prevotella-cerevisiae]
WP_252760927.1	Type1.BREX	DDE_Tnp_ISL3->XIS-HTH->?->AAA+-><-?|| wHTH+WYL+WCX ->HTH_3-> BrxA-DNA-binding -> BrxB-BrxF-iSTAND -> DUF6079 *-> N6-METHYLASE ->Schlafen+HATPase+wHTH-> PglZ -> SIGMA+OB-Fold+AAA-ATPase+ HKD-DNase -BrxL ->SIR2+SIR2->?->?-><-tRNA||HTH_31->	1206	Bacteria>FCB_group>Bacteroidetes/Chlorobi_group>Bacteroidota>Bacteroidia>Bacteroidales	GCF_024054555.1	Prevotella_cerevisiae	NG821_RS06905	6531..10151	2053716	BREX-system-P-loop-protein-BrxC-[Prevotella-cerevisiae]
WP_258327305.1	Type1.BREX	<-HHH_5+SF1-SF2-Helicase<-HIRAN<-RADICAL-SAM<-NADAR<- SIGMA+OB-Fold+AAA-ATPase+ HKD-DNase -BrxL <- PglZ <-Schlafen+HATPase+wHTH<-SIR2<- N6-METHYLASE <- DUF6079 *<- BrxB-BrxF-iSTAND <- BrxA-DNA-binding <-HTH_3||?->?->?->?-><-XIS-HTH<-TOPRIM	1217	Bacteria>FCB_group>Bacteroidetes/Chlorobi_group>Bacteroidota>Bacteroidia>Bacteroidales	GCF_024733785.1	Barnesiella_sp._ET7	NW211_RS06370	166743..170396	2972460	BREX-system-P-loop-protein-BrxC-[Barnesiella-sp.-ET7]
WP_258898999.1	Type1.BREX	<- SIGMA+OB-Fold+AAA-ATPase+ HKD-DNase -BrxL <- PglZ <-SWIM+DUF3670+SF1-SF2-Helicase<-?<-?<-?<-Peptidase_C39_2<-ACET+TPR<- N6-METHYLASE <- DUF6079 *<- BrxB-BrxF-iSTAND <- BrxA-DNA-binding <- wHTH+WYL+WCX <-HTH_3||?->?->?->NUDIX+NUDIX->DUF4365+REase->	1208	Bacteria>FCB_group>Bacteroidetes/Chlorobi_group>Bacteroidota>Bacteroidia>Bacteroidales	GCF_024759365.1	Bacteroides_ovatus	NXX44_RS13110	3467956..3471582	28116	BREX-system-P-loop-protein-BrxC-[Bacteroides-ovatus]
WP_258991265.1	Type1.BREX	<- SIGMA+OB-Fold+AAA-ATPase+ HKD-DNase -BrxL <- PglZ <-SWIM+DUF3670+SF1-SF2-Helicase<-?<-?<-?<-Peptidase_C39_2<-ACET+TPR<- N6-METHYLASE <- DUF6079 *<- BrxB-BrxF-iSTAND <- BrxA-DNA-binding <- wHTH+WYL+WCX <-HTH_3||?->?->?->NUDIX+NUDIX->	1208	Bacteria>FCB_group>Bacteroidetes/Chlorobi_group>Bacteroidota>Bacteroidia>Bacteroidales	GCF_024790585.1	Phocaeicola_vulgatus	NXX11_RS15345	3784105..3787731	821	BREX-system-P-loop-protein-BrxC-[Phocaeicola-vulgatus]
WP_259011403.1	Type1.BREX	BrxA-DNA-binding -> BrxB-BrxF-iSTAND -> DUF6079 *-> N6-METHYLASE ->ParB->OLD-ABC->?->Schlafen+HATPase+wHTH-> PglZ -> SIGMA+OB-Fold+AAA-ATPase+ HKD-DNase -BrxL -><-35exo+SF1-SF2-Helicase	1202	Bacteria>FCB_group>Bacteroidetes/Chlorobi_group>Bacteroidota>Bacteroidia>Bacteroidales	GCF_024791585.1	Bacteroides_sp._BFG-638	NXX54_RS14145	3701947..3705555	2972765	BREX-system-P-loop-protein-BrxC-[Bacteroides-sp.-BFG-638]
WP_258973412.1	Type1.BREX	Polbeta+HEPN->D5ATPase+DUF3874-><-?||ADH_N+ADH_zinc_N+METHYLASE->SPOUT-><- SIGMA+OB-Fold+AAA-ATPase+ HKD-DNase -BrxL <- PglZ <-HTH+REase<- N6-METHYLASE <- DUF6079 *<-?<- BrxB-BrxF-iSTAND <- BrxA-DNA-binding <-HTH_3||CR-ATPase5+REase->?->?-><-RVT	1133	Bacteria>FCB_group>Bacteroidetes/Chlorobi_group>Bacteroidota>Bacteroidia>Bacteroidales	GCF_024793205.1	Bacteroides_thetaiotaomicron	NXX32_RS11560	3197619..3201020	818	BREX-system-P-loop-protein-BrxC-[Bacteroides-thetaiotaomicron]
WP_258979859.1	Type1.BREX	BrxA-DNA-binding -> BrxB-BrxF-iSTAND -> DUF6079 -p*-> DUF6079 -p-> N6-METHYLASE ->ParB->OLD-ABC->?->Schlafen+HATPase+wHTH-> PglZ -> SIGMA+OB-Fold+AAA-ATPase+ HKD-DNase -BrxL ->	780	Bacteria>FCB_group>Bacteroidetes/Chlorobi_group>Bacteroidota>Bacteroidia>Bacteroidales	GCF_024793605.1	Bacteroides_sp._BFG-637	NXX38_RS14295	3701972..3704314	2972764	BREX-system-P-loop-protein-BrxC-[Bacteroides-sp.-BFG-637]
WP_009000708.1	Type1.BREX	BrxA-DNA-binding -> BrxB-BrxF-iSTAND -> DUF6079 *-> N6-METHYLASE ->?->?->Schlafen+HATPase+wHTH-> PglZ -> SIGMA+OB-Fold+AAA-ATPase+ HKD-DNase -BrxL ->?-><-35exo+SF1-SF2-Helicase||BACON+Peptidase_M64->	1202	Bacteria>FCB_group>Bacteroidetes/Chlorobi_group>Bacteroidota>Bacteroidia>Bacteroidales	GCF_025147085.1	Bacteroides	NQ505_RS12870	3648371..3651979	816	MULTISPECIES:-BREX-system-P-loop-protein-BrxC-[Bacteroides]
WP_195661285.1	Type1.BREX	BrxA-DNA-binding -> BrxB-BrxF-iSTAND -> DUF6079 *-> N6-METHYLASE ->SIR2+SIR2->Schlafen+HATPase+wHTH-> PglZ -> SIGMA+OB-Fold+AAA-ATPase+ HKD-DNase -BrxL ->	1202	Bacteria>FCB_group>Bacteroidetes/Chlorobi_group>Bacteroidota>Bacteroidia>Bacteroidales	GCF_025914175.1	Bacteroides_nordii	KQP55_RS20540	5436855..5440463	291645	BREX-system-P-loop-protein-BrxC-[Bacteroides-nordii]
WP_264912090.1	Type1.BREX	<-HIRAN<-RADICAL-SAM<-ARG<-?<-?||HTH_3-> wHTH+WYL+WCX -> BrxA-DNA-binding -> BrxB-BrxF-iSTAND -> DUF6079 *->DOC+HTH-> N6-METHYLASE ->SIR2+SIR2-> PglZ -> SIGMA+OB-Fold+AAA-ATPase+ HKD-DNase -BrxL ->McrB-AAA->McrC-NTD+REase->	1206	Bacteria>FCB_group>Bacteroidetes/Chlorobi_group>Bacteroidota>Bacteroidia>Bacteroidales	GCF_026015575.1	Prevotella_copri	ONS98_RS12175	17740..21360	165179	BREX-system-P-loop-protein-BrxC-[Prevotella-copri]
WP_264965401.1	Type1.BREX	ABC-ATPase+ABC-ATPase+ABC-ATPase+SbcC->Peptidase_M13_N+Peptidase_M13-><- SIGMA+OB-Fold+AAA-ATPase+ HKD-DNase -BrxL <- PglZ <- N6-METHYLASE -p<-Schlafen+HATPase+wHTH<- N6-METHYLASE <- DUF6079 *<- BrxB-BrxF-iSTAND <- BrxA-DNA-binding <-?<-?||?-><-?<-?<-?<-ABC-ATPase+SbcC	1194	Bacteria>FCB_group>Bacteroidetes/Chlorobi_group>Bacteroidota>Bacteroidia>Bacteroidales	GCF_026015685.1	Prevotella_copri	ONT16_RS03155	729578..733162	165179	BREX-system-P-loop-protein-BrxC-[Prevotella-copri]
WP_270558549.1	Type1.BREX	<-HISKIN<-?<-Polbeta<-MarR-HTH<-?||HTH_3-> wHTH+WYL+WCX -> BrxA-DNA-binding -> BrxB-BrxF-iSTAND -> DUF6079 *-> N6-METHYLASE ->DOC+HTH->SIR2+SIR2-> N6-METHYLASE -> PglZ -> SIGMA+OB-Fold+AAA-ATPase+ HKD-DNase -BrxL ->CASPASE->SLATT_4->	1216	Bacteria>FCB_group>Bacteroidetes/Chlorobi_group>Bacteroidota>Bacteroidia>Bacteroidales	GCF_027686315.1	Bacteroides_uniformis	PG022_RS08930	563554..567204	820	BREX-system-P-loop-protein-BrxC-[Bacteroides-uniformis]
WP_271687043.1	Type1.BREX	<- SIGMA+OB-Fold+AAA-ATPase+ HKD-DNase -BrxL <- PglZ <-SWIM+DUF3670+SF1-SF2-Helicase<-?<-?<-?<-Peptidase_C39_2<-ACET+TPR<- N6-METHYLASE <- DUF6079 *<- BrxB-BrxF-iSTAND <- BrxA-DNA-binding <- wHTH+WYL+WCX <-HTH_3||?->?->?->NUDIX+NUDIX->	1208	Bacteria>FCB_group>Bacteroidetes/Chlorobi_group>Bacteroidota>Bacteroidia>Bacteroidales	GCF_028199265.1	Bacteroides_xylanisolvens	PL435_RS07875	93897..97523	371601	BREX-system-P-loop-protein-BrxC-[Bacteroides-xylanisolvens]
WP_271704578.1	Type1.BREX	HTH_3->MORC+DUF3684+REase->SF1-SF2-Helicase+PIN->HTH_3-> BrxA-DNA-binding -> BrxB-BrxF-iSTAND -> DUF6079 *-> N6-METHYLASE ->SIR2+SIR2->Schlafen+HATPase+wHTH-> PglZ -> SIGMA+OB-Fold+AAA-ATPase+ HKD-DNase -BrxL ->	1209	Bacteria>FCB_group>Bacteroidetes/Chlorobi_group>Bacteroidota>Bacteroidia>Bacteroidales	GCF_028200085.1	Bacteroides_xylanisolvens	PL493_RS08910	20513..24142	371601	BREX-system-P-loop-protein-BrxC-[Bacteroides-xylanisolvens]
WP_272709339.1	Type1.BREX	<-DUF4365+REase<-NUDIX+NUDIX<-?<-?<-?||HTH_3-> wHTH+WYL+WCX -> BrxA-DNA-binding -> BrxB-BrxF-iSTAND -> DUF6079 *-> N6-METHYLASE ->Peptidase_C39_2->SIR2-> PglZ -> SIGMA+OB-Fold+AAA-ATPase+ HKD-DNase -BrxL ->	1208	Bacteria>FCB_group>Bacteroidetes/Chlorobi_group>Bacteroidota>Bacteroidia>Bacteroidales	GCF_028528025.1	Bacteroides	PQG94_RS11850	55098..58724	816	MULTISPECIES:-BREX-system-P-loop-protein-BrxC-[Bacteroides]
WP_272706625.1	Type6.BREX	XIS-HTH-><-?<-?||?->?->HTH_3-> BrxE -> BrxA-DNA-binding -> BrxB-BrxF-iSTAND -> DUF6079 -p*-> DUF6079 -p-> DUF6079 -p-> N6-METHYLASE -> PglZ -> SIGMA+OB-Fold+AAA-ATPase+ HKD-DNase -BrxL ->KAP-NTPase->KAP-glycosylase->?->TatD_DNase->	191	Bacteria>FCB_group>Bacteroidetes/Chlorobi_group>Bacteroidota>Bacteroidia>Bacteroidales	GCF_028528065.1	Bacteroides_faecis	PQH00_RS23500	20443..21015	674529	DUF6079-family-protein,-partial-[Bacteroides-faecis]
WP_272706631.1	Type6.BREX	HTH_3-> BrxE -> BrxA-DNA-binding -> BrxB-BrxF-iSTAND -> DUF6079 -p-> DUF6079 -p*-> DUF6079 -p-> N6-METHYLASE -> PglZ -> SIGMA+OB-Fold+AAA-ATPase+ HKD-DNase -BrxL ->KAP-NTPase->KAP-glycosylase->?->TatD_DNase->	862	Bacteria>FCB_group>Bacteroidetes/Chlorobi_group>Bacteroidota>Bacteroidia>Bacteroidales	GCF_028528065.1	Bacteroides_faecis	PQH00_RS23505	21026..23611	674529	BREX-system-P-loop-protein-BrxC,-partial-[Bacteroides-faecis]
WP_274990748.1	Type6.BREX	Arm-DNA-bind_5-><-UDG<-?<-ARG||?-><- SIGMA+OB-Fold+AAA-ATPase+ HKD-DNase -BrxL <- PglZ <- N6-METHYLASE <- DUF6079 -p*<- DUF6079 -p<- BrxB-BrxF-iSTAND <- BrxA-DNA-binding <- BrxE <-HTH_3<-?||?->?-><-XIS-HTH	772	Bacteria>FCB_group>Bacteroidetes/Chlorobi_group>Bacteroidota>Bacteroidia>Bacteroidales	GCF_028993715.1	Bacteroides_uniformis	PX688_RS13675	79793..82111	820	hypothetical-protein-[Bacteroides-uniformis]
WP_274990749.1	Type6.BREX	Arm-DNA-bind_5-><-UDG<-?<-ARG||?-><- SIGMA+OB-Fold+AAA-ATPase+ HKD-DNase -BrxL <- PglZ <- N6-METHYLASE <- DUF6079 -p<- DUF6079 -p*<- BrxB-BrxF-iSTAND <- BrxA-DNA-binding <- BrxE <-HTH_3<-?||?->?-><-XIS-HTH<-AbiEii	411	Bacteria>FCB_group>Bacteroidetes/Chlorobi_group>Bacteroidota>Bacteroidia>Bacteroidales	GCF_028993715.1	Bacteroides_uniformis	PX688_RS13680	82199..83434	820	DUF6079-family-protein-[Bacteroides-uniformis]
WP_032560932.1	Type1.BREX	SF1-SF2-Helicase->?-><-DOC+HTH<-URI-DNase+RAMA<-?<- SIGMA+OB-Fold+AAA-ATPase+ HKD-DNase -BrxL <- PglZ <-Schlafen+HATPase+wHTH<- N6-METHYLASE <- DUF6079 *<- BrxB-BrxF-iSTAND <- BrxA-DNA-binding <-HTH_3||HEPN-><-?<-?<-STYKinase-like||Phage_int_SAM_5+Phage_integrase->	1211	Bacteria>FCB_group>Bacteroidetes/Chlorobi_group>Bacteroidota>Bacteroidia>Bacteroidales	GCF_029872615.1	Bacteroides_fragilis	M068_RS14675	124538..128173	817	BREX-system-P-loop-protein-BrxC-[Bacteroides-fragilis]
WP_074557116.1	Type1.BREX	<-HAD+PRTase+PRTase+REase+SF1-SF2-Helicase<-?<-TPR+TPR<-?<-Schlafen+HATPase+wHTH<-TrwF|| BrxA-DNA-binding -> BrxB-BrxF-iSTAND -> DUF6079 *-> N6-METHYLASE ->SIR2->Schlafen+HATPase+wHTH-> PglZ -> SIGMA+OB-Fold+AAA-ATPase+ HKD-DNase -BrxL -><-35exo+SF1-SF2-Helicase||BACON+Peptidase_M64->	1202	Bacteria>FCB_group>Bacteroidetes/Chlorobi_group>Bacteroidota>Bacteroidia>Bacteroidales	GCF_900102645.1	Bacteroides_ovatus	BLR14_RS05840	106864..110472	28116	BREX-system-P-loop-protein-BrxC-[Bacteroides-ovatus]
WP_072542576.1	Type1.BREX	<-AAA-ATPase<-HTH+rve<-?<- SIGMA+OB-Fold+AAA-ATPase+ HKD-DNase -BrxL <- PglZ <-SIR2+SIR2<- N6-METHYLASE <-TOPRIM<-OLD-ABC<- DUF6079 *<- BrxB-BrxF-iSTAND <- BrxA-DNA-binding ||?->?->?->?-><-?<-?<-OLD-ABC	1195	Bacteria>FCB_group>Bacteroidetes/Chlorobi_group>Bacteroidota>Bacteroidia>Bacteroidales	GCF_900128455.1	Bacteroides_mediterraneensis	BQ7480_RS08760	2129051..2132638	1841856	BREX-system-P-loop-protein-BrxC-[Bacteroides-mediterraneensis]
WP_254795118.1	Type1.BREX	<-Classical-AAA<-?||tRNA-><-?<-?<-HTH+DOC||?->HTH_3-> BrxA-DNA-binding -> BrxB-BrxF-iSTAND -> DUF6079 *-> N6-METHYLASE ->SbcC+SbcC->?->SIR2+SIR2-> PglZ -> SIGMA+OB-Fold+AAA-ATPase+ HKD-DNase -BrxL ->?->?->ParB+HNH->	1209	Bacteria>FCB_group>Bacteroidetes/Chlorobi_group>Bacteroidota>Bacteroidia>Bacteroidales	GCF_900142525.1	Prevotella_ruminicola	BUB86_RS07050	46256..49885	839	BREX-system-P-loop-protein-BrxC-[Prevotella-ruminicola]
WP_125105113.1	Type1.BREX	<-HHH_5+SF1-SF2-Helicase<-HIRAN<-RADICAL-SAM<-NADAR<- SIGMA+OB-Fold+AAA-ATPase+ HKD-DNase -BrxL <- PglZ <-Schlafen+HATPase+wHTH<-SIR2<- N6-METHYLASE <- DUF6079 *<- BrxB-BrxF-iSTAND <- BrxA-DNA-binding <-HTH_3||?->?->?->?-><-XIS-HTH<-TOPRIM	1217	Bacteria>FCB_group>Bacteroidetes/Chlorobi_group>Bacteroidota>Bacteroidia>Bacteroidales	GCF_900625065.1	Prevotella_marseillensis	EJL04_RS12230	3058927..3062580	2479840	BREX-system-P-loop-protein-BrxC-[Prevotella-marseillensis]
WP_144153273.1	Type1.BREX	<-HIRAN<-RADICAL-SAM<-ARG<-?<-?||HTH_3-> wHTH+WYL+WCX -> BrxA-DNA-binding -> BrxB-BrxF-iSTAND -> DUF6079 *-> N6-METHYLASE ->SIR2+SIR2-> PglZ -> SIGMA+OB-Fold+AAA-ATPase+ HKD-DNase -BrxL ->McrB-AAA->McrC-NTD+REase->	1194	Bacteria>FCB_group>Bacteroidetes/Chlorobi_group>Bacteroidota>Bacteroidia>Bacteroidales	GCF_902167755.1	Prevotella_copri	BIL0145876EF_RS09275	107988..111572	165179	BREX-system-P-loop-protein-BrxC-[Prevotella-copri]
WP_032543214.1	Type1.BREX	<-METHYLASE<-ACET<-DOC+HTH<-URI-DNase+RAMA<-?<- SIGMA+OB-Fold+AAA-ATPase+ HKD-DNase -BrxL <- PglZ <-Schlafen+HATPase+wHTH<- N6-METHYLASE <- DUF6079 *<- BrxB-BrxF-iSTAND <- BrxA-DNA-binding <-HTH_3<-?<-?<-TraJ-RHH<-ZnR+TOPRIM<-CR-ATPase4<-XIS-HTH	1211	Bacteria>FCB_group>Bacteroidetes/Chlorobi_group>Bacteroidota>Bacteroidia>Bacteroidales	GCF_902364655.1	Bacteroides_fragilis	PDN68_RS18445	100181..103816	817	BREX-system-P-loop-protein-BrxC-[Bacteroides-fragilis]
WP_008770354.1	Type6.BREX	DDE_Tnp-><-?<- SIGMA+OB-Fold+AAA-ATPase+ HKD-DNase -BrxL <- PglZ <-HNH<-OLD-ABC<- N6-METHYLASE <- DUF6079 *<- BrxB-BrxF-iSTAND <- BrxA-DNA-binding <- BrxE <-HTH_3<-?<-?<-TraJ-RHH<-ZnR+TOPRIM<-CR-ATPase4	1219	Bacteria>FCB_group>Bacteroidetes/Chlorobi_group>Bacteroidota>Bacteroidia>Bacteroidales	GCF_902374095.1	Bacteroides	BSAG_RS19905	423901..427560	816	MULTISPECIES:-BREX-system-P-loop-protein-BrxC-[Bacteroides]
WP_060407611.1	Type1.BREX	<- SIGMA+OB-Fold+AAA-ATPase+ HKD-DNase -BrxL <- PglZ <-Schlafen+HATPase+wHTH<-SIR2<- N6-METHYLASE -p<- N6-METHYLASE <- DUF6079 *<- BrxB-BrxF-iSTAND <- BrxA-DNA-binding <-HTH_3<-?<-?<-?||SIGMA-HTH->	1205	Bacteria>FCB_group>Bacteroidetes/Chlorobi_group>Bacteroidota>Bacteroidia>Bacteroidales	GCF_902387305.1	Bacteroides_cellulosilyticus	FYC30_RS03960	75039..78656	246787	BREX-system-P-loop-protein-BrxC-[Bacteroides-cellulosilyticus]
WP_174646033.1	Type6.BREX	AbiEii->XIS-HTH-><-?||?->HTH_3-> BrxE -> BrxA-DNA-binding -> BrxB-BrxF-iSTAND -> DUF6079 *-> N6-METHYLASE -> N6-METHYLASE -> PglZ -> SIGMA+OB-Fold+AAA-ATPase+ HKD-DNase -BrxL -><-?<-RHH+MobC||ARG->?->UDG->	1215	Bacteria>FCB_group>Bacteroidetes/Chlorobi_group>Bacteroidota>Bacteroidia>Bacteroidales	GCF_902825465.1	Bacteroides_sp._Marseille-P8574	HUX74_RS00900	196439..200086	2697504	BREX-system-P-loop-protein-BrxC-[Bacteroides-sp.-Marseille-P8574]
WP_118419043.1	Type1.BREX	BrxA-DNA-binding -> BrxB-BrxF-iSTAND -> DUF6079 *-> N6-METHYLASE ->SIR2+SIR2->Schlafen+HATPase+wHTH-> PglZ -> SIGMA+OB-Fold+AAA-ATPase+ HKD-DNase -BrxL -><-35exo+SF1-SF2-Helicase||BACON+Peptidase_M64->	1202	Bacteria>FCB_group>Bacteroidetes/Chlorobi_group>Bacteroidota>Bacteroidia>Bacteroidales	GCF_918279245.1	Bacteroides_ovatus	PO203_RS09380	101158..104766	28116	BREX-system-P-loop-protein-BrxC-[Bacteroides-ovatus]
WP_117985926.1	Type1.BREX	XIS-HTH->SMS-KaiC-><-SF1-SF2-Helicase<- SIGMA+OB-Fold+AAA-ATPase+ HKD-DNase -BrxL <- PglZ <-Schlafen+HATPase+wHTH<-SF1-SF2-Helicase<-OLD-ABC+TOPRIM<- N6-METHYLASE <- DUF6079 *<- BrxB-BrxF-iSTAND <- BrxA-DNA-binding <-HTH_3<-AbiEi_4+AbiEi_1<-STYKinase-like<-TRPR-HTH||SbcC->DEDD_Tnp_IS110+Transposase_20->	1210	Bacteria>FCB_group>Bacteroidetes/Chlorobi_group>Bacteroidota>Bacteroidia>Bacteroidales	GCF_934726555.1	Bacteroides	O1446_RS12920	68574..72206	816	MULTISPECIES:-BREX-system-P-loop-protein-BrxC-[Bacteroides]
WP_158582848.1	Type1.BREX	ACET-><-?<-Cysteine-Desulfurase||tRNA-><-?<-?<-?<-?|| BrxA-DNA-binding -> BrxB-BrxF-iSTAND -> DUF6079 *-> N6-METHYLASE ->Schlafen+HATPase+wHTH-> PglZ -> SIGMA+OB-Fold+AAA-ATPase+ HKD-DNase -BrxL -><-?||?->PolB->HEPN->	1210	Bacteria>FCB_group>Bacteroidetes/Chlorobi_group>Bacteroidota>Bacteroidia>Bacteroidales	GCF_938033175.1	Parabacteroides_sp._AF17-28	DWW90_RS15090	22936..26568	2292241	BREX-system-P-loop-protein-BrxC-[Parabacteroides-sp.-AF17-28]
WP_046149424.1	Type6.BREX	AbiEii->?-><-?||XIS-HTH->HTH_3-> BrxE -> BrxA-DNA-binding -> BrxB-BrxF-iSTAND -> DUF6079 *-> N6-METHYLASE -> PglZ -> SIGMA+OB-Fold+AAA-ATPase+ HKD-DNase -BrxL ->KAP-NTPase->KAP-glycosylase->?->TatD_DNase->McrB-AAA->McrC-NTD+REase->	1217	Bacteria>FCB_group>Bacteroidetes/Chlorobi_group>Bacteroidota>Bacteroidia>Bacteroidales	-	Parabacteroides_sp._HGS0025	HMPREF1212_RS26675	2885204..2888857	1078087	BREX-system-P-loop-protein-BrxC-[Parabacteroides-sp.-HGS0025]
WP_118139864.1	Type1.BREX	<-HIRAN<-RADICAL-SAM<-ARG<-?<-?||HTH_3-> wHTH+WYL+WCX -> BrxA-DNA-binding -> BrxB-BrxF-iSTAND -> DUF6079 *->OLD-ABC->TOPRIM-> N6-METHYLASE -> N6-METHYLASE -p->?->SF1-SF2-Helicase-> PglZ -> SIGMA+OB-Fold+AAA-ATPase+ HKD-DNase -BrxL ->McrB-AAA->	1202	Bacteria>FCB_group>Bacteroidetes/Chlorobi_group>Bacteroidota>Bacteroidia>Bacteroidales	-	Prevotella_copri	DWV60_RS07145	31878..35486	165179	BREX-system-P-loop-protein-BrxC-[Prevotella-copri]
WP_117695280.1	Type6.BREX	<-SLATT_4<- SIGMA+OB-Fold+AAA-ATPase+ HKD-DNase -BrxL <- PglZ -p<- PglZ <-Schlafen+HATPase+wHTH<- N6-METHYLASE <- DUF6079 *<- BrxB-BrxF-iSTAND <- BrxA-DNA-binding <- BrxE <-HTH_3<-RNasePH+RNasePH+KH+S1||DUF4369+Thioredoxin_8->?->NUDIX->TOPRIM->	1216	Bacteria>FCB_group>Bacteroidetes/Chlorobi_group>Bacteroidota>Bacteroidia>Bacteroidales	-	Prevotella_copri	DXC61_RS12020	26690..30340	165179	BREX-system-P-loop-protein-BrxC-[Prevotella-copri]
WP_233339205.1	Type6.BREX	<-SLATT_4<- SIGMA+OB-Fold+AAA-ATPase+ HKD-DNase -BrxL <- PglZ <-Schlafen+HATPase+wHTH<- N6-METHYLASE <- DUF6079 *<- BrxB-BrxF-iSTAND <- BrxA-DNA-binding <- BrxE <-HTH_3<-RNasePH+RNasePH+KH+S1||DUF4369+Thioredoxin_8->?->NUDIX->TOPRIM->	1216	Bacteria>FCB_group>Bacteroidetes/Chlorobi_group>Bacteroidota>Bacteroidia>Bacteroidales	-	Prevotella_copri	LYY06_RS08410	34942..38592	165179	BREX-system-P-loop-protein-BrxC-[Prevotella-copri]
WP_264908465.1	Type1.BREX	<-McrC-NTD+REase<-McrB-AAA<- SIGMA+OB-Fold+AAA-ATPase+ HKD-DNase -BrxL <- PglZ <-SIR2+SIR2<- N6-METHYLASE <- DUF6079 *<- BrxB-BrxF-iSTAND <- BrxA-DNA-binding <- wHTH+WYL+WCX <-HTH_3||?->?->ARG->RADICAL-SAM->HIRAN->	1206	Bacteria>FCB_group>Bacteroidetes/Chlorobi_group>Bacteroidota>Bacteroidia>Bacteroidales	-	Prevotella_copri	ONT12_RS10110	2444120..2447740	165179	BREX-system-P-loop-protein-BrxC-[Prevotella-copri]
WP_264904325.1	Type1.BREX	<-HIRAN<-RADICAL-SAM<-ARG<-?<-?||HTH_3-> wHTH+WYL+WCX -> BrxA-DNA-binding -> BrxB-BrxF-iSTAND -> DUF6079 *-> N6-METHYLASE -p-><-DEDD_Tnp_IS110+Transposase_20<-DEDD_Tnp_IS110+Transposase_20|| N6-METHYLASE ->SIR2+SIR2-> PglZ -> SIGMA+OB-Fold+AAA-ATPase+ HKD-DNase -BrxL ->McrB-AAA->McrC-NTD+REase->	1206	Bacteria>FCB_group>Bacteroidetes/Chlorobi_group>Bacteroidota>Bacteroidia>Bacteroidales	-	Prevotella_copri	ONT18_RS04485	1079147..1082767	165179	BREX-system-P-loop-protein-BrxC-[Prevotella-copri]
WP_073207711.1	Type1.BREX	<-Classical-AAA<-?||tRNA-><-?<-?<-HTH+DOC||?->HTH_3-> BrxA-DNA-binding -> BrxB-BrxF-iSTAND -> DUF6079 *-> N6-METHYLASE ->SbcC+SbcC->?->SIR2+SIR2-> PglZ -> SIGMA+OB-Fold+AAA-ATPase+ HKD-DNase -BrxL ->?->ParB+HNH->ParB+HNH->	1206	Bacteria>FCB_group>Bacteroidetes/Chlorobi_group>Bacteroidota>Bacteroidia>Bacteroidales	-	Prevotella_ruminicola	SAMN05216463_10942	46265..49885	839	BREX-system-P-loop-protein-BrxC-[Prevotella-ruminicola]
WP_181980669.1	Type1.BREX	TOPRIM->OLD-ABC+TOPRIM->SF1-SF2-Helicase->MORC+DUF3684+REase-><- SIGMA+OB-Fold+AAA-ATPase+ HKD-DNase -BrxL <- PglZ <- N6-METHYLASE <-REase<-OLD-ABC<- DUF6079 *<- BrxB-BrxF-iSTAND <- BrxA-DNA-binding <-HTH_3<-SMS-KaiC<-XIS-HTH<-?<-Phage_int_SAM_5+Phage_integrase<-XIS-HTH+XIS-HTH+XIS-HTH+XIS-HTH<-sGTP	1192	Bacteria>FCB_group>Bacteroidetes/Chlorobi_group>Bacteroidota>Bacteroidia	GCF_015558535.1	Bacteroidales	QH473_RS20245	26845..30423	171549	MULTISPECIES:-BREX-system-P-loop-protein-BrxC-[Bacteroidales]
BBE20443.1	Type1.BREX	TraJ-RHH->TraI-VirD2-RCRE-><- wHTH+WYL+WCX ||?->SMODS->?->HTH_3-> BrxA-DNA-binding -> BrxB-BrxF-iSTAND -> DUF6079 *-> N6-METHYLASE -> PglZ -> HKD-DNase ->AbiEi_4->AbiEii-> SIGMA+OB-Fold+AAA-ATPase+ HKD-DNase -BrxL ->	1173	Bacteria>FCB_group>Bacteroidetes/Chlorobi_group>Bacteroidota>Bacteroidia>Marinilabiliales	-	Aquipluma_nitroreducens	AQPE_4635	5186015..5189536	2010828	hypothetical-protein-AQPE_4635-[Aquipluma-nitroreducens]
MCF8362389.1	Type1.BREX	<- SIGMA+OB-Fold+AAA-ATPase+ HKD-DNase -BrxL <- PglZ <- PglZ <-Schlafen+HATPase+wHTH<- N6-METHYLASE <- N6-METHYLASE -p<- DUF6079 *<- BrxB-BrxF-iSTAND <- BrxA-DNA-binding <-HTH_3<-Schlafen+HATPase+wHTH-p	1180	Bacteria>FCB_group>Bacteroidetes/Chlorobi_group>Bacteroidota>Bacteroidia>Marinilabiliales	GCA_021740345.1	Prolixibacteraceae_bacterium	K9G70_07170	11489..15031	2053594	BREX-system-P-loop-protein-BrxC-[Prolixibacteraceae-bacterium]
MCK9411439.1	Type1.BREX	Peptidase_A8->Peptidase_A8-><- wHTH+WYL+WCX ||?->SMODS->?->HTH_3-> BrxA-DNA-binding -> BrxB-BrxF-iSTAND -> DUF6079 *-> N6-METHYLASE ->Schlafen+HATPase+wHTH->?-> PglZ ->AbiEi_4-> SIGMA+OB-Fold+AAA-ATPase+ HKD-DNase -BrxL ->?->REC+LYTTR->	1173	Bacteria>FCB_group>Bacteroidetes/Chlorobi_group>Bacteroidota>Bacteroidia>Marinilabiliales	GCA_023229575.1	Prolixibacteraceae_bacterium	MOQ53_04000	25065..28586	2053594	BREX-system-P-loop-protein-BrxC-[Prolixibacteraceae-bacterium]
MCU4173933.1	Type1.BREX	<-REase+RNase||?->?->?->?->?->HTH_3-> BrxA-DNA-binding -> BrxB-BrxF-iSTAND -> DUF6079 *-> N6-METHYLASE -> PglZ -> SIGMA+OB-Fold+AAA-ATPase+ HKD-DNase -BrxL ->AbiEii->?-><-?||VWA+REase->	1167	Bacteria>FCB_group>Bacteroidetes/Chlorobi_group>Bacteroidota>Bacteroidia>Marinilabiliales	GCA_025499625.1	Marinilabiliaceae_bacterium_N1Y90	J1N11_02510	10462..13965	2816964	BREX-system-P-loop-protein-BrxC-[Marinilabiliaceae-bacterium-N1Y90]
WP_062122677.1	Type1.BREX	ABhydrolase->tRNA-><- SIGMA+OB-Fold+AAA-ATPase+ HKD-DNase -BrxL <- PglZ <-Schlafen+HATPase+wHTH<- N6-METHYLASE <- DUF6079 -p<-DUF499<- DUF6079 -p*<- BrxB-BrxF-iSTAND <- BrxA-DNA-binding <-HTH_3||?-><-?<-?<-?<-SIGMA-HTH	329	Bacteria>FCB_group>Bacteroidetes/Chlorobi_group>Bacteroidota>Bacteroidia>Marinilabiliales	GCF_000974365.1	Geofilum_rubicundum	JCM15548_RS04470	156242..157231	472113	hypothetical-protein-[Geofilum-rubicundum]
WP_107823954.1	Type1.BREX	<-STYKinase-like<-Couple_hipA<-HTH_3<- SIGMA+OB-Fold+AAA-ATPase+ HKD-DNase -BrxL <- PglZ <- N6-METHYLASE <-Schlafen+HATPase+wHTH<- DUF6079 *<- BrxB-BrxF-iSTAND <- BrxA-DNA-binding <-HTH_3	1168	Bacteria>FCB_group>Bacteroidetes/Chlorobi_group>Bacteroidota>Bacteroidia>Marinilabiliales	GCF_003046625.1	Mangrovibacterium_marinum	C8N47_RS17935	11546..15052	1639118	BREX-system-P-loop-protein-BrxC-[Mangrovibacterium-marinum]
WP_163710191.1	Type1.BREX	HTH_3-> BrxA-DNA-binding -> BrxB-BrxF-iSTAND -> DUF6079 *->Schlafen+HATPase+wHTH->SbcC->?-> N6-METHYLASE ->SF1-SF2-Helicase-> PglZ -> SIGMA+OB-Fold+AAA-ATPase+ HKD-DNase -BrxL ->	1168	Bacteria>FCB_group>Bacteroidetes/Chlorobi_group>Bacteroidota>Bacteroidia>Marinilabiliales	GCF_010882175.1	Mangrovibacterium_lignilyticum	GJV22_RS06145	1519093..1522599	2668052	BREX-system-P-loop-protein-BrxC-[Mangrovibacterium-lignilyticum]
WP_250725046.1	Type1.BREX	DDE_Tnp_ISL3->DUF4372+DDE_Tnp_1-><- SIGMA+OB-Fold+AAA-ATPase+ HKD-DNase -BrxL <- PglZ <-Couple_hipA+HipA_C<-HTH_3<- N6-METHYLASE -p||DDE_Tnp_1_4-><- N6-METHYLASE <- DUF6079 *<- BrxB-BrxF-iSTAND <- BrxA-DNA-binding <-HTH_3||ZnR->DDE_Tnp_ISL3-><-sGTP||?->SIR2->	1173	Bacteria>FCB_group>Bacteroidetes/Chlorobi_group>Bacteroidota>Bacteroidia>Marinilabiliales	GCF_023674465.1	Alkaliflexus_sp._Ai-910	M9189_RS04830	1155477..1158998	2949635	BREX-system-P-loop-protein-BrxC-[Alkaliflexus-sp.-Ai-910]
OJV92258.1	Type1.BREX	RNase->?->TatD_DNase->?->tRNA-><- SIGMA+OB-Fold+AAA-ATPase+ HKD-DNase -BrxL <- PglZ <-?<- N6-METHYLASE <- DUF6079 *<- BrxB-BrxF-iSTAND <- BrxA-DNA-binding <-HTH_3	1200	Bacteria>FCB_group>Bacteroidetes/Chlorobi_group>Bacteroidota>Bacteroidia>unclassified_Bacteroidia	GCA_001897595.1	Bacteroidia_bacterium_44-10	BGO34_14870	43076..46678	1895721	hypothetical-protein-BGO34_14870-[Bacteroidia-bacterium-44-10]
PJE46487.1	Type1.BREX	<-DOC+HTH<-?<-?<-HerA/FtsK|| wHTH+WYL+WCX ->?->REase+RNase-> BrxA-DNA-binding -> BrxB-BrxF-iSTAND -> DUF6079 *-> N6-METHYLASE -> PglZ -> SIGMA+OB-Fold+AAA-ATPase+ HKD-DNase -BrxL ->?->?->STYKIN->SF1-SF2-Helicase->HTH+HEPN->McrB-AAA->	1169	Bacteria>FCB_group>Bacteroidetes/Chlorobi_group>Bacteroidota>Chitinophagia>Chitinophagales	GCA_002786355.1	Sediminibacterium_sp._FEMGT703S	CUR34_07570	66784..70293	2080766	MAG:-hypothetical-protein-CUR34_07570-[Sediminibacterium-sp.]
QEC55281.1	Type1.BREX	<-AbiEi_4<-MORC+SF1-SF2-Helicase<-TatD_DNase<-?<-KAP-glycosylase<-KAP-NTPase<- SIGMA+OB-Fold+AAA-ATPase+ HKD-DNase -BrxL <- PglZ <- N6-METHYLASE <- DUF6079 *<- BrxB-BrxF-iSTAND <- BrxA-DNA-binding <-?<- wHTH+WYL+WCX	1177	Bacteria>FCB_group>Bacteroidetes/Chlorobi_group>Bacteroidota>Chitinophagia>Chitinophagales	GCA_007970805.1	Flavisolibacter_ginsenosidimutans	FSB75_04955	1196460..1199993	661481	BREX-system-P-loop-protein-BrxC-[Flavisolibacter-ginsenosidimutans]
NTS43012.1	Type1.BREX	<- SIGMA+OB-Fold+AAA-ATPase+ HKD-DNase -BrxL <- PglZ <- N6-METHYLASE <- DUF6079 *<- BrxB-BrxF-iSTAND <- BrxA-DNA-binding <-CASPASE<-?<-?<-?<- wHTH+WYL+WCX	1173	Bacteria>FCB_group>Bacteroidetes/Chlorobi_group>Bacteroidota>Chitinophagia>Chitinophagales	GCA_013327885.1	Flavisolibacter_longurius	HRG84_19150	28734..32255	2740583	BREX-system-P-loop-protein-BrxC-[Flavisolibacter-longurius]
WP_172623061.1	Type1.BREX	<-AbiEi_4<-MORC+SF1-SF2-Helicase<-TatD_DNase<-?<-KAP-glycosylase<-KAP-NTPase<- SIGMA+OB-Fold+AAA-ATPase+ HKD-DNase -BrxL <- PglZ <- N6-METHYLASE <- DUF6079 *<- BrxB-BrxF-iSTAND <- BrxA-DNA-binding <-?<- wHTH+WYL+WCX	1174	Bacteria>FCB_group>Bacteroidetes/Chlorobi_group>Bacteroidota>Chitinophagia>Chitinophagales	GCF_007970805.1	Flavisolibacter_ginsenosidimutans	FSB75_RS04955	1196460..1199984	661481	BREX-system-P-loop-protein-BrxC-[Flavisolibacter-ginsenosidimutans]
ADB40822.1	Type1.BREX	GDResolvase+HIN-HTH-><-?||?-><-?||?-><-?||URI-DNase-> BrxA-DNA-binding -> BrxB-BrxF-iSTAND -> DUF6079 *->MarR-HTH->AbiEii-> N6-METHYLASE -> PglZ -> SIGMA+OB-Fold+AAA-ATPase+ HKD-DNase -BrxL -><-DUF4096+DDE_Tnp_1+DDE_Tnp_1_2||HTH+rve->AAA-ATPase->	1186	Bacteria>FCB_group>Bacteroidetes/Chlorobi_group>Bacteroidota>Cytophagia>Cytophagales	GCA_000024525.1	Spirosoma_linguale_DSM_74	Slin_4844	5894993..5898553	504472	hypothetical-protein-Slin_4844-[Spirosoma-linguale-DSM-74]
UOQ83254.1	Type1.BREX	<-pPIWI+pPIWI||HEPN-><-REase<-?||Phage_integrase+Phage_integrase->NUMOD4+HNH->HTH_3-> BrxA-DNA-binding -> BrxB-BrxF-iSTAND -> DUF6079 *-> N6-METHYLASE -p-> N6-METHYLASE -p-> PglZ -> SIGMA+OB-Fold+AAA-ATPase+ HKD-DNase -BrxL -><-SF1-SF2-Helicase+REase||NtrC-AAA+FIS-HTH->LexA-protease->	1148	Bacteria>FCB_group>Bacteroidetes/Chlorobi_group>Bacteroidota>Cytophagia>Cytophagales	GCA_022919455.1	Hymenobacter_sp._5414T-23	MUN83_21225	87571..91017	2932252	BREX-system-P-loop-protein-BrxC-[Hymenobacter-sp.-5414T-23]
WP_028665035.1	Type1.BREX	DSBH+AraC-HTH->?-><-?<-CNMP+CRP-HTH<-?|| BrxA-DNA-binding -> BrxB-BrxF-iSTAND -> DUF6079 *->AbiEi_4+AbiEi_1->AbiEii-> N6-METHYLASE -> PglZ -> SIGMA+OB-Fold+AAA-ATPase+ HKD-DNase -BrxL ->TPR+TPR+TPR-><-?<-SIGMA-HTH	1171	Bacteria>FCB_group>Bacteroidetes/Chlorobi_group>Bacteroidota>Cytophagia>Cytophagales	GCF_000423565.1	Runella_zeae	G563_RS0108985	25803..29318	94255	BREX-system-P-loop-protein-BrxC-[Runella-zeae]
WP_053405044.1	Type1.BREX	<- SIGMA+OB-Fold+AAA-ATPase+ HKD-DNase -BrxL <- PglZ ||ParB+HNH-><- N6-METHYLASE <- DUF6079 *<- BrxB-BrxF-iSTAND <- BrxA-DNA-binding <-AAA-ToxA||?->?-><-ParB<-ParA-Soj-PloopNTPase<-?<-AAA-ATPase	1179	Bacteria>FCB_group>Bacteroidetes/Chlorobi_group>Bacteroidota>Cytophagia>Cytophagales	GCF_001274635.1	Persicobacter_sp._CCB-QB2	NG22_RS06825	1502189..1505728	1561025	BREX-system-P-loop-protein-BrxC-[Persicobacter-sp.-CCB-QB2]
WP_073850520.1	Type1.BREX	<-HNH+SAVED<-?<-?<-?<-?<- SIGMA+OB-Fold+AAA-ATPase+ HKD-DNase -BrxL <- PglZ <- N6-METHYLASE <- DUF6079 *<- BrxB-BrxF-iSTAND <- BrxA-DNA-binding <-HTH_3||?->ZnR+TPR->TPR-><-HTH_3<-HTH_36<-XIS-HTH	1179	Bacteria>FCB_group>Bacteroidetes/Chlorobi_group>Bacteroidota>Cytophagia>Cytophagales	GCF_001907195.1	Pontibacter_flavimaris	A3841_RS01550	388287..391826	1797110	BREX-system-P-loop-protein-BrxC-[Pontibacter-flavimaris]
WP_146899771.1	Type1.BREX	<- SIGMA+OB-Fold+AAA-ATPase+ HKD-DNase -BrxL <- PglZ <- N6-METHYLASE <- DUF6079 *<- BrxB-BrxF-iSTAND <- BrxA-DNA-binding <-?<-?||?->DNA-glycosylase->?-><-TPR	1182	Bacteria>FCB_group>Bacteroidetes/Chlorobi_group>Bacteroidota>Cytophagia>Cytophagales	GCF_007991635.1	Adhaeribacter_aerolatus	AAE02nite_RS16235	111241..114789	670289	BREX-system-P-loop-protein-BrxC-[Adhaeribacter-aerolatus]
WP_168675169.1	Type1.BREX	<-CASPASE<-DDE_Tnp_4+DDE_Tnp_1_2||Phage_integrase+Phage_integrase-><-McrC-NTD+REase<-McrB-AAA<-?<- SIGMA+OB-Fold+AAA-ATPase+ HKD-DNase -BrxL <- PglZ <- N6-METHYLASE <- DUF6079 *<- BrxB-BrxF-iSTAND <- BrxA-DNA-binding ||NtrC-AAA+FIS-HTH->SMODS->?->Classical-AAA->?->LexA-protease->	1172	Bacteria>FCB_group>Bacteroidetes/Chlorobi_group>Bacteroidota>Cytophagia>Cytophagales	GCF_012275535.1	Hymenobacter_artigasi	HBN54_RS20840	55199..58717	2719616	BREX-system-P-loop-protein-BrxC-[Hymenobacter-artigasi]
WP_185195977.1	Type1.BREX	<-TIR<-?<-HTH+MPTase<-?||AbiEi_4->AbiEii-><- SIGMA+OB-Fold+AAA-ATPase+ HKD-DNase -BrxL <- PglZ <- N6-METHYLASE <- DUF6079 *<- BrxB-BrxF-iSTAND <- BrxA-DNA-binding <-HTH_3||?->ZnR+TPR->ParB+HNH-><-GDResolvase+Recombinase	1179	Bacteria>FCB_group>Bacteroidetes/Chlorobi_group>Bacteroidota>Cytophagia>Cytophagales	GCF_014212495.1	Pontibacter_sp._Tf4	H7F15_RS05560	22760..26299	2761620	BREX-system-P-loop-protein-BrxC-[Pontibacter-sp.-Tf4]
WP_187455529.1	Type1.BREX	<- N6-METHYLASE <-?||XIS-HTH-><- SIGMA+OB-Fold+AAA-ATPase+ HKD-DNase -BrxL <- PglZ <- N6-METHYLASE <- DUF6079 *<- BrxB-BrxF-iSTAND <- BrxA-DNA-binding <-?<-?<-HEPN<-?<-?<-HNH	1178	Bacteria>FCB_group>Bacteroidetes/Chlorobi_group>Bacteroidota>Cytophagia>Cytophagales	GCF_014349165.1	Hymenobacter_sp._BT491	H9I52_RS21275	49708..53244	2766779	BREX-system-P-loop-protein-BrxC-[Hymenobacter-sp.-BT491]
WP_188933335.1	Type1.BREX	<- wHTH+WYL+WCX ||?->TIR-><-HTH_28+HTH_32+rve+rve_3<-SF1-SF2-Helicase<- SIGMA+OB-Fold+AAA-ATPase+ HKD-DNase -BrxL <- PglZ <- N6-METHYLASE <- DUF6079 *<- BrxB-BrxF-iSTAND <- BrxA-DNA-binding <-HTH_3||Preader17-><-ABhydrolase<-Arm-DNA-bind_5+Phage_int_SAM_5+Phage_integrase	1165	Bacteria>FCB_group>Bacteroidetes/Chlorobi_group>Bacteroidota>Cytophagia>Cytophagales	GCF_014641595.1	Dyadobacter_endophyticus	IEY24_RS14735	618400..621897	1749036	BREX-system-P-loop-protein-BrxC-[Dyadobacter-endophyticus]
WP_191042738.1	Type1.BREX	TraJ-RHH->TraI-VirD2-RCRE-><-NtrC-AAA+FIS-HTH||35exo+wHTH+SF1-SF2-Helicase+HTH-> N6-METHYLASE ->?->?-> BrxA-DNA-binding -> BrxB-BrxF-iSTAND -> DUF6079 *-> N6-METHYLASE ->HigB->HTH_3->?->?->?->?-> PglZ -> SIGMA+OB-Fold+AAA-ATPase+ HKD-DNase -BrxL ->	1206	Bacteria>FCB_group>Bacteroidetes/Chlorobi_group>Bacteroidota>Cytophagia>Cytophagales	GCF_014699035.1	Spirosoma_validum	IC230_RS29845	51060..54680	2771355	BREX-system-P-loop-protein-BrxC-[Spirosoma-validum]
WP_191006191.1	Type6.BREX	HEPN->Phage_integrase+Phage_integrase-><-?<-HEPN<-TIR||?-> BrxE -> BrxA-DNA-binding -> BrxB-BrxF-iSTAND -> DUF6079 *-> N6-METHYLASE ->HEPN-> PglZ -> SIGMA+OB-Fold+AAA-ATPase+ HKD-DNase -BrxL ->N6-N4-METHYLASE->MORC+HISKIN->?-><-DOC+HTH	1204	Bacteria>FCB_group>Bacteroidetes/Chlorobi_group>Bacteroidota>Cytophagia>Cytophagales	GCF_014699115.1	Hymenobacter_montanus	IC235_RS15945	31815..35429	2771359	BREX-system-P-loop-protein-BrxC-[Hymenobacter-montanus]

Protein-ID	System	Gene-Neighbourhoods	Length	Taxonomy	Assembly-ID	Strain	Locus-Tag	Genomic-coordinates	TaxID	Fasta-define
WP_204660265.1	Type6.BREX	<-HTH_3||?->?->CopG/RHH->CR-ATPase5+REase->AbiEi->BrxE->BrxA-DNA-binding->BrxB-BrxF-iSTAND->DUF6079*->N6-METHYLASE->PglZ->SIGMA+OB-Fold+AAA-ATPase+HKD-DNase-BrxL->McrB-AAA->McrC-NTD+REase-><-MORC+DUF3684+REase<-DOC+zhth<-?||HEPN->	1216	Bacteria>FCB_group>Bacteroidetes/Chlorobi_group>Bacteroidota>Cytophagia>Cytophagales	GCF_016894445.1	Dyadobacter_sandarakinus	HWI92_RS00525	139788..143438	2747268	BREX-system-P-loop-protein-BrxC-[Dyadobacter-sandarakinus]
WP_205503392.1	Type1.BREX	<-wHTH+WYL+WCX||SF1-SF2-Helicase->RVT->?-><-SIGMA+OB-Fold+AAA-ATPase+HKD-DNase-BrxL<-PglZ<-N6-METHYLASE<-DUF6079*<-BrxB-BrxF-iSTAND<-BrxA-DNA-binding<-McrC-NTD+REase<-McrB-AAA||?->DDE_Tnp_4+DDE_Tnp_1_2-><-tRNA||HAD+HAD->Peptidase_M50->	1180	Bacteria>FCB_group>Bacteroidetes/Chlorobi_group>Bacteroidota>Cytophagia>Cytophagales	GCF_016924645.1	Rufibacter_sp._SYSU_D00308	JT718_RS14320	179098..182640	2812556	BREX-system-P-loop-protein-BrxC-[Rufibacter-sp.-SYSU-D00308]
WP_234861365.1	Type1.BREX	STYKIN-><-?||?->GDResolvase-><-?||TPR+TPR->BrxA-DNA-binding->BrxB-BrxF-iSTAND->DUF6079*->N6-METHYLASE->OLD-ABC+TOPRIM->PglZ->SIGMA+OB-Fold+AAA-ATPase+HKD-DNase-BrxL->TIR->?->?->?->ParB+N6_N4_METHYLASE->	1174	Bacteria>FCB_group>Bacteroidetes/Chlorobi_group>Bacteroidota>Cytophagia>Cytophagales	GCF_021491425.1	Mariniradius_sp._RY-2	L0U89_RS09865	251856..255380	2909237	BREX-system-P-loop-protein-BrxC-[Mariniradius-sp.-RY-2]
WP_241348547.1	Type1.BREX	<-ParB+N6_N4_METHYLASE<-?<-?<-SIGMA+OB-Fold+AAA-ATPase+HKD-DNase-BrxL<-PglZ<-CR-Kinase+CR-Kinase<-N6-METHYLASE<-?<-DUF6079*<-BrxB-BrxF-iSTAND<-BrxA-DNA-binding<-TPR+TPR<-MarR-HTH||?->?-><-?<-GDResolvase	1174	Bacteria>FCB_group>Bacteroidetes/Chlorobi_group>Bacteroidota>Cytophagia>Cytophagales	GCF_022549775.1	Belliella_sp._DSM_111904	MM239_RS12315	24692..28216	2923435	BREX-system-P-loop-protein-BrxC-[Belliella-sp.-DSM-111904]
WP_243520943.1	Type6.BREX	Glyoxalase-><-?<-HEPN<-?||?->?->BrxE->BrxA-DNA-binding->BrxB-BrxF-iSTAND->DUF6079*->N6-METHYLASE->HEPN->PglZ->SIGMA+OB-Fold+AAA-ATPase+HKD-DNase-BrxL->?->HKD-DNase->HTH+REase-><-CopG_antitoxin	1204	Bacteria>FCB_group>Bacteroidetes/Chlorobi_group>Bacteroidota>Cytophagia>Cytophagales	GCF_022811645.1	Hymenobacter_monticola	MTP16_RS25545	68480..72094	1705399	BREX-system-P-loop-protein-BrxC-[Hymenobacter-monticola]
WP_262922299.1	Type1.BREX	<-pPIWI+pPIWI||HEPN-><-REase<-?||Phage_integrase+Phage_integrase->NUMOD4+HNH->HTH_3->BrxA-DNA-binding->BrxB-BrxF-iSTAND->DUF6079*->N6-METHYLASE-p->N6-METHYLASE->N6-METHYLASE-p->PglZ->SIGMA+OB-Fold+AAA-ATPase+HKD-DNase-BrxL-><-SF1-SF2-Helicase+REase||NtrC-AAA+FIS-HTH->LexA-protease->	1164	Bacteria>FCB_group>Bacteroidetes/Chlorobi_group>Bacteroidota>Cytophagia>Cytophagales	GCF_022919455.1	Hymenobacter_sp._5414T-23	MUN83_RS21225	87523..91017	2932252	BREX-system-P-loop-protein-BrxC-[Hymenobacter-sp.-5414T-23]
WP_235164443.1	Type6.BREX	<-STYKIN<-?<-?<-?<-McrC-NTD+REase<-HTH+McrB-AAA<-SIGMA+OB-Fold+AAA-ATPase+HKD-DNase-BrxL<-PglZ<-N6-METHYLASE<-DUF6079*<-BrxB-BrxF-iSTAND<-BrxA-DNA-binding<-BrxE<-AbiEi||NERD+SF1-SF2-Helicase->HTH_21+rve+rve_3->?->wHTH+HNH->REase+N-glycosylase->	1216	Bacteria>FCB_group>Bacteroidetes/Chlorobi_group>Bacteroidota>Cytophagia>Cytophagales	GCF_023821985.2	Dyadobacter_chenhuakuii	NFI80_RS01415	331734..335384	2909339	BREX-system-P-loop-protein-BrxC-[Dyadobacter-chenhuakuii]
WP_269011923.1	Type1.BREX	HTH_3->Couple_hipA+HipA_C-><-?<-HNH+Polbeta<-SIGMA+OB-Fold+AAA-ATPase+HKD-DNase-BrxL<-PglZ<-N6-METHYLASE<-DUF6079*<-BrxB-BrxF-iSTAND<-BrxA-DNA-binding<-?<-?<-?<-?<-?<-wHTH+WYL+WCX||TPR+TPR+TPR->	1166	Bacteria>FCB_group>Bacteroidetes/Chlorobi_group>Bacteroidota>Cytophagia>Cytophagales	GCF_026938185.1	Aquirufa_antheringensis	G9H55_RS04755	131184..134684	2516559	BREX-system-P-loop-protein-BrxC-[Aquirufa-antheringensis]
WP_244679416.1	Type1.BREX	<-pPIWI+pPIWI||HEPN-><-REase<-?||Phage_integrase+Phage_integrase->NUMOD4+HNH->HTH_3->BrxA-DNA-binding->BrxB-BrxF-iSTAND->DUF6079*->N6-METHYLASE-p->N6-METHYLASE->N6-METHYLASE-p->PglZ->SIGMA+OB-Fold+AAA-ATPase+HKD-DNase-BrxL-><-SF1-SF2-Helicase+REase||NtrC-AAA+FIS-HTH->LexA-protease->	1148	Bacteria>FCB_group>Bacteroidetes/Chlorobi_group>Bacteroidota>Cytophagia>Cytophagales	-	Hymenobacter_sp._5414T-23	MUN83_21225	87571..91017	2932252	BREX-system-P-loop-protein-BrxC-[Hymenobacter-sp.-5414T-23]
OIQ21186.1	Type1.BREX	CHTH+LexA-protease-><-GDResolvase+Recombinase+ZnR<-?||?-><-?||BrxA-DNA-binding->BrxB-BrxF-iSTAND->DUF6079*->N6-METHYLASE->SF1-SF2-Helicase->?->PglZ->SIGMA+OB-Fold+AAA-ATPase+HKD-DNase-BrxL->ParB+HNH->URI-DNase->?->GDResolvase->	1165	Bacteria>FCB_group>Bacteroidetes/Chlorobi_group>Bacteroidota>Flavobacteriia>Flavobacteriales	GCA_001874145.1	Lacinutrix_sp._MedPE-SW	BM549_09430	19615..23112	1860087	MAG:-hypothetical-protein-BM549_09430-[Lacinutrix-sp.-MedPE-SW]
MAM30277.1	Type1.BREX	<-ParB+N6_N4_METHYLASE||XIS-HTH-><-SIGMA+OB-Fold+AAA-ATPase+HKD-DNase-BrxL<-PglZ<-TPR+S1<-NERD+SF1-SF2-Helicase<-N6-METHYLASE<-ParB+HNH<-DUF6079*<-BrxB-BrxF-iSTAND<-BrxA-DNA-binding<-HTH_3||GDResolvase->?->?->ABC-ATPase+SbcC->N6-METHYLASE->	1165	Bacteria>FCB_group>Bacteroidetes/Chlorobi_group>Bacteroidota>Flavobacteriia>Flavobacteriales	GCA_002693005.1	Flavobacteriaceae_bacterium	CMC13_14765	19860..23357	1871037	hypothetical-protein-[Flavobacteriaceae-bacterium]
ROI04325.1	Type1.BREX	<-HTH_3<-?||?->?-><-?<-?<-SIGMA+OB-Fold+AAA-ATPase+HKD-DNase-BrxL<-PglZ<-N6-METHYLASE<-DUF6079*<-BrxB-BrxF-iSTAND<-BrxA-DNA-binding<-Schlafen+HATPase+wHTH||?->ChlI+Lon_C+AAA-><-?||?->SLOG->	1174	Bacteria>FCB_group>Bacteroidetes/Chlorobi_group>Bacteroidota>Flavobacteriia>Flavobacteriales	GCA_003729975.1	Kaistella_haifensis	EGH90_10140	55406..58930	421525	BREX-system-P-loop-protein-BrxC-[Kaistella-haifensis]
AZB21330.1	Type1.BREX	<-SLOG<-?||?-><-ChlI+Lon_C+AAA||BrxA-DNA-binding->BrxB-BrxF-iSTAND->DUF6079-p->DUF6079-p*->N6-METHYLASE->PglZ->SIGMA+OB-Fold+AAA-ATPase+HKD-DNase-BrxL->?->?->ZnR->DDE_Tnp_ISL3->	808	Bacteria>FCB_group>Bacteroidetes/Chlorobi_group>Bacteroidota>Flavobacteriia>Flavobacteriales	GCA_003816015.1	Kaistella_haifensis	EG338_03940	799177..801603	421525	BREX-system-P-loop-protein-BrxC-[Kaistella-haifensis]
RZJ34503.1	Type1.BREX	<-GDResolvase||HTH_3->BrxA-DNA-binding->BrxB-BrxF-iSTAND->DUF6079*->SF1-SF2-Helicase->?->PglZ->SIGMA+OB-Fold+AAA-ATPase+HKD-DNase-BrxL-><-URI-DNase||tRNA-><-?<-REC<-AraC-HTH	1165	Bacteria>FCB_group>Bacteroidetes/Chlorobi_group>Bacteroidota>Flavobacteriia>Flavobacteriales	GCA_004210845.1	Flavobacterium_sp.	EO051_09215	4934..8431	239	MAG:-BREX-system-P-loop-protein-BrxC-[Flavobacterium-sp.]
MBW7869163.1	Type1.BREX	HTH_3->BrxA-DNA-binding->BrxB-BrxF-iSTAND->DUF6079*->N6-METHYLASE->?->Schlafen+HATPase+wHTH->KAP-NTPase->PglZ->SIGMA+OB-Fold+AAA-ATPase+HKD-DNase-BrxL->	1166	Bacteria>FCB_group>Bacteroidetes/Chlorobi_group>Bacteroidota>Flavobacteriia>Flavobacteriales	GCA_019455205.1	Brumimicrobium_sp.	H3C31_12675	1666..5166	2029867	BREX-system-P-loop-protein-BrxC-[Brumimicrobium-sp.]
MBZ0328278.1	Type1.BREX	[illegible]	1164	Bacteria>FCB_group>Bacteroidetes/Chlorobi_group>Bacteroidota>Flavobacteriia>Flavobacteriales	GCA_019913785.1	Altibacter_sp.	K8F54_11775	128036..131530	2024823	BREX-system-P-loop-protein-BrxC-[Altibacter-sp.]
WP_051224566.1	Type1.BREX	[illegible]	1168	Bacteria>FCB_group>Bacteroidetes/Chlorobi_group>Bacteroidota>Flavobacteriia>Flavobacteriales	GCF_000425485.1	Flavobacterium_tegetincola	H602_RS18955	62387..65893	150172	BREX-system-P-loop-protein-BrxC-[Flavobacterium-tegetincola]
WP_027137224.1	Type6.BREX	[illegible]	1196	Bacteria>FCB_group>Bacteroidetes/Chlorobi_group>Bacteroidota>Flavobacteriia>Flavobacteriales	GCF_000425645.1	Gaetbulibacter_saemankumensis	H611_RS0104475	476638..480228	311208	BREX-system-P-loop-protein-BrxC-[Gaetbulibacter-saemankumensis]
WP_028123425.1	Type1.BREX	[illegible]	1188	Bacteria>FCB_group>Bacteroidetes/Chlorobi_group>Bacteroidota>Flavobacteriia>Flavobacteriales	GCF_000428485.1	Epilithonimonas_tenax	F564_RS0115480	6701..10267	191577	BREX-system-P-loop-protein-BrxC-[Epilithonimonas-tenax]
WP_034657463.1	Type1.BREX	[illegible]	1160	Bacteria>FCB_group>Bacteroidetes/Chlorobi_group>Bacteroidota>Flavobacteriia>Flavobacteriales	GCF_000468595.1	Cellulophaga_baltica	M665_RS0118760	90424..93906	76594	BREX-system-P-loop-protein-BrxC-[Cellulophaga-baltica]
WP_088323652.1	Type1.BREX	[illegible]	1158	Bacteria>FCB_group>Bacteroidetes/Chlorobi_group>Bacteroidota>Flavobacteriia>Flavobacteriales	GCF_002201235.1	Polaribacter_tangerinus	CCS00_RS05195	350003..353479	1920034	BREX-system-P-loop-protein-BrxC-[Polaribacter-tangerinus]
WP_108879107.1	Type1.BREX	[illegible]	1166	Bacteria>FCB_group>Bacteroidetes/Chlorobi_group>Bacteroidota>Flavobacteriia>Flavobacteriales	GCF_003095655.1	Dokdonia_sp._Dokd-P16	DCS32_RS15465	3477371..3480871	2173169	BREX-system-P-loop-protein-BrxC-[Dokdonia-sp.-Dokd-P16]
WP_129366655.1	Type1.BREX	[illegible]	1168	Bacteria>FCB_group>Bacteroidetes/Chlorobi_group>Bacteroidota>Flavobacteriia>Flavobacteriales	GCF_004121075.1	Lutibacter_sp._HS1-25	EC396_RS10485	12877..16383	2485000	BREX-system-P-loop-protein-BrxC-[Lutibacter-sp.-HS1-25]
WP_132215847.1	Type1.BREX	[illegible]	1169	Bacteria>FCB_group>Bacteroidetes/Chlorobi_group>Bacteroidota>Flavobacteriia>Flavobacteriales	GCF_004341235.1	Mariniflexile_fucanivorans	EV196_RS05490	443037..446546	264023	BREX-system-P-loop-protein-BrxC-[Mariniflexile-fucanivorans]
WP_133546801.1	Type1.BREX	[illegible]	1167	Bacteria>FCB_group>Bacteroidetes/Chlorobi_group>Bacteroidota>Flavobacteriia>Flavobacteriales	GCF_004362395.1	Salegentibacter_sp._24	DET49_RS03950	90042..93545	2183986	BREX-system-P-loop-protein-BrxC-[Salegentibacter-sp.-24]
WP_136564624.1	Type1.BREX	[illegible]	1164	Bacteria>FCB_group>Bacteroidetes/Chlorobi_group>Bacteroidota>Flavobacteriia>Flavobacteriales	GCF_004916895.1	Muricauda_alvinocaridis	EZV76_RS00375	77026..80520	2530200	BREX-system-P-loop-protein-BrxC-[Muricauda-alvinocaridis]
WP_141421442.1	Type1.BREX	[illegible]	1165	Bacteria>FCB_group>Bacteroidetes/Chlorobi_group>Bacteroidota>Flavobacteriia>Flavobacteriales	GCF_006546625.1	Haloflavibacter_putidus	FKR84_RS06225	21354..24851	2576776	BREX-system-P-loop-protein-BrxC-[Haloflavibacter-putidus]
WP_019974718.1	Type1.BREX	[illegible]	1191	Bacteria>FCB_group>Bacteroidetes/Chlorobi_group>Bacteroidota>Flavobacteriia>Flavobacteriales	GCF_007990795.1	Empedobacter_brevis	EB1_RS03060	16539..20114	247	BREX-system-P-loop-protein-BrxC-[Empedobacter-brevis]
WP_153266434.1	Type1.BREX	[illegible]	1157	Bacteria>FCB_group>Bacteroidetes/Chlorobi_group>Bacteroidota>Flavobacteriia>Flavobacteriales	GCF_009498295.1	Oceanihabitans_sp._IOP_32	FEZ18_RS00165	31374..34847	2529032	BREX-system-P-loop-protein-BrxC-[Oceanihabitans-sp.-IOP_32]
WP_158973850.1	Type1.BREX	[illegible]	1170	Bacteria>FCB_group>Bacteroidetes/Chlorobi_group>Bacteroidota>Flavobacteriia>Flavobacteriales	GCF_009797025.1	Cellulophaga_sp._L1A9	GQR94_RS02145	525105..528617	2686362	BREX-system-P-loop-protein-BrxC-[Cellulophaga-sp.-L1A9]
WP_166386804.1	Type6.BREX	[illegible]	1195	Bacteria>FCB_group>Bacteroidetes/Chlorobi_group>Bacteroidota>Flavobacteriia>Flavobacteriales	GCF_011379025.1	Polaribacter_sp._11A2H	GQS58_RS13590	79017..82604	2687290	BREX-system-P-loop-protein-BrxC-[Polaribacter-sp.-11A2H]
WP_073117772.1	Type1.BREX	[illegible]	1170	Bacteria>FCB_group>Bacteroidetes/Chlorobi_group>Bacteroidota>Flavobacteriia>Flavobacteriales	GCF_014636515.1	Wenyingzhuangia_marina	IEV03_RS11465	186450..189962	1195760	BREX-system-P-loop-protein-BrxC-[Wenyingzhuangia-marina]
WP_193813001.1	Type6.BREX	[illegible]	1217	Bacteria>FCB_group>Bacteroidetes/Chlorobi_group>Bacteroidota>Flavobacteriia>Flavobacteriales	GCF_015191005.1	Kaistella_flava_(ex_Peng_et_al._2021)	Q73A0000_05090	1135416..1139069	2038776	BREX-system-P-loop-protein-BrxC-[Kaistella-flava-(ex-Peng-et-al.-2021)]
WP_194850146.1	Type1.BREX	[illegible]	1160	Bacteria>FCB_group>Bacteroidetes/Chlorobi_group>Bacteroidota>Flavobacteriia>Flavobacteriales	GCF_015356755.1	Nonlabens_antarcticus	IQ282_RS01355	76947..80429	392714	BREX-system-P-loop-protein-BrxC-[Nonlabens-antarcticus]
WP_199600307.1	Type1.BREX	[illegible]	1160	Bacteria>FCB_group>Bacteroidetes/Chlorobi_group>Bacteroidota>Flavobacteriia>Flavobacteriales	GCF_016469035.1	Gelidibacter_salicanalis	JEM65_RS13235	170248..173730	291193	BREX-system-P-loop-protein-BrxC-[Gelidibacter-salicanalis]
WP_203057453.1	Type1.BREX	[illegible]	1178	Bacteria>FCB_group>Bacteroidetes/Chlorobi_group>Bacteroidota>Flavobacteriia>Flavobacteriales	GCF_016786255.1	Robertkochia_sediminum	IS446_RS01110	192082..195618	2785326	BREX-system-P-loop-protein-BrxC-[Robertkochia-sediminum]
WP_203251454.1	Type1.BREX	[illegible]	1160	Bacteria>FCB_group>Bacteroidetes/Chlorobi_group>Bacteroidota>Flavobacteriia>Flavobacteriales	GCF_016811105.1	Aurantibacter_crassamenti	JQC67_RS03370	810968..814450	1837375	BREX-system-P-loop-protein-BrxC-[Aurantibacter-crassamenti]
WP_224137560.1	Type1.BREX	[illegible]	1174	Bacteria>FCB_group>Bacteroidetes/Chlorobi_group>Bacteroidota>Flavobacteriia>Flavobacteriales	GCF_020084865.1	Candidatus_Kaistella_beijingensis	J4771_RS06115	1314125..1317649	2820270	BREX-system-P-loop-protein-BrxC-[Candidatus-Kaistella-beijingensis]
WP_224474755.1	Type1.BREX	[illegible]	1167	Bacteria>FCB_group>Bacteroidetes/Chlorobi_group>Bacteroidota>Flavobacteriia>Flavobacteriales	GCF_020164485.1	Salegentibacter_tibetensis	LB467_RS14420	15605..19108	2873600	BREX-system-P-loop-protein-BrxC-[Salegentibacter-tibetensis]
WP_226063322.1	Type1.BREX	[illegible]	1175	Bacteria>FCB_group>Bacteroidetes/Chlorobi_group>Bacteroidota>Flavobacteriia>Flavobacteriales	GCF_020410745.1	Kaistella_sp._GW4-15	LC814_RS07510	1609850..1613377	2878534	BREX-system-P-loop-protein-BrxC-[Kaistella-sp.-GW4-15]
WP_232121385.1	Type1.BREX	[illegible]	1176	Bacteria>FCB_group>Bacteroidetes/Chlorobi_group>Bacteroidota>Flavobacteriia>Flavobacteriales	GCF_021205915.1	Tenacibaculum_dicentrarchi	LNI89_RS11375	20484..24014	669041	BREX-system-P-loop-protein-BrxC-[Tenacibaculum-dicentrarchi]
WP_232125551.1	Type1.BREX	[illegible]	1176	Bacteria>FCB_group>Bacteroidetes/Chlorobi_group>Bacteroidota>Flavobacteriia>Flavobacteriales	GCF_021206215.1	Tenacibaculum_finnmarkense	LNJ08_RS11655	18832..22362	2781243	BREX-system-P-loop-protein-BrxC-[Tenacibaculum-finnmarkense]
WP_233899778.1	Type1.BREX	[illegible]	1176	Bacteria>FCB_group>Bacteroidetes/Chlorobi_group>Bacteroidota>Flavobacteriia>Flavobacteriales	GCF_021390795.1	Tenacibaculum_piscium	LOU78_RS02170	464400..467930	1458515	BREX-system-P-loop-protein-BrxC-[Tenacibaculum-piscium]
WP_232123632.1	Type1.BREX	[illegible]	1176	Bacteria>FCB_group>Bacteroidetes/Chlorobi_group>Bacteroidota>Flavobacteriia>Flavobacteriales	GCF_022381915.1	Tenacibaculum_finnmarkense	G1K59_RS11550	3814..7344	2781243	BREX-system-P-loop-protein-BrxC-[Tenacibaculum-finnmarkense]
WP_193709240.1	Type1.BREX	[illegible]	1176	Bacteria>FCB_group>Bacteroidetes/Chlorobi_group>Bacteroidota>Flavobacteriia>Flavobacteriales	GCF_022382115.1	Tenacibaculum_finnmarkense	PJJ26_RS00320	69854..73384	2781243	BREX-system-P-loop-protein-BrxC-[Tenacibaculum-finnmarkense]
WP_249986967.1	Type1.BREX	[illegible]	1176	Bacteria>FCB_group>Bacteroidetes/Chlorobi_group>Bacteroidota>Flavobacteriia>Flavobacteriales	GCF_023555435.1	Polaribacter_sp._Z014	MPF19_RS04515	308529..312059	2927126	BREX-system-P-loop-protein-BrxC-[Polaribacter-sp.-Z014]
WP_264521759.1	Type1.BREX	[illegible]	1166	Bacteria>FCB_group>Bacteroidetes/Chlorobi_group>Bacteroidota>Flavobacteriia>Flavobacteriales	GCF_025947145.1	Flavobacterium_sp._N1994	OLM53_RS03950	842577..846077	2986827	BREX-system-P-loop-protein-BrxC-[Flavobacterium-sp.-N1994]
WP_267609146.1	Type1.BREX	[illegible]	1173	Bacteria>FCB_group>Bacteroidetes/Chlorobi_group>Bacteroidota>Flavobacteriia>Flavobacteriales	GCF_026626535.1	Chryseobacterium_sp._CY353	OUO28_RS14320	10693..14214	2997334	BREX-system-P-loop-protein-BrxC-[Chryseobacterium-sp.-CY353]
WP_089756260.1	Type1.BREX	[illegible]	1176	Bacteria>FCB_group>Bacteroidetes/Chlorobi_group>Bacteroidota>Flavobacteriia>Flavobacteriales	GCF_900100115.1	Chryseobacterium_soldanellicola	BLT04_RS13255	831929..835459	311333	BREX-system-P-loop-protein-BrxC-[Chryseobacterium-soldanellicola]
WP_089663492.1	Type1.BREX	[illegible]	1167	Bacteria>FCB_group>Bacteroidetes/Chlorobi_group>Bacteroidota>Flavobacteriia>Flavobacteriales	GCF_900105035.1	Gramella_echinicola	BLT93_RS14455	3168257..3171760	279359	BREX-system-P-loop-protein-BrxC-[Christiangramia-echinicola]
WP_103999283.1	Type1.BREX	[illegible]	1170	Bacteria>FCB_group>Bacteroidetes/Chlorobi_group>Bacteroidota>Flavobacteriia>Flavobacteriales	GCF_900108015.1	Flavobacterium_urumqiense	C4S35_RS05860	195084..198596	935224	BREX-system-P-loop-protein-BrxC-[Flavobacterium-urumqiense]
WP_091208763.1	Type1.BREX	[illegible]	1172	Bacteria>FCB_group>Bacteroidetes/Chlorobi_group>Bacteroidota>Flavobacteriia>Flavobacteriales	GCF_900112975.1	Flavobacterium_xueshanense	BMZ76_RS14825	46469..49987	935223	BREX-system-P-loop-protein-BrxC-[Flavobacterium-xueshanense]
WP_089819303.1	Type1.BREX	[illegible]	1174	Bacteria>FCB_group>Bacteroidetes/Chlorobi_group>Bacteroidota>Flavobacteriia>Flavobacteriales	GCF_900114045.1	Kaistella_treverensis	BMZ55_RS05400	1141726..1145250	631455	BREX-system-P-loop-protein-BrxC-[Kaistella-treverensis]
WP_159447525.1	Type1.BREX	[illegible]	719	Bacteria>FCB_group>Bacteroidetes/Chlorobi_group>Bacteroidota>Flavobacteriia>Flavobacteriales	GCF_900167935.1	Maribacter_arcticus	B5X55_RS04985	472839..474998	561365	BREX-system-P-loop-protein-BrxC-[Maribacter-arcticus]
WP_095070899.1	Type1.BREX	[illegible]	1178	Bacteria>FCB_group>Bacteroidetes/Chlorobi_group>Bacteroidota>Flavobacteriia>Flavobacteriales	GCF_900187185.1	Chryseobacterium_taklimakanense	CKV81_RS04675	977692..981228	536441	BREX-system-P-loop-protein-BrxC-[Chryseobacterium-taklimakanense]
WP_089379946.1	Type1.BREX	[illegible]	1168	Bacteria>FCB_group>Bacteroidetes/Chlorobi_group>Bacteroidota>Flavobacteriia>Flavobacteriales	GCF_900188235.1	Lutibacter_agarilyticus	SAMN06265371_101285	359460..362966	1109740	BREX-system-P-loop-protein-BrxC-[Lutibacter-agarilyticus]
WP_213195478.1	Type1.BREX	[illegible]	1176	Bacteria>FCB_group>Bacteroidetes/Chlorobi_group>Bacteroidota>Flavobacteriia>Flavobacteriales	GCF_907163105.1	Cloacibacterium_caeni	KKQ76_RS01235	263131..266661	2004710	BREX-system-P-loop-protein-BrxC-[Cloacibacterium-caeni]
WP_271393637.1	Type1.BREX	[illegible]	1168	Bacteria>FCB_group>Bacteroidetes/Chlorobi_group>Bacteroidota>Flavobacteriia>Flavobacteriales	GCF_947492035.1	Aequorivita_sinensis	POR58_RS10295	126765..130271	1382458	BREX-system-P-loop-protein-BrxC-[Aequorivita-sinensis]
WP_271393758.1	Type1.BREX	[illegible]	1165	Bacteria>FCB_group>Bacteroidetes/Chlorobi_group>Bacteroidota>Flavobacteriia>Flavobacteriales	GCF_947492035.1	Aequorivita_sinensis	POR58_RS11110	23754..27251	1382458	BREX-system-P-loop-protein-BrxC-[Aequorivita-sinensis]
MCB0517804.1	Type1.BREX	[illegible]	1174	Bacteria>FCB_group>Bacteroidetes/Chlorobi_group>Bacteroidota>Saprospiria>Saprospirales	GCA_020436325.1	Saprospiraceae_bacterium	KDD27_02635	9612..13136	2202734	BREX-system-P-loop-protein-BrxC-[Saprospiraceae-bacterium]
MCB0524505.1	Type1.BREX	[illegible]	1094	Bacteria>FCB_group>Bacteroidetes/Chlorobi_group>Bacteroidota>Saprospiria>Saprospirales	GCA_020436385.1	Saprospiraceae_bacterium	KDC86_08270	14975..18259	2202734	BREX-system-P-loop-protein-BrxC-[Saprospiraceae-bacterium]
WP_020534907.1	Type1.BREX	[illegible]	1159	Bacteria>FCB_group>Bacteroidetes/Chlorobi_group>Bacteroidota>Saprospiria>Saprospirales	GCF_000379805.1	Lewinella_cohaerens	A3EU_RS29375	116498..119977	70995	BREX-system-P-loop-protein-BrxC-[Lewinella-cohaerens]
WP_168040578.1	Type1.BREX	[illegible]	1211	Bacteria>FCB_group>Bacteroidetes/Chlorobi_group>Bacteroidota>Saprospiria>Saprospirales	GCF_011927835.1	Neolewinella_antarctica	GGR27_RS19565	12164..15799	442734	BREX-system-P-loop-protein-BrxC-[Neolewinella-antarctica]
WP_236977640.1	Type1.BREX	[illegible]	1159	Bacteria>FCB_group>Bacteroidetes/Chlorobi_group>Bacteroidota>Saprospiria>Saprospirales	GCF_021729145.1	Membranihabitans_sp._C281	LVD47_RS14080	21527..25006	2904244	BREX-system-P-loop-protein-BrxC-[Membranihabitans-maritimus]
OYD42847.1	Type1.BREX	[illegible]	1175	Bacteria>FCB_group>Bacteroidetes/Chlorobi_group>Bacteroidota>Sphingobacteriia>Sphingobacteriales	GCA_002245855.1	Sphingobacterium_cellulitidis	CHT99_08530	557163..560690	1768011	hypothetical-protein-CHT99_08530-[Sphingobacterium-cellulitidis]
WP_157278947.1	Type1.BREX	[illegible]	1169	Bacteria>FCB_group>Bacteroidetes/Chlorobi_group>Bacteroidota>Sphingobacteriia>Sphingobacteriales	GCF_000427965.1	Olivibacter_sitiensis	A37S_RS0120635	7227..10736	376470	BREX-system-P-loop-protein-BrxC-[Olivibacter-sitiensis]
WP_094282244.1	Type1.BREX	[illegible]	1175	Bacteria>FCB_group>Bacteroidetes/Chlorobi_group>Bacteroidota>Sphingobacteriia>Sphingobacteriales	GCF_002245765.1	Sphingobacterium_cellulitidis	CHU00_RS15775	18381..21908	1768011	BREX-system-P-loop-protein-BrxC-[Sphingobacterium-cellulitidis]
WP_070561019.1	Type1.BREX	[illegible]	1187	Bacteria>FCB_group>Bacteroidetes/Chlorobi_group>Bacteroidota>Sphingobacteriia>Sphingobacteriales	GCF_002339225.1	Sphingobacterium_sp._HMSC13C05	HMPREF3127_RS01230	95716..99279	1581095	BREX-system-P-loop-protein-BrxC-[Sphingobacterium-sp.-HMSC13C05]
WP_165305702.1	Type1.BREX	[illegible]	1187	Bacteria>FCB_group>Bacteroidetes/Chlorobi_group>Bacteroidota>Sphingobacteriia>Sphingobacteriales	GCF_011064245.1	Sphingobacterium_sp._DR205	G6053_RS05210	1244063..1247626	2713573	BREX-system-P-loop-protein-BrxC-[Sphingobacterium-sp.-DR205]
WP_090698254.1	Type1.BREX	[illegible]	1169	Bacteria>FCB_group>Bacteroidetes/Chlorobi_group>Bacteroidota>Sphingobacteriia>Sphingobacteriales	GCF_900103545.1	Daejeonella_rubra	BLR27_RS01455	316504..320013	990371	BREX-system-P-loop-protein-BrxC-[Daejeonella-rubra]
KPP94735.1	Type1.BREX	[illegible]	1203	Bacteria>FCB_group>Bacteroidetes/Chlorobi_group>Bacteroidota>unclassified_Bacteroidota	GCA_001314545.1	Bacteroidetes_bacterium_HLUCCA01	HLUCCA01_08750	188157..191768	1666909	Brex-phage-defense-system-ATPase-BrxC-[Bacteroidetes-bacterium-HLUCCA01]
MBK7566196.1	Type6.BREX	[illegible]	1193	Bacteria>FCB_group>Bacteroidetes/Chlorobi_group>Bacteroidota>unclassified_Bacteroidota	GCA_016706865.1	Bacteroidota_bacterium	IPI31_00060	18734..22315	1898104	BREX-system-P-loop-protein-BrxC-[Bacteroidota-bacterium]
MCH7965547.1	Type1.BREX	[illegible]	1161	Bacteria>FCB_group>Bacteroidetes/Chlorobi_group>Bacteroidota>unclassified_Bacteroidota	GCA_022564295.1	Bacteroidota_bacterium	IH852_16610	28479..31964	1898104	BREX-system-P-loop-protein-BrxC-[Bacteroidota-bacterium]
MAO64836.1	Type1.BREX	[illegible]	1168	Bacteria>FCB_group>Bacteroidetes/Chlorobi_group>Balneolota>Balneolia>Balneolales	GCA_002696035.1	Balneola_sp.	CL666_07525	664531..668037	2024824	hypothetical-protein-[Balneola-sp.]
MCC5907043.1	Type1.BREX	[illegible]	1167	Bacteria>FCB_group>Bacteroidetes/Chlorobi_group>Balneolota>Balneolia>Balneolales	GCA_020832285.1	Balneolaceae_bacterium	JJU13_12585	29362..32865	2053516	BREX-system-P-loop-protein-BrxC-[Balneolaceae-bacterium]
MCH2449130.1	Type1.BREX	[illegible]	1151	Bacteria>FCB_group>Bacteroidetes/Chlorobi_group>Balneolota>Balneolia>Balneolales	GCA_022450045.1	Gracilimonas_sp.	MK198_03190	295077..298532	1974203	BREX-system-P-loop-protein-BrxC-[Gracilimonas-sp.]
WP_237852616.1	Type1.BREX	[illegible]	1164	Bacteria>FCB_group>Bacteroidetes/Chlorobi_group>Balneolota>Balneolia>Balneolales	GCF_022012475.1	Rhodohalobacter_sp._WB101	L6773_RS04280	217944..221438	2911366	BREX-system-P-loop-protein-BrxC-[Rhodohalobacter-sp.-WB101]
WP_265763895.1	Type1.BREX	[illegible]	1165	Bacteria>FCB_group>Bacteroidetes/Chlorobi_group>Balneolota>Balneolia>Balneolales	GCF_026229185.1	Aliifodinibius_sp._1BSP15-2V2	J6I44_RS00130	34405..37902	2820877	BREX-system-P-loop-protein-BrxC-[Aliifodinibius-sp.-1BSP15-2V2]
KAA6232806.1	Type1.BREX	[illegible]	1204	Bacteria>FCB_group>Bacteroidetes/Chlorobi_group>Chlorobiota>Chlorobiia>Chlorobiales	GCA_008634005.1	Chlorobium_phaeovibrioides	FP507_06840	1409593..1413207	1094	BREX-system-P-loop-protein-BrxC-[Chlorobium-phaeovibrioides]
NMW22669.1	Type6.BREX	[illegible]	1210	Bacteria>FCB_group>Bacteroidetes/Chlorobi_group>Chlorobiota>Chlorobiia>Chlorobiales	GCA_012972835.1	Chlorobiaceae_bacterium	HKK67_13830	33011..36643	2067991	BREX-system-P-loop-protein-BrxC-[Chlorobiaceae-bacterium]
WP_204208384.1	Type1.BREX	[illegible]	997	Bacteria>FCB_group>Bacteroidetes/Chlorobi_group>Chlorobiota>Chlorobiia>Chlorobiales	GCF_001687065.1	Prosthecochloris_sp._CIB_2401	Ptc2401_RS05035	1057250..1060243	1868325	BREX-system-P-loop-protein-BrxC-[Prosthecochloris-sp.-CIB-2401]
WP_076791024.1	Type1.BREX	[illegible]	1179	Bacteria>FCB_group>Bacteroidetes/Chlorobi_group>Chlorobiota>Chlorobiia>Chlorobiales	GCF_001975465.1	Chlorobium_sp._KB01	BOO20_RS04865	12313..15852	1917528	BREX-system-P-loop-protein-BrxC-[Chlorobium-sp.-KB01]
WP_192496514.1	Type1.BREX	[illegible]	1203	Bacteria>FCB_group>Bacteroidetes/Chlorobi_group>Chlorobiota>Chlorobiia>Chlorobiales	GCF_008634005.1	Chlorobium_phaeovibrioides	FP507_RS07315	1409593..1413204	1094	BREX-system-P-loop-protein-BrxC-[Chlorobium-phaeovibrioides]
WP_151419733.1	Type1.BREX	[illegible]	1183	Bacteria>FCB_group>Bacteroidetes/Chlorobi_group>Chlorobiota>Chlorobiia>Chlorobiales	GCF_008634005.1	Chlorobium_phaeovibrioides	FP507_RS10470	13780..17331	1094	BREX-system-P-loop-protein-BrxC-[Chlorobium-phaeovibrioides]
WP_098060820.1	Type1.BREX	[illegible]	1206	Bacteria>FCB_group>Bacteroidetes/Chlorobi_group>Rhodothermota>Rhodothermia>Rhodothermales	GCF_002554705.1	Longimonas_halophila	CRI93_RS01465	364524..368144	1469170	BREX-system-P-loop-protein-BrxC-[Longimonas-halophila]
OQC05381.1	Type1.BREX	[illegible]	1162	Bacteria>FCB_group>Candidatus_Cloacimonetes>unclassified_Candidatus_Cloacimonetes	GCA_002070875.1	Candidatus_Cloacimonetes_bacterium_ADurb.Bin089	BWX76_00750	1812..5300	1852836	MAG:-hypothetical-protein-BWX76_00750-[Candidatus-Cloacimonetes-bacterium-ADurb.Bin089]
MCB5271576.1	Type1.BREX	[illegible]	1171	Bacteria>FCB_group>Candidatus_Cloacimonetes>unclassified_Candidatus_Cloacimonetes	GCA_020532545.1	Candidatus_Cloacimonetes_bacterium	LHW56_07005	18816..22331	2030808	BREX-system-P-loop-protein-BrxC-[Candidatus-Cloacimonadota-bacterium]
NQV48959.1	Type6.BREX	[illegible]	1210	Bacteria>FCB_group>Candidatus_Marinimicrobia	GCA_013202385.1	Candidatus_Marinimicrobia_bacterium	HQ507_00570	3171..6803	2026760	BREX-system-P-loop-protein-BrxC-[Candidatus-Marinimicrobia-bacterium]
MBT4361892.1	Type1.BREX	[illegible]	1148	Bacteria>FCB_group>Candidatus_Marinimicrobia	GCA_018653465.1	Candidatus_Marinimicrobia_bacterium	HOD11_13085	1..3448	2026760	BREX-system-P-loop-protein-BrxC-[Candidatus-Marinimicrobia-bacterium]
NLW30564.1	Type1.BREX	[illegible]	1185	Bacteria>FCB_group>Fibrobacterota>Fibrobacteria>Fibrobacterales>Fibrobacteraceae	GCA_012728995.1	Fibrobacter_sp.	GXY77_03805	4326..7883	35828	BREX-system-P-loop-protein-BrxC-[Fibrobacter-sp.]
WP_073424902.1	Type1.BREX	[illegible]	1212	Bacteria>FCB_group>Fibrobacterota>Fibrobacteria>Fibrobacterales>Fibrobacteraceae	GCF_900142945.1	Fibrobacter_sp._UWB7	BUC30_RS10995	309469..313107	1896206	BREX-system-P-loop-protein-BrxC-[Fibrobacter-sp.-UWB7]
WP_240631539.1	Type6.BREX	[illegible]	1234	Bacteria	GCF_003671915.1	Pseudomonadota	D3M96_RS03425	754727..758431	1224	MULTISPECIES:-BREX-system-P-loop-protein-BrxC-[Pseudomonadota]
WP_203328719.1	Type6.BREX	[illegible]	1213	Bacteria	GCF_016836415.1	Pseudomonadota	JQ482_RS25705	5512214..5515855	1224	MULTISPECIES:-BREX-system-P-loop-protein-BrxC-[Pseudomonadota]
WP_240685338.1	Type6.BREX	[illegible]	1213	Bacteria	GCF_025461165.1	Pseudomonadota	JAK52_RS02505	287897..291538	1224	MULTISPECIES:-BREX-system-P-loop-protein-BrxC-[Pseudomonadota]
WP_172437661.1	Type1.BREX	[illegible]	1203	Bacteria>Pseudomonadota>Acidithiobacillia>Acidithiobacillales>Acidithiobacillaceae>Acidithiobacillus	-	Acidithiobacillus_sp._SH	HF670_09010	8231..11842	187490	MULTISPECIES:-BREX-system-P-loop-protein-BrxC-[Acidithiobacillus]
MBU2839699.1	Type1.BREX	[illegible]	1203	Bacteria>Pseudomonadota>Acidithiobacillia>Acidithiobacillales>Acidithiobacillaceae>Acidithiobacillus	GCA_018854655.1	Acidithiobacillus_thiooxidans	HF670_09010	8231..11842	930	BREX-system-P-loop-protein-BrxC-[Acidithiobacillus-thiooxidans]
WP_014029717.1	Type1.BREX	[illegible]	1179	Bacteria>Pseudomonadota>Acidithiobacillia>Acidithiobacillales>Acidithiobacillaceae>Acidithiobacillus	GCF_000214095.2	Acidithiobacillus_ferrivorans	ACIFE_RS11315	2362677..2366216	160808	BREX-system-P-loop-protein-BrxC-[Acidithiobacillus-ferrivorans]
WP_064218004.1	Type1.BREX	[illegible]	1187	Bacteria>Pseudomonadota>Acidithiobacillia>Acidithiobacillales>Acidithiobacillaceae>Acidithiobacillus	GCF_001652185.1	Acidithiobacillus_ferrooxidans	A4H96_RS01790	31399..34962	920	BREX-system-P-loop-protein-BrxC-[Acidithiobacillus-ferrooxidans]
WP_163056479.1	Type1.BREX	[illegible]	1203	Bacteria>Pseudomonadota>Acidithiobacillia>Acidithiobacillales>Acidithiobacillaceae>Acidithiobacillus	GCF_010577825.1	Acidithiobacillus_ferrooxidans	X857_RS08730	38026..41637	920	BREX-system-P-loop-protein-BrxC-[Acidithiobacillus-ferrooxidans]
WP_271780036.1	Type1.BREX	[illegible]	1179	Bacteria>Pseudomonadota>Acidithiobacillia>Acidithiobacillales>Acidithiobacillaceae>Acidithiobacillus	GCF_028215975.1	Acidithiobacillus_ferriphilus	PJU76_RS00595	119550..123089	1689834	BREX-system-P-loop-protein-BrxC-[Acidithiobacillus-ferriphilus]
WP_101538034.1	Type1.BREX	[illegible]	1204	Bacteria>Pseudomonadota>Acidithiobacillia>Acidithiobacillales>Acidithiobacillaceae	GCF_018854655.1	Acidithiobacillus	HF670_RS09000	8228..11842	119977	MULTISPECIES:-BREX-system-P-loop-protein-BrxC-[Acidithiobacillus]
WP_023449856.1	Type1.BREX	[illegible]	1177	Bacteria>Pseudomonadota>Alphaproteobacteria>Caulobacterales>Caulobacteraceae>Asticcacaulis	GCF_000495835.1	Asticcacaulis_sp._AC402	ABAC402_RS31845	123615..127148	1282361	BREX-system-P-loop-protein-BrxC-[Asticcacaulis-sp.-AC402]
WP_114817812.1	Type1.BREX	[illegible]	1179	Bacteria>Pseudomonadota>Alphaproteobacteria>Caulobacterales>Caulobacteraceae>Brevundimonas	GCF_003350205.1	Brevundimonas_bullata	DXK21_RS10945	61535..65074	13160	BREX-system-P-loop-protein-BrxC-[Brevundimonas-bullata]
MAS04597.1	Type1.BREX	[illegible]	1153	Bacteria>Pseudomonadota>Alphaproteobacteria>Hyphomicrobiales>Ahrensiaceae>Ahrensia	GCA_002698425.1	Ahrensia_sp.	CL534_07875	15621..19082	1872419	ATP-binding-protein-[Ahrensia-sp.]
WP_061974428.1	Type1.BREX	[illegible]	1181	Bacteria>Pseudomonadota>Alphaproteobacteria>Hyphomicrobiales>Aurantimonadaceae>Aureimonas	GCF_001463725.1	Aureimonas_sp._AU20	AUL37_RS03985	274964..278509	1349819	BREX-system-P-loop-protein-BrxC-[Aureimonas-sp.-AU20]
WP_268883456.1	Type1.BREX	[illegible]	617	Bacteria>Pseudomonadota>Alphaproteobacteria>Hyphomicrobiales>Aurantimonadaceae>Jiella	GCF_026898155.1	Jiella_sp._HL-NP1	OH818_RS13650	2639917..2641770	2986949	BREX-system-P-loop-protein-BrxC-[Jiella-sp.-HL-NP1]

Protein-ID	System	Gene-Neighbourhoods	Length	Taxonomy	Assembly-ID	Strain	Locus-Tag	Genomic-coordinates	TaxID	Fasta-define
WP_230722685.1	Type1.BREX	<-ArdCN+MPTase<-DEDD_Tnp_IS110+Transposase_20<-Small-B-barrel+SF1-SF2-Helicase+RdgC+REase<-SF1-SF2-Helicase+REase<-35Exo+REase<-?|| wHTH+WYL+WCX -> BrxA-DNA-binding -> BrxB-BrxF-iSTAND -> DUF6079 *->OLD-ABC->NERD-> N6-METHYLASE -> PglZ -> SIGMA+OB-Fold+AAA-ATPase+ HKD-DNase -BrxL -><-HHH_4+HTH+wHTH+SF1-SF2-Helicase+REase	1177	Bacteria>Pseudomonadota>Alphaproteobacteria>Hyphomicrobiales>Aurantimonadaceae>Martelella	GCF_021021415.1	Martelella_mediterranea	K7H91_RS23870	16033..19566	293089	BREX-system-P-loop-protein-BrxC-[Martelella-mediterranea]
WP_094540671.1	Type1.BREX	HTH_3->Recombinase+ZnR-><-SF1-SF2-Helicase<- HKD-DNase + HKD-DNase <-wHTH<-SF1-SF2-Helicase+REase<- SIGMA+OB-Fold+AAA-ATPase+ HKD-DNase -BrxL <- PglZ <- N6-METHYLASE <- DUF6079 *<- BrxB-BrxF-iSTAND <- BrxA-DNA-binding <- wHTH+WYL+WCX ||Calcineurin+Calcineurin-SbcD->SbcC+SbcC-><-MODE-HTH+PBPII||TetR-HTH-><-RHH<-VirB10	1179	Bacteria>Pseudomonadota>Alphaproteobacteria>Hyphomicrobiales>Brucellaceae>Brucella/Ochrobactrum_group	GCF_002252505.1	Brucella_grignonensis	CEV33_RS11715	211751..215290	94627	BREX-system-P-loop-protein-BrxC-[Brucella-grignonensis]
WP_113731560.1	Type1.BREX	VirB8->VirB9->VirB10->RHH-><-TetR-HTH||MODE-HTH+PBPII-> wHTH+WYL+WCX -> BrxA-DNA-binding -> BrxB-BrxF-iSTAND -> DUF6079 *-> N6-METHYLASE -> PglZ -> SIGMA+OB-Fold+AAA-ATPase+ HKD-DNase -BrxL ->SF1-SF2-Helicase+REase->SF1-SF2-Helicase+REase->wHTH-> HKD-DNase + HKD-DNase ->SF1-SF2-Helicase->	1179	Bacteria>Pseudomonadota>Alphaproteobacteria>Hyphomicrobiales>Brucellaceae>Brucella/Ochrobactrum_group	GCF_003261015.1	Brucella_anthropi	F9L04_RS18285	14553..18092	529	BREX-system-P-loop-protein-BrxC-[Brucella-anthropi]
WP_151664132.1	Type1.BREX	ParA-Soj-PloopNTPase->ParB+RepB->ZDNA-HTH+HTH->CHTH-><-SbcC+CCDC158<-Calcineurin-SbcD|| wHTH+WYL+WCX -> BrxA-DNA-binding -> BrxB-BrxF-iSTAND -> DUF6079 *-> N6-METHYLASE -> PglZ -> SIGMA+OB-Fold+AAA-ATPase+ HKD-DNase -BrxL ->HTH_3-><-ParB+HNH<-HEPN<-SF1-SF2-Helicase+REase<-SF1-SF2-Helicase+REase<-REase	1179	Bacteria>Pseudomonadota>Alphaproteobacteria>Hyphomicrobiales>Brucellaceae>Brucella/Ochrobactrum_group	GCF_008932705.1	Brucella_anthropi	F9L04_RS20265	51555..55094	529	BREX-system-P-loop-protein-BrxC-[Brucella-anthropi]
WP_151665645.1	Type1.BREX	ParB+RepB->ZDNA-HTH+HTH->?->?->LuxR-HTH-><-?<- SIGMA+OB-Fold+AAA-ATPase+ HKD-DNase -BrxL <- PglZ <- N6-METHYLASE <- DUF6079 *<- BrxB-BrxF-iSTAND <- BrxA-DNA-binding <- wHTH+WYL+WCX ||Calcineurin-SbcD->SbcC+CCDC158->	1180	Bacteria>Pseudomonadota>Alphaproteobacteria>Hyphomicrobiales>Brucellaceae>Brucella/Ochrobactrum_group	GCF_008932825.1	Brucella_anthropi	F9K96_RS24475	19243..22785	529	BREX-system-P-loop-protein-BrxC-[Brucella-anthropi]
WP_204157651.1	Type6.BREX	<-TUDOR+SF1-SF2-Helicase+iREase+CTD<- SIGMA+OB-Fold+AAA-ATPase+ HKD-DNase -BrxL <- PglZ <- N6-METHYLASE -p<-AAA-ATPase<-rve<- N6-METHYLASE -p<- N6-METHYLASE <- DUF6079 *<- BrxB-BrxF-iSTAND <- BrxE <-PhdYeFM_antitox<-TraI-VirD2-RCRE<-TraJ-RHH||?-><-DOC+HTH	1208	Bacteria>Pseudomonadota>Alphaproteobacteria>Hyphomicrobiales>Brucellaceae>Brucella/Ochrobactrum_group	GCF_016887905.1	Brucella_anthropi	JQC79_RS05930	1236117..1239743	529	BREX-system-P-loop-protein-BrxC-[Brucella-anthropi]
WP_109366634.1	Type1.BREX	PIN+OST-HTH->DOC+HTH->?-><-SbcC<-SbcC+CCDC158<-Calcineurin-SbcD|| wHTH+WYL+WCX -> BrxA-DNA-binding -> BrxB-BrxF-iSTAND -> DUF6079 *-> N6-METHYLASE -> PglZ -> SIGMA+OB-Fold+AAA-ATPase+ HKD-DNase -BrxL -><-HIN-HTH<-SF1-SF2-Helicase+REase<-SF1-SF2-Helicase+REase<-REase	1179	Bacteria>Pseudomonadota>Alphaproteobacteria>Hyphomicrobiales>Brucellaceae>Brucella/Ochrobactrum_group	GCF_900243435.1	[Ochrobactrum]_soli	OHAE_RS05910	16979..20518	2448455	BREX-system-P-loop-protein-BrxC-[[Ochrobactrum]
WP_184220677.1	Type1.BREX	<-ParB||?->?->?-><-?<- SIGMA+OB-Fold+AAA-ATPase+ HKD-DNase -BrxL <- PglZ <- N6-METHYLASE <- DUF6079 *<- BrxB-BrxF-iSTAND <- BrxA-DNA-binding ||?->?->NTP_transferase+MannoseP_isomer->?-><-?||?->SAM-METHYLASE->	1179	Bacteria>Pseudomonadota>Alphaproteobacteria>Hyphomicrobiales>Brucellaceae>Paenochrobactrum	GCF_014205685.1	Paenochrobactrum_gallinarii	FHS77_RS04490	13414..16953	643673	BREX-system-P-loop-protein-BrxC-[Paenochrobactrum-gallinarii]
WP_075657534.1	Type1.BREX	VirB8->VirB9->VirB10->RHH-><-TetR-HTH||MODE-HTH+PBPII-> wHTH+WYL+WCX -> BrxA-DNA-binding -> BrxB-BrxF-iSTAND -> DUF6079 *-> N6-METHYLASE -> PglZ -> SIGMA+OB-Fold+AAA-ATPase+ HKD-DNase -BrxL ->SF1-SF2-Helicase+REase->SF1-SF2-Helicase+REase->wHTH-> HKD-DNase + HKD-DNase ->SF1-SF2-Helicase->	1179	Bacteria>Pseudomonadota>Alphaproteobacteria>Hyphomicrobiales>Brucellaceae>Pseudochrobactrum	GCF_001939785.1	Pseudochrobactrum_sp._B5	BKD14_RS17970	55973..59512	1289478	BREX-system-P-loop-protein-BrxC-[Pseudochrobactrum-sp.-B5]
WP_113944570.1	Type1.BREX	VirB8->VirB9->VirB10->RHH-><-TetR-HTH||MODE-HTH+PBPII-> wHTH+WYL+WCX -> BrxA-DNA-binding -> BrxB-BrxF-iSTAND -> DUF6079 *-> N6-METHYLASE -> PglZ -> SIGMA+OB-Fold+AAA-ATPase+ HKD-DNase -BrxL ->SF1-SF2-Helicase+REase->SF1-SF2-Helicase+REase->wHTH-> HKD-DNase + HKD-DNase ->SF1-SF2-Helicase->	1179	Bacteria>Pseudomonadota>Alphaproteobacteria>Hyphomicrobiales>Brucellaceae>Pseudochrobactrum	GCF_003314995.1	Pseudochrobactrum_asaccharolyticum	DFR47_RS11470	41745..45284	354351	BREX-system-P-loop-protein-BrxC-[Pseudochrobactrum-asaccharolyticum]
MBX3547065.1	Type1.BREX	<-HIN-HTH<-REase<-SF1-SF2-Helicase<-OLD-ABC+TOPRIM<-AbiEii<-AbiEi_4<- SIGMA+OB-Fold+AAA-ATPase+ HKD-DNase -BrxL <- PglZ <- N6-METHYLASE <- DUF6079 -p*	777	Bacteria>Pseudomonadota>Alphaproteobacteria>Hyphomicrobiales>Chelatococcaceae>Chelatococcus	GCA_019635905.1	Chelatococcus_sp.	KF818_30290	46824..49157	1953771	BREX-system-P-loop-protein-BrxC-[Chelatococcus-sp.]
WP_046141798.1	Type6.BREX	<-XIS-HTH<-?||RAMA->GDResolvase+ZnR->ZnR->?-> BrxE -> BrxA-DNA-binding -> BrxB-BrxF-iSTAND -> DUF6079 *-> N6-METHYLASE -> PglZ -> SIGMA+OB-Fold+AAA-ATPase+ HKD-DNase -BrxL -><-tRNA<-ADH_N+ADH_zinc_N	1208	Bacteria>Pseudomonadota>Alphaproteobacteria>Hyphomicrobiales>Devosiaceae>Devosia	-	Devosia_soli	VW35_RS04250	28883..32509	361041	BREX-system-P-loop-protein-BrxC-[Devosia-soli]
MBL8596415.1	Type1.BREX	ANK+ANK-> wHTH+WYL+WCX -> BrxA-DNA-binding -> BrxB-BrxF-iSTAND -> DUF6079 *-> N6-METHYLASE -> PglZ -> SIGMA+OB-Fold+AAA-ATPase+ HKD-DNase -BrxL ->?-><-?<-?||HTH_Tnp_1->?->MPTase->	1166	Bacteria>Pseudomonadota>Alphaproteobacteria>Hyphomicrobiales>Devosiaceae>Devosia	GCA_016793625.1	Devosia_sp.	JNL14_01620	65385..68885	1871048	BREX-system-P-loop-protein-BrxC-[Devosia-sp.]
WP_240230050.1	Type1.BREX	<-rve<-REase<-?<-Peptidase<-Classical-AAA||?-><- SIGMA+OB-Fold+AAA-ATPase+ HKD-DNase -BrxL <- PglZ <- N6-METHYLASE <- DUF6079 *<- BrxB-BrxF-iSTAND <- BrxA-DNA-binding <- wHTH+WYL+WCX <-ANK+ANK||HEPN-><-tRNA||ICLR-HTH->	1166	Bacteria>Pseudomonadota>Alphaproteobacteria>Hyphomicrobiales>Devosiaceae>Devosia	GCF_022429625.1	Devosia_sp._JXJ_CY_41	MF606_RS14450	2929921..2933421	2917991	BREX-system-P-loop-protein-BrxC-[Devosia-sp.-JXJ-CY-41]
WP_203065852.1	Type1.BREX	VirB10->RHH-><-TetR-HTH||MODE-HTH+PBPII-><-SbcC+SbcC<-Calcineurin+Calcineurin-SbcD|| wHTH+WYL+WCX -> BrxA-DNA-binding -> BrxB-BrxF-iSTAND -> DUF6079 *-> N6-METHYLASE -> PglZ -> SIGMA+OB-Fold+AAA-ATPase+ HKD-DNase -BrxL ->OLD-ABC+TOPRIM->SF1-SF2-Helicase-><-?<-Arm-DNA-bind_3+Phage_int_SAM_3+Phage_integrase	1179	Bacteria>Pseudomonadota>Alphaproteobacteria>Hyphomicrobiales>Devosiaceae>Paradevosia	GCF_016801025.1	Paradevosia_shaoguanensis	JNE37_RS07680	1630850..1634389	1335043	BREX-system-P-loop-protein-BrxC-[Paradevosia-shaoguanensis]
WP_266332120.1	Type1.BREX	<-SF1-SF2-Helicase<- HKD-DNase + HKD-DNase <-wHTH<-SF1-SF2-Helicase+REase<-SF1-SF2-Helicase+REase<- SIGMA+OB-Fold+AAA-ATPase+ HKD-DNase -BrxL <- PglZ <- N6-METHYLASE <- DUF6079 *<- BrxB-BrxF-iSTAND <- BrxA-DNA-binding <- wHTH+WYL+WCX <-MODE-HTH+PBPII||TetR-HTH-><-RHH<-VirB10<-VirB9<-VirB8	1179	Bacteria>Pseudomonadota>Alphaproteobacteria>Hyphomicrobiales>Kaistiaceae>Kaistia	GCF_026343955.1	Kaistia_defluvii	OSH10_RS12655	370045..373584	410841	BREX-system-P-loop-protein-BrxC-[Kaistia-defluvii]
KFG67132.1	Type6.BREX	<-AbiGi+seqA+RAMA|| BrxE -> BrxA-DNA-binding -> BrxB-BrxF-iSTAND -> DUF6079 *-> N6-METHYLASE -p-> PglZ -> SIGMA+OB-Fold+AAA-ATPase+ HKD-DNase -BrxL ->HNH->HNH->HNH->?-><-HEPN<-Polbeta	1210	Bacteria>Pseudomonadota>Alphaproteobacteria>Hyphomicrobiales>Methylobacteriaceae>Microvirga	GCA_000739015.1	Microvirga_sp._BSC39	JH26_23850	105316..108948	1549810	ATPase-[Microvirga-sp.-BSC39]
WP_036357316.1	Type6.BREX	<-AbiGi+seqA+RAMA<-?||PhdYeFM_antitox-> BrxE -> BrxA-DNA-binding -> BrxB-BrxF-iSTAND -> DUF6079 *-> N6-METHYLASE -> PglZ -> SIGMA+OB-Fold+AAA-ATPase+ HKD-DNase -BrxL ->HNH->HNH->HNH->?-><-HEPN<-Polbeta	1208	Bacteria>Pseudomonadota>Alphaproteobacteria>Hyphomicrobiales>Methylobacteriaceae>Microvirga	GCF_000739015.1	Microvirga_sp._BSC39	MA07_RS22775	105322..108948	1549810	BREX-system-P-loop-protein-BrxC-[Microvirga-sp.-BSC39]
WP_199050625.1	Type6.BREX	<-ParB+HNH<-ARG||?-><-?<-SF1-SF2-Helicase+DUF1998<-?|| BrxE -> BrxA-DNA-binding -> BrxB-BrxF-iSTAND -> DUF6079 *-> N6-METHYLASE -> N6-METHYLASE -p-> PglZ -> SIGMA+OB-Fold+AAA-ATPase+ HKD-DNase -BrxL -><-Phage_integrase	1206	Bacteria>Pseudomonadota>Alphaproteobacteria>Hyphomicrobiales>Methylobacteriaceae>Microvirga	GCF_016427565.1	Microvirga_splendida	JAO75_RS18515	48792..52412	2795727	BREX-system-P-loop-protein-BrxC-[Microvirga-splendida]
WP_202057660.1	Type1.BREX	Phage_integrase+Phage_integrase->REase->DnaK->?->?->?-> BrxA-DNA-binding -> BrxB-BrxF-iSTAND -> DUF6079 *-> N6-METHYLASE -> PglZ -> SIGMA+OB-Fold+AAA-ATPase+ HKD-DNase -BrxL ->?->?->?-><-?<-tRNA<-?<-CDP-OH_P_transf	1176	Bacteria>Pseudomonadota>Alphaproteobacteria>Hyphomicrobiales>Methylobacteriaceae>Microvirga	GCF_016743775.1	Microvirga_aerilata	JKG68_RS07685	69600..73130	670292	BREX-system-P-loop-protein-BrxC-[Microvirga-aerilata]
WP_036280608.1	Type1.BREX	HKD-DNase ->SF1-SF2-Helicase-><-Small-B-barrel+SF1-SF2-Helicase+RdgC+REase<-SF1-SF2-Helicase+REase<-35Exo+REase|| wHTH+WYL+WCX -> BrxA-DNA-binding -> BrxB-BrxF-iSTAND -> DUF6079 *->OLD-ABC->REase-> N6-METHYLASE -> PglZ -> SIGMA+OB-Fold+AAA-ATPase+ HKD-DNase -BrxL ->HIN-HTH->?->LZ_Tnp_IS66+zf-IS66+DDE_Tnp_IS66+DDE_Tnp_IS66_C->	1178	Bacteria>Pseudomonadota>Alphaproteobacteria>Hyphomicrobiales>Methylocystaceae>Methylocystis	GCF_000188155.2	Methylocystis_sp._ATCC_49242	MET49242_RS03150	256459..259995	622637	BREX-system-P-loop-protein-BrxC-[Methylocystis-sp.-ATCC-49242]
WP_253589372.1	Type6.BREX	<-HIRAN<-BirA-HTH<-SF1-SF2-Helicase+REase<-35Exo+REase<-?|| wHTH+WYL+WCX -> BrxE -> BrxA-DNA-binding -> BrxB-BrxF-iSTAND -> DUF6079 *-> N6-METHYLASE -> N6-METHYLASE -> PglZ -> SIGMA+OB-Fold+AAA-ATPase+ HKD-DNase -BrxL ->?-><-?||?->?->HNH->	1208	Bacteria>Pseudomonadota>Alphaproteobacteria>Hyphomicrobiales>Nitrobacteraceae>Bradyrhizobium	-	Bradyrhizobium_elkanii	J2R96_RS28580	6233220..6236846	29448	BREX-system-P-loop-protein-BrxC-[Bradyrhizobium-elkanii]
WP_212207485.1	Type6.BREX	<-PIWI<- SIGMA+OB-Fold+AAA-ATPase+ HKD-DNase -BrxL <- PglZ <-ParB+HNH<- N6-METHYLASE -p<- N6-METHYLASE <- DUF6079 *<- BrxB-BrxF-iSTAND <- BrxA-DNA-binding <- BrxE <- wHTH+WYL+WCX ||35Exo+REase->SF1-SF2-Helicase+REase->	1208	Bacteria>Pseudomonadota>Alphaproteobacteria>Hyphomicrobiales>Nitrobacteraceae>Bradyrhizobium	-	Bradyrhizobium_sp._AUGA_SZCCT0274	JQ620_09165	74241..77867	2807670	BREX-system-P-loop-protein-BrxC-[Bradyrhizobium-sp.-AUGA-SZCCT0274]
MBR1240293.1	Type6.BREX	<-PIWI<- SIGMA+OB-Fold+AAA-ATPase+ HKD-DNase -BrxL <- PglZ <-ParB+HNH<- N6-METHYLASE -p<- N6-METHYLASE <- DUF6079 *<- BrxB-BrxF-iSTAND <- BrxA-DNA-binding <- BrxE <- wHTH+WYL+WCX ||35Exo+REase->SF1-SF2-Helicase+REase->	1208	Bacteria>Pseudomonadota>Alphaproteobacteria>Hyphomicrobiales>Nitrobacteraceae>Bradyrhizobium	GCA_018130325.1	Bradyrhizobium_sp._AUGA_SZCCT0274	JQ620_09165	74241..77867	2807670	BREX-system-P-loop-protein-BrxC-[Bradyrhizobium-sp.-AUGA-SZCCT0274]
WP_130224749.1	Type1.BREX	<-ArdCN+MPTase<-HEPN<-Small-B-barrel+SF1-SF2-Helicase+RdgC+REase<-35Exo+REase<-?|| wHTH+WYL+WCX -> BrxA-DNA-binding -> BrxB-BrxF-iSTAND -> DUF6079 *-> N6-METHYLASE -> PglZ -> SIGMA+OB-Fold+AAA-ATPase+ HKD-DNase -BrxL ->AbiEi_4->AbiEii->OLD-ABC+TOPRIM->SF1-SF2-Helicase->REase-><-ABhydrolase	1181	Bacteria>Pseudomonadota>Alphaproteobacteria>Hyphomicrobiales>Nitrobacteraceae>Bradyrhizobium	GCF_004212675.1	Bradyrhizobium_sp._Leo121	CWO90_RS07090	48469..52014	1571195	BREX-system-P-loop-protein-BrxC-[Bradyrhizobium-sp.-Leo121]
WP_157343672.1	Type1.BREX	ABC-ATPase+SbcC->?->?-><-tRNA<-Small-B-barrel+SF1-SF2-Helicase+RdgC+REase<-SF1-SF2-Helicase+REase<-35Exo+REase|| wHTH+WYL+WCX -> BrxA-DNA-binding -> BrxB-BrxF-iSTAND -> DUF6079 *-> N6-METHYLASE -> PglZ -> SIGMA+OB-Fold+AAA-ATPase+ HKD-DNase -BrxL -><-?<-HTH+REase<-Arm-DNA-bind_3+Phage_integrase<-?||?-><-Peptidase_M24	1181	Bacteria>Pseudomonadota>Alphaproteobacteria>Hyphomicrobiales>Nitrobacteraceae>Bradyrhizobium	GCF_009759865.1	Bradyrhizobium_pachyrhizi	GPL21_13485	267907..271452	280333	BREX-system-P-loop-protein-BrxC-[Bradyrhizobium-pachyrhizi]
WP_249144787.1	Type6.BREX	<-PIWI<- SIGMA+OB-Fold+AAA-ATPase+ HKD-DNase -BrxL <- PglZ <-ParB+HNH<- N6-METHYLASE -p<- N6-METHYLASE <- DUF6079 *<- BrxB-BrxF-iSTAND <- BrxA-DNA-binding <- BrxE <- wHTH+WYL+WCX ||35Exo+REase->SF1-SF2-Helicase+REase->	1203	Bacteria>Pseudomonadota>Alphaproteobacteria>Hyphomicrobiales>Nitrobacteraceae>Bradyrhizobium	GCF_018130325.1	Bradyrhizobium_sp._AUGA_SZCCT0274	JQ620_RS09170	74241..77852	2807670	BREX-system-P-loop-protein-BrxC-[Bradyrhizobium-sp.-AUGA-SZCCT0274]
WP_212056797.1	Type1.BREX	<-SF1-SF2-Helicase<-MORC+DUF3684+REase<-HHH_4+SF1-SF2-Helicase+REase<-AbiEi_4<- SIGMA+OB-Fold+AAA-ATPase+ HKD-DNase -BrxL <- PglZ <- N6-METHYLASE <- DUF6079 *<- BrxB-BrxF-iSTAND <- BrxA-DNA-binding <- wHTH+WYL+WCX ||35Exo+REase->SF1-SF2-Helicase+REase->Small-B-barrel+SF1-SF2-Helicase+RdgC+REase-><-?||GDResolvase+Recombinase+ZnR->	1180	Bacteria>Pseudomonadota>Alphaproteobacteria>Hyphomicrobiales>Nitrobacteraceae>Bradyrhizobium	GCF_018131045.1	Bradyrhizobium_elkanii	JQ569_RS15725	68040..71582	29448	BREX-system-P-loop-protein-BrxC-[Bradyrhizobium-elkanii]
WP_213286691.1	Type6.BREX	Phage_integrase->MarR-HTH->AbiEii->?->?-> BrxE -> BrxA-DNA-binding -> BrxB-BrxF-iSTAND -> DUF6079 *-> N6-METHYLASE -> N6-METHYLASE ->ParB+HNH-> PglZ -> SIGMA+OB-Fold+AAA-ATPase+ HKD-DNase -BrxL -><-TPR<-?<-?|| wHTH+WYL+WCX ->	1208	Bacteria>Pseudomonadota>Alphaproteobacteria>Hyphomicrobiales>Nitrobacteraceae>Bradyrhizobium	GCF_018398875.1	Bradyrhizobium_sp._sGM-13	KJQ96_RS11300	145012..148638	2831781	BREX-system-P-loop-protein-BrxC-[Bradyrhizobium-sp.-sGM-13]
WP_225161486.1	Type1.BREX	<-ParB+HNH+Schlafen<-?<- SIGMA+OB-Fold+AAA-ATPase+ HKD-DNase -BrxL <- PglZ <- N6-METHYLASE <- DUF6079 *<- BrxB-BrxF-iSTAND <- BrxA-DNA-binding <- wHTH+WYL+WCX ||?->35Exo+REase->SF1-SF2-Helicase+REase->35exo+SF1-SF2-Helicase-><-Phage_integrase	1183	Bacteria>Pseudomonadota>Alphaproteobacteria>Hyphomicrobiales>Nitrobacteraceae>Bradyrhizobium	GCF_020194035.1	Bradyrhizobium_sp._BRP56	I6F38_19500	73459..77010	2793819	BREX-system-P-loop-protein-BrxC-[Bradyrhizobium-sp.-BRP56]
WP_247477718.1	Type6.BREX	BrxE -> BrxA-DNA-binding -> BrxB-BrxF-iSTAND -> DUF6079 *-> N6-METHYLASE -> PglZ -> SIGMA+OB-Fold+AAA-ATPase+ HKD-DNase -BrxL ->SbcC->HNH-><-?<-?||TPR+TPR+TPR+TPR->	1206	Bacteria>Pseudomonadota>Alphaproteobacteria>Hyphomicrobiales>Nitrobacteraceae>Bradyrhizobium	GCF_023072855.1	Bradyrhizobium_sp._CW9	IVB57_RS09580	204980..208600	2782689	BREX-system-P-loop-protein-BrxC-[Bradyrhizobium-sp.-CW9]
WP_259215308.1	Type6.BREX	DSBH->?->tRNA-> BrxE -> BrxA-DNA-binding -> BrxB-BrxF-iSTAND -> DUF6079 *-> N6-METHYLASE -> PglZ -> SIGMA+OB-Fold+AAA-ATPase+ HKD-DNase -BrxL ->SbcC->HNH-><-?<-?<-?<-SIGMA-HTH+HIN-HTH	1207	Bacteria>Pseudomonadota>Alphaproteobacteria>Hyphomicrobiales>Nitrobacteraceae>Bradyrhizobium	GCF_024809395.1	Bradyrhizobium_centrosematis	GGE24_RS17830	223880..227503	1300039	BREX-system-P-loop-protein-BrxC-[Bradyrhizobium-centrosematis]
WP_260373497.1	Type1.BREX	<-ParB+HNH+Schlafen<-?<- SIGMA+OB-Fold+AAA-ATPase+ HKD-DNase -BrxL <- PglZ <- N6-METHYLASE <- DUF6079 *<- BrxB-BrxF-iSTAND <- BrxA-DNA-binding <- wHTH+WYL+WCX ||?->35Exo+REase->SF1-SF2-Helicase+REase->35exo+SF1-SF2-Helicase-><-Phage_integrase	1183	Bacteria>Pseudomonadota>Alphaproteobacteria>Hyphomicrobiales>Nitrobacteraceae>Bradyrhizobium	GCF_025200925.1	Bradyrhizobium_sp._CB1015	N2604_RS01610	347161..350712	2976822	BREX-system-P-loop-protein-BrxC-[Bradyrhizobium-sp.-CB1015]
WP_110780661.1	Type1.BREX	<-DOC+HTH<-SF1-SF2-Helicase<- HKD-DNase + HKD-DNase <-wHTH<-SF1-SF2-Helicase+REase<-SF1-SF2-Helicase+REase<- SIGMA+OB-Fold+AAA-ATPase+ HKD-DNase -BrxL <- PglZ <- N6-METHYLASE <- DUF6079 *<- BrxB-BrxF-iSTAND <- BrxA-DNA-binding <- wHTH+WYL+WCX <-MODE-HTH+PBPII||TetR-HTH-><-RHH<-VirB10<-VirB9<-VirB8	1179	Bacteria>Pseudomonadota>Alphaproteobacteria>Hyphomicrobiales>Nitrobacteraceae>Rhodopseudomonas	GCF_003217325.1	Rhodopseudomonas_faecalis	BJ122_RS08545	91514..95053	99655	BREX-system-P-loop-protein-BrxC-[Rhodopseudomonas-faecalis]
MAM94519.1	Type1.BREX	VirB10->RHH-><-TetR-HTH||MODE-HTH+PBPII-><-SbcC+SbcC<-Calcineurin+Calcineurin-SbcD|| wHTH+WYL+WCX -> BrxA-DNA-binding -> BrxB-BrxF-iSTAND -> DUF6079 *-> N6-METHYLASE -> PglZ -> SIGMA+OB-Fold+AAA-ATPase+ HKD-DNase -BrxL ->OLD-ABC+TOPRIM->SF1-SF2-Helicase-><-Arm-DNA-bind_3+Phage_int_SAM_3+Phage_integrase<-?<-?<-MerR-HTH	1179	Bacteria>Pseudomonadota>Alphaproteobacteria>Hyphomicrobiales>Parvibaculaceae>Parvibaculum	GCA_002694985.1	Parvibaculum_sp.	CMI61_07805	145242..148781	2024848	hypothetical-protein-[Parvibaculum-sp.]
WP_110030326.1	Type1.BREX	RAMPs->RAMPs->?->RVT->CRISPR_Cas6->?-><-?|| BrxA-DNA-binding -> BrxB-BrxF-iSTAND -> DUF6079 *-> N6-METHYLASE -> PglZ -> SIGMA+OB-Fold+AAA-ATPase+ HKD-DNase -BrxL ->?-> HKD-DNase + HKD-DNase ->SF1-SF2-Helicase->MORC+DUF3684+REase->REase+SF1-SF2-Helicase->	1173	Bacteria>Pseudomonadota>Alphaproteobacteria>Hyphomicrobiales>Phyllobacteriaceae>Hoeflea	GCF_003182275.1	Hoeflea_marina	DFR52_RS02490	542434..545955	274592	BREX-system-P-loop-protein-BrxC-[Hoeflea-marina]
ESX24129.1	Type1.BREX	<- SIGMA+OB-Fold+AAA-ATPase+ HKD-DNase -BrxL <- PglZ <- N6-METHYLASE <- DUF6079 *<- BrxB-BrxF-iSTAND <- BrxA-DNA-binding <-?||REase->HIN-HTH->?->LZ_Tnp_IS66+zf-IS66+DDE_Tnp_IS66+DDE_Tnp_IS66_C->	1186	Bacteria>Pseudomonadota>Alphaproteobacteria>Hyphomicrobiales>Phyllobacteriaceae>Mesorhizobium	GCA_000502755.1	Mesorhizobium_sp._LSJC264A00	X767_13205	150936..154496	1287321	hypothetical-protein-X767_13205-[Mesorhizobium-sp.-LSJC264A00]
ESX84801.1	Type1.BREX	PIWI->HIN-HTH->HTH_21+rve+rve_3->Y1_Tnp-><- SIGMA+OB-Fold+AAA-ATPase+ HKD-DNase -BrxL <- PglZ <- N6-METHYLASE <-NERD<-OLD-ABC<- DUF6079 *<- BrxB-BrxF-iSTAND <- BrxA-DNA-binding ||?->?-><-HEPN<-HTH_37	1177	Bacteria>Pseudomonadota>Alphaproteobacteria>Hyphomicrobiales>Phyllobacteriaceae>Mesorhizobium	GCA_000503035.1	Mesorhizobium_sp._LSHC412B00	X756_23895	63877..67410	1287285	hypothetical-protein-X756_23895-[Mesorhizobium-sp.-LSHC412B00]
RWC29997.1	Type1.BREX	SbcC+CCDC158->?->REase+PNTD1+PNTD2->PIWI-><- SIGMA+OB-Fold+AAA-ATPase+ HKD-DNase -BrxL <- PglZ <-HNH<-OLD-ABC<- N6-METHYLASE <- DUF6079 *<- BrxB-BrxF-iSTAND <- BrxA-DNA-binding ||REase-><-?||?-><-?||REase->?->rve->	1181	Bacteria>Pseudomonadota>Alphaproteobacteria>Hyphomicrobiales>Phyllobacteriaceae>Mesorhizobium	GCA_004017395.1	Mesorhizobium_sp.	EOS27_14215	67406..70951	1871066	BREX-system-P-loop-protein-BrxC-[Mesorhizobium-sp.]
RWM07956.1	Type1.BREX	CHTH->Phage_integrase-><-?<-TnsC-ATPase<-rve+Mu-transpos_C<-?||?-> BrxA-DNA-binding -> BrxB-BrxF-iSTAND -> DUF6079 *-> N6-METHYLASE -> PglZ -> SIGMA+OB-Fold+AAA-ATPase+ HKD-DNase -BrxL ->GreA_GreB+SF1-SF2-Helicase-><- wHTH+WYL+WCX ||?->?->?-><-HEPN	1184	Bacteria>Pseudomonadota>Alphaproteobacteria>Hyphomicrobiales>Phyllobacteriaceae>Mesorhizobium	GCA_004020545.1	Mesorhizobium_sp.	EOR71_14460	11856..15410	1871066	BREX-system-P-loop-protein-BrxC-[Mesorhizobium-sp.]
RWD20271.1	Type1.BREX	AbiEi_4-><-DEDD_Tnp_IS110+Transposase_20<- SIGMA+OB-Fold+AAA-ATPase+ HKD-DNase -BrxL <- PglZ <- N6-METHYLASE <-NERD<-OLD-ABC<- DUF6079 *<- BrxB-BrxF-iSTAND <- BrxA-DNA-binding <- wHTH+WYL+WCX ||35Exo+REase->SF1-SF2-Helicase+REase->Small-B-barrel+SF1-SF2-Helicase+RdgC+REase->NUDIX-><-?||NACHT+SF1-SF2-Helicase+ HKD-DNase ->	1178	Bacteria>Pseudomonadota>Alphaproteobacteria>Hyphomicrobiales>Phyllobacteriaceae>Mesorhizobium	GCA_004021465.1	Mesorhizobium_sp.	EOS09_27860	18398..21934	1871066	BREX-system-P-loop-protein-BrxC-[Mesorhizobium-sp.]
TIL20465.1	Type6.BREX	BrxE -> BrxA-DNA-binding -> BrxB-BrxF-iSTAND -> DUF6079 *-> N6-METHYLASE -> N6-METHYLASE ->ParB+HNH-> PglZ -> SIGMA+OB-Fold+AAA-ATPase+ HKD-DNase -BrxL ->	1208	Bacteria>Pseudomonadota>Alphaproteobacteria>Hyphomicrobiales>Phyllobacteriaceae>Mesorhizobium	GCA_004961195.1	Mesorhizobium_sp.	E5Y88_32415	5019..8645	1871066	BREX-system-P-loop-protein-BrxC-[Mesorhizobium-sp.]
TIV88924.1	Type1.BREX	<-35Exo+REase|| wHTH+WYL+WCX -> BrxA-DNA-binding -> BrxB-BrxF-iSTAND -> DUF6079 *->OLD-ABC->NERD-> N6-METHYLASE -> PglZ -> SIGMA+OB-Fold+AAA-ATPase+ HKD-DNase -BrxL -><-HEPN<-RVT<-AbiEi_4	1179	Bacteria>Pseudomonadota>Alphaproteobacteria>Hyphomicrobiales>Phyllobacteriaceae>Mesorhizobium	GCA_004963965.1	Mesorhizobium_sp.	E5V78_03705	4955..8494	1871066	BREX-system-P-loop-protein-BrxC-[Mesorhizobium-sp.]
WP_156951459.1	Type1.BREX	<- SIGMA+OB-Fold+AAA-ATPase+ HKD-DNase -BrxL <- PglZ <- N6-METHYLASE <- DUF6079 *<- BrxB-BrxF-iSTAND <- BrxA-DNA-binding <-?||REase->HIN-HTH->?->LZ_Tnp_IS66+zf-IS66+DDE_Tnp_IS66+DDE_Tnp_IS66_C->	1188	Bacteria>Pseudomonadota>Alphaproteobacteria>Hyphomicrobiales>Phyllobacteriaceae>Mesorhizobium	GCF_000502755.1	Mesorhizobium_sp._LSJC264A00	X768_RS154485	150936..154502	1287321	BREX-system-P-loop-protein-BrxC-[Mesorhizobium-sp.-LSJC264A00]
WP_156933469.1	Type1.BREX	REase+PNTD1+PNTD2->PIWI->Y1_Tnp-><- SIGMA+OB-Fold+AAA-ATPase+ HKD-DNase -BrxL <- PglZ <- N6-METHYLASE <-NERD<-OLD-ABC<- DUF6079 *<- BrxB-BrxF-iSTAND <- BrxA-DNA-binding ||?->RVT-><-HEPN<-HTH_37	1179	Bacteria>Pseudomonadota>Alphaproteobacteria>Hyphomicrobiales>Phyllobacteriaceae>Mesorhizobium	GCF_000503035.1	Mesorhizobium_sp._LSHC412B00	X757_RS142950	63877..67416	1287285	BREX-system-P-loop-protein-BrxC-[Mesorhizobium-sp.-LSHC412B00]
WP_128286158.1	Type1.BREX	ParB+HNH-><- SIGMA+OB-Fold+AAA-ATPase+ HKD-DNase -BrxL <- PglZ <-HNH<-OLD-ABC<- N6-METHYLASE <- DUF6079 *<- BrxB-BrxF-iSTAND <- BrxA-DNA-binding <-RNaseH	1181	Bacteria>Pseudomonadota>Alphaproteobacteria>Hyphomicrobiales>Phyllobacteriaceae>Mesorhizobium	GCF_004023115.1	Mesorhizobium_sp._M6A.T.Cr.TU.017.01.1.1	EOB36_RS28010	14606..18151	2496774	BREX-system-P-loop-protein-BrxC-[Mesorhizobium-sp.-M6A.T.Cr.TU.017.01.1.1]
WP_136091391.1	Type1.BREX	REase+PNTD1+PNTD2->PIWI->Y1_Tnp-><- SIGMA+OB-Fold+AAA-ATPase+ HKD-DNase -BrxL <- PglZ <-HNH<-OLD-ABC<- N6-METHYLASE <- DUF6079 *<- BrxB-BrxF-iSTAND <- BrxA-DNA-binding ||REase->?-><-?||REase->rve->TnsC-ATPase->TnsC-ATPase->	1181	Bacteria>Pseudomonadota>Alphaproteobacteria>Hyphomicrobiales>Phyllobacteriaceae>Mesorhizobium	GCF_004794715.1	Mesorhizobium_sp._M8A.F.Ca.ET.207.01.1.1	EN850_RS12645	56072..59617	2563968	BREX-system-P-loop-protein-BrxC-[Mesorhizobium-sp.-M8A.F.Ca.ET.207.01.1.1]
WP_140781888.1	Type6.BREX	<-D5ATPase<-XIS-HTH||MerR-HTH->?-> HKD-DNase +HKD-Rease->?-> BrxE -> BrxA-DNA-binding -> BrxB-BrxF-iSTAND -> DUF6079 *-> N6-METHYLASE -> PglZ -> SIGMA+OB-Fold+AAA-ATPase+ HKD-DNase -BrxL -><-?<-?<-CHTH	1211	Bacteria>Pseudomonadota>Alphaproteobacteria>Hyphomicrobiales>Phyllobacteriaceae>Mesorhizobium	GCF_006442585.1	Mesorhizobium_sp._B2-4-8	FJ947_RS26560	105800..109435	2589941	BREX-system-P-loop-protein-BrxC-[Mesorhizobium-sp.-B2-4-8]
WP_199202187.1	Type1.BREX	DOC+HTH-><-?||?-><-Small-B-barrel+SF1-SF2-Helicase+RdgC+REase<-SF1-SF2-Helicase+REase<-35Exo+REase|| wHTH+WYL+WCX -> BrxA-DNA-binding -> BrxB-BrxF-iSTAND -> DUF6079 *-> N6-METHYLASE -> PglZ -> SIGMA+OB-Fold+AAA-ATPase+ HKD-DNase -BrxL ->REase+PNTD1+PNTD2->PIWI-><-?<-?||RADICAL-SAM->	1185	Bacteria>Pseudomonadota>Alphaproteobacteria>Hyphomicrobiales>Phyllobacteriaceae>Mesorhizobium	GCF_016756495.1	Mesorhizobium_sp._113-3-3	MesoLj113b_71250	318356..321913	2744516	BREX-system-P-loop-protein-BrxC-[Mesorhizobium-sp.-113-3-3]
WP_092958042.1	Type1.BREX	<-Aminotran_1_2<-?||?->?-><-tRNA||Arm-DNA-bind_3+Phage_int_SAM_3+Phage_integrase-><-SF1-SF2-Helicase<- SIGMA+OB-Fold+AAA-ATPase+ HKD-DNase -BrxL <- PglZ <- N6-METHYLASE <- DUF6079 *<- BrxB-BrxF-iSTAND <- BrxA-DNA-binding <- wHTH+WYL+WCX <-MODE-HTH+PBPII||TetR-HTH-><-RHH<-VirB10<-VirB9<-VirB8	1179	Bacteria>Pseudomonadota>Alphaproteobacteria>Hyphomicrobiales>Phyllobacteriaceae>Mesorhizobium	GCF_020164785.1	Mesorhizobium_sp._ES1-1	LB513_RS22695	51971..55510	2876629	BREX-system-P-loop-protein-BrxC-[Mesorhizobium-sp.-ES1-1]
WP_224719979.1	Type6.BREX	<-Ntox49<-REase+HTH+35exo+Small-B-barrel+SF1-SF2-Helicase||wHTH+WYL-> wHTH+WYL+WCX ->?->GreA_GreB+SF1-SF2-Helicase-p-> BrxE -> BrxA-DNA-binding -> BrxB-BrxF-iSTAND -> DUF6079 *-> N6-METHYLASE -> N6-METHYLASE -> PglZ -> SIGMA+OB-Fold+AAA-ATPase+ HKD-DNase -BrxL ->	1208	Bacteria>Pseudomonadota>Alphaproteobacteria>Hyphomicrobiales>Phyllobacteriaceae>Mesorhizobium	GCF_020164885.1	Mesorhizobium_sp._CO1-1-7	LB516_RS16265	26347..29973	2876632	BREX-system-P-loop-protein-BrxC-[Mesorhizobium-sp.-CO1-1-7]
WP_256745318.1	Type1.BREX	<-D5ATPase<-XIS-HTH||MerR-HTH->?->?-> HKD-DNase +HKD-Rease->?-> BrxA-DNA-binding -> BrxB-BrxF-iSTAND -> DUF6079 *-> N6-METHYLASE -> N6-METHYLASE -> PglZ -> SIGMA+OB-Fold+AAA-ATPase+ HKD-DNase -BrxL -><-?<-?<-CHTH	1211	Bacteria>Pseudomonadota>Alphaproteobacteria>Hyphomicrobiales>Phyllobacteriaceae>Mesorhizobium	GCF_024518695.1	Mesorhizobium_sp._LMG17149	NP945_RS00755	156415..160050	2968497	BREX-system-P-loop-protein-BrxC-[Mesorhizobium-sp.-LMG17149]
WP_009450523.1	Type1.BREX	ACET-><-SF1-SF2-Helicase<- HKD-DNase + HKD-DNase <-wHTH<-SF1-SF2-Helicase+REase<-SF1-SF2-Helicase+REase<- SIGMA+OB-Fold+AAA-ATPase+ HKD-DNase -BrxL <- PglZ <- N6-METHYLASE <- DUF6079 *<- BrxB-BrxF-iSTAND <- BrxA-DNA-binding <- wHTH+WYL+WCX <-MODE-HTH+PBPII||TetR-HTH-><-RHH<-VirB10<-VirB9<-VirB8	1179	Bacteria>Pseudomonadota>Alphaproteobacteria>Hyphomicrobiales>Phyllobacteriaceae>Nitratireductor	GCF_000300515.1	Nitratireductor_indicus	NA8A_RS11845	137447..140986	721133	BREX-system-P-loop-protein-BrxC-[Nitratireductor-indicus]
WP_080921652.1	Type1.BREX	HEPN->HEPN->?-><-DEDD_Tnp_IS110+Transposase_20||<-?<-HEPN<- SIGMA+OB-Fold+AAA-ATPase+ HKD-DNase -BrxL <- PglZ <- N6-METHYLASE <- DUF6079 *<- BrxB-BrxF-iSTAND <- BrxA-DNA-binding <- wHTH+WYL+WCX ||Calcineurin-SbcD->SbcC+CCDC158->?->HTH_3->Peptidase+SF1-SF2-Helicase+REase->	1179	Bacteria>Pseudomonadota>Alphaproteobacteria>Hyphomicrobiales>Phyllobacteriaceae>Pseudaminobacter	GCF_002075885.1	Pseudaminobacter_manganicus	BFN67_RS22220	17195..20734	1873176	BREX-system-P-loop-protein-BrxC-[Pseudaminobacter-manganicus]
WP_150288342.1	Type1.BREX	Glyco_hydro_53+Phage-tail_3->?->?-><-URI-DNase+RAMA<- SIGMA+OB-Fold+AAA-ATPase+ HKD-DNase -BrxL <- PglZ <- N6-METHYLASE <- DUF6079 *<- BrxB-BrxF-iSTAND <- BrxA-DNA-binding <- wHTH+WYL+WCX <-XIS-HTH	1173	Bacteria>Pseudomonadota>Alphaproteobacteria>Hyphomicrobiales>Rhabdaerophilaceae>Rhabdaerophilum	GCF_008641065.1	Rhabdaerophilum_calidifontis	FWJ24_RS13435	109797..113318	2604328	BREX-system-P-loop-protein-BrxC-[Rhabdaerophilum-calidifontis]
WP_154722158.1	Type1.BREX	<-Classical-AAA<-?<-SMODS<-SF1-SF2-Helicase+REase<-35Exo+REase<-?|| wHTH+WYL+WCX -> BrxA-DNA-binding -> BrxB-BrxF-iSTAND -> DUF6079 *-> N6-METHYLASE -> PglZ -> SIGMA+OB-Fold+AAA-ATPase+ HKD-DNase -BrxL -><-REase<-REase||?->35exo->	1181	Bacteria>Pseudomonadota>Alphaproteobacteria>Hyphomicrobiales>Rhizobiaceae>Ciceribacter	GCF_900492205.1	Ciceribacter_naphthalenivorans	DYZ14_RS19075	12704..16249	1118451	BREX-system-P-loop-protein-BrxC-[Ciceribacter-naphthalenivorans]
WP_183807573.1	Type6.BREX	<- SIGMA+OB-Fold+AAA-ATPase+ HKD-DNase -BrxL <- PglZ <- N6-METHYLASE <- N6-METHYLASE <- DUF6079 *<- BrxB-BrxF-iSTAND <- BrxE <-PhdYeFM_antitox<-PIN+OST-HTH	1206	Bacteria>Pseudomonadota>Alphaproteobacteria>Hyphomicrobiales>Rhizobiaceae>Mycoplana	GCF_014196765.1	Mycoplana_azooxidifex	GGQ64_RS22460	165035..168655	1636188	BREX-system-P-loop-protein-BrxC-[Mycoplana-azooxidifex]
WP_225246472.1	Type1.BREX	<-DDE_Tnp_1_4<-GDResolvase+HIN-HTH<-?<-?<-?<-?|| wHTH+WYL+WCX -> BrxA-DNA-binding -> BrxB-BrxF-iSTAND -> DUF6079 *-> N6-METHYLASE -> PglZ -> SIGMA+OB-Fold+AAA-ATPase+ HKD-DNase -BrxL ->?-><-?<-LexA-protease||?-><-?<-HTH_3	1175	Bacteria>Pseudomonadota>Alphaproteobacteria>Hyphomicrobiales>Rhizobiaceae>Rhizobium/Agrobacterium_group	-	Agrobacterium_tumefaciens_complex	HW573_RS24905	44719..48246	1183400	MULTISPECIES:-BREX-system-P-loop-protein-BrxC-[Agrobacterium-tumefaciens-complex]
WP_174060722.1	Type1.BREX	DEDD_Tnp_IS110+Transposase_20-><-HTH_3||HTH_3-><-DEDD_Tnp_IS110+Transposase_20||Relaxase+SF1-SF2-Helicase->||EVE+HNH->REase-> BrxA-DNA-binding -> BrxB-BrxF-iSTAND -> DUF6079 *-> N6-METHYLASE -> PglZ -> SIGMA+OB-Fold+AAA-ATPase+ HKD-DNase -BrxL -><-?<-?<-REase<-MORC+DUF3684+DUF3883||?->||HIN-HTH->	1175	Bacteria>Pseudomonadota>Alphaproteobacteria>Hyphomicrobiales>Rhizobiaceae>Rhizobium/Agrobacterium_group	-	Agrobacterium_tumefaciens	G6M83_RS06120	12437..15964	358	BREX-system-P-loop-protein-BrxC-[Agrobacterium-tumefaciens]
WP_156535776.1	Type1.BREX	<-ParB<-ArdCN+MPTase<-Small-B-barrel+SF1-SF2-Helicase+RdgC+REase<-SF1-SF2-Helicase+REase<-35Exo+REase|| wHTH+WYL+WCX -> BrxA-DNA-binding -> BrxB-BrxF-iSTAND -> DUF6079 *-> N6-METHYLASE -> PglZ -> SIGMA+OB-Fold+AAA-ATPase+ HKD-DNase -BrxL ->?->Calcineurin+Calcineurin-SbcD->SbcC+CCDC158->?->?-><-HTH+REase	1181	Bacteria>Pseudomonadota>Alphaproteobacteria>Hyphomicrobiales>Rhizobiaceae>Rhizobium/Agrobacterium_group	-	Agrobacterium_vitis	GOZ98_21320	20620..24165	373	BREX-system-P-loop-protein-BrxC-[Agrobacterium-vitis]
PZU76796.1	Type1.BREX	XIS-HTH->?->?->?->TraJ-RHH->TraI-VirD2-RCRE->?-> BrxA-DNA-binding -> BrxB-BrxF-iSTAND -> DUF6079 *-> N6-METHYLASE -> PglZ -> SIGMA+OB-Fold+AAA-ATPase+ HKD-DNase -BrxL -><-tRNA<-?||?-><-?||PAS+HISKIN->	1188	Bacteria>Pseudomonadota>Alphaproteobacteria>Hyphomicrobiales>Rhizobiaceae>Rhizobium/Agrobacterium_group	GCA_003248675.1	Rhizobium_sp.	DI546_06985	39524..43090	391	BREX-system-P-loop-protein-BrxC-[Rhizobium-sp.]
TBF64186.1	Type6.BREX	<-XIS-HTH||HTH_19->?->Couple_hipA+HipA_C->CHTH-><- SIGMA+OB-Fold+AAA-ATPase+ HKD-DNase -BrxL <- PglZ <- N6-METHYLASE <- N6-METHYLASE <- DUF6079 *<- BrxB-BrxF-iSTAND <- BrxA-DNA-binding <- BrxE <-PhdYeFM_antitox<-VirD4-TraG+VirD4-FtsK<-TraI-VirD2-RCRE<-TraJ-RHH<-HTH_36||HIN-HTH->	1208	Bacteria>Pseudomonadota>Alphaproteobacteria>Hyphomicrobiales>Rhizobiaceae>Rhizobium/Agrobacterium_group	GCA_004306065.1	Rhizobium_leguminosarum	ELG90_15310	3002859..3006485	384	BREX-system-P-loop-protein-BrxC-[Rhizobium-leguminosarum]
TQN59072.1	Type1.BREX	<-SF1-SF2-Helicase<- HKD-DNase + HKD-DNase <-wHTH<-SF1-SF2-Helicase+REase<- SIGMA+OB-Fold+AAA-ATPase+ HKD-DNase -BrxL <- PglZ <- N6-METHYLASE <- DUF6079 *<- BrxB-BrxF-iSTAND <- BrxA-DNA-binding <- wHTH+WYL+WCX <-MODE-HTH+PBPII||TetR-HTH-><-RHH<-VirB10<-VirB9<-VirB8	1179	Bacteria>Pseudomonadota>Alphaproteobacteria>Hyphomicrobiales>Rhizobiaceae>Rhizobium/Agrobacterium_group	GCA_006757705.1	Agrobacterium_tumefaciens	FLX27_23795	22501..26040	358	BREX-system-P-loop-protein-BrxC-[Agrobacterium-tumefaciens]
NEH61390.1	Type1.BREX	<-REase<-Classical-AAA<-Classical-AAA<-REase+ZnR<-ParB||?->?-> BrxA-DNA-binding -> BrxB-BrxF-iSTAND -> DUF6079 *-> N6-METHYLASE -> PglZ -> SIGMA+OB-Fold+AAA-ATPase+ HKD-DNase -BrxL -><-SbcC<-ABC-3C<-?||NACHT-><-ParB+HNH<-ParB+HNH	1174	Bacteria>Pseudomonadota>Alphaproteobacteria>Hyphomicrobiales>Rhizobiaceae>Rhizobium/Agrobacterium_group	GCA_010668215.1	Rhizobium_leguminosarum	GR207_02220	29199..32723	384	BREX-system-P-loop-protein-BrxC-[Rhizobium-leguminosarum]
MBA8801280.1	Type6.BREX	<- SIGMA+OB-Fold+AAA-ATPase+ HKD-DNase -BrxL <- PglZ <- N6-METHYLASE <- N6-METHYLASE <- DUF6079 *<- BrxB-BrxF-iSTAND <- BrxA-DNA-binding <- BrxE ||?-><- wHTH+WYL+WCX ||?->35Exo+REase->SMODS->	1208	Bacteria>Pseudomonadota>Alphaproteobacteria>Hyphomicrobiales>Rhizobiaceae>Rhizobium/Agrobacterium_group	GCA_014138475.1	Agrobacterium_sp._RC10-4-1	FHW77_005035	9105..12731	2587039	hypothetical-protein-[Agrobacterium-sp.-RC10-4-1]
MBX5048875.1	Type6.BREX	TetR-HTH->?-><- SIGMA+OB-Fold+AAA-ATPase+ HKD-DNase -BrxL <- PglZ <-ParB+HNH<- N6-METHYLASE <- N6-METHYLASE <- DUF6079 *<- BrxB-BrxF-iSTAND <- BrxA-DNA-binding <- BrxE <-tRNA||?->Thioredoxin->	1213	Bacteria>Pseudomonadota>Alphaproteobacteria>Hyphomicrobiales>Rhizobiaceae>Rhizobium/Agrobacterium_group	GCA_019684595.1	Rhizobium_lentis	HJB64_19910	192083..195724	1138194	BREX-system-P-loop-protein-BrxC-[Rhizobium-lentis]
MBY3093307.1	Type1.BREX	Phage_integrase-><-HTH+Phage_integrase+Phage_integrase<-?<-Classical-AAA<-Classical-AAA<-REase+ZnR<-ParB|| BrxA-DNA-binding -> BrxB-BrxF-iSTAND -> DUF6079 *-> N6-METHYLASE -> PglZ -> SIGMA+OB-Fold+AAA-ATPase+ HKD-DNase -BrxL ->||HIN-HTH->?->LZ_Tnp_IS66+zf-IS66+DDE_Tnp_IS66+DDE_Tnp_IS66_C->?->?-><-SbcC	1174	Bacteria>Pseudomonadota>Alphaproteobacteria>Hyphomicrobiales>Rhizobiaceae>Rhizobium/Agrobacterium_group	GCA_019778505.1	Rhizobium_laguerreae	HFO72_21270	353189..356713	1076926	BREX-system-P-loop-protein-BrxC-[Rhizobium-laguerreae]
MBY5873817.1	Type1.BREX	DnaB+McrB-AAA->McrC-NTD+REase+ZnR->?->OLD-ABC+TOPRIM->SF1-SF2-Helicase->ParB+HNH+Schlafen-><- SIGMA+OB-Fold+AAA-ATPase+ HKD-DNase -BrxL <- PglZ <- N6-METHYLASE <- DUF6079 *<- BrxB-BrxF-iSTAND <- BrxA-DNA-binding ||ParB->REase+ZnR->?->Classical-AAA->Classical-AAA->REase->	1174	Bacteria>Pseudomonadota>Alphaproteobacteria>Hyphomicrobiales>Rhizobiaceae>Rhizobium/Agrobacterium_group	GCA_019799745.1	Rhizobium_leguminosarum	HFN53_17500	401377..404901	384	BREX-system-P-loop-protein-BrxC-[Rhizobium-leguminosarum]
WP_131614693.1	Type1.BREX	HKD-DNase ->SF1-SF2-Helicase->?->?-><- SIGMA+OB-Fold+AAA-ATPase+ HKD-DNase -BrxL <- PglZ <- N6-METHYLASE <-NERD<-OLD-ABC<- DUF6079 *<- BrxB-BrxF-iSTAND <- BrxA-DNA-binding <-?||ParB->REase+ZnR->Classical-AAA->Classical-AAA->REase->	1174	Bacteria>Pseudomonadota>Alphaproteobacteria>Hyphomicrobiales>Rhizobiaceae>Rhizobium/Agrobacterium_group	GCA_030820635.1	Rhizobium_leguminosarum	HFN52_RS21030	83963..87487	384	BREX-system-P-loop-protein-BrxC-[Rhizobium-leguminosarum]
CAD6617282.1	Type1.BREX	<-XIS-HTH<-Arm-DNA-bind_3+Phage_integrase<- SIGMA+OB-Fold+AAA-ATPase+ HKD-DNase -BrxL <- PglZ <- N6-METHYLASE <-REase<-OLD-ABC<- DUF6079 *<- BrxB-BrxF-iSTAND <- BrxA-DNA-binding <- wHTH+WYL+WCX ||GDResolvase+HIN-HTH->	1172	Bacteria>Pseudomonadota>Alphaproteobacteria>Hyphomicrobiales>Rhizobiaceae>Rhizobium/Agrobacterium_group	GCA_902502895.2	Rhizobium_sp._Khangiran2	RKHAN_03345	3387102..3390620	1435607	BREX-system-P-loop-protein-BrxC-[Rhizobium-sp.-Khangiran2]
WP_027666511.1	Type1.BREX	<-SbcC<-ABC-3C<-?<- SIGMA+OB-Fold+AAA-ATPase+ HKD-DNase -BrxL <- PglZ <- N6-METHYLASE <- DUF6079 *<- BrxB-BrxF-iSTAND <- BrxA-DNA-binding <-?<-?||ParB->REase+ZnR->?->Classical-AAA->Classical-AAA->	1174	Bacteria>Pseudomonadota>Alphaproteobacteria>Hyphomicrobiales>Rhizobiaceae>Rhizobium/Agrobacterium_group	GCF_000427945.1	Rhizobium_leguminosarum	A19Q_RS0117655	62384..65908	384	BREX-system-P-loop-protein-BrxC-[Rhizobium-leguminosarum]
WP_088677288.1	Type1.BREX	<-SPOUT||tRNA->tRNA->?->?->?->tRNA->?->Classical-AAA+S2P->HEPN-> BrxA-DNA-binding -> BrxB-BrxF-iSTAND -> DUF6079 *-> N6-METHYLASE -> PglZ -> SIGMA+OB-Fold+AAA-ATPase+ HKD-DNase -BrxL -><-?||?->?->MORC+REase->RNase->	1194	Bacteria>Pseudomonadota>Alphaproteobacteria>Hyphomicrobiales>Rhizobiaceae>Rhizobium/Agrobacterium_group	GCF_002211395.1	Rhizobium_sp._R339	ATY76_RS24585	353019..356603	1764273	BREX-system-P-loop-protein-BrxC-[Rhizobium-sp.-R339]
WP_126920114.1	Type1.BREX	<-ABC-3C<-?||35exo+SF1-SF2-Helicase-><-HHH_4+HTH+wHTH+SF1-SF2-Helicase+REase||MORC+SF1-SF2-Helicase-><-?<- SIGMA+OB-Fold+AAA-ATPase+ HKD-DNase -BrxL <- PglZ <- N6-METHYLASE <- DUF6079 *<- BrxB-BrxF-iSTAND <- BrxA-DNA-binding <-nSTAND1+HNH||ParB->REase+ZnR->?->Classical-AAA->Classical-AAA->REase->	1175	Bacteria>Pseudomonadota>Alphaproteobacteria>Hyphomicrobiales>Rhizobiaceae>Rhizobium/Agrobacterium_group	GCF_003985155.1	Rhizobium_vallis	EFQ99_RS06935	372119..375646	634290	BREX-system-P-loop-protein-BrxC-[Rhizobium-vallis]
WP_130750054.1	Type6.BREX	<-XIS-HTH||HTH_19->?->Couple_hipA+HipA_C->CHTH-><- SIGMA+OB-Fold+AAA-ATPase+ HKD-DNase -BrxL <- PglZ <- N6-METHYLASE <- N6-METHYLASE <- DUF6079 *<- BrxB-BrxF-iSTAND <- BrxA-DNA-binding <- BrxE <-PhdYeFM_antitox<-VirD4-TraG+VirD4-FtsK<-?<-?<-TraJ-RHH<-HTH_36	1208	Bacteria>Pseudomonadota>Alphaproteobacteria>Hyphomicrobiales>Rhizobiaceae>Rhizobium/Agrobacterium_group	GCF_004304245.1	Rhizobium_leguminosarum	ELI03_RS14940	2921080..2924706	384	BREX-system-P-loop-protein-BrxC-[Rhizobium-leguminosarum]
WP_130755622.1	Type1.BREX	35exo+SF1-SF2-Helicase->MarR-HTH->Polbeta->REase->SbcC-><-Phage_int_SAM_4<- SIGMA+OB-Fold+AAA-ATPase+ HKD-DNase -BrxL <- PglZ <- N6-METHYLASE <- DUF6079 *<- BrxB-BrxF-iSTAND <- BrxA-DNA-binding <-?||ParB->REase+ZnR->Classical-AAA->Classical-AAA->REase->	1174	Bacteria>Pseudomonadota>Alphaproteobacteria>Hyphomicrobiales>Rhizobiaceae>Rhizobium/Agrobacterium_group	GCF_004304705.1	Rhizobium_leguminosarum	ELH79_RS02355	502725..506249	384	BREX-system-P-loop-protein-BrxC-[Rhizobium-leguminosarum]
WP_197729861.1	Type6.BREX	<-XIS-HTH||HTH_19->?->Couple_hipA+HipA_C->CHTH-><- SIGMA+OB-Fold+AAA-ATPase+ HKD-DNase -BrxL <- PglZ <- N6-METHYLASE <- N6-METHYLASE <- DUF6079 *<- BrxB-BrxF-iSTAND <- BrxA-DNA-binding -p<- BrxE <-PhdYeFM_antitox<-VirD4-TraG+VirD4-FtsK<-?<-TraJ-RHH<-HTH_36	1210	Bacteria>Pseudomonadota>Alphaproteobacteria>Hyphomicrobiales>Rhizobiaceae>Rhizobium/Agrobacterium_group	GCF_004306065.1	Rhizobium_leguminosarum	ELG90_RS15310	3002859..3006491	384	BREX-system-P-loop-protein-BrxC-[Rhizobium-leguminosarum]
WP_131718742.1	Type1.BREX	URI-DNase->REase->?-><-SbcC+SbcC<-Calcineurin+Calcineurin-SbcD|| wHTH+WYL+WCX -> BrxA-DNA-binding -> BrxB-BrxF-iSTAND -> DUF6079 *-> N6-METHYLASE -> PglZ -> SIGMA+OB-Fold+AAA-ATPase+ HKD-DNase -BrxL -><-?<-GDResolvase<-?<-?<-tRNA||?->SAM-METHYLASE->	1178	Bacteria>Pseudomonadota>Alphaproteobacteria>Hyphomicrobiales>Rhizobiaceae>Rhizobium/Agrobacterium_group	GCF_004330655.1	Rhizobium_leguminosarum	EOH57_RS05710	350884..354420	384	BREX-system-P-loop-protein-BrxC-[Rhizobium-leguminosarum]
WP_132661711.1	Type6.BREX	Glyoxalase+ZnR->HipA_C->?-> HKD-DNase +HKD-Rease-><- SIGMA+OB-Fold+AAA-ATPase+ HKD-DNase -BrxL <- PglZ <- N6-METHYLASE <- N6-METHYLASE <- DUF6079 *<- BrxB-BrxF-iSTAND <- BrxA-DNA-binding <- BrxE <-PhdYeFM_antitox<- wHTH+WYL+WCX ||SMODS->?->Classical-AAA->Cap4_nuclease+SAVED->	1208	Bacteria>Pseudomonadota>Alphaproteobacteria>Hyphomicrobiales>Rhizobiaceae>Rhizobium/Agrobacterium_group	GCF_004342325.1	Rhizobium_azibense	EV130_RS24200	158902..162528	1136135	BREX-system-P-loop-protein-BrxC-[Rhizobium-azibense]
WP_168337993.1	Type1.BREX	<- SIGMA+OB-Fold+AAA-ATPase+ HKD-DNase -BrxL <- PglZ <- N6-METHYLASE <- DUF6079 *<- BrxB-BrxF-iSTAND <- BrxA-DNA-binding ||?->ParB->REase+ZnR->?->Classical-AAA->Classical-AAA->REase->	1174	Bacteria>Pseudomonadota>Alphaproteobacteria>Hyphomicrobiales>Rhizobiaceae>Rhizobium/Agrobacterium_group	GCF_012275885.1	Rhizobium_laguerreae	GFL43_RS28845	8439..11963	1076926	BREX-system-P-loop-protein-BrxC-[Rhizobium-laguerreae]
WP_168349960.1	Type1.BREX	<- SIGMA+OB-Fold+AAA-ATPase+ HKD-DNase -BrxL <- PglZ <-ParB+HNH<- N6-METHYLASE <- N6-METHYLASE <- DUF6079 -p*<-Classical-AAA<-REase+wHTH||?->PIN-><-ParB<-ParB<-GDResolvase+PPP1-HTH||CHTH->	190	Bacteria>Pseudomonadota>Alphaproteobacteria>Hyphomicrobiales>Rhizobiaceae>Rhizobium/Agrobacterium_group	GCF_012276085.1	Rhizobium_leguminosarum	GFM28_RS29310	8641..9213	384	hypothetical-protein-[Rhizobium-leguminosarum]
WP_136542766.1	Type1.BREX	ACET-><-SF1-SF2-Helicase<- HKD-DNase + HKD-DNase <-wHTH<-SF1-SF2-Helicase+REase<-SF1-SF2-Helicase+REase<- SIGMA+OB-Fold+AAA-ATPase+ HKD-DNase -BrxL <- PglZ <- N6-METHYLASE <- DUF6079 *<- BrxB-BrxF-iSTAND <- BrxA-DNA-binding <- wHTH+WYL+WCX <-MODE-HTH+PBPII||TetR-HTH-><-RHH<-VirB10<-VirB9<-VirB8	1179	Bacteria>Pseudomonadota>Alphaproteobacteria>Hyphomicrobiales>Rhizobiaceae>Rhizobium/Agrobacterium_group	GCF_014202175.1	Rhizobium_rosettiformans	FAA86_RS19555	64899..68438	1368430	BREX-system-P-loop-protein-BrxC-[Rhizobium-rosettiformans]
WP_192117159.1	Type1.BREX	<-REase<-Classical-AAA<-Classical-AAA<-?<-REase+ZnR<-ParB||nSTAND1+HNH-> BrxA-DNA-binding -> BrxB-BrxF-iSTAND -> DUF6079 *-> N6-METHYLASE -> PglZ -> SIGMA+OB-Fold+AAA-ATPase+ HKD-DNase -BrxL ->?->SF1-SF2-Helicase-><-?||||HTH_Tnp_1+HTH_21+rve+rve_3->?-> HKD-DNase +HKD-Rease->	1174	Bacteria>Pseudomonadota>Alphaproteobacteria>Hyphomicrobiales>Rhizobiaceae>Rhizobium/Agrobacterium_group	GCF_014841565.1	Rhizobium_sp._CFBP_8762	IFT84_RS03115	659482..663006	2775279	BREX-system-P-loop-protein-BrxC-[Rhizobium-sp.-CFBP-8762]
WP_215570403.1	Type1.BREX	HTH_3->?-><-?||LexA-protease->?-><-?<- SIGMA+OB-Fold+AAA-ATPase+ HKD-DNase -BrxL <- PglZ <- N6-METHYLASE <- DUF6079 *<- BrxB-BrxF-iSTAND <- BrxA-DNA-binding <- wHTH+WYL+WCX ||?->?->?->?->GDResolvase+HIN-HTH-><-DUF772+DDE_Tnp_1+DDE_Tnp_1_6	1175	Bacteria>Pseudomonadota>Alphaproteobacteria>Hyphomicrobiales>Rhizobiaceae>Rhizobium/Agrobacterium_group	GCF_018760785.1	Rhizobium_sp._CSW-27	KLP42_RS24160	30886..34413	2839985	BREX-system-P-loop-protein-BrxC-[Rhizobium-sp.-CSW-27]
WP_246638929.1	Type1.BREX	<-REase<-Classical-AAA<-Classical-AAA<-REase+ZnR<-ParB||?-> BrxA-DNA-binding -> BrxB-BrxF-iSTAND -> DUF6079 *-> N6-METHYLASE -> PglZ -> SIGMA+OB-Fold+AAA-ATPase+ HKD-DNase -BrxL ->||HIN-HTH->?->LZ_Tnp_IS66+zf-IS66+DDE_Tnp_IS66+DDE_Tnp_IS66_C->?->?-><-SbcC	1170	Bacteria>Pseudomonadota>Alphaproteobacteria>Hyphomicrobiales>Rhizobiaceae>Rhizobium/Agrobacterium_group	GCF_019778505.1	Rhizobium_laguerreae	HFO72_RS21050	353201..356713	1076926	BREX-system-P-loop-protein-BrxC-[Rhizobium-laguerreae]
WP_222362949.1	Type1.BREX	<-SF1-SF2-Helicase<-OLD-ABC+TOPRIM<- SIGMA+OB-Fold+AAA-ATPase+ HKD-DNase -BrxL <- PglZ <- N6-METHYLASE <- DUF6079 *<- BrxB-BrxF-iSTAND <- BrxA-DNA-binding <-?<-?||ParB->REase+ZnR->?->Classical-AAA->Classical-AAA->	1174	Bacteria>Pseudomonadota>Alphaproteobacteria>Hyphomicrobiales>Rhizobiaceae>Rhizobium/Agrobacterium_group	GCF_019798305.1	Rhizobium_leguminosarum	HFO99_RS19360	191490..195014	384	BREX-system-P-loop-protein-BrxC-[Rhizobium-leguminosarum]
WP_222342569.1	Type1.BREX	<-REase<-Classical-AAA<-Classical-AAA||?-><-REase+ZnR<-ParB|| BrxA-DNA-binding -> BrxB-BrxF-iSTAND -> DUF6079 *-> N6-METHYLASE -> PglZ -> SIGMA+OB-Fold+AAA-ATPase+ HKD-DNase -BrxL -><-SbcC<-ABC-3C	1174	Bacteria>Pseudomonadota>Alphaproteobacteria>Hyphomicrobiales>Rhizobiaceae>Rhizobium/Agrobacterium_group	GCF_019799465.1	Rhizobium_leguminosarum	HFO12_RS18165	9414..12938	384	BREX-system-P-loop-protein-BrxC-[Rhizobium-leguminosarum]
WP_221976415.1	Type1.BREX	ParB+HNH->?-><-NACHT||?->ABC-3C->SbcC-><- SIGMA+OB-Fold+AAA-ATPase+ HKD-DNase -BrxL <- PglZ <- N6-METHYLASE <- DUF6079 *<- BrxB-BrxF-iSTAND <- BrxA-DNA-binding <-?||ParB->REase+ZnR-><-?||Classical-AAA->Classical-AAA->REase->	1174	Bacteria>Pseudomonadota>Alphaproteobacteria>Hyphomicrobiales>Rhizobiaceae>Rhizobium/Agrobacterium_group	GCF_021391715.1	Rhizobium	LZK79_RS04540	933670..937194	379	MULTISPECIES:-BREX-system-P-loop-protein-BrxC-[Rhizobium]
WP_239619846.1	Type1.BREX	<-Classical-AAA||?->?-><-REase+ZnR<-REase+ZnR<-ParB<-?|| BrxA-DNA-binding -> BrxB-BrxF-iSTAND -> DUF6079 *-> N6-METHYLASE -> PglZ -p-> PglZ -> SIGMA+OB-Fold+AAA-ATPase+ HKD-DNase -BrxL ->?->ABC-3C->SbcC->||HIN-HTH->AAA-ATPase->	1174	Bacteria>Pseudomonadota>Alphaproteobacteria>Hyphomicrobiales>Rhizobiaceae>Rhizobium/Agrobacterium_group	GCF_022354505.1	Rhizobium_sp._C104	MF410_RS14985	2774142..2777666	2917727	BREX-system-P-loop-protein-BrxC-[Rhizobium-sp.-C104]
WP_250810295.1	Type6.BREX	<-GreA_GreB+SF1-SF2-Helicase<-PIWI<-REase+TIR<-?|| wHTH+WYL+WCX -> BrxE -> BrxA-DNA-binding -> BrxB-BrxF-iSTAND -> DUF6079 *-> N6-METHYLASE -> PglZ -> SIGMA+OB-Fold+AAA-ATPase+ HKD-DNase -BrxL -><-?<-ParB+HNH||GDResolvase+HIN-HTH-><-HTH_3	1208	Bacteria>Pseudomonadota>Alphaproteobacteria>Hyphomicrobiales>Rhizobiaceae>Rhizobium/Agrobacterium_group	GCF_023667605.1	Neorhizobium_tomejilense	L1082_RS18485	51181..54807	2093828	BREX-system-P-loop-protein-BrxC-[Neorhizobium-tomejilense]
WP_245134299.1	Type1.BREX	<-Peptidase_M29<-?||?-><- SIGMA+OB-Fold+AAA-ATPase+ HKD-DNase -BrxL <- PglZ <- N6-METHYLASE <- DUF6079 *<- BrxB-BrxF-iSTAND <- BrxA-DNA-binding <- wHTH+WYL+WCX ||35Exo+REase->SF1-SF2-Helicase+REase->Small-B-barrel+SF1-SF2-Helicase+RdgC+REase->?-> HKD-DNase ->SF1-SF2-Helicase->	1177	Bacteria>Pseudomonadota>Alphaproteobacteria>Hyphomicrobiales>Rhizobiaceae>Rhizobium/Agrobacterium_group	GCF_030369675.1	Rhizobium_sp._SSM4.3	MKJ03_RS01550	316527..320060	2919917	BREX-system-P-loop-protein-BrxC-[Rhizobium-sp.-SSM4.3]
WP_077987199.1	Type1.BREX	HTH_3->Couple_hipA+HipA_C-><-?||OLD-ABC+TOPRIM->SF1-SF2-Helicase-><- SIGMA+OB-Fold+AAA-ATPase+ HKD-DNase -BrxL <- PglZ <- N6-METHYLASE <- DUF6079 *<- BrxB-BrxF-iSTAND <- BrxA-DNA-binding <-REase||ParB->REase+ZnR->REase+ZnR->?->Classical-AAA->Classical-AAA->	1175	Bacteria>Pseudomonadota>Alphaproteobacteria>Hyphomicrobiales>Rhizobiaceae>Rhizobium/Agrobacterium_group	GCF_030440405.1	Rhizobium_pusense	BTE56_RS17195	18928..22455	648995	BREX-system-P-loop-protein-BrxC-[Rhizobium-pusense]
WP_149748055.1	Type1.BREX	nSTAND1+HNH->REase-> BrxA-DNA-binding -> BrxB-BrxF-iSTAND -> DUF6079 *-> N6-METHYLASE -> PglZ -> SIGMA+OB-Fold+AAA-ATPase+ HKD-DNase -BrxL ->?->SF1-SF2-Helicase+DUF1998->	1175	Bacteria>Pseudomonadota>Alphaproteobacteria>Hyphomicrobiales>Rhizobiaceae>Rhizobium/Agrobacterium_group	GCF_900156005.1	Rhizobium_sp._RU35A	F3H95_RS25745	3768..7295	1907414	BREX-system-P-loop-protein-BrxC-[Rhizobium-sp.-RU35A]
WP_245278427.1	Type1.BREX	<-REase<-Classical-AAA<-Classical-AAA<-REase+ZnR<-ParB||?-> BrxA-DNA-binding -> BrxB-BrxF-iSTAND -> DUF6079 *-> N6-METHYLASE -> PglZ -> SIGMA+OB-Fold+AAA-ATPase+ HKD-DNase -BrxL -><-?<-?<-?||?-><-McrC-NTD+REase+ZnR<-DnaB+McrB-AAA	1170	Bacteria>Pseudomonadota>Alphaproteobacteria>Hyphomicrobiales>Rhizobiaceae>Rhizobium/Agrobacterium_group	-	Rhizobium_leguminosarum	A3A3_RS0116705	192694..196206	384	BREX-system-P-loop-protein-BrxC-[Rhizobium-leguminosarum]
WP_131707092.1	Type1.BREX	<-REase<-Classical-AAA<-Classical-AAA<-REase+ZnR<-ParB||?-> BrxA-DNA-binding -> BrxB-BrxF-iSTAND -> DUF6079 *-> N6-METHYLASE -> PglZ -> SIGMA+OB-Fold+AAA-ATPase+ HKD-DNase -BrxL -><-?<-?<-SF1-SF2-Helicase<- HKD-DNase <-?<-Peptidase_S8	1174	Bacteria>Pseudomonadota>Alphaproteobacteria>Hyphomicrobiales>Rhizobiaceae>Rhizobium/Agrobacterium_group	-	Rhizobium_leguminosarum	EOH59_RS18255	300646..304170	384	BREX-system-P-loop-protein-BrxC-[Rhizobium-leguminosarum]
WP_024321352.1	Type1.BREX	DnaB+McrB-AAA->McrC-NTD+REase+ZnR-><-?||?->?->?-><- SIGMA+OB-Fold+AAA-ATPase+ HKD-DNase -BrxL <- PglZ <- N6-METHYLASE <- DUF6079 *<- BrxB-BrxF-iSTAND <- BrxA-DNA-binding <-?||ParB->REase+ZnR->Classical-AAA->Classical-AAA->REase->	1174	Bacteria>Pseudomonadota>Alphaproteobacteria>Hyphomicrobiales>Rhizobiaceae>Rhizobium/Agrobacterium_group	-	Rhizobium_leguminosarum	EOJ20_RS04835	93871..97395	384	BREX-system-P-loop-protein-BrxC-[Rhizobium-leguminosarum]
WP_130784075.1	Type1.BREX	<-LZ_Tnp_IS66+zf-IS66+DDE_Tnp_IS66+DDE_Tnp_IS66_C<-?<-HIN-HTH<- SIGMA+OB-Fold+AAA-ATPase+ HKD-DNase -BrxL <- PglZ <- N6-METHYLASE <- DUF6079 *<- BrxB-BrxF-iSTAND <- BrxA-DNA-binding <- wHTH+WYL+WCX ||Calcineurin+Calcineurin-SbcD->SbcC+SbcC-><-?<-REase<-URI-DNase	1178	Bacteria>Pseudomonadota>Alphaproteobacteria>Hyphomicrobiales>Rhizobiaceae>Rhizobium/Agrobacterium_group	-	Rhizobium_leguminosarum	ELG96_RS28915	11184..14720	384	BREX-system-P-loop-protein-BrxC-[Rhizobium-leguminosarum]
WP_130764093.1	Type1.BREX	<-REase<-Classical-AAA<-Classical-AAA||?-><-REase+ZnR<-ParB||<-?|| BrxA-DNA-binding -> BrxB-BrxF-iSTAND -> DUF6079 *-> N6-METHYLASE -> PglZ -> SIGMA+OB-Fold+AAA-ATPase+ HKD-DNase -BrxL ->?->ABC-3C->SbcC->SbcC-><-?<-McrC-NTD+REase+ZnR	1174	Bacteria>Pseudomonadota>Alphaproteobacteria>Hyphomicrobiales>Rhizobiaceae>Rhizobium/Agrobacterium_group	-	Rhizobium_leguminosarum	ELI00_RS29085	22850..26374	384	BREX-system-P-loop-protein-BrxC-[Rhizobium-leguminosarum]
WP_245488667.1	Type1.BREX	DADA-Ligase->||?->?->SbcC-><- SIGMA+OB-Fold+AAA-ATPase+ HKD-DNase -BrxL <- PglZ <- N6-METHYLASE <- DUF6079 *<- BrxB-BrxF-iSTAND <- BrxA-DNA-binding <-?||ParB->REase+ZnR->?->Classical-AAA->Classical-AAA->REase->	1170	Bacteria>Pseudomonadota>Alphaproteobacteria>Hyphomicrobiales>Rhizobiaceae>Rhizobium/Agrobacterium_group	-	Rhizobium_leguminosarum	ELI09_RS02630	538702..542214	384	BREX-system-P-loop-protein-BrxC-[Rhizobium-leguminosarum]
WP_130672003.1	Type1.BREX	DADA-Ligase->||?->?->SbcC-><- SIGMA+OB-Fold+AAA-ATPase+ HKD-DNase -BrxL <- PglZ <- N6-METHYLASE <- DUF6079 *<- BrxB-BrxF-iSTAND <- BrxA-DNA-binding <-?||ParB->REase+ZnR->?->Classical-AAA->Classical-AAA->REase->	1174	Bacteria>Pseudomonadota>Alphaproteobacteria>Hyphomicrobiales>Rhizobiaceae>Rhizobium/Agrobacterium_group	-	Rhizobium_leguminosarum	ELI10_RS02625	537164..540688	384	BREX-system-P-loop-protein-BrxC-[Rhizobium-leguminosarum]
WP_168316672.1	Type1.BREX	<-Small-B-barrel+SF1-SF2-Helicase+RdgC+REase<-SF1-SF2-Helicase+REase<-35Exo+REase<-?|| wHTH+WYL+WCX -> BrxA-DNA-binding -> BrxB-BrxF-iSTAND -> DUF6079 *-> N6-METHYLASE -> PglZ -> SIGMA+OB-Fold+AAA-ATPase+ HKD-DNase -BrxL ->	1181	Bacteria>Pseudomonadota>Alphaproteobacteria>Hyphomicrobiales>Rhizobiaceae>Rhizobium/Agrobacterium_group	-	Rhizobium_leguminosarum	GFL49_RS25835	227535..231080	384	BREX-system-P-loop-protein-BrxC-[Rhizobium-leguminosarum]
WP_168308740.1	Type1.BREX	<-ParB<-Small-B-barrel+SF1-SF2-Helicase+RdgC+REase<-SF1-SF2-Helicase+REase<-35Exo+REase<-?|| wHTH+WYL+WCX -> BrxA-DNA-binding -> BrxB-BrxF-iSTAND -> DUF6079 *-> N6-METHYLASE -> PglZ -> SIGMA+OB-Fold+AAA-ATPase+ HKD-DNase -BrxL -><-SIR2<-HTH+REase<-Arm-DNA-bind_3+Phage_integrase||?->Peptidase->	1181	Bacteria>Pseudomonadota>Alphaproteobacteria>Hyphomicrobiales>Rhizobiaceae>Rhizobium/Agrobacterium_group	-	Rhizobium_leguminosarum	GFM13_RS06520	103411..106956	384	BREX-system-P-loop-protein-BrxC-[Rhizobium-leguminosarum]
WP_246708550.1	Type1.BREX	<-Classical-AAA<-Classical-AAA||?-><-REase+ZnR<-ParB||?->?-> BrxA-DNA-binding -> BrxB-BrxF-iSTAND -> DUF6079 *-> N6-METHYLASE -> PglZ -> SIGMA+OB-Fold+AAA-ATPase+ HKD-DNase -BrxL -><-SbcC<-ABC-3C<-?||NACHT-><-ParB+HNH<-ParB+HNH	1170	Bacteria>Pseudomonadota>Alphaproteobacteria>Hyphomicrobiales>Rhizobiaceae>Rhizobium/Agrobacterium_group	-	Rhizobium_leguminosarum	GR207_RS02230	29211..32723	384	BREX-system-P-loop-protein-BrxC-[Rhizobium-leguminosarum]
WP_221145756.1	Type6.BREX	TetR-HTH->?-><- SIGMA+OB-Fold+AAA-ATPase+ HKD-DNase -BrxL <- PglZ <-ParB+HNH<- N6-METHYLASE <- N6-METHYLASE <- DUF6079 *<- BrxB-BrxF-iSTAND <- BrxA-DNA-binding <- BrxE <-tRNA||?->Thioredoxin->	1213	Bacteria>Pseudomonadota>Alphaproteobacteria>Hyphomicrobiales>Rhizobiaceae>Rhizobium/Agrobacterium_group	-	Rhizobium_lentis	HJB64_19910	192083..195724	1138194	BREX-system-P-loop-protein-BrxC-[Rhizobium-lentis]
WP_192067374.1	Type6.BREX	<- SIGMA+OB-Fold+AAA-ATPase+ HKD-DNase -BrxL <- PglZ <- N6-METHYLASE <- N6-METHYLASE <- DUF6079 *<- BrxB-BrxF-iSTAND <- BrxA-DNA-binding <- BrxE <-Phage_integrase<-XIS-HTH<-Phage_integrase||REase->wHTH+REase->MPTase->	1208	Bacteria>Pseudomonadota>Alphaproteobacteria>Hyphomicrobiales>Rhizobiaceae>Rhizobium/Agrobacterium_group	-	unclassified_Rhizobium	IFT53_20370	8816..12442	2613769	MULTISPECIES:-BREX-system-P-loop-protein-BrxC-[unclassified-Rhizobium]
PZU19146.1	Type1.BREX	HTH_3->?-><-?||LexA-protease->?-><- SIGMA+OB-Fold+AAA-ATPase+ HKD-DNase -BrxL <- PglZ <- N6-METHYLASE <- DUF6079 *<- BrxB-BrxF-iSTAND <- BrxA-DNA-binding <- wHTH+WYL+WCX ||?->?->?->?->GDResolvase+HIN-HTH->	1175	Bacteria>Pseudomonadota>Alphaproteobacteria>Hyphomicrobiales>Rhizobiaceae>Shinella	GCA_003248545.1	Shinella_sp.	DI589_22170	12827..16354	1870904	MAG:-BREX-system-P-loop-protein-BrxC-[Shinella-sp.]
KSV61619.1	Type6.BREX	<- SIGMA+OB-Fold+AAA-ATPase+ HKD-DNase -BrxL <- PglZ <- N6-METHYLASE <- DUF6079 *<- BrxB-BrxF-iSTAND <- BrxA-DNA-binding <- BrxE <-tRNA||?->?->ACET->ACET-><-?||sGTP->	1203	Bacteria>Pseudomonadota>Alphaproteobacteria>Hyphomicrobiales>Rhizobiaceae>Sinorhizobium/Ensifer_group	GCA_001461715.1	Sinorhizobium_sp._GL2	N182_13660	115788..119399	1358416	hypothetical-protein-N182_13660-[Sinorhizobium-sp.-GL2]

Protein-ID	System	Gene-Neighbourhoods	Length	Taxonomy	Assembly-ID	Strain	Locus-Tag	Genomic-coordinates	TaxID	Fasta-define
WP_004434288.1	Type1.BREX	<-PIN<-PhdYeFM_antitox<- SIGMA+OB-Fold+AAA-ATPase+HKD-DNase -BrxL <- PglZ <- N6-METHYLASE <-REase<-OLD-ABC<- DUF6079 *<- BrxB-BrxF-iSTAND <- BrxA-DNA-binding <- wHTH+WYL+WCX ||35Exo+REase->SF1-SF2-Helicase+REase->Small-B-barrel+SF1-SF2-Helicase+RdgC+REase-><-SF1-SF2-Helicase<- HKD-DNase	1177	Bacteria>Pseudomonadota>Alphaproteobacteria>Hyphomicrobiales>Rhizobiaceae>Sinorhizobium/Ensifer_group	GCF_000236945.1	Sinorhizobium_meliloti	SM0020_RS29775	166866..170399	382	BREX-system-P-loop-protein-BrxC-[Sinorhizobium-meliloti]
WP_037424884.1	Type1.BREX	<-GDResolvase+HIN-HTH<-Small-B-barrel+SF1-SF2-Helicase+RdgC+REase<-35Exo+REase<-?|| wHTH+WYL+WCX -> BrxA-DNA-binding -> BrxB-BrxF-iSTAND -> DUF6079 *-> N6-METHYLASE -> PglZ -> SIGMA+OB-Fold+AAA-ATPase+HKD-DNase -BrxL -><-REase||?->?->?-><-?||URI-DNase->	1181	Bacteria>Pseudomonadota>Alphaproteobacteria>Hyphomicrobiales>Rhizobiaceae>Sinorhizobium/Ensifer_group	GCF_000261005.1	Sinorhizobium_sp._CCBAU_05631	SS05631_RS30325	18322..21867	794846	BREX-system-P-loop-protein-BrxC-[Sinorhizobium-sp.-CCBAU-05631]
WP_042775876.1	Type6.BREX	<-HTH_Tnp<-?<-TUDOR+SF1-SF2-Helicase+iREase+CTD<- SIGMA+OB-Fold+AAA-ATPase+HKD-DNase -BrxL <- PglZ <- N6-METHYLASE <- N6-METHYLASE <- DUF6079 *<- BrxB-BrxF-iSTAND <- BrxA-DNA-binding <- BrxE <-?||?-><-HIN-HTH	1208	Bacteria>Pseudomonadota>Alphaproteobacteria>Hyphomicrobiales>Rhizobiaceae>Sinorhizobium/Ensifer_group	GCF_000261925.1	Sinorhizobium_fredii	IC54_RS06660	41741..45367	380	BREX-system-P-loop-protein-BrxC-[Sinorhizobium-fredii]
WP_209439290.1	Type6.BREX	Calcineurin->SbcC+SbcC->TUDOR+SF1-SF2-Helicase+iREase+CTD->?->?-><- SIGMA+OB-Fold+AAA-ATPase+HKD-DNase -BrxL <- PglZ <- N6-METHYLASE <- N6-METHYLASE <- DUF6079 *<- BrxB-BrxF-iSTAND <- BrxA-DNA-binding <- BrxE <-PhdYeFM_antitox||?->PhdYeFM_antitox->PIN-><-PhdYeFM_antitox||Phage_integrase->	1210	Bacteria>Pseudomonadota>Alphaproteobacteria>Hyphomicrobiales>Rhizobiaceae>Sinorhizobium/Ensifer_group	GCF_000427465.1	Sinorhizobium_arboris	SINAR_RS0100665	116577..120209	76745	BREX-system-P-loop-protein-BrxC-[Sinorhizobium-arboris]
WP_127638472.1	Type1.BREX	BrxA-DNA-binding -> BrxB-BrxF-iSTAND -> DUF6079 *-> N6-METHYLASE -> PglZ -> SIGMA+OB-Fold+AAA-ATPase+HKD-DNase -BrxL -><-REase+HTH+35exo+Small-B-barrel+SF1-SF2-Helicase<-EVE+REase||?->?->HEPN+RES-><-Thermonuclease	1188	Bacteria>Pseudomonadota>Alphaproteobacteria>Hyphomicrobiales>Rhizobiaceae>Sinorhizobium/Ensifer_group	GCF_004003915.1	Sinorhizobium_meliloti	CN087_RS12760	106891..110457	382	BREX-system-P-loop-protein-BrxC-[Sinorhizobium-meliloti]
WP_035540926.1	Type1.BREX	<-SbcC+SbcC||?-><-HTH_3||SIGMA-HTH->SIGMA-HTH->?-><- SIGMA+OB-Fold+AAA-ATPase+HKD-DNase -BrxL <- PglZ <- N6-METHYLASE <- DUF6079 *<- BrxB-BrxF-iSTAND <- BrxA-DNA-binding ||ArdA->	1157	Bacteria>Pseudomonadota>Alphaproteobacteria>Hyphomonadales>Hyphomonadaceae>Hyphomonas	GCF_000685295.1	Hyphomonas_oceanitis	HOC_RS17370	29720..33193	81033	BREX-system-P-loop-protein-BrxC-[Hyphomonas-oceanitis]
MCE2892634.1	Type1.BREX	<- SIGMA+OB-Fold+AAA-ATPase+HKD-DNase -BrxL <- PglZ <- N6-METHYLASE <- DUF6079 *<- BrxB-BrxF-iSTAND <- BrxA-DNA-binding <-HTH_3<-?<-?<-Peptidase<-VirB10<-VirB9<-VirB8	1195	Bacteria>Pseudomonadota>Alphaproteobacteria>Hyphomonadales>Hyphomonadaceae>unclassified_Hyphomonadaceae	GCA_021299265.1	Hyphomonadaceae_bacterium	LW848_16090	9073..12660	2026748	BREX-system-P-loop-protein-BrxC-[Hyphomonadaceae-bacterium]
ADM09203.1	Type1.BREX	DUF736->?->XIS-HTH->Peptidase_S26->TraI-VirD2-RCRE-><- SIGMA+OB-Fold+AAA-ATPase+HKD-DNase -BrxL <- PglZ <- N6-METHYLASE <- DUF6079 *<- BrxB-BrxF-iSTAND <- BrxA-DNA-binding <- wHTH+WYL+WCX ||VirD4-TraG+VirD4-FtsK->?->GreA_GreB+SF1-SF2-Helicase->HTH_21+rve+rve_3->VirB11+GTPase-AIG->VirB2->	1171	Bacteria>Pseudomonadota>Alphaproteobacteria>Parvularculales>Parvularculaceae>Parvularcula	GCA_000152825.2	Parvularcula_bermudensis_HTCC2503	PB2503_05652	1076655..1080170	314260	hypothetical-protein-PB2503_05652-[Parvularcula-bermudensis-HTCC2503]
WP_238525821.1	Type1.BREX	ParB->DUF736->?->XIS-HTH->Peptidase_S26->TraI-VirD2-RCRE-><- SIGMA+OB-Fold+AAA-ATPase+HKD-DNase -BrxL <- PglZ <- N6-METHYLASE <- DUF6079 *<- BrxB-BrxF-iSTAND <- BrxA-DNA-binding <- wHTH+WYL+WCX ||VirD4-TraG+VirD4-FtsK->?->GreA_GreB+SF1-SF2-Helicase->HIN-HTH->VirB11+GTPase-AIG->VirB2->	1168	Bacteria>Pseudomonadota>Alphaproteobacteria>Parvularculales>Parvularculaceae>Parvularcula	GCF_000152825.2	Parvularcula_bermudensis	PB2503_RS05170	1076655..1080161	208216	BREX-system-P-loop-protein-BrxC-[Parvularcula-bermudensis]
WP_212538477.1	Type6.BREX	<-TetR-HTH||?-> wHTH+WYL+WCX -> BrxE -> BrxA-DNA-binding -> BrxB-BrxF-iSTAND -> DUF6079 *-> N6-METHYLASE -> N6-METHYLASE -p->ParB+HNH-> PglZ -> SIGMA+OB-Fold+AAA-ATPase+HKD-DNase -BrxL -><-XIS-HTH<-?||HTH+LexA-protease->UDG->	1208	Bacteria>Pseudomonadota>Alphaproteobacteria>Rhodobacterales>Paracoccaceae>Aestuariicoccus	GCF_018224955.1	Aestuariicoccus_sp._KMU-90	KB874_RS20730	16568..20194	2827239	BREX-system-P-loop-protein-BrxC-[Aestuariicoccus-sp.-KMU-90]
WP_217361198.1	Type1.BREX	<-HATPase_c||Arm-DNA-bind_3+Phage_integrase->LON-AAA->?->?-><-?|| wHTH+WYL+WCX -> BrxA-DNA-binding -> BrxB-BrxF-iSTAND -> DUF6079 *-> N6-METHYLASE -> PglZ -> SIGMA+OB-Fold+AAA-ATPase+HKD-DNase -BrxL ->?->HEPN+PAS+DUF4011+SF1-SF2-Helicase+REase->ParB+HNH+Schlafen->	1168	Bacteria>Pseudomonadota>Alphaproteobacteria>Rhodobacterales>Paracoccaceae>Aliiroseovarius	GCF_013283115.1	unclassified_Aliiroseovarius	XMV209_RS00060	11614..15120	2623558	MULTISPECIES:-BREX-system-P-loop-protein-BrxC-[unclassified-Aliiroseovarius]
WP_173486905.1	Type1.BREX	<-REase+iREase<- SIGMA+OB-Fold+AAA-ATPase+HKD-DNase -BrxL <- PglZ <- N6-METHYLASE <- DUF6079 *<- BrxB-BrxF-iSTAND <- BrxA-DNA-binding <- wHTH+WYL+WCX ||?->?->?-><-?<-ANK+ANK+ANK	1173	Bacteria>Pseudomonadota>Alphaproteobacteria>Rhodobacterales>Paracoccaceae>Aliiroseovarius	GCF_013283525.1	unclassified_Aliiroseovarius	XMM309_RS11965	259901..263422	2623558	MULTISPECIES:-BREX-system-P-loop-protein-BrxC-[unclassified-Aliiroseovarius]
WP_263337868.1	Type6.BREX	Peptidase-M15->?-><- SIGMA+OB-Fold+AAA-ATPase+HKD-DNase -BrxL <- PglZ <- N6-METHYLASE <- N6-METHYLASE <- DUF6079 *<- BrxB-BrxF-iSTAND <- BrxA-DNA-binding <- BrxE <-XIS-HTH<-Arm-DNA-bind_3+Phage_int_SAM_3+Phage_integrase||MerR-HTH->	1203	Bacteria>Pseudomonadota>Alphaproteobacteria>Rhodobacterales>Paracoccaceae>Defluviimonas	GCF_025673475.1	Defluviimonas_sp._WL0024	OEZ60_RS15020	60055..63666	2984153	BREX-system-P-loop-protein-BrxC-[Defluviimonas-sp.-WL0024]
WP_146284416.1	Type1.BREX	Glyco_hydro_53+Phage-tail_3->?-><- SIGMA+OB-Fold+AAA-ATPase+HKD-DNase -BrxL <- PglZ <- N6-METHYLASE <- DUF6079 *<- BrxB-BrxF-iSTAND <- BrxA-DNA-binding <- wHTH+WYL+WCX <-Arm-DNA-bind_3+Phage_int_SAM_3+Phage_integrase||SWIM-><-HTH_21+rve+rve_3	1173	Bacteria>Pseudomonadota>Alphaproteobacteria>Rhodobacterales>Paracoccaceae>Gemmobacter	GCF_007859075.1	Gemmobacter_aquaticus	FQU74_RS00760	54180..57701	490185	BREX-system-P-loop-protein-BrxC-[Gemmobacter-aquaticus]
WP_076528450.1	Type1.BREX	Arm-DNA-bind_3+Phage_int_SAM_3+Phage_integrase-><-SF1-SF2-Helicase<-OLD-ABC+TOPRIM<- SIGMA+OB-Fold+AAA-ATPase+HKD-DNase -BrxL <- PglZ <- N6-METHYLASE <- DUF6079 *<- BrxB-BrxF-iSTAND <- BrxA-DNA-binding <- wHTH+WYL+WCX ||Calcineurin+Calcineurin-SbcD->SbcC+SbcC-><-MODE-HTH+PBPII||TetR-HTH-><-RHH<-VirB10	1179	Bacteria>Pseudomonadota>Alphaproteobacteria>Rhodobacterales>Paracoccaceae>Gemmobacter	GCF_014636395.1	Gemmobacter_megaterium	IEV79_RS02805	604470..608009	1086013	BREX-system-P-loop-protein-BrxC-[Gemmobacter-megaterium]
WP_229704463.1	Type1.BREX	<-SWIM||Arm-DNA-bind_3+Phage_int_SAM_3+Phage_integrase-> wHTH+WYL+WCX -> BrxA-DNA-binding -> BrxB-BrxF-iSTAND -> DUF6079 *-> N6-METHYLASE -> PglZ -> SIGMA+OB-Fold+AAA-ATPase+HKD-DNase -BrxL -><-?<-Glyco_hydro_53+Phage-tail_3	1169	Bacteria>Pseudomonadota>Alphaproteobacteria>Rhodobacterales>Paracoccaceae>Gemmobacter	GCF_014645295.1	Gemmobacter_aquaticus	IEX45_RS17475	6610..10119	490185	BREX-system-P-loop-protein-BrxC-[Gemmobacter-aquaticus]
WP_131389735.1	Type1.BREX	VirB8->VirB9->VirB10->RHH-><-TetR-HTH||MODE-HTH+PBPII-> wHTH+WYL+WCX -> BrxA-DNA-binding -> BrxB-BrxF-iSTAND -> DUF6079 *-> N6-METHYLASE -> PglZ -> SIGMA+OB-Fold+AAA-ATPase+HKD-DNase -BrxL ->SF1-SF2-Helicase+REase->SF1-SF2-Helicase+REase->wHTH-> HKD-DNase + HKD-DNase ->SF1-SF2-Helicase->	1179	Bacteria>Pseudomonadota>Alphaproteobacteria>Rhodobacterales>Paracoccaceae>Haematobacter	GCF_004328555.1	Haematobacter_massiliensis	HmaOT1_RS15910	65338..68877	195105	BREX-system-P-loop-protein-BrxC-[Haematobacter-massiliensis]
WP_039019257.1	Type1.BREX	<-VirD4-TraG+VirD4-FtsK<-TraI-VirD2-RCRE<-HIN-HTH+TraJ-RHH<-?<-FurR-HTH<-ArdCN+MPTase|| wHTH+WYL+WCX -> BrxA-DNA-binding -> BrxB-BrxF-iSTAND -> DUF6079 *-> N6-METHYLASE -> PglZ -> SIGMA+OB-Fold+AAA-ATPase+HKD-DNase -BrxL ->REase+iREase-><-?<-PIN<-XIS-HTH<-HTH_36<-Phage_integrase	1172	Bacteria>Pseudomonadota>Alphaproteobacteria>Rhodobacterales>Paracoccaceae>Halocynthiibacter	GCF_000812685.1	Halocynthiibacter_namhaensis	RJ38_RS12670	13538..17056	1290553	BREX-system-P-loop-protein-BrxC-[Halocynthiibacter-namhaensis]
WP_263953721.1	Type1.BREX	<-PAS|| wHTH+WYL+WCX -> BrxA-DNA-binding -> BrxB-BrxF-iSTAND -> DUF6079 *-> N6-METHYLASE -> PglZ -> SIGMA+OB-Fold+AAA-ATPase+HKD-DNase -BrxL -><-SLOG<-HAD<-GP+REase||Phage_integrase->	1172	Bacteria>Pseudomonadota>Alphaproteobacteria>Rhodobacterales>Paracoccaceae>Halocynthiibacter	GCF_025802015.1	unclassified_Halocynthiibacter	OH136_RS09920	459600..463118	2640401	MULTISPECIES:-BREX-system-P-loop-protein-BrxC-[unclassified-Halocynthiibacter]
WP_274653192.1	Type1.BREX	<-ParB+HNH+Schlafen<-?<- SIGMA+OB-Fold+AAA-ATPase+HKD-DNase -BrxL <- PglZ <- N6-METHYLASE <- N6-METHYLASE <-NERD<-OLD-ABC<- DUF6079 *<- BrxB-BrxF-iSTAND <- BrxA-DNA-binding <- wHTH+WYL+WCX ||ArdCN+MPTase->?->?->FurR-HTH->?->HIN-HTH+TraJ-RHH->	1172	Bacteria>Pseudomonadota>Alphaproteobacteria>Rhodobacterales>Paracoccaceae>Halocynthiibacter	GCF_028823805.1	Halocynthiibacter_sp._C4	OU789_RS03095	642215..645733	2992758	BREX-system-P-loop-protein-BrxC-[Halocynthiibacter-sp.-C4]
WP_269398497.1	Type1.BREX	<-Integrase_DNA+Phage_integrase||?->SF1-SF2-Helicase+DUF1998-><- SIGMA+OB-Fold+AAA-ATPase+HKD-DNase -BrxL <- PglZ <- N6-METHYLASE <- DUF6079 *<- BrxB-BrxF-iSTAND <- BrxA-DNA-binding <-?<-DnaB||XIS-HTH->XIS-HTH->Phage_integrase->Phage_integrase->	1171	Bacteria>Pseudomonadota>Alphaproteobacteria>Rhodobacterales>Paracoccaceae>Limimaricola	GCF_027213785.1	Limimaricola_sp._G21655-S1	O4G76_RS17300	20007..23522	3014768	BREX-system-P-loop-protein-BrxC-[Limimaricola-sp.-G21655-S1]
WP_094023246.1	Type1.BREX	<-Phage_integrase<-XIS-HTH||HIN-HTH->AAA-ATPase->DEOR-HTH-> wHTH+WYL+WCX -p->wHTH+WYL-> BrxA-DNA-binding -> BrxB-BrxF-iSTAND -> DUF6079 *-> N6-METHYLASE -> PglZ ->?-> SIGMA+OB-Fold+AAA-ATPase+HKD-DNase -BrxL ->SF1-SF2-Helicase->	1175	Bacteria>Pseudomonadota>Alphaproteobacteria>Rhodobacterales>Paracoccaceae>Maliponia	GCF_900184945.1	Maliponia_aquimaris	MAA8898_RS22355	47898..51425	1673631	BREX-system-P-loop-protein-BrxC-[Maliponia-aquimaris]
MBO6774806.1	Type1.BREX	<-HHH_5+SF1-SF2-Helicase<-Thermonuclease<-DOC+HTH||RNase_T+35exo-><-?<-?|| wHTH+WYL+WCX -> BrxA-DNA-binding -> BrxB-BrxF-iSTAND -> DUF6079 *-> N6-METHYLASE -> PglZ -> SIGMA+OB-Fold+AAA-ATPase+HKD-DNase -BrxL ->REase+iREase->?->?-><-RADICAL-SAM	1172	Bacteria>Pseudomonadota>Alphaproteobacteria>Rhodobacterales>Paracoccaceae>Marinibacterium	GCA_017643305.1	Marinibacterium_sp.	JJ897_04735	11284..14802	1931201	BREX-system-P-loop-protein-BrxC-[Marinibacterium-sp.]
AP088218.1	Type1.BREX	<-HEPN+PAS+DUF4011+SF1-SF2-Helicase+REase<-?<- SIGMA+OB-Fold+AAA-ATPase+HKD-DNase -BrxL <- PglZ <- N6-METHYLASE <- DUF6079 *<- BrxB-BrxF-iSTAND <- BrxA-DNA-binding <- wHTH+WYL+WCX ||?-><-LON-AAA||<-tRNA||PRTase->?->REC+OmpR-HTH->HPT+HISKIN+CheW+REC->	1166	Bacteria>Pseudomonadota>Alphaproteobacteria>Rhodobacterales>Paracoccaceae>Marivivens_group	GCA_001908835.1	Marivivens_sp._JLT3646	BSK21_01620	313072..316572	1920883	hypothetical-protein-BSK21_01620-[Marivivens-sp.-JLT3646]
WP_017998950.1	Type1.BREX	<-HIN-HTH<-RNasePH<-LZ_Tnp_IS66+zf-IS66+DDE_Tnp_IS66+DDE_Tnp_IS66_C<-?<-HIN-HTH<- SIGMA+OB-Fold+AAA-ATPase+HKD-DNase -BrxL <- PglZ <- N6-METHYLASE <- DUF6079 *<- BrxB-BrxF-iSTAND <- BrxA-DNA-binding <- wHTH+WYL+WCX <-AAA+HTH<-?||?->?->Classical-AAA->	1177	Bacteria>Pseudomonadota>Alphaproteobacteria>Rhodobacterales>Paracoccaceae>Paracoccus	GCF_000371965.1	Paracoccus_sp._N5	PARN5_RS0106405	1284773..1288306	1101189	BREX-system-P-loop-protein-BrxC-[Paracoccus-sp.-N5]
WP_042245816.1	Type1.BREX	DDE_Tnp->DDE_Tnp-><- HKD-DNase +Preader14<- SIGMA+OB-Fold+AAA-ATPase+HKD-DNase -BrxL <- PglZ <- PglZ -p<- N6-METHYLASE -p<- N6-METHYLASE <- DUF6079 *<- BrxB-BrxF-iSTAND <- BrxA-DNA-binding <-35exo<-RelE-ParE<-MetJarc-HTH||?-><-DDE_3	1174	Bacteria>Pseudomonadota>Alphaproteobacteria>Rhodobacterales>Paracoccaceae>Paracoccus	GCF_000787695.1	Paracoccus_sp._PAMC_22219	TY51_RS03260	14723..18247	1569209	BREX-system-P-loop-protein-BrxC-[Paracoccus-sp.-PAMC-22219]
WP_046001801.1	Type1.BREX	TarH+HAMP+MA-><-Glyoxalase<-?<-ARG||WXG-><-PIN<- SIGMA+OB-Fold+AAA-ATPase+HKD-DNase -BrxL <- PglZ <- N6-METHYLASE <- DUF6079 *<- BrxB-BrxF-iSTAND <- BrxA-DNA-binding <-35exo<-RelE-ParE<-MetJarc-HTH|| HKD-DNase +DUF3150->HTH_Tnp_1->	1174	Bacteria>Pseudomonadota>Alphaproteobacteria>Rhodobacterales>Paracoccaceae>Paracoccus	GCF_000967825.1	Paracoccus_sp._228	SY26_RS18685	30060..33584	1192054	BREX-system-P-loop-protein-BrxC-[Paracoccus-sp.-228]
WP_199257821.1	Type1.BREX	GDResolvase+Recombinase+ZnR-><-SF1-SF2-Helicase+REase<-?<- HKD-DNase + HKD-DNase <-wHTH<-SF1-SF2-Helicase+REase<-SF1-SF2-Helicase+REase<- SIGMA+OB-Fold+AAA-ATPase+HKD-DNase -BrxL <- PglZ <- N6-METHYLASE <- DUF6079 *<- BrxB-BrxF-iSTAND <- BrxA-DNA-binding <- wHTH+WYL+WCX <-MODE-HTH+PBPII||TetR-HTH-><-RHH<-VirB10<-VirB9<-VirB8	1179	Bacteria>Pseudomonadota>Alphaproteobacteria>Rhodobacterales>Paracoccaceae>Paracoccus	GCF_016446475.1	Paracoccus_binzhouensis	I9P22_RS02450	23714..27253	2796149	BREX-system-P-loop-protein-BrxC-[Paracoccus-binzhouensis]
WP_260276663.1	Type1.BREX	URI-DNase+RAMA->XIS-HTH-> wHTH+WYL+WCX -> BrxA-DNA-binding -> BrxB-BrxF-iSTAND -> DUF6079 *-> N6-METHYLASE -> PglZ -> SIGMA+OB-Fold+AAA-ATPase+HKD-DNase -BrxL ->URI-DNase+RAMA-><-?<-Glyco_hydro_53+Phage-tail_3	1173	Bacteria>Pseudomonadota>Alphaproteobacteria>Rhodobacterales>Paracoccaceae>Paracoccus	GCF_025195095.1	Paracoccus_sp._YLB-12	MU516_RS07900	40235..43756	2933292	BREX-system-P-loop-protein-BrxC-[Paracoccus-sp.-YLB-12]
WP_271118256.1	Type1.BREX	HTH_3->?-><-TrbM<-Thermonuclease<-?|| wHTH+WYL+WCX -> BrxA-DNA-binding -> BrxB-BrxF-iSTAND -> DUF6079 *-> N6-METHYLASE -> PglZ -> SIGMA+OB-Fold+AAA-ATPase+HKD-DNase -BrxL ->HIN-HTH->AAA-ATPase->	1173	Bacteria>Pseudomonadota>Alphaproteobacteria>Rhodobacterales>Paracoccaceae>Paracoccus	GCF_027912835.1	Paracoccus_sp._SCSIO_76264	PAF18_RS16795	74012..77533	3017783	BREX-system-P-loop-protein-BrxC-[Paracoccus-sp.-SCSIO-76264]
WP_149766716.1	Type1.BREX	<-HTH<-Classical-AAA<-?<-?||?->AAA+HTH-> wHTH+WYL+WCX -> BrxA-DNA-binding -> BrxB-BrxF-iSTAND -> DUF6079 *-> PglZ -> SIGMA+OB-Fold+AAA-ATPase+HKD-DNase -BrxL ->?->ParB->	1177	Bacteria>Pseudomonadota>Alphaproteobacteria>Rhodobacterales>Paracoccaceae>Paracoccus	GCF_900156255.1	Paracoccus_thiocyanatus	F3H32_RS16120	13661..17194	34006	BREX-system-P-loop-protein-BrxC-[Paracoccus-thiocyanatus]
WP_085502591.1	Type1.BREX	<-ABhydrolase+ABhydrolase<-?||MODE-HTH+PBPII-><-SbcC+SbcC<-Calcineurin+Calcineurin-SbcD|| wHTH+WYL+WCX -> BrxA-DNA-binding -> BrxB-BrxF-iSTAND -> DUF6079 *-> N6-METHYLASE -> PglZ -> SIGMA+OB-Fold+AAA-ATPase+HKD-DNase -BrxL ->?-><-?<-?||SF1-SF2-Helicase+DUF1998->?->RelE-ParE->	1179	Bacteria>Pseudomonadota>Alphaproteobacteria>Rhodobacterales>Paracoccaceae>Paracoccus	GCF_900177635.1	Paracoccus_sp._J56	B9Y79_RS14640	24785..28324	935850	BREX-system-P-loop-protein-BrxC-[Paracoccus-sp.-J56]
WP_113913307.1	Type1.BREX	SIR2-><-?<-REase<-Immank+ANK+ANK<-GreA_GreB+SF1-SF2-Helicase<- SIGMA+OB-Fold+AAA-ATPase+HKD-DNase -BrxL <- PglZ <- N6-METHYLASE <- DUF6079 *<- BrxB-BrxF-iSTAND <- BrxA-DNA-binding <- wHTH+WYL+WCX ||Calcineurin+Calcineurin-SbcD->SbcC+SbcC->	1179	Bacteria>Pseudomonadota>Alphaproteobacteria>Rhodobacterales>Paracoccaceae>Pelagivirga	GCF_003316635.1	Pelagivirga_dicentrarchi	DQ230_RS14395	19757..23296	2250573	BREX-system-P-loop-protein-BrxC-[Pelagivirga-dicentrarchi]
RBP80165.1	Type1.BREX	XIS-HTH-> wHTH+WYL+WCX -> BrxA-DNA-binding -> BrxB-BrxF-iSTAND -> DUF6079 *->OLD-ABC-> N6-METHYLASE -> PglZ -> SIGMA+OB-Fold+AAA-ATPase+HKD-DNase -BrxL -><-?<-Glyco_hydro_53+Phage-tail_3	1172	Bacteria>Pseudomonadota>Alphaproteobacteria>Rhodobacterales>Paracoccaceae>Rhodobacter	GCA_003315475.1	Rhodobacter_sp._140A	DFO80_1418	4623..8141	2183910	hypothetical-protein-DFO80_1418-[Rhodobacter-sp.-140A]
WP_223637900.1	Type1.BREX	MMR_HSR1+sGTP+sGTP->?-><-?||?->?->SF1-SF2-Helicase+DUF1998-> BrxA-DNA-binding -> BrxB-BrxF-iSTAND -> DUF6079 *-> N6-METHYLASE -> PglZ -> SIGMA+OB-Fold+AAA-ATPase+HKD-DNase -BrxL ->	1175	Bacteria>Pseudomonadota>Alphaproteobacteria>Rhodobacterales>Paracoccaceae>Rhodobacter	GCF_020023995.1	Rhodobacter_sp._TJ_12	CKO11_RS10405	86480..90007	2029399	BREX-system-P-loop-protein-BrxC-[Rhodobacter-sp.-TJ_12]
WP_249060201.1	Type6.BREX	RelE-ParE->TPR->RNase_T+35exo->XIS-HTH-> BrxE -> BrxA-DNA-binding -> BrxB-BrxF-iSTAND -> DUF6079 *-> N6-METHYLASE -> N6-METHYLASE -> PglZ -> SIGMA+OB-Fold+AAA-ATPase+HKD-DNase -BrxL ->?->DUF4236+TPR->McrB-AAA->McrC-NTD+REase->	1206	Bacteria>Pseudomonadota>Alphaproteobacteria>Rhodobacterales>Paracoccaceae>Roseibaca	GCF_023336755.1	Roseibaca_sp._V10	M3N55_RS14200	5935..9555	2940293	BREX-system-P-loop-protein-BrxC-[Roseibaca-sp.-V10]
WP_121096034.1	Type1.BREX	Glyco_hydro_53+Phage-tail_3->?->?->?-><- SIGMA+OB-Fold+AAA-ATPase+HKD-DNase -BrxL <- PglZ <- N6-METHYLASE <- DUF6079 *<- BrxB-BrxF-iSTAND <- BrxA-DNA-binding <- wHTH+WYL+WCX <-RNase_T+35exo<-TPR<-Couple_hipA+HipA_C<-HTH_3||Arm-DNA-bind_3+Phage_int_SAM_3+Phage_integrase->	1173	Bacteria>Pseudomonadota>Alphaproteobacteria>Rhodobacterales>Paracoccaceae>Roseibaca	GCF_900499075.1	Roseibaca_ekhonensis	ROE7235_RS13515	24046..27567	254356	BREX-system-P-loop-protein-BrxC-[Roseibaca-ekhonensis]
WP_218631289.1	Type1.BREX	<- SIGMA+OB-Fold+AAA-ATPase+HKD-DNase -BrxL <- PglZ <- N6-METHYLASE <- DUF6079 *<- BrxB-BrxF-iSTAND <- BrxA-DNA-binding <-?<-ZnR+TOPRIM+AAA-helicase+wHTH<-TOPRIM<-Phage_integrase<-tRNA||?-><-?<-PALP	1173	Bacteria>Pseudomonadota>Alphaproteobacteria>Rhodobacterales>Paracoccaceae>Sedimentimonas	GCF_019264375.1	Sedimentimonas_flavescens	KTQ37_RS14800	8221..11742	2851012	BREX-system-P-loop-protein-BrxC-[Sedimentimonas-flavescens]
WP_233675470.1	Type5.BREX	<-TPR+REase<-?<-TUDOR+SF1-SF2-Helicase+iREase+CTD<- SIGMA+OB-Fold+AAA-ATPase+HKD-DNase -BrxL <- PglZ <- N6-METHYLASE <-?<- N6-METHYLASE <- DUF6079 *<- BrxB-BrxF-iSTAND <- BrxA-DNA-binding <- wHTH+WYL+WCX <-Phage_integrase<-tRNA||?-><-?<-PALP	1173	Bacteria>Pseudomonadota>Alphaproteobacteria>Rhodobacterales>Paracoccaceae>Sinirhodobacter	-	Sinirhodobacter_sp._WL0062	LZA78_02955	53668..57189	2907145	BREX-system-P-loop-protein-BrxC-[Sinirhodobacter-sp.-WL0062]
NTT88404.1	Type6.BREX	<-SWIM||Arm-DNA-bind_3+Phage_int_SAM_3+Phage_integrase-> wHTH+WYL+WCX -> BrxE -> BrxA-DNA-binding -> BrxB-BrxF-iSTAND -> DUF6079 *-> N6-METHYLASE -> PglZ -> SIGMA+OB-Fold+AAA-ATPase+HKD-DNase -BrxL ->	1208	Bacteria>Pseudomonadota>Alphaproteobacteria>Rhodobacterales>Paracoccaceae>Tabrizicola	GCA_013328815.1	Tabrizicola_sp._SY72	HS053_21140	11855..15481	2741673	BREX-system-P-loop-protein-BrxC-[Tabrizicola-sp.-SY72]
WP_194245628.1	Type6.BREX	<-SWIM||Arm-DNA-bind_3+Phage_int_SAM_3+Phage_integrase-> wHTH+WYL+WCX -> BrxE -> BrxA-DNA-binding -> BrxB-BrxF-iSTAND -> DUF6079 *-> N6-METHYLASE -> N6-METHYLASE -p-> PglZ -> SIGMA+OB-Fold+AAA-ATPase+HKD-DNase -BrxL ->	1213	Bacteria>Pseudomonadota>Alphaproteobacteria>Rhodobacterales>Paracoccaceae>Tabrizicola	GCA_013328815.1	Tabrizicola_sp._SY72	HS053_RS21145	11840..15481	2741673	BREX-system-P-loop-protein-BrxC-[Tabrizicola-sp.-SY72]
WP_108641830.1	Type1.BREX	<-Schlafen<-?<-ParB+HNH+Schlafen<- SIGMA+OB-Fold+AAA-ATPase+HKD-DNase -BrxL <- PglZ <- N6-METHYLASE <- DUF6079 *<- BrxB-BrxF-iSTAND <- BrxA-DNA-binding <- wHTH+WYL+WCX ||?->Thermonuclease->TrbM-><-?<-HTH_3	1173	Bacteria>Pseudomonadota>Alphaproteobacteria>Rhodobacterales>Paracoccaceae>Thalassorhabdomicrobium	GCF_003072065.1	Thalassorhabdomicrobium_marinisediminis	DC363_RS14250	78584..82105	2170577	BREX-system-P-loop-protein-BrxC-[Thalassorhabdomicrobium-marinisediminis]
WP_058289795.1	Type1.BREX	<-TraI-VirD2-RCRE<-HIN-HTH+TraJ-RHH<-?<-FurR-HTH<-?<-ArdCN+MPTase|| wHTH+WYL+WCX -> BrxA-DNA-binding -> BrxB-BrxF-iSTAND -> DUF6079 *->OLD-ABC->NERD-> N6-METHYLASE -> PglZ -> SIGMA+OB-Fold+AAA-ATPase+HKD-DNase -BrxL ->ParB+HNH+Schlafen-><-?<-Couple_hipA+HipA_C<-HTH_3	1172	Bacteria>Pseudomonadota>Alphaproteobacteria>Rhodobacterales>Paracoccaceae>Tritonibacter	GCF_001458415.1	Tritonibacter_multivorans	PGB32_RS12460	15411..18929	928856	BREX-system-P-loop-protein-BrxC-[Tritonibacter-multivorans]
WP_065322029.1	Type1.BREX	XIS-HTH->PIN-><-AbiEi_4||HTH_3->?->?-><-REase+iREase<- SIGMA+OB-Fold+AAA-ATPase+HKD-DNase -BrxL <- PglZ <- N6-METHYLASE <- DUF6079 *<- BrxB-BrxF-iSTAND <- BrxA-DNA-binding <- wHTH+WYL+WCX ||ArdCN+MPTase->?->?->FurR-HTH-><-?||Phage_integrase->	1172	Bacteria>Pseudomonadota>Alphaproteobacteria>Rhodobacterales>Paracoccaceae>Tritonibacter	GCF_001681255.1	Tritonibacter_mobilis	UB66_RS09365	554629..558147	379347	BREX-system-P-loop-protein-BrxC-[Tritonibacter-mobilis]
WP_225262956.1	Type1.BREX	<-HEPN+OST-HTH<-ParB+HNH<-Phage_integrase+Phage_integrase<-?<-Phage_int_SAM_1+Phage_integrase<-ArdCN+MPTase|| wHTH+WYL+WCX -> BrxA-DNA-binding -> BrxB-BrxF-iSTAND -> DUF6079 *-> N6-METHYLASE -> PglZ -> SIGMA+OB-Fold+AAA-ATPase+HKD-DNase -BrxL ->S1COLD->?-><-AAA-ATPase<-HIN-HTH	1172	Bacteria>Pseudomonadota>Alphaproteobacteria>Rhodobacterales>Paracoccaceae>Tritonibacter	GCF_020216085.1	Tritonibacter_mobilis	LC038_RS07430	133716..137234	379347	BREX-system-P-loop-protein-BrxC-[Tritonibacter-mobilis]
PHO04850.1	Type1.BREX	<- SIGMA+OB-Fold+AAA-ATPase+HKD-DNase -BrxL <- PglZ <- N6-METHYLASE <- DUF6079 *<- BrxB-BrxF-iSTAND <- BrxA-DNA-binding ||?->Classical-AAA->?-><-?<-?<-TraI-VirD2-RCRE<-CopG/RHH	1166	Bacteria>Pseudomonadota>Alphaproteobacteria>Rhodobacterales>Paracoccaceae>unclassified_Paracoccaceae	GCA_002700045.1	Rhodobacteraceae_bacterium_4F10	CSC82_04805	8196..11696	2045445	hypothetical-protein-CSC82_04805-[Rhodobacteraceae-bacterium-4F10]
NKX29277.1	Type1.BREX	<-McrC-NTD+REase<-HTH+McrB-AAA<-SbcC<-SbcC-p<-Calcineurin-SbcD<- SIGMA+OB-Fold+AAA-ATPase+HKD-DNase -BrxL <- PglZ <- N6-METHYLASE <- DUF6079 *<- BrxB-BrxF-iSTAND <- BrxA-DNA-binding <- wHTH+WYL+WCX ||ArdCN+MPTase->Phage_integrase->	1168	Bacteria>Pseudomonadota>Alphaproteobacteria>Rhodobacterales>Paracoccaceae>unclassified_Paracoccaceae	GCA_012395685.1	Rhodobacteraceae_bacterium_R_SAG6	HGE68_05220	14041..17547	2719008	BREX-system-P-loop-protein-BrxC-[Rhodobacteraceae-bacterium-R_SAG6]
MBD1206697.1	Type1.BREX	<-SIGMA-HTH+HIN-HTH<- SIGMA+OB-Fold+AAA-ATPase+HKD-DNase -BrxL <- PglZ <- N6-METHYLASE <- DUF6079 *<- BrxB-BrxF-iSTAND <- BrxA-DNA-binding <-REase<- wHTH+WYL+WCX <-GDResolvase+HIN-HTH	1181	Bacteria>Pseudomonadota>Alphaproteobacteria>Rhodobacterales>Paracoccaceae>unclassified_Paracoccaceae	GCA_014654775.1	Paracoccaceae_bacterium	H9533_21535	9124..12669	1904441	BREX-system-P-loop-protein-BrxC-[Paracoccaceae-bacterium]
MBL4542464.1	Type1.BREX	Glyco_hydro_53+Phage-tail_3->?-><-URI-DNase+RAMA<- SIGMA+OB-Fold+AAA-ATPase+HKD-DNase -BrxL <- PglZ <- N6-METHYLASE <- DUF6079 *<- BrxB-BrxF-iSTAND <- BrxA-DNA-binding <- wHTH+WYL+WCX <-Arm-DNA-bind_3+Phage_int_SAM_3+Phage_integrase||<-?||?->ABC-ATPase+SbcC->	1173	Bacteria>Pseudomonadota>Alphaproteobacteria>Rhodobacterales>Paracoccaceae>unclassified_Paracoccaceae	GCA_016762795.1	Paracoccaceae_bacterium	JKP97_11775	15111..18632	1904441	BREX-system-P-loop-protein-BrxC-[Paracoccaceae-bacterium]
MCR9111160.1	Type1.BREX	DDE_Tnp_IS66+DDE_Tnp_IS66_C-><-?||<-HTH_3||?-> HKD-DNase +DUF3150-> wHTH+WYL+WCX -> BrxA-DNA-binding -> BrxB-BrxF-iSTAND -> DUF6079 *-> N6-METHYLASE -> PglZ -> SIGMA+OB-Fold+AAA-ATPase+HKD-DNase -BrxL ->?->CHTH->?-><-Arm-DNA-bind_3+Phage_integrase	1176	Bacteria>Pseudomonadota>Alphaproteobacteria>Rhodobacterales>Paracoccaceae>unclassified_Paracoccaceae	GCA_024742895.1	Paracoccaceae_bacterium	NXH94_20060	7296..10826	1904441	BREX-system-P-loop-protein-BrxC-[Paracoccaceae-bacterium]
MBD3677805.1	Type1.BREX	<-SbcC+SbcC<-Calcineurin|| wHTH+WYL+WCX ->?->?-><- SIGMA+OB-Fold+AAA-ATPase+HKD-DNase -BrxL <- PglZ <- N6-METHYLASE <- DUF6079 *<- BrxB-BrxF-iSTAND <- BrxA-DNA-binding <- wHTH+WYL+WCX ||?->Thermonuclease->TrbM-><-?<-HTH_3	1173	Bacteria>Pseudomonadota>Alphaproteobacteria>Rhodobacterales>Paracoccaceae>unclassified_Paracoccaceae	-	Paracoccaceae_bacterium	HUJ27_05300	15046..18567	1904441	BREX-system-P-loop-protein-BrxC-[Paracoccaceae-bacterium]
WP_089279695.1	Type1.BREX	Glyco_hydro_53+Phage-tail_3->?->?->?-><- SIGMA+OB-Fold+AAA-ATPase+HKD-DNase -BrxL <- PglZ <- N6-METHYLASE <- DUF6079 *<- BrxB-BrxF-iSTAND <- BrxA-DNA-binding <- wHTH+WYL+WCX <-35exo<-TPR<-Couple_hipA+HipA_C<-HTH_3	1173	Bacteria>Pseudomonadota>Alphaproteobacteria>Rhodobacterales>Roseobacteraceae>Antarctobacter	GCF_900188425.1	Antarctobacter_heliothermus	CHB90_RS20470	19687..23208	74033	BREX-system-P-loop-protein-BrxC-[Antarctobacter-heliothermus]
WP_106262543.1	Type1.BREX	<-Phage_int_SAM_1+Phage_integrase<-?<-?||SKI->?-><-HEPN|| wHTH+WYL+WCX -> BrxA-DNA-binding -> BrxB-BrxF-iSTAND -> DUF6079 *->OLD-ABC->REase-> N6-METHYLASE -> PglZ -> SIGMA+OB-Fold+AAA-ATPase+HKD-DNase -BrxL -><-?||HTH_Tnp_1->HTH_Tnp_1+HTH_21+rve+rve_3-><-HIN-HTH	1172	Bacteria>Pseudomonadota>Alphaproteobacteria>Rhodobacterales>Roseobacteraceae>Donghicola	GCF_003003355.1	Donghicola_tyrosinivorans	CLV74_RS02110	436162..439680	1652492	BREX-system-P-loop-protein-BrxC-[Donghicola-tyrosinivorans]
WP_027259561.1	Type1.BREX	<-TraI-VirD2-RCRE<-HIN-HTH+TraJ-RHH<-?<-FurR-HTH<-?<-ArdCN+MPTase|| wHTH+WYL+WCX -> BrxA-DNA-binding -> BrxB-BrxF-iSTAND -> DUF6079 *-> N6-METHYLASE -> PglZ -> SIGMA+OB-Fold+AAA-ATPase+HKD-DNase -BrxL ->REase+iREase-><-?<-HTH_3||AbiEi_4->AbiEii-><-HTH_36	1172	Bacteria>Pseudomonadota>Alphaproteobacteria>Rhodobacterales>Roseobacteraceae>Leisingera	GCF_000473165.1	Leisingera_aquimarina	AQUI_RS0122640	3713027..3716545	476529	BREX-system-P-loop-protein-BrxC-[Leisingera-aquimarina]
WP_263764345.1	Type1.BREX	<-HTH_3<-?||DEDD_Tnp_IS110+Transposase_20-><-?<-?|| wHTH+WYL+WCX -> BrxA-DNA-binding -> BrxB-BrxF-iSTAND -> DUF6079 *-> N6-METHYLASE -> PglZ -> SIGMA+OB-Fold+AAA-ATPase+HKD-DNase -BrxL -><-?||HNH->	1175	Bacteria>Pseudomonadota>Alphaproteobacteria>Rhodobacterales>Roseobacteraceae>Lentibacter	GCF_025751535.1	Lentibacter_sp._XHP0401	OE750_RS11445	67112..70639	2984334	BREX-system-P-loop-protein-BrxC-[Lentibacter-sp.-XHP0401]
WP_263765218.1	Type1.BREX	<-REase<-Immank+ANK+ANK<-GreA_GreB+SF1-SF2-Helicase<- SIGMA+OB-Fold+AAA-ATPase+HKD-DNase -BrxL <- PglZ <- N6-METHYLASE <- DUF6079 *<- BrxB-BrxF-iSTAND <- BrxA-DNA-binding <- wHTH+WYL+WCX ||Calcineurin+Calcineurin-SbcD->SbcC+SbcC->?->AAA+HTH-><-?||LON-AAA->	1167	Bacteria>Pseudomonadota>Alphaproteobacteria>Rhodobacterales>Roseobacteraceae>Lentibacter	GCF_025751535.1	Lentibacter_sp._XHP0401	OE750_RS15480	13619..17122	2984334	BREX-system-P-loop-protein-BrxC-[Lentibacter-sp.-XHP0401]
WP_107845004.1	Type6.BREX	<-TraI-VirD2-RCRE<-HIN-HTH+TraJ-RHH<-?<-FurR-HTH<-ArdCN+MPTase|| wHTH+WYL+WCX -> BrxE -> BrxA-DNA-binding -> BrxB-BrxF-iSTAND -> DUF6079 *-> N6-METHYLASE -> N6-METHYLASE -p-> PglZ -> SIGMA+OB-Fold+AAA-ATPase+HKD-DNase -BrxL -><-?<-Couple_hipA+HipA_C<-HTH_3<-HTH_36	1208	Bacteria>Pseudomonadota>Alphaproteobacteria>Rhodobacterales>Roseobacteraceae>Litoreibacter	GCF_003054285.1	Litoreibacter_ponti	C8N43_RS07515	1489253..1492879	1510457	BREX-system-P-loop-protein-BrxC-[Litoreibacter-ponti]
WP_088668915.1	Type1.BREX	VirB10->RHH-><-TetR-HTH||MODE-HTH+PBPII-><-SbcC+SbcC<-Calcineurin+Calcineurin-SbcD|| wHTH+WYL+WCX -> BrxA-DNA-binding -> BrxB-BrxF-iSTAND -> DUF6079 *-> N6-METHYLASE -> PglZ -> SIGMA+OB-Fold+AAA-ATPase+HKD-DNase -BrxL -><-Zn_ribbon_recom||?->ParA-Soj-PloopNTPase->	1179	Bacteria>Pseudomonadota>Alphaproteobacteria>Rhodobacterales>Roseobacteraceae>Mameliella	GCF_002211215.1	Mameliella_alba	CDZ95_RS03685	62213..65752	561184	BREX-system-P-loop-protein-BrxC-[Mameliella-alba]
WP_167646419.1	Type1.BREX	BrxA-DNA-binding -> BrxB-BrxF-iSTAND -> DUF6079 *-> N6-METHYLASE -> PglZ -> SIGMA+OB-Fold+AAA-ATPase+HKD-DNase -BrxL ->?->DUF4096+DDE_Tnp_1+DDE_Tnp_1_2->	1175	Bacteria>Pseudomonadota>Alphaproteobacteria>Rhodobacterales>Roseobacteraceae>Mameliella	GCF_011858205.1	Mameliella_alba	DAA47_RS08650	638377..641904	561184	BREX-system-P-loop-protein-BrxC-[Mameliella-alba]
WP_091845786.1	Type1.BREX	<-Phage_integrase<-?<-ArdCN+MPTase|| wHTH+WYL+WCX -> BrxA-DNA-binding -> BrxB-BrxF-iSTAND -> DUF6079 *-> N6-METHYLASE -> PglZ -> SIGMA+OB-Fold+AAA-ATPase+HKD-DNase -BrxL ->REase+iREase-><-?<-?<-HORMA<-STYKinase-like<-TRPR-HTH	1172	Bacteria>Pseudomonadota>Alphaproteobacteria>Rhodobacterales>Roseobacteraceae>Maribius	GCF_900110115.1	Maribius_pelagius	BMX96_RS12425	183654..187172	387096	BREX-system-P-loop-protein-BrxC-[Maribius-pelagius]
WP_274591702.1	Type1.BREX	XIS-HTH-> wHTH+WYL+WCX -> BrxA-DNA-binding -> BrxB-BrxF-iSTAND -> DUF6079 *->OLD-ABC->REase-> N6-METHYLASE -> PglZ -> SIGMA+OB-Fold+AAA-ATPase+HKD-DNase -BrxL ->	1173	Bacteria>Pseudomonadota>Alphaproteobacteria>Rhodobacterales>Roseobacteraceae>Marinovum	GCF_028807375.1	Marinovum_sp._PR37	PVW49_RS22970	3287..6808	3028382	BREX-system-P-loop-protein-BrxC-[Marinovum-sp.-PR37]
WP_188483502.1	Type6.BREX	Glyco_hydro_53+Phage-tail_3->?->?->Peptidase-M15->?->?-><- SIGMA+OB-Fold+AAA-ATPase+HKD-DNase -BrxL <- PglZ <- N6-METHYLASE <- N6-METHYLASE <- DUF6079 *<- BrxB-BrxF-iSTAND <- BrxA-DNA-binding <- BrxE <- wHTH+WYL+WCX <-McrC-NTD+REase<-EVE+McrB-AAA<-?<-ParB<-ParB	1208	Bacteria>Pseudomonadota>Alphaproteobacteria>Rhodobacterales>Roseobacteraceae>Marivita	GCF_014637725.1	Marivita_lacus	IEV82_RS18225	47405..51031	1323742	BREX-system-P-loop-protein-BrxC-[Marivita-lacus]
WP_123792783.1	Type1.BREX	<-ParB+HNH+Schlafen<- SIGMA+OB-Fold+AAA-ATPase+HKD-DNase -BrxL <- PglZ <- N6-METHYLASE <-REase<-OLD-ABC<- N6-METHYLASE <- DUF6079 *<- BrxB-BrxF-iSTAND <- BrxA-DNA-binding <-HIN-HTH<-HTH_36<-?<-?<-Arm-DNA-bind_3+Phage_integrase	1172	Bacteria>Pseudomonadota>Alphaproteobacteria>Rhodobacterales>Roseobacteraceae>Pacificibacter	GCF_003814915.1	Pacificibacter_maritimus	EDD53_RS08420	394668..398186	762213	BREX-system-P-loop-protein-BrxC-[Pacificibacter-maritimus]
WP_260112390.1	Type1.BREX	<-TraI-VirD2-RCRE<-HIN-HTH+TraJ-RHH<-?<-FurR-HTH<-?<-ArdCN+MPTase|| wHTH+WYL+WCX -> BrxA-DNA-binding -> BrxB-BrxF-iSTAND -> DUF6079 *->OLD-ABC->NERD-> N6-METHYLASE -> PglZ -> SIGMA+OB-Fold+AAA-ATPase+HKD-DNase -BrxL ->REase+iREase-><-?<-HTH_3||AbiEi_4->	1172	Bacteria>Pseudomonadota>Alphaproteobacteria>Rhodobacterales>Roseobacteraceae>Phaeobacter	GCF_025142595.1	Phaeobacter_inhibens	K4F86_RS11215	2363506..2367024	221822	BREX-system-P-loop-protein-BrxC-[Phaeobacter-inhibens]
WP_076451079.1	Type6.BREX	ABhydrolase-><-?<-Phage_integrase||XIS-HTH-> wHTH+WYL+WCX -> BrxE -> BrxA-DNA-binding -> BrxB-BrxF-iSTAND -> DUF6079 *-> N6-METHYLASE -> N6-METHYLASE -> PglZ -> SIGMA+OB-Fold+AAA-ATPase+HKD-DNase -BrxL -><-?<-?<-Peptidase-M15	1208	Bacteria>Pseudomonadota>Alphaproteobacteria>Rhodobacterales>Roseobacteraceae>Roseivivax	GCF_900156805.1	Roseivivax_lentus	BW990_RS19885	19994..23620	633194	BREX-system-P-loop-protein-BrxC-[Roseivivax-lentus]
WP_009810044.1	Type1.BREX	<-HARE-HTH+REase<- SIGMA+OB-Fold+AAA-ATPase+HKD-DNase -BrxL <- PglZ <- N6-METHYLASE <- DUF6079 *<- BrxB-BrxF-iSTAND <- BrxA-DNA-binding <- wHTH+WYL+WCX ||?->?->?-><-RNase_T+35exo<-?||DOC+HTH->	1172	Bacteria>Pseudomonadota>Alphaproteobacteria>Rhodobacterales>Roseobacteraceae>Roseobacter	GCF_000152965.1	Roseobacter_sp._MED193	MED193_RS12240	2047846..2051364	314262	BREX-system-P-loop-protein-BrxC-[Roseobacter-sp.-MED193]
KJS41393.1	Type1.BREX	<-SbcC+SbcC<-Calcineurin+Calcineurin-SbcD|| wHTH+WYL+WCX -> BrxA-DNA-binding -> BrxB-BrxF-iSTAND -> DUF6079 *-> N6-METHYLASE -> PglZ -> SIGMA+OB-Fold+AAA-ATPase+HKD-DNase -BrxL ->?->SF1-SF2-Helicase+DUF1998->CR-ATPase9->HEPN-><-DOC+HTH	1178	Bacteria>Pseudomonadota>Alphaproteobacteria>Rhodobacterales>Roseobacteraceae>Roseovarius	GCA_000961715.1	Roseovarius_sp._BRH_c41	VR71_18660	26779..30315	1629709	hypothetical-protein-VR71_18660-[Roseovarius-sp.-BRH_c41]
WP_268956598.1	Type6.BREX	<- SIGMA+OB-Fold+AAA-ATPase+HKD-DNase -BrxL <- PglZ <- N6-METHYLASE <- DUF6079 *<- BrxB-BrxF-iSTAND <- BrxA-DNA-binding <- BrxE <-XIS-HTH<-HTH_3||?->?->?->wHTH+HNH->	1203	Bacteria>Pseudomonadota>Alphaproteobacteria>Rhodobacterales>Roseobacteraceae>Roseovarius	-	Roseovarius_sp._EGI_FJ00037	N5A93_RS18625	8608..12219	2978479	BREX-system-P-loop-protein-BrxC-[Roseovarius-sp.-EGI-FJ00037]
WP_139076871.1	Type1.BREX	WXG-><-?<-?||RAMA->?->?-> BrxA-DNA-binding -> BrxB-BrxF-iSTAND -> DUF6079 *-> N6-METHYLASE -> PglZ -> SIGMA+OB-Fold+AAA-ATPase+HKD-DNase -BrxL ->	1175	Bacteria>Pseudomonadota>Alphaproteobacteria>Rhodobacterales>Roseobacteraceae>Rubellimicrobium	GCF_006152115.1	Rubellimicrobium_rubrum	FHG66_11370	28349..31876	2585369	BREX-system-P-loop-protein-BrxC-[Rubellimicrobium-rubrum]
WP_254429641.1	Type1.BREX	HORMA-><-?||CopG/RHH->TraI-VirD2-RCRE-><-Classical-AAA||?-> BrxA-DNA-binding -> BrxB-BrxF-iSTAND -> DUF6079 *-> N6-METHYLASE -> PglZ -> SIGMA+OB-Fold+AAA-ATPase+HKD-DNase -BrxL -><-?||?-><-tRNA<-?<-RNASE-EG	1170	Bacteria>Pseudomonadota>Alphaproteobacteria>Rhodobacterales>Roseobacteraceae>Ruegeria	GCF_013031165.1	Ruegeria_sp._HKCCD7251	GOX75_RS12475	203228..206740	2683011	BREX-system-P-loop-protein-BrxC-[Ruegeria-sp.-HKCCD7251]
WP_171123550.1	Type1.BREX	RNASE-EG->?->tRNA-><-?||?-><- SIGMA+OB-Fold+AAA-ATPase+HKD-DNase -BrxL <- PglZ <- N6-METHYLASE <- DUF6079 *<- BrxB-BrxF-iSTAND <- BrxA-DNA-binding <-?||Classical-AAA-><-TraI-VirD2-RCRE<-CopG/RHH||?-><-HORMA	1174	Bacteria>Pseudomonadota>Alphaproteobacteria>Rhodobacterales>Roseobacteraceae>Ruegeria	GCF_013031185.1	Ruegeria_sp._HKCCD7239	GOX74_RS13410	208876..212400	2683010	BREX-system-P-loop-protein-BrxC-[Ruegeria-sp.-HKCCD7239]
WP_170339699.1	Type1.BREX	TraI-VirD2-RCRE->?->?-><-Glyco_hydro_3<-Classical-AAA<-?||?-> BrxA-DNA-binding -> BrxB-BrxF-iSTAND -> DUF6079 *-> N6-METHYLASE -> PglZ -> SIGMA+OB-Fold+AAA-ATPase+HKD-DNase -BrxL ->?-><-RVT<-?<-CR-ATPase4	1173	Bacteria>Pseudomonadota>Alphaproteobacteria>Rhodobacterales>Roseobacteraceae>Ruegeria	GCF_013031325.1	Ruegeria_arenilitoris	GOX56_RS07985	128142..131663	1173585	BREX-system-P-loop-protein-BrxC-[Ruegeria-arenilitoris]
WP_254433969.1	Type1.BREX	CopG/RHH-><-?<-?<-?<-Classical-AAA||?-> BrxA-DNA-binding -> BrxB-BrxF-iSTAND -> DUF6079 *-> N6-METHYLASE -> PglZ -> SIGMA+OB-Fold+AAA-ATPase+HKD-DNase -BrxL -><-?<-tRNA<-?<-RNASE-EG<-?<-?<-CN_hydrolase	1170	Bacteria>Pseudomonadota>Alphaproteobacteria>Rhodobacterales>Roseobacteraceae>Ruegeria	GCF_013032735.1	Ruegeria_arenilitoris	GOW80_RS09865	349565..353077	1173585	BREX-system-P-loop-protein-BrxC-[Ruegeria-arenilitoris]
WP_170509651.1	Type1.BREX	<-Classical-AAA||?-> BrxA-DNA-binding -> BrxB-BrxF-iSTAND -> DUF6079 *-> N6-METHYLASE -> PglZ -> SIGMA+OB-Fold+AAA-ATPase+HKD-DNase -BrxL -><-?<-tRNA<-?<-RNASE-EG<-?<-?<-CN_hydrolase	1174	Bacteria>Pseudomonadota>Alphaproteobacteria>Rhodobacterales>Roseobacteraceae>Ruegeria	GCF_013032765.1	Ruegeria_arenilitoris	GOW79_RS10365	805076..808600	1173585	BREX-system-P-loop-protein-BrxC-[Ruegeria-arenilitoris]
WP_222457132.1	Type1.BREX	CopG/RHH-><-?<-?<-?<-Classical-AAA||?-> BrxA-DNA-binding -> BrxB-BrxF-iSTAND -> DUF6079 *-> N6-METHYLASE -> PglZ -> SIGMA+OB-Fold+AAA-ATPase+HKD-DNase -BrxL -><-?<-?<-tRNA<-?<-RNASE-EG	1174	Bacteria>Pseudomonadota>Alphaproteobacteria>Rhodobacterales>Roseobacteraceae>Ruegeria	GCF_019801065.1	Ruegeria_arenilitoris	KUW05_RS05645	1133713..1137237	1173585	BREX-system-P-loop-protein-BrxC-[Ruegeria-arenilitoris]
WP_150282504.1	Type1.BREX	MPTase-><-HTH+MPTase<-?<- SIGMA+OB-Fold+AAA-ATPase+HKD-DNase -BrxL <- PglZ <- N6-METHYLASE <-REase<-OLD-ABC<- DUF6079 *<- BrxB-BrxF-iSTAND <- BrxA-DNA-binding <- wHTH+WYL+WCX ||?->Thermonuclease->TrbM->	1173	Bacteria>Pseudomonadota>Alphaproteobacteria>Rhodobacterales>Roseobacteraceae>Salipiger	GCF_008692285.1	Salipiger_aestuarii	AL036_RS18780	13068..16589	568098	BREX-system-P-loop-protein-BrxC-[Salipiger-aestuarii]
WP_089852186.1	Type1.BREX	GDResolvase+HIN-HTH->HTH->ParA-Soj-PloopNTPase->?->?->?-> BrxA-DNA-binding -> BrxB-BrxF-iSTAND -> DUF6079 *-> N6-METHYLASE -> PglZ -> SIGMA+OB-Fold+AAA-ATPase+HKD-DNase -BrxL ->	1173	Bacteria>Pseudomonadota>Alphaproteobacteria>Rhodobacterales>Roseobacteraceae>Salipiger	GCF_900100085.1	Salipiger_marinus	BLR51_RS25320	13015..16536	555512	BREX-system-P-loop-protein-BrxC-[Salipiger-marinus]
PSL17302.1	Type6.BREX	<-HAMP+HISKIN||?->tRNA-><-SIR2<- SIGMA+OB-Fold+AAA-ATPase+HKD-DNase -BrxL <- PglZ <- N6-METHYLASE <- N6-METHYLASE <- DUF6079 *<- BrxB-BrxF-iSTAND <- BrxA-DNA-binding <- BrxE <-XIS-HTH<-HEPN<-Polbeta<-wHTH+AbiEi_2||REase->	1216	Bacteria>Pseudomonadota>Alphaproteobacteria>Rhodobacterales>Roseobacteraceae>Shimia	GCA_003014475.1	Shimia_abyssi	CLV88_11975	80664..84314	1662395	hypothetical-protein-CLV88_11975-[Shimia-abyssi]
WP_207797113.1	Type6.BREX	<-HAMP+HISKIN||?->tRNA-><-SIR2<- SIGMA+OB-Fold+AAA-ATPase+HKD-DNase -BrxL <- PglZ <- N6-METHYLASE <- N6-METHYLASE <- DUF6079 *<- BrxB-BrxF-iSTAND <- BrxA-DNA-binding <- BrxE <-XIS-HTH<-HEPN<-Polbeta<-wHTH+AbiEi_2||REase->	1207	Bacteria>Pseudomonadota>Alphaproteobacteria>Rhodobacterales>Roseobacteraceae>Shimia	GCF_003014475.1	Shimia_abyssi	CLV88_RS20200	80664..84287	1662395	BREX-system-P-loop-protein-BrxC-[Shimia-abyssi]
WP_203210578.1	Type1.BREX	STYKinase-like->?-><-McrC-NTD+REase<-HTH+McrB-AAA<-SbcC+CCDC158<-Calcineurin-SbcD<- SIGMA+OB-Fold+AAA-ATPase+HKD-DNase -BrxL <- PglZ <- N6-METHYLASE <- DUF6079 *<- BrxB-BrxF-iSTAND <- BrxA-DNA-binding <- wHTH+WYL+WCX ||ArdCN+MPTase->?->FurR-HTH->?->HIN-HTH+TraJ-RHH->TraI-VirD2-RCRE->	1172	Bacteria>Pseudomonadota>Alphaproteobacteria>Rhodobacterales>Roseobacteraceae>Sulfitobacter	GCF_016801775.1	Sulfitobacter_mediterraneus	JNX03_RS00635	149782..153300	83219	BREX-system-P-loop-protein-BrxC-[Sulfitobacter-mediterraneus]
WP_259953659.1	Type1.BREX	XIS-HTH->?-><-?<-?|| BrxA-DNA-binding -> BrxB-BrxF-iSTAND -> DUF6079 *-> N6-METHYLASE -> PglZ -> SIGMA+OB-Fold+AAA-ATPase+HKD-DNase -BrxL ->REase+iREase->Calcineurin-SbcD->SbcC+CCDC158->	1174	Bacteria>Pseudomonadota>Alphaproteobacteria>Rhodobacterales>Roseobacteraceae>Sulfitobacter	GCF_025140475.1	Sulfitobacter_mediterraneus	K3753_RS16990	3485906..3489430	83219	BREX-system-P-loop-protein-BrxC-[Sulfitobacter-mediterraneus]
WP_267167638.1	Type1.BREX	Phage_integrase->Arm-DNA-bind_3+Phage_integrase-><-?<-HAD+PRTase+PRTase+REase+SF1-SF2-Helicase||?->?->?-> BrxA-DNA-binding -> BrxB-BrxF-iSTAND -> DUF6079 *-> N6-METHYLASE -> PglZ -> SIGMA+OB-Fold+AAA-ATPase+HKD-DNase -BrxL ->TraJ-RHH-><-?||?-><-?<-ZnR	1173	Bacteria>Pseudomonadota>Alphaproteobacteria>Rhodobacterales>Roseobacteraceae>Sulfitobacter	GCF_026410285.1	Sulfitobacter_sp._F26169L	OS189_RS12380	41979..45500	2996015	BREX-system-P-loop-protein-BrxC-[Sulfitobacter-sp.-F26169L]
WP_220429122.1	Type6.BREX	<-DDE_Tnp_1_6||OLD-ABC->HNH-><-?<-HIN-HTH||XIS-HTH-> BrxE -> BrxA-DNA-binding -> BrxB-BrxF-iSTAND -> DUF6079 *-> N6-METHYLASE -> PglZ -> SIGMA+OB-Fold+AAA-ATPase+HKD-DNase -BrxL ->tRNA->ABC-ATPase+SbcC->?->?->ABhydrolase-><-Phosphodiest	1207	Bacteria>Pseudomonadota>Alphaproteobacteria>Rhodobacterales>Roseobacteraceae>Sulfitobacter	GCF_900313045.1	Sulfitobacter_mediterraneus	D9A10_RS06915	36169..39792	83219	BREX-system-P-loop-protein-BrxC-[Sulfitobacter-mediterraneus]
WP_058243041.1	Type1.BREX	<-AbiEii<-AbiEi_4||HTH_3->?->?-><-REase+iREase<- SIGMA+OB-Fold+AAA-ATPase+HKD-DNase -BrxL <- PglZ <- N6-METHYLASE <- DUF6079 *<- BrxB-BrxF-iSTAND <- BrxA-DNA-binding <- wHTH+WYL+WCX ||ArdCN+MPTase->?->FurR-HTH->?->HIN-HTH+TraJ-RHH->TraI-VirD2-RCRE->	1172	Bacteria>Pseudomonadota>Alphaproteobacteria>Rhodobacterales>Roseobacteraceae>Thalassobius	GCF_001458275.1	Thalassobius_autumnalis	TL5120_RS07660	24794..28312	2072972	BREX-system-P-loop-protein-BrxC-[Thalassobius-autumnalis]
OHC57392.1	Type1.BREX	HIN-HTH->?->Calcineurin+Calcineurin-SbcD->SbcC+SbcC-><-?<-?|| wHTH+WYL+WCX -> BrxA-DNA-binding -> BrxB-BrxF-iSTAND -> DUF6079 *-> N6-METHYLASE -> PglZ -> SIGMA+OB-Fold+AAA-ATPase+HKD-DNase -BrxL ->	1173	Bacteria>Pseudomonadota>Alphaproteobacteria>Rhodobacterales>unclassified_Rhodobacterales	GCA_001828855.1	Rhodobacterales_bacterium_RIFCSPHIGHO2_02_FULL_62_130	A3D16_23615	14989..18510	1802014	hypothetical-protein-A3D16_23615-[Rhodobacterales-bacterium-RIFCSPHIGHO2_02_FULL_62_130]
OUS21258.1	Type1.BREX	HTH_3->?-><-TrbM<-Thermonuclease<-?|| wHTH+WYL+WCX -> BrxA-DNA-binding -> BrxB-BrxF-iSTAND -> DUF6079 *->OLD-ABC->REase-> N6-METHYLASE -> PglZ -> SIGMA+OB-Fold+AAA-ATPase+HKD-DNase -BrxL ->REase+iREase->	1173	Bacteria>Pseudomonadota>Alphaproteobacteria>Rhodobacterales>unclassified_Rhodobacterales	GCA_002162885.1	Rhodobacterales_bacterium_59_46_T64	A9Q95_07015	5961..9482	1856300	hypothetical-protein-A9Q95_07015-[Rhodobacterales-bacterium-59_46_T64]
WP_061508432.1	Type1.BREX	<-sGTP<-SF1-SF2-Helicase+REase<-35Exo+REase|| wHTH+WYL+WCX -> BrxA-DNA-binding -> BrxB-BrxF-iSTAND -> DUF6079 *-> N6-METHYLASE -> PglZ -> SIGMA+OB-Fold+AAA-ATPase+HKD-DNase -BrxL -><-DOC+HTH<-?<-RNase	1177	Bacteria>Pseudomonadota>Alphaproteobacteria>Rhodospirillales>Acetobacteraceae>Acetobacter	GCF_001580695.1	Acetobacter_malorum	AD933_RS09485	34256..37789	178901	BREX-system-P-loop-protein-BrxC-[Acetobacter-malorum]
WP_212335128.1	Type1.BREX	DOC+HTH-><- SIGMA+OB-Fold+AAA-ATPase+HKD-DNase -BrxL <- PglZ <- N6-METHYLASE <- DUF6079 *<- BrxB-BrxF-iSTAND <- BrxA-DNA-binding <- wHTH+WYL+WCX ||35Exo+REase->	1177	Bacteria>Pseudomonadota>Alphaproteobacteria>Rhodospirillales>Acetobacteraceae>Acetobacter	GCF_018256855.1	Acetobacter_thailandicus	JK172_RS08930	62967..66500	1502842	BREX-system-P-loop-protein-BrxC-[Acetobacter-thailandicus]
CAG4901729.1	Type1.BREX	RHH->TraJ-RHH->ACET+ACET-> BrxA-DNA-binding -p-> DUF6079 ->HIN-HTH->?->LZ_Tnp_IS66+zf-IS66+DDE_Tnp_IS66+DDE_Tnp_IS66_C->?-> DUF6079 -p*-> N6-METHYLASE -> N6-METHYLASE ->HTH_29+HTH_32+HTH_33+DDE_3-> PglZ -> SIGMA+OB-Fold+AAA-ATPase+HKD-DNase -BrxL -><-Arm-DNA-bind_3+Phage_int_SAM_3+Phage_integrase<-?||DUF4096+DDE_Tnp_1+DDE_Tnp_1_2->	459	Bacteria>Pseudomonadota>Alphaproteobacteria>Rhodospirillales>Acetobacteraceae>Acidocella	GCA_907164655.1	Acidocella_sp._C78	ACIDOCELLA78_258	233780..235159	1671486	unnamed-protein-product,-partial-[Acidocella-sp.-C78]
WP_201727547.1	Type6.BREX	VirB8->VirB9->VirB10->RHH->TraJ-RHH->ACET+ACET-> BrxE -> BrxA-DNA-binding -> BrxB-BrxF-iSTAND -> DUF6079 -p*->HIN-HTH->?->LZ_Tnp_IS66+zf-IS66+DDE_Tnp_IS66+DDE_Tnp_IS66_C->?-> DUF6079 -p-> N6-METHYLASE -> N6-METHYLASE -p->ParB+HNH->HTH_29+HTH_32+HTH_33+DDE_3->	650	Bacteria>Pseudomonadota>Alphaproteobacteria>Rhodospirillales>Acetobacteraceae>Acidocella	GCF_907164655.1	Acidocella_sp._C78	ACIDOCELLA78_RS32075	228724..230676	1671486	BREX-system-P-loop-protein-BrxC-[Acidocella-sp.-C78]
WP_201727548.1	Type6.BREX	ACET+ACET-> BrxE -> BrxA-DNA-binding -> BrxB-BrxF-iSTAND -> DUF6079 -p->HIN-HTH->?->LZ_Tnp_IS66+zf-IS66+DDE_Tnp_IS66+DDE_Tnp_IS66_C->?-> DUF6079 -p*-> N6-METHYLASE -> N6-METHYLASE -p->ParB+HNH->HTH_29+HTH_32+HTH_33+DDE_3->ParB+HNH-> PglZ -> SIGMA+OB-Fold+AAA-ATPase+HKD-DNase -BrxL -><-Arm-DNA-bind_3+Phage_int_SAM_3+Phage_integrase	495	Bacteria>Pseudomonadota>Alphaproteobacteria>Rhodospirillales>Acetobacteraceae>Acidocella	GCF_907164655.1	Acidocella_sp._C78	ACIDOCELLA78_RS32100	233672..235159	1671486	hypothetical-protein-[Acidocella-sp.-C78]
KAA0676972.1	Type6.BREX	<-DDE_Tnp_IS240<-DDE_Tnp_1_5+DDE_Tnp_1<-Phage_integrase+Phage_integrase||?->?-><-REC+LuxR-HTH<-?<- SIGMA+OB-Fold+AAA-ATPase+HKD-DNase -BrxL <- PglZ <-ParB+HNH<- DUF6079 *<- BrxB-BrxF-iSTAND <- BrxA-DNA-binding <- BrxE <-?<-HHH_4+HTH+wHTH+SF1-SF2-Helicase+REase<-SF1-SF2-Helicase<-MORC+DUF3684+REase||XIS-HTH->NtrC-AAA->	1207	Bacteria>Pseudomonadota>Alphaproteobacteria>Rhodospirillales>Acetobacteraceae>Roseomonas	GCA_008365375.1	Roseomonas_genomospecies_6	DS843_25180	43725..47348	214106	BREX-system-P-loop-protein-BrxC-[Roseomonas-genomospecies-6]
MCA3353385.1	Type1.BREX	<- SIGMA+OB-Fold+AAA-ATPase+HKD-DNase -BrxL <- PglZ <- N6-METHYLASE <-NERD<-OLD-ABC<- DUF6079 *<- BrxB-BrxF-iSTAND <- BrxA-DNA-binding <- wHTH+WYL+WCX ||35Exo+REase->SF1-SF2-Helicase+REase->Small-B-barrel+SF1-SF2-Helicase+RdgC+REase-><-SF1-SF2-Helicase<- HKD-DNase	1178	Bacteria>Pseudomonadota>Alphaproteobacteria>Rhodospirillales>Acetobacteraceae>Roseomonas	GCA_020249745.1	Roseomonas_sp.	INF80_03760	34583..38119	1869190	BREX-system-P-loop-protein-BrxC-[Roseomonas-sp.]
WP_209351251.1	Type1.BREX	HKD-DNase ->SF1-SF2-Helicase-><-Small-B-barrel+SF1-SF2-Helicase+RdgC+REase<-SF1-SF2-Helicase+REase<-35Exo+REase|| wHTH+WYL+WCX -> BrxA-DNA-binding -> BrxB-BrxF-iSTAND -> DUF6079 *->OLD-ABC->NERD-> N6-METHYLASE -> PglZ -> SIGMA+OB-Fold+AAA-ATPase+HKD-DNase -BrxL ->?->?-><-?<-Glyoxalase	1177	Bacteria>Pseudomonadota>Alphaproteobacteria>Rhodospirillales>Acetobacteraceae>Roseomonas	GCF_017813355.1	Roseomonas_sp._PWR1	J5Y09_RS08145	78258..81791	2820810	BREX-system-P-loop-protein-BrxC-[Roseomonas-sp.-PWR1]
WP_239419500.1	Type1.BREX	SbcC-><-PIN-><-?||ParA-Soj-PloopNTPase->?->HTH_36-><-?|| BrxA-DNA-binding -> BrxB-BrxF-iSTAND -> DUF6079 *-> N6-METHYLASE -> PglZ -> SIGMA+OB-Fold+AAA-ATPase+HKD-DNase -BrxL -><-?<-MerR-HTH<-Relaxase+SF1-SF2-Helicase||?->VirD4-TraG+VirD4-FtsK->	1170	Bacteria>Pseudomonadota>Alphaproteobacteria>Rhodospirillales>Acetobacteraceae>Roseomonas	-	Roseomonas_sp._ACRSG	MHZ93_RS20975	7121..10633	2918212	BREX-system-P-loop-protein-BrxC-[Roseomonas-sp.-ACRSG]
MBI1207569.1	Type6.BREX	ACT->?-><-?||?-> BrxE -> BrxA-DNA-binding -> BrxB-BrxF-iSTAND -> DUF6079 *-> N6-METHYLASE -> PglZ -> SIGMA+OB-Fold+AAA-ATPase+HKD-DNase -BrxL -><-?<-SAM-METHYLASE<-?<-?<-NUDIX	1222	Bacteria>Pseudomonadota>Alphaproteobacteria>Rhodospirillales>Azospirillaceae>Azospirillum	GCA_016124535.1	Azospirillum_sp.	GC191_09820	259214..262882	34012	BREX-system-P-loop-protein-BrxC-[Azospirillum-sp.]
WP_200477164.1	Type6.BREX	Phage_integrase-><-HHH_4+HTH+wHTH+SF1-SF2-Helicase+REase<-SF1-SF2-Helicase<-MORC+DUF3684+REase<- SIGMA+OB-Fold+AAA-ATPase+HKD-DNase -BrxL <- PglZ <- N6-METHYLASE <- N6-METHYLASE <- DUF6079 *<- BrxB-BrxF-iSTAND <- BrxA-DNA-binding <- BrxE	1208	Bacteria>Pseudomonadota>Alphaproteobacteria>Rhodospirillales>Azospirillaceae>Azospirillum	GCF_016622105.1	Azospirillum_brasilense	GAY29_RS06575	31945..35571	192	BREX-system-P-loop-protein-BrxC-[Azospirillum-brasilense]
WP_089290578.1	Type1.BREX	<-ABhydrolase||?-><-?<-TetR-HTH||?->?-> BrxA-DNA-binding -> BrxB-BrxF-iSTAND -> DUF6079 *-> N6-METHYLASE -> PglZ -> SIGMA+OB-Fold+AAA-ATPase+HKD-DNase -BrxL ->	1202	Bacteria>Pseudomonadota>Alphaproteobacteria>Rhodospirillales>Azospirillaceae>Azospirillum	GCF_900188385.1	unclassified_Azospirillum	CHB97_RS24120	34901..38509	2630922	MULTISPECIES:-BREX-system-P-loop-protein-BrxC-[unclassified-Azospirillum]
HCW69913.1	Type6.BREX	VirB11->VirD4-TraG+VirD4-FtsK->?->ArdCN+MPTase+TOPRIM->TraJ-RHH->TraI-VirD2-RCRE-><- SIGMA+OB-Fold+AAA-ATPase+HKD-DNase -BrxL <- PglZ <-ParB+HNH<- DUF6079 *<- BrxB-BrxF-iSTAND <- BrxA-DNA-binding <- BrxE ||GDResolvase+Recombinase->?->?-><-?<-HTH_36	1208	Bacteria>Pseudomonadota>Alphaproteobacteria>Rhodospirillales>Thalassospiraceae>Thalassospira	GCA_003539675.1	Thalassospira_lucentensis	DHR80_22445	45804..49430	168935	BREX-system-P-loop-protein-BrxC-[Thalassospira-lucentensis]
MBL4843424.1	Type1.BREX	<-SF1-SF2-Helicase<- HKD-DNase + HKD-DNase <-wHTH<-SF1-SF2-Helicase+REase<-SF1-SF2-Helicase+REase<- SIGMA+OB-Fold+AAA-ATPase+HKD-DNase -BrxL <- PglZ <- N6-METHYLASE <- DUF6079 *<- BrxB-BrxF-iSTAND <- BrxA-DNA-binding <- wHTH+WYL+WCX ||Calcineurin+Calcineurin-SbcD->SbcC+SbcC-><-MODE-HTH+PBPII||TetR-HTH-><-RHH||HTH_Tnp_1+HTH_21+rve+rve_3->	1179	Bacteria>Pseudomonadota>Alphaproteobacteria>Rhodospirillales>Thalassospiraceae>Thalassospira	GCA_016765055.1	Thalassospira_sp.	JKY47_21635	20483..24022	1912094	BREX-system-P-loop-protein-BrxC-[Thalassospira-sp.]
WP_085595207.1	Type1.BREX	XIS-HTH-> BrxA-DNA-binding -> BrxB-BrxF-iSTAND -> DUF6079 *->OLD-ABC->REase-> N6-METHYLASE ->HTH+REase-> PglZ -> SIGMA+OB-Fold+AAA-ATPase+HKD-DNase -BrxL -><-SF1-SF2-Helicase	1182	Bacteria>Pseudomonadota>Alphaproteobacteria>Rhodospirillales>Thalassospiraceae>Thalassospira	GCF_002115825.1	Thalassospira_sp._MCCC_1A01428	THS27_RS23975	1729..5277	1470575	BREX-system-P-loop-protein-BrxC-[Thalassospira-sp.-MCCC-1A01428]
TVR84541.1	Type6.BREX	<- SIGMA+OB-Fold+AAA-ATPase+HKD-DNase -BrxL <- PglZ <-ParB+HNH<- N6-METHYLASE <- DUF6079 *<- BrxB-BrxF-iSTAND <- BrxA-DNA-binding <- BrxE	1206	Bacteria>Pseudomonadota>Alphaproteobacteria>Rhodospirillales>unclassified_Rhodospirillales	GCF_007695045.1	Rhodospirillales_bacterium	EA405_01435	12860..16480	2026786	BREX-system-P-loop-protein-BrxC-[Rhodospirillales-bacterium]
WP_193715186.1	Type6.BREX	<-Arm-DNA-bind_2+Phage_int_SAM_3+Phage_integrase<-Phage_AlpA<-GreA_GreB+SF1-SF2-Helicase<-SbcC+SbcC<-Calcineurin|| wHTH+WYL+WCX -> BrxE -> BrxA-DNA-binding -> BrxB-BrxF-iSTAND -> DUF6079 *-> N6-METHYLASE ->ParB+HNH-> PglZ -> SIGMA+OB-Fold+AAA-ATPase+HKD-DNase -BrxL -><-tRNA||?->?-><-?<-ABC-ATPase+SbcC	1215	Bacteria>Pseudomonadota>Alphaproteobacteria>Sneathiellales>Sneathiellaceae>Sneathiella	GCF_015143595.1	Sneathiella_sp._P13V-1	GUA87_RS03840	775657..779304	2697366	BREX-system-P-loop-protein-BrxC-[Sneathiella-sp.-P13V-1]
WP_160484443.1	Type1.BREX	TraJ-RHH->VirD4-TraG+VirD4-FtsK->MobF->EVE+HNH-><-LexA-protease|| BrxA-DNA-binding -> BrxB-BrxF-iSTAND -> DUF6079 *->OLD-ABC->NERD-> N6-METHYLASE -> PglZ -> SIGMA+OB-Fold+AAA-ATPase+HKD-DNase -BrxL ->REase+iREase-><-Couple_hipA+HipA_C<-HTH_3	1172	Bacteria>Pseudomonadota>Alphaproteobacteria>Sphingomonadales>Erythrobacteraceae>Aurantiacibacter	GCF_009807005.1	Aurantiacibacter_rhizosphaerae	GRF63_RS02615	556566..560084	2691582	BREX-system-P-loop-protein-BrxC-[Aurantiacibacter-rhizosphaerae]
RNJ61798.1	Type1.BREX	TraN->Thioredoxin->TraH->?-><-?|| wHTH+WYL+WCX -> BrxA-DNA-binding -> BrxB-BrxF-iSTAND -> DUF6079 *-> N6-METHYLASE -> PglZ -> SIGMA+OB-Fold+AAA-ATPase+HKD-DNase -BrxL -><-?<-SF1-SF2-Helicase+DUF1998<-?<-SWIM+DUF3670+SF1-SF2-Helicase	1173	Bacteria>Pseudomonadota>Alphaproteobacteria>Sphingomonadales>Erythrobacteraceae>Erythrobacter/Porphyrobacter_group	GCA_003724185.1	Porphyrobacter_sp._IPPAS_B-1204	EDM03_09690	47855..51376	2483793	BREX-system-P-loop-protein-BrxC-[Porphyrobacter-sp.-IPPAS-B-1204]
WP_011415860.1	Type1.BREX	SSB-><-?||?->TraJ-RHH->VirD4-TraG+VirD4-FtsK->MobF-><-LexA-protease|| BrxA-DNA-binding -> BrxB-BrxF-iSTAND -> DUF6079 *->OLD-ABC->REase-> N6-METHYLASE -> PglZ -> SIGMA+OB-Fold+AAA-ATPase+HKD-DNase -BrxL ->REase+iREase-><-Couple_hipA+HipA_C<-HTH_3	1172	Bacteria>Pseudomonadota>Alphaproteobacteria>Sphingomonadales>Erythrobacteraceae>Erythrobacter/Porphyrobacter_group	GCF_000013005.1	Erythrobacter_litoralis	EL2594_RS14475	2964416..2967934	39960	BREX-system-P-loop-protein-BrxC-[Erythrobacter-litoralis]
WP_069309464.1	Type6.BREX	HD+Tox-RelA-><-?<-?||tRNA-><- SIGMA+OB-Fold+AAA-ATPase+HKD-DNase -BrxL <- PglZ <- N6-METHYLASE -p<- N6-METHYLASE -p<- N6-METHYLASE <- DUF6079 *<- BrxB-BrxF-iSTAND <- BrxA-DNA-binding <- BrxE <- wHTH+WYL+WCX <-?<-GDResolvase+ZnR<-RAMA<-HD	1208	Bacteria>Pseudomonadota>Alphaproteobacteria>Sphingomonadales>Erythrobacteraceae>Erythrobacter/Porphyrobacter_group	GCF_001720465.1	Porphyrobacter_sp._LM_6	BG023_RS04915	1019092..1022718	1896196	BREX-system-P-loop-protein-BrxC-[Porphyrobacter-sp.-LM-6]

Protein-ID	System	Gene-Neighbourhoods	Length	Taxonomy	Assembly-ID	Strain	Locus-Tag	Genomic-coordinates	TaxID	Fasta-defline
WP_196847550.1	Type1.BREX	SWIM+DUF3670+SF1-SF2-Helicase->?->?->SF1-SF2-Helicase+DUF1998->BrxA-DNA-binding->BrxB-BrxF-iSTAND->DUF6079*->N6-METHYLASE->PglZ->SIGMA+OB-Fold+AAA-ATPase+HKD-DNase-BrxL->REase+iREase-><-Couple_hipA+HipA_C<-HTH_3||?-><-?<-TraH	1172	Bacteria>Pseudomonadota>Alphaproteobacteria>Sphingomonadales>Erythrobacteraceae>Erythrobacter/Porphyrobacter_group	GCF_015767595.1	Erythrobacter_sp._A30-3	IT881_RS05450	1101811..1105329	2785907	BREX-system-P-loop-protein-BrxC-[Erythrobacter-sp.-A30-3]
WP_226699433.1	Type1.BREX	<-Cysteine-Desulfurase<-ADH_N+ADH_zinc_N||?->GDResolvase+HIN-HTH-><-?||BrxA-DNA-binding->BrxB-BrxF-iSTAND->DUF6079*->N6-METHYLASE->PglZ->SIGMA+OB-Fold+AAA-ATPase+HKD-DNase-BrxL-><-?<-Phage_int_SAM_5+Phage_integrase	1170	Bacteria>Pseudomonadota>Alphaproteobacteria>Sphingomonadales>Erythrobacteraceae>Qipengyuania	GCF_020524765.1	Qipengyuania_gaetbuli	LC083_RS04940	959412..962924	266952	BREX-system-P-loop-protein-BrxC-[Qipengyuania-gaetbuli]
MBB3862674.1	Type6.BREX	<-BrxD<-BrxD-AAA<-?<-RapA+SF1-SF2-Helicase+APATPase||?->XIS-HTH->BrxE->BrxA-DNA-binding-p->BrxB-BrxF-iSTAND->DUF6079*->N6-METHYLASE->PglZ->SIGMA+OB-Fold+AAA-ATPase+HKD-DNase-BrxL->LexA-protease-><-EVE+HNH<-MobF<-VirD4-TraG+VirD4-FtsK<-TraJ-RHH	1208	Bacteria>Pseudomonadota>Alphaproteobacteria>Sphingomonadales>Sphingomonadaceae>Novosphingobium	GCA_014196055.1	Novosphingobium_hassiacum	GGQ88_003976	14858..18484	173676	hypothetical-protein-[Novosphingobium-hassiacum]
WP_246386386.1	Type6.BREX	<-BrxD<-BrxD-AAA<-?<-RapA+SF1-SF2-Helicase+APATPase||?->XIS-HTH->BrxE->BrxA-DNA-binding->BrxB-BrxF-iSTAND->DUF6079*->N6-METHYLASE->PglZ->SIGMA+OB-Fold+AAA-ATPase+HKD-DNase-BrxL-><-HTH||LexA-protease-><-nSTAND1+HNH<-MobF<-VirD4-TraG+VirD4-FtsK<-TraJ-RHH	1213	Bacteria>Pseudomonadota>Alphaproteobacteria>Sphingomonadales>Sphingomonadaceae>Novosphingobium	GCF_014196055.1	Novosphingobium_hassiacum	GGQ88_RS19485	14843..18484	173676	BREX-system-P-loop-protein-BrxC-[Novosphingobium-hassiacum]
WP_243992543.1	Type1.BREX	<-SIGMA-HTH<-SIGMA+OB-Fold+AAA-ATPase+HKD-DNase-BrxL<-PglZ<-N6-METHYLASE<-NERD<-OLD-ABC<-DUF6079*<-BrxB-BrxF-iSTAND<-BrxA-DNA-binding<-?<-XIS-HTH<-?<-Arm-DNA-bind_3+Phage_integrase	1189	Bacteria>Pseudomonadota>Alphaproteobacteria>Sphingomonadales>Sphingomonadaceae>Novosphingobium	GCF_022832385.1	Novosphingobium_sp._B2580	MTR64_RS07820	57412..60981	2930093	BREX-system-P-loop-protein-BrxC-[Novosphingobium-sp.-B2580]
WP_243995499.1	Type1.BREX	<-PIN<-?<-?<-?<-SIGMA+OB-Fold+AAA-ATPase+HKD-DNase-BrxL<-PglZ<-N6-METHYLASE<-DUF6079*<-BrxB-BrxF-iSTAND<-BrxA-DNA-binding<-wHTH+WYL+WCX||35Exo+REase->SF1-SF2-Helicase+REase->GreA_GreB+SF1-SF2-Helicase->?-><-PIN<-N6-METHYLASE	1179	Bacteria>Pseudomonadota>Alphaproteobacteria>Sphingomonadales>Sphingomonadaceae>Novosphingobium	GCF_022832385.1	Novosphingobium_sp._B2580	MTR64_RS16290	56132..59671	2930093	BREX-system-P-loop-protein-BrxC-[Novosphingobium-sp.-B2580]
WP_022673601.1	Type1.BREX	HTH_3->Couple_hipA+HipA_C->HNH-><-REase+HTH+35exo+Small-B-barrel+SF1-SF2-Helicase<-SIGMA+OB-Fold+AAA-ATPase+HKD-DNase-BrxL<-PglZ<-N6-METHYLASE<-REase<-OLD-ABC<-DUF6079*<-BrxB-BrxF-iSTAND<-BrxA-DNA-binding<-wHTH+WYL+WCX<-HTH||LexA-protease-><-EVE+HNH<-MobF<-VirD4-TraG+VirD4-FtsK<-TraJ-RHH	1189	Bacteria>Pseudomonadota>Alphaproteobacteria>Sphingomonadales>Sphingomonadaceae>Novosphingopyxis	GCF_000420305.1	Novosphingopyxis_baekryungensis	G410_RS0110420	254465..258034	279369	BREX-system-P-loop-protein-BrxC-[Novosphingopyxis-baekryungensis]
ARR56525.1	Type1.BREX	<-OBFOLD-NUCLEASE<-LexA-protease||HTH->wHTH+WYL+WCX->BrxA-DNA-binding->BrxB-BrxF-iSTAND->DUF6079*->OLD-ABC->NERD->Zn_Tnp_IS1595+DDE_Tnp_IS1595->N6-METHYLASE-p->PglZ->SIGMA+OB-Fold+AAA-ATPase+HKD-DNase-BrxL-><-?||DDE_Tnp_1_4->HTH+HTH+SF1-SF2-Helicase-p->	1189	Bacteria>Pseudomonadota>Alphaproteobacteria>Sphingomonadales>Sphingomonadaceae>Rhizorhabdus	GCA_000715175.2	Rhizorhabdus_wittichii_DC-6	HY78_25365	5371615..5375184	1283312	hypothetical-protein-HY78_25365-[Rhizorhabdus-wittichii-DC-6]
WP_209047668.1	Type1.BREX	MODE-HTH+PBPII->?-><-?<-HTH_3<-SIGMA+OB-Fold+AAA-ATPase+HKD-DNase-BrxL<-PglZ<-N6-METHYLASE<-DUF6079*<-BrxB-BrxF-iSTAND<-BrxA-DNA-binding<-wHTH+WYL+WCX||35Exo+REase->SF1-SF2-Helicase+REase->GreA_GreB+SF1-SF2-Helicase->?-><-PIN<-N6-METHYLASE	1179	Bacteria>Pseudomonadota>Alphaproteobacteria>Sphingomonadales>Sphingomonadaceae>Rhizorhabdus	GCA_029543145.1	Rhizorhabdus_histidinilytica	P7B04_RS17610	19171..22710	439228	MULTISPECIES:-BREX-system-P-loop-protein-BrxC-[Sphingomonadaceae]
WP_010336176.1	Type1.BREX	VirB8->VirB9->VirB10->RHH-><-TetR-HTH||MODE-HTH+PBPII->wHTH+WYL+WCX->BrxA-DNA-binding->BrxB-BrxF-iSTAND->DUF6079*->N6-METHYLASE->PglZ->SIGMA+OB-Fold+AAA-ATPase+HKD-DNase-BrxL->SF1-SF2-Helicase+REase->SF1-SF2-Helicase+REase->wHTH->HKD-DNase+HKD-DNase->SF1-SF2-Helicase->	1179	Bacteria>Pseudomonadota>Alphaproteobacteria>Sphingomonadales>Sphingomonadaceae>Sphingobium	GCF_000224695.1	Sphingobium_yanoikuyae	SYA_RS0104815	38393..41932	13690	BREX-system-P-loop-protein-BrxC-[Sphingobium-yanoikuyae]
WP_007688323.1	Type1.BREX	MobF->?-><-?<-?<-OBFOLD-NUCLEASE<-LexA-protease||wHTH+WYL+WCX->BrxA-DNA-binding->BrxB-BrxF-iSTAND->DUF6079*->OLD-ABC->NERD->N6-METHYLASE->PglZ->SIGMA+OB-Fold+AAA-ATPase+HKD-DNase-BrxL->MORC+SF1-SF2-Helicase->?-><-?<-TraH	1189	Bacteria>Pseudomonadota>Alphaproteobacteria>Sphingomonadales>Sphingomonadaceae>Sphingobium	GCF_000264945.2	Sphingobium_indicum	SIDU_RS03565	700467..704036	332055	BREX-system-P-loop-protein-BrxC-[Sphingobium-indicum]
WP_021240096.1	Type1.BREX	HKD-DNase->SF1-SF2-Helicase-><-Small-B-barrel+SF1-SF2-Helicase+RdgC+REase<-SF1-SF2-Helicase+REase<-35Exo+REase<-?||wHTH+WYL+WCX->BrxA-DNA-binding->BrxB-BrxF-iSTAND->DUF6079*->OLD-ABC->REase->N6-METHYLASE->PglZ->SIGMA+OB-Fold+AAA-ATPase+HKD-DNase-BrxL-><-?<-Arm-DNA-bind_3+Phage_integrase	1177	Bacteria>Pseudomonadota>Alphaproteobacteria>Sphingomonadales>Sphingomonadaceae>Sphingobium	GCF_000445065.1	Sphingobium_quisquiliarum	L288_RS41575	32905..36438	538379	BREX-system-P-loop-protein-BrxC-[Sphingobium-quisquiliarum]
WP_264583946.1	Type1.BREX	ParB->HipA_C-><-ParB+HNH+Schlafen<-GreA_GreB+SF1-SF2-Helicase<-SF1-SF2-Helicase+REase<-35Exo+REase||wHTH+WYL+WCX->BrxA-DNA-binding->BrxB-BrxF-iSTAND->DUF6079*->N6-METHYLASE->PglZ->SIGMA+OB-Fold+AAA-ATPase+HKD-DNase-BrxL-><-?<-?<-?<-NUDIX	1179	Bacteria>Pseudomonadota>Alphaproteobacteria>Sphingomonadales>Sphingomonadaceae>Sphingobium	GCF_025961895.1	Sphingobium_sp._B7D2B	M2341_RS01365	293056..296595	2940583	BREX-system-P-loop-protein-BrxC-[Sphingobium-sp.-B7D2B]
NJC05753.1	Type6.BREX	TraI-VirD2-RCRE->?->BrxE->BrxA-DNA-binding->BrxB-BrxF-iSTAND->DUF6079*->N6-METHYLASE->PglZ->SIGMA+OB-Fold+AAA-ATPase+HKD-DNase-BrxL-><-URI-DNase||?->?->REase+HTH+35exo+Small-B-barrel+SF1-SF2-Helicase->?->HD+PspB->	1209	Bacteria>Pseudomonadota>Alphaproteobacteria>Sphingomonadales>Sphingomonadaceae>Sphingomonas	GCA_011927725.1	Sphingomonas_kaistensis	GGQ97_001546	1560422..1564051	298708	hypothetical-protein-[Sphingomonas-kaistensis]
WP_066478880.1	Type1.BREX	Peptidase_S26->DUF736->?->TraI-VirD2-RCRE->||MODE-HTH+PBPII->wHTH+WYL+WCX->BrxA-DNA-binding->BrxB-BrxF-iSTAND->DUF6079*->N6-METHYLASE->PglZ->SIGMA+OB-Fold+AAA-ATPase+HKD-DNase-BrxL->SF1-SF2-Helicase+REase->SF1-SF2-Helicase+REase->wHTH->HKD-DNase+HKD-DNase->SF1-SF2-Helicase->	1179	Bacteria>Pseudomonadota>Alphaproteobacteria>Sphingomonadales>Sphingomonadaceae>Sphingomonas	GCF_001555785.1	unclassified_Sphingomonas	AX667_RS15610	42451..45990	196159	MULTISPECIES:-BREX-system-P-loop-protein-BrxC-[unclassified-Sphingomonas]
WP_107968397.1	Type1.BREX	<-OBFOLD-NUCLEASE<-LexA-protease||HTH->wHTH+WYL+WCX->BrxA-DNA-binding->BrxB-BrxF-iSTAND->DUF6079*->N6-METHYLASE->PglZ->SIGMA+OB-Fold+AAA-ATPase+HKD-DNase-BrxL->MORC+DUF3684+REase->SF1-SF2-Helicase->?-><-?<-TraH<-Thioredoxin	1186	Bacteria>Pseudomonadota>Alphaproteobacteria>Sphingomonadales>Sphingomonadaceae>Sphingomonas	GCF_003050615.1	Sphingomonas_oleivorans	CLG96_RS13035	363272..366832	1735121	BREX-system-P-loop-protein-BrxC-[Sphingomonas-oleivorans]
WP_192976950.1	Type1.BREX	Phage_integrase->HEPN-><-HKD-DNase+Preader14<-?||PIWI->REase-><-SIGMA+OB-Fold+AAA-ATPase+HKD-DNase-BrxL<-PglZ<-N6-METHYLASE<-DUF6079*<-BrxB-BrxF-iSTAND<-BrxA-DNA-binding<-wHTH+WYL+WCX<-?<-SWACOS||OLD-ABC+TOPRIM->SF1-SF2-Helicase-><-SbcC+CCDC158<-Calcineurin	1177	Bacteria>Pseudomonadota>Alphaproteobacteria>Sphingomonadales>Sphingomonadaceae>Sphingomonas	GCF_014892665.1	Sphingomonas_sp._CFBP_13603	IFR23_RS17335	87025..90558	2774040	BREX-system-P-loop-protein-BrxC-[Sphingomonas-sp.-CFBP-13603]
WP_089221081.1	Type1.BREX	<-Small-B-barrel+SF1-SF2-Helicase+RdgC+REase<-SF1-SF2-Helicase+REase<-35Exo+REase||wHTH+WYL+WCX->BrxA-DNA-binding->BrxB-BrxF-iSTAND->DUF6079*->N6-METHYLASE->PglZ->SIGMA+OB-Fold+AAA-ATPase+HKD-DNase-BrxL->	1179	Bacteria>Pseudomonadota>Alphaproteobacteria>Sphingomonadales>Sphingomonadaceae>Sphingomonas	GCF_900188165.1	Sphingomonas_laterariae	CHB74_RS18985	16083..19622	861865	BREX-system-P-loop-protein-BrxC-[Sphingomonas-laterariae]
WP_133494816.1	Type1.BREX	MobF->?-><-?<-?<-OBFOLD-NUCLEASE<-LexA-protease||wHTH+WYL+WCX->BrxA-DNA-binding->BrxB-BrxF-iSTAND->DUF6079*->OLD-ABC->NERD->N6-METHYLASE->PglZ->SIGMA+OB-Fold+AAA-ATPase+HKD-DNase-BrxL->MORC+SF1-SF2-Helicase->?-><-?<-TraH	1189	Bacteria>Pseudomonadota>Alphaproteobacteria>Sphingomonadales>Sphingomonadaceae>Stakelama	GCF_014645395.1	Stakelama_pacifica	IEX20_RS07270	305060..308629	517720	BREX-system-P-loop-protein-BrxC-[Stakelama-pacifica]
WP_121052748.1	Type1.BREX	<-GDResolvase+Recombinase+ZnR<-?||XIS-HTH->Tox-RES-><-SIGMA+OB-Fold+AAA-ATPase+HKD-DNase-BrxL<-PglZ<-N6-METHYLASE<-NERD<-OLD-ABC<-DUF6079*<-BrxB-BrxF-iSTAND<-BrxA-DNA-binding<-wHTH+WYL+WCX||?->35Exo+REase->SF1-SF2-Helicase+REase->Small-B-barrel+SF1-SF2-Helicase+RdgC+REase->ArdCN+MPTase->SAM-METHYLASE+SF1-SF2-Helicase->	1179	Bacteria>Pseudomonadota>Alphaproteobacteria>Sphingomonadales>Sphingosinicellaceae>Sphingosinicella	GCF_003967095.1	Sphingosinicella_microcystinivorans	SmB9_RS16135	3426933..3430472	335406	BREX-system-P-loop-protein-BrxC-[Sphingosinicella-microcystinivorans]
OYY96513.1	Type1.BREX	wHTH+WYL+WCX->BrxA-DNA-binding->BrxB-BrxF-iSTAND->DUF6079*->N6-METHYLASE->PglZ->SIGMA+OB-Fold+AAA-ATPase+HKD-DNase-BrxL->REase+iREase->	1189	Bacteria>Pseudomonadota>Alphaproteobacteria>Sphingomonadales>unclassified_Sphingomonadales	GCA_002281585.1	Sphingomonadales_bacterium_28-56-43	B7Y38_10875	3957..7526	1970590	MAG:-hypothetical-protein-B7Y38_10875-[Sphingomonadales-bacterium-28-56-43]
OYZ13690.1	Type5.BREX	<-MarR-HTH<-?<-?||TUDOR+SF1-SF2-Helicase+iREase+CTD->?->BrxA-DNA-binding->BrxB-BrxF-iSTAND->DUF6079*->N6-METHYLASE->PglZ->SIGMA+OB-Fold+AAA-ATPase+HKD-DNase-BrxL-><-Phage_integrase<-tRNA||?-><-?<-?||SAM-METHYLASE->	1197	Bacteria>Pseudomonadota>Alphaproteobacteria>Sphingomonadales>unclassified_Sphingomonadales	GCA_002281615.1	Sphingomonadales_bacterium_28-64-96	B7Y35_07475	14573..18166	1970591	MAG:-hypothetical-protein-B7Y35_07475-[Sphingomonadales-bacterium-28-64-96]
TNE62528.1	Type1.BREX	REase+wHTH->rve->TnsC-ATPase->LON-AAA->?-><-Schlafen+REase<-SIGMA+OB-Fold+AAA-ATPase+HKD-DNase-BrxL<-PglZ<-N6-METHYLASE<-DUF6079*<-BrxB-BrxF-iSTAND<-BrxA-DNA-binding<-wHTH+WYL+WCX||REase+wHTH->HTH_28+rve+Mu-transpos_C->	1175	Bacteria>Pseudomonadota>Alphaproteobacteria>Sphingomonadales>unclassified_Sphingomonadales	GCA_006226385.1	Sphingomonadales_bacterium	EP341_00225	33441..36968	1978525	MAG:-BREX-system-P-loop-protein-BrxC-[Sphingomonadales-bacterium]
WP_015740180.1	Type1.BREX	VirB10->RHH-><-TetR-HTH||MODE-HTH+PBPII-><-SbcC+SbcC<-Calcineurin+Calcineurin-SbcD||wHTH+WYL+WCX->BrxA-DNA-binding->BrxB-BrxF-iSTAND->DUF6079*->N6-METHYLASE->PglZ->SIGMA+OB-Fold+AAA-ATPase+HKD-DNase-BrxL->SF1-SF2-Helicase+REase->?->?-><-MODE-HTH+PBPII<-ArdCN||GNTR-HTH+Aminotran_1_2->	1179	Bacteria>Pseudomonadota>Alphaproteobacteria>Sphingomonadales>Zymomonadaceae>Zymomonas	GCF_000024245.1	Zymomonas_mobilis	Za10_1374	1591366..1594905	542	BREX-system-P-loop-protein-BrxC-[Zymomonas-mobilis]
RYG90169.1	Type6.BREX	ArdCN+MPTase->REase+RNase-><-SIGMA+OB-Fold+AAA-ATPase+HKD-DNase-BrxL<-PglZ<-N6-METHYLASE<-N6-METHYLASE<-DUF6079*<-BrxB-BrxF-iSTAND<-BrxA-DNA-binding<-BrxE<-wHTH+WYL+WCX||Ntox49->TUDOR+SF1-SF2-Helicase+iREase+CTD->	1208	Bacteria>Pseudomonadota>Alphaproteobacteria>unclassified_Alphaproteobacteria	GCA_004145145.1	Alphaproteobacteria_bacterium	EON59_00500	24262..27888	1913988	BREX-system-P-loop-protein-BrxC-[Alphaproteobacteria-bacterium]
NCC22319.1	Type6.BREX	HEPN-><-?<-SIGMA+OB-Fold+AAA-ATPase+HKD-DNase-BrxL<-PglZ<-N6-METHYLASE<-DUF6079*<-BrxB-BrxF-iSTAND<-BrxA-DNA-binding<-BrxE<-?<-HTH_3||Phage_integrase->Phage_integrase->Phage_integrase->	1212	Bacteria>Pseudomonadota>Alphaproteobacteria>unclassified_Alphaproteobacteria	GCA_009929775.1	Alphaproteobacteria_bacterium	EOM26_07635	14398..18036	1913988	BREX-system-P-loop-protein-BrxC-[Alphaproteobacteria-bacterium]
MBN9530327.1	Type1.BREX	<-ICLR-HTH<-?||?->HTH_21+rve+rve_3->HTH_21+rve+rve_3-><-SIGMA+OB-Fold+AAA-ATPase+HKD-DNase-BrxL<-PglZ<-N6-METHYLASE<-DUF6079*<-BrxB-BrxF-iSTAND<-BrxA-DNA-binding<-wHTH+WYL+WCX||35Exo+REase->SF1-SF2-Helicase+REase->Small-B-barrel+SF1-SF2-Helicase+RdgC+REase-><-?||?-><-HTH_3	1180	Bacteria>Pseudomonadota>Alphaproteobacteria>unclassified_Alphaproteobacteria	GCA_017307375.1	Alphaproteobacteria_bacterium	JOH82_29215	188733..192275	1913988	BREX-system-P-loop-protein-BrxC-[Alphaproteobacteria-bacterium]
MBQ8347028.1	Type1.BREX	VirD4-TraG+VirD4-FtsK->?-><-?<-TraI-VirD2-RCRE<-?<-?<-SIGMA+OB-Fold+AAA-ATPase+HKD-DNase-BrxL<-PglZ<-N6-METHYLASE<-DUF6079*<-BrxB-BrxF-iSTAND<-BrxA-DNA-binding||?-><-?<-ArdCN+MPTase||SF1-SF2-Helicase->	1150	Bacteria>Pseudomonadota>Alphaproteobacteria>unclassified_Alphaproteobacteria	GCA_017462585.1	Alphaproteobacteria_bacterium	IJY17_05445	46698..50150	1913988	BREX-system-P-loop-protein-BrxC-[Alphaproteobacteria-bacterium]
MBN8451986.1	Type6.BREX	<-KAP-glycosylase<-KAP-NTPase<-SIGMA+OB-Fold+AAA-ATPase+HKD-DNase-BrxL<-PglZ<-TOPRIM<-OLD-ABC<-N6-METHYLASE<-N6-METHYLASE<-GreA_GreB+SF1-SF2-Helicase<-DUF6079*<-BrxB-BrxF-iSTAND<-BrxA-DNA-binding<-BrxE<-wHTH+WYL+WCX||?->SMODS->MODE-HTH+PBPII-><-HTH_3||LysR-HTH->	1213	Bacteria>Pseudomonadota>Betaproteobacteria>Betaproteobacteria_incertae_sedis>Candidatus_Accumulibacter>unclassified_Candidatus_Accumulibacter	GCA_017302415.1	Candidatus_Accumulibacter_sp.	JOM31_02680	99081..102722	2053492	BREX-system-P-loop-protein-BrxC-[Candidatus-Accumulibacter-sp.]
WP_042797405.1	Type6.BREX	ZnR->NERD+SF1-SF2-Helicase->Exonuc_V_gamma+REase->SF1-SF2-Helicase+REase->sGTP->?->BrxE->BrxA-DNA-binding->BrxB-BrxF-iSTAND->DUF6079*->OLD-ABC->ParB+HNH->PglZ->SIGMA+OB-Fold+AAA-ATPase+HKD-DNase-BrxL-><-HipA_C<-Couple_hipA+HipA_C||VirB5->	1207	Bacteria>Pseudomonadota>Betaproteobacteria>Burkholderiales>Alcaligenaceae>Achromobacter	GCF_000783435.2	Achromobacter_insolitus	MC81_20460	4405602..4409225	217204	BREX-system-P-loop-protein-BrxC-[Achromobacter-insolitus]
WP_076471941.1	Type6.BREX	<-DUF3262<-?<-?<-?<-JetA||wHTH+WYL+WCX->BrxE->BrxA-DNA-binding->BrxB-BrxF-iSTAND->DUF6079*->OLD-ABC->PIN->N6-METHYLASE->N6-METHYLASE->ParB+HNH->PglZ->SIGMA+OB-Fold+AAA-ATPase+HKD-DNase-BrxL-><-JAB	1207	Bacteria>Pseudomonadota>Betaproteobacteria>Burkholderiales>Alcaligenaceae>Achromobacter	GCF_001957055.1	Achromobacter_xylosoxidans	BIZ53_RS00410	81330..84953	85698	BREX-system-P-loop-protein-BrxC-[Achromobacter-xylosoxidans]
WP_212312045.1	Type6.BREX	HTH_3-><-MODE-HTH+PBPII||wHTH+WYL+WCX->BrxE->BrxA-DNA-binding->BrxB-BrxF-iSTAND->DUF6079*->GreA_GreB+SF1-SF2-Helicase->N6-METHYLASE->N6-METHYLASE->?->ParB+HNH->DOC+HTH->PglZ->SIGMA+OB-Fold+AAA-ATPase+HKD-DNase-BrxL-><-HDRnase	1217	Bacteria>Pseudomonadota>Betaproteobacteria>Burkholderiales>Alcaligenaceae>Achromobacter	GCF_018195695.1	Achromobacter_sp._Marseille-Q0513	KDH83_RS00260	59261..62914	2829161	BREX-system-P-loop-protein-BrxC-[Achromobacter-sp.-Marseille-Q0513]
WP_233436978.1	Type6.BREX	HTH_3-><-HIN-HTH<-SIGMA+OB-Fold+AAA-ATPase+HKD-DNase-BrxL<-PglZ<-ParB+HNH<-N6-METHYLASE<-N6-METHYLASE<-GreA_GreB+SF1-SF2-Helicase<-DUF6079*<-BrxB-BrxF-iSTAND<-BrxA-DNA-binding<-BrxE<-sGTP<-SF1-SF2-Helicase+REase<-Exonuc_V_gamma+REase<-NERD+SF1-SF2-Helicase<-?<-wHTH+WYL+WCX	1234	Bacteria>Pseudomonadota>Betaproteobacteria>Burkholderiales>Alcaligenaceae>Achromobacter	GCF_022976495.1	Achromobacter_xylosoxidans	DBA06_RS11680	120350..124054	85698	BREX-system-P-loop-protein-BrxC-[Achromobacter-xylosoxidans]
WP_259250627.1	Type6.BREX	SF1-SF2-Helicase+REase->?->?->?->HKD-DNase+HKD-DNase->SF1-SF2-Helicase->BrxE->BrxA-DNA-binding->BrxB-BrxF-iSTAND->DUF6079*->GreA_GreB+SF1-SF2-Helicase->N6-METHYLASE->N6-METHYLASE->ParB+HNH->PglZ->SIGMA+OB-Fold+AAA-ATPase+HKD-DNase-BrxL-><-Couple_hipA+HipA_C<-HTH_3	1213	Bacteria>Pseudomonadota>Betaproteobacteria>Burkholderiales>Alcaligenaceae>Achromobacter	GCF_024806975.1	Achromobacter_sp._JUb104	M2361_RS16380	989592..993233	2940590	BREX-system-P-loop-protein-BrxC-[Achromobacter-sp.-JUb104]
WP_076518288.1	Type1.BREX	Arm-DNA-bind_3+Phage_integrase-><-SIGMA+OB-Fold+AAA-ATPase+HKD-DNase-BrxL<-PglZ<-N6-METHYLASE-p<-?<-REase<-OLD-ABC<-N6-METHYLASE<-DUF6079*<-BrxB-BrxF-iSTAND<-BrxA-DNA-binding<-XIS-HTH<-Phage_int_SAM_2+Integrase_1<-Phage_rep<-DOC+HTH||ACET-><-?||REC+OmpR-HTH->	1181	Bacteria>Pseudomonadota>Betaproteobacteria>Burkholderiales>Alcaligenaceae>Achromobacter	GCF_900156745.1	Achromobacter_sp._MFA1_R4	BXA00_RS09440	2091384..2094929	1881016	BREX-system-P-loop-protein-BrxC-[Achromobacter-sp.-MFA1-R4]
WP_175212942.1	Type1.BREX	Arm-DNA-bind_3+Phage_integrase-><-SIGMA+OB-Fold+AAA-ATPase+HKD-DNase-BrxL<-PglZ<-N6-METHYLASE<-?<-NERD<-OLD-ABC<-N6-METHYLASE<-?<-DUF6079*<-BrxB-BrxF-iSTAND<-BrxA-DNA-binding<-XIS-HTH<-Phage_int_SAM_2+Integrase_1<-Phage_rep<-MarR-HTH||?-><-?||ACET->	1181	Bacteria>Pseudomonadota>Betaproteobacteria>Burkholderiales>Alcaligenaceae>Achromobacter	GCF_902859965.1	Achromobacter_aegrifaciens	HV793_RS07505	1613364..1616909	1287736	BREX-system-P-loop-protein-BrxC-[Achromobacter-aegrifaciens]
WP_180097564.1	Type6.BREX	<-SIGMA+OB-Fold+AAA-ATPase+HKD-DNase-BrxL<-PglZ<-ParB+HNH<-N6-METHYLASE<-N6-METHYLASE<-GreA_GreB+SF1-SF2-Helicase<-DUF6079*<-BrxB-BrxF-iSTAND<-BrxA-DNA-binding<-BrxE<-?<-sGTP<-SF1-SF2-Helicase+REase<-Exonuc_V_gamma+REase<-NERD+SF1-SF2-Helicase	1213	Bacteria>Pseudomonadota>Betaproteobacteria>Burkholderiales>Alcaligenaceae>Achromobacter	GCF_902859995.1	Achromobacter_mucicolens	H0520_RS02915	616832..620473	1389922	BREX-system-P-loop-protein-BrxC-[Achromobacter-mucicolens]
WP_200235209.1	Type1.BREX	GDResolvase+HIN-HTH->?->?-><-SIGMA+OB-Fold+AAA-ATPase+HKD-DNase-BrxL<-PglZ<-Schlafen+HATPase+wHTH<-N6-METHYLASE-p<-RVT<-N6-METHYLASE<-DUF6079*<-BrxB-BrxF-iSTAND<-BrxA-DNA-binding<-XIS-HTH<-wHTH+HNH-p||N6-METHYLASE->?->LexA-protease->?->ParB+HNH->	1190	Bacteria>Pseudomonadota>Betaproteobacteria>Burkholderiales>Alcaligenaceae>Advenella	GCF_016595155.1	Advenella_mandrilli	JHL22_RS06635	201531..205103	2800330	BREX-system-P-loop-protein-BrxC-[Advenella-mandrilli]
AYN22647.1	Type6.BREX	<-Tox-RES<-XIS-HTH||HDRnase-><-SIGMA+OB-Fold+AAA-ATPase+HKD-DNase-BrxL<-PglZ<-DOC+HTH<-N6-METHYLASE<-GreA_GreB+SF1-SF2-Helicase<-DUF6079*<-BrxB-BrxF-iSTAND<-BrxA-DNA-binding<-BrxE<-wHTH+WYL+WCX<-?||MODE-HTH+PBPII-><-HTH_3||LysR-HTH->TRPR-HTH->	1213	Bacteria>Pseudomonadota>Betaproteobacteria>Burkholderiales>Alcaligenaceae>Alcaligenes	GCA_003671915.1	Alcaligenes_aquatilis	D3M96_03425	754727..758368	323284	BREX-system-P-loop-protein-BrxC-[Alcaligenes-aquatilis]
WP_121740016.1	Type1.BREX	Phage_integrase-><-RHH_1||DOC+HTH->HTH_36->DUF772+DDE_Tnp_1_6-><-CHTH||XIS-HTH->BrxA-DNA-binding->BrxB-BrxF-iSTAND->DUF6079*->N6-METHYLASE->?->RVT->N6-METHYLASE-p->Schlafen+HATPase+wHTH->PglZ->SIGMA+OB-Fold+AAA-ATPase+HKD-DNase-BrxL->REase->	1190	Bacteria>Pseudomonadota>Betaproteobacteria>Burkholderiales>Alcaligenaceae>Alcaligenes	GCF_003671915.1	Alcaligenes_aquatilis	D3M96_RS02075	458513..462085	323284	BREX-system-P-loop-protein-BrxC-[Alcaligenes-aquatilis]
WP_199265315.1	Type1.BREX	Arm-DNA-bind_3+Phage_integrase-><-SIGMA+OB-Fold+AAA-ATPase+HKD-DNase-BrxL<-PglZ<-ParB+HNH<-N6-METHYLASE<-GreA_GreB+SF1-SF2-Helicase<-DUF6079*<-BrxB-BrxF-iSTAND<-BrxA-DNA-binding<-BrxE<-wHTH+WYL+WCX||?->?->?->?-><-HTH_3	1213	Bacteria>Pseudomonadota>Betaproteobacteria>Burkholderiales>Alcaligenaceae>Alcaligenes	GCF_016446305.1	Alcaligenes_faecalis	GTU60_RS02720	606582..610223	511	BREX-system-P-loop-protein-BrxC-[Alcaligenes-faecalis]
CCJ47860.1	Type1.BREX	<-FtsK+VirB4-FtsK+A32<-?<-?<-TraK<-TraE<-TraM||?->BrxA-DNA-binding->BrxB-BrxF-iSTAND->DUF6079*->OLD-ABC->REase->SIR2->HerA/FtsK->PglZ->SIGMA+OB-Fold+AAA-ATPase+HKD-DNase-BrxL->SbcC+SbcC->Calcineurin+Calcineurin-SbcD->SbcC->	1194	Bacteria>Pseudomonadota>Betaproteobacteria>Burkholderiales>Alcaligenaceae>Bordetella	GCA_000317935.1	Bordetella_parapertussis_Bpp5	BN117_0527	555038..558622	1208660	conserved-hypothetical-protein-[Bordetella-parapertussis-Bpp5]
WP_164926424.1	Type1.BREX	<-TraK<-TraE<-TraM<-TraM<-DUF3262<-?||XIS-HTH->BrxA-DNA-binding->BrxB-BrxF-iSTAND->DUF6079*->N6-METHYLASE->OLD-ABC->REase->SIR2->HerA/FtsK->N6-METHYLASE->PglZ->SIGMA+OB-Fold+AAA-ATPase+HKD-DNase-BrxL->SbcC+SbcC->	1188	Bacteria>Pseudomonadota>Betaproteobacteria>Burkholderiales>Alcaligenaceae>Bordetella	GCF_000317935.1	Bordetella_parapertussis	BN117_RS02695	555056..558622	519	BREX-system-P-loop-protein-BrxC-[Bordetella-parapertussis]
WP_032972587.1	Type6.BREX	DUF4400-><-SbcC<-SIGMA+OB-Fold+AAA-ATPase+HKD-DNase-BrxL<-PglZ<-?<-ParB+HNH<-N6-METHYLASE<-N6-METHYLASE<-GreA_GreB+SF1-SF2-Helicase<-DUF6079*<-BrxB-BrxF-iSTAND<-BrxA-DNA-binding<-BrxE<-wHTH+WYL+WCX||?->SMODS->?->DUF3262->TraM->	1213	Bacteria>Pseudomonadota>Betaproteobacteria>Burkholderiales>Alcaligenaceae>Bordetella	GCF_006770345.1	Bordetella_hinzii	CBR67_RS06420	1279803..1283444	103855	BREX-system-P-loop-protein-BrxC-[Bordetella-hinzii]
WP_165720894.1	Type1.BREX	sGTP->Arm-DNA-bind_3+Phage_integrase-><-SIGMA+OB-Fold+AAA-ATPase+HKD-DNase-BrxL<-PglZ<-N6-METHYLASE-p<-RVT<-N6-METHYLASE<-?<-DUF6079*<-BrxB-BrxF-iSTAND<-BrxA-DNA-binding<-XIS-HTH<-Phage_int_SAM_2+Integrase_1<-HTH_3<-Phage_rep	1181	Bacteria>Pseudomonadota>Betaproteobacteria>Burkholderiales>Alcaligenaceae>Bordetella	GCF_011386965.1	Bordetella_trematum	G6Z49_RS19625	4277130..4280675	123899	BREX-system-P-loop-protein-BrxC-[Bordetella-trematum]
WP_068373657.1	Type1.BREX	<-TetR-HTH||?->Phage_rep->HTH_3->Phage_int_SAM_2+Integrase_1->XIS-HTH->BrxA-DNA-binding->BrxB-BrxF-iSTAND->DUF6079*->?->N6-METHYLASE->OLD-ABC->REase->?->N6-METHYLASE-p->PglZ->SIGMA+OB-Fold+AAA-ATPase+HKD-DNase-BrxL-><-Arm-DNA-bind_3+Phage_integrase	1176	Bacteria>Pseudomonadota>Betaproteobacteria>Burkholderiales>Alcaligenaceae>Kerstersia	GCF_000988095.1	Kerstersia_gyiorum	AAV32_RS14310	103983..107513	206506	BREX-system-P-loop-protein-BrxC-[Kerstersia-gyiorum]
WP_115075260.1	Type1.BREX	MODE-HTH+PBPII-><-SIGMA+OB-Fold+AAA-ATPase+HKD-DNase-BrxL<-PglZ<-N6-METHYLASE-p<-NERD<-OLD-ABC<-REase<-N6-METHYLASE<-DUF6079*<-BrxB-BrxF-iSTAND<-BrxA-DNA-binding	1187	Bacteria>Pseudomonadota>Betaproteobacteria>Burkholderiales>Alcaligenaceae>Oligella	GCF_900454255.1	Oligella_urethralis	DX091_RS10510	2175019..2178582	90245	BREX-system-P-loop-protein-BrxC-[Oligella-urethralis]
WP_166406662.1	Type1.BREX	Integrase_1-><-?||SbcC->GDResolvase+HIN-HTH-><-SIGMA+OB-Fold+AAA-ATPase+HKD-DNase-BrxL<-PglZ<-Schlafen+HATPase+wHTH<-N6-METHYLASE<-DUF6079*<-BrxB-BrxF-iSTAND<-BrxA-DNA-binding<-XIS-HTH||Arm-DNA-bind_3+Phage_integrase+Phage_integr_3->?->?->?-><-?<-REase+SF1-SF2-Helicase	1190	Bacteria>Pseudomonadota>Betaproteobacteria>Burkholderiales>Alcaligenaceae>Paenalcaligenes	GCF_011290505.1	Paenalcaligenes_suwonensis	GY634_RS00685	163356..166928	1202713	BREX-system-P-loop-protein-BrxC-[Paenalcaligenes-suwonensis]
MCK9506949.1	Type1.BREX	DUF6079*->N6-METHYLASE->PglZ->SIGMA+OB-Fold+AAA-ATPase+HKD-DNase-BrxL->SF1-SF2-Helicase+REase->SF1-SF2-Helicase+REase->wHTH->HKD-DNase+HKD-DNase->SF1-SF2-Helicase->	1069	Bacteria>Pseudomonadota>Betaproteobacteria>Burkholderiales>Alcaligenaceae>Pigmentiphaga	GCA_023228545.1	Pigmentiphaga_sp.	MOQ54_00780	41..3250	1977564	BREX-system-P-loop-protein-BrxC-[Pigmentiphaga-sp.]
WP_132980217.1	Type1.BREX	RelE-ParE->?->SF1-SF2-Helicase->Phage_rep->HTH_3->Phage_int_SAM_2+Integrase_1->XIS-HTH->BrxA-DNA-binding->BrxB-BrxF-iSTAND->DUF6079*->?->RVT->N6-METHYLASE->OLD-ABC->REase->?->N6-METHYLASE-p->PglZ->SIGMA+OB-Fold+AAA-ATPase+HKD-DNase-BrxL-><-Arm-DNA-bind_3+Phage_integrase	1181	Bacteria>Pseudomonadota>Betaproteobacteria>Burkholderiales>Alcaligenaceae>Pigmentiphaga	GCF_004352985.1	Pigmentiphaga_sp._D-2	EZY13_RS19720	185799..189344	1002116	BREX-system-P-loop-protein-BrxC-[Pigmentiphaga-sp.-D-2]
WP_102070953.1	Type1.BREX	<-AbiEii<-AbiEi_3_N+AbiEi_3<-DOC+HTH<-XIS-HTH<-HTH_19<-?<-SIGMA+OB-Fold+AAA-ATPase+HKD-DNase-BrxL<-PglZ<-N6-METHYLASE<-DUF6079*<-BrxB-BrxF-iSTAND<-BrxA-DNA-binding<-XIS-HTH<-HerA/FtsK<-SIR2||?->?->?->SSB->	1181	Bacteria>Pseudomonadota>Betaproteobacteria>Burkholderiales>Alcaligenaceae>Pollutimonas	GCF_002849615.1	Pollutimonas_nitritireducens	CR155_RS15505	30043..33588	2045209	BREX-system-P-loop-protein-BrxC-[Pollutimonas-nitritireducens]
MAO51251.1	Type6.BREX	<-TraM||Peptidase_C39->wHTH+WYL+WCX->BrxE->BrxA-DNA-binding->BrxB-BrxF-iSTAND->DUF6079*->SF1-SF2-Helicase+RNase+RNase->TPR->N6-METHYLASE->N6-METHYLASE-p->DOC+HTH->PglZ->SIGMA+OB-Fold+AAA-ATPase+HKD-DNase-BrxL->DDE_Tnp_ISL3->	1209	Bacteria>Pseudomonadota>Betaproteobacteria>Burkholderiales>Alcaligenaceae>Pusillimonas	GCA_002693665.1	Pusillimonas_sp._(ex_Stolz_et_al._2005)	CML16_10275	63709..67338	1979962	ATPase-[Pusillimonas-sp.-(ex-Stolz-et-al.-2005)]
NYT45622.1	Type6.BREX	<-AbiEii<-AbiEi_3_N+AbiEi_3<-DOC+HTH<-XIS-HTH<-HTH_19||wHTH+WYL+WCX->BrxE->BrxA-DNA-binding->BrxB-BrxF-iSTAND->DUF6079*->N6-METHYLASE->N6-METHYLASE->DOC+HTH->PglZ->SIGMA+OB-Fold+AAA-ATPase+HKD-DNase-BrxL->KAP-NTPase-><-?||ParB+HNH->ParB+HNH->	1214	Bacteria>Pseudomonadota>Betaproteobacteria>Burkholderiales>Alcaligenaceae>unclassified_Alcaligenaceae	GCA_013416345.1	Alcaligenaceae_bacterium	HOA64_12395	218321..221965	2021368	BREX-system-P-loop-protein-BrxC-[Alcaligenaceae-bacterium]
NYT60502.1	Type1.BREX	<-SIGMA+OB-Fold+AAA-ATPase+HKD-DNase-BrxL<-PglZ<-N6-METHYLASE-p<-Schlafen+HATPase+wHTH<-N6-METHYLASE<-DUF6079*<-BrxB-BrxF-iSTAND<-BrxA-DNA-binding<-XIS-HTH||?-><-tRNA||?-><-RelE-ParE<-CHTH<-RRXRR+HNH	1188	Bacteria>Pseudomonadota>Betaproteobacteria>Burkholderiales>Alcaligenaceae>unclassified_Alcaligenaceae	GCA_013416405.1	Alcaligenaceae_bacterium	HOA65_16390	10015..13581	2021368	BREX-system-P-loop-protein-BrxC-[Alcaligenaceae-bacterium]
NYT63370.1	Type1.BREX	<-SIGMA+OB-Fold+AAA-ATPase+HKD-DNase-BrxL<-PglZ<-ParB+HNH<-N6-METHYLASE-p<-HerA/FtsK<-SIR2<-NERD<-OLD-ABC<-N6-METHYLASE<-DUF6079*<-BrxB-BrxF-iSTAND<-BrxA-DNA-binding<-XIS-HTH||?->?->SSB->?-><-ORC-CDC6<-HEPN	1191	Bacteria>Pseudomonadota>Betaproteobacteria>Burkholderiales>Alcaligenaceae>unclassified_Alcaligenaceae	GCA_013416445.1	Alcaligenaceae_bacterium	HOA66_13705	63657..67232	2021368	BREX-system-P-loop-protein-BrxC-[Alcaligenaceae-bacterium]
NYT66107.1	Type1.BREX	YdaS_antitoxin-><-SIGMA+OB-Fold+AAA-ATPase+HKD-DNase-BrxL<-PglZ<-?<-N6-METHYLASE-p<-PadR-HTH<-NERD<-OLD-ABC<-N6-METHYLASE-p<-DUF6079*<-BrxB-BrxF-iSTAND<-BrxA-DNA-binding<-XIS-HTH||?-><-HTH_Tnp_1+HTH_21+rve+rve_3	1193	Bacteria>Pseudomonadota>Betaproteobacteria>Burkholderiales>Alcaligenaceae>unclassified_Alcaligenaceae	GCA_013416485.1	Alcaligenaceae_bacterium	HOA58_09005	172517..176098	2021368	BREX-system-P-loop-protein-BrxC-[Alcaligenaceae-bacterium]
MCA8264434.1	Type6.BREX	<-SIGMA+OB-Fold+AAA-ATPase+HKD-DNase-BrxL<-PglZ<-?<-N6-METHYLASE<-N6-METHYLASE<-DUF6079*<-BrxB-BrxF-iSTAND<-BrxA-DNA-binding<-BrxE<-wHTH+WYL+WCX||ARG->	1210	Bacteria>Pseudomonadota>Betaproteobacteria>Burkholderiales>Burkholderiaceae>Burkholderia	GCA_020420005.1	Burkholderia_multivorans	LGM79_28445	10767..14399	87883	BREX-system-P-loop-protein-BrxC-[Burkholderia-multivorans]
WP_059944780.1	Type6.BREX	<-wHTH+WYL+WCX||?-><-SIGMA+OB-Fold+AAA-ATPase+HKD-DNase-BrxL<-PglZ<-ParB+HNH<-N6-METHYLASE<-N6-METHYLASE<-GreA_GreB+SF1-SF2-Helicase<-DUF6079*<-BrxB-BrxF-iSTAND<-BrxA-DNA-binding<-BrxE	1213	Bacteria>Pseudomonadota>Betaproteobacteria>Burkholderiales>Burkholderiaceae>Burkholderia	GCF_001527205.1	Burkholderia_territorii	WT00_RS19595	122446..126087	1503055	BREX-system-P-loop-protein-BrxC-[Burkholderia-territorii]
WP_060189440.1	Type1.BREX	<-ABhydrolase||?-><-?<-?||?->Schlafen+HATPase+wHTH->wHTH+WYL+WCX->BrxA-DNA-binding->BrxB-BrxF-iSTAND->DUF6079*->N6-METHYLASE->PglZ->SIGMA+OB-Fold+AAA-ATPase+HKD-DNase-BrxL->?-><-AAA-ATPase<-Phage_integrase	1186	Bacteria>Pseudomonadota>Betaproteobacteria>Burkholderiales>Burkholderiaceae>Burkholderia	GCF_001533155.1	Burkholderia_diffusa	WL85_RS21680	47133..50693	488732	BREX-system-P-loop-protein-BrxC-[Burkholderia-diffusa]
WP_124661146.1	Type6.BREX	<-SIGMA+OB-Fold+AAA-ATPase+HKD-DNase-BrxL<-PglZ<-N6-METHYLASE-p<-N6-METHYLASE<-DUF6079*<-BrxB-BrxF-iSTAND<-BrxA-DNA-binding<-BrxE<-NACHT+GTPase-AIG+sGTP<-?<-?<-?<-HTH_1+PBP_like	1210	Bacteria>Pseudomonadota>Betaproteobacteria>Burkholderiales>Burkholderiaceae>Burkholderia	GCF_003854235.1	Burkholderia_sp._Bp8998	DIE06_RS09175	82371..86003	2184557	BREX-system-P-loop-protein-BrxC-[Burkholderia-sp.-Bp8998]
WP_226255239.1	Type6.BREX	HTH_1+PBP_like->?->?->XIS-HTH->NACHT+GTPase-AIG+sGTP->BrxE->BrxA-DNA-binding->BrxB-BrxF-iSTAND->DUF6079*->N6-METHYLASE->N6-METHYLASE->ParB+HNH->PglZ->SIGMA+OB-Fold+AAA-ATPase+HKD-DNase-BrxL->	1208	Bacteria>Pseudomonadota>Betaproteobacteria>Burkholderiales>Burkholderiaceae>Burkholderia	GCF_020419005.1	Burkholderia_cepacia	LGM39_RS24930	366713..370339	292	BREX-system-P-loop-protein-BrxC-[Burkholderia-cepacia]
WP_175041065.1	Type6.BREX	<-SIGMA+OB-Fold+AAA-ATPase+HKD-DNase-BrxL<-PglZ<-N6-METHYLASE<-DUF6079*<-BrxB-BrxF-iSTAND<-BrxA-DNA-binding<-BrxE<-MORC+DNA_gyrase+TOPRIM<-?<-RFC-AAA+DNAA-HTH||?->RNaseP->	1215	Bacteria>Pseudomonadota>Betaproteobacteria>Burkholderiales>Burkholderiaceae>Burkholderia	GCF_902499225.1	Burkholderia_contaminans	BLA3211_RS35460	10621..14268	488447	BREX-system-P-loop-protein-BrxC-[Burkholderia-contaminans]
WP_175704189.1	Type6.BREX	<-RNaseP<-?||RFC-AAA+DNAA-HTH->?->MORC+DNA_gyrase+TOPRIM->BrxE->BrxA-DNA-binding->BrxB-BrxF-iSTAND->DUF6079*->N6-METHYLASE->N6-METHYLASE->PglZ->SIGMA+OB-Fold+AAA-ATPase+HKD-DNase-BrxL->	1215	Bacteria>Pseudomonadota>Betaproteobacteria>Burkholderiales>Burkholderiaceae>Burkholderia	GCF_902829775.1	Burkholderia_ambifaria	HWW86_RS26205	192524..196171	152480	BREX-system-P-loop-protein-BrxC-[Burkholderia-ambifaria]
WP_186043215.1	Type6.BREX	Phage_holin->Phage_lysozyme-><-SIGMA+OB-Fold+AAA-ATPase+HKD-DNase-BrxL<-PglZ<-ParB+HNH<-N6-METHYLASE<-GreA_GreB+SF1-SF2-Helicase<-DUF6079*<-BrxB-BrxF-iSTAND<-BrxA-DNA-binding<-BrxE<-wHTH+WYL+WCX||SF1-SF2-Helicase->PIN->?->ClpABC-AAA->	1213	Bacteria>Pseudomonadota>Betaproteobacteria>Burkholderiales>Burkholderiaceae>Burkholderia	GCF_902830235.1	Burkholderia_gladioli	H9C96_RS14825	15196..18837	28095	BREX-system-P-loop-protein-BrxC-[Burkholderia-gladioli]
WP_186236015.1	Type6.BREX	ParB+HNH->ParB+HNH->HNH->Phage_integrase-><-SIGMA+OB-Fold+AAA-ATPase+HKD-DNase-BrxL<-PglZ<-N6-METHYLASE<-DUF6079*<-BrxB-BrxF-iSTAND<-BrxA-DNA-binding<-BrxE<-MORC+DNA_gyrase+TOPRIM<-?<-RFC-AAA+DNAA-HTH||?->RNaseP->	1215	Bacteria>Pseudomonadota>Betaproteobacteria>Burkholderiales>Burkholderiaceae>Burkholderia	GCF_902832475.1	Burkholderia_gladioli	H9G27_RS00755	169921..173568	28095	BREX-system-P-loop-protein-BrxC-[Burkholderia-gladioli]
WP_075360437.1	Type6.BREX	McrB-AAA->McrC-NTD+REase-><-RVT<-SIGMA+OB-Fold+AAA-ATPase+HKD-DNase-BrxL<-PglZ<-N6-METHYLASE<-N6-METHYLASE-p<-N6-METHYLASE<-DUF6079*<-BrxB-BrxF-iSTAND<-BrxA-DNA-binding<-BrxE<-tRNA<-AA_kinase+ACT+ACT_7<-?<-?<-?<-?||TPR+TPR->	1210	Bacteria>Pseudomonadota>Betaproteobacteria>Burkholderiales>Burkholderiaceae>Caballeronia	GCF_001935975.1	Caballeronia_sordidicola	BSU03_RS34875	87497..91129	196367	BREX-system-P-loop-protein-BrxC-[Caballeronia-sordidicola]
WP_187608173.1	Type6.BREX	XIS-HTH->?->GDResolvase+HIN-HTH->SF1-SF2-Helicase->?->HTH_26->BrxE->BrxA-DNA-binding->BrxB-BrxF-iSTAND->DUF6079*->N6-METHYLASE->N6-METHYLASE->PglZ->SIGMA+OB-Fold+AAA-ATPase+HKD-DNase-BrxL-><-?<-?<-?||Glyoxalase->	1215	Bacteria>Pseudomonadota>Betaproteobacteria>Burkholderiales>Burkholderiaceae>Caballeronia	GCF_014384845.1	Caballeronia_sp._EK	IAG25_RS30620	154529..158176	2767469	BREX-system-P-loop-protein-BrxC-[Caballeronia-sp.-EK]
WP_091908362.1	Type6.BREX	<-REase<-?||?->?-><-MODE-HTH+PBPII||wHTH+WYL+WCX->BrxE->BrxA-DNA-binding->BrxB-BrxF-iSTAND->DUF6079*->GreA_GreB+SF1-SF2-Helicase->N6-METHYLASE->ParB+HNH->PglZ->SIGMA+OB-Fold+AAA-ATPase+HKD-DNase-BrxL->	1213	Bacteria>Pseudomonadota>Betaproteobacteria>Burkholderiales>Burkholderiaceae>Chitinasiproducens	GCF_900095735.1	Chitinasiproducens_palmae	BLU54_RS11860	213511..217152	1770053	BREX-system-P-loop-protein-BrxC-[Chitinasiproducens-palmae]
WP_013958181.1	Type6.BREX	<-SIGMA+OB-Fold+AAA-ATPase+HKD-DNase-BrxL<-PglZ<-?<-N6-METHYLASE<-?<-GreA_GreB+SF1-SF2-Helicase<-DUF6079*<-BrxB-BrxF-iSTAND<-BrxA-DNA-binding<-BrxE	1214	Bacteria>Pseudomonadota>Betaproteobacteria>Burkholderiales>Burkholderiaceae>Cupriavidus	GCF_000219215.1	Cupriavidus_necator	CNE_1c33280	3480419..3484063	106590	BREX-system-P-loop-protein-BrxC-[Cupriavidus-necator]
WP_174424431.1	Type6.BREX	<-RNaseP<-?||RFC-AAA+DNAA-HTH->?->MORC+DNA_gyrase+TOPRIM->HTH_26->BrxE->BrxA-DNA-binding->BrxB-BrxF-iSTAND->DUF6079*->N6-METHYLASE->N6-METHYLASE-p->PglZ->SIGMA+OB-Fold+AAA-ATPase+HKD-DNase-BrxL->	1215	Bacteria>Pseudomonadota>Betaproteobacteria>Burkholderiales>Burkholderiaceae>Cupriavidus	GCF_013337645.1	Cupriavidus_basilensis	FCJ59_RS11575	146108..149755	68895	BREX-system-P-loop-protein-BrxC-[Cupriavidus-basilensis]
WP_059038859.1	Type6.BREX	<-SIGMA+OB-Fold+AAA-ATPase+HKD-DNase-BrxL<-PglZ<-N6-METHYLASE<-DUF6079*<-BrxB-BrxF-iSTAND<-BrxA-DNA-binding<-BrxE<-MORC+DNA_gyrase+TOPRIM<-?<-RFC-AAA+DNAA-HTH||?->RNaseP->	1215	Bacteria>Pseudomonadota>Betaproteobacteria>Burkholderiales>Burkholderiaceae	GCF_001485395.1	Ralstonia	SAMD00023378_RS10640	9198..12845	48736	MULTISPECIES:-BREX-system-P-loop-protein-BrxC-[Ralstonia]
WP_220363411.1	Type6.BREX	Phage_integrase-><-SIGMA+OB-Fold+AAA-ATPase+HKD-DNase-BrxL<-PglZ<-N6-METHYLASE<-DUF6079*<-BrxB-BrxF-iSTAND<-BrxA-DNA-binding<-BrxE<-HTH_26<-?<-ParB+HNH	1215	Bacteria>Pseudomonadota>Betaproteobacteria>Burkholderiales>Burkholderiaceae>Paraburkholderia	GCF_019511605.1	Paraburkholderia_phenoliruptrix	JNB89_RS20360	10910..14557	252970	BREX-system-P-loop-protein-BrxC-[Paraburkholderia-phenoliruptrix]
WP_230560751.1	Type6.BREX	Phage_integrase-><-SIGMA+OB-Fold+AAA-ATPase+HKD-DNase-BrxL<-PglZ<-N6-METHYLASE<-N6-METHYLASE<-DUF6079*<-BrxB-BrxF-iSTAND<-BrxA-DNA-binding<-BrxE<-HTH_26<-Phage_lambda_P<-HTH_36	1215	Bacteria>Pseudomonadota>Betaproteobacteria>Burkholderiales>Burkholderiaceae>Paraburkholderia	GCF_020881035.1	Paraburkholderia_sp._MMS20-SJTN17	LJ655_RS08170	67515..71162	2886945	BREX-system-P-loop-protein-BrxC-[Paraburkholderia-sp.-MMS20-SJTN17]
WP_167306478.1	Type1.BREX	<-PAS+STAS<-?<-?<-SIGMA+OB-Fold+AAA-ATPase+HKD-DNase-BrxL<-PglZ<-N6-METHYLASE<-REase<-OLD-ABC<-N6-METHYLASE<-DUF6079*<-BrxB-BrxF-iSTAND<-BrxA-DNA-binding<-XIS-HTH<-SF1-SF2-Helicase<-HKD-DNase+HKD-Rease	1182	Bacteria>Pseudomonadota>Betaproteobacteria>Burkholderiales>Burkholderiaceae>Paraburkholderia	GCF_900100565.1	Paraburkholderia_steynii	BLT65_RS12240	289685..293233	1245441	BREX-system-P-loop-protein-BrxC-[Paraburkholderia-steynii]
WP_201107905.1	Type1.BREX	HTH_36->Phage_lambda_P->?->GDResolvase+HIN-HTH->BrxA-DNA-binding->BrxB-BrxF-iSTAND->DUF6079*->N6-METHYLASE->PglZ->SIGMA+OB-Fold+AAA-ATPase+HKD-DNase-BrxL-><-Phage_integrase<-AraC-HTH||?->?-><-TarH+HAMP+MA||TetR-HTH->	1179	Bacteria>Pseudomonadota>Betaproteobacteria>Burkholderiales>Burkholderiaceae>Paraburkholderia	GCF_905220855.1	Paraburkholderia_domus	KH036_RS10755	51203..54742	2793075	BREX-system-P-loop-protein-BrxC-[Paraburkholderia-domus]
WP_183964658.1	Type6.BREX	<-SWIM<-?||NUDIX+NAAR1+NAAR2->?->?->?->HEPN->BrxE->BrxB-BrxF-iSTAND->DUF6079*->N6-METHYLASE->N6-METHYLASE-p->PglZ->SIGMA+OB-Fold+AAA-ATPase+HKD-DNase-BrxL-><-RVT<-?<-SAM-METHYLASE+wHTH+HNH<-BirA-HTH	1210	Bacteria>Pseudomonadota>Betaproteobacteria>Burkholderiales>Burkholderiaceae>Quisquiliibacterium	GCF_014202215.1	Quisquiliibacterium_transsilvanicum	HNQ70_RS04390	209103..212735	1549638	BREX-system-P-loop-protein-BrxC-[Quisquiliibacterium-transsilvanicum]
MBP6902893.1	Type6.BREX	HTH_26->BrxE->BrxA-DNA-binding->BrxB-BrxF-iSTAND->DUF6079*->N6-METHYLASE->N6-METHYLASE->?->?->PglZ->SIGMA+OB-Fold+AAA-ATPase+HKD-DNase-BrxL->TUDOR+SF1-SF2-Helicase+iREase+CTD->?->REase+iREase->	1219	Bacteria>Pseudomonadota>Betaproteobacteria>Burkholderiales>Burkholderiaceae>unclassified_Burkholderiaceae	GCA_017992055.1	Burkholderiaceae_bacterium	KBC73_22560	2680..6339	2030806	BREX-system-P-loop-protein-BrxC-[Burkholderiaceae-bacterium]
MCP5271536.1	Type6.BREX	HTH_3-><-URI-DNase<-?<-Arm-DNA-bind_2+Phage_int_SAM_3+Phage_integrase<-Phage_AlpA||HTH_26->BrxE->BrxA-DNA-binding->BrxB-BrxF-iSTAND->DUF6079*->N6-METHYLASE->N6-METHYLASE->?->?->PglZ->SIGMA+OB-Fold+AAA-ATPase+HKD-DNase-BrxL->TUDOR+SF1-SF2-Helicase+iREase+CTD->	1219	Bacteria>Pseudomonadota>Betaproteobacteria>Burkholderiales>Burkholderiaceae>unclassified_Burkholderiaceae	GCA_024235855.1	Burkholderiaceae_bacterium	H6932_09900	152250..155909	2030806	BREX-system-P-loop-protein-BrxC-[Burkholderiaceae-bacterium]
WP_143968626.1	Type6.BREX	PglZ->SIGMA+OB-Fold+AAA-ATPase+HKD-DNase-BrxL->||Phage_integrase->Phage_integrase->Phage_integrase->?->BrxE->BrxA-DNA-binding->BrxB-BrxF-iSTAND->DUF6079*->N6-METHYLASE->OLD-ABC+TOPRIM->N6-METHYLASE->PglZ->SIGMA+OB-Fold+AAA-ATPase+HKD-DNase-BrxL->	1210	Bacteria>Pseudomonadota>Betaproteobacteria>Burkholderiales>Burkholderiales_genera_incertae_sedis>Tepidimonas	GCF_007556755.1	Tepidimonas_fonticaldi	Tfont_RS05070	95231..98863	1101373	BREX-system-P-loop-protein-BrxC-[Tepidimonas-fonticaldi]
WP_054257718.1	Type6.BREX	HTH_19-><-SIGMA+OB-Fold+AAA-ATPase+HKD-DNase-BrxL<-PglZ<-?<-ParB+HNH<-N6-METHYLASE-p<-N6-METHYLASE<-DUF6079*<-BrxB-BrxF-iSTAND<-BrxA-DNA-binding<-BrxE<-HTH_21+rve+rve_3	1215	Bacteria>Pseudomonadota>Betaproteobacteria>Burkholderiales>Comamonadaceae>Acidovorax	GCF_001298675.1	Acidovorax_caeni	BN2503_RS16450	13891..17538	343013	BREX-system-P-loop-protein-BrxC-[Acidovorax-caeni]
WP_057202647.1	Type6.BREX	HTH+sGTP->?->?->BrxE->BrxA-DNA-binding->BrxB-BrxF-iSTAND->DUF6079*->GreA_GreB+SF1-SF2-Helicase->N6-METHYLASE->N6-METHYLASE->ParB+HNH->PglZ->SIGMA+OB-Fold+AAA-ATPase+HKD-DNase-BrxL->?->GGDEF-><-Peptidase_M48+TPR_9	1207	Bacteria>Pseudomonadota>Betaproteobacteria>Burkholderiales>Comamonadaceae>Acidovorax	GCF_001428645.1	Acidovorax_sp._Root217	ASE31_RS15170	91296..94919	1736492	BREX-system-P-loop-protein-BrxC-[Acidovorax-sp.-Root217]
GAO21740.1	Type6.BREX	HDRnase-><-TatD_DNase<-?<-KAP-glycosylase<-KAP-NTPase<-SIGMA+OB-Fold+AAA-ATPase+HKD-DNase-BrxL<-PglZ<-N6-METHYLASE<-GreA_GreB+SF1-SF2-Helicase<-DUF6079*<-BrxB-BrxF-iSTAND<-BrxA-DNA-binding<-BrxE<-wHTH+WYL+WCX||?-><-HTH_28+rve+rve_3||MODE-HTH+PBPII-><-HTH_3||LysR-HTH->	1213	Bacteria>Pseudomonadota>Betaproteobacteria>Burkholderiales>Comamonadaceae>Alicycliphilus	GCA_000974645.1	Alicycliphilus_sp._B1	ALISP_1560	91569..95210	1603291	ATPase-[Alicycliphilus-sp.-B1]
WP_034379112.1	Type6.BREX	Phage_holin->Phage_lysozyme-><-SIR2<-SIGMA+OB-Fold+AAA-ATPase+HKD-DNase-BrxL<-PglZ<-?<-ParB+HNH<-N6-METHYLASE-p<-N6-METHYLASE<-DUF6079*<-BrxB-BrxF-iSTAND<-BrxA-DNA-binding<-BrxE<-wHTH+WYL+WCX||JetA->JetB->SbcC->	1210	Bacteria>Pseudomonadota>Betaproteobacteria>Burkholderiales>Comamonadaceae>Comamonas	GCF_000761335.1	Comamonas_thiooxydans	P245_RS10570	85855..89487	363952	BREX-system-P-loop-protein-BrxC-[Comamonas-thiooxydans]
KEH09926.1	Type1.BREX	<-DUF932||XIS-HTH->OLD-ABC->BrxA-DNA-binding->BrxB-BrxF-iSTAND->DUF6079*->N6-METHYLASE->OLD-ABC->REase->SIR2->?->ParB+HNH->PglZ->SIGMA+OB-Fold+AAA-ATPase+HKD-DNase-BrxL->	1185	Bacteria>Pseudomonadota>Betaproteobacteria>Burkholderiales>Comamonadaceae>Delftia	GCA_000710165.1	Delftia_tsuruhatensis	GY14_08945	17729..21286	180282	hypothetical-protein-GY14_08945-[Delftia-tsuruhatensis]
OJX16153.1	Type1.BREX	<-SIGMA+OB-Fold+AAA-ATPase+HKD-DNase-BrxL<-PglZ<-Schlafen+HATPase+wHTH<-N6-METHYLASE-p<-?<-REase<-OLD-ABC<-N6-METHYLASE<-?<-DUF6079*<-BrxB-BrxF-iSTAND<-BrxA-DNA-binding<-Phage_int_SAM_2+Integrase_1<-Phage_rep||CNMP-><-PRTase||CBS+CBS->HAD+HAD->	1181	Bacteria>Pseudomonadota>Betaproteobacteria>Burkholderiales>Comamonadaceae>Delftia	GCA_001898745.1	Delftia_sp._67-8	BGO79_00315	75959..79504	1895749	hypothetical-protein-BGO79_00315-[Delftia-sp.-67-8]
WP_096915618.1	Type6.BREX	<-ABhydrolase+ABhydrolase<-?<-?<-SIGMA+OB-Fold+AAA-ATPase+HKD-DNase-BrxL<-PglZ<-N6-METHYLASE<-N6-METHYLASE<-DUF6079*<-BrxB-BrxF-iSTAND<-BrxA-DNA-binding<-BrxE<-AbiEii<-MarR-HTH<-GNTR-HTH||?->SIR2->	1224	Bacteria>Pseudomonadota>Betaproteobacteria>Burkholderiales>Comamonadaceae>Delftia	GCF_002443055.1	Delftia_acidovorans	CHL79_RS18790	4226898..4230572	80866	BREX-system-P-loop-protein-BrxC-[Delftia-acidovorans]
WP_234638190.1	Type6.BREX	MarR-HTH->AbiEii->BrxE->BrxA-DNA-binding->BrxB-BrxF-iSTAND->DUF6079*->N6-METHYLASE->N6-METHYLASE->PglZ->SIGMA+OB-Fold+AAA-ATPase+HKD-DNase-BrxL->	1219	Bacteria>Pseudomonadota>Betaproteobacteria>Burkholderiales>Comamonadaceae>Delftia	GCF_903684635.1	Delftia_tsuruhatensis	MJ845_RS20380	81444..85103	180282	BREX-system-P-loop-protein-BrxC-[Delftia-tsuruhatensis]
TFI47144.1	Type1.BREX	<-HTH+rve||?-><-SIGMA+OB-Fold+AAA-ATPase+HKD-DNase-BrxL<-PglZ<-N6-METHYLASE-p<-HTH+REase<-DUF6079*<-BrxB-BrxF-iSTAND<-BrxA-DNA-binding<-wHTH+WYL+WCX<-Phage_integrase+Phage_integrase<-?||HIN-HTH->	1188	Bacteria>Pseudomonadota>Betaproteobacteria>Burkholderiales>Comamonadaceae>Diaphorobacter	GCA_004563745.1	Diaphorobacter_sp._DS2	E4093_14250	10625..14191	1302548	BREX-system-P-loop-protein-BrxC-[Diaphorobacter-sp.-DS2]
WP_047349524.1	Type6.BREX	JAB-><-SIGMA+OB-Fold+AAA-ATPase+HKD-DNase-BrxL<-PglZ<-DUF488-s<-DUF488-l<-DUF4326<-N6-METHYLASE<-N6-METHYLASE<-GreA_GreB+SF1-SF2-Helicase<-DUF6079*<-BrxB-BrxF-iSTAND<-BrxA-DNA-binding<-BrxE<-wHTH+WYL+WCX||JetA->JetB->SbcC->	1213	Bacteria>Pseudomonadota>Betaproteobacteria>Burkholderiales>Comamonadaceae>Diaphorobacter	GCF_001023515.1	Diaphorobacter_sp._J5-51	OX89_RS03130	14085..17726	680496	BREX-system-P-loop-protein-BrxC-[Diaphorobacter-sp.-J5-51]
WP_106685100.1	Type6.BREX	GspA-ATPase->?-><-LysR-HTH||HTH_3-><-MODE-HTH+PBPII||wHTH+WYL+WCX->BrxE->BrxA-DNA-binding->BrxB-BrxF-iSTAND->DUF6079*->GreA_GreB+SF1-SF2-Helicase->N6-METHYLASE->N6-METHYLASE->DOC+HTH->PglZ->SIGMA+OB-Fold+AAA-ATPase+HKD-DNase-BrxL-><-HDRnase||XIS-HTH->PrlF_antitoxin->	1213	Bacteria>Pseudomonadota>Betaproteobacteria>Burkholderiales>Comamonadaceae>Melaminivora	GCF_003008575.1	Melaminivora_suipulveris	C6568_RS16475	3505730..3509371	2109913	BREX-system-P-loop-protein-BrxC-[Melaminivora-suipulveris]
CDS49862.1	Type6.BREX	HTH_29+HTH_32+HTH_33+DDE_3->ARG-><-?<-SIGMA+OB-Fold+AAA-ATPase+HKD-DNase-BrxL<-PglZ<-ParB+HNH<-N6-METHYLASE<-TPR<-SF1-SF2-Helicase+RNase+RNase<-DUF6079*<-BrxB-BrxF-iSTAND<-BrxA-DNA-binding<-BrxE-p<-?<-?<-AAA+HTH<-TOPRIM<-Arm-DNA-bind_3+Phage_integrase<-HAD+HAD	1210	Bacteria>Pseudomonadota>Betaproteobacteria>Burkholderiales>Comamonadaceae>Polaromonas	-	Polaromonas_sp._CG9_12	.	485322..488954	1504672	hypothetical-protein-[Polaromonas-sp.-CG9_12]
MBW7834374.1	Type6.BREX	<-RNaseP<-?||RFC-AAA+DNAA-HTH->?->MORC+DNA_gyrase+TOPRIM-><-SIGMA+OB-Fold+AAA-ATPase+HKD-DNase-BrxL<-PglZ<-DUF6079*<-BrxB-BrxF-iSTAND<-BrxA-DNA-binding<-BrxE<-XIS-HTH<-?<-HTH_3||HTH->	1210	Bacteria>Pseudomonadota>Betaproteobacteria>Burkholderiales>Comamonadaceae>Simplicispira	GCA_019455265.1	Simplicispira_suum	H3C29_14305	45952..49584	2109915	BREX-system-P-loop-protein-BrxC-[Simplicispira-suum]
WP_273364231.1	Type6.BREX	<-RNaseP<-?||RFC-AAA+DNAA-HTH->?->MORC+DNA_gyrase+TOPRIM-><-SIGMA+OB-Fold+AAA-ATPase+HKD-DNase-BrxL<-PglZ<-N6-METHYLASE<-DUF6079*<-BrxB-BrxF-iSTAND<-BrxA-DNA-binding<-BrxE<-XIS-HTH<-?<-HTH_3||HTH->	1210	Bacteria>Pseudomonadota>Betaproteobacteria>Burkholderiales>Comamonadaceae>Simplicispira	GCA_019455265.1	Simplicispira_suum	H3C29_RS14325	45952..49584	2109915	BREX-system-P-loop-protein-BrxC-[Simplicispira-suum]
WP_068673039.1	Type6.BREX	<-SbcC<-?<-?<-SIGMA+OB-Fold+AAA-ATPase+HKD-DNase-BrxL<-PglZ<-ParB+HNH<-N6-METHYLASE<-N6-METHYLASE<-DUF6079*<-BrxB-BrxF-iSTAND<-BrxA-DNA-binding<-BrxE<-wHTH+WYL+WCX	1212	Bacteria>Pseudomonadota>Betaproteobacteria>Burkholderiales>Comamonadaceae>Variovorax	GCF_001541225.1	unclassified_Variovorax	APY03_RS01000	202384..206022	663243	MULTISPECIES:-BREX-system-P-loop-protein-BrxC-[unclassified-Variovorax]
WP_218509735.1	Type6.BREX	CNMP+CRP-HTH-><-?||?->Arm-DNA-bind_3+Phage_integrase->?-><-SIGMA+OB-Fold+AAA-ATPase+HKD-DNase-BrxL<-PglZ<-N6-METHYLASE-p<-N6-METHYLASE<-DUF6079*<-BrxB-BrxF-iSTAND<-BrxA-DNA-binding<-BrxE||REase->GDResolvase+HIN-HTH-><-TIR	1213	Bacteria>Pseudomonadota>Betaproteobacteria>Burkholderiales>Comamonadaceae>Variovorax	GCF_019207085.1	Variovorax_sp._dw_308	HCU76_RS14255	36638..40279	2721546	BREX-system-P-loop-protein-BrxC-[Variovorax-sp.-dw_308]
WP_093162784.1	Type6.BREX	<-NUDIX<-?<-?<-?||?->RelE-ParE->BrxE->BrxA-DNA-binding->BrxB-BrxF-iSTAND->DUF6079*->N6-METHYLASE->N6-METHYLASE-p->PglZ->SIGMA+OB-Fold+AAA-ATPase+HKD-DNase-BrxL-><-HEPN+OST-HTH	1212	Bacteria>Pseudomonadota>Betaproteobacteria>Burkholderiales>Comamonadaceae>Variovorax	GCF_900107745.1	Variovorax_sp._YR216	BLW05_RS12080	465668..469306	1882828	BREX-system-P-loop-protein-BrxC-[Variovorax-sp.-YR216]
WP_186915942.1	Type1.BREX	wHTH+WYL+WCX->?->?->wHTH+WYL+WCX-p->ANK->XIS-HTH->BrxA-DNA-binding->BrxB-BrxF-iSTAND->DUF6079*->OLD-ABC->REase->N6-METHYLASE->ParB+HNH->PglZ->SIGMA+OB-Fold+AAA-ATPase+HKD-DNase-BrxL->	1183	Bacteria>Pseudomonadota>Betaproteobacteria>Burkholderiales>Oxalobacteraceae	GCF_014284115.1	Undibacterium	H8K35_RS08325	157325..160876	401469	MULTISPECIES:-BREX-system-P-loop-protein-BrxC-[Undibacterium]
WP_141835430.1	Type6.BREX	<-SIGMA+OB-Fold+AAA-ATPase+HKD-DNase-BrxL<-PglZ<-ParB+HNH<-N6-METHYLASE<-N6-METHYLASE<-GreA_GreB+SF1-SF2-Helicase<-TPR<-SF1-SF2-Helicase+RNase+RNase<-DUF6079*<-BrxB-BrxF-iSTAND<-BrxA-DNA-binding<-BrxE<-XIS-HTH<-?||Preader+URI-DNase-><-SF1-SF2-Helicase<-HKD-DNase	1209	Bacteria>Pseudomonadota>Betaproteobacteria>Burkholderiales>Oxalobacteraceae>Herbaspirillum	-	Herbaspirillum_sp._SJZ107	FBX97_RS19505	315565..319194	2572881	BREX-system-P-loop-protein-BrxC-[Herbaspirillum-sp.-SJZ107]
WP_152879050.1	Type6.BREX	<-DDE_Tnp<-?<-?<-sGTP||?->wHTH+WYL+WCX->BrxE->BrxB-BrxF-iSTAND->DUF6079*->N6-METHYLASE->ParB+HNH->PglZ->SIGMA+OB-Fold+AAA-ATPase+HKD-DNase-BrxL-><-LZ_Tnp_IS66+zf-IS66+DDE_Tnp_IS66+DDE_Tnp_IS66_C<-?<-HIN-HTH||<-AraC-HTH+AraC-HTH	1215	Bacteria>Pseudomonadota>Betaproteobacteria>Burkholderiales>Oxalobacteraceae>Massilia_group	GCF_009295655.1	Duganella_sp._FT27W	F8197_RS00385	74619..78266	2654636	BREX-system-P-loop-protein-BrxC-[Duganella-sp.-FT27W]
WP_258819858.1	Type1.BREX	Phage_integrase-><-?<-SbcC<-SIGMA+OB-Fold+AAA-ATPase+HKD-DNase-BrxL<-PglZ<-N6-METHYLASE-p<-REase<-OLD-ABC<-N6-METHYLASE<-DUF6079*<-BrxB-BrxF-iSTAND<-BrxA-DNA-binding<-wHTH+WYL+WCX	1186	Bacteria>Pseudomonadota>Betaproteobacteria>Burkholderiales>Oxalobacteraceae>Massilia_group	GCF_024753285.1	Massilia_pinisoli	NX784_RS27510	42185..45745	1772194	BREX-system-P-loop-protein-BrxC-[Massilia-pinisoli]
WP_182219587.1	Type1.BREX	<-RNaseP<-?||RFC-AAA+DNAA-HTH->?->MORC+DNA_gyrase+TOPRIM->wHTH+WYL+WCX->BrxA-DNA-binding->BrxB-BrxF-iSTAND->DUF6079*->N6-METHYLASE->OLD-ABC->NERD->N6-METHYLASE-p->PglZ->SIGMA+OB-Fold+AAA-ATPase+HKD-DNase-BrxL->PHP->?->REase->	1186	Bacteria>Pseudomonadota>Betaproteobacteria>Burkholderiales>Oxalobacteraceae>Rugamonas	GCF_014042365.1	Rugamonas_fusca	H3H36_RS18580	34208..37768	2758568	BREX-system-P-loop-protein-BrxC-[Rugamonas-fusca]
OGB35307.1	Type6.BREX	MORC+DNA_gyrase+TOPRIM->CARF+NtrC-AAA+FIS-HTH-><-HTH_32+DDE_3||<-HTH_23+HTH_32<-SIGMA+OB-Fold+AAA-ATPase+HKD-DNase-BrxL<-PglZ<-ParB+HNH<-N6-METHYLASE<-N6-METHYLASE<-DUF6079*<-BrxB-BrxF-iSTAND<-BrxA-DNA-binding<-BrxE<-HTH_26||?->TIR->SIR2-><-DDE_Tnp_1||REase->	1215	Bacteria>Pseudomonadota>Betaproteobacteria>Burkholderiales>unclassified_Burkholderiales	GCA_001770935.1	Burkholderiales_bacterium_RIFCSPLOWO2_12_FULL_61_40	A3F78_11695	46897..50544	1797566	ATPase-[Burkholderiales-bacterium-RIFCSPLOWO2_12_FULL_61_40]
MBN8486519.1	Type1.BREX	<-SIR2||?->wHTH+WYL+WCX->BrxA-DNA-binding->BrxB-BrxF-iSTAND->DUF6079*->N6-METHYLASE->OLD-ABC->NERD->RVT->N6-METHYLASE-p->HTH+REase->PglZ->SIGMA+OB-Fold+AAA-ATPase+HKD-DNase-BrxL-><-HISKIN	1186	Bacteria>Pseudomonadota>Betaproteobacteria>Burkholderiales>unclassified_Burkholderiales	GCA_017302485.1	Burkholderiales_bacterium	JOM20_02125	4400..7960	1891238	BREX-system-P-loop-protein-BrxC-[Burkholderiales-bacterium]
MCP5275500.1	Type1.BREX	PhdYeFM_antitox->RelE-ParE-><-HTH_32+DDE_3<-?<-BrnT_toxin<-REase+TOPRIM<-HEPN+OST-HTH||BrxA-DNA-binding->BrxB-BrxF-iSTAND->DUF6079*->?->ABC-ATPase->REase->N6-METHYLASE->ParB+HNH->OLD-ABC->?->PglZ->SIGMA+OB-Fold+AAA-ATPase+HKD-DNase-BrxL->	1176	Bacteria>Pseudomonadota>Betaproteobacteria>Burkholderiales>unclassified_Burkholderiales	GCA_024235795.1	Burkholderiales_bacterium	H6936_11775	653459..656989	1891238	BREX-system-P-loop-protein-BrxC-[Burkholderiales-bacterium]
WP_194117489.1	Type6.BREX	PIN-><-ACET+ACET<-TraJ-RHH<-AAA-ATPase||<-SIGMA+OB-Fold+AAA-ATPase+HKD-DNase-BrxL<-PglZ<-ParB+HNH<-N6-METHYLASE<-GreA_GreB+SF1-SF2-Helicase<-DUF6079*<-BrxB-BrxF-iSTAND<-BrxE<-wHTH+WYL+WCX||DDE_Tnp_1_5+DDE_Tnp_1-><-Phage_integrase+Phage_integrase	1213	Bacteria>Pseudomonadota>Betaproteobacteria>Neisseriales>Chromobacteriaceae>Chitinilyticum	GCF_015223195.1	Chitinilyticum_piscinae	INR99_RS16780	39673..43314	2866724	BREX-system-P-loop-protein-BrxC-[Chitinilyticum-piscinae]
WP_239907373.1	Type1.BREX	<-SIGMA+OB-Fold+AAA-ATPase+HKD-DNase-BrxL<-PglZ<-HTH+REase<-N6-METHYLASE-p<-HerA/FtsK<-SIR2<-REase<-OLD-ABC<-N6-METHYLASE<-DUF6079*<-BrxB-BrxF-iSTAND<-BrxA-DNA-binding<-wHTH+WYL+WCX<-Phage_integrase+Phage_integrase<-HTH_36<-XIS-HTH<-?<-Arm-DNA-bind_3+Phage_integrase<-sGTP	1185	Bacteria>Pseudomonadota>Betaproteobacteria>Neisseriales>Chromobacteriaceae>Laribacter	GCF_022394255.1	Laribacter_hongkongensis	LH425_05855	38844..42401	168471	BREX-system-P-loop-protein-BrxC-[Laribacter-hongkongensis]

Protein-ID	System	Gene-Neighbourhoods	Length	Taxonomy	Assembly-ID	Strain	Locus-Tag	Genomic-coordinates	TaxID	Fasta-define
WP_239909460.1	Type1.BREX	<-CopG/RHH<-?<-?||HTH_3->?->TraI-VirD2-RCRE->wHTH+WYL+WCX->BrxA-DNA-binding->BrxB-BrxF-iSTAND->DUF6079*->N6-METHYLASE->OLD-ABC->REase->SIR2->HerA/FtsK->N6-METHYLASE-p->OLD-ABC-> [illegible]	1186	Bacteria>Pseudomonadota>Betaproteobacteria>Neisseriales>Chromobacteriaceae>Laribacter	GCF_022394655.1	Laribacter_hongkongensis	LH423_RS01205	99182..102742	168471	BREX-system-P-loop-protein-BrxC-[Laribacter-hongkongensis]
WP_088965422.1	Type6.BREX	<-GTPase-AIG<-?||HTH_3-><-?<-?<-DUF932<-SIGMA+OB-Fold+AAA-ATPase+HKD-DNase-BrxL<-PglZ<-N6-METHYLASE<-DUF6079*<-BrxB-BrxF-iSTAND<-BrxA-DNA-binding<-BrxE<-wHTH+WYL+WCX<-ParB<- [illegible]	1215	Bacteria>Pseudomonadota>Betaproteobacteria>Neisseriales>Chromobacteriaceae>Vogesella	GCF_900090205.1	Vogesella_sp._LIG4	PSELUDRAFT_RS02905	629858..633505	1192162	BREX-system-P-loop-protein-BrxC-[Vogesella-sp.-LIG4]
WP_153715435.1	Type6.BREX	<-SIGMA+OB-Fold+AAA-ATPase+HKD-DNase-BrxL<-PglZ<-ParB+HNH<-N6-METHYLASE<-N6-METHYLASE<-N6-METHYLASE<-GreA_GreB+SF1-SF2-Helicase<-DUF6079*<-BrxB-BrxF-iSTAND<-BrxA-DNA-binding [illegible]	1213	Bacteria>Pseudomonadota>Betaproteobacteria>Neisseriales>Neisseriaceae>Eikenella	GCF_009650905.1	Eikenella_corrodens	GJ661_RS08680	18367..22008	539	BREX-system-P-loop-protein-BrxC-[Eikenella-corrodens]
MBK7364915.1	Type1.BREX	<-HIN-HTH+rve+rve_3||tRNA-><-SIGMA+OB-Fold+AAA-ATPase+HKD-DNase-BrxL<-PglZ<-N6-METHYLASE<-REase<-OLD-ABC<-DUF6079*<-BrxB-BrxF-iSTAND<-BrxA-DNA-binding<-XIS-HTH||ArdA->TRPR-HTH- [illegible]	1187	Bacteria>Pseudomonadota>Betaproteobacteria>Nitrosomonadales>Nitrosomonadaceae>Nitrosomonas	GCA_016705625.1	Nitrosomonas_sp.	IPI97_07925	1671776..1675339	42353	BREX-system-P-loop-protein-BrxC-[Nitrosomonas-sp.]
MBK7352718.1	Type1.BREX	TraI-VirD2-RCRE->HNH-><-?<-TRPR-HTH<-?<-ArdA||XIS-HTH->BrxA-DNA-binding->BrxB-BrxF-iSTAND->DUF6079-p*->DUF6079-p->?->?->OLD-ABC->N6-METHYLASE->PglZ->SIGMA+OB-Fold+AAA-ATPase+ [illegible]	705	Bacteria>Pseudomonadota>Betaproteobacteria>Nitrosomonadales>Nitrosomonadaceae>Nitrosomonas	GCA_016710135.1	Nitrosomonas_sp.	IPJ05_03020	595038..597155	42353	BREX-system-P-loop-protein-BrxC-[Nitrosomonas-sp.]
MBV6446594.1	Type1.BREX	TraI-VirD2-RCRE->HNH->?-><-Couple_hipA+HipA_C<-TRPR-HTH<-ArdA||XIS-HTH->BrxA-DNA-binding->BrxB-BrxF-iSTAND->DUF6079*->OLD-ABC->REase->N6-METHYLASE->PglZ->SIGMA+OB-Fold+AAA-ATPa [illegible]	1182	Bacteria>Pseudomonadota>Betaproteobacteria>Nitrosomonadales>Nitrosomonadaceae>Nitrosomonas	GCA_019187415.1	Nitrosomonas_sp.	FCKEOINB_00187	17095..20643	42353	hypothetical-protein-[Nitrosomonas-sp.]
MBX3639968.1	Type1.BREX	<-SIGMA+OB-Fold+AAA-ATPase+HKD-DNase-BrxL<-PglZ<-REase<-N6-METHYLASE<-?<-DUF6079*<-BrxB-BrxF-iSTAND<-BrxA-DNA-binding<-XIS-HTH||HIN-HTH->SIR2->	1182	Bacteria>Pseudomonadota>Betaproteobacteria>Nitrosomonadales>Nitrosomonadaceae>Nitrosomonas	GCA_019635175.1	Nitrosomonas_sp.	KF888_05565	120382..123930	42353	BREX-system-P-loop-protein-BrxC-[Nitrosomonas-sp.]
WP_181258202.1	Type1.BREX	<-REase+TOPRIM<-HEPN+OST-HTH||BrxA-DNA-binding->BrxB-BrxF-iSTAND->DUF6079*->N6-METHYLASE->ParB+HNH->OLD-ABC->?->PglZ->SIGMA+OB-Fold+AAA-ATPase+HKD-DNase-BrxL->SbcC->Calcineurin [illegible]	1179	Bacteria>Pseudomonadota>Betaproteobacteria>Nitrosomonadales>Nitrosomonadaceae>Nitrosomonas	GCF_003046585.1	Nitrosomonas_aestuarii	C8R11_107137	171439..174978	52441	BREX-system-P-loop-protein-BrxC-[Nitrosomonas-aestuarii]
WP_107790392.1	Type1.BREX	ParB+RepB->?-><-SbcC<-Calcineurin+Calcineurin-SbcD<-SbcC<-SIGMA+OB-Fold+AAA-ATPase+HKD-DNase-BrxL<-PglZ<-N6-METHYLASE<-Schlafen+HATPase+wHTH+HTH<-DUF6079*<-BrxB-BrxF-iSTAND<- [illegible]	1180	Bacteria>Pseudomonadota>Betaproteobacteria>Nitrosomonadales>Nitrosomonadaceae>Nitrosomonas	GCF_003051105.1	Nitrosomonas_nitrosa	C8R30_RS13785	18563..22105	52442	BREX-system-P-loop-protein-BrxC-[Nitrosomonas-nitrosa]
WP_204800510.1	Type1.BREX	<-SbcC<-Calcineurin+Calcineurin-SbcD<-SbcC<-SIGMA+OB-Fold+AAA-ATPase+HKD-DNase-BrxL<-PglZ<-HTH+REase<-?<-Schlafen+HATPase+wHTH+HTH<-DUF6079*<-BrxB-BrxF-iSTAND<-BrxA-DNA-binding [illegible]	1180	Bacteria>Pseudomonadota>Betaproteobacteria>Nitrosomonadales>Nitrosomonadaceae>Nitrosomonas	GCF_905220645.1	Nitrosomonas_nitrosa	NUZ5A_RS15530	137140..140682	52442	BREX-system-P-loop-protein-BrxC-[Nitrosomonas-nitrosa]
MBA0014163.1	Type6.BREX	<-SIR2<-SIGMA+OB-Fold+AAA-ATPase+HKD-DNase-BrxL<-PglZ<-N6-METHYLASE<-?<-DUF6079*<-BrxB-BrxF-iSTAND<-BrxA-DNA-binding<-BrxE||?->?->HIN-HTH->	1210	Bacteria>Pseudomonadota>Betaproteobacteria>Nitrosomonadales>Nitrosomonadaceae>Nitrosospira	GCA_013450215.1	Nitrosospira_sp.	HOZ53_00860	54117..57749	38080	BREX-system-P-loop-protein-BrxC-[Nitrosospira-sp.]
WP_090458040.1	Type6.BREX	Y1_Tnp-><-?<-?||tRNA->BrxE->BrxA-DNA-binding->BrxB-BrxF-iSTAND->DUF6079*->GreA_GreB+SF1-SF2-Helicase->N6-METHYLASE->N6-METHYLASE->PglZ->SIGMA+OB-Fold+AAA-ATPase+HKD-DNase-Brx [illegible]	1213	Bacteria>Pseudomonadota>Betaproteobacteria>Nitrosomonadales>Nitrosomonadaceae>Nitrosospira	GCF_900100985.1	Nitrosospira_sp._Nsp1	BLP78_RS07395	18423..22064	136547	BREX-system-P-loop-protein-BrxC-[Nitrosospira-sp.-Nsp1]
SFW23276.1	Type6.BREX	<-AAA-ATPase<-HIN-HTH||Phage_integrase-><-?<-SIGMA+OB-Fold+AAA-ATPase+HKD-DNase-BrxL<-PglZ<-ParB+HNH<-N6-METHYLASE<-GreA_GreB+SF1-SF2-Helicase<-DUF6079*<-BrxB-BrxF-iSTAND<-BrxA- [illegible]	1213	Bacteria>Pseudomonadota>Betaproteobacteria>Nitrosomonadales>Nitrosomonadaceae>Nitrosovibrio	GCA_900119085.1	Nitrosovibrio_sp._Nv17	SAMN05216414_10762	70156..73797	1855339	hypothetical-protein-SAMN05216414_10762-[Nitrosovibrio-sp.-Nv17]
WP_218184829.1	Type6.BREX	[illegible]	1234	Bacteria>Pseudomonadota>Betaproteobacteria>Nitrosomonadales>Nitrosomonadaceae>Nitrosovibrio	GCF_900119085.1	Nitrosovibrio_sp._Nv17	BT108_RS05915	70156..73860	1855339	BREX-system-P-loop-protein-BrxC-[Nitrosovibrio-sp.-Nv17]
WP_121241046.1	Type6.BREX	[illegible]	1202	Bacteria>Pseudomonadota>Betaproteobacteria>Nitrosomonadales>Sterolibacteriaceae>Sulfurisoma	GCF_003865015.1	Sulfurisoma_sediminicola	EJE94_RS09880	239721..243329	1381557	BREX-system-P-loop-protein-BrxC-[Sulfurisoma-sediminicola]
ODU51178.1	Type6.BREX	[illegible]	1213	Bacteria>Pseudomonadota>Betaproteobacteria>Nitrosomonadales>Thiobacillaceae>Thiobacillus	GCA_001725135.1	Thiobacillus_sp._SCN_63-374	ABS92_00645	9244..12885	1660149	MAG:-ATPase-[Thiobacillus-sp.-SCN-63-374]
MBW8470844.1	Type6.BREX	[illegible]	1215	Bacteria>Pseudomonadota>Betaproteobacteria>Nitrosomonadales>Thiobacillaceae>Thiobacillus	GCA_019459125.1	Thiobacillus_sp.	KOM67_21445	16263..19910	924	BREX-system-P-loop-protein-BrxC-[Thiobacillus-sp.]
NMG55067.1	Type1.BREX	[illegible]	1189	Bacteria>Pseudomonadota>Betaproteobacteria>Rhodocyclales>Rhodocyclaceae>Aromatoleum	GCA_012910915.1	Aromatoleum_aromaticum	GPA23_10435	14208..17777	551760	BREX-system-P-loop-protein-BrxC-[Aromatoleum-aromaticum]
WP_168953997.1	Type1.BREX	[illegible]	1188	Bacteria>Pseudomonadota>Betaproteobacteria>Rhodocyclales>Rhodocyclaceae>Aromatoleum	GCF_023704195.1	Aromatoleum_aromaticum	L8M16_RS08665	1808924..1812490	551760	BREX-system-P-loop-protein-BrxC-[Aromatoleum-aromaticum]
MBP6421402.1	Type1.BREX	[illegible]	923	Bacteria>Pseudomonadota>Betaproteobacteria>Rhodocyclales>Rhodocyclaceae>Propionivibrio	GCA_017988215.1	Propionivibrio_sp.	KA271_00765	12654..15427	2212460	BREX-system-P-loop-protein-BrxC-[Propionivibrio-sp.]
MBK6907823.1	Type6.BREX	[illegible]	1202	Bacteria>Pseudomonadota>Betaproteobacteria>Rhodocyclales>Rhodocyclaceae>unclassified_Rhodocyclaceae	GCA_016702335.1	Rhodocyclaceae_bacterium	IPH08_12425	301349..304957	1898103	BREX-system-P-loop-protein-BrxC-[Rhodocyclaceae-bacterium]
MCA3121411.1	Type6.BREX	[illegible]	1210	Bacteria>Pseudomonadota>Betaproteobacteria>Rhodocyclales>Rhodocyclaceae>unclassified_Rhodocyclaceae	GCA_020248305.1	Rhodocyclaceae_bacterium	ING90_02100	367371..371003	1898103	BREX-system-P-loop-protein-BrxC-[Rhodocyclaceae-bacterium]
TVT60730.1	Type6.BREX	[illegible]	1213	Bacteria>Pseudomonadota>Betaproteobacteria>Rhodocyclales>Zoogloeaceae>Azoarcus	GCA_007713545.1	Azoarcus_sp._PHD	FHK80_01725	174831..178472	1628740	MAG:-BREX-system-P-loop-protein-BrxC-[Azoarcus-sp.-PHD]
WP_141017182.1	Type6.BREX	[illegible]	1207	Bacteria>Pseudomonadota>Betaproteobacteria>Rhodocyclales>Zoogloeaceae>Azoarcus	GCF_006496635.1	Azoarcus_sp._DD4	CJ010_RS05935	1245819..1249442	2027405	BREX-system-P-loop-protein-BrxC-[Azoarcus-sp.-DD4]
NMG49986.1	Type1.BREX	[illegible]	1189	Bacteria>Pseudomonadota>Betaproteobacteria>Rhodocyclales>Zoogloeaceae>Parazoarcus	GCA_012910825.1	Parazoarcus_communis	GO613_17970	21745..25314	41977	BREX-system-P-loop-protein-BrxC-[Parazoarcus-communis]
WP_168942691.1	Type1.BREX	[illegible]	1188	Bacteria>Pseudomonadota>Betaproteobacteria>Rhodocyclales>Zoogloeaceae>Parazoarcus	GCF_012910825.1	Parazoarcus_communis	GO613_RS17995	21748..25314	41977	BREX-system-P-loop-protein-BrxC-[Parazoarcus-communis]
MBL8465274.1	Type6.BREX	[illegible]	1209	Bacteria>Pseudomonadota>Betaproteobacteria>Rhodocyclales>Zoogloeaceae>Thauera	GCA_016790365.1	Thauera_sp.	JNL37_10520	3981..7610	1905334	BREX-system-P-loop-protein-BrxC-[Thauera-sp.]
WP_148578091.1	Type1.BREX	[illegible]	1186	Bacteria>Pseudomonadota>Betaproteobacteria>Rhodocyclales>Zoogloeaceae>Zoogloea	GCF_008107625.1	Zoogloea_oleivorans	ETQ85_RS05700	193093..196653	1552750	BREX-system-P-loop-protein-BrxC-[Zoogloea-oleivorans]
GHT91750.1	Type6.BREX	[illegible]	1209	Bacteria>Pseudomonadota>Betaproteobacteria>unclassified_Betaproteobacteria	GCA_026000515.1	Betaproteobacteria_bacterium	FACS1894116_00820	42503..46132	1891241	hypothetical-protein-FACS1894116_00820-[Betaproteobacteria-bacterium]
AUZ77883.1	Type1.BREX	[illegible]	1183	Bacteria>Pseudomonadota>Gammaproteobacteria>Aeromonadales>Aeromonadaceae>Aeromonas	GCA_002934425.1	Aeromonas_sp._ASNIH4	C2U40_16585	3601638..3605189	1636609	BREX-system-P-loop-protein-BrxC-[Aeromonas-sp.-ASNIH4]
WP_043557612.1	Type1.BREX	[illegible]	1182	Bacteria>Pseudomonadota>Gammaproteobacteria>Aeromonadales>Aeromonadaceae>Aeromonas	GCF_000819745.1	Aeromonas_bestiarum	BN1118_RS19650	69736..73284	105751	BREX-system-P-loop-protein-BrxC-[Aeromonas-bestiarum]
WP_100653505.1	Type6.BREX	[illegible]	1216	Bacteria>Pseudomonadota>Gammaproteobacteria>Aeromonadales>Aeromonadaceae>Aeromonas	GCF_002803925.1	Aeromonas_veronii	CVS41_08490	1813618..1817268	654	BREX-system-P-loop-protein-BrxC-[Aeromonas-veronii]
WP_103244178.1	Type6.BREX	[illegible]	1210	Bacteria>Pseudomonadota>Gammaproteobacteria>Aeromonadales>Aeromonadaceae>Aeromonas	GCF_002902865.1	Aeromonas_sp._ASNIH5	C2U30_RS17315	3671715..3675347	1758179	BREX-system-P-loop-protein-BrxC-[Aeromonas-sp.-ASNIH5]
WP_216948840.1	Type6.BREX	[illegible]	1216	Bacteria>Pseudomonadota>Gammaproteobacteria>Aeromonadales>Aeromonadaceae>Aeromonas	GCF_019046985.1	Aeromonas_sp._FDAARGOS_1417	I6L47_RS05350	1228193..1231843	2778066	BREX-system-P-loop-protein-BrxC-[Aeromonas-sp.-FDAARGOS-1417]
WP_241417643.1	Type6.BREX	[illegible]	1216	Bacteria>Pseudomonadota>Gammaproteobacteria>Aeromonadales>Aeromonadaceae>Aeromonas	GCF_022557195.1	Aeromonas_hydrophila	MKW86_RS08070	1734303..1737953	644	BREX-system-P-loop-protein-BrxC-[Aeromonas-hydrophila]
WP_257706959.1	Type1.BREX	[illegible]	1182	Bacteria>Pseudomonadota>Gammaproteobacteria>Aeromonadales>Aeromonadaceae>Aeromonas	GCF_024649325.1	Aeromonas_caviae	NUK33_RS01495	150155..153703	648	BREX-system-P-loop-protein-BrxC-[Aeromonas-caviae]
WP_181014924.1	Type1.BREX	[illegible]	1182	Bacteria>Pseudomonadota>Gammaproteobacteria>Aeromonadales>Aeromonadaceae	GCF_002934425.1	Aeromonas	C2U40_RS16580	3601638..3605186	642	MULTISPECIES:-BREX-system-P-loop-protein-BrxC-[Aeromonas]
WP_019002750.1	Type1.BREX	[illegible]	1210	Bacteria>Pseudomonadota>Gammaproteobacteria>Aeromonadales>Succinivibrionaceae>Succinimonas	GCF_000378405.1	Succinimonas_amylolytica	F557_RS20035	169312..172944	83769	BREX-system-P-loop-protein-BrxC-[Succinimonas-amylolytica]
WP_219938189.1	Type1.BREX	[illegible]	1210	Bacteria>Pseudomonadota>Gammaproteobacteria>Aeromonadales>Succinivibrionaceae>Succinivibrio	GCF_019448115.1	Succinivibrio_faecicola	J5V48_RS08685	2494..6126	2820300	BREX-system-P-loop-protein-BrxC-[Succinivibrio-faecicola]
WP_265045927.1	Type1.BREX	[illegible]	1194	Bacteria>Pseudomonadota>Gammaproteobacteria>Alteromonadales>Alteromonadaceae>Alkalimarinus	GCF_026016465.1	Alkalimarinus_sp._A2M4	NKI27_RS10060	2208861..2212445	2961619	BREX-system-P-loop-protein-BrxC-[Alkalimarinus-sp.-A2M4]
WP_217358045.1	Type6.BREX	[illegible]	951	Bacteria>Pseudomonadota>Gammaproteobacteria>Alteromonadales>Alteromonadaceae>Alteromonas/Salinimonas_group	GCF_002499975.2	Alteromonas_pelagimontana	CA267_RS01545	356718..359573	1858656	BREX-system-P-loop-protein-BrxC-[Alteromonas-pelagimontana]
WP_006006589.1	Type1.BREX	[illegible]	1189	Bacteria>Pseudomonadota>Gammaproteobacteria>Alteromonadales>Alteromonadaceae>Glaciecola	GCF_000252165.1	Glaciecola_punicea	GPUN_RS10800	142812..146381	56804	BREX-system-P-loop-protein-BrxC-[Glaciecola-punicea]
WP_136550441.1	Type1.BREX	[illegible]	1193	Bacteria>Pseudomonadota>Gammaproteobacteria>Alteromonadales>Alteromonadaceae>Hydrocarboniclastica	GCF_004851605.1	Hydrocarboniclastica_marina	soil367_RS18210	4131404..4134985	2259620	BREX-system-P-loop-protein-BrxC-[Hydrocarboniclastica-marina]
NQY33786.1	Type1.BREX	[illegible]	1200	Bacteria>Pseudomonadota>Gammaproteobacteria>Alteromonadales>Alteromonadaceae>unclassified_Alteromonadaceae	GCA_013216015.1	Alteromonadaceae_bacterium	HRT37_02225	108462..112064	1916082	BREX-system-P-loop-protein-BrxC-[Alteromonadaceae-bacterium]
WP_220104111.1	Type6.BREX	[illegible]	1208	Bacteria>Pseudomonadota>Gammaproteobacteria>Alteromonadales>Alteromonadales_genera_incertae_sedis>Neiella	GCF_019457915.1	Neiella_holothuriorum	K0504_RS10305	24423..28049	2870530	BREX-system-P-loop-protein-BrxC-[Neiella-holothuriorum]
WP_130603913.1	Type1.BREX	[illegible]	1188	Bacteria>Pseudomonadota>Gammaproteobacteria>Alteromonadales>Colwelliaceae>Litorilituus	GCF_004295665.1	Litorilituus_sediminis	EMK97_RS16655	3755554..3759120	718192	BREX-system-P-loop-protein-BrxC-[Litorilituus-sediminis]
WP_112714387.1	Type1.BREX	[illegible]	1199	Bacteria>Pseudomonadota>Gammaproteobacteria>Alteromonadales>Moritellaceae>Moritella	GCF_900465055.1	Moritella_yayanosii	MORIYA_RS08605	1893116..1896715	69539	BREX-system-P-loop-protein-BrxC-[Moritella-yayanosii]
WP_010607320.1	Type1.BREX	[illegible]	1188	Bacteria>Pseudomonadota>Gammaproteobacteria>Alteromonadales>Pseudoalteromonadaceae	GCF_000276645.1	Pseudoalteromonas	UY7_RS0121200	90697..94263	53246	MULTISPECIES:-BREX-system-P-loop-protein-BrxC-[Pseudoalteromonas]
WP_024611453.1	Type1.BREX	[illegible]	1193	Bacteria>Pseudomonadota>Gammaproteobacteria>Alteromonadales>Pseudoalteromonadaceae>Pseudoalteromonas	GCF_000498095.1	Pseudoalteromonas_sp._TB64	L647_RS0112855	131987..135568	1938600	BREX-system-P-loop-protein-BrxC-[Pseudoalteromonas-sp.-TB64]
WP_167658112.1	Type1.BREX	[illegible]	1188	Bacteria>Pseudomonadota>Gammaproteobacteria>Alteromonadales>Pseudoalteromonadaceae>Pseudoalteromonas	GCF_011806445.1	Pseudoalteromonas_sp._HF66	HCZ11_RS07660	212811..216377	2721559	BREX-system-P-loop-protein-BrxC-[Pseudoalteromonas-sp.-HF66]
WP_200220630.1	Type1.BREX	[illegible]	1193	Bacteria>Pseudomonadota>Gammaproteobacteria>Alteromonadales>Pseudoalteromonadaceae>Pseudoalteromonas	GCF_016598755.1	Pseudoalteromonas_sp._LC2018020214	JG479_RS17805	301190..304771	2799564	BREX-system-P-loop-protein-BrxC-[Pseudoalteromonas-sp.-LC2018020214]
PKG74712.1	Type1.BREX	[illegible]	1191	Bacteria>Pseudomonadota>Gammaproteobacteria>Alteromonadales>Shewanellaceae>Shewanella	GCA_002836135.1	Shewanella_sp._GutCb	CXF86_11690	122253..125828	2058315	BREX-system-P-loop-protein-BrxC-[Shewanella-sp.-GutCb]
MBB1438394.1	Type1.BREX	[illegible]	1182	Bacteria>Pseudomonadota>Gammaproteobacteria>Alteromonadales>Shewanellaceae>Shewanella	GCA_014164505.1	Shewanella_sp._SG41-4	H5202_06785	3542..7090	2760976	BREX-system-P-loop-protein-BrxC-[Shewanella-sp.-SG41-4]
WP_257985331.1	Type1.BREX	[illegible]	1193	Bacteria>Pseudomonadota>Gammaproteobacteria>Alteromonadales>Shewanellaceae>Shewanella	GCF_002836135.1	Shewanella_sp._GutCb	CXF86_RS11730	122253..125834	2058315	BREX-system-P-loop-protein-BrxC-[Shewanella-sp.-GutCb]
WP_173496635.1	Type6.BREX	[illegible]	1211	Bacteria>Pseudomonadota>Gammaproteobacteria>Alteromonadales>Shewanellaceae>Shewanella	GCF_013283205.1	Shewanella_sp._ISTPL2	HUB64_RS06505	1443171..1446806	2699425	BREX-system-P-loop-protein-BrxC-[Shewanella-sp.-ISTPL2]
WP_259397220.1	Type1.BREX	[illegible]	1180	Bacteria>Pseudomonadota>Gammaproteobacteria>Alteromonadales>Shewanellaceae>Shewanella	GCF_014164505.1	Shewanella_sp._SG41-4	H5202_RS06785	3548..7090	2760976	BREX-system-P-loop-protein-BrxC-[Shewanella-sp.-SG41-4]
WP_188922770.1	Type6.BREX	[illegible]	1210	Bacteria>Pseudomonadota>Gammaproteobacteria>Alteromonadales>Shewanellaceae>Shewanella	GCF_014648295.1	Shewanella_saliphila	IEY48_RS17840	13004..16636	2282698	BREX-system-P-loop-protein-BrxC-[Shewanella-saliphila]
WP_219012197.1	Type6.BREX	[illegible]	1211	Bacteria>Pseudomonadota>Gammaproteobacteria>Alteromonadales>Shewanellaceae>Shewanella	GCF_019303235.1	Shewanella_algae	KOE59_RS06910	134402..138037	38313	BREX-system-P-loop-protein-BrxC-[Shewanella-algae]
WP_249554920.1	Type1.BREX	[illegible]	1188	Bacteria>Pseudomonadota>Gammaproteobacteria>Alteromonadales>Shewanellaceae>Shewanella	GCF_023475945.1	Shewanella_sp._8A	M0413_RS16385	25717..29283	2943323	BREX-system-P-loop-protein-BrxC-[Shewanella-sp.-8A]
WP_112352819.1	Type1.BREX	[illegible]	1197	Bacteria>Pseudomonadota>Gammaproteobacteria>Alteromonadales>Shewanellaceae>Shewanella	GCF_900476435.1	Shewanella_benthica	SHEWBE_RS12055	2634220..2637813	43661	BREX-system-P-loop-protein-BrxC-[Shewanella-benthica]
WP_102039083.1	Type1.BREX	[illegible]	1191	Bacteria>Pseudomonadota>Gammaproteobacteria>Alteromonadales>Shewanellaceae>Shewanella	-	Shewanella_sp._GutCb	CXF86_11690	122253..125828	2058315	BREX-system-P-loop-protein-BrxC-[Shewanella-sp.-GutCb]
WP_182771402.1	Type1.BREX	[illegible]	1182	Bacteria>Pseudomonadota>Gammaproteobacteria>Alteromonadales>Shewanellaceae>Shewanella	-	Shewanella_sp._SG41-4	H5202_06785	3542..7090	2760976	BREX-system-P-loop-protein-BrxC-[Shewanella-sp.-SG41-4]
MBV7434872.1	Type1.BREX	[illegible]	1175	Bacteria>Pseudomonadota>Gammaproteobacteria>Cardiobacteriales>Cardiobacteriaceae>unclassified_Cardiobacteriaceae	GCA_019218315.1	Cardiobacteriaceae_bacterium_TAE3-ERU3	KRX19_07510	264796..268323	2856340	BREX-system-P-loop-protein-BrxC-[Cardiobacteriaceae-bacterium-TAE3-ERU3]
WP_265366287.1	Type1.BREX	[illegible]	1193	Bacteria>Pseudomonadota>Gammaproteobacteria>Cellvibrionales>Cellvibrionaceae>Marinimicrobium	GCF_026153455.1	Marinimicrobium_koreense	OOT55_RS13025	3024461..3028042	306545	BREX-system-P-loop-protein-BrxC-[Marinimicrobium-sp.-C6131]
WP_009576096.1	Type1.BREX	[illegible]	1178	Bacteria>Pseudomonadota>Gammaproteobacteria>Cellvibrionales>Halieaceae>Aequoribacter	GCF_000204315.1	Aequoribacter_fuscus	EYZ66_RS04240	942285..945821	2518989	BREX-system-P-loop-protein-BrxC-[Aequoribacter-fuscus]
WP_084199902.1	Type1.BREX	[illegible]	1184	Bacteria>Pseudomonadota>Gammaproteobacteria>Cellvibrionales>Halieaceae>Halioglobus	GCF_014652255.1	Halioglobus_japonicus	C0029_RS05530	1195751..1199305	930805	BREX-system-P-loop-protein-BrxC-[Halioglobus-japonicus]
WP_148062597.1	Type1.BREX	[illegible]	1180	Bacteria>Pseudomonadota>Gammaproteobacteria>Cellvibrionales>Halieaceae>Parahaliea	GCF_008064635.1	Parahaliea_aestuarii	FVW59_RS02260	491803..495345	1852021	BREX-system-P-loop-protein-BrxC-[Parahaliea-aestuarii]
WP_036859475.1	Type1.BREX	[illegible]	1187	Bacteria>Pseudomonadota>Gammaproteobacteria>Cellvibrionales>Porticoccaceae>Porticoccus	GCF_000744735.1	Porticoccus_hydrocarbonoclasticus	U740_RS05135	1073957..1077520	1073414	BREX-system-P-loop-protein-BrxC-[Porticoccus-hydrocarbonoclasticus]
WP_273033648.1	Type1.BREX	[illegible]	1193	Bacteria>Pseudomonadota>Gammaproteobacteria>Cellvibrionales>Spongiibacteraceae>Spongiibacter	GCF_950061765.1	Spongiibacter_tropicus	B4C78_RS03795	102796..106377	454602	BREX-system-P-loop-protein-BrxC-[Spongiibacter-tropicus]
MTW21824.1	Type1.BREX	[illegible]	1173	Bacteria>Pseudomonadota>Gammaproteobacteria>Chromatiales>Chromatiaceae>Allochromatium	GCA_009720725.1	Allochromatium_palmeri	GJ668_12065	37225..40746	231048	BREX-system-P-loop-protein-BrxC-[Allochromatium-palmeri]
WP_186343071.1	Type1.BREX	[illegible]	1172	Bacteria>Pseudomonadota>Gammaproteobacteria>Chromatiales>Chromatiaceae>Allochromatium	GCF_009720725.1	Allochromatium_palmeri	GJ668_RS12065	37225..40743	231048	BREX-system-P-loop-protein-BrxC-[Allochromatium-palmeri]
NEX15944.1	Type1.BREX	[illegible]	1173	Bacteria>Pseudomonadota>Gammaproteobacteria>Chromatiales>Chromatiaceae>Halochromatium	GCA_010912705.1	Halochromatium_sp.	C1943_04720	67001..70522	2049430	BREX-system-P-loop-protein-BrxC-[Halochromatium-sp.]
WP_132582321.1	Type6.BREX	[illegible]	1208	Bacteria>Pseudomonadota>Gammaproteobacteria>Chromatiales>Chromatiaceae>Rheinheimera	GCF_004345025.1	Rheinheimera_sp._D18	E0Z06_RS04605	954479..958105	2545632	BREX-system-P-loop-protein-BrxC-[Rheinheimera-sp.-D18]
WP_120797099.1	Type1.BREX	[illegible]	1173	Bacteria>Pseudomonadota>Gammaproteobacteria>Chromatiales>Chromatiaceae>Thiocapsa	GCF_003634315.1	Thiocapsa_rosea	BDD21_RS10290	2320412..2323933	69360	BREX-system-P-loop-protein-BrxC-[Thiocapsa-rosea]
WP_015282553.1	Type6.BREX	[illegible]	1209	Bacteria>Pseudomonadota>Gammaproteobacteria>Chromatiales>Chromatiaceae>Thioflavicoccus	GCF_000327045.1	Thioflavicoccus_mobilis	THIMO_RS17980	83065..86694	80679	BREX-system-P-loop-protein-BrxC-[Thioflavicoccus-mobilis]
WP_058554449.1	Type6.BREX	[illegible]	1214	Bacteria>Pseudomonadota>Gammaproteobacteria>Chromatiales>Chromatiaceae>Thiohalocapsa	GCF_001469165.1	Thiohalocapsa_sp._ML1	ML01_RS05745	155025..158669	1431688	BREX-system-P-loop-protein-BrxC-[Thiohalocapsa-sp.-ML1]
WP_239679777.1	Type6.BREX	[illegible]	1223	Bacteria>Pseudomonadota>Gammaproteobacteria>Chromatiales>Ectothiorhodospiraceae>Ectothiorhodospira	GCF_022227625.1	Ectothiorhodospira_variabilis	LPW30_RS00090	25011..28682	505694	BREX-system-P-loop-protein-BrxC-[Ectothiorhodospira-variabilis]
WP_238639758.1	Type6.BREX	[illegible]	1207	Bacteria>Pseudomonadota>Gammaproteobacteria>Chromatiales>Ectothiorhodospiraceae>Halorhodospira	GCF_022229325.1	Halorhodospira_halochloris	LRD17_RS10705	4342..7965	1052	BREX-system-P-loop-protein-BrxC-[Halorhodospira-halochloris]
WP_253479345.1	Type6.BREX	[illegible]	1216	Bacteria>Pseudomonadota>Gammaproteobacteria>Chromatiales>Ectothiorhodospiraceae>Natronocella	GCF_024170285.1	Natronocella_acetinitrilica	J2T57_RS14030	326743..330393	414046	BREX-system-P-loop-protein-BrxC-[Natronocella-acetinitrilica]
WP_019611713.1	Type6.BREX	[illegible]	1216	Bacteria>Pseudomonadota>Gammaproteobacteria>Chromatiales>Ectothiorhodospiraceae>Thioalkalivibrio	GCF_000381705.1	Thioalkalivibrio_sp._AKL7	G315_RS0100950	222198..225848	1158155	BREX-system-P-loop-protein-BrxC-[Thioalkalivibrio-sp.-AKL7]
WP_167070220.1	Type1.BREX	[illegible]	1184	Bacteria>Pseudomonadota>Gammaproteobacteria>Enterobacterales>Erwiniaceae>Pantoea	GCF_011752565.1	Pantoea_sp._Ap-967	F3J45_27210	36650..40204	2608362	BREX-system-P-loop-protein-BrxC-[Pantoea-sp.-Ap-967]
QIG07578.1	Type1.BREX	[illegible]	1183	Bacteria>Pseudomonadota>Gammaproteobacteria>Enterobacterales>Morganellaceae>Proteus	GCA_011046025.1	Proteus_sp._ZN5	GTK47_19170	4181966..4185517	2697019	BREX-system-P-loop-protein-BrxC-[Proteus-sp.-ZN5]
WP_206535871.1	Type1.BREX	[illegible]	1185	Bacteria>Pseudomonadota>Gammaproteobacteria>Enterobacterales>Morganellaceae>Proteus	GCF_011046025.1	Proteus_sp._ZN5	GTK47_RS19230	4181960..4185517	2697019	BREX-system-P-loop-protein-BrxC-[Proteus-sp.-ZN5]
WP_272670012.1	Type1.BREX	[illegible]	1185	Bacteria>Pseudomonadota>Gammaproteobacteria>Enterobacterales>Morganellaceae>Providencia	GCF_028480605.1	unclassified_Providencia	PSE59_RS21125	8138..11695	2633465	MULTISPECIES:-BREX-system-P-loop-protein-BrxC-[unclassified-Providencia]
WP_068809899.1	Type6.BREX	[illegible]	1214	Bacteria>Pseudomonadota>Gammaproteobacteria>Gammaproteobacteria_incertae_sedis>Pseudohongiella	GCF_014642795.1	Pseudohongiella_nitratireducens	IEW51_RS03970	206813..210457	1768907	BREX-system-P-loop-protein-BrxC-[Pseudohongiella-nitratireducens]
MBT4669056.1	Type1.BREX	[illegible]	1167	Bacteria>Pseudomonadota>Gammaproteobacteria>Gammaproteobacteria_incertae_sedis>sulfur-oxidizing_symbionts>Candidatus_Ruthia	GCA_018657745.1	Candidatus_Ruthia_sp.	HN615_06440	8657..12160	2735566	BREX-system-P-loop-protein-BrxC-[Candidatus-Ruthturnera-sp.]
MCG7867606.1	Type1.BREX	[illegible]	1172	Bacteria>Pseudomonadota>Gammaproteobacteria>Gammaproteobacteria_incertae_sedis>sulfur-oxidizing_symbionts>Candidatus_Thiodiazotropha	GCA_016842585.1	Candidatus_Thiodiazotropha_taylori	JAY74_14755	23622..27140	2792791	BREX-system-P-loop-protein-BrxC-[Candidatus-Thiodiazotropha-taylori]
MCG7876672.1	Type6.BREX	[illegible]	1215	Bacteria>Pseudomonadota>Gammaproteobacteria>Gammaproteobacteria_incertae_sedis>sulfur-oxidizing_symbionts>Candidatus_Thiodiazotropha	GCA_016842655.1	Candidatus_Thiodiazotropha_taylori	JAY78_10080	4978..8625	2792791	BREX-system-P-loop-protein-BrxC-[Candidatus-Thiodiazotropha-taylori]
MCG7951159.1	Type6.BREX	[illegible]	1215	Bacteria>Pseudomonadota>Gammaproteobacteria>Gammaproteobacteria_incertae_sedis>sulfur-oxidizing_symbionts>Candidatus_Thiodiazotropha	GCA_016842765.1	Candidatus_Thiodiazotropha_taylori	JAZ09_08050	22496..26143	2792791	BREX-system-P-loop-protein-BrxC-[Candidatus-Thiodiazotropha-taylori]
MCG8102611.1	Type6.BREX	[illegible]	1215	Bacteria>Pseudomonadota>Gammaproteobacteria>Gammaproteobacteria_incertae_sedis>sulfur-oxidizing_symbionts>Candidatus_Thiodiazotropha	GCA_016842845.1	Candidatus_Thiodiazotropha_taylori	JAY70_09490	22526..26173	2792791	BREX-system-P-loop-protein-BrxC-[Candidatus-Thiodiazotropha-taylori]
MCG8058214.1	Type6.BREX	[illegible]	1215	Bacteria>Pseudomonadota>Gammaproteobacteria>Gammaproteobacteria_incertae_sedis>sulfur-oxidizing_symbionts>Candidatus_Thiodiazotropha	GCA_016843165.1	Candidatus_Thiodiazotropha_taylori	JAZ21_00070	27190..30837	2792791	BREX-system-P-loop-protein-BrxC-[Candidatus-Thiodiazotropha-taylori]
MCG7871843.1	Type1.BREX	[illegible]	1174	Bacteria>Pseudomonadota>Gammaproteobacteria>Gammaproteobacteria_incertae_sedis>sulfur-oxidizing_symbionts>Candidatus_Thiodiazotropha	GCA_016848925.1	Candidatus_Thiodiazotropha_lotti	JAZ11_07035	23024..26548	2792787	BREX-system-P-loop-protein-BrxC-[Candidatus-Thiodiazotropha-lotti]
MCG7872379.1	Type1.BREX	[illegible]	1170	Bacteria>Pseudomonadota>Gammaproteobacteria>Gammaproteobacteria_incertae_sedis>sulfur-oxidizing_symbionts>Candidatus_Thiodiazotropha	GCA_016848925.1	Candidatus_Thiodiazotropha_lotti	JAZ11_09800	178889..182401	2792787	BREX-system-P-loop-protein-BrxC-[Candidatus-Thiodiazotropha-lotti]
WP_207387236.1	Type1.BREX	[illegible]	1194	Bacteria>Pseudomonadota>Gammaproteobacteria>Legionellales>Legionellaceae>Fluoribacter	GCF_900640105.1	Fluoribacter_gormanii	E1P13_RS01655	70546..74130	464	BREX-system-P-loop-protein-BrxC-[Fluoribacter-gormanii]
TIG67286.1	Type1.BREX	[illegible]	1185	Bacteria>Pseudomonadota>Gammaproteobacteria>Legionellales>Legionellaceae>Legionella	GCA_004921705.1	Legionella_pneumophila	JAN10_12325	31417..34974	446	BREX-system-P-loop-protein-BrxC-[Legionella-pneumophila]
HAT1978996.1	Type1.BREX	[illegible]	1184	Bacteria>Pseudomonadota>Gammaproteobacteria>Legionellales>Legionellaceae>Legionella	GCA_015895665.1	Legionella_pneumophila	I8Z83_002252	23760..27314	446	BREX-system-P-loop-protein-BrxC-[Legionella-pneumophila]
HAT6348316.1	Type1.BREX	[illegible]	1185	Bacteria>Pseudomonadota>Gammaproteobacteria>Legionellales>Legionellaceae>Legionella	GCA_015945075.1	Legionella_pneumophila	JAJ33_001368	159147..162704	446	BREX-system-P-loop-protein-BrxC-[Legionella-pneumophila]
HAT6334489.1	Type1.BREX	[illegible]	1185	Bacteria>Pseudomonadota>Gammaproteobacteria>Legionellales>Legionellaceae>Legionella	GCA_015946305.1	Legionella_pneumophila	JAJ38_001743	27953..31510	446	BREX-system-P-loop-protein-BrxC-[Legionella-pneumophila]
HAT9211453.1	Type1.BREX	[illegible]	1185	Bacteria>Pseudomonadota>Gammaproteobacteria>Legionellales>Legionellaceae>Legionella	GCA_015973865.1	Legionella_pneumophila_subsp._pneumophila	JBB81_06050	33487..37044	91891	BREX-system-P-loop-protein-BrxC-[Legionella-pneumophila-subsp.-pneumophila]
HAU0774874.1	Type1.BREX	[illegible]	1185	Bacteria>Pseudomonadota>Gammaproteobacteria>Legionellales>Legionellaceae>Legionella	GCA_015983005.1	Legionella_pneumophila	JBH09_07400	2447..6004	446	BREX-system-P-loop-protein-BrxC-[Legionella-pneumophila]
HAU2155475.1	Type1.BREX	[illegible]	1185	Bacteria>Pseudomonadota>Gammaproteobacteria>Legionellales>Legionellaceae>Legionella	GCA_016020315.1	Legionella_pneumophila	JBK36_03075	164784..168341	446	BREX-system-P-loop-protein-BrxC-[Legionella-pneumophila]
HAU3861597.1	Type1.BREX	[illegible]	1185	Bacteria>Pseudomonadota>Gammaproteobacteria>Legionellales>Legionellaceae>Legionella	GCA_016062615.1	Legionella_pneumophila	F7Z80_07285	265287..268844	446	BREX-system-P-loop-protein-BrxC-[Legionella-pneumophila]
HCJ1122636.1	Type1.BREX	[illegible]	1184	Bacteria>Pseudomonadota>Gammaproteobacteria>Legionellales>Legionellaceae>Legionella	GCA_024486645.1	Legionella_pneumophila	NQN52_000248	14141..17695	446	BREX-system-P-loop-protein-BrxC-[Legionella-pneumophila]
WP_061693758.1	Type1.BREX	[illegible]	1184	Bacteria>Pseudomonadota>Gammaproteobacteria>Legionellales>Legionellaceae>Legionella	GCF_002002625.1	Legionella_pneumophila	LPM_RS06580	16051..19605	446	BREX-system-P-loop-protein-BrxC-[Legionella-pneumophila]
WP_192875104.1	Type1.BREX	[illegible]	1184	Bacteria>Pseudomonadota>Gammaproteobacteria>Legionellales>Legionellaceae>Legionella	GCF_003004175.1	Legionella_pneumophila	CA922_RS00900	198017..201571	446	BREX-system-P-loop-protein-BrxC-[Legionella-pneumophila]
WP_061721013.1	Type1.BREX	[illegible]	1184	Bacteria>Pseudomonadota>Gammaproteobacteria>Legionellales>Legionellaceae>Legionella	GCF_900054435.1	Legionella_pneumophila	A2E62_RS14865	12190..15744	446	BREX-system-P-loop-protein-BrxC-[Legionella-pneumophila]

Protein-ID	System	Gene-Neighbourhoods	Length	Taxonomy	Assembly-ID	Strain	Locus-Tag	Genomic-coordinates	TaxID	Fasta-defline
WP_061637858.1	Type1.BREX	<-GDResolvase+HIN-HTH<-?||REase->REase+RNase-><- SIGMA+OB-Fold+AAA-ATPase+ HKD-DNase -BrxL <- PglZ <- N6-METHYLASE <-?<- DUF6079 *<- BrxB-BrxF-iSTAND <- BrxA-DNA-binding <-XIS-HTH<-HTH+LexA- [illegible]	1184	Bacteria>Pseudomonadota>Gammaproteobacteria>Legionellales>Legionellaceae>Legionella	GCF_900061645.1	Legionella_pneumophila	A1L82_RS06820	180647..184201	446	BREX-system-P-loop-protein-BrxC-[Legionella-pneumophila]
WP_027222687.1	Type1.BREX	<-ACET||REase+RNase-><- SIGMA+OB-Fold+AAA-ATPase+ HKD-DNase -BrxL <- PglZ <- N6-METHYLASE <-?<- DUF6079 *<- BrxB-BrxF-iSTAND <- BrxA-DNA-binding <-XIS-HTH<-HTH+LexA-protease||Phage_rep-> [illegible]	1184	Bacteria>Pseudomonadota>Gammaproteobacteria>Legionellales>Legionellaceae>Legionella	GCF_900639945.1	Legionella_pneumophila	E1076_RS05405	246005..249559	446	BREX-system-P-loop-protein-BrxC-[Legionella-pneumophila]
WP_165474921.1	Type1.BREX	<-Phage_rep||HTH+LexA-protease->XIS-HTH-> BrxA-DNA-binding -> BrxB-BrxF-iSTAND -> DUF6079 *-> N6-METHYLASE -> PglZ -> SIGMA+OB-Fold+AAA-ATPase+ HKD-DNase -BrxL -><-REase+RNase||ACET-><- [illegible]	1184	Bacteria>Pseudomonadota>Gammaproteobacteria>Legionellales>Legionellaceae>Legionella	GCF_900640175.1	Legionella_sp._W10-070	E1065_RS06915	46772..50326	1117709	BREX-system-P-loop-protein-BrxC-[Legionella-sp.-W10-070]
WP_159653266.1	Type1.BREX	<-METHYLASE||?->Y1_Tnp-><-ABC-ATPase+SbcC+ABC-ATPase+SbcC<-?||?->XIS-HTH-> BrxA-DNA-binding -> BrxB-BrxF-iSTAND -> DUF6079 *->REase+AIPR-> N6-METHYLASE ->?-> PglZ ->?-> [illegible]	1184	Bacteria>Pseudomonadota>Gammaproteobacteria>Methylococcales>Methylococcaceae>Methylicorpusculum	GCF_009828925.2	Methylicorpusculum_oleiharenae	G0003_RS20515	4601128..4604682	1338687	BREX-system-P-loop-protein-BrxC-[Methylicorpusculum-oleiharenae]
WP_256360610.1	Type1.BREX	<- SIGMA+OB-Fold+AAA-ATPase+ HKD-DNase -BrxL <- PglZ <-REase<-REase<-HTH+REase<- N6-METHYLASE -p<- N6-METHYLASE -p<- N6-METHYLASE -p<- N6-METHYLASE -p<- DUF6079 *	1145	Bacteria>Pseudomonadota>Gammaproteobacteria>Methylococcales>Methylococcaceae>Methylomonas	GCF_001312005.1	Methylomonas_koyamae	JCM16701_RS06940	49293..52730	702114	BREX-system-P-loop-protein-BrxC-[Methylomonas-koyamae]
WP_096876853.1	Type1.BREX	GDResolvase+HIN-HTH->?->XIS-HTH-> BrxA-DNA-binding -> BrxB-BrxF-iSTAND -> DUF6079 *->OLD-ABC->REase-> N6-METHYLASE ->HTH+REase-> PglZ -> SIGMA+OB-Fold+AAA-ATPase+ HKD-DNase -BrxL ->Calcineur [illegible]	1180	Bacteria>Pseudomonadota>Gammaproteobacteria>Methylococcales>Methylococcaceae>Methylomonas	GCF_002441955.1	Methylomonas_koyamae	MKLM6_RS22285	58076..61618	702114	BREX-system-P-loop-protein-BrxC-[Methylomonas-koyamae]
WP_221054675.1	Type1.BREX	DUF6079 *-> N6-METHYLASE ->HTH+REase->REase-> PglZ -> SIGMA+OB-Fold+AAA-ATPase+ HKD-DNase -BrxL ->Calcineurin-SbcD->SbcC+CCDC158->OLD-ABC+TOPRIM->SF1-SF2-Helicase->	1176	Bacteria>Pseudomonadota>Gammaproteobacteria>Methylococcales>Methylococcaceae>Methylomonas	GCF_019669905.1	Methylomonas_koyamae	MKFW1EY_RS21895	239254..1338	702114	BREX-system-P-loop-protein-BrxC-[Methylomonas-koyamae]
WP_004984466.1	Type1.BREX	ZnR+ZnR->DUF932->?->?->XIS-HTH-> BrxA-DNA-binding -> BrxB-BrxF-iSTAND -> DUF6079 *->OLD-ABC->REase-> N6-METHYLASE -> PglZ -> SIGMA+OB-Fold+AAA-ATPase+ HKD-DNase -BrxL ->?->?->ABC-ATPase+SbcC [illegible]	1190	Bacteria>Pseudomonadota>Gammaproteobacteria>Moraxellales>Moraxellaceae>Acinetobacter	GCF_000368805.1	Acinetobacter_johnsonii	F946_RS15710	148915..152487	40214	BREX-system-P-loop-protein-BrxC-[Acinetobacter-johnsonii]
WP_005162499.1	Type6.BREX	<-DDE_Tnp_1<-DUF4096+DDE_Tnp_1+DDE_Tnp_1_2<- SIGMA+OB-Fold+AAA-ATPase+ HKD-DNase -BrxL <- PglZ <-ParB+HNH<- N6-METHYLASE <- N6-METHYLASE <-?||DDE_Tnp_1-><- DUF6079 *<- BrxB-BrxF-iSTAND <- [illegible]	1063	Bacteria>Pseudomonadota>Gammaproteobacteria>Moraxellales>Moraxellaceae>Acinetobacter	GCF_000369425.1	Acinetobacter_sp._NIPH_284	F908_RS12580	1400419..1403610	1217704	BREX-system-P-loop-protein-BrxC-[Acinetobacter-sp.-NIPH-284]
WP_131214141.1	Type1.BREX	ABC-ATPase+SbcC+ABC-ATPase+SbcC->?-><- SIGMA+OB-Fold+AAA-ATPase+ HKD-DNase -BrxL <- PglZ <- N6-METHYLASE <-REase<-OLD-ABC<- DUF6079 *<- BrxB-BrxF-iSTAND <- BrxA-DNA-binding <-XIS-HTH<- [illegible]	1190	Bacteria>Pseudomonadota>Gammaproteobacteria>Moraxellales>Moraxellaceae>Acinetobacter	GCF_004331285.1	Acinetobacter_sp._ANC_3781	E0H89_RS01980	72363..75935	2529835	BREX-system-P-loop-protein-BrxC-[Acinetobacter-sp.-ANC-3781]
WP_140963539.1	Type6.BREX	<-CR-Kinase+Classical-AAA<-?<-?<-?<- SIGMA+OB-Fold+AAA-ATPase+ HKD-DNase -BrxL <- PglZ <-ParB+HNH<- N6-METHYLASE <- N6-METHYLASE <- DUF6079 *<- BrxB-BrxF-iSTAND <- BrxA-DNA-binding <- BrxE [illegible]	1214	Bacteria>Pseudomonadota>Gammaproteobacteria>Moraxellales>Moraxellaceae>Acinetobacter	GCF_006494575.1	Acinetobacter_baumannii	FJU84_RS09090	25284..28928	470	BREX-system-P-loop-protein-BrxC-[Acinetobacter-baumannii]
WP_151834043.1	Type1.BREX	XIS-HTH-> BrxA-DNA-binding -> BrxB-BrxF-iSTAND -> DUF6079 *->OLD-ABC->REase-> N6-METHYLASE -> PglZ -> SIGMA+OB-Fold+AAA-ATPase+ HKD-DNase -BrxL ->?->HerA/FtsK->ParB+HNH->ParB+HNH->	1190	Bacteria>Pseudomonadota>Gammaproteobacteria>Moraxellales>Moraxellaceae>Acinetobacter	GCF_009012055.1	Acinetobacter_ursingii	GBX89_RS12550	5375..8947	108980	BREX-system-P-loop-protein-BrxC-[Acinetobacter-ursingii]
WP_180108075.1	Type1.BREX	<- SIGMA+OB-Fold+AAA-ATPase+ HKD-DNase -BrxL <- PglZ <- N6-METHYLASE <-REase<-OLD-ABC<- DUF6079 *<- BrxB-BrxF-iSTAND <-XIS-HTH<-?<-wHTH+HNH<-SF1-SF2-Helicase<-?<-SAM-METHYLASE||?-><-ParB+HN [illegible]	1190	Bacteria>Pseudomonadota>Gammaproteobacteria>Moraxellales>Moraxellaceae>Acinetobacter	GCF_013419415.1	Acinetobacter_sp._YH12085	FTU28_RS13915	49013..52585	2601077	BREX-system-P-loop-protein-BrxC-[Acinetobacter-sp.-YH12085]
WP_032068282.1	Type6.BREX	DUF932->?->?->XIS-HTH->SAM-METHYLASE->wHTH(SBD)+HNH-> BrxE -> BrxA-DNA-binding -> BrxB-BrxF-iSTAND -> DUF6079 *-> N6-METHYLASE -> N6-METHYLASE ->ParB+HNH-> PglZ -> SIGMA+OB-Fold+AAA-ATPase+ [illegible]	1214	Bacteria>Pseudomonadota>Gammaproteobacteria>Moraxellales>Moraxellaceae>Acinetobacter	GCF_014559915.1	Acinetobacter_baumannii	HPJ29_RS06280	177170..180814	470	BREX-system-P-loop-protein-BrxC-[Acinetobacter-baumannii]
WP_216936951.1	Type1.BREX	<-HTH_3||?-><-Phage_integrase<-Phage_integrase+Phage_integrase<-HTH||XIS-HTH-> BrxA-DNA-binding -> BrxB-BrxF-iSTAND -> DUF6079 *->OLD-ABC->REase-> N6-METHYLASE -> PglZ -> [illegible]	1190	Bacteria>Pseudomonadota>Gammaproteobacteria>Moraxellales>Moraxellaceae>Acinetobacter	GCF_019038255.1	unclassified_Acinetobacter	KUG40_RS10660	10626..14198	196816	MULTISPECIES:-BREX-system-P-loop-protein-BrxC-[unclassified-Acinetobacter]
WP_264453044.1	Type6.BREX	[illegible]	1214	Bacteria>Pseudomonadota>Gammaproteobacteria>Moraxellales>Moraxellaceae>Acinetobacter	GCF_025938795.1	Acinetobacter_baumannii	OLN40_RS03910	58425..62069	470	BREX-system-P-loop-protein-BrxC-[Acinetobacter-baumannii]
WP_134244656.1	Type6.BREX	[illegible]	1217	Bacteria>Pseudomonadota>Gammaproteobacteria>Moraxellales>Moraxellaceae>Alkanindiges	GCF_004377485.1	Alkanindiges_illinoisensis	E2B99_RS09020	10631..14284	197183	BREX-system-P-loop-protein-BrxC-[Alkanindiges-illinoisensis]
WP_151794804.1	Type6.BREX	[illegible]	1214	Bacteria>Pseudomonadota>Gammaproteobacteria>Moraxellales>Moraxellaceae	GCF_008997035.1	Acinetobacter	GBP95_RS04055	337470..341114	469	MULTISPECIES:-BREX-system-P-loop-protein-BrxC-[Acinetobacter]
WP_102077888.1	Type6.BREX	[illegible]	1219	Bacteria>Pseudomonadota>Gammaproteobacteria>Moraxellales>Moraxellaceae>Psychrobacter	GCF_001742065.1	unclassified_Psychrobacter	CXF69_RS01065	108247..111906	196806	MULTISPECIES:-BREX-system-P-loop-protein-BrxC-[unclassified-Psychrobacter]
WP_218690652.1	Type6.BREX	[illegible]	1213	Bacteria>Pseudomonadota>Gammaproteobacteria>Moraxellales>Moraxellaceae>Psychrobacter	GCF_019279065.1	Psychrobacter_sp._BF1	KZA12_RS14005	8359..12000	2821147	BREX-system-P-loop-protein-BrxC-[Psychrobacter-sp.-BF1]
WP_238051284.1	Type6.BREX	[illegible]	1213	Bacteria>Pseudomonadota>Gammaproteobacteria>Moraxellales>Moraxellaceae>Psychrobacter	GCF_022129405.1	Psychrobacter_sp._Ps2	I3254_RS03805	13291..16932	2790956	BREX-system-P-loop-protein-BrxC-[Psychrobacter-sp.-Ps2]
WP_201561160.1	Type6.BREX	[illegible]	1219	Bacteria>Pseudomonadota>Gammaproteobacteria>Moraxellales>Moraxellaceae>Psychrobacter	GCF_904846215.1	Psychrobacter_glacincola	JMW90_RS00425	116435..120094	56810	BREX-system-P-loop-protein-BrxC-[Psychrobacter-glacincola]
TQC95078.1	Type1.BREX	[illegible]	1192	Bacteria>Pseudomonadota>Gammaproteobacteria>Moraxellales>Moraxellaceae>unclassified_Moraxellaceae	GCA_006546595.1	Moraxellaceae_bacterium_AER2_44_116	FK216_14950	12152..15730	2591038	BREX-system-P-loop-protein-BrxC-[Moraxellaceae-bacterium-AER2_44_116]
MBC7753680.1	Type1.BREX	[illegible]	1163	Bacteria>Pseudomonadota>Gammaproteobacteria>Moraxellales>Moraxellaceae>unclassified_Moraxellaceae	GCA_014378775.1	Moraxellaceae_bacterium	H7Z71_05545	8991..12482	1889775	BREX-system-P-loop-protein-BrxC-[Moraxellaceae-bacterium]
MCP5177609.1	Type1.BREX	[illegible]	1192	Bacteria>Pseudomonadota>Gammaproteobacteria>Moraxellales>Moraxellaceae>unclassified_Moraxellaceae	GCA_024234655.1	Moraxellaceae_bacterium	H6997_08670	33703..37281	1889775	BREX-system-P-loop-protein-BrxC-[Moraxellaceae-bacterium]
WP_161382580.1	Type1.BREX	[illegible]	1193	Bacteria>Pseudomonadota>Gammaproteobacteria>Oceanospirillales>Alcanivoracaceae>Alcanivorax	-	Alcanivorax_sp._ZXX171	GN155_RS00640	132435..136016	2695858	BREX-system-P-loop-protein-BrxC-[Alcanivorax-sp.-ZXX171]
KAF0808603.1	Type1.BREX	[illegible]	1165	Bacteria>Pseudomonadota>Gammaproteobacteria>Oceanospirillales>Alcanivoracaceae>Alcanivorax	GCA_009846755.1	Alcanivorax_sp._S71-1-4	A167_02568	1..3498	1177159	ATPase,-partial-[Alcanivorax-sp.-S71-1-4]
MCH2557814.1	Type1.BREX	[illegible]	1193	Bacteria>Pseudomonadota>Gammaproteobacteria>Oceanospirillales>Alcanivoracaceae>Alcanivorax	GCA_022449455.1	Alcanivorax_sp.	MK005_11000	47715..51296	1872427	BREX-system-P-loop-protein-BrxC-[Alcanivorax-sp.]
WP_153499070.1	Type6.BREX	[illegible]	1214	Bacteria>Pseudomonadota>Gammaproteobacteria>Oceanospirillales>Alcanivoracaceae>Alcanivorax	GCF_009601165.1	Alcanivorax_sediminis	GFN93_RS03795	861896..865540	2663008	BREX-system-P-loop-protein-BrxC-[Alcanivorax-sediminis]
WP_159558342.1	Type6.BREX	[illegible]	1213	Bacteria>Pseudomonadota>Gammaproteobacteria>Oceanospirillales>Alcanivoracaceae>Alcanivorax	GCF_009846755.1	Alcanivorax_sp._S71-1-4	A167_RS05685	90732..94373	1177159	BREX-system-P-loop-protein-BrxC-[Alcanivorax-sp.-S71-1-4]
WP_159559845.1	Type1.BREX	[illegible]	1165	Bacteria>Pseudomonadota>Gammaproteobacteria>Oceanospirillales>Alcanivoracaceae>Alcanivorax	GCF_009846755.1	Alcanivorax_sp._S71-1-4	A167_RS13005	1..3498	1177159	BREX-system-P-loop-protein-BrxC,-partial-[Alcanivorax-sp.-S71-1-4]
WP_103916700.1	Type1.BREX	[illegible]	1193	Bacteria>Pseudomonadota>Gammaproteobacteria>Oceanospirillales>Alcanivoracaceae>Alcanivorax	GCF_900107995.1	Alcanivorax_sp._DSM_26293	C4S63_RS03510	780383..783964	1798238	BREX-system-P-loop-protein-BrxC-[Alcanivorax-sp.-DSM-26293]
WP_204429962.1	Type1.BREX	[illegible]	1193	Bacteria>Pseudomonadota>Gammaproteobacteria>Oceanospirillales>Alcanivoracaceae>Alloalcanivorax	GCF_016906305.1	Alloalcanivorax_marinus	IM760_RS12910	16365..19946	1177169	BREX-system-P-loop-protein-BrxC-[Alloalcanivorax-marinus]
WP_228232948.1	Type1.BREX	[illegible]	1193	Bacteria>Pseudomonadota>Gammaproteobacteria>Oceanospirillales>Alcanivoracaceae>Alloalcanivorax	GCF_020782135.1	Alloalcanivorax_marinus	LL252_RS03465	287759..291340	1177169	BREX-system-P-loop-protein-BrxC-[Alloalcanivorax-marinus]
WP_008734404.1	Type1.BREX	[illegible]	1192	Bacteria>Pseudomonadota>Gammaproteobacteria>Oceanospirillales>Alcanivoracaceae>Isoalcanivorax	GCF_000299335.2	Isoalcanivorax_pacificus	S7S_RS18615	4158589..4162167	1306787	BREX-system-P-loop-protein-BrxC-[Isoalcanivorax-pacificus]
RLP52821.1	Type1.BREX	[illegible]	1193	Bacteria>Pseudomonadota>Gammaproteobacteria>Oceanospirillales>Alcanivoracaceae>Ketobacter	GCA_003667185.1	Ketobacter_sp.	D6160_18810	15503..19084	2083498	MAG:-BREX-system-P-loop-protein-BrxC-[Ketobacter-sp.]
AZZ95337.1	Type1.BREX	[illegible]	1183	Bacteria>Pseudomonadota>Gammaproteobacteria>Oceanospirillales>Hahellaceae>Hahella	GCA_004014695.1	Hahella_sp._KA22	ENC22_30655	6892256..6895807	1628392	BREX-system-P-loop-protein-BrxC-[Hahella-sp.-KA22]
WP_164887423.1	Type1.BREX	[illegible]	1182	Bacteria>Pseudomonadota>Gammaproteobacteria>Oceanospirillales>Hahellaceae>Hahella	GCF_004014695.1	Hahella_sp._KA22	EUZ85_RS02365	526761..530309	1628392	BREX-system-P-loop-protein-BrxC-[Hahella-sp.-KA22]
MBR2514422.1	Type6.BREX	[illegible]	1224	Bacteria>Pseudomonadota>Gammaproteobacteria>Oceanospirillales>Halomonadaceae>Halomonas	GCA_017515565.1	Halomonas_sp.	IKE45_10465	23089..26763	1486246	BREX-system-P-loop-protein-BrxC-[Halomonas-sp.]
WP_133481548.1	Type1.BREX	[illegible]	1193	Bacteria>Pseudomonadota>Gammaproteobacteria>Oceanospirillales>Halomonadaceae>Halomonas	GCF_004361885.1	Halomonas_ventosae	DFO68_RS01985	17598..21179	229007	BREX-system-P-loop-protein-BrxC-[Halomonas-ventosae]
WP_159343441.1	Type1.BREX	[illegible]	1193	Bacteria>Pseudomonadota>Gammaproteobacteria>Oceanospirillales>Halomonadaceae>Halomonas	GCF_009846525.1	Halomonas_meridiana	CTT34_RS16815	3563093..3566674	29570	BREX-system-P-loop-protein-BrxC-[Halomonas-meridiana]
WP_161432258.1	Type6.BREX	[illegible]	1223	Bacteria>Pseudomonadota>Gammaproteobacteria>Oceanospirillales>Halomonadaceae>Halomonas	GCF_009902005.1	Halomonas_alimentaria	GRB96_RS11410	2397586..2401257	147248	BREX-system-P-loop-protein-BrxC-[Halomonas-alimentaria]
WP_169956446.1	Type1.BREX	[illegible]	1193	Bacteria>Pseudomonadota>Gammaproteobacteria>Oceanospirillales>Halomonadaceae>Halomonas	GCF_013000985.1	Halomonas_sp._PGE1	HIR79_RS02110	421842..425423	2730360	BREX-system-P-loop-protein-BrxC-[Halomonas-sp.-PGE1]
WP_019020105.1	Type1.BREX	[illegible]	1193	Bacteria>Pseudomonadota>Gammaproteobacteria>Oceanospirillales>Halomonadaceae>Halomonas	GCF_014651575.1	Halomonas_lutea	IEO67_RS16235	61170..64751	453962	BREX-system-P-loop-protein-BrxC-[Halomonas-lutea]
WP_103969332.1	Type1.BREX	[illegible]	1193	Bacteria>Pseudomonadota>Gammaproteobacteria>Oceanospirillales>Halomonadaceae>Halomonas	GCF_900108215.1	Halomonas_desiderata	C4S71_RS12465	87857..91438	52021	BREX-system-P-loop-protein-BrxC-[Halomonas-desiderata]
WP_159177832.1	Type1.BREX	[illegible]	1193	Bacteria>Pseudomonadota>Gammaproteobacteria>Oceanospirillales>Halomonadaceae>Halomonas	GCF_902506335.1	unclassified_Halomonas	JMX33_RS24540	5309589..5313170	2609666	MULTISPECIES:-BREX-system-P-loop-protein-BrxC-[unclassified-Halomonas]
WP_110669866.1	Type6.BREX	[illegible]	1225	Bacteria>Pseudomonadota>Gammaproteobacteria>Oceanospirillales>Halomonadaceae>Salinicola	GCF_003206155.1	Salinicola_halophilus	DOM33_RS13680	401271..404948	184065	BREX-system-P-loop-protein-BrxC-[Salinicola-halophilus]
WP_027854332.1	Type1.BREX	[illegible]	1193	Bacteria>Pseudomonadota>Gammaproteobacteria>Oceanospirillales>Oceanospirillaceae>Marinobacterium	GCF_000428985.1	Marinobacterium_litorale	G403_RS0112740	74962..78543	404770	BREX-system-P-loop-protein-BrxC-[Marinobacterium-litorale]
WP_254579406.1	Type6.BREX	[illegible]	1212	Bacteria>Pseudomonadota>Gammaproteobacteria>Oceanospirillales>Oceanospirillaceae>Marinobacterium	GCF_024267675.1	Marinobacterium_sedimentorum	MRS74_RS06180	150386..154024	2927804	BREX-system-P-loop-protein-BrxC-[Marinobacterium-sedimentorum]
AWY02455.1	Type1.BREX	[illegible]	1197	Bacteria>Pseudomonadota>Gammaproteobacteria>Oceanospirillales>Oceanospirillaceae>Marinomonas	GCA_003285205.1	Marinomonas_primoryensis	A8139_14855	3171018..3174611	178399	hypothetical-protein-A8139_14855-[Marinomonas-primoryensis]
WP_239495510.1	Type1.BREX	[illegible]	1199	Bacteria>Pseudomonadota>Gammaproteobacteria>Oceanospirillales>Oceanospirillaceae>Marinomonas	GCF_003285205.1	Marinomonas_primoryensis	A8139_RS14980	3171018..3174617	178399	BREX-system-P-loop-protein-BrxC-[Marinomonas-primoryensis]
WP_205113926.1	Type1.BREX	[illegible]	1191	Bacteria>Pseudomonadota>Gammaproteobacteria>Oceanospirillales>Oceanospirillaceae>Marinomonas	GCF_016906065.1	Marinomonas_foliarum	JSY38_RS15135	3246777..3250352	491950	BREX-system-P-loop-protein-BrxC-[Marinomonas-foliarum]
WP_138986811.1	Type1.BREX	[illegible]	1190	Bacteria>Pseudomonadota>Gammaproteobacteria>Oceanospirillales>Oceanospirillaceae>Neptunomonas	GCF_008630635.1	Neptunomonas_concharum	FOU83_RS12940	2741645..2745217	1031538	BREX-system-P-loop-protein-BrxC-[Neptunomonas-concharum]
QEW05875.1	Type6.BREX	[illegible]	1212	Bacteria>Pseudomonadota>Gammaproteobacteria>Oceanospirillales>Oceanospirillaceae>Nitrincola	GCA_008727795.1	Nitrincola_iocasae	F5I99_04885	1032629..1036267	2614693	BREX-system-P-loop-protein-BrxC-[Nitrincola-iocasae]
WP_225307559.1	Type6.BREX	[illegible]	1214	Bacteria>Pseudomonadota>Gammaproteobacteria>Oceanospirillales>Oceanospirillaceae>Nitrincola	GCF_008727795.1	Nitrincola_iocasae	F5I99_RS04885	1032623..1036267	2614693	BREX-system-P-loop-protein-BrxC-[Nitrincola-iocasae]
MAD46040.1	Type1.BREX	[illegible]	1189	Bacteria>Pseudomonadota>Gammaproteobacteria>Oceanospirillales>Oceanospirillaceae>unclassified_Oceanospirillaceae	GCA_002685055.1	Oceanospirillaceae_bacterium	CMH98_13650	9512..13081	1899355	hypothetical-protein-[Oceanospirillaceae-bacterium]
MAR01289.1	Type1.BREX	[illegible]	1185	Bacteria>Pseudomonadota>Gammaproteobacteria>Oceanospirillales>Oceanospirillaceae>unclassified_Oceanospirillaceae	GCA_002701445.1	Oceanospirillaceae_bacterium	CMI00_12230	13782..17339	1899355	hypothetical-protein-[Oceanospirillaceae-bacterium]
WP_058011517.1	Type6.BREX	[illegible]	1213	Bacteria>Pseudomonadota>Gammaproteobacteria>Pseudomonadales	GCF_901482535.1	Pseudomonadaceae	FQU16_RS01885	279271..282912	135621	MULTISPECIES:-BREX-system-P-loop-protein-BrxC-[Pseudomonadaceae]
MBE95557.1	Type1.BREX	[illegible]	1193	Bacteria>Pseudomonadota>Gammaproteobacteria>Pseudomonadales>Marinobacteraceae>Marinobacter	GCA_002714765.1	Marinobacter_sp.	CMG89_11180	696..4277	50741	hypothetical-protein-[Marinobacter-sp.]
WP_011784101.1	Type1.BREX	[illegible]	1193	Bacteria>Pseudomonadota>Gammaproteobacteria>Pseudomonadales>Marinobacteraceae>Marinobacter	GCF_000015365.1	Marinobacter_nauticus	MAQU_RS02805	628948..632529	2743	BREX-system-P-loop-protein-BrxC-[Marinobacter-nauticus]
WP_007349691.1	Type5.BREX	[illegible]	1194	Bacteria>Pseudomonadota>Gammaproteobacteria>Pseudomonadales>Marinobacteraceae>Marinobacter	GCF_000169375.1	Marinobacter_sp._ELB17	MELB17_RS08075	35418..39002	270374	BREX-system-P-loop-protein-BrxC-[Marinobacter-sp.-ELB17]
WP_018404435.1	Type1.BREX	[illegible]	1194	Bacteria>Pseudomonadota>Gammaproteobacteria>Pseudomonadales>Marinobacteraceae>Marinobacter	GCF_000372805.1	Marinobacter_gelidimuriae	A3OI_RS0111615	2203..5787	2739064	BREX-system-P-loop-protein-BrxC-[Marinobacter-gelidimuriae]
WP_022992169.1	Type1.BREX	[illegible]	1193	Bacteria>Pseudomonadota>Gammaproteobacteria>Pseudomonadales>Marinobacteraceae>Marinobacter	GCF_000475355.1	Marinobacter_sp._C1S70	Q669_RS10410	149187..152768	1396859	BREX-system-P-loop-protein-BrxC-[Marinobacter-sp.-C1S70]
WP_023008352.1	Type1.BREX	[illegible]	1193	Bacteria>Pseudomonadota>Gammaproteobacteria>Pseudomonadales>Marinobacteraceae>Marinobacter	GCF_000475375.1	Marinobacter_sp._EVN1	Q674_RS26680	52773..56354	1397532	BREX-system-P-loop-protein-BrxC-[Marinobacter-sp.-EVN1]
WP_104416052.1	Type1.BREX	[illegible]	1193	Bacteria>Pseudomonadota>Gammaproteobacteria>Pseudomonadales>Marinobacteraceae>Marinobacter	GCF_002934485.1	Marinobacter_persicus	BY455_RS08980	65339..68920	930118	BREX-system-P-loop-protein-BrxC-[Marinobacter-persicus]
WP_113863603.1	Type1.BREX	[illegible]	1193	Bacteria>Pseudomonadota>Gammaproteobacteria>Pseudomonadales>Marinobacteraceae>Marinobacter	GCF_003315345.1	Marinobacter_pelagius	DET50_RS14950	40642..44223	379482	BREX-system-P-loop-protein-BrxC-[Marinobacter-pelagius]
WP_141068072.1	Type1.BREX	[illegible]	1193	Bacteria>Pseudomonadota>Gammaproteobacteria>Pseudomonadales>Marinobacteraceae>Marinobacter	GCF_006516615.1	Marinobacter_nauticus	FH712_RS17655	33566..37147	2743	BREX-system-P-loop-protein-BrxC-[Marinobacter-nauticus]
WP_247116152.1	Type1.BREX	[illegible]	1189	Bacteria>Pseudomonadota>Gammaproteobacteria>Pseudomonadales>Marinobacteraceae>Marinobacter	GCF_023016045.1	Marinobacter_sp._S6332	MWU62_RS17920	19915..23484	2926403	BREX-system-P-loop-protein-BrxC-[Marinobacter-sp.-S6332]
WP_092033189.1	Type1.BREX	[illegible]	1193	Bacteria>Pseudomonadota>Gammaproteobacteria>Pseudomonadales>Marinobacteraceae>Marinobacter	GCF_900112835.1	Marinobacter_sp._DSM_26671	BM035_RS16335	75345..78926	1761793	BREX-system-P-loop-protein-BrxC-[Marinobacter-sp.-DSM-26671]
BBJ05051.1	Type1.BREX	[illegible]	1193	Bacteria>Pseudomonadota>Gammaproteobacteria>Pseudomonadales>Marinobacteraceae>Marinobacter	-	Marinobacter_nauticus	YBY_29000	3185607..3189188	2743	hypothetical-protein-YBY_29000-[Marinobacter-nauticus]
WP_116918937.1	Type1.BREX	[illegible]	1193	Bacteria>Pseudomonadota>Gammaproteobacteria>Pseudomonadales>Marinobacteraceae>Tamilnaduibacter	GCF_003096655.1	Tamilnaduibacter_salinus	C8D92_104115	123558..127139	1484056	BREX-system-P-loop-protein-BrxC-[Tamilnaduibacter-salinus]
WP_022966741.1	Type1.BREX	[illegible]	1184	Bacteria>Pseudomonadota>Gammaproteobacteria>Pseudomonadales>Pseudomonadaceae>Denitrificimonas	GCF_000421765.1	Denitrificimonas_caeni	H591_RS0109630	17243..20797	521720	BREX-system-P-loop-protein-BrxC-[Denitrificimonas-caeni]
WP_269819144.1	Type6.BREX	[illegible]	1219	Bacteria>Pseudomonadota>Gammaproteobacteria>Pseudomonadales>Pseudomonadaceae>Denitrificimonas	GCF_027498055.1	Denitrificimonas_caeni	O6P33_RS05150	1067699..1071358	521720	BREX-system-P-loop-protein-BrxC-[Denitrificimonas-caeni]
WP_019470415.1	Type6.BREX	[illegible]	1212	Bacteria>Pseudomonadota>Gammaproteobacteria>Pseudomonadales>Pseudomonadaceae	GCF_000292285.1	Pseudomonas	N805_RS25690	5809538..5813176	286	MULTISPECIES:-BREX-system-P-loop-protein-BrxC-[Pseudomonas]
WP_033898350.1	Type1.BREX	[illegible]	1185	Bacteria>Pseudomonadota>Gammaproteobacteria>Pseudomonadales>Pseudomonadaceae	GCF_000510895.2	Pseudomonas	GIW75_RS00865	8119..11676	286	MULTISPECIES:-BREX-system-P-loop-protein-BrxC-[Pseudomonas]
WP_096426782.1	Type1.BREX	[illegible]	1185	Bacteria>Pseudomonadota>Gammaproteobacteria>Pseudomonadales>Pseudomonadaceae	GCF_002356095.1	Pseudomonas	J3A71_RS03870	842777..846334	286	MULTISPECIES:-BREX-system-P-loop-protein-BrxC-[Pseudomonas]
WP_105523318.1	Type1.BREX	[illegible]	1185	Bacteria>Pseudomonadota>Gammaproteobacteria>Pseudomonadales>Pseudomonadaceae	GCF_002975295.1	Pseudomonas	CQ048_RS15780	97047..100604	286	MULTISPECIES:-BREX-system-P-loop-protein-BrxC-[Pseudomonas]
WP_170037970.1	Type1.BREX	[illegible]	1185	Bacteria>Pseudomonadota>Gammaproteobacteria>Pseudomonadales>Pseudomonadaceae	GCF_012985445.1	Pseudomonas	HBO05_RS12455	196790..200347	286	MULTISPECIES:-BREX-system-P-loop-protein-BrxC-[Pseudomonas]
WP_218430053.1	Type1.BREX	[illegible]	1189	Bacteria>Pseudomonadota>Gammaproteobacteria>Pseudomonadales>Pseudomonadaceae	GCF_019219185.1	Pseudomonas	KW849_RS14820	325397..328966	286	MULTISPECIES:-BREX-system-P-loop-protein-BrxC-[Pseudomonas]
WP_033986129.1	Type6.BREX	[illegible]	1212	Bacteria>Pseudomonadota>Gammaproteobacteria>Pseudomonadales>Pseudomonadaceae	GCF_020180035.1	Pseudomonas	AO894_RS16050	13512..17150	286	MULTISPECIES:-BREX-system-P-loop-protein-BrxC-[Pseudomonas]
WP_060765715.1	Type1.BREX	[illegible]	1186	Bacteria>Pseudomonadota>Gammaproteobacteria>Pseudomonadales>Pseudomonadaceae	GCF_029959765.1	Pseudomonas	PFL603g_RS03160	383988..387548	286	MULTISPECIES:-BREX-system-P-loop-protein-BrxC-[Pseudomonas]
WP_104739193.1	Type1.BREX	[illegible]	1180	Bacteria>Pseudomonadota>Gammaproteobacteria>Pseudomonadales>Pseudomonadaceae>Halopseudomonas	GCF_014641295.1	Halopseudomonas_oceani	IEX06_RS08390	366595..370137	1708783	BREX-system-P-loop-protein-BrxC-[Halopseudomonas-oceani]
PPB00131.1	Type1.BREX	[illegible]	1194	Bacteria>Pseudomonadota>Gammaproteobacteria>Pseudomonadales>Pseudomonadaceae>Pseudomonas	GCA_002927135.1	Pseudomonas_aeruginosa	HV84_25295	2273516..2277100	287	BREX-system-P-loop-protein-BrxC-[Pseudomonas-aeruginosa]
RPD95236.1	Type1.BREX	[illegible]	1191	Bacteria>Pseudomonadota>Gammaproteobacteria>Pseudomonadales>Pseudomonadaceae>Pseudomonas	GCA_003813925.1	Pseudomonas_monteilii	EGN69_04400	958521..962096	76759	BREX-system-P-loop-protein-BrxC-[Pseudomonas-monteilii]
MBG3983980.1	Type6.BREX	[illegible]	1212	Bacteria>Pseudomonadota>Gammaproteobacteria>Pseudomonadales>Pseudomonadaceae>Pseudomonas	GCA_015733145.1	Pseudomonas_aeruginosa	I4919_20885	86674..90312	287	BREX-system-P-loop-protein-BrxC-[Pseudomonas-aeruginosa]
MBG4530231.1	Type1.BREX	[illegible]	1181	Bacteria>Pseudomonadota>Gammaproteobacteria>Pseudomonadales>Pseudomonadaceae>Pseudomonas	GCA_015734885.1	Pseudomonas_aeruginosa	I4952_20115	27108..30653	287	BREX-system-P-loop-protein-BrxC-[Pseudomonas-aeruginosa]
MBG4863325.1	Type1.BREX	[illegible]	1184	Bacteria>Pseudomonadota>Gammaproteobacteria>Pseudomonadales>Pseudomonadaceae>Pseudomonas	GCA_015735985.1	Pseudomonas_aeruginosa	GHU23_28970	1726..5280	287	BREX-system-P-loop-protein-BrxC-[Pseudomonas-aeruginosa]
MBG6730505.1	Type1.BREX	[illegible]	1184	Bacteria>Pseudomonadota>Gammaproteobacteria>Pseudomonadales>Pseudomonadaceae>Pseudomonas	GCA_015764285.1	Pseudomonas_aeruginosa	I5I92_11320	4371..7925	287	BREX-system-P-loop-protein-BrxC-[Pseudomonas-aeruginosa]
MBH3980671.1	Type6.BREX	[illegible]	1213	Bacteria>Pseudomonadota>Gammaproteobacteria>Pseudomonadales>Pseudomonadaceae>Pseudomonas	GCA_016011075.1	Pseudomonas_aeruginosa	I5Q70_02570	503331..506972	287	BREX-system-P-loop-protein-BrxC-[Pseudomonas-aeruginosa]
MBL7230767.1	Type6.BREX	[illegible]	1214	Bacteria>Pseudomonadota>Gammaproteobacteria>Pseudomonadales>Pseudomonadaceae>Pseudomonas	GCA_016785205.1	Pseudomonas_sp.	JJK56_22660	12252..15896	306	BREX-system-P-loop-protein-BrxC-[Pseudomonas-sp.]
MBN0299744.1	Type6.BREX	[illegible]	1213	Bacteria>Pseudomonadota>Gammaproteobacteria>Pseudomonadales>Pseudomonadaceae>Pseudomonas	GCA_016922145.1	Pseudomonas_aeruginosa	JTM78_25120	125675..129316	287	BREX-system-P-loop-protein-BrxC-[Pseudomonas-aeruginosa]
MBN0536373.1	Type6.BREX	[illegible]	1206	Bacteria>Pseudomonadota>Gammaproteobacteria>Pseudomonadales>Pseudomonadaceae>Pseudomonas	GCA_016922535.1	Pseudomonas_aeruginosa	JTM59_25840	125679..129299	287	BREX-system-P-loop-protein-BrxC-[Pseudomonas-aeruginosa]
MBV5724753.1	Type1.BREX	[illegible]	1184	Bacteria>Pseudomonadota>Gammaproteobacteria>Pseudomonadales>Pseudomonadaceae>Pseudomonas	GCA_019166825.1	Pseudomonas_aeruginosa	KUT37_26485	44544..48098	287	BREX-system-P-loop-protein-BrxC-[Pseudomonas-aeruginosa]
HBN8119635.1	Type6.BREX	[illegible]	1213	Bacteria>Pseudomonadota>Gammaproteobacteria>Pseudomonadales>Pseudomonadaceae>Pseudomonas	GCA_021586615.1	Pseudomonas_aeruginosa	L3655_000128	111220..114861	287	BREX-system-P-loop-protein-BrxC-[Pseudomonas-aeruginosa]
HBO2945375.1	Type1.BREX	[illegible]	1184	Bacteria>Pseudomonadota>Gammaproteobacteria>Pseudomonadales>Pseudomonadaceae>Pseudomonas	GCA_021651875.1	Pseudomonas_aeruginosa	L4R51_003866	17556..21110	287	BREX-system-P-loop-protein-BrxC-[Pseudomonas-aeruginosa]
HBO3309314.1	Type6.BREX	[illegible]	1188	Bacteria>Pseudomonadota>Gammaproteobacteria>Pseudomonadales>Pseudomonadaceae>Pseudomonas	GCA_021652015.1	Pseudomonas_aeruginosa	L4R58_004351	30228..33794	287	BREX-system-P-loop-protein-BrxC-[Pseudomonas-aeruginosa]
HBO4843314.1	Type6.BREX	[illegible]	1207	Bacteria>Pseudomonadota>Gammaproteobacteria>Pseudomonadales>Pseudomonadaceae>Pseudomonas	GCA_021660595.1	Pseudomonas_aeruginosa	L4V37_002405	61513..65136	287	BREX-system-P-loop-protein-BrxC-[Pseudomonas-aeruginosa]
HBO7186446.1	Type1.BREX	[illegible]	978	Bacteria>Pseudomonadota>Gammaproteobacteria>Pseudomonadales>Pseudomonadaceae>Pseudomonas	GCA_021674675.1	Pseudomonas_aeruginosa	L4968_002122	1..2938	287	BREX-system-P-loop-protein-BrxC-[Pseudomonas-aeruginosa]
HBP1093904.1	Type6.BREX	[illegible]	1213	Bacteria>Pseudomonadota>Gammaproteobacteria>Pseudomonadales>Pseudomonadaceae>Pseudomonas	GCA_021687815.1	Pseudomonas_aeruginosa	L5S32_002755	180884..184525	287	BREX-system-P-loop-protein-BrxC-[Pseudomonas-aeruginosa]
HBP5527123.1	Type6.BREX	[illegible]	1212	Bacteria>Pseudomonadota>Gammaproteobacteria>Pseudomonadales>Pseudomonadaceae>Pseudomonas	GCA_021712095.1	Pseudomonas_aeruginosa	L6B52_09510	5147..8785	287	BREX-system-P-loop-protein-BrxC-[Pseudomonas-aeruginosa]
HCE6004670.1	Type6.BREX	[illegible]	1213	Bacteria>Pseudomonadota>Gammaproteobacteria>Pseudomonadales>Pseudomonadaceae>Pseudomonas	GCA_023904815.1	Pseudomonas_aeruginosa	NHK94_000107	121075..124716	287	BREX-system-P-loop-protein-BrxC-[Pseudomonas-aeruginosa]
MCO2922446.1	Type1.BREX	[illegible]	1189	Bacteria>Pseudomonadota>Gammaproteobacteria>Pseudomonadales>Pseudomonadaceae>Pseudomonas	GCA_023909145.1	Pseudomonas_aeruginosa	FA260_04940	145579..149148	287	BREX-system-P-loop-protein-BrxC-[Pseudomonas-aeruginosa]
HCF1683404.1	Type6.BREX	[illegible]	1213	Bacteria>Pseudomonadota>Gammaproteobacteria>Pseudomonadales>Pseudomonadaceae>Pseudomonas	GCA_023941775.1	Pseudomonas_aeruginosa	NH867_002024	49404..53045	287	BREX-system-P-loop-protein-BrxC-[Pseudomonas-aeruginosa]
HCF1762884.1	Type1.BREX	[illegible]	1168	Bacteria>Pseudomonadota>Gammaproteobacteria>Pseudomonadales>Pseudomonadaceae>Pseudomonas	GCA_023941915.1	Pseudomonas_aeruginosa	NH889_002155	1..3508	287	BREX-system-P-loop-protein-BrxC-[Pseudomonas-aeruginosa]
HCF5867680.1	Type1.BREX	[illegible]	1181	Bacteria>Pseudomonadota>Gammaproteobacteria>Pseudomonadales>Pseudomonadaceae>Pseudomonas	GCA_023959235.1	Pseudomonas_aeruginosa	NIH49_002287	171515..175060	287	BREX-system-P-loop-protein-BrxC-[Pseudomonas-aeruginosa]
MCO7642712.1	Type6.BREX	[illegible]	1204	Bacteria>Pseudomonadota>Gammaproteobacteria>Pseudomonadales>Pseudomonadaceae>Pseudomonas	GCA_024128275.1	Pseudomonas_sp._S_311-6	NJI34_38735	21195..24809	2956727	BREX-system-P-loop-protein-BrxC-[Pseudomonas-sp.-S-311-6]

Protein-ID	System	Gene-Neighbourhoods	Length	Taxonomy	Assembly-ID	Strain	Locus-Tag	Genomic-coordinates	TaxID	Fasta-define
WCY64468.1	Type6.BREX	JAB-><-SIGMA+OB-Fold+AAA-ATPase+HKD-DNase-BrxL<-PglZ<-?<-ParB+HNH<-N6-METHYLASE<-N6-METHYLASE<-GreA_GreB+SF1-SF2-Helicase<-DUF6079*<-BrxB-BrxF-iSTAND<-BrxA-DNA-binding<-BrxE [illegible]	1213	Bacteria>Pseudomonadota>Gammaproteobacteria>Pseudomonadales>Pseudomonadaceae>Pseudomonas	GCA_028610105.1	Pseudomonas_aeruginosa	NHK94_000107	121075..124716	287	BREX-system-P-loop-protein-BrxC-[Pseudomonas-aeruginosa]
WP_257648015.1	Type1.BREX	<-SIGMA+OB-Fold+AAA-ATPase+HKD-DNase-BrxL<-PglZ<-N6-METHYLASE-p<-?<-REase<-SbcC-p<-N6-METHYLASE<-DUF6079*	1073	Bacteria>Pseudomonadota>Gammaproteobacteria>Pseudomonadales>Pseudomonadaceae>Pseudomonas	GCA_030820635.1	Pseudomonas_aeruginosa	K3Z95_RS14360	10117..13340	287	BREX-system-P-loop-protein-BrxC,-partial-[Pseudomonas-aeruginosa]
WP_013692225.1	Type6.BREX	<-RHH||XIS-HTH->?-><-?||HTH_3->wHTH+WYL+WCX->BrxE->BrxA-DNA-binding->BrxB-BrxF-iSTAND->DUF6079*->N6-METHYLASE->ParB+HNH->PglZ->SIGMA+OB-Fold+AAA-ATPase+HKD-DNase-BrxL-><-HNH [illegible]	1213	Bacteria>Pseudomonadota>Gammaproteobacteria>Pseudomonadales>Pseudomonadaceae>Pseudomonas	GCF_000194805.1	Pseudomonas_brassicacearum	PSEBR_RS03955	941705..945346	930166	BREX-system-P-loop-protein-BrxC-[Pseudomonas-brassicacearum]
WP_007159175.1	Type6.BREX	PIN+OST-HTH->?-><-SIGMA+OB-Fold+AAA-ATPase+HKD-DNase-BrxL<-PglZ<-ParB+HNH<-N6-METHYLASE<-N6-METHYLASE<-DUF6079*<-BrxB-BrxF-iSTAND<-BrxA-DNA-binding<-BrxE<-SF1-SF2-Helicase<- [illegible]	1212	Bacteria>Pseudomonadota>Gammaproteobacteria>Pseudomonadales>Pseudomonadaceae>Pseudomonas	GCF_000236825.1	Pseudomonas_psychrotolerans	PPL19_RS03910	152166..155804	237610	BREX-system-P-loop-protein-BrxC-[Pseudomonas-psychrotolerans]
WP_010463595.1	Type1.BREX	<-SIGMA+OB-Fold+AAA-ATPase+HKD-DNase-BrxL<-PglZ<-ParB+HNH<-N6-METHYLASE-p<-REase<-OLD-ABC<-N6-METHYLASE-p<-DUF6079*<-BrxB-BrxF-iSTAND<-BrxA-DNA-binding<-XIS-HTH<-ARG<-?||wHTH [illegible]	1185	Bacteria>Pseudomonadota>Gammaproteobacteria>Pseudomonadales>Pseudomonadaceae>Pseudomonas	GCF_000257545.3	Pseudomonas_mandelii	OU5_RS13450	3003724..3007281	75612	BREX-system-P-loop-protein-BrxC-[Pseudomonas-mandelii]
WP_019485333.1	Type1.BREX	<-TraE<-TraM<-TraM<-DUF3262<-?||XIS-HTH->BrxA-DNA-binding->BrxB-BrxF-iSTAND->DUF6079*->N6-METHYLASE->OLD-ABC->REase->SIR2->HerA/FtsK->N6-METHYLASE-p->PglZ->SIGMA+OB-Fold+AAA-AT [illegible]	1193	Bacteria>Pseudomonadota>Gammaproteobacteria>Pseudomonadales>Pseudomonadaceae>Pseudomonas	GCF_000290555.2	Pseudomonas_aeruginosa	E4028_RS01350	3998..7579	287	BREX-system-P-loop-protein-BrxC-[Pseudomonas-aeruginosa]
WP_017337797.1	Type6.BREX	MarR-HTH-><-RHH||XIS-HTH-><-HIN-HTH||?->wHTH+WYL+WCX->BrxE->BrxA-DNA-binding->BrxB-BrxF-iSTAND->DUF6079*->N6-METHYLASE->N6-METHYLASE->ParB+HNH->PglZ->SIGMA+OB-Fold+AAA-ATPase [illegible]	1213	Bacteria>Pseudomonadota>Gammaproteobacteria>Pseudomonadales>Pseudomonadaceae>Pseudomonas	GCF_000293885.2	Pseudomonas_fluorescens	B723_RS16820	3631858..3635499	294	BREX-system-P-loop-protein-BrxC-[Pseudomonas-fluorescens]
WP_042135808.1	Type1.BREX	<-MarR-HTH<-SIGMA+OB-Fold+AAA-ATPase+HKD-DNase-BrxL<-PglZ<-ParB+HNH<-N6-METHYLASE-p<-RVT<-NERD<-OLD-ABC<-N6-METHYLASE<-DUF6079*<-BrxB-BrxF-iSTAND<-BrxA-DNA-binding<-XIS-HTH<- [illegible]	1184	Bacteria>Pseudomonadota>Gammaproteobacteria>Pseudomonadales>Pseudomonadaceae>Pseudomonas	GCF_000730625.1	Pseudomonas_oryzihabitans	POR01S_RS10925	121811..125365	47885	BREX-system-P-loop-protein-BrxC-[Pseudomonas-oryzihabitans]
WP_034081877.1	Type1.BREX	MerR-HTH->tRNA->XIS-HTH->BrxA-DNA-binding->BrxB-BrxF-iSTAND->DUF6079*->N6-METHYLASE->OLD-ABC->REase->SIR2->HerA/FtsK->N6-METHYLASE-p->PglZ->SIGMA+OB-Fold+AAA-ATPase+HKD-DNase [illegible]	1184	Bacteria>Pseudomonadota>Gammaproteobacteria>Pseudomonadales>Pseudomonadaceae>Pseudomonas	GCF_000793185.1	Pseudomonas_aeruginosa	NT71_RS08285	193065..196619	287	BREX-system-P-loop-protein-BrxC-[Pseudomonas-aeruginosa]
WP_044305280.1	Type6.BREX	JAB-><-SIGMA+OB-Fold+AAA-ATPase+HKD-DNase-BrxL<-PglZ<-?<-ParB+HNH<-N6-METHYLASE<-N6-METHYLASE<-GreA_GreB+SF1-SF2-Helicase<-DUF6079*<-BrxB-BrxF-iSTAND<-BrxA-DNA-binding<-BrxE [illegible]	1213	Bacteria>Pseudomonadota>Gammaproteobacteria>Pseudomonadales>Pseudomonadaceae>Pseudomonas	GCF_000793505.1	Pseudomonas_aeruginosa	NT87_RS20770	137629..141270	287	BREX-system-P-loop-protein-BrxC-[Pseudomonas-aeruginosa]
WP_041506104.1	Type1.BREX	DUF4969-><-SIGMA+OB-Fold+AAA-ATPase+HKD-DNase-BrxL<-PglZ<-ParB+HNH<-N6-METHYLASE-p<-?<-N6-METHYLASE<-DUF6079*<-BrxB-BrxF-iSTAND<-BrxA-DNA-binding<-XIS-HTH<-?||wHTH+WYL+WCX [illegible]	1184	Bacteria>Pseudomonadota>Gammaproteobacteria>Pseudomonadales>Pseudomonadaceae>Pseudomonas	GCF_000831585.1	Pseudomonas_plecoglossicida	RK21_RS13525	2976546..2980100	70775	BREX-system-P-loop-protein-BrxC-[Pseudomonas-plecoglossicida]
WP_044727109.1	Type1.BREX	RelE-ParE-><-?<-wHTH+WYL+WCX||?->Peptidase_M15->?->XIS-HTH->BrxA-DNA-binding->BrxB-BrxF-iSTAND->DUF6079*->N6-METHYLASE->OLD-ABC->NERD->RVT->N6-METHYLASE-p->PglZ->SIGMA+OB-Fold [illegible]	1184	Bacteria>Pseudomonadota>Gammaproteobacteria>Pseudomonadales>Pseudomonadaceae>Pseudomonas	GCF_000937465.2	Pseudomonas_aeruginosa	TN45_RS09710	568754..572308	287	BREX-system-P-loop-protein-BrxC-[Pseudomonas-aeruginosa]
WP_047306816.1	Type1.BREX	<-SIGMA+OB-Fold+AAA-ATPase+HKD-DNase-BrxL<-PglZ<-HNH<-OLD-ABC<-N6-METHYLASE-p<-RVT<-N6-METHYLASE-p<-DUF6079*<-BrxB-BrxF-iSTAND<-BrxA-DNA-binding<-XIS-HTH<-?||wHTH+WYL+WCX [illegible]	1185	Bacteria>Pseudomonadota>Gammaproteobacteria>Pseudomonadales>Pseudomonadaceae>Pseudomonas	GCF_001020715.1	Pseudomonas_fluorescens	AA061_RS32150	16461..20018	294	BREX-system-P-loop-protein-BrxC-[Pseudomonas-fluorescens]
WP_048385719.1	Type1.BREX	<-SIGMA+OB-Fold+AAA-ATPase+HKD-DNase-BrxL<-PglZ<-ParB+HNH<-N6-METHYLASE-p<-REase<-OLD-ABC<-N6-METHYLASE-p<-DUF6079*<-BrxB-BrxF-iSTAND<-BrxA-DNA-binding<-XIS-HTH<-ARG<-?||wHTH [illegible]	1185	Bacteria>Pseudomonadota>Gammaproteobacteria>Pseudomonadales>Pseudomonadaceae>Pseudomonas	GCF_001043065.1	Pseudomonas_helleri	TU85_RS02975	88982..92539	1608996	BREX-system-P-loop-protein-BrxC-[Pseudomonas-helleri]
WP_058181876.1	Type6.BREX	<-SbcC<-JetB<-JetA||wHTH+WYL+WCX->BrxE->BrxA-DNA-binding->BrxB-BrxF-iSTAND->DUF6079*->GreA_GreB+SF1-SF2-Helicase->N6-METHYLASE->N6-METHYLASE->ParB+HNH->?->PglZ->SIGMA+OB-Fold [illegible]	1213	Bacteria>Pseudomonadota>Gammaproteobacteria>Pseudomonadales>Pseudomonadaceae>Pseudomonas	GCF_001450675.1	Pseudomonas_aeruginosa	AO933_RS04770	131954..135595	287	BREX-system-P-loop-protein-BrxC-[Pseudomonas-aeruginosa]
WP_031639867.1	Type1.BREX	LZ_Tnp_IS66+zf-IS66+DDE_Tnp_IS66+DDE_Tnp_IS66_C-><- [illegible]	1184	Bacteria>Pseudomonadota>Gammaproteobacteria>Pseudomonadales>Pseudomonadaceae>Pseudomonas	GCF_001548335.1	Pseudomonas_aeruginosa	IOMTU133_RS23320	5069423..5072977	287	BREX-system-P-loop-protein-BrxC-[Pseudomonas-aeruginosa]
WP_069953193.1	Type1.BREX	DUF4969-><-SIGMA+OB-Fold+AAA-ATPase+HKD-DNase [illegible]	828	Bacteria>Pseudomonadota>Gammaproteobacteria>Pseudomonadales>Pseudomonadaceae>Pseudomonas	GCF_001750225.1	Pseudomonas_aeruginosa	A7R77_RS00815	21562..24049	287	BREX-system-P-loop-protein-BrxC,-partial-[Pseudomonas-aeruginosa]
WP_074859114.1	Type1.BREX	MerR-HTH->tRNA->XIS-HTH->?-><-?<-wHTH+WYL+WCX||?-> [illegible]	1184	Bacteria>Pseudomonadota>Gammaproteobacteria>Pseudomonadales>Pseudomonadaceae>Pseudomonas	GCF_002091815.1	Pseudomonas_oleovorans	POL01S_RS18595	25960..29514	301	BREX-system-P-loop-protein-BrxC-[Pseudomonas-oleovorans]
WP_084929168.1	Type1.BREX	Arm-DNA-bind_3+Phage_integrase-><- [illegible]	1184	Bacteria>Pseudomonadota>Gammaproteobacteria>Pseudomonadales>Pseudomonadaceae>Pseudomonas	GCF_002094805.1	Pseudomonas_aeruginosa	B7H20_RS24080	14270..17824	287	BREX-system-P-loop-protein-BrxC-[Pseudomonas-aeruginosa]
WP_086230732.1	Type1.BREX	<-wHTH+WYL+WCX||?->Peptidase_M15->Peptidase_M15-> [illegible]	1184	Bacteria>Pseudomonadota>Gammaproteobacteria>Pseudomonadales>Pseudomonadaceae>Pseudomonas	GCF_002135805.1	Pseudomonas_aeruginosa	CAZ03_RS13145	128061..131615	287	BREX-system-P-loop-protein-BrxC-[Pseudomonas-aeruginosa]
WP_101196767.1	Type1.BREX	ZnR-><-wHTH+WYL+WCX<-AAA-ToxA+Classical-AAA||?-> [illegible]	1180	Bacteria>Pseudomonadota>Gammaproteobacteria>Pseudomonadales>Pseudomonadaceae>Pseudomonas	GCF_002835905.1	Pseudomonas_monteilii	CXB65_RS25690	173341..176883	76759	BREX-system-P-loop-protein-BrxC-[Pseudomonas-monteilii]
WP_101152285.1	Type6.BREX	CopG/RHH->TrwC+SF1-SF2-Helicase->HNH-><- [illegible]	1211	Bacteria>Pseudomonadota>Gammaproteobacteria>Pseudomonadales>Pseudomonadaceae>Pseudomonas	GCF_002836195.1	Pseudomonas_sp._Choline-3u-10	CXF92_RS00360	72818..76453	2058311	BREX-system-P-loop-protein-BrxC-[Pseudomonas-sp.-Choline-3u-10]
WP_102082564.1	Type1.BREX	DUF4969-><-SIGMA+OB-Fold+AAA-ATPase+HKD-DNase [illegible]	1184	Bacteria>Pseudomonadota>Gammaproteobacteria>Pseudomonadales>Pseudomonadaceae>Pseudomonas	GCF_002864915.1	Pseudomonas_guariconensis	CXG53_RS23735	60064..63618	1288410	BREX-system-P-loop-protein-BrxC-[Pseudomonas-guariconensis]
WP_102368059.1	Type1.BREX	<-CopG/RHH<-HTH_Tnp_1+HTH_21+rve+rve_3<-CopG/RHH|| [illegible]	1188	Bacteria>Pseudomonadota>Gammaproteobacteria>Pseudomonadales>Pseudomonadaceae>Pseudomonas	GCF_002874965.1	Pseudomonas_sp._NC02	C0058_RS03795	845324..848890	2067572	BREX-system-P-loop-protein-BrxC-[Pseudomonas-sp.-NC02]
WP_102886089.1	Type1.BREX	<-SIGMA+OB-Fold+AAA-ATPase+HKD-DNase-BrxL<-PglZ [illegible]	1184	Bacteria>Pseudomonadota>Gammaproteobacteria>Pseudomonadales>Pseudomonadaceae>Pseudomonas	GCF_002891885.1	Pseudomonas_putida	CBL13_04161	1040459..1044013	303	BREX-system-P-loop-protein-BrxC-[Pseudomonas-putida]
WP_116801381.1	Type1.BREX	DUF4969-><-SIGMA+OB-Fold+AAA-ATPase+HKD-DNase [illegible]	1184	Bacteria>Pseudomonadota>Gammaproteobacteria>Pseudomonadales>Pseudomonadaceae>Pseudomonas	GCF_003410555.2	Pseudomonas_aeruginosa	DZ982_RS28380	35413..38967	287	BREX-system-P-loop-protein-BrxC-[Pseudomonas-aeruginosa]
WP_121398877.1	Type1.BREX	Arm-DNA-bind_3+Phage_integrase-><- [illegible]	1180	Bacteria>Pseudomonadota>Gammaproteobacteria>Pseudomonadales>Pseudomonadaceae>Pseudomonas	GCF_003631245.1	Pseudomonas_aeruginosa	DQH61_RS02890	22908..26450	287	BREX-system-P-loop-protein-BrxC-[Pseudomonas-aeruginosa]
WP_128551186.1	Type1.BREX	Arm-DNA-bind_3+Phage_integrase-><- [illegible]	1184	Bacteria>Pseudomonadota>Gammaproteobacteria>Pseudomonadales>Pseudomonadaceae>Pseudomonas	GCF_003698635.1	Pseudomonas_aeruginosa	IPC82_RS03385	45130..48684	287	BREX-system-P-loop-protein-BrxC-[Pseudomonas-aeruginosa]
WP_124126664.1	Type6.BREX	<-LON-AAA||HTH_19-><-?<-ARG||wHTH+WYL+WCX->BrxE-> [illegible]	1210	Bacteria>Pseudomonadota>Gammaproteobacteria>Pseudomonadales>Pseudomonadaceae>Pseudomonas	GCF_003836525.1	Pseudomonas_aeruginosa	IPC776_RS06145	34793..38425	287	BREX-system-P-loop-protein-BrxC-[Pseudomonas-aeruginosa]
WP_126190092.1	Type1.BREX	<-RNase<-?<-JetB<-?<-wHTH+WYL+WCX||XIS-HTH-> [illegible]	1185	Bacteria>Pseudomonadota>Gammaproteobacteria>Pseudomonadales>Pseudomonadaceae>Pseudomonas	GCF_003957495.3	Pseudomonas_songnenensis	EJA06_RS16615	27316..30873	1176259	BREX-system-P-loop-protein-BrxC-[Pseudomonas-songnenensis]
WP_128704208.1	Type1.BREX	<-HTH_20||Arm-DNA-bind_3+Phage_integrase-><- [illegible]	1184	Bacteria>Pseudomonadota>Gammaproteobacteria>Pseudomonadales>Pseudomonadaceae>Pseudomonas	GCF_003974605.1	Pseudomonas_aeruginosa	IPC1390_RS17360	217632..221186	287	BREX-system-P-loop-protein-BrxC-[Pseudomonas-aeruginosa]
WP_126632346.1	Type6.BREX	<-Small-B-barrel+SF1-SF2-Helicase+RdgC+REase<- [illegible]	1213	Bacteria>Pseudomonadota>Gammaproteobacteria>Pseudomonadales>Pseudomonadaceae>Pseudomonas	GCF_003975645.1	Pseudomonas_aeruginosa	IPC447_RS09565	370705..374346	287	BREX-system-P-loop-protein-BrxC-[Pseudomonas-aeruginosa]
WP_023094202.1	Type6.BREX	<-SbcC<-JetB<-JetA||wHTH+WYL+WCX->BrxE-> [illegible]	1213	Bacteria>Pseudomonadota>Gammaproteobacteria>Pseudomonadales>Pseudomonadaceae>Pseudomonas	GCF_004346985.1	Pseudomonas_aeruginosa	C9I70_RS03025	613762..617403	287	BREX-system-P-loop-protein-BrxC-[Pseudomonas-aeruginosa]
WP_134249510.1	Type6.BREX	<-KAP-glycosylase<-KAP-NTPase<- [illegible]	1213	Bacteria>Pseudomonadota>Gammaproteobacteria>Pseudomonadales>Pseudomonadaceae>Pseudomonas	GCF_004374205.1	Pseudomonas_aeruginosa	IPC115_RS16040	120938..124579	287	BREX-system-P-loop-protein-BrxC-[Pseudomonas-aeruginosa]
WP_134295820.1	Type6.BREX	<-KAP-glycosylase<-KAP-NTPase<- [illegible]	1213	Bacteria>Pseudomonadota>Gammaproteobacteria>Pseudomonadales>Pseudomonadaceae>Pseudomonas	GCF_004374605.1	Pseudomonas_aeruginosa	IPC112_RS05880	126222..129863	287	BREX-system-P-loop-protein-BrxC-[Pseudomonas-aeruginosa]
WP_143505426.1	Type6.BREX	HAD+HAD->?->?->?->?-><-wHTH+WYL+WCX||BrxE-> [illegible]	1212	Bacteria>Pseudomonadota>Gammaproteobacteria>Pseudomonadales>Pseudomonadaceae>Pseudomonas	GCF_007049795.1	Pseudomonas_mendocina	EQ826_RS09455	197604..201242	300	BREX-system-P-loop-protein-BrxC-[Pseudomonas-mendocina]
WP_150073350.1	Type1.BREX	XIS-HTH->?->XIS-HTH->BrxA-DNA-binding-> [illegible]	1186	Bacteria>Pseudomonadota>Gammaproteobacteria>Pseudomonadales>Pseudomonadaceae>Pseudomonas	GCF_008632325.1	Pseudomonas_lactis	F3K52_RS19340	4156..7716	1615674	BREX-system-P-loop-protein-BrxC-[Pseudomonas-lactis]
WP_151183841.1	Type1.BREX	35exo->AbiEi_3_N+AbiEi_3->AbiEii->?->?-> [illegible]	1181	Bacteria>Pseudomonadota>Gammaproteobacteria>Pseudomonadales>Pseudomonadaceae>Pseudomonas	GCF_008807395.1	Pseudomonas_sp._C27(2019)	FXF61_RS02795	588989..592534	2604941	BREX-system-P-loop-protein-BrxC-[Pseudomonas-sp.-C27(2019)]
WP_153329486.1	Type1.BREX	<-SIGMA+OB-Fold+AAA-ATPase+HKD-DNase-BrxL<-PglZ [illegible]	1184	Bacteria>Pseudomonadota>Gammaproteobacteria>Pseudomonadales>Pseudomonadaceae>Pseudomonas	GCF_009600225.1	Pseudomonas_helleri	GHO39_RS16765	34385..37939	1608996	BREX-system-P-loop-protein-BrxC-[Pseudomonas-helleri]
WP_153386820.1	Type1.BREX	Pkinase+Pkinase->?-><-?<-?||XIS-HTH-> [illegible]	1183	Bacteria>Pseudomonadota>Gammaproteobacteria>Pseudomonadales>Pseudomonadaceae>Pseudomonas	GCF_009600315.1	Pseudomonas_helleri	GHN86_RS14975	48582..52133	1608996	BREX-system-P-loop-protein-BrxC-[Pseudomonas-helleri]
WP_153384138.1	Type1.BREX	MerR-HTH->tRNA-><-RHH||XIS-HTH->?->XIS-HTH-> [illegible]	1186	Bacteria>Pseudomonadota>Gammaproteobacteria>Pseudomonadales>Pseudomonadaceae>Pseudomonas	GCF_009600565.1	unclassified_Pseudomonas	GHO24_RS06625	35218..38778	196821	MULTISPECIES:-BREX-system-P-loop-protein-BrxC-[unclassified-Pseudomonas]
WP_161928857.1	Type6.BREX	<-Arm-DNA-bind_2+Phage_int_SAM_3+Phage_integrase|| [illegible]	1212	Bacteria>Pseudomonadota>Gammaproteobacteria>Pseudomonadales>Pseudomonadaceae>Pseudomonas	GCF_009932815.1	Pseudomonas_yangonensis	GYH25_RS19705	87173..90811	2579922	BREX-system-P-loop-protein-BrxC-[Pseudomonas-yangonensis]
WP_169989748.1	Type1.BREX	<-SIGMA+OB-Fold+AAA-ATPase+HKD-DNase-BrxL<-PglZ [illegible]	1186	Bacteria>Pseudomonadota>Gammaproteobacteria>Pseudomonadales>Pseudomonadaceae>Pseudomonas	GCF_012985575.1	Pseudomonas_sp._WS_5413	HBO21_RS11490	97465..101025	2717488	BREX-system-P-loop-protein-BrxC-[Pseudomonas-sp.-WS-5413]
WP_169883492.1	Type6.BREX	HNH-><-SIGMA+OB-Fold+AAA-ATPase+HKD-DNase-BrxL<- [illegible]	1213	Bacteria>Pseudomonadota>Gammaproteobacteria>Pseudomonadales>Pseudomonadaceae>Pseudomonas	GCF_012986805.1	Pseudomonas_fragi	HBN91_RS17750	10864..14505	296	BREX-system-P-loop-protein-BrxC-[Pseudomonas-fragi]
WP_177031785.1	Type1.BREX	sGTP-><-wHTH+WYL+WCX||?->ARG->XIS-HTH-> [illegible]	1185	Bacteria>Pseudomonadota>Gammaproteobacteria>Pseudomonadales>Pseudomonadaceae>Pseudomonas	GCF_013386345.1	Pseudomonas_veronii	HX878_RS08640	6154..9711	76761	BREX-system-P-loop-protein-BrxC-[Pseudomonas-veronii]
WP_178910878.1	Type1.BREX	<-SIGMA+OB-Fold+AAA-ATPase+HKD-DNase-BrxL<-PglZ [illegible]	1186	Bacteria>Pseudomonadota>Gammaproteobacteria>Pseudomonadales>Pseudomonadaceae>Pseudomonas	GCF_013386495.1	Pseudomonas_tolaasii	HX799_RS03165	122791..126351	29442	BREX-system-P-loop-protein-BrxC-[Pseudomonas-tolaasii]
WP_180216146.1	Type6.BREX	<-DUF3262<-?<-?<-SMODS<-?<-?||wHTH+WYL+WCX->BrxE [illegible]	1213	Bacteria>Pseudomonadota>Gammaproteobacteria>Pseudomonadales>Pseudomonadaceae>Pseudomonas	GCF_013421145.1	Pseudomonas_aeruginosa	H0B52_RS17845	402112..405753	287	BREX-system-P-loop-protein-BrxC-[Pseudomonas-aeruginosa]
WP_180689220.1	Type1.BREX	ABC-ATPase+SbcC->?->?->?->?->DUF772+DDE_Tnp_1-> [illegible]	1185	Bacteria>Pseudomonadota>Gammaproteobacteria>Pseudomonadales>Pseudomonadaceae>Pseudomonas	GCF_013425825.1	Pseudomonas_putida	H0H12_RS03375	759269..762826	303	BREX-system-P-loop-protein-BrxC-[Pseudomonas-putida]
WP_181088903.1	Type6.BREX	TPR->wHTH->Integrase_1->Phage_integrase-><- [illegible]	1214	Bacteria>Pseudomonadota>Gammaproteobacteria>Pseudomonadales>Pseudomonadaceae>Pseudomonas	GCF_013523035.1	Pseudomonas_plecoglossicida	G7011_RS14160	93028..96672	70775	BREX-system-P-loop-protein-BrxC-[Pseudomonas-plecoglossicida]
WP_185818333.1	Type6.BREX	MerR-HTH->tRNA-><-CopG/RHH||BirA-HTH->?->XIS-HTH-> [illegible]	1214	Bacteria>Pseudomonadota>Gammaproteobacteria>Pseudomonadales>Pseudomonadaceae>Pseudomonas	GCF_014236655.1	Pseudomonas_kielensis	H7995_RS10625	125852..129496	2762577	BREX-system-P-loop-protein-BrxC-[Pseudomonas-kielensis]
WP_186598283.1	Type6.BREX	<-sGTP<-GTPase-AIG<-?<-?||<-CopG/RHH||XIS-HTH->?-> [illegible]	1212	Bacteria>Pseudomonadota>Gammaproteobacteria>Pseudomonadales>Pseudomonadaceae>Pseudomonas	GCF_014269085.1	Pseudomonas_taiwanensis	HU747_RS04800	431376..435014	470150	BREX-system-P-loop-protein-BrxC-[Pseudomonas-taiwanensis]
WP_198790924.1	Type1.BREX	<-CopG/RHH||XIS-HTH->?->?->XIS-HTH-> [illegible]	1185	Bacteria>Pseudomonadota>Gammaproteobacteria>Pseudomonadales>Pseudomonadaceae>Pseudomonas	GCF_016405175.1	Pseudomonas_sp._MF7451	JFT65_RS10900	194530..198087	2797538	BREX-system-P-loop-protein-BrxC-[Pseudomonas-sp.-MF7451]
WP_198832884.1	Type1.BREX	<-SIGMA+OB-Fold+AAA-ATPase+HKD-DNase-BrxL<-PglZ [illegible]	1185	Bacteria>Pseudomonadota>Gammaproteobacteria>Pseudomonadales>Pseudomonadaceae>Pseudomonas	GCF_016405445.1	Pseudomonas_haemolytica	JFT42_RS28230	10084..13641	2600065	BREX-system-P-loop-protein-BrxC-[Pseudomonas-haemolytica]
WP_198849248.1	Type1.BREX	wHTH+WYL+WCX-><-SIGMA+OB-Fold+AAA-ATPase+ [illegible]	1186	Bacteria>Pseudomonadota>Gammaproteobacteria>Pseudomonadales>Pseudomonadaceae>Pseudomonas	GCF_016405505.1	Pseudomonas_sp._MF6772	JFT60_RS22275	24346..27906	2797533	BREX-system-P-loop-protein-BrxC-[Pseudomonas-sp.-MF6772]
WP_273507518.1	Type6.BREX	<-CopG/RHH||CITB-HTH->XIS-HTH->?->wHTH+WYL+WCX-> [illegible]	1214	Bacteria>Pseudomonadota>Gammaproteobacteria>Pseudomonadales>Pseudomonadaceae>Pseudomonas	GCF_016785205.1	Pseudomonas_sp.	JJK56_22660	12252..15896	306	BREX-system-P-loop-protein-BrxC-[Pseudomonas-sp.]
WP_203328783.1	Type6.BREX	<-LysR-HTH||HTH_3-><-MODE-HTH+PBPII||wHTH+WYL+WCX [illegible]	1213	Bacteria>Pseudomonadota>Gammaproteobacteria>Pseudomonadales>Pseudomonadaceae>Pseudomonas	GCF_016836545.1	Pseudomonas_aeruginosa	JQ483_RS07485	1620720..1624361	287	BREX-system-P-loop-protein-BrxC-[Pseudomonas-aeruginosa]
WP_209939193.1	Type6.BREX	<-SbcC<-JetB<-JetA||wHTH+WYL+WCX->BrxE-> [illegible]	1213	Bacteria>Pseudomonadota>Gammaproteobacteria>Pseudomonadales>Pseudomonadaceae>Pseudomonas	GCF_017838075.1	Pseudomonas_aeruginosa	JOA89_RS01675	341330..344971	287	BREX-system-P-loop-protein-BrxC-[Pseudomonas-aeruginosa]
WP_223023201.1	Type1.BREX	<-REase||DNA_METHYLASE-><-CopG/RHH||XIS-HTH->?-> [illegible]	1183	Bacteria>Pseudomonadota>Gammaproteobacteria>Pseudomonadales>Pseudomonadaceae>Pseudomonas	GCF_019891425.1	Pseudomonas_carnis	J1G36_06600	89435..92986	2487355	BREX-system-P-loop-protein-BrxC-[Pseudomonas-carnis]
WP_223717957.1	Type6.BREX	MerR-HTH->tRNA->BrxE->BrxB-BrxF-iSTAND->DUF6079 [illegible]	1213	Bacteria>Pseudomonadota>Gammaproteobacteria>Pseudomonadales>Pseudomonadaceae>Pseudomonas	GCF_020036535.1	Pseudomonas_aeruginosa	LC626_RS08340	482522..486163	287	BREX-system-P-loop-protein-BrxC-[Pseudomonas-aeruginosa]
WP_227364598.1	Type6.BREX	VirD4-TraG->DUF4400-><-SbcC<- [illegible]	1213	Bacteria>Pseudomonadota>Gammaproteobacteria>Pseudomonadales>Pseudomonadaceae>Pseudomonas	GCF_020639995.1	Pseudomonas_aeruginosa	LJG07_RS13375	107262..110903	287	BREX-system-P-loop-protein-BrxC-[Pseudomonas-aeruginosa]
WP_229605965.1	Type6.BREX	AlcRN+AraC-HTH-><-?||HNH-><- [illegible]	1212	Bacteria>Pseudomonadota>Gammaproteobacteria>Pseudomonadales>Pseudomonadaceae>Pseudomonas	GCF_020831405.1	Pseudomonas_oryzagri	LMJ29_RS13110	91421..95059	2892443	BREX-system-P-loop-protein-BrxC-[Pseudomonas-oryzagri]
WP_033990403.1	Type6.BREX	SMODS->?->JAB-><-SIGMA+OB-Fold+AAA-ATPase+ [illegible]	1213	Bacteria>Pseudomonadota>Gammaproteobacteria>Pseudomonadales>Pseudomonadaceae>Pseudomonas	GCF_020907765.1	Pseudomonas_aeruginosa	LOH47_RS10865	120991..124632	287	BREX-system-P-loop-protein-BrxC-[Pseudomonas-aeruginosa]
WP_196706085.1	Type1.BREX	<-SIGMA+OB-Fold+AAA-ATPase+HKD-DNase-BrxL<-PglZ [illegible]	1184	Bacteria>Pseudomonadota>Gammaproteobacteria>Pseudomonadales>Pseudomonadaceae>Pseudomonas	GCF_021460775.1	Pseudomonas_putida	LH705_RS23300	5093270..5096824	303	BREX-system-P-loop-protein-BrxC-[Pseudomonas-putida]
WP_236238652.1	Type1.BREX	<-HIN-HTH<-SIGMA+OB-Fold+AAA-ATPase+HKD-DNase [illegible]	1185	Bacteria>Pseudomonadota>Gammaproteobacteria>Pseudomonadales>Pseudomonadaceae>Pseudomonas	GCF_021603345.1	Pseudomonas_rhodesiae	L6E14_RS13730	266364..269921	76760	BREX-system-P-loop-protein-BrxC-[Pseudomonas-rhodesiae]
WP_236165768.1	Type6.BREX	DUF932->?->?->HNH-><-SIGMA+OB-Fold+AAA-ATPase+ [illegible]	1212	Bacteria>Pseudomonadota>Gammaproteobacteria>Pseudomonadales>Pseudomonadaceae>Pseudomonas	GCF_021603865.1	Pseudomonas_fulva	L6D10_RS06765	41152..44790	47880	BREX-system-P-loop-protein-BrxC-[Pseudomonas-fulva]
WP_247325109.1	Type6.BREX	<-DUF3262<-?<-?<-SMODS<-?||wHTH+WYL+WCX->BrxE-> [illegible]	371	Bacteria>Pseudomonadota>Gammaproteobacteria>Pseudomonadales>Pseudomonadaceae>Pseudomonas	GCF_023101305.1	Pseudomonas_aeruginosa	MX621_RS22735	4826578..4827693	287	DUF6079-family-protein-[Pseudomonas-aeruginosa]
WP_124171099.1	Type1.BREX	RelE-ParE-><-?<-wHTH+WYL+WCX||?->Peptidase_M15->?- [illegible]	1184	Bacteria>Pseudomonadota>Gammaproteobacteria>Pseudomonadales>Pseudomonadaceae>Pseudomonas	GCF_025697325.1	Pseudomonas_aeruginosa	IPC368_RS11155	506506..510060	287	BREX-system-P-loop-protein-BrxC-[Pseudomonas-aeruginosa]
WP_265072431.1	Type1.BREX	<-SIGMA+OB-Fold+AAA-ATPase+HKD-DNase-BrxL<-PglZ [illegible]	1186	Bacteria>Pseudomonadota>Gammaproteobacteria>Pseudomonadales>Pseudomonadaceae>Pseudomonas	GCF_026016265.1	Pseudomonas_viciae	LOY66_RS25465	5798638..5802198	2505979	BREX-system-P-loop-protein-BrxC-[Pseudomonas-viciae]
WP_010792139.1	Type6.BREX	SMODS->?->JAB-><-SIGMA+OB-Fold+AAA-ATPase+ [illegible]	1210	Bacteria>Pseudomonadota>Gammaproteobacteria>Pseudomonadales>Pseudomonadaceae>Pseudomonas	GCF_026126725.1	Pseudomonas_aeruginosa	K3Z93_RS02355	20406..24038	287	BREX-system-P-loop-protein-BrxC-[Pseudomonas-aeruginosa]
WP_265535081.1	Type1.BREX	<-SIGMA+OB-Fold+AAA-ATPase+HKD-DNase-BrxL<-PglZ [illegible]	1184	Bacteria>Pseudomonadota>Gammaproteobacteria>Pseudomonadales>Pseudomonadaceae>Pseudomonas	GCF_026173695.1	Pseudomonas_saponiphila	ORM81_RS30140	14715..18269	556534	BREX-system-P-loop-protein-BrxC-[Pseudomonas-saponiphila]
WP_265980027.1	Type1.BREX	Phage_integrase-><-SIGMA+OB-Fold+AAA-ATPase+ [illegible]	1184	Bacteria>Pseudomonadota>Gammaproteobacteria>Pseudomonadales>Pseudomonadaceae>Pseudomonas	GCF_026241005.1	Pseudomonas_sp._DCB_BG	OO254_RS23990	64105..67659	2993595	BREX-system-P-loop-protein-BrxC-[Pseudomonas-sp.-DCB_BG]
WP_057380122.1	Type6.BREX	LZ_Tnp_IS66+zf-IS66+DDE_Tnp_IS66+DDE_Tnp_IS66_C-><- [illegible]	1213	Bacteria>Pseudomonadota>Gammaproteobacteria>Pseudomonadales>Pseudomonadaceae>Pseudomonas	GCF_026727755.1	Pseudomonas_aeruginosa	PAC42_RS26725	5539866..5543507	287	BREX-system-P-loop-protein-BrxC-[Pseudomonas-aeruginosa]
WP_196620444.1	Type1.BREX	<-XIS-HTH<-SIGMA+OB-Fold+AAA-ATPase+HKD-DNase [illegible]	1181	Bacteria>Pseudomonadota>Gammaproteobacteria>Pseudomonadales>Pseudomonadaceae>Pseudomonas	GCF_027857235.1	Pseudomonas_aeruginosa	OFL78_RS02500	39831..43376	287	BREX-system-P-loop-protein-BrxC-[Pseudomonas-aeruginosa]
WP_273916456.1	Type1.BREX	XIS-HTH->BrxA-DNA-binding->BrxB-BrxF-iSTAND-> [illegible]	1186	Bacteria>Pseudomonadota>Gammaproteobacteria>Pseudomonadales>Pseudomonadaceae>Pseudomonas	GCF_028656995.1	Pseudomonas_sp._TNT2022_ID642	M5G26_RS21745	58505..62065	2942632	BREX-system-P-loop-protein-BrxC-[Pseudomonas-sp.-TNT2022-ID642]
WP_058409982.1	Type6.BREX	<-LuxR-HTH<-?<-ZnR+TOPRIM+AAA-helicase+wHTH|| [illegible]	1212	Bacteria>Pseudomonadota>Gammaproteobacteria>Pseudomonadales>Pseudomonadaceae>Pseudomonas	GCF_029959515.1	Pseudomonas_sp._ICMP_19500	AO239_RS04345	8658..12296	1208102	BREX-system-P-loop-protein-BrxC-[Pseudomonas-sp.-ICMP-19500]
WP_176947249.1	Type1.BREX	<-TraK<-TraE<-TraM<-TraM<-DUF3262<-?||XIS-HTH-> [illegible]	1188	Bacteria>Pseudomonadota>Gammaproteobacteria>Pseudomonadales>Pseudomonadaceae>Pseudomonas	GCF_900100495.1	Pseudomonas_benzenivorans	BLS63_RS10310	86902..90468	556533	BREX-system-P-loop-protein-BrxC-[Pseudomonas-benzenivorans]
WP_034038617.1	Type6.BREX	MerR-HTH->tRNA->BrxE->BrxB-BrxF-iSTAND->DUF6079 [illegible]	1213	Bacteria>Pseudomonadota>Gammaproteobacteria>Pseudomonadales>Pseudomonadaceae>Pseudomonas	GCF_900147165.1	Pseudomonas_aeruginosa	K3Z85_RS02500	4652..8293	287	BREX-system-P-loop-protein-BrxC-[Pseudomonas-aeruginosa]
WP_076583447.1	Type1.BREX	DUF4969-><-SIGMA+OB-Fold+AAA-ATPase+HKD-DNase [illegible]	1184	Bacteria>Pseudomonadota>Gammaproteobacteria>Pseudomonadales>Pseudomonadaceae>Pseudomonas	GCF_900156545.1	Pseudomonas_alcaligenes	BW984_RS17050	273058..276612	43263	BREX-system-P-loop-protein-BrxC-[Pseudomonas-alcaligenes]
WP_031684509.1	Type1.BREX	PhdYeFM_antitox->?-><-wHTH+WYL+WCX||?->Peptidase_ [illegible]	1184	Bacteria>Pseudomonadota>Gammaproteobacteria>Pseudomonadales>Pseudomonadaceae>Pseudomonas	GCF_900708485.1	Pseudomonas_aeruginosa	ID019_RS13300	2848972..2852526	287	BREX-system-P-loop-protein-BrxC-[Pseudomonas-aeruginosa]
WP_150756947.1	Type6.BREX	wHTH+WYL+WCX-><-?<-Phage_AlpA<-?<-?||tRNA->BrxE-> [illegible]	1210	Bacteria>Pseudomonadota>Gammaproteobacteria>Pseudomonadales>Pseudomonadaceae>Pseudomonas	GCF_902498135.1	Pseudomonas_fluorescens	F8N72_RS03340	145208..148840	294	BREX-system-P-loop-protein-BrxC-[Pseudomonas-fluorescens]
WP_151213669.1	Type6.BREX	SIR2->?->?-><-?<-TUDOR+SF1-SF2-Helicase+iREase+CTD [illegible]	1210	Bacteria>Pseudomonadota>Gammaproteobacteria>Pseudomonadales>Pseudomonadaceae>Pseudomonas	GCF_911283405.1	Pseudomonas_fluorescens	F9263_RS07600	503497..507129	294	BREX-system-P-loop-protein-BrxC-[Pseudomonas-fluorescens]
WP_033944490.1	Type1.BREX	Arm-DNA-bind_3+Phage_integrase-><- [illegible]	1184	Bacteria>Pseudomonadota>Gammaproteobacteria>Pseudomonadales>Pseudomonadaceae>Pseudomonas	GCF_947387505.1	Pseudomonas_aeruginosa	GPV34_RS03115	17071..20625	287	BREX-system-P-loop-protein-BrxC-[Pseudomonas-aeruginosa]
WP_095027056.1	Type6.BREX	<-LZ_Tnp_IS66+zf-IS66+DDE_Tnp_IS66+DDE_Tnp_IS66_C<- [illegible]	1213	Bacteria>Pseudomonadota>Gammaproteobacteria>Pseudomonadales>Pseudomonadaceae	-	Pseudomonas	JFT63_RS16080	178886..182527	286	MULTISPECIES:-BREX-system-P-loop-protein-BrxC-[Pseudomonas]
WP_121740032.1	Type6.BREX	<-Tox-RES<-XIS-HTH||HDRnase-><- [illegible]	1213	Bacteria>Pseudomonadota>Gammaproteobacteria>Pseudomonadales>Pseudomonadaceae>Pseudomonas	-	Pseudomonas_aeruginosa	D3M96_03425	754727..758368	287	MULTISPECIES:-BREX-system-P-loop-protein-BrxC-[Proteobacteria]
WP_150298006.1	Type6.BREX	<-Arm-DNA-bind_2+Phage_int_SAM_3+Phage_integrase|| [illegible]	1212	Bacteria>Pseudomonadota>Gammaproteobacteria>Pseudomonadales>Pseudomonadaceae>Pseudomonas	-	Pseudomonas_aeruginosa	F3G60_11255	510071..513709	287	BREX-system-P-loop-protein-BrxC-[Pseudomonas-aeruginosa]
EIU4198817.1	Type1.BREX	DUF932->?->?->?-><-SIGMA+OB-Fold+AAA-ATPase+ [illegible]	1180	Bacteria>Pseudomonadota>Gammaproteobacteria>Pseudomonadales>Pseudomonadaceae>Pseudomonas	-	Pseudomonas_aeruginosa	L4V49_002912	27945..31487	287	BREX-system-P-loop-protein-BrxC-[Pseudomonas-aeruginosa]
WP_046164318.1	Type1.BREX	<-SIGMA+OB-Fold+AAA-ATPase+HKD-DNase-BrxL<-PglZ [illegible]	1186	Bacteria>Pseudomonadota>Gammaproteobacteria>Pseudomonadales>Pseudomonadaceae>Pseudomonas	-	Pseudomonas_sp._10B238	RT21_RS21355	15645..19205	1586417	BREX-system-P-loop-protein-BrxC-[Pseudomonas-sp.-10B238]
WP_223843585.1	Type6.BREX	JAB-><-wHTH+WYL+WCX||?->?->BrxE->BrxA-DNA-binding [illegible]	858	Bacteria>Pseudomonadota>Gammaproteobacteria>Pseudomonadales>Pseudomonadaceae>Pseudomonas	-	Pseudomonas_sp._CFA	F7661_RS08255	1849794..1852370	2651048	BREX-system-P-loop-protein-BrxC-[Pseudomonas-sp.-CFA]
WP_223843584.1	Type6.BREX	JAB-><-wHTH+WYL+WCX||?->?->BrxE->BrxA-DNA-binding [illegible]	372	Bacteria>Pseudomonadota>Gammaproteobacteria>Pseudomonadales>Pseudomonadaceae>Pseudomonas	-	Pseudomonas_sp._CFA	F7661_RS27875	1848730..1849848	2651048	hypothetical-protein-[Pseudomonas-sp.-CFA]
EPL61008.1	Type1.BREX	<-Phage-HTH<-SIGMA+OB-Fold+AAA-ATPase+HKD-DNase [illegible]	1188	Bacteria>Pseudomonadota>Gammaproteobacteria>Pseudomonadales>Pseudomonadaceae>Stutzerimonas	GCA_000416345.1	Stutzerimonas_stutzeri_B1SMN1	B382_18428	65079..68645	1212549	hypothetical-protein-B382_18428-[Stutzerimonas-stutzeri-B1SMN1]
OWG36865.1	Type1.BREX	<-RNase<-?<-JetB<-?<-wHTH+WYL+WCX||XIS-HTH-> [illegible]	1185	Bacteria>Pseudomonadota>Gammaproteobacteria>Pseudomonadales>Pseudomonadaceae>Stutzerimonas	GCA_002193135.1	Stutzerimonas_stutzeri	CAQ69_18770	20186..23743	316	hypothetical-protein-CAQ69_18770-[Stutzerimonas-stutzeri]
PZR87477.1	Type1.BREX	RHH-><-TetR-HTH||MODE-HTH+PBPII->wHTH+WYL+WCX-> [illegible]	1179	Bacteria>Pseudomonadota>Gammaproteobacteria>Pseudomonadales>Pseudomonadaceae>Stutzerimonas	GCA_003243385.1	Stutzerimonas_stutzeri	DI537_26065	4408..7947	316	MAG:-BREX-system-P-loop-protein-BrxC-[Stutzerimonas-stutzeri]
MCQ2029875.1	Type1.BREX	<-sGTP<-sGTP<-sGTP<-wHTH+WYL+WCX||?-><-?||XIS-HTH- [illegible]	1186	Bacteria>Pseudomonadota>Gammaproteobacteria>Pseudomonadales>Pseudomonadaceae>Stutzerimonas	GCA_024397535.1	[Pseudomonas]_zhaodongensis	NA613_09780	17231..20791	1176257	BREX-system-P-loop-protein-BrxC-[[Pseudomonas]
WP_014819601.1	Type1.BREX	<-Phage-HTH<-SIGMA+OB-Fold+AAA-ATPase+HKD-DNase [illegible]	1188	Bacteria>Pseudomonadota>Gammaproteobacteria>Pseudomonadales>Pseudomonadaceae>Stutzerimonas	GCF_000267545.1	Stutzerimonas_stutzeri_subgroup	A458_RS28085	1395712..1399278	578833	MULTISPECIES:-BREX-system-P-loop-protein-BrxC-[Stutzerimonas-stutzeri-subgroup]
WP_023443891.1	Type6.BREX	<-SbcC<-JetB<-JetA||wHTH+WYL+WCX->BrxE-> [illegible]	1213	Bacteria>Pseudomonadota>Gammaproteobacteria>Pseudomonadales>Pseudomonadaceae>Stutzerimonas	GCF_000495915.1	Stutzerimonas_stutzeri_group	F753_RS26950	57885..61526	136846	MULTISPECIES:-BREX-system-P-loop-protein-BrxC-[Stutzerimonas-stutzeri-group]
WP_120997717.1	Type6.BREX	TrwC+SF1-SF2-Helicase->35exo->?-><-?<- [illegible]	1212	Bacteria>Pseudomonadota>Gammaproteobacteria>Pseudomonadales>Pseudomonadaceae>Stutzerimonas	GCF_003640395.1	[Pseudomonas]_urumqiensis	D7325_RS17370	36612..40250	638269	BREX-system-P-loop-protein-BrxC-[[Pseudomonas]
WP_125866641.1	Type6.BREX	JAB-><-SIGMA+OB-Fold+AAA-ATPase+HKD-DNase-BrxL<- [illegible]	1213	Bacteria>Pseudomonadota>Gammaproteobacteria>Pseudomonadales>Pseudomonadaceae>Stutzerimonas	GCF_003935625.1	Stutzerimonas_stutzeri	EGJ12_RS17010	223453..227094	316	BREX-system-P-loop-protein-BrxC-[Stutzerimonas-stutzeri]
WP_212739636.1	Type6.BREX	<-SbcC<-JetB<-JetA||wHTH+WYL+WCX->BrxE-> [illegible]	1207	Bacteria>Pseudomonadota>Gammaproteobacteria>Pseudomonadales>Pseudomonadaceae>Stutzerimonas	GCF_018316915.2	Stutzerimonas_balearica	D3P44_RS15315	3232724..3236347	74829	BREX-system-P-loop-protein-BrxC-[Stutzerimonas-balearica]
WP_255830664.1	Type1.BREX	<-sGTP<-sGTP<-sGTP<-wHTH+WYL+WCX||?-><-?||XIS-HTH- [illegible]	1186	Bacteria>Pseudomonadota>Gammaproteobacteria>Pseudomonadales>Pseudomonadaceae>Stutzerimonas	GCF_024397535.1	[Pseudomonas]_zhaodongensis	NA613_RS09805	17231..20791	1176257	BREX-system-P-loop-protein-BrxC-[[Pseudomonas]
WP_238586968.1	Type1.BREX	<-TraK<-TraE<-TraM<-TraM<-DUF3262<-?||XIS-HTH-> [illegible]	718	Bacteria>Pseudomonadota>Gammaproteobacteria>Pseudomonadales>Pseudomonadaceae>Stutzerimonas	-	Stutzerimonas_degradans	.	57411..59567	2968968	BREX-system-P-loop-protein-BrxC-[Stutzerimonas-degradans]
BBJ02099.1	Type1.BREX	<-TraK<-TraE<-TraM<-TraM<-DUF3262<-?||XIS-HTH-> [illegible]	718	Bacteria>Pseudomonadota>Gammaproteobacteria>Pseudomonadales>Pseudomonadaceae>Stutzerimonas	-	Stutzerimonas_degradans	.	57411..59567	2968968	uncharacterized-protein-sporadically-distributed-in-bacteria-and-archaea-[Stutzerimonas-degradans]
WP_020308058.1	Type1.BREX	<-Phage-HTH<-SIGMA+OB-Fold+AAA-ATPase+HKD-DNase [illegible]	1188	Bacteria>Pseudomonadota>Gammaproteobacteria>Pseudomonadales>Pseudomonadaceae>Stutzerimonas	-	Stutzerimonas_stutzeri	B382_18428	65079..68645	316	BREX-system-P-loop-protein-BrxC-[Pseudomonas-stutzeri]
WP_045633496.1	Type1.BREX	<-RNase<-?<-JetB<-?<-wHTH+WYL+WCX||XIS-HTH-> [illegible]	1185	Bacteria>Pseudomonadota>Gammaproteobacteria>Pseudomonadales>Pseudomonadaceae>Stutzerimonas	-	Stutzerimonas_stutzeri	CAQ69_18770	20186..23743	316	MULTISPECIES:-BREX-system-P-loop-protein-BrxC-[Gammaproteobacteria]
WP_041106841.1	Type6.BREX	<-SbcC<-JetB<-JetA||wHTH+WYL+WCX->BrxE-> [illegible]	1213	Bacteria>Pseudomonadota>Gammaproteobacteria>Pseudomonadales>Pseudomonadaceae>Stutzerimonas	-	Stutzerimonas_stutzeri	QX25_RS10015	148024..151665	316	BREX-system-P-loop-protein-BrxC-[Stutzerimonas-stutzeri]
WP_007146327.1	Type1.BREX	UbiA-><-HNH<-tRNA<-?<-?<-SIGMA+OB-Fold+AAA-ATPase+ [illegible]	1179	Bacteria>Pseudomonadota>Gammaproteobacteria>Thiotrichales>Piscirickettsiaceae>Methylophaga	GCF_000214595.1	Methylophaga_aminisulfidivorans	MAMP_RS12010	87136..90675	230105	BREX-system-P-loop-protein-BrxC-[Methylophaga-aminisulfidivorans]
WP_210225661.1	Type1.BREX	BrxA-DNA-binding->BrxB-BrxF-iSTAND->DUF6079*-> [illegible]	1173	Bacteria>Pseudomonadota>Gammaproteobacteria>Thiotrichales>Thiotrichaceae>Thiothrix	GCF_017901155.1	Candidatus_Thiothrix_anitrata	J8380_RS10820	2094889..2098410	2823902	BREX-system-P-loop-protein-BrxC-[Candidatus-Thiothrix-anitrata]

Protein-ID	System	Gene-Neighbourhoods	Length	Taxonomy	Assembly-ID	Strain	Locus-Tag	Genomic-coordinates	TaxID	Fasta-define
WP_210226002.1	Type6.BREX	<-PAS||?->?->BrxE->BrxA-DNA-binding->BrxB-BrxF-iSTAND->DUF6079*->N6-METHYLASE->OLD-ABC->TOPRIM->PglZ->SIGMA+OB-Fold+AAA-ATPase+HKD-DNase-BrxL-><-?<-?<-?<-SAM-METHYLASE	1210	Bacteria>Pseudomonadota>Gammaproteobacteria>Thiotrichales>Thiotrichaceae>Thiothrix	GCF_017901155.1	Candidatus_Thiothrix_anitrata	J8380_RS12830	2543544..2547176	2823902	BREX-system-P-loop-protein-BrxC-[Candidatus-Thiothrix-anitrata]
PCJ88241.1	Type1.BREX	<-RelE-ParE<-MetJarc-HTH||?-><-HKD-DNase+DUF3150<-SIGMA+OB-Fold+AAA-ATPase+HKD-DNase-BrxL<-PglZ<-N6-METHYLASE<-REase<-OLD-ABC<-DUF6079*<-BrxB-BrxF-iSTAND<-BrxA-DNA-binding<-XIS-HTH<-?<-?||RHH_5->?-><-HISKIN	1177	Bacteria>Pseudomonadota>Gammaproteobacteria>Thiotrichales>Thiotrichaceae>unclassified_Thiotrichaceae	GCA_002746365.1	Thiotrichaceae_bacterium	COA54_02955	15853..19386	2030882	hypothetical-protein-COA54_02955-[Thiotrichaceae-bacterium]
MBD3820859.1	Type1.BREX	wHTH+WYL+WCX-p->BrxA-DNA-binding->BrxB-BrxF-iSTAND->DUF6079*->N6-METHYLASE->HTH+REase->REase->PglZ->SIGMA+OB-Fold+AAA-ATPase+HKD-DNase-BrxL->	1191	Bacteria>Pseudomonadota>Gammaproteobacteria>Thiotrichales>unclassified_Thiotrichales	GCA_014764155.1	Thiotrichales_bacterium	IE914_01145	1632..5207	2026796	BREX-system-P-loop-protein-BrxC-[Thiotrichales-bacterium]
MBT3348501.1	Type1.BREX	<-SIGMA+OB-Fold+AAA-ATPase+HKD-DNase-BrxL<-PglZ<-DOC+HTH<-N6-METHYLASE-p<-HerA/FtsK<-SIR2<-Schlafen+HATPase+wHTH<-N6-METHYLASE<-DUF6079*<-BrxB-BrxF-iSTAND<-BrxA-DNA-binding	1170	Bacteria>Pseudomonadota>Gammaproteobacteria>Thiotrichales>unclassified_Thiotrichales	GCA_018648495.1	Thiotrichales_bacterium	HN398_08700	13875..17387	2026796	BREX-system-P-loop-protein-BrxC-[Thiotrichales-bacterium]
TAK52340.1	Type6.BREX	Phage_holin->Phage_lysozyme->N6-N4-METHYLASE-><-Cas4+SF1-SF2-Helicase<-SIGMA+OB-Fold+AAA-ATPase+HKD-DNase-BrxL<-PglZ<-N6-METHYLASE<-N6-METHYLASE<-DUF6079*<-BrxB-BrxF-iSTAND<-BrxA-DNA-binding<-BrxE<-wHTH+WYL+WCX||HNH->AbiEi_4->AbiEii->	1210	Bacteria>Pseudomonadota>Gammaproteobacteria>unclassified_Gammaproteobacteria	GCA_004297335.1	Gammaproteobacteria_bacterium	EP025_13990	58344..61976	1913989	BREX-system-P-loop-protein-BrxC-[Gammaproteobacteria-bacterium]
QFY90721.2	Type1.BREX	TRD+TRD->DOC+HTH->?->RNase->SF1-SF2-Helicase-><-rve_3||XIS-HTH->BrxA-DNA-binding->BrxB-BrxF-iSTAND->DUF6079*->?->REase->N6-METHYLASE->HTH+REase->PglZ->REase->SIGMA+OB-Fold+AAA-ATPase+HKD-DNase-BrxL->SbcC->Calcineurin+Calcineurin-SbcD->	1179	Bacteria>Pseudomonadota>Gammaproteobacteria>unclassified_Gammaproteobacteria	GCA_009497875.2	gamma_proteobacterium_SS-5	D5125_15330	2185771..2189310	947516	BREX-system-P-loop-protein-BrxC-[gamma-proteobacterium-SS-5]
NPU94904.1	Type6.BREX	<-DnaK||35exo-><-SIGMA+OB-Fold+AAA-ATPase+HKD-DNase-BrxL<-PglZ<-TOPRIM<-OLD-ABC<-N6-METHYLASE<-N6-METHYLASE<-GreA_GreB+SF1-SF2-Helicase<-DUF6079*<-BrxB-BrxF-iSTAND<-BrxA-DNA-binding<-BrxE<-wHTH+WYL+WCX||REase->rve->TnsC-ATPase->	1213	Bacteria>Pseudomonadota>Gammaproteobacteria>unclassified_Gammaproteobacteria	GCA_013178375.1	Gammaproteobacteria_bacterium	HPY82_23615	18761..22402	1913989	BREX-system-P-loop-protein-BrxC-[Gammaproteobacteria-bacterium]
NVK29385.1	Type1.BREX	MetJarc-HTH-><-SIGMA+OB-Fold+AAA-ATPase+HKD-DNase-BrxL<-PglZ<-N6-METHYLASE<-DUF6079*<-BrxB-BrxF-iSTAND<-BrxA-DNA-binding<-wHTH+WYL+WCX||?->SF1-SF2-Helicase+DUF1998-><-PIN<-MazE_antitoxin	1180	Bacteria>Pseudomonadota>Gammaproteobacteria>unclassified_Gammaproteobacteria	GCA_013373515.1	Gammaproteobacteria_bacterium	HWE20_00180	17762..21304	1913989	BREX-system-P-loop-protein-BrxC-[Gammaproteobacteria-bacterium]
MBT4196271.1	Type1.BREX	<-SIGMA+OB-Fold+AAA-ATPase+HKD-DNase-BrxL<-REase<-Immank+ANK+ANK<-PglZ<-N6-METHYLASE<-SLOG+BirA-HTH<-SF1-SF2-Helicase+PRTase<-DUF6079*<-BrxB-BrxF-iSTAND<-BrxA-DNA-binding<-HTH_26||?->?->REase->rve+Mu-transpos_C->TnsC-ATPase->ZnR->	1178	Bacteria>Pseudomonadota>Gammaproteobacteria>unclassified_Gammaproteobacteria	GCA_018651435.1	Gammaproteobacteria_bacterium	HOE12_18240	31044..34580	1913989	BREX-system-P-loop-protein-BrxC-[Gammaproteobacteria-bacterium]
MBU0810851.1	Type1.BREX	DUF4969-><-SIGMA+OB-Fold+AAA-ATPase+HKD-DNase-BrxL<-PglZ<-N6-METHYLASE-p<-RVT<-NERD<-OLD-ABC<-N6-METHYLASE<-DUF6079*<-BrxB-BrxF-iSTAND<-BrxA-DNA-binding<-XIS-HTH<-?||wHTH+WYL+WCX->?-><-?<-XIS-HTH<-tRNA<-MerR-HTH	1184	Bacteria>Pseudomonadota>Gammaproteobacteria>unclassified_Gammaproteobacteria	GCA_018815345.1	Gammaproteobacteria_bacterium	KKH69_04595	28957..32511	1913989	BREX-system-P-loop-protein-BrxC-[Gammaproteobacteria-bacterium]
PSU45803.1	Type1.BREX	HTH_8->XIS-HTH->BrxA-DNA-binding->BrxB-BrxF-iSTAND->DUF6079*->N6-METHYLASE->PglZ->SIGMA+OB-Fold+AAA-ATPase+HKD-DNase-BrxL-><-DDE_Tnp<-?||?->?-><-?<-LuxR-HTH	1185	Bacteria>Pseudomonadota>Gammaproteobacteria>Vibrionales>Vibrionaceae>Photobacterium	GCA_003025615.1	Photobacterium_frigidiphilum	C9J12_20970	5140..8697	264736	BREX-system-P-loop-protein-BrxC-[Photobacterium-frigidiphilum]
WP_006647488.1	Type1.BREX	HTH_Tnp_1+HTH_21+rve+rve_3-><-SIGMA+OB-Fold+AAA-ATPase+HKD-DNase-BrxL<-PglZ<-ParB+HNH<-N6-METHYLASE<-REase<-OLD-ABC<-HTH_Tnp_1+HTH_21+rve+rve_3<-OLD-ABC<-DUF6079*<-BrxB-BrxF-iSTAND<-BrxA-DNA-binding<-XIS-HTH<-?<-TnsC-ATPase||HTH_Tnp_1+HTH_21+rve+rve_3-><-rve+Mu-transpos_C<-REase+wHTH	1193	Bacteria>Pseudomonadota>Gammaproteobacteria>Vibrionales>Vibrionaceae>Photobacterium	GCF_000153325.1	Photobacterium_sp._SKA34	SKA34_RS21260	87534..91115	121723	BREX-system-P-loop-protein-BrxC-[Photobacterium-sp.-SKA34]
WP_058119139.1	Type6.BREX	<-McrC-NTD+REase+ZnR<-DnaB+McrB-AAA||HNH-><-SIGMA+OB-Fold+AAA-ATPase+HKD-DNase-BrxL<-PglZ<-N6-METHYLASE<-N6-METHYLASE<-DUF6079*<-BrxB-BrxF-iSTAND<-BrxA-DNA-binding<-BrxE<-XIS-HTH<-XIS-HTH||?->?->DUF2787->wHTH+WYL+WCX->	1208	Bacteria>Pseudomonadota>Gammaproteobacteria>Vibrionales>Vibrionaceae>Photobacterium	GCF_001455895.1	Photobacterium_kishitanii	AT442_RS02840	630344..633970	318456	BREX-system-P-loop-protein-BrxC-[Photobacterium-kishitanii]
WP_227876424.1	Type1.BREX	HTH_8->XIS-HTH->BrxA-DNA-binding->BrxB-BrxF-iSTAND->DUF6079*->SbcC->N6-METHYLASE->PglZ->SIGMA+OB-Fold+AAA-ATPase+HKD-DNase-BrxL-><-?||?-><-?<-LuxR-HTH	1187	Bacteria>Pseudomonadota>Gammaproteobacteria>Vibrionales>Vibrionaceae>Photobacterium	GCF_003025615.1	Photobacterium_frigidiphilum	C9J12_RS20995	5134..8697	264736	BREX-system-P-loop-protein-BrxC-[Photobacterium-frigidiphilum]
ASG04983.1	Type1.BREX	<-DeoR-HTH+ISOCOT<-DeoR-HTH+ISOCOT<-DUF2787<-?<-?||XIS-HTH->XIS-HTH->BrxA-DNA-binding->BrxB-BrxF-iSTAND->DUF6079*->OLD-ABC->REase->N6-METHYLASE->PglZ->SIGMA+OB-Fold+AAA-ATPase+HKD-DNase-BrxL-><-Arm-DNA-bind_3+Phage_integrase	1185	Bacteria>Pseudomonadota>Gammaproteobacteria>Vibrionales>Vibrionaceae>Vibrio	GCA_002211985.1	Vibrio_anguillarum	CEJ46_04105	862780..866337	55601	hypothetical-protein-CEJ46_04105-[Vibrio-anguillarum]
ASI98027.1	Type1.BREX	<-SIGMA+OB-Fold+AAA-ATPase+HKD-DNase-BrxL<-PglZ<-N6-METHYLASE<-NERD<-OLD-ABC<-DUF6079*<-BrxB-BrxF-iSTAND<-BrxA-DNA-binding<-XIS-HTH<-RNase<-DUF3584+DUF5401<-JetB<-MukF||wHTH+WYL+WCX->	1183	Bacteria>Pseudomonadota>Gammaproteobacteria>Vibrionales>Vibrionaceae>Vibrio	GCA_002214395.1	Vibrio_rotiferianus	BSZ04_17745	1918666..1922217	190895	hypothetical-protein-BSZ04_17745-[Vibrio-rotiferianus]
NAZ69809.1	Type1.BREX	<-SIGMA+OB-Fold+AAA-ATPase+HKD-DNase-BrxL<-PglZ<-N6-METHYLASE<-REase<-OLD-ABC<-DUF6079*<-BrxB-BrxF-iSTAND<-BrxA-DNA-binding<-XIS-HTH<-HNH<-?<-XIS-HTH||?->DDE_Tnp_1_5+DDE_Tnp_1->	1185	Bacteria>Pseudomonadota>Gammaproteobacteria>Vibrionales>Vibrionaceae>Vibrio	GCA_009906175.1	Vibrio_toranzoniae	GL180_08045	10464..14021	1194427	BREX-system-P-loop-protein-BrxC-[Vibrio-toranzoniae]
EGQ8448095.1	Type1.BREX	<-SIGMA+OB-Fold+AAA-ATPase+HKD-DNase-BrxL<-PglZ<-N6-METHYLASE<-REase<-OLD-ABC<-DUF6079*<-BrxB-BrxF-iSTAND<-BrxA-DNA-binding<-XIS-HTH<-HNH<-XIS-HTH||?->?->DUF2787->wHTH+WYL+WCX->	1185	Bacteria>Pseudomonadota>Gammaproteobacteria>Vibrionales>Vibrionaceae>Vibrio	GCA_015775815.1	Vibrio_alginolyticus	GTP59_14930	64166..67723	663	BREX-system-P-loop-protein-BrxC-[Vibrio-alginolyticus]
MBH9740666.1	Type1.BREX	SF1-SF2-Helicase+HTH->MukF->JetB->XIS-HTH->BrxA-DNA-binding->BrxB-BrxF-iSTAND->DUF6079*->OLD-ABC->REase->N6-METHYLASE->PglZ->SIGMA+OB-Fold+AAA-ATPase+HKD-DNase-BrxL->	1185	Bacteria>Pseudomonadota>Gammaproteobacteria>Vibrionales>Vibrionaceae>Vibrio	GCA_016094345.1	Vibrio_navarrensis	CJ419_13180	152917..156474	29495	hypothetical-protein-[Vibrio-navarrensis]
MBT0011870.1	Type1.BREX	<-SIGMA+OB-Fold+AAA-ATPase+HKD-DNase-BrxL<-PglZ<-N6-METHYLASE<-REase<-OLD-ABC<-DUF6079*<-BrxB-BrxF-iSTAND<-BrxA-DNA-binding<-XIS-HTH<-HNH<-XIS-HTH||?->?->DUF2787->wHTH+WYL+WCX->	1187	Bacteria>Pseudomonadota>Gammaproteobacteria>Vibrionales>Vibrionaceae>Vibrio	GCA_018457755.1	Vibrio_alginolyticus	N7I24_002738	64007..67570	663	BREX-system-P-loop-protein-BrxC-[Vibrio-alginolyticus]
EIZ110574.1	Type1.BREX	HTH_3->Couple_hipA+HipA_C->SAM-METHYLASE->HKD-DNase+SF1-SF2-Helicase+HTH+HTH+SRA-><-SIGMA+OB-Fold+AAA-ATPase+HKD-DNase-BrxL<-PglZ<-N6-METHYLASE<-REase<-OLD-ABC<-DUF6079*<-BrxB-BrxF-iSTAND<-BrxA-DNA-binding<-XIS-HTH<-RNase<-DUF3584+DUF5401<-JetB<-MukF||wHTH+WYL+WCX-><-TPR	1187	Bacteria>Pseudomonadota>Gammaproteobacteria>Vibrionales>Vibrionaceae>Vibrio	GCA_022609845.1	Vibrio_vulnificus	MOS09_002154	288384..291947	672	BREX-system-P-loop-protein-BrxC-[Vibrio-vulnificus]
GIB72257.1	Type6.BREX	<-LexA-protease||35exo->?->wHTH+WYL+WCX->wHTH+WYL+WCX-p->BrxE->BrxA-DNA-binding->BrxB-BrxF-iSTAND->DUF6079*->N6-METHYLASE->DOC+HTH->PglZ->SIGMA+OB-Fold+AAA-ATPase+HKD-DNase-BrxL-><-KAP-NTPase->HDRnase->VirD4-TraG+VirB4-FtsK->	1211	Bacteria>Pseudomonadota>Gammaproteobacteria>Vibrionales>Vibrionaceae>Vibrio	GCA_023164365.1	Vibrio_cholerae	VCSRD19_0643	268485..272120	666	ATPase-[Vibrio-cholerae]
HCG7393723.1	Type1.BREX	McrB-AAA->McrC-NTD+REase->?->HNH->?-><-HEPN||XIS-HTH->BrxA-DNA-binding->BrxB-BrxF-iSTAND->DUF6079*->OLD-ABC->REase->N6-METHYLASE->PglZ->SIGMA+OB-Fold+AAA-ATPase+HKD-DNase-BrxL->Phage_integrase-><-ParB<-GDResolvase+HIN-HTH||OLD-ABC->	1187	Bacteria>Pseudomonadota>Gammaproteobacteria>Vibrionales>Vibrionaceae>Vibrio	GCA_024044075.1	Vibrio_parahaemolyticus	NJ383_004129	13694..17257	670	BREX-system-P-loop-protein-BrxC-[Vibrio-parahaemolyticus]
UTZ28051.1	Type1.BREX	ABhydrolase-><-HTH_18+HTH_AraC<-?<-SIGMA+OB-Fold+AAA-ATPase+HKD-DNase-BrxL<-PglZ<-N6-METHYLASE<-NERD<-OLD-ABC<-DUF6079*<-BrxB-BrxF-iSTAND<-BrxA-DNA-binding<-XIS-HTH<-RNase<-JetB<-MukF||wHTH+WYL+WCX-><-tRNA<-?<-IONCHANNEL+CACHE+HISKIN+REC	1183	Bacteria>Pseudomonadota>Gammaproteobacteria>Vibrionales>Vibrionaceae>Vibrio	GCA_024442175.1	Vibrio_campbellii	HB761_02515	550346..553897	680	BREX-system-P-loop-protein-BrxC-[Vibrio-campbellii]
EJV5950193.1	Type1.BREX	<-SIGMA+OB-Fold+AAA-ATPase+HKD-DNase-BrxL<-PglZ<-N6-METHYLASE<-REase<-OLD-ABC<-DUF6079*<-BrxB-BrxF-iSTAND<-BrxA-DNA-binding<-XIS-HTH<-HNH<-XIS-HTH||?->?->DUF2787->wHTH+WYL+WCX->	1187	Bacteria>Pseudomonadota>Gammaproteobacteria>Vibrionales>Vibrionaceae>Vibrio	GCA_025320405.1	Vibrio_alginolyticus	N7I24_002738	64007..67570	663	BREX-system-P-loop-protein-BrxC-[Vibrio-alginolyticus]
GLT15886.1	Type1.BREX	<-wHTH+WYL+WCX||MukF->JetB->DUF3584+DUF5401->RNase->XIS-HTH->BrxA-DNA-binding->BrxB-BrxF-iSTAND->DUF6079*->OLD-ABC->REase->N6-METHYLASE->PglZ->SIGMA+OB-Fold+AAA-ATPase+HKD-DNase-BrxL->?->HTH_3->Couple_hipA+HipA_C->	1186	Bacteria>Pseudomonadota>Gammaproteobacteria>Vibrionales>Vibrionaceae>Vibrio	GCA_030161215.1	Vibrio_algivorus	GCM10007931_28610	496776..500336	1667024	hypothetical-protein-GCM10007931_28610-[Vibrio-algivorus]
WP_198300681.1	Type1.BREX	<-DeoR-HTH+ISOCOT<-DeoR-HTH+ISOCOT<-?<-?||XIS-HTH->XIS-HTH->BrxA-DNA-binding->BrxB-BrxF-iSTAND->DUF6079*->OLD-ABC->REase->N6-METHYLASE->PglZ->SIGMA+OB-Fold+AAA-ATPase+HKD-DNase-BrxL-><-Arm-DNA-bind_3+Phage_integrase	1187	Bacteria>Pseudomonadota>Gammaproteobacteria>Vibrionales>Vibrionaceae>Vibrio	GCF_002211985.1	Vibrio_anguillarum	CEJ46_RS04105	862774..866337	55601	BREX-system-P-loop-protein-BrxC-[Vibrio-anguillarum]
WP_197697128.1	Type1.BREX	<-SIGMA+OB-Fold+AAA-ATPase+HKD-DNase-BrxL<-PglZ<-N6-METHYLASE<-NERD<-OLD-ABC<-DUF6079*<-BrxB-BrxF-iSTAND<-BrxA-DNA-binding<-XIS-HTH<-RNase<-DUF3584+DUF5401<-JetB<-MukF||wHTH+WYL+WCX->	1185	Bacteria>Pseudomonadota>Gammaproteobacteria>Vibrionales>Vibrionaceae>Vibrio	GCF_002214395.1	Vibrio_rotiferianus	BSZ04_RS17965	1918666..1922223	190895	BREX-system-P-loop-protein-BrxC-[Vibrio-rotiferianus]
WP_089123599.1	Type1.BREX	<-Couple_hipA+HipA_C<-HTH_3<-?<-SIGMA+OB-Fold+AAA-ATPase+HKD-DNase-BrxL<-PglZ<-N6-METHYLASE<-REase<-OLD-ABC<-DUF6079*<-BrxB-BrxF-iSTAND<-BrxA-DNA-binding<-XIS-HTH<-RNase<-DUF3584+DUF5401<-JetB<-MukF||wHTH+WYL+WCX->	1184	Bacteria>Pseudomonadota>Gammaproteobacteria>Vibrionales>Vibrionaceae>Vibrio	GCF_002218005.2	Vibrio_algivorus	AVR_RS07075	1502603..1506157	1667024	BREX-system-P-loop-protein-BrxC-[Vibrio-algivorus]
WP_202601719.1	Type1.BREX	<-SIGMA+OB-Fold+AAA-ATPase+HKD-DNase-BrxL<-PglZ<-N6-METHYLASE<-REase<-OLD-ABC<-DUF6079*<-BrxB-BrxF-iSTAND<-BrxA-DNA-binding<-XIS-HTH<-HNH<-SF1-SF2-Helicase+HTH<-SF1-SF2-Helicase<-?<-XIS-HTH	1187	Bacteria>Pseudomonadota>Gammaproteobacteria>Vibrionales>Vibrionaceae>Vibrio	GCF_009906175.1	Vibrio_toranzoniae	GL180_RS08040	10464..14027	1194427	BREX-system-P-loop-protein-BrxC-[Vibrio-toranzoniae]
WP_198106729.1	Type1.BREX	SF1-SF2-Helicase+HTH->MukF->JetB->DUF3584+DUF5401->RNase->XIS-HTH->BrxA-DNA-binding->BrxB-BrxF-iSTAND->DUF6079*->OLD-ABC->REase->N6-METHYLASE->PglZ->SIGMA+OB-Fold+AAA-ATPase+HKD-DNase-BrxL->Phage_integrase->	1187	Bacteria>Pseudomonadota>Gammaproteobacteria>Vibrionales>Vibrionaceae>Vibrio	GCF_016094345.1	Vibrio_navarrensis	CJ419_RS12980	152911..156474	29495	BREX-system-P-loop-protein-BrxC-[Vibrio-navarrensis]
WP_210499744.1	Type1.BREX	NUDIX->SF1-SF2-Helicase+HTH->HNH->XIS-HTH->BrxA-DNA-binding->BrxB-BrxF-iSTAND->DUF6079*->OLD-ABC->NERD->N6-METHYLASE->PglZ->SIGMA+OB-Fold+AAA-ATPase+HKD-DNase-BrxL-><-wHTH+WYL+WCX||TOPRIM->JetC->	1185	Bacteria>Pseudomonadota>Gammaproteobacteria>Vibrionales>Vibrionaceae>Vibrio	GCF_017916775.1	Vibrio_crassostreae	KB594_RS01450	322447..326004	246167	BREX-system-P-loop-protein-BrxC-[Vibrio-crassostreae]
WP_250156418.1	Type1.BREX	<-wHTH+WYL+WCX<-DUF2787<-?<-?||XIS-HTH->HNH->XIS-HTH->BrxA-DNA-binding->BrxB-BrxF-iSTAND->DUF6079*->OLD-ABC->REase->N6-METHYLASE->PglZ->SIGMA+OB-Fold+AAA-ATPase+HKD-DNase-BrxL->	1185	Bacteria>Pseudomonadota>Gammaproteobacteria>Vibrionales>Vibrionaceae>Vibrio	GCF_018457845.1	Vibrio_alginolyticus	J4H60_RS12980	321648..325205	663	BREX-system-P-loop-protein-BrxC-[Vibrio-alginolyticus]
WP_228097414.1	Type6.BREX	ParB-><-?<-Phage_integrase<-HNH<-OLD-ABC<-REase<-SIGMA+OB-Fold+AAA-ATPase+HKD-DNase-BrxL<-PglZ<-N6-METHYLASE<-DUF6079*<-BrxB-BrxF-iSTAND<-BrxA-DNA-binding<-BrxE<-RNase<-DUF3584+DUF5401<-JetB<-MukF||wHTH+WYL+WCX-><-ANK+ANK	1208	Bacteria>Pseudomonadota>Gammaproteobacteria>Vibrionales>Vibrionaceae>Vibrio	GCF_020741135.1	Vibrio_parahaemolyticus	IB300_RS08390	552360..555986	670	BREX-system-P-loop-protein-BrxC-[Vibrio-parahaemolyticus]
WP_255937424.1	Type1.BREX	ABhydrolase-><-HTH_18+HTH_AraC<-?<-SIGMA+OB-Fold+AAA-ATPase+HKD-DNase-BrxL<-PglZ<-N6-METHYLASE<-NERD<-OLD-ABC<-DUF6079*<-BrxB-BrxF-iSTAND<-BrxA-DNA-binding<-XIS-HTH<-RNase<-?<-DUF3584+DUF5401<-JetB<-MukF||wHTH+WYL+WCX->	1185	Bacteria>Pseudomonadota>Gammaproteobacteria>Vibrionales>Vibrionaceae>Vibrio	GCF_024442175.1	Vibrio_campbellii	HB761_RS02525	550346..553903	680	BREX-system-P-loop-protein-BrxC-[Vibrio-campbellii]
WP_267363047.1	Type1.BREX	<-XIS-HTH<-Arm-DNA-bind_3+Phage_int_SAM_3+Phage_integrase||?->?->||?-><-HEPN||XIS-HTH->BrxA-DNA-binding->BrxB-BrxF-iSTAND->DUF6079*->OLD-ABC->REase->N6-METHYLASE->PglZ->SIGMA+OB-Fold+AAA-ATPase+HKD-DNase-BrxL->	1187	Bacteria>Pseudomonadota>Gammaproteobacteria>Vibrionales>Vibrionaceae>Vibrio	GCF_026537625.1	Vibrio_parahaemolyticus	NOL37_RS24175	9284..12847	670	BREX-system-P-loop-protein-BrxC-[Vibrio-parahaemolyticus]
WP_268670710.1	Type1.BREX	MODE-HTH+PBPII-><-?||XIS-HTH->?->SF1-SF2-Helicase+HTH->HNH->XIS-HTH->BrxA-DNA-binding->BrxB-BrxF-iSTAND->DUF6079*->OLD-ABC->REase->N6-METHYLASE->PglZ->SIGMA+OB-Fold+AAA-ATPase+HKD-DNase-BrxL-><-PALP<-?<-MODE-HTH+PBPII	1185	Bacteria>Pseudomonadota>Gammaproteobacteria>Vibrionales>Vibrionaceae>Vibrio	GCF_026798035.1	Vibrio_coralliirubri	OTK49_RS06670	229636..233193	1516159	BREX-system-P-loop-protein-BrxC-[Vibrio-coralliirubri]
WP_274674022.1	Type1.BREX	<-HAMP+GGDEF<-?<-SIGMA+OB-Fold+AAA-ATPase+HKD-DNase-BrxL<-PglZ<-N6-METHYLASE<-REase<-OLD-ABC<-DUF6079*<-BrxB-BrxF-iSTAND<-BrxA-DNA-binding<-XIS-HTH	1187	Bacteria>Pseudomonadota>Gammaproteobacteria>Vibrionales>Vibrionaceae>Vibrio	GCF_028857845.1	Vibrio_aestuarianus	L9W73_RS08980	42250..45813	28171	BREX-system-P-loop-protein-BrxC-[Vibrio-aestuarianus]
WP_213868350.1	Type1.BREX	<-SIGMA+OB-Fold+AAA-ATPase+HKD-DNase-BrxL<-PglZ<-N6-METHYLASE<-REase<-OLD-ABC<-DUF6079*<-BrxB-BrxF-iSTAND<-BrxA-DNA-binding<-XIS-HTH<-HNH<-XIS-HTH||?->?->DUF2787->wHTH+WYL+WCX->	1187	Bacteria>Pseudomonadota>Gammaproteobacteria>Vibrionales>Vibrionaceae>Vibrio	-	Vibrio_alginolyticus	N7I24_002738	64007..67570	663	BREX-system-P-loop-protein-BrxC-[Vibrio-alginolyticus]
WP_017462916.1	Type6.BREX	<-SbcC<-JetB<-JetA||wHTH+WYL+WCX->BrxE->BrxA-DNA-binding->BrxB-BrxF-iSTAND->DUF6079*->GreA_GreB+SF1-SF2-Helicase->N6-METHYLASE->N6-METHYLASE->ParB+HNH->?->PglZ->SIGMA+OB-Fold+AAA-ATPase+HKD-DNase-BrxL-><-JAB	1213	Bacteria>Pseudomonadota>Gammaproteobacteria>Xanthomonadales>Rhodanobacteraceae>Dyella	GCF_000334915.1	Dyella_ginsengisoli	C693_RS0114050	37588..41229	363848	BREX-system-P-loop-protein-BrxC-[Dyella-ginsengisoli]
WP_049622247.1	Type6.BREX	<-SbcC<-JetB<-JetA||wHTH+WYL+WCX->BrxE->BrxA-DNA-binding->BrxB-BrxF-iSTAND->DUF6079*->N6-METHYLASE->PglZ->SIGMA+OB-Fold+AAA-ATPase+HKD-DNase-BrxL->DEDD_Tnp_IS110+Transposase_20->DUF772+DDE_Tnp_1->	1210	Bacteria>Pseudomonadota>Gammaproteobacteria>Xanthomonadales>Rhodanobacteraceae>Frateuria	GCF_001182895.1	Frateuria_defendens	ACH65_RS08940	10628..14260	2219559	BREX-system-P-loop-protein-BrxC-[Frateuria-defendens]
WP_024958028.1	Type6.BREX	PIN-><-?<-SbcC<-JetB<-JetA||wHTH+WYL+WCX->BrxE->BrxA-DNA-binding->BrxB-BrxF-iSTAND->DUF6079*->N6-METHYLASE->N6-METHYLASE->DUF4326->DUF488-1->DUF488-s->PglZ->SIGMA+OB-Fold+AAA-ATPase+HKD-DNase-BrxL-><-Phage_lysozyme<-Phage_holin	1210	Bacteria>Pseudomonadota>Gammaproteobacteria>Xanthomonadales>Xanthomonadaceae	GCF_001591205.1	Stenotrophomonas	SMA02S_RS05105	16595..20227	40323	MULTISPECIES:-BREX-system-P-loop-protein-BrxC-[Stenotrophomonas]
WP_070426722.1	Type6.BREX	METHYLASE->RNaseH->35exo-><-?||tRNA->wHTH+WYL+WCX->BrxE->BrxA-DNA-binding->BrxB-BrxF-iSTAND->DUF6079*->SF1-SF2-Helicase+RNase+RNase->TPR->N6-METHYLASE->N6-METHYLASE-p->ParB+HNH->PglZ->SIGMA+OB-Fold+AAA-ATPase+HKD-DNase-BrxL->DUF932->	1209	Bacteria>Pseudomonadota>Gammaproteobacteria>Xanthomonadales>Xanthomonadaceae	GCF_900766255.1	Stenotrophomonas	BIZ42_RS13545	3079736..3083365	40323	MULTISPECIES:-BREX-system-P-loop-protein-BrxC-[Stenotrophomonas]
WP_194339884.1	Type1.BREX	35exo->REase-><-SIGMA+OB-Fold+AAA-ATPase+HKD-DNase-BrxL<-PglZ<-N6-METHYLASE-p<-NERD<-OLD-ABC<-N6-METHYLASE-p<-?<-DUF6079*<-BrxB-BrxF-iSTAND<-BrxA-DNA-binding<-XIS-HTH<-?<-SbcC+SbcC<-Calcineurin+Calcineurin-SbcD	1190	Bacteria>Pseudomonadota>Gammaproteobacteria>Xanthomonadales>Xanthomonadaceae>Lysobacter	GCF_015277535.1	Lysobacter_sp._H21R4	INQ40_RS07385	1620041..1623613	2781021	BREX-system-P-loop-protein-BrxC-[Lysobacter-sp.-H21R4]
WP_162203842.1	Type1.BREX	AbiEi_4->Phage_lysozyme-><-SIGMA+OB-Fold+AAA-ATPase+HKD-DNase-BrxL<-PglZ<-?<-N6-METHYLASE<-N6-METHYLASE<-GreA_GreB+SF1-SF2-Helicase<-DUF6079*<-BrxB-BrxF-iSTAND<-BrxA-DNA-binding<-BrxE<-wHTH+WYL+WCX	1213	Bacteria>Pseudomonadota>Gammaproteobacteria>Xanthomonadales>Xanthomonadaceae>Pseudoxanthomonas	GCA_010093235.1	Pseudoxanthomonas_suwonensis	CSC68_RS12285	54892..58533	314722	BREX-system-P-loop-protein-BrxC-[Pseudoxanthomonas-suwonensis]
MBH1753233.1	Type6.BREX	<-DUF932<-SIGMA+OB-Fold+AAA-ATPase+HKD-DNase-BrxL<-PglZ<-ParB+HNH<-N6-METHYLASE-p<-N6-METHYLASE<-TPR<-SF1-SF2-Helicase+RNase+RNase<-DUF6079*<-BrxB-BrxF-iSTAND<-BrxA-DNA-binding<-BrxE<-wHTH+WYL+WCX<-tRNA||?-><-35exo<-RNaseH<-METHYLASE	1209	Bacteria>Pseudomonadota>Gammaproteobacteria>Xanthomonadales>Xanthomonadaceae>Stenotrophomonas	GCA_015998385.1	Stenotrophomonas_maltophilia	I5V37_01935	25775..29404	40324	BREX-system-P-loop-protein-BrxC-[Stenotrophomonas-maltophilia]
MBN5094965.1	Type6.BREX	<-DUF932<-SIGMA+OB-Fold+AAA-ATPase+HKD-DNase-BrxL<-PglZ<-N6-METHYLASE<-GreA_GreB+SF1-SF2-Helicase<-DUF6079*<-BrxB-BrxF-iSTAND<-BrxA-DNA-binding<-BrxE<-wHTH+WYL+WCX<-tRNA||?-><-35exo<-RNaseH<-METHYLASE	1213	Bacteria>Pseudomonadota>Gammaproteobacteria>Xanthomonadales>Xanthomonadaceae>Stenotrophomonas	GCA_017150075.1	Stenotrophomonas_maltophilia	JY450_00935	90079..93720	40324	BREX-system-P-loop-protein-BrxC-[Stenotrophomonas-maltophilia]
MBN5083090.1	Type6.BREX	<-DUF932<-SIGMA+OB-Fold+AAA-ATPase+HKD-DNase-BrxL<-PglZ<-ParB+HNH<-N6-METHYLASE<-PIN<-OLD-ABC<-DUF6079*<-BrxB-BrxF-iSTAND<-BrxA-DNA-binding<-BrxE<-wHTH+WYL+WCX<-Arm-DNA-bind_3+Phage_integrase<-tRNA<-SIGMA-HTH+SIGMA-HTH+SIGMA-HTH	1207	Bacteria>Pseudomonadota>Gammaproteobacteria>Xanthomonadales>Xanthomonadaceae>Stenotrophomonas	GCA_017150495.1	Stenotrophomonas_maltophilia	JY467_05250	61681..65304	40324	BREX-system-P-loop-protein-BrxC-[Stenotrophomonas-maltophilia]
WP_004146422.1	Type6.BREX	<-XIS-HTH||?->Phage_integrase->tRNA->Arm-DNA-bind_3+Phage_integrase->wHTH+WYL+WCX->BrxE->BrxA-DNA-binding->BrxB-BrxF-iSTAND->DUF6079*->OLD-ABC->PIN->N6-METHYLASE->N6-METHYLASE->ParB+HNH->PglZ->SIGMA+OB-Fold+AAA-ATPase+HKD-DNase-BrxL->DUF932->	1207	Bacteria>Pseudomonadota>Gammaproteobacteria>Xanthomonadales>Xanthomonadaceae>Stenotrophomonas	GCF_000355725.1	Stenotrophomonas_maltophilia	G5A34_RS07915	81973..85596	40324	BREX-system-P-loop-protein-BrxC-[Stenotrophomonas-maltophilia]
WP_088610961.1	Type6.BREX	<-DUF932<-SIGMA+OB-Fold+AAA-ATPase+HKD-DNase-BrxL<-PglZ<-N6-METHYLASE-p<-N6-METHYLASE<-REase+AIPR<-DUF6079*<-BrxB-BrxF-iSTAND<-BrxA-DNA-binding<-BrxE<-wHTH+WYL+WCX<-tRNA||?-><-35exo<-RNaseH<-METHYLASE	1209	Bacteria>Pseudomonadota>Gammaproteobacteria>Xanthomonadales>Xanthomonadaceae>Stenotrophomonas	GCF_002208885.2	Stenotrophomonas_maltophilia	CEQ03_RS09960	2076489..2080118	40324	BREX-system-P-loop-protein-BrxC-[Stenotrophomonas-maltophilia]
WP_099491466.1	Type6.BREX	METHYLASE->RNaseH->35exo-><-?||tRNA->wHTH+WYL+WCX->BrxE->BrxA-DNA-binding->BrxB-BrxF-iSTAND->DUF6079*->SF1-SF2-Helicase+RNase+RNase->TPR->N6-METHYLASE->N6-METHYLASE-p->ParB+HNH->PglZ->SIGMA+OB-Fold+AAA-ATPase+HKD-DNase-BrxL->DUF932->	1209	Bacteria>Pseudomonadota>Gammaproteobacteria>Xanthomonadales>Xanthomonadaceae>Stenotrophomonas	GCF_002739275.1	Stenotrophomonas_maltophilia	BRB91_RS07170	53995..57624	40324	BREX-system-P-loop-protein-BrxC-[Stenotrophomonas-maltophilia]
WP_099562737.1	Type6.BREX	METHYLASE->RNaseH->35exo-><-?||tRNA->wHTH+WYL+WCX->BrxE->BrxA-DNA-binding->BrxB-BrxF-iSTAND->DUF6079*->REase+AIPR->N6-METHYLASE->N6-METHYLASE-p->PglZ->SIGMA+OB-Fold+AAA-ATPase+HKD-DNase-BrxL->DUF932->	1209	Bacteria>Pseudomonadota>Gammaproteobacteria>Xanthomonadales>Xanthomonadaceae>Stenotrophomonas	GCF_002740395.1	Stenotrophomonas_maltophilia	BRC18_RS18230	18705..22334	40324	BREX-system-P-loop-protein-BrxC-[Stenotrophomonas-maltophilia]
WP_180875343.1	Type6.BREX	METHYLASE->RNaseH->35exo-><-?||tRNA->wHTH+WYL+WCX->BrxE->BrxA-DNA-binding->BrxB-BrxF-iSTAND->DUF6079*->SF1-SF2-Helicase+RNase+RNase->TPR->N6-METHYLASE->N6-METHYLASE-p->ParB+HNH->PglZ->SIGMA+OB-Fold+AAA-ATPase+HKD-DNase-BrxL->DUF932->	1209	Bacteria>Pseudomonadota>Gammaproteobacteria>Xanthomonadales>Xanthomonadaceae>Stenotrophomonas	GCF_013463485.1	Stenotrophomonas_maltophilia	D7U74_RS02160	10322..13951	40324	BREX-system-P-loop-protein-BrxC-[Stenotrophomonas-maltophilia]
WP_049450109.1	Type6.BREX	<-DUF932<-SIGMA+OB-Fold+AAA-ATPase+HKD-DNase-BrxL<-PglZ<-ParB+HNH<-N6-METHYLASE-p<-N6-METHYLASE<-TPR<-SF1-SF2-Helicase+RNase+RNase<-DUF6079*<-BrxB-BrxF-iSTAND<-BrxA-DNA-binding<-BrxE<-wHTH+WYL+WCX<-tRNA||?-><-35exo<-RNaseH<-METHYLASE	1209	Bacteria>Pseudomonadota>Gammaproteobacteria>Xanthomonadales>Xanthomonadaceae>Stenotrophomonas	GCF_030549525.1	Stenotrophomonas_maltophilia	MT454_RS13320	40759..44388	40324	BREX-system-P-loop-protein-BrxC-[Stenotrophomonas-maltophilia]
WP_111722876.1	Type6.BREX	METHYLASE->RNaseH->35exo-><-?||tRNA->wHTH+WYL+WCX->BrxE->BrxA-DNA-binding->BrxB-BrxF-iSTAND->DUF6079*->SF1-SF2-Helicase+RNase+RNase->TPR->N6-METHYLASE->N6-METHYLASE-p->ParB+HNH->PglZ->SIGMA+OB-Fold+AAA-ATPase+HKD-DNase-BrxL->DUF932->	1209	Bacteria>Pseudomonadota>Gammaproteobacteria>Xanthomonadales>Xanthomonadaceae>Stenotrophomonas	GCF_900475685.1	Stenotrophomonas_maltophilia	DQN96_RS04535	962467..966096	40324	BREX-system-P-loop-protein-BrxC-[Stenotrophomonas-maltophilia]
WP_225763940.1	Type1.BREX	<-TRPR-HTH||?->PRTase->HTH_Tnp_1+HTH_21+rve+rve_3->XIS-HTH->BrxA-DNA-binding->BrxB-BrxF-iSTAND->DUF6079*->?->N6-METHYLASE->OLD-ABC->REase->?->N6-METHYLASE-p->PglZ->SIGMA+OB-Fold+AAA-ATPase+HKD-DNase-BrxL->	1181	Bacteria>Pseudomonadota>Gammaproteobacteria>Xanthomonadales>Xanthomonadaceae>Stenotrophomonas	GCF_916618915.1	Stenotrophomonas_sp._Marseille-Q4652	LG380_RS05370	1171221..1174766	2866595	BREX-system-P-loop-protein-BrxC-[Stenotrophomonas-sp.-Marseille-Q4652]
WP_180834305.1	Type6.BREX	<-DUF932<-SIGMA+OB-Fold+AAA-ATPase+HKD-DNase-BrxL<-PglZ<-N6-METHYLASE-p<-N6-METHYLASE<-REase+AIPR<-DUF6079*<-BrxB-BrxF-iSTAND<-BrxA-DNA-binding<-BrxE<-wHTH+WYL+WCX<-Arm-DNA-bind_3+Phage_integrase<-tRNA<-?<-SIGMA-HTH+SIGMA-HTH+SIGMA-HTH	1209	Bacteria>Pseudomonadota>Gammaproteobacteria>Xanthomonadales>Xanthomonadaceae>Stenotrophomonas	-	Stenotrophomonas_maltophilia	D7U93_RS19145	16028..19657	40324	BREX-system-P-loop-protein-BrxC-[Stenotrophomonas-maltophilia]
WP_262214630.1	Type6.BREX	METHYLASE->RNaseH->35exo-><-?||tRNA->wHTH+WYL+WCX->BrxE->BrxA-DNA-binding->BrxB-BrxF-iSTAND->DUF6079*->GreA_GreB+SF1-SF2-Helicase->N6-METHYLASE->N6-METHYLASE->ParB+HNH->PglZ->SIGMA+OB-Fold+AAA-ATPase+HKD-DNase-BrxL->DUF932->?-><-HTH_3	1213	Bacteria>Pseudomonadota>Gammaproteobacteria>Xanthomonadales>Xanthomonadaceae>Stenotrophomonas	-	Stenotrophomonas_maltophilia	JAK45_RS19240	75512..79153	40324	BREX-system-P-loop-protein-BrxC-[Stenotrophomonas-maltophilia]
VUJ46804.1	Type6.BREX	<-LexA-protease||?-><-Phage_integrase||Arm-DNA-bind_3+Phage_integrase->wHTH+WYL+WCX->BrxE->BrxA-DNA-binding->BrxB-BrxF-iSTAND->DUF6079*->OLD-ABC->PIN->N6-METHYLASE->N6-METHYLASE->ParB+HNH->PglZ->SIGMA+OB-Fold+AAA-ATPase+HKD-DNase-BrxL->DUF932->	1207	Bacteria>Pseudomonadota>Gammaproteobacteria>Xanthomonadales>Xanthomonadaceae>Stenotrophomonas	-	Stenotrophomonas_maltophilia	N5D45_RS14200	24687..28310	40324	hypothetical-protein-NGKLMNPD_00401-[Stenotrophomonas-maltophilia]
MBS3958360.1	Type6.BREX	wHTH+WYL+WCX->BrxE->BrxA-DNA-binding->BrxB-BrxF-iSTAND->DUF6079*->GreA_GreB+SF1-SF2-Helicase->N6-METHYLASE->N6-METHYLASE-p->DUF4326->DUF488-1->DUF488-s->PglZ->SIGMA+OB-Fold+AAA-ATPase+HKD-DNase-BrxL-><-Phage_lysozyme	1213	Bacteria>Pseudomonadota>Gammaproteobacteria>Xanthomonadales>Xanthomonadaceae>unclassified_Xanthomonadaceae	GCA_018333835.1	Xanthomonadaceae_bacterium	KGZ52_03015	3241..6882	1926873	BREX-system-P-loop-protein-BrxC-[Xanthomonadaceae-bacterium]
WP_218196661.1	Type1.BREX	Arm-DNA-bind_3+Phage_integrase-><-SIGMA+OB-Fold+AAA-ATPase+HKD-DNase-BrxL<-PglZ<-N6-METHYLASE-p<-?<-REase<-OLD-ABC<-N6-METHYLASE<-?<-DUF6079*<-BrxB-BrxF-iSTAND<-BrxA-DNA-binding<-XIS-HTH<-Phage_int_SAM_2+Integrase_1<-Phage_rep	1182	Bacteria>Pseudomonadota>Gammaproteobacteria>Xanthomonadales>Xanthomonadaceae>Xanthomonas	GCF_019193005.1	Xanthomonas_euvesicatoria	KV691_RS15985	182541..186089	456327	BREX-system-P-loop-protein-BrxC-[Xanthomonas-euvesicatoria]
WP_069709528.1	Type1.BREX	VirB8->VirB9->VirB10->RHH-><-TetR-HTH||MODE-HTH+PBPII->wHTH+WYL+WCX->BrxA-DNA-binding->BrxB-BrxF-iSTAND->DUF6079*->N6-METHYLASE->PglZ->SIGMA+OB-Fold+AAA-ATPase+HKD-DNase-BrxL->SF1-SF2-Helicase+REase->SF1-SF2-Helicase+REase->wHTH->?-><-AAA-ATPase<-HIN-HTH	1179	Bacteria>Pseudomonadota	GCF_001742225.1	Alphaproteobacteria	BES08_RS24075	1524283..1527822	28211	MULTISPECIES:-BREX-system-P-loop-protein-BrxC-[Alphaproteobacteria]
WP_066700609.1	Type1.BREX	VirB8->VirB9->VirB10->RHH-><-TetR-HTH||MODE-HTH+PBPII->wHTH+WYL+WCX->BrxA-DNA-binding->BrxB-BrxF-iSTAND->DUF6079*->N6-METHYLASE->PglZ->SIGMA+OB-Fold+AAA-ATPase+HKD-DNase-BrxL->SF1-SF2-Helicase+REase->SF1-SF2-Helicase+REase->wHTH->HKD-DNase+HKD-DNase->SF1-SF2-Helicase->ABC-ATPase+SbcC->	1179	Bacteria>Pseudomonadota	GCF_003967075.1	Alphaproteobacteria	SAMIE_RS17570	3625371..3628910	28211	MULTISPECIES:-BREX-system-P-loop-protein-BrxC-[Alphaproteobacteria]
WP_142804161.1	Type6.BREX	TPR+TPR-><-?||BrxE->BrxA-DNA-binding->BrxB-BrxF-iSTAND->DUF6079*->N6-METHYLASE->AbrB-HTH->PIN->N6-METHYLASE->DOC+HTH->PglZ->SIGMA+OB-Fold+AAA-ATPase+HKD-DNase-BrxL->	1210	Bacteria>Pseudomonadota>Hydrogenophilia>Hydrogenophilales>Hydrogenophilaceae>Tepidiphilus	GCF_006980785.1	Tepidiphilus_sp._J10	EPD81_RS04405	897239..900871	2502185	BREX-system-P-loop-protein-BrxC-[Tepidiphilus-sp.-J10]
WP_169116165.1	Type6.BREX	Glyoxalase->?->?->?->?->?-><-?||BrxE->BrxB-BrxF-iSTAND->DUF6079*->N6-METHYLASE->AbrB-HTH->PIN->N6-METHYLASE->DOC+HTH->PglZ->SIGMA+OB-Fold+AAA-ATPase+HKD-DNase-BrxL->	1210	Bacteria>Pseudomonadota>Hydrogenophilia>Hydrogenophilales>Hydrogenophilaceae>Tepidiphilus	GCF_012911495.1	Tepidiphilus_baoligensis	GV368_RS08140	10579..14211	2698687	BREX-system-P-loop-protein-BrxC-[Tepidiphilus-baoligensis]
MBU0673651.1	Type1.BREX	RADICAL-SAM-><-?||?->BrxA-DNA-binding->BrxB-BrxF-iSTAND->DUF6079*->Schlafen+HATPase+wHTH->?->N6-METHYLASE->REase->ParB+HNH->OLD-ABC->?->PglZ->SIGMA+OB-Fold+AAA-ATPase+HKD-DNase-BrxL->	1167	Bacteria>Pseudomonadota>unclassified_Pseudomonadota	GCA_018814305.1	Pseudomonadota_bacterium	KJ950_03300	48865..52368	1977087	BREX-system-P-loop-protein-BrxC-[Pseudomonadota-bacterium]
MBU0973505.1	Type6.BREX	BrxE->BrxA-DNA-binding->BrxB-BrxF-iSTAND->DUF6079*->N6-METHYLASE->N6-METHYLASE->DOC+HTH->PglZ->SIGMA+OB-Fold+AAA-ATPase+HKD-DNase-BrxL-><-?<-?<-?<-DnaB	1209	Bacteria>Pseudomonadota>unclassified_Pseudomonadota	GCA_018816605.1	Pseudomonadota_bacterium	KKC20_22890	2683..6312	1977087	BREX-system-P-loop-protein-BrxC-[Pseudomonadota-bacterium]
MBU4294441.1	Type1.BREX	<-HAMP+MA+MA<-?<-HPT+HISKIN+CheW+REC<-?<-?<-?||XIS-HTH->BrxA-DNA-binding->BrxB-BrxF-iSTAND->DUF6079*->N6-METHYLASE->Schlafen+HATPase+wHTH->PglZ->SIGMA+OB-Fold+AAA-ATPase+HKD-DNase-BrxL->	1176	Bacteria>Pseudomonadota>unclassified_Pseudomonadota	GCA_018897105.1	Pseudomonadota_bacterium	KJ862_00965	66976..70506	1977087	BREX-system-P-loop-protein-BrxC-[Pseudomonadota-bacterium]
WP_053322564.1	Type1.BREX	HNH->?->?->LON-AAA->Calcineurin->?->BrxA-DNA-binding->BrxB-BrxF-iSTAND->DUF6079*->N6-METHYLASE-p->Schlafen+HATPase+wHTH->HEPN->REase->N6-METHYLASE-p->HerA/FtsK->PglZ->SIGMA+OB-Fold+AAA-ATPase+HKD-DNase-BrxL->	1181	Bacteria>Pseudomonadota>Zetaproteobacteria>Mariprofundales>Mariprofundaceae>Mariprofundus	GCF_001273615.1	Mariprofundus_ferrooxydans	AL013_RS02410	88551..92096	314344	BREX-system-P-loop-protein-BrxC-[Mariprofundus-ferrooxydans]
PIX55556.1	Type6.BREX	BrxE->BrxA-DNA-binding->BrxB-BrxF-iSTAND->DUF6079*->N6-METHYLASE->ParB+HNH->?->PglZ->SIGMA+OB-Fold+AAA-ATPase+HKD-DNase-BrxL->ZNR+RmuC->XIS-HTH-><-HTH_26||Phage_AlpA->	1215	Bacteria>Pseudomonadota>Zetaproteobacteria>unclassified_Zetaproteobacteria	GCA_002782825.1	Zetaproteobacteria_bacterium_CG_4_10_14_3_um_filter_54_28	COZ50_02095	2235..5882	1974114	BREX-system-P-loop-protein-BrxC-[Zetaproteobacteria-bacterium-CG_4_10_14_3_um_filter_54_28]
MBQ6007751.1	Type1.BREX	<-SIGMA+OB-Fold+AAA-ATPase+HKD-DNase-BrxL<-PglZ<-?<-N6-METHYLASE<-DUF6079*<-BrxB-BrxF-iSTAND<-BrxA-DNA-binding	1146	Bacteria>PVC_group>Kiritimatiellota>Kiritimatiellia>unclassified_Kiritimatiellia	GCA_017427485.1	Kiritimatiellia_bacterium	IJL17_04385	19209..22649	2212473	BREX-system-P-loop-protein-BrxC-[Kiritimatiellia-bacterium]
MBQ7190494.1	Type1.BREX	BrxA-DNA-binding->BrxB-BrxF-iSTAND->DUF6079*->N6-METHYLASE->Y1_Tnp->PglZ->SIGMA+OB-Fold+AAA-ATPase+HKD-DNase-BrxL->?->?->?->?-><-ArsR-HTH	1172	Bacteria>PVC_group>Kiritimatiellota>Kiritimatiellia>unclassified_Kiritimatiellia	GCA_017450695.1	Kiritimatiellia_bacterium	IJR99_13895	2360..5878	2212473	BREX-system-P-loop-protein-BrxC-[Kiritimatiellia-bacterium]
MCF7790541.1	Type1.BREX	<-HEPN<-SIGMA+OB-Fold+AAA-ATPase+HKD-DNase-BrxL<-PglZ<-TOPRIM<-OLD-ABC<-?<-?<-?<-DUF6079*<-BrxB-BrxF-iSTAND<-BrxA-DNA-binding<-wHTH+WYL+WCX<-?||?-><-?<-?||S2P+PDZ->	1176	Bacteria>PVC_group>Lentisphaerota>Lentisphaeria>Victivallales>unclassified_Victivallales	GCA_021733985.1	Victivallales_bacterium	K9M56_00960	14578..18108	2212475	BREX-system-P-loop-protein-BrxC-[Victivallales-bacterium]
MCC7337722.1	Type1.BREX	SF1-SF2-Helicase+wHTH+Small-B-barrel+DUF1998->?->?->PIN->?->BrxA-DNA-binding->BrxB-BrxF-iSTAND->DUF6079*->?->N6-METHYLASE->REase+TPR+GreAB-C+PIN->MORC+DUF3684+REase->SF1-SF2-Helicase->?->PIN->PglZ->SIGMA+OB-Fold+AAA-ATPase+HKD-DNase-BrxL->	1171	Bacteria>PVC_group>Planctomycetota>Planctomycetia>Pirellulales>Pirellulaceae	GCA_020852965.1	Pirellulaceae_bacterium	IT422_21750	7905..11420	2699754	BREX-system-P-loop-protein-BrxC-[Pirellulaceae-bacterium]
WP_145350724.1	Type1.BREX	<-SIR2<-?<-SIGMA+OB-Fold+AAA-ATPase+HKD-DNase-BrxL<-PglZ<-REase+TPR+GreAB-C+PIN<-N6-METHYLASE<-Schlafen+HATPase+wHTH<-REase<-OLD-ABC<-DUF6079*<-BrxB-BrxF-iSTAND<-BrxA-DNA-binding<-?||TrwC+SF1-SF2-Helicase->?-><-?<-?<-ArdC+MPTase	1169	Bacteria>PVC_group>Planctomycetota>Planctomycetia>Pirellulales>Pirellulaceae	GCF_007741495.1	Roseimaritima_multifibrata	FF011L_RS05780	1542279..1545788	1930274	BREX-system-P-loop-protein-BrxC-[Roseimaritima-multifibrata]
WP_068129828.1	Type1.BREX	<-SIGMA+OB-Fold+AAA-ATPase+HKD-DNase-BrxL<-Four-helical-protein<-PglZ<-PIN<-HTH+MPTase<-N6-METHYLASE<-DUF6079*<-BrxB-BrxF-iSTAND<-BrxA-DNA-binding<-XIS-HTH<-URI-DNase||?-><-Vrr-REase	1169	Bacteria>PVC_group>Planctomycetota>Planctomycetia>Pirellulales>Pirellulaceae	GCF_008065135.1	Roseimaritima_ulvae	UC8_RS27055	7584147..7587656	980254	BREX-system-P-loop-protein-BrxC-[Roseimaritima-ulvae]
WP_184301662.1	Type1.BREX	<-nSTAND1+HNH<-URI-DNase+RAMA<-SIGMA+OB-Fold+AAA-ATPase+HKD-DNase-BrxL<-PglZ<-REase+TPR+GreAB-C+PIN<-N6-METHYLASE<-?<-ABC-ATPase<-DUF6079*<-BrxB-BrxF-iSTAND<-BrxA-DNA-binding<-XIS-HTH||OLD-ABC+TOPRIM->SF1-SF2-Helicase->	1169	Bacteria>PVC_group>Planctomycetota>Planctomycetia>Pirellulales>Pirellulaceae	GCF_014192335.1	Rhodopirellula_rubra	FHS27_RS03210	253910..257419	980271	BREX-system-P-loop-protein-BrxC-[Rhodopirellula-rubra]
HBJ35663.1	Type1.BREX	<-SIGMA+OB-Fold+AAA-ATPase+HKD-DNase-BrxL<-PglZ<-PIN<-HTH+MPTase||ZDNA-HTH-><-?<-Pkinase+CR-Kinase<-N6-METHYLASE<-Schlafen+HATPase+wHTH<-DUF6079*<-Four-helical-protein<-BrxB-BrxF-iSTAND<-BrxA-DNA-binding<-XIS-HTH	1188	Bacteria>PVC_group>Planctomycetota>Planctomycetia>Planctomycetales>Planctomycetaceae	GCA_003506095.1	Planctomycetaceae_bacterium	DDZ51_13145	16532..20098	2026779	BREX-system-P-loop-protein-BrxC-[Planctomycetaceae-bacterium]
MBW8002946.1	Type1.BREX	BrxA-DNA-binding->BrxB-BrxF-iSTAND->DUF6079*->N6-METHYLASE->Ntox45->HEPN->PglZ->SIGMA+OB-Fold+AAA-ATPase+HKD-DNase-BrxL-><-HEPN<-DDE_Tnp_ISAZ013<-DDE_Tnp_ISAZ013	1164	Bacteria>PVC_group>Planctomycetota>unclassified_Planctomycetota	GCA_019456255.1	Planctomycetota_bacterium	FVQ80_13155	4626..8120	2026780	BREX-system-P-loop-protein-BrxC-[Planctomycetota-bacterium]
RRJ96051.1	Type6.BREX	<-TrwC+SF1-SF2-Helicase||?-><-DNA_METHYLASE<-SIGMA+OB-Fold+AAA-ATPase+HKD-DNase-BrxL<-PglZ<-?<-OLD-ABC<-ParB<-DUF6079*<-BrxB-BrxF-iSTAND<-BrxA-DNA-binding<-BrxE<-REase<-?<-Phage_int_SAM_1+Phage_integrase<-HEPN<-ZnR+ATPcone	1206	Bacteria>PVC_group>Verrucomicrobiota>Opitutae>Opitutales>Opitutaceae	GCA_001653825.2	Opitutaceae_bacterium_TAV4	Ga0100231_019065	2732930..2736550	278959	BREX-system-P-loop-protein-BrxC-[Opitutaceae-bacterium-TAV4]
MCC5808698.1	Type1.BREX	Phage_integrase-><-SIGMA+OB-Fold+AAA-ATPase+HKD-DNase-BrxL<-PglZ<-HNH<-OLD-ABC<-N6-METHYLASE<-REase<-OLD-ABC<-DUF6079*<-BrxB-BrxF-iSTAND<-BrxA-DNA-binding<-XIS-HTH	1183	Bacteria>PVC_group>Verrucomicrobiota>Opitutae>Opitutales>unclassified_Opitutales	GCA_020831525.1	Opitutales_bacterium	JJU00_20390	12178..15729	2026772	BREX-system-P-loop-protein-BrxC-[Opitutales-bacterium]
MBU3665708.1	Type1.BREX	ABhydrolase-><-?<-?<-SIGMA+OB-Fold+AAA-ATPase+HKD-DNase-BrxL<-PglZ<-KAP-NTPase<-N6-METHYLASE<-Polbeta<-MarR-HTH<-DUF6079*<-BrxB-BrxF-iSTAND<-BrxA-DNA-binding<-ZnR<-?<-?<-?<-?<-VIT+VWA<-MerR-HTH	1169	Bacteria>PVC_group>Verrucomicrobiota>Spartobacteria>Chthoniobacterales>unclassified_Chthoniobacterales	GCA_018883245.1	Chthoniobacterales_bacterium	FGM15_07520	29744..33253	2201154	BREX-system-P-loop-protein-BrxC-[Chthoniobacterales-bacterium]
PTY02769.1	Type1.BREX	RAMA->GDResolvase+ZnR->PglZ->SIGMA+OB-Fold+AAA-ATPase+HKD-DNase-BrxL-><-N6-METHYLASE<-Polbeta<-MarR-HTH<-DUF6079*<-BrxB-BrxF-iSTAND<-BrxA-DNA-binding||DUF4096+DDE_Tnp_1+DDE_Tnp_1_2->	1172	Bacteria>PVC_group>Verrucomicrobiota>unclassified_Verrucomicrobiota	GCA_003054695.1	Verrucomicrobia_bacterium_LW23	DB346_08320	46382..49900	2161867	BREX-system-P-loop-protein-BrxC-[Verrucomicrobia-bacterium-LW23]
TAF24165.1	Type6.BREX	<-ACYC<-?<-SIGMA+OB-Fold+AAA-ATPase+HKD-DNase-BrxL<-PglZ<-N6-METHYLASE<-DUF6079*<-BrxB-BrxF-iSTAND<-BrxA-DNA-binding<-BrxE<-?<-MPTase<-HTH_3||?->REase->	1215	Bacteria>PVC_group>Verrucomicrobiota>unclassified_Verrucomicrobiota	GCA_004293445.1	Verrucomicrobiota_bacterium	EAZ71_11350	54847..58494	2026799	BREX-system-P-loop-protein-BrxC-[Verrucomicrobiota-bacterium]
MCP5535434.1	Type6.BREX	<-XIS-HTH||?->Phage_integrase->?->?->?->BrxE->BrxA-DNA-binding->BrxB-BrxF-iSTAND->DUF6079*->N6-METHYLASE->DrHyd->Schlafen+HATPase+wHTH->PglZ->SIGMA+OB-Fold+AAA-ATPase+HKD-DNase-BrxL->?->?->?->RAMPs->	1186	Bacteria>PVC_group>Verrucomicrobiota>Verrucomicrobiae>Verrucomicrobiales>Akkermansiaceae	GCA_024233115.1	Akkermansiaceae_bacterium	H7A51_04270	18569..22129	2562705	BREX-system-P-loop-protein-BrxC-[Akkermansiaceae-bacterium]
MBE7502027.1	Type6.BREX	HTH_3-><-?<-?<-?<-GNAT+NERD+ZnR||BrxE->BrxA-DNA-binding->BrxB-BrxF-iSTAND->DUF6079*->N6-METHYLASE-><-DDE_Tnp_1||N6-METHYLASE->ParB+HNH->OLD-ABC->TOPRIM->DDE_Tnp_1->PglZ->SIGMA+OB-Fold+AAA-ATPase+HKD-DNase-BrxL->	1231	Bacteria>PVC_group>Verrucomicrobiota>Verrucomicrobiae>Verrucomicrobiales>unclassified_Verrucomicrobiales	GCA_015075445.1	Verrucomicrobiales_bacterium	HS113_17385	108161..111856	2026801	BREX-system-P-loop-protein-BrxC-[Verrucomicrobiales-bacterium]
MCH7225050.1	Type1.BREX	<-SIGMA+OB-Fold+AAA-ATPase+HKD-DNase-BrxL<-PglZ<-URI-DNase<-ZnR<-?<-N6-METHYLASE<-?<-DUF6079*<-BrxB-BrxF-iSTAND<-BrxA-DNA-binding<-TPR<-Peptidase||?->?->TPR+TPR+TPR+TPR+TPR->	1166	Bacteria>PVC_group>Verrucomicrobiota>Verrucomicrobiae>Verrucomicrobiales>Verrucomicrobiaceae	GCA_022548915.1	Verrucomicrobiaceae_bacterium_E54	MLD59_02825	188898..192398	2922395	BREX-system-P-loop-protein-BrxC-[Verrucomicrobiaceae-bacterium-E54]
MCH3907669.1	Type1.BREX	GspA-ATPase+REase-><-Peptidase_S58<-RHH<-?<-?<-?||XIS-HTH->BrxA-DNA-binding->BrxB-BrxF-iSTAND->DUF6079*->N6-METHYLASE->Schlafen+HATPase+wHTH->PglZ->SIGMA+OB-Fold+AAA-ATPase+HKD-DNase-BrxL-><-SF1-SF2-Helicase<-SbcC<-DDE_Tnp_1	1157	Bacteria>Spirochaetota>Spirochaetia>Spirochaetales>Sphaerochaetaceae>Sphaerochaeta	GCA_022482495.1	Sphaerochaeta_sp.	LKE28_05345	253773..257246	1972642	BREX-system-P-loop-protein-BrxC-[Sphaerochaeta-sp.]
WP_114628929.1	Type1.BREX	<-Thymidylate_kin||ParB+HNH->ParB+HNH-><-?<-D5N+D5ATPase||XIS-HTH->BrxA-DNA-binding->BrxB-BrxF-iSTAND->DUF6079*->OLD-ABC->REase->N6-METHYLASE->SIGMA+OB-Fold+AAA-ATPase+HKD-DNase-BrxL-><-AbiEii<-AbiEi_3_N+AbiEi_3	1173	Bacteria>Spirochaetota>Spirochaetia>Spirochaetales>Spirochaetaceae>Oceanispirochaeta	GCF_013166645.2	unclassified_Oceanispirochaeta	HNV09_RS06050	178318..181839	2635722	MULTISPECIES:-BREX-system-P-loop-protein-BrxC-[unclassified-Oceanispirochaeta]
OQB98767.1	Type6.BREX	ANK+ANK-><-?<-SIGMA+OB-Fold+AAA-ATPase+HKD-DNase-BrxL<-PglZ<-N6-METHYLASE<-?<-?<-N6-METHYLASE<-DUF6079*<-BrxB-BrxF-iSTAND<-BrxA-DNA-binding<-BrxE||?->SF1-SF2-Helicase+DUF1998-><-REase<-Peptidase_C14	1205	Bacteria>Spirochaetota>unclassified_Spirochaetota	GCA_002070755.1	Spirochaetes_bacterium_ADurb.Bin110	BWX81_00245	23321..26938	1852908	hypothetical-protein-BWX81_00245-[Spirochaetes-bacterium-ADurb.Bin110]
TXT43486.1	Type1.BREX	<-SIGMA+OB-Fold+AAA-ATPase+HKD-DNase-BrxL<-?||?-><-HTH+REase<-N6-METHYLASE-p<-N6-METHYLASE-p<-N6-METHYLASE-p<-Schlafen+HATPase+wHTH-p<-DUF6079*<-BrxB-BrxF-iSTAND<-BrxA-DNA-binding<-XIS-HTH	1162	Bacteria>Spirochaetota>unclassified_Spirochaetota	GCA_008080685.1	Spirochaetota_bacterium	FD137_1824	16028..19516	2202144	hypothetical-protein-FD137_1824-[Spirochaetota-bacterium]
WP_075374702.1	Type1.BREX	TetR-HTH->ABC-ATPase+SbcC->?->?-><-SIGMA+OB-Fold+AAA-ATPase+HKD-DNase-BrxL<-PglZ<-Schlafen+HATPase+wHTH<-SbcC<-N6-METHYLASE<-DUF6079*<-BrxB-BrxF-iSTAND<-BrxA-DNA-binding||PhoH->?->?->sGTP->?->MetJarc-HTH->PemK_toxin->	1172	Bacteria>Terrabacteria_group>Actinomycetota>Actinomycetes>Actinomycetales>Actinomycetaceae	-	Actinomyces_oris	BKH18_06090	96462..99980	544580	BREX-system-P-loop-protein-BrxC-[Actinomyces-oris]
WP_075392896.1	Type1.BREX	TetR-HTH->ABC-ATPase+SbcC->?->?-><-SIGMA+OB-Fold+AAA-ATPase+HKD-DNase-BrxL<-PglZ<-Schlafen+HATPase+wHTH<-N6-METHYLASE<-DUF6079*<-BrxB-BrxF-iSTAND<-BrxA-DNA-binding||PhoH->?->?->sGTP->?->MetJarc-HTH->PemK_toxin->	1172	Bacteria>Terrabacteria_group>Actinomycetota>Actinomycetes>Actinomycetales>Actinomycetaceae	-	Actinomyces_oris	BKH22_09300	69704..73222	544580	BREX-system-P-loop-protein-BrxC-[Actinomyces-oris]
WP_075371911.1	Type1.BREX	TetR-HTH->ABC-ATPase+SbcC->?->?-><-SIGMA+OB-Fold+AAA-ATPase+HKD-DNase-BrxL<-PglZ<-Schlafen+HATPase+wHTH<-SbcC<-N6-METHYLASE<-DUF6079*<-BrxB-BrxF-iSTAND<-BrxA-DNA-binding||PhoH->?->?->sGTP->?->MetJarc-HTH->PemK_toxin->	1172	Bacteria>Terrabacteria_group>Actinomycetota>Actinomycetes>Actinomycetales>Actinomycetaceae	-	Actinomyces_oris	BKH23_02505	283433..286951	544580	BREX-system-P-loop-protein-BrxC-[Actinomyces-oris]
WP_075400926.1	Type1.BREX	<-PemK_toxin<-MetJarc-HTH<-?<-sGTP<-?<-?<-PhoH||BrxA-DNA-binding->BrxB-BrxF-iSTAND->DUF6079*->N6-METHYLASE->SbcC->Schlafen+HATPase+wHTH->PglZ->SIGMA+OB-Fold+AAA-ATPase+HKD-DNase-BrxL-><-?<-?<-ABC-ATPase+SbcC<-TetR-HTH	1171	Bacteria>Terrabacteria_group>Actinomycetota>Actinomycetes>Actinomycetales>Actinomycetaceae	-	Actinomyces_oris	BKH24_00995	75611..79126	544580	BREX-system-P-loop-protein-BrxC-[Actinomyces-oris]
WP_075550941.1	Type1.BREX	TetR-HTH->ABC-ATPase+SbcC->?->?-><-SIGMA+OB-Fold+AAA-ATPase+HKD-DNase-BrxL<-PglZ<-Schlafen+HATPase+wHTH<-SbcC<-N6-METHYLASE<-DUF6079*<-BrxB-BrxF-iSTAND<-BrxA-DNA-binding||PhoH->?->?->sGTP->?->MetJarc-HTH->PemK_toxin->	1172	Bacteria>Terrabacteria_group>Actinomycetota>Actinomycetes>Actinomycetales>Actinomycetaceae	-	Actinomyces_oris	BKH26_09165	68268..71786	544580	BREX-system-P-loop-protein-BrxC-[Actinomyces-oris]
WP_075411517.1	Type1.BREX	TetR-HTH->ABC-ATPase+SbcC->?->?-><-SIGMA+OB-Fold+AAA-ATPase+HKD-DNase-BrxL<-PglZ<-Schlafen+HATPase+wHTH<-SbcC<-N6-METHYLASE<-DUF6079*<-BrxB-BrxF-iSTAND<-BrxA-DNA-binding||PhoH->?->?->sGTP->?->MetJarc-HTH->PemK_toxin->	1172	Bacteria>Terrabacteria_group>Actinomycetota>Actinomycetes>Actinomycetales>Actinomycetaceae	-	Actinomyces_oris	BKH31_05960	55043..58561	544580	BREX-system-P-loop-protein-BrxC-[Actinomyces-oris]
WP_141407194.1	Type1.BREX	HTH_3-><-PemK_toxin<-?<-sGTP<-?<-?<-PhoH||BrxA-DNA-binding->BrxB-BrxF-iSTAND->DUF6079*->N6-METHYLASE->SbcC->Schlafen+HATPase+wHTH->PglZ->SIGMA+OB-Fold+AAA-ATPase+HKD-DNase-BrxL-><-?<-?<-ABC-ATPase+SbcC<-TetR-HTH	1172	Bacteria>Terrabacteria_group>Actinomycetota>Actinomycetes>Actinomycetales>Actinomycetaceae	-	Actinomyces_oris	I6I08_06235	1496012..1499530	544580	BREX-system-P-loop-protein-BrxC-[Actinomyces-oris]
WP_119835580.1	Type1.BREX	HMA+HAD+HAD-><-MarR-HTH+PADR-HTH<-?||?->BrxA-DNA-binding->BrxB-BrxF-iSTAND->DUF6079*->N6-METHYLASE->SbcC->Schlafen+HATPase+wHTH->PglZ->SIGMA+OB-Fold+AAA-ATPase+HKD-DNase-BrxL->ABC-ATPase+SbcC->	1171	Bacteria>Terrabacteria_group>Actinomycetota>Actinomycetes>Actinomycetales>Actinomycetaceae	-	Actinomyces_sp._2119	D4740_02085	49121..52636	2321393	BREX-system-P-loop-protein-BrxC-[Actinomyces-sp.-2119]
AOS47539.1	Type1.BREX	<-LACI-HTH+PBPI<-?<-?||?->DOC+HTH->BrxA-DNA-binding->BrxB-BrxF-iSTAND->DUF6079*->N6-METHYLASE->OLD-ABC->TOPRIM->PglZ->SIGMA+OB-Fold+AAA-ATPase+HKD-DNase-BrxL-><-ABC-3<-ABC-ATPase+SbcC	1176	Bacteria>Terrabacteria_group>Actinomycetota>Actinomycetes>Actinomycetales>Actinomycetaceae	GCA_001746855.1	Pauljensenia_hongkongensis	BH719_06520	1584234..1587764	178339	hypothetical-protein-BH719_06520-[Pauljensenia-hongkongensis]
OFQ22233.1	Type1.BREX	<-ABC-ATPase+SbcC+ABC-ATPase+SbcC<-?<-SIGMA+OB-Fold+AAA-ATPase+HKD-DNase-BrxL<-PglZ<-TOPRIM<-OLD-ABC<-N6-METHYLASE<-DUF6079*<-BrxB-BrxF-iSTAND<-BrxA-DNA-binding	1173	Bacteria>Terrabacteria_group>Actinomycetota>Actinomycetes>Actinomycetales>Actinomycetaceae	GCA_001813985.1	Actinomyces_sp._HMSC062G12	HMPREF2946_04765	260177..263698	1739450	hypothetical-protein-HMPREF2946_04765-[Actinomyces-sp.-HMSC062G12]
OLO46937.1	Type1.BREX	TetR-HTH->ABC-ATPase+SbcC->?->?-><-SIGMA+OB-Fold+AAA-ATPase+HKD-DNase-BrxL<-PglZ<-Schlafen+HATPase+wHTH<-SbcC<-N6-METHYLASE<-DUF6079*<-BrxB-BrxF-iSTAND<-BrxA-DNA-binding||PhoH->?->?->sGTP->?->MetJarc-HTH->PemK_toxin->	1172	Bacteria>Terrabacteria_group>Actinomycetota>Actinomycetes>Actinomycetales>Actinomycetaceae	GCA_001937365.1	Actinomyces_oris	BKH31_05960	55043..58561	544580	hypothetical-protein-BKH31_05960-[Actinomyces-oris]
OLO54967.1	Type1.BREX	TetR-HTH->ABC-ATPase+SbcC->?->?-><-SIGMA+OB-Fold+AAA-ATPase+HKD-DNase-BrxL<-PglZ<-Schlafen+HATPase+wHTH<-SbcC<-N6-METHYLASE<-DUF6079*<-BrxB-BrxF-iSTAND<-BrxA-DNA-binding||PhoH->?->?->sGTP->?->MetJarc-HTH->PemK_toxin->	1172	Bacteria>Terrabacteria_group>Actinomycetota>Actinomycetes>Actinomycetales>Actinomycetaceae	GCA_001937415.1	Actinomyces_oris	BKH26_09165	68268..71786	544580	hypothetical-protein-BKH26_09165-[Actinomyces-oris]
OLO62906.1	Type1.BREX	<-PemK_toxin<-MetJarc-HTH<-?<-sGTP<-?<-?<-PhoH||BrxA-DNA-binding->BrxB-BrxF-iSTAND->DUF6079*->N6-METHYLASE->SbcC->Schlafen+HATPase+wHTH->PglZ->SIGMA+OB-Fold+AAA-ATPase+HKD-DNase-BrxL-><-?<-?<-ABC-ATPase+SbcC<-TetR-HTH	1171	Bacteria>Terrabacteria_group>Actinomycetota>Actinomycetes>Actinomycetales>Actinomycetaceae	GCA_001937425.1	Actinomyces_oris	BKH24_00995	75611..79126	544580	hypothetical-protein-BKH24_00995-[Actinomyces-oris]
OLO63895.1	Type1.BREX	TetR-HTH->ABC-ATPase+SbcC->?->?-><-SIGMA+OB-Fold+AAA-ATPase+HKD-DNase-BrxL<-PglZ<-Schlafen+HATPase+wHTH<-SbcC<-N6-METHYLASE<-DUF6079*<-BrxB-BrxF-iSTAND<-BrxA-DNA-binding||PhoH->?->?->sGTP->?->MetJarc-HTH->PemK_toxin->	1172	Bacteria>Terrabacteria_group>Actinomycetota>Actinomycetes>Actinomycetales>Actinomycetaceae	GCA_001937485.1	Actinomyces_oris	BKH23_02505	283433..286951	544580	hypothetical-protein-BKH23_02505-[Actinomyces-oris]
OLO77585.1	Type1.BREX	TetR-HTH->ABC-ATPase+SbcC->?->?-><-SIGMA+OB-Fold+AAA-ATPase+HKD-DNase-BrxL<-PglZ<-Schlafen+HATPase+wHTH<-SbcC<-N6-METHYLASE<-DUF6079*<-BrxB-BrxF-iSTAND<-BrxA-DNA-binding||PhoH->?->?->sGTP->?->MetJarc-HTH->PemK_toxin->	1172	Bacteria>Terrabacteria_group>Actinomycetota>Actinomycetes>Actinomycetales>Actinomycetaceae	GCA_001937535.1	Actinomyces_oris	BKH18_06090	96462..99980	544580	hypothetical-protein-BKH18_06090-[Actinomyces-oris]

<table>
<tr><th>Protein-ID</th><th>System</th><th>Gene-Neighbourhoods</th><th>Length</th><th>Taxonomy</th><th>Assembly-ID</th><th>Strain</th><th>Locus-Tag</th><th>Genomic-coordinates</th><th>TaxID</th><th>Fasta-defline</th></tr>
<tr><td>OLO63014.1</td><td>Type1.BREX</td><td>TetR-HTH->ABC-ATPase+SbcC->?->?-><-SIGMA+OB-Fold+AAA-ATPase+HKD-DNase-BrxL<-PglZ<-Schlafen+HATPase+wHTH<-N6-METHYLASE<-DUF6079*<-BrxB-BrxF-iSTAND<-BrxA-DNA-binding||PhoH->?->?->sGTP->?->MetJarc-HTH->PemK_toxin-></td><td>1172</td><td>Bacteria>Terrabacteria_group>Actinomycetota>Actinomycetes>Actinomycetales>Actinomycetaceae</td><td>GCA_001937725.1</td><td>Actinomyces_oris</td><td>BKH22_09300</td><td>69704..73222</td><td>544580</td><td>hypothetical-protein-BKH22_09300-[Actinomyces-oris]</td></tr>
<tr><td>RJF44065.1</td><td>Type1.BREX</td><td>HMA+HAD+HAD-><-MarR-HTH+PADR-HTH<-?||?->BrxA-DNA-binding->BrxB-BrxF-iSTAND->DUF6079*->N6-METHYLASE->SbcC->Schlafen+HATPase+wHTH->PglZ->SIGMA+OB-Fold+AAA-ATPase+HKD-DNase-BrxL->ABC-ATPase+SbcC-></td><td>1171</td><td>Bacteria>Terrabacteria_group>Actinomycetota>Actinomycetes>Actinomycetales>Actinomycetaceae</td><td>GCA_003591215.1</td><td>Actinomyces_sp._2119</td><td>D4740_02085</td><td>49121..52636</td><td>2321393</td><td>BREX-system-P-loop-protein-BrxC-[Actinomyces-sp.-2119]</td></tr>
<tr><td>MBF0967807.1</td><td>Type1.BREX</td><td>BrxB-BrxF-iSTAND->DUF6079*->N6-METHYLASE->OLD-ABC->TOPRIM->PglZ->SIGMA+OB-Fold+AAA-ATPase+HKD-DNase-BrxL-><-?||?->ABC-ATPase+SbcC+ABC-ATPase+SbcC-></td><td>1169</td><td>Bacteria>Terrabacteria_group>Actinomycetota>Actinomycetes>Actinomycetales>Actinomycetaceae</td><td>GCA_015259445.1</td><td>Actinomyces_sp.</td><td>HXK08_00790</td><td>718..4227</td><td>29317</td><td>BREX-system-P-loop-protein-BrxC-[Actinomyces-sp.]</td></tr>
<tr><td>QQC41011.1</td><td>Type1.BREX</td><td>HTH_3-><-PemK_toxin<-?<-sGTP<-?<-?<-PhoH||BrxA-DNA-binding->BrxB-BrxF-iSTAND->DUF6079*->N6-METHYLASE->SbcC->Schlafen+HATPase+wHTH->PglZ->SIGMA+OB-Fold+AAA-ATPase+HKD-DNase-BrxL-><-?<-?<-ABC-ATPase+SbcC<-TetR-HTH</td><td>1172</td><td>Bacteria>Terrabacteria_group>Actinomycetota>Actinomycetes>Actinomycetales>Actinomycetaceae</td><td>GCA_016127955.1</td><td>Actinomyces_oris</td><td>I6I08_06235</td><td>1496012..1499530</td><td>544580</td><td>BREX-system-P-loop-protein-BrxC-[Actinomyces-oris]</td></tr>
<tr><td>MBS6968676.1</td><td>Type1.BREX</td><td>DEOR-HTH+ISOCOT->?-><-SIGMA+OB-Fold+AAA-ATPase+HKD-DNase-BrxL<-PglZ<-TOPRIM<-OLD-ABC<-N6-METHYLASE<-DUF6079*<-BrxB-BrxF-iSTAND<-BrxA-DNA-binding</td><td>1169</td><td>Bacteria>Terrabacteria_group>Actinomycetota>Actinomycetes>Actinomycetales>Actinomycetaceae</td><td>GCA_018381115.1</td><td>Actinomyces_sp.</td><td>KH140_06985</td><td>13628..17137</td><td>29317</td><td>BREX-system-P-loop-protein-BrxC-[Actinomyces-sp.]</td></tr>
<tr><td>MCI6557834.1</td><td>Type1.BREX</td><td>ABhydrolase-><-?<-SIGMA+OB-Fold+AAA-ATPase+HKD-DNase-BrxL<-PglZ<-?<-N6-METHYLASE-p<-Phage_int_SAM_3+Phage_integrase||N6-METHYLASE-p-><-N6-METHYLASE<-DUF6079*<-BrxB-BrxF-iSTAND<-BrxA-DNA-binding||SF1-SF2-Helicase+DUF3418-></td><td>1160</td><td>Bacteria>Terrabacteria_group>Actinomycetota>Actinomycetes>Actinomycetales>Actinomycetaceae</td><td>GCA_022780845.1</td><td>Schaalia_hyovaginalis</td><td>MR422_09445</td><td>69432..72914</td><td>29316</td><td>BREX-system-P-loop-protein-BrxC-[Schaalia-hyovaginalis]</td></tr>
<tr><td>MCI7671412.1</td><td>Type1.BREX</td><td><-SIGMA+OB-Fold+AAA-ATPase+HKD-DNase-BrxL<-PglZ<-?<-N6-METHYLASE-p<-Phage_int_SAM_3+Phage_integrase||N6-METHYLASE-p->DDE_Tnp_IS240->ParA-Soj-PloopNTPase-><-N6-METHYLASE-p<-DUF6079*<-BrxB-BrxF-iSTAND<-BrxA-DNA-binding||SF1-SF2-Helicase+DUF3418-><-Glyco_hydro_36N+Melibiase<-?||?->?->?->LACI-HTH+PBPI-></td><td>1161</td><td>Bacteria>Terrabacteria_group>Actinomycetota>Actinomycetes>Actinomycetales>Actinomycetaceae</td><td>GCA_022786785.1</td><td>Schaalia_hyovaginalis</td><td>MSS20_03495</td><td>24103..27588</td><td>29316</td><td>BREX-system-P-loop-protein-BrxC-[Schaalia-hyovaginalis]</td></tr>
<tr><td>WP_009648402.1</td><td>Type1.BREX</td><td>REase-><-?<-DDE_Tnp<-DDE_Tnp<-?||?->BrxA-DNA-binding->BrxB-BrxF-iSTAND->DUF6079*->N6-METHYLASE->RVT->PglZ->SIGMA+OB-Fold+AAA-ATPase+HKD-DNase-BrxL->HEPN-></td><td>1169</td><td>Bacteria>Terrabacteria_group>Actinomycetota>Actinomycetes>Actinomycetales>Actinomycetaceae</td><td>GCF_000278725.1</td><td>Actinomyces_sp._ICM47</td><td>HMPREF1136_RS05845</td><td>15295..18804</td><td>936548</td><td>BREX-system-P-loop-protein-BrxC-[Actinomyces-sp.-ICM47]</td></tr>
<tr><td>WP_024332155.1</td><td>Type1.BREX</td><td>HTH+REase->?->35exo->BrxA-DNA-binding->BrxB-BrxF-iSTAND->DUF6079*->N6-METHYLASE->PglZ->SIGMA+OB-Fold+AAA-ATPase+HKD-DNase-BrxL->?->?->?-><-?||?-><-ABhydrolase</td><td>1184</td><td>Bacteria>Terrabacteria_group>Actinomycetota>Actinomycetes>Actinomycetales>Actinomycetaceae</td><td>GCF_000420405.1</td><td>Winkia_neuii</td><td>G437_RS0108590</td><td>20470..24024</td><td>33007</td><td>BREX-system-P-loop-protein-BrxC-[Winkia-neuii]</td></tr>
<tr><td>WP_026406950.1</td><td>Type1.BREX</td><td>TetR-HTH->ABC-ATPase+SbcC->?->?-><-SIGMA+OB-Fold+AAA-ATPase+HKD-DNase-BrxL<-PglZ<-Schlafen+HATPase+wHTH<-SbcC<-N6-METHYLASE<-DUF6079*<-BrxB-BrxF-iSTAND<-BrxA-DNA-binding||PhoH->?->?->sGTP->Peptidase_S9-></td><td>1174</td><td>Bacteria>Terrabacteria_group>Actinomycetota>Actinomycetes>Actinomycetales>Actinomycetaceae</td><td>GCF_000429265.1</td><td>Actinomyces_gerencseriae</td><td>G448_RS0104040</td><td>223321..226845</td><td>52769</td><td>BREX-system-P-loop-protein-BrxC-[Actinomyces-gerencseriae]</td></tr>
<tr><td>WP_173647185.1</td><td>Type1.BREX</td><td><-LACI-HTH+PBPI<-?<-?||?->DOC+HTH->BrxA-DNA-binding->BrxB-BrxF-iSTAND->DUF6079*->N6-METHYLASE->OLD-ABC->TOPRIM->PglZ->SIGMA+OB-Fold+AAA-ATPase+HKD-DNase-BrxL->?-><-ABC-3<-ABC-ATPase+SbcC</td><td>1174</td><td>Bacteria>Terrabacteria_group>Actinomycetota>Actinomycetes>Actinomycetales>Actinomycetaceae</td><td>GCF_001746855.1</td><td>Pauljensenia_hongkongensis</td><td>BH719_RS06520</td><td>1584240..1587764</td><td>178339</td><td>BREX-system-P-loop-protein-BrxC-[Pauljensenia-hongkongensis]</td></tr>
<tr><td>WP_256693306.1</td><td>Type1.BREX</td><td>TetR-HTH->ABC-ATPase+SbcC->?->?-><-SIGMA+OB-Fold+AAA-ATPase+HKD-DNase-BrxL<-PglZ<-Schlafen+HATPase+wHTH<-SbcC<-N6-METHYLASE<-DUF6079*<-BrxB-BrxF-iSTAND<-BrxA-DNA-binding||PhoH->?->?->sGTP->?->PemK_toxin-><-HTH_3</td><td>1175</td><td>Bacteria>Terrabacteria_group>Actinomycetota>Actinomycetes>Actinomycetales>Actinomycetaceae</td><td>GCF_001937365.1</td><td>Actinomyces_oris</td><td>BKH31_RS05960</td><td>55043..58570</td><td>544580</td><td>BREX-system-P-loop-protein-BrxC-[Actinomyces-oris]</td></tr>
<tr><td>WP_256693847.1</td><td>Type1.BREX</td><td>TetR-HTH->ABC-ATPase+SbcC->?->?-><-SIGMA+OB-Fold+AAA-ATPase+HKD-DNase-BrxL<-PglZ<-Schlafen+HATPase+wHTH<-SbcC<-N6-METHYLASE<-DUF6079*<-BrxB-BrxF-iSTAND<-BrxA-DNA-binding||PhoH->?->?->sGTP->?->MetJarc-HTH->PemK_toxin-></td><td>1175</td><td>Bacteria>Terrabacteria_group>Actinomycetota>Actinomycetes>Actinomycetales>Actinomycetaceae</td><td>GCF_001937415.1</td><td>Actinomyces_oris</td><td>BKH26_RS09145</td><td>68268..71795</td><td>544580</td><td>BREX-system-P-loop-protein-BrxC-[Actinomyces-oris]</td></tr>
<tr><td>WP_256699678.1</td><td>Type1.BREX</td><td>HTH_3-><-PemK_toxin<-?<-sGTP<-?<-?<-PhoH||BrxA-DNA-binding->BrxB-BrxF-iSTAND->DUF6079*->N6-METHYLASE->SbcC->Schlafen+HATPase+wHTH->PglZ->SIGMA+OB-Fold+AAA-ATPase+HKD-DNase-BrxL-><-?<-?<-ABC-ATPase+SbcC<-TetR-HTH</td><td>1174</td><td>Bacteria>Terrabacteria_group>Actinomycetota>Actinomycetes>Actinomycetales>Actinomycetaceae</td><td>GCF_001937425.1</td><td>Actinomyces_oris</td><td>BKH24_RS00995</td><td>75602..79126</td><td>544580</td><td>BREX-system-P-loop-protein-BrxC-[Actinomyces-oris]</td></tr>
<tr><td>WP_256699054.1</td><td>Type1.BREX</td><td>TetR-HTH->ABC-ATPase+SbcC->?->?-><-SIGMA+OB-Fold+AAA-ATPase+HKD-DNase-BrxL<-PglZ<-Schlafen+HATPase+wHTH<-SbcC<-N6-METHYLASE<-DUF6079*<-BrxB-BrxF-iSTAND<-BrxA-DNA-binding||PhoH->?->?->sGTP->?->PemK_toxin-><-HTH_3</td><td>1175</td><td>Bacteria>Terrabacteria_group>Actinomycetota>Actinomycetes>Actinomycetales>Actinomycetaceae</td><td>GCF_001937485.1</td><td>Actinomyces_oris</td><td>BKH23_RS02505</td><td>283433..286960</td><td>544580</td><td>BREX-system-P-loop-protein-BrxC-[Actinomyces-oris]</td></tr>
<tr><td>WP_256694375.1</td><td>Type1.BREX</td><td>TetR-HTH->ABC-ATPase+SbcC->?->?-><-SIGMA+OB-Fold+AAA-ATPase+HKD-DNase-BrxL<-PglZ<-Schlafen+HATPase+wHTH<-SbcC<-N6-METHYLASE<-DUF6079*<-BrxB-BrxF-iSTAND<-BrxA-DNA-binding||PhoH->?->?->sGTP->?->MetJarc-HTH->PemK_toxin-></td><td>1175</td><td>Bacteria>Terrabacteria_group>Actinomycetota>Actinomycetes>Actinomycetales>Actinomycetaceae</td><td>GCF_001937535.1</td><td>Actinomyces_oris</td><td>BKH18_RS06085</td><td>96462..99989</td><td>544580</td><td>BREX-system-P-loop-protein-BrxC-[Actinomyces-oris]</td></tr>
<tr><td>WP_256697766.1</td><td>Type1.BREX</td><td><-MetJarc-HTH||TetR-HTH->ABC-ATPase+SbcC->?->?-><-SIGMA+OB-Fold+AAA-ATPase+HKD-DNase-BrxL<-PglZ<-Schlafen+HATPase+wHTH<-N6-METHYLASE<-DUF6079*<-BrxB-BrxF-iSTAND<-BrxA-DNA-binding||PhoH->?->?->sGTP->?->PemK_toxin-><-HTH_3</td><td>1175</td><td>Bacteria>Terrabacteria_group>Actinomycetota>Actinomycetes>Actinomycetales>Actinomycetaceae</td><td>GCF_001937725.1</td><td>Actinomyces_oris</td><td>BKH22_RS09310</td><td>69704..73231</td><td>544580</td><td>BREX-system-P-loop-protein-BrxC-[Actinomyces-oris]</td></tr>
<tr><td>WP_076066523.1</td><td>Type1.BREX</td><td><-AraC-HTH+AraC-HTH||?->NAD_binding_10-><-SIGMA+OB-Fold+AAA-ATPase+HKD-DNase-BrxL<-PglZ<-Schlafen+HATPase+wHTH<-SbcC<-N6-METHYLASE<-DUF6079*<-BrxB-BrxF-iSTAND<-BrxA-DNA-binding||PhoH->?->?->sGTP->?-><-?||TetR-HTH-></td><td>1174</td><td>Bacteria>Terrabacteria_group>Actinomycetota>Actinomycetes>Actinomycetales>Actinomycetaceae</td><td>GCF_001956485.1</td><td>Actinomyces_naeslundii</td><td>BKH36_RS03225</td><td>74556..78080</td><td>1655</td><td>BREX-system-P-loop-protein-BrxC-[Actinomyces-naeslundii]</td></tr>
<tr><td>WP_108716712.1</td><td>Type1.BREX</td><td><-SIGMA+OB-Fold+AAA-ATPase+HKD-DNase-BrxL<-PglZ<-?<-Schlafen-p<-SbcC<-N6-METHYLASE-p<-N6-METHYLASE<-DUF6079*<-BrxB-BrxF-iSTAND<-BrxA-DNA-binding||Schlafen+HATPase+wHTH-p->Schlafen+HATPase+wHTH-p-><-tRNA<-?<-?<-ABC-ATPase+SbcC||Sensor+HisKA_3+HATPase_c->REC+LuxR-HTH-></td><td>1174</td><td>Bacteria>Terrabacteria_group>Actinomycetota>Actinomycetes>Actinomycetales>Actinomycetaceae</td><td>GCF_003073475.1</td><td>Actinobaculum_sp._313</td><td>DDD63_RS08615</td><td>1982694..1986218</td><td>2495645</td><td>BREX-system-P-loop-protein-BrxC-[Actinobaculum-sp.-313]</td></tr>
<tr><td>WP_109093701.1</td><td>Type1.BREX</td><td><-PIN<-PhdYeFM_antitox||?->REase-><-ParB+HNH<-SIGMA+OB-Fold+AAA-ATPase+HKD-DNase-BrxL<-PglZ<-Schlafen+HATPase+wHTH<-N6-METHYLASE<-DUF6079*<-BrxB-BrxF-iSTAND<-BrxA-DNA-binding||?-><-?<-?<-LACI-HTH+PBPI<-Glyco_hydro_1</td><td>1170</td><td>Bacteria>Terrabacteria_group>Actinomycetota>Actinomycetes>Actinomycetales>Actinomycetaceae</td><td>GCF_003123745.1</td><td>Ancrocorticia_populi</td><td>DD236_RS06730</td><td>275849..279361</td><td>2175228</td><td>BREX-system-P-loop-protein-BrxC-[Ancrocorticia-populi]</td></tr>
<tr><td>WP_259390601.1</td><td>Type1.BREX</td><td>HMA+HAD+HAD-><-MarR-HTH+PADR-HTH<-?||?->BrxA-DNA-binding->BrxB-BrxF-iSTAND->DUF6079*->N6-METHYLASE->SbcC->Schlafen+HATPase+wHTH->PglZ->SIGMA+OB-Fold+AAA-ATPase+HKD-DNase-BrxL->ABC-ATPase+SbcC-></td><td>1174</td><td>Bacteria>Terrabacteria_group>Actinomycetota>Actinomycetes>Actinomycetales>Actinomycetaceae</td><td>GCF_003591215.1</td><td>Actinomyces_sp._2119</td><td>D4740_RS02085</td><td>49112..52636</td><td>2321393</td><td>BREX-system-P-loop-protein-BrxC-[Actinomyces-sp.-2119]</td></tr>
<tr><td>WP_124851247.1</td><td>Type1.BREX</td><td><-SIGMA+OB-Fold+AAA-ATPase+HKD-DNase-BrxL<-PglZ<-HTH+REase<-N6-METHYLASE-p<-Phage_int_SAM_3+Phage_integrase||N6-METHYLASE-p-><-N6-METHYLASE<-DUF6079*<-BrxB-BrxF-iSTAND<-BrxA-DNA-binding<-?||?->?->?->SbcC+SbcC-></td><td>1170</td><td>Bacteria>Terrabacteria_group>Actinomycetota>Actinomycetes>Actinomycetales>Actinomycetaceae</td><td>GCF_003858495.1</td><td>Buchananella_hordeovulneris</td><td>EII12_RS02040</td><td>65131..68643</td><td>52770</td><td>BREX-system-P-loop-protein-BrxC-[Buchananella-hordeovulneris]</td></tr>
<tr><td>WP_126145915.1</td><td>Type1.BREX</td><td><-SIGMA+OB-Fold+AAA-ATPase+HKD-DNase-BrxL<-PglZ<-?<-SbcC<-N6-METHYLASE<-DUF6079*<-BrxB-BrxF-iSTAND<-BrxA-DNA-binding||Schlafen+HATPase+wHTH-><-MPTase<-HTH_3||HTH_3-></td><td>1174</td><td>Bacteria>Terrabacteria_group>Actinomycetota>Actinomycetes>Actinomycetales>Actinomycetaceae</td><td>GCF_003957265.1</td><td>Actinobaculum_sp._352</td><td>EKN07_RS06065</td><td>220063..223587</td><td>2490946</td><td>BREX-system-P-loop-protein-BrxC-[Actinobaculum-sp.-352]</td></tr>
<tr><td>WP_243118374.1</td><td>Type1.BREX</td><td><-ABC-ATPase+SbcC<-SIGMA+OB-Fold+AAA-ATPase+HKD-DNase-BrxL<-PglZ<-Schlafen+HATPase+wHTH<-?<-N6-METHYLASE<-DUF6079*<-BrxB-BrxF-iSTAND<-BrxA-DNA-binding<-?||?->MarR-HTH+PADR-HTH-><-HMA+HAD+HAD</td><td>1174</td><td>Bacteria>Terrabacteria_group>Actinomycetota>Actinomycetes>Actinomycetales>Actinomycetaceae</td><td>GCF_004010955.1</td><td>Actinomyces_wuliandei</td><td>CWS50_RS00410</td><td>93588..97112</td><td>2057743</td><td>BREX-system-P-loop-protein-BrxC-[Actinomyces-wuliandei]</td></tr>
<tr><td>WP_247595282.1</td><td>Type1.BREX</td><td>ADH_N+ADH_zinc_N->TetR-HTH->ABC-ATPase+SbcC->?-><-SIGMA+OB-Fold+AAA-ATPase+HKD-DNase-BrxL<-PglZ<-Schlafen+HATPase+wHTH<-REase<-N6-METHYLASE<-DUF6079*<-BrxB-BrxF-iSTAND<-BrxA-DNA-binding||?->PhoH->?->?->sGTP->Peptidase_S9-></td><td>1174</td><td>Bacteria>Terrabacteria_group>Actinomycetota>Actinomycetes>Actinomycetales>Actinomycetaceae</td><td>GCF_004796555.1</td><td>Actinomyces_procaprae</td><td>FAQ94_RS07530</td><td>102106..105630</td><td>2560010</td><td>BREX-system-P-loop-protein-BrxC-[Actinomyces-procaprae]</td></tr>
<tr><td>WP_167202365.1</td><td>Type1.BREX</td><td><-GNTR-HTH+UTRA||?->?->?->?->SIR2->BrxA-DNA-binding->BrxB-BrxF-iSTAND->DUF6079*->N6-METHYLASE->SbcC->Schlafen+HATPase+wHTH->PglZ->SIGMA+OB-Fold+AAA-ATPase+HKD-DNase-BrxL-></td><td>1171</td><td>Bacteria>Terrabacteria_group>Actinomycetota>Actinomycetes>Actinomycetales>Actinomycetaceae</td><td>GCF_011751985.1</td><td>Actinomyces_respiraculi</td><td>HCR20_RS03210</td><td>129007..132522</td><td>2744574</td><td>BREX-system-P-loop-protein-BrxC-[Actinomyces-respiraculi]</td></tr>
<tr><td>WP_184451418.1</td><td>Type1.BREX</td><td>Glyco_hydro_36N+Melibiase-><-SF1-SF2-Helicase+DUF3418||?->BrxA-DNA-binding->BrxB-BrxF-iSTAND->DUF6079*->N6-METHYLASE-><-N6-METHYLASE-p||Phage_int_SAM_3+Phage_integrase->N6-METHYLASE-p->?->PglZ->SIGMA+OB-Fold+AAA-ATPase+HKD-DNase-BrxL-></td><td>1160</td><td>Bacteria>Terrabacteria_group>Actinomycetota>Actinomycetes>Actinomycetales>Actinomycetaceae</td><td>GCF_014208035.1</td><td>Schaalia_hyovaginalis</td><td>HD592_RS01490</td><td>338165..341647</td><td>29316</td><td>BREX-system-P-loop-protein-BrxC-[Schaalia-hyovaginalis]</td></tr>
<tr><td>WP_187994004.1</td><td>Type1.BREX</td><td><-NAD_binding_10||?->ADH_N+ADH_zinc_N-><-?||TetR-HTH-><-HipA_C<-?||BrxA-DNA-binding->BrxB-BrxF-iSTAND->DUF6079*->N6-METHYLASE-><-N6-METHYLASE-p||Phage_int_SAM_3+Phage_integrase->N6-METHYLASE-p->Schlafen+HATPase+wHTH->HATPase+wHTH->PglZ->SIGMA+OB-Fold+AAA-ATPase+HKD-DNase-BrxL-></td><td>1173</td><td>Bacteria>Terrabacteria_group>Actinomycetota>Actinomycetes>Actinomycetales>Actinomycetaceae</td><td>GCF_014525425.1</td><td>Schaalia_sp._JY-X159</td><td>IDL90_RS00760</td><td>170975..174496</td><td>2758575</td><td>BREX-system-P-loop-protein-BrxC-[Schaalia-sp.-JY-X159]</td></tr>
<tr><td>WP_166855918.1</td><td>Type1.BREX</td><td><-SIGMA+OB-Fold+AAA-ATPase+HKD-DNase-BrxL<-PglZ<-Schlafen+HATPase+wHTH<-SbcC<-N6-METHYLASE<-DUF6079*<-BrxB-BrxF-iSTAND<-BrxA-DNA-binding<-SIR2<-?<-?<-?<-?||GNTR-HTH+UTRA-></td><td>1171</td><td>Bacteria>Terrabacteria_group>Actinomycetota>Actinomycetes>Actinomycetales>Actinomycetaceae</td><td>GCF_014595995.2</td><td>Actinomyces</td><td>ID810_RS09115</td><td>2190565..2194080</td><td>1654</td><td>MULTISPECIES:-BREX-system-P-loop-protein-BrxC-[Actinomyces]</td></tr>
<tr><td>WP_232622595.1</td><td>Type1.BREX</td><td>HTH_3-><-PemK_toxin<-?<-sGTP<-?<-?<-PhoH||BrxA-DNA-binding->BrxB-BrxF-iSTAND->DUF6079*->N6-METHYLASE->SbcC->Schlafen+HATPase+wHTH->PglZ->SIGMA+OB-Fold+AAA-ATPase+HKD-DNase-BrxL-><-?<-?<-ABC-ATPase+SbcC<-TetR-HTH</td><td>1175</td><td>Bacteria>Terrabacteria_group>Actinomycetota>Actinomycetes>Actinomycetales>Actinomycetaceae</td><td>GCF_016127955.1</td><td>Actinomyces_oris</td><td>I6I08_RS06235</td><td>1496003..1499530</td><td>544580</td><td>BREX-system-P-loop-protein-BrxC-[Actinomyces-oris]</td></tr>
<tr><td>WP_271289309.1</td><td>Type1.BREX</td><td>TetR-HTH->ABC-ATPase+SbcC->?->?-><-SIGMA+OB-Fold+AAA-ATPase+HKD-DNase-BrxL<-PglZ<-Schlafen+HATPase+wHTH<-?<-N6-METHYLASE<-DUF6079*<-BrxB-BrxF-iSTAND<-BrxA-DNA-binding||PhoH->?->?->sGTP->?->MetJarc-HTH->PemK_toxin-></td><td>1175</td><td>Bacteria>Terrabacteria_group>Actinomycetota>Actinomycetes>Actinomycetales>Actinomycetaceae</td><td>GCF_027945475.1</td><td>Actinomyces_oris</td><td>PGE45_RS05470</td><td>1261783..1265310</td><td>544580</td><td>BREX-system-P-loop-protein-BrxC-[Actinomyces-oris]</td></tr>
<tr><td>WP_272432709.1</td><td>Type1.BREX</td><td>ABhydrolase-><-?<-SIGMA+OB-Fold+AAA-ATPase+HKD-DNase-BrxL<-PglZ<-?<-N6-METHYLASE-p<-Phage_int_SAM_3+Phage_integrase||N6-METHYLASE-p-><-N6-METHYLASE<-DUF6079*<-BrxB-BrxF-iSTAND<-BrxA-DNA-binding||SF1-SF2-Helicase+DUF3418-><-Glyco_hydro_36N+Melibiase<-?||?->?->?->LACI-HTH+PBPI-></td><td>1160</td><td>Bacteria>Terrabacteria_group>Actinomycetota>Actinomycetes>Actinomycetales>Actinomycetaceae</td><td>GCF_028437565.1</td><td>Actinomyces_sp._B33</td><td>M3T53_RS01725</td><td>406019..409501</td><td>2942131</td><td>BREX-system-P-loop-protein-BrxC-[Actinomyces-sp.-B33]</td></tr>
<tr><td>WP_126382192.1</td><td>Type1.BREX</td><td><-SIGMA+OB-Fold+AAA-ATPase+HKD-DNase-BrxL<-PglZ<-Schlafen+HATPase+wHTH<-REase<-N6-METHYLASE<-DUF6079*<-BrxB-BrxF-iSTAND<-BrxA-DNA-binding<-SIR2<-?<-?<-?||GNTR-HTH+UTRA-></td><td>1170</td><td>Bacteria>Terrabacteria_group>Actinomycetota>Actinomycetes>Actinomycetales>Actinomycetaceae</td><td>GCF_900637165.1</td><td>Actinomyces_howellii</td><td>GYB74_RS04555</td><td>58123..61635</td><td>52771</td><td>BREX-system-P-loop-protein-BrxC-[Actinomyces-howellii]</td></tr>
<tr><td>WP_004808447.1</td><td>Type1.BREX</td><td><-RNase<-?||?->?->?->?->HTH+REase->BrxA-DNA-binding->BrxB-BrxF-iSTAND->DUF6079*->N6-METHYLASE->PglZ->SIGMA+OB-Fold+AAA-ATPase+HKD-DNase-BrxL-><-?<-?<-ABhydrolase<-DOC+HTH<-?||ZnR-></td><td>1184</td><td>Bacteria>Terrabacteria_group>Actinomycetota>Actinomycetes>Actinomycetales</td><td>GCF_028201595.1</td><td>Actinomycetaceae</td><td>PIG85_RS08980</td><td>1926978..1930532</td><td>2049</td><td>MULTISPECIES:-BREX-system-P-loop-protein-BrxC-[Actinomycetaceae]</td></tr>
<tr><td>WP_129869215.1</td><td>Type1.BREX</td><td>MODE-HTH+PBPII->YTCD-HTH-><-HTH_3<-RelE-ParE<-ACET+ACET<-ZnR<-ZnR||BrxA-DNA-binding->BrxB-BrxF-iSTAND->DUF6079*->N6-METHYLASE->PglZ->SIGMA+OB-Fold+AAA-ATPase+HKD-DNase-BrxL-><-PRTase</td><td>1214</td><td>Bacteria>Terrabacteria_group>Actinomycetota>Actinomycetes>Bifidobacteriales>Bifidobacteriaceae</td><td>-</td><td>Bifidobacterium_pseudolongum</td><td>PG1791B_RS04610</td><td>183618..187262</td><td>1694</td><td>BREX-system-P-loop-protein-BrxC-[Bifidobacterium-pseudolongum]</td></tr>
<tr><td>KFI62072.1</td><td>Type1.BREX</td><td><-XIS-HTH+GDResolvase||REase->?->BrxA-DNA-binding->Schlafen+HATPase+wHTH+HTH-><-XIS-HTH||BrxA-DNA-binding->BrxB-BrxF-iSTAND->DUF6079*->HEPN->N6-METHYLASE->PglZ->SIGMA+OB-Fold+AAA-ATPase+HKD-DNase-BrxL-><-?<-ZnR+GP<-?<-MPTase</td><td>1219</td><td>Bacteria>Terrabacteria_group>Actinomycetota>Actinomycetes>Bifidobacteriales>Bifidobacteriaceae</td><td>GCA_000741575.1</td><td>Bifidobacterium_cuniculi</td><td>BCUN_1388</td><td>51093..54752</td><td>1688</td><td>putative-ATP-binding-protein-[Bifidobacterium-cuniculi]</td></tr>
<tr><td>PJM72971.1</td><td>Type1.BREX</td><td>MACRODOMAIN-><-?<-SIGMA+OB-Fold+AAA-ATPase+HKD-DNase-BrxL<-PglZ<-?||OLD-ABC->TOPRIM-><-N6-METHYLASE<-DUF6079*<-BrxB-BrxF-iSTAND<-BrxA-DNA-binding||?->?-><-?<-?<-JetB</td><td>1243</td><td>Bacteria>Terrabacteria_group>Actinomycetota>Actinomycetes>Bifidobacteriales>Bifidobacteriaceae</td><td>GCA_002802875.1</td><td>Bifidobacterium_primatium</td><td>CS006_06875</td><td>170776..174507</td><td>2045438</td><td>hypothetical-protein-CS006_06875-[Bifidobacterium-primatium]</td></tr>
<tr><td>PKC83363.1</td><td>Type1.BREX</td><td><-HTH_Tnp_1||RelB->N6-METHYLASE->CR-ATPase5+REase-p-><-CR-Kinase<-?||BrxA-DNA-binding->BrxB-BrxF-iSTAND->DUF6079-p->DUF6079-p*->N6-METHYLASE->N6-METHYLASE->SLATT->SMODS->Schlafen+wHTH->PglZ->Lon_2->SIGMA+OB-Fold+AAA-ATPase+HKD-DNase-BrxL->TIR-></td><td>977</td><td>Bacteria>Terrabacteria_group>Actinomycetota>Actinomycetes>Bifidobacteriales>Bifidobacteriaceae</td><td>GCA_002832945.1</td><td>Bifidobacterium_longum</td><td>DPC6317_0876</td><td>237584..240517</td><td>216816</td><td>hypothetical-protein-DPC6317_0876-[Bifidobacterium-longum]</td></tr>
<tr><td>PKV03451.1</td><td>Type1.BREX</td><td><-DUF6079-p||DUF6079-p*->N6-METHYLASE->PglZ->SIGMA+OB-Fold+AAA-ATPase+HKD-DNase-BrxL-><-HEPN<-PRTase</td><td>839</td><td>Bacteria>Terrabacteria_group>Actinomycetota>Actinomycetes>Bifidobacteriales>Bifidobacteriaceae</td><td>GCA_002846775.1</td><td>Bifidobacterium_pseudolongum_subsp._globosum</td><td>CQR50_1159</td><td>592..3111</td><td>1690</td><td>hypothetical-protein-CQR50_1159-[Bifidobacterium-pseudolongum-subsp.-globosum]</td></tr>
<tr><td>PLS30252.1</td><td>Type1.BREX</td><td><-ABC-ATPase+SbcC<-?<-?<-?<-SIGMA+OB-Fold+AAA-ATPase+HKD-DNase-BrxL<-PglZ<-Schlafen+HATPase+wHTH<-N6-METHYLASE<-?<-DUF6079*<-BrxB-BrxF-iSTAND<-BrxA-DNA-binding<-?<-?<-?||Peptidase_A24-></td><td>1244</td><td>Bacteria>Terrabacteria_group>Actinomycetota>Actinomycetes>Bifidobacteriales>Bifidobacteriaceae</td><td>GCA_002860355.1</td><td>Bifidobacterium_margollesii</td><td>Uis1B_1922</td><td>37676..41410</td><td>2020964</td><td>hypothetical-protein-Uis1B_1922-[Bifidobacterium-margollesii]</td></tr>
<tr><td>RDX32761.1</td><td>Type1.BREX</td><td>RelB->N6-METHYLASE->REase-><-CR-Kinase<-?||BrxA-DNA-binding->BrxB-BrxF-iSTAND->DUF6079-p->DUF6079-p*->N6-METHYLASE->PglZ->PglZ-p->SIGMA+OB-Fold+AAA-ATPase+HKD-DNase-BrxL->TIR-></td><td>727</td><td>Bacteria>Terrabacteria_group>Actinomycetota>Actinomycetes>Bifidobacteriales>Bifidobacteriaceae</td><td>GCA_003370195.1</td><td>Bifidobacterium_breve</td><td>CE167_03785</td><td>16097..18282</td><td>1685</td><td>hypothetical-protein-CE167_03785,-partial-[Bifidobacterium-breve]</td></tr>
<tr><td>RGY77464.1</td><td>Type1.BREX</td><td>ABC-ATPase+SbcC->Pkinase+Pkinase->?->REC+LuxR-HTH->HISKIN-><-SbcD||BrxA-DNA-binding->DUF6079*->N6-METHYLASE->SIR2->HerA/FtsK->N6-METHYLASE-p->SIR2->PglZ->SIGMA+OB-Fold+AAA-ATPase+HKD-DNase-BrxL->ZnR+GP->ZnR-></td><td>1219</td><td>Bacteria>Terrabacteria_group>Actinomycetota>Actinomycetes>Bifidobacteriales>Bifidobacteriaceae</td><td>GCA_003463615.1</td><td>Bifidobacterium_pseudocatenulatum</td><td>DXA22_03235</td><td>61960..65619</td><td>28026</td><td>BREX-system-P-loop-protein-BrxC-[Bifidobacterium-pseudocatenulatum]</td></tr>
<tr><td>KAB5743305.1</td><td>Type1.BREX</td><td><-Abhydrolase<-ParB+HNH+RAMA<-ZnR<-ZnR+GP<-SIGMA+OB-Fold+AAA-ATPase+HKD-DNase-BrxL<-PglZ<-Schlafen+HATPase+wHTH<-SIR2<-N6-METHYLASE<-DUF6079*<-BrxB-BrxF-iSTAND<-BrxA-DNA-binding<-Pentapeptide+Pentapeptide<-RelE-ParE<-HAD||SbcD-><-?<-?<-ACET+ACET</td><td>1218</td><td>Bacteria>Terrabacteria_group>Actinomycetota>Actinomycetes>Bifidobacteriales>Bifidobacteriaceae</td><td>GCA_009075895.1</td><td>Bifidobacterium_adolescentis</td><td>GA749_07780</td><td>122966..126622</td><td>1680</td><td>BREX-system-P-loop-protein-BrxC-[Bifidobacterium-adolescentis]</td></tr>
<tr><td>KAB5930818.1</td><td>Type1.BREX</td><td>PemK_toxin->PolB->HEPN-><-?<-?||?->||Pentapeptide+Pentapeptide+Pentapeptide->BrxA-DNA-binding->BrxB-BrxF-iSTAND->DUF6079*->N6-METHYLASE->SIR2->Schlafen+HATPase+wHTH->PglZ->SIGMA+OB-Fold+AAA-ATPase+HKD-DNase-BrxL->?->?-><-REase</td><td>1219</td><td>Bacteria>Terrabacteria_group>Actinomycetota>Actinomycetes>Bifidobacteriales>Bifidobacteriaceae</td><td>GCA_009094485.1</td><td>Bifidobacterium_adolescentis</td><td>GA595_06005</td><td>91928..95587</td><td>1680</td><td>BREX-system-P-loop-protein-BrxC-[Bifidobacterium-adolescentis]</td></tr>
<tr><td>MBE5064977.1</td><td>Type1.BREX</td><td>ACET->ACET+ACET->?->Thioredoxin_3->?-><-Glycoside_Hydrolase||OLD-ABC->BrxA-DNA-binding->BrxB-BrxF-iSTAND->DUF6079*->N6-METHYLASE->REase->?->Schlafen+HATPase+wHTH->PglZ->SIGMA+OB-Fold+AAA-ATPase+HKD-DNase-BrxL-></td><td>1221</td><td>Bacteria>Terrabacteria_group>Actinomycetota>Actinomycetes>Bifidobacteriales>Bifidobacteriaceae</td><td>GCA_014983005.1</td><td>Bifidobacterium_pullorum_subsp._saeculare</td><td>INF27_02890</td><td>184023..187688</td><td>78257</td><td>BREX-system-P-loop-protein-BrxC-[Bifidobacterium-pullorum-subsp.-saeculare]</td></tr>
<tr><td>GDY90547.1</td><td>Type1.BREX</td><td><-Sigma-HTH<-ParB+HNH<-ZnR<-ZnR+GP<-SIGMA+OB-Fold+AAA-ATPase+HKD-DNase-BrxL<-PglZ<-Schlafen+HATPase+wHTH<-SIR2<-N6-METHYLASE<-DUF6079*<-BrxB-BrxF-iSTAND<-BrxA-DNA-binding<-HTH_28</td><td>1219</td><td>Bacteria>Terrabacteria_group>Actinomycetota>Actinomycetes>Bifidobacteriales>Bifidobacteriaceae</td><td>GCA_018326505.1</td><td>Bifidobacteriaceae_bacterium_MCC01971</td><td>MCC01971_21650</td><td>16049..19708</td><td>2569485</td><td>hypothetical-protein-MCC01971_21650-[Bifidobacteriaceae-bacterium-MCC01971]</td></tr>
<tr><td>GDY95791.1</td><td>Type1.BREX</td><td><-HTH_3<-tRNA<-ACET||Acyl_transf_3->?-><-?||?->BrxA-DNA-binding->BrxB-BrxF-iSTAND->DUF6079*->N6-METHYLASE->HEPN->Schlafen+HATPase+wHTH->PglZ->SIGMA+OB-Fold+AAA-ATPase+HKD-DNase-BrxL->ZnR+GP->ZnR->ParB+HNH+RAMA-></td><td>1223</td><td>Bacteria>Terrabacteria_group>Actinomycetota>Actinomycetes>Bifidobacteriales>Bifidobacteriaceae</td><td>GCA_018326565.1</td><td>Bifidobacteriaceae_bacterium_MCC01943</td><td>MCC01943_10780</td><td>5409..9080</td><td>2569488</td><td>hypothetical-protein-MCC01943_10780-[Bifidobacteriaceae-bacterium-MCC01943]</td></tr>
<tr><td>GDZ58121.1</td><td>Type1.BREX</td><td><-SIR2<-?||BrxA-DNA-binding->BrxB-BrxF-iSTAND->DUF6079*->N6-METHYLASE->?->Schlafen+HATPase+wHTH->PglZ->SIGMA+OB-Fold+AAA-ATPase+HKD-DNase-BrxL->?-><-?||GDResolvase+HIN-HTH-></td><td>1219</td><td>Bacteria>Terrabacteria_group>Actinomycetota>Actinomycetes>Bifidobacteriales>Bifidobacteriaceae</td><td>GCA_018327225.1</td><td>Bifidobacteriaceae_bacterium_MCC01967</td><td>MCC01967_09770</td><td>127200..130859</td><td>2569520</td><td>hypothetical-protein-MCC01967_09770-[Bifidobacteriaceae-bacterium-MCC01967]</td></tr>
<tr><td>MBS6717590.1</td><td>Type1.BREX</td><td>HTH_28->BrxA-DNA-binding->BrxB-BrxF-iSTAND->DUF6079*->REase->Schlafen+HATPase+wHTH->PglZ->SIGMA+OB-Fold+AAA-ATPase+HKD-DNase-BrxL->SLATT->SMODS-></td><td>1218</td><td>Bacteria>Terrabacteria_group>Actinomycetota>Actinomycetes>Bifidobacteriales>Bifidobacteriaceae</td><td>GCA_018378875.1</td><td>Bifidobacterium_longum</td><td>KH250_10240</td><td>6375..10031</td><td>216816</td><td>BREX-system-P-loop-protein-BrxC-[Bifidobacterium-longum]</td></tr>
<tr><td>MBS7047476.1</td><td>Type1.BREX</td><td>RelB->PemK_toxin->PolB->HEPN-><-?<-?<-REase||BrxA-DNA-binding->BrxB-BrxF-iSTAND->DUF6079*->N6-METHYLASE->SIR2->Schlafen+HATPase+wHTH->PglZ->SIGMA+OB-Fold+AAA-ATPase+HKD-DNase-BrxL->ZnR+GP->ZnR->ParB+HNH+RAMA->Abhydrolase-></td><td>1218</td><td>Bacteria>Terrabacteria_group>Actinomycetota>Actinomycetes>Bifidobacteriales>Bifidobacteriaceae</td><td>GCA_018381815.1</td><td>Bifidobacterium_adolescentis</td><td>KH096_03850</td><td>341650..345306</td><td>1680</td><td>BREX-system-P-loop-protein-BrxC-[Bifidobacterium-adolescentis]</td></tr>
<tr><td>MCH4189981.1</td><td>Type1.BREX</td><td><-SIGMA+OB-Fold+AAA-ATPase+HKD-DNase-BrxL<-PglZ<-N6-METHYLASE-p<-Phage_int_SAM_3+Phage_integrase||N6-METHYLASE-p-><-N6-METHYLASE<-?<-DUF6079*<-BrxB-BrxF-iSTAND<-BrxA-DNA-binding<-?<-ENDO3+NUDIX</td><td>1173</td><td>Bacteria>Terrabacteria_group>Actinomycetota>Actinomycetes>Bifidobacteriales>Bifidobacteriaceae</td><td>GCA_022486485.1</td><td>Bifidobacterium_tibiigranuli</td><td>LKF45_06440</td><td>46152..49673</td><td>2172043</td><td>BREX-system-P-loop-protein-BrxC-[Bifidobacterium-tibiigranuli]</td></tr>
<tr><td>MCK6131816.1</td><td>Type1.BREX</td><td>35exo+HRDC->?->35exo+BRCT-><-HTH||?-><-ACET||?->BrxA-DNA-binding->BrxB-BrxF-iSTAND->DUF6079*->N6-METHYLASE->?->Schlafen+HATPase+wHTH->PglZ->SIGMA+OB-Fold+AAA-ATPase+HKD-DNase-BrxL->ZnR-><-DSBH+AraC-HTH+AraC-HTH||Glyoxalase-></td><td>1217</td><td>Bacteria>Terrabacteria_group>Actinomycetota>Actinomycetes>Bifidobacteriales>Bifidobacteriaceae</td><td>GCA_023147995.1</td><td>Bifidobacterium_dentium</td><td>KZX63_03525</td><td>164396..168049</td><td>1689</td><td>BREX-system-P-loop-protein-BrxC-[Bifidobacterium-dentium]</td></tr>
<tr><td>WP_014697718.1</td><td>Type1.BREX</td><td><-MODE-HTH+PBPII<-HAD+HAD||REase->ARG->YTCD-HTH->NUDIX-><-ACET+ACET||BrxA-DNA-binding->BrxB-BrxF-iSTAND->DUF6079*->N6-METHYLASE->PglZ->SIGMA+OB-Fold+AAA-ATPase+HKD-DNase-BrxL->SIR2-><-PRTase</td><td>1212</td><td>Bacteria>Terrabacteria_group>Actinomycetota>Actinomycetes>Bifidobacteriales>Bifidobacteriaceae</td><td>GCF_000260715.1</td><td>Bifidobacterium_animalis</td><td>CNCMI4602_RS04575</td><td>1015719..1019357</td><td>28025</td><td>BREX-system-P-loop-protein-BrxC-[Bifidobacterium-animalis]</td></tr>
<tr><td>WP_003812955.1</td><td>Type1.BREX</td><td>ZnR->?->ABC-ATPase+SbcC->?->?->?->BrxA-DNA-binding->BrxB-BrxF-iSTAND->DUF6079*->N6-METHYLASE->SIR2->Schlafen+HATPase+wHTH->PglZ->SIGMA+OB-Fold+AAA-ATPase+HKD-DNase-BrxL->?-><-REase||?-><-Glyoxalase</td><td>1218</td><td>Bacteria>Terrabacteria_group>Actinomycetota>Actinomycetes>Bifidobacteriales>Bifidobacteriaceae</td><td>GCF_000273525.1</td><td>Bifidobacterium_bifidum</td><td>BBNG_RS0103505</td><td>833890..837546</td><td>1681</td><td>BREX-system-P-loop-protein-BrxC-[Bifidobacterium-bifidum]</td></tr>
<tr><td>WP_003819016.1</td><td>Type1.BREX</td><td>ZnR->?->ABC-ATPase+SbcC->?->?->?->BrxA-DNA-binding->BrxB-BrxF-iSTAND->DUF6079*->N6-METHYLASE->REase->Schlafen+HATPase+wHTH->PglZ->SIGMA+OB-Fold+AAA-ATPase+HKD-DNase-BrxL->?-><-REase||?-><-Glyoxalase</td><td>1217</td><td>Bacteria>Terrabacteria_group>Actinomycetota>Actinomycetes>Bifidobacteriales>Bifidobacteriaceae</td><td>GCF_000300215.1</td><td>Bifidobacterium_bifidum</td><td>IF1010_RS06520</td><td>82392..86045</td><td>1681</td><td>BREX-system-P-loop-protein-BrxC-[Bifidobacterium-bifidum]</td></tr>
<tr><td>WP_022857559.1</td><td>Type1.BREX</td><td>HAD+HAD->MODE-HTH+PBPII->YTCD-HTH-><-ACET+ACET<-ZnR||BrxA-DNA-binding->BrxB-BrxF-iSTAND->DUF6079*->N6-METHYLASE->PglZ->SIGMA+OB-Fold+AAA-ATPase+HKD-DNase-BrxL->SIR2-><-PRTase</td><td>1214</td><td>Bacteria>Terrabacteria_group>Actinomycetota>Actinomycetes>Bifidobacteriales>Bifidobacteriaceae</td><td>GCF_000421365.1</td><td>Bifidobacterium_pseudolongum</td><td>G627_RS0100670</td><td>128741..132385</td><td>1694</td><td>BREX-system-P-loop-protein-BrxC-[Bifidobacterium-pseudolongum]</td></tr>
<tr><td>WP_029679903.1</td><td>Type1.BREX</td><td>HTH_28->BrxA-DNA-binding->BrxB-BrxF-iSTAND->DUF6079*->N6-METHYLASE->SIR2->Schlafen+HATPase+wHTH->PglZ->SIGMA+OB-Fold+AAA-ATPase+HKD-DNase-BrxL->?-><-rve</td><td>1218</td><td>Bacteria>Terrabacteria_group>Actinomycetota>Actinomycetes>Bifidobacteriales>Bifidobacteriaceae</td><td>GCF_000421385.1</td><td>Bifidobacterium_longum</td><td>G629_RS0105465</td><td>6869..10525</td><td>216816</td><td>BREX-system-P-loop-protein-BrxC-[Bifidobacterium-longum]</td></tr>
<tr><td>WP_026645358.1</td><td>Type1.BREX</td><td>YTCD-HTH->?-><-?||ARG-><-ACET+ACET<-ZnR||BrxA-DNA-binding->BrxB-BrxF-iSTAND->DUF6079*->N6-METHYLASE->PglZ->SIGMA+OB-Fold+AAA-ATPase+HKD-DNase-BrxL->SIR2-><-HEPN<-PRTase</td><td>1221</td><td>Bacteria>Terrabacteria_group>Actinomycetota>Actinomycetes>Bifidobacteriales>Bifidobacteriaceae</td><td>GCF_000424225.1</td><td>Bifidobacterium_sp._AGR2158</td><td>G606_RS0106975</td><td>126292..129957</td><td>1280675</td><td>BREX-system-P-loop-protein-BrxC-[Bifidobacterium-sp.-AGR2158]</td></tr>
<tr><td>WP_032745990.1</td><td>Type1.BREX</td><td><-Sigma-HTH<-ParB+HNH<-ZnR<-ZnR+GP<-SIGMA+OB-Fold+AAA-ATPase+HKD-DNase-BrxL<-PglZ<-Schlafen+HATPase+wHTH<-SIR2<-N6-METHYLASE<-DUF6079*<-BrxB-BrxF-iSTAND<-BrxA-DNA-binding<-HTH_28</td><td>1219</td><td>Bacteria>Terrabacteria_group>Actinomycetota>Actinomycetes>Bifidobacteriales>Bifidobacteriaceae</td><td>GCF_000730035.1</td><td>Bifidobacterium_longum</td><td>BL171B_RS06775</td><td>278302..281961</td><td>216816</td><td>BREX-system-P-loop-protein-BrxC-[Bifidobacterium-longum]</td></tr>
<tr><td>WP_032738770.1</td><td>Type1.BREX</td><td>HTH_28->BrxA-DNA-binding->BrxB-BrxF-iSTAND->DUF6079*->N6-METHYLASE->HEPN->?->Schlafen+HATPase+wHTH->PglZ->SIGMA+OB-Fold+AAA-ATPase+HKD-DNase-BrxL-></td><td>1218</td><td>Bacteria>Terrabacteria_group>Actinomycetota>Actinomycetes>Bifidobacteriales>Bifidobacteriaceae</td><td>GCF_000730055.1</td><td>Bifidobacterium_longum</td><td>BL71B_RS01495</td><td>365977..369633</td><td>216816</td><td>BREX-system-P-loop-protein-BrxC-[Bifidobacterium-longum]</td></tr>
<tr><td>WP_032735726.1</td><td>Type1.BREX</td><td>HTH_28->BrxA-DNA-binding->BrxB-BrxF-iSTAND->DUF6079*->N6-METHYLASE->SIR2->Schlafen+HATPase+wHTH->PglZ->SIGMA+OB-Fold+AAA-ATPase+HKD-DNase-BrxL-></td><td>1218</td><td>Bacteria>Terrabacteria_group>Actinomycetota>Actinomycetes>Bifidobacteriales>Bifidobacteriaceae</td><td>GCF_000730105.1</td><td>Bifidobacterium_longum</td><td>BL15B_RS01745</td><td>289359..293015</td><td>216816</td><td>BREX-system-P-loop-protein-BrxC-[Bifidobacterium-longum]</td></tr>
<tr><td>WP_051922710.1</td><td>Type1.BREX</td><td><-His_Phos_1<-?||ZnR->?->ABC-ATPase+SbcC-><-?||BrxA-DNA-binding->BrxB-BrxF-iSTAND->DUF6079*->N6-METHYLASE->?->HEPN+RES->Schlafen+HATPase+wHTH->PglZ->SIGMA+OB-Fold+AAA-ATPase+HKD-DNase-BrxL-></td><td>1249</td><td>Bacteria>Terrabacteria_group>Actinomycetota>Actinomycetes>Bifidobacteriales>Bifidobacteriaceae</td><td>GCF_000741785.1</td><td>Bifidobacterium_stellenboschense</td><td>BSTEL_1465</td><td>61247..64996</td><td>762211</td><td>BREX-system-P-loop-protein-BrxC-[Bifidobacterium-stellenboschense]</td></tr>
<tr><td>WP_026646062.1</td><td>Type1.BREX</td><td><-RelB<-ParB+HNH+RAMA<-ZnR<-ZnR+GP<-SIGMA+OB-Fold+AAA-ATPase+HKD-DNase-BrxL<-PglZ<-Schlafen+HATPase+wHTH<-?<-N6-METHYLASE<-DUF6079*<-BrxB-BrxF-iSTAND<-BrxA-DNA-binding||REase->?->?-><-HEPN<-PolB<-PemK_toxin||SbcD-></td><td>1219</td><td>Bacteria>Terrabacteria_group>Actinomycetota>Actinomycetes>Bifidobacteriales>Bifidobacteriaceae</td><td>GCF_000770925.1</td><td>Bifidobacterium_ruminantium</td><td>T534_RS0102115</td><td>18145..21804</td><td>78346</td><td>BREX-system-P-loop-protein-BrxC-[Bifidobacterium-ruminantium]</td></tr>
<tr><td>WP_033509374.1</td><td>Type1.BREX</td><td><-SIGMA+OB-Fold+AAA-ATPase+HKD-DNase-BrxL<-PglZ<-Schlafen+HATPase+wHTH<-N6-METHYLASE-p<-HerA/FtsK<-SIR2<-N6-METHYLASE<-DUF6079*<-BrxB-BrxF-iSTAND<-BrxA-DNA-binding<-TOPRIM<-OLD-ABC||Glycoside_Hydrolase-><-?<-Thioredoxin_3<-?<-ACET+ACET</td><td>1221</td><td>Bacteria>Terrabacteria_group>Actinomycetota>Actinomycetes>Bifidobacteriales>Bifidobacteriaceae</td><td>GCF_000770965.1</td><td>Bifidobacterium_pullorum</td><td>OU28_RS04650</td><td>22611..26276</td><td>78448</td><td>BREX-system-P-loop-protein-BrxC-[Bifidobacterium-pullorum]</td></tr>
<tr><td>WP_160257632.1</td><td>Type1.BREX</td><td><-XIS-HTH+GDResolvase||REase->?->BrxA-DNA-binding->Schlafen+HATPase+wHTH+HTH-><-XIS-HTH||BrxA-DNA-binding->BrxB-BrxF-iSTAND->DUF6079*->HEPN->N6-METHYLASE->PglZ->SIGMA+OB-Fold+AAA-ATPase+HKD-DNase-BrxL-><-?<-ZnR+GP<-MPTase</td><td>1215</td><td>Bacteria>Terrabacteria_group>Actinomycetota>Actinomycetes>Bifidobacteriales>Bifidobacteriaceae</td><td>GCF_000771465.1</td><td>Bifidobacterium_cuniculi</td><td>BCUN_RS07325</td><td>51105..54752</td><td>1688</td><td>BREX-system-P-loop-protein-BrxC-[Bifidobacterium-cuniculi]</td></tr>
<tr><td>WP_051923722.1</td><td>Type1.BREX</td><td><-METHYLASE<-SIGMA+OB-Fold+AAA-ATPase+HKD-DNase-BrxL<-PglZ<-Schlafen+HATPase+wHTH<-SMODS<-ABhydrolase<-N6-METHYLASE<-CR-ATPase5+REase<-DUF6079*<-BrxB-BrxF-iSTAND<-BrxA-DNA-binding<-?||?-><-?<-?<-AlcRN+AraC-HTH</td><td>1247</td><td>Bacteria>Terrabacteria_group>Actinomycetota>Actinomycetes>Bifidobacteriales>Bifidobacteriaceae</td><td>GCF_000771645.1</td><td>Bifidobacterium_biavatii</td><td>OU23_RS06270</td><td>17800..21543</td><td>762212</td><td>BREX-system-P-loop-protein-BrxC-[Bifidobacterium-biavatii]</td></tr>
<tr><td>WP_038426289.1</td><td>Type1.BREX</td><td>HTH_28->BrxA-DNA-binding->BrxB-BrxF-iSTAND->DUF6079*->N6-METHYLASE->?->?->Schlafen+HATPase+wHTH->PglZ->SIGMA+OB-Fold+AAA-ATPase+HKD-DNase-BrxL->ZnR+GP->ZnR->ParB+HNH-></td><td>1218</td><td>Bacteria>Terrabacteria_group>Actinomycetota>Actinomycetes>Bifidobacteriales>Bifidobacteriaceae</td><td>GCF_000772485.1</td><td>Bifidobacterium_longum</td><td>BLGT_RS02155</td><td>511827..515483</td><td>216816</td><td>BREX-system-P-loop-protein-BrxC-[Bifidobacterium-longum]</td></tr>
<tr><td>WP_041080188.1</td><td>Type1.BREX</td><td>HTH_28->BrxA-DNA-binding->BrxB-BrxF-iSTAND->DUF6079*->N6-METHYLASE->?->Schlafen+HATPase+wHTH->PglZ->SIGMA+OB-Fold+AAA-ATPase+HKD-DNase-BrxL-></td><td>1219</td><td>Bacteria>Terrabacteria_group>Actinomycetota>Actinomycetes>Bifidobacteriales>Bifidobacteriaceae</td><td>GCF_000829295.1</td><td>Bifidobacterium_longum</td><td>BL105A_RS01810</td><td>418620..422279</td><td>216816</td><td>BREX-system-P-loop-protein-BrxC-[Bifidobacterium-longum]</td></tr>
<tr><td>WP_046999386.1</td><td>Type1.BREX</td><td>REase-><-ZnR<-ZnR+GP<-SIGMA+OB-Fold+AAA-ATPase+HKD-DNase-BrxL<-PglZ<-Schlafen+HATPase+wHTH<-REase<-N6-METHYLASE<-DUF6079*<-BrxB-BrxF-iSTAND<-BrxA-DNA-binding<-Pentapeptide+Pentapeptide+Pentapeptide<-HAD||SbcD-><-?||HTH-><-35exo+BRCT</td><td>1217</td><td>Bacteria>Terrabacteria_group>Actinomycetota>Actinomycetes>Bifidobacteriales>Bifidobacteriaceae</td><td>GCF_001010915.1</td><td>Bifidobacterium_adolescentis</td><td>AAX71_RS05490</td><td>58570..62223</td><td>1680</td><td>BREX-system-P-loop-protein-BrxC-[Bifidobacterium-adolescentis]</td></tr>
<tr><td>WP_047298571.1</td><td>Type1.BREX</td><td><-Sigma-HTH<-ParB+HNH<-?<-ZnR+GP<-SIGMA+OB-Fold+AAA-ATPase+HKD-DNase-BrxL<-PglZ<-Schlafen+HATPase+wHTH<-?<-N6-METHYLASE<-DUF6079*<-BrxB-BrxF-iSTAND<-BrxA-DNA-binding||REase-></td><td>1217</td><td>Bacteria>Terrabacteria_group>Actinomycetota>Actinomycetes>Bifidobacteriales>Bifidobacteriaceae</td><td>GCF_001020335.1</td><td>Bifidobacterium_bifidum</td><td>LMG11583_RS03370</td><td>14804..18457</td><td>1681</td><td>BREX-system-P-loop-protein-BrxC-[Bifidobacterium-bifidum]</td></tr>
<tr><td>WP_003826153.1</td><td>Type1.BREX</td><td><-SbcD||HAD->PolB->HEPN-><-?<-?<-REase||BrxA-DNA-binding->BrxB-BrxF-iSTAND->DUF6079*->N6-METHYLASE->SIR2->HerA/FtsK->N6-METHYLASE-p->Schlafen+HATPase+wHTH->PglZ->SIGMA+OB-Fold+AAA-ATPase+HKD-DNase-BrxL->?-><-REase</td><td>1218</td><td>Bacteria>Terrabacteria_group>Actinomycetota>Actinomycetes>Bifidobacteriales>Bifidobacteriaceae</td><td>GCF_001025155.1</td><td>Bifidobacterium_angulatum</td><td>BBAG_RS03465</td><td>863700..867356</td><td>1683</td><td>BREX-system-P-loop-protein-BrxC-[Bifidobacterium-angulatum]</td></tr>
<tr><td>WP_050824906.1</td><td>Type1.BREX</td><td><-SIGMA+OB-Fold+AAA-ATPase+HKD-DNase-BrxL<-PglZ<-Schlafen+wHTH<-Schlafen+wHTH<-SMODS<-SLATT<-N6-METHYLASE<-DUF6079*<-BrxB-BrxF-iSTAND<-BrxA-DNA-binding||?->CR-Kinase-><-CR-ATPase5+REase-p<-N6-METHYLASE<-RelB</td><td>1229</td><td>Bacteria>Terrabacteria_group>Actinomycetota>Actinomycetes>Bifidobacteriales>Bifidobacteriaceae</td><td>GCF_001189355.1</td><td>Bifidobacterium_breve</td><td>BBRI4_RS05550</td><td>137028..140717</td><td>1685</td><td>BREX-system-P-loop-protein-BrxC-[Bifidobacterium-breve]</td></tr>
<tr><td>WP_052828891.1</td><td>Type1.BREX</td><td><-TIR<-SIGMA+OB-Fold+AAA-ATPase+HKD-DNase-BrxL<-PglZ<-Schlafen+wHTH<-SMODS<-SLATT<-N6-METHYLASE<-DUF6079*<-BrxB-BrxF-iSTAND<-BrxA-DNA-binding||?->CR-Kinase-><-CR-ATPase5+REase-p<-N6-METHYLASE<-RelB</td><td>1229</td><td>Bacteria>Terrabacteria_group>Actinomycetota>Actinomycetes>Bifidobacteriales>Bifidobacteriaceae</td><td>GCF_001264045.1</td><td>Bifidobacterium_breve</td><td>BAD03_RS05925</td><td>144131..147820</td><td>1685</td><td>BREX-system-P-loop-protein-BrxC-[Bifidobacterium-breve]</td></tr>
<tr><td>WP_052792432.1</td><td>Type1.BREX</td><td><-SIR2||BrxA-DNA-binding->BrxB-BrxF-iSTAND->DUF6079*->N6-METHYLASE->SIR2->HerA/FtsK->N6-METHYLASE-p->SIR2->Schlafen+HATPase+wHTH->PglZ->SIGMA+OB-Fold+AAA-ATPase+HKD-DNase-BrxL-></td><td>1218</td><td>Bacteria>Terrabacteria_group>Actinomycetota>Actinomycetes>Bifidobacteriales>Bifidobacteriaceae</td><td>GCF_001264105.1</td><td>Bifidobacterium</td><td>BBM1340_RS06775</td><td>7664..11320</td><td>1678</td><td>MULTISPECIES:-BREX-system-P-loop-protein-BrxC-[Bifidobacterium]</td></tr>
<tr><td>WP_053824992.1</td><td>Type1.BREX</td><td>ZnR->?->ABC-ATPase+SbcC->?->?->?->BrxA-DNA-binding->BrxB-BrxF-iSTAND->DUF6079*->N6-METHYLASE->SIR2->Schlafen+HATPase+wHTH->PglZ->SIGMA+OB-Fold+AAA-ATPase+HKD-DNase-BrxL->?-><-REase||?-><-Glyoxalase</td><td>1217</td><td>Bacteria>Terrabacteria_group>Actinomycetota>Actinomycetes>Bifidobacteriales>Bifidobacteriaceae</td><td>GCF_001281345.1</td><td>Bifidobacterium_bifidum</td><td>RY70_RS04415</td><td>1112532..1116185</td><td>1681</td><td>BREX-system-P-loop-protein-BrxC-[Bifidobacterium-bifidum]</td></tr>
<tr><td>WP_253276104.1</td><td>Type1.BREX</td><td><-SbcD||RelB->PemK_toxin->PolB->HEPN-><-?<-AIPR||BrxA-DNA-binding->BrxB-BrxF-iSTAND->DUF6079-p*->DUF6079-p->N6-METHYLASE->SIR2->Schlafen+HATPase+wHTH->PglZ->SIGMA+OB-Fold+AAA-ATPase+HKD-DNase-BrxL->?-><-AAA-ATPase<-HTH+rve</td><td>561</td><td>Bacteria>Terrabacteria_group>Actinomycetota>Actinomycetes>Bifidobacteriales>Bifidobacteriaceae</td><td>GCF_001406455.1</td><td>Bifidobacterium_adolescentis</td><td>ARB73_RS10570</td><td>418734..420419</td><td>1680</td><td>BREX-system-P-loop-protein-BrxC-[Bifidobacterium-adolescentis]</td></tr>
<tr><td>WP_065465686.1</td><td>Type1.BREX</td><td><-TIR<-SIGMA+OB-Fold+AAA-ATPase+HKD-DNase-BrxL<-PglZ<-Schlafen+wHTH<-?<-N6-METHYLASE<-DUF6079*<-BrxB-BrxF-iSTAND<-BrxA-DNA-binding||?->CR-Kinase-><-CR-ATPase5+REase-p<-N6-METHYLASE<-RelB</td><td>1230</td><td>Bacteria>Terrabacteria_group>Actinomycetota>Actinomycetes>Bifidobacteriales>Bifidobacteriaceae</td><td>GCF_001685805.1</td><td>Bifidobacterium_breve</td><td>BAD00_RS06410</td><td>112826..116518</td><td>1685</td><td>BREX-system-P-loop-protein-BrxC-[Bifidobacterium-breve]</td></tr>
<tr><td>WP_065435300.1</td><td>Type1.BREX</td><td>Pkinase+Pkinase->REC+LuxR-HTH->HISKIN-><-?<-SbcD<-?<-REase||BrxA-DNA-binding->BrxB-BrxF-iSTAND->DUF6079*->N6-METHYLASE->SIR2->Schlafen+HATPase+wHTH->PglZ->SIGMA+OB-Fold+AAA-ATPase+HKD-DNase-BrxL->?-><-RNaseH||Abhydrolase-></td><td>1219</td><td>Bacteria>Terrabacteria_group>Actinomycetota>Actinomycetes>Bifidobacteriales>Bifidobacteriaceae</td><td>GCF_001686025.1</td><td>Bifidobacterium_pseudocatenulatum</td><td>BAD11_RS06840</td><td>26725..30384</td><td>28026</td><td>BREX-system-P-loop-protein-BrxC-[Bifidobacterium-pseudocatenulatum]</td></tr>
<tr><td>WP_065445635.1</td><td>Type1.BREX</td><td>HTH_28->BrxA-DNA-binding->BrxB-BrxF-iSTAND->DUF6079*->N6-METHYLASE->REase->Schlafen+HATPase+wHTH->PglZ->SIGMA+OB-Fold+AAA-ATPase+HKD-DNase-BrxL->ZnR+GP->ZnR->ParB+HNH->Sigma-HTH-></td><td>1219</td><td>Bacteria>Terrabacteria_group>Actinomycetota>Actinomycetes>Bifidobacteriales>Bifidobacteriaceae</td><td>GCF_001686165.1</td><td>Bifidobacterium_longum</td><td>MCC10077_RS10215</td><td>2512..6171</td><td>216816</td><td>BREX-system-P-loop-protein-BrxC-[Bifidobacterium-longum]</td></tr>
<tr><td>WP_065453246.1</td><td>Type1.BREX</td><td>HTH_28->BrxA-DNA-binding->BrxB-BrxF-iSTAND->DUF6079*->N6-METHYLASE->SIR2->Schlafen+HATPase+wHTH->PglZ->SIGMA+OB-Fold+AAA-ATPase+HKD-DNase-BrxL->ZnR+GP->ZnR->ParB+HNH->Sigma-HTH-></td><td>1219</td><td>Bacteria>Terrabacteria_group>Actinomycetota>Actinomycetes>Bifidobacteriales>Bifidobacteriaceae</td><td>GCF_001686185.1</td><td>Bifidobacterium_longum</td><td>BAD19_RS10570</td><td>392770..396429</td><td>216816</td><td>BREX-system-P-loop-protein-BrxC-[Bifidobacterium-longum]</td></tr>
<tr><td>WP_071474421.1</td><td>Type1.BREX</td><td><-Sigma-HTH<-ParB+HNH<-ZnR<-ZnR+GP<-SIGMA+OB-Fold+AAA-ATPase+HKD-DNase-BrxL<-PglZ<-Schlafen+HATPase+wHTH<-SIR2<-N6-METHYLASE<-DUF6079*<-BrxB-BrxF-iSTAND<-BrxA-DNA-binding<-DEDD_Tnp_IS110+Transposase_20</td><td>1218</td><td>Bacteria>Terrabacteria_group>Actinomycetota>Actinomycetes>Bifidobacteriales>Bifidobacteriaceae</td><td>GCF_001870705.1</td><td>Bifidobacterium_longum</td><td>BFS26_RS01875</td><td>17972..21628</td><td>216816</td><td>BREX-system-P-loop-protein-BrxC-[Bifidobacterium-longum]</td></tr>
<tr><td>WP_071477881.1</td><td>Type1.BREX</td><td><-SIGMA+OB-Fold+AAA-ATPase+HKD-DNase-BrxL<-PglZ<-RNase<-Schlafen+HATPase+wHTH<-SMODS<-SLATT<-N6-METHYLASE<-DUF6079*<-BrxB-BrxF-iSTAND<-BrxA-DNA-binding<-CR-ATPase5+REase-p<-?||?-><-?<-?||<-?<-MarR_2+ROK-HTH</td><td>1229</td><td>Bacteria>Terrabacteria_group>Actinomycetota>Actinomycetes>Bifidobacteriales>Bifidobacteriaceae</td><td>GCF_001870755.1</td><td>Bifidobacterium_longum</td><td>BFS25_RS03445</td><td>115725..119414</td><td>216816</td><td>BREX-system-P-loop-protein-BrxC-[Bifidobacterium-longum]</td></tr>
<tr><td>WP_094668334.1</td><td>Type1.BREX</td><td>DDE_Tnp_ISL3-><-ABhydrolase||BrxA-DNA-binding->BrxB-BrxF-iSTAND->DUF6079*->CR-ATPase5+REase->TPR+TPR->N6-METHYLASE->Schlafen+HATPase+wHTH->MazE_antitoxin->PemK_toxin->PglZ->SIGMA+OB-Fold+AAA-ATPase+HKD-DNase-BrxL->METHYLASE-></td><td>1270</td><td>Bacteria>Terrabacteria_group>Actinomycetota>Actinomycetes>Bifidobacteriales>Bifidobacteriaceae</td><td>GCF_002259745.1</td><td>Bifidobacterium_myosotis</td><td>BMYO_RS10065</td><td>3739..7551</td><td>1630166</td><td>BREX-system-P-loop-protein-BrxC-[Bifidobacterium-myosotis]</td></tr>
<tr><td>WP_095613737.1</td><td>Type1.BREX</td><td>MODE-HTH+PBPII->YTCD-HTH->?-><-?||ARG-><-ACET+ACET||?->BrxA-DNA-binding->BrxB-BrxF-iSTAND->DUF6079*->N6-METHYLASE-p->N6-METHYLASE->PglZ->SIGMA+OB-Fold+AAA-ATPase+HKD-DNase-BrxL->SIR2-><-OLD-ABC<-PRTase</td><td>1215</td><td>Bacteria>Terrabacteria_group>Actinomycetota>Actinomycetes>Bifidobacteriales>Bifidobacteriaceae</td><td>GCF_002286915.1</td><td>Bifidobacterium_italicum</td><td>B1400_RS07185</td><td>20228..23875</td><td>1960968</td><td>BREX-system-P-loop-protein-BrxC-[Bifidobacterium-italicum]</td></tr>
<tr><td>WP_100511074.1</td><td>Type1.BREX</td><td>MACRODOMAIN-><-?<-SIGMA+OB-Fold+AAA-ATPase+HKD-DNase-BrxL<-PglZ<-?||OLD-ABC->TOPRIM-><-N6-METHYLASE<-DUF6079*<-BrxB-BrxF-iSTAND<-BrxA-DNA-binding||?->?-><-?<-?<-JetB</td><td>1251</td><td>Bacteria>Terrabacteria_group>Actinomycetota>Actinomycetes>Bifidobacteriales>Bifidobacteriaceae</td><td>GCF_002802875.1</td><td>Bifidobacterium_primatium</td><td>CS006_RS06855</td><td>170776..174531</td><td>2045438</td><td>BREX-system-P-loop-protein-BrxC-[Bifidobacterium-primatium]</td></tr>
<tr><td>WP_101025546.1</td><td>Type1.BREX</td><td>HTH_28->BrxA-DNA-binding->BrxB-BrxF-iSTAND->DUF6079*->N6-METHYLASE->?->Schlafen+HATPase+wHTH->PglZ->SIGMA+OB-Fold+AAA-ATPase+HKD-DNase-BrxL->?-><-?<-?<-ABC-ATPase+SbcC</td><td>1217</td><td>Bacteria>Terrabacteria_group>Actinomycetota>Actinomycetes>Bifidobacteriales>Bifidobacteriaceae</td><td>GCF_002832945.1</td><td>Bifidobacterium_longum</td><td>DPC6317_RS01855</td><td>212185..215838</td><td>216816</td><td>BREX-system-P-loop-protein-BrxC-[Bifidobacterium-longum]</td></tr>
<tr><td>WP_106628753.1</td><td>Type1.BREX</td><td>rve->AAA-ATPase-><-?<-?<-?<-?<-SIR2||BrxA-DNA-binding->BrxB-BrxF-iSTAND->DUF6079*->N6-METHYLASE->SIR2->HerA/FtsK->N6-METHYLASE-p->SIR2->Schlafen+HATPase+wHTH->PglZ->SIGMA+OB-Fold+AAA-ATPase+HKD-DNase-BrxL-></td><td>1218</td><td>Bacteria>Terrabacteria_group>Actinomycetota>Actinomycetes>Bifidobacteriales>Bifidobacteriaceae</td><td>GCF_002838305.1</td><td>Bifidobacterium_breve</td><td>NRBB11_RS04990</td><td>1136892..1140548</td><td>1685</td><td>BREX-system-P-loop-protein-BrxC-[Bifidobacterium-breve]</td></tr>
<tr><td>WP_106620813.1</td><td>Type1.BREX</td><td><-rve||RelB->REase->N6-METHYLASE->CR-ATPase5+REase-p-><-CR-Kinase<-?||BrxA-DNA-binding->BrxB-BrxF-iSTAND->DUF6079*->N6-METHYLASE->SLATT->SMODS->Schlafen-p->Schlafen+wHTH->PglZ->SIGMA+OB-Fold+AAA-ATPase+HKD-DNase-BrxL->TIR-></td><td>1229</td><td>Bacteria>Terrabacteria_group>Actinomycetota>Actinomycetes>Bifidobacteriales>Bifidobacteriaceae</td><td>GCF_002838345.1</td><td>Bifidobacterium_breve</td><td>NRBB57_RS05005</td><td>1130879..1134568</td><td>1685</td><td>BREX-system-P-loop-protein-BrxC-[Bifidobacterium-breve]</td></tr>
<tr><td>WP_101428597.1</td><td>Type1.BREX</td><td>HAD+HAD->MODE-HTH+PBPII->YTCD-HTH-><-ACET+ACET<-ZnR||BrxA-DNA-binding->BrxB-BrxF-iSTAND->DUF6079*->N6-METHYLASE->PglZ->SIGMA+OB-Fold+AAA-ATPase+HKD-DNase-BrxL-><-PRTase</td><td>1212</td><td>Bacteria>Terrabacteria_group>Actinomycetota>Actinomycetes>Bifidobacteriales>Bifidobacteriaceae</td><td>GCF_002846755.1</td><td>Bifidobacterium_pseudolongum</td><td>CQR49_RS04725</td><td>132480..136118</td><td>1694</td><td>BREX-system-P-loop-protein-BrxC-[Bifidobacterium-pseudolongum]</td></tr>
<tr><td>WP_180326342.1</td><td>Type1.BREX</td><td>DUF6079-p*->N6-METHYLASE->PglZ->SIGMA+OB-Fold+AAA-ATPase+HKD-DNase-BrxL-><-HEPN<-PRTase</td><td>865</td><td>Bacteria>Terrabacteria_group>Actinomycetota>Actinomycetes>Bifidobacteriales>Bifidobacteriaceae</td><td>GCF_002846775.1</td><td>Bifidobacterium_pseudolongum</td><td>CQR50_RS05955</td><td>514..3111</td><td>1694</td><td>BREX-system-P-loop-protein-BrxC-[Bifidobacterium-pseudolongum]</td></tr>
<tr><td>WP_165782845.1</td><td>Type1.BREX</td><td><-SIGMA+OB-Fold+AAA-ATPase+HKD-DNase-BrxL<-PglZ<-Schlafen+HATPase+wHTH<-N6-METHYLASE<-?<-TPR+TPR<-DUF6079*<-BrxB-BrxF-iSTAND<-BrxA-DNA-binding<-?<-?<-?||Peptidase_A24-></td><td>1259</td><td>Bacteria>Terrabacteria_group>Actinomycetota>Actinomycetes>Bifidobacteriales>Bifidobacteriaceae</td><td>GCF_002860355.1</td><td>Bifidobacterium_margollesii</td><td>Uis1B_RS09895</td><td>37676..41455</td><td>2020964</td><td>BREX-system-P-loop-protein-BrxC-[Bifidobacterium-margollesii]</td></tr>
<tr><td>WP_101629209.1</td><td>Type1.BREX</td><td>ABC-ATPase+SbcC->?->?-><-?<-SIGMA+OB-Fold+AAA-ATPase+HKD-DNase-BrxL<-PglZ<-Schlafen+HATPase+wHTH<-?<-N6-METHYLASE<-DUF6079*<-BrxB-BrxF-iSTAND<-BrxA-DNA-binding<-HTH_28</td><td>1218</td><td>Bacteria>Terrabacteria_group>Actinomycetota>Actinomycetes>Bifidobacteriales>Bifidobacteriaceae</td><td>GCF_002861445.1</td><td>Bifidobacterium_longum</td><td>CYJ39_RS09195</td><td>68952..72608</td><td>216816</td><td>BREX-system-P-loop-protein-BrxC-[Bifidobacterium-longum]</td></tr>
<tr><td>WP_109056521.1</td><td>Type1.BREX</td><td><-ABhydrolase||BrxA-DNA-binding->BrxB-BrxF-iSTAND->DUF6079*->N6-METHYLASE->HEPN->Schlafen+HATPase+wHTH->CR-ATPase5+REase->PglZ->SIGMA+OB-Fold+AAA-ATPase+HKD-DNase-BrxL->METHYLASE-></td><td>1246</td><td>Bacteria>Terrabacteria_group>Actinomycetota>Actinomycetes>Bifidobacteriales>Bifidobacteriaceae</td><td>GCF_003129905.1</td><td>Bifidobacterium_callitrichidarum</td><td>DF196_RS03600</td><td>108404..112144</td><td>2052941</td><td>BREX-system-P-loop-protein-BrxC-[Bifidobacterium-callitrichidarum]</td></tr>
<tr><td>WP_134859198.1</td><td>Type1.BREX</td><td>RelB->N6-METHYLASE->REase->CR-ATPase5+REase-p-><-CR-Kinase<-?||BrxA-DNA-binding->BrxB-BrxF-iSTAND->DUF6079-p*->N6-METHYLASE->SLATT->SMODS->Schlafen-p->Schlafen+wHTH->PglZ-p->SIGMA+OB-Fold+AAA-ATPase+HKD-DNase-BrxL->TIR-></td><td>480</td><td>Bacteria>Terrabacteria_group>Actinomycetota>Actinomycetes>Bifidobacteriales>Bifidobacteriaceae</td><td>GCF_003370195.1</td><td>Bifidobacterium_breve</td><td>CE167_RS03750</td><td>14573..16012</td><td>1685</td><td>BREX-system-P-loop-protein-BrxC,-partial-[Bifidobacterium-breve]</td></tr>
<tr><td>WP_115955260.1</td><td>Type1.BREX</td><td><-OLD-ABC+TOPRIM<-ZnR<-ZnR+GP<-SIGMA+OB-Fold+AAA-ATPase+HKD-DNase-BrxL<-PglZ<-Schlafen+HATPase+wHTH<-?<-HEPN<-N6-METHYLASE<-DUF6079*<-BrxB-BrxF-iSTAND<-BrxA-DNA-binding<-?<-?<-?<-ABC-ATPase+SbcC<-?<-ZnR</td><td>1217</td><td>Bacteria>Terrabacteria_group>Actinomycetota>Actinomycetes>Bifidobacteriales>Bifidobacteriaceae</td><td>GCF_003390735.1</td><td>Bifidobacterium_bifidum</td><td>CJD48_RS06210</td><td>1417204..1420857</td><td>1681</td><td>BREX-system-P-loop-protein-BrxC-[Bifidobacterium-bifidum]</td></tr>
<tr><td>WP_117658188.1</td><td>Type1.BREX</td><td>Glyoxalase-><-?||REase-><-?<-SIGMA+OB-Fold+AAA-ATPase+HKD-DNase-BrxL<-PglZ<-Schlafen+HATPase+wHTH<-SIR2<-N6-METHYLASE<-DUF6079*<-BrxB-BrxF-iSTAND<-BrxA-DNA-binding<-?<-?<-?<-ABC-ATPase+SbcC<-?<-ZnR</td><td>1218</td><td>Bacteria>Terrabacteria_group>Actinomycetota>Actinomycetes>Bifidobacteriales>Bifidobacteriaceae</td><td>GCF_003436575.1</td><td>Bifidobacterium_bifidum</td><td>DXD34_RS01735</td><td>367842..371498</td><td>1681</td><td>BREX-system-P-loop-protein-BrxC-[Bifidobacterium-bifidum]</td></tr>
<tr><td>WP_117656287.1</td><td>Type1.BREX</td><td><-SIGMA+OB-Fold+AAA-ATPase+HKD-DNase-BrxL<-PglZ<-Schlafen+HATPase+wHTH<-SIR2<-N6-METHYLASE-p<-HerA/FtsK<-SIR2<-N6-METHYLASE<-DUF6079*<-BrxB-BrxF-iSTAND<-BrxA-DNA-binding<-Pentapeptide+Pentapeptide+Pentapeptide+Pentapeptide||?->?->SbcD-></td><td>1218</td><td>Bacteria>Terrabacteria_group>Actinomycetota>Actinomycetes>Bifidobacteriales>Bifidobacteriaceae</td><td>GCF_003437075.1</td><td>Bifidobacterium_pseudocatenulatum</td><td>DXC75_RS03300</td><td>120423..124079</td><td>28026</td><td>BREX-system-P-loop-protein-BrxC-[Bifidobacterium-pseudocatenulatum]</td></tr>
<tr><td>WP_117664462.1</td><td>Type1.BREX</td><td><-ZnR+GP<-SIGMA+OB-Fold+AAA-ATPase+HKD-DNase-BrxL<-PglZ<-Schlafen+HATPase+wHTH<-SIR2<-SIR2<-N6-METHYLASE-p<-N6-METHYLASE-p<-N6-METHYLASE-p<-DUF6079*<-BrxB-BrxF-iSTAND<-BrxA-DNA-binding<-Pentapeptide+Pentapeptide||SbcD-><-ACET+ACET||HTH-><-?<-35exo+HRDC</td><td>1218</td><td>Bacteria>Terrabacteria_group>Actinomycetota>Actinomycetes>Bifidobacteriales>Bifidobacteriaceae</td><td>GCF_003437835.1</td><td>Bifidobacterium_pseudocatenulatum</td><td>DXC45_RS01980</td><td>419525..423181</td><td>28026</td><td>BREX-system-P-loop-protein-BrxC-[Bifidobacterium-pseudocatenulatum]</td></tr>
<tr><td>WP_117759621.1</td><td>Type1.BREX</td><td><-ParB+HNH+RAMA<-ZnR<-ZnR+GP<-SIGMA+OB-Fold+AAA-ATPase+HKD-DNase-BrxL<-PglZ<-Schlafen+HATPase+wHTH<-HEPN<-N6-METHYLASE<-DUF6079*<-BrxB-BrxF-iSTAND<-BrxA-DNA-binding<-?||?-><-Acyl_transf_3||ACET->tRNA-><-PIN<-XIS-HTH</td><td>1223</td><td>Bacteria>Terrabacteria_group>Actinomycetota>Actinomycetes>Bifidobacteriales>Bifidobacteriaceae</td><td>GCF_003458805.1</td><td>Bifidobacterium_adolescentis</td><td>DWX79_RS04615</td><td>1022537..1026208</td><td>1680</td><td>BREX-system-P-loop-protein-BrxC-[Bifidobacterium-adolescentis]</td></tr>
<tr><td>WP_118279738.1</td><td>Type1.BREX</td><td>REase-><-?<-SIGMA+OB-Fold+AAA-ATPase+HKD-DNase-BrxL<-PglZ<-Schlafen+HATPase+wHTH<-REase<-N6-METHYLASE<-DUF6079*<-BrxB-BrxF-iSTAND<-BrxA-DNA-binding||REase->?->SbcD-><-?||?-><-HISKIN<-REC+LuxR-HTH</td><td>1219</td><td>Bacteria>Terrabacteria_group>Actinomycetota>Actinomycetes>Bifidobacteriales>Bifidobacteriaceae</td><td>GCF_003472685.1</td><td>Bifidobacterium_pseudocatenulatum</td><td>DW102_RS06345</td><td>114145..117804</td><td>28026</td><td>BREX-system-P-loop-protein-BrxC-[Bifidobacterium-pseudocatenulatum]</td></tr>
<tr><td>WP_118269378.1</td><td>Type1.BREX</td><td>Glyoxalase-><-?||REase-><-?<-SIGMA+OB-Fold+AAA-ATPase+HKD-DNase-BrxL<-PglZ<-Schlafen+HATPase+wHTH<-SIR2<-N6-METHYLASE<-DUF6079*<-BrxB-BrxF-iSTAND<-BrxA-DNA-binding<-?<-?<-?<-ABC-ATPase+SbcC<-?<-ZnR</td><td>1218</td><td>Bacteria>Terrabacteria_group>Actinomycetota>Actinomycetes>Bifidobacteriales>Bifidobacteriaceae</td><td>GCF_003473145.1</td><td>Bifidobacterium_bifidum</td><td>DW145_RS03305</td><td>289418..293074</td><td>1681</td><td>BREX-system-P-loop-protein-BrxC-[Bifidobacterium-bifidum]</td></tr>
<tr><td>WP_117641243.1</td><td>Type1.BREX</td><td>HTH_28->BrxA-DNA-binding->BrxB-BrxF-iSTAND->DUF6079*->N6-METHYLASE->?->Schlafen+HATPase+wHTH->PglZ->SIGMA+OB-Fold+AAA-ATPase+HKD-DNase-BrxL->ZnR+GP->ZnR->ParB+HNH->Sigma-HTH-></td><td>1219</td><td>Bacteria>Terrabacteria_group>Actinomycetota>Actinomycetes>Bifidobacteriales>Bifidobacteriaceae</td><td>GCF_003475835.1</td><td>Bifidobacterium_longum</td><td>DWZ18_RS09025</td><td>34331..37990</td><td>216816</td><td>BREX-system-P-loop-protein-BrxC-[Bifidobacterium-longum]</td></tr>
<tr><td>WP_124879770.1</td><td>Type1.BREX</td><td>RNasePH->?-><-?<-tRNA<-tRNA||?->HTH_28->BrxA-DNA-binding->BrxB-BrxF-iSTAND->DUF6079*->N6-METHYLASE->SIR2->Schlafen+HATPase+wHTH->PglZ->SIGMA+OB-Fold+AAA-ATPase+HKD-DNase-BrxL->ZnR+GP->ZnR->ParB+HNH->Sigma-HTH-></td><td>1219</td><td>Bacteria>Terrabacteria_group>Actinomycetota>Actinomycetes>Bifidobacteriales>Bifidobacteriaceae</td><td>GCF_003860285.1</td><td>Bifidobacterium_breve</td><td>EH245_RS01900</td><td>428223..431882</td><td>1685</td><td>BREX-system-P-loop-protein-BrxC-[Bifidobacterium-breve]</td></tr>
<tr><td>WP_126029122.1</td><td>Type1.BREX</td><td><-SIGMA+OB-Fold+AAA-ATPase+HKD-DNase-BrxL<-PglZ<-HEPN<-N6-METHYLASE<-DUF6079*<-BrxB-BrxF-iSTAND<-BrxA-DNA-binding<-?||SIGMA-HTH-><-?<-?<-?<-?<-METHYLASE</td><td>1235</td><td>Bacteria>Terrabacteria_group>Actinomycetota>Actinomycetes>Bifidobacteriales>Bifidobacteriaceae</td><td>GCF_003952005.1</td><td>Bifidobacterium_callimiconis</td><td>D2E23_RS00850</td><td>182499..186206</td><td>2306973</td><td>BREX-system-P-loop-protein-BrxC-[Bifidobacterium-callimiconis]</td></tr>
<tr><td>WP_126016404.1</td><td>Type1.BREX</td><td><-ParB+HNH+RAMA<-ParB+HNH<-ZnR<-ZnR+GP<-SIGMA+OB-Fold+AAA-ATPase+HKD-DNase-BrxL<-PglZ<-Schlafen+HATPase+wHTH<-REase<-N6-METHYLASE<-DUF6079*<-BrxB-BrxF-iSTAND<-BrxA-DNA-binding<-?||SbcD-><-ACET+ACET||HTH-><-?<-35exo+HRDC</td><td>1217</td><td>Bacteria>Terrabacteria_group>Actinomycetota>Actinomycetes>Bifidobacteriales>Bifidobacteriaceae</td><td>GCF_003952825.1</td><td>Bifidobacterium_pseudocatenulatum</td><td>CWS99_RS05770</td><td>1339669..1343322</td><td>28026</td><td>BREX-system-P-loop-protein-BrxC-[Bifidobacterium-pseudocatenulatum]</td></tr>
<tr><td>WP_129237752.1</td><td>Type1.BREX</td><td><-SIGMA+OB-Fold+AAA-ATPase+HKD-DNase-BrxL<-PglZ<-Schlafen+HATPase+wHTH<-?<-N6-METHYLASE<-DUF6079*<-BrxB-BrxF-iSTAND<-BrxA-DNA-binding||HEPN-><-AAA-ATPase<-HTH+rve<-SIR2||?->Glycoside_Hydrolase-><-ACET+ACET</td><td>1221</td><td>Bacteria>Terrabacteria_group>Actinomycetota>Actinomycetes>Bifidobacteriales>Bifidobacteriaceae</td><td>GCF_004135085.1</td><td>Bifidobacterium_pullorum</td><td>ESN35_RS07340</td><td>1680432..1684097</td><td>78448</td><td>BREX-system-P-loop-protein-BrxC-[Bifidobacterium-pullorum]</td></tr>
</table>

Protein-ID	System	Gene-Neighbourhoods	Length	Taxonomy	Assembly-ID	Strain	Locus-Tag	Genomic-coordinates	TaxID	Fasta-define
WP_130013100.1	Type1.BREX	MODE-HTH+PBPII->YTCD-HTH-><-HTH_3<-RelE-ParE<-ACET+ACET<-ZnR<-ZnR|| BrxA-DNA-binding -> BrxB-BrxF-iSTAND -> DUF6079 *-> PglZ -> SIGMA+OB-Fold+AAA-ATPase+ HKD-DNase -BrxL ->SIR2-><-PRTase	1214	Bacteria>Terrabacteria_group>Actinomycetota>Actinomycetes>Bifidobacteriales>Bifidobacteriaceae	GCF_004155015.1	Bifidobacterium_pseudolongum	PG2054B_RS04700	182910..186554	1694	BREX-system-P-loop-protein-BrxC-[Bifidobacterium-pseudolongum]
WP_129902658.1	Type1.BREX	HAD+HAD->MODE-HTH+PBPII->YTCD-HTH-><-HTH_3<-RelE-ParE<-ACET+ACET<-ZnR|| BrxA-DNA-binding -> BrxB-BrxF-iSTAND -> DUF6079 *-> N6-METHYLASE -> PglZ -> SIGMA+OB-Fold+AAA-ATPase+ HKD-DNase -BrxL -><-PRTase	1214	Bacteria>Terrabacteria_group>Actinomycetota>Actinomycetes>Bifidobacteriales>Bifidobacteriaceae	GCF_004155045.1	Bifidobacterium_pseudolongum	PG2083B_RS04940	90141..93785	1694	BREX-system-P-loop-protein-BrxC-[Bifidobacterium-pseudolongum]
WP_129867688.1	Type1.BREX	HAD+HAD->MODE-HTH+PBPII->YTCD-HTH-><-HTH_3<-RelE-ParE<-ACET+ACET<-ZnR|| BrxA-DNA-binding -> BrxB-BrxF-iSTAND -> DUF6079 *-> N6-METHYLASE -> PglZ -> SIGMA+OB-Fold+AAA-ATPase+ HKD-DNase -BrxL -><-PRTase	1212	Bacteria>Terrabacteria_group>Actinomycetota>Actinomycetes>Bifidobacteriales>Bifidobacteriaceae	GCF_004155405.1	Bifidobacterium_pseudolongum	PG102017_RS04060	111812..115450	1694	BREX-system-P-loop-protein-BrxC-[Bifidobacterium-pseudolongum]
WP_129914280.1	Type1.BREX	MODE-HTH+PBPII->YTCD-HTH-><-HTH_3<-RelE-ParE<-ACET+ACET<-ZnR<-ZnR|| BrxA-DNA-binding -> BrxB-BrxF-iSTAND -> DUF6079 *-> N6-METHYLASE -p-> N6-METHYLASE -> PglZ -> SIGMA+OB-Fold+AAA-ATPase+ HKD-DNase -BrxL ->SIR2-><-PRTase	1212	Bacteria>Terrabacteria_group>Actinomycetota>Actinomycetes>Bifidobacteriales>Bifidobacteriaceae	GCF_004155645.1	Bifidobacterium_pseudolongum	PG2001B_RS04910	52519..56157	1694	BREX-system-P-loop-protein-BrxC-[Bifidobacterium-pseudolongum]
WP_129869778.1	Type1.BREX	HAD+HAD->MODE-HTH+PBPII->YTCD-HTH-><-ACET+ACET<-ZnR|| BrxA-DNA-binding -> BrxB-BrxF-iSTAND -> DUF6079 *-> N6-METHYLASE -> PglZ -> SIGMA+OB-Fold+AAA-ATPase+ HKD-DNase -BrxL -><-PRTase	1214	Bacteria>Terrabacteria_group>Actinomycetota>Actinomycetes>Bifidobacteriales>Bifidobacteriaceae	GCF_004155805.1	Bifidobacterium_pseudolongum	PG1550B_RS04805	1102192..1105836	1694	BREX-system-P-loop-protein-BrxC-[Bifidobacterium-pseudolongum]
WP_129900679.1	Type1.BREX	<-HTH_3<-RelE-ParE<-ACET+ACET<-ZnR|| BrxA-DNA-binding -> BrxB-BrxF-iSTAND -> DUF6079 *-> N6-METHYLASE -> PglZ -> SIGMA+OB-Fold+AAA-ATPase+ HKD-DNase -BrxL -><-PRTase	1214	Bacteria>Terrabacteria_group>Actinomycetota>Actinomycetes>Bifidobacteriales>Bifidobacteriaceae	GCF_004155855.1	Bifidobacterium_pseudolongum	PG112206_RS04240	4644..8288	1694	BREX-system-P-loop-protein-BrxC-[Bifidobacterium-pseudolongum]
WP_129917395.1	Type1.BREX	BrxA-DNA-binding -> BrxB-BrxF-iSTAND -> DUF6079 *-> N6-METHYLASE -> PglZ -> SIGMA+OB-Fold+AAA-ATPase+ HKD-DNase -BrxL ->SIR2-><-PRTase	1214	Bacteria>Terrabacteria_group>Actinomycetota>Actinomycetes>Bifidobacteriales>Bifidobacteriaceae	GCF_004156175.1	Bifidobacterium_pseudolongum	PG2103B_RS04555	1564..5208	1694	BREX-system-P-loop-protein-BrxC-[Bifidobacterium-pseudolongum]
WP_131220402.1	Type1.BREX	HTH_28-> BrxA-DNA-binding -> BrxB-BrxF-iSTAND -> DUF6079 *-> N6-METHYLASE ->?->Schlafen+HATPase+wHTH-> PglZ -> SIGMA+OB-Fold+AAA-ATPase+ HKD-DNase -BrxL ->ZnR+GP->ZnR->ParB+HNH->Sigma-HTH->	1219	Bacteria>Terrabacteria_group>Actinomycetota>Actinomycetes>Bifidobacteriales>Bifidobacteriaceae	GCF_004334345.1	Bifidobacterium_longum	MCC10040_RS02080	100674..104333	216816	BREX-system-P-loop-protein-BrxC-[Bifidobacterium-longum]
WP_136510830.1	Type1.BREX	BrxA-DNA-binding -> BrxB-BrxF-iSTAND -> DUF6079 *-> N6-METHYLASE -> PglZ -> SIGMA+OB-Fold+AAA-ATPase+ HKD-DNase -BrxL ->SIR2-><-PRTase	1214	Bacteria>Terrabacteria_group>Actinomycetota>Actinomycetes>Bifidobacteriales>Bifidobacteriaceae	GCF_004801625.1	Bifidobacterium_pseudolongum	E5991_RS00930	3050..6694	1694	BREX-system-P-loop-protein-BrxC-[Bifidobacterium-pseudolongum]
WP_140556589.1	Type1.BREX	<-Cysteine-Desulfurase<-ABC-ATPase+SbcC<-?<-?<-?<- SIGMA+OB-Fold+AAA-ATPase+ HKD-DNase -BrxL <- PglZ <-Schlafen+HATPase+wHTH<- N6-METHYLASE <- DUF6079 *<- BrxB-BrxF-iSTAND <- BrxA-DNA-binding <-?<-Peptidase_M20<-?<-?<-?||Peptidase_A24->	1232	Bacteria>Terrabacteria_group>Actinomycetota>Actinomycetes>Bifidobacteriales>Bifidobacteriaceae	GCF_006408715.1	Bifidobacterium_sp._UTCIF-39	EP30_RS04700	102584..106282	1465359	BREX-system-P-loop-protein-BrxC-[Bifidobacterium-sp.-UTCIF-39]
WP_142421439.1	Type1.BREX	RNaseH-><-?<- SIGMA+OB-Fold+AAA-ATPase+ HKD-DNase -BrxL <- PglZ <-Schlafen+HATPase+wHTH<-REase<- N6-METHYLASE -p<- N6-METHYLASE <- DUF6079 *<- BrxB-BrxF-iSTAND <- BrxA-DNA-binding ||REase->?-><-ACET+ACET||HTH-><-?<-35exo+HRDC	1218	Bacteria>Terrabacteria_group>Actinomycetota>Actinomycetes>Bifidobacteriales>Bifidobacteriaceae	GCF_006828885.1	Bifidobacterium_pseudocatenulatum	GH753_RS08045	1981980..1985636	28026	BREX-system-P-loop-protein-BrxC-[Bifidobacterium-pseudocatenulatum]
WP_150379196.1	Type1.BREX	<-SbcD||?-><-ABhydrolase|| BrxA-DNA-binding -> BrxB-BrxF-iSTAND -> DUF6079 *->CR-ATPase5+REase->TPR+TPR-> N6-METHYLASE ->HEPN->MazE_antitoxin-> PglZ -> SIGMA+OB-Fold+AAA-ATPase+ HKD-DNase -BrxL ->METHYLASE->	1267	Bacteria>Terrabacteria_group>Actinomycetota>Actinomycetes>Bifidobacteriales>Bifidobacteriaceae	GCF_008698155.1	Bifidobacterium_myosotis	EM091_RS06000	83624..87427	1630166	BREX-system-P-loop-protein-BrxC-[Bifidobacterium-myosotis]
WP_150382415.1	Type1.BREX	ABC-ATPase+SbcC-><-CR-ATPase5+REase||?-><-ZnR||MazE_antitoxin->PemK_toxin-> BrxA-DNA-binding -> BrxB-BrxF-iSTAND -> DUF6079 *-> N6-METHYLASE ->?->?->?->HEPN+RES->MazE_antitoxin->PemK_toxin-> PglZ -> SIGMA+OB-Fold+AAA-ATPase+ HKD-DNase -BrxL ->	1244	Bacteria>Terrabacteria_group>Actinomycetota>Actinomycetes>Bifidobacteriales>Bifidobacteriaceae	GCF_008698235.1	Bifidobacterium_rousetti	CSQ85_RS00640	119339..123073	2045439	BREX-system-P-loop-protein-BrxC-[Bifidobacterium-rousetti]
WP_151916615.1	Type1.BREX	HNH->||DOC+HTH-><-sGTP||?->?->?-><-HAD+HAD|| BrxA-DNA-binding -> BrxB-BrxF-iSTAND -> DUF6079 *->CR-ATPase5+REase->TPR+TPR-> N6-METHYLASE -><-Tox-RelE<-RelB||CR-ATPase5+REase-> PglZ -> SIGMA+OB-Fold+AAA-ATPase+ HKD-DNase -BrxL ->METHYLASE->	1269	Bacteria>Terrabacteria_group>Actinomycetota>Actinomycetes>Bifidobacteriales>Bifidobacteriaceae	GCF_009078285.1	Bifidobacterium_jacchi	EHS19_RS04610	26586..30395	2490545	BREX-system-P-loop-protein-BrxC-[Bifidobacterium-jacchi]
WP_152233646.1	Type1.BREX	REase+Bacteriocin_IIc->?->?->?->?->?-><-?|| BrxA-DNA-binding -> BrxB-BrxF-iSTAND -> DUF6079 *-><-?||CR-ATPase5+REase-> N6-METHYLASE ->?->?->HEPN+RES->Schlafen+HATPase+wHTH-> PglZ -> SIGMA+OB-Fold+AAA-ATPase+ HKD-DNase -BrxL ->	1235	Bacteria>Terrabacteria_group>Actinomycetota>Actinomycetes>Bifidobacteriales>Bifidobacteriaceae	GCF_009193355.1	Bifidobacterium_leontopitheci	F7D09_RS01345	123122..126829	2650774	BREX-system-P-loop-protein-BrxC-[Bifidobacterium-leontopitheci]
WP_117762529.1	Type1.BREX	<-ParB+HNH+RAMA<-ZnR<-ZnR+GP<- SIGMA+OB-Fold+AAA-ATPase+ HKD-DNase -BrxL <- PglZ <-Schlafen+HATPase+wHTH<-HEPN<- N6-METHYLASE <- DUF6079 *<- BrxB-BrxF-iSTAND <- BrxA-DNA-binding <-?||?-><-Acyl_transf_3||ACET->	1223	Bacteria>Terrabacteria_group>Actinomycetota>Actinomycetes>Bifidobacteriales>Bifidobacteriaceae	GCF_009832825.1	Bifidobacterium_adolescentis	DWZ00_RS05085	1174565..1178236	1680	BREX-system-P-loop-protein-BrxC-[Bifidobacterium-adolescentis]
WP_163196475.1	Type1.BREX	<- SIGMA+OB-Fold+AAA-ATPase+ HKD-DNase -BrxL <- PglZ <-Schlafen+HATPase+wHTH<-?<- N6-METHYLASE <- DUF6079 *<- BrxB-BrxF-iSTAND <- BrxA-DNA-binding <-ABC-ATPase+SbcC<-?<-ZnR<-?<-Cas_Cas1	1248	Bacteria>Terrabacteria_group>Actinomycetota>Actinomycetes>Bifidobacteriales>Bifidobacteriaceae	GCF_010667645.1	Bifidobacterium_platyrrhinorum	GFD21_RS03310	757948..761694	2661628	BREX-system-P-loop-protein-BrxC-[Bifidobacterium-platyrrhinorum]
WP_163113202.1	Type1.BREX	MULE-TRANSPOSASE-><-ParB+HNH<-ZnR<-ZnR+GP<- SIGMA+OB-Fold+AAA-ATPase+ HKD-DNase -BrxL <- PglZ <-Schlafen+HATPase+wHTH<-SIR2<- N6-METHYLASE <- DUF6079 *<- BrxB-BrxF-iSTAND <- BrxA-DNA-binding ||REase-><-HTH+HisKin<-?<-?||?-><-REase	1218	Bacteria>Terrabacteria_group>Actinomycetota>Actinomycetes>Bifidobacteriales>Bifidobacteriaceae	GCF_011029205.1	Bifidobacterium_bifidum	G5T23_RS07350	43765..47421	1681	BREX-system-P-loop-protein-BrxC-[Bifidobacterium-bifidum]
WP_168445148.1	Type1.BREX	<-Glyoxalase||DSBH+AraC-HTH+AraC-HTH-><-ZnR<- SIGMA+OB-Fold+AAA-ATPase+ HKD-DNase -BrxL <- PglZ <- PglZ <-Schlafen+HATPase+wHTH<-?<- N6-METHYLASE <- DUF6079 *<- BrxB-BrxF-iSTAND <- BrxA-DNA-binding ||REase-><-HEPN<-?||HTH-><-35exo+BRCT<-?<-35exo+HRDC	1217	Bacteria>Terrabacteria_group>Actinomycetota>Actinomycetes>Bifidobacteriales>Bifidobacteriaceae	GCF_012310535.1	Bifidobacterium_dentium	PG133_RS04075	130679..134332	1689	BREX-system-P-loop-protein-BrxC-[Bifidobacterium-dentium]
WP_169274836.1	Type1.BREX	<-PIN<-RelB<-?||HTH_3->?->PIN-> BrxA-DNA-binding -> BrxB-BrxF-iSTAND -> DUF6079 *->CR-ATPase5+REase-> N6-METHYLASE ->Schlafen+HATPase+wHTH-> PglZ -> SIGMA+OB-Fold+AAA-ATPase+ HKD-DNase -BrxL ->REase->?-> BrxA-DNA-binding ->	1226	Bacteria>Terrabacteria_group>Actinomycetota>Actinomycetes>Bifidobacteriales>Bifidobacteriaceae	GCF_012932365.1	Bifidobacterium_sp._DSM_109958	G1C96_RS01295	288582..292262	2705430	BREX-system-P-loop-protein-BrxC-[Bifidobacterium-sp.-DSM-109958]
WP_193020253.1	Type1.BREX	HTH+MPTase-><-?<-HEPN<-?<-?||?-> BrxA-DNA-binding -> BrxB-BrxF-iSTAND -> DUF6079 *-> N6-METHYLASE ->REase->Schlafen+wHTH-> PglZ -> SIGMA+OB-Fold+AAA-ATPase+ HKD-DNase -BrxL ->?->?->RNase->	1229	Bacteria>Terrabacteria_group>Actinomycetota>Actinomycetes>Bifidobacteriales>Bifidobacteriaceae	GCF_014898135.1	Bifidobacterium_longum	BL5934_RS04380	976256..979945	216816	BREX-system-P-loop-protein-BrxC-[Bifidobacterium-longum]
WP_200406515.1	Type1.BREX	HTH_28+HTH_28->HTH_Tnp_1+HTH_21+rve+rve_3->HTH_28->DEDD_Tnp_IS110+Transposase_20-> BrxA-DNA-binding -> BrxB-BrxF-iSTAND -> DUF6079 *-> N6-METHYLASE ->?->Schlafen+HATPase+wHTH-> PglZ -> SIGMA+OB-Fold+AAA-ATPase+ HKD-DNase -BrxL ->?-><-?<-?<-ABC-ATPase+SbcC	1219	Bacteria>Terrabacteria_group>Actinomycetota>Actinomycetes>Bifidobacteriales>Bifidobacteriaceae	GCF_014898255.1	Bifidobacterium_longum	BL7055_RS03240	790763..794422	216816	BREX-system-P-loop-protein-BrxC-[Bifidobacterium-longum]
WP_227411048.1	Type1.BREX	ACET->ACET+ACET->?->Thioredoxin_3->ArsR-HTH-><-Glycoside_Hydrolase||OLD-ABC-> BrxA-DNA-binding -> BrxB-BrxF-iSTAND -> DUF6079 *-> N6-METHYLASE ->REase->?->Schlafen+HATPase+wHTH-> PglZ -> SIGMA+OB-Fold+AAA-ATPase+ HKD-DNase -BrxL ->	1242	Bacteria>Terrabacteria_group>Actinomycetota>Actinomycetes>Bifidobacteriales>Bifidobacteriaceae	GCF_014983005.1	Bifidobacterium_pullorum	INF27_RS02890	183960..187688	78448	BREX-system-P-loop-protein-BrxC-[Bifidobacterium-pullorum]
WP_195393981.1	Type1.BREX	PemK_toxin->?->PolB->HEPN-><-?<-?||?-> BrxA-DNA-binding -> BrxB-BrxF-iSTAND -> DUF6079 *-> N6-METHYLASE ->SIR2->Schlafen+HATPase+wHTH-> PglZ -> SIGMA+OB-Fold+AAA-ATPase+ HKD-DNase -BrxL ->ZnR+GP->ZnR->ParB+HNH+RAMA->	1218	Bacteria>Terrabacteria_group>Actinomycetota>Actinomycetes>Bifidobacteriales>Bifidobacteriaceae	GCF_015553845.1	Bifidobacterium_adolescentis	I2D91_RS01775	423737..427393	1680	BREX-system-P-loop-protein-BrxC-[Bifidobacterium-adolescentis]
WP_195451643.1	Type1.BREX	<-ABhydrolase<-?<-Glyoxalase||DSBH+AraC-HTH+AraC-HTH-><-ZnR<- SIGMA+OB-Fold+AAA-ATPase+ HKD-DNase -BrxL <- PglZ <-Schlafen+HATPase+wHTH<- N6-METHYLASE <- DUF6079 *<- BrxB-BrxF-iSTAND <- BrxA-DNA-binding <-?||ACET-><-?||HTH-><-35exo+BRCT<-35exo+HRDC	1217	Bacteria>Terrabacteria_group>Actinomycetota>Actinomycetes>Bifidobacteriales>Bifidobacteriaceae	GCF_015555105.1	Bifidobacterium_dentium	I2E42_RS03170	226129..229782	1689	BREX-system-P-loop-protein-BrxC-[Bifidobacterium-dentium]
WP_195482034.1	Type1.BREX	<-ParB+HNH+RAMA<-ZnR<-ZnR+GP<- SIGMA+OB-Fold+AAA-ATPase+ HKD-DNase -BrxL <- PglZ <-Schlafen+HATPase+wHTH<-SIR2<- N6-METHYLASE <- DUF6079 *<- BrxB-BrxF-iSTAND <- BrxA-DNA-binding <-?||?-><-?<-HEPN<-PolB<-PemK_toxin<-RelB	1218	Bacteria>Terrabacteria_group>Actinomycetota>Actinomycetes>Bifidobacteriales>Bifidobacteriaceae	GCF_015555595.1	Bifidobacterium_adolescentis	I2E65_RS01565	362593..366249	1680	BREX-system-P-loop-protein-BrxC-[Bifidobacterium-adolescentis]
WP_195936831.1	Type1.BREX	REase-><-?<- SIGMA+OB-Fold+AAA-ATPase+ HKD-DNase -BrxL <- PglZ <-Schlafen+HATPase+wHTH<-?<- N6-METHYLASE <- DUF6079 *<- BrxB-BrxF-iSTAND <- BrxA-DNA-binding <-?<-?<-ABC-ATPase+SbcC<-ZnR	1218	Bacteria>Terrabacteria_group>Actinomycetota>Actinomycetes>Bifidobacteriales>Bifidobacteriaceae	GCF_015667555.1	Bifidobacterium_bifidum	I4U71_RS07235	13396..17052	1681	BREX-system-P-loop-protein-BrxC-[Bifidobacterium-bifidum]
WP_195938980.1	Type1.BREX	35exo+HRDC-><-HTH||ACET+ACET-><-?<-SbcD<-?<-REase|| BrxA-DNA-binding -> BrxB-BrxF-iSTAND -> DUF6079 *-> N6-METHYLASE ->SIR2->Schlafen+HATPase+wHTH-> PglZ -> SIGMA+OB-Fold+AAA-ATPase+ HKD-DNase -BrxL ->ZnR+GP->ZnR->ParB+HNH+RAMA->	1217	Bacteria>Terrabacteria_group>Actinomycetota>Actinomycetes>Bifidobacteriales>Bifidobacteriaceae	GCF_015667675.1	Bifidobacterium_pseudocatenulatum	I4U64_RS06630	371599..375252	28026	BREX-system-P-loop-protein-BrxC-[Bifidobacterium-pseudocatenulatum]
WP_196336889.1	Type1.BREX	35exo+HRDC->35exo+BRCT-><-HTH||?-><-ACET||?-> BrxA-DNA-binding -> BrxB-BrxF-iSTAND -> DUF6079 *-> N6-METHYLASE ->HEPN->?->Schlafen+HATPase+wHTH-> PglZ -> SIGMA+OB-Fold+AAA-ATPase+ HKD-DNase -BrxL ->ZnR-><-DSBH+AraC-HTH+AraC-HTH||Glyoxalase->	1217	Bacteria>Terrabacteria_group>Actinomycetota>Actinomycetes>Bifidobacteriales>Bifidobacteriaceae	GCF_015703835.1	Bifidobacterium_dentium	IHQ82_RS06050	179848..183501	1689	BREX-system-P-loop-protein-BrxC-[Bifidobacterium-dentium]
WP_202574831.1	Type1.BREX	<-Peptidase_S26<-?||ABC-ATPase+SbcC->?-><-?<- SIGMA+OB-Fold+AAA-ATPase+ HKD-DNase -BrxL <- PglZ <-Schlafen+HATPase+wHTH<- N6-METHYLASE <- DUF6079 *<- BrxB-BrxF-iSTAND <- BrxA-DNA-binding <-HTH_28	1219	Bacteria>Terrabacteria_group>Actinomycetota>Actinomycetes>Bifidobacteriales>Bifidobacteriaceae	GCF_016759645.1	Bifidobacterium_longum	JL737_RS05465	24879..28538	216816	BREX-system-P-loop-protein-BrxC-[Bifidobacterium-longum]
WP_204464696.1	Type1.BREX	ACET-><-O6MGT-HTH||Phage_integrase-><-?<- SIGMA+OB-Fold+AAA-ATPase+ HKD-DNase -BrxL <- PglZ <-Schlafen+HATPase+wHTH<- N6-METHYLASE <- DUF6079 *<- BrxB-BrxF-iSTAND <- BrxA-DNA-binding <-?<-?<-?||Glycoside_Hydrolase-><-ArsR-HTH<-Thioredoxin_3	1221	Bacteria>Terrabacteria_group>Actinomycetota>Actinomycetes>Bifidobacteriales>Bifidobacteriaceae	GCF_016899675.1	Bifidobacterium_pullorum	H6A68_RS03865	164290..167955	78448	BREX-system-P-loop-protein-BrxC-[Bifidobacterium-pullorum]
WP_204389272.1	Type1.BREX	<-IstB_IS21||AAA-ATPase-><-CMP-hydrolase<- SIGMA+OB-Fold+AAA-ATPase+ HKD-DNase -BrxL <- PglZ <-Schlafen+HATPase+wHTH<- N6-METHYLASE <- DUF6079 *<- BrxB-BrxF-iSTAND <- BrxA-DNA-binding <-TOPRIM<-OLD-ABC||Glycoside_Hydrolase-><-ACET+ACET<-ACET	1221	Bacteria>Terrabacteria_group>Actinomycetota>Actinomycetes>Bifidobacteriales>Bifidobacteriaceae	GCF_016900615.1	Bifidobacterium_pullorum	H6A14_RS02700	186110..189775	78448	BREX-system-P-loop-protein-BrxC-[Bifidobacterium-pullorum]
WP_217054570.1	Type1.BREX	<- SIGMA+OB-Fold+AAA-ATPase+ HKD-DNase -BrxL <- PglZ <-HEPN+RES<-?<-?<-REase<- N6-METHYLASE <- DUF6079 *<- BrxB-BrxF-iSTAND <- BrxA-DNA-binding ||?->CR-Kinase-><-CR-ATPase5+REase-p<-REase<-HTH_3	1229	Bacteria>Terrabacteria_group>Actinomycetota>Actinomycetes>Bifidobacteriales>Bifidobacteriaceae	GCF_019041665.1	Bifidobacterium_longum	KTF47_RS04030	15252..18941	216816	BREX-system-P-loop-protein-BrxC-[Bifidobacterium-longum]
WP_219100165.1	Type1.BREX	<-ABhydrolase||?->MACRODOMAIN-><-?<- SIGMA+OB-Fold+AAA-ATPase+ HKD-DNase -BrxL <- PglZ <-?<-?<- N6-METHYLASE <- DUF6079 *<- BrxB-BrxF-iSTAND <- BrxA-DNA-binding ||?-><-ABhydrolase	1253	Bacteria>Terrabacteria_group>Actinomycetota>Actinomycetes>Bifidobacteriales>Bifidobacteriaceae	GCF_019331695.1	Bifidobacterium_pluvialisilvae	KIH77_RS05905	203323..207084	2834436	BREX-system-P-loop-protein-BrxC-[Bifidobacterium-pluvialisilvae]
WP_226586661.1	Type1.BREX	REC+LuxR-HTH->HISKIN->?->?-><-SbcD<-?<-REase|| BrxA-DNA-binding -> BrxB-BrxF-iSTAND -> DUF6079 *-> N6-METHYLASE ->?->Schlafen+HATPase+wHTH-> PglZ -> SIGMA+OB-Fold+AAA-ATPase+ HKD-DNase -BrxL ->SLATT->SMODS->ParB+HNH+RAMA->	1219	Bacteria>Terrabacteria_group>Actinomycetota>Actinomycetes>Bifidobacteriales>Bifidobacteriaceae	GCF_020525765.1	Bifidobacterium_pseudocatenulatum	PG776_RS01085	258368..262027	28026	BREX-system-P-loop-protein-BrxC-[Bifidobacterium-pseudocatenulatum]
WP_226650300.1	Type1.BREX	<- SIGMA+OB-Fold+AAA-ATPase+ HKD-DNase -BrxL <- PglZ <-Schlafen+HATPase+wHTH<-REase<- N6-METHYLASE <- DUF6079 *<- BrxB-BrxF-iSTAND <- BrxA-DNA-binding ||REase->?->SbcD-><-?||?-><-?||MerR-HTH->	1219	Bacteria>Terrabacteria_group>Actinomycetota>Actinomycetes>Bifidobacteriales>Bifidobacteriaceae	GCF_020526585.1	Bifidobacterium_pseudocatenulatum	KZP10_RS05695	1065058..1068717	28026	BREX-system-P-loop-protein-BrxC-[Bifidobacterium-pseudocatenulatum]
WP_235341612.1	Type1.BREX	METHYLASE->?->Glycos_transf_4-><-?||?->?-> BrxA-DNA-binding -> BrxB-BrxF-iSTAND -> DUF6079 *-> PglZ -> SIGMA+OB-Fold+AAA-ATPase+ HKD-DNase -BrxL ->?->?->35exo->HAD+S6PP->SF1-SF2-Helicase+RNase->	1238	Bacteria>Terrabacteria_group>Actinomycetota>Actinomycetes>Bifidobacteriales>Bifidobacteriaceae	GCF_021531155.1	Bifidobacterium_boum	I6E06_RS03585	150899..154615	78343	BREX-system-P-loop-protein-BrxC-[Bifidobacterium-boum]
WP_237919300.1	Type1.BREX	ACET+ACET->?-><-?||?-><-SbcD||?-><-?|| BrxA-DNA-binding -> BrxB-BrxF-iSTAND -> DUF6079 *-> N6-METHYLASE ->SIR2->Schlafen+HATPase+wHTH-> PglZ -> SIGMA+OB-Fold+AAA-ATPase+ HKD-DNase -BrxL ->	1219	Bacteria>Terrabacteria_group>Actinomycetota>Actinomycetes>Bifidobacteriales>Bifidobacteriaceae	GCF_022135125.1	Bifidobacterium_pseudocatenulatum	LOM88_RS00205	52697..56356	28026	BREX-system-P-loop-protein-BrxC-[Bifidobacterium-pseudocatenulatum]
WP_237970585.1	Type1.BREX	HTH_28-> BrxA-DNA-binding -> BrxB-BrxF-iSTAND -> DUF6079 *-> N6-METHYLASE ->?->Schlafen+HATPase+wHTH-> PglZ -> SIGMA+OB-Fold+AAA-ATPase+ HKD-DNase -BrxL ->ZnR+GP->ZnR->ParB+HNH->Sigma-HTH->	1218	Bacteria>Terrabacteria_group>Actinomycetota>Actinomycetes>Bifidobacteriales>Bifidobacteriaceae	GCF_022136875.1	Bifidobacterium_longum	LOO84_RS04690	81950..85606	216816	BREX-system-P-loop-protein-BrxC-[Bifidobacterium-longum]
WP_273192946.1	Type1.BREX	<-SF1-SF2-Helicase||?->?-><-SPOUT||ENDO3+NUDIX->?-> BrxA-DNA-binding -> BrxB-BrxF-iSTAND -> DUF6079 *->?-> N6-METHYLASE -><- N6-METHYLASE -p||Phage_int_SAM_3+Phage_integrase-> N6-METHYLASE -p-> PglZ -> SIGMA+OB-Fold+AAA-ATPase+ HKD-DNase -BrxL ->	1173	Bacteria>Terrabacteria_group>Actinomycetota>Actinomycetes>Bifidobacteriales>Bifidobacteriaceae	GCF_022487345.1	Bifidobacterium_tibiigranuli	LKF49_RS06765	121598..125119	2172043	BREX-system-P-loop-protein-BrxC-[Bifidobacterium-tibiigranuli]
WP_240833140.1	Type1.BREX	<-Abhydrolase<-ParB+HNH+RAMA<-ZnR<-ZnR+GP<- SIGMA+OB-Fold+AAA-ATPase+ HKD-DNase -BrxL <- PglZ <-Schlafen+HATPase+wHTH<-?<- N6-METHYLASE <- DUF6079 *<- BrxB-BrxF-iSTAND <- BrxA-DNA-binding ||REase->?-><-?||SbcD->	1217	Bacteria>Terrabacteria_group>Actinomycetota>Actinomycetes>Bifidobacteriales>Bifidobacteriaceae	GCF_022496395.1	Bifidobacterium_pseudocatenulatum	LF914_RS07635	54595..58248	28026	BREX-system-P-loop-protein-BrxC-[Bifidobacterium-pseudocatenulatum]
WP_242075534.1	Type1.BREX	HTH_28-> BrxA-DNA-binding -> BrxB-BrxF-iSTAND -> DUF6079 *-> N6-METHYLASE ->Schlafen+HATPase+wHTH-> PglZ -> SIGMA+OB-Fold+AAA-ATPase+ HKD-DNase -BrxL ->?-><-?<-?<-ABC-ATPase+SbcC	1218	Bacteria>Terrabacteria_group>Actinomycetota>Actinomycetes>Bifidobacteriales>Bifidobacteriaceae	GCF_022643745.1	Bifidobacterium_longum	G8B15_RS07005	1527026..1530682	216816	BREX-system-P-loop-protein-BrxC-[Bifidobacterium-longum]
WP_032684176.1	Type1.BREX	<- SIGMA+OB-Fold+AAA-ATPase+ HKD-DNase -BrxL <- PglZ <-Schlafen+HATPase+wHTH<- N6-METHYLASE -p<-HerA/FtsK<-SIR2<- N6-METHYLASE <- DUF6079 *<- BrxB-BrxF-iSTAND <- BrxA-DNA-binding <-HTH_28	1219	Bacteria>Terrabacteria_group>Actinomycetota>Actinomycetes>Bifidobacteriales>Bifidobacteriaceae	GCF_022701235.1	Bifidobacterium_longum	LMY38_RS03520	757375..761034	216816	BREX-system-P-loop-protein-BrxC-[Bifidobacterium-longum]
WP_248110663.1	Type1.BREX	35exo+HRDC->?->35exo+BRCT-><-HTH||?-><-ACET||?-> BrxA-DNA-binding -> BrxB-BrxF-iSTAND -> DUF6079 *-> N6-METHYLASE ->?->Schlafen+HATPase+wHTH-> PglZ -> SIGMA+OB-Fold+AAA-ATPase+ HKD-DNase -BrxL ->ZnR-><-DSBH+AraC-HTH+AraC-HTH||Glyoxalase->	1219	Bacteria>Terrabacteria_group>Actinomycetota>Actinomycetes>Bifidobacteriales>Bifidobacteriaceae	GCF_023147995.1	Bifidobacterium_dentium	KZX63_RS03535	164390..168049	1689	BREX-system-P-loop-protein-BrxC-[Bifidobacterium-dentium]
WP_269374243.1	Type1.BREX	<-Sigma-HTH<-ParB+HNH<-ZnR<-ZnR+GP<- SIGMA+OB-Fold+AAA-ATPase+ HKD-DNase -BrxL <- PglZ <-Schlafen+HATPase+wHTH<-SIR2<- N6-METHYLASE <- DUF6079 *<- BrxB-BrxF-iSTAND <- BrxA-DNA-binding	1219	Bacteria>Terrabacteria_group>Actinomycetota>Actinomycetes>Bifidobacteriales>Bifidobacteriaceae	GCF_027214485.1	Bifidobacterium_breve	O4C76_RS09020	75244..78903	1685	BREX-system-P-loop-protein-BrxC-[Bifidobacterium-breve]
WP_269355165.1	Type1.BREX	<-Sigma-HTH<-ParB+HNH<-ZnR<-ZnR+GP<- SIGMA+OB-Fold+AAA-ATPase+ HKD-DNase -BrxL <- PglZ <-Schlafen+HATPase+wHTH<-SIR2<- N6-METHYLASE <- DUF6079 *<- BrxB-BrxF-iSTAND <- BrxA-DNA-binding <-HTH_28<-?||tRNA->tRNA->?-><-?<-RNasePH	1218	Bacteria>Terrabacteria_group>Actinomycetota>Actinomycetes>Bifidobacteriales>Bifidobacteriaceae	GCF_027215185.1	Bifidobacterium_breve	O4C27_RS08995	67566..71222	1685	BREX-system-P-loop-protein-BrxC-[Bifidobacterium-breve]
WP_270294263.1	Type1.BREX	<- SIGMA+OB-Fold+AAA-ATPase+ HKD-DNase -BrxL <- PglZ <-Schlafen+HATPase+wHTH<-SIR2<- N6-METHYLASE -p<-HerA/FtsK<-SIR2<- N6-METHYLASE <- DUF6079 *<- BrxB-BrxF-iSTAND <- BrxA-DNA-binding ||REase->?->SbcD-><-?||?-><-HISKIN<-REC+LuxR-HTH	1219	Bacteria>Terrabacteria_group>Actinomycetota>Actinomycetes>Bifidobacteriales>Bifidobacteriaceae	GCF_027659585.1	Bifidobacterium_adolescentis	PGD90_RS01995	459377..463036	1680	BREX-system-P-loop-protein-BrxC-[Bifidobacterium-adolescentis]
WP_270267401.1	Type1.BREX	<- SIGMA+OB-Fold+AAA-ATPase+ HKD-DNase -BrxL <- PglZ <-RNase<-BirA-HTH<-Schlafen+HATPase+wHTH<-SMODS<-SLATT<- N6-METHYLASE <- DUF6079 *<- BrxB-BrxF-iSTAND <- BrxA-DNA-binding ||?->CR-Kinase-><-CR-ATPase5+REase-p<- N6-METHYLASE <-RelB	1229	Bacteria>Terrabacteria_group>Actinomycetota>Actinomycetes>Bifidobacteriales>Bifidobacteriaceae	GCF_027663845.1	Bifidobacterium_breve	PGQ90_RS07415	209217..212906	1685	BREX-system-P-loop-protein-BrxC-[Bifidobacterium-breve]
WP_117779802.1	Type1.BREX	<- SIGMA+OB-Fold+AAA-ATPase+ HKD-DNase -BrxL <- PglZ <-Schlafen+HATPase+wHTH<-SIR2<- N6-METHYLASE -p<-HerA/FtsK<-SIR2<- N6-METHYLASE <- DUF6079 *<- BrxB-BrxF-iSTAND <- BrxA-DNA-binding ||REase->?->?-><-RelE-ParE<-PhdYeFM_antitox||SbcD->	1219	Bacteria>Terrabacteria_group>Actinomycetota>Actinomycetes>Bifidobacteriales>Bifidobacteriaceae	GCF_027664445.1	Bifidobacterium_adolescentis	PGQ86_RS05915	1356414..1360073	1680	BREX-system-P-loop-protein-BrxC-[Bifidobacterium-adolescentis]
WP_270271788.1	Type1.BREX	REase-><-?||?->?->HTH+HisKin-><-?<-REase|| BrxA-DNA-binding -> BrxB-BrxF-iSTAND -> DUF6079 *-> N6-METHYLASE ->?->Schlafen+HATPase+wHTH-> PglZ -> SIGMA+OB-Fold+AAA-ATPase+ HKD-DNase -BrxL ->ZnR+GP->ZnR->ParB+HNH->	1218	Bacteria>Terrabacteria_group>Actinomycetota>Actinomycetes>Bifidobacteriales>Bifidobacteriaceae	GCF_027667785.1	Bifidobacterium_bifidum	PGS22_RS05345	127181..130837	1681	BREX-system-P-loop-protein-BrxC-[Bifidobacterium-bifidum]
WP_117715835.1	Type1.BREX	ZnR->?->ABC-ATPase+SbcC->?->?->?-> BrxA-DNA-binding -> BrxB-BrxF-iSTAND -> DUF6079 *-> N6-METHYLASE ->SIR2->Schlafen+HATPase+wHTH-> PglZ -> SIGMA+OB-Fold+AAA-ATPase+ HKD-DNase -BrxL ->?-><-REase||?-><-Glyoxalase	1217	Bacteria>Terrabacteria_group>Actinomycetota>Actinomycetes>Bifidobacteriales>Bifidobacteriaceae	GCF_027669765.1	Bifidobacterium_bifidum	PHA16_RS02375	586926..590579	1681	BREX-system-P-loop-protein-BrxC-[Bifidobacterium-bifidum]
WP_270200767.1	Type1.BREX	REC+LuxR-HTH->HISKIN-><-?<-SbcD<-?<-?||Pentapeptide+Pentapeptide+Pentapeptide+Pentapeptide-> BrxA-DNA-binding -> BrxB-BrxF-iSTAND -> DUF6079 -p*-> DUF6079 -p-> N6-METHYLASE ->REase->Schlafen+HATPase+wHTH-> PglZ -> SIGMA+OB-Fold+AAA-ATPase+ HKD-DNase -BrxL ->?-><-RNaseH	467	Bacteria>Terrabacteria_group>Actinomycetota>Actinomycetes>Bifidobacteriales>Bifidobacteriaceae	GCF_027671795.1	Bifidobacterium_pseudocatenulatum	PGT11_RS00265	67391..68794	28026	BREX-system-P-loop-protein-BrxC-[Bifidobacterium-pseudocatenulatum]
WP_270199955.1	Type1.BREX	<-SbcD<-?<-?||?->||Pentapeptide+Pentapeptide+Pentapeptide+Pentapeptide-> BrxA-DNA-binding -> BrxB-BrxF-iSTAND -> DUF6079 *-> DUF6079 -p-> N6-METHYLASE ->REase->Schlafen+HATPase+wHTH-> PglZ -> SIGMA+OB-Fold+AAA-ATPase+ HKD-DNase -BrxL ->?-><-RNaseH	1044	Bacteria>Terrabacteria_group>Actinomycetota>Actinomycetes>Bifidobacteriales>Bifidobacteriaceae	GCF_027675485.1	Bifidobacterium_pseudocatenulatum	PGV18_RS06405	41954..45088	28026	BREX-system-P-loop-protein-BrxC-[Bifidobacterium-pseudocatenulatum]
WP_270271488.1	Type1.BREX	<-ZnR<-ZnR+GP<- SIGMA+OB-Fold+AAA-ATPase+ HKD-DNase -BrxL <- PglZ <-Schlafen+HATPase+wHTH<-SIR2<- N6-METHYLASE <- DUF6079 *<- BrxB-BrxF-iSTAND <- BrxA-DNA-binding <-Pentapeptide+Pentapeptide+Pentapeptide<-HAD||SbcD-><-?<-ACET+ACET||HTH-><-35exo+BRCT	1219	Bacteria>Terrabacteria_group>Actinomycetota>Actinomycetes>Bifidobacteriales>Bifidobacteriaceae	GCF_027679525.1	Bifidobacterium_adolescentis	PGW19_RS08965	15037..18696	1680	BREX-system-P-loop-protein-BrxC-[Bifidobacterium-adolescentis]
WP_270447025.1	Type1.BREX	HISKIN-><-?||<-SbcD<-?<-?||?->||Pentapeptide+Pentapeptide+Pentapeptide+Pentapeptide-> BrxA-DNA-binding -> BrxB-BrxF-iSTAND -> DUF6079 -p*-> DUF6079 -p-> N6-METHYLASE ->SIR2->HerA/FtsK-> N6-METHYLASE -p->SIR2->Schlafen+HATPase+wHTH-> PglZ -> SIGMA+OB-Fold+AAA-ATPase+ HKD-DNase -BrxL ->	958	Bacteria>Terrabacteria_group>Actinomycetota>Actinomycetes>Bifidobacteriales>Bifidobacteriaceae	GCF_027682665.1	Bifidobacterium_pseudocatenulatum	PGX68_RS01375	343601..346477	28026	BREX-system-P-loop-protein-BrxC-[Bifidobacterium-pseudocatenulatum]
WP_270479593.1	Type1.BREX	<-ZnR+GP<- SIGMA+OB-Fold+AAA-ATPase+ HKD-DNase -BrxL <- PglZ <-Schlafen+HATPase+wHTH<-SIR2<- N6-METHYLASE -p<-HerA/FtsK<-SIR2<- N6-METHYLASE <- DUF6079 *<- BrxB-BrxF-iSTAND <- BrxA-DNA-binding ||?->HEPN-><-?||?->?-><-HEPN<-PolB	1219	Bacteria>Terrabacteria_group>Actinomycetota>Actinomycetes>Bifidobacteriales>Bifidobacteriaceae	GCF_027683145.1	Bifidobacterium_adolescentis	PGX62_RS02115	472627..476286	1680	BREX-system-P-loop-protein-BrxC-[Bifidobacterium-adolescentis]
WP_117726345.1	Type1.BREX	ZnR->?->ABC-ATPase+SbcC->?->?->?-> BrxA-DNA-binding -> BrxB-BrxF-iSTAND -> DUF6079 *-> N6-METHYLASE ->?->Schlafen+HATPase+wHTH-> PglZ -> SIGMA+OB-Fold+AAA-ATPase+ HKD-DNase -BrxL ->?-><-REase||?-><-Glyoxalase	1217	Bacteria>Terrabacteria_group>Actinomycetota>Actinomycetes>Bifidobacteriales>Bifidobacteriaceae	GCF_027685785.1	Bifidobacterium_bifidum	PG427_RS02260	555349..559002	1681	BREX-system-P-loop-protein-BrxC-[Bifidobacterium-bifidum]
WP_270266433.1	Type1.BREX	<- SIGMA+OB-Fold+AAA-ATPase+ HKD-DNase -BrxL <- PglZ <-Schlafen+HATPase+wHTH<-?<- N6-METHYLASE <- DUF6079 *<- BrxB-BrxF-iSTAND <- BrxA-DNA-binding <-?<-?<-ABC-ATPase+SbcC<-?<-ZnR	1219	Bacteria>Terrabacteria_group>Actinomycetota>Actinomycetes>Bifidobacteriales>Bifidobacteriaceae	GCF_027687815.1	Bifidobacterium_bifidum	PG972_RS08475	12443..16102	1681	BREX-system-P-loop-protein-BrxC-[Bifidobacterium-bifidum]
WP_117501722.1	Type1.BREX	REC+LuxR-HTH->HISKIN-><-?||?-><-SbcD<-?<-REase|| BrxA-DNA-binding -> BrxB-BrxF-iSTAND -> DUF6079 *-> N6-METHYLASE ->SIR2->Schlafen+HATPase+wHTH-> PglZ -> SIGMA+OB-Fold+AAA-ATPase+ HKD-DNase -BrxL ->	1219	Bacteria>Terrabacteria_group>Actinomycetota>Actinomycetes>Bifidobacteriales>Bifidobacteriaceae	GCF_027689935.1	Bifidobacterium_pseudocatenulatum	PG028_RS01370	345834..349493	28026	BREX-system-P-loop-protein-BrxC-[Bifidobacterium-pseudocatenulatum]
WP_270558503.1	Type1.BREX	<-ParB+HNH+RAMA<-ZnR<-ZnR+GP<- SIGMA+OB-Fold+AAA-ATPase+ HKD-DNase -BrxL <- PglZ <-Schlafen+HATPase+wHTH<-REase<- N6-METHYLASE <- DUF6079 *<- BrxB-BrxF-iSTAND <- BrxA-DNA-binding <-?<-?||?-><-Peptidase_M78<-HigA	1219	Bacteria>Terrabacteria_group>Actinomycetota>Actinomycetes>Bifidobacteriales>Bifidobacteriaceae	GCF_027690155.1	Bifidobacterium_adolescentis	PG057_RS05020	457971..461630	1680	BREX-system-P-loop-protein-BrxC-[Bifidobacterium-adolescentis]
WP_270554212.1	Type1.BREX	<-SbcD||HAD->PhdYeFM_antitox->RelE-ParE-><-?<-REase|| BrxA-DNA-binding -> BrxB-BrxF-iSTAND -> DUF6079 *-> N6-METHYLASE ->REase->Schlafen+HATPase+wHTH-> PglZ -> SIGMA+OB-Fold+AAA-ATPase+ HKD-DNase -BrxL ->ZnR+GP->ZnR->ParB+HNH+RAMA->	1219	Bacteria>Terrabacteria_group>Actinomycetota>Actinomycetes>Bifidobacteriales>Bifidobacteriaceae	GCF_027695195.1	Bifidobacterium_adolescentis	PG742_RS01120	252838..256497	1680	BREX-system-P-loop-protein-BrxC-[Bifidobacterium-adolescentis]
WP_270561184.1	Type1.BREX	35exo+HRDC->?->35exo+BRCT-><-HTH||?-><-ACET||?-> BrxA-DNA-binding -> BrxB-BrxF-iSTAND -> DUF6079 *->SIR2->HerA/FtsK-> N6-METHYLASE -p->HEPN->?->Schlafen+HATPase+wHTH-> PglZ -> SIGMA+OB-Fold+AAA-ATPase+ HKD-DNase -BrxL ->ZnR->	1217	Bacteria>Terrabacteria_group>Actinomycetota>Actinomycetes>Bifidobacteriales>Bifidobacteriaceae	GCF_027698005.1	Bifidobacterium_dentium	PG823_RS09640	38589..42242	1689	BREX-system-P-loop-protein-BrxC-[Bifidobacterium-dentium]
WP_118376243.1	Type1.BREX	RNaseH-><-?<- SIGMA+OB-Fold+AAA-ATPase+ HKD-DNase -BrxL <- PglZ <-Schlafen+HATPase+wHTH<-SIR2<- N6-METHYLASE <- DUF6079 *<- BrxB-BrxF-iSTAND <- BrxA-DNA-binding ||REase->?->?-><-?<-HEPN<-PolB	1219	Bacteria>Terrabacteria_group>Actinomycetota>Actinomycetes>Bifidobacteriales>Bifidobacteriaceae	GCF_027698785.1	Bifidobacterium_pseudocatenulatum	PG820_RS05645	117035..120694	28026	BREX-system-P-loop-protein-BrxC-[Bifidobacterium-pseudocatenulatum]
WP_271768971.1	Type1.BREX	<-SbcD<-?<-REase|| BrxA-DNA-binding -> BrxB-BrxF-iSTAND -> DUF6079 *-> N6-METHYLASE ->SIR2->Schlafen+HATPase+wHTH-> PglZ -> SIGMA+OB-Fold+AAA-ATPase+ HKD-DNase -BrxL ->ZnR+GP->ZnR->ParB+HNH+RAMA->	1219	Bacteria>Terrabacteria_group>Actinomycetota>Actinomycetes>Bifidobacteriales>Bifidobacteriaceae	GCF_028314485.1	Bifidobacterium_pseudocatenulatum	PMN34_RS05085	218989..222648	28026	BREX-system-P-loop-protein-BrxC-[Bifidobacterium-pseudocatenulatum]
WP_116463714.1	Type1.BREX	HTH_28-> BrxA-DNA-binding -> BrxB-BrxF-iSTAND -> DUF6079 *-> N6-METHYLASE ->?->Schlafen+HATPase+wHTH-> PglZ -> SIGMA+OB-Fold+AAA-ATPase+ HKD-DNase -BrxL ->?-><-?<-?<-ABC-ATPase+SbcC	1219	Bacteria>Terrabacteria_group>Actinomycetota>Actinomycetes>Bifidobacteriales>Bifidobacteriaceae	GCF_028898865.1	Bifidobacterium_longum	PWA56_RS02155	487318..490977	216816	BREX-system-P-loop-protein-BrxC-[Bifidobacterium-longum]
WP_077383013.1	Type1.BREX	ABC-ATPase+SbcC->?->?-><-?<- SIGMA+OB-Fold+AAA-ATPase+ HKD-DNase -BrxL <- PglZ <-Schlafen+HATPase+wHTH<-?<- N6-METHYLASE <- DUF6079 *<- BrxB-BrxF-iSTAND <- BrxA-DNA-binding <-HTH_28	1219	Bacteria>Terrabacteria_group>Actinomycetota>Actinomycetes>Bifidobacteriales>Bifidobacteriaceae	GCF_900157155.1	Bifidobacterium_longum	BQ8631_RS08415	45904..49563	216816	BREX-system-P-loop-protein-BrxC-[Bifidobacterium-longum]
WP_154050436.1	Type1.BREX	HAD+S6PP->?->?-><-?<-?<-?<-SIR2|| BrxA-DNA-binding -> BrxB-BrxF-iSTAND -> DUF6079 *-> N6-METHYLASE ->SIR2->Schlafen+HATPase+wHTH-> PglZ -> SIGMA+OB-Fold+AAA-ATPase+ HKD-DNase -BrxL ->?-><-ABhydrolase	1217	Bacteria>Terrabacteria_group>Actinomycetota>Actinomycetes>Bifidobacteriales>Bifidobacteriaceae	GCF_901212475.1	Bifidobacterium_longum	FGK88_RS06805	85798..89451	216816	BREX-system-P-loop-protein-BrxC-[Bifidobacterium-longum]
WP_143724686.1	Type1.BREX	<- SIGMA+OB-Fold+AAA-ATPase+ HKD-DNase -BrxL <- PglZ <-RNase<-Schlafen+HATPase+wHTH<-SMODS<-SLATT<- N6-METHYLASE <- DUF6079 *<- BrxB-BrxF-iSTAND <- BrxA-DNA-binding <-CR-ATPase5+REase-p<-?||?-><-?<-?||<-?<-MarR_2+ROK-HTH	1229	Bacteria>Terrabacteria_group>Actinomycetota>Actinomycetes>Bifidobacteriales>Bifidobacteriaceae	GCF_902167555.1	Bifidobacterium_longum	FP566_RS07260	1570713..1574402	216816	BREX-system-P-loop-protein-BrxC-[Bifidobacterium-longum]
WP_143246218.1	Type1.BREX	<-GDResolvase+HIN-HTH<-?<- SIGMA+OB-Fold+AAA-ATPase+ HKD-DNase -BrxL <- PglZ <-Schlafen+HATPase+wHTH<-SIR2<- N6-METHYLASE <- DUF6079 *<- BrxB-BrxF-iSTAND <- BrxA-DNA-binding	1218	Bacteria>Terrabacteria_group>Actinomycetota>Actinomycetes>Bifidobacteriales>Bifidobacteriaceae	GCF_902167575.1	Bifidobacterium_breve	FPG44_RS02615	449270..452926	1685	BREX-system-P-loop-protein-BrxC-[Bifidobacterium-breve]
WP_200406352.1	Type1.BREX	RelB-> N6-METHYLASE ->CR-ATPase5+REase-p-><-CR-Kinase<-?|| BrxA-DNA-binding -> BrxB-BrxF-iSTAND -> DUF6079 *->?-> N6-METHYLASE ->SLATT->SMODS->Schlafen-p->Schlafen+wHTH-> PglZ -> SIGMA+OB-Fold+AAA-ATPase+ HKD-DNase -BrxL ->TIR->	1229	Bacteria>Terrabacteria_group>Actinomycetota>Actinomycetes>Bifidobacteriales>Bifidobacteriaceae	GCF_934675445.1	Bifidobacterium_longum	BL7055_RS05750	1406658..1410347	216816	BREX-system-P-loop-protein-BrxC-[Bifidobacterium-longum]
WP_245206784.1	Type1.BREX	<-ZnR<- SIGMA+OB-Fold+AAA-ATPase+ HKD-DNase -BrxL <- PglZ <-Schlafen+HATPase+wHTH<-SIR2<- N6-METHYLASE <-AAA-ATPase<-HTH_7+rve+rve_3<- N6-METHYLASE -p<- DUF6079 *<- DUF6079 -p<- BrxB-BrxF-iSTAND <- BrxA-DNA-binding <-?||ACET-><-?||HTH-><-35exo+BRCT	1073	Bacteria>Terrabacteria_group>Actinomycetota>Actinomycetes>Bifidobacteriales>Bifidobacteriaceae	GCF_937997665.1	Bifidobacterium_dentium	J7M36_RS06065	1427241..1430462	1689	BREX-system-P-loop-protein-BrxC-[Bifidobacterium-dentium]
WP_250246699.1	Type1.BREX	MarR_2+ROK-HTH->?->||?->?-><-?||?->CR-ATPase5+REase-p-> BrxA-DNA-binding -> BrxB-BrxF-iSTAND -> DUF6079 *-> N6-METHYLASE ->SLATT->SMODS->Schlafen+HATPase+wHTH->RNase-> PglZ -> SIGMA+OB-Fold+AAA-ATPase+ HKD-DNase -BrxL ->	1229	Bacteria>Terrabacteria_group>Actinomycetota>Actinomycetes>Bifidobacteriales>Bifidobacteriaceae	GCF_939148185.1	Bifidobacterium_longum	NBW58_RS09955	18627..22316	216816	BREX-system-P-loop-protein-BrxC-[Bifidobacterium-longum]
WP_196330425.1	Type1.BREX	35exo+HRDC->?->35exo+BRCT-><-HTH||?-><-ACET||?-> BrxA-DNA-binding -> BrxB-BrxF-iSTAND -> DUF6079 *-> N6-METHYLASE ->?->Schlafen+HATPase+wHTH-> PglZ -> SIGMA+OB-Fold+AAA-ATPase+ HKD-DNase -BrxL ->ZnR-><-DSBH+AraC-HTH+AraC-HTH||Glyoxalase->	1217	Bacteria>Terrabacteria_group>Actinomycetota>Actinomycetes>Bifidobacteriales>Bifidobacteriaceae	GCF_958433885.1	Bifidobacterium_dentium	J7M35_RS04855	1219062..1222715	1689	BREX-system-P-loop-protein-BrxC-[Bifidobacterium-dentium]
WP_085380386.1	Type1.BREX	<-ParB+HNH+RAMA||MULE-TRANSPOSASE-><-ZnR<-ZnR+GP<- SIGMA+OB-Fold+AAA-ATPase+ HKD-DNase -BrxL <- PglZ <-Schlafen+HATPase+wHTH<-SIR2<- N6-METHYLASE <- DUF6079 *<- BrxB-BrxF-iSTAND <- BrxA-DNA-binding <-Pentapeptide+Pentapeptide+Pentapeptide<-HAD||SbcD-><-?<-ACET+ACET||HTH-><-35exo+BRCT	1219	Bacteria>Terrabacteria_group>Actinomycetota>Actinomycetes>Bifidobacteriales>Bifidobacteriaceae	GCF_959020145.1	Bifidobacterium_adolescentis	LMG10734_RS05600	489007..492666	1680	BREX-system-P-loop-protein-BrxC-[Bifidobacterium-adolescentis]
WP_196335743.1	Type1.BREX	<-Glyoxalase||DSBH+AraC-HTH+AraC-HTH-><-ZnR<- SIGMA+OB-Fold+AAA-ATPase+ HKD-DNase -BrxL <- PglZ <-Schlafen+HATPase+wHTH<-REase<- N6-METHYLASE <- DUF6079 *<- BrxB-BrxF-iSTAND <- BrxA-DNA-binding ||REase-><-HEPN<-?||HTH-><-35exo+BRCT<-35exo+HRDC	1217	Bacteria>Terrabacteria_group>Actinomycetota>Actinomycetes>Bifidobacteriales>Bifidobacteriaceae	GCF_959600175.1	Bifidobacterium_dentium	IHQ76_RS06180	228522..232175	1689	BREX-system-P-loop-protein-BrxC-[Bifidobacterium-dentium]
WP_096305156.1	Type5.BREX	<-rve+rve_3||?->?-><-?<-?||Peptidase->XIS-HTH-> BrxA-DNA-binding -> BrxB-BrxF-iSTAND -> DUF6079 *-> N6-METHYLASE -> PglZ ->SF1-SF2-Helicase-><-AbiEii<-?||?->TUDOR+SF1-SF2-Helicase+iREase+CTD+REase-p-><-HIN-HTH	1161	Bacteria>Terrabacteria_group>Actinomycetota>Actinomycetes>Jatrophihabitantales>Jatrophihabitantaceae	GCF_900230215.1	Jatrophihabitans_sp._GAS493	CPH63_RS13795	2948299..2951784	1907575	BREX-system-P-loop-protein-BrxC-[Jatrophihabitans-sp.-GAS493]
EST38900.1	Type1.BREX	Phage_int_SAM_3+Phage_integrase->DDE_Tnp_1_assoc+DDE_Tnp_1->DDE_Tnp_ISAZ013-><- SIGMA+OB-Fold+AAA-ATPase+ HKD-DNase -BrxL ||HTH_29+HTH_32+DDE_3-><- SIGMA+OB-Fold+AAA-ATPase+Lon-Protease-BrxL -p<-?<-HTH_29+HTH_32+DDE_3||zf-ISL3+HTH_Tnp_ISL3-><- DUF6079 -p*<- BrxB-BrxF-iSTAND <- BrxA-DNA-binding ||HTH_29+HTH_32+DDE_3-><-DDE_Tnp_1<- BrxA-DNA-binding <-XIS-HTH||HAD+HAD->	571	Bacteria>Terrabacteria_group>Actinomycetota>Actinomycetes>Kitasatosporales>Streptomycetaceae	GCA_000497405.1	Streptomycetaceae_bacterium_MP113-05	N566_04930	13788..15503	1380770	hypothetical-protein-N566_04930-[Streptomycetaceae-bacterium-MP113-05]
MCG7205148.1	Type1.BREX	<-DUF4096+DDE_Tnp_1+DDE_Tnp_1_2||?->?-><- SIGMA+OB-Fold+AAA-ATPase+ HKD-DNase -BrxL <- PglZ <- N6-METHYLASE <- DUF6079 *<- BrxB-BrxF-iSTAND <- BrxA-DNA-binding <-XIS-HTH|| N6-METHYLASE ->	1161	Bacteria>Terrabacteria_group>Actinomycetota>Actinomycetes>Kitasatosporales>Streptomycetaceae	GCA_022343545.1	Streptomyces_arenae	MB828_15195	11459..14944	29301	BREX-system-P-loop-protein-BrxC-[Streptomyces-arenae]
WP_030399158.1	Type1.BREX	OLD-ABC+TOPRIM->SF1-SF2-Helicase->?-><-DNA_glycosylase||REase-><- SIGMA+OB-Fold+AAA-ATPase+ HKD-DNase -BrxL <- PglZ <- N6-METHYLASE <- DUF6079 *<- BrxB-BrxF-iSTAND <- BrxA-DNA-binding <-XIS-HTH<-pPIWI+MID+RNaseH<-pPIWI+SF1-SF2-Helicase<-pPIWI+pPIWI<-HTH_3+HTH_3||?-><-DDE_Tnp_1_4	1161	Bacteria>Terrabacteria_group>Actinomycetota>Actinomycetes>Kitasatosporales>Streptomycetaceae	GCF_000719665.1	unclassified_Streptomyces	ADK87_RS19375	27273..30758	2593676	MULTISPECIES:-BREX-system-P-loop-protein-BrxC-[unclassified-Streptomyces]
WP_042419815.1	Type1.BREX	<-VirB6<-?<-TraM<-?||N6-N4-METHYLASE-><-REase+DUF5655||XIS-HTH-> BrxA-DNA-binding -> BrxB-BrxF-iSTAND -> DUF6079 *-> N6-METHYLASE -> PglZ -> SIGMA+OB-Fold+AAA-ATPase+ HKD-DNase -BrxL -><-PIWI<-REase+PNTD1+PNTD2<- HKD-DNase + HKD-DNase	1161	Bacteria>Terrabacteria_group>Actinomycetota>Actinomycetes>Kitasatosporales>Streptomycetaceae	GCF_000787855.1	Streptacidiphilus_anmyonensis	TR50_RS13090	101724..105209	405782	BREX-system-P-loop-protein-BrxC-[Streptacidiphilus-anmyonensis]
WP_062022136.1	Type1.BREX	METHYLASE->?->?-><-DNA_glycosylase||REase-><- SIGMA+OB-Fold+AAA-ATPase+ HKD-DNase -BrxL <- PglZ <- N6-METHYLASE <- DUF6079 *<- BrxB-BrxF-iSTAND <- BrxA-DNA-binding <-XIS-HTH<-pPIWI+MID+RNaseH<-pPIWI+SF1-SF2-Helicase<-pPIWI+pPIWI<-HTH_3+HTH_3||REase+DUF5655-><-N6-N4-METHYLASE	1161	Bacteria>Terrabacteria_group>Actinomycetota>Actinomycetes>Kitasatosporales>Streptomycetaceae	GCF_001513985.1	Streptomyces_griseorubiginosus	AQI84_07455	596850..600335	67304	BREX-system-P-loop-protein-BrxC-[Streptomyces-griseorubiginosus]
WP_129286571.1	Type1.BREX	PolB->RADICAL-SAM->?->HD->Glyco_trans_1_4->?<-?||XIS-HTH-> BrxA-DNA-binding -> BrxB-BrxF-iSTAND -> DUF6079 *-> N6-METHYLASE -> PglZ -> SIGMA+OB-Fold+AAA-ATPase+ HKD-DNase -BrxL -><-Aminotran_1_2||?->||METHYLASE->	1163	Bacteria>Terrabacteria_group>Actinomycetota>Actinomycetes>Kitasatosporales>Streptomycetaceae	GCF_004123705.1	Streptomyces_sp._GZWMJZ-114	EJA11_RS17595	541597..545088	2494734	BREX-system-P-loop-protein-BrxC-[Streptomyces-sp.-GZWMJZ-114]
WP_138352701.1	Type1.BREX	REase+wHTH->rve->TnsC-ATPase->?->?-><-?<- SIGMA+OB-Fold+AAA-ATPase+ HKD-DNase -BrxL <- PglZ <- N6-METHYLASE <- DUF6079 *<- BrxB-BrxF-iSTAND <- BrxA-DNA-binding <-XIS-HTH<-?||HTH_3->SF1-SF2-Helicase-><-?||?-><-TIR	1186	Bacteria>Terrabacteria_group>Actinomycetota>Actinomycetes>Kitasatosporales>Streptomycetaceae	GCF_005860785.2	Streptomyces_sp._So13.3	C8250_RS11580	2549516..2553076	2136173	BREX-system-P-loop-protein-BrxC-[Streptomyces-sp.-So13.3]
WP_161225978.1	Type1.BREX	<-MarR-HTH<-?<-Hiskin||?->METHYLASE-><-HTH_31+DUF5753<- SIGMA+OB-Fold+AAA-ATPase+ HKD-DNase -BrxL <- PglZ <- N6-METHYLASE <- DUF6079 *<- BrxB-BrxF-iSTAND <-tRNA||CR-Kinase+pi3KINASE-><-?<-?<-Glycos_transf_1+Glyco_trans_1_2<-?<-?||HTH_3->	1165	Bacteria>Terrabacteria_group>Actinomycetota>Actinomycetes>Kitasatosporales>Streptomycetaceae	GCF_009862265.1	Streptomyces_sp._SID8352	GTY84_RS10560	297618..301115	2690338	BREX-system-P-loop-protein-BrxC-[Streptomyces-sp.-SID8352]
WP_164340211.1	Type1.BREX	<-Pkinase+Pkinase<-ABhydrolase||?-><-?||<-TetR-HTH||?-><- SIGMA+OB-Fold+AAA-ATPase+ HKD-DNase -BrxL <- PglZ <- N6-METHYLASE <- DUF6079 *<- BrxB-BrxF-iSTAND <- BrxA-DNA-binding <-XIS-HTH||Acetyltransf_8->ABC-ATPase->ABC-ATPase-><-?||Peptidase_C25->	1163	Bacteria>Terrabacteria_group>Actinomycetota>Actinomycetes>Kitasatosporales>Streptomycetaceae	GCF_010548045.1	Streptomyces_anulatus	G3I47_RS13230	74640..78131	1892	BREX-system-P-loop-protein-BrxC-[Streptomyces-anulatus]
WP_182441002.1	Type1.BREX	<-PhdYeFM_antitox<-?||?-><-?||?-><-?<- SIGMA+OB-Fold+AAA-ATPase+ HKD-DNase -BrxL <- PglZ <- N6-METHYLASE <- DUF6079 *<- BrxB-BrxF-iSTAND <- BrxA-DNA-binding <-XIS-HTH||REase->ACET-><-?<-HAD+HAD<-PRTase	1159	Bacteria>Terrabacteria_group>Actinomycetota>Actinomycetes>Kitasatosporales>Streptomycetaceae	GCF_014084125.1	Streptacidiphilus_sp._PB12-B1b	GXW83_RS01100	242781..246260	2705012	BREX-system-P-loop-protein-BrxC-[Streptacidiphilus-sp.-PB12-B1b]
WP_073800314.1	Type1.BREX	<-Peptidase_C25||?-><-ABC-ATPase+SbcC<-ABC-ATPase<-Acetyltransf_8||XIS-HTH-> BrxA-DNA-binding -> BrxB-BrxF-iSTAND -> DUF6079 *-> N6-METHYLASE -> PglZ -> SIGMA+OB-Fold+AAA-ATPase+ HKD-DNase -BrxL -><-?||TetR-HTH-><-REC+OmpR-HTH	1163	Bacteria>Terrabacteria_group>Actinomycetota>Actinomycetes>Kitasatosporales>Streptomycetaceae	GCF_014495705.1	unclassified_Streptomyces	AMK20_RS03875	898838..902329	2593676	MULTISPECIES:-BREX-system-P-loop-protein-BrxC-[unclassified-Streptomyces]
WP_265293811.1	Type1.BREX	Glycos_transf_1+Glyco_trans_1_2->?->HTH_33+DDE_3->?-> BrxA-DNA-binding -> BrxB-BrxF-iSTAND -> DUF6079 *-> N6-METHYLASE -> PglZ -> SIGMA+OB-Fold+AAA-ATPase+ HKD-DNase -BrxL ->HTH_31+DUF5753-><-METHYLASE<-?||Hiskin->?->MarR-HTH->	1168	Bacteria>Terrabacteria_group>Actinomycetota>Actinomycetes>Kitasatosporales>Streptomycetaceae	GCF_026126445.1	Streptomyces_sp._SHP_1-2	IBX28_RS14860	97274..100780	2769489	BREX-system-P-loop-protein-BrxC-[Streptomyces-sp.-SHP-1-2]
WP_266948097.1	Type1.BREX	<-DUF397<-?||?->||?-><-ACET+ACET||?-><- SIGMA+OB-Fold+AAA-ATPase+ HKD-DNase -BrxL <- PglZ <- N6-METHYLASE <- DUF6079 *<- BrxB-BrxF-iSTAND <- BrxA-DNA-binding <-XIS-HTH||HIN-HTH->?-><-NUDIX<-HTH_31	1186	Bacteria>Terrabacteria_group>Actinomycetota>Actinomycetes>Kitasatosporales>Streptomycetaceae	GCF_026342255.1	Streptomyces_sp._NBC_00291	OG914_RS06430	1462666..1466226	2975704	BREX-system-P-loop-protein-BrxC-[Streptomyces-sp.-NBC_00291]
WP_093695822.1	Type1.BREX	<-ZDNA-HTH||ICLR-HTH->?->sGTP+sGTP+GTPase-AIG->sGTP-><-DNA_glycosylase<- SIGMA+OB-Fold+AAA-ATPase+ HKD-DNase -BrxL <- PglZ <- N6-METHYLASE <- DUF6079 *<- BrxB-BrxF-iSTAND <- BrxA-DNA-binding <-XIS-HTH<-?<-PRTase<-?||?-><-GNTR-HTH||SKI->	1162	Bacteria>Terrabacteria_group>Actinomycetota>Actinomycetes>Kitasatosporales>Streptomycetaceae	GCF_900105605.1	Streptomyces_sp._2231.1	BLV73_RS10010	2127135..2130623	1855347	BREX-system-P-loop-protein-BrxC-[Streptomyces-sp.-2231.1]
WP_005885100.1	Type1.BREX	<-ABC-ATPase+SbcC<-?<-?||ACET-><-OLD-ABC<- SIGMA+OB-Fold+AAA-ATPase+ HKD-DNase -BrxL <- PglZ <-HTH+REase<- N6-METHYLASE <- DUF6079 *<- BrxB-BrxF-iSTAND <- BrxA-DNA-binding <-XIS-HTH<-ABhydrolase||?-><-HTH	1178	Bacteria>Terrabacteria_group>Actinomycetota>Actinomycetes>Micrococcales>Brevibacteriaceae	GCF_000178455.1	Brevibacterium_mcbrellneri	HMPREF0183_RS08780	50551..54087	53363	BREX-system-P-loop-protein-BrxC-[Brevibacterium-mcbrellneri]
WP_125240568.1	Type1.BREX	<-ACET<-?<-?||ADH_N+ADH_zinc_N-><-HerA/FtsK<-SIR2+SIR2<- SIGMA+OB-Fold+AAA-ATPase+ HKD-DNase -BrxL <- PglZ <- N6-METHYLASE <- DUF6079 *<- BrxB-BrxF-iSTAND <- BrxA-DNA-binding <-?<-?<-?<-?<-?<-?||ICLR-HTH->	1170	Bacteria>Terrabacteria_group>Actinomycetota>Actinomycetes>Micrococcales>Brevibacteriaceae	GCF_003932995.1	Brevibacterium_aurantiacum	CXR25_RS11695	2538315..2541827	273384	BREX-system-P-loop-protein-BrxC-[Brevibacterium-aurantiacum]
WP_201670680.1	Type1.BREX	NUDIX-><-MACRODOMAIN+SIR2||Pkinase+STYKIN-><- SIGMA+OB-Fold+AAA-ATPase+ HKD-DNase -BrxL <- PglZ <- N6-METHYLASE -p<-Phage_int_SAM_3+Phage_integrase|| N6-METHYLASE -p-><- N6-METHYLASE <- DUF6079 *<- BrxB-BrxF-iSTAND <- BrxA-DNA-binding <-ADH_N+ADH_zinc_N||?->?->ACET->Aminotran_3->?->wHTH+WYL->	1160	Bacteria>Terrabacteria_group>Actinomycetota>Actinomycetes>Micrococcales>Brevibacteriaceae	GCF_016726245.1	Brevibacterium_casei	I6I57_RS09610	2195903..2199385	33889	BREX-system-P-loop-protein-BrxC-[Brevibacterium-casei]
WP_259801020.1	Type1.BREX	<-VirB11+MuB-ATPase||DNA_METHYLASE-><-HerA/FtsK<-SIR2<- SIGMA+OB-Fold+AAA-ATPase+ HKD-DNase -BrxL <- PglZ <-HTH+REase<- N6-METHYLASE <- DUF6079 *<- BrxB-BrxF-iSTAND <- BrxA-DNA-binding <-XIS-HTH<-ABhydrolase<-?<-MPTase<-HTH||ArdCN+MPTase->Primpol+PriCT1->	1173	Bacteria>Terrabacteria_group>Actinomycetota>Actinomycetes>Micrococcales>Brevibacteriaceae	GCF_025144065.1	Brevibacterium_casei	M3G43_RS08140	29205..32726	33889	BREX-system-P-loop-protein-BrxC-[Brevibacterium-casei]
WP_101598836.1	Type1.BREX	<-Sigma-HTH||NUDIX-><- SIGMA+OB-Fold+AAA-ATPase+ HKD-DNase -BrxL <- PglZ <- N6-METHYLASE <- N6-METHYLASE -p<- DUF6079 *<- BrxB-BrxF-iSTAND <- BrxA-DNA-binding <-ADH_N+ADH_zinc_N||?->?->ACET->Aminotran_3->?->wHTH+WYL->	1160	Bacteria>Terrabacteria_group>Actinomycetota>Actinomycetes>Micrococcales>Brevibacteriaceae	GCF_900169315.1	Brevibacterium_aurantiacum	BAURA63_03577	9324..12806	273384	BREX-system-P-loop-protein-BrxC-[Brevibacterium-aurantiacum]
WP_029253807.1	Type1.BREX	ABC-ATPase+SbcC-><-?<-HEPN<-ParB+HNH<- SIGMA+OB-Fold+AAA-ATPase+ HKD-DNase -BrxL <- PglZ <- N6-METHYLASE <- DUF6079 *<- BrxB-BrxF-iSTAND <- BrxA-DNA-binding <-AbiEii<-AbiEii<-REase	1160	Bacteria>Terrabacteria_group>Actinomycetota>Actinomycetes>Micrococcales>Cellulomonadaceae	GCF_000701465.1	Paraoerskovia_marina	Q343_RS0111945	114651..118133	545619	BREX-system-P-loop-protein-BrxC-[Paraoerskovia-marina]
WP_130780297.1	Type1.BREX	<-SPOUT<-AbiEi_4<-?<-?<-PhdYeFM_antitox<-?||XIS-HTH-> BrxA-DNA-binding -> BrxB-BrxF-iSTAND -> DUF6079 *-> N6-METHYLASE -p-> N6-METHYLASE -> PglZ -> SIGMA+OB-Fold+AAA-ATPase+ HKD-DNase -BrxL -><-?<-TRPR-HTH||Phage_int_SAM_1->DDE_Tnp_1_4->	1159	Bacteria>Terrabacteria_group>Actinomycetota>Actinomycetes>Micrococcales>Cellulomonadaceae	GCF_004306155.1	Cellulomonas_biazotea	CBZ_RS03705	43649..47128	1709	BREX-system-P-loop-protein-BrxC-[Cellulomonas-biazotea]
WP_010552220.1	Type1.BREX	<-REase<-ParB+HNH<-ParB+HNH||?-><-MULE-TRANSPOSASE|| BrxA-DNA-binding -> BrxB-BrxF-iSTAND -> DUF6079 *-> N6-METHYLASE -> PglZ -> SIGMA+OB-Fold+AAA-ATPase+ HKD-DNase -BrxL ->HIN-HTH->	1160	Bacteria>Terrabacteria_group>Actinomycetota>Actinomycetes>Micrococcales>Dermabacteraceae	GCF_000233655.1	Brachybacterium_paraconglomeratum	I7Y_RS0116025	54770..58252	173362	BREX-system-P-loop-protein-BrxC-[Brachybacterium-paraconglomeratum]
WP_114305671.1	Type1.BREX	<-SF1-SF2-Helicase<-MODE-HTH+PBPII||?-><-RNase||?-><-?|| BrxA-DNA-binding -> BrxB-BrxF-iSTAND -> DUF6079 *-> N6-METHYLASE -> PglZ -> SIGMA+OB-Fold+AAA-ATPase+ HKD-DNase -BrxL ->	1160	Bacteria>Terrabacteria_group>Actinomycetota>Actinomycetes>Micrococcales>Dermabacteraceae	GCF_003335355.1	Brachybacterium_alimentarium	CIK67_RS09225	107631..111113	47845	BREX-system-P-loop-protein-BrxC-[Brachybacterium-alimentarium]
WP_249738141.1	Type1.BREX	<-PTSEIIA1<-?<-HHH_5+SNF2_N+SF1-SF2-Helicase||CASPASE-><- SIGMA+OB-Fold+AAA-ATPase+ HKD-DNase -BrxL <- PglZ <-HTH+REase<- N6-METHYLASE <- DUF6079 *<- BrxB-BrxF-iSTAND <- BrxA-DNA-binding <-SAM-METHYLASE<-?||HIN-HTH->HTH_Tnp_1+HTH_21+rve+rve_3->	1173	Bacteria>Terrabacteria_group>Actinomycetota>Actinomycetes>Micrococcales>Dermabacteraceae	GCF_023509525.1	Brachybacterium_sp._JHP9	Bequi_RS11780	103104..106625	2910770	BREX-system-P-loop-protein-BrxC-[Brachybacterium-sp.-JHP9]
WP_179478704.1	Type1.BREX	ADH_N+ADH_zinc_N->?->Thymidylate_kin->ABhydrolase->tRNA-><-?<-?<-?||<- SIGMA+OB-Fold+AAA-ATPase+ HKD-DNase -BrxL <- N6-METHYLASE <- DUF6079 *<- BrxB-BrxF-iSTAND <- BrxA-DNA-binding ||SF1-SF2-Helicase+REase-p->SF1-SF2-Helicase+REase->REase->?-><-?||MACRODOMAIN+SIR2->	1156	Bacteria>Terrabacteria_group>Actinomycetota>Actinomycetes>Micrococcales>Dermacoccaceae	GCF_013410455.1	Allobranchiibius_huperziae	HNR15_RS01985	409066..412536	1874116	BREX-system-P-loop-protein-BrxC-[Allobranchiibius-huperziae]
WP_233763981.1	Type1.BREX	<-MACRODOMAIN+SIR2||?->?-><-SF1-SF2-Helicase+REase<-SF1-SF2-Helicase+REase|| BrxA-DNA-binding -> BrxB-BrxF-iSTAND -> DUF6079 *-> N6-METHYLASE -> PglZ -> SIGMA+OB-Fold+AAA-ATPase+ HKD-DNase -BrxL ->||HTH_Tnp_1+HTH_21+rve+rve_3->||?->?-><-?||?-><-tRNA<-ABhydrolase	1156	Bacteria>Terrabacteria_group>Actinomycetota>Actinomycetes>Micrococcales>Dermacoccaceae	GCF_021391335.1	Allobranchiibius_sp._GilTou73	LVQ62_RS01295	263639..267109	2904523	BREX-system-P-loop-protein-BrxC-[Allobranchiibius-sp.-GilTou73]
WP_040162373.1	Type1.BREX	35exo->tRNA-><-GDResolvase+Recombinase+ZnR<-?||HIN-HTH->HTH_Tnp_1+HTH_21+rve+rve_3->XIS-HTH-> BrxA-DNA-binding -> BrxB-BrxF-iSTAND -> DUF6079 *-> N6-METHYLASE ->?-> PglZ -> SIGMA+OB-Fold+AAA-ATPase+ HKD-DNase -BrxL ->OLD-ABC+TOPRIM->SF1-SF2-Helicase->GTPase-AIG->MULE-TRANSPOSASE->	1178	Bacteria>Terrabacteria_group>Actinomycetota>Actinomycetes>Micrococcales>Dermatophilaceae	GCF_902375455.1	Mobilicoccus_massiliensis	FX004_RS16815	289663..293199	1522310	BREX-system-P-loop-protein-BrxC-[Mobilicoccus-massiliensis]
WP_215519875.1	Type1.BREX	<-NERD||?->?-><-sGTP<-GTPase-AIG<- SIGMA+OB-Fold+AAA-ATPase+ HKD-DNase -BrxL <- SIGMA+OB-Fold+AAA-ATPase+Lon-Protease-BrxL -p<- PglZ <- N6-METHYLASE <- DUF6079 *<- BrxB-BrxF-iSTAND <- BrxA-DNA-binding <-?<-ZnR+ATPcone	1156	Bacteria>Terrabacteria_group>Actinomycetota>Actinomycetes>Micrococcales>Intrasporangiaceae	GCF_018729305.1	Phycicoccus_mangrovi	KMZ30_RS04765	132164..135634	2840470	BREX-system-P-loop-protein-BrxC-[Phycicoccus-mangrovi]
WP_265231741.1	Type1.BREX	<-HD<-DnaB<-DNA_glycosylase<-HNH<-NUDIX||?->XIS-HTH-> BrxA-DNA-binding -> BrxB-BrxF-iSTAND -> DUF6079 *-> N6-METHYLASE -> N6-METHYLASE -p-> PglZ -> N6-METHYLASE -p-> N6-METHYLASE -> SIGMA+OB-Fold+AAA-ATPase+ HKD-DNase -BrxL ->HTH_Tnp_1+HTH_21+rve+rve_3-><-?<-sGTP	1154	Bacteria>Terrabacteria_group>Actinomycetota>Actinomycetes>Micrococcales>Intrasporangiaceae	GCF_026114115.1	Janibacter_hoylei	ON003_RS16090	3001704..3005168	364298	BREX-system-P-loop-protein-BrxC-[Janibacter-hoylei]
WP_270886872.1	Type1.BREX	XIS-HTH-> BrxA-DNA-binding -> BrxB-BrxF-iSTAND -> DUF6079 *-> N6-METHYLASE -> PglZ -> SIGMA+OB-Fold+AAA-ATPase+ HKD-DNase -BrxL -><-HIN-HTH<-Phage_int_SAM_3+Phage_integrase<-MarR-HTH<-XIS-HTH<-?||Phage_int_SAM_1+Phage_integrase->	1154	Bacteria>Terrabacteria_group>Actinomycetota>Actinomycetes>Micrococcales>Intrasporangiaceae	GCF_027812955.1	Pedococcus_sp._5OH_020	PHA84_RS04350	172901..176365	2989814	BREX-system-P-loop-protein-BrxC-[Pedococcus-sp.-5OH_020]

Protein-ID	System	Gene-Neighbourhoods	Length	Taxonomy	Assembly-ID	Strain	Locus-Tag	Genomic-coordinates	TaxID	Fasta-define
WP_272461397.1	Type1.BREX	<-MODE-HTH+PBPII||XIS-HTH->BrxA-DNA-binding->BrxB-BrxF-iSTAND->DUF6079*->N6-METHYLASE->PglZ->SIGMA+OB-Fold+AAA-ATPase+HKD-DNase-BrxL->GTPase-AIG->sGTP->REase->DUF932-><-?<-VirB11+MuB-ATPase	1154	Bacteria>Terrabacteria_group>Actinomycetota>Actinomycetes>Micrococcales>Intrasporangiaceae	GCF_028446585.1	Intrasporangium_calvum	O0014_RS06100	11826..15290	53358	BREX-system-P-loop-protein-BrxC-[Intrasporangium-calvum]
TFB71537.1	Type1.BREX	<-URI-DNase<-VirB11+MuB-ATPase<-HEPN+REase<-REase<-SIGMA+OB-Fold+AAA-ATPase+HKD-DNase-BrxL<-PglZ<-N6-METHYLASE<-DUF6079*<-BrxB-BrxF-iSTAND<-BrxA-DNA-binding<-XIS-HTH<-ABhydrolase<-?<-MPTase<-HTH||ArdCN+MPTase->Primpol+PriCT1->	1170	Bacteria>Terrabacteria_group>Actinomycetota>Actinomycetes>Micrococcales>Microbacteriaceae	GCA_004402535.1	Cryobacterium_sp._HLT2-23	E3006_11880	40394..43906	1259145	BREX-system-P-loop-protein-BrxC-[Cryobacterium-glaciale]
QIM17270.1	Type1.BREX	<-SIGMA+OB-Fold+AAA-ATPase+HKD-DNase-BrxL<-?<-?<-SIR2<-PglZ<-?<-DUF6079*<-BrxB-BrxF-iSTAND<-BrxA-DNA-binding<-?<-?||TetR-HTH-><-PIN<-PhdYeFM_antitox<-ABC-ATPase+SbcC<-Sulfate_transp+STAS	1193	Bacteria>Terrabacteria_group>Actinomycetota>Actinomycetes>Micrococcales>Microbacteriaceae	GCA_011382965.1	Leucobacter_insecticola	G7067_02015	442468..446049	2714934	BREX-system-P-loop-protein-BrxC-[Leucobacter-insecticola]
MBX3116430.1	Type1.BREX	CR-ATPase5+REase-><-?||HTH_6+SIS->?->PolB->BrxA-DNA-binding->BrxB-BrxF-iSTAND->DUF6079*->N6-METHYLASE->OLD-ABC->TOPRIM->PglZ->SIGMA+OB-Fold+AAA-ATPase+HKD-DNase-BrxL->Pkinase+CR-Kinase-><-PIN<-DUF433||REase+HTH+35exo+Small-B-barrel+SF1-SF2-Helicase->	1182	Bacteria>Terrabacteria_group>Actinomycetota>Actinomycetes>Micrococcales>Microbacteriaceae	GCA_019638875.1	Cryobacterium_sp.	KF808_03680	24849..28397	1926290	BREX-system-P-loop-protein-BrxC-[Cryobacterium-sp.]
MBX3086848.1	Type1.BREX	<-VirB11+MuB-ATPase<-?<-SIGMA+OB-Fold+AAA-ATPase+HKD-DNase-BrxL<-PglZ<-N6-METHYLASE<-DUF6079*<-BrxB-BrxF-iSTAND<-BrxA-DNA-binding<-XIS-HTH<-ABhydrolase<-?<-MPTase<-HTH||ArdCN+MPTase->Primpol+PriCT1->	1160	Bacteria>Terrabacteria_group>Actinomycetota>Actinomycetes>Micrococcales>Microbacteriaceae	GCA_019638955.1	Cryobacterium_sp.	KF742_00030	12107..15589	1926290	BREX-system-P-loop-protein-BrxC-[Cryobacterium-sp.]
WP_029153784.1	Type1.BREX	<-REase+SF1-SF2-Helicase<-TRD+TRD||?->?->BrxA-DNA-binding->BrxB-BrxF-iSTAND->DUF6079*->N6-METHYLASE->PglZ->SIGMA+OB-Fold+AAA-ATPase+HKD-DNase-BrxL-><-?||?->?->?->HIN-HTH->	1160	Bacteria>Terrabacteria_group>Actinomycetota>Actinomycetes>Micrococcales>Microbacteriaceae	GCF_000422745.1	Microbacterium_gubbeenense	G510_RS0111780	9571..13053	159896	BREX-system-P-loop-protein-BrxC-[Microbacterium-gubbeenense]
WP_028245009.1	Type1.BREX	<-Thioredoxin||?-><-PALP||tRNA->35exo->tRNA->BrxA-DNA-binding->BrxB-BrxF-iSTAND->DUF6079*->N6-METHYLASE-><-N6-METHYLASE-p||N6-METHYLASE-p->Schlafen+HATPase+wHTH->PglZ->SIGMA+OB-Fold+AAA-ATPase+HKD-DNase-BrxL->SIR2->HerA/FtsK->PhdYeFM_antitox->	1170	Bacteria>Terrabacteria_group>Actinomycetota>Actinomycetes>Micrococcales>Microbacteriaceae	GCF_000423505.1	Pseudoclavibacter_soli	G557_RS0103810	190122..193634	452623	BREX-system-P-loop-protein-BrxC-[Pseudoclavibacter-soli]
WP_053098414.1	Type1.BREX	<-HEPN+SF1-SF2-Helicase+REase||UDG-><-?<-?<-?||BrxA-DNA-binding->BrxB-BrxF-iSTAND->DUF6079*->N6-METHYLASE->PglZ->SIGMA+OB-Fold+AAA-ATPase+HKD-DNase-BrxL->Pkinase->?-><-?<-MarR-HTH||ADH_N+ADH_zinc_N-><-McrC-NTD+REase	1186	Bacteria>Terrabacteria_group>Actinomycetota>Actinomycetes>Micrococcales>Microbacteriaceae	GCF_001266755.1	Microbacterium_sp._CGR1	AKG07_RS13635	2816924..2820484	1696072	BREX-system-P-loop-protein-BrxC-[Microbacterium-sp.-CGR1]
WP_053352376.1	Type1.BREX	<-MODE-HTH+PBPII||?->?->?->?->tRNA->XIS-HTH->BrxA-DNA-binding->BrxB-BrxF-iSTAND->DUF6079*->N6-METHYLASE->PglZ->SIGMA+OB-Fold+AAA-ATPase+HKD-DNase-BrxL-><-?<-?<-?||HNH+SAVED->ParB+HNH->	1182	Bacteria>Terrabacteria_group>Actinomycetota>Actinomycetes>Micrococcales>Microbacteriaceae	GCF_001273845.1	Leucobacter_musarum	CH42_RS06350	60565..64113	1930747	BREX-system-P-loop-protein-BrxC-[Leucobacter-musarum]
WP_085605911.1	Type1.BREX	<-HTH_Tnp_1||DUF6079*->N6-METHYLASE->PglZ->SIGMA+OB-Fold+AAA-ATPase+HKD-DNase-BrxL->?->VirB11+MuB-ATPase->	1160	Bacteria>Terrabacteria_group>Actinomycetota>Actinomycetes>Micrococcales>Microbacteriaceae	GCF_002114135.1	Microbacterium_sp._LEMMJ01	B7W94_RS08480	394..3876	1978350	BREX-system-P-loop-protein-BrxC-[Microbacterium-sp.-LEMMJ01]
WP_104291918.1	Type1.BREX	McrB-AAA->McrC-NTD+REase->GDResolvase+HIN-HTH-><-?||?->?->XIS-HTH->BrxA-DNA-binding->BrxB-BrxF-iSTAND->DUF6079*->N6-METHYLASE->PglZ->SIGMA+OB-Fold+AAA-ATPase+HKD-DNase-BrxL->REase+HTH+35exo+Small-B-barrel+SF1-SF2-Helicase-><-RNase	1170	Bacteria>Terrabacteria_group>Actinomycetota>Actinomycetes>Micrococcales>Microbacteriaceae	GCF_002930935.1	Rathayibacter_sp._AY1A2	C5B93_RS04570	113948..117460	2080520	BREX-system-P-loop-protein-BrxC-[Rathayibacter-sp.-AY1A2]
WP_104323594.1	Type1.BREX	MarR-HTH->?->?-><-?||?->?-><-SIGMA+OB-Fold+AAA-ATPase+HKD-DNase-BrxL<-PglZ<-N6-METHYLASE<-DUF6079*<-BrxB-BrxF-iSTAND<-BrxA-DNA-binding	1160	Bacteria>Terrabacteria_group>Actinomycetota>Actinomycetes>Micrococcales>Microbacteriaceae	GCF_002931415.1	unclassified_Rathayibacter	C5C74_RS01635	26455..29937	2609250	MULTISPECIES:-BREX-system-P-loop-protein-BrxC-[unclassified-Rathayibacter]
WP_104309935.1	Type1.BREX	TIR-><-?||?-><-TIR<-SIGMA+OB-Fold+AAA-ATPase+HKD-DNase-BrxL<-PglZ<-REase<-N6-METHYLASE<-DUF6079*<-BrxB-BrxF-iSTAND<-BrxA-DNA-binding<-XIS-HTH<-?||GDResolvase+HIN-HTH-><-Acyltransferase<-DSBH<-SIGMA-HTH	1162	Bacteria>Terrabacteria_group>Actinomycetota>Actinomycetes>Micrococcales>Microbacteriaceae	GCF_002932095.1	Rathayibacter_sp._AY1G1	C5C36_RS14015	21701..25189	2080564	BREX-system-P-loop-protein-BrxC-[Rathayibacter-sp.-AY1G1]
WP_104347518.1	Type1.BREX	<-ClpABN-AAA+ClpABC-AAA<-?<-SIGMA+OB-Fold+AAA-ATPase+HKD-DNase-BrxL<-PglZ<-HTH+REase<-N6-METHYLASE<-DUF6079*<-BrxB-BrxF-iSTAND<-BrxA-DNA-binding<-XIS-HTH<-ABhydrolase	1182	Bacteria>Terrabacteria_group>Actinomycetota>Actinomycetes>Micrococcales>Microbacteriaceae	GCF_002932695.1	Rathayibacter_sp._AY1D2	C5D04_RS07575	19648..23196	2080543	BREX-system-P-loop-protein-BrxC-[Rathayibacter-sp.-AY1D2]
WP_124973569.1	Type1.BREX	Aminotran_4-><-?<-NAD_synthase||?-><-tRNA||35exo->tRNA->XIS-HTH->BrxA-DNA-binding->BrxB-BrxF-iSTAND->DUF6079*->N6-METHYLASE->HTH+REase->PglZ->SIGMA+OB-Fold+AAA-ATPase+HKD-DNase-BrxL->SIR2->HerA/FtsK-><-DNA_METHYLASE	1170	Bacteria>Terrabacteria_group>Actinomycetota>Actinomycetes>Micrococcales>Microbacteriaceae	GCF_003865375.1	Gulosibacter_macacae	EG850_RS11335	21958..25470	2488791	BREX-system-P-loop-protein-BrxC-[Gulosibacter-macacae]
WP_237399358.1	Type1.BREX	<-Glycos_transf<-Glyco_transf_9<-HAD+HAD||Glyco_transf_9-><-SIGMA-HTH||?->?->tRNA->BrxA-DNA-binding->BrxB-BrxF-iSTAND->DUF6079-p*->N6-METHYLASE-p-><-N6-METHYLASE-p||Phage_int_SAM_3+Phage_integrase->N6-METHYLASE-p->Schlafen+HATPase+wHTH->PglZ->SIGMA+OB-Fold+AAA-ATPase+HKD-DNase-BrxL->	878	Bacteria>Terrabacteria_group>Actinomycetota>Actinomycetes>Micrococcales>Microbacteriaceae	GCF_004011115.1	Rathayibacter_iranicus	C7V51_RS01465	309166..311802	59737	BREX-system-P-loop-protein-BrxC-[Rathayibacter-iranicus]
WP_129387833.1	Type1.BREX	ABhydrolase->?->Peptidase_S15+PepX_C->?-><-?<-?<-Peptidase_S66+Peptidase_S66C||BrxA-DNA-binding->BrxB-BrxF-iSTAND->DUF6079*->N6-METHYLASE-><-N6-METHYLASE-p||Phage_int_SAM_3+Phage_integrase->N6-METHYLASE-p->Schlafen+HATPase+wHTH->PglZ->SIGMA+OB-Fold+AAA-ATPase+HKD-DNase-BrxL-><-PhdYeFM_antitox	1162	Bacteria>Terrabacteria_group>Actinomycetota>Actinomycetes>Micrococcales>Microbacteriaceae	GCF_004135285.1	Microbacterium_protaetiae	ET475_07150	1526355..1529843	2509458	BREX-system-P-loop-protein-BrxC-[Microbacterium-protaetiae]
WP_130108838.1	Type1.BREX	<-TetR-HTH||TrwC+SF1-SF2-Helicase+TOPRIM->METHYLASE-><-VirB11+MuB-ATPase<-HerA/FtsK<-SIR2+SIR2<-SIGMA+OB-Fold+AAA-ATPase+HKD-DNase-BrxL<-PglZ<-N6-METHYLASE<-DUF6079*<-BrxB-BrxF-iSTAND<-BrxA-DNA-binding<-XIS-HTH<-ABhydrolase<-?<-MPTase<-HTH||ArdCN+MPTase->Primpol+PriCT1->	1160	Bacteria>Terrabacteria_group>Actinomycetota>Actinomycetes>Micrococcales>Microbacteriaceae	GCF_004208635.1	Leucobacter_triazinivorans	EVS81_RS01585	346722..350204	1784719	BREX-system-P-loop-protein-BrxC-[Leucobacter-triazinivorans]
WP_134415956.1	Type1.BREX	<-HNH<-?<-?<-VirB11+MuB-ATPase<-HerA/FtsK<-SIR2+SIR2<-SIGMA+OB-Fold+AAA-ATPase+HKD-DNase-BrxL<-PglZ<-N6-METHYLASE<-DUF6079*<-BrxB-BrxF-iSTAND<-BrxA-DNA-binding<-XIS-HTH<-ABhydrolase<-?<-MPTase<-HTH||ArdCN+MPTase->Primpol+PriCT1->	1170	Bacteria>Terrabacteria_group>Actinomycetota>Actinomycetes>Micrococcales>Microbacteriaceae	GCF_004402465.1	Cryobacterium_sp._TMT2-14	E3028_RS10860	27948..31460	1259245	BREX-system-P-loop-protein-BrxC-[Cryobacterium-sp.-TMT2-14]
WP_241980122.1	Type1.BREX	<-URI-DNase<-VirB11+MuB-ATPase<-HEPN+REase<-REase<-SIGMA+OB-Fold+AAA-ATPase+HKD-DNase-BrxL<-PglZ<-N6-METHYLASE<-DUF6079*<-BrxB-BrxF-iSTAND<-BrxA-DNA-binding<-XIS-HTH<-ABhydrolase<-?<-MPTase<-HTH||ArdCN+MPTase->Primpol+PriCT1->	1172	Bacteria>Terrabacteria_group>Actinomycetota>Actinomycetes>Micrococcales>Microbacteriaceae	GCF_004402535.1	Cryobacterium_sp._HLT2-23	E3006_RS11880	40394..43912	1259145	BREX-system-P-loop-protein-BrxC-[Cryobacterium-glaciale]
WP_134434730.1	Type1.BREX	<-VirB11+MuB-ATPase<-SbcC<-?<-SIGMA+OB-Fold+AAA-ATPase+HKD-DNase-BrxL<-PglZ<-N6-METHYLASE<-DUF6079*<-BrxB-BrxF-iSTAND<-BrxA-DNA-binding<-XIS-HTH||AraC-HTH+AraC-HTH->ArdCN+MPTase->Primpol+PriCT1->DUF2637+HIN-HTH->	1164	Bacteria>Terrabacteria_group>Actinomycetota>Actinomycetes>Micrococcales>Microbacteriaceae	GCF_004403545.1	Cryobacterium_sp._TmT2-59	E3T24_RS13895	41136..44630	1259264	BREX-system-P-loop-protein-BrxC-[Cryobacterium-sp.-TmT2-59]
WP_146322974.1	Type1.BREX	DOC+HTH->?->BrxA-DNA-binding->BrxB-BrxF-iSTAND->DUF6079*->N6-METHYLASE-><-N6-METHYLASE-p||Phage_int_SAM_3+Phage_integrase->N6-METHYLASE-p->?->?->PglZ->SIGMA+OB-Fold+AAA-ATPase+HKD-DNase-BrxL->ParB+HNH->	1162	Bacteria>Terrabacteria_group>Actinomycetota>Actinomycetes>Micrococcales>Microbacteriaceae	GCF_007859675.1	Humibacter_ginsenosidimutans	FPZ11_RS17385	3786036..3789524	2599293	BREX-system-P-loop-protein-BrxC-[Humibacter-ginsenosidimutans]
WP_200838962.1	Type1.BREX	<-SIGMA+OB-Fold+AAA-ATPase+HKD-DNase-BrxL<-PglZ<-N6-METHYLASE-p<-Phage_int_SAM_3+Phage_integrase||N6-METHYLASE-p-><-N6-METHYLASE<-DUF6079*<-BrxB-BrxF-iSTAND<-BrxA-DNA-binding<-XIS-HTH||HTH_3-><-XIS-HTH<-tRNA<-ABC-ATPase+ABC-ATPase<-ZnR+TOPRIM+TOPRIM<-HD	1182	Bacteria>Terrabacteria_group>Actinomycetota>Actinomycetes>Micrococcales>Microbacteriaceae	GCF_008271365.1	Microbacterium	FY549_RS13635	2819312..2822860	33882	MULTISPECIES:-BREX-system-P-loop-protein-BrxC-[Microbacterium]
WP_163473715.1	Type1.BREX	Aminotran_4->?->tRNA->XIS-HTH->Phage_integrase->TrwC+SF1-SF2-Helicase+TOPRIM->XIS-HTH->BrxA-DNA-binding->BrxB-BrxF-iSTAND->DUF6079*->N6-METHYLASE->REase->PglZ->SIGMA+OB-Fold+AAA-ATPase+HKD-DNase-BrxL->N6-METHYLASE->MORC+HISKIN->?->PIN->PIN->	1162	Bacteria>Terrabacteria_group>Actinomycetota>Actinomycetes>Micrococcales>Microbacteriaceae	GCF_010686725.1	Galbitalea_soli	HNR17_RS04680	978511..981999	1268042	BREX-system-P-loop-protein-BrxC-[Galbitalea-soli]
WP_244301194.1	Type1.BREX	<-SIGMA+OB-Fold+AAA-ATPase+HKD-DNase-BrxL<-?<-?<-HerA/FtsK<-SIR2<-PglZ<-?<-N6-METHYLASE-p<-N6-METHYLASE<-DUF6079*<-BrxB-BrxF-iSTAND<-BrxA-DNA-binding<-?<-?||TetR-HTH-><-PIN<-PhdYeFM_antitox<-ABC-ATPase+SbcC<-Sulfate_transp+STAS	1194	Bacteria>Terrabacteria_group>Actinomycetota>Actinomycetes>Micrococcales>Microbacteriaceae	GCF_011382965.1	Leucobacter_insecticola	G7067_RS02010	442468..446052	2714934	BREX-system-P-loop-protein-BrxC-[Leucobacter-insecticola]
WP_166866044.1	Type1.BREX	<-REase<-SbcC<-JetB<-JetA<-SIGMA+OB-Fold+AAA-ATPase+HKD-DNase-BrxL<-PglZ<-N6-METHYLASE<-DUF6079*<-BrxB-BrxF-iSTAND<-BrxA-DNA-binding<-REase<-tRNA<-?<-?<-?||?-><-?<-Peptidase_M28	1160	Bacteria>Terrabacteria_group>Actinomycetota>Actinomycetes>Micrococcales>Microbacteriaceae	GCF_011751865.2	Salinibacterium_sp._ZJ70	HCR12_RS10200	2144642..2148124	2708084	BREX-system-P-loop-protein-BrxC-[Salinibacterium-sp.-ZJ70]
WP_169077984.1	Type1.BREX	BrxA-DNA-binding->BrxB-BrxF-iSTAND->DUF6079*->N6-METHYLASE->REase->PglZ->SIGMA+OB-Fold+AAA-ATPase+HKD-DNase-BrxL->SIR2->HerA/FtsK-><-Peptidase_M48||?->REase+HTH+35exo+Small-B-barrel+SF1-SF2-Helicase->	1162	Bacteria>Terrabacteria_group>Actinomycetota>Actinomycetes>Micrococcales>Microbacteriaceae	GCF_012845865.1	Yonghaparkia	HFV16_RS05520	206976..210464	355929	MULTISPECIES:-BREX-system-P-loop-protein-BrxC-[Yonghaparkia]
WP_121673638.1	Type1.BREX	Phage_integrase-><-HEPN<-ParB+HNH||VirB11+MuB-ATPase-><-sGTP<-GTPase-AIG<-SIGMA+OB-Fold+AAA-ATPase+HKD-DNase-BrxL<-PglZ<-N6-METHYLASE<-DUF6079*<-BrxB-BrxF-iSTAND<-BrxA-DNA-binding<-XIS-HTH<-?<-?||?->ORC-CDC6->?->N6-METHYLASE->	1161	Bacteria>Terrabacteria_group>Actinomycetota>Actinomycetes>Micrococcales>Microbacteriaceae	GCF_014651755.1	Mycetocola_manganoxydans	IE057_RS13450	51714..55199	699879	BREX-system-P-loop-protein-BrxC-[Mycetocola-manganoxydans]
WP_197132648.1	Type1.BREX	REase-><-?<-sGTP<-GTPase-AIG<-SIGMA+OB-Fold+AAA-ATPase+HKD-DNase-BrxL<-N6-METHYLASE<-N6-METHYLASE<-DUF6079*<-BrxB-BrxF-iSTAND<-BrxA-DNA-binding<-XIS-HTH||Phage_integrase->Phage_integrase->DDE_Tnp_1+DDE_Tnp_1->	1171	Bacteria>Terrabacteria_group>Actinomycetota>Actinomycetes>Micrococcales>Microbacteriaceae	GCF_015864675.1	Salinibacterium_sp._SWN1162	I6E58_RS09540	2021923..2025438	2792053	BREX-system-P-loop-protein-BrxC-[Salinibacterium-sp.-SWN1162]
WP_206395457.1	Type1.BREX	ParB+HNH-><-ABhydrolase<-?<-SIGMA+OB-Fold+AAA-ATPase+HKD-DNase-BrxL<-PglZ<-N6-METHYLASE-p<-Phage_int_SAM_3+Phage_integrase||N6-METHYLASE-p-><-N6-METHYLASE<-DUF6079*<-BrxB-BrxF-iSTAND<-BrxA-DNA-binding<-tRNA||?->?->?->?->?->?->Pkinase+TPR+TPR->	1158	Bacteria>Terrabacteria_group>Actinomycetota>Actinomycetes>Micrococcales>Microbacteriaceae	GCF_017167395.1	Pseudoclavibacter_alba	JRG19_RS09065	120280..123756	272241	BREX-system-P-loop-protein-BrxC-[Pseudoclavibacter-alba]
WP_240240880.1	Type1.BREX	<-VirB11+MuB-ATPase||DNA_METHYLASE-><-HerA/FtsK<-SIR2+SIR2<-SIGMA+OB-Fold+AAA-ATPase+HKD-DNase-BrxL<-PglZ<-?<-N6-METHYLASE<-DUF6079*<-BrxB-BrxF-iSTAND<-BrxA-DNA-binding<-XIS-HTH<-ABhydrolase<-?<-MPTase<-HTH||ArdCN+MPTase->Primpol+PriCT1->	1178	Bacteria>Terrabacteria_group>Actinomycetota>Actinomycetes>Micrococcales>Microbacteriaceae	GCF_022436485.1	Agrococcus_sp._ARC_14	MKD51_RS07980	1621402..1624938	2919927	BREX-system-P-loop-protein-BrxC-[Agrococcus-sp.-ARC_14]
WP_242086581.1	Type1.BREX	XIS-HTH->BrxA-DNA-binding->BrxB-BrxF-iSTAND->DUF6079*->N6-METHYLASE-><-N6-METHYLASE-p||Phage_int_SAM_3+Phage_integrase->N6-METHYLASE-p->PglZ->SIGMA+OB-Fold+AAA-ATPase+HKD-DNase-BrxL->	1160	Bacteria>Terrabacteria_group>Actinomycetota>Actinomycetes>Micrococcales>Microbacteriaceae	GCF_022662255.1	Microbacterium_lacticum	L8F16_RS04085	1822..5304	33885	BREX-system-P-loop-protein-BrxC-[Microbacterium-lacticum]
WP_156999272.1	Type1.BREX	<-HEPN<-PolB<-HEPN+SF1-SF2-Helicase+REase||?->CBS+HEPN+HEPN->BrxA-DNA-binding->BrxB-BrxF-iSTAND->DUF6079*->N6-METHYLASE->PglZ->SIGMA+OB-Fold+AAA-ATPase+HKD-DNase-BrxL->sGTP+sGTP+GTPase-AIG->?-><-ParB+HNH||Exonuc_V_gamma+REase->REase->REase->	1173	Bacteria>Terrabacteria_group>Actinomycetota>Actinomycetes>Micrococcales>Microbacteriaceae	GCF_024172065.1	Agromyces_terreus	BJ978_RS07845	1788329..1791850	424795	BREX-system-P-loop-protein-BrxC-[Agromyces-terreus]
WP_257523879.1	Type1.BREX	<-PIN<-URI-DNase||?->?-><-?||XIS-HTH->BrxA-DNA-binding->BrxB-BrxF-iSTAND->DUF6079*->N6-METHYLASE->PglZ->SIGMA+OB-Fold+AAA-ATPase+HKD-DNase-BrxL->CMP-hydrolase->	1160	Bacteria>Terrabacteria_group>Actinomycetota>Actinomycetes>Micrococcales>Microbacteriaceae	GCF_024623505.1	Microbacterium_sp._zg.B48	NQ152_RS10060	193646..197128	2969408	BREX-system-P-loop-protein-BrxC-[Microbacterium-sp.-zg.B48]
WP_259980691.1	Type1.BREX	MerR-HTH-><-?||N6-METHYLASE-p-><-HerA/FtsK<-SIR2+SIR2<-SIGMA+OB-Fold+AAA-ATPase+HKD-DNase-BrxL<-PglZ<-N6-METHYLASE<-DUF6079*<-BrxB-BrxF-iSTAND<-BrxA-DNA-binding<-XIS-HTH<-DnaK||HTH_3-><-DUF6361<-HNH	1162	Bacteria>Terrabacteria_group>Actinomycetota>Actinomycetes>Micrococcales>Microbacteriaceae	GCF_025151145.1	Microbacterium_paraoxydans	M3D59_RS02760	52380..55868	199592	BREX-system-P-loop-protein-BrxC-[Microbacterium-paraoxydans]
WP_261607502.1	Type1.BREX	SIR2->ADH_N+ADH_zinc_N-><-?||tRNA->?-><-Phage_int_SAM_3+Phage_integrase<-?<-?||BrxA-DNA-binding->BrxB-BrxF-iSTAND->DUF6079*->N6-METHYLASE->PglZ->SIGMA+OB-Fold+AAA-ATPase+HKD-DNase-BrxL->Pkinase-><-SF1-SF2-Helicase	1186	Bacteria>Terrabacteria_group>Actinomycetota>Actinomycetes>Micrococcales>Microbacteriaceae	GCF_025398275.1	Microbacterium_sp._PMIC_1C1B	N4R40_RS11380	57181..60741	2978350	BREX-system-P-loop-protein-BrxC-[Microbacterium-sp.-PMIC_1C1B]
WP_263798144.1	Type1.BREX	<-Phage_int_SAM_3+Phage_integrase||?->BrxA-DNA-binding->BrxB-BrxF-iSTAND->DUF6079*->N6-METHYLASE->PglZ->SIGMA+OB-Fold+AAA-ATPase+HKD-DNase-BrxL->Pkinase-><-?<-REase+HTH+35exo+Small-B-barrel+SF1-SF2-Helicase<-URI-DNase+RAMA||TrwC+SF1-SF2-Helicase->REase->	1186	Bacteria>Terrabacteria_group>Actinomycetota>Actinomycetes>Micrococcales>Microbacteriaceae	GCF_027943945.1	Microbacterium_terricola	OAU46_RS04855	990438..993998	344163	BREX-system-P-loop-protein-BrxC-[Microbacterium-terricola]
WP_257508277.1	Type1.BREX	<-METHYLASE<-?||LACI-HTH+PBPI->?->?->?-><-?||tRNA->||BrxA-DNA-binding->BrxB-BrxF-iSTAND->DUF6079*->N6-METHYLASE->PglZ->SIGMA+OB-Fold+AAA-ATPase+HKD-DNase-BrxL->Pkinase-><-?||?-><-REase+HTH+35exo+Small-B-barrel+SF1-SF2-Helicase<-?||AAA-ATPase->	1186	Bacteria>Terrabacteria_group>Actinomycetota>Actinomycetes>Micrococcales>Microbacteriaceae	GCF_030246865.1	Microbacterium_sp._zg.B96	NQ154_RS06000	561655..565215	2969409	BREX-system-P-loop-protein-BrxC-[Microbacterium-sp.-zg.B96]
WP_257505179.1	Type1.BREX	<-McrC-NTD+REase<-McrB-AAA<-?||HTH_Tnp_1+HTH_21+rve+rve_3->ACET-><-SIGMA+OB-Fold+AAA-ATPase+HKD-DNase-BrxL<-PglZ<-N6-METHYLASE<-DUF6079*<-BrxB-BrxF-iSTAND<-BrxA-DNA-binding<-HIN-HTH<-HIRAN||REase-><-tRNA||?-><-REase	1162	Bacteria>Terrabacteria_group>Actinomycetota>Actinomycetes>Micrococcales>Microbacteriaceae	GCF_030246925.1	Microbacterium_sp._zg.Y625	NQ156_RS01310	276922..280410	2969411	BREX-system-P-loop-protein-BrxC-[Microbacterium-sp.-zg.Y625]
WP_028495577.1	Type1.BREX	VirB11+MuB-ATPase->?-><-?||XIS-HTH->BrxA-DNA-binding->BrxB-BrxF-iSTAND->DUF6079*->N6-METHYLASE->PglZ->SIGMA+OB-Fold+AAA-ATPase+HKD-DNase-BrxL->?->tRNA->?-><-?<-ADH_N+ADH_zinc_N<-DSBH<-HAD+HAD	1160	Bacteria>Terrabacteria_group>Actinomycetota>Actinomycetes>Micrococcales>Microbacteriaceae	GCF_900113885.1	Microbacterium	BM226_RS05185	1039412..1042894	33882	MULTISPECIES:-BREX-system-P-loop-protein-BrxC-[Microbacterium]
WP_201614919.1	Type1.BREX	Aminotran_4-><-?<-Peptidase<-NAD_synthase<-tRNA||35exo->tRNA->BrxA-DNA-binding->BrxB-BrxF-iSTAND->DUF6079*->N6-METHYLASE-><-N6-METHYLASE-p||Phage_int_SAM_3+Phage_integrase->N6-METHYLASE-p->HTH+REase->PglZ->SIGMA+OB-Fold+AAA-ATPase+HKD-DNase-BrxL->	1170	Bacteria>Terrabacteria_group>Actinomycetota>Actinomycetes>Micrococcales>Microbacteriaceae	GCF_904848685.1	Gulosibacter_hominis	JMX86_RS10045	19055..22567	2770504	BREX-system-P-loop-protein-BrxC-[Gulosibacter-hominis]
WP_230672563.1	Type1.BREX	ABC-ATPase+SbcC->?->?-><-?<-?||MACRODOMAIN+SIR2->BrxA-DNA-binding->BrxB-BrxF-iSTAND->DUF6079*->N6-METHYLASE->HTH+REase->HTH+REase->PglZ->SIGMA+OB-Fold+AAA-ATPase+HKD-DNase-BrxL-><-?||?->HEPN+SF1-SF2-Helicase+REase->	1176	Bacteria>Terrabacteria_group>Actinomycetota>Actinomycetes>Micrococcales>Microbacteriaceae	GCF_920939495.1	Rathayibacter_sp._Leaf248	LRG51_RS10050	2075326..2078856	2876555	BREX-system-P-loop-protein-BrxC-[Rathayibacter-sp.-Leaf248]
WP_219885258.1	Type1.BREX	HIN-HTH-><-SIGMA+OB-Fold+AAA-ATPase+HKD-DNase-BrxL<-PglZ<-N6-METHYLASE<-DUF6079*<-BrxB-BrxF-iSTAND<-XIS-HTH<-?<-?||XIS-HTH->?->HNH->	1210	Bacteria>Terrabacteria_group>Actinomycetota>Actinomycetes>Micrococcales>Micrococcaceae	-	Arthrobacter_sp._PAMC25284	KY499_09320	2016646..2020278	2861279	BREX-system-P-loop-protein-BrxC-[Arthrobacter-sp.-PAMC25284]
AFR34584.1	Type1.BREX	<-SIGMA+OB-Fold+AAA-ATPase+HKD-DNase-BrxL<-PglZ<-N6-METHYLASE<-DUF6079*<-BrxB-BrxF-iSTAND<-BrxA-DNA-binding||ParA-Soj-PloopNTPase->?->?->?->VirB11->	1166	Bacteria>Terrabacteria_group>Actinomycetota>Actinomycetes>Micrococcales>Micrococcaceae	GCA_000294695.2	Arthrobacter_sp._Rue61a	ARUE_113p00760	81229..84729	1118963	putative-ATP-binding-protein-[Arthrobacter-sp.-Rue61a]
ERI35655.1	Type1.BREX	<-Radical-SAM<-?||XIS-HTH->BrxA-DNA-binding->BrxB-BrxF-iSTAND->DUF6079*->N6-METHYLASE->PglZ->SIGMA+OB-Fold+AAA-ATPase+HKD-DNase-BrxL-><-?||URI-DNase->?->?-><-ParB+HNH	1161	Bacteria>Terrabacteria_group>Actinomycetota>Actinomycetes>Micrococcales>Micrococcaceae	GCA_000465895.2	Arthrobacter_sp._AK-YN10	M707_20590	5564..9049	1349820	ATP-binding-protein-[Arthrobacter-sp.-AK-YN10]
ALV40633.1	Type1.BREX	<-HEPN+SF1-SF2-Helicase+REase<-?<-?<-ParB+HNH<-SIGMA+OB-Fold+AAA-ATPase+HKD-DNase-BrxL<-PglZ<-N6-METHYLASE<-DUF6079*<-BrxB-BrxF-iSTAND<-BrxA-DNA-binding<-XIS-HTH<-HKD-DNase+SF1-SF2-Helicase+HTH+HTH+SRA||?->?->?->McrB-AAA->McrC-NTD+REase->	1172	Bacteria>Terrabacteria_group>Actinomycetota>Actinomycetes>Micrococcales>Micrococcaceae	GCA_001484605.1	Pseudarthrobacter_sulfonivorans	AU252_05160	1179330..1182848	121292	hypothetical-protein-AU252_05160-[Pseudarthrobacter-sulfonivorans]
AOY70771.1	Type1.BREX	RNase+DSRBD->?-><-?||XIS-HTH->BrxA-DNA-binding->BrxB-BrxF-iSTAND->DUF6079*->N6-METHYLASE->PglZ->SIGMA+OB-Fold+AAA-ATPase+HKD-DNase-BrxL->?-><-HTH_21+rve+rve_3<-HIN-HTH||?->NERD->	1161	Bacteria>Terrabacteria_group>Actinomycetota>Actinomycetes>Micrococcales>Micrococcaceae	GCA_001854365.1	Arthrobacter_sp._ZXY-2	ARZXY2_1218	1271114..1274599	1806905	ATP-binding-protein-[Arthrobacter-sp.-ZXY-2]
ASN39993.1	Type1.BREX	<-Peptidase_M16+Peptidase_M16_C<-RNasePH+RNasePH+KH+S1||RNase+PHP-><-?<-TRD<-N6-METHYLASE<-SIGMA+OB-Fold+AAA-ATPase+HKD-DNase-BrxL<-PglZ<-N6-METHYLASE<-DUF6079*<-BrxB-BrxF-iSTAND<-BrxA-DNA-binding<-XIS-HTH||Schlafen->	1170	Bacteria>Terrabacteria_group>Actinomycetota>Actinomycetes>Micrococcales>Micrococcaceae	GCA_002224445.1	Arthrobacter_sp._7749	CGQ24_13870	3050415..3053927	2020130	ATP-binding-protein-[Arthrobacter-sp.-7749]
THE19344.1	Type1.BREX	<-TIR||XIS-HTH->BrxA-DNA-binding->BrxB-BrxF-iSTAND->DUF6079*->N6-METHYLASE->PglZ->SIGMA+OB-Fold+AAA-ATPase+HKD-DNase-BrxL-><-?||REase->HTH+REase->URI-DNase->DUF6361->HKD-DNase->	1166	Bacteria>Terrabacteria_group>Actinomycetota>Actinomycetes>Micrococcales>Micrococcaceae	GCA_004799725.1	Kocuria_rosea	E1J17_01420	4081..7581	1275	BREX-system-P-loop-protein-BrxC-[Kocuria-rosea]
NHQ58332.1	Type1.BREX	ICLR-HTH-><-?<-?||?-><-?||?->BrxA-DNA-binding->BrxB-BrxF-iSTAND->DUF6079*->N6-METHYLASE->PglZ->SIGMA+OB-Fold+AAA-ATPase+HKD-DNase-BrxL-><-?||?-><-RADICAL-SAM<-?<-ACET<-XIS-HTH	1174	Bacteria>Terrabacteria_group>Actinomycetota>Actinomycetes>Micrococcales>Micrococcaceae	GCA_011602345.1	Micrococcus_luteus	G9B47_07855	17274..20798	1270	BREX-system-P-loop-protein-BrxC-[Micrococcus-luteus]
MBB4882886.1	Type1.BREX	35exo->tRNA-><-Recombinase+ZnR||ABhydrolase->XIS-HTH->BrxA-DNA-binding->BrxB-BrxF-iSTAND->DUF6079*->N6-METHYLASE->HTH+REase->PglZ->SIGMA+OB-Fold+AAA-ATPase+HKD-DNase-BrxL->SIR2->HIN-HTH->HTH_Tnp_1+HTH_21+rve+rve_3->	1173	Bacteria>Terrabacteria_group>Actinomycetota>Actinomycetes>Micrococcales>Micrococcaceae	GCA_014204815.1	Micrococcus_flavus	BJ976_001237	1342727..1346248	384602	hypothetical-protein-[Micrococcus-flavus]
MBR9643072.1	Type1.BREX	<-DNA-glycosylase<-URI-DNase<-MACRODOMAIN+SIR2<-REase||HKD-DNase+SF1-SF2-Helicase+HTH+HTH+SRA-><-SIGMA+OB-Fold+AAA-ATPase+HKD-DNase-BrxL<-PglZ<-N6-METHYLASE<-DUF6079*<-BrxB-BrxF-iSTAND<-BrxA-DNA-binding||McrB-AAA->McrC-NTD+REase->ABhydrolase+ABhydrolase->	1173	Bacteria>Terrabacteria_group>Actinomycetota>Actinomycetes>Micrococcales>Micrococcaceae	GCA_018215265.1	Paenarthrobacter_sp._GOM3	IRJ34_14045	199455..202976	2782567	BREX-system-P-loop-protein-BrxC-[Paenarthrobacter-sp.-GOM3]
QYF88488.1	Type1.BREX	HIN-HTH-><-SIGMA+OB-Fold+AAA-ATPase+HKD-DNase-BrxL<-PglZ<-N6-METHYLASE<-DUF6079*<-BrxB-BrxF-iSTAND<-XIS-HTH<-?<-?||XIS-HTH->?->HNH->	1210	Bacteria>Terrabacteria_group>Actinomycetota>Actinomycetes>Micrococcales>Micrococcaceae	GCA_019443425.1	Arthrobacter_sp._PAMC25284	KY499_09320	2016646..2020278	2861279	BREX-system-P-loop-protein-BrxC-[Arthrobacter-sp.-PAMC25284]
WP_019309257.1	Type1.BREX	<-MPTase<-HTH_3||SMODS->?->?-><-SIGMA+OB-Fold+AAA-ATPase+HKD-DNase-BrxL<-PglZ<-N6-METHYLASE<-DUF6079*<-BrxB-BrxF-iSTAND<-BrxA-DNA-binding<-XIS-HTH<-HTH_3<-?<-?<-?<-?||ABhydrolase->	1159	Bacteria>Terrabacteria_group>Actinomycetota>Actinomycetes>Micrococcales>Micrococcaceae	GCF_000214115.1	Kocuria_rhizophila	GGK_RS0101750	388045..391524	72000	BREX-system-P-loop-protein-BrxC-[Kocuria-rhizophila]
WP_017834907.1	Type1.BREX	<-SIGMA+OB-Fold+AAA-ATPase+HKD-DNase-BrxL<-PglZ<-N6-METHYLASE<-DUF6079*<-BrxB-BrxF-iSTAND<-BrxA-DNA-binding<-XIS-HTH<-?<-McrC-NTD+REase<-McrB-AAA<-REase<-?||<-Schlafen+HATPase+wHTH	1190	Bacteria>Terrabacteria_group>Actinomycetota>Actinomycetes>Micrococcales>Micrococcaceae	GCF_000349605.1	Kocuria_sp._UCD-OTCP	H488_RS34905	37795..41367	1292021	BREX-system-P-loop-protein-BrxC-[Kocuria-sp.-UCD-OTCP]
WP_155853802.1	Type1.BREX	<-Primpol+PriCT1<-ArdCN+MPTase||HTH->MPTase->?->ABhydrolase->XIS-HTH->BrxA-DNA-binding->BrxB-BrxF-iSTAND->DUF6079*->N6-METHYLASE->PglZ->SIGMA+OB-Fold+AAA-ATPase+HKD-DNase-BrxL->?->VirB11+MuB-ATPase->	1170	Bacteria>Terrabacteria_group>Actinomycetota>Actinomycetes>Micrococcales>Micrococcaceae	GCF_000520595.1	Arthrobacter_sp._H5	Y708_RS0109785	51778..55290	1267973	BREX-system-P-loop-protein-BrxC-[Arthrobacter-sp.-H5]
WP_062292549.1	Type1.BREX	VirB11+MuB-ATPase->?-><-SIGMA+OB-Fold+AAA-ATPase+HKD-DNase-BrxL<-PglZ<-N6-METHYLASE-p<-Phage_int_SAM_3+Phage_integrase||N6-METHYLASE-p-><-N6-METHYLASE<-DUF6079*<-BrxB-BrxF-iSTAND<-BrxA-DNA-binding<-XIS-HTH<-ArdCN+MPTase<-?<-VirD4-TraG+VirD4-FtsK<-nSTAND3	1169	Bacteria>Terrabacteria_group>Actinomycetota>Actinomycetes>Micrococcales>Micrococcaceae	GCF_001445575.1	Arthrobacter_alpinus	AS189_RS18910	4268679..4272188	656366	BREX-system-P-loop-protein-BrxC-[Arthrobacter-alpinus]
WP_099093371.1	Type1.BREX	<-HEPN+SF1-SF2-Helicase+REase<-?||?-><-ParB+HNH<-SIGMA+OB-Fold+AAA-ATPase+HKD-DNase-BrxL<-PglZ<-N6-METHYLASE<-DUF6079*<-BrxB-BrxF-iSTAND<-BrxA-DNA-binding<-XIS-HTH<-HKD-DNase+SF1-SF2-Helicase+HTH+HTH+SRA||?->?->McrB-AAA->McrC-NTD+REase->	1173	Bacteria>Terrabacteria_group>Actinomycetota>Actinomycetes>Micrococcales>Micrococcaceae	GCF_001484605.1	Pseudarthrobacter_sulfonivorans	AU252_RS05160	1179330..1182851	121292	BREX-system-P-loop-protein-BrxC-[Pseudarthrobacter-sulfonivorans]
WP_078028155.1	Type1.BREX	<-EVE+HNH||?->?->CHTH->?->?-><-SIGMA+OB-Fold+AAA-ATPase+HKD-DNase-BrxL<-PglZ<-N6-METHYLASE<-DUF6079*<-BrxB-BrxF-iSTAND<-BrxA-DNA-binding<-XIS-HTH||?-><-?||?-><-Phage_integrase<-XIS-HTH<-AAA-ATPase	1176	Bacteria>Terrabacteria_group>Actinomycetota>Actinomycetes>Micrococcales>Micrococcaceae	GCF_002008315.2	Arthrobacter_sp._KBS0703	B1A87_RS03145	655510..659040	1955698	BREX-system-P-loop-protein-BrxC-[Arthrobacter-sp.-KBS0703]
WP_104138811.1	Type5.BREX	NUDIX->ARPP2-><-MACRODOMAIN+SIR2<-URI-DNase||HKD-DNase+SF1-SF2-Helicase+HTH+HTH+SRA->XIS-HTH->BrxA-DNA-binding->BrxB-BrxF-iSTAND->DUF6079*->N6-METHYLASE->PglZ->N6-METHYLASE-p->N6-METHYLASE->SIGMA+OB-Fold+AAA-ATPase+HKD-DNase-BrxL->?->ParB+HNH->?->35exo+TUDOR+SF1-SF2-Helicase+iREase+CTD->	1173	Bacteria>Terrabacteria_group>Actinomycetota>Actinomycetes>Micrococcales>Micrococcaceae	GCF_002929275.1	Arthrobacter_sp._ZGTC131	CXQ90_RS09900	131121..134642	2058898	BREX-system-P-loop-protein-BrxC-[Arthrobacter-sp.-ZGTC131]
WP_242630351.1	Type1.BREX	ParA-Soj-PloopNTPase->?-><-TIR||XIS-HTH->BrxA-DNA-binding->BrxB-BrxF-iSTAND->DUF6079*->N6-METHYLASE->PglZ->SIGMA+OB-Fold+AAA-ATPase+HKD-DNase-BrxL-><-?||REase->HTH+REase->URI-DNase->DUF6361->HKD-DNase->	1169	Bacteria>Terrabacteria_group>Actinomycetota>Actinomycetes>Micrococcales>Micrococcaceae	GCF_004799725.1	Kocuria_rosea	E1J17_RS01420	4072..7581	1275	BREX-system-P-loop-protein-BrxC-[Kocuria-rosea]
WP_142936644.1	Type1.BREX	<-NERD<-?<-PIN<-?<-NAD_binding_10<-SIGMA+OB-Fold+AAA-ATPase+HKD-DNase-BrxL<-PglZ<-N6-METHYLASE<-DUF6079*<-BrxB-BrxF-iSTAND<-BrxA-DNA-binding<-XIS-HTH||?->XIS-HTH->Phage_int_SAM_3->Phage_integrase->	1161	Bacteria>Terrabacteria_group>Actinomycetota>Actinomycetes>Micrococcales>Micrococcaceae	GCF_006964045.1	Arthrobacter_sp._TS-15	EU811_RS00880	63009..66494	2510797	BREX-system-P-loop-protein-BrxC-[Arthrobacter-sp.-TS-15]
WP_208011566.1	Type1.BREX	ICLR-HTH-><-?<-?||?-><-?||?->BrxA-DNA-binding->BrxB-BrxF-iSTAND->DUF6079*->N6-METHYLASE->PglZ->SIGMA+OB-Fold+AAA-ATPase+HKD-DNase-BrxL-><-?||?-><-RADICAL-SAM<-?<-ACET<-XIS-HTH	1172	Bacteria>Terrabacteria_group>Actinomycetota>Actinomycetes>Micrococcales>Micrococcaceae	GCF_011602345.1	Micrococcus_luteus	G9B47_RS07850	17280..20798	1270	BREX-system-P-loop-protein-BrxC-[Micrococcus-luteus]
WP_171585597.1	Type1.BREX	Peptidase_M20-><-Peptidase_M20||DNA_METHYLASE-><-?<-?<-?||?-><-SIGMA+OB-Fold+AAA-ATPase+HKD-DNase-BrxL<-PglZ<-N6-METHYLASE<-DUF6079*<-BrxB-BrxF-iSTAND<-XIS-HTH<-?||?-><-?<-HNH||Peptidase->	1171	Bacteria>Terrabacteria_group>Actinomycetota>Actinomycetes>Micrococcales>Micrococcaceae	GCF_013116745.1	Arthrobacter_sp._260	HMI57_RS08915	135623..139138	2735314	BREX-system-P-loop-protein-BrxC-[Arthrobacter-sp.-260]
WP_135027971.1	Type1.BREX	35exo->tRNA-><-Recombinase+ZnR||ABhydrolase->XIS-HTH->BrxA-DNA-binding->BrxB-BrxF-iSTAND->DUF6079*->N6-METHYLASE->HTH+REase->PglZ->SIGMA+OB-Fold+AAA-ATPase+HKD-DNase-BrxL->SIR2->HTH_Tnp_1+HTH_21+rve+rve_3-><-?||?-><-HAD+HAD	1170	Bacteria>Terrabacteria_group>Actinomycetota>Actinomycetes>Micrococcales>Micrococcaceae	GCF_014644875.1	Micrococcus_flavus	E4A47_RS01340	286387..289899	384602	BREX-system-P-loop-protein-BrxC-[Micrococcus-flavus]
WP_193340403.1	Type1.BREX	<-SIGMA+OB-Fold+AAA-ATPase+HKD-DNase-BrxL<-PglZ<-REase<-N6-METHYLASE<-DUF6079*<-BrxB-BrxF-iSTAND<-BrxA-DNA-binding<-XIS-HTH||?-><-EVE+REase	1153	Bacteria>Terrabacteria_group>Actinomycetota>Actinomycetes>Micrococcales>Micrococcaceae	GCF_014926485.1	Pseudarthrobacter_sp._AB1	DAD99_RS03725	12045..15506	2138309	BREX-system-P-loop-protein-BrxC-[Pseudarthrobacter-sp.-AB1]
WP_258190683.1	Type1.BREX	HIN-HTH-><-SIGMA+OB-Fold+AAA-ATPase+HKD-DNase-BrxL<-PglZ<-N6-METHYLASE<-DUF6079*<-BrxA-DNA-binding<-XIS-HTH<-?<-?||?->HNH->	1170	Bacteria>Terrabacteria_group>Actinomycetota>Actinomycetes>Micrococcales>Micrococcaceae	GCF_019443425.1	Arthrobacter_sp._PAMC25284	KY499_RS09320	2016646..2020158	2861279	BREX-system-P-loop-protein-BrxC-[Arthrobacter-sp.-PAMC25284]
WP_142938938.1	Type1.BREX	ParB+HNH-p->ParB+HNH-><-?<-?<-URI-DNase||?-><-SIGMA+OB-Fold+AAA-ATPase+HKD-DNase-BrxL<-PglZ<-N6-METHYLASE<-DUF6079*<-BrxB-BrxF-iSTAND<-BrxA-DNA-binding<-XIS-HTH||?->Radical-SAM-><-METHYLASE<-Phage_integrase+Phage_integrase||Phage_integrase-><-HTH_3	1162	Bacteria>Terrabacteria_group>Actinomycetota>Actinomycetes>Micrococcales>Micrococcaceae	GCF_019976975.1	unclassified_Arthrobacter	LDN90_RS14640	3248673..3252161	235627	MULTISPECIES:-BREX-system-P-loop-protein-BrxC-[unclassified-Arthrobacter]
WP_224048794.1	Type1.BREX	NUDIX->wHTH(SBD)+HNH-><-?||?-><-SF1-SF2-Helicase||XIS-HTH->BrxA-DNA-binding->BrxB-BrxF-iSTAND->DUF6079*->N6-METHYLASE->PglZ->N6-METHYLASE->SIGMA+OB-Fold+AAA-ATPase+HKD-DNase-BrxL-><-ParB+HNH||HEPN+SF1-SF2-Helicase+REase->	1173	Bacteria>Terrabacteria_group>Actinomycetota>Actinomycetes>Micrococcales>Micrococcaceae	GCF_019977335.1	Arthrobacter_sp._NicSoilB4	LDO13_RS04105	882114..885635	2830997	BREX-system-P-loop-protein-BrxC-[Arthrobacter-sp.-NicSoilB4]
WP_226761965.1	Type1.BREX	LexA-protease->?->?->?->XIS-HTH->BrxA-DNA-binding->BrxB-BrxF-iSTAND->DUF6079*->N6-METHYLASE->PglZ->SIGMA+OB-Fold+AAA-ATPase+HKD-DNase-BrxL-><-?<-?<-?||HEPN->GDResolvase+HIN-HTH->	1161	Bacteria>Terrabacteria_group>Actinomycetota>Actinomycetes>Micrococcales>Micrococcaceae	GCF_020532825.1	Arthrobacter_sp._SO3	BJQ90_RS20165	19380..22865	1897057	BREX-system-P-loop-protein-BrxC-[Arthrobacter-sp.-SO3]
WP_235519134.1	Type5.BREX	<-MarR-HTH+DTXR-HTH||?-><-HKD-DNase+HKD-Rease<-?<-?||HKD-DNase+SF1-SF2-Helicase+HTH+HTH+SRA-><-SF1-SF2-Helicase<-35exo+TUDOR+SF1-SF2-Helicase+iREase+CTD||BrxA-DNA-binding->BrxB-BrxF-iSTAND->DUF6079*->N6-METHYLASE->PglZ->SIGMA+OB-Fold+AAA-ATPase+HKD-DNase-BrxL-><-?||?-><-REase	1162	Bacteria>Terrabacteria_group>Actinomycetota>Actinomycetes>Micrococcales>Micrococcaceae	GCF_021538675.1	Paenarthrobacter_sp._AR_02	LRQ04_RS07575	297017..300505	2899821	BREX-system-P-loop-protein-BrxC-[Paenarthrobacter-sp.-AR-02]
WP_241470046.1	Type1.BREX	DNA_glycosylase->DNA_glycosylase->?->?-><-SIGMA+OB-Fold+AAA-ATPase+HKD-DNase-BrxL<-PglZ<-N6-METHYLASE<-DUF6079*<-BrxB-BrxF-iSTAND<-BrxA-DNA-binding||?->HTH_26->?-><-ABC-ATPase+SbcC	1168	Bacteria>Terrabacteria_group>Actinomycetota>Actinomycetes>Micrococcales>Micrococcaceae	GCF_022565835.1	Nesterenkonia_sp._DZ6	LTH93_RS03075	681136..684642	2901229	BREX-system-P-loop-protein-BrxC-[Nesterenkonia-sp.-DZ6]
WP_241707532.1	Type1.BREX	REase->ChlI+Lon_C+AAA->?->?->?->?->XIS-HTH->BrxA-DNA-binding->BrxB-BrxF-iSTAND->DUF6079*->N6-METHYLASE->PglZ->SIGMA+OB-Fold+AAA-ATPase+HKD-DNase-BrxL-><-?<-DNA-glycosylase||?->NACHT->	1171	Bacteria>Terrabacteria_group>Actinomycetota>Actinomycetes>Micrococcales>Micrococcaceae	GCF_022606295.1	Arthrobacter_bambusae	KNN17_RS03605	221894..225409	1338426	BREX-system-P-loop-protein-BrxC-[Arthrobacter-bambusae]
WP_247040359.1	Type1.BREX	HTH_3-><-Phage_integrase||Phage_integrase+Phage_integrase->METHYLASE-><-Radical-SAM<-?||XIS-HTH->BrxA-DNA-binding->BrxB-BrxF-iSTAND->DUF6079*->N6-METHYLASE->PglZ->SIGMA+OB-Fold+AAA-ATPase+HKD-DNase-BrxL-><-?||URI-DNase->?->?-><-ParB+HNH	1168	Bacteria>Terrabacteria_group>Actinomycetota>Actinomycetes>Micrococcales>Micrococcaceae	GCF_023062595.1	Arthrobacter_rhizosphaerae	MU551_RS01475	284158..287664	2855490	BREX-system-P-loop-protein-BrxC-[Arthrobacter-rhizosphaerae]
WP_248193800.1	Type1.BREX	<-SIGMA+OB-Fold+AAA-ATPase+HKD-DNase-BrxL<-PglZ<-?<-N6-METHYLASE<-DUF6079*<-BrxB-BrxF-iSTAND<-BrxA-DNA-binding<-XIS-HTH||?-><-HAD+HAD	1173	Bacteria>Terrabacteria_group>Actinomycetota>Actinomycetes>Micrococcales>Micrococcaceae	GCF_023147525.1	Micrococcus_endophyticus	KZX39_RS06505	8911..12432	455343	BREX-system-P-loop-protein-BrxC-[Micrococcus-endophyticus]
WP_073116219.1	Type1.BREX	ICLR-HTH-><-?<-?||?-><-?||?->BrxA-DNA-binding->BrxB-BrxF-iSTAND->DUF6079*->N6-METHYLASE->PglZ->SIGMA+OB-Fold+AAA-ATPase+HKD-DNase-BrxL-><-?||?-><-RADICAL-SAM<-?<-ACET<-XIS-HTH	1174	Bacteria>Terrabacteria_group>Actinomycetota>Actinomycetes>Micrococcales>Micrococcaceae	GCF_900142795.1	Micrococcus	BUB74_RS03185	94466..97990	1269	MULTISPECIES:-BREX-system-P-loop-protein-BrxC-[Micrococcus]
WP_237187176.1	Type1.BREX	<-HHH_5+SNF2_N+SF1-SF2-Helicase||SWACOS-><-?<-SIGMA+OB-Fold+AAA-ATPase+HKD-DNase-BrxL<-PglZ<-Schlafen+HATPase+wHTH<-N6-METHYLASE<-DUF6079*<-BrxB-BrxF-iSTAND<-BrxA-DNA-binding||?-><-Aminotran_4<-?<-ABC-ATPase+SbcC+ABC-ATPase+SbcC	1190	Bacteria>Terrabacteria_group>Actinomycetota>Actinomycetes>Micrococcales>Micrococcaceae	GCF_928381705.1	Rothia_nasimurium	L6762_RS10435	40591..44163	85336	BREX-system-P-loop-protein-BrxC-[Rothia-nasimurium]
WP_211713005.1	Type1.BREX	<-DNA-glycosylase<-URI-DNase<-MACRODOMAIN+SIR2<-REase||HKD-DNase+SF1-SF2-Helicase+HTH+HTH+SRA-><-SIGMA+OB-Fold+AAA-ATPase+HKD-DNase-BrxL<-PglZ<-N6-METHYLASE<-DUF6079*<-BrxB-BrxF-iSTAND<-BrxA-DNA-binding||McrB-AAA->McrC-NTD+REase->ABhydrolase+ABhydrolase->	1173	Bacteria>Terrabacteria_group>Actinomycetota>Actinomycetes>Micrococcales>Micrococcaceae	-	Paenarthrobacter_sp._GOM3	IRJ34_14045	199455..202976	2782567	BREX-system-P-loop-protein-BrxC-[Paenarthrobacter-sp.-GOM3]
WP_100350249.1	Type1.BREX	HEPN+SF1-SF2-Helicase+REase->?-><-Pkinase+CR-Kinase<-SIGMA+OB-Fold+AAA-ATPase+HKD-DNase-BrxL<-PglZ<-N6-METHYLASE-p<-Phage_int_SAM_3+Phage_integrase||N6-METHYLASE-p-><-N6-METHYLASE<-DUF6079*<-BrxB-BrxF-iSTAND<-BrxA-DNA-binding||?->ZNR+RmuC->	1179	Bacteria>Terrabacteria_group>Actinomycetota>Actinomycetes>Micrococcales>Micrococcales_incertae_sedis	GCF_002798015.1	Luteimicrobium_subarcticum	CLV34_RS10655	413171..416710	620910	BREX-system-P-loop-protein-BrxC-[Luteimicrobium-subarcticum]
SDF46422.1	Type1.BREX	ABC-ATPase+SbcC->Cysteine-Desulfurase->?->?->?->BrxA-DNA-binding->BrxB-BrxF-iSTAND->DUF6079*->N6-METHYLASE->PglZ->SIGMA+OB-Fold+AAA-ATPase+HKD-DNase-BrxL->REase->PAIREDC-HTH->	1166	Bacteria>Terrabacteria_group>Actinomycetota>Actinomycetes>Micrococcales>Promicromonosporaceae	GCA_900102445.1	Cellulosimicrobium_cellulans	SAMN04487781_1443	34344..37844	1710	hypothetical-protein-SAMN04487781_1443-[Cellulosimicrobium-cellulans]
WP_162290006.1	Type1.BREX	PIWI-><-HerA/FtsK<-SIR2+SIR2<-SIGMA+OB-Fold+AAA-ATPase+HKD-DNase-BrxL<-PglZ<-N6-METHYLASE<-DUF6079*<-BrxB-BrxF-iSTAND<-BrxA-DNA-binding<-XIS-HTH<-tRNA<-?<-GNTR-HTH||DSBH+AraC-HTH+AraC-HTH-><-HTH_42<-ABhydrolase	1167	Bacteria>Terrabacteria_group>Actinomycetota>Actinomycetes>Micrococcales>Promicromonosporaceae	GCF_010287905.1	Cellulosimicrobium_fucosivorans	GYH36_RS12555	12661..16164	2850388	BREX-system-P-loop-protein-BrxC-[Cellulosimicrobium-fucosivorans]
WP_253873967.1	Type1.BREX	<-URI-DNase||?->SIR2->EVE-><-SIGMA+OB-Fold+AAA-ATPase+HKD-DNase-BrxL<-PglZ<-N6-METHYLASE<-DUF6079*<-BrxB-BrxF-iSTAND<-BrxA-DNA-binding||?-><-CHTH||?-><-REase<-REase<-ANK+ANK+ANK<-REase	1157	Bacteria>Terrabacteria_group>Actinomycetota>Actinomycetes>Micrococcales>Promicromonosporaceae	GCF_024171995.1	Promicromonospora_umidemergens	APR04_RS20365	11407..14880	629679	BREX-system-P-loop-protein-BrxC-[Promicromonospora-umidemergens]
WP_142121348.1	Type1.BREX	CR-ATPase5+REase-><-REase+SF1-SF2-Helicase-p||BrxA-DNA-binding->BrxB-BrxF-iSTAND->DUF6079*->N6-METHYLASE->?->PglZ->RVT->SIGMA+OB-Fold+AAA-ATPase+HKD-DNase-BrxL-><-?||SF1-SF2-Helicase->wHTH-><-CR-ATPase5+REase	1170	Bacteria>Terrabacteria_group>Actinomycetota>Actinomycetes>Micrococcales>Rarobacteraceae	GCF_006716265.1	Rarobacter_faecitabidus	FB461_RS08150	23162..26674	13243	BREX-system-P-loop-protein-BrxC-[Rarobacter-faecitabidus]
WP_142113745.1	Type1.BREX	<-VirB11+MuB-ATPase||DNA_METHYLASE-><-HerA/FtsK<-SIR2<-SIGMA+OB-Fold+AAA-ATPase+HKD-DNase-BrxL<-PglZ<-Schlafen+HATPase+wHTH<-?<-N6-METHYLASE<-DUF6079*<-BrxB-BrxF-iSTAND<-BrxA-DNA-binding<-XIS-HTH<-ABhydrolase<-ABhydrolase<-?<-MPTase<-HTH||ArdCN+MPTase->	1173	Bacteria>Terrabacteria_group>Actinomycetota>Actinomycetes>Micrococcales>Rarobacteraceae	-	Rarobacter_incanus	FB389_RS10090	2435944..2439465	153494	BREX-system-P-loop-protein-BrxC-[Rarobacter-incanus]
SNU02231.1	Type1.BREX	<-SbcC<-?<-SIGMA+OB-Fold+AAA-ATPase+HKD-DNase-BrxL<-PglZ<-N6-METHYLASE-p<-Phage_int_SAM_3+Phage_integrase||?->N6-METHYLASE-p-><-N6-METHYLASE<-DUF6079*<-BrxB-BrxF-iSTAND<-BrxA-DNA-binding||SF1-SF2-Helicase+PhospholipaseD-><-?<-?||Peptidase_M23-><-?||TraM->	1159	Bacteria>Terrabacteria_group>Actinomycetota>Actinomycetes>Micrococcales>Ruaniaceae	GCA_900199505.1	Ruaniaceae_bacterium_KH17	SAMN06298212_12910	14626..18105	1945888	hypothetical-protein-SAMN06298212_12910-[Ruaniaceae-bacterium-KH17]
WP_147917199.1	Type1.BREX	HicA-><-?||?->DNA_glycosylase-><-?<-?<-?||BrxA-DNA-binding->BrxB-BrxF-iSTAND->DUF6079*->N6-METHYLASE->PglZ->SIGMA+OB-Fold+AAA-ATPase+HKD-DNase-BrxL->HTH_3-><-?||?->?->?->ABC-ATPase+SbcC->	1152	Bacteria>Terrabacteria_group>Actinomycetota>Actinomycetes>Micrococcales>Ruaniaceae	GCF_008000995.1	Ruania_zhangjianzhongii	FU260_RS11560	2487086..2490544	2603206	BREX-system-P-loop-protein-BrxC-[Ruania-zhangjianzhongii]
WP_163542003.1	Type1.BREX	<-HIN-HTH||SbcC->?-><-?<-SIGMA+OB-Fold+AAA-ATPase+HKD-DNase-BrxL<-PglZ<-REase<-N6-METHYLASE<-DUF6079*<-BrxB-BrxF-iSTAND<-BrxA-DNA-binding<-XIS-HTH<-?<-?||CN_hydrolase+NAD_synthase->?-><-Pkinase<-RelE-ParE	1154	Bacteria>Terrabacteria_group>Actinomycetota>Actinomycetes>Micrococcales>Ruaniaceae	GCF_010994285.1	Occultella_kanbiaonis	G7B41_RS06055	118944..122408	2675754	BREX-system-P-loop-protein-BrxC-[Occultella-kanbiaonis]
MCL2802837.1	Type1.BREX	<-RADICAL-SAM<-sGTP<-?||SWACOS->TPR->?-><-SIGMA+OB-Fold+AAA-ATPase+HKD-DNase-BrxL<-PglZ<-N6-METHYLASE<-DUF6079*<-BrxB-BrxF-iSTAND<-BrxA-DNA-binding||?-><-?||?-><-PhdYeFM_antitox	1162	Bacteria>Terrabacteria_group>Actinomycetota>Actinomycetes>Micrococcales>unclassified_Micrococcales	GCA_009784895.1	Micrococcales_bacterium	FWD29_02600	43259..46747	2026762	BREX-system-P-loop-protein-BrxC-[Micrococcales-bacterium]
WP_183221872.1	Type1.BREX	<-STYKIN+Pkinase<-?<-?||?-><-?||BrxA-DNA-binding->BrxB-BrxF-iSTAND->DUF6079*->N6-METHYLASE->PglZ->SIGMA+OB-Fold+AAA-ATPase+HKD-DNase-BrxL-><-GGDEF+EAL<-?||ABhydrolase->?->?->METHYLASE->	1164	Bacteria>Terrabacteria_group>Actinomycetota>Actinomycetes>Micromonosporales>Micromonosporaceae	GCF_014647795.1	Actinoplanes_campanulatus	IEZ13_RS15515	158515..162009	113559	BREX-system-P-loop-protein-BrxC-[Actinoplanes-campanulatus]
WP_203833901.1	Type1.BREX	<-METHYLASE<-?<-?<-ABhydrolase||?->GGDEF+EAL-><-SIGMA+OB-Fold+AAA-ATPase+HKD-DNase-BrxL<-PglZ<-N6-METHYLASE<-DUF6079*<-BrxB-BrxF-iSTAND<-BrxA-DNA-binding||?-><-?||?->?->STYKIN+Pkinase->	1164	Bacteria>Terrabacteria_group>Actinomycetota>Actinomycetes>Micromonosporales>Micromonosporaceae	GCF_016862035.1	Actinoplanes_campanulatus	Aca09nite_RS24855	101506..105000	113559	BREX-system-P-loop-protein-BrxC-[Actinoplanes-campanulatus]
WP_188126500.1	Type1.BREX	<-METHYLASE<-?<-?<-ABhydrolase||GGDEF+EAL-><-SIGMA+OB-Fold+AAA-ATPase+HKD-DNase-BrxL<-PglZ<-N6-METHYLASE<-DUF6079*<-BrxB-BrxF-iSTAND<-BrxA-DNA-binding||?-><-?||?->?->STYKIN+Pkinase->	1164	Bacteria>Terrabacteria_group>Actinomycetota>Actinomycetes>Micromonosporales>Micromonosporaceae	GCF_016862255.1	Actinoplanes_lobatus	IEZ29_RS29630	68673..72167	113568	BREX-system-P-loop-protein-BrxC-[Actinoplanes-lobatus]
WP_203928003.1	Type1.BREX	TPR-><-SIGMA+OB-Fold+AAA-ATPase+HKD-DNase-BrxL<-PglZ<-N6-METHYLASE<-DUF6079*<-BrxB-BrxF-iSTAND<-BrxA-DNA-binding<-APATPase+TPR+TPR+TPR+TPR+TPR+TPR||NAD_binding_10->?-><-NACHT||SIGMA-HTH->	1154	Bacteria>Terrabacteria_group>Actinomycetota>Actinomycetes>Micromonosporales>Micromonosporaceae	GCF_016863655.1	Virgisporangium_ochraceum	Voc01_RS14900	8921..12385	65505	BREX-system-P-loop-protein-BrxC-[Virgisporangium-ochraceum]
WP_260726425.1	Type1.BREX	<-SIGMA-HTH<-?<-SAM-METHYLASE||?->?->?-><-SIGMA+OB-Fold+AAA-ATPase+HKD-DNase-BrxL<-PglZ<-N6-METHYLASE<-DUF6079*<-BrxB-BrxF-iSTAND<-BrxA-DNA-binding<-XIS-HTH||HAMP+MA+MA->?->SIGMA-HTH-><-Glycos_transf_1+Glyco_trans_1_2<-Glycos_transf_1+Glyco_trans_1_2<-SF1-SF2-Helicase	1154	Bacteria>Terrabacteria_group>Actinomycetota>Actinomycetes>Micromonosporales>Micromonosporaceae	GCF_025264685.1	Dactylosporangium_roseum	Drose_RS01775	392440..395904	47989	BREX-system-P-loop-protein-BrxC-[Dactylosporangium-roseum]
WP_091673594.1	Type1.BREX	RADICAL-SAM->?->?-><-HAD||XIS-HTH->Phage_int_SAM_3+Phage_integrase->XIS-HTH->BrxA-DNA-binding->BrxB-BrxF-iSTAND->DUF6079*->N6-METHYLASE->PglZ->SIGMA+OB-Fold+AAA-ATPase+HKD-DNase-BrxL->DNA_glycosylase-><-?<-SF1-SF2-Helicase<-HKD-DNase<-DUF6361<-RNase	1156	Bacteria>Terrabacteria_group>Actinomycetota>Actinomycetes>Micromonosporales>Micromonosporaceae	GCF_900089595.1	Micromonospora_auratinigra	GA0070611_RS28700	6259973..6263443	261654	BREX-system-P-loop-protein-BrxC-[Micromonospora-auratinigra]
WP_091348932.1	Type1.BREX	XIS-HTH->BrxA-DNA-binding->BrxB-BrxF-iSTAND->DUF6079*->N6-METHYLASE->PglZ->SIGMA+OB-Fold+AAA-ATPase+HKD-DNase-BrxL->HIN-HTH-><-HTH_32<-HTH_32<-SF1-SF2-Helicase<-HKD-DNase+HKD-Rease	1153	Bacteria>Terrabacteria_group>Actinomycetota>Actinomycetes>Micromonosporales>Micromonosporaceae	GCF_900091465.1	Micromonospora_rhizosphaerae	GA0070624_RS15965	3332590..3336051	568872	BREX-system-P-loop-protein-BrxC-[Micromonospora-rhizosphaerae]
HJG42479.1	Type1.BREX	BrxA-DNA-binding->BrxB-BrxF-iSTAND->DUF6079*->N6-METHYLASE->PglZ->SIGMA+OB-Fold+AAA-ATPase+HKD-DNase-BrxL->	1176	Bacteria>Terrabacteria_group>Actinomycetota>Actinomycetes>Mycobacteriales>Corynebacteriaceae	GCA_020743465.1	Corynebacterium_phoceense	K8V34_00330	34206..37736	1686286	BREX-system-P-loop-protein-BrxC-[Corynebacterium-phoceense]
WP_102213741.1	Type1.BREX	XIS-HTH->BrxA-DNA-binding->BrxB-BrxF-iSTAND->DUF6079*->N6-METHYLASE->HTH+REase->PglZ->SIGMA+OB-Fold+AAA-ATPase+HKD-DNase-BrxL-><-ParB+HNH+METHYLASE	1173	Bacteria>Terrabacteria_group>Actinomycetota>Actinomycetes>Mycobacteriales>Corynebacteriaceae	GCF_002871715.1	Corynebacterium_xerosis	CJ204_09375	1693..5214	1725	BREX-system-P-loop-protein-BrxC-[Corynebacterium-xerosis]
WP_220313057.1	Type1.BREX	<-REC+OmpR-HTH<-MerR-HTH+TipAS<-?<-SIR2||?->TetR-HTH->?->BrxA-DNA-binding->BrxB-BrxF-iSTAND->DUF6079*->N6-METHYLASE-><-N6-METHYLASE-p||Phage_int_SAM_3+Phage_integrase->N6-METHYLASE-p->PglZ->SIGMA+OB-Fold+AAA-ATPase+HKD-DNase-BrxL-><-?<-?<-ADH_N+ADH_zinc_N	1160	Bacteria>Terrabacteria_group>Actinomycetota>Actinomycetes>Mycobacteriales>Corynebacteriaceae	GCF_019551735.1	Corynebacterium_glutamicum	JJQ73_RS12320	2669346..2672828	1718	BREX-system-P-loop-protein-BrxC-[Corynebacterium-glutamicum]
WP_259898411.1	Type1.BREX	<-HIN-HTH<-ParB+HNH<-SIGMA+OB-Fold+AAA-ATPase+HKD-DNase-BrxL<-PglZ<-N6-METHYLASE<-DUF6079*<-BrxB-BrxF-iSTAND<-BrxA-DNA-binding<-XIS-HTH	1160	Bacteria>Terrabacteria_group>Actinomycetota>Actinomycetes>Mycobacteriales>Corynebacteriaceae	GCF_025152505.1	Corynebacterium_sanguinis	M3G46_RS10600	10062..13544	2594913	BREX-system-P-loop-protein-BrxC-[Corynebacterium-sanguinis]
WP_271034470.1	Type1.BREX	<-HTH_23+rve+rve_3||?->BrxA-DNA-binding->BrxB-BrxF-iSTAND->DUF6079*->N6-METHYLASE-><-N6-METHYLASE-p||Phage_int_SAM_3+Phage_integrase->N6-METHYLASE-p->PglZ->SIGMA+OB-Fold+AAA-ATPase+HKD-DNase-BrxL->	1159	Bacteria>Terrabacteria_group>Actinomycetota>Actinomycetes>Mycobacteriales>Corynebacteriaceae	GCF_027886475.1	Corynebacterium_sp._SCR221107	PAB09_RS02255	466456..469935	3017361	BREX-system-P-loop-protein-BrxC-[Corynebacterium-sp.-SCR221107]
WP_245802167.1	Type1.BREX	<-ARG<-SIR2<-SIGMA+OB-Fold+AAA-ATPase+HKD-DNase-BrxL<-PglZ<-Schlafen+HATPase+wHTH<-N6-METHYLASE<-DUF6079*<-BrxB-BrxF-iSTAND<-BrxA-DNA-binding	1179	Bacteria>Terrabacteria_group>Actinomycetota>Actinomycetes>Mycobacteriales>Corynebacteriaceae	GCF_900169525.1	Corynebacterium_pacaense	B7E02_RS05025	1051363..1054902	1816684	BREX-system-P-loop-protein-BrxC-[Corynebacterium-pacaense]
WP_047036580.1	Type1.BREX	<-Phage_integrase||ACET-><-HerA/FtsK<-SIR2+SIR2<-SIGMA+OB-Fold+AAA-ATPase+HKD-DNase-BrxL<-PglZ<-N6-METHYLASE<-DUF6079*<-BrxB-BrxF-iSTAND<-BrxA-DNA-binding<-XIS-HTH<-TetR+HTH<-Phage_integrase+Phage_integrase<-Phage_integrase+Phage_integrase<-Phage_int_SAM_1+Phage_integrase<-DNA_glycosylase	1156	Bacteria>Terrabacteria_group>Actinomycetota>Actinomycetes>Mycobacteriales	GCF_900141005.1	Mycobacteriaceae	ABW05_RS31210	104572..108042	1762	MULTISPECIES:-BREX-system-P-loop-protein-BrxC-[Mycobacteriaceae]
PZT90276.1	Type1.BREX	HAD+HAD-><-?||XIS-HTH->BrxA-DNA-binding->BrxB-BrxF-iSTAND->DUF6079*->N6-METHYLASE->HTH+REase->PglZ->SIGMA+OB-Fold+AAA-ATPase+HKD-DNase-BrxL->REase->	1137	Bacteria>Terrabacteria_group>Actinomycetota>Actinomycetes>Mycobacteriales>Gordoniaceae	GCA_003248405.1	Gordonia_sp._(in:_high_G+C_Gram-positive_bacteria)	DI630_31230	6915..10328	84139	MAG:-BREX-system-P-loop-protein-BrxC-[Gordonia-sp.-(in:-high-G+C-Gram-positive-bacteria)]
WP_042377474.1	Type1.BREX	TetR-HTH->?-><-?<-?<-SIGMA+OB-Fold+AAA-ATPase+HKD-DNase-BrxL<-PglZ<-N6-METHYLASE<-DUF6079*<-BrxB-BrxF-iSTAND<-BrxA-DNA-binding||Exonuc_V_gamma+REase->SF1-SF2-Helicase+REase-><-?<-DNA_glycosylase<-HNH+HNH	1161	Bacteria>Terrabacteria_group>Actinomycetota>Actinomycetes>Mycobacteriales>Gordoniaceae	GCF_000529675.1	Gordonia_alkanivorans	TY17_RS15305	47194..50679	84096	BREX-system-P-loop-protein-BrxC-[Gordonia-alkanivorans]
WP_159370828.1	Type1.BREX	<-DNA_glycosylase||DUF6361->HKD-DNase->SF1-SF2-Helicase-><-SIGMA+OB-Fold+AAA-ATPase+HKD-DNase-BrxL<-PglZ<-N6-METHYLASE<-DUF6079*<-BrxB-BrxF-iSTAND<-BrxA-DNA-binding<-?<-?<-ARG<-?||?-><-SAM-METHYLASE	1174	Bacteria>Terrabacteria_group>Actinomycetota>Actinomycetes>Mycobacteriales>Gordoniaceae	GCF_009856645.1	Gordonia_sp._JH63	GR168_RS12095	2701909..2705433	2698900	BREX-system-P-loop-protein-BrxC-[Gordonia-sp.-JH63]
WP_213244950.1	Type1.BREX	<-SIGMA+OB-Fold+AAA-ATPase+HKD-DNase-BrxL<-PglZ<-HTH+REase<-N6-METHYLASE-p<-?<-Phage_int_SAM_3+Phage_integrase<-Phage_int_SAM_3+Phage_integrase||N6-METHYLASE-p-><-N6-METHYLASE<-DUF6079*<-BrxB-BrxF-iSTAND<-BrxA-DNA-binding	1174	Bacteria>Terrabacteria_group>Actinomycetota>Actinomycetes>Mycobacteriales>Gordoniaceae	GCF_009914535.1	Gordonia_pseudamarae	GII33_RS19265	4440551..4444075	2831662	BREX-system-P-loop-protein-BrxC-[Gordonia-pseudamarae]

Protein-ID	System	Gene-Neighbourhoods	Length	Taxonomy	Assembly-ID	Strain	Locus-Tag	Genomic-coordinates	TaxID	Fasta-define
WP_223374200.1	Type1.BREX	REase->sGTP+sGTP+GTPase-AIG->sGTP-><-PHP+SbcC<-?<-SIGMA+OB-Fold+AAA-ATPase+HKD-DNase-BrxL<-PglZ<-N6-METHYLASE<-DUF6079*<-BrxB-BrxF-iSTAND<-BrxA-DNA-binding<-XIS-HTH<-?<-TnsC-ATPase||PAIREDC-HTH-><-rve||HTH_29+HTH_32+DDE_3-><-REase+wHTH	1154	Bacteria>Terrabacteria_group>Actinomycetota>Actinomycetes>Mycobacteriales>Gordoniaceae	GCF_019930645.1	Gordonia_bronchialis	K8093_RS05575	1152614..1156078	2054	BREX-system-P-loop-protein-BrxC-[Gordonia-bronchialis]
WP_226512485.1	Type1.BREX	MACRODOMAIN+SIR2-><-?<-?||DUF6361->HKD-DNase->SF1-SF2-Helicase->URI-DNase->BrxA-DNA-binding->BrxB-BrxF-iSTAND->DUF6079-p*->DUF6079-p->XIS-HTH->PIN->N6-METHYLASE-p->N6-METHYLASE->PglZ->?->AbiEi_4+AbiEi_1->SIGMA+OB-Fold+AAA-ATPase+HKD-DNase-BrxL->	435	Bacteria>Terrabacteria_group>Actinomycetota>Actinomycetes>Mycobacteriales>Gordoniaceae	GCF_020520305.1	Gordonia_sp._WA4-43	LEL84_RS18910	4162672..4163979	2878678	BREX-system-P-loop-protein-BrxC-[Gordonia-sp.-WA4-43]
WP_226512486.1	Type1.BREX	DUF6361->HKD-DNase->SF1-SF2-Helicase->URI-DNase->BrxA-DNA-binding->BrxB-BrxF-iSTAND->DUF6079-p->DUF6079-p*->XIS-HTH->PIN->N6-METHYLASE-p->N6-METHYLASE->PglZ->?->AbiEi_4+AbiEi_1->SIGMA+OB-Fold+AAA-ATPase+HKD-DNase-BrxL-><-ABhydrolase+ABhydrolase	785	Bacteria>Terrabacteria_group>Actinomycetota>Actinomycetes>Mycobacteriales>Gordoniaceae	GCF_020520305.1	Gordonia_sp._WA4-43	LEL84_RS18915	4163880..4166237	2878678	hypothetical-protein-[Gordonia-sp.-WA4-43]
WP_246991513.1	Type1.BREX	<-SIGMA+OB-Fold+AAA-ATPase+HKD-DNase-BrxL<-PglZ<-?<-?<-N6-METHYLASE-p<-Phage_int_SAM_3+Phage_integrase||N6-METHYLASE-p-><-N6-METHYLASE<-DUF6079*<-BrxB-BrxF-iSTAND<-BrxA-DNA-binding<-sGTP<-?||?->O6MGT-HTH->	1175	Bacteria>Terrabacteria_group>Actinomycetota>Actinomycetes>Mycobacteriales>Gordoniaceae	GCF_023017165.1	Gordonia_alkaliphila	MUG78_01460	307013..310540	1053547	BREX-system-P-loop-protein-BrxC-[Gordonia-alkaliphila]
OKH76423.1	Type1.BREX	<-NUDIX||ARG-><-SbcC<-?<-SIGMA+OB-Fold+AAA-ATPase+HKD-DNase-BrxL<-PglZ<-N6-METHYLASE<-DUF6079*<-BrxB-BrxF-iSTAND<-BrxA-DNA-binding<-XIS-HTH	1156	Bacteria>Terrabacteria_group>Actinomycetota>Actinomycetes>Mycobacteriales>Mycobacteriaceae	GCA_001905565.1	Mycobacterium_sp._SWH-M1	EB72_01455	44646..48116	1490485	ATP-binding-protein-[Mycobacterium-sp.-SWH-M1]
OKH79002.1	Type1.BREX	XIS-HTH->BrxA-DNA-binding->BrxB-BrxF-iSTAND->DUF6079*->N6-METHYLASE->PglZ->SIGMA+OB-Fold+AAA-ATPase+HKD-DNase-BrxL->?->SbcC-><-MPTase<-?<-?<-Phage_integrase	1156	Bacteria>Terrabacteria_group>Actinomycetota>Actinomycetes>Mycobacteriales>Mycobacteriaceae	GCA_001905655.1	Mycobacterium_sp._ST-F2	EB75_25595	1846..5316	1490484	ATP-binding-protein-[Mycobacterium-sp.-ST-F2]
WP_018600838.1	Type1.BREX	<-ABhydrolase||?-><-?<-?||?-><-HTH_3||BrxA-DNA-binding->BrxB-BrxF-iSTAND->DUF6079*->N6-METHYLASE->PglZ->SIGMA+OB-Fold+AAA-ATPase+HKD-DNase-BrxL-><-?<-?||?-><-?<-ACT<-SIR2	1152	Bacteria>Terrabacteria_group>Actinomycetota>Actinomycetes>Mycobacteriales>Mycobacteriaceae	GCF_000373905.1	Mycobacterium_sp._155	B133_RS0110195	2156875..2160333	1157943	BREX-system-P-loop-protein-BrxC-[Mycobacterium-sp.-155]
WP_020822248.1	Type1.BREX	Pkinase+TPR+TPR-><-?||TetR-HTH-><-?||Glyoxalase-><-SIGMA+OB-Fold+AAA-ATPase+HKD-DNase-BrxL<-RVT<-PglZ<-N6-METHYLASE<-DUF6079*<-BrxB-BrxF-iSTAND<-BrxA-DNA-binding<-XIS-HTH<-zf-ISL3+HTH_Tnp_ISL3+DDE_Tnp_ISL3	1152	Bacteria>Terrabacteria_group>Actinomycetota>Actinomycetes>Mycobacteriales>Mycobacteriaceae	GCF_000418535.1	Mycobacterium_intracellulare	OEM_RS09395	2016549..2020007	1767	BREX-system-P-loop-protein-BrxC-[Mycobacterium-intracellulare]
WP_036372476.1	Type5.BREX	<-TRD+TRD<-?<-HTH_Tnp_1+HTH_21+rve+rve_3<-AAA-ATPase<-HEPN+DUF499<-SIR2+SIR2<-SIGMA+OB-Fold+AAA-ATPase+HKD-DNase-BrxL<-PglZ<-N6-METHYLASE<-DUF6079*<-BrxB-BrxF-iSTAND<-BrxA-DNA-binding<-XIS-HTH<-TetR+HTH<-Phage_integrase+Phage_integrase<-Phage_integrase+Phage_integrase<-Phage_integrase<-TUDOR+SF1-SF2-Helicase+iREase+CTD+REase<-XIS-HTH	1152	Bacteria>Terrabacteria_group>Actinomycetota>Actinomycetes>Mycobacteriales>Mycobacteriaceae	GCF_000612725.1	Mycolicibacterium_austroafricanum	BN976_RS17355	59223..62681	39687	BREX-system-P-loop-protein-BrxC-[Mycolicibacterium-austroafricanum]
WP_052621181.1	Type1.BREX	<-HerA/FtsK<-?<-?<-SF1-SF2-Helicase+REase<-HerA/FtsK<-SIR2<-SIGMA+OB-Fold+AAA-ATPase+HKD-DNase-BrxL<-PglZ<-N6-METHYLASE<-DUF6079*<-BrxB-BrxF-iSTAND<-BrxA-DNA-binding<-XIS-HTH<-TetR+HTH<-Phage_integrase+Phage_integrase<-Phage_integrase+Phage_integrase<-Phage_int_SAM_1+Phage_integrase||HEPN->	1156	Bacteria>Terrabacteria_group>Actinomycetota>Actinomycetes>Mycobacteriales>Mycobacteriaceae	GCF_001214625.1	Mycobacteroides_abscessus	B5319_RS07405	1475995..1479465	36809	BREX-system-P-loop-protein-BrxC-[Mycobacteroides-abscessus]
WP_065289569.1	Type1.BREX	DUF5086-><-ANK+ANK<-ZnR||XIS-HTH->BrxA-DNA-binding->BrxB-BrxF-iSTAND->DUF6079*->N6-METHYLASE->PglZ->SIGMA+OB-Fold+AAA-ATPase+HKD-DNase-BrxL->	1152	Bacteria>Terrabacteria_group>Actinomycetota>Actinomycetes>Mycobacteriales>Mycobacteriaceae	GCF_001679965.1	Mycolicibacter_kumamotonensis	ACT18_RS22010	4536..7994	354243	BREX-system-P-loop-protein-BrxC-[Mycolicibacter-kumamotonensis]
WP_070953149.1	Type1.BREX	SAM-METHYLASE-><-SopB+HNH<-?<-?||XIS-HTH->BrxA-DNA-binding->BrxB-BrxF-iSTAND->DUF6079*->N6-METHYLASE->PglZ->SIGMA+OB-Fold+AAA-ATPase+HKD-DNase-BrxL->Acyl_transf_3->?->?-><-ACET	1156	Bacteria>Terrabacteria_group>Actinomycetota>Actinomycetes>Mycobacteriales>Mycobacteriaceae	GCF_001853775.1	Mycobacteroides_chelonae	BKG71_RS10405	2100910..2104380	1774	BREX-system-P-loop-protein-BrxC-[Mycobacteroides-chelonae]
WP_234813880.1	Type1.BREX	<-ParB+HNH<-SIGMA+OB-Fold+AAA-ATPase+HKD-DNase-BrxL<-PglZ<-N6-METHYLASE<-DUF6079-p<-DUF6079-p*<-BrxB-BrxF-iSTAND<-BrxA-DNA-binding	682	Bacteria>Terrabacteria_group>Actinomycetota>Actinomycetes>Mycobacteriales>Mycobacteriaceae	GCF_002101705.1	Mycolicibacterium_iranicum	AWC12_RS31570	96008..98056	912594	BREX-system-P-loop-protein-BrxC-[Mycolicibacterium-iranicum]
WP_119635996.1	Type1.BREX	<-XIS-HTH<-?<-?<-SF1-SF2-Helicase+REase<-HerA/FtsK<-SIR2+SIR2<-SIGMA+OB-Fold+AAA-ATPase+HKD-DNase-BrxL<-PglZ<-N6-METHYLASE<-DUF6079*<-BrxB-BrxF-iSTAND<-BrxA-DNA-binding<-XIS-HTH<-TetR+HTH<-Phage_integrase+Phage_integrase<-Phage_integrase+Phage_integrase<-Phage_int_SAM_1+Phage_integrase||HEPN->	1156	Bacteria>Terrabacteria_group>Actinomycetota>Actinomycetes>Mycobacteriales>Mycobacteriaceae	GCF_003582985.1	Mycobacteroides_abscessus	D2E92_RS23075	16528..19998	36809	BREX-system-P-loop-protein-BrxC-[Mycobacteroides-abscessus]
WP_134206037.1	Type1.BREX	<-REase<-MORC<-HEPN+DUF499<-METHYLASE-p<-HerA/FtsK<-SIR2+SIR2<-SIGMA+OB-Fold+AAA-ATPase+HKD-DNase-BrxL<-PglZ<-N6-METHYLASE<-DUF6079*<-BrxB-BrxF-iSTAND<-BrxA-DNA-binding<-XIS-HTH<-TetR+HTH<-Phage_integrase+Phage_integrase<-Phage_integrase+Phage_integrase<-Phage_int_SAM_1+Phage_integrase<-REase||DNA_METHYLASE->	1155	Bacteria>Terrabacteria_group>Actinomycetota>Actinomycetes>Mycobacteriales>Mycobacteriaceae	GCF_004366875.1	Mycobacteroides_salmoniphilum	CCUG62472_RS09145	418307..421774	404941	BREX-system-P-loop-protein-BrxC-[Mycobacteroides-salmoniphilum]
WP_085074024.1	Type1.BREX	<-sGTP<-?<-sGTP<-HerA/FtsK<-SIR2+SIR2<-SIGMA+OB-Fold+AAA-ATPase+HKD-DNase-BrxL<-PglZ<-N6-METHYLASE<-DUF6079*<-BrxB-BrxF-iSTAND<-BrxA-DNA-binding<-XIS-HTH	1154	Bacteria>Terrabacteria_group>Actinomycetota>Actinomycetes>Mycobacteriales>Mycobacteriaceae	GCF_010723135.1	Mycobacterium_kubicae	H7H99_RS20140	22484..25948	120959	BREX-system-P-loop-protein-BrxC-[Mycobacterium-kubicae]
WP_163778779.1	Type1.BREX	GGDEF+EAL->?-><-Glyco_tran_28_C<-?||?-><-?<-tRNA||XIS-HTH->BrxA-DNA-binding->BrxB-BrxF-iSTAND->DUF6079*->N6-METHYLASE->PglZ->SIGMA+OB-Fold+AAA-ATPase+HKD-DNase-BrxL->ParB+HNH->?->AAA-ATPase->ZnR->?->Phage_integrase->	1156	Bacteria>Terrabacteria_group>Actinomycetota>Actinomycetes>Mycobacteriales>Mycobacteriaceae	GCF_010727945.1	Mycobacterium_cookii	G6N27_RS18550	3937772..3941242	1775	BREX-system-P-loop-protein-BrxC-[Mycobacterium-cookii]
WP_085152401.1	Type1.BREX	<-ABhydrolase+ABhydrolase<-Preader7<-HEPN+SF1-SF2-Helicase+REase<-?<-SIGMA+OB-Fold+AAA-ATPase+HKD-DNase-BrxL<-PglZ<-N6-METHYLASE<-DUF6079*<-BrxB-BrxF-iSTAND<-BrxA-DNA-binding<-XIS-HTH<-HKD-DNase+SF1-SF2-Helicase+HTH+HTH+SRA||35exo+SF1-SF2-Helicase->SF1-SF2-Helicase->	1156	Bacteria>Terrabacteria_group>Actinomycetota>Actinomycetes>Mycobacteriales>Mycobacteriaceae	GCF_010729895.1	Mycolicibacterium_confluentis	H7K00_RS24370	129281..132751	28047	BREX-system-P-loop-protein-BrxC-[Mycolicibacterium-confluentis]
WP_196326342.1	Type1.BREX	ABhydrolase->?-><-tRNA||?-><-?||XIS-HTH->XIS-HTH->BrxA-DNA-binding->BrxB-BrxF-iSTAND->DUF6079*->N6-METHYLASE->PglZ->SIGMA+OB-Fold+AAA-ATPase+HKD-DNase-BrxL->SIR2->HerA/FtsK-><-NUDIX||?->?->HerA/FtsK->	1156	Bacteria>Terrabacteria_group>Actinomycetota>Actinomycetes>Mycobacteriales>Mycobacteriaceae	GCF_015700725.1	Mycobacteroides_chelonae	HA135_RS11130	2312216..2315686	1774	BREX-system-P-loop-protein-BrxC-[Mycobacteroides-chelonae]
WP_074292199.1	Type1.BREX	<-HerA/FtsK<-?<-?||NUDIX-><-HerA/FtsK<-SIR2<-SIGMA+OB-Fold+AAA-ATPase+HKD-DNase-BrxL<-PglZ<-N6-METHYLASE<-DUF6079*<-BrxB-BrxF-iSTAND<-BrxA-DNA-binding<-XIS-HTH<-PIN<-XIS-HTH	1156	Bacteria>Terrabacteria_group>Actinomycetota>Actinomycetes>Mycobacteriales>Mycobacteriaceae	GCF_017183935.1	Mycobacteroides_abscessus	I3U31_RS04170	833809..837279	36809	BREX-system-P-loop-protein-BrxC-[Mycobacteroides-abscessus]
WP_241204715.1	Type1.BREX	<-DnaK<-?||?->?-><-HerA/FtsK<-SIR2<-SIGMA+OB-Fold+AAA-ATPase+HKD-DNase-BrxL<-PglZ<-N6-METHYLASE<-DUF6079*<-BrxB-BrxF-iSTAND<-BrxA-DNA-binding<-XIS-HTH||HEPN-><-AAA-ATPase<-HTH_7+rve+rve_3||HEPN->	1156	Bacteria>Terrabacteria_group>Actinomycetota>Actinomycetes>Mycobacteriales>Mycobacteriaceae	GCF_022348085.1	Mycobacterium_sp._PSTR-4-N	MHN84_RS11260	180742..184212	2917745	BREX-system-P-loop-protein-BrxC-[Mycobacterium-sp.-PSTR-4-N]
WP_260940163.1	Type1.BREX	XIS-HTH->BrxA-DNA-binding->BrxB-BrxF-iSTAND->DUF6079*->N6-METHYLASE->PglZ->SIGMA+OB-Fold+AAA-ATPase+HKD-DNase-BrxL->?->ParB+HNH-p->HIN-HTH-><-wHTH||MORC+DUF3684+DUF3883-><-REase+HTH+35exo+Small-B-barrel+SF1-SF2-Helicase	1156	Bacteria>Terrabacteria_group>Actinomycetota>Actinomycetes>Mycobacteriales>Mycobacteriaceae	GCF_025331195.1	Mycolicibacterium_llatzerense	A7G45_RS21390	50451..53921	280871	BREX-system-P-loop-protein-BrxC-[Mycolicibacterium-llatzerense]
WP_066911619.1	Type1.BREX	<-UDG-conflict<-HerA/FtsK<-SIR2+SIR2<-SIGMA+OB-Fold+AAA-ATPase+HKD-DNase-BrxL<-PglZ<-N6-METHYLASE<-DUF6079*<-BrxB-BrxF-iSTAND<-BrxA-DNA-binding<-XIS-HTH<-Classical-AAA<-Peptidase_S8	1161	Bacteria>Terrabacteria_group>Actinomycetota>Actinomycetes>Mycobacteriales>Mycobacteriaceae	GCF_025821415.1	Mycobacterium_interjectum	H7H80_RS25830	12521..16006	33895	BREX-system-P-loop-protein-BrxC-[Mycobacterium-interjectum]
WP_264033192.1	Type1.BREX	Classical-AAA->HTH_3-><-SF1-SF2-Helicase+REase<-SF1-SF2-Helicase+REase<-HerA/FtsK<-SIR2<-SIGMA+OB-Fold+AAA-ATPase+HKD-DNase-BrxL<-PglZ<-N6-METHYLASE<-DUF6079*<-BrxB-BrxF-iSTAND	1156	Bacteria>Terrabacteria_group>Actinomycetota>Actinomycetes>Mycobacteriales>Mycobacteriaceae	GCF_025821885.1	Mycolicibacterium_gilvum	H7I56_RS29610	57941..61411	1804	BREX-system-P-loop-protein-BrxC-[Mycolicibacterium-gilvum]
WP_265123865.1	Type5.BREX	EVE-><-nSTAND<-SIGMA+OB-Fold+AAA-ATPase+HKD-DNase-BrxL<-HerA/FtsK<-SIR2<-PglZ<-N6-METHYLASE<-DUF6079*<-BrxB-BrxF-iSTAND<-BrxA-DNA-binding<-XIS-HTH||ParB+HNH->?->35exo+TUDOR+SF1-SF2-Helicase+iREase+CTD->SF1-SF2-Helicase->?->ABhydrolase->	1159	Bacteria>Terrabacteria_group>Actinomycetota>Actinomycetes>Mycobacteriales>Mycobacteriaceae	GCF_026041215.1	Hoyosella_sp._YIM_151337	ONR57_RS12750	17833..21312	2992742	BREX-system-P-loop-protein-BrxC-[Hoyosella-sp.-YIM-151337]
WP_074325944.1	Type1.BREX	<-HEPN||Phage_int_SAM_1+Phage_integrase->Phage_integrase+Phage_integrase->Phage_integrase+Phage_integrase->TetR+HTH->XIS-HTH->BrxA-DNA-binding->BrxB-BrxF-iSTAND->DUF6079*->N6-METHYLASE->PglZ->SIGMA+OB-Fold+AAA-ATPase+HKD-DNase-BrxL->SIR2+SIR2->HerA/FtsK->?->?->XIS-HTH->Phage_int_SAM_3+Phage_integrase->	1152	Bacteria>Terrabacteria_group>Actinomycetota>Actinomycetes>Mycobacteriales>Mycobacteriaceae	GCF_900133325.1	Mycobacteroides_abscessus	BUU36_RS03865	789941..793399	36809	BREX-system-P-loop-protein-BrxC-[Mycobacteroides-abscessus]
OFE08162.1	Type1.BREX	<-Pkinase+TPR+TPR<-?<-?<-?<-?<-?||tRNA-><-?||BrxA-DNA-binding->BrxB-BrxF-iSTAND->DUF6079*->PglZ->SIGMA+OB-Fold+AAA-ATPase+HKD-DNase-BrxL->?->?->?-><-ParB+HNH||?->?->LZ_Tnp_IS481+HTH_32+rve+rve_3->	1175	Bacteria>Terrabacteria_group>Actinomycetota>Actinomycetes>Mycobacteriales>Nocardiaceae	GCA_001757665.1	Rhodococcus_sp._1139	A5N83_14165	58033..61560	1833762	ATP-binding-protein-[Rhodococcus-sp.-1139]
ORL42035.1	Type1.BREX	<-ANK+ANK<-SIGMA+OB-Fold+AAA-ATPase+HKD-DNase-BrxL<-AbiEii+ZnR<-AbiEi_4+AbiEi_1<-sGTP<-sGTP+sGTP+GTPase-AIG<-PglZ<-N6-METHYLASE<-DUF6079*<-BrxB-BrxF-iSTAND<-BrxA-DNA-binding	1188	Bacteria>Terrabacteria_group>Actinomycetota>Actinomycetes>Mycobacteriales>Nocardiaceae	GCA_002094315.1	Prescottella_equi	A6F59_07450	52967..56533	43767	hypothetical-protein-A6F59_07450-[Prescottella-equi]
OUS97312.1	Type5.BREX	<-MULE-TRANSPOSASE<-TPR+TPR+TPR+TPR+TPR<-?||BrxA-DNA-binding->BrxB-BrxF-iSTAND->DUF6079*->N6-METHYLASE->HTH+REase->PglZ->SIGMA+OB-Fold+AAA-ATPase+HKD-DNase-BrxL-><-DNA_glycosylase||?-><-TUDOR+SF1-SF2-Helicase+iREase+CTD+REase||SF1-SF2-Helicase->	1175	Bacteria>Terrabacteria_group>Actinomycetota>Actinomycetes>Mycobacteriales>Nocardiaceae	GCA_002165735.1	Rhodococcus_sp._NCIMB_12038	CA951_02905	126594..130121	933800	ATP-binding-protein-[Rhodococcus-sp.-NCIMB-12038]
RGP48319.1	Type1.BREX	<-GDResolvase+HIN-HTH<-SIGMA+OB-Fold+AAA-ATPase+HKD-DNase-BrxL<-PglZ<-N6-METHYLASE<-DUF6079*<-BrxB-BrxF-iSTAND-p	1175	Bacteria>Terrabacteria_group>Actinomycetota>Actinomycetes>Mycobacteriales>Nocardiaceae	GCA_003444715.1	Rhodococcus_erythropolis	AWH04_08905	69911..73438	1833	ATP-binding-protein-[Rhodococcus-erythropolis]
MBM4589636.1	Type1.BREX	<-ANK+ANK<-SIGMA+OB-Fold+AAA-ATPase+HKD-DNase-BrxL<-AbiEii+ZnR<-AbiEi_4+AbiEi_1<-sGTP<-sGTP<-sGTP<-PglZ<-N6-METHYLASE<-DUF6079*<-BrxB-BrxF-iSTAND<-BrxA-DNA-binding<-?||?-><-Glyoxalase||?->?->Calcineurin->MalT+LuxR-HTH->	1188	Bacteria>Terrabacteria_group>Actinomycetota>Actinomycetes>Mycobacteriales>Nocardiaceae	GCA_016879295.1	Prescottella_equi	GS507_15715	26092..29658	43767	BREX-system-P-loop-protein-BrxC-[Prescottella-equi]
MBM4523030.1	Type1.BREX	<-MalT+LuxR-HTH<-Calcineurin<-?<-?||Glyoxalase-><-?||?->BrxA-DNA-binding->BrxB-BrxF-iSTAND->DUF6079*->N6-METHYLASE->PglZ->sGTP+sGTP+GTPase-AIG->sGTP->AbiEi_4+AbiEi_1->AbiEii+ZnR->SIGMA+OB-Fold+AAA-ATPase+HKD-DNase-BrxL->ANK+ANK->	1188	Bacteria>Terrabacteria_group>Actinomycetota>Actinomycetes>Mycobacteriales>Nocardiaceae	GCA_016879495.1	Prescottella_equi	GS442_03230	599834..603400	43767	BREX-system-P-loop-protein-BrxC-[Prescottella-equi]
MBP1050938.1	Type1.BREX	<-REase<-?<-?<-ARG||NUDIX->BrxA-DNA-binding->BrxB-BrxF-iSTAND->DUF6079*->N6-METHYLASE->PglZ->SIGMA+OB-Fold+AAA-ATPase+HKD-DNase-BrxL-><-?<-?||GDResolvase+HIN-HTH-><-ZnR||HISKIN->	1171	Bacteria>Terrabacteria_group>Actinomycetota>Actinomycetes>Mycobacteriales>Nocardiaceae	GCA_017831275.1	Rhodococcus_qingshengii	J6397_12385	68679..72194	334542	BREX-system-P-loop-protein-BrxC-[Rhodococcus-qingshengii]
MBT2268266.1	Type1.BREX	<-SIGMA+OB-Fold+AAA-ATPase+HKD-DNase-BrxL<-PglZ<-N6-METHYLASE-p<-N6-METHYLASE-p<-DUF6079*<-BrxB-BrxF-iSTAND<-BrxA-DNA-binding<-?<-TIR-like	1170	Bacteria>Terrabacteria_group>Actinomycetota>Actinomycetes>Mycobacteriales>Nocardiaceae	GCA_018615075.1	Rhodococcus_erythropolis	J7E74_25040	11077..14589	1833	BREX-system-P-loop-protein-BrxC-[Rhodococcus-erythropolis]
BDU00126.1	Type1.BREX	<-ABhydrolase||Recombinase+ZnR->BrxA-DNA-binding->BrxB-BrxF-iSTAND->DUF6079*->N6-METHYLASE->?->PglZ->SIGMA+OB-Fold+AAA-ATPase+HKD-DNase-BrxL-><-HTH_21+rve+rve_3||HIN-HTH->HTH_Tnp_1+HTH_21+rve+rve_3-><-HTH_21+rve+rve_3<-HIN-HTH	1173	Bacteria>Terrabacteria_group>Actinomycetota>Actinomycetes>Mycobacteriales>Nocardiaceae	GCA_027924405.1	Nocardia_sp._ON39_IFM12276	IFM12276_31540	3396523..3400044	2984338	hypothetical-protein-IFM12276_31540-[Nocardia-sp.-ON39_IFM12276]
WP_020907622.1	Type1.BREX	<-GDResolvase||?-><-PIN<-?<-GDResolvase+HIN-HTH<-?<-SIGMA+OB-Fold+AAA-ATPase+HKD-DNase-BrxL<-PglZ<-N6-METHYLASE<-DUF6079*<-BrxB-BrxF-iSTAND<-BrxA-DNA-binding<-XIS-HTH<-Phage_integrase	1168	Bacteria>Terrabacteria_group>Actinomycetota>Actinomycetes>Mycobacteriales>Nocardiaceae	GCF_000010105.1	Rhodococcus_erythropolis	RER_RS16440	3107254..3110760	1833	BREX-system-P-loop-protein-BrxC-[Rhodococcus-erythropolis]
WP_039798161.1	Type1.BREX	HTH+REase->?->?-><-DDE_Tnp_ISL3<-DDE_Tnp_ISL3<-DEDD_Tnp_IS110+Transposase_20||BrxA-DNA-binding->BrxB-BrxF-iSTAND->DUF6079*->N6-METHYLASE->?->PglZ->SIGMA+OB-Fold+AAA-ATPase+HKD-DNase-BrxL-><-?<-?||NUDIX-><-?<-RADICAL-SAM	1173	Bacteria>Terrabacteria_group>Actinomycetota>Actinomycetes>Mycobacteriales>Nocardiaceae	GCF_000308435.1	Nocardia_araoensis	ON21_RS13315	16578..20099	228600	BREX-system-P-loop-protein-BrxC-[Nocardia-araoensis]
WP_029256831.1	Type1.BREX	ParB+HNH-><-?<-?<-SIGMA+OB-Fold+AAA-ATPase+HKD-DNase-BrxL<-PglZ<-N6-METHYLASE<-DUF6079*<-BrxB-BrxF-iSTAND<-BrxA-DNA-binding||?-><-tRNA||?->?->?->?->?->Pkinase+TPR+TPR->	1173	Bacteria>Terrabacteria_group>Actinomycetota>Actinomycetes>Mycobacteriales>Nocardiaceae	GCF_000696675.2	Rhodococcus_erythropolis	H351_RS02855	635383..638904	1833	BREX-system-P-loop-protein-BrxC-[Rhodococcus-erythropolis]
WP_032384849.1	Type1.BREX	<-EVE+REase<-ParB+HNH||SF1-SF2-Helicase+REase->SF1-SF2-Helicase+HTH-><-HerA/FtsK<-SIR2<-SIGMA+OB-Fold+AAA-ATPase+HKD-DNase-BrxL<-PglZ<-N6-METHYLASE<-DUF6079*<-BrxB-BrxF-iSTAND<-BrxA-DNA-binding<-XIS-HTH<-REC+OmpR-HTH<-HisKA_3+HATPase_c||DUF4352->?->URI-DNase->	1155	Bacteria>Terrabacteria_group>Actinomycetota>Actinomycetes>Mycobacteriales>Nocardiaceae	GCF_000760875.1	Rhodococcus_fascians	BH91_RS0103225	150682..154149	1828	BREX-system-P-loop-protein-BrxC-[Rhodococcus-fascians]
WP_045066308.1	Type1.BREX	IG+IG-><-?||Acyltransferase->?-><-MORC+DUF3684+REase<-?||BrxA-DNA-binding->BrxB-BrxF-iSTAND->DUF6079*->N6-METHYLASE->PglZ->SIGMA+OB-Fold+AAA-ATPase+HKD-DNase-BrxL->?-><-?<-?<-?||HAD+HAD->	1173	Bacteria>Terrabacteria_group>Actinomycetota>Actinomycetes>Mycobacteriales>Nocardiaceae	GCF_000949305.1	unclassified_Rhodococcus_(in:_high_G+C_Gram-positive_bacteria)	SZ00_RS11305	2127210..2130731	192944	MULTISPECIES:-BREX-system-P-loop-protein-BrxC-[unclassified-Rhodococcus-(in:-high-G+C-Gram-positive-bacteria)]
WP_062991956.1	Type1.BREX	<-MACRODOMAIN<-?||HTH_3->ACET->SWACOS->Cysteine-Desulfurase->Pkinase->BrxA-DNA-binding->BrxB-BrxF-iSTAND->DUF6079*->N6-METHYLASE->?->PglZ->SIGMA+OB-Fold+AAA-ATPase+HKD-DNase-BrxL-><-URI-DNase+RAMA<-?<-AbiEii	1173	Bacteria>Terrabacteria_group>Actinomycetota>Actinomycetes>Mycobacteriales>Nocardiaceae	GCF_001613085.1	Nocardia_salmonicida	NS1_RS36345	21477..24998	53431	BREX-system-P-loop-protein-BrxC-[Nocardia-salmonicida]
WP_154100274.1	Type1.BREX	BrxA-DNA-binding->BrxB-BrxF-iSTAND->DUF6079*->N6-METHYLASE->PglZ->SIGMA+OB-Fold+AAA-ATPase+HKD-DNase-BrxL->?->?-><-?<-RAMA<-?<-?||GDResolvase+HIN-HTH->	1172	Bacteria>Terrabacteria_group>Actinomycetota>Actinomycetes>Mycobacteriales>Nocardiaceae	GCF_001653035.1	Rhodococcus_sp._YH3-3	YH3_RS02030	410612..414130	1803579	BREX-system-P-loop-protein-BrxC-[Rhodococcus-sp.-YH3-3]
WP_070161347.1	Type1.BREX	<-Pkinase+TPR+TPR<-?<-?<-?<-?<-?||tRNA-><-?||BrxA-DNA-binding->BrxB-BrxF-iSTAND->DUF6079*->N6-METHYLASE->N6-METHYLASE-p->PglZ->SIGMA+OB-Fold+AAA-ATPase+HKD-DNase-BrxL->?->?-><-ParB+HNH	1175	Bacteria>Terrabacteria_group>Actinomycetota>Actinomycetes>Mycobacteriales>Nocardiaceae	GCF_001757665.1	Rhodococcus_sp._1139	A5N83_RS14165	58033..61560	1833762	BREX-system-P-loop-protein-BrxC-[Rhodococcus-sp.-1139]
WP_032407000.1	Type1.BREX	<-DUF4352||HisKA_3+HATPase_c->REC+OmpR-HTH->REase->?->XIS-HTH->BrxA-DNA-binding->BrxB-BrxF-iSTAND->DUF6079*->N6-METHYLASE->PglZ->SIGMA+OB-Fold+AAA-ATPase+HKD-DNase-BrxL->SIR2->HerA/FtsK-><-SF1-SF2-Helicase+HTH<-SF1-SF2-Helicase+REase||ParB+HNH->EVE+REase->	1155	Bacteria>Terrabacteria_group>Actinomycetota>Actinomycetes>Mycobacteriales>Nocardiaceae	GCF_001894785.1	Rhodococcus_fascians	BI05_RS0109965	97728..101195	1828	BREX-system-P-loop-protein-BrxC-[Rhodococcus-fascians]
WP_080909365.1	Type1.BREX	<-GDResolvase+HIN-HTH||?->?->RAMA->?->?-><-SIGMA+OB-Fold+AAA-ATPase+HKD-DNase-BrxL<-PglZ<-N6-METHYLASE<-DUF6079*<-BrxB-BrxF-iSTAND<-BrxA-DNA-binding||?-><-tRNA||?->?->?->?->?->Pkinase+TPR+TPR->	1173	Bacteria>Terrabacteria_group>Actinomycetota>Actinomycetes>Mycobacteriales>Nocardiaceae	GCF_002076325.1	Rhodococcus_sp._66b	BOE55_RS07060	288553..292074	1945511	BREX-system-P-loop-protein-BrxC-[Rhodococcus-sp.-66b]
WP_081557823.1	Type1.BREX	<-PIN<-?<-GDResolvase+HIN-HTH<-?<-SIGMA+OB-Fold+AAA-ATPase+HKD-DNase-BrxL<-PglZ<-N6-METHYLASE-p<-N6-METHYLASE<-DUF6079*<-BrxB-BrxF-iSTAND<-BrxA-DNA-binding<-XIS-HTH<-Phage_integrase	1168	Bacteria>Terrabacteria_group>Actinomycetota>Actinomycetes>Mycobacteriales>Nocardiaceae	GCF_002079265.1	Rhodococcus_sp._BH4	AOW34_RS13850	2965873..2969379	1807790	BREX-system-P-loop-protein-BrxC-[Rhodococcus-sp.-BH4]
WP_256973250.1	Type5.BREX	<-MULE-TRANSPOSASE<-TPR+TPR+TPR+TPR+TPR+TPR||BrxA-DNA-binding->BrxB-BrxF-iSTAND->DUF6079*->N6-METHYLASE->HTH+REase->PglZ->SIGMA+OB-Fold+AAA-ATPase+HKD-DNase-BrxL-><-DNA_glycosylase||?-><-TUDOR+SF1-SF2-Helicase+iREase+CTD+REase||SF1-SF2-Helicase->	1213	Bacteria>Terrabacteria_group>Actinomycetota>Actinomycetes>Mycobacteriales>Nocardiaceae	GCF_002165735.1	Rhodococcus_sp._NCIMB_12038	CA951_RS02920	126480..130121	933800	BREX-system-P-loop-protein-BrxC-[Rhodococcus-sp.-NCIMB-12038]
WP_088896782.1	Type1.BREX	ParB+HNH->nSTAND1+HNH-><-?||XIS-HTH->BrxA-DNA-binding->BrxB-BrxF-iSTAND->DUF6079*->N6-METHYLASE->PglZ->SIGMA+OB-Fold+AAA-ATPase+HKD-DNase-BrxL->?-><-HTH_21+rve+rve_3	1154	Bacteria>Terrabacteria_group>Actinomycetota>Actinomycetes>Mycobacteriales>Nocardiaceae	GCF_002215235.1	Rhodococcus_sp._BUPNP1	B9C99_RS01850	433431..436895	1432786	BREX-system-P-loop-protein-BrxC-[Rhodococcus-sp.-BUPNP1]
WP_094721084.1	Type1.BREX	SF1-SF2-Helicase+REase->SF1-SF2-Helicase+HTH-><-N6-METHYLASE||MORC+HISKIN->?->?-><-SIGMA+OB-Fold+AAA-ATPase+HKD-DNase-BrxL<-PglZ<-N6-METHYLASE<-DUF6079*<-BrxB-BrxF-iSTAND<-BrxA-DNA-binding<-HEPN+SF1-SF2-Helicase+REase||HIRAN->?-><-TIR<-HD<-?<-MarR-HTH	1174	Bacteria>Terrabacteria_group>Actinomycetota>Actinomycetes>Mycobacteriales>Nocardiaceae	GCF_002259415.1	Rhodococcus_sp._14-2470-1b	CH293_RS18620	24333..27857	2023149	BREX-system-P-loop-protein-BrxC-[Rhodococcus-sp.-14-2470-1b]
WP_198387782.1	Type1.BREX	HEPN+SF1-SF2-Helicase+REase->HTH_36-><-SIGMA+OB-Fold+AAA-ATPase+HKD-DNase-BrxL<-PglZ<-HTH+REase<-N6-METHYLASE-p||<-N6-METHYLASE<-DUF6079*<-BrxB-BrxF-iSTAND<-BrxA-DNA-binding<-DNA_METHYLASE<-DrmB<-SF1-SF2-Helicase<-SF1-SF2-Helicase<-MORC+SF1-SF2-Helicase||ParB+HNH->SF1-SF2-Helicase->	1173	Bacteria>Terrabacteria_group>Actinomycetota>Actinomycetes>Mycobacteriales>Nocardiaceae	GCF_002269365.1	Rhodococcus_pyridinivorans	CJU69_RS21710	4620785..4624306	103816	BREX-system-P-loop-protein-BrxC-[Rhodococcus-pyridinivorans]
WP_122191117.1	Type1.BREX	<-ABhydrolase||?->Glyoxalase-><-?<-SIGMA+OB-Fold+AAA-ATPase+HKD-DNase-BrxL<-PglZ<-?<-N6-METHYLASE<-DUF6079*<-BrxB-BrxF-iSTAND<-BrxA-DNA-binding||?-><-STYKIN<-TPR	1173	Bacteria>Terrabacteria_group>Actinomycetota>Actinomycetes>Mycobacteriales>Nocardiaceae	GCF_003696265.1	Nocardia_stercoris	EBN03_RS27825	64799..68320	2483361	BREX-system-P-loop-protein-BrxC-[Nocardia-stercoris]
WP_149431764.1	Type1.BREX	<-SIGMA+OB-Fold+AAA-ATPase+HKD-DNase-BrxL<-AbiEii+ZnR<-AbiEi_4+AbiEi_1<-PglZ<-N6-METHYLASE<-?<-DUF6079*<-BrxB-BrxF-iSTAND<-BrxA-DNA-binding	1184	Bacteria>Terrabacteria_group>Actinomycetota>Actinomycetes>Mycobacteriales>Nocardiaceae	GCF_008297975.1	Rhodococcus_cavernicola	FOY51_RS18630	121957..125511	2495913	BREX-system-P-loop-protein-BrxC-[Rhodococcus-cavernicola]
WP_225320218.1	Type1.BREX	<-Pkinase+TPR+TPR<-?<-?<-?<-?<-?||tRNA-><-?||BrxA-DNA-binding->BrxB-BrxF-iSTAND->DUF6079-p*->DUF6079-p->N6-METHYLASE->PglZ->SIGMA+OB-Fold+AAA-ATPase+HKD-DNase-BrxL->?->?-><-AAA+<-rve	435	Bacteria>Terrabacteria_group>Actinomycetota>Actinomycetes>Mycobacteriales>Nocardiaceae	GCF_011611865.1	Rhodococcus_erythropolis	G9444_RS32380	46132..47439	1833	BREX-system-P-loop-protein-BrxC-[Rhodococcus-erythropolis]
WP_195040827.1	Type1.BREX	McrB-AAA->McrC-NTD+REase-><-?<-N6-METHYLASE<-?<-?<-SIGMA+OB-Fold+AAA-ATPase+HKD-DNase-BrxL<-PglZ<-N6-METHYLASE<-DUF6079*<-BrxB-BrxF-iSTAND<-BrxA-DNA-binding<-XIS-HTH<-?<-HTH_31+DUF5753	1159	Bacteria>Terrabacteria_group>Actinomycetota>Actinomycetes>Mycobacteriales>Nocardiaceae	GCF_015477975.1	Nocardia_cyriacigeorgica	IU440_RS19520	298293..301772	135487	BREX-system-P-loop-protein-BrxC-[Nocardia-cyriacigeorgica]
WP_238424144.1	Type1.BREX	HTH_3->?-><-?<-HTH_36<-?||BrxA-DNA-binding->BrxB-BrxF-iSTAND->DUF6079*->N6-METHYLASE->HTH+REase->PglZ->SIGMA+OB-Fold+AAA-ATPase+HKD-DNase-BrxL->zf-ISL3+HTH_Tnp_ISL3-><-SF1-SF2-Helicase<-HKD-DNase<-DUF6361||TetR-HTH->	1175	Bacteria>Terrabacteria_group>Actinomycetota>Actinomycetes>Mycobacteriales>Nocardiaceae	GCF_016804345.1	Rhodococcus_ruber	F1734_RS10025	2133745..2137272	1830	BREX-system-P-loop-protein-BrxC-[Rhodococcus-ruber]
WP_245219430.1	Type1.BREX	<-REase<-?<-?||BrxA-DNA-binding->BrxB-BrxF-iSTAND->DUF6079*->N6-METHYLASE->PglZ->SIGMA+OB-Fold+AAA-ATPase+HKD-DNase-BrxL-><-?<-?||GDResolvase+HIN-HTH-><-ZnR||HISKIN->	1173	Bacteria>Terrabacteria_group>Actinomycetota>Actinomycetes>Mycobacteriales>Nocardiaceae	GCF_017831275.1	Rhodococcus_qingshengii	J6397_RS12385	68673..72194	334542	BREX-system-P-loop-protein-BrxC-[Rhodococcus-qingshengii]
WP_222635591.1	Type1.BREX	RAMA->wHTH+REase->HNH->SF1-SF2-Helicase+REase->SF1-SF2-Helicase+HTH->BrxA-DNA-binding->BrxB-BrxF-iSTAND->DUF6079*->N6-METHYLASE->PglZ->SIGMA+OB-Fold+AAA-ATPase+HKD-DNase-BrxL-><-?<-HEPN+SF1-SF2-Helicase+REase||CMP-hydrolase->	1188	Bacteria>Terrabacteria_group>Actinomycetota>Actinomycetes>Mycobacteriales>Nocardiaceae	GCF_019834785.1	unclassified_Rhodococcus_(in:_high_G+C_Gram-positive_bacteria)	HQ363_RS22195	12044..15610	192944	MULTISPECIES:-BREX-system-P-loop-protein-BrxC-[unclassified-Rhodococcus-(in:-high-G+C-Gram-positive-bacteria)]
WP_230793610.1	Type1.BREX	<-GDResolvase+HIN-HTH<-?<-REase<-?<-Polbeta||BrxA-DNA-binding->BrxB-BrxF-iSTAND->DUF6079*->N6-METHYLASE->PglZ->SIGMA+OB-Fold+AAA-ATPase+HKD-DNase-BrxL-><-?<-?||GDResolvase+HIN-HTH->URI-DNase->	1173	Bacteria>Terrabacteria_group>Actinomycetota>Actinomycetes>Mycobacteriales>Nocardiaceae	GCF_021026075.1	Rhodococcus_qingshengii	LQ424_RS30730	10749..14270	334542	BREX-system-P-loop-protein-BrxC-[Rhodococcus-qingshengii]
WP_231782753.1	Type1.BREX	<-Pkinase+TPR+TPR<-?<-?<-?<-?<-?||tRNA-><-?||BrxA-DNA-binding->BrxB-BrxF-iSTAND->DUF6079*->N6-METHYLASE->PglZ->SIGMA+OB-Fold+AAA-ATPase+HKD-DNase-BrxL->?->?->?-><-ParB+HNH||?-><-MULE-TRANSPOSASE	1173	Bacteria>Terrabacteria_group>Actinomycetota>Actinomycetes>Mycobacteriales>Nocardiaceae	GCF_021165975.1	Rhodococcus_qingshengii	LRL17_RS24605	5269427..5272948	334542	BREX-system-P-loop-protein-BrxC-[Rhodococcus-qingshengii]
WP_233062445.1	Type1.BREX	<-SAM-METHYLASE<-?<-DSBH<-?||BrxA-DNA-binding->BrxB-BrxF-iSTAND->DUF6079*->N6-METHYLASE->PglZ->SIGMA+OB-Fold+AAA-ATPase+HKD-DNase-BrxL->nSTAND3->	1173	Bacteria>Terrabacteria_group>Actinomycetota>Actinomycetes>Mycobacteriales>Nocardiaceae	GCF_021183645.1	Nocardia_gipuzkoensis	LTT66_RS33135	7242905..7246426	2749991	BREX-system-P-loop-protein-BrxC-[Nocardia-gipuzkoensis]
WP_242324362.1	Type1.BREX	<-SIGMA+OB-Fold+AAA-ATPase+HKD-DNase-BrxL<-AbiEii+ZnR<-AbiEi_4+AbiEi_1<-PglZ<-N6-METHYLASE<-DUF6079*<-BrxB-BrxF-iSTAND<-BrxA-DNA-binding<-RAMA<-?||?-><-REase<-?||TIR->	1188	Bacteria>Terrabacteria_group>Actinomycetota>Actinomycetes>Mycobacteriales>Nocardiaceae	GCF_022655485.1	Rhodococcus_opacus	MOD23_RS40495	105196..108762	37919	BREX-system-P-loop-protein-BrxC-[Rhodococcus-opacus]
WP_064077541.1	Type1.BREX	LuxR-HTH-><-ANK+ANK<-SIGMA+OB-Fold+AAA-ATPase+HKD-DNase-BrxL<-AbiEii+ZnR<-AbiEi_4+AbiEi_1<-sGTP<-PglZ<-N6-METHYLASE<-DUF6079*<-BrxB-BrxF-iSTAND<-BrxA-DNA-binding<-?||?-><-Glyoxalase||?->?->Calcineurin->MalT+LuxR-HTH->	1188	Bacteria>Terrabacteria_group>Actinomycetota>Actinomycetes>Mycobacteriales>Nocardiaceae	GCF_022691665.1	Prescottella_equi	MPC39_RS12500	2698129..2701695	43767	BREX-system-P-loop-protein-BrxC-[Prescottella-equi]
WP_242421152.1	Type1.BREX	SF1-SF2-Helicase+DUF1998->REase-><-DNA_METHYLASE||REase-><-SIGMA+OB-Fold+AAA-ATPase+HKD-DNase-BrxL<-PglZ<-HTH+REase<-N6-METHYLASE<-DUF6079*<-BrxB-BrxF-iSTAND<-BrxA-DNA-binding<-?||?-><-?||?->RNase->	1172	Bacteria>Terrabacteria_group>Actinomycetota>Actinomycetes>Mycobacteriales>Nocardiaceae	GCF_022691685.1	Prescottella_equi	MPC38_RS08825	1859263..1862781	43767	BREX-system-P-loop-protein-BrxC-[Prescottella-equi]
WP_156525037.1	Type1.BREX	<-GDResolvase+HIN-HTH||?->?-><-?<-Polbeta||BrxA-DNA-binding->BrxB-BrxF-iSTAND->DUF6079*->N6-METHYLASE->PglZ->SIGMA+OB-Fold+AAA-ATPase+HKD-DNase-BrxL-><-REase<-?<-?||GDResolvase+HIN-HTH->Phage_integrase->Phage_integrase->	1173	Bacteria>Terrabacteria_group>Actinomycetota>Actinomycetes>Mycobacteriales>Nocardiaceae	GCF_023221595.1	Rhodococcus	M0639_RS29930	74142..77663	1827	MULTISPECIES:-BREX-system-P-loop-protein-BrxC-[Rhodococcus]
WP_253659814.1	Type1.BREX	<-REase<-SIGMA+OB-Fold+AAA-ATPase+HKD-DNase-BrxL<-PglZ<-N6-METHYLASE<-DUF6079*<-BrxB-BrxF-iSTAND<-BrxA-DNA-binding||DNA_glycosylase->?->REase-><-?<-?||PhdYeFM_antitox->	1156	Bacteria>Terrabacteria_group>Actinomycetota>Actinomycetes>Mycobacteriales>Nocardiaceae	GCF_024171815.1	Williamsia_maris	LX13_RS03015	647894..651364	72806	BREX-system-P-loop-protein-BrxC-[Williamsia-maris]
WP_155277929.1	Type1.BREX	<-Pkinase+TPR+TPR<-?<-?<-?<-?<-?||tRNA-><-?||BrxA-DNA-binding->BrxB-BrxF-iSTAND->DUF6079*->N6-METHYLASE->PglZ->SIGMA+OB-Fold+AAA-ATPase+HKD-DNase-BrxL->?->?-><-ParB+HNH	1173	Bacteria>Terrabacteria_group>Actinomycetota>Actinomycetes>Mycobacteriales>Nocardiaceae	GCF_024349805.1	Rhodococcus	RE2895_RS00215	46904..50425	1827	MULTISPECIES:-BREX-system-P-loop-protein-BrxC-[Rhodococcus]
WP_256520207.1	Type1.BREX	<-Pkinase+TPR+TPR<-?<-?<-?<-?<-?||tRNA-><-?||BrxA-DNA-binding->BrxB-BrxF-iSTAND->DUF6079*->N6-METHYLASE->PglZ->SIGMA+OB-Fold+AAA-ATPase+HKD-DNase-BrxL->?->?->?-><-ParB+HNH	1174	Bacteria>Terrabacteria_group>Actinomycetota>Actinomycetes>Mycobacteriales>Nocardiaceae	GCF_024498635.1	Rhodococcus_qingshengii	LRQ08_RS00215	46197..49721	334542	BREX-system-P-loop-protein-BrxC-[Rhodococcus-qingshengii]
WP_269577378.1	Type1.BREX	<-Pkinase+TPR+TPR<-?<-?<-?<-?<-?||tRNA-><-?||BrxA-DNA-binding->BrxB-BrxF-iSTAND->DUF6079*->N6-METHYLASE->PglZ->SIGMA+OB-Fold+AAA-ATPase+HKD-DNase-BrxL->?->?-><-?||?->GDResolvase+HIN-HTH-><-ZnR||HISKIN->	1173	Bacteria>Terrabacteria_group>Actinomycetota>Actinomycetes>Mycobacteriales>Nocardiaceae	GCF_027270845.1	Rhodococcus_erythropolis	O4216_RS13595	117955..121476	1833	BREX-system-P-loop-protein-BrxC-[Rhodococcus-erythropolis]
WP_118604627.1	Type1.BREX	<-GDResolvase+HIN-HTH<-SIGMA+OB-Fold+AAA-ATPase+HKD-DNase-BrxL<-PglZ<-N6-METHYLASE<-DUF6079*<-BrxB-BrxF-iSTAND-p	1173	Bacteria>Terrabacteria_group>Actinomycetota>Actinomycetes>Mycobacteriales>Nocardiaceae	-	Rhodococcus_erythropolis	AWH04_RS14655	69911..73432	1833	BREX-system-P-loop-protein-BrxC-[Rhodococcus-erythropolis]
WP_214887135.1	Type1.BREX	<-SIGMA+OB-Fold+AAA-ATPase+HKD-DNase-BrxL<-PglZ<-N6-METHYLASE-p<-N6-METHYLASE-p<-DUF6079*<-BrxB-BrxF-iSTAND<-BrxA-DNA-binding<-?<-TIR-like	1170	Bacteria>Terrabacteria_group>Actinomycetota>Actinomycetes>Mycobacteriales>Nocardiaceae	-	Rhodococcus_erythropolis	J7E74_25040	11077..14589	1833	BREX-system-P-loop-protein-BrxC-[Rhodococcus-erythropolis]
TWS24839.1	Type1.BREX	<-Glyoxalase+Glyoxalase<-TPR||?-><-?<-?<-?<-SIGMA+OB-Fold+AAA-ATPase+HKD-DNase-BrxL<-PglZ<-N6-METHYLASE<-DUF6079*<-BrxB-BrxF-iSTAND<-BrxA-DNA-binding||HTH_3->JetA->JetB->SbcC->?->TetR-HTH->	1298	Bacteria>Terrabacteria_group>Actinomycetota>Actinomycetes>Mycobacteriales>Tsukamurellaceae	GCA_007858445.1	Tsukamurella_sputi	FK268_06235	209185..213081	2591848	BREX-system-P-loop-protein-BrxC-[Tsukamurella-sputi]
NKY16937.1	Type1.BREX	<-TetR-HTH<-?<-SbcC<-JetB<-JetA<-HTH_3||BrxA-DNA-binding->BrxB-BrxF-iSTAND->DUF6079*->N6-METHYLASE->PglZ->SIGMA+OB-Fold+AAA-ATPase+HKD-DNase-BrxL-><-?||<-MoxR-AAA<-?<-?||?->TPR->	1182	Bacteria>Terrabacteria_group>Actinomycetota>Actinomycetes>Mycobacteriales>Tsukamurellaceae	GCA_012396015.1	Tsukamurella_spumae	HF999_00885	181603..185151	44753	BREX-system-P-loop-protein-BrxC-[Tsukamurella-spumae]
WP_068569480.1	Type1.BREX	<-TetR-HTH<-?<-SbcC<-JetB<-JetA<-HTH_3||BrxA-DNA-binding->BrxB-BrxF-iSTAND->DUF6079*->N6-METHYLASE->PglZ->SIGMA+OB-Fold+AAA-ATPase+HKD-DNase-BrxL->HTH+REase->?-><-?<-TraB<-MoxR-AAA	1182	Bacteria>Terrabacteria_group>Actinomycetota>Actinomycetes>Mycobacteriales>Tsukamurellaceae	GCF_001575195.1	Tsukamurella_pseudospumae	AXK60_RS03070	654047..657595	239498	BREX-system-P-loop-protein-BrxC-[Tsukamurella-pseudospumae]
WP_068743364.1	Type1.BREX	MoxR-AAA->TraB->?-><-HTH+REase<-HTH+REase<-SIGMA+OB-Fold+AAA-ATPase+HKD-DNase-BrxL<-PglZ<-N6-METHYLASE<-DUF6079*<-BrxB-BrxF-iSTAND<-BrxA-DNA-binding||HTH_3->JetA->JetB->SbcC->?->TetR-HTH->	1180	Bacteria>Terrabacteria_group>Actinomycetota>Actinomycetes>Mycobacteriales>Tsukamurellaceae	GCF_001575205.1	Tsukamurella_pseudospumae	AXK61_RS01225	254752..258294	239498	BREX-system-P-loop-protein-BrxC-[Tsukamurella-pseudospumae]
WP_158636061.1	Type1.BREX	<-Glyoxalase+Glyoxalase<-TPR||?-><-?<-?<-?<-SIGMA+OB-Fold+AAA-ATPase+HKD-DNase-BrxL<-PglZ<-N6-METHYLASE<-DUF6079*<-BrxB-BrxF-iSTAND<-BrxA-DNA-binding||JetA->JetB->SbcC->?->TetR-HTH->	1180	Bacteria>Terrabacteria_group>Actinomycetota>Actinomycetes>Mycobacteriales>Tsukamurellaceae	GCF_007858445.1	Tsukamurella_sputi	FK268_RS06235	209185..212727	2591848	BREX-system-P-loop-protein-BrxC-[Tsukamurella-sputi]
WP_246207064.1	Type1.BREX	<-TetR-HTH<-?<-SbcC<-JetB<-JetA<-HTH_3||BrxA-DNA-binding->BrxB-BrxF-iSTAND->DUF6079*->N6-METHYLASE->SIGMA+OB-Fold+AAA-ATPase+HKD-DNase-BrxL->HTH+REase-><-?<-TraB<-MoxR-AAA	1179	Bacteria>Terrabacteria_group>Actinomycetota>Actinomycetes>Mycobacteriales>Tsukamurellaceae	GCF_012396015.1	Tsukamurella_spumae	HF999_RS00885	181612..185151	44753	BREX-system-P-loop-protein-BrxC-[Tsukamurella-spumae]
TCC44056.1	Type1.BREX	ArsR-HTH->?-><-Aminotran_1_2<-?||ABhydrolase->ACET->XIS-HTH->BrxA-DNA-binding->BrxB-BrxF-iSTAND->DUF6079*->N6-METHYLASE->PglZ->SIGMA+OB-Fold+AAA-ATPase+HKD-DNase-BrxL->	1159	Bacteria>Terrabacteria_group>Actinomycetota>Actinomycetes>Propionibacteriales>Kribbellaceae	GCA_004331485.1	Kribbella_capetownensis	EOH75_37810	267915..271394	1572659	BREX-system-P-loop-protein-BrxC-[Kribbella-capetownensis]
WP_238163516.1	Type1.BREX	ArsR-HTH->?-><-Aminotran_1_2<-?||ABhydrolase->ACET->XIS-HTH->BrxA-DNA-binding->BrxB-BrxF-iSTAND->DUF6079*->N6-METHYLASE->PglZ->SIGMA+OB-Fold+AAA-ATPase+HKD-DNase-BrxL->	1162	Bacteria>Terrabacteria_group>Actinomycetota>Actinomycetes>Propionibacteriales>Kribbellaceae	GCF_004331485.1	Kribbella_capetownensis	EOH75_RS37805	267906..271394	1572659	BREX-system-P-loop-protein-BrxC-[Kribbella-capetownensis]
WP_010833228.1	Type1.BREX	<-CHTH<-HerA/FtsK<-SIR2<-SIGMA+OB-Fold+AAA-ATPase+HKD-DNase-BrxL<-PglZ<-N6-METHYLASE<-DUF6079*<-BrxB-BrxF-iSTAND<-BrxA-DNA-binding<-XIS-HTH<-VirB11+MuB-ATPase||?->?->?->?->VirB11->	1160	Bacteria>Terrabacteria_group>Actinomycetota>Actinomycetes>Propionibacteriales>Nocardioidaceae	GCF_000389985.1	Nocardioides_sp._CF8	CF8_RS11160	2288637..2292119	110319	BREX-system-P-loop-protein-BrxC-[Nocardioides-sp.-CF8]
WP_028707702.1	Type1.BREX	HEPN-><-SIGMA+OB-Fold+AAA-ATPase+HKD-DNase-BrxL<-PglZ<-Schlafen+HATPase+wHTH<-N6-METHYLASE-p<-Phage_int_SAM_3+Phage_integrase||N6-METHYLASE-p->N6-METHYLASE-p-><-N6-METHYLASE<-DUF6079*<-BrxB-BrxF-iSTAND<-BrxA-DNA-binding<-?<-?<-?<-?||?->AbiEi_4->	1159	Bacteria>Terrabacteria_group>Actinomycetota>Actinomycetes>Propionibacteriales>Nocardioidaceae	GCF_000423465.1	Propionicicella_superfundia	G555_RS0102905	100593..104072	348582	BREX-system-P-loop-protein-BrxC-[Propionicicella-superfundia]
WP_068111667.1	Type1.BREX	VirB11+MuB-ATPase->?-><-?||XIS-HTH->BrxA-DNA-binding->BrxB-BrxF-iSTAND->DUF6079*->N6-METHYLASE->REase->PglZ->SIGMA+OB-Fold+AAA-ATPase+HKD-DNase-BrxL->OLD-ABC+TOPRIM->SF1-SF2-Helicase->?->HTH+MPTase->Phage_int_SAM_1+Phage_integrase->	1154	Bacteria>Terrabacteria_group>Actinomycetota>Actinomycetes>Propionibacteriales>Nocardioidaceae	GCF_001653335.1	Nocardioides_dokdonensis	I601_RS15600	3293021..3296485	450734	BREX-system-P-loop-protein-BrxC-[Nocardioides-dokdonensis]
WP_107767053.1	Type1.BREX	LZ_Tnp_IS481+HTH_32+rve+rve_3-><-?<-?||XIS-HTH->BrxA-DNA-binding->BrxB-BrxF-iSTAND->DUF6079*->N6-METHYLASE->PglZ->SIGMA+OB-Fold+AAA-ATPase+HKD-DNase-BrxL->	1154	Bacteria>Terrabacteria_group>Actinomycetota>Actinomycetes>Propionibacteriales>Nocardioidaceae	GCF_003047265.1	Nocardioides_terrigena	DAE25_RS15680	407524..410988	424797	BREX-system-P-loop-protein-BrxC-[Nocardioides-terrigena]
WP_116571233.1	Type1.BREX	VirB11+MuB-ATPase->?-><-?||XIS-HTH->BrxA-DNA-binding->BrxB-BrxF-iSTAND->DUF6079*->N6-METHYLASE->PglZ->SIGMA+OB-Fold+AAA-ATPase+HKD-DNase-BrxL->SIR2->HerA/FtsK->DUF4326->DUF488-1->DUF488-s->	1152	Bacteria>Terrabacteria_group>Actinomycetota>Actinomycetes>Propionibacteriales>Nocardioidaceae	GCF_003076135.1	Nocardioides_gansuensis	DDE18_RS05460	245415..248873	2138300	BREX-system-P-loop-protein-BrxC-[Nocardioides-gansuensis]
WP_147685627.1	Type1.BREX	ACET->?-><-SF1-SF2-Helicase<-SIGMA+OB-Fold+AAA-ATPase+HKD-DNase-BrxL<-PglZ<-N6-METHYLASE<-DUF6079*<-BrxB-BrxF-iSTAND<-BrxA-DNA-binding	1154	Bacteria>Terrabacteria_group>Actinomycetota>Actinomycetes>Propionibacteriales>Nocardioidaceae	GCF_008039565.1	Aeromicrobium_terrae	FHP06_RS08160	302941..306405	2498846	BREX-system-P-loop-protein-BrxC-[Aeromicrobium-terrae]
WP_148615130.1	Type1.BREX	XIS-HTH->Phage_int_SAM_3+Phage_integrase-><-REase||?-><-SIGMA+OB-Fold+AAA-ATPase+HKD-DNase-BrxL<-PglZ<-N6-METHYLASE<-DUF6079*<-BrxB-BrxF-iSTAND<-BrxA-DNA-binding<-XIS-HTH	1154	Bacteria>Terrabacteria_group>Actinomycetota>Actinomycetes>Propionibacteriales>Nocardioidaceae	GCF_008087085.1	Nocardioides_rubriscoriae	FZC61_RS15785	254232..257696	642762	BREX-system-P-loop-protein-BrxC-[Nocardioides-rubriscoriae]
WP_153653111.1	Type5.BREX	<-HTH_3||?->SF1-SF2-Helicase+REase->SF1-SF2-Helicase+REase-><-ParB+HNH<-SIGMA+OB-Fold+AAA-ATPase+HKD-DNase-BrxL<-PglZ<-N6-METHYLASE<-DUF6079*<-BrxB-BrxF-iSTAND<-BrxA-DNA-binding||GTPase-AIG->sGTP-><-?<-SF1-SF2-Helicase<-35exo+TUDOR+SF1-SF2-Helicase+iREase+CTD	1160	Bacteria>Terrabacteria_group>Actinomycetota>Actinomycetes>Propionibacteriales>Nocardioidaceae	GCF_009649075.1	Aeromicrobium_yanjiei	GEV26_RS10980	2228820..2232302	2662028	BREX-system-P-loop-protein-BrxC-[Aeromicrobium-yanjiei]
WP_166189200.1	Type1.BREX	XIS-HTH->BrxA-DNA-binding->BrxB-BrxF-iSTAND->DUF6079*->N6-METHYLASE->PglZ->SIGMA+OB-Fold+AAA-ATPase+HKD-DNase-BrxL->REase-><-Phage_int_SAM_3+Phage_integrase<-XIS-HTH	1154	Bacteria>Terrabacteria_group>Actinomycetota>Actinomycetes>Propionibacteriales>Nocardioidaceae	GCF_011250565.1	Nocardioides_sp._IC4_145	G6553_RS03445	61321..64785	2714037	BREX-system-P-loop-protein-BrxC-[Nocardioides-sp.-IC4_145]
WP_179652935.1	Type1.BREX	<-LPD36+METHYLASE+SF1-SF2-Helicase||REase->XIS-HTH->BrxA-DNA-binding->BrxB-BrxF-iSTAND->DUF6079*->N6-METHYLASE->PglZ->SIGMA+OB-Fold+AAA-ATPase+HKD-DNase-BrxL->AAA-ATPase-><-?<-Peptidase_M48	1154	Bacteria>Terrabacteria_group>Actinomycetota>Actinomycetes>Propionibacteriales>Nocardioidaceae	GCF_013408525.1	Nocardioides_aromaticivorans	BJ993_RS25170	157779..161243	200618	BREX-system-P-loop-protein-BrxC-[Nocardioides-aromaticivorans]
BAK36822.1	Type1.BREX	HTH_3->ADH_N+ADH_zinc_N->?->?->?->BrxA-DNA-binding->BrxB-BrxF-iSTAND->DUF6079*->N6-METHYLASE-><-N6-METHYLASE-p||Phage_int_SAM_3+Phage_integrase->N6-METHYLASE-p->Schlafen+HATPase+wHTH->PglZ->SIGMA+OB-Fold+AAA-ATPase+HKD-DNase-BrxL->SIR2-><-DDE_Tnp_1	1176	Bacteria>Terrabacteria_group>Actinomycetota>Actinomycetes>Propionibacteriales>Propionibacteriaceae	GCA_000270245.1	Microlunatus_phosphovorus_NM-1	MLP_38080	4107257..4110787	1032480	hypothetical-protein-MLP_38080-[Microlunatus-phosphovorus-NM-1]
NLI84511.1	Type1.BREX	HIN-HTH-><-SMODS<-SLATT<-?<-AbiEii<-REase<-SIGMA+OB-Fold+AAA-ATPase+HKD-DNase-BrxL<-PglZ<-N6-METHYLASE<-DUF6079*<-BrxB-BrxF-iSTAND<-BrxA-DNA-binding||?->TIR->?-><-DUF4096+DDE_Tnp_1_2	1160	Bacteria>Terrabacteria_group>Actinomycetota>Actinomycetes>Propionibacteriales>Propionibacteriaceae	GCA_012517405.1	Propionibacterium_sp.	GX440_03735	112337..115819	1977903	BREX-system-P-loop-protein-BrxC-[Propionibacterium-sp.]
MBK7820961.1	Type1.BREX	<-DUF2637+HIN-HTH<-ArdCN+MPTase||HTH->MPTase->XIS-HTH->BrxA-DNA-binding->BrxB-BrxF-iSTAND->DUF6079*->N6-METHYLASE->HTH+REase->PglZ->SIGMA+OB-Fold+AAA-ATPase+HKD-DNase-BrxL->OLD-ABC+TOPRIM->SF1-SF2-Helicase->?->DDE_Tnp_1-><-DNA_METHYLASE	1176	Bacteria>Terrabacteria_group>Actinomycetota>Actinomycetes>Propionibacteriales>Propionibacteriaceae	GCA_016714275.1	Tessaracoccus_sp.	IPJ61_07760	1693709..1697239	1971211	BREX-system-P-loop-protein-BrxC-[Tessaracoccus-sp.]
MCI1746855.1	Type1.BREX	sGTP->?->?->?->tRNA->tRNA->BrxA-DNA-binding->BrxB-BrxF-iSTAND->DUF6079*->?->N6-METHYLASE-><-N6-METHYLASE-p||Phage_int_SAM_3+Phage_integrase->N6-METHYLASE-p->Schlafen+HATPase+wHTH-p->PglZ->SIGMA+OB-Fold+AAA-ATPase+HKD-DNase-BrxL->Schlafen->	1159	Bacteria>Terrabacteria_group>Actinomycetota>Actinomycetes>Propionibacteriales>Propionibacteriaceae	GCA_022647765.1	Acidipropionibacterium_sp.	LKI24_01350	263130..266609	1912218	BREX-system-P-loop-protein-BrxC-[Acidipropionibacterium-sp.]
WP_041792941.1	Type1.BREX	Glyco_hydro_3+Glyco_hydro_3_C+Fn3-like->ADH_N+ADH_zinc_N->?->HTH_3->ADH_N+ADH_zinc_N->?->?->BrxA-DNA-binding->BrxB-BrxF-iSTAND->DUF6079*->N6-METHYLASE->Phage_int_SAM_3+Phage_integrase->N6-METHYLASE-p->Schlafen+HATPase+wHTH->PglZ->SIGMA+OB-Fold+AAA-ATPase+HKD-DNase-BrxL->SIR2-><-DDE_Tnp_1||HIN-HTH->	1173	Bacteria>Terrabacteria_group>Actinomycetota>Actinomycetes>Propionibacteriales>Propionibacteriaceae	GCF_000270245.1	Microlunatus_phosphovorus	MLP_RS18400	4107266..4110787	29405	BREX-system-P-loop-protein-BrxC-[Microlunatus-phosphovorus]
WP_028706416.1	Type1.BREX	<-SIGMA-HTH+SIGMA-HTH<-SF1-SF2-Helicase+HTH<-SIGMA+OB-Fold+AAA-ATPase+HKD-DNase-BrxL<-PglZ<-Schlafen+HATPase+wHTH-p<-?<-HerA/FtsK<-SIR2<-N6-METHYLASE<-DUF6079*<-BrxB-BrxF-iSTAND<-BrxA-DNA-binding||Glyco_hydro_36N+Melibiase+Glyco_hydro_36C->?->?->?->?-><-XIS-HTH	1171	Bacteria>Terrabacteria_group>Actinomycetota>Actinomycetes>Propionibacteriales>Propionibacteriaceae	GCF_000426605.1	Propionibacterium_acidifaciens	K336_RS0104645	45562..49077	556499	BREX-system-P-loop-protein-BrxC-[Propionibacterium-acidifaciens]
WP_021796710.1	Type1.BREX	XIS-HTH-><-?<-?<-?<-?<-Glyco_hydro_36N+Melibiase+Glyco_hydro_36C||BrxA-DNA-binding->BrxB-BrxF-iSTAND->DUF6079*->N6-METHYLASE->REase->?->HATPase+wHTH->PglZ->SIGMA+OB-Fold+AAA-ATPase+HKD-DNase-BrxL->SF1-SF2-Helicase+HTH->	1171	Bacteria>Terrabacteria_group>Actinomycetota>Actinomycetes>Propionibacteriales>Propionibacteriaceae	GCF_000478805.1	Propionibacterium_acidifaciens	HMPREF0682_RS02920	11275..14790	556499	BREX-system-P-loop-protein-BrxC-[Propionibacterium-acidifaciens]
WP_048768895.1	Type1.BREX	<-DUF2637+HIN-HTH->Primpol+PriCT1<-ArdCN+MPTase<-AraC-HTH||XIS-HTH->BrxA-DNA-binding->BrxB-BrxF-iSTAND->DUF6079*->N6-METHYLASE->REase->?->Schlafen+HATPase+wHTH->PglZ->SIGMA+OB-Fold+AAA-ATPase+HKD-DNase-BrxL->SIR2->HerA/FtsK->VirB11+MuB-ATPase->	1170	Bacteria>Terrabacteria_group>Actinomycetota>Actinomycetes>Propionibacteriales>Propionibacteriaceae	GCF_001050955.1	Propionibacterium_freudenreichii	PFCIRM1025_RS01850	33525..37037	1744	BREX-system-P-loop-protein-BrxC-[Propionibacterium-freudenreichii]
WP_055346094.1	Type1.BREX	<-DUF2637+HIN-HTH->Primpol+PriCT1<-ArdCN+MPTase<-AraC-HTH||XIS-HTH->BrxA-DNA-binding->BrxB-BrxF-iSTAND->DUF6079*->N6-METHYLASE->REase->?->Schlafen+HATPase+wHTH->PglZ->SIGMA+OB-Fold+AAA-ATPase+HKD-DNase-BrxL->SIR2->HerA/FtsK->VirB11+MuB-ATPase->	1170	Bacteria>Terrabacteria_group>Actinomycetota>Actinomycetes>Propionibacteriales>Propionibacteriaceae	GCF_001369055.1	Propionibacterium_freudenreichii	PFCIRM516_RS05500	48312..51824	1744	BREX-system-P-loop-protein-BrxC-[Propionibacterium-freudenreichii]
WP_094364310.1	Type1.BREX	DNA_METHYLASE-><-sGTP<-GTPase-AIG<-HerA/FtsK<-SIR2+SIR2<-SIGMA+OB-Fold+AAA-ATPase+HKD-DNase-BrxL<-PglZ<-HTH+REase<-N6-METHYLASE<-DUF6079*<-BrxB-BrxF-iSTAND<-BrxA-DNA-binding<-XIS-HTH<-ABhydrolase<-?<-MPTase<-HTH||ArdCN+MPTase->Primpol+PriCT1->	1173	Bacteria>Terrabacteria_group>Actinomycetota>Actinomycetes>Propionibacteriales>Propionibacteriaceae	GCF_002250625.1	Enemella_dayhoffiae	CGZ93_RS11755	175760..179281	2016507	BREX-system-P-loop-protein-BrxC-[Enemella-dayhoffiae]
WP_097828824.1	Type1.BREX	<-ABC-ATPase+SbcC+ABC-ATPase+SbcC<-?<-?<-tRNA||35exo->tRNA->BrxA-DNA-binding->BrxB-BrxF-iSTAND->DUF6079*->N6-METHYLASE-><-N6-METHYLASE-p||Phage_int_SAM_3+Phage_integrase->N6-METHYLASE-p->Schlafen+HATPase+wHTH->PglZ->SIGMA+OB-Fold+AAA-ATPase+HKD-DNase-BrxL-><-?<-HTH_Tnp_1+HTH_21+rve+rve_3	1173	Bacteria>Terrabacteria_group>Actinomycetota>Actinomycetes>Propionibacteriales>Propionibacteriaceae	GCF_004011075.1	Acidipropionibacterium_jensenii	COZ11_RS09870	2230980..2234501	1749	BREX-system-P-loop-protein-BrxC-[Acidipropionibacterium-jensenii]
WP_182782739.1	Type1.BREX	Peptidase_C14->?->?->?->PIN-><-URI-DNase<-TrwC+SF1-SF2-Helicase+TOPRIM||BrxA-DNA-binding->BrxB-BrxF-iSTAND->DUF6079*->N6-METHYLASE->N6-METHYLASE-p->PglZ->SIGMA+OB-Fold+AAA-ATPase+HKD-DNase-BrxL-><-ParB+HNH||NACHT->?->?-><-REase	1159	Bacteria>Terrabacteria_group>Actinomycetota>Actinomycetes>Propionibacteriales>Propionibacteriaceae	GCF_014164725.1	Tessaracoccus_sp._MC1627	H5399_RS09110	126520..129999	2760312	BREX-system-P-loop-protein-BrxC-[Tessaracoccus-sp.-MC1627]
WP_182799522.1	Type1.BREX	<-DOC+HTH<-RelE-ParE<-PhdYeFM_antitox<-Calcineurin<-ABhydrolase||XIS-HTH->BrxA-DNA-binding->BrxB-BrxF-iSTAND->DUF6079*->N6-METHYLASE->?->PglZ->SIGMA+OB-Fold+AAA-ATPase+HKD-DNase-BrxL->OLD-ABC+TOPRIM->SF1-SF2-Helicase->GTPase-AIG->sGTP->SF1-SF2-Helicase->	1173	Bacteria>Terrabacteria_group>Actinomycetota>Actinomycetes>Propionibacteriales>Propionibacteriaceae	GCF_014164775.1	Tessaracoccus_sp._MC1679	H5398_RS07140	376087..379608	2760313	BREX-system-P-loop-protein-BrxC-[Tessaracoccus-sp.-MC1679]
WP_232547416.1	Type1.BREX	<-OLD-ABC||?->?->?->nSTAND3->nSTAND3->?->BrxA-DNA-binding->BrxB-BrxF-iSTAND->DUF6079*->N6-METHYLASE->PglZ->SIGMA+OB-Fold+AAA-ATPase+HKD-DNase-BrxL->?->VirD4-TraG+VirD4-FtsK->?-><-Peptidase_A24	1159	Bacteria>Terrabacteria_group>Actinomycetota>Actinomycetes>Propionibacteriales>Propionibacteriaceae	GCF_014858005.1	Propioniciclava_soli	IFY26_RS02480	540114..543593	2775081	BREX-system-P-loop-protein-BrxC-[Propioniciclava-soli]
WP_204918880.1	Type1.BREX	<-sGTP<-GTPase-AIG<-SF1-SF2-Helicase<-OLD-ABC+TOPRIM<-SIGMA+OB-Fold+AAA-ATPase+HKD-DNase-BrxL<-PglZ<-?<-N6-METHYLASE<-DUF6079*<-BrxB-BrxF-iSTAND<-BrxA-DNA-binding<-XIS-HTH<-ABhydrolase||Recombinase-><-tRNA<-35exo<-?||tRNA-><-MarR-HTH	1173	Bacteria>Terrabacteria_group>Actinomycetota>Actinomycetes>Propionibacteriales>Propionibacteriaceae	GCF_016907535.1	Microlunatus_panaciterrae	JOE57_RS13940	3049076..3052597	400768	BREX-system-P-loop-protein-BrxC-[Microlunatus-panaciterrae]
WP_073185198.1	Type1.BREX	GDResolvase+HIN-HTH->Peptidase_A24-><-?<-VirD4-TraG+VirD4-FtsK<-?<-SIGMA+OB-Fold+AAA-ATPase+HKD-DNase-BrxL<-PglZ<-N6-METHYLASE<-DUF6079*<-BrxB-BrxF-iSTAND<-BrxA-DNA-binding<-?<-nSTAND3	1159	Bacteria>Terrabacteria_group>Actinomycetota>Actinomycetes>Propionibacteriales>Propionibacteriaceae	GCF_900141915.1	Tessaracoccus_bendigoensis	BUC15_RS00075	21322..24801	72764	BREX-system-P-loop-protein-BrxC-[Tessaracoccus-bendigoensis]
WP_073189906.1	Type1.BREX	XIS-HTH->BrxA-DNA-binding->BrxB-BrxF-iSTAND->DUF6079*->N6-METHYLASE->Schlafen+HATPase+wHTH->PglZ->SIGMA+OB-Fold+AAA-ATPase+HKD-DNase-BrxL->SIR2+SIR2->HerA/FtsK-><-?||GTPase-AIG->sGTP->	1176	Bacteria>Terrabacteria_group>Actinomycetota>Actinomycetes>Propionibacteriales>Propionibacteriaceae	GCF_900141915.1	Tessaracoccus_bendigoensis	BUC15_RS15345	1766..5296	72764	BREX-system-P-loop-protein-BrxC-[Tessaracoccus-bendigoensis]
WP_130875595.1	Type1.BREX	<-Peptidase_M20||?-><-SIGMA+OB-Fold+AAA-ATPase+HKD-DNase-BrxL<-PglZ<-Schlafen+HATPase+wHTH<-N6-METHYLASE<-DUF6079*<-BrxB-BrxF-iSTAND<-BrxA-DNA-binding<-?<-HAD<-Trypsin+PDZ||?-><-?<-METHYLASE||ACET->	1171	Bacteria>Terrabacteria_group>Actinomycetota>Actinomycetes>Propionibacteriales>Propionibacteriaceae	GCF_900607225.1	[Pseudopropionibacterium]_massiliense	EOE39_RS01895	450669..454184	2220000	BREX-system-P-loop-protein-BrxC-[[Pseudopropionibacterium]

<table>
<tr><th>Protein-ID</th><th>System</th><th>Gene-Neighbourhoods</th><th>Length</th><th>Taxonomy</th><th>Assembly-ID</th><th>Strain</th><th>Locus-Tag</th><th>Genomic-coordinates</th><th>TaxID</th><th>Fasta-defline</th></tr>
<tr><td>CRK59369.1</td><td>Type1.BREX</td><td><-SIGMA-HTH<-?<-?||HTH_31+DUF5753->?-><-SIGMA+OB-Fold+AAA-ATPase+HKD-DNase-BrxL<-PglZ<-N6-METHYLASE<-DUF6079*<-BrxB-BrxF-iSTAND<-BrxA-DNA-binding<-XIS-HTH<-?||?->?->?->?-><-Pkinase+pi3KINASE</td><td>1156</td><td>Bacteria>Terrabacteria_group>Actinomycetota>Actinomycetes>Pseudonocardiales>Pseudonocardiaceae</td><td>GCA_900070365.1</td><td>Alloactinosynnema_sp._L-07</td><td>.</td><td>4548794..4552264</td><td>1653480</td><td>ATPase-like-protein-[Alloactinosynnema-sp.-L-07]</td></tr>
<tr><td>WP_027928180.1</td><td>Type1.BREX</td><td><-SF1-SF2-Helicase<-?<-REase<-?||Calcineurin->SbcC+SbcC->?->BrxA-DNA-binding->BrxB-BrxF-iSTAND->DUF6079*->N6-METHYLASE->PglZ->SIGMA+OB-Fold+AAA-ATPase+HKD-DNase-BrxL-><-REase<-GDResolvase+Recombinase+ZnR<-?<-BirA-HTH<-?||HIN-HTH-></td><td>1164</td><td>Bacteria>Terrabacteria_group>Actinomycetota>Actinomycetes>Pseudonocardiales>Pseudonocardiaceae</td><td>GCF_000383915.1</td><td>Amycolatopsis_benzoatilytica</td><td>AMYBE_RS0130435</td><td>6563032..6566526</td><td>346045</td><td>BREX-system-P-loop-protein-BrxC-[Amycolatopsis-benzoatilytica]</td></tr>
<tr><td>WP_116112927.1</td><td>Type1.BREX</td><td>HTH_3->?->HEPN-><-HIN-HTH<-?<-GDResolvase+Recombinase||XIS-HTH->BrxA-DNA-binding->BrxB-BrxF-iSTAND->DUF6079*->N6-METHYLASE->PglZ->SIGMA+OB-Fold+AAA-ATPase+HKD-DNase-BrxL-><-?<-?<-?||HTH_3->MPTase-><-HIN-HTH</td><td>1159</td><td>Bacteria>Terrabacteria_group>Actinomycetota>Actinomycetes>Pseudonocardiales>Pseudonocardiaceae</td><td>GCF_023376025.1</td><td>Amycolatopsis</td><td>L1857_RS08640</td><td>1752725..1756204</td><td>1813</td><td>MULTISPECIES:-BREX-system-P-loop-protein-BrxC-[Amycolatopsis]</td></tr>
<tr><td>WP_259630250.1</td><td>Type1.BREX</td><td><-SIGMA+OB-Fold+AAA-ATPase+HKD-DNase-BrxL<-PglZ<-N6-METHYLASE<-DUF6079*<-BrxB-BrxF-iSTAND<-BrxA-DNA-binding<-XIS-HTH||N6-METHYLASE-><-Pkinase+TPR+TPR||REase->pPIWI+pPIWI->pPIWI+SF1-SF2-Helicase->pPIWI+MID+RNaseH-></td><td>1159</td><td>Bacteria>Terrabacteria_group>Actinomycetota>Actinomycetes>Pseudonocardiales>Pseudonocardiaceae</td><td>GCF_025061645.1</td><td>Umezawaea_endophytica</td><td>NZH93_RS48935</td><td>8817..12296</td><td>1654476</td><td>BREX-system-P-loop-protein-BrxC-[Umezawaea-endophytica]</td></tr>
<tr><td>WP_231949657.1</td><td>Type1.BREX</td><td><-SIGMA-HTH<-?<-?||HTH_31+DUF5753->DUF397-><-SIGMA+OB-Fold+AAA-ATPase+HKD-DNase-BrxL<-PglZ<-N6-METHYLASE<-DUF6079*<-BrxB-BrxF-iSTAND<-BrxA-DNA-binding<-XIS-HTH<-Pkinase+pi3KINASE||REase->pPIWI+pPIWI->pPIWI+SF1-SF2-Helicase->pPIWI+MID+RNaseH-><-TPR+TPR</td><td>1159</td><td>Bacteria>Terrabacteria_group>Actinomycetota>Actinomycetes>Pseudonocardiales>Pseudonocardiaceae</td><td>GCF_900070365.1</td><td>Alloactinosynnema_sp._L-07</td><td>BN1701_RS20310</td><td>4548794..4552273</td><td>1653480</td><td>BREX-system-P-loop-protein-BrxC-[Alloactinosynnema-sp.-L-07]</td></tr>
<tr><td>MBU1800642.1</td><td>Type1.BREX</td><td>DUF6079-p*->N6-METHYLASE->PglZ->SIGMA+OB-Fold+AAA-ATPase+HKD-DNase-BrxL->GTPase-AIG-></td><td>728</td><td>Bacteria>Terrabacteria_group>Actinomycetota>Actinomycetes>unclassified_Actinomycetes</td><td>GCA_018823685.1</td><td>Actinomycetia_bacterium</td><td>KKA97_00020</td><td>1..2189</td><td>1883427</td><td>BREX-system-P-loop-protein-BrxC-[Actinomycetia-bacterium]</td></tr>
<tr><td>MCA0378127.1</td><td>Type1.BREX</td><td>TraJ-RHH->?-><-?<-MACRODOMAIN||MerR-HTH->MerR-HTH->XIS-HTH->BrxA-DNA-binding->BrxB-BrxF-iSTAND->DUF6079*->N6-METHYLASE-><-N6-METHYLASE-p||Phage_int_SAM_3+Phage_integrase->N6-METHYLASE-p->Schlafen+HATPase+wHTH->PglZ->RVT->SIGMA+OB-Fold+AAA-ATPase+HKD-DNase-BrxL-><-SMODS</td><td>1183</td><td>Bacteria>Terrabacteria_group>Actinomycetota>Actinomycetes>unclassified_Actinomycetes</td><td>GCA_020161315.1</td><td>Actinomycetia_bacterium</td><td>LCH36_01625</td><td>63135..66686</td><td>1883427</td><td>BREX-system-P-loop-protein-BrxC-[Actinomycetia-bacterium]</td></tr>
<tr><td>MBR3160180.1</td><td>Type1.BREX</td><td><-ACET<-?<-SF1-SF2-Helicase<-HTH_3||?-><-?||BrxA-DNA-binding->BrxB-BrxF-iSTAND->DUF6079*->N6-METHYLASE-p->N6-METHYLASE->PglZ->SF1-SF2-Helicase->SIGMA+OB-Fold+AAA-ATPase+HKD-DNase-BrxL->AbiGi->ParB-></td><td>1186</td><td>Bacteria>Terrabacteria_group>Actinomycetota>Coriobacteriia>Coriobacteriales>Atopobiaceae</td><td>GCA_017533825.1</td><td>Atopobiaceae_bacterium</td><td>IKF14_13935</td><td>9053..12613</td><td>2485926</td><td>BREX-system-P-loop-protein-BrxC-[Atopobiaceae-bacterium]</td></tr>
<tr><td>MCC6106578.1</td><td>Type1.BREX</td><td>HTH_Tnp_1+HTH_21+rve+rve_3->BrxA-DNA-binding->BrxB-BrxF-iSTAND->DUF6079*->PglZ->N6-METHYLASE->SIGMA+OB-Fold+AAA-ATPase+HKD-DNase-BrxL->SF1-SF2-Helicase->?-><-?<-SF1-SF2-Helicase+wHTH+Small-B-barrel+DUF1998<-BrxD-AAA</td><td>1195</td><td>Bacteria>Terrabacteria_group>Actinomycetota>Coriobacteriia>Coriobacteriales>Atopobiaceae</td><td>GCA_020844585.1</td><td>Atopobium_sp.</td><td>LIV26_02205</td><td>3285..6872</td><td>1872650</td><td>BREX-system-P-loop-protein-BrxC-[Atopobium-sp.]</td></tr>
<tr><td>MCC6107938.1</td><td>Type1.BREX</td><td><-SIGMA+OB-Fold+AAA-ATPase+HKD-DNase-BrxL<-PglZ<-N6-METHYLASE<-DUF6079*<-BrxB-BrxF-iSTAND<-BrxA-DNA-binding<-TraJ-RHH<-PAIREDC-HTH</td><td>1196</td><td>Bacteria>Terrabacteria_group>Actinomycetota>Coriobacteriia>Coriobacteriales>Atopobiaceae</td><td>GCA_020844585.1</td><td>Atopobium_sp.</td><td>LIV26_09295</td><td>8478..12068</td><td>1872650</td><td>BREX-system-P-loop-protein-BrxC-[Atopobium-sp.]</td></tr>
<tr><td>MCH4081490.1</td><td>Type1.BREX</td><td>BrxD-AAA->SF1-SF2-Helicase+wHTH+Small-B-barrel+DUF1998->?-><-?<-SF1-SF2-Helicase<-MULE-TRANSPOSASE<-SIGMA+OB-Fold+AAA-ATPase+HKD-DNase-BrxL<-N6-METHYLASE<-PglZ<-DUF6079*<-BrxB-BrxF-iSTAND<-BrxA-DNA-binding||URI-DNase-><-HTH_6+SIS</td><td>1194</td><td>Bacteria>Terrabacteria_group>Actinomycetota>Coriobacteriia>Coriobacteriales>Atopobiaceae</td><td>GCA_022483965.1</td><td>Atopobiaceae_bacterium</td><td>LKE92_05045</td><td>1068955..1072539</td><td>2485926</td><td>BREX-system-P-loop-protein-BrxC-[Atopobiaceae-bacterium]</td></tr>
<tr><td>MCI1665141.1</td><td>Type1.BREX</td><td>ST-Hiskin-><-?<-tRNA<-tRNA||?->tRNA->XIS-HTH-><-?||REase-><-SIGMA+OB-Fold+AAA-ATPase+HKD-DNase-BrxL<-PglZ<-N6-METHYLASE<-DUF6079*<-BrxB-BrxF-iSTAND<-BrxA-DNA-binding<-PhdYeFM_antitox||?->REase->McrB-AAA->McrC-NTD+REase->?->TraJ-RHH-></td><td>1166</td><td>Bacteria>Terrabacteria_group>Actinomycetota>Coriobacteriia>Coriobacteriales>Atopobiaceae</td><td>GCA_022648095.1</td><td>Atopobiaceae_bacterium</td><td>LKI25_02075</td><td>510347..513847</td><td>2485926</td><td>BREX-system-P-loop-protein-BrxC-[Atopobiaceae-bacterium]</td></tr>
<tr><td>SFW99921.1</td><td>Type1.BREX</td><td><-DOC+HTH||?->?-><-HTH+REase<-?<-SIGMA+OB-Fold+AAA-ATPase+HKD-DNase-BrxL<-N6-METHYLASE<-PglZ<-DUF6079*<-BrxB-BrxF-iSTAND<-BrxA-DNA-binding<-TraJ-RHH||ParB+HNH->Phage-portal-><-?<-Glyco_hydro_1<-PTSEIIC</td><td>1199</td><td>Bacteria>Terrabacteria_group>Actinomycetota>Coriobacteriia>Coriobacteriales>Atopobiaceae</td><td>GCA_900119625.1</td><td>Olsenella_sp._kh2p3</td><td>SAMN04487823_101110</td><td>126088..129687</td><td>1797112</td><td>hypothetical-protein-SAMN04487823_101110-[Olsenella-sp.-kh2p3]</td></tr>
<tr><td>WP_204537521.1</td><td>Type1.BREX</td><td>Cysteine-Desulfurase->?-><-SIGMA+OB-Fold+AAA-ATPase+HKD-DNase-BrxL<-PglZ<-Schlafen+HATPase+wHTH<-N6-METHYLASE<-HEPN<-DUF6079*<-BrxB-BrxF-iSTAND<-BrxA-DNA-binding<-TraJ-RHH</td><td>1171</td><td>Bacteria>Terrabacteria_group>Actinomycetota>Coriobacteriia>Coriobacteriales>Coriobacteriaceae</td><td>-</td><td>Collinsella_tanakaei</td><td>H6A33_RS00145</td><td>33492..37007</td><td>626935</td><td>BREX-system-P-loop-protein-BrxC-[Collinsella-tanakaei]</td></tr>
<tr><td>RRF95308.1</td><td>Type1.BREX</td><td><-TraI-VirD2-RCRE<-TraJ-RHH||TraJ-RHH->BrxA-DNA-binding->BrxB-BrxF-iSTAND->DUF6079*->PglZ->N6-METHYLASE->?->SIGMA+OB-Fold+AAA-ATPase+HKD-DNase-BrxL-><-REase<-XIS-HTH<-Phage_int_SAM_5+Phage_integrase</td><td>1196</td><td>Bacteria>Terrabacteria_group>Actinomycetota>Coriobacteriia>Coriobacteriales>Coriobacteriaceae</td><td>GCA_003862165.1</td><td>Coriobacteriaceae_bacterium</td><td>DUD31_01665</td><td>3961..7551</td><td>2011094</td><td>BREX-system-P-loop-protein-BrxC-[Coriobacteriaceae-bacterium]</td></tr>
<tr><td>HJG30547.1</td><td>Type1.BREX</td><td>ACET-><-REase<-SbcC<-JetB<-JetA||?->TraJ-RHH->BrxA-DNA-binding->BrxB-BrxF-iSTAND->DUF6079*->PglZ->N6-METHYLASE->?->SIGMA+OB-Fold+AAA-ATPase+HKD-DNase-BrxL->?->?->?->TetR-HTH->ABC-ATPase+SbcC-></td><td>1187</td><td>Bacteria>Terrabacteria_group>Actinomycetota>Coriobacteriia>Coriobacteriales>Coriobacteriaceae</td><td>GCA_020743295.1</td><td>Collinsella_ihumii</td><td>K8U80_04025</td><td>25860..29423</td><td>1720204</td><td>BREX-system-P-loop-protein-BrxC-[Collinsella-ihumii]</td></tr>
<tr><td>WP_204559883.1</td><td>Type1.BREX</td><td>Cysteine-Desulfurase->?-><-SIGMA+OB-Fold+AAA-ATPase+HKD-DNase-BrxL<-PglZ<-Schlafen+HATPase+wHTH<-REase<-N6-METHYLASE<-HEPN<-DUF6079*<-BrxB-BrxF-iSTAND<-BrxA-DNA-binding<-TraJ-RHH</td><td>1171</td><td>Bacteria>Terrabacteria_group>Actinomycetota>Coriobacteriia>Coriobacteriales>Coriobacteriaceae</td><td>GCF_016902615.1</td><td>Collinsella_tanakaei</td><td>H6A21_RS01790</td><td>55502..59017</td><td>626935</td><td>BREX-system-P-loop-protein-BrxC-[Collinsella-tanakaei]</td></tr>
<tr><td>WP_012797473.1</td><td>Type1.BREX</td><td>ParB+HNH-><-SLATT<-SMODS<-SIGMA+OB-Fold+AAA-ATPase+HKD-DNase-BrxL<-?<-N6-METHYLASE<-N6-METHYLASE<-PglZ<-DUF6079*<-BrxB-BrxF-iSTAND<-BrxA-DNA-binding<-TraJ-RHH<-?<-?<-?<-ParB+HNH||SAM-METHYLASE->wHTH+HNH-></td><td>1180</td><td>Bacteria>Terrabacteria_group>Actinomycetota>Coriobacteriia>Eggerthellales>Eggerthellaceae</td><td>GCF_000023885.1</td><td>Slackia_heliotrinireducens</td><td>EL225_RS01045</td><td>248773..252315</td><td>84110</td><td>BREX-system-P-loop-protein-BrxC-[Slackia-heliotrinireducens]</td></tr>
<tr><td>WP_251178638.1</td><td>Type1.BREX</td><td><-ABhydrolase<-?<-?||MerR-HTH->?->CHTH-><-SIGMA+OB-Fold+AAA-ATPase+HKD-DNase-BrxL<-N6-METHYLASE<-PglZ<-DUF6079*<-BrxB-BrxF-iSTAND<-BrxA-DNA-binding<-PhdYeFM_antitox||?->?->HTH_3->Peptidase_C14->TIR->Peptidase_C14-></td><td>1185</td><td>Bacteria>Terrabacteria_group>Actinomycetota>Coriobacteriia>Eggerthellales>Eggerthellaceae</td><td>GCF_943193125.1</td><td>Adlercreutzia_agrestimuris</td><td>NFR28_RS04520</td><td>318927..322484</td><td>2941324</td><td>BREX-system-P-loop-protein-BrxC-[Adlercreutzia-agrestimuris]</td></tr>
<tr><td>WP_263326060.1</td><td>Type6.BREX</td><td>GDResolvase+Recombinase->AAA-ATPase->REase->BrxE->BrxA-DNA-binding->BrxB-BrxF-iSTAND->DUF6079*->N6-METHYLASE->PglZ->SIGMA+OB-Fold+AAA-ATPase+HKD-DNase-BrxL->Trypsin->GDResolvase+HIN-HTH->ParB+HNH->HEPN-></td><td>1207</td><td>Bacteria>Terrabacteria_group>Bacillota>Bacilli>Bacillales>Bacillaceae</td><td>GCF_946151075.1</td><td>Neobacillus_sp._Marseille-Q6967</td><td>OGZ30_RS15230</td><td>466591..470214</td><td>2963105</td><td>BREX-system-P-loop-protein-BrxC-[Neobacillus-sp.-Marseille-Q6967]</td></tr>
<tr><td>NLN64679.1</td><td>Type1.BREX</td><td><-REase+DUF5655<-URI-DNase+RAMA||HTH_3->BrxA-DNA-binding->BrxB-BrxF-iSTAND->DUF6079*->N6-METHYLASE->PglZ->SIGMA+OB-Fold+AAA-ATPase+HKD-DNase-BrxL->SF1-SF2-Helicase-><-?<-HIT<-?<-ABhydrolase</td><td>1174</td><td>Bacteria>Terrabacteria_group>Bacillota>Clostridia>Eubacteriales>Clostridiaceae</td><td>GCA_012522505.1</td><td>Clostridiaceae_bacterium</td><td>GX144_04605</td><td>4627..8151</td><td>1898204</td><td>BREX-system-P-loop-protein-BrxC-[Clostridiaceae-bacterium]</td></tr>
<tr><td>MBR4608207.1</td><td>Type1.BREX</td><td><-REase<-Phage_integrase<-Phage_integrase||?->MobA_MobL->?->?->BrxA-DNA-binding->BrxB-BrxF-iSTAND->DUF6079*->N6-METHYLASE-p->N6-METHYLASE->OLD-ABC->TOPRIM->PglZ->SIGMA+OB-Fold+AAA-ATPase+HKD-DNase-BrxL-><-HEPN<-ParB+HNH<-DNA_METHYLASE</td><td>1155</td><td>Bacteria>Terrabacteria_group>Bacillota>Clostridia>Eubacteriales>Lachnospiraceae</td><td>GCA_017536085.1</td><td>Lachnospiraceae_bacterium</td><td>IKO41_18520</td><td>18020..21487</td><td>1898203</td><td>BREX-system-P-loop-protein-BrxC-[Lachnospiraceae-bacterium]</td></tr>
<tr><td>KKM11538.1</td><td>Type6.BREX</td><td><-PAS+NtrC-AAA+FIS-HTH<-DDE_Tnp_ISAZ013||MPTase->PIN-><-SIGMA+OB-Fold+AAA-ATPase+HKD-DNase-BrxL<-PglZ<-N6-METHYLASE<-DUF6079*<-BrxB-BrxF-iSTAND<-BrxA-DNA-binding<-BrxE<-TPR<-TIR<-?<-?<-?||DDE_Tnp_1-></td><td>1207</td><td>Bacteria>Terrabacteria_group>Bacillota>Clostridia>Eubacteriales>unclassified_Eubacteriales</td><td>GCA_000987045.1</td><td>Clostridiales_bacterium_PH28_bin88</td><td>SY88_07480</td><td>24699..28322</td><td>1605376</td><td>ATPase-[Clostridiales-bacterium-PH28_bin88]</td></tr>
<tr><td>MBJ7595965.1</td><td>Type1.BREX</td><td><-SIGMA+OB-Fold+AAA-ATPase+HKD-DNase-BrxL<-PglZ<-N6-METHYLASE<-DUF6079*<-BrxB-BrxF-iSTAND<-BrxA-DNA-binding</td><td>1162</td><td>Bacteria>Terrabacteria_group>Candidatus_Dormibacteraeota>unclassified_Candidatus_Dormibacteraeota</td><td>GCA_016464765.1</td><td>Candidatus_Dormibacteraeota_bacterium</td><td>JF886_14125</td><td>8709..12197</td><td>2052315</td><td>BREX-system-P-loop-protein-BrxC-[Candidatus-Dormibacteraeota-bacterium]</td></tr>
<tr><td>MAT43664.1</td><td>Type1.BREX</td><td><-AAA-ATPase<-TPR+TPR+TPR+TPR<-?<-?<-?<-?<-SIGMA+OB-Fold+AAA-ATPase+HKD-DNase-BrxL<-PglZ<-N6-METHYLASE<-DUF6079*<-BrxB-BrxF-iSTAND<-BrxA-DNA-binding<-CBS+HEPN||?->SF1-SF2-Helicase+DUF1998->ParB-><-?<-rve</td><td>1169</td><td>Bacteria>Terrabacteria_group>Chloroflexota>Anaerolineae>Anaerolineales>Anaerolineaceae</td><td>GCA_002702705.1</td><td>Anaerolineaceae_bacterium</td><td>CL609_15105</td><td>30474..33983</td><td>2024896</td><td>hypothetical-protein-[Anaerolineaceae-bacterium]</td></tr>
<tr><td>MAT45245.1</td><td>Type1.BREX</td><td><-SIGMA+OB-Fold+AAA-ATPase+HKD-DNase-BrxL<-PglZ<-HTH+MPTase<-HigB_toxin<-RADICAL-SAM<-?<-NACHT<-N6-METHYLASE<-DUF6079*<-BrxB-BrxF-iSTAND<-BrxA-DNA-binding<-XIS-HTH<-?<-TniQ<-TnsC-ATPase<-rve+Mu-transpos_C<-REase</td><td>1172</td><td>Bacteria>Terrabacteria_group>Chloroflexota>Anaerolineae>Anaerolineales>Anaerolineaceae</td><td>GCA_002702705.1</td><td>Anaerolineaceae_bacterium</td><td>CL609_23195</td><td>14888..18406</td><td>2024896</td><td>hypothetical-protein-[Anaerolineaceae-bacterium]</td></tr>
<tr><td>MBE2233048.1</td><td>Type1.BREX</td><td><-SIGMA+OB-Fold+AAA-ATPase+HKD-DNase-BrxL<-PglZ<-HTH+MPTase<-HigB_toxin<-N6-METHYLASE<-SF1-SF2-Helicase<-DUF6079*</td><td>1166</td><td>Bacteria>Terrabacteria_group>Chloroflexota>Anaerolineae>Anaerolineales>Anaerolineaceae</td><td>GCA_014879765.1</td><td>Anaerolinea_sp.</td><td>IAE85_06095</td><td>14605..18105</td><td>1872519</td><td>BREX-system-P-loop-protein-BrxC-[Anaerolinea-sp.]</td></tr>
<tr><td>MBQ6502300.1</td><td>Type1.BREX</td><td>DOC+HTH-><-?<-?||?->?->BrxA-DNA-binding->BrxB-BrxF-iSTAND->DUF6079*->OLD-ABC->TOPRIM->?->?->N6-METHYLASE->PglZ->SIGMA+OB-Fold+AAA-ATPase+HKD-DNase-BrxL->ARG->ParB+HNH-></td><td>1194</td><td>Bacteria>Terrabacteria_group>Chloroflexota>Anaerolineae>Anaerolineales>Anaerolineaceae</td><td>GCA_017432685.1</td><td>Flexilinea_sp.</td><td>IJI57_00125</td><td>8965..12549</td><td>2017390</td><td>BREX-system-P-loop-protein-BrxC-[Flexilinea-sp.]</td></tr>
<tr><td>WP_062419291.1</td><td>Type6.BREX</td><td>METHYLASE+SF1-SF2-Helicase-><-?<-SIGMA+OB-Fold+AAA-ATPase+HKD-DNase-BrxL<-PglZ<-TOPRIM<-OLD-ABC<-N6-METHYLASE<-N6-METHYLASE<-DUF6079*<-BrxB-BrxF-iSTAND<-BrxA-DNA-binding<-BrxE<-XIS-HTH<-CHTH<-LuxR-HTH||?->XIS-HTH->Phage_integrase-></td><td>1206</td><td>Bacteria>Terrabacteria_group>Chloroflexota>Anaerolineae>Anaerolineales>Anaerolineaceae</td><td>GCF_001050255.2</td><td>Levilinea_saccharolytica</td><td>LSAC_RS18205</td><td>3303565..3307185</td><td>229921</td><td>BREX-system-P-loop-protein-BrxC-[Levilinea-saccharolytica]</td></tr>
<tr><td>WP_062280889.1</td><td>Type6.BREX</td><td><-Phage_int_SAM_1+Phage_integrase<-XIS-HTH<-?||LuxR-HTH->CHTH->XIS-HTH->BrxE->BrxA-DNA-binding->BrxB-BrxF-iSTAND->DUF6079*->N6-METHYLASE->PglZ->SIGMA+OB-Fold+AAA-ATPase+HKD-DNase-BrxL->?->HerA/FtsK-><-METHYLASE+SF1-SF2-Helicase<-?||SIGMA-HTH-></td><td>1206</td><td>Bacteria>Terrabacteria_group>Chloroflexota>Anaerolineae>Anaerolineales>Anaerolineaceae</td><td>GCF_001192795.1</td><td>Flexilinea_flocculi</td><td>ATC1_RS09365</td><td>1036215..1039835</td><td>1678840</td><td>BREX-system-P-loop-protein-BrxC-[Flexilinea-flocculi]</td></tr>
<tr><td>MCA9933330.1</td><td>Type1.BREX</td><td>HTH_28+rve->XIS-HTH-><-?||BrxA-DNA-binding->BrxB-BrxF-iSTAND->DUF6079*->N6-METHYLASE->HEPN->PglZ->SIGMA+OB-Fold+AAA-ATPase+HKD-DNase-BrxL-><-CopG_antitoxin<-BrnT_toxin<-GDResolvase+HIN-HTH||?->TIR-></td><td>1174</td><td>Bacteria>Terrabacteria_group>Chloroflexota>Anaerolineae>Anaerolineales>unclassified_Anaerolineales</td><td>GCA_020432585.1</td><td>Anaerolineales_bacterium</td><td>KC415_05370</td><td>4959..8483</td><td>2073117</td><td>BREX-system-P-loop-protein-BrxC-[Anaerolineales-bacterium]</td></tr>
<tr><td>MCJ7529874.1</td><td>Type1.BREX</td><td>rve->?->?->PIN->?->PemK_toxin->BrxA-DNA-binding->BrxB-BrxF-iSTAND->DUF6079*->?->Schlafen->N6-METHYLASE->HigB_toxin->HTH+MPTase->PglZ->SIGMA+OB-Fold+AAA-ATPase+HKD-DNase-BrxL-><-HTH_37</td><td>1170</td><td>Bacteria>Terrabacteria_group>Chloroflexota>Anaerolineae>Anaerolineales>unclassified_Anaerolineales</td><td>GCA_022865645.1</td><td>Anaerolineales_bacterium</td><td>MUO64_02430</td><td>4866..8378</td><td>2073117</td><td>BREX-system-P-loop-protein-BrxC-[Anaerolineales-bacterium]</td></tr>
<tr><td>NPV77432.1</td><td>Type6.BREX</td><td>LuxR-HTH->CHTH->XIS-HTH->BrxE->BrxA-DNA-binding->BrxB-BrxF-iSTAND->DUF6079*->N6-METHYLASE->SIGMA+OB-Fold+AAA-ATPase+HKD-DNase-BrxL->?-><-METHYLASE+SF1-SF2-Helicase<-?||wHTH+AbiEi_2->AbiEii-><-DNA_pol3_beta+DNA_pol3_beta_2+DNA_pol3_beta_3</td><td>1206</td><td>Bacteria>Terrabacteria_group>Chloroflexota>Anaerolineae>unclassified_Anaerolineae</td><td>GCA_013178185.1</td><td>Anaerolineae_bacterium</td><td>HPY59_13805</td><td>3635..7255</td><td>2052143</td><td>BREX-system-P-loop-protein-BrxC-[Anaerolineae-bacterium]</td></tr>
<tr><td>MCL4264723.1</td><td>Type6.BREX</td><td><-SIGMA-HTH<-SIGMA-HTH||?->?->TPR+TPR-><-SIGMA+OB-Fold+AAA-ATPase+HKD-DNase-BrxL<-PglZ<-N6-METHYLASE<-DUF6079*<-BrxB-BrxF-iSTAND<-BrxA-DNA-binding<-BrxE||?->TPR+TPR+TPR->?-><-TPR+TPR+TPR+TPR<-CASPASE</td><td>1209</td><td>Bacteria>Terrabacteria_group>Chloroflexota>Anaerolineae>unclassified_Anaerolineae</td><td>GCA_023382655.1</td><td>Anaerolineae_bacterium</td><td>KJ069_15985</td><td>16020..19649</td><td>2052143</td><td>BREX-system-P-loop-protein-BrxC-[Anaerolineae-bacterium]</td></tr>
<tr><td>ADJ26904.1</td><td>Type1.BREX</td><td><-MULE-TRANSPOSASE<-SF1-SF2-Helicase<-?<-SLOG+BirA-HTH<-?<-SIGMA+OB-Fold+AAA-ATPase+HKD-DNase-BrxL<-PglZ<-N6-METHYLASE<-DUF6079*<-BrxB-BrxF-iSTAND<-BrxA-DNA-binding<-XIS-HTH<-?<-?<-?<-wHTH</td><td>1208</td><td>Bacteria>Terrabacteria_group>Chloroflexota>Dehalococcoidia>Dehalogenimonas>Dehalogenimonas_lykanthroporepellens</td><td>GCA_000143165.1</td><td>Dehalogenimonas_lykanthroporepellens_BL-DC-9</td><td>Dehly_1626</td><td>1598643..1602269</td><td>552811</td><td>conserved-hypothetical-protein-[Dehalogenimonas-lykanthroporepellens-BL-DC-9]</td></tr>
<tr><td>OJX40202.1</td><td>Type6.BREX</td><td><-MerR-HTH||?->METHYLASE+SF1-SF2-Helicase-><-SIGMA+OB-Fold+AAA-ATPase+HKD-DNase-BrxL<-PglZ<-N6-METHYLASE<-DUF6079*<-BrxB-BrxF-iSTAND<-BrxA-DNA-binding<-BrxE<-XIS-HTH<-CHTH<-LuxR-HTH||?->XIS-HTH->Phage_integrase-></td><td>1199</td><td>Bacteria>Terrabacteria_group>Chloroflexota>unclassified_Chloroflexota</td><td>GCA_001899005.1</td><td>Chloroflexi_bacterium_44-23</td><td>BGO78_14595</td><td>64174..67773</td><td>1895927</td><td>ATPase-[Chloroflexi-bacterium-44-23]</td></tr>
<tr><td>HDQ70548.1</td><td>Type1.BREX</td><td><-SIGMA+OB-Fold+AAA-ATPase+HKD-DNase-BrxL<-PglZ<-N6-METHYLASE<-?<-DUF6079*<-BrxB-BrxF-iSTAND<-BrxA-DNA-binding</td><td>1168</td><td>Bacteria>Terrabacteria_group>Chloroflexota>unclassified_Chloroflexota</td><td>GCA_011046405.1</td><td>Chloroflexota_bacterium</td><td>ENN19_00445</td><td>9344..12850</td><td>2026724</td><td>BREX-system-P-loop-protein-BrxC-[Chloroflexota-bacterium]</td></tr>
<tr><td>MBK8989553.1</td><td>Type1.BREX</td><td>PIN->CRISPR_Cas6->HTH_Tnp_1+HTH_21+rve+rve_3-><-?<-SIGMA+OB-Fold+AAA-ATPase+HKD-DNase-BrxL<-PglZ||RHH-><-?<-N6-METHYLASE<-DUF6079*<-BrxB-BrxF-iSTAND<-BrxA-DNA-binding<-?||wHTH+WYL+WCX->RNase_T+35exo->?->ABhydrolase+ABhydrolase->?->BrxB-BrxF-iSTAND-></td><td>1173</td><td>Bacteria>Terrabacteria_group>Chloroflexota>unclassified_Chloroflexota</td><td>GCA_016716065.1</td><td>Chloroflexota_bacterium</td><td>IPM39_26430</td><td>84319..87840</td><td>2026724</td><td>BREX-system-P-loop-protein-BrxC-[Chloroflexota-bacterium]</td></tr>
<tr><td>MBX9724057.1</td><td>Type1.BREX</td><td><-SIGMA+OB-Fold+AAA-ATPase+HKD-DNase-BrxL||BrxA-DNA-binding->BrxB-BrxF-iSTAND->DUF6079*->OLD-ABC->REase->N6-METHYLASE->PglZ-p-></td><td>1175</td><td>Bacteria>Terrabacteria_group>Cyanobacteriota/Melainabacteria_group>Candidatus_Melainabacteria>Candidatus_Obscuribacterales>unclassified_Candidatus_Obscuribacterales</td><td>GCA_019747345.1</td><td>Candidatus_Obscuribacterales_bacterium</td><td>K2X81_21805</td><td>3793..7320</td><td>2364082</td><td>BREX-system-P-loop-protein-BrxC-[Candidatus-Obscuribacterales-bacterium]</td></tr>
<tr><td>MBT9546703.1</td><td>Type1.BREX</td><td>Peptidase_M2->BrxA-DNA-binding->BrxB-BrxF-iSTAND->DUF6079*->N6-METHYLASE->RVT->N6-METHYLASE-p->OLD-ABC->HNH->PglZ->SIGMA+OB-Fold+AAA-ATPase+HKD-DNase-BrxL-></td><td>1179</td><td>Bacteria>Terrabacteria_group>Cyanobacteriota/Melainabacteria_group>Candidatus_Sericytochromatia>unclassified_Candidatus_Sericytochromatia</td><td>GCA_018780035.1</td><td>Candidatus_Sericytochromatia_bacterium</td><td>IV090_15030</td><td>94995..98534</td><td>2762020</td><td>BREX-system-P-loop-protein-BrxC-[Candidatus-Sericytochromatia-bacterium]</td></tr>
<tr><td>BAZ90345.1</td><td>Type1.BREX</td><td><-SIGMA+OB-Fold+AAA-ATPase+HKD-DNase-BrxL<-PglZ<-HTH+REase<-N6-METHYLASE<-REase<-OLD-ABC<-PIN<-DUF433<-DUF6079*<-BrxB-BrxF-iSTAND||?->?-><-?<-?||?-><-?<-ABC-ATPase+ABC-ATPase<-NTP_transferase+MannoseP_isomer</td><td>1166</td><td>Bacteria>Terrabacteria_group>Cyanobacteriota/Melainabacteria_group>Cyanobacteriota>Cyanophyceae>Nostocales</td><td>GCA_002368135.1</td><td>Raphidiopsis_curvata_NIES-932</td><td>NIES932_18440</td><td>1957862..1961362</td><td>1973482</td><td>hypothetical-protein-NIES932_18440-[Raphidiopsis-curvata-NIES-932]</td></tr>
<tr><td>WP_006277878.1</td><td>Type1.BREX</td><td><-REase||?-><-SIGMA+OB-Fold+AAA-ATPase+HKD-DNase-BrxL<-PglZ<-HTH+REase<-N6-METHYLASE-p<-OLD-ABC<-PIN<-DUF433<-DUF6079*<-BrxB-BrxF-iSTAND||?->?-><-?<-?<-?<-ParA-Soj-PloopNTPase<-?||ACT-></td><td>1166</td><td>Bacteria>Terrabacteria_group>Cyanobacteriota/Melainabacteria_group>Cyanobacteriota>Cyanophyceae>Nostocales</td><td>GCF_000175835.1</td><td>Cylindrospermopsis_raciborskii</td><td>CRC_RS10895</td><td>11801..15301</td><td>77022</td><td>BREX-system-P-loop-protein-BrxC-[Cylindrospermopsis-raciborskii]</td></tr>
<tr><td>WP_057176814.1</td><td>Type1.BREX</td><td><-ACT||?->ParA-Soj-PloopNTPase->?->?->?-><-?<-?||BrxB-BrxF-iSTAND->DUF6079*->DUF433->PIN->OLD-ABC->REase->REase->N6-METHYLASE->HTH+REase->PglZ->SIGMA+OB-Fold+AAA-ATPase+HKD-DNase-BrxL-></td><td>1166</td><td>Bacteria>Terrabacteria_group>Cyanobacteriota/Melainabacteria_group>Cyanobacteriota>Cyanophyceae>Nostocales</td><td>GCF_001432185.1</td><td>Cylindrospermopsis_sp._CR12</td><td>ASL19_RS00210</td><td>43411..46911</td><td>1747196</td><td>BREX-system-P-loop-protein-BrxC-[Cylindrospermopsis-sp.-CR12]</td></tr>
<tr><td>WP_071242349.1</td><td>Type1.BREX</td><td><-ACT||?->ParA-Soj-PloopNTPase->?->?-><-?<-?||BrxB-BrxF-iSTAND->DUF6079*->DUF433->PIN->OLD-ABC->REase->N6-METHYLASE->HTH+REase->PglZ->SIGMA+OB-Fold+AAA-ATPase+HKD-DNase-BrxL->REase-></td><td>1166</td><td>Bacteria>Terrabacteria_group>Cyanobacteriota/Melainabacteria_group>Cyanobacteriota>Cyanophyceae>Nostocales</td><td>GCF_001858115.1</td><td>Cylindrospermopsis_raciborskii</td><td>BCV63_RS11865</td><td>45142..48642</td><td>77022</td><td>BREX-system-P-loop-protein-BrxC-[Cylindrospermopsis-raciborskii]</td></tr>
<tr><td>WP_071248143.1</td><td>Type1.BREX</td><td><-REase<-REase<-REase<-REase||?->BrxA-DNA-binding->BrxB-BrxF-iSTAND->DUF6079*->OLD-ABC->REase->N6-METHYLASE->HTH+REase->PglZ->SIGMA+OB-Fold+AAA-ATPase+HKD-DNase-BrxL-><-DsbD<-Thioredoxin</td><td>1172</td><td>Bacteria>Terrabacteria_group>Cyanobacteriota/Melainabacteria_group>Cyanobacteriota>Cyanophyceae>Nostocales</td><td>GCF_001858125.1</td><td>Cylindrospermopsis_raciborskii</td><td>CENA302_RS09780</td><td>17155..20673</td><td>77022</td><td>BREX-system-P-loop-protein-BrxC-[Cylindrospermopsis-raciborskii]</td></tr>
<tr><td>WP_102947438.1</td><td>Type1.BREX</td><td><-SIGMA+OB-Fold+AAA-ATPase+HKD-DNase-BrxL<-PglZ<-HTH+REase<-N6-METHYLASE<-REase<-OLD-ABC<-PIN<-DUF433<-DUF6079*<-BrxB-BrxF-iSTAND||?->?-><-?<-?<-?<-ParA-Soj-PloopNTPase<-?||ACT-></td><td>1166</td><td>Bacteria>Terrabacteria_group>Cyanobacteriota/Melainabacteria_group>Cyanobacteriota>Cyanophyceae>Nostocales</td><td>GCF_002893285.1</td><td>Cylindrospermopsis_raciborskii</td><td>CEP07_RS06090</td><td>15682..19182</td><td>77022</td><td>BREX-system-P-loop-protein-BrxC-[Cylindrospermopsis-raciborskii]</td></tr>
<tr><td>WP_187705577.1</td><td>Type1.BREX</td><td>NTP_transferase+MannoseP_isomer->ABC-ATPase+ABC-ATPase->?-><-?||?->?-><-?<-?||BrxB-BrxF-iSTAND->DUF6079*->DUF433->PIN->OLD-ABC->REase->N6-METHYLASE->HTH+REase->PglZ->SIGMA+OB-Fold+AAA-ATPase+HKD-DNase-BrxL-></td><td>1166</td><td>Bacteria>Terrabacteria_group>Cyanobacteriota/Melainabacteria_group>Cyanobacteriota>Cyanophyceae>Nostocales</td><td>GCF_014489415.1</td><td>Cylindrospermopsis_curvispora</td><td>IAR63_RS13115</td><td>2945663..2949163</td><td>747548</td><td>BREX-system-P-loop-protein-BrxC-[Cylindrospermopsis-curvispora]</td></tr>
<tr><td>WP_236308453.1</td><td>Type1.BREX</td><td><-SIGMA+OB-Fold+AAA-ATPase+HKD-DNase-BrxL<-PglZ<-HTH+REase<-N6-METHYLASE<-REase<-OLD-ABC<-PIN<-DUF433<-DUF6079*<-BrxB-BrxF-iSTAND||?->?-><-?<-?<-?<-ParA-Soj-PloopNTPase<-?||ACT-></td><td>1166</td><td>Bacteria>Terrabacteria_group>Cyanobacteriota/Melainabacteria_group>Cyanobacteriota>Cyanophyceae>Nostocales</td><td>GCF_021650815.1</td><td>Cylindrospermopsis_raciborskii</td><td>L3I90_RS00125</td><td>24709..28209</td><td>77022</td><td>BREX-system-P-loop-protein-BrxC-[Cylindrospermopsis-raciborskii]</td></tr>
<tr><td>WP_240869109.1</td><td>Type1.BREX</td><td>ART-DarT-><-SIGMA+OB-Fold+AAA-ATPase+HKD-DNase-BrxL<-PglZ<-HTH+REase<-N6-METHYLASE<-OLD-ABC<-PIN<-DUF433<-DUF6079*<-BrxB-BrxF-iSTAND||?->?-><-?<-?<-?<-ParA-Soj-PloopNTPase<-?||ACT-><-Peptidase_M17_N+Peptidase_M17</td><td>1166</td><td>Bacteria>Terrabacteria_group>Cyanobacteriota/Melainabacteria_group>Cyanobacteriota>Cyanophyceae>Nostocales</td><td>GCF_022496815.1</td><td>Cylindrospermopsis_raciborskii</td><td>GSN00_13735</td><td>14003..17503</td><td>77022</td><td>BREX-system-P-loop-protein-BrxC-[Cylindrospermopsis-raciborskii]</td></tr>
<tr><td>WP_156114218.1</td><td>Type1.BREX</td><td>DUF932->?-><-?<-?<-sGTP<-SIGMA+OB-Fold+AAA-ATPase+HKD-DNase-BrxL<-PglZ<-N6-METHYLASE<-DUF6079-p*<-?<-DUF6079-p<-BrxB-BrxF-iSTAND<-BrxA-DNA-binding||?->ParB+HNH->Thermonuclease->?->MazE_antitoxin-></td><td>835</td><td>Bacteria>Terrabacteria_group>Cyanobacteriota/Melainabacteria_group>Cyanobacteriota>Cyanophyceae>Pleurocapsales</td><td>GCF_000756305.1</td><td>Myxosarcina_sp._GI1</td><td>KV40_RS27950</td><td>18823..21330</td><td>1541065</td><td>BREX-system-P-loop-protein-BrxC-[Myxosarcina-sp.-GI1]</td></tr>
<tr><td>KPQ32454.1</td><td>Type1.BREX</td><td>BrxA-DNA-binding->BrxB-BrxF-iSTAND->DUF6079*->OLD-ABC->NERD->N6-METHYLASE->OLD-ABC->HNH->PglZ->SIGMA+OB-Fold+AAA-ATPase+HKD-DNase-BrxL-><-?||DDE_Tnp-></td><td>1200</td><td>Bacteria>Terrabacteria_group>Cyanobacteriota/Melainabacteria_group>Cyanobacteriota>Cyanophyceae>Pseudanabaenales</td><td>GCA_001314865.1</td><td>Phormidesmis_priestleyi_Ana</td><td>HLUCCA11_21420</td><td>1901..5503</td><td>1666911</td><td>Brex-phage-defense-system-ATPase-BrxC-[Phormidesmis-priestleyi-Ana]</td></tr>
<tr><td>WP_009625661.1</td><td>Type1.BREX</td><td><-CARF+HEPN<-ABhydrolase<-SAM-METHYLASE<-SIGMA+OB-Fold+AAA-ATPase+HKD-DNase-BrxL<-PglZ<-ParB+HNH<-N6-METHYLASE<-NERD<-OLD-ABC<-DUF6079*<-BrxB-BrxF-iSTAND<-BrxA-DNA-binding<-wHTH+REase+ZnR</td><td>1178</td><td>Bacteria>Terrabacteria_group>Cyanobacteriota/Melainabacteria_group>Cyanobacteriota>Cyanophyceae>Pseudanabaenales</td><td>GCF_000332215.1</td><td>Pseudanabaena_biceps</td><td>PSE7429_RS03435</td><td>33637..37173</td><td>927669</td><td>MULTISPECIES:-BREX-system-P-loop-protein-BrxC-[Pseudanabaena]</td></tr>
<tr><td>WP_121969249.1</td><td>Type6.BREX</td><td>CHTH->MazG->BrxE->BrxA-DNA-binding->BrxB-BrxF-iSTAND->DUF6079*->N6-METHYLASE->?->PIN->N6-METHYLASE-p->?->PglZ->SIGMA+OB-Fold+AAA-ATPase+HKD-DNase-BrxL-></td><td>1213</td><td>Bacteria>Terrabacteria_group>Cyanobacteriota/Melainabacteria_group>Cyanobacteriota>Cyanophyceae>Pseudanabaenales</td><td>GCF_002286735.1</td><td>Leptolyngbya_sp._BC1307</td><td>CKL79_RS08920</td><td>112124..115765</td><td>2029589</td><td>BREX-system-P-loop-protein-BrxC-[Leptolyngbya-sp.-BC1307]</td></tr>
<tr><td>WP_190797467.1</td><td>Type1.BREX</td><td><-SIGMA+OB-Fold+AAA-ATPase+HKD-DNase-BrxL<-PglZ<-ParB+HNH<-N6-METHYLASE<-NERD<-OLD-ABC<-DUF6079*<-BrxB-BrxF-iSTAND<-BrxA-DNA-binding<-ParB+HTH<-ParA-Soj-PloopNTPase||HTH+REase->?-><-?<-PrimPol<-RHH</td><td>1186</td><td>Bacteria>Terrabacteria_group>Cyanobacteriota/Melainabacteria_group>Cyanobacteriota>Cyanophyceae>Pseudanabaenales</td><td>GCF_014695875.1</td><td>Leptolyngbya_sp._FACHB-541</td><td>H6G00_RS15685</td><td>19029..22589</td><td>2692810</td><td>BREX-system-P-loop-protein-BrxC-[Leptolyngbya-sp.-FACHB-541]</td></tr>
<tr><td>WP_094560560.1</td><td>Type1.BREX</td><td><-SIGMA-HTH||HicB_lk_antitox->Unstab_antitox->wHTH+HNH+HIT->EVE+HNH->BrxA-DNA-binding->BrxB-BrxF-iSTAND->DUF6079*->N6-METHYLASE-><-?||PglZ->PhdYeFM_antitox->PIN->SIGMA+OB-Fold+AAA-ATPase+HKD-DNase-BrxL->?-><-SF1-SF2-Helicase<-HEPN+PAS+DUF4011+SF1-SF2-Helicase+REase</td><td>1161</td><td>Bacteria>Terrabacteria_group>Cyanobacteriota/Melainabacteria_group>Cyanobacteriota>Cyanophyceae>Synechococcales</td><td>GCF_002252665.1</td><td>Synechococcus_sp._8F6</td><td>CJZ78_RS08320</td><td>43413..46898</td><td>2025606</td><td>BREX-system-P-loop-protein-BrxC-[Synechococcus-sp.-8F6]</td></tr>
<tr><td>WP_188020741.1</td><td>Type1.BREX</td><td><-DUF4236||DOC+HTH->?->?-><-Sak4+WYL||?->?->BrxA-DNA-binding->BrxB-BrxF-iSTAND->DUF6079*->MNS+REase->N6-METHYLASE->PglZ->SIGMA+OB-Fold+AAA-ATPase+HKD-DNase-BrxL->HTH+REase-><-ZnR||?-><-SF1-SF2-Helicase<-sGTP+sGTP</td><td>1163</td><td>Bacteria>Terrabacteria_group>Cyanobacteriota/Melainabacteria_group>Cyanobacteriota>Cyanophyceae>Synechococcales</td><td>GCF_014279615.1</td><td>Synechococcus_sp._RS9907</td><td>SynRS9907_RS08975</td><td>1616845..1620336</td><td>221350</td><td>BREX-system-P-loop-protein-BrxC-[Synechococcus-sp.-RS9907]</td></tr>
<tr><td>WP_186471764.1</td><td>Type1.BREX</td><td>Peptidase_M28->?->?-><-Sak4+WYL||?->?->BrxA-DNA-binding->BrxB-BrxF-iSTAND->DUF6079*->N6-METHYLASE->N6-METHYLASE-><-SWACOS+DTXR-HTH||PglZ->SIGMA+OB-Fold+AAA-ATPase+HKD-DNase-BrxL->HTH+REase-><-ZnR||?-><-SF1-SF2-Helicase</td><td>1163</td><td>Bacteria>Terrabacteria_group>Cyanobacteriota/Melainabacteria_group>Cyanobacteriota>Cyanophyceae>Synechococcales</td><td>GCF_014279955.1</td><td>Synechococcus_sp._BMK-MC-1</td><td>SynBMKMC1_RS11005</td><td>1938101..1941592</td><td>1442551</td><td>BREX-system-P-loop-protein-BrxC-[Synechococcus-sp.-BMK-MC-1]</td></tr>
<tr><td>WP_186513675.1</td><td>Type1.BREX</td><td><-AAA+wHTH+WYL||?->?->BrxA-DNA-binding->BrxB-BrxF-iSTAND->DUF6079*->MNS+REase->N6-METHYLASE->?->HTH_3->Couple_hipA+HipA_C->PglZ->SIGMA+OB-Fold+AAA-ATPase+HKD-DNase-BrxL->HTH+REase-><-ZnR</td><td>1163</td><td>Bacteria>Terrabacteria_group>Cyanobacteriota/Melainabacteria_group>Cyanobacteriota>Cyanophyceae>Synechococcales</td><td>GCF_014280115.1</td><td>Synechococcus_sp._A15-44</td><td>SynA1544_02490</td><td>2002733..2006224</td><td>1050646</td><td>BREX-system-P-loop-protein-BrxC-[Synechococcus-sp.-A15-44]</td></tr>
<tr><td>WP_186696561.1</td><td>Type1.BREX</td><td>HIN-HTH->HAD+CHTH->?->ArdCN+MPTase->BrxA-DNA-binding->BrxB-BrxF-iSTAND->DUF6079*->N6-METHYLASE->PglZ->SIGMA+OB-Fold+AAA-ATPase+HKD-DNase-BrxL-><-REase||DUF4236+TPR-><-ABhydrolase<-PIN</td><td>1164</td><td>Bacteria>Terrabacteria_group>Cyanobacteriota/Melainabacteria_group>Cyanobacteriota>Cyanophyceae>Synechococcales</td><td>GCF_014280235.1</td><td>Cyanobium_sp._NS01</td><td>CyaNS01_RS07595</td><td>1454207..1457701</td><td>261284</td><td>BREX-system-P-loop-protein-BrxC-[Cyanobium-sp.-NS01]</td></tr>
<tr><td>WP_197170384.1</td><td>Type1.BREX</td><td><-Sak4+WYL||?->PIN->PIN-><-DUF772+DDE_Tnp_1+DDE_Tnp_1_6<-?||?->BrxA-DNA-binding->BrxB-BrxF-iSTAND->DUF6079*->N6-METHYLASE->GTPase-AIG->sGTP-><-SWACOS+DTXR-HTH||PglZ->PhdYeFM_antitox->PIN->SIGMA+OB-Fold+AAA-ATPase+HKD-DNase-BrxL-><-REase</td><td>1161</td><td>Bacteria>Terrabacteria_group>Cyanobacteriota/Melainabacteria_group>Cyanobacteriota>Cyanophyceae>Synechococcales</td><td>GCF_015840915.1</td><td>Synechococcus_sp._CBW1002</td><td>H8F24_RS17545</td><td>3558842..3562327</td><td>1353134</td><td>BREX-system-P-loop-protein-BrxC-[Synechococcus-sp.-CBW1002]</td></tr>
<tr><td>WP_250398658.1</td><td>Type1.BREX</td><td>Peptidase_S26->?-><-?||?->BrxA-DNA-binding->BrxB-BrxF-iSTAND->DUF6079*->N6-METHYLASE->PglZ->SIGMA+OB-Fold+AAA-ATPase+HKD-DNase-BrxL-></td><td>1163</td><td>Bacteria>Terrabacteria_group>Cyanobacteriota/Melainabacteria_group>Cyanobacteriota>Cyanophyceae>Synechococcales</td><td>GCF_023630615.1</td><td>Synechococcus_sp._MU1648</td><td>NCN06_RS03450</td><td>583154..586645</td><td>2508351</td><td>BREX-system-P-loop-protein-BrxC-[Synechococcus-sp.-MU1648]</td></tr>
<tr><td>WP_255087766.1</td><td>Type1.BREX</td><td>REase-><-SIGMA+OB-Fold+AAA-ATPase+HKD-DNase-BrxL<-PglZ<-sGTP<-GTPase-AIG<-N6-METHYLASE<-MNS+REase<-DUF6079*<-BrxB-BrxF-iSTAND<-BrxA-DNA-binding</td><td>1161</td><td>Bacteria>Terrabacteria_group>Cyanobacteriota/Melainabacteria_group>Cyanobacteriota>Cyanophyceae>Synechococcales</td><td>GCF_024345415.1</td><td>unclassified_Synechococcus</td><td>KBY92_RS03480</td><td>114966..118451</td><td>2626047</td><td>MULTISPECIES:-BREX-system-P-loop-protein-BrxC-[unclassified-Synechococcus]</td></tr>
<tr><td>WP_254967828.1</td><td>Type1.BREX</td><td><-PIN<-MazE_antitoxin||?-><-SIGMA+OB-Fold+AAA-ATPase+HKD-DNase-BrxL<-PIN<-PhdYeFM_antitox<-PglZ<-?<-N6-METHYLASE<-DUF6079*<-BrxB-BrxF-iSTAND<-BrxA-DNA-binding||DnaJ->?->?->?-><-?||?-><-PIN</td><td>1161</td><td>Bacteria>Terrabacteria_group>Cyanobacteriota/Melainabacteria_group>Cyanobacteriota>Cyanophyceae>Synechococcales</td><td>GCF_024346135.1</td><td>Cyanobium_sp._CH-040</td><td>KBY90_RS07090</td><td>13099..16584</td><td>2823708</td><td>BREX-system-P-loop-protein-BrxC-[Cyanobium-sp.-CH-040]</td></tr>
<tr><td>WP_255005473.1</td><td>Type1.BREX</td><td>PIN->PIN->?-><-DOC||?->ArdCN+MPTase->BrxA-DNA-binding->BrxB-BrxF-iSTAND->DUF6079*->N6-METHYLASE->PglZ->PhdYeFM_antitox->PIN->SIGMA+OB-Fold+AAA-ATPase+HKD-DNase-BrxL-><-REase<-TPR<-Peptidase</td><td>1161</td><td>Bacteria>Terrabacteria_group>Cyanobacteriota/Melainabacteria_group>Cyanobacteriota>Cyanophyceae>Synechococcales</td><td>GCF_024346315.1</td><td>Cyanobium_sp._ATX_6F1</td><td>KBZ13_RS02100</td><td>369855..373340</td><td>2823702</td><td>BREX-system-P-loop-protein-BrxC-[Cyanobium-sp.-ATX-6F1]</td></tr>
<tr><td>WP_254995750.1</td><td>Type1.BREX</td><td><-Sak4+WYL||?->?->?-><-SIGMA+OB-Fold+AAA-ATPase+HKD-DNase-BrxL<-PglZ<-Schlafen+HATPase+wHTH<-N6-METHYLASE<-DUF6079*<-BrxB-BrxF-iSTAND<-BrxA-DNA-binding<-ZnR||?->PolB->?->?->Peptidase->HicB-></td><td>1161</td><td>Bacteria>Terrabacteria_group>Cyanobacteriota/Melainabacteria_group>Cyanobacteriota>Cyanophyceae>Synechococcales</td><td>GCF_024346415.1</td><td>Cyanobium_sp._Aljojuca_7D2</td><td>KBY57_RS10265</td><td>32496..35981</td><td>2823698</td><td>BREX-system-P-loop-protein-BrxC-[Cyanobium-sp.-Aljojuca-7D2]</td></tr>
<tr><td>MBD1853918.1</td><td>Type1.BREX</td><td>HTH+REase->DDE_Tnp_IS240-><-HTH+REase||ParA-Soj-PloopNTPase->ParB+HTH->BrxA-DNA-binding->BrxB-BrxF-iSTAND->DUF6079*->OLD-ABC->NERD->N6-METHYLASE->ParB+HNH->PglZ->SIGMA+OB-Fold+AAA-ATPase+HKD-DNase-BrxL-></td><td>1186</td><td>Bacteria>Terrabacteria_group>Cyanobacteriota/Melainabacteria_group>Cyanobacteriota>unclassified_Cyanobacteriota</td><td>GCA_014695325.1</td><td>Cyanobacteria_bacterium_FACHB-502</td><td>H6F87_28810</td><td>26444..30004</td><td>2692765</td><td>BREX-system-P-loop-protein-BrxC-[Cyanobacteria-bacterium-FACHB-502]</td></tr>
<tr><td>MBU6197215.1</td><td>Type1.BREX</td><td>BrxA-DNA-binding->BrxB-BrxF-iSTAND->DUF6079*->OLD-ABC->N6-METHYLASE->PglZ->SIGMA+OB-Fold+AAA-ATPase+HKD-DNase-BrxL-></td><td>1179</td><td>Bacteria>Terrabacteria_group>Cyanobacteriota/Melainabacteria_group>Cyanobacteriota>unclassified_Cyanobacteriota</td><td>GCA_018970325.1</td><td>Cyanobacteria_bacterium_REEB446</td><td>KGO93_06600</td><td>1417..4956</td><td>2800105</td><td>BREX-system-P-loop-protein-BrxC-[Cyanobacteria-bacterium-REEB446]</td></tr>
<tr><td>WP_080803995.1</td><td>Type6.BREX</td><td>MerR-HTH->?-><-SIGMA+OB-Fold+AAA-ATPase+HKD-DNase-BrxL<-HTH_3<-?<-PglZ<-N6-METHYLASE<-?<-BrnT_toxin<-DUF6079*<-BrxB-BrxF-iSTAND<-BrxA-DNA-binding<-BrxE||?-><-?<-REC+PAS+HISKIN<-Shikimate_dh_N+SDH_C<-REC+OmpR-HTH<-BirA-HTH</td><td>1205</td><td>Bacteria>Thermodesulfobacteriota>Desulfobacteria>Desulfobacterales>Desulfobacteraceae>Desulfamplus</td><td>GCF_900170035.1</td><td>Desulfamplus_magnetovallimortis</td><td>MTBBW1_RS12175</td><td>100549..104166</td><td>1246637</td><td>BREX-system-P-loop-protein-BrxC-[Desulfamplus-magnetovallimortis]</td></tr>
<tr><td>WP_163354568.1</td><td>Type1.BREX</td><td><-35exo||GDResolvase+HIN-HTH-><-?<-HHH_5+SF1-SF2-Helicase||XIS-HTH->BrxA-DNA-binding->BrxB-BrxF-iSTAND->DUF6079*->OLD-ABC->REase->N6-METHYLASE->PglZ->SIGMA+OB-Fold+AAA-ATPase+HKD-DNase-BrxL-><-Zn_Tnp_IS91+Y2_Tnp</td><td>1187</td><td>Bacteria>Thermodesulfobacteriota>Desulfobacteria>Desulfobacterales>Desulfobacteraceae>Desulfobacter</td><td>-</td><td>Desulfobacter_hydrogenophilus</td><td>G3N28_17830</td><td>17749..21312</td><td>2291</td><td>BREX-system-P-loop-protein-BrxC-[Desulfobacter-hydrogenophilus]</td></tr>
<tr><td>NDY73873.1</td><td>Type1.BREX</td><td><-35exo||GDResolvase+HIN-HTH-><-?<-HHH_5+SF1-SF2-Helicase||XIS-HTH->BrxA-DNA-binding->BrxB-BrxF-iSTAND->DUF6079*->OLD-ABC->REase->N6-METHYLASE->PglZ->SIGMA+OB-Fold+AAA-ATPase+HKD-DNase-BrxL-><-Zn_Tnp_IS91+Y2_Tnp</td><td>1187</td><td>Bacteria>Thermodesulfobacteriota>Desulfobacteria>Desulfobacterales>Desulfobacteraceae>Desulfobacter</td><td>GCA_010499545.1</td><td>Desulfobacter_hydrogenophilus</td><td>G3N28_17830</td><td>17749..21312</td><td>2291</td><td>BREX-system-P-loop-protein-BrxC-[Desulfobacter-hydrogenophilus]</td></tr>
<tr><td>WP_035236115.1</td><td>Type1.BREX</td><td><-SIGMA+OB-Fold+AAA-ATPase+HKD-DNase-BrxL<-PglZ<-ParB+HNH<-N6-METHYLASE<-REase<-OLD-ABC<-DUF6079*<-BrxB-BrxF-iSTAND<-BrxA-DNA-binding<-XIS-HTH||?->?->EAL->S1+GTPase-AIG-></td><td>1189</td><td>Bacteria>Thermodesulfobacteriota>Desulfobacteria>Desulfobacterales>Desulfobacteraceae>Desulfobacter</td><td>GCF_000745975.1</td><td>Desulfobacter_vibrioformis</td><td>Q366_RS02915</td><td>253933..257502</td><td>34031</td><td>BREX-system-P-loop-protein-BrxC-[Desulfobacter-vibrioformis]</td></tr>
<tr><td>WP_111959628.1</td><td>Type1.BREX</td><td>Zn_Tnp_IS91+Y2_Tnp-><-SIGMA+OB-Fold+AAA-ATPase+HKD-DNase-BrxL<-PglZ<-N6-METHYLASE<-REase<-OLD-ABC<-DUF6079*<-BrxB-BrxF-iSTAND<-BrxA-DNA-binding<-XIS-HTH||HHH_5+SF1-SF2-Helicase->?-><-GDResolvase+HIN-HTH||35exo-></td><td>1188</td><td>Bacteria>Thermodesulfobacteriota>Desulfobacteria>Desulfobacterales>Desulfobacteraceae>Desulfobacter</td><td>GCF_004319545.1</td><td>Desulfobacter_hydrogenophilus</td><td>DO021_RS19095</td><td>11943..15509</td><td>2291</td><td>BREX-system-P-loop-protein-BrxC-[Desulfobacter-hydrogenophilus]</td></tr>
<tr><td>WP_178365089.1</td><td>Type1.BREX</td><td><-DDE_Tnp||?->?->PRTase->RADICAL-SAM->BrxA-DNA-binding->BrxB-BrxF-iSTAND->DUF6079*->N6-METHYLASE->OLD-ABC->HNH->PglZ->SIGMA+OB-Fold+AAA-ATPase+HKD-DNase-BrxL->tRNA-><-?<-PAS+NtrC-AAA+FIS-HTH||?->REase-></td><td>1189</td><td>Bacteria>Thermodesulfobacteriota>Desulfobacteria>Desulfobacterales>Desulfobacteraceae>Desulfobacter</td><td>GCF_013389365.1</td><td>Desulfobacter_latus</td><td>HXW94_RS01255</td><td>9015..12584</td><td>2292</td><td>BREX-system-P-loop-protein-BrxC-[Desulfobacter-latus]</td></tr>
<tr><td>MBT7628744.1</td><td>Type1.BREX</td><td>Phage_holin->BrxA-DNA-binding->BrxB-BrxF-iSTAND->DUF6079*->N6-METHYLASE->RAMA->PglZ->SIGMA+OB-Fold+AAA-ATPase+HKD-DNase-BrxL-></td><td>1196</td><td>Bacteria>Thermodesulfobacteriota>Desulfobacteria>Desulfobacterales>Desulfobacteraceae>Desulfobacula</td><td>GCA_018675165.1</td><td>Desulfobacula_sp.</td><td>HN597_03445</td><td>17169..20759</td><td>2593537</td><td>BREX-system-P-loop-protein-BrxC-[Desulfobacula-sp.]</td></tr>
<tr><td>WP_014958234.1</td><td>Type1.BREX</td><td><-SIGMA+OB-Fold+AAA-ATPase+HKD-DNase-BrxL<-PglZ<-?<-OLD-ABC<-ParB<-N6-METHYLASE<-DUF6079*<-BrxB-BrxF-iSTAND<-BrxA-DNA-binding||?->GspA-ATPase-></td><td>1193</td><td>Bacteria>Thermodesulfobacteriota>Desulfobacteria>Desulfobacterales>Desulfobacteraceae>Desulfobacula</td><td>GCF_000307105.1</td><td>Desulfobacula_toluolica</td><td>TOL2_RS15330</td><td>3369625..3373206</td><td>28223</td><td>BREX-system-P-loop-protein-BrxC-[Desulfobacula-toluolica]</td></tr>
<tr><td>WP_092229947.1</td><td>Type1.BREX</td><td><-Phage_integrase||?->DSBH->?-><-?||BrxA-DNA-binding->BrxB-BrxF-iSTAND->DUF6079*->N6-METHYLASE->ParB->OLD-ABC->?->PglZ->SIGMA+OB-Fold+AAA-ATPase+HKD-DNase-BrxL-></td><td>1193</td><td>Bacteria>Thermodesulfobacteriota>Desulfobacteria>Desulfobacterales>Desulfobacteraceae>Desulfobacula</td><td>GCF_900105645.1</td><td>Desulfobacula_phenolica</td><td>BLT94_RS02150</td><td>503008..506589</td><td>90732</td><td>BREX-system-P-loop-protein-BrxC-[Desulfobacula-phenolica]</td></tr>
<tr><td>WP_139446530.1</td><td>Type1.BREX</td><td><-SIGMA+OB-Fold+AAA-ATPase+Lon-Protease-BrxL||DiS_P_DiS+Peptidase_A24-><-?||?-><-SIGMA+OB-Fold+AAA-ATPase+HKD-DNase-BrxL<-PglZ<-HNH<-OLD-ABC<-N6-METHYLASE<-DUF6079*<-BrxB-BrxF-iSTAND<-BrxA-DNA-binding<-tRNA||?->?-><-?<-?<-HDRnase</td><td>1199</td><td>Bacteria>Thermodesulfobacteriota>Desulfobacteria>Desulfobacterales>Desulfobacteraceae>Desulfobotulus</td><td>GCF_006175995.1</td><td>Desulfobotulus_mexicanus</td><td>FIM25_RS03945</td><td>130812..134411</td><td>2586642</td><td>BREX-system-P-loop-protein-BrxC-[Desulfobotulus-mexicanus]</td></tr>
<tr><td>MCF8089162.1</td><td>Type6.BREX</td><td>ZetaToxin->?->?->BrxE->BrxA-DNA-binding->BrxB-BrxF-iSTAND->DUF6079*->N6-METHYLASE->OLD-ABC->PglZ->SIGMA+OB-Fold+AAA-ATPase+HKD-DNase-BrxL->?->SbcC-></td><td>1219</td><td>Bacteria>Thermodesulfobacteriota>Desulfobacteria>Desulfobacterales>Desulfobacteraceae>Desulfotignum</td><td>GCA_021737465.1</td><td>Desulfotignum_sp.</td><td>K9L23_14045</td><td>5260..8919</td><td>2049434</td><td>BREX-system-P-loop-protein-BrxC-[Desulfotignum-sp.]</td></tr>
<tr><td>RZB38310.1</td><td>Type1.BREX</td><td><-TPR+TPR<-?||?->?->?-><-?||?->BrxA-DNA-binding->BrxB-BrxF-iSTAND->DUF6079*->N6-METHYLASE-p->N6-METHYLASE->OLD-ABC->HNH->PglZ->SIGMA+OB-Fold+AAA-ATPase+HKD-DNase-BrxL-><-?<-?<-ABC-ATPase+SbcC</td><td>1173</td><td>Bacteria>Thermodesulfobacteriota>Desulfobacteria>Desulfobacterales>Desulfobacteraceae>unclassified_Desulfobacteraceae</td><td>GCA_004193595.1</td><td>Desulfobacteraceae_bacterium_Eth-SRB2</td><td>SRB2_00058</td><td>60586..64107</td><td>2528438</td><td>hypothetical-protein-SRB2_00058-[Desulfobacteraceae-bacterium-Eth-SRB2]</td></tr>
<tr><td>MCB9481003.1</td><td>Type1.BREX</td><td>HAMP+MA+MA->wHTH+WYL+WCX->?->?->?->?->SF1-SF2-Helicase+DUF1998->BrxA-DNA-binding->BrxB-BrxF-iSTAND->DUF6079*->N6-METHYLASE->PglZ-p->PglZ->SIGMA+OB-Fold+AAA-ATPase+HKD-DNase-BrxL->?->?->?-><-DOC+HTH<-Phage_integrase</td><td>1181</td><td>Bacteria>Thermodesulfobacteriota>Desulfobacteria>Desulfobacterales>Desulfobacteraceae>unclassified_Desulfobacteraceae</td><td>GCA_020633955.1</td><td>Desulfobacteraceae_bacterium</td><td>H6680_04155</td><td>92278..95823</td><td>2049433</td><td>BREX-system-P-loop-protein-BrxC-[Desulfobacteraceae-bacterium]</td></tr>
<tr><td>CBX26795.1</td><td>Type6.BREX</td><td><-rve<-SIGMA+OB-Fold+AAA-ATPase+HKD-DNase-BrxL<-PglZ<-Schlafen+HATPase+wHTH<-DrHyd<-N6-METHYLASE<-REase<-REase<-OLD-ABC<-DUF6079*<-BrxB-BrxF-iSTAND<-BrxA-DNA-binding<-BrxE||URI-DNase-><-tRNA<-?||?-><-PRTase<-DnaJ</td><td>1208</td><td>Bacteria>Thermodesulfobacteriota>Desulfobacteria>Desulfobacterales>Desulfobacteriaceae>Desulfobacterium</td><td>-</td><td>uncultured_Desulfobacterium_sp.</td><td>N47_A08240</td><td>98686..102312</td><td>201089</td><td>hypothetical-protein-N47_A08240-[uncultured-Desulfobacterium-sp.]</td></tr>
<tr><td>WP_236888848.1</td><td>Type1.BREX</td><td>DUF3670+SF1-SF2-Helicase->?->?->?->?->?->ZDNA-HTH->BrxA-DNA-binding->BrxB-BrxF-iSTAND->DUF6079*->N6-METHYLASE->PglZ->SIGMA+OB-Fold+AAA-ATPase+HKD-DNase-BrxL->GTPase-AIG->sGTP->?->JetB->SbcC+SidE-></td><td>1177</td><td>Bacteria>Thermodesulfobacteriota>Desulfobacteria>Desulfobacterales>Desulfolunaceae>Desulfoluna</td><td>GCF_021654575.1</td><td>Desulfoluna_limicola</td><td>L6434_RS14765</td><td>3845772..3849305</td><td>2810562</td><td>BREX-system-P-loop-protein-BrxC-[Desulfoluna-limicola]</td></tr>
<tr><td>WP_205228200.1</td><td>Type1.BREX</td><td><-ParB+HNH<-SbcC<-Calcineurin+Calcineurin-SbcD<-SbcC+SbcC<-SIGMA+OB-Fold+AAA-ATPase+HKD-DNase-BrxL<-PglZ<-N6-METHYLASE<-REase<-ABC-ATPase<-DUF6079*<-BrxB-BrxF-iSTAND<-BrxA-DNA-binding<-XIS-HTH<-?<-?<-?<-?<-wHTH</td><td>1182</td><td>Bacteria>Thermodesulfobacteriota>Desulfobulbia>Desulfobulbales>Desulfobulbaceae>Desulfobulbus</td><td>GCF_016919065.1</td><td>Desulfobulbus_rhabdoformis</td><td>JWJ90_RS15890</td><td>30219..33767</td><td>34032</td><td>BREX-system-P-loop-protein-BrxC-[Desulfobulbus-rhabdoformis]</td></tr>
<tr><td>WP_028584971.1</td><td>Type1.BREX</td><td><-ZnR<-SIGMA+OB-Fold+AAA-ATPase+HKD-DNase-BrxL<-PglZ<-PadR-HTH<-Schlafen+HATPase+wHTH<-N6-METHYLASE<-REase<-OLD-ABC<-DUF6079*<-BrxB-BrxF-iSTAND<-BrxA-DNA-binding<-?<-VirB11<-?<-PCMT+METHYLASE</td><td>1201</td><td>Bacteria>Thermodesulfobacteriota>Desulfobulbia>Desulfobulbales>Desulfobulbaceae>Desulfogranum</td><td>GCF_000429965.1</td><td>Desulfogranum_mediterraneum</td><td>G494_RS0113505</td><td>11738..15343</td><td>160661</td><td>BREX-system-P-loop-protein-BrxC-[Desulfogranum-mediterraneum]</td></tr>
<tr><td>OQX18971.1</td><td>Type6.BREX</td><td>ABC-ATPase+SbcC-><-?||?->PadR-HTH-><-SIGMA+OB-Fold+AAA-ATPase+HKD-DNase-BrxL<-PglZ<-N6-METHYLASE<-HATPase+wHTH<-DUF6079*<-BrxB-BrxF-iSTAND<-BrxA-DNA-binding<-BrxE-p</td><td>1229</td><td>Bacteria>Thermodesulfobacteriota>Desulfobulbia>Desulfobulbales>Desulfobulbaceae>unclassified_Desulfobulbaceae</td><td>GCA_002083975.1</td><td>Desulfobulbaceae_bacterium_A2</td><td>BWK76_05990</td><td>20716..24405</td><td>1934250</td><td>hypothetical-protein-BWK76_05990-[Desulfobulbaceae-bacterium-A2]</td></tr>
<tr><td>TKB07808.1</td><td>Type1.BREX</td><td><-HEPN<-SbcC<-Calcineurin+Calcineurin-SbcD<-SbcC+SbcC<-SIGMA+OB-Fold+AAA-ATPase+HKD-DNase-BrxL<-PglZ<-N6-METHYLASE<-REase<-OLD-ABC<-DUF6079*<-BrxB-BrxF-iSTAND<-BrxA-DNA-binding<-XIS-HTH<-?<-?<-?<-?<-wHTH</td><td>1183</td><td>Bacteria>Thermodesulfobacteriota>Desulfobulbia>Desulfobulbales>Desulfocapsaceae>Desulforhopalus</td><td>GCA_005116575.1</td><td>Desulforhopalus_sp._IMCC35007</td><td>FCL48_15775</td><td>80281..83832</td><td>2569543</td><td>BREX-system-P-loop-protein-BrxC-[Desulforhopalus-sp.-IMCC35007]</td></tr>
<tr><td>WP_169309248.1</td><td>Type1.BREX</td><td><-HEPN<-SbcC<-Calcineurin+Calcineurin-SbcD<-SbcC+SbcC<-SIGMA+OB-Fold+AAA-ATPase+HKD-DNase-BrxL<-PglZ<-N6-METHYLASE<-REase<-OLD-ABC<-DUF6079*<-BrxB-BrxF-iSTAND<-BrxA-DNA-binding<-XIS-HTH<-?<-?<-?<-?<-wHTH</td><td>1182</td><td>Bacteria>Thermodesulfobacteriota>Desulfobulbia>Desulfobulbales>Desulfocapsaceae>Desulforhopalus</td><td>GCA_005116575.1</td><td>Desulforhopalus_sp._IMCC35007</td><td>FCL48_RS15780</td><td>80281..83829</td><td>2569543</td><td>BREX-system-P-loop-protein-BrxC-[Desulforhopalus-sp.-IMCC35007]</td></tr>
<tr><td>BDV01246.1</td><td>Type6.BREX</td><td><-DTXR-HTH||?->BrxE->BrxA-DNA-binding->BrxB-BrxF-iSTAND->DUF6079*->N6-METHYLASE->N6-METHYLASE->OLD-ABC->TOPRIM->PglZ->SIGMA+OB-Fold+AAA-ATPase+HKD-DNase-BrxL->?-><-?||HAMP+MA+MA-></td><td>1210</td><td>Bacteria>Thermodesulfobacteriota>Desulfovibrionia>Desulfovibrionales>Desulfomicrobiaceae>Thermodesulfomicrobium</td><td>GCA_027925145.1</td><td>Thermodesulfomicrobium_sp._WS</td><td>TDMWS_13310</td><td>1427384..1431016</td><td>3004129</td><td>hypothetical-protein-TDMWS_13310-[Thermodesulfomicrobium-sp.-WS]</td></tr>
<tr><td>WP_035067990.1</td><td>Type1.BREX</td><td><-35exo<-?<-SbcC+SbcC<-SIGMA+OB-Fold+AAA-ATPase+HKD-DNase-BrxL<-PglZ<-N6-METHYLASE<-REase<-OLD-ABC<-DUF6079*<-BrxB-BrxF-iSTAND<-BrxA-DNA-binding<-wHTH+WYL+WCX||RHH_1->RelE-ParE-><-?||Phd-YefM->PIN-></td><td>1182</td><td>Bacteria>Thermodesulfobacteriota>Desulfovibrionia>Desulfovibrionales>Desulfovibrionaceae>Cupidesulfovibrio</td><td>GCF_000504305.1</td><td>Cupidesulfovibrio_termitidis</td><td>DESTE_RS12450</td><td>3095808..3099356</td><td>42252</td><td>BREX-system-P-loop-protein-BrxC-[Cupidesulfovibrio-termitidis]</td></tr>
<tr><td>AAS96500.1</td><td>Type1.BREX</td><td><-GGDEF<-ZnR+HTH_Tnp_ISL3+DDE_Tnp_ISL3<-SIGMA+OB-Fold+AAA-ATPase+HKD-DNase-BrxL<-PglZ<-REase<-N6-METHYLASE<-REase<-OLD-ABC<-DUF6079*<-BrxB-BrxF-iSTAND||?-><-wHTH+WYL+WCX-p||HIN-HTH->HIN-HTH-><-?||?->35exo-></td><td>1183</td><td>Bacteria>Thermodesulfobacteriota>Desulfovibrionia>Desulfovibrionales>Desulfovibrionaceae>Desulfovibrio</td><td>GCA_000195755.1</td><td>Desulfovibrio_vulgaris_str._Hildenborough</td><td>DVU_2025</td><td>2106300..2109851</td><td>882</td><td>conserved-hypothetical-protein-[Desulfovibrio-vulgaris-str.-Hildenborough]</td></tr>
<tr><td>ATD82493.1</td><td>Type1.BREX</td><td>zf-ISL3+HTH_Tnp_ISL3+DDE_Tnp_ISL3->?->?->?->BrxB-BrxF-iSTAND->BrxA-DNA-binding->BrxB-BrxF-iSTAND->DUF6079*->?->N6-METHYLASE->PglZ->SIGMA+OB-Fold+AAA-ATPase+HKD-DNase-BrxL->SbcC+SbcC->Calcineurin+Calcineurin-SbcD->SbcC->?->HIN-HTH+rve+rve_3-></td><td>1183</td><td>Bacteria>Thermodesulfobacteriota>Desulfovibrionia>Desulfovibrionales>Desulfovibrionaceae>Desulfovibrio</td><td>GCA_002334685.1</td><td>Desulfovibrio_sp._G11</td><td>CNY67_14735</td><td>3238216..3241767</td><td>631220</td><td>hypothetical-protein-CNY67_14735-[Desulfovibrio-sp.-G11]</td></tr>
<tr><td>GFH62752.1</td><td>Type6.BREX</td><td>HISKIN->?->?->SIGMA-HTH->ncRNA->?->?->BrxE->BrxA-DNA-binding->BrxB-BrxF-iSTAND->DUF6079*->N6-METHYLASE->OLD-ABC->NERD->PglZ->SIGMA+OB-Fold+AAA-ATPase+HKD-DNase-BrxL-></td><td>1209</td><td>Bacteria>Thermodesulfobacteriota>Desulfovibrionia>Desulfovibrionales>Desulfovibrionaceae>Desulfovibrio</td><td>GCA_013374015.1</td><td>Candidatus_Desulfovibrio_kirbyi</td><td>ZNDK_0523</td><td>67387..71016</td><td>2696086</td><td>BREX-system-P-loop-protein-[Candidatus-Desulfovibrio-kirbyi]</td></tr>
<tr><td>MCQ4861473.1</td><td>Type1.BREX</td><td>PAS+PAS+GGDEF-><-?<-?||PhdYeFM_antitox->BrxA-DNA-binding->BrxB-BrxF-iSTAND->DUF6079*->?->N6-METHYLASE->PglZ->SIGMA+OB-Fold+AAA-ATPase+HKD-DNase-BrxL->SbcC+SbcC->Calcineurin+Calcineurin-SbcD->SbcC->?->35exo-></td><td>1179</td><td>Bacteria>Thermodesulfobacteriota>Desulfovibrionia>Desulfovibrionales>Desulfovibrionaceae>Desulfovibrio</td><td>GCA_024460775.1</td><td>Desulfovibrio_desulfuricans</td><td>NE637_09955</td><td>25210..28749</td><td>876</td><td>BREX-system-P-loop-protein-BrxC-[Desulfovibrio-desulfuricans]</td></tr>
<tr><td>WP_014524353.1</td><td>Type1.BREX</td><td><-GGDEF<-ZnR+HTH_Tnp_ISL3+DDE_Tnp_ISL3<-SbcC<-SIGMA+OB-Fold+AAA-ATPase+HKD-DNase-BrxL<-PglZ<-REase<-N6-METHYLASE<-REase<-OLD-ABC<-DUF6079*<-BrxB-BrxF-iSTAND<-BrxA-DNA-binding<-wHTH+WYL+WCX-p||HIN-HTH-><-wHTH+WYL+WCX||?->35exo->?-><-RelE-ParE</td><td>1182</td><td>Bacteria>Thermodesulfobacteriota>Desulfovibrionia>Desulfovibrionales>Desulfovibrionaceae>Desulfovibrio</td><td>GCF_000195755.1</td><td>Desulfovibrio_vulgaris</td><td>DVU_RS09565</td><td>2106300..2109848</td><td>881</td><td>BREX-system-P-loop-protein-BrxC-[Desulfovibrio-vulgaris]</td></tr>
<tr><td>WP_043638458.1</td><td>Type1.BREX</td><td>NTP_transferase-><-?||GAF+GAF+HISKIN-><-?<-?||ParB+N6_N4_METHYLASE->?->BrxA-DNA-binding->BrxB-BrxF-iSTAND->DUF6079*->OLD-ABC->REase->N6-METHYLASE->PglZ->SIGMA+OB-Fold+AAA-ATPase+HKD-DNase-BrxL-></td><td>1182</td><td>Bacteria>Thermodesulfobacteriota>Desulfovibrionia>Desulfovibrionales>Desulfovibrionaceae>Desulfovibrio</td><td>GCF_000801335.2</td><td>Desulfovibrio_sp._TomC</td><td>NY78_RS16925</td><td>95476..99024</td><td>1562888</td><td>BREX-system-P-loop-protein-BrxC-[Desulfovibrio-sp.-TomC]</td></tr>
<tr><td>WP_256267712.1</td><td>Type1.BREX</td><td>PAS+PAS+GGDEF-><-?<-?||PhdYeFM_antitox->BrxA-DNA-binding->BrxB-BrxF-iSTAND->DUF6079*->?->N6-METHYLASE->PglZ->SIGMA+OB-Fold+AAA-ATPase+HKD-DNase-BrxL->SbcC+SbcC->Calcineurin+Calcineurin-SbcD->SbcC->?->35exo-></td><td>1179</td><td>Bacteria>Thermodesulfobacteriota>Desulfovibrionia>Desulfovibrionales>Desulfovibrionaceae>Desulfovibrio</td><td>GCF_900079715.1</td><td>Desulfovibrio_desulfuricans</td><td>NE637_09955</td><td>25210..28749</td><td>876</td><td>BREX-system-P-loop-protein-BrxC-[Desulfovibrio-desulfuricans]</td></tr>
<tr><td>WP_187008409.1</td><td>Type1.BREX</td><td><-HIN-HTH||zf-ISL3+HTH_Tnp_ISL3+DDE_Tnp_ISL3->?->?->?->BrxB-BrxF-iSTAND->DUF6079-p*->XIS-HTH->BrxA-DNA-binding->BrxB-BrxF-iSTAND->DUF6079*->?->N6-METHYLASE->PglZ->SIGMA+OB-Fold+AAA-ATPase+HKD-DNase-BrxL->SbcC+SbcC-></td><td>191</td><td>Bacteria>Thermodesulfobacteriota>Desulfovibrionia>Desulfovibrionales>Desulfovibrionaceae>Desulfovibrio</td><td>GCF_900243745.1</td><td>Desulfovibrio_sp._G11</td><td>DSVG11_RS05805</td><td>1310698..1311273</td><td>631220</td><td>DUF6079-family-protein-[Desulfovibrio-sp.-G11]</td></tr>
<tr><td>WP_072312019.1</td><td>Type1.BREX</td><td><-HIN-HTH||zf-ISL3+HTH_Tnp_ISL3+DDE_Tnp_ISL3->?->?->?->BrxB-BrxF-iSTAND->XIS-HTH->BrxA-DNA-binding->BrxB-BrxF-iSTAND->DUF6079*->?->N6-METHYLASE->PglZ->SIGMA+OB-Fold+AAA-ATPase+HKD-DNase-BrxL->SbcC+SbcC->Calcineurin+Calcineurin-SbcD->SbcC->?->HIN-HTH+rve+rve_3-></td><td>1182</td><td>Bacteria>Thermodesulfobacteriota>Desulfovibrionia>Desulfovibrionales>Desulfovibrionaceae</td><td>GCF_002334685.1</td><td>Desulfovibrio</td><td>CNY67_RS14735</td><td>3238219..3241767</td><td>872</td><td>MULTISPECIES:-BREX-system-P-loop-protein-BrxC-[Desulfovibrio]</td></tr>
<tr><td>PLX83270.1</td><td>Type1.BREX</td><td><-STYKinase-like||Thermonuclease-><-SIGMA+OB-Fold+AAA-ATPase+HKD-DNase-BrxL<-ZnR<-PglZ<-TOPRIM<-OLD-ABC<-N6-METHYLASE<-DUF6079*<-BrxB-BrxF-iSTAND<-BrxA-DNA-binding||?->?->?-><-?||?->GDResolvase+HIN-HTH-></td><td>1175</td><td>Bacteria>Thermodesulfobacteriota>Desulfuromonadia>Desulfuromonadales>Desulfuromonadaceae>Desulfuromonas</td><td>GCA_002868845.1</td><td>Desulfuromonas_sp.</td><td>C0617_12115</td><td>157475..161002</td><td>892</td><td>MAG:-BREX-system-P-loop-protein-BrxC-[Desulfuromonas-sp.]</td></tr>
<tr><td>KIH76252.1</td><td>Type6.BREX</td><td><-REase+wHTH||?-><-SIGMA+OB-Fold+AAA-ATPase+HKD-DNase-BrxL<-PglZ<-N6-METHYLASE<-DUF6079*<-BrxB-BrxF-iSTAND<-BrxA-DNA-binding<-BrxE<-ANK+ANK<-SIR2</td><td>1206</td><td>Bacteria>Thermodesulfobacteriota>Desulfuromonadia>Desulfuromonadales>Geoalkalibacteraceae>Geoalkalibacter</td><td>GCF_000820505.1</td><td>Geoalkalibacter_ferrihydriticus_DSM_17813</td><td>BLS07_RS00630</td><td>142762..146382</td><td>1121915</td><td>hypothetical-protein-GFER_11575-[Geoalkalibacter-ferrihydriticus-DSM-17813]</td></tr>
<tr><td>WP_040099782.1</td><td>Type6.BREX</td><td><-REase+wHTH||?-><-SIGMA+OB-Fold+AAA-ATPase+HKD-DNase-BrxL<-PglZ<-N6-METHYLASE<-DUF6079*<-BrxB-BrxF-iSTAND<-BrxA-DNA-binding<-BrxE<-ANK+ANK<-SIR2</td><td>1206</td><td>Bacteria>Thermodesulfobacteriota>Desulfuromonadia>Desulfuromonadales>Geoalkalibacteraceae>Geoalkalibacter</td><td>GCF_900103065.1</td><td>Geoalkalibacter_ferrihydriticus</td><td>BLS07_RS00630</td><td>142762..146382</td><td>392333</td><td>BREX-system-P-loop-protein-BrxC-[Geoalkalibacter-ferrihydriticus]</td></tr>
<tr><td>MBE0574417.1</td><td>Type1.BREX</td><td><-VirD4-TraG+VirD4-FtsK<-?<-?||XIS-HTH->BrxA-DNA-binding->BrxB-BrxF-iSTAND->DUF6079*->OLD-ABC->REase->N6-METHYLASE->REase->PglZ->SIGMA+OB-Fold+AAA-ATPase+HKD-DNase-BrxL->SbcC+APATPase+SbcC->Calcineurin+Calcineurin-SbcD->SbcC-></td><td>1183</td><td>Bacteria>Thermodesulfobacteriota>Desulfuromonadia>Desulfuromonadales>unclassified_Desulfuromonadales</td><td>GCA_014860225.1</td><td>Desulfuromonadales_bacterium</td><td>IH613_00815</td><td>71489..75040</td><td>2099678</td><td>BREX-system-P-loop-protein-BrxC-[Desulfuromonadales-bacterium]</td></tr>
<tr><td>AJY71182.1</td><td>Type1.BREX</td><td>NTP_transf_8->Calcineurin+Calcineurin-SbcD->SbcC+CCDC158->PIN-><-DUF4372+DDE_Tnp_1||HTH_Tnp_1->HTH_Tnp_1+HTH_21+rve+rve_3->BrxA-DNA-binding->BrxB-BrxF-iSTAND->DUF6079*->?->N6-METHYLASE->ParB+HNH->PglZ->SIGMA+OB-Fold+AAA-ATPase+HKD-DNase-BrxL->?->35exo+SF1-SF2-Helicase-><-HTH_3</td><td>1183</td><td>Bacteria>Thermodesulfobacteriota>Desulfuromonadia>Geobacterales>Geobacteraceae>Geobacter</td><td>GCA_000961035.1</td><td>Geobacter_sulfurreducens</td><td>RW64_17280</td><td>3775110..3778661</td><td>35554</td><td>hypothetical-protein-RW64_17280-[Geobacter-sulfurreducens]</td></tr>
<tr><td>WP_207162791.1</td><td>Type1.BREX</td><td><-Peptidase_C14<-TIR<-SIGMA+OB-Fold+AAA-ATPase+HKD-DNase-BrxL<-PglZ<-TOPRIM<-OLD-ABC<-N6-METHYLASE<-REase<-OLD-ABC<-DUF6079*<-BrxB-BrxF-iSTAND<-BrxA-DNA-binding<-AbiEi_4+AbiEi_1<-ParB<-ParB<-GDResolvase+PPP1-HTH||?-><-Arm-DNA-bind_3+Phage_integrase</td><td>1175</td><td>Bacteria>Thermodesulfobacteriota>Desulfuromonadia>Geobacterales>Geobacteraceae>Geobacter</td><td>GCF_017338855.1</td><td>Geobacter_benzoatilyticus</td><td>JZM60_RS12610</td><td>2741421..2744948</td><td>2815309</td><td>BREX-system-P-loop-protein-BrxC-[Geobacter-benzoatilyticus]</td></tr>
<tr><td>MBJ6802709.1</td><td>Type1.BREX</td><td>BrxA-DNA-binding->BrxB-BrxF-iSTAND->DUF6079*->OLD-ABC->REase->N6-METHYLASE->PglZ->SIGMA+OB-Fold+AAA-ATPase+HKD-DNase-BrxL->?-><-?<-NERD+ZnR<-wHTH+SAD+HNH</td><td>1183</td><td>Bacteria>Thermodesulfobacteriota>Desulfuromonadia>Geobacterales>Geobacteraceae>Geomonas</td><td>GCA_016458235.1</td><td>Geomonas_propionica</td><td>JFN90_21475</td><td>2703..6254</td><td>2798582</td><td>BREX-system-P-loop-protein-BrxC-[Geomonas-propionica]</td></tr>
<tr><td>WP_236019113.1</td><td>Type1.BREX</td><td>BrxA-DNA-binding->BrxB-BrxF-iSTAND->DUF6079*->OLD-ABC->REase->N6-METHYLASE->PglZ->SIGMA+OB-Fold+AAA-ATPase+HKD-DNase-BrxL->?-><-?<-NERD+ZnR<-wHTH+SAD+HNH</td><td>1191</td><td>Bacteria>Thermodesulfobacteriota>Desulfuromonadia>Geobacterales>Geobacteraceae>Geomonas</td><td>GCF_016458235.1</td><td>Geomonas_propionica</td><td>JFN90_RS21475</td><td>2679..6254</td><td>2798582</td><td>BREX-system-P-loop-protein-BrxC-[Geomonas-propionica]</td></tr>
</table>

Protein-ID	System	Gene-Neighbourhoods	Length	Taxonomy	Assembly-ID	Strain	Locus-Tag	Genomic-coordinates	TaxID	Fasta-defline
WP_216500608.1	Type6.BREX	Phage_integrase-><-?<- SIGMA+OB-Fold+AAA-ATPase+HKD-DNase-BrxL <- PglZ <- N6-METHYLASE <- DUF6079 *<- BrxB-BrxF-iSTAND <- BrxA-DNA-binding <- BrxE <-MORC+REase<-wHTH+REase+ZnR<-McrB-AAA<-HTH+HEPN<-SF1-SF2-Helicase<-STYKIN+CR-Kinase	1206	Bacteria>Thermodesulfobacteriota>Desulfuromonadia>Geobacterales>Geobacteraceae>Geomonas	GCF_018919385.1	Geomonas_diazotrophica	KOM00_RS19070	11745..15365	2843197	BREX-system-P-loop-protein-BrxC-[Geomonas-diazotrophica]
OPY81706.1	Type1.BREX	HIN-HTH->AAA-ATPase-><-DnaB+TnsC-ATPase<-?<-?<-XIS-HTH<-?|| BrxA-DNA-binding -> BrxB-BrxF-iSTAND -> DUF6079 *->Schlafen+HATPase+wHTH+HTH-> N6-METHYLASE ->OLD-ABC->TOPRIM-> PglZ -> SIGMA+OB-Fold+AAA-ATPase+HKD-DNase-BrxL ->RVT->HTH_Tnp_1->HTH_Tnp_1+HTH_21+rve+rve_3->	1200	Bacteria>Thermodesulfobacteriota>Syntrophia>Syntrophales>Syntrophaceae>Syntrophus	GCF_002067745.1	Syntrophus_sp._PtaU1.Bin005	A4E70_01072	12725..16327	1811712	MAG:-hypothetical-protein-A4E70_01072-[Syntrophus-sp.-PtaU1.Bin005]
KUO61378.1	Type1.BREX	<-Schlafen+HATPase+wHTH<- SIGMA+OB-Fold+AAA-ATPase+HKD-DNase-BrxL <- PglZ <-HTH+REase<-?<- N6-METHYLASE <- DUF6079 *<- BrxB-BrxF-iSTAND <- BrxA-DNA-binding <-HTH_Tnp_1+HTH_21+rve+rve_3	1157	Bacteria>unclassified_Bacteria	GCA_001516025.1	bacterium_BRH_c32	APF79_11995	11772..15245	1734400	hypothetical-protein-APF79_11995-[bacterium-BRH_c32]
TGQ70838.1	Type6.BREX	HKD-DNase+HKD-Rease->?-> BrxE -> BrxA-DNA-binding -> BrxB-BrxF-iSTAND -> DUF6079 *-> N6-METHYLASE -> N6-METHYLASE -> PglZ -> SIGMA+OB-Fold+AAA-ATPase+HKD-DNase-BrxL -><-?<-?<-CHTH	1211	Bacteria>unclassified_Bacteria	GCA_004791255.1	bacterium_M00.F.Ca.ET.205.01.1.1	EN848_08645	8202..11837	2563914	BREX-system-P-loop-protein-BrxC-[bacterium-M00.F.Ca.ET.205.01.1.1]
TGU46786.1	Type1.BREX	<- SIGMA+OB-Fold+AAA-ATPase+HKD-DNase-BrxL <- PglZ <- N6-METHYLASE <- DUF6079 *<- BrxB-BrxF-iSTAND <- BrxA-DNA-binding <- wHTH+WYL+WCX ||35Exo+REase->SF1-SF2-Helicase+REase->Small-B-barrel+SF1-SF2-Helicase+RdgC+REase->DUF6361->HKD-DNase->SF1-SF2-Helicase->	1179	Bacteria>unclassified_Bacteria	GCA_004791775.1	bacterium_M00.F.Ca.ET.152.01.1.1	EN795_30760	8346..11885	2563900	BREX-system-P-loop-protein-BrxC-[bacterium-M00.F.Ca.ET.152.01.1.1]
NCP08807.1	Type1.BREX	HTH_3-><-PIN<-wHTH<-?||CR-ATPase5+REase-> BrxA-DNA-binding -> BrxB-BrxF-iSTAND -> DUF6079 *->Schlafen+HATPase+wHTH+HTH->?->?->HTH+REase-> PglZ ->?-> SIGMA+OB-Fold+AAA-ATPase+HKD-DNase-BrxL ->	1186	Bacteria>unclassified_Bacteria	GCA_009993525.1	bacterium	GW860_07900	18171..21731	1869227	BREX-system-P-loop-protein-BrxC-[bacterium]
MBD5384945.1	Type1.BREX	<-Acyltransferase<-?<-?||HISKIN+MORC->REC+OmpR-HTH-> BrxA-DNA-binding -> BrxB-BrxF-iSTAND -> DUF6079 -p*-> N6-METHYLASE ->OLD-ABC->TOPRIM-> PglZ -> SIGMA+OB-Fold+AAA-ATPase+HKD-DNase-BrxL ->	843	Bacteria>unclassified_Bacteria	GCA_014802625.1	bacterium	HDR69_00765	93850..96381	1869227	BREX-system-P-loop-protein-BrxC-[bacterium]
MBI1293571.1	Type1.BREX	HHH_5+SF1-SF2-Helicase->?->?-><-Glyco_transf_28+UDPGT<-TetR-HTH<-PhdYeFM_antitox|| BrxA-DNA-binding -> BrxB-BrxF-iSTAND -> DUF6079 *->Y1_Tnp-> N6-METHYLASE ->REase->?->HTH_3->?-> PglZ -> SIGMA+OB-Fold+AAA-ATPase+HKD-DNase-BrxL -><-SF1-SF2-Helicase+REase	1173	Bacteria>unclassified_Bacteria	GCA_016124945.1	bacterium	GC175_01265	40562..44083	1869227	BREX-system-P-loop-protein-BrxC-[bacterium]
MBI9030472.1	Type1.BREX	REase+DUF5655-><-SF1-SF2-Helicase+PhospholipaseD<-?<- SIGMA+OB-Fold+AAA-ATPase+HKD-DNase-BrxL <- PglZ <-Schlafen+HATPase+wHTH<- N6-METHYLASE <- DUF6079 *<- BrxB-BrxF-iSTAND <- BrxA-DNA-binding	1162	Bacteria>unclassified_Bacteria	GCA_016342615.1	bacterium	JEZ13_00510	21679..25167	1869227	BREX-system-P-loop-protein-BrxC-[bacterium]
MBP7862612.1	Type1.BREX	HTH+MPTase->SLOG+BirA-HTH->?->Glycos_transf-><-?||?-><- SIGMA+OB-Fold+AAA-ATPase+HKD-DNase-BrxL <- PglZ <- N6-METHYLASE <- DUF6079 *<- BrxB-BrxF-iSTAND <- BrxA-DNA-binding <-?<-ABhydrolase||?->?-><-TPR+TPR+TPR+TPR+TPR<-Trypsin+PDZ||URI-DNase->	1181	Bacteria>unclassified_Bacteria	GCA_018001945.1	bacterium	KA183_13090	24829..28374	1869227	BREX-system-P-loop-protein-BrxC-[bacterium]
MBQ0129310.1	Type1.BREX	<- SIGMA+OB-Fold+AAA-ATPase+HKD-DNase-BrxL <-Schlafen+HATPase+wHTH<- PglZ <-?<-?<- N6-METHYLASE -p<-Schlafen+HATPase+wHTH<-SIR2<- N6-METHYLASE <- DUF6079 *<- BrxB-BrxF-iSTAND <- BrxA-DNA-binding	1169	Bacteria>unclassified_Bacteria	GCA_018065885.1	bacterium	KBT57_05675	15436..18945	1869227	BREX-system-P-loop-protein-BrxC-[bacterium]
MBS5529915.1	Type1.BREX	<- SIGMA+OB-Fold+AAA-ATPase+HKD-DNase-BrxL <- PglZ <-?<- N6-METHYLASE <-HD<- N6-METHYLASE -p<- DUF6079 *<- BrxB-BrxF-iSTAND <- BrxA-DNA-binding <-XIS-HTH	1169	Bacteria>unclassified_Bacteria	GCA_018369055.1	bacterium	KH017_03920	10993..14502	1869227	BREX-system-P-loop-protein-BrxC-[bacterium]
MCP3686167.1	Type5.BREX	SF1-SF2-Helicase->?-><- SIGMA+OB-Fold+AAA-ATPase+HKD-DNase-BrxL <- PglZ <- N6-METHYLASE <- DUF6079 *<- BrxB-BrxF-iSTAND <- BrxA-DNA-binding <-ANK+ANK<-?<-TUDOR+SF1-SF2-Helicase+iREase+CTD||?->SIR2-><-PRTase+PRTase<-HAD	1183	Bacteria>unclassified_Bacteria	GCA_024206195.1	bacterium	GY861_26265	21131..24682	1869227	BREX-system-P-loop-protein-BrxC-[bacterium]
MCH8487209.1	Type1.BREX	BrxA-DNA-binding -> BrxB-BrxF-iSTAND -> DUF6079 *-> N6-METHYLASE ->AbiGi+seqA+RAMA->?->SF1-SF2-Helicase->?-> PglZ -> SIGMA+OB-Fold+AAA-ATPase+HKD-DNase-BrxL ->HigB_toxin->HTH_3->	1177	-	GCA_022565315.1	-	LAT75_10090	1579..5112	2875933	BREX-system-P-loop-protein-BrxC-[Cyclonatronum-sp.]
--										
-- HKD-DNase+OGG-like										
--										
MCI0392588.1	Type1.BREX	NtrC-AAA+FIS-HTH-> BrxA-DNA-binding -> BrxB-BrxF-iSTAND -> DUF6079 *-> HKD-DNase+DUF3150-> N6-METHYLASE ->	1160	Bacteria>Acidobacteriota>unclassified_Acidobacteriota	GCA_022614935.1	Acidobacteriota_bacterium	MOB07_27970	3246..6728	1978231	BREX-system-P-loop-protein-BrxC-[Acidobacteriota-bacterium]
WP_183972373.1	Type1.BREX	D5ATPase->?->?-> HKD-DNase -><-?<-?<-?|| BrxA-DNA-binding -> BrxB-BrxF-iSTAND -> DUF6079 *->REase-> N6-METHYLASE -> HKD-DNase ->?->?->STYKIN->?->?->Schlafen+HATPase+wHTH->	1202	Bacteria>FCB_group>Bacteroidetes/Chlorobi_group>Bacteroidota>Cytophagia>Cytophagales	GCF_014195535.1	Runella_defluvii	FHS57_RS08095	111516..115124	370973	BREX-system-P-loop-protein-BrxC-[Runella-defluvii]
MBX6362419.1	Type6.BREX	Peptidase-><-?|| BrxE -> BrxA-DNA-binding -> BrxB-BrxF-iSTAND -> DUF6079 *-> N6-METHYLASE -> HKD-DNase ->?-> N6-METHYLASE -p->OLD-ABC->TOPRIM->?-> N6-METHYLASE -> PglZ ->	1201	Bacteria>FCB_group>Gemmatimonadota>unclassified_Gemmatimonadota	GCA_019689775.1	Gemmatimonadota_bacterium	IRZ00_01015	11407..15012	2026742	BREX-system-P-loop-protein-BrxC-[Gemmatimonadota-bacterium]
WP_036025094.1	Type6.BREX	<-BirA-HTH<-MetJarc-HTH<-UDG<-Ntox49<-Phage_AlpA|| BrxE -> BrxA-DNA-binding -> BrxB-BrxF-iSTAND -> DUF6079 *-> N6-METHYLASE -> N6-METHYLASE -> PglZ -> SIGMA+OB-Fold+AAA-ATPase+Lon-Protease-BrxL -> HKD-DNase -><-?<-?||?->CR-Trypsin1+HKD-DNase ->	1179	Bacteria>Pseudomonadota>Alphaproteobacteria>Hyphomicrobiales>Nitrobacteraceae>Bradyrhizobium	GCF_000261805.1	Bradyrhizobium_yuanmingense	IC68_RS15665	22684..26223	108015	BREX-system-P-loop-protein-BrxC-[Bradyrhizobium-yuanmingense]
WP_225048696.1	Type6.BREX	<-UDG<-Ntox49<-Phage_AlpA|| BrxE -> BrxA-DNA-binding -> BrxB-BrxF-iSTAND -><- N6-METHYLASE <-HTH_Tnp_IS630<- N6-METHYLASE <- DUF6079 -p*|| PglZ -> SIGMA+OB-Fold+AAA-ATPase+Lon-Protease-BrxL -> HKD-DNase -><-?<-?<-?||HTH_29+HTH_32+DDE_3->?->CR-Trypsin1+HKD-DNase ->	404	Bacteria>Pseudomonadota>Alphaproteobacteria>Hyphomicrobiales>Nitrobacteraceae>Bradyrhizobium	GCF_017565685.3	Bradyrhizobium_barranii	J4G48_RS15880	3299543..3300757	2992140	BREX-system-P-loop-protein-BrxC-[Bradyrhizobium-barranii]
WP_271557646.1	Type6.BREX	HKD-DNase+HKD-Rease->?-> BrxE -> BrxA-DNA-binding -> BrxB-BrxF-iSTAND -> DUF6079 *-> N6-METHYLASE -> PglZ -> SIGMA+OB-Fold+AAA-ATPase+Lon-Protease-BrxL ->	1176	Bacteria>Pseudomonadota>Alphaproteobacteria>Hyphomicrobiales>Nitrobacteraceae>Bradyrhizobium	GCF_028128655.1	Bradyrhizobium_sp._CCBAU_45394	WN73_RS39375	411495..415025	1325087	BREX-system-P-loop-protein-BrxC-[Bradyrhizobium-sp.-CCBAU-45394]
WP_012494683.1	Type6.BREX	<-TnpB_IS66<-?<-?<-?||?->CopG/RHH-> BrxE -> BrxA-DNA-binding -> BrxB-BrxF-iSTAND -> DUF6079 *-> N6-METHYLASE -> PglZ -> SIGMA+OB-Fold+AAA-ATPase+Lon-Protease-BrxL -> HKD-DNase -><-HisKin<-?<-HIRAN<-BirA-HTH	1177	Bacteria>Pseudomonadota>Alphaproteobacteria>Hyphomicrobiales>Nitrobacteraceae	GCF_000020445.1	Rhodopseudomonas	RPAL_RS05615	1188010..1191543	1073	MULTISPECIES:-BREX-system-P-loop-protein-BrxC-[Rhodopseudomonas]
RWM88364.1	Type6.BREX	<- N6-METHYLASE <- DUF6079 *<- BrxB-BrxF-iSTAND <- BrxA-DNA-binding <- BrxE <-?<- HKD-DNase+HKD-Rease<-Peptidase-M15<-REC+NtrC-AAA+FIS-HTH||Peptidase_M3_N+Peptidase_M3->	1211	Bacteria>Pseudomonadota>Alphaproteobacteria>Hyphomicrobiales>Phyllobacteriaceae>Mesorhizobium	GCA_004020365.1	Mesorhizobium_sp.	EOR84_28155	2131..5766	1871066	BREX-system-P-loop-protein-BrxC-[Mesorhizobium-sp.]
WP_068504545.1	Type1.BREX	<- HKD-DNase <-?<-?<- SIGMA+OB-Fold+AAA-ATPase+Lon-Protease-BrxL <- PglZ <- N6-METHYLASE <- DUF6079 *<- BrxB-BrxF-iSTAND <- BrxA-DNA-binding ||?->?->?->?->ZnR-><-Phage_integrase||ParA-Soj-PloopNTPase->	1205	Bacteria>Pseudomonadota>Alphaproteobacteria>Rhodospirillales>Rhodospirillaceae>Magnetospirillum	GCF_001650635.1	Magnetospirillum_moscoviense	A6A05_RS18805	69930..73547	1437059	BREX-system-P-loop-protein-BrxC-[Magnetospirillum-moscoviense]
MCG9059785.1	Type1.BREX	DDE_Tnp_1-><-?||ARG-><- SIGMA+OB-Fold+AAA-ATPase+Lon-Protease-BrxL <- PglZ <-TOPRIM<-OLD-ABC<- N6-METHYLASE <- HKD-DNase <- DUF6079 *<- BrxB-BrxF-iSTAND <- BrxA-DNA-binding <- wHTH+WYL+WCX ||||?->DOC-><-DUF772+DDE_Tnp_1||?->||AAA-ATPase-><-CRISPR_Cas6	1217	Bacteria>Pseudomonadota>Betaproteobacteria>Neisseriales>Chromobacteriaceae>Laribacter	GCA_022394335.1	Laribacter_hongkongensis	LH452_12755	30373..34026	168471	BREX-system-P-loop-protein-BrxC-[Laribacter-hongkongensis]
WP_239898705.1	Type1.BREX	DDE_Tnp_1-><-?||ARG-><- SIGMA+OB-Fold+AAA-ATPase+Lon-Protease-BrxL <- PglZ <-TOPRIM<-OLD-ABC<- N6-METHYLASE <- HKD-DNase <- DUF6079 *<- BrxB-BrxF-iSTAND <- BrxA-DNA-binding <- wHTH+WYL+WCX ||||?->DOC-><-DUF772+DDE_Tnp_1||?->||AAA-ATPase-><-Cas_Cas1	1222	Bacteria>Pseudomonadota>Betaproteobacteria>Neisseriales>Chromobacteriaceae>Laribacter	GCF_022394335.1	Laribacter_hongkongensis	LH452_RS12745	30373..34041	168471	BREX-system-P-loop-protein-BrxC-[Laribacter-hongkongensis]
WP_239891840.1	Type1.BREX	DDE_Tnp_1-><-?||ARG-><- SIGMA+OB-Fold+AAA-ATPase+Lon-Protease-BrxL <- PglZ <-TOPRIM<-OLD-ABC<- N6-METHYLASE <- HKD-DNase <- DUF6079 *<- BrxB-BrxF-iSTAND <- BrxA-DNA-binding <- wHTH+WYL+WCX ||||?->DOC-><-DUF772+DDE_Tnp_1||?->||AAA-ATPase-><-CRISPR_Cas6	1217	Bacteria>Pseudomonadota>Betaproteobacteria>Neisseriales>Chromobacteriaceae>Laribacter	-	Laribacter_hongkongensis	LH452_12755	30373..34026	168471	BREX-system-P-loop-protein-BrxC-[Laribacter-hongkongensis]
MBL8410223.1	Type1.BREX	TOPRIM->?-><-ZnR+TPR<- SIGMA+OB-Fold+AAA-ATPase+Lon-Protease-BrxL <- PglZ <-?<- HKD-DNase <- N6-METHYLASE <-Couple_hipA+HipA_C<-HTH_3<- DUF6079 *<- BrxB-BrxF-iSTAND <- BrxA-DNA-binding <-?||NACHT->?->HD+Tox-RelA->	1154	Bacteria>Pseudomonadota>Betaproteobacteria>Rhodocyclales>Azonexaceae>Dechloromonas	GCA_016791065.1	Dechloromonas_sp.	JNM15_02850	32710..36174	1917218	BREX-system-P-loop-protein-BrxC-[Dechloromonas-sp.]
CAH1748211.1	Type1.BREX	<-DUF3584+DUF5401<-JetB<-MukF<-?||Terminase_5+HTH_33+DDE_3-><-?|| wHTH+WYL+WCX -> BrxA-DNA-binding -> BrxB-BrxF-iSTAND -> DUF6079 *-> HKD-DNase -> N6-METHYLASE ->TIR->SbcC+NACHT->OLD-ABC->TOPRIM->PIWI-> PglZ -> SIGMA+OB-Fold+AAA-ATPase+Lon-Protease-BrxL ->	1203	Bacteria>Pseudomonadota>Betaproteobacteria>Rhodocyclales>Zoogloeaceae>Thauera	GCA_932126575.1	Thauera_sp._PIV-1	C4PIVTH_3071	3140142..3143753	204461	ATPase-like-protein-[Thauera-sp.-PIV-1]
WP_141354347.1	Type1.BREX	wHTH+WYL+WCX -> BrxA-DNA-binding -> BrxB-BrxF-iSTAND -> DUF6079 *-><-PIN<-RHH_6|| N6-METHYLASE -p-> HKD-DNase -> N6-METHYLASE ->HTH+REase->ZnR+TOPRIM-> PglZ -> SIGMA+OB-Fold+AAA-ATPase+Lon-Protease-BrxL ->	1203	Bacteria>Pseudomonadota>Betaproteobacteria>Rhodocyclales>Zoogloeaceae>Zoogloea	GCF_006539865.1	Zoogloea_ramigera	ZRA01_RS16820	2482..6093	350	BREX-system-P-loop-protein-BrxC-[Zoogloea-ramigera]
HCL6754282.1	Type1.BREX	<-HerA/FtsK+A32<-?||tRNA->Arm-DNA-bind_3+Phage_integrase-><- HKD-DNase+OGG-like <- SIGMA+OB-Fold+AAA-ATPase+Lon-Protease-BrxL <- PglZ <- N6-METHYLASE <- DUF6079 *<- BrxB-BrxF-iSTAND <- BrxA-DNA-binding <-ZnR<-?<-XIS-HTH<-?||RHH_antitoxin-><-URI-DNase<-Glyoxalase_8	1213	Bacteria>Pseudomonadota>Gammaproteobacteria>Enterobacterales>Enterobacteriaceae>Citrobacter	GCA_025039155.1	Citrobacter_freundii	N2R09_003052	112932..116573	546	BREX-system-P-loop-protein-BrxC-[Citrobacter-freundii]
WP_046487315.1	Type1.BREX	<-HIN-HTH||<-?||?->?-> BrxA-DNA-binding -> BrxB-BrxF-iSTAND -> DUF6079 *-> PglZ -> SIGMA+OB-Fold+AAA-ATPase+Lon-Protease-BrxL -> HKD-DNase+OGG-like ->||ParA-Soj-PloopNTPase->?->DnaB+TnsC-ATPase->ParB+BAM->HTH+HTH->HTH_36->	1213	Bacteria>Pseudomonadota>Gammaproteobacteria>Enterobacterales>Enterobacteriaceae>Citrobacter	GCF_000981805.1	Citrobacter_amalonaticus	F384_RS16675	3635289..3638930	35703	BREX-system-P-loop-protein-BrxC-[Citrobacter-amalonaticus]
WP_135912646.1	Type1.BREX	<-PAS+AraC-HTH+AraC-HTH||?->?->?-><-?->SymE_toxin-><-Glyoxalase_8|| BrxA-DNA-binding -> BrxB-BrxF-iSTAND -> DUF6079 *-> N6-METHYLASE -> PglZ -> SIGMA+OB-Fold+AAA-ATPase+Lon-Protease-BrxL -> HKD-DNase+OGG-like -><-REase<-?<-tRNA||wHTH+HNH->SAM-METHYLASE->	1213	Bacteria>Pseudomonadota>Gammaproteobacteria>Enterobacterales>Enterobacteriaceae>Citrobacter	GCF_004792475.1	Citrobacter_freundii	E5284_RS20140	4206675..4210316	546	BREX-system-P-loop-protein-BrxC-[Citrobacter-freundii]
WP_192466357.1	Type1.BREX	<-HerA/FtsK+A32<-?||tRNA->Arm-DNA-bind_3+Phage_integrase-><- HKD-DNase+OGG-like <- SIGMA+OB-Fold+AAA-ATPase+Lon-Protease-BrxL <- PglZ <- N6-METHYLASE <- DUF6079 *<- BrxB-BrxF-iSTAND <- BrxA-DNA-binding <-ZnR<-?<-XIS-HTH<-?||RHH_antitoxin-><-URI-DNase<-Glyoxalase_8	1213	Bacteria>Pseudomonadota>Gammaproteobacteria>Enterobacterales>Enterobacteriaceae>Citrobacter	GCF_014855915.1	Citrobacter_koseri	FOT44_RS18785	338007..341648	545	BREX-system-P-loop-protein-BrxC-[Citrobacter-koseri]
WP_200025358.1	Type1.BREX	Glyoxalase_8->URI-DNase-><-RHH_antitoxin||?->XIS-HTH->?->ZnR-> BrxA-DNA-binding -> BrxB-BrxF-iSTAND -> DUF6079 *-> N6-METHYLASE -> PglZ -> SIGMA+OB-Fold+AAA-ATPase+Lon-Protease-BrxL -> HKD-DNase+OGG-like -><-Arm-DNA-bind_3+Phage_integrase<-tRNA||?->HerA/FtsK+A32->	1213	Bacteria>Pseudomonadota>Gammaproteobacteria>Enterobacterales>Enterobacteriaceae>Citrobacter	GCF_016500865.1	Citrobacter_freundii	I5388_RS15310	359245..362886	546	BREX-system-P-loop-protein-BrxC-[Citrobacter-freundii]
WP_241511105.1	Type1.BREX	<-HerA/FtsK+A32<-?||tRNA->Arm-DNA-bind_3+Phage_integrase-><- HKD-DNase+OGG-like <- SIGMA+OB-Fold+AAA-ATPase+Lon-Protease-BrxL <- PglZ <-TOPRIM<-OLD-ABC<- N6-METHYLASE <- DUF6079 *<- BrxB-BrxF-iSTAND <- BrxA-DNA-binding ||NACHT+SF1-SF2-Helicase+HKD-DNase -><-?<-ZnR<-XIS-HTH	1197	Bacteria>Pseudomonadota>Gammaproteobacteria>Enterobacterales>Enterobacteriaceae>Citrobacter	GCF_022585175.1	Citrobacter_freundii	MMQ01_RS10405	144077..147670	546	BREX-system-P-loop-protein-BrxC-[Citrobacter-freundii]
WP_196010428.1	Type1.BREX	Glyoxalase_8->URI-DNase-><-RHH_antitoxin||?->XIS-HTH->?->ZnR-> BrxA-DNA-binding -> BrxB-BrxF-iSTAND -> DUF6079 *-> N6-METHYLASE -> PglZ -> SIGMA+OB-Fold+AAA-ATPase+Lon-Protease-BrxL -> HKD-DNase+OGG-like ->||<-REase<-Arm-DNA-bind_3+Phage_integrase	1213	Bacteria>Pseudomonadota>Gammaproteobacteria>Enterobacterales>Enterobacteriaceae>Citrobacter	GCF_029717585.1	Citrobacter_amalonaticus	NFJ54_RS22965	4694172..4697813	35703	MULTISPECIES:-BREX-system-P-loop-protein-BrxC-[Enterobacteriaceae]
WP_007729758.1	Type1.BREX	<-REC+LuxR-HTH<-SAM-METHYLASE<-wHTH+HNH||tRNA->Arm-DNA-bind_3+Phage_integrase-><- HKD-DNase+OGG-like <- SIGMA+OB-Fold+AAA-ATPase+Lon-Protease-BrxL <- PglZ <- N6-METHYLASE <- DUF6079 *<- BrxB-BrxF-iSTAND <- BrxA-DNA-binding <-HIN-HTH||REase->HTH_19->	1213	Bacteria>Pseudomonadota>Gammaproteobacteria>Enterobacterales>Enterobacteriaceae>Cronobacter	GCF_000409225.1	Cronobacter_dublinensis	AFK67_RS02785	592165..595806	413497	BREX-system-P-loop-protein-BrxC-[Cronobacter-dublinensis]
WP_105547575.1	Type1.BREX	Phage_Cox->XIS-HTH->AAA+wHTH->?-><-Zn_Tnp_IS1+HTH_Tnp_IS1+DDE_Tnp_IS1<- HKD-DNase+OGG-like <- SIGMA+OB-Fold+AAA-ATPase+Lon-Protease-BrxL <- PglZ <- N6-METHYLASE <- DUF6079 *<- BrxB-BrxF-iSTAND <- BrxA-DNA-binding ||nSTAND1+GTPase-AIG+sGTP->DUF932->wHTH+WYL->?->?->?->DUF905->	1213	Bacteria>Pseudomonadota>Gammaproteobacteria>Enterobacterales>Enterobacteriaceae>Cronobacter	GCF_002974775.1	Cronobacter_sakazakii	C5959_RS02070	327806..331447	28141	BREX-system-P-loop-protein-BrxC-[Cronobacter-sakazakii]
WP_163363286.1	Type1.BREX	Arm-DNA-bind_3+Phage_integrase-><- HKD-DNase+OGG-like <- SIGMA+OB-Fold+AAA-ATPase+Lon-Protease-BrxL <- PglZ <-TOPRIM<-OLD-ABC<- N6-METHYLASE <- DUF6079 *<- BrxB-BrxF-iSTAND <- BrxA-DNA-binding ||NACHT+SF1-SF2-Helicase+HKD-DNase -><-?<-ZnR<-ZnR<-XIS-HTH	1197	Bacteria>Pseudomonadota>Gammaproteobacteria>Enterobacterales>Enterobacteriaceae>Enterobacter	-	Enterobacter_cloacae	G4556_RS12910	16910..20503	550	BREX-system-P-loop-protein-BrxC-[Enterobacter-cloacae]
WP_203465577.1	Type1.BREX	nSTAND1+HNH->RNaseH->NACHT+SF1-SF2-Helicase+HKD-DNase ->REase-><- HKD-DNase+OGG-like <- SIGMA+OB-Fold+AAA-ATPase+Lon-Protease-BrxL <- PglZ <- N6-METHYLASE <- DUF6079 *<- BrxB-BrxF-iSTAND <- BrxA-DNA-binding <-SAM-METHYLASE<-wHTH+HNH||SymE_toxin-><-?||ASCH+Glyoxalase->	1213	Bacteria>Pseudomonadota>Gammaproteobacteria>Enterobacterales>Enterobacteriaceae>Enterobacter	-	Enterobacter_cloacae	GEA59_RS02615	551852..555493	550	BREX-system-P-loop-protein-BrxC-[Enterobacter-cloacae]
PWG69505.1	Type1.BREX	NACHT+SF1-SF2-Helicase+HKD-DNase ->REase-><- HKD-DNase+OGG-like <- SIGMA+OB-Fold+AAA-ATPase+Lon-Protease-BrxL <- PglZ <- N6-METHYLASE <- DUF6079 *<- BrxB-BrxF-iSTAND <- BrxA-DNA-binding <-SAM-METHYLASE<-wHTH+HNH||?->?->?->SymE_toxin->	1213	Bacteria>Pseudomonadota>Gammaproteobacteria>Enterobacterales>Enterobacteriaceae>Enterobacter	GCA_003129945.1	Enterobacter_mori	DEM28_19810	168758..172399	539813	BREX-system-P-loop-protein-BrxC-[Enterobacter-mori]
WP_021240656.1	Type1.BREX	<-SymE_toxin<-?<-?<-?||wHTH+HNH->SAM-METHYLASE-> BrxA-DNA-binding -> BrxB-BrxF-iSTAND -> DUF6079 *-> PglZ -> SIGMA+OB-Fold+AAA-ATPase+Lon-Protease-BrxL -> HKD-DNase+OGG-like -><-REase||SF1-SF2-Helicase->	1213	Bacteria>Pseudomonadota>Gammaproteobacteria>Enterobacterales>Enterobacteriaceae>Enterobacter	GCF_000467655.2	Enterobacter_roggenkampii	L799_RS145635	328329..331970	1812935	BREX-system-P-loop-protein-BrxC-[Enterobacter-roggenkampii]
WP_095453563.1	Type1.BREX	<-SymE_toxin<-?<-?<-?||wHTH+HNH->SAM-METHYLASE-> BrxA-DNA-binding -> BrxB-BrxF-iSTAND -> DUF6079 *-> N6-METHYLASE ->OLD-ABC->TOPRIM-> PglZ -> SIGMA+OB-Fold+AAA-ATPase+Lon-Protease-BrxL -> HKD-DNase+OGG-like -><-REase||SF1-SF2-Helicase->	1213	Bacteria>Pseudomonadota>Gammaproteobacteria>Enterobacterales>Enterobacteriaceae>Enterobacter	GCF_002278355.1	Enterobacter_cloacae	CIW57_RS16410	135960..139601	550	BREX-system-P-loop-protein-BrxC-[Enterobacter-cloacae]
WP_057055966.1	Type1.BREX	Arm-DNA-bind_3+Phage_integrase-><- HKD-DNase+OGG-like <- SIGMA+OB-Fold+AAA-ATPase+Lon-Protease-BrxL <- PglZ <-TOPRIM<-OLD-ABC<- N6-METHYLASE <- DUF6079 *<- BrxB-BrxF-iSTAND <- BrxA-DNA-binding ||NACHT+SF1-SF2-Helicase+HKD-DNase -><-?<-ZnR<-ZnR<-XIS-HTH	1197	Bacteria>Pseudomonadota>Gammaproteobacteria>Enterobacterales>Enterobacteriaceae>Enterobacter	GCF_014169295.1	Enterobacter_cloacae_complex	H7R61_RS03370	684757..688350	354276	MULTISPECIES:-BREX-system-P-loop-protein-BrxC-[Enterobacter-cloacae-complex]
WP_248201800.1	Type1.BREX	Glyoxalase_8->URI-DNase-><-RHH_antitoxin||?->XIS-HTH->?->ZnR-> BrxA-DNA-binding -> BrxB-BrxF-iSTAND -> DUF6079 *-> N6-METHYLASE -> PglZ -> SIGMA+OB-Fold+AAA-ATPase+Lon-Protease-BrxL -> HKD-DNase+OGG-like -><-Arm-DNA-bind_3+Phage_integrase	1213	Bacteria>Pseudomonadota>Gammaproteobacteria>Enterobacterales>Enterobacteriaceae>Enterobacter	GCF_023151575.1	Enterobacter_asburiae	L8T09_RS18420	27571..31212	61645	BREX-system-P-loop-protein-BrxC-[Enterobacter-asburiae]
WP_192468115.1	Type1.BREX	URI-DNase-><-HIN-HTH<-RHH_antitoxin||?->XIS-HTH->?->ZnR-> BrxA-DNA-binding -> BrxB-BrxF-iSTAND -> DUF6079 *-> N6-METHYLASE -> PglZ -> SIGMA+OB-Fold+AAA-ATPase+Lon-Protease-BrxL -> HKD-DNase+OGG-like -><-Arm-DNA-bind_3+Phage_integrase	1213	Bacteria>Pseudomonadota>Gammaproteobacteria>Enterobacterales>Enterobacteriaceae>Enterobacter	GCF_023749245.1	Enterobacter_asburiae	M8N38_RS13285	26000..29641	61645	BREX-system-P-loop-protein-BrxC-[Enterobacter-asburiae]
WP_257246766.1	Type1.BREX	<-ASCH+Glyoxalase||?->DUF2754->?-><-SymE_toxin||wHTH+SAD+HNH-><-NACHT+SF1-SF2-Helicase+HKD-DNase || BrxA-DNA-binding -> BrxB-BrxF-iSTAND -> DUF6079 *-> N6-METHYLASE ->OLD-ABC->TOPRIM-> PglZ -> SIGMA+OB-Fold+AAA-ATPase+Lon-Protease-BrxL -> HKD-DNase+OGG-like ->	1197	Bacteria>Pseudomonadota>Gammaproteobacteria>Enterobacterales>Enterobacteriaceae>Enterobacter	GCF_024607805.1	unclassified_Enterobacter	NR293_RS15365	69785..73378	2608935	MULTISPECIES:-BREX-system-P-loop-protein-BrxC-[unclassified-Enterobacter]
WP_058688926.1	Type1.BREX	nSTAND1+HNH->RNaseH->NACHT+SF1-SF2-Helicase+HKD-DNase ->REase-><- HKD-DNase+OGG-like <- SIGMA+OB-Fold+AAA-ATPase+Lon-Protease-BrxL <- PglZ <- N6-METHYLASE <- DUF6079 *<- BrxB-BrxF-iSTAND <- BrxA-DNA-binding <-SAM-METHYLASE<-wHTH+HNH||SymE_toxin-><-?<-?<-PAAR_motif+Pyocin_S+ColE7+HNH	1213	Bacteria>Pseudomonadota>Gammaproteobacteria>Enterobacterales>Enterobacteriaceae>Enterobacter	GCF_024655335.1	Enterobacter_hormaechei	ASU86_RS07715	183720..187361	158836	BREX-system-P-loop-protein-BrxC-[Enterobacter-hormaechei]
WP_274416986.1	Type1.BREX	XIS-HTH->ZnR->ZnR->?-><-NACHT+SF1-SF2-Helicase+HKD-DNase || BrxA-DNA-binding -> BrxB-BrxF-iSTAND -> DUF6079 *-> N6-METHYLASE ->OLD-ABC->TOPRIM-> PglZ -> SIGMA+OB-Fold+AAA-ATPase+Lon-Protease-BrxL -> HKD-DNase+OGG-like -><-Arm-DNA-bind_3+Phage_integrase<-Arm-DNA-bind_3+Phage_integrase	1204	Bacteria>Pseudomonadota>Gammaproteobacteria>Enterobacterales>Enterobacteriaceae>Enterobacter	GCF_028765355.1	Enterobacter_kobei	PVM09_RS00190	32375..35989	208224	BREX-system-P-loop-protein-BrxC-[Enterobacter-kobei]
WP_227303300.1	Type1.BREX	<-Phage_integrase<-Phage_integrase||?->?->?->||wHTH+SAD+HNH-><-REase+iREase|| BrxA-DNA-binding -> BrxB-BrxF-iSTAND -> DUF6079 *-> N6-METHYLASE -> PglZ -> SIGMA+OB-Fold+AAA-ATPase+Lon-Protease-BrxL -> HKD-DNase+OGG-like ->||AbiEi_3_N+AbiEi_3->AbiEii->?-><-DUF6216	1213	Bacteria>Pseudomonadota>Gammaproteobacteria>Enterobacterales>Enterobacteriaceae>Escherichia	-	Escherichia_coli	LJD42_RS04270	217680..221321	562	BREX-system-P-loop-protein-BrxC-[Escherichia-coli]
EHW21311.1	Type1.BREX	<-AbiEii<-AbiEi_3_N+AbiEi_3<- HKD-DNase+OGG-like ||?-><- SIGMA+OB-Fold+AAA-ATPase+Lon-Protease-BrxL <-Zn_Tnp_IS91+Y2_Tnp<- N6-METHYLASE <- DUF6079 *||HIN-HTH->HTH_Tnp_1+HTH_21+rve+rve_3-><- BrxA-DNA-binding ||REase+iREase->REase-><-wHTH+SAD+HNH||HNH->	1001	Bacteria>Pseudomonadota>Gammaproteobacteria>Enterobacterales>Enterobacteriaceae>Escherichia	GCA_000249875.2	Escherichia_coli_DEC8E	ECDEC8E_5320	144024..147029	868174	putative-DNA-repair-ATPase-[Escherichia-coli-DEC8E]
EHX52156.1	Type1.BREX	DUF6216-><-?<-AbiEii<-AbiEi_3_N+AbiEi_3<- HKD-DNase+OGG-like <- SIGMA+OB-Fold+AAA-ATPase+Lon-Protease-BrxL <- PglZ <- N6-METHYLASE -p<- N6-METHYLASE <- DUF6079 -p*<- DUF6079 -p<- BrxB-BrxF-iSTAND <- BrxA-DNA-binding ||?->REase+iREase-><-wHTH+SAD+HNH||HNH->	788	Bacteria>Pseudomonadota>Gammaproteobacteria>Enterobacterales>Enterobacteriaceae>Escherichia	GCA_000250315.2	Escherichia_coli_DEC13A	ECDEC13A_0072	76997..79363	868196	putative-DNA-repair-ATPase-[Escherichia-coli-DEC13A]
EHX55090.1	Type1.BREX	<-AbiEii<-AbiEi_3_N+AbiEi_3<- HKD-DNase+OGG-like <- SIGMA+OB-Fold+AAA-ATPase+Lon-Protease-BrxL <- PglZ <- N6-METHYLASE -p<- N6-METHYLASE -p<- N6-METHYLASE <- DUF6079 -p*<- DUF6079 -p<- BrxB-BrxF-iSTAND <- BrxA-DNA-binding ||REase+iREase-><-wHTH+SAD+HNH||HNH->	804	Bacteria>Pseudomonadota>Gammaproteobacteria>Enterobacterales>Enterobacteriaceae>Escherichia	GCA_000250335.2	Escherichia_coli_DEC13B	ECDEC13B_4632	100761..103175	868197	putative-DNA-repair-ATPase-[Escherichia-coli-DEC13B]
CTT26978.1	Type1.BREX	DUF6079 *-> N6-METHYLASE -> N6-METHYLASE -p-> PglZ -> SIGMA+OB-Fold+AAA-ATPase+Lon-Protease-BrxL -> HKD-DNase+OGG-like ->AbiEi_3_N+AbiEi_3->AbiEii-><-DUF6216<-Arm-DNA-bind_3	1088	Bacteria>Pseudomonadota>Gammaproteobacteria>Enterobacterales>Enterobacteriaceae>Escherichia	GCA_001283645.1	Escherichia_coli	ERS085343_03077	214..3480	562	ATPase-like-protein-[Escherichia-coli]
CTW38723.1	Type1.BREX	DUF6079 -p*-> N6-METHYLASE -> PglZ -> SIGMA+OB-Fold+AAA-ATPase+Lon-Protease-BrxL -> HKD-DNase+OGG-like ->AbiEi_3_N+AbiEi_3->AbiEii-><-DUF6216<-Arm-DNA-bind_3	788	Bacteria>Pseudomonadota>Gammaproteobacteria>Enterobacterales>Enterobacteriaceae>Escherichia	GCA_001283765.1	Escherichia_coli	ERS085413_04497	243..2609	562	ATPase-like-protein-[Escherichia-coli]
CUA46950.1	Type1.BREX	DUF6079 *-> N6-METHYLASE -> N6-METHYLASE -p-> PglZ -> SIGMA+OB-Fold+AAA-ATPase+Lon-Protease-BrxL -> HKD-DNase+OGG-like ->AbiEi_3_N+AbiEi_3->AbiEii-><-DUF6216<-Arm-DNA-bind_3	1088	Bacteria>Pseudomonadota>Gammaproteobacteria>Enterobacterales>Enterobacteriaceae>Escherichia	GCA_001286505.1	Escherichia_coli	ERS139261_03500	135..3401	562	ATPase-like-protein-[Escherichia-coli]
EEG9579271.1	Type1.BREX	DUF6079 *-> N6-METHYLASE -> PglZ -> SIGMA+OB-Fold+AAA-ATPase+Lon-Protease-BrxL -> HKD-DNase+OGG-like ->||AbiEi_3_N+AbiEi_3->AbiEii->?-><-DUF6216<-Arm-DNA-bind_3	1111	Bacteria>Pseudomonadota>Gammaproteobacteria>Enterobacterales>Enterobacteriaceae>Escherichia	GCA_003736085.2	Escherichia_coli	EB576_RS15540	1..3336	562	BREX-system-P-loop-protein-BrxC-[Escherichia-coli]
EED0771052.1	Type1.BREX	BrxA-DNA-binding -> BrxB-BrxF-iSTAND -> DUF6079 *-> N6-METHYLASE -> PglZ -> SIGMA+OB-Fold+AAA-ATPase+Lon-Protease-BrxL -> HKD-DNase+OGG-like ->||AbiEi_3_N+AbiEi_3->AbiEii-><-DUF6216<-Arm-DNA-bind_3	1213	Bacteria>Pseudomonadota>Gammaproteobacteria>Enterobacterales>Enterobacteriaceae>Escherichia	GCA_003747865.2	Escherichia_coli	ED219_RS15230	935..4576	562	BREX-system-P-loop-protein-BrxC-[Escherichia-coli]
EED1762068.1	Type1.BREX	DUF6079 *-> PglZ -> SIGMA+OB-Fold+AAA-ATPase+Lon-Protease-BrxL -> HKD-DNase+OGG-like ->||AbiEi_3_N+AbiEi_3->AbiEii-><-DUF6216<-Arm-DNA-bind_3<-tRNA||?->HerA/FtsK+A32->	1073	Bacteria>Pseudomonadota>Gammaproteobacteria>Enterobacterales>Enterobacteriaceae>Escherichia	GCA_003786715.2	Escherichia_coli	EEP34_RS13370	1..3223	562	BREX-system-P-loop-protein-BrxC-[Escherichia-coli]
EEC9685943.1	Type1.BREX	DUF6079 -p*-> N6-METHYLASE -> PglZ -> SIGMA+OB-Fold+AAA-ATPase+Lon-Protease-BrxL -> HKD-DNase+OGG-like ->||AbiEi_3_N+AbiEi_3->AbiEii-><-DUF6216<-Arm-DNA-bind_3	989	Bacteria>Pseudomonadota>Gammaproteobacteria>Enterobacterales>Enterobacteriaceae>Escherichia	GCA_004231765.2	Escherichia_coli	D8K54_RS17535	1..2971	562	BREX-system-P-loop-protein-BrxC-[Escherichia-coli]
EEC7405250.1	Type1.BREX	<-Phage_integrase||?->?->?-><-HNH||wHTH+SAD+HNH-><-REase+iREase|| BrxA-DNA-binding -> BrxB-BrxF-iSTAND -> DUF6079 *-> N6-METHYLASE -> PglZ -> SIGMA+OB-Fold+AAA-ATPase+Lon-Protease-BrxL -> HKD-DNase+OGG-like ->||AbiEi_3_N+AbiEi_3->AbiEii-><-DUF6216<-Arm-DNA-bind_3	1213	Bacteria>Pseudomonadota>Gammaproteobacteria>Enterobacterales>Enterobacteriaceae>Escherichia	GCA_004272605.2	Escherichia_coli	D4L89_RS08005	71644..75285	562	BREX-system-P-loop-protein-BrxC-[Escherichia-coli]
KAA9812768.1	Type1.BREX	DUF6079 -p*-> N6-METHYLASE -> PglZ -> SIGMA+OB-Fold+AAA-ATPase+Lon-Protease-BrxL -> HKD-DNase+OGG-like ->||AbiEi_3_N+AbiEi_3->AbiEii-><-DUF6216<-Arm-DNA-bind_3	891	Bacteria>Pseudomonadota>Gammaproteobacteria>Enterobacterales>Enterobacteriaceae>Escherichia	GCA_008755545.1	Escherichia_coli	F7F73_23785	1..2676	562	BREX-system-P-loop-protein-BrxC,-partial-[Escherichia-coli]
EEQ1797639.1	Type1.BREX	DUF6079 -p*-> N6-METHYLASE -> PglZ -> SIGMA+OB-Fold+AAA-ATPase+Lon-Protease-BrxL -> HKD-DNase+OGG-like ->||AbiEi_3_N+AbiEi_3->AbiEii-><-DUF6216<-Arm-DNA-bind_3	861	Bacteria>Pseudomonadota>Gammaproteobacteria>Enterobacterales>Enterobacteriaceae>Escherichia	GCA_011802225.1	Escherichia_coli	GUY43_002901	1..2587	562	BREX-system-P-loop-protein-BrxC-[Escherichia-coli]
EEQ2509736.1	Type1.BREX	BrxA-DNA-binding -> BrxB-BrxF-iSTAND -> DUF6079 *-> N6-METHYLASE -> PglZ -> SIGMA+OB-Fold+AAA-ATPase+Lon-Protease-BrxL -> HKD-DNase+OGG-like ->||AbiEi_3_N+AbiEi_3->AbiEii-><-DUF6216<-Arm-DNA-bind_3	1213	Bacteria>Pseudomonadota>Gammaproteobacteria>Enterobacterales>Enterobacteriaceae>Escherichia	GCA_011806325.1	Escherichia_coli	GSE75_002859	1085..4726	562	BREX-system-P-loop-protein-BrxC-[Escherichia-coli]
EEQ7780471.1	Type1.BREX	<-HNH||wHTH+SAD+HNH-><-REase+iREase|| BrxA-DNA-binding -> BrxB-BrxF-iSTAND -> DUF6079 *-> N6-METHYLASE -> PglZ -> SIGMA+OB-Fold+AAA-ATPase+Lon-Protease-BrxL -> HKD-DNase+OGG-like ->||AbiEi_3_N+AbiEi_3->AbiEii-><-DUF6216<-Arm-DNA-bind_3	1213	Bacteria>Pseudomonadota>Gammaproteobacteria>Enterobacterales>Enterobacteriaceae>Escherichia	GCA_011830195.1	Escherichia_coli	GLS89_002414	7714..11355	562	BREX-system-P-loop-protein-BrxC-[Escherichia-coli]
EER7097196.1	Type1.BREX	DUF6079 *-> N6-METHYLASE -> PglZ -> SIGMA+OB-Fold+AAA-ATPase+Lon-Protease-BrxL -> HKD-DNase+OGG-like ->||AbiEi_3_N+AbiEi_3->AbiEii-><-DUF6216<-Arm-DNA-bind_3	1064	Bacteria>Pseudomonadota>Gammaproteobacteria>Enterobacterales>Enterobacteriaceae>Escherichia	GCA_011867305.1	Escherichia_coli	F2Z67_003270	1..3197	562	BREX-system-P-loop-protein-BrxC-[Escherichia-coli]
EER7518149.1	Type1.BREX	DUF6079 -p*-> N6-METHYLASE -> PglZ -> SIGMA+OB-Fold+AAA-ATPase+Lon-Protease-BrxL -> HKD-DNase+OGG-like ->||AbiEi_3_N+AbiEi_3->AbiEii-><-DUF6216<-Arm-DNA-bind_3	990	Bacteria>Pseudomonadota>Gammaproteobacteria>Enterobacterales>Enterobacteriaceae>Escherichia	GCA_011869725.1	Escherichia_coli	F1Z98_003251	1..2974	562	BREX-system-P-loop-protein-BrxC-[Escherichia-coli]
EER8632679.1	Type1.BREX	DUF6079 *-> N6-METHYLASE -> PglZ -> SIGMA+OB-Fold+AAA-ATPase+Lon-Protease-BrxL -> HKD-DNase+OGG-like ->||AbiEi_3_N+AbiEi_3->AbiEii-><-DUF6216<-Arm-DNA-bind_3	1080	Bacteria>Pseudomonadota>Gammaproteobacteria>Enterobacterales>Enterobacteriaceae>Escherichia	GCA_011873745.1	Escherichia_coli	F0E18_004248	1..3245	562	BREX-system-P-loop-protein-BrxC-[Escherichia-coli]
EES3817132.1	Type1.BREX	DUF6079 -p*-> N6-METHYLASE -> PglZ -> SIGMA+OB-Fold+AAA-ATPase+Lon-Protease-BrxL -> HKD-DNase+OGG-like ->||AbiEi_3_N+AbiEi_3->AbiEii-><-DUF6216<-Arm-DNA-bind_3	952	Bacteria>Pseudomonadota>Gammaproteobacteria>Enterobacterales>Enterobacteriaceae>Escherichia	GCA_011896505.1	Escherichia_coli	FS433_003751	1..2860	562	BREX-system-P-loop-protein-BrxC-[Escherichia-coli]
EES8430772.1	Type1.BREX	DUF6079 -p*-> N6-METHYLASE -> PglZ -> SIGMA+OB-Fold+AAA-ATPase+Lon-Protease-BrxL -> HKD-DNase+OGG-like ->||AbiEi_3_N+AbiEi_3->AbiEii-><-DUF6216<-Arm-DNA-bind_3	988	Bacteria>Pseudomonadota>Gammaproteobacteria>Enterobacterales>Enterobacteriaceae>Escherichia	GCA_011915225.1	Escherichia_coli	FU162_003513	1..2968	562	BREX-system-P-loop-protein-BrxC-[Escherichia-coli]
EET5967869.1	Type1.BREX	DUF6079 *-> N6-METHYLASE -> PglZ -> SIGMA+OB-Fold+AAA-ATPase+Lon-Protease-BrxL -> HKD-DNase+OGG-like ->||AbiEi_3_N+AbiEi_3->AbiEii-><-DUF6216<-Arm-DNA-bind_3	1065	Bacteria>Pseudomonadota>Gammaproteobacteria>Enterobacterales>Enterobacteriaceae>Escherichia	GCA_011942865.1	Escherichia_coli	FE570_001648	1..3198	562	BREX-system-P-loop-protein-BrxC-[Escherichia-coli]
EEU4094847.1	Type1.BREX	DUF6079 -p*-> N6-METHYLASE -> PglZ -> SIGMA+OB-Fold+AAA-ATPase+Lon-Protease-BrxL -> HKD-DNase+OGG-like ->||AbiEi_3_N+AbiEi_3->AbiEii-><-DUF6216<-Arm-DNA-bind_3	779	Bacteria>Pseudomonadota>Gammaproteobacteria>Enterobacterales>Enterobacteriaceae>Escherichia	GCA_011974935.1	Escherichia_coli	E8H53_003141	1..2340	562	BREX-system-P-loop-protein-BrxC-[Escherichia-coli]
EEV0352856.1	Type1.BREX	DUF6079 *-> N6-METHYLASE -> PglZ -> SIGMA+OB-Fold+AAA-ATPase+Lon-Protease-BrxL -> HKD-DNase+OGG-like ->||AbiEi_3_N+AbiEi_3->AbiEii-><-DUF6216<-Arm-DNA-bind_3	1194	Bacteria>Pseudomonadota>Gammaproteobacteria>Enterobacterales>Enterobacteriaceae>Escherichia	GCA_012011485.1	Escherichia_coli	EYE11_003099	1..3587	562	BREX-system-P-loop-protein-BrxC-[Escherichia-coli]
EEV8657615.1	Type1.BREX	DUF6079 *-> N6-METHYLASE -> PglZ -> SIGMA+OB-Fold+AAA-ATPase+Lon-Protease-BrxL -> HKD-DNase+OGG-like ->||AbiEi_3_N+AbiEi_3->AbiEii-><-DUF6216<-Arm-DNA-bind_3	1180	Bacteria>Pseudomonadota>Gammaproteobacteria>Enterobacterales>Enterobacteriaceae>Escherichia	GCA_012058765.1	Escherichia_coli	EHJ50_001183	1..3543	562	BREX-system-P-loop-protein-BrxC-[Escherichia-coli]
EEW0980710.1	Type1.BREX	<-Phage_integrase||?->?->?-><-HNH||wHTH+SAD+HNH-><-REase+iREase|| BrxA-DNA-binding -> BrxB-BrxF-iSTAND -> DUF6079 *-> N6-METHYLASE -> PglZ -> SIGMA+OB-Fold+AAA-ATPase+Lon-Protease-BrxL -> HKD-DNase+OGG-like ->||AbiEi_3_N+AbiEi_3->AbiEii-><-DUF6216<-Arm-DNA-bind_3	1213	Bacteria>Pseudomonadota>Gammaproteobacteria>Enterobacterales>Enterobacteriaceae>Escherichia	GCA_012068555.1	Escherichia_coli	ED289_06180	111258..114899	562	BREX-system-P-loop-protein-BrxC-[Escherichia-coli]
EEW3089662.1	Type1.BREX	<-Phage_integrase<-Phage_integrase||?->?->?-><-HNH||wHTH(SBD)+HNH->|| BrxA-DNA-binding -> BrxB-BrxF-iSTAND -> DUF6079 *-> N6-METHYLASE -> PglZ -> SIGMA+OB-Fold+AAA-ATPase+Lon-Protease-BrxL -> HKD-DNase+OGG-like ->||AbiEi_3_N+AbiEi_3->AbiEii-><-DUF6216<-Arm-DNA-bind_3	1213	Bacteria>Pseudomonadota>Gammaproteobacteria>Enterobacterales>Enterobacteriaceae>Escherichia	GCA_012078325.1	Escherichia_coli	EBA98_002639	14697..18338	562	BREX-system-P-loop-protein-BrxC-[Escherichia-coli]
EEW3885152.1	Type1.BREX	<-HNH||wHTH(SBD)+HNH->|| BrxA-DNA-binding -> BrxB-BrxF-iSTAND -> DUF6079 *-> N6-METHYLASE -> PglZ -> SIGMA+OB-Fold+AAA-ATPase+Lon-Protease-BrxL -> HKD-DNase+OGG-like ->||AbiEi_3_N+AbiEi_3->AbiEii-><-DUF6216<-Arm-DNA-bind_3	1213	Bacteria>Pseudomonadota>Gammaproteobacteria>Enterobacterales>Enterobacteriaceae>Escherichia	GCA_012080795.1	Escherichia_coli	D9Y07_001392	7166..10807	562	BREX-system-P-loop-protein-BrxC-[Escherichia-coli]
EEW4281070.1	Type1.BREX	DUF6079 *-> N6-METHYLASE -> PglZ -> SIGMA+OB-Fold+AAA-ATPase+Lon-Protease-BrxL -> HKD-DNase+OGG-like ->||AbiEi_3_N+AbiEi_3->AbiEii-><-DUF6216<-Arm-DNA-bind_3	1085	Bacteria>Pseudomonadota>Gammaproteobacteria>Enterobacterales>Enterobacteriaceae>Escherichia	GCA_012082755.1	Escherichia_coli	D9T01_23345	1..3259	562	BREX-system-P-loop-protein-BrxC-[Escherichia-coli]
EEW6030677.1	Type1.BREX	DUF6079 *-> PglZ -> SIGMA+OB-Fold+AAA-ATPase+Lon-Protease-BrxL -> HKD-DNase+OGG-like ->||AbiEi_3_N+AbiEi_3->AbiEii-><-DUF6216<-Arm-DNA-bind_3<-tRNA||?->HerA/FtsK+A32->	1178	Bacteria>Pseudomonadota>Gammaproteobacteria>Enterobacterales>Enterobacteriaceae>Escherichia	GCA_012088895.1	Escherichia_coli	D7V62_16715	1..3539	562	BREX-system-P-loop-protein-BrxC-[Escherichia-coli]
EEX1719682.1	Type1.BREX	<-HNH||wHTH(SBD)+HNH->|| BrxA-DNA-binding -> BrxB-BrxF-iSTAND -> DUF6079 *-> N6-METHYLASE -> PglZ -> SIGMA+OB-Fold+AAA-ATPase+Lon-Protease-BrxL -> HKD-DNase+OGG-like ->||AbiEi_3_N+AbiEi_3->AbiEii-><-DUF6216<-Arm-DNA-bind_3	1213	Bacteria>Pseudomonadota>Gammaproteobacteria>Enterobacterales>Enterobacteriaceae>Escherichia	GCA_012115095.1	Escherichia_coli	D3S79_003407	6685..10326	562	BREX-system-P-loop-protein-BrxC-[Escherichia-coli]
EEX4954053.1	Type1.BREX	BrxB-BrxF-iSTAND -> DUF6079 *-> N6-METHYLASE -> PglZ -> SIGMA+OB-Fold+AAA-ATPase+Lon-Protease-BrxL -> HKD-DNase+OGG-like ->||AbiEi_3_N+AbiEi_3->AbiEii->	1213	Bacteria>Pseudomonadota>Gammaproteobacteria>Enterobacterales>Enterobacteriaceae>Escherichia	GCA_012126795.1	Escherichia_coli	D0791_003833	606..4247	562	BREX-system-P-loop-protein-BrxC-[Escherichia-coli]
EEX6773445.1	Type1.BREX	DUF6079 -p*-> N6-METHYLASE -> PglZ -> SIGMA+OB-Fold+AAA-ATPase+Lon-Protease-BrxL -> HKD-DNase+OGG-like ->||AbiEi_3_N+AbiEi_3->AbiEii-><-DUF6216<-Arm-DNA-bind_3	987	Bacteria>Pseudomonadota>Gammaproteobacteria>Enterobacterales>Enterobacteriaceae>Escherichia	GCA_012133985.1	Escherichia_coli	DYL55_002403	1..2965	562	BREX-system-P-loop-protein-BrxC-[Escherichia-coli]
EEX7673116.1	Type1.BREX	DUF6079 *-> N6-METHYLASE -> PglZ -> SIGMA+OB-Fold+AAA-ATPase+Lon-Protease-BrxL -> HKD-DNase+OGG-like ->||AbiEi_3_N+AbiEi_3->AbiEii-><-DUF6216<-Arm-DNA-bind_3	1156	Bacteria>Pseudomonadota>Gammaproteobacteria>Enterobacterales>Enterobacteriaceae>Escherichia	GCA_012137685.1	Escherichia_coli	DX034_003680	1..3471	562	BREX-system-P-loop-protein-BrxC-[Escherichia-coli]
EEY1277168.1	Type1.BREX	DUF6079 *-> N6-METHYLASE -> PglZ -> SIGMA+OB-Fold+AAA-ATPase+Lon-Protease-BrxL -> HKD-DNase+OGG-like ->||AbiEi_3_N+AbiEi_3->AbiEii->?-><-DUF6216<-Arm-DNA-bind_3	1189	Bacteria>Pseudomonadota>Gammaproteobacteria>Enterobacterales>Enterobacteriaceae>Escherichia	GCA_012153105.1	Escherichia_coli	BFU70_002920	1..3572	562	BREX-system-P-loop-protein-BrxC-[Escherichia-coli]
EEY1600790.1	Type1.BREX	<-Phage_integrase||?->?->?-><-HNH||wHTH+SAD+HNH-><-REase+iREase|| BrxA-DNA-binding -> BrxB-BrxF-iSTAND -> DUF6079 *-> N6-METHYLASE -> PglZ -> SIGMA+OB-Fold+AAA-ATPase+Lon-Protease-BrxL -> HKD-DNase+OGG-like ->||AbiEi_3_N+AbiEi_3->AbiEii-><-DUF6216<-Arm-DNA-bind_3	1213	Bacteria>Pseudomonadota>Gammaproteobacteria>Enterobacterales>Enterobacteriaceae>Escherichia	GCA_012154025.1	Escherichia_coli	DP662_003727	9743..13384	562	BREX-system-P-loop-protein-BrxC-[Escherichia-coli]
EEY2596885.1	Type1.BREX	DUF6079 *-> N6-METHYLASE -> PglZ -> SIGMA+OB-Fold+AAA-ATPase+Lon-Protease-BrxL -> HKD-DNase+OGG-like ->||AbiEi_3_N+AbiEi_3->AbiEii-><-DUF6216<-tRNA||?->HerA/FtsK+A32->	1032	Bacteria>Pseudomonadota>Gammaproteobacteria>Enterobacterales>Enterobacteriaceae>Escherichia	GCA_012158085.1	Escherichia_coli	E9W20_002908	1..3101	562	BREX-system-P-loop-protein-BrxC-[Escherichia-coli]
EEY5880446.1	Type1.BREX	<-Phage_integrase||?->?->?-><-HNH||wHTH+SAD+HNH-><-REase+iREase|| BrxA-DNA-binding -> BrxB-BrxF-iSTAND -> DUF6079 *-> N6-METHYLASE -> PglZ -> SIGMA+OB-Fold+AAA-ATPase+Lon-Protease-BrxL -> HKD-DNase+OGG-like ->||AbiEi_3_N+AbiEi_3->AbiEii-><-DUF6216<-Arm-DNA-bind_3	1213	Bacteria>Pseudomonadota>Gammaproteobacteria>Enterobacterales>Enterobacteriaceae>Escherichia	GCA_012171595.1	Escherichia_coli	EU637_10820	71590..75231	562	BREX-system-P-loop-protein-BrxC-[Escherichia-coli]
EEZ3165536.1	Type1.BREX	DUF6079 *-> N6-METHYLASE -> PglZ -> SIGMA+OB-Fold+AAA-ATPase+Lon-Protease-BrxL -> HKD-DNase+OGG-like ->||AbiEi_3_N+AbiEi_3->AbiEii-><-DUF6216<-Arm-DNA-bind_3	1136	Bacteria>Pseudomonadota>Gammaproteobacteria>Enterobacterales>Enterobacteriaceae>Escherichia	GCA_012200425.1	Escherichia_coli	DIN68_003954	1..3411	562	BREX-system-P-loop-protein-BrxC-[Escherichia-coli]
EEZ5289151.1	Type1.BREX	<-Phage_integrase||?->?->?-><-HNH||wHTH+SAD+HNH-><-REase+iREase|| BrxA-DNA-binding -> BrxB-BrxF-iSTAND -> DUF6079 *-> N6-METHYLASE -> PglZ -> SIGMA+OB-Fold+AAA-ATPase+Lon-Protease-BrxL -> HKD-DNase+OGG-like ->||AbiEi_3_N+AbiEi_3->AbiEii-><-DUF6216<-Arm-DNA-bind_3	1213	Bacteria>Pseudomonadota>Gammaproteobacteria>Enterobacterales>Enterobacteriaceae>Escherichia	GCA_012210255.1	Escherichia_coli	DDL85_000894	111149..114790	562	BREX-system-P-loop-protein-BrxC-[Escherichia-coli]
EEZ5688036.1	Type1.BREX	<-Phage_integrase||?->?->?-><-HNH||wHTH+SAD+HNH-><-REase+iREase|| BrxA-DNA-binding -> BrxB-BrxF-iSTAND -> DUF6079 *-> N6-METHYLASE -> PglZ -> SIGMA+OB-Fold+AAA-ATPase+Lon-Protease-BrxL -> HKD-DNase+OGG-like ->||AbiEi_3_N+AbiEi_3->AbiEii-><-DUF6216<-Arm-DNA-bind_3	1213	Bacteria>Pseudomonadota>Gammaproteobacteria>Enterobacterales>Enterobacteriaceae>Escherichia	GCA_012215265.1	Escherichia_coli_O25	DBU11_001474	113366..117007	1095709	BREX-system-P-loop-protein-BrxC-[Escherichia-coli-O25]
EEZ7212406.1	Type1.BREX	<-REase+iREase|| BrxA-DNA-binding -> BrxB-BrxF-iSTAND -> DUF6079 *-> N6-METHYLASE -> PglZ -> SIGMA+OB-Fold+AAA-ATPase+Lon-Protease-BrxL -> HKD-DNase+OGG-like ->||AbiEi_3_N+AbiEi_3->AbiEii-><-DUF6216<-Arm-DNA-bind_3	1213	Bacteria>Pseudomonadota>Gammaproteobacteria>Enterobacterales>Enterobacteriaceae>Escherichia	GCA_012218605.1	Escherichia_coli	DAB40_000420	2198..5839	562	BREX-system-P-loop-protein-BrxC-[Escherichia-coli]
EEZ9156819.1	Type1.BREX	DUF6079 *-> N6-METHYLASE -> PglZ -> SIGMA+OB-Fold+AAA-ATPase+Lon-Protease-BrxL -> HKD-DNase+OGG-like ->||AbiEi_3_N+AbiEi_3->AbiEii-><-DUF6216<-Arm-DNA-bind_3	1010	Bacteria>Pseudomonadota>Gammaproteobacteria>Enterobacterales>Enterobacteriaceae>Escherichia	GCA_012228465.1	Escherichia_coli	DDB94_002970	1..3035	562	BREX-system-P-loop-protein-BrxC-[Escherichia-coli]
EEZ8952063.1	Type1.BREX	<-Phage_integrase||?->?->?-><-HNH||wHTH+SAD+HNH-><-REase+iREase|| BrxA-DNA-binding -> BrxB-BrxF-iSTAND -> DUF6079 *-> PglZ -> SIGMA+OB-Fold+AAA-ATPase+Lon-Protease-BrxL -> HKD-DNase+OGG-like ->||AbiEi_3_N+AbiEi_3->AbiEii-><-DUF6216<-Arm-DNA-bind_3<-TOPRIM-DNAG+D5N+D5ATPase+P4PRIMASE-HTH	1171	Bacteria>Pseudomonadota>Gammaproteobacteria>Enterobacterales>Enterobacteriaceae>Escherichia	GCA_012229475.1	Escherichia_coli_O35	C2D51_003183	9758..13273	2072460	BREX-system-P-loop-protein-BrxC-[Escherichia-coli-O35]
EFA5158822.1	Type1.BREX	<-Phage_integrase||?->?->?-><-HNH||wHTH+SAD+HNH-><-REase+iREase|| BrxA-DNA-binding -> BrxB-BrxF-iSTAND -> DUF6079 *-> N6-METHYLASE -> PglZ -> SIGMA+OB-Fold+AAA-ATPase+Lon-Protease-BrxL -> HKD-DNase+OGG-like ->||AbiEi_3_N+AbiEi_3->AbiEii-><-DUF6216<-Arm-DNA-bind_3	1213	Bacteria>Pseudomonadota>Gammaproteobacteria>Enterobacterales>Enterobacteriaceae>Escherichia	GCA_012250845.1	Escherichia_coli	E2930_09975	121051..124692	562	BREX-system-P-loop-protein-BrxC-[Escherichia-coli]
EFB2626049.1	Type1.BREX	<- HKD-DNase+OGG-like <- SIGMA+OB-Fold+AAA-ATPase+Lon-Protease-BrxL <- PglZ <- N6-METHYLASE <- DUF6079 *<- BrxB-BrxF-iSTAND <- BrxA-DNA-binding ||REase+iREase-><-wHTH+SAD+HNH||HNH-><-?<-?<-?||Phage_integrase->	1213	Bacteria>Pseudomonadota>Gammaproteobacteria>Enterobacterales>Enterobacteriaceae>Escherichia	GCA_012293805.1	Escherichia_coli	FHL63_20525	10185..13826	562	BREX-system-P-loop-protein-BrxC-[Escherichia-coli]
EFC1641769.1	Type1.BREX	<-HerA/FtsK+A32<-?||tRNA->DUF6216-><-AbiEii<-AbiEi_3_N+AbiEi_3||<- HKD-DNase+OGG-like <- SIGMA+OB-Fold+AAA-ATPase+Lon-Protease-BrxL <- N6-METHYLASE <- DUF6079 *	1140	Bacteria>Pseudomonadota>Gammaproteobacteria>Enterobacterales>Enterobacteriaceae>Escherichia	GCA_012339895.1	Escherichia_coli	F2I36_20020	26288..29710	562	BREX-system-P-loop-protein-BrxC-[Escherichia-coli]
MBC0786087.1	Type1.BREX	<-Phage_integrase||?->?->?-><-HNH||wHTH+SAD+HNH-><-REase+iREase|| BrxA-DNA-binding -> BrxB-BrxF-iSTAND -> DUF6079 *-> N6-METHYLASE -> PglZ -> SIGMA+OB-Fold+AAA-ATPase+Lon-Protease-BrxL -> HKD-DNase+OGG-like ->||AbiEi_3_N+AbiEi_3->AbiEii->?-><-DUF6216<-Arm-DNA-bind_3	1213	Bacteria>Pseudomonadota>Gammaproteobacteria>Enterobacterales>Enterobacteriaceae>Escherichia	GCA_012382335.2	Escherichia_coli	FSG57_002365	111024..114665	562	BREX-system-P-loop-protein-BrxC-[Escherichia-coli]
EFD1659139.1	Type1.BREX	<-Phage_integrase||?->?->?-><-HNH||wHTH+SAD+HNH-><-REase+iREase|| BrxA-DNA-binding -> BrxB-BrxF-iSTAND -> DUF6079 *-> N6-METHYLASE -> PglZ -> SIGMA+OB-Fold+AAA-ATPase+Lon-Protease-BrxL -> HKD-DNase+OGG-like ->||AbiEi_3_N+AbiEi_3->AbiEii-><-DUF6216<-Arm-DNA-bind_3	1213	Bacteria>Pseudomonadota>Gammaproteobacteria>Enterobacterales>Enterobacteriaceae>Escherichia	GCA_012382975.1	Escherichia_coli	FGU32_03035	69619..73260	562	BREX-system-P-loop-protein-BrxC-[Escherichia-coli]
EFE1965719.1	Type1.BREX	<-Phage_integrase<-Phage_integrase||?->?->?-><-HNH||wHTH(SBD)+HNH->|| BrxA-DNA-binding -> BrxB-BrxF-iSTAND -> DUF6079 *-> N6-METHYLASE -> PglZ -> SIGMA+OB-Fold+AAA-ATPase+Lon-Protease-BrxL -> HKD-DNase+OGG-like ->||AbiEi_3_N+AbiEi_3->AbiEii-><-DUF6216<-Arm-DNA-bind_3	1213	Bacteria>Pseudomonadota>Gammaproteobacteria>Enterobacterales>Enterobacteriaceae>Escherichia	GCA_012428445.1	Escherichia_coli	CJV81_002667	14696..18337	562	BREX-system-P-loop-protein-BrxC-[Escherichia-coli]
EFF3839660.1	Type1.BREX	DUF6079 -p*-> N6-METHYLASE -> PglZ -> SIGMA+OB-Fold+AAA-ATPase+Lon-Protease-BrxL -> HKD-DNase+OGG-like ->||AbiEi_3_N+AbiEi_3->AbiEii-><-DUF6216<-Arm-DNA-bind_3	739	Bacteria>Pseudomonadota>Gammaproteobacteria>Enterobacterales>Enterobacteriaceae>Escherichia	GCA_012482765.1	Escherichia_coli	B6310_003615	1..2221	562	BREX-system-P-loop-protein-BrxC-[Escherichia-coli]
HAH2520952.1	Type1.BREX	<-Phage_integrase||?->?->?-><-HNH||wHTH+SAD+HNH-><-REase+iREase|| BrxA-DNA-binding -> BrxB-BrxF-iSTAND -> DUF6079 *-> PglZ -> SIGMA+OB-Fold+AAA-ATPase+Lon-Protease-BrxL -> HKD-DNase+OGG-like ->||AbiEi_3_N+AbiEi_3->AbiEii->	1162	Bacteria>Pseudomonadota>Gammaproteobacteria>Enterobacterales>Enterobacteriaceae>Escherichia	GCA_012530295.1	Escherichia_coli	GF659_15740	71446..74934	562	BREX-system-P-loop-protein-BrxC-[Escherichia-coli]
EFG3005928.1	Type1.BREX	DUF6079 -p*-> N6-METHYLASE -> PglZ -> SIGMA+OB-Fold+AAA-ATPase+Lon-Protease-BrxL -> HKD-DNase+OGG-like ->||AbiEi_3_N+AbiEi_3->AbiEii-><-DUF6216<-Arm-DNA-bind_3	897	Bacteria>Pseudomonadota>Gammaproteobacteria>Enterobacterales>Enterobacteriaceae>Escherichia	GCA_012550755.1	Escherichia_coli	BOZ89_003087	1..2694	562	BREX-system-P-loop-protein-BrxC-[Escherichia-coli]
EFG4404338.1	Type1.BREX	<-Phage_integrase<-Phage_integrase||?->?->?-><-HNH||wHTH(SBD)+HNH->|| BrxA-DNA-binding -> BrxB-BrxF-iSTAND -> DUF6079 *-> N6-METHYLASE -> PglZ -> SIGMA+OB-Fold+AAA-ATPase+Lon-Protease-BrxL -> HKD-DNase+OGG-like ->||AbiEi_3_N+AbiEi_3->AbiEii-><-DUF6216<-Arm-DNA-bind_3	1213	Bacteria>Pseudomonadota>Gammaproteobacteria>Enterobacterales>Enterobacteriaceae>Escherichia	GCA_012556825.1	Escherichia_coli	BM772_002472	14697..18338	562	BREX-system-P-loop-protein-BrxC-[Escherichia-coli]
EFH0548056.1	Type1.BREX	DUF6079 -p*-> N6-METHYLASE -> PglZ -> SIGMA+OB-Fold+AAA-ATPase+Lon-Protease-BrxL -> HKD-DNase+OGG-like ->||AbiEi_3_N+AbiEi_3->AbiEii-><-DUF6216<-Arm-DNA-bind_3	789	Bacteria>Pseudomonadota>Gammaproteobacteria>Enterobacterales>Enterobacteriaceae>Escherichia	GCA_012582395.1	Escherichia_coli	BJE23_002820	1..2370	562	BREX-system-P-loop-protein-BrxC-[Escherichia-coli]
EFH4936891.1	Type1.BREX	<-Phage_integrase||?->?->?-><-HNH||wHTH+SAD+HNH-><-REase+iREase|| BrxA-DNA-binding -> BrxB-BrxF-iSTAND -> DUF6079 *-> N6-METHYLASE -> PglZ -> SIGMA+OB-Fold+AAA-ATPase+Lon-Protease-BrxL -> HKD-DNase+OGG-like ->||AbiEi_3_N+AbiEi_3->AbiEii-><-DUF6216<-Arm-DNA-bind_3	1213	Bacteria>Pseudomonadota>Gammaproteobacteria>Enterobacterales>Enterobacteriaceae>Escherichia	GCA_012603315.1	Escherichia_coli	F9408_05585	110160..113801	562	BREX-system-P-loop-protein-BrxC-[Escherichia-coli]
EFI1580322.1	Type1.BREX	DUF6079 *-> N6-METHYLASE -> PglZ -> SIGMA+OB-Fold+AAA-ATPase+Lon-Protease-BrxL -> HKD-DNase+OGG-like ->||AbiEi_3_N+AbiEi_3->AbiEii-><-DUF6216<-Arm-DNA-bind_3	1071	Bacteria>Pseudomonadota>Gammaproteobacteria>Enterobacterales>Enterobacteriaceae>Escherichia	GCA_012634445.1	Escherichia_coli	BFN79_002999	1..3216	562	BREX-system-P-loop-protein-BrxC-[Escherichia-coli]
EFI3320066.1	Type1.BREX	DDE_3-><- HKD-DNase+OGG-like <- SIGMA+OB-Fold+AAA-ATPase+Lon-Protease-BrxL <- PglZ <- N6-METHYLASE <- DUF6079 *<- BrxB-BrxF-iSTAND <- BrxA-DNA-binding ||REase+iREase-><-wHTH+SAD+HNH||HNH-><-?<-?<-?||Phage_integrase->	1213	Bacteria>Pseudomonadota>Gammaproteobacteria>Enterobacterales>Enterobacteriaceae>Escherichia	GCA_012643725.1	Escherichia_coli	BCC72_001616	10313..13954	562	BREX-system-P-loop-protein-BrxC-[Escherichia-coli]

Protein-ID	System	Gene-Neighbourhoods	Length	Taxonomy	Assembly-ID	Strain	Locus-Tag	Genomic-coordinates	TaxID	Fasta-defline
EFJ5167524.1	Type1.BREX	DUF6079 *-> N6-METHYLASE -> PglZ -> SIGMA+OB-Fold+AAA-ATPase+Lon-Protease-BrxL -> HKD-DNase +OGG-like ->||AbiEi_3_N+AbiEi_3->AbiEii-><-DUF6216<-Arm-DNA-bind_3	1181	Bacteria>Pseudomonadota>Gammaproteobacteria>Enterobacterales>Enterobacteriaceae>Escherichia	GCA_012707625.1	Escherichia_coli	A5T96_003401	1..3546	562	BREX-system-P-loop-protein-BrxC-[Escherichia-coli]
HAH8430180.1	Type1.BREX	DUF6079 -p*-> N6-METHYLASE -> PglZ -> SIGMA+OB-Fold+AAA-ATPase+Lon-Protease-BrxL -> HKD-DNase +OGG-like ->||AbiEi_3_N+AbiEi_3->AbiEii-><-DUF6216<-Arm-DNA-bind_3	846	Bacteria>Pseudomonadota>Gammaproteobacteria>Enterobacterales>Enterobacteriaceae>Escherichia	GCA_012716735.1	Escherichia_coli	HIR43_003168	1..2542	562	BREX-system-P-loop-protein-BrxC-[Escherichia-coli]
HAH9940666.1	Type1.BREX	<-Phage_integrase||?->?->?-><-HNH||wHTH+SAD+HNH-><-REase+iREase|| BrxA-DNA-binding -> BrxB-BrxF-iSTAND -> DUF6079 *-> N6-METHYLASE -> PglZ -> SIGMA+OB-Fold+AAA-ATPase+Lon-Protease-BrxL -> HKD-DNase +OGG-like ->||AbiEi_3_N+AbiEi_3->AbiEii-><-DUF6216<-Arm-DNA-bind_3<-REase	1213	Bacteria>Pseudomonadota>Gammaproteobacteria>Enterobacterales>Enterobacteriaceae>Escherichia	GCA_012753595.1	Escherichia_coli	HIF13_004044	9744..13385	562	BREX-system-P-loop-protein-BrxC-[Escherichia-coli]
EFL0544862.1	Type1.BREX	DUF6079 -p*-> N6-METHYLASE -> PglZ -> SIGMA+OB-Fold+AAA-ATPase+Lon-Protease-BrxL -> HKD-DNase +OGG-like ->||AbiEi_3_N+AbiEi_3->AbiEii-><-DUF6216<-Arm-DNA-bind_3<-TOPRIM-DNAG+D5N+D5ATPase+P4PRIMASE-HTH	803	Bacteria>Pseudomonadota>Gammaproteobacteria>Enterobacterales>Enterobacteriaceae>Escherichia	GCA_012789875.1	Escherichia_coli	HIZ25_002081	1..2412	562	BREX-system-P-loop-protein-BrxC-[Escherichia-coli]
EFL4011089.1	Type1.BREX	<-Phage_integrase||?->?->?-><-HNH||wHTH+SAD+HNH-><-REase+iREase|| BrxA-DNA-binding -> BrxB-BrxF-iSTAND -> DUF6079 *-> N6-METHYLASE -> PglZ -> SIGMA+OB-Fold+AAA-ATPase+Lon-Protease-BrxL -> HKD-DNase +OGG-like ->||AbiEi_3_N+AbiEi_3->AbiEii-><-DUF6216<-Arm-DNA-bind_3	1213	Bacteria>Pseudomonadota>Gammaproteobacteria>Enterobacterales>Enterobacteriaceae>Escherichia	GCA_012809955.1	Escherichia_coli	AC312_001861	91319..94960	562	BREX-system-P-loop-protein-BrxC-[Escherichia-coli]
EFM7055934.1	Type1.BREX	DUF6079 *-> N6-METHYLASE -> PglZ -> SIGMA+OB-Fold+AAA-ATPase+Lon-Protease-BrxL -> HKD-DNase +OGG-like ->||AbiEi_3_N+AbiEi_3->AbiEii-><-DUF6216<-Arm-DNA-bind_3	1050	Bacteria>Pseudomonadota>Gammaproteobacteria>Enterobacterales>Enterobacteriaceae>Escherichia	GCA_012894495.1	Escherichia_coli	BAU66_002360	1..3155	562	BREX-system-P-loop-protein-BrxC-[Escherichia-coli]
HAI9090450.1	Type1.BREX	DUF6079 *-> N6-METHYLASE -> PglZ -> SIGMA+OB-Fold+AAA-ATPase+Lon-Protease-BrxL -> HKD-DNase +OGG-like ->||AbiEi_3_N+AbiEi_3->AbiEii-><-DUF6216<-Arm-DNA-bind_3	1180	Bacteria>Pseudomonadota>Gammaproteobacteria>Enterobacterales>Enterobacteriaceae>Escherichia	GCA_012925565.1	Escherichia_coli	HKC72_003106	1..3544	562	BREX-system-P-loop-protein-BrxC-[Escherichia-coli]
EFN5734792.1	Type1.BREX	<-Phage_integrase||?->?->?-><-HNH||wHTH+SAD+HNH-><-REase+iREase|| BrxA-DNA-binding -> BrxB-BrxF-iSTAND -> DUF6079 *-> N6-METHYLASE -> PglZ -> SIGMA+OB-Fold+AAA-ATPase+Lon-Protease-BrxL -> HKD-DNase +OGG-like ->||AbiEi_3_N+AbiEi_3->AbiEii-><-DUF6216<-Arm-DNA-bind_3	1213	Bacteria>Pseudomonadota>Gammaproteobacteria>Enterobacterales>Enterobacteriaceae>Escherichia	GCA_012995705.1	Escherichia_coli	FQJ28_11185	111254..114895	562	BREX-system-P-loop-protein-BrxC-[Escherichia-coli]
HAJ1412079.1	Type1.BREX	DUF6079 *-> PglZ -> SIGMA+OB-Fold+AAA-ATPase+Lon-Protease-BrxL -> HKD-DNase +OGG-like ->||AbiEi_3_N+AbiEi_3->AbiEii->	1046	Bacteria>Pseudomonadota>Gammaproteobacteria>Enterobacterales>Enterobacteriaceae>Escherichia	GCA_013000285.1	Escherichia_coli	HMG43_22065	1..3142	562	BREX-system-P-loop-protein-BrxC-[Escherichia-coli]
EFO2091244.1	Type1.BREX	<-35exo||?->?->?-><-HNH||wHTH+SAD+HNH-><-REase+iREase|| BrxA-DNA-binding -> BrxB-BrxF-iSTAND -> DUF6079 *-> N6-METHYLASE -> PglZ -> SIGMA+OB-Fold+AAA-ATPase+Lon-Protease-BrxL -> HKD-DNase +OGG-like ->||AbiEi_3_N+AbiEi_3->AbiEii-><-DUF6216<-Arm-DNA-bind_3	1213	Bacteria>Pseudomonadota>Gammaproteobacteria>Enterobacterales>Enterobacteriaceae>Escherichia	GCA_013066245.1	Escherichia_coli_O54	DP047_19880	9767..13408	2184075	BREX-system-P-loop-protein-BrxC-[Escherichia-coli-O54]
EFR2049109.1	Type1.BREX	DUF6079 -p*-> N6-METHYLASE -> PglZ -> SIGMA+OB-Fold+AAA-ATPase+Lon-Protease-BrxL -> HKD-DNase +OGG-like ->||AbiEi_3_N+AbiEi_3->AbiEii-><-DUF6216<-Arm-DNA-bind_3	884	Bacteria>Pseudomonadota>Gammaproteobacteria>Enterobacterales>Enterobacteriaceae>Escherichia	GCA_013546455.1	Escherichia_coli	HZO98_003737	1..2655	562	BREX-system-P-loop-protein-BrxC-[Escherichia-coli]
EFR9656371.1	Type1.BREX	DUF6079 -p*-> N6-METHYLASE -> PglZ -> SIGMA+OB-Fold+AAA-ATPase+Lon-Protease-BrxL -> HKD-DNase +OGG-like ->||AbiEi_3_N+AbiEi_3->AbiEii-><-DUF6216<-Arm-DNA-bind_3	991	Bacteria>Pseudomonadota>Gammaproteobacteria>Enterobacterales>Enterobacteriaceae>Escherichia	GCA_013588925.1	Escherichia_coli	H2N11_003246	1..2976	562	BREX-system-P-loop-protein-BrxC-[Escherichia-coli]
EFS8463113.1	Type1.BREX	<-Phage_integrase||?->?->?-><-HNH||wHTH+SAD+HNH-><-REase+iREase|| BrxA-DNA-binding -> BrxB-BrxF-iSTAND -> DUF6079 *-> N6-METHYLASE -> PglZ -> SIGMA+OB-Fold+AAA-ATPase+Lon-Protease-BrxL -> HKD-DNase +OGG-like ->||AbiEi_3_N+AbiEi_3->AbiEii-><-DUF6216<-Arm-DNA-bind_3	1213	Bacteria>Pseudomonadota>Gammaproteobacteria>Enterobacterales>Enterobacteriaceae>Escherichia	GCA_013669455.1	Escherichia_coli	HWT39_002319	97196..100837	562	BREX-system-P-loop-protein-BrxC-[Escherichia-coli]
EFU0098527.1	Type1.BREX	DUF6079 *-> N6-METHYLASE -> PglZ -> SIGMA+OB-Fold+AAA-ATPase+Lon-Protease-BrxL -> HKD-DNase +OGG-like ->||AbiEi_3_N+AbiEi_3->AbiEii->?-><-DUF6216<-Arm-DNA-bind_3	1009	Bacteria>Pseudomonadota>Gammaproteobacteria>Enterobacterales>Enterobacteriaceae>Escherichia	GCA_013728855.1	Escherichia_coli	HVV04_003689	1..3030	562	BREX-system-P-loop-protein-BrxC-[Escherichia-coli]
HAL9997354.1	Type1.BREX	DUF6079 *-> N6-METHYLASE -> PglZ -> SIGMA+OB-Fold+AAA-ATPase+Lon-Protease-BrxL -> HKD-DNase +OGG-like ->||AbiEi_3_N+AbiEi_3->AbiEii-><-DUF6216<-Arm-DNA-bind_3	1081	Bacteria>Pseudomonadota>Gammaproteobacteria>Enterobacterales>Enterobacteriaceae>Escherichia	GCA_014189205.1	Escherichia_coli	H7W48_003266	1..3246	562	BREX-system-P-loop-protein-BrxC-[Escherichia-coli]
EGD5155863.1	Type1.BREX	[illegible]	1213	Bacteria>Pseudomonadota>Gammaproteobacteria>Enterobacterales>Enterobacteriaceae>Escherichia	GCA_014387005.1	Escherichia_coli	DTY31_07100	71746..75387	562	BREX-system-P-loop-protein-BrxC-[Escherichia-coli]
EGF7347429.1	Type1.BREX	[illegible]	1213	Bacteria>Pseudomonadota>Gammaproteobacteria>Enterobacterales>Enterobacteriaceae>Escherichia	GCA_014521725.1	Escherichia_coli	HJV04_003560	44..3685	562	BREX-system-P-loop-protein-BrxC-[Escherichia-coli]
HAO2821705.1	Type1.BREX	[illegible]	1111	Bacteria>Pseudomonadota>Gammaproteobacteria>Enterobacterales>Enterobacteriaceae>Escherichia	GCA_014823275.1	Escherichia_coli	IHL91_001055	1..3336	562	BREX-system-P-loop-protein-BrxC-[Escherichia-coli]
EGK3603428.1	Type1.BREX	[illegible]	1179	Bacteria>Pseudomonadota>Gammaproteobacteria>Enterobacterales>Enterobacteriaceae>Escherichia	GCA_014957425.1	Escherichia_coli	IOT28_003449	1..3540	562	BREX-system-P-loop-protein-BrxC-[Escherichia-coli]
EGY5636086.1	Type1.BREX	[illegible]	1171	Bacteria>Pseudomonadota>Gammaproteobacteria>Enterobacterales>Enterobacteriaceae>Escherichia	GCA_016519105.1	Escherichia_coli	JI450_003022	1..3517	562	BREX-system-P-loop-protein-BrxC-[Escherichia-coli]
EGY5620863.1	Type1.BREX	[illegible]	1213	Bacteria>Pseudomonadota>Gammaproteobacteria>Enterobacterales>Enterobacteriaceae>Escherichia	GCA_016519195.1	Escherichia_coli	JI433_002890	14696..18337	562	BREX-system-P-loop-protein-BrxC-[Escherichia-coli]
EGY8947056.1	Type1.BREX	[illegible]	763	Bacteria>Pseudomonadota>Gammaproteobacteria>Enterobacterales>Enterobacteriaceae>Escherichia	GCA_016541865.1	Escherichia_coli	JI613_002965	1..2292	562	BREX-system-P-loop-protein-BrxC-[Escherichia-coli]
EGY9082468.1	Type1.BREX	[illegible]	1175	Bacteria>Pseudomonadota>Gammaproteobacteria>Enterobacterales>Enterobacteriaceae>Escherichia	GCA_016542525.1	Escherichia_coli	JI646_002303	1..3528	562	BREX-system-P-loop-protein-BrxC-[Escherichia-coli]
EGY9336114.1	Type1.BREX	[illegible]	1177	Bacteria>Pseudomonadota>Gammaproteobacteria>Enterobacterales>Enterobacteriaceae>Escherichia	GCA_016543525.1	Escherichia_coli	HZO78_003642	1..3535	562	BREX-system-P-loop-protein-BrxC-[Escherichia-coli]
EGZ0051356.1	Type1.BREX	[illegible]	834	Bacteria>Pseudomonadota>Gammaproteobacteria>Enterobacterales>Enterobacteriaceae>Escherichia	GCA_016546855.1	Escherichia_coli	JJA24_002218	1..2505	562	BREX-system-P-loop-protein-BrxC-[Escherichia-coli]
EGZ1391640.1	Type1.BREX	[illegible]	789	Bacteria>Pseudomonadota>Gammaproteobacteria>Enterobacterales>Enterobacteriaceae>Escherichia	GCA_016552825.1	Escherichia_coli	FG160_002854	1..2370	562	BREX-system-P-loop-protein-BrxC-[Escherichia-coli]
EGZ1415768.1	Type1.BREX	[illegible]	1213	Bacteria>Pseudomonadota>Gammaproteobacteria>Enterobacterales>Enterobacteriaceae>Escherichia	GCA_016552885.1	Escherichia_coli	FFU33_001578	154175..157816	562	BREX-system-P-loop-protein-BrxC-[Escherichia-coli]
EGZ1559467.1	Type1.BREX	[illegible]	768	Bacteria>Pseudomonadota>Gammaproteobacteria>Enterobacterales>Enterobacteriaceae>Escherichia	GCA_016553465.1	Escherichia_coli	FAV16_003764	1..2307	562	BREX-system-P-loop-protein-BrxC-[Escherichia-coli]
EGZ1869331.1	Type1.BREX	[illegible]	796	Bacteria>Pseudomonadota>Gammaproteobacteria>Enterobacterales>Enterobacteriaceae>Escherichia	GCA_016554585.1	Escherichia_coli	EYE54_001992	1..2391	562	BREX-system-P-loop-protein-BrxC-[Escherichia-coli]
EGZ2354229.1	Type1.BREX	[illegible]	1213	Bacteria>Pseudomonadota>Gammaproteobacteria>Enterobacterales>Enterobacteriaceae>Escherichia	GCA_016556665.1	Escherichia_coli	D7J47_002044	14697..18338	562	BREX-system-P-loop-protein-BrxC-[Escherichia-coli]
EGZ2497683.1	Type1.BREX	[illegible]	969	Bacteria>Pseudomonadota>Gammaproteobacteria>Enterobacterales>Enterobacteriaceae>Escherichia	GCA_016557105.1	Escherichia_coli	D6W78_003631	1..2912	562	BREX-system-P-loop-protein-BrxC-[Escherichia-coli]
EGZ2674120.1	Type1.BREX	[illegible]	1096	Bacteria>Pseudomonadota>Gammaproteobacteria>Enterobacterales>Enterobacteriaceae>Escherichia	GCA_016557745.1	Escherichia_coli	D1H08_002340	1..3291	562	BREX-system-P-loop-protein-BrxC-[Escherichia-coli]
EGZ2777080.1	Type1.BREX	[illegible]	1213	Bacteria>Pseudomonadota>Gammaproteobacteria>Enterobacterales>Enterobacteriaceae>Escherichia	GCA_016558225.1	Escherichia_coli	DVO41_002389	14697..18338	562	BREX-system-P-loop-protein-BrxC-[Escherichia-coli]
EGZ3242782.1	Type1.BREX	[illegible]	1213	Bacteria>Pseudomonadota>Gammaproteobacteria>Enterobacterales>Enterobacteriaceae>Escherichia	GCA_016560025.1	Escherichia_coli	C5N70_002404	39991..43632	562	BREX-system-P-loop-protein-BrxC-[Escherichia-coli]
EGZ4145352.1	Type1.BREX	[illegible]	1095	Bacteria>Pseudomonadota>Gammaproteobacteria>Enterobacterales>Enterobacteriaceae>Escherichia	GCA_016563925.1	Escherichia_coli	CKY95_002785	1..3290	562	BREX-system-P-loop-protein-BrxC-[Escherichia-coli]
EGZ5520004.1	Type1.BREX	[illegible]	815	Bacteria>Pseudomonadota>Gammaproteobacteria>Enterobacterales>Enterobacteriaceae>Escherichia	GCA_016569715.1	Escherichia_coli	LF100_002181	1..2450	562	BREX-system-P-loop-protein-BrxC-[Escherichia-coli]
EGZ7391807.1	Type1.BREX	[illegible]	970	Bacteria>Pseudomonadota>Gammaproteobacteria>Enterobacterales>Enterobacteriaceae>Escherichia	GCA_016578925.1	Escherichia_coli	BAU88_004031	1..2913	562	BREX-system-P-loop-protein-BrxC-[Escherichia-coli]
EGZ7370259.1	Type1.BREX	[illegible]	880	Bacteria>Pseudomonadota>Gammaproteobacteria>Enterobacterales>Enterobacteriaceae>Escherichia	GCA_016578935.1	Escherichia_coli	BAU61_002957	1..2643	562	BREX-system-P-loop-protein-BrxC-[Escherichia-coli]
EGZ7468561.1	Type1.BREX	[illegible]	931	Bacteria>Pseudomonadota>Gammaproteobacteria>Enterobacterales>Enterobacteriaceae>Escherichia	GCA_016579325.1	Escherichia_coli	A6F02_004042	1..2798	562	BREX-system-P-loop-protein-BrxC-[Escherichia-coli]
EGZ7821797.1	Type1.BREX	[illegible]	729	Bacteria>Pseudomonadota>Gammaproteobacteria>Enterobacterales>Enterobacteriaceae>Escherichia	GCA_016581105.1	Escherichia_coli	AWC87_001490	1..2190	562	BREX-system-P-loop-protein-BrxC-[Escherichia-coli]
EGZ8838308.1	Type1.BREX	[illegible]	1213	Bacteria>Pseudomonadota>Gammaproteobacteria>Enterobacterales>Enterobacteriaceae>Escherichia	GCA_016590495.1	Escherichia_coli	AT823_000493	8719..12360	562	BREX-system-P-loop-protein-BrxC-[Escherichia-coli]
EGZ9024440.1	Type1.BREX	[illegible]	963	Bacteria>Pseudomonadota>Gammaproteobacteria>Enterobacterales>Enterobacteriaceae>Escherichia	GCA_016591155.1	Escherichia_coli	AFE50_004176	1..2892	562	BREX-system-P-loop-protein-BrxC-[Escherichia-coli]
EHA0949388.1	Type1.BREX	[illegible]	762	Bacteria>Pseudomonadota>Gammaproteobacteria>Enterobacterales>Enterobacteriaceae>Escherichia	GCA_016610545.1	Escherichia_coli	JI650_002930	1..2291	562	BREX-system-P-loop-protein-BrxC-[Escherichia-coli]
HAW1448903.1	Type1.BREX	[illegible]	1213	Bacteria>Pseudomonadota>Gammaproteobacteria>Enterobacterales>Enterobacteriaceae>Escherichia	GCA_016674355.1	Escherichia_coli	JLM49_000126	135704..139345	562	BREX-system-P-loop-protein-BrxC-[Escherichia-coli]
EHA9087556.1	Type1.BREX	[illegible]	1213	Bacteria>Pseudomonadota>Gammaproteobacteria>Enterobacterales>Enterobacteriaceae>Escherichia	GCA_016846225.1	Escherichia_coli	D4094_002230	14699..18340	562	BREX-system-P-loop-protein-BrxC-[Escherichia-coli]
HAX4031531.1	Type1.BREX	[illegible]	1213	Bacteria>Pseudomonadota>Gammaproteobacteria>Enterobacterales>Enterobacteriaceae>Escherichia	GCA_017003755.1	Escherichia_coli	JV397_001890	160694..164335	562	BREX-system-P-loop-protein-BrxC-[Escherichia-coli]
EHC2076257.1	Type1.BREX	[illegible]	1213	Bacteria>Pseudomonadota>Gammaproteobacteria>Enterobacterales>Enterobacteriaceae>Escherichia	GCA_017031715.1	Escherichia_coli	JT651_000259	8773..12414	562	BREX-system-P-loop-protein-BrxC-[Escherichia-coli]
HAX9732773.1	Type1.BREX	[illegible]	1213	Bacteria>Pseudomonadota>Gammaproteobacteria>Enterobacterales>Enterobacteriaceae>Escherichia	GCA_017073235.1	Escherichia_coli	JS163_001163	104185..107826	562	BREX-system-P-loop-protein-BrxC-[Escherichia-coli]
HBA3375039.1	Type1.BREX	[illegible]	1213	Bacteria>Pseudomonadota>Gammaproteobacteria>Enterobacterales>Enterobacteriaceae>Escherichia	GCA_017728515.1	Escherichia_coli	J8Z50_001951	111486..115127	562	BREX-system-P-loop-protein-BrxC-[Escherichia-coli]
HBA6912638.1	Type1.BREX	[illegible]	1213	Bacteria>Pseudomonadota>Gammaproteobacteria>Enterobacterales>Enterobacteriaceae>Escherichia	GCA_017762865.1	Escherichia_coli	J1V61_002563	8737..12378	562	BREX-system-P-loop-protein-BrxC-[Escherichia-coli]
HBB4280095.1	Type1.BREX	[illegible]	1060	Bacteria>Pseudomonadota>Gammaproteobacteria>Enterobacterales>Enterobacteriaceae>Escherichia	GCA_017899135.1	Escherichia_coli	KAW34_004647	1..3185	562	BREX-system-P-loop-protein-BrxC-[Escherichia-coli]
EHJ8640897.1	Type1.BREX	[illegible]	1213	Bacteria>Pseudomonadota>Gammaproteobacteria>Enterobacterales>Enterobacteriaceae>Escherichia	GCA_017945505.1	Escherichia_coli	KB366_000307	330961..334602	562	BREX-system-P-loop-protein-BrxC-[Escherichia-coli]
EHK0633846.1	Type1.BREX	[illegible]	1213	Bacteria>Pseudomonadota>Gammaproteobacteria>Enterobacterales>Enterobacteriaceae>Escherichia	GCA_017971565.1	Escherichia_coli	KCG30_002478	111267..114908	562	BREX-system-P-loop-protein-BrxC-[Escherichia-coli]
EHK3416136.1	Type1.BREX	[illegible]	1126	Bacteria>Pseudomonadota>Gammaproteobacteria>Enterobacterales>Enterobacteriaceae>Escherichia	GCA_018020775.1	Escherichia_coli	JYE89_003262	1..3383	562	BREX-system-P-loop-protein-BrxC-[Escherichia-coli]
EHL0363096.1	Type1.BREX	[illegible]	921	Bacteria>Pseudomonadota>Gammaproteobacteria>Enterobacterales>Enterobacteriaceae>Escherichia	GCA_018090865.1	Escherichia_coli	D1H14_003269	1..2766	562	BREX-system-P-loop-protein-BrxC-[Escherichia-coli]
EHL0920537.1	Type1.BREX	[illegible]	767	Bacteria>Pseudomonadota>Gammaproteobacteria>Enterobacterales>Enterobacteriaceae>Escherichia	GCA_018093285.1	Escherichia_coli	CFF49_003158	1..2306	562	BREX-system-P-loop-protein-BrxC-[Escherichia-coli]
EHL1132628.1	Type1.BREX	[illegible]	752	Bacteria>Pseudomonadota>Gammaproteobacteria>Enterobacterales>Enterobacteriaceae>Escherichia	GCA_018094045.1	Escherichia_coli	BIB01_002904	1..2259	562	BREX-system-P-loop-protein-BrxC-[Escherichia-coli]
EHL1162648.1	Type1.BREX	[illegible]	1213	Bacteria>Pseudomonadota>Gammaproteobacteria>Enterobacterales>Enterobacteriaceae>Escherichia	GCA_018094265.1	Escherichia_coli	BE892_002471	14698..18339	562	BREX-system-P-loop-protein-BrxC-[Escherichia-coli]
EHM5922455.1	Type1.BREX	[illegible]	1213	Bacteria>Pseudomonadota>Gammaproteobacteria>Enterobacterales>Enterobacteriaceae>Escherichia	GCA_018270935.1	Escherichia_coli	KGR67_002708	6748..10389	562	BREX-system-P-loop-protein-BrxC-[Escherichia-coli]
EHN3342070.1	Type1.BREX	[illegible]	1213	Bacteria>Pseudomonadota>Gammaproteobacteria>Enterobacterales>Enterobacteriaceae>Escherichia	GCA_018356405.1	Escherichia_coli	KIR71_003047	14697..18338	562	BREX-system-P-loop-protein-BrxC-[Escherichia-coli]
HBD5250199.1	Type1.BREX	[illegible]	1060	Bacteria>Pseudomonadota>Gammaproteobacteria>Enterobacterales>Enterobacteriaceae>Escherichia	GCA_018460085.1	Escherichia_coli	KKL26_003784	28719..31903	562	BREX-system-P-loop-protein-BrxC-[Escherichia-coli]
EHQ7823790.1	Type1.BREX	[illegible]	927	Bacteria>Pseudomonadota>Gammaproteobacteria>Enterobacterales>Enterobacteriaceae>Escherichia	GCA_018975885.1	Escherichia_coli	KSS40_002990	1..2786	562	BREX-system-P-loop-protein-BrxC-[Escherichia-coli]
EHW5636696.1	Type1.BREX	[illegible]	1213	Bacteria>Pseudomonadota>Gammaproteobacteria>Enterobacterales>Enterobacteriaceae>Escherichia	GCA_019482305.1	Escherichia_coli	K2955_003127	35708..39349	562	BREX-system-P-loop-protein-BrxC-[Escherichia-coli]
EHX2153194.1	Type1.BREX	[illegible]	1213	Bacteria>Pseudomonadota>Gammaproteobacteria>Enterobacterales>Enterobacteriaceae>Escherichia	GCA_019516635.1	Escherichia_coli	K3P68_001530	1396..5037	562	BREX-system-P-loop-protein-BrxC-[Escherichia-coli]
EHX8392339.1	Type1.BREX	[illegible]	1213	Bacteria>Pseudomonadota>Gammaproteobacteria>Enterobacterales>Enterobacteriaceae>Escherichia	GCA_019550135.1	Escherichia_coli	K3368_004098	15146..18787	562	BREX-system-P-loop-protein-BrxC-[Escherichia-coli]
EHX8476217.1	Type1.BREX	[illegible]	1213	Bacteria>Pseudomonadota>Gammaproteobacteria>Enterobacterales>Enterobacteriaceae>Escherichia	GCA_019550495.1	Escherichia_coli	K3324_002225	23193..26834	562	BREX-system-P-loop-protein-BrxC-[Escherichia-coli]
EHY5298658.1	Type1.BREX	[illegible]	861	Bacteria>Pseudomonadota>Gammaproteobacteria>Enterobacterales>Enterobacteriaceae>Escherichia	GCA_019588505.1	Escherichia_coli	K4I57_002534	1..2588	562	BREX-system-P-loop-protein-BrxC-[Escherichia-coli]
EHY5690081.1	Type1.BREX	[illegible]	1213	Bacteria>Pseudomonadota>Gammaproteobacteria>Enterobacterales>Enterobacteriaceae>Escherichia	GCA_019590565.1	Escherichia_coli	K4J54_002559	14696..18337	562	BREX-system-P-loop-protein-BrxC-[Escherichia-coli]
EHZ2564701.1	Type1.BREX	[illegible]	1130	Bacteria>Pseudomonadota>Gammaproteobacteria>Enterobacterales>Enterobacteriaceae>Escherichia	GCA_019681855.1	Escherichia_coli	K5N16_003075	1..3394	562	BREX-system-P-loop-protein-BrxC-[Escherichia-coli]
HBJ0235562.1	Type1.BREX	[illegible]	1213	Bacteria>Pseudomonadota>Gammaproteobacteria>Enterobacterales>Enterobacteriaceae>Escherichia	GCA_019980105.1	Escherichia_coli	LAM28_003686	10586..14227	562	BREX-system-P-loop-protein-BrxC-[Escherichia-coli]
EID3638522.1	Type1.BREX	[illegible]	1213	Bacteria>Pseudomonadota>Gammaproteobacteria>Enterobacterales>Enterobacteriaceae>Escherichia	GCA_020041725.1	Escherichia_coli	LB206_001712	80689..84330	562	BREX-system-P-loop-protein-BrxC-[Escherichia-coli]
EID8685857.1	Type1.BREX	[illegible]	949	Bacteria>Pseudomonadota>Gammaproteobacteria>Enterobacterales>Enterobacteriaceae>Escherichia	GCA_020094635.1	Escherichia_coli	LCI88_002092	151835..154685	562	BREX-system-P-loop-protein-BrxC-[Escherichia-coli]
EIG2625391.1	Type1.BREX	[illegible]	771	Bacteria>Pseudomonadota>Gammaproteobacteria>Enterobacterales>Enterobacteriaceae>Escherichia	GCA_020303335.1	Escherichia_coli	GLS16_002890	1..2318	562	BREX-system-P-loop-protein-BrxC-[Escherichia-coli]
EIG7860374.1	Type1.BREX	[illegible]	1077	Bacteria>Pseudomonadota>Gammaproteobacteria>Enterobacterales>Enterobacteriaceae>Escherichia	GCA_020324895.1	Escherichia_coli	D0584_004201	1..3234	562	BREX-system-P-loop-protein-BrxC-[Escherichia-coli]
EIH7117202.1	Type1.BREX	[illegible]	1174	Bacteria>Pseudomonadota>Gammaproteobacteria>Enterobacterales>Enterobacteriaceae>Escherichia	GCA_020376505.1	Escherichia_coli	BB077_003959	1..3527	562	BREX-system-P-loop-protein-BrxC-[Escherichia-coli]
EIO0019318.1	Type1.BREX	[illegible]	816	Bacteria>Pseudomonadota>Gammaproteobacteria>Enterobacterales>Enterobacteriaceae>Escherichia	GCA_020946425.1	Escherichia_coli	LO099_004443	1..2453	562	BREX-system-P-loop-protein-BrxC-[Escherichia-coli]
HBM8747261.1	Type1.BREX	[illegible]	1213	Bacteria>Pseudomonadota>Gammaproteobacteria>Enterobacterales>Enterobacteriaceae>Escherichia	GCA_021417505.1	Escherichia_coli	L0V31_003260	39874..43515	562	BREX-system-P-loop-protein-BrxC-[Escherichia-coli]
HBN0573063.1	Type1.BREX	[illegible]	1213	Bacteria>Pseudomonadota>Gammaproteobacteria>Enterobacterales>Enterobacteriaceae>Escherichia	GCA_021454505.1	Escherichia_coli	L1N12_002093	35071..38712	562	BREX-system-P-loop-protein-BrxC-[Escherichia-coli]
HBP9919082.1	Type1.BREX	[illegible]	1213	Bacteria>Pseudomonadota>Gammaproteobacteria>Enterobacterales>Enterobacteriaceae>Escherichia	GCA_021766545.1	Escherichia_coli	L7G70_002290	14696..18337	562	BREX-system-P-loop-protein-BrxC-[Escherichia-coli]
EIX9838476.1	Type1.BREX	[illegible]	1208	Bacteria>Pseudomonadota>Gammaproteobacteria>Enterobacterales>Enterobacteriaceae>Escherichia	GCA_022433775.1	Escherichia_coli	ML439_002959	1..3628	562	BREX-system-P-loop-protein-BrxC-[Escherichia-coli]
EJA4563483.1	Type1.BREX	[illegible]	1213	Bacteria>Pseudomonadota>Gammaproteobacteria>Enterobacterales>Enterobacteriaceae>Escherichia	GCA_022888875.1	Escherichia_coli	MVR17_003830	10074..13715	562	BREX-system-P-loop-protein-BrxC-[Escherichia-coli]
EJH0789763.1	Type1.BREX	[illegible]	1107	Bacteria>Pseudomonadota>Gammaproteobacteria>Enterobacterales>Enterobacteriaceae>Escherichia	GCA_023637565.1	Escherichia_coli	NCI57_002418	1..3324	562	BREX-system-P-loop-protein-BrxC-[Escherichia-coli]
EJM6441909.1	Type1.BREX	[illegible]	1006	Bacteria>Pseudomonadota>Gammaproteobacteria>Enterobacterales>Enterobacteriaceae>Escherichia	GCA_024340725.1	Escherichia_coli	NOR68_003993	1..3023	562	BREX-system-P-loop-protein-BrxC-[Escherichia-coli]
MCQ4517261.1	Type1.BREX	[illegible]	1213	Bacteria>Pseudomonadota>Gammaproteobacteria>Enterobacterales>Enterobacteriaceae>Escherichia	GCA_024450325.1	Escherichia_coli	NO030_20220	28424..32065	562	BREX-system-P-loop-protein-BrxC-[Escherichia-coli]
EJN8021038.1	Type1.BREX	[illegible]	1213	Bacteria>Pseudomonadota>Gammaproteobacteria>Enterobacterales>Enterobacteriaceae>Escherichia	GCA_024524615.1	Escherichia_coli	NRW59_000779	2207..5848	562	BREX-system-P-loop-protein-BrxC-[Escherichia-coli]
EJO9498073.1	Type1.BREX	[illegible]	1213	Bacteria>Pseudomonadota>Gammaproteobacteria>Enterobacterales>Enterobacteriaceae>Escherichia	GCA_024612475.1	Escherichia_coli	NUZ09_002875	7894..11535	562	BREX-system-P-loop-protein-BrxC-[Escherichia-coli]
HCN4662824.1	Type1.BREX	[illegible]	1213	Bacteria>Pseudomonadota>Gammaproteobacteria>Enterobacterales>Enterobacteriaceae>Escherichia	GCA_025276155.1	Escherichia_coli	N6W02_002919	2176..5817	562	BREX-system-P-loop-protein-BrxC-[Escherichia-coli]
HCO6583160.1	Type1.BREX	[illegible]	1213	Bacteria>Pseudomonadota>Gammaproteobacteria>Enterobacterales>Enterobacteriaceae>Escherichia	GCA_025412985.1	Escherichia_coli	OBR12_001368	111262..114903	562	BREX-system-P-loop-protein-BrxC-[Escherichia-coli]
HCP1392420.1	Type1.BREX	[illegible]	1213	Bacteria>Pseudomonadota>Gammaproteobacteria>Enterobacterales>Enterobacteriaceae>Escherichia	GCA_025491965.1	Escherichia_coli	OCX38_000210	218312..221953	562	BREX-system-P-loop-protein-BrxC-[Escherichia-coli]
CEK08497.1	Type1.BREX	[illegible]	1218	Bacteria>Pseudomonadota>Gammaproteobacteria>Enterobacterales>Enterobacteriaceae>Escherichia	GCA_900000205.1	Escherichia_coli_O26:H11	ECO26H__800033	35711..39367	244319	conserved-hypothetical-protein-[Escherichia-coli-O26:H11]
WP_001019642.1	Type1.BREX	[illegible]	1188	Bacteria>Pseudomonadota>Gammaproteobacteria>Enterobacterales>Enterobacteriaceae>Escherichia	GCF_000194355.1	Escherichia_coli	ECJB195_RS15905	80241..83807	562	BREX-system-P-loop-protein-BrxC-[Escherichia-coli]
WP_001704019.1	Type1.BREX	[illegible]	1213	Bacteria>Pseudomonadota>Gammaproteobacteria>Enterobacterales>Enterobacteriaceae>Escherichia	GCF_000354275.2	Escherichia_coli	EC2875000_RS00745	314498..318139	562	BREX-system-P-loop-protein-BrxC-[Escherichia-coli]
WP_001704755.1	Type1.BREX	[illegible]	1213	Bacteria>Pseudomonadota>Gammaproteobacteria>Enterobacterales>Enterobacteriaceae>Escherichia	GCF_000354455.1	Escherichia_coli	EC2872800_RS00650	314338..317979	562	BREX-system-P-loop-protein-BrxC-[Escherichia-coli]
WP_001707406.1	Type1.BREX	[illegible]	1213	Bacteria>Pseudomonadota>Gammaproteobacteria>Enterobacterales>Enterobacteriaceae>Escherichia	GCF_000354815.2	Escherichia_coli	EC2867750_RS24725	314336..317977	562	BREX-system-P-loop-protein-BrxC-[Escherichia-coli]
WP_003969750.1	Type1.BREX	[illegible]	1213	Bacteria>Pseudomonadota>Gammaproteobacteria>Enterobacterales>Enterobacteriaceae>Escherichia	GCF_000354935.2	Escherichia_coli	EC2866450_RS25180	218299..221940	562	BREX-system-P-loop-protein-BrxC-[Escherichia-coli]
WP_001709521.1	Type1.BREX	[illegible]	1213	Bacteria>Pseudomonadota>Gammaproteobacteria>Enterobacterales>Enterobacteriaceae>Escherichia	GCF_000354955.1	Escherichia_coli	EC2866550_RS00550	55545..59186	562	BREX-system-P-loop-protein-BrxC-[Escherichia-coli]
WP_001713779.1	Type1.BREX	[illegible]	1213	Bacteria>Pseudomonadota>Gammaproteobacteria>Enterobacterales>Enterobacteriaceae>Escherichia	GCF_000355015.2	Escherichia_coli	EC2851500_RS25090	314221..317862	562	BREX-system-P-loop-protein-BrxC-[Escherichia-coli]
WP_001715918.1	Type1.BREX	[illegible]	1213	Bacteria>Pseudomonadota>Gammaproteobacteria>Enterobacterales>Enterobacteriaceae>Escherichia	GCF_000355055.1	Escherichia_coli	EC2850400_RS01425	314375..318016	562	BREX-system-P-loop-protein-BrxC-[Escherichia-coli]
WP_001714681.1	Type1.BREX	[illegible]	1213	Bacteria>Pseudomonadota>Gammaproteobacteria>Enterobacterales>Enterobacteriaceae>Escherichia	GCF_000355335.2	Escherichia_coli	EC2850750_RS25235	55686..59327	562	BREX-system-P-loop-protein-BrxC-[Escherichia-coli]
WP_001462984.1	Type1.BREX	[illegible]	1213	Bacteria>Pseudomonadota>Gammaproteobacteria>Enterobacterales>Enterobacteriaceae>Escherichia	GCF_000357005.2	Escherichia_coli	ECP02994383_RS24020	76495..80136	562	BREX-system-P-loop-protein-BrxC-[Escherichia-coli]
WP_001464881.1	Type1.BREX	[illegible]	1213	Bacteria>Pseudomonadota>Gammaproteobacteria>Enterobacterales>Enterobacteriaceae>Escherichia	GCF_000357045.2	Escherichia_coli	ECP02994386_RS23895	144466..148107	562	BREX-system-P-loop-protein-BrxC-[Escherichia-coli]
WP_001466204.1	Type1.BREX	[illegible]	1213	Bacteria>Pseudomonadota>Gammaproteobacteria>Enterobacterales>Enterobacteriaceae>Escherichia	GCF_000357245.1	Escherichia_coli	ECP02994387_RS14340	10081..13722	562	BREX-system-P-loop-protein-BrxC-[Escherichia-coli]
WP_001505004.1	Type1.BREX	[illegible]	1213	Bacteria>Pseudomonadota>Gammaproteobacteria>Enterobacterales>Enterobacteriaceae>Escherichia	GCF_000446625.2	Escherichia_coli	ECC52794_RS10870	64245..67886	562	BREX-system-P-loop-protein-BrxC-[Escherichia-coli]
WP_024222704.1	Type1.BREX	[illegible]	1213	Bacteria>Pseudomonadota>Gammaproteobacteria>Enterobacterales>Enterobacteriaceae>Escherichia	GCF_000446725.2	Escherichia_coli	CTH77_RS23215	13991..17632	562	BREX-system-P-loop-protein-BrxC-[Escherichia-coli]
WP_024236748.1	Type1.BREX	[illegible]	1129	Bacteria>Pseudomonadota>Gammaproteobacteria>Enterobacterales>Enterobacteriaceae>Escherichia	GCF_000446745.2	Escherichia_coli	ECC71710_RS03330	1..3392	562	BREX-system-P-loop-protein-BrxC,-partial-[Escherichia-coli]
WP_024243520.1	Type1.BREX	[illegible]	1213	Bacteria>Pseudomonadota>Gammaproteobacteria>Enterobacterales>Enterobacteriaceae>Escherichia	GCF_000446965.2	Escherichia_coli	C3I80_RS17935	35165..38806	562	BREX-system-P-loop-protein-BrxC-[Escherichia-coli]

Protein-ID	System	Gene-Neighbourhoods	Length	Taxonomy	Assembly-ID	Strain	Locus-Tag	Genomic-coordinates	TaxID	Fasta-define
WP_029488486.1	Type1.BREX	[illegible]	1213	Bacteria>Pseudomonadota>Gammaproteobacteria>Enterobacterales>Enterobacteriaceae>Escherichia	GCF_000511545.1	Escherichia_coli	U124_RS0103885	150968..154609	562	BREX-system-P-loop-protein-BrxC-[Escherichia-coli]
WP_032271764.1	Type1.BREX	[illegible]	996	Bacteria>Pseudomonadota>Gammaproteobacteria>Enterobacterales>Enterobacteriaceae>Escherichia	GCF_000614035.2	Escherichia_coli	BX93_RS13740	78498..81489	562	BREX-system-P-loop-protein-BrxC,-partial-[Escherichia-coli]
WP_032272074.1	Type1.BREX	[illegible]	1188	Bacteria>Pseudomonadota>Gammaproteobacteria>Enterobacterales>Enterobacteriaceae>Escherichia	GCF_000615605.2	Escherichia_coli	BX59_RS22390	1..3568	562	BREX-system-P-loop-protein-BrxC,-partial-[Escherichia-coli]
WP_032353829.1	Type1.BREX	[illegible]	1213	Bacteria>Pseudomonadota>Gammaproteobacteria>Enterobacterales>Enterobacteriaceae>Escherichia	GCF_000632675.2	Escherichia_coli	BX99_RS21625	78780..82421	562	BREX-system-P-loop-protein-BrxC-[Escherichia-coli]
WP_033804924.1	Type1.BREX	[illegible]	862	Bacteria>Pseudomonadota>Gammaproteobacteria>Enterobacterales>Enterobacteriaceae>Escherichia	GCF_000752535.1	Escherichia_coli	MY85_RS14095	76836..79424	562	BREX-system-P-loop-protein-BrxC,-partial-[Escherichia-coli]
WP_053916754.1	Type1.BREX	[illegible]	1213	Bacteria>Pseudomonadota>Gammaproteobacteria>Enterobacterales>Enterobacteriaceae>Escherichia	GCF_001283285.1	Escherichia_coli	AOH25_RS20055	35212..38853	562	BREX-system-P-loop-protein-BrxC-[Escherichia-coli]
WP_053892086.1	Type1.BREX	[illegible]	1213	Bacteria>Pseudomonadota>Gammaproteobacteria>Enterobacterales>Enterobacteriaceae>Escherichia	GCF_001285285.1	Escherichia_coli	AOH78_RS17135	37162..40803	562	BREX-system-P-loop-protein-BrxC-[Escherichia-coli]
WP_021568966.1	Type1.BREX	[illegible]	1213	Bacteria>Pseudomonadota>Gammaproteobacteria>Enterobacterales>Enterobacteriaceae>Escherichia	GCF_001285965.1	Escherichia_coli	AOJ37_RS12845	35181..38822	562	BREX-system-P-loop-protein-BrxC-[Escherichia-coli]
WP_032257286.1	Type1.BREX	[illegible]	1213	Bacteria>Pseudomonadota>Gammaproteobacteria>Enterobacterales>Enterobacteriaceae>Escherichia	GCF_001286385.1	Escherichia_coli	AOJ18_RS01745	37207..40848	562	BREX-system-P-loop-protein-BrxC-[Escherichia-coli]
WP_053897162.1	Type1.BREX	[illegible]	1132	Bacteria>Pseudomonadota>Gammaproteobacteria>Enterobacterales>Enterobacteriaceae>Escherichia	GCF_001286505.1	Escherichia_coli	AOJ58_RS17615	1..3401	562	BREX-system-P-loop-protein-BrxC,-partial-[Escherichia-coli]
WP_062863778.1	Type1.BREX	[illegible]	998	Bacteria>Pseudomonadota>Gammaproteobacteria>Enterobacterales>Enterobacteriaceae>Escherichia	GCF_001607105.1	Escherichia_coli	AML45_RS08440	1..2999	562	BREX-system-P-loop-protein-BrxC,-partial-[Escherichia-coli]
WP_074525756.1	Type1.BREX	[illegible]	1213	Bacteria>Pseudomonadota>Gammaproteobacteria>Enterobacterales>Enterobacteriaceae>Escherichia	GCF_001911555.1	Escherichia_coli	ACN68_RS20915	50623..54264	562	BREX-system-P-loop-protein-BrxC-[Escherichia-coli]
WP_089613509.1	Type1.BREX	[illegible]	1213	Bacteria>Pseudomonadota>Gammaproteobacteria>Enterobacterales>Enterobacteriaceae>Escherichia	GCF_002231555.1	Escherichia_coli	ATB14_RS09425	78080..81721	562	BREX-system-P-loop-protein-BrxC-[Escherichia-coli]
WP_097426405.1	Type1.BREX	[illegible]	1213	Bacteria>Pseudomonadota>Gammaproteobacteria>Enterobacterales>Enterobacteriaceae>Escherichia	GCF_002510325.1	Escherichia_coli	A7660_RS13795	13148..16789	562	BREX-system-P-loop-protein-BrxC-[Escherichia-coli]
WP_244587252.1	Type1.BREX	[illegible]	1137	Bacteria>Pseudomonadota>Gammaproteobacteria>Enterobacterales>Enterobacteriaceae>Escherichia	GCF_002519325.1	Escherichia_coli	A7034_RS17685	1..3416	562	BREX-system-P-loop-protein-BrxC,-partial-[Escherichia-coli]
WP_099139404.1	Type1.BREX	[illegible]	1213	Bacteria>Pseudomonadota>Gammaproteobacteria>Enterobacterales>Enterobacteriaceae>Escherichia	GCF_002631765.2	Escherichia_coli	CQR85_RS13645	71476..75117	562	BREX-system-P-loop-protein-BrxC-[Escherichia-coli]
WP_096974508.1	Type1.BREX	[illegible]	1213	Bacteria>Pseudomonadota>Gammaproteobacteria>Enterobacterales>Enterobacteriaceae>Escherichia	GCF_002741355.1	Escherichia_coli	BFZ27_RS06875	15053..18694	562	BREX-system-P-loop-protein-BrxC-[Escherichia-coli]
WP_106879201.1	Type1.BREX	[illegible]	1213	Bacteria>Pseudomonadota>Gammaproteobacteria>Enterobacterales>Enterobacteriaceae>Escherichia	GCF_003018215.1	Escherichia_coli	C6P67_RS24165	4665409..4669050	562	BREX-system-P-loop-protein-BrxC-[Escherichia-coli]
WP_044805810.1	Type1.BREX	[illegible]	1213	Bacteria>Pseudomonadota>Gammaproteobacteria>Enterobacterales>Enterobacteriaceae>Escherichia	GCF_003018495.1	Escherichia_coli	C6953_RS06925	1324145..1327786	562	BREX-system-P-loop-protein-BrxC-[Escherichia-coli]
WP_109548991.1	Type1.BREX	[illegible]	1213	Bacteria>Pseudomonadota>Gammaproteobacteria>Enterobacterales>Enterobacteriaceae>Escherichia	GCF_003145675.1	Escherichia_coli	B6S34_RS22850	50490..54131	562	BREX-system-P-loop-protein-BrxC-[Escherichia-coli]
WP_121351411.1	Type1.BREX	[illegible]	1213	Bacteria>Pseudomonadota>Gammaproteobacteria>Enterobacterales>Enterobacteriaceae>Escherichia	GCF_003651285.1	Escherichia_coli	EAO63_RS07885	97180..100821	562	BREX-system-P-loop-protein-BrxC-[Escherichia-coli]
WP_044782573.1	Type1.BREX	[illegible]	1213	Bacteria>Pseudomonadota>Gammaproteobacteria>Enterobacterales>Enterobacteriaceae>Escherichia	GCF_005390485.1	Escherichia_coli	FFG15_RS13655	95922..99563	562	BREX-system-P-loop-protein-BrxC-[Escherichia-coli]
WP_097512272.1	Type1.BREX	[illegible]	1213	Bacteria>Pseudomonadota>Gammaproteobacteria>Enterobacterales>Enterobacteriaceae>Escherichia	GCF_005391145.1	Escherichia_coli	A7301_RS09385	138963..142604	562	BREX-system-P-loop-protein-BrxC-[Escherichia-coli]
WP_137536909.1	Type1.BREX	[illegible]	1213	Bacteria>Pseudomonadota>Gammaproteobacteria>Enterobacterales>Enterobacteriaceae>Escherichia	GCF_005392025.1	Escherichia_coli	FFI00_RS00495	95917..99558	562	BREX-system-P-loop-protein-BrxC-[Escherichia-coli]
WP_063115883.1	Type1.BREX	[illegible]	1213	Bacteria>Pseudomonadota>Gammaproteobacteria>Enterobacterales>Enterobacteriaceae>Escherichia	GCF_005393265.1	Escherichia_coli	MMU60_RS13225	76316..79957	562	BREX-system-P-loop-protein-BrxC-[Escherichia-coli]
WP_137551577.1	Type1.BREX	[illegible]	1213	Bacteria>Pseudomonadota>Gammaproteobacteria>Enterobacterales>Enterobacteriaceae>Escherichia	GCF_005394845.1	Escherichia_coli	FFJ65_RS02650	22335..25976	562	BREX-system-P-loop-protein-BrxC-[Escherichia-coli]
WP_137585959.1	Type1.BREX	[illegible]	1213	Bacteria>Pseudomonadota>Gammaproteobacteria>Enterobacterales>Enterobacteriaceae>Escherichia	GCF_005399505.1	Escherichia_coli	FFL73_RS10880	97640..101281	562	BREX-system-P-loop-protein-BrxC-[Escherichia-coli]
WP_137590371.1	Type1.BREX	[illegible]	1213	Bacteria>Pseudomonadota>Gammaproteobacteria>Enterobacterales>Enterobacteriaceae>Escherichia	GCF_005399685.1	Escherichia_coli	FFM00_RS14520	19836..23477	562	BREX-system-P-loop-protein-BrxC-[Escherichia-coli]
WP_050939406.1	Type1.BREX	[illegible]	1213	Bacteria>Pseudomonadota>Gammaproteobacteria>Enterobacterales>Enterobacteriaceae>Escherichia	GCF_009618015.1	Escherichia_coli	DTB71_RS01510	297715..301356	562	BREX-system-P-loop-protein-BrxC-[Escherichia-coli]
WP_160518859.1	Type1.BREX	[illegible]	1213	Bacteria>Pseudomonadota>Gammaproteobacteria>Enterobacterales>Enterobacteriaceae>Escherichia	GCF_009820915.1	Escherichia_coli	FQ032_RS06170	111352..114993	562	BREX-system-P-loop-protein-BrxC-[Escherichia-coli]
WP_164476769.1	Type1.BREX	[illegible]	1213	Bacteria>Pseudomonadota>Gammaproteobacteria>Enterobacterales>Enterobacteriaceae>Escherichia	GCF_011045235.1	Escherichia_coli	G6Z93_RS19880	4188029..4191670	562	BREX-system-P-loop-protein-BrxC-[Escherichia-coli]
WP_250854521.1	Type1.BREX	[illegible]	887	Bacteria>Pseudomonadota>Gammaproteobacteria>Enterobacterales>Enterobacteriaceae>Escherichia	GCF_012556305.1	Escherichia_coli	MLIZ_RS15475	1..2666	562	BREX-system-P-loop-protein-BrxC,-partial-[Escherichia-coli]
WP_025653408.1	Type1.BREX	[illegible]	1213	Bacteria>Pseudomonadota>Gammaproteobacteria>Enterobacterales>Enterobacteriaceae>Escherichia	GCF_014169595.1	Escherichia_coli	H7R13_RS19900	4165197..4168838	562	BREX-system-P-loop-protein-BrxC-[Escherichia-coli]
WP_197364841.1	Type1.BREX	[illegible]	1213	Bacteria>Pseudomonadota>Gammaproteobacteria>Enterobacterales>Enterobacteriaceae>Escherichia	GCF_015912555.1	Escherichia_coli	F2X22_RS13815	740..4381	562	BREX-system-P-loop-protein-BrxC-[Escherichia-coli]
WP_205937296.1	Type1.BREX	[illegible]	1213	Bacteria>Pseudomonadota>Gammaproteobacteria>Enterobacterales>Enterobacteriaceae>Escherichia	GCF_017117165.1	Escherichia_coli	C3E82_RS10505	78550..82191	562	BREX-system-P-loop-protein-BrxC-[Escherichia-coli]
WP_207516242.1	Type1.BREX	[illegible]	1213	Bacteria>Pseudomonadota>Gammaproteobacteria>Enterobacterales>Enterobacteriaceae>Escherichia	GCF_017356105.1	Escherichia_coli	J4Q20_RS14625	71539..75180	562	BREX-system-P-loop-protein-BrxC-[Escherichia-coli]
WP_210173219.1	Type1.BREX	[illegible]	1213	Bacteria>Pseudomonadota>Gammaproteobacteria>Enterobacterales>Enterobacteriaceae>Escherichia	GCF_017897155.1	Escherichia_coli	J8352_RS05700	22726..26367	562	BREX-system-P-loop-protein-BrxC-[Escherichia-coli]
WP_186240487.1	Type1.BREX	[illegible]	1213	Bacteria>Pseudomonadota>Gammaproteobacteria>Enterobacterales>Enterobacteriaceae>Escherichia	GCF_018141145.1	Escherichia_coli	HUS69_RS19530	4048381..4052022	562	BREX-system-P-loop-protein-BrxC-[Escherichia-coli]
WP_216317187.1	Type1.BREX	[illegible]	1213	Bacteria>Pseudomonadota>Gammaproteobacteria>Enterobacterales>Enterobacteriaceae>Escherichia	GCF_018884345.1	Escherichia_coli	EXA04_RS21875	4479425..4483066	562	BREX-system-P-loop-protein-BrxC-[Escherichia-coli]
WP_236936469.1	Type1.BREX	[illegible]	1213	Bacteria>Pseudomonadota>Gammaproteobacteria>Enterobacterales>Enterobacteriaceae>Escherichia	GCF_021698335.1	Escherichia_coli	IHV39_RS14485	12915..16556	562	BREX-system-P-loop-protein-BrxC-[Escherichia-coli]
WP_255944649.1	Type1.BREX	[illegible]	1213	Bacteria>Pseudomonadota>Gammaproteobacteria>Enterobacterales>Enterobacteriaceae>Escherichia	GCF_024450325.1	Escherichia_coli	NO030_RS20220	28424..32065	562	BREX-system-P-loop-protein-BrxC-[Escherichia-coli]
WP_257249285.1	Type1.BREX	[illegible]	1213	Bacteria>Pseudomonadota>Gammaproteobacteria>Enterobacterales>Enterobacteriaceae>Escherichia	GCF_024608215.1	Escherichia_coli	NO078_RS04965	41836..45477	562	BREX-system-P-loop-protein-BrxC-[Escherichia-coli]
WP_263767786.1	Type1.BREX	[illegible]	1072	Bacteria>Pseudomonadota>Gammaproteobacteria>Enterobacterales>Enterobacteriaceae>Escherichia	GCF_025750965.1	Escherichia_coli	OHF01_RS16785	78632..81850	562	BREX-system-P-loop-protein-BrxC,-partial-[Escherichia-coli]
WP_270658579.1	Type1.BREX	[illegible]	1213	Bacteria>Pseudomonadota>Gammaproteobacteria>Enterobacterales>Enterobacteriaceae>Escherichia	GCF_027696005.1	Escherichia_coli	PG805_RS04695	85075..88716	562	BREX-system-P-loop-protein-BrxC-[Escherichia-coli]
WP_152072595.1	Type1.BREX	[illegible]	1213	Bacteria>Pseudomonadota>Gammaproteobacteria>Enterobacterales>Enterobacteriaceae>Escherichia	GCF_902501575.1	Escherichia_coli	GDK01_RS16655	24181..27822	562	BREX-system-P-loop-protein-BrxC-[Escherichia-coli]
WP_096955310.1	Type1.BREX	[illegible]	1213	Bacteria>Pseudomonadota>Gammaproteobacteria>Enterobacterales>Enterobacteriaceae>Escherichia	GCF_904831825.1	Escherichia_coli	A7198_RS13140	15059..18700	562	BREX-system-P-loop-protein-BrxC-[Escherichia-coli]
WP_096964704.1	Type1.BREX	[illegible]	1213	Bacteria>Pseudomonadota>Gammaproteobacteria>Enterobacterales>Enterobacteriaceae>Escherichia	GCF_948144475.1	Escherichia_coli	A7257_RS18210	50280..53921	562	BREX-system-P-loop-protein-BrxC-[Escherichia-coli]
WP_160450150.1	Type1.BREX	[illegible]	1213	Bacteria>Pseudomonadota>Gammaproteobacteria>Enterobacterales>Enterobacteriaceae	-	Escherichia	H1X95_RS06210	181594..185235	561	MULTISPECIES:-BREX-system-P-loop-protein-BrxC-[Escherichia]
WP_052939500.1	Type1.BREX	[illegible]	1204	Bacteria>Pseudomonadota>Gammaproteobacteria>Enterobacterales>Enterobacteriaceae	GCF_001569545.1	Salmonella	MA624_RS20100	4127586..4131200	590	MULTISPECIES:-BREX-system-P-loop-protein-BrxC-[Salmonella]
WP_063308429.1	Type1.BREX	[illegible]	1213	Bacteria>Pseudomonadota>Gammaproteobacteria>Enterobacterales>Enterobacteriaceae	GCF_001623605.1	Enterobacter	A4308_RS00205	35188..38829	547	MULTISPECIES:-BREX-system-P-loop-protein-BrxC-[Enterobacter]
WP_070534016.1	Type1.BREX	[illegible]	1213	Bacteria>Pseudomonadota>Gammaproteobacteria>Enterobacterales>Enterobacteriaceae	GCF_024104775.1	Enterobacter	KGY84_RS21865	326949..330590	547	MULTISPECIES:-BREX-system-P-loop-protein-BrxC-[Enterobacter]
WP_048999825.1	Type1.BREX	[illegible]	1204	Bacteria>Pseudomonadota>Gammaproteobacteria>Enterobacterales>Enterobacteriaceae	GCF_900775105.1	Klebsiella/Raoultella_group	E7V87_RS13635	63633..67247	2890311	MULTISPECIES:-BREX-system-P-loop-protein-BrxC-[Klebsiella/Raoultella-group]
QJK24913.1	Type1.BREX	[illegible]	1213	Bacteria>Pseudomonadota>Gammaproteobacteria>Enterobacterales>Enterobacteriaceae>Klebsiella/Raoultella_group	GCA_012967125.1	Klebsiella_pneumoniae	HJX28_24000	4748106..4751747	573	BREX-system-P-loop-protein-BrxC-[Klebsiella-pneumoniae]
MBL3233196.1	Type1.BREX	[illegible]	1213	Bacteria>Pseudomonadota>Gammaproteobacteria>Enterobacterales>Enterobacteriaceae>Klebsiella/Raoultella_group	GCA_016755095.1	Klebsiella_pneumoniae	ELP31_24695	10226..13867	573	BREX-system-P-loop-protein-BrxC-[Klebsiella-pneumoniae]
HBQ6953848.1	Type1.BREX	[illegible]	1043	Bacteria>Pseudomonadota>Gammaproteobacteria>Enterobacterales>Enterobacteriaceae>Klebsiella/Raoultella_group	GCA_021875055.1	Klebsiella_pneumoniae	L8O79_001073	40057..43187	573	BREX-system-P-loop-protein-BrxC-[Klebsiella-pneumoniae]
HBR1265003.1	Type1.BREX	[illegible]	1213	Bacteria>Pseudomonadota>Gammaproteobacteria>Enterobacterales>Enterobacteriaceae>Klebsiella/Raoultella_group	GCA_021909255.1	Klebsiella_quasipneumoniae_subsp._quasipneumoniae	L9L24_003166	12201..15842	1667327	BREX-system-P-loop-protein-BrxC-[Klebsiella-quasipneumoniae-subsp.-quasipneumoniae]
HBR1743014.1	Type1.BREX	[illegible]	1213	Bacteria>Pseudomonadota>Gammaproteobacteria>Enterobacterales>Enterobacteriaceae>Klebsiella/Raoultella_group	GCA_021911065.1	Klebsiella_pneumoniae	L9M23_004127	37623..41264	573	BREX-system-P-loop-protein-BrxC-[Klebsiella-pneumoniae]
HBR4416044.1	Type1.BREX	[illegible]	1213	Bacteria>Pseudomonadota>Gammaproteobacteria>Enterobacterales>Enterobacteriaceae>Klebsiella/Raoultella_group	GCA_021925925.1	Klebsiella_pneumoniae	L9V39_001889	16089..19730	573	BREX-system-P-loop-protein-BrxC-[Klebsiella-pneumoniae]
HBR6062602.1	Type1.BREX	[illegible]	1213	Bacteria>Pseudomonadota>Gammaproteobacteria>Enterobacterales>Enterobacteriaceae>Klebsiella/Raoultella_group	GCA_021932975.1	Klebsiella_pneumoniae	L9O24_005225	4087..7728	573	BREX-system-P-loop-protein-BrxC-[Klebsiella-pneumoniae]
EIV7918660.1	Type1.BREX	[illegible]	1213	Bacteria>Pseudomonadota>Gammaproteobacteria>Enterobacterales>Enterobacteriaceae>Klebsiella/Raoultella_group	GCA_022009395.1	Klebsiella_pneumoniae	MA315_005123	14682..18323	573	BREX-system-P-loop-protein-BrxC-[Klebsiella-pneumoniae]
HBU3475135.1	Type1.BREX	[illegible]	1213	Bacteria>Pseudomonadota>Gammaproteobacteria>Enterobacterales>Enterobacteriaceae>Klebsiella/Raoultella_group	GCA_022085545.1	Klebsiella_pneumoniae	MCW21_004534	37275..40916	573	BREX-system-P-loop-protein-BrxC-[Klebsiella-pneumoniae]
HBV1956328.1	Type1.BREX	[illegible]	1213	Bacteria>Pseudomonadota>Gammaproteobacteria>Enterobacterales>Enterobacteriaceae>Klebsiella/Raoultella_group	GCA_022126105.1	Klebsiella_pneumoniae	MDQ85_004847	2358..5999	573	BREX-system-P-loop-protein-BrxC-[Klebsiella-pneumoniae]
HBV8971411.1	Type1.BREX	[illegible]	1213	Bacteria>Pseudomonadota>Gammaproteobacteria>Enterobacterales>Enterobacteriaceae>Klebsiella/Raoultella_group	GCA_022162485.1	Klebsiella_oxytoca	MD887_005073	22560..26201	571	BREX-system-P-loop-protein-BrxC-[Klebsiella-oxytoca]
HBW3476175.1	Type1.BREX	[illegible]	1162	Bacteria>Pseudomonadota>Gammaproteobacteria>Enterobacterales>Enterobacteriaceae>Klebsiella/Raoultella_group	GCA_022184305.1	Klebsiella_pneumoniae	MEZ45_004510	80809..84297	573	BREX-system-P-loop-protein-BrxC-[Klebsiella-pneumoniae]
HBW3608486.1	Type1.BREX	[illegible]	1213	Bacteria>Pseudomonadota>Gammaproteobacteria>Enterobacterales>Enterobacteriaceae>Klebsiella/Raoultella_group	GCA_022184865.1	Klebsiella_pneumoniae	MEZ81_004956	16088..19729	573	BREX-system-P-loop-protein-BrxC-[Klebsiella-pneumoniae]
HBW7740799.1	Type1.BREX	[illegible]	1213	Bacteria>Pseudomonadota>Gammaproteobacteria>Enterobacterales>Enterobacteriaceae>Klebsiella/Raoultella_group	GCA_022200625.1	Klebsiella_pneumoniae	MFE66_003732	16104..19745	573	BREX-system-P-loop-protein-BrxC-[Klebsiella-pneumoniae]
HBX0790077.1	Type1.BREX	[illegible]	1213	Bacteria>Pseudomonadota>Gammaproteobacteria>Enterobacterales>Enterobacteriaceae>Klebsiella/Raoultella_group	GCA_022212165.1	Klebsiella_pneumoniae	MF115_18880	50016..53657	573	BREX-system-P-loop-protein-BrxC-[Klebsiella-pneumoniae]
HBX5767913.1	Type1.BREX	[illegible]	1213	Bacteria>Pseudomonadota>Gammaproteobacteria>Enterobacterales>Enterobacteriaceae>Klebsiella/Raoultella_group	GCA_022248915.1	Klebsiella_pneumoniae	MH553_25875	12227..15868	573	BREX-system-P-loop-protein-BrxC-[Klebsiella-pneumoniae]
HBY1687637.1	Type1.BREX	[illegible]	1213	Bacteria>Pseudomonadota>Gammaproteobacteria>Enterobacterales>Enterobacteriaceae>Klebsiella/Raoultella_group	GCA_022272455.1	Klebsiella_pneumoniae	MIQ44_23625	54198..57839	573	BREX-system-P-loop-protein-BrxC-[Klebsiella-pneumoniae]
EIX9551660.1	Type1.BREX	[illegible]	1213	Bacteria>Pseudomonadota>Gammaproteobacteria>Enterobacterales>Enterobacteriaceae>Klebsiella/Raoultella_group	GCA_022431205.1	Klebsiella_pneumoniae	ML061_004130	17308..20949	573	BREX-system-P-loop-protein-BrxC-[Klebsiella-pneumoniae]
HBZ8012775.1	Type1.BREX	[illegible]	1213	Bacteria>Pseudomonadota>Gammaproteobacteria>Enterobacterales>Enterobacteriaceae>Klebsiella/Raoultella_group	GCA_022500545.1	Klebsiella_pneumoniae	MM158_002941	16412..20053	573	BREX-system-P-loop-protein-BrxC-[Klebsiella-pneumoniae]
HCA0942642.1	Type1.BREX	[illegible]	1213	Bacteria>Pseudomonadota>Gammaproteobacteria>Enterobacterales>Enterobacteriaceae>Klebsiella/Raoultella_group	GCA_022528725.1	Klebsiella_pneumoniae	MNJ11_004335	17307..20948	573	BREX-system-P-loop-protein-BrxC-[Klebsiella-pneumoniae]
HCB0434648.1	Type1.BREX	[illegible]	1213	Bacteria>Pseudomonadota>Gammaproteobacteria>Enterobacterales>Enterobacteriaceae>Klebsiella/Raoultella_group	GCA_023030065.1	Klebsiella_variicola_subsp._variicola	MYM82_004336	18617..22258	2590157	BREX-system-P-loop-protein-BrxC-[Klebsiella-variicola-subsp.-variicola]
MCP6627038.1	Type1.BREX	[illegible]	1213	Bacteria>Pseudomonadota>Gammaproteobacteria>Enterobacterales>Enterobacteriaceae>Klebsiella/Raoultella_group	GCA_024240675.1	Klebsiella_pneumoniae	NL507_17490	188287..191928	573	BREX-system-P-loop-protein-BrxC-[Klebsiella-pneumoniae]
HCI5816641.1	Type1.BREX	[illegible]	1213	Bacteria>Pseudomonadota>Gammaproteobacteria>Enterobacterales>Enterobacteriaceae>Klebsiella/Raoultella_group	GCA_024431185.1	Klebsiella_pneumoniae	NPN32_003719	27788..31429	573	BREX-system-P-loop-protein-BrxC-[Klebsiella-pneumoniae]
GKN17816.1	Type1.BREX	[illegible]	725	Bacteria>Pseudomonadota>Gammaproteobacteria>Enterobacterales>Enterobacteriaceae>Klebsiella/Raoultella_group	GCA_026006635.1	Klebsiella_pneumoniae	NUBL17184_45010	12144..14321	573	hypothetical-protein-NUBL17184_45010-[Klebsiella-pneumoniae]
GKN17819.1	Type1.BREX	[illegible]	453	Bacteria>Pseudomonadota>Gammaproteobacteria>Enterobacterales>Enterobacteriaceae>Klebsiella/Raoultella_group	GCA_026006635.1	Klebsiella_pneumoniae	NUBL17184_45020	14424..15785	573	hypothetical-protein-NUBL17184_45020-[Klebsiella-pneumoniae]
STR42120.1	Type1.BREX	[illegible]	800	Bacteria>Pseudomonadota>Gammaproteobacteria>Enterobacterales>Enterobacteriaceae>Klebsiella/Raoultella_group	GCA_900451375.1	Klebsiella_michiganensis	NCTC11694_03329	2640700..2643102	1134687	Uncharacterised-protein-[Klebsiella-michiganensis]
VTO17843.1	Type1.BREX	[illegible]	864	Bacteria>Pseudomonadota>Gammaproteobacteria>Enterobacterales>Enterobacteriaceae>Klebsiella/Raoultella_group	GCA_901421915.1	Klebsiella_variicola	NCTC9178_02481	2086491..2089085	244366	Uncharacterised-protein-[Klebsiella-variicola]
WP_038432196.1	Type1.BREX	[illegible]	1213	Bacteria>Pseudomonadota>Gammaproteobacteria>Enterobacterales>Enterobacteriaceae>Klebsiella/Raoultella_group	GCF_000742755.1	Klebsiella_pneumoniae	PF543_RS25520	27597..31238	573	BREX-system-P-loop-protein-BrxC-[Klebsiella-pneumoniae]
WP_064406990.1	Type1.BREX	[illegible]	1213	Bacteria>Pseudomonadota>Gammaproteobacteria>Enterobacterales>Enterobacteriaceae>Klebsiella/Raoultella_group	GCF_001598695.1	Klebsiella_oxytoca	KO1_RS15010	40025..43666	571	BREX-system-P-loop-protein-BrxC-[Klebsiella-oxytoca]
WP_094487773.1	Type1.BREX	[illegible]	1213	Bacteria>Pseudomonadota>Gammaproteobacteria>Enterobacterales>Enterobacteriaceae>Klebsiella/Raoultella_group	GCF_002251875.2	Klebsiella_pneumoniae	CI667_RS22335	4584289..4587930	573	BREX-system-P-loop-protein-BrxC-[Klebsiella-pneumoniae]
WP_096925291.1	Type1.BREX	[illegible]	1213	Bacteria>Pseudomonadota>Gammaproteobacteria>Enterobacterales>Enterobacteriaceae>Klebsiella/Raoultella_group	GCF_002411925.1	Klebsiella_pneumoniae	CP905_RS22210	5854..9495	573	BREX-system-P-loop-protein-BrxC-[Klebsiella-pneumoniae]
WP_105178007.1	Type1.BREX	[illegible]	1213	Bacteria>Pseudomonadota>Gammaproteobacteria>Enterobacterales>Enterobacteriaceae>Klebsiella/Raoultella_group	GCF_002934865.1	Klebsiella_pneumoniae	CSC91_RS11390	28681..32322	573	BREX-system-P-loop-protein-BrxC-[Klebsiella-pneumoniae]
WP_023313883.1	Type1.BREX	[illegible]	1213	Bacteria>Pseudomonadota>Gammaproteobacteria>Enterobacterales>Enterobacteriaceae>Klebsiella/Raoultella_group	GCF_003427195.1	Klebsiella_pneumoniae	D2N15_RS14250	17171..20812	573	BREX-system-P-loop-protein-BrxC-[Klebsiella-pneumoniae]
WP_087652989.1	Type1.BREX	[illegible]	1213	Bacteria>Pseudomonadota>Gammaproteobacteria>Enterobacterales>Enterobacteriaceae>Klebsiella/Raoultella_group	GCF_003429625.1	Klebsiella_variicola	BC497_RS18300	3693589..3697230	244366	BREX-system-P-loop-protein-BrxC-[Klebsiella-variicola]
WP_049088891.1	Type1.BREX	[illegible]	1213	Bacteria>Pseudomonadota>Gammaproteobacteria>Enterobacterales>Enterobacteriaceae>Klebsiella/Raoultella_group	GCF_003851825.1	Klebsiella	KPPSC5_RS01070	215107..218748	570	MULTISPECIES:-BREX-system-P-loop-protein-BrxC-[Klebsiella]
WP_087867252.1	Type1.BREX	[illegible]	1213	Bacteria>Pseudomonadota>Gammaproteobacteria>Enterobacterales>Enterobacteriaceae>Klebsiella/Raoultella_group	GCF_004311705.1	Klebsiella_pneumoniae	EOM06_RS18340	199992..203633	573	BREX-system-P-loop-protein-BrxC-[Klebsiella-pneumoniae]
WP_131033948.1	Type1.BREX	[illegible]	1213	Bacteria>Pseudomonadota>Gammaproteobacteria>Enterobacterales>Enterobacteriaceae>Klebsiella/Raoultella_group	GCF_004312565.1	Klebsiella_variicola	EON46_RS24005	106854..110495	244366	BREX-system-P-loop-protein-BrxC-[Klebsiella-variicola]
WP_032410729.1	Type1.BREX	[illegible]	1213	Bacteria>Pseudomonadota>Gammaproteobacteria>Enterobacterales>Enterobacteriaceae>Klebsiella/Raoultella_group	GCF_004312665.1	Klebsiella_pneumoniae	EON31_RS20140	33140..36781	573	BREX-system-P-loop-protein-BrxC-[Klebsiella-pneumoniae]
WP_048334010.1	Type1.BREX	[illegible]	1213	Bacteria>Pseudomonadota>Gammaproteobacteria>Enterobacterales>Enterobacteriaceae>Klebsiella/Raoultella_group	GCF_004313665.1	Klebsiella_pneumoniae	EOO19_RS25945	16153..19794	573	BREX-system-P-loop-protein-BrxC-[Klebsiella-pneumoniae]
WP_032425511.1	Type1.BREX	[illegible]	1213	Bacteria>Pseudomonadota>Gammaproteobacteria>Enterobacterales>Enterobacteriaceae>Klebsiella/Raoultella_group	GCF_013735155.1	Klebsiella_pneumoniae	HV247_RS22800	4632201..4635842	573	BREX-system-P-loop-protein-BrxC-[Klebsiella-pneumoniae]
WP_196942032.1	Type1.BREX	[illegible]	1213	Bacteria>Pseudomonadota>Gammaproteobacteria>Enterobacterales>Enterobacteriaceae>Klebsiella/Raoultella_group	GCF_015773165.1	Klebsiella_michiganensis	I5M64_RS05730	22758..26399	1134687	BREX-system-P-loop-protein-BrxC-[Klebsiella-michiganensis]
WP_200794272.1	Type1.BREX	[illegible]	1213	Bacteria>Pseudomonadota>Gammaproteobacteria>Enterobacterales>Enterobacteriaceae>Klebsiella/Raoultella_group	GCF_016635835.1	Klebsiella_pneumoniae	HZ910_RS23410	16106..19747	573	BREX-system-P-loop-protein-BrxC-[Klebsiella-pneumoniae]
WP_099719181.1	Type1.BREX	[illegible]	1213	Bacteria>Pseudomonadota>Gammaproteobacteria>Enterobacterales>Enterobacteriaceae>Klebsiella/Raoultella_group	GCF_018137755.1	Klebsiella_pneumoniae	KFB18_RS20840	34480..38121	573	BREX-system-P-loop-protein-BrxC-[Klebsiella-pneumoniae]
WP_211586105.1	Type1.BREX	[illegible]	1213	Bacteria>Pseudomonadota>Gammaproteobacteria>Enterobacterales>Enterobacteriaceae>Klebsiella/Raoultella_group	GCF_018137855.1	Klebsiella_pneumoniae	G3W03_RS23040	37519..41160	573	BREX-system-P-loop-protein-BrxC-[Klebsiella-pneumoniae]
WP_223098448.1	Type1.BREX	[illegible]	1213	Bacteria>Pseudomonadota>Gammaproteobacteria>Enterobacterales>Enterobacteriaceae>Klebsiella/Raoultella_group	GCF_019900805.2	Klebsiella_pneumoniae	KZ657_RS22900	4647952..4651593	573	BREX-system-P-loop-protein-BrxC-[Klebsiella-pneumoniae]
WP_053064557.1	Type1.BREX	[illegible]	1213	Bacteria>Pseudomonadota>Gammaproteobacteria>Enterobacterales>Enterobacteriaceae>Klebsiella/Raoultella_group	GCF_019930185.1	Klebsiella	KFB82_RS19625	81811..85452	570	MULTISPECIES:-BREX-system-P-loop-protein-BrxC-[Klebsiella]
WP_101858977.1	Type1.BREX	[illegible]	1213	Bacteria>Pseudomonadota>Gammaproteobacteria>Enterobacterales>Enterobacteriaceae>Klebsiella/Raoultella_group	GCF_020479405.1	Klebsiella_variicola	B6I40_RS13715	18030..21671	244366	BREX-system-P-loop-protein-BrxC-[Klebsiella-variicola]
WP_100751493.1	Type1.BREX	[illegible]	1213	Bacteria>Pseudomonadota>Gammaproteobacteria>Enterobacterales>Enterobacteriaceae>Klebsiella/Raoultella_group	GCF_021261545.1	Klebsiella	CWM56_RS26125	34746..38387	570	MULTISPECIES:-BREX-system-P-loop-protein-BrxC-[Klebsiella]
WP_241187802.1	Type1.BREX	[illegible]	1213	Bacteria>Pseudomonadota>Gammaproteobacteria>Enterobacterales>Enterobacteriaceae>Klebsiella/Raoultella_group	GCF_022543825.1	Klebsiella_michiganensis	MOA98_RS25025	250948..254589	1134687	BREX-system-P-loop-protein-BrxC-[Klebsiella-michiganensis]
WP_241506431.1	Type1.BREX	[illegible]	1213	Bacteria>Pseudomonadota>Gammaproteobacteria>Enterobacterales>Enterobacteriaceae>Klebsiella/Raoultella_group	GCF_022585755.1	Klebsiella_pneumoniae	MMP84_RS15875	154032..157673	573	BREX-system-P-loop-protein-BrxC-[Klebsiella-pneumoniae]
WP_157261982.1	Type1.BREX	[illegible]	1213	Bacteria>Pseudomonadota>Gammaproteobacteria>Enterobacterales>Enterobacteriaceae>Klebsiella/Raoultella_group	GCF_022759445.1	Klebsiella	GSE35_RS20710	46310..49951	570	MULTISPECIES:-BREX-system-P-loop-protein-BrxC-[Klebsiella]
WP_202181851.1	Type1.BREX	[illegible]	1213	Bacteria>Pseudomonadota>Gammaproteobacteria>Enterobacterales>Enterobacteriaceae>Klebsiella/Raoultella_group	GCF_023703955.1	Klebsiella_pneumoniae	KU670_RS11590	2422249..2425890	573	BREX-system-P-loop-protein-BrxC-[Klebsiella-pneumoniae]
WP_255075067.1	Type1.BREX	[illegible]	1213	Bacteria>Pseudomonadota>Gammaproteobacteria>Enterobacterales>Enterobacteriaceae>Klebsiella/Raoultella_group	GCF_024357365.1	Klebsiella_pneumoniae	L1C80_RS20005	166541..170182	573	BREX-system-P-loop-protein-BrxC-[Klebsiella-pneumoniae]
WP_101881469.1	Type1.BREX	[illegible]	1213	Bacteria>Pseudomonadota>Gammaproteobacteria>Enterobacterales>Enterobacteriaceae>Klebsiella/Raoultella_group	GCF_024813835.1	Klebsiella_quasipneumoniae	B6J71_RS18320	31229..34870	1463165	BREX-system-P-loop-protein-BrxC-[Klebsiella-quasipneumoniae]
WP_259574830.1	Type1.BREX	[illegible]	1213	Bacteria>Pseudomonadota>Gammaproteobacteria>Enterobacterales>Enterobacteriaceae>Klebsiella/Raoultella_group	GCF_024998145.1	Klebsiella_pneumoniae	KQ276_RS10525	179571..183212	573	BREX-system-P-loop-protein-BrxC-[Klebsiella-pneumoniae]
WP_265815522.1	Type1.BREX	[illegible]	1213	Bacteria>Pseudomonadota>Gammaproteobacteria>Enterobacterales>Enterobacteriaceae>Klebsiella/Raoultella_group	GCF_026224285.1	Klebsiella_oxytoca	ORM16_RS17370	107113..110754	571	BREX-system-P-loop-protein-BrxC-[Klebsiella-oxytoca]

Protein-ID	System	Gene-Neighbourhoods	Length	Taxonomy	Assembly-ID	Strain	Locus-Tag	Genomic-coordinates	TaxID	Fasta-defline
WP_274901739.1	Type1.BREX	[illegible]	1213	Bacteria>Pseudomonadota>Gammaproteobacteria>Enterobacterales>Enterobacteriaceae>Klebsiella/Raoultella_group	GCF_028894275.1	Klebsiella_pneumoniae	PXW84_RS25015	34732..38373	573	BREX-system-P-loop-protein-BrxC-[Klebsiella-pneumoniae]
WP_275268565.1	Type1.BREX	[illegible]	1196	Bacteria>Pseudomonadota>Gammaproteobacteria>Enterobacterales>Enterobacteriaceae>Klebsiella/Raoultella_group	GCF_029079245.1	Klebsiella_variicola	PZY30_RS10230	1..3591	244366	BREX-system-P-loop-protein-BrxC,-partial-[Klebsiella-variicola]
WP_064171377.1	Type1.BREX	[illegible]	1213	Bacteria>Pseudomonadota>Gammaproteobacteria>Enterobacterales>Enterobacteriaceae>Klebsiella/Raoultella_group	GCF_900084795.1	Klebsiella_variicola	GJ687_RS21830	17638..21279	244366	BREX-system-P-loop-protein-BrxC-[Klebsiella-variicola]
WP_065808708.1	Type1.BREX	[illegible]	1213	Bacteria>Pseudomonadota>Gammaproteobacteria>Enterobacterales>Enterobacteriaceae>Klebsiella/Raoultella_group	GCF_900093245.1	Klebsiella_pneumoniae	BC433_RS15565	37858..41499	573	BREX-system-P-loop-protein-BrxC-[Klebsiella-pneumoniae]
WP_080916293.1	Type1.BREX	[illegible]	1213	Bacteria>Pseudomonadota>Gammaproteobacteria>Enterobacterales>Enterobacteriaceae>Klebsiella/Raoultella_group	GCF_900172605.1	Klebsiella_pneumoniae	B7F35_RS00110	14289..17930	573	BREX-system-P-loop-protein-BrxC-[Klebsiella-pneumoniae]
WP_109220871.1	Type1.BREX	[illegible]	1213	Bacteria>Pseudomonadota>Gammaproteobacteria>Enterobacterales>Enterobacteriaceae>Klebsiella/Raoultella_group	GCF_900407115.1	Klebsiella_oxytoca	DK336_RS07845	45020..48661	571	BREX-system-P-loop-protein-BrxC-[Klebsiella-oxytoca]
WP_115658225.1	Type1.BREX	[illegible]	1213	Bacteria>Pseudomonadota>Gammaproteobacteria>Enterobacterales>Enterobacteriaceae>Klebsiella/Raoultella_group	GCF_900498955.1	Klebsiella_pneumoniae	DY137_RS22260	323150..326791	573	BREX-system-P-loop-protein-BrxC-[Klebsiella-pneumoniae]
WP_117104235.1	Type1.BREX	[illegible]	1213	Bacteria>Pseudomonadota>Gammaproteobacteria>Enterobacterales>Enterobacteriaceae>Klebsiella/Raoultella_group	GCF_900502405.1	Klebsiella_pneumoniae	D2Z44_RS21380	236554..240195	573	BREX-system-P-loop-protein-BrxC-[Klebsiella-pneumoniae]
WP_117044181.1	Type1.BREX	[illegible]	1213	Bacteria>Pseudomonadota>Gammaproteobacteria>Enterobacterales>Enterobacteriaceae>Klebsiella/Raoultella_group	GCF_900503165.1	Klebsiella_pneumoniae	D2094_RS08665	16725..20366	573	BREX-system-P-loop-protein-BrxC-[Klebsiella-pneumoniae]
WP_117133599.1	Type1.BREX	[illegible]	1213	Bacteria>Pseudomonadota>Gammaproteobacteria>Enterobacterales>Enterobacteriaceae>Klebsiella/Raoultella_group	GCF_900509525.1	Klebsiella_variicola	D2R55_RS21945	231466..235107	244366	BREX-system-P-loop-protein-BrxC-[Klebsiella-variicola]
WP_117082329.1	Type1.BREX	[illegible]	1213	Bacteria>Pseudomonadota>Gammaproteobacteria>Enterobacterales>Enterobacteriaceae>Klebsiella/Raoultella_group	GCF_900514375.1	Klebsiella_pneumoniae	SAMEA3515241_04816	29985..33626	573	BREX-system-P-loop-protein-BrxC-[Klebsiella-pneumoniae]
WP_122533088.1	Type1.BREX	[illegible]	1213	Bacteria>Pseudomonadota>Gammaproteobacteria>Enterobacterales>Enterobacteriaceae>Klebsiella/Raoultella_group	GCF_900576735.1	Klebsiella_variicola	EES49_RS00110	18527..22168	244366	BREX-system-P-loop-protein-BrxC-[Klebsiella-variicola]
WP_085803383.1	Type1.BREX	[illegible]	1213	Bacteria>Pseudomonadota>Gammaproteobacteria>Enterobacterales>Enterobacteriaceae>Klebsiella/Raoultella_group	GCF_900774735.1	Klebsiella_pneumoniae	EF470_RS14885	16936..20577	573	BREX-system-P-loop-protein-BrxC-[Klebsiella-pneumoniae]
WP_031591770.1	Type1.BREX	[illegible]	1213	Bacteria>Pseudomonadota>Gammaproteobacteria>Enterobacterales>Enterobacteriaceae>Klebsiella/Raoultella_group	GCF_900774955.1	Klebsiella_pneumoniae	P3L21_RS14210	37429..41070	573	BREX-system-P-loop-protein-BrxC-[Klebsiella-pneumoniae]
WP_114690464.1	Type1.BREX	[illegible]	1213	Bacteria>Pseudomonadota>Gammaproteobacteria>Enterobacterales>Enterobacteriaceae>Klebsiella/Raoultella_group	GCF_900775385.1	Klebsiella_pneumoniae	MC274_RS12705	209352..212993	573	BREX-system-P-loop-protein-BrxC-[Klebsiella-pneumoniae]
WP_064153723.1	Type1.BREX	[illegible]	1213	Bacteria>Pseudomonadota>Gammaproteobacteria>Enterobacterales>Enterobacteriaceae>Klebsiella/Raoultella_group	GCF_900775555.1	Klebsiella_pneumoniae	E7W23_RS23250	82036..85677	573	BREX-system-P-loop-protein-BrxC-[Klebsiella-pneumoniae]
WP_135723013.1	Type1.BREX	[illegible]	1204	Bacteria>Pseudomonadota>Gammaproteobacteria>Enterobacterales>Enterobacteriaceae>Klebsiella/Raoultella_group	GCF_900775875.1	Klebsiella_pneumoniae	E7X40_RS22910	78837..82451	573	BREX-system-P-loop-protein-BrxC-[Klebsiella-pneumoniae]
WP_136086191.1	Type1.BREX	[illegible]	1213	Bacteria>Pseudomonadota>Gammaproteobacteria>Enterobacterales>Enterobacteriaceae>Klebsiella/Raoultella_group	GCF_900978435.1	Klebsiella_variicola	E9559_RS11315	84067..87708	244366	BREX-system-P-loop-protein-BrxC-[Klebsiella-variicola]
WP_142470399.1	Type1.BREX	[illegible]	1213	Bacteria>Pseudomonadota>Gammaproteobacteria>Enterobacterales>Enterobacteriaceae>Klebsiella/Raoultella_group	GCF_902158685.1	Klebsiella_pasteurii	SB6424_04698	319733..323374	2587529	BREX-system-P-loop-protein-BrxC-[Klebsiella-pasteurii]
WP_048266247.1	Type1.BREX	[illegible]	1213	Bacteria>Pseudomonadota>Gammaproteobacteria>Enterobacterales>Enterobacteriaceae>Klebsiella/Raoultella_group	GCF_902161325.1	Klebsiella_pneumoniae	FN306_RS22675	100097..103738	573	BREX-system-P-loop-protein-BrxC-[Klebsiella-pneumoniae]
WP_085820485.1	Type1.BREX	[illegible]	1213	Bacteria>Pseudomonadota>Gammaproteobacteria>Enterobacterales>Enterobacteriaceae>Klebsiella/Raoultella_group	GCF_902508285.1	Klebsiella_pneumoniae	GVM87_RS22970	29420..33061	573	BREX-system-P-loop-protein-BrxC-[Klebsiella-pneumoniae]
WP_225377085.1	Type1.BREX	[illegible]	1204	Bacteria>Pseudomonadota>Gammaproteobacteria>Enterobacterales>Enterobacteriaceae>Klebsiella/Raoultella_group	GCF_916172235.1	Klebsiella_pneumoniae	LF666_RS16195	10713..14327	573	BREX-system-P-loop-protein-BrxC-[Klebsiella-pneumoniae]
WP_225386868.1	Type1.BREX	[illegible]	1213	Bacteria>Pseudomonadota>Gammaproteobacteria>Enterobacterales>Enterobacteriaceae>Klebsiella/Raoultella_group	GCF_916173145.1	Klebsiella_variicola	LF736_RS25380	17904..21545	244366	BREX-system-P-loop-protein-BrxC-[Klebsiella-variicola]
EIV5715275.1	Type1.BREX	[illegible]	1213	Bacteria>Pseudomonadota>Gammaproteobacteria>Enterobacterales>Enterobacteriaceae>Klebsiella/Raoultella_group	-	Klebsiella_pneumoniae	L9F37_004806	56067..59708	573	BREX-system-P-loop-protein-BrxC-[Klebsiella-pneumoniae]
HCA5579377.1	Type1.BREX	[illegible]	1213	Bacteria>Pseudomonadota>Gammaproteobacteria>Enterobacterales>Enterobacteriaceae>Klebsiella/Raoultella_group	-	Klebsiella_pneumoniae	MXD08_004593	70758..74399	573	BREX-system-P-loop-protein-BrxC-[Klebsiella-pneumoniae]
WP_168234029.1	Type1.BREX	[illegible]	1213	Bacteria>Pseudomonadota>Gammaproteobacteria>Enterobacterales>Enterobacteriaceae>Klebsiella/Raoultella_group	-	Klebsiella_variicola	HGK55_RS15775	337116..340757	244366	BREX-system-P-loop-protein-BrxC-[Klebsiella-variicola]
WP_144838631.1	Type1.BREX	[illegible]	1213	Bacteria>Pseudomonadota>Gammaproteobacteria>Enterobacterales>Enterobacteriaceae>Leclercia	GCF_007680555.1	Leclercia_adecarboxylata	FS784_RS12375	119273..122914	83655	BREX-system-P-loop-protein-BrxC-[Leclercia-adecarboxylata]
WP_102104498.1	Type1.BREX	[illegible]	1204	Bacteria>Pseudomonadota>Gammaproteobacteria>Enterobacterales>Enterobacteriaceae>Lelliottia	GCF_002870005.1	unclassified_Lelliottia	F159LOC_RS08780	168198..171812	2642424	MULTISPECIES:-BREX-system-P-loop-protein-BrxC-[unclassified-Lelliottia]
ESG75014.1	Type1.BREX	[illegible]	1142	Bacteria>Pseudomonadota>Gammaproteobacteria>Enterobacterales>Enterobacteriaceae>Salmonella	GCA_000487055.1	Salmonella_enterica_subsp._enterica_serovar_Kentucky_str._ATCC_9263	SEEK9263_16857	19218..22646	984236	ATPase-like-protein-[Salmonella-enterica-subsp.-enterica-serovar-Kentucky-str.-ATCC-9263]
AZT73891.1	Type1.BREX	[illegible]	1204	Bacteria>Pseudomonadota>Gammaproteobacteria>Enterobacterales>Enterobacteriaceae>Salmonella	GCA_003994735.1	Salmonella_enterica_subsp._enterica_serovar_Waycross	ELZ91_19520	3951400..3955014	58101	BREX-system-P-loop-protein-BrxC-[Salmonella-enterica-subsp.-enterica-serovar-Waycross]
EAA5580610.1	Type1.BREX	[illegible]	1213	Bacteria>Pseudomonadota>Gammaproteobacteria>Enterobacterales>Enterobacteriaceae>Salmonella	GCA_004185805.1	Salmonella_enterica_subsp._enterica	DQD90_06840	58937..62578	59201	BREX-system-P-loop-protein-BrxC-[Salmonella-enterica-subsp.-enterica]
EAA8875705.1	Type1.BREX	[illegible]	1213	Bacteria>Pseudomonadota>Gammaproteobacteria>Enterobacterales>Enterobacteriaceae>Salmonella	GCA_004223005.1	Salmonella_enterica	CIU45_09625	381857..385498	28901	BREX-system-P-loop-protein-BrxC-[Salmonella-enterica]
EAB2831578.1	Type1.BREX	[illegible]	1213	Bacteria>Pseudomonadota>Gammaproteobacteria>Enterobacterales>Enterobacteriaceae>Salmonella	GCA_004240605.1	Salmonella_enterica	EAV48_05270	157994..161635	28901	BREX-system-P-loop-protein-BrxC-[Salmonella-enterica]
EAB6843525.1	Type1.BREX	[illegible]	1213	Bacteria>Pseudomonadota>Gammaproteobacteria>Enterobacterales>Enterobacteriaceae>Salmonella	GCA_004258725.1	Salmonella_enterica_subsp._salamae	XT66_14185	50997..54638	59202	BREX-system-P-loop-protein-BrxC-[Salmonella-enterica-subsp.-salamae]
EAB9078796.1	Type1.BREX	[illegible]	1213	Bacteria>Pseudomonadota>Gammaproteobacteria>Enterobacterales>Enterobacteriaceae>Salmonella	GCA_004265565.1	Salmonella_enterica_subsp._enterica	AAL03_01510	52372..56013	59201	BREX-system-P-loop-protein-BrxC-[Salmonella-enterica-subsp.-enterica]
EAM3013482.1	Type1.BREX	[illegible]	1213	Bacteria>Pseudomonadota>Gammaproteobacteria>Enterobacterales>Enterobacteriaceae>Salmonella	GCA_005412095.1	Salmonella_enterica	CBJ93_01865	394707..398348	28901	BREX-system-P-loop-protein-BrxC-[Salmonella-enterica]
EAM3141100.1	Type1.BREX	[illegible]	1204	Bacteria>Pseudomonadota>Gammaproteobacteria>Enterobacterales>Enterobacteriaceae>Salmonella	GCA_005412435.1	Salmonella_enterica	DMT78_01470	307802..311416	28901	BREX-system-P-loop-protein-BrxC-[Salmonella-enterica]
EAT1639063.1	Type1.BREX	[illegible]	1213	Bacteria>Pseudomonadota>Gammaproteobacteria>Enterobacterales>Enterobacteriaceae>Salmonella	GCA_005720135.1	Salmonella_enterica	EKF56_11740	25006..28647	28901	BREX-system-P-loop-protein-BrxC-[Salmonella-enterica]
EAT2430707.1	Type1.BREX	[illegible]	1213	Bacteria>Pseudomonadota>Gammaproteobacteria>Enterobacterales>Enterobacteriaceae>Salmonella	GCA_005726115.1	Salmonella_enterica	EI207_04325	177142..180783	28901	BREX-system-P-loop-protein-BrxC-[Salmonella-enterica]
EAW3206019.1	Type1.BREX	[illegible]	1204	Bacteria>Pseudomonadota>Gammaproteobacteria>Enterobacterales>Enterobacteriaceae>Salmonella	GCA_005905825.1	Salmonella_enterica	FEL99_04750	232863..236477	28901	BREX-system-P-loop-protein-BrxC-[Salmonella-enterica]
EAW9396387.1	Type1.BREX	[illegible]	1204	Bacteria>Pseudomonadota>Gammaproteobacteria>Enterobacterales>Enterobacteriaceae>Salmonella	GCA_005940865.1	Salmonella_enterica	QE38_19735	48409..52023	28901	BREX-system-P-loop-protein-BrxC-[Salmonella-enterica]
EAY5508618.1	Type1.BREX	[illegible]	1204	Bacteria>Pseudomonadota>Gammaproteobacteria>Enterobacterales>Enterobacteriaceae>Salmonella	GCA_006012725.1	Salmonella_enterica	NE47_11015	49392..53006	28901	BREX-system-P-loop-protein-BrxC-[Salmonella-enterica]
EAZ9311961.1	Type1.BREX	[illegible]	1213	Bacteria>Pseudomonadota>Gammaproteobacteria>Enterobacterales>Enterobacteriaceae>Salmonella	GCA_006073635.1	Salmonella_enterica	CBJ28_03025	158675..162316	28901	BREX-system-P-loop-protein-BrxC-[Salmonella-enterica]
EBF4455779.1	Type1.BREX	[illegible]	1204	Bacteria>Pseudomonadota>Gammaproteobacteria>Enterobacterales>Enterobacteriaceae>Salmonella	GCA_006343165.1	Salmonella_enterica	FH443_09905	412115..415729	28901	BREX-system-P-loop-protein-BrxC-[Salmonella-enterica]
EBM6187520.1	Type1.BREX	[illegible]	1213	Bacteria>Pseudomonadota>Gammaproteobacteria>Enterobacterales>Enterobacteriaceae>Salmonella	GCA_006738745.1	Salmonella_enterica	D1943_04230	100576..104217	28901	BREX-system-P-loop-protein-BrxC-[Salmonella-enterica]
EBR0226079.1	Type1.BREX	[illegible]	1204	Bacteria>Pseudomonadota>Gammaproteobacteria>Enterobacterales>Enterobacteriaceae>Salmonella	GCA_006936115.1	Salmonella_enterica_subsp._enterica_serovar_Monschaui	GPN34_002360	410647..414261	29480	BREX-system-P-loop-protein-BrxC-[Salmonella-enterica-subsp.-enterica-serovar-Monschaui]
EBR5149893.1	Type1.BREX	[illegible]	1204	Bacteria>Pseudomonadota>Gammaproteobacteria>Enterobacterales>Enterobacteriaceae>Salmonella	GCA_006958505.1	Salmonella_enterica	B5W64_09845	350811..354425	28901	BREX-system-P-loop-protein-BrxC-[Salmonella-enterica]
EBU7641088.1	Type1.BREX	[illegible]	1213	Bacteria>Pseudomonadota>Gammaproteobacteria>Enterobacterales>Enterobacteriaceae>Salmonella	GCA_007142065.1	Salmonella_enterica_subsp._enterica_serovar_Brevik	DK709_13715	59395..63036	2564275	BREX-system-P-loop-protein-BrxC-[Salmonella-enterica-subsp.-enterica-serovar-Brevik]
EBU9347433.1	Type1.BREX	[illegible]	1204	Bacteria>Pseudomonadota>Gammaproteobacteria>Enterobacterales>Enterobacteriaceae>Salmonella	GCA_007149485.1	Salmonella_enterica_subsp._enterica_serovar_Angoda	DMU95_04690	163241..166855	2564159	BREX-system-P-loop-protein-BrxC-[Salmonella-enterica-subsp.-enterica-serovar-Angoda]
EBU9775078.1	Type1.BREX	[illegible]	1213	Bacteria>Pseudomonadota>Gammaproteobacteria>Enterobacterales>Enterobacteriaceae>Salmonella	GCA_007151525.1	Salmonella_enterica_subsp._enterica_serovar_Kumasi	DM304_21645	3074..6715	2493647	BREX-system-P-loop-protein-BrxC-[Salmonella-enterica-subsp.-enterica-serovar-Kumasi]
EBV2735692.1	Type1.BREX	[illegible]	1213	Bacteria>Pseudomonadota>Gammaproteobacteria>Enterobacterales>Enterobacteriaceae>Salmonella	GCA_007164145.1	Salmonella_enterica_subsp._enterica_serovar_Stourbridge	DOA65_09445	58944..62585	1160766	BREX-system-P-loop-protein-BrxC-[Salmonella-enterica-subsp.-enterica-serovar-Stourbridge]
EBV4609756.1	Type1.BREX	[illegible]	1213	Bacteria>Pseudomonadota>Gammaproteobacteria>Enterobacterales>Enterobacteriaceae>Salmonella	GCA_007170635.1	Salmonella_enterica_subsp._enterica_serovar_Solt	DOX24_02175	3073..6714	2565040	BREX-system-P-loop-protein-BrxC-[Salmonella-enterica-subsp.-enterica-serovar-Solt]
EBX0519400.1	Type1.BREX	[illegible]	1213	Bacteria>Pseudomonadota>Gammaproteobacteria>Enterobacterales>Enterobacteriaceae>Salmonella	GCA_007236875.1	Salmonella_enterica_subsp._enterica_serovar_Saintpaul	AHL42_03320	144059..147700	90105	BREX-system-P-loop-protein-BrxC-[Salmonella-enterica-subsp.-enterica-serovar-Saintpaul]
EBY1767815.1	Type1.BREX	[illegible]	1213	Bacteria>Pseudomonadota>Gammaproteobacteria>Enterobacterales>Enterobacteriaceae>Salmonella	GCA_007283275.1	Salmonella_enterica_subsp._enterica_serovar_Georgia	DU102_16105	50651..54292	2564467	BREX-system-P-loop-protein-BrxC-[Salmonella-enterica-subsp.-enterica-serovar-Georgia]
EBZ6491792.1	Type1.BREX	[illegible]	1213	Bacteria>Pseudomonadota>Gammaproteobacteria>Enterobacterales>Enterobacteriaceae>Salmonella	GCA_007345015.1	Salmonella_enterica_subsp._enterica_serovar_Dugbe	ED480_20060	16934..20575	2564376	BREX-system-P-loop-protein-BrxC-[Salmonella-enterica-subsp.-enterica-serovar-Dugbe]
ECB3805264.1	Type1.BREX	[illegible]	1213	Bacteria>Pseudomonadota>Gammaproteobacteria>Enterobacterales>Enterobacteriaceae>Salmonella	GCA_007422765.1	Salmonella_enterica_subsp._enterica_serovar_Fufu	EW367_11360	52142..55783	2564449	BREX-system-P-loop-protein-BrxC-[Salmonella-enterica-subsp.-enterica-serovar-Fufu]
ECC1667299.1	Type1.BREX	[illegible]	1213	Bacteria>Pseudomonadota>Gammaproteobacteria>Enterobacterales>Enterobacteriaceae>Salmonella	GCA_007463295.1	Salmonella_enterica_subsp._salamae	FNN95_01255	267827..271468	59202	BREX-system-P-loop-protein-BrxC-[Salmonella-enterica-subsp.-salamae]
ECD2821735.1	Type1.BREX	[illegible]	1213	Bacteria>Pseudomonadota>Gammaproteobacteria>Enterobacterales>Enterobacteriaceae>Salmonella	GCA_007513645.1	Salmonella_enterica_subsp._enterica_serovar_Fanti	EXA45_11150	52318..55959	2564418	BREX-system-P-loop-protein-BrxC-[Salmonella-enterica-subsp.-enterica-serovar-Fanti]
ECD9608662.1	Type1.BREX	[illegible]	1213	Bacteria>Pseudomonadota>Gammaproteobacteria>Enterobacterales>Enterobacteriaceae>Salmonella	GCA_007542335.1	Salmonella_enterica_subsp._salamae	FNZ18_04560	256517..260158	59202	BREX-system-P-loop-protein-BrxC-[Salmonella-enterica-subsp.-salamae]
ECF0142189.1	Type1.BREX	[illegible]	1204	Bacteria>Pseudomonadota>Gammaproteobacteria>Enterobacterales>Enterobacteriaceae>Salmonella	GCA_007595955.1	Salmonella_enterica_subsp._enterica_serovar_Monschaui	EX931_14155	69682..73296	29480	BREX-system-P-loop-protein-BrxC-[Salmonella-enterica-subsp.-enterica-serovar-Monschaui]
ECG8301814.1	Type1.BREX	[illegible]	1213	Bacteria>Pseudomonadota>Gammaproteobacteria>Enterobacterales>Enterobacteriaceae>Salmonella	GCA_007694625.1	Salmonella_enterica_subsp._enterica_serovar_Glostrup	E1853_02135	58935..62576	1151180	BREX-system-P-loop-protein-BrxC-[Salmonella-enterica-subsp.-enterica-serovar-Glostrup]
ECG9327787.1	Type1.BREX	[illegible]	1213	Bacteria>Pseudomonadota>Gammaproteobacteria>Enterobacterales>Enterobacteriaceae>Salmonella	GCA_007700105.1	Salmonella_enterica	FO199_03415	131756..135397	28901	BREX-system-P-loop-protein-BrxC-[Salmonella-enterica]
ECJ6092446.1	Type1.BREX	[illegible]	1213	Bacteria>Pseudomonadota>Gammaproteobacteria>Enterobacterales>Enterobacteriaceae>Salmonella	GCA_007834095.1	Salmonella_enterica	GR78_06760	372350..375991	28901	BREX-system-P-loop-protein-BrxC-[Salmonella-enterica]
ECY3255615.1	Type1.BREX	[illegible]	1204	Bacteria>Pseudomonadota>Gammaproteobacteria>Enterobacterales>Enterobacteriaceae>Salmonella	GCA_008731355.1	Salmonella_enterica_subsp._enterica_serovar_Alachua	AU577_03140	17059..20673	913240	BREX-system-P-loop-protein-BrxC-[Salmonella-enterica-subsp.-enterica-serovar-Alachua]
EDB0845244.1	Type1.BREX	[illegible]	1213	Bacteria>Pseudomonadota>Gammaproteobacteria>Enterobacterales>Enterobacteriaceae>Salmonella	GCA_008888525.1	Salmonella_enterica	F9C59_12685	144464..148105	28901	BREX-system-P-loop-protein-BrxC-[Salmonella-enterica]
EDN6133533.1	Type1.BREX	[illegible]	1204	Bacteria>Pseudomonadota>Gammaproteobacteria>Enterobacterales>Enterobacteriaceae>Salmonella	GCA_010008765.1	Salmonella_enterica_subsp._enterica_serovar_Kentucky	GJQ78_12610	62378..65992	192955	BREX-system-P-loop-protein-BrxC-[Salmonella-enterica-subsp.-enterica-serovar-Kentucky]
EDQ2405063.1	Type1.BREX	[illegible]	1213	Bacteria>Pseudomonadota>Gammaproteobacteria>Enterobacterales>Enterobacteriaceae>Salmonella	GCA_010391095.1	Salmonella_enterica_subsp._enterica_serovar_Saintpaul	DQ96_001225	144464..148105	90105	BREX-system-P-loop-protein-BrxC-[Salmonella-enterica-subsp.-enterica-serovar-Saintpaul]
EDS3575399.1	Type1.BREX	[illegible]	1204	Bacteria>Pseudomonadota>Gammaproteobacteria>Enterobacterales>Enterobacteriaceae>Salmonella	GCA_010490095.1	Salmonella_enterica_subsp._enterica	GR179_002518	3402..7016	59201	BREX-system-P-loop-protein-BrxC-[Salmonella-enterica-subsp.-enterica]
EDT7223673.1	Type1.BREX	[illegible]	1204	Bacteria>Pseudomonadota>Gammaproteobacteria>Enterobacterales>Enterobacteriaceae>Salmonella	GCA_010559045.1	Salmonella_enterica_subsp._enterica	AH268_003334	169288..172902	59201	BREX-system-P-loop-protein-BrxC-[Salmonella-enterica-subsp.-enterica]
EDV5116990.1	Type1.BREX	[illegible]	1213	Bacteria>Pseudomonadota>Gammaproteobacteria>Enterobacterales>Enterobacteriaceae>Salmonella	GCA_010665025.1	Salmonella_enterica_subsp._enterica	ZJ36_000825	28924..32565	59201	BREX-system-P-loop-protein-BrxC-[Salmonella-enterica-subsp.-enterica]
EDV9109398.1	Type1.BREX	[illegible]	1213	Bacteria>Pseudomonadota>Gammaproteobacteria>Enterobacterales>Enterobacteriaceae>Salmonella	GCA_010691445.1	Salmonella_enterica_subsp._enterica	ZK68_001600	51998..55639	59201	BREX-system-P-loop-protein-BrxC-[Salmonella-enterica-subsp.-enterica]
EDW4488615.1	Type1.BREX	[illegible]	1213	Bacteria>Pseudomonadota>Gammaproteobacteria>Enterobacterales>Enterobacteriaceae>Salmonella	GCA_010711495.1	Salmonella_enterica_subsp._enterica	YV39_000487	166105..169746	59201	BREX-system-P-loop-protein-BrxC-[Salmonella-enterica-subsp.-enterica]
EDX0369803.1	Type1.BREX	[illegible]	1204	Bacteria>Pseudomonadota>Gammaproteobacteria>Enterobacterales>Enterobacteriaceae>Salmonella	GCA_010742735.1	Salmonella_enterica	GTL21_000361	386606..390247	28901	BREX-system-P-loop-protein-BrxC-[Salmonella-enterica]
EEE1152714.1	Type1.BREX	[illegible]	1213	Bacteria>Pseudomonadota>Gammaproteobacteria>Enterobacterales>Enterobacteriaceae>Salmonella	GCA_011117415.1	Salmonella_enterica_subsp._enterica_serovar_Teko	CAE49_001975	330756..334397	2079198	BREX-system-P-loop-protein-BrxC-[Salmonella-enterica-subsp.-enterica-serovar-Teko]
EEE6742962.1	Type1.BREX	[illegible]	1213	Bacteria>Pseudomonadota>Gammaproteobacteria>Enterobacterales>Enterobacteriaceae>Salmonella	GCA_011144815.1	Salmonella_enterica_subsp._enterica_serovar_Westhampton	CBJ05_002020	408792..412433	356621	BREX-system-P-loop-protein-BrxC-[Salmonella-enterica-subsp.-enterica-serovar-Westhampton]
EEH2707246.1	Type1.BREX	[illegible]	1213	Bacteria>Pseudomonadota>Gammaproteobacteria>Enterobacterales>Enterobacteriaceae>Salmonella	GCA_011274245.1	Salmonella_enterica_subsp._enterica	GYE71_000437	54012..57653	59201	BREX-system-P-loop-protein-BrxC-[Salmonella-enterica-subsp.-enterica]
EEI5362885.1	Type1.BREX	[illegible]	1204	Bacteria>Pseudomonadota>Gammaproteobacteria>Enterobacterales>Enterobacteriaceae>Salmonella	GCA_011357245.1	Salmonella_enterica_subsp._enterica_serovar_Kentucky	GXM95_001563	173373..176987	192955	BREX-system-P-loop-protein-BrxC-[Salmonella-enterica-subsp.-enterica-serovar-Kentucky]
EEM2455575.1	Type1.BREX	[illegible]	1204	Bacteria>Pseudomonadota>Gammaproteobacteria>Enterobacterales>Enterobacteriaceae>Salmonella	GCA_011558015.1	Salmonella_enterica_subsp._enterica_serovar_Kentucky	CWG70_02775	173706..177320	192955	BREX-system-P-loop-protein-BrxC-[Salmonella-enterica-subsp.-enterica-serovar-Kentucky]
EEO3564960.1	Type1.BREX	[illegible]	1213	Bacteria>Pseudomonadota>Gammaproteobacteria>Enterobacterales>Enterobacteriaceae>Salmonella	GCA_011665275.1	Salmonella_enterica_subsp._enterica_serovar_Poona	DEI68_08995	52368..56009	436295	BREX-system-P-loop-protein-BrxC-[Salmonella-enterica-subsp.-enterica-serovar-Poona]
EGB5697881.1	Type1.BREX	[illegible]	1213	Bacteria>Pseudomonadota>Gammaproteobacteria>Enterobacterales>Enterobacteriaceae>Salmonella	GCA_014213495.1	Salmonella_enterica_subsp._enterica	H7531_003071	17313..20927	59201	BREX-system-P-loop-protein-BrxC-[Salmonella-enterica-subsp.-enterica]
MBH5277084.1	Type1.BREX	[illegible]	1213	Bacteria>Pseudomonadota>Gammaproteobacteria>Enterobacterales>Enterobacteriaceae>Salmonella	GCA_016019715.1	Salmonella_enterica	I3A81_01650	358597..362238	28901	BREX-system-P-loop-protein-BrxC-[Salmonella-enterica]
EHJ7117733.1	Type1.BREX	[illegible]	1204	Bacteria>Pseudomonadota>Gammaproteobacteria>Enterobacterales>Enterobacteriaceae>Salmonella	GCA_017926475.1	Salmonella_enterica_subsp._enterica_serovar_Kentucky	KAP12_002965	412329..415943	192955	BREX-system-P-loop-protein-BrxC-[Salmonella-enterica-subsp.-enterica-serovar-Kentucky]
EHO5648342.1	Type1.BREX	[illegible]	1213	Bacteria>Pseudomonadota>Gammaproteobacteria>Enterobacterales>Enterobacteriaceae>Salmonella	GCA_018535125.1	Salmonella_enterica	KLR08_000690	144464..148105	28901	BREX-system-P-loop-protein-BrxC-[Salmonella-enterica]
EHS4662101.1	Type1.BREX	[illegible]	1213	Bacteria>Pseudomonadota>Gammaproteobacteria>Enterobacterales>Enterobacteriaceae>Salmonella	GCA_019144395.1	Salmonella_enterica	KV604_003674	73553..77194	28901	BREX-system-P-loop-protein-BrxC-[Salmonella-enterica]
EHU7518552.1	Type1.BREX	[illegible]	1213	Bacteria>Pseudomonadota>Gammaproteobacteria>Enterobacterales>Enterobacteriaceae>Salmonella	GCA_019318345.1	Salmonella_enterica	KZL07_001983	273071..276712	28901	BREX-system-P-loop-protein-BrxC-[Salmonella-enterica]
EHX4782365.1	Type1.BREX	[illegible]	1213	Bacteria>Pseudomonadota>Gammaproteobacteria>Enterobacterales>Enterobacteriaceae>Salmonella	GCA_019533715.1	Salmonella_enterica	K3Y02_002640	144286..147927	28901	BREX-system-P-loop-protein-BrxC-[Salmonella-enterica]
EIJ6143992.1	Type1.BREX	[illegible]	1213	Bacteria>Pseudomonadota>Gammaproteobacteria>Enterobacterales>Enterobacteriaceae>Salmonella	GCA_020548845.1	Salmonella_enterica_subsp._enterica_serovar_4:a:-	LI678_001014	394746..398387	2885150	BREX-system-P-loop-protein-BrxC-[Salmonella-enterica-subsp.-enterica-serovar-4:a:-]
EJC3638306.1	Type1.BREX	[illegible]	1213	Bacteria>Pseudomonadota>Gammaproteobacteria>Enterobacterales>Enterobacteriaceae>Salmonella	GCA_023047235.1	Salmonella_enterica	MYX88_003393	151139..154780	28901	BREX-system-P-loop-protein-BrxC-[Salmonella-enterica]
UTZ95782.1	Type1.BREX	[illegible]	1204	Bacteria>Pseudomonadota>Gammaproteobacteria>Enterobacterales>Enterobacteriaceae>Salmonella	GCA_024442275.1	Salmonella_enterica_subsp._enterica_serovar_Kentucky	NOQ95_21560	4448475..4452089	192955	BREX-system-P-loop-protein-BrxC-[Salmonella-enterica-subsp.-enterica-serovar-Kentucky]
EJU7772063.1	Type1.BREX	[illegible]	1213	Bacteria>Pseudomonadota>Gammaproteobacteria>Enterobacterales>Enterobacteriaceae>Salmonella	GCA_025208445.1	Salmonella_enterica_subsp._salamae_serovar_4,12:e,n,x:1,6	KAB52_003008	279421..283062	2978379	BREX-system-P-loop-protein-BrxC-[Salmonella-enterica-subsp.-salamae-serovar-4,12:e,n,x:1,6]
WP_111761031.1	Type1.BREX	[illegible]	1213	Bacteria>Pseudomonadota>Gammaproteobacteria>Enterobacterales>Enterobacteriaceae>Salmonella	GCA_030820635.1	Salmonella_enterica	KXD81_RS02520	21979..25620	28901	BREX-system-P-loop-protein-BrxC-[Salmonella-enterica]
WP_001523601.1	Type1.BREX	[illegible]	1204	Bacteria>Pseudomonadota>Gammaproteobacteria>Enterobacterales>Enterobacteriaceae>Salmonella	GCF_000170235.1	Salmonella_enterica	SESPB_RS10230	40505..44146	28901	BREX-system-P-loop-protein-BrxC-[Salmonella-enterica]
WP_071055527.1	Type1.BREX	[illegible]	1213	Bacteria>Pseudomonadota>Gammaproteobacteria>Enterobacterales>Enterobacteriaceae>Salmonella	GCF_000486165.2	Salmonella_enterica	SES26_RS19315	4009469..4013110	28901	BREX-system-P-loop-protein-BrxC-[Salmonella-enterica]
WP_061383284.1	Type1.BREX	[illegible]	1213	Bacteria>Pseudomonadota>Gammaproteobacteria>Enterobacterales>Enterobacteriaceae>Salmonella	GCF_001570045.1	Salmonella_enterica	AW140_RS06880	353892..357506	28901	BREX-system-P-loop-protein-BrxC-[Salmonella-enterica]
WP_064013329.1	Type1.BREX	[illegible]	1204	Bacteria>Pseudomonadota>Gammaproteobacteria>Enterobacterales>Enterobacteriaceae>Salmonella	GCF_001643815.1	Salmonella_enterica	IJ96_RS19570	3320..6934	28901	BREX-system-P-loop-protein-BrxC-[Salmonella-enterica]
WP_070793518.1	Type1.BREX	[illegible]	1213	Bacteria>Pseudomonadota>Gammaproteobacteria>Enterobacterales>Enterobacteriaceae>Salmonella	GCF_001832595.1	Salmonella_enterica	A7T54_RS11885	259865..263506	28901	BREX-system-P-loop-protein-BrxC-[Salmonella-enterica]
WP_001729064.1	Type1.BREX	[illegible]	1213	Bacteria>Pseudomonadota>Gammaproteobacteria>Enterobacterales>Enterobacteriaceae>Salmonella	GCF_002034005.1	Salmonella_enterica	AJH59_RS23280	9917..13558	28901	BREX-system-P-loop-protein-BrxC-[Salmonella-enterica]
WP_079808688.1	Type1.BREX	[illegible]	1204	Bacteria>Pseudomonadota>Gammaproteobacteria>Enterobacterales>Enterobacteriaceae>Salmonella	GCF_002035875.1	Salmonella_enterica	Y985_RS08730	120043..123684	28901	BREX-system-P-loop-protein-BrxC-[Salmonella-enterica]
WP_079897969.1	Type1.BREX	[illegible]	1213	Bacteria>Pseudomonadota>Gammaproteobacteria>Enterobacterales>Enterobacteriaceae>Salmonella	GCF_002045805.1	Salmonella_enterica	VY62_RS13065	181728..185342	28901	BREX-system-P-loop-protein-BrxC-[Salmonella-enterica]
WP_079965457.1	Type1.BREX	[illegible]	1213	Bacteria>Pseudomonadota>Gammaproteobacteria>Enterobacterales>Enterobacteriaceae>Salmonella	GCF_002046745.1	Salmonella_enterica	VX54_RS10650	52174..55815	28901	BREX-system-P-loop-protein-BrxC-[Salmonella-enterica]
WP_080094962.1	Type1.BREX	[illegible]	1204	Bacteria>Pseudomonadota>Gammaproteobacteria>Enterobacterales>Enterobacteriaceae>Salmonella	GCF_002052895.1	Salmonella_enterica	DL104_05730	29230..32871	28901	BREX-system-P-loop-protein-BrxC-[Salmonella-enterica]
WP_079905548.1	Type1.BREX	[illegible]	1213	Bacteria>Pseudomonadota>Gammaproteobacteria>Enterobacterales>Enterobacteriaceae>Salmonella	GCF_002065435.1	Salmonella_enterica	SN48_RS13425	18385..21999	28901	BREX-system-P-loop-protein-BrxC-[Salmonella-enterica]
WP_080250285.1	Type1.BREX	[illegible]	1213	Bacteria>Pseudomonadota>Gammaproteobacteria>Enterobacterales>Enterobacteriaceae>Salmonella	GCF_002066965.1	Salmonella_enterica	RZ13_RS02295	164882..168523	28901	BREX-system-P-loop-protein-BrxC-[Salmonella-enterica]
WP_086805524.1	Type1.BREX	[illegible]	1204	Bacteria>Pseudomonadota>Gammaproteobacteria>Enterobacterales>Enterobacteriaceae>Salmonella	GCF_002156225.1	Salmonella_enterica	CCL33_RS10730	52147..55761	28901	BREX-system-P-loop-protein-BrxC-[Salmonella-enterica]
WP_247258912.1	Type1.BREX	[illegible]	1204	Bacteria>Pseudomonadota>Gammaproteobacteria>Enterobacterales>Enterobacteriaceae>Salmonella	GCF_023007725.1	Salmonella_enterica	MA804_RS14880	357716..361330	28901	BREX-system-P-loop-protein-BrxC-[Salmonella-enterica]
WP_255929194.1	Type1.BREX	[illegible]	1204	Bacteria>Pseudomonadota>Gammaproteobacteria>Enterobacterales>Enterobacteriaceae>Salmonella	GCF_024442275.1	Salmonella_enterica	NOQ95_RS21560	4448475..4452089	28901	BREX-system-P-loop-protein-BrxC-[Salmonella-enterica]
WP_088524188.1	Type1.BREX	[illegible]	1204	Bacteria>Pseudomonadota>Gammaproteobacteria>Enterobacterales>Enterobacteriaceae>Salmonella	GCF_903931065.1	Salmonella_enterica	LFZ40_19410	3810154..3813768	28901	BREX-system-P-loop-protein-BrxC-[Salmonella-enterica]

Protein-ID	System	Gene-Neighbourhoods	Length	Taxonomy	Assembly-ID	Strain	Locus-Tag	Genomic-coordinates	TaxID	Fasta-defline
ECG0705015.1	Type1.BREX	<-HEPN<-REase+iREase<-?||||YjhX_toxin->SAM-METHYLASE->?->|| BrxA-DNA-binding -> BrxB-BrxF-iSTAND -> DUF6079 *-> N6-METHYLASE ->OLD-ABC->TOPRIM-> PglZ -> SIGMA+OB-Fold+AAA-ATPase+Lon-Protease-BrxL -> HKD-DNase +OGG-like -><-REase<-NACHT+SF1-SF2-Helicase+ HKD-DNase <-nSTAND1+HNH	1213	Bacteria>Pseudomonadota>Gammaproteobacteria>Enterobacterales>Enterobacteriaceae>Salmonella	-	Salmonella_enterica_subsp._salamae	DL122_04700	35596..39237	59202	BREX-system-P-loop-protein-BrxC-[Salmonella-enterica-subsp.-salamae]
WP_222233110.1	Type1.BREX	<- HKD-DNase +OGG-like <- SIGMA+OB-Fold+AAA-ATPase+Lon-Protease-BrxL <- PglZ <-TOPRIM<-OLD-ABC<- N6-METHYLASE <- DUF6079 *<- BrxB-BrxF-iSTAND <- BrxA-DNA-binding <-?<-?<-?<-?<-PRTase<-DADA-Ligase	1202	Bacteria>Pseudomonadota>Gammaproteobacteria>Enterobacterales>Erwiniaceae>Pantoea	GCF_019795975.1	Pantoea_sp._DY-17	K5Y33_RS01225	252070..255678	2871490	BREX-system-P-loop-protein-BrxC-[Pantoea-sp.-DY-17]
WP_060426782.1	Type1.BREX	<-RNase||?->?->SAD+HNH->HTH+REase-> BrxA-DNA-binding -> BrxB-BrxF-iSTAND -> DUF6079 *-> N6-METHYLASE -> PglZ -> SIGMA+OB-Fold+AAA-ATPase+Lon-Protease-BrxL -> HKD-DNase +OGG-like -><-?<-?<-tRNA||?->DEOR-HTH+ISOCOT->	1213	Bacteria>Pseudomonadota>Gammaproteobacteria>Enterobacterales>Yersiniaceae	GCF_001537885.1	Serratia	AWX06_RS15845	182718..186359	613	MULTISPECIES:-BREX-system-P-loop-protein-BrxC-[Serratia]
WP_112197427.1	Type1.BREX	<-CopG/RHH||?->XIS-HTH->ZnR->ZnR->?->?-> BrxA-DNA-binding -> BrxB-BrxF-iSTAND -> DUF6079 *-> N6-METHYLASE ->OLD-ABC->TOPRIM-> PglZ -> SIGMA+OB-Fold+AAA-ATPase+Lon-Protease-BrxL -> HKD-DNase +OGG-like -><-Arm-DNA-bind_3+Phage_integrase	1204	Bacteria>Pseudomonadota>Gammaproteobacteria>Enterobacterales>Yersiniaceae>Rahnella	GCF_003263525.1	Rahnella_sp._NRRL_B-41462	UC14_RS06670	31474..35088	1610579	BREX-system-P-loop-protein-BrxC-[Rahnella-sp.-NRRL-B-41462]
WP_197785670.1	Type1.BREX	Glyco_hydro_1-><-?<-?||?->?->SAD+HNH->HTH+REase-> BrxA-DNA-binding -> BrxB-BrxF-iSTAND -> DUF6079 *-> N6-METHYLASE -> PglZ -> SIGMA+OB-Fold+AAA-ATPase+Lon-Protease-BrxL -> HKD-DNase +OGG-like ->	1213	Bacteria>Pseudomonadota>Gammaproteobacteria>Enterobacterales>Yersiniaceae>Serratia	GCF_016007555.1	Serratia_ureilytica	I5P92_RS12860	24369..28010	300181	BREX-system-P-loop-protein-BrxC-[Serratia-ureilytica]
WP_212602139.1	Type1.BREX	<-AraC-HTH<-?||?->Aminotran_3->wHTH+SAD+HNH-><-?<-REase|| BrxA-DNA-binding -> BrxB-BrxF-iSTAND -> DUF6079 *-> N6-METHYLASE -> PglZ -> SIGMA+OB-Fold+AAA-ATPase+Lon-Protease-BrxL -> HKD-DNase +OGG-like -><-?<-?<-tRNA||DEOR-HTH+ISOCOT->	1213	Bacteria>Pseudomonadota>Gammaproteobacteria>Enterobacterales>Yersiniaceae>Serratia	GCF_018257315.1	Serratia_rubidaea	JK228_RS02460	34162..37803	61652	BREX-system-P-loop-protein-BrxC-[Serratia-rubidaea]
WP_244757920.1	Type1.BREX	<- HKD-DNase +OGG-like <- SIGMA+OB-Fold+AAA-ATPase+Lon-Protease-BrxL <- PglZ <- N6-METHYLASE <- DUF6079 *<- BrxB-BrxF-iSTAND <- BrxA-DNA-binding <-HTH+REase<-SAD+HNH<-?||?->?-><-Glyco_hydro_1	1213	Bacteria>Pseudomonadota>Gammaproteobacteria>Enterobacterales>Yersiniaceae>Serratia	GCF_022870585.1	Serratia_marcescens	HW877_RS02460	535877..539518	615	BREX-system-P-loop-protein-BrxC-[Serratia-marcescens]
WP_261139451.1	Type1.BREX	DOC+HTH-><-LACI-HTH+PBPI||?->Glyco_hydro_1->?->SAD+HNH-> BrxA-DNA-binding -> BrxB-BrxF-iSTAND -> DUF6079 *-> PglZ -> SIGMA+OB-Fold+AAA-ATPase+Lon-Protease-BrxL -> HKD-DNase +OGG-like -><-Couple_hipA+HipA_C<-HTH_3<-?||HIN-HTH-><-HTH_21+rve+rve_3<-rve	1213	Bacteria>Pseudomonadota>Gammaproteobacteria>Enterobacterales>Yersiniaceae>Serratia	GCF_946226675.1	Serratia_liquefaciens	N8M82_RS16050	322435..326076	614	BREX-system-P-loop-protein-BrxC-[Serratia-liquefaciens]
WP_261135874.1	Type1.BREX	DOC+HTH-><-LACI-HTH+PBPI||?->Glyco_hydro_1->?->SAD+HNH-> BrxA-DNA-binding -> BrxB-BrxF-iSTAND -> DUF6079 *-> N6-METHYLASE -> PglZ -> SIGMA+OB-Fold+AAA-ATPase+Lon-Protease-BrxL -> HKD-DNase +OGG-like -><-Couple_hipA+HipA_C<-HTH_3	1213	Bacteria>Pseudomonadota>Gammaproteobacteria>Enterobacterales>Yersiniaceae>Serratia	GCF_946226775.1	Serratia_liquefaciens	N8M94_RS14775	322685..326326	614	BREX-system-P-loop-protein-BrxC-[Serratia-liquefaciens]
WP_261427666.1	Type1.BREX	Glyco_hydro_1-><-?<-?||?->?->SAD+HNH-> BrxA-DNA-binding -> BrxB-BrxF-iSTAND -> DUF6079 *-> N6-METHYLASE -> PglZ -> SIGMA+OB-Fold+AAA-ATPase+Lon-Protease-BrxL -> HKD-DNase +OGG-like -><-Couple_hipA+HipA_C<-HTH_3	1213	Bacteria>Pseudomonadota>Gammaproteobacteria>Enterobacterales>Yersiniaceae>Serratia	GCF_946227925.1	Serratia_proteamaculans	OCH17_RS14295	357572..361213	28151	BREX-system-P-loop-protein-BrxC-[Serratia-proteamaculans]
WP_195869502.1	Type1.BREX	<-Peptidase_M17_N+Peptidase_M17||?->tRNA->Arm-DNA-bind_3+Phage_integrase-><- HKD-DNase +OGG-like <- SIGMA+OB-Fold+AAA-ATPase+Lon-Protease-BrxL <- PglZ <-TOPRIM<-OLD-ABC<- N6-METHYLASE <- DUF6079 *<- BrxB-BrxF-iSTAND <- BrxA-DNA-binding <-?<-?||<-ZnR<-XIS-HTH<-?||OLD-ABC+TOPRIM->	1202	Bacteria>Pseudomonadota>Gammaproteobacteria>Enterobacterales>Yersiniaceae>Serratia	-	Serratia_liquefaciens	I1A46_RS14725	213767..217375	614	BREX-system-P-loop-protein-BrxC-[Serratia-liquefaciens]
WP_053011787.1	Type1.BREX	XIS-HTH->?->ZnR->XIS-HTH->ZnR->?->HTH+MPTase->|| BrxA-DNA-binding -> BrxB-BrxF-iSTAND -> DUF6079 *-> N6-METHYLASE ->OLD-ABC->TOPRIM-> PglZ -> SIGMA+OB-Fold+AAA-ATPase+Lon-Protease-BrxL -> HKD-DNase +OGG-like -><-Arm-DNA-bind_3+Phage_integrase<-tRNA||?-><-HTH_3	1202	Bacteria>Pseudomonadota>Gammaproteobacteria>Enterobacterales>Yersiniaceae>Yersinia	GCF_001244675.1	Yersinia_intermedia	ALC37_RS11575	459968..463576	631	BREX-system-P-loop-protein-BrxC-[Yersinia-intermedia]
WP_044865655.1	Type1.BREX	<-RHH_antitoxin||?->XIS-HTH->?->ZnR-> BrxA-DNA-binding -> BrxB-BrxF-iSTAND -> DUF6079 *-> N6-METHYLASE -> PglZ -> SIGMA+OB-Fold+AAA-ATPase+Lon-Protease-BrxL -> HKD-DNase +OGG-like -><-Arm-DNA-bind_3+Phage_integrase	1213	Bacteria>Pseudomonadota>Gammaproteobacteria	GCF_001540705.1	Enterobacterales	TJ11_RS03570	21952..25593	91347	MULTISPECIES:-BREX-system-P-loop-protein-BrxC-[Enterobacterales]
WP_031438510.1	Type1.BREX	HD->?->?->||Phage_integrase->Phage_integrase->?-> wHTH+WYL+WCX -> BrxA-DNA-binding -> BrxB-BrxF-iSTAND -> DUF6079 *-> HKD-DNase +DUF3150-> N6-METHYLASE ->OLD-ABC->TOPRIM->DOC+HTH-> PglZ -> SIGMA+OB-Fold+AAA-ATPase+Lon-Protease-BrxL ->SMODS->SLATT->	1218	Bacteria>Pseudomonadota>Gammaproteobacteria>Methylococcales>Methylococcaceae>Methylobacter	GCF_000733835.1	Methylobacter_tundripaludum	T449_RS0121480	4638570..4642226	173365	BREX-system-P-loop-protein-BrxC-[Methylobacter-tundripaludum]
WP_136143645.1	Type1.BREX	SMODS->?->Classical-AAA->ABhydrolase->?-><- SIGMA+OB-Fold+AAA-ATPase+Lon-Protease-BrxL <- PglZ <- HKD-DNase +DUF3150<- N6-METHYLASE <- DUF6079 *<- BrxB-BrxF-iSTAND <- BrxA-DNA-binding <- wHTH+WYL+WCX ||DUF4236-><-DOC+HTH<-?<-ParB+HNH<-Phage_integrase+Phage_integrase<-Phage_integrase+Phage_integrase	1226	Bacteria>Pseudomonadota>Gammaproteobacteria>Moraxellales>Moraxellaceae>Acinetobacter	GCA_030820635.1	Acinetobacter_towneri	EQG52_RS02450	14374..18054	202956	BREX-system-P-loop-protein-BrxC-[Acinetobacter-towneri]
WP_023013360.1	Type1.BREX	<- SIGMA+OB-Fold+AAA-ATPase+Lon-Protease-BrxL <- PglZ <- HKD-DNase +DUF3150<- N6-METHYLASE <- DUF6079 *<- BrxB-BrxF-iSTAND <- BrxA-DNA-binding <- wHTH+WYL+WCX <-wHTH+HNH<-SF1-SF2-Helicase<-?<-SAM-METHYLASE<-JAB	1226	Bacteria>Pseudomonadota>Gammaproteobacteria>Moraxellales>Moraxellaceae>Acinetobacter	GCF_000475335.1	Acinetobacter_sp._COS3	IO68_RS25385	11690..15370	1397525	BREX-system-P-loop-protein-BrxC-[Acinetobacter-sp.-COS3]
WP_039048213.1	Type1.BREX	<-GDResolvase+HIN-HTH||<-SbcC+ABC-ATPase<-?<-?<-?||<- SIGMA+OB-Fold+AAA-ATPase+Lon-Protease-BrxL <- PglZ <- HKD-DNase +DUF3150<- N6-METHYLASE <- DUF6079 *<- BrxB-BrxF-iSTAND <- BrxA-DNA-binding <- wHTH+WYL+WCX ||DUF4236-><-CR-ATPase5+REase<-ParB+HNH<-Phage_integrase+Phage_integrase<-Phage_integrase+Phage_integrase	1226	Bacteria>Pseudomonadota>Gammaproteobacteria>Moraxellales>Moraxellaceae>Acinetobacter	GCF_000813495.1	Acinetobacter_junii	RP26_RS13815	17614..21294	40215	BREX-system-P-loop-protein-BrxC-[Acinetobacter-junii]
WP_180041457.1	Type1.BREX	ParB+HNH-><-?||SAM-METHYLASE->?->SF1-SF2-Helicase->wHTH+HNH-> wHTH+WYL+WCX -> BrxA-DNA-binding -> BrxB-BrxF-iSTAND -> DUF6079 *-> N6-METHYLASE -> HKD-DNase +DUF3150-> PglZ -> SIGMA+OB-Fold+AAA-ATPase+Lon-Protease-BrxL ->SF1-SF2-Helicase+REase->	1221	Bacteria>Pseudomonadota>Gammaproteobacteria>Moraxellales>Moraxellaceae>Acinetobacter	GCF_013417925.1	Acinetobacter_sp._YH12218	FTV03_RS11390	13993..17658	2601152	BREX-system-P-loop-protein-BrxC-[Acinetobacter-sp.-YH12218]
WP_180131186.1	Type1.BREX	Phage_integrase-><-HTH_OrfB_IS605+OrfB_IS605+OrfB_Zn_ribbon||Y1_Tnp->ParB+HNH->CR-ATPase5+REase-><-DUF4236|| wHTH+WYL+WCX -> BrxA-DNA-binding -> BrxB-BrxF-iSTAND -> DUF6079 *-> N6-METHYLASE -> HKD-DNase +DUF3150-> PglZ -> SIGMA+OB-Fold+AAA-ATPase+Lon-Protease-BrxL ->	1226	Bacteria>Pseudomonadota>Gammaproteobacteria>Moraxellales>Moraxellaceae>Acinetobacter	GCF_013419565.1	Acinetobacter_sp._YH12072	FTU19_RS11080	35259..38939	2601068	BREX-system-P-loop-protein-BrxC-[Acinetobacter-sp.-YH12072]
WP_218698253.1	Type1.BREX	<-MarR-HTH<-SF1-SF2-Helicase<-STYKIN+CR-Kinase<-?<- SIGMA+OB-Fold+AAA-ATPase+Lon-Protease-BrxL <- PglZ <- HKD-DNase +DUF3150<-ParB+HNH<- N6-METHYLASE <- DUF6079 *<- BrxB-BrxF-iSTAND <- BrxA-DNA-binding <- wHTH+WYL+WCX ||DUF4236-><-RelE-ParE<-ParD_antitoxin||<-?<-HIN-HTH	1224	Bacteria>Pseudomonadota>Gammaproteobacteria>Moraxellales>Moraxellaceae>Acinetobacter	GCF_019279015.1	Acinetobacter_harbinensis	KZA13_RS08660	34272..37946	1353941	BREX-system-P-loop-protein-BrxC-[Acinetobacter-harbinensis]
WP_234115082.1	Type1.BREX	Phage_integrase+Phage_integrase->Phage_integrase+Phage_integrase->ParB+HNH->?->DOC+HTH-><-DUF4236|| wHTH+WYL+WCX -> BrxA-DNA-binding -> BrxB-BrxF-iSTAND -> DUF6079 *-> N6-METHYLASE -> HKD-DNase +DUF3150-> PglZ -> SIGMA+OB-Fold+AAA-ATPase+Lon-Protease-BrxL -><-?<-ABhydrolase<-Classical-AAA<-?<-SMODS	1226	Bacteria>Pseudomonadota>Gammaproteobacteria>Moraxellales>Moraxellaceae>Acinetobacter	GCF_021432045.1	Acinetobacter_towneri	LZG54_RS14455	23931..27611	202956	BREX-system-P-loop-protein-BrxC-[Acinetobacter-towneri]
WP_234693498.1	Type1.BREX	Phage_integrase+Phage_integrase->Phage_integrase+Phage_integrase->ParB+HNH->?->DOC+HTH-><-DUF4236|| wHTH+WYL+WCX -> BrxA-DNA-binding -> BrxB-BrxF-iSTAND -> DUF6079 *-> N6-METHYLASE -> HKD-DNase +DUF3150-> PglZ -> SIGMA+OB-Fold+AAA-ATPase+Lon-Protease-BrxL -><-?<-ABhydrolase<-Classical-AAA<-?<-SMODS	1226	Bacteria>Pseudomonadota>Gammaproteobacteria>Moraxellales>Moraxellaceae>Acinetobacter	GCF_021460555.1	Acinetobacter_baumannii	LH676_RS19735	26787..30467	470	BREX-system-P-loop-protein-BrxC-[Acinetobacter-baumannii]
WP_262597505.1	Type1.BREX	Zn_Tnp_IS1595+DDE_Tnp_IS1595-><-TIR<-?<-SLOG+BirA-HTH<-SF1-SF2-Helicase+PRTase<- N6-METHYLASE ||?-><-?<- HKD-DNase +DUF3150<- DUF6079 *<- BrxB-BrxF-iSTAND <- BrxA-DNA-binding <-XIS-HTH||?-><-?||?->GspA-ATPase-><-?||RVT->	1227	Bacteria>Pseudomonadota>Gammaproteobacteria>Oceanospirillales>Endozoicomonadaceae>Endozoicomonas	GCF_025562755.1	Endozoicomonas_euniceicola	NX720_RS21900	5400416..5404099	1234143	BREX-system-P-loop-protein-BrxC-[Endozoicomonas-euniceicola]
WP_125460588.1	Type1.BREX	<- PglZ <-AbiEii<-AbiEi_3_N+AbiEi_3<-SF1-SF2-Helicase<- HKD-DNase +HKD-Rease<-?<-?<- N6-METHYLASE <-AbiGi+seqA+RAMA<- DUF6079 *<- BrxB-BrxF-iSTAND <- BrxA-DNA-binding <-DUF772+DDE_Tnp_1||?->Arm-DNA-bind_2+Phage_int_SAM_3+Phage_integrase->	1220	Bacteria>Pseudomonadota>Gammaproteobacteria>Pseudomonadales>Pseudomonadaceae>Stutzerimonas	GCF_003936745.1	Stutzerimonas_stutzeri	EGJ43_RS19065	24827..28489	316	BREX-system-P-loop-protein-BrxC-[Stutzerimonas-stutzeri]
MCP5179014.1	Type1.BREX	<-DUF772+DDE_Tnp_1+DDE_Tnp_1_6<- PglZ <-ParB+HNH<-?<-viral-capsid<-ZnR<- N6-METHYLASE <- HKD-DNase <-sGTP+GTPase-AIG<- DUF6079 *<- BrxB-BrxF-iSTAND <- BrxA-DNA-binding <-?||?-><-rve<-rve<-Couple_hipA+HipA_C<-?<-HTH_Tnp_1+HTH_21+rve+rve_3	1153	Bacteria>Pseudomonadota>Gammaproteobacteria>Pseudomonadales>unclassified_Pseudomonadales	-	Pseudomonadales_bacterium	H6994_03785	340860..344321	1891229	BREX-system-P-loop-protein-BrxC-[Pseudomonadales-bacterium]
MCF7788313.1	Type1.BREX	BrxA-DNA-binding -> BrxB-BrxF-iSTAND -> DUF6079 *-> HKD-DNase +DUF3150->MarR-HTH->Polbeta-> N6-METHYLASE ->?->?->SbcC-> PglZ -p->REase->	1167	Bacteria>PVC_group>Verrucomicrobiota>Verrucomicrobiae>Verrucomicrobiales>Verrucomicrobiaceae	GCA_021733925.1	Prosthecobacter_sp.	K9N47_19475	2530..6033	1965333	BREX-system-P-loop-protein-BrxC-[Prosthecobacter-sp.]
MCO4261480.1	Type1.BREX	<-SbcC<-JetB<-JetA<-HEPN<-SF1-SF2-Helicase<-REase<- SIGMA+OB-Fold+AAA-ATPase+Lon-Protease-BrxL <- PglZ <- N6-METHYLASE <- DUF6079 *<- BrxB-BrxF-iSTAND <- BrxA-DNA-binding <- HKD-DNase +SF1-SF2-Helicase+HTH+HTH+SRA||URI-DNase->MACRODOMAIN+SIR2->URI-DNase-><-?<-NUDIX	1151	Bacteria>Terrabacteria_group>Actinomycetota>Actinomycetes>Micrococcales>Micrococcaceae	GCA_023913835.1	Pseudarthrobacter_sp._MDT3-26	NG697_00745	166559..170014	2953651	BREX-system-P-loop-protein-BrxC-[Pseudarthrobacter-sp.-MDT3-26]
WP_252613231.1	Type1.BREX	<-SbcC<-JetB<-JetA<-HEPN<-SF1-SF2-Helicase<-REase<- SIGMA+OB-Fold+AAA-ATPase+Lon-Protease-BrxL <- PglZ <- N6-METHYLASE <- DUF6079 *<- BrxB-BrxF-iSTAND <- BrxA-DNA-binding <- HKD-DNase +SF1-SF2-Helicase+HTH+HTH+SRA||URI-DNase->MACRODOMAIN+SIR2->URI-DNase-><-?<-NUDIX	1151	Bacteria>Terrabacteria_group>Actinomycetota>Actinomycetes>Micrococcales>Micrococcaceae	-	Pseudarthrobacter_sp._MDT3-26	NG697_00745	166559..170014	2953651	BREX-system-P-loop-protein-BrxC-[Pseudarthrobacter-sp.-MDT3-26]
OBF48784.1	Type6.BREX	HEPN+SF1-SF2-Helicase+REase->HIRAN-><- HKD-DNase + HKD-DNase <-?|| BrxE -> BrxA-DNA-binding -> BrxB-BrxF-iSTAND -> DUF6079 *-> PglZ -> SIGMA+OB-Fold+AAA-ATPase+Lon-Protease-BrxL -><-SF1-SF2-Helicase+REase||?-><-SF1-SF2-Helicase<-SF1-SF2-Helicase+REase	1143	Bacteria>Terrabacteria_group>Actinomycetota>Actinomycetes>Mycobacteriales>Mycobacteriaceae	GCA_001667465.1	Mycolicibacterium_monacense	A5778_22445	215738..219169	85693	hypothetical-protein-A5778_22445-[Mycolicibacterium-monacense]
WP_067214457.1	Type1.BREX	Calcineurin->SbcC+SbcC->?-><-REase||MACRODOMAIN+SIR2-><-?<- SIGMA+OB-Fold+AAA-ATPase+Lon-Protease-BrxL <- PglZ <- N6-METHYLASE <- DUF6079 *<- BrxB-BrxF-iSTAND <- BrxA-DNA-binding ||?-> HKD-DNase + HKD-DNase -><-Peptidase_M48+TerB<-HIRAN<-?<-HEPN+SF1-SF2-Helicase+REase<-Peptidase_M23+SLT	1155	Bacteria>Terrabacteria_group>Actinomycetota>Actinomycetes>Mycobacteriales>Mycobacteriaceae	GCF_001667065.1	Mycolicibacterium_celeriflavum	A5765_RS01610	25848..29315	1249101	BREX-system-P-loop-protein-BrxC-[Mycolicibacterium-celeriflavum]
WP_120314218.1	Type6.BREX	HEPN+SF1-SF2-Helicase+REase->HIRAN-><- HKD-DNase + HKD-DNase <-?|| BrxE -> BrxA-DNA-binding -> BrxB-BrxF-iSTAND -> DUF6079 *-> N6-METHYLASE -> PglZ -> SIGMA+OB-Fold+AAA-ATPase+Lon-Protease-BrxL -><-SF1-SF2-Helicase+REase<-SF1-SF2-Helicase<-SF1-SF2-Helicase+REase<-?<-?<-SbcC+SbcC	1163	Bacteria>Terrabacteria_group>Actinomycetota>Actinomycetes>Mycobacteriales>Mycobacteriaceae	GCF_001667465.1	Mycolicibacterium_monacense	A5778_RS22240	215678..219169	85693	BREX-system-P-loop-protein-BrxC-[Mycolicibacterium-monacense]
WP_230497381.1	Type1.BREX	<-SIR2||?->?->?-> BrxA-DNA-binding -> BrxB-BrxF-iSTAND -> DUF6079 *-> N6-METHYLASE -> PglZ -> SIGMA+OB-Fold+AAA-ATPase+Lon-Protease-BrxL -><- HKD-DNase +DUF3150||ZnR+HARE-HTH->?-><-Glyoxalase	1193	Bacteria>Terrabacteria_group>Bacillota>Bacilli>Bacillales>Bacillaceae	GCF_917563885.1	Pseudoneobacillus_rhizosphaerae	NEOCIP111885_02886	60307..63888	2880968	BREX-system-P-loop-protein-BrxC-[Pseudoneobacillus-rhizosphaerae]
WP_012369225.1	Type1.BREX	<-PadR-HTH||?->Phage_holin-><-RNase||?-> HKD-DNase +SF1-SF2-Helicase+HTH+HTH+SRA->?-> BrxA-DNA-binding -> BrxB-BrxF-iSTAND -> DUF6079 *-> N6-METHYLASE -> PglZ -> SIGMA+OB-Fold+AAA-ATPase+Lon-Protease-BrxL ->OLD-ABC->HNH-><-Glyoxalase||LACI-HTH+PBPI-><-HTH_OrfB_IS605+OrfB_IS605+OrfB_Zn_ribbon	1192	Bacteria>Terrabacteria_group>Bacillota>Bacilli>Bacillales>Bacillales_incertae_sedis	GCF_000019905.1	Exiguobacterium_sibiricum	Exig_0314	327084..330662	332410	BREX-system-P-loop-protein-BrxC-[Exiguobacterium-sibiricum]
WP_225047865.1	Type1.BREX	Thioredoxin->?->?-><-?||ABC-ATPase+SbcC->?-> BrxA-DNA-binding -> BrxB-BrxF-iSTAND -> DUF6079 *-> N6-METHYLASE -><-Arm-DNA-bind_4+Phage_int_SAM_5+Phage_integrase|| PglZ -> SIGMA+OB-Fold+AAA-ATPase+Lon-Protease-BrxL -> HKD-DNase +SF1-SF2-Helicase+HTH+HTH+SRA->	1221	Bacteria>Terrabacteria_group>Bacillota>Bacilli>Lactobacillales>Lactobacillaceae	GCF_020180945.1	Lacticaseibacillus_kribbianus	LE416_RS07415	6539..10204	2926292	BREX-system-P-loop-protein-BrxC-[Lacticaseibacillus-kribbianus]
KJS18733.1	Type1.BREX	HNH->?->HEPN->Peptidase->?->HTH_26-> BrxA-DNA-binding -> BrxB-BrxF-iSTAND -> DUF6079 *-> N6-METHYLASE ->?-> PglZ -> SIGMA+OB-Fold+AAA-ATPase+Lon-Protease-BrxL -> HKD-DNase ->?->?->Calcineurin->SbcC->	1210	Bacteria>Terrabacteria_group>Bacillota>Clostridia>Eubacteriales>Clostridiaceae	GCA_000961765.1	Clostridiaceae_bacterium_BRH_c20a	VR72_21175	10275..13907	1629719	ATPase-[Clostridiaceae-bacterium-BRH_c20a]
MPQ42518.1	Type1.BREX	<- SIGMA+OB-Fold+AAA-ATPase+Lon-Protease-BrxL <- PglZ <-HTH+ HKD-DNase <-Cas4+SF1-SF2-Helicase<-?<-?<-?<-HTH_OrfB_IS605+OrfB_IS605+OrfB_Zn_ribbon<- N6-METHYLASE <- DUF6079 -p*<-HTH_OrfB_IS605+OrfB_IS605+OrfB_Zn_ribbon<- BrxB-BrxF-iSTAND <- BrxA-DNA-binding <-Ntox45<-HEPN	995	Bacteria>Terrabacteria_group>Bacillota>Clostridia>Eubacteriales>Clostridiaceae	GCA_009295725.1	Clostridium_tarantellae	GBZ86_01880	24585..27572	39493	BREX-system-P-loop-protein-BrxC-[Clostridium-tarantellae]
WP_043662653.1	Type1.BREX	<-McrC-NTD+REase<-wHTH+McrB-AAA<- SIGMA+OB-Fold+AAA-ATPase+Lon-Protease-BrxL <-?<- PglZ <-HNH<-OLD-ABC<- N6-METHYLASE <- DUF6079 *<- BrxB-BrxF-iSTAND <- BrxA-DNA-binding <- HKD-DNase +SF1-SF2-Helicase+HTH+HTH+SRA<-SAM-METHYLASE<-RNase<-HNH+NERD+ZnR<-?<-?<-Amidohydro_3	1216	Bacteria>Terrabacteria_group>Bacillota>Clostridia>Eubacteriales>Clostridiaceae	GCF_000785185.1	Clostridium_butyricum	AWN73_RS08010	18592..22242	1492	BREX-system-P-loop-protein-BrxC-[Clostridium-butyricum]
WP_156211829.1	Type1.BREX	<-wHTH+McrB-AAA<-?<- PglZ <-?<-?<-OLD-ABC<-ParB+HNH<- N6-METHYLASE <-CR-ATPase9+REase<- DUF6079 *<- BrxB-BrxF-iSTAND <- BrxA-DNA-binding <- HKD-DNase +SF1-SF2-Helicase+HTH+HTH+SRA<-SAM-METHYLASE<-NERD+HRDC<-Ntox45<-HEPN	1216	Bacteria>Terrabacteria_group>Bacillota>Clostridia>Eubacteriales>Clostridiaceae	GCF_009735365.2	Clostridium_butyricum	GND98_RS05145	54704..58354	1492	BREX-system-P-loop-protein-BrxC-[Clostridium-butyricum]
WP_164958329.1	Type1.BREX	<- HKD-DNase <- N6-METHYLASE <-?<-GDResolvase<-?<-?<-GDResolvase+HIN-HTH<-SIGMA+OB-Fold+AAA-ATPase-BrxL<- PglZ <- DUF6079 *<- BrxB-BrxF-iSTAND <-DUF4007<- N6-METHYLASE	1363	Bacteria>Terrabacteria_group>Bacillota>Clostridia>Eubacteriales>Clostridiaceae	GCF_011065935.2	Clostridium_estertheticum	G9F72_RS19190	4031148..4035239	238834	hypothetical-protein-[Clostridium-estertheticum]
MCI6150868.1	Type1.BREX	<-wHTH+WYL<-?<-HTH+MPTase<-ZnR<- SIGMA+OB-Fold+AAA-ATPase+Lon-Protease-BrxL <- PglZ <-HEPN<- HKD-DNase <- N6-METHYLASE <- DUF6079 *<- BrxB-BrxF-iSTAND <- BrxA-DNA-binding <-HMG+NLPC+NLPC<-VirB4-FtsK<-?||?-><-TrwI<-TraM<-VirD4-TraG+VirD4-FtsK	1179	Bacteria>Terrabacteria_group>Bacillota>Clostridia>Eubacteriales>Eubacteriales_incertae_sedis	GCA_022772165.1	Flintibacter_sp.	MR705_10640	43131..46670	1918624	BREX-system-P-loop-protein-BrxC-[Flintibacter-sp.]
MCC2230802.1	Type1.BREX	<-HAMP+HISKIN<-REC+OmpR-HTH<- HKD-DNase + HKD-DNase <- HKD-DNase + HKD-DNase <- SIGMA+OB-Fold+AAA-ATPase+Lon-Protease-BrxL <- PglZ <-Schlafen+HATPase+wHTH<-?<- N6-METHYLASE <- DUF6079 *<- BrxB-BrxF-iSTAND <- BrxA-DNA-binding <-XIS-HTH<-?<-?<-TraM<-TraJ-RHH<-LPD32+LPD36+N6_Mtase+SF1-SF2-Helicase	1217	Bacteria>Terrabacteria_group>Bacillota>Clostridia>Eubacteriales>Lachnospiraceae	GCA_020687485.1	Lachnospiraceae_bacterium_CLA-AA-H215	LKD81_07295	25894..29547	2885348	BREX-system-P-loop-protein-BrxC-[Lachnospiraceae-bacterium-CLA-AA-H215]
WP_090167800.1	Type1.BREX	<- HKD-DNase <-?<- SIGMA+OB-Fold+AAA-ATPase+Lon-Protease-BrxL <-BrxD-AAA<-?<- PglZ <-HEPN<- N6-METHYLASE <- N6-METHYLASE <- DUF6079 *<- BrxB-BrxF-iSTAND <- BrxA-DNA-binding <-HTH_26<-?<-?<- wHTH+WYL+WCX <-HNH<-?<-ARG	1201	Bacteria>Terrabacteria_group>Bacillota>Clostridia>Eubacteriales>Lachnospiraceae	GCF_900100545.1	Pseudobutyrivibrio_sp._49	BLP54_RS06100	185622..189227	1855344	BREX-system-P-loop-protein-BrxC-[Pseudobutyrivibrio-sp.-49]
WP_090545210.1	Type1.BREX	<- HKD-DNase <-?<- SIGMA+OB-Fold+AAA-ATPase+Lon-Protease-BrxL <-BrxD-AAA<-?<- PglZ <-HEPN<- N6-METHYLASE <- N6-METHYLASE <- DUF6079 *<- BrxB-BrxF-iSTAND <- BrxA-DNA-binding <-HTH_26<-?<-?<- wHTH+WYL+WCX <-HNH<-?<-ARG	1201	Bacteria>Terrabacteria_group>Bacillota>Clostridia>Eubacteriales>Lachnospiraceae	GCF_900113655.1	Pseudobutyrivibrio_sp._OR37	BM097_RS01535	134498..138103	1798186	BREX-system-P-loop-protein-BrxC-[Pseudobutyrivibrio-sp.-OR37]
WP_061213686.1	Type1.BREX	RelE-ParE->?->Classical-AAA->CR-ATPase5+REase-><-HEPN|| BrxA-DNA-binding -> BrxB-BrxF-iSTAND -> DUF6079 *-> N6-METHYLASE -> PglZ -> SIGMA+OB-Fold+AAA-ATPase+Lon-Protease-BrxL ->?-> HKD-DNase +SF1-SF2-Helicase+HTH+HTH+SRA-><-PIN<-?||?->RAMPs->	1199	Bacteria>Terrabacteria_group>Bacillota>Clostridia>Eubacteriales>Syntrophomonadaceae	GCF_001570625.1	Syntrophomonas_wolfei	AWB54_RS05890	82988..86587	863	BREX-system-P-loop-protein-BrxC-[Syntrophomonas-wolfei]
MBN1450028.1	Type1.BREX	<- SIGMA+OB-Fold+AAA-ATPase+Lon-Protease-BrxL <- PglZ <-REase+DUF5655<- N6-METHYLASE <-HTH+ HKD-DNase <-Pkinase+SF1-SF2-Helicase<-?<-NERD+Pkinase<- DUF6079 *<- BrxB-BrxF-iSTAND <- BrxA-DNA-binding	1209	Bacteria>Terrabacteria_group>Chloroflexota>Anaerolineae>Anaerolineales>unclassified_Anaerolineales	GCA_016928525.1	Anaerolineales_bacterium	JW963_03360	18534..22163	2073117	BREX-system-P-loop-protein-BrxC-[Anaerolineales-bacterium]
MBI4322558.1	Type6.BREX	<- DUF6079 -p*<- BrxB-BrxF-iSTAND <- BrxA-DNA-binding <- BrxE <-Phage_integrase<-?||?-> HKD-DNase +HKD-Rease->REase->	427	Bacteria>Terrabacteria_group>Chloroflexota>unclassified_Chloroflexota	GCA_016210495.1	Chloroflexota_bacterium	HY675_29045	1..1283	2026724	BREX-system-P-loop-protein-BrxC-[Chloroflexota-bacterium]
MCL4487040.1	Type1.BREX	BrxA-DNA-binding -> BrxB-BrxF-iSTAND ->Peptidase_M91-> N6-METHYLASE -> HKD-DNase -> DUF6079 *->HEPN->PhdYeFM_antitox->PIN-> PglZ ->?->?->?->MerR-HTH+TipAS->	1221	Bacteria>Terrabacteria_group>Chloroflexota>unclassified_Chloroflexota	GCA_023381515.1	Chloroflexota_bacterium	M1570_02770	10289..13954	2026724	BREX-system-P-loop-protein-BrxC-[Chloroflexota-bacterium]
BBD57221.1	Type1.BREX	<-MerR-HTH||RHH-><-?<-?||?->TPR+TPR+TPR-><-Phage_integrase<-?|| BrxB-BrxF-iSTAND -> DUF6079 *-><-?<-REase||?->PIN-> N6-METHYLASE ->HEPN-> PglZ +Phosphodiest -> HKD-DNase -> SIGMA+OB-Fold+AAA-ATPase+Lon-Protease-BrxL ->	1176	Bacteria>Terrabacteria_group>Cyanobacteriota/Melainabacteria_group>Cyanobacteriota>Cyanophyceae>Oscillatoriophycideae	GCA_003609755.1	Planktothrix_agardhii_NIES-204	NIES204_45570	14633..18163	282423	hypothetical-protein-NIES204_45570-[Planktothrix-agardhii-NIES-204]
WP_275522152.1	Type1.BREX	ACET-> BrxA-DNA-binding -><- SIGMA+OB-Fold+AAA-ATPase+Lon-Protease-BrxL <- HKD-DNase <- PglZ +Phosphodiest <- N6-METHYLASE <- DUF6079 *<- BrxB-BrxF-iSTAND <-AbiEii<-AbiEi_4<-?||?-><-TOPRIM||?->RVT+HNH->	1181	Bacteria>Terrabacteria_group>Cyanobacteriota/Melainabacteria_group>Cyanobacteriota>Cyanophyceae>Oscillatoriophycideae	GCF_029167235.1	Kamptonema_sp._UHCC_0994	PY364_RS09785	12149..15694	3031329	BREX-system-P-loop-protein-BrxC-[Kamptonema-sp.-UHCC-0994]
WP_254034082.1	Type1.BREX	TPR+TPR+TPR+TPR-><-?|| BrxA-DNA-binding -><- SIGMA+OB-Fold+AAA-ATPase+Lon-Protease-BrxL <- HKD-DNase <- PglZ +Phosphodiest <- N6-METHYLASE <-PIN<-?<- DUF6079 *<- BrxB-BrxF-iSTAND <-?||?->?->Phage_integrase->	1176	Bacteria>Terrabacteria_group>Cyanobacteriota/Melainabacteria_group>Cyanobacteriota>Cyanophyceae>Oscillatoriophycideae	GCF_904830845.1	Planktothrix_agardhii	NMG87_RS21740	24173..27703	1160	BREX-system-P-loop-protein-BrxC-[Planktothrix-agardhii]
WP_015862248.1	Type1.BREX	<-Phage_integrase<-?<-?||ParB+N6_N4_METHYLASE->PHP+SbcC-><- SIGMA+OB-Fold+AAA-ATPase+Lon-Protease-BrxL <- PglZ <-HEPN<- N6-METHYLASE <- DUF6079 *<- BrxB-BrxF-iSTAND <- BrxA-DNA-binding <- HKD-DNase <-?<-SbcC<-HAD<-?||?->Radical-SAM->	1188	Bacteria>Thermodesulfobacteriota>Desulfovibrionia>Desulfovibrionales>Desulfovibrionaceae>Solidesulfovibrio	GCF_000010665.1	Solidesulfovibrio_magneticus	DMR_RS17110	4099313..4102879	184917	BREX-system-P-loop-protein-BrxC-[Solidesulfovibrio-magneticus]
WP_086519900.1	Type1.BREX	<-PIN||nSTAND1+HNH->RNaseH->NACHT+SF1-SF2-Helicase+ HKD-DNase ->REase-><- HKD-DNase +OGG-like <- SIGMA+OB-Fold+AAA-ATPase+Lon-Protease-BrxL <- PglZ <- N6-METHYLASE <- DUF6079 *<- BrxB-BrxF-iSTAND <- BrxA-DNA-binding <-?<-ColE7+HNH<-ACET||NUDIX->?-><-DUF2754	1213	-	GCF_900775425.1	Bacteria	E7W97_RS22710	357533..361174	2	MULTISPECIES:-BREX-system-P-loop-protein-BrxC-[Bacteria]

Protein-ID	System	Gene-Neighbourhoods	Length	Taxonomy	Assembly-ID	Strain	Locus-Tag	Genomic-coordinates	TaxID	Fasta-define
--										
-- ParB + HNH -(GmrSD)										
--										
WP_103992123.1	Type5.BREX	<-Calcineurin<-HKD-DNase||CARF+HTH->?-><-?<-?<-?|| ParB + HNH -> BrxA-DNA-binding -> DUF6079 *-> BrxB-BrxF-iSTAND -> DUF6079 -><-OLD-ABC+TOPRIM|| N6-METHYLASE -><-REase|| PglZ ->REase+METHYLASE->TUDOR+SF1-SF2-Helicase+iREase+CTD->	1227	Archaea>Euryarchaeota>Stenosarchaea_group>Halobacteria>Haloferacales>Haloferacaceae	GCF_004799685.1	Halobellus_limi	DV707_RS08430	1701258..1704941	699433	hypothetical-protein-[Halobellus-limi]
WP_103992125.1	Type5.BREX	CARF+HTH->?-><-?<-?<-?|| ParB + HNH -> BrxA-DNA-binding -> DUF6079 -> BrxB-BrxF-iSTAND -> DUF6079 *-><-OLD-ABC+TOPRIM|| N6-METHYLASE -><-REase|| PglZ ->REase+METHYLASE->TUDOR+SF1-SF2-Helicase+iREase+CTD->	1284	Archaea>Euryarchaeota>Stenosarchaea_group>Halobacteria>Haloferacales>Haloferacaceae	GCF_004799685.1	Halobellus_limi	DV707_RS08440	1705516..1709370	699433	hypothetical-protein-[Halobellus-limi]
WP_076580859.1	Type6.BREX	REase-> BrxE -> BrxA-DNA-binding -> BrxB-BrxF-iSTAND -> DUF6079 *-><-REase+METHYLASE|| N6-METHYLASE -><- ParB + HNH || PglZ -> SIGMA+OB-Fold+AAA-ATPase+Lon-Protease-BrxL -><-?<-tRNA<- N6-METHYLASE	1206	Archaea>Euryarchaeota>Stenosarchaea_group>Halobacteria>Natrialbales>Natrialbaceae	GCF_001971705.1	Natronorubrum_daqingense	BW960_RS07735	340245..343865	588898	BREX-system-P-loop-protein-BrxC-[Natronorubrum-daqingense]
WP_253430473.1	Type6.BREX	Phage_integrase+Phage_integr_3-><-?||?-><-REase||?-> BrxE -> BrxA-DNA-binding -> BrxB-BrxF-iSTAND -> DUF6079 *->?-> N6-METHYLASE ->?-><- ParB + HNH || PglZ -> SIGMA+OB-Fold+AAA-ATPase+Lon-Protease-BrxL ->	1206	Archaea>Euryarchaeota>Stenosarchaea_group>Halobacteria>Natrialbales>Natrialbaceae	GCF_024138145.1	Halovivax_sp._AGai3-5	NGM15_RS10380	2104102..2107722	2953746	BREX-system-P-loop-protein-BrxC-[Halovivax-sp.-AGai3-5]
WP_229395640.1	Type1.BREX	BrxA-DNA-binding -> BrxB-BrxF-iSTAND -> DUF6079 *-> N6-METHYLASE -> ParB + HNH -> PglZ -> SIGMA+OB-Fold+AAA-ATPase+Lon-Protease-BrxL -><-DUF488<-Glycos_transf_1+Glyco_trans_1_2<-?<-DSBH	1143	Archaea>Euryarchaeota>Stenosarchaea_group>Methanomicrobia>Methanosarcinales>Methanosarcinaceae	GCF_020804205.1	Methanosarcina_sp._DH1	FXW07_RS12025	174817..178248	2605695	BREX-system-P-loop-protein-BrxC-[Methanosarcina-sp.-DH1]
MBD0371455.1	Type1.BREX	VirB11->PSBP->?-><- SIGMA+OB-Fold+AAA-ATPase+Lon-Protease-BrxL <- PglZ <- ParB + HNH <-NERD<- N6-METHYLASE <-HEPN<- DUF6079 *<- BrxB-BrxF-iSTAND <- BrxA-DNA-binding	1218	Bacteria>Acidobacteriota>Blastocatellia>Blastocatellales>Pyrinomonadaceae>unclassified_Pyrinomonadaceae	GCA_014534425.1	Pyrinomonadaceae_bacterium	ICV60_11505	29495..33151	2283092	BREX-system-P-loop-protein-BrxC-[Pyrinomonadaceae-bacterium]
MBK9963544.1	Type6.BREX	HTH+MPTase-><-SF1-SF2-Helicase+wHTH+Small-B-barrel+DUF1998<- ParB + HNH <- PglZ <- N6-METHYLASE <-OLD-ABC+TOPRIM<-ASCH<-PRTase<-?<- DUF6079 *<- BrxB-BrxF-iSTAND <- BrxA-DNA-binding <- BrxE ||?-><-NUDIX<-SAM-METHYLASE	1207	Bacteria>Acidobacteriota>Holophagae>Holophagales>unclassified_Holophagales	GCA_016722225.1	Holophagales_bacterium	IPP07_01030	234150..237773	2478486	BREX-system-P-loop-protein-BrxC-[Holophagales-bacterium]
MCG3192298.1	Type6.BREX	<-DUF932<- ParB + HNH <-?<- SIGMA+OB-Fold+AAA-ATPase+Lon-Protease-BrxL <- PglZ <-TOPRIM<-OLD-ABC<- N6-METHYLASE <- DUF6079 *<- BrxB-BrxF-iSTAND <- BrxA-DNA-binding <- BrxE <-?<-?<-?<-HTH+LexA-protease	1183	Bacteria>Acidobacteriota>Thermoanaerobaculia>unclassified_Thermoanaerobaculia	GCA_022071945.1	Thermoanaerobaculia_bacterium	DIJKHBIC_01536	22288..25839	2651171	hypothetical-protein-[Thermoanaerobaculia-bacterium]
MBO7131877.1	Type1.BREX	ZnR->?-><-?||DOC+HTH-><- SIGMA+OB-Fold+AAA-ATPase+Lon-Protease-BrxL <- PglZ <- N6-METHYLASE <- DUF6079 *<- BrxB-BrxF-iSTAND <- BrxA-DNA-binding <- ParB + HNH <-?||HD->	1109	Bacteria>Bacteria_incertae_sedis>Bacteria_candidate_phyla>Candidatus_Saccharibacteria>unclassified_Candidatus_Saccharibacteria	GCA_017645955.1	Candidatus_Saccharibacteria_bacterium	J6V85_01240	41463..44792	2026720	BREX-system-P-loop-protein-BrxC-[Candidatus-Saccharibacteria-bacterium]
WP_146981109.1	Type6.BREX	Peptidase_S8-><-SF1-SF2-Helicase+wHTH+Small-B-barrel+DUF1998<-BrxD-AAA<- PglZ <- ParB + HNH <-TIR<-?<- N6-METHYLASE <-?<- DUF6079 *<- BrxB-BrxF-iSTAND <- BrxA-DNA-binding <- BrxE ||?->?->?-><-?<-?<-ABhydrolase	1216	Bacteria>delta/epsilon_subdivisions>Deltaproteobacteria>Bradymonadales>Lujinxingiaceae>Lujinxingia	GCF_007997015.1	Lujinxingia_vulgaris	FRC98_RS09600	57836..61486	2600176	BREX-system-P-loop-protein-BrxC-[Lujinxingia-vulgaris]
WP_251951637.1	Type5.BREX	<-Calcineurin-SbcD<-SF1-SF2-Helicase<-OLD-ABC+TOPRIM<-TUDOR+SF1-SF2-Helicase+iREase+CTD<-?<-DDE_Tnp_1<- PglZ <- ParB + HNH <- N6-METHYLASE <- DUF6079 *<- BrxB-BrxF-iSTAND <- DUF6079 <- BrxA-DNA-binding <-REase+RNase<-viral-protein<-?<-RHH<-TraJ-RHH||HTH_3+Peptidase_S24->	1108	Bacteria>FCB_group>Bacteroidetes/Chlorobi_group>Bacteroidota>Bacteroidetes_incertae_sedis>Rhodothermaceae	GCF_943908255.1	Salinibacter_ruber	NGL47_RS11295	22718..26044	146919	hypothetical-protein-[Salinibacter-ruber]
WP_251951639.1	Type5.BREX	<-OLD-ABC+TOPRIM<-TUDOR+SF1-SF2-Helicase+iREase+CTD<-?<-DDE_Tnp_1<- PglZ <- ParB + HNH <- N6-METHYLASE <- DUF6079 <- BrxB-BrxF-iSTAND <- DUF6079 *<- BrxA-DNA-binding <-REase+RNase<-viral-protein<-?<-RHH<-TraJ-RHH||HTH_3+Peptidase_S24->	1245	Bacteria>FCB_group>Bacteroidetes/Chlorobi_group>Bacteroidota>Bacteroidetes_incertae_sedis>Rhodothermaceae	GCF_943908255.1	Salinibacter_ruber	NGL47_RS11305	26687..30424	146919	hypothetical-protein-[Salinibacter-ruber]
MBS6238336.1	Type1.BREX	<-PAS+HISKIN||?-><-AraC-HTH+AraC-HTH<- SIGMA+OB-Fold+AAA-ATPase+HKD-DNase-BrxL <- PglZ <-Schlafen+HATPase+wHTH<- ParB + HNH <- ParB + HNH <- N6-METHYLASE <- DUF6079 *<- BrxB-BrxF-iSTAND <- BrxA-DNA-binding <-HTH_3<-?<-?<-?<-?||SIGMA-HTH->	1205	Bacteria>FCB_group>Bacteroidetes/Chlorobi_group>Bacteroidota>Bacteroidia>Bacteroidales	GCA_018374845.1	Bacteroides_sp.	KH897_08200	204452..208069	29523	BREX-system-P-loop-protein-BrxC-[Bacteroides-sp.]
SHF69941.1	Type1.BREX	<-RADICAL-SAM<-?||tRNA-><- SIGMA+OB-Fold+AAA-ATPase+Lon-Protease-BrxL <- PglZ <-Schlafen+HATPase+wHTH<- ParB + HNH <- N6-METHYLASE <-?<- DUF6079 *<- BrxB-BrxF-iSTAND <- BrxA-DNA-binding	1182	Bacteria>FCB_group>Bacteroidetes/Chlorobi_group>Bacteroidota>Bacteroidia>Bacteroidales	GCA_900128905.1	Bacteroides_luti	SAMN05444405_11263	81108..84656	1297750	hypothetical-protein-SAMN05444405_11263-[Bacteroides-luti]
SHK68061.1	Type1.BREX	<-Classical-AAA<-?||tRNA-><-?<-?<-HTH+DOC||?->HTH_3-> BrxA-DNA-binding -> BrxB-BrxF-iSTAND -> DUF6079 *-> N6-METHYLASE ->SbcC+SbcC->?->SIR2+SIR2-> PglZ -> SIGMA+OB-Fold+AAA-ATPase+HKD-DNase-BrxL ->?-> ParB + HNH -> ParB + HNH ->	1206	Bacteria>FCB_group>Bacteroidetes/Chlorobi_group>Bacteroidota>Bacteroidia>Bacteroidales	GCA_900142525.1	Prevotella_ruminicola	SAMN05216463_10942	46265..49885	839	hypothetical-protein-SAMN05216463_10942-[Prevotella-ruminicola]
WP_073402639.1	Type1.BREX	<-RADICAL-SAM<-?||tRNA-><- SIGMA+OB-Fold+AAA-ATPase+Lon-Protease-BrxL <- PglZ <-Schlafen+HATPase+wHTH<- ParB + HNH <- N6-METHYLASE <-?<- DUF6079 *<- BrxB-BrxF-iSTAND <- BrxA-DNA-binding	1188	Bacteria>FCB_group>Bacteroidetes/Chlorobi_group>Bacteroidota>Bacteroidia>Bacteroidales	GCF_900128905.1	Bacteroides_luti	BUC21_RS11790	81108..84674	1297750	BREX-system-P-loop-protein-BrxC-[Bacteroides-luti]
WP_254795118.1	Type1.BREX	<-Classical-AAA<-?||tRNA-><-?<-?<-HTH+DOC||?->HTH_3-> BrxA-DNA-binding -> BrxB-BrxF-iSTAND -> DUF6079 *-> N6-METHYLASE ->SbcC+SbcC->?->SIR2+SIR2-> PglZ -> SIGMA+OB-Fold+AAA-ATPase+HKD-DNase-BrxL ->?->?-> ParB + HNH ->	1209	Bacteria>FCB_group>Bacteroidetes/Chlorobi_group>Bacteroidota>Bacteroidia>Bacteroidales	GCF_900142525.1	Prevotella_ruminicola	BUB86_RS07050	46256..49885	839	BREX-system-P-loop-protein-BrxC-[Prevotella-ruminicola]
WP_176134075.1	Type1.BREX	<-Phage_holin||?->tRNA-><-?||?->?->ABC-ATPase+SbcC-><-?|| BrxA-DNA-binding -> BrxB-BrxF-iSTAND -> DUF6079 *-> N6-METHYLASE ->OLD-ABC+TOPRIM->SF1-SF2-Helicase->?-> N6-METHYLASE -p-> ParB + HNH -> ParB + HNH -> PglZ -> SIGMA+OB-Fold+AAA-ATPase+Lon-Protease-BrxL ->	1192	Bacteria>FCB_group>Bacteroidetes/Chlorobi_group>Bacteroidota>Bacteroidia>Bacteroidales	GCF_900168155.1	Parabacteroides_chartae	B5X57_RS12575	48804..52382	1037355	BREX-system-P-loop-protein-BrxC-[Parabacteroides-chartae]
WP_073207711.1	Type1.BREX	<-Classical-AAA<-?||tRNA-><-?<-?<-HTH+DOC||?->HTH_3-> BrxA-DNA-binding -> BrxB-BrxF-iSTAND -> DUF6079 *-> N6-METHYLASE ->SbcC+SbcC->?->SIR2+SIR2-> PglZ -> SIGMA+OB-Fold+AAA-ATPase+HKD-DNase-BrxL ->?-> ParB + HNH -> ParB + HNH ->	1206	Bacteria>FCB_group>Bacteroidetes/Chlorobi_group>Bacteroidota>Bacteroidia>Bacteroidales	-	Prevotella_ruminicola	SAMN05216463_10942	46265..49885	839	BREX-system-P-loop-protein-BrxC-[Prevotella-ruminicola]
WP_075603947.1	Type1.BREX	TPR+TPR+TPR+TPR->?->XIS-HTH->?->CHC2+TOPRIM-DNAG->?-> BrxA-DNA-binding -> BrxB-BrxF-iSTAND -> DUF6079 *-> N6-METHYLASE ->?->?-> ParB + HNH -> PglZ -> SIGMA+OB-Fold+AAA-ATPase+Lon-Protease-BrxL ->ZDNA-HTH->	1204	Bacteria>FCB_group>Bacteroidetes/Chlorobi_group>Bacteroidota>Bacteroidia>Marinilabiliales	GCF_001660705.2	Saccharicrinis_aurantiacus	A8B35_RS21005	65274..68888	1849719	BREX-system-P-loop-protein-BrxC-[Saccharicrinis-aurantiacus]
WP_053405044.1	Type1.BREX	<- SIGMA+OB-Fold+AAA-ATPase+HKD-DNase-BrxL <- PglZ || ParB + HNH -><- N6-METHYLASE <- DUF6079 *<- BrxB-BrxF-iSTAND <- BrxA-DNA-binding <-AAA-ToxA||?->?-><- ParB <-ParA-Soj-PloopNTPase<-?<-AAA-ATPase	1179	Bacteria>FCB_group>Bacteroidetes/Chlorobi_group>Bacteroidota>Cytophagia>Cytophagales	GCF_001274635.1	Persicobacter_sp._CCB-QB2	NG22_RS06825	1502189..1505728	1561025	BREX-system-P-loop-protein-BrxC-[Persicobacter-sp.-CCB-QB2]
OIQ21186.1	Type1.BREX	CHTH+LexA-protease-><-GDResolvase+Recombinase+ZnR<-?||?-><-?|| BrxA-DNA-binding -> BrxB-BrxF-iSTAND -> DUF6079 *-> N6-METHYLASE ->SF1-SF2-Helicase->?-> PglZ -> SIGMA+OB-Fold+AAA-ATPase+HKD-DNase-BrxL -> ParB + HNH ->URI-DNase->?->GDResolvase->	1165	Bacteria>FCB_group>Bacteroidetes/Chlorobi_group>Bacteroidota>Flavobacteriia>Flavobacteriales	GCA_001874145.1	Lacinutrix_sp._MedPE-SW	BM549_09430	19615..23112	1860087	MAG:-hypothetical-protein-BM549_09430-[Lacinutrix-sp.-MedPE-SW]
MAM30277.1	Type1.BREX	<- ParB +N6_N4_METHYLASE||XIS-HTH-><- SIGMA+OB-Fold+AAA-ATPase+HKD-DNase-BrxL <- PglZ <-TPR+S1<-NERD+SF1-SF2-Helicase<- N6-METHYLASE <- ParB + HNH <- DUF6079 *<- BrxB-BrxF-iSTAND <- BrxA-DNA-binding <-HTH_3||GDResolvase->?->?->ABC-ATPase+SbcC-> N6-METHYLASE ->	1165	Bacteria>FCB_group>Bacteroidetes/Chlorobi_group>Bacteroidota>Flavobacteriia>Flavobacteriales	GCA_002693005.1	Flavobacteriaceae_bacterium	CMC13_14765	19860..23357	1871037	hypothetical-protein-[Flavobacteriaceae-bacterium]
GEM55955.1	Type1.BREX	<- SIGMA+OB-Fold+AAA-ATPase+Lon-Protease-BrxL <- PglZ <-AbiEii<-AbiEi_4<- ParB + HNH <- ParB + HNH <- N6-METHYLASE <- DUF6079 *<- BrxB-BrxF-iSTAND <- BrxA-DNA-binding <-tRNA||Arm-DNA-bind_5+Phage_int_SAM_5+Phage_integrase->?->XIS-HTH->?->DUF488->GDResolvase->	1216	Bacteria>FCB_group>Bacteroidetes/Chlorobi_group>Bacteroidota>Flavobacteriia>Flavobacteriales	GCA_007990815.1	Flavobacterium_branchiophilum_NBRC_15030_=_ATCC_35035	FB1_21760	14247..17897	1218110	hypothetical-protein-FB1_21760-[Flavobacterium-branchiophilum-NBRC-15030-=-ATCC-35035]
WP_027137224.1	Type6.BREX	DarT+Peptidase->MACRODOMAIN->TPR+TPR->?-><- SIGMA+OB-Fold+AAA-ATPase+HKD-DNase-BrxL <- PglZ <- ParB + HNH <- N6-METHYLASE <- DUF6079 *<- BrxB-BrxF-iSTAND <- BrxA-DNA-binding <- BrxE <-?<- wHTH+WYL+WCX ||?-><-HEPN<-ARG	1196	Bacteria>FCB_group>Bacteroidetes/Chlorobi_group>Bacteroidota>Flavobacteriia>Flavobacteriales	GCF_000425645.1	Gaetbulibacter_saemankumensis	H611_RS0104475	476638..480228	311208	BREX-system-P-loop-protein-BrxC-[Gaetbulibacter-saemankumensis]
WP_059014619.1	Type1.BREX	<-HEPN<- ParB + HNH <- N6-METHYLASE <- DUF6079 *<- BrxB-BrxF-iSTAND <- BrxA-DNA-binding <-HTH_3<-Classical-AAA<-HTH_36<-HTH_36<-XIS-HTH<-Arm-DNA-bind_5+Phage_int_SAM_5+Phage_integrase	1169	Bacteria>FCB_group>Bacteroidetes/Chlorobi_group>Bacteroidota>Flavobacteriia>Flavobacteriales	GCF_001485515.1	Flavobacterium_psychrophilum	FPK15_RS07995	6803..10312	96345	BREX-system-P-loop-protein-BrxC-[Flavobacterium-psychrophilum]
WP_076662677.1	Type1.BREX	DOC+HTH-><-?<-?<-?<- SIGMA+OB-Fold+AAA-ATPase+Lon-Protease-BrxL <- PglZ <- ParB + HNH <-?<- N6-METHYLASE <- DUF6079 *<- BrxB-BrxF-iSTAND <- BrxA-DNA-binding <- HNH <-sGTP<-GTPase-AIG<-?<-?|| wHTH+WYL+WCX ->	1201	Bacteria>FCB_group>Bacteroidetes/Chlorobi_group>Bacteroidota>Flavobacteriia>Flavobacteriales	GCF_001975575.1	Mangrovimonas_sp._DI_80	BKM32_RS04295	828004..831609	1779330	BREX-system-P-loop-protein-BrxC-[Mangrovimonas-sp.-DI-80]
WP_132215847.1	Type1.BREX	<-CARF+REase<-CARF<-HKD-DNase+HKD-DNase<-?<-S1+SF1-SF2-Helicase+S1<- SIGMA+OB-Fold+AAA-ATPase+HKD-DNase-BrxL <- PglZ <- ParB + HNH <- N6-METHYLASE <- DUF6079 *<- BrxB-BrxF-iSTAND <- BrxA-DNA-binding <-ZnR+CC+REase<- wHTH+WYL+WCX <-Arm-DNA-bind_5+Phage_int_SAM_5+Phage_integrase<-ZnR||Phage_integrase+Phage_integrase-><-Zn_Tnp_IS1595+DDE_Tnp_IS1595<-REC+LuxR-HTH	1169	Bacteria>FCB_group>Bacteroidetes/Chlorobi_group>Bacteroidota>Flavobacteriia>Flavobacteriales	GCF_004341235.1	Mariniflexile_fucanivorans	EV196_RS05490	443037..446546	264023	BREX-system-P-loop-protein-BrxC-[Mariniflexile-fucanivorans]
WP_131908313.1	Type1.BREX	<-XIS-HTH<-?||?-><- SIGMA+OB-Fold+AAA-ATPase+Lon-Protease-BrxL <- PglZ <-AbiEii<-AbiEi_4<- ParB + HNH <- N6-METHYLASE <- DUF6079 *<- BrxB-BrxF-iSTAND <- BrxA-DNA-binding ||?-><-?||HTH_18+HTH_AraC->	1203	Bacteria>FCB_group>Bacteroidetes/Chlorobi_group>Bacteroidota>Flavobacteriia>Flavobacteriales	GCF_004349085.1	Flavobacterium_caseinilyticum	E0F89_RS02690	598586..602197	2541732	BREX-system-P-loop-protein-BrxC-[Flavobacterium-caseinilyticum]
WP_141421442.1	Type1.BREX	<-HAD+PRTase+PRTase+REase+SF1-SF2-Helicase<-?<- SIGMA+OB-Fold+AAA-ATPase+HKD-DNase-BrxL <- PglZ <-TPR+S1<-NERD+SF1-SF2-Helicase<- N6-METHYLASE <- ParB + HNH <- DUF6079 *<- BrxB-BrxF-iSTAND <- BrxA-DNA-binding <-TPR+TPR<-?<-?<-?<-Phage_int+DNA-glycosylase<- HNH <-OLD-ABC	1165	Bacteria>FCB_group>Bacteroidetes/Chlorobi_group>Bacteroidota>Flavobacteriia>Flavobacteriales	GCF_006546625.1	Haloflavibacter_putidus	FKR84_RS06225	21354..24851	2576776	BREX-system-P-loop-protein-BrxC-[Haloflavibacter-putidus]
WP_089081509.1	Type1.BREX	<- SIGMA+OB-Fold+AAA-ATPase+Lon-Protease-BrxL <- PglZ <-AbiEii<-AbiEi_4<- ParB + HNH <- ParB + HNH <- N6-METHYLASE <- DUF6079 *<- BrxB-BrxF-iSTAND <- BrxA-DNA-binding <-tRNA||Arm-DNA-bind_5+Phage_int_SAM_5+Phage_integrase->?->XIS-HTH->?->DUF488->GDResolvase->	1199	Bacteria>FCB_group>Bacteroidetes/Chlorobi_group>Bacteroidota>Flavobacteriia>Flavobacteriales	GCF_007990815.1	Flavobacterium_branchiophilum	FB1_RS11100	14247..17846	55197	BREX-system-P-loop-protein-BrxC-[Flavobacterium-branchiophilum]
WP_153266434.1	Type1.BREX	HTH_36->Classical-AAA->TRPR-HTH->STYKinase-like->HTH_3->?->HTH_3-> BrxA-DNA-binding -> BrxB-BrxF-iSTAND -> DUF6079 *-> ParB + HNH -> N6-METHYLASE -> PglZ -> SIGMA+OB-Fold+AAA-ATPase+HKD-DNase-BrxL ->HigB->HTH_3->?-><-XIS-HTH	1157	Bacteria>FCB_group>Bacteroidetes/Chlorobi_group>Bacteroidota>Flavobacteriia>Flavobacteriales	GCF_009498295.1	Oceanihabitans_sp._IOP_32	FEZ18_RS00165	31374..34847	2529032	BREX-system-P-loop-protein-BrxC-[Oceanihabitans-sp.-IOP_32]
WP_165150638.1	Type1.BREX	<-ABhydrolase<-REase+RNase<-?<- wHTH+WYL+WCX ||?->?->?-> BrxA-DNA-binding -> BrxB-BrxF-iSTAND -> DUF6079 *-> N6-METHYLASE -> ParB + HNH -> PglZ -> SIGMA+OB-Fold+AAA-ATPase+Lon-Protease-BrxL ->HEPN->?->?-><-FGase<-tRNA||SKI->	1198	Bacteria>FCB_group>Bacteroidetes/Chlorobi_group>Bacteroidota>Flavobacteriia>Flavobacteriales	GCF_011065475.1	Aequorivita_sp._KMM_9714	G3A46_RS13570	21223..24819	2707173	BREX-system-P-loop-protein-BrxC-[Aequorivita-sp.-KMM-9714]
WP_193813001.1	Type6.BREX	<- wHTH+WYL+WCX ||ZnR+CC+REase->?-><-?<- SIGMA+OB-Fold+AAA-ATPase+HKD-DNase-BrxL <- PglZ <- ParB + HNH <- N6-METHYLASE <- DUF6079 *<- BrxB-BrxF-iSTAND <- BrxA-DNA-binding <- BrxE <-?<-FGase<-HEPN	1217	Bacteria>FCB_group>Bacteroidetes/Chlorobi_group>Bacteroidota>Flavobacteriia>Flavobacteriales	GCF_015191005.1	Kaistella_flava_(ex_Peng_et_al._2021)	Q73A0000_05090	1135416..1139069	2038776	BREX-system-P-loop-protein-BrxC-[Kaistella-flava-(ex-Peng-et-al.-2021)]
WP_198639224.1	Type1.BREX	REase->SF1-SF2-Helicase+REase->NERD+SF1-SF2-Helicase-> HNH -><-?<- SIGMA+OB-Fold+AAA-ATPase+Lon-Protease-BrxL <- PglZ <- ParB + HNH <- N6-METHYLASE <- DUF6079 *<- BrxB-BrxF-iSTAND <- BrxA-DNA-binding <-?<-?<-?<-?<-?<-?<- wHTH+WYL+WCX	1201	Bacteria>FCB_group>Bacteroidetes/Chlorobi_group>Bacteroidota>Flavobacteriia>Flavobacteriales	GCF_016236975.1	Salegentibacter_maritimus	I6U50_RS13470	35086..38691	2794347	BREX-system-P-loop-protein-BrxC-[Salegentibacter-maritimus]
WP_179314673.1	Type1.BREX	<- ParB + HNH <- ParB + HNH <- PglZ <-METHYLASE<-?<-SF1-SF2-Helicase<-?<-?<- N6-METHYLASE <- DUF6079 *<- BrxB-BrxF-iSTAND <- BrxA-DNA-binding <-HTH_3<-Classical-AAA<-HTH_36<-XIS-HTH	1160	Bacteria>FCB_group>Bacteroidetes/Chlorobi_group>Bacteroidota>Flavobacteriia>Flavobacteriales	GCF_019278425.1	Winogradskyella	HOI32_RS04080	939044..942526	286104	MULTISPECIES:-BREX-system-P-loop-protein-BrxC-[Winogradskyella]
WP_244340081.1	Type1.BREX	<- PglZ <-Couple_hipA+HipA_C<-HTH_3<-?<-URI-DNase<- ParB + HNH <- N6-METHYLASE <-?<- N6-METHYLASE <- DUF6079 *<- BrxB-BrxF-iSTAND <- BrxA-DNA-binding <-HTH_3||TraJ-RHH->?->?->?->Peptidase_S9+Hydrolase_4->	1169	Bacteria>FCB_group>Bacteroidetes/Chlorobi_group>Bacteroidota>Flavobacteriia>Flavobacteriales	GCF_022836875.1	Flavobacterium	LZF87_RS14585	3214137..3217646	237	MULTISPECIES:-BREX-system-P-loop-protein-BrxC-[Flavobacterium]
WP_089756260.1	Type1.BREX	<-RHS+RHS<-?<-?||NUMOD4+ HNH ->NUMOD4+ HNH -><- SIGMA+OB-Fold+AAA-ATPase+HKD-DNase-BrxL <- PglZ <- ParB + HNH <- N6-METHYLASE <- DUF6079 *<- BrxB-BrxF-iSTAND <- BrxA-DNA-binding <-HTH_3<-HTH_19||SIGMA-HTH-><-REase<- ParB + HNH <-?<-TraI-VirD2-RCRE	1176	Bacteria>FCB_group>Bacteroidetes/Chlorobi_group>Bacteroidota>Flavobacteriia>Flavobacteriales	GCF_900100115.1	Chryseobacterium_soldanellicola	BLT04_RS13255	831929..835459	311333	BREX-system-P-loop-protein-BrxC-[Chryseobacterium-soldanellicola]
WP_103999283.1	Type1.BREX	<-GDResolvase+Recombinase+ZnR||?-><-?||?-><-?||HTH_3-> BrxA-DNA-binding -> BrxB-BrxF-iSTAND -> DUF6079 *-> N6-METHYLASE -> ParB + HNH ->URI-DNase->RADICAL-SAM->METHYLASE+RNA-Helicase->HTH_3-> PglZ -> SIGMA+OB-Fold+AAA-ATPase+HKD-DNase-BrxL -><-Arm-DNA-bind_5+Phage_int_SAM_5+Phage_integrase	1170	Bacteria>FCB_group>Bacteroidetes/Chlorobi_group>Bacteroidota>Flavobacteriia>Flavobacteriales	GCF_900108015.1	Flavobacterium_urumqiense	C4S35_RS05860	195084..198596	395224	BREX-system-P-loop-protein-BrxC-[Flavobacterium-urumqiense]
WP_090838887.1	Type1.BREX	<-SAM-METHYLASE<-?<- ParB + HNH <- ParB + HNH <- PglZ <-TPR+S1<-NERD+SF1-SF2-Helicase<- N6-METHYLASE <- DUF6079 *<- BrxB-BrxF-iSTAND <- BrxA-DNA-binding <-HTH_3	1160	Bacteria>FCB_group>Bacteroidetes/Chlorobi_group>Bacteroidota>Flavobacteriia>Flavobacteriales	GCF_900114005.1	Olleya_namhaensis	BMZ09_RS07050	330310..333792	1144750	BREX-system-P-loop-protein-BrxC-[Olleya-namhaensis]
WP_159447525.1	Type1.BREX	<-HISKIN+REC<-?<-?|| wHTH+WYL+WCX ->ZnR+CC+REase-> BrxA-DNA-binding -> BrxB-BrxF-iSTAND -> DUF6079 -p*-> N6-METHYLASE -> ParB + HNH ->HTH_3->Couple_hipA+HipA_C-> PglZ -> SIGMA+OB-Fold+AAA-ATPase+HKD-DNase-BrxL ->	719	Bacteria>FCB_group>Bacteroidetes/Chlorobi_group>Bacteroidota>Flavobacteriia>Flavobacteriales	GCF_900167935.1	Maribacter_arcticus	B5X55_RS04985	472839..474998	561365	BREX-system-P-loop-protein-BrxC-[Maribacter-arcticus]
WP_271393758.1	Type1.BREX	<- SIGMA+OB-Fold+AAA-ATPase+HKD-DNase-BrxL <- PglZ <-?<-SF1-SF2-Helicase<-?<- N6-METHYLASE <- ParB + HNH <- DUF6079 *<- BrxB-BrxF-iSTAND <- BrxA-DNA-binding <-?<-STYKinase-like<-TRPR-HTH<-?<-Classical-AAA<-HTH_36	1165	Bacteria>FCB_group>Bacteroidetes/Chlorobi_group>Bacteroidota>Flavobacteriia>Flavobacteriales	GCF_947492035.1	Aequorivita_sinensis	PQR58_RS11110	23754..27251	1382458	BREX-system-P-loop-protein-BrxC-[Aequorivita-sinensis]
WP_272023608.1	Type1.BREX	<- ParB + HNH <- PglZ <-METHYLASE<-?<-SF1-SF2-Helicase<-?<-TPR+REase<-?<- N6-METHYLASE <- DUF6079 *<- BrxB-BrxF-iSTAND <- BrxA-DNA-binding <-HTH_3<-?<-?<-?<-ParA-Soj-PloopNTPase<-Relaxase	1160	Bacteria>FCB_group>Bacteroidetes/Chlorobi_group>Bacteroidota>Flavobacteriia>Flavobacteriales	GCF_947496445.1	Olleya_namhaensis	PQG05_RS12495	26845..30327	1144750	BREX-system-P-loop-protein-BrxC-[Olleya-namhaensis]
OYD42847.1	Type1.BREX	wHTH+WYL+WCX -><-?<-?||?-><-?||sGTP->HTH_3-> BrxA-DNA-binding -> BrxB-BrxF-iSTAND -> DUF6079 *-> N6-METHYLASE -> ParB + HNH -> PglZ -> SIGMA+OB-Fold+AAA-ATPase+HKD-DNase-BrxL ->	1175	Bacteria>FCB_group>Bacteroidetes/Chlorobi_group>Bacteroidota>Sphingobacteriia>Sphingobacteriales	GCA_002245855.1	Sphingobacterium_cellulitidis	CHT99_08530	557163..560690	1768011	hypothetical-protein-CHT99_08530-[Sphingobacterium-cellulitidis]
WP_094282244.1	Type1.BREX	<- SIGMA+OB-Fold+AAA-ATPase+HKD-DNase-BrxL <- PglZ <- ParB + HNH <- N6-METHYLASE <- N6-METHYLASE -p<- DUF6079 *<- BrxB-BrxF-iSTAND <- BrxA-DNA-binding <-HTH_3<-sGTP||?-><-?||SIGMA-HTH->	1175	Bacteria>FCB_group>Bacteroidetes/Chlorobi_group>Bacteroidota>Sphingobacteriia>Sphingobacteriales	GCF_002245765.1	Sphingobacterium_cellulitidis	CHU00_RS15775	18381..21908	1768011	BREX-system-P-loop-protein-BrxC-[Sphingobacterium-cellulitidis]
MBS1911241.1	Type1.BREX	<- SIGMA+OB-Fold+AAA-ATPase+Lon-Protease-BrxL <- PglZ <- HNH <-OLD-ABC <- ParB + HNH <- N6-METHYLASE <- DUF6079 *<- BrxB-BrxF-iSTAND <- BrxA-DNA-binding <-tRNA<-?||?-><-?||SIGMA-HTH->	1145	Bacteria>FCB_group>Bacteroidetes/Chlorobi_group>Bacteroidota>unclassified_Bacteroidota	GCA_018266795.1	Bacteroidota_bacterium	JST22_04585	55920..59357	1898104	BREX-system-P-loop-protein-BrxC-[Bacteroidota-bacterium]
MBV5326252.1	Type1.BREX	<- ParB + HNH <- N6-METHYLASE -p<-RVT<-Schlafen+HATPase+wHTH+HTH<- DUF6079 *<- BrxB-BrxF-iSTAND <- BrxA-DNA-binding <-XIS-HTH<-?<-tRNA<-HsIUClpX-AAA<-?<-?<-?<-His_Phos_1	1179	Bacteria>FCB_group>Bacteroidetes/Chlorobi_group>Chlorobiota>Chlorobiia>Chlorobiales	GCA_019162945.1	Chlorobium_sp.	JZU65_01250	9464..13003	1095	BREX-system-P-loop-protein-BrxC-[Chlorobium-sp.]
WP_204208384.1	Type1.BREX	ABhydrolase->?->Aminotran_1_2-><-?||DUF772+DDE_Tnp_1_2-> BrxA-DNA-binding -> BrxB-BrxF-iSTAND -> DUF6079 -p*->?->Schlafen+HATPase+wHTH+HTH-> N6-METHYLASE ->DUF4372+DDE_Tnp_1-> N6-METHYLASE -p-> ParB + HNH ->RVT-> PglZ -> SIGMA+OB-Fold+AAA-ATPase+HKD-DNase-BrxL ->	997	Bacteria>FCB_group>Bacteroidetes/Chlorobi_group>Chlorobiota>Chlorobiia>Chlorobiales	GCF_001687065.1	Prosthecochloris_sp._CIB_2401	Ptc2401_RS05035	1057250..1060243	1868325	BREX-system-P-loop-protein-BrxC-[Prosthecochloris-sp.-CIB-2401]
WP_139626761.1	Type1.BREX	<- PglZ <- ParB + HNH <- N6-METHYLASE <- N6-METHYLASE -p<-Schlafen+HATPase+wHTH+HTH<- DUF6079 *<- BrxB-BrxF-iSTAND <- BrxA-DNA-binding ||<-Aminotran_1_2<-?<-ABhydrolase	1204	Bacteria>FCB_group>Bacteroidetes/Chlorobi_group>Chlorobiota>Chlorobiia>Chlorobiales	GCF_006265245.1	Prosthecochloris_vibrioformis	FGF68_RS08505	9816..13430	1098	BREX-system-P-loop-protein-BrxC-[Prosthecochloris-vibrioformis]
NLW30564.1	Type1.BREX	HNH ->?-> BrxA-DNA-binding -> BrxB-BrxF-iSTAND -> DUF6079 *->?-> ParB + HNH -> PglZ -> SIGMA+OB-Fold+AAA-ATPase+HKD-DNase-BrxL ->	1185	Bacteria>FCB_group>Fibrobacterota>Fibrobacteria>Fibrobacterales>Fibrobacteraceae	GCA_012728995.1	Fibrobacter_sp.	GXY77_03805	4326..7883	35828	BREX-system-P-loop-protein-BrxC-[Fibrobacter-sp.]
SHK42502.1	Type1.BREX	<-VirE<-?<-XIS-HTH||?->?->?-> BrxA-DNA-binding -> BrxB-BrxF-iSTAND -> DUF6079 *-> ParB + HNH -> N6-METHYLASE ->Schlafen+HATPase+wHTH-> PglZ -> SIGMA+OB-Fold+AAA-ATPase+Lon-Protease-BrxL ->TIR->?->REase->Phage_int_SAM_1+Phage_integrase->	1186	Bacteria>FCB_group>Fibrobacterota>Fibrobacteria>Fibrobacterales>Fibrobacteraceae	GCA_900142505.1	Fibrobacter_sp._UWH5	SAMN05720764_101395	444346..447906	1896211	hypothetical-protein-SAMN05720764_101395-[Fibrobacter-sp.-UWH5]
WP_212668349.1	Type1.BREX	<-VirE<-?<-XIS-HTH||?->?->?-> BrxA-DNA-binding -> BrxB-BrxF-iSTAND -> DUF6079 *-> ParB + HNH -> N6-METHYLASE ->Schlafen+HATPase+wHTH-> PglZ -> SIGMA+OB-Fold+AAA-ATPase+Lon-Protease-BrxL ->REase->REase->Phage_int_SAM_1+Phage_integrase->	1186	Bacteria>FCB_group>Fibrobacterota>Fibrobacteria>Fibrobacterales>Fibrobacteraceae	GCF_002210355.1	Fibrobacter_sp._UWH3	B7993_RS04325	153736..157296	1964353	BREX-system-P-loop-protein-BrxC-[Fibrobacter-sp.-UWH3]
WP_073283173.1	Type1.BREX	<-VirE<-?<-XIS-HTH||?->?->?-> BrxA-DNA-binding -> BrxB-BrxF-iSTAND -> DUF6079 *-> ParB + HNH -> N6-METHYLASE ->Schlafen+HATPase+wHTH-> PglZ -> SIGMA+OB-Fold+AAA-ATPase+Lon-Protease-BrxL ->REase->REase->Phage_int_SAM_1+Phage_integrase->	1184	Bacteria>FCB_group>Fibrobacterota>Fibrobacteria>Fibrobacterales>Fibrobacteraceae	GCF_900142465.1	Fibrobacter_sp._UWH6	BUB73_RS01535	351031..354585	1896212	BREX-system-P-loop-protein-BrxC-[Fibrobacter-sp.-UWH6]
WP_073156756.1	Type1.BREX	<-VirE<-?<-XIS-HTH||?->?->?-> BrxA-DNA-binding -> BrxB-BrxF-iSTAND -> DUF6079 *-> ParB + HNH -> N6-METHYLASE ->Schlafen+HATPase+wHTH-> PglZ -> SIGMA+OB-Fold+AAA-ATPase+Lon-Protease-BrxL ->TIR->?->REase->Phage_int_SAM_1+Phage_integrase->	1184	Bacteria>FCB_group>Fibrobacterota>Fibrobacteria>Fibrobacterales>Fibrobacteraceae	GCF_900142505.1	Fibrobacter_sp._UWH5	BUB38_RS01950	444352..447906	1896211	BREX-system-P-loop-protein-BrxC-[Fibrobacter-sp.-UWH5]
WP_005903411.1	Type1.BREX	<- PglZ <- ParB + HNH +RAMA<-OLD-ABC+TOPRIM<- N6-METHYLASE <-Schlafen+HATPase+wHTH<-?<-RNase+PHP<-?<-OLD-ABC<- DUF6079 *||?->?-><-?<-ABC-ATPase+SbcC<-?<-?<- wHTH+WYL+WCX <-JAB+sGTP<-SKI	1250	Bacteria>Fusobacteriota>Fusobacteriia>Fusobacteriales>Fusobacteriaceae>Fusobacterium	GCF_000178895.1	Fusobacterium_nucleatum	C4N14_RS08075	1667383..1671135	851	BREX-system-P-loop-protein-BrxC-[Fusobacterium-nucleatum]
WP_124794840.1	Type1.BREX	wHTH+WYL+WCX ->?->?->ABC-ATPase+SbcC->?-><-?<-?<-?|| DUF6079 *->OLD-ABC->TOPRIM-> N6-METHYLASE ->?->OLD-ABC+TOPRIM->CR-ATPase8+REase-> ParB + HNH +RAMA-> PglZ -> SIGMA+OB-Fold+AAA-ATPase+Lon-Protease-BrxL ->	1250	Bacteria>Fusobacteriota>Fusobacteriia>Fusobacteriales>Fusobacteriaceae>Fusobacterium	GCF_003859915.1	Fusobacterium_canifelinum	EII27_RS00780	173024..176776	285729	BREX-system-P-loop-protein-BrxC-[Fusobacterium-canifelinum]
WP_005980883.1	Type1.BREX	PHP->Sulfatase+DUF229+DUF4976->JetA->?->?->CR-ATPase8+REase-> DUF6079 *-> N6-METHYLASE -> ParB + HNH ->RAMA-> PglZ -> SIGMA+OB-Fold+AAA-ATPase+Lon-Protease-BrxL -> BrxA-DNA-binding -> BrxB-BrxF-iSTAND ->	1242	Bacteria>Fusobacteriota>Fusobacteriia>Fusobacteriales>Fusobacteriaceae>Fusobacterium	GCF_902373815.1	Fusobacterium_ulcerans	FXX38_RS11815	320598..324326	861	BREX-system-P-loop-protein-BrxC-[Fusobacterium-ulcerans]
WP_008800858.1	Type1.BREX	PrmA+METHYLASE->?->?->METHYLASE->?-> DUF6079 *->Schlafen+HATPase+wHTH-> N6-METHYLASE -> ParB + HNH +RAMA-> PglZ -> BrxA-DNA-binding -> BrxB-BrxF-iSTAND ->	1240	Bacteria>Fusobacteriota>Fusobacteriia>Fusobacteriales>Fusobacteriaceae>Fusobacterium	GCF_902373895.1	Fusobacterium_gonidiaformans	FY288_RS06315	242664..246386	849	BREX-system-P-loop-protein-BrxC-[Fusobacterium-gonidiaformans]
WP_203328719.1	Type6.BREX	<-TraM<-DUF3262<-?<-?<-SMODS<-?|| wHTH+WYL+WCX -> BrxE -> BrxB-BrxF-iSTAND -> DUF6079 *->GreA_GreB+SF1-SF2-Helicase-> N6-METHYLASE -> N6-METHYLASE ->?-> ParB + HNH -> PglZ -> SIGMA+OB-Fold+AAA-ATPase+HKD-DNase-BrxL -><-JAB<-?<-SMODS	1213	Bacteria	GCF_016836415.1	Pseudomonadota	JQ482_RS25705	5512214..5515855	1224	MULTISPECIES:-BREX-system-P-loop-protein-BrxC-[Pseudomonadota]
WP_240685338.1	Type6.BREX	SMODS->?->JAB-><- SIGMA+OB-Fold+AAA-ATPase+HKD-DNase-BrxL <- PglZ <- ParB + HNH <- N6-METHYLASE <-GreA_GreB+SF1-SF2-Helicase<- DUF6079 *<- BrxB-BrxF-iSTAND <- BrxA-DNA-binding <- BrxE <- wHTH+WYL+WCX ||JetA->JetB->SbcC->	1213	Bacteria	GCF_025461165.1	Pseudomonadota	JAK52_RS02505	287897..291538	1224	MULTISPECIES:-BREX-system-P-loop-protein-BrxC-[Pseudomonadota]
MBA3548104.1	Type1.BREX	Thioredoxin-><-?||?-><-Pkinase<-?<- SIGMA+OB-Fold+AAA-ATPase+Lon-Protease-BrxL <- PglZ <-?<- N6-METHYLASE <- DUF6079 *<- BrxB-BrxF-iSTAND <- BrxA-DNA-binding || ParB + HNH ->?->AAA-ATPase->?->?-><-?<-Trypsin+PDZ	1231	Bacteria>Myxococcota>Polyangia>Nannocystales>Nannocystaceae>Nannocystis	GCA_013812955.1	Nannocystis_sp.	HOT76_16605	30110..33805	1962667	BREX-system-P-loop-protein-BrxC-[Nannocystis-sp.]
MBK8262770.1	Type6.BREX	<-DnaJ||?-><-?|| BrxE -> BrxA-DNA-binding -> BrxB-BrxF-iSTAND -> DUF6079 *->OLD-ABC->TOPRIM-> N6-METHYLASE -> PglZ ->BrxD-AAA-> ParB + HNH ->SF1-SF2-Helicase+wHTH+Small-B-barrel+35exo-><-HEPN	1208	Bacteria>Myxococcota>Polyangia>Nannocystales>Nannocystaceae>Nannocystis	GCA_016712315.1	Nannocystis_sp.	IPK80_15725	176860..180486	1962667	BREX-system-P-loop-protein-BrxC-[Nannocystis-sp.]
MCK6512397.1	Type1.BREX	<- SIGMA+OB-Fold+AAA-ATPase+Lon-Protease-BrxL <- PglZ <- ParB + HNH <- N6-METHYLASE <- DUF6079 *<- BrxB-BrxF-iSTAND <- BrxA-DNA-binding <-PemK_toxin	1145	Bacteria>Myxococcota>unclassified_Myxococcota	GCA_023150835.1	Myxococcota_bacterium	L6R29_20905	15899..19336	2818507	BREX-system-P-loop-protein-BrxC-[Myxococcota-bacterium]
WP_123104890.1	Type6.BREX	SIGMA-HTH->tRNA-><- SIGMA+OB-Fold+AAA-ATPase+Lon-Protease-BrxL <- PglZ <- ParB + HNH <-TOPRIM<-OLD-ABC<- N6-METHYLASE <- N6-METHYLASE <- DUF6079 *<- BrxB-BrxF-iSTAND <- BrxA-DNA-binding <- BrxE ||<-?<-XIS-HTH||?->?->TOPRIM->	1210	Bacteria>Pseudomonadota>Acidithiobacillia>Acidithiobacillales>Acidithiobacillaceae>Acidithiobacillus	GCF_003721225.1	Acidithiobacillus_sulfuriphilus	EC580_RS10670	12374..16006	1867749	BREX-system-P-loop-protein-BrxC-[Acidithiobacillus-sulfuriphilus]
WP_215855738.1	Type6.BREX	<-SF1-SF2-Helicase||tRNA->Phage_integrase-> BrxE -> BrxA-DNA-binding -> BrxB-BrxF-iSTAND -> DUF6079 *->?-> N6-METHYLASE -> ParB + HNH -> PglZ -> SIGMA+OB-Fold+AAA-ATPase+Lon-Protease-BrxL ->?-><-DDE_Tnp_IS1595	1210	Bacteria>Pseudomonadota>Acidithiobacillia>Acidithiobacillales>Acidithiobacillaceae>Acidithiobacillus	GCF_018854635.1	Acidithiobacillus_ferridurans	HF641_RS00655	27295..30927	1232575	BREX-system-P-loop-protein-BrxC-[Acidithiobacillus-ferridurans]
WP_023449856.1	Type1.BREX	Ntox45->?-><-Couple_hipA+HipA_C<-HTH_3||UDG-conflict-><- ParB + HNH <- SIGMA+OB-Fold+AAA-ATPase+HKD-DNase-BrxL <- PglZ <- N6-METHYLASE <- DUF6079 *<- BrxB-BrxF-iSTAND <- BrxA-DNA-binding <-RelE-ParE<-MetJarc-HTH<-?||AbiEi_4->AbiEii->SWIM-> wHTH+WYL+WCX ->	1177	Bacteria>Pseudomonadota>Alphaproteobacteria>Caulobacterales>Caulobacteraceae>Asticcacaulis	GCF_000495835.1	Asticcacaulis_sp._AC402	ABAC402_RS31845	123615..127148	1282361	BREX-system-P-loop-protein-BrxC-[Asticcacaulis-sp.-AC402]
WP_212207485.1	Type6.BREX	<-PIWI<- SIGMA+OB-Fold+AAA-ATPase+HKD-DNase-BrxL <- PglZ <- ParB + HNH <- N6-METHYLASE -p<- N6-METHYLASE <- DUF6079 *<- BrxB-BrxF-iSTAND <- BrxA-DNA-binding <- BrxE <- wHTH+WYL+WCX ||35Exo+REase->SF1-SF2-Helicase+REase->	1208	Bacteria>Pseudomonadota>Alphaproteobacteria>Hyphomicrobiales>Nitrobacteraceae>Bradyrhizobium	-	Bradyrhizobium_sp._AUGA_SZCCT0274	JQ620_09165	74241..77867	2807670	BREX-system-P-loop-protein-BrxC-[Bradyrhizobium-sp.-AUGA-SZCCT0274]
MBR1240293.1	Type6.BREX	<-PIWI<- SIGMA+OB-Fold+AAA-ATPase+HKD-DNase-BrxL <- PglZ <- ParB + HNH <- N6-METHYLASE -p<- N6-METHYLASE <- DUF6079 *<- BrxB-BrxF-iSTAND <- BrxA-DNA-binding <- BrxE <- wHTH+WYL+WCX ||35Exo+REase->SF1-SF2-Helicase+REase->	1208	Bacteria>Pseudomonadota>Alphaproteobacteria>Hyphomicrobiales>Nitrobacteraceae>Bradyrhizobium	GCA_018130325.1	Bradyrhizobium_sp._AUGA_SZCCT0274	JQ620_09165	74241..77867	2807670	BREX-system-P-loop-protein-BrxC-[Bradyrhizobium-sp.-AUGA-SZCCT0274]
WP_249144787.1	Type6.BREX	<-PIWI<- SIGMA+OB-Fold+AAA-ATPase+HKD-DNase-BrxL <- PglZ <- ParB + HNH <- N6-METHYLASE -p<- N6-METHYLASE <- DUF6079 *<- BrxB-BrxF-iSTAND <- BrxA-DNA-binding <- BrxE <- wHTH+WYL+WCX ||35Exo+REase->SF1-SF2-Helicase+REase->	1203	Bacteria>Pseudomonadota>Alphaproteobacteria>Hyphomicrobiales>Nitrobacteraceae>Bradyrhizobium	GCF_018130325.1	Bradyrhizobium_sp._AUGA_SZCCT0274	JQ620_RS09170	74241..77852	2807670	BREX-system-P-loop-protein-BrxC-[Bradyrhizobium-sp.-AUGA-SZCCT0274]
WP_213286691.1	Type6.BREX	Phage_integrase->MarR-HTH->AbiEii->?->?-> BrxE -> BrxA-DNA-binding -> BrxB-BrxF-iSTAND -> DUF6079 *-> N6-METHYLASE -> N6-METHYLASE -> ParB + HNH -> PglZ -> SIGMA+OB-Fold+AAA-ATPase+HKD-DNase-BrxL -><-TPR<-?<-?|| wHTH+WYL+WCX ->	1208	Bacteria>Pseudomonadota>Alphaproteobacteria>Hyphomicrobiales>Nitrobacteraceae>Bradyrhizobium	GCF_018398875.1	Bradyrhizobium_sp._sGM-13	KJQ96_RS11300	145012..148638	2831781	BREX-system-P-loop-protein-BrxC-[Bradyrhizobium-sp.-sGM-13]
WP_225161486.1	Type1.BREX	<- ParB + HNH +Schlafen<-?<- SIGMA+OB-Fold+AAA-ATPase+HKD-DNase-BrxL <- PglZ <- N6-METHYLASE <- DUF6079 *<- BrxB-BrxF-iSTAND <- BrxA-DNA-binding <- wHTH+WYL+WCX ||?->35Exo+REase->SF1-SF2-Helicase+REase->35exo+SF1-SF2-Helicase-><-Phage_integrase	1183	Bacteria>Pseudomonadota>Alphaproteobacteria>Hyphomicrobiales>Nitrobacteraceae>Bradyrhizobium	GCF_020194035.1	Bradyrhizobium_sp._BRP56	I6F38_19500	73459..77010	2793819	BREX-system-P-loop-protein-BrxC-[Bradyrhizobium-sp.-BRP56]
WP_260373497.1	Type1.BREX	<- ParB + HNH +Schlafen<-?<- SIGMA+OB-Fold+AAA-ATPase+HKD-DNase-BrxL <- PglZ <- N6-METHYLASE <- DUF6079 *<- BrxB-BrxF-iSTAND <- BrxA-DNA-binding <- wHTH+WYL+WCX ||?->35Exo+REase->SF1-SF2-Helicase+REase->35exo+SF1-SF2-Helicase-><-Phage_integrase	1183	Bacteria>Pseudomonadota>Alphaproteobacteria>Hyphomicrobiales>Nitrobacteraceae>Bradyrhizobium	GCF_025200925.1	Bradyrhizobium_sp._CB1015	N2604_RS01610	347161..350712	2976822	BREX-system-P-loop-protein-BrxC-[Bradyrhizobium-sp.-CB1015]
RWD70309.1	Type6.BREX	Glycos_transf->?-><-SAM-METHYLASE<-RADICAL-SAM<- SIGMA+OB-Fold+AAA-ATPase+Lon-Protease-BrxL <- PglZ <- N6-METHYLASE <- N6-METHYLASE <-Tox-RelA<- DUF6079 *<- BrxB-BrxF-iSTAND <- BrxA-DNA-binding <- BrxE || ParB + HNH ->?->GDResolvase+HIN-HTH-><-?<-Primpol	1181	Bacteria>Pseudomonadota>Alphaproteobacteria>Hyphomicrobiales>Phyllobacteriaceae>Mesorhizobium	GCA_004018245.1	Mesorhizobium_sp.	EOS37_15285	26400..29945	1871066	BREX-system-P-loop-protein-BrxC-[Mesorhizobium-sp.]
TIL20465.1	Type6.BREX	BrxE -> BrxA-DNA-binding -> BrxB-BrxF-iSTAND -> DUF6079 *-> N6-METHYLASE -> N6-METHYLASE -> ParB + HNH -> PglZ -> SIGMA+OB-Fold+AAA-ATPase+HKD-DNase-BrxL ->	1208	Bacteria>Pseudomonadota>Alphaproteobacteria>Hyphomicrobiales>Phyllobacteriaceae>Mesorhizobium	GCA_004961195.1	Mesorhizobium_sp.	E5Y88_32415	5019..8645	1871066	BREX-system-P-loop-protein-BrxC-[Mesorhizobium-sp.]
WP_023809232.1	Type1.BREX	<- ParB + HNH <-?<- ParB + HNH -p<- N6-METHYLASE || BrxA-DNA-binding -> BrxB-BrxF-iSTAND -> DUF6079 *-> PglZ -> SIGMA+OB-Fold+AAA-ATPase+Lon-Protease-BrxL -><-GDResolvase+Recombinase<- ParB <-?<-MULE-TRANSPOSASE	1150	Bacteria>Pseudomonadota>Alphaproteobacteria>Hyphomicrobiales>Phyllobacteriaceae>Mesorhizobium	GCF_000502875.1	Mesorhizobium_sp._L2C084A000	X735_RS161770	861519..864971	1287116	BREX-system-P-loop-protein-BrxC-[Mesorhizobium-sp.-L2C084A000]
WP_128286158.1	Type1.BREX	ParB + HNH -><- SIGMA+OB-Fold+AAA-ATPase+HKD-DNase-BrxL <- PglZ <- HNH <-OLD-ABC <- N6-METHYLASE <- DUF6079 *<- BrxB-BrxF-iSTAND <- BrxA-DNA-binding <-RNaseH	1181	Bacteria>Pseudomonadota>Alphaproteobacteria>Hyphomicrobiales>Phyllobacteriaceae>Mesorhizobium	GCF_004023115.1	Mesorhizobium_sp._M6A.T.Cr.TU.017.01.1.1	EOB36_RS28010	14606..18151	2496774	BREX-system-P-loop-protein-BrxC-[Mesorhizobium-sp.-M6A.T.Cr.TU.017.01.1.1]
MBX5048875.1	Type6.BREX	TetR-HTH->?-><- SIGMA+OB-Fold+AAA-ATPase+HKD-DNase-BrxL <- PglZ <- ParB + HNH <- N6-METHYLASE <- N6-METHYLASE <- DUF6079 *<- BrxB-BrxF-iSTAND <- BrxA-DNA-binding <- BrxE <-tRNA||?->Thioredoxin->	1213	Bacteria>Pseudomonadota>Alphaproteobacteria>Hyphomicrobiales>Rhizobiaceae>Rhizobium/Agrobacterium_group	GCA_019684595.1	Rhizobium_lentis	HJB64_19910	192083..195724	1138194	BREX-system-P-loop-protein-BrxC-[Rhizobium-lentis]
MBY5873817.1	Type1.BREX	DnaB+McrB-AAA->McrC-NTD+REase+ZnR->?->OLD-ABC+TOPRIM->SF1-SF2-Helicase-> ParB + HNH +Schlafen-><- SIGMA+OB-Fold+AAA-ATPase+HKD-DNase-BrxL <- PglZ <- N6-METHYLASE <- DUF6079 *<- BrxB-BrxF-iSTAND <- BrxA-DNA-binding || ParB ->REase+ZnR->?->Classical-AAA->Classical-AAA->REase->	1174	Bacteria>Pseudomonadota>Alphaproteobacteria>Hyphomicrobiales>Rhizobiaceae>Rhizobium/Agrobacterium_group	GCA_019799745.1	Rhizobium_leguminosarum	HFN53_17500	401377..404901	384	BREX-system-P-loop-protein-BrxC-[Rhizobium-leguminosarum]
WP_168349960.1	Type1.BREX	<- SIGMA+OB-Fold+AAA-ATPase+HKD-DNase-BrxL <- PglZ <- ParB + HNH <- N6-METHYLASE <- DUF6079 -p*<-Classical-AAA<-REase+wHTH||?->PIN-><- ParB <- ParB <-GDResolvase+PPP1-HTH||CHTH->	190	Bacteria>Pseudomonadota>Alphaproteobacteria>Hyphomicrobiales>Rhizobiaceae>Rhizobium/Agrobacterium_group	GCF_012276085.1	Rhizobium_leguminosarum	GFM28_RS29310	8641..9213	384	hypothetical-protein-[Rhizobium-leguminosarum]
WP_250810295.1	Type6.BREX	<-GreA_GreB+SF1-SF2-Helicase<-PIWI<-REase+TIR<-?|| wHTH+WYL+WCX -> BrxE -> BrxA-DNA-binding -> BrxB-BrxF-iSTAND -> DUF6079 *-> N6-METHYLASE -> PglZ -> SIGMA+OB-Fold+AAA-ATPase+HKD-DNase-BrxL -><-?<- ParB + HNH ||GDResolvase+HIN-HTH-><-HTH_3	1208	Bacteria>Pseudomonadota>Alphaproteobacteria>Hyphomicrobiales>Rhizobiaceae>Rhizobium/Agrobacterium_group	GCF_023667605.1	Neorhizobium_tomejilense	L1082_RS18485	51181..54807	2093828	BREX-system-P-loop-protein-BrxC-[Neorhizobium-tomejilense]
WP_221145756.1	Type6.BREX	TetR-HTH->?-><- SIGMA+OB-Fold+AAA-ATPase+HKD-DNase-BrxL <- PglZ <- ParB + HNH <- N6-METHYLASE <- N6-METHYLASE <- DUF6079 *<- BrxB-BrxF-iSTAND <- BrxA-DNA-binding <- BrxE <-tRNA||?->Thioredoxin->	1213	Bacteria>Pseudomonadota>Alphaproteobacteria>Hyphomicrobiales>Rhizobiaceae>Rhizobium/Agrobacterium_group	-	Rhizobium_lentis	HJB64_19910	192083..195724	1138194	BREX-system-P-loop-protein-BrxC-[Rhizobium-lentis]
WP_027288600.1	Type1.BREX	TPR+TPR+TPR->?->tRNA->Phage_integrase-> BrxA-DNA-binding -> BrxB-BrxF-iSTAND -> DUF6079 *-> N6-METHYLASE -> PglZ -> SIGMA+OB-Fold+AAA-ATPase+Lon-Protease-BrxL -><- ParB + HNH ||?->||?->?->SbcC->	1150	Bacteria>Pseudomonadota>Alphaproteobacteria>Hyphomicrobiales>Rhodovibrionaceae>Rhodovibrio	GCF_000515255.1	Rhodovibrio_salinarum	RHOSA_RS0110320	2222437..2225889	1087	BREX-system-P-loop-protein-BrxC-[Rhodovibrio-salinarum]
WP_212538477.1	Type6.BREX	<-TetR-HTH||?-> wHTH+WYL+WCX -> BrxE -> BrxA-DNA-binding -> BrxB-BrxF-iSTAND -> DUF6079 *-> N6-METHYLASE -> N6-METHYLASE -p-> ParB + HNH -> PglZ -> SIGMA+OB-Fold+AAA-ATPase+HKD-DNase-BrxL -><-XIS-HTH<-?||HTH+LexA-protease->UDG->	1208	Bacteria>Pseudomonadota>Alphaproteobacteria>Rhodobacterales>Paracoccaceae>Aestuariicoccus	GCF_018224955.1	Aestuariicoccus_sp._KMU-90	KB874_RS20730	16568..20194	2827239	BREX-system-P-loop-protein-BrxC-[Aestuariicoccus-sp.-KMU-90]
WP_274653192.1	Type1.BREX	<- ParB + HNH +Schlafen<-?<- SIGMA+OB-Fold+AAA-ATPase+HKD-DNase-BrxL <- PglZ <- N6-METHYLASE <- N6-METHYLASE <-NERD<-OLD-ABC<- DUF6079 *<- BrxB-BrxF-iSTAND <- BrxA-DNA-binding <- wHTH+WYL+WCX ||ArdCN+MPTase->?->?->FurR-HTH->?->HIN-HTH+TraJ-RHH->	1172	Bacteria>Pseudomonadota>Alphaproteobacteria>Rhodobacterales>Paracoccaceae>Halocynthiibacter	GCF_028823805.1	Halocynthiibacter_sp._C4	OU789_RS03095	642215..645733	2992758	BREX-system-P-loop-protein-BrxC-[Halocynthiibacter-sp.-C4]
WP_108641830.1	Type1.BREX	<-Schlafen<-?<- ParB + HNH +Schlafen<-?<- SIGMA+OB-Fold+AAA-ATPase+HKD-DNase-BrxL <- PglZ <- N6-METHYLASE <- DUF6079 *<- BrxB-BrxF-iSTAND <- BrxA-DNA-binding <- wHTH+WYL+WCX ||?->Thermonuclease->TrbM-><-?<-HTH_3	1173	Bacteria>Pseudomonadota>Alphaproteobacteria>Rhodobacterales>Paracoccaceae>Thalassorhabdomicrobium	GCF_003072065.1	Thalassorhabdomicrobium_marinisediminis	DC363_RS14250	78584..82105	2170577	BREX-system-P-loop-protein-BrxC-[Thalassorhabdomicrobium-marinisediminis]
WP_058289795.1	Type1.BREX	<-TraI-VirD2-RCRE<-HIN-HTH+TraJ-RHH<-?<-FurR-HTH<-?<-ArdCN+MPTase|| wHTH+WYL+WCX -> BrxA-DNA-binding -> BrxB-BrxF-iSTAND -> DUF6079 *->OLD-ABC->NERD-> N6-METHYLASE -> PglZ -> SIGMA+OB-Fold+AAA-ATPase+HKD-DNase-BrxL -> ParB + HNH +Schlafen-><-?<-Couple_hipA+HipA_C<-HTH_3	1172	Bacteria>Pseudomonadota>Alphaproteobacteria>Rhodobacterales>Paracoccaceae>Tritonibacter	GCF_001458415.1	Tritonibacter_multivorans	PGB32_RS12460	15411..18929	928856	BREX-system-P-loop-protein-BrxC-[Tritonibacter-multivorans]
WP_089279817.1	Type1.BREX	<-RelE-ParE<-ParD_antitoxin<-CHTH||?->GDResolvase+PPP1-HTH-> ParB -> wHTH+WYL+WCX -> BrxA-DNA-binding -> BrxB-BrxF-iSTAND -> DUF6079 *-> N6-METHYLASE -> PglZ -> SIGMA+OB-Fold+AAA-ATPase+Lon-Protease-BrxL -> ParB + HNH -><-Peptidase-M15<-?<-?<-?<-Glyco_hydro_53+Phage-tail_3	1147	Bacteria>Pseudomonadota>Alphaproteobacteria>Rhodobacterales>Roseobacteraceae>Antarctobacter	GCF_900188425.1	Antarctobacter_heliothermus	CHB90_RS21205	7452..10895	74033	BREX-system-P-loop-protein-BrxC-[Antarctobacter-heliothermus]
WP_123792783.1	Type1.BREX	<- ParB + HNH +Schlafen<- SIGMA+OB-Fold+AAA-ATPase+HKD-DNase-BrxL <- PglZ <- N6-METHYLASE <-REase<-OLD-ABC<- DUF6079 *<- BrxB-BrxF-iSTAND <- BrxA-DNA-binding <-HIN-HTH<-HTH_36<-?<-?<-?<-Arm-DNA-bind_3+Phage_integrase	1172	Bacteria>Pseudomonadota>Alphaproteobacteria>Rhodobacterales>Roseobacteraceae>Pacificibacter	GCF_003814915.1	Pacificibacter_maritimus	EDD53_RS08420	394668..398186	762213	BREX-system-P-loop-protein-BrxC-[Pacificibacter-maritimus]
MAE92765.1	Type1.BREX	<-Thioredoxin_4|| wHTH+WYL+WCX -> BrxA-DNA-binding -> BrxB-BrxF-iSTAND -> DUF6079 *-> N6-METHYLASE -> ParB + HNH -> PglZ -> SIGMA+OB-Fold+AAA-ATPase+Lon-Protease-BrxL ->REase+PNTD1+PNTD2->PIWI->REase->	1219	Bacteria>Pseudomonadota>Alphaproteobacteria>Rhodobacterales>Roseobacteraceae>Pelagibaca	GCA_002687285.1	Pelagibaca_sp.	CMI67_24850	4770..8429	1979400	ATPase-[Pelagibaca-sp.]
WP_201727547.1	Type6.BREX	VirB8->VirB9->VirB10->RHH->TraJ-RHH->ACET+ACET-> BrxE -> BrxA-DNA-binding -> BrxB-BrxF-iSTAND -> DUF6079 -p*->HIN-HTH->?->LZ_Tnp_IS66+zf-IS66+DDE_Tnp_IS66+DDE_Tnp_IS66_C->?-> DUF6079 -p-> N6-METHYLASE -> N6-METHYLASE -p-> ParB + HNH ->HTH_29+HTH_32+HTH_33+DDE_3->	650	Bacteria>Pseudomonadota>Alphaproteobacteria>Rhodospirillales>Acetobacteraceae>Acidocella	GCF_907164655.1	Acidocella_sp._C78	ACIDOCELLA78_RS32075	228724..230676	1671486	BREX-system-P-loop-protein-BrxC-[Acidocella-sp.-C78]
WP_201727548.1	Type6.BREX	ACET+ACET-> BrxE -> BrxA-DNA-binding -> BrxB-BrxF-iSTAND -> DUF6079 -p->HIN-HTH->?->LZ_Tnp_IS66+zf-IS66+DDE_Tnp_IS66+DDE_Tnp_IS66_C->?-> DUF6079 -p*-> N6-METHYLASE -> N6-METHYLASE -p-> ParB + HNH ->HTH_29+HTH_32+HTH_33+DDE_3-> ParB + HNH -> PglZ -> SIGMA+OB-Fold+AAA-ATPase+HKD-DNase-BrxL -><-Arm-DNA-bind_3+Phage_int_SAM_3+Phage_integrase	495	Bacteria>Pseudomonadota>Alphaproteobacteria>Rhodospirillales>Acetobacteraceae>Acidocella	GCF_907164655.1	Acidocella_sp._C78	ACIDOCELLA78_RS32100	233672..235159	1671486	hypothetical-protein-[Acidocella-sp.-C78]
HCW69913.1	Type1.BREX	VirB11->VirD4-TraG+VirD4-FtsK->?->ArdCN+MPTase+TOPRIM->TraJ-RHH->TraI-VirD2-RCRE-><- SIGMA+OB-Fold+AAA-ATPase+HKD-DNase-BrxL <- PglZ <- ParB + HNH <- DUF6079 *<- BrxB-BrxF-iSTAND <- BrxA-DNA-binding <- BrxE ||GDResolvase+Recombinase->?->?-><-?<-HTH_36	1208	Bacteria>Pseudomonadota>Alphaproteobacteria>Rhodospirillales>Thalassospiraceae>Thalassospira	GCA_003539675.1	Thalassospira_lucentensis	DHR80_22445	45804..49430	168935	BREX-system-P-loop-protein-BrxC-[Thalassospira-lucentensis]
TVR84541.1	Type6.BREX	<- SIGMA+OB-Fold+AAA-ATPase+HKD-DNase-BrxL <- PglZ <- ParB + HNH <- N6-METHYLASE <- DUF6079 *<- BrxB-BrxF-iSTAND <- BrxA-DNA-binding <- BrxE	1206	Bacteria>Pseudomonadota>Alphaproteobacteria>Rhodospirillales>unclassified_Rhodospirillales	GCA_007695045.1	Rhodospirillales_bacterium	EA405_01435	12860..16480	2026786	BREX-system-P-loop-protein-BrxC-[Rhodospirillales-bacterium]
WP_193715186.1	Type6.BREX	<-Arm-DNA-bind_2+Phage_int_SAM_3+Phage_integrase<-Phage_AlpA<-GreA_GreB+SF1-SF2-Helicase<-SbcC+SbcC<-Calcineurin|| wHTH+WYL+WCX -> BrxE -> BrxA-DNA-binding -> BrxB-BrxF-iSTAND -> DUF6079 *-> N6-METHYLASE -> ParB + HNH -> PglZ -> SIGMA+OB-Fold+AAA-ATPase+HKD-DNase-BrxL -><-tRNA||?->?-><-?<-ABC-ATPase+SbcC	1215	Bacteria>Pseudomonadota>Alphaproteobacteria>Sneathiellales>Sneathiellaceae>Sneathiella	GCF_015143595.1	Sneathiella_sp._P13V-1	GUA87_RS03840	775657..779304	2697366	BREX-system-P-loop-protein-BrxC-[Sneathiella-sp.-P13V-1]
MBN8436950.1	Type1.BREX	<- SIGMA+OB-Fold+AAA-ATPase+Lon-Protease-BrxL <- PglZ <- ParB + HNH <-DUF932<- N6-METHYLASE <- DUF6079 *<- BrxB-BrxF-iSTAND <- BrxA-DNA-binding <-?<-RepB_primase<-XIS-HTH<-?<-HTH<-MqsA_antitoxin<-RNase	1146	Bacteria>Pseudomonadota>Betaproteobacteria>Betaproteobacteria_incertae_sedis>Candidatus_Accumulibacter>unclassified_Candidatus_Accumulibacter	GCA_017302555.1	Candidatus_Accumulibacter_sp.	JOL95_02725	187324..190764	2053492	BREX-system-P-loop-protein-BrxC-[Candidatus-Accumulibacter-sp.]
WP_042797405.1	Type6.BREX	ZnR->NERD+SF1-SF2-Helicase->Exonuc_V_gamma+REase->SF1-SF2-Helicase+REase->sGTP->?-> BrxE -> BrxA-DNA-binding -> BrxB-BrxF-iSTAND -> DUF6079 *->OLD-ABC-> ParB + HNH -> PglZ -> SIGMA+OB-Fold+AAA-ATPase+HKD-DNase-BrxL -><-HipA_C<-Couple_hipA+HipA_C||VirB5->	1207	Bacteria>Pseudomonadota>Betaproteobacteria>Burkholderiales>Alcaligenaceae>Achromobacter	GCF_000783435.2	Achromobacter_insolitus	MC81_20460	4405602..4409225	217204	BREX-system-P-loop-protein-BrxC-[Achromobacter-insolitus]
WP_050447538.1	Type1.BREX	XIS-HTH->?-><-Phage_integrase||RHH_5-> BrxA-DNA-binding -> BrxB-BrxF-iSTAND -> DUF6079 *->?-> N6-METHYLASE ->PHP+SbcC-> ParB + HNH -> PglZ -> SIGMA+OB-Fold+AAA-ATPase+Lon-Protease-BrxL -><-ANK|| ParB ->	1153	Bacteria>Pseudomonadota>Betaproteobacteria>Burkholderiales>Alcaligenaceae>Achromobacter	GCF_001189595.1	Achromobacter_spanius	AFM18_RS14295	68240..71701	217203	BREX-system-P-loop-protein-BrxC-[Achromobacter-spanius]
WP_063584995.1	Type1.BREX	<-HTH+REase<-?||HTH_36->Phage_int_SAM_2+Integrase_1-><- SIGMA+OB-Fold+AAA-ATPase+Lon-Protease-BrxL <- PglZ <- ParB + HNH <-DUF3644<- N6-METHYLASE <- DUF6079 *<- BrxB-BrxF-iSTAND <- BrxA-DNA-binding <-DOC+HTH<-Arm-DNA-bind_3+Phage_integrase<-sGTP	1146	Bacteria>Pseudomonadota>Betaproteobacteria>Burkholderiales>Alcaligenaceae>Achromobacter	GCF_001637105.1	Achromobacter_ruhlandii	AOT39_RS22745	36149..39589	72557	BREX-system-P-loop-protein-BrxC-[Achromobacter-ruhlandii]
WP_076471941.1	Type6.BREX	<-DUF3262<-?<-?<-?<-JetA|| wHTH+WYL+WCX -> BrxE -> BrxA-DNA-binding -> BrxB-BrxF-iSTAND -> DUF6079 *->OLD-ABC->PIN-> N6-METHYLASE -> N6-METHYLASE -> ParB + HNH -> PglZ -> SIGMA+OB-Fold+AAA-ATPase+HKD-DNase-BrxL -><-JAB	1207	Bacteria>Pseudomonadota>Betaproteobacteria>Burkholderiales>Alcaligenaceae>Achromobacter	GCF_001957055.1	Achromobacter_xylosoxidans	BIZ53_RS00410	81330..84953	85698	BREX-system-P-loop-protein-BrxC-[Achromobacter-xylosoxidans]
WP_212312045.1	Type6.BREX	HTH_3-><-MODE-HTH+PBPII|| wHTH+WYL+WCX -> BrxE -> BrxA-DNA-binding -> BrxB-BrxF-iSTAND -> DUF6079 *->GreA_GreB+SF1-SF2-Helicase-> N6-METHYLASE -> N6-METHYLASE ->?-> ParB + HNH ->DOC+HTH-> PglZ -> SIGMA+OB-Fold+AAA-ATPase+HKD-DNase-BrxL -><-HDRnase	1217	Bacteria>Pseudomonadota>Betaproteobacteria>Burkholderiales>Alcaligenaceae>Achromobacter	GCF_018195695.1	Achromobacter_sp._Marseille-Q0513	KDH83_RS00260	59261..62914	2829161	BREX-system-P-loop-protein-BrxC-[Achromobacter-sp.-Marseille-Q0513]
WP_233436978.1	Type6.BREX	HTH_3-><-HIN-HTH<- SIGMA+OB-Fold+AAA-ATPase+HKD-DNase-BrxL <- PglZ <- ParB + HNH <- N6-METHYLASE <- N6-METHYLASE <-GreA_GreB+SF1-SF2-Helicase<- DUF6079 *<- BrxB-BrxF-iSTAND <- BrxA-DNA-binding <- BrxE <-sGTP<-SF1-SF2-Helicase+REase<-Exonuc_V_gamma+REase<-NERD+SF1-SF2-Helicase<-?<- wHTH+WYL+WCX	1234	Bacteria>Pseudomonadota>Betaproteobacteria>Burkholderiales>Alcaligenaceae>Achromobacter	GCF_022976495.1	Achromobacter_xylosoxidans	DBA06_RS11680	120350..124054	85698	BREX-system-P-loop-protein-BrxC-[Achromobacter-xylosoxidans]
WP_259250627.1	Type6.BREX	SF1-SF2-Helicase+REase->?->?->?->HKD-DNase+HKD-DNase->SF1-SF2-Helicase-> BrxE -> BrxA-DNA-binding -> BrxB-BrxF-iSTAND -> DUF6079 *->GreA_GreB+SF1-SF2-Helicase-> N6-METHYLASE -> N6-METHYLASE -> ParB + HNH -> PglZ -> SIGMA+OB-Fold+AAA-ATPase+HKD-DNase-BrxL -><-Couple_hipA+HipA_C<-HTH_3	1213	Bacteria>Pseudomonadota>Betaproteobacteria>Burkholderiales>Alcaligenaceae>Achromobacter	GCF_024806975.1	Achromobacter_sp._JUb104	M2361_RS16380	989592..993233	2940590	BREX-system-P-loop-protein-BrxC-[Achromobacter-sp.-JUb104]
WP_180097564.1	Type6.BREX	<- SIGMA+OB-Fold+AAA-ATPase+HKD-DNase-BrxL <- PglZ <- ParB + HNH <- N6-METHYLASE <- N6-METHYLASE <-GreA_GreB+SF1-SF2-Helicase<- DUF6079 *<- BrxB-BrxF-iSTAND <- BrxA-DNA-binding <- BrxE <-?<-sGTP<-SF1-SF2-Helicase+REase<-Exonuc_V_gamma+REase<-NERD+SF1-SF2-Helicase	1213	Bacteria>Pseudomonadota>Betaproteobacteria>Burkholderiales>Alcaligenaceae>Achromobacter	GCF_902859995.1	Achromobacter_mucicolens	H0520_RS02915	616832..620473	1389922	BREX-system-P-loop-protein-BrxC-[Achromobacter-mucicolens]
WP_200235209.1	Type1.BREX	GDResolvase+HIN-HTH->?-><- SIGMA+OB-Fold+AAA-ATPase+HKD-DNase-BrxL <- PglZ <-Schlafen+HATPase+wHTH<- N6-METHYLASE -p<-RVT<- N6-METHYLASE <- DUF6079 *<- BrxB-BrxF-iSTAND <- BrxA-DNA-binding <-XIS-HTH<-wHTH+ HNH -p|| N6-METHYLASE ->?->LexA-protease->?-> ParB + HNH ->	1190	Bacteria>Pseudomonadota>Betaproteobacteria>Burkholderiales>Alcaligenaceae>Advenella	GCF_016595155.1	Advenella_mandrilli	JHL22_RS06635	201531..205103	2800330	BREX-system-P-loop-protein-BrxC-[Advenella-mandrilli]
WP_109088944.1	Type1.BREX	<-nSTAND<-URI-DNase<-ACET+ACET<-?||Phage_integrase-><- SIGMA+OB-Fold+AAA-ATPase+Lon-Protease-BrxL <- PglZ <- ParB + HNH <- N6-METHYLASE <- DUF6079 *<- BrxB-BrxF-iSTAND <- BrxA-DNA-binding <- wHTH+WYL+WCX <-?||Phage_integrase->Phage_holin-><-TIR<-TIR	1146	Bacteria>Pseudomonadota>Betaproteobacteria>Burkholderiales>Alcaligenaceae>Alcaligenes	GCF_003122065.1	Alcaligenes_faecalis	DF183_RS09485	797094..800534	511	BREX-system-P-loop-protein-BrxC-[Alcaligenes-faecalis]
WP_199265315.1	Type6.BREX	Arm-DNA-bind_3+Phage_integrase-><- SIGMA+OB-Fold+AAA-ATPase+HKD-DNase-BrxL <- PglZ <- ParB + HNH <- N6-METHYLASE <-GreA_GreB+SF1-SF2-Helicase<- DUF6079 *<- BrxB-BrxF-iSTAND <- BrxA-DNA-binding <- BrxE <- wHTH+WYL+WCX ||?->?->?->?-><-HTH_3	1213	Bacteria>Pseudomonadota>Betaproteobacteria>Burkholderiales>Alcaligenaceae>Alcaligenes	GCF_016446305.1	Alcaligenes_faecalis	GTU60_RS02720	606582..610223	511	BREX-system-P-loop-protein-BrxC-[Alcaligenes-faecalis]
WP_197022064.1	Type1.BREX	MODE-HTH+PBPII-><-?<-?||MODE-HTH+PBPII-><- SIGMA+OB-Fold+AAA-ATPase+Lon-Protease-BrxL <- PglZ <- ParB + HNH <-PHP+SbcC<- N6-METHYLASE <- DUF6079 *<- BrxB-BrxF-iSTAND <- BrxA-DNA-binding <- wHTH+WYL+WCX ||ARG->	1150	Bacteria>Pseudomonadota>Betaproteobacteria>Burkholderiales>Alcaligenaceae>Bordetella	GCF_000518845.1	Bordetella_petrii	L636_RS0102150	157001..160453	94624	BREX-system-P-loop-protein-BrxC-[Bordetella-petrii]
WP_032972587.1	Type6.BREX	DUF4400-><-SbcC<- SIGMA+OB-Fold+AAA-ATPase+HKD-DNase-BrxL <- PglZ <-?<- ParB + HNH <- N6-METHYLASE <- N6-METHYLASE <-GreA_GreB+SF1-SF2-Helicase<- DUF6079 *<- BrxB-BrxF-iSTAND <- BrxA-DNA-binding <- BrxE <- wHTH+WYL+WCX ||?->SMODS->?->DUF3262->TraM->	1213	Bacteria>Pseudomonadota>Betaproteobacteria>Burkholderiales>Alcaligenaceae>Bordetella	GCF_006770345.1	Bordetella_hinzii	CBR67_RS06420	1279803..1283444	103855	BREX-system-P-loop-protein-BrxC-[Bordetella-hinzii]
WP_148812759.1	Type1.BREX	<-METHYLASE<-?<-?||tRNA->RHH_5-> BrxA-DNA-binding -> BrxB-BrxF-iSTAND -> DUF6079 *-> N6-METHYLASE ->PHP+SbcC-> ParB + HNH -> PglZ -> SIGMA+OB-Fold+AAA-ATPase+Lon-Protease-BrxL -><-AbiEi_4	1153	Bacteria>Pseudomonadota>Betaproteobacteria>Burkholderiales>Alcaligenaceae>Pigmentiphaga	GCF_008119665.1	Pigmentiphaga_aceris	FXN63_RS03140	736142..739603	1940612	BREX-system-P-loop-protein-BrxC-[Pigmentiphaga-aceris]
RIY39966.1	Type1.BREX	XIS-HTH-> BrxA-DNA-binding -> BrxB-BrxF-iSTAND -> DUF6079 *-> N6-METHYLASE ->PHP+SbcC-> ParB + HNH -> PglZ -> SIGMA+OB-Fold+AAA-ATPase+Lon-Protease-BrxL ->HARE-HTH+REase->?->SF1-SF2-Helicase->	1145	Bacteria>Pseudomonadota>Betaproteobacteria>Burkholderiales>Alcaligenaceae>Pusillimonas	GCA_003585905.1	Pusillimonas_maritima	CJP73_12365	2891..6328	2026239	hypothetical-protein-CJP73_12365-[Pusillimonas-maritima]
WP_218972830.1	Type1.BREX	XIS-HTH-> BrxA-DNA-binding -> BrxB-BrxF-iSTAND -> DUF6079 *-> N6-METHYLASE ->PHP+SbcC-> ParB + HNH -> PglZ -> SIGMA+OB-Fold+AAA-ATPase+Lon-Protease-BrxL ->HARE-HTH+REase->?->SF1-SF2-Helicase->	1146	Bacteria>Pseudomonadota>Betaproteobacteria>Burkholderiales>Alcaligenaceae>Pusillimonas	GCF_003585905.1	Pusillimonas_maritima	CJP73_RS12395	2888..6328	2026239	BREX-system-P-loop-protein-BrxC-[Pusillimonas-maritima]
NYT63370.1	Type1.BREX	<- SIGMA+OB-Fold+AAA-ATPase+HKD-DNase-BrxL <- PglZ <- ParB + HNH <- N6-METHYLASE -p<-HerA/FtsK<-SIR2<-NERD<-OLD-ABC<- N6-METHYLASE <- DUF6079 *<- BrxB-BrxF-iSTAND <- BrxA-DNA-binding <-XIS-HTH||?->?->SSB->?-><-ORC-CDC6<-HEPN	1191	Bacteria>Pseudomonadota>Betaproteobacteria>Burkholderiales>Alcaligenaceae>unclassified_Alcaligenaceae	GCA_013416445.1	Alcaligenaceae_bacterium	HOA66_13705	63657..67232	2021368	BREX-system-P-loop-protein-BrxC-[Alcaligenaceae-bacterium]
MCA8485082.1	Type1.BREX	NUDIX-><-?<-?||?-><- SIGMA+OB-Fold+AAA-ATPase+Lon-Protease-BrxL <- PglZ <- ParB + HNH <-PHP+SbcC<- N6-METHYLASE <- DUF6079 -p*<- DUF6079 -p<- BrxB-BrxF-iSTAND <- BrxA-DNA-binding <- wHTH+WYL+WCX	879	Bacteria>Pseudomonadota>Betaproteobacteria>Burkholderiales>Burkholderiaceae>Burkholderia	GCA_020421385.1	Burkholderia_multivorans	LGM85_14180	202982..205621	87883	BREX-system-P-loop-protein-BrxC-[Burkholderia-multivorans]
MCA8485083.1	Type1.BREX	<- SIGMA+OB-Fold+AAA-ATPase+Lon-Protease-BrxL <- PglZ <- ParB + HNH <-PHP+SbcC<- N6-METHYLASE <- DUF6079 -p<- DUF6079 -p*<- BrxB-BrxF-iSTAND <- BrxA-DNA-binding <- wHTH+WYL+WCX ||<-?<-?<-?<-?<-?||HTH_36->	266	Bacteria>Pseudomonadota>Betaproteobacteria>Burkholderiales>Burkholderiaceae>Burkholderia	GCA_020421385.1	Burkholderia_multivorans	LGM85_14185	205622..206422	87883	hypothetical-protein-[Burkholderia-multivorans]
WP_059944780.1	Type6.BREX	<- wHTH+WYL+WCX ||?-><- SIGMA+OB-Fold+AAA-ATPase+HKD-DNase-BrxL <- PglZ <- ParB + HNH <- N6-METHYLASE <- N6-METHYLASE <-GreA_GreB+SF1-SF2-Helicase<- DUF6079 *<- BrxB-BrxF-iSTAND <- BrxA-DNA-binding <- BrxE	1213	Bacteria>Pseudomonadota>Betaproteobacteria>Burkholderiales>Burkholderiaceae>Burkholderia	GCF_001527205.1	Burkholderia_territorii	WTO0_RS19595	122446..126087	1503055	BREX-system-P-loop-protein-BrxC-[Burkholderia-territorii]
WP_059936887.1	Type6.BREX	<-HIN-HTH<- PglZ <- ParB + HNH <- N6-METHYLASE <-GreA_GreB+SF1-SF2-Helicase<- DUF6079 *<- BrxB-BrxF-iSTAND <- BrxA-DNA-binding <- BrxE <- wHTH+WYL+WCX ||ARG->?-><-HTH_19||LON-AAA->	1213	Bacteria>Pseudomonadota>Betaproteobacteria>Burkholderiales>Burkholderiaceae>Burkholderia	GCF_001528605.1	Burkholderia_multivorans	WK33_RS03230	12913..16554	87883	BREX-system-P-loop-protein-BrxC-[Burkholderia-multivorans]
WP_060225079.1	Type1.BREX	REase->SIS_2+SIS->?->?->tRNA->Arm-DNA-bind_3-><-HIN-HTH|| wHTH+WYL+WCX -> BrxA-DNA-binding -> BrxB-BrxF-iSTAND -> DUF6079 *-> N6-METHYLASE -> ParB + HNH -> PglZ -> SIGMA+OB-Fold+AAA-ATPase+Lon-Protease-BrxL -><-?||?->PspA->	1146	Bacteria>Pseudomonadota>Betaproteobacteria>Burkholderiales>Burkholderiaceae>Burkholderia	GCF_001531525.1	Burkholderia_cepacia	WL34_RS17275	238513..241953	292	BREX-system-P-loop-protein-BrxC-[Burkholderia-cepacia]
WP_226255239.1	Type6.BREX	HTH_1+PBP_like->?->?->XIS-HTH->NACHT+GTPase-AIG+sGTP-> BrxE -> BrxA-DNA-binding -> BrxB-BrxF-iSTAND -> DUF6079 *-> N6-METHYLASE -> ParB + HNH -> PglZ -> SIGMA+OB-Fold+AAA-ATPase+HKD-DNase-BrxL ->	1208	Bacteria>Pseudomonadota>Betaproteobacteria>Burkholderiales>Burkholderiaceae>Burkholderia	GCF_020419005.1	Burkholderia_cepacia	LGM39_RS24930	366713..370339	292	BREX-system-P-loop-protein-BrxC-[Burkholderia-cepacia]
WP_186043215.1	Type6.BREX	Phage_holin->Phage_lysozyme-><- SIGMA+OB-Fold+AAA-ATPase+HKD-DNase-BrxL <- PglZ <- ParB + HNH <- N6-METHYLASE <-GreA_GreB+SF1-SF2-Helicase<- DUF6079 *<- BrxB-BrxF-iSTAND <- BrxA-DNA-binding <- BrxE <- wHTH+WYL+WCX ||SF1-SF2-Helicase->PIN->?->ClpABC-AAA->	1213	Bacteria>Pseudomonadota>Betaproteobacteria>Burkholderiales>Burkholderiaceae>Burkholderia	GCF_902830235.1	Burkholderia_gladioli	H9C96_RS14825	15196..18837	28095	BREX-system-P-loop-protein-BrxC-[Burkholderia-gladioli]
WP_186236015.1	Type6.BREX	ParB + HNH -> ParB + HNH -> HNH ->Phage_integrase-><- SIGMA+OB-Fold+AAA-ATPase+HKD-DNase-BrxL <- PglZ <- N6-METHYLASE <- DUF6079 *<- BrxB-BrxF-iSTAND <- BrxA-DNA-binding <- BrxE <-MORC+DNA_gyrase+TOPRIM<-?<-RFC-AAA+DNAA-HTH||?->RNaseP->	1215	Bacteria>Pseudomonadota>Betaproteobacteria>Burkholderiales>Burkholderiaceae>Burkholderia	GCF_902832475.1	Burkholderia_gladioli	H9G27_RS00755	169921..173568	28095	BREX-system-P-loop-protein-BrxC-[Burkholderia-gladioli]
WP_091908362.1	Type6.BREX	<-REase<-?||?->?-><-MODE-HTH+PBPII|| wHTH+WYL+WCX -> BrxE -> BrxA-DNA-binding -> BrxB-BrxF-iSTAND -> DUF6079 *->GreA_GreB+SF1-SF2-Helicase-> N6-METHYLASE -> ParB + HNH -> PglZ -> SIGMA+OB-Fold+AAA-ATPase+HKD-DNase-BrxL ->	1213	Bacteria>Pseudomonadota>Betaproteobacteria>Burkholderiales>Burkholderiaceae>Chitinasiproducens	GCF_900095735.1	Chitinasiproducens_palmae	BLU54_RS11860	213511..217152	1770053	BREX-system-P-loop-protein-BrxC-[Chitinasiproducens-palmae]
WP_125094459.1	Type1.BREX	wHTH+WYL+WCX -> BrxA-DNA-binding -> BrxB-BrxF-iSTAND -> DUF6079 *-> N6-METHYLASE -> ParB + HNH -> PglZ -> SIGMA+OB-Fold+AAA-ATPase+Lon-Protease-BrxL ->?->?->?->?-><-HAD+HAD	1146	Bacteria>Pseudomonadota>Betaproteobacteria>Burkholderiales>Burkholderiaceae>Lautropia	GCF_003892345.1	Lautropia_dentalis	EHV23_RS01850	480599..484039	2490857	BREX-system-P-loop-protein-BrxC-[Lautropia-dentalis]
WP_275272077.1	Type1.BREX	TIR-><-Tox-RES<-CHTH|| BrxA-DNA-binding -> BrxB-BrxF-iSTAND -> DUF6079 *-> ParB + HNH -> N6-METHYLASE ->HEPN+RES->	1193	Bacteria>Pseudomonadota>Betaproteobacteria>Burkholderiales>Burkholderiaceae>Limnobacter	GCF_029087225.1	Limnobacter_sp._P1	PY990_RS13680	12633..16214	3031298	BREX-system-P-loop-protein-BrxC-[Limnobacter-sp.-P1]
MCK6421971.1	Type1.BREX	NERD+SF1-SF2-Helicase-><- SIGMA+OB-Fold+AAA-ATPase+Lon-Protease-BrxL <- PglZ <- ParB + HNH <-?<-viral-capsid<-ZnR<- N6-METHYLASE <-sGTP+GTPase-AIG<- DUF6079 *<- BrxB-BrxF-iSTAND <- BrxA-DNA-binding <-RHH_5	1153	Bacteria>Pseudomonadota>Betaproteobacteria>Burkholderiales>Burkholderiales_genera_incertae_sedis>Aquabacterium	GCA_023150345.1	Aquabacterium_sp.	L6Q73_13845	24265..27726	1872578	BREX-system-P-loop-protein-BrxC-[Aquabacterium-sp.]
WP_054257718.1	Type6.BREX	HTH_19-><- SIGMA+OB-Fold+AAA-ATPase+HKD-DNase-BrxL <- PglZ <-?<- ParB + HNH <- N6-METHYLASE -p<- N6-METHYLASE <- DUF6079 *<- BrxB-BrxF-iSTAND <- BrxA-DNA-binding <- BrxE <-HTH_21+rve+rve_3	1215	Bacteria>Pseudomonadota>Betaproteobacteria>Burkholderiales>Comamonadaceae>Acidovorax	GCF_001298675.1	Acidovorax_caeni	BN2503_RS16450	13891..17538	343013	BREX-system-P-loop-protein-BrxC-[Acidovorax-caeni]
WP_057202647.1	Type1.BREX	HTH+sGTP->?->?-> BrxE -> BrxA-DNA-binding -> BrxB-BrxF-iSTAND -> DUF6079 *->GreA_GreB+SF1-SF2-Helicase-> N6-METHYLASE -> N6-METHYLASE -> ParB + HNH -> PglZ -> SIGMA+OB-Fold+AAA-ATPase+HKD-DNase-BrxL ->?->GGDEF-><-Peptidase_M48+TPR_9	1207	Bacteria>Pseudomonadota>Betaproteobacteria>Burkholderiales>Comamonadaceae>Acidovorax	GCF_001428645.1	Acidovorax_sp._Root217	ASE31_RS15170	91296..94919	1736492	BREX-system-P-loop-protein-BrxC-[Acidovorax-sp.-Root217]
WP_086927320.1	Type1.BREX	<- SIGMA+OB-Fold+AAA-ATPase+Lon-Protease-BrxL <- PglZ <- ParB + HNH <-SF1-SF2-Helicase<-OLD-ABC+TOPRIM<- N6-METHYLASE <-HEPN<- DUF6079 *<- BrxB-BrxF-iSTAND <- BrxA-DNA-binding <-RHH_5<-?<-XIS-HTH<-tRNA<-tRNA<-35exo<-?<-?<-DnaJ	1153	Bacteria>Pseudomonadota>Betaproteobacteria>Burkholderiales>Comamonadaceae>Acidovorax	GCF_002157125.1	Acidovorax_carolinensis	CBP36_RS10055	2162910..2166371	553814	BREX-system-P-loop-protein-BrxC-[Acidovorax-carolinensis]
WP_204247869.1	Type1.BREX	DnaJ->?->?->tRNA->tRNA->XIS-HTH->?->RHH_5-> BrxA-DNA-binding -> BrxB-BrxF-iSTAND -> DUF6079 -p*-> DUF6079 -p->HEPN-> N6-METHYLASE ->OLD-ABC+TOPRIM->SF1-SF2-Helicase-> ParB + HNH -> PglZ -> SIGMA+OB-Fold+AAA-ATPase+Lon-Protease-BrxL ->	590	Bacteria>Pseudomonadota>Betaproteobacteria>Burkholderiales>Comamonadaceae>Acidovorax	GCF_002157185.1	Acidovorax_carolinensis	CBP35_RS21920	1914645..1916417	553814	BREX-system-P-loop-protein-BrxC-[Acidovorax-carolinensis]
WP_034379112.1	Type6.BREX	Phage_holin->Phage_lysozyme-><-SIR2<- SIGMA+OB-Fold+AAA-ATPase+HKD-DNase-BrxL <- PglZ <-?<- ParB + HNH <- N6-METHYLASE -p<- N6-METHYLASE <- DUF6079 *<- BrxB-BrxF-iSTAND <- BrxA-DNA-binding <- BrxE <- wHTH+WYL+WCX ||JetA->JetB->SbcC->	1210	Bacteria>Pseudomonadota>Betaproteobacteria>Burkholderiales>Comamonadaceae>Comamonas	GCF_000761335.1	Comamonas_thiooxydans	P245_RS10570	85855..89487	363952	BREX-system-P-loop-protein-BrxC-[Comamonas-thiooxydans]
KEH09926.1	Type1.BREX	<-DUF932||XIS-HTH->OLD-ABC-> BrxA-DNA-binding -> BrxB-BrxF-iSTAND -> DUF6079 *-> N6-METHYLASE ->OLD-ABC->REase->SIR2->?-> ParB + HNH -> PglZ -> SIGMA+OB-Fold+AAA-ATPase+HKD-DNase-BrxL ->	1185	Bacteria>Pseudomonadota>Betaproteobacteria>Burkholderiales>Comamonadaceae>Delftia	GCA_000710165.1	Delftia_tsuruhatensis	GY14_08945	17729..21286	180282	hypothetical-protein-GY14_08945-[Delftia-tsuruhatensis]

Protein-ID	System	Gene-Neighbourhoods	Length	Taxonomy	Assembly-ID	Strain	Locus-Tag	Genomic-coordinates	TaxID	Fasta-define
WP_101103324.1	Type1.BREX	[illegible]	1153	Bacteria>Pseudomonadota>Betaproteobacteria>Burkholderiales>Comamonadaceae>Macromonas	GCF_002837135.1	Macromonas_bipunctata	CON76_RS10585	3906..7367	183670	BREX-system-P-loop-protein-BrxC-[Macromonas-bipunctata]
CDS49862.1	Type6.BREX	[illegible]	1210	Bacteria>Pseudomonadota>Betaproteobacteria>Burkholderiales>Comamonadaceae>Polaromonas	-	Polaromonas_sp._CG9_12	.	485322..488954	1504672	hypothetical-protein-[Polaromonas-sp.-CG9_12]
WP_169420224.1	Type1.BREX	[illegible]	1153	Bacteria>Pseudomonadota>Betaproteobacteria>Burkholderiales>Comamonadaceae>Ramlibacter	GCF_012927085.1	Ramlibacter_agri	HHL11_RS19385	189581..193042	2728837	BREX-system-P-loop-protein-BrxC-[Ramlibacter-agri]
WP_068673039.1	Type6.BREX	[illegible]	1212	Bacteria>Pseudomonadota>Betaproteobacteria>Burkholderiales>Comamonadaceae>Variovorax	GCF_001541225.1	unclassified_Variovorax	APY03_RS01000	202384..206022	663243	MULTISPECIES:-BREX-system-P-loop-protein-BrxC-[unclassified-Variovorax]
WP_186915942.1	Type1.BREX	[illegible]	1183	Bacteria>Pseudomonadota>Betaproteobacteria>Burkholderiales>Oxalobacteraceae	GCF_014284115.1	Undibacterium	H8K35_RS08325	157325..160876	401469	MULTISPECIES:-BREX-system-P-loop-protein-BrxC-[Undibacterium]
WP_141835430.1	Type6.BREX	[illegible]	1209	Bacteria>Pseudomonadota>Betaproteobacteria>Burkholderiales>Oxalobacteraceae>Herbaspirillum	-	Herbaspirillum_sp._SJZ107	FBX97_RS19505	315565..319194	2572881	BREX-system-P-loop-protein-BrxC-[Herbaspirillum-sp.-SJZ107]
WP_106758218.1	Type1.BREX	[illegible]	1154	Bacteria>Pseudomonadota>Betaproteobacteria>Burkholderiales>Oxalobacteraceae>Massilia_group	GCF_003011895.2	Massilia_glaciei	C7C56_RS15200	27208..30672	1524097	BREX-system-P-loop-protein-BrxC-[Massilia-glaciei]
WP_152879050.1	Type6.BREX	[illegible]	1215	Bacteria>Pseudomonadota>Betaproteobacteria>Burkholderiales>Oxalobacteraceae>Massilia_group	GCF_009295655.1	Duganella_sp._FT27W	F8197_RS00385	74619..78266	2654636	BREX-system-P-loop-protein-BrxC-[Duganella-sp.-FT27W]
OGB35307.1	Type6.BREX	[illegible]	1215	Bacteria>Pseudomonadota>Betaproteobacteria>Burkholderiales>unclassified_Burkholderiales	GCA_001770935.1	Burkholderiales_bacterium_RIFCSPLOWO2_12_FULL_61_40	A3F78_11695	46897..50544	1797566	ATPase-[Burkholderiales-bacterium-RIFCSPLOWO2_12_FULL_61_40]
MCP5275500.1	Type1.BREX	[illegible]	1176	Bacteria>Pseudomonadota>Betaproteobacteria>Burkholderiales>unclassified_Burkholderiales	GCA_024235795.1	Burkholderiales_bacterium	H6936_11775	653459..656989	1891238	BREX-system-P-loop-protein-BrxC-[Burkholderiales-bacterium]
WP_194117489.1	Type6.BREX	[illegible]	1213	Bacteria>Pseudomonadota>Betaproteobacteria>Neisseriales>Chromobacteriaceae>Chitinilyticum	GCF_015223195.1	Chitinilyticum_piscinae	INR99_RS16780	39673..43314	2866724	BREX-system-P-loop-protein-BrxC-[Chitinilyticum-piscinae]
WP_153715435.1	Type6.BREX	[illegible]	1213	Bacteria>Pseudomonadota>Betaproteobacteria>Neisseriales>Neisseriaceae>Eikenella	GCF_009650905.1	Eikenella_corrodens	GJ661_RS08680	18367..22008	539	BREX-system-P-loop-protein-BrxC-[Eikenella-corrodens]
ROH85757.1	Type1.BREX	[illegible]	1146	Bacteria>Pseudomonadota>Betaproteobacteria>Nitrosomonadales>Methylophilaceae>Pseudomethylobacillus	GCA_003730115.1	Pseudomethylobacillus_aquaticus	ED236_08405	68439..71879	2676064	BREX-system-P-loop-protein-BrxC-[Pseudomethylobacillus-aquaticus]
WP_205125664.1	Type1.BREX	[illegible]	1150	Bacteria>Pseudomonadota>Betaproteobacteria>Nitrosomonadales>Methylophilaceae>Pseudomethylobacillus	GCF_003730115.1	Pseudomethylobacillus_aquaticus	ED236_RS08405	68439..71891	2676064	BREX-system-P-loop-protein-BrxC-[Pseudomethylobacillus-aquaticus]
SDW37048.1	Type1.BREX	[illegible]	1145	Bacteria>Pseudomonadota>Betaproteobacteria>Nitrosomonadales>Nitrosomonadaceae>Nitrosomonas	GCA_900106545.1	Nitrosomonas_communis	SAMN05421882_100921	53532..56969	44574	hypothetical-protein-SAMN05421882_100921-[Nitrosomonas-communis]
WP_181258202.1	Type1.BREX	[illegible]	1179	Bacteria>Pseudomonadota>Betaproteobacteria>Nitrosomonadales>Nitrosomonadaceae>Nitrosomonas	GCF_003046585.1	Nitrosomonas_aestuarii	C8R11_107137	171439..174978	52441	BREX-system-P-loop-protein-BrxC-[Nitrosomonas-aestuarii]
WP_211752264.1	Type1.BREX	[illegible]	1146	Bacteria>Pseudomonadota>Betaproteobacteria>Nitrosomonadales>Nitrosomonadaceae>Nitrosomonas	GCF_900106545.1	Nitrosomonas_communis	BLV21_RS05435	53532..56972	44574	BREX-system-P-loop-protein-BrxC-[Nitrosomonas-communis]
SFW23276.1	Type6.BREX	[illegible]	1213	Bacteria>Pseudomonadota>Betaproteobacteria>Nitrosomonadales>Nitrosomonadaceae>Nitrosovibrio	GCA_900119085.1	Nitrosovibrio_sp._Nv17	SAMN05216414_10762	70156..73797	1855339	hypothetical-protein-SAMN05216414_10762-[Nitrosovibrio-sp.-Nv17]
WP_218184829.1	Type6.BREX	[illegible]	1234	Bacteria>Pseudomonadota>Betaproteobacteria>Nitrosomonadales>Nitrosomonadaceae>Nitrosovibrio	GCF_900119085.1	Nitrosovibrio_sp._Nv17	BT108_RS05915	70156..73860	1855339	BREX-system-P-loop-protein-BrxC-[Nitrosovibrio-sp.-Nv17]
MBW8470844.1	Type6.BREX	[illegible]	1215	Bacteria>Pseudomonadota>Betaproteobacteria>Nitrosomonadales>Thiobacillaceae>Thiobacillus	GCA_019459125.1	Thiobacillus_sp.	KOM67_21445	16263..19910	924	BREX-system-P-loop-protein-BrxC-[Thiobacillus-sp.]
MBK9161130.1	Type1.BREX	[illegible]	1146	Bacteria>Pseudomonadota>Betaproteobacteria>Nitrosomonadales>unclassified_Nitrosomonadales	GCA_016716325.1	Nitrosomonadales_bacterium	IPM27_06155	1292283..1295723	2026761	BREX-system-P-loop-protein-BrxC-[Nitrosomonadales-bacterium]
MBP6421402.1	Type1.BREX	[illegible]	923	Bacteria>Pseudomonadota>Betaproteobacteria>Rhodocyclales>Rhodocyclaceae>Propionivibrio	GCA_017988215.1	Propionivibrio_sp.	KA271_00765	12654..15427	2212460	BREX-system-P-loop-protein-BrxC-[Propionivibrio-sp.]
MCA3121411.1	Type1.BREX	[illegible]	1210	Bacteria>Pseudomonadota>Betaproteobacteria>Rhodocyclales>Rhodocyclaceae>unclassified_Rhodocyclaceae	GCA_020248305.1	Rhodocyclaceae_bacterium	ING90_02100	367371..371003	1898103	BREX-system-P-loop-protein-BrxC-[Rhodocyclaceae-bacterium]
WP_141017182.1	Type6.BREX	[illegible]	1207	Bacteria>Pseudomonadota>Betaproteobacteria>Rhodocyclales>Zoogloeaceae>Azoarcus	GCF_006496635.1	Azoarcus_sp._DD4	CJ010_RS05935	1245819..1249442	2027405	BREX-system-P-loop-protein-BrxC-[Azoarcus-sp.-DD4]
WP_148578351.1	Type1.BREX	[illegible]	1146	Bacteria>Pseudomonadota>Betaproteobacteria>Rhodocyclales>Zoogloeaceae>Zoogloea	GCF_008107625.1	Zoogloea_oleivorans	ETQ85_RS07180	242245..245685	1552750	BREX-system-P-loop-protein-BrxC-[Zoogloea-oleivorans]
AUZ77883.1	Type1.BREX	[illegible]	1183	Bacteria>Pseudomonadota>Gammaproteobacteria>Aeromonadales>Aeromonadaceae>Aeromonas	GCA_002934425.1	Aeromonas_sp._ASNIH4	C2U40_16585	3601638..3605189	1636609	BREX-system-P-loop-protein-BrxC-[Aeromonas-sp.-ASNIH4]
BBR09393.1	Type1.BREX	[illegible]	1226	Bacteria>Pseudomonadota>Gammaproteobacteria>Aeromonadales>Aeromonadaceae>Aeromonas	GCA_014158455.1	Aeromonas_caviae	WP3S18E02_10540	1118346..1122026	648	hypothetical-protein-WP3S18E02_10540-[Aeromonas-caviae]
EGX6956813.1	Type1.BREX	[illegible]	1218	Bacteria>Pseudomonadota>Gammaproteobacteria>Aeromonadales>Aeromonadaceae>Aeromonas	GCA_016414985.1	Aeromonas_hydrophila	JFQ85_000577	650804..654460	644	BREX-system-P-loop-protein-BrxC-[Aeromonas-hydrophila]
MBS4697267.1	Type1.BREX	[illegible]	1226	Bacteria>Pseudomonadota>Gammaproteobacteria>Aeromonadales>Aeromonadaceae>Aeromonas	GCA_018359965.1	Aeromonas_allosaccharophila	J4G65_17610	87511..91191	656	BREX-system-P-loop-protein-BrxC-[Aeromonas-allosaccharophila]
BDO08311.1	Type1.BREX	[illegible]	1089	Bacteria>Pseudomonadota>Gammaproteobacteria>Aeromonadales>Aeromonadaceae>Aeromonas	GCA_024347775.1	Aeromonas_caviae	KAM643c_18840	2028863..2032132	648	hypothetical-protein-KAM643c_18840-[Aeromonas-caviae]
WP_010634561.1	Type1.BREX	[illegible]	1157	Bacteria>Pseudomonadota>Gammaproteobacteria>Aeromonadales>Aeromonadaceae>Aeromonas	GCF_000315195.1	Aeromonas_dhakensis	AQU_RS10070	136801..140274	196024	BREX-system-P-loop-protein-BrxC-[Aeromonas-dhakensis]
WP_043557612.1	Type1.BREX	[illegible]	1182	Bacteria>Pseudomonadota>Gammaproteobacteria>Aeromonadales>Aeromonadaceae>Aeromonas	GCF_000819745.1	Aeromonas_bestiarum	BN1118_RS19650	69736..73284	105751	BREX-system-P-loop-protein-BrxC-[Aeromonas-bestiarum]
WP_087756130.1	Type1.BREX	[illegible]	1231	Bacteria>Pseudomonadota>Gammaproteobacteria>Aeromonadales>Aeromonadaceae>Aeromonas	GCF_002180335.1	Aeromonas_salmonicida	O23A_RS10590	2337193..2340888	645	BREX-system-P-loop-protein-BrxC-[Aeromonas-salmonicida]
WP_095593101.1	Type1.BREX	[illegible]	1231	Bacteria>Pseudomonadota>Gammaproteobacteria>Aeromonadales>Aeromonadaceae>Aeromonas	GCF_002285935.1	Aeromonas_dhakensis	CK627_RS18865	4072109..4075804	196024	BREX-system-P-loop-protein-BrxC-[Aeromonas-dhakensis]
WP_103244178.1	Type6.BREX	[illegible]	1210	Bacteria>Pseudomonadota>Gammaproteobacteria>Aeromonadales>Aeromonadaceae>Aeromonas	GCF_002902865.1	Aeromonas_sp._ASNIH5	C2U30_RS17315	3671715..3675347	1758179	BREX-system-P-loop-protein-BrxC-[Aeromonas-sp.-ASNIH5]
WP_120415687.1	Type1.BREX	[illegible]	1231	Bacteria>Pseudomonadota>Gammaproteobacteria>Aeromonadales>Aeromonadaceae>Aeromonas	GCF_003611985.1	Aeromonas_veronii	D6R50_RS16910	827539..831234	654	BREX-system-P-loop-protein-BrxC-[Aeromonas-veronii]
WP_139442435.1	Type5.BREX	[illegible]	1231	Bacteria>Pseudomonadota>Gammaproteobacteria>Aeromonadales>Aeromonadaceae>Aeromonas	GCF_006246325.1	Aeromonas_veronii	CF108_RS03265	124178..127873	654	BREX-system-P-loop-protein-BrxC-[Aeromonas-veronii]
WP_159144201.1	Type1.BREX	[illegible]	1231	Bacteria>Pseudomonadota>Gammaproteobacteria>Aeromonadales>Aeromonadaceae>Aeromonas	GCF_009834065.1	Aeromonas_veronii	GRF56_RS18105	3933894..3937589	654	BREX-system-P-loop-protein-BrxC-[Aeromonas-veronii]
WP_171280306.1	Type1.BREX	[illegible]	1157	Bacteria>Pseudomonadota>Gammaproteobacteria>Aeromonadales>Aeromonadaceae>Aeromonas	GCF_013085505.1	Aeromonas_sp._2692-1	E5E97_RS12155	2531425..2534898	2560029	BREX-system-P-loop-protein-BrxC-[Aeromonas-sp.-2692-1]
WP_232090117.1	Type1.BREX	[illegible]	1281	Bacteria>Pseudomonadota>Gammaproteobacteria>Aeromonadales>Aeromonadaceae>Aeromonas	GCF_014158455.1	Aeromonas_caviae	H7X73_RS05535	1118181..1122026	648	BREX-system-P-loop-protein-BrxC-[Aeromonas-caviae]
WP_193000848.1	Type1.BREX	[illegible]	1226	Bacteria>Pseudomonadota>Gammaproteobacteria>Aeromonadales>Aeromonadaceae>Aeromonas	GCF_014892695.1	Aeromonas_simiae	FE240_RS10745	2272968..2276648	218936	BREX-system-P-loop-protein-BrxC-[Aeromonas-simiae]
WP_244842130.1	Type1.BREX	[illegible]	1281	Bacteria>Pseudomonadota>Gammaproteobacteria>Aeromonadales>Aeromonadaceae>Aeromonas	GCF_018359965.1	Aeromonas_allosaccharophila	J4G65_RS17605	87511..91356	656	BREX-system-P-loop-protein-BrxC-[Aeromonas-allosaccharophila]
WP_203133976.1	Type1.BREX	[illegible]	1156	Bacteria>Pseudomonadota>Gammaproteobacteria>Aeromonadales>Aeromonadaceae>Aeromonas	GCF_019348745.1	Aeromonas_hydrophila	PVL97_RS11265	2527654..2531124	644	BREX-system-P-loop-protein-BrxC-[Aeromonas-hydrophila]
WP_223931722.1	Type1.BREX	[illegible]	1231	Bacteria>Pseudomonadota>Gammaproteobacteria>Aeromonadales>Aeromonadaceae>Aeromonas	GCF_019972495.1	Aeromonas_caviae	LDN23_RS02850	18313..22008	648	BREX-system-P-loop-protein-BrxC-[Aeromonas-caviae]
WP_241871100.1	Type1.BREX	[illegible]	1157	Bacteria>Pseudomonadota>Gammaproteobacteria>Aeromonadales>Aeromonadaceae>Aeromonas	GCF_022631175.1	Aeromonas_hydrophila	MOO40_RS17050	3752340..3755813	644	BREX-system-P-loop-protein-BrxC-[Aeromonas-hydrophila]
WP_261925329.1	Type1.BREX	[illegible]	1089	Bacteria>Pseudomonadota>Gammaproteobacteria>Aeromonadales>Aeromonadaceae>Aeromonas	GCF_024347775.1	Aeromonas_caviae	OCX50_RS09760	2028863..2032132	648	BREX-system-P-loop-protein-BrxC-[Aeromonas-caviae]
WP_257706959.1	Type1.BREX	[illegible]	1182	Bacteria>Pseudomonadota>Gammaproteobacteria>Aeromonadales>Aeromonadaceae>Aeromonas	GCF_024649325.1	Aeromonas_caviae	NUK33_RS01495	150155..153703	648	BREX-system-P-loop-protein-BrxC-[Aeromonas-caviae]
WP_270809144.1	Type1.BREX	[illegible]	1157	Bacteria>Pseudomonadota>Gammaproteobacteria>Aeromonadales>Aeromonadaceae>Aeromonas	GCF_027705685.1	Aeromonas_sp._QDB08	OKB79_RS12620	11744..15217	2990480	BREX-system-P-loop-protein-BrxC-[Aeromonas-sp.-QDB08]
WP_181014924.1	Type1.BREX	[illegible]	1182	Bacteria>Pseudomonadota>Gammaproteobacteria>Aeromonadales>Aeromonadaceae	GCF_002934425.1	Aeromonas	C2U40_RS16580	3601638..3605186	642	MULTISPECIES:-BREX-system-P-loop-protein-BrxC-[Aeromonas]
WP_201934227.1	Type1.BREX	[illegible]	1231	Bacteria>Pseudomonadota>Gammaproteobacteria>Aeromonadales>Aeromonadaceae	GCF_019972675.1	Aeromonas	MWG22_RS17445	1818..5513	642	MULTISPECIES:-BREX-system-P-loop-protein-BrxC-[Aeromonas]
WP_270796656.1	Type1.BREX	[illegible]	1157	Bacteria>Pseudomonadota>Gammaproteobacteria>Aeromonadales>Aeromonadaceae	GCF_029024985.1	Aeromonas	OKC40_RS19420	7580..11053	642	MULTISPECIES:-BREX-system-P-loop-protein-BrxC-[Aeromonas]
WP_069785631.1	Type1.BREX	[illegible]	1157	Bacteria>Pseudomonadota>Gammaproteobacteria>Aeromonadales>Aeromonadaceae	GCF_029773025.1	Aeromonas	QBS29_RS17430	3038195..3041668	642	MULTISPECIES:-BREX-system-P-loop-protein-BrxC-[Aeromonas]
MCR4553833.1	Type1.BREX	[illegible]	1184	Bacteria>Pseudomonadota>Gammaproteobacteria>Aeromonadales>Succinivibrionaceae>unclassified_Succinivibrionaceae	GCA_024683965.1	Succinivibrionaceae_bacterium	K5752_02815	10310..13864	2053620	BREX-system-P-loop-protein-BrxC-[Succinivibrionaceae-bacterium]
WP_057786312.1	Type1.BREX	[illegible]	1157	Bacteria>Pseudomonadota>Gammaproteobacteria>Alteromonadales>Alteromonadaceae>Alishewanella	GCF_001441695.1	Alishewanella_sp._WH16-1	AAY72_RS06860	5881..9354	1651088	BREX-system-P-loop-protein-BrxC-[Alishewanella-sp.-WH16-1]
WP_207611650.1	Type1.BREX	[illegible]	1157	Bacteria>Pseudomonadota>Gammaproteobacteria>Alteromonadales>Alteromonadaceae>Alteromonas/Salinimonas_group	GCF_017368475.1	Alteromonas_sp._5E99-2	J3L16_RS04210	79358..82831	2817683	BREX-system-P-loop-protein-BrxC-[Alteromonas-sp.-5E99-2]
WP_240919947.1	Type1.BREX	[illegible]	1180	Bacteria>Pseudomonadota>Gammaproteobacteria>Alteromonadales>Alteromonadaceae>Paraglaciecola	GCF_011378905.1	Paraglaciecola_sp._20A4	GQS54_RS13935	91436..94978	2687288	BREX-system-P-loop-protein-BrxC-[Paraglaciecola-sp.-20A4]
AWB59509.1	Type1.BREX	[illegible]	1232	Bacteria>Pseudomonadota>Gammaproteobacteria>Alteromonadales>Colwelliaceae>Colwellia	GCA_003061515.1	Colwellia_sp._Arc7-D	DBO93_09830	2239405..2243103	2161872	BREX-system-P-loop-protein-BrxC-[Colwellia-sp.-Arc7-D]
WP_239058953.1	Type1.BREX	[illegible]	1235	Bacteria>Pseudomonadota>Gammaproteobacteria>Alteromonadales>Colwelliaceae>Colwellia	GCF_003061515.1	Colwellia_sp._Arc7-D	DBO93_RS09830	2239405..2243112	2161872	BREX-system-P-loop-protein-BrxC-[Colwellia-sp.-Arc7-D]
WP_085284213.1	Type1.BREX	[illegible]	1243	Bacteria>Pseudomonadota>Gammaproteobacteria>Alteromonadales>Colwelliaceae>Colwellia	GCF_900109795.1	Colwellia_chukchiensis	B6K83_RS06980	108494..112225	641665	BREX-system-P-loop-protein-BrxC-[Colwellia-chukchiensis]
KXS36208.1	Type1.BREX	[illegible]	1150	Bacteria>Pseudomonadota>Gammaproteobacteria>Alteromonadales>Idiomarinaceae>Idiomarina	GCA_001577655.1	Idiomarina_sp._T82-3	AWU56_323	88359..91811	1794809	hypothetical-protein-AWU56_323-[Idiomarina-sp.-T82-3]
WP_112714387.1	Type1.BREX	[illegible]	1199	Bacteria>Pseudomonadota>Gammaproteobacteria>Alteromonadales>Moritellaceae>Moritella	GCF_900465055.1	Moritella_yayanosii	MORIYA_RS08605	1893116..1896715	69539	BREX-system-P-loop-protein-BrxC-[Moritella-yayanosii]
WP_010607320.1	Type1.BREX	[illegible]	1188	Bacteria>Pseudomonadota>Gammaproteobacteria>Alteromonadales>Pseudoalteromonadaceae	GCF_000276645.1	Pseudoalteromonas	UY7_RS0121200	90697..94263	53246	MULTISPECIES:-BREX-system-P-loop-protein-BrxC-[Pseudoalteromonas]
WP_054983666.1	Type1.BREX	[illegible]	1159	Bacteria>Pseudomonadota>Gammaproteobacteria>Alteromonadales>Pseudoalteromonadaceae>Pseudoalteromonas	GCF_001400005.1	Pseudoalteromonas_sp._P1-30	AN395_RS16640	61256..64735	1723760	BREX-system-P-loop-protein-BrxC-[Pseudoalteromonas-sp.-P1-30]
WP_064663367.1	Type1.BREX	[illegible]	1242	Bacteria>Pseudomonadota>Gammaproteobacteria>Alteromonadales>Pseudoalteromonadaceae>Pseudoalteromonas	GCF_001661605.1	Pseudoalteromonas_sp._MQS005	A9479_RS10285	16730..20458	1854052	BREX-system-P-loop-protein-BrxC-[Pseudoalteromonas-sp.-MQS005]
WP_234000034.1	Type1.BREX	[illegible]	1252	Bacteria>Pseudomonadota>Gammaproteobacteria>Alteromonadales>Pseudoalteromonadaceae>Pseudoalteromonas	GCF_002964725.1	Pseudoalteromonas_sp._T1lg76	C2M35_RS06890	48486..52244	2077103	BREX-system-P-loop-protein-BrxC-[Pseudoalteromonas-sp.-T1lg76]
WP_130052623.1	Type1.BREX	[illegible]	1244	Bacteria>Pseudomonadota>Gammaproteobacteria>Alteromonadales>Pseudoalteromonadaceae>Pseudoalteromonas	GCF_004195145.1	Pseudoalteromonas_sp._MEBiC_03485	EVU92_RS18430	351830..355564	2571103	BREX-system-P-loop-protein-BrxC-[Pseudoalteromonas-sp.-MEBiC-03485]
WP_096038338.1	Type1.BREX	[illegible]	1159	Bacteria>Pseudomonadota>Gammaproteobacteria>Alteromonadales>Pseudoalteromonadaceae>Pseudoalteromonas	GCF_007991235.1	Pseudoalteromonas_tetraodonis	PTE01_RS15665	26152..29631	43659	BREX-system-P-loop-protein-BrxC-[Pseudoalteromonas-tetraodonis]
WP_089368387.1	Type1.BREX	[illegible]	1229	Bacteria>Pseudomonadota>Gammaproteobacteria>Alteromonadales>Pseudoalteromonadaceae>Pseudoalteromonas	GCF_007991255.1	Pseudoalteromonas_nigrifaciens	PNIG_RS09950	2060327..2064016	28109	BREX-system-P-loop-protein-BrxC-[Pseudoalteromonas-nigrifaciens]
WP_167658112.1	Type1.BREX	[illegible]	1188	Bacteria>Pseudomonadota>Gammaproteobacteria>Alteromonadales>Pseudoalteromonadaceae>Pseudoalteromonas	GCF_011806445.1	Pseudoalteromonas_sp._HF66	HCZ11_RS07660	212811..216377	2721559	BREX-system-P-loop-protein-BrxC-[Pseudoalteromonas-sp.-HF66]
WP_193038371.1	Type1.BREX	[illegible]	1159	Bacteria>Pseudomonadota>Gammaproteobacteria>Alteromonadales>Pseudoalteromonadaceae>Pseudoalteromonas	GCF_014897925.1	Pseudoalteromonas_nigrifaciens	INT11_RS00030	7982..11461	28109	BREX-system-P-loop-protein-BrxC-[Pseudoalteromonas-nigrifaciens]
WP_074988793.1	Type1.BREX	[illegible]	1242	Bacteria>Pseudomonadota>Gammaproteobacteria>Alteromonadales>Pseudoalteromonadaceae>Pseudoalteromonas	GCF_014925285.1	Pseudoalteromonas_lipolytica	PLIP_RS19215	618195..621923	570156	BREX-system-P-loop-protein-BrxC-[Pseudoalteromonas-lipolytica]
WP_037429580.1	Type1.BREX	[illegible]	1157	Bacteria>Pseudomonadota>Gammaproteobacteria>Alteromonadales>Shewanellaceae	GCF_000282755.1	Shewanella	SHEWPOL2_RS15310	7136..10609	22	MULTISPECIES:-BREX-system-P-loop-protein-BrxC-[Shewanella]
PKG74712.1	Type1.BREX	[illegible]	1191	Bacteria>Pseudomonadota>Gammaproteobacteria>Alteromonadales>Shewanellaceae>Shewanella	GCA_002836135.1	Shewanella_sp._GutCb	CXF86_11690	122253..125828	2058315	BREX-system-P-loop-protein-BrxC-[Shewanella-sp.-GutCb]
HAY94623.1	Type1.BREX	[illegible]	1224	Bacteria>Pseudomonadota>Gammaproteobacteria>Alteromonadales>Shewanellaceae>Shewanella	GCA_003486485.1	Shewanella_sp.	DCY70_12125	4933..8607	50422	BREX-system-P-loop-protein-BrxC-[Shewanella-sp.]
WP_011622483.1	Type1.BREX	[illegible]	1157	Bacteria>Pseudomonadota>Gammaproteobacteria>Alteromonadales>Shewanellaceae>Shewanella	GCF_000014685.1	Shewanella_sp._MR-4	SHEWMR4_RS09030	2019364..2022837	60480	BREX-system-P-loop-protein-BrxC-[Shewanella-sp.-MR-4]
WP_011716828.1	Type1.BREX	[illegible]	1157	Bacteria>Pseudomonadota>Gammaproteobacteria>Alteromonadales>Shewanellaceae>Shewanella	GCF_000203935.1	Shewanella_sp._ANA-3	SHEWANA3_RS09590	2144277..2147750	94122	BREX-system-P-loop-protein-BrxC-[Shewanella-sp.-ANA-3]
WP_006086465.1	Type1.BREX	[illegible]	1229	Bacteria>Pseudomonadota>Gammaproteobacteria>Alteromonadales>Shewanellaceae>Shewanella	GCF_000231345.1	Shewanella_baltica	SBAL625DRAFT_RS15525	212954..216643	62322	BREX-system-P-loop-protein-BrxC-[Shewanella-baltica]
WP_028764576.1	Type1.BREX	[illegible]	1157	Bacteria>Pseudomonadota>Gammaproteobacteria>Alteromonadales>Shewanellaceae>Shewanella	GCF_000518705.1	Shewanella_colwelliana	L876_RS0115155	4861..8334	23	BREX-system-P-loop-protein-BrxC-[Shewanella-colwelliana]
WP_231570521.1	Type1.BREX	[illegible]	941	Bacteria>Pseudomonadota>Gammaproteobacteria>Alteromonadales>Shewanellaceae>Shewanella	GCF_000798835.1	Shewanella_sp._ZOR0012	L976_RS20155	4209796..4212621	1339231	BREX-system-P-loop-protein-BrxC-[Shewanella-sp.-ZOR0012]
WP_055648628.1	Type1.BREX	[illegible]	1157	Bacteria>Pseudomonadota>Gammaproteobacteria>Alteromonadales>Shewanellaceae>Shewanella	GCF_001308045.1	Shewanella_sp._Sh95	AEA42_RS19845	36695..40168	1689868	BREX-system-P-loop-protein-BrxC-[Shewanella-sp.-Sh95]
WP_080916542.1	Type1.BREX	[illegible]	1234	Bacteria>Pseudomonadota>Gammaproteobacteria>Alteromonadales>Shewanellaceae>Shewanella	GCF_002075795.1	Shewanella_japonica	SJ2017_RS16905	3936240..3939944	93973	BREX-system-P-loop-protein-BrxC-[Shewanella-japonica]
WP_257985331.1	Type1.BREX	[illegible]	1193	Bacteria>Pseudomonadota>Gammaproteobacteria>Alteromonadales>Shewanellaceae>Shewanella	GCF_002836135.1	Shewanella_sp._GutCb	CXF86_RS11730	122253..125834	2058315	BREX-system-P-loop-protein-BrxC-[Shewanella-sp.-GutCb]
WP_108947326.1	Type1.BREX	[illegible]	1225	Bacteria>Pseudomonadota>Gammaproteobacteria>Alteromonadales>Shewanellaceae>Shewanella	GCF_003112715.1	Shewanella_halifaxensis	C6V78_RS24360	248833..252510	271098	BREX-system-P-loop-protein-BrxC-[Shewanella-halifaxensis]
WP_252977707.1	Type1.BREX	[illegible]	261	Bacteria>Pseudomonadota>Gammaproteobacteria>Alteromonadales>Shewanellaceae>Shewanella	GCF_013307415.1	Shewanella_sp._ZOR0012	HT094_RS07805	1783246..1784031	1339231	hypothetical-protein-[Shewanella-sp.-ZOR0012]
WP_252977708.1	Type1.BREX	[illegible]	746	Bacteria>Pseudomonadota>Gammaproteobacteria>Alteromonadales>Shewanellaceae>Shewanella	GCF_013307415.1	Shewanella_sp._ZOR0012	HT094_RS07815	1785170..1787410	1339231	BREX-system-P-loop-protein-BrxC-[Shewanella-sp.-ZOR0012]
WP_208124435.1	Type1.BREX	[illegible]	1157	Bacteria>Pseudomonadota>Gammaproteobacteria>Alteromonadales>Shewanellaceae>Shewanella	GCF_017570045.1	Shewanella_algae	JKK45_RS07515	1709365..1712838	38313	BREX-system-P-loop-protein-BrxC-[Shewanella-algae]
WP_101059463.1	Type1.BREX	[illegible]	1157	Bacteria>Pseudomonadota>Gammaproteobacteria>Alteromonadales>Shewanellaceae>Shewanella	GCF_017580385.1	Shewanella_algae	CX652_RS12220	2312..5785	38313	BREX-system-P-loop-protein-BrxC-[Shewanella-algae]
WP_144191359.1	Type1.BREX	[illegible]	1157	Bacteria>Pseudomonadota>Gammaproteobacteria>Alteromonadales>Shewanellaceae>Shewanella	GCF_017580415.1	Shewanella_algae	I6M45_RS07395	495996..499469	38313	BREX-system-P-loop-protein-BrxC-[Shewanella-algae]
WP_208223521.1	Type1.BREX	[illegible]	1157	Bacteria>Pseudomonadota>Gammaproteobacteria>Alteromonadales>Shewanellaceae>Shewanella	GCF_017580425.1	Shewanella_algae	I6M46_RS07445	1788..5261	38313	BREX-system-P-loop-protein-BrxC-[Shewanella-algae]
WP_220062217.1	Type1.BREX	[illegible]	1157	Bacteria>Pseudomonadota>Gammaproteobacteria>Alteromonadales>Shewanellaceae>Shewanella	GCF_019457515.1	Shewanella_sp._FJAT-53870	KOI73_RS17055	3880275..3883748	2864208	BREX-system-P-loop-protein-BrxC-[Shewanella-sp.-FJAT-53870]
WP_220768073.1	Type1.BREX	[illegible]	1225	Bacteria>Pseudomonadota>Gammaproteobacteria>Alteromonadales>Shewanellaceae>Shewanella	GCF_019654915.1	Shewanella_sp._MBTL60-007	K5Q92_RS05655	54742..58419	2815911	BREX-system-P-loop-protein-BrxC-[Shewanella-sp.-MBTL60-007]
WP_221058246.1	Type1.BREX	[illegible]	1199	Bacteria>Pseudomonadota>Gammaproteobacteria>Alteromonadales>Shewanellaceae>Shewanella	GCF_019670685.1	Shewanella_algae	K5631_RS12960	2926333..2929932	38313	BREX-system-P-loop-protein-BrxC-[Shewanella-algae]
WP_224020606.1	Type1.BREX	[illegible]	1157	Bacteria>Pseudomonadota>Gammaproteobacteria>Alteromonadales>Shewanellaceae>Shewanella	GCF_019973655.1	Shewanella_xiamenensis	LDO18_RS12250	2746465..2749938	332186	BREX-system-P-loop-protein-BrxC-[Shewanella-xiamenensis]
WP_248084385.1	Type1.BREX	[illegible]	1231	Bacteria>Pseudomonadota>Gammaproteobacteria>Alteromonadales>Shewanellaceae>Shewanella	GCF_023159095.1	unclassified_Shewanella	MOD77_RS02670	216752..220447	196818	MULTISPECIES:-BREX-system-P-loop-protein-BrxC-[unclassified-Shewanella]
WP_259476695.1	Type1.BREX	[illegible]	1225	Bacteria>Pseudomonadota>Gammaproteobacteria>Alteromonadales>Shewanellaceae>Shewanella	GCF_024972795.1	Shewanella_xiamenensis	K5M76_RS09815	2212502..2216179	332186	BREX-system-P-loop-protein-BrxC-[Shewanella-xiamenensis]
WP_259506670.1	Type1.BREX	[illegible]	1157	Bacteria>Pseudomonadota>Gammaproteobacteria>Alteromonadales>Shewanellaceae>Shewanella	GCF_024999425.1	Shewanella_baltica	G3479_RS14595	93624..97097	62322	BREX-system-P-loop-protein-BrxC-[Shewanella-baltica]
WP_263145911.1	Type1.BREX	[illegible]	1224	Bacteria>Pseudomonadota>Gammaproteobacteria>Alteromonadales>Shewanellaceae>Shewanella	GCF_025653395.1	Shewanella_sp._SW24	L5M17_RS05810	187685..191359	2912815	BREX-system-P-loop-protein-BrxC-[Shewanella-sp.-SW24]
WP_263177453.1	Type1.BREX	[illegible]	1225	Bacteria>Pseudomonadota>Gammaproteobacteria>Alteromonadales>Shewanellaceae>Shewanella	GCF_025654415.1	unclassified_Shewanella	L5M16_RS21425	11352..15029	196818	MULTISPECIES:-BREX-system-P-loop-protein-BrxC-[unclassified-Shewanella]
WP_264921054.1	Type1.BREX	[illegible]	1157	Bacteria>Pseudomonadota>Gammaproteobacteria>Alteromonadales>Shewanellaceae>Shewanella	GCF_026013945.1	Shewanella_algae	OLL83_RS07695	1703466..1706939	38313	BREX-system-P-loop-protein-BrxC-[Shewanella-algae]
WP_275482470.1	Type1.BREX	[illegible]	1225	Bacteria>Pseudomonadota>Gammaproteobacteria>Alteromonadales>Shewanellaceae>Shewanella	GCF_029167175.1	Shewanella_sp._A32	PY479_RS00080	16338..20015	3031327	BREX-system-P-loop-protein-BrxC-[Shewanella-sp.-A32]
WP_102039083.1	Type1.BREX	[illegible]	1191	Bacteria>Pseudomonadota>Gammaproteobacteria>Alteromonadales>Shewanellaceae>Shewanella	-	Shewanella_sp._GutCb	CXF86_11690	122253..125828	2058315	BREX-system-P-loop-protein-BrxC-[Shewanella-sp.-GutCb]
MBV7434872.1	Type1.BREX	[illegible]	1175	Bacteria>Pseudomonadota>Gammaproteobacteria>Cardiobacteriales>Cardiobacteriaceae>unclassified_Cardiobacteriaceae	GCA_019218315.1	Cardiobacteriaceae_bacterium_TAE3-ERU3	KRX19_07510	264796..268323	2856340	BREX-system-P-loop-protein-BrxC-[Cardiobacteriaceae-bacterium-TAE3-ERU3]
WP_265366287.1	Type1.BREX	[illegible]	1193	Bacteria>Pseudomonadota>Gammaproteobacteria>Cellvibrionales>Cellvibrionaceae>Marinimicrobium	GCF_026153455.1	Marinimicrobium_koreense	OOT55_RS13025	3024461..3028042	306545	BREX-system-P-loop-protein-BrxC-[Marinimicrobium-sp.-C6131]
WP_187410606.1	Type1.BREX	[illegible]	1146	Bacteria>Pseudomonadota>Gammaproteobacteria>Cellvibrionales>Cellvibrionaceae>Saccharophagus	GCF_014336955.1	Saccharophagus_sp._K07	DWB84_RS14900	71821..75261	2283636	BREX-system-P-loop-protein-BrxC-[Saccharophagus-sp.-K07]
WP_043319021.1	Type1.BREX	[illegible]	1145	Bacteria>Pseudomonadota>Gammaproteobacteria>Cellvibrionales>Microbulbiferaceae>Microbulbifer	GCF_000708675.1	Microbulbifer_sp._HZ11	AU11_RS09640	451911..455348	1453501	BREX-system-P-loop-protein-BrxC-[Microbulbifer-sp.-HZ11]
WP_036859475.1	Type1.BREX	[illegible]	1187	Bacteria>Pseudomonadota>Gammaproteobacteria>Cellvibrionales>Porticoccaceae>Porticoccus	GCF_000744735.1	Porticoccus_hydrocarbonoclasticus	U740_RS05135	1073957..1077520	1073414	BREX-system-P-loop-protein-BrxC-[Porticoccus-hydrocarbonoclasticus]
MAT50029.1	Type6.BREX	[illegible]	1215	Bacteria>Pseudomonadota>Gammaproteobacteria>Cellvibrionales>Porticoccaceae>unclassified_Porticoccaceae	GCA_002702725.1	Porticoccaceae_bacterium	CMK32_02455	2974..6621	2026782	ATPase-[Porticoccaceae-bacterium]
WP_268905395.1	Type1.BREX	[illegible]	1220	Bacteria>Pseudomonadota>Gammaproteobacteria>Cellvibrionales>Spongiibacteraceae>Dasania	GCF_026891035.1	Dasania_sp._GY-19	OOV09_RS18305	21121..24783	2965066	BREX-system-P-loop-protein-BrxC-[Dasania-sp.-GY-19]
WP_190766547.1	Type1.BREX	[illegible]	1192	Bacteria>Pseudomonadota>Gammaproteobacteria>Cellvibrionales>Spongiibacteraceae>Spongiibacter	GCF_014705705.1	Spongiibacter_pelagi	IB286_RS13890	27960..31538	2760804	BREX-system-P-loop-protein-BrxC-[Spongiibacter-pelagi]
WP_273033648.1	Type1.BREX	[illegible]	1193	Bacteria>Pseudomonadota>Gammaproteobacteria>Cellvibrionales>Spongiibacteraceae>Spongiibacter	GCF_950061765.1	Spongiibacter_tropicus	B4C78_RS03795	102796..106377	454602	BREX-system-P-loop-protein-BrxC-[Spongiibacter-tropicus]
MTW21824.1	Type1.BREX	[illegible]	1173	Bacteria>Pseudomonadota>Gammaproteobacteria>Chromatiales>Chromatiaceae>Allochromatium	GCA_009720725.1	Allochromatium_palmeri	GJ668_12065	37225..40746	231048	BREX-system-P-loop-protein-BrxC-[Allochromatium-palmeri]
WP_186343071.1	Type1.BREX	[illegible]	1172	Bacteria>Pseudomonadota>Gammaproteobacteria>Chromatiales>Chromatiaceae>Allochromatium	GCF_009720725.1	Allochromatium_palmeri	GJ668_RS12065	37225..40743	231048	BREX-system-P-loop-protein-BrxC-[Allochromatium-palmeri]
NEX15944.1	Type1.BREX	[illegible]	1173	Bacteria>Pseudomonadota>Gammaproteobacteria>Chromatiales>Chromatiaceae>Halochromatium	GCA_010912705.1	Halochromatium_sp.	C1943_04720	67001..70522	2049430	BREX-system-P-loop-protein-BrxC-[Halochromatium-sp.]
WP_201222419.1	Type1.BREX	[illegible]	1174	Bacteria>Pseudomonadota>Gammaproteobacteria>Chromatiales>Chromatiaceae>Halochromatium	GCF_016653315.1	Halochromatium_roseum	CCR96_RS19410	137258..140782	391920	BREX-system-P-loop-protein-BrxC-[Halochromatium-roseum]
WP_264346732.1	Type1.BREX	[illegible]	1157	Bacteria>Pseudomonadota>Gammaproteobacteria>Chromatiales>Chromatiaceae>Rheinheimera	GCF_947090785.1	Rheinheimera_sp.	OM978_RS09960	2096767..2100240	1869214	BREX-system-P-loop-protein-BrxC-[Rheinheimera-sp.-MM224]
WP_120797099.1	Type1.BREX	[illegible]	1173	Bacteria>Pseudomonadota>Gammaproteobacteria>Chromatiales>Chromatiaceae>Thiocapsa	GCF_003634315.1	Thiocapsa_rosea	BDD21_RS10290	2320412..2323933	69360	BREX-system-P-loop-protein-BrxC-[Thiocapsa-rosea]
WP_018716649.1	Type1.BREX	[illegible]	1144	Bacteria>Pseudomonadota>Gammaproteobacteria>Chromatiales>Ectothiorhodospiraceae>Arhodomonas	GCF_000374645.1	Arhodomonas_aquaeolei	C516_RS0101825	422257..425691	2369	BREX-system-P-loop-protein-BrxC-[Arhodomonas-aquaeolei]
WP_239679777.1	Type6.BREX	[illegible]	1223	Bacteria>Pseudomonadota>Gammaproteobacteria>Chromatiales>Ectothiorhodospiraceae>Ectothiorhodospira	GCF_022227625.1	Ectothiorhodospira_variabilis	LPW30_RS00090	25011..28682	505694	BREX-system-P-loop-protein-BrxC-[Ectothiorhodospira-variabilis]
MBK5942672.1	Type1.BREX	[illegible]	1146	Bacteria>Pseudomonadota>Gammaproteobacteria>Chromatiales>Ectothiorhodospiraceae>Halorhodospira	GCA_016653405.1	Halorhodospira_halophila	CCR79_02095	7976..11416	1053	hypothetical-protein-[Halorhodospira-halophila]
WP_242510795.1	Type1.BREX	[illegible]	1148	Bacteria>Pseudomonadota>Gammaproteobacteria>Chromatiales>Ectothiorhodospiraceae>Halorhodospira	GCF_016653405.1	Halorhodospira_halophila	CCR79_RS02135	7976..11422	1053	BREX-system-P-loop-protein-BrxC-[Halorhodospira-halophila]
WP_253479345.1	Type6.BREX	[illegible]	1216	Bacteria>Pseudomonadota>Gammaproteobacteria>Chromatiales>Ectothiorhodospiraceae>Natronocella	GCF_024170285.1	Natronocella_acetinitrilica	J2T57_RS14030	326743..330393	414046	BREX-system-P-loop-protein-BrxC-[Natronocella-acetinitrilica]
AGM41053.1	Type1.BREX	[illegible]	1145	Bacteria>Pseudomonadota>Gammaproteobacteria>Chromatiales>Ectothiorhodospiraceae>Spiribacter	GCA_000319575.2	Spiribacter_salinus_M19-40	SPISAL_04795	979011..982448	1260251	hypothetical-protein-SPISAL_04795-[Spiribacter-salinus-M19-40]
KAF0285942.1	Type1.BREX	[illegible]	1145	Bacteria>Pseudomonadota>Gammaproteobacteria>Chromatiales>Ectothiorhodospiraceae>Spiribacter	GCA_009805925.1	Spiribacter_sp._SSL99	BA899_01280	102096..105533	1866884	hypothetical-protein-BA899_01280-[Spiribacter-sp.-SSL99]
WP_245539889.1	Type1.BREX	[illegible]	1151	Bacteria>Pseudomonadota>Gammaproteobacteria>Chromatiales>Ectothiorhodospiraceae>Spiribacter	GCF_000319575.2	Spiribacter_salinus	SPISAL_RS04875	979011..982466	1335746	BREX-system-P-loop-protein-BrxC-[Spiribacter-salinus]
WP_154297058.1	Type1.BREX	[illegible]	1145	Bacteria>Pseudomonadota>Gammaproteobacteria>Chromatiales>Ectothiorhodospiraceae>Spiribacter	GCF_009676705.1	Spiribacter_sp._2438	GJ672_RS05635	1128117..1131554	2666185	BREX-system-P-loop-protein-BrxC-[Spiribacter-sp.-2438]
WP_235013293.1	Type1.BREX	[illegible]	1151	Bacteria>Pseudomonadota>Gammaproteobacteria>Chromatiales>Ectothiorhodospiraceae>Spiribacter	GCF_009805925.1	Spiribacter_sp._SSL99	BA899_RS04165	102078..105533	1866884	BREX-system-P-loop-protein-BrxC-[Spiribacter-sp.-SSL99]
WP_019570748.1	Type1.BREX	[illegible]	1225	Bacteria>Pseudomonadota>Gammaproteobacteria>Chromatiales>Ectothiorhodospiraceae>Thioalkalivibrio	GCF_000381205.1	Thioalkalivibrio_sp._ALE11	F620_RS0112040	26049..29726	1265494	BREX-system-P-loop-protein-BrxC-[Thioalkalivibrio-sp.-ALE11]
WP_019611713.1	Type6.BREX	[illegible]	1216	Bacteria>Pseudomonadota>Gammaproteobacteria>Chromatiales>Ectothiorhodospiraceae>Thioalkalivibrio	GCF_000381705.1	Thioalkalivibrio_sp._AKL7	G315_RS0100950	222198..225848	1158155	BREX-system-P-loop-protein-BrxC-[Thioalkalivibrio-sp.-AKL7]
RJT19525.1	Type1.BREX	[illegible]	1233	Bacteria>Pseudomonadota>Gammaproteobacteria>Enterobacterales>Enterobacteriaceae>Buttiauxella	GCA_003601925.1	Buttiauxella_izardii	D6029_18525	11062..14763	82991	BREX-system-P-loop-protein-BrxC-[Buttiauxella-izardii]
WP_244212427.1	Type1.BREX	[illegible]	1237	Bacteria>Pseudomonadota>Gammaproteobacteria>Enterobacterales>Enterobacteriaceae>Buttiauxella	GCF_003601925.1	Buttiauxella_izardii	D6029_RS18475	11062..14775	82991	BREX-system-P-loop-protein-BrxC-[Buttiauxella-izardii]
WP_124023337.1	Type1.BREX	[illegible]	1157	Bacteria>Pseudomonadota>Gammaproteobacteria>Enterobacterales>Enterobacteriaceae>Buttiauxella	GCF_003818135.1	Buttiauxella_warmboldiae	EHN07_RS06350	61248..64721	82993	BREX-system-P-loop-protein-BrxC-[Buttiauxella-warmboldiae]
WP_094760992.1	Type1.BREX	[illegible]	1233	Bacteria>Pseudomonadota>Gammaproteobacteria>Enterobacterales>Enterobacteriaceae>Citrobacter	-	Citrobacter_braakii	B9P84_05295	62196..65897	57706	MULTISPECIES:-BREX-system-P-loop-protein-BrxC-[Citrobacter]

Protein-ID	System	Gene-Neighbourhoods	Length	Taxonomy	Assembly-ID	Strain	Locus-Tag	Genomic-coordinates	TaxID	Fasta-defline
WP_182347709.1	Type1.BREX	<-Schlafen+HATPase+wHTH<-PHP+SbcC<-?||BrxA-DNA-binding->BrxB-BrxF-iSTAND->DUF6079*->ParB+HNH->N6-METHYLASE->PglZ->SIGMA+OB-Fold+AAA-ATPase+Lon-Protease-BrxL-><-Arm-DNA-bind_3+Phage_integrase<-tRNA||?-><-SKI||METHYLASE->	1233	Bacteria>Pseudomonadota>Gammaproteobacteria>Enterobacterales>Enterobacteriaceae>Citrobacter	-	Citrobacter_sp._RHBSTW-00013	HV034_17810	3734345..3738046	2742628	BREX-system-P-loop-protein-BrxC-[Citrobacter-sp.-RHBSTW-00013]
WP_181630678.1	Type1.BREX	<-Sulfatase<-?<-?<-?<-Schlafen+HATPase+wHTH<-PHP+SbcC<-?||BrxA-DNA-binding->BrxB-BrxF-iSTAND->DUF6079*->ParB+HNH->N6-METHYLASE->HEPN-><-HIN-HTH||N6-METHYLASE-p->N6-METHYLASE-p->PglZ->SIGMA+OB-Fold+AAA-ATPase+Lon-Protease-BrxL-><-HTH_23+HTH_32+HTH_33+DDE_3	1233	Bacteria>Pseudomonadota>Gammaproteobacteria>Enterobacterales>Enterobacteriaceae>Citrobacter	-	Citrobacter_sp._RHBSTW-01013	HV350_21470	4470372..4474073	2742677	BREX-system-P-loop-protein-BrxC-[Citrobacter-sp.-RHBSTW-01013]
OXU12831.1	Type1.BREX	XIS-HTH->?-><-Sulfatase<-?<-?<-?<-PHP+SbcC||BrxA-DNA-binding->BrxB-BrxF-iSTAND->DUF6079*->ParB+HNH->N6-METHYLASE->PglZ->SIGMA+OB-Fold+AAA-ATPase+Lon-Protease-BrxL-><-Arm-DNA-bind_3+Phage_integrase<-tRNA||?-><-SKI||METHYLASE->	1233	Bacteria>Pseudomonadota>Gammaproteobacteria>Enterobacterales>Enterobacteriaceae>Citrobacter	GCA_002239605.1	Citrobacter_braakii	B9P84_05295	62196..65897	57706	DNA-repair-protein-[Citrobacter-braakii]
QLR26611.1	Type1.BREX	<-Sulfatase<-?<-?<-?<-Schlafen+HATPase+wHTH<-PHP+SbcC<-?||BrxA-DNA-binding->BrxB-BrxF-iSTAND->DUF6079*->ParB+HNH->N6-METHYLASE->HEPN-><-HIN-HTH||N6-METHYLASE-p->N6-METHYLASE-p->PglZ->SIGMA+OB-Fold+AAA-ATPase+Lon-Protease-BrxL-><-HTH_23+HTH_32+HTH_33+DDE_3	1233	Bacteria>Pseudomonadota>Gammaproteobacteria>Enterobacterales>Enterobacteriaceae>Citrobacter	GCA_013781985.1	Citrobacter_sp._RHBSTW-01013	HV350_21470	4470372..4474073	2742677	BREX-system-P-loop-protein-BrxC-[Citrobacter-sp.-RHBSTW-01013]
MBA8131541.1	Type1.BREX	<-Schlafen+HATPase+wHTH<-PHP+SbcC<-?||BrxA-DNA-binding->BrxB-BrxF-iSTAND->DUF6079*->ParB+HNH->N6-METHYLASE->PglZ->SIGMA+OB-Fold+AAA-ATPase+Lon-Protease-BrxL-><-Arm-DNA-bind_3+Phage_integrase<-tRNA||?-><-SKI||METHYLASE->	1233	Bacteria>Pseudomonadota>Gammaproteobacteria>Enterobacterales>Enterobacteriaceae>Citrobacter	GCA_014104375.1	Citrobacter_sp._RHBSTW-00013	HV034_17810	3734345..3738046	2742628	BREX-system-P-loop-protein-BrxC-[Citrobacter-sp.-RHBSTW-00013]
MBM9568541.1	Type1.BREX	<-wHTH+WYL<-DUF932<-nSTAND1+GTPase-AIG+sGTP<-CopG/RHH||XIS-HTH->?->?->BrxA-DNA-binding->BrxB-BrxF-iSTAND->DUF6079*->ParB+HNH->N6-METHYLASE->HEPN->RVT->N6-METHYLASE->PglZ->SIGMA+OB-Fold+AAA-ATPase+Lon-Protease-BrxL-><-Arm-DNA-bind_3+Phage_integrase	1233	Bacteria>Pseudomonadota>Gammaproteobacteria>Enterobacterales>Enterobacteriaceae>Citrobacter	GCA_014931625.2	Citrobacter_sedlakii	INP82_14060	158441..162142	67826	BREX-system-P-loop-protein-BrxC-[Citrobacter-sedlakii]
EGT0661408.1	Type1.BREX	REase+SPF-cons->?->?-><-?<-Schlafen+HATPase+wHTH<-PHP+SbcC<-?||BrxA-DNA-binding->BrxB-BrxF-iSTAND->DUF6079*->ParB+HNH->N6-METHYLASE->N6-METHYLASE-p->PglZ->SIGMA+OB-Fold+AAA-ATPase+Lon-Protease-BrxL-><-Arm-DNA-bind_3+Phage_integrase<-tRNA||?->HerA/FtsK+A32->	1233	Bacteria>Pseudomonadota>Gammaproteobacteria>Enterobacterales>Enterobacteriaceae>Citrobacter	GCA_015942525.1	Citrobacter_werkmanii	JAF83_001575	366383..370084	67827	BREX-system-P-loop-protein-BrxC-[Citrobacter-werkmanii]
HAT7499716.1	Type1.BREX	<-Peptidase_M17_N+Peptidase_M17||?->?-><-HerA/FtsK+A32<-?||tRNA->Arm-DNA-bind_3+Phage_integrase->wHTH+WYL+WCX->BrxA-DNA-binding->BrxB-BrxF-iSTAND->DUF6079*->N6-METHYLASE->ParB+HNH->PglZ-><-SF1-SF2-Helicase+REase<-SF1-SF2-Helicase+REase||NACHT+SF1-SF2-Helicase+HKD-DNase-><-?<-Glyoxalase<-ZnR	1157	Bacteria>Pseudomonadota>Gammaproteobacteria>Enterobacterales>Enterobacteriaceae>Citrobacter	GCA_015956985.1	Citrobacter_freundii	JAV71_002653	147091..150564	546	BREX-system-P-loop-protein-BrxC-[Citrobacter-freundii]
SYX30363.1	Type1.BREX	GDResolvase+HIN-HTH->MPTase-><-SIGMA+OB-Fold+AAA-ATPase+Lon-Protease-BrxL<-PglZ<-PglZ<-?<-N6-METHYLASE<-ParB+HNH<-DUF6079*<-BrxB-BrxF-iSTAND<-BrxA-DNA-binding<-ZnR<-?<-?<-?<-XIS-HTH<-?||?->nSTAND1+GTPase-AIG+sGTP->	1233	Bacteria>Pseudomonadota>Gammaproteobacteria>Enterobacterales>Enterobacteriaceae>Citrobacter	GCA_900520375.1	Citrobacter_freundii	CFU2785_04435	4465123..4468824	546	Uncharacterised-protein-[Citrobacter-freundii]
WP_231587318.1	Type1.BREX	<-wHTH+WYL<-nSTAND1+sGTP<-?||XIS-HTH->?->?->BrxA-DNA-binding->BrxB-BrxF-iSTAND->DUF6079*->ParB+HNH->N6-METHYLASE->N6-METHYLASE-p->PglZ->SIGMA+OB-Fold+AAA-ATPase+Lon-Protease-BrxL-><-Arm-DNA-bind_3+Phage_integrase<-tRNA||?->HerA/FtsK+A32->	1240	Bacteria>Pseudomonadota>Gammaproteobacteria>Enterobacterales>Enterobacteriaceae>Citrobacter	GCF_001037515.1	Citrobacter_sp._MGH110	SK38_RS14780	3143856..3147578	1686383	BREX-system-P-loop-protein-BrxC-[Citrobacter-sp.-MGH110]
WP_249418019.1	Type1.BREX	<-Sulfatase<-?<-?<-?<-Schlafen+HATPase+wHTH<-PHP+SbcC<-?||BrxA-DNA-binding->BrxB-BrxF-iSTAND->DUF6079*->ParB+HNH->N6-METHYLASE->HEPN->RVT-><-HIN-HTH||RVT->N6-METHYLASE-p->N6-METHYLASE-p->PglZ->	1240	Bacteria>Pseudomonadota>Gammaproteobacteria>Enterobacterales>Enterobacteriaceae>Citrobacter	GCF_013781985.1	Citrobacter_sp._RHBSTW-01013	HV350_RS21465	4470351..4474073	2742677	BREX-system-P-loop-protein-BrxC-[Citrobacter-sp.-RHBSTW-01013]
WP_249424276.1	Type1.BREX	<-Schlafen+HATPase+wHTH<-PHP+SbcC<-?||BrxA-DNA-binding->BrxB-BrxF-iSTAND->DUF6079*->ParB+HNH->N6-METHYLASE->PglZ->SIGMA+OB-Fold+AAA-ATPase+Lon-Protease-BrxL-><-Arm-DNA-bind_3+Phage_integrase<-tRNA||?-><-SKI||METHYLASE->	1240	Bacteria>Pseudomonadota>Gammaproteobacteria>Enterobacterales>Enterobacteriaceae>Citrobacter	GCF_014104375.1	Citrobacter_sp._RHBSTW-00013	HV034_RS17810	3734324..3738046	2742628	BREX-system-P-loop-protein-BrxC-[Citrobacter-sp.-RHBSTW-00013]
WP_200054816.1	Type1.BREX	<-Peptidase_M17_N+Peptidase_M17||?->?-><-HerA/FtsK+A32<-?||tRNA->Arm-DNA-bind_3+Phage_integrase->wHTH+WYL+WCX->BrxA-DNA-binding->BrxB-BrxF-iSTAND->DUF6079*->N6-METHYLASE->ParB+HNH->PglZ-><-SF1-SF2-Helicase+REase<-SF1-SF2-Helicase+REase||NACHT+SF1-SF2-Helicase+HKD-DNase-><-ZnR<-XIS-HTH	1157	Bacteria>Pseudomonadota>Gammaproteobacteria>Enterobacterales>Enterobacteriaceae>Citrobacter	GCF_016504925.1	Citrobacter_freundii	GHT45_RS09240	237227..240700	546	BREX-system-P-loop-protein-BrxC-[Citrobacter-freundii]
WP_205688206.1	Type1.BREX	<-MAD-NTD+HNH<-HTH_Tnp_1+HTH_21+rve+rve_3||?->?->SF1-SF2-Helicase+REase->SF1-SF2-Helicase+REase-><-PglZ<-ParB+HNH<-N6-METHYLASE<-DUF6079*<-BrxB-BrxF-iSTAND<-BrxA-DNA-binding<-Arm-DNA-bind_3+Phage_integrase<-tRNA||?->HerA/FtsK+A32-><-?<-?<-?||Peptidase_M17_N+Peptidase_M17->	1157	Bacteria>Pseudomonadota>Gammaproteobacteria>Enterobacterales>Enterobacteriaceae>Citrobacter	GCF_017086505.1	Citrobacter_freundii	GHT30_RS20265	4262904..4266377	546	BREX-system-P-loop-protein-BrxC-[Citrobacter-freundii]
WP_275379687.1	Type1.BREX	Arm-DNA-bind_3+Phage_integrase-><-SIGMA+OB-Fold+AAA-ATPase+Lon-Protease-BrxL<-PglZ<-N6-METHYLASE-p<-RVT<-HEPN<-N6-METHYLASE<-ParB+HNH<-DUF6079*<-BrxB-BrxF-iSTAND<-BrxA-DNA-binding<-?<-?<-?<-XIS-HTH<-?||?->nSTAND1+GTPase-AIG+sGTP->DUF932->	1240	Bacteria>Pseudomonadota>Gammaproteobacteria>Enterobacterales>Enterobacteriaceae>Citrobacter	GCF_029104295.1	Citrobacter_freundii	L2W90_RS09965	166217..169939	546	BREX-system-P-loop-protein-BrxC-[Citrobacter-freundii]
PUV47226.1	Type1.BREX	<-wHTH+WYL<-sGTP<-?||XIS-HTH->?->?->BrxA-DNA-binding->BrxB-BrxF-iSTAND->DUF6079*->ParB+HNH->N6-METHYLASE->PglZ->SIGMA+OB-Fold+AAA-ATPase+Lon-Protease-BrxL-><-Arm-DNA-bind_3+Phage_integrase<-?<-tRNA||wHTH(SBD)+HNH->SAM-METHYLASE->	1233	Bacteria>Pseudomonadota>Gammaproteobacteria>Enterobacterales>Enterobacteriaceae>Cronobacter	GCA_002532375.1	Cronobacter_sakazakii	CDU02_01545	336773..340474	28141	BREX-system-P-loop-protein-BrxC-[Cronobacter-sakazakii]
KAB0815149.1	Type1.BREX	<-wHTH(SBD)+HNH||tRNA->Arm-DNA-bind_3+Phage_integrase-><-SF1-SF2-Helicase||OLD-ABC+TOPRIM->SF1-SF2-Helicase-><-SIGMA+OB-Fold+AAA-ATPase+Lon-Protease-BrxL<-PglZ<-N6-METHYLASE<-ParB+HNH<-DUF6079*<-BrxB-BrxF-iSTAND<-BrxA-DNA-binding||TIR-><-?<-XIS-HTH||?->nSTAND1+GTPase-AIG+sGTP->wHTH+WYL->	1233	Bacteria>Pseudomonadota>Gammaproteobacteria>Enterobacterales>Enterobacteriaceae>Cronobacter	GCA_008805175.1	Cronobacter_sakazakii	FZI24_17905	37972..41673	28141	BREX-system-P-loop-protein-BrxC-[Cronobacter-sakazakii]
WP_239984508.1	Type1.BREX	<-wHTH+WYL<-GTPase-AIG<-?||XIS-HTH->?-><-TIR||BrxA-DNA-binding->BrxB-BrxF-iSTAND->DUF6079*->ParB+HNH->N6-METHYLASE->PglZ->SIGMA+OB-Fold+AAA-ATPase+Lon-Protease-BrxL-><-SF1-SF2-Helicase<-OLD-ABC+TOPRIM||SF1-SF2-Helicase-><-?<-HIN-HTH	1240	Bacteria>Pseudomonadota>Gammaproteobacteria>Enterobacterales>Enterobacteriaceae>Cronobacter	GCF_002942265.1	Cronobacter_sakazakii	C3D67_RS10955	271432..275154	28141	BREX-system-P-loop-protein-BrxC-[Cronobacter-sakazakii]
WP_161575571.1	Type1.BREX	<-Peptidase_M17_N+Peptidase_M17||?->?->?->?-><-?||tRNA->Arm-DNA-bind_3+Phage_integrase->BrxA-DNA-binding->BrxB-BrxF-iSTAND->DUF6079*->N6-METHYLASE->ParB+HNH->PglZ-><-SF1-SF2-Helicase+REase<-SF1-SF2-Helicase+REase<-?<-XIS-HTH	1157	Bacteria>Pseudomonadota>Gammaproteobacteria>Enterobacterales>Enterobacteriaceae>Cronobacter	GCF_009938355.1	Cronobacter_sakazakii	EHJ08_RS11755	205127..208600	28141	BREX-system-P-loop-protein-BrxC-[Cronobacter-sakazakii]
WP_239978191.1	Type1.BREX	<-SAM-METHYLASE<-wHTH(SBD)+HNH||tRNA->Arm-DNA-bind_3+Phage_integrase-><-SIGMA+OB-Fold+AAA-ATPase+Lon-Protease-BrxL<-PglZ<-N6-METHYLASE<-ParB+HNH<-DUF6079*<-BrxB-BrxF-iSTAND<-BrxA-DNA-binding<-?<-?<-XIS-HTH||?->sGTP->wHTH+WYL->	1240	Bacteria>Pseudomonadota>Gammaproteobacteria>Enterobacterales>Enterobacteriaceae>Cronobacter	GCF_017845055.1	Cronobacter_sakazakii	KAR72_RS09245	217726..221448	28141	BREX-system-P-loop-protein-BrxC-[Cronobacter-sakazakii]
CAF2998376.1	Type1.BREX	Arm-DNA-bind_3+Phage_integrase-><-SIGMA+OB-Fold+AAA-ATPase+Lon-Protease-BrxL<-PglZ<-N6-METHYLASE-p<-RVT<-HEPN<-N6-METHYLASE<-ParB+HNH<-DUF6079*<-BrxB-BrxF-iSTAND<-BrxA-DNA-binding<-Glyoxalase<-ZnR<-SAM-METHYLASE<-YjhX_toxin<-Glyoxalase<-?<-Glycoside_Hydrolase	1240	Bacteria>Pseudomonadota>Gammaproteobacteria>Enterobacterales>Enterobacteriaceae>Enterobacter	-	Enterobacter_cloacae	AI2972V1_0508	557695..561417	550	hypothetical-protein-AI2972V1_0508-[Enterobacter-cloacae]
WP_129258922.1	Type1.BREX	<-CopG/RHH||XIS-HTH->?->?->SF1-SF2-Helicase+REase->SF1-SF2-Helicase+REase-><-PglZ<-ParB+HNH<-N6-METHYLASE<-DUF6079*<-BrxB-BrxF-iSTAND<-BrxA-DNA-binding<-wHTH+WYL+WCX<-Arm-DNA-bind_3+Phage_integrase	1157	Bacteria>Pseudomonadota>Gammaproteobacteria>Enterobacterales>Enterobacteriaceae>Enterobacter	-	Enterobacter_cloacae_complex_sp._GF14B	DD606_17375	24450..27923	2511982	BREX-system-P-loop-protein-BrxC-[Enterobacter-cloacae-complex-sp.-GF14B]
WP_039268698.1	Type1.BREX	Arm-DNA-bind_3+Phage_integrase-><-SIGMA+OB-Fold+AAA-ATPase+Lon-Protease-BrxL<-PglZ<-N6-METHYLASE-p<-RVT<-HEPN<-ParB+HNH<-DUF6079*<-BrxB-BrxF-iSTAND<-BrxA-DNA-binding<-Glyoxalase<-ZnR<-SAM-METHYLASE<-YjhX_toxin<-Glyoxalase<-?<-Glycoside_Hydrolase	1233	Bacteria>Pseudomonadota>Gammaproteobacteria>Enterobacterales>Enterobacteriaceae>Enterobacter	-	Enterobacter_hormaechei	LI66_02640	557969..561670	158836	MULTISPECIES:-BREX-system-P-loop-protein-BrxC-[Enterobacter]
WP_181507052.1	Type1.BREX	REase+SPF-cons->?->?-><-?<-Schlafen+HATPase+wHTH<-PHP+SbcC<-?||BrxA-DNA-binding->BrxB-BrxF-iSTAND->DUF6079*->ParB+HNH->N6-METHYLASE->N6-METHYLASE-p->PglZ->SIGMA+OB-Fold+AAA-ATPase+Lon-Protease-BrxL-><-Arm-DNA-bind_3+Phage_integrase<-tRNA||?->HerA/FtsK+A32->	1233	Bacteria>Pseudomonadota>Gammaproteobacteria>Enterobacterales>Enterobacteriaceae>Enterobacter	-	Enterobacter_roggenkampii	JAF83_001575	366383..370084	1812935	MULTISPECIES:-BREX-system-P-loop-protein-BrxC-[Enterobacteriaceae]
AFP68425.1	Type1.BREX	Arm-DNA-bind_3+Phage_integrase-><-SIGMA+OB-Fold+AAA-ATPase+Lon-Protease-BrxL<-PglZ<-N6-METHYLASE-p<-RVT<-HEPN<-ParB+HNH<-DUF6079*<-BrxB-BrxF-iSTAND<-BrxA-DNA-binding||?->?-><-?<-?<-Glyoxalase<-ZnR<-METHYLASE	1233	Bacteria>Pseudomonadota>Gammaproteobacteria>Enterobacterales>Enterobacteriaceae>Enterobacter	GCA_000286275.1	Enterobacter_kobei	ECENHK_02660	581253..584954	208224	hypothetical-protein-ECENHK_02660-[Enterobacter-kobei]
AJB80307.1	Type1.BREX	Arm-DNA-bind_3+Phage_integrase-><-SIGMA+OB-Fold+AAA-ATPase+Lon-Protease-BrxL<-PglZ<-N6-METHYLASE-p<-RVT<-HEPN<-ParB+HNH<-DUF6079*<-BrxB-BrxF-iSTAND<-BrxA-DNA-binding<-Glyoxalase<-ZnR<-SAM-METHYLASE<-YjhX_toxin<-Glyoxalase<-?<-Glycoside_Hydrolase	1233	Bacteria>Pseudomonadota>Gammaproteobacteria>Enterobacterales>Enterobacteriaceae>Enterobacter	GCA_000814225.1	Enterobacter_hormaechei_subsp._xiangfangensis	LI66_02640	557969..561670	1296536	DNA-repair-protein-[Enterobacter-hormaechei-subsp.-xiangfangensis]
KZR28106.1	Type1.BREX	<-wHTH+WYL<-sGTP<-?||XIS-HTH->?->?-><-?||BrxA-DNA-binding->BrxB-BrxF-iSTAND->DUF6079*->ParB+HNH->N6-METHYLASE->N6-METHYLASE-p->PglZ->SIGMA+OB-Fold+AAA-ATPase+Lon-Protease-BrxL-><-Arm-DNA-bind_3+Phage_integrase	1233	Bacteria>Pseudomonadota>Gammaproteobacteria>Enterobacterales>Enterobacteriaceae>Enterobacter	GCA_001631925.1	Enterobacter_hormaechei_subsp._steigerwaltii	A3N67_04030	129053..132754	299766	DNA-repair-protein-[Enterobacter-hormaechei-subsp.-steigerwaltii]
AWF32098.1	Type1.BREX	Arm-DNA-bind_3+Phage_integrase-><-SIGMA+OB-Fold+AAA-ATPase+Lon-Protease-BrxL<-PglZ<-N6-METHYLASE-p<-N6-METHYLASE<-ParB+HNH<-DUF6079*<-BrxA-DNA-binding<-?<-?||?-><-?||?->sGTP->wHTH+WYL->	1233	Bacteria>Pseudomonadota>Gammaproteobacteria>Enterobacterales>Enterobacteriaceae>Enterobacter	GCA_003073995.1	Enterobacter_hormaechei	CSC19_3711	3849441..3853142	158836	hypothetical-protein-CSC19_3711-[Enterobacter-hormaechei]
MBG0523800.1	Type1.BREX	Arm-DNA-bind_3+Phage_integrase-><-SIGMA+OB-Fold+AAA-ATPase+Lon-Protease-BrxL<-PglZ<-N6-METHYLASE-p<-RVT<-HEPN<-N6-METHYLASE<-ParB+HNH<-DUF6079*<-BrxB-BrxF-iSTAND<-BrxA-DNA-binding<-HIN-HTH<-REase<-wHTH(SBD)+HNH||SymE_toxin->	1233	Bacteria>Pseudomonadota>Gammaproteobacteria>Enterobacterales>Enterobacteriaceae>Enterobacter	GCA_015704905.1	Enterobacter_cloacae	IOR24_17915	3774454..3778155	550	BREX-system-P-loop-protein-BrxC-[Enterobacter-cloacae]
MBK3145473.1	Type1.BREX	Glyoxalase->ACET->YjhX_toxin->SAM-METHYLASE->ZnR->Glyoxalase->BrxA-DNA-binding->BrxB-BrxF-iSTAND->DUF6079*->ParB+HNH->N6-METHYLASE->HEPN->RVT->N6-METHYLASE-p->PglZ->SIGMA+OB-Fold+AAA-ATPase+Lon-Protease-BrxL-><-Arm-DNA-bind_3+Phage_integrase	1233	Bacteria>Pseudomonadota>Gammaproteobacteria>Enterobacterales>Enterobacteriaceae>Enterobacter	GCA_016616795.1	Enterobacter_hormaechei	FKK11_12155	273388..277089	158836	BREX-system-P-loop-protein-BrxC-[Enterobacter-hormaechei]
HBL8846881.1	Type1.BREX	DUF6079*->ParB+HNH->N6-METHYLASE->PglZ->SIGMA+OB-Fold+AAA-ATPase+Lon-Protease-BrxL->	1089	Bacteria>Pseudomonadota>Gammaproteobacteria>Enterobacterales>Enterobacteriaceae>Enterobacter	GCA_021095225.1	Enterobacter_hormaechei	LTW48_004335	1..3271	158836	BREX-system-P-loop-protein-BrxC-[Enterobacter-hormaechei]
HCM9559898.1	Type1.BREX	Glyco_hydro_43+GH43_C2-><-ICLR-HTH<-?||?->?->?->tRNA->BrxA-DNA-binding->BrxB-BrxF-iSTAND->DUF6079*->N6-METHYLASE->ParB+HNH->PglZ-><-SF1-SF2-Helicase+REase<-SF1-SF2-Helicase+REase||HTH_Tnp_1+HTH_21+rve+rve_3->?->REase->	1157	Bacteria>Pseudomonadota>Gammaproteobacteria>Enterobacterales>Enterobacteriaceae>Enterobacter	GCA_025214055.1	Enterobacter_cloacae_subsp._cloacae	N5D13_002966	176473..179946	336306	BREX-system-P-loop-protein-BrxC-[Enterobacter-cloacae-subsp.-cloacae]
WP_228371847.1	Type1.BREX	Arm-DNA-bind_3+Phage_integrase-><-SIGMA+OB-Fold+AAA-ATPase+Lon-Protease-BrxL<-PglZ<-N6-METHYLASE-p<-RVT<-HEPN<-N6-METHYLASE<-ParB+HNH<-DUF6079*<-BrxB-BrxF-iSTAND<-BrxA-DNA-binding||?-><-?<-Glyoxalase<-ZnR<-METHYLASE<-YjhX_toxin	1240	Bacteria>Pseudomonadota>Gammaproteobacteria>Enterobacterales>Enterobacteriaceae>Enterobacter	GCF_000286275.1	Enterobacter_kobei	ECENHK_RS02760	581253..584975	208224	BREX-system-P-loop-protein-BrxC-[Enterobacter-kobei]
WP_234704204.1	Type1.BREX	Arm-DNA-bind_3+Phage_integrase-><-SIGMA+OB-Fold+AAA-ATPase+Lon-Protease-BrxL<-PglZ<-N6-METHYLASE-p<-RVT<-HEPN<-N6-METHYLASE<-ParB+HNH<-DUF6079*<-BrxB-BrxF-iSTAND<-BrxA-DNA-binding<-Glyoxalase<-ZnR<-SAM-METHYLASE<-YjhX_toxin<-ACET<-Glyoxalase	1240	Bacteria>Pseudomonadota>Gammaproteobacteria>Enterobacterales>Enterobacteriaceae>Enterobacter	GCF_000814225.1	Enterobacter_cloacae_complex	LI66_RS02640	557969..561691	354276	MULTISPECIES:-BREX-system-P-loop-protein-BrxC-[Enterobacter-cloacae-complex]
WP_230373248.1	Type1.BREX	<-wHTH+WYL<-sGTP<-?||XIS-HTH->?->?->BrxA-DNA-binding->BrxB-BrxF-iSTAND->DUF6079*->ParB+HNH->N6-METHYLASE->N6-METHYLASE-p->PglZ->SIGMA+OB-Fold+AAA-ATPase+Lon-Protease-BrxL-><-Arm-DNA-bind_3+Phage_integrase	1240	Bacteria>Pseudomonadota>Gammaproteobacteria>Enterobacterales>Enterobacteriaceae>Enterobacter	GCF_001631925.1	Enterobacter_hormaechei	A3N67_RS02925	129032..132754	158836	BREX-system-P-loop-protein-BrxC-[Enterobacter-hormaechei]
WP_237764241.1	Type1.BREX	Arm-DNA-bind_3+Phage_integrase-><-SIGMA+OB-Fold+AAA-ATPase+Lon-Protease-BrxL<-PglZ<-N6-METHYLASE-p<-N6-METHYLASE<-ParB+HNH<-DUF6079*<-BrxB-BrxF-iSTAND<-BrxA-DNA-binding<-?<-?<-XIS-HTH||?->sGTP->wHTH+WYL->	1240	Bacteria>Pseudomonadota>Gammaproteobacteria>Enterobacterales>Enterobacteriaceae>Enterobacter	GCF_003073995.1	Enterobacter_hormaechei	CSC19_RS19005	3849441..3853163	158836	BREX-system-P-loop-protein-BrxC-[Enterobacter-hormaechei]
WP_110875386.1	Type1.BREX	<-CopG/RHH||XIS-HTH->?->?->SF1-SF2-Helicase+REase->SF1-SF2-Helicase+REase-><-PglZ<-ParB+HNH<-N6-METHYLASE<-DUF6079*<-BrxB-BrxF-iSTAND<-BrxA-DNA-binding<-wHTH+WYL+WCX<-Arm-DNA-bind_3+Phage_integrase	1157	Bacteria>Pseudomonadota>Gammaproteobacteria>Enterobacterales>Enterobacteriaceae>Enterobacter	GCF_003207215.1	Enterobacter_hormaechei	DOM70_RS06250	341163..344636	158836	BREX-system-P-loop-protein-BrxC-[Enterobacter-hormaechei]
WP_125357895.1	Type1.BREX	<-SIGMA+OB-Fold+AAA-ATPase+Lon-Protease-BrxL<-PglZ<-N6-METHYLASE<-ParB+HNH<-DUF6079*<-BrxB-BrxF-iSTAND<-BrxA-DNA-binding<-wHTH+WYL+WCX<-Phage_integrase<-?<-ArdCN+MPTase<-?||HEPN-><-Phage_int_SAM_2+Integrase_1	1225	Bacteria>Pseudomonadota>Gammaproteobacteria>Enterobacterales>Enterobacteriaceae>Enterobacter	GCF_003937295.2	Enterobacter_hormaechei	EGK38_RS19825	4108658..4112335	158836	BREX-system-P-loop-protein-BrxC-[Enterobacter-hormaechei]
WP_126349337.1	Type1.BREX	Glyco_hydro_43+GH43_C2-><-ICLR-HTH<-?<-?||?->?->?->tRNA->BrxA-DNA-binding->BrxB-BrxF-iSTAND->DUF6079*->N6-METHYLASE->ParB+HNH->PglZ-><-SF1-SF2-Helicase+REase<-SF1-SF2-Helicase+REase	1157	Bacteria>Pseudomonadota>Gammaproteobacteria>Enterobacterales>Enterobacteriaceae>Enterobacter	GCF_003965695.1	Enterobacter_roggenkampii	EKN52_RS20255	23415..26888	1812935	BREX-system-P-loop-protein-BrxC-[Enterobacter-roggenkampii]
WP_126800556.1	Type1.BREX	SF1-SF2-Helicase+REase->SF1-SF2-Helicase+REase-><-PglZ<-ParB+HNH<-N6-METHYLASE<-DUF6079*<-BrxB-BrxF-iSTAND<-BrxA-DNA-binding<-Arm-DNA-bind_3+Phage_integrase<-tRNA<-?<-?<-?||?->?->ICLR-HTH->	1157	Bacteria>Pseudomonadota>Gammaproteobacteria>Enterobacterales>Enterobacteriaceae>Enterobacter	GCF_003986875.1	Enterobacter_sp._WCHEn090032	EKN83_RS06700	53011..56484	2497435	BREX-system-P-loop-protein-BrxC-[Enterobacter-sp.-WCHEn090032]
WP_129694025.1	Type1.BREX	<-REase<-?<-HTH_Tnp_1+HTH_21+rve+rve_3||SF1-SF2-Helicase+REase->SF1-SF2-Helicase+REase-><-PglZ<-ParB+HNH<-N6-METHYLASE<-DUF6079*<-BrxB-BrxF-iSTAND<-BrxA-DNA-binding<-tRNA<-?<-?<-?||?->?->ICLR-HTH-><-Glyco_hydro_43+GH43_C2	1157	Bacteria>Pseudomonadota>Gammaproteobacteria>Enterobacterales>Enterobacteriaceae>Enterobacter	GCF_004138605.1	Enterobacter_roggenkampii	D8675_RS06390	1085829..1089302	1812935	BREX-system-P-loop-protein-BrxC-[Enterobacter-roggenkampii]
WP_164474334.1	Type1.BREX	SF1-SF2-Helicase+REase->SF1-SF2-Helicase+REase-><-PglZ<-ParB+HNH<-N6-METHYLASE<-N6-METHYLASE<-DUF6079*<-BrxB-BrxF-iSTAND<-BrxA-DNA-binding<-Arm-DNA-bind_3+Phage_integrase	1157	Bacteria>Pseudomonadota>Gammaproteobacteria>Enterobacterales>Enterobacteriaceae>Enterobacter	GCF_011045335.1	Enterobacter_hormaechei	G5C52_RS10105	2135134..2138607	158836	BREX-system-P-loop-protein-BrxC-[Enterobacter-hormaechei]
WP_230863208.1	Type1.BREX	Arm-DNA-bind_3+Phage_integrase-><-SIGMA+OB-Fold+AAA-ATPase+Lon-Protease-BrxL<-PglZ<-N6-METHYLASE-p<-RVT<-HEPN<-N6-METHYLASE<-ParB+HNH<-DUF6079*<-BrxB-BrxF-iSTAND<-BrxA-DNA-binding<-REase<-wHTH(SBD)+HNH||SymE_toxin->	1240	Bacteria>Pseudomonadota>Gammaproteobacteria>Enterobacterales>Enterobacteriaceae>Enterobacter	GCF_015704905.1	Enterobacter_cloacae	IOR24_RS17915	3774454..3778176	550	BREX-system-P-loop-protein-BrxC-[Enterobacter-cloacae]
WP_234450174.1	Type1.BREX	Glycoside_Hydrolase->?->Glyoxalase->YjhX_toxin->SAM-METHYLASE->ZnR->Glyoxalase->BrxA-DNA-binding->BrxB-BrxF-iSTAND->DUF6079*->ParB+HNH->N6-METHYLASE->HEPN->RVT->N6-METHYLASE-p->PglZ->SIGMA+OB-Fold+AAA-ATPase+Lon-Protease-BrxL-><-Arm-DNA-bind_3+Phage_integrase	1240	Bacteria>Pseudomonadota>Gammaproteobacteria>Enterobacterales>Enterobacteriaceae>Enterobacter	GCF_016616795.1	Enterobacter_hormaechei	FKK11_RS12010	273367..277089	158836	BREX-system-P-loop-protein-BrxC-[Enterobacter-hormaechei]
WP_211449384.1	Type1.BREX	<-SymE_toxin||wHTH(SBD)+HNH-><-NACHT+SF1-SF2-Helicase+HKD-DNase||SF1-SF2-Helicase+REase->SF1-SF2-Helicase+REase-><-PglZ<-ParB+HNH<-N6-METHYLASE<-DUF6079*<-BrxB-BrxF-iSTAND<-BrxA-DNA-binding<-wHTH+WYL+WCX||ParA-Soj-PloopNTPase->?->DnaB+TnsC-ATPase->ParB+BAM->HTH+HTH->HTH_36->	1157	Bacteria>Pseudomonadota>Gammaproteobacteria>Enterobacterales>Enterobacteriaceae>Enterobacter	GCF_018299605.1	Enterobacter_mori	GUY16_RS21845	58984..62457	539813	BREX-system-P-loop-protein-BrxC-[Enterobacter-mori]
WP_230238963.1	Type1.BREX	Glyco_hydro_43+GH43_C2-><-ICLR-HTH<-?||?->?->?->tRNA->BrxA-DNA-binding->BrxB-BrxF-iSTAND->DUF6079*->N6-METHYLASE->ParB+HNH->PglZ-><-SF1-SF2-Helicase+REase<-SF1-SF2-Helicase+REase||?-><-REase<-wHTH(SBD)+HNH||SymE_toxin->	1157	Bacteria>Pseudomonadota>Gammaproteobacteria>Enterobacterales>Enterobacteriaceae>Enterobacter	GCF_020971765.1	Enterobacter_sp._AN-K1	LOC22_RS13480	2816173..2819646	2894203	BREX-system-P-loop-protein-BrxC-[Enterobacter-sp.-AN-K1]
WP_241512123.1	Type1.BREX	Arm-DNA-bind_3+Phage_integrase-><-SIGMA+OB-Fold+AAA-ATPase+Lon-Protease-BrxL<-PglZ<-N6-METHYLASE-p<-N6-METHYLASE<-ParB+HNH<-DUF6079*<-BrxB-BrxF-iSTAND<-BrxA-DNA-binding<-Glyoxalase<-ZnR<-METHYLASE<-YjhX_toxin<-ACET<-Glyoxalase	1240	Bacteria>Pseudomonadota>Gammaproteobacteria>Enterobacterales>Enterobacteriaceae>Enterobacter	GCF_022585395.1	Enterobacter_hormaechei	MM050_RS10500	125167..128889	158836	BREX-system-P-loop-protein-BrxC-[Enterobacter-hormaechei]
WP_248076370.1	Type1.BREX	YjhX_toxin->SAM-METHYLASE->ZnR->Glyoxalase->BrxA-DNA-binding->BrxB-BrxF-iSTAND->DUF6079*->ParB+HNH->N6-METHYLASE->N6-METHYLASE-p->PglZ->SIGMA+OB-Fold+AAA-ATPase+Lon-Protease-BrxL-><-Arm-DNA-bind_3+Phage_integrase||Phage_integrase->REase->DNA_METHYLASE->	1240	Bacteria>Pseudomonadota>Gammaproteobacteria>Enterobacterales>Enterobacteriaceae>Enterobacter	GCF_023153855.1	Enterobacter_cloacae	L8P05_RS09265	348723..352445	550	BREX-system-P-loop-protein-BrxC-[Enterobacter-cloacae]
WP_095526333.1	Type1.BREX	<-35exo<-SIGMA+OB-Fold+AAA-ATPase+Lon-Protease-BrxL<-PglZ<-N6-METHYLASE<-ParB+HNH<-DUF6079*<-BrxB-BrxF-iSTAND<-BrxA-DNA-binding<-wHTH+WYL+WCX<-XIS-HTH<-ZnR||?-><-?||?->ArdB->	1225	Bacteria>Pseudomonadota>Gammaproteobacteria>Enterobacterales>Enterobacteriaceae>Escherichia	-	Escherichia_coli	CDN88_RS21130	10739..14416	562	BREX-system-P-loop-protein-BrxC-[Escherichia-coli]
WP_121868993.1	Type1.BREX	<-HerA/FtsK+A32<-?||tRNA->Arm-DNA-bind_3+Phage_integrase-><-SIGMA+OB-Fold+AAA-ATPase+Lon-Protease-BrxL<-PglZ<-N6-METHYLASE-p<-N6-METHYLASE<-ParB+HNH<-DUF6079*<-BrxB-BrxF-iSTAND<-BrxA-DNA-binding<-?<-?<-XIS-HTH||?->nSTAND1+sGTP->wHTH+WYL->	1233	Bacteria>Pseudomonadota>Gammaproteobacteria>Enterobacterales>Enterobacteriaceae>Escherichia	-	Escherichia_coli	EAI39_15065	84115..87816	562	BREX-system-P-loop-protein-BrxC-[Escherichia-coli]
WP_174580485.1	Type1.BREX	DDE_Tnp_IS1->?->ZnR->BrxA-DNA-binding->BrxB-BrxF-iSTAND->DUF6079*->ParB+HNH->N6-METHYLASE->HEPN->RVT->N6-METHYLASE-p->PglZ->SIGMA+OB-Fold+AAA-ATPase+Lon-Protease-BrxL-><-Arm-DNA-bind_3+Phage_integrase||Phage_integrase->	1233	Bacteria>Pseudomonadota>Gammaproteobacteria>Enterobacterales>Enterobacteriaceae>Escherichia	-	Escherichia_coli	HUC40_15465	2945..6646	562	BREX-system-P-loop-protein-BrxC-[Escherichia-coli]
MCN3919819.1	Type1.BREX	<-METHYLASE||SKI-><-?||tRNA->Arm-DNA-bind_3+Phage_integrase-><-SIGMA+OB-Fold+AAA-ATPase+Lon-Protease-BrxL<-PglZ<-N6-METHYLASE<-ParB+HNH<-DUF6079*<-BrxB-BrxF-iSTAND<-BrxA-DNA-binding<-?<-?<-XIS-HTH||?->wHTH+WYL->	1240	Bacteria>Pseudomonadota>Gammaproteobacteria>Enterobacterales>Enterobacteriaceae>Escherichia	-	Escherichia_coli	MLZ57_18615	66742..70464	562	BREX-system-P-loop-protein-BrxC-[Escherichia-coli]
KEM88219.1	Type1.BREX	<-HTH_Tnp_1+HTH_21+rve+rve_3<-HTH_Tnp_1||wHTH+WYL+WCX->BrxA-DNA-binding->BrxB-BrxF-iSTAND->DUF6079-p->DUF6079-p*->ParB+HNH->N6-METHYLASE->PglZ->SIGMA+OB-Fold+AAA-ATPase+Lon-Protease-BrxL->35exo->?-><-HTH_21+rve+rve_3<-HIN-HTH||DDE_Tnp_IS1->	947	Bacteria>Pseudomonadota>Gammaproteobacteria>Enterobacterales>Enterobacteriaceae>Escherichia	GCA_000713255.1	Escherichia_coli_6-537-08_S4_C1	AC92_4028	7171..10014	1444227	putative-aTPase-like-protein-[Escherichia-coli-6-537-08_S4_C1]
OWF25643.1	Type1.BREX	DUF6079-p*->ParB+HNH->N6-METHYLASE-p->PglZ->SIGMA+OB-Fold+AAA-ATPase+Lon-Protease-BrxL-><-HTH_Tnp_1+HTH_21+rve+rve_3	902	Bacteria>Pseudomonadota>Gammaproteobacteria>Enterobacterales>Enterobacteriaceae>Escherichia	GCA_002190095.1	Escherichia_coli	A8M76_10400	1..2709	562	DNA-repair-protein,-partial-[Escherichia-coli]
POU04217.1	Type1.BREX	HNH-><-?||?->XIS-HTH->SF1-SF2-Helicase+REase->SF1-SF2-Helicase+REase-><-PglZ<-ParB+HNH<-N6-METHYLASE<-DUF6079*<-BrxB-BrxF-iSTAND<-BrxA-DNA-binding<-Arm-DNA-bind_3+Phage_integrase<-tRNA||wHTH(SBD)+HNH->SAM-METHYLASE->	1157	Bacteria>Pseudomonadota>Gammaproteobacteria>Enterobacterales>Enterobacteriaceae>Escherichia	GCA_002918705.1	Escherichia_sp._ESNIH1	C3369_02380	500495..503968	1985876	BREX-system-P-loop-protein-BrxC-[Escherichia-sp.-ESNIH1]
RLY30110.1	Type1.BREX	<-HerA/FtsK+A32<-?||tRNA->Arm-DNA-bind_3+Phage_integrase-><-SIGMA+OB-Fold+AAA-ATPase+Lon-Protease-BrxL<-PglZ<-N6-METHYLASE-p<-N6-METHYLASE<-ParB+HNH<-DUF6079*<-BrxB-BrxF-iSTAND<-BrxA-DNA-binding<-?<-?<-XIS-HTH||?->nSTAND1+sGTP->wHTH+WYL->	1233	Bacteria>Pseudomonadota>Gammaproteobacteria>Enterobacterales>Enterobacteriaceae>Escherichia	GCA_003687125.1	Escherichia_coli	EAI39_15065	84115..87816	562	BREX-system-P-loop-protein-BrxC-[Escherichia-coli]
TZF09961.1	Type1.BREX	RVT->DEDD_Tnp_IS110+Transposase_20-><-SIGMA+OB-Fold+AAA-ATPase+Lon-Protease-BrxL<-PglZ<-N6-METHYLASE<-ParB+HNH<-DUF6079*	1149	Bacteria>Pseudomonadota>Gammaproteobacteria>Enterobacterales>Enterobacteriaceae>Escherichia	GCA_008273745.1	Escherichia_coli	EOI42_22665	16149..19600	562	BREX-system-P-loop-protein-BrxC,-partial-[Escherichia-coli]
TZE05560.1	Type1.BREX	<-CopG/RHH||<-RHH||?->wHTH+WYL+WCX->BrxA-DNA-binding->BrxB-BrxF-iSTAND->DUF6079*->ParB+HNH->N6-METHYLASE->PglZ->SIGMA+OB-Fold+AAA-ATPase+Lon-Protease-BrxL-><-DEDD_Tnp_IS110+Transposase_20<-RVT	1225	Bacteria>Pseudomonadota>Gammaproteobacteria>Enterobacterales>Enterobacteriaceae>Escherichia	GCA_008273905.1	Escherichia_coli	EOI14_22605	9953..13630	562	BREX-system-P-loop-protein-BrxC-[Escherichia-coli]
EEW8735903.1	Type1.BREX	<-HIN-HTH||<-35exo<-SIGMA+OB-Fold+AAA-ATPase+Lon-Protease-BrxL<-PglZ<-N6-METHYLASE<-ParB+HNH<-DUF6079*<-BrxB-BrxF-iSTAND<-BrxA-DNA-binding<-wHTH+WYL+WCX<-ZnR	1221	Bacteria>Pseudomonadota>Gammaproteobacteria>Enterobacterales>Enterobacteriaceae>Escherichia	GCA_012099745.1	Escherichia_coli	D5Q61_02350	121702..125367	562	BREX-system-P-loop-protein-BrxC-[Escherichia-coli]
EEZ5714529.1	Type1.BREX	<-wHTH+WYL<-nSTAND1+sGTP<-?||XIS-HTH->?->?->ZnR->BrxA-DNA-binding->BrxB-BrxF-iSTAND->DUF6079*->ParB+HNH->N6-METHYLASE->HEPN->RVT->N6-METHYLASE-p->PglZ->SIGMA+OB-Fold+AAA-ATPase+Lon-Protease-BrxL-><-Arm-DNA-bind_3+Phage_integrase	1233	Bacteria>Pseudomonadota>Gammaproteobacteria>Enterobacterales>Enterobacteriaceae>Escherichia	GCA_012215525.1	Escherichia_coli_O25	DBU27_000026	20065..23766	1095709	BREX-system-P-loop-protein-BrxC-[Escherichia-coli-O25]
EFB3218130.1	Type1.BREX	<-Zn_Tnp_IS1+HTH_Tnp_IS1||wHTH+WYL+WCX->BrxA-DNA-binding->BrxB-BrxF-iSTAND->DUF6079*->ParB+HNH->N6-METHYLASE->PglZ->SIGMA+OB-Fold+AAA-ATPase+Lon-Protease-BrxL-><-HTH_Tnp_1+HTH_21+rve+rve_3<-HIN-HTH<-PIN<-MazE_antitoxin	1225	Bacteria>Pseudomonadota>Gammaproteobacteria>Enterobacterales>Enterobacteriaceae>Escherichia	GCA_012299345.1	Escherichia_coli	FLO28_20235	2574..6251	562	BREX-system-P-loop-protein-BrxC-[Escherichia-coli]
EFB6629151.1	Type1.BREX	<-SIGMA-HTH<-?<-ArsR-HTH||?-><-DDE_Tnp_1_5+DDE_Tnp_1||||wHTH+WYL+WCX->BrxA-DNA-binding->BrxB-BrxF-iSTAND->DUF6079*->ParB+HNH->N6-METHYLASE->PglZ->SIGMA+OB-Fold+AAA-ATPase+Lon-Protease-BrxL-><-HTH_Tnp_1+HTH_21+rve+rve_3<-HIN-HTH<-PIN<-MazE_antitoxin	1225	Bacteria>Pseudomonadota>Gammaproteobacteria>Enterobacterales>Enterobacteriaceae>Escherichia	GCA_012316585.1	Escherichia_coli	FQF26_18830	72071..75748	562	BREX-system-P-loop-protein-BrxC-[Escherichia-coli]
EFD0312067.1	Type1.BREX	GDResolvase+HIN-HTH-><-SIGMA+OB-Fold+AAA-ATPase+Lon-Protease-BrxL<-PglZ<-N6-METHYLASE-p<-RVT<-HEPN<-N6-METHYLASE<-ParB+HNH<-DUF6079*<-BrxB-BrxF-iSTAND<-BrxA-DNA-binding<-?<-?<-XIS-HTH<-?||?->nSTAND1+GTPase-AIG+sGTP->DUF932->	1233	Bacteria>Pseudomonadota>Gammaproteobacteria>Enterobacterales>Enterobacteriaceae>Escherichia	GCA_012380475.1	Escherichia_coli	E6D44_20535	19474..23175	562	BREX-system-P-loop-protein-BrxC-[Escherichia-coli]
EFH7369082.1	Type1.BREX	<-RelE-ParE<-CopG/RHH||<-RHH||?->wHTH+WYL+WCX->BrxA-DNA-binding->BrxB-BrxF-iSTAND->DUF6079*->ParB+HNH->N6-METHYLASE-p->	1225	Bacteria>Pseudomonadota>Gammaproteobacteria>Enterobacterales>Enterobacteriaceae>Escherichia	GCA_012614485.1	Escherichia_coli	GKQ19_21210	5665..9342	562	BREX-system-P-loop-protein-BrxC-[Escherichia-coli]
HAI3857478.1	Type1.BREX	DEDD_Tnp_IS110+Transposase_20-><-RHH||?->wHTH+WYL+WCX->BrxA-DNA-binding->BrxB-BrxF-iSTAND->DUF6079*->ParB+HNH->N6-METHYLASE->PglZ->SIGMA+OB-Fold+AAA-ATPase+Lon-Protease-BrxL-><-DEDD_Tnp_IS110+Transposase_20<-PIN<-MazE_antitoxin	1225	Bacteria>Pseudomonadota>Gammaproteobacteria>Enterobacterales>Enterobacteriaceae>Escherichia	GCA_012860255.1	Escherichia_coli	HJN25_004326	5261..8938	562	BREX-system-P-loop-protein-BrxC-[Escherichia-coli]
EFN5198232.1	Type1.BREX	<-TIR||BrxA-DNA-binding->BrxB-BrxF-iSTAND->DUF6079*->ParB+HNH->N6-METHYLASE->HEPN->RVT->N6-METHYLASE-p->PglZ->SIGMA+OB-Fold+AAA-ATPase+Lon-Protease-BrxL->wHTH+WYL+WCX->	1233	Bacteria>Pseudomonadota>Gammaproteobacteria>Enterobacterales>Enterobacteriaceae>Escherichia	GCA_012993025.1	Escherichia_coli	FQG10_00765	2767..6468	562	BREX-system-P-loop-protein-BrxC-[Escherichia-coli]
HAJ7467856.1	Type1.BREX	<-RelE-ParE<-CopG/RHH||<-RHH||?->wHTH+WYL+WCX->BrxA-DNA-binding->BrxB-BrxF-iSTAND->DUF6079*->ParB+HNH->N6-METHYLASE->PglZ->SIGMA+OB-Fold+AAA-ATPase+Lon-Protease-BrxL-><-RVT<-PIN<-MazE_antitoxin||?->Phage_holin->	1225	Bacteria>Pseudomonadota>Gammaproteobacteria>Enterobacterales>Enterobacteriaceae>Escherichia	GCA_013077315.1	Escherichia_coli	HNV92_21025	13565..17242	562	BREX-system-P-loop-protein-BrxC-[Escherichia-coli]
NUC65042.1	Type1.BREX	DDE_Tnp_IS1->?->ZnR->BrxA-DNA-binding->BrxB-BrxF-iSTAND->DUF6079*->ParB+HNH->N6-METHYLASE->HEPN->RVT->N6-METHYLASE-p->PglZ->SIGMA+OB-Fold+AAA-ATPase+Lon-Protease-BrxL-><-Arm-DNA-bind_3+Phage_integrase||Phage_integrase->	1233	Bacteria>Pseudomonadota>Gammaproteobacteria>Enterobacterales>Enterobacteriaceae>Escherichia	GCA_013342565.1	Escherichia_coli	HUC40_15465	2945..6646	562	BREX-system-P-loop-protein-BrxC-[Escherichia-coli]
EFT2808154.1	Type1.BREX	ZnR->wHTH+WYL+WCX->BrxA-DNA-binding->BrxB-BrxF-iSTAND->DUF6079*->ParB+HNH->N6-METHYLASE->PglZ->SIGMA+OB-Fold+AAA-ATPase+Lon-Protease-BrxL->35exo->	1221	Bacteria>Pseudomonadota>Gammaproteobacteria>Enterobacterales>Enterobacteriaceae>Escherichia	GCA_013688275.1	Escherichia_coli	HVL53_003849	3457..7122	562	BREX-system-P-loop-protein-BrxC-[Escherichia-coli]
EGK3607993.1	Type1.BREX	Arm-DNA-bind_3+Phage_integrase->XIS-HTH->?->?->?->BrxA-DNA-binding->BrxB-BrxF-iSTAND->DUF6079*->ParB+HNH->N6-METHYLASE->SIR2->HerA/FtsK->N6-METHYLASE-p->	1238	Bacteria>Pseudomonadota>Gammaproteobacteria>Enterobacterales>Enterobacteriaceae>Escherichia	GCA_014957385.1	Escherichia_coli	IOT31_003182	92049..95765	562	BREX-system-P-loop-protein-BrxC-[Escherichia-coli]
HAX1741551.1	Type1.BREX	DUF6079-p*->ParB+HNH->N6-METHYLASE->PglZ->SIGMA+OB-Fold+AAA-ATPase+Lon-Protease-BrxL->SbcC->	793	Bacteria>Pseudomonadota>Gammaproteobacteria>Enterobacterales>Enterobacteriaceae>Escherichia	GCA_016984655.1	Escherichia_coli	JXS76_004485	1..2383	562	BREX-system-P-loop-protein-BrxC-[Escherichia-coli]
EHK3830299.1	Type1.BREX	HTH_Tnp_1+HTH_21+rve+rve_3->wHTH+WYL+WCX->BrxA-DNA-binding->BrxB-BrxF-iSTAND->DUF6079*->ParB+HNH->N6-METHYLASE->PglZ->SIGMA+OB-Fold+AAA-ATPase+Lon-Protease-BrxL-><-DEDD_Tnp_IS110+Transposase_20	1225	Bacteria>Pseudomonadota>Gammaproteobacteria>Enterobacterales>Enterobacteriaceae>Escherichia	GCA_018024135.1	Escherichia_coli	JR760_003915	2875..6552	562	BREX-system-P-loop-protein-BrxC-[Escherichia-coli]
HBC6897266.1	Type1.BREX	DEDD_Tnp_IS110+Transposase_20-><-RHH||?->wHTH+WYL+WCX->BrxA-DNA-binding->BrxB-BrxF-iSTAND->DUF6079*->ParB+HNH->N6-METHYLASE->PglZ-p->	1225	Bacteria>Pseudomonadota>Gammaproteobacteria>Enterobacterales>Enterobacteriaceae>Escherichia	GCA_018163035.1	Escherichia_coli	KEZ94_003915	5261..8938	562	BREX-system-P-loop-protein-BrxC-[Escherichia-coli]
HBD3449422.1	Type1.BREX	<-N6-METHYLASE-p<-ParB+HNH<-DUF6079*<-BrxB-BrxF-iSTAND<-BrxA-DNA-binding<-wHTH+WYL+WCX<-Arm-DNA-bind_3+Phage_integrase||?-><-?||?->METHYLASE->	1225	Bacteria>Pseudomonadota>Gammaproteobacteria>Enterobacterales>Enterobacteriaceae>Escherichia	GCA_018390165.1	Escherichia_coli	KMB60_001824	3370..7047	562	BREX-system-P-loop-protein-BrxC-[Escherichia-coli]
EHY5030619.1	Type1.BREX	<-wHTH+WYL<-?||XIS-HTH->?->BrxA-DNA-binding->BrxB-BrxF-iSTAND->DUF6079*->ParB+HNH->N6-METHYLASE->PglZ->SIGMA+OB-Fold+AAA-ATPase+Lon-Protease-BrxL-><-Arm-DNA-bind_3+Phage_integrase||Phage_integrase->	1233	Bacteria>Pseudomonadota>Gammaproteobacteria>Enterobacterales>Enterobacteriaceae>Escherichia	GCA_019582855.1	Escherichia_coli	K4I20_002754	15002..18703	562	BREX-system-P-loop-protein-BrxC-[Escherichia-coli]
EID8792051.1	Type1.BREX	wHTH+WYL+WCX->BrxA-DNA-binding->BrxB-BrxF-iSTAND->DUF6079*->ParB+HNH->N6-METHYLASE->PglZ->SIGMA+OB-Fold+AAA-ATPase+Lon-Protease-BrxL-><-HTH_Tnp_1+HTH_21+rve+rve_3<-HIN-HTH<-PIN<-MazE_antitoxin	1225	Bacteria>Pseudomonadota>Gammaproteobacteria>Enterobacterales>Enterobacteriaceae>Escherichia	GCA_020095335.1	Escherichia_coli	LCJ17_004431	3670..7347	562	BREX-system-P-loop-protein-BrxC-[Escherichia-coli]
HBN0250039.1	Type1.BREX	<-DUF932<-ArdB<-?||?->XIS-HTH->ZnR->wHTH+WYL+WCX->BrxA-DNA-binding->BrxB-BrxF-iSTAND->DUF6079*->ParB+HNH->N6-METHYLASE->PglZ->SIGMA+OB-Fold+AAA-ATPase+Lon-Protease-BrxL->35exo->DUF772+DDE_Tnp_1->OBFOLD-NUCLEASE->	1225	Bacteria>Pseudomonadota>Gammaproteobacteria>Enterobacterales>Enterobacteriaceae>Escherichia	GCA_021454405.1	Escherichia_coli	L1L95_003624	57124..60801	562	BREX-system-P-loop-protein-BrxC-[Escherichia-coli]
MCK3244657.1	Type1.BREX	<-HIN-HTH<-RelE-ParE<-CopG/RHH||<-RHH||?->wHTH+WYL+WCX->BrxA-DNA-binding->BrxB-BrxF-iSTAND->DUF6079*->ParB+HNH->	1225	Bacteria>Pseudomonadota>Gammaproteobacteria>Enterobacterales>Enterobacteriaceae>Escherichia	GCA_023098955.1	Escherichia_coli	MZH07_24520	5688..9365	562	BREX-system-P-loop-protein-BrxC-[Escherichia-coli]
EJE3980761.1	Type1.BREX	<-RelE-ParE<-CopG/RHH||<-RHH||?->wHTH+WYL+WCX->BrxA-DNA-binding->BrxB-BrxF-iSTAND->DUF6079*->ParB+HNH->N6-METHYLASE->PglZ->SIGMA+OB-Fold+AAA-ATPase+Lon-Protease-BrxL-><-DEDD_Tnp_IS110+Transposase_20<-RVT	1225	Bacteria>Pseudomonadota>Gammaproteobacteria>Enterobacterales>Enterobacteriaceae>Escherichia	GCA_023310495.1	Escherichia_coli	M4B16_004238	5667..9344	562	BREX-system-P-loop-protein-BrxC-[Escherichia-coli]
EJN1844348.1	Type1.BREX	<-RHH||?->wHTH+WYL+WCX->BrxA-DNA-binding->BrxB-BrxF-iSTAND->DUF6079*->ParB+HNH->N6-METHYLASE->PglZ->SIGMA+OB-Fold+AAA-ATPase+Lon-Protease-BrxL-><-DEDD_Tnp_IS110+Transposase_20	1225	Bacteria>Pseudomonadota>Gammaproteobacteria>Enterobacterales>Enterobacteriaceae>Escherichia	GCA_024422675.1	Escherichia_coli	NPJ45_004376	3256..6933	562	BREX-system-P-loop-protein-BrxC-[Escherichia-coli]
EJU9647232.1	Type1.BREX	<-DDE_Tnp_1_5+DDE_Tnp_1||||wHTH+WYL+WCX->BrxA-DNA-binding->BrxB-BrxF-iSTAND->DUF6079*->ParB+HNH->N6-METHYLASE->PglZ->SIGMA+OB-Fold+AAA-ATPase+Lon-Protease-BrxL-><-HTH_Tnp_1+HTH_21+rve+rve_3<-HIN-HTH<-PIN<-MazE_antitoxin	1225	Bacteria>Pseudomonadota>Gammaproteobacteria>Enterobacterales>Enterobacteriaceae>Escherichia	GCA_025224895.1	Escherichia_coli	N5N25_003986	5462..9139	562	BREX-system-P-loop-protein-BrxC-[Escherichia-coli]
HCO5071633.1	Type1.BREX	<-RelE-ParE<-CopG/RHH||<-RHH||?->wHTH+WYL+WCX->BrxA-DNA-binding->BrxB-BrxF-iSTAND->DUF6079*->ParB+HNH->	1225	Bacteria>Pseudomonadota>Gammaproteobacteria>Enterobacterales>Enterobacteriaceae>Escherichia	GCA_025390635.1	Escherichia_coli	N8039_004383	5665..9342	562	BREX-system-P-loop-protein-BrxC-[Escherichia-coli]
WP_250907975.1	Type1.BREX	DDE_Tnp_IS1->?->ZnR->BrxA-DNA-binding->BrxB-BrxF-iSTAND->DUF6079*->ParB+HNH->N6-METHYLASE->HEPN->RVT->N6-METHYLASE-p->PglZ->SIGMA+OB-Fold+AAA-ATPase+Lon-Protease-BrxL-><-Arm-DNA-bind_3+Phage_integrase||Phage_integrase->	1240	Bacteria>Pseudomonadota>Gammaproteobacteria>Enterobacterales>Enterobacteriaceae>Escherichia	GCA_030820635.1	Escherichia_coli	HUC40_RS15465	2924..6646	562	BREX-system-P-loop-protein-BrxC-[Escherichia-coli]
STN20139.1	Type1.BREX	DDE_Tnp_1_5+DDE_Tnp_1-><-?||HTH_21+rve+rve_3-><-RHH||?->wHTH+WYL+WCX->BrxA-DNA-binding->BrxB-BrxF-iSTAND->DUF6079-p->DUF6079-p*->DUF6079-p->DUF6079-p->?->DUF6079-p->ParB+HNH->ParB+HNH->N6-METHYLASE->?->PglZ-p->	353	Bacteria>Pseudomonadota>Gammaproteobacteria>Enterobacterales>Enterobacteriaceae>Escherichia	GCA_900450565.1	Escherichia_fergusonii	NCTC12128_00054	38373..39434	564	ATPase-(modular-protein)-[Escherichia-fergusonii]
WP_001698869.1	Type1.BREX	RVT->DEDD_Tnp_IS110+Transposase_20-><-SIGMA+OB-Fold+AAA-ATPase+Lon-Protease-BrxL<-PglZ<-N6-METHYLASE<-ParB+HNH<-DUF6079*<-BrxB-BrxF-iSTAND<-BrxA-DNA-binding<-wHTH+WYL+WCX<-?||RHH-><-DEDD_Tnp_IS110+Transposase_20<-?||MerR-HTH+ParA-Soj-PloopNTPase->ParB->	1225	Bacteria>Pseudomonadota>Gammaproteobacteria>Enterobacterales>Enterobacteriaceae>Escherichia	GCF_000340255.1	Escherichia_coli	C201_RS01205	14823..18500	562	BREX-system-P-loop-protein-BrxC-[Escherichia-coli]
WP_032216088.1	Type1.BREX	XIS-HTH-><-HTH_Tnp_1+HTH_21+rve+rve_3||wHTH+WYL+WCX->BrxA-DNA-binding->BrxB-BrxF-iSTAND->DUF6079*->ParB+HNH->N6-METHYLASE->PglZ->SIGMA+OB-Fold+AAA-ATPase+Lon-Protease-BrxL->35exo->||HIN-HTH->	1225	Bacteria>Pseudomonadota>Gammaproteobacteria>Enterobacterales>Enterobacteriaceae>Escherichia	GCF_000713175.1	Escherichia_coli	AD46_RS16460	5265..8942	562	BREX-system-P-loop-protein-BrxC-[Escherichia-coli]
WP_032305083.1	Type1.BREX	XIS-HTH-><-HTH_Tnp_1+HTH_21+rve+rve_3||wHTH+WYL+WCX->BrxA-DNA-binding->BrxB-BrxF-iSTAND->DUF6079*->ParB+HNH->N6-METHYLASE->PglZ->SIGMA+OB-Fold+AAA-ATPase+Lon-Protease-BrxL->35exo->	1225	Bacteria>Pseudomonadota>Gammaproteobacteria>Enterobacterales>Enterobacteriaceae>Escherichia	GCF_000714055.1	Escherichia_coli	AC36_RS01690	6338..10015	562	BREX-system-P-loop-protein-BrxC-[Escherichia-coli]
WP_032222683.1	Type1.BREX	XIS-HTH-><-HTH_Tnp_1+HTH_21+rve+rve_3||wHTH+WYL+WCX->BrxA-DNA-binding->BrxB-BrxF-iSTAND->DUF6079*->ParB+HNH->N6-METHYLASE->PglZ->SIGMA+OB-Fold+AAA-ATPase+Lon-Protease-BrxL->35exo->	1225	Bacteria>Pseudomonadota>Gammaproteobacteria>Enterobacterales>Enterobacteriaceae>Escherichia	GCF_000714385.1	Escherichia_coli	AC91_RS16385	5217..8894	562	BREX-system-P-loop-protein-BrxC-[Escherichia-coli]
WP_072752451.1	Type1.BREX	DDE_Tnp_1_5+DDE_Tnp_1-><-35exo<-SIGMA+OB-Fold+AAA-ATPase+Lon-Protease-BrxL<-PglZ<-N6-METHYLASE<-ParB+HNH<-DUF6079*<-BrxB-BrxF-iSTAND<-BrxA-DNA-binding<-wHTH+WYL+WCX	1225	Bacteria>Pseudomonadota>Gammaproteobacteria>Enterobacterales>Enterobacteriaceae>Escherichia	GCF_001894415.1	Escherichia_coli	BK380_RS19185	23163..26840	562	BREX-system-P-loop-protein-BrxC-[Escherichia-coli]
WP_096969144.1	Type1.BREX	<-METHYLASE<-?||?-><-?||Arm-DNA-bind_3+Phage_integrase->wHTH+WYL+WCX->BrxA-DNA-binding->BrxB-BrxF-iSTAND->DUF6079*->ParB+HNH->N6-METHYLASE->PglZ->SIGMA+OB-Fold+AAA-ATPase+Lon-Protease-BrxL->35exo-><-DDE_Tnp_1_5+DDE_Tnp_1	1225	Bacteria>Pseudomonadota>Gammaproteobacteria>Enterobacterales>Enterobacteriaceae>Escherichia	GCF_002466035.1	Escherichia_coli	BSN44_RS10850	73237..76914	562	BREX-system-P-loop-protein-BrxC-[Escherichia-coli]
WP_252363318.1	Type1.BREX	<-HerA/FtsK+A32||tRNA->Arm-DNA-bind_3+Phage_integrase-><-SIGMA+OB-Fold+AAA-ATPase+Lon-Protease-BrxL<-PglZ<-N6-METHYLASE-p<-N6-METHYLASE<-ParB+HNH<-DUF6079*<-BrxB-BrxF-iSTAND<-BrxA-DNA-binding<-?<-?<-XIS-HTH||?->nSTAND1+sGTP->wHTH+WYL->	1240	Bacteria>Pseudomonadota>Gammaproteobacteria>Enterobacterales>Enterobacteriaceae>Escherichia	GCF_003687125.1	Escherichia_coli	EAI39_RS15070	84115..87837	562	BREX-system-P-loop-protein-BrxC-[Escherichia-coli]
WP_147697439.1	Type1.BREX	DEDD_Tnp_IS110+Transposase_20-><-SIGMA+OB-Fold+AAA-ATPase+Lon-Protease-BrxL<-PglZ<-N6-METHYLASE<-ParB+HNH<-DUF6079*<-BrxB-BrxF-iSTAND<-BrxA-DNA-binding<-wHTH+WYL+WCX<-?||RHH->||CopG/RHH->RelE-ParE->HIN-HTH->	1225	Bacteria>Pseudomonadota>Gammaproteobacteria>Enterobacterales>Enterobacteriaceae>Escherichia	GCF_008041475.1	Escherichia_coli	FV285_RS21415	12602..16279	562	BREX-system-P-loop-protein-BrxC-[Escherichia-coli]
WP_149488190.1	Type1.BREX	DDE_Tnp_1_5+DDE_Tnp_1-><-35exo<-SIGMA+OB-Fold+AAA-ATPase+Lon-Protease-BrxL<-PglZ<-N6-METHYLASE<-ParB+HNH<-DUF6079*<-BrxB-BrxF-iSTAND<-BrxA-DNA-binding<-wHTH+WYL+WCX<-Arm-DNA-bind_3+Phage_integrase||?-><-?||?->METHYLASE->	1225	Bacteria>Pseudomonadota>Gammaproteobacteria>Enterobacterales>Enterobacteriaceae>Escherichia	GCF_008364435.1	Escherichia_coli	F0318_RS09425	23572..27249	562	BREX-system-P-loop-protein-BrxC-[Escherichia-coli]
WP_023063795.1	Type1.BREX	<-35exo<-SIGMA+OB-Fold+AAA-ATPase+Lon-Protease-BrxL<-PglZ<-N6-METHYLASE<-ParB+HNH<-DUF6079*<-BrxB-BrxF-iSTAND<-BrxA-DNA-binding<-wHTH+WYL+WCX	1225	Bacteria>Pseudomonadota>Gammaproteobacteria>Enterobacterales>Enterobacteriaceae>Escherichia	GCF_008370755.1	Escherichia_coli	L913_RS00645	11831..15508	562	BREX-system-P-loop-protein-BrxC-[Escherichia-coli]
WP_136516625.1	Type1.BREX	<-DUF772+DDE_Tnp_1<-35exo<-SIGMA+OB-Fold+AAA-ATPase+Lon-Protease-BrxL<-PglZ<-N6-METHYLASE<-ParB+HNH<-DUF6079*<-BrxB-BrxF-iSTAND<-BrxA-DNA-binding<-wHTH+WYL+WCX<-XIS-HTH<-ZnR||?-><-XIS-HTH	1225	Bacteria>Pseudomonadota>Gammaproteobacteria>Enterobacterales>Enterobacteriaceae>Escherichia	GCF_011045875.1	Escherichia_coli	EIZ78_RS19775	10945..14622	562	BREX-system-P-loop-protein-BrxC-[Escherichia-coli]
WP_072657276.1	Type1.BREX	wHTH+WYL+WCX->BrxA-DNA-binding->BrxB-BrxF-iSTAND->DUF6079*->ParB+HNH->N6-METHYLASE->PglZ->SIGMA+OB-Fold+AAA-ATPase+Lon-Protease-BrxL->35exo->||<-ParB+HNH	1225	Bacteria>Pseudomonadota>Gammaproteobacteria>Enterobacterales>Enterobacteriaceae>Escherichia	GCF_011077045.1	Escherichia_coli	G6Q90_RS23590	2674..6351	562	BREX-system-P-loop-protein-BrxC-[Escherichia-coli]
WP_032228177.1	Type1.BREX	wHTH+WYL+WCX->BrxA-DNA-binding->BrxB-BrxF-iSTAND->DUF6079*->ParB+HNH->N6-METHYLASE->PglZ->SIGMA+OB-Fold+AAA-ATPase+Lon-Protease-BrxL->35exo->||HIN-HTH->||HIN-HTH+PAIREDC-HTH-><-Arm-DNA-bind_3+Phage_integrase<-tRNA<-TetR-HTH	1225	Bacteria>Pseudomonadota>Gammaproteobacteria>Enterobacterales>Enterobacteriaceae>Escherichia	GCF_013424245.1	Escherichia_coli	H0P07_RS08595	2755..6432	562	BREX-system-P-loop-protein-BrxC-[Escherichia-coli]
WP_087888065.1	Type1.BREX	ABhydrolase-><-LZ_Tnp_IS66+zf-IS66+DDE_Tnp_IS66+DDE_Tnp_IS66_C||?-><-Zn_Tnp_IS1+HTH_Tnp_IS1+DDE_Tnp_IS1||wHTH+WYL+WCX->BrxA-DNA-binding->BrxB-BrxF-iSTAND->DUF6079*->ParB+HNH->N6-METHYLASE->PglZ->SIGMA+OB-Fold+AAA-ATPase+Lon-Protease-BrxL-><-DEDD_Tnp_IS110+Transposase_20<-RVT	1225	Bacteria>Pseudomonadota>Gammaproteobacteria>Enterobacterales>Enterobacteriaceae>Escherichia	GCF_015229345.1	Escherichia_coli	GVI22_RS02065	20896..24573	562	BREX-system-P-loop-protein-BrxC-[Escherichia-coli]
WP_224452732.1	Type1.BREX	<-HTH_OrfB_IS605+OrfB_IS605+OrfB_Zn_ribbon<-ParB<-MerR-HTH+ParA-Soj-PloopNTPase||?->DEDD_Tnp_IS110+Transposase_20->||wHTH+WYL+WCX->BrxA-DNA-binding->BrxB-BrxF-iSTAND->DUF6079*->ParB+HNH->N6-METHYLASE->PglZ->SIGMA+OB-Fold+AAA-ATPase+Lon-Protease-BrxL->	1225	Bacteria>Pseudomonadota>Gammaproteobacteria>Enterobacterales>Enterobacteriaceae>Escherichia	GCF_020162075.2	Escherichia_coli	KD159_RS16650	41518..45195	562	BREX-system-P-loop-protein-BrxC-[Escherichia-coli]
WP_231833501.1	Type1.BREX	<-ParB+HNH<-DUF6079*<-BrxB-BrxF-iSTAND<-BrxA-DNA-binding<-wHTH+WYL+WCX||DUF4158+DDE_Tnp_Tn3->	1225	Bacteria>Pseudomonadota>Gammaproteobacteria>Enterobacterales>Enterobacteriaceae>Escherichia	GCF_021168085.1	Escherichia_coli	LTT78_RS21790	2950..6627	562	BREX-system-P-loop-protein-BrxC-[Escherichia-coli]
WP_247149149.1	Type1.BREX	<-METHYLASE<-?||?-><-?||<-AAA-ATPase<-HIN-HTH||wHTH+WYL+WCX->BrxA-DNA-binding->BrxB-BrxF-iSTAND->DUF6079*->ParB+HNH->N6-METHYLASE->PglZ->SIGMA+OB-Fold+AAA-ATPase+Lon-Protease-BrxL->35exo-><-DDE_Tnp_1_5+DDE_Tnp_1	1225	Bacteria>Pseudomonadota>Gammaproteobacteria>Enterobacterales>Enterobacteriaceae>Escherichia	GCF_022975115.1	Escherichia_coli	MZS66_RS10030	63457..67134	562	BREX-system-P-loop-protein-BrxC-[Escherichia-coli]
WP_249573915.1	Type1.BREX	wHTH+WYL+WCX->BrxA-DNA-binding->BrxB-BrxF-iSTAND->DUF6079*->ParB+HNH->N6-METHYLASE->PglZ->SIGMA+OB-Fold+AAA-ATPase+Lon-Protease-BrxL->35exo->	1225	Bacteria>Pseudomonadota>Gammaproteobacteria>Enterobacterales>Enterobacteriaceae>Escherichia	GCF_023483525.1	Escherichia_coli	M5X65_RS22540	2590..6267	562	BREX-system-P-loop-protein-BrxC-[Escherichia-coli]
WP_250239414.1	Type1.BREX	MobI-><-?<-LexA-protease||35exo->?->wHTH+WYL+WCX->BrxA-DNA-binding->BrxB-BrxF-iSTAND->DUF6079*->ParB+HNH->N6-METHYLASE->PglZ->SIGMA+OB-Fold+AAA-ATPase+Lon-Protease-BrxL->SbcC->HDRnase->VirD4-TraG+VirB4-FtsK->	1225	Bacteria>Pseudomonadota>Gammaproteobacteria>Enterobacterales>Enterobacteriaceae>Escherichia	GCF_023599595.1	Escherichia_coli	NFF73_RS04910	234938..238615	562	MULTISPECIES:-BREX-system-P-loop-protein-BrxC-[Enterobacterales]
WP_261629031.1	Type1.BREX	<-HIN-HTH<-RelE-ParE<-CopG/RHH||<-RHH||?->wHTH+WYL+WCX->BrxA-DNA-binding->BrxB-BrxF-iSTAND->DUF6079*->ParB+HNH->N6-METHYLASE->PglZ->SIGMA+OB-Fold+AAA-ATPase+Lon-Protease-BrxL-><-DEDD_Tnp_IS110+Transposase_20||Zn_Tnp_IS1+HTH_Tnp_IS1+DDE_Tnp_IS1->	1225	Bacteria>Pseudomonadota>Gammaproteobacteria>Enterobacterales>Enterobacteriaceae>Escherichia	GCF_025404235.1	Escherichia_coli	J0W24_RS20355	5362..9039	562	BREX-system-P-loop-protein-BrxC-[Escherichia-coli]
WP_268646541.1	Type1.BREX	<-VirD4-TraG+VirB4-FtsK<-HDRnase<-SbcC<-SIGMA+OB-Fold+AAA-ATPase+Lon-Protease-BrxL<-PglZ<-N6-METHYLASE<-ParB+HNH<-DUF6079*<-BrxB-BrxF-iSTAND<-BrxA-DNA-binding<-wHTH+WYL+WCX<-?<-35exo||LexA-protease->?-><-?<-MobI	1225	Bacteria>Pseudomonadota>Gammaproteobacteria>Enterobacterales>Enterobacteriaceae>Escherichia	GCF_026801415.1	Escherichia_coli	OZI01_RS14450	44846..48523	562	BREX-system-P-loop-protein-BrxC-[Escherichia-coli]
WP_272434363.1	Type1.BREX	<-DUF772+DDE_Tnp_1<-HIN-HTH<-RelE-ParE<-CopG/RHH||<-RHH||?->wHTH+WYL+WCX->BrxA-DNA-binding->BrxB-BrxF-iSTAND->DUF6079*->ParB+HNH->N6-METHYLASE->PglZ->SIGMA+OB-Fold+AAA-ATPase+Lon-Protease-BrxL-><-DEDD_Tnp_IS110+Transposase_20||Zn_Tnp_IS1+HTH_Tnp_IS1+DDE_Tnp_IS1->?->?->HEPN+SF1-SF2-Helicase+RNase+RNase->	1225	Bacteria>Pseudomonadota>Gammaproteobacteria>Enterobacterales>Enterobacteriaceae>Escherichia	GCF_028436275.1	Escherichia_coli	IXW67_RS19930	12892..16569	562	BREX-system-P-loop-protein-BrxC-[Escherichia-coli]
WP_087894339.1	Type1.BREX	<-35exo<-SIGMA+OB-Fold+AAA-ATPase+Lon-Protease-BrxL<-PglZ<-N6-METHYLASE<-ParB+HNH<-DUF6079*<-BrxB-BrxF-iSTAND<-BrxA-DNA-binding<-wHTH+WYL+WCX	1225	Bacteria>Pseudomonadota>Gammaproteobacteria>Enterobacterales>Enterobacteriaceae>Escherichia	GCF_030716655.1	Escherichia_coli	A8C62_RS24175	11745..15422	562	BREX-system-P-loop-protein-BrxC-[Escherichia-coli]
WP_096317297.1	Type1.BREX	HTH_21+rve+rve_3->||?->wHTH+WYL+WCX->BrxA-DNA-binding->BrxB-BrxF-iSTAND->DUF6079*->ParB+HNH->N6-METHYLASE->PglZ->SIGMA+OB-Fold+AAA-ATPase+Lon-Protease-BrxL-><-RVT	1225	Bacteria>Pseudomonadota>Gammaproteobacteria>Enterobacterales>Enterobacteriaceae>Escherichia	GCF_900196545.1	Escherichia_coli	LLQ47_RS24465	3245..6922	562	BREX-system-P-loop-protein-BrxC-[Escherichia-coli]
WP_244586021.1	Type1.BREX	<-wHTH+WYL<-?||XIS-HTH->?->BrxA-DNA-binding->BrxB-BrxF-iSTAND->DUF6079*->ParB+HNH->N6-METHYLASE->PglZ->	1240	Bacteria>Pseudomonadota>Gammaproteobacteria>Enterobacterales>Enterobacteriaceae>Escherichia	GCF_948144585.1	Escherichia_coli	A7Y91_RS22675	15059..18781	562	BREX-system-P-loop-protein-BrxC-[Escherichia-coli]
WP_241755053.1	Type1.BREX	<-METHYLASE||SKI-><-?||tRNA->Arm-DNA-bind_3+Phage_integrase-><-SIGMA+OB-Fold+AAA-ATPase+Lon-Protease-BrxL<-PglZ<-N6-METHYLASE<-ParB+HNH<-DUF6079*<-BrxB-BrxF-iSTAND<-BrxA-DNA-binding||?->PHP+SbcC->Schlafen+HATPase+wHTH->?->?->?->Sulfatase->	1240	Bacteria>Pseudomonadota>Gammaproteobacteria>Enterobacterales>Enterobacteriaceae	GCF_013791465.1	Citrobacter	HV084_RS03275	673516..677238	544	MULTISPECIES:-BREX-system-P-loop-protein-BrxC-[Citrobacter]
WP_233470897.1	Type1.BREX	<-DUF932<-nSTAND1+GTPase-AIG+sGTP<-CopG/RHH||?->XIS-HTH->?->?->BrxA-DNA-binding->BrxB-BrxF-iSTAND->DUF6079*->ParB+HNH->N6-METHYLASE->HEPN->RVT->N6-METHYLASE->PglZ->SIGMA+OB-Fold+AAA-ATPase+Lon-Protease-BrxL-><-Arm-DNA-bind_3+Phage_integrase	1240	Bacteria>Pseudomonadota>Gammaproteobacteria>Enterobacterales>Enterobacteriaceae	GCF_014931625.2	Citrobacter	INP82_RS14360	158420..162142	544	MULTISPECIES:-BREX-system-P-loop-protein-BrxC-[Citrobacter]
WP_225990544.1	Type1.BREX	Glyoxalase->ACET->YjhX_toxin->SAM-METHYLASE->ZnR->Glyoxalase->BrxA-DNA-binding->BrxB-BrxF-iSTAND->DUF6079*->ParB+HNH->N6-METHYLASE->HEPN->RVT->N6-METHYLASE-p->PglZ->SIGMA+OB-Fold+AAA-ATPase+Lon-Protease-BrxL-><-Arm-DNA-bind_3+Phage_integrase	1240	Bacteria>Pseudomonadota>Gammaproteobacteria>Enterobacterales>Enterobacteriaceae	GCF_954736865.1	Enterobacter	CU081_RS00925	173954..177676	547	MULTISPECIES:-BREX-system-P-loop-protein-BrxC-[Enterobacter]
PLL44193.1	Type1.BREX	<-N6-METHYLASE-p<-N6-METHYLASE<-ParB+HNH<-DUF6079*<-BrxB-BrxF-iSTAND<-BrxA-DNA-binding||<-XIS-HTH	1233	Bacteria>Pseudomonadota>Gammaproteobacteria>Enterobacterales>Enterobacteriaceae>Klebsiella/Raoultella_group	GCA_002856085.1	Klebsiella_michiganensis	CWN50_01600	4530..8231	1134687	BREX-system-P-loop-protein-BrxC-[Klebsiella-michiganensis]
AUN75366.1	Type1.BREX	Arm-DNA-bind_3+Phage_integrase-><-SIGMA+OB-Fold+AAA-ATPase+Lon-Protease-BrxL<-PglZ<-N6-METHYLASE-p<-RVT<-HEPN<-N6-METHYLASE<-ParB+HNH<-DUF6079*<-BrxB-BrxF-iSTAND<-BrxA-DNA-binding<-?<-?<-XIS-HTH||?->nSTAND1+sGTP->wHTH+WYL->	1233	Bacteria>Pseudomonadota>Gammaproteobacteria>Enterobacterales>Enterobacteriaceae>Klebsiella/Raoultella_group	GCA_002870985.1	Klebsiella_pneumoniae	C0077_01580	326913..330614	573	BREX-system-P-loop-protein-BrxC-[Klebsiella-pneumoniae]
QHX00137.1	Type1.BREX	YjhX_toxin->ACET->METHYLASE->ZnR->Glyoxalase->BrxA-DNA-binding->BrxB-BrxF-iSTAND->DUF6079*->ParB+HNH->N6-METHYLASE->HEPN->RVT->N6-METHYLASE-p->PglZ->SIGMA+OB-Fold+AAA-ATPase+Lon-Protease-BrxL-><-Arm-DNA-bind_3+Phage_integrase	1233	Bacteria>Pseudomonadota>Gammaproteobacteria>Enterobacterales>Enterobacteriaceae>Klebsiella/Raoultella_group	GCA_010365565.1	Klebsiella_variicola	GZS05_24295	4949707..4953408	244366	BREX-system-P-loop-protein-BrxC-[Klebsiella-variicola]
QJM45197.1	Type1.BREX	<-wHTH+WYL<-sGTP<-?||XIS-HTH->?->?->BrxA-DNA-binding->BrxB-BrxF-iSTAND->DUF6079*->ParB+HNH->N6-METHYLASE->HEPN->RVT->N6-METHYLASE-p->PglZ->SIGMA+OB-Fold+AAA-ATPase+Lon-Protease-BrxL-><-Arm-DNA-bind_3+Phage_integrase	1233	Bacteria>Pseudomonadota>Gammaproteobacteria>Enterobacterales>Enterobacteriaceae>Klebsiella/Raoultella_group	GCA_012970665.1	Klebsiella_pneumoniae	HJW71_23035	4620464..4624165	573	BREX-system-P-loop-protein-BrxC-[Klebsiella-pneumoniae]
EGT0044780.1	Type1.BREX	<-DUF905<-DUF932<-CopG/RHH||?->XIS-HTH-><-TIR<-TIR||BrxA-DNA-binding->BrxB-BrxF-iSTAND->DUF6079*->ParB+HNH->N6-METHYLASE->N6-METHYLASE-p->PglZ->SIGMA+OB-Fold+AAA-ATPase+Lon-Protease-BrxL->wHTH+WYL+WCX->?->HNH->	1233	Bacteria>Pseudomonadota>Gammaproteobacteria>Enterobacterales>Enterobacteriaceae>Klebsiella/Raoultella_group	GCA_015929385.1	Klebsiella_oxytoca	I9Y61_001542	37432..41133	571	BREX-system-P-loop-protein-BrxC-[Klebsiella-oxytoca]
EJG2380286.1	Type1.BREX	<-DUF905<-DUF932<-CopG/RHH||?->XIS-HTH-><-TIR<-TIR||BrxA-DNA-binding->BrxB-BrxF-iSTAND->DUF6079*->ParB+HNH->N6-METHYLASE->HEPN->RVT->N6-METHYLASE-p->PglZ->SIGMA+OB-Fold+AAA-ATPase+Lon-Protease-BrxL->wHTH+WYL+WCX->	1233	Bacteria>Pseudomonadota>Gammaproteobacteria>Enterobacterales>Enterobacteriaceae>Klebsiella/Raoultella_group	GCA_016088515.2	Raoultella_ornithinolytica	AM381_RS08835	19475..23176	54291	BREX-system-P-loop-protein-BrxC-[Raoultella-ornithinolytica]
HAV2047149.1	Type1.BREX	<-Calcineurin<-?<-?<-?||tRNA-><-SIGMA+OB-Fold+AAA-ATPase+Lon-Protease-BrxL<-PglZ<-N6-METHYLASE-p<-N6-METHYLASE<-ParB+HNH<-DUF6079*<-BrxB-BrxF-iSTAND<-BrxA-DNA-binding||?-><-Glyco_hydro_43+GH43_C2<-?||AP_endonuc_2->	1233	Bacteria>Pseudomonadota>Gammaproteobacteria>Enterobacterales>Enterobacteriaceae>Klebsiella/Raoultella_group	GCA_016445365.1	Raoultella_ornithinolytica	JHJ87_003574	41911..45612	54291	BREX-system-P-loop-protein-BrxC-[Raoultella-ornithinolytica]
MBN7733855.1	Type1.BREX	<-wHTH+WYL<-sGTP<-?||XIS-HTH->?->?->BrxA-DNA-binding->BrxB-BrxF-iSTAND->DUF6079*->ParB+HNH->N6-METHYLASE->HEPN->RVT->N6-METHYLASE-p->PglZ->SIGMA+OB-Fold+AAA-ATPase+Lon-Protease-BrxL-><-Arm-DNA-bind_3+Phage_integrase	1233	Bacteria>Pseudomonadota>Gammaproteobacteria>Enterobacterales>Enterobacteriaceae>Klebsiella/Raoultella_group	GCA_017255495.1	Klebsiella_pneumoniae	JX616_18220	31914..35615	573	BREX-system-P-loop-protein-BrxC-[Klebsiella-pneumoniae]
MBZ6545107.1	Type1.BREX	<-wHTH+WYL<-nSTAND1+sGTP<-?||XIS-HTH->?->?->BrxA-DNA-binding->BrxB-BrxF-iSTAND->DUF6079*->ParB+HNH->	1233	Bacteria>Pseudomonadota>Gammaproteobacteria>Enterobacterales>Enterobacteriaceae>Klebsiella/Raoultella_group	GCA_020115175.1	Klebsiella_pneumoniae	FMJ66_23200	19841..23542	573	BREX-system-P-loop-protein-BrxC-[Klebsiella-pneumoniae]
MBZ7196032.1	Type1.BREX	ACET->?->YjhX_toxin->ACET->METHYLASE->ZnR->Glyoxalase->BrxA-DNA-binding->BrxB-BrxF-iSTAND->DUF6079*->ParB+HNH->N6-METHYLASE->N6-METHYLASE-p->PglZ->SIGMA+OB-Fold+AAA-ATPase+Lon-Protease-BrxL-><-Arm-DNA-bind_3+Phage_integrase	1233	Bacteria>Pseudomonadota>Gammaproteobacteria>Enterobacterales>Enterobacteriaceae>Klebsiella/Raoultella_group	GCA_020118595.1	Klebsiella_oxytoca	FMK61_04485	39995..43696	571	BREX-system-P-loop-protein-BrxC-[Klebsiella-oxytoca]
EIV3098057.1	Type1.BREX	<-wHTH+WYL<-nSTAND1+sGTP<-?||XIS-HTH->?->?->BrxA-DNA-binding->BrxB-BrxF-iSTAND->DUF6079*->ParB+HNH->N6-METHYLASE->HEPN->RVT->N6-METHYLASE-p->PglZ->SIGMA+OB-Fold+AAA-ATPase+Lon-Protease-BrxL-><-Arm-DNA-bind_3+Phage_integrase	1233	Bacteria>Pseudomonadota>Gammaproteobacteria>Enterobacterales>Enterobacteriaceae>Klebsiella/Raoultella_group	GCA_021854435.1	Klebsiella_pneumoniae	L7W77_004838	45573..49274	573	BREX-system-P-loop-protein-BrxC-[Klebsiella-pneumoniae]
HBQ4939450.1	Type1.BREX	XIS-HTH->SF1-SF2-Helicase+REase->SF1-SF2-Helicase+REase-><-PglZ<-ParB+HNH<-N6-METHYLASE<-DUF6079*<-BrxB-BrxF-iSTAND<-BrxA-DNA-binding<-Arm-DNA-bind_3+Phage_integrase	1157	Bacteria>Pseudomonadota>Gammaproteobacteria>Enterobacterales>Enterobacteriaceae>Klebsiella/Raoultella_group	GCA_021861305.1	Klebsiella_pneumoniae	L7366_003904	52372..55845	573	BREX-system-P-loop-protein-BrxC-[Klebsiella-pneumoniae]
HBR1749211.1	Type1.BREX	REase+iREase-><-RHH||?->wHTH+WYL+WCX->BrxA-DNA-binding->BrxB-BrxF-iSTAND->DUF6079*->ParB+HNH->N6-METHYLASE->PglZ->SIGMA+OB-Fold+AAA-ATPase+Lon-Protease-BrxL-><-DEDD_Tnp_IS110+Transposase_20<-PIN<-MazE_antitoxin||?-><-EAL	1225	Bacteria>Pseudomonadota>Gammaproteobacteria>Enterobacterales>Enterobacteriaceae>Klebsiella/Raoultella_group	GCA_021911085.1	Klebsiella_pneumoniae	L9M14_005022	6747..10424	573	BREX-system-P-loop-protein-BrxC-[Klebsiella-pneumoniae]
HBR1784209.1	Type1.BREX	<-wHTH+WYL<-sGTP<-?||XIS-HTH->?->?->BrxA-DNA-binding->BrxB-BrxF-iSTAND->DUF6079*->ParB+HNH->N6-METHYLASE->HEPN->RVT->N6-METHYLASE-p->PglZ->SIGMA+OB-Fold+AAA-ATPase+Lon-Protease-BrxL-><-Arm-DNA-bind_3+Phage_integrase	1233	Bacteria>Pseudomonadota>Gammaproteobacteria>Enterobacterales>Enterobacteriaceae>Klebsiella/Raoultella_group	GCA_021911385.1	Klebsiella_quasipneumoniae_subsp._quasipneumoniae	L9M28_003408	20306..24007	1667327	BREX-system-P-loop-protein-BrxC-[Klebsiella-quasipneumoniae-subsp.-quasipneumoniae]
HBS2746978.1	Type1.BREX	<-wHTH+WYL<-nSTAND1+sGTP<-?||XIS-HTH->?->?->BrxA-DNA-binding->BrxB-BrxF-iSTAND->DUF6079*->ParB+HNH->	1233	Bacteria>Pseudomonadota>Gammaproteobacteria>Enterobacterales>Enterobacteriaceae>Klebsiella/Raoultella_group	GCA_021960165.1	Klebsiella_quasipneumoniae_subsp._quasipneumoniae	MAE70_005431	15539..19240	1667327	BREX-system-P-loop-protein-BrxC-[Klebsiella-quasipneumoniae-subsp.-quasipneumoniae]
HBS5818882.1	Type1.BREX	Arm-DNA-bind_3+Phage_integrase->BrxA-DNA-binding->BrxB-BrxF-iSTAND->DUF6079*->N6-METHYLASE->ParB+HNH->PglZ-><-SF1-SF2-Helicase+REase<-SF1-SF2-Helicase+REase<-XIS-HTH	1157	Bacteria>Pseudomonadota>Gammaproteobacteria>Enterobacterales>Enterobacteriaceae>Klebsiella/Raoultella_group	GCA_021971955.1	Klebsiella_pneumoniae	MAK71_003676	3132..6605	573	BREX-system-P-loop-protein-BrxC-[Klebsiella-pneumoniae]
HBY8309079.1	Type1.BREX	DUF6079*->ParB+HNH->N6-METHYLASE->PglZ->SIGMA+OB-Fold+AAA-ATPase+Lon-Protease-BrxL-><-HTH_Tnp_1+HTH_21+rve+rve_3	1167	Bacteria>Pseudomonadota>Gammaproteobacteria>Enterobacterales>Enterobacteriaceae>Klebsiella/Raoultella_group	GCA_022301875.1	Klebsiella_pneumoniae	MI837_25465	1..3506	573	BREX-system-P-loop-protein-BrxC-[Klebsiella-pneumoniae]
EIX9370752.1	Type1.BREX	<-wHTH+WYL<-nSTAND1+sGTP<-?||XIS-HTH->?->?->BrxA-DNA-binding->BrxB-BrxF-iSTAND->DUF6079*->ParB+HNH->N6-METHYLASE->PglZ->SIGMA+OB-Fold+AAA-ATPase+Lon-Protease-BrxL-><-Arm-DNA-bind_3+Phage_integrase	1233	Bacteria>Pseudomonadota>Gammaproteobacteria>Enterobacterales>Enterobacteriaceae>Klebsiella/Raoultella_group	GCA_022430905.1	Klebsiella_pneumoniae	L7649_004474	53291..56992	573	BREX-system-P-loop-protein-BrxC-[Klebsiella-pneumoniae]
HCD1392381.1	Type1.BREX	<-wHTH+WYL+WCX<-SIGMA+OB-Fold+AAA-ATPase+Lon-Protease-BrxL<-PglZ<-N6-METHYLASE-p<-RVT<-HEPN<-N6-METHYLASE<-ParB+HNH<-DUF6079*<-BrxB-BrxF-iSTAND<-BrxA-DNA-binding||TIR->TIR-><-XIS-HTH<-?||CopG/RHH->DUF932->DUF905->	1233	Bacteria>Pseudomonadota>Gammaproteobacteria>Enterobacterales>Enterobacteriaceae>Klebsiella/Raoultella_group	GCA_023530865.1	Klebsiella_pneumoniae_subsp._pneumoniae	M9022_004645	23383..27084	72407	BREX-system-P-loop-protein-BrxC-[Klebsiella-pneumoniae-subsp.-pneumoniae]
MCM6196114.1	Type1.BREX	<-Calcineurin<-?<-?<-?<-?<-?||tRNA->Arm-DNA-bind_3+Phage_integrase->BrxA-DNA-binding->BrxB-BrxF-iSTAND->DUF6079*->N6-METHYLASE->ParB+HNH->PglZ-><-SF1-SF2-Helicase+REase<-SF1-SF2-Helicase+REase<-XIS-HTH	1157	Bacteria>Pseudomonadota>Gammaproteobacteria>Enterobacterales>Enterobacteriaceae>Klebsiella/Raoultella_group	GCA_023741115.1	Klebsiella_pneumoniae	M4C47_13300	309323..312796	573	BREX-system-P-loop-protein-BrxC-[Klebsiella-pneumoniae]

<table>
<tr><th>Protein-ID</th><th>System</th><th>Gene-Neighbourhoods</th><th>Length</th><th>Taxonomy</th><th>Assembly-ID</th><th>Strain</th><th>Locus-Tag</th><th>Genomic-coordinates</th><th>TaxID</th><th>Fasta-define</th></tr>
<tr><td>MCS5967892.1</td><td>Type1.BREX</td><td><-nSTAND1+sGTP<-?<-?||?-> BrxA-DNA-binding -> BrxB-BrxF-iSTAND -> DUF6079 -p*-> DUF6079 -p->?-> DUF6079 -p-> ParB + HNH -> ParB + HNH -> N6-METHYLASE ->HEPN->?->RVT-></td><td>389</td><td>Bacteria>Pseudomonadota>Gammaproteobacteria>Enterobacterales>Enterobacteriaceae>Klebsiella/Raoultella_group</td><td>GCA_024990005.1</td><td>Klebsiella_variicola_subsp._variicola</td><td>LNP26_07865</td><td>1516930..1518099</td><td>2590157</td><td>BREX-system-P-loop-protein-BrxC-[Klebsiella-variicola-subsp.-variicola]</td></tr>
<tr><td>WEJ89011.1</td><td>Type1.BREX</td><td><- wHTH(SBD)+HNH ||tRNA->Arm-DNA-bind_3+Phage_integrase-><- SIGMA+OB-Fold+AAA-ATPase+Lon-Protease-BrxL <- PglZ <- N6-METHYLASE -p<-RVT<-HEPN<- N6-METHYLASE <- ParB + HNH <- DUF6079 [illegible]</td><td>1240</td><td>Bacteria>Pseudomonadota>Gammaproteobacteria>Enterobacterales>Enterobacteriaceae>Klebsiella/Raoultella_group</td><td>GCA_029202365.1</td><td>Klebsiella_huaxiensis</td><td>P0Y63_27795</td><td>5833350..5837072</td><td>2153354</td><td>MAG:-BREX-system-P-loop-protein-BrxC-[Klebsiella-huaxiensis]</td></tr>
<tr><td>WP_228134609.1</td><td>Type1.BREX</td><td>Glyoxalase->ACET->YjhX_toxin->METHYLASE->ZnR->Glyoxalase-> BrxA-DNA-binding -> BrxB-BrxF-iSTAND -> DUF6079 *-> ParB + HNH -> N6-METHYLASE -> N6-METHYLASE -> PglZ -> [illegible]</td><td>1240</td><td>Bacteria>Pseudomonadota>Gammaproteobacteria>Enterobacterales>Enterobacteriaceae>Klebsiella/Raoultella_group</td><td>GCF_000632415.1</td><td>Klebsiella_michiganensis</td><td>J415_RS00260</td><td>45760..49482</td><td>1134687</td><td>BREX-system-P-loop-protein-BrxC-[Klebsiella-michiganensis]</td></tr>
<tr><td>WP_256991583.1</td><td>Type1.BREX</td><td><-DUF905<-DUF932<-CopG/RHH||?->XIS-HTH->ZnR-><-TIR|| BrxA-DNA-binding -> BrxB-BrxF-iSTAND -> DUF6079 *-> ParB + HNH -> N6-METHYLASE ->HEPN->RVT-> N6-METHYLASE -p-> PglZ -> [illegible]</td><td>1240</td><td>Bacteria>Pseudomonadota>Gammaproteobacteria>Enterobacterales>Enterobacteriaceae>Klebsiella/Raoultella_group</td><td>GCF_002264145.1</td><td>Raoultella_planticola</td><td>CIG23_RS11860</td><td>59081..62803</td><td>575</td><td>BREX-system-P-loop-protein-BrxC-[Raoultella-planticola]</td></tr>
<tr><td>WP_264757344.1</td><td>Type1.BREX</td><td><-wHTH+WYL<-nSTAND1+sGTP<-?||XIS-HTH->?->?-> BrxA-DNA-binding -> BrxB-BrxF-iSTAND -><-DDE_Tnp_1_5+DDE_Tnp_1|| DUF6079 -p*-> ParB + HNH -> N6-METHYLASE ->HEPN->RVT-> N6-METHYLASE [illegible]</td><td>995</td><td>Bacteria>Pseudomonadota>Gammaproteobacteria>Enterobacterales>Enterobacteriaceae>Klebsiella/Raoultella_group</td><td>GCF_003660185.1</td><td>Klebsiella_pneumoniae</td><td>D9K63_RS23205</td><td>4667642..4670629</td><td>573</td><td>BREX-system-P-loop-protein-BrxC-[Klebsiella-pneumoniae]</td></tr>
<tr><td>WP_130973675.1</td><td>Type1.BREX</td><td><- SIGMA+OB-Fold+AAA-ATPase+Lon-Protease-BrxL <- PglZ <- N6-METHYLASE <- ParB + HNH <- DUF6079 *<- BrxB-BrxF-iSTAND <- BrxA-DNA-binding <- wHTH+WYL+WCX <-?||RHH-></td><td>1225</td><td>Bacteria>Pseudomonadota>Gammaproteobacteria>Enterobacterales>Enterobacteriaceae>Klebsiella/Raoultella_group</td><td>GCF_004311945.1</td><td>Klebsiella_pneumoniae</td><td>EOM14_RS25420</td><td>10722..14399</td><td>573</td><td>BREX-system-P-loop-protein-BrxC-[Klebsiella-pneumoniae]</td></tr>
<tr><td>WP_223814682.1</td><td>Type1.BREX</td><td><-wHTH+WYL<-nSTAND1+sGTP<-?||XIS-HTH->?->?-> BrxA-DNA-binding -> BrxB-BrxF-iSTAND -> DUF6079 *-> ParB + HNH -> N6-METHYLASE ->HEPN->RVT-> N6-METHYLASE -p-> PglZ -> [illegible]</td><td>1240</td><td>Bacteria>Pseudomonadota>Gammaproteobacteria>Enterobacterales>Enterobacteriaceae>Klebsiella/Raoultella_group</td><td>GCF_004313365.1</td><td>Klebsiella</td><td>EOO18_RS24630</td><td>24494..28216</td><td>570</td><td>MULTISPECIES:-BREX-system-P-loop-protein-BrxC-[Klebsiella]</td></tr>
<tr><td>WP_096812545.1</td><td>Type1.BREX</td><td>Arm-DNA-bind_3+Phage_integrase-> BrxA-DNA-binding -> BrxB-BrxF-iSTAND -> DUF6079 *-> N6-METHYLASE -> ParB + HNH -> PglZ -><-SF1-SF2-Helicase+REase<-SF1-SF2-Helicase+REase<-XIS-HTH [illegible]</td><td>1157</td><td>Bacteria>Pseudomonadota>Gammaproteobacteria>Enterobacterales>Enterobacteriaceae>Klebsiella/Raoultella_group</td><td>GCF_009907625.1</td><td>Klebsiella_pneumoniae</td><td>GV742_RS00800</td><td>3194..6667</td><td>573</td><td>BREX-system-P-loop-protein-BrxC-[Klebsiella-pneumoniae]</td></tr>
<tr><td>WP_227665788.1</td><td>Type1.BREX</td><td>XIS-HTH->ZnR-><-TIR<-TIR|| BrxA-DNA-binding -> BrxB-BrxF-iSTAND -> DUF6079 *-> ParB + HNH -> N6-METHYLASE ->?-> N6-METHYLASE -p-> PglZ -> SIGMA+OB-Fold+AAA-ATPase+Lon-Protease-BrxL -> [illegible]</td><td>1240</td><td>Bacteria>Pseudomonadota>Gammaproteobacteria>Enterobacterales>Enterobacteriaceae>Klebsiella/Raoultella_group</td><td>GCF_010602405.1</td><td>Klebsiella_variicola</td><td>G4552_RS17215</td><td>23636..27358</td><td>244366</td><td>BREX-system-P-loop-protein-BrxC-[Klebsiella-variicola]</td></tr>
<tr><td>WP_182012819.1</td><td>Type1.BREX</td><td>HIN-HTH-><- SIGMA+OB-Fold+AAA-ATPase+Lon-Protease-BrxL <- PglZ <- N6-METHYLASE <- ParB + HNH <- DUF6079 *<- BrxB-BrxF-iSTAND <- BrxA-DNA-binding <- wHTH+WYL+WCX <-Phage_int_SAM_4+Phag[illegible]</td><td>1225</td><td>Bacteria>Pseudomonadota>Gammaproteobacteria>Enterobacterales>Enterobacteriaceae>Klebsiella/Raoultella_group</td><td>GCF_013926025.1</td><td>Klebsiella_michiganensis</td><td>HV264_RS11965</td><td>2488431..2492108</td><td>1134687</td><td>BREX-system-P-loop-protein-BrxC-[Klebsiella-michiganensis]</td></tr>
<tr><td>WP_187950301.1</td><td>Type1.BREX</td><td>wHTH+WYL+WCX -> BrxA-DNA-binding -> BrxB-BrxF-iSTAND -> DUF6079 *-> ParB + HNH -> N6-METHYLASE -> PglZ -> SIGMA+OB-Fold+AAA-ATPase+Lon-Protease-BrxL -></td><td>1225</td><td>Bacteria>Pseudomonadota>Gammaproteobacteria>Enterobacterales>Enterobacteriaceae>Klebsiella/Raoultella_group</td><td>GCF_014500865.1</td><td>Klebsiella_pneumoniae</td><td>HKB44_RS26575</td><td>2539..6216</td><td>573</td><td>BREX-system-P-loop-protein-BrxC-[Klebsiella-pneumoniae]</td></tr>
<tr><td>WP_202707962.1</td><td>Type1.BREX</td><td>wHTH+WYL+WCX -> BrxA-DNA-binding -> BrxB-BrxF-iSTAND -> DUF6079 *-> ParB + HNH -> N6-METHYLASE -> PglZ -> SIGMA+OB-Fold+AAA-ATPase+Lon-Protease-BrxL -></td><td>1225</td><td>Bacteria>Pseudomonadota>Gammaproteobacteria>Enterobacterales>Enterobacteriaceae>Klebsiella/Raoultella_group</td><td>GCF_016761975.1</td><td>Klebsiella_pneumoniae</td><td>H9R49_RS24845</td><td>3004..6681</td><td>573</td><td>BREX-system-P-loop-protein-BrxC-[Klebsiella-pneumoniae]</td></tr>
<tr><td>WP_049123912.1</td><td>Type1.BREX</td><td>Arm-DNA-bind_3+Phage_integrase-> BrxA-DNA-binding -> BrxB-BrxF-iSTAND -> DUF6079 *-> N6-METHYLASE -> ParB + HNH -> PglZ -><-?<-XIS-HTH||CopG/RHH->nSTAND1+sGTP->?->DUF932-></td><td>1157</td><td>Bacteria>Pseudomonadota>Gammaproteobacteria>Enterobacterales>Enterobacteriaceae>Klebsiella/Raoultella_group</td><td>GCF_018223925.1</td><td>Klebsiella</td><td>KHQ26_RS17845</td><td>3287..6760</td><td>570</td><td>MULTISPECIES:-BREX-system-P-loop-protein-BrxC-[Klebsiella]</td></tr>
<tr><td>WP_217074485.1</td><td>Type1.BREX</td><td><-REase+iREase|| wHTH(SBD)+HNH -><-?<-?<- SIGMA+OB-Fold+AAA-ATPase+Lon-Protease-BrxL <- PglZ <-TOPRIM<-OLD-ABC<- N6-METHYLASE <- DUF6079 *<- BrxB-BrxF-iSTAND <- BrxA-DNA-binding || [illegible]</td><td>1204</td><td>Bacteria>Pseudomonadota>Gammaproteobacteria>Enterobacterales>Enterobacteriaceae>Klebsiella/Raoultella_group</td><td>GCF_019048885.1</td><td>Klebsiella_aerogenes</td><td>KSL85_RS22185</td><td>4652934..4656548</td><td>548</td><td>BREX-system-P-loop-protein-BrxC-[Klebsiella-aerogenes]</td></tr>
<tr><td>WP_219665441.1</td><td>Type1.BREX</td><td><-Phage_holin<-?||MazE_antitoxin->PIN->DEDD_Tnp_IS110+Transposase_20-><- SIGMA+OB-Fold+AAA-ATPase+Lon-Protease-BrxL <- PglZ <- N6-METHYLASE <- ParB + HNH <- DUF6079 *<- BrxB-BrxF-i[illegible]</td><td>1225</td><td>Bacteria>Pseudomonadota>Gammaproteobacteria>Enterobacterales>Enterobacteriaceae>Klebsiella/Raoultella_group</td><td>GCF_019401235.1</td><td>Klebsiella_pneumoniae</td><td>GQQ64_RS25415</td><td>25134..28811</td><td>573</td><td>BREX-system-P-loop-protein-BrxC-[Klebsiella-pneumoniae]</td></tr>
<tr><td>WP_023329155.1</td><td>Type1.BREX</td><td><-SAM-METHYLASE<- wHTH(SBD)+HNH ||tRNA->Arm-DNA-bind_3+Phage_integrase-> wHTH+WYL+WCX -> BrxA-DNA-binding -> BrxB-BrxF-iSTAND -> DUF6079 *-> N6-METHYLASE -> ParB + HNH -> PglZ -><-SF[illegible]</td><td>1157</td><td>Bacteria>Pseudomonadota>Gammaproteobacteria>Enterobacterales>Enterobacteriaceae>Klebsiella/Raoultella_group</td><td>GCF_021264605.1</td><td>Klebsiella_oxytoca</td><td>LVW29_RS13105</td><td>76515..79988</td><td>571</td><td>BREX-system-P-loop-protein-BrxC-[Klebsiella-oxytoca]</td></tr>
<tr><td>WP_235410418.1</td><td>Type1.BREX</td><td><-wHTH+WYL<-sGTP<-?||XIS-HTH->?->?-> BrxA-DNA-binding -> BrxB-BrxF-iSTAND -> DUF6079 *-> ParB + HNH -> N6-METHYLASE ->HEPN->RVT-> N6-METHYLASE -p-> PglZ -> SIGMA+OB-Fold+AAA-ATPase+L[illegible]</td><td>1240</td><td>Bacteria>Pseudomonadota>Gammaproteobacteria>Enterobacterales>Enterobacteriaceae>Klebsiella/Raoultella_group</td><td>GCF_021532695.1</td><td>Klebsiella_pneumoniae</td><td>LZ602_RS12600</td><td>31915..35637</td><td>573</td><td>BREX-system-P-loop-protein-BrxC-[Klebsiella-pneumoniae]</td></tr>
<tr><td>WP_233435617.1</td><td>Type1.BREX</td><td>PIWI-><-Phage_integrase||tRNA-><- SIGMA+OB-Fold+AAA-ATPase+Lon-Protease-BrxL <- PglZ <- N6-METHYLASE -p<- ParB + HNH <- DUF6079 *<- BrxB-BrxF-iSTAND <- BrxA-DNA-binding ||?-><-Glyco_[illegible]</td><td>1240</td><td>Bacteria>Pseudomonadota>Gammaproteobacteria>Enterobacterales>Enterobacteriaceae>Klebsiella/Raoultella_group</td><td>GCF_021699015.1</td><td>Raoultella_ornithinolytica</td><td>INS59_RS15575</td><td>205995..209717</td><td>54291</td><td>BREX-system-P-loop-protein-BrxC-[Raoultella-ornithinolytica]</td></tr>
<tr><td>WP_238640446.1</td><td>Type1.BREX</td><td>Arm-DNA-bind_3+Phage_integrase-><- SIGMA+OB-Fold+AAA-ATPase+Lon-Protease-BrxL <- PglZ <- N6-METHYLASE <- ParB + HNH <- DUF6079 *<- BrxB-BrxF-iSTAND <- BrxA-DNA-binding <-?<-XIS-HTH||[illegible]</td><td>1240</td><td>Bacteria>Pseudomonadota>Gammaproteobacteria>Enterobacterales>Enterobacteriaceae>Klebsiella/Raoultella_group</td><td>GCF_022229165.1</td><td>Klebsiella_pneumoniae</td><td>LLG23_RS24005</td><td>30146..33868</td><td>573</td><td>BREX-system-P-loop-protein-BrxC-[Klebsiella-pneumoniae]</td></tr>
<tr><td>WP_223339928.1</td><td>Type1.BREX</td><td>[illegible]</td><td>1240</td><td>Bacteria>Pseudomonadota>Gammaproteobacteria>Enterobacterales>Enterobacteriaceae>Klebsiella/Raoultella_group</td><td>GCF_022353805.1</td><td>Klebsiella_pneumoniae</td><td>JX616_RS18220</td><td>31893..35615</td><td>573</td><td>BREX-system-P-loop-protein-BrxC-[Klebsiella-pneumoniae]</td></tr>
<tr><td>WP_254541266.1</td><td>Type1.BREX</td><td>[illegible]</td><td>1157</td><td>Bacteria>Pseudomonadota>Gammaproteobacteria>Enterobacterales>Enterobacteriaceae>Klebsiella/Raoultella_group</td><td>GCF_024224875.1</td><td>Klebsiella_pneumoniae</td><td>NLZ12_RS02805</td><td>582600..586073</td><td>573</td><td>BREX-system-P-loop-protein-BrxC-[Klebsiella-pneumoniae]</td></tr>
<tr><td>WP_064161342.1</td><td>Type1.BREX</td><td>[illegible]</td><td>1225</td><td>Bacteria>Pseudomonadota>Gammaproteobacteria>Enterobacterales>Enterobacteriaceae>Klebsiella/Raoultella_group</td><td>GCF_027577885.1</td><td>Klebsiella</td><td>OEE45_RS29300</td><td>43964..47641</td><td>570</td><td>MULTISPECIES:-BREX-system-P-loop-protein-BrxC-[Klebsiella]</td></tr>
<tr><td>WP_064182275.1</td><td>Type1.BREX</td><td>[illegible]</td><td>1225</td><td>Bacteria>Pseudomonadota>Gammaproteobacteria>Enterobacterales>Enterobacteriaceae>Klebsiella/Raoultella_group</td><td>GCF_900084835.1</td><td>Klebsiella_pneumoniae</td><td>A8U42_RS24700</td><td>50900..54577</td><td>573</td><td>BREX-system-P-loop-protein-BrxC-[Klebsiella-pneumoniae]</td></tr>
<tr><td>WP_227643422.1</td><td>Type1.BREX</td><td>[illegible]</td><td>1240</td><td>Bacteria>Pseudomonadota>Gammaproteobacteria>Enterobacterales>Enterobacteriaceae>Klebsiella/Raoultella_group</td><td>GCF_900173735.1</td><td>Klebsiella_pneumoniae</td><td>B7E22_RS15515</td><td>109572..113294</td><td>573</td><td>BREX-system-P-loop-protein-BrxC-[Klebsiella-pneumoniae]</td></tr>
<tr><td>WP_224385469.1</td><td>Type1.BREX</td><td>[illegible]</td><td>1240</td><td>Bacteria>Pseudomonadota>Gammaproteobacteria>Enterobacterales>Enterobacteriaceae>Klebsiella/Raoultella_group</td><td>GCF_902158715.1</td><td>Klebsiella</td><td>FN110_RS17010</td><td>11054..14776</td><td>570</td><td>MULTISPECIES:-BREX-system-P-loop-protein-BrxC-[Klebsiella]</td></tr>
<tr><td>WP_256682067.1</td><td>Type1.BREX</td><td>[illegible]</td><td>1240</td><td>Bacteria>Pseudomonadota>Gammaproteobacteria>Enterobacterales>Enterobacteriaceae>Klebsiella/Raoultella_group</td><td>GCF_902158845.1</td><td>Klebsiella_michiganensis</td><td>FN096_RS13265</td><td>22370..26092</td><td>1134687</td><td>BREX-system-P-loop-protein-BrxC-[Klebsiella-michiganensis]</td></tr>
<tr><td>WP_227635281.1</td><td>Type1.BREX</td><td>[illegible]</td><td>1240</td><td>Bacteria>Pseudomonadota>Gammaproteobacteria>Enterobacterales>Enterobacteriaceae>Klebsiella/Raoultella_group</td><td>GCF_902159715.1</td><td>Klebsiella</td><td>PIK69_RS17535</td><td>2737..6459</td><td>570</td><td>MULTISPECIES:-BREX-system-P-loop-protein-BrxC-[Klebsiella]</td></tr>
<tr><td>WP_101867996.1</td><td>Type1.BREX</td><td>[illegible]</td><td>1157</td><td>Bacteria>Pseudomonadota>Gammaproteobacteria>Enterobacterales>Enterobacteriaceae>Klebsiella/Raoultella_group</td><td>-</td><td>Klebsiella_michiganensis</td><td>CWN04_RS12955</td><td>12917..16390</td><td>1134687</td><td>BREX-system-P-loop-protein-BrxC-[Klebsiella-michiganensis]</td></tr>
<tr><td>WP_154904474.1</td><td>Type1.BREX</td><td>[illegible]</td><td>1233</td><td>Bacteria>Pseudomonadota>Gammaproteobacteria>Enterobacterales>Enterobacteriaceae>Klebsiella/Raoultella_group</td><td>-</td><td>Klebsiella_michiganensis</td><td>CWN50_01600</td><td>4530..8231</td><td>1134687</td><td>BREX-system-P-loop-protein-BrxC-[Klebsiella-michiganensis]</td></tr>
<tr><td>WP_227627429.1</td><td>Type1.BREX</td><td>[illegible]</td><td>1240</td><td>Bacteria>Pseudomonadota>Gammaproteobacteria>Enterobacterales>Enterobacteriaceae>Klebsiella/Raoultella_group</td><td>-</td><td>Klebsiella_michiganensis</td><td>G4403_RS10440</td><td>98242..101964</td><td>1134687</td><td>BREX-system-P-loop-protein-BrxC-[Klebsiella-michiganensis]</td></tr>
<tr><td>WP_097365540.1</td><td>Type1.BREX</td><td>[illegible]</td><td>1233</td><td>Bacteria>Pseudomonadota>Gammaproteobacteria>Enterobacterales>Enterobacteriaceae>Klebsiella/Raoultella_group</td><td>-</td><td>Klebsiella_oxytoca</td><td>I9Y61_001542</td><td>37432..41133</td><td>571</td><td>MULTISPECIES:-BREX-system-P-loop-protein-BrxC-[Klebsiella]</td></tr>
<tr><td>WP_117042583.1</td><td>Type1.BREX</td><td>[illegible]</td><td>1233</td><td>Bacteria>Pseudomonadota>Gammaproteobacteria>Enterobacterales>Enterobacteriaceae>Klebsiella/Raoultella_group</td><td>-</td><td>Klebsiella_pneumoniae</td><td>AM381_RS08835</td><td>19475..23176</td><td>573</td><td>MULTISPECIES:-BREX-system-P-loop-protein-BrxC-[Klebsiella]</td></tr>
<tr><td>WP_114263049.1</td><td>Type1.BREX</td><td>[illegible]</td><td>1233</td><td>Bacteria>Pseudomonadota>Gammaproteobacteria>Enterobacterales>Enterobacteriaceae>Klebsiella/Raoultella_group</td><td>-</td><td>Klebsiella_pneumoniae</td><td>AN2353V1_3685</td><td>3967252..3970953</td><td>573</td><td>MULTISPECIES:-BREX-system-P-loop-protein-BrxC-[Enterobacteriaceae]</td></tr>
<tr><td>WP_062729754.1</td><td>Type1.BREX</td><td>[illegible]</td><td>1233</td><td>Bacteria>Pseudomonadota>Gammaproteobacteria>Enterobacterales>Enterobacteriaceae>Klebsiella/Raoultella_group</td><td>-</td><td>Klebsiella_pneumoniae</td><td>C0077_01580</td><td>326913..330614</td><td>573</td><td>MULTISPECIES:-BREX-system-P-loop-protein-BrxC-[Enterobacteriaceae]</td></tr>
<tr><td>WP_073511310.1</td><td>Type1.BREX</td><td>[illegible]</td><td>1233</td><td>Bacteria>Pseudomonadota>Gammaproteobacteria>Enterobacterales>Enterobacteriaceae>Klebsiella/Raoultella_group</td><td>-</td><td>Klebsiella_pneumoniae</td><td>L7V03_004584</td><td>32186..35887</td><td>573</td><td>BREX-system-P-loop-protein-BrxC-[Klebsiella-pneumoniae]</td></tr>
<tr><td>WP_117247358.1</td><td>Type1.BREX</td><td>[illegible]</td><td>1233</td><td>Bacteria>Pseudomonadota>Gammaproteobacteria>Enterobacterales>Enterobacteriaceae>Klebsiella/Raoultella_group</td><td>-</td><td>Klebsiella_pneumoniae</td><td>L7W77_004838</td><td>45573..49274</td><td>573</td><td>MULTISPECIES:-BREX-system-P-loop-protein-BrxC-[Klebsiella]</td></tr>
<tr><td>WP_117076897.1</td><td>Type1.BREX</td><td>[illegible]</td><td>1233</td><td>Bacteria>Pseudomonadota>Gammaproteobacteria>Enterobacterales>Enterobacteriaceae>Klebsiella/Raoultella_group</td><td>-</td><td>Klebsiella_pneumoniae</td><td>ML041_004566</td><td>31807..35508</td><td>573</td><td>BREX-system-P-loop-protein-BrxC-[Klebsiella-pneumoniae]</td></tr>
<tr><td>EJN1576646.1</td><td>Type1.BREX</td><td>[illegible]</td><td>1233</td><td>Bacteria>Pseudomonadota>Gammaproteobacteria>Enterobacterales>Enterobacteriaceae>Klebsiella/Raoultella_group</td><td>-</td><td>Klebsiella_variicola</td><td>NPH96_004932</td><td>32414..36115</td><td>244366</td><td>BREX-system-P-loop-protein-BrxC-[Klebsiella-variicola]</td></tr>
<tr><td>URQ56457.1</td><td>Type1.BREX</td><td>[illegible]</td><td>769</td><td>Bacteria>Pseudomonadota>Gammaproteobacteria>Enterobacterales>Enterobacteriaceae>Klebsiella/Raoultella_group</td><td>-</td><td>Raoultella_ornithinolytica</td><td>.</td><td>79835..82144</td><td>54291</td><td>Hypothetical-protein-[Raoultella-ornithinolytica]</td></tr>
<tr><td>EHT9899814.1</td><td>Type1.BREX</td><td>[illegible]</td><td>1233</td><td>Bacteria>Pseudomonadota>Gammaproteobacteria>Enterobacterales>Enterobacteriaceae>Klebsiella/Raoultella_group</td><td>-</td><td>Raoultella_ornithinolytica</td><td>KY209_001651</td><td>277271..280972</td><td>54291</td><td>BREX-system-P-loop-protein-BrxC-[Raoultella-ornithinolytica]</td></tr>
<tr><td>WP_094898295.1</td><td>Type1.BREX</td><td>[illegible]</td><td>1233</td><td>Bacteria>Pseudomonadota>Gammaproteobacteria>Enterobacterales>Enterobacteriaceae>Klebsiella/Raoultella_group</td><td>-</td><td>Raoultella_planticola</td><td>FQG10_00765</td><td>2767..6468</td><td>575</td><td>BREX-system-P-loop-protein-BrxC-[Raoultella-planticola]</td></tr>
<tr><td>HAT1572918.1</td><td>Type1.BREX</td><td>[illegible]</td><td>1225</td><td>Bacteria>Pseudomonadota>Gammaproteobacteria>Enterobacterales>Enterobacteriaceae>Kluyvera</td><td>GCA_015892965.1</td><td>Kluyvera_cryocrescens</td><td>I8Y10_004331</td><td>41743..45420</td><td>580</td><td>BREX-system-P-loop-protein-BrxC-[Kluyvera-cryocrescens]</td></tr>
<tr><td>QXB23949.1</td><td>Type1.BREX</td><td>[illegible]</td><td>1233</td><td>Bacteria>Pseudomonadota>Gammaproteobacteria>Enterobacterales>Enterobacteriaceae>Lelliottia</td><td>GCA_019048185.1</td><td>Lelliottia_amnigena</td><td>I6L76_15755</td><td>3242307..3246008</td><td>61646</td><td>BREX-system-P-loop-protein-BrxC-[Lelliottia-amnigena]</td></tr>
<tr><td>WP_254208058.1</td><td>Type1.BREX</td><td>[illegible]</td><td>1237</td><td>Bacteria>Pseudomonadota>Gammaproteobacteria>Enterobacterales>Enterobacteriaceae>Lelliottia</td><td>GCF_019048185.1</td><td>Lelliottia_amnigena</td><td>I6L76_RS15755</td><td>3242295..3246008</td><td>61646</td><td>BREX-system-P-loop-protein-BrxC-[Lelliottia-amnigena]</td></tr>
<tr><td>WP_216981896.1</td><td>Type1.BREX</td><td>[illegible]</td><td>1233</td><td>Bacteria>Pseudomonadota>Gammaproteobacteria>Enterobacterales>Enterobacteriaceae>Lelliottia</td><td>-</td><td>Lelliottia_amnigena</td><td>I6L76_15755</td><td>3242307..3246008</td><td>61646</td><td>BREX-system-P-loop-protein-BrxC-[Lelliottia-amnigena]</td></tr>
<tr><td>WP_265846293.1</td><td>Type1.BREX</td><td>[illegible]</td><td>1238</td><td>Bacteria>Pseudomonadota>Gammaproteobacteria>Enterobacterales>Enterobacteriaceae>Plesiomonas</td><td>GCF_026240155.1</td><td>Plesiomonas_shigelloides</td><td>OQ486_RS15170</td><td>39648..43364</td><td>703</td><td>BREX-system-P-loop-protein-BrxC-[Plesiomonas-shigelloides]</td></tr>
<tr><td>EHC84522.1</td><td>Type1.BREX</td><td>[illegible]</td><td>1247</td><td>Bacteria>Pseudomonadota>Gammaproteobacteria>Enterobacterales>Enterobacteriaceae>Salmonella</td><td>GCA_000231705.2</td><td>Salmonella_enterica_subsp._enterica_serovar_Uganda_str._R8-3404</td><td>LTSEUGA_5994</td><td>10868..14611</td><td>913083</td><td>hypothetical-protein-LTSEUGA_5994-[Salmonella-enterica-subsp.-enterica-serovar-Uganda-str.-R8-3404]</td></tr>
<tr><td>APV85542.1</td><td>Type1.BREX</td><td>[illegible]</td><td>1233</td><td>Bacteria>Pseudomonadota>Gammaproteobacteria>Enterobacterales>Enterobacteriaceae>Salmonella</td><td>GCA_000487155.2</td><td>Salmonella_enterica_subsp._enterica_serovar_Inverness_str._ATCC_10720</td><td>SEEI0720_021515</td><td>4182897..4186598</td><td>941187</td><td>DNA-repair-protein-[Salmonella-enterica-subsp.-enterica-serovar-Inverness-str.-ATCC-10720]</td></tr>
<tr><td>AXD95438.1</td><td>Type1.BREX</td><td>[illegible]</td><td>1233</td><td>Bacteria>Pseudomonadota>Gammaproteobacteria>Enterobacterales>Enterobacteriaceae>Salmonella</td><td>GCA_003325015.1</td><td>Salmonella_enterica</td><td>CHC75_12745</td><td>2557798..2561499</td><td>28901</td><td>BREX-system-P-loop-protein-BrxC-[Salmonella-enterica]</td></tr>
<tr><td>EAA4435938.1</td><td>Type1.BREX</td><td>[illegible]</td><td>1233</td><td>Bacteria>Pseudomonadota>Gammaproteobacteria>Enterobacterales>Enterobacteriaceae>Salmonella</td><td>GCA_004179155.1</td><td>Salmonella_enterica_subsp._salamae</td><td>DPE44_05505</td><td>71371..75072</td><td>59202</td><td>BREX-system-P-loop-protein-BrxC-[Salmonella-enterica-subsp.-salamae]</td></tr>
<tr><td>EAU0181515.1</td><td>Type1.BREX</td><td>[illegible]</td><td>1233</td><td>Bacteria>Pseudomonadota>Gammaproteobacteria>Enterobacterales>Enterobacteriaceae>Salmonella</td><td>GCA_005756755.1</td><td>Salmonella_enterica</td><td>A4I02_04465</td><td>211497..215198</td><td>28901</td><td>BREX-system-P-loop-protein-BrxC-[Salmonella-enterica]</td></tr>
<tr><td>EAY5330993.1</td><td>Type1.BREX</td><td>[illegible]</td><td>1233</td><td>Bacteria>Pseudomonadota>Gammaproteobacteria>Enterobacterales>Enterobacteriaceae>Salmonella</td><td>GCA_006011505.1</td><td>Salmonella_enterica</td><td>LS33_08700</td><td>4073..7774</td><td>28901</td><td>BREX-system-P-loop-protein-BrxC-[Salmonella-enterica]</td></tr>
<tr><td>EAY8201055.1</td><td>Type1.BREX</td><td>[illegible]</td><td>1233</td><td>Bacteria>Pseudomonadota>Gammaproteobacteria>Enterobacterales>Enterobacteriaceae>Salmonella</td><td>GCA_006024175.1</td><td>Salmonella_enterica</td><td>ATQ84_14875</td><td>10975..14676</td><td>28901</td><td>BREX-system-P-loop-protein-BrxC-[Salmonella-enterica]</td></tr>
<tr><td>EBF3214239.1</td><td>Type1.BREX</td><td>[illegible]</td><td>1233</td><td>Bacteria>Pseudomonadota>Gammaproteobacteria>Enterobacterales>Enterobacteriaceae>Salmonella</td><td>GCA_006336125.1</td><td>Salmonella_enterica</td><td>FHK34_03265</td><td>183037..186738</td><td>28901</td><td>BREX-system-P-loop-protein-BrxC-[Salmonella-enterica]</td></tr>
<tr><td>EBS7807126.1</td><td>Type1.BREX</td><td>[illegible]</td><td>1233</td><td>Bacteria>Pseudomonadota>Gammaproteobacteria>Enterobacterales>Enterobacteriaceae>Salmonella</td><td>GCA_007023485.1</td><td>Salmonella_enterica</td><td>CD664_12540</td><td>43837..47538</td><td>28901</td><td>BREX-system-P-loop-protein-BrxC-[Salmonella-enterica]</td></tr>
<tr><td>EBW8181804.1</td><td>Type1.BREX</td><td>[illegible]</td><td>1233</td><td>Bacteria>Pseudomonadota>Gammaproteobacteria>Enterobacterales>Enterobacteriaceae>Salmonella</td><td>GCA_007227235.1</td><td>Salmonella_enterica_subsp._enterica_serovar_Uganda</td><td>AR874_12565</td><td>406191..409892</td><td>487004</td><td>BREX-system-P-loop-protein-BrxC-[Salmonella-enterica-subsp.-enterica-serovar-Uganda]</td></tr>
<tr><td>EBZ8208146.1</td><td>Type1.BREX</td><td>[illegible]</td><td>1233</td><td>Bacteria>Pseudomonadota>Gammaproteobacteria>Enterobacterales>Enterobacteriaceae>Salmonella</td><td>GCA_007351255.1</td><td>Salmonella_enterica_subsp._enterica_serovar_Zanzibar</td><td>EGK94_05900</td><td>411162..414863</td><td>399588</td><td>BREX-system-P-loop-protein-BrxC-[Salmonella-enterica-subsp.-enterica-serovar-Zanzibar]</td></tr>
<tr><td>ECD4420808.1</td><td>Type1.BREX</td><td>[illegible]</td><td>1233</td><td>Bacteria>Pseudomonadota>Gammaproteobacteria>Enterobacterales>Enterobacteriaceae>Salmonella</td><td>GCA_007522325.1</td><td>Salmonella_enterica_subsp._enterica_serovar_Berkeley</td><td>E0910_10430</td><td>8115..11816</td><td>1965103</td><td>BREX-system-P-loop-protein-BrxC-[Salmonella-enterica-subsp.-enterica-serovar-Berkeley]</td></tr>
<tr><td>ECJ2539588.1</td><td>Type1.BREX</td><td>[illegible]</td><td>1157</td><td>Bacteria>Pseudomonadota>Gammaproteobacteria>Enterobacterales>Enterobacteriaceae>Salmonella</td><td>GCA_007808255.1</td><td>Salmonella_enterica_subsp._salamae</td><td>FNI64_03990</td><td>384975..388448</td><td>59202</td><td>BREX-system-P-loop-protein-BrxC-[Salmonella-enterica-subsp.-salamae]</td></tr>
<tr><td>ECK3339581.1</td><td>Type1.BREX</td><td>[illegible]</td><td>1233</td><td>Bacteria>Pseudomonadota>Gammaproteobacteria>Enterobacterales>Enterobacteriaceae>Salmonella</td><td>GCA_007873945.1</td><td>Salmonella_enterica</td><td>FRH68_09850</td><td>220275..223976</td><td>28901</td><td>BREX-system-P-loop-protein-BrxC-[Salmonella-enterica]</td></tr>
<tr><td>EDB2044587.1</td><td>Type1.BREX</td><td>[illegible]</td><td>1233</td><td>Bacteria>Pseudomonadota>Gammaproteobacteria>Enterobacterales>Enterobacteriaceae>Salmonella</td><td>GCA_008894145.1</td><td>Salmonella_enterica_subsp._enterica</td><td>F9P84_01850</td><td>405984..409685</td><td>59201</td><td>BREX-system-P-loop-protein-BrxC-[Salmonella-enterica-subsp.-enterica]</td></tr>
<tr><td>EHE5127202.1</td><td>Type1.BREX</td><td>[illegible]</td><td>1233</td><td>Bacteria>Pseudomonadota>Gammaproteobacteria>Enterobacterales>Enterobacteriaceae>Salmonella</td><td>GCA_017241415.1</td><td>Salmonella_enterica</td><td>JMY69_002120</td><td>181649..185350</td><td>28901</td><td>BREX-system-P-loop-protein-BrxC-[Salmonella-enterica]</td></tr>
<tr><td>EIB7574746.1</td><td>Type1.BREX</td><td>[illegible]</td><td>1233</td><td>Bacteria>Pseudomonadota>Gammaproteobacteria>Enterobacterales>Enterobacteriaceae>Salmonella</td><td>GCA_019905755.1</td><td>Salmonella_enterica</td><td>K9I12_000373</td><td>406371..410072</td><td>28901</td><td>BREX-system-P-loop-protein-BrxC-[Salmonella-enterica]</td></tr>
<tr><td>EJV9378859.1</td><td>Type1.BREX</td><td>[illegible]</td><td>1225</td><td>Bacteria>Pseudomonadota>Gammaproteobacteria>Enterobacterales>Enterobacteriaceae>Salmonella</td><td>GCA_025367915.1</td><td>Salmonella_enterica</td><td>N7887_004537</td><td>2923..6600</td><td>28901</td><td>BREX-system-P-loop-protein-BrxC-[Salmonella-enterica]</td></tr>
<tr><td>WP_077956997.1</td><td>Type1.BREX</td><td>[illegible]</td><td>1240</td><td>Bacteria>Pseudomonadota>Gammaproteobacteria>Enterobacterales>Enterobacteriaceae>Salmonella</td><td>GCF_000487155.2</td><td>Salmonella_enterica</td><td>A7T10_RS09345</td><td>418937..422659</td><td>28901</td><td>BREX-system-P-loop-protein-BrxC-[Salmonella-enterica]</td></tr>
<tr><td>WP_079798429.1</td><td>Type1.BREX</td><td>[illegible]</td><td>1240</td><td>Bacteria>Pseudomonadota>Gammaproteobacteria>Enterobacterales>Enterobacteriaceae>Salmonella</td><td>GCF_003325015.1</td><td>Salmonella_enterica</td><td>HFU03_RS19925</td><td>4101513..4105235</td><td>28901</td><td>BREX-system-P-loop-protein-BrxC-[Salmonella-enterica]</td></tr>
<tr><td>WP_079805580.1</td><td>Type1.BREX</td><td>[illegible]</td><td>1240</td><td>Bacteria>Pseudomonadota>Gammaproteobacteria>Enterobacterales>Enterobacteriaceae>Salmonella</td><td>GCF_016903075.1</td><td>Salmonella_enterica</td><td>AJH70_RS01330</td><td>291078..294800</td><td>28901</td><td>BREX-system-P-loop-protein-BrxC-[Salmonella-enterica]</td></tr>
<tr><td>WP_259423574.1</td><td>Type1.BREX</td><td>[illegible]</td><td>921</td><td>Bacteria>Pseudomonadota>Gammaproteobacteria>Enterobacterales>Enterobacteriaceae>Salmonella</td><td>GCF_024919285.1</td><td>Salmonella_enterica</td><td>NXM87_RS15320</td><td>123096..125861</td><td>28901</td><td>BREX-system-P-loop-protein-BrxC-[Salmonella-enterica]</td></tr>
<tr><td>WP_274877667.1</td><td>Type1.BREX</td><td>[illegible]</td><td>806</td><td>Bacteria>Pseudomonadota>Gammaproteobacteria>Enterobacterales>Enterobacteriaceae>Salmonella</td><td>GCF_028892685.1</td><td>Salmonella_enterica</td><td>PQP98_RS20365</td><td>4148316..4150736</td><td>28901</td><td>BREX-system-P-loop-protein-BrxC-[Salmonella-enterica]</td></tr>
<tr><td>WP_274894925.1</td><td>Type1.BREX</td><td>[illegible]</td><td>1240</td><td>Bacteria>Pseudomonadota>Gammaproteobacteria>Enterobacterales>Enterobacteriaceae>Salmonella</td><td>GCF_028892745.1</td><td>Salmonella_enterica</td><td>PQQ04_RS20380</td><td>4148528..4152250</td><td>28901</td><td>BREX-system-P-loop-protein-BrxC-[Salmonella-enterica]</td></tr>
<tr><td>WP_261983090.1</td><td>Type1.BREX</td><td>[illegible]</td><td>1240</td><td>Bacteria>Pseudomonadota>Gammaproteobacteria>Enterobacterales>Enterobacteriaceae>Salmonella</td><td>GCF_901212535.1</td><td>Salmonella_enterica</td><td>ECOMC1_RS14180</td><td>234231..237953</td><td>28901</td><td>BREX-system-P-loop-protein-BrxC-[Salmonella-enterica]</td></tr>
<tr><td>WP_154023496.1</td><td>Type1.BREX</td><td>[illegible]</td><td>1233</td><td>Bacteria>Pseudomonadota>Gammaproteobacteria>Enterobacterales>Enterobacteriaceae>Salmonella</td><td>-</td><td>Salmonella_enterica</td><td>A4I02_04465</td><td>211497..215198</td><td>28901</td><td>BREX-system-P-loop-protein-BrxC-[Salmonella-enterica]</td></tr>
<tr><td>WP_097363546.1</td><td>Type1.BREX</td><td>[illegible]</td><td>1233</td><td>Bacteria>Pseudomonadota>Gammaproteobacteria>Enterobacterales>Enterobacteriaceae>Salmonella</td><td>-</td><td>Salmonella_enterica</td><td>CHC75_12745</td><td>2557798..2561499</td><td>28901</td><td>BREX-system-P-loop-protein-BrxC-[Salmonella-enterica]</td></tr>
<tr><td>WP_023227805.1</td><td>Type1.BREX</td><td>[illegible]</td><td>1233</td><td>Bacteria>Pseudomonadota>Gammaproteobacteria>Enterobacterales>Enterobacteriaceae>Salmonella</td><td>-</td><td>Salmonella_enterica</td><td>SEEI0720_021515</td><td>4182897..4186598</td><td>28901</td><td>BREX-system-P-loop-protein-BrxC-[Salmonella-enterica]</td></tr>
<tr><td>MRS92356.1</td><td>Type1.BREX</td><td>[illegible]</td><td>1157</td><td>Bacteria>Pseudomonadota>Gammaproteobacteria>Enterobacterales>Enterobacteriaceae>unclassified_Enterobacteriaceae</td><td>GCA_009668015.1</td><td>Enterobacteriaceae_bacterium_RIT714</td><td>GJV04_20305</td><td>30577..34050</td><td>2666193</td><td>BREX-system-P-loop-protein-BrxC-[Enterobacteriaceae-bacterium-RIT714]</td></tr>
<tr><td>UCB31815.1</td><td>Type1.BREX</td><td>[illegible]</td><td>1240</td><td>Bacteria>Pseudomonadota>Gammaproteobacteria>Enterobacterales>Erwiniaceae>Duffyella</td><td>GCA_020342335.1</td><td>Duffyella_gerundensis</td><td>J9874_02366</td><td>2569295..2573017</td><td>1619313</td><td>hypothetical-protein-J9874_02366-[Duffyella-gerundensis]</td></tr>
<tr><td>WP_225700910.1</td><td>Type1.BREX</td><td>[illegible]</td><td>1237</td><td>Bacteria>Pseudomonadota>Gammaproteobacteria>Enterobacterales>Erwiniaceae>Duffyella</td><td>GCF_020342335.1</td><td>Duffyella_gerundensis</td><td>J9874_RS11820</td><td>2569304..2573017</td><td>1619313</td><td>BREX-system-P-loop-protein-BrxC-[Duffyella-gerundensis]</td></tr>
<tr><td>HCP27301.1</td><td>Type1.BREX</td><td>[illegible]</td><td>1233</td><td>Bacteria>Pseudomonadota>Gammaproteobacteria>Enterobacterales>Erwiniaceae>Pantoea</td><td>GCA_003533915.1</td><td>Pantoea_ananatis</td><td>DIT85_12780</td><td>28453..32154</td><td>553</td><td>BREX-system-P-loop-protein-BrxC-[Pantoea-ananatis]</td></tr>
<tr><td>NEG80386.1</td><td>Type1.BREX</td><td>[illegible]</td><td>1233</td><td>Bacteria>Pseudomonadota>Gammaproteobacteria>Enterobacterales>Erwiniaceae>Pantoea</td><td>GCA_010667945.1</td><td>Pantoea_agglomerans</td><td>GQQ17_05160</td><td>300399..304100</td><td>549</td><td>BREX-system-P-loop-protein-BrxC-[Pantoea-agglomerans]</td></tr>
<tr><td>WP_242462783.1</td><td>Type1.BREX</td><td>[illegible]</td><td>1240</td><td>Bacteria>Pseudomonadota>Gammaproteobacteria>Enterobacterales>Erwiniaceae>Pantoea</td><td>GCF_010667945.1</td><td>Pantoea_agglomerans</td><td>GQQ17_RS05165</td><td>300399..304121</td><td>549</td><td>BREX-system-P-loop-protein-BrxC-[Pantoea-agglomerans]</td></tr>
<tr><td>WP_167070220.1</td><td>Type1.BREX</td><td>[illegible]</td><td>1184</td><td>Bacteria>Pseudomonadota>Gammaproteobacteria>Enterobacterales>Erwiniaceae>Pantoea</td><td>GCF_011752565.1</td><td>Pantoea_sp._Ap-967</td><td>F3J45_27210</td><td>36650..40204</td><td>2608362</td><td>BREX-system-P-loop-protein-BrxC-[Pantoea-sp.-Ap-967]</td></tr>
<tr><td>WP_187492467.1</td><td>Type1.BREX</td><td>[illegible]</td><td>1157</td><td>Bacteria>Pseudomonadota>Gammaproteobacteria>Enterobacterales>Erwiniaceae>Pantoea</td><td>GCF_014353895.1</td><td>Pantoea_agglomerans</td><td>IBU46_RS01740</td><td>23077..26550</td><td>549</td><td>BREX-system-P-loop-protein-BrxC-[Pantoea-agglomerans]</td></tr>
<tr><td>WP_272844016.1</td><td>Type1.BREX</td><td>[illegible]</td><td>1157</td><td>Bacteria>Pseudomonadota>Gammaproteobacteria>Enterobacterales>Erwiniaceae>Pantoea</td><td>GCF_028555165.1</td><td>Pantoea_ananatis</td><td>SZ66_RS15300</td><td>342532..346005</td><td>553</td><td>BREX-system-P-loop-protein-BrxC-[Pantoea-ananatis]</td></tr>
<tr><td>WP_000687847.1</td><td>Type1.BREX</td><td>[illegible]</td><td>1225</td><td>Bacteria>Pseudomonadota>Gammaproteobacteria>Enterobacterales</td><td>GCF_000026225.1</td><td>Enterobacteriaceae</td><td>EFER_RS00085</td><td>15017..18694</td><td>543</td><td>MULTISPECIES:-BREX-system-P-loop-protein-BrxC-[Enterobacteriaceae]</td></tr>
<tr><td>WP_006788201.1</td><td>Type1.BREX</td><td>[illegible]</td><td>1225</td><td>Bacteria>Pseudomonadota>Gammaproteobacteria>Enterobacterales</td><td>GCF_000388555.1</td><td>Enterobacteriaceae</td><td>MUG00_RS24960</td><td>10799..14476</td><td>543</td><td>MULTISPECIES:-BREX-system-P-loop-protein-BrxC-[Enterobacteriaceae]</td></tr>
<tr><td>WP_032187199.1</td><td>Type1.BREX</td><td>[illegible]</td><td>1225</td><td>Bacteria>Pseudomonadota>Gammaproteobacteria>Enterobacterales</td><td>GCF_000703445.1</td><td>Enterobacteriaceae</td><td>AD25_RS20800</td><td>23140..26817</td><td>543</td><td>MULTISPECIES:-BREX-system-P-loop-protein-BrxC-[Enterobacteriaceae]</td></tr>
<tr><td>WP_054528932.1</td><td>Type1.BREX</td><td>[illegible]</td><td>1225</td><td>Bacteria>Pseudomonadota>Gammaproteobacteria>Enterobacterales</td><td>GCF_002788295.1</td><td>Enterobacteriaceae</td><td>AM338_RS26100</td><td>11174..14851</td><td>543</td><td>MULTISPECIES:-BREX-system-P-loop-protein-BrxC-[Enterobacteriaceae]</td></tr>
<tr><td>WP_223177211.1</td><td>Type1.BREX</td><td>[illegible]</td><td>1240</td><td>Bacteria>Pseudomonadota>Gammaproteobacteria>Enterobacterales</td><td>GCF_004312085.1</td><td>Enterobacteriaceae</td><td>EOM53_RS14900</td><td>53286..57008</td><td>543</td><td>MULTISPECIES:-BREX-system-P-loop-protein-BrxC-[Enterobacteriaceae]</td></tr>
<tr><td>WP_117259785.1</td><td>Type1.BREX</td><td>[illegible]</td><td>1157</td><td>Bacteria>Pseudomonadota>Gammaproteobacteria>Enterobacterales</td><td>GCF_013395115.1</td><td>Enterobacteriaceae</td><td>HYG96_RS13000</td><td>239421..242894</td><td>543</td><td>MULTISPECIES:-BREX-system-P-loop-protein-BrxC-[Enterobacteriaceae]</td></tr>
<tr><td>WP_232051962.1</td><td>Type1.BREX</td><td>[illegible]</td><td>1240</td><td>Bacteria>Pseudomonadota>Gammaproteobacteria>Enterobacterales</td><td>GCF_018421715.1</td><td>Enterobacteriaceae</td><td>L6492_RS02235</td><td>461320..465042</td><td>543</td><td>MULTISPECIES:-BREX-system-P-loop-protein-BrxC-[Enterobacteriaceae]</td></tr>
<tr><td>WP_224243997.1</td><td>Type1.BREX</td><td>[illegible]</td><td>1157</td><td>Bacteria>Pseudomonadota>Gammaproteobacteria>Enterobacterales</td><td>GCF_023752605.1</td><td>Enterobacteriaceae</td><td>M8S83_RS11470</td><td>342574..346047</td><td>543</td><td>MULTISPECIES:-BREX-system-P-loop-protein-BrxC-[Enterobacteriaceae]</td></tr>
<tr><td>WP_224293199.1</td><td>Type1.BREX</td><td>[illegible]</td><td>1240</td><td>Bacteria>Pseudomonadota>Gammaproteobacteria>Enterobacterales</td><td>GCF_029226505.1</td><td>Enterobacteriaceae</td><td>PVK22_RS25565</td><td>5368784..5372506</td><td>543</td><td>MULTISPECIES:-BREX-system-P-loop-protein-BrxC-[Enterobacteriaceae]</td></tr>
<tr><td>WP_063149682.1</td><td>Type1.BREX</td><td>[illegible]</td><td>1225</td><td>Bacteria>Pseudomonadota>Gammaproteobacteria>Enterobacterales</td><td>GCF_900075035.1</td><td>Enterobacteriaceae</td><td>K1J36_RS23220</td><td>10587..14264</td><td>543</td><td>MULTISPECIES:-BREX-system-P-loop-protein-BrxC-[Enterobacteriaceae]</td></tr>
<tr><td>WP_045345932.1</td><td>Type1.BREX</td><td>[illegible]</td><td>1157</td><td>Bacteria>Pseudomonadota>Gammaproteobacteria>Enterobacterales</td><td>GCF_902160145.1</td><td>Enterobacteriaceae</td><td>FN179_RS14130</td><td>343492..346965</td><td>543</td><td>MULTISPECIES:-BREX-system-P-loop-protein-BrxC-[Enterobacteriaceae]</td></tr>
<tr><td>WP_047929056.1</td><td>Type1.BREX</td><td>[illegible]</td><td>1225</td><td>Bacteria>Pseudomonadota>Gammaproteobacteria>Enterobacterales</td><td>GCF_904799765.1</td><td>Enterobacteriaceae</td><td>PCN061_RS24650</td><td>104442..108119</td><td>543</td><td>MULTISPECIES:-BREX-system-P-loop-protein-BrxC-[Enterobacteriaceae]</td></tr>
<tr><td>WP_175320436.1</td><td>Type1.BREX</td><td>[illegible]</td><td>1233</td><td>Bacteria>Pseudomonadota>Gammaproteobacteria>Enterobacterales>Hafniaceae>Hafnia</td><td>GCF_013360205.1</td><td>Hafnia_paralvei</td><td>G4F99_RS14350</td><td>55379..59080</td><td>546367</td><td>BREX-system-P-loop-protein-BrxC-[Hafnia-paralvei]</td></tr>
<tr><td>WP_234619586.1</td><td>Type1.BREX</td><td>[illegible]</td><td>1237</td><td>Bacteria>Pseudomonadota>Gammaproteobacteria>Enterobacterales>Hafniaceae>Hafnia</td><td>GCF_021440285.1</td><td>Hafnia_alvei</td><td>LZ667_RS17495</td><td>58169..61882</td><td>569</td><td>BREX-system-P-loop-protein-BrxC-[Hafnia-alvei]</td></tr>
<tr><td>WP_108479632.1</td><td>Type1.BREX</td><td>[illegible]</td><td>1225</td><td>Bacteria>Pseudomonadota>Gammaproteobacteria>Enterobacterales>Morganellaceae</td><td>GCF_003057415.1</td><td>Providencia</td><td>C6581_RS17855</td><td>39720..43397</td><td>586</td><td>MULTISPECIES:-BREX-system-P-loop-protein-BrxC-[Providencia]</td></tr>
<tr><td>WP_241542954.1</td><td>Type1.BREX</td><td>[illegible]</td><td>1225</td><td>Bacteria>Pseudomonadota>Gammaproteobacteria>Enterobacterales>Morganellaceae>Moellerella</td><td>GCF_022592015.1</td><td>Moellerella_wisconsensis</td><td>MNY72_RS15945</td><td>14916..18593</td><td>158849</td><td>BREX-system-P-loop-protein-BrxC-[Moellerella-wisconsensis]</td></tr>
<tr><td>AUR32853.1</td><td>Type1.BREX</td><td>[illegible]</td><td>1233</td><td>Bacteria>Pseudomonadota>Gammaproteobacteria>Enterobacterales>Morganellaceae>Morganella</td><td>GCA_002891475.1</td><td>Morganella_morganii</td><td>C1O70_15730</td><td>3342273..3345974</td><td>582</td><td>BREX-system-P-loop-protein-BrxC-[Morganella-morganii]</td></tr>
<tr><td>MBS5193172.1</td><td>Type1.BREX</td><td>[illegible]</td><td>1157</td><td>Bacteria>Pseudomonadota>Gammaproteobacteria>Enterobacterales>Morganellaceae>Morganella</td><td>GCA_018366325.1</td><td>Morganella_morganii</td><td>KHY92_05180</td><td>18454..21927</td><td>582</td><td>BREX-system-P-loop-protein-BrxC-[Morganella-morganii]</td></tr>
<tr><td>WP_225973345.1</td><td>Type1.BREX</td><td>[illegible]</td><td>1240</td><td>Bacteria>Pseudomonadota>Gammaproteobacteria>Enterobacterales>Morganellaceae>Morganella</td><td>GCF_002891475.1</td><td>Morganella_morganii</td><td>C1O70_RS15730</td><td>3342252..3345974</td><td>582</td><td>BREX-system-P-loop-protein-BrxC-[Morganella-morganii]</td></tr>
<tr><td>WP_214179407.1</td><td>Type1.BREX</td><td>[illegible]</td><td>1225</td><td>Bacteria>Pseudomonadota>Gammaproteobacteria>Enterobacterales>Morganellaceae>Morganella</td><td>GCF_018474625.1</td><td>Morganella_morganii</td><td>ISO84_RS18050</td><td>5738..9415</td><td>582</td><td>BREX-system-P-loop-protein-BrxC-[Morganella-morganii]</td></tr>
<tr><td>WP_274765494.1</td><td>Type1.BREX</td><td>[illegible]</td><td>1157</td><td>Bacteria>Pseudomonadota>Gammaproteobacteria>Enterobacterales>Morganellaceae>Morganella</td><td>GCF_028867915.1</td><td>Morganella_morganii</td><td>PS169_RS13665</td><td>158962..162435</td><td>582</td><td>BREX-system-P-loop-protein-BrxC-[Morganella-morganii]</td></tr>
<tr><td>WP_081119725.1</td><td>Type1.BREX</td><td>[illegible]</td><td>1225</td><td>Bacteria>Pseudomonadota>Gammaproteobacteria>Enterobacterales>Morganellaceae>Morganella</td><td>-</td><td>Morganella_morganii</td><td>B2H99_RS12750</td><td>8810..12487</td><td>582</td><td>BREX-system-P-loop-protein-BrxC-[Morganella-morganii]</td></tr>
<tr><td>HAT2164885.1</td><td>Type1.BREX</td><td>[illegible]</td><td>1225</td><td>Bacteria>Pseudomonadota>Gammaproteobacteria>Enterobacterales>Morganellaceae>Proteus</td><td>GCA_015896365.1</td><td>Proteus_mirabilis</td><td>I8052_002910</td><td>10079..13756</td><td>584</td><td>BREX-system-P-loop-protein-BrxC-[Proteus-mirabilis]</td></tr>
<tr><td>MBI6417160.1</td><td>Type1.BREX</td><td>[illegible]</td><td>1225</td><td>Bacteria>Pseudomonadota>Gammaproteobacteria>Enterobacterales>Morganellaceae>Proteus</td><td>GCA_016238675.1</td><td>Proteus_mirabilis</td><td>JEP37_15655</td><td>69055..72732</td><td>584</td><td>BREX-system-P-loop-protein-BrxC-[Proteus-mirabilis]</td></tr>
<tr><td>WP_152118569.1</td><td>Type1.BREX</td><td>[illegible]</td><td>1225</td><td>Bacteria>Pseudomonadota>Gammaproteobacteria>Enterobacterales>Morganellaceae>Proteus</td><td>GCF_009183735.1</td><td>Proteus_mirabilis</td><td>GBN13_RS03935</td><td>69067..72744</td><td>584</td><td>BREX-system-P-loop-protein-BrxC-[Proteus-mirabilis]</td></tr>
<tr><td>WP_220699168.1</td><td>Type1.BREX</td><td>[illegible]</td><td>1225</td><td>Bacteria>Pseudomonadota>Gammaproteobacteria>Enterobacterales>Morganellaceae>Proteus</td><td>GCF_019660825.1</td><td>Proteus_mirabilis</td><td>K2V98_RS14125</td><td>5608..9285</td><td>584</td><td>BREX-system-P-loop-protein-BrxC-[Proteus-mirabilis]</td></tr>
<tr><td>WP_210849185.1</td><td>Type1.BREX</td><td>[illegible]</td><td>1225</td><td>Bacteria>Pseudomonadota>Gammaproteobacteria>Enterobacterales>Morganellaceae>Proteus</td><td>GCF_022349905.1</td><td>Proteus_mirabilis</td><td>MJ636_RS02915</td><td>47390..51067</td><td>584</td><td>BREX-system-P-loop-protein-BrxC-[Proteus-mirabilis]</td></tr>
<tr><td>WP_243213747.1</td><td>Type1.BREX</td><td>[illegible]</td><td>1225</td><td>Bacteria>Pseudomonadota>Gammaproteobacteria>Enterobacterales>Morganellaceae>Proteus</td><td>GCF_022802355.1</td><td>Proteus_mirabilis</td><td>JNG69_RS16550</td><td>64025..67702</td><td>584</td><td>BREX-system-P-loop-protein-BrxC-[Proteus-mirabilis]</td></tr>
<tr><td>WP_261598777.1</td><td>Type1.BREX</td><td>[illegible]</td><td>1225</td><td>Bacteria>Pseudomonadota>Gammaproteobacteria>Enterobacterales>Morganellaceae>Proteus</td><td>GCF_025399735.1</td><td>Proteus_mirabilis</td><td>NMC40_RS18460</td><td>15597..19274</td><td>584</td><td>BREX-system-P-loop-protein-BrxC-[Proteus-mirabilis]</td></tr>
<tr><td>UVX22921.1</td><td>Type1.BREX</td><td>[illegible]</td><td>789</td><td>Bacteria>Pseudomonadota>Gammaproteobacteria>Enterobacterales>Morganellaceae>Proteus</td><td>-</td><td>Proteus_terrae_subsp._cibarius</td><td>.</td><td>73397..75766</td><td>626774</td><td>hypothetical-protein-[Proteus-terrae-subsp.-cibarius]</td></tr>
<tr><td>OBY34180.1</td><td>Type1.BREX</td><td>[illegible]</td><td>1225</td><td>Bacteria>Pseudomonadota>Gammaproteobacteria>Enterobacterales>Morganellaceae>Providencia</td><td>GCA_001680125.1</td><td>Providencia_rettgeri</td><td>PR729_03900</td><td>44936..48613</td><td>587</td><td>DNA-repair-protein-[Providencia-rettgeri]</td></tr>
<tr><td>EJD6671300.1</td><td>Type1.BREX</td><td>[illegible]</td><td>1225</td><td>Bacteria>Pseudomonadota>Gammaproteobacteria>Enterobacterales>Morganellaceae>Providencia</td><td>GCA_020680725.2</td><td>Providencia_rettgeri</td><td>M0K80_RS10540</td><td>473799..477476</td><td>587</td><td>BREX-system-P-loop-protein-BrxC-[Providencia-rettgeri]</td></tr>
<tr><td>MCL0017528.1</td><td>Type1.BREX</td><td>[illegible]</td><td>1225</td><td>Bacteria>Pseudomonadota>Gammaproteobacteria>Enterobacterales>Morganellaceae>Providencia</td><td>GCA_022387615.2</td><td>Providencia_rettgeri</td><td>MKT11_000360</td><td>78084..81761</td><td>587</td><td>BREX-system-P-loop-protein-BrxC-[Providencia-rettgeri]</td></tr>
<tr><td>MCK9997621.1</td><td>Type1.BREX</td><td>[illegible]</td><td>1225</td><td>Bacteria>Pseudomonadota>Gammaproteobacteria>Enterobacterales>Morganellaceae>Providencia</td><td>GCA_022387885.2</td><td>Providencia_rettgeri</td><td>MKT39_000560</td><td>19282..22959</td><td>587</td><td>BREX-system-P-loop-protein-BrxC-[Providencia-rettgeri]</td></tr>
<tr><td>MCL0006618.1</td><td>Type1.BREX</td><td>[illegible]</td><td>1225</td><td>Bacteria>Pseudomonadota>Gammaproteobacteria>Enterobacterales>Morganellaceae>Providencia</td><td>GCA_022388475.2</td><td>Providencia_rettgeri</td><td>MKT60_007090</td><td>64410..68087</td><td>587</td><td>BREX-system-P-loop-protein-BrxC-[Providencia-rettgeri]</td></tr>
<tr><td>WP_269723554.1</td><td>Type1.BREX</td><td>[illegible]</td><td>1225</td><td>Bacteria>Pseudomonadota>Gammaproteobacteria>Enterobacterales>Morganellaceae>Providencia</td><td>GCF_027362595.1</td><td>Providencia_sp._21OH12SH02B-Prov</td><td>O7C57_RS02370</td><td>500730..504407</td><td>3015951</td><td>BREX-system-P-loop-protein-BrxC-[Providencia-sp.-21OH12SH02B-Prov]</td></tr>
<tr><td>WP_272668877.1</td><td>Type1.BREX</td><td>[illegible]</td><td>1225</td><td>Bacteria>Pseudomonadota>Gammaproteobacteria>Enterobacterales>Morganellaceae>Providencia</td><td>GCF_028481005.1</td><td>Providencia_sp._PROV151</td><td>PSE52_RS00990</td><td>220703..224380</td><td>2949861</td><td>BREX-system-P-loop-protein-BrxC-[Providencia-sp.-PROV151]</td></tr>
<tr><td>WP_272687949.1</td><td>Type1.BREX</td><td>[illegible]</td><td>1225</td><td>Bacteria>Pseudomonadota>Gammaproteobacteria>Enterobacterales>Morganellaceae>Providencia</td><td>GCF_028484835.1</td><td>Providencia_sp._PROV035</td><td>PSF71_RS08200</td><td>5027..8704</td><td>2949766</td><td>BREX-system-P-loop-protein-BrxC-[Providencia-sp.-PROV035]</td></tr>
<tr><td>EIP8110719.1</td><td>Type1.BREX</td><td>[illegible]</td><td>1225</td><td>Bacteria>Pseudomonadota>Gammaproteobacteria>Enterobacterales>Morganellaceae>Providencia</td><td>-</td><td>Providencia_rettgeri</td><td>LT185_001364</td><td>559434..563111</td><td>587</td><td>BREX-system-P-loop-protein-BrxC-[Providencia-rettgeri]</td></tr>
<tr><td>GKV76958.1</td><td>Type1.BREX</td><td>[illegible]</td><td>1157</td><td>Bacteria>Pseudomonadota>Gammaproteobacteria>Enterobacterales>Pectobacteriaceae>Pectobacterium</td><td>GCA_024342785.1</td><td>Pectobacterium_carotovorum_subsp._carotovorum</td><td>PEC106568_21320</td><td>196499..199972</td><td>555</td><td>hypothetical-protein-PEC106568_21320-[Pectobacterium-carotovorum-subsp.-carotovorum]</td></tr>
<tr><td>WP_211024717.1</td><td>Type1.BREX</td><td>[illegible]</td><td>1157</td><td>Bacteria>Pseudomonadota>Gammaproteobacteria>Enterobacterales>Pectobacteriaceae>Pectobacterium</td><td>GCF_018094745.1</td><td>Pectobacterium_versatile</td><td>F9U42_RS09295</td><td>89992..93465</td><td>2488639</td><td>BREX-system-P-loop-protein-BrxC-[Pectobacterium-versatile]</td></tr>
<tr><td>WP_249681848.1</td><td>Type1.BREX</td><td>[illegible]</td><td>1157</td><td>Bacteria>Pseudomonadota>Gammaproteobacteria>Enterobacterales>Pectobacteriaceae>Pectobacterium</td><td>-</td><td>Pectobacterium_polaris</td><td>EXT50_03115</td><td>82893..86366</td><td>2042057</td><td>BREX-system-P-loop-protein-BrxC-[Pectobacterium-polaris]</td></tr>
<tr><td>WP_050135880.1</td><td>Type1.BREX</td><td>[illegible]</td><td>1225</td><td>Bacteria>Pseudomonadota>Gammaproteobacteria>Enterobacterales>Yersiniaceae</td><td>GCF_025758435.1</td><td>Yersinia</td><td>N4W04_RS19100</td><td>4124889..4128566</td><td>629</td><td>MULTISPECIES:-BREX-system-P-loop-protein-BrxC-[Yersinia]</td></tr>
<tr><td>WP_217147154.1</td><td>Type1.BREX</td><td>[illegible]</td><td>1157</td><td>Bacteria>Pseudomonadota>Gammaproteobacteria>Enterobacterales>Yersiniaceae>Rahnella</td><td>GCF_019049515.1</td><td>Rahnella_aceris</td><td>J1789_RS04120</td><td>11901..15374</td><td>2703885</td><td>BREX-system-P-loop-protein-BrxC-[Rahnella-aceris]</td></tr>
<tr><td>WP_232770162.1</td><td>Type1.BREX</td><td>[illegible]</td><td>1157</td><td>Bacteria>Pseudomonadota>Gammaproteobacteria>Enterobacterales>Yersiniaceae>Rahnella</td><td>GCF_021276285.1</td><td>Rahnella_victoriana</td><td>J9880_RS04875</td><td>1051789..1055262</td><td>1510570</td><td>BREX-system-P-loop-protein-BrxC-[Rahnella-victoriana]</td></tr>
<tr><td>WP_266057125.1</td><td>Type1.BREX</td><td>[illegible]</td><td>1157</td><td>Bacteria>Pseudomonadota>Gammaproteobacteria>Enterobacterales>Yersiniaceae>Rahnella</td><td>GCF_026241995.1</td><td>Rahnella_perminowiae</td><td>ORG37_RS00070</td><td>21089..24562</td><td>2816244</td><td>BREX-system-P-loop-protein-BrxC-[Rahnella-perminowiae]</td></tr>
<tr><td>NYA44684.1</td><td>Type1.BREX</td><td>[illegible]</td><td>1158</td><td>Bacteria>Pseudomonadota>Gammaproteobacteria>Enterobacterales>Yersiniaceae>Serratia</td><td>GCA_013401715.1</td><td>Serratia_fonticola</td><td>HZI31_15400</td><td>48979..52455</td><td>47917</td><td>BREX-system-P-loop-protein-BrxC-[Serratia-fonticola]</td></tr>
<tr><td>EGS9996488.1</td><td>Type1.BREX</td><td>[illegible]</td><td>1233</td><td>Bacteria>Pseudomonadota>Gammaproteobacteria>Enterobacterales>Yersiniaceae>Serratia</td><td>GCA_015929585.1</td><td>Serratia_marcescens</td><td>I9Y15_003567</td><td>178923..182624</td><td>615</td><td>BREX-system-P-loop-protein-BrxC-[Serratia-marcescens]</td></tr>
</table>

Protein-ID	System	Gene-Neighbourhoods	Length	Taxonomy	Assembly-ID	Strain	Locus-Tag	Genomic-coordinates	TaxID	Fasta-define
HBK4794038.1	Type1.BREX	HIN-HTH-><-?<-?<-sGTP<-AAA-ATPase<-rve|| BrxA-DNA-binding -> BrxB-BrxF-iSTAND -> DUF6079 *-> ParB + HNH -> N6-METHYLASE ->?-> N6-METHYLASE -p-> PglZ -> SIGMA+OB-Fold+AAA-ATPase+Lon-P[illegible]	1233	Bacteria>Pseudomonadota>Gammaproteobacteria>Enterobacterales>Yersiniaceae>Serratia	GCA_020682225.1	Serratia_marcescens	LLT15_004323	35577..39278	615	BREX-system-P-loop-protein-BrxC-[Serratia-marcescens]
WP_218833283.1	Type1.BREX	<-REase|| wHTH+WYL+WCX ->?->REase+SPF-cons-><- PglZ <- ParB + HNH <- N6-METHYLASE <- DUF6079 *<- BrxB-BrxF-iSTAND <- BrxA-DNA-binding <-SF1-SF2-Helicase+REase<-SF1-SF2-Helicase+REase[illegible]	1197	Bacteria>Pseudomonadota>Gammaproteobacteria>Enterobacterales>Yersiniaceae>Serratia	GCA_013401715.1	Serratia_fonticola	HZI31_RS15400	48979..52572	47917	BREX-system-P-loop-protein-BrxC-[Serratia-fonticola]
WP_206196473.1	Type1.BREX	<-ACET<-?<-?<-Peptidase_M17_N+Peptidase_M17||?->?->tRNA->Arm-DNA-bind_3+Phage_integrase-> BrxA-DNA-binding -> BrxB-BrxF-iSTAND -> DUF6079 *-> N6-METHYLASE -> ParB + HNH -> PglZ -><-[illegible]	1157	Bacteria>Pseudomonadota>Gammaproteobacteria>Enterobacterales>Yersiniaceae>Serratia	GCF_017152235.1	Serratia_ureilytica	JY544_RS23375	37448..40921	300181	BREX-system-P-loop-protein-BrxC-[Serratia-ureilytica]
WP_223536794.1	Type1.BREX	<-REase+HTH<-?<-?||tRNA->Arm-DNA-bind_3+Phage_integrase->SF1-SF2-Helicase+REase->SF1-SF2-Helicase+REase-> BrxA-DNA-binding -> BrxB-BrxF-iSTAND -> DUF6079 *-> N6-METHYLASE -> ParB[illegible]	1158	Bacteria>Pseudomonadota>Gammaproteobacteria>Enterobacterales>Yersiniaceae>Serratia	GCF_019968705.1	unclassified_Serratia_(in:_enterobacteria)	KGP26_RS00385	95452..98928	2647522	MULTISPECIES:-BREX-system-P-loop-protein-BrxC-[unclassified-Serratia-(in:-enterobacteria)]
WP_260965787.1	Type1.BREX	Arm-DNA-bind_3+Phage_integrase-><- SIGMA+OB-Fold+AAA-ATPase+Lon-Protease-BrxL <- PglZ <- N6-METHYLASE -p<-RVT<-HEPN<- N6-METHYLASE <- ParB + HNH <- DUF6079 *<- BrxB-BrxF-iSTAND <- Br[illegible]	1240	Bacteria>Pseudomonadota>Gammaproteobacteria>Enterobacterales>Yersiniaceae>Serratia	GCF_025336405.1	Serratia_marcescens	N7751_RS05105	1087639..1091361	615	BREX-system-P-loop-protein-BrxC-[Serratia-marcescens]
WP_273866014.1	Type1.BREX	<-Peptidase_M17_N+Peptidase_M17||?->?->?->tRNA->Arm-DNA-bind_3+Phage_integrase-> BrxA-DNA-binding -> BrxB-BrxF-iSTAND -> DUF6079 *-> N6-METHYLASE -> ParB + HNH -> PglZ -><-SF1-SF2-H[illegible]	1157	Bacteria>Pseudomonadota>Gammaproteobacteria>Enterobacterales>Yersiniaceae>Serratia	GCF_028616515.1	Serratia_ureilytica	PVU68_RS17145	216123..219596	300181	BREX-system-P-loop-protein-BrxC-[Serratia-ureilytica]
WP_262254721.1	Type1.BREX	<-GTPase-AIG<-CopG/RHH||?->XIS-HTH-><-?<-?|| BrxA-DNA-binding -> BrxB-BrxF-iSTAND -> DUF6079 *-> ParB + HNH -> N6-METHYLASE ->HEPN->RVT-> N6-METHYLASE -p-> PglZ -> SIGMA+OB-Fold+AAA-[illegible]	1240	Bacteria>Pseudomonadota>Gammaproteobacteria>Enterobacterales>Yersiniaceae>Serratia	GCF_946228755.1	Serratia_proteamaculans	OC575_RS10145	2133622..2137344	28151	BREX-system-P-loop-protein-BrxC-[Serratia-proteamaculans]
WP_046051668.1	Type1.BREX	XIS-HTH->?->SF1-SF2-Helicase+REase->SF1-SF2-Helicase+REase-><- PglZ <- ParB + HNH <- N6-METHYLASE <- DUF6079 *<- BrxB-BrxF-iSTAND <- BrxA-DNA-binding <- wHTH+WYL+WCX <-Arm-DNA-bind_3[illegible]	1157	Bacteria>Pseudomonadota>Gammaproteobacteria>Enterobacterales>Yersiniaceae>Yersinia	GCF_000968115.1	Yersinia_enterocolitica	YE5303_RS19630	4225906..4229379	630	BREX-system-P-loop-protein-BrxC-[Yersinia-enterocolitica]
WP_050073003.1	Type1.BREX	<-Fimbrial||<-?<-?<-HIN-HTH||SF1-SF2-Helicase+REase->SF1-SF2-Helicase+REase-><- PglZ <- ParB + HNH <- N6-METHYLASE <- DUF6079 *<- BrxB-BrxF-iSTAND <- BrxA-DNA-binding <-Arm-DNA-bind[illegible]	1157	Bacteria>Pseudomonadota>Gammaproteobacteria>Enterobacterales>Yersiniaceae>Yersinia	GCF_001088505.1	Yersinia_intermedia	AE858_RS05265	31386..34859	631	BREX-system-P-loop-protein-BrxC-[Yersinia-intermedia]
WP_068809899.1	Type6.BREX	Arm-DNA-bind_3+Phage_integrase-><-?<-XIS-HTH<-?<- SIGMA+OB-Fold+AAA-ATPase+HKD-DNase-BrxL <- PglZ <- ParB + HNH <- N6-METHYLASE <- N6-METHYLASE <- DUF6079 *<- BrxB-BrxF-iSTAND <- Brx[illegible]	1214	Bacteria>Pseudomonadota>Gammaproteobacteria>Gammaproteobacteria_incertae_sedis>Pseudohongiella	GCF_014642795.1	Pseudohongiella_nitratireducens	IEW51_RS03970	206813..210457	1768907	BREX-system-P-loop-protein-BrxC-[Pseudohongiella-nitratireducens]
MCG7876672.1	Type6.BREX	TraG_N-><-?<-?<-?|| wHTH+WYL+WCX -> BrxE -> BrxA-DNA-binding -> BrxB-BrxF-iSTAND -> DUF6079 *-> N6-METHYLASE -> ParB + HNH -> PglZ -> SIGMA+OB-Fold+AAA-ATPase+HKD-DNase-BrxL ->Exonuc_[illegible]	1215	Bacteria>Pseudomonadota>Gammaproteobacteria>Gammaproteobacteria_incertae_sedis>sulfur-oxidizing_symbionts>Candidatus_Thiodiazotropha	GCA_016842655.1	Candidatus_Thiodiazotropha_taylori	JAY78_10080	4978..8625	2792791	BREX-system-P-loop-protein-BrxC-[Candidatus-Thiodiazotropha-taylori]
MCG7951159.1	Type6.BREX	TraH->?->TraG_N-><-?<-?|| wHTH+WYL+WCX -> BrxE -> BrxA-DNA-binding -> BrxB-BrxF-iSTAND -> DUF6079 *-> N6-METHYLASE -> ParB + HNH -> PglZ -> SIGMA+OB-Fold+AAA-ATPase+HKD-DNase-BrxL ->Ex[illegible]	1215	Bacteria>Pseudomonadota>Gammaproteobacteria>Gammaproteobacteria_incertae_sedis>sulfur-oxidizing_symbionts>Candidatus_Thiodiazotropha	GCA_016842765.1	Candidatus_Thiodiazotropha_taylori	JAZ09_08050	22496..26143	2792791	BREX-system-P-loop-protein-BrxC-[Candidatus-Thiodiazotropha-taylori]
MCG8102611.1	Type6.BREX	TraH->?->TraG_N-><-?<-?|| wHTH+WYL+WCX -> BrxE -> BrxA-DNA-binding -> BrxB-BrxF-iSTAND -> DUF6079 *-> N6-METHYLASE -> ParB + HNH -> PglZ -> SIGMA+OB-Fold+AAA-ATPase+HKD-DNase-BrxL ->Ex[illegible]	1215	Bacteria>Pseudomonadota>Gammaproteobacteria>Gammaproteobacteria_incertae_sedis>sulfur-oxidizing_symbionts>Candidatus_Thiodiazotropha	GCA_016842845.1	Candidatus_Thiodiazotropha_taylori	JAY70_09490	22526..26173	2792791	BREX-system-P-loop-protein-BrxC-[Candidatus-Thiodiazotropha-taylori]
MCG8058214.1	Type6.BREX	<-SbcC<-Calcineurin+Calcineurin-SbcD<-SbcC<-SF1-SF2-Helicase+REase<-Exonuc_V_gamma+REase<- SIGMA+OB-Fold+AAA-ATPase+HKD-DNase-BrxL <- PglZ <- ParB + HNH <- N6-METHYLASE <- DUF6079 [illegible]	1215	Bacteria>Pseudomonadota>Gammaproteobacteria>Gammaproteobacteria_incertae_sedis>sulfur-oxidizing_symbionts>Candidatus_Thiodiazotropha	GCA_016843165.1	Candidatus_Thiodiazotropha_taylori	JAZ21_00070	27190..30837	2792791	BREX-system-P-loop-protein-BrxC-[Candidatus-Thiodiazotropha-taylori]
WP_223374968.1	Type1.BREX	Arm-DNA-bind_3+Phage_integrase-><- SIGMA+OB-Fold+AAA-ATPase+Lon-Protease-BrxL <- PglZ <- N6-METHYLASE -p<-RVT<-HEPN<- N6-METHYLASE <- ParB + HNH <- DUF6079 *<- BrxB-BrxF-iSTAND <- Br[illegible]	1240	Bacteria>Pseudomonadota>Gammaproteobacteria	GCF_003051945.2	Enterobacterales	N5D94_RS08195	82368..86090	91347	MULTISPECIES:-BREX-system-P-loop-protein-BrxC-[Enterobacterales]
WP_052921406.1	Type1.BREX	MobI->?-><-?<-LexA-protease||35exo->?-> wHTH+WYL+WCX -> BrxA-DNA-binding -> BrxB-BrxF-iSTAND -> DUF6079 *-> ParB + HNH -> N6-METHYLASE -> PglZ -> SIGMA+OB-Fold+AAA-ATPase+Lon-Proteas[illegible]	1225	Bacteria>Pseudomonadota>Gammaproteobacteria	GCF_003402955.1	Enterobacterales	DS732_RS04425	849664..853341	91347	MULTISPECIES:-BREX-system-P-loop-protein-BrxC-[Enterobacterales]
WP_099433763.1	Type1.BREX	<-Schlafen+HATPase+wHTH<-PHP+SbcC<-?|| BrxA-DNA-binding -> BrxB-BrxF-iSTAND ->DUF772+DDE_Tnp_1-> DUF6079 *-> ParB + HNH -> N6-METHYLASE ->HEPN->RVT-> N6-METHYLASE -p-> PglZ -> SIGMA+[illegible]	1233	Bacteria>Pseudomonadota>Gammaproteobacteria	GCF_014168815.1	Enterobacterales	H7R56_RS21645	4484852..4488553	91347	MULTISPECIES:-BREX-system-P-loop-protein-BrxC-[Enterobacterales]
WP_099476278.1	Type1.BREX	Phage_integrase+Phage_integrase->Phage_integrase+Phage_integrase-> ParB + HNH ->?->DOC+HTH-><-DUF4236|| wHTH+WYL+WCX -> BrxA-DNA-binding -> BrxB-BrxF-iSTAND -> DUF6079 *-> N6-METHY[illegible]	1226	Bacteria>Pseudomonadota>Gammaproteobacteria>Moraxellales>Moraxellaceae>Acinetobacter	-	Acinetobacter_pittii	HPG40_RS00205	37613..41293	48296	BREX-system-P-loop-protein-BrxC-[Acinetobacter-pittii]
WP_019836725.1	Type1.BREX	RelB-><-TPR+TPR<-?||<- SIGMA+OB-Fold+AAA-ATPase+Lon-Protease-BrxL <- PglZ <- ParB + HNH <- N6-METHYLASE <- DUF6079 *<- BrxB-BrxF-iSTAND <- BrxA-DNA-binding <- wHTH+WYL+WCX ||DUF4236->[illegible]	1226	Bacteria>Pseudomonadota>Gammaproteobacteria>Moraxellales>Moraxellaceae>Acinetobacter	-	Acinetobacter_sp._MDS7A	H048_RS0104285	13386..17066	1209355	BREX-system-P-loop-protein-BrxC-[Acinetobacter-sp.-MDS7A]
WP_005303500.1	Type1.BREX	ZnR+ZnR->DUF932->?->?-><-?<-TetR-HTH|| wH[illegible]	1226	Bacteria>Pseudomonadota>Gammaproteobacteria>Moraxellales>Moraxellaceae>Acinetobacter	-	Acinetobacter_sp._NIPH_542	F886_RS07830	464704..468384	1217688	BREX-system-P-loop-protein-BrxC-[Acinetobacter-sp.-NIPH-542]
ENX11167.1	Type1.BREX	CR-ATPase5+REase-><-DUF4236|| wHTH+WYL+WC[illegible]	911	Bacteria>Pseudomonadota>Gammaproteobacteria>Moraxellales>Moraxellaceae>Acinetobacter	GCA_000369645.1	Acinetobacter_sp._CIP_64.2	F895_03860	13889..16624	1217694	hypothetical-protein-F895_03860-[Acinetobacter-sp.-CIP-64.2]
HAV3580163.1	Type1.BREX	<-MACRODOMAIN<-DarT+Peptidase<-SF1-SF2-He[illegible]	1226	Bacteria>Pseudomonadota>Gammaproteobacteria>Moraxellales>Moraxellaceae>Acinetobacter	GCA_016485765.1	Acinetobacter_baumannii	JIE63_001443	180845..184525	470	BREX-system-P-loop-protein-BrxC-[Acinetobacter-baumannii]
HAV4979216.1	Type1.BREX	<-HTH_Tnp_1+HTH_21+rve+rve_3||<-HTH_3<-Hi[illegible]	1226	Bacteria>Pseudomonadota>Gammaproteobacteria>Moraxellales>Moraxellaceae>Acinetobacter	GCA_016507565.1	Acinetobacter_baumannii	JIZ05_14385	7251..10931	470	BREX-system-P-loop-protein-BrxC-[Acinetobacter-baumannii]
HAV6134262.1	Type1.BREX	ZnR+DUF3482+ZnR-><-PIN+OST-HTH||DUF932->[illegible]	1226	Bacteria>Pseudomonadota>Gammaproteobacteria>Moraxellales>Moraxellaceae>Acinetobacter	GCA_016514395.1	Acinetobacter_baumannii	JI295_12625	9155..12835	470	BREX-system-P-loop-protein-BrxC-[Acinetobacter-baumannii]
BCZ16231.1	Type1.BREX	<-HTH_21+rve+rve_3<- SIGMA+OB-Fold+AAA-AT[illegible]	1142	Bacteria>Pseudomonadota>Gammaproteobacteria>Moraxellales>Moraxellaceae>Acinetobacter	GCA_019703285.1	Acinetobacter_baumannii	OCUAc18_37710	57364..60792	470	hypothetical-protein-OCUAc18_37710-[Acinetobacter-baumannii]
HCA4923662.1	Type1.BREX	Peptidase_M48-><-HIN-HTH<-?||<- SIGMA+OB-[illegible]	1226	Bacteria>Pseudomonadota>Gammaproteobacteria>Moraxellales>Moraxellaceae>Acinetobacter	GCA_022931145.1	Acinetobacter_baumannii	MW112_001902	190856..194536	470	BREX-system-P-loop-protein-BrxC-[Acinetobacter-baumannii]
USI88235.1	Type1.BREX	<- ParB <-ParA-Soj-PloopNTPase||?->CR-ATPa[illegible]	1226	Bacteria>Pseudomonadota>Gammaproteobacteria>Moraxellales>Moraxellaceae>Acinetobacter	GCA_023822225.1	Acinetobacter_johnsonii	LZ086_18665	10226..13906	40214	BREX-system-P-loop-protein-BrxC-[Acinetobacter-johnsonii]
WP_005401825.1	Type1.BREX	<- SIGMA+OB-Fold+AAA-ATPase+Lon-Protease-[illegible]	1226	Bacteria>Pseudomonadota>Gammaproteobacteria>Moraxellales>Moraxellaceae>Acinetobacter	GCF_000162055.1	Acinetobacter_johnsonii	HMPREF0016_RS15525	47644..51324	40214	BREX-system-P-loop-protein-BrxC-[Acinetobacter-johnsonii]
WP_010116441.1	Type1.BREX	<- SIGMA+OB-Fold+AAA-ATPase+Lon-Protease-[illegible]	1226	Bacteria>Pseudomonadota>Gammaproteobacteria>Moraxellales>Moraxellaceae>Acinetobacter	GCF_000214135.1	Acinetobacter_sp._P8-3-8	GGG_RS0115320	12069..15749	1029823	BREX-system-P-loop-protein-BrxC-[Acinetobacter-sp.-P8-3-8]
WP_001021843.1	Type1.BREX	<-MACRODOMAIN<-DarT+Peptidase<-SF1-SF2-He[illegible]	1226	Bacteria>Pseudomonadota>Gammaproteobacteria>Moraxellales>Moraxellaceae>Acinetobacter	GCF_000305235.1	Acinetobacter_baumannii	ACIN5098_RS09930	47279..50959	470	BREX-system-P-loop-protein-BrxC-[Acinetobacter-baumannii]
WP_004811070.1	Type1.BREX	HTH_Tnp_4+DDE_Tnp_4-><-?<-SF1-SF2-Helicas[illegible]	1225	Bacteria>Pseudomonadota>Gammaproteobacteria>Moraxellales>Moraxellaceae>Acinetobacter	GCF_000368465.1	Acinetobacter_schindleri	F965_RS04725	393977..397654	108981	BREX-system-P-loop-protein-BrxC-[Acinetobacter-schindleri]
WP_005162499.1	Type6.BREX	<-DDE_Tnp_1<-DUF4096+DDE_Tnp_1+DDE_Tnp_1_[illegible]	1063	Bacteria>Pseudomonadota>Gammaproteobacteria>Moraxellales>Moraxellaceae>Acinetobacter	GCF_000369425.1	Acinetobacter_sp._NIPH_284	F908_RS12580	1400419..1403610	1217704	BREX-system-P-loop-protein-BrxC-[Acinetobacter-sp.-NIPH-284]
WP_031946304.1	Type1.BREX	DUF932->?->?->?-><-?<-TetR-HTH|| wHTH+WYL[illegible]	1226	Bacteria>Pseudomonadota>Gammaproteobacteria>Moraxellales>Moraxellaceae>Acinetobacter	GCF_000516575.2	Acinetobacter_baumannii	UN97_RS12380	246428..250108	470	BREX-system-P-loop-protein-BrxC-[Acinetobacter-baumannii]
WP_034686569.1	Type1.BREX	CR-ATPase5+REase-><-DUF4236|| wHTH+WYL+WC[illegible]	1226	Bacteria>Pseudomonadota>Gammaproteobacteria>Moraxellales>Moraxellaceae>Acinetobacter	GCF_000581035.1	Acinetobacter_sp._869535	J520_RS12320	4406..8086	1310621	BREX-system-P-loop-protein-BrxC-[Acinetobacter-sp.-869535]
WP_025095052.1	Type1.BREX	<-RelE-ParE<-PhdYeFM_antitox|| wHTH+WYL+W[illegible]	1226	Bacteria>Pseudomonadota>Gammaproteobacteria>Moraxellales>Moraxellaceae>Acinetobacter	GCF_000633005.1	Acinetobacter_soli	EG58_RS0106955	8205..11885	487316	BREX-system-P-loop-protein-BrxC-[Acinetobacter-soli]
WP_045544683.1	Type1.BREX	DDE_Tnp_IS240-><-ADH_N+ADH_zinc_N+METHYL[illegible]	1226	Bacteria>Pseudomonadota>Gammaproteobacteria>Moraxellales>Moraxellaceae>Acinetobacter	GCF_000828935.1	Acinetobacter_baumannii	IOMTU433_RS19345	66852..70532	470	BREX-system-P-loop-protein-BrxC-[Acinetobacter-baumannii]
WP_043972752.1	Type1.BREX	ParB -> ParB + HNH ->CR-ATPase5+REase-><-D[illegible]	1226	Bacteria>Pseudomonadota>Gammaproteobacteria>Moraxellales>Moraxellaceae>Acinetobacter	GCF_000876115.1	Acinetobacter_sp._NBRC_110496	Aba26S_RS13795	7444..11124	1550715	BREX-system-P-loop-protein-BrxC-[Acinetobacter-sp.-NBRC-110496]
WP_032059948.1	Type1.BREX	DUF932->?->?->?-><-?<-TetR-HTH|| wHTH+WYL[illegible]	1226	Bacteria>Pseudomonadota>Gammaproteobacteria>Moraxellales>Moraxellaceae>Acinetobacter	GCF_001077555.2	Acinetobacter_baumannii	AB895_RS13070	2715747..2719427	470	BREX-system-P-loop-protein-BrxC-[Acinetobacter-baumannii]
WP_058952662.1	Type1.BREX	<-Phage_integrase<-?||<-ParA-Soj-PloopNT[illegible]	1220	Bacteria>Pseudomonadota>Gammaproteobacteria>Moraxellales>Moraxellaceae>Acinetobacter	GCF_001484935.1	Acinetobacter_johnsonii	EHS42_RS19740	21243..24905	40214	BREX-system-P-loop-protein-BrxC-[Acinetobacter-johnsonii]
WP_065184483.1	Type1.BREX	<-Phage_GPD+Phage_base_V<-TetR-HTH<-?<- S[illegible]	1226	Bacteria>Pseudomonadota>Gammaproteobacteria>Moraxellales>Moraxellaceae>Acinetobacter	GCF_001674695.1	Acinetobacter_baumannii	GKP36_RS06470	16105..19785	470	BREX-system-P-loop-protein-BrxC-[Acinetobacter-baumannii]
WP_069122561.1	Type1.BREX	ZnR+DUF3482+ZnR-><-PIN+OST-HTH||DUF932->[illegible]	1226	Bacteria>Pseudomonadota>Gammaproteobacteria>Moraxellales>Moraxellaceae>Acinetobacter	GCF_001716945.1	Acinetobacter_pittii	AXH23_RS05625	247550..251230	48296	BREX-system-P-loop-protein-BrxC-[Acinetobacter-pittii]
WP_075040289.1	Type1.BREX	DUF932->?->?->XIS-HTH->SAM-METHYLASE-> wH[illegible]	1226	Bacteria>Pseudomonadota>Gammaproteobacteria>Moraxellales>Moraxellaceae>Acinetobacter	GCF_001917365.1	Acinetobacter_radioresistens	SA188iCDA_RS03905	54372..58052	40216	BREX-system-P-loop-protein-BrxC-[Acinetobacter-radioresistens]
WP_086231303.1	Type1.BREX	<- SIGMA+OB-Fold+AAA-ATPase+Lon-Protease-[illegible]	1226	Bacteria>Pseudomonadota>Gammaproteobacteria>Moraxellales>Moraxellaceae>Acinetobacter	GCF_002137255.1	Acinetobacter_baumannii	B9Y01_RS02195	14367..18047	470	BREX-system-P-loop-protein-BrxC-[Acinetobacter-baumannii]
WP_096902445.1	Type1.BREX	<-ACET<-?||<- SIGMA+OB-Fold+AAA-ATPase+Lo[illegible]	1222	Bacteria>Pseudomonadota>Gammaproteobacteria>Moraxellales>Moraxellaceae>Acinetobacter	GCF_002412355.1	Acinetobacter_sp._YT-02	CF596_RS10765	15331..18999	2018564	BREX-system-P-loop-protein-BrxC-[Acinetobacter-sp.-YT-02]
WP_099475573.1	Type1.BREX	Phage_integrase+Phage_integrase->Phage_in[illegible]	1226	Bacteria>Pseudomonadota>Gammaproteobacteria>Moraxellales>Moraxellaceae>Acinetobacter	GCF_002740745.1	Acinetobacter_pittii	CT134_RS17990	28430..32110	48296	BREX-system-P-loop-protein-BrxC-[Acinetobacter-pittii]
WP_104442411.1	Type1.BREX	<-DDE_Tnp_1||<-?<-HTH_3<-?<- SIGMA+OB-Fol[illegible]	1226	Bacteria>Pseudomonadota>Gammaproteobacteria>Moraxellales>Moraxellaceae>Acinetobacter	GCF_002934965.1	Acinetobacter_sp._ACNIH2	C3F34_RS18690	72475..76155	1758189	BREX-system-P-loop-protein-BrxC-[Acinetobacter-sp.-ACNIH2]
WP_104500994.1	Type1.BREX	<- SIGMA+OB-Fold+AAA-ATPase+Lon-Protease-[illegible]	1226	Bacteria>Pseudomonadota>Gammaproteobacteria>Moraxellales>Moraxellaceae>Acinetobacter	GCF_002938745.1	Acinetobacter_indicus	C5505_RS10055	19022..22702	756892	BREX-system-P-loop-protein-BrxC-[Acinetobacter-indicus]
WP_109291743.1	Type1.BREX	<-DDE_Tnp_1_5+DDE_Tnp_1||CR-ATPase5+REase[illegible]	1226	Bacteria>Pseudomonadota>Gammaproteobacteria>Moraxellales>Moraxellaceae>Acinetobacter	GCF_003131095.1	Acinetobacter_baumannii	DF172_RS10505	4712..8392	470	BREX-system-P-loop-protein-BrxC-[Acinetobacter-baumannii]
WP_111281982.1	Type1.BREX	DUF932->?->?->XIS-HTH->SAM-METHYLASE-> wH[illegible]	1226	Bacteria>Pseudomonadota>Gammaproteobacteria>Moraxellales>Moraxellaceae>Acinetobacter	GCF_003258335.1	Acinetobacter_radioresistens	NVV25_RS10895	13399..17079	40216	BREX-system-P-loop-protein-BrxC-[Acinetobacter-radioresistens]
WP_119025213.1	Type1.BREX	<-PhdYeFM_antitox|| wHTH+WYL+WCX -> BrxA-D[illegible]	1226	Bacteria>Pseudomonadota>Gammaproteobacteria>Moraxellales>Moraxellaceae>Acinetobacter	GCF_003569585.1	Acinetobacter_soli	D5I70_RS01315	8436..12116	487316	BREX-system-P-loop-protein-BrxC-[Acinetobacter-soli]
WP_119047908.1	Type1.BREX	ZnR+DUF3482+ZnR-><-PIN+OST-HTH||DUF932->[illegible]	1226	Bacteria>Pseudomonadota>Gammaproteobacteria>Moraxellales>Moraxellaceae>Acinetobacter	GCF_003569645.1	Acinetobacter_baumannii	D5I76_RS05685	52847..56527	470	BREX-system-P-loop-protein-BrxC-[Acinetobacter-baumannii]
WP_123785432.1	Type1.BREX	<-DDE_Tnp_IS240||?-><-?<- SIGMA+OB-Fold+A[illegible]	1226	Bacteria>Pseudomonadota>Gammaproteobacteria>Moraxellales>Moraxellaceae>Acinetobacter	GCF_003812065.1	Acinetobacter_baumannii	LH406_RS10280	68046..71726	470	BREX-system-P-loop-protein-BrxC-[Acinetobacter-baumannii]
WP_131273640.1	Type1.BREX	<- SIGMA+OB-Fold+AAA-ATPase+Lon-Protease-[illegible]	1154	Bacteria>Pseudomonadota>Gammaproteobacteria>Moraxellales>Moraxellaceae>Acinetobacter	GCF_004331195.1	Acinetobacter_sp._ANC_4216	E0H88_RS07250	81102..84566	2529840	BREX-system-P-loop-protein-BrxC-[Acinetobacter-sp.-ANC-4216]
WP_138000997.1	Type1.BREX	<- PglZ <- ParB + HNH <- N6-METHYLASE <- DUF[illegible]	1226	Bacteria>Pseudomonadota>Gammaproteobacteria>Moraxellales>Moraxellaceae>Acinetobacter	GCF_005519305.1	Acinetobacter_radioresistens	E3H47_RS14125	6771..10451	40216	BREX-system-P-loop-protein-BrxC-[Acinetobacter-radioresistens]
WP_140948309.1	Type1.BREX	<-MACRODOMAIN<-DarT+Peptidase<-SF1-SF2-He[illegible]	1226	Bacteria>Pseudomonadota>Gammaproteobacteria>Moraxellales>Moraxellaceae>Acinetobacter	GCF_006491975.1	Acinetobacter_baumannii	FJV17_RS05945	24470..28150	470	BREX-system-P-loop-protein-BrxC-[Acinetobacter-baumannii]
WP_140963539.1	Type6.BREX	<-CR-Kinase+Classical-AAA<-?<-?<-?<- SIGM[illegible]	1214	Bacteria>Pseudomonadota>Gammaproteobacteria>Moraxellales>Moraxellaceae>Acinetobacter	GCF_006494575.1	Acinetobacter_baumannii	FJU84_RS09090	25284..28928	470	BREX-system-P-loop-protein-BrxC-[Acinetobacter-baumannii]
WP_144583841.1	Type1.BREX	<-HTH_Tnp_1<-DUF4236|| wHTH+WYL+WCX -> Brx[illegible]	1226	Bacteria>Pseudomonadota>Gammaproteobacteria>Moraxellales>Moraxellaceae>Acinetobacter	GCF_007713425.1	Acinetobacter_colistiniresistens	FPV60_RS17750	3413..7093	280145	BREX-system-P-loop-protein-BrxC-[Acinetobacter-colistiniresistens]
WP_153389475.1	Type1.BREX	HTH_3-><-?<-?<-?<-?<- SIGMA+OB-Fold+AAA-A[illegible]	1224	Bacteria>Pseudomonadota>Gammaproteobacteria>Moraxellales>Moraxellaceae>Acinetobacter	GCF_009601085.1	Acinetobacter_wanghuae	GHJ48_RS13160	17934..21608	2662362	BREX-system-P-loop-protein-BrxC-[Acinetobacter-wanghuae]
WP_160255370.1	Type1.BREX	<- SIGMA+OB-Fold+AAA-ATPase+Lon-Protease-[illegible]	1226	Bacteria>Pseudomonadota>Gammaproteobacteria>Moraxellales>Moraxellaceae>Acinetobacter	GCF_009914395.1	Acinetobacter_indicus	GA646_RS00530	100361..104041	756892	BREX-system-P-loop-protein-BrxC-[Acinetobacter-indicus]
WP_163071913.1	Type1.BREX	<- SIGMA+OB-Fold+AAA-ATPase+Lon-Protease-[illegible]	1226	Bacteria>Pseudomonadota>Gammaproteobacteria>Moraxellales>Moraxellaceae>Acinetobacter	GCF_010499775.1	Acinetobacter_baumannii	G3N14_RS16470	10817..14497	470	BREX-system-P-loop-protein-BrxC-[Acinetobacter-baumannii]
WP_168370063.1	Type1.BREX	DUF932->?->?->XIS-HTH->SAM-METHYLASE-> wH[illegible]	1224	Bacteria>Pseudomonadota>Gammaproteobacteria>Moraxellales>Moraxellaceae>Acinetobacter	GCF_012278715.1	Acinetobacter_indicus	HHC64_RS10945	2259038..2262712	756892	BREX-system-P-loop-protein-BrxC-[Acinetobacter-indicus]
WP_169025139.1	Type1.BREX	Phage_integrase+Phage_integrase->Phage_in[illegible]	1226	Bacteria>Pseudomonadota>Gammaproteobacteria>Moraxellales>Moraxellaceae>Acinetobacter	GCF_012931665.1	Acinetobacter_baumannii	HIN88_RS18590	12016..15696	470	BREX-system-P-loop-protein-BrxC-[Acinetobacter-baumannii]
WP_171530296.1	Type1.BREX	<-ACET<-?||<- SIGMA+OB-Fold+AAA-ATPase+Lo[illegible]	1226	Bacteria>Pseudomonadota>Gammaproteobacteria>Moraxellales>Moraxellaceae>Acinetobacter	GCF_013072655.1	Acinetobacter_defluvii	A7P53_RS01880	14398..18078	1871111	BREX-system-P-loop-protein-BrxC-[Acinetobacter-defluvii]
WP_174727826.1	Type1.BREX	BrxA-DNA-binding -> BrxB-BrxF-iSTAND -> DU[illegible]	1224	Bacteria>Pseudomonadota>Gammaproteobacteria>Moraxellales>Moraxellaceae>Acinetobacter	GCF_013344765.1	Acinetobacter_lactucae	HUN22_RS07895	1711..5385	1785128	BREX-system-P-loop-protein-BrxC-[Acinetobacter-lactucae]
WP_179994566.1	Type1.BREX	Arm-DNA-bind_3+Phage_integrase-><-?<-?<-?[illegible]	1226	Bacteria>Pseudomonadota>Gammaproteobacteria>Moraxellales>Moraxellaceae>Acinetobacter	GCF_013416255.1	Acinetobacter_baumannii	HLG75_RS04210	863150..866830	470	BREX-system-P-loop-protein-BrxC-[Acinetobacter-baumannii]
WP_180045992.1	Type1.BREX	LZ_Tnp_IS66+zf-IS66+DDE_Tnp_IS66+DDE_Tnp_[illegible]	1218	Bacteria>Pseudomonadota>Gammaproteobacteria>Moraxellales>Moraxellaceae>Acinetobacter	GCF_013418865.1	Acinetobacter_sp._YH12117	FTU55_RS01900	159420..163076	2601104	BREX-system-P-loop-protein-BrxC-[Acinetobacter-sp.-YH12117]
WP_185218776.1	Type1.BREX	CR-ATPase5+REase-><-DUF4236|| wHTH+WYL+WC[illegible]	1226	Bacteria>Pseudomonadota>Gammaproteobacteria>Moraxellales>Moraxellaceae>Acinetobacter	GCF_014218985.1	Acinetobacter_baumannii	H6W49_RS17735	6511..10191	470	BREX-system-P-loop-protein-BrxC-[Acinetobacter-baumannii]
WP_032068282.1	Type6.BREX	DUF932->?->?->XIS-HTH->SAM-METHYLASE-> wH[illegible]	1214	Bacteria>Pseudomonadota>Gammaproteobacteria>Moraxellales>Moraxellaceae>Acinetobacter	GCF_014559915.1	Acinetobacter_baumannii	HPJ29_RS06280	177170..180814	470	BREX-system-P-loop-protein-BrxC-[Acinetobacter-baumannii]
WP_193893631.1	Type1.BREX	HigB_toxin->HTH_3->CR-ATPase5+REase-><-DU[illegible]	1226	Bacteria>Pseudomonadota>Gammaproteobacteria>Moraxellales>Moraxellaceae>Acinetobacter	GCF_015209685.1	Acinetobacter_albensis	IQR32_RS12760	7183..10863	1673609	BREX-system-P-loop-protein-BrxC-[Acinetobacter-albensis]
WP_136512845.1	Type1.BREX	<- SIGMA+OB-Fold+AAA-ATPase+Lon-Protease-[illegible]	1226	Bacteria>Pseudomonadota>Gammaproteobacteria>Moraxellales>Moraxellaceae>Acinetobacter	GCF_015265775.1	Acinetobacter_baumannii	O9X70_RS06810	133723..137403	470	BREX-system-P-loop-protein-BrxC-[Acinetobacter-baumannii]
WP_196075355.1	Type1.BREX	AbiEii->|| ParB + HNH ->CR-ATPase5+REase->[illegible]	1204	Bacteria>Pseudomonadota>Gammaproteobacteria>Moraxellales>Moraxellaceae>Acinetobacter	GCF_015676315.1	unclassified_Acinetobacter	IMO39_RS19270	9799..13413	196816	MULTISPECIES:-BREX-system-P-loop-protein-BrxC-[unclassified-Acinetobacter]
WP_198080379.1	Type1.BREX	<-SF1-SF2-Helicase+REase<- SIGMA+OB-Fold+[illegible]	1222	Bacteria>Pseudomonadota>Gammaproteobacteria>Moraxellales>Moraxellaceae>Acinetobacter	GCF_016082245.1	Acinetobacter_calcoaceticus	BL242_RS20750	15792..19460	471	BREX-system-P-loop-protein-BrxC-[Acinetobacter-calcoaceticus]
WP_199939733.1	Type1.BREX	<-Phage_GPD+Phage_base_V+T6SS_Vgr+DUF2345[illegible]	1226	Bacteria>Pseudomonadota>Gammaproteobacteria>Moraxellales>Moraxellaceae>Acinetobacter	GCF_016506315.1	Acinetobacter_baumannii	I5555_RS13010	139911..143591	470	BREX-system-P-loop-protein-BrxC-[Acinetobacter-baumannii]
WP_203080037.1	Type1.BREX	TRD->Cap4_nuclease->?->?->?-><-TetR-HTH||[illegible]	1226	Bacteria>Pseudomonadota>Gammaproteobacteria>Moraxellales>Moraxellaceae>Acinetobacter	GCF_016804045.1	Acinetobacter_pittii	HRJ47_RS04290	884929..888609	48296	BREX-system-P-loop-protein-BrxC-[Acinetobacter-pittii]
WP_205779680.1	Type1.BREX	Phage_integrase+Phage_integrase->Phage_in[illegible]	1226	Bacteria>Pseudomonadota>Gammaproteobacteria>Moraxellales>Moraxellaceae>Acinetobacter	GCF_017132735.1	Acinetobacter_indicus	JYB86_RS06365	1326455..1330135	756892	BREX-system-P-loop-protein-BrxC-[Acinetobacter-indicus]
WP_109426631.1	Type1.BREX	DUF932->?->?->?-><-?<-TetR-HTH|| wHTH+WYL[illegible]	1226	Bacteria>Pseudomonadota>Gammaproteobacteria>Moraxellales>Moraxellaceae>Acinetobacter	GCF_017726575.1	Acinetobacter_baumannii	J6I88_RS04260	873693..877373	470	BREX-system-P-loop-protein-BrxC-[Acinetobacter-baumannii]
WP_216174886.1	Type1.BREX	DUF932->?->?->?-><-?<-TetR-HTH|| wHTH+WYL[illegible]	1226	Bacteria>Pseudomonadota>Gammaproteobacteria>Moraxellales>Moraxellaceae>Acinetobacter	GCF_018863255.1	Acinetobacter_baumannii	KPE97_RS15710	83323..87003	470	BREX-system-P-loop-protein-BrxC-[Acinetobacter-baumannii]
WP_216116819.1	Type1.BREX	Peptidase_M48-><-MACRODOMAIN<-DarT+Peptid[illegible]	1226	Bacteria>Pseudomonadota>Gammaproteobacteria>Moraxellales>Moraxellaceae>Acinetobacter	GCF_018883565.1	Acinetobacter_baumannii	KPF69_RS05265	35520..39200	470	BREX-system-P-loop-protein-BrxC-[Acinetobacter-baumannii]
WP_218698253.1	Type1.BREX	<-MarR-HTH<-SF1-SF2-Helicase<-STYKIN+CR-K[illegible]	1224	Bacteria>Pseudomonadota>Gammaproteobacteria>Moraxellales>Moraxellaceae>Acinetobacter	GCF_019279015.1	Acinetobacter_harbinensis	KZA13_RS08660	34272..37946	1353941	BREX-system-P-loop-protein-BrxC-[Acinetobacter-harbinensis]
WP_221260378.1	Type1.BREX	<-sGTP<- wHTH+WYL+WCX <-?<-?<-?<- SIGMA+OB[illegible]	1226	Bacteria>Pseudomonadota>Gammaproteobacteria>Moraxellales>Moraxellaceae>Acinetobacter	GCF_019702905.1	Acinetobacter_sp._Tol_5	TOL5_RS12615	2696813..2700493	710648	BREX-system-P-loop-protein-BrxC-[Acinetobacter-sp.-Tol-5]
WP_207272916.1	Type1.BREX	<-MACRODOMAIN<-DarT+Peptidase<-SF1-SF2-He[illegible]	1226	Bacteria>Pseudomonadota>Gammaproteobacteria>Moraxellales>Moraxellaceae>Acinetobacter	GCF_019921105.1	Acinetobacter_baumannii	HLH91_RS14165	18027..21707	470	BREX-system-P-loop-protein-BrxC-[Acinetobacter-baumannii]
WP_005236280.1	Type1.BREX	<-DUF4096+DDE_Tnp_1+DDE_Tnp_1_2||?->?->Sb[illegible]	1226	Bacteria>Pseudomonadota>Gammaproteobacteria>Moraxellales>Moraxellaceae>Acinetobacter	GCF_020099375.1	Acinetobacter_variabilis	LA331_RS04655	963646..967326	70346	BREX-system-P-loop-protein-BrxC-[Acinetobacter-variabilis]
WP_225554349.1	Type1.BREX	DUF6079 *-> N6-METHYLASE -> ParB + HNH -> P[illegible]	1226	Bacteria>Pseudomonadota>Gammaproteobacteria>Moraxellales>Moraxellaceae>Acinetobacter	GCF_020271765.1	Acinetobacter_pseudolwoffii	LDD52_RS14245	193616..1867	2053287	BREX-system-P-loop-protein-BrxC-[Acinetobacter-pseudolwoffii]
WP_228414014.1	Type1.BREX	ZnR+DUF3482+ZnR->||DUF932-> wHTH+WYL+WCX [illegible]	1226	Bacteria>Pseudomonadota>Gammaproteobacteria>Moraxellales>Moraxellaceae>Acinetobacter	GCF_020810655.1	Acinetobacter_baumannii	NLHDIDDJ_RS06545	1352098..1355778	470	BREX-system-P-loop-protein-BrxC-[Acinetobacter-baumannii]
WP_233945509.1	Type1.BREX	<-SF1-SF2-Helicase+REase<- SIGMA+OB-Fold+[illegible]	1226	Bacteria>Pseudomonadota>Gammaproteobacteria>Moraxellales>Moraxellaceae>Acinetobacter	GCF_021391555.1	Acinetobacter_sp._SH20PTE14	LXF01_RS17045	120215..123895	2905879	BREX-system-P-loop-protein-BrxC-[Acinetobacter-sp.-SH20PTE14]
WP_236095241.1	Type1.BREX	<- PglZ <- ParB + HNH <- N6-METHYLASE <- DUF[illegible]	1226	Bacteria>Pseudomonadota>Gammaproteobacteria>Moraxellales>Moraxellaceae>Acinetobacter	GCF_021560715.1	Acinetobacter_sp._SCLZS86	LZP46_RS14905	6362..10042	2908637	BREX-system-P-loop-protein-BrxC-[Acinetobacter-sp.-SCLZS86]
WP_236163089.1	Type1.BREX	Phage_integrase-> ParB + HNH ->HTH_Tnp_1+H[illegible]	1226	Bacteria>Pseudomonadota>Gammaproteobacteria>Moraxellales>Moraxellaceae>Acinetobacter	GCF_021602685.1	unclassified_Acinetobacter	L6B80_RS11000	14595..18275	196816	MULTISPECIES:-BREX-system-P-loop-protein-BrxC-[unclassified-Acinetobacter]
WP_241338465.1	Type1.BREX	<- SIGMA+OB-Fold+AAA-ATPase+Lon-Protease-[illegible]	1226	Bacteria>Pseudomonadota>Gammaproteobacteria>Moraxellales>Moraxellaceae>Acinetobacter	GCF_022549435.1	Acinetobacter_sp._ANC_5659	MMP64_RS09425	122256..125936	2923427	BREX-system-P-loop-protein-BrxC-[Acinetobacter-sp.-ANC-5659]
WP_128202440.1	Type1.BREX	<-TPR+TPR<-?<-MACRODOMAIN<-DarT+Peptidase[illegible]	1226	Bacteria>Pseudomonadota>Gammaproteobacteria>Moraxellales>Moraxellaceae>Acinetobacter	GCF_023896505.1	Acinetobacter_baumannii	O9X56_RS14280	107190..110870	470	BREX-system-P-loop-protein-BrxC-[Acinetobacter-baumannii]
WP_257259040.1	Type1.BREX	<-MACRODOMAIN<-DarT+Peptidase<-SF1-SF2-He[illegible]	1226	Bacteria>Pseudomonadota>Gammaproteobacteria>Moraxellales>Moraxellaceae>Acinetobacter	GCF_024589115.1	Acinetobacter_baumannii	L2615_RS07865	180845..184525	470	BREX-system-P-loop-protein-BrxC-[Acinetobacter-baumannii]
WP_257935145.1	Type1.BREX	<-TPR+TPR<-MACRODOMAIN<-DarT+Peptidase<-S[illegible]	1226	Bacteria>Pseudomonadota>Gammaproteobacteria>Moraxellales>Moraxellaceae>Acinetobacter	GCF_024705725.1	Acinetobacter_baumannii	MSH04_RS15115	32588..36268	470	BREX-system-P-loop-protein-BrxC-[Acinetobacter-baumannii]
WP_238640059.1	Type1.BREX	<- SIGMA+OB-Fold+AAA-ATPase+Lon-Protease-[illegible]	1226	Bacteria>Pseudomonadota>Gammaproteobacteria>Moraxellales>Moraxellaceae>Acinetobacter	GCF_025264005.1	Acinetobacter_baumannii	NT399_RS17670	10742..14422	470	BREX-system-P-loop-protein-BrxC-[Acinetobacter-baumannii]
WP_262579272.1	Type1.BREX	<- SIGMA+OB-Fold+AAA-ATPase+Lon-Protease-[illegible]	1226	Bacteria>Pseudomonadota>Gammaproteobacteria>Moraxellales>Moraxellaceae>Acinetobacter	GCF_025562355.1	Acinetobacter_junii	KTH64_RS13010	10561..14241	40215	BREX-system-P-loop-protein-BrxC-[Acinetobacter-junii]
WP_264453044.1	Type6.BREX	DUF932->?->?->XIS-HTH->SAM-METHYLASE-> wH[illegible]	1214	Bacteria>Pseudomonadota>Gammaproteobacteria>Moraxellales>Moraxellaceae>Acinetobacter	GCF_025938795.1	Acinetobacter_baumannii	OLN40_RS03910	58425..62069	470	BREX-system-P-loop-protein-BrxC-[Acinetobacter-baumannii]
WP_265466015.1	Type1.BREX	<-Calcineurin|| ParB + HNH ->SAM-METHYLASE[illegible]	1226	Bacteria>Pseudomonadota>Gammaproteobacteria>Moraxellales>Moraxellaceae>Acinetobacter	GCF_026168575.1	Acinetobacter_sp._BIT-DXN8	OKC24_RS16365	19397..23077	2989714	BREX-system-P-loop-protein-BrxC-[Acinetobacter-sp.-BIT-DXN8]
WP_071225618.1	Type1.BREX	ADH_N+ADH_zinc_N-><-MODE-HTH+PBPII||MarR-[illegible]	1226	Bacteria>Pseudomonadota>Gammaproteobacteria>Moraxellales>Moraxellaceae>Acinetobacter	GCF_027854895.1	Acinetobacter_baumannii	MJO31_RS19545	43789..47469	470	BREX-system-P-loop-protein-BrxC-[Acinetobacter-baumannii]
WP_274579352.1	Type1.BREX	<-DDE_Tnp_1_5+DDE_Tnp_1||?->DOC+HTH-><-DU[illegible]	1226	Bacteria>Pseudomonadota>Gammaproteobacteria>Moraxellales>Moraxellaceae>Acinetobacter	GCF_028774645.1	Acinetobacter_lactucae	MO054_RS17975	24360..28040	1785128	BREX-system-P-loop-protein-BrxC-[Acinetobacter-lactucae]
WP_275290158.1	Type1.BREX	<-HTH_Tnp_1+HTH_21+rve+rve_3||<-HTH_3<-Hi[illegible]	1226	Bacteria>Pseudomonadota>Gammaproteobacteria>Moraxellales>Moraxellaceae>Acinetobacter	GCF_029100495.1	Acinetobacter_nosocomialis	JIZ05_14385	7251..10931	106654	BREX-system-P-loop-protein-BrxC-[Acinetobacter-nosocomialis]
WP_161409476.1	Type1.BREX	wHTH+WYL+WCX -> BrxA-DNA-binding -> BrxB-B[illegible]	1226	Bacteria>Pseudomonadota>Gammaproteobacteria>Moraxellales>Moraxellaceae>Acinetobacter	GCF_030329445.1	Acinetobacter_haemolyticus	GPS61_RS07925	49241..52921	29430	BREX-system-P-loop-protein-BrxC-[Acinetobacter-haemolyticus]
WP_005115116.1	Type1.BREX	<- SIGMA+OB-Fold+AAA-ATPase+Lon-Protease-[illegible]	1226	Bacteria>Pseudomonadota>Gammaproteobacteria>Moraxellales>Moraxellaceae>Acinetobacter	GCF_030413705.1	Acinetobacter_baumannii	AWM09_RS06130	10901..14581	470	BREX-system-P-loop-protein-BrxC-[Acinetobacter-baumannii]
WP_002010901.1	Type1.BREX	<-MACRODOMAIN<-DarT+Peptidase<-SF1-SF2-He[illegible]	1226	Bacteria>Pseudomonadota>Gammaproteobacteria>Moraxellales>Moraxellaceae	GCF_000214965.1	Acinetobacter	ACINNAV18_RS19255	65599..69279	469	MULTISPECIES:-BREX-system-P-loop-protein-BrxC-[Acinetobacter]
WP_111885889.1	Type1.BREX	<- SIGMA+OB-Fold+AAA-ATPase+Lon-Protease-[illegible]	1226	Bacteria>Pseudomonadota>Gammaproteobacteria>Moraxellales>Moraxellaceae	GCF_002339435.1	Acinetobacter	NVV26_RS12530	20211..23891	469	MULTISPECIES:-BREX-system-P-loop-protein-BrxC-[Acinetobacter]
WP_108675357.1	Type1.BREX	Arm-DNA-bind_3+Phage_integrase-><-?||?->Z[illegible]	1225	Bacteria>Pseudomonadota>Gammaproteobacteria>Moraxellales>Moraxellaceae	GCF_003072585.2	Acinetobacter	CO119_RS05575	1118908..1122585	469	MULTISPECIES:-BREX-system-P-loop-protein-BrxC-[Acinetobacter]
WP_151794804.1	Type6.BREX	<-CR-Kinase+Classical-AAA<-?<-?<-?<- SIGM[illegible]	1214	Bacteria>Pseudomonadota>Gammaproteobacteria>Moraxellales>Moraxellaceae	GCF_008997035.1	Acinetobacter	GBP95_RS04055	337470..341114	469	MULTISPECIES:-BREX-system-P-loop-protein-BrxC-[Acinetobacter]
WP_178319334.1	Type1.BREX	<-DUF4096+DDE_Tnp_1+DDE_Tnp_1_2||DDE_5+DD[illegible]	1226	Bacteria>Pseudomonadota>Gammaproteobacteria>Moraxellales>Moraxellaceae	GCF_013390985.1	Acinetobacter	HX743_RS11980	35327..39007	469	MULTISPECIES:-BREX-system-P-loop-protein-BrxC-[Acinetobacter]
ABE75386.1	Type1.BREX	ABC-ATPase+ABC-ATPase->?->?->?->tRNA->tR[illegible]	1201	Bacteria>Pseudomonadota>Gammaproteobacteria>Moraxellales>Moraxellaceae>Psychrobacter	GCA_000013905.1	Psychrobacter_cryohalolentis_K5	Pcryo_1609	1979457..1983062	335284	conserved-hypothetical-protein-[Psychrobacter-cryohalolentis-K5]
MCG3880112.1	Type1.BREX	<-wHTH+REase+ZnR<-McrB-AAA<-MarR-HTH<-SF1[illegible]	1154	Bacteria>Pseudomonadota>Gammaproteobacteria>Moraxellales>Moraxellaceae>Psychrobacter	GCA_022129235.1	Psychrobacter_sp._Ps6	I3255_11455	48655..52119	2790960	BREX-system-P-loop-protein-BrxC-[Psychrobacter-sp.-Ps6]
WP_226939352.1	Type1.BREX	ABC-ATPase+ABC-ATPase->?->?->?->tRNA->tR[illegible]	1221	Bacteria>Pseudomonadota>Gammaproteobacteria>Moraxellales>Moraxellaceae>Psychrobacter	GCF_000013905.1	Psychrobacter_cryohalolentis	CEP87_RS02910	676881..680546	330922	BREX-system-P-loop-protein-BrxC-[Psychrobacter-cryohalolentis]
WP_261869530.1	Type1.BREX	Phage_integrase+Phage_integrase->Phage_in[illegible]	642	Bacteria>Pseudomonadota>Gammaproteobacteria>Moraxellales>Moraxellaceae>Psychrobacter	GCF_000586435.1	Psychrobacter_sp._JCM_18901	TT11_RS05300	11820..13748	1298609	BREX-system-P-loop-protein-BrxC-[Psychrobacter-sp.-JCM-18901]
WP_089554755.1	Type1.BREX	Phage_integrase->?->XIS-HTH->AAA+HTH-><-?[illegible]	1223	Bacteria>Pseudomonadota>Gammaproteobacteria>Moraxellales>Moraxellaceae>Psychrobacter	GCF_002224105.1	Psychrobacter_sp._DAB_AL32B	CAN34_RS05290	13543..17214	1028414	BREX-system-P-loop-protein-BrxC-[Psychrobacter-sp.-DAB_AL32B]
WP_191691193.1	Type1.BREX	<-Phage_integrase<-Phage_integrase+Phage_[illegible]	1222	Bacteria>Pseudomonadota>Gammaproteobacteria>Moraxellales>Moraxellaceae>Psychrobacter	GCF_014836505.1	Psychrobacter_communis	H9653_RS05990	24340..28008	2762238	BREX-system-P-loop-protein-BrxC-[Psychrobacter-communis]
WP_207991848.1	Type1.BREX	<-TOPRIM<-SbcC<-REase<-REase||ZnR-> BrxA-[illegible]	1221	Bacteria>Pseudomonadota>Gammaproteobacteria>Moraxellales>Moraxellaceae>Psychrobacter	GCF_017498085.1	Psychrobacter_coccoides	J3492_RS09680	48612..52277	2818440	BREX-system-P-loop-protein-BrxC-[Psychrobacter-coccoides]
WP_218690652.1	Type6.BREX	<-AAA+HTH|| ParB + HNH -> BrxE -> BrxA-DNA-b[illegible]	1213	Bacteria>Pseudomonadota>Gammaproteobacteria>Moraxellales>Moraxellaceae>Psychrobacter	GCF_019279065.1	Psychrobacter_sp._BF1	KZA12_RS14005	8359..12000	2821147	BREX-system-P-loop-protein-BrxC-[Psychrobacter-sp.-BF1]
WP_238038933.1	Type1.BREX	<-wHTH+REase+ZnR<-McrB-AAA<-MarR-HTH<-SF1[illegible]	1162	Bacteria>Pseudomonadota>Gammaproteobacteria>Moraxellales>Moraxellaceae>Psychrobacter	GCF_022129235.1	Psychrobacter_sp._Ps6	I3255_RS11455	48631..52119	2790960	BREX-system-P-loop-protein-BrxC-[Psychrobacter-sp.-Ps6]
WP_238051284.1	Type6.BREX	<-DDE_3<-HTH_Tnp_IS630<-AAA+HTH||?-> ParB[illegible]	1213	Bacteria>Pseudomonadota>Gammaproteobacteria>Moraxellales>Moraxellaceae>Psychrobacter	GCF_022129405.1	Psychrobacter_sp._Ps2	I3254_RS03805	13291..16932	2790956	BREX-system-P-loop-protein-BrxC-[Psychrobacter-sp.-Ps2]
WP_199774478.1	Type1.BREX	<-REase<-HKD-DNase<-?<-?|| BrxA-DNA-bindi[illegible]	1164	Bacteria>Pseudomonadota>Gammaproteobacteria>Moraxellales>Moraxellaceae>Psychrobacter	GCF_900291405.1	Psychrobacter_sp._Marseille-P5312	C6W44_RS10120	2338090..2341584	2086574	BREX-system-P-loop-protein-BrxC-[Psychrobacter-sp.-Marseille-P5312]
WP_201558729.1	Type1.BREX	Arm-DNA-bind_3+Phage_integrase-><-?<-?<-[illegible]	1201	Bacteria>Pseudomonadota>Gammaproteobacteria>Moraxellales>Moraxellaceae>Psychrobacter	GCF_904846085.1	Psychrobacter_immobilis	JMW74_RS07725	23770..27375	498	BREX-system-P-loop-protein-BrxC-[Psychrobacter-immobilis]
WP_201625912.1	Type1.BREX	HTH_Tnp_1+HTH_21+rve+rve_3-><- SIGMA+OB-F[illegible]	1146	Bacteria>Pseudomonadota>Gammaproteobacteria>Moraxellales>Moraxellaceae>Psychrobacter	GCF_904846275.1	Psychrobacter_immobilis	JMX31_RS11870	11441..14881	498	BREX-system-P-loop-protein-BrxC-[Psychrobacter-immobilis]
WP_198334111.1	Type1.BREX	DnaB+TnsC-ATPase-> ParB ->Phage_integrase[illegible]	1210	Bacteria>Pseudomonadota>Gammaproteobacteria>Moraxellales>Moraxellaceae>Psychrobacter	GCF_904846355.1	Psychrobacter_namhaensis	JMX36_RS06945	21523..25155	292734	BREX-system-P-loop-protein-BrxC-[Psychrobacter-namhaensis]
WP_201542137.1	Type1.BREX	<- SIGMA+OB-Fold+AAA-ATPase+Lon-Protease-[illegible]	1210	Bacteria>Pseudomonadota>Gammaproteobacteria>Moraxellales>Moraxellaceae>Psychrobacter	GCF_904846515.1	Psychrobacter_sanguinis	LK453_RS13670	3248348..3251980	861445	BREX-system-P-loop-protein-BrxC-[Psychrobacter-sanguinis]
WP_201579699.1	Type1.BREX	<-ZnR||HTH_26-> ParB ->Phage_integrase+Ph[illegible]	1223	Bacteria>Pseudomonadota>Gammaproteobacteria>Moraxellales>Moraxellaceae>Psychrobacter	GCF_904846705.1	Psychrobacter_sp._Pi2-52	JMY00_RS07535	19023..22694	2774133	BREX-system-P-loop-protein-BrxC-[Psychrobacter-sp.-Pi2-52]
WP_011513935.1	Type1.BREX	ABC-ATPase+ABC-ATPase->?->?->?->tRNA->tR[illegible]	1201	Bacteria>Pseudomonadota>Gammaproteobacteria>Moraxellales>Moraxellaceae>Psychrobacter	-	Psychrobacter_cryohalolentis	Pcryo_1609	1979457..1983062	330922	BREX-system-P-loop-protein-BrxC-[Psychrobacter-cryohalolentis]
WP_010129447.1	Type1.BREX	<-Phage_holin<-?||MazE_antitoxin->PIN->D[illegible]	1225	Bacteria>Pseudomonadota	-	Gammaproteobacteria	MTT88_RS00175	38411..42088	1236	MULTISPECIES:-BREX-system-P-loop-protein-BrxC-[Gammaproteobacteria]
WP_228349567.1	Type1.BREX	<-DUF772+DDE_Tnp_1||?-><-DUF772+DDE_Tnp_1[illegible]	1203	Bacteria>Pseudomonadota>Gammaproteobacteria>Nevskiales>Sinobacteraceae>Flagellatimonas	GCF_016918765.2	Flagellatimonas_centrodinii	JN531_RS14475	2992880..2996491	2806210	BREX-system-P-loop-protein-BrxC-[Flagellatimonas-centrodinii]
WP_161384457.1	Type1.BREX	<- wHTH+WYL+WCX ||?-><-RelE-ParE<-RHH_1||[illegible]	1192	Bacteria>Pseudomonadota>Gammaproteobacteria>Oceanospirillales>Alcanivoracaceae>Alcanivorax	-	Alcanivorax_sp._ZXX171	GN155_RS10555	118232..121810	2695858	BREX-system-P-loop-protein-BrxC-[Alcanivorax-sp.-ZXX171]
MBA3979207.1	Type1.BREX	PhdYeFM_antitox->?-><-Trypsin+PDZ<-Mrr-N|[illegible]	1145	Bacteria>Pseudomonadota>Gammaproteobacteria>Oceanospirillales>Alcanivoracaceae>Alcanivorax	GCA_013823245.1	Alcanivorax_sp.	C0462_01260	7976..11413	1872427	BREX-system-P-loop-protein-BrxC-[Alcanivorax-sp.]
WP_153499070.1	Type6.BREX	<-PHP<- wHTH+WYL+WCX ||?->?->HEPN-p-> BrxE[illegible]	1214	Bacteria>Pseudomonadota>Gammaproteobacteria>Oceanospirillales>Alcanivoracaceae>Alcanivorax	GCF_009601165.1	Alcanivorax_sediminis	GFN93_RS03795	861896..865540	2663008	BREX-system-P-loop-protein-BrxC-[Alcanivorax-sediminis]
WP_103916700.1	Type1.BREX	<- SIGMA+OB-Fold+AAA-ATPase+HKD-DNase-Brx[illegible]	1193	Bacteria>Pseudomonadota>Gammaproteobacteria>Oceanospirillales>Alcanivoracaceae>Alcanivorax	GCF_900107995.1	Alcanivorax_sp._DSM_26293	C4S63_RS03510	780383..783964	1798238	BREX-system-P-loop-protein-BrxC-[Alcanivorax-sp.-DSM-26293]
WP_101745536.1	Type1.BREX	ABC-ATPase+SbcC+ABC-ATPase+SbcC-><-?<-?||[illegible]	1220	Bacteria>Pseudomonadota>Gammaproteobacteria>Oceanospirillales>Endozoicomonadaceae>Endozoicomonas	GCF_002864045.1	Endozoicomonas_acroporae	CX664_RS04280	14964..18626	1701104	BREX-system-P-loop-protein-BrxC-[Endozoicomonas-acroporae]

Protein-ID	System	Gene-Neighbourhoods	Length	Taxonomy	Assembly-ID	Strain	Locus-Tag	Genomic-coordinates	TaxID	Fasta-defline
WP_153766763.1	Type1.BREX	[illegible]	1223	Bacteria>Pseudomonadota>Gammaproteobacteria>Oceanospirillales>Endozoicomonadaceae>Endozoicomonas	GCF_009653635.1	Endozoicomonas_sp._OPT23	EOPP23_14760	167593..171264	2072845	BREX-system-P-loop-protein-BrxC-[Endozoicomonas-sp.-OPT23]
WP_163371285.1	Type1.BREX	[illegible]	1220	Bacteria>Pseudomonadota>Gammaproteobacteria>Oceanospirillales>Endozoicomonadaceae>Endozoicomonas	GCF_010994335.1	Endozoicomonas_acroporae	EO230_RS03420	3343..7005	1701104	BREX-system-P-loop-protein-BrxC-[Endozoicomonas-acroporae]
MCP5206320.1	Type1.BREX	[illegible]	910	Bacteria>Pseudomonadota>Gammaproteobacteria>Oceanospirillales>Hahellaceae>unclassified_Hahellaceae	GCA_024234475.1	Hahellaceae_bacterium	H7A01_03865	876629..879361	2048971	BREX-system-P-loop-protein-BrxC-[Hahellaceae-bacterium]
MCP5206322.1	Type1.BREX	[illegible]	261	Bacteria>Pseudomonadota>Gammaproteobacteria>Oceanospirillales>Hahellaceae>unclassified_Hahellaceae	GCA_024234475.1	Hahellaceae_bacterium	H7A01_03875	880608..881393	2048971	hypothetical-protein-[Hahellaceae-bacterium]
WP_040239271.1	Type1.BREX	[illegible]	1144	Bacteria>Pseudomonadota>Gammaproteobacteria>Oceanospirillales>Halomonadaceae>Chromohalobacter	GCF_000821045.2	Chromohalobacter_japonicus	BN995_RS00415	38131..41565	223900	BREX-system-P-loop-protein-BrxC-[Chromohalobacter-japonicus]
PHR03208.1	Type1.BREX	[illegible]	1144	Bacteria>Pseudomonadota>Gammaproteobacteria>Oceanospirillales>Halomonadaceae>Halomonas	GCA_002733025.1	Halomonas_sp.	COB32_05665	22826..26260	1486246	hypothetical-protein-COB32_05665-[Halomonas-sp.]
NQY75752.1	Type1.BREX	[illegible]	1227	Bacteria>Pseudomonadota>Gammaproteobacteria>Oceanospirillales>Halomonadaceae>Halomonas	GCA_013213865.1	Halomonas_sp.	HRT78_01215	164704..168387	1486246	BREX-system-P-loop-protein-BrxC-[Halomonas-sp.]
WP_017428908.1	Type1.BREX	[illegible]	1247	Bacteria>Pseudomonadota>Gammaproteobacteria>Oceanospirillales>Halomonadaceae>Halomonas	GCF_000334215.1	Halomonas_jeotgali	C793_RS0100125	27130..30873	553386	BREX-system-P-loop-protein-BrxC-[Halomonas-jeotgali]
WP_035558509.1	Type1.BREX	[illegible]	1236	Bacteria>Pseudomonadota>Gammaproteobacteria>Oceanospirillales>Halomonadaceae>Halomonas	GCF_000734975.2	Halomonas_sp._KO116	KO116_RS11715	2523126..2526836	1504981	BREX-system-P-loop-protein-BrxC-[Halomonas-sp.-KO116]
WP_159343441.1	Type1.BREX	[illegible]	1193	Bacteria>Pseudomonadota>Gammaproteobacteria>Oceanospirillales>Halomonadaceae>Halomonas	GCF_009846525.1	Halomonas_meridiana	CTT34_RS16815	3563093..3566674	29570	BREX-system-P-loop-protein-BrxC-[Halomonas-meridiana]
WP_161432258.1	Type6.BREX	[illegible]	1223	Bacteria>Pseudomonadota>Gammaproteobacteria>Oceanospirillales>Halomonadaceae>Halomonas	GCF_009902005.1	Halomonas_alimentaria	GRB96_RS11410	2397586..2401257	147248	BREX-system-P-loop-protein-BrxC-[Halomonas-alimentaria]
WP_172515133.1	Type1.BREX	[illegible]	1193	Bacteria>Pseudomonadota>Gammaproteobacteria>Oceanospirillales>Halomonadaceae>Halomonas	GCF_011764625.1	Halomonas_meridiana	HMEPL2_RS11780	2496852..2500433	29570	BREX-system-P-loop-protein-BrxC-[Halomonas-meridiana]
WP_179915949.1	Type1.BREX	[illegible]	1227	Bacteria>Pseudomonadota>Gammaproteobacteria>Oceanospirillales>Halomonadaceae>Halomonas	GCF_013415125.1	Halomonas_glaciei	HZS80_RS09710	42867..46550	186761	BREX-system-P-loop-protein-BrxC-[Halomonas-glaciei]
WP_188638083.1	Type1.BREX	[illegible]	1154	Bacteria>Pseudomonadota>Gammaproteobacteria>Oceanospirillales>Halomonadaceae>Halomonas	GCF_014640815.1	Halomonas_lutescens	IEW65_RS03380	721900..725364	1602943	BREX-system-P-loop-protein-BrxC-[Halomonas-lutescens]
WP_019020105.1	Type1.BREX	[illegible]	1193	Bacteria>Pseudomonadota>Gammaproteobacteria>Oceanospirillales>Halomonadaceae>Halomonas	GCF_014651575.1	Halomonas_lutea	IE067_RS16235	61170..64751	453962	BREX-system-P-loop-protein-BrxC-[Halomonas-lutea]
WP_263591023.1	Type1.BREX	[illegible]	1144	Bacteria>Pseudomonadota>Gammaproteobacteria>Oceanospirillales>Halomonadaceae>Halomonas	GCF_025725685.1	Halomonas_sp._M4R1S46	OCT48_RS01560	327087..330521	2982692	BREX-system-P-loop-protein-BrxC-[Halomonas-sp.-M4R1S46]
WP_089705394.1	Type1.BREX	[illegible]	1189	Bacteria>Pseudomonadota>Gammaproteobacteria>Oceanospirillales>Halomonadaceae>Halomonas	GCF_900103865.1	Halomonas_arcis	BLR97_RS09380	247223..250792	416873	BREX-system-P-loop-protein-BrxC-[Halomonas-arcis]
WP_081193131.1	Type1.BREX	[illegible]	1154	Bacteria>Pseudomonadota>Gammaproteobacteria>Oceanospirillales>Halomonadaceae>Halomonas	-	Halomonas_sp._BC1	B7L72_RS05665	10416..13880	1670448	BREX-system-P-loop-protein-BrxC-[Halomonas-sp.-BC1]
WP_086901154.1	Type1.BREX	[illegible]	1237	Bacteria>Pseudomonadota>Gammaproteobacteria>Oceanospirillales>Halomonadaceae>Kushneria	GCF_002157205.1	Kushneria_marisflavi	B9H00_RS13900	3054889..3058602	157779	BREX-system-P-loop-protein-BrxC-[Kushneria-marisflavi]
WP_189514712.1	Type1.BREX	[illegible]	1144	Bacteria>Pseudomonadota>Gammaproteobacteria>Oceanospirillales>Halomonadaceae>Kushneria	GCF_014652795.1	Kushneria_pakistanensis	IE123_RS02380	531699..535133	1508770	BREX-system-P-loop-protein-BrxC-[Kushneria-pakistanensis]
WP_110669866.1	Type6.BREX	[illegible]	1225	Bacteria>Pseudomonadota>Gammaproteobacteria>Oceanospirillales>Halomonadaceae>Salinicola	GCF_003206155.1	Salinicola_halophilus	DOM33_RS13680	401271..404948	184065	BREX-system-P-loop-protein-BrxC-[Salinicola-halophilus]
WP_261843643.1	Type1.BREX	[illegible]	1225	Bacteria>Pseudomonadota>Gammaproteobacteria>Oceanospirillales>Oceanospirillaceae>Aliamphritea	GCF_024347215.1	Aliamphritea_ceti	OCU49_RS03510	764184..767861	1524258	BREX-system-P-loop-protein-BrxC-[Aliamphritea-ceti]
WP_027853089.1	Type1.BREX	[illegible]	1154	Bacteria>Pseudomonadota>Gammaproteobacteria>Oceanospirillales>Oceanospirillaceae>Marinobacterium	GCF_000428985.1	Marinobacterium_litorale	G403_RS0105385	191863..195327	404770	BREX-system-P-loop-protein-BrxC-[Marinobacterium-litorale]
WP_181736357.1	Type1.BREX	[illegible]	1154	Bacteria>Pseudomonadota>Gammaproteobacteria>Oceanospirillales>Oceanospirillaceae>Marinobacterium	GCF_013868415.1	Marinobacterium_sp._3-1745	H1S06_RS00670	30655..34119	2756129	BREX-system-P-loop-protein-BrxC-[Marinobacterium-sp.-3-1745]
AWY02455.1	Type1.BREX	[illegible]	1197	Bacteria>Pseudomonadota>Gammaproteobacteria>Oceanospirillales>Oceanospirillaceae>Marinomonas	GCA_003285205.1	Marinomonas_primoryensis	A8139_14855	3171018..3174611	178399	hypothetical-protein-A8139_14855-[Marinomonas-primoryensis]
WP_100635909.1	Type1.BREX	[illegible]	1157	Bacteria>Pseudomonadota>Gammaproteobacteria>Oceanospirillales>Oceanospirillaceae>Marinomonas	GCF_002806845.1	Marinomonas_sp._ef1	CCS02_RS04315	398110..401583	2005043	BREX-system-P-loop-protein-BrxC-[Marinomonas-sp.-ef1]
WP_239495510.1	Type1.BREX	[illegible]	1199	Bacteria>Pseudomonadota>Gammaproteobacteria>Oceanospirillales>Oceanospirillaceae>Marinomonas	GCF_003285205.1	Marinomonas_primoryensis	A8139_RS14980	3171018..3174617	178399	BREX-system-P-loop-protein-BrxC-[Marinomonas-primoryensis]
QEW05875.1	Type6.BREX	[illegible]	1212	Bacteria>Pseudomonadota>Gammaproteobacteria>Oceanospirillales>Oceanospirillaceae>Nitrincola	GCA_008727795.1	Nitrincola_iocasae	F5I99_04885	1032629..1036267	2614693	BREX-system-P-loop-protein-BrxC-[Nitrincola-iocasae]
WP_225307559.1	Type6.BREX	[illegible]	1214	Bacteria>Pseudomonadota>Gammaproteobacteria>Oceanospirillales>Oceanospirillaceae>Nitrincola	GCF_008727795.1	Nitrincola_iocasae	F5I99_RS04885	1032623..1036267	2614693	BREX-system-P-loop-protein-BrxC-[Nitrincola-iocasae]
WP_260997651.1	Type1.BREX	[illegible]	1156	Bacteria>Pseudomonadota>Gammaproteobacteria>Oceanospirillales>Oceanospirillaceae>Thalassolituus	GCF_025345565.1	Thalassolituus_hydrocarbonoclasticus	HUF19_RS16715	3723205..3726675	2742796	BREX-system-P-loop-protein-BrxC-[Thalassolituus-hydrocarbonoclasticus]
MAS25385.1	Type1.BREX	[illegible]	1145	Bacteria>Pseudomonadota>Gammaproteobacteria>Oceanospirillales>Oceanospirillaceae>unclassified_Oceanospirillaceae	GCA_002698675.1	Oceanospirillaceae_bacterium	CMI08_10750	15208..18645	1899355	hypothetical-protein-[Oceanospirillaceae-bacterium]
WP_116688140.1	Type1.BREX	[illegible]	1236	Bacteria>Pseudomonadota>Gammaproteobacteria>Oceanospirillales>Oceanospirillales_incertae_sedis>Pelagibaculum	GCF_003097315.1	Pelagibaculum_spongiae	DC094_RS16040	158438..162148	2080658	BREX-system-P-loop-protein-BrxC-[Pelagibaculum-spongiae]
WP_100257386.1	Type1.BREX	[illegible]	1215	Bacteria>Pseudomonadota>Gammaproteobacteria>Oceanospirillales>Saccharospirillaceae>Reinekea	GCF_002795845.1	Reinekea_forsetii	REIFOR_RS09825	2117568..2121215	1336806	BREX-system-P-loop-protein-BrxC-[Reinekea-forsetii]
MBR9867112.1	Type1.BREX	[illegible]	1225	Bacteria>Pseudomonadota>Gammaproteobacteria>Oceanospirillales>unclassified_Oceanospirillales	GCA_018223265.1	Oceanospirillales_bacterium	GYB18_08415	21169..24846	1914300	BREX-system-P-loop-protein-BrxC-[Oceanospirillales-bacterium]
WP_241468927.1	Type1.BREX	[illegible]	1225	Bacteria>Pseudomonadota>Gammaproteobacteria>Pasteurellales>Pasteurellaceae>Pasteurella	GCF_022559605.1	Pasteurella_multocida	MMZ64_RS09105	1895707..1899384	747	BREX-system-P-loop-protein-BrxC-[Pasteurella-multocida]
WP_119518392.1	Type1.BREX	[illegible]	1154	Bacteria>Pseudomonadota>Gammaproteobacteria>Pseudomonadales	GCF_021610005.1	Pseudomonadaceae	L1776_RS13375	2929669..2933133	135621	MULTISPECIES:-BREX-system-P-loop-protein-BrxC-[Pseudomonadaceae]
WP_058011517.1	Type6.BREX	[illegible]	1213	Bacteria>Pseudomonadota>Gammaproteobacteria>Pseudomonadales	GCF_901482535.1	Pseudomonadaceae	FQU16_RS01885	279271..282912	135621	MULTISPECIES:-BREX-system-P-loop-protein-BrxC-[Pseudomonadaceae]
MBE95557.1	Type1.BREX	[illegible]	1193	Bacteria>Pseudomonadota>Gammaproteobacteria>Pseudomonadales>Marinobacteraceae>Marinobacter	GCA_002714765.1	Marinobacter_sp.	CMG89_11180	696..4277	50741	hypothetical-protein-[Marinobacter-sp.]
WP_048384700.1	Type1.BREX	[illegible]	1145	Bacteria>Pseudomonadota>Gammaproteobacteria>Pseudomonadales>Marinobacteraceae>Marinobacter	GCF_001043175.1	Marinobacter_psychrophilus	ABA45_RS05800	1298938..1302375	330734	BREX-system-P-loop-protein-BrxC-[Marinobacter-psychrophilus]
WP_071262841.1	Type1.BREX	[illegible]	1217	Bacteria>Pseudomonadota>Gammaproteobacteria>Pseudomonadales>Marinobacteraceae>Marinobacter	GCF_001858325.1	Marinobacter_sp._AC-23	BCA33_RS00460	104149..107802	1879031	BREX-system-P-loop-protein-BrxC-[Marinobacter-sp.-AC-23]
WP_141068072.1	Type1.BREX	[illegible]	1193	Bacteria>Pseudomonadota>Gammaproteobacteria>Pseudomonadales>Marinobacteraceae>Marinobacter	GCF_006516615.1	Marinobacter_nauticus	FH712_RS17655	33566..37147	2743	BREX-system-P-loop-protein-BrxC-[Marinobacter-nauticus]
WP_213480159.1	Type1.BREX	[illegible]	1145	Bacteria>Pseudomonadota>Gammaproteobacteria>Pseudomonadales>Marinobacteraceae>Marinobacter	GCF_018424725.1	Marinobacter_salarius	DYI41_RS19390	15297..18734	1420917	BREX-system-P-loop-protein-BrxC-[Marinobacter-salarius]
WP_216008134.1	Type1.BREX	[illegible]	1217	Bacteria>Pseudomonadota>Gammaproteobacteria>Pseudomonadales>Marinobacteraceae>Marinobacter	GCF_018860765.1	Marinobacter_salexigens	KO508_RS09770	175903..179556	1925763	BREX-system-P-loop-protein-BrxC-[Marinobacter-salexigens]
WP_247116152.1	Type1.BREX	[illegible]	1189	Bacteria>Pseudomonadota>Gammaproteobacteria>Pseudomonadales>Marinobacteraceae>Marinobacter	GCF_023016045.1	Marinobacter_sp._S6332	MWU62_RS17920	19915..23484	2926403	BREX-system-P-loop-protein-BrxC-[Marinobacter-sp.-S6332]
WP_248236652.1	Type1.BREX	[illegible]	1145	Bacteria>Pseudomonadota>Gammaproteobacteria>Pseudomonadales>Marinobacteraceae>Marinobacter	GCF_023156245.1	Marinobacter_bryozoorum	MLC59_RS17300	17294..20731	256324	BREX-system-P-loop-protein-BrxC-[Marinobacter-bryozoorum]
WP_249013861.1	Type1.BREX	[illegible]	1217	Bacteria>Pseudomonadota>Gammaproteobacteria>Pseudomonadales>Marinobacteraceae>Marinobacter	GCF_023311895.1	Marinobacter_sp._M3C	MIH18_RS02775	634198..637851	2917715	BREX-system-P-loop-protein-BrxC-[Marinobacter-sp.-M3C]
WP_092021229.1	Type1.BREX	[illegible]	1219	Bacteria>Pseudomonadota>Gammaproteobacteria>Pseudomonadales>Marinobacteraceae>Marinobacter	GCF_900114775.1	Marinobacter_zhejiangensis	BMZ67_RS06955	334318..337977	488535	BREX-system-P-loop-protein-BrxC-[Marinobacter-zhejiangensis]
WP_019470415.1	Type6.BREX	[illegible]	1212	Bacteria>Pseudomonadota>Gammaproteobacteria>Pseudomonadales>Pseudomonadaceae	GCF_000292285.1	Pseudomonas	N805_RS25690	5809538..5813176	286	MULTISPECIES:-BREX-system-P-loop-protein-BrxC-[Pseudomonas]
WP_057024539.1	Type1.BREX	[illegible]	1154	Bacteria>Pseudomonadota>Gammaproteobacteria>Pseudomonadales>Pseudomonadaceae	GCF_002091795.1	Pseudomonas	TU77_RS22280	179418..182882	286	MULTISPECIES:-BREX-system-P-loop-protein-BrxC-[Pseudomonas]
WP_096426782.1	Type1.BREX	[illegible]	1185	Bacteria>Pseudomonadota>Gammaproteobacteria>Pseudomonadales>Pseudomonadaceae	GCF_002356095.1	Pseudomonas	J3A71_RS03870	842777..846334	286	MULTISPECIES:-BREX-system-P-loop-protein-BrxC-[Pseudomonas]
WP_196184087.1	Type1.BREX	[illegible]	1154	Bacteria>Pseudomonadota>Gammaproteobacteria>Pseudomonadales>Pseudomonadaceae	GCF_015680805.1	Pseudomonas	IR013_RS15060	51443..54907	286	MULTISPECIES:-BREX-system-P-loop-protein-BrxC-[Pseudomonas]
WP_033986129.1	Type6.BREX	[illegible]	1212	Bacteria>Pseudomonadota>Gammaproteobacteria>Pseudomonadales>Pseudomonadaceae	GCF_020180035.1	Pseudomonas	AO894_RS16050	13512..17150	286	MULTISPECIES:-BREX-system-P-loop-protein-BrxC-[Pseudomonas]
WP_064314265.1	Type1.BREX	[illegible]	1154	Bacteria>Pseudomonadota>Gammaproteobacteria>Pseudomonadales>Pseudomonadaceae	GCF_021601465.1	Pseudomonas	L6D68_RS06035	31411..34875	286	MULTISPECIES:-BREX-system-P-loop-protein-BrxC-[Pseudomonas]
WP_021219096.1	Type1.BREX	[illegible]	1220	Bacteria>Pseudomonadota>Gammaproteobacteria>Pseudomonadales>Pseudomonadaceae	GCF_023093875.1	Pseudomonas	MYO55_RS15275	3320590..3324252	286	MULTISPECIES:-BREX-system-P-loop-protein-BrxC-[Pseudomonas]
WP_060765715.1	Type1.BREX	[illegible]	1186	Bacteria>Pseudomonadota>Gammaproteobacteria>Pseudomonadales>Pseudomonadaceae	GCF_029959765.1	Pseudomonas	PFL603g_RS03160	383988..387548	286	MULTISPECIES:-BREX-system-P-loop-protein-BrxC-[Pseudomonas]
WP_023115268.1	Type1.BREX	[illegible]	1154	Bacteria>Pseudomonadota>Gammaproteobacteria>Pseudomonadales>Pseudomonadaceae	GCF_900707185.1	Pseudomonas	E4Y87_RS04340	6868..10332	286	MULTISPECIES:-BREX-system-P-loop-protein-BrxC-[Pseudomonas]
WP_198417420.1	Type1.BREX	[illegible]	1159	Bacteria>Pseudomonadota>Gammaproteobacteria>Pseudomonadales>Pseudomonadaceae	GCF_902329585.1	Pseudomonas	PPTH39_RS22285	114113..117592	286	MULTISPECIES:-BREX-system-P-loop-protein-BrxC-[Pseudomonas]
WP_104739193.1	Type1.BREX	[illegible]	1180	Bacteria>Pseudomonadota>Gammaproteobacteria>Pseudomonadales>Pseudomonadaceae>Halopseudomonas	GCF_014641295.1	Halopseudomonas_oceani	IEX06_RS08390	366595..370137	1708783	BREX-system-P-loop-protein-BrxC-[Halopseudomonas-oceani]
EQL43544.1	Type6.BREX	[illegible]	1213	Bacteria>Pseudomonadota>Gammaproteobacteria>Pseudomonadales>Pseudomonadaceae>Pseudomonas	GCA_000467675.1	Pseudomonas_aeruginosa_VRFPA03	M770_31820	18788..22429	1350465	ATPase-[Pseudomonas-aeruginosa-VRFPA03]
ARQ76275.1	Type1.BREX	[illegible]	1156	Bacteria>Pseudomonadota>Gammaproteobacteria>Pseudomonadales>Pseudomonadaceae>Pseudomonas	GCA_002128325.1	Pseudomonas_fragi	B6D87_19590	4227178..4230648	296	hypothetical-protein-B6D87_19590-[Pseudomonas-fragi]
PAA41060.1	Type1.BREX	[illegible]	1156	Bacteria>Pseudomonadota>Gammaproteobacteria>Pseudomonadales>Pseudomonadaceae>Pseudomonas	GCA_002269445.1	Pseudomonas_fragi	CJU80_12345	107560..111030	296	hypothetical-protein-CJU80_12345-[Pseudomonas-fragi]
ATN11139.1	Type1.BREX	[illegible]	1222	Bacteria>Pseudomonadota>Gammaproteobacteria>Pseudomonadales>Pseudomonadaceae>Pseudomonas	GCA_002591235.1	Pseudomonas_sp._FDAARGOS_380	CRN80_16460	3507093..3510761	2018067	hypothetical-protein-CRN80_16460-[Pseudomonas-sp.-FDAARGOS_380]
TKK14312.1	Type1.BREX	[illegible]	1222	Bacteria>Pseudomonadota>Gammaproteobacteria>Pseudomonadales>Pseudomonadaceae>Pseudomonas	GCA_005233695.1	Pseudomonas_fluorescens	PflCFBP13510_04710	495926..499594	294	BREX-system-P-loop-protein-BrxC-[Pseudomonas-fluorescens]
MVW77192.1	Type1.BREX	[illegible]	1221	Bacteria>Pseudomonadota>Gammaproteobacteria>Pseudomonadales>Pseudomonadaceae>Pseudomonas	GCA_009763245.1	Pseudomonas_xionganensis	GJV18_17880	24794..28459	2654845	BREX-system-P-loop-protein-BrxC-[Pseudomonas-xionganensis]
NLU01326.1	Type1.BREX	[illegible]	1222	Bacteria>Pseudomonadota>Gammaproteobacteria>Pseudomonadales>Pseudomonadaceae>Pseudomonas	GCA_012720005.1	Pseudomonas_lundensis	GXW88_11975	7910..11578	86185	BREX-system-P-loop-protein-BrxC-[Pseudomonas-lundensis]
NMY25069.1	Type1.BREX	[illegible]	1222	Bacteria>Pseudomonadota>Gammaproteobacteria>Pseudomonadales>Pseudomonadaceae>Pseudomonas	GCA_012985535.1	Pseudomonas_sp._WS_5021	HBO19_03685	794090..797758	2717490	BREX-system-P-loop-protein-BrxC-[Pseudomonas-sp.-WS-5021]
NWF08290.1	Type1.BREX	[illegible]	1222	Bacteria>Pseudomonadota>Gammaproteobacteria>Pseudomonadales>Pseudomonadaceae>Pseudomonas	GCA_013386885.1	Pseudomonas_salomonii	HX810_11520	509017..512685	191391	BREX-system-P-loop-protein-BrxC-[Pseudomonas-salomonii]
MBF9242733.1	Type1.BREX	[illegible]	1222	Bacteria>Pseudomonadota>Gammaproteobacteria>Pseudomonadales>Pseudomonadaceae>Pseudomonas	GCA_015694365.1	Pseudomonas_syringae_pv._tomato	I4I80_00295	46308..49976	323	BREX-system-P-loop-protein-BrxC-[Pseudomonas-syringae-pv.-tomato]
MBG3983980.1	Type6.BREX	[illegible]	1212	Bacteria>Pseudomonadota>Gammaproteobacteria>Pseudomonadales>Pseudomonadaceae>Pseudomonas	GCA_015733145.1	Pseudomonas_aeruginosa	I4919_20885	86674..90312	287	BREX-system-P-loop-protein-BrxC-[Pseudomonas-aeruginosa]
MBG4373384.1	Type1.BREX	[illegible]	1154	Bacteria>Pseudomonadota>Gammaproteobacteria>Pseudomonadales>Pseudomonadaceae>Pseudomonas	GCA_015734445.1	Pseudomonas_aeruginosa	I5F50_23085	8082..11546	287	BREX-system-P-loop-protein-BrxC-[Pseudomonas-aeruginosa]
MBG4395801.1	Type1.BREX	[illegible]	1154	Bacteria>Pseudomonadota>Gammaproteobacteria>Pseudomonadales>Pseudomonadaceae>Pseudomonas	GCA_015734545.1	Pseudomonas_aeruginosa	GHT73_12640	7113..10577	287	BREX-system-P-loop-protein-BrxC-[Pseudomonas-aeruginosa]
MBG4463093.1	Type1.BREX	[illegible]	1154	Bacteria>Pseudomonadota>Gammaproteobacteria>Pseudomonadales>Pseudomonadaceae>Pseudomonas	GCA_015734755.1	Pseudomonas_aeruginosa	GHU50_17045	10342..13806	287	BREX-system-P-loop-protein-BrxC-[Pseudomonas-aeruginosa]
MBG5216128.1	Type1.BREX	[illegible]	1154	Bacteria>Pseudomonadota>Gammaproteobacteria>Pseudomonadales>Pseudomonadaceae>Pseudomonas	GCA_015737185.1	Pseudomonas_aeruginosa	GHT75_27125	7080..10544	287	BREX-system-P-loop-protein-BrxC-[Pseudomonas-aeruginosa]
MBG6382130.1	Type6.BREX	[illegible]	1210	Bacteria>Pseudomonadota>Gammaproteobacteria>Pseudomonadales>Pseudomonadaceae>Pseudomonas	GCA_015762815.1	Pseudomonas_aeruginosa	I5I12_28760	1691..5323	287	BREX-system-P-loop-protein-BrxC-[Pseudomonas-aeruginosa]
MBH3980671.1	Type6.BREX	[illegible]	1213	Bacteria>Pseudomonadota>Gammaproteobacteria>Pseudomonadales>Pseudomonadaceae>Pseudomonas	GCA_016011075.1	Pseudomonas_aeruginosa	I5Q70_02570	503331..506972	287	BREX-system-P-loop-protein-BrxC-[Pseudomonas-aeruginosa]
MBI8737743.1	Type1.BREX	[illegible]	1154	Bacteria>Pseudomonadota>Gammaproteobacteria>Pseudomonadales>Pseudomonadaceae>Pseudomonas	GCA_016342945.1	Pseudomonas_aeruginosa	JEZ70_02820	55146..58610	287	BREX-system-P-loop-protein-BrxC-[Pseudomonas-aeruginosa]
MBM1201834.1	Type1.BREX	[illegible]	1154	Bacteria>Pseudomonadota>Gammaproteobacteria>Pseudomonadales>Pseudomonadaceae>Pseudomonas	GCA_016812215.1	Pseudomonas_fragi	GYM96_18820	91266..94730	296	BREX-system-P-loop-protein-BrxC-[Pseudomonas-fragi]
MBN0299744.1	Type6.BREX	[illegible]	1213	Bacteria>Pseudomonadota>Gammaproteobacteria>Pseudomonadales>Pseudomonadaceae>Pseudomonas	GCA_016922145.1	Pseudomonas_aeruginosa	JTM78_25120	125675..129316	287	BREX-system-P-loop-protein-BrxC-[Pseudomonas-aeruginosa]
MBN0536373.1	Type6.BREX	[illegible]	1206	Bacteria>Pseudomonadota>Gammaproteobacteria>Pseudomonadales>Pseudomonadaceae>Pseudomonas	GCA_016922535.1	Pseudomonas_aeruginosa	JTM59_25840	125679..129299	287	BREX-system-P-loop-protein-BrxC-[Pseudomonas-aeruginosa]
MBV5724753.1	Type1.BREX	[illegible]	1184	Bacteria>Pseudomonadota>Gammaproteobacteria>Pseudomonadales>Pseudomonadaceae>Pseudomonas	GCA_019166825.1	Pseudomonas_aeruginosa	KUT37_26485	44544..48098	287	BREX-system-P-loop-protein-BrxC-[Pseudomonas-aeruginosa]
MBX6150345.1	Type1.BREX	[illegible]	1154	Bacteria>Pseudomonadota>Gammaproteobacteria>Pseudomonadales>Pseudomonadaceae>Pseudomonas	GCA_019686265.1	Pseudomonas_aeruginosa	ISD36_12550	248185..251649	287	BREX-system-P-loop-protein-BrxC-[Pseudomonas-aeruginosa]
MBX5915342.1	Type1.BREX	[illegible]	1154	Bacteria>Pseudomonadota>Gammaproteobacteria>Pseudomonadales>Pseudomonadaceae>Pseudomonas	GCA_019688355.1	Pseudomonas_aeruginosa	ISE50_29115	6954..10418	287	BREX-system-P-loop-protein-BrxC-[Pseudomonas-aeruginosa]
MBY8973665.1	Type1.BREX	[illegible]	1222	Bacteria>Pseudomonadota>Gammaproteobacteria>Pseudomonadales>Pseudomonadaceae>Pseudomonas	GCA_019891455.1	Pseudomonas_sp._P867	J1G33_25110	13708..17376	2816050	BREX-system-P-loop-protein-BrxC-[Pseudomonas-sp.-P867]
HBN8119635.1	Type6.BREX	[illegible]	1213	Bacteria>Pseudomonadota>Gammaproteobacteria>Pseudomonadales>Pseudomonadaceae>Pseudomonas	GCA_021586615.1	Pseudomonas_aeruginosa	L3655_000128	111220..114861	287	BREX-system-P-loop-protein-BrxC-[Pseudomonas-aeruginosa]
HBN9708921.1	Type1.BREX	[illegible]	1154	Bacteria>Pseudomonadota>Gammaproteobacteria>Pseudomonadales>Pseudomonadaceae>Pseudomonas	GCA_021591785.1	Pseudomonas_aeruginosa	L3920_001787	275283..278747	287	BREX-system-P-loop-protein-BrxC-[Pseudomonas-aeruginosa]
HBO1619179.1	Type1.BREX	[illegible]	1154	Bacteria>Pseudomonadota>Gammaproteobacteria>Pseudomonadales>Pseudomonadaceae>Pseudomonas	GCA_021598085.1	Pseudomonas_aeruginosa	L4F62_005712	16995..20459	287	BREX-system-P-loop-protein-BrxC-[Pseudomonas-aeruginosa]
EIU3464059.1	Type1.BREX	[illegible]	1154	Bacteria>Pseudomonadota>Gammaproteobacteria>Pseudomonadales>Pseudomonadaceae>Pseudomonas	GCA_021644165.1	Pseudomonas_aeruginosa	L4P80_001287	38375..41839	287	BREX-system-P-loop-protein-BrxC-[Pseudomonas-aeruginosa]
HBO3309314.1	Type6.BREX	[illegible]	1188	Bacteria>Pseudomonadota>Gammaproteobacteria>Pseudomonadales>Pseudomonadaceae>Pseudomonas	GCA_021652015.1	Pseudomonas_aeruginosa	L4R58_004351	30228..33794	287	BREX-system-P-loop-protein-BrxC-[Pseudomonas-aeruginosa]
HBO6336105.1	Type1.BREX	[illegible]	1154	Bacteria>Pseudomonadota>Gammaproteobacteria>Pseudomonadales>Pseudomonadaceae>Pseudomonas	GCA_021670735.1	Pseudomonas_aeruginosa	L4747_005548	6948..10412	287	BREX-system-P-loop-protein-BrxC-[Pseudomonas-aeruginosa]
HBP6330513.1	Type1.BREX	[illegible]	1154	Bacteria>Pseudomonadota>Gammaproteobacteria>Pseudomonadales>Pseudomonadaceae>Pseudomonas	GCA_021710255.1	Pseudomonas_aeruginosa	L6A37_21805	72354..75818	287	BREX-system-P-loop-protein-BrxC-[Pseudomonas-aeruginosa]
HBP5527123.1	Type6.BREX	[illegible]	1212	Bacteria>Pseudomonadota>Gammaproteobacteria>Pseudomonadales>Pseudomonadaceae>Pseudomonas	GCA_021712095.1	Pseudomonas_aeruginosa	L6B52_09510	5147..8785	287	BREX-system-P-loop-protein-BrxC-[Pseudomonas-aeruginosa]
MCK9534930.1	Type1.BREX	[illegible]	1154	Bacteria>Pseudomonadota>Gammaproteobacteria>Pseudomonadales>Pseudomonadaceae>Pseudomonas	GCA_023228505.1	Pseudomonas_sp.	M0Q98_09595	5981..9445	306	BREX-system-P-loop-protein-BrxC-[Pseudomonas-sp.]
HCE6004670.1	Type6.BREX	[illegible]	1213	Bacteria>Pseudomonadota>Gammaproteobacteria>Pseudomonadales>Pseudomonadaceae>Pseudomonas	GCA_023904815.1	Pseudomonas_aeruginosa	NHK94_000107	121075..124716	287	BREX-system-P-loop-protein-BrxC-[Pseudomonas-aeruginosa]
MCO2467177.1	Type1.BREX	[illegible]	1146	Bacteria>Pseudomonadota>Gammaproteobacteria>Pseudomonadales>Pseudomonadaceae>Pseudomonas	GCA_023907445.1	Pseudomonas_aeruginosa	FA343_15170	67465..70905	287	BREX-system-P-loop-protein-BrxC-[Pseudomonas-aeruginosa]
HCE7261574.1	Type1.BREX	[illegible]	1154	Bacteria>Pseudomonadota>Gammaproteobacteria>Pseudomonadales>Pseudomonadaceae>Pseudomonas	GCA_023919205.1	Pseudomonas_aeruginosa	NHQ13_005985	6842..10306	287	BREX-system-P-loop-protein-BrxC-[Pseudomonas-aeruginosa]
HCF1683404.1	Type6.BREX	[illegible]	1213	Bacteria>Pseudomonadota>Gammaproteobacteria>Pseudomonadales>Pseudomonadaceae>Pseudomonas	GCA_023941775.1	Pseudomonas_aeruginosa	NH867_002024	49404..53045	287	BREX-system-P-loop-protein-BrxC-[Pseudomonas-aeruginosa]
HCH0453160.1	Type1.BREX	[illegible]	1154	Bacteria>Pseudomonadota>Gammaproteobacteria>Pseudomonadales>Pseudomonadaceae>Pseudomonas	GCA_024072695.1	Pseudomonas_aeruginosa	NK007_001951	228071..231535	287	BREX-system-P-loop-protein-BrxC-[Pseudomonas-aeruginosa]
BDM24922.1	Type1.BREX	[illegible]	1154	Bacteria>Pseudomonadota>Gammaproteobacteria>Pseudomonadales>Pseudomonadaceae>Pseudomonas	GCA_024349685.1	Pseudomonas_sp._LRP2-20	KMS_R46790	5328463..5331927	2944234	BREX-system-P-loop-protein-BrxC-[Pseudomonas-sp.-LRP2-20]
WCY64468.1	Type6.BREX	[illegible]	1213	Bacteria>Pseudomonadota>Gammaproteobacteria>Pseudomonadales>Pseudomonadaceae>Pseudomonas	GCA_028610105.1	Pseudomonas_aeruginosa	NHK94_000107	121075..124716	287	BREX-system-P-loop-protein-BrxC-[Pseudomonas-aeruginosa]
WP_132538218.1	Type1.BREX	[illegible]	1154	Bacteria>Pseudomonadota>Gammaproteobacteria>Pseudomonadales>Pseudomonadaceae>Pseudomonas	GCA_030820635.1	Pseudomonas_aeruginosa	EQ548_RS01360	6947..10411	287	BREX-system-P-loop-protein-BrxC-[Pseudomonas-aeruginosa]
WP_230691811.1	Type1.BREX	[illegible]	1225	Bacteria>Pseudomonadota>Gammaproteobacteria>Pseudomonadales>Pseudomonadaceae>Pseudomonas	GCA_030820635.1	Pseudomonas_syringae_group_genomosp._3	I4I80_RS00295	46299..49976	251701	BREX-system-P-loop-protein-BrxC-[Pseudomonas-syringae-group-genomosp.-3]
WP_241090907.1	Type1.BREX	[illegible]	1225	Bacteria>Pseudomonadota>Gammaproteobacteria>Pseudomonadales>Pseudomonadaceae>Pseudomonas	GCA_030820635.1	Pseudomonas_viridiflava	EE220_RS21960	64975..68652	33069	BREX-system-P-loop-protein-BrxC-[Pseudomonas-viridiflava]
SBW85146.1	Type1.BREX	[illegible]	1154	Bacteria>Pseudomonadota>Gammaproteobacteria>Pseudomonadales>Pseudomonadaceae>Pseudomonas	GCA_900092355.1	Pseudomonas_veronii_1YdBTEX2	PVE_P0103	88310..91774	1295141	hypothetical-protein-PVE_P0103-[Pseudomonas-veronii-1YdBTEX2]
VVQ23891.1	Type5.BREX	[illegible]	1225	Bacteria>Pseudomonadota>Gammaproteobacteria>Pseudomonadales>Pseudomonadaceae>Pseudomonas	GCA_902498245.1	Pseudomonas_fluorescens	PS928_05643	91300..94977	294	hypothetical-protein-PS928_05643-[Pseudomonas-fluorescens]
WP_013692225.1	Type6.BREX	[illegible]	1213	Bacteria>Pseudomonadota>Gammaproteobacteria>Pseudomonadales>Pseudomonadaceae>Pseudomonas	GCF_000194805.1	Pseudomonas_brassicacearum	PSEBR_RS03955	941705..945346	930166	BREX-system-P-loop-protein-BrxC-[Pseudomonas-brassicacearum]
WP_007159175.1	Type6.BREX	[illegible]	1212	Bacteria>Pseudomonadota>Gammaproteobacteria>Pseudomonadales>Pseudomonadaceae>Pseudomonas	GCF_000236825.1	Pseudomonas_psychrotolerans	PPL19_RS03910	152166..155804	237610	BREX-system-P-loop-protein-BrxC-[Pseudomonas-psychrotolerans]
WP_010463595.1	Type1.BREX	[illegible]	1185	Bacteria>Pseudomonadota>Gammaproteobacteria>Pseudomonadales>Pseudomonadaceae>Pseudomonas	GCF_000257545.3	Pseudomonas_mandelii	OU5_RS13450	3003724..3007281	75612	BREX-system-P-loop-protein-BrxC-[Pseudomonas-mandelii]
WP_017337797.1	Type6.BREX	[illegible]	1213	Bacteria>Pseudomonadota>Gammaproteobacteria>Pseudomonadales>Pseudomonadaceae>Pseudomonas	GCF_000293885.2	Pseudomonas_fluorescens	B723_RS16820	3631858..3635499	294	BREX-system-P-loop-protein-BrxC-[Pseudomonas-fluorescens]
WP_023113968.1	Type1.BREX	[illegible]	1146	Bacteria>Pseudomonadota>Gammaproteobacteria>Pseudomonadales>Pseudomonadaceae>Pseudomonas	GCF_000481245.1	Pseudomonas_aeruginosa	Q036_RS16830	2151526..2154966	287	BREX-system-P-loop-protein-BrxC-[Pseudomonas-aeruginosa]
WP_042135808.1	Type1.BREX	[illegible]	1184	Bacteria>Pseudomonadota>Gammaproteobacteria>Pseudomonadales>Pseudomonadaceae>Pseudomonas	GCF_000730625.1	Pseudomonas_oryzihabitans	POR01S_RS10925	121811..125365	47885	BREX-system-P-loop-protein-BrxC-[Pseudomonas-oryzihabitans]
WP_033954069.1	Type1.BREX	[illegible]	1154	Bacteria>Pseudomonadota>Gammaproteobacteria>Pseudomonadales>Pseudomonadaceae>Pseudomonas	GCF_000793385.1	Pseudomonas_aeruginosa	NT88_RS29575	15006..18470	287	BREX-system-P-loop-protein-BrxC-[Pseudomonas-aeruginosa]
WP_044305280.1	Type6.BREX	[illegible]	1213	Bacteria>Pseudomonadota>Gammaproteobacteria>Pseudomonadales>Pseudomonadaceae>Pseudomonas	GCF_000793505.1	Pseudomonas_aeruginosa	NT87_RS20770	137629..141270	287	BREX-system-P-loop-protein-BrxC-[Pseudomonas-aeruginosa]
WP_202808417.1	Type1.BREX	[illegible]	937	Bacteria>Pseudomonadota>Gammaproteobacteria>Pseudomonadales>Pseudomonadaceae>Pseudomonas	GCF_000794515.1	Pseudomonas_aeruginosa	NQ84_RS28370	16579..19392	287	BREX-system-P-loop-protein-BrxC-[Pseudomonas-aeruginosa]
WP_228480203.1	Type1.BREX	[illegible]	619	Bacteria>Pseudomonadota>Gammaproteobacteria>Pseudomonadales>Pseudomonadaceae>Pseudomonas	GCF_000829885.1	Pseudomonas_aeruginosa	EG09_RS07505	1715440..1717299	287	BREX-system-P-loop-protein-BrxC-[Pseudomonas-aeruginosa]
WP_041506104.1	Type1.BREX	[illegible]	1184	Bacteria>Pseudomonadota>Gammaproteobacteria>Pseudomonadales>Pseudomonadaceae>Pseudomonas	GCF_000831585.1	Pseudomonas_plecoglossicida	RK21_RS13525	2976546..2980100	70775	BREX-system-P-loop-protein-BrxC-[Pseudomonas-plecoglossicida]
WP_047283135.1	Type1.BREX	[illegible]	1154	Bacteria>Pseudomonadota>Gammaproteobacteria>Pseudomonadales>Pseudomonadaceae>Pseudomonas	GCF_001020725.2	Pseudomonas_lundensis	AA042_RS07115	1582722..1586186	86185	BREX-system-P-loop-protein-BrxC-[Pseudomonas-lundensis]
WP_048385719.1	Type1.BREX	[illegible]	1185	Bacteria>Pseudomonadota>Gammaproteobacteria>Pseudomonadales>Pseudomonadaceae>Pseudomonas	GCF_001043065.1	Pseudomonas_helleri	TU85_RS02975	88982..92539	1608996	BREX-system-P-loop-protein-BrxC-[Pseudomonas-helleri]
WP_057429193.1	Type1.BREX	[illegible]	1156	Bacteria>Pseudomonadota>Gammaproteobacteria>Pseudomonadales>Pseudomonadaceae>Pseudomonas	GCF_001401385.1	Pseudomonas_syringae_group_genomosp._3	AL087_RS04320	127987..131457	251701	BREX-system-P-loop-protein-BrxC-[Pseudomonas-syringae-group-genomosp.-3]
WP_058181876.1	Type6.BREX	[illegible]	1213	Bacteria>Pseudomonadota>Gammaproteobacteria>Pseudomonadales>Pseudomonadaceae>Pseudomonas	GCF_001450675.1	Pseudomonas_aeruginosa	AO933_RS04770	131954..135595	287	BREX-system-P-loop-protein-BrxC-[Pseudomonas-aeruginosa]
WP_058174899.1	Type1.BREX	[illegible]	1184	Bacteria>Pseudomonadota>Gammaproteobacteria>Pseudomonadales>Pseudomonadaceae>Pseudomonas	GCF_001452485.1	Pseudomonas_aeruginosa	APA89_RS03075	652482..656036	287	BREX-system-P-loop-protein-BrxC-[Pseudomonas-aeruginosa]
WP_070624003.1	Type1.BREX	[illegible]	1154	Bacteria>Pseudomonadota>Gammaproteobacteria>Pseudomonadales>Pseudomonadaceae>Pseudomonas	GCF_001810465.1	Pseudomonas_aeruginosa	HMPREF2746_RS21475	11855..15319	287	BREX-system-P-loop-protein-BrxC-[Pseudomonas-aeruginosa]
WP_079783554.1	Type1.BREX	[illegible]	1154	Bacteria>Pseudomonadota>Gammaproteobacteria>Pseudomonadales>Pseudomonadaceae>Pseudomonas	GCF_002037565.1	Pseudomonas_sp._LPH1	BHQ29_RS09350	2029984..2033448	1898684	BREX-system-P-loop-protein-BrxC-[Pseudomonas-sp.-LPH1]
WP_205881761.1	Type1.BREX	[illegible]	1159	Bacteria>Pseudomonadota>Gammaproteobacteria>Pseudomonadales>Pseudomonadaceae>Pseudomonas	GCF_002128325.1	Pseudomonas_fragi	B6D87_RS19610	4227178..4230657	296	BREX-system-P-loop-protein-BrxC-[Pseudomonas-fragi]
WP_086230732.1	Type1.BREX	[illegible]	1184	Bacteria>Pseudomonadota>Gammaproteobacteria>Pseudomonadales>Pseudomonadaceae>Pseudomonas	GCF_002135805.1	Pseudomonas_aeruginosa	CAZ03_RS13145	128061..131615	287	BREX-system-P-loop-protein-BrxC-[Pseudomonas-aeruginosa]
WP_095025594.1	Type1.BREX	[illegible]	1154	Bacteria>Pseudomonadota>Gammaproteobacteria>Pseudomonadales>Pseudomonadaceae>Pseudomonas	GCF_002269515.1	Pseudomonas_fragi	CJU79_RS17655	17310..20774	296	BREX-system-P-loop-protein-BrxC-[Pseudomonas-fragi]
WP_095626344.1	Type1.BREX	[illegible]	1154	Bacteria>Pseudomonadota>Gammaproteobacteria>Pseudomonadales>Pseudomonadaceae>Pseudomonas	GCF_002287035.1	Pseudomonas_sp._WN033	CK507_RS15715	130328..133792	2032629	BREX-system-P-loop-protein-BrxC-[Pseudomonas-sp.-WN033]
WP_256588486.1	Type1.BREX	[illegible]	1225	Bacteria>Pseudomonadota>Gammaproteobacteria>Pseudomonadales>Pseudomonadaceae>Pseudomonas	GCF_002591235.1	Pseudomonas_sp._FDAARGOS_380	CRN80_RS16460	3507084..3510761	2018067	BREX-system-P-loop-protein-BrxC-[Pseudomonas-sp.-FDAARGOS_380]
WP_101152285.1	Type6.BREX	[illegible]	1211	Bacteria>Pseudomonadota>Gammaproteobacteria>Pseudomonadales>Pseudomonadaceae>Pseudomonas	GCF_002836195.1	Pseudomonas_sp._Choline-3u-10	CXF92_RS00360	72818..76453	2058311	BREX-system-P-loop-protein-BrxC-[Pseudomonas-sp.-Choline-3u-10]
WP_102082564.1	Type1.BREX	[illegible]	1184	Bacteria>Pseudomonadota>Gammaproteobacteria>Pseudomonadales>Pseudomonadaceae>Pseudomonas	GCF_002864915.1	Pseudomonas_guariconensis	CXG53_RS23735	60064..63618	1288410	BREX-system-P-loop-protein-BrxC-[Pseudomonas-guariconensis]
WP_102684491.1	Type1.BREX	[illegible]	1222	Bacteria>Pseudomonadota>Gammaproteobacteria>Pseudomonadales>Pseudomonadaceae>Pseudomonas	GCF_002883975.1	Pseudomonas_sp._FW305-42	C1X79_RS19065	47194..50862	2070677	BREX-system-P-loop-protein-BrxC-[Pseudomonas-sp.-FW305-42]
WP_109933363.1	Type1.BREX	[illegible]	1154	Bacteria>Pseudomonadota>Gammaproteobacteria>Pseudomonadales>Pseudomonadaceae>Pseudomonas	GCF_003176655.1	Pseudomonas_sp._RW407	DK254_RS08115	332506..335970	2202894	BREX-system-P-loop-protein-BrxC-[Pseudomonas-sp.-RW407]

Protein-ID	System	Gene-Neighbourhoods	Length	Taxonomy	Assembly-ID	Strain	Locus-Tag	Genomic-coordinates	TaxID	Fasta-defline
WP_116805555.1	Type1.BREX	<- wHTH+WYL+WCX ||?->Peptidase_M15->?-> BrxA-DNA-binding -> BrxB-BrxF-iSTAND -> DUF6079 *-> N6-METHYLASE ->?-> HNH +GCN1->?-> ParB + HNH -> PglZ -> [illegible]	1154	Bacteria>Pseudomonadota>Gammaproteobacteria>Pseudomonadales>Pseudomonadaceae>Pseudomonas	GCF_003412045.2	Pseudomonas_aeruginosa	L4E78_005908	8239..11703	287	BREX-system-P-loop-protein-BrxC-[Pseudomonas-aeruginosa]
WP_119519035.1	Type1.BREX	<-AbiEii<-AbiEi_4<-?<-?<-REase+Bacteriocin_IIc<-?<- N6-METHYLASE <-DNA-glycosylase<- ParB + HNH <- DUF6079 *<- BrxB-BrxF-iSTAND <- BrxA-DNA-binding <-?<-RNase<-?<-JetB<-?|| [illegible]	1220	Bacteria>Pseudomonadota>Gammaproteobacteria>Pseudomonadales>Pseudomonadaceae>Pseudomonas	GCF_003585225.1	Pseudomonas_aeruginosa	AXX04_RS18775	17983..21645	287	BREX-system-P-loop-protein-BrxC-[Pseudomonas-aeruginosa]
WP_128551186.1	Type1.BREX	Arm-DNA-bind_3+Phage_integrase-><- SIGMA+OB-Fold+AAA-ATPase+HKD-DNase-BrxL <- PglZ <- ParB + HNH <- N6-METHYLASE -p<-RVT<-NERD<-OLD-ABC<- N6-METHYLASE <- DUF6079 *<- [illegible]	1184	Bacteria>Pseudomonadota>Gammaproteobacteria>Pseudomonadales>Pseudomonadaceae>Pseudomonas	GCF_003698635.1	Pseudomonas_aeruginosa	IPC82_RS03385	45130..48684	287	BREX-system-P-loop-protein-BrxC-[Pseudomonas-aeruginosa]
WP_124187492.1	Type1.BREX	Arm-DNA-bind_3+Phage_integrase->GreA_GreB+SF1-SF2-Helicase-><- SIGMA+OB-Fold+AAA-ATPase+Lon-Protease-BrxL <- PglZ <- ParB + HNH <-?<-MarR-HTH<-MNS+ORC-CDC6<- N6-METHYLASE <- [illegible]	1154	Bacteria>Pseudomonadota>Gammaproteobacteria>Pseudomonadales>Pseudomonadaceae>Pseudomonas	GCF_003840445.1	Pseudomonas_aeruginosa	IPC460_RS14135	71105..74569	287	BREX-system-P-loop-protein-BrxC-[Pseudomonas-aeruginosa]
WP_126525533.1	Type1.BREX	<-LexA-protease<- wHTH+WYL+WCX ||ARG->?->?-> wHTH+WYL+WCX -> BrxA-DNA-binding -> BrxB-BrxF-iSTAND -> DUF6079 *-> N6-METHYLASE ->DUF932-> ParB + HNH -> PglZ -> [illegible]	1154	Bacteria>Pseudomonadota>Gammaproteobacteria>Pseudomonadales>Pseudomonadaceae>Pseudomonas	GCF_003969495.1	Pseudomonas_aeruginosa	DZA06_RS08835	36272..39736	287	BREX-system-P-loop-protein-BrxC-[Pseudomonas-aeruginosa]
WP_126632346.1	Type6.BREX	<-Small-B-barrel+SF1-SF2-Helicase+RdgC+REase<-35exo+SF1-SF2-Helicase<-XIS-HTH<- SIGMA+OB-Fold+AAA-ATPase+HKD-DNase-BrxL <- PglZ <- ParB + HNH <- N6-METHYLASE <- N6-METHYLASE <- [illegible]	1213	Bacteria>Pseudomonadota>Gammaproteobacteria>Pseudomonadales>Pseudomonadaceae>Pseudomonas	GCF_003975645.1	Pseudomonas_aeruginosa	IPC447_RS09565	370705..374346	287	BREX-system-P-loop-protein-BrxC-[Pseudomonas-aeruginosa]
WP_129388875.1	Type1.BREX	XIS-HTH->?-> wHTH+WYL+WCX -> BrxA-DNA-binding -> BrxB-BrxF-iSTAND -> DUF6079 *-> N6-METHYLASE -> ParB + HNH -> PglZ -> SIGMA+OB-Fold+AAA-ATPase+Lon-Protease-BrxL -><-DUF4969<-?<-?|| [illegible]	1156	Bacteria>Pseudomonadota>Gammaproteobacteria>Pseudomonadales>Pseudomonadaceae>Pseudomonas	GCF_004124275.1	Pseudomonas_sp._DTU12.3	C2E19_RS05070	1143497..1146967	2073078	BREX-system-P-loop-protein-BrxC-[Pseudomonas-sp.-DTU12.3]
WP_130899395.1	Type1.BREX	<-HAMP+MA<-ACET<-?<-RHH||XIS-HTH->?-> wHTH+WYL+WCX -> BrxA-DNA-binding -> BrxB-BrxF-iSTAND -> DUF6079 *-> N6-METHYLASE -> ParB + HNH -> PglZ -> [illegible]	1154	Bacteria>Pseudomonadota>Gammaproteobacteria>Pseudomonadales>Pseudomonadaceae>Pseudomonas	GCF_004307505.1	Pseudomonas_sp._Sample_11	D8U60_RS02310	26340..29804	2448261	BREX-system-P-loop-protein-BrxC-[Pseudomonas-sp.-Sample_11]
WP_023094202.1	Type6.BREX	<-SbcC<-JetB<-JetA|| wHTH+WYL+WCX -> BrxE -> BrxA-DNA-binding -> BrxB-BrxF-iSTAND -> DUF6079 *->GreA_GreB+SF1-SF2-Helicase-> N6-METHYLASE -> N6-METHYLASE -> ParB + HNH ->?-> PglZ -> [illegible]	1213	Bacteria>Pseudomonadota>Gammaproteobacteria>Pseudomonadales>Pseudomonadaceae>Pseudomonas	GCF_004346985.1	Pseudomonas_aeruginosa	C9I70_RS03025	613762..617403	287	BREX-system-P-loop-protein-BrxC-[Pseudomonas-aeruginosa]
WP_134242485.1	Type1.BREX	HTH_Tnp_1+HTH_21+rve+rve_3-><-REase<- SIGMA+OB-Fold+AAA-ATPase+Lon-Protease-BrxL <- PglZ <- ParB + HNH <-?<- HNH +GCN1<-?<- N6-METHYLASE <- DUF6079 *<- BrxB-BrxF-iSTAND <- [illegible]	1154	Bacteria>Pseudomonadota>Gammaproteobacteria>Pseudomonadales>Pseudomonadaceae>Pseudomonas	GCF_004370105.1	Pseudomonas_aeruginosa	IPC1592_RS30135	15141..18605	287	BREX-system-P-loop-protein-BrxC-[Pseudomonas-aeruginosa]
WP_202876546.1	Type1.BREX	<-REase<- SIGMA+OB-Fold+AAA-ATPase+Lon-Protease-BrxL <- PglZ <- ParB + HNH <-?<- HNH +GCN1<-?<- N6-METHYLASE <- DUF6079 -p<- DUF6079 -p*<- BrxB-BrxF-iSTAND <- BrxA-DNA-binding <-?<- [illegible]	531	Bacteria>Pseudomonadota>Gammaproteobacteria>Pseudomonadales>Pseudomonadaceae>Pseudomonas	GCF_004373095.1	Pseudomonas_aeruginosa	IPC174_RS30040	16693..18288	287	BREX-system-P-loop-protein-BrxC-[Pseudomonas-aeruginosa]
WP_202876713.1	Type1.BREX	HTH_Tnp_1+HTH_21+rve+rve_3-><-REase<- SIGMA+OB-Fold+AAA-ATPase+Lon-Protease-BrxL <- PglZ <- ParB + HNH <-?<- HNH +GCN1<-?<- N6-METHYLASE <- DUF6079 -p*<- DUF6079 -p<- [illegible]	539	Bacteria>Pseudomonadota>Gammaproteobacteria>Pseudomonadales>Pseudomonadaceae>Pseudomonas	GCF_004373095.1	Pseudomonas_aeruginosa	IPC175_RS30340	15284..16903	287	hypothetical-protein-[Pseudomonas-aeruginosa]
WP_134282343.1	Type1.BREX	<-REase<- SIGMA+OB-Fold+AAA-ATPase+Lon-Protease-BrxL <- PglZ <- ParB + HNH <-?<- HNH +GCN1<-?<- N6-METHYLASE -p<- N6-METHYLASE <- DUF6079 *<- BrxB-BrxF-iSTAND <- BrxA-DNA-binding <-?<- [illegible]	1154	Bacteria>Pseudomonadota>Gammaproteobacteria>Pseudomonadales>Pseudomonadaceae>Pseudomonas	GCF_004373575.1	Pseudomonas_aeruginosa	IPC135_RS30160	15285..18749	287	BREX-system-P-loop-protein-BrxC-[Pseudomonas-aeruginosa]
WP_202876545.1	Type1.BREX	HTH_Tnp_1+HTH_21+rve+rve_3-><-REase<- SIGMA+OB-Fold+AAA-ATPase+Lon-Protease-BrxL <- PglZ <- ParB + HNH <-?<- HNH +GCN1<- N6-METHYLASE -p<- N6-METHYLASE <- DUF6079 -p*<- DUF6079 -p<- [illegible]	624	Bacteria>Pseudomonadota>Gammaproteobacteria>Pseudomonadales>Pseudomonadaceae>Pseudomonas	GCF_004373665.1	Pseudomonas_aeruginosa	IPC174_RS30035	14826..16700	287	hypothetical-protein-[Pseudomonas-aeruginosa]
WP_134249510.1	Type6.BREX	<-KAP-glycosylase<-[illegible]	1213	Bacteria>Pseudomonadota>Gammaproteobacteria>Pseudomonadales>Pseudomonadaceae>Pseudomonas	GCF_004374205.1	Pseudomonas_aeruginosa	IPC115_RS16040	120938..124579	287	BREX-system-P-loop-protein-BrxC-[Pseudomonas-aeruginosa]
WP_134295820.1	Type6.BREX	<-KAP-glycosylase<-[illegible]	1213	Bacteria>Pseudomonadota>Gammaproteobacteria>Pseudomonadales>Pseudomonadaceae>Pseudomonas	GCF_004374605.1	Pseudomonas_aeruginosa	IPC112_RS05880	126222..129863	287	BREX-system-P-loop-protein-BrxC-[Pseudomonas-aeruginosa]
WP_136489742.1	Type1.BREX	<-MarR-HTH<- ParB + HNH <-?<-[illegible]	1154	Bacteria>Pseudomonadota>Gammaproteobacteria>Pseudomonadales>Pseudomonadaceae>Pseudomonas	GCF_004801855.1	Pseudomonas_sp._A-1	E5198_RS09425	20467..23931	1821274	BREX-system-P-loop-protein-BrxC-[Pseudomonas-sp.-A-1]
WP_155677773.1	Type1.BREX	<-AraC-HTH+AraC-HTH||?-><-[illegible]	1155	Bacteria>Pseudomonadota>Gammaproteobacteria>Pseudomonadales>Pseudomonadaceae>Pseudomonas	GCF_004919535.4	Pseudomonas_veronii	E4167_12595	2651456..2654923	76761	BREX-system-P-loop-protein-BrxC-[Pseudomonas-veronii]
WP_155678129.1	Type1.BREX	MerR-HTH->tRNA-><- [illegible]	1221	Bacteria>Pseudomonadota>Gammaproteobacteria>Pseudomonadales>Pseudomonadaceae>Pseudomonas	GCF_004919535.4	Pseudomonas_veronii	E4167_RS17855	3896046..3899711	76761	BREX-system-P-loop-protein-BrxC-[Pseudomonas-veronii]
WP_256658147.1	Type1.BREX	NERD-><- wHTH+WYL+WCX ||?->[illegible]	1220	Bacteria>Pseudomonadota>Gammaproteobacteria>Pseudomonadales>Pseudomonadaceae>Pseudomonas	GCF_005502555.1	Pseudomonas_sp._D(2018)	FFS60_RS25250	8931..12593	2502238	BREX-system-P-loop-protein-BrxC-[Pseudomonas-sp.-D(2018)]
WP_138763800.1	Type5.BREX	<- ParB + HNH <- ParB + HNH [illegible]	1223	Bacteria>Pseudomonadota>Gammaproteobacteria>Pseudomonadales>Pseudomonadaceae>Pseudomonas	GCF_005930675.1	Pseudomonas_lactis	FHL14_RS16420	47911..51582	1615674	BREX-system-P-loop-protein-BrxC-[Pseudomonas-lactis]
WP_143505426.1	Type6.BREX	HAD+HAD->?->?->?->?-><- [illegible]	1212	Bacteria>Pseudomonadota>Gammaproteobacteria>Pseudomonadales>Pseudomonadaceae>Pseudomonas	GCF_007049795.1	Pseudomonas_mendocina	EQ826_RS09455	197604..201242	300	BREX-system-P-loop-protein-BrxC-[Pseudomonas-mendocina]
WP_150073350.1	Type1.BREX	XIS-HTH->?->XIS-HTH-> [illegible]	1186	Bacteria>Pseudomonadota>Gammaproteobacteria>Pseudomonadales>Pseudomonadaceae>Pseudomonas	GCF_008632325.1	Pseudomonas_lactis	F3K52_RS19340	4156..7716	1615674	BREX-system-P-loop-protein-BrxC-[Pseudomonas-lactis]
WP_153329486.1	Type1.BREX	<- SIGMA+OB-Fold+AAA-ATPase[illegible]	1184	Bacteria>Pseudomonadota>Gammaproteobacteria>Pseudomonadales>Pseudomonadaceae>Pseudomonas	GCF_009600225.1	Pseudomonas_helleri	GHO39_RS16765	34385..37939	1608996	BREX-system-P-loop-protein-BrxC-[Pseudomonas-helleri]
WP_153384138.1	Type1.BREX	MerR-HTH->tRNA-><-RHH||[illegible]	1186	Bacteria>Pseudomonadota>Gammaproteobacteria>Pseudomonadales>Pseudomonadaceae>Pseudomonas	GCF_009600565.1	unclassified_Pseudomonas	GHO24_RS06625	35218..38778	196821	MULTISPECIES:-BREX-system-P-loop-protein-BrxC-[unclassified-Pseudomonas]
WP_233280221.1	Type1.BREX	<- PglZ <-AbiEii<-[illegible]	1216	Bacteria>Pseudomonadota>Gammaproteobacteria>Pseudomonadales>Pseudomonadaceae>Pseudomonas	GCF_009763245.1	Pseudomonas_xionganensis	GJV18_RS17875	24794..28444	2654845	BREX-system-P-loop-protein-BrxC-[Pseudomonas-xionganensis]
WP_161928857.1	Type6.BREX	<-Arm-DNA-bind_2+[illegible]	1212	Bacteria>Pseudomonadota>Gammaproteobacteria>Pseudomonadales>Pseudomonadaceae>Pseudomonas	GCF_009932815.1	Pseudomonas_yangonensis	GYH25_RS19705	87173..90811	2579922	BREX-system-P-loop-protein-BrxC-[Pseudomonas-yangonensis]
WP_262386226.1	Type1.BREX	PIN+OST-HTH-> HNH ->?->?->[illegible]	1225	Bacteria>Pseudomonadota>Gammaproteobacteria>Pseudomonadales>Pseudomonadaceae>Pseudomonas	GCF_012985535.1	Pseudomonas_sp._WS_5021	HBO19_RS03675	794081..797758	2717490	BREX-system-P-loop-protein-BrxC-[Pseudomonas-sp.-WS-5021]
WP_170050473.1	Type1.BREX	<-JetB<-MukF<- [illegible]	1154	Bacteria>Pseudomonadota>Gammaproteobacteria>Pseudomonadales>Pseudomonadaceae>Pseudomonas	GCF_012985775.1	Pseudomonas_sp._WS_5011	HBN63_RS18875	35814..39278	2717477	BREX-system-P-loop-protein-BrxC-[Pseudomonas-sp.-WS-5011]
WP_094987826.1	Type1.BREX	DUF4969-><- SIGMA+OB-Fold+[illegible]	1154	Bacteria>Pseudomonadota>Gammaproteobacteria>Pseudomonadales>Pseudomonadaceae>Pseudomonas	GCF_012986325.1	Pseudomonas_lundensis	HBN80_RS14475	101799..105263	86185	BREX-system-P-loop-protein-BrxC-[Pseudomonas-lundensis]
WP_172792831.1	Type1.BREX	NERD->PhdYeFM_antitox-><-[illegible]	1154	Bacteria>Pseudomonadota>Gammaproteobacteria>Pseudomonadales>Pseudomonadaceae>Pseudomonas	GCF_013260445.1	Pseudomonas_aeruginosa	HQN50_RS11915	2493948..2497412	287	BREX-system-P-loop-protein-BrxC-[Pseudomonas-aeruginosa]
WP_173406509.1	Type1.BREX	<-ABhydrolase+METHYLASE<-[illegible]	1154	Bacteria>Pseudomonadota>Gammaproteobacteria>Pseudomonadales>Pseudomonadaceae>Pseudomonas	GCF_013283895.1	Pseudomonas_sp._MPDS	HQ912_RS04605	1048127..1051591	2762896	BREX-system-P-loop-protein-BrxC-[Pseudomonas-sp.-MPDS]
WP_180216146.1	Type6.BREX	<-DUF3262<-?<-?<-SMODS<-[illegible]	1213	Bacteria>Pseudomonadota>Gammaproteobacteria>Pseudomonadales>Pseudomonadaceae>Pseudomonas	GCF_013421145.1	Pseudomonas_aeruginosa	HOB52_RS17845	402112..405753	287	BREX-system-P-loop-protein-BrxC-[Pseudomonas-aeruginosa]
WP_180689220.1	Type1.BREX	ABC-ATPase+SbcC->?->?->?->[illegible]	1185	Bacteria>Pseudomonadota>Gammaproteobacteria>Pseudomonadales>Pseudomonadaceae>Pseudomonas	GCF_013425825.1	Pseudomonas_putida	HOH12_RS03375	759269..762826	303	BREX-system-P-loop-protein-BrxC-[Pseudomonas-putida]
WP_181088903.1	Type6.BREX	TPR->wHTH->Integrase_1->[illegible]	1214	Bacteria>Pseudomonadota>Gammaproteobacteria>Pseudomonadales>Pseudomonadaceae>Pseudomonas	GCF_013523035.1	Pseudomonas_plecoglossicida	G7011_RS14160	93028..96672	70775	BREX-system-P-loop-protein-BrxC-[Pseudomonas-plecoglossicida]
WP_182367101.1	Type1.BREX	Arm-DNA-bind_3+[illegible]	1154	Bacteria>Pseudomonadota>Gammaproteobacteria>Pseudomonadales>Pseudomonadaceae>Pseudomonas	GCF_014109765.1	Pseudomonas_sp._MSPm1	HS968_RS15945	3439076..3442540	2726956	BREX-system-P-loop-protein-BrxC-[Pseudomonas-sp.-MSPm1]
WP_186598283.1	Type6.BREX	<-sGTP<-GTPase-AIG<-?<-?||[illegible]	1212	Bacteria>Pseudomonadota>Gammaproteobacteria>Pseudomonadales>Pseudomonadaceae>Pseudomonas	GCF_014269085.1	Pseudomonas_taiwanensis	HU747_RS04800	431376..435014	470150	BREX-system-P-loop-protein-BrxC-[Pseudomonas-taiwanensis]
WP_192985630.1	Type1.BREX	HTH_19-><- SIGMA+OB-Fold+[illegible]	1220	Bacteria>Pseudomonadota>Gammaproteobacteria>Pseudomonadales>Pseudomonadaceae>Pseudomonas	GCF_014892995.1	Pseudomonas_sp._EggHat1	INS93_RS22765	65820..69482	2761624	BREX-system-P-loop-protein-BrxC-[Pseudomonas-sp.-EggHat1]
WP_230091331.1	Type1.BREX	<- SIGMA+OB-Fold+AAA-ATPase[illegible]	1215	Bacteria>Pseudomonadota>Gammaproteobacteria>Pseudomonadales>Pseudomonadaceae>Pseudomonas	GCF_015666575.1	Pseudomonas_aeruginosa	I1957_RS22560	16962..20609	287	BREX-system-P-loop-protein-BrxC-[Pseudomonas-aeruginosa]
WP_196476665.1	Type1.BREX	PhdYeFM_antitox->RelE-ParE[illegible]	1154	Bacteria>Pseudomonadota>Gammaproteobacteria>Pseudomonadales>Pseudomonadaceae>Pseudomonas	GCF_015712105.2	Pseudomonas_sp._LMG_31766	H3221_RS24535	30166..33630	2758695	BREX-system-P-loop-protein-BrxC-[Pseudomonas-chaetocerotis]
WP_198726217.1	Type1.BREX	<-TetR-HTH<- wHTH+WYL+WCX [illegible]	1154	Bacteria>Pseudomonadota>Gammaproteobacteria>Pseudomonadales>Pseudomonadaceae>Pseudomonas	GCF_016307475.1	Pseudomonas_corrugata	YA0788_RS19970	31466..34930	47879	BREX-system-P-loop-protein-BrxC-[Pseudomonas-corrugata]
WP_198790924.1	Type1.BREX	<-CopG/RHH||XIS-HTH->?->?[illegible]	1185	Bacteria>Pseudomonadota>Gammaproteobacteria>Pseudomonadales>Pseudomonadaceae>Pseudomonas	GCF_016405175.1	Pseudomonas_sp._MF7451	JFT65_RS10900	194530..198087	2797538	BREX-system-P-loop-protein-BrxC-[Pseudomonas-sp.-MF7451]
WP_203327703.1	Type1.BREX	XIS-HTH->?-> wHTH+WYL+WCX [illegible]	1157	Bacteria>Pseudomonadota>Gammaproteobacteria>Pseudomonadales>Pseudomonadaceae>Pseudomonas	GCF_016812215.1	Pseudomonas_fragi	GYM96_RS18895	91257..94730	296	BREX-system-P-loop-protein-BrxC-[Pseudomonas-fragi]
WP_108110611.1	Type1.BREX	<-HIN-HTH<-REase<-?<- [illegible]	1154	Bacteria>Pseudomonadota>Gammaproteobacteria>Pseudomonadales>Pseudomonadaceae>Pseudomonas	GCF_016820125.1	Pseudomonas_aeruginosa	JQS52_RS11310	2388468..2391932	287	BREX-system-P-loop-protein-BrxC-[Pseudomonas-aeruginosa]
WP_203542487.1	Type1.BREX	RHH_5->SbcC->[illegible]	1146	Bacteria>Pseudomonadota>Gammaproteobacteria>Pseudomonadales>Pseudomonadaceae>Pseudomonas	GCF_016865555.1	Pseudomonas_arcuscaelestis	IIE18_RS12195	346577..350017	2710591	BREX-system-P-loop-protein-BrxC-[Pseudomonas-arcuscaelestis]
WP_205518899.1	Type1.BREX	<- PglZ <-AbiEii<-[illegible]	1107	Bacteria>Pseudomonadota>Gammaproteobacteria>Pseudomonadales>Pseudomonadaceae>Pseudomonas	GCF_016925675.1	Pseudomonas_sp._SDM007	JTY93_RS19480	4366691..4370014	2812000	BREX-system-P-loop-protein-BrxC-[Pseudomonas-sp.-SDM007]
WP_219730400.1	Type1.BREX	METHYLASE-> [illegible]	1225	Bacteria>Pseudomonadota>Gammaproteobacteria>Pseudomonadales>Pseudomonadaceae>Pseudomonas	GCF_017350895.1	unclassified_Pseudomonas	J4E12_RS18510	3053..6730	196821	MULTISPECIES:-BREX-system-P-loop-protein-BrxC-[unclassified-Pseudomonas]
WP_209939193.1	Type6.BREX	<-SbcC<-JetB<-JetA|| [illegible]	1213	Bacteria>Pseudomonadota>Gammaproteobacteria>Pseudomonadales>Pseudomonadaceae>Pseudomonas	GCF_017838075.1	Pseudomonas_aeruginosa	J0A89_RS01675	341330..344971	287	BREX-system-P-loop-protein-BrxC-[Pseudomonas-aeruginosa]
WP_125970731.1	Type1.BREX	DUF4969-><-SLOG+BirA-HTH<-[illegible]	1154	Bacteria>Pseudomonadota>Gammaproteobacteria>Pseudomonadales>Pseudomonadaceae>Pseudomonas	GCF_018336155.1	Pseudomonas_fluorescens_group	EJJ21_RS24495	5255523..5258987	136843	MULTISPECIES:-BREX-system-P-loop-protein-BrxC-[Pseudomonas-fluorescens-group]
WP_213936039.1	Type1.BREX	DUF4969-><- SIGMA+OB-Fold+[illegible]	1157	Bacteria>Pseudomonadota>Gammaproteobacteria>Pseudomonadales>Pseudomonadaceae>Pseudomonas	GCF_018449005.1	Pseudomonas_lundensis	KIY13_RS15530	3400509..3403982	86185	BREX-system-P-loop-protein-BrxC-[Pseudomonas-lundensis]
WP_262416944.1	Type1.BREX	<- SIGMA+OB-Fold+AAA-ATPase[illegible]	1225	Bacteria>Pseudomonadota>Gammaproteobacteria>Pseudomonadales>Pseudomonadaceae>Pseudomonas	GCF_019891455.1	Pseudomonas_sp._P867	J1G33_RS25105	13708..17385	2816050	BREX-system-P-loop-protein-BrxC-[Pseudomonas-sp.-P867]
WP_223717957.1	Type6.BREX	MerR-HTH->tRNA-> BrxE -> [illegible]	1213	Bacteria>Pseudomonadota>Gammaproteobacteria>Pseudomonadales>Pseudomonadaceae>Pseudomonas	GCF_020036535.1	Pseudomonas_aeruginosa	LC626_RS08340	482522..486163	287	BREX-system-P-loop-protein-BrxC-[Pseudomonas-aeruginosa]
WP_227364598.1	Type6.BREX	VirD4-TraG->DUF4400-><-[illegible]	1213	Bacteria>Pseudomonadota>Gammaproteobacteria>Pseudomonadales>Pseudomonadaceae>Pseudomonas	GCF_020639995.1	Pseudomonas_aeruginosa	LJG07_RS13375	107262..110903	287	BREX-system-P-loop-protein-BrxC-[Pseudomonas-aeruginosa]
WP_229605965.1	Type6.BREX	AlcRN+AraC-HTH-><-?|| HNH [illegible]	1212	Bacteria>Pseudomonadota>Gammaproteobacteria>Pseudomonadales>Pseudomonadaceae>Pseudomonas	GCF_020831405.1	Pseudomonas_oryzagri	LMJ29_RS13110	91421..95059	2892443	BREX-system-P-loop-protein-BrxC-[Pseudomonas-oryzagri]
WP_033990403.1	Type6.BREX	SMODS->?->JAB-><- [illegible]	1213	Bacteria>Pseudomonadota>Gammaproteobacteria>Pseudomonadales>Pseudomonadaceae>Pseudomonas	GCF_020907765.1	Pseudomonas_aeruginosa	LOH47_RS10865	120991..124632	287	BREX-system-P-loop-protein-BrxC-[Pseudomonas-aeruginosa]
WP_231661141.1	Type1.BREX	<-RHH||XIS-HTH->?-> [illegible]	1156	Bacteria>Pseudomonadota>Gammaproteobacteria>Pseudomonadales>Pseudomonadaceae>Pseudomonas	GCF_021147865.1	Pseudomonas_sp._CDFA_610	KDX39_RS16865	240745..244215	2829825	BREX-system-P-loop-protein-BrxC-[Pseudomonas-sp.-CDFA-610]
WP_236166438.1	Type1.BREX	DUF4969-><- SIGMA+OB-Fold+[illegible]	1154	Bacteria>Pseudomonadota>Gammaproteobacteria>Pseudomonadales>Pseudomonadaceae>Pseudomonas	GCF_021601865.1	Pseudomonas_juntendi	L6D32_RS09730	148894..152358	2666183	BREX-system-P-loop-protein-BrxC-[Pseudomonas-juntendi]
WP_236238652.1	Type1.BREX	<-HIN-HTH<- SIGMA+OB-Fold+[illegible]	1185	Bacteria>Pseudomonadota>Gammaproteobacteria>Pseudomonadales>Pseudomonadaceae>Pseudomonas	GCF_021603345.1	Pseudomonas_rhodesiae	L6E14_RS13730	266364..269921	76760	BREX-system-P-loop-protein-BrxC-[Pseudomonas-rhodesiae]
WP_236165768.1	Type6.BREX	DUF932->?->?-> HNH -><- [illegible]	1212	Bacteria>Pseudomonadota>Gammaproteobacteria>Pseudomonadales>Pseudomonadaceae>Pseudomonas	GCF_021603865.1	Pseudomonas_fulva	L6D10_RS06765	41152..44790	47880	BREX-system-P-loop-protein-BrxC-[Pseudomonas-fulva]
WP_236471270.1	Type1.BREX	XIS-HTH->?-><-REase+ZnR||[illegible]	1156	Bacteria>Pseudomonadota>Gammaproteobacteria>Pseudomonadales>Pseudomonadaceae>Pseudomonas	GCF_021606115.1	Pseudomonas_syringae	GIW32_RS04305	7736..11206	317	BREX-system-P-loop-protein-BrxC-[Pseudomonas-syringae]
WP_243242998.1	Type1.BREX	Amidohydro_2->ABhydrolase[illegible]	1154	Bacteria>Pseudomonadota>Gammaproteobacteria>Pseudomonadales>Pseudomonadaceae>Pseudomonas	GCF_022802935.1	Pseudomonas_atacamensis	PSD_RS21520	46330..49794	2565368	BREX-system-P-loop-protein-BrxC-[Pseudomonas-atacamensis]
WP_247325109.1	Type6.BREX	<-DUF3262<-?<-?<-SMODS<-?[illegible]	371	Bacteria>Pseudomonadota>Gammaproteobacteria>Pseudomonadales>Pseudomonadaceae>Pseudomonas	GCF_023101305.1	Pseudomonas_aeruginosa	MX621_RS22735	4826578..4827693	287	DUF6079-family-protein-[Pseudomonas-aeruginosa]
WP_253132516.1	Type1.BREX	XIS-HTH->?-><-?<-[illegible]	1184	Bacteria>Pseudomonadota>Gammaproteobacteria>Pseudomonadales>Pseudomonadaceae>Pseudomonas	GCF_024128115.1	Pseudomonas_guariconensis	NJC29_RS17685	128726..132280	1288410	BREX-system-P-loop-protein-BrxC-[Pseudomonas-guariconensis]
WP_043087434.1	Type1.BREX	<-HIN-HTH<- wHTH+WYL+WCX <-[illegible]	1154	Bacteria>Pseudomonadota>Gammaproteobacteria>Pseudomonadales>Pseudomonadaceae>Pseudomonas	GCF_024300845.1	Pseudomonas_aeruginosa	MUG61_RS11500	2420957..2424421	287	BREX-system-P-loop-protein-BrxC-[Pseudomonas-aeruginosa]
WP_261941654.1	Type1.BREX	DUF932->?->?->?->DUF4969->[illegible]	1154	Bacteria>Pseudomonadota>Gammaproteobacteria>Pseudomonadales>Pseudomonadaceae>Pseudomonas	GCF_024349685.1	Pseudomonas_sp._LRP2-20	OCX61_RS23895	5328463..5331927	2944234	BREX-system-P-loop-protein-BrxC-[Pseudomonas-sp.-LRP2-20]
WP_257780136.1	Type1.BREX	<-LZ_Tnp_IS66+zf-IS66+[illegible]	1225	Bacteria>Pseudomonadota>Gammaproteobacteria>Pseudomonadales>Pseudomonadaceae>Pseudomonas	GCF_024655625.1	Pseudomonas_sp._18.1.10	NUV89_RS02215	18654..22331	2969302	BREX-system-P-loop-protein-BrxC-[Pseudomonas-sp.-18.1.10]
WP_258640408.1	Type1.BREX	ABC-ATPase+SbcC->?->?-><-[illegible]	1154	Bacteria>Pseudomonadota>Gammaproteobacteria>Pseudomonadales>Pseudomonadaceae>Pseudomonas	GCF_024748715.1	Pseudomonas_moraviensis	LOY57_RS03675	870131..873595	321662	BREX-system-P-loop-protein-BrxC-[Pseudomonas-moraviensis]
WP_258626742.1	Type1.BREX	<- SIGMA+OB-Fold+AAA-ATPase[illegible]	1225	Bacteria>Pseudomonadota>Gammaproteobacteria>Pseudomonadales>Pseudomonadaceae>Pseudomonas	GCF_024749045.1	Pseudomonas_sp._B21-028	LOY35_RS21325	4957302..4960979	2895480	BREX-system-P-loop-protein-BrxC-[Pseudomonas-sp.-B21-028]
WP_259155464.1	Type1.BREX	DUF932->?->?->?->DUF4969->[illegible]	1154	Bacteria>Pseudomonadota>Gammaproteobacteria>Pseudomonadales>Pseudomonadaceae>Pseudomonas	GCF_024809425.1	Pseudomonas_sp._JAI111	HNR03_RS20420	285382..288846	2735913	BREX-system-P-loop-protein-BrxC-[Pseudomonas-sp.-JAI111]
WP_260092499.1	Type1.BREX	<-SF1-SF2-Helicase<-REase[illegible]	1154	Bacteria>Pseudomonadota>Gammaproteobacteria>Pseudomonadales>Pseudomonadaceae>Pseudomonas	GCF_025152895.1	Pseudomonas_aeruginosa	MNU23_RS16440	110369..113833	287	BREX-system-P-loop-protein-BrxC-[Pseudomonas-aeruginosa]
WP_265072431.1	Type1.BREX	<- SIGMA+OB-Fold+AAA-ATPase[illegible]	1186	Bacteria>Pseudomonadota>Gammaproteobacteria>Pseudomonadales>Pseudomonadaceae>Pseudomonas	GCF_026016265.1	Pseudomonas_viciae	LOY66_RS25465	5798638..5802198	2505979	BREX-system-P-loop-protein-BrxC-[Pseudomonas-viciae]
WP_010792139.1	Type6.BREX	SMODS->?->JAB-><- [illegible]	1210	Bacteria>Pseudomonadota>Gammaproteobacteria>Pseudomonadales>Pseudomonadaceae>Pseudomonas	GCF_026126725.1	Pseudomonas_aeruginosa	K3Z93_RS02355	20406..24038	287	BREX-system-P-loop-protein-BrxC-[Pseudomonas-aeruginosa]
WP_265980027.1	Type1.BREX	Phage_integrase-><- [illegible]	1184	Bacteria>Pseudomonadota>Gammaproteobacteria>Pseudomonadales>Pseudomonadaceae>Pseudomonas	GCF_026241005.1	Pseudomonas_sp._DCB_BG	OO254_RS23990	64105..67659	2993595	BREX-system-P-loop-protein-BrxC-[Pseudomonas-sp.-DCB_BG]
WP_057380122.1	Type6.BREX	LZ_Tnp_IS66+zf-IS66+[illegible]	1213	Bacteria>Pseudomonadota>Gammaproteobacteria>Pseudomonadales>Pseudomonadaceae>Pseudomonas	GCF_026727755.1	Pseudomonas_aeruginosa	PAC42_RS26725	5539866..5543507	287	BREX-system-P-loop-protein-BrxC-[Pseudomonas-aeruginosa]
WP_272218090.1	Type1.BREX	RelE-ParE-><-?<- [illegible]	932	Bacteria>Pseudomonadota>Gammaproteobacteria>Pseudomonadales>Pseudomonadaceae>Pseudomonas	GCF_028404085.1	Pseudomonas_aeruginosa	PMJ90_RS12960	2696481..2699279	287	BREX-system-P-loop-protein-BrxC-[Pseudomonas-aeruginosa]
WP_023131618.1	Type1.BREX	<- wHTH+WYL+WCX ||?-> [illegible]	1223	Bacteria>Pseudomonadota>Gammaproteobacteria>Pseudomonadales>Pseudomonadaceae>Pseudomonas	GCF_029842935.1	Pseudomonas_aeruginosa	N5I19_RS16015	16585..20256	287	MULTISPECIES:-BREX-system-P-loop-protein-BrxC-[Pseudomonas]
WP_121324126.1	Type1.BREX	<-REase<- wHTH+WYL+WCX ||?[illegible]	1154	Bacteria>Pseudomonadota>Gammaproteobacteria>Pseudomonadales>Pseudomonadaceae>Pseudomonas	GCF_030121855.1	Pseudomonas_aeruginosa	DQH07_RS08025	7041..10505	287	BREX-system-P-loop-protein-BrxC-[Pseudomonas-aeruginosa]
WP_065945478.1	Type1.BREX	<-AAA+wHTH<-?<- [illegible]	1154	Bacteria>Pseudomonadota>Gammaproteobacteria>Pseudomonadales>Pseudomonadaceae>Pseudomonas	GCF_900004795.1	Pseudomonas_sp._24_R_17	BFP98_RS06365	99601..103065	1844096	BREX-system-P-loop-protein-BrxC-[Pseudomonas-sp.-24-R-17]
WP_092146146.1	Type1.BREX	<-AAA+wHTH<-?<- [illegible]	1154	Bacteria>Pseudomonadota>Gammaproteobacteria>Pseudomonadales>Pseudomonadaceae>Pseudomonas	GCF_900102655.1	unclassified_Pseudomonas	BLP71_RS14905	103193..106657	196821	MULTISPECIES:-BREX-system-P-loop-protein-BrxC-[unclassified-Pseudomonas]
WP_090347579.1	Type1.BREX	<- wHTH+WYL+WCX <-?||ARG->[illegible]	1154	Bacteria>Pseudomonadota>Gammaproteobacteria>Pseudomonadales>Pseudomonadaceae>Pseudomonas	GCF_900104805.1	Pseudomonas_oryzae	BLT78_RS03155	660554..664018	1392877	BREX-system-P-loop-protein-BrxC-[Pseudomonas-oryzae]
WP_090214592.1	Type1.BREX	REC+PP2C->STAS-><-?<-?<-[illegible]	1218	Bacteria>Pseudomonadota>Gammaproteobacteria>Pseudomonadales>Pseudomonadaceae>Pseudomonas	GCF_900105885.1	Pseudomonas_guangdongensis	BLU22_RS11505	2422793..2426449	1245526	BREX-system-P-loop-protein-BrxC-[Pseudomonas-guangdongensis]
WP_034038617.1	Type6.BREX	MerR-HTH->tRNA-> BrxE -> [illegible]	1213	Bacteria>Pseudomonadota>Gammaproteobacteria>Pseudomonadales>Pseudomonadaceae>Pseudomonas	GCF_900147165.1	Pseudomonas_aeruginosa	K3Z85_RS02500	4652..8293	287	BREX-system-P-loop-protein-BrxC-[Pseudomonas-aeruginosa]
WP_076583447.1	Type1.BREX	DUF4969-><- SIGMA+OB-Fold+[illegible]	1184	Bacteria>Pseudomonadota>Gammaproteobacteria>Pseudomonadales>Pseudomonadaceae>Pseudomonas	GCF_900156545.1	Pseudomonas_alcaligenes	BW984_RS17050	273058..276612	43263	BREX-system-P-loop-protein-BrxC-[Pseudomonas-alcaligenes]
WP_095181954.1	Type1.BREX	<- SIGMA+OB-Fold+AAA-ATPase[illegible]	1222	Bacteria>Pseudomonadota>Gammaproteobacteria>Pseudomonadales>Pseudomonadaceae>Pseudomonas	GCF_900187595.1	Pseudomonas_sp._Irchel_3F6	CKW30_RS22375	287368..291036	2009003	BREX-system-P-loop-protein-BrxC-[Pseudomonas-sp.-Irchel-3F6]
WP_115284295.1	Type1.BREX	AAA-ATPase->?->?->DUF4969[illegible]	1154	Bacteria>Pseudomonadota>Gammaproteobacteria>Pseudomonadales>Pseudomonadaceae>Pseudomonas	GCF_900455465.1	Pseudomonas_fluorescens	DYC60_RS18930	4157779..4161243	294	BREX-system-P-loop-protein-BrxC-[Pseudomonas-fluorescens]
WP_122521120.1	Type1.BREX	<-RHH||XIS-HTH->?-> [illegible]	1154	Bacteria>Pseudomonadota>Gammaproteobacteria>Pseudomonadales>Pseudomonadaceae>Pseudomonas	GCF_900576635.1	Pseudomonas_viridiflava	EET02_RS10950	375629..379093	33069	BREX-system-P-loop-protein-BrxC-[Pseudomonas-viridiflava]
WP_127881958.1	Type1.BREX	DUF932->?->?->?-><- [illegible]	1146	Bacteria>Pseudomonadota>Gammaproteobacteria>Pseudomonadales>Pseudomonadaceae>Pseudomonas	GCF_900618835.1	Pseudomonas_carnis	PCSC_RS23730	94995..98435	2487355	BREX-system-P-loop-protein-BrxC-[Pseudomonas-carnis]
WP_150549236.1	Type1.BREX	<-DUF3584+DUF5401<-JetB<-[illegible]	1154	Bacteria>Pseudomonadota>Gammaproteobacteria>Pseudomonadales>Pseudomonadaceae>Pseudomonas	GCF_900682675.2	Pseudomonas_marincola	PMYSY11_RS11215	2416219..2419683	437900	BREX-system-P-loop-protein-BrxC-[Pseudomonas-marincola]
WP_043107115.1	Type1.BREX	GreA_GreB+SF1-SF2-Helicase[illegible]	1154	Bacteria>Pseudomonadota>Gammaproteobacteria>Pseudomonadales>Pseudomonadaceae>Pseudomonas	GCF_900706985.1	Pseudomonas_aeruginosa_group	E4Z08_RS29645	1460204..1463668	136841	MULTISPECIES:-BREX-system-P-loop-protein-BrxC-[Pseudomonas-aeruginosa-group]
WP_134576235.1	Type1.BREX	<-AbiEi_4<-?<-?<- [illegible]	769	Bacteria>Pseudomonadota>Gammaproteobacteria>Pseudomonadales>Pseudomonadaceae>Pseudomonas	GCF_900707355.1	Pseudomonas_aeruginosa	E4Z10_RS26780	10282..12591	287	BREX-system-P-loop-protein-BrxC,-partial-[Pseudomonas-aeruginosa]
WP_150597441.1	Type1.BREX	DUF4969-><- SIGMA+OB-Fold+[illegible]	1154	Bacteria>Pseudomonadota>Gammaproteobacteria>Pseudomonadales>Pseudomonadaceae>Pseudomonas	GCF_902497985.1	Pseudomonas_fluorescens	F8N16_RS17980	132187..135651	294	BREX-system-P-loop-protein-BrxC-[Pseudomonas-fluorescens]
WP_150787878.1	Type5.BREX	TUDOR+SF1-SF2-Helicase+[illegible]	1222	Bacteria>Pseudomonadota>Gammaproteobacteria>Pseudomonadales>Pseudomonadaceae>Pseudomonas	GCF_902498245.1	Pseudomonas_fluorescens	F8N86_RS29045	91300..94968	294	BREX-system-P-loop-protein-BrxC-[Pseudomonas-fluorescens]
WP_229999483.1	Type1.BREX	XIS-HTH->?->URI-DNase+[illegible]	1156	Bacteria>Pseudomonadota>Gammaproteobacteria>Pseudomonadales>Pseudomonadaceae>Pseudomonas	GCF_918697895.1	Pseudomonas_carnis	LQ224_RS08485	110382..113852	2487355	BREX-system-P-loop-protein-BrxC-[Pseudomonas-carnis]
WP_033944490.1	Type1.BREX	Arm-DNA-bind_3+[illegible]	1184	Bacteria>Pseudomonadota>Gammaproteobacteria>Pseudomonadales>Pseudomonadaceae>Pseudomonas	GCF_947387505.1	Pseudomonas_aeruginosa	GPV34_RS03115	17071..20625	287	BREX-system-P-loop-protein-BrxC-[Pseudomonas-aeruginosa]
WP_095027056.1	Type6.BREX	<-LZ_Tnp_IS66+zf-IS66+[illegible]	1213	Bacteria>Pseudomonadota>Gammaproteobacteria>Pseudomonadales>Pseudomonadaceae	-	Pseudomonas	JFT63_RS16080	178886..182527	286	MULTISPECIES:-BREX-system-P-loop-protein-BrxC-[Pseudomonas]
WP_150298006.1	Type6.BREX	<-Arm-DNA-bind_2+[illegible]	1212	Bacteria>Pseudomonadota>Gammaproteobacteria>Pseudomonadales>Pseudomonadaceae>Pseudomonas	-	Pseudomonas_aeruginosa	F3G60_11255	510071..513709	287	BREX-system-P-loop-protein-BrxC-[Pseudomonas-aeruginosa]
EIU5588796.1	Type1.BREX	PhdYeFM_antitox->RelE-ParE[illegible]	1154	Bacteria>Pseudomonadota>Gammaproteobacteria>Pseudomonadales>Pseudomonadaceae>Pseudomonas	-	Pseudomonas_aeruginosa	L4689_001050	82930..86394	287	BREX-system-P-loop-protein-BrxC-[Pseudomonas-aeruginosa]
EIU3153299.1	Type1.BREX	GreA_GreB+SF1-SF2-Helicase[illegible]	512	Bacteria>Pseudomonadota>Gammaproteobacteria>Pseudomonadales>Pseudomonadaceae>Pseudomonas	-	Pseudomonas_aeruginosa	L4P27_006629	14238..15778	287	hypothetical-protein-[Pseudomonas-aeruginosa]
EIU4198817.1	Type1.BREX	DUF932->?->?->?-><- [illegible]	1180	Bacteria>Pseudomonadota>Gammaproteobacteria>Pseudomonadales>Pseudomonadaceae>Pseudomonas	-	Pseudomonas_aeruginosa	L4V49_002912	27945..31487	287	BREX-system-P-loop-protein-BrxC-[Pseudomonas-aeruginosa]
WP_154949571.1	Type1.BREX	DUF932->?->?->DUF4969-><- [illegible]	1154	Bacteria>Pseudomonadota>Gammaproteobacteria>Pseudomonadales>Pseudomonadaceae>Pseudomonas	-	Pseudomonas_fluorescens	F8N22_RS18775	172273..175737	294	BREX-system-P-loop-protein-BrxC-[Pseudomonas-fluorescens]
WP_032902483.1	Type1.BREX	<-AAA+wHTH<-?<- [illegible]	1154	Bacteria>Pseudomonadota>Gammaproteobacteria>Pseudomonadales>Pseudomonadaceae>Pseudomonas	-	Pseudomonas_fluorescens	H098_RS08890	14970..18434	294	BREX-system-P-loop-protein-BrxC-[Pseudomonas-fluorescens]
WP_241666011.1	Type1.BREX	<-TraI-VirD2-RCRE<-[illegible]	1225	Bacteria>Pseudomonadota>Gammaproteobacteria>Pseudomonadales>Pseudomonadaceae>Pseudomonas	-	Pseudomonas_fluorescens	PflCFBP13510_RS04715	495917..499594	294	BREX-system-P-loop-protein-BrxC-[Pseudomonas-fluorescens]
WP_112249959.1	Type1.BREX	wHTH+WYL+WCX -> [illegible]	1154	Bacteria>Pseudomonadota>Gammaproteobacteria>Pseudomonadales>Pseudomonadaceae>Pseudomonas	-	Pseudomonas_putida	GT37_RS03100	3180..6644	303	BREX-system-P-loop-protein-BrxC-[Pseudomonas-putida]
WP_046164318.1	Type1.BREX	<- SIGMA+OB-Fold+AAA-ATPase[illegible]	1186	Bacteria>Pseudomonadota>Gammaproteobacteria>Pseudomonadales>Pseudomonadaceae>Pseudomonas	-	Pseudomonas_sp._10B238	RT21_RS21355	15645..19205	1586417	BREX-system-P-loop-protein-BrxC-[Pseudomonas-sp.-10B238]
WP_099493679.1	Type1.BREX	<- SIGMA+OB-Fold+AAA-ATPase[illegible]	1222	Bacteria>Pseudomonadota>Gammaproteobacteria>Pseudomonadales>Pseudomonadaceae>Pseudomonas	-	Pseudomonas_sp._2588-5	AOA57_22445	341678..345346	1712676	BREX-system-P-loop-protein-BrxC-[Pseudomonas-sp.-2588-5]
WP_098950596.1	Type1.BREX	<-TraI-VirD2-RCRE<-[illegible]	1222	Bacteria>Pseudomonadota>Gammaproteobacteria>Pseudomonadales>Pseudomonadaceae>Pseudomonas	-	Pseudomonas_sp._FDAARGOS_380	CRN80_16460	3507093..3510761	2018067	BREX-system-P-loop-protein-BrxC-[Pseudomonas-sp.-FDAARGOS_380]
WP_223019012.1	Type1.BREX	<- SIGMA+OB-Fold+AAA-ATPase[illegible]	1222	Bacteria>Pseudomonadota>Gammaproteobacteria>Pseudomonadales>Pseudomonadaceae>Pseudomonas	-	Pseudomonas_sp._P867	J1G33_25110	13708..17376	2816050	BREX-system-P-loop-protein-BrxC-[Pseudomonas-sp.-P867]
WP_170056913.1	Type1.BREX	PIN+OST-HTH-> HNH ->?->?->[illegible]	1222	Bacteria>Pseudomonadota>Gammaproteobacteria>Pseudomonadales>Pseudomonadaceae>Pseudomonas	-	Pseudomonas_sp._WS_5021	HBO19_03685	794090..797758	2717490	BREX-system-P-loop-protein-BrxC-[Pseudomonas-sp.-WS-5021]
WP_240033455.1	Type1.BREX	<- wHTH+WYL+WCX ||?->ARG-> [illegible]	1225	Bacteria>Pseudomonadota>Gammaproteobacteria>Pseudomonadales>Pseudomonadaceae>Pseudomonas	-	Pseudomonas_viridiflava	EE173_RS11530	5062..8739	33069	BREX-system-P-loop-protein-BrxC-[Pseudomonas-viridiflava]
OWG36865.1	Type1.BREX	<-RNase<-?<-JetB<-?<- [illegible]	1185	Bacteria>Pseudomonadota>Gammaproteobacteria>Pseudomonadales>Pseudomonadaceae>Stutzerimonas	GCA_002193135.1	Stutzerimonas_stutzeri	CAQ69_18770	20186..23743	316	hypothetical-protein-CAQ69_18770-[Stutzerimonas-stutzeri]
MCQ2029875.1	Type1.BREX	<-sGTP<-sGTP<-sGTP<- [illegible]	1186	Bacteria>Pseudomonadota>Gammaproteobacteria>Pseudomonadales>Pseudomonadaceae>Stutzerimonas	GCA_024397535.1	[Pseudomonas]_zhaodongensis	NA613_09780	17231..20791	1176257	BREX-system-P-loop-protein-BrxC-[[Pseudomonas]
MCQ4256509.1	Type1.BREX	<-SF1-SF2-Helicase<-REase[illegible]	1154	Bacteria>Pseudomonadota>Gammaproteobacteria>Pseudomonadales>Pseudomonadaceae>Stutzerimonas	GCA_024448895.1	Stutzerimonas_stutzeri	NA619_00645	147157..150621	316	BREX-system-P-loop-protein-BrxC-[Stutzerimonas-stutzeri]
WP_017245161.1	Type1.BREX	<- PglZ <-AbiEii<-AbiEi_4<-[illegible]	1220	Bacteria>Pseudomonadota>Gammaproteobacteria>Pseudomonadales>Pseudomonadaceae>Stutzerimonas	GCF_000280555.1	Stutzerimonas_stutzeri	A598_RS0110895	41008..44670	316	BREX-system-P-loop-protein-BrxC-[Stutzerimonas-stutzeri]
WP_023443891.1	Type6.BREX	<-SbcC<-JetB<-JetA|| [illegible]	1213	Bacteria>Pseudomonadota>Gammaproteobacteria>Pseudomonadales>Pseudomonadaceae>Stutzerimonas	GCF_000495915.1	Stutzerimonas_stutzeri_group	F753_RS26950	57885..61526	136846	MULTISPECIES:-BREX-system-P-loop-protein-BrxC-[Stutzerimonas-stutzeri-group]
WP_120997717.1	Type6.BREX	TrwC+SF1-SF2-Helicase->[illegible]	1212	Bacteria>Pseudomonadota>Gammaproteobacteria>Pseudomonadales>Pseudomonadaceae>Stutzerimonas	GCF_003640395.1	[Pseudomonas]_urumqiensis	D7325_RS17370	36612..40250	638269	BREX-system-P-loop-protein-BrxC-[[Pseudomonas]
WP_125866641.1	Type6.BREX	JAB-><- [illegible]	1213	Bacteria>Pseudomonadota>Gammaproteobacteria>Pseudomonadales>Pseudomonadaceae>Stutzerimonas	GCF_003935625.1	Stutzerimonas_stutzeri	EGJ12_RS17010	223453..227094	316	BREX-system-P-loop-protein-BrxC-[Stutzerimonas-stutzeri]
WP_125856771.1	Type1.BREX	<-LexA-protease<- [illegible]	1154	Bacteria>Pseudomonadota>Gammaproteobacteria>Pseudomonadales>Pseudomonadaceae>Stutzerimonas	GCF_003940515.1	Stutzerimonas_stutzeri	EGJ36_RS18105	6035..9499	316	BREX-system-P-loop-protein-BrxC-[Stutzerimonas-stutzeri]
WP_212739636.1	Type6.BREX	<-SbcC<-JetB<-JetA|| [illegible]	1207	Bacteria>Pseudomonadota>Gammaproteobacteria>Pseudomonadales>Pseudomonadaceae>Stutzerimonas	GCF_018316915.2	Stutzerimonas_balearica	D3P44_RS15315	3232724..3236347	74829	BREX-system-P-loop-protein-BrxC-[Stutzerimonas-balearica]
WP_218422415.1	Type1.BREX	<-HTH_Tnp_1+HTH_21+rve+[illegible]	1154	Bacteria>Pseudomonadota>Gammaproteobacteria>Pseudomonadales>Pseudomonadaceae>Stutzerimonas	GCF_019203165.1	Stutzerimonas_stutzeri	KUD99_RS07220	1491661..1495125	316	BREX-system-P-loop-protein-BrxC-[Stutzerimonas-stutzeri]
WP_255830664.1	Type1.BREX	<-sGTP<-sGTP<-sGTP<- [illegible]	1186	Bacteria>Pseudomonadota>Gammaproteobacteria>Pseudomonadales>Pseudomonadaceae>Stutzerimonas	GCF_024397535.1	[Pseudomonas]_zhaodongensis	NA613_RS09805	17231..20791	1176257	BREX-system-P-loop-protein-BrxC-[[Pseudomonas]
WP_255958979.1	Type1.BREX	<-SF1-SF2-Helicase<-REase[illegible]	1154	Bacteria>Pseudomonadota>Gammaproteobacteria>Pseudomonadales>Pseudomonadaceae>Stutzerimonas	GCF_024448895.1	Stutzerimonas_stutzeri	NA619_RS00645	147157..150621	316	BREX-system-P-loop-protein-BrxC-[Stutzerimonas-stutzeri]
WP_045633496.1	Type1.BREX	<-RNase<-?<-JetB<-?<- [illegible]	1185	Bacteria>Pseudomonadota>Gammaproteobacteria>Pseudomonadales>Pseudomonadaceae>Stutzerimonas	-	Stutzerimonas_stutzeri	CAQ69_18770	20186..23743	316	MULTISPECIES:-BREX-system-P-loop-protein-BrxC-[Gammaproteobacteria]
WP_041106841.1	Type6.BREX	<-SbcC<-JetB<-JetA|| [illegible]	1213	Bacteria>Pseudomonadota>Gammaproteobacteria>Pseudomonadales>Pseudomonadaceae>Stutzerimonas	-	Stutzerimonas_stutzeri	QX25_RS10015	148024..151665	316	BREX-system-P-loop-protein-BrxC-[Stutzerimonas-stutzeri]
WP_053105412.1	Type1.BREX	METHYLASE->?->?-><-?<-?<-[illegible]	1155	Bacteria>Pseudomonadota>Gammaproteobacteria>Pseudomonadales>Pseudomonadaceae>Thiopseudomonas	GCF_001267275.1	Thiopseudomonas_alkaliphila	AKN90_RS00615	123321..126788	1697053	BREX-system-P-loop-protein-BrxC-[Thiopseudomonas-alkaliphila]
MAB42111.1	Type1.BREX	<- SIGMA+OB-Fold+AAA-ATPase[illegible]	1154	Bacteria>Pseudomonadota>Gammaproteobacteria>Pseudomonadales>unclassified_Pseudomonadales	GCA_002684155.1	Pseudomonadales_bacterium	CMK77_07490	13688..17152	1891229	hypothetical-protein-[Pseudomonadales-bacterium]
MAQ23221.1	Type1.BREX	<-Calcineurin+Calcineurin[illegible]	1193	Bacteria>Pseudomonadota>Gammaproteobacteria>Pseudomonadales>unclassified_Pseudomonadales	GCA_002701315.1	Pseudomonadales_bacterium	CMK83_03295	18015..21596	1891229	hypothetical-protein-[Pseudomonadales-bacterium]
MCP5171278.1	Type1.BREX	TraG_N-><-?<-?<-?<-?<-?|| [illegible]	1216	Bacteria>Pseudomonadota>Gammaproteobacteria>Pseudomonadales>unclassified_Pseudomonadales	GCA_024234435.1	Pseudomonadales_bacterium	H7A02_03290	695235..698885	1891229	BREX-system-P-loop-protein-BrxC-[Pseudomonadales-bacterium]
MCP5179014.1	Type1.BREX	<-DUF772+DDE_Tnp_1+[illegible]	1153	Bacteria>Pseudomonadota>Gammaproteobacteria>Pseudomonadales>unclassified_Pseudomonadales	-	Pseudomonadales_bacterium	H6994_03785	340860..344321	1891229	BREX-system-P-loop-protein-BrxC-[Pseudomonadales-bacterium]
WP_007146327.1	Type1.BREX	UbiA-><- HNH <-tRNA<-?<-?<-[illegible]	1179	Bacteria>Pseudomonadota>Gammaproteobacteria>Thiotrichales>Piscirickettsiaceae>Methylophaga	GCF_000214595.1	Methylophaga_aminisulfidivorans	MAMP_RS12010	87136..90675	230105	BREX-system-P-loop-protein-BrxC-[Methylophaga-aminisulfidivorans]
CAA6806547.1	Type1.BREX	<- ParB + HNH <- [illegible]	849	Bacteria>Pseudomonadota>Gammaproteobacteria>Thiotrichales>Thiotrichaceae>environmental_samples	-	uncultured_Thiotrichaceae_bacterium	HELGO_WM31114	6431..8980	298394	FIG00607287:-hypothetical-protein,-partial-[uncultured-Thiotrichaceae-bacterium]
NLY14765.1	Type1.BREX	<- SIGMA+OB-Fold+AAA-ATPase[illegible]	438	Bacteria>Pseudomonadota>Gammaproteobacteria>unclassified_Gammaproteobacteria	GCA_012728415.1	Gammaproteobacteria_bacterium	GXZ05_00075	12795..14111	1913989	hypothetical-protein-[Gammaproteobacteria-bacterium]
MBU1286063.1	Type1.BREX	BrxA-DNA-binding -> [illegible]	1222	Bacteria>Pseudomonadota>Gammaproteobacteria>unclassified_Gammaproteobacteria	GCA_018819505.1	Gammaproteobacteria_bacterium	KJ794_21910	1033..4701	1913989	BREX-system-P-loop-protein-BrxC-[Gammaproteobacteria-bacterium]
WP_005419548.1	Type1.BREX	Peptidase_M32->[illegible]	1157	Bacteria>Pseudomonadota>Gammaproteobacteria>Vibrionales>Vibrionaceae>Aliivibrio	GCF_000241785.1	Aliivibrio_fischeri	VFSR5_RS07520	1604629..1608102	668	BREX-system-P-loop-protein-BrxC-[Aliivibrio-fischeri]

Protein-ID	System	Gene-Neighbourhoods	Length	Taxonomy	Assembly-ID	Strain	Locus-Tag	Genomic-coordinates	TaxID	Fasta-define
WP_065641602.1	Type1.BREX	[illegible]	[illegible]	[illegible]	[illegible]	[illegible]	[illegible]	[illegible]	[illegible]	[illegible]
WP_130049303.1	Type1.BREX	[illegible]	[illegible]	[illegible]	[illegible]	[illegible]	[illegible]	[illegible]	[illegible]	[illegible]
WP_133416081.1	Type1.BREX	[illegible]	[illegible]	[illegible]	[illegible]	[illegible]	[illegible]	[illegible]	[illegible]	[illegible]
WP_155683402.1	Type1.BREX	[illegible]	[illegible]	[illegible]	[illegible]	[illegible]	[illegible]	[illegible]	[illegible]	[illegible]
WP_102260244.1	Type1.BREX	[illegible]	[illegible]	[illegible]	[illegible]	[illegible]	[illegible]	[illegible]	[illegible]	[illegible]
WP_025612391.1	Type1.BREX	[illegible]	[illegible]	[illegible]	[illegible]	[illegible]	[illegible]	[illegible]	[illegible]	[illegible]
WP_159406353.1	Type1.BREX	[illegible]	[illegible]	[illegible]	[illegible]	[illegible]	[illegible]	[illegible]	[illegible]	[illegible]
WP_228075537.1	Type1.BREX	[illegible]	[illegible]	[illegible]	[illegible]	[illegible]	[illegible]	[illegible]	[illegible]	[illegible]
WP_180208765.1	Type1.BREX	[illegible]	[illegible]	[illegible]	[illegible]	[illegible]	[illegible]	[illegible]	[illegible]	[illegible]
WP_086713494.1	Type1.BREX	[illegible]	[illegible]	[illegible]	[illegible]	[illegible]	[illegible]	[illegible]	[illegible]	[illegible]
WP_198107480.1	Type1.BREX	[illegible]	[illegible]	[illegible]	[illegible]	[illegible]	[illegible]	[illegible]	[illegible]	[illegible]
WP_130066623.1	Type1.BREX	[illegible]	[illegible]	[illegible]	[illegible]	[illegible]	[illegible]	[illegible]	[illegible]	[illegible]
WP_006647488.1	Type1.BREX	[illegible]	[illegible]	[illegible]	[illegible]	[illegible]	[illegible]	[illegible]	[illegible]	[illegible]
WP_115426815.1	Type1.BREX	[illegible]	[illegible]	[illegible]	[illegible]	[illegible]	[illegible]	[illegible]	[illegible]	[illegible]
WP_138222247.1	Type1.BREX	[illegible]	[illegible]	[illegible]	[illegible]	[illegible]	[illegible]	[illegible]	[illegible]	[illegible]
WP_138976661.1	Type1.BREX	[illegible]	[illegible]	[illegible]	[illegible]	[illegible]	[illegible]	[illegible]	[illegible]	[illegible]
WP_207103965.1	Type1.BREX	[illegible]	[illegible]	[illegible]	[illegible]	[illegible]	[illegible]	[illegible]	[illegible]	[illegible]
WP_232580803.1	Type1.BREX	[illegible]	[illegible]	[illegible]	[illegible]	[illegible]	[illegible]	[illegible]	[illegible]	[illegible]
WP_239972118.1	Type1.BREX	[illegible]	[illegible]	[illegible]	[illegible]	[illegible]	[illegible]	[illegible]	[illegible]	[illegible]
WP_101767576.1	Type1.BREX	[illegible]	[illegible]	[illegible]	[illegible]	[illegible]	[illegible]	[illegible]	[illegible]	[illegible]
WP_087820331.1	Type1.BREX	[illegible]	[illegible]	[illegible]	[illegible]	[illegible]	[illegible]	[illegible]	[illegible]	[illegible]
GAJ78762.1	Type1.BREX	[illegible]	[illegible]	[illegible]	[illegible]	[illegible]	[illegible]	[illegible]	[illegible]	[illegible]
GAJ78763.1	Type1.BREX	[illegible]	[illegible]	[illegible]	[illegible]	[illegible]	[illegible]	[illegible]	[illegible]	[illegible]
MVB73516.1	Type1.BREX	[illegible]	[illegible]	[illegible]	[illegible]	[illegible]	[illegible]	[illegible]	[illegible]	[illegible]
MVC74394.1	Type1.BREX	[illegible]	[illegible]	[illegible]	[illegible]	[illegible]	[illegible]	[illegible]	[illegible]	[illegible]
MBE3824873.1	Type1.BREX	[illegible]	[illegible]	[illegible]	[illegible]	[illegible]	[illegible]	[illegible]	[illegible]	[illegible]
MBE4310404.1	Type1.BREX	[illegible]	[illegible]	[illegible]	[illegible]	[illegible]	[illegible]	[illegible]	[illegible]	[illegible]
MBE4432200.1	Type1.BREX	[illegible]	[illegible]	[illegible]	[illegible]	[illegible]	[illegible]	[illegible]	[illegible]	[illegible]
MBE5192784.1	Type1.BREX	[illegible]	[illegible]	[illegible]	[illegible]	[illegible]	[illegible]	[illegible]	[illegible]	[illegible]
EGQ7646156.1	Type1.BREX	[illegible]	[illegible]	[illegible]	[illegible]	[illegible]	[illegible]	[illegible]	[illegible]	[illegible]
HAS6187654.1	Type1.BREX	[illegible]	[illegible]	[illegible]	[illegible]	[illegible]	[illegible]	[illegible]	[illegible]	[illegible]
HAS6259595.1	Type1.BREX	[illegible]	[illegible]	[illegible]	[illegible]	[illegible]	[illegible]	[illegible]	[illegible]	[illegible]
HAS6362912.1	Type1.BREX	[illegible]	[illegible]	[illegible]	[illegible]	[illegible]	[illegible]	[illegible]	[illegible]	[illegible]
HAS6345170.1	Type1.BREX	[illegible]	[illegible]	[illegible]	[illegible]	[illegible]	[illegible]	[illegible]	[illegible]	[illegible]
HAS6333998.1	Type1.BREX	[illegible]	[illegible]	[illegible]	[illegible]	[illegible]	[illegible]	[illegible]	[illegible]	[illegible]
HAS6310370.1	Type1.BREX	[illegible]	[illegible]	[illegible]	[illegible]	[illegible]	[illegible]	[illegible]	[illegible]	[illegible]
EGQ8898350.1	Type1.BREX	[illegible]	[illegible]	[illegible]	[illegible]	[illegible]	[illegible]	[illegible]	[illegible]	[illegible]
EGQ9599053.1	Type1.BREX	[illegible]	[illegible]	[illegible]	[illegible]	[illegible]	[illegible]	[illegible]	[illegible]	[illegible]
EGR0072241.1	Type1.BREX	[illegible]	[illegible]	[illegible]	[illegible]	[illegible]	[illegible]	[illegible]	[illegible]	[illegible]
EGR0489752.1	Type1.BREX	[illegible]	[illegible]	[illegible]	[illegible]	[illegible]	[illegible]	[illegible]	[illegible]	[illegible]
EGR0719595.1	Type1.BREX	[illegible]	[illegible]	[illegible]	[illegible]	[illegible]	[illegible]	[illegible]	[illegible]	[illegible]
EGR0983685.1	Type1.BREX	[illegible]	[illegible]	[illegible]	[illegible]	[illegible]	[illegible]	[illegible]	[illegible]	[illegible]
EGR3003176.1	Type1.BREX	[illegible]	[illegible]	[illegible]	[illegible]	[illegible]	[illegible]	[illegible]	[illegible]	[illegible]
EGR4188113.1	Type1.BREX	[illegible]	[illegible]	[illegible]	[illegible]	[illegible]	[illegible]	[illegible]	[illegible]	[illegible]
EJG0966117.1	Type1.BREX	[illegible]	[illegible]	[illegible]	[illegible]	[illegible]	[illegible]	[illegible]	[illegible]	[illegible]
EJG0997619.1	Type1.BREX	[illegible]	[illegible]	[illegible]	[illegible]	[illegible]	[illegible]	[illegible]	[illegible]	[illegible]
EJG0638704.1	Type1.BREX	[illegible]	[illegible]	[illegible]	[illegible]	[illegible]	[illegible]	[illegible]	[illegible]	[illegible]
EGR4671255.1	Type1.BREX	[illegible]	[illegible]	[illegible]	[illegible]	[illegible]	[illegible]	[illegible]	[illegible]	[illegible]
EJG1873897.1	Type1.BREX	[illegible]	[illegible]	[illegible]	[illegible]	[illegible]	[illegible]	[illegible]	[illegible]	[illegible]
HAS8442986.1	Type1.BREX	[illegible]	[illegible]	[illegible]	[illegible]	[illegible]	[illegible]	[illegible]	[illegible]	[illegible]
HAS8502529.1	Type1.BREX	[illegible]	[illegible]	[illegible]	[illegible]	[illegible]	[illegible]	[illegible]	[illegible]	[illegible]
MBM4838763.1	Type1.BREX	[illegible]	[illegible]	[illegible]	[illegible]	[illegible]	[illegible]	[illegible]	[illegible]	[illegible]
MBM4875012.1	Type1.BREX	[illegible]	[illegible]	[illegible]	[illegible]	[illegible]	[illegible]	[illegible]	[illegible]	[illegible]
EHD2253057.1	Type1.BREX	[illegible]	[illegible]	[illegible]	[illegible]	[illegible]	[illegible]	[illegible]	[illegible]	[illegible]
EHH1264392.1	Type1.BREX	[illegible]	[illegible]	[illegible]	[illegible]	[illegible]	[illegible]	[illegible]	[illegible]	[illegible]
EHK0048698.1	Type1.BREX	[illegible]	[illegible]	[illegible]	[illegible]	[illegible]	[illegible]	[illegible]	[illegible]	[illegible]
EHK9043270.1	Type1.BREX	[illegible]	[illegible]	[illegible]	[illegible]	[illegible]	[illegible]	[illegible]	[illegible]	[illegible]
HBC3376470.1	Type1.BREX	[illegible]	[illegible]	[illegible]	[illegible]	[illegible]	[illegible]	[illegible]	[illegible]	[illegible]
HBC3399177.1	Type1.BREX	[illegible]	[illegible]	[illegible]	[illegible]	[illegible]	[illegible]	[illegible]	[illegible]	[illegible]
HBC3481459.1	Type1.BREX	[illegible]	[illegible]	[illegible]	[illegible]	[illegible]	[illegible]	[illegible]	[illegible]	[illegible]
HBC3540120.1	Type1.BREX	[illegible]	[illegible]	[illegible]	[illegible]	[illegible]	[illegible]	[illegible]	[illegible]	[illegible]
HBC3921009.1	Type1.BREX	[illegible]	[illegible]	[illegible]	[illegible]	[illegible]	[illegible]	[illegible]	[illegible]	[illegible]
HBC3861549.1	Type1.BREX	[illegible]	[illegible]	[illegible]	[illegible]	[illegible]	[illegible]	[illegible]	[illegible]	[illegible]
HBC3986622.1	Type1.BREX	[illegible]	[illegible]	[illegible]	[illegible]	[illegible]	[illegible]	[illegible]	[illegible]	[illegible]
HBC3940910.1	Type1.BREX	[illegible]	[illegible]	[illegible]	[illegible]	[illegible]	[illegible]	[illegible]	[illegible]	[illegible]
EHW0635156.1	Type1.BREX	[illegible]	[illegible]	[illegible]	[illegible]	[illegible]	[illegible]	[illegible]	[illegible]	[illegible]
EHW0654650.1	Type1.BREX	[illegible]	[illegible]	[illegible]	[illegible]	[illegible]	[illegible]	[illegible]	[illegible]	[illegible]
MBY8044957.1	Type1.BREX	[illegible]	[illegible]	[illegible]	[illegible]	[illegible]	[illegible]	[illegible]	[illegible]	[illegible]
MBY8148861.1	Type1.BREX	[illegible]	[illegible]	[illegible]	[illegible]	[illegible]	[illegible]	[illegible]	[illegible]	[illegible]
MBY8207859.1	Type1.BREX	[illegible]	[illegible]	[illegible]	[illegible]	[illegible]	[illegible]	[illegible]	[illegible]	[illegible]
EII3725635.1	Type1.BREX	[illegible]	[illegible]	[illegible]	[illegible]	[illegible]	[illegible]	[illegible]	[illegible]	[illegible]
HBK3330408.1	Type1.BREX	[illegible]	[illegible]	[illegible]	[illegible]	[illegible]	[illegible]	[illegible]	[illegible]	[illegible]
EIU7849849.1	Type1.BREX	[illegible]	[illegible]	[illegible]	[illegible]	[illegible]	[illegible]	[illegible]	[illegible]	[illegible]
EIY9800890.1	Type1.BREX	[illegible]	[illegible]	[illegible]	[illegible]	[illegible]	[illegible]	[illegible]	[illegible]	[illegible]
GHW31175.1	Type1.BREX	[illegible]	[illegible]	[illegible]	[illegible]	[illegible]	[illegible]	[illegible]	[illegible]	[illegible]
EJE8545413.1	Type1.BREX	[illegible]	[illegible]	[illegible]	[illegible]	[illegible]	[illegible]	[illegible]	[illegible]	[illegible]
EJE8564157.1	Type1.BREX	[illegible]	[illegible]	[illegible]	[illegible]	[illegible]	[illegible]	[illegible]	[illegible]	[illegible]
EJF4458718.1	Type1.BREX	[illegible]	[illegible]	[illegible]	[illegible]	[illegible]	[illegible]	[illegible]	[illegible]	[illegible]
HCD5120999.1	Type1.BREX	[illegible]	[illegible]	[illegible]	[illegible]	[illegible]	[illegible]	[illegible]	[illegible]	[illegible]
HCE2679243.1	Type1.BREX	[illegible]	[illegible]	[illegible]	[illegible]	[illegible]	[illegible]	[illegible]	[illegible]	[illegible]
HCG5108676.1	Type1.BREX	[illegible]	[illegible]	[illegible]	[illegible]	[illegible]	[illegible]	[illegible]	[illegible]	[illegible]
HCG6132052.1	Type1.BREX	[illegible]	[illegible]	[illegible]	[illegible]	[illegible]	[illegible]	[illegible]	[illegible]	[illegible]
HCG7048824.1	Type1.BREX	[illegible]	[illegible]	[illegible]	[illegible]	[illegible]	[illegible]	[illegible]	[illegible]	[illegible]
HCG7769728.1	Type1.BREX	[illegible]	[illegible]	[illegible]	[illegible]	[illegible]	[illegible]	[illegible]	[illegible]	[illegible]
HCG8025121.1	Type1.BREX	[illegible]	[illegible]	[illegible]	[illegible]	[illegible]	[illegible]	[illegible]	[illegible]	[illegible]
HCG8269540.1	Type1.BREX	[illegible]	[illegible]	[illegible]	[illegible]	[illegible]	[illegible]	[illegible]	[illegible]	[illegible]
HCH5919840.1	Type1.BREX	[illegible]	[illegible]	[illegible]	[illegible]	[illegible]	[illegible]	[illegible]	[illegible]	[illegible]
EJL6432478.1	Type1.BREX	[illegible]	[illegible]	[illegible]	[illegible]	[illegible]	[illegible]	[illegible]	[illegible]	[illegible]
EJO3860683.1	Type1.BREX	[illegible]	[illegible]	[illegible]	[illegible]	[illegible]	[illegible]	[illegible]	[illegible]	[illegible]
HCJ6872077.1	Type1.BREX	[illegible]	[illegible]	[illegible]	[illegible]	[illegible]	[illegible]	[illegible]	[illegible]	[illegible]
HCJ7299597.1	Type1.BREX	[illegible]	[illegible]	[illegible]	[illegible]	[illegible]	[illegible]	[illegible]	[illegible]	[illegible]
EJU9535832.1	Type1.BREX	[illegible]	[illegible]	[illegible]	[illegible]	[illegible]	[illegible]	[illegible]	[illegible]	[illegible]
WP_114867813.1	Type1.BREX	[illegible]	[illegible]	[illegible]	[illegible]	[illegible]	[illegible]	[illegible]	[illegible]	[illegible]
WP_178326691.1	Type1.BREX	[illegible]	[illegible]	[illegible]	[illegible]	[illegible]	[illegible]	[illegible]	[illegible]	[illegible]
WP_005376166.1	Type1.BREX	[illegible]	[illegible]	[illegible]	[illegible]	[illegible]	[illegible]	[illegible]	[illegible]	[illegible]
WP_000934396.1	Type1.BREX	[illegible]	[illegible]	[illegible]	[illegible]	[illegible]	[illegible]	[illegible]	[illegible]	[illegible]
WP_016790631.1	Type1.BREX	[illegible]	[illegible]	[illegible]	[illegible]	[illegible]	[illegible]	[illegible]	[illegible]	[illegible]
WP_005444576.1	Type1.BREX	[illegible]	[illegible]	[illegible]	[illegible]	[illegible]	[illegible]	[illegible]	[illegible]	[illegible]
WP_001963844.1	Type1.BREX	[illegible]	[illegible]	[illegible]	[illegible]	[illegible]	[illegible]	[illegible]	[illegible]	[illegible]
WP_023585137.1	Type1.BREX	[illegible]	[illegible]	[illegible]	[illegible]	[illegible]	[illegible]	[illegible]	[illegible]	[illegible]
WP_025605138.1	Type1.BREX	[illegible]	[illegible]	[illegible]	[illegible]	[illegible]	[illegible]	[illegible]	[illegible]	[illegible]
WP_025525716.1	Type1.BREX	[illegible]	[illegible]	[illegible]	[illegible]	[illegible]	[illegible]	[illegible]	[illegible]	[illegible]
WP_029810016.1	Type1.BREX	[illegible]	[illegible]	[illegible]	[illegible]	[illegible]	[illegible]	[illegible]	[illegible]	[illegible]
WP_029830481.1	Type1.BREX	[illegible]	[illegible]	[illegible]	[illegible]	[illegible]	[illegible]	[illegible]	[illegible]	[illegible]
WP_029805255.1	Type1.BREX	[illegible]	[illegible]	[illegible]	[illegible]	[illegible]	[illegible]	[illegible]	[illegible]	[illegible]
WP_024701189.1	Type1.BREX	[illegible]	[illegible]	[illegible]	[illegible]	[illegible]	[illegible]	[illegible]	[illegible]	[illegible]
WP_025796292.1	Type1.BREX	[illegible]	[illegible]	[illegible]	[illegible]	[illegible]	[illegible]	[illegible]	[illegible]	[illegible]
WP_032482144.1	Type1.BREX	[illegible]	[illegible]	[illegible]	[illegible]	[illegible]	[illegible]	[illegible]	[illegible]	[illegible]
WP_033931157.1	Type1.BREX	[illegible]	[illegible]	[illegible]	[illegible]	[illegible]	[illegible]	[illegible]	[illegible]	[illegible]
WP_045378182.1	Type1.BREX	[illegible]	[illegible]	[illegible]	[illegible]	[illegible]	[illegible]	[illegible]	[illegible]	[illegible]
WP_047110486.1	Type1.BREX	[illegible]	[illegible]	[illegible]	[illegible]	[illegible]	[illegible]	[illegible]	[illegible]	[illegible]
WP_050646850.1	Type1.BREX	[illegible]	[illegible]	[illegible]	[illegible]	[illegible]	[illegible]	[illegible]	[illegible]	[illegible]
WP_061065627.1	Type1.BREX	[illegible]	[illegible]	[illegible]	[illegible]	[illegible]	[illegible]	[illegible]	[illegible]	[illegible]
WP_065090484.1	Type1.BREX	[illegible]	[illegible]	[illegible]	[illegible]	[illegible]	[illegible]	[illegible]	[illegible]	[illegible]
WP_069530140.1	Type1.BREX	[illegible]	[illegible]	[illegible]	[illegible]	[illegible]	[illegible]	[illegible]	[illegible]	[illegible]
WP_074189993.1	Type1.BREX	[illegible]	[illegible]	[illegible]	[illegible]	[illegible]	[illegible]	[illegible]	[illegible]	[illegible]
WP_080540649.1	Type1.BREX	[illegible]	[illegible]	[illegible]	[illegible]	[illegible]	[illegible]	[illegible]	[illegible]	[illegible]
WP_088731321.1	Type1.BREX	[illegible]	[illegible]	[illegible]	[illegible]	[illegible]	[illegible]	[illegible]	[illegible]	[illegible]
WP_095475146.1	Type1.BREX	[illegible]	[illegible]	[illegible]	[illegible]	[illegible]	[illegible]	[illegible]	[illegible]	[illegible]
WP_095470311.1	Type1.BREX	[illegible]	[illegible]	[illegible]	[illegible]	[illegible]	[illegible]	[illegible]	[illegible]	[illegible]
WP_096070868.1	Type1.BREX	[illegible]	[illegible]	[illegible]	[illegible]	[illegible]	[illegible]	[illegible]	[illegible]	[illegible]
WP_100267429.1	Type1.BREX	[illegible]	[illegible]	[illegible]	[illegible]	[illegible]	[illegible]	[illegible]	[illegible]	[illegible]
WP_100754281.1	Type1.BREX	[illegible]	[illegible]	[illegible]	[illegible]	[illegible]	[illegible]	[illegible]	[illegible]	[illegible]
WP_102465509.1	Type1.BREX	[illegible]	[illegible]	[illegible]	[illegible]	[illegible]	[illegible]	[illegible]	[illegible]	[illegible]
WP_102402203.1	Type1.BREX	[illegible]	[illegible]	[illegible]	[illegible]	[illegible]	[illegible]	[illegible]	[illegible]	[illegible]
WP_102420196.1	Type1.BREX	[illegible]	[illegible]	[illegible]	[illegible]	[illegible]	[illegible]	[illegible]	[illegible]	[illegible]
WP_102376359.1	Type1.BREX	[illegible]	[illegible]	[illegible]	[illegible]	[illegible]	[illegible]	[illegible]	[illegible]	[illegible]
WP_102582639.1	Type1.BREX	[illegible]	[illegible]	[illegible]	[illegible]	[illegible]	[illegible]	[illegible]	[illegible]	[illegible]
WP_102573392.1	Type1.BREX	[illegible]	[illegible]	[illegible]	[illegible]	[illegible]	[illegible]	[illegible]	[illegible]	[illegible]
WP_102456769.1	Type1.BREX	[illegible]	[illegible]	[illegible]	[illegible]	[illegible]	[illegible]	[illegible]	[illegible]	[illegible]
WP_102887270.1	Type1.BREX	[illegible]	[illegible]	[illegible]	[illegible]	[illegible]	[illegible]	[illegible]	[illegible]	[illegible]
WP_104040811.1	Type1.BREX	[illegible]	[illegible]	[illegible]	[illegible]	[illegible]	[illegible]	[illegible]	[illegible]	[illegible]
WP_040122842.1	Type1.BREX	[illegible]	[illegible]	[illegible]	[illegible]	[illegible]	[illegible]	[illegible]	[illegible]	[illegible]
WP_108187618.1	Type1.BREX	[illegible]	[illegible]	[illegible]	[illegible]	[illegible]	[illegible]	[illegible]	[illegible]	[illegible]

Protein-ID	System	Gene-Neighbourhoods	Length	Taxonomy	Assembly-ID	Strain	Locus-Tag	Genomic-coordinates	TaxID	Fasta-defline
WP_113603075.1	Type1.BREX	<-VirD4-TraG+VirB4-FtsK<-HDRnase<-SbcC<- SIGMA+OB-Fold+AAA-ATPase+Lon-Protease-BrxL <- PglZ <- N6-METHYLASE <- ParB + HNH <- DUF6079 *<- BrxB-BrxF-iSTAND <- BrxA-DNA-binding <- wHTH+WYL+WCX <-?<-35exo<-?||LexA-protease->?-><-MobI	1225	Bacteria>Pseudomonadota>Gammaproteobacteria>Vibrionales>Vibrionaceae>Vibrio	GCF_003311885.1	Vibrio_tarriae	DLR65_RS02205	60103..63780	2014742	BREX-system-P-loop-protein-BrxC-[Vibrio-tarriae]
WP_122015998.1	Type1.BREX	CR-ATPase4->?-><- SIGMA+OB-Fold+AAA-ATPase+Lon-Protease-BrxL <- PglZ <-?<-?<- ParB + HNH <- N6-METHYLASE <- DUF6079 *<- BrxB-BrxF-iSTAND <- BrxA-DNA-binding <- wHTH+WYL+WCX ||SF1-SF2-Helicase->MarR-HTH->HKD-DNase+HKD-DNase->CARF+NtrC-AAA->PIN+OST-HTH->	1248	Bacteria>Pseudomonadota>Gammaproteobacteria>Vibrionales>Vibrionaceae>Vibrio	GCF_003691525.1	Vibrio_parahaemolyticus	D0871_RS08265	1554661..1558407	670	BREX-system-P-loop-protein-BrxC-[Vibrio-parahaemolyticus]
WP_130200308.1	Type1.BREX	AbiEi->viral-protein->SF1-SF2-Helicase+REase->SF1-SF2-Helicase+REase-><-?<-ZetaToxin<- PglZ <- ParB + HNH <- N6-METHYLASE <- DUF6079 *<- BrxB-BrxF-iSTAND <- BrxA-DNA-binding <- wHTH+WYL+WCX <-DDE_Tnp_1_5+DDE_Tnp_1||<-?<-CR-ATPase4||SbcC->	1157	Bacteria>Pseudomonadota>Gammaproteobacteria>Vibrionales>Vibrionaceae>Vibrio	GCF_004214625.1	Vibrio_vulnificus	D8T51_RS18945	13637..17110	672	BREX-system-P-loop-protein-BrxC-[Vibrio-vulnificus]
WP_137027181.1	Type1.BREX	SF1-SF2-Helicase->AbiEi->viral-protein-> wHTH+WYL+WCX -> BrxA-DNA-binding -> BrxB-BrxF-iSTAND -> DUF6079 *-> N6-METHYLASE -> ParB + HNH ->GrpE->?-> PglZ -> SIGMA+OB-Fold+AAA-ATPase+Lon-Protease-BrxL -><-wHTH||DUF4113->	1225	Bacteria>Pseudomonadota>Gammaproteobacteria>Vibrionales>Vibrionaceae>Vibrio	GCF_005145785.1	Vibrio_kanaloae	FCV44_20755	6017..9694	170673	BREX-system-P-loop-protein-BrxC-[Vibrio-kanaloae]
WP_140128967.1	Type1.BREX	AbiEi->viral-protein-> wHTH+WYL+WCX -> BrxA-DNA-binding -> BrxB-BrxF-iSTAND -> DUF6079 *-> N6-METHYLASE -> ParB + HNH ->GrpE->?-> PglZ -> SIGMA+OB-Fold+AAA-ATPase+Lon-Protease-BrxL -><-wHTH<-?<-CR-ATPase4	1225	Bacteria>Pseudomonadota>Gammaproteobacteria>Vibrionales>Vibrionaceae>Vibrio	GCF_006368845.1	Vibrio_parahaemolyticus	CGJ25_RS23760	3581..7258	670	BREX-system-P-loop-protein-BrxC-[Vibrio-parahaemolyticus]
WP_140247448.1	Type1.BREX	AbiEi->viral-protein->SF1-SF2-Helicase+REase->SF1-SF2-Helicase+REase-><-?<-ZetaToxin<- PglZ <- ParB + HNH <- N6-METHYLASE <- DUF6079 *<- BrxB-BrxF-iSTAND <- BrxA-DNA-binding <- wHTH+WYL+WCX ||<-?<-CR-ATPase4||PIN+OST-HTH-><-REase+SPF-cons	1157	Bacteria>Pseudomonadota>Gammaproteobacteria>Vibrionales>Vibrionaceae>Vibrio	GCF_006371495.1	Vibrio_parahaemolyticus	CGJ41_RS23680	13693..17166	670	BREX-system-P-loop-protein-BrxC-[Vibrio-parahaemolyticus]
WP_140344322.1	Type1.BREX	CR-ATPase4->?->|| wHTH+WYL+WCX -> BrxA-DNA-binding -> BrxB-BrxF-iSTAND -> DUF6079 *-> N6-METHYLASE -> ParB + HNH -> PglZ ->SF1-SF2-Helicase->	1157	Bacteria>Pseudomonadota>Gammaproteobacteria>Vibrionales>Vibrionaceae>Vibrio	GCF_006372045.1	Vibrio_parahaemolyticus	CGJ19_RS21455	3824..7297	670	BREX-system-P-loop-protein-BrxC-[Vibrio-parahaemolyticus]
WP_140256559.1	Type1.BREX	MobI-><-?<-LexA-protease||35exo->?-> wHTH+WYL+WCX -> BrxA-DNA-binding -> BrxB-BrxF-iSTAND -> DUF6079 *-> ParB + HNH -> N6-METHYLASE -> PglZ -> SIGMA+OB-Fold+AAA-ATPase+Lon-Protease-BrxL ->SbcC->HDRnase->VirD4-TraG+VirB4-FtsK->	1225	Bacteria>Pseudomonadota>Gammaproteobacteria>Vibrionales>Vibrionaceae>Vibrio	GCF_006372155.1	Vibrio_parahaemolyticus	D9A28_03775	149461..153138	670	BREX-system-P-loop-protein-BrxC-[Vibrio-parahaemolyticus]
WP_140272583.1	Type1.BREX	CR-ATPase4->?-><- SIGMA+OB-Fold+AAA-ATPase+Lon-Protease-BrxL <- PglZ <-?<-?<- ParB + HNH <- N6-METHYLASE <- DUF6079 *<- BrxB-BrxF-iSTAND <- BrxA-DNA-binding <- wHTH+WYL+WCX ||SF1-SF2-Helicase->MarR-HTH->HKD-DNase+HKD-DNase->CARF+NtrC-AAA->PIN+OST-HTH->	1248	Bacteria>Pseudomonadota>Gammaproteobacteria>Vibrionales>Vibrionaceae>Vibrio	GCF_006372715.1	Vibrio_parahaemolyticus	CGI87_RS22525	16069..19815	670	BREX-system-P-loop-protein-BrxC-[Vibrio-parahaemolyticus]
WP_148513618.1	Type1.BREX	AbiEi->viral-protein->SF1-SF2-Helicase+REase->SF1-SF2-Helicase+REase-><-TPR<-HEPN+SF1-SF2-Helicase+RNase+RNase<- PglZ <- ParB + HNH <- N6-METHYLASE <- DUF6079 *<- BrxB-BrxF-iSTAND <- BrxA-DNA-binding <- wHTH+WYL+WCX ||DDE_Tnp_1_5+DDE_Tnp_1->	1157	Bacteria>Pseudomonadota>Gammaproteobacteria>Vibrionales>Vibrionaceae>Vibrio	GCF_008083315.1	Vibrio_cholerae	FXF10_RS00340	29414..32887	666	BREX-system-P-loop-protein-BrxC-[Vibrio-cholerae]
WP_148532770.1	Type1.BREX	<-VirD4-TraG+VirB4-FtsK<-HDRnase<-SbcC<- SIGMA+OB-Fold+AAA-ATPase+Lon-Protease-BrxL <- PglZ <- N6-METHYLASE <- ParB + HNH <- DUF6079 *<- BrxB-BrxF-iSTAND <- BrxA-DNA-binding <- wHTH+WYL+WCX <-?<-35exo<-?||LexA-protease->	1225	Bacteria>Pseudomonadota>Gammaproteobacteria>Vibrionales>Vibrionaceae>Vibrio	GCF_008084025.1	Vibrio_cholerae	FXE74_RS08220	132887..136564	666	BREX-system-P-loop-protein-BrxC-[Vibrio-cholerae]
WP_148490856.1	Type1.BREX	<-DDE_Tnp_1_5+DDE_Tnp_1||HTH_Tnp_1+HTH_21+rve+rve_3-><-SbcC||CR-ATPase4->?-><-DUF4113|| wHTH+WYL+WCX -> BrxA-DNA-binding -> BrxB-BrxF-iSTAND -> DUF6079 *-> N6-METHYLASE -> ParB + HNH -> PglZ ->HEPN+SF1-SF2-Helicase+RNase+RNase->TPR-><-SF1-SF2-Helicase+REase<-SF1-SF2-Helicase+REase<-viral-protein<-AbiEi	1157	Bacteria>Pseudomonadota>Gammaproteobacteria>Vibrionales>Vibrionaceae>Vibrio	GCF_008084645.1	Vibrio_cholerae	FXE54_RS16715	26974..30447	666	BREX-system-P-loop-protein-BrxC-[Vibrio-cholerae]
WP_148536810.1	Type1.BREX	<-CARF+NtrC-AAA+FIS-HTH<- SIGMA+OB-Fold+AAA-ATPase+Lon-Protease-BrxL <- PglZ <- N6-METHYLASE <- ParB + HNH <- DUF6079 *<- BrxB-BrxF-iSTAND <- BrxA-DNA-binding <- wHTH+WYL+WCX <-?<-35exo||LexA-protease->?-><-MobI	1225	Bacteria>Pseudomonadota>Gammaproteobacteria>Vibrionales>Vibrionaceae>Vibrio	GCF_008085615.1	Vibrio_cholerae	FXE25_RS13680	35779..39456	666	BREX-system-P-loop-protein-BrxC-[Vibrio-cholerae]
WP_123161578.1	Type1.BREX	MobI-><-?<-LexA-protease||35exo->?-> wHTH+WYL+WCX -> BrxA-DNA-binding -> BrxB-BrxF-iSTAND -> DUF6079 *-> ParB + HNH -> N6-METHYLASE -> PglZ -> SIGMA+OB-Fold+AAA-ATPase+Lon-Protease-BrxL ->SbcC->HDRnase->VirD4-TraG+VirB4-FtsK->	1225	Bacteria>Pseudomonadota>Gammaproteobacteria>Vibrionales>Vibrionaceae>Vibrio	GCF_009763665.1	Vibrio_cholerae	GPY01_RS14950	2265324..2269001	666	BREX-system-P-loop-protein-BrxC-[Vibrio-cholerae]
WP_172566988.1	Type1.BREX	CR-ATPase4->?-><-?<-LexA-protease|| wHTH+WYL+WCX -> BrxA-DNA-binding -> BrxB-BrxF-iSTAND -> DUF6079 *-> N6-METHYLASE -> ParB + HNH -> PglZ ->ZetaToxin->?-><-SF1-SF2-Helicase+REase<-SF1-SF2-Helicase+REase<-viral-protein<-AbiEi	1157	Bacteria>Pseudomonadota>Gammaproteobacteria>Vibrionales>Vibrionaceae>Vibrio	GCF_012274945.1	Vibrio_fluvialis	HF303_RS09150	434217..437690	676	BREX-system-P-loop-protein-BrxC-[Vibrio-fluvialis]
WP_169592914.1	Type1.BREX	MobI-><-Glyoxalase_8<-?<-LexA-protease||35exo->?-> wHTH+WYL+WCX -> BrxA-DNA-binding -> BrxB-BrxF-iSTAND -> DUF6079 *-> ParB + HNH -> N6-METHYLASE -> PglZ -> SIGMA+OB-Fold+AAA-ATPase+Lon-Protease-BrxL ->SbcC->HDRnase->VirD4-TraG+VirB4-FtsK->	1225	Bacteria>Pseudomonadota>Gammaproteobacteria>Vibrionales>Vibrionaceae>Vibrio	GCF_012956215.1	Vibrio_parahaemolyticus	HKB29_RS26535	221026..224703	670	BREX-system-P-loop-protein-BrxC-[Vibrio-parahaemolyticus]
WP_171375390.1	Type1.BREX	<-ZnR<-?||?->SF1-SF2-Helicase->AbiEi->viral-protein-> wHTH+WYL+WCX -> BrxA-DNA-binding -> BrxB-BrxF-iSTAND -> DUF6079 *-> N6-METHYLASE -> ParB + HNH ->GrpE->?-> PglZ -> SIGMA+OB-Fold+AAA-ATPase+Lon-Protease-BrxL -><-TPR<-HEPN+SF1-SF2-Helicase+RNase+RNase||LexA-protease->	1225	Bacteria>Pseudomonadota>Gammaproteobacteria>Vibrionales>Vibrionaceae>Vibrio	GCF_013114495.1	Vibrio_aestuarianus	F0230_RS00325	78960..82637	28171	BREX-system-P-loop-protein-BrxC-[Vibrio-aestuarianus]
WP_182291541.1	Type1.BREX	REase+SPF-cons-><-PIN+OST-HTH||CR-ATPase4->?->|| wHTH+WYL+WCX -> BrxA-DNA-binding -> BrxB-BrxF-iSTAND -> DUF6079 *-> N6-METHYLASE -> ParB + HNH -> PglZ ->ZetaToxin->?-><-SF1-SF2-Helicase+REase<-SF1-SF2-Helicase+REase<-viral-protein<-AbiEi	1157	Bacteria>Pseudomonadota>Gammaproteobacteria>Vibrionales>Vibrionaceae>Vibrio	GCF_014107515.1	Vibrio_vulnificus	F6X00_RS18205	373031..376504	672	BREX-system-P-loop-protein-BrxC-[Vibrio-vulnificus]
WP_238930029.1	Type1.BREX	HTH_20-><-SbcC||CR-ATPase4->?->|| wHTH+WYL+WCX -> BrxA-DNA-binding -> BrxB-BrxF-iSTAND -> DUF6079 -p*-> DUF6079 -p-> N6-METHYLASE -> ParB + HNH -> PglZ ->ZetaToxin->?-><-SF1-SF2-Helicase+REase<-SF1-SF2-Helicase+REase<-Tnp_DNA_bind+DDE_Tnp_1+Dimer_Tnp_Tn5	779	Bacteria>Pseudomonadota>Gammaproteobacteria>Vibrionales>Vibrionaceae>Vibrio	GCF_014525155.1	Vibrio_vulnificus	GD186_RS04075	575963..578302	672	BREX-system-P-loop-protein-BrxC-[Vibrio-vulnificus]
WP_238930031.1	Type1.BREX	HTH_20-><-SbcC||CR-ATPase4->?->|| wHTH+WYL+WCX -> BrxA-DNA-binding -> BrxB-BrxF-iSTAND -> DUF6079 -p-> DUF6079 -p*-> N6-METHYLASE -> ParB + HNH -> PglZ ->ZetaToxin->?-><-SF1-SF2-Helicase+REase<-Tnp_DNA_bind+DDE_Tnp_1+Dimer_Tnp_Tn5<-AbiEi	366	Bacteria>Pseudomonadota>Gammaproteobacteria>Vibrionales>Vibrionaceae>Vibrio	GCF_014525155.1	Vibrio_vulnificus	GD186_RS25670	578332..579432	672	hypothetical-protein-[Vibrio-vulnificus]
WP_191116552.1	Type1.BREX	CR-ATPase4->?-><- SIGMA+OB-Fold+AAA-ATPase+Lon-Protease-BrxL <- PglZ <-?<-?<- ParB + HNH <- N6-METHYLASE <- DUF6079 *<- BrxB-BrxF-iSTAND <- BrxA-DNA-binding <- wHTH+WYL+WCX ||SF1-SF2-Helicase->MarR-HTH->HKD-DNase+HKD-DNase->CARF+NtrC-AAA->PIN+OST-HTH-><-AAA-ATPase	1248	Bacteria>Pseudomonadota>Gammaproteobacteria>Vibrionales>Vibrionaceae>Vibrio	GCF_014769095.1	Vibrio_parahaemolyticus	EUV06_RS14935	117914..121660	670	BREX-system-P-loop-protein-BrxC-[Vibrio-parahaemolyticus]
WP_054579585.1	Type1.BREX	AbiEi->viral-protein->SF1-SF2-Helicase+REase->SF1-SF2-Helicase+REase-><-?<-ZetaToxin<- PglZ <- ParB + HNH <- N6-METHYLASE <- DUF6079 *<- BrxB-BrxF-iSTAND <- BrxA-DNA-binding <- wHTH+WYL+WCX ||<-?<-CR-ATPase4||HEPN-><-sGTP	1157	Bacteria>Pseudomonadota>Gammaproteobacteria>Vibrionales>Vibrionaceae>Vibrio	GCF_014769135.1	Vibrio_alginolyticus	AOR09_RS18625	85180..88653	663	BREX-system-P-loop-protein-BrxC-[Vibrio-alginolyticus]
WP_206630456.1	Type1.BREX	GTPase-AIG->GTPase-AIG-><-HEPN||?->?-><- SIGMA+OB-Fold+AAA-ATPase+Lon-Protease-BrxL <- PglZ <- ParB + HNH <- N6-METHYLASE <- DUF6079 *<- BrxB-BrxF-iSTAND <- BrxA-DNA-binding <- wHTH+WYL+WCX <-viral-protein||?->ZnR-><-SF1-SF2-Helicase<-Couple_hipA+HipA_C<-HTH_3	1232	Bacteria>Pseudomonadota>Gammaproteobacteria>Vibrionales>Vibrionaceae>Vibrio	GCF_017291155.1	Vibrio_vulnificus	JOJ25_RS14690	90356..94054	672	BREX-system-P-loop-protein-BrxC-[Vibrio-vulnificus]
WP_206946493.1	Type1.BREX	sGTP-><-HEPN<-?||CR-ATPase4->?->|| wHTH+WYL+WCX -> BrxA-DNA-binding -> BrxB-BrxF-iSTAND -> DUF6079 *-> N6-METHYLASE -> ParB + HNH -> PglZ ->ZetaToxin->?-><-SF1-SF2-Helicase+REase<-SF1-SF2-Helicase+REase<-viral-protein<-AbiEi	1157	Bacteria>Pseudomonadota>Gammaproteobacteria>Vibrionales>Vibrionaceae>Vibrio	GCF_017313505.1	Vibrio_sp._Vb2424	J0679_RS01025	227908..231381	2816074	BREX-system-P-loop-protein-BrxC-[Vibrio-sp.-Vb2424]
WP_210942219.1	Type1.BREX	<- wHTH+WYL+WCX ||SF1-SF2-Helicase+REase->?->XIS-HTH-> wHTH+WYL+WCX -> BrxA-DNA-binding -> BrxB-BrxF-iSTAND -> DUF6079 *-> N6-METHYLASE -> ParB + HNH -> PglZ ->ZetaToxin->?-><-SF1-SF2-Helicase+REase<-SF1-SF2-Helicase+REase<-Arm-DNA-bind_3+Phage_integrase	1157	Bacteria>Pseudomonadota>Gammaproteobacteria>Vibrionales>Vibrionaceae>Vibrio	GCF_018035165.1	Vibrio_paracholerae	GSG51_RS14590	9402..12875	650003	BREX-system-P-loop-protein-BrxC-[Vibrio-paracholerae]
WP_213968024.1	Type1.BREX	<-PIN+OST-HTH<-CARF+NtrC-AAA<-HKD-DNase+HKD-DNase<-MarR-HTH<-SF1-SF2-Helicase|| wHTH+WYL+WCX -> BrxA-DNA-binding -> BrxB-BrxF-iSTAND -> DUF6079 *-> N6-METHYLASE -> ParB + HNH ->ZnR+3C-ABC+TPR->?->?-> PglZ -> SIGMA+OB-Fold+AAA-ATPase+Lon-Protease-BrxL -><-?<-CR-ATPase4	1248	Bacteria>Pseudomonadota>Gammaproteobacteria>Vibrionales>Vibrionaceae>Vibrio	GCF_018458215.1	Vibrio_alginolyticus	J4H41_RS21340	12665..16411	663	BREX-system-P-loop-protein-BrxC-[Vibrio-alginolyticus]
WP_213970810.1	Type1.BREX	AbiEi->viral-protein->SF1-SF2-Helicase+REase->SF1-SF2-Helicase+REase-><-?<-ZetaToxin<- PglZ <- ParB + HNH <- N6-METHYLASE <- DUF6079 *<- BrxB-BrxF-iSTAND <- BrxA-DNA-binding <- wHTH+WYL+WCX ||DUF4113-><-?<-CR-ATPase4	1157	Bacteria>Pseudomonadota>Gammaproteobacteria>Vibrionales>Vibrionaceae>Vibrio	GCF_018458405.1	Vibrio_alginolyticus	J4H58_RS14535	108443..111916	663	BREX-system-P-loop-protein-BrxC-[Vibrio-alginolyticus]
WP_213899843.1	Type1.BREX	AbiEi->viral-protein->SF1-SF2-Helicase+REase->SF1-SF2-Helicase+REase-><-TPR<-HEPN+SF1-SF2-Helicase+RNase+RNase<- PglZ <- ParB + HNH <- N6-METHYLASE <- DUF6079 *<- BrxB-BrxF-iSTAND <- BrxA-DNA-binding <- wHTH+WYL+WCX ||<-?<-CR-ATPase4||SbcC->HEPN-><-HTH_20	1157	Bacteria>Pseudomonadota>Gammaproteobacteria>Vibrionales>Vibrionaceae>Vibrio	GCF_018458525.1	Vibrio_alginolyticus	J4H25_RS19085	22843..26316	663	BREX-system-P-loop-protein-BrxC-[Vibrio-alginolyticus]
WP_213874127.1	Type1.BREX	sGTP-><-HEPN<-?||CR-ATPase4->?->|| wHTH+WYL+WCX -> BrxA-DNA-binding -> BrxB-BrxF-iSTAND -> DUF6079 *-> N6-METHYLASE -> ParB + HNH -> PglZ ->ZetaToxin->?-><-SF1-SF2-Helicase+REase<-SF1-SF2-Helicase+REase<-viral-protein<-AbiEi	1157	Bacteria>Pseudomonadota>Gammaproteobacteria>Vibrionales>Vibrionaceae>Vibrio	GCF_018458545.1	Vibrio_alginolyticus	J4H28_RS04435	1018248..1021721	663	BREX-system-P-loop-protein-BrxC-[Vibrio-alginolyticus]
WP_217555808.1	Type1.BREX	AbiEi->viral-protein->SF1-SF2-Helicase+REase->SF1-SF2-Helicase+REase-><-?<-ZetaToxin<- PglZ <- ParB + HNH <- N6-METHYLASE <- DUF6079 *<- BrxB-BrxF-iSTAND <- BrxA-DNA-binding <- wHTH+WYL+WCX ||LexA-protease->	1157	Bacteria>Pseudomonadota>Gammaproteobacteria>Vibrionales>Vibrionaceae>Vibrio	GCF_019091765.1	Vibrio_metschnikovii	KVT01_RS15810	26769..30242	28172	BREX-system-P-loop-protein-BrxC-[Vibrio-metschnikovii]
WP_222133201.1	Type1.BREX	<-PIN+OST-HTH<-CARF+NtrC-AAA<-HKD-DNase+HKD-DNase<-MarR-HTH<-SF1-SF2-Helicase|| wHTH+WYL+WCX -> BrxA-DNA-binding -> BrxB-BrxF-iSTAND -> DUF6079 *-> N6-METHYLASE -> ParB + HNH ->?->?-> PglZ -> SIGMA+OB-Fold+AAA-ATPase+Lon-Protease-BrxL ->	1248	Bacteria>Pseudomonadota>Gammaproteobacteria>Vibrionales>Vibrionaceae>Vibrio	GCF_019793245.1	Vibrio_alginolyticus	K6L11_RS14305	27139..30885	663	BREX-system-P-loop-protein-BrxC-[Vibrio-alginolyticus]
WP_222134563.1	Type1.BREX	<-VirD4-TraG+VirB4-FtsK<-HDRnase<-NACHT+HEAT+REase<- SIGMA+OB-Fold+AAA-ATPase+Lon-Protease-BrxL <- PglZ <- N6-METHYLASE <- ParB + HNH <- DUF6079 *<- BrxB-BrxF-iSTAND <- BrxA-DNA-binding <- wHTH+WYL+WCX <-?<-35exo||LexA-protease->?-><-MobI	1225	Bacteria>Pseudomonadota>Gammaproteobacteria>Vibrionales>Vibrionaceae>Vibrio	GCF_019793275.1	Vibrio_cholerae	NB536_RS17220	131235..134912	666	BREX-system-P-loop-protein-BrxC-[Vibrio-cholerae]
WP_224640811.1	Type1.BREX	MobI-><-?<-LexA-protease||?->35exo->?-> wHTH+WYL+WCX -> BrxA-DNA-binding -> BrxB-BrxF-iSTAND -> DUF6079 *-> ParB + HNH -> N6-METHYLASE -> PglZ -> SIGMA+OB-Fold+AAA-ATPase+Lon-Protease-BrxL ->NACHT+HEAT+REase->HDRnase->VirD4-TraG+VirB4-FtsK->	1225	Bacteria>Pseudomonadota>Gammaproteobacteria>Vibrionales>Vibrionaceae>Vibrio	GCF_020169625.1	Vibrio_vulnificus	KUO11_RS09480	205814..209491	672	BREX-system-P-loop-protein-BrxC-[Vibrio-vulnificus]
WP_225482458.1	Type1.BREX	<- SIGMA+OB-Fold+AAA-ATPase+Lon-Protease-BrxL <- PglZ ||RADICAL-SAM->?-><-?<-?<- ParB + HNH <- N6-METHYLASE <-?<- DUF6079 *<- BrxB-BrxF-iSTAND <- BrxA-DNA-binding <- wHTH+WYL+WCX ||?->?-><-viral-protein||?-><-SF1-SF2-Helicase<-Couple_hipA+HipA_C	1231	Bacteria>Pseudomonadota>Gammaproteobacteria>Vibrionales>Vibrionaceae>Vibrio	GCF_020251885.1	Vibrio_vulnificus	JKP23_RS02685	250869..254564	672	BREX-system-P-loop-protein-BrxC-[Vibrio-vulnificus]
WP_225454267.1	Type1.BREX	TraJ-RHH->TraI-VirD2-RCRE->REase-><-AbiEi_4||?->|| wHTH+WYL+WCX -> BrxA-DNA-binding -> BrxB-BrxF-iSTAND -> DUF6079 *-> N6-METHYLASE -> ParB + HNH ->ANK+ANK+ANK->ZnR+3C-ABC+TPR->?->?-> PglZ -> SIGMA+OB-Fold+AAA-ATPase+Lon-Protease-BrxL ->	1248	Bacteria>Pseudomonadota>Gammaproteobacteria>Vibrionales>Vibrionaceae>Vibrio	GCF_020252125.1	Vibrio_vulnificus	JKP08_RS08925	222135..225881	672	BREX-system-P-loop-protein-BrxC-[Vibrio-vulnificus]
WP_228096077.1	Type1.BREX	<-HTH_Tnp_1||SF1-SF2-Helicase+REase->SF1-SF2-Helicase+REase-><-?<-ZetaToxin<- PglZ <- ParB + HNH <- N6-METHYLASE <- DUF6079 *<- BrxB-BrxF-iSTAND <- BrxA-DNA-binding <- wHTH+WYL+WCX ||DUF4113-><-?<-CR-ATPase4||SbcC->HEPN-><-HTH_20	1157	Bacteria>Pseudomonadota>Gammaproteobacteria>Vibrionales>Vibrionaceae>Vibrio	GCF_020741035.1	Vibrio_parahaemolyticus	IB294_RS01075	246074..249547	670	BREX-system-P-loop-protein-BrxC-[Vibrio-parahaemolyticus]
WP_236110294.1	Type1.BREX	<-GspA-ATPase||SF1-SF2-Helicase->?->HKD-DNase+HKD-DNase->CARF+NtrC-AAA-> wHTH+WYL+WCX -> BrxA-DNA-binding -> BrxB-BrxF-iSTAND -> DUF6079 *-> N6-METHYLASE -> ParB + HNH -> PglZ ->ZetaToxin->?-><-SF1-SF2-Helicase+REase<-SF1-SF2-Helicase+REase	1157	Bacteria>Pseudomonadota>Gammaproteobacteria>Vibrionales>Vibrionaceae>Vibrio	GCF_021568915.1	Vibrio_sp._McD22-P3	L2W36_RS13785	194928..198401	2724880	BREX-system-P-loop-protein-BrxC-[Vibrio-sp.-McD22-P3]
WP_238795743.1	Type1.BREX	MobI-><-?<-LexA-protease||35exo->?-> wHTH+WYL+WCX -> BrxA-DNA-binding -> BrxB-BrxF-iSTAND -> DUF6079 *-> ParB + HNH -> N6-METHYLASE -> PglZ -> SIGMA+OB-Fold+AAA-ATPase+Lon-Protease-BrxL ->CARF+NtrC-AAA+FIS-HTH->OBFOLD-NUCLEASE->	1225	Bacteria>Pseudomonadota>Gammaproteobacteria>Vibrionales>Vibrionaceae>Vibrio	GCF_022289115.1	Vibrio_furnissii	K6U56_RS11800	143910..147587	29494	BREX-system-P-loop-protein-BrxC-[Vibrio-furnissii]
WP_140273790.1	Type1.BREX	<-AAA-ATPase-p<-HEPN||CR-ATPase4->?->|| wHTH+WYL+WCX -> BrxA-DNA-binding -> BrxB-BrxF-iSTAND -> DUF6079 *-> N6-METHYLASE -> ParB + HNH -> PglZ ->ZetaToxin->?-><-SF1-SF2-Helicase+REase<-SF1-SF2-Helicase+REase<-viral-protein<-AbiEi	1157	Bacteria>Pseudomonadota>Gammaproteobacteria>Vibrionales>Vibrionaceae>Vibrio	GCF_022291015.1	Vibrio_parahaemolyticus	K6U15_RS27495	32395..35868	670	BREX-system-P-loop-protein-BrxC-[Vibrio-parahaemolyticus]
WP_238967171.1	Type1.BREX	sGTP-><-HEPN<-?||CR-ATPase4->?->|| wHTH+WYL+WCX -> BrxA-DNA-binding -> BrxB-BrxF-iSTAND -> DUF6079 *-> N6-METHYLASE -> ParB + HNH -> PglZ ->ZetaToxin->?-><-SF1-SF2-Helicase+REase<-SF1-SF2-Helicase+REase<-viral-protein<-AbiEi	1157	Bacteria>Pseudomonadota>Gammaproteobacteria>Vibrionales>Vibrionaceae>Vibrio	GCF_022343145.1	Vibrio_alginolyticus	K6750_RS21880	1365022..1368495	663	BREX-system-P-loop-protein-BrxC-[Vibrio-alginolyticus]
WP_243579240.1	Type1.BREX	<-BrxD<- SIGMA+OB-Fold+AAA-ATPase+Lon-Protease-BrxL <- PglZ <-?<-?<-ZnR+3C-ABC+TPR<-ANK+ANK+ANK<- ParB + HNH <- N6-METHYLASE <- DUF6079 *<- BrxB-BrxF-iSTAND <- BrxA-DNA-binding <- wHTH+WYL+WCX ||SF1-SF2-Helicase+HTH-><-?<-?||?-><-Calcineurin-SbcD<-REase	1247	Bacteria>Pseudomonadota>Gammaproteobacteria>Vibrionales>Vibrionaceae>Vibrio	GCF_022812045.1	Vibrio_splendidus	LTQ02_RS02805	621543..625286	29497	BREX-system-P-loop-protein-BrxC-[Vibrio-splendidus]
WP_253644856.1	Type1.BREX	CR-ATPase4->?-><- SIGMA+OB-Fold+AAA-ATPase+Lon-Protease-BrxL <- PglZ <-?<- ParB + HNH <- N6-METHYLASE <- DUF6079 *<- BrxB-BrxF-iSTAND <- BrxA-DNA-binding <- wHTH+WYL+WCX	1248	Bacteria>Pseudomonadota>Gammaproteobacteria>Vibrionales>Vibrionaceae>Vibrio	GCF_024181785.1	Vibrio_sp._Y42_MX_L11	NL643_RS16680	61256..65002	2957765	BREX-system-P-loop-protein-BrxC-[Vibrio-sp.-Y42_MX_L11]
WP_253658043.1	Type1.BREX	<- SIGMA+OB-Fold+AAA-ATPase+Lon-Protease-BrxL <- PglZ <-?<-?<-?<-?<-ZnR+3C-ABC+TPR<- ParB + HNH <- N6-METHYLASE <- DUF6079 *<- BrxB-BrxF-iSTAND <- BrxA-DNA-binding <- wHTH+WYL+WCX ||SF1-SF2-Helicase->MarR-HTH->HKD-DNase+HKD-DNase->CARF+NtrC-AAA->PIN+OST-HTH->MORC->	1248	Bacteria>Pseudomonadota>Gammaproteobacteria>Vibrionales>Vibrionaceae>Vibrio	GCF_024181795.1	unclassified_Vibrio	NL644_RS04175	947816..951562	2614977	MULTISPECIES:-BREX-system-P-loop-protein-BrxC-[unclassified-Vibrio]
WP_261872676.1	Type1.BREX	<- wHTH+WYL+WCX ||SF1-SF2-Helicase+REase->SF1-SF2-Helicase+REase-><-?<-ZetaToxin<- PglZ <- ParB + HNH <- N6-METHYLASE <- DUF6079 *<- BrxB-BrxF-iSTAND <- BrxA-DNA-binding <-tRNA||?-><-?<-TPR+TPR+TPR	1151	Bacteria>Pseudomonadota>Gammaproteobacteria>Vibrionales>Vibrionaceae>Vibrio	GCF_024347075.1	Vibrio_rarus	OCU56_RS07815	1705601..1709056	413403	BREX-system-P-loop-protein-BrxC-[Vibrio-rarus]
WP_256897643.1	Type1.BREX	<-TrkH<-REC+OmpR-HTH<-HISKIN<-?<-?<-?|| wHTH+WYL+WCX -> BrxA-DNA-binding -> BrxB-BrxF-iSTAND -> DUF6079 *-> N6-METHYLASE -> ParB + HNH ->?->?-> PglZ -> SIGMA+OB-Fold+AAA-ATPase+Lon-Protease-BrxL -><-?<-CR-ATPase4	1248	Bacteria>Pseudomonadota>Gammaproteobacteria>Vibrionales>Vibrionaceae>Vibrio	GCF_024530955.1	Vibrio_parahaemolyticus	H2C82_RS04830	209368..213114	670	BREX-system-P-loop-protein-BrxC-[Vibrio-parahaemolyticus]
WP_258495358.1	Type1.BREX	AbiEi->viral-protein->SF1-SF2-Helicase+REase->SF1-SF2-Helicase+REase-><-?<-ZetaToxin<- PglZ <- ParB + HNH <- N6-METHYLASE <- DUF6079 *<- BrxB-BrxF-iSTAND <- BrxA-DNA-binding <- wHTH+WYL+WCX ||<-?<-CR-ATPase4||HEPN-><-sGTP	1157	Bacteria>Pseudomonadota>Gammaproteobacteria>Vibrionales>Vibrionaceae>Vibrio	GCF_024744595.1	Vibrio_alginolyticus	NB477_RS23070	13774..17247	663	BREX-system-P-loop-protein-BrxC-[Vibrio-alginolyticus]
WP_248354663.1	Type1.BREX	AbiEi->viral-protein->SF1-SF2-Helicase+REase->SF1-SF2-Helicase+REase-><-TPR<-HEPN+SF1-SF2-Helicase+RNase+RNase<- PglZ <- ParB + HNH <- N6-METHYLASE <- DUF6079 *<- BrxB-BrxF-iSTAND <- BrxA-DNA-binding <- wHTH+WYL+WCX ||DDE_Tnp_1_5+DDE_Tnp_1->PIN+OST-HTH-><-?<-HTH_20	1157	Bacteria>Pseudomonadota>Gammaproteobacteria>Vibrionales>Vibrionaceae>Vibrio	GCF_024745175.1	Vibrio_parahaemolyticus	DDM99_15775	19819..23292	670	BREX-system-P-loop-protein-BrxC-[Vibrio-parahaemolyticus]
WP_258484886.1	Type1.BREX	MobI-><-?<-LexA-protease||35exo->?-> wHTH+WYL+WCX -> BrxA-DNA-binding -> BrxB-BrxF-iSTAND -> DUF6079 *-> ParB + HNH -> N6-METHYLASE -> PglZ -> SIGMA+OB-Fold+AAA-ATPase+Lon-Protease-BrxL ->CARF+NtrC-AAA+FIS-HTH->OBFOLD-NUCLEASE->	1225	Bacteria>Pseudomonadota>Gammaproteobacteria>Vibrionales>Vibrionaceae>Vibrio	GCF_024745305.1	Vibrio_parahaemolyticus	NB589_RS16770	223237..226914	670	BREX-system-P-loop-protein-BrxC-[Vibrio-parahaemolyticus]
WP_258517485.1	Type1.BREX	AbiEi->viral-protein-> wHTH+WYL+WCX -> BrxA-DNA-binding -> BrxB-BrxF-iSTAND -> DUF6079 *-> N6-METHYLASE -> ParB + HNH ->GrpE->?-> PglZ -> SIGMA+OB-Fold+AAA-ATPase+Lon-Protease-BrxL -><-wHTH	1225	Bacteria>Pseudomonadota>Gammaproteobacteria>Vibrionales>Vibrionaceae>Vibrio	GCF_024745395.1	Vibrio_parahaemolyticus	NB556_RS01245	3462..7139	670	BREX-system-P-loop-protein-BrxC-[Vibrio-parahaemolyticus]
WP_264912148.1	Type1.BREX	<- SIGMA+OB-Fold+AAA-ATPase+Lon-Protease-BrxL <- PglZ <-?<-?<-?<-?<-ZnR+3C-ABC+TPR<- ParB + HNH <- N6-METHYLASE <- DUF6079 *<- BrxB-BrxF-iSTAND <- BrxA-DNA-binding <- wHTH+WYL+WCX ||SF1-SF2-Helicase->MarR-HTH->HKD-DNase+HKD-DNase->CARF+NtrC-AAA->PIN+OST-HTH->MORC->	1248	Bacteria>Pseudomonadota>Gammaproteobacteria>Vibrionales>Vibrionaceae>Vibrio	GCF_026000495.1	Vibrio_sp._STUT-A16	OO778_RS24845	1838056..1841802	2976237	BREX-system-P-loop-protein-BrxC-[Vibrio-sp.-STUT-A16]
WP_267365291.1	Type1.BREX	<-ACET<-ZnR<-?|| wHTH+WYL+WCX -> BrxA-DNA-binding -> BrxB-BrxF-iSTAND -> DUF6079 *-> N6-METHYLASE -> ParB + HNH ->ANK+ANK+ANK->ZnR+3C-ABC+TPR->?->?->?->?-> PglZ ->	1248	Bacteria>Pseudomonadota>Gammaproteobacteria>Vibrionales>Vibrionaceae>Vibrio	GCF_026537625.1	Vibrio_parahaemolyticus	NOL37_RS23780	19146..22892	670	BREX-system-P-loop-protein-BrxC-[Vibrio-parahaemolyticus]
WP_269646266.1	Type1.BREX	MobI-><-?<-LexA-protease||35exo->?-> wHTH+WYL+WCX -> BrxA-DNA-binding -> BrxB-BrxF-iSTAND -> DUF6079 *-> ParB + HNH -> N6-METHYLASE -> PglZ -> SIGMA+OB-Fold+AAA-ATPase+Lon-Protease-BrxL ->SbcC->HDRnase->VirD4-TraG+VirB4-FtsK->	1225	Bacteria>Pseudomonadota>Gammaproteobacteria>Vibrionales>Vibrionaceae>Vibrio	GCF_027277845.1	Vibrio_parahaemolyticus	D4N70_RS12810	220363..224040	670	BREX-system-P-loop-protein-BrxC-[Vibrio-parahaemolyticus]
WP_269665659.1	Type1.BREX	MobI-><-?<-LexA-protease||35exo->?-> wHTH+WYL+WCX -> BrxA-DNA-binding -> BrxB-BrxF-iSTAND -> DUF6079 *-> ParB + HNH -> N6-METHYLASE -> PglZ -> SIGMA+OB-Fold+AAA-ATPase+Lon-Protease-BrxL ->NACHT+HEAT+REase->HDRnase->VirD4-TraG+VirB4-FtsK->	1225	Bacteria>Pseudomonadota>Gammaproteobacteria>Vibrionales>Vibrionaceae>Vibrio	GCF_027277905.1	Vibrio_parahaemolyticus	D4M94_RS09585	134179..137856	670	BREX-system-P-loop-protein-BrxC-[Vibrio-parahaemolyticus]
WP_274020835.1	Type1.BREX	<- PglZ <-?<-?<-?<-?<-ZnR+3C-ABC+TPR<- ParB + HNH <- N6-METHYLASE -p<- N6-METHYLASE <- DUF6079 *<- BrxB-BrxF-iSTAND <- BrxA-DNA-binding <- wHTH+WYL+WCX ||SF1-SF2-Helicase->MarR-HTH->HKD-DNase+HKD-DNase->CARF+NtrC-AAA->PIN+OST-HTH->MORC->	1248	Bacteria>Pseudomonadota>Gammaproteobacteria>Vibrionales>Vibrionaceae>Vibrio	GCF_028617275.1	Vibrio_parahaemolyticus	O8254_RS13605	106668..110414	670	BREX-system-P-loop-protein-BrxC-[Vibrio-parahaemolyticus]
WP_069535824.1	Type1.BREX	REase+SPF-cons-><-PIN+OST-HTH||CR-ATPase4->?->|| wHTH+WYL+WCX -> BrxA-DNA-binding -> BrxB-BrxF-iSTAND -> DUF6079 *-> N6-METHYLASE -> ParB + HNH -> PglZ ->ZetaToxin->?-><-SF1-SF2-Helicase+REase<-SF1-SF2-Helicase+REase<-viral-protein<-AbiEi	1157	Bacteria>Pseudomonadota>Gammaproteobacteria>Vibrionales>Vibrionaceae>Vibrio	GCF_905331795.1	Vibrio_parahaemolyticus	LPR62_RS04710	409258..412731	670	BREX-system-P-loop-protein-BrxC-[Vibrio-parahaemolyticus]
WP_229611425.1	Type1.BREX	REase+SPF-cons-><-PIN+OST-HTH||CR-ATPase4->?->|| wHTH+WYL+WCX -> BrxA-DNA-binding -> BrxB-BrxF-iSTAND -> DUF6079 *-> N6-METHYLASE -> ParB + HNH -> PglZ ->HEPN+SF1-SF2-Helicase+RNase+RNase->TPR-><-SF1-SF2-Helicase+REase<-SF1-SF2-Helicase+REase<-viral-protein<-AbiEi	1157	Bacteria>Pseudomonadota>Gammaproteobacteria>Vibrionales>Vibrionaceae>Vibrio	GCF_905331955.1	Vibrio_parahaemolyticus	LPR99_RS16740	18511..21984	670	BREX-system-P-loop-protein-BrxC-[Vibrio-parahaemolyticus]
WP_237468615.1	Type1.BREX	wHTH+WYL+WCX -> BrxA-DNA-binding -> BrxB-BrxF-iSTAND -> DUF6079 *-> N6-METHYLASE -> ParB + HNH -> ParB + HNH -> PglZ -> SIGMA+OB-Fold+AAA-ATPase+Lon-Protease-BrxL -><-Acetate_kinase	1231	Bacteria>Pseudomonadota>Gammaproteobacteria>Vibrionales>Vibrionaceae>Vibrio	GCF_921293875.1	Vibrio_stylophorae	L9P36_RS15955	492812..496507	659351	BREX-system-P-loop-protein-BrxC-[Vibrio-stylophorae]
WP_168780466.1	Type1.BREX	<- SIGMA+OB-Fold+AAA-ATPase+Lon-Protease-BrxL <- PglZ <-?<-?<-?<-GrpE<-ANK+ANK+ANK<- ParB + HNH <- N6-METHYLASE <- DUF6079 *<- BrxB-BrxF-iSTAND <- BrxA-DNA-binding <- wHTH+WYL+WCX <-?||AbiEi_4-><-REase<-TraI-VirD2-RCRE<-TraJ-RHH<-SbcC	1248	Bacteria>Pseudomonadota>Gammaproteobacteria>Vibrionales>Vibrionaceae>Vibrio	GCF_944738875.1	Vibrio_aestuarianus	OCX59_RS00150	25401..29147	28171	BREX-system-P-loop-protein-BrxC-[Vibrio-aestuarianus]
WP_272166069.1	Type1.BREX	CR-ATPase4->?-><- SIGMA+OB-Fold+AAA-ATPase+Lon-Protease-BrxL <- PglZ <-?<-?<- ParB + HNH <- N6-METHYLASE <- DUF6079 *<- BrxB-BrxF-iSTAND <- BrxA-DNA-binding <- wHTH+WYL+WCX ||SF1-SF2-Helicase->MarR-HTH->HKD-DNase+HKD-DNase->CARF+NtrC-AAA->Vrr-REase->HEPN+PAS+DUF4011+SF1-SF2-Helicase+REase->	1248	Bacteria>Pseudomonadota>Gammaproteobacteria>Vibrionales>Vibrionaceae>Vibrio	GCF_947503745.1	Vibrio_diabolicus	PQF93_RS21015	28188..31934	50719	BREX-system-P-loop-protein-BrxC-[Vibrio-diabolicus]
WP_142738823.1	Type1.BREX	MobI-><-?<-LexA-protease||?-> wHTH+WYL+WCX -> BrxA-DNA-binding -> BrxB-BrxF-iSTAND -> DUF6079 *-> ParB + HNH -> N6-METHYLASE ->OLD-ABC->TOPRIM-> PglZ -> SIGMA+OB-Fold+AAA-ATPase+Lon-Protease-BrxL ->SbcC->HDRnase->VirD4-TraG+VirB4-FtsK->	1224	Bacteria>Pseudomonadota>Gammaproteobacteria>Vibrionales>Vibrionaceae>Vibrio	-	Vibrio_cholerae	FLL79_RS01410	36024..39698	666	BREX-system-P-loop-protein-BrxC-[Vibrio-cholerae]
WP_226420703.1	Type1.BREX	<-MORC<-PIN+OST-HTH<-CARF+NtrC-AAA<-HKD-DNase+HKD-DNase<-MarR-HTH<-SF1-SF2-Helicase|| wHTH+WYL+WCX -> BrxA-DNA-binding -> BrxB-BrxF-iSTAND -> DUF6079 *-> N6-METHYLASE ->?-> ParB + HNH ->ZnR+3C-ABC+TPR->?->?->?->?-> PglZ ->	1248	Bacteria>Pseudomonadota>Gammaproteobacteria>Vibrionales>Vibrionaceae>Vibrio	-	Vibrio_sp._E14	LIX84_RS04085	940499..944245	2849869	BREX-system-P-loop-protein-BrxC-[Vibrio-sp.-E14]
WP_017462916.1	Type6.BREX	<-SbcC<-JetB<-JetA|| wHTH+WYL+WCX -> BrxE -> BrxA-DNA-binding -> BrxB-BrxF-iSTAND -> DUF6079 *->GreA_GreB+SF1-SF2-Helicase-> N6-METHYLASE -> N6-METHYLASE -> ParB + HNH ->?-> PglZ -> SIGMA+OB-Fold+AAA-ATPase+HKD-DNase-BrxL -><-JAB	1213	Bacteria>Pseudomonadota>Gammaproteobacteria>Xanthomonadales>Rhodanobacteraceae>Dyella	GCF_000334915.1	Dyella_ginsengisoli	C693_RS0114050	37588..41229	363848	BREX-system-P-loop-protein-BrxC-[Dyella-ginsengisoli]
MBK7210551.1	Type1.BREX	<-HDRnase<-Arm-DNA-bind_2+Phage_int_SAM_3+Phage_integrase<-Phage_AlpA||HTH_26->SLOG+BirA-HTH-> ParB + HNH -><-?|| BrxA-DNA-binding -> BrxB-BrxF-iSTAND -> DUF6079 *->?-> PglZ -> SIGMA+OB-Fold+AAA-ATPase+Lon-Protease-BrxL -><-tRNA||NUDIX->?->?-><-HISKIN+REC+HPT<-REC+GGDEF+EAL	1144	Bacteria>Pseudomonadota>Gammaproteobacteria>Xanthomonadales>unclassified_Xanthomonadales	GCA_016707485.1	Xanthomonadales_bacterium	IPH99_09390	1022709..1026143	2006849	BREX-system-P-loop-protein-BrxC-[Xanthomonadales-bacterium]
WP_070426722.1	Type6.BREX	METHYLASE->RNaseH->35exo-><-?||tRNA-> wHTH+WYL+WCX -> BrxE -> BrxA-DNA-binding -> BrxB-BrxF-iSTAND -> DUF6079 *->SF1-SF2-Helicase+RNase+RNase->TPR-> N6-METHYLASE -> N6-METHYLASE -p-> ParB + HNH -> PglZ -> SIGMA+OB-Fold+AAA-ATPase+HKD-DNase-BrxL ->DUF932->	1209	Bacteria>Pseudomonadota>Gammaproteobacteria>Xanthomonadales>Xanthomonadaceae	GCF_900766255.1	Stenotrophomonas	BIZ42_RS13545	3079736..3083365	40323	MULTISPECIES:-BREX-system-P-loop-protein-BrxC-[Stenotrophomonas]
WP_162376315.1	Type1.BREX	<-NTP_transf_3+Hexapep+Hexapep||?-><-?<- SIGMA+OB-Fold+AAA-ATPase+Lon-Protease-BrxL <- PglZ <-Polbeta<-MarR-HTH<- ParB + HNH <- N6-METHYLASE <- DUF6079 *<- BrxB-BrxF-iSTAND <- BrxA-DNA-binding ||REase-><-?<-?<-TOPRIM<-XIS-HTH	1148	Bacteria>Pseudomonadota>Gammaproteobacteria>Xanthomonadales>Xanthomonadaceae>Pseudoxanthomonas	GCF_010211765.1	Pseudoxanthomonas_kaohsiungensis	CSC66_RS05120	191406..194852	283923	BREX-system-P-loop-protein-BrxC-[Pseudoxanthomonas-kaohsiungensis]
WP_271250641.1	Type1.BREX	<- SIGMA+OB-Fold+AAA-ATPase+Lon-Protease-BrxL <- PglZ <-Polbeta<-MarR-HTH<- ParB + HNH <- N6-METHYLASE <- DUF6079 *<- BrxB-BrxF-iSTAND <- BrxA-DNA-binding ||REase-><-?<-?<-?<-TOPRIM<-?<-XIS-HTH	1148	Bacteria>Pseudomonadota>Gammaproteobacteria>Xanthomonadales>Xanthomonadaceae>Pseudoxanthomonas	GCF_027941875.1	Pseudoxanthomonas_mexicana	PE064_RS03375	689805..693251	128785	BREX-system-P-loop-protein-BrxC-[Pseudoxanthomonas-mexicana]
MBH1702634.1	Type1.BREX	<-RFC-AAA+RuvB-AAA||tRNA->?->AAA+wHTH-><- SIGMA+OB-Fold+AAA-ATPase+Lon-Protease-BrxL <- PglZ <- HNH <-OLD-ABC <- ParB + HNH <- N6-METHYLASE <- DUF6079 *<- BrxB-BrxF-iSTAND <- BrxA-DNA-binding <- wHTH+WYL+WCX <-?<-?<-?<-DUF932||XIS-HTH->	1146	Bacteria>Pseudomonadota>Gammaproteobacteria>Xanthomonadales>Xanthomonadaceae>Stenotrophomonas	GCA_015998095.1	Stenotrophomonas_maltophilia	I5U93_05090	24102..27542	40324	BREX-system-P-loop-protein-BrxC-[Stenotrophomonas-maltophilia]
MBH1753233.1	Type6.BREX	<-DUF932<- SIGMA+OB-Fold+AAA-ATPase+HKD-DNase-BrxL <- PglZ <- ParB + HNH <- N6-METHYLASE -p<- N6-METHYLASE <-TPR<-SF1-SF2-Helicase+RNase+RNase<- DUF6079 *<- BrxB-BrxF-iSTAND <- BrxA-DNA-binding <- BrxE <- wHTH+WYL+WCX <-tRNA||?-><-35exo<-RNaseH<-METHYLASE	1209	Bacteria>Pseudomonadota>Gammaproteobacteria>Xanthomonadales>Xanthomonadaceae>Stenotrophomonas	GCA_015998385.1	Stenotrophomonas_maltophilia	I5V37_01935	25775..29404	40324	BREX-system-P-loop-protein-BrxC-[Stenotrophomonas-maltophilia]
MBN5083090.1	Type6.BREX	<-DUF932<- SIGMA+OB-Fold+AAA-ATPase+HKD-DNase-BrxL <- PglZ <- ParB + HNH <- N6-METHYLASE <- N6-METHYLASE <-PIN<-OLD-ABC<- DUF6079 *<- BrxB-BrxF-iSTAND <- BrxA-DNA-binding <- BrxE <- wHTH+WYL+WCX <-Arm-DNA-bind_3+Phage_integrase<-tRNA<-SIGMA-HTH+SIGMA-HTH+SIGMA-HTH	1207	Bacteria>Pseudomonadota>Gammaproteobacteria>Xanthomonadales>Xanthomonadaceae>Stenotrophomonas	GCA_017150495.1	Stenotrophomonas_maltophilia	JY467_05250	61681..65304	40324	BREX-system-P-loop-protein-BrxC-[Stenotrophomonas-maltophilia]
WP_004146422.1	Type6.BREX	<-XIS-HTH||?->Phage_integrase->tRNA->Arm-DNA-bind_3+Phage_integrase-> wHTH+WYL+WCX -> BrxE -> BrxA-DNA-binding -> BrxB-BrxF-iSTAND -> DUF6079 *->OLD-ABC->PIN-> N6-METHYLASE -> N6-METHYLASE -> ParB + HNH -> PglZ -> SIGMA+OB-Fold+AAA-ATPase+HKD-DNase-BrxL ->DUF932->	1207	Bacteria>Pseudomonadota>Gammaproteobacteria>Xanthomonadales>Xanthomonadaceae>Stenotrophomonas	GCF_000355725.1	Stenotrophomonas_maltophilia	G5A34_RS07915	81973..85596	40324	BREX-system-P-loop-protein-BrxC-[Stenotrophomonas-maltophilia]
WP_099491466.1	Type6.BREX	METHYLASE->RNaseH->35exo-><-?||tRNA-> wHTH+WYL+WCX -> BrxE -> BrxA-DNA-binding -> BrxB-BrxF-iSTAND -> DUF6079 *->SF1-SF2-Helicase+RNase+RNase->TPR-> N6-METHYLASE -> N6-METHYLASE -p-> ParB + HNH -> PglZ -> SIGMA+OB-Fold+AAA-ATPase+HKD-DNase-BrxL ->DUF932->	1209	Bacteria>Pseudomonadota>Gammaproteobacteria>Xanthomonadales>Xanthomonadaceae>Stenotrophomonas	GCF_002739275.1	Stenotrophomonas_maltophilia	BRB91_RS07170	53995..57624	40324	BREX-system-P-loop-protein-BrxC-[Stenotrophomonas-maltophilia]
WP_125428654.1	Type1.BREX	<-RFC-AAA+RuvB-AAA||tRNA->?->AAA+wHTH-><- SIGMA+OB-Fold+AAA-ATPase+Lon-Protease-BrxL <- PglZ <- HNH <-OLD-ABC <- ParB + HNH <- N6-METHYLASE <- DUF6079 *<- BrxB-BrxF-iSTAND <- BrxA-DNA-binding <- wHTH+WYL+WCX <-?<-?<-?<-DUF932||XIS-HTH->	1146	Bacteria>Pseudomonadota>Gammaproteobacteria>Xanthomonadales>Xanthomonadaceae>Stenotrophomonas	GCF_003935615.1	Stenotrophomonas_maltophilia	EGJ89_RS09210	127004..130444	40324	BREX-system-P-loop-protein-BrxC-[Stenotrophomonas-maltophilia]
WP_164085131.1	Type6.BREX	METHYLASE->RNaseH->35exo-><-?||tRNA-> wHTH+WYL+WCX -> BrxE -> BrxA-DNA-binding -> BrxB-BrxF-iSTAND -> DUF6079 *->SF1-SF2-Helicase+RNase+RNase->TPR-> N6-METHYLASE -> N6-METHYLASE -p-> ParB + HNH ->	1209	Bacteria>Pseudomonadota>Gammaproteobacteria>Xanthomonadales>Xanthomonadaceae>Stenotrophomonas	GCF_010605045.1	Stenotrophomonas_maltophilia	G5A32_RS08645	68360..71989	40324	BREX-system-P-loop-protein-BrxC-[Stenotrophomonas-maltophilia]
WP_180875343.1	Type6.BREX	METHYLASE->RNaseH->35exo-><-?||tRNA-> wHTH+WYL+WCX -> BrxE -> BrxA-DNA-binding -> BrxB-BrxF-iSTAND -> DUF6079 *->SF1-SF2-Helicase+RNase+RNase->TPR-> N6-METHYLASE -> N6-METHYLASE -p-> ParB + HNH -> PglZ -> SIGMA+OB-Fold+AAA-ATPase+HKD-DNase-BrxL ->DUF932->	1209	Bacteria>Pseudomonadota>Gammaproteobacteria>Xanthomonadales>Xanthomonadaceae>Stenotrophomonas	GCF_013463485.1	Stenotrophomonas_maltophilia	D7U74_RS02160	10322..13951	40324	BREX-system-P-loop-protein-BrxC-[Stenotrophomonas-maltophilia]
WP_192288052.1	Type1.BREX	SF1-SF2-Helicase->tRNA->tRNA-> BrxA-DNA-binding -> BrxB-BrxF-iSTAND -> DUF6079 *-> N6-METHYLASE -> ParB + HNH ->MarR-HTH->Polbeta-> PglZ -> SIGMA+OB-Fold+AAA-ATPase+Lon-Protease-BrxL ->?->?-><-HTH_3||TPR->	1148	Bacteria>Pseudomonadota>Gammaproteobacteria>Xanthomonadales>Xanthomonadaceae>Stenotrophomonas	GCF_014852135.1	Stenotrophomonas_sp._STM01	IB227_RS14890	150260..153706	2769278	BREX-system-P-loop-protein-BrxC-[Stenotrophomonas-sp.-STM01]
WP_049450109.1	Type6.BREX	<-DUF932<- SIGMA+OB-Fold+AAA-ATPase+HKD-DNase-BrxL <- PglZ <- ParB + HNH <- N6-METHYLASE -p<- N6-METHYLASE <-TPR<-SF1-SF2-Helicase+RNase+RNase<- DUF6079 *<- BrxB-BrxF-iSTAND <- BrxA-DNA-binding <- BrxE <- wHTH+WYL+WCX <-tRNA||?-><-35exo<-RNaseH<-METHYLASE	1209	Bacteria>Pseudomonadota>Gammaproteobacteria>Xanthomonadales>Xanthomonadaceae>Stenotrophomonas	GCF_030549525.1	Stenotrophomonas_maltophilia	MT454_RS13320	40759..44388	40324	BREX-system-P-loop-protein-BrxC-[Stenotrophomonas-maltophilia]
WP_111722876.1	Type6.BREX	METHYLASE->RNaseH->35exo-><-?||tRNA-> wHTH+WYL+WCX -> BrxE -> BrxA-DNA-binding -> BrxB-BrxF-iSTAND -> DUF6079 *->SF1-SF2-Helicase+RNase+RNase->TPR-> N6-METHYLASE -> N6-METHYLASE -p-> ParB + HNH -> PglZ -> SIGMA+OB-Fold+AAA-ATPase+HKD-DNase-BrxL ->DUF932->	1209	Bacteria>Pseudomonadota>Gammaproteobacteria>Xanthomonadales>Xanthomonadaceae>Stenotrophomonas	GCF_900475685.1	Stenotrophomonas_maltophilia	DQN96_RS04535	962467..966096	40324	BREX-system-P-loop-protein-BrxC-[Stenotrophomonas-maltophilia]
WP_262214630.1	Type6.BREX	METHYLASE->RNaseH->35exo-><-?||tRNA-> wHTH+WYL+WCX -> BrxE -> BrxA-DNA-binding -> BrxB-BrxF-iSTAND -> DUF6079 *->GreA_GreB+SF1-SF2-Helicase-> N6-METHYLASE -> N6-METHYLASE -> ParB + HNH -> PglZ -> SIGMA+OB-Fold+AAA-ATPase+HKD-DNase-BrxL ->DUF932->?-><-HTH_3	1213	Bacteria>Pseudomonadota>Gammaproteobacteria>Xanthomonadales>Xanthomonadaceae>Stenotrophomonas	-	Stenotrophomonas_maltophilia	JAK45_RS19240	75512..79153	40324	BREX-system-P-loop-protein-BrxC-[Stenotrophomonas-maltophilia]
VUJ46804.1	Type6.BREX	<-LexA-protease||?-><-Phage_integrase||Arm-DNA-bind_3+Phage_integrase-> wHTH+WYL+WCX -> BrxE -> BrxA-DNA-binding -> BrxB-BrxF-iSTAND -> DUF6079 *->OLD-ABC->PIN-> N6-METHYLASE -> N6-METHYLASE -> ParB + HNH -> PglZ -> SIGMA+OB-Fold+AAA-ATPase+HKD-DNase-BrxL ->DUF932->	1207	Bacteria>Pseudomonadota>Gammaproteobacteria>Xanthomonadales>Xanthomonadaceae>Stenotrophomonas	-	Stenotrophomonas_maltophilia	N5D45_RS14200	24687..28310	40324	hypothetical-protein-NGKLMNPD_00401-[Stenotrophomonas-maltophilia]
EGD07603.1	Type1.BREX	<-RFC-AAA+RuvB-AAA||tRNA->?->AAA+wHTH-><- SIGMA+OB-Fold+AAA-ATPase+Lon-Protease-BrxL <- PglZ <- ParB + HNH <- N6-METHYLASE <- DUF6079 *<- BrxB-BrxF-iSTAND <- BrxA-DNA-binding <- wHTH+WYL+WCX <-DUF772+DDE_Tnp_1||XIS-HTH->	1152	Bacteria>Pseudomonadota>Gammaproteobacteria>Xanthomonadales>Xanthomonadaceae>Xanthomonas	GCA_000192025.2	Xanthomonas_vesicatoria_ATCC_35937	XVE_4193	13272..16730	925775	hypothetical-protein-XVE_4193-[Xanthomonas-vesicatoria-ATCC-35937]
WP_042828568.1	Type1.BREX	<-RFC-AAA+RuvB-AAA||tRNA->?->AAA+wHTH-><- SIGMA+OB-Fold+AAA-ATPase+Lon-Protease-BrxL <- PglZ <- ParB + HNH <- N6-METHYLASE <- DUF6079 *<- BrxB-BrxF-iSTAND <- BrxA-DNA-binding <- wHTH+WYL+WCX ||<-DUF772+DDE_Tnp_1||XIS-HTH->	1146	Bacteria>Pseudomonadota>Gammaproteobacteria>Xanthomonadales>Xanthomonadaceae>Xanthomonas	GCF_000192025.1	Xanthomonas_vesicatoria	XVE_RS20000	13272..16712	56460	BREX-system-P-loop-protein-BrxC-[Xanthomonas-vesicatoria]
WP_260816965.1	Type1.BREX	DnaJ->?->?->?-><-?<-XIS-HTH|| wHTH+WYL+WCX -> BrxA-DNA-binding -> BrxB-BrxF-iSTAND -> DUF6079 *-> N6-METHYLASE -> ParB + HNH -> PglZ -> SIGMA+OB-Fold+AAA-ATPase+Lon-Protease-BrxL -><-AAA+wHTH<-?<-tRNA||RFC-AAA+RuvB-AAA->?->HHH_5+RecR+TOPRIM->	1146	Bacteria>Pseudomonadota>Gammaproteobacteria>Xanthomonadales>Xanthomonadaceae>Xanthomonas	GCF_025263745.1	Xanthomonas_prunicola	MOD45_RS16865	3940549..3943989	2053930	BREX-system-P-loop-protein-BrxC-[Xanthomonas-prunicola]
WP_000687848.1	Type1.BREX	<-VirD4-TraG+VirB4-FtsK<-HDRnase<-SbcC<- SIGMA+OB-Fold+AAA-ATPase+Lon-Protease-BrxL <- PglZ <- N6-METHYLASE <- ParB + HNH <- DUF6079 *<- BrxB-BrxF-iSTAND <- BrxA-DNA-binding <- wHTH+WYL+WCX <-?<-35exo<-?||LexA-protease->?->Phage_integrase->	1225	Bacteria>Pseudomonadota	GCF_000022585.1	Gammaproteobacteria	ALD23_RS14425	130597..134274	1236	MULTISPECIES:-BREX-system-P-loop-protein-BrxC-[Gammaproteobacteria]
WP_156734107.1	Type1.BREX	<-LexA-protease||?->35exo->?-> wHTH+WYL+WCX -> BrxA-DNA-binding -> BrxB-BrxF-iSTAND -> DUF6079 *-> ParB + HNH -> N6-METHYLASE -> PglZ -> SIGMA+OB-Fold+AAA-ATPase+Lon-Protease-BrxL ->NACHT+HEAT+REase->HDRnase->VirD4-TraG+VirB4-FtsK->	1225	Bacteria>Pseudomonadota	GCF_009740265.1	Gammaproteobacteria	GTH23_RS00050	5219..8896	1236	MULTISPECIES:-BREX-system-P-loop-protein-BrxC-[Gammaproteobacteria]
WP_119567031.1	Type1.BREX	<-OBFOLD-NUCLEASE<-CARF+NtrC-AAA+FIS-HTH<- SIGMA+OB-Fold+AAA-ATPase+Lon-Protease-BrxL <- PglZ <- N6-METHYLASE <- ParB + HNH <- DUF6079 *<- BrxB-BrxF-iSTAND <- BrxA-DNA-binding <- wHTH+WYL+WCX <-?<-35exo||LexA-protease->?-><-MobI	1225	Bacteria>Pseudomonadota	GCF_013602615.1	Gammaproteobacteria	G9G97_RS25355	26642..30319	1236	MULTISPECIES:-BREX-system-P-loop-protein-BrxC-[Gammaproteobacteria]
WP_125941663.1	Type1.BREX	GDResolvase+HIN-HTH-><-?<-?<- SIGMA+OB-Fold+AAA-ATPase+Lon-Protease-BrxL <- PglZ <- ParB + HNH <- N6-METHYLASE -p<-HTH_23+HTH_32+HTH_33+DDE_3<- N6-METHYLASE <- DUF6079 *<- BrxB-BrxF-iSTAND <- BrxA-DNA-binding <- wHTH+WYL+WCX ||XIS-HTH->?->?-><-?<-Phage_int_SAM_5+Phage_integrase<-sGTP	1154	Bacteria>Pseudomonadota	GCF_019046925.1	Gammaproteobacteria	I6L32_RS07295	1524921..1528385	1236	MULTISPECIES:-BREX-system-P-loop-protein-BrxC-[Gammaproteobacteria]
WP_110076276.1	Type1.BREX	<-VirD4-TraG+VirB4-FtsK<-HDRnase<-SbcC<- SIGMA+OB-Fold+AAA-ATPase+Lon-Protease-BrxL <- PglZ <- N6-METHYLASE <- ParB + HNH <- DUF6079 *<- BrxB-BrxF-iSTAND <- BrxA-DNA-binding <- wHTH+WYL+WCX <-?<-35exo||LexA-protease->||?->?->Pkinase->	1225	Bacteria>Pseudomonadota	GCF_021431945.1	Gammaproteobacteria	LY388_RS16340	2424979..2428656	1236	MULTISPECIES:-BREX-system-P-loop-protein-BrxC-[Gammaproteobacteria]
WP_111805008.1	Type1.BREX	<-VirD4-TraG+VirB4-FtsK<-HDRnase<-SbcC<- SIGMA+OB-Fold+AAA-ATPase+Lon-Protease-BrxL <- PglZ <- N6-METHYLASE <- ParB + HNH <- DUF6079 *<- BrxB-BrxF-iSTAND <- BrxA-DNA-binding <- wHTH+WYL+WCX <-?<-35exo||LexA-protease->?-><-MobI	1225	Bacteria>Pseudomonadota	GCF_028482435.1	Gammaproteobacteria	DR130_RS11140	144958..148635	1236	MULTISPECIES:-BREX-system-P-loop-protein-BrxC-[Gammaproteobacteria]
PIX55556.1	Type6.BREX	BrxE -> BrxA-DNA-binding -> BrxB-BrxF-iSTAND -> DUF6079 *-> N6-METHYLASE -> ParB + HNH ->?->?-> PglZ -> SIGMA+OB-Fold+AAA-ATPase+HKD-DNase-BrxL ->ZNR+RmuC->XIS-HTH-><-HTH_26||Phage_AlpA->	1215	Bacteria>Pseudomonadota>Zetaproteobacteria>unclassified_Zetaproteobacteria	GCA_002782825.1	Zetaproteobacteria_bacterium_CG_4_10_14_3_um_filter_54_28	COZ50_02095	2235..5882	1974114	BREX-system-P-loop-protein-BrxC-[Zetaproteobacteria-bacterium-CG_4_10_14_3_um_filter_54_28]
MBX3380229.1	Type1.BREX	Pkinase+pi3KINASE->?->?-><-?<- SIGMA+OB-Fold+AAA-ATPase+Lon-Protease-BrxL <- PglZ <- ParB + HNH <- ParB + HNH <- N6-METHYLASE <- DUF6079 *<- BrxB-BrxF-iSTAND <- BrxA-DNA-binding <- BrxE <-?<-Phage_integrase	1207	Bacteria>PVC_group>Planctomycetota>Phycisphaerae>Phycisphaerales>Phycisphaeraceae	GCA_019636565.1	Phycisphaeraceae_bacterium	KF805_09030	72021..75644	2026777	BREX-system-P-loop-protein-BrxC-[Phycisphaeraceae-bacterium]
WP_145095869.1	Type1.BREX	<-TPR+TPR<-Peptidase<-?<-?<- SIGMA+OB-Fold+AAA-ATPase+Lon-Protease-BrxL <- PglZ <-RAMA<- N6-METHYLASE <- ParB + HNH <- DUF6079 *<- BrxB-BrxF-iSTAND <- BrxA-DNA-binding <-XIS-HTH||Thermonuclease->OLD-ABC->?->ABhydrolase->	1192	Bacteria>PVC_group>Planctomycetota>Planctomycetia>Pirellulales>Pirellulaceae	GCF_007753095.1	Rosistilla_carotiformis	Poly24_RS13225	3653187..3656765	2528017	BREX-system-P-loop-protein-BrxC-[Rosistilla-carotiformis]
WP_207398110.1	Type1.BREX	<-Phage_int_SAM_1+Phage_integrase<-?<-RFC-AAA<-?<-PIWI<-REase+PNTD1+PNTD2||XIS-HTH-> BrxA-DNA-binding -> BrxB-BrxF-iSTAND -> DUF6079 *-> ParB + HNH -> N6-METHYLASE ->REase->?->REase->?-> PglZ -> SIGMA+OB-Fold+AAA-ATPase+Lon-Protease-BrxL ->KAP-NTPase->	1192	Bacteria>PVC_group>Planctomycetota>Planctomycetia>Pirellulales>Pirellulaceae	GCF_013618625.1	Bremerella_alba	HOV93_RS18975	110238..113816	980252	BREX-system-P-loop-protein-BrxC-[Bremerella-alba]
TET34868.1	Type1.BREX	<- HNH ||?-><-?||XIS-HTH->DUF4007->LON-AAA-> N6-METHYLASE -> ParB + HNH ->HEPN-> DUF6079 *-> PglZ ->SIGMA+OB-Fold+AAA-ATPase-BrxL->SbcC->HISKIN->wHTH+WYL->RADICAL-SAM->DEOR-HTH-><-GDResolvase+Recombinase+Zn_ribbon_recom	1347	Bacteria>PVC_group>Planctomycetota>unclassified_Planctomycetota	GCA_004376375.1	Planctomycetota_bacterium	E3J72_12085	41890..45933	2026780	MAG:-hypothetical-protein-E3J72_12085-[Planctomycetota-bacterium]
TWU09954.1	Type1.BREX	<-ABhydrolase<-?||?->Calcineurin->SbcC+SbcC->?->XIS-HTH-> BrxA-DNA-binding -> BrxB-BrxF-iSTAND -> DUF6079 *-> ParB + HNH -> N6-METHYLASE ->REase-> PglZ ->SKI->?-> SIGMA+OB-Fold+AAA-ATPase+Lon-Protease-BrxL ->	1205	Bacteria>PVC_group>Planctomycetota>unclassified_Planctomycetota	GCA_007860105.1	Planctomycetes_bacterium_Poly21	Poly21_52830	31284..34901	2528014	hypothetical-protein-Poly21_52830-[Planctomycetes-bacterium-Poly21]
MCD6405191.1	Type1.BREX	<-Schlafen+HATPase+wHTH-p<-PHP+SbcC<- SIGMA+OB-Fold+AAA-ATPase+Lon-Protease-BrxL <- PglZ <- ParB + HNH <- N6-METHYLASE <- DUF6079 *<- BrxB-BrxF-iSTAND <- BrxA-DNA-binding <-XIS-HTH	1143	Bacteria>PVC_group>Planctomycetota>unclassified_Planctomycetota	GCA_021160625.1	Planctomycetota_bacterium	J7M19_05155	12563..15994	2026780	BREX-system-P-loop-protein-BrxC-[Planctomycetota-bacterium]
WP_215427518.1	Type1.BREX	ANK+ANK+ANK-> HNH ->HTH_3->Couple_hipA->HipA_C->?->XIS-HTH-> BrxA-DNA-binding -> BrxB-BrxF-iSTAND -> DUF6079 *-> N6-METHYLASE -> PglZ -> SIGMA+OB-Fold+AAA-ATPase+Lon-Protease-BrxL -> HNH -><- ParB + HNH ||DUF932->PadR-HTH->	1141	Bacteria>PVC_group>Verrucomicrobiota>Verrucomicrobiae>Verrucomicrobiales>Akkermansiaceae	GCF_018709685.1	Akkermansia_muciniphila	HHJ06_RS03145	88477..91902	239935	BREX-system-P-loop-protein-BrxC-[Akkermansia-muciniphila]
MBE7502027.1	Type6.BREX	HTH_3-><-?<-?<-?<-GNAT<-NERD+ZnR|| BrxE -> BrxA-DNA-binding -> BrxB-BrxF-iSTAND -> DUF6079 *-> N6-METHYLASE -><-DDE_Tnp_1|| N6-METHYLASE -> ParB + HNH ->OLD-ABC->TOPRIM->DDE_Tnp_1-> PglZ -> SIGMA+OB-Fold+AAA-ATPase+HKD-DNase-BrxL ->	1231	Bacteria>PVC_group>Verrucomicrobiota>Verrucomicrobiae>Verrucomicrobiales>unclassified_Verrucomicrobiales	GCA_015075445.1	Verrucomicrobiales_bacterium	HS113_17385	108161..111856	2026801	BREX-system-P-loop-protein-BrxC-[Verrucomicrobiales-bacterium]
WP_015675681.1	Type6.BREX	<-SF1-SF2-Helicase+wHTH+Small-B-barrel+DUF1998<-BrxD-AAA<- PglZ <- N6-METHYLASE -p<-AbiEii<-AbiEi_3_N+AbiEi_3<- ParB + HNH <- N6-METHYLASE <- DUF6079 *<- BrxB-BrxF-iSTAND <- BrxA-DNA-binding <- BrxE <-TPR+TPR<-TPR<-MarR-HTH<- ParB <-ParA-Soj-PloopNTPase	1203	Bacteria>Spirochaetota>Spirochaetia>Leptospirales>Leptospiraceae>Leptospira	GCF_000332475.2	Leptospira_yanagawae	LEP1GSC202_0423	26532..30143	293069	BREX-system-P-loop-protein-BrxC-[Leptospira-yanagawae]
WP_135627473.1	Type1.BREX	DUF6079 *-> N6-METHYLASE -> ParB + HNH -> PglZ -> SIGMA+OB-Fold+AAA-ATPase+Lon-Protease-BrxL ->	1122	Bacteria>Spirochaetota>Spirochaetia>Leptospirales>Leptospiraceae>Leptospira	GCF_004770835.1	Leptospira_selangorensis	EHQ82_RS10885	1..3369	2484982	BREX-system-P-loop-protein-BrxC,-partial-[Leptospira-selangorensis]
WP_238754851.1	Type6.BREX	ANK->ParA-Soj-PloopNTPase-> ParB ->MarR-HTH->TPR+TPR-> BrxE -> BrxA-DNA-binding -> BrxB-BrxF-iSTAND -> DUF6079 *-> N6-METHYLASE ->?->?->?-> ParB + HNH ->AbiEi_3_N+AbiEi_3->DOC+HTH-> N6-METHYLASE -p-> PglZ ->	1201	Bacteria>Spirochaetota>Spirochaetia>Leptospirales>Leptospiraceae>Leptospira	GCF_022267385.1	Leptospira_bandrabouensis	LFX13_RS16565	34041..37646	2484903	BREX-system-P-loop-protein-BrxC-[Leptospira-bandrabouensis]
OQY41116.1	Type1.BREX	REase-> BrxA-DNA-binding -> BrxB-BrxF-iSTAND -> DUF6079 *->OLD-ABC->TOPRIM-> N6-METHYLASE -> ParB + HNH -> ParB + HNH -> SIGMA+OB-Fold+AAA-ATPase+Lon-Protease-BrxL ->	1207	Bacteria>Spirochaetota>Spirochaetia>Spirochaetales>Spirochaetaceae>unclassified_Spirochaetaceae	GCA_002084785.1	Spirochaetaceae_bacterium_4572_7	B6229_00020	2034..5657	1972456	hypothetical-protein-B6229_00020-[Spirochaetaceae-bacterium-4572_7]
WP_109093701.1	Type1.BREX	<-PIN<-PhdYeFM_antitox||?->REase-><- ParB + HNH <- SIGMA+OB-Fold+AAA-ATPase+HKD-DNase-BrxL <- PglZ <-Schlafen+HATPase+wHTH<- N6-METHYLASE <- DUF6079 *<- BrxB-BrxF-iSTAND <- BrxA-DNA-binding ||?-><-?<-?<-LACI-HTH+PBPI<-Glyco_hydro_1	1170	Bacteria>Terrabacteria_group>Actinomycetota>Actinomycetes>Actinomycetales>Actinomycetaceae	GCF_003123745.1	Ancrocorticia_populi	DD236_RS06730	275849..279361	2175228	BREX-system-P-loop-protein-BrxC-[Ancrocorticia-populi]
QZE24962.1	Type1.BREX	HTH+MPTase->?->Small-B-barrel+SF1-SF2-Helicase+RdgC+REase-><-?||<-zf-ISL3+HTH_Tnp_ISL3<- ParB + HNH <- SIGMA+OB-Fold+AAA-ATPase+Lon-Protease-BrxL <- PglZ <- N6-METHYLASE <- DUF6079 *<- BrxB-BrxF-iSTAND <- BrxA-DNA-binding <-XIS-HTH<-ABhydrolase<-?<-MPTase<-HTH||ArdCN+MPTase->DUF2637+HIN-HTH->	1152	Bacteria>Terrabacteria_group>Actinomycetota>Actinomycetes>Micrococcales>Brevibacteriaceae	GCA_019720815.1	Brevibacterium_casei	K4X33_12630	2739675..2743133	33889	BREX-system-P-loop-protein-BrxC-[Brevibacterium-casei]
WP_186372589.1	Type1.BREX	<-VirB11+MuB-ATPase||DNA_METHYLASE->DNA_METHYLASE+DCM->DNA_METHYLASE-><- ParB + HNH <- SIGMA+OB-Fold+AAA-ATPase+Lon-Protease-BrxL <- PglZ <- N6-METHYLASE <- DUF6079 *<- BrxB-BrxF-iSTAND <- BrxA-DNA-binding <-XIS-HTH<-ABhydrolase<-?<-MPTase<-HTH||ArdCN+MPTase->Primpol+PriCT1->	1152	Bacteria>Terrabacteria_group>Actinomycetota>Actinomycetes>Micrococcales>Brevibacteriaceae	GCF_002339175.1	Brevibacterium_casei	B3D11_RS04595	80759..84217	33889	BREX-system-P-loop-protein-BrxC-[Brevibacterium-casei]
WP_029253807.1	Type1.BREX	ABC-ATPase+SbcC-><-?<-HEPN<- ParB + HNH <- SIGMA+OB-Fold+AAA-ATPase+HKD-DNase-BrxL <- PglZ <- N6-METHYLASE <- DUF6079 *<- BrxB-BrxF-iSTAND <- BrxA-DNA-binding <-AbiEii<-AbiEii<-REase	1160	Bacteria>Terrabacteria_group>Actinomycetota>Actinomycetes>Micrococcales>Cellulomonadaceae	GCF_000701465.1	Paraoerskovia_marina	Q343_RS0111945	114651..118133	545619	BREX-system-P-loop-protein-BrxC-[Paraoerskovia-marina]
QAY61399.1	Type1.BREX	<-VirB11+MuB-ATPase||DNA_METHYLASE-><- ParB + HNH <- SIGMA+OB-Fold+AAA-ATPase+Lon-Protease-BrxL <- PglZ <- N6-METHYLASE <- DUF6079 *<- BrxB-BrxF-iSTAND <- BrxA-DNA-binding <-XIS-HTH<-ABhydrolase<-?<-MPTase<-HTH||ArdCN+MPTase->Primpol+PriCT1->	1176	Bacteria>Terrabacteria_group>Actinomycetota>Actinomycetes>Micrococcales>Microbacteriaceae	GCA_004135285.1	Microbacterium_protaetiae	ET475_16440	3529806..3533336	2509458	BREX-system-P-loop-protein-BrxC-[Microbacterium-protaetiae]
MBY6061973.1	Type1.BREX	TetR-HTH->?-><-?<-Glyoxalase||OLD-ABC+TOPRIM->?-> BrxA-DNA-binding -> BrxB-BrxF-iSTAND -> DUF6079 *-> N6-METHYLASE -> PglZ -> SIGMA+OB-Fold+AAA-ATPase+Lon-Protease-BrxL -><- ParB + HNH ||?->MAD-NTD->	1152	Bacteria>Terrabacteria_group>Actinomycetota>Actinomycetes>Micrococcales>Microbacteriaceae	GCA_019801485.1	Microbacterium_esteraromaticum	KUV70_11015	269249..272707	57043	BREX-system-P-loop-protein-BrxC-[Microbacterium-esteraromaticum]
WP_053352376.1	Type1.BREX	<-MODE-HTH+PBPII||?->?->?->?->tRNA->XIS-HTH-> BrxA-DNA-binding -> BrxB-BrxF-iSTAND -> DUF6079 *-> N6-METHYLASE -> PglZ -> SIGMA+OB-Fold+AAA-ATPase+HKD-DNase-BrxL -><-?<-?<-?|| HNH +SAVED-> ParB + HNH ->	1182	Bacteria>Terrabacteria_group>Actinomycetota>Actinomycetes>Micrococcales>Microbacteriaceae	GCF_001273845.1	Leucobacter_musarum	CH42_RS06350	60565..64113	1930747	BREX-system-P-loop-protein-BrxC-[Leucobacter-musarum]
WP_165310971.1	Type1.BREX	<-VirB11+MuB-ATPase||DNA_METHYLASE->DNA_METHYLASE+DCM->DNA_METHYLASE-><- ParB + HNH <- SIGMA+OB-Fold+AAA-ATPase+Lon-Protease-BrxL <- PglZ <- N6-METHYLASE <- DUF6079 *<- BrxB-BrxF-iSTAND <- BrxA-DNA-binding <-XIS-HTH<-ABhydrolase<-?<-MPTase<-HTH||ArdCN+MPTase->Primpol+PriCT1->	1152	Bacteria>Terrabacteria_group>Actinomycetota>Actinomycetes>Micrococcales>Microbacteriaceae	GCF_004135285.1	Microbacterium_protaetiae	ET475_RS16440	3529806..3533264	2509458	BREX-system-P-loop-protein-BrxC-[Microbacterium-protaetiae]
WP_146322974.1	Type1.BREX	DOC+HTH->?-> BrxA-DNA-binding -> BrxB-BrxF-iSTAND -> DUF6079 *-> N6-METHYLASE -><- N6-METHYLASE -p||Phage_int_SAM_3+Phage_integrase-> N6-METHYLASE -p->?->?-> PglZ -> SIGMA+OB-Fold+AAA-ATPase+HKD-DNase-BrxL -> ParB + HNH ->	1162	Bacteria>Terrabacteria_group>Actinomycetota>Actinomycetes>Micrococcales>Microbacteriaceae	GCF_007859675.1	Humibacter_ginsenosidimutans	FPZ11_RS17385	3786036..3789524	2599293	BREX-system-P-loop-protein-BrxC-[Humibacter-ginsenosidimutans]
WP_261393916.1	Type1.BREX	TetR-HTH->?-><-?<-Glyoxalase||OLD-ABC+TOPRIM->?-> BrxA-DNA-binding -> BrxB-BrxF-iSTAND -> DUF6079 *-> N6-METHYLASE -> PglZ -> SIGMA+OB-Fold+AAA-ATPase+Lon-Protease-BrxL -><- ParB + HNH ||?->MAD-NTD->	1146	Bacteria>Terrabacteria_group>Actinomycetota>Actinomycetes>Micrococcales>Microbacteriaceae	GCF_019801485.1	Microbacterium_esteraromaticum	KUV70_RS11015	269267..272707	57043	BREX-system-P-loop-protein-BrxC-[Microbacterium-esteraromaticum]
WP_222446684.1	Type1.BREX	TetR-HTH->?-><-?<-Glyoxalase||OLD-ABC+TOPRIM->?-> BrxA-DNA-binding -> BrxB-BrxF-iSTAND -> DUF6079 *-> N6-METHYLASE -> PglZ -> SIGMA+OB-Fold+AAA-ATPase+Lon-Protease-BrxL -><- ParB + HNH ||?->MAD-NTD->	1152	Bacteria>Terrabacteria_group>Actinomycetota>Actinomycetes>Micrococcales>Microbacteriaceae	-	Microbacterium_esteraromaticum	KUV70_11015	269249..272707	57043	BREX-system-P-loop-protein-BrxC-[Microbacterium-esteraromaticum]
ALV40633.1	Type1.BREX	<-HEPN+SF1-SF2-Helicase+REase<-?<-?<- ParB + HNH <- SIGMA+OB-Fold+AAA-ATPase+HKD-DNase-BrxL <- PglZ <- N6-METHYLASE <- DUF6079 *<- BrxB-BrxF-iSTAND <- BrxA-DNA-binding <-XIS-HTH<-HKD-DNase+SF1-SF2-Helicase+HTH+HTH+SRA||?->?->?->McrB-AAA->McrC-NTD+REase->	1172	Bacteria>Terrabacteria_group>Actinomycetota>Actinomycetes>Micrococcales>Micrococcaceae	GCA_001484605.1	Pseudarthrobacter_sulfonivorans	AU252_05160	1179330..1182848	121292	hypothetical-protein-AU252_05160-[Pseudarthrobacter-sulfonivorans]
BBE21856.1	Type1.BREX	<-SF1-SF2-Helicase+REase<-SF1-SF2-Helicase+REase-p||?->LZ_Tnp_IS481+HTH_32+rve+rve_3-><-HTH_Tnp_1+HTH_21+rve+rve_3<-HIN-HTH|| BrxA-DNA-binding -> BrxB-BrxF-iSTAND -> DUF6079 *-> N6-METHYLASE -> PglZ -> SIGMA+OB-Fold+AAA-ATPase+Lon-Protease-BrxL -> ParB + HNH -><-?<-DDE_Tnp_ISL3<-?||SF1-SF2-Helicase->	1144	Bacteria>Terrabacteria_group>Actinomycetota>Actinomycetes>Micrococcales>Micrococcaceae	GCA_004001285.1	Arthrobacter_sp._MN05-02	MN0502_07390	781393..784827	1571833	hypothetical-protein-MN0502_07390-[Arthrobacter-sp.-MN05-02]
QOD04369.1	Type1.BREX	NUDIX-><-URI-DNase<-MACRODOMAIN+SIR2<-REase<-?||HKD-DNase+SF1-SF2-Helicase+HTH+HTH+SRA-><- ParB + HNH || BrxA-DNA-binding -> BrxB-BrxF-iSTAND -> DUF6079 *-> N6-METHYLASE -> N6-METHYLASE -> PglZ -> SIGMA+OB-Fold+AAA-ATPase+Lon-Protease-BrxL ->SF1-SF2-Helicase->Sigma-HTH->Sigma-HTH->rve->HTH+rve->	1145	Bacteria>Terrabacteria_group>Actinomycetota>Actinomycetes>Micrococcales>Micrococcaceae	GCA_014764445.1	Pseudarthrobacter_sp._BIM_B-2242	IDT60_04725	994235..997672	2772401	BREX-system-P-loop-protein-BrxC-[Pseudarthrobacter-sp.-BIM-B-2242]
WP_099093371.1	Type1.BREX	<-HEPN+SF1-SF2-Helicase+REase<-?||?-><- ParB + HNH <- SIGMA+OB-Fold+AAA-ATPase+HKD-DNase-BrxL <- PglZ <- N6-METHYLASE <- DUF6079 *<- BrxB-BrxF-iSTAND <- BrxA-DNA-binding <-XIS-HTH<-HKD-DNase+SF1-SF2-Helicase+HTH+HTH+SRA||?->McrB-AAA->McrC-NTD+REase->	1173	Bacteria>Terrabacteria_group>Actinomycetota>Actinomycetes>Micrococcales>Micrococcaceae	GCF_001484605.1	Pseudarthrobacter_sulfonivorans	AU252_RS05160	1179330..1182851	121292	BREX-system-P-loop-protein-BrxC-[Pseudarthrobacter-sulfonivorans]
WP_104138811.1	Type5.BREX	NUDIX->ARPP2-><-MACRODOMAIN+SIR2<-URI-DNase||HKD-DNase+SF1-SF2-Helicase+HTH+HTH+SRA->XIS-HTH-> BrxA-DNA-binding -> BrxB-BrxF-iSTAND -> DUF6079 *-> N6-METHYLASE -> PglZ -> N6-METHYLASE -p-> N6-METHYLASE -> SIGMA+OB-Fold+AAA-ATPase+HKD-DNase-BrxL ->?-> ParB + HNH ->?->35exo+TUDOR+SF1-SF2-Helicase+iREase+CTD->	1173	Bacteria>Terrabacteria_group>Actinomycetota>Actinomycetes>Micrococcales>Micrococcaceae	GCF_002929275.1	Arthrobacter_sp._ZGTC131	CXQ90_RS09900	131121..134642	2058898	BREX-system-P-loop-protein-BrxC-[Arthrobacter-sp.-ZGTC131]
WP_223883883.1	Type1.BREX	NUDIX-><-URI-DNase<-MACRODOMAIN+SIR2<-REase<-?||HKD-DNase+SF1-SF2-Helicase+HTH+HTH+SRA-><- ParB + HNH || BrxA-DNA-binding -> BrxB-BrxF-iSTAND -> DUF6079 *-> N6-METHYLASE -> N6-METHYLASE -> PglZ -> SIGMA+OB-Fold+AAA-ATPase+Lon-Protease-BrxL ->SF1-SF2-Helicase->Sigma-HTH->HTH+rve->AAA-ATPase->	1144	Bacteria>Terrabacteria_group>Actinomycetota>Actinomycetes>Micrococcales>Micrococcaceae	GCF_014764445.1	Pseudarthrobacter_sp._BIM_B-2242	IDT60_RS04725	994238..997672	2772401	BREX-system-P-loop-protein-BrxC-[Pseudarthrobacter-sp.-BIM-B-2242]
WP_224048794.1	Type1.BREX	NUDIX-> wHTH(SBD)+ HNH -><-?||?-><-SF1-SF2-Helicase||XIS-HTH-> BrxA-DNA-binding -> BrxB-BrxF-iSTAND -> DUF6079 *-> N6-METHYLASE -> PglZ -> N6-METHYLASE -> SIGMA+OB-Fold+AAA-ATPase+HKD-DNase-BrxL -><- ParB + HNH ||HEPN+SF1-SF2-Helicase+REase->	1173	Bacteria>Terrabacteria_group>Actinomycetota>Actinomycetes>Micrococcales>Micrococcaceae	GCF_019977335.1	Arthrobacter_sp._NicSoilB4	LDO13_RS04105	882114..885635	2830997	BREX-system-P-loop-protein-BrxC-[Arthrobacter-sp.-NicSoilB4]
WP_075958588.1	Type1.BREX	<-MazG<-nSTAND1+ HNH <-REase+SF1-SF2-Helicase-p<-REase||XIS-HTH-> BrxA-DNA-binding -> BrxB-BrxF-iSTAND -> DUF6079 -p*-> DUF6079 -p-> N6-METHYLASE -> SIGMA+OB-Fold+AAA-ATPase+Lon-Protease-BrxL -> ParB + HNH -><-REase<-SF1-SF2-Helicase+REase<-REase<-REase<-REase	295	Bacteria>Terrabacteria_group>Actinomycetota>Actinomycetes>Micrococcales>Ornithinimicrobiaceae	GCF_001942405.1	Ornithinimicrobium_sp._CNJ-824	BJF81_RS12330	99001..99888	1904966	hypothetical-protein-[Ornithinimicrobium-sp.-CNJ-824]
WP_075958589.1	Type1.BREX	<-MazG<-nSTAND1+ HNH <-REase+SF1-SF2-Helicase-p<-REase||XIS-HTH-> BrxA-DNA-binding -> BrxB-BrxF-iSTAND -> DUF6079 -p-> DUF6079 -p*-> N6-METHYLASE -> SIGMA+OB-Fold+AAA-ATPase+Lon-Protease-BrxL -> ParB + HNH -><-REase<-SF1-SF2-Helicase+REase<-REase<-REase<-REase	882	Bacteria>Terrabacteria_group>Actinomycetota>Actinomycetes>Micrococcales>Ornithinimicrobiaceae	GCF_001942405.1	Ornithinimicrobium_sp._CNJ-824	BJF81_RS12335	99810..102458	1904966	BREX-system-P-loop-protein-BrxC-[Ornithinimicrobium-sp.-CNJ-824]
WP_102213741.1	Type1.BREX	XIS-HTH-> BrxA-DNA-binding -> BrxB-BrxF-iSTAND -> DUF6079 *-> N6-METHYLASE ->HTH+REase-> PglZ -> SIGMA+OB-Fold+AAA-ATPase+HKD-DNase-BrxL -><- ParB + HNH +METHYLASE	1173	Bacteria>Terrabacteria_group>Actinomycetota>Actinomycetes>Mycobacteriales>Corynebacteriaceae	GCF_002871715.1	Corynebacterium_xerosis	CJ204_09375	1693..5214	1725	BREX-system-P-loop-protein-BrxC-[Corynebacterium-xerosis]
WP_259898411.1	Type1.BREX	<-HIN-HTH<- ParB + HNH <- SIGMA+OB-Fold+AAA-ATPase+HKD-DNase-BrxL <- PglZ <- N6-METHYLASE <- DUF6079 *<- BrxB-BrxF-iSTAND <- BrxA-DNA-binding <-XIS-HTH	1160	Bacteria>Terrabacteria_group>Actinomycetota>Actinomycetes>Mycobacteriales>Corynebacteriaceae	GCF_025152505.1	Corynebacterium_sanguinis	M3G46_RS10600	10062..13544	2594913	BREX-system-P-loop-protein-BrxC-[Corynebacterium-sanguinis]
WP_064441930.1	Type1.BREX	<- PglZ -p<- N6-METHYLASE <- DUF6079 *<- BrxB-BrxF-iSTAND <- BrxA-DNA-binding || ParB + HNH -> ParB + HNH ->?->DndD->HEPN->?->REase->	1148	Bacteria>Terrabacteria_group>Actinomycetota>Actinomycetes>Mycobacteriales>Mycobacteriaceae	GCF_001653095.1	Hoyosella_altamirensis	HAL02_RS18845	3788..7234	616997	BREX-system-P-loop-protein-BrxC-[Hoyosella-altamirensis]
WP_068042721.1	Type6.BREX	<-Phage_integrase<-?<-ZnR<-AAA-ATPase<- ParB + HNH ||?-><- SIGMA+OB-Fold+AAA-ATPase+Lon-Protease-BrxL <- PglZ <- N6-METHYLASE <- DUF6079 *<- BrxB-BrxF-iSTAND <- BrxA-DNA-binding <- BrxE ||ABhydrolase+ACYC->	1161	Bacteria>Terrabacteria_group>Actinomycetota>Actinomycetes>Mycobacteriales>Mycobacteriaceae	GCF_001667585.1	Mycobacterium_sp._E2733	A5678_RS12510	65198..68683	1834138	BREX-system-P-loop-protein-BrxC-[Mycobacterium-sp.-E2733]
WP_234813880.1	Type1.BREX	<- ParB + HNH <- SIGMA+OB-Fold+AAA-ATPase+HKD-DNase-BrxL <- PglZ <- N6-METHYLASE <- DUF6079 -p<- DUF6079 -p*<- BrxB-BrxF-iSTAND <- BrxA-DNA-binding	682	Bacteria>Terrabacteria_group>Actinomycetota>Actinomycetes>Mycobacteriales>Mycobacteriaceae	GCF_002101705.1	Mycolicibacterium_iranicum	AWC12_RS31570	96008..98056	912594	BREX-system-P-loop-protein-BrxC-[Mycolicibacterium-iranicum]
WP_163778779.1	Type1.BREX	GGDEF+EAL->?-><-Glyco_tran_28_C<-?||?-><-?<-tRNA||XIS-HTH-> BrxA-DNA-binding -> BrxB-BrxF-iSTAND -> DUF6079 *-> N6-METHYLASE -> PglZ -> SIGMA+OB-Fold+AAA-ATPase+HKD-DNase-BrxL -> ParB + HNH ->?->AAA-ATPase->ZnR->?->Phage_integrase->	1156	Bacteria>Terrabacteria_group>Actinomycetota>Actinomycetes>Mycobacteriales>Mycobacteriaceae	GCF_010727945.1	Mycobacterium_cookii	G6N27_RS18550	3937772..3941242	1775	BREX-system-P-loop-protein-BrxC-[Mycobacterium-cookii]
WP_260940163.1	Type1.BREX	XIS-HTH-> BrxA-DNA-binding -> BrxB-BrxF-iSTAND -> DUF6079 *-> N6-METHYLASE -> PglZ -> SIGMA+OB-Fold+AAA-ATPase+HKD-DNase-BrxL ->?-> ParB + HNH -p->HIN-HTH-><-wHTH||MORC+DUF3684+DUF3883-><-REase+HTH+35exo+Small-B-barrel+SF1-SF2-Helicase	1156	Bacteria>Terrabacteria_group>Actinomycetota>Actinomycetes>Mycobacteriales>Mycobacteriaceae	GCF_025331195.1	Mycolicibacterium_llatzerense	A7G45_RS21390	50451..53921	280871	BREX-system-P-loop-protein-BrxC-[Mycolicibacterium-llatzerense]
WP_267319448.1	Type1.BREX	wHTH(SBD)+ HNH -><-HEPN<-DndD||RAMA-><- ParB + HNH <- SIGMA+OB-Fold+AAA-ATPase+Lon-Protease-BrxL <- PglZ <- N6-METHYLASE <- DUF6079 *<- BrxB-BrxF-iSTAND <- BrxA-DNA-binding ||DEDD_Tnp_IS110+Transposase_20->	1149	Bacteria>Terrabacteria_group>Actinomycetota>Actinomycetes>Mycobacteriales>Mycobacteriaceae	GCF_026501225.1	Mycolicibacterium_mucogenicum	OS122_RS24315	502363..505812	56689	BREX-system-P-loop-protein-BrxC-[Mycolicibacterium-mucogenicum]
OFE08162.1	Type1.BREX	<-Pkinase+TPR+TPR<-?<-?<-?<-?<-?||tRNA-><-?|| BrxA-DNA-binding -> BrxB-BrxF-iSTAND -> DUF6079 *-> PglZ -> SIGMA+OB-Fold+AAA-ATPase+HKD-DNase-BrxL ->?->?->?-><- ParB + HNH ||?->?->LZ_Tnp_IS481+HTH_32+rve+rve_3->	1175	Bacteria>Terrabacteria_group>Actinomycetota>Actinomycetes>Mycobacteriales>Nocardiaceae	GCA_001757665.1	Rhodococcus_sp._1139	A5N83_14165	58033..61560	1833762	ATP-binding-protein-[Rhodococcus-sp.-1139]
MBY6706229.1	Type1.BREX	<-SF1-SF2-Helicase+DUF1998<-wHTH<-LON-AAA<-REase||?-><-ABhydrolase+ABhydrolase<- HNH || BrxA-DNA-binding -> BrxB-BrxF-iSTAND -> DUF6079 *-> N6-METHYLASE -> PglZ -> SIGMA+OB-Fold+AAA-ATPase+Lon-Protease-BrxL -> ParB + HNH ->||Exonuc_V_gamma+REase->SF1-SF2-Helicase+REase-><-SF1-SF2-Helicase+Classical-AAA<-HD	1147	Bacteria>Terrabacteria_group>Actinomycetota>Actinomycetes>Mycobacteriales>Nocardiaceae	GCA_019836145.1	Rhodococcus_sp._BP-241	HQ308_05385	442671..446114	2739441	BREX-system-P-loop-protein-BrxC-[Rhodococcus-sp.-BP-241]
WP_029256831.1	Type1.BREX	ParB + HNH -><-?<-?<- SIGMA+OB-Fold+AAA-ATPase+HKD-DNase-BrxL <- PglZ <- N6-METHYLASE <- DUF6079 *<- BrxB-BrxF-iSTAND <- BrxA-DNA-binding ||?-><-tRNA||?->?->?->?->?->Pkinase+TPR+TPR->	1173	Bacteria>Terrabacteria_group>Actinomycetota>Actinomycetes>Mycobacteriales>Nocardiaceae	GCF_000696675.2	Rhodococcus_erythropolis	H351_RS02855	635383..638904	1833	BREX-system-P-loop-protein-BrxC-[Rhodococcus-erythropolis]
WP_070161347.1	Type1.BREX	<-Pkinase+TPR+TPR<-?<-?<-?<-?<-?||tRNA-><-?|| BrxA-DNA-binding -> BrxB-BrxF-iSTAND -> DUF6079 *-> N6-METHYLASE -> N6-METHYLASE -p-> PglZ -> SIGMA+OB-Fold+AAA-ATPase+HKD-DNase-BrxL ->?->?-><- ParB + HNH	1175	Bacteria>Terrabacteria_group>Actinomycetota>Actinomycetes>Mycobacteriales>Nocardiaceae	GCF_001757665.1	Rhodococcus_sp._1139	A5N83_RS14165	58033..61560	1833762	BREX-system-P-loop-protein-BrxC-[Rhodococcus-sp.-1139]

Protein-ID	System	Gene-Neighbourhoods	Length	Taxonomy	Assembly-ID	Strain	Locus-Tag	Genomic-coordinates	TaxID	Fasta-defline
WP_261775798.1	Type1.BREX	<-SF1-SF2-Helicase+DUF1998<-wHTH<-LON-AAA<-REase||?-><-ABhydrolase+ABhydrolase<- HNH || BrxA-DNA-binding -> BrxB-BrxF-iSTAND -> DUF6079 *-> N6-METHYLASE -> SIGMA+OB-Fold+AAA-ATPase [illegible]	1142	Bacteria>Terrabacteria_group>Actinomycetota>Actinomycetes>Mycobacteriales>Nocardiaceae	GCF_019836145.1	Rhodococcus_sp._BP-241	HQ308_RS05380	442686..446114	2739441	BREX-system-P-loop-protein-BrxC-[Rhodococcus-sp.-BP-241]
WP_231782753.1	Type1.BREX	<-Pkinase+TPR+TPR<-?<-?<-?<-?<-?||tRNA-><-?|| BrxA-DNA-binding -> BrxB-BrxF-iSTAND -> DUF6079 *-> N6-METHYLASE -> PglZ -> SIGMA+OB-Fold+AAA-ATPase+HKD-DNase-BrxL ->?->?->?-><- ParB [illegible]	1173	Bacteria>Terrabacteria_group>Actinomycetota>Actinomycetes>Mycobacteriales>Nocardiaceae	GCF_021165975.1	Rhodococcus_qingshengii	LRL17_RS24605	5269427..5272948	334542	BREX-system-P-loop-protein-BrxC-[Rhodococcus-qingshengii]
WP_155277929.1	Type1.BREX	<-Pkinase+TPR+TPR<-?<-?<-?<-?<-?||tRNA-><-?|| BrxA-DNA-binding -> BrxB-BrxF-iSTAND -> DUF6079 *-> N6-METHYLASE -> PglZ -> SIGMA+OB-Fold+AAA-ATPase+HKD-DNase-BrxL ->?->?-><- ParB + [illegible]	1173	Bacteria>Terrabacteria_group>Actinomycetota>Actinomycetes>Mycobacteriales>Nocardiaceae	GCF_024349805.1	Rhodococcus	RE2895_RS00215	46904..50425	1827	MULTISPECIES:-BREX-system-P-loop-protein-BrxC-[Rhodococcus]
WP_256520207.1	Type1.BREX	<-Pkinase+TPR+TPR<-?<-?<-?<-?<-?||tRNA-><-?|| BrxA-DNA-binding -> BrxB-BrxF-iSTAND -> DUF6079 *-> N6-METHYLASE -> PglZ -> SIGMA+OB-Fold+AAA-ATPase+HKD-DNase-BrxL ->?->?->?-><- ParB [illegible]	1174	Bacteria>Terrabacteria_group>Actinomycetota>Actinomycetes>Mycobacteriales>Nocardiaceae	GCF_024498635.1	Rhodococcus_qingshengii	LRQ08_RS00215	46197..49721	334542	BREX-system-P-loop-protein-BrxC-[Rhodococcus-qingshengii]
WP_222645759.1	Type1.BREX	<-SF1-SF2-Helicase+DUF1998<-wHTH<-LON-AAA<-REase||?-><-ABhydrolase+ABhydrolase<- HNH || BrxA-DNA-binding -> BrxB-BrxF-iSTAND -> DUF6079 *-> N6-METHYLASE -> PglZ -> SIGMA+OB-Fold+AAA [illegible]	1147	Bacteria>Terrabacteria_group>Actinomycetota>Actinomycetes>Mycobacteriales>Nocardiaceae	-	Rhodococcus_sp._BP-241	HQ308_05385	442671..446114	2739441	BREX-system-P-loop-protein-BrxC-[Rhodococcus-sp.-BP-241]
WP_153653111.1	Type5.BREX	<-HTH_3||?->SF1-SF2-Helicase+REase->SF1-SF2-Helicase+REase->?-><- ParB + HNH <- SIGMA+OB-Fold+AAA-ATPase+HKD-DNase-BrxL <- PglZ <- N6-METHYLASE <- DUF6079 *<- BrxB-BrxF-iSTAND <- [illegible]	1160	Bacteria>Terrabacteria_group>Actinomycetota>Actinomycetes>Propionibacteriales>Nocardioidaceae	GCF_009649075.1	Aeromicrobium_yanjiei	GEV26_RS10980	2228820..2232302	2662028	BREX-system-P-loop-protein-BrxC-[Aeromicrobium-yanjiei]
WP_182782739.1	Type1.BREX	Peptidase_C14->?->?->?->PIN-><-URI-DNase<-TrwC+SF1-SF2-Helicase+TOPRIM|| BrxA-DNA-binding -> BrxB-BrxF-iSTAND -> DUF6079 *-> N6-METHYLASE -> N6-METHYLASE -p-> PglZ -> SIGMA+OB-Fold [illegible]	1159	Bacteria>Terrabacteria_group>Actinomycetota>Actinomycetes>Propionibacteriales>Propionibacteriaceae	GCF_014164725.1	Tessaracoccus_sp._MC1627	H5399_RS09110	126520..129999	2760312	BREX-system-P-loop-protein-BrxC-[Tessaracoccus-sp.-MC1627]
WP_255521255.1	Type1.BREX	<-ABC-ATPase+SbcC<-ABC-ATPase+SbcC<-?<-?<-?||tRNA-><-?<- SIGMA+OB-Fold+AAA-ATPase+Lon-Protease-BrxL <- PglZ <- N6-METHYLASE <- DUF6079 *<- BrxB-BrxF-iSTAND <- BrxA-DNA-binding || [illegible]	1146	Bacteria>Terrabacteria_group>Actinomycetota>Actinomycetes>Propionibacteriales>Propionibacteriaceae	GCF_017355265.1	Tessaracoccus_sp._SD287	IPV09_RS05845	30832..34272	2782008	BREX-system-P-loop-protein-BrxC-[Tessaracoccus-sp.-SD287]
WP_217922216.1	Type1.BREX	ABC-ATPase+SbcC->ABC-ATPase+SbcC-> ParB + HNH -><-?<-?<- SIGMA+OB-Fold+AAA-ATPase+Lon-Protease-BrxL <- PglZ <- N6-METHYLASE <- N6-METHYLASE <- DUF6079 *<- BrxB-BrxF-iSTAND <- [illegible]	1154	Bacteria>Terrabacteria_group>Actinomycetota>Thermoleophilia>Miltoncostaeales>Miltoncostaeaceae	GCF_018141545.1	Miltoncostaea_oceani	IU369_RS17235	3377817..3381281	2843216	BREX-system-P-loop-protein-BrxC-[Miltoncostaea-oceani]
MBJ7470365.1	Type1.BREX	BrxA-DNA-binding -> BrxB-BrxF-iSTAND -> DUF6079 *-> N6-METHYLASE -> PglZ -> SIGMA+OB-Fold+AAA-ATPase+Lon-Protease-BrxL -> ParB + HNH -><-?<-SF1-SF2-Helicase	1157	Bacteria>Terrabacteria_group>Actinomycetota>Thermoleophilia>Solirubrobacterales>Solirubrobacteraceae	GCA_016463515.1	Solirubrobacteraceae_bacterium	JHD16_03650	3440..6913	2586742	BREX-system-P-loop-protein-BrxC-[Solirubrobacteraceae-bacterium]
PFG07777.1	Type5.BREX	<-HKD-DNase+SF1-SF2-Helicase+HTH+HTH+SRA<-NUDIX|| wHTH(SBD)+ HNH -><-?||HTH_3->?-><-TUDOR+SF1-SF2-Helicase+iREase+CTD+REase<- PglZ <- N6-METHYLASE <- DUF6079 *<- BrxB-BrxF-iSTAND [illegible]	1195	Bacteria>Terrabacteria_group>Bacillota>Bacilli>Bacillales>Bacillaceae	GCA_002563655.1	Bacillus_sp._es.034	ATG71_4686	4473829..4477416	1761763	hypothetical-protein-ATG71_4686-[Bacillus-sp.-es.034]
WP_042224724.1	Type1.BREX	Cas9+ HNH +RuvC->Cas_Cas1->CRISPR_Cas2-> BrxA-DNA-binding -> BrxB-BrxF-iSTAND -> DUF6079 *-> N6-METHYLASE -> PglZ -> SIGMA+OB-Fold+AAA-ATPase+Lon-Protease-BrxL -> ParB + HNH ->?->NTP_ [illegible]	1180	Bacteria>Terrabacteria_group>Bacillota>Bacilli>Bacillales>Bacillaceae	GCF_000724085.1	Oceanobacillus_manasiensis	BN985_RS16170	528907..532449	586413	BREX-system-P-loop-protein-BrxC-[Oceanobacillus-manasiensis]
WP_095320972.1	Type1.BREX	<-HISKIN<-REC+OmpR-HTH||?-><-?<-OLD-ABC<- ParB + HNH <- SIGMA+OB-Fold+AAA-ATPase+Lon-Protease-BrxL <- PglZ <- N6-METHYLASE <- DUF6079 *<- BrxB-BrxF-iSTAND <- BrxA-DNA-binding [illegible]	1194	Bacteria>Terrabacteria_group>Bacillota>Bacilli>Bacillales>Bacillaceae	GCF_002271995.1	Niallia_circulans	CHH55_RS22000	53589..57173	1397	BREX-system-P-loop-protein-BrxC-[Niallia-circulans]
WP_108713788.1	Type1.BREX	TPR-><-?<-Phage_CRI+Phage_X<- ParB + HNH <- SIGMA+OB-Fold+AAA-ATPase+Lon-Protease-BrxL <- PglZ <-HEPN<-AbiGi+seqA+RAMA<- N6-METHYLASE <- DUF6079 *<- BrxB-BrxF-iSTAND <- [illegible]	1191	Bacteria>Terrabacteria_group>Bacillota>Bacilli>Bacillales>Bacillaceae	GCF_003073375.1	unclassified_Lysinibacillus	DCE79_RS14920	3050129..3053704	2636778	MULTISPECIES:-BREX-system-P-loop-protein-BrxC-[unclassified-Lysinibacillus]
WP_144538762.1	Type1.BREX	[illegible]	1191	Bacteria>Terrabacteria_group>Bacillota>Bacilli>Bacillales>Bacillaceae	GCF_007677425.1	Bacillus_sp._FJAT-22090	FS503_RS11600	136183..139758	1581038	BREX-system-P-loop-protein-BrxC-[Bacillus-sp.-FJAT-22090]
WP_166025629.1	Type1.BREX	[illegible]	1191	Bacteria>Terrabacteria_group>Bacillota>Bacilli>Bacillales>Bacillaceae	GCF_011393025.1	Neobacillus_terrae	G8761_RS08595	43782..47357	3034837	BREX-system-P-loop-protein-BrxC-[Neobacillus-terrae]
WP_216486703.1	Type1.BREX	[illegible]	1194	Bacteria>Terrabacteria_group>Bacillota>Bacilli>Bacillales>Bacillaceae	GCF_018918075.1	Virgibacillus_sp._MSJ-26	KQI49_RS03110	240564..244148	2841522	BREX-system-P-loop-protein-BrxC-[Virgibacillus-sp.-MSJ-26]
WP_237075536.1	Type1.BREX	[illegible]	1192	Bacteria>Terrabacteria_group>Bacillota>Bacilli>Bacillales>Bacillaceae	GCF_021864615.1	Priestia_megaterium	L4R70_RS26975	37253..40831	1404	BREX-system-P-loop-protein-BrxC-[Priestia-megaterium]
WP_259420301.1	Type1.BREX	[illegible]	1191	Bacteria>Terrabacteria_group>Bacillota>Bacilli>Bacillales>Bacillaceae	GCF_024919535.1	Lysinibacillus_sp._A4	NY607_RS20580	136984..140559	2976269	BREX-system-P-loop-protein-BrxC-[Lysinibacillus-sp.-A4]
WP_010494807.1	Type1.BREX	[illegible]	1187	Bacteria>Terrabacteria_group>Bacillota>Bacilli>Bacillales>Paenibacillaceae	GCF_000213315.1	Paenibacillus_elgii	MGF69_RS0111725	12079..15642	189691	BREX-system-P-loop-protein-BrxC-[Paenibacillus-elgii]
WP_038596865.1	Type1.BREX	[illegible]	1189	Bacteria>Terrabacteria_group>Bacillota>Bacilli>Bacillales>Paenibacillaceae	GCF_000758525.1	Paenibacillus_sp._FSL_H7-0357	H70357_RS30035	6820533..6824102	1536774	BREX-system-P-loop-protein-BrxC-[Paenibacillus-sp.-FSL-H7-0357]
WP_272441147.1	Type5.BREX	[illegible]	1201	Bacteria>Terrabacteria_group>Bacillota>Bacilli>Bacillales>Paenibacillaceae	GCF_028421645.1	Aneurinibacillus_sp._Ricciae_BoGa-3	PP175_RS01545	299012..302617	3022697	BREX-system-P-loop-protein-BrxC-[Aneurinibacillus-sp.-Ricciae_BoGa-3]
WP_108587666.1	Type5.BREX	[illegible]	1191	Bacteria>Terrabacteria_group>Bacillota>Bacilli>Bacillales>Planococcaceae	GCF_003058165.1	Paenisporosarcina_sp._OV554	C8K15_RS17245	15921..19496	2135694	BREX-system-P-loop-protein-BrxC-[Paenisporosarcina-sp.-OV554]
OZT77510.1	Type1.BREX	[illegible]	1183	Bacteria>Terrabacteria_group>Bacillota>Bacilli>Bacillales>Staphylococcaceae	GCA_002265785.1	Salinicoccus_roseus	CFN03_06110	209519..213070	45670	hypothetical-protein-CFN03_06110-[Salinicoccus-roseus]
WP_256974727.1	Type1.BREX	[illegible]	1178	Bacteria>Terrabacteria_group>Bacillota>Bacilli>Bacillales>Staphylococcaceae	GCF_002265785.1	Salinicoccus_roseus	CFN03_RS06110	209534..213070	45670	BREX-system-P-loop-protein-BrxC-[Salinicoccus-roseus]
WP_094906236.1	Type1.BREX	[illegible]	1183	Bacteria>Terrabacteria_group>Bacillota>Bacilli>Bacillales>Staphylococcaceae	-	Salinicoccus_roseus	CFN03_06110	209519..213070	45670	BREX-system-P-loop-protein-BrxC-[Salinicoccus-roseus]
HAJ70155.1	Type1.BREX	[illegible]	1213	Bacteria>Terrabacteria_group>Bacillota>Bacilli>Lactobacillales>Carnobacteriaceae	GCA_003454485.1	Alkalibacterium_sp.	DCO62_02885	43231..46872	1872447	BREX-system-P-loop-protein-BrxC-[Alkalibacterium-sp.]
WP_091633361.1	Type1.BREX	[illegible]	1209	Bacteria>Terrabacteria_group>Bacillota>Bacilli>Lactobacillales>Carnobacteriaceae	GCF_900109085.1	Alkalibacterium_gilvum	BMX83_RS03335	22133..25762	1130080	BREX-system-P-loop-protein-BrxC-[Alkalibacterium-gilvum]
WP_061100912.1	Type1.BREX	[illegible]	1221	Bacteria>Terrabacteria_group>Bacillota>Bacilli>Lactobacillales>Enterococcaceae	GCF_001563075.1	Enterococcus_faecalis	AW733_RS05605	279027..282692	1351	BREX-system-P-loop-protein-BrxC-[Enterococcus-faecalis]
WP_144771940.1	Type1.BREX	[illegible]	1223	Bacteria>Terrabacteria_group>Bacillota>Bacilli>Lactobacillales>Enterococcaceae	GCF_007682015.1	Enterococcus_casseliflavus	FS854_RS14505	69459..73130	37734	BREX-system-P-loop-protein-BrxC-[Enterococcus-casseliflavus]
WP_156237125.1	Type1.BREX	[illegible]	1223	Bacteria>Terrabacteria_group>Bacillota>Bacilli>Lactobacillales>Enterococcaceae	GCF_009734725.1	Enterococcus_casseliflavus	EZ072_RS09880	26969..30640	37734	BREX-system-P-loop-protein-BrxC-[Enterococcus-casseliflavus]
WP_194194888.1	Type1.BREX	[illegible]	1223	Bacteria>Terrabacteria_group>Bacillota>Bacilli>Lactobacillales>Enterococcaceae	GCF_015230945.1	Enterococcus_casseliflavus	HAX40_RS05295	101566..105237	37734	BREX-system-P-loop-protein-BrxC-[Enterococcus-casseliflavus]
WP_251851849.1	Type1.BREX	[illegible]	1202	Bacteria>Terrabacteria_group>Bacillota>Bacilli>Lactobacillales>Enterococcaceae	GCF_023744155.1	Enterococcus_italicus	NE261_RS07355	549628..553236	246144	BREX-system-P-loop-protein-BrxC-[Enterococcus-italicus]
ALJ23836.1	Type1.BREX	[illegible]	1242	Bacteria>Terrabacteria_group>Bacillota>Bacilli>Lactobacillales>Lactobacillaceae	GCA_001314245.2	Lactobacillus_gallinarum	AO203_08530	1678493..1682221	52242	ATPase-[Lactobacillus-gallinarum]
AUI77142.1	Type1.BREX	[illegible]	1240	Bacteria>Terrabacteria_group>Bacillota>Bacilli>Lactobacillales>Lactobacillaceae	GCA_002849955.1	Lactobacillus_helveticus	Lh22155_05455	1074208..1077930	1587	ATPase-[Lactobacillus-helveticus]
MBT8803912.1	Type1.BREX	[illegible]	1178	Bacteria>Terrabacteria_group>Bacillota>Bacilli>Lactobacillales>Lactobacillaceae	GCA_018719835.1	Lactobacillus_delbrueckii_subsp._bulgaricus	BTJ20_08000	3864..7400	1585	ATPase-[Lactobacillus-delbrueckii-subsp.-bulgaricus]
MBT8900174.1	Type1.BREX	[illegible]	1178	Bacteria>Terrabacteria_group>Bacillota>Bacilli>Lactobacillales>Lactobacillaceae	GCA_018720625.1	Lactobacillus_delbrueckii_subsp._bulgaricus	BTI57_06430	8367..11903	1585	ATPase-[Lactobacillus-delbrueckii-subsp.-bulgaricus]
MBT8901771.1	Type1.BREX	[illegible]	1178	Bacteria>Terrabacteria_group>Bacillota>Bacilli>Lactobacillales>Lactobacillaceae	GCA_018720695.1	Lactobacillus_delbrueckii_subsp._bulgaricus	BTI52_06840	21471..25007	1585	ATPase-[Lactobacillus-delbrueckii-subsp.-bulgaricus]
MBT8914213.1	Type1.BREX	[illegible]	1178	Bacteria>Terrabacteria_group>Bacillota>Bacilli>Lactobacillales>Lactobacillaceae	GCA_018720815.1	Lactobacillus_delbrueckii_subsp._bulgaricus	BTI61_07605	4087..7623	1585	ATPase-[Lactobacillus-delbrueckii-subsp.-bulgaricus]
MCH3996392.1	Type1.BREX	[illegible]	1244	Bacteria>Terrabacteria_group>Bacillota>Bacilli>Lactobacillales>Lactobacillaceae	GCA_022483225.1	Lactobacillus_amylovorus	LKE67_02050	413910..417644	1604	BREX-system-P-loop-protein-BrxC-[Lactobacillus-amylovorus]
MCI7713737.1	Type1.BREX	[illegible]	1244	Bacteria>Terrabacteria_group>Bacillota>Bacilli>Lactobacillales>Lactobacillaceae	GCA_022786405.1	Lactobacillus_johnsonii	MSL49_00240	14783..18517	33959	BREX-system-P-loop-protein-BrxC-[Lactobacillus-johnsonii]
WP_014569899.1	Type1.BREX	[illegible]	1210	Bacteria>Terrabacteria_group>Bacillota>Bacilli>Lactobacillales>Lactobacillaceae	GCF_000026505.1	Lacticaseibacillus_rhamnosus	LGG_RS10100	2165561..2169193	47715	BREX-system-P-loop-protein-BrxC-[Lacticaseibacillus-rhamnosus]
WP_014563593.1	Type1.BREX	[illegible]	1238	Bacteria>Terrabacteria_group>Bacillota>Bacilli>Lactobacillales>Lactobacillaceae	GCF_000189515.1	Lactobacillus_helveticus	LBHH_RS05435	1072223..1075939	1587	BREX-system-P-loop-protein-BrxC-[Lactobacillus-helveticus]
WP_005715305.1	Type1.BREX	[illegible]	1210	Bacteria>Terrabacteria_group>Bacillota>Bacilli>Lactobacillales>Lactobacillaceae	GCF_000311945.1	Lacticaseibacillus_rhamnosus	LRHMDP2_RS10615	154290..157922	47715	BREX-system-P-loop-protein-BrxC-[Lacticaseibacillus-rhamnosus]
WP_003602979.1	Type1.BREX	[illegible]	1241	Bacteria>Terrabacteria_group>Bacillota>Bacilli>Lactobacillales>Lactobacillaceae	GCF_000376145.1	Lacticaseibacillus	LCAUW4_RS03725	15865..19590	2759736	MULTISPECIES:-BREX-system-P-loop-protein-BrxC-[Lacticaseibacillus]
WP_015638640.1	Type1.BREX	[illegible]	1230	Bacteria>Terrabacteria_group>Bacillota>Bacilli>Lactobacillales>Lactobacillaceae	GCF_000397165.1	Limosilactobacillus_fermentum	LBFF_RS02010	427124..430816	1613	BREX-system-P-loop-protein-BrxC-[Limosilactobacillus-fermentum]
WP_027828098.1	Type1.BREX	[illegible]	1241	Bacteria>Terrabacteria_group>Bacillota>Bacilli>Lactobacillales>Lactobacillaceae	GCF_000425885.1	Schleiferilactobacillus_harbinensis	FC91_RS07095	17902..21627	304207	BREX-system-P-loop-protein-BrxC-[Schleiferilactobacillus-harbinensis]
WP_023192364.1	Type1.BREX	[illegible]	1179	Bacteria>Terrabacteria_group>Bacillota>Bacilli>Lactobacillales>Lactobacillaceae	GCF_000493335.1	Lactobacillus_helveticus	LHE_RS17595	1223279..1226818	1587	BREX-system-P-loop-protein-BrxC-[Lactobacillus-helveticus]
WP_023192226.1	Type1.BREX	[illegible]	1243	Bacteria>Terrabacteria_group>Bacillota>Bacilli>Lactobacillales>Lactobacillaceae	GCF_000493555.1	Lactobacillus_helveticus	LHCIRMBIA104_RS09710	11167..14898	1587	BREX-system-P-loop-protein-BrxC-[Lactobacillus-helveticus]
WP_025283578.1	Type1.BREX	[illegible]	1243	Bacteria>Terrabacteria_group>Bacillota>Bacilli>Lactobacillales>Lactobacillaceae	GCF_000525715.1	Lactobacillus_helveticus	LBH_0939	971853..975584	1587	BREX-system-P-loop-protein-BrxC-[Lactobacillus-helveticus]
WP_047678144.1	Type1.BREX	[illegible]	1121	Bacteria>Terrabacteria_group>Bacillota>Bacilli>Lactobacillales>Lactobacillaceae	GCF_000801045.1	Lacticaseibacillus_rhamnosus	LaR116_RS11810	1..3366	47715	BREX-system-P-loop-protein-BrxC,-partial-[Lacticaseibacillus-rhamnosus]
WP_060598044.1	Type1.BREX	[illegible]	1178	Bacteria>Terrabacteria_group>Bacillota>Bacilli>Lactobacillales>Lactobacillaceae	GCF_001469775.1	Lactobacillus_delbrueckii	AT236_RS05345	1068532..1072068	1584	BREX-system-P-loop-protein-BrxC-[Lactobacillus-delbrueckii]
WP_060416898.1	Type1.BREX	[illegible]	1226	Bacteria>Terrabacteria_group>Bacillota>Bacilli>Lactobacillales>Lactobacillaceae	GCF_001540905.1	Levilactobacillus_brevis	ABB39_RS11500	6540..10220	1580	BREX-system-P-loop-protein-BrxC-[Levilactobacillus-brevis]
WP_046782810.1	Type1.BREX	[illegible]	1241	Bacteria>Terrabacteria_group>Bacillota>Bacilli>Lactobacillales>Lactobacillaceae	GCF_001656585.1	Lacticaseibacillus_rhamnosus	PY74_RS00235	14729..18454	47715	BREX-system-P-loop-protein-BrxC-[Lacticaseibacillus-rhamnosus]
WP_070065450.1	Type1.BREX	[illegible]	1241	Bacteria>Terrabacteria_group>Bacillota>Bacilli>Lactobacillales>Lactobacillaceae	GCF_001752545.1	Oenococcus_kitaharae	NT95_RS03550	13261..16986	336988	BREX-system-P-loop-protein-BrxC-[Oenococcus-kitaharae]
WP_070489741.1	Type1.BREX	[illegible]	1188	Bacteria>Terrabacteria_group>Bacillota>Bacilli>Lactobacillales>Lactobacillaceae	GCF_001808415.1	Lactobacillus_sp._HMSC08B12	HMPREF3168_RS09245	1333..4899	1581136	BREX-system-P-loop-protein-BrxC-[Lactobacillus-sp.-HMSC08B12]
WP_070792499.1	Type1.BREX	[illegible]	1227	Bacteria>Terrabacteria_group>Bacillota>Bacilli>Lactobacillales>Lactobacillaceae	GCF_001831225.1	Lacticaseibacillus_rhamnosus	BKP38_RS08735	14861..18544	47715	BREX-system-P-loop-protein-BrxC-[Lacticaseibacillus-rhamnosus]
WP_081371217.1	Type1.BREX	[illegible]	1243	Bacteria>Terrabacteria_group>Bacillota>Bacilli>Lactobacillales>Lactobacillaceae	GCF_001895375.1	Lactobacillus_helveticus	BEL10_RS03665	22378..26109	1587	BREX-system-P-loop-protein-BrxC-[Lactobacillus-helveticus]
WP_085769594.1	Type1.BREX	[illegible]	1241	Bacteria>Terrabacteria_group>Bacillota>Bacilli>Lactobacillales>Lactobacillaceae	GCF_002117345.1	Levilactobacillus_brevis	AZI11_RS13305	1..3726	1580	BREX-system-P-loop-protein-BrxC-[Levilactobacillus-brevis]
WP_086355469.1	Type1.BREX	[illegible]	1183	Bacteria>Terrabacteria_group>Bacillota>Bacilli>Lactobacillales>Lactobacillaceae	GCF_002142575.1	Lactobacillus_delbrueckii	B9N98_RS00590	112113..115664	1584	BREX-system-P-loop-protein-BrxC-[Lactobacillus-delbrueckii]
WP_087236056.1	Type1.BREX	[illegible]	1244	Bacteria>Terrabacteria_group>Bacillota>Bacilli>Lactobacillales>Lactobacillaceae	GCF_002160635.1	Lactobacillus_gallinarum	B5E95_07165	9727..13461	52242	BREX-system-P-loop-protein-BrxC-[Lactobacillus-gallinarum]
WP_087363710.1	Type1.BREX	[illegible]	1244	Bacteria>Terrabacteria_group>Bacillota>Bacilli>Lactobacillales>Lactobacillaceae	GCF_002160975.1	Lactobacillus_gallinarum	B5E63_RS08425	13791..17525	52242	BREX-system-P-loop-protein-BrxC-[Lactobacillus-gallinarum]
WP_095342391.1	Type1.BREX	[illegible]	1246	Bacteria>Terrabacteria_group>Bacillota>Bacilli>Lactobacillales>Lactobacillaceae	GCF_002276565.1	Lactobacillus_kefiranofaciens	B8W86_RS09305	2234..5974	267818	BREX-system-P-loop-protein-BrxC-[Lactobacillus-kefiranofaciens]
WP_049166573.1	Type1.BREX	[illegible]	1210	Bacteria>Terrabacteria_group>Bacillota>Bacilli>Lactobacillales>Lactobacillaceae	GCF_002287945.1	Lacticaseibacillus_rhamnosus	N507_RS04265	896062..899694	47715	BREX-system-P-loop-protein-BrxC-[Lacticaseibacillus-rhamnosus]
WP_097550471.1	Type1.BREX	[illegible]	1243	Bacteria>Terrabacteria_group>Bacillota>Bacilli>Lactobacillales>Lactobacillaceae	GCF_002532085.1	Lactobacillus_helveticus	CR094_RS03640	2555..6286	1587	BREX-system-P-loop-protein-BrxC-[Lactobacillus-helveticus]
WP_101812672.1	Type1.BREX	[illegible]	1241	Bacteria>Terrabacteria_group>Bacillota>Bacilli>Lactobacillales>Lactobacillaceae	GCF_002849955.1	Lactobacillus_helveticus	Lh22155_RS05635	1074208..1077933	1587	BREX-system-P-loop-protein-BrxC-[Lactobacillus-helveticus]
WP_102116084.1	Type1.BREX	[illegible]	1230	Bacteria>Terrabacteria_group>Bacillota>Bacilli>Lactobacillales>Lactobacillaceae	GCF_002869825.2	Limosilactobacillus_fermentum	C0965_RS02310	470388..474080	1613	BREX-system-P-loop-protein-BrxC-[Limosilactobacillus-fermentum]
WP_106904923.1	Type1.BREX	[illegible]	1241	Bacteria>Terrabacteria_group>Bacillota>Bacilli>Lactobacillales>Lactobacillaceae	GCF_003020005.1	Lactiplantibacillus_plantarum	B5G53_RS16135	18984..22709	1590	BREX-system-P-loop-protein-BrxC-[Lactiplantibacillus-plantarum]
WP_225367119.1	Type1.BREX	[illegible]	1182	Bacteria>Terrabacteria_group>Bacillota>Bacilli>Lactobacillales>Lactobacillaceae	GCF_003046115.1	Lacticaseibacillus_rhamnosus	DA801_RS04680	68737..72285	47715	BREX-system-P-loop-protein-BrxC-[Lacticaseibacillus-rhamnosus]
WP_114788341.1	Type1.BREX	[illegible]	1241	Bacteria>Terrabacteria_group>Bacillota>Bacilli>Lactobacillales>Lactobacillaceae	GCF_003347455.1	Lactiplantibacillus_plantarum	DB726_RS11995	25864..29589	1590	BREX-system-P-loop-protein-BrxC-[Lactiplantibacillus-plantarum]
WP_125770444.1	Type1.BREX	[illegible]	1225	Bacteria>Terrabacteria_group>Bacillota>Bacilli>Lactobacillales>Lactobacillaceae	GCF_003946325.1	Companilactobacillus_furfuricola	EKM78_RS06175	4477..8154	1462575	BREX-system-P-loop-protein-BrxC-[Companilactobacillus-furfuricola]
WP_125762723.1	Type1.BREX	[illegible]	1225	Bacteria>Terrabacteria_group>Bacillota>Bacilli>Lactobacillales>Lactobacillaceae	GCF_003946555.1	Companilactobacillus_hulinensis	EKM66_RS06280	16382..20059	2486007	BREX-system-P-loop-protein-BrxC-[Companilactobacillus-hulinensis]
WP_125647605.1	Type1.BREX	[illegible]	1230	Bacteria>Terrabacteria_group>Bacillota>Bacilli>Lactobacillales>Lactobacillaceae	GCF_003946675.1	Lapidilactobacillus_gannanensis	EKM81_RS01890	14683..18375	2486002	BREX-system-P-loop-protein-BrxC-[Lapidilactobacillus-gannanensis]
WP_014918808.1	Type1.BREX	[illegible]	1244	Bacteria>Terrabacteria_group>Bacillota>Bacilli>Lactobacillales>Lactobacillaceae	GCF_003955865.1	Lactobacillus_helveticus	LH5_RS02000	414775..418509	1587	BREX-system-P-loop-protein-BrxC-[Lactobacillus-helveticus]
WP_128537187.1	Type1.BREX	[illegible]	1241	Bacteria>Terrabacteria_group>Bacillota>Bacilli>Lactobacillales>Lactobacillaceae	GCF_004055415.1	Lactiplantibacillus_plantarum	EQG58_RS16175	99358..103083	1590	BREX-system-P-loop-protein-BrxC-[Lactiplantibacillus-plantarum]
WP_128529840.1	Type1.BREX	[illegible]	1241	Bacteria>Terrabacteria_group>Bacillota>Bacilli>Lactobacillales>Lactobacillaceae	GCF_004078645.1	Lactiplantibacillus_plantarum	EQJ06_RS15675	35919..39644	1590	BREX-system-P-loop-protein-BrxC-[Lactiplantibacillus-plantarum]
WP_128485902.1	Type1.BREX	[illegible]	1241	Bacteria>Terrabacteria_group>Bacillota>Bacilli>Lactobacillales>Lactobacillaceae	GCF_004101645.1	Lactiplantibacillus_plantarum	EQK45_RS16695	37408..41133	1590	BREX-system-P-loop-protein-BrxC-[Lactiplantibacillus-plantarum]
WP_129335566.1	Type1.BREX	[illegible]	1188	Bacteria>Terrabacteria_group>Bacillota>Bacilli>Lactobacillales>Lactobacillaceae	GCF_004123065.1	Lactobacillus_delbrueckii	EST31_RS04460	19918..23484	1584	BREX-system-P-loop-protein-BrxC-[Lactobacillus-delbrueckii]
WP_064613173.1	Type1.BREX	[illegible]	1219	Bacteria>Terrabacteria_group>Bacillota>Bacilli>Lactobacillales>Lactobacillaceae	GCF_006151905.1	Lacticaseibacillus_rhamnosus	FG342_RS13010	2567642..2571301	47715	BREX-system-P-loop-protein-BrxC-[Lacticaseibacillus-rhamnosus]
WP_153110820.1	Type1.BREX	[illegible]	1241	Bacteria>Terrabacteria_group>Bacillota>Bacilli>Lactobacillales>Lactobacillaceae	GCF_009495575.1	Lentilactobacillus_buchneri	FIU75_RS13270	20367..24092	1581	BREX-system-P-loop-protein-BrxC-[Lentilactobacillus-buchneri]
WP_095662164.1	Type1.BREX	[illegible]	1243	Bacteria>Terrabacteria_group>Bacillota>Bacilli>Lactobacillales>Lactobacillaceae	GCF_011392295.1	Lactobacillus_helveticus	ERJ65_RS00195	7386..11117	1587	BREX-system-P-loop-protein-BrxC-[Lactobacillus-helveticus]
WP_172993980.1	Type1.BREX	[illegible]	1243	Bacteria>Terrabacteria_group>Bacillota>Bacilli>Lactobacillales>Lactobacillaceae	GCF_013280765.1	Lactobacillus_helveticus	IMAU60206_RS01575	25352..29083	1587	BREX-system-P-loop-protein-BrxC-[Lactobacillus-helveticus]
WP_172993225.1	Type1.BREX	[illegible]	597	Bacteria>Terrabacteria_group>Bacillota>Bacilli>Lactobacillales>Lactobacillaceae	GCF_013280775.1	Lactobacillus_helveticus	IMAU60211_RS07170	7274..9066	1587	BREX-system-P-loop-protein-BrxC,-partial-[Lactobacillus-helveticus]
WP_172993587.1	Type1.BREX	[illegible]	1243	Bacteria>Terrabacteria_group>Bacillota>Bacilli>Lactobacillales>Lactobacillaceae	GCF_013280875.1	Lactobacillus_helveticus	IMAU60201_RS07155	4819..8550	1587	BREX-system-P-loop-protein-BrxC-[Lactobacillus-helveticus]
WP_172987666.1	Type1.BREX	[illegible]	1243	Bacteria>Terrabacteria_group>Bacillota>Bacilli>Lactobacillales>Lactobacillaceae	GCF_013280955.1	Lactobacillus_helveticus	IMAU50022_RS01295	3445..7176	1587	BREX-system-P-loop-protein-BrxC-[Lactobacillus-helveticus]
WP_172982106.1	Type1.BREX	[illegible]	1243	Bacteria>Terrabacteria_group>Bacillota>Bacilli>Lactobacillales>Lactobacillaceae	GCF_013281265.1	Lactobacillus_helveticus	IMAU30078_RS05865	2723..6454	1587	BREX-system-P-loop-protein-BrxC-[Lactobacillus-helveticus]
WP_173006741.1	Type1.BREX	[illegible]	1241	Bacteria>Terrabacteria_group>Bacillota>Bacilli>Lactobacillales>Lactobacillaceae	GCF_013281475.1	Lactobacillus_helveticus	IMAU30014_RS01855	25052..28777	1587	BREX-system-P-loop-protein-BrxC-[Lactobacillus-helveticus]
WP_173001654.1	Type1.BREX	[illegible]	1241	Bacteria>Terrabacteria_group>Bacillota>Bacilli>Lactobacillales>Lactobacillaceae	GCF_013281675.1	Lactobacillus_helveticus	IMAU10149_RS03700	20557..24282	1587	BREX-system-P-loop-protein-BrxC-[Lactobacillus-helveticus]
WP_182611612.1	Type1.BREX	[illegible]	1241	Bacteria>Terrabacteria_group>Bacillota>Bacilli>Lactobacillales>Lactobacillaceae	GCF_014155845.1	Lacticaseibacillus_rhamnosus	FOS38_RS12550	14856..18581	47715	BREX-system-P-loop-protein-BrxC-[Lacticaseibacillus-rhamnosus]
WP_188891961.1	Type1.BREX	[illegible]	1241	Bacteria>Terrabacteria_group>Bacillota>Bacilli>Lactobacillales>Lactobacillaceae	GCF_014656585.1	Lactobacillus_kefiranofaciens	ICI50_RS03490	670073..673798	267818	BREX-system-P-loop-protein-BrxC-[Lactobacillus-kefiranofaciens]
WP_064657275.1	Type1.BREX	[illegible]	1210	Bacteria>Terrabacteria_group>Bacillota>Bacilli>Lactobacillales>Lactobacillaceae	GCF_015558325.1	Lacticaseibacillus_rhamnosus	LW144_RS03870	70650..74282	47715	BREX-system-P-loop-protein-BrxC-[Lacticaseibacillus-rhamnosus]
WP_195631091.1	Type1.BREX	[illegible]	1241	Bacteria>Terrabacteria_group>Bacillota>Bacilli>Lactobacillales>Lactobacillaceae	GCF_015561225.1	Lactiplantibacillus_plantarum	I2H95_RS14900	2281..6006	1590	BREX-system-P-loop-protein-BrxC-[Lactiplantibacillus-plantarum]
WP_193820257.1	Type1.BREX	[illegible]	1219	Bacteria>Terrabacteria_group>Bacillota>Bacilli>Lactobacillales>Lactobacillaceae	GCF_016653515.1	Lacticaseibacillus_rhamnosus	JIO02_RS10990	2305265..2308924	47715	BREX-system-P-loop-protein-BrxC-[Lacticaseibacillus-rhamnosus]
WP_064533504.1	Type1.BREX	[illegible]	1210	Bacteria>Terrabacteria_group>Bacillota>Bacilli>Lactobacillales>Lactobacillaceae	GCF_016887865.1	Lacticaseibacillus_rhamnosus	JNW95_RS08680	79348..82980	47715	BREX-system-P-loop-protein-BrxC-[Lacticaseibacillus-rhamnosus]
WP_103658548.1	Type1.BREX	[illegible]	1243	Bacteria>Terrabacteria_group>Bacillota>Bacilli>Lactobacillales>Lactobacillaceae	GCF_017565805.1	Lactobacillus_helveticus	MVU88_RS02210	7396..11127	1587	BREX-system-P-loop-protein-BrxC-[Lactobacillus-helveticus]
WP_220028697.1	Type1.BREX	[illegible]	1244	Bacteria>Terrabacteria_group>Bacillota>Bacilli>Lactobacillales>Lactobacillaceae	GCF_019455965.1	Lactobacillus_helveticus	E0712_RS03715	165606..169340	1587	BREX-system-P-loop-protein-BrxC-[Lactobacillus-helveticus]
WP_220023636.1	Type1.BREX	[illegible]	1241	Bacteria>Terrabacteria_group>Bacillota>Bacilli>Lactobacillales>Lactobacillaceae	GCF_019455975.1	Lactobacillus_helveticus	E0715_RS08290	19434..23159	1587	BREX-system-P-loop-protein-BrxC-[Lactobacillus-helveticus]
WP_032957101.1	Type1.BREX	[illegible]	1210	Bacteria>Terrabacteria_group>Bacillota>Bacilli>Lactobacillales>Lactobacillaceae	GCF_020826335.1	Lacticaseibacillus_rhamnosus	LL671_RS03770	826686..830318	47715	BREX-system-P-loop-protein-BrxC-[Lacticaseibacillus-rhamnosus]
WP_231523637.1	Type1.BREX	[illegible]	1183	Bacteria>Terrabacteria_group>Bacillota>Bacilli>Lactobacillales>Lactobacillaceae	GCF_021134645.1	Lactobacillus_delbrueckii	LOB39_RS07020	33020..36571	1584	BREX-system-P-loop-protein-BrxC-[Lactobacillus-delbrueckii]
WP_231524175.1	Type1.BREX	[illegible]	1188	Bacteria>Terrabacteria_group>Bacillota>Bacilli>Lactobacillales>Lactobacillaceae	GCF_021134795.1	Lactobacillus_delbrueckii	LOB43_RS04555	15910..19476	1584	BREX-system-P-loop-protein-BrxC-[Lactobacillus-delbrueckii]
WP_231529866.1	Type1.BREX	[illegible]	1188	Bacteria>Terrabacteria_group>Bacillota>Bacilli>Lactobacillales>Lactobacillaceae	GCF_021135055.1	Lactobacillus_delbrueckii	LOB49_05200	20821..24387	1584	BREX-system-P-loop-protein-BrxC-[Lactobacillus-delbrueckii]
WP_231535729.1	Type1.BREX	[illegible]	1188	Bacteria>Terrabacteria_group>Bacillota>Bacilli>Lactobacillales>Lactobacillaceae	GCF_021135595.1	Lactobacillus_delbrueckii	LOC07_RS06205	4796..8362	1584	BREX-system-P-loop-protein-BrxC-[Lactobacillus-delbrueckii]
WP_191671322.1	Type1.BREX	[illegible]	1230	Bacteria>Terrabacteria_group>Bacillota>Bacilli>Lactobacillales>Lactobacillaceae	GCF_021278125.1	Limosilactobacillus_fermentum	LR965_RS05320	12718..16410	1613	BREX-system-P-loop-protein-BrxC-[Limosilactobacillus-fermentum]
WP_236153484.1	Type1.BREX	[illegible]	1183	Bacteria>Terrabacteria_group>Bacillota>Bacilli>Lactobacillales>Lactobacillaceae	GCF_021600265.1	Lactobacillus_delbrueckii	ME802_14840	21460..25011	1584	BREX-system-P-loop-protein-BrxC-[Lactobacillus-delbrueckii]
WP_236149793.1	Type1.BREX	[illegible]	1188	Bacteria>Terrabacteria_group>Bacillota>Bacilli>Lactobacillales>Lactobacillaceae	GCF_021600925.1	Lactobacillus_delbrueckii	L5981_RS02870	31328..34894	1584	BREX-system-P-loop-protein-BrxC-[Lactobacillus-delbrueckii]
WP_050952512.1	Type1.BREX	[illegible]	1188	Bacteria>Terrabacteria_group>Bacillota>Bacilli>Lactobacillales>Lactobacillaceae	GCF_021601025.1	Lactobacillus_delbrueckii	L5988_RS00790	158168..161734	1584	BREX-system-P-loop-protein-BrxC-[Lactobacillus-delbrueckii]
WP_238064446.1	Type1.BREX	[illegible]	1241	Bacteria>Terrabacteria_group>Bacillota>Bacilli>Lactobacillales>Lactobacillaceae	GCF_022131015.1	Lacticaseibacillus_paracasei	K9853_RS00445	1326..5051	1597	BREX-system-P-loop-protein-BrxC-[Lacticaseibacillus-paracasei]
WP_242073729.1	Type1.BREX	[illegible]	1188	Bacteria>Terrabacteria_group>Bacillota>Bacilli>Lactobacillales>Lactobacillaceae	GCF_022642945.1	Lactobacillus_delbrueckii	G8B25_RS07935	1514865..1518431	1584	BREX-system-P-loop-protein-BrxC-[Lactobacillus-delbrueckii]
WP_249905369.1	Type1.BREX	[illegible]	1244	Bacteria>Terrabacteria_group>Bacillota>Bacilli>Lactobacillales>Lactobacillaceae	GCF_023523445.1	Lactobacillus_amylovorus	LOB74_RS05625	1115195..1118929	1604	BREX-system-P-loop-protein-BrxC-[Lactobacillus-amylovorus]
WP_250825484.1	Type1.BREX	[illegible]	1247	Bacteria>Terrabacteria_group>Bacillota>Bacilli>Lactobacillales>Lactobacillaceae	GCF_023674385.1	Lactobacillus_kefiranofaciens	MU859_RS01790	311336..315079	267818	BREX-system-P-loop-protein-BrxC-[Lactobacillus-kefiranofaciens]
WP_261809685.1	Type1.BREX	[illegible]	1211	Bacteria>Terrabacteria_group>Bacillota>Bacilli>Lactobacillales>Lactobacillaceae	GCF_024345185.1	Levilactobacillus_humaensis	OCU14_RS02680	59918..63553	2950375	BREX-system-P-loop-protein-BrxC-[Levilactobacillus-humaensis]
WP_249391181.1	Type1.BREX	[illegible]	1241	Bacteria>Terrabacteria_group>Bacillota>Bacilli>Lactobacillales>Lactobacillaceae	GCF_025122345.1	Lactiplantibacillus_plantarum	M4S29_RS14340	1936..5661	1590	BREX-system-P-loop-protein-BrxC-[Lactiplantibacillus-plantarum]
WP_259693422.1	Type1.BREX	[illegible]	1242	Bacteria>Terrabacteria_group>Bacillota>Bacilli>Lactobacillales>Lactobacillaceae	GCF_025129275.1	Lactobacillus_helveticus	EFM10_RS03595	12781..16509	1587	BREX-system-P-loop-protein-BrxC-[Lactobacillus-helveticus]
WP_259685554.1	Type1.BREX	[illegible]	1240	Bacteria>Terrabacteria_group>Bacillota>Bacilli>Lactobacillales>Lactobacillaceae	GCF_025129335.1	Lactobacillus_helveticus	EFM06_RS11120	12796..16518	1587	BREX-system-P-loop-protein-BrxC-[Lactobacillus-helveticus]
WP_260182661.1	Type1.BREX	[illegible]	1227	Bacteria>Terrabacteria_group>Bacillota>Bacilli>Lactobacillales>Lactobacillaceae	GCF_025189415.1	Lacticaseibacillus_rhamnosus	EFO73_RS10425	40181..43864	47715	BREX-system-P-loop-protein-BrxC-[Lacticaseibacillus-rhamnosus]
WP_260274304.1	Type1.BREX	[illegible]	1243	Bacteria>Terrabacteria_group>Bacillota>Bacilli>Lactobacillales>Lactobacillaceae	GCF_025191325.1	Lactobacillus_helveticus	EFP83_RS06575	9420..13151	1587	BREX-system-P-loop-protein-BrxC-[Lactobacillus-helveticus]
WP_260307210.1	Type1.BREX	[illegible]	1243	Bacteria>Terrabacteria_group>Bacillota>Bacilli>Lactobacillales>Lactobacillaceae	GCF_025191425.1	Lactobacillus_helveticus	EFP97_RS06690	9418..13149	1587	BREX-system-P-loop-protein-BrxC-[Lactobacillus-helveticus]
WP_260212031.1	Type1.BREX	[illegible]	1244	Bacteria>Terrabacteria_group>Bacillota>Bacilli>Lactobacillales>Lactobacillaceae	GCF_025194565.1	Lactobacillus_amylovorus	EFR95_RS04430	29331..33065	1604	BREX-system-P-loop-protein-BrxC-[Lactobacillus-amylovorus]
WP_260565630.1	Type1.BREX	[illegible]	1227	Bacteria>Terrabacteria_group>Bacillota>Bacilli>Lactobacillales>Lactobacillaceae	GCF_025215665.1	Lacticaseibacillus_paracasei	EOK76_RS04770	1022969..1026652	1597	BREX-system-P-loop-protein-BrxC-[Lacticaseibacillus-paracasei]
WP_267160700.1	Type1.BREX	[illegible]	1241	Bacteria>Terrabacteria_group>Bacillota>Bacilli>Lactobacillales>Lactobacillaceae	GCF_026410225.1	Levilactobacillus_brevis	OSJ94_RS09260	5042..8767	1580	BREX-system-P-loop-protein-BrxC-[Levilactobacillus-brevis]
WP_268886389.1	Type1.BREX	[illegible]	1183	Bacteria>Terrabacteria_group>Bacillota>Bacilli>Lactobacillales>Lactobacillaceae	GCF_026891475.1	Lactobacillus_delbrueckii	O0549_RS05810	14866..18417	1584	BREX-system-P-loop-protein-BrxC-[Lactobacillus-delbrueckii]
WP_271876485.1	Type1.BREX	[illegible]	1233	Bacteria>Terrabacteria_group>Bacillota>Bacilli>Lactobacillales>Lactobacillaceae	GCF_028308365.1	Lactobacillus_amylovorus	ODV29_RS04110	28769..32470	1604	BREX-system-P-loop-protein-BrxC-[Lactobacillus-amylovorus]
WP_271882935.1	Type1.BREX	[illegible]	1227	Bacteria>Terrabacteria_group>Bacillota>Bacilli>Lactobacillales>Lactobacillaceae	GCF_028308535.1	Lactobacillus_amylovorus	ODV81_RS08115	8502..12185	1604	BREX-system-P-loop-protein-BrxC-[Lactobacillus-amylovorus]
WP_271875350.1	Type1.BREX	[illegible]	1233	Bacteria>Terrabacteria_group>Bacillota>Bacilli>Lactobacillales>Lactobacillaceae	GCF_028308735.1	Lactobacillus_amylovorus	ODV82_RS03950	34428..38129	1604	BREX-system-P-loop-protein-BrxC-[Lactobacillus-amylovorus]
WP_271873858.1	Type1.BREX	[illegible]	1244	Bacteria>Terrabacteria_group>Bacillota>Bacilli>Lactobacillales>Lactobacillaceae	GCF_028308865.1	Lactobacillus_amylovorus	ODV08_RS03455	54390..58124	1604	BREX-system-P-loop-protein-BrxC-[Lactobacillus-amylovorus]
WP_271897403.1	Type1.BREX	[illegible]	1233	Bacteria>Terrabacteria_group>Bacillota>Bacilli>Lactobacillales>Lactobacillaceae	GCF_028308945.1	Lactobacillus_amylovorus	ODV12_RS07245	16097..19798	1604	BREX-system-P-loop-protein-BrxC-[Lactobacillus-amylovorus]
WP_271862779.1	Type1.BREX	[illegible]	1244	Bacteria>Terrabacteria_group>Bacillota>Bacilli>Lactobacillales>Lactobacillaceae	GCF_028309095.1	Lactobacillus_amylovorus	ODU71_RS08740	22834..26568	1604	BREX-system-P-loop-protein-BrxC-[Lactobacillus-amylovorus]
WP_274784940.1	Type1.BREX	[illegible]	1239	Bacteria>Terrabacteria_group>Bacillota>Bacilli>Lactobacillales>Lactobacillaceae	GCF_028878205.1	Lacticaseibacillus_casei	LTZ17_RS09610	16306..20025	1582	BREX-system-P-loop-protein-BrxC-[Lacticaseibacillus-casei]
WP_101511690.1	Type1.BREX	[illegible]	1243	Bacteria>Terrabacteria_group>Bacillota>Bacilli>Lactobacillales>Lactobacillaceae	GCF_900248185.1	Lactobacillus_helveticus	JM182_RS07250	23706..27437	1587	BREX-system-P-loop-protein-BrxC-[Lactobacillus-helveticus]
WP_048488336.1	Type1.BREX	[illegible]	1227	Bacteria>Terrabacteria_group>Bacillota>Bacilli>Lactobacillales>Lactobacillaceae	GCF_901971785.1	Lacticaseibacillus_rhamnosus	LRHP344_RS03410	15330..19013	47715	BREX-system-P-loop-protein-BrxC-[Lacticaseibacillus-rhamnosus]
WP_021350006.1	Type1.BREX	[illegible]	1230	Bacteria>Terrabacteria_group>Bacillota>Bacilli>Lactobacillales>Lactobacillaceae	GCF_925284415.1	Limosilactobacillus_fermentum	QFK68_RS03250	14373..18065	1613	BREX-system-P-loop-protein-BrxC-[Limosilactobacillus-fermentum]
WP_064081740.1	Type1.BREX	[illegible]	1178	Bacteria>Terrabacteria_group>Bacillota>Bacilli>Lactobacillales>Lactobacillaceae	GCF_925300675.1	Lactobacillus_delbrueckii	NGL01_RS07850	3981..7517	1584	BREX-system-P-loop-protein-BrxC-[Lactobacillus-delbrueckii]
WP_060472123.1	Type1.BREX	[illegible]	1242	Bacteria>Terrabacteria_group>Bacillota>Bacilli>Lactobacillales>Lactobacillaceae	-	Lactobacillus_gallinarum	AO203_08530	1678493..1682221	52242	BREX-system-P-loop-protein-BrxC-[Lactobacillus-gallinarum]

Protein-ID	System	Gene-Neighbourhoods	Length	Taxonomy	Assembly-ID	Strain	Locus-Tag	Genomic-coordinates	TaxID	Fasta-defline
WP_236656790.1	Type1.BREX	ASCH->Glyoxalase->ParB+HNH->?->ParB+HNH->BrxA-DNA-binding->BrxB-BrxF-iSTAND->DUF6079*->N6-METHYLASE->nSTAND3-><-MULE-TRANSPOSASE	1159	Bacteria>Terrabacteria_group>Bacillota>Bacilli>Lactobacillales>Lactobacillaceae	-	Lactobacillus_helveticus	LHEW6_RS10210	11901..15380	1587	BREX-system-P-loop-protein-BrxC-[Lactobacillus-helveticus]
WP_168924054.1	Type1.BREX	McrB-AAA->McrC-NTD+REase->?->BrxA-DNA-binding->BrxB-BrxF-iSTAND->DUF6079*-><-N6-METHYLASE-p<-?||PglZ->SIGMA+OB-Fold+AAA-ATPase+Lon-Protease-BrxL->ParB+HNH->	1191	Bacteria>Terrabacteria_group>Bacillota>Bacilli>Lactobacillales>Lactobacillaceae	-	Secundilactobacillus_angelensis	L3V66_04100	164372..167947	2722706	BREX-system-P-loop-protein-BrxC-[Secundilactobacillus-angelensis]
MCH4057848.1	Type1.BREX	<-HTH_28+HTH_32||<-?<-ParB+HNH<-SIGMA+OB-Fold+AAA-ATPase+Lon-Protease-BrxL<-PglZ||N6-METHYLASE->Arm-DNA-bind_4+Phage_int_SAM_5+Phage_integrase-><-N6-METHYLASE<-DUF6079*<-BrxB-BrxF-iSTAND<-BrxA-DNA-binding	1214	Bacteria>Terrabacteria_group>Bacillota>Bacilli>Lactobacillales>unclassified_Lactobacillales	GCA_022483825.1	Lactobacillaceae_bacterium	LKE89_08070	16697..20341	2291323	BREX-system-P-loop-protein-BrxC-[Lactobacillaceae-bacterium]
WP_076917462.1	Type1.BREX	AT-HTH-HYD2->Tox-ART-HYD2->AT-HTH-HYD2-><-SbcC<-ParB+HNH<-SIGMA+OB-Fold+AAA-ATPase+Lon-Protease-BrxL<-PglZ<-N6-METHYLASE<-TIR<-DUF6079*<-BrxB-BrxF-iSTAND<-BrxA-DNA-binding<-HTH_26<-HTH||?->Peptidase-><-?<-?<-TraJ-RHH	1178	Bacteria>Terrabacteria_group>Bacillota>Bacillota_sensu_stricto_incertae_sedis>Negativibacillus	GCF_900148495.1	Negativibacillus_massiliensis	BQ5375_RS04300	340320..343856	1871035	BREX-system-P-loop-protein-BrxC-[Negativibacillus-massiliensis]
NFG40378.1	Type1.BREX	Calcineurin->?->BrxA-DNA-binding->BrxB-BrxF-iSTAND->DUF6079*->N6-METHYLASE->ParB+HNH->OLD-ABC->HNH->PglZ-><-REase||SIGMA+OB-Fold+AAA-ATPase+Lon-Protease-BrxL->Peptidase_M42->TIR->	1215	Bacteria>Terrabacteria_group>Bacillota>Clostridia>Eubacteriales>Clostridiaceae	GCA_011013445.1	Clostridium_botulinum	FC789_04035	879792..883439	1491	BREX-system-P-loop-protein-BrxC-[Clostridium-botulinum]
NFI95403.1	Type1.BREX	<-Peptidase_M42<-SIGMA+OB-Fold+AAA-ATPase+Lon-Protease-BrxL||REase-><-PglZ<-HNH<-OLD-ABC<-ParB+HNH<-N6-METHYLASE<-DUF6079*<-BrxB-BrxF-iSTAND<-BrxA-DNA-binding	1215	Bacteria>Terrabacteria_group>Bacillota>Clostridia>Eubacteriales>Clostridiaceae	GCA_011014625.1	Clostridium_botulinum	FC961_13560	15228..18875	1491	BREX-system-P-loop-protein-BrxC-[Clostridium-botulinum]
NFO12999.1	Type1.BREX	Calcineurin->?->BrxA-DNA-binding->BrxB-BrxF-iSTAND->DUF6079*->CR-ATPase9+REase->N6-METHYLASE->ParB+HNH->OLD-ABC->?->PglZ-><-REase||SIGMA+OB-Fold+AAA-ATPase+Lon-Protease-BrxL->Peptidase_M42->	1215	Bacteria>Terrabacteria_group>Bacillota>Clostridia>Eubacteriales>Clostridiaceae	GCA_011016315.1	Clostridium_botulinum	FDB34_02025	196714..200361	1491	BREX-system-P-loop-protein-BrxC-[Clostridium-botulinum]
WP_089984090.1	Type1.BREX	<-SIGMA-HTH<-?||HNH->?->SbcC->HTH_26->BrxA-DNA-binding->BrxB-BrxF-iSTAND->DUF6079*->N6-METHYLASE->PglZ->SIGMA+OB-Fold+AAA-ATPase+Lon-Protease-BrxL->ParB+HNH->?-><-?<-?<-HTH_32+rve	1183	Bacteria>Terrabacteria_group>Bacillota>Clostridia>Eubacteriales>Clostridiaceae	GCF_001517425.1	Clostridium_sp._C105KSO13	BN3456_RS10470	25187..28738	1776045	BREX-system-P-loop-protein-BrxC-[Clostridium-sp.-C105KSO13]
WP_156211829.1	Type1.BREX	<-wHTH+McrB-AAA<-?<-PglZ<-?<-?<-OLD-ABC<-ParB+HNH<-N6-METHYLASE<-CR-ATPase9+REase<-DUF6079*<-BrxB-BrxF-iSTAND<-BrxA-DNA-binding<-HKD-DNase+SF1-SF2-Helicase+HTH+HTH+SRA<-SAM-METHYLASE<-NERD+HRDC<-Ntox45<-HEPN	1216	Bacteria>Terrabacteria_group>Bacillota>Clostridia>Eubacteriales>Clostridiaceae	GCF_009735365.2	Clostridium_butyricum	GND98_RS05145	54704..58354	1492	BREX-system-P-loop-protein-BrxC-[Clostridium-butyricum]
WP_185162822.1	Type1.BREX	NTP_transf-><-SIGMA+OB-Fold+AAA-ATPase+Lon-Protease-BrxL<-?<-PglZ<-Couple_hipA<-N6-METHYLASE<-?<-DUF6079*<-BrxB-BrxF-iSTAND<-BrxA-DNA-binding||HEPN-><-N6-METHYLASE<-?<-ParB+HNH	1215	Bacteria>Terrabacteria_group>Bacillota>Clostridia>Eubacteriales>Clostridiaceae	GCF_014212415.1	Clostridium_algidicarnis	H7D93_RS12595	17014..20661	37659	BREX-system-P-loop-protein-BrxC-[Clostridium-algidicarnis]
WP_207130137.1	Type1.BREX	<-SIGMA+OB-Fold+AAA-ATPase+Lon-Protease-BrxL<-PglZ<-AT-HTH-HYD2-2+AT-HTH-HYD2-1<-?<-ParB+HNH<-N6-METHYLASE<-DUF6079*<-BrxB-BrxF-iSTAND<-BrxA-DNA-binding<-ParB C<-?<-Cysteine-Desulfurase<-?<-?||?-><-HISKIN	1215	Bacteria>Terrabacteria_group>Bacillota>Clostridia>Eubacteriales>Clostridiaceae	GCF_017330945.1	Clostridium_botulinum	EXQ34_RS04235	14589..18236	1491	BREX-system-P-loop-protein-BrxC-[Clostridium-botulinum]
WP_226104726.1	Type1.BREX	<-SIGMA+OB-Fold+AAA-ATPase+Lon-Protease-BrxL<-?<-PglZ<-SLOG+BirA-HTH<-SF1-SF2-Helicase+PRTase<-?<-ParB+HNH<-N6-METHYLASE<-?<-DUF6079*<-BrxB-BrxF-iSTAND<-BrxA-DNA-binding||NTP_transf-><-?<-HTH_3	1215	Bacteria>Terrabacteria_group>Bacillota>Clostridia>Eubacteriales>Clostridiaceae	GCF_020443655.1	Clostridium_algidicarnis	LGK99_RS11195	54237..57884	37659	BREX-system-P-loop-protein-BrxC-[Clostridium-algidicarnis]
WP_252235883.1	Type1.BREX	<-PglZ<-?<-?<-OLD-ABC<-ParB+HNH<-N6-METHYLASE<-?<-Tox-ART-HYD2<-DUF6079*<-BrxB-BrxF-iSTAND<-BrxA-DNA-binding<-?<-Calcineurin	1215	Bacteria>Terrabacteria_group>Bacillota>Clostridia>Eubacteriales>Clostridiaceae	GCF_023846115.1	Clostridium_sp._ZS1	NG923_RS15480	30630..34277	2949989	BREX-system-P-loop-protein-BrxC-[Clostridium-sp.-ZS1]
WP_066893479.1	Type1.BREX	<-SIGMA+OB-Fold+AAA-ATPase+Lon-Protease-BrxL||REase-><-PglZ<-DOC+HTH<-SLOG+BirA-HTH<-SF1-SF2-Helicase+PRTase<-ParB+HNH<-?<-N6-METHYLASE<-DUF6079*<-BrxB-BrxF-iSTAND<-BrxA-DNA-binding<-?<-SF1-SF2-Helicase||?->?-><-?<-ABC-ATPase+SbcC	1215	Bacteria>Terrabacteria_group>Bacillota>Clostridia>Eubacteriales>Clostridiaceae	GCF_900086595.1	Clostridium_nigeriense	BN6774_RS10780	2270723..2274370	1805470	BREX-system-P-loop-protein-BrxC-[Clostridium-nigeriense]
WP_118637679.1	Type1.BREX	<-LexA-protease<-MNS+REase<-wHTH+WYL+WCX<-N6-METHYLASE<-HEPN<-ParB+HNH<-SIGMA+OB-Fold+AAA-ATPase+Lon-Protease-BrxL<-PglZ<-N6-METHYLASE<-DUF6079*<-BrxB-BrxF-iSTAND<-BrxA-DNA-binding<-HTH_26||TraJ-RHH->TraI-VirD2-RCRE->	1215	Bacteria>Terrabacteria_group>Bacillota>Clostridia>Eubacteriales	GCF_027924705.1	Oscillospiraceae	H6A32_RS13625	30472..34119	216572	MULTISPECIES:-BREX-system-P-loop-protein-BrxC-[Oscillospiraceae]
TGX97927.1	Type1.BREX	<-OLD-ABC+TOPRIM-p<-OLD-ABC+TOPRIM-p<-PglZ<-HNH<-OLD-ABC<-N6-METHYLASE<-HEPN<-ParB+HNH<-N6-METHYLASE<-DUF6079*<-BrxB-BrxF-iSTAND<-BrxA-DNA-binding<-HTH_26<-ZnR+ZnR+ZnR<-35exo+ADA-DBD<-?<-MACRODOMAIN	1201	Bacteria>Terrabacteria_group>Bacillota>Clostridia>Eubacteriales>Lachnospiraceae	GCA_004793585.1	Lachnospiraceae_bacterium	E5357_10825	35909..39514	1898203	BREX-system-P-loop-protein-BrxC-[Lachnospiraceae-bacterium]
MBR3171576.1	Type1.BREX	<-ParB+HNH<-SIGMA+OB-Fold+AAA-ATPase+Lon-Protease-BrxL<-PglZ<-RAMA<-N6-METHYLASE<-DUF6079*<-BrxB-BrxF-iSTAND<-BrxA-DNA-binding<-HTH_26<-OLD-ABC||SMODS->?->?-><-AbiEii<-AbiEi_4	1178	Bacteria>Terrabacteria_group>Bacillota>Clostridia>Eubacteriales>Lachnospiraceae	GCA_017514845.1	Lachnospiraceae_bacterium	IKF22_10080	15258..18794	1898203	BREX-system-P-loop-protein-BrxC-[Lachnospiraceae-bacterium]
MCM1125306.1	Type1.BREX	MoxR-AAA->LRR_5->Glyco_hydro_3_C+Fn3-like+Glyco_hydro_3->Peptidase_C26->tRNA->tRNA-><-?<-MerR-HTH||HTH_26->BrxA-DNA-binding->BrxB-BrxF-iSTAND->DUF6079*->ParB+HNH->HEPN->N6-METHYLASE-p->?-><-tRNA||?->HAD+HAD->	1205	Bacteria>Terrabacteria_group>Bacillota>Clostridia>Eubacteriales>Lachnospiraceae	GCA_023663945.1	Lachnospiraceae_bacterium	NC429_02425	28301..31918	1898203	BREX-system-P-loop-protein-BrxC-[Lachnospiraceae-bacterium]
SFG07145.1	Type1.BREX	ArdCN+MPTase-inact+LPD19->TOPRIM+Topoisom_bac->LPD2->TraJ-RHH->?->HTH_26->BrxA-DNA-binding->BrxB-BrxF-iSTAND->DUF6079*->N6-METHYLASE->?->PglZ->AbiGi->SIGMA+OB-Fold+AAA-ATPase+Lon-Protease-BrxL->ParB+HNH->HEPN->	1182	Bacteria>Terrabacteria_group>Bacillota>Clostridia>Eubacteriales>Lachnospiraceae	GCA_900113385.1	Lachnospiraceae_bacterium_C7	SAMN04487761_10376	78580..82128	1792306	hypothetical-protein-SAMN04487761_10376-[Lachnospiraceae-bacterium-C7]
WP_027207205.1	Type1.BREX	XIS-HTH-><-?<-ParB+HNH<-SIGMA+OB-Fold+AAA-ATPase+Lon-Protease-BrxL<-HEPN<-PglZ<-N6-METHYLASE<-DUF6079*<-BrxB-BrxF-iSTAND<-BrxA-DNA-binding<-HTH_26<-SF1-SF2-Helicase<-?<-HEPN+PAS+DUF4011+SF1-SF2-Helicase+REase<-ParB<-DDE_Tnp_1_4	1196	Bacteria>Terrabacteria_group>Bacillota>Clostridia>Eubacteriales>Lachnospiraceae	GCF_000423985.1	Butyrivibrio_fibrisolvens	G591_RS0114720	29938..33528	831	BREX-system-P-loop-protein-BrxC-[Butyrivibrio-fibrisolvens]
WP_027204781.1	Type1.BREX	ArdCN+MPTase-inact+LPD19->CHTH->TOPRIM+Topoisom_bac->LPD2->TraJ-RHH->?->HTH_26->BrxA-DNA-binding->BrxB-BrxF-iSTAND->DUF6079*->N6-METHYLASE->RAMA->?->PglZ->SIGMA+OB-Fold+AAA-ATPase+Lon-Protease-BrxL->?->?->ParB+HNH->HNH->	1181	Bacteria>Terrabacteria_group>Bacillota>Clostridia>Eubacteriales>Lachnospiraceae	GCF_000424425.1	Butyrivibrio_fibrisolvens	G624_RS0113140	43921..47466	831	BREX-system-P-loop-protein-BrxC-[Butyrivibrio-fibrisolvens]
WP_029067206.1	Type1.BREX	HTH_26->BrxA-DNA-binding->BrxB-BrxF-iSTAND->DUF6079*->N6-METHYLASE->PglZ->ParB+HNH->SIGMA+OB-Fold+AAA-ATPase+Lon-Protease-BrxL->RVT+HEPN->HTH_38->HTH_26->BrxA-DNA-binding->BrxB-BrxF-iSTAND->	1182	Bacteria>Terrabacteria_group>Bacillota>Clostridia>Eubacteriales>Lachnospiraceae	GCF_000687515.1	Lachnobacterium_bovis	CC91_RS0108435	378442..381990	140626	BREX-system-P-loop-protein-BrxC-[Lachnobacterium-bovis]
WP_029067213.1	Type1.BREX	N6-METHYLASE->PglZ->ParB+HNH->SIGMA+OB-Fold+AAA-ATPase+Lon-Protease-BrxL->RVT+HEPN->HTH_38->HTH_26->BrxA-DNA-binding->BrxB-BrxF-iSTAND->DUF6079*->N6-METHYLASE->RAMA->PglZ->?->SIGMA+OB-Fold+AAA-ATPase+Lon-Protease-BrxL->KAP-NTPase->KAP-glycosylase->?->TatD_DNase->	1182	Bacteria>Terrabacteria_group>Bacillota>Clostridia>Eubacteriales>Lachnospiraceae	GCF_000687515.1	Lachnobacterium_bovis	CC91_RS0108490	397340..400888	140626	BREX-system-P-loop-protein-BrxC-[Lachnobacterium-bovis]
WP_059085682.1	Type1.BREX	MACRODOMAIN->?->ZnR+ZnR+ZnR->HTH_26->BrxA-DNA-binding->BrxB-BrxF-iSTAND->DUF6079*->N6-METHYLASE->ParB+HNH->OLD-ABC->?->OLD-ABC->HNH->PglZ->	1196	Bacteria>Terrabacteria_group>Bacillota>Clostridia>Eubacteriales>Lachnospiraceae	GCF_001487165.1	Blautia_massiliensis	BN3261_RS05710	1028364..1031954	1737424	BREX-system-P-loop-protein-BrxC-[Blautia-massiliensis]
WP_114002767.1	Type1.BREX	<-PglZ<-HNH<-OLD-ABC<-?<-OLD-ABC<-ParB+HNH<-N6-METHYLASE<-DUF6079*<-BrxB-BrxF-iSTAND<-BrxA-DNA-binding<-HTH_26<-ZnR+ZnR+ZnR<-?<-MACRODOMAIN	1196	Bacteria>Terrabacteria_group>Bacillota>Clostridia>Eubacteriales>Lachnospiraceae	GCF_003324155.1	Blautia_obeum	C4886_RS16030	22477..26067	40520	BREX-system-P-loop-protein-BrxC-[Blautia-obeum]
WP_195251421.1	Type1.BREX	MACRODOMAIN->?->35exo+ADA-DBD->HTH_26->BrxA-DNA-binding->BrxB-BrxF-iSTAND->DUF6079*->N6-METHYLASE->ParB+HNH->HEPN->	1201	Bacteria>Terrabacteria_group>Bacillota>Clostridia>Eubacteriales>Lachnospiraceae	GCF_015550485.1	Coprococcus_comes	I2A60_RS13430	4839..8444	410072	BREX-system-P-loop-protein-BrxC-[Coprococcus-comes]
WP_118278843.1	Type1.BREX	<-ParB+HNH<-?<-SIGMA+OB-Fold+AAA-ATPase+Lon-Protease-BrxL<-?<-PglZ<-N6-METHYLASE<-Four-helical-protein<-wHTH+ZnR<-DUF6079*<-BrxB-BrxF-iSTAND<-BrxA-DNA-binding<-HTH_26<-HTH_3	1205	Bacteria>Terrabacteria_group>Bacillota>Clostridia>Eubacteriales>Lachnospiraceae	GCF_027662525.1	[Ruminococcus]_lactaris	DW116_RS04365	20004..23621	46228	BREX-system-P-loop-protein-BrxC-[[Ruminococcus]]
WP_271824191.1	Type1.BREX	MPTase->AT-HTH-HYD2->Tox-ART-HYD2->AT-HTH-HYD2-><-SbcC<-ParB+HNH<-SIGMA+OB-Fold+AAA-ATPase+Lon-Protease-BrxL<-PglZ<-N6-METHYLASE<-DUF6079*<-BrxB-BrxF-iSTAND<-BrxA-DNA-binding<-HTH_26<-HTH||?->Peptidase-><-?<-?<-TraJ-RHH	1178	Bacteria>Terrabacteria_group>Bacillota>Clostridia>Eubacteriales>Lachnospiraceae	GCF_028210655.1	Enterocloster_clostridioformis	PMX39_RS12925	18239..21775	1531	BREX-system-P-loop-protein-BrxC-[Enterocloster-clostridioformis]
WP_090815514.1	Type1.BREX	<-SF1-SF2-Helicase<-SF1-SF2-Helicase<-SF1-SF2-Helicase+TOPC<-ParB+HNH<-?<-?<-SIGMA+OB-Fold+AAA-ATPase+Lon-Protease-BrxL<-PglZ<-N6-METHYLASE<-DUF6079*<-BrxB-BrxF-iSTAND<-BrxA-DNA-binding<-HTH_26<-?<-?<-?<-TraJ-RHH<-ArdCN+MPTase-inact+LPD19	1182	Bacteria>Terrabacteria_group>Bacillota>Clostridia>Eubacteriales>Lachnospiraceae	GCF_900107835.1	Oribacterium_sp._KHPX15	BLV86_RS05660	48724..52272	1855342	BREX-system-P-loop-protein-BrxC-[Oribacterium-sp.-KHPX15]
WP_091825279.1	Type1.BREX	AbiEii->?->?->TraJ-RHH->Integrase_DNA+Phage_integrase->ZnR+TOPRIM->XIS-HTH->BrxA-DNA-binding->BrxB-BrxF-iSTAND->DUF6079*->N6-METHYLASE->PglZ->SIGMA+OB-Fold+AAA-ATPase+Lon-Protease-BrxL->?->ParB+HNH->UDG-conflict->?->?->HTH_28->	1145	Bacteria>Terrabacteria_group>Bacillota>Clostridia>Eubacteriales>Lachnospiraceae	GCF_900109475.1	Butyrivibrio_sp._ob235	BMW61_RS19080	17910..21347	1761780	BREX-system-P-loop-protein-BrxC-[Butyrivibrio-sp.-ob235]
MCR4638957.1	Type1.BREX	<-HTH_3<-SIGMA+OB-Fold+AAA-ATPase+Lon-Protease-BrxL<-PglZ<-AbiGi<-N6-METHYLASE<-ParB+HNH<-DUF6079*<-BrxB-BrxF-iSTAND<-BrxA-DNA-binding<-?<-?<-?<-?<-?<-HIRAN<-DnaJ	1175	Bacteria>Terrabacteria_group>Bacillota>Clostridia>Eubacteriales>Oscillospiraceae	GCA_024684275.1	Ruminococcus_sp.	K5677_07000	11947..15474	41978	BREX-system-P-loop-protein-BrxC-[Ruminococcus-sp.]
UYJ00845.1	Type1.BREX	<-NUDIX<-?<-HKD-DNase+SF1-SF2-Helicase+HTH+HTH+SRA||XIS-HTH->BrxA-DNA-binding->BrxB-BrxF-iSTAND->DUF6079*->HTH+REase->N6-METHYLASE->TIR->PglZ->SIGMA+OB-Fold+AAA-ATPase+Lon-Protease-BrxL->ParB+HNH->STYKinase-like->	1218	Bacteria>Terrabacteria_group>Bacillota>Clostridia>Eubacteriales>Oscillospiraceae	GCA_025757765.1	Faecalibacterium_prausnitzii	OGM65_10405	2149952..2153608	853	MAG:-BREX-system-P-loop-protein-BrxC-[Faecalibacterium-prausnitzii]
UYJ11714.1	Type1.BREX	<-CR-ATPase8+REase<-?<-CR-ATPase5+REase<-?<-?||DOC+HTH->BrxA-DNA-binding->BrxB-BrxF-iSTAND->DUF6079*->?->?->N6-METHYLASE->OLD-ABC->REase->PglZ->SIGMA+OB-Fold+AAA-ATPase+Lon-Protease-BrxL->ParB+HNH->REase+SF1-SF2-Helicase->	1182	Bacteria>Terrabacteria_group>Bacillota>Clostridia>Eubacteriales>Oscillospiraceae	GCA_025757845.1	Oscillospiraceae_bacterium	OGM78_02700	567882..571430	2485925	MAG:-BREX-system-P-loop-protein-BrxC-[Oscillospiraceae-bacterium]
WP_024861712.1	Type1.BREX	Phage_int_SAM_3->?->GDResolvase+HIN-HTH->?->?->ParB+HNH->BrxA-DNA-binding->BrxB-BrxF-iSTAND->DUF6079*->N6-METHYLASE-p->N6-METHYLASE-p->PglZ->SIGMA+OB-Fold+AAA-ATPase+Lon-Protease-BrxL-><-?<-?<-tRNA<-?||SF1-SF2-Helicase+TUDOR->	1171	Bacteria>Terrabacteria_group>Bacillota>Clostridia>Eubacteriales>Oscillospiraceae	GCF_000526795.1	Ruminococcus_flavefaciens	N774_RS0113310	3002422..3005937	1265	BREX-system-P-loop-protein-BrxC-[Ruminococcus-flavefaciens]
WP_097782652.1	Type1.BREX	<-DUF932<-NUDIX<-HKD-DNase+SF1-SF2-Helicase+HTH+HTH+SRA||XIS-HTH->BrxA-DNA-binding->BrxB-BrxF-iSTAND->DUF6079*->Trypsin->N6-METHYLASE->TIR->PglZ->SIGMA+OB-Fold+AAA-ATPase+Lon-Protease-BrxL->ParB+HNH->HKD-DNase+SF1-SF2-Helicase+HTH+HTH+SRA->STYKinase-like->HNH->	1220	Bacteria>Terrabacteria_group>Bacillota>Clostridia>Eubacteriales>Oscillospiraceae	GCF_002549935.1	Faecalibacterium_prausnitzii	CGS55_RS03365	35885..39547	853	BREX-system-P-loop-protein-BrxC-[Faecalibacterium-prausnitzii]
WP_113992229.1	Type1.BREX	<-CR-ATPase5+REase<-?<-?<-DUF932<-NUDIX<-HKD-DNase+SF1-SF2-Helicase+HTH+HTH+SRA||XIS-HTH->BrxA-DNA-binding->BrxB-BrxF-iSTAND->DUF6079*->wHTH+ZnR->Four-helical-protein->N6-METHYLASE->HEPN->PglZ->SIGMA+OB-Fold+AAA-ATPase+Lon-Protease-BrxL->ParB+HNH->HKD-DNase+SF1-SF2-Helicase+HTH+HTH+SRA->nSTAND->	1218	Bacteria>Terrabacteria_group>Bacillota>Clostridia>Eubacteriales>Oscillospiraceae	GCF_003324185.1	Faecalibacterium_prausnitzii	C7J97_RS04695	32898..36554	853	BREX-system-P-loop-protein-BrxC-[Faecalibacterium-prausnitzii]
WP_117554516.1	Type1.BREX	<-PIN<-HTH+MPTase<-STYKinase-like<-ParB+HNH<-SIGMA+OB-Fold+AAA-ATPase+Lon-Protease-BrxL<-PglZ<-?<-N6-METHYLASE<-Trypsin<-DUF6079*<-BrxB-BrxF-iSTAND<-BrxA-DNA-binding<-XIS-HTH||HKD-DNase+SF1-SF2-Helicase+HTH+HTH+SRA->NUDIX->DUF932->?->?->CR-ATPase5+REase->	1217	Bacteria>Terrabacteria_group>Bacillota>Clostridia>Eubacteriales>Oscillospiraceae	GCF_003434135.1	Faecalibacterium_prausnitzii	DW855_RS10430	56056..59709	853	BREX-system-P-loop-protein-BrxC-[Faecalibacterium-prausnitzii]
WP_215675194.1	Type1.BREX	<-CR-ATPase8+REase<-?<-CR-ATPase5+REase<-?<-?<-DUF932||DOC+HTH->BrxA-DNA-binding->BrxB-BrxF-iSTAND->DUF6079*->TIR->N6-METHYLASE->?->PglZ->SIGMA+OB-Fold+AAA-ATPase+Lon-Protease-BrxL->ParB+HNH->HKD-DNase+SF1-SF2-Helicase+HTH+HTH+SRA->SF1-SF2-Helicase+TOPC->STYKinase-like->	1182	Bacteria>Terrabacteria_group>Bacillota>Clostridia>Eubacteriales>Oscillospiraceae	GCF_018784645.1	Faecalibacterium_prausnitzii	GPK62_RS07855	35115..38663	853	BREX-system-P-loop-protein-BrxC-[Faecalibacterium-prausnitzii]
WP_235255096.1	Type1.BREX	<-SbcC+SbcC<-?<-JetA||XIS-HTH->BrxA-DNA-binding->BrxB-BrxF-iSTAND->DUF6079*->HNH+SAVED->SMODS->?->N6-METHYLASE->ParB+HNH->sGTP->?->HerA/FtsK->HNH->	1199	Bacteria>Terrabacteria_group>Bacillota>Clostridia>Eubacteriales>Oscillospiraceae	GCF_021531255.1	Fournierella_massiliensis	JQM64_RS00320	59255..62854	1650663	BREX-system-P-loop-protein-BrxC-[Fournierella-massiliensis]
WP_275252236.1	Type1.BREX	<-DUF932<-NUDIX<-?<-HKD-DNase+SF1-SF2-Helicase+HTH+HTH+SRA||XIS-HTH->BrxA-DNA-binding->BrxB-BrxF-iSTAND->DUF6079*->wHTH+ZnR->Four-helical-protein->N6-METHYLASE->OLD-ABC->NERD->PglZ->SIGMA+OB-Fold+AAA-ATPase+Lon-Protease-BrxL->ParB+HNH-><-VirD4-TraG+VirD4-FtsK	1218	Bacteria>Terrabacteria_group>Bacillota>Clostridia>Eubacteriales>Oscillospiraceae	GCF_029076575.1	Faecalibacterium_sp._DFI.5.82	POG09_RS06330	132484..136140	3031725	BREX-system-P-loop-protein-BrxC-[Faecalibacterium-sp.-DFI.5.82]
WP_158398728.1	Type1.BREX	<-ParB+HNH<-SIGMA+OB-Fold+AAA-ATPase+Lon-Protease-BrxL<-PglZ<-HEPN<-N6-METHYLASE<-TIR<-?<-DUF6079*<-BrxB-BrxF-iSTAND<-BrxA-DNA-binding<-HTH_3<-?<-RepA_N+HTH||<-Peptidase_S51||SIGMA-HTH+SIGMA-HTH->ncRNA->RFC-AAA+RuvB-AAA->	1182	Bacteria>Terrabacteria_group>Bacillota>Clostridia>Eubacteriales>Oscillospiraceae	GCF_902167845.1	Faecalibacterium_prausnitzii	FQ260_RS07280	12413..15961	853	BREX-system-P-loop-protein-BrxC-[Faecalibacterium-prausnitzii]
MBD9283724.1	Type1.BREX	<-ParB+HNH<-DUF6079*<-BrxB-BrxF-iSTAND<-BrxA-DNA-binding<-RADICAL-SAM<-METHYLASE<-HTH_26<-?||HKD-DNase->||OLD-ABC->NERD->	1202	Bacteria>Terrabacteria_group>Bacillota>Clostridia>Eubacteriales>unclassified_Eubacteriales	GCA_014848695.1	Clostridiales_bacterium	EGR96_02215	1987..5595	1898207	BREX-system-P-loop-protein-BrxC-[Clostridiales-bacterium]
MCD8148190.1	Type1.BREX	DUF6079-p*->N6-METHYLASE->RAMA->Peptidase_U49->PglZ->ParB+HNH-p->?->SbcC->HNH->Schlafen+HATPase+wHTH->	773	Bacteria>Terrabacteria_group>Bacillota>Clostridia>Eubacteriales>unclassified_Eubacteriales	GCA_021203005.1	Clostridiales_bacterium	LUE92_01205	1..2322	1898207	BREX-system-P-loop-protein-BrxC-[Clostridiales-bacterium]
WP_271628813.1	Type1.BREX	<-DNA_METHYLASE<-SF1-SF2-Helicase<-OLD-ABC+TOPRIM<-ParB+HNH<-?<-SIGMA+OB-Fold+AAA-ATPase+Lon-Protease-BrxL<-PglZ<-N6-METHYLASE<-DUF6079*<-BrxB-BrxF-iSTAND<-BrxA-DNA-binding<-?<-nSTAND<-NERD+ZnR<-?<-?<-PIN	1186	Bacteria>Terrabacteria_group>Bacillota>Clostridia>Thermoanaerobacterales>Thermoanaerobacterales_Family_III._Incertae_Sedis	GCF_028198085.1	Caldicellulosiruptor_sp._DIB_104C	PGC14_RS09960	2010036..2013596	3019889	BREX-system-P-loop-protein-BrxC-[Caldicellulosiruptor-sp.-DIB-104C]
MBQ8616934.1	Type1.BREX	<-N6-METHYLASE<-ParB+HNH<-DUF6079*<-BrxB-BrxF-iSTAND<-BrxA-DNA-binding<-HTH_26<-?<-ARG<-MACRODOMAIN	1186	Bacteria>Terrabacteria_group>Bacillota>Clostridia>unclassified_Clostridia	GCA_017472845.1	Clostridia_bacterium	IJ418_05400	5468..9028	2044939	BREX-system-P-loop-protein-BrxC-[Clostridia-bacterium]
MBR6253006.1	Type1.BREX	<-NUDIX<-HEPN<-?<-REase<-SbcC<-ParB+HNH<-PglZ<-N6-METHYLASE<-DUF6079*<-BrxB-BrxF-iSTAND<-BrxA-DNA-binding<-FGase<-?<-?<-?<-HTH_3	1184	Bacteria>Terrabacteria_group>Bacillota>Clostridia>unclassified_Clostridia	GCA_017539905.1	Clostridia_bacterium	IKR04_04115	193581..197135	2044939	BREX-system-P-loop-protein-BrxC-[Clostridia-bacterium]
MCR5783583.1	Type1.BREX	<-ABhydrolase+ABhydrolase<-?<-GDResolvase+Recombinase<-ParB+HNH<-SIGMA+OB-Fold+AAA-ATPase+Lon-Protease-BrxL<-PglZ<-Schlafen+HATPase+wHTH<-Schlafen+HATPase+wHTH<-N6-METHYLASE<-DUF6079*<-BrxB-BrxF-iSTAND<-BrxA-DNA-binding<-HTH_26||Cysteine-Desulfurase->HIN-HTH->DNA_METHYLASE->?->VirD4-TraG+VirD4-FtsK->ArdCN+MPTase-inact+LPD19->	1177	Bacteria>Terrabacteria_group>Bacillota>Clostridia>unclassified_Clostridia	GCA_024699005.1	Clostridia_bacterium	K6G90_12720	47894..51427	2044939	BREX-system-P-loop-protein-BrxC-[Clostridia-bacterium]
WP_270396150.1	Type1.BREX	BrxA-DNA-binding->BrxB-BrxF-iSTAND->DUF6079*->N6-METHYLASE->ParB+HNH->HEPN+HEPN->PglZ->SbcC->SIGMA+OB-Fold+AAA-ATPase+Lon-Protease-BrxL->REase->	1177	Bacteria>Terrabacteria_group>Bacillota>Erysipelotrichia>Erysipelotrichales>Coprobacillaceae	GCF_027659655.1	Catenibacterium_mitsuokai	PGO85_RS08450	1355..4888	100886	BREX-system-P-loop-protein-BrxC-[Catenibacterium-mitsuokai]
NLG80270.1	Type1.BREX	ABC-ATPase+SbcC->ABC-ATPase+SbcC-><-REase+NACHT<-PglZ<-N6-METHYLASE<-N6-METHYLASE<-DUF6079*<-BrxB-BrxF-iSTAND<-BrxA-DNA-binding<-SIGMA+OB-Fold+AAA-ATPase+Lon-Protease-BrxL<-ParB+HNH<-AAA-ATPase-p<-DEDD_Tnp_IS110+Transposase_20||?->DDE_Tnp_ISL3->	1185	Bacteria>Terrabacteria_group>Bacillota	GCA_012516125.1	Bacillota_bacterium	GX492_10705	20631..24188	1879010	BREX-system-P-loop-protein-BrxC-[Bacillota-bacterium]
WP_072743893.1	Type1.BREX	HTH_26->?->DOC+HTH->HAD+HAD->ParB+HNH->BrxA-DNA-binding->BrxB-BrxF-iSTAND->DUF6079*->N6-METHYLASE->OLD-ABC->TOPRIM->PglZ->SIGMA+OB-Fold+AAA-ATPase+Lon-Protease-BrxL->?->?->HDRnase->	1192	Bacteria>Terrabacteria_group>Bacillota>Tissierellia>Tissierellales>Sporanaerobacteraceae	GCF_900130025.1	Sporanaerobacter_acetigenes	BUA21_RS05940	73351..76929	165813	BREX-system-P-loop-protein-BrxC-[Sporanaerobacter-acetigenes]
WP_132026817.1	Type1.BREX	<-EVE+McrB-AAA<-?||?-><-HKD-DNase<-ParB+HNH<-SIGMA+OB-Fold+AAA-ATPase+Lon-Protease-BrxL<-PglZ<-N6-METHYLASE<-N6-METHYLASE<-DUF6079*<-BrxB-BrxF-iSTAND<-BrxA-DNA-binding<-?<-?<-?<-CRISPR_Cas2<-Cas_Cas1<-Cas9+HNH+RuvC_III	1197	Bacteria>Terrabacteria_group>Bacillota>Tissierellia>Tissierellales>Tepidimicrobiaceae	GCF_016767175.1	Keratinibaculum_paraultunense	JL105_RS06905	1394896..1398489	1278232	BREX-system-P-loop-protein-BrxC-[Keratinibaculum-paraultunense]
MBQ6518172.1	Type1.BREX	Recombinase->HTH_26->BrxA-DNA-binding->BrxB-BrxF-iSTAND->DUF6079*->ParB+HNH->?->N6-METHYLASE->MORC+REase->Schlafen+HATPase+wHTH+HTH->PglZ->TIR->SIGMA+OB-Fold+AAA-ATPase+Lon-Protease-BrxL->KAP-NTPase->	1176	Bacteria>Terrabacteria_group>Chloroflexota>Anaerolineae>Anaerolineales>Anaerolineaceae	GCA_017433515.1	Anaerolineaceae_bacterium	IJI14_05585	5340..8870	2024896	BREX-system-P-loop-protein-BrxC-[Anaerolineaceae-bacterium]
NLF02862.1	Type1.BREX	Phage_integrase->LuxR-HTH->Phage_integrase->?->BrxA-DNA-binding->BrxB-BrxF-iSTAND->DUF6079*->N6-METHYLASE->ParB+HNH->PglZ->SIGMA+OB-Fold+AAA-ATPase+Lon-Protease-BrxL->	1157	Bacteria>Terrabacteria_group>Chloroflexota>Anaerolineae>Anaerolineales>unclassified_Anaerolineales	GCA_012514695.1	Anaerolineales_bacterium	GX601_18015	11185..14658	2073117	BREX-system-P-loop-protein-BrxC-[Anaerolineales-bacterium]
MCB0100628.1	Type1.BREX	PTSEIIA1->MarR-HTH->?->BrxA-DNA-binding->BrxB-BrxF-iSTAND->DUF6079*->HEPN->PglZ->SIGMA+OB-Fold+AAA-ATPase+Lon-Protease-BrxL->ParB+HNH->?->HTH_3->	1152	Bacteria>Terrabacteria_group>Chloroflexota>Anaerolineae>Anaerolineales>unclassified_Anaerolineales	GCA_020433725.1	Anaerolineales_bacterium	KDD74_01185	15066..18524	2073117	BREX-system-P-loop-protein-BrxC-[Anaerolineales-bacterium]
MBK9096490.1	Type1.BREX	<-SIGMA+OB-Fold+AAA-ATPase+Lon-Protease-BrxL<-PglZ<-ParB+HNH<-HEPN+REase<-?<-N6-METHYLASE<-TOPRIM<-OLD-ABC<-DUF6079-p*<-DUF6079-p<-BrxB-BrxF-iSTAND<-BrxA-DNA-binding<-?||?->N6-METHYLASE-p->RVT->	900	Bacteria>Terrabacteria_group>Chloroflexota>Anaerolineae>unclassified_Anaerolineae	GCA_016719785.1	Anaerolineae_bacterium	IPM84_27780	244922..247624	2052143	hypothetical-protein-[Anaerolineae-bacterium]
WP_190817045.1	Type1.BREX	HTH_28+HTH_32+rve+Mu-transpos_C->TnsC-ATPase->BrxA-DNA-binding->BrxB-BrxF-iSTAND->DUF6079*->DOC+HTH->N6-METHYLASE->ParB+HNH->HEPN->PglZ->SIGMA+OB-Fold+AAA-ATPase+Lon-Protease-BrxL->?-><-TPR+TPR+TPR<-AAA-ATPase	1234	Bacteria>Terrabacteria_group>Cyanobacteriota/Melainabacteria_group>Cyanobacteriota>Cyanophyceae>Oscillatoriophycideae	GCF_014698845.1	Coleofasciculus_sp._FACHB-1120	H6H02_RS09830	171769..175473	2692783	BREX-system-P-loop-protein-BrxC-[Coleofasciculus-sp.-FACHB-1120]
WP_156114218.1	Type1.BREX	DUF932->?-><-?<-?<-sGTP<-SIGMA+OB-Fold+AAA-ATPase+HKD-DNase-BrxL<-PglZ<-N6-METHYLASE<-DUF6079-p*<-DUF6079-p<-BrxB-BrxF-iSTAND<-BrxA-DNA-binding||?->ParB+HNH->Thermonuclease->?->MazE_antitoxin->	835	Bacteria>Terrabacteria_group>Cyanobacteriota/Melainabacteria_group>Cyanobacteriota>Cyanophyceae>Pleurocapsales	GCF_000756305.1	Myxosarcina_sp._GI1	KV40_RS27950	18823..21330	1541065	BREX-system-P-loop-protein-BrxC-[Myxosarcina-sp.-GI1]
WP_009625661.1	Type1.BREX	<-CARF+HEPN<-ABhydrolase<-SAM-METHYLASE<-SIGMA+OB-Fold+AAA-ATPase+HKD-DNase-BrxL<-PglZ<-ParB+HNH<-N6-METHYLASE<-NERD<-OLD-ABC<-DUF6079*<-BrxB-BrxF-iSTAND<-BrxA-DNA-binding<-wHTH+REase+ZnR	1178	Bacteria>Terrabacteria_group>Cyanobacteriota/Melainabacteria_group>Cyanobacteriota>Cyanophyceae>Pseudanabaenales	GCF_000332215.1	Pseudanabaena_biceps	PSE7429_RS03435	33637..37173	927669	MULTISPECIES:-BREX-system-P-loop-protein-BrxC-[Pseudanabaena]
WP_094532951.1	Type1.BREX	STYKIN+CR-Kinase->SF1-SF2-Helicase->HTH+HEPN->McrB-AAA->wHTH+REase+ZnR->BrxA-DNA-binding->BrxB-BrxF-iSTAND->DUF6079*->N6-METHYLASE->ParB+HNH->	1181	Bacteria>Terrabacteria_group>Cyanobacteriota/Melainabacteria_group>Cyanobacteriota>Cyanophyceae>Pseudanabaenales	GCF_002251945.1	Pseudanabaena_sp._SR411	B9G53_RS13005	14836..18381	1980935	BREX-system-P-loop-protein-BrxC-[Pseudanabaena-sp.-SR411]
WP_190797467.1	Type1.BREX	<-SIGMA+OB-Fold+AAA-ATPase+HKD-DNase-BrxL<-PglZ<-ParB+HNH<-N6-METHYLASE<-NERD<-OLD-ABC<-DUF6079*<-BrxB-BrxF-iSTAND<-BrxA-DNA-binding<-ParB+HTH<-ParA-Soj-PloopNTPase||HTH+REase->?-><-?<-PrimPol<-RHH	1186	Bacteria>Terrabacteria_group>Cyanobacteriota/Melainabacteria_group>Cyanobacteriota>Cyanophyceae>Pseudanabaenales	GCF_014695875.1	Leptolyngbya_sp._FACHB-541	H6G00_RS15685	19029..22589	2692810	BREX-system-P-loop-protein-BrxC-[Leptolyngbya-sp.-FACHB-541]
MBD1853918.1	Type1.BREX	HTH+REase->DDE_Tnp_IS240-><-HTH+REase||ParA-Soj-PloopNTPase->ParB+HTH->BrxA-DNA-binding->BrxB-BrxF-iSTAND->DUF6079*->OLD-ABC->NERD->N6-METHYLASE->ParB+HNH->PglZ->SIGMA+OB-Fold+AAA-ATPase+HKD-DNase-BrxL->	1186	Bacteria>Terrabacteria_group>Cyanobacteriota/Melainabacteria_group>Cyanobacteriota>unclassified_Cyanobacteriota	GCA_014695325.1	Cyanobacteria_bacterium_FACHB-502	H6F87_28810	26444..30004	2692765	BREX-system-P-loop-protein-BrxC-[Cyanobacteria-bacterium-FACHB-502]
HBT89391.1	Type1.BREX	<-REC+GAF+HDGYP||?->?->?->?->BrxA-DNA-binding->BrxB-BrxF-iSTAND->DUF6079*->ABC-ATPase->REase->?->ParB+HNH->PglZ->?->?->PAS+NtrC-AAA+FIS-HTH->	1187	Bacteria>Thermodesulfobacteriota>Desulfobacteria>Desulfobacterales>Desulfobacteraceae>Desulfobacter	GCA_003510365.1	Desulfobacter_sp.	DEB50_13295	35068..38631	2294	BREX-system-P-loop-protein-BrxC-[Desulfobacter-sp.]
WP_035236115.1	Type1.BREX	<-SIGMA+OB-Fold+AAA-ATPase+HKD-DNase-BrxL<-PglZ<-ParB+HNH<-N6-METHYLASE<-REase<-OLD-ABC<-DUF6079*<-BrxB-BrxF-iSTAND<-BrxA-DNA-binding<-XIS-HTH||?->?->EAL->S1+GTPase-AIG->	1189	Bacteria>Thermodesulfobacteriota>Desulfobacteria>Desulfobacterales>Desulfobacteraceae>Desulfobacter	GCF_000745975.1	Desulfobacter_vibrioformis	Q366_RS02915	253933..257502	34031	BREX-system-P-loop-protein-BrxC-[Desulfobacter-vibrioformis]
WP_024336643.1	Type1.BREX	<-DDE_Tnp_ISAZ013<-DDE_Tnp_ISAZ013||AIPR->BrxA-DNA-binding->BrxB-BrxF-iSTAND->DUF6079*->Schlafen+HATPase+wHTH->N6-METHYLASE->PHP+SbcC->ParB+HNH->DUF932->OLD-ABC->HNH->PglZ->SIGMA+OB-Fold+AAA-ATPase+Lon-Protease-BrxL->	1144	Bacteria>Thermodesulfobacteriota>Desulfobacteria>Desulfobacterales>Desulfobacteraceae>Desulfotignum	GCF_000421285.1	Desulfotignum_balticum	K365_RS0111330	2237205..2240639	115781	BREX-system-P-loop-protein-BrxC-[Desulfotignum-balticum]
PIP37810.1	Type1.BREX	<-DUF6079*<-BrxB-BrxF-iSTAND<-BrxA-DNA-binding<-ParB+HNH<-?<-?<-REase+HTH+35exo+Small-B-barrel+SF1-SF2-Helicase<-URI-DNase	1174	Bacteria>Thermodesulfobacteriota>Desulfobacteria>Desulfobacterales>unclassified_Desulfobacterales	GCA_002772195.1	Desulfobacterales_bacterium_CG23_combo_of_CG06-09_8_20_14_all_51_8	CDX19_15630	1..3524	1973983	hypothetical-protein-CDX19_15630,-partial-[Desulfobacterales-bacterium-CG23_combo_of_CG06-09_8_20_14_all_51_8]
MBC8432684.1	Type6.BREX	<-REase<-SF1-SF2-Helicase+REase<-DNA_METHYLASE<-SIGMA+OB-Fold+AAA-ATPase+Lon-Protease-BrxL<-TOPRIM<-OLD-ABC<-PglZ<-ParB+HNH+Schlafen<-N6-METHYLASE<-DUF6079*<-BrxB-BrxF-iSTAND<-BrxA-DNA-binding<-BrxE<-XIS-HTH||TatD_DNase-><-?<-HEPN<-PolB	1227	Bacteria>Thermodesulfobacteriota>Desulfobacteria>Desulfobacterales>unclassified_Desulfobacterales	GCA_014383215.1	Desulfobacterales_bacterium	H8D96_12300	20226..23909	2044940	BREX-system-P-loop-protein-BrxC-[Desulfobacterales-bacterium]
KGO34922.1	Type1.BREX	<-PglZ<-ParB+HNH<-N6-METHYLASE<-DUF6079*<-BrxB-BrxF-iSTAND<-BrxA-DNA-binding	1193	Bacteria>Thermodesulfobacteriota>Desulfobulbia>Desulfobulbales>Desulfobulbaceae>Desulfobulbus	GCA_000769715.1	Desulfobulbus_sp._Tol-SR	JT06_05465	8780..12361	1536652	hypothetical-protein-JT06_05465-[Desulfobulbus-sp.-Tol-SR]
WP_092376845.1	Type1.BREX	Phage_integrase->TOPRIM->?-><-TRPR-HTH||?-><-HTH_3||AbrB-HTH->BrxA-DNA-binding->BrxB-BrxF-iSTAND->DUF6079*->ParB+HNH->N6-METHYLASE->?->?->PglZ->HEPN+SH3-like+SF1-SF2-Helicase+wHTH->MORC+DUF3684+HNH->SIGMA+OB-Fold+AAA-ATPase+Lon-Protease-BrxL->	1183	Bacteria>Thermodesulfobacteriota>Desulfovibrionia>Desulfovibrionales>Desulfomicrobiaceae>Desulfomicrobium	GCF_900114115.1	Desulfomicrobium_apsheronum	BMZ40_RS13600	74396..77947	52560	BREX-system-P-loop-protein-BrxC-[Desulfomicrobium-apsheronum]
MBI4803787.1	Type1.BREX	<-LuxR-HTH<-SIGMA+OB-Fold+AAA-ATPase+Lon-Protease-BrxL<-PglZ<-ParB+HNH<-PHP+SbcC<-HTH_21+rve<-PIN<-OLD-ABC<-DUF6079*<-BrxB-BrxF-iSTAND<-BrxA-DNA-binding<-XIS-HTH<-?||TraJ-RHH->TraI-VirD2-RCRE->SF1-SF2-Helicase->?-><-DUF4372+DDE_Tnp_1	1145	Bacteria>Thermodesulfobacteriota>Desulfovibrionia>Desulfovibrionales>Desulfovibrionaceae>Desulfovibrio	GCA_016208105.1	Desulfovibrio_sp.	HY795_00975	45852..49289	885	BREX-system-P-loop-protein-BrxC-[Desulfovibrio-sp.]
MBL3581670.1	Type1.BREX	<-KAP-glycosylase<-KAP-NTPase<-HARE-HTH+REase<-SIGMA+OB-Fold+AAA-ATPase+Lon-Protease-BrxL<-PglZ<-ParB+HNH<-PHP+SbcC<-N6-METHYLASE<-DUF6079*<-BrxB-BrxF-iSTAND<-BrxA-DNA-binding<-XIS-HTH<-?<-?<-?<-wHTH	1145	Bacteria>Thermodesulfobacteriota>Desulfovibrionia>Desulfovibrionales>Desulfovibrionaceae>Oleidesulfovibrio	GCA_016756815.1	Oleidesulfovibrio_alaskensis	JMJ96_04565	108279..111716	58180	BREX-system-P-loop-protein-BrxC-[Oleidesulfovibrio-alaskensis]
WP_273192722.1	Type1.BREX	<-KAP-glycosylase<-KAP-NTPase<-HARE-HTH+REase<-SIGMA+OB-Fold+AAA-ATPase+Lon-Protease-BrxL<-PglZ<-ParB+HNH<-PHP+SbcC<-N6-METHYLASE<-DUF6079*<-BrxB-BrxF-iSTAND<-BrxA-DNA-binding<-XIS-HTH<-?<-?<-?<-?<-wHTH	1146	Bacteria>Thermodesulfobacteriota>Desulfovibrionia>Desulfovibrionales>Desulfovibrionaceae>Oleidesulfovibrio	GCF_016756815.1	Oleidesulfovibrio_alaskensis	JMJ96_RS04575	108279..111719	58180	BREX-system-P-loop-protein-BrxC-[Oleidesulfovibrio-alaskensis]
AJY71182.1	Type1.BREX	NTP_transf_8->Calcineurin+Calcineurin-SbcD->SbcC+CCDC158->PIN-><-DUF4372+DDE_Tnp_1||HTH_Tnp_1->HTH_Tnp_1+HTH_21+rve+rve_3->BrxA-DNA-binding->BrxB-BrxF-iSTAND->DUF6079*->?->N6-METHYLASE->ParB+HNH->PglZ->SIGMA+OB-Fold+AAA-ATPase+HKD-DNase-BrxL->?->35exo+SF1-SF2-Helicase-><-HTH_3	1183	Bacteria>Thermodesulfobacteriota>Desulfuromonadia>Geobacterales>Geobacteraceae>Geobacter	GCA_000961035.1	Geobacter_sulfurreducens	RW64_17280	3775110..3778661	35554	hypothetical-protein-RW64_17280-[Geobacter-sulfurreducens]
OPY81323.1	Type1.BREX	<-ART-DarT<-SF1-SF2-Helicase<-HARE-HTH<-SIGMA+OB-Fold+AAA-ATPase+Lon-Protease-BrxL<-PglZ<-ParB+HNH<-HTH+REase<-N6-METHYLASE<-HATPase+wHTH<-DUF6079*<-BrxB-BrxF-iSTAND<-BrxA-DNA-binding	1144	Bacteria>Thermodesulfobacteriota>Syntrophia>Syntrophales>Syntrophaceae>Syntrophus	GCA_002067745.1	Syntrophus_sp._PtaU1.Bin005	A4E70_01341	26340..29774	1811712	MAG:-hypothetical-protein-A4E70_01341-[Syntrophus-sp.-PtaU1.Bin005]
WP_103134081.1	Type1.BREX	<-SIGMA+OB-Fold+AAA-ATPase+Lon-Protease-BrxL<-PglZ<-ParB+HNH<-N6-METHYLASE<-DUF6079*<-BrxB-BrxF-iSTAND<-BrxA-DNA-binding||?->HTH_36->HTH_36->AAA+->	1167	Bacteria>Thermotogota>Thermotogae>Kosmotogales>Kosmotogaceae>Mesotoga	GCF_002896535.1	Mesotoga_sp._B105.6.4	RJ60_RS04180	254841..258344	1582224	BREX-system-P-loop-protein-BrxC-[Mesotoga-sp.-B105.6.4]
WP_110990429.1	Type1.BREX	<-CASPASE<-?<-SIGMA+OB-Fold+AAA-ATPase+Lon-Protease-BrxL<-PglZ<-ParB+HNH<-N6-METHYLASE<-DUF6079*<-BrxB-BrxF-iSTAND<-BrxA-DNA-binding<-PAS+NtrC-AAA+FIS-HTH<-METHYLASE<-tRNA||?->nSTAND1+SbcC+ABC-ATPase+SbcC->	1163	Bacteria>Thermotogota>Thermotogae>Kosmotogales>Kosmotogaceae>Mesotoga	GCF_003228205.1	Mesotoga_sp._TolDC	LH53_RS05220	29090..32581	1389250	BREX-system-P-loop-protein-BrxC-[Mesotoga-sp.-TolDC]
WP_121509576.1	Type1.BREX	<-DnaB<-HTH_36<-?||BrxA-DNA-binding->BrxB-BrxF-iSTAND->DUF6079*->N6-METHYLASE->PglZ->SIGMA+OB-Fold+AAA-ATPase+Lon-Protease-BrxL->HNH->?->ParB+HNH->REase->AbiEi->	1162	Bacteria>Thermotogota>Thermotogae>Kosmotogales>Kosmotogaceae>Mesotoga	GCF_003664935.1	Mesotoga_sp._H07pep.5.4	Y696_RS00810	21677..25165	1463664	BREX-system-P-loop-protein-BrxC-[Mesotoga-sp.-H07pep.5.4]
MBI1292832.1	Type1.BREX	DDE_Tnp->?-><-D5N+viral-AAA+CDC6-HTH<-?<-?<-?||BrxA-DNA-binding->BrxB-BrxF-iSTAND->DUF6079*->?->N6-METHYLASE->DOC+HTH->ParB+HNH->PglZ->SIGMA+OB-Fold+AAA-ATPase+Lon-Protease-BrxL-><-?||?->Recombinase+ZnR->	1147	Bacteria>unclassified_Bacteria	GCA_016125005.1	bacterium	GC173_16580	8915..12358	1869227	BREX-system-P-loop-protein-BrxC-[bacterium]
EGR4851343.1	Type1.BREX	<-ZnR<-?||?->SF1-SF2-Helicase->AbiEi->viral-protein->wHTH+WYL+WCX->BrxA-DNA-binding->BrxB-BrxF-iSTAND->DUF6079*->N6-METHYLASE->ParB+HNH->GrpE->?->PglZ->SIGMA+OB-Fold+AAA-ATPase+Lon-Protease-BrxL-><-wHTH||DUF4113->	1225	-	-	-	C4G37_RS13070	147789..151466	-	DNA-repair-protein-[Vibrio-parahaemolyticus]
EGR4808858.1	Type1.BREX	<-ZnR<-?||?->SF1-SF2-Helicase->AbiEi->viral-protein->wHTH+WYL+WCX->BrxA-DNA-binding->BrxB-BrxF-iSTAND->DUF6079*->N6-METHYLASE->ParB+HNH->GrpE->?->PglZ->SIGMA+OB-Fold+AAA-ATPase+Lon-Protease-BrxL-><-wHTH||DUF4113->	1225	-	-	-	C4G65_RS20915	24307..27984	-	DNA-repair-protein-[Vibrio-parahaemolyticus]
EIL2714655.1	Type1.BREX	<-wHTH+WYL<-?<-?||?->XIS-HTH->ZnR->wHTH+WYL+WCX->BrxA-DNA-binding->BrxB-BrxF-iSTAND->DUF6079*->ParB+HNH->N6-METHYLASE->PglZ->SIGMA+OB-Fold+AAA-ATPase+Lon-Protease-BrxL->CARF+NtrC-AAA+FIS-HTH->	1225	-	-	-	M0K80_RS10540	473799..477476	-	BREX-system-P-loop-protein-BrxC-[Providencia-rettgeri]
--										
-- OLD-ABC->HNH										
--										
AGC72568.1	Type6.BREX	METHYLASE-><-SIGMA+OB-Fold+AAA-ATPase+Lon-Protease-BrxL<-PglZ<-HNH<-OLD-ABC<-N6-METHYLASE<-Schlafen+HATPase+wHTH<-DUF6079*<-BrxB-BrxF-iSTAND<-BrxA-DNA-binding<-BrxE||glycosylase->?->?->?-><-HNH<-OLD-ABC	1181	Bacteria>environmental_samples	-	uncultured_bacterium_A1Q1_fos_1870	.	12880..16425	1256554	hypothetical-protein-[uncultured-bacterium-A1Q1_fos_1870]
EFG14417.1	Type6.BREX	PRTase->?->?->tRNA->tRNA-><-?<-SIGMA+OB-Fold+AAA-ATPase+HKD-DNase-BrxL<-PglZ<-HNH<-OLD-ABC<-N6-METHYLASE<-DUF6079-p*<-DUF6079-p<-BrxB-BrxF-iSTAND<-BrxA-DNA-binding<-BrxE<-HTH_3<-?<-TraJ-RHH<-ZnR+TOPRIM<-CR-ATPase4	762	Bacteria>FCB_group>Bacteroidetes/Chlorobi_group>Bacteroidota>Bacteroidia>Bacteroidales	GCA_000178215.1	Bacteroides_xylanisolvens_SD_CC_1b	CW3_0497	100900..103188	702447	hypothetical-protein-CW3_0497-[Bacteroides-xylanisolvens-SD-CC-1b]
EFF57551.1	Type6.BREX	CR-ATPase4->ZnR+TOPRIM->TraJ-RHH->?->HTH_3->BrxE->BrxA-DNA-binding->BrxB-BrxF-iSTAND->DUF6079-p->DUF6079-p*->N6-METHYLASE->OLD-ABC->HNH->PglZ->SIGMA+OB-Fold+AAA-ATPase+HKD-DNase-BrxL->	706	Bacteria>FCB_group>Bacteroidetes/Chlorobi_group>Bacteroidota>Bacteroidia>Bacteroidales	GCA_000178295.1	Bacteroides_xylanisolvens_SD_CC_2a	CW1_2295	25339..27459	702444	hypothetical-protein-CW1_2295-[Bacteroides-xylanisolvens-SD-CC-2a]
CDM00531.1	Type6.BREX	PRTase->?->?->tRNA->tRNA-><-?<-SIGMA+OB-Fold+AAA-ATPase+HKD-DNase-BrxL<-PglZ<-HNH<-OLD-ABC<-N6-METHYLASE<-DUF6079-p*<-DUF6079-p<-BrxB-BrxF-iSTAND<-BrxA-DNA-binding<-BrxE<-HTH_3<-?<-TraJ-RHH<-ZnR+TOPRIM	689	Bacteria>FCB_group>Bacteroidetes/Chlorobi_group>Bacteroidota>Bacteroidia>Bacteroidales	GCA_000577955.1	Bacteroides_xylanisolvens_SD_CC_2a	BN891_34560	156968..159037	702444	hypothetical-protein-BN891_34560-[Bacteroides-xylanisolvens-SD-CC-2a]
HAM28416.1	Type6.BREX	ChlI+Lon_C+AAA-><-RNase<-AraC-HTH||?-><-?||HTH_3->BrxE->BrxA-DNA-binding->BrxB-BrxF-iSTAND->DUF6079*->N6-METHYLASE->OLD-ABC->HNH->PglZ->SIGMA+OB-Fold+AAA-ATPase+HKD-DNase-BrxL->?->?->35exo->	1218	Bacteria>FCB_group>Bacteroidetes/Chlorobi_group>Bacteroidota>Bacteroidia>Bacteroidales	GCA_003453655.1	Prevotella_sp.	DCP79_00675	66224..69880	59823	BREX-system-P-loop-protein-BrxC-[Prevotella-sp.]
MBQ2852763.1	Type1.BREX	AbiEi_4->AbiEii-><-Response_reg||HTH_3->BrxA-DNA-binding->BrxB-BrxF-iSTAND->DUF6079*->SbcC->N6-METHYLASE->OLD-ABC->HNH->PglZ->SIGMA+OB-Fold+AAA-ATPase+Lon-Protease-BrxL-><-?<-?<-XIS-HTH	1215	Bacteria>FCB_group>Bacteroidetes/Chlorobi_group>Bacteroidota>Bacteroidia>Bacteroidales	GCA_017409275.1	Bacteroidales_bacterium	IJE76_05160	110084..113731	2030927	hypothetical-protein-[Bacteroidales-bacterium]
MCI6342424.1	Type6.BREX	DnaB+TnsC-ATPase->?-><-SIGMA+OB-Fold+AAA-ATPase+HKD-DNase-BrxL<-PglZ<-HNH<-OLD-ABC<-N6-METHYLASE<-DUF6079*<-BrxB-BrxF-iSTAND<-BrxA-DNA-binding<-BrxE<-HTH_3	1217	Bacteria>FCB_group>Bacteroidetes/Chlorobi_group>Bacteroidota>Bacteroidia>Bacteroidales	GCA_022777695.1	Prevotella_sp.	MR788_07720	61686..65339	59823	BREX-system-P-loop-protein-BrxC-[Prevotella-sp.]
MCI6336457.1	Type1.BREX	PIWI->?->BrxA-DNA-binding->BrxB-BrxF-iSTAND->DUF6079*->ParB->OLD-ABC->?->N6-METHYLASE->OLD-ABC->HNH->PglZ->SIGMA+OB-Fold+AAA-ATPase+Lon-Protease-BrxL->	1215	Bacteria>FCB_group>Bacteroidetes/Chlorobi_group>Bacteroidota>Bacteroidia>Bacteroidales	GCA_022777825.1	Prevotella_sp.	MR802_00705	5343..8990	59823	BREX-system-P-loop-protein-BrxC-[Prevotella-sp.]
WP_232506819.1	Type6.BREX	PRTase->?->?->tRNA->tRNA-><-?<-SIGMA+OB-Fold+AAA-ATPase+HKD-DNase-BrxL<-PglZ<-HNH<-OLD-ABC<-N6-METHYLASE<-DUF6079-p*<-DUF6079-p<-BrxB-BrxF-iSTAND<-BrxA-DNA-binding<-BrxE<-HTH_3<-?<-TraJ-RHH<-ZnR+TOPRIM	794	Bacteria>FCB_group>Bacteroidetes/Chlorobi_group>Bacteroidota>Bacteroidia>Bacteroidales	GCF_000178215.1	Bacteroides_xylanisolvens	CW3_RS0123000	100900..103284	371601	hypothetical-protein-[Bacteroides-xylanisolvens]
WP_008016782.1	Type6.BREX	<-SIGMA+OB-Fold+AAA-ATPase+HKD-DNase-BrxL<-PglZ<-HNH<-OLD-ABC<-N6-METHYLASE<-DUF6079-p<-DUF6079-p*<-BrxB-BrxF-iSTAND<-BrxA-DNA-binding<-BrxE<-HTH_3<-?<-TraJ-RHH<-ZnR+TOPRIM<-CR-ATPase4	425	Bacteria>FCB_group>Bacteroidetes/Chlorobi_group>Bacteroidota>Bacteroidia>Bacteroidales	GCF_000178215.1	Bacteroides_xylanisolvens	CW3_RS26225	103281..104558	371601	DUF6079-family-protein-[Bacteroides-xylanisolvens]
WP_004314649.1	Type6.BREX	XIS-HTH->CR-ATPase4->ZnR+TOPRIM->TraJ-RHH->?->?->BrxE->BrxA-DNA-binding->BrxB-BrxF-iSTAND->DUF6079-p*->DUF6079-p->N6-METHYLASE->OLD-ABC->HNH->PglZ->SIGMA+OB-Fold+AAA-ATPase+HKD-DNase-BrxL->	515	Bacteria>FCB_group>Bacteroidetes/Chlorobi_group>Bacteroidota>Bacteroidia>Bacteroidales	GCF_000178295.1	Bacteroides_xylanisolvens	CW1_RS26610	23799..25346	371601	BREX-system-P-loop-protein-BrxC-[Bacteroides-xylanisolvens]
WP_232505074.1	Type6.BREX	CR-ATPase4->ZnR+TOPRIM->TraJ-RHH->?->?->BrxE->BrxA-DNA-binding->BrxB-BrxF-iSTAND->DUF6079-p->DUF6079-p*->N6-METHYLASE->OLD-ABC->HNH->PglZ->SIGMA+OB-Fold+AAA-ATPase+HKD-DNase-BrxL->	711	Bacteria>FCB_group>Bacteroidetes/Chlorobi_group>Bacteroidota>Bacteroidia>Bacteroidales	GCF_000178295.1	Bacteroides_xylanisolvens	CW1_RS26615	25324..27459	371601	hypothetical-protein-[Bacteroides-xylanisolvens]
WP_044064719.1	Type6.BREX	<-REase<-?<-SIGMA+OB-Fold+AAA-ATPase+HKD-DNase-BrxL<-PglZ<-HNH<-OLD-ABC<-N6-METHYLASE<-DUF6079*<-BrxB-BrxF-iSTAND<-BrxA-DNA-binding<-BrxE<-HTH_3<-AAA+<-?<-XIS-HTH<-?<-Phage_int_SAM_5+Phage_integrase	1217	Bacteria>FCB_group>Bacteroidetes/Chlorobi_group>Bacteroidota>Bacteroidia>Bacteroidales	GCF_000833995.1	Prevotella_pectinovora	ST45_RS02505	69518..73171	1602169	BREX-system-P-loop-protein-BrxC-[Prevotella-pectinovora]
WP_215758559.1	Type6.BREX	Phage_int_SAM_5+Phage_integrase->?->XIS-HTH->?->AAA+->HTH_3->BrxE->BrxA-DNA-binding->BrxB-BrxF-iSTAND->DUF6079*->N6-METHYLASE->OLD-ABC->HNH->PglZ->SIGMA+OB-Fold+AAA-ATPase+HKD-DNase-BrxL-><-?||ParA-Soj-PloopNTPase->	1217	Bacteria>FCB_group>Bacteroidetes/Chlorobi_group>Bacteroidota>Bacteroidia>Bacteroidales	GCF_018789675.1	Hallella_faecis	KM934_00340	78197..81850	2841596	BREX-system-P-loop-protein-BrxC-[Hallella-faecis]
WP_008770354.1	Type6.BREX	DDE_Tnp-><-?<-SIGMA+OB-Fold+AAA-ATPase+HKD-DNase-BrxL<-PglZ<-HNH<-OLD-ABC<-N6-METHYLASE<-DUF6079*<-BrxB-BrxF-iSTAND<-BrxA-DNA-binding<-BrxE<-HTH_3<-?<-TraJ-RHH<-ZnR+TOPRIM<-CR-ATPase4	1219	Bacteria>FCB_group>Bacteroidetes/Chlorobi_group>Bacteroidota>Bacteroidia>Bacteroidales	GCF_902374095.1	Bacteroides	BSAG_RS19905	423901..427560	816	MULTISPECIES:-BREX-system-P-loop-protein-BrxC-[Bacteroides]
WP_273212250.1	Type1.BREX	<-AbiEii<-wHTH+AbiEi_2<-SIGMA+OB-Fold+AAA-ATPase+Lon-Protease-BrxL<-PglZ<-Schlafen+HATPase+wHTH<-REase<-N6-METHYLASE<-HNH<-OLD-ABC<-DUF6079*<-BrxB-BrxF-iSTAND<-BrxA-DNA-binding<-?<-?<-D5ATPase<-XIS-HTH<-?<-Arm-DNA-bind_5+Phage_int_SAM_5+Phage_integrase	1173	Bacteria>FCB_group>Bacteroidetes/Chlorobi_group>Bacteroidota>Cytophagia>Cytophagales	GCF_002483185.1	Runella_zeae	B4E63_RS12460	307458..310979	94255	BREX-system-P-loop-protein-BrxC-[Runella-zeae]
WP_159471214.1	Type1.BREX	<-XIS-HTH<-AbiEii<-AbiEi_4+AbiEi_1<-REase<-SIGMA+OB-Fold+AAA-ATPase+Lon-Protease-BrxL<-PglZ<-N6-METHYLASE<-HNH<-OLD-ABC<-DUF6079*<-BrxB-BrxF-iSTAND<-BrxA-DNA-binding||CHTH->?->?->?-><-CopG/RHH	1179	Bacteria>FCB_group>Bacteroidetes/Chlorobi_group>Bacteroidota>Cytophagia>Cytophagales	GCF_009801115.1	Dyadobacter_sp._3J3	FZF08_RS17845	89009..92548	2606600	BREX-system-P-loop-protein-BrxC-[Dyadobacter-sp.-3J3]
MBN8706499.1	Type1.BREX	Phage_int_SAM_4+Phage_integrase->35exo+URI-DNase->tRNA->HTH->BrxA-DNA-binding->BrxB-BrxF-iSTAND->DUF6079*->OLD-ABC->HNH->N6-METHYLASE->?->Schlafen+HATPase+wHTH->PglZ->SIGMA+OB-Fold+AAA-ATPase+Lon-Protease-BrxL->SbcC->	1173	Bacteria>FCB_group>Bacteroidetes/Chlorobi_group>Bacteroidota>unclassified_Bacteroidota	GCA_017303995.1	Bacteroidota_bacterium	J0L62_11545	8364..11885	1898104	BREX-system-P-loop-protein-BrxC-[Bacteroidota-bacterium]
WP_201422032.1	Type6.BREX	<-glycosylase<-SIGMA+OB-Fold+AAA-ATPase+Lon-Protease-BrxL<-PglZ<-HNH<-OLD-ABC<-N6-METHYLASE<-N6-METHYLASE<-Tox-RelA<-DUF6079*<-BrxB-BrxF-iSTAND<-BrxA-DNA-binding<-BrxE<-XIS-HTH||<-ACET||?->?->REC+NtrC-AAA+FIS-HTH->	1188	Bacteria>Myxococcota>Myxococcia>Myxococcales>Cystobacterineae>Myxococcaceae	GCF_016698685.1	Myxococcus_xanthus	JKA73_RS29390	7346078..7349644	34	BREX-system-P-loop-protein-BrxC-[Myxococcus-xanthus]
WP_267556343.1	Type6.BREX	<-rve_3<-SIGMA+OB-Fold+AAA-ATPase+Lon-Protease-BrxL<-PglZ<-HNH<-OLD-ABC<-N6-METHYLASE<-Tox-RelA<-DUF6079*<-BrxB-BrxF-iSTAND<-BrxA-DNA-binding<-BrxE<-XIS-HTH||?-><-?<-wHTH(SBD)+HNH<-?<-rve_3	1189	Bacteria>Myxococcota>Myxococcia>Myxococcales>Cystobacterineae>Myxococcaceae	GCF_026626625.1	Corallococcus_sp._BB11-1	OV207_RS17695	240885..244454	2996783	BREX-system-P-loop-protein-BrxC-[Corallococcus-sp.-BB11-1]
WP_020733584.1	Type6.BREX	ABC-ATPase+SbcC-><-SIGMA+OB-Fold+AAA-ATPase+Lon-Protease-BrxL<-PglZ<-HNH<-OLD-ABC<-N6-METHYLASE<-N6-METHYLASE<-Schlafen+HATPase+wHTH<-DUF6079*<-BrxB-BrxF-iSTAND<-BrxA-DNA-binding<-BrxE<-XIS-HTH||?->?->ACET->	1182	Bacteria>Myxococcota>Polyangia>Polyangiales>Polyangiaceae>Sorangium	GCF_000418325.1	Sorangium_cellulosum	SCE1572_RS08150	2251441..2254989	56	BREX-system-P-loop-protein-BrxC-[Sorangium-cellulosum]
MCK6536698.1	Type1.BREX	<-TetR-HTH||MerR-HTH->?->?->?-><-?||XIS-HTH->BrxA-DNA-binding->BrxB-BrxF-iSTAND->DUF6079*->N6-METHYLASE->OLD-ABC->HNH->PglZ->SIGMA+OB-Fold+AAA-ATPase+Lon-Protease-BrxL->REase+PNTD1+PNTD2->	1149	Bacteria>Myxococcota>Polyangia>Polyangiales>Polyangiaceae>unclassified_Polyangiaceae	GCA_023150915.1	Polyangiaceae_bacterium	L6Q84_27330	8687..12136	2268199	BREX-system-P-loop-protein-BrxC-[Polyangiaceae-bacterium]
MCK6527600.1	Type6.BREX	RADICAL-SAM->?-><-SIGMA+OB-Fold+AAA-ATPase+Lon-Protease-BrxL<-?<-?<-?<-HNH<-OLD-ABC<-N6-METHYLASE<-DUF6079*<-BrxB-BrxF-iSTAND<-BrxA-DNA-binding<-BrxE||?->GDResolvase+Recombinase+ZnR->	1184	Bacteria>Myxococcota>unclassified_Myxococcota	GCA_023150875.1	Myxococcota_bacterium	L6R50_08565	57228..60782	2818507	BREX-system-P-loop-protein-BrxC-[Myxococcota-bacterium]
WP_225487034.1	Type1.BREX	<-HTH_58<-TrwC+SF1-SF2-Helicase||GNAT+DDE_Tnp_1_assoc->DDE_Tnp_1_assoc->Tox-RelE->HTH_3->wHTH+WYL+WCX-p->BrxA-DNA-binding->BrxB-BrxF-iSTAND->DUF6079*->N6-METHYLASE->OLD-ABC->HNH->PglZ->SIGMA+OB-Fold+AAA-ATPase+Lon-Protease-BrxL-><-tRNA<-FURR-HTH	1146	Bacteria>Pseudomonadota>Acidithiobacillia>Acidithiobacillales>Acidithiobacillaceae>Acidithiobacillus	-	Acidithiobacillus_ferrooxidans	LDB30_08025	1563873..1567313	920	BREX-system-P-loop-protein-BrxC-[Acidithiobacillus-ferrooxidans]
RWC29997.1	Type1.BREX	SbcC+CCDC158->?->REase+PNTD1+PNTD2->PIWI-><-SIGMA+OB-Fold+AAA-ATPase+HKD-DNase-BrxL<-PglZ<-HNH<-OLD-ABC<-N6-METHYLASE<-DUF6079*<-BrxB-BrxF-iSTAND<-BrxA-DNA-binding||REase-><-?||?-><-?||REase->?->rve->	1181	Bacteria>Pseudomonadota>Alphaproteobacteria>Hyphomicrobiales>Phyllobacteriaceae>Mesorhizobium	GCA_004017395.1	Mesorhizobium_sp.	EOS27_14215	67406..70951	1871066	BREX-system-P-loop-protein-BrxC-[Mesorhizobium-sp.]
WP_136091391.1	Type1.BREX	REase+PNTD1+PNTD2->PIWI->Y1_Tnp-><-SIGMA+OB-Fold+AAA-ATPase+HKD-DNase-BrxL<-PglZ<-HNH<-OLD-ABC<-N6-METHYLASE<-DUF6079*<-BrxB-BrxF-iSTAND<-BrxA-DNA-binding||REase->?-><-?||REase->rve->TnsC-ATPase->TnsC-ATPase->	1181	Bacteria>Pseudomonadota>Alphaproteobacteria>Hyphomicrobiales>Phyllobacteriaceae>Mesorhizobium	GCA_004794715.1	Mesorhizobium_sp._M8A.F.Ca.ET.207.01.1.1	EN850_RS12645	56072..59617	2563968	BREX-system-P-loop-protein-BrxC-[Mesorhizobium-sp.-M8A.F.Ca.ET.207.01.1.1]
MBF0399382.1	Type1.BREX	BrxA-DNA-binding->BrxB-BrxF-iSTAND->DUF6079*->HicB_lk_antitox->HicA_toxin->N6-METHYLASE->OLD-ABC->HNH->PglZ->SIGMA+OB-Fold+AAA-ATPase+Lon-Protease-BrxL->?-><-STAS	1144	Bacteria>Pseudomonadota>Alphaproteobacteria>Magnetococcales>unclassified_Magnetococcales	GCA_015232635.1	Magnetococcales_bacterium	HQL90_01290	18375..21809	2026759	BREX-system-P-loop-protein-BrxC-[Magnetococcales-bacterium]
QKS29579.1	Type6.BREX	<-SIGMA+OB-Fold+AAA-ATPase+Lon-Protease-BrxL<-PglZ<-HNH<-OLD-ABC<-N6-METHYLASE<-ORC-CDC6<-ParA-Soj-PloopNTPase<-HEPN<-N6-METHYLASE<-DUF6079*<-BrxB-BrxF-iSTAND<-BrxA-DNA-binding<-BrxE<-XIS-HTH	1180	Bacteria>Pseudomonadota>Betaproteobacteria>Betaproteobacteria_incertae_sedis>Candidatus_Accumulibacter	GCA_013347225.1	Candidatus_Accumulibacter_similis	HT579_12075	2666587..2670129	2954388	MAG:-BREX-system-P-loop-protein-BrxC-[Candidatus-Accumulibacter-similis]
MBK8385355.1	Type1.BREX	<-SIGMA+OB-Fold+AAA-ATPase+Lon-Protease-BrxL<-PglZ<-N6-METHYLASE<-HNH<-OLD-ABC<-DUF6079*<-BrxB-BrxF-iSTAND<-BrxA-DNA-binding<-BrxA-DNA-binding<-Polbeta<-wHTH+AbiEi_2	1154	Bacteria>Pseudomonadota>Betaproteobacteria>Betaproteobacteria_incertae_sedis>Candidatus_Accumulibacter>unclassified_Candidatus_Accumulibacter	GCA_016711725.1	Candidatus_Accumulibacter_sp.	IPL11_06655	113668..117132	2053492	BREX-system-P-loop-protein-BrxC-[Candidatus-Accumulibacter-sp.]
MBN8436515.1	Type1.BREX	wHTH+AbiEi_2-><-?||?->wHTH+AbiEi_2->Polbeta->BrxA-DNA-binding->BrxA-DNA-binding->BrxB-BrxF-iSTAND->DUF6079*->N6-METHYLASE->OLD-ABC->HNH->PglZ->SIGMA+OB-Fold+AAA-ATPase+Lon-Protease-BrxL->	1154	Bacteria>Pseudomonadota>Betaproteobacteria>Betaproteobacteria_incertae_sedis>Candidatus_Accumulibacter>unclassified_Candidatus_Accumulibacter	GCA_017302555.1	Candidatus_Accumulibacter_sp.	JOL95_00470	105300..108764	2053492	BREX-system-P-loop-protein-BrxC-[Candidatus-Accumulibacter-sp.]
WP_043683814.1	Type1.BREX	DDE_Tnp_1_4->?->VirB5-><-DUF772+DDE_Tnp_1_6<-?||wHTH+WYL+WCX->BrxA-DNA-binding->BrxB-BrxF-iSTAND->DUF6079*->HEPN->N6-METHYLASE->OLD-ABC->HNH->DUF772+DDE_Tnp_1+DDE_Tnp_1_6->HNH->PglZ->SIGMA+OB-Fold+AAA-ATPase+Lon-Protease-BrxL-><-YdaS_antitoxin	1153	Bacteria>Pseudomonadota>Betaproteobacteria>Burkholderiales>Alcaligenaceae>Castellaniella	GCF_000612685.1	Castellaniella_defragrans	BN940_RS13240	2967883..2971344	75697	BREX-system-P-loop-protein-BrxC-[Castellaniella-defragrans]
WP_085318435.1	Type1.BREX	<-DiS_P_DiS+Peptidase_A24<-?<-VirB11||RHH_5->BrxA-DNA-binding->BrxB-BrxF-iSTAND->DUF6079*->?->N6-METHYLASE->OLD-ABC->HNH->PglZ->SIGMA+OB-Fold+AAA-ATPase+Lon-Protease-BrxL->	1153	Bacteria>Pseudomonadota>Betaproteobacteria>Burkholderiales>Alcaligenaceae>Derxia	GCF_002105195.1	Derxia_lacustris	B9P64_RS23165	16077..19538	764842	BREX-system-P-loop-protein-BrxC-[Derxia-lacustris]
WP_115063824.1	Type1.BREX	Phage_AlpA->?->TraJ-RHH-><-REase+NACHT<-?<-RADICAL-SAM<-DUF772+DDE_Tnp_1||BrxA-DNA-binding->BrxB-BrxF-iSTAND->DUF6079*->N6-METHYLASE->DUF3644->OLD-ABC->TOPRIM->OLD-ABC->HNH->PglZ->SIGMA+OB-Fold+AAA-ATPase+Lon-Protease-BrxL-><-Y1_Tnp	1155	Bacteria>Pseudomonadota>Betaproteobacteria>Burkholderiales>Alcaligenaceae>Oligella	GCF_900454315.1	Oligella_ureolytica	NCTC11998_02420	2456151..2459618	90244	BREX-system-P-loop-protein-BrxC-[Oligella-ureolytica]
WP_108330269.1	Type1.BREX	<-SIGMA+OB-Fold+AAA-ATPase+Lon-Protease-BrxL<-PglZ<-HNH<-OLD-ABC<-N6-METHYLASE<-REase<-OLD-ABC<-DUF6079*<-BrxB-BrxF-iSTAND<-BrxA-DNA-binding<-PIN<-ZnR+TPR	1154	Bacteria>Pseudomonadota>Betaproteobacteria>Burkholderiales>Comamonadaceae>Limnohabitans	GCF_003063435.1	Limnohabitans_sp._2KL-1	B9Z47_RS05555	224189..227653	1100699	BREX-system-P-loop-protein-BrxC-[Limnohabitans-sp.-2KL-1]
WP_108311897.1	Type1.BREX	<-SF1-SF2-Helicase<-SIGMA+OB-Fold+AAA-ATPase+Lon-Protease-BrxL<-PglZ<-HNH<-OLD-ABC<-N6-METHYLASE<-DUF6079*<-BrxB-BrxF-iSTAND<-BrxA-DNA-binding<-PIN<-ZnR+TPR	1153	Bacteria>Pseudomonadota>Betaproteobacteria>Burkholderiales>Comamonadaceae>Limnohabitans	GCF_003063455.1	Limnohabitans_parvus	B9Z37_RS03875	837390..840851	540061	BREX-system-P-loop-protein-BrxC-[Limnohabitans-parvus]
MBH2043138.1	Type1.BREX	RHH_5->BrxA-DNA-binding->BrxB-BrxF-iSTAND->DUF6079*->MarR-HTH+Polbeta->HEPN->OLD-ABC->HNH->PglZ->SIGMA+OB-Fold+AAA-ATPase+Lon-Protease-BrxL->	1153	Bacteria>Pseudomonadota>Betaproteobacteria>Burkholderiales>Comamonadaceae>unclassified_Comamonadaceae	GCA_016000285.1	Comamonadaceae_bacterium	I8H87_06315	1878..5339	1871071	BREX-system-P-loop-protein-BrxC-[Comamonadaceae-bacterium]
WP_071078524.1	Type1.BREX	Phage_integrase-><-?<-SIGMA+OB-Fold+AAA-ATPase+Lon-Protease-BrxL<-PglZ<-HNH<-OLD-ABC<-N6-METHYLASE<-DUF6079*<-BrxB-BrxF-iSTAND<-BrxA-DNA-binding<-RHH_5<-?<-HEPN+OST-HTH	1153	Bacteria>Pseudomonadota>Betaproteobacteria>Burkholderiales>Oxalobacteraceae>Janthinobacterium	GCF_001854915.1	Janthinobacterium_lividum	AKG95_RS19180	51113..54574	29581	BREX-system-P-loop-protein-BrxC-[Janthinobacterium-lividum]
WP_046167751.1	Type1.BREX	BrxA-DNA-binding->BrxB-BrxF-iSTAND->DUF6079*->N6-METHYLASE->OLD-ABC->HNH->PglZ->SIGMA+OB-Fold+AAA-ATPase+Lon-Protease-BrxL->?->?-><-AraC-HTH+AraC-HTH	1153	Bacteria>Pseudomonadota>Betaproteobacteria>Neisseriales>Chromobacteriaceae>Chromobacterium_group	GCF_000971355.1	Chromobacterium_vaccinii	VL68_RS13365	2032..5493	1108595	BREX-system-P-loop-protein-BrxC-[Chromobacterium-vaccinii]
WP_096450985.1	Type6.BREX	<-SIGMA+OB-Fold+AAA-ATPase+Lon-Protease-BrxL<-PglZ<-HNH<-OLD-ABC<-N6-METHYLASE<-ORC-CDC6<-ParA-Soj-PloopNTPase<-HEPN<-N6-METHYLASE<-DUF6079*<-BrxB-BrxF-iSTAND<-BrxA-DNA-binding<-BrxE<-XIS-HTH<-HTH_3||?-><-ParB<-?||HTH_3->	1180	Bacteria>Pseudomonadota>Betaproteobacteria>Rhodocyclales>Zoogloeaceae>Thauera	GCF_002354895.1	Thauera_sp._K11	CCZ27_RS19540	4450815..4454357	2005884	BREX-system-P-loop-protein-BrxC-[Thauera-sp.-K11]

<table>
<tr><th>Protein-ID</th><th>System</th><th>Gene-Neighbourhoods</th><th>Length</th><th>Taxonomy</th><th>Assembly-ID</th><th>Strain</th><th>Locus-Tag</th><th>Genomic-coordinates</th><th>TaxID</th><th>Fasta-define</th></tr>
<tr><td>EJN6954191.1</td><td>Type1.BREX</td><td><-LexA-protease||?-><- wHTH+WYL+WCX ||ARG->?->ZnR->RHS-> BrxA-DNA-binding -> BrxB-BrxF-iSTAND -> DUF6079 *-> N6-METHYLASE -> OLD-ABC-> HNH -> PglZ ->HORMA-><-SF1-SF2-Helicase+REase [illegible]</td><td>1157</td><td>Bacteria>Pseudomonadota>Gammaproteobacteria>Aeromonadales>Aeromonadaceae>Aeromonas</td><td>GCA_024506355.1</td><td>Aeromonas_hydrophila</td><td>NRD12_000732</td><td>206979..210452</td><td>644</td><td>BREX-system-P-loop-protein-BrxC-[Aeromonas-hydrophila]</td></tr>
<tr><td>WP_043124789.1</td><td>Type1.BREX</td><td>SF1-SF2-Helicase+REase->SF1-SF2-Helicase+REase-><-HORMA<- PglZ <- HNH <-OLD-ABC <- N6-METHYLASE <- DUF6079 *<- BrxB-BrxF-iSTAND <- BrxA-DNA-binding <-URI-DNase<-?||LexA-protease-> [illegible]</td><td>1157</td><td>Bacteria>Pseudomonadota>Gammaproteobacteria>Aeromonadales>Aeromonadaceae>Aeromonas</td><td>GCF_000724965.1</td><td>Aeromonas_hydrophila</td><td>HR52_RS13935</td><td>3102820..3106293</td><td>644</td><td>BREX-system-P-loop-protein-BrxC-[Aeromonas-hydrophila]</td></tr>
<tr><td>WP_113721905.1</td><td>Type1.BREX</td><td>SF1-SF2-Helicase+REase->SF1-SF2-Helicase+REase-><-HEPN+Sel1+SEC-C<- PglZ <- HNH <-OLD-ABC <- N6-METHYLASE <- DUF6079 *<- BrxB-BrxF-iSTAND <- BrxA-DNA-binding <-RHS<-?<-ARG|| [illegible]</td><td>1157</td><td>Bacteria>Pseudomonadota>Gammaproteobacteria>Aeromonadales>Aeromonadaceae>Aeromonas</td><td>GCF_003265515.1</td><td>Aeromonas_salmonicida</td><td>CPA05_RS19945</td><td>20643..24116</td><td>645</td><td>BREX-system-P-loop-protein-BrxC-[Aeromonas-salmonicida]</td></tr>
<tr><td>WP_114523382.1</td><td>Type1.BREX</td><td><-LexA-protease||?->URI-DNase-> BrxA-DNA-binding -> BrxB-BrxF-iSTAND -> DUF6079 *-> N6-METHYLASE -> OLD-ABC-> HNH -> PglZ -></td><td>1157</td><td>Bacteria>Pseudomonadota>Gammaproteobacteria>Aeromonadales>Aeromonadaceae>Aeromonas</td><td>GCF_003344485.1</td><td>Aeromonas_sp._ARM81</td><td>ASJ36_RS21100</td><td>4447..7920</td><td>1747384</td><td>BREX-system-P-loop-protein-BrxC-[Aeromonas-sp.-ARM81]</td></tr>
<tr><td>WP_182951579.1</td><td>Type1.BREX</td><td><-LexA-protease||?->REase+N6_METHYLASE->TRD+TRD->REase+SF1-SF2-Helicase->zf-ISL3+HTH_Tnp_ISL3+DDE_Tnp_ISL3->URI-DNase-> BrxA-DNA-binding -> BrxB-BrxF-iSTAND -> DUF6079 *-> [illegible]</td><td>1157</td><td>Bacteria>Pseudomonadota>Gammaproteobacteria>Aeromonadales>Aeromonadaceae>Aeromonas</td><td>GCF_014168615.1</td><td>Aeromonas_sp._WP2-W18-CRE-05</td><td>WP2W18C05_13360</td><td>1426228..1429701</td><td>2675707</td><td>BREX-system-P-loop-protein-BrxC-[Aeromonas-sp.-WP2-W18-CRE-05]</td></tr>
<tr><td>WP_203130882.1</td><td>Type1.BREX</td><td><- wHTH+WYL+WCX ||ZnR+ZnR-><-?<-LexA-protease||?->URI-DNase-> BrxA-DNA-binding -> BrxB-BrxF-iSTAND -> DUF6079 -p*-> DUF6079 -p-> N6-METHYLASE -> OLD-ABC-> HNH -> PglZ ->HORMA-><-SF1- [illegible]</td><td>935</td><td>Bacteria>Pseudomonadota>Gammaproteobacteria>Aeromonadales>Aeromonadaceae>Aeromonas</td><td>GCF_016805325.1</td><td>Aeromonas_jandaei</td><td>GNZ06_RS06060</td><td>150251..153058</td><td>650</td><td>BREX-system-P-loop-protein-BrxC-[Aeromonas-jandaei]</td></tr>
<tr><td>WP_203130883.1</td><td>Type1.BREX</td><td><- wHTH+WYL+WCX ||ZnR+ZnR-><-?<-LexA-protease||?->URI-DNase-> BrxA-DNA-binding -> BrxB-BrxF-iSTAND -> DUF6079 -p-> DUF6079 -p*-> N6-METHYLASE -> OLD-ABC-> HNH -> PglZ ->HORMA-><-SF1- [illegible]</td><td>226</td><td>Bacteria>Pseudomonadota>Gammaproteobacteria>Aeromonadales>Aeromonadaceae>Aeromonas</td><td>GCF_016805325.1</td><td>Aeromonas_jandaei</td><td>GNZ06_RS06065</td><td>153058..153738</td><td>650</td><td>hypothetical-protein-[Aeromonas-jandaei]</td></tr>
<tr><td>WP_203133261.1</td><td>Type1.BREX</td><td>SF1-SF2-Helicase+REase->SF1-SF2-Helicase+REase-><-HORMA<- PglZ <- HNH <-OLD-ABC <- N6-METHYLASE <- DUF6079 *<- BrxB-BrxF-iSTAND <- BrxA-DNA-binding <-URI-DNase<-?||LexA-protease->? [illegible]</td><td>1157</td><td>Bacteria>Pseudomonadota>Gammaproteobacteria>Aeromonadales>Aeromonadaceae>Aeromonas</td><td>GCF_016805365.1</td><td>Aeromonas_jandaei</td><td>G4224_RS08640</td><td>376570..380043</td><td>650</td><td>BREX-system-P-loop-protein-BrxC-[Aeromonas-jandaei]</td></tr>
<tr><td>WP_213387487.1</td><td>Type1.BREX</td><td><-LexA-protease||?-><- wHTH+WYL+WCX ||ARG->?->ZnR->RHS-> BrxA-DNA-binding -> BrxB-BrxF-iSTAND -> DUF6079 *-> N6-METHYLASE -> OLD-ABC-> HNH -> PglZ ->HEPN+Sel1+SEC-C->HIN-HTH-><-SF1- [illegible]</td><td>1157</td><td>Bacteria>Pseudomonadota>Gammaproteobacteria>Aeromonadales>Aeromonadaceae>Aeromonas</td><td>GCF_018359765.1</td><td>Aeromonas_veronii</td><td>J4G64_RS11995</td><td>290568..294041</td><td>654</td><td>BREX-system-P-loop-protein-BrxC-[Aeromonas-veronii]</td></tr>
<tr><td>WP_215805224.1</td><td>Type1.BREX</td><td><-HORMA<-DDE_Tnp_1<- PglZ -p<- PglZ -p<- PglZ <- HNH <- HNH <-OLD-ABC <- N6-METHYLASE <- DUF6079 *<- BrxB-BrxF-iSTAND -p<- BrxB-BrxF-iSTAND <- BrxA-DNA-binding ||HIN-HTH-><-URI-DNase<- [illegible]</td><td>1157</td><td>Bacteria>Pseudomonadota>Gammaproteobacteria>Aeromonadales>Aeromonadaceae>Aeromonas</td><td>GCF_018802365.1</td><td>Aeromonas_jandaei</td><td>HQ398_RS10760</td><td>2353585..2357058</td><td>650</td><td>BREX-system-P-loop-protein-BrxC-[Aeromonas-jandaei]</td></tr>
<tr><td>WP_216986928.1</td><td>Type1.BREX</td><td>HDRnase-><-?<-LexA-protease||ARG->ZnR->||?->RHS-> BrxA-DNA-binding -> BrxB-BrxF-iSTAND -> DUF6079 *-> N6-METHYLASE -> OLD-ABC-> HNH -> PglZ ->HORMA-><-SF1-SF2-Helicase+REase<-SF1-S [illegible]</td><td>1157</td><td>Bacteria>Pseudomonadota>Gammaproteobacteria>Aeromonadales>Aeromonadaceae>Aeromonas</td><td>GCF_019048565.1</td><td>Aeromonas_sp._FDAARGOS_1406</td><td>I6L36_RS00705</td><td>137070..140543</td><td>2778055</td><td>BREX-system-P-loop-protein-BrxC-[Aeromonas-sp.-FDAARGOS-1406]</td></tr>
<tr><td>WP_216990395.1</td><td>Type1.BREX</td><td>DnaJ->SF1-SF2-Helicase+REase->SF1-SF2-Helicase+REase-><-HORMA<- PglZ <- HNH <-OLD-ABC <- N6-METHYLASE <- DUF6079 *<- BrxB-BrxF-iSTAND <- BrxA-DNA-binding <-RHS<-?<-?<-RNase<-DUF358 [illegible]</td><td>1157</td><td>Bacteria>Pseudomonadota>Gammaproteobacteria>Aeromonadales>Aeromonadaceae>Aeromonas</td><td>GCF_019048705.1</td><td>Aeromonas_sp._FDAARGOS_1409</td><td>I6L39_RS17660</td><td>3801324..3804797</td><td>2778058</td><td>BREX-system-P-loop-protein-BrxC-[Aeromonas-sp.-FDAARGOS-1409]</td></tr>
<tr><td>WP_216992842.1</td><td>Type1.BREX</td><td>SF1-SF2-Helicase+REase->SF1-SF2-Helicase+REase-><-HORMA<- PglZ <- HNH <-OLD-ABC <- N6-METHYLASE <- DUF6079 *<- BrxB-BrxF-iSTAND <- BrxA-DNA-binding <-?||LexA-protease->HTH+rve->AAA [illegible]</td><td>1157</td><td>Bacteria>Pseudomonadota>Gammaproteobacteria>Aeromonadales>Aeromonadaceae>Aeromonas</td><td>GCF_019048725.1</td><td>Aeromonas_sp._FDAARGOS_1407</td><td>I6L37_20730</td><td>4426496..4429969</td><td>2778056</td><td>BREX-system-P-loop-protein-BrxC-[Aeromonas-sp.-FDAARGOS-1407]</td></tr>
<tr><td>WP_223938541.1</td><td>Type1.BREX</td><td><-SAM-METHYLASE<-Tnp_DNA_bind+DDE_Tnp_1+Dimer_Tnp_Tn5||SF1-SF2-Helicase+REase->SF1-SF2-Helicase+REase-><-HORMA<- PglZ <- HNH <-OLD-ABC <- N6-METHYLASE <- DUF6079 *<- BrxB-BrxF-iST [illegible]</td><td>1157</td><td>Bacteria>Pseudomonadota>Gammaproteobacteria>Aeromonadales>Aeromonadaceae>Aeromonas</td><td>GCF_019971155.1</td><td>Aeromonas_caviae</td><td>LDN24_RS08690</td><td>33954..37427</td><td>648</td><td>BREX-system-P-loop-protein-BrxC-[Aeromonas-caviae]</td></tr>
<tr><td>WP_235462874.1</td><td>Type1.BREX</td><td>XIS-HTH->?->?-><- wHTH+WYL+WCX ||ARG->?->RHS-> BrxA-DNA-binding -> BrxB-BrxF-iSTAND -> DUF6079 *-> N6-METHYLASE -> OLD-ABC-> HNH -> PglZ ->HEPN+Sel1+SEC-C-><-SF1-SF2-Helicase+REase- [illegible]</td><td>1157</td><td>Bacteria>Pseudomonadota>Gammaproteobacteria>Aeromonadales>Aeromonadaceae>Aeromonas</td><td>GCF_021538905.1</td><td>Aeromonas_australiensis</td><td>EHZ86_RS12350</td><td>8704..12177</td><td>1114880</td><td>BREX-system-P-loop-protein-BrxC-[Aeromonas-australiensis]</td></tr>
<tr><td>WP_241325388.1</td><td>Type1.BREX</td><td>SF1-SF2-Helicase+REase-p->SF1-SF2-Helicase+REase-p->SF1-SF2-Helicase+REase-p-><-HEPN+Sel1+SEC-C<- PglZ <- HNH <-OLD-ABC <- N6-METHYLASE <- DUF6079 *<- BrxB-BrxF-iSTAND <- BrxA-DNA- [illegible]</td><td>1157</td><td>Bacteria>Pseudomonadota>Gammaproteobacteria>Aeromonadales>Aeromonadaceae>Aeromonas</td><td>GCF_022549295.1</td><td>Aeromonas_sp._MR7</td><td>MM192_RS09020</td><td>75622..79095</td><td>2923419</td><td>BREX-system-P-loop-protein-BrxC-[Aeromonas-sp.-MR7]</td></tr>
<tr><td>WP_247824022.1</td><td>Type1.BREX</td><td>DOC+HTH-><-?<-LexA-protease||?-><- wHTH+WYL+WCX ||ZnR+ZnR->RHS-> BrxA-DNA-binding -> BrxB-BrxF-iSTAND -> DUF6079 *-> N6-METHYLASE -> OLD-ABC-> HNH -> PglZ ->HORMA-><-SF1-SF2-Helicas [illegible]</td><td>1157</td><td>Bacteria>Pseudomonadota>Gammaproteobacteria>Aeromonadales>Aeromonadaceae>Aeromonas</td><td>GCF_023100745.1</td><td>Aeromonas_veronii</td><td>MYF86_09055</td><td>1896227..1899700</td><td>654</td><td>BREX-system-P-loop-protein-BrxC-[Aeromonas-veronii]</td></tr>
<tr><td>WP_264787731.1</td><td>Type1.BREX</td><td><-LexA-protease<-?||RHS->ARG->||RHS-> BrxA-DNA-binding -> BrxB-BrxF-iSTAND -> DUF6079 *-> N6-METHYLASE -> OLD-ABC-> HNH -> PglZ ->DDE_Tnp_1->HORMA-><-?<-zf-IS66+DDE_Tnp_IS66<-zf-IS [illegible]</td><td>1157</td><td>Bacteria>Pseudomonadota>Gammaproteobacteria>Aeromonadales>Aeromonadaceae>Aeromonas</td><td>GCF_026000055.1</td><td>Aeromonas_caviae</td><td>OPI30_RS10860</td><td>2213781..2217254</td><td>648</td><td>BREX-system-P-loop-protein-BrxC-[Aeromonas-caviae]</td></tr>
<tr><td>WP_270670318.1</td><td>Type1.BREX</td><td><-HTH+rve||<-HEPN+Sel1+SEC-C<- PglZ <- HNH <-OLD-ABC <- N6-METHYLASE <- DUF6079 *<- BrxB-BrxF-iSTAND <- BrxA-DNA-binding <-RHS<-?||<-ZnR<-ARG</td><td>1157</td><td>Bacteria>Pseudomonadota>Gammaproteobacteria>Aeromonadales>Aeromonadaceae>Aeromonas</td><td>GCF_027705165.1</td><td>Aeromonas_sp._QDB04</td><td>OKC37_RS13865</td><td>9577..13050</td><td>2990477</td><td>BREX-system-P-loop-protein-BrxC-[Aeromonas-sp.-QDB04]</td></tr>
<tr><td>WP_270659881.1</td><td>Type1.BREX</td><td>SF1-SF2-Helicase+REase->SF1-SF2-Helicase+REase-><-HEPN+Sel1+SEC-C<- PglZ <- HNH <-OLD-ABC <- N6-METHYLASE <- DUF6079 *<- BrxB-BrxF-iSTAND <- BrxA-DNA-binding <-URI-DNase<-?||LexA-p [illegible]</td><td>1157</td><td>Bacteria>Pseudomonadota>Gammaproteobacteria>Aeromonadales>Aeromonadaceae>Aeromonas</td><td>GCF_027705965.1</td><td>Aeromonas_sp._Y301-2</td><td>OKB83_RS15925</td><td>143279..146752</td><td>2990505</td><td>BREX-system-P-loop-protein-BrxC-[Aeromonas-sp.-Y301-2]</td></tr>
<tr><td>GGW57088.1</td><td>Type1.BREX</td><td><- wHTH+WYL+WCX -p||SIR2->?-><-?||HEPN-p->JAB-> BrxA-DNA-binding -> BrxB-BrxF-iSTAND -> DUF6079 *-> N6-METHYLASE -> OLD-ABC-> HNH -> PglZ ->HEPN+Sel1+SEC-C->Immank+ANK+ANK->REase-> [illegible]</td><td>1114</td><td>Bacteria>Pseudomonadota>Gammaproteobacteria>Alteromonadales>Alteromonadaceae>Alishewanella</td><td>GCA_014652375.1</td><td>Alishewanella_tabrizica</td><td>GCM10008111_11430</td><td>80462..83806</td><td>671278</td><td>hypothetical-protein-GCM10008111_11430-[Alishewanella-tabrizica]</td></tr>
<tr><td>WP_229796961.1</td><td>Type1.BREX</td><td><- wHTH+WYL+WCX -p||SIR2->?-><-?||HEPN-p->JAB-> BrxA-DNA-binding -> BrxB-BrxF-iSTAND -> DUF6079 *-> N6-METHYLASE -> OLD-ABC-> HNH -> PglZ ->HEPN+Sel1+SEC-C->Immank+ANK+ANK->REase-> [illegible]</td><td>1125</td><td>Bacteria>Pseudomonadota>Gammaproteobacteria>Alteromonadales>Alteromonadaceae>Alishewanella</td><td>GCF_014652375.1</td><td>Alishewanella_tabrizica</td><td>IE070_RS05825</td><td>80429..83806</td><td>671278</td><td>BREX-system-P-loop-protein-BrxC-[Alishewanella-tabrizica]</td></tr>
<tr><td>WP_089368479.1</td><td>Type1.BREX</td><td><-Phage_integrase<-DUF2787<-?||XIS-HTH-><-?<-?|| wHTH+WYL+WCX -> BrxA-DNA-binding -> BrxB-BrxF-iSTAND -> DUF6079 *-> N6-METHYLASE -> OLD-ABC-> HNH -> PglZ ->NADAR->rve->AAA-ATPase-> [illegible]</td><td>1169</td><td>Bacteria>Pseudomonadota>Gammaproteobacteria>Alteromonadales>Pseudoalteromonadaceae>Pseudoalteromonas</td><td>GCF_007991255.1</td><td>Pseudoalteromonas_nigrifaciens</td><td>PNIG_RS10730</td><td>2243615..2247124</td><td>28109</td><td>BREX-system-P-loop-protein-BrxC-[Pseudoalteromonas-nigrifaciens]</td></tr>
<tr><td>WP_182752860.1</td><td>Type1.BREX</td><td><-Phage_integrase<-DUF2787<-?||XIS-HTH-><-?|| wHTH+WYL+WCX -> BrxA-DNA-binding -> BrxB-BrxF-iSTAND -> DUF6079 *-> N6-METHYLASE -> OLD-ABC-> HNH -> PglZ ->NADAR->ART-DarT->TPR+TPR->Z [illegible]</td><td>1159</td><td>Bacteria>Pseudomonadota>Gammaproteobacteria>Alteromonadales>Pseudoalteromonadaceae>Pseudoalteromonas</td><td>GCF_014163875.1</td><td>Pseudoalteromonas_sp._SR43-3</td><td>H5088_RS10925</td><td>129286..132765</td><td>2760943</td><td>BREX-system-P-loop-protein-BrxC-[Pseudoalteromonas-sp.-SR43-3]</td></tr>
<tr><td>WP_200158841.1</td><td>Type1.BREX</td><td><- SIGMA+OB-Fold+AAA-ATPase+Lon-Protease-BrxL <- PglZ <- HNH <-OLD-ABC <-SF1-SF2-Helicase<-OLD-ABC+TOPRIM<- N6-METHYLASE <-PIN<-OLD-ABC<- DUF6079 *<- BrxB-BrxF-iSTAND <- BrxA-DNA-bi [illegible]</td><td>1154</td><td>Bacteria>Pseudomonadota>Gammaproteobacteria>Chromatiales>Chromatiaceae>Allochromatium</td><td>GCF_016584135.1</td><td>Allochromatium_vinosum</td><td>CKO29_RS16480</td><td>18892..22356</td><td>1049</td><td>BREX-system-P-loop-protein-BrxC-[Allochromatium-vinosum]</td></tr>
<tr><td>WP_200385867.1</td><td>Type1.BREX</td><td>DUF6079 *->?->PIN-><-?|| N6-METHYLASE -> OLD-ABC-> HNH -> PglZ ->AbrB-HTH->RHH-></td><td>1179</td><td>Bacteria>Pseudomonadota>Gammaproteobacteria>Chromatiales>Chromatiaceae>Thiocapsa</td><td>GCF_016583985.1</td><td>Thiocapsa_imhoffii</td><td>CKO25_RS00005</td><td>26..3565</td><td>382777</td><td>BREX-system-P-loop-protein-BrxC-[Thiocapsa-imhoffii]</td></tr>
<tr><td>MCC7280699.1</td><td>Type6.BREX</td><td><- SIGMA+OB-Fold+AAA-ATPase+Lon-Protease-BrxL <- PglZ <- HNH <-OLD-ABC <-RHH<- N6-METHYLASE <-Schlafen+HATPase+wHTH+HTH<- DUF6079 *<- BrxB-BrxF-iSTAND <- BrxA-DNA-binding <- BrxE</td><td>1181</td><td>Bacteria>Pseudomonadota>Gammaproteobacteria>Chromatiales>Chromatiaceae>unclassified_Chromatiaceae</td><td>GCA_020851955.1</td><td>Chromatiaceae_bacterium</td><td>IT487_20620</td><td>13992..17537</td><td>2049432</td><td>BREX-system-P-loop-protein-BrxC-[Chromatiaceae-bacterium]</td></tr>
<tr><td>WP_238620557.1</td><td>Type1.BREX</td><td>sGTP-><-35exo+T7DNApol||?->?-> BrxA-DNA-binding -> BrxB-BrxF-iSTAND -> DUF6079 *-> N6-METHYLASE -> OLD-ABC-> HNH -> OLD-ABC-> HNH -> PglZ -> SIGMA+OB-Fold+AAA-ATPase+Lon-Protease-Br [illegible]</td><td>1154</td><td>Bacteria>Pseudomonadota>Gammaproteobacteria>Chromatiales>Ectothiorhodospiraceae>Ectothiorhodospira</td><td>GCF_022227785.1</td><td>Ectothiorhodospira_sp._B14B</td><td>LQ952_00335</td><td>78448..81912</td><td>2899127</td><td>BREX-system-P-loop-protein-BrxC-[Ectothiorhodospira-sp.-B14B]</td></tr>
<tr><td>QEP43020.1</td><td>Type1.BREX</td><td><-DDE_Tnp_1<-HTH_Tnp_1+HTH_21+rve+rve_3<-HTH_Tnp_1<-TOPRIM<-OLD-ABC<-RNase<- N6-METHYLASE <- HNH <-OLD-ABC <- DUF6079 *<- BrxB-BrxF-iSTAND <- BrxA-DNA-binding <-Phage_holin<-?<-?| [illegible]</td><td>1145</td><td>Bacteria>Pseudomonadota>Gammaproteobacteria>Chromatiales>Ectothiorhodospiraceae>unclassified_Ectothiorhodospiraceae</td><td>GCA_008375315.1</td><td>Ectothiorhodospiraceae_bacterium_BW-2</td><td>D5085_07760</td><td>1613185..1616622</td><td>947515</td><td>BREX-system-P-loop-protein-BrxC-[Ectothiorhodospiraceae-bacterium-BW-2]</td></tr>
<tr><td>WP_132538615.1</td><td>Type6.BREX</td><td><-LexA-protease||?-><-?<- SIGMA+OB-Fold+AAA-ATPase+Lon-Protease-BrxL <- PglZ <- HNH <-OLD-ABC <- N6-METHYLASE <-?<- DUF6079 *<- BrxB-BrxF-iSTAND <- BrxA-DNA-binding <- BrxE <-RHH_5|| [illegible]</td><td>1179</td><td>Bacteria>Pseudomonadota>Gammaproteobacteria>Gammaproteobacteria_incertae_sedis>Candidatus_Competibacteraceae>Plasticicumulans</td><td>GCF_004341245.1</td><td>Plasticicumulans_lactativorans</td><td>EV699_RS02475</td><td>34554..38093</td><td>1133106</td><td>BREX-system-P-loop-protein-BrxC-[Plasticicumulans-lactativorans]</td></tr>
<tr><td>WP_132542053.1</td><td>Type1.BREX</td><td><- SIGMA+OB-Fold+AAA-ATPase+Lon-Protease-BrxL <- PglZ <- HNH <-OLD-ABC <- N6-METHYLASE <-HEPN<-PolB<- DUF6079 *<- BrxB-BrxF-iSTAND <- BrxA-DNA-binding ||ABhydrolase-><-Acyl_transf_3 [illegible]</td><td>1153</td><td>Bacteria>Pseudomonadota>Gammaproteobacteria>Gammaproteobacteria_incertae_sedis>Candidatus_Competibacteraceae>Plasticicumulans</td><td>GCF_004341245.1</td><td>Plasticicumulans_lactativorans</td><td>EV699_RS09620</td><td>184945..188406</td><td>1133106</td><td>BREX-system-P-loop-protein-BrxC-[Plasticicumulans-lactativorans]</td></tr>
<tr><td>WP_071159674.1</td><td>Type1.BREX</td><td><- SIGMA+OB-Fold+AAA-ATPase+Lon-Protease-BrxL <- PglZ <- HNH <-OLD-ABC <- N6-METHYLASE <- DUF6079 *<- BrxB-BrxF-iSTAND <- BrxA-DNA-binding <-XIS-HTH||?-><-?<-?<-?<-?<-JAB+sGTP</td><td>1153</td><td>Bacteria>Pseudomonadota>Gammaproteobacteria>Methylococcales>Methylococcaceae>Methylomonas</td><td>GCF_001856095.1</td><td>Methylomonas_sp._LWB</td><td>BJL95_RS17210</td><td>210511..213972</td><td>1905845</td><td>BREX-system-P-loop-protein-BrxC-[Methylomonas-sp.-LWB]</td></tr>
<tr><td>WP_262567770.1</td><td>Type1.BREX</td><td><-zf-ISL3+HTH_Tnp_ISL3+DDE_Tnp_ISL3||?-><-Integrase_1<- SIGMA+OB-Fold+AAA-ATPase+Lon-Protease-BrxL <- PglZ <- HNH <-OLD-ABC <- N6-METHYLASE <- DUF6079 *<- BrxB-BrxF-iSTAND <- BrxA- [illegible]</td><td>1221</td><td>Bacteria>Pseudomonadota>Gammaproteobacteria>Oceanospirillales>Endozoicomonadaceae>Endozoicomonas</td><td>GCF_025562715.2</td><td>Endozoicomonas_gorgoniicola</td><td>NX722_RS09445</td><td>2085702..2089367</td><td>1234144</td><td>BREX-system-P-loop-protein-BrxC-[Endozoicomonas-gorgoniicola]</td></tr>
<tr><td>WP_031385055.1</td><td>Type1.BREX</td><td><- PglZ <- HNH <-OLD-ABC <- N6-METHYLASE -p<-HerA/FtsK<-SIR2<-REase<- N6-METHYLASE <-Schlafen+HATPase+wHTH+HTH<- DUF6079 *<- BrxB-BrxF-iSTAND <- BrxA-DNA-binding <-XIS-HTH<-MobI||XI [illegible]</td><td>1193</td><td>Bacteria>Pseudomonadota>Gammaproteobacteria>Oceanospirillales>Halomonadaceae>Halomonas</td><td>GCF_000731955.1</td><td>Halomonas_zincidurans</td><td>HALZIN_RS0115275</td><td>3254805..3258386</td><td>1178777</td><td>BREX-system-P-loop-protein-BrxC-[Halomonas-zincidurans]</td></tr>
<tr><td>WP_107181354.1</td><td>Type1.BREX</td><td><- SIGMA+OB-Fold+AAA-ATPase+Lon-Protease-BrxL <- PglZ <- HNH <-OLD-ABC <-PHP+SbcC<- N6-METHYLASE <- DUF6079 *<- BrxB-BrxF-iSTAND <- BrxA-DNA-binding <-XIS-HTH</td><td>1147</td><td>Bacteria>Pseudomonadota>Gammaproteobacteria>Oceanospirillales>Halomonadaceae>Halomonas</td><td>GCF_003030865.2</td><td>Halomonas_sp._SL1</td><td>C9J49_RS09500</td><td>199072..202515</td><td>2137478</td><td>BREX-system-P-loop-protein-BrxC-[Halomonas-sp.-SL1]</td></tr>
<tr><td>WP_087719602.1</td><td>Type1.BREX</td><td><-ICLR-HTH<-?||?->Amidohydro_1->GNTR-HTH->XIS-HTH-> BrxA-DNA-binding -> BrxB-BrxF-iSTAND -> DUF6079 *->Schlafen+HATPase+wHTH-> N6-METHYLASE ->PHP+SbcC-> OLD-ABC-> HNH -> PglZ -> [illegible]</td><td>1145</td><td>Bacteria>Pseudomonadota>Gammaproteobacteria>Oceanospirillales>Halomonadaceae>Salinicola</td><td>GCF_002179555.1</td><td>Salinicola_salarius</td><td>CCD76_RS11235</td><td>10059..13496</td><td>430457</td><td>BREX-system-P-loop-protein-BrxC-[Salinicola-salarius]</td></tr>
<tr><td>WP_206838922.1</td><td>Type1.BREX</td><td>MODE-HTH+PBPII-><-ADH_N+ADH_zinc_N<-?||?->?-> wHTH+WYL+WCX -> BrxA-DNA-binding -> BrxB-BrxF-iSTAND -> DUF6079 *-> N6-METHYLASE ->DUF932->OLD-ABC->TOPRIM-> OLD-ABC-> HNH -> PglZ -> [illegible]</td><td>1154</td><td>Bacteria>Pseudomonadota>Gammaproteobacteria>Oceanospirillales>Oceanospirillaceae>Marinobacterium</td><td>GCF_017310015.1</td><td>Marinobacterium_georgiense</td><td>CFI10_RS02405</td><td>480710..484174</td><td>48076</td><td>BREX-system-P-loop-protein-BrxC-[Marinobacterium-iners]</td></tr>
<tr><td>WP_217336191.1</td><td>Type1.BREX</td><td>DUF772+DDE_Tnp_1+DDE_Tnp_1_6-> wHTH+WYL+WCX -> BrxA-DNA-binding -> BrxB-BrxF-iSTAND -> DUF6079 *-> N6-METHYLASE ->DUF932-> OLD-ABC-> HNH -> OLD-ABC-> HNH -> PglZ -> SIGMA+OB-Fold+AAA [illegible]</td><td>1154</td><td>Bacteria>Pseudomonadota>Gammaproteobacteria>Oceanospirillales>Oceanospirillaceae>Marinobacterium</td><td>GCF_019061305.1</td><td>Marinobacterium_sp._A346</td><td>KTN04_RS15750</td><td>3266..6730</td><td>2851016</td><td>BREX-system-P-loop-protein-BrxC-[Marinobacterium-sp.-A346]</td></tr>
<tr><td>WP_119395059.1</td><td>Type1.BREX</td><td>SF1-SF2-Helicase->HTH+HEPN->McrB-AAA->wHTH+REase+ZnR->HKD-DNase-> OLD-ABC-> HNH -> BrxA-DNA-binding -> BrxB-BrxF-iSTAND -> DUF6079 *-> N6-METHYLASE ->sGTP->sGTP->?->HerA/FtsK-> [illegible]</td><td>1155</td><td>Bacteria>Pseudomonadota>Gammaproteobacteria>Oceanospirillales>Saccharospirillaceae>Salinibius</td><td>GCF_003577475.1</td><td>Salinibius_halmophilus</td><td>DUW71_RS09715</td><td>145555..149022</td><td>1853216</td><td>BREX-system-P-loop-protein-BrxC-[Salinibius-halmophilus]</td></tr>
<tr><td>MBC7192650.1</td><td>Type1.BREX</td><td><- SIGMA+OB-Fold+AAA-ATPase+Lon-Protease-BrxL <- PglZ <- HNH <-OLD-ABC <-TOPRIM<-OLD-ABC<-HTH+REase<- N6-METHYLASE <-Schlafen+HATPase+wHTH<- DUF6079 *<- BrxB-BrxF-iSTAND <- BrxA-DN [illegible]</td><td>1145</td><td>Bacteria>Pseudomonadota>Gammaproteobacteria>Pseudomonadales>Marinobacteraceae>Marinobacter</td><td>GCA_014359475.1</td><td>Marinobacter_sp.</td><td>H5U31_09890</td><td>22634..26071</td><td>50741</td><td>BREX-system-P-loop-protein-BrxC-[Marinobacter-sp.]</td></tr>
<tr><td>WP_007349691.1</td><td>Type5.BREX</td><td><-Glycos_transf_2||tRNA->?->?-><- SIGMA+OB-Fold+AAA-ATPase+HKD-DNase-BrxL <- PglZ <- HNH <-OLD-ABC <- N6-METHYLASE <-Schlafen+HATPase+wHTH+HTH<- DUF6079 *<- BrxB-BrxF-iSTAND <- Brx [illegible]</td><td>1194</td><td>Bacteria>Pseudomonadota>Gammaproteobacteria>Pseudomonadales>Marinobacteraceae>Marinobacter</td><td>GCF_000169375.1</td><td>Marinobacter_sp._ELB17</td><td>MELB17_RS08075</td><td>35418..39002</td><td>270374</td><td>BREX-system-P-loop-protein-BrxC-[Marinobacter-sp.-ELB17]</td></tr>
<tr><td>WP_018404435.1</td><td>Type1.BREX</td><td>OrfB_Zn_ribbon-> BrxA-DNA-binding -> BrxB-BrxF-iSTAND -> DUF6079 *->Schlafen+HATPase+wHTH+HTH-> N6-METHYLASE -> OLD-ABC-> HNH -> PglZ -> SIGMA+OB-Fold+AAA-ATPase+HKD-DNase-BrxL -> [illegible]</td><td>1194</td><td>Bacteria>Pseudomonadota>Gammaproteobacteria>Pseudomonadales>Marinobacteraceae>Marinobacter</td><td>GCF_000372805.1</td><td>Marinobacter_gelidimuriae</td><td>A3OI_RS0111615</td><td>2203..5787</td><td>2739064</td><td>BREX-system-P-loop-protein-BrxC-[Marinobacter-gelidimuriae]</td></tr>
<tr><td>WP_033898350.1</td><td>Type1.BREX</td><td><- wHTH+WYL+WCX ||?->ARG->XIS-HTH-> BrxA-DNA-binding -> BrxB-BrxF-iSTAND -> DUF6079 *-> N6-METHYLASE -p->?-><-?|| N6-METHYLASE -p-> OLD-ABC-> HNH -> SIGMA+OB-Fold+AAA-ATPase+HKD-DNa [illegible]</td><td>1185</td><td>Bacteria>Pseudomonadota>Gammaproteobacteria>Pseudomonadales>Pseudomonadaceae</td><td>GCF_000510895.2</td><td>Pseudomonas</td><td>GIW75_RS00865</td><td>8119..11676</td><td>286</td><td>MULTISPECIES:-BREX-system-P-loop-protein-BrxC-[Pseudomonas]</td></tr>
<tr><td>WP_269686934.1</td><td>Type1.BREX</td><td>SF1-SF2-Helicase->SF1-SF2-Helicase->?-><-RADICAL-SAM<-?<-MazE_antitoxin|| wHTH+WYL+WCX -> BrxA-DNA-binding -> BrxB-BrxF-iSTAND -> DUF6079 *-> N6-METHYLASE ->?->?-> OLD-ABC-> HNH -> [illegible]</td><td>1223</td><td>Bacteria>Pseudomonadota>Gammaproteobacteria>Pseudomonadales>Pseudomonadaceae>Halopseudomonas</td><td>GCF_030295685.1</td><td>Halopseudomonas_sp._MFKK-1</td><td>O5O48_RS10320</td><td>2203460..2207131</td><td>3015994</td><td>BREX-system-P-loop-protein-BrxC-[Halopseudomonas-sp.-MFKK-1]</td></tr>
<tr><td>TXH92079.1</td><td>Type1.BREX</td><td><-RelE-ParE<-RHH_1<- PglZ <- HNH <-OLD-ABC <- N6-METHYLASE -p<-NERD<-OLD-ABC<- N6-METHYLASE <- DUF6079 *<- BrxB-BrxF-iSTAND <- BrxA-DNA-binding <-XIS-HTH<-?|| wHTH+WYL+WCX -></td><td>1186</td><td>Bacteria>Pseudomonadota>Gammaproteobacteria>Pseudomonadales>Pseudomonadaceae>Pseudomonas</td><td>GCA_008015405.1</td><td>Pseudomonas_sp.</td><td>E6Q71_01680</td><td>13269..16829</td><td>306</td><td>BREX-system-P-loop-protein-BrxC-[Pseudomonas-sp.]</td></tr>
<tr><td>MBX5763959.1</td><td>Type1.BREX</td><td>Phage_integrase-><-?<- SIGMA+OB-Fold+AAA-ATPase+Lon-Protease-BrxL <- PglZ <- HNH <-OLD-ABC <-?<-?<- N6-METHYLASE <- DUF6079 *<- BrxB-BrxF-iSTAND <- BrxA-DNA-binding <- wHTH+WYL+WCX [illegible]</td><td>1223</td><td>Bacteria>Pseudomonadota>Gammaproteobacteria>Pseudomonadales>Pseudomonadaceae>Pseudomonas</td><td>GCA_019687095.1</td><td>Pseudomonas_aeruginosa</td><td>ISD89_03975</td><td>862812..866483</td><td>287</td><td>BREX-system-P-loop-protein-BrxC-[Pseudomonas-aeruginosa]</td></tr>
<tr><td>HBN8454461.1</td><td>Type1.BREX</td><td>XIS-HTH-><-?<-tRNA||HTH_36->Phage_int_SAM_2+Integrase_1-><- SIGMA+OB-Fold+AAA-ATPase+Lon-Protease-BrxL <- PglZ <- HNH <-OLD-ABC <- N6-METHYLASE <- DUF6079 *<- BrxB-BrxF-iSTAND <- [illegible]</td><td>1146</td><td>Bacteria>Pseudomonadota>Gammaproteobacteria>Pseudomonadales>Pseudomonadaceae>Pseudomonas</td><td>GCA_021587585.1</td><td>Pseudomonas_aeruginosa</td><td>L3711_005718</td><td>39513..42953</td><td>287</td><td>BREX-system-P-loop-protein-BrxC-[Pseudomonas-aeruginosa]</td></tr>
<tr><td>HBO3110869.1</td><td>Type1.BREX</td><td>Tox-RelE->HTH_3-><-tRNA||HTH_36->Phage_int_SAM_2+Integrase_1-><- SIGMA+OB-Fold+AAA-ATPase+Lon-Protease-BrxL <- PglZ <- HNH <-OLD-ABC <- N6-METHYLASE <- DUF6079 *<- BrxB-BrxF-iSTAND [illegible]</td><td>1146</td><td>Bacteria>Pseudomonadota>Gammaproteobacteria>Pseudomonadales>Pseudomonadaceae>Pseudomonas</td><td>GCA_021651555.1</td><td>Pseudomonas_aeruginosa</td><td>L4R01_001445</td><td>31462..34902</td><td>287</td><td>BREX-system-P-loop-protein-BrxC-[Pseudomonas-aeruginosa]</td></tr>
<tr><td>HBP6726062.1</td><td>Type1.BREX</td><td>SF1-SF2-Helicase-><-HTH_3||?->CHTH-><-?|| wHTH+WYL+WCX -> BrxA-DNA-binding -> BrxB-BrxF-iSTAND -> DUF6079 *-> N6-METHYLASE ->?->?-> OLD-ABC-> HNH -> PglZ -> SIGMA+OB-Fold+AAA-ATPase- [illegible]</td><td>1223</td><td>Bacteria>Pseudomonadota>Gammaproteobacteria>Pseudomonadales>Pseudomonadaceae>Pseudomonas</td><td>GCA_021711795.1</td><td>Pseudomonas_aeruginosa</td><td>L6B36_03955</td><td>73272..76943</td><td>287</td><td>BREX-system-P-loop-protein-BrxC-[Pseudomonas-aeruginosa]</td></tr>
<tr><td>CAB5551672.1</td><td>Type1.BREX</td><td>DUF4969-><- SIGMA+OB-Fold+AAA-ATPase+Lon-Protease-BrxL <- PglZ <- HNH <-OLD-ABC <-?<-DUF932<- N6-METHYLASE <- DUF6079 *<- BrxB-BrxF-iSTAND <- BrxA-DNA-binding <- wHTH+WYL+WCX <-?<-XI [illegible]</td><td>1154</td><td>Bacteria>Pseudomonadota>Gammaproteobacteria>Pseudomonadales>Pseudomonadaceae>Pseudomonas</td><td>GCA_903684265.1</td><td>Pseudomonas_putida</td><td>GHA_03446</td><td>127342..130806</td><td>303</td><td>Uncharacterised-protein-[Pseudomonas-putida]</td></tr>
<tr><td>WP_047306816.1</td><td>Type1.BREX</td><td><- SIGMA+OB-Fold+AAA-ATPase+HKD-DNase-BrxL <- PglZ <- HNH <-OLD-ABC <- N6-METHYLASE -p<-RVT<- N6-METHYLASE -p<- DUF6079 *<- BrxB-BrxF-iSTAND <- BrxA-DNA-binding <-XIS-HTH<-?|| wHTH+W [illegible]</td><td>1185</td><td>Bacteria>Pseudomonadota>Gammaproteobacteria>Pseudomonadales>Pseudomonadaceae>Pseudomonas</td><td>GCF_001020715.1</td><td>Pseudomonas_fluorescens</td><td>AA061_RS32150</td><td>16461..20018</td><td>294</td><td>BREX-system-P-loop-protein-BrxC-[Pseudomonas-fluorescens]</td></tr>
<tr><td>WP_169839752.1</td><td>Type1.BREX</td><td>DUF4969-><- SIGMA+OB-Fold+AAA-ATPase+Lon-Protease-BrxL <- PglZ <- HNH <-OLD-ABC <- N6-METHYLASE <- DUF6079 *<- BrxB-BrxF-iSTAND <- BrxA-DNA-binding <- wHTH+WYL+WCX <-McrC-NTD+REase+Z [illegible]</td><td>1154</td><td>Bacteria>Pseudomonadota>Gammaproteobacteria>Pseudomonadales>Pseudomonadaceae>Pseudomonas</td><td>GCF_012986505.1</td><td>Pseudomonas_lundensis</td><td>HBN72_RS14625</td><td>129337..132801</td><td>86185</td><td>BREX-system-P-loop-protein-BrxC-[Pseudomonas-lundensis]</td></tr>
<tr><td>WP_196169752.1</td><td>Type1.BREX</td><td>DUF4969-><- SIGMA+OB-Fold+AAA-ATPase+Lon-Protease-BrxL <- PglZ <- HNH <-OLD-ABC <-DUF932<- N6-METHYLASE <- DUF6079 *<- BrxB-BrxF-iSTAND <- BrxA-DNA-binding <- wHTH+WYL+WCX <-?<-XIS-H [illegible]</td><td>1154</td><td>Bacteria>Pseudomonadota>Gammaproteobacteria>Pseudomonadales>Pseudomonadaceae>Pseudomonas</td><td>GCF_015680295.1</td><td>Pseudomonas_guariconensis</td><td>IRZ59_RS14965</td><td>185250..188714</td><td>1288410</td><td>BREX-system-P-loop-protein-BrxC-[Pseudomonas-guariconensis]</td></tr>
<tr><td>WP_198849248.1</td><td>Type1.BREX</td><td>wHTH+WYL+WCX -><- SIGMA+OB-Fold+AAA-ATPase+HKD-DNase-BrxL <- HNH <-OLD-ABC <- N6-METHYLASE -p<-HerA/FtsK<-SIR2<- N6-METHYLASE -p<- DUF6079 *<- BrxB-BrxF-iSTAND <- BrxA-DNA-binding [illegible]</td><td>1186</td><td>Bacteria>Pseudomonadota>Gammaproteobacteria>Pseudomonadales>Pseudomonadaceae>Pseudomonas</td><td>GCF_016405505.1</td><td>Pseudomonas_sp._MF6772</td><td>JFT60_RS22275</td><td>24346..27906</td><td>2797533</td><td>BREX-system-P-loop-protein-BrxC-[Pseudomonas-sp.-MF6772]</td></tr>
<tr><td>WP_270945767.1</td><td>Type1.BREX</td><td>MerR-HTH->||?->?->DUF772+DDE_Tnp_1-><-?|| wHTH+WYL+WCX -> BrxA-DNA-binding -> BrxB-BrxF-iSTAND -> DUF6079 *-> N6-METHYLASE ->?->?-> OLD-ABC-> HNH -> PglZ -> SIGMA+OB-Fold+AAA-ATPase- [illegible]</td><td>1223</td><td>Bacteria>Pseudomonadota>Gammaproteobacteria>Pseudomonadales>Pseudomonadaceae>Pseudomonas</td><td>GCF_027856115.1</td><td>Pseudomonas_aeruginosa</td><td>KKQ36_RS16450</td><td>3486481..3490152</td><td>287</td><td>BREX-system-P-loop-protein-BrxC-[Pseudomonas-aeruginosa]</td></tr>
<tr><td>WP_053526945.1</td><td>Type1.BREX</td><td><-HTH_3<-?<- SIGMA+OB-Fold+AAA-ATPase+Lon-Protease-BrxL <- PglZ <- HNH <-OLD-ABC <-?<-?<- N6-METHYLASE <- DUF6079 *<- BrxB-BrxF-iSTAND <- BrxA-DNA-binding <- wHTH+WYL+WCX ||?->RVT->? [illegible]</td><td>1223</td><td>Bacteria>Pseudomonadota>Gammaproteobacteria>Pseudomonadales>Pseudomonadaceae>Stutzerimonas</td><td>GCF_001276475.1</td><td>Stutzerimonas_stutzeri</td><td>ABW53_RS00980</td><td>14361..18032</td><td>316</td><td>BREX-system-P-loop-protein-BrxC-[Stutzerimonas-stutzeri]</td></tr>
<tr><td>WP_150138110.1</td><td>Type1.BREX</td><td>wHTH+WYL+WCX -> BrxA-DNA-binding -> BrxB-BrxF-iSTAND -> DUF6079 *-> N6-METHYLASE -> OLD-ABC-> HNH -> PglZ -></td><td>1159</td><td>Bacteria>Pseudomonadota>Gammaproteobacteria>Vibrionales>Vibrionaceae>Enterovibrio</td><td>GCF_008692425.1</td><td>Candidatus_Enterovibrio_escacola</td><td>EDM10_RS06800</td><td>2982..6461</td><td>1927127</td><td>BREX-system-P-loop-protein-BrxC-[Candidatus-Enterovibrio-escacola]</td></tr>
<tr><td>HAS6390651.1</td><td>Type1.BREX</td><td><-HTH||RNase_H-><-DUF2787<-?<-?||XIS-HTH-> wHTH+WYL+WCX -> BrxA-DNA-binding -> BrxB-BrxF-iSTAND -> DUF6079 *-> N6-METHYLASE -> OLD-ABC-> HNH -> PglZ ->ZetaToxin-><-SF1-SF2-Helicase+ [illegible]</td><td>1157</td><td>Bacteria>Pseudomonadota>Gammaproteobacteria>Vibrionales>Vibrionaceae>Vibrio</td><td>GCA_015777605.1</td><td>Vibrio_vulnificus</td><td>I7251_16610</td><td>90517..93990</td><td>672</td><td>BREX-system-P-loop-protein-BrxC-[Vibrio-vulnificus]</td></tr>
<tr><td>ODU42543.1</td><td>Type1.BREX</td><td><- SIGMA+OB-Fold+AAA-ATPase+Lon-Protease-BrxL <- PglZ <-Polbeta<-MarR-HTH<- HNH <-OLD-ABC <- N6-METHYLASE <- DUF6079 *<- BrxB-BrxF-iSTAND <- BrxA-DNA-binding ||?-><-?||HKD-DNase->?- [illegible]</td><td>1148</td><td>Bacteria>Pseudomonadota>Gammaproteobacteria>Xanthomonadales>Xanthomonadaceae>unclassified_Xanthomonadaceae</td><td>GCA_001725645.1</td><td>Xanthomonadaceae_bacterium_SCN_69-123</td><td>ABS96_27585</td><td>191713..195159</td><td>1660170</td><td>hypothetical-protein-ABS96_27585-[Xanthomonadaceae-bacterium-SCN-69-123]</td></tr>
<tr><td>PIQ12823.1</td><td>Type1.BREX</td><td><-HARE-HTH+REase<- SIGMA+OB-Fold+AAA-ATPase+Lon-Protease-BrxL <- PglZ <- HNH <-OLD-ABC <- N6-METHYLASE <- DUF6079 *<- BrxB-BrxF-iSTAND <- BrxA-DNA-binding <-SLOG+BirA-HTH<-SF1-SF2-H [illegible]</td><td>1146</td><td>Bacteria>Pseudomonadota>Hydrogenophilia>Hydrogenophilales>unclassified_Hydrogenophilales</td><td>GCA_002770925.1</td><td>Hydrogenophilales_bacterium_CG18_big_fil_WC_8_21_14_2_50_58_12</td><td>COW70_07940</td><td>10882..14322</td><td>1974036</td><td>hypothetical-protein-COW70_07940-[Hydrogenophilales-bacterium-CG18_big_fil_WC_8_21_14_2_50_58_12]</td></tr>
<tr><td>NLF73010.1</td><td>Type1.BREX</td><td>XIS-HTH-> BrxA-DNA-binding -> BrxB-BrxF-iSTAND -> DUF6079 *-> N6-METHYLASE -> OLD-ABC-> HNH -> PglZ -> SIGMA+OB-Fold+AAA-ATPase+Lon-Protease-BrxL -></td><td>1145</td><td>Bacteria>PVC_group>Planctomycetota>Planctomycetia>Pirellulales>Pirellulaceae</td><td>GCA_012515235.1</td><td>Candidatus_Anammoximicrobium_sp.</td><td>GX575_28570</td><td>2105..5542</td><td>2699742</td><td>BREX-system-P-loop-protein-BrxC-[Candidatus-Anammoximicrobium-sp.]</td></tr>
<tr><td>MCC5808698.1</td><td>Type1.BREX</td><td>Phage_integrase-><- SIGMA+OB-Fold+AAA-ATPase+HKD-DNase-BrxL <- PglZ <- HNH <-OLD-ABC <- N6-METHYLASE <-REase<-OLD-ABC<- DUF6079 *<- BrxB-BrxF-iSTAND <- BrxA-DNA-binding <-XIS-HTH</td><td>1183</td><td>Bacteria>PVC_group>Verrucomicrobiota>Opitutae>Opitutales>unclassified_Opitutales</td><td>GCA_020831525.1</td><td>Opitutales_bacterium</td><td>JJU00_20390</td><td>12178..15729</td><td>2026772</td><td>BREX-system-P-loop-protein-BrxC-[Opitutales-bacterium]</td></tr>
<tr><td>OPA28160.1</td><td>Type1.BREX</td><td>PIN->REase-> OLD-ABC-> HNH ->?-> BrxA-DNA-binding -> BrxB-BrxF-iSTAND -> DUF6079 *-> N6-METHYLASE -> PglZ -> SIGMA+OB-Fold+AAA-ATPase+Lon-Protease-BrxL ->MarR-HTH->?->?->BZIP+SF1-SF [illegible]</td><td>1190</td><td>Bacteria>Terrabacteria_group>Bacillota>Bacilli>Bacillales>Bacillaceae</td><td>GCA_002021465.1</td><td>Bacillus_cereus</td><td>BHL47_19745</td><td>10940..14512</td><td>1396</td><td>hypothetical-protein-BHL47_19745-[Bacillus-cereus]</td></tr>
<tr><td>ATI52289.1</td><td>Type1.BREX</td><td>GDResolvase+HIN-HTH->TOPRIM+Topoisom_bac-> OLD-ABC-> HNH ->?-> N6-METHYLASE -p->?-> BrxA-DNA-binding -> BrxB-BrxF-iSTAND -> DUF6079 *-> N6-METHYLASE -> PglZ -> SIGMA+OB-Fold+AAA-ATP [illegible]</td><td>1190</td><td>Bacteria>Terrabacteria_group>Bacillota>Bacilli>Bacillales>Bacillaceae</td><td>GCA_002504225.1</td><td>Bacillus_cereus</td><td>CPZ32_18870</td><td>3450635..3454207</td><td>1396</td><td>hypothetical-protein-CPZ32_18870-[Bacillus-cereus]</td></tr>
<tr><td>WP_078386197.1</td><td>Type1.BREX</td><td>PIN->REase-> OLD-ABC-> HNH ->?-> BrxA-DNA-binding -> BrxB-BrxF-iSTAND -> DUF6079 *-> N6-METHYLASE -> PglZ -> SIGMA+OB-Fold+AAA-ATPase+Lon-Protease-BrxL ->MarR-HTH->?->?->BZIP+SF1-SF [illegible]</td><td>1190</td><td>Bacteria>Terrabacteria_group>Bacillota>Bacilli>Bacillales>Bacillaceae</td><td>GCF_002021465.1</td><td>Bacillus_cereus</td><td>BHL47_19745</td><td>10940..14512</td><td>1396</td><td>BREX-system-P-loop-protein-BrxC-[Bacillus-cereus]</td></tr>
<tr><td>WP_061531636.1</td><td>Type1.BREX</td><td>HTH_3-><- ParB ||SAD-DUF3427C+SbcC-> HNH -><-REase+DUF5655||EVE+ HNH -> BrxA-DNA-binding -> BrxB-BrxF-iSTAND -> DUF6079 *-> N6-METHYLASE -> PglZ -> SIGMA+OB-Fold+AAA-ATPase+Lon-Prote [illegible]</td><td>1195</td><td>Bacteria>Terrabacteria_group>Bacillota>Bacilli>Bacillales>Bacillaceae</td><td>GCF_029504955.1</td><td>Bacillus_cereus_group</td><td>M2246_RS18875</td><td>193407..196994</td><td>86661</td><td>MULTISPECIES:-BREX-system-P-loop-protein-BrxC-[Bacillus]</td></tr>
<tr><td>MBN1067552.1</td><td>Type1.BREX</td><td>Calcineurin->?-> BrxA-DNA-binding -> BrxB-BrxF-iSTAND -> DUF6079 *->CR-ATPase9+REase-> N6-METHYLASE -> OLD-ABC-> HNH -> PglZ -><-REase|| SIGMA+OB-Fold+AAA-ATPase+Lon-Protease-BrxL [illegible]</td><td>1215</td><td>Bacteria>Terrabacteria_group>Bacillota>Clostridia>Eubacteriales>Clostridiaceae</td><td>GCA_016925955.1</td><td>Clostridium_botulinum</td><td>DVW02_06040</td><td>97050..100697</td><td>1491</td><td>BREX-system-P-loop-protein-BrxC-[Clostridium-botulinum]</td></tr>
<tr><td>WP_043662653.1</td><td>Type1.BREX</td><td><-McrC-NTD+REase<-wHTH+McrB-AAA<- SIGMA+OB-Fold+AAA-ATPase+Lon-Protease-BrxL <-?<- PglZ <- HNH <-OLD-ABC <- N6-METHYLASE <- DUF6079 *<- BrxB-BrxF-iSTAND <- BrxA-DNA-binding <-HKD-DN [illegible]</td><td>1216</td><td>Bacteria>Terrabacteria_group>Bacillota>Clostridia>Eubacteriales>Clostridiaceae</td><td>GCF_000785185.1</td><td>Clostridium_butyricum</td><td>AWN73_RS08010</td><td>18592..22242</td><td>1492</td><td>BREX-system-P-loop-protein-BrxC-[Clostridium-butyricum]</td></tr>
<tr><td>WP_077851369.1</td><td>Type1.BREX</td><td><- SIGMA+OB-Fold+AAA-ATPase+Lon-Protease-BrxL <-?<- PglZ <- HNH <-OLD-ABC <- N6-METHYLASE <- DUF6079 *<- BrxB-BrxF-iSTAND <- BrxA-DNA-binding <-?<-?<-?<-?<-?<-?<-Aminotran_1_2</td><td>1216</td><td>Bacteria>Terrabacteria_group>Bacillota>Clostridia>Eubacteriales>Clostridiaceae</td><td>GCF_002006235.2</td><td>Clostridium_felsineum</td><td>CLAUR_RS09645</td><td>2191072..2194722</td><td>36839</td><td>BREX-system-P-loop-protein-BrxC-[Clostridium-felsineum]</td></tr>
<tr><td>WP_118513467.1</td><td>Type1.BREX</td><td><-Tox-ART-HYD2<-TIR<- PglZ <-Schlafen+HATPase+wHTH<-AbiGi+seqA+RAMA<- N6-METHYLASE <- HNH <-OLD-ABC <- DUF6079 *<- BrxB-BrxF-iSTAND <- BrxA-DNA-binding <-HTH_26<-SIGMA-HTH+SIGMA-HT [illegible]</td><td>1183</td><td>Bacteria>Terrabacteria_group>Bacillota>Clostridia>Eubacteriales>Clostridiaceae</td><td>GCF_003477775.1</td><td>Clostridium_sp._AF37-5AT</td><td>DW020_RS00895</td><td>209396..212947</td><td>2292007</td><td>BREX-system-P-loop-protein-BrxC-[Clostridium-sp.-AF37-5AT]</td></tr>
<tr><td>WP_164949146.1</td><td>Type1.BREX</td><td><-HEPN<- SIGMA+OB-Fold+AAA-ATPase+Lon-Protease-BrxL <- PglZ <- HNH <-OLD-ABC <- N6-METHYLASE <- DUF6079 *<- BrxB-BrxF-iSTAND <- BrxA-DNA-binding <-?<-CR-ATPase8+REase||?->?-><-?<-?| [illegible]</td><td>1215</td><td>Bacteria>Terrabacteria_group>Bacillota>Clostridia>Eubacteriales>Clostridiaceae</td><td>GCF_011065955.2</td><td>Clostridium_sp._FP2</td><td>G9F71_RS17965</td><td>3736090..3739737</td><td>2724481</td><td>BREX-system-P-loop-protein-BrxC-[Clostridium-sp.-FP2]</td></tr>
<tr><td>WP_171786495.1</td><td>Type1.BREX</td><td><-D5N+D5ATPase<- SIGMA+OB-Fold+AAA-ATPase+Lon-Protease-BrxL <- PglZ <- HNH <-OLD-ABC <-PIWI<- N6-METHYLASE <- DUF6079 *<- BrxB-BrxF-iSTAND <- BrxA-DNA-binding <-?<-HTH_3<-?<-?||tRNA [illegible]</td><td>1216</td><td>Bacteria>Terrabacteria_group>Bacillota>Clostridia>Eubacteriales>Clostridiaceae</td><td>GCF_013149405.1</td><td>Clostridium_beijerinckii</td><td>BOH39_RS13645</td><td>3014066..3017716</td><td>1520</td><td>BREX-system-P-loop-protein-BrxC-[Clostridium-beijerinckii]</td></tr>
<tr><td>WP_171780276.1</td><td>Type1.BREX</td><td>rve+rve_3->AAA-ATPase-><-D5N+D5ATPase<- SIGMA+OB-Fold+AAA-ATPase+Lon-Protease-BrxL <- PglZ <- HNH <-OLD-ABC <-PIWI<- N6-METHYLASE <- DUF6079 *<- BrxB-BrxF-iSTAND <- BrxA-DNA-binding [illegible]</td><td>1216</td><td>Bacteria>Terrabacteria_group>Bacillota>Clostridia>Eubacteriales>Clostridiaceae</td><td>GCF_014230655.1</td><td>Clostridium_beijerinckii</td><td>HGI39_RS06505</td><td>40889..44539</td><td>1520</td><td>BREX-system-P-loop-protein-BrxC-[Clostridium-beijerinckii]</td></tr>
<tr><td>WP_216171751.1</td><td>Type1.BREX</td><td><- SIGMA+OB-Fold+AAA-ATPase+Lon-Protease-BrxL <- PglZ <- HNH <-OLD-ABC <- N6-METHYLASE <- DUF6079 *<- BrxB-BrxF-iSTAND <- BrxA-DNA-binding ||Phage_int_SAM_3-><-Phage_int_SAM_1+Phage [illegible]</td><td>1215</td><td>Bacteria>Terrabacteria_group>Bacillota>Clostridia>Eubacteriales>Clostridiaceae</td><td>GCF_026650905.1</td><td>Clostridium_estertheticum</td><td>LL037_RS21145</td><td>4418214..4421861</td><td>238834</td><td>BREX-system-P-loop-protein-BrxC-[Clostridium-estertheticum]</td></tr>
<tr><td>WP_270487117.1</td><td>Type1.BREX</td><td><- SIGMA+OB-Fold+AAA-ATPase+Lon-Protease-BrxL <-?<-PIN<-OLD-ABC<- PglZ <- HNH <-OLD-ABC <-TIR<- N6-METHYLASE <- DUF6079 *<- BrxB-BrxF-iSTAND <- BrxA-DNA-binding <-HTH_26<-PIN<-?<-MA [illegible]</td><td>1201</td><td>Bacteria>Terrabacteria_group>Bacillota>Clostridia>Eubacteriales>Clostridiaceae</td><td>GCF_027681635.1</td><td>Clostridium_fessum</td><td>PGX92_RS02760</td><td>620693..624298</td><td>2126740</td><td>BREX-system-P-loop-protein-BrxC-[Clostridium-fessum]</td></tr>
<tr><td>WP_115641994.1</td><td>Type1.BREX</td><td>NTP_transf->HEPN-><- PglZ <- HNH <-OLD-ABC <- N6-METHYLASE <-?<- DUF6079 *<- BrxB-BrxF-iSTAND <- BrxA-DNA-binding ||HEPN-><-?<-ABC-ATPase+SbcC<-RADICAL-SAM<-?||DDE_Tnp_IS240+rve_3- [illegible]</td><td>1215</td><td>Bacteria>Terrabacteria_group>Bacillota>Clostridia>Eubacteriales>Clostridiaceae</td><td>GCF_900461105.1</td><td>Clostridium_putrefaciens</td><td>DY168_RS12220</td><td>2676627..2680274</td><td>99675</td><td>BREX-system-P-loop-protein-BrxC-[Clostridium-putrefaciens]</td></tr>
<tr><td>WP_154780312.1</td><td>Type1.BREX</td><td>Peptidase->?->?->MACRODOMAIN->?->ZnR+ZnR+ZnR->HTH_26-> BrxA-DNA-binding -> BrxB-BrxF-iSTAND -> DUF6079 *-> N6-METHYLASE ->RVT->?-> OLD-ABC-> HNH ->?-> PglZ ->HEPN-> SIGMA+OB-Fold+A [illegible]</td><td>1201</td><td>Bacteria>Terrabacteria_group>Bacillota>Clostridia>Eubacteriales>Lachnospiraceae</td><td>GCF_009707925.1</td><td>Blautia_luti</td><td>GKZ57_RS08760</td><td>74022..77627</td><td>89014</td><td>BREX-system-P-loop-protein-BrxC-[Blautia-luti]</td></tr>
<tr><td>WP_207739085.1</td><td>Type1.BREX</td><td>METHYLASE->?->?->?->HTH_26-> BrxA-DNA-binding -> BrxB-BrxF-iSTAND -> DUF6079 *-> N6-METHYLASE -> OLD-ABC-> HNH -> PglZ -> SIGMA+OB-Fold+AAA-ATPase+Lon-Protease-BrxL ->?->Arm-DNA-bin [illegible]</td><td>1173</td><td>Bacteria>Terrabacteria_group>Bacillota>Clostridia>Eubacteriales>Oscillospiraceae</td><td>GCF_015558275.1</td><td>Massiliimalia_timonensis</td><td>I2G46_RS07880</td><td>51772..55293</td><td>1987501</td><td>BREX-system-P-loop-protein-BrxC-[Massiliimalia-timonensis]</td></tr>
<tr><td>WP_215659170.1</td><td>Type1.BREX</td><td><-TIR<- SIGMA+OB-Fold+AAA-ATPase+Lon-Protease-BrxL <- PglZ <-DUF4404<-ZnR+ZnR<- N6-METHYLASE <-?<- HNH <-OLD-ABC <- DUF6079 *<- BrxB-BrxF-iSTAND <- BrxA-DNA-binding <-HTH_26<-MazE_a [illegible]</td><td>1184</td><td>Bacteria>Terrabacteria_group>Bacillota>Clostridia>Eubacteriales>Oscillospiraceae</td><td>GCF_018783905.1</td><td>Ruminococcus_sp._MCC718</td><td>GPK60_RS07265</td><td>26956..30510</td><td>2592649</td><td>BREX-system-P-loop-protein-BrxC-[Ruminococcus-sp.-MCC718]</td></tr>
<tr><td>CCX63523.1</td><td>Type1.BREX</td><td>Phage_integrase-><-RelE-ParE<-MazE_antitoxin<- SIGMA+OB-Fold+AAA-ATPase+Lon-Protease-BrxL <- PglZ <- HNH <-OLD-ABC <- N6-METHYLASE <- DUF6079 *<- BrxB-BrxF-iSTAND <- BrxA-DNA-bindi [illegible]</td><td>1217</td><td>Bacteria>Terrabacteria_group>Bacillota>environmental_samples</td><td>GCA_000431495.1</td><td>Firmicutes_bacterium_CAG:791</td><td>BN785_00943</td><td>24533..28186</td><td>1262993</td><td>uncharacterized-protein-BN785_00943-[Firmicutes-bacterium-CAG:791]</td></tr>
<tr><td>MBR3457117.1</td><td>Type1.BREX</td><td><- PglZ -p<- HNH <-OLD-ABC <- N6-METHYLASE -p<-?<-PIN<- N6-METHYLASE <- DUF6079 *<- BrxB-BrxF-iSTAND -p</td><td>1215</td><td>Bacteria>Terrabacteria_group>Bacillota>Negativicutes>Selenomonadales>Selenomonadaceae</td><td>GCA_017509225.1</td><td>Selenomonadaceae_bacterium</td><td>IKH16_03040</td><td>6412..10059</td><td>2652289</td><td>BREX-system-P-loop-protein-BrxC-[Selenomonadaceae-bacterium]</td></tr>
<tr><td>WP_037354735.1</td><td>Type1.BREX</td><td>DDE_Tnp_1-><-?<-?<-?||?-> BrxA-DNA-binding -> BrxB-BrxF-iSTAND -> DUF6079 *-> N6-METHYLASE -> OLD-ABC-> HNH ->?-> PglZ -> SIGMA+OB-Fold+AAA-ATPase+Lon-Protease-BrxL ->HTH_3-></td><td>1182</td><td>Bacteria>Terrabacteria_group>Bacillota>Negativicutes>Selenomonadales>Selenomonadaceae</td><td>GCF_000701565.1</td><td>Selenomonas_sp._FC4001</td><td>T320_RS08130</td><td>15634..19182</td><td>1408313</td><td>BREX-system-P-loop-protein-BrxC-[Selenomonas-sp.-FC4001]</td></tr>
<tr><td>RGH23082.1</td><td>Type1.BREX</td><td>PemK_toxin->?->?-> BrxA-DNA-binding -> BrxB-BrxF-iSTAND -> DUF6079 *-> N6-METHYLASE -> OLD-ABC-> HNH -> PglZ -><-?||?->?-><-ZnR</td><td>1180</td><td>Bacteria>Terrabacteria_group>Bacillota>unclassified_Bacillota</td><td>GCA_003461705.1</td><td>Firmicutes_bacterium_AF12-30</td><td>DWV72_07055</td><td>48691..52233</td><td>2292884</td><td>BREX-system-P-loop-protein-BrxC-[Firmicutes-bacterium-AF12-30]</td></tr>
<tr><td>MBE9475406.1</td><td>Type1.BREX</td><td>BrxB-BrxF-iSTAND -p-> BrxA-DNA-binding -> BrxB-BrxF-iSTAND -> DUF6079 *-> N6-METHYLASE ->REase+AIPR-> OLD-ABC-> HNH -> PglZ -> SIGMA+OB-Fold+AAA-ATPase+Lon-Protease-BrxL -></td><td>1145</td><td>Bacteria>Terrabacteria_group>Chloroflexota>unclassified_Chloroflexota</td><td>GCA_015222395.1</td><td>Chloroflexota_bacterium</td><td>IMY85_10995</td><td>2370..5807</td><td>2026724</td><td>BREX-system-P-loop-protein-BrxC-[Chloroflexota-bacterium]</td></tr>
<tr><td>MBI4317923.1</td><td>Type1.BREX</td><td>Radical_SAM->?->?->?-> BrxA-DNA-binding -> BrxB-BrxF-iSTAND -> DUF6079 *-> N6-METHYLASE -> OLD-ABC-> HNH -> PglZ -> SIGMA+OB-Fold+AAA-ATPase+Lon-Protease-BrxL -></td><td>1145</td><td>Bacteria>Terrabacteria_group>Chloroflexota>unclassified_Chloroflexota</td><td>GCA_016210495.1</td><td>Chloroflexota_bacterium</td><td>HY675_05490</td><td>15525..18962</td><td>2026724</td><td>BREX-system-P-loop-protein-BrxC-[Chloroflexota-bacterium]</td></tr>
<tr><td>MBT9546703.1</td><td>Type1.BREX</td><td>Peptidase_M2-> BrxA-DNA-binding -> BrxB-BrxF-iSTAND -> DUF6079 *-> N6-METHYLASE ->RVT-> N6-METHYLASE -p-> OLD-ABC-> HNH -> PglZ -> SIGMA+OB-Fold+AAA-ATPase+HKD-DNase-BrxL -></td><td>1179</td><td>Bacteria>Terrabacteria_group>Cyanobacteriota/Melainabacteria_group>Candidatus_Sericytochromatia>unclassified_Candidatus_Sericytochromatia</td><td>GCA_018780035.1</td><td>Candidatus_Sericytochromatia_bacterium</td><td>IV090_15030</td><td>94995..98534</td><td>2762020</td><td>BREX-system-P-loop-protein-BrxC-[Candidatus-Sericytochromatia-bacterium]</td></tr>
<tr><td>WP_069067979.1</td><td>Type1.BREX</td><td>CHTH->?->?-> BrxA-DNA-binding -> BrxB-BrxF-iSTAND -> DUF6079 *->?-> N6-METHYLASE -> OLD-ABC-> HNH -> PglZ -> SIGMA+OB-Fold+AAA-ATPase+Lon-Protease-BrxL -><-?<-HTH_23</td><td>1220</td><td>Bacteria>Terrabacteria_group>Cyanobacteriota/Melainabacteria_group>Cyanobacteriota>Cyanophyceae>Nostocales</td><td>GCF_001712795.1</td><td>Nostoc_sp._KVJ20</td><td>A4S05_RS00980</td><td>24778..28440</td><td>457944</td><td>BREX-system-P-loop-protein-BrxC-[Nostoc-sp.-KVJ20]</td></tr>
<tr><td>WP_190429845.1</td><td>Type1.BREX</td><td>Pkinase->HTH+HKD-DNase-><- SIGMA+OB-Fold+AAA-ATPase+Lon-Protease-BrxL <- PglZ <- HNH <-OLD-ABC <- N6-METHYLASE <-?<- DUF6079 *<- BrxB-BrxF-iSTAND <- BrxA-DNA-binding ||ParA-Soj-Ploo [illegible]</td><td>1222</td><td>Bacteria>Terrabacteria_group>Cyanobacteriota/Melainabacteria_group>Cyanobacteriota>Cyanophyceae>Oscillatoriophycideae</td><td>GCF_014695635.1</td><td>Coleofasciculus_sp._FACHB-712</td><td>H6F50_RS18615</td><td>18748..22416</td><td>2692789</td><td>BREX-system-P-loop-protein-BrxC-[Coleofasciculus-sp.-FACHB-712]</td></tr>
<tr><td>KPQ32454.1</td><td>Type1.BREX</td><td>BrxA-DNA-binding -> BrxB-BrxF-iSTAND -> DUF6079 *->OLD-ABC->NERD-> N6-METHYLASE -> OLD-ABC-> HNH -> PglZ -> SIGMA+OB-Fold+AAA-ATPase+HKD-DNase-BrxL -><-?||DDE_Tnp-></td><td>1200</td><td>Bacteria>Terrabacteria_group>Cyanobacteriota/Melainabacteria_group>Cyanobacteriota>Cyanophyceae>Pseudanabaenales</td><td>GCA_001314865.1</td><td>Phormidesmis_priestleyi_Ana</td><td>HLUCCA11_21420</td><td>1901..5503</td><td>1666911</td><td>Brex-phage-defense-system-ATPase-BrxC-[Phormidesmis-priestleyi-Ana]</td></tr>
<tr><td>HBH74800.1</td><td>Type1.BREX</td><td>PIN->?-> BrxA-DNA-binding -> BrxB-BrxF-iSTAND -> DUF6079 *-> N6-METHYLASE ->SAM-METHYLASE->ParA-Soj-PloopNTPase->?->?-> PglZ -> OLD-ABC-> HNH -> SIGMA+OB-Fold+AAA-ATPase+Lon-Proteas [illegible]</td><td>1163</td><td>Bacteria>Terrabacteria_group>Cyanobacteriota/Melainabacteria_group>Cyanobacteriota>Cyanophyceae>Synechococcales</td><td>GCA_003508015.1</td><td>Synechococcales_bacterium_UBA10510</td><td>DDY43_15550</td><td>2736..6227</td><td>2055795</td><td>BREX-system-P-loop-protein-BrxC-[Synechococcales-bacterium-UBA10510]</td></tr>
<tr><td>WP_178365089.1</td><td>Type1.BREX</td><td><-DDE_Tnp||?->?->PRTase->RADICAL-SAM-> BrxA-DNA-binding -> BrxB-BrxF-iSTAND -> DUF6079 *-> N6-METHYLASE -> OLD-ABC-> HNH -> PglZ -> SIGMA+OB-Fold+AAA-ATPase+HKD-DNase-BrxL ->tRNA-> [illegible]</td><td>1189</td><td>Bacteria>Thermodesulfobacteriota>Desulfobacteria>Desulfobacterales>Desulfobacteraceae>Desulfobacter</td><td>GCF_013389365.1</td><td>Desulfobacter_latus</td><td>HXW94_RS01255</td><td>9015..12584</td><td>2292</td><td>BREX-system-P-loop-protein-BrxC-[Desulfobacter-latus]</td></tr>
<tr><td>WP_139446530.1</td><td>Type1.BREX</td><td><- SIGMA+OB-Fold+AAA-ATPase+Lon-Protease-BrxL ||DiS_P_DiS+Peptidase_A24-><-?||?-><- SIGMA+OB-Fold+AAA-ATPase+HKD-DNase-BrxL <- PglZ <- HNH <-OLD-ABC <- N6-METHYLASE <- DUF6079 *<- [illegible]</td><td>1199</td><td>Bacteria>Thermodesulfobacteriota>Desulfobacteria>Desulfobacterales>Desulfobacteraceae>Desulfobotulus</td><td>GCF_006175995.1</td><td>Desulfobotulus_mexicanus</td><td>FIM25_RS03945</td><td>130812..134411</td><td>2586642</td><td>BREX-system-P-loop-protein-BrxC-[Desulfobotulus-mexicanus]</td></tr>
<tr><td>RZB38310.1</td><td>Type1.BREX</td><td><-TPR+TPR<-?||?->?->?-><-?||?-> BrxA-DNA-binding -> BrxB-BrxF-iSTAND -> DUF6079 *-> N6-METHYLASE -p-> N6-METHYLASE -> OLD-ABC-> HNH -> PglZ -> SIGMA+OB-Fold+AAA-ATPase+HKD-DNase-Brx [illegible]</td><td>1173</td><td>Bacteria>Thermodesulfobacteriota>Desulfobacteria>Desulfobacterales>Desulfobacteraceae>unclassified_Desulfobacteraceae</td><td>GCA_004193595.1</td><td>Desulfobacteraceae_bacterium_Eth-SRB2</td><td>SRB2_00058</td><td>60586..64107</td><td>2528438</td><td>hypothetical-protein-SRB2_00058-[Desulfobacteraceae-bacterium-Eth-SRB2]</td></tr>
<tr><td>WP_155308948.1</td><td>Type1.BREX</td><td>VirB10->?->DnaB+HerA/FtsK->TraH->TraG_N-><-?||XIS-HTH-> BrxA-DNA-binding -> BrxB-BrxF-iSTAND -> DUF6079 *-> OLD-ABC-> HNH -> N6-METHYLASE -> N6-METHYLASE ->REase+AIPR-> PglZ -> SIGM [illegible]</td><td>1145</td><td>Bacteria>Thermodesulfobacteriota>Desulfobacteria>Desulfobacterales>Desulfosarcinaceae>Desulfosarcina</td><td>GCF_009689005.1</td><td>Desulfosarcina_ovata</td><td>GN112_RS03415</td><td>763655..767092</td><td>83564</td><td>BREX-system-P-loop-protein-BrxC-[Desulfosarcina-ovata]</td></tr>
<tr><td>KGO34873.1</td><td>Type1.BREX</td><td>MlaE->ABC-ATPase+SbcC->?-><-?||XIS-HTH-> BrxA-DNA-binding -> BrxB-BrxF-iSTAND -> DUF6079 *->OLD-ABC->REase-> N6-METHYLASE ->?-> OLD-ABC-> HNH -> PglZ -> SIGMA+OB-Fold+AAA-ATPase+Lo [illegible]</td><td>1146</td><td>Bacteria>Thermodesulfobacteriota>Desulfobulbia>Desulfobulbales>Desulfobulbaceae>Desulfobulbus</td><td>GCA_000769715.1</td><td>Desulfobulbus_sp._Tol-SR</td><td>JT06_05895</td><td>26559..29999</td><td>1536652</td><td>hypothetical-protein-JT06_05895-[Desulfobulbus-sp.-Tol-SR]</td></tr>
<tr><td>WP_066605667.1</td><td>Type1.BREX</td><td>HDRnase->?-><-?<-tRNA<-?||?->?->?-> BrxA-DNA-binding -> BrxB-BrxF-iSTAND -> DUF6079 *-> N6-METHYLASE -> OLD-ABC-> HNH -> PglZ -> SIGMA+OB-Fold+AAA-ATPase+Lon-Protease-BrxL -><-RNA_M [illegible]</td><td>1145</td><td>Bacteria>Thermodesulfobacteriota>Desulfovibrionia>Desulfovibrionales>Desulfomicrobiaceae>Desulfomicrobium</td><td>GCF_001553625.1</td><td>Desulfomicrobium_orale</td><td>AXF15_RS07915</td><td>1725501..1728938</td><td>132132</td><td>BREX-system-P-loop-protein-BrxC-[Desulfomicrobium-orale]</td></tr>
<tr><td>MCB6991564.1</td><td>Type1.BREX</td><td><-ACET<-?<-ABC-ATPase+SbcC<-ABC-ATPase+SbcC<-MarR-HTH||?->HTH_26-> BrxA-DNA-binding -> BrxB-BrxF-iSTAND -> DUF6079 *-> OLD-ABC-> HNH -> N6-METHYLASE ->KAP-NTPase-></td><td>1183</td><td>Bacteria>unclassified_Bacteria</td><td>GCA_020558425.1</td><td>bacterium_210820-DFI.6.38</td><td>LI187_13870</td><td>12055..15606</td><td>2883293</td><td>BREX-system-P-loop-protein-BrxC-[bacterium-210820-DFI.6.38]</td></tr>
</table>

--

-- wHTH(SBD)+ HNH

--

<table>
<tr><td>WP_032621388.1</td><td>Type1.BREX</td><td><-SymE_toxin|| wHTH(SBD)+ HNH -><- SIGMA+OB-Fold+AAA-ATPase+Lon-Protease-BrxL <- PglZ <-TOPRIM<-OLD-ABC<- N6-METHYLASE <- DUF6079 *<- BrxB-BrxF-iSTAND <- BrxA-DNA-binding ||ZnR-><-t [illegible]</td><td>1204</td><td>Bacteria>Pseudomonadota>Gammaproteobacteria>Enterobacterales>Enterobacteriaceae>Enterobacter</td><td>GCF_023734955.1</td><td>Enterobacter_hormaechei</td><td>NVR78_RS04785</td><td>326171..329785</td><td>158836</td><td>BREX-system-P-loop-protein-BrxC-[Enterobacter-hormaechei]</td></tr>
<tr><td>WP_180264800.1</td><td>Type1.BREX</td><td><-HTH_36<-HTH+HTH<- ParB +BAM<-XIS-HTH<-wHTH<-ZnR|| BrxA-DNA-binding -> BrxB-BrxF-iSTAND -> DUF6079 *-> N6-METHYLASE ->OLD-ABC->TOPRIM-> PglZ -> SIGMA+OB-Fold+AAA-ATPase+Lon-Proteas [illegible]</td><td>1204</td><td>Bacteria>Pseudomonadota>Gammaproteobacteria>Enterobacterales>Enterobacteriaceae>Enterobacter</td><td>GCF_900558725.1</td><td>Enterobacter_hormaechei</td><td>H0848_RS18555</td><td>27730..31344</td><td>158836</td><td>BREX-system-P-loop-protein-BrxC-[Enterobacter-hormaechei]</td></tr>
<tr><td>WP_063851018.1</td><td>Type1.BREX</td><td><-HTH_36<-HTH+HTH<- ParB +BAM<-XIS-HTH<-wHTH<-ZnR|| BrxA-DNA-binding -> BrxB-BrxF-iSTAND -> DUF6079 *-> N6-METHYLASE ->OLD-ABC->TOPRIM-> PglZ -> SIGMA+OB-Fold+AAA-ATPase+Lon-Proteas [illegible]</td><td>1204</td><td>Bacteria>Pseudomonadota>Gammaproteobacteria>Enterobacterales>Enterobacteriaceae>Enterobacter</td><td>GCF_902160625.1</td><td>Enterobacter_hormaechei</td><td>FN214_RS22220</td><td>26549..30163</td><td>158836</td><td>BREX-system-P-loop-protein-BrxC-[Enterobacter-hormaechei]</td></tr>
<tr><td>GCK20492.1</td><td>Type1.BREX</td><td><-Fimbrial<-Phage_integrase<-Phage_integrase||?->?->?-> wHTH(SBD)+ HNH -> BrxA-DNA-binding -> BrxB-BrxF-iSTAND -> DUF6079 -p*-></td><td>621</td><td>Bacteria>Pseudomonadota>Gammaproteobacteria>Enterobacterales>Enterobacteriaceae>Escherichia</td><td>GCA_005401165.1</td><td>Escherichia_coli</td><td>BvCmsA58A_02120</td><td>111143..113008</td><td>562</td><td>ATPase-like-protein-[Escherichia-coli]</td></tr>
<tr><td>EEU4096206.1</td><td>Type1.BREX</td><td><- DUF6079 -p*<- BrxB-BrxF-iSTAND <- BrxA-DNA-binding ||<- wHTH(SBD)+ HNH || HNH -><-?<-?<-?||Phage_integrase->Phage_integrase-></td><td>433</td><td>Bacteria>Pseudomonadota>Gammaproteobacteria>Enterobacterales>Enterobacteriaceae>Escherichia</td><td>GCA_011974935.1</td><td>Escherichia_coli</td><td>E8H53_004589</td><td>1..1299</td><td>562</td><td>BREX-system-P-loop-protein-BrxC-[Escherichia-coli]</td></tr>
<tr><td>EEV1146578.1</td><td>Type1.BREX</td><td><-Phage_integrase||?->?->?-><- HNH || wHTH(SBD)+ HNH ->|| BrxA-DNA-binding -> BrxB-BrxF-iSTAND -> DUF6079 *-> N6-METHYLASE -></td><td>1213</td><td>Bacteria>Pseudomonadota>Gammaproteobacteria>Enterobacterales>Enterobacteriaceae>Escherichia</td><td>GCA_012013845.1</td><td>Escherichia_coli</td><td>EUB49_004354</td><td>8713..12354</td><td>562</td><td>BREX-system-P-loop-protein-BrxC-[Escherichia-coli]</td></tr>
<tr><td>EEW3089662.1</td><td>Type1.BREX</td><td><-Phage_integrase<-Phage_integrase||?->?->?-><- HNH || wHTH(SBD)+ HNH ->|| BrxA-DNA-binding -> BrxB-BrxF-iSTAND -> DUF6079 *-> N6-METHYLASE -> PglZ -> SIGMA+OB-Fold+AAA-ATPase+Lon-P [illegible]</td><td>1213</td><td>Bacteria>Pseudomonadota>Gammaproteobacteria>Enterobacterales>Enterobacteriaceae>Escherichia</td><td>GCA_012078325.1</td><td>Escherichia_coli</td><td>EBA98_002639</td><td>14697..18338</td><td>562</td><td>BREX-system-P-loop-protein-BrxC-[Escherichia-coli]</td></tr>
<tr><td>EEW3885152.1</td><td>Type1.BREX</td><td><- HNH || wHTH(SBD)+ HNH ->|| BrxA-DNA-binding -> BrxB-BrxF-iSTAND -> DUF6079 *-> N6-METHYLASE -> PglZ -> SIGMA+OB-Fold+AAA-ATPase+Lon-Protease-BrxL ->HKD-DNase+OGG-like->||AbiEi_3_N [illegible]</td><td>1213</td><td>Bacteria>Pseudomonadota>Gammaproteobacteria>Enterobacterales>Enterobacteriaceae>Escherichia</td><td>GCA_012080795.1</td><td>Escherichia_coli</td><td>D9Y07_001392</td><td>7166..10807</td><td>562</td><td>BREX-system-P-loop-protein-BrxC-[Escherichia-coli]</td></tr>
</table>

<table>
<tr><th>Protein-ID</th><th>System</th><th>Gene-Neighbourhoods</th><th>Length</th><th>Taxonomy</th><th>Assembly-ID</th><th>Strain</th><th>Locus-Tag</th><th>Genomic-coordinates</th><th>TaxID</th><th>Fasta-define</th></tr>
<tr><td>EEX1719682.1</td><td>Type1.BREX</td><td><-HNH||wHTH(SBD)+HNH->||BrxA-DNA-binding->BrxB-BrxF-iSTAND->DUF6079*->N6-METHYLASE->PglZ->SIGMA+OB-Fold+AAA-ATPase+Lon-Protease-BrxL->HKD-DNase+OGG-like->||AbiEi_3_N+AbiEi_3->AbiEii-><-DUF6216<-Arm-DNA-bind_3</td><td>1213</td><td>Bacteria>Pseudomonadota>Gammaproteobacteria>Enterobacterales>Enterobacteriaceae>Escherichia</td><td>GCA_012115095.1</td><td>Escherichia_coli</td><td>D3S79_003407</td><td>6685..10326</td><td>562</td><td>BREX-system-P-loop-protein-BrxC-[Escherichia-coli]</td></tr>
<tr><td>EEX6733972.1</td><td>Type1.BREX</td><td><-DUF6079*<-BrxB-BrxF-iSTAND<-BrxA-DNA-binding||<-wHTH(SBD)+HNH||HNH-><-?<-?<-?||Phage_integrase->Phage_integrase-></td><td>1175</td><td>Bacteria>Pseudomonadota>Gammaproteobacteria>Enterobacterales>Enterobacteriaceae>Escherichia</td><td>GCA_012135265.1</td><td>Escherichia_coli</td><td>L0765_004693</td><td>1..3524</td><td>562</td><td>BREX-system-P-loop-protein-BrxC-[Escherichia-coli]</td></tr>
<tr><td>EEZ2582139.1</td><td>Type1.BREX</td><td><-DUF6079-p*<-BrxB-BrxF-iSTAND<-BrxA-DNA-binding||<-wHTH(SBD)+HNH||HNH-><-?<-?<-?||Phage_integrase->Phage_integrase-></td><td>800</td><td>Bacteria>Pseudomonadota>Gammaproteobacteria>Enterobacterales>Enterobacteriaceae>Escherichia</td><td>GCA_012201865.1</td><td>Escherichia_coli</td><td>DMH54_003958</td><td>1..2402</td><td>562</td><td>BREX-system-P-loop-protein-BrxC-[Escherichia-coli]</td></tr>
<tr><td>EEZ3215399.1</td><td>Type1.BREX</td><td><-HNH||wHTH(SBD)+HNH->||BrxA-DNA-binding->BrxB-BrxF-iSTAND->DUF6079*->N6-METHYLASE-></td><td>1213</td><td>Bacteria>Pseudomonadota>Gammaproteobacteria>Enterobacterales>Enterobacteriaceae>Escherichia</td><td>GCA_012202545.1</td><td>Escherichia_coli</td><td>DKV48_004298</td><td>6695..10336</td><td>562</td><td>BREX-system-P-loop-protein-BrxC-[Escherichia-coli]</td></tr>
<tr><td>EFC3446201.1</td><td>Type1.BREX</td><td><-DUF6079-p*<-BrxB-BrxF-iSTAND<-BrxA-DNA-binding||<-wHTH(SBD)+HNH||HNH-><-?<-?<-?||Phage_integrase->Phage_integrase-></td><td>844</td><td>Bacteria>Pseudomonadota>Gammaproteobacteria>Enterobacterales>Enterobacteriaceae>Escherichia</td><td>GCA_012346015.1</td><td>Escherichia_coli</td><td>C0751_004598</td><td>1..2534</td><td>562</td><td>BREX-system-P-loop-protein-BrxC-[Escherichia-coli]</td></tr>
<tr><td>EFD3459874.1</td><td>Type1.BREX</td><td><-HNH||wHTH(SBD)+HNH->||BrxA-DNA-binding->BrxB-BrxF-iSTAND->DUF6079*->N6-METHYLASE->PglZ->SIGMA+OB-Fold+AAA-ATPase+Lon-Protease-BrxL-></td><td>1213</td><td>Bacteria>Pseudomonadota>Gammaproteobacteria>Enterobacterales>Enterobacteriaceae>Escherichia</td><td>GCA_012389335.1</td><td>Escherichia_coli</td><td>CL211_004302</td><td>6759..10400</td><td>562</td><td>BREX-system-P-loop-protein-BrxC-[Escherichia-coli]</td></tr>
<tr><td>EFE1965719.1</td><td>Type1.BREX</td><td><-Phage_integrase<-Phage_integrase||?->?->?-><-HNH||wHTH(SBD)+HNH->||BrxA-DNA-binding->BrxB-BrxF-iSTAND->DUF6079*->N6-METHYLASE->PglZ->SIGMA+OB-Fold+AAA-ATPase+Lon-Protease-BrxL->HKD-DNase+OGG-like->||AbiEi_3_N+AbiEi_3->AbiEii-><-DUF6216<-Arm-DNA-bind_3</td><td>1213</td><td>Bacteria>Pseudomonadota>Gammaproteobacteria>Enterobacterales>Enterobacteriaceae>Escherichia</td><td>GCA_012428445.1</td><td>Escherichia_coli</td><td>CJV81_002667</td><td>14696..18337</td><td>562</td><td>BREX-system-P-loop-protein-BrxC-[Escherichia-coli]</td></tr>
<tr><td>EFG4404338.1</td><td>Type1.BREX</td><td><-Phage_integrase<-Phage_integrase||?->?->?-><-HNH||wHTH(SBD)+HNH->||BrxA-DNA-binding->BrxB-BrxF-iSTAND->DUF6079*->N6-METHYLASE->PglZ->SIGMA+OB-Fold+AAA-ATPase+Lon-Protease-BrxL->HKD-DNase+OGG-like->||AbiEi_3_N+AbiEi_3->AbiEii-><-DUF6216<-Arm-DNA-bind_3</td><td>1213</td><td>Bacteria>Pseudomonadota>Gammaproteobacteria>Enterobacterales>Enterobacteriaceae>Escherichia</td><td>GCA_012556825.1</td><td>Escherichia_coli</td><td>BM772_002472</td><td>14697..18338</td><td>562</td><td>BREX-system-P-loop-protein-BrxC-[Escherichia-coli]</td></tr>
<tr><td>EFH0549024.1</td><td>Type1.BREX</td><td><-Phage_integrase<-Phage_integrase||?->?->?-><-HNH||wHTH(SBD)+HNH->||BrxA-DNA-binding->BrxB-BrxF-iSTAND->DUF6079-p*-></td><td>422</td><td>Bacteria>Pseudomonadota>Gammaproteobacteria>Enterobacterales>Enterobacteriaceae>Escherichia</td><td>GCA_012582395.1</td><td>Escherichia_coli</td><td>BJE23_003837</td><td>39992..41258</td><td>562</td><td>BREX-system-P-loop-protein-BrxC-[Escherichia-coli]</td></tr>
<tr><td>EGY5620863.1</td><td>Type1.BREX</td><td><-Phage_integrase<-Phage_integrase||?->?->?-><-HNH||wHTH(SBD)+HNH->||BrxA-DNA-binding->BrxB-BrxF-iSTAND->DUF6079*->N6-METHYLASE->PglZ->SIGMA+OB-Fold+AAA-ATPase+Lon-Protease-BrxL->HKD-DNase+OGG-like->||AbiEi_3_N+AbiEi_3->AbiEii-><-DUF6216<-Arm-DNA-bind_3</td><td>1213</td><td>Bacteria>Pseudomonadota>Gammaproteobacteria>Enterobacterales>Enterobacteriaceae>Escherichia</td><td>GCA_016519195.1</td><td>Escherichia_coli</td><td>JI433_002890</td><td>14696..18337</td><td>562</td><td>BREX-system-P-loop-protein-BrxC-[Escherichia-coli]</td></tr>
<tr><td>EGY7451904.1</td><td>Type1.BREX</td><td><-DUF6079-p*<-BrxB-BrxF-iSTAND<-BrxA-DNA-binding||<-wHTH(SBD)+HNH||HNH-><-?<-?<-?||Phage_integrase->Phage_integrase-></td><td>968</td><td>Bacteria>Pseudomonadota>Gammaproteobacteria>Enterobacterales>Enterobacteriaceae>Escherichia</td><td>GCA_016533805.1</td><td>Escherichia_coli</td><td>JI519_004377</td><td>1..2904</td><td>562</td><td>BREX-system-P-loop-protein-BrxC-[Escherichia-coli]</td></tr>
<tr><td>EGY8948619.1</td><td>Type1.BREX</td><td><-DUF6079-p*<-BrxB-BrxF-iSTAND<-BrxA-DNA-binding||<-wHTH(SBD)+HNH||HNH-><-?<-?<-?||Phage_integrase->Phage_integrase-></td><td>450</td><td>Bacteria>Pseudomonadota>Gammaproteobacteria>Enterobacterales>Enterobacteriaceae>Escherichia</td><td>GCA_016541865.1</td><td>Escherichia_coli</td><td>JI613_004635</td><td>1..1349</td><td>562</td><td>BREX-system-P-loop-protein-BrxC-[Escherichia-coli]</td></tr>
<tr><td>EGY9306082.1</td><td>Type1.BREX</td><td><-HNH||wHTH(SBD)+HNH->||BrxA-DNA-binding->BrxB-BrxF-iSTAND->DUF6079*->N6-METHYLASE->PglZ-></td><td>1213</td><td>Bacteria>Pseudomonadota>Gammaproteobacteria>Enterobacterales>Enterobacteriaceae>Escherichia</td><td>GCA_016543505.1</td><td>Escherichia_coli</td><td>HZ096_004640</td><td>4089..7730</td><td>562</td><td>BREX-system-P-loop-protein-BrxC-[Escherichia-coli]</td></tr>
<tr><td>EGZ1560300.1</td><td>Type1.BREX</td><td><-DUF6079-p*<-BrxB-BrxF-iSTAND<-BrxA-DNA-binding||<-wHTH(SBD)+HNH||HNH-><-?<-?<-?||Phage_integrase->Phage_integrase-></td><td>444</td><td>Bacteria>Pseudomonadota>Gammaproteobacteria>Enterobacterales>Enterobacteriaceae>Escherichia</td><td>GCA_016553465.1</td><td>Escherichia_coli</td><td>FAV16_004661</td><td>1..1332</td><td>562</td><td>BREX-system-P-loop-protein-BrxC-[Escherichia-coli]</td></tr>
<tr><td>EGZ1871551.1</td><td>Type1.BREX</td><td><-DUF6079-p*<-BrxB-BrxF-iSTAND<-BrxA-DNA-binding||<-wHTH(SBD)+HNH||HNH-><-?<-?<-?||Phage_integrase->Phage_integrase-></td><td>416</td><td>Bacteria>Pseudomonadota>Gammaproteobacteria>Enterobacterales>Enterobacteriaceae>Escherichia</td><td>GCA_016554585.1</td><td>Escherichia_coli</td><td>EYE54_004341</td><td>1..1248</td><td>562</td><td>BREX-system-P-loop-protein-BrxC-[Escherichia-coli]</td></tr>
<tr><td>EGZ2189929.1</td><td>Type1.BREX</td><td><-DUF6079-p*<-BrxB-BrxF-iSTAND<-BrxA-DNA-binding||<-wHTH(SBD)+HNH||HNH-><-?<-?<-?||Phage_integrase-></td><td>628</td><td>Bacteria>Pseudomonadota>Gammaproteobacteria>Enterobacterales>Enterobacteriaceae>Escherichia</td><td>GCA_016555885.1</td><td>Escherichia_coli</td><td>ED609_004465</td><td>1..1884</td><td>562</td><td>BREX-system-P-loop-protein-BrxC-[Escherichia-coli]</td></tr>
<tr><td>EGZ2354229.1</td><td>Type1.BREX</td><td><-Phage_integrase<-Phage_integrase||?->?->?-><-HNH||wHTH(SBD)+HNH->||BrxA-DNA-binding->BrxB-BrxF-iSTAND->DUF6079*->N6-METHYLASE->PglZ->SIGMA+OB-Fold+AAA-ATPase+Lon-Protease-BrxL->HKD-DNase+OGG-like->||AbiEi_3_N+AbiEi_3->AbiEii-><-DUF6216<-Arm-DNA-bind_3</td><td>1213</td><td>Bacteria>Pseudomonadota>Gammaproteobacteria>Enterobacterales>Enterobacteriaceae>Escherichia</td><td>GCA_016556665.1</td><td>Escherichia_coli</td><td>D7J47_002044</td><td>14697..18338</td><td>562</td><td>BREX-system-P-loop-protein-BrxC-[Escherichia-coli]</td></tr>
<tr><td>EGZ2777080.1</td><td>Type1.BREX</td><td><-Phage_integrase<-Phage_integrase||?->?->?-><-HNH||wHTH(SBD)+HNH->||BrxA-DNA-binding->BrxB-BrxF-iSTAND->DUF6079*->N6-METHYLASE->PglZ->SIGMA+OB-Fold+AAA-ATPase+Lon-Protease-BrxL->HKD-DNase+OGG-like->||AbiEi_3_N+AbiEi_3->AbiEii-><-DUF6216<-Arm-DNA-bind_3</td><td>1213</td><td>Bacteria>Pseudomonadota>Gammaproteobacteria>Enterobacterales>Enterobacteriaceae>Escherichia</td><td>GCA_016558225.1</td><td>Escherichia_coli</td><td>DV041_002389</td><td>14697..18338</td><td>562</td><td>BREX-system-P-loop-protein-BrxC-[Escherichia-coli]</td></tr>
<tr><td>EGZ3242782.1</td><td>Type1.BREX</td><td><-Phage_integrase<-Phage_integrase||?->?->?-><-HNH||wHTH(SBD)+HNH->||BrxA-DNA-binding->BrxB-BrxF-iSTAND->DUF6079*->N6-METHYLASE->PglZ->SIGMA+OB-Fold+AAA-ATPase+Lon-Protease-BrxL->HKD-DNase+OGG-like->||AbiEi_3_N+AbiEi_3->AbiEii-><-DUF6216<-Arm-DNA-bind_3</td><td>1213</td><td>Bacteria>Pseudomonadota>Gammaproteobacteria>Enterobacterales>Enterobacteriaceae>Escherichia</td><td>GCA_016560025.1</td><td>Escherichia_coli</td><td>C5N70_002404</td><td>39991..43632</td><td>562</td><td>BREX-system-P-loop-protein-BrxC-[Escherichia-coli]</td></tr>
<tr><td>EGZ5166758.1</td><td>Type1.BREX</td><td><-DUF6079-p*<-BrxB-BrxF-iSTAND<-BrxA-DNA-binding||<-wHTH(SBD)+HNH||HNH-><-?<-?<-?||Phage_integrase->Phage_integrase-></td><td>624</td><td>Bacteria>Pseudomonadota>Gammaproteobacteria>Enterobacterales>Enterobacteriaceae>Escherichia</td><td>GCA_016568175.1</td><td>Escherichia_coli</td><td>CDS35_004366</td><td>1..1874</td><td>562</td><td>BREX-system-P-loop-protein-BrxC-[Escherichia-coli]</td></tr>
<tr><td>EGZ5591347.1</td><td>Type1.BREX</td><td><-DUF6079-p*<-BrxB-BrxF-iSTAND<-BrxA-DNA-binding||<-wHTH(SBD)+HNH||HNH-><-?<-?<-?||Phage_integrase->Phage_integrase-></td><td>707</td><td>Bacteria>Pseudomonadota>Gammaproteobacteria>Enterobacterales>Enterobacteriaceae>Escherichia</td><td>GCA_016569835.1</td><td>Escherichia_coli</td><td>BZZ56_004473</td><td>1..2121</td><td>562</td><td>BREX-system-P-loop-protein-BrxC-[Escherichia-coli]</td></tr>
<tr><td>EGZ5731917.1</td><td>Type1.BREX</td><td><-DUF6079-p*<-BrxB-BrxF-iSTAND<-BrxA-DNA-binding||<-wHTH(SBD)+HNH||HNH-><-?<-?<-?||Phage_integrase->Phage_integrase-></td><td>885</td><td>Bacteria>Pseudomonadota>Gammaproteobacteria>Enterobacterales>Enterobacteriaceae>Escherichia</td><td>GCA_016570515.1</td><td>Escherichia_coli</td><td>BL334_004670</td><td>1..2655</td><td>562</td><td>BREX-system-P-loop-protein-BrxC-[Escherichia-coli]</td></tr>
<tr><td>EGZ5978150.1</td><td>Type1.BREX</td><td><-Phage_integrase<-Phage_integrase||?->?->?-><-HNH||wHTH(SBD)+HNH->||BrxA-DNA-binding->BrxB-BrxF-iSTAND->DUF6079-p*-></td><td>421</td><td>Bacteria>Pseudomonadota>Gammaproteobacteria>Enterobacterales>Enterobacteriaceae>Escherichia</td><td>GCA_016571515.1</td><td>Escherichia_coli</td><td>BIB10_004104</td><td>39992..41255</td><td>562</td><td>BREX-system-P-loop-protein-BrxC-[Escherichia-coli]</td></tr>
<tr><td>EGZ8838308.1</td><td>Type1.BREX</td><td><-Phage_integrase||?->?->?-><-HNH||wHTH(SBD)+HNH->||BrxA-DNA-binding->BrxB-BrxF-iSTAND->DUF6079*->N6-METHYLASE->PglZ->SIGMA+OB-Fold+AAA-ATPase+Lon-Protease-BrxL->HKD-DNase+OGG-like->||AbiEi_3_N+AbiEi_3->AbiEii-><-DUF6216<-Arm-DNA-bind_3</td><td>1213</td><td>Bacteria>Pseudomonadota>Gammaproteobacteria>Enterobacterales>Enterobacteriaceae>Escherichia</td><td>GCA_016590495.1</td><td>Escherichia_coli</td><td>AT823_000493</td><td>8719..12360</td><td>562</td><td>BREX-system-P-loop-protein-BrxC-[Escherichia-coli]</td></tr>
<tr><td>EHA9087556.1</td><td>Type1.BREX</td><td><-Phage_integrase<-Phage_integrase||?->?->?-><-HNH||wHTH(SBD)+HNH->||BrxA-DNA-binding->BrxB-BrxF-iSTAND->DUF6079*->N6-METHYLASE->PglZ->SIGMA+OB-Fold+AAA-ATPase+Lon-Protease-BrxL->HKD-DNase+OGG-like->||AbiEi_3_N+AbiEi_3->AbiEii-><-DUF6216<-Arm-DNA-bind_3</td><td>1213</td><td>Bacteria>Pseudomonadota>Gammaproteobacteria>Enterobacterales>Enterobacteriaceae>Escherichia</td><td>GCA_016846225.1</td><td>Escherichia_coli</td><td>D4094_002230</td><td>14699..18340</td><td>562</td><td>BREX-system-P-loop-protein-BrxC-[Escherichia-coli]</td></tr>
<tr><td>EHJ8640897.1</td><td>Type1.BREX</td><td><-Phage_integrase<-Phage_integrase||?->?->?-><-HNH||wHTH(SBD)+HNH->||BrxA-DNA-binding->BrxB-BrxF-iSTAND->DUF6079*->N6-METHYLASE->PglZ->SIGMA+OB-Fold+AAA-ATPase+Lon-Protease-BrxL->HKD-DNase+OGG-like->||AbiEi_3_N+AbiEi_3->AbiEii->?-><-DUF6216<-Arm-DNA-bind_3</td><td>1213</td><td>Bacteria>Pseudomonadota>Gammaproteobacteria>Enterobacterales>Enterobacteriaceae>Escherichia</td><td>GCA_017945505.1</td><td>Escherichia_coli</td><td>KB366_000307</td><td>330961..334602</td><td>562</td><td>BREX-system-P-loop-protein-BrxC-[Escherichia-coli]</td></tr>
<tr><td>EHL0175571.1</td><td>Type1.BREX</td><td><-DUF6079*<-BrxB-BrxF-iSTAND<-BrxA-DNA-binding||<-wHTH(SBD)+HNH||HNH-><-?<-?<-?||Phage_integrase->Phage_integrase-></td><td>1016</td><td>Bacteria>Pseudomonadota>Gammaproteobacteria>Enterobacterales>Enterobacteriaceae>Escherichia</td><td>GCA_018090545.1</td><td>Escherichia_coli</td><td>D7K57_004368</td><td>1..3050</td><td>562</td><td>BREX-system-P-loop-protein-BrxC-[Escherichia-coli]</td></tr>
<tr><td>EHL0676199.1</td><td>Type1.BREX</td><td><-Phage_integrase<-Phage_integrase||?->?->?-><-HNH||wHTH(SBD)+HNH->||BrxA-DNA-binding->BrxB-BrxF-iSTAND->DUF6079*->N6-METHYLASE-></td><td>1213</td><td>Bacteria>Pseudomonadota>Gammaproteobacteria>Enterobacterales>Enterobacteriaceae>Escherichia</td><td>GCA_018092345.1</td><td>Escherichia_coli</td><td>CP667_004253</td><td>9829..13470</td><td>562</td><td>BREX-system-P-loop-protein-BrxC-[Escherichia-coli]</td></tr>
<tr><td>EHL1134570.1</td><td>Type1.BREX</td><td><-DUF6079-p*<-BrxB-BrxF-iSTAND<-BrxA-DNA-binding||<-wHTH(SBD)+HNH||HNH-><-?<-?<-?||Phage_integrase->Phage_integrase-></td><td>460</td><td>Bacteria>Pseudomonadota>Gammaproteobacteria>Enterobacterales>Enterobacteriaceae>Escherichia</td><td>GCA_018094045.1</td><td>Escherichia_coli</td><td>BIB01_004961</td><td>1..1380</td><td>562</td><td>BREX-system-P-loop-protein-BrxC-[Escherichia-coli]</td></tr>
<tr><td>EHL1162648.1</td><td>Type1.BREX</td><td><-Phage_integrase<-Phage_integrase||?->?->?-><-HNH||wHTH(SBD)+HNH->||BrxA-DNA-binding->BrxB-BrxF-iSTAND->DUF6079*->N6-METHYLASE->PglZ->SIGMA+OB-Fold+AAA-ATPase+Lon-Protease-BrxL->HKD-DNase+OGG-like->||AbiEi_3_N+AbiEi_3->AbiEii->?-><-DUF6216<-Arm-DNA-bind_3</td><td>1213</td><td>Bacteria>Pseudomonadota>Gammaproteobacteria>Enterobacterales>Enterobacteriaceae>Escherichia</td><td>GCA_018094265.1</td><td>Escherichia_coli</td><td>BE892_002471</td><td>14698..18339</td><td>562</td><td>BREX-system-P-loop-protein-BrxC-[Escherichia-coli]</td></tr>
<tr><td>EHN3342070.1</td><td>Type1.BREX</td><td><-Phage_integrase<-Phage_integrase||?->?->?-><-HNH||wHTH(SBD)+HNH->||BrxA-DNA-binding->BrxB-BrxF-iSTAND->DUF6079*->N6-METHYLASE->PglZ->SIGMA+OB-Fold+AAA-ATPase+Lon-Protease-BrxL->HKD-DNase+OGG-like->||AbiEi_3_N+AbiEi_3->AbiEii->?-><-DUF6216<-Arm-DNA-bind_3</td><td>1213</td><td>Bacteria>Pseudomonadota>Gammaproteobacteria>Enterobacterales>Enterobacteriaceae>Escherichia</td><td>GCA_018356405.1</td><td>Escherichia_coli</td><td>KIR71_003047</td><td>14697..18338</td><td>562</td><td>BREX-system-P-loop-protein-BrxC-[Escherichia-coli]</td></tr>
<tr><td>EHY5690081.1</td><td>Type1.BREX</td><td><-Phage_integrase<-Phage_integrase||?->?->?-><-HNH||wHTH(SBD)+HNH->||BrxA-DNA-binding->BrxB-BrxF-iSTAND->DUF6079*->N6-METHYLASE->PglZ->SIGMA+OB-Fold+AAA-ATPase+Lon-Protease-BrxL->HKD-DNase+OGG-like->||AbiEi_3_N+AbiEi_3->AbiEii->?-><-DUF6216<-Arm-DNA-bind_3</td><td>1213</td><td>Bacteria>Pseudomonadota>Gammaproteobacteria>Enterobacterales>Enterobacteriaceae>Escherichia</td><td>GCA_019590565.1</td><td>Escherichia_coli</td><td>K4J54_002559</td><td>14696..18337</td><td>562</td><td>BREX-system-P-loop-protein-BrxC-[Escherichia-coli]</td></tr>
<tr><td>EIB0293606.1</td><td>Type1.BREX</td><td><-DUF6079-p*<-BrxB-BrxF-iSTAND<-BrxA-DNA-binding||<-wHTH(SBD)+HNH||HNH-><-?<-?<-?||Phage_integrase->Phage_integrase-></td><td>529</td><td>Bacteria>Pseudomonadota>Gammaproteobacteria>Enterobacterales>Enterobacteriaceae>Escherichia</td><td>GCA_019848435.1</td><td>Escherichia_coli</td><td>K7644_004610</td><td>1..1589</td><td>562</td><td>BREX-system-P-loop-protein-BrxC-[Escherichia-coli]</td></tr>
<tr><td>EIG6470759.1</td><td>Type1.BREX</td><td><-DUF6079-p*<-BrxB-BrxF-iSTAND<-BrxA-DNA-binding||<-wHTH(SBD)+HNH||HNH-><-?<-?<-?||Phage_integrase->Phage_integrase-></td><td>634</td><td>Bacteria>Pseudomonadota>Gammaproteobacteria>Enterobacterales>Enterobacteriaceae>Escherichia</td><td>GCA_020319175.1</td><td>Escherichia_coli</td><td>EG665_005071</td><td>1..1903</td><td>562</td><td>BREX-system-P-loop-protein-BrxC-[Escherichia-coli]</td></tr>
<tr><td>HBP9919082.1</td><td>Type1.BREX</td><td><-Phage_integrase<-Phage_integrase||?->?->?-><-HNH||wHTH(SBD)+HNH->||BrxA-DNA-binding->BrxB-BrxF-iSTAND->DUF6079*->N6-METHYLASE->PglZ->SIGMA+OB-Fold+AAA-ATPase+Lon-Protease-BrxL->HKD-DNase+OGG-like->||AbiEi_3_N+AbiEi_3->AbiEii->?-><-DUF6216<-Arm-DNA-bind_3</td><td>1213</td><td>Bacteria>Pseudomonadota>Gammaproteobacteria>Enterobacterales>Enterobacteriaceae>Escherichia</td><td>GCA_021766545.1</td><td>Escherichia_coli</td><td>L7G70_002290</td><td>14696..18337</td><td>562</td><td>BREX-system-P-loop-protein-BrxC-[Escherichia-coli]</td></tr>
<tr><td>EJW3212697.1</td><td>Type1.BREX</td><td><-DUF6079-p*<-BrxB-BrxF-iSTAND<-BrxA-DNA-binding||<-wHTH(SBD)+HNH||HNH-><-?<-?<-?||Phage_integrase->Phage_integrase-></td><td>418</td><td>Bacteria>Pseudomonadota>Gammaproteobacteria>Enterobacterales>Enterobacteriaceae>Escherichia</td><td>GCA_025418235.1</td><td>Escherichia_coli</td><td>OBZ04_004372</td><td>1..1254</td><td>562</td><td>BREX-system-P-loop-protein-BrxC-[Escherichia-coli]</td></tr>
<tr><td>WP_109548991.1</td><td>Type1.BREX</td><td><-Fimbrial<-Phage_integrase<-Phage_integrase||?->?->?->||wHTH(SBD)+HNH->||BrxA-DNA-binding->BrxB-BrxF-iSTAND->DUF6079*->N6-METHYLASE->PglZ->SIGMA+OB-Fold+AAA-ATPase+Lon-Protease-BrxL->HKD-DNase+OGG-like->||AbiEi_3_N+AbiEi_3->AbiEii-><-DUF6216</td><td>1213</td><td>Bacteria>Pseudomonadota>Gammaproteobacteria>Enterobacterales>Enterobacteriaceae>Escherichia</td><td>GCF_003145675.1</td><td>Escherichia_coli</td><td>B6S34_RS22850</td><td>50490..54131</td><td>562</td><td>BREX-system-P-loop-protein-BrxC-[Escherichia-coli]</td></tr>
<tr><td>EIY5117082.1</td><td>Type1.BREX</td><td><-Calcineurin<-?<-?<-?<-?<-?||tRNA-><-ZnR||BrxA-DNA-binding->BrxB-BrxF-iSTAND->DUF6079*->N6-METHYLASE->OLD-ABC->TOPRIM->PglZ->SIGMA+OB-Fold+AAA-ATPase+Lon-Protease-BrxL-><-wHTH(SBD)+HNH||MAD-NTD+HNH-><-Glyco_hydro_43+GH43_C2</td><td>1197</td><td>Bacteria>Pseudomonadota>Gammaproteobacteria>Enterobacterales>Enterobacteriaceae>Klebsiella/Raoultella_group</td><td>GCA_022503905.1</td><td>Klebsiella_quasipneumoniae</td><td>ML076_004996</td><td>20031..23624</td><td>1463165</td><td>BREX-system-P-loop-protein-BrxC-[Klebsiella-quasipneumoniae]</td></tr>
<tr><td>EAY5508618.1</td><td>Type1.BREX</td><td><-wHTH||wHTH+WYL+WCX-><-MarR-wHTH<-?<-SIGMA-HTH+REase<-wHTH(SBD)+HNH||?->BrxA-DNA-binding->BrxB-BrxF-iSTAND->DUF6079*->N6-METHYLASE->OLD-ABC->TOPRIM->PglZ->SIGMA+OB-Fold+AAA-ATPase+Lon-Protease-BrxL->HKD-DNase+OGG-like-><-REase<-NACHT+SF1-SF2-Helicase+HKD-DNase<-Arm-DNA-bind_3</td><td>1204</td><td>Bacteria>Pseudomonadota>Gammaproteobacteria>Enterobacterales>Enterobacteriaceae>Salmonella</td><td>GCA_006012725.1</td><td>Salmonella_enterica</td><td>NE47_11015</td><td>49392..53006</td><td>28901</td><td>BREX-system-P-loop-protein-BrxC-[Salmonella-enterica]</td></tr>
<tr><td>EIC8525255.1</td><td>Type1.BREX</td><td><-REase+iREase<-?||YjhX_toxin->SAM-METHYLASE->?-><-wHTH(SBD)+HNH||?->BrxA-DNA-binding->BrxB-BrxF-iSTAND->DUF6079*->N6-METHYLASE->OLD-ABC->TOPRIM->PglZ->SIGMA+OB-Fold+AAA-ATPase+Lon-Protease-BrxL-><-REase<-NACHT+SF1-SF2-Helicase+HKD-DNase<-Arm-DNA-bind_3</td><td>1204</td><td>Bacteria>Pseudomonadota>Gammaproteobacteria>Enterobacterales>Enterobacteriaceae>Salmonella</td><td>GCA_019993965.1</td><td>Salmonella_enterica</td><td>LAR10_002215</td><td>30447..34061</td><td>28901</td><td>BREX-system-P-loop-protein-BrxC-[Salmonella-enterica]</td></tr>
<tr><td>WP_040031759.1</td><td>Type1.BREX</td><td><-HIN-HTH||DUF4096+DDE_Tnp_1+DDE_Tnp_1_2->?->tRNA->tRNA->Arm-DNA-bind_3+Phage_integrase-><-SIGMA+OB-Fold+AAA-ATPase+Lon-Protease-BrxL<-PglZ<-TOPRIM<-OLD-ABC<-N6-METHYLASE<-DUF6079*<-BrxB-BrxF-iSTAND<-BrxA-DNA-binding<-wHTH(SBD)+HNH<-LuxR-HTH<-?<-TPR</td><td>1202</td><td>Bacteria>Pseudomonadota>Gammaproteobacteria>Enterobacterales>Pectobacteriaceae</td><td>GCF_026723725.1</td><td>Pectobacterium</td><td>OWC53_RS08230</td><td>1782306..1785914</td><td>122277</td><td>MULTISPECIES:-BREX-system-P-loop-protein-BrxC-[Pectobacterium]</td></tr>
<tr><td>GKX39595.1</td><td>Type1.BREX</td><td>Arm-DNA-bind_3+Phage_integrase-><-SIGMA+OB-Fold+AAA-ATPase+Lon-Protease-BrxL<-PglZ<-TOPRIM<-OLD-ABC<-N6-METHYLASE<-DUF6079*<-BrxB-BrxF-iSTAND<-BrxA-DNA-binding<-wHTH(SBD)+HNH<-LuxR-HTH<-?<-TPR</td><td>1202</td><td>Bacteria>Pseudomonadota>Gammaproteobacteria>Enterobacterales>Pectobacteriaceae>Pectobacterium</td><td>GCA_024343445.1</td><td>Pectobacterium_carotovorum_subsp._carotovorum</td><td>SOASR014_33340</td><td>124830..128438</td><td>555</td><td>hypothetical-protein-SOASR014_33340-[Pectobacterium-carotovorum-subsp.-carotovorum]</td></tr>
<tr><td>WP_103199124.1</td><td>Type1.BREX</td><td><-ParA-Soj-PloopNTPase<-XIS-HTH<-MODE-HTH+PBPII||tRNA->tRNA->Arm-DNA-bind_3+Phage_integrase-><-SIGMA+OB-Fold+AAA-ATPase+Lon-Protease-BrxL<-PglZ<-TOPRIM<-OLD-ABC<-N6-METHYLASE<-DUF6079*<-BrxB-BrxF-iSTAND<-BrxA-DNA-binding<-wHTH(SBD)+HNH<-LuxR-HTH</td><td>1202</td><td>Bacteria>Pseudomonadota>Gammaproteobacteria>Enterobacterales>Pectobacteriaceae>Pectobacterium</td><td>GCF_002904085.1</td><td>Pectobacterium_odoriferum</td><td>BV921_RS15390</td><td>113976..117584</td><td>78398</td><td>BREX-system-P-loop-protein-BrxC-[Pectobacterium-odoriferum]</td></tr>
<tr><td>WP_116155987.1</td><td>Type1.BREX</td><td>LuxR-HTH->wHTH(SBD)+HNH->BrxA-DNA-binding->BrxB-BrxF-iSTAND->DUF6079*->N6-METHYLASE->OLD-ABC->TOPRIM->PglZ->SIGMA+OB-Fold+AAA-ATPase+Lon-Protease-BrxL-><-Arm-DNA-bind_3+Phage_integrase<-tRNA<-tRNA<-?<-URI-DNase+UVR+RNase+HHH<-REC+OmpR-HTH</td><td>1202</td><td>Bacteria>Pseudomonadota>Gammaproteobacteria>Enterobacterales>Pectobacteriaceae>Pectobacterium</td><td>GCF_003382585.2</td><td>Pectobacterium_aquaticum</td><td>DMB81_RS10735</td><td>4900..8508</td><td>2204145</td><td>BREX-system-P-loop-protein-BrxC-[Pectobacterium-aquaticum]</td></tr>
<tr><td>WP_181847885.1</td><td>Type1.BREX</td><td>REC+OmpR-HTH->?->tRNA->tRNA->Arm-DNA-bind_3+Phage_integrase-><-SIGMA+OB-Fold+AAA-ATPase+Lon-Protease-BrxL<-PglZ<-TOPRIM<-OLD-ABC<-N6-METHYLASE<-DUF6079*<-BrxB-BrxF-iSTAND<-BrxA-DNA-binding<-wHTH(SBD)+HNH<-LuxR-HTH<-?<-?<-?<-ZnR<-XIS-HTH</td><td>1204</td><td>Bacteria>Pseudomonadota>Gammaproteobacteria>Enterobacterales>Pectobacteriaceae>Pectobacterium</td><td>GCF_013913645.1</td><td>Pectobacterium_aroidearum</td><td>H2Y54_RS11285</td><td>236602..240216</td><td>1201031</td><td>BREX-system-P-loop-protein-BrxC-[Pectobacterium-aroidearum]</td></tr>
<tr><td>HAT4275166.1</td><td>Type1.BREX</td><td><-Glyco_hydro<-?||SbcC->?->wHTH(SBD)+HNH->BrxA-DNA-binding->BrxB-BrxF-iSTAND->DUF6079*->?->N6-METHYLASE->Cas9+HNH+RuvC->Cas_Cas1->CRISPR_Cas2->PglZ->SIGMA+OB-Fold+AAA-ATPase+Lon-Protease-BrxL-><-SH3+SH3+SH3+SH3+SH3+SH3+SH3</td><td>1221</td><td>Bacteria>Terrabacteria_group>Bacillota>Clostridia>Eubacteriales>Clostridiaceae</td><td>GCA_015933065.1</td><td>Clostridium_perfringens</td><td>I9084_000700</td><td>10095..13760</td><td>1502</td><td>BREX-system-P-loop-protein-BrxC-[Clostridium-perfringens]</td></tr>
<tr><td>EGT3599215.1</td><td>Type1.BREX</td><td><-Glyco_hydro||?-><-?||SbcC->?->wHTH(SBD)+HNH->BrxA-DNA-binding->BrxB-BrxF-iSTAND->DUF6079*->?->N6-METHYLASE->Cas9+HNH+RuvC->Cas_Cas1->CRISPR_Cas2->PglZ->SIGMA+OB-Fold+AAA-ATPase+Lon-Protease-BrxL-><-SH3+SH3+SH3+SH3+SH3+SH3+SH3</td><td>1221</td><td>Bacteria>Terrabacteria_group>Bacillota>Clostridia>Eubacteriales>Clostridiaceae</td><td>GCA_016068655.1</td><td>Clostridium_perfringens</td><td>FT888_03085</td><td>92913..96578</td><td>1502</td><td>BREX-system-P-loop-protein-BrxC-[Clostridium-perfringens]</td></tr>
<tr><td>EHK2362606.1</td><td>Type1.BREX</td><td>SH3+SH3+SH3+SH3+SH3+SH3+SH3-><-SIGMA+OB-Fold+AAA-ATPase+Lon-Protease-BrxL<-PglZ<-CRISPR_Cas2<-Cas_Cas1<-Cas9+HNH+RuvC<-N6-METHYLASE<-DUF6079*<-BrxB-BrxF-iSTAND<-BrxA-DNA-binding<-wHTH(SBD)+HNH<-SLATT<-SLATT_4<-?<-HTH_OrfB_IS605+OrfB_IS605+OrfB_Zn_ribbon</td><td>1221</td><td>Bacteria>Terrabacteria_group>Bacillota>Clostridia>Eubacteriales>Clostridiaceae</td><td>GCA_018011955.1</td><td>Clostridium_perfringens</td><td>KCL45_000419</td><td>449510..453175</td><td>1502</td><td>BREX-system-P-loop-protein-BrxC-[Clostridium-perfringens]</td></tr>
<tr><td>MBS5995886.1</td><td>Type1.BREX</td><td>SH3+SH3+SH3+SH3+SH3+SH3+SH3-><-SIGMA+OB-Fold+AAA-ATPase+Lon-Protease-BrxL<-PglZ<-CRISPR_Cas2<-Cas_Cas1<-Cas9+HNH+RuvC<-N6-METHYLASE<-?<-DUF6079*<-BrxB-BrxF-iSTAND<-BrxA-DNA-binding<-wHTH(SBD)+HNH<-VirB8||?-><-?||Glyco_hydro-><-?<-Amidohydro_1</td><td>1221</td><td>Bacteria>Terrabacteria_group>Bacillota>Clostridia>Eubacteriales>Clostridiaceae</td><td>GCA_018372935.1</td><td>Clostridium_perfringens</td><td>KIB05_13835</td><td>52036..55701</td><td>1502</td><td>BREX-system-P-loop-protein-BrxC-[Clostridium-perfringens]</td></tr>
<tr><td>HCG3022573.1</td><td>Type1.BREX</td><td>SH3+SH3+SH3+SH3+SH3+SH3+SH3-><-SIGMA+OB-Fold+AAA-ATPase+Lon-Protease-BrxL<-PglZ<-CRISPR_Cas2<-Cas_Cas1<-Cas9+HNH+RuvC<-N6-METHYLASE<-?<-DUF6079*<-BrxB-BrxF-iSTAND<-BrxA-DNA-binding<-wHTH(SBD)+HNH<-VirB8||?-><-?||Glyco_hydro-><-?<-Amidohydro_1</td><td>1221</td><td>Bacteria>Terrabacteria_group>Bacillota>Clostridia>Eubacteriales>Clostridiaceae</td><td>GCA_024023955.1</td><td>Clostridium_perfringens</td><td>NJU98_002507</td><td>52406..56071</td><td>1502</td><td>BREX-system-P-loop-protein-BrxC-[Clostridium-perfringens]</td></tr>
<tr><td>EJT5935287.1</td><td>Type1.BREX</td><td><-Cas9+HNH+RuvC<-SF1-SF2-Helicase<-MarR-HTH<-?<-HerA/FtsK<-?<-sGTP<-N6-METHYLASE<-?<-DUF6079*<-BrxB-BrxF-iSTAND<-BrxA-DNA-binding<-wHTH(SBD)+HNH<-?<-VirB8||?-><-?||Glyco_hydro-></td><td>1221</td><td>Bacteria>Terrabacteria_group>Bacillota>Clostridia>Eubacteriales>Clostridiaceae</td><td>GCA_025090725.1</td><td>Clostridium_perfringens</td><td>N2W52_000012</td><td>19602..23267</td><td>1502</td><td>BREX-system-P-loop-protein-BrxC-[Clostridium-perfringens]</td></tr>
<tr><td>EJT6164547.1</td><td>Type1.BREX</td><td><-Glyco_hydro||?-><-?||VirB8->?->wHTH(SBD)+HNH->BrxA-DNA-binding->BrxB-BrxF-iSTAND->DUF6079*->?->N6-METHYLASE->Cas9+HNH+RuvC->Cas_Cas1->CRISPR_Cas2->PglZ->SIGMA+OB-Fold+AAA-ATPase+Lon-Protease-BrxL-><-SH3+SH3+SH3+SH3+SH3+SH3+SH3</td><td>1221</td><td>Bacteria>Terrabacteria_group>Bacillota>Clostridia>Eubacteriales>Clostridiaceae</td><td>GCA_025091785.1</td><td>Clostridium_perfringens</td><td>N2W22_000999</td><td>1099443..1103108</td><td>1502</td><td>BREX-system-P-loop-protein-BrxC-[Clostridium-perfringens]</td></tr>
<tr><td>EJT6169494.1</td><td>Type1.BREX</td><td>SH3+SH3+SH3+SH3+SH3+SH3+SH3-><-SIGMA+OB-Fold+AAA-ATPase+Lon-Protease-BrxL<-PglZ<-CRISPR_Cas2<-Cas_Cas1<-Cas9+HNH+RuvC<-N6-METHYLASE<-?<-DUF6079*<-BrxB-BrxF-iSTAND<-BrxA-DNA-binding<-wHTH(SBD)+HNH<-VirB8||?-><-?||Glyco_hydro-><-?<-Amidohydro_1</td><td>1221</td><td>Bacteria>Terrabacteria_group>Bacillota>Clostridia>Eubacteriales>Clostridiaceae</td><td>GCA_025091845.1</td><td>Clostridium_perfringens</td><td>N2W53_000036</td><td>52483..56148</td><td>1502</td><td>BREX-system-P-loop-protein-BrxC-[Clostridium-perfringens]</td></tr>
<tr><td>EJT6540217.1</td><td>Type1.BREX</td><td>SH3+SH3+SH3+SH3+SH3+SH3+SH3-><-SIGMA+OB-Fold+AAA-ATPase+Lon-Protease-BrxL<-PglZ<-CRISPR_Cas2<-Cas_Cas1<-Cas9+HNH+RuvC<-N6-METHYLASE<-?<-DUF6079*<-BrxB-BrxF-iSTAND<-BrxA-DNA-binding<-wHTH(SBD)+HNH<-VirB8||?-><-?||Glyco_hydro-><-?<-Amidohydro_1</td><td>1221</td><td>Bacteria>Terrabacteria_group>Bacillota>Clostridia>Eubacteriales>Clostridiaceae</td><td>GCA_025095895.1</td><td>Clostridium_perfringens</td><td>N2W31_000036</td><td>52406..56071</td><td>1502</td><td>BREX-system-P-loop-protein-BrxC-[Clostridium-perfringens]</td></tr>
<tr><td>WP_003452047.1</td><td>Type1.BREX</td><td><-Glyco_hydro||?-><-?||VirB8->?->Y1_Tnp->wHTH(SBD)+HNH->BrxA-DNA-binding->BrxB-BrxF-iSTAND->DUF6079*->N6-METHYLASE->HIN-HTH+PAIREDC-HTH->Cas9+HNH+RuvC->Cas_Cas1->CRISPR_Cas2->PglZ->SIGMA+OB-Fold+AAA-ATPase+Lon-Protease-BrxL-><-SH3+SH3+SH3+SH3+SH3+SH3+SH3</td><td>1221</td><td>Bacteria>Terrabacteria_group>Bacillota>Clostridia>Eubacteriales>Clostridiaceae</td><td>GCF_000171135.1</td><td>Clostridium_perfringens</td><td>CPC_RS09775</td><td>28796..32461</td><td>1502</td><td>BREX-system-P-loop-protein-BrxC-[Clostridium-perfringens]</td></tr>
<tr><td>WP_003473497.1</td><td>Type1.BREX</td><td><-Cas_Cas1<-Cas9+HNH+RuvC<-MarR-HTH<-?<-HerA/FtsK<-?<-sGTP<-N6-METHYLASE<-?<-DUF6079*<-BrxB-BrxF-iSTAND<-BrxA-DNA-binding<-wHTH(SBD)+HNH<-?<-SbcC||?-><-?||Glyco_hydro-></td><td>1221</td><td>Bacteria>Terrabacteria_group>Bacillota>Clostridia>Eubacteriales>Clostridiaceae</td><td>GCF_000172455.1</td><td>Clostridium_perfringens</td><td>CJD_RS01695</td><td>75268..78933</td><td>1502</td><td>BREX-system-P-loop-protein-BrxC-[Clostridium-perfringens]</td></tr>
<tr><td>WP_060670433.1</td><td>Type1.BREX</td><td><-Cas_Cas1<-Cas9+HNH+RuvC<-MarR-HTH<-?<-HerA/FtsK<-?<-sGTP<-N6-METHYLASE<-?<-DUF6079*<-BrxB-BrxF-iSTAND<-BrxA-DNA-binding<-wHTH(SBD)+HNH<-?<-SbcC||?-><-?||Glyco_hydro-></td><td>1221</td><td>Bacteria>Terrabacteria_group>Bacillota>Clostridia>Eubacteriales>Clostridiaceae</td><td>GCF_001304735.1</td><td>Clostridium_perfringens</td><td>FORC3_RS07365</td><td>1728066..1731731</td><td>1502</td><td>BREX-system-P-loop-protein-BrxC-[Clostridium-perfringens]</td></tr>
<tr><td>WP_115613438.1</td><td>Type1.BREX</td><td><-Glyco_hydro||?-><-?||SbcC->?->wHTH(SBD)+HNH->BrxA-DNA-binding->BrxB-BrxF-iSTAND->DUF6079*->?->N6-METHYLASE->Cas9+HNH+RuvC->Cas_Cas1->CRISPR_Cas2->PglZ->SIGMA+OB-Fold+AAA-ATPase+Lon-Protease-BrxL-></td><td>1221</td><td>Bacteria>Terrabacteria_group>Bacillota>Clostridia>Eubacteriales>Clostridiaceae</td><td>GCF_016027155.1</td><td>Clostridium_perfringens</td><td>I6G61_RS10175</td><td>2178205..2181870</td><td>1502</td><td>BREX-system-P-loop-protein-BrxC-[Clostridium-perfringens]</td></tr>
<tr><td>WP_198605367.1</td><td>Type1.BREX</td><td><-Glyco_hydro||?-><-?||SbcC->?->wHTH(SBD)+HNH->BrxA-DNA-binding->BrxB-BrxF-iSTAND->DUF6079*->?->N6-METHYLASE->sGTP->?->HerA/FtsK->?->MarR-HTH->Cas_Cas1->CRISPR_Cas2-></td><td>1221</td><td>Bacteria>Terrabacteria_group>Bacillota>Clostridia>Eubacteriales>Clostridiaceae</td><td>GCF_016236125.1</td><td>Clostridium_perfringens</td><td>H8J62_RS02875</td><td>34894..38559</td><td>1502</td><td>BREX-system-P-loop-protein-BrxC-[Clostridium-perfringens]</td></tr>
<tr><td>WP_198621144.1</td><td>Type1.BREX</td><td><-CRISPR_Cas2<-Cas_Cas1<-MarR-HTH<-?<-HerA/FtsK<-?<-sGTP<-N6-METHYLASE<-?<-DUF6079*<-BrxB-BrxF-iSTAND<-BrxA-DNA-binding<-wHTH(SBD)+HNH<-?<-SbcC||?-><-?||Glyco_hydro-></td><td>1221</td><td>Bacteria>Terrabacteria_group>Bacillota>Clostridia>Eubacteriales>Clostridiaceae</td><td>GCF_016236545.1</td><td>Clostridium_perfringens</td><td>H8J92_RS02870</td><td>79478..83143</td><td>1502</td><td>BREX-system-P-loop-protein-BrxC-[Clostridium-perfringens]</td></tr>
<tr><td>WP_198616415.1</td><td>Type1.BREX</td><td><-Glyco_hydro<-?||VirB8->?->wHTH(SBD)+HNH->BrxA-DNA-binding->BrxB-BrxF-iSTAND->DUF6079*->?->N6-METHYLASE->sGTP->?->HerA/FtsK->?->MarR-HTH->Cas_Cas1->CRISPR_Cas2-></td><td>1221</td><td>Bacteria>Terrabacteria_group>Bacillota>Clostridia>Eubacteriales>Clostridiaceae</td><td>GCF_016236635.1</td><td>Clostridium_perfringens</td><td>H8J95_RS08945</td><td>20074..23739</td><td>1502</td><td>BREX-system-P-loop-protein-BrxC-[Clostridium-perfringens]</td></tr>
<tr><td>WP_164804802.1</td><td>Type1.BREX</td><td>Amidohydro_1->?-><-Glyco_hydro||<-?||SbcC->?->wHTH(SBD)+HNH->BrxA-DNA-binding->BrxB-BrxF-iSTAND->DUF6079*->?->N6-METHYLASE->sGTP->?->HerA/FtsK->?->MarR-HTH->Cas_Cas1->CRISPR_Cas2-></td><td>1221</td><td>Bacteria>Terrabacteria_group>Bacillota>Clostridia>Eubacteriales>Clostridiaceae</td><td>GCF_018135545.1</td><td>Clostridium_perfringens</td><td>G6Z22_RS06045</td><td>21931..25596</td><td>1502</td><td>BREX-system-P-loop-protein-BrxC-[Clostridium-perfringens]</td></tr>
<tr><td>WP_198609318.1</td><td>Type1.BREX</td><td>SH3+SH3+SH3+SH3+SH3+SH3+SH3-><-SIGMA+OB-Fold+AAA-ATPase+Lon-Protease-BrxL<-PglZ<-CRISPR_Cas2<-Cas_Cas1<-Cas9+HNH+RuvC<-N6-METHYLASE<-?<-DUF6079*<-BrxB-BrxF-iSTAND<-BrxA-DNA-binding<-wHTH(SBD)+HNH<-?<-SbcC||?-><-?||Glyco_hydro-></td><td>1221</td><td>Bacteria>Terrabacteria_group>Bacillota>Clostridia>Eubacteriales>Clostridiaceae</td><td>GCF_020139275.1</td><td>Clostridium_perfringens</td><td>KLF33_RS07605</td><td>1732833..1736498</td><td>1502</td><td>BREX-system-P-loop-protein-BrxC-[Clostridium-perfringens]</td></tr>
<tr><td>WP_228572337.1</td><td>Type1.BREX</td><td>Amidohydro_1->?-><-Glyco_hydro||<-?||VirB8->?->wHTH(SBD)+HNH->BrxA-DNA-binding->BrxB-BrxF-iSTAND->DUF6079*->?->N6-METHYLASE->Cas_Cas1->CRISPR_Cas2->PglZ->SIGMA+OB-Fold+AAA-ATPase+Lon-Protease-BrxL-><-SH3+SH3+SH3+SH3+SH3+SH3+SH3</td><td>1221</td><td>Bacteria>Terrabacteria_group>Bacillota>Clostridia>Eubacteriales>Clostridiaceae</td><td>GCF_020819925.1</td><td>Clostridium_perfringens</td><td>LMS36_RS03155</td><td>130857..134522</td><td>1502</td><td>BREX-system-P-loop-protein-BrxC-[Clostridium-perfringens]</td></tr>
<tr><td>WP_111744218.1</td><td>Type1.BREX</td><td>SbcC->?->wHTH(SBD)+HNH->BrxA-DNA-binding->BrxB-BrxF-iSTAND->DUF6079*->?->N6-METHYLASE->Cas9+HNH+RuvC->Cas_Cas1->CRISPR_Cas2->PglZ->SIGMA+OB-Fold+AAA-ATPase+Lon-Protease-BrxL-></td><td>1221</td><td>Bacteria>Terrabacteria_group>Bacillota>Clostridia>Eubacteriales>Clostridiaceae</td><td>GCF_900475545.1</td><td>Clostridium_perfringens</td><td>Cp4442_RS12820</td><td>4585..8250</td><td>1502</td><td>BREX-system-P-loop-protein-BrxC-[Clostridium-perfringens]</td></tr>
<tr><td>WP_142710734.1</td><td>Type1.BREX</td><td><-Cas9+HNH+RuvC<-SF1-SF2-Helicase<-MarR-HTH<-?<-HerA/FtsK<-?<-sGTP<-N6-METHYLASE<-?<-DUF6079*<-BrxB-BrxF-iSTAND<-BrxA-DNA-binding<-wHTH(SBD)+HNH<-SLATT<-SLATT_4</td><td>1221</td><td>Bacteria>Terrabacteria_group>Bacillota>Clostridia>Eubacteriales>Clostridiaceae</td><td>GCF_902166005.1</td><td>Clostridium_perfringens</td><td>FN719_RS05040</td><td>228419..232084</td><td>1502</td><td>BREX-system-P-loop-protein-BrxC-[Clostridium-perfringens]</td></tr>
<tr><td>WP_142975592.1</td><td>Type1.BREX</td><td>SH3+SH3+SH3+SH3+SH3+SH3+SH3-><-SIGMA+OB-Fold+AAA-ATPase+Lon-Protease-BrxL<-PglZ<-CRISPR_Cas2<-Cas_Cas1<-Cas9+HNH+RuvC<-N6-METHYLASE<-?<-DUF6079*<-BrxB-BrxF-iSTAND<-BrxA-DNA-binding<-wHTH(SBD)+HNH<-?<-SbcC||?-><-?||Glyco_hydro-></td><td>1221</td><td>Bacteria>Terrabacteria_group>Bacillota>Clostridia>Eubacteriales>Clostridiaceae</td><td>GCF_902166025.1</td><td>Clostridium_perfringens</td><td>FN715_RS03295</td><td>751975..755640</td><td>1502</td><td>BREX-system-P-loop-protein-BrxC-[Clostridium-perfringens]</td></tr>
<tr><td>WP_195381616.1</td><td>Type1.BREX</td><td>Amidohydro_1->?-><-Glyco_hydro<-?||SbcC->?->wHTH(SBD)+HNH->BrxA-DNA-binding->BrxB-BrxF-iSTAND->DUF6079*->?->N6-METHYLASE->Cas_Cas1->CRISPR_Cas2->PglZ->SIGMA+OB-Fold+AAA-ATPase+Lon-Protease-BrxL-><-SH3+SH3+SH3+SH3+SH3+SH3+SH3</td><td>1221</td><td>Bacteria>Terrabacteria_group>Bacillota>Clostridia>Eubacteriales>Clostridiaceae</td><td>GCF_951336195.1</td><td>Clostridium_perfringens</td><td>H8J86_RS06960</td><td>66718..70383</td><td>1502</td><td>BREX-system-P-loop-protein-BrxC-[Clostridium-perfringens]</td></tr>
<tr><td>--</td><td></td><td></td><td></td><td></td><td></td><td></td><td></td><td></td><td></td><td></td></tr>
<tr><td>--HNH</td><td></td><td></td><td></td><td></td><td></td><td></td><td></td><td></td><td></td><td></td></tr>
<tr><td>--</td><td></td><td></td><td></td><td></td><td></td><td></td><td></td><td></td><td></td><td></td></tr>
<tr><td>MBP7797364.1</td><td>Type6.BREX</td><td><-RNase<-ANK+ANK<-REase<-SbcC<-?<-HNH<-SIGMA+OB-Fold+AAA-ATPase+Lon-Protease-BrxL<-PglZ<-N6-METHYLASE<-DUF6079*<-BrxB-BrxF-iSTAND<-BrxA-DNA-binding<-BrxE||?-><-Phage_integrase<-Phage_integrase<-HerA/FtsK<-?<-CHTH</td><td>1177</td><td>Bacteria>Acidobacteriota>Thermoanaerobaculia>Thermoanaerobaculales>Thermoanaerobaculaceae>unclassified_Thermoanaerobaculaceae</td><td>GCA_018000955.1</td><td>Thermoanaerobaculaceae_bacterium</td><td>KA072_01920</td><td>40724..44257</td><td>2699758</td><td>BREX-system-P-loop-protein-BrxC-[Thermoanaerobaculaceae-bacterium]</td></tr>
<tr><td>PKP62156.1</td><td>Type1.BREX</td><td>SbcC+SbcC->BrxA-DNA-binding->BrxB-BrxF-iSTAND->DUF6079*->N6-METHYLASE->PglZ->SIGMA+OB-Fold+AAA-ATPase+Lon-Protease-BrxL->HNH->STYKIN->TPR+TPR->TPR-></td><td>1193</td><td>Bacteria>Atribacterota>unclassified_Atribacterota</td><td>GCA_002841055.1</td><td>Candidatus_Atribacteria_bacterium_HGW-Atribacteria-1</td><td>CVT91_00360</td><td>3425..7006</td><td>2013676</td><td>BREX-system-P-loop-protein-BrxC-[Candidatus-Atribacteria-bacterium-HGW-Atribacteria-1]</td></tr>
<tr><td>MBI2566519.1</td><td>Type1.BREX</td><td>NtrC-AAA+FIS-HTH->?->?-><-?<-?||BrxA-DNA-binding->BrxB-BrxF-iSTAND->DUF6079*->?->N6-METHYLASE->HEPN->ParA-Soj-PloopNTPase->?->PglZ->HNH-><-?<-NtrC-AAA+FIS-HTH</td><td>1109</td><td>Bacteria>Bacteria_incertae_sedis>Bacteria_candidate_phyla>Candidatus_Schekmanbacteria</td><td>GCA_016188575.1</td><td>Candidatus_Schekmanbacteria_bacterium</td><td>HYV63_05760</td><td>102186..105515</td><td>2419984</td><td>BREX-system-P-loop-protein-BrxC-[Candidatus-Schekmanbacteria-bacterium]</td></tr>
<tr><td>MCM1267126.1</td><td>Type1.BREX</td><td><-SbcC<-?<-HNH<-SIGMA+OB-Fold+AAA-ATPase+Lon-Protease-BrxL<-PglZ<-DOC+HTH<-?<-N6-METHYLASE<-DUF6079*<-BrxB-BrxF-iSTAND<-BrxA-DNA-binding<-?<-?<-?<-CHTH</td><td>1173</td><td>Bacteria>FCB_group>Bacteroidetes/Chlorobi_group>Bacteroidota>Bacteroidia>Bacteroidales</td><td>GCA_023665065.1</td><td>Bacteroidales_bacterium</td><td>NC302_04400</td><td>49162..52683</td><td>2030927</td><td>BREX-system-P-loop-protein-BrxC-[Bacteroidales-bacterium]</td></tr>
<tr><td>MCB0517804.1</td><td>Type1.BREX</td><td><-SIGMA+OB-Fold+AAA-ATPase+HKD-DNase-BrxL<-PglZ<-N6-METHYLASE<-DUF6079*<-BrxB-BrxF-iSTAND<-BrxA-DNA-binding<-?<-HNH||REC-><-?<-SF1-SF2-Helicase+RNase+RNase<-?<-RVT</td><td>1174</td><td>Bacteria>FCB_group>Bacteroidetes/Chlorobi_group>Bacteroidota>Saprospiria>Saprospirales</td><td>GCA_020436325.1</td><td>Saprospiraceae_bacterium</td><td>KDD27_02635</td><td>9612..13136</td><td>2202734</td><td>BREX-system-P-loop-protein-BrxC-[Saprospiraceae-bacterium]</td></tr>
<tr><td>WP_147932457.1</td><td>Type1.BREX</td><td>Calcineurin->RADICAL-SAM->METHYLASE+RNA-Helicase->?->?->DnaJ->BrxA-DNA-binding->BrxB-BrxF-iSTAND->DUF6079*->N6-METHYLASE->?->?->HNH->?->URI-DNase->wHTH+AbiEi_2->AbiEii->PglZ-></td><td>1198</td><td>Bacteria>FCB_group>Bacteroidetes/Chlorobi_group>Bacteroidota>Saprospiria>Saprospirales</td><td>GCF_008014675.1</td><td>Neolewinella_aurantiaca</td><td>FUA23_RS19525</td><td>18155..21751</td><td>2602767</td><td>BREX-system-P-loop-protein-BrxC-[Neolewinella-aurantiaca]</td></tr>
<tr><td>WP_098973854.1</td><td>Type1.BREX</td><td>SKI->JAB+sGTP->wHTH+WYL+WCX->?->?->ABC-ATPase+SbcC->?-><-?||DUF6079*->OLD-ABC->TOPRIM->N6-METHYLASE->N6-METHYLASE->MORC+HISKIN->?->HNH->PglZ->SIGMA+OB-Fold+AAA-ATPase+Lon-Protease-BrxL-></td><td>1250</td><td>Bacteria>Fusobacteriota>Fusobacteriia>Fusobacteriales>Fusobacteriaceae>Fusobacterium</td><td>GCF_002591515.1</td><td>Fusobacterium_nucleatum</td><td>CBG58_RS00340</td><td>77641..81393</td><td>851</td><td>BREX-system-P-loop-protein-BrxC-[Fusobacterium-polymorphum]</td></tr>
<tr><td>HCF56393.1</td><td>Type6.BREX</td><td><-Schlafen+HATPase+wHTH<-N6-METHYLASE-p||?->PIN-><-TOPRIM<-OLD-ABC<-DUF6079-p<-APATPase<-DUF6079-p*<-DUF6079-p<-HNH<-BrxB-BrxF-iSTAND<-BrxA-DNA-binding<-BrxA-DNA-binding<-BrxE</td><td>508</td><td>Bacteria>Myxococcota>Myxococcia>Myxococcales>unclassified_Myxococcales</td><td>GCA_003534605.1</td><td>Myxococcales_bacterium</td><td>DFS52_00150</td><td>7726..9251</td><td>2026763</td><td>BREX-system-P-loop-protein-BrxC-[Myxococcales-bacterium]</td></tr>
<tr><td>HCF56394.1</td><td>Type6.BREX</td><td><-Schlafen+HATPase+wHTH<-N6-METHYLASE-p||?->PIN-><-TOPRIM<-OLD-ABC<-DUF6079-p<-APATPase<-DUF6079-p*<-HNH<-BrxB-BrxF-iSTAND<-BrxA-DNA-binding<-BrxA-DNA-binding<-BrxE</td><td>363</td><td>Bacteria>Myxococcota>Myxococcia>Myxococcales>unclassified_Myxococcales</td><td>GCA_003534605.1</td><td>Myxococcales_bacterium</td><td>DFS52_00155</td><td>9470..10559</td><td>2026763</td><td>BREX-system-P-loop-protein-BrxC-[Myxococcales-bacterium]</td></tr>
<tr><td>KFG67132.1</td><td>Type6.BREX</td><td><-AbiGi+seqA+RAMA||BrxE->BrxA-DNA-binding->BrxB-BrxF-iSTAND->DUF6079*->N6-METHYLASE-p->PglZ->SIGMA+OB-Fold+AAA-ATPase+HKD-DNase-BrxL->HNH->HNH->HNH->?-><-HEPN<-Polbeta</td><td>1210</td><td>Bacteria>Pseudomonadota>Alphaproteobacteria>Hyphomicrobiales>Methylobacteriaceae>Microvirga</td><td>GCA_000739015.1</td><td>Microvirga_sp._BSC39</td><td>JH26_23850</td><td>105316..108948</td><td>1549810</td><td>ATPase-[Microvirga-sp.-BSC39]</td></tr>
<tr><td>WP_036357316.1</td><td>Type6.BREX</td><td><-AbiGi+seqA+RAMA<-?||PhdYeFM_antitox->BrxE->BrxA-DNA-binding->BrxB-BrxF-iSTAND->DUF6079*->N6-METHYLASE->PglZ->SIGMA+OB-Fold+AAA-ATPase+HKD-DNase-BrxL->HNH->HNH->HNH->?-><-HEPN<-Polbeta</td><td>1208</td><td>Bacteria>Pseudomonadota>Alphaproteobacteria>Hyphomicrobiales>Methylobacteriaceae>Microvirga</td><td>GCF_000739015.1</td><td>Microvirga_sp._BSC39</td><td>MA07_RS22775</td><td>105322..108948</td><td>1549810</td><td>BREX-system-P-loop-protein-BrxC-[Microvirga-sp.-BSC39]</td></tr>
<tr><td>MBK8578271.1</td><td>Type1.BREX</td><td><-SIGMA+OB-Fold+AAA-ATPase+Lon-Protease-BrxL<-PglZ<-N6-METHYLASE<-HNH<-?<-SbcC+NACHT<-DUF6079*<-BrxB-BrxF-iSTAND<-BrxA-DNA-binding<-BrxA-DNA-binding<-Polbeta<-wHTH+AbiEi_2<-?||?-><-wHTH+AbiEi_2</td><td>1154</td><td>Bacteria>Pseudomonadota>Betaproteobacteria>Betaproteobacteria_incertae_sedis>Candidatus_Accumulibacter>unclassified_Candidatus_Accumulibacter</td><td>GCA_016714935.1</td><td>Candidatus_Accumulibacter_sp.</td><td>IPN78_08405</td><td>116670..120134</td><td>2053492</td><td>BREX-system-P-loop-protein-BrxC-[Candidatus-Accumulibacter-sp.]</td></tr>
<tr><td>ABO55019.1</td><td>Type1.BREX</td><td><-HNH<-SIGMA+OB-Fold+AAA-ATPase+Lon-Protease-BrxL<-PglZ<-HTH_29+HTH_32+HTH_33+DDE_3||HIN-HTH+PAIREDC-HTH-><-DUF6079*<-BrxB-BrxF-iSTAND<-BrxA-DNA-binding||?-><-ANK-><-?||ANK+ANK->?->?-><-ANK</td><td>1210</td><td>Bacteria>Pseudomonadota>Betaproteobacteria>Burkholderiales>Burkholderiaceae>Burkholderia</td><td>GCA_000016205.1</td><td>Burkholderia_vietnamiensis_G4</td><td>Bcep1808_2017</td><td>2211669..2215301</td><td>269482</td><td>ATPase-like-protein-[Burkholderia-vietnamiensis-G4]</td></tr>
<tr><td>WP_013697442.1</td><td>Type1.BREX</td><td>ZnR+TOPRIM+AAA-helicase+wHTH->?->?->?->SAM-METHYLASE->ACET-><-ANK+ANK||BrxA-DNA-binding->BrxB-BrxF-iSTAND->DUF6079*->N6-METHYLASE->PglZ->SIGMA+OB-Fold+AAA-ATPase+Lon-Protease-BrxL->HNH-><-?||ANK-><-?||wHTH(SBD)+HNH-><-REase</td><td>1223</td><td>Bacteria>Pseudomonadota>Betaproteobacteria>Burkholderiales>Burkholderiaceae>Burkholderia</td><td>GCF_000194745.1</td><td>Burkholderia_gladioli</td><td>BGLA_RS07070</td><td>1576041..1579712</td><td>28095</td><td>BREX-system-P-loop-protein-BrxC-[Burkholderia-gladioli]</td></tr>
<tr><td>WP_124831269.1</td><td>Type1.BREX</td><td><-MORC||?-><-HNH<-SIGMA+OB-Fold+AAA-ATPase+Lon-Protease-BrxL<-PglZ<-N6-METHYLASE<-DUF6079*<-BrxB-BrxF-iSTAND<-BrxA-DNA-binding||?-><-?||?-><-?<-?<-REase<-Phage_GPD+Phage_base_V+T6SS_Vgr+DUF2345</td><td>1200</td><td>Bacteria>Pseudomonadota>Betaproteobacteria>Burkholderiales>Burkholderiaceae>Burkholderia</td><td>GCF_003857875.1</td><td>Burkholderia_stagnalis</td><td>DF113_RS32940</td><td>12280..15882</td><td>1503054</td><td>BREX-system-P-loop-protein-BrxC-[Burkholderia-stagnalis]</td></tr>
<tr><td>WP_122833867.1</td><td>Type1.BREX</td><td>ANK-><-?<-?<-?<-ANK+ANK||?-><-ANK||BrxA-DNA-binding->BrxB-BrxF-iSTAND->DUF6079*->N6-METHYLASE->PglZ->SIGMA+OB-Fold+AAA-ATPase+Lon-Protease-BrxL->HNH-></td><td>1223</td><td>Bacteria>Pseudomonadota>Betaproteobacteria>Burkholderiales>Burkholderiaceae>Burkholderia</td><td>GCF_900597175.1</td><td>Burkholderia_pseudomallei</td><td>EFT75_RS19485</td><td>195449..199120</td><td>28450</td><td>BREX-system-P-loop-protein-BrxC-[Burkholderia-pseudomallei]</td></tr>
<tr><td>WP_121308231.1</td><td>Type1.BREX</td><td><-MORC<-?||?->?-><-HNH<-SIGMA+OB-Fold+AAA-ATPase+Lon-Protease-BrxL<-PglZ<-N6-METHYLASE<-DUF6079*<-BrxB-BrxF-iSTAND<-BrxA-DNA-binding<-?||?->wHTH+WYL+WCX->?->?-><-?||ANK+ANK-></td><td>1204</td><td>Bacteria>Pseudomonadota>Betaproteobacteria>Burkholderiales>Burkholderiaceae>Paraburkholderia</td><td>GCF_003634175.1</td><td>Paraburkholderia_sp._BL17N1</td><td>BOG82_RS17815</td><td>3937370..3940984</td><td>1938798</td><td>BREX-system-P-loop-protein-BrxC-[Paraburkholderia-sp.-BL17N1]</td></tr>
<tr><td>WP_201659285.1</td><td>Type1.BREX</td><td>SbcC->HTH_3-><-NACHT<-HNH<-SIGMA+OB-Fold+AAA-ATPase+Lon-Protease-BrxL<-PglZ<-N6-METHYLASE<-DUF6079*<-BrxB-BrxF-iSTAND<-BrxA-DNA-binding||PhdYeFM_antitox->RelE-ParE-><-REC<-REC+NtrC-AAA+FIS-HTH||?-><-GGDEF||REC+REC-></td><td>1218</td><td>Bacteria>Pseudomonadota>Betaproteobacteria>Burkholderiales>Burkholderiaceae>Paraburkholderia</td><td>GCF_904848645.1</td><td>Paraburkholderia_sabiae</td><td>JMY25_RS35995</td><td>30018..33674</td><td>273251</td><td>BREX-system-P-loop-protein-BrxC-[Paraburkholderia-sabiae]</td></tr>
<tr><td>WP_094097616.1</td><td>Type1.BREX</td><td><-Peptidase_C39_2<-?||?-><-?||XIS-HTH->Phage_X->BrxA-DNA-binding->BrxB-BrxF-iSTAND->DUF6079*->HNH->HEPN->ParD_antitoxin->RelE-ParE->N6-METHYLASE->OLD-ABC->TOPRIM->ZnR+TOPRIM->PglZ-></td><td>1214</td><td>Bacteria>Pseudomonadota>Betaproteobacteria>Burkholderiales>Comamonadaceae>Acidovorax</td><td>GCF_002157145.1</td><td>Acidovorax_carolinensis</td><td>CBP34_RS06480</td><td>1379414..1383058</td><td>553814</td><td>BREX-system-P-loop-protein-BrxC-[Acidovorax-carolinensis]</td></tr>
<tr><td>WP_086912102.1</td><td>Type1.BREX</td><td>HTH_Tnp_1+HTH_21+rve+rve_3-><-PIN<-MazE_antitoxin||?-><-HEPN<-HNH<-DUF6079*<-BrxB-BrxF-iSTAND<-BrxA-DNA-binding<-GNTR-HTH||PBPI-></td><td>1213</td><td>Bacteria>Pseudomonadota>Betaproteobacteria>Burkholderiales>Comamonadaceae>Acidovorax</td><td>GCF_002157165.1</td><td>Acidovorax_carolinensis</td><td>CBP33_RS08115</td><td>1704915..1708556</td><td>553814</td><td>BREX-system-P-loop-protein-BrxC-[Acidovorax-carolinensis]</td></tr>
<tr><td>GAO25248.1</td><td>Type1.BREX</td><td>HTH_3-><-SIGMA+OB-Fold+AAA-ATPase+Lon-Protease-BrxL<-PglZ<-ZnR+TOPRIM<-TOPRIM<-OLD-ABC<-N6-METHYLASE<-HEPN<-HNH<-DUF6079*<-BrxB-BrxF-iSTAND<-BrxA-DNA-binding<-HAD+CHTH<-CHTH||?->AAA-ATPase-p->?-><-AAA-ATPase</td><td>1205</td><td>Bacteria>Pseudomonadota>Betaproteobacteria>Burkholderiales>Comamonadaceae>Alicycliphilus</td><td>GCA_000974645.1</td><td>Alicycliphilus_sp._B1</td><td>ALISP_5068</td><td>1538304..1541921</td><td>1603291</td><td>ATPase-like-protein-[Alicycliphilus-sp.-B1]</td></tr>
<tr><td>WP_172207178.1</td><td>Type1.BREX</td><td><-OLD-ABC||<-N6-METHYLASE-p<-RelE-ParE<-ParD_antitoxin<-PIN<-MazE_antitoxin||?-><-HEPN<-HNH<-DUF6079*<-BrxB-BrxF-iSTAND<-BrxA-DNA-binding<-Phage_X<-XIS-HTH||?-><-?||?->Peptidase_C39_2-></td><td>1213</td><td>Bacteria>Pseudomonadota>Betaproteobacteria>Burkholderiales>Comamonadaceae>Diaphorobacter</td><td>GCF_013154915.1</td><td>Diaphorobacter_sp._JS3050</td><td>HND92_RS11875</td><td>2478331..2481972</td><td>2735554</td><td>BREX-system-P-loop-protein-BrxC-[Diaphorobacter-sp.-JS3050]</td></tr>
<tr><td>MCG9118882.1</td><td>Type1.BREX</td><td><-SIGMA+OB-Fold+AAA-ATPase+Lon-Protease-BrxL<-PglZ<-HNH<-?<-?<-N6-METHYLASE<-?<-DUF6079*<-BrxB-BrxF-iSTAND<-BrxA-DNA-binding<-wHTH+WYL+WCX<-?<-?<-?||?->HTH_Tnp_1+HTH_21+rve+rve_3->Phage_int_SAM_1+Phage_integrase-></td><td>1221</td><td>Bacteria>Pseudomonadota>Betaproteobacteria>Neisseriales>Chromobacteriaceae>Laribacter</td><td>GCA_022394685.1</td><td>Laribacter_hongkongensis</td><td>LH456_09760</td><td>59894..63559</td><td>168471</td><td>BREX-system-P-loop-protein-BrxC-[Laribacter-hongkongensis]</td></tr>
<tr><td>WP_275667170.1</td><td>Type1.BREX</td><td><-SIGMA+OB-Fold+AAA-ATPase+Lon-Protease-BrxL<-PglZ<-HNH<-?<-?<-N6-METHYLASE<-?<-DUF6079*<-BrxB-BrxF-iSTAND<-BrxA-DNA-binding<-wHTH+WYL+WCX<-?<-?<-?||?->HTH_Tnp_1+HTH_21+rve+rve_3->Phage_int_SAM_1+Phage_integrase-></td><td>1216</td><td>Bacteria>Pseudomonadota>Betaproteobacteria>Neisseriales>Chromobacteriaceae>Laribacter</td><td>GCA_022394685.1</td><td>Laribacter_hongkongensis</td><td>LH456_RS09740</td><td>59894..63544</td><td>168471</td><td>BREX-system-P-loop-protein-BrxC-[Laribacter-hongkongensis]</td></tr>
<tr><td>WP_239901116.1</td><td>Type1.BREX</td><td><-SIGMA+OB-Fold+AAA-ATPase+Lon-Protease-BrxL<-PglZ<-HNH<-?<-?<-N6-METHYLASE<-?<-DUF6079*<-BrxB-BrxF-iSTAND<-BrxA-DNA-binding<-wHTH+WYL+WCX<-?<-?<-?||?->HTH_Tnp_1+HTH_21+rve+rve_3->Phage_int_SAM_1+Phage_integrase-></td><td>1221</td><td>Bacteria>Pseudomonadota>Betaproteobacteria>Neisseriales>Chromobacteriaceae>Laribacter</td><td>-</td><td>Laribacter_hongkongensis</td><td>LH456_09760</td><td>59894..63559</td><td>168471</td><td>BREX-system-P-loop-protein-BrxC-[Laribacter-hongkongensis]</td></tr>
<tr><td>PZQ93954.1</td><td>Type1.BREX</td><td><-HNH<-DUF6079*<-BrxB-BrxF-iSTAND||HIN-HTH->IstB_IS21-><-HTH_Tnp_1+HTH_21+rve+rve_3</td><td>1218</td><td>Bacteria>Pseudomonadota>Gammaproteobacteria>Aeromonadales>Aeromonadaceae>Aeromonas</td><td>GCA_003243215.1</td><td>Aeromonas_media</td><td>DI541_18955</td><td>3274..6930</td><td>651</td><td>BREX-system-P-loop-protein-BrxC-[Aeromonas-media]</td></tr>
<tr><td>GJA32139.1</td><td>Type1.BREX</td><td><-Peptidase_C39_2<-?<-N6-METHYLASE-p<-HEPN<-N6-METHYLASE-p||DDE_Tnp_1_assoc+DDE_Tnp_1-><-N6-METHYLASE<-?<-HNH<-DUF6079*<-BrxB-BrxF-iSTAND<-BrxA-DNA-binding<-RHS<-?<-Ntox49||wHTH+WYL+WCX-><-XIS-HTH||?-><-HTH_26</td><td>1227</td><td>Bacteria>Pseudomonadota>Gammaproteobacteria>Aeromonadales>Aeromonadaceae>Aeromonas</td><td>GCA_019971155.1</td><td>Aeromonas_caviae</td><td>KAM347_17720</td><td>32751..36434</td><td>648</td><td>hypothetical-protein-KAM341_18170-[Aeromonas-caviae]</td></tr>
<tr><td>WP_059169685.1</td><td>Type1.BREX</td><td><-PglZ<-N6-METHYLASE-p<-HEPN<-RVT<-N6-METHYLASE<-?<-HNH<-TOPRIM<-OLD-ABC<-DUF6079*<-BrxB-BrxF-iSTAND<-BrxA-DNA-binding<-RHS<-ARG<-RHS||LexA-protease->?-><-HDRnase<-Arm-DNA-bind_2+Phage_int_SAM_3+Phage_integrase</td><td>1240</td><td>Bacteria>Pseudomonadota>Gammaproteobacteria>Aeromonadales>Aeromonadaceae>Aeromonas</td><td>GCF_001481535.2</td><td>Aeromonas_salmonicida</td><td>VO70_RS19205</td><td>18975..22697</td><td>645</td><td>BREX-system-P-loop-protein-BrxC-[Aeromonas-salmonicida]</td></tr>
<tr><td>WP_210557479.1</td><td>Type1.BREX</td><td>DEDD_Tnp_IS110+Transposase_20->||BrxA-DNA-binding->BrxB-BrxF-iSTAND->DUF6079*->HNH->?->N6-METHYLASE->N6-METHYLASE-p->HEPN->N6-METHYLASE-p-></td><td>1218</td><td>Bacteria>Pseudomonadota>Gammaproteobacteria>Aeromonadales>Aeromonadaceae>Aeromonas</td><td>GCF_017939245.1</td><td>Aeromonas_sp._PrichA-15</td><td>J9889_RS17255</td><td>4362..8018</td><td>2823360</td><td>BREX-system-P-loop-protein-BrxC-[Aeromonas-sp.-PrichA-15]</td></tr>
<tr><td>WP_270805301.1</td><td>Type1.BREX</td><td><-N6-METHYLASE-p<-HEPN<-N6-METHYLASE-p<-N6-METHYLASE<-?<-HNH<-TOPRIM<-OLD-ABC<-DUF6079*<-BrxB-BrxF-iSTAND<-BrxA-DNA-binding<-RHS<-ZnR</td><td>1240</td><td>Bacteria>Pseudomonadota>Gammaproteobacteria>Aeromonadales>Aeromonadaceae>Aeromonas</td><td>GCF_027705505.1</td><td>Aeromonas_sp._QDB56</td><td>OKB81_RS14905</td><td>8131..11853</td><td>2990495</td><td>BREX-system-P-loop-protein-BrxC-[Aeromonas-sp.-QDB56]</td></tr>
<tr><td>WP_182788683.1</td><td>Type1.BREX</td><td>HDRnase-><-?<-LexA-protease||RHS->ARG->ZnR->RHS->BrxA-DNA-binding->BrxB-BrxF-iSTAND->DUF6079*->OLD-ABC->TOPRIM->HNH->?->N6-METHYLASE->SIR2->HerA/FtsK->N6-METHYLASE-p-></td><td>1236</td><td>Bacteria>Pseudomonadota>Gammaproteobacteria>Aeromonadales>Aeromonadaceae</td><td>GCF_014161425.1</td><td>Aeromonas</td><td>H7K58_RS09750</td><td>2083810..2087520</td><td>642</td><td>MULTISPECIES:-BREX-system-P-loop-protein-BrxC-[Aeromonas]</td></tr>
<tr><td>WP_223915718.1</td><td>Type1.BREX</td><td><-Peptidase_C39_2<-?<-N6-METHYLASE-p<-HEPN<-N6-METHYLASE-p||DDE_Tnp_1_assoc+DDE_Tnp_1-><-N6-METHYLASE<-?<-HNH<-DUF6079*<-BrxB-BrxF-iSTAND<-BrxA-DNA-binding<-RHS<-?<-Ntox49||wHTH+WYL+WCX-><-XIS-HTH||?-><-HTH_26</td><td>1218</td><td>Bacteria>Pseudomonadota>Gammaproteobacteria>Aeromonadales>Aeromonadaceae</td><td>GCF_019972515.1</td><td>Aeromonas</td><td>LDN24_RS09390</td><td>22833..26489</td><td>642</td><td>MULTISPECIES:-BREX-system-P-loop-protein-BrxC-[Aeromonas]</td></tr>
<tr><td>MBO2546554.1</td><td>Type1.BREX</td><td><-SIGMA+OB-Fold+AAA-ATPase+Lon-Protease-BrxL<-PglZ<-?<-N6-METHYLASE-p<-RVT<-N6-METHYLASE<-?<-HNH<-DUF6079*<-BrxB-BrxF-iSTAND<-BrxA-DNA-binding<-wHTH+WYL+WCX<-?<-?<-XIS-HTH<-XIS-HTH<-?<-Arm-DNA-bind_3+Phage_integrase</td><td>1225</td><td>Bacteria>Pseudomonadota>Gammaproteobacteria>Alteromonadales>Shewanellaceae>Shewanella</td><td>GCA_017580215.1</td><td>Shewanella_algae</td><td>I6M36_02045</td><td>117246..120923</td><td>38313</td><td>BREX-system-P-loop-protein-BrxC-[Shewanella-algae]</td></tr>
<tr><td>MCD8561045.1</td><td>Type1.BREX</td><td><-N6-METHYLASE-p<-HEPN<-N6-METHYLASE-p<-N6-METHYLASE<-?<-HNH<-DUF6079*<-BrxB-BrxF-iSTAND<-BrxA-DNA-binding<-wHTH+WYL+WCX<-XIS-HTH</td><td>1231</td><td>Bacteria>Pseudomonadota>Gammaproteobacteria>Alteromonadales>Shewanellaceae>Shewanella</td><td>GCA_021209305.1</td><td>Shewanella_xiamenensis</td><td>LRY75_19995</td><td>6155..9850</td><td>332186</td><td>BREX-system-P-loop-protein-BrxC-[Shewanella-xiamenensis]</td></tr>
<tr><td>WP_244159705.1</td><td>Type1.BREX</td><td><-HDRnase<-DDE_Tnp_1<-Arm-DNA-bind_2+Phage_int_SAM_3+Phage_integrase<-Phage_AlpA||HTH_26->wHTH+WYL+WCX->BrxA-DNA-binding->BrxB-BrxF-iSTAND->DUF6079*->HNH->?->N6-METHYLASE->N6-METHYLASE-p->HEPN->N6-METHYLASE-p->MULE-TRANSPOSASE->N6-METHYLASE-p-></td><td>1254</td><td>Bacteria>Pseudomonadota>Gammaproteobacteria>Alteromonadales>Shewanellaceae>Shewanella</td><td>GCF_001723195.1</td><td>Shewanella_xiamenensis</td><td>ABT47_RS18205</td><td>4085889..4089653</td><td>332186</td><td>BREX-system-P-loop-protein-BrxC-[Shewanella-xiamenensis]</td></tr>
<tr><td>WP_069454895.1</td><td>Type1.BREX</td><td><-HDRnase<-Arm-DNA-bind_2+Phage_int_SAM_3+Phage_integrase<-Phage_AlpA||HTH_26->wHTH+WYL+WCX->BrxA-DNA-binding->BrxB-BrxF-iSTAND->DUF6079*->HNH->?->N6-METHYLASE->N6-METHYLASE-p->HEPN->N6-METHYLASE-p->MULE-TRANSPOSASE->N6-METHYLASE-p-></td><td>1240</td><td>Bacteria>Pseudomonadota>Gammaproteobacteria>Alteromonadales>Shewanellaceae>Shewanella</td><td>GCF_002074855.1</td><td>Shewanella_xiamenensis</td><td>VO00_RS12940</td><td>4702..8424</td><td>332186</td><td>BREX-system-P-loop-protein-BrxC-[Shewanella-xiamenensis]</td></tr>
<tr><td>WP_107947297.1</td><td>Type1.BREX</td><td><-Phage_integrase<-DUF2787<-DUF2787<-CbeA_antitoxin<-?<-?||wHTH+WYL+WCX->BrxA-DNA-binding->BrxB-BrxF-iSTAND->DUF6079*->HNH->?->N6-METHYLASE->SIR2->HerA/FtsK->N6-METHYLASE-p->PglZ->SIGMA+OB-Fold+AAA-ATPase+Lon-Protease-BrxL-></td><td>1246</td><td>Bacteria>Pseudomonadota>Gammaproteobacteria>Alteromonadales>Shewanellaceae>Shewanella</td><td>GCF_003052765.1</td><td>Shewanella_baltica</td><td>C7T95_RS09475</td><td>2180761..2184501</td><td>62322</td><td>BREX-system-P-loop-protein-BrxC-[Shewanella-baltica]</td></tr>
<tr><td>WP_173294709.1</td><td>Type1.BREX</td><td>wHTH(SBD)+HNH->BrxA-DNA-binding->BrxB-BrxF-iSTAND->DUF6079*->N6-METHYLASE->PglZ-><-SF1-SF2-Helicase+REase<-SF1-SF2-Helicase+REase||?-><-PAIREDC-HTH</td><td>1157</td><td>Bacteria>Pseudomonadota>Gammaproteobacteria>Alteromonadales>Shewanellaceae>Shewanella</td><td>GCF_013248905.1</td><td>Shewanella_sp._VB17</td><td>HQQ94_RS12350</td><td>2876249..2879722</td><td>2739432</td><td>BREX-system-P-loop-protein-BrxC-[Shewanella-sp.-VB17]</td></tr>
<tr><td>WP_243891830.1</td><td>Type1.BREX</td><td><-SIGMA+OB-Fold+AAA-ATPase+Lon-Protease-BrxL<-PglZ<-?<-N6-METHYLASE-p<-RVT<-N6-METHYLASE<-?<-HNH<-DUF6079*<-BrxB-BrxF-iSTAND<-BrxA-DNA-binding<-wHTH+WYL+WCX<-?<-?<-XIS-HTH<-?<-Arm-DNA-bind_3+Phage_integrase</td><td>1226</td><td>Bacteria>Pseudomonadota>Gammaproteobacteria>Alteromonadales>Shewanellaceae>Shewanella</td><td>GCF_017580215.1</td><td>Shewanella_algae</td><td>I6M36_RS02045</td><td>117246..120926</td><td>38313</td><td>BREX-system-P-loop-protein-BrxC-[Shewanella-algae]</td></tr>
<tr><td>WP_219012197.1</td><td>Type6.BREX</td><td>Arm-DNA-bind_3+Phage_integrase-><-HTH_3||?-><-SF1-SF2-Helicase<-REase||HNH-><-SIGMA+OB-Fold+AAA-ATPase+HKD-DNase-BrxL<-PglZ<-N6-METHYLASE<-DUF6079*<-BrxB-BrxF-iSTAND<-BrxA-DNA-binding<-BrxE<-XIS-HTH||?->?->DUF2787->Phage_integrase-></td><td>1211</td><td>Bacteria>Pseudomonadota>Gammaproteobacteria>Alteromonadales>Shewanellaceae>Shewanella</td><td>GCF_019303235.1</td><td>Shewanella_algae</td><td>KOE59_RS06910</td><td>134402..138037</td><td>38313</td><td>BREX-system-P-loop-protein-BrxC-[Shewanella-algae]</td></tr>
<tr><td>WP_263171306.1</td><td>Type1.BREX</td><td><-Phage_integrase<-DUF2787<-DUF2787<-CbeA_antitoxin<-?<-?||wHTH+WYL+WCX->BrxA-DNA-binding->BrxB-BrxF-iSTAND->DUF6079*->HNH->?->N6-METHYLASE->SIR2->HerA/FtsK->N6-METHYLASE-p->PglZ->SIGMA+OB-Fold+AAA-ATPase+Lon-Protease-BrxL-></td><td>1235</td><td>Bacteria>Pseudomonadota>Gammaproteobacteria>Alteromonadales>Shewanellaceae>Shewanella</td><td>GCF_025653795.1</td><td>Shewanella_sp._SM73</td><td>L5M51_RS01900</td><td>416485..420192</td><td>2912806</td><td>BREX-system-P-loop-protein-BrxC-[Shewanella-sp.-SM73]</td></tr>
<tr><td>WP_263197229.1</td><td>Type1.BREX</td><td><-SIGMA+OB-Fold+AAA-ATPase+Lon-Protease-BrxL<-PglZ<-N6-METHYLASE-p<-HEPN<-N6-METHYLASE-p<-N6-METHYLASE<-?<-HNH<-DUF6079*<-BrxB-BrxF-iSTAND<-BrxA-DNA-binding<-wHTH+WYL+WCX<-HTH_26||Phage_AlpA->Arm-DNA-bind_2+Phage_int_SAM_3+Phage_integrase->HDRnase-></td><td>1246</td><td>Bacteria>Pseudomonadota>Gammaproteobacteria>Alteromonadales>Shewanellaceae>Shewanella</td><td>GCF_025654545.1</td><td>Shewanella_sp._SM20</td><td>L5M18_RS13095</td><td>42119..45859</td><td>2912792</td><td>BREX-system-P-loop-protein-BrxC-[Shewanella-sp.-SM20]</td></tr>
<tr><td>WP_132582321.1</td><td>Type6.BREX</td><td>Phage_integrase->Phage_integrase->?->HerA/FtsK->TrwC+SF1-SF2-Helicase->?->BrxE->BrxA-DNA-binding->BrxB-BrxF-iSTAND->DUF6079*->N6-METHYLASE->PglZ->SIGMA+OB-Fold+AAA-ATPase+HKD-DNase-BrxL-><-HNH||?->MarR-HTH->AbiEii-></td><td>1208</td><td>Bacteria>Pseudomonadota>Gammaproteobacteria>Chromatiales>Chromatiaceae>Rheinheimera</td><td>GCF_004345025.1</td><td>Rheinheimera_sp._D18</td><td>E0Z06_RS04605</td><td>954479..958105</td><td>2545632</td><td>BREX-system-P-loop-protein-BrxC-[Rheinheimera-sp.-D18]</td></tr>
<tr><td>WP_099719181.1</td><td>Type1.BREX</td><td>nSTAND1+HNH->RNaseH->NACHT+SF1-SF2-Helicase+HKD-DNase->REase-><-HKD-DNase+OGG-like<-SIGMA+OB-Fold+AAA-ATPase+Lon-Protease-BrxL<-PglZ<-N6-METHYLASE<-DUF6079*<-BrxB-BrxF-iSTAND<-BrxA-DNA-binding<-?<-HNH<-ACET||?-><-DUF2754</td><td>1213</td><td>Bacteria>Pseudomonadota>Gammaproteobacteria>Enterobacterales>Enterobacteriaceae>Klebsiella/Raoultella_group</td><td>GCF_018137755.1</td><td>Klebsiella_pneumoniae</td><td>KFB18_RS20840</td><td>34480..38121</td><td>573</td><td>BREX-system-P-loop-protein-BrxC-[Klebsiella-pneumoniae]</td></tr>
<tr><td>WP_261139451.1</td><td>Type1.BREX</td><td>DOC+HTH-><-LACI-HTH+PBPI||?->Glyco_hydro_1->?->SAD+HNH->BrxA-DNA-binding->BrxB-BrxF-iSTAND->DUF6079*->PglZ->SIGMA+OB-Fold+AAA-ATPase+Lon-Protease-BrxL->HKD-DNase+OGG-like-><-Couple_hipA+HipA_C<-HTH_3<-?||HIN-HTH-><-HTH_21+rve+rve_3<-rve</td><td>1213</td><td>Bacteria>Pseudomonadota>Gammaproteobacteria>Enterobacterales>Yersiniaceae>Serratia</td><td>GCF_946226675.1</td><td>Serratia_liquefaciens</td><td>N8M82_RS16050</td><td>322435..326076</td><td>614</td><td>BREX-system-P-loop-protein-BrxC-[Serratia-liquefaciens]</td></tr>
</table>

Protein-ID	System	Gene-Neighbourhoods	Length	Taxonomy	Assembly-ID	Strain	Locus-Tag	Genomic-coordinates	TaxID	Fasta-defline
WP_261135874.1	Type1.BREX	DOC+HTH-><-LACI-HTH+PBPI||?->Glyco_hydro_1->?-> SAD+ HNH -> BrxA-DNA-binding -> BrxB-BrxF-iSTAND -> DUF6079 *-> N6-METHYLASE -> PglZ -> SIGMA+OB-Fold+AAA-ATPase+Lon-Protease-BrxL ->HKD-DNase+OGG-like-><-Couple_hipA+HipA_C<-HTH_3	1213	Bacteria>Pseudomonadota>Gammaproteobacteria>Enterobacterales>Yersiniaceae>Serratia	GCF_946226775.1	Serratia_liquefaciens	N8M94_RS14775	322685..326326	614	BREX-system-P-loop-protein-BrxC-[Serratia-liquefaciens]
WP_261427666.1	Type1.BREX	Glyco_hydro_1-><-?<-?||?->?-> SAD+ HNH -> BrxA-DNA-binding -> BrxB-BrxF-iSTAND -> DUF6079 *-> N6-METHYLASE -> PglZ -> SIGMA+OB-Fold+AAA-ATPase+Lon-Protease-BrxL ->HKD-DNase+OGG-like-><-Couple_hipA+HipA_C<-HTH_3	1213	Bacteria>Pseudomonadota>Gammaproteobacteria>Enterobacterales>Yersiniaceae>Serratia	GCF_946227925.1	Serratia_proteamaculans	OCH17_RS14295	357572..361213	28151	BREX-system-P-loop-protein-BrxC-[Serratia-proteamaculans]
WP_104852217.1	Type1.BREX	<-AAA-ATPase<-HIN-HTH||DDE_Tnp_1_3-><-?<-?||HIN-HTH-><- PglZ <- N6-METHYLASE <- DUF6079 *<- BrxB-BrxF-iSTAND <- BrxA-DNA-binding <- wHTH(SBD)+ HNH <-SAM-METHYLASE<-XIS-HTH<-?<-?<-DUF932	1247	Bacteria>Pseudomonadota>Gammaproteobacteria>Moraxellales>Moraxellaceae>Acinetobacter	GCF_002946595.1	Acinetobacter_sp._SWBY1	C3Y93_RS04635	951564..955307	2079596	BREX-system-P-loop-protein-BrxC-[Acinetobacter-sp.-SWBY1]
WP_201567145.1	Type1.BREX	<-REase<- wHTH+WYL+WCX <-?||?->SIR2-><- SIGMA+OB-Fold+AAA-ATPase+Lon-Protease-BrxL <- PglZ <- N6-METHYLASE <- N6-METHYLASE <- DUF6079 *<- BrxB-BrxF-iSTAND <- BrxA-DNA-binding <- wHTH(SBD)+ HNH <-XIS-HTH||35exo-><-?<-HTH_Tnp_1+HTH_21+rve+rve_3	1221	Bacteria>Pseudomonadota>Gammaproteobacteria>Moraxellales>Moraxellaceae>Psychrobacter	GCF_904846115.1	Psychrobacter_immobilis	JMW93_RS11045	31356..35021	498	BREX-system-P-loop-protein-BrxC-[Psychrobacter-immobilis]
WP_201591398.1	Type1.BREX	Arm-DNA-bind_3+Phage_integrase->Phage_integrase-><- HNH <-?<-?<- SIGMA+OB-Fold+AAA-ATPase+Lon-Protease-BrxL <- PglZ <- N6-METHYLASE <- DUF6079 *<- BrxB-BrxF-iSTAND <- BrxA-DNA-binding <-ZnR||REase->REase-><-?<-?<-GNTR-HTH	1221	Bacteria>Pseudomonadota>Gammaproteobacteria>Moraxellales>Moraxellaceae>Psychrobacter	GCF_904846465.1	Psychrobacter_pulmonis	JMX70_RS09025	249655..253320	228654	BREX-system-P-loop-protein-BrxC-[Psychrobacter-pulmonis]
NRB78692.1	Type1.BREX	<-sGTP<-sGTP<-?<- SIGMA+OB-Fold+AAA-ATPase+Lon-Protease-BrxL <- PglZ <- N6-METHYLASE <- DUF6079 *<- BrxB-BrxF-iSTAND <- BrxA-DNA-binding <- HNH <-?||?->?-><-SF1-SF2-Helicase<-HKD-DNase+HKD-Rease	1233	Bacteria>Pseudomonadota>Gammaproteobacteria>Oceanospirillales>Saccharospirillaceae>unclassified_Saccharospirillaceae	GCA_013214075.1	Saccharospirillaceae_bacterium	HRU38_08495	18482..22183	2497634	BREX-system-P-loop-protein-BrxC-[Saccharospirillaceae-bacterium]
KPX73888.1	Type1.BREX	PIN+OST-HTH-> HNH ->?->?-> DUF6079 -p*-> PglZ -> SIGMA+OB-Fold+AAA-ATPase+Lon-Protease-BrxL ->HIN-HTH->HTH_Tnp_1+HTH_21+rve+rve_3->	889	Bacteria>Pseudomonadota>Gammaproteobacteria>Pseudomonadales>Pseudomonadaceae>Pseudomonas	GCA_001400855.1	Pseudomonas_syringae_pv._maculicola	ALO84_01403	56181..58850	59511	Uncharacterized-protein-ALO84_01403-[Pseudomonas-syringae-pv.-maculicola]
MBL7230767.1	Type6.BREX	<-CopG/RHH||CITB-HTH->XIS-HTH->?-> wHTH+WYL+WCX -> BrxE -> BrxA-DNA-binding -> BrxB-BrxF-iSTAND -> DUF6079 *-> PglZ -> SIGMA+OB-Fold+AAA-ATPase+HKD-DNase-BrxL -><- HNH <-DUF4969	1214	Bacteria>Pseudomonadota>Gammaproteobacteria>Pseudomonadales>Pseudomonadaceae>Pseudomonas	GCA_016785205.1	Pseudomonas_sp.	JJK56_22660	12252..15896	306	BREX-system-P-loop-protein-BrxC-[Pseudomonas-sp.]
WP_052507173.1	Type1.BREX	PIN+OST-HTH-> HNH ->?->?->|| BrxB-BrxF-iSTAND -> DUF6079 -p-> DUF6079 -p*->AbiEii-> PglZ -> SIGMA+OB-Fold+AAA-ATPase+Lon-Protease-BrxL ->	886	Bacteria>Pseudomonadota>Gammaproteobacteria>Pseudomonadales>Pseudomonadaceae>Pseudomonas	GCF_000935725.1	Pseudomonas_syringae_group_genomosp._3	AC506_RS17885	8350..11010	251701	BREX-system-P-loop-protein-BrxC-[Pseudomonas-syringae-group-genomosp.-3]
WP_169883492.1	Type6.BREX	HNH -><- SIGMA+OB-Fold+AAA-ATPase+HKD-DNase-BrxL <- PglZ <- N6-METHYLASE <- N6-METHYLASE <- DUF6079 *<- BrxB-BrxF-iSTAND <- BrxE <- wHTH+WYL+WCX <-?<-XIS-HTH||RHH-><-tRNA<-MerR-HTH	1213	Bacteria>Pseudomonadota>Gammaproteobacteria>Pseudomonadales>Pseudomonadaceae>Pseudomonas	GCF_012986805.1	Pseudomonas_fragi	HBN91_RS17750	10864..14505	296	BREX-system-P-loop-protein-BrxC-[Pseudomonas-fragi]
WP_244972677.1	Type1.BREX	<- SIGMA+OB-Fold+AAA-ATPase+Lon-Protease-BrxL <- PglZ <-AbiEii<-AbiEi_3_N+AbiEi_3<- HNH <- N6-METHYLASE <-DNA-glycosylase<- DUF6079 *<- BrxB-BrxF-iSTAND <- BrxA-DNA-binding <-?|| wHTH+WYL+WCX -><-tRNA<-MerR-HTH	1225	Bacteria>Pseudomonadota>Gammaproteobacteria>Pseudomonadales>Pseudomonadaceae>Pseudomonas	GCF_013386885.1	Pseudomonas_salomonii	HX810_RS11525	509017..512694	191391	BREX-system-P-loop-protein-BrxC-[Pseudomonas-salomonii]
WP_273507518.1	Type6.BREX	<-CopG/RHH||CITB-HTH->XIS-HTH->?-> wHTH+WYL+WCX -> BrxE -> BrxA-DNA-binding -> BrxB-BrxF-iSTAND -> DUF6079 *-> PglZ -> SIGMA+OB-Fold+AAA-ATPase+HKD-DNase-BrxL -><- HNH <-DUF4969	1214	Bacteria>Pseudomonadota>Gammaproteobacteria>Pseudomonadales>Pseudomonadaceae>Pseudomonas	GCF_016785205.1	Pseudomonas_sp.	JJK56_22660	12252..15896	306	BREX-system-P-loop-protein-BrxC-[Pseudomonas-sp.]
WP_058409982.1	Type6.BREX	<-LuxR-HTH<-?<-ZnR+TOPRIM+AAA-helicase+wHTH||XIS-HTH->?-> wHTH+WYL+WCX -> BrxE -> BrxA-DNA-binding -> BrxB-BrxF-iSTAND -> DUF6079 *-> N6-METHYLASE -> PglZ -> SIGMA+OB-Fold+AAA-ATPase+HKD-DNase-BrxL -><- HNH <-?<-?||HTH_3-><-ZnR+ZnR<-DUF4969	1212	Bacteria>Pseudomonadota>Gammaproteobacteria>Pseudomonadales>Pseudomonadaceae>Pseudomonas	GCF_029959515.1	Pseudomonas_sp._ICMP_19500	AO239_RS04345	8658..12296	1208102	BREX-system-P-loop-protein-BrxC-[Pseudomonas-sp.-ICMP-19500]
WP_033991202.1	Type1.BREX	<- HNH <- SIGMA+OB-Fold+AAA-ATPase+Lon-Protease-BrxL <- PglZ <-MNS+REase<-?<- HNH +GCN1<-?<- N6-METHYLASE <- DUF6079 *<- BrxB-BrxF-iSTAND <- BrxA-DNA-binding <-RNase<-?<-JetB<-?<-Peptidase_M15<-?|| wHTH+WYL+WCX ->	1154	Bacteria>Pseudomonadota>Gammaproteobacteria>Pseudomonadales>Pseudomonadaceae>Pseudomonas	GCF_929614685.1	Pseudomonas_aeruginosa	MJP43_RS27275	28461..31925	287	BREX-system-P-loop-protein-BrxC-[Pseudomonas-aeruginosa]
WP_125870426.1	Type1.BREX	<-DUF932<-?||NERD->REase-> BrxA-DNA-binding -> BrxB-BrxF-iSTAND -> DUF6079 *-> N6-METHYLASE ->?-> HNH +GCN1->?-> PglZ -> SIGMA+OB-Fold+AAA-ATPase+Lon-Protease-BrxL ->REase->SF1-SF2-Helicase-><-Glycos_transf	1154	Bacteria>Pseudomonadota>Gammaproteobacteria>Pseudomonadales>Pseudomonadaceae>Stutzerimonas	GCF_024199075.1	Stutzerimonas_stutzeri	NLG33_RS12680	207505..210969	316	BREX-system-P-loop-protein-BrxC-[Stutzerimonas-stutzeri]
MCJ8311755.1	Type1.BREX	<-sGTP<-sGTP<-?<- SIGMA+OB-Fold+AAA-ATPase+Lon-Protease-BrxL <- PglZ <- N6-METHYLASE <- DUF6079 *<- BrxB-BrxF-iSTAND <- BrxA-DNA-binding <- HNH <-?||?->?-><-SF1-SF2-Helicase<-HKD-DNase+HKD-Rease	1233	Bacteria>Pseudomonadota>Gammaproteobacteria>Pseudomonadales>unclassified_Pseudomonadales	GCA_022968985.1	Pseudomonadales_bacterium	HRU38_08495	18482..22183	1891229	BREX-system-P-loop-protein-BrxC-[Pseudomonadales-bacterium]
MBS1228526.1	Type1.BREX	Arm-DNA-bind_3+Phage_integrase-><-Phage_int_SAM_2+Integrase_1||?->?->?->?-> wHTH+WYL+WCX -> BrxA-DNA-binding -> BrxB-BrxF-iSTAND -> DUF6079 *-> N6-METHYLASE -> HNH ->?->HTH+REase->DUF3644-> PglZ -> SIGMA+OB-Fold+AAA-ATPase+Lon-Protease-BrxL ->DOC+HTH->	1213	Bacteria>Pseudomonadota>unclassified_Pseudomonadota	GCA_018262095.1	Pseudomonadota_bacterium	H6R17_1803	75999..79640	1977087	ATPase-like-protein-[Pseudomonadota-bacterium]
MBY0113214.1	Type6.BREX	<-GDResolvase+ZnR<-RAMA<-HARE-HTH||TIR-><- SIGMA+OB-Fold+AAA-ATPase+Lon-Protease-BrxL <- PglZ <- N6-METHYLASE -p<- N6-METHYLASE -p<- N6-METHYLASE <- DUF6079 *<- BrxB-BrxF-iSTAND <- BrxA-DNA-binding <- BrxE <-EVE+ HNH <-?<-LuxR-HTH<- ParB +N6_N4_METHYLASE<-NtrC-AAA	1179	Bacteria>PVC_group>Planctomycetota>Phycisphaerae>Phycisphaerales>unclassified_Phycisphaerales	GCA_019744815.1	Phycisphaerales_bacterium	K2Y21_10355	73058..76597	2052180	BREX-system-P-loop-protein-BrxC-[Phycisphaerales-bacterium]
WP_100495666.1	Type1.BREX	<-ABhydrolase||TPR+TPR+TPR+TPR-> BrxA-DNA-binding -> BrxB-BrxF-iSTAND -> DUF6079 *-><-?<-?<-?|| HNH ->?->SbcC-> N6-METHYLASE ->HEPN->Schlafen+HATPase+wHTH->	1240	Bacteria>Terrabacteria_group>Actinomycetota>Actinomycetes>Bifidobacteriales>Bifidobacteriaceae	GCF_002802865.1	Bifidobacterium_scaligerum	CUU80_RS01790	434556..438278	2052656	BREX-system-P-loop-protein-BrxC-[Bifidobacterium-scaligerum]
WP_079914137.1	Type1.BREX	REase->?->REase+DUF4268->?-> BrxA-DNA-binding -> BrxB-BrxF-iSTAND -> DUF6079 *-> N6-METHYLASE -> PglZ -> SIGMA+OB-Fold+AAA-ATPase+Lon-Protease-BrxL -> HNH ->?->HTH+MPTase->?->?->Schlafen+HATPase+wHTH->	1184	Bacteria>Terrabacteria_group>Bacillota>Bacilli>Bacillales>Paenibacillaceae	GCF_002042965.1	Paenibacillus_sp._32352	B1728_RS30170	158863..162417	1969111	BREX-system-P-loop-protein-BrxC-[Paenibacillus-sp.-32352]
WP_022757624.1	Type1.BREX	<- HNH +NERD+ZnR||GDResolvase-><-?<-?<- SIGMA+OB-Fold+AAA-ATPase+Lon-Protease-BrxL <- HNH <- PglZ <-?<- N6-METHYLASE <- DUF6079 *<- BrxB-BrxF-iSTAND <- BrxA-DNA-binding <-HTH_26<-?<-TraI-VirD2-RCRE<-TraJ-RHH<-?<-HTH_3||ParA-Soj-PloopNTPase->	1181	Bacteria>Terrabacteria_group>Bacillota>Clostridia>Eubacteriales>Lachnospiraceae	GCF_000420985.1	Butyrivibrio_fibrisolvens	G616_RS0108215	23876..27421	831	BREX-system-P-loop-protein-BrxC-[Butyrivibrio-fibrisolvens]
WP_103203921.1	Type1.BREX	RHH_8-><-?<-ZnR+TOPRIM||?->SymE_toxin->?-> wHTH(SBD)+ HNH -> BrxA-DNA-binding -> BrxB-BrxF-iSTAND -> DUF6079 *-> N6-METHYLASE -> PglZ ->?-> SIGMA+OB-Fold+AAA-ATPase+Lon-Protease-BrxL ->RAMA->ARG->	1186	Bacteria>Terrabacteria_group>Bacillota>Clostridia>Eubacteriales>Lachnospiraceae	GCF_002904165.1	Herbinix_hemicellulosilytica	HHT355_RS13290	82989..86549	1564487	BREX-system-P-loop-protein-BrxC-[Herbinix-hemicellulosilytica]
WP_215631393.1	Type1.BREX	<-HTH+REase<- wHTH+WYL+WCX <- SIGMA+OB-Fold+AAA-ATPase+Lon-Protease-BrxL <-?<- PglZ <- HNH +CXC3<- N6-METHYLASE <-Four-helical-protein<-wHTH+ZnR<- DUF6079 *<- BrxB-BrxF-iSTAND <- BrxA-DNA-binding <-HTH_26<-HTH_3||?-><-Lysozyme+NLPC<-?<-?<-VirB4-FtsK	1205	Bacteria>Terrabacteria_group>Bacillota>Clostridia>Eubacteriales>Lachnospiraceae	GCF_018785175.1	Coprococcus_catus	GPK82_RS01090	19804..23421	116085	BREX-system-P-loop-protein-BrxC-[Coprococcus-catus]
MCC3659975.1	Type1.BREX	AraC-HTH+AraC-HTH-><-IONCHANNEL+HISKIN+REC<- SAD+ HNH <-?<- PglZ <-?<- N6-METHYLASE <- DUF6079 *<- BrxB-BrxF-iSTAND <- BrxA-DNA-binding <-HTH_26<-?<-?<-?<-?||?-><-68TM-wHTH+CACHE+GGDEF	1184	Bacteria>Terrabacteria_group>Bacillota>Clostridia>Eubacteriales>Oscillospiraceae	GCA_020740265.1	Ruminococcus_albus	LLP35_08975	12774..16328	1264	BREX-system-P-loop-protein-BrxC-[Ruminococcus-albus]
SCI31347.1	Type1.BREX	68TM-wHTH+CACHE+GGDEF->?->?->?->?->?->HTH_26-> BrxA-DNA-binding -> BrxB-BrxF-iSTAND -> DUF6079 *-> N6-METHYLASE ->?-> PglZ -> SIGMA+OB-Fold+AAA-ATPase+Lon-Protease-BrxL ->?-> SAD+ HNH ->IONCHANNEL+HISKIN+REC-><-DSBH+AraC-HTH+AraC-HTH	1184	Bacteria>Terrabacteria_group>Bacillota>Clostridia>Eubacteriales>Oscillospiraceae	GCA_900066635.1	uncultured_Ruminococcus_sp.	SAMEA3545357_00481	41099..44653	165186	Uncharacterised-protein-[uncultured-Ruminococcus-sp.]
WP_014256029.1	Type1.BREX	<-Glyoxalase<- N6-METHYLASE <-Peptidase<-?<-?<- SIGMA+OB-Fold+AAA-ATPase+Lon-Protease-BrxL <- PglZ <-Schlafen+HATPase+wHTH<- N6-METHYLASE <- DUF6079 *<- BrxB-BrxF-iSTAND <- BrxA-DNA-binding <- wHTH(SBD)+ HNH <-?<-?<- N6-METHYLASE	1186	Bacteria>Terrabacteria_group>Bacillota>Clostridia>Eubacteriales>Oscillospiraceae	GCF_000237085.1	Acetivibrio_clariflavus	CLOCL_RS14360	3415236..3418796	288965	BREX-system-P-loop-protein-BrxC-[Acetivibrio-clariflavus]
WP_027621802.1	Type1.BREX	<-Glyoxalase<- N6-METHYLASE <-Peptidase<-?<-?<- SIGMA+OB-Fold+AAA-ATPase+Lon-Protease-BrxL <- PglZ <-Schlafen+HATPase+wHTH<- N6-METHYLASE <- DUF6079 *<- BrxB-BrxF-iSTAND <- BrxA-DNA-binding <- wHTH(SBD)+ HNH ||DDE_5+DDE_Tnp_1-><-?<-?<- N6-METHYLASE	1186	Bacteria>Terrabacteria_group>Bacillota>Clostridia>Eubacteriales>Oscillospiraceae	GCF_000519985.1	Acetivibrio_clariflavus	CLOCLA_RS0107445	1195913..1199473	288965	BREX-system-P-loop-protein-BrxC-[Acetivibrio-clariflavus]
RJX18832.1	Type1.BREX	<-Thermonuclease<-?<-?<-PRTase<-?<- HNH <- SIGMA+OB-Fold+AAA-ATPase+Lon-Protease-BrxL <- PglZ <- N6-METHYLASE <- DUF6079 *<- BrxB-BrxF-iSTAND <- BrxA-DNA-binding ||HIN-HTH->AAA-ATPase->	1147	Bacteria>Terrabacteria_group>Bacillota>Clostridia>Thermoanaerobacterales>Thermoanaerobacteraceae	GCA_003604985.1	Ammonifex_sp.	C4570_06155	13420..16863	1872493	MAG:-BREX-system-P-loop-protein-BrxC-[Ammonifex-sp.]
MBP3871947.1	Type1.BREX	<- N6-METHYLASE -p<- PglZ <- DUF6079 *<- BrxB-BrxF-iSTAND <- BrxA-DNA-binding <-?<- HNH <-?<- HNH	1175	Bacteria>Terrabacteria_group>Bacillota>Erysipelotrichia>Erysipelotrichales>Erysipelotrichaceae	GCA_017941505.1	Faecalicoccus_sp.	J6E46_13345	3304..6831	1971758	BREX-system-P-loop-protein-BrxC-[Faecalicoccus-sp.]
MCU0565455.1	Type6.BREX	<-SF1-SF2-Helicase+wHTH+Small-B-barrel+DUF1998<-BrxD-AAA<- PglZ ||?-> HNH -><- N6-METHYLASE <-?<-SKI<- DUF6079 *<- BrxB-BrxF-iSTAND <- BrxA-DNA-binding <- BrxE	1209	Bacteria>Terrabacteria_group>Cyanobacteriota/Melainabacteria_group>Cyanobacteriota>Cyanophyceae>Pseudanabaenales	GCA_025454315.1	Oculatellaceae_cyanobacterium_Prado106	MUF49_02535	23046..26675	2929566	BREX-system-P-loop-protein-BrxC-[Oculatellaceae-cyanobacterium-Prado106]
WP_254937144.1	Type6.BREX	<-SIGMA-HTH||REase-> HNH -><- SIGMA+OB-Fold+AAA-ATPase+Lon-Protease-BrxL <- PglZ <- N6-METHYLASE <- DUF6079 *<- BrxB-BrxF-iSTAND <- BrxA-DNA-binding <- BrxE <-?||Sak4+WYL->?->PemK_toxin-><-?<-SAM-METHYLASE	1178	Bacteria>Terrabacteria_group>Cyanobacteriota/Melainabacteria_group>Cyanobacteriota>Cyanophyceae>Synechococcales	GCF_024345395.1	Cyanobium_sp._Tous-M-B4	KBY73_RS11030	158493..162029	2823724	BREX-system-P-loop-protein-BrxC-[Cyanobium-sp.-Tous-M-B4]
WP_221587161.1	Type1.BREX	<- SIGMA+OB-Fold+AAA-ATPase+Lon-Protease-BrxL <- PglZ ||Herpes_LMP1+REase-><-NADAR<-HTH+ART-DarT<- HNH <- N6-METHYLASE <- DUF6079 *<- BrxB-BrxF-iSTAND <- BrxA-DNA-binding <-CHTH||HKD-DNase+SF1-SF2-Helicase+HTH+HTH+SRA-><-?||?->?->?->Phage_integrase->	1145	Bacteria>Terrabacteria_group>Deinococcota>Deinococci>Deinococcales>Deinococcaceae	-	Deinococcus_sp._RIT780	K5D85_00055	17975..21412	2870472	BREX-system-P-loop-protein-BrxC-[Deinococcus-sp.-RIT780]
GGM57141.1	Type1.BREX	<- SIGMA+OB-Fold+AAA-ATPase+Lon-Protease-BrxL <- PglZ ||Herpes_LMP1+REase-><-NADAR<-fvmX2+ART-DarT<- HNH <- N6-METHYLASE <- DUF6079 *<- BrxB-BrxF-iSTAND <- BrxA-DNA-binding <-CHTH<-?<-?<-?<-REase	1153	Bacteria>Terrabacteria_group>Deinococcota>Deinococci>Deinococcales>Deinococcaceae	GCA_014647995.1	Deinococcus_arenae	GCM10008956_36020	13025..16486	1452751	hypothetical-protein-GCM10008956_36020-[Deinococcus-arenae]
MBX8463533.1	Type1.BREX	<- SIGMA+OB-Fold+AAA-ATPase+Lon-Protease-BrxL <- PglZ ||Herpes_LMP1+REase-><-NADAR<-HTH+ART-DarT<- HNH <- N6-METHYLASE <- DUF6079 *<- BrxB-BrxF-iSTAND <- BrxA-DNA-binding <-CHTH||HKD-DNase+SF1-SF2-Helicase+HTH+HTH+SRA-><-?||?->?->?->Phage_integrase->	1145	Bacteria>Terrabacteria_group>Deinococcota>Deinococci>Deinococcales>Deinococcaceae	GCA_019718715.1	Deinococcus_sp._RIT780	K5D85_00055	17975..21412	2870472	BREX-system-P-loop-protein-BrxC-[Deinococcus-sp.-RIT780]
WP_135229998.1	Type1.BREX	RNase->?-><-SF1-SF2-Helicase<-REase||<-RNase||?-><-SF1-SF2-Helicase+wHTH+Small-B-barrel+DUF1998|| BrxA-DNA-binding -> BrxB-BrxF-iSTAND -> DUF6079 *->RelE-ParE+SF1-SF2-Helicase->?-> N6-METHYLASE -> HNH ->HTH+ART-DarT->NADAR->REase->PhdYeFM_antitox->PIN->	1141	Bacteria>Terrabacteria_group>Deinococcota>Deinococci>Deinococcales>Deinococcaceae	GCF_004634215.1	Deinococcus_fonticola	E5Z01_RS14400	28954..32379	2528713	BREX-system-P-loop-protein-BrxC-[Deinococcus-fonticola]
WP_143721449.1	Type1.BREX	SF1-SF2-Helicase+wHTH+Small-B-barrel+DUF1998-><-McrC-NTD+REase<-AAA-ATPase<- SIGMA+OB-Fold+AAA-ATPase+Lon-Protease-BrxL <- PglZ ||?-><- HNH <- N6-METHYLASE <- DUF6079 *<- BrxB-BrxF-iSTAND <- BrxA-DNA-binding <- wHTH+WYL+WCX <-?<-SF1-SF2-Helicase+REase<-Exonuc_V_gamma+REase<-RNase<-?<-MerR-HTH	1148	Bacteria>Terrabacteria_group>Deinococcota>Deinococci>Deinococcales>Deinococcaceae	GCF_007280555.1	Deinococcus_detaillensis	FNU79_RS14110	42957..46403	2592048	BREX-system-P-loop-protein-BrxC-[Deinococcus-detaillensis]
WP_189062792.1	Type5.BREX	<- SIGMA+OB-Fold+AAA-ATPase+Lon-Protease-BrxL <- PglZ ||Herpes_LMP1+REase-><-NADAR<-fvmX2+ART-DarT<- HNH <- N6-METHYLASE <- DUF6079 *<- BrxB-BrxF-iSTAND <- BrxA-DNA-binding <-CHTH<-?<-REase||?->?->?-><-TUDOR+SF1-SF2-Helicase+iREase+CTD+REase	1145	Bacteria>Terrabacteria_group>Deinococcota>Deinococci>Deinococcales>Deinococcaceae	GCF_014647995.1	Deinococcus_arenae	IEY66_RS18050	13025..16462	1452751	BREX-system-P-loop-protein-BrxC-[Deinococcus-arenae]
WP_255578705.1	Type1.BREX	<- SIGMA+OB-Fold+AAA-ATPase+Lon-Protease-BrxL <- PglZ ||Herpes_LMP1+REase-><-NADAR<-ART-DarT<- HNH <- N6-METHYLASE <- DUF6079 *<- BrxB-BrxF-iSTAND <- BrxA-DNA-binding <-CHTH||HKD-DNase+SF1-SF2-Helicase+HTH+HTH+SRA-><-?||?->?->?->Phage_integrase->	1153	Bacteria>Terrabacteria_group>Deinococcota>Deinococci>Deinococcales>Deinococcaceae	GCF_019718715.1	Deinococcus_sp._RIT780	K5D85_RS00055	17975..21436	2870472	BREX-system-P-loop-protein-BrxC-[Deinococcus-sp.-RIT780]
WP_092265648.1	Type1.BREX	<-HIN-HTH<- SIGMA+OB-Fold+AAA-ATPase+Lon-Protease-BrxL <- PglZ <-?<-wHTH||URI-DNase-><- HNH <- N6-METHYLASE <- DUF6079 *<- BrxB-BrxF-iSTAND <- BrxA-DNA-binding <-SF1-SF2-Helicase+REase<-Exonuc_V_gamma+REase<-RNase<-?<-DDE_3	1160	Bacteria>Terrabacteria_group>Deinococcota>Deinococci>Deinococcales>Deinococcaceae	GCF_900109185.1	Deinococcus_reticulitermitis	BMY43_RS15290	11757..15239	856736	BREX-system-P-loop-protein-BrxC-[Deinococcus-reticulitermitis]
WP_254867722.1	Type1.BREX	<-HIN-HTH|| BrxA-DNA-binding -> BrxB-BrxF-iSTAND -> DUF6079 *-> N6-METHYLASE -> PglZ -> SIGMA+OB-Fold+AAA-ATPase+Lon-Protease-BrxL -> HNH ->CR-ATPase-><-?<-?||ABhydrolase->	1163	Bacteria>Thermotogota>Thermotogae>Petrotogales>Petrotogaceae>Marinitoga	GCF_013363285.1	Marinitoga_sp._1154	XO12_RS09410	1913..5404	1643335	BREX-system-P-loop-protein-BrxC-[Marinitoga-sp.-1154]
MBU4348826.1	Type1.BREX	BrxB-BrxF-iSTAND -> DUF6079 *-> N6-METHYLASE -> N6-METHYLASE -p-> PglZ -> SIGMA+OB-Fold+AAA-ATPase+Lon-Protease-BrxL -> HNH ->?->SF1-SF2-Helicase->?->REase->	1193	Bacteria>unclassified_Bacteria	GCA_018896635.1	bacterium	KJ599_00710	356..3937	1869227	BREX-system-P-loop-protein-BrxC-[bacterium]

Protein-ID	System	Gene-Neighbourhoods	Length	Taxonomy	Assembly-ID	Strain	Locus-Tag	Genomic-coordinates	TaxID	Fasta-defline
--										
--PIN-EndoRNase										
--										
MCL2156391.1	Type1.BREX	BrxA-DNA-binding -> BrxB-BrxF-iSTAND -> DUF6079 *->Phage_integrase+Phage_integrase-><- N6-METHYLASE || N6-METHYLASE ->?-> PIN -> PglZ -> SIGMA+OB-Fold+AAA-ATPase+Lon-Protease-BrxL ->	1194	Archaea>Euryarchaeota>Methanomada_group>Methanobacteria>Methanobacteriales>Methanobacteriaceae	GCA_009776495.1	Methanobrevibacter_sp.	FWH54_00015	1416..5000	66852	BREX-system-P-loop-protein-BrxC-[Methanobrevibacter-sp.]
ELZ19699.1	Type1.BREX	<- N6-METHYLASE -p||?->?->?-><- PIN ||?-><-REase<-?||?-><- DUF6079 *<- BrxB-BrxF-iSTAND <- DUF6079 <- BrxA-DNA-binding <-CR-ATPase9+REase<-?<-?||REase->SF1-SF2-Helicase->REase->	1198	Archaea>Euryarchaeota>Stenosarchaea_group>Halobacteria>Halobacteriales>Haloarculaceae	GCA_000337455.1	Halosimplex_carlsbadense_2-9-1	C475_22179	59263..62859	797114	hypothetical-protein-C475_22179-[Halosimplex-carlsbadense-2-9-1]
ELZ19701.1	Type1.BREX	<- PIN ||?-><-REase<-?||?-><- DUF6079 <- BrxB-BrxF-iSTAND <- DUF6079 *<- BrxA-DNA-binding <-CR-ATPase9+REase<-?<-?||REase->SF1-SF2-Helicase->REase->SF1-SF2-Helicase->	1248	Archaea>Euryarchaeota>Stenosarchaea_group>Halobacteria>Halobacteriales>Haloarculaceae	GCA_000337455.1	Halosimplex_carlsbadense_2-9-1	C475_22189	63665..67411	797114	hypothetical-protein-C475_22189-[Halosimplex-carlsbadense-2-9-1]
WP_135304847.1	Type5.BREX	<-REase||?-><-MORC<- TUDOR+SF1-SF2-Helicase+iREase+CTD <- N6-METHYLASE <- PglZ <- N6-METHYLASE <- PIN <-?<- DUF6079 *<- BrxB-BrxF-iSTAND <- DUF6079 <- BrxA-DNA-binding ||?->?->?-><-?||McrB-AAA->wHTH+REase->	1273	Archaea>Euryarchaeota>Stenosarchaea_group>Halobacteria>Halobacteriales>Haloarculaceae	GCF_004681185.1	Halomicroarcula_amylolytica	E4S61_RS15390	199089..202910	2562280	hypothetical-protein-[Halomicroarcula-amylolytica]
WP_135304849.1	Type5.BREX	<-MORC<- TUDOR+SF1-SF2-Helicase+iREase+CTD <- N6-METHYLASE <- PglZ <- N6-METHYLASE <- PIN <-?<- DUF6079 <- BrxB-BrxF-iSTAND <- DUF6079 *<- BrxA-DNA-binding ||?->?->?-><-?||McrB-AAA->wHTH+REase-><-SF1-SF2-Helicase	1250	Archaea>Euryarchaeota>Stenosarchaea_group>Halobacteria>Halobacteriales>Haloarculaceae	GCF_004681185.1	Halomicroarcula_amylolytica	E4S61_RS15400	203494..207246	2562280	hypothetical-protein-[Halomicroarcula-amylolytica]
WP_170076589.1	Type6.BREX	<-SbcC<-Calcineurin<-REase<-?<-Calcineurin-SbcD|| BrxE -> BrxA-DNA-binding -> BrxB-BrxF-iSTAND -> DUF6079 *-> N6-METHYLASE ->?->SbcC-> PglZ -> SIGMA+OB-Fold+AAA-ATPase+Lon-Protease-BrxL -> PIN ->ARG->	1214	Archaea>Euryarchaeota>Stenosarchaea_group>Halobacteria>Haloferacales>Haloferacaceae	GCF_012726105.1	Haloferax_alexandrinus	GOC85_RS10615	2063512..2067156	114529	BREX-system-P-loop-protein-BrxC-[Haloferax-alexandrinus]
WP_188786134.1	Type5.BREX	<-ABC-ATPase+SbcC<-?<-?||tRNA->tRNA-><- TUDOR+SF1-SF2-Helicase+iREase+CTD <- PglZ <- N6-METHYLASE <- PIN <-?<-?<- DUF6079 *<- BrxB-BrxF-iSTAND <- DUF6079 <- BrxA-DNA-binding <-Phage_integrase<-REase<-PAS||?-><-DSBH	1265	Archaea>Euryarchaeota>Stenosarchaea_group>Halobacteria>Haloferacales>Haloferacaceae	GCF_014646875.1	Halobellus_salinus	IEX72_RS04225	173567..177364	931585	hypothetical-protein-[Halobellus-salinus]
WP_188786136.1	Type5.BREX	<- TUDOR+SF1-SF2-Helicase+iREase+CTD <- PglZ <- N6-METHYLASE <- PIN <-?<-?<- DUF6079 <- BrxB-BrxF-iSTAND <- DUF6079 *<- BrxA-DNA-binding <-Phage_integrase<-REase<-PAS||?-><-DSBH<-?<-Sulfatase+Phosphodiest+DUF4976<-NUDIX+DUF429	1239	Archaea>Euryarchaeota>Stenosarchaea_group>Halobacteria>Haloferacales>Haloferacaceae	GCF_014646875.1	Halobellus_salinus	IEX72_RS04235	177948..181667	931585	hypothetical-protein-[Halobellus-salinus]
WP_015911586.1	Type1.BREX	<- N6-METHYLASE -p||MazE_antitoxin-> PIN -><- DUF6079 *<- BrxB-BrxF-iSTAND <- DUF6079 <- BrxA-DNA-binding <-SF1-SF2-Helicase+REase<-SF1-SF2-Helicase+REase	1270	Archaea>Euryarchaeota>Stenosarchaea_group>Halobacteria>Haloferacales>Halorubraceae	GCF_000022205.1	Halorubrum_lacusprofundi	CF318_RS14700	1993..5805	2247	hypothetical-protein-[Halorubrum-lacusprofundi]
WP_015911588.1	Type1.BREX	<- TUDOR+SF1-SF2-Helicase+iREase+CTD <- PglZ <- N6-METHYLASE ||DDE_Tnp_1-><- N6-METHYLASE -p||MazE_antitoxin-> PIN -><- DUF6079 <- BrxB-BrxF-iSTAND <- DUF6079 *<- BrxA-DNA-binding <-SF1-SF2-Helicase+REase<-SF1-SF2-Helicase+REase<-?||DDE_Tnp_1-><-?<-MazE_antitoxin	1239	Archaea>Euryarchaeota>Stenosarchaea_group>Halobacteria>Haloferacales>Halorubraceae	GCF_000022205.1	Halorubrum_lacusprofundi	CF318_RS14710	6389..10108	2247	hypothetical-protein-[Halorubrum-lacusprofundi]
WP_256418943.1	Type5.BREX	<-ORC-CDC6+HTH||?-><- TUDOR+SF1-SF2-Helicase+iREase+CTD <- PglZ <-?<- N6-METHYLASE || PIN -><- DUF6079 <- BrxB-BrxF-iSTAND <- DUF6079 *<- BrxA-DNA-binding ||SF1-SF2-Helicase+REase->SF1-SF2-Helicase+REase->ParA-Soj-PloopNTPase->	1248	Archaea>Euryarchaeota>Stenosarchaea_group>Halobacteria>Haloferacales>Halorubraceae	GCF_024494655.1	Halorubrum_laminariae	NO887_RS14550	91131..94877	1433523	hypothetical-protein-[Halorubrum-laminariae]
AEN07515.1	Type5.BREX	MazE_antitoxin->?-><-DDE_Tnp_1||?->SF1-SF2-Helicase+REase->SF1-SF2-Helicase+REase->?-> BrxA-DNA-binding -> DUF6079 *-> BrxB-BrxF-iSTAND -> DUF6079 -><- PIN <-MazE_antitoxin|| N6-METHYLASE ->?-> PglZ -> TUDOR+SF1-SF2-Helicase+iREase+CTD ->	1239	Archaea>Euryarchaeota>Stenosarchaea_group>Halobacteria>Haloferacales>unclassified_Haloferacales	GCA_000224475.1	halophilic_archaeon_DL31	Halar_0255	246678..250397	756883	hypothetical-protein-Halar_0255-[halophilic-archaeon-DL31]
AEN07517.1	Type5.BREX	<-DDE_Tnp_1||?->SF1-SF2-Helicase+REase->SF1-SF2-Helicase+REase->?-> BrxA-DNA-binding -> DUF6079 -> BrxB-BrxF-iSTAND -> DUF6079 *-><- PIN <-MazE_antitoxin|| N6-METHYLASE ->?-> PglZ -> TUDOR+SF1-SF2-Helicase+iREase+CTD -><-?<-SF1-SF2-Helicase	1270	Archaea>Euryarchaeota>Stenosarchaea_group>Halobacteria>Haloferacales>unclassified_Haloferacales	GCA_000224475.1	halophilic_archaeon_DL31	Halar_0257	250981..254793	756883	hypothetical-protein-Halar_0257-[halophilic-archaeon-DL31]
WP_179261303.1	Type6.BREX	<-HD<-?||ABhydrolase->?-><- SIGMA+OB-Fold+AAA-ATPase+Lon-Protease-BrxL <- PglZ <- N6-METHYLASE <- DUF6079 *<- BrxB-BrxF-iSTAND <- BrxA-DNA-binding <- BrxE <-?<- PIN <-MazE_antitoxin	1214	Archaea>Euryarchaeota>Stenosarchaea_group>Halobacteria>Natrialbales>Natrialbaceae	GCF_013402815.2	Natrinema_halophilum	HYG82_RS33105	2537554..2541198	1699371	BREX-system-P-loop-protein-BrxC-[Natrinema-halophilum]
MCI0624094.1	Type1.BREX	<-HHH_5+RecR+TOPRIM<-?<-RFC-AAA+RuvB-AAA<-ncRNA<- PglZ <- PIN <-PhdYeFM_antitox<-REase<- DUF6079 *<- BrxB-BrxF-iSTAND <- BrxA-DNA-binding ||?->?->?-><-AA_kinase+ACT+ACT_7<-NAD_binding_3+Homoserine_dh<-REC+NtrC-AAA+FIS-HTH<-GAF+PAS+HISKIN	1197	Bacteria>Acidobacteriota>unclassified_Acidobacteriota	GCA_022615935.1	Acidobacteriota_bacterium	LO387_20995	11986..15579	1978231	BREX-system-P-loop-protein-BrxC-[Acidobacteriota-bacterium]
PXF57197.1	Type1.BREX	<-PemK_toxin<-MetJarc-HTH<- PglZ <-TOPRIM<-OLD-ABC<- N6-METHYLASE <- PIN <-Phd-YefM<- DUF6079 *<- BrxB-BrxF-iSTAND <- BrxA-DNA-binding <- PIN <-?||?->?->?->?->His_biosynth->	1215	Bacteria>delta/epsilon_subdivisions>Deltaproteobacteria>unclassified_Deltaproteobacteria	GCA_003194495.1	Deltaproteobacteria_bacterium	C4B58_10725	19842..23489	2026735	BREX-system-P-loop-protein-BrxC-[Deltaproteobacteria-bacterium]
MBI2378669.1	Type6.BREX	PIN -><-?<- PglZ <-Schlafen+HATPase+wHTH<- PglZ -p<-?<-RHH<- PIN <-?<- DUF6079 *<- BrxB-BrxF-iSTAND <- BrxA-DNA-binding <- BrxE || PIN ->?-><-?<-RADICAL-SAM	1207	Bacteria>delta/epsilon_subdivisions>Deltaproteobacteria>unclassified_Deltaproteobacteria	GCA_016183035.1	Deltaproteobacteria_bacterium	HYV07_32040	22898..26521	2026735	BREX-system-P-loop-protein-BrxC-[Deltaproteobacteria-bacterium]
MBI4818899.1	Type6.BREX	<- PglZ -p<-TOPRIM<-OLD-ABC<-Schlafen+HATPase+wHTH<-?<-Schlafen+HATPase+wHTH<- N6-METHYLASE <- PIN <-?<- DUF6079 *<- BrxB-BrxF-iSTAND <- BrxA-DNA-binding <- BrxE <- SF1-SF2-Helicase+wHTH+Small-B-barrel+DUF1998 <- BrxD-AAA <- PglZ	1207	Bacteria>delta/epsilon_subdivisions>Deltaproteobacteria>unclassified_Deltaproteobacteria	GCA_016208175.1	Deltaproteobacteria_bacterium	HY791_21690	12157..15780	2026735	BREX-system-P-loop-protein-BrxC-[Deltaproteobacteria-bacterium]
MBI5606167.1	Type1.BREX	<-Schlafen+HATPase+wHTH<- DUF6079 *<- BrxB-BrxF-iSTAND <- BrxA-DNA-binding ||?-><- PIN <-?||HicA_toxin->HicB_lk_antitox->ACET->	1147	Bacteria>delta/epsilon_subdivisions>Deltaproteobacteria>unclassified_Deltaproteobacteria	GCA_016219225.1	Deltaproteobacteria_bacterium	HY879_22775	11994..15437	2026735	BREX-system-P-loop-protein-BrxC-[Deltaproteobacteria-bacterium]
MCD6306396.1	Type1.BREX	<- N6-METHYLASE -p||PhdYeFM_antitox-> PIN -><- DUF6079 *<- BrxB-BrxF-iSTAND <- BrxA-DNA-binding || wHTH+WYL+WCX -p->	1148	Bacteria>delta/epsilon_subdivisions>Deltaproteobacteria>unclassified_Deltaproteobacteria	GCA_021159525.1	Deltaproteobacteria_bacterium	J7M32_08925	1514..4960	2026735	BREX-system-P-loop-protein-BrxC-[Deltaproteobacteria-bacterium]
WP_259087839.1	Type1.BREX	URI-DNase-><- SIGMA+OB-Fold+AAA-ATPase+HKD-DNase-BrxL <- PglZ <- PIN <-XIS-HTH<-?<-?<- N6-METHYLASE <- DUF6079 *<- BrxB-BrxF-iSTAND <- BrxA-DNA-binding <-?||Phage_integrase-><-TOPRIM<-?||XIS-HTH->Phage_int_SAM_4+Phage_integrase-><-tRNA||BetaPropeller+ABhydrolase->	1168	Bacteria>FCB_group>Bacteroidetes/Chlorobi_group>Bacteroidota>Bacteroidetes_incertae_sedis>Rhodothermaceae	GCF_024809725.1	Salinibacter_ruber	GGQ07_RS07865	19343..22849	146919	BREX-system-P-loop-protein-BrxC-[Salinibacter-ruber]
HAH90337.1	Type1.BREX	<-D5ATPase+DUF3874<-?|| BrxA-DNA-binding -> BrxB-BrxF-iSTAND -> DUF6079 *->HTH+MPTase-> PIN ->?->?->?-> N6-METHYLASE -> PglZ -> SIGMA+OB-Fold+AAA-ATPase+Lon-Protease-BrxL ->HAD+HAD->	1222	Bacteria>FCB_group>Bacteroidetes/Chlorobi_group>Bacteroidota>Bacteroidia>Bacteroidales	GCA_003450425.1	Prevotella_sp.	DCL96_01170	7620..11288	59823	BREX-system-P-loop-protein-BrxC-[Prevotella-sp.]
MBR4647494.1	Type1.BREX	MNS+CR-ATPase5-><-?||?->?->Calcineurin->SbcC+CCDC158-> BrxA-DNA-binding -> BrxB-BrxF-iSTAND -> DUF6079 *-> OLD-ABC-> PIN -> N6-METHYLASE -> PglZ -> SIGMA+OB-Fold+AAA-ATPase+Lon-Protease-BrxL ->?->?->?->Cysteine-Desulfurase->	1174	Bacteria>FCB_group>Bacteroidetes/Chlorobi_group>Bacteroidota>Bacteroidia>Bacteroidales	GCA_017525245.1	Bacteroidales_bacterium	IK075_10320	20247..23771	2030927	BREX-system-P-loop-protein-BrxC-[Bacteroidales-bacterium]
WP_234146092.1	Type1.BREX	Arm-DNA-bind_5+Phage_int_SAM_5+Phage_integrase-><-Schlafen+HATPase+wHTH<- SIGMA+OB-Fold+AAA-ATPase+Lon-Protease-BrxL <- PglZ <- N6-METHYLASE <- PIN <-OLD-ABC <- DUF6079 *<- BrxB-BrxF-iSTAND <- BrxA-DNA-binding <-HTH_3||?->?-> N6-METHYLASE -><-AAA+<-HTH_36<-XIS-HTH	1180	Bacteria>FCB_group>Bacteroidetes/Chlorobi_group>Bacteroidota>Bacteroidia>Bacteroidales	GCF_024760085.1	Bacteroides_uniformis	J8872_RS07820	80453..83995	820	BREX-system-P-loop-protein-BrxC-[Bacteroides-uniformis]
WP_264909062.1	Type1.BREX	<-HAD+HAD<- SIGMA+OB-Fold+AAA-ATPase+Lon-Protease-BrxL <- PglZ <- N6-METHYLASE <-?<-?<-?<- PIN <-HTH+MPTase<- DUF6079 *<- BrxB-BrxF-iSTAND <- BrxA-DNA-binding ||?->D5ATPase+DUF3874-><-?<-CRISPR_Cas2<-Cas_Cas1<-Cas9+HNH+RuvC_III<-HTH+MPTase	1222	Bacteria>FCB_group>Bacteroidetes/Chlorobi_group>Bacteroidota>Bacteroidia>Bacteroidales	-	Prevotella_copri	ONT11_RS09740	2183354..2187022	165179	BREX-system-P-loop-protein-BrxC-[Prevotella-copri]
WP_264951499.1	Type1.BREX	SLOG+BirA-HTH-><-?||AT-HTH-HYD2->AT-HTH-HYD2-><-HNH<-OLD-ABC<-?|| BrxA-DNA-binding -> BrxB-BrxF-iSTAND -> DUF6079 *->HTH+MPTase-> PIN ->?->?->?-> N6-METHYLASE -> PglZ -> SIGMA+OB-Fold+AAA-ATPase+Lon-Protease-BrxL ->HAD+HAD->	1222	Bacteria>FCB_group>Bacteroidetes/Chlorobi_group>Bacteroidota>Bacteroidia>Bacteroidales	-	Prevotella_copri	ONT17_RS13510	3302536..3306204	165179	BREX-system-P-loop-protein-BrxC-[Prevotella-copri]
WP_094415998.1	Type1.BREX	<- PIN <-?<- SIGMA+OB-Fold+AAA-ATPase+Lon-Protease-BrxL <- PglZ <- N6-METHYLASE <-Schlafen+HATPase+wHTH<- DUF6079 *<- BrxB-BrxF-iSTAND <- BrxA-DNA-binding <-HTH<-?<-XIS-HTH	1197	Bacteria>FCB_group>Bacteroidetes/Chlorobi_group>Bacteroidota>Flavobacteriia>Flavobacteriales	GCF_002251835.1	Flavobacterium_cyanobacteriorum	CHU92_RS12285	18327..21920	2022802	BREX-system-P-loop-protein-BrxC-[Flavobacterium-cyanobacteriorum]
WP_210512160.1	Type1.BREX	Phage_int_SAM_5+Phage_integrase->?->?->REase->?->REase-> BrxA-DNA-binding -> BrxB-BrxF-iSTAND -> DUF6079 *-> N6-METHYLASE -><-AbiEii<-AbiEi_4||XIS-HTH-> PIN -> PglZ -> SIGMA+OB-Fold+AAA-ATPase+Lon-Protease-BrxL ->	1141	Bacteria>FCB_group>Bacteroidetes/Chlorobi_group>Balneolota>Balneolia>Balneolales	GCF_017921895.1	Natronogracilivirga_saccharolytica	NATSA_RS09880	86175..89600	2812953	BREX-system-P-loop-protein-BrxC-[Natronogracilivirga-saccharolytica]
MCB5286841.1	Type1.BREX	<-IG+IG||?->?-><-tRNA||?->?->XIS-HTH-> BrxA-DNA-binding -> BrxB-BrxF-iSTAND -> DUF6079 *-> OLD-ABC-> PIN -> N6-METHYLASE ->BirA-HTH-> PglZ -> SIGMA+OB-Fold+AAA-ATPase+Lon-Protease-BrxL ->	1145	Bacteria>FCB_group>Candidatus_Cloacimonetes>unclassified_Candidatus_Cloacimonetes	GCA_020532145.1	Candidatus_Cloacimonetes_bacterium	LHW64_03450	9843..13280	2030808	BREX-system-P-loop-protein-BrxC-[Candidatus-Cloacimonadota-bacterium]
HCF56393.1	Type6.BREX	<-Schlafen+HATPase+wHTH<- N6-METHYLASE -p||?-> PIN -><-TOPRIM<-OLD-ABC<- DUF6079 -p<-APATPase<- DUF6079 -p*<- DUF6079 -p<-HNH<- BrxB-BrxF-iSTAND <- BrxA-DNA-binding <- BrxA-DNA-binding <- BrxE	508	Bacteria>Myxococcota>Myxococcia>Myxococcales>unclassified_Myxococcales	GCA_003534605.1	Myxococcales_bacterium	DFS52_00150	7726..9251	2026763	BREX-system-P-loop-protein-BrxC-[Myxococcales-bacterium]
HCF56394.1	Type6.BREX	<-Schlafen+HATPase+wHTH<- N6-METHYLASE -p||?-> PIN -><-TOPRIM<-OLD-ABC<- DUF6079 -p<-APATPase<- DUF6079 -p<- DUF6079 -p*<-HNH<- BrxB-BrxF-iSTAND <- BrxA-DNA-binding <- BrxA-DNA-binding <- BrxE	363	Bacteria>Myxococcota>Myxococcia>Myxococcales>unclassified_Myxococcales	GCA_003534605.1	Myxococcales_bacterium	DFS52_00155	9470..10559	2026763	BREX-system-P-loop-protein-BrxC-[Myxococcales-bacterium]
MBK6517532.1	Type6.BREX	SF1-SF2-Helicase+REase->SF1-SF2-Helicase+REase->REase-><-TOPRIM<-OLD-ABC<-?|| BrxE -> BrxA-DNA-binding -> BrxB-BrxF-iSTAND -> DUF6079 *-><-PemK_toxin<-?|| N6-METHYLASE -> N6-METHYLASE -p->?->?-> PIN -> PglZ -> BrxD-AAA ->	1209	Bacteria>Myxococcota>Polyangia>Polyangiales>Polyangiaceae>unclassified_Polyangiaceae	GCA_016704335.1	Polyangiaceae_bacterium	IPG04_26305	282797..286426	2268199	BREX-system-P-loop-protein-BrxC-[Polyangiaceae-bacterium]
MBK8937968.1	Type6.BREX	<- BrxD-AAA <- PglZ <- PIN <-?<-?<- N6-METHYLASE -p<- N6-METHYLASE ||?->PemK_toxin-><- DUF6079 *<- BrxB-BrxF-iSTAND <- BrxA-DNA-binding <- BrxE ||OLD-ABC->TOPRIM-><-REase<-SF1-SF2-Helicase+REase<-SF1-SF2-Helicase<-SF1-SF2-Helicase+REase-p	1016	Bacteria>Myxococcota>Polyangia>Polyangiales>Polyangiaceae>unclassified_Polyangiaceae	GCA_016719945.1	Polyangiaceae_bacterium	IPM79_10070	425737..428787	2268199	BREX-system-P-loop-protein-BrxC-[Polyangiaceae-bacterium]
MCB9617769.1	Type1.BREX	RAMA->GDResolvase+ZnR-><- SIGMA+OB-Fold+AAA-ATPase+Lon-Protease-BrxL <- PglZ <-Schlafen+HATPase+wHTH<- N6-METHYLASE <- PIN <-?<- DUF6079 *<- BrxB-BrxF-iSTAND <- BrxA-DNA-binding <-tRNA||?-><-ADH_N+ADH_zinc_N+METHYLASE	1156	Bacteria>Myxococcota>Polyangia>Polyangiales>Sandaracinaceae>Sandaracinus	GCA_020633155.1	Sandaracinus_sp.	H6722_35485	14055..17525	2024858	BREX-system-P-loop-protein-BrxC-[Sandaracinus-sp.]
WP_240530317.1	Type1.BREX	N6-METHYLASE -> PIN -><-?<-GreA_GreB+SF1-SF2-Helicase<-SF1-SF2-Helicase+REase<-35Exo+REase|| wHTH+WYL+WCX -> BrxA-DNA-binding -> BrxB-BrxF-iSTAND -> DUF6079 -p*->	735	Bacteria>Pseudomonadota>Alphaproteobacteria>Sphingomonadales>Sphingomonadaceae>Novosphingobium	GCF_002556635.1	Novosphingobium_sp._PC22D	B2G71_RS22885	26272..28479	1962403	BREX-system-P-loop-protein-BrxC-[Novosphingobium-sp.-PC22D]
WP_243995499.1	Type1.BREX	<- PIN <-?<-?<-?<- SIGMA+OB-Fold+AAA-ATPase+HKD-DNase-BrxL <- PglZ <- N6-METHYLASE <- DUF6079 *<- BrxB-BrxF-iSTAND <- BrxA-DNA-binding <- wHTH+WYL+WCX ||35Exo+REase->SF1-SF2-Helicase+REase->GreA_GreB+SF1-SF2-Helicase->?-><- PIN <- N6-METHYLASE	1179	Bacteria>Pseudomonadota>Alphaproteobacteria>Sphingomonadales>Sphingomonadaceae>Novosphingobium	GCF_022832385.1	Novosphingobium_sp._B2580	MTR64_RS16290	56132..59671	2930093	BREX-system-P-loop-protein-BrxC-[Novosphingobium-sp.-B2580]
MBQ2886288.1	Type1.BREX	<-wHTH+WYL<-REase<- SIGMA+OB-Fold+AAA-ATPase+Lon-Protease-BrxL <- PglZ <-Schlafen+HATPase+wHTH<- N6-METHYLASE <- PIN <-OLD-ABC <- DUF6079 *<- BrxB-BrxF-iSTAND <- BrxA-DNA-binding <-HTH_3	1180	Bacteria>Pseudomonadota>Alphaproteobacteria>unclassified_Alphaproteobacteria	GCA_017409945.1	Alphaproteobacteria_bacterium	IJE43_21410	13139..16681	1913988	BREX-system-P-loop-protein-BrxC-[Alphaproteobacteria-bacterium]
EXI84705.1	Type1.BREX	DDE_Tnp_1-><-?<-MqsA_antitoxin<-HTH<-?<-Arm-DNA-bind_3+Phage_integrase|| BrxA-DNA-binding -> BrxB-BrxF-iSTAND -> DUF6079 *-> N6-METHYLASE ->HTH+REase-> PglZ -> SIGMA+OB-Fold+AAA-ATPase+Lon-Protease-BrxL -> PIN +HTH->	1204	Bacteria>Pseudomonadota>Betaproteobacteria>Betaproteobacteria_incertae_sedis>Candidatus_Accumulibacter	GCA_000585075.1	Candidatus_Accumulibacter_regalis	AW11_03760	6873..10487	522306	MAG:-hypothetical-protein-AW11_03760-[Candidatus-Accumulibacter-regalis]
EXI72915.1	Type1.BREX	<- PIN +OST-HTH<-?||?->?->?-><-?|| BrxA-DNA-binding -> BrxB-BrxF-iSTAND -> DUF6079 *->HTH_3->DOC+HTH-> N6-METHYLASE -><- PIN <-MazE_antitoxin||SF1-SF2-Helicase+PRTase-><-?||BrxL->	1155	Bacteria>Pseudomonadota>Betaproteobacteria>Betaproteobacteria_incertae_sedis>Candidatus_Accumulibacter>unclassified_Candidatus_Accumulibacter	GCA_000584995.1	Candidatus_Accumulibacter_sp._SK-11	AW07_03051	55405..58872	1454000	MAG:-hypothetical-protein-AW07_03051-[Candidatus-Accumulibacter-sp.-SK-11]
HAY27737.1	Type1.BREX	BrxA-DNA-binding -> DUF6079 *->HTH_3->DOC+HTH-> N6-METHYLASE -><- PIN <-MazE_antitoxin||SF1-SF2-Helicase+PRTase->SLOG+BirA-HTH->	1286	Bacteria>Pseudomonadota>Betaproteobacteria>Betaproteobacteria_incertae_sedis>Candidatus_Accumulibacter>unclassified_Candidatus_Accumulibacter	GCA_003487685.1	Candidatus_Accumulibacter_sp.	DCY47_09605	6303..10163	2053492	BREX-system-P-loop-protein-BrxC-[Candidatus-Accumulibacter-sp.]
WP_076471941.1	Type6.BREX	<-DUF3262<-?<-?<-?<-JetA|| wHTH+WYL+WCX -> BrxE -> BrxA-DNA-binding -> BrxB-BrxF-iSTAND -> DUF6079 *-> OLD-ABC-> PIN -> N6-METHYLASE -> N6-METHYLASE ->ParB+HNH-> PglZ -> SIGMA+OB-Fold+AAA-ATPase+HKD-DNase-BrxL -><-JAB	1207	Bacteria>Pseudomonadota>Betaproteobacteria>Burkholderiales>Alcaligenaceae>Achromobacter	GCF_001957055.1	Achromobacter_xylosoxidans	BIZ53_RS00410	81330..84953	85698	BREX-system-P-loop-protein-BrxC-[Achromobacter-xylosoxidans]
WP_250462416.1	Type6.BREX	<-MODE-HTH+PBPII||?-><-ADH_N+ADH_zinc_N||?-><-?|| BrxE -> BrxA-DNA-binding -> BrxB-BrxF-iSTAND -> DUF6079 *->Tox-RelA-> N6-METHYLASE -> PglZ -> SIGMA+OB-Fold+AAA-ATPase+Lon-Protease-BrxL -> PIN ->	1181	Bacteria>Pseudomonadota>Betaproteobacteria>Burkholderiales>Burkholderiaceae>Caballeronia	GCF_023631485.1	Caballeronia_sp._GAFFF2	NCT04_RS00125	29887..33432	2921741	BREX-system-P-loop-protein-BrxC-[Caballeronia-sp.-GAFFF2]
WP_108330269.1	Type1.BREX	<- SIGMA+OB-Fold+AAA-ATPase+Lon-Protease-BrxL <- PglZ <-HNH<-OLD-ABC<- N6-METHYLASE <-REase<-OLD-ABC<- DUF6079 *<- BrxB-BrxF-iSTAND <- BrxA-DNA-binding <- PIN <-ZnR+TPR	1154	Bacteria>Pseudomonadota>Betaproteobacteria>Burkholderiales>Comamonadaceae>Limnohabitans	GCF_003063435.1	Limnohabitans_sp._2KL-1	B9Z47_RS05555	224189..227653	1100699	BREX-system-P-loop-protein-BrxC-[Limnohabitans-sp.-2KL-1]
WP_108311897.1	Type1.BREX	<-SF1-SF2-Helicase<- SIGMA+OB-Fold+AAA-ATPase+Lon-Protease-BrxL <- PglZ <-HNH<-OLD-ABC<- N6-METHYLASE <- DUF6079 *<- BrxB-BrxF-iSTAND <- BrxA-DNA-binding <- PIN <-ZnR+TPR	1153	Bacteria>Pseudomonadota>Betaproteobacteria>Burkholderiales>Comamonadaceae>Limnohabitans	GCF_003063455.1	Limnohabitans_parvus	B9Z37_RS03875	837390..840851	540061	BREX-system-P-loop-protein-BrxC-[Limnohabitans-parvus]
PIQ50876.1	Type1.BREX	dCache_1+HisKA+HATPase_c-><- SIGMA+OB-Fold+AAA-ATPase+Lon-Protease-BrxL <- PglZ <-Schlafen+HATPase+wHTH<-SbcC<- N6-METHYLASE <- PIN <-MazE_antitoxin<- DUF6079 *<- BrxB-BrxF-iSTAND <- BrxA-DNA-binding <-RHH_5||?->?->SF1-SF2-Helicase+REase->?->RelE-ParE->	1153	Bacteria>Pseudomonadota>Betaproteobacteria>Burkholderiales>Comamonadaceae>unclassified_Comamonadaceae	GCA_002786915.1	Comamonadaceae_bacterium_CG12_big_fil_rev_8_21_14_0_65_59_15	COW02_17785	48153..51614	1973952	hypothetical-protein-COW02_17785-[Comamonadaceae-bacterium-CG12_big_fil_rev_8_21_14_0_65_59_15]
ODV10849.1	Type1.BREX	<-RelE-ParE<-?<-?<-DOC<-MazE_antitoxin||PhdYeFM_antitox-> PIN -> BrxA-DNA-binding -> DUF6079 -p*->	451	Bacteria>Pseudomonadota>Betaproteobacteria>Nitrosomonadales>Thiobacillaceae>Thiobacillus	GCA_001725535.1	Thiobacillus_sp._SCN_64-317	ABT22_10740	12066..13421	1660143	MAG:-hypothetical-protein-ABT22_10740-[Thiobacillus-sp.-SCN-64-317]
MBN8763784.1	Type1.BREX	<-PRTase||?->?-><- N6-METHYLASE -p<- DUF6079 -p*<- PIN <-PhdYeFM_antitox||MazE_antitoxin->DOC->?->RelE-ParE->	865	Bacteria>Pseudomonadota>Betaproteobacteria>Nitrosomonadales>Thiobacillaceae>Thiobacillus	GCA_017309235.1	Thiobacillus_sp.	JOJO2_08935	6734..9331	924	hypothetical-protein-[Thiobacillus-sp.]
WP_011238768.1	Type1.BREX	<-Peptidase_M48_N+Peptidase_M48||35exo-><-?<-?<-?||?-> wHTH+WYL+WCX -> BrxA-DNA-binding -> BrxB-BrxF-iSTAND -> DUF6079 *-><- PIN <-RHH_6||?-> N6-METHYLASE ->OLD-ABC->HNH->OLD-ABC->TOPRIM->OLD-ABC->	1203	Bacteria>Pseudomonadota>Betaproteobacteria>Rhodocyclales>Rhodocyclaceae>Aromatoleum	GCF_000025965.1	Aromatoleum_aromaticum	EBN1_RS14750	3092506..3096117	551760	BREX-system-P-loop-protein-BrxC-[Aromatoleum-aromaticum]
WP_141017182.1	Type6.BREX	PhdYeFM_antitox->RelE-ParE-><-DOC+HTH<-Phage_integrase<-?|| wHTH+WYL+WCX -> BrxE -> BrxA-DNA-binding -> BrxB-BrxF-iSTAND -> DUF6079 *-> OLD-ABC-> PIN -> N6-METHYLASE -> N6-METHYLASE ->?->ParB+HNH-> PglZ -> SIGMA+OB-Fold+AAA-ATPase+HKD-DNase-BrxL -><-XIS-HTH	1207	Bacteria>Pseudomonadota>Betaproteobacteria>Rhodocyclales>Zoogloeaceae>Azoarcus	GCF_006496635.1	Azoarcus_sp._DD4	CJ010_RS05935	1245819..1249442	2027405	BREX-system-P-loop-protein-BrxC-[Azoarcus-sp.-DD4]
TXH82461.1	Type1.BREX	PhdYeFM_antitox-> PIN -> BrxA-DNA-binding -> BrxB-BrxF-iSTAND -> DUF6079 *->OLD-ABC->REase-> N6-METHYLASE -p->	1153	Bacteria>Pseudomonadota>Betaproteobacteria>Rhodocyclales>Zoogloeaceae>Thauera	GCA_008015255.1	Thauera_aminoaromatica	E6Q80_15435	2474..5935	164330	BREX-system-P-loop-protein-BrxC-[Thauera-aminoaromatica]
WP_037984790.1	Type1.BREX	wHTH+WYL+WCX -> BrxA-DNA-binding -> BrxB-BrxF-iSTAND -> DUF6079 *-><- PIN <-RHH_6|| N6-METHYLASE ->UDG->REase->?-> PglZ ->	1203	Bacteria>Pseudomonadota>Betaproteobacteria>Rhodocyclales>Zoogloeaceae>Thauera	GCF_000310145.2	Thauera_sp._28	C662_RS14985	3144..6755	303682	BREX-system-P-loop-protein-BrxC-[Thauera-sp.-28]
WP_247724881.1	Type1.BREX	<- PglZ -p<-REase+AIPR<- N6-METHYLASE <- PIN <-MazE_antitoxin<- DUF6079 *<- BrxB-BrxF-iSTAND <- BrxA-DNA-binding <-SPOUT<-RnaseHII	1153	Bacteria>Pseudomonadota>Betaproteobacteria>Rhodocyclales>Zoogloeaceae>Thauera	GCF_023093595.1	Thauera_aromatica	MXC99_RS07615	6533..9994	59405	BREX-system-P-loop-protein-BrxC-[Thauera-aromatica]
WP_247725420.1	Type6.BREX	<-wHTH<-ORC-CDC6<-ParA-Soj-PloopNTPase<-HEPN<- N6-METHYLASE -p<- PIN <-?<- N6-METHYLASE <-MNS+MarR-HTH<- DUF6079 *<- BrxB-BrxF-iSTAND <- BrxA-DNA-binding <- BrxE <-?||ABC-ATPase+SbcC+ABC-ATPase+SbcC->HAD->	1209	Bacteria>Pseudomonadota>Betaproteobacteria>Rhodocyclales>Zoogloeaceae>Thauera	GCF_023093595.1	Thauera_aromatica	MXC99_RS10610	105350..108979	59405	BREX-system-P-loop-protein-BrxC-[Thauera-aromatica]
WP_247725480.1	Type1.BREX	<- SIGMA+OB-Fold+AAA-ATPase+Lon-Protease-BrxL <- PglZ <-PIWI<-TOPRIM<-OLD-ABC<- N6-METHYLASE ||RHH_6-> PIN -><- DUF6079 *<- BrxB-BrxF-iSTAND <- BrxA-DNA-binding <- wHTH+WYL+WCX	1203	Bacteria>Pseudomonadota>Betaproteobacteria>Rhodocyclales>Zoogloeaceae>Thauera	GCF_023093595.1	Thauera_aromatica	MXC99_RS10910	53320..56931	59405	BREX-system-P-loop-protein-BrxC-[Thauera-aromatica]
MBK6655412.1	Type1.BREX	HDRnase->?-><-HTH_19||?->?->PhdYeFM_antitox-> PIN -> BrxA-DNA-binding -> BrxB-BrxF-iSTAND -> DUF6079 *->OLD-ABC->REase-> N6-METHYLASE ->ZnR->viral-capsid->?->?->TPR+REase->	1153	Bacteria>Pseudomonadota>Betaproteobacteria>Rhodocyclales>Zoogloeaceae>Zoogloea	GCA_016703645.1	Zoogloea_sp.	IPG40_16580	748210..751671	49181	BREX-system-P-loop-protein-BrxC-[Zoogloea-sp.]
MBK7848388.1	Type1.BREX	Arm-DNA-bind_2+Phage_int_SAM_3+Phage_integrase->?-><-HTH_19||?->?->PhdYeFM_antitox-> PIN -> BrxA-DNA-binding -> BrxB-BrxF-iSTAND -> DUF6079 *->REase->ZnR->viral-capsid->?->?->TPR+REase->?-> PglZ -> SIGMA+OB-Fold+AAA-ATPase+Lon-Protease-BrxL ->	1153	Bacteria>Pseudomonadota>Betaproteobacteria>Rhodocyclales>Zoogloeaceae>Zoogloea	GCA_016714095.1	Zoogloea_sp.	IPJ73_15250	34710..38171	49181	BREX-system-P-loop-protein-BrxC-[Zoogloea-sp.]
WP_141354347.1	Type1.BREX	wHTH+WYL+WCX -> BrxA-DNA-binding -> BrxB-BrxF-iSTAND -> DUF6079 *-><- PIN <-RHH_6|| N6-METHYLASE -p->HKD-DNase-> N6-METHYLASE ->HTH+REase->ZnR+TOPRIM-> PglZ -> SIGMA+OB-Fold+AAA-ATPase+Lon-Protease-BrxL ->	1203	Bacteria>Pseudomonadota>Betaproteobacteria>Rhodocyclales>Zoogloeaceae>Zoogloea	GCF_006539865.1	Zoogloea_ramigera	ZRA01_RS16820	2482..6093	350	BREX-system-P-loop-protein-BrxC-[Zoogloea-ramigera]
WP_144225094.1	Type1.BREX	<-Phage_integrase<-DUF2787<-?<-?||XIS-HTH->XIS-HTH-> BrxA-DNA-binding -> BrxB-BrxF-iSTAND -p-> DUF6079 *-> OLD-ABC-> PIN -> N6-METHYLASE -> PglZ -> SIGMA+OB-Fold+AAA-ATPase+Lon-Protease-BrxL ->?-><-Arm-DNA-bind_3+Phage_integrase<-?||RNasePH->	1145	Bacteria>Pseudomonadota>Gammaproteobacteria>Alteromonadales>Shewanellaceae>Shewanella	GCF_017580345.1	Shewanella_algae	I6M43_RS01845	49386..52823	38313	BREX-system-P-loop-protein-BrxC-[Shewanella-algae]
WP_200158841.1	Type1.BREX	<- SIGMA+OB-Fold+AAA-ATPase+Lon-Protease-BrxL <- PglZ <-HNH<-OLD-ABC<-SF1-SF2-Helicase<-OLD-ABC+TOPRIM<- N6-METHYLASE <- PIN <-OLD-ABC <- DUF6079 *<- BrxB-BrxF-iSTAND <- BrxA-DNA-binding <-GDResolvase+HIN-HTH<-ZnR	1154	Bacteria>Pseudomonadota>Gammaproteobacteria>Chromatiales>Chromatiaceae>Allochromatium	GCF_016584135.1	Allochromatium_vinosum	CK029_RS16480	18892..22356	1049	BREX-system-P-loop-protein-BrxC-[Allochromatium-vinosum]
MBV5272983.1	Type1.BREX	<- PglZ <-?<-REase+AIPR<- N6-METHYLASE -p<- N6-METHYLASE -p<- N6-METHYLASE -p<- PIN <-MazE_antitoxin<- DUF6079 *<- BrxB-BrxF-iSTAND <- BrxA-DNA-binding <-Aminotran_3<-FURR-HTH||?-><-?<-?<-?<-Classical-AAA	1143	Bacteria>Pseudomonadota>Gammaproteobacteria>Chromatiales>Chromatiaceae>Lamprocystis	GCA_019163055.1	Lamprocystis_purpurea	JZU52_04840	9550..12981	61598	BREX-system-P-loop-protein-BrxC-[Lamprocystis-purpurea]
WP_020507127.1	Type1.BREX	XIS-HTH-> BrxE -> BrxA-DNA-binding -> BrxB-BrxF-iSTAND -> DUF6079 *->Schlafen+HATPase+wHTH+HTH-><-?|| N6-METHYLASE ->?-> PIN -> N6-METHYLASE ->AbrB-HTH->	1179	Bacteria>Pseudomonadota>Gammaproteobacteria>Chromatiales>Chromatiaceae>Lamprocystis	GCF_000379525.1	Lamprocystis_purpurea	A390_RS0123535	17517..21056	61598	BREX-system-P-loop-protein-BrxC-[Lamprocystis-purpurea]
WP_062271501.1	Type1.BREX	<-OLD-ABC<-RHH<-SF1-SF2-Helicase<-OLD-ABC+TOPRIM<- N6-METHYLASE <- PIN <-OLD-ABC <- PIN <-?<- DUF6079 *<- BrxB-BrxF-iSTAND <- BrxA-DNA-binding <-tRNA<-Aminotran_1_2||SF1-SF2-Helicase->	1153	Bacteria>Pseudomonadota>Gammaproteobacteria>Chromatiales>Chromatiaceae>Marichromatium	GCF_001583505.1	Marichromatium_gracile	AY586_RS00820	23071..26532	1048	BREX-system-P-loop-protein-BrxC-[Marichromatium-gracile]
QVL48954.1	Type1.BREX	<- PglZ <- PIN <-ParD_antitoxin<-wHTH<-TOPRIM<-SbcC<- N6-METHYLASE <-ACET+ACET<-TraJ-RHH<- DUF6079 *<- BrxB-BrxF-iSTAND <- BrxA-DNA-binding <-tRNA||RADICAL-SAM->Glycos_transf_2-><-?||?-><-tRNA||NTP_transf+HDRnase->	1136	Bacteria>Pseudomonadota>Gammaproteobacteria>Chromatiales>Chromatiaceae>Thiocapsa	GCA_018399035.1	Thiocapsa_sp.	KFB96_26095	5699625..5703035	2024551	MAG:-BREX-system-P-loop-protein-BrxC-[Thiocapsa-sp.]
WP_200385867.1	Type1.BREX	DUF6079 *->?-> PIN -><-?|| N6-METHYLASE ->OLD-ABC->HNH-> PglZ ->AbrB-HTH->RHH->	1179	Bacteria>Pseudomonadota>Gammaproteobacteria>Chromatiales>Chromatiaceae>Thiocapsa	GCF_016583985.1	Thiocapsa_imhoffii	CKO25_RS00005	26..3565	382777	BREX-system-P-loop-protein-BrxC-[Thiocapsa-imhoffii]
WP_100920354.1	Type6.BREX	<-HMA+HAD+HAD||?->?->?-> BrxE -> BrxA-DNA-binding -> BrxB-BrxF-iSTAND ->?->|| DUF6079 *->Schlafen+HATPase+wHTH+HTH-><-?|| N6-METHYLASE ->?-> PIN -> N6-METHYLASE ->RHH-> PIN ->RHH->	1178	Bacteria>Pseudomonadota>Gammaproteobacteria>Chromatiales>Chromatiaceae>Thiodictyon	GCF_002813775.1	Candidatus_Thiodictyon_syntrophicum	THSYN_17920	4302311..4305847	1166950	BREX-system-P-loop-protein-BrxC-[Candidatus-Thiodictyon-syntrophicum]
WP_100920747.1	Type1.BREX	BrxA-DNA-binding -> BrxB-BrxF-iSTAND ->?-> DUF6079 *->PhdYeFM_antitox-> PIN ->MqsA_antitoxin-> N6-METHYLASE -p-> N6-METHYLASE ->Transposase_31->OLD-ABC->Polbeta-> PglZ ->	1194	Bacteria>Pseudomonadota>Gammaproteobacteria>Chromatiales>Chromatiaceae>Thiodictyon	GCF_002813775.1	Candidatus_Thiodictyon_syntrophicum	THSYN_RS20415	4833326..4836910	1166950	BREX-system-P-loop-protein-BrxC-[Candidatus-Thiodictyon-syntrophicum]
WP_015282553.1	Type6.BREX	<- SIGMA+OB-Fold+AAA-ATPase+HKD-DNase-BrxL <- PglZ <- PIN <-RHH<- N6-METHYLASE <-?<-SF1-SF2-Helicase<- N6-METHYLASE <- DUF6079 *<- BrxB-BrxF-iSTAND <- BrxA-DNA-binding <- BrxE	1209	Bacteria>Pseudomonadota>Gammaproteobacteria>Chromatiales>Chromatiaceae>Thioflavicoccus	GCF_000327045.1	Thioflavicoccus_mobilis	THIMO_RS17980	83065..86694	80679	BREX-system-P-loop-protein-BrxC-[Thioflavicoccus-mobilis]
MCG6940983.1	Type1.BREX	<- N6-METHYLASE -p<- PIN <-VapB_antitoxin<- DUF6079 -p*<-tRNA<-?<-AA_kinase+ACT+ACT_7	251	Bacteria>Pseudomonadota>Gammaproteobacteria>Chromatiales>Chromatiaceae>Thiohalocapsa	GCA_022340605.1	Thiohalocapsa_sp.	LJE69_07005	1995..2750	2497641	hypothetical-protein-[Thiohalocapsa-sp.]
WP_058554449.1	Type6.BREX	TIR->?->?->?-> BrxE -> BrxA-DNA-binding -> BrxB-BrxF-iSTAND -> DUF6079 *-> N6-METHYLASE ->?->Schlafen+HATPase+wHTH-> N6-METHYLASE ->?-> PIN -> PglZ -> SIGMA+OB-Fold+AAA-ATPase+HKD-DNase-BrxL -><-tRNA||HHH_5+SF1-SF2-Helicase->	1214	Bacteria>Pseudomonadota>Gammaproteobacteria>Chromatiales>Chromatiaceae>Thiohalocapsa	GCF_001469165.1	Thiohalocapsa_sp._ML1	ML01_RS05745	155025..158669	1431688	BREX-system-P-loop-protein-BrxC-[Thiohalocapsa-sp.-ML1]
WP_058554877.1	Type1.BREX	DDE_Tnp_1->?->?->?->?->AA_kinase+ACT+ACT_7->tRNA-> BrxA-DNA-binding -> BrxB-BrxF-iSTAND -> DUF6079 *->VapB_antitoxin->VapB_antitoxin-> PIN -><-?|| N6-METHYLASE ->	1152	Bacteria>Pseudomonadota>Gammaproteobacteria>Chromatiales>Chromatiaceae>Thiohalocapsa	GCF_001469165.1	Thiohalocapsa_sp._ML1	ML01_RS08025	156057..159515	1431688	BREX-system-P-loop-protein-BrxC-[Thiohalocapsa-sp.-ML1]
WP_200239121.1	Type1.BREX	<- PglZ <- PIN <-PhdYeFM_antitox<-HicA_toxin<-HicB_lk_antitox<-MazE_antitoxin<- PIN <-MazE_antitoxin<- DUF6079 *<- BrxB-BrxF-iSTAND <- BrxA-DNA-binding <-?<-ABhydrolase<-?<-?||?->?-><-REase	1197	Bacteria>Pseudomonadota>Gammaproteobacteria>Chromatiales>Chromatiaceae>Thiohalocapsa	GCF_016583825.1	Thiohalocapsa_halophila	CKO31_RS15145	13443..17036	69359	BREX-system-P-loop-protein-BrxC-[Thiohalocapsa-halophila]
WP_164452822.1	Type6.BREX	<-NtrC-AAA+FIS-HTH||?->?->SF1-SF2-Helicase+DUF1998->GrpE->?-> BrxE -> BrxA-DNA-binding -> BrxB-BrxF-iSTAND -> DUF6079 *->?-> N6-METHYLASE -p->?-> PIN ->AbrB-HTH->?-> N6-METHYLASE ->OLD-ABC->TOPRIM->	1214	Bacteria>Pseudomonadota>Gammaproteobacteria>Chromatiales>Chromatiaceae>Thiorhodococcus	GCF_010820565.1	Thiorhodococcus_minor	G3446_RS10685	55754..59398	57489	BREX-system-P-loop-protein-BrxC-[Thiorhodococcus-minor]
EIC19850.1	Type1.BREX	<- SIGMA+OB-Fold+AAA-ATPase+Lon-Protease-BrxL <- PglZ <-RelE-ParE<-PhdYeFM_antitox<-Schlafen+HATPase+wHTH<- N6-METHYLASE <- PIN <-VapB_antitoxin<- DUF6079 *<- BrxB-BrxF-iSTAND <- BrxA-DNA-binding <-?<-HISKIN<-?<-?<-?<-Thioredoxin	1152	Bacteria>Pseudomonadota>Gammaproteobacteria>Chromatiales>Chromatiaceae>Thiorhodovibrio	GCA_000228725.3	Thiorhodovibrio_sp._970	Thi970DRAFT_03455	369329..372787	631362	hypothetical-protein-Thi970DRAFT_03455-[Thiorhodovibrio-sp.-970]
WP_009149681.1	Type6.BREX	<-BrxD<-?<-?<-?<-?|| BrxE -> BrxA-DNA-binding -> BrxB-BrxF-iSTAND -> DUF6079 *->?->RelE-ParE-> N6-METHYLASE ->?-> PIN -> N6-METHYLASE -p->?-> N6-METHYLASE -p->RHH->	1208	Bacteria>Pseudomonadota>Gammaproteobacteria>Chromatiales>Chromatiaceae>Thiorhodovibrio	GCF_000228725.2	Thiorhodovibrio_sp._970	THI970DRAFT_RS14125	1743457..1747083	631362	BREX-system-P-loop-protein-BrxC-[Thiorhodovibrio-sp.-970]
WP_223296802.1	Type1.BREX	<- SIGMA+OB-Fold+AAA-ATPase+Lon-Protease-BrxL <- PglZ <-RelE-ParE<-PhdYeFM_antitox<-Schlafen+HATPase+wHTH<- N6-METHYLASE <- PIN <-VapB_antitoxin<- DUF6079 *<- BrxB-BrxF-iSTAND <- BrxA-DNA-binding <-?<-HISKIN<-?<-?<-Thioredoxin||?->REC+GGDEF+EAL->	1154	Bacteria>Pseudomonadota>Gammaproteobacteria>Chromatiales>Chromatiaceae>Thiorhodovibrio	GCF_000228725.2	Thiorhodovibrio_sp._970	THI970DRAFT_RS16355	369329..372793	631362	BREX-system-P-loop-protein-BrxC-[Thiorhodovibrio-sp.-970]
WP_201065314.1	Type6.BREX	CR-ATPase5+REase->?-><-?<-HicB<-?||?-><- N6-METHYLASE <- PIN <-wHTH<- DUF6079 *<- BrxB-BrxF-iSTAND <- BrxA-DNA-binding <- BrxE ||?->?->?->?->?->BrxD->	1212	Bacteria>Pseudomonadota>Gammaproteobacteria>Chromatiales>Chromatiaceae>Thiorhodovibrio	GCF_016653415.1	Thiorhodovibrio_winogradskyi	CCR91_RS06145	56528..60166	77007	BREX-system-P-loop-protein-BrxC-[Thiorhodovibrio-winogradskyi]
WP_201066887.1	Type1.BREX	<-wHTH<-?<- PIN <-?<- N6-METHYLASE <-HicB_lk_antitox<-MazE_antitoxin<-?<- PIN <- DUF6079 *<- BrxB-BrxF-iSTAND <- BrxA-DNA-binding <-tRNA<-Sulfate_transp+STAS	1203	Bacteria>Pseudomonadota>Gammaproteobacteria>Chromatiales>Chromatiaceae>Thiorhodovibrio	GCF_016653415.1	Thiorhodovibrio_winogradskyi	CCR91_RS10560	25140..28751	77007	BREX-system-P-loop-protein-BrxC-[Thiorhodovibrio-winogradskyi]
TVQ85671.1	Type6.BREX	<- N6-METHYLASE -p<- PIN <-?<-?<-?<- N6-METHYLASE <- DUF6079 *<- BrxB-BrxF-iSTAND <- BrxA-DNA-binding <- BrxE <-?<-Aminotran_1_2<-?<-ABC-ATPase+SbcC	1212	Bacteria>Pseudomonadota>Gammaproteobacteria>Chromatiales>Chromatiaceae>unclassified_Chromatiaceae	GCA_007693535.1	Chromatiaceae_bacterium	EA400_15015	6841..10479	2049432	BREX-system-P-loop-protein-BrxC-[Chromatiaceae-bacterium]
MBK8638067.1	Type1.BREX	AAA-ATPase->?->ZnR->?->?->?-> DUF6079 *->PhdYeFM_antitox->AbrB-HTH-> PIN -> N6-METHYLASE -> PIN ->?->?-> PglZ -> SIGMA+OB-Fold+AAA-ATPase+Lon-Protease-BrxL -><-SIR2	1067	Bacteria>Pseudomonadota>Gammaproteobacteria>Chromatiales>Chromatiaceae>unclassified_Chromatiaceae	GCA_016714645.1	Chromatiaceae_bacterium	IPN92_07155	42706..45909	2049432	BREX-system-P-loop-protein-BrxC-[Chromatiaceae-bacterium]
MBK8639641.1	Type6.BREX	REC-><-?|| BrxE -> BrxA-DNA-binding -> BrxB-BrxF-iSTAND -> DUF6079 *->?-> N6-METHYLASE ->RHH-> PIN -> PglZ ->PolB->HEPN-> SIGMA+OB-Fold+AAA-ATPase+Lon-Protease-BrxL ->	1178	Bacteria>Pseudomonadota>Gammaproteobacteria>Chromatiales>Chromatiaceae>unclassified_Chromatiaceae	GCA_016714645.1	Chromatiaceae_bacterium	IPN92_15725	846355..849891	2049432	BREX-system-P-loop-protein-BrxC-[Chromatiaceae-bacterium]
MBP6733459.1	Type1.BREX	BrxA-DNA-binding -> BrxB-BrxF-iSTAND -> DUF6079 *-><- BrxB-BrxF-iSTAND ||?->PhdYeFM_antitox-> PIN -> N6-METHYLASE ->HTH+REase->REase-> N6-METHYLASE -p->	1171	Bacteria>Pseudomonadota>Gammaproteobacteria>Chromatiales>Chromatiaceae>unclassified_Chromatiaceae	GCA_017990755.1	Chromatiaceae_bacterium	KA142_01990	1791..5306	2049432	BREX-system-P-loop-protein-BrxC-[Chromatiaceae-bacterium]
MBP6807296.1	Type1.BREX	BrxA-DNA-binding -> BrxB-BrxF-iSTAND -> DUF6079 *->MazE_antitoxin-> PIN -> N6-METHYLASE -p->AbrB-HTH->	1194	Bacteria>Pseudomonadota>Gammaproteobacteria>Chromatiales>Chromatiaceae>unclassified_Chromatiaceae	GCA_017991215.1	Chromatiaceae_bacterium	KA125_03275	984..4568	2049432	BREX-system-P-loop-protein-BrxC-[Chromatiaceae-bacterium]
MCF7978332.1	Type1.BREX	<-AbrB-HTH<- PIN <-AbrB-HTH<- N6-METHYLASE <-DOC<-MazE_antitoxin<-MqsA_antitoxin<-?<- PIN <- DUF6079 *	1223	Bacteria>Pseudomonadota>Gammaproteobacteria>Chromatiales>Chromatiaceae>unclassified_Chromatiaceae	GCA_021736685.1	Chromatiaceae_bacterium	K9L82_09985	9865..13536	2049432	BREX-system-P-loop-protein-BrxC-[Chromatiaceae-bacterium]
MCF8016509.1	Type1.BREX	<-AbrB-HTH<- PIN <-AbrB-HTH<- N6-METHYLASE <-DOC<-MazE_antitoxin<-MqsA_antitoxin<-?<- PIN <- DUF6079 -p*	835	Bacteria>Pseudomonadota>Gammaproteobacteria>Chromatiales>Chromatiaceae>unclassified_Chromatiaceae	GCA_021736865.1	Chromatiaceae_bacterium	K9L65_12595	6416..8923	2049432	BREX-system-P-loop-protein-BrxC-[Chromatiaceae-bacterium]
MCP5407916.1	Type1.BREX	ABhydrolase->?->TraU->?->TraG_N-><-?|| BrxA-DNA-binding -> BrxB-BrxF-iSTAND -> DUF6079 *->MazE_antitoxin-> PIN -> N6-METHYLASE ->SIR2->?-> PglZ -> SIGMA+OB-Fold+AAA-ATPase+Lon-Protease-BrxL ->XIS-HTH->	1194	Bacteria>Pseudomonadota>Gammaproteobacteria>Chromatiales>Chromatiaceae>unclassified_Chromatiaceae	GCA_024235335.1	Chromatiaceae_bacterium	H6963_01295	248686..252270	2049432	BREX-system-P-loop-protein-BrxC-[Chromatiaceae-bacterium]
MCK7579547.1	Type1.BREX	<-OLD-ABC+TOPRIM<-?<-?<- N6-METHYLASE <- PIN <-OLD-ABC ||?-><- DUF6079 -p<- DUF6079 -p*<-?<- DUF6079 -p<- BrxB-BrxF-iSTAND <- BrxA-DNA-binding -p<-ZnR	299	Bacteria>Pseudomonadota>Gammaproteobacteria>Chromatiales>unclassified_Chromatiales	GCA_023156155.1	Chromatiales_bacterium	MZV65_30115	14491..15390	2026725	hypothetical-protein-[Chromatiales-bacterium]
MCK7579549.1	Type1.BREX	<- N6-METHYLASE <- PIN <-OLD-ABC ||?-><- DUF6079 -p<- DUF6079 -p<-?<- DUF6079 -p*<- BrxB-BrxF-iSTAND <- BrxA-DNA-binding -p<-ZnR	648	Bacteria>Pseudomonadota>Gammaproteobacteria>Chromatiales>unclassified_Chromatiales	GCA_023156155.1	Chromatiales_bacterium	MZV65_30125	15595..17541	2026725	BREX-system-P-loop-protein-BrxC-[Chromatiales-bacterium]
MBZ4194014.1	Type1.BREX	<- N6-METHYLASE <- PIN <-HTH+MPTase<- DUF6079 *<- BrxB-BrxF-iSTAND <- BrxA-DNA-binding <-?<-TPR	1138	Bacteria>Pseudomonadota>Gammaproteobacteria>Gammaproteobacteria_incertae_sedis>Candidatus_Competibacteraceae>Candidatus_Contendobacter	GCA_020035205.1	Candidatus_Contendobacter_sp.	LAE24_06875	5150..8566	2651161	BREX-system-P-loop-protein-BrxC-[Candidatus-Contendobacter-sp.]
MBK8752263.1	Type1.BREX	<-STYKinase-like<-?<-HTH_37||?-><-RHH_5||?->|| BrxA-DNA-binding -> BrxB-BrxF-iSTAND -> DUF6079 *->HTH+MPTase-> PIN -> N6-METHYLASE ->?->PemK_toxin-><-?<-?|| PglZ -> SIGMA+OB-Fold+AAA-ATPase+Lon-Protease-BrxL ->	1139	Bacteria>Pseudomonadota>Gammaproteobacteria>Gammaproteobacteria_incertae_sedis>Candidatus_Competibacteraceae>unclassified_Candidatus_Competibacteraceae	GCA_016710535.1	Candidatus_Competibacteraceae_bacterium	IPL99_11760	9123..12542	2053538	BREX-system-P-loop-protein-BrxC-[Candidatus-Competibacteraceae-bacterium]
MCB1769904.1	Type1.BREX	<- N6-METHYLASE -p<- PIN <-RHH<-MetJarc-HTH<-HicA_toxin<- N6-METHYLASE -p<- PIN <-?<- N6-METHYLASE <- DUF6079 -p*	760	Bacteria>Pseudomonadota>Gammaproteobacteria>Gammaproteobacteria_incertae_sedis>Candidatus_Competibacteraceae>unclassified_Candidatus_Competibacteraceae	GCA_020445005.1	Candidatus_Competibacteraceae_bacterium	KDJ31_09455	11685..13969	2053538	BREX-system-P-loop-protein-BrxC-[Candidatus-Competibacteraceae-bacterium]
WP_177417917.1	Type1.BREX	BrxB-BrxF-iSTAND -> DUF6079 *->MazE_antitoxin-> PIN -> N6-METHYLASE ->OLD-ABC->TOPRIM-> PglZ -> SIGMA+OB-Fold+AAA-ATPase+Lon-Protease-BrxL ->	1188	Bacteria>Pseudomonadota>Gammaproteobacteria>Gammaproteobacteria_incertae_sedis>sulfur-oxidizing_symbionts	GCF_011947365.2	endosymbiont_of_Lamellibrachia_barhami	G9Q73_RS00010	458..4024	205975	BREX-system-P-loop-protein-BrxC-[endosymbiont-of-Lamellibrachia-barhami]
MBS3952424.1	Type1.BREX	XIS-HTH-> BrxA-DNA-binding -> BrxB-BrxF-iSTAND -> DUF6079 *->OLD-ABC->REase-> N6-METHYLASE ->PhdYeFM_antitox-> PIN -> N6-METHYLASE -p->HTH+REase->Schlafen+HATPase+wHTH->SIR2->	1178	Bacteria>Pseudomonadota>Gammaproteobacteria>Methylococcales>Methylococcaceae>Methylomicrobium	GCA_018333785.1	Methylomicrobium_sp.	KGZ88_05695	2093..5629	2303549	BREX-system-P-loop-protein-BrxC-[Methylomicrobium-sp.]
WP_024296674.1	Type1.BREX	GDResolvase+HIN-HTH->?-> BrxA-DNA-binding -> BrxB-BrxF-iSTAND -> DUF6079 *->ParD_antitoxin-> PIN -> N6-METHYLASE ->SbcC->Schlafen+HATPase+wHTH-> PglZ -> SIGMA+OB-Fold+AAA-ATPase+Lon-Protease-BrxL ->	1202	Bacteria>Pseudomonadota>Gammaproteobacteria>Methylococcales>Methylococcaceae>Methylomicrobium	GCF_000527095.1	Methylomicrobium_lacus	METLA_RS0100460	95992..99600	136992	BREX-system-P-loop-protein-BrxC-[Methylomicrobium-lacus]
WP_255188315.1	Type1.BREX	<- PIN <- PIN <- SIGMA+OB-Fold+AAA-ATPase+Lon-Protease-BrxL <- PglZ <-TOPRIM<-OLD-ABC<- N6-METHYLASE <- PIN <-ParD_antitoxin<- DUF6079 *<- BrxB-BrxF-iSTAND <- BrxA-DNA-binding ||?-><-?<-HIN-HTH||?-><-ACET+ACET<-TraJ-RHH	1206	Bacteria>Pseudomonadota>Gammaproteobacteria>Methylococcales>Methylococcaceae>Methylomonas	GCF_024360925.2	Methylomonas_rapida	NM686_RS13235	2825288..2828908	2963939	BREX-system-P-loop-protein-BrxC-[Methylomonas-rapida]
WP_192375581.1	Type1.BREX	<-KAP-glycosylase<-KAP-NTPase<- PIN <- SIGMA+OB-Fold+AAA-ATPase+Lon-Protease-BrxL <- SIGMA+OB-Fold+AAA-ATPase+Lon-Protease-BrxL <- PglZ <- N6-METHYLASE <- PIN <-ParD_antitoxin<- DUF6079 *<- BrxB-BrxF-iSTAND <- BrxA-DNA-binding ||RNase-><-?<-GDResolvase+HIN-HTH	1207	Bacteria>Pseudomonadota>Gammaproteobacteria>Methylococcales>Methylococcaceae>Methylomonas	GCF_903064715.1	Methylomonas_albis	EBA_RS15675	3420343..3423966	1854563	BREX-system-P-loop-protein-BrxC-[Methylomonas-albis]
WP_080523593.1	Type1.BREX	<- N6-METHYLASE -p||HTH_Tnp_1+HTH_21+rve+rve_3-><- N6-METHYLASE <- PIN <-?<-ACET+ACET<- BrxB-BrxF-iSTAND <- DUF6079 *<- BrxB-BrxF-iSTAND <- BrxA-DNA-binding <-?<-?||GSDH-><-?<- BrxB-BrxF-iSTAND <-?<- PIN	1206	Bacteria>Pseudomonadota>Gammaproteobacteria>Methylococcales>Methylococcaceae>Methyloprofundus	GCF_002072955.1	Methyloprofundus_sedimenti	AU255_RS14210	277762..281382	1420851	BREX-system-P-loop-protein-BrxC-[Methyloprofundus-sedimenti]
MCF8007296.1	Type1.BREX	BrxA-DNA-binding -> BrxB-BrxF-iSTAND -> DUF6079 -p*-> N6-METHYLASE -p->?-> PIN -> PglZ -p-><-Aminotran_1_2	781	Bacteria>Pseudomonadota>Gammaproteobacteria>Methylococcales>Methylococcaceae>Methylovulum	GCA_021736885.1	Methylovulum_sp.	K9K84_07785	12351..14696	1916980	BREX-system-P-loop-protein-BrxC-[Methylovulum-sp.]
WP_088619247.1	Type1.BREX	<-Glycos_transf<-?<- SIGMA+OB-Fold+AAA-ATPase+Lon-Protease-BrxL <- PglZ <-Schlafen+HATPase+wHTH<-PHP+SbcC<- N6-METHYLASE <- PIN <-ParD_antitoxin<- DUF6079 *<- BrxB-BrxF-iSTAND <- BrxA-DNA-binding <- wHTH+WYL+WCX	1201	Bacteria>Pseudomonadota>Gammaproteobacteria>Methylococcales>Methylococcaceae>Methylovulum	GCF_002209385.1	Methylovulum_psychrotolerans	CEK71_RS09965	2189788..2193393	1704499	BREX-system-P-loop-protein-BrxC-[Methylovulum-psychrotolerans]
WP_103975698.1	Type1.BREX	<- SIGMA+OB-Fold+AAA-ATPase+Lon-Protease-BrxL <- PglZ <-Schlafen+HATPase+wHTH<-PHP+SbcC<-?<-?<- N6-METHYLASE <- PIN <-ParD_antitoxin<- DUF6079 *<- BrxB-BrxF-iSTAND <- BrxA-DNA-binding <- wHTH+WYL+WCX ||?->SAM-METHYLASE->?->SbcC->SAM-METHYLASE->SAM-METHYLASE->	1201	Bacteria>Pseudomonadota>Gammaproteobacteria>Methylococcales>Methylococcaceae>Methylovulum	GCF_002923755.1	Methylovulum_psychrotolerans	AADEFJLK_RS21345	25888..29493	1704499	BREX-system-P-loop-protein-BrxC-[Methylovulum-psychrotolerans]
WP_215479507.1	Type1.BREX	wHTH+WYL+WCX -p-> wHTH+WYL+WCX -> BrxA-DNA-binding -> BrxB-BrxF-iSTAND -> DUF6079 *->ParD_antitoxin-> PIN -> N6-METHYLASE ->?->?->PHP+SbcC->Schlafen+HATPase+wHTH-> PglZ -> SIGMA+OB-Fold+AAA-ATPase+Lon-Protease-BrxL ->	1201	Bacteria>Pseudomonadota>Gammaproteobacteria>Methylococcales>Methylococcaceae>Methylovulum	GCF_018722605.1	Methylovulum_psychrotolerans	KFZ76_RS21360	105012..108617	1704499	BREX-system-P-loop-protein-BrxC-[Methylovulum-psychrotolerans]
TSA38613.1	Type1.BREX	BrxA-DNA-binding -> BrxB-BrxF-iSTAND -> DUF6079 *->PRTase->?-> PIN ->?-> N6-METHYLASE -p->?->?-> PIN -> N6-METHYLASE -p->	1218	Bacteria>Pseudomonadota>Gammaproteobacteria>Methylococcales>Methylococcaceae>unclassified_Methylococcaceae	GCA_007280895.1	Methylococcaceae_bacterium	D4R63_10680	1521..5177	1933926	BREX-system-P-loop-protein-BrxC-[Methylococcaceae-bacterium]
WP_040239271.1	Type1.BREX	<-sGTP<-?<-?||?->tRNA-> BrxA-DNA-binding -> BrxB-BrxF-iSTAND -> DUF6079 *-> OLD-ABC-> PIN -> N6-METHYLASE ->ParB+HNH->?->?->?-> PglZ -> SIGMA+OB-Fold+AAA-ATPase+Lon-Protease-BrxL ->	1144	Bacteria>Pseudomonadota>Gammaproteobacteria>Oceanospirillales>Halomonadaceae>Chromohalobacter	GCF_000821045.2	Chromohalobacter_japonicus	BN995_RS00415	38131..41565	223900	BREX-system-P-loop-protein-BrxC-[Chromohalobacter-japonicus]
WP_189514712.1	Type1.BREX	<-DUF4096+DDE_Tnp_1+DDE_Tnp_1_2<-?||?->DUF932->?->?->?-> BrxA-DNA-binding -> BrxB-BrxF-iSTAND -> DUF6079 *-> OLD-ABC-> PIN -> N6-METHYLASE ->ParB+HNH-> PglZ -> SIGMA+OB-Fold+AAA-ATPase+Lon-Protease-BrxL ->McrB-AAA->wHTH+REase->	1144	Bacteria>Pseudomonadota>Gammaproteobacteria>Oceanospirillales>Halomonadaceae>Kushneria	GCF_014652795.1	Kushneria_pakistanensis	IE123_RS02380	531699..535133	1508770	BREX-system-P-loop-protein-BrxC-[Kushneria-pakistanensis]
WP_163650581.1	Type1.BREX	<- N6-METHYLASE -p<- PIN <-OLD-ABC <- DUF6079 *<- BrxB-BrxF-iSTAND <- BrxA-DNA-binding <-tRNA<-?||?->?->sGTP->	1144	Bacteria>Pseudomonadota>Gammaproteobacteria>Oceanospirillales>Halomonadaceae>Modicisalibacter	GCF_010993675.1	Modicisalibacter_sp._'Wilcox'	G7A98_RS15465	3721..7155	2679914	BREX-system-P-loop-protein-BrxC-[Modicisalibacter-sp.-'Wilcox']
WP_224416377.1	Type1.BREX	<- PglZ <-?<-?<-?<- N6-METHYLASE -p<-HTH_Tnp_1+HTH_21+rve+rve_3<- N6-METHYLASE <- PIN <-OLD-ABC <- DUF6079 *<- BrxB-BrxF-iSTAND <- BrxA-DNA-binding <-tRNA<-?||?->?->sGTP->	1144	Bacteria>Pseudomonadota>Gammaproteobacteria>Oceanospirillales>Halomonadaceae>Modicisalibacter	GCF_020147965.1	Modicisalibacter_tunisiensis	KGQ90_RS13860	2977413..2980847	390637	BREX-system-P-loop-protein-BrxC-[Modicisalibacter-tunisiensis]
WP_202806908.1	Type1.BREX	<- PglZ <-TOPRIM<-OLD-ABC<-SbcC<- N6-METHYLASE -p<- N6-METHYLASE <- N6-METHYLASE -p<- PIN <-OLD-ABC <- DUF6079 -p*<- DUF6079 -p<- BrxB-BrxF-iSTAND <- BrxA-DNA-binding <-XIS-HTH<-?<-HDRnase<-Phage_int_SAM<-Integrase_1||XIS-HTH->	460	Bacteria>Pseudomonadota>Gammaproteobacteria>Oceanospirillales>Oceanospirillaceae>Nitrincola	GCF_000585235.1	Nitrincola_nitratireducens	D791_RS20495	12347..13729	1229521	hypothetical-protein-[Nitrincola-nitratireducens]
WP_202806909.1	Type1.BREX	<-TOPRIM<-OLD-ABC<-SbcC<- N6-METHYLASE -p<- N6-METHYLASE <- N6-METHYLASE -p<- PIN <-OLD-ABC <- DUF6079 -p<- DUF6079 -p*<- BrxB-BrxF-iSTAND <- BrxA-DNA-binding <-XIS-HTH<-?<-HDRnase<-Phage_int_SAM<-Integrase_1||XIS-HTH->Phage_integrase->	697	Bacteria>Pseudomonadota>Gammaproteobacteria>Oceanospirillales>Oceanospirillaceae>Nitrincola	GCF_000585235.1	Nitrincola_nitratireducens	D791_RS20500	13689..15782	1229521	BREX-system-P-loop-protein-BrxC-[Nitrincola-nitratireducens]
HBO4843314.1	Type6.BREX	<-ACET<-YjhX_toxin||?->?-><-MODE-HTH+PBPII|| wHTH+WYL+WCX -> BrxE -> BrxA-DNA-binding -> BrxB-BrxF-iSTAND -> DUF6079 *-> OLD-ABC-> PIN -> N6-METHYLASE ->?-> PglZ -> SIGMA+OB-Fold+AAA-ATPase+HKD-DNase-BrxL -><-HDRnase||PrlF_antitoxin->	1207	Bacteria>Pseudomonadota>Gammaproteobacteria>Pseudomonadales>Pseudomonadaceae>Pseudomonas	GCA_021660595.1	Pseudomonas_aeruginosa	L4V37_002405	61513..65136	287	BREX-system-P-loop-protein-BrxC-[Pseudomonas-aeruginosa]
HFD80332.1	Type1.BREX	<- SIGMA+OB-Fold+AAA-ATPase+Lon-Protease-BrxL <- PglZ <-Schlafen+HATPase+wHTH<-PHP+SbcC<- N6-METHYLASE <- PIN <- DUF6079 *<- BrxB-BrxF-iSTAND <- BrxA-DNA-binding	1156	Bacteria>Pseudomonadota>Gammaproteobacteria>unclassified_Gammaproteobacteria	GCA_011321775.1	Gammaproteobacteria_bacterium	ENK05_08090	12367..15837	1913989	BREX-system-P-loop-protein-BrxC-[Gammaproteobacteria-bacterium]
MBT5223355.1	Type1.BREX	BrxA-DNA-binding -> BrxB-BrxF-iSTAND -> DUF6079 *->MazE_antitoxin-> PIN -> N6-METHYLASE -> N6-METHYLASE ->HEPN->GrpE->?->?->RelE-ParE->	1192	Bacteria>Pseudomonadota>Gammaproteobacteria>unclassified_Gammaproteobacteria	GCA_018661125.1	Gammaproteobacteria_bacterium	HDM15_11165	1377..4955	1913989	BREX-system-P-loop-protein-BrxC-[Gammaproteobacteria-bacterium]
MBN5083090.1	Type6.BREX	<-DUF932<- SIGMA+OB-Fold+AAA-ATPase+HKD-DNase-BrxL <- PglZ <-ParB+HNH<- N6-METHYLASE <- N6-METHYLASE <- PIN <-OLD-ABC <- DUF6079 *<- BrxB-BrxF-iSTAND <- BrxA-DNA-binding <- BrxE <- wHTH+WYL+WCX <-Arm-DNA-bind_3+Phage_integrase<-tRNA<-SIGMA-HTH+SIGMA-HTH+SIGMA-HTH	1207	Bacteria>Pseudomonadota>Gammaproteobacteria>Xanthomonadales>Xanthomonadaceae>Stenotrophomonas	GCA_017150495.1	Stenotrophomonas_maltophilia	JY467_05250	61681..65304	40324	BREX-system-P-loop-protein-BrxC-[Stenotrophomonas-maltophilia]
WP_004146422.1	Type6.BREX	<-XIS-HTH||?->Phage_integrase->tRNA->Arm-DNA-bind_3+Phage_integrase-> wHTH+WYL+WCX -> BrxE -> BrxA-DNA-binding -> BrxB-BrxF-iSTAND -> DUF6079 *-> OLD-ABC-> PIN -> N6-METHYLASE -> N6-METHYLASE ->ParB+HNH-> PglZ -> SIGMA+OB-Fold+AAA-ATPase+HKD-DNase-BrxL ->DUF932->	1207	Bacteria>Pseudomonadota>Gammaproteobacteria>Xanthomonadales>Xanthomonadaceae>Stenotrophomonas	GCF_000355725.1	Stenotrophomonas_maltophilia	G5A34_RS07915	81973..85596	40324	BREX-system-P-loop-protein-BrxC-[Stenotrophomonas-maltophilia]

Protein-ID	System	Gene-Neighbourhoods	Length	Taxonomy	Assembly-ID	Strain	Locus-Tag	Genomic-coordinates	TaxID	Fasta-defline
VUJ46804.1	Type6.BREX	<-LexA-protease||?-><-Phage_integrase||Arm-DNA-bind_3+Phage_integrase-> wHTH+WYL+WCX -> BrxE -> BrxA-DNA-binding -> BrxB-BrxF-iSTAND -> DUF6079 *-> OLD-ABC-> PIN -> N6-METHYLASE -> N6-METHYLASE ->ParB+HNH-> PglZ -> SIGMA+OB-Fold+AAA-ATPase+HKD-DNase-BrxL ->DUF932->	1207	Bacteria>Pseudomonadota>Gammaproteobacteria>Xanthomonadales>Xanthomonadaceae>Stenotrophomonas	-	Stenotrophomonas_maltophilia	N5D45_RS14200	24687..28310	40324	hypothetical-protein-NGKLMNPD_00401-[Stenotrophomonas-maltophilia]
WP_142804161.1	Type6.BREX	TPR+TPR-><-?|| BrxE -> BrxA-DNA-binding -> BrxB-BrxF-iSTAND -> DUF6079 *-> N6-METHYLASE ->AbrB-HTH-> PIN -> N6-METHYLASE ->DOC+HTH-> PglZ -> SIGMA+OB-Fold+AAA-ATPase+HKD-DNase-BrxL ->	1210	Bacteria>Pseudomonadota>Hydrogenophilia>Hydrogenophilales>Hydrogenophilaceae>Tepidiphilus	GCF_006980785.1	Tepidiphilus_sp._J10	EPD81_RS04405	897239..900871	2502185	BREX-system-P-loop-protein-BrxC-[Tepidiphilus-sp.-J10]
WP_169116165.1	Type6.BREX	Glyoxalase->?->?->?->?->?-><-?|| BrxE -> BrxB-BrxF-iSTAND -> DUF6079 *-> N6-METHYLASE ->AbrB-HTH-> PIN -> N6-METHYLASE ->DOC+HTH-> PglZ -> SIGMA+OB-Fold+AAA-ATPase+HKD-DNase-BrxL ->	1210	Bacteria>Pseudomonadota>Hydrogenophilia>Hydrogenophilales>Hydrogenophilaceae>Tepidiphilus	GCF_012911495.1	Tepidiphilus_baoligensis	GV368_RS08140	10579..14211	2698687	BREX-system-P-loop-protein-BrxC-[Tepidiphilus-baoligensis]
MCC7337722.1	Type1.BREX	SF1-SF2-Helicase+wHTH+Small-B-barrel+DUF1998 ->?->?-> PIN ->?-> BrxA-DNA-binding -> BrxB-BrxF-iSTAND -> DUF6079 *->?-> N6-METHYLASE ->REase+TPR+GreAB-C+ PIN ->MORC+DUF3684+REase->SF1-SF2-Helicase->?-> PIN -> PglZ -> SIGMA+OB-Fold+AAA-ATPase+HKD-DNase-BrxL ->	1171	Bacteria>PVC_group>Planctomycetota>Planctomycetia>Pirellulales>Pirellulaceae	GCA_020852965.1	Pirellulaceae_bacterium	IT422_21750	7905..11420	2699754	BREX-system-P-loop-protein-BrxC-[Pirellulaceae-bacterium]
WP_068129828.1	Type1.BREX	<- SIGMA+OB-Fold+AAA-ATPase+HKD-DNase-BrxL <-Four-helical-protein<- PglZ <- PIN <-HTH+MPTase<- N6-METHYLASE <- DUF6079 *<- BrxB-BrxF-iSTAND <- BrxA-DNA-binding <-XIS-HTH<-URI-DNase||?-><-Vrr-REase	1169	Bacteria>PVC_group>Planctomycetota>Planctomycetia>Pirellulales>Pirellulaceae	GCF_008065135.1	Roseimaritima_ulvae	UC8_RS27055	7584147..7587656	980254	BREX-system-P-loop-protein-BrxC-[Roseimaritima-ulvae]
WP_259631594.1	Type1.BREX	<-SF1-SF2-Helicase<-?<-?<-?||XIS-HTH-> BrxA-DNA-binding -> BrxB-BrxF-iSTAND -> DUF6079 *-> N6-METHYLASE -> PIN ->ParB+HNH->TIR->MarR-HTH->Polbeta-> PglZ -> SIGMA+OB-Fold+AAA-ATPase+Lon-Protease-BrxL ->	1147	Bacteria>PVC_group>Planctomycetota>Planctomycetia>Pirellulales>Pirellulaceae	GCF_025060895.1	Stieleria_sedimenti	NZK35_RS05520	42893..46336	2976331	BREX-system-P-loop-protein-BrxC-[Stieleria-sedimenti]
HBJ35663.1	Type1.BREX	<- SIGMA+OB-Fold+AAA-ATPase+HKD-DNase-BrxL <- PglZ <- PIN <-HTH+MPTase||ZDNA-HTH-><-?<-Pkinase+CR-Kinase<- N6-METHYLASE <-Schlafen+HATPase+wHTH<- DUF6079 *<-Four-helical-protein<- BrxB-BrxF-iSTAND <- BrxA-DNA-binding <-XIS-HTH	1188	Bacteria>PVC_group>Planctomycetota>Planctomycetia>Planctomycetales>Planctomycetaceae	GCA_003506095.1	Planctomycetaceae_bacterium	DDZ51_13145	16532..20098	2026779	BREX-system-P-loop-protein-BrxC-[Planctomycetaceae-bacterium]
MBI3097943.1	Type1.BREX	<- SIGMA+OB-Fold+AAA-ATPase+Lon-Protease-BrxL <- PglZ <-DOC+HTH<- N6-METHYLASE <- PIN <- DUF6079 *<- BrxB-BrxF-iSTAND <- BrxA-DNA-binding ||?->?-><-tRNA||ParA-Soj-PloopNTPase->	1104	Bacteria>PVC_group>Planctomycetota>unclassified_Planctomycetota	GCA_016192525.1	Planctomycetota_bacterium	HYY93_06795	72529..75843	2026780	BREX-system-P-loop-protein-BrxC-[Planctomycetota-bacterium]
NBB80453.1	Type1.BREX	HTH_3-> BrxA-DNA-binding -> BrxB-BrxF-iSTAND -> DUF6079 *-> OLD-ABC-> PIN -> N6-METHYLASE ->OLD-ABC->HNH->ParB+HNH->Schlafen+HATPase+wHTH-> PglZ -> SIGMA+OB-Fold+AAA-ATPase+Lon-Protease-BrxL ->	1147	Bacteria>PVC_group>Verrucomicrobiota>unclassified_Verrucomicrobiota	GCA_009908325.1	Verrucomicrobiota_bacterium	GVY36_13595	2227..5670	2026799	BREX-system-P-loop-protein-BrxC-[Verrucomicrobiota-bacterium]
MBL9184319.1	Type1.BREX	DUF932-> BrxA-DNA-binding -> BrxB-BrxF-iSTAND -> DUF6079 *-> PIN ->TIR-> N6-METHYLASE ->?-> PglZ -> SIGMA+OB-Fold+AAA-ATPase+Lon-Protease-BrxL ->REase+PNTD1+PNTD2->PIWI->	1153	Bacteria>PVC_group>Verrucomicrobiota>Verrucomicrobiae>Verrucomicrobiales>Verrucomicrobiaceae	GCA_016795465.1	Verrucomicrobiaceae_bacterium	JNN17_19395	2113..5574	2026800	BREX-system-P-loop-protein-BrxC-[Verrucomicrobiaceae-bacterium]
MCB0987052.1	Type1.BREX	<- N6-METHYLASE -p<- DUF6079 *<- BrxB-BrxF-iSTAND <- BrxA-DNA-binding <-?||?-><-Phage_integrase<- PIN <-HTH+MPTase<-?<-PolB	1154	Bacteria>Terrabacteria_group>Actinomycetota>Acidimicrobiia>Acidimicrobiales>unclassified_Acidimicrobiales	GCA_020439505.1	Acidimicrobiales_bacterium	KDB09_04685	1462..4926	2201156	BREX-system-P-loop-protein-BrxC-[Acidimicrobiales-bacterium]
WP_169274836.1	Type1.BREX	<- PIN <-RelB<-?||HTH_3->?-> PIN -> BrxA-DNA-binding -> BrxB-BrxF-iSTAND -> DUF6079 *->CR-ATPase5+REase-> N6-METHYLASE ->Schlafen+HATPase+wHTH-> PglZ -> SIGMA+OB-Fold+AAA-ATPase+HKD-DNase-BrxL ->REase->?-> BrxA-DNA-binding ->	1226	Bacteria>Terrabacteria_group>Actinomycetota>Actinomycetes>Bifidobacteriales>Bifidobacteriaceae	GCF_012932365.1	Bifidobacterium_sp._DSM_109958	G1C96_RS01295	288582..292262	2705430	BREX-system-P-loop-protein-BrxC-[Bifidobacterium-sp.-DSM-109958]
WP_226512485.1	Type1.BREX	MACRODOMAIN+SIR2-><-?<-?||DUF6361->HKD-DNase->SF1-SF2-Helicase->URI-DNase-> BrxA-DNA-binding -> BrxB-BrxF-iSTAND -> DUF6079 -p*-> DUF6079 -p->XIS-HTH-> PIN -> N6-METHYLASE -p-> N6-METHYLASE -> PglZ ->?->AbiEi_4+AbiEi_1-> SIGMA+OB-Fold+AAA-ATPase+HKD-DNase-BrxL ->	435	Bacteria>Terrabacteria_group>Actinomycetota>Actinomycetes>Mycobacteriales>Gordoniaceae	GCF_020520305.1	Gordonia_sp._WA4-43	LEL84_RS18910	4162672..4163979	2878678	BREX-system-P-loop-protein-BrxC-[Gordonia-sp.-WA4-43]
WP_226512486.1	Type1.BREX	DUF6361->HKD-DNase->SF1-SF2-Helicase->URI-DNase-> BrxA-DNA-binding -> BrxB-BrxF-iSTAND -> DUF6079 -p-> DUF6079 -p*->XIS-HTH-> PIN -> N6-METHYLASE -p-> N6-METHYLASE -> PglZ ->?->AbiEi_4+AbiEi_1-> SIGMA+OB-Fold+AAA-ATPase+HKD-DNase-BrxL -><-ABhydrolase+ABhydrolase	785	Bacteria>Terrabacteria_group>Actinomycetota>Actinomycetes>Mycobacteriales>Gordoniaceae	GCF_020520305.1	Gordonia_sp._WA4-43	LEL84_RS18915	4163880..4166237	2878678	hypothetical-protein-[Gordonia-sp.-WA4-43]
QIN77519.1	Type1.BREX	Thermonuclease->DOC+HTH->?->N6_N4_METHYLASE->SF1-SF2-Helicase-> BrxA-DNA-binding -> BrxB-BrxF-iSTAND -> DUF6079 *->?-> N6-METHYLASE ->REase->?-> PIN -> PglZ -> SIGMA+OB-Fold+AAA-ATPase+Lon-Protease-BrxL -><-?<-BirA-HTH	1167	Bacteria>Terrabacteria_group>Actinomycetota>Rubrobacteria>Rubrobacterales>Rubrobacteraceae	GCA_011492965.1	Rubrobacter_marinus	GBA65_02255	450674..454177	2653852	BREX-system-P-loop-protein-BrxC-[Rubrobacter-marinus]
MBA3426223.1	Type1.BREX	<- DUF6079 -p*<- BrxB-BrxF-iSTAND <- BrxA-DNA-binding <-?<-?<- PIN <-?<-ParA-Soj-PloopNTPase	773	Bacteria>Terrabacteria_group>Actinomycetota>Rubrobacteria>Rubrobacterales>Rubrobacteraceae	GCA_013812245.1	Rubrobacter_sp.	HOU04_17185	1..2321	2036010	BREX-system-P-loop-protein-BrxC-[Rubrobacter-sp.]
WP_166395201.1	Type1.BREX	Thermonuclease->DOC+HTH->?->N6_N4_METHYLASE->SF1-SF2-Helicase-> BrxA-DNA-binding -> BrxB-BrxF-iSTAND -> DUF6079 *->?-> N6-METHYLASE ->REase->?-> PIN -> PglZ -> SIGMA+OB-Fold+AAA-ATPase+Lon-Protease-BrxL -><-?<-BirA-HTH	1151	Bacteria>Terrabacteria_group>Actinomycetota>Rubrobacteria>Rubrobacterales>Rubrobacteraceae	GCA_011492965.1	Rubrobacter_marinus	GBA65_RS02320	450722..454177	2653852	BREX-system-P-loop-protein-BrxC-[Rubrobacter-marinus]
MBB2481351.1	Type1.BREX	REase+DUF4268-><-?||ZnR+RHH_7->XIS-HTH->MerR-HTH->?->?-> BrxA-DNA-binding -> BrxB-BrxF-iSTAND -> DUF6079 *-> N6-METHYLASE -> PglZ -> SIGMA+OB-Fold+AAA-ATPase+Lon-Protease-BrxL -> PIN ->HEPN->HEPN->?->MarR-HTH->	1187	Bacteria>Terrabacteria_group>Bacillota>Bacilli>Bacillales>Bacillaceae	GCA_014174455.1	Bacillus_sp._APMAM	H5P36_14260	24010..27573	2761103	BREX-system-P-loop-protein-BrxC-[Bacillus-sp.-APMAM]
MBO1513528.1	Type1.BREX	ZnR+RHH_7->XIS-HTH->MerR-HTH->?-><-?||?-> BrxA-DNA-binding -> BrxB-BrxF-iSTAND -> DUF6079 *-> N6-METHYLASE -> PglZ -> SIGMA+OB-Fold+AAA-ATPase+Lon-Protease-BrxL ->?-> PIN ->HEPN->HEPN->?->MarR-HTH->	1187	Bacteria>Terrabacteria_group>Bacillota>Bacilli>Bacillales>Bacillaceae	GCF_017497975.1	Metabacillus_bambusae	I7822_17955	21833..25396	2795218	BREX-system-P-loop-protein-BrxC-[Metabacillus-bambusae]
WP_243459544.1	Type1.BREX	ZnR+RHH_7->XIS-HTH->MerR-HTH->?-><-?||?-> BrxA-DNA-binding -> BrxB-BrxF-iSTAND -> DUF6079 *-> N6-METHYLASE -> PglZ -> SIGMA+OB-Fold+AAA-ATPase+Lon-Protease-BrxL ->?-> PIN ->HEPN->HEPN->?->MarR-HTH->	1182	Bacteria>Terrabacteria_group>Bacillota>Bacilli>Bacillales>Bacillaceae	GCF_017497975.1	Metabacillus_bambusae	I7822_RS17965	21848..25396	2795218	BREX-system-P-loop-protein-BrxC-[Metabacillus-bambusae]
WP_259548277.1	Type1.BREX	<-MarR-HTH<-?<-?<-HEPN<-HEPN<- PIN <- SIGMA+OB-Fold+AAA-ATPase+Lon-Protease-BrxL <- PglZ <- N6-METHYLASE <- DUF6079 *<- BrxB-BrxF-iSTAND <- BrxA-DNA-binding <-?<-?<-MerR-HTH<-XIS-HTH<-ZnR+RHH_7||?->UDG->	1187	Bacteria>Terrabacteria_group>Bacillota>Bacilli>Bacillales>Bacillaceae	GCF_024971915.1	Heyndrickxia_oleronia	KXF32_RS03880	741080..744643	38875	BREX-system-P-loop-protein-BrxC-[Heyndrickxia-oleronia]
CDD75188.1	Type1.BREX	MACRODOMAIN->?-> PIN ->ZnR+ZnR+ZnR->HTH_26-> BrxA-DNA-binding -> BrxB-BrxF-iSTAND -> DUF6079 *-> OLD-ABC-> PIN -> N6-METHYLASE ->OLD-ABC->NERD->HEPN->Schlafen+HATPase+wHTH-> PglZ ->DUF4365+REase->	1200	Bacteria>Terrabacteria_group>Bacillota>Clostridia>Eubacteriales>Clostridiaceae	GCA_000436175.1	Clostridium_sp._CAG:62	BN737_01375	69547..73149	1262828	uncharacterized-protein-BN737_01375-[Clostridium-sp.-CAG:62]
WP_118744885.1	Type1.BREX	ACET->REase->?->?->?->?->HTH_26-> BrxA-DNA-binding -> BrxB-BrxF-iSTAND -> DUF6079 *-> OLD-ABC-> PIN -> N6-METHYLASE ->HEPN->?->OLD-ABC->HNH->?->TIR->	1203	Bacteria>Terrabacteria_group>Bacillota>Clostridia>Eubacteriales>Clostridiaceae	GCF_003480745.1	Clostridium_sp._AM27-31LB	DW721_RS03885	61324..64935	2293026	BREX-system-P-loop-protein-BrxC-[Clostridium-sp.-AM27-31LB]
WP_173680574.1	Type1.BREX	<-Cap4_nuclease<-?<-HTH_3||REase-><- SIGMA+OB-Fold+AAA-ATPase+Lon-Protease-BrxL <-?<- PglZ <- PIN <- N6-METHYLASE <- DUF6079 *<- BrxB-BrxF-iSTAND <- BrxA-DNA-binding ||PADR-HTH+MarR-HTH-><-D5ATPase<-?<-?<-?<-?||LexA-protease->	1216	Bacteria>Terrabacteria_group>Bacillota>Clostridia>Eubacteriales>Clostridiaceae	GCF_014230485.1	Clostridium_tetanomorphum	FHX96_RS20165	4177507..4181157	1553	BREX-system-P-loop-protein-BrxC-[Clostridium-tetanomorphum]
WP_221260245.1	Type1.BREX	<-HISKIN||RADICAL-SAM->?->?->HEPN->Ntox45->|| BrxA-DNA-binding -> BrxB-BrxF-iSTAND -> DUF6079 *-> N6-METHYLASE ->?-> PglZ -><-REase|| SIGMA+OB-Fold+AAA-ATPase+Lon-Protease-BrxL ->?-> PIN ->Peptidase_M42->	1218	Bacteria>Terrabacteria_group>Bacillota>Clostridia>Eubacteriales>Clostridiaceae	GCF_019705275.1	Clostridium_chauvoei	K4H38_08460	40416..44072	46867	BREX-system-P-loop-protein-BrxC-[Clostridium-chauvoei]
WP_231016614.1	Type1.BREX	PolB->TOPRIM->?->TrwC+SF1-SF2-Helicase-> BrxA-DNA-binding -> BrxB-BrxF-iSTAND -> DUF6079 *-> N6-METHYLASE ->HTH_3->MPTase-> PIN -> PglZ -> SIGMA+OB-Fold+AAA-ATPase+Lon-Protease-BrxL -><-CopG/RHH	1189	Bacteria>Terrabacteria_group>Bacillota>Clostridia>Eubacteriales>Clostridiaceae	GCF_021043395.1	Clostridium_guangxiense	LQK79_RS11195	7642..11211	1662055	BREX-system-P-loop-protein-BrxC-[Clostridium-guangxiense]
WP_252217802.1	Type1.BREX	<-Peptidase_M42<- PIN <-?<- SIGMA+OB-Fold+AAA-ATPase+Lon-Protease-BrxL ||REase-><- PglZ <-?<- N6-METHYLASE <- DUF6079 *<- BrxB-BrxF-iSTAND <- BrxA-DNA-binding ||<-Ntox45<-HEPN<-HEPN<-?<-?<-RADICAL-SAM||HISKIN->	1218	Bacteria>Terrabacteria_group>Bacillota>Clostridia>Eubacteriales>Clostridiaceae	GCF_023846215.1	Clostridium_chauvoei	J9878_RS07560	18587..22243	46867	BREX-system-P-loop-protein-BrxC-[Clostridium-chauvoei]
WP_270487117.1	Type1.BREX	<- SIGMA+OB-Fold+AAA-ATPase+Lon-Protease-BrxL <-?<- PIN <-SbcC<- PglZ <-HNH<-OLD-ABC<-TIR<- N6-METHYLASE <- DUF6079 *<- BrxB-BrxF-iSTAND <- BrxA-DNA-binding <-HTH_26<- PIN <-?<-MACRODOMAIN	1201	Bacteria>Terrabacteria_group>Bacillota>Clostridia>Eubacteriales>Clostridiaceae	GCF_027681635.1	Clostridium_fessum	PGX92_RS02760	620693..624298	2126740	BREX-system-P-loop-protein-BrxC-[Clostridium-fessum]
WP_270807575.1	Type1.BREX	<-LRR_5+LRR_8<- SIGMA+OB-Fold+AAA-ATPase+Lon-Protease-BrxL <-?<- PIN <-SbcC<- PglZ <-HEPN<- N6-METHYLASE <- DUF6079 *<- BrxB-BrxF-iSTAND <- BrxA-DNA-binding <-HTH_26<- PIN <-?<-MACRODOMAIN	1201	Bacteria>Terrabacteria_group>Bacillota>Clostridia>Eubacteriales>Clostridiaceae	GCF_027723425.1	Clostridium_fessum	PG965_RS01510	334129..337734	2126740	BREX-system-P-loop-protein-BrxC-[Clostridium-fessum]
WP_021876221.1	Type1.BREX	<-Peptidase_M42<- PIN <-?<- SIGMA+OB-Fold+AAA-ATPase+Lon-Protease-BrxL ||REase-><- PglZ <-?<- N6-METHYLASE <- DUF6079 *<- BrxB-BrxF-iSTAND <- BrxA-DNA-binding ||<-Ntox45<-HEPN<-?<-?<-RADICAL-SAM||HISKIN->	1218	Bacteria>Terrabacteria_group>Bacillota>Clostridia>Eubacteriales>Clostridiaceae	GCF_900037355.1	Clostridium_chauvoei	C6H62_RS09310	1987009..1990665	46867	BREX-system-P-loop-protein-BrxC-[Clostridium-chauvoei]
RGI65884.1	Type1.BREX	HTH_26-> BrxA-DNA-binding -> BrxB-BrxF-iSTAND -> DUF6079 *-> OLD-ABC-> PIN -> N6-METHYLASE ->TIR->HEPN->Schlafen+HATPase+wHTH-> PglZ ->Tox-ART-HYD3-> SIGMA+OB-Fold+AAA-ATPase+Lon-Protease-BrxL ->	1196	Bacteria>Terrabacteria_group>Bacillota>Clostridia>Eubacteriales>Lachnospiraceae	GCA_003436035.1	[Eubacterium]_rectale	DXD95_13285	1619..5209	39491	BREX-system-P-loop-protein-BrxC-[[Eubacterium]
HCI18472.1	Type1.BREX	<- PIN <-?<- SIGMA+OB-Fold+AAA-ATPase+Lon-Protease-BrxL <-OLD-ABC+TOPRIM<- PglZ <-Schlafen+HATPase+wHTH<-REase<- N6-METHYLASE <- DUF6079 -p*<- DUF6079 -p<- BrxB-BrxF-iSTAND <- BrxA-DNA-binding <-HTH_26	799	Bacteria>Terrabacteria_group>Bacillota>Clostridia>Eubacteriales>Lachnospiraceae	GCA_003521585.1	Lachnospiraceae_bacterium	DFH32_07645	14441..16840	1898203	BREX-system-P-loop-protein-BrxC-[Lachnospiraceae-bacterium]
HCX41706.1	Type1.BREX	SKI->?->tRNA->?->?->?->?->HTH_26-> BrxA-DNA-binding -> BrxB-BrxF-iSTAND -> DUF6079 *-> N6-METHYLASE ->REase->Schlafen+HATPase+wHTH-> PglZ ->OLD-ABC+TOPRIM-> SIGMA+OB-Fold+AAA-ATPase+Lon-Protease-BrxL ->?-> PIN ->MACRODOMAIN->	1196	Bacteria>Terrabacteria_group>Bacillota>Clostridia>Eubacteriales>Lachnospiraceae	GCA_003539645.1	Lachnospiraceae_bacterium	DHH50_05105	179559..183149	1898203	BREX-system-P-loop-protein-BrxC-[Lachnospiraceae-bacterium]
MBR4345194.1	Type1.BREX	RelB-> PIN -> BrxA-DNA-binding -> BrxB-BrxF-iSTAND -> DUF6079 *->VbhA+DOC+HTH-> N6-METHYLASE -> N6-METHYLASE -p->HEPN->SbcC+SbcC->Schlafen+HATPase+wHTH-> PglZ -> SIGMA+OB-Fold+AAA-ATPase+Lon-Protease-BrxL ->TIR->	1173	Bacteria>Terrabacteria_group>Bacillota>Clostridia>Eubacteriales>Lachnospiraceae	GCA_017542245.1	Lachnospiraceae_bacterium	IKP88_21230	56947..60468	1898203	BREX-system-P-loop-protein-BrxC-[Lachnospiraceae-bacterium]
MCI7121458.1	Type1.BREX	HATPase+wHTH->?->MACRODOMAIN->?->35exo+ADA-DBD->ZnR+ZnR+ZnR->HTH_26-> BrxA-DNA-binding -> BrxB-BrxF-iSTAND -> DUF6079 *-> OLD-ABC-> PIN -> N6-METHYLASE ->?-> PglZ ->Schlafen+HATPase+wHTH-> SIGMA+OB-Fold+AAA-ATPase+Lon-Protease-BrxL ->LRR_5+LRR_8->Cas9+REC+HNH+Cas9->	1201	Bacteria>Terrabacteria_group>Bacillota>Clostridia>Eubacteriales>Lachnospiraceae	GCA_022775325.1	[Ruminococcus]_gnavus	MR941_06720	10105..13710	33038	BREX-system-P-loop-protein-BrxC-[[Ruminococcus]
WP_173865463.1	Type1.BREX	<-STYKIN<-?<-LRR_5+LRR_8<- SIGMA+OB-Fold+AAA-ATPase+Lon-Protease-BrxL <-Schlafen+HATPase+wHTH<- PglZ <- N6-METHYLASE <- PIN <-OLD-ABC <- DUF6079 *<- BrxB-BrxF-iSTAND <- BrxA-DNA-binding <-HTH_26<-ZnR+ZnR+ZnR<-35exo+ADA-DBD<-?<-MACRODOMAIN<-?<-DOC+zhth	1201	Bacteria>Terrabacteria_group>Bacillota>Clostridia>Eubacteriales>Lachnospiraceae	GCF_013300965.1	Faecalicatena_fissicatena	HFN12_RS00390	75728..79333	290055	BREX-system-P-loop-protein-BrxC-[Faecalicatena-fissicatena]
WP_270645287.1	Type1.BREX	HTH_19-><- SIGMA+OB-Fold+AAA-ATPase+Lon-Protease-BrxL <-?<- PglZ <- PIN <-OLD-ABC <-OLD-ABC+TOPRIM<- N6-METHYLASE <- DUF6079 *<- BrxB-BrxF-iSTAND <- BrxA-DNA-binding <-HTH_26<-?<-?<-?<-ArdCN+MPTase-inact+LPD19<-?<-TOPRIM+Topoisom_bac	1205	Bacteria>Terrabacteria_group>Bacillota>Clostridia>Eubacteriales>Lachnospiraceae	GCF_027692785.1	Merdimonas_faecis	PG729_RS15130	28205..31822	1653435	BREX-system-P-loop-protein-BrxC-[Merdimonas-faecis]
MCI8835830.1	Type1.BREX	ABC-ATPase->ABC-ATPase+SbcC->?-> BrxA-DNA-binding -> BrxB-BrxF-iSTAND -> DUF6079 *->Schlafen+HATPase+wHTH-> N6-METHYLASE -> OLD-ABC-> PIN -> PglZ ->	1205	Bacteria>Terrabacteria_group>Bacillota>Clostridia>Eubacteriales>Oscillospiraceae	GCA_022795995.1	Ruminococcus_sp.	HFH24_10725	31305..34922	41978	BREX-system-P-loop-protein-BrxC-[Ruminococcus-sp.]
WP_226924212.1	Type1.BREX	AraC-HTH-><-?|| BrxA-DNA-binding -> BrxB-BrxF-iSTAND -> DUF6079 *-> N6-METHYLASE ->REase-> PIN ->?-> PglZ -> SIGMA+OB-Fold+AAA-ATPase+Lon-Protease-BrxL ->	1190	Bacteria>Terrabacteria_group>Bacillota>Clostridia>Eubacteriales>Peptostreptococcaceae	GCF_020537215.1	Intestinibacter_bartlettii	LIP50_RS08300	25949..29521	261299	BREX-system-P-loop-protein-BrxC-[Intestinibacter-bartlettii]
MBQ3415902.1	Type1.BREX	<- SIGMA+OB-Fold+AAA-ATPase+Lon-Protease-BrxL <- PglZ <-?<- PIN <-OLD-ABC <- N6-METHYLASE <-?<- DUF6079 *<- BrxB-BrxF-iSTAND <- BrxA-DNA-binding <-?<-?<-RHH_8	1216	Bacteria>Terrabacteria_group>Bacillota>Clostridia>unclassified_Clostridia	GCA_017404025.1	Clostridia_bacterium	IJH39_11340	11283..14933	2044939	BREX-system-P-loop-protein-BrxC-[Clostridia-bacterium]
MBE6098022.1	Type1.BREX	<- SIGMA+OB-Fold+AAA-ATPase+Lon-Protease-BrxL <- PglZ <-REase<-OLD-ABC<- N6-METHYLASE <- DUF6079 *<- BrxB-BrxF-iSTAND <- BrxA-DNA-binding <- PIN <-?<-Peptidase_M15	1220	Bacteria>Terrabacteria_group>Bacillota>Negativicutes>Selenomonadales>Selenomonadaceae	GCA_015058685.1	Schwartzia_succinivorans	E7198_09540	51689..55351	55507	BREX-system-P-loop-protein-BrxC-[Schwartzia-succinivorans]
MBR3457117.1	Type1.BREX	<- PglZ -p<-HNH<-OLD-ABC<- N6-METHYLASE -p<-?<- PIN <- N6-METHYLASE <- DUF6079 *<- BrxB-BrxF-iSTAND -p	1215	Bacteria>Terrabacteria_group>Bacillota>Negativicutes>Selenomonadales>Selenomonadaceae	GCA_017509225.1	Selenomonadaceae_bacterium	IKH16_03040	6412..10059	2652289	BREX-system-P-loop-protein-BrxC-[Selenomonadaceae-bacterium]
SEH31389.1	Type1.BREX	<- PglZ <- PIN <-?<- N6-METHYLASE <- DUF6079 *<- BrxB-BrxF-iSTAND <- BrxA-DNA-binding <-OLD-ABC<-ParB<-ParA-Soj-PloopNTPase<-?<-HTH_3||?->ParA-Soj-PloopNTPase->	1188	Bacteria>Terrabacteria_group>Bacillota>Negativicutes>Selenomonadales>Selenomonadaceae	GCA_900108415.1	Selenomonas_ruminantium	SAMN05216583_11711	10616..14182	971	hypothetical-protein-SAMN05216583_11711-[Selenomonas-ruminantium]
WP_273038291.1	Type1.BREX	<- SIGMA+OB-Fold+AAA-ATPase+Lon-Protease-BrxL <- PglZ <-REase<-OLD-ABC<- N6-METHYLASE <- DUF6079 *<- BrxB-BrxF-iSTAND <- BrxA-DNA-binding <- PIN <-?<-Peptidase_M15	1220	Bacteria>Terrabacteria_group>Bacillota>Negativicutes>Selenomonadales>Selenomonadaceae	GCF_015058685.1	Schwartzia_succinivorans	E7198_RS09640	51689..55351	55507	BREX-system-P-loop-protein-BrxC-[Schwartzia-succinivorans]
WP_273698075.1	Type1.BREX	<-REase<- PglZ <- N6-METHYLASE <- PIN <-OLD-ABC <-HNH<-OLD-ABC<- DUF6079 *<- BrxB-BrxF-iSTAND <- BrxA-DNA-binding <-?<-?<-?<-?<-SKI<-?||O6MGT-HTH->	1181	Bacteria>Terrabacteria_group>Bacillota>Negativicutes>Selenomonadales>Selenomonadaceae	GCF_905371815.1	Selenomonas_noxia	NW174_RS07850	36953..40498	135083	BREX-system-P-loop-protein-BrxC-[Selenomonas-noxia]
WP_273079788.1	Type1.BREX	BrxA-DNA-binding -> BrxB-BrxF-iSTAND -> DUF6079 *->OLD-ABC->HNH-> OLD-ABC-> PIN -> N6-METHYLASE -> PglZ ->SIR2->HerA/FtsK->	1187	Bacteria>Terrabacteria_group>Bacillota>Negativicutes>Selenomonadales>Selenomonadaceae	GCF_905371945.1	Selenomonas_felix	NW177_RS10645	2029..5592	1944634	BREX-system-P-loop-protein-BrxC-[Selenomonas-felix]
MBQ6343447.1	Type1.BREX	BrxA-DNA-binding -> BrxB-BrxF-iSTAND -> DUF6079 *-><-?<-?||PhdYeFM_antitox-> PIN ->?->?->?-> N6-METHYLASE ->	1175	Bacteria>Terrabacteria_group>Chloroflexota>Anaerolineae>Anaerolineales>Anaerolineaceae	GCA_017432985.1	Anaerolineaceae_bacterium	IJI41_10010	1911..5438	2024896	BREX-system-P-loop-protein-BrxC-[Anaerolineaceae-bacterium]
NJN66674.1	Type1.BREX	<- N6-METHYLASE -p<- PIN <-CopG/RHH<- N6-METHYLASE -p<- DUF6079 -p*<- DUF6079 -p<- BrxB-BrxF-iSTAND <- BrxA-DNA-binding <-HTH_3||?->?->?-><-DDE_Tnp||DUF4158->	607	Bacteria>Terrabacteria_group>Chloroflexota>Chloroflexia>Chloroflexales>Chloroflexineae	GCA_012032475.1	Chloroflexaceae_bacterium	HC884_08105	4075..5898	2562117	hypothetical-protein-[Chloroflexaceae-bacterium]
WP_097651212.1	Type1.BREX	<- PglZ <- PIN <-?<- N6-METHYLASE -p<- PIN <-CopG/RHH<-?<-PolB<- N6-METHYLASE <- DUF6079 *<- BrxB-BrxF-iSTAND <- BrxA-DNA-binding <-?||?-><-?<-?<-ParA-Soj-PloopNTPase+ParA-Soj-PloopNTPase	1204	Bacteria>Terrabacteria_group>Chloroflexota>Chloroflexia>Chloroflexales>Chloroflexineae	GCF_002532075.1	Candidatus_Chloroploca_asiatica	A9Q02_RS04750	75845..79459	1506545	BREX-system-P-loop-protein-BrxC-[Candidatus-Chloroploca-asiatica]
MBI2864205.1	Type1.BREX	<-ABC-ATPase+SbcC<-ABC-ATPase+SbcC||PolB-><-PhdYeFM_antitox||?-> PIN -> BrxA-DNA-binding -> BrxB-BrxF-iSTAND -> DUF6079 -p*->	773	Bacteria>Terrabacteria_group>Chloroflexota>unclassified_Chloroflexota	GCA_016190625.1	Chloroflexota_bacterium	HYX94_06540	21104..23423	2026724	BREX-system-P-loop-protein-BrxC-[Chloroflexota-bacterium]
MBK7180413.1	Type1.BREX	<-REase<-SF1-SF2-Helicase+DUF1998<-?<-?<- wHTH+WYL+WCX <-?||?-> BrxA-DNA-binding -> BrxB-BrxF-iSTAND -> DUF6079 *-> N6-METHYLASE ->?-><- PIN <-RHH|| PglZ ->	1173	Bacteria>Terrabacteria_group>Chloroflexota>unclassified_Chloroflexota	GCA_016707865.1	Chloroflexota_bacterium	IPH82_25035	72782..76303	2026724	BREX-system-P-loop-protein-BrxC-[Chloroflexota-bacterium]
MCC6629583.1	Type1.BREX	HIN-HTH-> BrxA-DNA-binding -> BrxB-BrxF-iSTAND -> DUF6079 *->?-> PIN -> N6-METHYLASE ->PolB->HEPN->?-> PglZ -> SIGMA+OB-Fold+AAA-ATPase+Lon-Protease-BrxL ->	1204	Bacteria>Terrabacteria_group>Chloroflexota>unclassified_Chloroflexota	GCA_020849725.1	Chloroflexota_bacterium	IT340_19565	31214..34828	2026724	BREX-system-P-loop-protein-BrxC-[Chloroflexota-bacterium]
MCL4487040.1	Type1.BREX	BrxA-DNA-binding -> BrxB-BrxF-iSTAND ->Peptidase_M91-> N6-METHYLASE ->HKD-DNase-> DUF6079 *->HEPN->PhdYeFM_antitox-> PIN -> PglZ ->?->?->?->MerR-HTH+TipAS->	1221	Bacteria>Terrabacteria_group>Chloroflexota>unclassified_Chloroflexota	GCA_023381515.1	Chloroflexota_bacterium	M1570_02770	10289..13954	2026724	BREX-system-P-loop-protein-BrxC-[Chloroflexota-bacterium]
WAS06096.1	Type1.BREX	<- PglZ <- PIN <-PhdYeFM_antitox<-?<- PIN <-PhdYeFM_antitox<- N6-METHYLASE <- PIN <-PhdYeFM_antitox<- DUF6079 *<- BrxB-BrxF-iSTAND <- BrxA-DNA-binding ||?-><-NTP_transferase+MannoseP_isomer<-?<-TP_METHYLASE<-NTP_transf_3+Hexapep+Hexapep<-?<-RNase+DSRBD	1221	Bacteria>Terrabacteria_group>Cyanobacteriota/Melainabacteria_group>Cyanobacteriota>Cyanophyceae>Gloeomargaritales	GCA_022206215.1	Gloeomargaritales_cyanobacterium_VI4D9	LQF76_04175	903259..906924	2898145	BREX-system-P-loop-protein-BrxC-[Gloeomargaritales-cyanobacterium-VI4D9]
MBW4670333.1	Type6.BREX	<-Glycos_transf<-TPR||REase-><-Acetate_kinase|| BrxE -> BrxA-DNA-binding -> BrxB-BrxF-iSTAND -> DUF6079 *->?-> PIN ->?-><-REase<-?|| N6-METHYLASE ->	1211	Bacteria>Terrabacteria_group>Cyanobacteriota/Melainabacteria_group>Cyanobacteriota>Cyanophyceae>Nostocales	GCA_019359595.1	Cyanomargarita_calcarea_GSE-NOS-MK-12-04C	KME60_23685	99014..102649	2839659	BREX-system-P-loop-protein-BrxC-[Cyanomargarita-calcarea-GSE-NOS-MK-12-04C]
WP_127084959.1	Type1.BREX	CASPASE->?-><-?<-TOPRIM<-OLD-ABC<-Schlafen+HATPase+wHTH|| BrxA-DNA-binding -> BrxB-BrxF-iSTAND -> DUF6079 -p*->?->?-> PIN ->CopG/RHH-> N6-METHYLASE -p->OLD-ABC->HNH->	725	Bacteria>Terrabacteria_group>Cyanobacteriota/Melainabacteria_group>Cyanobacteriota>Cyanophyceae>Nostocales	GCF_003991905.1	Dulcicalothrix_desertica	DSM106972_RS34665	70206..72383	32056	BREX-system-P-loop-protein-BrxC-[Dulcicalothrix-desertica]
WP_224095538.1	Type1.BREX	BrxA-DNA-binding -><- SIGMA+OB-Fold+AAA-ATPase+Lon-Protease-BrxL <- PglZ +Phosphodiest || PIN ->?-><- N6-METHYLASE -p<-HTH_23+HTH_32<- N6-METHYLASE -p<- N6-METHYLASE -p<- DUF6079 *<- BrxB-BrxF-iSTAND <-?<-REase<-?||ArsR-HTH-><-?||?-><-?||HTH_32+DDE_3->	1178	Bacteria>Terrabacteria_group>Cyanobacteriota/Melainabacteria_group>Cyanobacteriota>Cyanophyceae>Nostocales	GCF_019976755.1	Nostoc_sp._MS1	NSMS1_RS32170	256782..260318	2764711	BREX-system-P-loop-protein-BrxC-[Nostoc-sp.-MS1]
WP_227788897.1	Type1.BREX	DUF6079 *-> PIN ->CopG/RHH->?->?->?-> PglZ -> BrxD-AAA -> SF1-SF2-Helicase+wHTH+Small-B-barrel+DUF1998 ->RHH_9->	1169	Bacteria>Terrabacteria_group>Cyanobacteriota/Melainabacteria_group>Cyanobacteriota>Cyanophyceae>Nostocales	GCF_020702635.1	Nodularia_sp._LEGE_04288	IQ240_RS09380	506..4015	1828639	BREX-system-P-loop-protein-BrxC-[Nodularia-sp.-LEGE-04288]
WP_162398350.1	Type1.BREX	BrxA-DNA-binding -><- SIGMA+OB-Fold+AAA-ATPase+Lon-Protease-BrxL <- PglZ +Phosphodiest ||REase-> PIN -><- N6-METHYLASE ||MetJarc-HTH->PemK_toxin-><- DUF6079 *<- BrxB-BrxF-iSTAND ||<-tRNA<-?<-?||?->?-><-?||?-><-Peptidase_M28	1178	Bacteria>Terrabacteria_group>Cyanobacteriota/Melainabacteria_group>Cyanobacteriota>Cyanophyceae>Nostocales	-	Nostoc_sp._B(2019)	GS682_RS23175	25673..29209	2690446	BREX-system-P-loop-protein-BrxC-[Nostoc-sp.-B(2019)]
BBD57221.1	Type1.BREX	<-MerR-HTH||RHH-><-?<-?||?->TPR+TPR+TPR-><-Phage_integrase<-?|| BrxB-BrxF-iSTAND -> DUF6079 *-><-?<-REase||?-> PIN -> N6-METHYLASE ->HEPN-> PglZ +Phosphodiest ->HKD-DNase-> SIGMA+OB-Fold+AAA-ATPase+Lon-Protease-BrxL ->	1176	Bacteria>Terrabacteria_group>Cyanobacteriota/Melainabacteria_group>Cyanobacteriota>Cyanophyceae>Oscillatoriophycideae	GCA_003609755.1	Planktothrix_agardhii_NIES-204	NIES204_45570	14633..18163	282423	hypothetical-protein-NIES204_45570-[Planktothrix-agardhii-NIES-204]
WP_235758970.1	Type1.BREX	<-Pkinase+CR-Kinase|| BrxB-BrxF-iSTAND -> DUF6079 *->?-> PIN -> N6-METHYLASE ->?->?-> PglZ +Phosphodiest -> PIN -> SIGMA+OB-Fold+AAA-ATPase+Lon-Protease-BrxL -><- BrxA-DNA-binding	1176	Bacteria>Terrabacteria_group>Cyanobacteriota/Melainabacteria_group>Cyanobacteriota>Cyanophyceae>Oscillatoriophycideae	GCA_030820635.1	Planktothrix_agardhii	L2E81_RS22490	2176..5706	1160	BREX-system-P-loop-protein-BrxC-[Planktothrix-agardhii]
WP_027255660.1	Type1.BREX	<-MerR-HTH||?->PAS-><-?<-?<-?||?-> BrxB-BrxF-iSTAND -> DUF6079 *->?-> PIN -> N6-METHYLASE -p-> N6-METHYLASE ->HEPN-> PglZ +Phosphodiest ->HTH_Tnp_IS630+DDE_3-><-DDE_Tnp_1||HKD-DNase->	1176	Bacteria>Terrabacteria_group>Cyanobacteriota/Melainabacteria_group>Cyanobacteriota>Cyanophyceae>Oscillatoriophycideae	GCF_000464825.1	Planktothrix_agardhii	N316_RS0124710	8429..11959	1160	BREX-system-P-loop-protein-BrxC-[Planktothrix-agardhii]
WP_254034082.1	Type1.BREX	TPR+TPR+TPR+TPR-><-?|| BrxA-DNA-binding -><- SIGMA+OB-Fold+AAA-ATPase+Lon-Protease-BrxL <-HKD-DNase<- PglZ +Phosphodiest <- N6-METHYLASE <- PIN <-?<- DUF6079 *<- BrxB-BrxF-iSTAND <-?||?->?->Phage_integrase->	1176	Bacteria>Terrabacteria_group>Cyanobacteriota/Melainabacteria_group>Cyanobacteriota>Cyanophyceae>Oscillatoriophycideae	GCF_904830845.1	Planktothrix_agardhii	NMG87_RS21740	24173..27703	1160	BREX-system-P-loop-protein-BrxC-[Planktothrix-agardhii]
KPQ33031.1	Type6.BREX	<- SF1-SF2-Helicase+wHTH+Small-B-barrel+DUF1998 <- BrxD-AAA <- PglZ <- PIN <-?<- N6-METHYLASE <-DUF433<- DUF6079 *<- BrxB-BrxF-iSTAND <- BrxA-DNA-binding <- BrxE <-?<-AAA-ATPase	1209	Bacteria>Terrabacteria_group>Cyanobacteriota/Melainabacteria_group>Cyanobacteriota>Cyanophyceae>Pseudanabaenales	GCA_001314865.1	Phormidesmis_priestleyi_Ana	HLUCCA11_19925	26535..30164	1666911	hypothetical-protein-HLUCCA11_19925-[Phormidesmis-priestleyi-Ana]
MBW4580237.1	Type6.BREX	<-ABC-ATPase+SbcC||?-><-SAM-METHYLASE<-?||CHTH-> BrxE -> BrxA-DNA-binding -> BrxB-BrxF-iSTAND -> DUF6079 *->?->REase->DUF29->AbrB-HTH-> PIN -> PglZ -> BrxD-AAA -> SF1-SF2-Helicase+wHTH+Small-B-barrel+DUF1998 ->	1217	Bacteria>Terrabacteria_group>Cyanobacteriota/Melainabacteria_group>Cyanobacteriota>Cyanophyceae>Pseudanabaenales	GCA_019358895.1	Tildeniella_nuda_ZEHNDER_1965/U140	KME42_11780	246570..250223	2136405	BREX-system-P-loop-protein-BrxC-[Tildeniella-nuda-ZEHNDER-1965/U140]
WP_121969249.1	Type6.BREX	CHTH->MazG-> BrxE -> BrxA-DNA-binding -> BrxB-BrxF-iSTAND -> DUF6079 *-> N6-METHYLASE ->?-> PIN -> N6-METHYLASE -p->?-> PglZ -> SIGMA+OB-Fold+AAA-ATPase+HKD-DNase-BrxL ->	1213	Bacteria>Terrabacteria_group>Cyanobacteriota/Melainabacteria_group>Cyanobacteriota>Cyanophyceae>Pseudanabaenales	GCF_002286735.1	Leptolyngbya_sp._BC1307	CKL79_RS08920	112124..115765	2029589	BREX-system-P-loop-protein-BrxC-[Leptolyngbya-sp.-BC1307]
HBH74800.1	Type1.BREX	PIN ->?-> BrxA-DNA-binding -> BrxB-BrxF-iSTAND -> DUF6079 *-> N6-METHYLASE ->SAM-METHYLASE->ParA-Soj-PloopNTPase->?->?-> PglZ ->OLD-ABC->HNH-> SIGMA+OB-Fold+AAA-ATPase+Lon-Protease-BrxL ->	1163	Bacteria>Terrabacteria_group>Cyanobacteriota/Melainabacteria_group>Cyanobacteriota>Cyanophyceae>Synechococcales	GCA_003508015.1	Synechococcales_bacterium_UBA10510	DDY43_15550	2736..6227	2055795	BREX-system-P-loop-protein-BrxC-[Synechococcales-bacterium-UBA10510]
NJL99823.1	Type1.BREX	<-SAM-METHYLASE||?-> BrxA-DNA-binding -> BrxB-BrxF-iSTAND -> DUF6079 *-><- PIN <-PhdYeFM_antitox|| N6-METHYLASE -p-> N6-METHYLASE ->	1145	Bacteria>Terrabacteria_group>Cyanobacteriota/Melainabacteria_group>Cyanobacteriota>Cyanophyceae>Synechococcales	GCA_012031565.1	Synechococcaceae_cyanobacterium_SM2_3_2	HC924_13960	2310..5747	2720471	BREX-system-P-loop-protein-BrxC-[Synechococcaceae-cyanobacterium-SM2_3_2]
WP_094560560.1	Type1.BREX	<-SIGMA-HTH||HicB_lk_antitox->Unstab_antitox->wHTH+HNH+HIT->EVE+HNH-> BrxA-DNA-binding -> BrxB-BrxF-iSTAND -> DUF6079 *-> N6-METHYLASE -><-?|| PglZ ->PhdYeFM_antitox-> PIN -> SIGMA+OB-Fold+AAA-ATPase+HKD-DNase-BrxL ->?-><-SF1-SF2-Helicase<-HEPN+PAS+DUF4011+SF1-SF2-Helicase+REase	1161	Bacteria>Terrabacteria_group>Cyanobacteriota/Melainabacteria_group>Cyanobacteriota>Cyanophyceae>Synechococcales	GCF_002252665.1	Synechococcus_sp._8F6	CJZ78_RS08320	43413..46898	2025606	BREX-system-P-loop-protein-BrxC-[Synechococcus-sp.-8F6]
WP_197170384.1	Type1.BREX	<-Sak4+WYL||?-> PIN -> PIN -><-DUF772+DDE_Tnp_1+DDE_Tnp_1_6<-?||?-> BrxA-DNA-binding -> BrxB-BrxF-iSTAND -> DUF6079 *-> N6-METHYLASE ->GTPase-AIG->sGTP-><-SWACOS+DTXR-HTH|| PglZ ->PhdYeFM_antitox-> PIN -> SIGMA+OB-Fold+AAA-ATPase+HKD-DNase-BrxL -><-REase	1161	Bacteria>Terrabacteria_group>Cyanobacteriota/Melainabacteria_group>Cyanobacteriota>Cyanophyceae>Synechococcales	GCF_015840915.1	Synechococcus_sp._CBW1002	H8F24_RS17545	3558842..3562327	1353134	BREX-system-P-loop-protein-BrxC-[Synechococcus-sp.-CBW1002]
WP_254967828.1	Type1.BREX	<- PIN <-MazE_antitoxin||?-><- SIGMA+OB-Fold+AAA-ATPase+HKD-DNase-BrxL <- PIN <-PhdYeFM_antitox<- PglZ <-?<- N6-METHYLASE <- DUF6079 *<- BrxB-BrxF-iSTAND <- BrxA-DNA-binding ||DnaJ->?->?->?-><-?||?-><- PIN	1161	Bacteria>Terrabacteria_group>Cyanobacteriota/Melainabacteria_group>Cyanobacteriota>Cyanophyceae>Synechococcales	GCF_024346135.1	Cyanobium_sp._CH-040	KBY90_RS07090	13099..16584	2823708	BREX-system-P-loop-protein-BrxC-[Cyanobium-sp.-CH-040]
WP_254970971.1	Type1.BREX	<- PglZ <-Couple_hipA+HipA_C<-HTH_3<-sGTP<-GTPase-AIG<- N6-METHYLASE <-MNS+REase|| PIN -> PIN -><- DUF6079 *<- BrxB-BrxF-iSTAND <- BrxA-DNA-binding ||Shikimate_dh_N+SDH_C->?-><-?||?->?-><-Peptidase_M1_N+Peptidase_M1	1161	Bacteria>Terrabacteria_group>Cyanobacteriota/Melainabacteria_group>Cyanobacteriota>Cyanophyceae>Synechococcales	GCF_024346155.1	Cyanobium_sp._Candia_9D4	KBZ08_RS02055	407526..411011	2823707	BREX-system-P-loop-protein-BrxC-[Cyanobium-sp.-Candia-9D4]
WP_255005473.1	Type1.BREX	PIN -> PIN ->?-><-DOC||?->ArdCN+MPTase-> BrxA-DNA-binding -> BrxB-BrxF-iSTAND -> DUF6079 *-> N6-METHYLASE -> PglZ ->PhdYeFM_antitox-> PIN -> SIGMA+OB-Fold+AAA-ATPase+HKD-DNase-BrxL -><-REase<-TPR<-Peptidase	1161	Bacteria>Terrabacteria_group>Cyanobacteriota/Melainabacteria_group>Cyanobacteriota>Cyanophyceae>Synechococcales	GCF_024346315.1	Cyanobium_sp._ATX_6F1	KBZ13_RS02100	369855..373340	2823702	BREX-system-P-loop-protein-BrxC-[Cyanobium-sp.-ATX-6F1]
KPA14790.1	Type1.BREX	<- N6-METHYLASE <-Schlafen+HATPase+wHTH<- DUF6079 *<- PIN <-?<- BrxB-BrxF-iSTAND <- BrxA-DNA-binding <-?<-?<-HD||?->AAA-ATPase-p->	1187	Bacteria>Thermodesulfobacteriota>Desulfobacteria>Desulfobacterales>Candidatus_Magnetomoraceae>Candidatus_Magnetomorum	GCA_001292585.1	Candidatus_Magnetomorum_sp._HK-1	MHK_004993	4620..8183	1509431	DNA-repair-ATPase-[Candidatus-Magnetomorum-sp.-HK-1]
HJA77858.1	Type1.BREX	BrxB-BrxF-iSTAND -p-><-?||ACET-> BrxA-DNA-binding -> BrxB-BrxF-iSTAND -> DUF6079 *-> OLD-ABC-> PIN -> N6-METHYLASE ->PHP+SbcC-> PglZ -> SIGMA+OB-Fold+AAA-ATPase+Lon-Protease-BrxL ->SIR2->	1145	Bacteria>Thermodesulfobacteriota>Desulfovibrionia>Desulfovibrionales>Desulfovibrionaceae>Desulfovibrio	GCA_019118745.1	Candidatus_Desulfovibrio_gallistercoris	H9774_13535	7700..11137	2838533	BREX-system-P-loop-protein-BrxC-[Candidatus-Desulfovibrio-gallistercoris]
OQA19974.1	Type1.BREX	BrxA-DNA-binding -> BrxB-BrxF-iSTAND -> DUF6079 *->?->RelE-ParE->VapB_antitoxin-> PIN -> N6-METHYLASE ->REase-> PglZ -> SIGMA+OB-Fold+AAA-ATPase+Lon-Protease-BrxL ->	1164	Bacteria>unclassified_Bacteria	GCA_002069435.1	bacterium_ADurb.Bin363	BWY64_00492	2230..5724	1866927	hypothetical-protein-BWY64_00492-[bacterium-ADurb.Bin363]
GBE48762.1	Type1.BREX	METHYLASE->REC+OmpR-HTH->REC+OmpR-HTH->?->URI-DNase+UVR+RNase+HHH->?->tRNA->tRNA->tRNA->?-> BrxB-BrxF-iSTAND -> DUF6079 *-> PIN -> N6-METHYLASE ->PHP+SbcC->Schlafen+HATPase+wHTH-> PglZ -> SIGMA+OB-Fold+AAA-ATPase+Lon-Protease-BrxL ->	1155	Bacteria>unclassified_Bacteria	GCA_002897795.1	bacterium_BMS3Bbin12	BMS3Bbin12_01950	17892..21359	2005735	hypothetical-protein-BMS3Bbin12_01950-[bacterium-BMS3Bbin12]

Protein-ID	System	Gene-Neighbourhoods	Length	Taxonomy	Assembly-ID	Strain	Locus-Tag	Genomic-coordinates	TaxID	Fasta-defline
--										
-- TUDOR+SF1-SF2-Helicase+i REase +CTD + REase										
--										
WP_007349691.1	Type5.BREX	<-Glycos_transf_2||tRNA->?->?-><- SIGMA+OB-Fold+AAA-ATPase+HKD-DNase-BrxL <- PglZ <-HNH<-OLD-ABC<- N6-METHYLASE <-Schlafen+HATPase+wHTH+HTH<- DUF6079 *<- BrxB-BrxF-iSTAND <- BrxA-DNA-binding <- TUDOR+SF1-SF2-Helicase+i REase +CTD + REase <-TPR	1194	Bacteria>Pseudomonadota>Gammaproteobacteria>Pseudomonadales>Marinobacteraceae>Marinobacter	GCF_000169375.1	Marinobacter_sp._ELB17	MELB17_RS08075	35418..39002	270374	BREX-system-P-loop-protein-BrxC-[Marinobacter-sp.-ELB17]
OUS97312.1	Type5.BREX	<-MULE-TRANSPOSASE<-TPR+TPR+TPR+TPR+TPR+TPR<-?|| BrxA-DNA-binding -> BrxB-BrxF-iSTAND -> DUF6079 *-> N6-METHYLASE -> HTH+ REase -> PglZ -> SIGMA+OB-Fold+AAA-ATPase+HKD-DNase-BrxL -><-DNA_glycosylase||?-><- TUDOR+SF1-SF2-Helicase+i REase +CTD + REase ||SF1-SF2-Helicase->	1175	Bacteria>Terrabacteria_group>Actinomycetota>Actinomycetes>Mycobacteriales>Nocardiaceae	GCA_002165735.1	Rhodococcus_sp._NCIMB_12038	CA951_02905	126594..130121	933800	ATP-binding-protein-[Rhodococcus-sp.-NCIMB-12038]
WP_256973250.1	Type5.BREX	<-MULE-TRANSPOSASE<-TPR+TPR+TPR+TPR+TPR+TPR|| BrxA-DNA-binding -> BrxB-BrxF-iSTAND -> DUF6079 *-> N6-METHYLASE -> HTH+ REase -> PglZ -> SIGMA+OB-Fold+AAA-ATPase+HKD-DNase-BrxL -><-DNA_glycosylase||?-><- TUDOR+SF1-SF2-Helicase+i REase +CTD + REase ||SF1-SF2-Helicase->	1213	Bacteria>Terrabacteria_group>Actinomycetota>Actinomycetes>Mycobacteriales>Nocardiaceae	GCF_002165735.1	Rhodococcus_sp._NCIMB_12038	CA951_RS02920	126480..130121	933800	BREX-system-P-loop-protein-BrxC-[Rhodococcus-sp.-NCIMB-12038]
WP_154708534.1	Type5.BREX	<- REase <- TUDOR+SF1-SF2-Helicase+i REase +CTD + REase <- PglZ <- N6-METHYLASE <- DUF6079 *<- BrxB-BrxF-iSTAND <- BrxA-DNA-binding <-?||?->wHTH+HNH->?-><-?<-HTH_36||HTH_3->	1187	Bacteria>Terrabacteria_group>Actinomycetota>Actinomycetes>Propionibacteriales>Propionibacteriaceae	-	Cutibacterium_acnes	CP883_05580	10794..14357	1747	BREX-system-P-loop-protein-BrxC-[Cutibacterium-acnes]
REB77450.1	Type5.BREX	<- REase <- TUDOR+SF1-SF2-Helicase+i REase +CTD + REase <- PglZ <- N6-METHYLASE <- DUF6079 *<- BrxB-BrxF-iSTAND <- BrxA-DNA-binding <-?||?->wHTH+HNH->?-><-?<-HTH_36||HTH_3->	1187	Bacteria>Terrabacteria_group>Actinomycetota>Actinomycetes>Propionibacteriales>Propionibacteriaceae	GCA_003384685.1	Cutibacterium_acnes	CP883_05580	10794..14357	1747	hypothetical-protein-CP883_05580-[Cutibacterium-acnes]
WP_029421470.1	Type5.BREX	<-ACET<-?<- REase <- TUDOR+SF1-SF2-Helicase+i REase +CTD + REase <- N6-METHYLASE -p<- N6-METHYLASE <- N6-METHYLASE <- PglZ <- DUF6079 *<- BrxB-BrxF-iSTAND <- BrxA-DNA-binding <-tRNA<-?<-?<-?<-35exo+T7DNApol	1205	Bacteria>Terrabacteria_group>Bacillota>Bacilli>Bacillales>Alicyclobacillaceae	GCF_000702485.1	Alicyclobacillus_macrosporangiidus	N687_RS0108610	1729200..1732817	392015	BREX-system-P-loop-protein-BrxC-[Alicyclobacillus-macrosporangiidus]
WP_002116626.1	Type5.BREX	<-PAS+NtrC-AAA+FIS-HTH||?->?-><-?<-HTH_Tnp_1+HTH_21+rve+rve_3<-HTH_Tnp_1||SF1-SF2-Helicase-> BrxA-DNA-binding -> BrxB-BrxF-iSTAND -> DUF6079 *-> N6-METHYLASE -> PglZ -> TUDOR+SF1-SF2-Helicase+i REase +CTD + REase ->?-> wHTH+WYL+WCX ->NUDIX->HKD-DNase+SF1-SF2-Helicase+HTH+HTH+SRA-> N6-METHYLASE ->	1188	Bacteria>Terrabacteria_group>Bacillota>Bacilli>Bacillales>Bacillaceae	-	Bacillus_cereus	IEQ_RS03950	791262..794828	1396	BREX-system-P-loop-protein-BrxC-[Bacillus-cereus]
OHR66989.1	Type5.BREX	GDResolvase+HIN-HTH-><-?<-?<-?||GDResolvase+HIN-HTH-> BrxA-DNA-binding -> BrxB-BrxF-iSTAND -> DUF6079 *-> N6-METHYLASE -> PglZ -> TUDOR+SF1-SF2-Helicase+i REase +CTD + REase ->?->SF1-SF2-Helicase-><-?||TetR-HTH->	1195	Bacteria>Terrabacteria_group>Bacillota>Bacilli>Bacillales>Bacillaceae	GCA_001838545.1	Bacillus_sp._HMSC76G11	HMPREF3291_01045	70062..73649	1608910	hypothetical-protein-HMPREF3291_01045-[Bacillus-sp.-HMSC76G11]
ASF41331.1	Type5.BREX	<- REase <- TUDOR+SF1-SF2-Helicase+i REase +CTD + REase <- PglZ <- N6-METHYLASE <- DUF6079 *<- BrxB-BrxF-iSTAND <- BrxA-DNA-binding <-?||ABC-ATPase+SbcC->?-><-?<-ZnR	1188	Bacteria>Terrabacteria_group>Bacillota>Bacilli>Bacillales>Bacillaceae	GCA_002209425.1	Halobacillus_halophilus	CEH05_20035	4059294..4062860	1570	hypothetical-protein-CEH05_20035-[Halobacillus-halophilus]
ASS85704.1	Type5.BREX	McrC-NTD+ REase ->?->?->?->HTH_3->?->SF1-SF2-Helicase-> BrxA-DNA-binding -> BrxB-BrxF-iSTAND -> DUF6079 *-> N6-METHYLASE -> PglZ -> TUDOR+SF1-SF2-Helicase+i REase +CTD + REase ->?-><-DDE_5+DDE_Tnp_2||?->HTH_32+rve+rve_3+Mu-transpos_C->GspA-ATPase->	1183	Bacteria>Terrabacteria_group>Bacillota>Bacilli>Bacillales>Bacillaceae	GCA_002243605.1	Geobacillus_lituanicus	GLN3_00240	44558..48109	169283	hypothetical-protein-GLN3_00240-[Geobacillus-lituanicus]
PFG07777.1	Type5.BREX	<-HKD-DNase+SF1-SF2-Helicase+HTH+HTH+SRA<-NUDIX||wHTH(SBD)+HNH-><-?||HTH_3->?-><- TUDOR+SF1-SF2-Helicase+i REase +CTD + REase <- PglZ <- N6-METHYLASE <- DUF6079 *<- BrxB-BrxF-iSTAND <- BrxA-DNA-binding <-ParB+HNH<-?<-HEPN+HTH	1195	Bacteria>Terrabacteria_group>Bacillota>Bacilli>Bacillales>Bacillaceae	GCA_002563655.1	Bacillus_sp._es.034	ATG71_4686	4473829..4477416	1761763	hypothetical-protein-ATG71_4686-[Bacillus-sp.-es.034]
PGR68857.1	Type5.BREX	<- McrC-NTD+ REase <-wHTH+McrB-AAA<-?<- wHTH+WYL+WCX <- TUDOR+SF1-SF2-Helicase+i REase +CTD + REase <- PglZ <- N6-METHYLASE <- DUF6079 *<- BrxB-BrxF-iSTAND <- BrxA-DNA-binding <-SF1-SF2-Helicase<-?<-NERD+SF1-SF2-Helicase<- REase <-?<-?<-REC+CITB-HTH+ZDNA-HTH	1191	Bacteria>Terrabacteria_group>Bacillota>Bacilli>Bacillales>Bacillaceae	GCA_002576445.1	Bacillus_cereus	COC49_05330	39030..42605	1396	hypothetical-protein-COC49_05330-[Bacillus-cereus]
PRT22691.1	Type5.BREX	REC+CITB-HTH+ZDNA-HTH->?->?-> REase ->NERD+SF1-SF2-Helicase->?->SF1-SF2-Helicase-> BrxA-DNA-binding -> BrxB-BrxF-iSTAND -> DUF6079 *-> N6-METHYLASE -> PglZ -> TUDOR+SF1-SF2-Helicase+i REase +CTD + REase ->URI-DNase+RAMA-> wHTH+WYL+WCX -><-rve	1188	Bacteria>Terrabacteria_group>Bacillota>Bacilli>Bacillales>Bacillaceae	GCA_002998815.1	Bacillus_thuringiensis	C6351_31305	13182..16748	1428	BREX-system-P-loop-protein-BrxC-[Bacillus-thuringiensis]
MBU9710538.1	Type5.BREX	RNase->?->?->?->?->ParB-> SF1-SF2-Helicase+ REase +HTH-> BrxA-DNA-binding -> BrxB-BrxF-iSTAND -> DUF6079 *-> N6-METHYLASE -> PglZ -> TUDOR+SF1-SF2-Helicase+i REase +CTD + REase ->?->SF1-SF2-Helicase+Z1->?->MazF-><-ABhydrolase	1188	Bacteria>Terrabacteria_group>Bacillota>Bacilli>Bacillales>Bacillaceae	GCA_019042215.1	Evansella_tamaricis	KS419_02125	46685..50251	2069301	BREX-system-P-loop-protein-BrxC-[Evansella-tamaricis]
WP_019720353.1	Type5.BREX	BrxA-DNA-binding -> BrxB-BrxF-iSTAND -> DUF6079 *-> N6-METHYLASE -> PglZ -> TUDOR+SF1-SF2-Helicase+i REase +CTD + REase ->	1187	Bacteria>Terrabacteria_group>Bacillota>Bacilli>Bacillales>Bacillaceae	GCF_000223155.1	Weizmannia_coagulans	IOE_RS0101945	1303..4866	1398	BREX-system-P-loop-protein-BrxC-[Weizmannia-coagulans]
WP_003250543.1	Type5.BREX	OLD-ABC+TOPRIM->SF1-SF2-Helicase-><-?<-?<-?||HTH-> BrxA-DNA-binding -> BrxB-BrxF-iSTAND -> DUF6079 *-> N6-METHYLASE -> PglZ -> TUDOR+SF1-SF2-Helicase+i REase +CTD + REase ->HTH_Tnp+HTH_Tnp->?->Phage_integrase-><-Phage_int_SAM_1+Phage_integrase	1194	Bacteria>Terrabacteria_group>Bacillota>Bacilli>Bacillales>Bacillaceae	GCF_000258725.1	Parageobacillus_thermoglucosidasius	GT20_RS09290	1817833..1821417	1426	BREX-system-P-loop-protein-BrxC-[Parageobacillus-thermoglucosidasius]
WP_223254242.1	Type5.BREX	<- REase <- TUDOR+SF1-SF2-Helicase+i REase +CTD + REase <- PglZ <- N6-METHYLASE <- DUF6079 *<- BrxB-BrxF-iSTAND <- BrxA-DNA-binding <-?||ABC-ATPase+SbcC->?-><-?<-ZnR	1203	Bacteria>Terrabacteria_group>Bacillota>Bacilli>Bacillales>Bacillaceae	GCF_000284515.1	Halobacillus_halophilus	HBHAL_RS19750	4059450..4063061	1570	BREX-system-P-loop-protein-BrxC-[Halobacillus-halophilus]
WP_026590665.1	Type5.BREX	<-PAS+NtrC-AAA+FIS-HTH<-NERD<-?<- wHTH+WYL+WCX <- TUDOR+SF1-SF2-Helicase+i REase +CTD + REase <- PglZ <- N6-METHYLASE <- DUF6079 *<- BrxB-BrxF-iSTAND <- BrxA-DNA-binding <-SF1-SF2-Helicase||<-ABhydrolase<- REase ||?-><-?<-McrB-AAA	1185	Bacteria>Terrabacteria_group>Bacillota>Bacilli>Bacillales>Bacillaceae	GCF_000482345.1	Bacillus_sp._UNC437CL72CviS29	M014_RS0104560	312641..316198	1340430	BREX-system-P-loop-protein-BrxC-[Bacillus-sp.-UNC437CL72CviS29]
WP_026908399.1	Type5.BREX	<-ParB+HNH<- SF1-SF2-Helicase+ REase <-?<-?<-?<- REase <- TUDOR+SF1-SF2-Helicase+i REase +CTD + REase <- PglZ <- N6-METHYLASE <- DUF6079 *<- BrxB-BrxF-iSTAND <- BrxA-DNA-binding <-?||wHTH->?->wHTH+HNH->?-><-?<-HTH_36	1187	Bacteria>Terrabacteria_group>Bacillota>Bacilli>Bacillales>Bacillaceae	GCF_000482485.1	Paucisalibacillus_globulus	K332_RS0116125	154598..158161	351095	BREX-system-P-loop-protein-BrxC-[Paucisalibacillus-globulus]
WP_048718293.1	Type5.BREX	HTH_36->?-><-?<-wHTH+HNH<-?<-wHTH||?-> BrxA-DNA-binding -> BrxB-BrxF-iSTAND -> DUF6079 *-> N6-METHYLASE -> PglZ -> TUDOR+SF1-SF2-Helicase+i REase +CTD + REase -> REase ->?->TIR->Schlafen+HATPase+wHTH->	1187	Bacteria>Terrabacteria_group>Bacillota>Bacilli>Bacillales>Bacillaceae	GCF_001076885.1	Bacillus_sp._522_BSPC	ADJ83_RS15690	17567..21130	1579338	BREX-system-P-loop-protein-BrxC-[Bacillus-sp.-522_BSPC]
WP_061570112.1	Type5.BREX	ACET-><-?|| N6-METHYLASE ->?-> BrxA-DNA-binding -> BrxB-BrxF-iSTAND -> DUF6079 *-> N6-METHYLASE -> PglZ -> TUDOR+SF1-SF2-Helicase+i REase +CTD + REase ->?-><-?||?->?->MerR-HTH->	1201	Bacteria>Terrabacteria_group>Bacillota>Bacilli>Bacillales>Bacillaceae	GCF_001587535.1	Caldibacillus_debilis	B4135_RS15850	22498..26103	301148	BREX-system-P-loop-protein-BrxC-[Caldibacillus-debilis]
WP_066159884.1	Type5.BREX	<-OLD-ABC+TOPRIM<-?<-?<- TUDOR+SF1-SF2-Helicase+i REase +CTD + REase <- PglZ <- N6-METHYLASE <- DUF6079 *<- BrxB-BrxF-iSTAND <- BrxA-DNA-binding <- SF1-SF2-Helicase+ REase -p<-SF1-SF2-Helicase<-SF1-SF2-Helicase<-ParB<-MerR-HTH	1205	Bacteria>Terrabacteria_group>Bacillota>Bacilli>Bacillales>Bacillaceae	GCF_001591945.1	Halalkalibacter_krulwichiae	BKR01S_RS20150	13948..17565	199441	BREX-system-P-loop-protein-BrxC-[Halalkalibacter-krulwichiae]
WP_077428558.1	Type5.BREX	<-SMODS<-?<-?<-SLATT<-ParB+HNH<-?<- TUDOR+SF1-SF2-Helicase+i REase +CTD + REase <- PglZ <- N6-METHYLASE <- DUF6079 *<- BrxB-BrxF-iSTAND <- BrxA-DNA-binding <-SF1-SF2-Helicase<- N6-METHYLASE <-HKD-DNase+SF1-SF2-Helicase+HTH+HTH+SRA<-?<-?||?-><-SAM-METHYLASE	1179	Bacteria>Terrabacteria_group>Bacillota>Bacilli>Bacillales>Bacillaceae	GCF_001996285.1	Anoxybacillus_karvacharensis	BO219_RS04435	413742..417281	1919277	BREX-system-P-loop-protein-BrxC-[Anoxybacillus-karvacharensis]
WP_047753295.1	Type5.BREX	<-DEDD_Tnp_IS110+Transposase_20<-DDE_3||?->DDE_Tnp_1-><-DDE_5+DDE_Tnp_2<-SAD+ REase <- TUDOR+SF1-SF2-Helicase+i REase +CTD + REase <- PglZ <- N6-METHYLASE <- DUF6079 *<- BrxB-BrxF-iSTAND <- BrxA-DNA-binding <-SF1-SF2-Helicase||DDE_Tnp_1-><-?<-SF1-SF2-Helicase<-HTH_3<-HigB	1183	Bacteria>Terrabacteria_group>Bacillota>Bacilli>Bacillales>Bacillaceae	GCF_002243665.1	Geobacillus	GT3921_RS13000	2562536..2566087	129337	MULTISPECIES:-BREX-system-P-loop-protein-BrxC-[Geobacillus]
WP_098359294.1	Type5.BREX	<-PAS+NtrC-AAA+FIS-HTH<-NERD<-?<- wHTH+WYL+WCX <- TUDOR+SF1-SF2-Helicase+i REase +CTD + REase <- PglZ <- N6-METHYLASE <- DUF6079 *<- BrxB-BrxF-iSTAND <- BrxA-DNA-binding <-SF1-SF2-Helicase<-ORC-CDC6<-HEPN<- McrC-NTD+ REase <-wHTH+McrB-AAA||ParB->	1186	Bacteria>Terrabacteria_group>Bacillota>Bacilli>Bacillales>Bacillaceae	GCF_002561295.1	Bacillus_cereus	CN373_RS10800	80983..84543	1396	BREX-system-P-loop-protein-BrxC-[Bacillus-cereus]
WP_098613368.1	Type5.BREX	<- McrC-NTD+ REase <-wHTH+McrB-AAA<-?<- wHTH+WYL+WCX <-?<- TUDOR+SF1-SF2-Helicase+i REase +CTD + REase <- PglZ <- N6-METHYLASE <- N6-METHYLASE <- DUF6079 *<- BrxB-BrxF-iSTAND <- BrxA-DNA-binding <-SF1-SF2-Helicase<-?<-NERD+SF1-SF2-Helicase<- REase <-?<-?<-REC+CITB-HTH+ZDNA-HTH	1188	Bacteria>Terrabacteria_group>Bacillota>Bacilli>Bacillales>Bacillaceae	GCF_002567495.1	Bacillus_cereus	COK05_RS16060	61384..64950	1396	BREX-system-P-loop-protein-BrxC-[Bacillus-cereus]
WP_098794042.1	Type5.BREX	<-HKD-DNase<-DrmB<-DrmA||SF1-SF2-Helicase->SF1-SF2-Helicase->DNA_METHYLASE->SF1-SF2-Helicase-> BrxA-DNA-binding -> BrxB-BrxF-iSTAND -> DUF6079 *-> N6-METHYLASE -> PglZ -> TUDOR+SF1-SF2-Helicase+i REase +CTD + REase ->?->?->?->?-><-?||ACET->TPR->	1188	Bacteria>Terrabacteria_group>Bacillota>Bacilli>Bacillales>Bacillaceae	GCF_002576235.1	Priestia_megaterium	COA18_RS28020	1940977..1944543	1404	BREX-system-P-loop-protein-BrxC-[Priestia-megaterium]
WP_098878446.1	Type5.BREX	<- McrC-NTD+ REase <-wHTH+McrB-AAA<-?<- wHTH+WYL+WCX <-?<- TUDOR+SF1-SF2-Helicase+i REase +CTD + REase <- PglZ <- N6-METHYLASE <- DUF6079 *<- BrxB-BrxF-iSTAND <- BrxA-DNA-binding <-SF1-SF2-Helicase<-?<-NERD+SF1-SF2-Helicase<- REase <-?<-?<-REC+CITB-HTH+ZDNA-HTH	1191	Bacteria>Terrabacteria_group>Bacillota>Bacilli>Bacillales>Bacillaceae	GCF_002576445.1	Bacillus_cereus	COC49_RS05330	39030..42605	1396	BREX-system-P-loop-protein-BrxC-[Bacillus-cereus]
WP_100332651.1	Type5.BREX	ANK->?-><-PolB||<-?||?-><- TUDOR+SF1-SF2-Helicase+i REase +CTD + REase <- PglZ <- N6-METHYLASE <- DUF6079 *<- BrxB-BrxF-iSTAND <- BrxA-DNA-binding <-?<-AAA+<-HTH_36||?->?->GDResolvase+Recombinase+ZnR-><-PolB	1187	Bacteria>Terrabacteria_group>Bacillota>Bacilli>Bacillales>Bacillaceae	GCF_002797355.1	Bacillus_xiapuensis	CEF20_RS14915	671484..675047	2014075	BREX-system-P-loop-protein-BrxC-[Bacillus-xiapuensis]
WP_066188676.1	Type5.BREX	SPOUT-><-?<-wHTH+HNH<-?<-wHTH||?-> BrxA-DNA-binding -> BrxB-BrxF-iSTAND -> DUF6079 *-> N6-METHYLASE -> PglZ -> TUDOR+SF1-SF2-Helicase+i REase +CTD + REase -> REase ->ZetaToxin->Classical-AAA->Peptidase_S8->	1187	Bacteria>Terrabacteria_group>Bacillota>Bacilli>Bacillales>Bacillaceae	GCF_002835735.1	Cytobacillus_horneckiae	CWS20_RS08115	27127..30690	549687	BREX-system-P-loop-protein-BrxC-[Cytobacillus-horneckiae]
WP_106082566.1	Type5.BREX	REC+CITB-HTH+ZDNA-HTH->?->?-> REase ->NERD+SF1-SF2-Helicase->?->SF1-SF2-Helicase-> BrxA-DNA-binding -> BrxB-BrxF-iSTAND -> DUF6079 *-> N6-METHYLASE -> PglZ -> TUDOR+SF1-SF2-Helicase+i REase +CTD + REase ->URI-DNase+RAMA-> wHTH+WYL+WCX -><-rve	1188	Bacteria>Terrabacteria_group>Bacillota>Bacilli>Bacillales>Bacillaceae	GCF_002998815.1	Bacillus_thuringiensis	C6351_31305	13182..16748	1428	BREX-system-P-loop-protein-BrxC-[Bacillus-thuringiensis]
WP_117327463.1	Type5.BREX	ABC-ATPase+SbcC-><-?||Phage_int_SAM_1+Phage_integrase-><-Phage_integrase||HIN-HTH-><- TUDOR+SF1-SF2-Helicase+i REase +CTD + REase <- PglZ <- N6-METHYLASE <- DUF6079 *<- BrxB-BrxF-iSTAND <- BrxA-DNA-binding <-GDResolvase+HIN-HTH<-Phage_integrase<-Phage_integrase<-Phage_integrase<-NERD+ZnR<-SbcC	1195	Bacteria>Terrabacteria_group>Bacillota>Bacilli>Bacillales>Bacillaceae	GCF_003429095.1	Peribacillus_saganii	D0469_RS14520	34367..37954	2303992	BREX-system-P-loop-protein-BrxC-[Peribacillus-saganii]
WP_132948756.1	Type5.BREX	NUDIX->ACET-><-?||McrB-NTD+McrB-AAA-> McrC-NTD+ REase ->?->SF1-SF2-Helicase-> BrxA-DNA-binding -> BrxB-BrxF-iSTAND -> DUF6079 *-> N6-METHYLASE -> PglZ -> TUDOR+SF1-SF2-Helicase+i REase +CTD + REase ->OLD-ABC+TOPRIM->HIN-HTH-><-?<-?<-ParA-Soj-PloopNTPase	1183	Bacteria>Terrabacteria_group>Bacillota>Bacilli>Bacillales>Bacillaceae	GCF_004341205.1	Thermolongibacillus_altinsuensis	EDD69_RS09635	59871..63422	575256	BREX-system-P-loop-protein-BrxC-[Thermolongibacillus-altinsuensis]
WP_136831108.1	Type5.BREX	SPOUT-><-GDResolvase+Recombinase+ZnR<-GDResolvase+Recombinase+ZnR<-?||NUDIX->GDResolvase+HIN-HTH-> BrxA-DNA-binding -> BrxB-BrxF-iSTAND -> DUF6079 *-> N6-METHYLASE -> PglZ -> TUDOR+SF1-SF2-Helicase+i REase +CTD + REase -><-PadR-HTH<-?<-?<-HTH_Tnp+HTH_Tnp||HKD-DNase->	1195	Bacteria>Terrabacteria_group>Bacillota>Bacilli>Bacillales>Bacillaceae	GCF_005116465.1	Robertmurraya_kyonggiensis	FA727_RS11510	51719..55306	1037680	BREX-system-P-loop-protein-BrxC-[Robertmurraya-kyonggiensis]
WP_144565381.1	Type5.BREX	<-GTPase-AIG<-GTPase-AIG<-?<- wHTH+WYL+WCX <-URI-DNase+RAMA<- TUDOR+SF1-SF2-Helicase+i REase +CTD + REase <- PglZ <- N6-METHYLASE <- N6-METHYLASE <- DUF6079 *<- BrxB-BrxF-iSTAND <- BrxA-DNA-binding <-SF1-SF2-Helicase<-JAB<-SF1-SF2-Helicase+Z1<-?<- REase <-?<-SF1-SF2-Helicase	1186	Bacteria>Terrabacteria_group>Bacillota>Bacilli>Bacillales>Bacillaceae	GCF_007681185.1	Bacillus_mobilis	FS794_RS23165	89317..92877	2026190	BREX-system-P-loop-protein-BrxC-[Bacillus-mobilis]
WP_144543246.1	Type5.BREX	Phage_int_SAM_3+Phage_integrase-><-?<-?<-?<- REase <- TUDOR+SF1-SF2-Helicase+i REase +CTD + REase <- PglZ <- N6-METHYLASE <- DUF6079 *<- BrxB-BrxF-iSTAND <- BrxA-DNA-binding <-?||wHTH->?->wHTH+HNH->?-><-AAA-ATPase-p	1192	Bacteria>Terrabacteria_group>Bacillota>Bacilli>Bacillales>Bacillaceae	GCF_007830235.1	Cytobacillus_oceanisediminis	IQ19_RS15315	231804..235382	665099	BREX-system-P-loop-protein-BrxC-[Cytobacillus-oceanisediminis]
WP_093855906.1	Type5.BREX	<-HNH<- REase <-?<- SF1-SF2-Helicase+ REase <-ParB+HNH<- TUDOR+SF1-SF2-Helicase+i REase +CTD + REase <- PglZ <- N6-METHYLASE <- DUF6079 *<- BrxB-BrxF-iSTAND <- BrxA-DNA-binding <-?||wHTH->?->wHTH+HNH->?-><-?<-HTH_36	1187	Bacteria>Terrabacteria_group>Bacillota>Bacilli>Bacillales>Bacillaceae	GCF_007989445.1	Tenuibacillus_multivorans	BLP77_RS04470	353994..357557	237069	BREX-system-P-loop-protein-BrxC-[Tenuibacillus-multivorans]
WP_155112638.1	Type5.BREX	<-Peptidase_S8<-Classical-AAA<-?<- REase <- TUDOR+SF1-SF2-Helicase+i REase +CTD + REase <- PglZ <- N6-METHYLASE -p<- N6-METHYLASE <- DUF6079 *<- BrxB-BrxF-iSTAND <- BrxA-DNA-binding <-?||wHTH->?->wHTH+HNH->?->AAA-ATPase->	1186	Bacteria>Terrabacteria_group>Bacillota>Bacilli>Bacillales>Bacillaceae	GCF_009711125.1	Metabacillus_mangrovi	GKZ89_RS11910	46455..50015	1491830	BREX-system-P-loop-protein-BrxC-[Metabacillus-mangrovi]
WP_167560822.1	Type5.BREX	<- TUDOR+SF1-SF2-Helicase+i REase +CTD + REase <- PglZ <- N6-METHYLASE <- DUF6079 *<- BrxB-BrxF-iSTAND <- BrxA-DNA-binding <-GDResolvase+HIN-HTH||?->Recombinase->GDResolvase->Recombinase+ZnR->?-><-SPOUT	1192	Bacteria>Terrabacteria_group>Bacillota>Bacilli>Bacillales>Bacillaceae	GCF_012030395.1	Bacillus_subtilis	HC742_RS20760	3449689..3453267	1423	BREX-system-P-loop-protein-BrxC-[Bacillus-subtilis]
WP_184528635.1	Type5.BREX	<-HEPN<-?<-?<- TUDOR+SF1-SF2-Helicase+i REase +CTD + REase <- PglZ <- N6-METHYLASE <- DUF6079 *<- BrxA-DNA-binding <-?||GDResolvase->?->GDResolvase+Zn_ribbon_recom-><-TRAM+METHYLASE	1192	Bacteria>Terrabacteria_group>Bacillota>Bacilli>Bacillales>Bacillaceae	GCF_014207535.1	Bacillus_benzoevorans	HNR53_RS18175	67020..70598	1456	BREX-system-P-loop-protein-BrxC-[Bacillus-benzoevorans]
WP_188383791.1	Type5.BREX	<-RVT<-ZnR<-?<- REase <- TUDOR+SF1-SF2-Helicase+i REase +CTD + REase <- PglZ <- N6-METHYLASE <- DUF6079 *<- BrxB-BrxF-iSTAND <- BrxA-DNA-binding <-?<-AAA+<-HTH_36||?->?->GDResolvase+Recombinase+ZnR-><-PolB	1187	Bacteria>Terrabacteria_group>Bacillota>Bacilli>Bacillales>Bacillaceae	GCF_014637405.1	Ornithinibacillus_halotolerans	IEV35_RS05945	32497..36060	1274357	BREX-system-P-loop-protein-BrxC-[Ornithinibacillus-halotolerans]
WP_188386543.1	Type5.BREX	Pkinase-><-?||?->?-> McrC-NTD+ REase ->SF1-SF2-Helicase-> BrxA-DNA-binding -> BrxB-BrxF-iSTAND -> DUF6079 *-> N6-METHYLASE -> PglZ -> TUDOR+SF1-SF2-Helicase+i REase +CTD + REase -> REase -> wHTH+WYL+WCX ->	1187	Bacteria>Terrabacteria_group>Bacillota>Bacilli>Bacillales>Bacillaceae	GCF_014638355.1	Priestia_taiwanensis	IEV41_RS00670	120045..123608	1347902	BREX-system-P-loop-protein-BrxC-[Priestia-taiwanensis]
WP_199714161.1	Type5.BREX	IONCHANNEL+PAS+HISKIN+MORC->REC+CITB-HTH+ZDNA-HTH->?-> REase ->NERD+SF1-SF2-Helicase->?->SF1-SF2-Helicase-> BrxA-DNA-binding -> BrxB-BrxF-iSTAND -> DUF6079 *-> N6-METHYLASE -> PglZ -> TUDOR+SF1-SF2-Helicase+i REase +CTD + REase ->?-> wHTH+WYL+WCX ->?->wHTH+McrB-AAA-> McrC-NTD+ REase -><- REase +ZnR	1191	Bacteria>Terrabacteria_group>Bacillota>Bacilli>Bacillales>Bacillaceae	GCF_016483555.1	Bacillus_cereus_group	JDS82_RS26190	13772..17347	86661	MULTISPECIES:-BREX-system-P-loop-protein-BrxC-[Bacillus-cereus-group]
WP_202778095.1	Type5.BREX	PadR-HTH-><-TatD_DNase<-?<-KAP-glycosylase<-KAP-NTPase<- TUDOR+SF1-SF2-Helicase+i REase +CTD + REase <- PglZ <- N6-METHYLASE <- N6-METHYLASE <- DUF6079 *<- BrxB-BrxF-iSTAND <- BrxA-DNA-binding <-GDResolvase+HIN-HTH<-?<-NUDIX||?->GDResolvase+Zn_ribbon_recom->GDResolvase+Zn_ribbon_recom-><-TRAM+METHYLASE	1192	Bacteria>Terrabacteria_group>Bacillota>Bacilli>Bacillales>Bacillaceae	GCF_016772275.1	Heyndrickxia_vini	I5776_RS19095	3841015..3844593	1476025	BREX-system-P-loop-protein-BrxC-[Heyndrickxia-vini]
WP_205168141.1	Type5.BREX	XIS-HTH->Phage_integrase->?->?->Beta-lactamase-><- TUDOR+SF1-SF2-Helicase+i REase +CTD + REase <- PglZ <- N6-METHYLASE <- DUF6079 *<- BrxB-BrxF-iSTAND <- BrxA-DNA-binding <-GDResolvase+HIN-HTH||?->?-><-XIS-HTH	1189	Bacteria>Terrabacteria_group>Bacillota>Bacilli>Bacillales>Bacillaceae	GCF_016908495.1	Bacillus_pakistanensis	JOC86_RS02215	441948..445517	992288	BREX-system-P-loop-protein-BrxC-[Bacillus-pakistanensis]
WP_212956143.1	Type5.BREX	<-sGTP<- McrC-NTD+ REase <-wHTH+McrB-AAA<- REase <-?<- wHTH+WYL+WCX <- TUDOR+SF1-SF2-Helicase+i REase +CTD + REase <- PglZ <- N6-METHYLASE <- DUF6079 *<- BrxB-BrxF-iSTAND <- BrxA-DNA-binding <-SF1-SF2-Helicase<-JAB<-SF1-SF2-Helicase+Z1<-?<-?<-REC+CITB-HTH+ZDNA-HTH<-IONCHANNEL+PAS+HISKIN+MORC	1188	Bacteria>Terrabacteria_group>Bacillota>Bacilli>Bacillales>Bacillaceae	GCF_018332475.1	Bacillus	KI369_RS12665	38598..42164	1386	MULTISPECIES:-BREX-system-P-loop-protein-BrxC-[Bacillus]
WP_214723414.1	Type5.BREX	<-GDResolvase+Recombinase+ZnR||?->?-><-GDResolvase+Recombinase+ZnR<-?||?->GDResolvase+HIN-HTH-> BrxA-DNA-binding -> BrxB-BrxF-iSTAND -> DUF6079 *-> N6-METHYLASE -> PglZ -> TUDOR+SF1-SF2-Helicase+i REase +CTD + REase ->	1189	Bacteria>Terrabacteria_group>Bacillota>Bacilli>Bacillales>Bacillaceae	GCF_018613175.1	Bacillus_sp._ISL-47	J7I93_RS03705	56511..60080	2819130	BREX-system-P-loop-protein-BrxC-[Bacillus-sp.-ISL-47]
WP_215562746.1	Type5.BREX	IONCHANNEL+PAS+HISKIN+MORC->REC+CITB-HTH+ZDNA-HTH->?->?->NERD+SF1-SF2-Helicase->?->SF1-SF2-Helicase-> BrxA-DNA-binding -> BrxB-BrxF-iSTAND -> DUF6079 *-> N6-METHYLASE -> PglZ -> TUDOR+SF1-SF2-Helicase+i REase +CTD + REase ->URI-DNase+RAMA-> wHTH+WYL+WCX ->?->GTPase-AIG->GTPase-AIG->wHTH+McrB-AAA->	1185	Bacteria>Terrabacteria_group>Bacillota>Bacilli>Bacillales>Bacillaceae	GCF_018741585.1	Bacillus_wiedmannii	EXW48_RS05020	1017548..1021105	1890302	BREX-system-P-loop-protein-BrxC-[Bacillus-wiedmannii]
WP_221874921.1	Type5.BREX	<-DnaB+TnsC-ATPase<-?<-?||GDResolvase+HIN-HTH-> BrxA-DNA-binding -> BrxB-BrxF-iSTAND -> DUF6079 *-> N6-METHYLASE -> PglZ ->|| TUDOR+SF1-SF2-Helicase+i REase +CTD + REase ->HNH->?->SbcC->	1192	Bacteria>Terrabacteria_group>Bacillota>Bacilli>Bacillales>Bacillaceae	GCF_019749255.1	Mesobacillus_maritimus	H0185_RS18235	45712..49290	1643336	BREX-system-P-loop-protein-BrxC-[Mesobacillus-maritimus]
WP_224936976.1	Type5.BREX	<-RVT+HEPN||?-><-Pkinase<-?<- REase <- TUDOR+SF1-SF2-Helicase+i REase +CTD + REase <- PglZ <- N6-METHYLASE <- DUF6079 *<- BrxB-BrxF-iSTAND <- BrxA-DNA-binding <-?||wHTH->?->wHTH+HNH->?-><-SPOUT	1187	Bacteria>Terrabacteria_group>Bacillota>Bacilli>Bacillales>Bacillaceae	GCF_020171845.1	Cytobacillus_kochii	LCM23_RS10855	44257..47820	859143	BREX-system-P-loop-protein-BrxC-[Cytobacillus-kochii]
WP_226516206.1	Type5.BREX	<-Peptidase_S8<-Classical-AAA<- REase <- TUDOR+SF1-SF2-Helicase+i REase +CTD + REase <- PglZ <- N6-METHYLASE <- DUF6079 *<- BrxB-BrxF-iSTAND <- BrxA-DNA-binding <-?||?->?->wHTH+HNH->?-><-SPOUT	1187	Bacteria>Terrabacteria_group>Bacillota>Bacilli>Bacillales>Bacillaceae	GCF_020519665.1	Bacillus_shivajii	LGQ02_RS20940	4386157..4389720	1983719	BREX-system-P-loop-protein-BrxC-[Bacillus-shivajii]
WP_229143299.1	Type5.BREX	<- REase +ZnR<-McrB-AAA<-?<- REase <- wHTH+WYL+WCX <-URI-DNase+RAMA<- TUDOR+SF1-SF2-Helicase+i REase +CTD + REase <- PglZ <- N6-METHYLASE <- DUF6079 *<- BrxB-BrxF-iSTAND <- BrxA-DNA-binding <-SF1-SF2-Helicase<-JAB<-SF1-SF2-Helicase+Z1<-?<- REase <-?<-REC+CITB-HTH+ZDNA-HTH	1188	Bacteria>Terrabacteria_group>Bacillota>Bacilli>Bacillales>Bacillaceae	GCF_020686245.1	Bacillus_paranthracis	LKL84_RS22410	92907..96473	2026186	BREX-system-P-loop-protein-BrxC-[Bacillus-paranthracis]
WP_048558547.1	Type5.BREX	IONCHANNEL+PAS+HISKIN+MORC->REC+CITB-HTH+ZDNA-HTH->?->?->SF1-SF2-Helicase+Z1->JAB->SF1-SF2-Helicase-> BrxA-DNA-binding -> BrxB-BrxF-iSTAND -> DUF6079 *-> N6-METHYLASE -> N6-METHYLASE -p-> N6-METHYLASE ->nSTAND3-> PglZ -> TUDOR+SF1-SF2-Helicase+i REase +CTD + REase ->URI-DNase+RAMA-> wHTH+WYL+WCX ->	1185	Bacteria>Terrabacteria_group>Bacillota>Bacilli>Bacillales>Bacillaceae	GCF_021655055.1	Bacillus_cereus	L6497_RS04770	943584..947141	1396	BREX-system-P-loop-protein-BrxC-[Bacillus-cereus]
WP_200152411.1	Type5.BREX	IONCHANNEL+PAS+HISKIN+MORC->REC+CITB-HTH+ZDNA-HTH->?->?->SF1-SF2-Helicase+Z1->JAB->SF1-SF2-Helicase-> BrxA-DNA-binding -> BrxB-BrxF-iSTAND -> DUF6079 *-> N6-METHYLASE -> N6-METHYLASE -p-> N6-METHYLASE ->nSTAND3-> PglZ -> TUDOR+SF1-SF2-Helicase+i REase +CTD + REase ->URI-DNase+RAMA-> wHTH+WYL+WCX ->	1185	Bacteria>Terrabacteria_group>Bacillota>Bacilli>Bacillales>Bacillaceae	GCF_021655215.1	Bacillus_cereus	L6484_RS04895	983694..987251	1396	BREX-system-P-loop-protein-BrxC-[Bacillus-cereus]
WP_242261892.1	Type5.BREX	<-wHTH+McrB-AAA<-GTPase-AIG<-GTPase-AIG<-?<- wHTH+WYL+WCX <-URI-DNase+RAMA<- TUDOR+SF1-SF2-Helicase+i REase +CTD + REase <- PglZ <- N6-METHYLASE <- DUF6079 *<- BrxB-BrxF-iSTAND <- BrxA-DNA-binding <-SF1-SF2-Helicase<-?<-NERD+SF1-SF2-Helicase<- REase <-?<-REC+CITB-HTH+ZDNA-HTH<-IONCHANNEL+PAS+HISKIN+MORC	1188	Bacteria>Terrabacteria_group>Bacillota>Bacilli>Bacillales>Bacillaceae	GCF_022662915.1	Bacillus_cereus_group_sp._BfR-BA-01453	MR070_RS20010	124720..128286	2920355	BREX-system-P-loop-protein-BrxC-[Bacillus-cereus-group-sp.-BfR-BA-01453]
WP_242225824.1	Type5.BREX	<- REase <- McrC-NTD+ REase <-wHTH+McrB-AAA<-?<- wHTH+WYL+WCX <-URI-DNase+RAMA<- TUDOR+SF1-SF2-Helicase+i REase +CTD + REase <- PglZ <- N6-METHYLASE <- DUF6079 *<- BrxB-BrxF-iSTAND <- BrxA-DNA-binding <-SF1-SF2-Helicase<-?<-NERD+SF1-SF2-Helicase<- REase <-?<-?<-REC+CITB-HTH+ZDNA-HTH	1185	Bacteria>Terrabacteria_group>Bacillota>Bacilli>Bacillales>Bacillaceae	GCF_022663655.1	Bacillus_cereus_group_sp._BfR-BA-01358	MR078_RS05890	38308..41865	2920320	BREX-system-P-loop-protein-BrxC-[Bacillus-cereus-group-sp.-BfR-BA-01358]
WP_242320708.1	Type5.BREX	IONCHANNEL+PAS+HISKIN+MORC->REC+CITB-HTH+ZDNA-HTH->?-> REase ->NERD+SF1-SF2-Helicase->?->SF1-SF2-Helicase-> BrxA-DNA-binding -> BrxB-BrxF-iSTAND -> DUF6079 *-> N6-METHYLASE -> PglZ -> TUDOR+SF1-SF2-Helicase+i REase +CTD + REase ->URI-DNase+RAMA-> wHTH+WYL+WCX ->?->GTPase-AIG->GTPase-AIG->SAD-DUF3427C+SbcC->	1186	Bacteria>Terrabacteria_group>Bacillota>Bacilli>Bacillales>Bacillaceae	GCF_022663735.1	Bacillus_cereus_group_sp._BfR-BA-01354	MR162_RS10425	194779..198339	2920317	BREX-system-P-loop-protein-BrxC-[Bacillus-cereus-group-sp.-BfR-BA-01354]
WP_243571747.1	Type5.BREX	<- TUDOR+SF1-SF2-Helicase+i REase +CTD + REase <- PglZ <- N6-METHYLASE <- DUF6079 *<- BrxB-BrxF-iSTAND <- BrxA-DNA-binding <-GDResolvase+HIN-HTH||GDResolvase->Recombinase+ZnR->?-><-?||?-><-ADH_N+ADH_zinc_N	1192	Bacteria>Terrabacteria_group>Bacillota>Bacilli>Bacillales>Bacillaceae	GCF_022814905.1	Bacillus_stercoris	MSS26_RS04990	1003415..1006993	2054641	BREX-system-P-loop-protein-BrxC-[Bacillus-stercoris]
WP_251625212.1	Type5.BREX	MazE_antitoxin+SpoVT_C-><-GDResolvase+HIN-HTH||?-> BrxA-DNA-binding -> BrxB-BrxF-iSTAND -> DUF6079 *-> N6-METHYLASE -> PglZ -> TUDOR+SF1-SF2-Helicase+i REase +CTD + REase -><-?||RVT->?-><-?<-LexA-protease	1192	Bacteria>Terrabacteria_group>Bacillota>Bacilli>Bacillales>Bacillaceae	GCF_023712125.1	Metabacillus_idriensis	M4D55_RS07610	46091..49669	324768	BREX-system-P-loop-protein-BrxC-[Metabacillus-idriensis]
WP_251646840.1	Type5.BREX	<-Phage_int_SAM_3+Phage_integrase||Glyoxalase->?-><-?<-Arm-DNA-bind_4+Phage_int_SAM_3+Phage_integrase||?-> BrxA-DNA-binding -> BrxB-BrxF-iSTAND -> DUF6079 *-> N6-METHYLASE -> PglZ -> TUDOR+SF1-SF2-Helicase+i REase +CTD + REase ->	1195	Bacteria>Terrabacteria_group>Bacillota>Bacilli>Bacillales>Bacillaceae	GCF_023714395.1	Niallia_sp._MER_6	M3600_17125	94088..97675	2939567	BREX-system-P-loop-protein-BrxC-[Niallia-sp.-MER-6]
WP_251339432.1	Type5.BREX	<-HTH_Tnp_1+HTH_21+rve+rve_3<-DndB<-Tox-RelA<-?<-TPR||SLATT<-?<- TUDOR+SF1-SF2-Helicase+i REase +CTD + REase <- PglZ <- N6-METHYLASE <- DUF6079 *<- BrxB-BrxF-iSTAND <- BrxA-DNA-binding <-GDResolvase+HIN-HTH||Phage_int_SAM_5+Phage_integrase->	1192	Bacteria>Terrabacteria_group>Bacillota>Bacilli>Bacillales>Bacillaceae	GCF_023714665.1	Heyndrickxia_oleronia	M3589_RS18030	387742..391320	38875	BREX-system-P-loop-protein-BrxC-[Heyndrickxia-oleronia]
WP_258922098.1	Type5.BREX	DDE_Tnp_1_4-><- TUDOR+SF1-SF2-Helicase+i REase +CTD + REase <- PglZ <- N6-METHYLASE <- DUF6079 *<- BrxB-BrxF-iSTAND <- BrxA-DNA-binding <-DDE_Tnp_1_4<-?<-?<-?||PadR-HTH->	1187	Bacteria>Terrabacteria_group>Bacillota>Bacilli>Bacillales>Bacillaceae	GCF_024758645.1	Weizmannia_coagulans	L1879_RS05770	1225557..1229120	1398	BREX-system-P-loop-protein-BrxC-[Weizmannia-coagulans]
WP_260358530.1	Type5.BREX	<-DNA_METHYLASE<-SF1-SF2-Helicase<-SF1-SF2-Helicase||DrmA->DrmB->HKD-DNase-><- TUDOR+SF1-SF2-Helicase+i REase +CTD + REase <- PglZ <- N6-METHYLASE <- DUF6079 *<- BrxB-BrxF-iSTAND <- BrxA-DNA-binding <-SF1-SF2-Helicase||NUDIX-><- McrC-NTD+ REase <-McrB-AAA||SF1-SF2-Helicase-><- REase +SPF-cons	1177	Bacteria>Terrabacteria_group>Bacillota>Bacilli>Bacillales>Bacillaceae	GCF_025209795.1	Peribacillus_frigoritolerans	NOU24_RS18785	747765..751298	450367	BREX-system-P-loop-protein-BrxC-[Peribacillus-frigoritolerans]
WP_268298773.1	Type5.BREX	SPOUT-><-Zn_ribbon_recom<-Recombinase<-GDResolvase<-?<-?||GDResolvase+HIN-HTH-> BrxA-DNA-binding -> BrxB-BrxF-iSTAND -> DUF6079 *-> N6-METHYLASE -> PglZ -> TUDOR+SF1-SF2-Helicase+i REase +CTD + REase -><-?<-GNTR-HTH+UTRA||?->Glyco_hydro_1->	1192	Bacteria>Terrabacteria_group>Bacillota>Bacilli>Bacillales>Bacillaceae	GCF_026789485.1	Bacillus_inaquosorum	MOB75_RS13790	96491..100069	483913	BREX-system-P-loop-protein-BrxC-[Bacillus-inaquosorum]
WP_269897765.1	Type5.BREX	<-HEPN+HTH<-?<-JAB<-?<- TUDOR+SF1-SF2-Helicase+i REase +CTD + REase <-?<- PglZ <-?<- N6-METHYLASE <- DUF6079 *<- BrxB-BrxF-iSTAND <- BrxA-DNA-binding ||?->ZnR+TOPRIM+AAA-helicase+wHTH->XIS-HTH->?->Phage_int_SAM_3+Phage_integrase->Arm-DNA-bind_4+Phage_int_SAM_3+Phage_integrase-><-HNH	1196	Bacteria>Terrabacteria_group>Bacillota>Bacilli>Bacillales>Bacillaceae	GCF_027557635.1	Lysinibacillus_sp._JK80	HXV90_RS18610	3707833..3711423	2749809	BREX-system-P-loop-protein-BrxC-[Lysinibacillus-sp.-JK80]
WP_231419114.1	Type5.BREX	TRAM+METHYLASE->?-> BrxA-DNA-binding -> BrxB-BrxF-iSTAND -> DUF6079 *-> N6-METHYLASE -> PglZ -> TUDOR+SF1-SF2-Helicase+i REase +CTD + REase ->HNH-><- REase +DUF5655<-SF1-SF2-Helicase||URI-DNase->TIR->	1188	Bacteria>Terrabacteria_group>Bacillota>Bacilli>Bacillales>Bacillaceae	GCF_030166655.1	Pontibacillus_sp._HN14	LQ226_RS16640	43379..46945	2898421	BREX-system-P-loop-protein-BrxC-[Pontibacillus-sp.-HN14]
WP_098147710.1	Type5.BREX	NERD+SF1-SF2-Helicase->?->SF1-SF2-Helicase-> BrxA-DNA-binding -> BrxB-BrxF-iSTAND -> DUF6079 *-> N6-METHYLASE -> PglZ -> TUDOR+SF1-SF2-Helicase+i REase +CTD + REase -> wHTH+WYL+WCX ->?->wHTH+McrB-AAA-> McrC-NTD+ REase ->?-> REase ->	1186	Bacteria>Terrabacteria_group>Bacillota>Bacilli>Bacillales>Bacillaceae	GCF_030676405.1	Bacillus_wiedmannii	CN685_RS18690	6510..10070	1890302	BREX-system-P-loop-protein-BrxC-[Bacillus-wiedmannii]
WP_073518919.1	Type5.BREX	SF1-SF2-Helicase->?-> REase ->?->SF1-SF2-Helicase+Z1->JAB->SF1-SF2-Helicase-> BrxA-DNA-binding -> BrxB-BrxF-iSTAND -> DUF6079 *-> N6-METHYLASE -> PglZ -> TUDOR+SF1-SF2-Helicase+i REase +CTD + REase -> wHTH+WYL+WCX ->?->GTPase-AIG->GTPase-AIG-><-NACHT	1185	Bacteria>Terrabacteria_group>Bacillota>Bacilli>Bacillales>Bacillaceae	GCF_900094815.1	Bacillus_cereus_group	CCY53_RS04725	210220..213777	86661	MULTISPECIES:-BREX-system-P-loop-protein-BrxC-[Bacillus-cereus-group]
WP_099159038.1	Type5.BREX	<-wHTH+HNH<-?<-wHTH<-?<-wHTH||?-> BrxA-DNA-binding -> BrxB-BrxF-iSTAND -> DUF6079 *-> N6-METHYLASE -> PglZ -> TUDOR+SF1-SF2-Helicase+i REase +CTD + REase -> REase ->?->?->?-> SF1-SF2-Helicase+ REase ->ParB+HNH->	1187	Bacteria>Terrabacteria_group>Bacillota>Bacilli>Bacillales>Bacillaceae	GCF_900187325.1	Virgibacillus_ndiopensis	CS937_RS13675	175761..179324	2004408	BREX-system-P-loop-protein-BrxC-[Virgibacillus-ndiopensis]
WP_163530739.1	Type5.BREX	ArdCN+MPTase-inact->TOPRIM->?-> BrxA-DNA-binding -> BrxB-BrxF-iSTAND -> DUF6079 *-> N6-METHYLASE -> PglZ -> TUDOR+SF1-SF2-Helicase+i REase +CTD + REase ->?->McrB-NTD+HNH->Phage_integrase-><-HEPN<-AAA-ATPase<-HTH_22+rve	1188	Bacteria>Terrabacteria_group>Bacillota>Bacilli>Bacillales>Bacillaceae	GCF_902726645.1	Halobacillus_ihumii	G6R08_RS20435	4081488..4085054	2686092	BREX-system-P-loop-protein-BrxC-[Halobacillus-ihumii]
WP_026834104.1	Type5.BREX	<-ABC-ATPase+SbcC<-LYTTR||PAIREDC-HTH+HIN-HTH->?->?-> SF1-SF2-Helicase+ REase -><- TUDOR+SF1-SF2-Helicase+i REase +CTD + REase <- PglZ <- N6-METHYLASE <- DUF6079 *<- BrxB-BrxF-iSTAND <- BrxA-DNA-binding <-HKD-DNase+SF1-SF2-Helicase+HTH+HTH+SRA<-?||RNase-><- HARE-HTH <-?||?->Peptidase->	1192	Bacteria>Terrabacteria_group>Bacillota>Bacilli>Bacillales>Bacillales_incertae_sedis	GCF_000620825.1	Exiguobacterium_undae	P399_RS0115220	2864676..2868254	169177	BREX-system-P-loop-protein-BrxC-[Exiguobacterium-undae]
WP_026826561.1	Type5.BREX	<- HARE-HTH <-Preader17<-HKD-DNase+SF1-SF2-Helicase+HTH+HTH+SRA<-?<- TUDOR+SF1-SF2-Helicase+i REase +CTD + REase <- PglZ <- N6-METHYLASE <- DUF6079 *<- BrxB-BrxF-iSTAND <- BrxA-DNA-binding <-?<-?<-?<-?<-?<-HHH+DNA_pol_B+RNase	1191	Bacteria>Terrabacteria_group>Bacillota>Bacilli>Bacillales>Bacillales_incertae_sedis	GCF_000620845.1	Exiguobacterium_marinum	P400_RS0112700	2404557..2408132	273528	BREX-system-P-loop-protein-BrxC-[Exiguobacterium-marinum]
WP_078145973.1	Type5.BREX	Trypsin+PDZ->?->SPOUT->?->?-><-PAIREDC-HTH+HIN-HTH||?-> BrxA-DNA-binding -> BrxB-BrxF-iSTAND -> DUF6079 *-> N6-METHYLASE -> PglZ -> TUDOR+SF1-SF2-Helicase+i REase +CTD + REase ->?-><-AAA-ATPase<-rve	1187	Bacteria>Terrabacteria_group>Bacillota>Bacilli>Bacillales>Bacillales_incertae_sedis	GCF_002009685.1	Exiguobacterium_sp._HVEsp1	BV910_RS00630	122796..126359	1934003	BREX-system-P-loop-protein-BrxC-[Exiguobacterium-sp.-HVEsp1]
WP_131508621.1	Type5.BREX	McrC-NTD+ REase -><-NERD+SF1-SF2-Helicase||?->?->?->?-><-wHTH+HNH|| BrxA-DNA-binding -> BrxB-BrxF-iSTAND -> DUF6079 *-> N6-METHYLASE -> PglZ -> TUDOR+SF1-SF2-Helicase+i REase +CTD + REase ->	1200	Bacteria>Terrabacteria_group>Bacillota>Bacilli>Bacillales>Bacillales_incertae_sedis	GCF_004337165.1	Exiguobacterium_sp._SH0S1	EVJ20_RS13610	61897..65499	2510949	BREX-system-P-loop-protein-BrxC-[Exiguobacterium-sp.-SH0S1]
WP_089525998.1	Type5.BREX	REase +wHTH-><-zf-IS66|| BrxA-DNA-binding -> BrxB-BrxF-iSTAND -> DUF6079 *-> N6-METHYLASE -> PglZ -> TUDOR+SF1-SF2-Helicase+i REase +CTD + REase ->Calcineurin->SbcC+SbcC->?->RVT->RNase->	1208	Bacteria>Terrabacteria_group>Bacillota>Bacilli>Bacillales>Paenibacillaceae	GCF_002233675.1	Paenibacillus_herberti	CGZ75_RS19630	18692..22318	1619309	BREX-system-P-loop-protein-BrxC-[Paenibacillus-herberti]
WP_272441147.1	Type5.BREX	<-Phage_int_SAM_5+Phage_integrase||?->?-><-XIS-HTH<-Phage_GPA|| BrxA-DNA-binding -> BrxB-BrxF-iSTAND -> DUF6079 *-> N6-METHYLASE ->ParB+HNH-> PglZ -> TUDOR+SF1-SF2-Helicase+i REase +CTD + REase ->?->DnaB+HINT+DnaB+HINT->ParB+HNH->?-><-XIS-HTH	1201	Bacteria>Terrabacteria_group>Bacillota>Bacilli>Bacillales>Paenibacillaceae	GCF_028421645.1	Aneurinibacillus_sp._Ricciae_BoGa-3	PP175_RS01545	299012..302617	3022697	BREX-system-P-loop-protein-BrxC-[Aneurinibacillus-sp.-Ricciae_BoGa-3]
WP_066463066.1	Type5.BREX	BrxA-DNA-binding -> BrxB-BrxF-iSTAND -> DUF6079 *-> N6-METHYLASE ->?-> PglZ -><-?<-?<-?|| TUDOR+SF1-SF2-Helicase+i REase +CTD + REase ->	1200	Bacteria>Terrabacteria_group>Bacillota>Bacilli>Bacillales>Planococcaceae	GCF_001700325.1	Caryophanon_latum	A6K76_RS05925	1324..4926	33977	BREX-system-P-loop-protein-BrxC-[Caryophanon-latum]
WP_107933601.1	Type5.BREX	HTH-><-?<-AAA+<-HTH_36<- TUDOR+SF1-SF2-Helicase+i REase +CTD + REase <- PglZ <- N6-METHYLASE <- DUF6079 *<- BrxB-BrxF-iSTAND <- BrxA-DNA-binding ||?->?-><-?||?->XIS-HTH->Phage_int_SAM_3+Phage_integrase->Arm-DNA-bind_4+Phage_int_SAM_3+Phage_integrase->	1192	Bacteria>Terrabacteria_group>Bacillota>Bacilli>Bacillales>Planococcaceae	GCF_003049615.1	Ureibacillus_chungkukjangi	C9J33_RS05745	69413..72991	1202712	BREX-system-P-loop-protein-BrxC-[Ureibacillus-chungkukjangi]
WP_108587666.1	Type5.BREX	wHTH+HNH-p->?->MORC+DUF3684+ REase ->?-><-ParB+HNH|| BrxA-DNA-binding -> BrxB-BrxF-iSTAND -> DUF6079 *-> N6-METHYLASE -> PglZ -> TUDOR+SF1-SF2-Helicase+i REase +CTD + REase ->GNTR-HTH+UTRA->?->HNH->?->?->ABC-ATPase+SbcC->	1191	Bacteria>Terrabacteria_group>Bacillota>Bacilli>Bacillales>Planococcaceae	GCF_003058165.1	Paenisporosarcina_sp._OV554	C8K15_RS17245	15921..19496	2135694	BREX-system-P-loop-protein-BrxC-[Paenisporosarcina-sp.-OV554]
WP_274307983.1	Type5.BREX	<- REase <-HEPN+HTH<-JAB<-?<- TUDOR+SF1-SF2-Helicase+i REase +CTD + REase <-?<- PglZ <- N6-METHYLASE <- DUF6079 *<- BrxB-BrxF-iSTAND <- BrxA-DNA-binding ||XIS-HTH->Phage_int_SAM_3+Phage_integrase->Arm-DNA-bind_4+Phage_int_SAM_3+Phage_integrase-><-?<-?<-RhoN+RhoN+RNase+S1<-ABhydrolase	1196	Bacteria>Terrabacteria_group>Bacillota>Bacilli>Bacillales>Planococcaceae	GCF_028747805.1	Solibacillus_sp._ZS111008	O7776_RS16115	3287543..3291133	2912187	BREX-system-P-loop-protein-BrxC-[Solibacillus-sp.-ZS111008]
WP_092984062.1	Type5.BREX	HTH_7+rve-><-HTH_Tnp_1+HTH_21+rve+rve_3<-HIN-HTH||IstB_IS21->?<-?<- TUDOR+SF1-SF2-Helicase+i REase +CTD + REase <- PglZ <- N6-METHYLASE <- DUF6079 *<- BrxA-DNA-binding <-?<-?||?-><-RelB	1184	Bacteria>Terrabacteria_group>Bacillota>Bacilli>Bacillales>Staphylococcaceae	GCF_900101075.1	Salinicoccus_qingdaonensis	BLR15_RS01740	53033..56587	576118	BREX-system-P-loop-protein-BrxC-[Salinicoccus-qingdaonensis]
WP_271398742.1	Type5.BREX	<-ABC-ATPase+SbcC||?->ParB+HNH->HNH->?-><- TUDOR+SF1-SF2-Helicase+i REase +CTD + REase <- PglZ <- N6-METHYLASE <- DUF6079 *<- BrxB-BrxF-iSTAND <- BrxA-DNA-binding <-?||SIGMA-HTH+SIGMA-HTH->35exo+wHTH+SF1-SF2-Helicase->	1184	Bacteria>Terrabacteria_group>Bacillota>Bacilli>Bacillales>Staphylococcaceae	GCF_947502135.1	Salinicoccus_roseus	POR62_RS08110	202088..205642	45670	BREX-system-P-loop-protein-BrxC-[Salinicoccus-roseus]
KKE80057.1	Type5.BREX	<-GNTR-HTH||?-><-?||?->?-><- TUDOR+SF1-SF2-Helicase+i REase +CTD + REase <- PglZ <- N6-METHYLASE <- DUF6079 *<- BrxB-BrxF-iSTAND <- BrxA-DNA-binding <-?||?->Phage_int_SAM_5+Phage_integrase-><-tRNA<-tRNA<-tRNA<-tRNA<-tRNA<-tRNA<-tRNA<-tRNA<-tRNA<-tRNA<-tRNA<-?||?-><-?<-MarR-HTH	1187	Bacteria>Terrabacteria_group>Bacillota>Bacilli>unclassified_Bacilli	GCA_000974285.1	Bacilli_bacterium_VT-13-104	WH51_03830	66407..69970	1637974	hypothetical-protein-WH51_03830-[Bacilli-bacterium-VT-13-104]
WP_164950923.1	Type5.BREX	<-GDResolvase<-?<-?<- TUDOR+SF1-SF2-Helicase+i REase +CTD + REase <- PglZ <-TOPRIM<-OLD-ABC<-HEPN<- N6-METHYLASE <- DUF6079 *<- BrxB-BrxF-iSTAND <- BrxA-DNA-binding <-?<-Calcineurin<-PARG+MACRODOMAIN<-Calcineurin<-HAD<-LRR+LRR	1191	Bacteria>Terrabacteria_group>Bacillota>Clostridia>Eubacteriales>Clostridiaceae	GCF_011065955.2	Clostridium_sp._FP2	G9F71_RS16320	3398773..3402348	2724481	BREX-system-P-loop-protein-BrxC-[Clostridium-sp.-FP2]
WP_185649263.1	Type5.BREX	RADICAL-SAM-><-?||?->?-><-?<-?<-ABhydrolase<- TUDOR+SF1-SF2-Helicase+i REase +CTD + REase <- N6-METHYLASE <- DUF6079 *<- BrxB-BrxF-iSTAND <- BrxA-DNA-binding <-HTH_36<-SMS-KaiC<-Peptidase-C39<-?<-?<-HEPN<-DndB	1189	Bacteria>Terrabacteria_group>Bacillota>Clostridia>Eubacteriales>Clostridiaceae	GCF_014231265.1	Clostridium_sp._DJ247	HGI79_RS03445	40571..44140	2726188	BREX-system-P-loop-protein-BrxC-[Clostridium-sp.-DJ247]
KJS50653.1	Type5.BREX	HKD-DNase-><-?<-Polbeta<- TUDOR+SF1-SF2-Helicase+i REase +CTD + REase <- PglZ <- N6-METHYLASE <- DUF6079 *<- BrxB-BrxF-iSTAND <- BrxA-DNA-binding <- SF1-SF2-Helicase+ REase <-?<-?<-HNH<-OLD-ABC<-?<-HATPase_c	1187	Bacteria>Terrabacteria_group>Bacillota>Clostridia>Eubacteriales>Peptococcaceae	GCA_000961805.1	Peptococcaceae_bacterium_BRH_c23	VR66_01655	19236..22799	1629714	hypothetical-protein-VR66_01655-[Peptococcaceae-bacterium-BRH_c23]
WP_146889206.1	Type5.BREX	<-HNH<-?<-ARSR-HTH<- TUDOR+SF1-SF2-Helicase+i REase +CTD + REase <-?||Peptidase_M48+TerB-> SF1-SF2-Helicase+ REase -> BrxA-DNA-binding -> BrxB-BrxF-iSTAND -> DUF6079 *-> N6-METHYLASE ->HicB-> OLD-ABC-> REase -> PglZ +Phosphodiest -> SIGMA+OB-Fold+AAA-ATPase+Lon-Protease-BrxL ->SF1-SF2-Helicase->?->SF1-SF2-Helicase->	1186	Bacteria>Terrabacteria_group>Deinococcota>Deinococci>Deinococcales>Deinococcaceae	GCF_007990775.1	Deinococcus_cellulosilyticus	DC3_RS23585	23663..27223	401558	BREX-system-P-loop-protein-BrxC-[Deinococcus-cellulosilyticus]
WP_189062792.1	Type5.BREX	<- SIGMA+OB-Fold+AAA-ATPase+Lon-Protease-BrxL <- PglZ ||Herpes_LMP1+ REase -><-NADAR<-fvmX2+ART-DarT<-HNH<- N6-METHYLASE <- DUF6079 *<- BrxB-BrxF-iSTAND <- BrxA-DNA-binding <-CHTH<-?<- REase ||?->?->?-><- TUDOR+SF1-SF2-Helicase+i REase +CTD + REase	1145	Bacteria>Terrabacteria_group>Deinococcota>Deinococci>Deinococcales>Deinococcaceae	GCF_014647995.1	Deinococcus_arenae	IEY66_RS18050	13025..16462	1452751	BREX-system-P-loop-protein-BrxC-[Deinococcus-arenae]
--										
--CR-ATPase+ REase										
--										
ELZ19699.1	Type1.BREX	<- N6-METHYLASE -p||?->?->?-><-PIN||?-><- REase <-?||?-><- DUF6079 <- BrxB-BrxF-iSTAND <- DUF6079 <- BrxA-DNA-binding <- CR-ATPase9 + REase <-?<-?|| REase ->SF1-SF2-Helicase-> REase ->	1198	Archaea>Euryarchaeota>Stenosarchaea_group>Halobacteria>Halobacteriales>Haloarculaceae	GCA_000337455.1	Halosimplex_carlsbadense_2-9-1	C475_22179	59263..62859	797114	hypothetical-protein-C475_22179-[Halosimplex-carlsbadense-2-9-1]
ELZ19701.1	Type1.BREX	<-PIN||?-><- REase <-?||?-><- DUF6079 <- BrxB-BrxF-iSTAND <- DUF6079 *<- BrxA-DNA-binding <- CR-ATPase9 + REase <-?<-?|| REase ->SF1-SF2-Helicase-> REase ->SF1-SF2-Helicase->	1248	Archaea>Euryarchaeota>Stenosarchaea_group>Halobacteria>Halobacteriales>Haloarculaceae	GCA_000337455.1	Halosimplex_carlsbadense_2-9-1	C475_22189	63665..67411	797114	hypothetical-protein-C475_22189-[Halosimplex-carlsbadense-2-9-1]
WP_049931199.1	Type1.BREX	<- PglZ <- N6-METHYLASE <-PIN<-?||?-><-Peptidase_M1<- REase <-?||?-><- DUF6079 *<- BrxB-BrxF-iSTAND <- DUF6079 <- BrxA-DNA-binding <- CR-ATPase9 + REase <-?<-?|| REase ->SF1-SF2-Helicase-> REase ->SF1-SF2-Helicase->	1272	Archaea>Euryarchaeota>Stenosarchaea_group>Halobacteria>Halobacteriales>Haloarculaceae	GCF_000337455.1	Halosimplex_carlsbadense	C475_RS21845	59263..63081	171164	hypothetical-protein-[Halosimplex-carlsbadense]
WP_049931237.1	Type1.BREX	<-PIN<-?||?-><-Peptidase_M1<- REase <-?||?-><- DUF6079 <- BrxB-BrxF-iSTAND <- DUF6079 *<- BrxA-DNA-binding <- CR-ATPase9 + REase <-?|| REase ->SF1-SF2-Helicase-> REase ->SF1-SF2-Helicase-><-?<-RNase	1239	Archaea>Euryarchaeota>Stenosarchaea_group>Halobacteria>Halobacteriales>Haloarculaceae	GCF_000337455.1	Halosimplex_carlsbadense	C475_RS21855	63665..67384	171164	hypothetical-protein-[Halosimplex-carlsbadense]
WP_013460546.1	Type1.BREX	Phage_integrase-><-XIS-HTH<-HTH_36<-?||HTH_3-><-XIS-HTH|| BrxA-DNA-binding -> BrxB-BrxF-iSTAND -> DUF6079 *-><-?|| N6-METHYLASE -p->?->?->HEPN->BrxD-> N6-METHYLASE -p-> CR-ATPase5 + REase -> PglZ ->	1163	Bacteria>Campylobacterota>Epsilonproteobacteria>Campylobacterales>Thiovulaceae>Sulfuricurvum	GCF_000183725.1	Sulfuricurvum_kujiense	SULKU_RS08475	1692937..1696428	148813	BREX-system-P-loop-protein-BrxC-[Sulfuricurvum-kujiense]
WP_250702187.1	Type1.BREX	<-GDResolvase+HIN-HTH<- HTH+ REase <-SF1-SF2-Helicase||ADH_N+ADH_zinc_N+METHYLASE->?-><-SPOUT|| CR-ATPase5 + REase -> BrxA-DNA-binding -> BrxB-BrxF-iSTAND -> DUF6079 *-> N6-METHYLASE -> PglZ -> SIGMA+OB-Fold+AAA-ATPase+Lon-Protease-BrxL -><-?<-?<-TraJ-RHH<-ZnR+TOPRIM<-CR-ATPase4<-XIS-HTH	1215	Bacteria>FCB_group>Bacteroidetes/Chlorobi_group>Bacteroidota>Bacteroidia>Bacteroidales	GCF_023668595.1	Bacteroides_ovatus	NCZ26_RS08955	97357..101004	28116	BREX-system-P-loop-protein-BrxC-[Bacteroides-ovatus]
MCB0524505.1	Type1.BREX	SF1-SF2-Helicase+RNase+RNase->?-> REase +35exo->?->?-> BrxA-DNA-binding -> BrxB-BrxF-iSTAND -> DUF6079 -p-> DUF6079 *-> N6-METHYLASE ->?->McrB-NTD+HNH->Polbeta->?-> CR-ATPase5 + REase -> PglZ -> SIGMA+OB-Fold+AAA-ATPase+HKD-DNase-BrxL ->	1094	Bacteria>FCB_group>Bacteroidetes/Chlorobi_group>Bacteroidota>Saprospiria>Saprospirales	GCA_020436385.1	Saprospiraceae_bacterium	KDC86_08270	14975..18259	2202734	BREX-system-P-loop-protein-BrxC-[Saprospiraceae-bacterium]
CDA09060.1	Type1.BREX	<- SIGMA+OB-Fold+AAA-ATPase+Lon-Protease-BrxL <- PglZ <- CR-ATPase8 + REase <-OLD-ABC +TOPRIM<-?<-?<- N6-METHYLASE <-TOPRIM<-OLD-ABC<- DUF6079 *<-?<-SWIM+DUF3670+SF1-SF2-Helicase<-?<-?<-?<-SF1-SF2-Helicase	1250	Bacteria>Fusobacteriota>Fusobacteriia>Fusobacteriales>Fusobacteriaceae>Fusobacterium	GCA_000433695.1	Fusobacterium_sp._CAG:649	BN748_00884	23068..26820	1262900	putative-uncharacterized-protein-[Fusobacterium-sp.-CAG:649]
MCF2639759.1	Type1.BREX	DUF6079 *-> N6-METHYLASE -> PglZ -> BrxA-DNA-binding -> BrxB-BrxF-iSTAND -> CR-ATPase5 + REase ->DOC->Calcineurin->HIN-HTH->	1244	Bacteria>Fusobacteriota>Fusobacteriia>Fusobacteriales>Fusobacteriaceae>Fusobacterium	GCA_021531615.1	Fusobacterium_varium	I6E31_07220	5895..9629	856	BREX-system-P-loop-protein-BrxC-[Fusobacterium-varium]
WP_008692035.1	Type1.BREX	DUF3670+SF1-SF2-Helicase->?-><-?<-?<-?|| DUF6079 *->OLD-ABC+TOPRIM-> N6-METHYLASE ->?->OLD-ABC+TOPRIM-> CR-ATPase8 + REase -> PglZ -> SIGMA+OB-Fold+AAA-ATPase+Lon-Protease-BrxL -> BrxA-DNA-binding ->	1250	Bacteria>Fusobacteriota>Fusobacteriia>Fusobacteriales>Fusobacteriaceae>Fusobacterium	GCF_000218655.1	Fusobacterium_nucleatum	HMPREF0401_RS10120	557323..561075	851	BREX-system-P-loop-protein-BrxC-[Fusobacterium-animalis]
WP_029493951.1	Type1.BREX	<- BrxB-BrxF-iSTAND <- BrxA-DNA-binding <- SIGMA+OB-Fold+AAA-ATPase+Lon-Protease-BrxL <- PglZ <- CR-ATPase8 + REase <-RVT<- N6-METHYLASE <-Schlafen+HATPase+wHTH<- DUF6079 *||?->?-><-?<-ABC-ATPase+SbcC<-?<-?<- wHTH+WYL+WCX <-JAB+sGTP<-SKI	1250	Bacteria>Fusobacteriota>Fusobacteriia>Fusobacteriales>Fusobacteriaceae>Fusobacterium	GCF_000455885.1	Fusobacterium_hwasookii	J143_RS0110850	68335..72087	1583098	BREX-system-P-loop-protein-BrxC-[Fusobacterium-hwasookii]
WP_032838665.1	Type1.BREX	<- PglZ <- CR-ATPase8 + REase <-RVT<- N6-METHYLASE <-Schlafen+HATPase+wHTH<- REase <-CR-ATPase4<-?<-?<- N6-METHYLASE <- DUF6079 *||?->?->?-><-Peptidase_M20<-?||35exo+T7DNApol->	1250	Bacteria>Fusobacteriota>Fusobacteriia>Fusobacteriales>Fusobacteriaceae>Fusobacterium	GCF_000524215.1	Fusobacterium_sp._CM1	HMPREF1498_RS02575	43304..47056	936561	BREX-system-P-loop-protein-BrxC-[Fusobacterium-sp.-CM1]
WP_032836039.1	Type1.BREX	<- PglZ <- CR-ATPase8 + REase <-HEPN<-HEPN<-MORC<- N6-METHYLASE <-Schlafen+HATPase+wHTH<-TOPRIM<-OLD-ABC<- DUF6079 *||?-><-?<-ABC-ATPase+SbcC<-?<-?<- wHTH+WYL+WCX <-JAB+sGTP<-SKI	1250	Bacteria>Fusobacteriota>Fusobacteriia>Fusobacteriales>Fusobacteriaceae>Fusobacterium	GCF_000524235.1	Fusobacterium_sp._OBRC1	HMPREF1501_RS01100	63475..67227	1032505	BREX-system-P-loop-protein-BrxC-[Fusobacterium-sp.-OBRC1]
WP_100024172.1	Type1.BREX	<-Peptidase_M20<-?<-?<-?<-RNaseH||Peptidase_M3_N+Peptidase_M3->?->?->?<-?|| DUF6079 *->OLD-ABC+TOPRIM->HEPN-> N6-METHYLASE -> CR-ATPase8 + REase -> PglZ -> SIGMA+OB-Fold+AAA-ATPase+Lon-Protease-BrxL -> BrxA-DNA-binding -> BrxB-BrxF-iSTAND ->	1250	Bacteria>Fusobacteriota>Fusobacteriia>Fusobacteriales>Fusobacteriaceae>Fusobacterium	GCF_002763625.1	Fusobacterium_pseudoperiodonticum	CTM72_RS01085	199877..203629	2663009	BREX-system-P-loop-protein-BrxC-[Fusobacterium-pseudoperiodonticum]
WP_099987862.1	Type1.BREX	<- BrxA-DNA-binding <- SIGMA+OB-Fold+AAA-ATPase+Lon-Protease-BrxL <- PglZ <- CR-ATPase8 + REase <-OLD-ABC +TOPRIM<-?<-?<- N6-METHYLASE <-TOPRIM<-OLD-ABC<- DUF6079 *||?->?->OrfB_IS605+OrfB_Zn_ribbon-><-URI-DNase+SF1-SF2-Helicase<-URI-DNase+SF1-SF2-Helicase<-?<-?<-Peptidase_M3_N+Peptidase_M3||RNaseH->	1250	Bacteria>Fusobacteriota>Fusobacteriia>Fusobacteriales>Fusobacteriaceae>Fusobacterium	GCF_002763695.1	Fusobacterium_pseudoperiodonticum	CTM74_RS09005	1721055..1724807	2663009	BREX-system-P-loop-protein-BrxC-[Fusobacterium-pseudoperiodonticum]
WP_238959505.1	Type1.BREX	<- BrxB-BrxF-iSTAND <- BrxA-DNA-binding <- SIGMA+OB-Fold+AAA-ATPase+Lon-Protease-BrxL <- PglZ <- CR-ATPase8 + REase <-HEPN<-MORC<- N6-METHYLASE <-Schlafen+HATPase+wHTH<- DUF6079 *||?->?-><-?<-ABC-ATPase+SbcC<-?<-?<- wHTH+WYL+WCX <-JAB+sGTP<-SKI	1250	Bacteria>Fusobacteriota>Fusobacteriia>Fusobacteriales>Fusobacteriaceae>Fusobacterium	GCF_022340045.1	Fusobacterium_nucleatum	K5I20_00280	65514..69266	851	BREX-system-P-loop-protein-BrxC-[Fusobacterium-nucleatum]
WP_238972693.1	Type1.BREX	SKI->JAB+sGTP-> wHTH+WYL+WCX ->?->ABC-ATPase+SbcC->?-><-?<-?|| DUF6079 *->OLD-ABC->TOPRIM->Schlafen+HATPase+wHTH-> N6-METHYLASE ->MORC->HEPN-> CR-ATPase8 + REase -> PglZ -> SIGMA+OB-Fold+AAA-ATPase+Lon-Protease-BrxL ->	1250	Bacteria>Fusobacteriota>Fusobacteriia>Fusobacteriales>Fusobacteriaceae>Fusobacterium	GCF_022340165.1	Fusobacterium_nucleatum	K5I19_RS02505	127932..131684	851	BREX-system-P-loop-protein-BrxC-[Fusobacterium-nucleatum]

Protein-ID	System	Gene-Neighbourhoods	Length	Taxonomy	Assembly-ID	Strain	Locus-Tag	Genomic-coordinates	TaxID	Fasta-defline
WP_060798221.1	Type1.BREX	<- BrxA-DNA-binding <- SIGMA+OB-Fold+AAA-ATPase+Lon-Protease-BrxL <- PglZ <- CR-ATPase8 + REase <-HEPN<-MORC<- N6-METHYLASE <-TOPRIM<-OLD-ABC<- DUF6079 *<-?<-SWIM+DUF3670+SF1-SF2-Helicase<-?<-?<-?<-SF1-SF2-Helicase	1250	Bacteria>Fusobacteriota>Fusobacteriia>Fusobacteriales>Fusobacteriaceae>Fusobacterium	GCF_023383525.1	Fusobacterium_nucleatum	YWH7054_RS02310	21716..25468	851	BREX-system-P-loop-protein-BrxC-[Fusobacterium-nucleatum]
WP_022069638.1	Type1.BREX	<- BrxA-DNA-binding <- SIGMA+OB-Fold+AAA-ATPase+Lon-Protease-BrxL <- PglZ <- CR-ATPase8 + REase <-OLD-ABC +TOPRIM<-?<- N6-METHYLASE <-TOPRIM<-OLD-ABC<- DUF6079 *	1250	Bacteria>Fusobacteriota>Fusobacteriia>Fusobacteriales>Fusobacteriaceae>Fusobacterium	GCF_023383545.1	Fusobacterium_nucleatum	YWH7053_RS01985	23867..27619	851	BREX-system-P-loop-protein-BrxC-[Fusobacterium-nucleatum]
WP_249524183.1	Type1.BREX	<- BrxA-DNA-binding <- SIGMA+OB-Fold+AAA-ATPase+Lon-Protease-BrxL <- PglZ <- CR-ATPase8 + REase <-HEPN<-MORC<- N6-METHYLASE <-TOPRIM<-OLD-ABC<- DUF6079 *<-?<-SWIM+DUF3670+SF1-SF2-Helicase<-?<-?<-?<-SF1-SF2-Helicase	1250	Bacteria>Fusobacteriota>Fusobacteriia>Fusobacteriales>Fusobacteriaceae>Fusobacterium	GCF_023383605.1	Fusobacterium_nucleatum	YWH7056_RS07045	28639..32391	851	BREX-system-P-loop-protein-BrxC-[Fusobacterium-nucleatum]
WP_271333596.1	Type1.BREX	<- PglZ <- CR-ATPase8 + REase <-?<- N6-METHYLASE <-Schlafen+HATPase+wHTH<- REase <-CR-ATPase4<-?<- N6-METHYLASE <- DUF6079 *||?-><-?<-ABC-ATPase+SbcC<-?<-?<- wHTH+WYL+WCX <-JAB+sGTP<-SKI	1250	Bacteria>Fusobacteriota>Fusobacteriia>Fusobacteriales>Fusobacteriaceae>Fusobacterium	GCF_027947475.1	Fusobacterium_nucleatum	PGW91_RS03610	747072..750824	851	BREX-system-P-loop-protein-BrxC-[Fusobacterium-nucleatum]
WP_005980883.1	Type1.BREX	PHP->Sulfatase+DUF229+DUF4976->JetA->?->?->?-> CR-ATPase8 + REase -> DUF6079 *-> N6-METHYLASE ->ParB+HNH->RAMA-> PglZ -> SIGMA+OB-Fold+AAA-ATPase+Lon-Protease-BrxL -> BrxA-DNA-binding -> BrxB-BrxF-iSTAND ->	1242	Bacteria>Fusobacteriota>Fusobacteriia>Fusobacteriales>Fusobacteriaceae>Fusobacterium	GCF_902373815.1	Fusobacterium_ulcerans	FXX38_RS11815	320598..324326	861	BREX-system-P-loop-protein-BrxC-[Fusobacterium-ulcerans]
WP_008793539.1	Type1.BREX	<-RNaseH||Peptidase_M3_N+Peptidase_M3->?->?-><-?<-HTH_OrfB_IS605+OrfB_IS605+OrfB_Zn_ribbon|| DUF6079 *->OLD-ABC->RNase+PHP->Schlafen+HATPase+wHTH-> N6-METHYLASE ->HEPN-> CR-ATPase8 + REase -> PglZ -> SIGMA+OB-Fold+AAA-ATPase+Lon-Protease-BrxL -> BrxA-DNA-binding ->	1250	Bacteria>Fusobacteriota>Fusobacteriia>Fusobacteriales>Fusobacteriaceae>Fusobacterium	GCF_902373915.1	Fusobacterium_periodonticum	FY302_RS02975	615184..618936	860	BREX-system-P-loop-protein-BrxC-[Fusobacterium-periodonticum]
NMA29242.1	Type6.BREX	HTH_3->MetJarc-HTH->PemK_toxin-><-?|| REase -><- CR-ATPase5 + REase <- REase <- DUF6079 *<- BrxB-BrxF-iSTAND <- BrxA-DNA-binding <- BrxE	1208	Bacteria>Pseudomonadota>Betaproteobacteria>Burkholderiales>unclassified_Burkholderiales	GCA_012799975.1	Burkholderiales_bacterium	GX934_15915	6559..10185	1891238	BREX-system-P-loop-protein-BrxC-[Burkholderiales-bacterium]
WP_109291743.1	Type1.BREX	<-DDE_Tnp_1_5+DDE_Tnp_1|| CR-ATPase5 + REase -><-DUF4236|| wHTH+WYL+WCX -> BrxA-DNA-binding -> BrxB-BrxF-iSTAND -> DUF6079 *-> N6-METHYLASE ->ParB+HNH-> PglZ -> SIGMA+OB-Fold+AAA-ATPase+Lon-Protease-BrxL -> SF1-SF2-Helicase+ REase -><-?<-CHTH<-RelE	1226	Bacteria>Pseudomonadota>Gammaproteobacteria>Moraxellales>Moraxellaceae>Acinetobacter	GCF_003131095.1	Acinetobacter_baumannii	DF172_RS10505	4712..8392	470	BREX-system-P-loop-protein-BrxC-[Acinetobacter-baumannii]
WP_200291399.1	Type1.BREX	Peptidase_A8-><-?<-?||LexA-protease->?-><-Phage_int_SAM<-Integrase_1|| BrxA-DNA-binding -> BrxB-BrxF-iSTAND -> DUF6079 *-> CR-ATPase5 + REase -> N6-METHYLASE ->GP+ REase -> N6-METHYLASE -p->SIR2->HerA/FtsK->?-> N6-METHYLASE -p->S1COLD->	1223	Bacteria>Pseudomonadota>Gammaproteobacteria>Pseudomonadales>Pseudomonadaceae>Stutzerimonas	GCF_016599775.1	Stutzerimonas_balearica	I6H70_RS19230	4083458..4087129	74829	BREX-system-P-loop-protein-BrxC-[Stutzerimonas-balearica]
WP_002710590.1	Type1.BREX	HAMP+PAS+HISKIN->REC+NtrC-AAA+FIS-HTH-> BrxA-DNA-binding -> BrxB-BrxF-iSTAND -> DUF6079 *->AbiEi_4+AbiEi_1->AbiEii->OLD-ABC->TOPRIM-> N6-METHYLASE -> CR-ATPase8 + REase -> PglZ -> SIGMA+OB-Fold+AAA-ATPase+Lon-Protease-BrxL ->	1209	Bacteria>Pseudomonadota>Gammaproteobacteria>Thiotrichales>Thiotrichaceae>Thiothrix	GCF_000260135.1	Thiothrix_nivea	THINI_RS21415	4300442..4304071	1031	BREX-system-P-loop-protein-BrxC-[Thiothrix-nivea]
MCP5494239.1	Type1.BREX	CR-ATPase8 + REase -><- SIGMA+OB-Fold+AAA-ATPase+Lon-Protease-BrxL <-?<-?<-?<- PglZ <- N6-METHYLASE <- REase +HTH<- DUF6079 *<- BrxB-BrxF-iSTAND <- BrxA-DNA-binding ||ArdCN->SecA+SF1-SF2-Helicase->	1148	Bacteria>Spirochaetota>Spirochaetia>Leptospirales>Leptospiraceae>unclassified_Leptospiraceae	GCA_024233865.1	Leptospiraceae_bacterium	H7A23_06750	342984..346430	2026757	BREX-system-P-loop-protein-BrxC-[Leptospiraceae-bacterium]
MBR5915376.1	Type1.BREX	<-SH3||Peptidase_M20-><-?||?-> REase -><- SIGMA+OB-Fold+AAA-ATPase+Lon-Protease-BrxL <- PglZ <-Schlafen+HATPase+wHTH<- N6-METHYLASE <- DUF6079 *<- BrxB-BrxF-iSTAND <- BrxA-DNA-binding <- CR-ATPase5 + REase <-ACET<-?<-?<-?<-HD	1170	Bacteria>Spirochaetota>Spirochaetia>unclassified_Spirochaetia	GCA_017554185.1	Spirochaetia_bacterium	IKZ57_02880	16752..20264	2053615	BREX-system-P-loop-protein-BrxC-[Spirochaetia-bacterium]
MBS6134848.1	Type1.BREX	<- N6-METHYLASE -p<-HerA/FtsK<-SIR2<- N6-METHYLASE <- DUF6079 *<- BrxB-BrxF-iSTAND <- BrxA-DNA-binding <- CR-ATPase5 + REase -p<-?<-ARG	1229	Bacteria>Terrabacteria_group>Actinomycetota>Actinomycetes>Bifidobacteriales>Bifidobacteriaceae	GCA_018374015.1	Bifidobacterium_longum	KH705_09795	10639..14328	216816	BREX-system-P-loop-protein-BrxC-[Bifidobacterium-longum]
MCI2117722.1	Type1.BREX	<-RNase<-BirA-HTH<-Schlafen+HATPase+wHTH<-SMODS<-SLATT<- N6-METHYLASE -p<-HerA/FtsK<-SIR2<- N6-METHYLASE <- DUF6079 *<- BrxB-BrxF-iSTAND <- BrxA-DNA-binding <- CR-ATPase5 + REase -p<-?||?-><-?<-?<-HAD	1229	Bacteria>Terrabacteria_group>Actinomycetota>Actinomycetes>Bifidobacteriales>Bifidobacteriaceae	GCA_022653935.1	Bifidobacterium_breve	LKK17_00135	29818..33507	1685	BREX-system-P-loop-protein-BrxC-[Bifidobacterium-breve]
WP_051923722.1	Type1.BREX	<-METHYLASE<- SIGMA+OB-Fold+AAA-ATPase+HKD-DNase-BrxL <- PglZ <-Schlafen+HATPase+wHTH<-SMODS<-ABhydrolase<- CR-ATPase5 + REase <- DUF6079 *<- BrxB-BrxF-iSTAND <- BrxA-DNA-binding <-?||?-><-?<-AlcRN+AraC-HTH	1247	Bacteria>Terrabacteria_group>Actinomycetota>Actinomycetes>Bifidobacteriales>Bifidobacteriaceae	GCF_000771645.1	Bifidobacterium_biavatii	OU23_RS06270	17800..21543	762212	BREX-system-P-loop-protein-BrxC-[Bifidobacterium-biavatii]
WP_052382006.1	Type1.BREX	<- N6-METHYLASE <-?<-?<-TPR+TPR<- CR-ATPase5 + REase <- DUF6079 *<- BrxB-BrxF-iSTAND <- BrxA-DNA-binding ||ABhydrolase->?-><-?<-HAD+S6PP<-ABC-ATPase+SbcC	1264	Bacteria>Terrabacteria_group>Actinomycetota>Actinomycetes>Bifidobacteriales>Bifidobacteriaceae	GCF_000771685.1	Bifidobacterium_reuteri	OU25_RS00505	118725..122519	983706	BREX-system-P-loop-protein-BrxC-[Bifidobacterium-reuteri]
WP_050824906.1	Type1.BREX	<- SIGMA+OB-Fold+AAA-ATPase+HKD-DNase-BrxL <- PglZ <-Schlafen+wHTH<-Schlafen+wHTH<-SMODS<-SLATT<- N6-METHYLASE <- DUF6079 *<- BrxB-BrxF-iSTAND <- BrxA-DNA-binding ||?->CR-Kinase-><- CR-ATPase5 + REase -p<- N6-METHYLASE <-RelB	1229	Bacteria>Terrabacteria_group>Actinomycetota>Actinomycetes>Bifidobacteriales>Bifidobacteriaceae	GCF_001189355.1	Bifidobacterium_breve	BBRI4_RS05550	137028..140717	1685	BREX-system-P-loop-protein-BrxC-[Bifidobacterium-breve]
WP_053495781.1	Type1.BREX	<-CbiA<-?<-?<-ZDNA-HTH<-?||ACET-><-?<-HTH_3||HTH_3-> DUF6079 -p*<- BrxB-BrxF-iSTAND <- BrxA-DNA-binding ||?->CR-Kinase-><- CR-ATPase5 + REase -p<- N6-METHYLASE <-RelB	531	Bacteria>Terrabacteria_group>Actinomycetota>Actinomycetes>Bifidobacteriales>Bifidobacteriaceae	GCF_001275745.1	Bifidobacterium_longum	BLOI2_RS03170	185000..186595	216816	BREX-system-P-loop-protein-BrxC-[Bifidobacterium-longum]
WP_071477881.1	Type1.BREX	<- SIGMA+OB-Fold+AAA-ATPase+HKD-DNase-BrxL <- PglZ <-RNase<-Schlafen+HATPase+wHTH<-SMODS<-SLATT<- N6-METHYLASE <- DUF6079 *<- BrxB-BrxF-iSTAND <- BrxA-DNA-binding <- CR-ATPase5 + REase -p<-?||?-><-?<-?||<-?<-MarR_2+ROK-HTH	1229	Bacteria>Terrabacteria_group>Actinomycetota>Actinomycetes>Bifidobacteriales>Bifidobacteriaceae	GCF_001870755.1	Bifidobacterium_longum	BFS25_RS03445	115725..119414	216816	BREX-system-P-loop-protein-BrxC-[Bifidobacterium-longum]
WP_094668334.1	Type1.BREX	DDE_Tnp_ISL3-><-ABhydrolase|| BrxA-DNA-binding -> BrxB-BrxF-iSTAND -> DUF6079 *-> CR-ATPase5 + REase ->TPR+TPR-> N6-METHYLASE ->Schlafen+HATPase+wHTH->MazE_antitoxin->PemK_toxin-> PglZ -> SIGMA+OB-Fold+AAA-ATPase+HKD-DNase-BrxL ->METHYLASE->	1270	Bacteria>Terrabacteria_group>Actinomycetota>Actinomycetes>Bifidobacteriales>Bifidobacteriaceae	GCF_002259745.1	Bifidobacterium_myosotis	BMYO_RS10065	3739..7551	1630166	BREX-system-P-loop-protein-BrxC-[Bifidobacterium-myosotis]
WP_109056521.1	Type1.BREX	<-ABhydrolase|| BrxA-DNA-binding -> BrxB-BrxF-iSTAND -> DUF6079 *-> N6-METHYLASE ->HEPN->Schlafen+wHTH-> CR-ATPase5 + REase -> PglZ -> SIGMA+OB-Fold+AAA-ATPase+HKD-DNase-BrxL ->METHYLASE->	1246	Bacteria>Terrabacteria_group>Actinomycetota>Actinomycetes>Bifidobacteriales>Bifidobacteriaceae	GCF_003129905.1	Bifidobacterium_callitrichidarum	DF196_RS03600	108404..112144	2052941	BREX-system-P-loop-protein-BrxC-[Bifidobacterium-callitrichidarum]
WP_131226808.1	Type1.BREX	<-METHYLASE-p<- McrC-NTD+ REase <-EVE+McrB-AAA<-?<- DUF6079 *<- BrxB-BrxF-iSTAND <- BrxA-DNA-binding <- CR-ATPase5 + REase -p<-?||HTH_38->?->HEPN->?-><-HTH+MPTase	1229	Bacteria>Terrabacteria_group>Actinomycetota>Actinomycetes>Bifidobacteriales>Bifidobacteriaceae	GCF_004333385.1	Bifidobacterium_longum	MCC10066_RS04685	5338..9027	216816	BREX-system-P-loop-protein-BrxC-[Bifidobacterium-longum]
WP_131210356.1	Type1.BREX	<- DUF6079 -p*<- BrxB-BrxF-iSTAND <- BrxA-DNA-binding <- CR-ATPase5 + REase -p<-?||HTH_38->?->HEPN->?-><-HTH+MPTase	442	Bacteria>Terrabacteria_group>Actinomycetota>Actinomycetes>Bifidobacteriales>Bifidobacteriaceae	GCF_004333565.1	Bifidobacterium_longum	MCC10048_RS04670	1..1326	216816	BREX-system-P-loop-protein-BrxC,-partial-[Bifidobacterium-longum]
WP_140489867.1	Type1.BREX	ABC-ATPase+SbcC->HAD+S6PP->?-><-?<-ABhydrolase|| BrxA-DNA-binding -> BrxB-BrxF-iSTAND -> DUF6079 *-> CR-ATPase5 + REase ->TPR+TPR->	1264	Bacteria>Terrabacteria_group>Actinomycetota>Actinomycetes>Bifidobacteriales>Bifidobacteriaceae	GCF_006408855.1	Bifidobacterium_sp._UTBIF-68	BW14_RS07205	86138..89932	1465262	BREX-system-P-loop-protein-BrxC-[Bifidobacterium-sp.-UTBIF-68]
WP_140558979.1	Type1.BREX	ABC-ATPase+SbcC->HAD+S6PP->?-><-?<-ABhydrolase|| BrxA-DNA-binding -> BrxB-BrxF-iSTAND -> DUF6079 *-> CR-ATPase5 + REase ->TPR+TPR->?->?->?-> N6-METHYLASE ->	1264	Bacteria>Terrabacteria_group>Actinomycetota>Actinomycetes>Bifidobacteriales>Bifidobacteriaceae	GCF_006408865.1	Bifidobacterium_sp._UTBIF-56	BW10_RS07695	47972..51766	1465261	BREX-system-P-loop-protein-BrxC-[Bifidobacterium-sp.-UTBIF-56]
WP_140463094.1	Type1.BREX	ABC-ATPase+SbcC->HAD+S6PP->?-><-?<-ABhydrolase|| BrxA-DNA-binding -> BrxB-BrxF-iSTAND -> DUF6079 *-> CR-ATPase5 + REase ->TPR+TPR-><-?||HTH_3->MPTase->	1264	Bacteria>Terrabacteria_group>Actinomycetota>Actinomycetes>Bifidobacteriales>Bifidobacteriaceae	GCF_006408915.1	unclassified_Bifidobacterium	BW09_RS08245	76569..80363	2608897	MULTISPECIES:-BREX-system-P-loop-protein-BrxC-[unclassified-Bifidobacterium]
WP_150335795.1	Type1.BREX	<-TPR+TPR<- CR-ATPase5 + REase <- DUF6079 *<- BrxB-BrxF-iSTAND <- BrxA-DNA-binding ||ABhydrolase->?-><-?<-HAD+S6PP<-ABC-ATPase+SbcC	1264	Bacteria>Terrabacteria_group>Actinomycetota>Actinomycetes>Bifidobacteriales>Bifidobacteriaceae	GCF_008698125.1	Bifidobacterium_reuteri	EM092_RS08290	2843..6637	983706	BREX-system-P-loop-protein-BrxC-[Bifidobacterium-reuteri]
WP_150379196.1	Type1.BREX	<-SbcD||?-><-ABhydrolase|| BrxA-DNA-binding -> BrxB-BrxF-iSTAND -> DUF6079 *-> CR-ATPase5 + REase ->TPR+TPR-> N6-METHYLASE ->HEPN->MazE_antitoxin-> PglZ -> SIGMA+OB-Fold+AAA-ATPase+HKD-DNase-BrxL ->METHYLASE->	1267	Bacteria>Terrabacteria_group>Actinomycetota>Actinomycetes>Bifidobacteriales>Bifidobacteriaceae	GCF_008698155.1	Bifidobacterium_myosotis	EM091_RS06000	83624..87427	1630166	BREX-system-P-loop-protein-BrxC-[Bifidobacterium-myosotis]
WP_151916615.1	Type1.BREX	HNH->||DOC+HTH-><-sGTP||?->?->?-><-HAD+HAD|| BrxA-DNA-binding -> BrxB-BrxF-iSTAND -> DUF6079 *-> CR-ATPase5 + REase ->TPR+TPR-> N6-METHYLASE -><-?<-Tox-RelE<-RelB|| CR-ATPase5 + REase -> PglZ -> SIGMA+OB-Fold+AAA-ATPase+HKD-DNase-BrxL ->METHYLASE->	1269	Bacteria>Terrabacteria_group>Actinomycetota>Actinomycetes>Bifidobacteriales>Bifidobacteriaceae	GCF_009078285.1	Bifidobacterium_jacchi	EHS19_RS04610	26586..30395	2490545	BREX-system-P-loop-protein-BrxC-[Bifidobacterium-jacchi]
WP_152233646.1	Type1.BREX	REase +Bacteriocin_IIc->?->?->?->?-><-?|| BrxA-DNA-binding -> BrxB-BrxF-iSTAND -> DUF6079 *-><-?|| CR-ATPase5 + REase -> N6-METHYLASE ->?->?->HEPN+RES->Schlafen+HATPase+wHTH-> PglZ -> SIGMA+OB-Fold+AAA-ATPase+HKD-DNase-BrxL ->	1235	Bacteria>Terrabacteria_group>Actinomycetota>Actinomycetes>Bifidobacteriales>Bifidobacteriaceae	GCF_009193355.1	Bifidobacterium_leontopitheci	F7D09_RS01345	123122..126829	2650774	BREX-system-P-loop-protein-BrxC-[Bifidobacterium-leontopitheci]
WP_169274836.1	Type1.BREX	<-PIN<-RelB<-?||HTH_3->?->PIN-> BrxA-DNA-binding -> BrxB-BrxF-iSTAND -> DUF6079 *-> CR-ATPase5 + REase -> N6-METHYLASE ->Schlafen+HATPase+wHTH-> PglZ -> SIGMA+OB-Fold+AAA-ATPase+HKD-DNase-BrxL -> REase ->?-> BrxA-DNA-binding ->	1226	Bacteria>Terrabacteria_group>Actinomycetota>Actinomycetes>Bifidobacteriales>Bifidobacteriaceae	GCF_012932365.1	Bifidobacterium_sp._DSM_109958	G1C96_RS01295	288582..292262	2705430	BREX-system-P-loop-protein-BrxC-[Bifidobacterium-sp.-DSM-109958]
WP_077384433.1	Type1.BREX	HTH+MPTase-><-?<-HEPN<-?<-HTH_38||?-> CR-ATPase5 + REase -p-> BrxA-DNA-binding -> BrxB-BrxF-iSTAND -> DUF6079 *->?->EVE+McrB-AAA-> McrC-NTD+ REase ->METHYLASE-p->	1229	Bacteria>Terrabacteria_group>Actinomycetota>Actinomycetes>Bifidobacteriales>Bifidobacteriaceae	GCF_017312625.1	Bifidobacterium_longum	MCC10017_RS04835	46545..50234	216816	BREX-system-P-loop-protein-BrxC-[Bifidobacterium-longum]
WP_214374600.1	Type1.BREX	ZnR->?->ABC-ATPase+SbcC-><-?||?-><-ABhydrolase|| BrxA-DNA-binding -> BrxB-BrxF-iSTAND -> DUF6079 *-> CR-ATPase5 + REase -> N6-METHYLASE ->?->?->HEPN+RES->Schlafen+HATPase+wHTH-> PglZ ->MIT_C->METHYLASE->	1249	Bacteria>Terrabacteria_group>Actinomycetota>Actinomycetes>Bifidobacteriales>Bifidobacteriaceae	GCF_018555335.1	Bifidobacterium	JS541_RS11260	52363..56112	1678	MULTISPECIES:-BREX-system-P-loop-protein-BrxC-[Bifidobacterium]
WP_217054570.1	Type1.BREX	<- SIGMA+OB-Fold+AAA-ATPase+HKD-DNase-BrxL <- PglZ <-HEPN+RES<-?<-?<- REase <- N6-METHYLASE <- DUF6079 *<- BrxB-BrxF-iSTAND <- BrxA-DNA-binding ||?->CR-Kinase-><- CR-ATPase5 + REase -p<- REase <-HTH_3	1229	Bacteria>Terrabacteria_group>Actinomycetota>Actinomycetes>Bifidobacteriales>Bifidobacteriaceae	GCF_019041665.1	Bifidobacterium_longum	KTF47_RS04030	15252..18941	216816	BREX-system-P-loop-protein-BrxC-[Bifidobacterium-longum]
WP_269360645.1	Type1.BREX	<-RNase<-BirA-HTH<-Schlafen+HATPase+wHTH<-SMODS<-SLATT<- N6-METHYLASE -p<-HerA/FtsK<-SIR2<- N6-METHYLASE <- DUF6079 *<- BrxB-BrxF-iSTAND <- BrxA-DNA-binding <- CR-ATPase5 + REase -p<-?||?-><-?<-HAD+S6PP<-ABC-ATPase+SbcC	1229	Bacteria>Terrabacteria_group>Actinomycetota>Actinomycetes>Bifidobacteriales>Bifidobacteriaceae	GCF_027215095.1	Bifidobacterium_breve	O4D08_RS00920	188382..192071	1685	BREX-system-P-loop-protein-BrxC-[Bifidobacterium-breve]
WP_269338054.1	Type1.BREX	<-MULE-TRANSPOSASE||?-><-?||?-> CR-ATPase5 + REase -p-> BrxA-DNA-binding -> BrxB-BrxF-iSTAND -> DUF6079 -p*-> N6-METHYLASE ->SIR2->HerA/FtsK-> N6-METHYLASE -p->SLATT->SMODS->Schlafen+HATPase+wHTH->BirA-HTH->	924	Bacteria>Terrabacteria_group>Actinomycetota>Actinomycetes>Bifidobacteriales>Bifidobacteriaceae	GCF_027215525.1	Bifidobacterium_breve	O4C30_RS07905	20359..23133	1685	BREX-system-P-loop-protein-BrxC-[Bifidobacterium-breve]
WP_270267401.1	Type1.BREX	<- SIGMA+OB-Fold+AAA-ATPase+HKD-DNase-BrxL <- PglZ <-RNase<-BirA-HTH<-Schlafen+HATPase+wHTH<-SMODS<-SLATT<- N6-METHYLASE <- DUF6079 *<- BrxB-BrxF-iSTAND <- BrxA-DNA-binding ||?->CR-Kinase-><- CR-ATPase5 + REase -p<- N6-METHYLASE <-RelB	1229	Bacteria>Terrabacteria_group>Actinomycetota>Actinomycetes>Bifidobacteriales>Bifidobacteriaceae	GCF_027663845.1	Bifidobacterium_breve	PGQ90_RS07415	209217..212906	1685	BREX-system-P-loop-protein-BrxC-[Bifidobacterium-breve]
WP_143724686.1	Type1.BREX	<- SIGMA+OB-Fold+AAA-ATPase+HKD-DNase-BrxL <- PglZ <-RNase<-Schlafen+HATPase+wHTH<-SMODS<-SLATT<- N6-METHYLASE <- DUF6079 *<- BrxB-BrxF-iSTAND <- BrxA-DNA-binding <- CR-ATPase5 + REase -p<-?||?-><-?<-?||<-?<-MarR_2+ROK-HTH	1229	Bacteria>Terrabacteria_group>Actinomycetota>Actinomycetes>Bifidobacteriales>Bifidobacteriaceae	GCF_902167555.1	Bifidobacterium_longum	FP566_RS07260	1570713..1574402	216816	BREX-system-P-loop-protein-BrxC-[Bifidobacterium-longum]
WP_250246699.1	Type1.BREX	MarR_2+ROK-HTH->?->||?->?-><-?||?-> CR-ATPase5 + REase -p-> BrxA-DNA-binding -> BrxB-BrxF-iSTAND -> DUF6079 *-> N6-METHYLASE ->SLATT->SMODS->Schlafen+HATPase+wHTH->RNase-> PglZ -> SIGMA+OB-Fold+AAA-ATPase+HKD-DNase-BrxL ->	1229	Bacteria>Terrabacteria_group>Actinomycetota>Actinomycetes>Bifidobacteriales>Bifidobacteriaceae	GCF_939148185.1	Bifidobacterium_longum	NBW58_RS09955	18627..22316	216816	BREX-system-P-loop-protein-BrxC-[Bifidobacterium-longum]
WP_251163982.1	Type1.BREX	Nif11-> BrxA-DNA-binding -> BrxB-BrxF-iSTAND -> DUF6079 *-> N6-METHYLASE ->?-> N6-METHYLASE -p-> PglZ -> SIGMA+OB-Fold+AAA-ATPase+Lon-Protease-BrxL -> CR-ATPase5 + REase ->	1193	Bacteria>Terrabacteria_group>Actinomycetota>Coriobacteriia>Coriobacteriales>Atopobiaceae	-	Caniella_muris	IM763_04355	91958..95539	2941502	BREX-system-P-loop-protein-BrxC-[Caniella-muris]
MBF0599289.1	Type1.BREX	Nif11-> BrxA-DNA-binding -> BrxB-BrxF-iSTAND -> DUF6079 *-> N6-METHYLASE ->?-> N6-METHYLASE -p-> PglZ -> SIGMA+OB-Fold+AAA-ATPase+Lon-Protease-BrxL -> CR-ATPase5 + REase ->	1193	Bacteria>Terrabacteria_group>Actinomycetota>Coriobacteriia>Coriobacteriales>Atopobiaceae	GCA_015234195.1	Atopobiaceae_bacterium_FL090493	IM763_04355	91958..95539	2661918	BREX-system-P-loop-protein-BrxC-[Atopobiaceae-bacterium-FL090493]
MBR2835999.1	Type1.BREX	<-ACET<- CR-ATPase5 + REase <- PglZ <- DUF6079 *<- BrxB-BrxF-iSTAND <- BrxA-DNA-binding <- CR-ATPase8 + REase <- PglZ	1194	Bacteria>Terrabacteria_group>Actinomycetota>Coriobacteriia>Coriobacteriales>unclassified_Coriobacteriales	GCA_017515595.1	Coriobacteriales_bacterium	IKE43_09885	4830..8414	2478480	BREX-system-P-loop-protein-BrxC-[Coriobacteriales-bacterium]
WP_251231669.1	Type1.BREX	<- SIGMA+OB-Fold+AAA-ATPase+Lon-Protease-BrxL <- PglZ <-?<-?<- N6-METHYLASE -p<-SIGMA-HTH+SIGMA-HTH<-HEPN<- REase <- N6-METHYLASE <- DUF6079 *<- BrxB-BrxF-iSTAND <- BrxA-DNA-binding <-Nif11<- CR-ATPase5 + REase <-?<-Pkinase<-?<-PspA	1213	Bacteria>Terrabacteria_group>Actinomycetota>Coriobacteriia>Eggerthellales>Eggerthellaceae	GCF_943193145.1	Adlercreutzia_aquisgranensis	NFR41_RS09570	19774..23415	2941323	BREX-system-P-loop-protein-BrxC-[Adlercreutzia-aquisgranensis]
WP_251851849.1	Type1.BREX	DSBH+AraC-HTH-><-ParB+HNH<- PglZ <- N6-METHYLASE -p||Phage_int_SAM_5+Phage_integrase-> N6-METHYLASE -p-><- N6-METHYLASE -p<- CR-ATPase5 + REase <- N6-METHYLASE <- DUF6079 *<- BrxB-BrxF-iSTAND <- BrxA-DNA-binding ||MULE-TRANSPOSASE->?->?-><-DDE_Tnp<-?||DDE_Tnp_IS240->	1202	Bacteria>Terrabacteria_group>Bacillota>Bacilli>Lactobacillales>Enterococcaceae	GCF_023744155.1	Enterococcus_italicus	NE261_RS07355	549628..553236	246144	BREX-system-P-loop-protein-BrxC-[Enterococcus-italicus]
NF012999.1	Type1.BREX	Calcineurin->?-> BrxA-DNA-binding -> BrxB-BrxF-iSTAND -> DUF6079 *-> CR-ATPase9 + REase -> N6-METHYLASE ->ParB+HNH->OLD-ABC->?-> PglZ -><- REase || SIGMA+OB-Fold+AAA-ATPase+Lon-Protease-BrxL ->Peptidase_M42->	1215	Bacteria>Terrabacteria_group>Bacillota>Clostridia>Eubacteriales>Clostridiaceae	GCA_011016315.1	Clostridium_botulinum	FDB34_02025	196714..200361	1491	BREX-system-P-loop-protein-BrxC-[Clostridium-botulinum]
MBN1067552.1	Type1.BREX	Calcineurin->?-> BrxA-DNA-binding -> BrxB-BrxF-iSTAND -> DUF6079 *-> CR-ATPase9 + REase -> N6-METHYLASE ->OLD-ABC->HNH-> PglZ -><- REase || SIGMA+OB-Fold+AAA-ATPase+Lon-Protease-BrxL ->Peptidase_M42->TIR->	1215	Bacteria>Terrabacteria_group>Bacillota>Clostridia>Eubacteriales>Clostridiaceae	GCA_016925955.1	Clostridium_botulinum	DVW02_06040	97050..100697	1491	BREX-system-P-loop-protein-BrxC-[Clostridium-botulinum]
WP_039258635.1	Type1.BREX	<-Calcineurin+LysM+LysM<- PglZ <-CRISPR_Cas2<-Cas_Cas1<-Cas9+HNH+RuvC<- N6-METHYLASE <- CR-ATPase9 + REase <- DUF6079 *<- BrxB-BrxF-iSTAND <- BrxA-DNA-binding <-?<-?<-?<-ClpABN-AAA+ClpABC-AAA||Cysteine-Desulfurase->	1214	Bacteria>Terrabacteria_group>Bacillota>Clostridia>Eubacteriales>Clostridiaceae	GCF_000768655.1	Clostridium_botulinum	ADU88_RS01575	72725..76369	1491	BREX-system-P-loop-protein-BrxC-[Clostridium-botulinum]
WP_156211829.1	Type1.BREX	<-wHTH+McrB-AAA<-?<- PglZ <-?<-?<-OLD-ABC<-ParB+HNH<- N6-METHYLASE <- CR-ATPase9 + REase <- DUF6079 *<- BrxB-BrxF-iSTAND <- BrxA-DNA-binding <-HKD-DNase+SF1-SF2-Helicase+HTH+HTH+SRA<-SAM-METHYLASE<-NERD+HRDC<-Ntox45<-HEPN	1216	Bacteria>Terrabacteria_group>Bacillota>Clostridia>Eubacteriales>Clostridiaceae	GCF_009735365.2	Clostridium_butyricum	GND98_RS05145	54704..58354	1492	BREX-system-P-loop-protein-BrxC-[Clostridium-butyricum]
WP_164949146.1	Type1.BREX	<-HEPN<- SIGMA+OB-Fold+AAA-ATPase+Lon-Protease-BrxL <- PglZ <-HNH<-OLD-ABC<- N6-METHYLASE <- DUF6079 *<- BrxB-BrxF-iSTAND <- BrxA-DNA-binding <-?<- CR-ATPase8 + REase ||?->?-><-?<-?||METHYLASE->	1215	Bacteria>Terrabacteria_group>Bacillota>Clostridia>Eubacteriales>Clostridiaceae	GCF_011065955.2	Clostridium_sp._FP2	G9F71_RS17965	3736090..3739737	2724481	BREX-system-P-loop-protein-BrxC-[Clostridium-sp.-FP2]
WP_257411542.1	Type1.BREX	<- PglZ <- N6-METHYLASE <- DUF6079 *<- BrxB-BrxF-iSTAND <- BrxA-DNA-binding ||?->?-><- CR-ATPase8 + REase <-MODE-HTH+PBPII	1195	Bacteria>Terrabacteria_group>Bacillota>Clostridia>Eubacteriales>Clostridiaceae	GCF_024622885.1	Clostridium_sporogenes	NSA52_RS02830	6440..10027	1509	BREX-system-P-loop-protein-BrxC-[Clostridium-sporogenes]
MBF1566405.1	Type1.BREX	<-CHTH||?-><- CR-ATPase8 + REase <-MNS+ REase <- SIGMA+OB-Fold+AAA-ATPase+Lon-Protease-BrxL <-?<- PglZ <-OLD-ABC<- N6-METHYLASE <- DUF6079 *<- BrxB-BrxF-iSTAND <- BrxA-DNA-binding <-SMODS<-?<-?<- CR-ATPase8 + REase	1178	Bacteria>Terrabacteria_group>Bacillota>Clostridia>Eubacteriales>Oscillospiraceae	GCA_017848115.1	Oscillospiraceae_bacterium	J6A58_11685	59197..62733	2485925	BREX-system-P-loop-protein-BrxC-[Oscillospiraceae-bacterium]
UYJ11714.1	Type1.BREX	<- CR-ATPase8 + REase <-?<- CR-ATPase5 + REase <-?<-?||DOC+HTH-> BrxA-DNA-binding -> BrxB-BrxF-iSTAND -> DUF6079 *->?->?-> N6-METHYLASE -> OLD-ABC-> REase -> PglZ -> SIGMA+OB-Fold+AAA-ATPase+Lon-Protease-BrxL ->ParB+HNH-> REase +SF1-SF2-Helicase ->	1182	Bacteria>Terrabacteria_group>Bacillota>Clostridia>Eubacteriales>Oscillospiraceae	GCA_025757845.1	Oscillospiraceae_bacterium	OGM78_02700	567882..571430	2485925	MAG:-BREX-system-P-loop-protein-BrxC-[Oscillospiraceae-bacterium]
MCR4615485.1	Type1.BREX	<- SIGMA+OB-Fold+AAA-ATPase+Lon-Protease-BrxL <- CR-ATPase5 + REase <- REase <-AbiGi+seqA+RAMA<- N6-METHYLASE <- PglZ <-?<- N6-METHYLASE <-?<- DUF6079 *<- BrxB-BrxF-iSTAND <- BrxA-DNA-binding <-?<- wHTH+WYL+WCX ||LRR_5->?->HTH_3->Couple_hipA->HipA_C->	1161	Bacteria>Terrabacteria_group>Bacillota>Clostridia>Eubacteriales>unclassified_Eubacteriales	GCA_024684435.1	Clostridiales_bacterium	K5756_04990	82244..85729	1898207	BREX-system-P-loop-protein-BrxC-[Clostridiales-bacterium]
MBQ6380318.1	Type1.BREX	N6-METHYLASE ->TRD->SbcC->?->STYKinase-like-><-?<-SAM-METHYLASE|| BrxA-DNA-binding -> BrxB-BrxF-iSTAND -> DUF6079 *-> CR-ATPase5 + REase -> N6-METHYLASE -p->	1174	Bacteria>Terrabacteria_group>Bacillota>Clostridia>unclassified_Clostridia	GCA_017430965.1	Clostridia_bacterium	IJJ41_01780	27986..31510	2044939	BREX-system-P-loop-protein-BrxC-[Clostridia-bacterium]
MBF0120841.1	Type1.BREX	<- SIGMA+OB-Fold+AAA-ATPase+Lon-Protease-BrxL <- PglZ <- N6-METHYLASE <- CR-ATPase8 + REase <- DUF6079 *<- BrxB-BrxF-iSTAND <- BrxA-DNA-binding <-HEPN	1201	Bacteria>Thermodesulfobacteriota>Desulfobacteria>Desulfobacterales>unclassified_Desulfobacterales	GCA_015228685.1	Desulfobacterales_bacterium	HQK79_18595	27077..30682	2044940	BREX-system-P-loop-protein-BrxC-[Desulfobacterales-bacterium]
NQX01743.1	Type6.BREX	BrxE -> BrxA-DNA-binding -> BrxB-BrxF-iSTAND -> DUF6079 *-> CR-ATPase5 + REase ->DOC+HTH->Four-helical-protein-> N6-METHYLASE -p->	1210	Bacteria>unclassified_Bacteria	GCA_013205625.1	bacterium	HQ447_13895	2028..5660	1869227	BREX-system-P-loop-protein-BrxC-[bacterium]
--										
-- HTH+ REase										
-- w HTH+ REase										
-- HARE-HTH + REase										
--										
WP_255191907.1	Type1.BREX	<-REC+CheB-Methylesterase<-REC+OmpR-HTH<-?||?-><-?||HTH_23+HTH_32+HTH_33-><- McrC-NTD+ REase <-?|| BrxA-DNA-binding -> DUF6079 *-> BrxB-BrxF-iSTAND -> DUF6079 -> N6-METHYLASE ->?-> PglZ -><-?||?-><-?||HTH+PADR-HTH->	1261	Archaea>Euryarchaeota>Stenosarchaea_group>Halobacteria>Natrialbales>Natrialbaceae	GCF_024362485.1	Halovivax_sp._WLHSJ27	NMQ09_RS17725	3493167..3496952	2963433	hypothetical-protein-[Halovivax-sp.-WLHSJ27]
WP_255191909.1	Type1.BREX	HTH_23+HTH_32+HTH_33-> McrC-NTD+ REase <-?|| BrxA-DNA-binding -> DUF6079 -> BrxB-BrxF-iSTAND -> DUF6079 *-> N6-METHYLASE ->?-> PglZ -><-?||?-><-?||HTH+PADR-HTH-> TUDOR+SF1-SF2-Helicase+i REase +CTD ->	1273	Archaea>Euryarchaeota>Stenosarchaea_group>Halobacteria>Natrialbales>Natrialbaceae	GCF_024362485.1	Halovivax_sp._WLHSJ27	NMQ09_RS17735	3497545..3501366	2963433	hypothetical-protein-[Halovivax-sp.-WLHSJ27]
WP_273738559.1	Type6.BREX	McrB-AAA-> McrC-NTD+ REase -> BrxE -> BrxA-DNA-binding -> BrxB-BrxF-iSTAND -> DUF6079 *-> N6-METHYLASE -p->	1214	Archaea>Euryarchaeota>Stenosarchaea_group>Halobacteria>Natrialbales>Natrialbaceae	GCF_028618695.1	Natrinema_soli	PGX04_RS11075	6409..10053	1930624	BREX-system-P-loop-protein-BrxC-[Natrinema-soli]
WP_004077401.1	Type1.BREX	wHTH+WYL-><- SIGMA+OB-Fold+AAA-ATPase+Lon-Protease-BrxL <- PglZ <- N6-METHYLASE -p<- HTH+ REase <-?<-?<- N6-METHYLASE <- DUF6079 *<- BrxB-BrxF-iSTAND	1180	Archaea>Euryarchaeota>Stenosarchaea_group>Methanomicrobia>Methanomicrobiales>Methanomicrobiaceae	GCF_000243255.1	Methanoplanus_limicola	METLIM_RS07750	1605385..1608927	2315	BREX-system-P-loop-protein-BrxC-[Methanoplanus-limicola]
WP_004078487.1	Type1.BREX	<- CR-ATPase5 + REase ||MORC+ REase -p-><- SIGMA+OB-Fold+AAA-ATPase+Lon-Protease-BrxL <- PglZ <-?<- HTH+ REase <- N6-METHYLASE <- DUF6079 *<- BrxB-BrxF-iSTAND <- BrxA-DNA-binding <-Peptidase_M42||?-><-PIN<-?||?->HicB_lk_antitox->	1155	Archaea>Euryarchaeota>Stenosarchaea_group>Methanomicrobia>Methanomicrobiales>Methanomicrobiaceae	GCF_000243255.1	Methanoplanus_limicola	METLIM_RS10810	2304713..2308180	2315	BREX-system-P-loop-protein-BrxC-[Methanoplanus-limicola]
WP_257742634.1	Type1.BREX	<- REase ||PIN-><-?||Peptidase_M42-> BrxA-DNA-binding -> BrxB-BrxF-iSTAND -> DUF6079 *-> N6-METHYLASE -> HTH+ REase ->DDE_Tnp_1->?-> PglZ -> SIGMA+OB-Fold+AAA-ATPase+Lon-Protease-BrxL ->?-><-DDE_Tnp	1155	Archaea>Euryarchaeota>Stenosarchaea_group>Methanomicrobia>Methanomicrobiales>Methanomicrobiaceae	GCF_024662215.1	Methanoplanus_endosymbiosus	L6E24_RS14385	3075748..3079215	33865	BREX-system-P-loop-protein-BrxC-[Methanoplanus-endosymbiosus]
MBN1165665.1	Type1.BREX	TPR+TPR+TPR-> BrxA-DNA-binding -> BrxB-BrxF-iSTAND -> DUF6079 *-> N6-METHYLASE -p-> N6-METHYLASE ->?->?->ParA-Soj-PloopNTPase->?-> HTH+ REase -> PglZ -> SIGMA+OB-Fold+AAA-ATPase+Lon-Protease-BrxL ->	1218	Archaea>Euryarchaeota>Stenosarchaea_group>Methanomicrobia>Methanomicrobiales>Methanospirillaceae	GCA_016926505.1	Methanospirillaceae_archaeon	JXA44_00885	48354..52010	2813030	BREX-system-P-loop-protein-BrxC-[Methanospirillaceae-archaeon]
WP_011448873.1	Type1.BREX	CR-ATPase8 + REase -><-TPR+TPR<-TPR<-TOPRIM<-OLD-ABC<-?|| BrxB-BrxF-iSTAND -> DUF6079 *-> N6-METHYLASE ->?->?->?->HATPase+wHTH-> HTH+ REase -> N6-METHYLASE -p-><-DDE_Tnp|| N6-METHYLASE -p->	1178	Archaea>Euryarchaeota>Stenosarchaea_group>Methanomicrobia>Methanomicrobiales>Methanospirillaceae	GCF_000013445.1	Methanospirillum_hungatei	MHUN_RS09850	2137740..2141276	2203	BREX-system-P-loop-protein-BrxC-[Methanospirillum-hungatei]
WP_214418671.1	Type1.BREX	<- SIGMA+OB-Fold+AAA-ATPase+Lon-Protease-BrxL <- PglZ <- N6-METHYLASE -p<- HTH+ REase <-Schlafen+HATPase+wHTH<- N6-METHYLASE <- DUF6079 *<- BrxB-BrxF-iSTAND <-zf-IS66<- N6-METHYLASE <-?<-RNase<-?<- REase ||EVE->	1171	Archaea>Euryarchaeota>Stenosarchaea_group>Methanomicrobia>Methanomicrobiales>Methanospirillaceae	GCF_018502485.1	Methanospirillum_sp._J.3.6.1-F.2.7.3	KHC33_RS10925	2269426..2272941	2834276	BREX-system-P-loop-protein-BrxC-[Methanospirillum-sp.-J.3.6.1-F.2.7.3]
KXS44097.1	Type1.BREX	<- SIGMA+OB-Fold+AAA-ATPase+Lon-Protease-BrxL <- PglZ <-TOPRIM<-OLD-ABC<-?<- N6-METHYLASE -p<-Schlafen+HATPase+wHTH<- N6-METHYLASE <- DUF6079 *<- BrxB-BrxF-iSTAND <- McrC-NTD+ REase <-EVE+McrB-AAA<-?|| McrC-NTD+ REase ->MNS+ REase ->RADICAL-SAM->	1185	Archaea>Euryarchaeota>Stenosarchaea_group>Methanomicrobia>Methanosarcinales>Methanosarcinaceae	GCA_001577565.1	Methanolobus_sp._T82-4	AWU59_730	16022..19579	1794908	MAG:-hypothetical-protein-AWU59_730-[Methanolobus-sp.-T82-4]
WP_048162601.1	Type1.BREX	Glyco_hydro_130->GT4+DSBH-><-?<-?||tRNA-><-NUDIX+DUF429||?-> BrxB-BrxF-iSTAND -> DUF6079 *-> N6-METHYLASE -> HTH+ REase -> N6-METHYLASE -p->?->Schlafen+HATPase+wHTH->?->ACET->Mrr_N+ACET-><-TPR+TPR+TPR+TPR	1223	Archaea>Euryarchaeota>Stenosarchaea_group>Methanomicrobia>Methanosarcinales>Methanosarcinaceae	GCF_000979975.1	Methanosarcina_sp._1.H.A.2.2	EO93_RS11285	63696..67367	1483601	BREX-system-P-loop-protein-BrxC-[Methanosarcina-sp.-1.H.A.2.2]
WP_141050577.1	Type1.BREX	<-DnaB+TnsC-ATPase<-ParB<-ParA-Soj-PloopNTPase+ORC-CDC6-like<-DDE_5+DDE_Tnp_1||BirA-HTH->?-> wHTH+WYL+WCX -> BrxA-DNA-binding -> BrxB-BrxF-iSTAND -> DUF6079 *-> HTH+ REase -> N6-METHYLASE -p->?-> N6-METHYLASE -p->AbiEi_4->AbiEii->Schlafen+HATPase+wHTH-> REase +SF1-SF2-Helicase ->	1162	Bacteria>Campylobacterota>Epsilonproteobacteria>Campylobacterales>Arcobacteraceae>Aliarcobacter	GCF_006503615.1	Aliarcobacter_cryaerophilus	AV936_RS08795	11744..15232	28198	BREX-system-P-loop-protein-BrxC-[Aliarcobacter-cryaerophilus]
RXJ95785.1	Type1.BREX	wHTH+WYL+WCX ->?->?->RADICAL-SAM->?->HIN-HTH-> BrxA-DNA-binding -> BrxB-BrxF-iSTAND -> DUF6079 *-> N6-METHYLASE -p->?->?-> HTH+ REase -> N6-METHYLASE -p->AbiEi_4->AbiEii-> PglZ -> SIGMA+OB-Fold+AAA-ATPase+Lon-Protease-BrxL ->	1162	Bacteria>Campylobacterota>Epsilonproteobacteria>Campylobacterales>Arcobacteraceae>Arcobacter	GCA_004116575.1	Arcobacter_sp._CECT_9299	CRU94_03995	49858..53346	2044506	hypothetical-protein-CRU94_03995-[Arcobacter-sp.-CECT-9299]
WP_129105531.1	Type1.BREX	wHTH+WYL+WCX ->?->?->RADICAL-SAM->?-> BrxA-DNA-binding -> BrxB-BrxF-iSTAND -> DUF6079 *-> N6-METHYLASE -p->?->?-> HTH+ REase -> N6-METHYLASE -p->AbiEi_4->AbiEii-> PglZ -> SIGMA+OB-Fold+AAA-ATPase+Lon-Protease-BrxL ->	1162	Bacteria>Campylobacterota>Epsilonproteobacteria>Campylobacterales>Arcobacteraceae>Arcobacter	GCF_004116575.1	Arcobacter_sp._CECT_9299	CRU94_RS03995	49858..53346	2044506	BREX-system-P-loop-protein-BrxC-[Arcobacter-sp.-CECT-9299]
WP_164968523.1	Type1.BREX	BrxA-DNA-binding -> BrxB-BrxF-iSTAND -> DUF6079 *-> N6-METHYLASE -p->?->?-> HTH+ REase -> N6-METHYLASE -p->AbiEi_4->AbiEii-> PglZ -> SIGMA+OB-Fold+AAA-ATPase+Lon-Protease-BrxL ->	1160	Bacteria>Campylobacterota>Epsilonproteobacteria>Campylobacterales>Arcobacteraceae>Arcobacter	GCF_013201725.1	Arcobacter_defluvii	ADFLV_RS07175	1422397..1425879	873191	BREX-system-P-loop-protein-BrxC-[Arcobacter-defluvii]
PHS59105.1	Type1.BREX	<-Methyltransf_30<-?<-?<-?<-?<-?||?-> BrxA-DNA-binding -> BrxB-BrxF-iSTAND -> DUF6079 *->PemK_toxin-> N6-METHYLASE -p->?->?-> N6-METHYLASE -p->?-> HTH+ REase -> PglZ -> SIGMA+OB-Fold+AAA-ATPase+Lon-Protease-BrxL ->	1158	Bacteria>Campylobacterota>Epsilonproteobacteria>Campylobacterales>Thiovulaceae>Sulfurimonas	GCA_002733155.1	Sulfurimonas_sp.	COB17_00805	100943..104419	2022749	MAG:-hypothetical-protein-COB17_00805-[Sulfurimonas-sp.]
MBZ4644075.1	Type1.BREX	<-TPR<-ARG<-?||McrB-AAA-> McrC-NTD+ REase -> BrxA-DNA-binding -> BrxB-BrxF-iSTAND -> DUF6079 *-> N6-METHYLASE -p->HEPN-> N6-METHYLASE -> PglZ -> SIGMA+OB-Fold+AAA-ATPase+Lon-Protease-BrxL ->?->SF1-SF2-Helicase->	1168	Bacteria>Deferribacterota>Deferribacteres>Deferribacterales>unclassified_Deferribacteraceae	GCA_020054865.1	Deferribacteraceae_bacterium	JG767_1684	7812..11318	2053543	hypothetical-protein-[Deferribacteraceae-bacterium]
WP_242820841.1	Type1.BREX	NUDIX-><-TPR+S1<-ARG<-?||McrB-AAA-> McrC-NTD+ REase -> BrxA-DNA-binding -> BrxB-BrxF-iSTAND -> DUF6079 *-> N6-METHYLASE -p->HEPN-> N6-METHYLASE -> PglZ -> SIGMA+OB-Fold+AAA-ATPase+Lon-Protease-BrxL ->?->SF1-SF2-Helicase->	1168	Bacteria>Deferribacterota>Deferribacteres>Deferribacterales>Deferrivibrionaceae>Deferrivibrio	GCF_020480685.1	Deferrivibrio_essentukiensis	LF845_RS09815	29861..33367	2880922	BREX-system-P-loop-protein-BrxC-[Deferrivibrio-essentukiensis]
MBF0105032.1	Type1.BREX	<- SIGMA+OB-Fold+AAA-ATPase+Lon-Protease-BrxL <- PglZ <- HTH+ REase <- N6-METHYLASE <- DUF6079 *<- BrxB-BrxF-iSTAND <- BrxA-DNA-binding	1166	Bacteria>delta/epsilon_subdivisions>Deltaproteobacteria>unclassified_Deltaproteobacteria	GCA_015228875.1	Deltaproteobacteria_bacterium	HQM16_06860	10380..13880	2026735	BREX-system-P-loop-protein-BrxC-[Deltaproteobacteria-bacterium]
MBF0105437.1	Type1.BREX	<- CR-ATPase5 + REase ||?-><-?<-?||?-> BrxA-DNA-binding -> BrxB-BrxF-iSTAND -> DUF6079 *-> N6-METHYLASE -> HTH+ REase ->?-> PglZ -> SIGMA+OB-Fold+AAA-ATPase+Lon-Protease-BrxL ->RADICAL-SAM->	1155	Bacteria>delta/epsilon_subdivisions>Deltaproteobacteria>unclassified_Deltaproteobacteria	GCA_015228875.1	Deltaproteobacteria_bacterium	HQM16_08940	21511..24978	2026735	BREX-system-P-loop-protein-BrxC-[Deltaproteobacteria-bacterium]
MCP4577326.1	Type1.BREX	<-Thioredoxin||?->?-><-?||?-><-?|| BrxA-DNA-binding -> BrxB-BrxF-iSTAND -> DUF6079 *->RNase->?-> HTH+ REase -> N6-METHYLASE ->Schlafen+HATPase+wHTH-> PglZ -> SIGMA+OB-Fold+AAA-ATPase+Lon-Protease-BrxL ->?->ABC-ATPase+SbcC->	1196	Bacteria>delta/epsilon_subdivisions>Deltaproteobacteria>unclassified_Deltaproteobacteria	GCA_024231575.1	Deltaproteobacteria_bacterium	GY846_13700	13452..17042	2026735	BREX-system-P-loop-protein-BrxC-[Deltaproteobacteria-bacterium]
NLA16135.1	Type1.BREX	<-HTH_3|| HTH+ REase -><- SIGMA+OB-Fold+AAA-ATPase+HKD-DNase-BrxL <- PglZ <- N6-METHYLASE <- DUF6079 *<- BrxB-BrxF-iSTAND <- BrxA-DNA-binding <-HTH_3<-?||?->tRNA-><-?<-?||?->LuxR-HTH->	1174	Bacteria>FCB_group>Bacteroidetes/Chlorobi_group>Bacteroidota>Bacteroidia>Bacteroidales	GCA_012510445.1	Bacteroidales_bacterium	GX877_06335	9723..13247	2030927	BREX-system-P-loop-protein-BrxC-[Bacteroidales-bacterium]
MBQ9588226.1	Type1.BREX	Peptidase->?-><-HD|| BrxA-DNA-binding -> BrxB-BrxF-iSTAND -> DUF6079 *-> HTH+ REase -> N6-METHYLASE -p-> PglZ -> SIGMA+OB-Fold+AAA-ATPase+HKD-DNase-BrxL ->	1183	Bacteria>FCB_group>Bacteroidetes/Chlorobi_group>Bacteroidota>Bacteroidia>Bacteroidales	GCA_017446745.1	Bacteroidales_bacterium	IJR26_10275	19779..23330	2030927	BREX-system-P-loop-protein-BrxC-[Bacteroidales-bacterium]
MBW6491485.1	Type1.BREX	<-HTH_3||XIS-HTH->?->?->?-> BrxA-DNA-binding -> BrxB-BrxF-iSTAND -> DUF6079 *-> HTH+ REase -> N6-METHYLASE ->AbiEii-> PglZ -> SIGMA+OB-Fold+AAA-ATPase+Lon-Protease-BrxL -> REase ->HEPN->ClpABN-AAA+ClpABC-AAA->	1144	Bacteria>FCB_group>Bacteroidetes/Chlorobi_group>Bacteroidota>Bacteroidia>Bacteroidales	GCA_019429275.1	Lentimicrobium_sp.	KOB15_09875	47933..51367	2034841	BREX-system-P-loop-protein-BrxC-[Lentimicrobium-sp.]
MCB9017748.1	Type1.BREX	<-ACET||?-><- SIGMA+OB-Fold+AAA-ATPase+HKD-DNase-BrxL <- PglZ <- N6-METHYLASE -p<- HTH+ REase <-?<-Schlafen+HATPase+wHTH<- DUF6079 *<- BrxB-BrxF-iSTAND <- BrxA-DNA-binding <-?<-PALP	1207	Bacteria>FCB_group>Bacteroidetes/Chlorobi_group>Bacteroidota>Bacteroidia>Bacteroidales	GCA_020636675.1	Prevotellaceae_bacterium	H6544_03995	62697..66320	2049047	BREX-system-P-loop-protein-BrxC-[Prevotellaceae-bacterium]
MCC8119638.1	Type1.BREX	<- PglZ <-Schlafen+HATPase+wHTH<-?<-ParB+HNH<-AAA-ATPase-p<-Schlafen+HATPase+wHTH<- N6-METHYLASE <- HTH+ REase <- DUF6079 *<- BrxB-BrxF-iSTAND <- BrxA-DNA-binding <-tRNA<-35exo+T7DNApol	1189	Bacteria>FCB_group>Bacteroidetes/Chlorobi_group>Bacteroidota>Bacteroidia>Bacteroidales	GCA_020860515.1	Bacteroidales_bacterium	LIP09_12950	29133..32702	2030927	BREX-system-P-loop-protein-BrxC-[Bacteroidales-bacterium]
MCI1742488.1	Type1.BREX	<-HTH_31||tRNA-><-?<-SIR2+SIR2<- SIGMA+OB-Fold+AAA-ATPase+HKD-DNase-BrxL <- PglZ <- HTH+ REase <-Schlafen+HATPase+wHTH<- N6-METHYLASE <- DUF6079 *<- BrxB-BrxF-iSTAND <- BrxA-DNA-binding <-HTH_3<- wHTH+WYL+WCX ||?-><-AAA+<-?<-XIS-HTH	1206	Bacteria>FCB_group>Bacteroidetes/Chlorobi_group>Bacteroidota>Bacteroidia>Bacteroidales	GCA_022647785.1	Prevotella_sp.	LKI18_09155	39395..43015	59823	BREX-system-P-loop-protein-BrxC-[Prevotella-sp.]
MCI2087046.1	Type1.BREX	<- SIGMA+OB-Fold+AAA-ATPase+HKD-DNase-BrxL <- PglZ <- HTH+ REase <-Schlafen+HATPase+wHTH<- N6-METHYLASE <- DUF6079 *<- BrxB-BrxF-iSTAND <- BrxA-DNA-binding <-HTH_3<- wHTH+WYL+WCX ||?-><-AAA+<-?<-XIS-HTH	1198	Bacteria>FCB_group>Bacteroidetes/Chlorobi_group>Bacteroidota>Bacteroidia>Bacteroidales	GCA_022653375.1	Prevotella_sp.	LKK10_02020	11419..15015	59823	BREX-system-P-loop-protein-BrxC-[Prevotella-sp.]
WP_008760280.1	Type1.BREX	D5ATPase+DUF3874-><-?||ADH_N+ADH_zinc_N+METHYLASE->SPOUT-><- SIGMA+OB-Fold+AAA-ATPase+HKD-DNase-BrxL <- PglZ <- HTH+ REase <- N6-METHYLASE -p<- N6-METHYLASE <- DUF6079 *<- BrxB-BrxF-iSTAND <- BrxA-DNA-binding <-HTH_3<-?<-?<-?<-TIR<-?<-TIR	1193	Bacteria>FCB_group>Bacteroidetes/Chlorobi_group>Bacteroidota>Bacteroidia>Bacteroidales	GCF_000162515.1	Bacteroides_thetaiotaomicron	HMPREF9007_RS20215	181866..185447	818	MULTISPECIES:-BREX-system-P-loop-protein-BrxC-[Bacteroides]
WP_130041244.1	Type1.BREX	Polbeta+HEPN->D5ATPase+DUF3874-><-?||ADH_N+ADH_zinc_N+METHYLASE->SPOUT-><- SIGMA+OB-Fold+AAA-ATPase+HKD-DNase-BrxL <- PglZ <- HTH+ REase <- N6-METHYLASE <- DUF6079 *<- BrxB-BrxF-iSTAND <- BrxA-DNA-binding <-HTH_3<-sGTP||?->Response_reg->NAD_binding_10->	1175	Bacteria>FCB_group>Bacteroidetes/Chlorobi_group>Bacteroidota>Bacteroidia>Bacteroidales	GCF_019733625.1	Bacteroides_thetaiotaomicron	EAJ10_RS04945	124743..128270	818	BREX-system-P-loop-protein-BrxC-[Bacteroides-thetaiotaomicron]
WP_225659484.1	Type1.BREX	Polbeta+HEPN->D5ATPase+DUF3874-><-?||ADH_N+ADH_zinc_N+METHYLASE->SPOUT-><- SIGMA+OB-Fold+AAA-ATPase+HKD-DNase-BrxL <- PglZ <- HTH+ REase <- N6-METHYLASE <- DUF6079 *<- BrxB-BrxF-iSTAND <- BrxA-DNA-binding <-HTH_3<-sGTP||?->Response_reg->NAD_binding_10->	1175	Bacteria>FCB_group>Bacteroidetes/Chlorobi_group>Bacteroidota>Bacteroidia>Bacteroidales	GCF_020313445.1	Bacteroides_thetaiotaomicron	KFX61_RS09580	34477..38004	818	BREX-system-P-loop-protein-BrxC-[Bacteroides-thetaiotaomicron]
WP_258973412.1	Type1.BREX	Polbeta+HEPN->D5ATPase+DUF3874-><-?||ADH_N+ADH_zinc_N+METHYLASE->SPOUT-><- SIGMA+OB-Fold+AAA-ATPase+HKD-DNase-BrxL <- PglZ <- HTH+ REase <- N6-METHYLASE <- DUF6079 *<-?<- BrxB-BrxF-iSTAND <- BrxA-DNA-binding <-HTH_3|| CR-ATPase5 + REase ->?->?-><-RVT	1133	Bacteria>FCB_group>Bacteroidetes/Chlorobi_group>Bacteroidota>Bacteroidia>Bacteroidales	GCF_024793205.1	Bacteroides_thetaiotaomicron	NXX32_RS11560	3197619..3201020	818	BREX-system-P-loop-protein-BrxC-[Bacteroides-thetaiotaomicron]
OSZ81159.1	Type1.BREX	BrxA-DNA-binding -> BrxB-BrxF-iSTAND -> DUF6079 *-> N6-METHYLASE ->McrB-AAA-> McrC-NTD+ REase -> PglZ -> SIGMA+OB-Fold+AAA-ATPase+Lon-Protease-BrxL -><-Arm-DNA-bind_5+Phage_int_SAM_5+Phage_integrase<-?<-REC+OmpR-HTH<-REC+CITB-HTH	1144	Bacteria>FCB_group>Bacteroidetes/Chlorobi_group>Bacteroidota>Chitinophagia>Chitinophagales	GCA_002127295.1	Chitinophagaceae_bacterium_IBVUCB2	CAP36_07965	1792100..1795534	1985174	hypothetical-protein-CAP36_07965-[Chitinophagaceae-bacterium-IBVUCB2]
MBL7764603.1	Type1.BREX	HTH-> BrxA-DNA-binding -> BrxB-BrxF-iSTAND -> DUF6079 *-> N6-METHYLASE ->McrB-AAA-> McrC-NTD+ REase -> PglZ -> SIGMA+OB-Fold+AAA-ATPase+Lon-Protease-BrxL ->	1146	Bacteria>FCB_group>Bacteroidetes/Chlorobi_group>Bacteroidota>Chitinophagia>Chitinophagales	GCA_016786845.1	Chitinophagaceae_bacterium	JNJ58_00790	47471..50911	1869212	BREX-system-P-loop-protein-BrxC-[Chitinophagaceae-bacterium]
MCK9404665.1	Type1.BREX	<- HARE-HTH + REase <- SIGMA+OB-Fold+AAA-ATPase+Lon-Protease-BrxL <- PglZ <-?<- N6-METHYLASE <- DUF6079 *<- BrxB-BrxF-iSTAND <- BrxA-DNA-binding <- REase	1144	Bacteria>FCB_group>Bacteroidetes/Chlorobi_group>Bacteroidota>Chitinophagia>Chitinophagales	GCA_023229465.1	Chitinophagaceae_bacterium	MOQ26_14830	11931..15365	1869212	BREX-system-P-loop-protein-BrxC-[Chitinophagaceae-bacterium]
WP_072366579.1	Type1.BREX	<-HTH_Tnp_1||HTH_3-> BrxA-DNA-binding -> BrxB-BrxF-iSTAND -> DUF6079 *->?-> N6-METHYLASE ->McrB-AAA-> McrC-NTD+ REase -> PglZ -> SIGMA+OB-Fold+AAA-ATPase+Lon-Protease-BrxL ->	1142	Bacteria>FCB_group>Bacteroidetes/Chlorobi_group>Bacteroidota>Chitinophagia>Chitinophagales	GCF_900119105.1	Chitinophaga_sancti	BT118_RS32550	2428..5856	1004	BREX-system-P-loop-protein-BrxC-[Chitinophaga-sancti]
WP_008625904.1	Type1.BREX	Calcineurin->?->SbcC->?-><-SF1-SF2-Helicase||?->?-> BrxA-DNA-binding -> BrxB-BrxF-iSTAND -> DUF6079 *-> N6-METHYLASE ->McrB-AAA-> McrC-NTD+ REase -> PglZ -> SIGMA+OB-Fold+AAA-ATPase+Lon-Protease-BrxL -><-URI-DNase<-?<-?<-SF1-SF2-Helicase+RNase+RNase	1146	Bacteria>FCB_group>Bacteroidetes/Chlorobi_group>Bacteroidota>Cytophagia>Cytophagales	GCF_000330725.2	Mariniradius_saccharolyticus	C943_RS07085	84904..88344	1245591	BREX-system-P-loop-protein-BrxC-[Mariniradius-saccharolyticus]
WP_122934661.1	Type1.BREX	<-ParB<-ParA-Soj-PloopNTPase||AbiEi_4->AbiEii-> BrxA-DNA-binding -> BrxB-BrxF-iSTAND -> DUF6079 *-> N6-METHYLASE -> McrC-NTD+ REase -> PglZ -> SIGMA+OB-Fold+AAA-ATPase+Lon-Protease-BrxL ->?->?->Peptidase_S8->	1142	Bacteria>FCB_group>Bacteroidetes/Chlorobi_group>Bacteroidota>Cytophagia>Cytophagales	GCF_003711225.1	Runella_sp._SP2	DTQ70_RS30140	43566..46994	2268026	BREX-system-P-loop-protein-BrxC-[Runella-sp.-SP2]
WP_189584875.1	Type1.BREX	AbiGi+seqA+RAMA-> BrxA-DNA-binding -> BrxB-BrxF-iSTAND -> DUF6079 *-> N6-METHYLASE -> McrC-NTD+ REase -> PglZ -> SIGMA+OB-Fold+AAA-ATPase+Lon-Protease-BrxL -><-?<-?<-URI-DNase<-TPR+TPR	1146	Bacteria>FCB_group>Bacteroidetes/Chlorobi_group>Bacteroidota>Cytophagia>Cytophagales	GCF_014652135.1	Mongoliitalea_lutea	IE080_RS15850	2275..5715	849756	BREX-system-P-loop-protein-BrxC-[Mongoliitalea-lutea]
WP_244713422.1	Type1.BREX	<-XIS-HTH||HAD+PRTase+PRTase+ REase +SF1-SF2-Helicase ->?-><-?<- SIGMA+OB-Fold+AAA-ATPase+Lon-Protease-BrxL <-Preader17<- PglZ <- N6-METHYLASE <- DUF6079 *<- BrxB-BrxF-iSTAND <- BrxA-DNA-binding ||?->?-><- McrC-NTD+ REase <-McrB-AAA||HKD-DNase->HNH->Phage_integrase+Phage_integrase->	1145	Bacteria>FCB_group>Bacteroidetes/Chlorobi_group>Bacteroidota>Cytophagia>Cytophagales	GCF_022919135.1	Hymenobacter_sp._5116S-27	MUN80_RS13030	3065695..3069132	2932249	BREX-system-P-loop-protein-BrxC-[Hymenobacter-sp.-5116S-27]
WP_084121639.1	Type1.BREX	<-TraJ-RHH||?->TPR+TPR->MazG-><-?<- SIGMA+OB-Fold+AAA-ATPase+Lon-Protease-BrxL <- PglZ <- McrC-NTD+ REase <- N6-METHYLASE <- DUF6079 *<- BrxB-BrxF-iSTAND <- BrxA-DNA-binding <-MPTase<-?<-?||SF1-SF2-Helicase->	1146	Bacteria>FCB_group>Bacteroidetes/Chlorobi_group>Bacteroidota>Cytophagia>Cytophagales	GCF_900176595.1	Aquiflexum_balticum	B9A52_RS17175	4064780..4068220	280473	BREX-system-P-loop-protein-BrxC-[Aquiflexum-balticum]
MCB0520339.1	Type1.BREX	URI-DNase-><- SIGMA+OB-Fold+AAA-ATPase+Lon-Protease-BrxL <- PglZ <- McrC-NTD+ REase <-McrB-AAA<- N6-METHYLASE || CR-ATPase5 + REase -><- DUF6079 *<- BrxB-BrxF-iSTAND <- BrxA-DNA-binding <-tRNA||?-><-?<-DTXR-HTH<-?<-Peptidase_M29	1145	Bacteria>FCB_group>Bacteroidetes/Chlorobi_group>Bacteroidota>Saprospiria>Saprospirales	GCA_020436325.1	Saprospiraceae_bacterium	KDD27_15480	21267..24704	2202734	BREX-system-P-loop-protein-BrxC-[Saprospiraceae-bacterium]
WP_013769021.1	Type1.BREX	CR-Kinase+Classical-AAA->?-><- SIGMA+OB-Fold+AAA-ATPase+Lon-Protease-BrxL <- PglZ <- McrC-NTD+ REase <-McrB-AAA<- N6-METHYLASE <- DUF6079 *<- BrxB-BrxF-iSTAND <- BrxA-DNA-binding <-?<-McrB-NTD<-MACRODOMAIN<-DarT+Peptidase	1144	Bacteria>FCB_group>Bacteroidetes/Chlorobi_group>Bacteroidota>Saprospiria>Saprospirales	GCF_000212735.1	Haliscomenobacter_hydrossis	HALHY_RS33450	27488..30922	2350	BREX-system-P-loop-protein-BrxC-[Haliscomenobacter-hydrossis]
WP_070561019.1	Type1.BREX	TPR+TPR+TPR+TPR+TPR->?->ANK->ANK+ANK+ANK-><-?<- SIGMA+OB-Fold+AAA-ATPase+HKD-DNase-BrxL <- PglZ <- HTH+ REase <- N6-METHYLASE <- DUF6079 *<- BrxB-BrxF-iSTAND <- BrxA-DNA-binding <-HTH_3<-?||?->XIS-HTH->HTH_36->?->Relaxase->	1187	Bacteria>FCB_group>Bacteroidetes/Chlorobi_group>Bacteroidota>Sphingobacteriia>Sphingobacteriales	GCF_002339225.1	Sphingobacterium_sp._HMSC13C05	HMPREF3127_RS01230	95716..99279	1581095	BREX-system-P-loop-protein-BrxC-[Sphingobacterium-sp.-HMSC13C05]
WP_165305702.1	Type1.BREX	SLATT_1->?->HEPN+ REase ->?->?->HTH_3-> BrxA-DNA-binding -> BrxB-BrxF-iSTAND -> DUF6079 *-> N6-METHYLASE -> HTH+ REase -> PglZ -> SIGMA+OB-Fold+AAA-ATPase+HKD-DNase-BrxL ->?-><-HTH_3	1187	Bacteria>FCB_group>Bacteroidetes/Chlorobi_group>Bacteroidota>Sphingobacteriia>Sphingobacteriales	GCF_011064245.1	Sphingobacterium_sp._DR205	G6053_RS05210	1244063..1247626	2713573	BREX-system-P-loop-protein-BrxC-[Sphingobacterium-sp.-DR205]
[truncated: 9,595,720 more chars]
